# A High-Resolution InDel (Insertion–Deletion) Markers-Anchored Consensus Genetic Map Identifies Major QTLs Governing Pod Number and Seed Yield in Chickpea

**DOI:** 10.3389/fpls.2016.01362

**Published:** 2016-09-16

**Authors:** Rishi Srivastava, Mohar Singh, Deepak Bajaj, Swarup K. Parida

**Affiliations:** ^1^National Institute of Plant Genome ResearchNew Delhi, India; ^2^National Bureau of Plant Genetic Resources Regional StationShimla, India

**Keywords:** chickpea, genetic map, InDel, pod number, QTL, seed yield

## Abstract

Development and large-scale genotyping of user-friendly informative genome/gene-derived InDel markers in natural and mapping populations is vital for accelerating genomics-assisted breeding applications of chickpea with minimal resource expenses. The present investigation employed a high-throughput whole genome next-generation resequencing strategy in low and high pod number parental accessions and homozygous individuals constituting the bulks from each of two inter-specific mapping populations [(Pusa 1103 × ILWC 46) and (Pusa 256 × ILWC 46)] to develop non-erroneous InDel markers at a genome-wide scale. Comparing these high-quality genomic sequences, 82,360 InDel markers with reference to *kabuli* genome and 13,891 InDel markers exhibiting differentiation between low and high pod number parental accessions and bulks of aforementioned mapping populations were developed. These informative markers were structurally and functionally annotated in diverse coding and non-coding sequence components of genome/genes of *kabuli* chickpea. The functional significance of regulatory and coding (frameshift and large-effect mutations) InDel markers for establishing marker-trait linkages through association/genetic mapping was apparent. The markers detected a greater amplification (97%) and intra-specific polymorphic potential (58–87%) among a diverse panel of cultivated *desi, kabuli*, and wild accessions even by using a simpler cost-efficient agarose gel-based assay implicating their utility in large-scale genetic analysis especially in domesticated chickpea with narrow genetic base. Two high-density inter-specific genetic linkage maps generated using aforesaid mapping populations were integrated to construct a consensus 1479 InDel markers-anchored high-resolution (inter-marker distance: 0.66 cM) genetic map for efficient molecular mapping of major QTLs governing pod number and seed yield per plant in chickpea. Utilizing these high-density genetic maps as anchors, three major genomic regions harboring each of pod number and seed yield robust QTLs (15–28% phenotypic variation explained) were identified on chromosomes 2, 4, and 6. The integration of genetic and physical maps at these QTLs mapped on chromosomes scaled-down the long major QTL intervals into high-resolution short pod number and seed yield robust QTL physical intervals (0.89–2.94 Mb) which were essentially got validated in multiple genetic backgrounds of two chickpea mapping populations. The genome-wide InDel markers including natural allelic variants and genomic loci/genes delineated at major six especially in one colocalized novel congruent robust pod number and seed yield robust QTLs mapped on a high-density consensus genetic map were found most promising in chickpea. These functionally relevant molecular tags can drive marker-assisted genetic enhancement to develop high-yielding cultivars with increased seed/pod number and yield in chickpea.

## Introduction

Insertion–deletions (InDels) are the preferred ideal sequence-based marker for driving genomics-assisted breeding applications in multiple crop plants. This is due to its myriad of desirable inherent genetic attributes in conjunction with other genetic markers like simple sequence repeats (SSRs) and single nucleotide polymorphisms (SNPs; Li et al., 2014; Moghaddam et al., 2014; Wang et al., 2014; Das et al., 2015). The available draft whole genome sequences and next-generation sequencing (NGS) genome/transcriptome resequences of diverse *desi, kabuli*, and wild accessions are found expedient to develop genome-wide including gene-derived InDel markers *in-silico* with minimal resource expenses in chickpea (Agarwal et al., 2012; Hiremath et al., 2012; Jain et al., 2013; Varshney et al., 2013; Deokar et al., 2014; Kudapa et al., 2014). Recently, the advantages of InDel markers structurally/functionally annotated at a whole genome and gene level are well-demonstrated in various large-scale genotyping applications of chickpea (Das et al., 2015). Essentially, this involves understanding the genetic diversity and phylogeny among cultivated *desi, kabuli*, and wild accessions, constructing high-density genetic linkage maps and molecular mapping of major QTLs governing various important agronomic traits like flowering and maturity time in chickpea (Das et al., 2015). Despite these efforts, hitherto none of the informative InDel markers tightly linked to the major genes/QTLs regulating a/biotic stress tolerance and yield component traits has been validated in multiple genetic backgrounds and delineated by genetic/association mapping to be exploited for marker-assisted genetic improvement of chickpea. The narrow genetic base, including low marker genetic polymorphism especially among mapping and natural populations coupled with inadequate accessibility of high-density genetic linkage maps are the major bottlenecks in identification and fine mapping/map-based cloning of trait-associated QTLs in chickpea. In these perspectives, development and high-throughput genotyping of numerous genome-wide informative InDel markers in mapping populations and natural germplasm lines (association panel) for generating high-density genetic linkage maps, high-resolution QTL mapping (fine mapping/positional cloning), and genetic association analysis are essential in chickpea. This will essentially assist us to delineate functionally relevant genes/QTLs and natural allelic variants governing vital agronomic traits for genomics-assisted crop improvement of chickpea with narrow genetic base.

In light of the above, the present study has made efforts to develop large-scale high-quality InDel markers at a genome-wide scale by employing a high-throughput NGS resequencing strategy in low and high pod number parental accessions and bulks (homozygous mapping individuals representing extreme pod number phenotypic trait values) constituted from two F_5_ mapping populations of *Cicer arietinum desi* and *Cicer reticulatum* wild inter-specific crosses. These genome-wide InDel markers were further utilized to detect potential of intra-/inter-specific polymorphism among cultivated (*desi* and *kabuli*) and wild chickpea accessions. The significance of InDel markers was further assessed to construct a high-density consensus inter-specific genetic linkage map and for efficient high-resolution molecular mapping of major genes/QTLs regulating vital agronomic traits including pod number and seed yield per plant with a prime objective of accelerating marker-assisted genetic enhancement in chickpea.

## Materials and methods

### Development of whole genome resequencing-based InDel markers

Two inter-specific F_5_ mapping populations [(*desi* Pusa 1103 × wild ILWC 46, 102 individuals) and (*desi* Pusa 256 × ILWC 46, 98 individuals)] derived from inter-crosses between *C. arietinum desi* and *C. reticulatum* wild accessions were developed. To identify more robust InDels, the high-quality mappable pair-end (100-bp read length), and normalized NGS genome resequencing data of parental accessions were acquired from the afore-mentioned two mapping populations individually as per our previous study (Das et al., 2016). Like-wise, genome resequences from 10 of each low and high pod number homozygous mapping individuals (representing two utmost ends of pod number normal frequency distribution curve) constituting the low pod number bulk (LPNB) and high pod number bulk (HPNB), respectively were obtained. The sequence reads of parental accessions as well as bulks (HPNB and LPNB) obtained from two inter-specific mapping populations were mapped onto the chromosome pseudomolecules and unanchored scaffolds of reference *kabuli* (CDC Frontier) genome (Varshney et al., 2013). Subsequently, high-quality (minimal false-positive) InDels among mapping parents and bulks/individuals were detected following Das et al. (2015). To develop genome-wide InDel markers, forward and reverse primers from the CDC Frontier *kabuli* genomic sequences flanking the InDels were designed using Primer3 interface module of MISA (http://pgrc.ipk-gatersleben.de/misa/primer3.html). The developed InDel markers were structurally and functionally annotated with respect to *kabuli* genome as per Das et al. (2015) and Kujur et al. (2015a). The KOG (eukaryotic orthologous groups of proteins, ftp://ftp.ncbi.nih.gov/pub/COG/KOG) and transcription factor (TF) gene-based functional annotation of InDel markers were performed in accordance with Das et al. (2015) and Kujur et al. (2015a).

### Experimental validation and polymorphic potential of InDel markers

To evaluate the amplification and polymorphic potential of InDel markers developed from two mapping populations, the InDel markers exhibiting ≥4 bp *in-silico* fragment length polymorphism between parental accessions and bulks (LPNB/HPNB) of two inter-specific mapping populations were selected. These markers were PCR amplified and genotyped by agarose gel- and PCR amplicon resequencing-based assays using the genomic DNA of 24 cultivated and wild chickpea accessions. This included three mapping parental accessions (Pusa 256, Pusa 1103, and ILWC 46) from which the InDel markers were originally identified, and 21 additional *desi* (4) and *kabuli* (3) and wild (14) chickpea accessions. The genotyping data of experimentally validated InDel markers were utilized to measure the average polymorphic alleles per marker, percent polymorphism and polymorphism information content (PIC) among chickpea accessions employing PowerMarker v3.51 (http://statgen.ncsu.edu/powermarker).

### Genetic linkage map construction

The InDel markers exhibiting polymorphism between parental accessions (Pusa 1103 vs. ILWC 46 and Pusa 256 vs. ILWC 46) were PCR amplified and genotyped using 102 and 98 individuals derived from two F_5_ inter-specific mapping populations of Pusa 1103 × ILWC 46 and Pusa 256 × ILWC 46, respectively using agarose gel- and PCR amplicon resequencing-based assays. The marker genotyping data were analyzed with the χ^2^-test (*P* < 0.05) to evaluate their goodness-of-fit to the expected Mendelian 1:1 segregation ratio. The JoinMap 4.1 (http://www.kyazma.nl/index.php/mc.JoinMap) at a higher logarithm of odds (LOD) threshold (4.0–8.0) with Kosambi mapping function was used to measure the linkage analysis among InDel markers. The InDel markers were assigned into defined LGs (linkage groups; designated as LG1 to LG8)/chromosomes of two inter-specific genetic maps according to their centiMorgan (cM) genetic distances and corresponding marker physical positions (bp) on the chromosomes. A consensus high-density genetic linkage map derived from two inter-specific genetic maps was constructed using JoinMap 4.1 (following Bohra et al., 2012; Varshney et al., 2014) and visualized using Circos as per Kujur et al. (2015a).

### QTL mapping

The mapping individuals and parental accessions of two F_5_ inter-specific mapping populations [(Pusa 1103 × ILWC 46) and (Pusa 256 × ILWC 46)] were grown in the field as per random complete block design (RCBD) with at least two replications and phenotyped at two diverse geographical locations of India (CSKHPKV, Palampur: latitude 32.1°N and longitude 76.5°E and NBPGR, New Delhi: 28.6°N and 77.2°E) for two consecutive years (2013 and 2014). For precise phenotyping, 10–15 representative plants from each mapping individual and parental accession of both mapping populations were selected to estimate the pod number and seed yield (g) per plant. The pod number (PN) was measured by counting the average number of fully developed pods per plant at maturity stage whereas seed yield per plant (SYP) was estimated by taking average weight (g) of fully matured dried seeds (at 10% moisture content) harvested from all representative plants belonging to each mapping individual and parental accession of aforesaid both populations. The inheritance pattern of PN and SYP based on diverse statistical parameters including mean, standard deviation, coefficient of variation (CV), broad-sense heritability (H^2^), Pearson's correlation coefficient and frequency distribution was measured in both mapping populations individually following Bajaj et al. (2015a) and Das et al. (2016).

For molecular mapping of major PN and SYP QTLs, the genotyping data of InDel markers genetically mapped on two individual and/or a consensus high-density chickpea genetic linkage map (comprising of eight LGs/chromosomes) was integrated with PN and SYP field phenotypic data of mapping individuals and parental accessions using a composite interval mapping (CIM) function of MapQTL 6 (Van Ooijen, 2009) as per Varshney et al. (2014) and Das et al. (2015). The LOD threshold score >4.0 with 1000 permutations at a *p* < 0.05 significance was used as major criteria in CIM for QTL mapping. The phenotypic variation explained (PVE) and additive effect specified by each major PN and SYP QTL at a significant LOD were measured in accordance with Bajaj et al. (2015a).

## Results

### Whole-genome resequencing of low and high pod number parental accessions and homozygous bulks from mapping populations

We generated on an average 81.5 million high-quality sequence reads (with a ~11.6-fold sequencing depth coverage) by high-throughput whole-genome NGS resequencing of two low and high pod number parental accessions as well as bulks (LPNB and HPNB) from each of two inter-specific mapping populations [(Pusa 1103 × ILWC 46) and (Pusa 256 × ILWC 46)]. To reduce the potential biasness of read depth in the examined samples, the high-quality uniquely mapped non-redundant sequence reads (69% mean coverage on *kabuli* reference genome) generated from parental accessions and bulks (LPNB and HPNB) of two mapping populations were normalized based on depth of read coverage. This analysis revealed ~11.6-fold average sequencing depth coverage and 64.1% (474.2 Mb) mean genome coverage (%) of *kabuli* chickpea (with an estimated genome size of ~740 Mb) in mapping parents and bulks. All these sequencing data were submitted to NCBI-sequence read archive (SRA) database (http://www.ncbi.nlm.nih.gov/sra) with accession number SRR2228974 for unrestricted public access. The normalized sequence reads of parents and bulks from each of two inter-specific mapping populations were compared individually with reference *kabuli* genomic sequences (pseudomolecules and unanchored scaffolds) to discover the high-quality non-erroneous InDels.

### Development of genome-wide informative InDel markers in a mapping population of Pusa 1103 × ILWC 46

**Figure 1:**
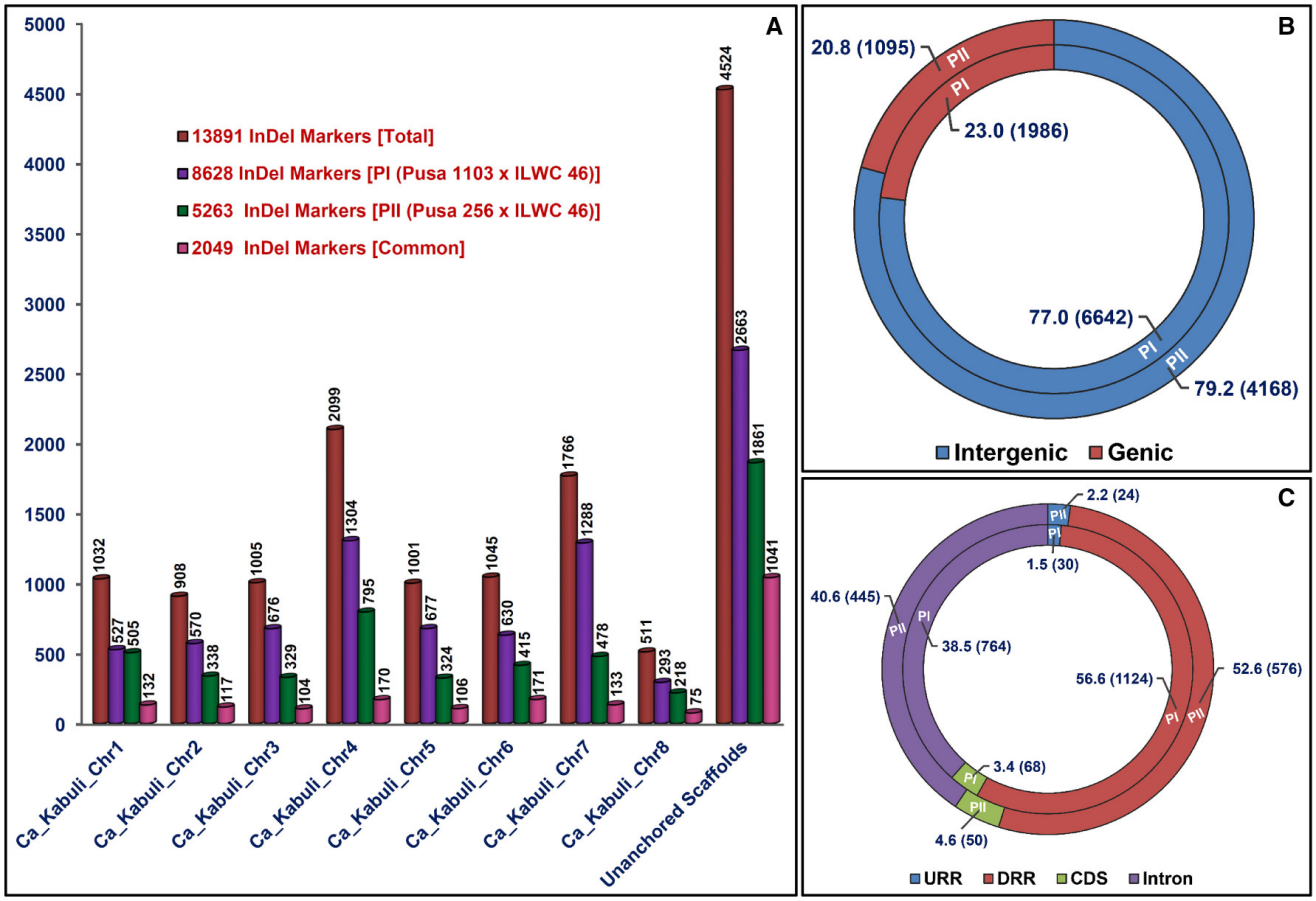
**Genetic constitution and genome|-wide distribution pattern of InDel markers exhibiting differentiation between high and low pod number parental accessions and homozygous bulks from each of two mapping populations [PI: (Pusa 1103 × ILWC 46) and PII: (Pusa 256 × ILWC 46)] with respect to ***kabuli*** chickpea (CDC Frontier) genome. (A)** These InDel markers were physically mapped on eight chromosomes and unanchored scaffolds of *kabuli* chickpea genome, which are illustrated by bar diagrams. **(B,C)** Relative frequency (proportionate distribution) of InDel markers designed from the intergenic as well as diverse coding (CDS) and non-coding (introns, URRs, and DRRs) sequence components of genes annotated from *kabuli* chickpea genome. Parenthesis designates the number of InDel markers developed from each sequence regions of *kabuli* genome. The CDS (coding DNA sequences), URR (upstream regulatory region), and DRR (downstream regulatory region) of genes were defined as per the gene annotation of *kabuli* chickpea genome (v).

**Figure 2:**
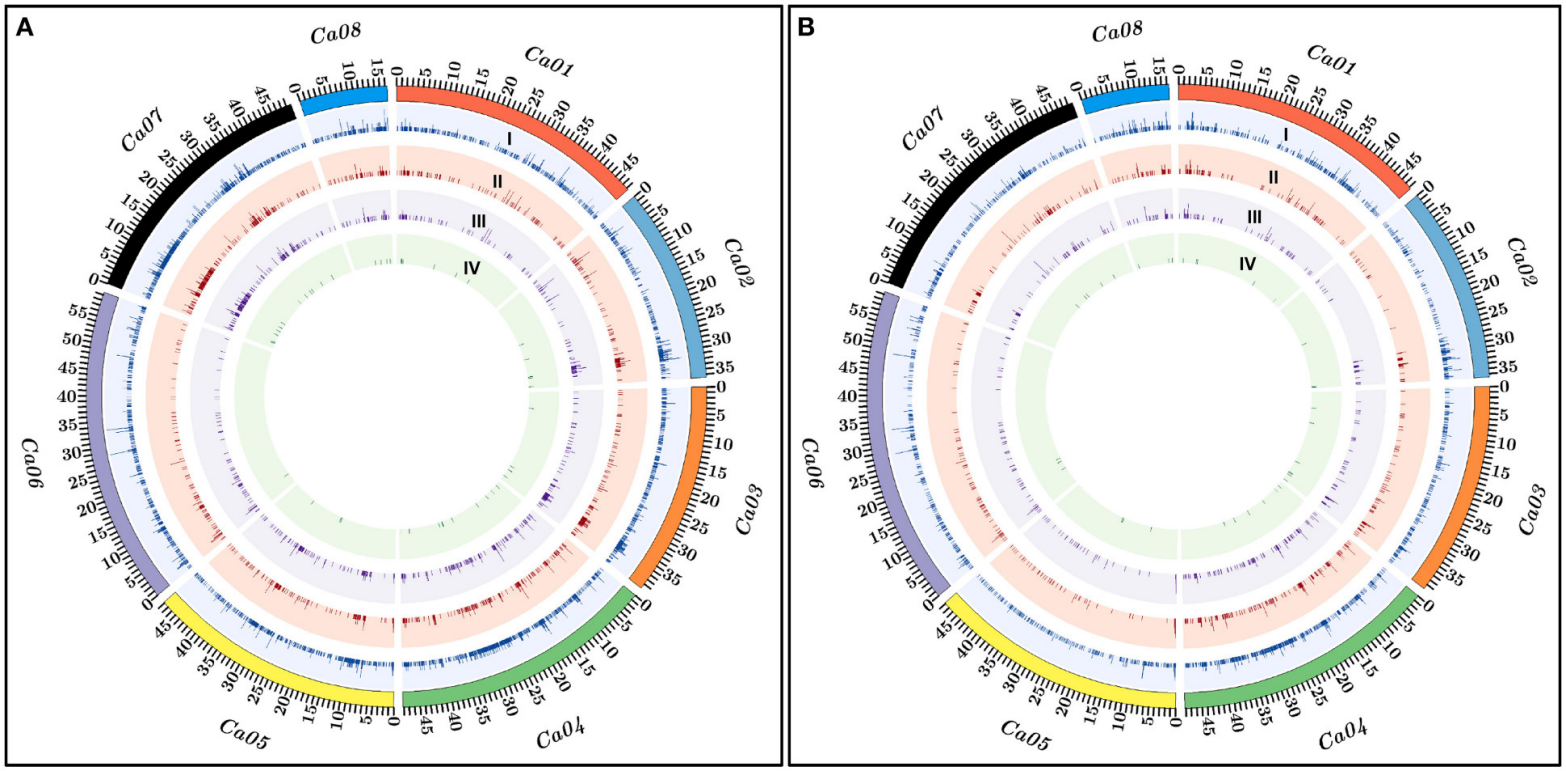
**The relative genomic distribution of PI (Pusa 1103 × ILWC 46) (A) and PII (Pusa 256 × ILWC 46) (B) mapping populations-derived polymorphic InDel markers physically mapped on eight chromosomes of ***kabuli*** chickpea genome are depicted by the Circos circular ideograms**. The outermost circles represent the different physical sizes (Mb) of eight chromosomes coded with multiple colors as per the pseudomolecule sizes documented in *kabuli* chickpea genome (Varshney et al., 2013). Total InDel markers (I) including gene-derived (II), regulatory (III), and coding (IV) markers polymorphic between high and low pod number parental accessions and homozygous bulks of two inter-specific mapping populations—PI (Pusa 1103 × ILWC 46) **(A)** and PII (Pusa 256 × ILWC 46) **(B)** are indicated.

The comparison of NGS genome resequences of high (Pusa 1103 and HPNB) and low (ILWC 46 and LPNB) pod number mapping parental accessions and bulks with reference *kabuli* (CDC Frontier) genomic sequence discovered 25,477 and 24,166 high-quality InDel markers (Tables S1, S2). This included 8628 InDel markers exhibiting polymorphism between high (Pusa 1103 and HPNB) and low (ILWC 46 and LPNB) pod number mapping parents and bulks according to their congruent physical positions (bp) on the reference *kabuli* genome (Figures 1A, 2A, Table S3). Notably, *in-silico* fragment length polymorphism detected by markers based on their size (bp) of InDels varied from 1 to 18 bp with a mean of 3.1 bp. More than 73.1% (6306) InDel markers exhibited 1–3 bp *in-silico* fragment length polymorphism while remaining 26.9% (2322) markers revealed 4–18 bp fragment length polymorphism (Table S3).

The 5965 and 2663 markers of the total designed 8628 InDel markers were physically mapped on eight chromosomes and unanchored scaffolds of *kabuli* chickpea genome with an average map density of 63.1 kb [varying from 37.7 (chromosome 4) to 94.4 (chromosome 6) kb] (Figures 1A, 2A, Table S3). Highest and lowest number of InDel markers were mapped on chromosomes 4 (1304 markers with a mean map density: 37.7 kb) and 8 (293 markers with a mean map density: 56.2 kb), respectively (Figure 2A, Table S3). The structural annotation of 8628 InDel markers revealed the occurrence of 6642 (77%) and 1986 (23%) markers in the intergenic regions and different sequence components of 1523 protein-coding genes, respectively (Figures 1B, 2A, Table S3). The average frequency of InDel markers within genes was estimated as 1.3 markers/gene. A maximum of 1124 (56.6%) gene-derived InDel markers were designed from the DRRs (downstream regulatory regions) of 945 genes and minimum of 30 (1.5%) markers derived from the URRs (upstream regulatory regions) of 23 genes (Figures 1C, 2A, Table S3). Remarkably, 33 and 35 coding InDel markers developed from the 33 and 34 genes caused frameshift mutations and affected initiation/stop codons (large-effect mutations), respectively.

The KOG-based functional annotation of 1523 genes with 1986 InDel markers exhibited primary roles of 1095 (55.1%) markers-carrying 815 genes in multiple cellular, biological, and molecular processes in crop plants (Table S3). This revealed enrichment of InDel markers-containing genes basically involved in post-translational modification, protein turnover, and chaperones (O, 111 markers in 81 genes, 10.1%), transcription (K, 74 markers in 49 genes, 6.7%), and signal transduction mechanisms (T, 70 markers in 55 genes, 6.4%), beside general function prediction (R, 200 markers in 158 genes, 18.3%; Table S3). Of the 799 (40.2%) InDel markers developed from 603 TF-encoding genes (representing 50 TF gene families), the genes belonging to *MYB* (90 markers in 62 genes, 11.3%), *bHLH* (78 markers in 61 genes, 9.8%), C2H2 zinc finger (51 markers in 36 genes, 6.4%), and *NAC* (48 markers in 36 genes, 6%) TF families were abundant (Table S3).

### Development of genome-wide informative InDel markers in a mapping population of Pusa 256 × ILWC 46

We developed 15,640 and 17,077 high-quality InDel markers by comparing the NGS genome resequences of high (Pusa 256 and HPNB) and low (ILWC 46 and LPNB) pod number mapping parental accessions and bulks with reference *kabuli* (CDC Frontier) genomic sequence (Tables S4, S5). This included 5263 InDel markers revealing polymorphism between high (Pusa 256 and HPNB) and low (ILWC 46 and LPNB) pod number mapping parents and bulks according to their congruent physical positions (bp) on the reference *kabuli* genome (Figures 1A, 2B, Table S6). Notably, *in-silico* fragment length polymorphism detected by markers based on their size (bp) of InDels varied from 1 to 18 bp with a mean of 3.1 bp. More than 73% (3842) InDel markers showed 1–3 bp *in-silico* fragment length polymorphism while remaining 27% (1421) markers revealed 4–18 bp fragment length polymorphism (Table S6). The 3402 and 1861 markers of the total designed 5263 InDel markers were physically mapped on eight chromosomes and unanchored scaffolds of *kabuli* chickpea genome with an average map density of 103.5 kb [ranging from 61.9 (chromosome 4) to 148.7 (chromosome 5) kb] (Figures 1A, 2B, Table S6). Highest and lowest number of InDel markers were mapped on chromosomes 4 (795 markers with a mean map density: 61.9 kb) and 8 (218 markers with a mean map density: 75.6 kb), respectively. The structural annotation of 5263 InDel markers revealed the presence of 4168 (79.2%) and 1095 (20.8%) markers in the intergenic regions and different sequence components of 868 protein-coding genes, respectively (Figures 1B, 2B, Table S6). The mean frequency of InDel markers within genes was measured as 1.3 markers/gene. A maximum of 576 (52.6%) gene-derived InDel markers were designed from the DRRs of 487 genes and minimum of 24 (2.2%) markers derived from the URRs of 19 genes (Figures 1C, 2B, Table S6). Remarkably, 24 and 26 coding InDel markers developed from the 24 and 26 genes caused frameshift mutations and affected initiation/stop codons (large-effect mutations), respectively. A total of 13,891 including 8628 and 5263 polymorphic InDel markers identified from two inter-specific mapping populations of Pusa 1103 × ILWC 46 and Pusa 256 × ILWC 46, respectively were compared/correlated. This included 2049 markers common between these two mapping populations based on congruent marker physical positions on the *kabuli* genome (Figure 1A, Tables S3, S6, S7).

The KOG-based functional annotation of 868 genes with 1095 InDel markers exhibited primary roles of 597 (54.5%) markers-carrying 466 genes in multiple cellular, biological, and molecular processes in crop plants (Table S6). This revealed enrichment of InDel markers-containing genes basically involved in post-translational modification, protein turnover, and chaperones (O, 59 markers in 46 genes, 9.9%) and signal transduction mechanisms (T, 37 markers in 30 genes, 6.2%), beside general function prediction (R, 87 markers in 75 genes, 14.6%) and unknown function (S, 35 markers in 29 genes, 5.9%; Table S6). Of the 440 (40.2%) InDel markers developed from 337 TF-encoding genes (representing 44 TF gene families), the genes belonging to *MYB* (55 markers in 36 genes, 12.5%), *NAC* (40 markers in 31 genes, 9.1%), *bHLH* (39 markers in 31 genes, 8.9%), C2H2 zinc finger (29 markers in 17 genes, 6.6%), and *B3* (24 markers in 21 genes, 5.4%) TF families were predominant (Table S6).

### Experimental validation of InDel markers to access their amplification and polymorphic potential among cultivated and wild chickpea accessions

**Figure 3:**
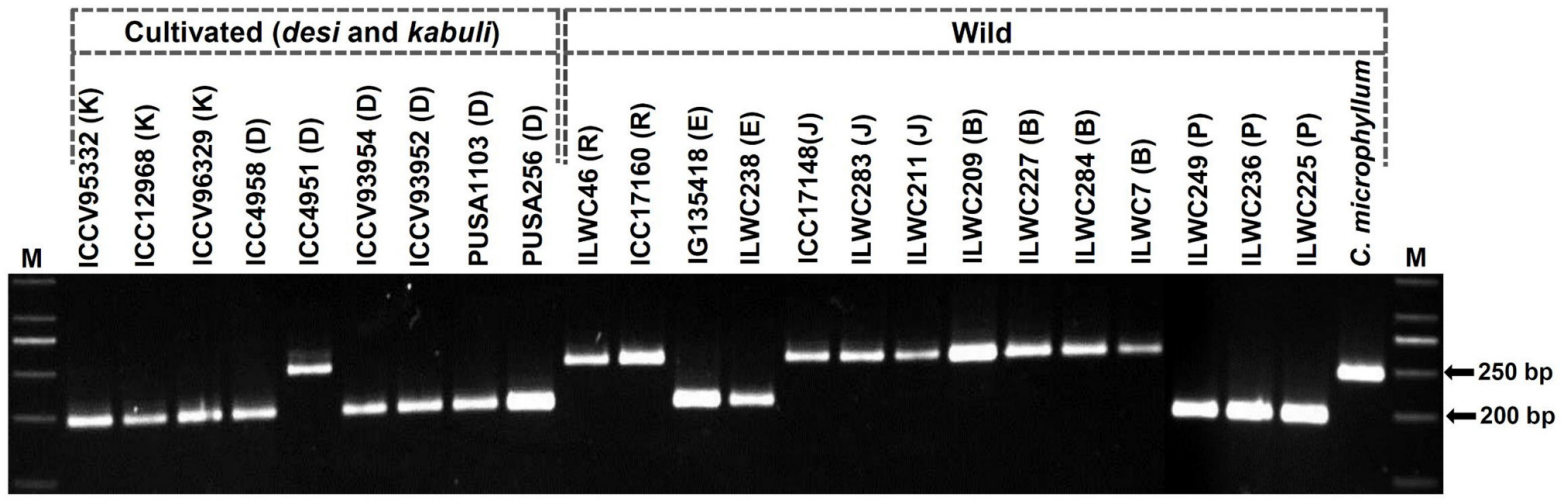
**Allelic polymorphism detected among 24 ***desi***, ***kabuli***, and wild chickpea accessions along with three mapping parental accessions (Pusa 256, Pusa 1103, and ILWC 46; from which the InDel markers were originally identified) using a representative InDel marker in agarose gel-based assay**. The amplified fragment sizes (bp) of two polymorphic alleles detected among accessions are specified. M: 50 bp DNA ladder size standard. Cultivated *C. arietinum* (K: *kabuli* and D: *desi*), and wild *C. reticulatum* (R), *C. echinospermum* (E), *C. judaicum* (J), *C. bijugum* (B), *C. pinnatifidum* (P), and *C. microphyllum* accessions utilized for polymorphism study are indicated.

To access the amplification and polymorphic potential of designed InDel markers, 3743 markers exhibiting ≥4 bp *in-silico* fragment length polymorphism between the parental accessions and bulks of two inter-specific mapping populations, were selected for experimental validation using the gel- and PCR amplicons resequencing-based assays. These markers were PCR amplified and genotyped using the genomic DNA of three mapping parental chickpea accessions (Pusa 1103, Pusa 256, and ILWC 46), from which the InDel markers were originally discovered. Notably, 3612 of 3743 markers produced single reproducible PCR amplicons in agarose gel with a mean amplification success rate of 96.5% (Figure 3). Of these, 3413 (94.5%) amplified markers revealing *in-silico* polymorphism at least between two combination of mapping parental chickpea accessions were got validated experimentally using both agarose gel- and PCR amplicons resequencing-based assays. The PCR amplicons resequencing-led validation and genotyping of InDel markers ascertained the presence of expected InDels, which further corresponded well with their *in-silico* fragment length polymorphism detected among three mapping parental chickpea accessions.

To evaluate the potential of InDel markers for detecting polymorphism among accessions, large-scale genotyping of 3413 polymorphic InDel markers were performed in a diverse set of 24 *desi, kabuli* and wild chickpea accessions (Figure 3). These markers overall generated 6849 alleles among accessions with an average PIC of 0.71. Three thousand three hundred-two (96.7%, mean PIC: 0.65) of 3413 markers were found to be polymorphic among cultivated and wild chickpea accessions, whereas 2831 (83%, mean PIC: 0.60) markers exhibited polymorphism among cultivated *desi* and *kabuli* accessions. Interestingly, 2355 (69%) markers exhibited polymorphism among six *desi* accessions (1–2 alleles with a mean PIC: 0.57), whereas 1980 (58%) markers revealed polymorphism among three *kabuli* accessions (1–2 alleles with a mean PIC: 0.51). A set of 2969 (87%) InDel markers exhibited polymorphism among 15 accessions belonging to six annual/perennial wild species—*Cicer reticulatum, C. echinospermum, C. judaicum, C. bijugum, C. pinnatifidum*, and *C. microphyllum* of primary, secondary, and tertiary gene-pools.

### Generation of a consensus high-density inter-specific chickpea genetic linkage map

**Figure 4:**
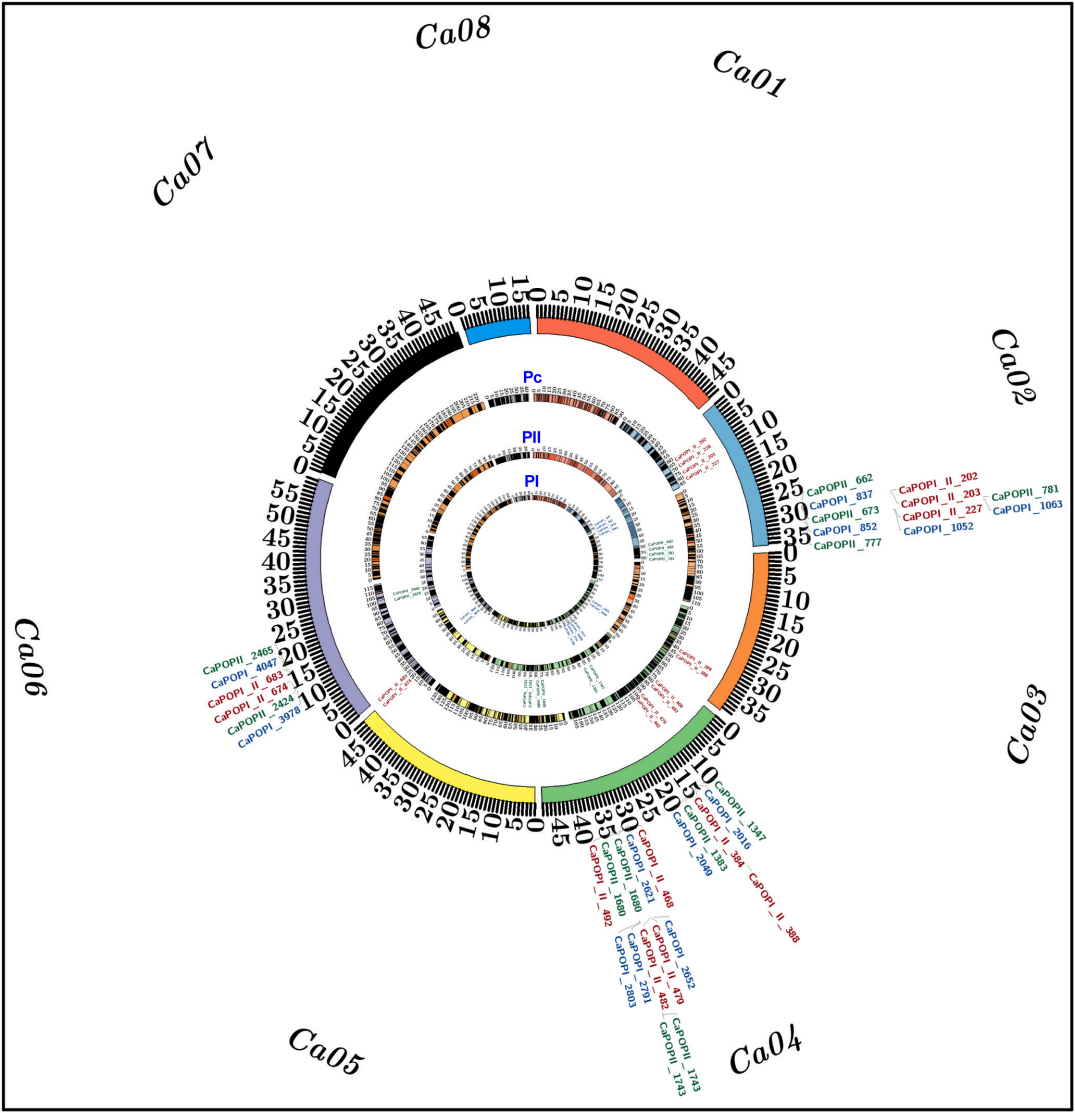
**The identified three of each major PN and SYP QTLs mapped on chromosomes 2, 4, and 6 of two high-density 1059 and 594 InDel markers-anchored inter-specific genetic linkage maps (PI: Pusa 1103 × ILWC 46) and (PII: Pusa 256 × ILWC 46) and a consensus 1479 InDel markers-led high-resolution genetic map (Pc) of chickpea, are illustrated by the Circos circular ideograms (PI, PII, and Pc)**. The circles represent the different genetic map length (cM) (spanning 5–10 cM uniform genetic distance intervals between bins) of eight LGs/chromosomes coded with multiple colors. The integration of a consensus genetic map (Pc) with physical map at the identified three of each major PN and SYP QTLs scaled-down the long genomic regions harboring these major QTLs into short PN and SYP robust QTL physical intervals (indicated with red color InDel markers) mapped on *kabuli* chromosomes 2, 4, and 6. The InDel markers flanking the six major PN and SYP QTLs mapped on chromosomes 2, 4, and 6 of high-resolution genetic maps—PI, PII, and Pc are highlighted with blue, green, and red color, respectively. The detail information on QTLs and InDel markers are provided in the Table 2. The outermost circles denote the various physical sizes (Mb) of eight chromosomes coded with multiple colors as per the pseudomolecule sizes documented in *kabuli* chickpea genome (Varshney et al., 2013).

For constructing high-resolution inter-specific genetic linkage maps, 1059 and 594 InDel markers revealing polymorphism between high and low pod number parental accessions (Pusa 1103 vs. ILWC 46 and Pusa 256 vs. ILWC 46) and bulks (HPNB vs. LPNB) were genotyped among 102 and 98 individuals of two F_5_ mapping populations—PI (Pusa 1103 × ILWC 46) and PII (Pusa 256 × ILWC 46), respectively. The linkage analysis using these marker genotyping data mapped 1059 and 594 InDel markers across eight LGs of two PI and PII mapping populations-derived inter-specific chickpea genetic maps, respectively (Figure 4, Table 1). In a PI mapping population-derived genetic map, highest and lowest numbers of InDel markers were mapped on CaLG07 (235 markers) and CaLG08 (47 markers), respectively (Table 1). In another PII mapping population-derived genetic map, the CaLG04 (128 markers) and CaLG08 (31 markers) contained maximum and minimum number of mapped InDel makers, respectively (Table 1). The eight LGs-based two inter-specific genetic maps generated from PI and PII mapping populations spanned total map-lengths of 978.21 and 603.26 cM, with the mean inter-marker distances of 0.92 and 1.01 cM, respectively (Table 1). Longest map-lengths spanning 221.34 and 122.28 cM were observed in CaLG07 and CaLG04 of PI and PII mapping populations-derived genetic linkage maps, respectively. The CaLG01 and CaLG04 of genetic linkage maps constructed from PI and PII mapping populations had most saturated genetic maps with the mean inter-marker distances of 0.81 and 0.99 cM, respectively (Table 1).

**Table 1:** **InDel markers mapped on eight chromosomes of two inter-specific genetic linkage maps [(Pusa 1103 × ILWC 46) and (Pusa 256 × ILWC 46)] and a consensus inter-specific chickpea genetic linkage map of chickpea**.

**Linkage groups (LGs)/chromosomes (Chr)**	**InDel markers mapped**			**Map length covered (cM)**			**Map-density [mean inter-marker distance (cM)]**		
	**(Pusa 1103 × ILWC 46)**	**(Pusa 256 × ILWC 46)**	**A consensus genetic linkage map**	**(Pusa 1103 × ILWC 46)**	**(Pusa 256 × ILWC 46)**	**A consensus genetic linkage map**	**(Pusa 1103 × ILWC 46)**	**(Pusa 256 × ILWC 46)**	**A consensus genetic linkage map**
LG(Chr)01	111	91	180	89.94	90.34	90.34	0.81	0.99	0.50
LG(Chr)02	88	59	127	87.79	60.74	87.79	1.0	1.03	0.69
LG(Chr)03	128	67	178	110.98	70.74	110.98	0.87	1.05	0.62
LG(Chr)04	185	128	288	169.91	122.28	169.91	0.92	0.95	0.59
LG(Chr)05	134	62	170	138.71	68.07	138.71	1.03	1.10	0.81
LG(Chr)06	132	67	172	117.95	67.61	117.95	0.90	1.01	0.68
LG(Chr)07	235	89	297	221.34	90.35	221.34	0.94	1.01	0.74
LG(Chr)08	47	31	67	41.59	33.13	41.59	0.88	1.07	0.62
Total	1059	594	1479	978.21	603.26	978.61	0.92	1.01	0.66

Combining the genotyping information of 1653 including 1059 and 594 InDel markers mapped genetically on two PI and PII mapping populations-derived inter-specific genetic linkage maps, respectively, we constructed a consensus high-resolution genetic linkage map of chickpea. A set of 174 InDel markers found common between two inter-specific genetic linkage maps were served as the anchor markers for integration and defining the linkage groups/chromosomes of these genetic maps. A consensus 1479 InDel markers-anchored inter-specific genetic linkage map was generated, which covered a total map-length of 978.61 cM with an average inter-marker distance (map-density) of 0.66 cM (Figure 4, Table 1). The map-density of a consensus inter-specific genetic map varied from 0.50 cM (CaLG01) to 0.81 cM (CaLG05). Highest (297) and lowest (67) number of InDel markers were mapped on CaLG07 and CaLG08 spanning longest and shortest map-lengths of 221.34 and 41.59 cM, respectively (Table 1).

### Molecular mapping of major pod number and seed yield QTLs

For molecular mapping of pod number and seed yield per plant QTLs, primarily the genetic inheritance pattern of PN and SYP traits in two inter-specific mapping populations was determined. A significant difference of PN (5–237) with 13–14.8% CV and 80–81% H^2^ in 102 and 98 individuals and parental accessions of two inter-specific F_5_ mapping populations of Pusa 1103 (PN: 129) × ILWC 46 (29) and Pusa 256 (125) × ILWC 46 (29) was observed. Moreover, the parental accessions and individuals belonging to these two mapping populations of Pusa 1103 (SYP: 38.3 g) × ILWC 46 (19.2 g) and Pusa 256 (37.4 g) × ILWC 46 (19.2 g) exhibited a significant difference of SYP (16.7–54.3 g) with 9.7–10.3% CV and 78–80% H^2^. The continuous variation as well as normal frequency distribution along with bi-directional transgressive segregation of PN and SYP were observed in these both mapping populations reflecting the quantitative genetic inheritance pattern of traits under study. A significant positive correlation between PN and SYP traits based on Pearson's correlation coefficient (*r* = 69–72%) was evident.

Two years multi-location replicated field phenotyping data of PN and genotyping information of 1059 and 594 InDel markers genetically mapped on eight chickpea chromosomes of two inter-specific genetic linkage maps constructed from PI (Pusa 1103 × ILWC 46) and PII (Pusa 256 × ILWC 46) mapping populations, respectively were integrated for molecular mapping of major PN QTLs. This analysis detected three major genomic regions harboring three robust QTLs associated with PN trait, which were mapped on chromosomes 2 and 4 of each PI and PII mapping populations-derived inter-specific genetic maps (Figure 4, Table 2). For PI mapping population-led high-resolution genetic linkage map, three major genomic regions underlying three PN QTLs (*Caq*^*a*^*PN2.1, Caq*^*a*^*PN4.1*, and *Caq*^*a*^*PN4.2*) spanned (7.55–8.99 cM on chromosome 4) with 51 InDel markers, were mapped on chromosomes 2 and 4 (Figure 4, Table 2). The individual major PN QTL explained 18–25% phenotypic variation (R^2^) for pod number trait at an 8.5–12.7 LOD. The PVE (phenotypic variation explained) measured for all three major PN QTLs in combination was 38.4%. All three major PN QTLs exhibited positive additive gene effect (2.7–4.3) of pod number trait with major allelic contribution from a high pod number parental chickpea accession Pusa 1103. For PII mapping population-derived high-density genetic linkage map, three major genomic regions underlying three PN QTLs (*Caq*^*b*^*PN2.1, Caq*^*b*^*PN4.1*, and *Caq*^*b*^*PN4.2*) spanned (5.45–7.71 cM on chromosome 4) with 33 InDel markers, were mapped on chromosomes 2 and 4 (Figure 4, Table 2). The individual major PN QTL explained 15–22% phenotypic variation (R^2^) for pod number trait at a 6.7–11.4 LOD. The PVE measured for all three major PN QTLs in combination was 34.1%. All three major PN QTLs exhibited positive additive gene effect (3.3–4.7) of pod number trait with major allelic contribution from a high pod number parental chickpea accession Pusa 256. Further, a high-density consensus genetic linkage map constructed by integrating two inter-specific genetic linkage maps was utilized as an anchor for molecular mapping of major PN QTLs in chickpea. This identified three major genomic regions underlying three PN QTLs (*Caq*^*c*^*PN2.1, Caq*^*c*^*PN4.1*, and *Caq*^*c*^*PN4.2*) spanned (2.7–5.7 cM on chromosome 4) with 33 InDel markers, which were mapped on three different genomic regions on chromosomes 2 and 4 (Figure 4, Table 2). The individual major PN QTL explained 20–28% phenotypic variation (R^2^) for pod number trait at a 9.4–13.8 LOD. The PVE measured for all three major PN QTLs in combination was 39.7%. All three major PN QTLs exhibited positive additive gene effect (3.8–4.5) of pod number trait with major allelic contribution from the high pod number parental chickpea accessions Pusa 1103/Pusa 256.

**Table 2:** **Molecular mapping of major pod number and seed yield per plant QTLs in chickpea**.

**InDel markers-anchored genetic linkage maps**	^*^QTLs	**LGs/Chromosomes**	**Marker intervals with genetic positions (cM)**	**QTL physical intervals (bp)**	**LOD**	**PVE (%)**	**A**
PI: (Pusa 1103 × ILWC 46)	*Caq^a^PN2.1*	CaLG(Chr)2	CaPOPI_837 (73.96)–CaPOPI_1052 (81.99)	CaPOPI_837 (29,220,253)–CaPOPI_1052 (32,393,633)	8.5	18	2.7
	*^#^Caq^a^PN4.1*	CaLG(Chr)4	CaPOPI_2016 (45.19)–CaPOPI_2049 (52.74)	CaPOPI_2016 (13,068,436)–CaPOPI_2049 (15,252,487)	12.7	25	3.9
	*^#^Caq^a^PN4.2*	CaLG(Chr)4	CaPOPI_2652 (108.09)–CaPOPI_2803 (117.08)	CaPOPI_2652 (31,259,145)–CaPOPI_2803 (33,857,426)	11.5	21	4.3
	*Caq^a^SYP2.1*	CaLG(Chr)2	CaPOPI_852 (75.50)–CaPOPI_1063 (82.80)	CaPOPI_852 (29,829,940)–CaPOPI_1063 (32,713,895)	8.0	16	2.5
	*Caq^a^SYP4.1*	CaLG(Chr)4	CaPOPI_2621 (106.11)–CaPOPI_2791 (116.58)	CaPOPI_2621 (30,685,617)–CaPOPI_2791 (33,714,297)	11.8	23	3.0
	*Caq^a^SYP6.1*	CaLG(Chr)6	CaPOPI_3978 (24.42)–CaPOPI_4047 (34.05)	CaPOPI_3978 (12,253,200)–CaPOPI_4047 (17,086,650)	12.3	20	4.1
PII: (Pusa 256 × ILWC 46)	*Caq^b^PN2.1*	CaLG(Chr)2	CaPOPII_673 (49.17)–CaPOPII_777 (54.07)	CaPOPII_673 (29,445,927)–CaPOPII_777 (32,379,585)	6.7	15	3.3
	*^#^Caq^b^PN4.1*	CaLG(Chr)4	CaPOPII_1347 (30.69)–CaPOPII_1383 (36.14)	CaPOPII_1347 (12,225,286)–CaPOPII_1383 (14,397,300)	11.4	22	4.7
	*^#^Caq^b^PN4.2*	CaLG(Chr)4	CaPOPII_1680 (78.06)–CaPOPII_1743 (85.77)	CaPOPII_1680 (31,101,573)–CaPOPII_1743 (34,169,705)	9.8	19	4.2
	*Caq^b^SYP2.1*	CaLG(Chr)2	CaPOPII_662 (48.55)–CaPOPII_781 (54.10)	CaPOPII_662 (29,072,040)–CaPOPII_781 (32,393,633)	7.5	17	3.1
	*Caq^b^SYP4.1*	CaLG(Chr)4	CaPOPII_1680 (78.06)–CaPOPII_1743 (85.77)	CaPOPII_1680 (31,101,573)–CaPOPII_1743 (34,169,705)	10.6	23	4.0
	*Caq^b^SYP6.1*	CaLG(Chr)6	CaPOPII_2424 (14.81)–CaPOPII_2465 (21.08)	CaPOPII_2424 (12,877,302)–CaPOPII_2465 (18,331,022)	9.5	20	3.8
Pc: [PI (Pusa 1103 × ILWC 46) and PII (Pusa 256 × ILWC 46)]	*Caq^c^PN2.1*	CaLG(Chr)2	CaPOPI_II_202 (61.8)–CaPOPI_II_228 (68.3)	CaPOPI_II_202 (29,445,927)–CaPOPI_II_228 (32,393,633)	9.4	20	3.8
	*^#^Caq^c^PN4.1*	CaLG(Chr)4	CaPOPI_II_384 (40.3)–CaPOPI_II_388 (43.0)	CaPOPI_II_384 (13,509,527)–CaPOPI_II_388 (14,397,300)	13.8	28	4.5
	*^#^Caq^c^PN4.2*	CaLG(Chr)4	CaPOPI_II_479 (94.9)–CaPOPI_II_492 (100.6)	CaPOPI_II_479 (31,806,633)–CaPOPI_II_492 (33,714,267)	11.7	24	4.0
	*Caq^c^SYP2.1*	CaLG(Chr)2	CaPOPI_II_203 (75.50)–CaPOPI_II_227 (81.95)	CaPOPI_II_203 (29,829,940)–CaPOPI_II_227 (32,379,585)	10.2	22	4.1
	*Caq^c^SYP4.1*	CaLG(Chr)4	CaPOPI_II_468 (76.64)–CaPOPI_II_482 (80.89)	CaPOPI_II_468 (30,535,832)–CaPOPI_II_482 (32,227,293)	14.5	27	4.3
	*Caq^c^SYP6.1*	CaLG(Chr)6	CaPOPI_II_674 (20.2)–CaPOPI_II_683 (26.0)	CaPOPI_II_674 (12,877,302)–CaPOPI_II_683 (16,547,931)	12.4	25	4.0

* Caq^a^PN2.1 (C. arietinum PI-derived pod number QTL on chromosome 2 number 1), Caq^b^PN2.1 (C. arietinum PII-derived pod number QTL on chromosome 2 number 1) and Caq^c^PN2.1 (C. arietinum Pc-derived pod number QTL on chromosome 2 number 1). Caq^a^SYP2.1 (C. arietinum PI-derived seed yield per plant QTL on chromosome 2 number 1), Caq^b^SPN2.1 (C. arietinum PII-derived seed yield per plant QTL on chromosome 2 number 1) and Caq^c^SPN2.1 (C. arietinum Pc-derived seed yield per plant QTL on chromosome 2 number 1). PVE, Proportion of phenotypic variation explained by QTLs, A, additive effect; positive additive effect infers alleles from high PN and SYP mapping parental chickpea accessions (Pusa 1103 and Pusa 256). Details regarding InDel markers are mentioned in the Tables S1–S8.

# documented previously by Das et al. (2016).

The integration of two individual and consensus inter-specific genetic maps with that of physical maps of *kabuli* genome exhibited the common occurrences of five and six InDel markers at the identified three major PN QTL regions of chromosomes 2 and 4, respectively, among these genetic maps based on congruent physical positions on the *kabuli* genome (Figure 4, Table 2). These consensus three major PN QTL regions spanning short physical intervals (*Caq*^*c*^*PN2.1*: 29,445,927–32,393,633 bp, *Caq*^*c*^*PN4.1*: 13,509,527–14,397,300 bp, and *Caq*^*c*^*PN4.2*: 31,806,633–33,714,267 bp) were got validated in two diverse inter-specific mapping populations. These were thus considered as promising major candidate genomic regions underlying robust QTLs governing pod number to be deployed for marker-assisted genetic enhancement of chickpea (Figure 4, Table 2, Tables S1–S6). The structural and functional annotation of these delineated three major short PN QTL intervals of 2.94 (*Caq*^*c*^*PN2.1*), 0.89 (*Caq*^*c*^*PN4.1*), and 1.91 (*Caq*^*c*^*PN4.2*) Mb exhibited the presence of a total 27, 5, and 14 InDel markers including 19, 4, and 13 markers in the intergenic regions and 8, 1, and 1 markers in the different sequence components of 7, 1, 1 genes annotated from *kabuli* chickpea genome, respectively (Tables S1–S4). At these three major PN robust QTL intervals, especially nine regulatory InDel markers-containing genes corresponding to diverse transcription factors (TFs; like DUF1677, LBD, WRKY, and C2H2 zinc finger) and multiple cellular metabolism-related proteins such as cytochrome P450 and ubiquitin were identified, which can possibly serve as candidates for quantitative dissection of complex pod number trait in chickpea (Table S8).

In order to evaluate the efficacy of identified major PN QTLs in governing seed yield, multi-location/years replicated field phenotyping data of SYP trait were integrated with genotyping information of InDel markers genetically mapped on chromosomes of PI (Pusa 1103 × ILWC 46) and PII (Pusa 256 × ILWC 46) mapping populations-derived two inter-specific genetic linkage maps for molecular mapping of major SYP QTLs in chickpea. This identified three major genomic regions underlying three robust QTLs associated with SYP trait which were mapped on chromosomes 2, 4, and 6 of each PI and PII mapping populations-led inter-specific genetic maps (Figure 4, Table 2). For PI mapping population-based high-resolution genetic linkage map, three major genomic regions underlying three robust SYP QTLs (*Caq*^*a*^*SYP2.1, Caq*^*a*^*SYP4.1*, and *Caq*^*a*^*SYP6.1*) covered (7.3 cM on chromosome 2–10.5 cM on chromosome 4) with 57 InDel markers were detected (Figure 4, Table 2). The individual major SYP QTL explained 16–23% phenotypic variation (R^2^) for seed yield trait at an 8.0–12.3 LOD. The PVE estimated for all three major SYP QTLs in combination was 30%. All of these identified three SYP QTLs exhibited positive additive gene effect (2.5–4.1) of seed yield trait with major allelic contribution from a high SYP parental chickpea accession Pusa 1103. For PII mapping population-led high-density genetic map, three major genomic regions harboring three robust YP QTLs (*Caq*^*b*^*SYP2.1, Caq*^*b*^*SYP4.1*, and *Caq*^*b*^*SYP6.1*) spanned (4.9 cM on chromosome 2–7.7 cM on chromosome 4) with 38 InDel markers were identified (Figure 4, Table 2). The individual major SYP QTL explained 17–23% phenotypic variation (R^2^) for yield trait at a 7.5–10.6 LOD. The PVE measured for all three major SYP QTLs in combination was 32%. All of these three major SYP QTLs exhibited positive additive gene effect (3.1–4.0) of seed yield trait with major allelic contribution from a high SYP parental chickpea accession Pusa 256. The use of a high-density consensus genetic linkage map (constructed by integrating two inter-specific genetic linkage maps) as an anchor identified three major genomic regions underlying three robust SYP QTLs (*Caq*^*c*^*SYP2.1, Caq*^*c*^*SYP4.1*, and *Caq*^*c*^*SYP6.1*) covered (4.2 cM on chromosome 4–6.4 cM on chromosome 2) with 50 InDel markers which were mapped on three different genomic regions on chromosomes 2, 4, and 6 (Figure 4, Table 2). The individual major SYP QTL explained 22–27% phenotypic variation (R^2^) for seed yield trait at a 10.2–14.5 LOD. The PVE estimated for all three major SYP QTLs in combination was 35%. All of these three major SYP QTLs exhibited positive additive gene effect (4.0–4.3) of seed yield trait with major allelic contribution from the high SYP parental chickpea accessions Pusa 1103/Pusa 256.

We integrated two individual and consensus inter-specific genetic maps with that of physical maps of *kabuli* genome which exhibited common occurrence of 25, 15, and 10 InDel markers at the identified three major SYP robust QTL regions of chromosomes 2, 4, and 6, respectively, among these genetic maps based on congruent physical positions on the *kabuli* genome (Figure 4, Table 2). These consensus three major SYP QTL regions spanning short physical intervals (*Caq*^*c*^*SYP2.1*: 29,829,940–32,379,585 bp, *Caq*^*c*^*SYP4.1*: 30,535,832–32,227,293 bp, and *Caq*^*c*^*SPN6.1*: 12,877,302–16,547,931 bp) were got validated in two diverse inter-specific mapping populations. Therefore, we considered these QTL intervals as promising major candidate genomic regions underlying robust QTLs regulating seed yield trait which could be deployed for marker-assisted genetic enhancement of chickpea (Figure 4, Table 2, Tables S1–S6). The structural and functional annotation of these delineated three major short SYP QTL intervals of 2.55 (*Caq*^*c*^*SYP2.1*), 1.69 (*Caq*^*c*^*SYP4.1*), and 3.67 (*Caq*^*c*^*SYP6.1*) Mb revealed the presence of 25, 15, and 10 InDel markers including 17, 11, and 9 markers in the intergenic regions and 8, 4, and 1 markers in the different sequence components of genes annotated from *kabuli* chickpea genome, respectively (Tables S1–S4). At these three major SYP robust QTL regions, especially three regulatory and one coding nonsense non-synoymous InDel markers-containing genes corresponding to diverse TFs such as *bHLH, LBD, WRKY*, and *NAC* were identified. These molecular tags can be considered as candidates for dissection of complex seed yield quantitative trait in chickpea (Table S8).

## Discussion

The pod number is a complex yield component quantitative trait, which is known to be regulated by multiple genes/QTLs in chickpea (Kujur et al., 2015a,b; Das et al., 2016). For more efficient dissection of this complex trait, the present study essentially utilized two inter-specific mapping populations [(Pusa 1103 × ILWC 46) and (Pusa 256 × ILWC 46)] with contrasting PN trait to construct a high-density InDel markers-anchored consensus inter-specific genetic linkage map for molecular mapping of major PN QTLs in chickpea. To attain these major objectives, high-throughput whole genome NGS resequencing data with a high mean *kabuli* genome (64.1%, 474 Mb) and sequencing depth (~11.6-fold) coverage, generated from high and low pod number parental accessions as well as bulks of two inter-specific mapping populations, were normalized/compared to develop high-quality accurate InDel markers at a genome-wide scale. The reliability of these identified InDels was ascertained by their potential to differentiate both high and low pod number mapping parental accessions as well as bulks constituted from two studied inter-specific mapping populations. This implicates the robustness of strategy developed in our study for mining and developing valid non-erroneous InDel markers at a genome-wide scale by comparing the resequences among parents and bulks of mapping populations. Consequently, 82,360 markers targeting these non-spurious informative InDels, discriminating the *desi* (Pusa 1103 and Pusa 256), *kabuli* (CDC Frontier) and wild (ILWC 46) accessions from each other including 13,891 markers differentiating the high and low PN—mapping parental accessions (Pusa 1103 vs. ILWC 46 and Pusa 256 vs. ILWC 46) and bulks, were developed in chickpea. These informative InDel markers were structurally and functionally annotated in diverse sequence components of genome/genes (TFs), thereby can be deployed for manifold genomics-assisted breeding applications in chickpea. The observed InDel marker-based genetic polymorphism expectedly infers close evolutionary relatedness of *desi* rather than *kabuli* with wild chickpea (Abbo et al., 2005; Berger et al., 2005; Toker, 2009; Jain et al., 2013; Varshney et al., 2013; Saxena et al., 2014a; Bajaj et al., 2015b; Das et al., 2015; Kujur et al., 2015a,b). We detected almost an identical range (1–18 bp) and mean (3.1 bp) level of InDel-based *in-silico* fragment length polymorphism between two inter-specific mapping populations [(Pusa 1103 × ILWC 46) and (Pusa 256 × ILWC 46)] developed by using a common (wild *C. reticulatum* accession: ILWC 46) as well as two different (*desi* accessions: Pusa 1103 and Pusa 256) parental accessions of chickpea. This is possibly because of common ancestry between Pusa 1103 and Pusa 256, since Pusa 1103 has been developed from the multiple inter-cross between wild *C. reticulatum* and *desi* chickpea accessions involving Pusa 256 as one of the parent (Bharadwaj et al., 2011). Therefore, close progenitor relatedness and similar genetic backgrounds of these two different parental accessions that were used to develop mapping populations could have influenced the detection of identical InDel polymorphism level (bp) between two studied inter-specific mapping populations of chickpea. The cost-efficient user-friendly InDel markers especially developed from the regulatory and coding (frameshift/large-effect mutations) regions of genes/TFs possibly have a greater impact on transcriptional gene regulation (expression) resulting alteration of gene functions in chickpea. These functionally relevant InDel markers thus have a broader practical application in establishing efficient marker-trait linkages and quick identification of potential genes/QTLs governing vital agronomic traits in chickpea.

The inter (97%)- and intra (58–87%)-specific polymorphic potential detected by the InDel markers among *desi, kabuli*, and wild chickpea accessions is much higher/comparable to that estimated especially with the sequence-based SSR, SNP, and InDel markers (Nayak et al., 2010; Bharadwaj et al., 2011; Gujaria et al., 2011; Agarwal et al., 2012; Hiremath et al., 2012; Kujur et al., 2013, 2015a,b,c; Deokar et al., 2014; Saxena et al., 2014a,b; Bajaj et al., 2015a,b,c; Das et al., 2015). The informative genome-wide InDel markers, especially those resolved/genotyped by a simpler cost-effective agarose gel-based assay, exhibiting high intra-specific polymorphic potential among accessions belonging to cultivated and wild chickpea are highly significant. These informative markers could thus serve as a beneficial genomic resource for their immense use in high-throughput genetic analysis including marker-assisted introgression breeding and genetic enhancement of chickpea.

We generated two high-resolution (mean inter-marker distances: 0.92 and 1.01 cM) 1059 and 594 InDel markers-anchored inter-specific genetic linkage maps [(Pusa 1103 × ILWC 46) and (Pusa 256 × ILWC 46)] and a high-resolution (0.66 cM) 1479 InDel markers-led consensus genetic map of chickpea. The map-densities estimated for these genetic maps are comparable with that documented so far in multiple intra- and inter-specific mapping populations-derived genetic maps of chickpea (Nayak et al., 2010; Gujaria et al., 2011; Hiremath et al., 2012; Kujur et al., 2013, 2015c; Deokar et al., 2014; Saxena et al., 2014b; Bajaj et al., 2015a; Das et al., 2015). Therefore, the genetic linkage maps constructed by us have potential to identify and map major QTLs governing various stress tolerance and yield component traits including pod number in chickpea.

The two inter-specific mapping populations utilized in the present study for major PN and SYP QTL mapping revealed a wider phenotypic variability and higher heritability (consistent phenotypic expression) across geographical locations/years for pod number and seed yield per plant trait. These mapping populations thus can serve as a useful genetic resource for molecular mapping of major PN and SYP QTLs in chickpea. The quantitative genetic inheritance pattern of PN and SYP trait was evident from its continuous variation and transgressive segregation as well as normal frequency distribution in the two studied inter-specific mapping populations. This infers the involvement of multiple genes/QTLs in controlling PN and SYP trait in these two diverse mapping populations of chickpea. The identification of strong trait-associated robust QTLs that are well-validated in multiple genetic backgrounds (inter-specific mapping populations in the present study) is essential for efficient deployment of informative markers tightly linked to these QTLs in marker-assisted genetic enhancement of chickpea. To detect well-validated robust PN and SYP QTLs, three major genomic regions underlying each of PN and SYP QTLs mapped individually on two inter-specific genetic maps derived from two mapping populations [(Pusa 1103 × ILWC 46) and (Pusa 256 × ILWC 46)] were compared and correlated. This led to identification of three redundant major genomic regions with short physical intervals of 2.94 (*Caq*^*c*^*PN2.1*), 0.89 (*Caq*^*c*^*PN4.1*), and 1.91 (*Caq*^*c*^*PN4.2*) Mb for PN as well as 2.55 (*Caq*^*c*^*SYP2.1*), 1.69 (*Caq*^*c*^*SYP4.1*), and 3.67 Mb (*Caq*^*c*^*SYP6.1*) for SYP mapped on chromosomes 2, 4, and 6 of a high-density consensus inter-specific genetic linkage map. The validation of three major PN and SYP QTLs across two diverse inter-specific mapping populations indicated their significance as robust QTLs to be utilized in marker-assisted selection and genetic enhancement of chickpea. Three of each long major PN (2.17–3.17 Mb) and SYP (2.88–5.45 Mb) QTL intervals mapped on two individual inter-specific genetic linkage maps were scaled-down into three short major genomic regions underlying robust PN (0.89–2.94 Mb) and SYP (1.69–3.67 Mb) QTLs on a high-density consensus genetic map. This implicates the potential utility of a high-density consensus inter-specific genetic linkage map for high-resolution molecular mapping/fine mapping of robust QTLs and delineation of potential candidate genes governing PN and SYP trait in chickpea.

To ascertain the novelty of three detected robust PN QTLs, the major genomic regions underlying these QTLs were compared with that reported in previous QTL mapping studies employing diverse inter- and intra-specific chickpea mapping populations. Based on congruent physical positions on chickpea chromosomes, two robust PN QTLs (*Caq*^*c*^*PN4.1* and *Caq*^*c*^*PN4.2*) exhibited correspondence with two known major PN QTLs (*CaqPN4.1* and *CaqPN4.2*) identified earlier from similar two inter-specific mapping populations using the mQTL-seq strategy in chickpea (Das et al., 2016). The remaining one robust PN QTL (*Caq*^*c*^*PN2.1*) identified by us has not been reported so far by any QTL mapping studies and thus considered as a novel QTL regulating pod number in chickpea. This could be due to use of genome-wide InDel markers in the present investigation for traditional QTL mapping vis-à-vis whole genome SNP markers for mQTL-seq analysis in the past study (Das et al., 2016). Notably, two robust PN QTLs (*Caq*^*c*^*PN4.1* and *Caq*^*c*^*PN4.2*) and one novel PN QTL (*Caq*^*c*^*PN2.1*) spanning 0.89–2.94 Mb physical intervals, mapped on the chromosomes 2 and 4 of a high-density inter-specific consensus genetic map, were targeted by us to delineate potential candidate genes regulating pod number in chickpea. Interestingly, one identified novel major PN robust QTL (*Caq*^*c*^*PN2.1*) revealing correspondence with a major SYP QTL (*Caq*^*c*^*SYP2.1*), was colocalized based on their congruent physical positions on chickpea chromosome 2. The structural and functional annotation of these short physical PN and SYP QTL regions with available *kabuli* genome sequence especially identified 12 informative InDel markers-led regulatory and one nonsense non-synoymous natural allelic variants in multiple candidate genes/TFs which are known to be the key players of growth and development in diverse crop plants (Moon et al., 2004; Libault et al., 2009; Agarwal et al., 2011; Bartel, 2012; Sadanandom et al., 2012; Bajaj et al., 2015c; Xu et al., 2015). These functionally relevant molecular tags possibly regulating pod number and seed yield delineated by us can be deployed in marker-assisted genetic enhancement to develop high seed and pod-yielding cultivars with increased pod/seed number and yield in chickpea.

Summarily, the current investigation was able to provide multiple novel outcomes vis-à-vis our previous study (Das et al., 2016) that utilizes similar two inter-specific mapping populations for pod number QTL mapping in chickpea. Our study optimized a strategy to develop high-quality informative InDel markers especially from the low coverage NGS genome resequencing data of multiple mapping parental accessions and homozygous bulks/individuals at a genome-wide scale in chickpea with limited resource expenses. In addition, more than three thousand InDel markers exhibiting high intra-/inter-specific polymorphic potential among cultivated (*desi* and *kabuli*) and wild accessions even by a simpler economical agarose gel-based assay were screened for their effective utilization in genomics-assisted breeding applications of chickpea with narrow genetic base. The efficacy of InDel markers for construction of a high-density inter-specific consensus genetic linkage map and molecular mapping of high-resolution major pod number robust QTLs was demonstrated. Despite using similar mapping populations, we scanned two alike and an additional novel major QTL governing pod number between our present and past studies in chickpea. The detection of novel major pod number QTL is possibly due to deployment of high-resolution InDel markers-based traditional QTL mapping in the current study by genotyping of genome-wide InDel markers individually among segregating lines of two mapping populations of chickpea. Efforts have been made to establish efficient correlation between our identified major pod number and seed yield robust QTLs to ascertain the efficacy of these PN QTLs in marker-assisted genetic enhancement for developing high seed and pod-yielding chickpea cultivars. Essentially, the user-friendly InDel markers tightly linked to the genes underlying three (two previously reported and one novel in this study) major pod number as well as three novel seed yield robust QTLs delineated by us can be utilized in marker-assisted foreground selection for efficient screening of numerous back-cross mapping individuals especially by a cost-effective agarose gel-based assay in order to complement the ongoing chickpea molecular breeding program. Among these, especially the InDel markers developed from the genes colocalized at both novel pod number and seed yield robust QTL regions exhibiting increased major allelic effect for combined high PN and SYP traits appear much promising to be utilized in chickpea genomics-assisted breeding. This will eventually drive marker-aided genetic enhancement to develop chickpea cultivars with high seed and pod number and yield in laboratories with minimal infrastructural facilities.

## Author contributions

RS conducted all experiments, bioinformatics analysis and drafted the manuscript. MS and DB involved in InDel markers genotyping and allelic diversity data analysis. MS developed mapping populations and helped in their multilocation phenotyping. SP conceived and designed the study, guided data analysis and interpretation, participated in drafting and correcting the manuscript critically and gave the final approval of the version to be published. All authors have read and approved the final manuscript.

### Conflict of interest statement

The authors declare that the research was conducted in the absence of any commercial or financial relationships that could be construed as a potential conflict of interest.
