# Supplementary material for: A High-Resolution InDel (Insertion–Deletion) Markers-Anchored Consensus Genetic Map Identifies Major QTLs Governing Pod Number and Seed Yield in Chickpea
Source: Front Plant Sci. 2016 Sep 16;7:1362. doi: 10.3389/fpls.2016.01362 (PMC5025440; doi:10.3389/fpls.2016.01362)
Supplement: Supplementary file 3 [file Table3.PDF]

**Table S3. InDel markers (8628) exhibiting differentiation between high (Pusa 1103 and HPNB) and low (ILWC 46 and LPNB) pod number-containing parental accessions and homozygous bulks of a mapping population (Pusa 1103 x ILWC 46) with reference to *kabuli* (CDC Frontier) genome**

| INDEL marker IDs | Chromosomes/ unanchored scaffolds | Physical positions (bp) | InDels (*Kabuli* reference genome-CDC Frontier/PI) | Forward primers (5'-3') | Reverse primers (5'-3') | Anneling temperature (0C) | Expected amplified product size (bp) | Structural annotation | | Functional annotation | | |
|---|---|---|---|---|---|---|---|---|---|---|---|---|
| | | | | | | | | Sequence components of *kabuli* genome | *Kabuli* gene accession IDs | NCBI-KOG | TFs | NCBI-nr database |
| CaPOPI_8625 | C11060010 | 682 | AATTATT/AATTATTATT | TCCGATATGCACCAATGAAA | AGGCGTCGGTTTTTCTTTCT | 59.9 | 413 | INTERGENIC | | | | |
| CaPOPI_8626 | C11155258 | 5339 | TTAAT/TT | CCCACATTTCATGATTTCCC | GCTTCAATGAAATCGCAATACA | 60.0 | 456 | INTERGENIC | | | | |
| CaPOPI_8627 | C11169592 | 3333 | CTTTT/CTTTTT | TCTTTTTCCGATGTTTTCACG | GGGTGCCATCAACACATACA | 60.1 | 125 | INTERGENIC | | | | |
| CaPOPI_8628 | C11178328 | 12753 | CAT/CATTAT | CCATCTTTTAGGCCTCCTCC | GAAGGCAACCATTCTAGGCA | 60.0 | 925 | DRR | Ca_28266 | T | M-type | Disease resistance protein |
| CaPOPI_1 | Ca1 | 18088 | TA/TAA | CCGCTTGTTTTTCTTTGCAG | TTGATTGCTTGTTGCTCCAG | 60.9 | 543 | INTERGENIC | | | | |
| CaPOPI_2 | Ca1 | 19168 | GCTCTCT/GCTCT | CAGAACCGAGGAATTAAGCG | AGGCTTGAAGCGTGGTAATG | 59.8 | 637 | INTERGENIC | | | | |
| CaPOPI_3 | Ca1 | 186383 | T/TC | TTCTGCATCACCATCTGAGG | TGATGAAGATCCCCCAAGAG | 59.8 | 783 | INTRON | Ca_00014 | | | Protein of unknown function DUF707 |
| CaPOPI_4 | Ca1 | 236262 | TAAAAAAAAA/TAAAAAAAA | TGAAAGAACAATATTCCACTAAACCA | GCAGCAATTGATGGTGTGAT | 60.2 | 129 | INTERGENIC | | | | |
| CaPOPI_5 | Ca1 | 831015 | T/TCTCTTATAAATGA | GCCGGATTAAATATAAATCCACA | TGACCTAATAAGCCTGTTTGTTT | 59.2 | 595 | DRR | Ca_00101 | | | |
| CaPOPI_6 | Ca1 | 1084300 | A/AC | TCCCAAATGACTTAATCCGC | ATCGTCGGAGAAATTTGACG | 59.9 | 608 | INTRON | Ca_00134 | DO | LBD | Peptidase C14, caspase catalytic |
| CaPOPI_7 | Ca1 | 1084940 | CTTT/CTTTT | CGTCAAATTTCTCCGACGAT | CTGCGAGTGTTTTCTGGTCA | 60.1 | 290 | INTRON | Ca_00134 | DO | LBD | Peptidase C14, caspase catalytic |
| CaPOPI_8 | Ca1 | 1088478 | A/AC | GGCATCACATTTGTGTCGAA | TCACCCCACTATCCCGTTTA | 60.5 | 717 | DRR | Ca_00135 | | C2H2 | Methyl-CpG DNA binding |
| CaPOPI_9 | Ca1 | 1094498 | AC/A | TTGAGGAATGGTGCAAGATG | TGTACTTGCTATTCATTCTCGTTGA | 59.6 | 254 | INTERGENIC | | | | |
| CaPOPI_10 | Ca1 | 1160199 | GATATATATATATATATATATATATA/GATATATATATATATATATATATATATATA | ATATTCGGGGAATCACCTCC | AGCCGACCTTCCCACTTAAT | 60.0 | 578 | DRR | Ca_00145 | | | |

| INDEL marker IDs | Chromosomes/ unanchored scaffolds | Physical positions (bp) | InDels (*Kabuli* reference genome-CDC Frontier/PI) | Forward primers (5'-3') | Reverse primers (5'-3') | Anneling temperature (0C) | Expected amplified product size (bp) | Structural annotation | | Functional annotation | | |
|---|---|---|---|---|---|---|---|---|---|---|---|---|
| | | | | | | | | Sequence components of *kabuli* genome | *Kabuli* gene accession IDs | NCBI-KOG | TFs | NCBI-nr database |
| CaPOPI_11 | Ca1 | 1237664 | GTC/G | CATCATCAATGAACGAGCCA | CGTAACTTGTGTTTTGCATGAGA | 60.6 | 626 | INTERGENIC | | | | |
| CaPOPI_12 | Ca1 | 1280971 | TTATATATATATATATATATATA/TTATATATATATATATATATATATATA | GGGGAAAACCTTGCTCCTAC | AGGGGGAGCAAAACAAAAAT | 59.9 | 379 | INTRON | Ca_00158 | | | Golgi apparatus membrane protein TVP15 |
| CaPOPI_13 | Ca1 | 1397435 | CA/C | TGGAAACAGCAACGTGGTAG | CAAAGGGTTGGATTTTCCAC | 59.8 | 555 | INTERGENIC | | | | |
| CaPOPI_14 | Ca1 | 1427442 | TTATATATATATATATATATA/TTATATATATATATATATA | TTTGCGTAATTGTTTGTGTCAA | TCCACCCTCACCTCACTACC | 59.2 | 554 | DRR | Ca_00173 | O | | Heat shock protein Hsp70 |
| CaPOPI_15 | Ca1 | 1540452 | CTTTTTTT/CTTTTTTTTTTTT | AGTCTAAAGCCCCCGATTGT | ATTTGGCGGGTCTTGTGTAG | 60.0 | 476 | INTERGENIC | | | | |
| CaPOPI_16 | Ca1 | 1617458 | AC/ACC | TGAAATCAAGTTTGGAAGAGTCA | TAAAAAGGAAATTGCGTCCG | 58.9 | 350 | INTERGENIC | | | | |
| CaPOPI_17 | Ca1 | 1723303 | TTGTAT/TT | ATGTCATCTGTGACCAAGCG | TGGATGAGTGAAAATTGATTGG | 59.7 | 420 | DRR | Ca_00216 | R | | NADPH-dependent FMN reductase |
| CaPOPI_18 | Ca1 | 1729960 | AAATAATAATAATAATAATAATAATAATAATAATAATAATAATAATAATAATAATAATAA/AAATAATAATAATAATAATAATAATAATAATAATAATAATAATAATAATAATAATAAT | GGAACGACCGTTGACTGATT | TTAACTCAGAGGAGCGCAGC | 60.0 | 618 | DRR | Ca_00217 | | TALE | Protein kinase, catalytic domain |
| CaPOPI_19 | Ca1 | 1745242 | AG/A | ATTGTGATTGCATTGCATGG | GAGTAGGTGCCTTCCCACAA | 60.4 | 548 | INTRON | Ca_00219 | | TALE | Protein kinase, catalytic domain |
| CaPOPI_20 | Ca1 | 1800007 | AGTT/A | TGTTGCACAAGGTCCAGAAG | TTTTGGCCAAGAGATGGATG | 59.9 | 601 | DRR | Ca_00227 | P | | Cation/H+ exchanger |
| CaPOPI_21 | Ca1 | 1820515 | AT/ATT | TGTTGACATTAAGGAAGACCGTT | GCTGTTACTACATGCTCTTCCAA | 59.9 | 823 | INTERGENIC | | | | |
| CaPOPI_22 | Ca1 | 1835247 | ATGAC/A | TCATAAGCCAGAACCCAAGC | GGTGGTGGAGATTCCAAGAA | 60.2 | 448 | INTERGENIC | | | | |
| CaPOPI_23 | Ca1 | 1836845 | TATTTTAATT/TATTTTAATTTTAATT | CATTTGCATACCGTTTCACG | AGCACCACTTCTTCATTCAAAA | 60.0 | 704 | INTERGENIC | | | | |
| CaPOPI_24 | Ca1 | 1841034 | TTTCTTCT/TTTCT | TCAAGGTGTTGATTCGGTAGG | TTTTGCAACCCGGTCTATTC | 60.0 | 595 | INTERGENIC | | | | |
| CaPOPI_25 | Ca1 | 1841061 | TAAAA/TAAAAAAAA | TCAAGGTGTTGATTCGGTAGG | TTTTGCAACCCGGTCTATTC | 60.0 | 595 | INTERGENIC | | | | |

| INDEL marker IDs | Chromosomes/ unanchored scaffolds | Physical positions (bp) | InDels (*Kabuli* reference genome-CDC Frontier/PI) | Forward primers (5'-3') | Reverse primers (5'-3') | Anneling temperature (0C) | Expected amplified product size (bp) | Structural annotation | | Functional annotation | | |
|---|---|---|---|---|---|---|---|---|---|---|---|---|
| | | | | | | | | Sequence components of *kabuli* genome | *Kabuli* gene accession IDs | NCBI-KOG | TFs | NCBI-nr database |
| CaPOPI_26 | Ca1 | 1841424 | A/AT | GAATAGACCGGGTTGCAAAA | CCTCTTTCCGGACAACACAT | 59.9 | 302 | INTERGENIC | | | | |
| CaPOPI_27 | Ca1 | 1843387 | ATATT/AT | CAACCAAAAATGCGACTTGA | GCCAAGCTAAGCTGACGAGT | 59.7 | 526 | INTERGENIC | | | | |
| CaPOPI_28 | Ca1 | 1849075 | AATTATTATTATTATTATTATTAT/AATTATTATTATTATTATTAT | GAATTCAACGGTGTGGGACT | TGTTTGAGCCAAATGTGGAA | 59.8 | 271 | CDS (large-effect mutations) | Ca_00232 | K | MYB | |
| CaPOPI_29 | Ca1 | 1850030 | CAATTAATTA/CAATTA | GTTGTCCGTCCTTCCTGGTA | GGACAACCGAAGAGGATCAA | 60.0 | 563 | INTRON | Ca_00232 | K | MYB | |
| CaPOPI_30 | Ca1 | 1876420 | AT/ATACGTAGTTCGGTTGAGT | CGGGTCTTCAAAATGGAATG | CAAAGGAAACGTGCTTCCAG | 60.3 | 861 | INTERGENIC | | | | |
| CaPOPI_31 | Ca1 | 1882571 | TC/T | ATATCGTTTTTCACACCCGC | GGTGCAAATGCAAGAGTCAA | 59.8 | 282 | INTERGENIC | | | | |
| CaPOPI_32 | Ca1 | 1882903 | GT/GTCAGCTTCTAT | TTGACTCTTGCATTTGCACC | ACATATGCGATTGGGACACA | 59.8 | 581 | DRR | Ca_00237 | A | | SPla/RYanodine receptor SPRY |
| CaPOPI_33 | Ca1 | 1883092 | C/CT | GCACGTGGCCCACTTATACT | ACATATGCGATTGGGACACA | 60.0 | 244 | DRR | Ca_00237 | A | | SANT domain, DNA binding |
| CaPOPI_34 | Ca1 | 1883148 | G/GA | GCACGTGGCCCACTTATACT | ACATATGCGATTGGGACACA | 60.0 | 244 | DRR | Ca_00237 | A | | SANT domain, DNA binding |
| CaPOPI_35 | Ca1 | 1888179 | TAAAAAAA/TAAAAAAAA | CCTCACCCCATTTTCCTTTT | ACCATGAACCATTGCCAAGT | 60.2 | 455 | INTERGENIC | | | | |
| CaPOPI_36 | Ca1 | 1915229 | CTCAT/CTCATCTGTTTCAT | TGGATACACAAAAACCGAACA | CACCTTTTCCATCCATCGAC | 58.9 | 709 | INTERGENIC | | | | |
| CaPOPI_37 | Ca1 | 1917237 | GAAAAA/GAAAA | TGGTTGATGAAAGAGAGATGGA | TGTAACAAGCAGATGGAATCTTG | 59.7 | 247 | INTERGENIC | | | | |
| CaPOPI_38 | Ca1 | 1930262 | ATTTTTTTT/ATTTTTTTTTT | GGCAACATTTTACAAAAGTTAAGG | AGTTTGATTAATGTCGGGCG | 58.3 | 318 | INTERGENIC | | | | |
| CaPOPI_39 | Ca1 | 1963997 | ATATTTATTT/ATATTTATTTATTT | AGGCAAACAACTTTTGCACC | TTACAATGTGCATAGCGGGA | 60.2 | 317 | DRR | Ca_00244 | | B3 | Transcriptional factor B3 |
| CaPOPI_40 | Ca1 | 1966252 | TTCTCT/TTCTCTCT | GGCAAGAGAGCATCAAAAGG | CAAGTGAAGTTTTGATTCAAGGG | 60.0 | 703 | DRR | Ca_00245 | U | bZIP | Target SNARE coiled-coil domain |

| INDEL marker IDs | Chromosomes/ unanchored scaffolds | Physical positions (bp) | InDels (*Kabuli* reference genome-CDC Frontier/PI) | Forward primers (5'-3') | Reverse primers (5'-3') | Anneling temperature (0C) | Expected amplified product size (bp) | Structural annotation | | Functional annotation | | |
|---|---|---|---|---|---|---|---|---|---|---|---|---|
| | | | | | | | | Sequence components of *kabuli* genome | *Kabuli* gene accession IDs | NCBI-KOG | TFs | NCBI-nr database |
| CaPOPI_41 | Ca1 | 1968368 | CAAAGAAAGAAAGAAAGA/CAAAGAAAGAAAGA | TCCAATCCATTCCACCATTT | TGCATTCCATTTAGCAGCAG | 60.0 | 775 | INTERGENIC | | | | |
| CaPOPI_42 | Ca1 | 1988980 | TC/TCCC | ACCTGCCAAAAGCAGAAGAA | CACTGAGTTTTCGGGGAGAA | 60.0 | 776 | INTRON | Ca_00248 | T | | Protein of unknown function DUF300 |
| CaPOPI_43 | Ca1 | 1990497 | TAA/TA | TGGGTCAACGTTGGTGTCTA | CCACTCAAAGGCTCACACAA | 60.0 | 769 | INTERGENIC | | | | |
| CaPOPI_44 | Ca1 | 1991447 | GATATATATATATATATATATATATATAT/GATATATATATATATATATATATAT | CCAACCCTTAGGGGACAAAT | CATGTGCCAGAAAGCTCAAA | 60.0 | 505 | DRR | Ca_00249 | CR | HB-other | Alcohol dehydrogenase superfamily, zinc-containing |
| CaPOPI_45 | Ca1 | 2015582 | TTGT/TT | CTCTAGAACCCCCAACTCCC | CTCCAATGTTGGGAGACTACG | 59.9 | 499 | DRR | Ca_00253 | E | | Amino acid transporter, transmembrane |
| CaPOPI_46 | Ca1 | 2023898 | TAA/TAAA | TAATGAGGGCTTGAATTCGC | TCCGTTGTTGAGGATGATGA | 60.2 | 386 | DRR | Ca_00255 | | | |
| CaPOPI_47 | Ca1 | 2057962 | GAAAAGTTA/GA | TGTGCATGAAAGCAAAATTGA | GGCCGTGAAAGATATTTGGA | 60.2 | 712 | DRR | Ca_00261 | | | Protein of unknown function DUF707 |
| CaPOPI_48 | Ca1 | 2073961 | CTTTT/CTTT | AGAGGCACGAGATTTCCAGA | AAATATCCCCGCAATAAGGG | 60.0 | 336 | CDS (FRAME SHIFT) | Ca_00263 | T | M-type | Disease resistance protein |
| CaPOPI_49 | Ca1 | 2085633 | CTTT/CTT | TGGGTTTGTAAAATGAAAAGTGA | TTGAGATTTTCCAAGCTCAAAG | 58.6 | 737 | INTERGENIC | | | | |
| CaPOPI_50 | Ca1 | 2108793 | GTT/GTTTT | GTTTTATCTGCCGAACCTGC | CAATGGCAGTGATCTGCTCT | 59.7 | 678 | INTRON | Ca_00267 | D | NAC | WD40 repeat |
| CaPOPI_51 | Ca1 | 2135161 | AGTGTG/AGTGTGTG | AGAATTTACTCCACGTCCCG | TGAACCTCGTGCATGTAATTCT | 59.1 | 437 | INTERGENIC | | | | |
| CaPOPI_52 | Ca1 | 2223979 | TATAATAATAATAATAATAATAATAATAATAATAA/TATAATAATAATAATAATAATAATAATAATAATAA | AATAGGTCAGGCCAGGCTTT | TATGGATGGGAATAGGCTCG | 60.1 | 659 | INTERGENIC | | | | |
| CaPOPI_53 | Ca1 | 2239989 | AATATATATATATATATATATAT/AATATATATATATATATAT | ATGGTGGCAACACAACTTGA | TGGTTTTGATTTGAGCTTTCC | 60.0 | 775 | DRR | Ca_00280 | I | | Fatty acid desaturase, type 1 |
| CaPOPI_54 | Ca1 | 2299530 | AATATATATATATATATATATATATATATA/AATATATATATATATATATATATATATATATATATA | CAGCAACTGCGATGAGATGT | TCACGGCGAGTCAATAAAAA | 60.0 | 835 | DRR | Ca_00288 | R | | Ribonuclease Zc3h12a-like |
| CaPOPI_55 | Ca1 | 2655633 | AGG/AG | TCCGTGGAAATGAACCTAGC | CATGGACTCAAAAACAGCCA | 60.1 | 812 | INTERGENIC | | | | |

| INDEL marker IDs | Chromosomes/ unanchored scaffolds | Physical positions (bp) | InDels (*Kabuli* reference genome-CDC Frontier/PI) | Forward primers (5'-3') | Reverse primers (5'-3') | Anneling temperature (0C) | Expected amplified product size (bp) | Structural annotation | | Functional annotation | | |
|---|---|---|---|---|---|---|---|---|---|---|---|---|
| | | | | | | | | Sequence components of *kabuli* genome | *Kabuli* gene accession IDs | NCBI-KOG | TFs | NCBI-nr database |
| CaPOPI_56 | Ca1 | 2663715 | TGAAAAGAAAA/TGAAAA | GTTGCCATGCCTATAACGGT | TGAAGACCATGGACACCAAC | 59.9 | 547 | INTERGENIC | | | | |
| CaPOPI_57 | Ca1 | 2674516 | CTAGATTGGAT/CTAGATTGGATTAGATTGGAT | TGTCGGAATATGGGAAGCTC | GCCGTTGTTTTCAGATCCAT | 60.0 | 934 | INTERGENIC | | | | |
| CaPOPI_58 | Ca1 | 2686910 | AGTATTTGGGT/AGT | AATGTGATGCACGTTTTGGA | CAAATTTCACGCAACAATCG | 60.0 | 444 | DRR | Ca_00328 | | MIKC | |
| CaPOPI_59 | Ca1 | 2702474 | CTT/CTTATT | CTGGCAGCATTGTTGTTGTT | ACTTCCTTCCACCACCACTG | 59.8 | 228 | CDS (large-effect mutations) | Ca_00331 | | | |
| CaPOPI_60 | Ca1 | 2766077 | CTTATGTT/CTTATGTTATGTT | AAAACAATTGATTGGGCTCC | CCAATCATACGCAGGTGAAA | 58.9 | 561 | INTERGENIC | | | | |
| CaPOPI_61 | Ca1 | 2796283 | T/TG | GGTGGACAGAGGCATCAACT | AAAAAGCGAGTTTCAGGGTG | 60.1 | 530 | INTERGENIC | | | | |
| CaPOPI_62 | Ca1 | 2800448 | ATTTTTTTTT/ATTTTTTT | GAGAGCAAGAGCGAGCAAGT | ACCACATGAGGGGCATTTTA | 60.0 | 439 | INTERGENIC | | | | |
| CaPOPI_63 | Ca1 | 2800724 | TTTT/TTTTTTTATTT | GAGAGCAAGAGCGAGCAAGT | TCCATTTTCTCCTTCAAGCG | 60.0 | 754 | INTRON | Ca_00344 | | NAC | No apical meristem (NAM) protein |
| CaPOPI_64 | Ca1 | 2837552 | TTCTCTCTCTCTCTCTCTCTCTCT/TTCTCTCTCTCTCTCTCT | CAGCACAAGCATGCCATAGT | TGCATCTGTTCCTGCTTCAC | 59.9 | 671 | INTERGENIC | | | | |
| CaPOPI_65 | Ca1 | 2851127 | TAT/TATAAT | TGTCGTTTTCAATGGACTGG | AGAGAGTATGGGGCAGTCAAA | 59.5 | 226 | INTERGENIC | | | | |
| CaPOPI_66 | Ca1 | 2862989 | CAA/CA | TAATGGCCAAATTCTCTCCG | GCTTTCACCAATTCCACGTT | 60.0 | 691 | INTRON | Ca_00348 | R | | Optic atrophy 3-like |
| CaPOPI_67 | Ca1 | 2885377 | ATTTTTTT/ATTTTTTTT | GCCATTGTTAACTCCGCAAT | CATTTGGAGAGGGCATAGGA | 60.0 | 360 | DRR | Ca_00351 | T | M-type | Disease resistance protein |
| CaPOPI_68 | Ca1 | 2901135 | TTCTGAGAAAGTTATCT/TTCTGAGAAAGTTATCTGAGAAAGTTATCT | AGCTTTCCACGAAAATCAGC | GCACATTTACAGTTGGGGCT | 59.5 | 565 | INTRON | Ca_00354 | | bHLH | Protein kinase, catalytic domain |
| CaPOPI_69 | Ca1 | 2921755 | A/ATATG | CAACTGGGCCAAATGCTTAT | TGATGATCACACGAGGGAGA | 60.0 | 570 | INTERGENIC | | | | |
| CaPOPI_70 | Ca1 | 2946069 | TA/TAA | ATTCCATCGCCATAGCATTC | GGGACCAACAAATTCAAACAA | 59.9 | 553 | INTERGENIC | | | | |

| INDEL marker IDs | Chromosomes/ unanchored scaffolds | Physical positions (bp) | InDels (*Kabuli* reference genome-CDC Frontier/PI) | Forward primers (5'-3') | Reverse primers (5'-3') | Anneling temperature (0C) | Expected amplified product size (bp) | Structural annotation | | Functional annotation | | |
|---|---|---|---|---|---|---|---|---|---|---|---|---|
| | | | | | | | | Sequence components of *kabuli* genome | *Kabuli* gene accession IDs | NCBI-KOG | TFs | NCBI-nr database |
| CaPOPI_71 | Ca1 | 2965539 | CAAAA/CAAA | ACATGCTGTGATTTGGGTCA | AAAGCGAATTTACAGACGTGG | 60.0 | 873 | INTERGENIC | | | | |
| CaPOPI_72 | Ca1 | 2973113 | TATA/TATAATA | TTTGATGTGGCGGCATAGTA | TGTTTGCCAATCACAGCTTC | 60.1 | 281 | INTRON | Ca_00361 | | | Lipase, GDSL |
| CaPOPI_73 | Ca1 | 2979509 | A/ATTATTT | GGAATTCCAAAACTGATCCAA | TGCCAACAAATCCTGAACAA | 58.9 | 653 | INTRON | Ca_00361 | | | Lipase, GDSL |
| CaPOPI_74 | Ca1 | 3003710 | AATATATATATATATATA/AATATATATATATATATATATATATA | GAAAGGCATGCAATATGGGT | AATCTGCCGACGCAAACTAT | 59.8 | 440 | DRR | Ca_00364 | A | bHLH | Helicase, C-terminal |
| CaPOPI_75 | Ca1 | 3020354 | AGG/AGGG | CAAGCAACATGCTGAAGGAA | ATGTCCTTAGTGTCACGGGG | 60.0 | 808 | INTERGENIC | | | | |
| CaPOPI_76 | Ca1 | 3042228 | ATTT/ATTTT | GAGCCAAGATCGATGGGAT | TTTGTATGAACACAACCCCAAA | 60.0 | 502 | INTERGENIC | | | | |
| CaPOPI_77 | Ca1 | 3043596 | GTATATATATATATAT/GTATATATATATATATAT | GGACCGAAATGTTGCCTAAA | GACTTGGTCAATTTGCCGAT | 59.9 | 531 | INTERGENIC | | | | |
| CaPOPI_78 | Ca1 | 3045718 | TCAC/TCACTACAC | CGTAATCACATACATAGGGCTCA | CATTGCACGGTCAATTCTTG | 59.0 | 479 | DRR | Ca_00371 | I | | Lipase, GDSL |
| CaPOPI_79 | Ca1 | 3049357 | ATT/AT | ATCCTTAAAGAGAGGGGCCA | AAACCCAGGAAAGGGTCACT | 60.0 | 888 | INTRON | Ca_00371 | I | | Lipase, GDSL |
| CaPOPI_80 | Ca1 | 3073093 | CTTTTT/CTTTTTT | CCTCCTTACGGCGAAACATA | CGTTGGAACTCCCAATGACT | 60.1 | 234 | INTRON | Ca_00375 | | bHLH | Lipase, GDSL |
| CaPOPI_81 | Ca1 | 3083516 | AT/ATT | TCATTTGACTGAGGCTGCAT | GGACGAGGAGTTGGATTTGA | 59.4 | 884 | INTRON | Ca_00376 | | bHLH | Lipase, GDSL |
| CaPOPI_82 | Ca1 | 3089369 | AT/ATT | AAAATCGGTCTTGGGCTCTT | CCGTGTTAGTCGTCTATTTTTACCA | 60.1 | 713 | DRR | Ca_00378 | I | | Lipase, class 3 |
| CaPOPI_83 | Ca1 | 3094552 | TA/T | GGTGCTCTTAGAGGCAAAGG | TTGGAACAATGACGGTGTTG | 59.1 | 633 | INTERGENIC | | | | |
| CaPOPI_84 | Ca1 | 3103121 | AT/ATT | GGGTGCAATGCTGGATTATT | GTTAAGTGAGGTCCGTCCCA | 59.8 | 462 | DRR | Ca_00380 | | | Glyoxal oxidase, N-terminal |
| CaPOPI_85 | Ca1 | 3113743 | GATATATATATAT/GATATATATAT | TGATTACAATCCTTTAAGCACG | GGAATCGAACTCGAGACTGC | 57.1 | 564 | DRR | Ca_00381 | | | Glyoxal oxidase, N-terminal |

| INDEL marker IDs | Chromosomes/ unanchored scaffolds | Physical positions (bp) | InDels (*Kabuli* reference genome-CDC Frontier/PI) | Forward primers (5'-3') | Reverse primers (5'-3') | Anneling temperature (0C) | Expected amplified product size (bp) | Structural annotation | | Functional annotation | | |
|---|---|---|---|---|---|---|---|---|---|---|---|---|
| | | | | | | | | Sequence components of *kabuli* genome | *Kabuli* gene accession IDs | NCBI-KOG | TFs | NCBI-nr database |
| CaPOPI_86 | Ca1 | 3131301 | TATA/TATACTCATA | GGCCAGACTCAGACCTTTCA | ACCCATTTTTCTGACAACCG | 60.4 | 506 | INTERGENIC | | | | |
| CaPOPI_87 | Ca1 | 3147144 | AATATATATATATATATATATATA/AATATATATATATATA | TCCCAATGCTGCATGTTTTA | TGGGTTGAGTTGTGAGTTCG | 60.1 | 650 | INTRON | Ca_00385 | | | LPPG:FO 2-phospho-L-lactate transferase CofD/UPF0052 |
| CaPOPI_88 | Ca1 | 3194081 | ACGAATGGTCAG/A | GCAAGGCTTGATTCCAAAAA | GGTTGCACCTTCCATGAACT | 60.2 | 799 | DRR | Ca_00390 | O | E2F/DP | 14-3-3 protein |
| CaPOPI_89 | Ca1 | 3783626 | TATTTGCATTTG/TATTTG | TTCACAATTACCACAAAGATCG | GCGACTATCCAGGAAATCCA | 57.7 | 433 | INTERGENIC | | | | |
| CaPOPI_90 | Ca1 | 3872384 | TTATATATATATATATATATATATA/TTATATATATATATATATATATATA | GGGCAGAGCAAGAAAACAAG | CTGACCGTTTTTCGATTTCC | 60.0 | 393 | INTERGENIC | | | | |
| CaPOPI_91 | Ca1 | 4009762 | AT/ATT | CGCACACACTTTCTACGCAT | TTGATCTGGTCAAATAAGCCA | 59.9 | 841 | INTERGENIC | | | | |
| CaPOPI_92 | Ca1 | 4105789 | A/AGCACT | AAAAGTTTGCTGAGGAGCCA | AAAGGGCAAGGATGTGTTTG | 60.0 | 163 | INTRON | Ca_00493 | D | TCP | Cullin, N-terminal |
| CaPOPI_93 | Ca1 | 4131582 | AAAATAAATAAATAAATAAATAAATAAA/AAAATAAATAAATAAATAAATAAATAAATAAATAAATAAATAAA | TCCGAATAAATCGCATACCC | CCAGAGAGACAGATGCACCA | 59.8 | 631 | INTERGENIC | | | | |
| CaPOPI_94 | Ca1 | 4157854 | TA/T | GTGGACACGCGTATGATTTG | TAACAGGCGATGCAATTGAG | 60.0 | 307 | INTERGENIC | | | | |
| CaPOPI_95 | Ca1 | 4211915 | TTATATATATATATATATATA/TTATATATATATATATATATATA | ATCTCTCACGCGACGAAGTT | CCAAATCCGTTCCGCTAGTA | 60.0 | 276 | INTERGENIC | | | | |
| CaPOPI_96 | Ca1 | 4233360 | TTTAAAATTAAAATTAAAATT/TTTAAAATTAAAATT | TAATGGGCTTCGTGGGTTAG | GTGCAGCACATTCGAGAGAA | 60.0 | 683 | DRR | Ca_00504 | | | Protein of unknown function DUF642 |
| CaPOPI_97 | Ca1 | 4444544 | AATATATATATATATATATATA/AATATATATATATATATA | AATCGATTGGCTGAAATCCA | GACATTTAACAGCGCCCATT | 60.4 | 663 | INTERGENIC | | | | |
| CaPOPI_98 | Ca1 | 4551372 | GTGAATTT/GT | AACCACGGACTAATTGTCTCA | AGTGTTCAACGTGGCTGTTG | 57.2 | 187 | INTERGENIC | | | | |
| CaPOPI_99 | Ca1 | 4742142 | TAAA/TAA | GACCGCGTAGATGTGGAACT | GGATGAGACCTCCTACTTGCC | 60.1 | 677 | INTERGENIC | | | | |
| CaPOPI_100 | Ca1 | 5106336 | CT/CTCTAT | CGAGTGATTTGCTTCATTGG | TGTTACGCATTGTTTGGGAA | 59.3 | 651 | INTERGENIC | | | | |

| INDEL marker IDs | Chromosomes/ unanchored scaffolds | Physical positions (bp) | InDels (*Kabuli* reference genome-CDC Frontier/PI) | Forward primers (5'-3') | Reverse primers (5'-3') | Anneling temperature (0C) | Expected amplified product size (bp) | Structural annotation | | Functional annotation | | |
|---|---|---|---|---|---|---|---|---|---|---|---|---|
| | | | | | | | | Sequence components of *kabuli* genome | *Kabuli* gene accession IDs | NCBI-KOG | TFs | NCBI-nr database |
| CaPOPI_101 | Ca1 | 5376097 | ATGGTGGTG/ATGGTG | AAAAGATTAGTGGCTGCCGA | TTTGTGGTGGTCAAAGTCCA | 59.8 | 219 | INTERGENIC | | | | |
| CaPOPI_102 | Ca1 | 5403862 | C/CTTTATTATT | ACGGTTGACTAATTGTGCCA | TCGCATCCCTACGATACGAT | 59.1 | 629 | DRR | Ca_00627 | | | |
| CaPOPI_103 | Ca1 | 5423307 | CTTTT/CTTTTT | CGGGGTAGTTTTTGTATTCCC | GGTGACGCCTTTTAATTTGC | 59.6 | 640 | INTERGENIC | | | | |
| CaPOPI_104 | Ca1 | 5425033 | TTTTATTT/TTTTATTTATTTATTT | TCACGGTGAAAGTTCTGTGC | AAATCCCAATAAGCAAGGCA | 59.9 | 427 | INTERGENIC | | | | |
| CaPOPI_105 | Ca1 | 5484796 | TAA/TAAA | CGCGCGTTGTGTTTATAAGT | TTACCAAGATTGCCCCTCTG | 58.9 | 444 | INTERGENIC | | | | |
| CaPOPI_106 | Ca1 | 5500920 | CTAT/CTATAAACGTCATAT | GGAAGGGAATCGATGAGAAA | TATTCATTGGTTGGGGGAAA | 59.1 | 456 | INTERGENIC | | | | |
| CaPOPI_107 | Ca1 | 5611256 | ATTTTTTTTT/ATTTTTTTT | TCTCAGAACCGACACTGTTCA | TGAATTCAGGTGAGCGTGAG | 59.5 | 886 | INTRON | Ca_00645 | | G2-like | Myb, DNA-binding |
| CaPOPI_108 | Ca1 | 5803481 | TTAAGATAAGAT/TTAAGAT | GCTCATGAGTTCCCTACCCA | GTCAGGGGCTCGTGTATGAT | 60.1 | 560 | INTRON | Ca_00671 | O | FAR1 | ATPase, AAA+ type, core |
| CaPOPI_109 | Ca1 | 5804081 | AT/ATT | AATAACACGGGCACAAGAGG | AGGTCCACCAGGTTTGTCAG | 60.0 | 457 | INTRON | Ca_00671 | O | FAR1 | ATPase, AAA+ type, core |
| CaPOPI_110 | Ca1 | 5955465 | AATATATATATATATATATATATATA/AATATATATATATATATATATATATATATA | TGCAGTGGAAGTGTTGTTGA | GAACGAGGTCGGCAAAAGTA | 58.8 | 686 | INTERGENIC | | | | |
| CaPOPI_111 | Ca1 | 6137600 | T/TC | TCTCATAGAAGGCGACGGTT | CATTTCCGGAGCCAGTAAAA | 59.8 | 412 | INTERGENIC | | | | |
| CaPOPI_112 | Ca1 | 6174612 | TTAT/TTATAT | CCTCTCATCCACATCCATCC | GATATCCATCGGCTTACGACA | 60.3 | 538 | INTERGENIC | | | | |
| CaPOPI_113 | Ca1 | 6176982 | A/AAT | GGTGGTAATAGTTCAGCCCG | AATCTTTGCCAATGGTTTGT | 59.5 | 564 | INTERGENIC | | | | |
| CaPOPI_114 | Ca1 | 6202713 | CTTTTT/CTTTTTT | CGCTTCCTTCTTCACACACA | AAAAGGCAATACATCCAATCAA | 60.0 | 640 | INTERGENIC | | | | |
| CaPOPI_115 | Ca1 | 6203281 | GTTTTTTTTT/GTTTTTTTTTT | CTCCTTGAGTTTTGAGACACGA | TCGATTATGCGTAGGGAAGG | 59.5 | 453 | INTERGENIC | | | | |

| INDEL marker IDs | Chromosomes/ unanchored scaffolds | Physical positions (bp) | InDels (*Kabuli* reference genome-CDC Frontier/PI) | Forward primers (5'-3') | Reverse primers (5'-3') | Anneling temperature (0C) | Expected amplified product size (bp) | Structural annotation | | Functional annotation | | |
|---|---|---|---|---|---|---|---|---|---|---|---|---|
| | | | | | | | | Sequence components of *kabuli* genome | *Kabuli* gene accession IDs | NCBI-KOG | TFs | NCBI-nr database |
| CaPOPI_116 | Ca1 | 6204822 | AAGA/AA | TGGTTTGGTTGCATCACTTT | TCGAAATTTGTGTTCCAAAAGA | 59.0 | 543 | DRR | Ca_07880 | T | | DOMON domain |
| CaPOPI_117 | Ca1 | 6205233 | CAAAAAAAAAAA/CAAAAAAAAAAAA | GGGTATTGGATAAAAATTTCAAAAA | TGAAAATTAAGGGACGGGTTT | 58.7 | 604 | DRR | Ca_07880 | T | | DOMON domain |
| CaPOPI_118 | Ca1 | 6206568 | CTTTTTT/CTTTTTTTT | TTATGCTTTCAACCAGTGCG | TGTCGGATAAGTGTGATATATTCG | 59.9 | 747 | INTERGENIC | | | | |
| CaPOPI_119 | Ca1 | 6212978 | AATATATATATAT/AATATATATATATAT | GTGCGCAGTACAATTCTCCA | CAACCGCTCCCAAATAGAAA | 59.9 | 573 | INTERGENIC | | | | |
| CaPOPI_120 | Ca1 | 6219100 | TTACGGTAT/TT | TGGGAAAACTTGTTCCATGAG | TTGATCTCCACATCACCAAGA | 60.0 | 587 | INTERGENIC | | | | |
| CaPOPI_121 | Ca1 | 6267078 | GAAA/GA | TTTCTTGCACTGGTTTGTGC | TGGGTCCCTCATTGTACTCC | 59.9 | 560 | INTERGENIC | | | | |
| CaPOPI_122 | Ca1 | 6293780 | TAAA/TAAAA | ACACACTGATCGATGATCCC | TATCTTTTTGGTGGGCCAAG | 58.3 | 716 | INTERGENIC | | | | |
| CaPOPI_123 | Ca1 | 6317430 | TTAAAG/TTAAAGCCATAAATAATAAAG | GCTGAGCATGTTGGAACAAA | AAGATCCCCCATGGAAACTC | 59.8 | 606 | INTRON | Ca_07892 | B | MYB_related | Zinc finger, ZZ-type |
| CaPOPI_124 | Ca1 | 6338411 | AAATTAA/AAA | CAACGTTGATTGGGTCAAAA | CCCTTCATACACGGACCATC | 59.4 | 662 | INTERGENIC | | | | |
| CaPOPI_125 | Ca1 | 6453816 | AT/ATT | GGTCTTGCTGACCTTGAAGC | CACGTTAAAATCTCTAACGATGAA | 60.0 | 644 | DRR | Ca_07909 | | Trihelix | Plant lipid transfer protein/seed storage/trypsin-alpha amylase inhibitor |
| CaPOPI_126 | Ca1 | 6467793 | GGAG/GGAGAG | AAAGGGCAAGATTTGTCCAC | AAGGGATAATGTGTTCCCCC | 59.0 | 197 | DRR | Ca_07910 | S | | tRNA (guanine-N1-)-methyltransferase, eukaryotic |
| CaPOPI_127 | Ca1 | 6535430 | GTTATAGTTTTTATAGTT/GTTATAGTT | GCGATGATCGATGACACTCTT | CAAAGTCGAGTATCACCGCC | 60.2 | 236 | INTERGENIC | | | | |
| CaPOPI_128 | Ca1 | 6965841 | CTTTTTT/CTTTTTTT | GTTTTCGTTCTCTCCTCCCC | TGTCATCAGGGAAACCACAA | 60.1 | 759 | INTERGENIC | | | | |
| CaPOPI_129 | Ca1 | 6999458 | TTATATATATATATATATATATATAT/TTATATATATATATATATATATATATAT | AACAACGGCTTTCATTGACTTT | TTTTTCCTGAAACAGGCAGT | 60.0 | 795 | INTERGENIC | | | | |
| CaPOPI_130 | Ca1 | 7215414 | AA/AACA | AACCCCCAACTACAAGGTCC | CAATTTGACAAGCCCCCTAA | 60.1 | 639 | INTERGENIC | | | | |

| INDEL marker IDs | Chromosomes/ unanchored scaffolds | Physical positions (bp) | InDels (*Kabuli* reference genome-CDC Frontier/PI) | Forward primers (5'-3') | Reverse primers (5'-3') | Anneling temperature (0C) | Expected amplified product size (bp) | Structural annotation | | Functional annotation | | |
|---|---|---|---|---|---|---|---|---|---|---|---|---|
| | | | | | | | | Sequence components of *kabuli* genome | *Kabuli* gene accession IDs | NCBI-KOG | TFs | NCBI-nr database |
| CaPOPI_131 | Ca1 | 7423449 | CTTTTTTTTTTTT/CTTTTTTTTTTT | CCATTTGGTTGCTTGGTTTT | TTTTGCTTATATGTTTGGTTCTTGA | 59.8 | 118 | INTERGENIC | | | | |
| CaPOPI_132 | Ca1 | 7634571 | GT/GTT | AAAGTGAATTGCTCAATTTCGTC | AATTGACACGTGAAGATTTGTTT | 59.7 | 777 | INTERGENIC | | | | |
| CaPOPI_133 | Ca1 | 7634633 | ATTTTTTTT/ATTTTTTTTTT | TCCAGCACAAATACCTTTTTACC | TGAATTCGCGTTGTTTGTGT | 59.4 | 666 | INTERGENIC | | | | |
| CaPOPI_134 | Ca1 | 7677905 | CTATTATTATTATTATTATTATT/CTATTATTATTATTATTATTATT | ATCGGAATCAAACCACTTCG | TGAGCCGGAATATTGAGATTG | 59.9 | 567 | INTERGENIC | | | | |
| CaPOPI_135 | Ca1 | 7775058 | C/CCTTAAT | AGCCCAACCCTTGACCTAAT | GCACGGAAACAACATGTGAC | 59.8 | 838 | INTERGENIC | | | | |
| CaPOPI_136 | Ca1 | 7778630 | CTTT/C | CCACACACATCAACCACCAC | GGGCGATTGACTTGAGACAT | 60.8 | 444 | INTERGENIC | | | | |
| CaPOPI_137 | Ca1 | 7790700 | AAAA/AAAACTGCAAA | TCCATAATGTCAAGTTTTGAGCC | ACATCCACAGCACTTGCATC | 60.4 | 949 | INTERGENIC | | | | |
| CaPOPI_138 | Ca1 | 7810637 | G/GAA | AACGATTGGTTGAGACCCAG | ACCTTGTGTCTATCTGGCCG | 60.0 | 505 | DRR | Ca_08031 | | FAR1 | Protein kinase, catalytic domain |
| CaPOPI_139 | Ca1 | 7895366 | GAAA/GAA | TTGCCACAAGCTAAAAGGCT | TGGTCAGTTTGGCCTATTCC | 60.0 | 514 | DRR | Ca_08038 | | | |
| CaPOPI_140 | Ca1 | 7947587 | AC/ACC | TCTTCCTCCAAATTCCAATCA | TTTTTATTCTCGTGGGAGTGAG | 59.5 | 578 | INTERGENIC | | | | |
| CaPOPI_141 | Ca1 | 7995324 | ATAGTAGT/ATAGTAGTAGT | CGGATTGTTTTCGATATGGG | AAGGGATTTTTATTCACAAAACAA | 60.1 | 527 | INTERGENIC | | | | |
| CaPOPI_142 | Ca1 | 8000857 | ATGT/AT | CGCCATCTTCACGTCACTTA | TCCAACCCAAATTTCAAACA | 59.9 | 699 | INTERGENIC | | | | |
| CaPOPI_143 | Ca1 | 8006710 | TG/TGG | TCTTCCCTCCTTCCTTGGAT | TAACACAAGTAGTCGCCCCC | 60.0 | 748 | INTERGENIC | | | | |
| CaPOPI_144 | Ca1 | 8035534 | T/TTA | CAAATGCAAATGCAATGACC | AGGCATCTTTTCAGGTTCCC | 59.9 | 782 | INTRON | Ca_08051 | M | FAR1 | NAD-dependent epimerase/dehydratase |
| CaPOPI_145 | Ca1 | 8125888 | G/GACATATTAAAA | TGTTGGGTTACGAGGGAGTC | TTGCGGGTTTTTAACCTGAG | 60.0 | 526 | INTERGENIC | | | | |

| INDEL marker IDs | Chromosomes/ unanchored scaffolds | Physical positions (bp) | InDels (*Kabuli* reference genome-CDC Frontier/PI) | Forward primers (5'-3') | Reverse primers (5'-3') | Anneling temperature (0C) | Expected amplified product size (bp) | Structural annotation | | Functional annotation | | |
|---|---|---|---|---|---|---|---|---|---|---|---|---|
| | | | | | | | | Sequence components of *kabuli* genome | *Kabuli* gene accession IDs | NCBI-KOG | TFs | NCBI-nr database |
| CaPOPI_146 | Ca1 | 8127338 | CTT/CTTT | CCCTCCTTCCAGAAAACCTT | AATCCTGCAGTGAAACCACC | 59.5 | 708 | INTERGENIC | | | | |
| CaPOPI_147 | Ca1 | 8403563 | TTAATAATAATAATAATAATAATAATAATAATAATAATAATAATAATAATAATA/TTAATAATAATAATAATAATAATAATAATAATAATAATAATA | CCCCTAAAAATTAGTCGAACTCC | GAGTCTTTGTCCCCAGCAAG | 59.4 | 474 | INTRON | Ca_08076 | | | |
| CaPOPI_148 | Ca1 | 8415420 | AG/A | ATGGGTGAAACGAGCAACTC | TTTTCCTTTTGCATCATCTTTG | 60.1 | 910 | INTERGENIC | | | | |
| CaPOPI_149 | Ca1 | 8830262 | TTATATATATATATATATATATATATA/TTATATATATATATATATATATATA | GCTACCTTAGCCTCTCCATGC | GGAATGCCATAGAAACACCAA | 60.4 | 811 | INTERGENIC | | | | |
| CaPOPI_150 | Ca1 | 8909387 | T/TCC | AGGCAAAAGGTTTTTGGGTT | GTGAGGCGTGAAGTGGTTTT | 59.9 | 806 | DRR | Ca_02914 | | | |
| CaPOPI_151 | Ca1 | 9233850 | GTTTTTTTTTTTTT/GTTTTTTTTTT | TGAGTTCTTTTGAATTTGCGTG | GTTGCGTCTAGCAACTTTCTG | 60.3 | 637 | INTERGENIC | | | | |
| CaPOPI_152 | Ca1 | 9871519 | ATT/A | CTTAGTCCACGTGCAAACGA | AAAGTGCGGGCCAATATCTA | 59.9 | 600 | INTERGENIC | | | | |
| CaPOPI_153 | Ca1 | 10040103 | ATTT/ATT | ATGCATCGAAAACCAACACA | TGCAATCCGGGCTTACTATC | 60.0 | 378 | INTRON | Ca_02784 | LK | | |
| CaPOPI_154 | Ca1 | 10057638 | CATATATATATATATATAT/CATATATATATATAT | TGATGGGAGCAATGAATGAA | TCAAATTCCGACTTGAGGGA | 60.0 | 542 | INTERGENIC | | | | |
| CaPOPI_155 | Ca1 | 10064752 | TTATATATATATATATATA/TTATATATATATATATATATATATATA | ATGCAAATTCTCCCCCTCTT | TCATTTGATTTTAATTGGACGA | 59.9 | 750 | INTERGENIC | | | | |
| CaPOPI_156 | Ca1 | 10323177 | G/GAGCT | TTGAGTGCTTCACCATTTGC | GTTGTATTGGCTCTCCTCGC | 59.8 | 463 | INTRON | Ca_02746 | B | C2H2 | WD40 repeat |
| CaPOPI_157 | Ca1 | 10414677 | CAGGAATNNNNNNNNNNAGGAAT/CAGGAAT | TTCTAAACGAGGCTTTTCAGTTG | GCATGTTGCTGATGATGGTC | 59.9 | 489 | DRR | Ca_02735 | R | ERF | 3-hydroxyisobutyrate dehydrogenase-related, conserved site |
| CaPOPI_158 | Ca1 | 10458891 | TACACACA/TACACA | CCAATTTCAGTAGGCCCAAA | AGCATTGAAACCAAACCAGC | 59.9 | 583 | DRR | Ca_02729 | | HSF | Survival protein SurE-like phosphatase/nucleotidase |
| CaPOPI_159 | Ca1 | 10471260 | TTATATATATATATATATATA/TTATATATATATATATATATA | TTCTCCATTCGCACTCACAC | TCAAATTTGGAGGCTGGAAC | 59.8 | 312 | INTERGENIC | | | | |
| CaPOPI_160 | Ca1 | 10513925 | CTTATTTATTTATTTATTTATTTATTTATTTATTTATTTATT/CTTATTTATTTATTTATTTATTTATTTATTTATT | CCAGATTTCCTTTTCCCCTC | GGGTAGGCCACAGACTGTCTTA | 59.9 | 634 | INTERGENIC | | | | |

| INDEL marker IDs | Chromosomes/ unanchored scaffolds | Physical positions (bp) | InDels (*Kabuli* reference genome-CDC Frontier/PI) | Forward primers (5'-3') | Reverse primers (5'-3') | Anneling temperature (0C) | Expected amplified product size (bp) | Structural annotation | | Functional annotation | | |
|---|---|---|---|---|---|---|---|---|---|---|---|---|
| | | | | | | | | Sequence components of *kabuli* genome | *Kabuli* gene accession IDs | NCBI-KOG | TFs | NCBI-nr database |
| CaPOPI_161 | Ca1 | 10944660 | TAAAAAAAAAA/TAAAAAAAAAAA | GCGACCTCGTGAATGAAAAA | GGCATAATTGTTCCCACAAA | 61.1 | 534 | INTERGENIC | | | | |
| CaPOPI_162 | Ca1 | 11102279 | ATTTTTTTTTT/ATTTTTTTTT | TGCCGCGAATTTAGATCTTT | TCGGTGTTGGTGTCGTTATC | 59.8 | 255 | DRR | Ca_02649 | C | | Mitochondrial carrier protein |
| CaPOPI_163 | Ca1 | 11145101 | TTATATATATATATATATATATATATAT/TTATATATATATATATATATATAT | TTTGAGTCCAAATTGAAGGCT | CGGATCAAAATTCGCACAAT | 58.8 | 837 | INTERGENIC | | | | |
| CaPOPI_164 | Ca1 | 11148737 | CAATAATAATAAT/CAATAATAAT | CGTGTAATTGGTAATGTGGCA | GGCTAACTTGAAGTCGTGGC | 59.4 | 693 | INTERGENIC | | | | |
| CaPOPI_165 | Ca1 | 11170203 | CATA/CATATATA | ATGAGGGTATCCGAGCCATA | CCACAAGAGTTTTGCCATTG | 59.4 | 378 | DRR | Ca_02641 | | | Protein of unknown function DUF3527 |
| CaPOPI_166 | Ca1 | 11190216 | ATTACT/ATTACTTACT | TATGGCTAGCTGGTGAAGCA | TAGAATCCCACGCAGCTCTT | 59.6 | 304 | INTRON | Ca_02639 | | | |
| CaPOPI_167 | Ca1 | 11324975 | GAAGA/GAAGAAAGA | CTGCATCGGGGATACTTCAC | TTTTGACCGAGCCAGATACC | 60.5 | 317 | INTRON | Ca_02622 | | | |
| CaPOPI_168 | Ca1 | 11456505 | TTATATATATATATATATATA/TTATATATATATATATATATATA | AACCGAGTGCCCTTTAACAA | TTGTTTTGCATTTTGGGACA | 59.6 | 273 | INTERGENIC | | | | |
| CaPOPI_169 | Ca1 | 11868314 | TTATATATATATATATAT/TTATATATATATATATATATATAT | GCAAGTATGCAACAACCTTCC | TTGCATATATTGTCACACCGC | 59.6 | 346 | INTERGENIC | | | | |
| CaPOPI_170 | Ca1 | 11977187 | CAAAAAA/CAAAAAAA | GCAATTGGATGACATTGCATA | ACAGACAGGGCACAGGATCT | 59.4 | 479 | INTERGENIC | | | | |
| CaPOPI_171 | Ca1 | 12195477 | CTTTTTTTTT/CTTTTTTTTTT | TTGTTTGTTTCCTTGTGGCA | AGAAAAATGCGATGGTTCTTT | 60.1 | 250 | INTERGENIC | | | | |
| CaPOPI_172 | Ca1 | 12420239 | CAAAAAAAAA/CAAAAAAAAAA | AAATGCAATCTTGGTTTCCA | TTTTGGTAATGTGCCCATGATA | 58.1 | 595 | INTERGENIC | | | | |
| CaPOPI_173 | Ca1 | 12550318 | ATTTTTTTTTTT/ATTTTTTTTTT | CGTCTTTATTTTATGCGAGCG | TAATGCCGATGGTGTCAAAA | 59.9 | 252 | INTERGENIC | | | | |
| CaPOPI_174 | Ca1 | 12580190 | TAAAAAAAAAA/TAAAAAAAAAAA | TAATTCACTCACCATCGCCA | TTCATGTTTGGTGTGGCACT | 60.1 | 476 | INTERGENIC | | | | |
| CaPOPI_175 | Ca1 | 12727627 | TTATATATATATATATATATATAT/TTATATATATATATATATAT | CCTTACGGACAACCAAGCAT | GCAAAAGAATTTGCAACAAAGA | 60.0 | 419 | INTERGENIC | | | | |

| INDEL marker IDs | Chromosomes/ unanchored scaffolds | Physical positions (bp) | InDels (*Kabuli* reference genome-CDC Frontier/PI) | Forward primers (5'-3') | Reverse primers (5'-3') | Anneling temperature (0C) | Expected amplified product size (bp) | Structural annotation | | Functional annotation | | |
|---|---|---|---|---|---|---|---|---|---|---|---|---|
| | | | | | | | | Sequence components of *kabuli* genome | *Kabuli* gene accession IDs | NCBI-KOG | TFs | NCBI-nr database |
| CaPOPI_176 | Ca1 | 12947634 | ATAAATTTAAA/ATAAATTTAAAATTTAAATTTAAA | AACTTTGTGGTCGTTTTGGC | TGCTTATTGTGGCTGCTTTG | 60.0 | 647 | INTERGENIC | | | | |
| CaPOPI_177 | Ca1 | 12956423 | CAAAAAAA/CAAAAA | TTGTCGGATACCTGCTCAGT | TTGCAAATTCGGACAAACAA | 58.3 | 455 | INTERGENIC | | | | |
| CaPOPI_178 | Ca1 | 12988934 | C/CGGG | ATCGGCATCCTTCCTTCTTT | GACGCTAAGCCCCTTTCTCT | 60.0 | 354 | INTERGENIC | | | | |
| CaPOPI_179 | Ca1 | 13005887 | TAAAAAAAAAAAAA/TAAAAAAAAAAA | TCGTCATCTAGCACCAGCAC | TCCCTCGTAAAGACCCATCT | 60.0 | 347 | INTERGENIC | | | | |
| CaPOPI_180 | Ca1 | 13007507 | TTAT/TTATATAT | CACGGGTCCAGATGGTTAGT | CCAAGCATCTTGGTGCATAA | 59.8 | 322 | INTERGENIC | | | | |
| CaPOPI_181 | Ca1 | 13117590 | TAATAAAAATAAAAATAAAA/TAATAAAAATAAAA | TGTTGTTTACGGAATTTGGTTTT | TTTCGTTGTATATTAAAGCACGG | 59.7 | 547 | INTERGENIC | | | | |
| CaPOPI_182 | Ca1 | 13134589 | TAAAAAAA/TAAAAAAAA | CGATGGATGACTTCCCGTAT | TTTACGTTGACGCACGAGAC | 59.8 | 587 | INTERGENIC | | | | |
| CaPOPI_183 | Ca1 | 13674092 | ATTTT/ATTT | AACTGGTTCGATTCGGTTTTT | TGAAAATTTCGCATTCACCA | 59.9 | 382 | INTERGENIC | | | | |
| CaPOPI_184 | Ca1 | 13676261 | ACT/ACTCT | CCGCTACAAGGCGATAGAAG | GGGTGTCATCATAGGTGGCT | 60.0 | 856 | DRR | Ca_14132 | | | |
| CaPOPI_185 | Ca1 | 13680810 | ATTTTT/ATTTT | TGGTTGAACTGTGAACAGGC | CCAATTTCCCCTACCCAAAT | 59.7 | 559 | INTERGENIC | | | | |
| CaPOPI_186 | Ca1 | 13686356 | T/TCTC | TTGTACGGGTGGTGTCAAAA | GAGGGTGACTCTGCTGGAAC | 59.9 | 655 | INTRON | Ca_14134 | | MYB_related | Glycoside hydrolase, family 16 |
| CaPOPI_187 | Ca1 | 13690477 | CAA/CAATCGGTTTTATAA | CCGGTTATTTAACCATAACCAA | TGCAAGGAACAAATGGCATA | 57.9 | 200 | INTERGENIC | | | | |
| CaPOPI_188 | Ca1 | 13692764 | CTTTT/CTTTTT | CGCCAGTGGTTATGCTCTTA | AAGGAACCACACTTGAACCG | 58.9 | 875 | INTERGENIC | | | | |
| CaPOPI_189 | Ca1 | 13694461 | GTA/GTAATA | CCACTTCGCCACGTTTTTAT | CGGTTTATCGTCAAAGCGAT | 60.0 | 379 | INTERGENIC | | | | |
| CaPOPI_190 | Ca1 | 13696144 | TAAAAA/TAAAA | TTTGCGTTACGAATTTGATGA | AGAAGTTGAACTCACGAATCACA | 59.2 | 757 | INTERGENIC | | | | |

| **INDEL marker IDs** | **Chromosomes/ unanchored scaffolds** | **Physical positions (bp)** | **InDels (*Kabuli* reference genome-CDC Frontier/PI)** | **Forward primers (5'-3')** | **Reverse primers (5'-3')** | **Anneling temperature (0C)** | **Expected amplified product size (bp)** | **Structural annotation** | | **Functional annotation** | | |
|---|---|---|---|---|---|---|---|---|---|---|---|---|
| | | | | | | | | **Sequence components of *kabuli* genome** | ***Kabuli* gene accession IDs** | **NCBI-KOG** | **TFs** | **NCBI-nr database** |
| CaPOPI_191 | Ca1 | 14385063 | CAA/CAAA | AATCGTCTCCGTCACACACA | GCGAATTGCAGACTGAAACA | 60.2 | 662 | DRR | Ca_07096 | | SAP | Aminotransferase-like, plant mobile domain |
| CaPOPI_192 | Ca1 | 14682464 | CTTTT/CTTT | TCCATTGTTGAGCGAAAACA | TGGATGAAGGATCAACCACA | 60.2 | 659 | INTERGENIC | | | | |
| CaPOPI_193 | Ca1 | 14777698 | TAAAAAAAA/TAAAAAAA | CAATGACCTTGGTCCTCCTG | TTAACTGCAGCCATGCATCT | 60.5 | 799 | DRR | Ca_07052 | R | | Phosphatidylethanolamine-binding, conserved site |
| CaPOPI_194 | Ca1 | 14893455 | GTTTTTTTT/GTTTTTTT | AGGGACAAGGTTGGGTTTTT | TTAAAACTTTGCACGGAGGG | 59.7 | 291 | INTERGENIC | | | | |
| CaPOPI_195 | Ca1 | 14995847 | ACATGCATGCA/ACATGCA | CAGCGCTTTTCCCAAATAAG | CCAAAAAGGGTGGAGATTGA | 59.8 | 359 | INTERGENIC | | | | |
| CaPOPI_196 | Ca1 | 15326926 | CATATATATATAT/CATATATATAT | GTTAAGCATCCCAAGGTGGA | AGCCAAGAGTTTGTGGAGTTG | 59.9 | 626 | INTERGENIC | | | | |
| CaPOPI_197 | Ca1 | 15811388 | TAAAAAAA/TAAAAAAAA | GCGAGATTTTCGACTTCTGC | TGCGGCAAACGTAATTGTAA | 60.1 | 590 | INTERGENIC | | | | |
| CaPOPI_198 | Ca1 | 15835234 | TTTATTATTATTATTATTATTATTATTATTATT/TTTATTATTATTATTATTATTATTATTATT | TCGTTTGCCGTTAACTAGGG | TTGGCCTAACCTGAAAGCAT | 60.1 | 768 | INTERGENIC | | | | |
| CaPOPI_199 | Ca1 | 15849587 | AATATATATATATATATATATATATATATATATATA/AATATATATATATATATATATATATATATATATATA | CCCCATTTTGTTTCTCTCCA | ATTCTTGGAGGAGCGGGTAT | 59.9 | 872 | INTERGENIC | | | | |
| CaPOPI_200 | Ca1 | 16078538 | CTTTTTTTTT/CTTTTTTTT | TGCATGTTGTTGGTAGAGTCAA | GCACAAGGTATGCCAAATGA | 59.2 | 632 | INTERGENIC | | | | |
| CaPOPI_201 | Ca1 | 16096244 | TG/TGG | ATGTAAGATTGGAAGCGGCA | CAATTGTTCCCCACTTGCTT | 60.6 | 298 | INTERGENIC | | | | |
| CaPOPI_202 | Ca1 | 16116426 | GA/GAA | AATGCAACGGTTGGTTTGAT | GCACAAATGAAAAGCAGAACA | 60.2 | 826 | DRR | Ca_06940 | R | | General substrate transporter |
| CaPOPI_203 | Ca1 | 16155793 | ATT/AT | GGCAACTCCATTGAATTCGT | AATCCCCACCCTAAATGACC | 59.9 | 333 | INTERGENIC | | | | |
| CaPOPI_204 | Ca1 | 16159274 | AAAAA/AAAAACAAAA | ATGGTTTTAAGCATTCCCCA | TTAAGTTGCGAGGTTGCTCA | 59.3 | 457 | INTERGENIC | | | | |
| CaPOPI_205 | Ca1 | 16160389 | TT/TTCT | CATGTGGATTTGTCTTTGCG | GCACAAGATCCTCAAAACTGG | 60.1 | 238 | INTERGENIC | | | | |

| INDEL marker IDs | Chromosomes/ unanchored scaffolds | Physical positions (bp) | InDels (*Kabuli* reference genome-CDC Frontier/PI) | Forward primers (5'-3') | Reverse primers (5'-3') | Anneling temperature (0C) | Expected amplified product size (bp) | Structural annotation | | Functional annotation | | |
|---|---|---|---|---|---|---|---|---|---|---|---|---|
| | | | | | | | | Sequence components of *kabuli* genome | *Kabuli* gene accession IDs | NCBI-KOG | TFs | NCBI-nr database |
| CaPOPI_206 | Ca1 | 16162054 | CTT/CTTT | TCCTCATGGCTTTTCATTCC | TCAATCTTCATAGGCTGAACGA | 60.0 | 796 | INTRON | Ca_06938 | R | | General substrate transporter |
| CaPOPI_207 | Ca1 | 16167140 | TAAAA/TAAAAA | TTTTTAGCATGGCACGTTTT | CGGGTTGGATTAGATTTCAAG | 58.4 | 378 | INTERGENIC | | | | |
| CaPOPI_208 | Ca1 | 16187503 | AGTTTATGACG/AG | GTGTCACCATTGGCAATACG | TTCCTTCGACCTCACAATCC | 59.8 | 382 | CDS (large-effect mutations) | Ca_06937 | | | GH3 auxin-responsive promoter |
| CaPOPI_209 | Ca1 | 16203089 | TC/T | CTTGTGTGGGGAAGGTCACT | TGTGTGACCCCTGCTGAATA | 60.0 | 685 | INTERGENIC | | | | |
| CaPOPI_210 | Ca1 | 16583164 | AATATATATATATATATATATATA/AATATATATATATATATATA | TTTGGCACTTTCTCATTCCC | GCCATGTATGGTCCAAGGTC | 60.1 | 793 | INTERGENIC | | | | |
| CaPOPI_211 | Ca1 | 16629521 | TTTAATTAATTAATTAATT/TTTAATTAATTAATTAATTAATTAATT | AACGTTTGAGGATTCCGTTG | GAAACTCCAGCTCCAAAACG | 60.0 | 370 | INTERGENIC | | | | |
| CaPOPI_212 | Ca1 | 16707141 | TTATATATATATATATATATATATATATAT/TTATATATATATATATATATATATAT | CTTTACAACCGCCACAAACC | CGAACAAAGTGCTTGTGGAA | 60.4 | 755 | INTERGENIC | | | | |
| CaPOPI_213 | Ca1 | 17185713 | TTTGATATTTGATTAT/TTTGATATTTGATTATTGATATTTGATTAT | GCGTTAAAGTTGGAAACCCA | TGACGTGACATCGAGAGTCTG | 60.0 | 594 | INTERGENIC | | | | |
| CaPOPI_214 | Ca1 | 18181716 | AATATATATATATATATATATATATATA/AATATATATATATATATATATATATATATATA | CGAATACTCATGGTTTTTGATCTG | ACCCAACGTAGCACGTTACC | 59.9 | 626 | INTERGENIC | | | | |
| CaPOPI_215 | Ca1 | 18300800 | ATTT/ATT | AGCAAGAAAATTGAAGGCCA | GGAAGCTTTAAAGGGAGCAAA | 59.8 | 161 | INTERGENIC | | | | |
| CaPOPI_216 | Ca1 | 18301437 | AATT/A | TGATAAGCTAACAGGCCAAAAA | ACATGGGCTAGCAAGAAACC | 58.9 | 417 | INTERGENIC | | | | |
| CaPOPI_217 | Ca1 | 18317638 | T/TG | TCATTTTCAACCCTTAGCAACA | TGGTAAGGTATACGCGGGAG | 59.6 | 690 | INTERGENIC | | | | |
| CaPOPI_218 | Ca1 | 18325523 | AGG/AG | CGGATCATTACACACTTGCG | GCCAAAGCCATGTTTTCAAT | 60.1 | 324 | INTERGENIC | | | | |
| CaPOPI_219 | Ca1 | 18328170 | GTTTTTT/GTTTTTTT | TTAAAACCACAAAATGGGGC | GAGGCAAAGCTTCCAAATCA | 59.7 | 475 | INTERGENIC | | | | |
| CaPOPI_220 | Ca1 | 18364053 | GAAA/GAAAA | CCCGTAGCAAGACTTGAAGC | TTGGAGAAACTTTTCCGCTG | 60.0 | 627 | INTERGENIC | | | | |

| INDEL marker IDs | Chromosomes/ unanchored scaffolds | Physical positions (bp) | InDels (*Kabuli* reference genome-CDC Frontier/PI) | Forward primers (5'-3') | Reverse primers (5'-3') | Anneling temperature (0C) | Expected amplified product size (bp) | Structural annotation | | Functional annotation | | |
|---|---|---|---|---|---|---|---|---|---|---|---|---|
| | | | | | | | | Sequence components of *kabuli* genome | *Kabuli* gene accession IDs | NCBI-KOG | TFs | NCBI-nr database |
| CaPOPI_221 | Ca1 | 18441371 | CACAAGAGAC/CAC | CCCTCTGTATGCCAGTCGAT | TGGCATTGATGATGGCTTTA | 60.1 | 771 | INTERGENIC | | | | |
| CaPOPI_222 | Ca1 | 18472041 | CAAAAAAAAAA/CAAAAAAAAAAA | CCTACATTCGCACGAGTTCA | TTTTTCTCCAGTTTTGGTCCT | 59.9 | 404 | INTERGENIC | | | | |
| CaPOPI_223 | Ca1 | 18688865 | TTGT/TT | TAGCAGCTGGAGGAACATGA | GCACCCAGTGTAAGACCCAT | 59.5 | 301 | INTERGENIC | | | | |
| CaPOPI_224 | Ca1 | 18868658 | CGG/CGGGAATGG | AATCGATTGATTCAGCCCAG | CGTTTTTCAAAGCGAGGGTA | 60.0 | 707 | INTERGENIC | | | | |
| CaPOPI_225 | Ca1 | 18899804 | CTATATATATATATATATATATAT/CTATATATATATATATATATATAT | GCTTCAATGTGCACGATACG | TTCTTTGTTGGTAGGGCACC | 60.3 | 237 | INTERGENIC | | | | |
| CaPOPI_226 | Ca1 | 18928985 | CTTATTTATTTATTTATTTATTTAT/CTTATTTATTTATTTATTTATTTATTTAT | TTTACCCTATCCCACACCCC | TCCAATGATTCCTGTTTTTGC | 60.8 | 613 | INTERGENIC | | | | |
| CaPOPI_227 | Ca1 | 19150973 | CTT/CT | AACTAGCCCGTCTTTGGGTT | GAAAACAAAAGCTTTACGGTCA | 60.0 | 488 | INTERGENIC | | | | |
| CaPOPI_228 | Ca1 | 19498678 | TTTTGATCCTTTGAT/TTTTGAT | GCCCGAGTTAACAACAAACG | ATCGATTTCCTCAGCACTGG | 60.5 | 374 | INTERGENIC | | | | |
| CaPOPI_229 | Ca1 | 19903627 | TA/TAATTGGCAGCA | TGGGCTGAAAAGCAACTTCT | ACCAGTGCTGTTCCCAAATC | 60.0 | 233 | INTRON | Ca_08847 | K | | RNA polymerase I associated factor, A49-like |
| CaPOPI_230 | Ca1 | 19909063 | TTATATATATATATATATATATATATATATATAT/TTATATATATATATATATATATATATAT | CCATAATTTTTGGATCCTCCT | TTTGCTTATGAGAAAAAGGAGATGT | 57.1 | 235 | INTERGENIC | | | | |
| CaPOPI_231 | Ca1 | 20097334 | CNNNNNNNNNNNNNN/CNNN | TTTCGTTTGCGATAGTGATCC | TCATTTCAAGATCCTGGTTCC | 60.1 | 250 | INTERGENIC | | | | |
| CaPOPI_232 | Ca1 | 21746056 | AC/ACACATATCAGCTTTC | CTATTGCCCCCTCCTCCTAC | TACCGATACCTCCAAGTGCC | 59.9 | 585 | INTRON | Ca_22116 | S | | Uncharacterised protein family FPL |
| CaPOPI_233 | Ca1 | 21772275 | CATATATATATATATAT/CATATATATATATATATAT | CTTTTGTCTCCCCATCCTCA | AAAACTGAGACGAAAAAGTTGTCC | 60.0 | 342 | DRR | Ca_22115 | | MYB | |
| CaPOPI_234 | Ca1 | 22131328 | AATATATAT/AATATATATAT | GTGTGGACATGCCTAAAACG | GTTGCAATTGAGGTGCTGAA | 59.1 | 403 | INTERGENIC | | | | |
| CaPOPI_235 | Ca1 | 22131513 | GAAAAA/GAAAA | GTGTGGACATGCCTAAAACG | GTTGCAATTGAGGTGCTGAA | 59.1 | 403 | INTERGENIC | | | | |

| INDEL marker IDs | Chromosomes/ unanchored scaffolds | Physical positions (bp) | InDels (*Kabuli* reference genome-CDC Frontier/PI) | Forward primers (5'-3') | Reverse primers (5'-3') | Anneling temperature (0C) | Expected amplified product size (bp) | Structural annotation | | Functional annotation | | |
|---|---|---|---|---|---|---|---|---|---|---|---|---|
| | | | | | | | | Sequence components of *kabuli* genome | *Kabuli* gene accession IDs | NCBI-KOG | TFs | NCBI-nr database |
| CaPOPI_236 | Ca1 | 22132039 | G/GC | GGATGGTAGTTCAGCACCTCA | GAGCATTAGTGCTTGGCACA | 60.1 | 774 | INTERGENIC | | | | |
| CaPOPI_237 | Ca1 | 22384776 | G/GAACAAGTGTGT | CTTCAGATCCTTGAGCTGCC | TGAATCTTTGTGTCCCACCC | 60.1 | 539 | INTERGENIC | | | | |
| CaPOPI_238 | Ca1 | 22745256 | AATATATATATATATATATATATATATATA/AAAATATATATATATATATATATATATATATATA | CTCCAGAAATTTTGCCTCCA | GTTAGGCCGAGGCTCTCTTT | 60.2 | 480 | INTRON | Ca_20613 | BD | bHLH | Nucleosome assembly protein (NAP) |
| CaPOPI_239 | Ca1 | 22886112 | GAA/GAAA | TGATTTGGGGTTTTGGGTAA | GGGTGAGTTCTTTGAGGGTG | 60.0 | 531 | INTERGENIC | | | | |
| CaPOPI_240 | Ca1 | 22896758 | CTTTTTTTTTTTTTTT/CTTTTTTTTTTT | GCACCGTTTACAACGCTTTT | GATGAAACCGAGGACCAGAA | 60.2 | 400 | INTERGENIC | | | | |
| CaPOPI_241 | Ca1 | 23192284 | TTAATAATAATAATAATAATAATAATAATAATAATAATAATAATAATAATAATAATAA/TTAATAATAATAATAATAATAATAATAATAATAATAATAATAATAATAATAATAATA | TTCGAAACTTTTGGCCACTT | TTGGATTTTGGCCACATTTT | 59.7 | 527 | INTERGENIC | | | | |
| CaPOPI_242 | Ca1 | 23386171 | GTTATTATTATTATTATTATTATTATTATTATTATTATTATTAT/GTTATTATTATTATTATTATTATTATTATTATTATTATTAT | ACAAGAATGGCAAATGGAGC | TTTGCCCCACTAACTCTTGG | 60.1 | 537 | INTERGENIC | | | | |
| CaPOPI_243 | Ca1 | 23496241 | CT/CTTT | AGGGTGGATCAGTTCGATTG | TTATATTAATTGGGTGCGCGT | 59.9 | 376 | DRR | Ca_19314 | | | |
| CaPOPI_244 | Ca1 | 23516838 | CAAAAAA/CAAAAA | GGAATCCAAACCAAAACCCT | TTGGTGTGGTTTTTGTGGAA | 60.0 | 634 | INTERGENIC | | | | |
| CaPOPI_245 | Ca1 | 23571644 | AA/AAGTCTTCCGATCTTTTGA | TGTGTTGGCTTAAAACATTTGAT | GGGCAATGTTGGTGGTACTC | 58.6 | 810 | INTERGENIC | | | | |
| CaPOPI_246 | Ca1 | 23784964 | ATTTTTTTT/ATTTTTT | GTCAACCAATTGACCCCAAC | TGCCAAGAAAACACCATGAA | 60.1 | 663 | INTERGENIC | | | | |
| CaPOPI_247 | Ca1 | 23838777 | ATGTGTGTGTGT/ATGTGTGT | AATGCCCACCCATAATTTCA | TGTACGAGAAGGTCGTGTGC | 60.0 | 593 | INTERGENIC | | | | |
| CaPOPI_248 | Ca1 | 23966814 | GA/GAA | AACTGTGGAGGAATCCATGC | GCTTCAAGTCTTGCTACGGG | 59.9 | 490 | INTERGENIC | | | | |
| CaPOPI_249 | Ca1 | 24030518 | CG/CGTGTTG | CCAATTCAAAAGCTCCCAAA | CAGATCCTCCTTTCCCAACA | 60.0 | 424 | DRR | Ca_19291 | OK | | E3 ubiquitin ligase, domain of unknown function DUF908 |
| CaPOPI_250 | Ca1 | 24098971 | GATATATATATATATATATATATATATATATA/GATATATATATATATATATATATATATATATA | AGGCATGGTGTTGGAGTCAT | TGCATTTTTCCACGTTCAAG | 60.4 | 526 | INTERGENIC | | | | |

| INDEL marker IDs | Chromosomes/ unanchored scaffolds | Physical positions (bp) | InDels (*Kabuli* reference genome-CDC Frontier/PI) | Forward primers (5'-3') | Reverse primers (5'-3') | Anneling temperature (0C) | Expected amplified product size (bp) | Structural annotation | | Functional annotation | | |
|---|---|---|---|---|---|---|---|---|---|---|---|---|
| | | | | | | | | Sequence components of *kabuli* genome | *Kabuli* gene accession IDs | NCBI-KOG | TFs | NCBI-nr database |
| CaPOPI_251 | Ca1 | 24154735 | ATTTTT/ATTTTTT | GGGTGCCATCATAAGCAAGT | CAGATACTGCAGCCTCCACA | 60.0 | 516 | INTERGENIC | | | | |
| CaPOPI_252 | Ca1 | 24242300 | TAAAAAAAAAAA/TAAAAAAAAAA | ACCAACTCATCCCTTTCCCT | ATGATTGCAGCATTTCACGA | 59.8 | 638 | INTERGENIC | | | | |
| CaPOPI_253 | Ca1 | 24611579 | AATATATATATATATATA/AATATATATATATATA | TGCCCACTTGATACAACAAAA | CATCGCCTTTGACGAATACA | 59.1 | 330 | INTERGENIC | | | | |
| CaPOPI_254 | Ca1 | 25058286 | AATATATATATATATATATATATATATAT/AATATATATATATATATATATATATATATATATAT | CATCCTGCAACACATGCTCT | TCATGCAAATTCAAAAGAAATGA | 59.9 | 794 | DRR | Ca_18316 | | | Pectinesterase, catalytic |
| CaPOPI_255 | Ca1 | 25382145 | AC/A | GAATCACTAGGCTTGCCAGG | GGGGTGAGAGAACGTTTGAA | 59.8 | 714 | INTERGENIC | | | | |
| CaPOPI_256 | Ca1 | 25458751 | AATATATATATATATATATATATATATATATATA/AATATATATATATATATATATATATATATATATATATATATATA | GACGTCGCTTCCGAAGTTAG | TCACTCAGGTTGCCAAAACTT | 60.0 | 538 | INTERGENIC | | | | |
| CaPOPI_257 | Ca1 | 25507843 | CAATAATAATAATAATAATAATAATAATAATAATAATA/CAATAATAATAATAATAATAATAATAATAATAATA | TCTCTGGCAACCGTTCTTCT | GAATGAAGTCCAAGGGTCCA | 60.0 | 341 | INTERGENIC | | | | |
| CaPOPI_258 | Ca1 | 25526955 | A/ACG | TTCGTAATGGACGGGATGAT | GGGTTTTGCATGTTGAGCTT | 60.2 | 510 | INTERGENIC | | | | |
| CaPOPI_259 | Ca1 | 25688981 | TAAAAAAA/TAAAAAA | AATGTCCCCAACAGAGTTGC | TCGTATCGAACTTGCCCATT | 60.0 | 542 | INTERGENIC | | | | |
| CaPOPI_260 | Ca1 | 26142349 | TTAC/T | CTTCGCACAACCATTGACAC | TTCCTGCTGTTGCTGATTTG | 60.2 | 206 | CDS (large-effect mutations) | Ca_18596 | R | ZF-HD | Pentatricopeptide repeat |
| CaPOPI_261 | Ca1 | 26208589 | CATATATATATATATATATATATATATATATA/CATATATATATATATATATATATA | CAGCCACCCCAAACTTTTTA | TCACCACCAGAGACAAACCA | 60.0 | 419 | INTERGENIC | | | | |
| CaPOPI_262 | Ca1 | 26263650 | ATATTATTATTATTATTATTATTATTA/ATATTATTATTATTATTATTATTA | TCACCCAACAAGATCGTGAA | CCAAGCCCAATATCCAACAT | 60.1 | 780 | INTERGENIC | | | | |
| CaPOPI_263 | Ca1 | 26358637 | TTTTAGT/TTTTAGTTTAGT | TGTGTTCATCCTTTCCACCA | GGGTTCAACTTCAAAAGCCA | 59.9 | 754 | INTRON | Ca_18604 | U | LSD | Snf7 |
| CaPOPI_264 | Ca1 | 26589205 | ATTTTTTTTT/ATTTTTTTT | TTCCTAACATTCTCCGCTGG | TGATGTTGCATCAACCATTTT | 60.2 | 168 | INTERGENIC | | | | |
| CaPOPI_265 | Ca1 | 26592081 | C/CTGACAGTGA | ACACACAAATTTTGACCGCA | GCAATTTTCTCCAATTGCGT | 60.0 | 801 | INTERGENIC | | | | |

| **INDEL marker IDs** | **Chromosomes/ unanchored scaffolds** | **Physical positions (bp)** | **InDels (*Kabuli* reference genome-CDC Frontier/PI)** | **Forward primers (5'-3')** | **Reverse primers (5'-3')** | **Anneling temperature (0C)** | **Expected amplified product size (bp)** | **Structural annotation** | | **Functional annotation** | | |
|---|---|---|---|---|---|---|---|---|---|---|---|---|
| | | | | | | | | **Sequence components of *kabuli* genome** | ***Kabuli* gene accession IDs** | **NCBI-KOG** | **TFs** | **NCBI-nr database** |
| CaPOPI_266 | Ca1 | 26683611 | TG/T | CAATTGTGTAAAACGTGCGG | TTGCATTTGGTTTGACTCCA | 60.0 | 538 | INTERGENIC | | | | |
| CaPOPI_267 | Ca1 | 26774824 | TCTTTCCNNNNNNNNNNCTTTCC/TCTTTCC | TTGCACCCTTCTTAGCACCT | ACAATGAGTCGTCGCACAAA | 59.9 | 212 | INTERGENIC | | | | |
| CaPOPI_268 | Ca1 | 26866750 | ATTTTTTTTTT/ATTTTTTTTTTT | TCTCAGTGGAAAACGAACCC | TCTCCACCATCTTCCTCTTCA | 60.1 | 679 | INTERGENIC | | | | |
| CaPOPI_269 | Ca1 | 26876468 | C/CG | TTGTGCATCCGTCTATCCAC | TCGGTGGAAATTGTAATGAGAG | 59.5 | 937 | INTERGENIC | | | | |
| CaPOPI_270 | Ca1 | 26958726 | GTTT/GTTTT | TGAGCGTGAAAAATTGTGGT | CCGACCAATAGACTTTTGTTGA | 59.2 | 204 | INTERGENIC | | | | |
| CaPOPI_271 | Ca1 | 27282873 | GTATA/GTATATA | AAAATCCAACAATCTCCCCC | CCCTGAGACTCACGAAGAGG | 60.0 | 561 | INTERGENIC | | | | |
| CaPOPI_272 | Ca1 | 28322869 | GAAAAAAAAAA/GAAAAAAAAA | GAGAACAACAACTTGACCCAAA | GTTACCAGCCCATCAATGCT | 59.1 | 376 | INTERGENIC | | | | |
| CaPOPI_273 | Ca1 | 28449282 | ACGAATATTTTCCGAATATTTTCCGAATATTT/ACGAATATTTTCCGAATATTT | TGGATTGGTTGAAGGAGGTC | CTCTGTCACTGGCGTCACAT | 59.9 | 695 | DRR | Ca_18508 | | | |
| CaPOPI_274 | Ca1 | 28550132 | ATTTTTTTTTT/ATTTTTT | TGGAAGCACGCTTAAGGACT | TTTCCCAATCGATTTGCTTC | 60.0 | 664 | INTERGENIC | | | | |
| CaPOPI_275 | Ca1 | 28550236 | T/TG | CTGACACAGGGGGAGAAAAA | TTTCCCAATCGATTTGCTTC | 60.1 | 159 | INTERGENIC | | | | |
| CaPOPI_276 | Ca1 | 28713213 | TTA/TTAGAAAGAGTGTA | TTCATTTCTCTGATTCCCCG | TCCGCATTATAACCTCTGCC | 60.0 | 425 | INTERGENIC | | | | |
| CaPOPI_277 | Ca1 | 28906702 | TAAAAAAA/TAAAAAA | TATGGAGAGCAGCGACTCAA | TGTTGGGTTGCACAACATTC | 59.7 | 184 | INTERGENIC | | | | |
| CaPOPI_278 | Ca1 | 28910299 | CATT/CATTAATT | GAAAATGATCGGTGGAGGAA | CTTGCGTGTTGCACGAGTAT | 59.9 | 620 | DRR | Ca_18486 | R | | Sugar/inositol transporter |
| CaPOPI_279 | Ca1 | 28910424 | TA/T | GAGTCATTGGTGCCTTGGTT | CTTGCGTGTTGCACGAGTAT | 60.0 | 385 | DRR | Ca_18486 | R | | Sugar/inositol transporter |
| CaPOPI_280 | Ca1 | 28910492 | GATTAA/GATTAATTAA | GAGTCATTGGTGCCTTGGTT | CTTGCGTGTTGCACGAGTAT | 60.0 | 385 | DRR | Ca_18486 | R | | Sugar/inositol transporter |

| INDEL marker IDs | Chromosomes/ unanchored scaffolds | Physical positions (bp) | InDels (*Kabuli* reference genome-CDC Frontier/PI) | Forward primers (5'-3') | Reverse primers (5'-3') | Anneling temperature (0C) | Expected amplified product size (bp) | Structural annotation | | Functional annotation | | |
|---|---|---|---|---|---|---|---|---|---|---|---|---|
| | | | | | | | | Sequence components of *kabuli* genome | *Kabuli* gene accession IDs | NCBI-KOG | TFs | NCBI-nr database |
| CaPOPI_281 | Ca1 | 28930954 | A/ATT | TGAAAAGTTTCTTTGTCC CTTTG | TGGTGTTGCCTTCAAAT TCA | 59.7 | 349 | INTERGENIC | | | | |
| CaPOPI_282 | Ca1 | 28935215 | ATTTTTTTT/ATTTTTTTTT | TTTTGTTGGCAGTTTGTT TG | CAGTCAACATTGATGTG ACGTTAT | 57.3 | 490 | INTERGENIC | | | | |
| CaPOPI_283 | Ca1 | 28936099 | ATTTTTTTT/ATTTTTTTTTTT | GAAATTTGACCAGAGCT GCC | TCGGTCACCGTTTTTAG TTT | 59.8 | 465 | INTERGENIC | | | | |
| CaPOPI_284 | Ca1 | 28936233 | AT/ATGTAATT | GAAATTTGACCAGAGCT GCC | GGACCAATTACTCTGGT GGAA | 59.8 | 650 | INTERGENIC | | | | |
| CaPOPI_285 | Ca1 | 28939471 | AAAATAAAACAAA/AAAA | CCATGTGTGAACCCTAA TTGTT | GTTTTGGCGTTGGTTTC ACT | 58.7 | 226 | INTERGENIC | | | | |
| CaPOPI_286 | Ca1 | 28941503 | CAA/CAAAA | TGGGGTTTAGGGTGTCA CAT | CTTTTGTAAAGGCAGCT CCG | 60.1 | 207 | INTERGENIC | | | | |
| CaPOPI_287 | Ca1 | 28943560 | GG/GGTG | ACTTGGGACAAATCACA TGG | TGATCACCAGCTTTCCA ATG | 58.3 | 252 | INTERGENIC | | | | |
| CaPOPI_288 | Ca1 | 28944496 | TTATATATAT/TTATATAT | TTGGCACCAGACTTCAT ATTG | AAATGTACTTTGCGGCA CAT | 58.6 | 775 | INTERGENIC | | | | |
| CaPOPI_289 | Ca1 | 28944803 | AACTA/AA | TTTCAATTGCATTAATCC GTTC | CAATAAAAATTGCGGTC CGA | 59.0 | 963 | INTERGENIC | | | | |
| CaPOPI_290 | Ca1 | 29124803 | ATTT/ATTTT | AGTGGCATTGGGCAAAT TAC | ACCAAATATACGCGTCT GCC | 59.8 | 350 | INTERGENIC | | | | |
| CaPOPI_291 | Ca1 | 29636835 | AA/AAGA | TCACAAATCTCAACCCT CCC | ATCAGTTGGAGCCTGGT TGT | 59.9 | 497 | INTRON | Ca_24382 | AR | | K Homology, type 1 |
| CaPOPI_292 | Ca1 | 29637072 | GAAAAAAAAA/GAAAAAAAAAAA | ACAACCAGGCTCCAACT GAT | ATGCTGCCAGCCTACAA AAT | 59.6 | 503 | INTRON | Ca_24382 | AR | | K Homology, type 1 |
| CaPOPI_293 | Ca1 | 29709028 | ACC/AC | AAAGATTTGTTGAGGCG TGG | GACGAGAATGGAGATGG GAA | 60.1 | 580 | INTERGENIC | | | | |
| CaPOPI_294 | Ca1 | 29738380 | GTATATATATATATATATAT/GTATAT ATATATATATATATAT | TATTTTGGATTTTGGCTC GG | AAACCGGTAAAATCTCC GCT | 59.9 | 699 | INTERGENIC | | | | |
| CaPOPI_295 | Ca1 | 29791416 | GAAAA/GAAAAA | TCCGATAAAATCTCCGTT CG | AAAAACACGTTCCCCTC CTT | 60.0 | 323 | INTERGENIC | | | | |

| INDEL marker IDs | Chromosomes/ unanchored scaffolds | Physical positions (bp) | InDels (*Kabuli* reference genome-CDC Frontier/PI) | Forward primers (5'-3') | Reverse primers (5'-3') | Anneling temperature (0C) | Expected amplified product size (bp) | Structural annotation | | Functional annotation | | |
|---|---|---|---|---|---|---|---|---|---|---|---|---|
| | | | | | | | | Sequence components of *kabuli* genome | *Kabuli* gene accession IDs | NCBI-KOG | TFs | NCBI-nr database |
| CaPOPI_296 | Ca1 | 29842423 | AC/A | TCATCAAAGGCATGCAAATC | GACCAAAACACAACGCAATG | 59.6 | 359 | INTERGENIC | | | | |
| CaPOPI_297 | Ca1 | 29846530 | CTTTT/CTTTTT | TGGATTGGGGATCATTCATT | TCAACATTTGGAAGCACAAGA | 60.0 | 897 | DRR | Ca_24388 | | | |
| CaPOPI_298 | Ca1 | 29846735 | A/AATATATATATAT | AGAACGGTTTTCTGTCCGTG | TCAACATTTGGAAGCACAAGA | 60.1 | 689 | DRR | Ca_24388 | | | |
| CaPOPI_299 | Ca1 | 29846941 | TAAAAAAAAAA/TAAAAAAAA | TGTTGCCAACTGAAAATCGT | ATACAGACACCGAATGGGGA | 59.2 | 828 | DRR | Ca_24388 | | | |
| CaPOPI_300 | Ca1 | 29848026 | ATGT/AT | TCTTTTCATCAGGCCCAATC | GGAAAGTGAGGGAACCAATG | 60.0 | 228 | INTRON | Ca_24388 | | | |
| CaPOPI_301 | Ca1 | 29853119 | TAA/TA | TCCACGAGATTTTCCTTTGG | TGTCATCATTCTCTGCTGGC | 60.0 | 357 | INTERGENIC | | | | |
| CaPOPI_302 | Ca1 | 29853730 | GTGGAAGTCT/GTGGAAGTCTGGAAGTCT | TTGCATTCCTAGGTTCAGCC | TATCGCTCGACCTCTGCTTT | 60.2 | 602 | INTERGENIC | | | | |
| CaPOPI_303 | Ca1 | 29856647 | CTTT/CTTTT | GCATTCCCTGCTCAAGACTC | AGACAAGGGAGAGTCCCACA | 60.0 | 812 | INTERGENIC | | | | |
| CaPOPI_304 | Ca1 | 30432201 | CTTTTTTT/CTTTTTT | CCACAATATCAAAGGGCCAC | AGAGAAAACAAAAGGCGCAA | 60.2 | 471 | INTERGENIC | | | | |
| CaPOPI_305 | Ca1 | 30475519 | ATTT/ATTTT | AGGATGCATGGGATGAAAAG | AAACTTCTACTCGGGCCCAT | 59.9 | 766 | INTERGENIC | | | | |
| CaPOPI_306 | Ca1 | 30627831 | AATATATATATATATATAT/AATATATATATATATATATATATATATAT | CTTGGGAACTGGAGAGCAAG | CCAACGTCACTTCCAAGGTT | 60.0 | 797 | INTERGENIC | | | | |
| CaPOPI_307 | Ca1 | 30682856 | CATATATATATATATATATATAT/CATATATATATATATATATAT | GTTGGTTCCCTAGCACCGTA | TCCGCCTAGGAGATAGCAGA | 60.0 | 672 | INTERGENIC | | | | |
| CaPOPI_308 | Ca1 | 30740126 | CTGT/CT | TCGGAGCACAACCTACACAA | ACATTTGCCACCGTTAGAGG | 60.3 | 901 | DRR | Ca_22454 | OE | WRKY | Peptidase S10, serine carboxypeptidase |
| CaPOPI_309 | Ca1 | 30745547 | C/CT | TTTTTGGGTTTCCTTTTCCC | TGCAATATGTATCGTGGATGG | 60.1 | 817 | INTERGENIC | | | | |
| CaPOPI_310 | Ca1 | 30770184 | TAAA/TAAAA | AGTGGCTTGGAGGAGTTGGT | CGTTCGGCTTTCTTCTTCTG | 61.1 | 535 | INTERGENIC | | | | |

| INDEL marker IDs | Chromosomes/ unanchored scaffolds | Physical positions (bp) | InDels (*Kabuli* reference genome-CDC Frontier/PI) | Forward primers (5'-3') | Reverse primers (5'-3') | Anneling temperature (0C) | Expected amplified product size (bp) | Structural annotation | | Functional annotation | | |
|---|---|---|---|---|---|---|---|---|---|---|---|---|
| | | | | | | | | Sequence components of *kabuli* genome | *Kabuli* gene accession IDs | NCBI-KOG | TFs | NCBI-nr database |
| CaPOPI_311 | Ca1 | 30790839 | TGTAAC/T | TTTTTGATGGTTCAACGAGAA | GGTTGAATAATTGAAAAGAGGTGG | 58.3 | 250 | INTERGENIC | | | | |
| CaPOPI_312 | Ca1 | 30874605 | A/AT | TGTGATGCAGTTGCTCCATT | CAAGCGTCATGCCAAACTTA | 60.3 | 624 | INTERGENIC | | | | |
| CaPOPI_313 | Ca1 | 30993040 | AAAAATTAAAATTAAAATTAA/AAAAATTAAAATTAAAATTAAAATTAA | TCCTTCCGATCGAGTAGTGG | AAACATGAAATGGACAATACAAAAA | 60.2 | 398 | INTERGENIC | | | | |
| CaPOPI_314 | Ca1 | 31090559 | CAA/CA | CGCTCGCTCATTGAATAACA | CTCCACGACCATCCTGAAAT | 60.0 | 417 | INTRON | Ca_25826 | S | | Cysteamine dioxygenase |
| CaPOPI_315 | Ca1 | 31141128 | C/CA | ATTATTGAGGTTGGCCCCTC | TCCACTTTTTGTGCTTTAGGC | 60.2 | 376 | INTERGENIC | | | | |
| CaPOPI_316 | Ca1 | 31162887 | T/TG | CCGGGGATTGCCATATAACT | CTAATAGGCTGCATCGGAGG | 60.9 | 342 | INTERGENIC | | | | |
| CaPOPI_317 | Ca1 | 31163466 | TGG/TAGAGG | ACTTTTGCGGAGAAGCACAT | CGTTCGTCTTTTCCTCTTGC | 59.9 | 360 | INTERGENIC | | | | |
| CaPOPI_318 | Ca1 | 31238518 | ATT/AT | CATCGTAAGCATGTGGTTGG | TAATGGCCCCATAGCAGAAG | 60.0 | 861 | DRR | Ca_21848 | R | FAR1 | Strictosidine synthase, conserved region |
| CaPOPI_319 | Ca1 | 31428294 | ATATTTTTATTTTTATTTTTATTTTTAT/ATATTTTTATTTTTATTTTTAT | TCAACAGGTTGATGGGTGAA | TGAGATGGAGGTGATGGTGA | 59.9 | 370 | INTERGENIC | | | | |
| CaPOPI_320 | Ca1 | 31592114 | CT/CTTT | TCAGCTGCAGTTCCAACATC | GCACAAGTCCTTGAGGAAGC | 60.0 | 375 | INTRON | Ca_21868 | F | | Orotidine 5'-phosphate decarboxylase domain |
| CaPOPI_321 | Ca1 | 31600651 | AC/A | GGATGGAAGAGAAATTGGGA | AGGATCATGTTTGTGGTTGC | 58.9 | 899 | INTERGENIC | | | | |
| CaPOPI_322 | Ca1 | 31716302 | A/AC | TCGGTATTTGCCATGAGACA | AAACAACCTTCATGCCTTGG | 60.1 | 482 | INTERGENIC | | | | |
| CaPOPI_323 | Ca1 | 31768486 | TTATA/TTTTTTATATA | ACACATTATATTAAATGCGCGA | TGCCAAACGATAGGTTGTTG | 57.4 | 646 | INTERGENIC | | | | |
| CaPOPI_324 | Ca1 | 31783488 | AATATATATATATATATATATATAT/AATATATATATATATATATAT | TGGATTGGGCTGGAATTAAA | TATAACCGACGAGGGACGAG | 60.3 | 466 | INTERGENIC | | | | |
| CaPOPI_325 | Ca1 | 31803627 | TAAA/TAAAA | CTTACGGTGAAGGAGAACGG | ATCATTCTTTTATGCGTTTGACT | 59.7 | 793 | INTERGENIC | | | | |

| INDEL marker IDs | Chromosomes/ unanchored scaffolds | Physical positions (bp) | InDels (*Kabuli* reference genome-CDC Frontier/PI) | Forward primers (5'-3') | Reverse primers (5'-3') | Anneling temperature (0C) | Expected amplified product size (bp) | Structural annotation | | Functional annotation | | |
|---|---|---|---|---|---|---|---|---|---|---|---|---|
| | | | | | | | | Sequence components of *kabuli* genome | *Kabuli* gene accession IDs | NCBI-KOG | TFs | NCBI-nr database |
| CaPOPI_326 | Ca1 | 31995451 | T/TCAC | TGCTTTGAAGACCATGTGGA | AAAAGGATTCATGCGTGTCC | 60.2 | 354 | INTERGENIC | | | | |
| CaPOPI_327 | Ca1 | 32030892 | AATATATATATATATATATATATATATATATATATAT/AATATATATATATATATATATATATATATATATATATATAT | CATGGATGGGTTTTGTACCC | TTGGAAGGAGCCCTTACCTT | 59.9 | 771 | INTERGENIC | | | | |
| CaPOPI_328 | Ca1 | 32037255 | CA/CAA | GGGAAAACTTTTGTGTGGGA | TTGGCAAATCGATTAGACTGG | 59.8 | 802 | INTERGENIC | | | | |
| CaPOPI_329 | Ca1 | 32037334 | CTTTTT/CTTTT | TCCAGTCATGTGGCTTCTCA | CTGAGCTTGTGACCAAATCG | 60.4 | 559 | INTERGENIC | | | | |
| CaPOPI_330 | Ca1 | 32037356 | CTTTTTT/CTTTTT | TCCAGTCATGTGGCTTCTCA | CTGAGCTTGTGACCAAATCG | 60.4 | 559 | INTERGENIC | | | | |
| CaPOPI_331 | Ca1 | 32080106 | T/TC | TTCCCCATGTCGATCTAAGC | AACCCTGAACAGATTGTCGG | 60.0 | 612 | INTRON | Ca_26405 | | | Uncharacterised protein family Ycf68 |
| CaPOPI_332 | Ca1 | 32080183 | C/CGGCGATTACT | TTCCCCATGTCGATCTAAGC | AACCCTGAACAGATTGTCGG | 60.0 | 612 | INTRON | Ca_26405 | | | Uncharacterised protein family Ycf68 |
| CaPOPI_333 | Ca1 | 32151710 | TAAAAAAAAAAA/TAAAAAAAAAAAAAAAA | TGAAGGTGTGAGCAGATTCG | TCGGGTGGAGAATTCCTATG | 60.0 | 593 | INTERGENIC | | | | |
| CaPOPI_334 | Ca1 | 32153452 | TTTATTTTAT/TTTATTTTATATTATTTTAT | GTTTTGGCGTTGGTTTCACT | TGGGTTTGACCCTATGTGTG | 60.0 | 353 | INTERGENIC | | | | |
| CaPOPI_335 | Ca1 | 32153737 | TTATATATATATAT/TTATATATATATATAT | CACACATAGGGTCAAACCCA | ATACATGGATTGGTTGGGGA | 59.3 | 585 | INTERGENIC | | | | |
| CaPOPI_336 | Ca1 | 32222272 | ATTTT/ATTTTT | ACAACTTCTCATGCTCGCAA | GGTGGCATACCTCAACGAGT | 59.6 | 620 | INTRON | Ca_26127 | | Trihelix | |
| CaPOPI_337 | Ca1 | 32267954 | T/TG | AAGCTATGTCAAGGAATGTGATTT | CCGTGTTTTTGAAAATTGACAC | 58.3 | 378 | INTERGENIC | | | | |
| CaPOPI_338 | Ca1 | 32454365 | A/AG | AGGAAACACATGAAGGCACC | TCTTCGTTGTCACTCCATGC | 60.0 | 572 | INTERGENIC | | | | |
| CaPOPI_339 | Ca1 | 32911601 | AAATAATAATAATAA/AAATAATAATAATAATAA | CAAAATGGAGCAAGAAACAGG | CAAGTTCAACGAGCGCCTAT | 59.7 | 892 | INTERGENIC | | | | |
| CaPOPI_340 | Ca1 | 33105635 | TC/T | TGCCAAGAACGATAAAAGCA | TTCTGCATTTTCCTTGAGCA | 59.4 | 101 | DRR | Ca_19438 | | | |

| INDEL marker IDs | Chromosomes/ unanchored scaffolds | Physical positions (bp) | InDels (*Kabuli* reference genome-CDC Frontier/PI) | Forward primers (5'-3') | Reverse primers (5'-3') | Anneling temperature (0C) | Expected amplified product size (bp) | Structural annotation | | Functional annotation | | |
|---|---|---|---|---|---|---|---|---|---|---|---|---|
| | | | | | | | | Sequence components of *kabuli* genome | *Kabuli* gene accession IDs | NCBI-KOG | TFs | NCBI-nr database |
| CaPOPI_341 | Ca1 | 33336930 | GAAAAA/GAAAA | CAATGGCAACAAGGTCAATG | TCACATCTGTTGGTAACGGG | 60.0 | 856 | INTRON | Ca_19441 | | MIKC | Transcription factor, K-box |
| CaPOPI_342 | Ca1 | 33746473 | GTATATATATATATATAT/GTATATATATAT | CCAATCGATTTCCTCCAAAA | TTTCATCAAAAACCCCCTTG | 59.9 | 779 | INTERGENIC | | | | |
| CaPOPI_343 | Ca1 | 33765021 | AAATAAAATTAATAAATAATAAAAT/AAATAAAAT | GCGACCGAATATGGAACAGT | TAGGCTAGGTCGTCCGTTTG | 60.0 | 301 | INTERGENIC | | | | |
| CaPOPI_344 | Ca1 | 33777775 | ATTT/ATT | CATGCTCAATACACGGATGG | AACGTTCTTGAGTGGCTTGG | 59.9 | 870 | INTERGENIC | | | | |
| CaPOPI_345 | Ca1 | 34057382 | C/CA | GGGGATTAAGTGCTTGGACA | AAAGAAAGGCACTAAGGCCC | 59.9 | 156 | INTERGENIC | | | | |
| CaPOPI_346 | Ca1 | 34147315 | C/CTG | TCAAATCGATTGGGACATCA | TACTCGCCAAACTCTCACGA | 59.9 | 900 | INTERGENIC | | | | |
| CaPOPI_347 | Ca1 | 34286273 | ATATTTTTTATTTTTTATTTTTTT/ATATTTTTTATTTTTT | TATGCATGCGGACAAGAGTC | TTGGAAAAGCCCACCTACAC | 59.8 | 448 | INTERGENIC | | | | |
| CaPOPI_348 | Ca1 | 34332252 | GA/G | AATTGGAAGCAGGGGTTTTT | GGATTGTACCCTTGGGGTTT | 59.8 | 849 | INTERGENIC | | | | |
| CaPOPI_349 | Ca1 | 34371294 | AAAGAAG/AAAGAAGAAG | AAACGGGTGCAAGAAGATTG | TCTTTACGACCTTTGTGGGC | 60.1 | 233 | INTERGENIC | | | | |
| CaPOPI_350 | Ca1 | 34386272 | GTTT/GTTTT | AATCTGGAGGGCGGTAAGTT | ATATCGGTCAAACGCACACA | 60.0 | 413 | INTERGENIC | | | | |
| CaPOPI_351 | Ca1 | 34647577 | CCA/CCATTAACA | GGGAGCAAGAAGTTGGTTGA | TAGTCCAACAATGGCCAACA | 60.2 | 132 | INTERGENIC | | | | |
| CaPOPI_352 | Ca1 | 34666828 | TAAAAAAAAA/TAAAAAAAA | TGTTTCAGGATGTCGACCAA | GTTACCGCAGAGGGACGTAA | 60.1 | 640 | INTERGENIC | | | | |
| CaPOPI_353 | Ca1 | 34667053 | ATTTTTTTTTT/ATTTTTTTTT | AGACTGCTCGGTCCAAAGAA | GTTACCGCAGAGGGACGTAA | 60.0 | 264 | INTERGENIC | | | | |
| CaPOPI_354 | Ca1 | 34812970 | ATTTTTTTTTTT/ATTTTTTTTTT | CACAACAATGTGGGTTGAGG | ACCAAGAACAAAATGGTGCC | 59.8 | 428 | INTERGENIC | | | | |
| CaPOPI_355 | Ca1 | 34971020 | CAAAAAAA/CAAAAA | AAATACCCCTTTTTCCCACG | TGAAAACGCTCAATGCCATA | 60.0 | 689 | INTERGENIC | | | | |

| INDEL marker IDs | Chromosomes/ unanchored scaffolds | Physical positions (bp) | InDels (*Kabuli* reference genome-CDC Frontier/PI) | Forward primers (5'-3') | Reverse primers (5'-3') | Anneling temperature (0C) | Expected amplified product size (bp) | Structural annotation | | Functional annotation | | |
|---|---|---|---|---|---|---|---|---|---|---|---|---|
| | | | | | | | | Sequence components of *kabuli* genome | *Kabuli* gene accession IDs | NCBI-KOG | TFs | NCBI-nr database |
| CaPOPI_356 | Ca1 | 34978712 | GT/GTT | ATGGCATGGTGGATTTTGTT | CTAGAAAGTTGCAGCCCAGG | 60.1 | 525 | INTRON | Ca_21407 | | | |
| CaPOPI_357 | Ca1 | 35096364 | AT/A | TTGATGTGGTCGGAGATTGA | ACAATTTTCAGCAACCCGAG | 60.0 | 588 | INTERGENIC | | | | |
| CaPOPI_358 | Ca1 | 35153435 | GTTTTTTTTTTTTT/GTTTTTT | TTTGGTTTCCGATTGCTAGG | GGTTGTATGGCTCCCAAGAA | 60.1 | 409 | INTERGENIC | | | | |
| CaPOPI_359 | Ca1 | 35165919 | GTTTTTT/GTTTTTTTTTTTT | TCATCGCAGGTGTACCATGT | CACCTAAGGGAATCGGTCAA | 60.0 | 377 | INTERGENIC | | | | |
| CaPOPI_360 | Ca1 | 35261821 | ATT/AT | TGGATTGTCATGGTCTGGAA | AATGGTCTTGAATTTTGCCG | 59.9 | 640 | INTERGENIC | | | | |
| CaPOPI_361 | Ca1 | 35266220 | ATAT/ATATTAT | CGCGGTAGAGTTATTGGAAAA | TGGGGATACGTAGGAGCAAG | 59.3 | 682 | INTERGENIC | | | | |
| CaPOPI_362 | Ca1 | 35281570 | TC/T | ATGACACGTCATTCGTTTCG | GGTTCGAGACATCCATAATTCA | 59.6 | 733 | INTRON | Ca_26099 | QI | bHLH | Cytochrome P450 |
| CaPOPI_363 | Ca1 | 35281940 | TA/TAA | AAACGTGATCTATACAGTTTCCAAAA | TTCACATCTGGAAACAACGC | 59.4 | 529 | INTRON | Ca_26099 | QI | bHLH | Cytochrome P450 |
| CaPOPI_364 | Ca1 | 35283797 | TGGGGG/TGGGG | GGGTGTGTTTGATAGGAGGG | CTCCCCTTCCATCTCCTCTT | 59.3 | 139 | INTERGENIC | | | | |
| CaPOPI_365 | Ca1 | 35284017 | ATTT/ATT | GAGATGGAAGGGGAGGAAAG | GCACAAAAGAAAAATACAAGTCCA | 60.0 | 273 | INTERGENIC | | | | |
| CaPOPI_366 | Ca1 | 35284253 | TT/TTGT | GAGATGGAAGGGGAGGAAAG | TCTGATCAAATATGTGGCAGAAA | 60.0 | 657 | INTERGENIC | | | | |
| CaPOPI_367 | Ca1 | 35321165 | GTTT/GTTTT | CATCATTCGTTTCAAATTTGTTG | ACGAAGGTTTGAGACATCCA | 59.4 | 586 | INTRON | Ca_26101 | QI | bHLH | Cytochrome P450 |
| CaPOPI_368 | Ca1 | 35540299 | AA/AATA | GGGGTGCTCAACTTGTTTGT | GGCCAAGGTAAGGGTGAGAT | 60.0 | 254 | INTERGENIC | | | | |
| CaPOPI_369 | Ca1 | 35679606 | CAAAAAAAAAAA/CAAAAAAAAAA | GTGCACAACCTTGTGGTGTC | TAGCCAACATGCCATACGTC | 60.1 | 604 | INTERGENIC | | | | |
| CaPOPI_370 | Ca1 | 35899211 | TTTTAGACAC/TTTTAGACACATTTAGACAC | CACCTCCCCTGAGTTCGTTA | GCAAGCTCCAAATGGATTACA | 60.1 | 404 | INTERGENIC | | | | |

| INDEL marker IDs | Chromosomes/ unanchored scaffolds | Physical positions (bp) | InDels (*Kabuli* reference genome-CDC Frontier/PI) | Forward primers (5'-3') | Reverse primers (5'-3') | Anneling temperature (0C) | Expected amplified product size (bp) | Structural annotation | | Functional annotation | | |
|---|---|---|---|---|---|---|---|---|---|---|---|---|
| | | | | | | | | Sequence components of *kabuli* genome | *Kabuli* gene accession IDs | NCBI-KOG | TFs | NCBI-nr database |
| CaPOPI_371 | Ca1 | 35977319 | TA/TAA | AACTCGTTGGAACTTCCCTCT | GCAAGCAGCCTTGTTGACTA | 59.2 | 540 | INTERGENIC | | | | |
| CaPOPI_372 | Ca1 | 36044248 | CGG/CG | CGATTTCCCTGCATGTTTTT | GCCTGTGATAGGCGGTACAT | 59.9 | 540 | INTERGENIC | | | | |
| CaPOPI_373 | Ca1 | 36063199 | TCC/TTTTTCCCC | CGTTCGTGGTCCCTTAACTT | TTGCGTTACGGATTTGATGA | 59.1 | 227 | INTERGENIC | | | | |
| CaPOPI_374 | Ca1 | 36166710 | CATTATTATTATTATTATTATTATTATTA/CATTATTATTATTATTATTATTATTA | TCGTTGGCCTATGGTACTCC | CCAACGATTCATACAGGGCT | 60.0 | 574 | INTERGENIC | | | | |
| CaPOPI_375 | Ca1 | 36726460 | GAAA/GAAAA | ACAGGAACGACCGAGAGAAA | ATAGTTTTTGGGCGGGCTAC | 59.8 | 502 | INTERGENIC | | | | |
| CaPOPI_376 | Ca1 | 36727739 | TCC/TC | AGGCTGCATTTTAGTCCACG | ACGACCACTGCAAACACAAA | 60.3 | 218 | INTERGENIC | | | | |
| CaPOPI_377 | Ca1 | 37075088 | TTAAA/T | GCCCTCAGATGAGAGTCAGG | ATTCTGCAGCACGGATCTCT | 59.9 | 830 | INTERGENIC | | | | |
| CaPOPI_378 | Ca1 | 37078511 | TTAT/TTATAT | CCATTCCAAAAAGGAGTTGG | TGAAAATGTTGGAGTGATGTGA | 59.4 | 938 | INTERGENIC | | | | |
| CaPOPI_379 | Ca1 | 37078904 | TTAAATAAATAAATA/TTAAATAAATAAATAAATAAATA | TGTGACACCCTAAACCCTAAGAA | TTGATTGCTTGCGTGTTCTC | 59.9 | 499 | INTERGENIC | | | | |
| CaPOPI_380 | Ca1 | 37122051 | CAAAAAAAA/CAAAAAAA | CGTATCTCGTATCACCGACG | AGTTGCGCTCCTCCTAATCA | 59.2 | 873 | INTERGENIC | | | | |
| CaPOPI_381 | Ca1 | 37122104 | CAAAAAAAAAA/CAAAAAAAAAAAAA | CGTATCTCGTATCACCGACG | AGTTGCGCTCCTCCTAATCA | 59.2 | 873 | INTERGENIC | | | | |
| CaPOPI_382 | Ca1 | 37122520 | AGG/AG | TGCAATCATTGGTGCTTAGG | AAAAAGCGCCGTAAAATGAG | 59.7 | 710 | INTERGENIC | | | | |
| CaPOPI_383 | Ca1 | 37122699 | GTTT/GTT | GGAGGAGCGCAACTTAATCA | AAAAAGCGCCGTAAAATGAG | 60.4 | 372 | INTERGENIC | | | | |
| CaPOPI_384 | Ca1 | 37122810 | TACAAC/TACAACAAC | GGAGGAGCGCAACTTAATCA | AAAAAGCGCCGTAAAATGAG | 60.4 | 372 | INTERGENIC | | | | |
| CaPOPI_385 | Ca1 | 37125192 | GATGGAGATGGTTTGCAATGGAGATG/GATGGAGATG | AGCGGTTTAGGGTTTAGGGA | CCAAATGTGCATACGAACCA | 60.0 | 113 | INTERGENIC | | | | |

| INDEL marker IDs | Chromosomes/ unanchored scaffolds | Physical positions (bp) | InDels (*Kabuli* reference genome-CDC Frontier/PI) | Forward primers (5'-3') | Reverse primers (5'-3') | Anneling temperature (0C) | Expected amplified product size (bp) | Structural annotation | | Functional annotation | | |
|---|---|---|---|---|---|---|---|---|---|---|---|---|
| | | | | | | | | Sequence components of *kabuli* genome | *Kabuli* gene accession IDs | NCBI-KOG | TFs | NCBI-nr database |
| CaPOPI_386 | Ca1 | 37128515 | ATTTTTTT/ATTTT | CAATTCCCACAGGTACGCTT | TTGGTCTCATCAATGATTGTAAAAA | 60.0 | 568 | INTERGENIC | | | | |
| CaPOPI_387 | Ca1 | 37391477 | TTATATATATATATATATATATAT/TTATATATATATATATATAT | CTGTCAATTGAGATGCGACG | GCGATTACGGTACCACGAGT | 60.4 | 600 | INTERGENIC | | | | |
| CaPOPI_388 | Ca1 | 37410363 | GAAAAAA/GAAAAAAA | TGAATGAGGAGCACGTCTTG | CTGTTGTTGCCAACGGTTTT | 60.0 | 445 | INTERGENIC | | | | |
| CaPOPI_389 | Ca1 | 37412282 | CCATG/CCATGCATG | CGTGGATGATGCTTTTCTCA | TGCTTCTCATCTGCTTGGTG | 59.8 | 580 | DRR | Ca_21842 | R | GRAS | Pentatricopeptide repeat |
| CaPOPI_390 | Ca1 | 37420598 | ATTTT/ATTT | GGGAAAAGGAAACCCAAAAA | TGAGTGAGGGAGACAAAGGG | 60.1 | 950 | INTERGENIC | | | | |
| CaPOPI_391 | Ca1 | 37421082 | CCTCACTCACTCACTCACTCAC/CCTCACTCACTCACTCAC | ACTTGACGGGGACTCCACTA | ACATGTTCCTTGTGCTCGTG | 59.6 | 637 | INTERGENIC | | | | |
| CaPOPI_392 | Ca1 | 37424329 | CATATATATATATATATATATAT/CATATATATATATATATAT | AAAAAGTATTGAACAAACACGCT | TGAAATCCAAATTAAATTGCTGAA | 57.2 | 549 | INTERGENIC | | | | |
| CaPOPI_393 | Ca1 | 37424983 | AC/A | GCAATTTAATTTGGATTTCATGG | TCACATCCATCCACGGTACA | 59.6 | 761 | INTERGENIC | | | | |
| CaPOPI_394 | Ca1 | 37425153 | G/GAT | GCAATTTAATTTGGATTTCATGG | TCACATCCATCCACGGTACA | 59.6 | 761 | INTERGENIC | | | | |
| CaPOPI_395 | Ca1 | 37425620 | ACG/A | TGTACCGTGGATGGATGTGA | TATGCATCCTCACGGTTTTG | 60.8 | 675 | INTERGENIC | | | | |
| CaPOPI_396 | Ca1 | 37426537 | ATT/AT | CTTCAAAACCGTGAGGATGC | TCTGAGCTTCTGATGCTTGC | 60.6 | 916 | INTERGENIC | | | | |
| CaPOPI_397 | Ca1 | 37433380 | CAA/CAACGTAAA | AATAAATTAGTGCAGCAAACCAA | GGCAAAGAGAGGAAAGTGGA | 58.0 | 756 | INTERGENIC | | | | |
| CaPOPI_398 | Ca1 | 37433540 | TATAATAGATAATAGATAATA/TATAATAGATAATA | TGAAAAACATCTCTCTAATAGGCG | ACCACTGCACCAAGGGTAAG | 59.0 | 532 | INTERGENIC | | | | |
| CaPOPI_399 | Ca1 | 37489959 | AT/ATT | ATTGTCCAGTTTCCTCACCG | CGATTGGTAAATCGATTGGG | 60.0 | 594 | INTERGENIC | | | | |
| CaPOPI_400 | Ca1 | 37490066 | AT/AATGTATT | ATTGTCCAGTTTCCTCACCG | TGAACTCTGAGCGTGTGACC | 60.0 | 775 | INTERGENIC | | | | |

| INDEL marker IDs | Chromosomes/ unanchored scaffolds | Physical positions (bp) | InDels (*Kabuli* reference genome-CDC Frontier/PI) | Forward primers (5'-3') | Reverse primers (5'-3') | Anneling temperature (0C) | Expected amplified product size (bp) | Structural annotation | | Functional annotation | | |
|---|---|---|---|---|---|---|---|---|---|---|---|---|
| | | | | | | | | Sequence components of *kabuli* genome | *Kabuli* gene accession IDs | NCBI-KOG | TFs | NCBI-nr database |
| CaPOPI_401 | Ca1 | 37516914 | TAAAAAA/TAAAAA | AGGGAGGACGAAAATGACCT | ACCAATCTCGTGCATCCAAT | 59.9 | 567 | INTERGENIC | | | | |
| CaPOPI_402 | Ca1 | 37712787 | A/ACATGTG | GGTGTTGGAGGTGTAATGGG | CATGTCCCGTATTCGCTTTT | 60.1 | 639 | INTERGENIC | | | | |
| CaPOPI_403 | Ca1 | 37724930 | CCACACACAC/CCACACAC | ACCCTGTTGCTGCCAAATAC | GCTCCAATTCCCTCAGATCA | 60.0 | 664 | INTRON | Ca_23534 | | | Protein of unknown function DUF620 |
| CaPOPI_404 | Ca1 | 37758491 | TA/TAA | TGACGAGGTTCGTGATGATG | AGCGAATGATTTCAACCACC | 60.7 | 489 | INTERGENIC | | | | |
| CaPOPI_405 | Ca1 | 37771437 | CTTTTT/CTTTT | CTGTGGCCCGACAAAGTATT | TCGTATATGCAGCAAGTGGC | 60.0 | 627 | INTERGENIC | | | | |
| CaPOPI_406 | Ca1 | 37809250 | ACTTTTCCTTTTCCT/ACTTTTCCT | TGCCTTTGGGGATTTTATCA | TTTTGCATTAAAAATGGATGAA | 60.3 | 579 | INTERGENIC | | | | |
| CaPOPI_407 | Ca1 | 37849378 | G/GA | CCTAAGGGCCGTAGTAACCTG | AGGATCCACCAAGCAAATCA | 60.0 | 154 | INTRON | Ca_23538 | T | E2F/DP | Zinc finger, RING-type |
| CaPOPI_408 | Ca1 | 37913733 | TAAA/TAA | ACGTTCGCCACCTATCTCAT | CCTTATGCGGTAATCCCTGA | 59.6 | 563 | INTERGENIC | | | | |
| CaPOPI_409 | Ca1 | 37917967 | ATATGACAGTTA/ATA | TCCACAAGTTATTCGACGAAAC | TTCCATTGATCTACGTTAATGTTTG | 59.1 | 558 | INTERGENIC | | | | |
| CaPOPI_410 | Ca1 | 37927331 | TAAA/TAAATTGTATCAAA | ATTTTGATCGCGGAAGTTTG | TCAATCAACCAGATTTTGCATT | 60.1 | 553 | INTERGENIC | | | | |
| CaPOPI_411 | Ca1 | 37927862 | G/GTA | TGCAAAATCTGGTTGATTGAG | TCCCAGATCAAAATTGGGTT | 58.8 | 745 | INTERGENIC | | | | |
| CaPOPI_412 | Ca1 | 37928853 | CT/CTGAAT | CCGTGCAACAATGTCAATTC | GACCACCATATTAACCGTCACA | 60.0 | 571 | INTERGENIC | | | | |
| CaPOPI_413 | Ca1 | 37929646 | TAAAAAAAA/TAAAAAAAAAA | TGTGACGGTTAATATGGTGGTC | TTGACTTGTTTTGCGGATTG | 59.6 | 817 | INTERGENIC | | | | |
| CaPOPI_414 | Ca1 | 37993665 | CAAAAAA/CAAAAA | GTGAGCCCTTTACCAATCCA | CAATTCCCACAGGTACGCTT | 59.9 | 654 | INTERGENIC | | | | |
| CaPOPI_415 | Ca1 | 38024874 | TATGAATGAAT/TATGAATGAATGAAT | GCAAGCTCAGTTCCAGTTCC | TGCATCAATTATGGCTCCAA | 60.0 | 655 | INTRON | Ca_27443 | S | | Zinc finger, GRF-type |

| INDEL marker IDs | Chromosomes/ unanchored scaffolds | Physical positions (bp) | InDels (*Kabuli* reference genome-CDC Frontier/PI) | Forward primers (5'-3') | Reverse primers (5'-3') | Anneling temperature (0C) | Expected amplified product size (bp) | Structural annotation | | Functional annotation | | |
|---|---|---|---|---|---|---|---|---|---|---|---|---|
| | | | | | | | | Sequence components of *kabuli* genome | *Kabuli* gene accession IDs | NCBI-KOG | TFs | NCBI-nr database |
| CaPOPI_416 | Ca1 | 38030335 | TTAAAATAAAATAAAATAAAA/TTAAAATAAAATAAAATAAAATAAAATAAAA | AGCTCGAAACATGGTATCCAA | CTAGCCATCCTCAGCGAGAG | 59.6 | 662 | INTERGENIC | | | | |
| CaPOPI_417 | Ca1 | 38060326 | CGG/CG | GGTTTGTCCGTTCTTCTCCA | CTTGTTGCGACGATGAAAAA | 60.1 | 310 | INTERGENIC | | | | |
| CaPOPI_418 | Ca1 | 38070666 | T/TA | AACCATTGGGGGAGAATAGAA | TTGTTAGAACCATTGGGGGA | 59.7 | 362 | INTERGENIC | | | | |
| CaPOPI_419 | Ca1 | 38070895 | ATGTGTGT/ATGTGT | CTCCCCCAATGGTTCTAACA | CACTGGAATCCGTTTCTTTTG | 59.8 | 587 | INTERGENIC | | | | |
| CaPOPI_420 | Ca1 | 38071534 | TACACACAC/TACACAC | CTCCTCCTCATTCACCCAAC | ACACACCCACTCTCCTTTGC | 59.5 | 189 | INTERGENIC | | | | |
| CaPOPI_421 | Ca1 | 38110776 | AT/ATTTT | GGGAGCATCCATAACATTGC | GCCATTTCAAACACATCACG | 60.3 | 188 | INTERGENIC | | | | |
| CaPOPI_422 | Ca1 | 38131605 | C/CT | CGTAACTCGTGTTGGGTGAA | GAAGCAAGAGGCAACTCCAC | 59.6 | 379 | INTERGENIC | | | | |
| CaPOPI_423 | Ca1 | 38193154 | TAAAAAAAAAAA/TAAAAAAAAA | CGAACAAACTCACCGGATCT | CCAATGGAGTCCCAAAAAGA | 60.1 | 724 | INTERGENIC | | | | |
| CaPOPI_424 | Ca1 | 38273940 | CAAAAAAAAAAA/CAAAAAAAAAA | TATTTGGGGACCAAAACTGC | CATGGTTTGAATGCGAGTTG | 59.8 | 637 | INTERGENIC | | | | |
| CaPOPI_425 | Ca1 | 38284193 | TC/T | GCCAACACATGGTGAAAGTG | AACAAAGTTTGCATTTGGGC | 60.0 | 939 | INTERGENIC | | | | |
| CaPOPI_426 | Ca1 | 38476810 | TTAT/TTATAT | TTGCAAGTACGTTCTCCAAAA | GTGTTACACGCCCTTCATCC | 58.5 | 280 | INTERGENIC | | | | |
| CaPOPI_427 | Ca1 | 38479463 | AATATATATATATATATATATATATATATA/AAAAAAATATATATATATATATATATATATATATATA | AGCGTGAATGTGACGTCTTG | TTAAAGAGATTCGCAGCGGT | 59.9 | 100 | INTERGENIC | | | | |
| CaPOPI_428 | Ca1 | 38487463 | ATTTTTTTTTT/ATTTTTTTTT | TGCTACCTGCAACCAGTGAG | GTCAACTTTAAGGGGGCGTC | 60.0 | 872 | DRR | Ca_13962 | | | Proteasome assembly chaperone 3 |
| CaPOPI_429 | Ca1 | 38495552 | AATTATTATTATTATTATTATTATTATTATTATTATTATTGTTATTATTATTATTATTA/AATTATTATTATTATTATTATTATTATTATTATTATTATTATTATTGTTATTATTAT | AACACCTTCCTGATCCTCCC | TGGCGAACTTTTAAGAATGGA | 60.3 | 659 | INTERGENIC | | | | |
| CaPOPI_430 | Ca1 | 38577185 | GTTTTT/GTTTT | CCATTTTCTGTCCCCTTTCA | GCATTGGCTAAGCATGGATT | 59.9 | 760 | INTERGENIC | | | | |

| INDEL marker IDs | Chromosomes/ unanchored scaffolds | Physical positions (bp) | InDels (*Kabuli* reference genome-CDC Frontier/PI) | Forward primers (5'-3') | Reverse primers (5'-3') | Anneling temperature (0C) | Expected amplified product size (bp) | Structural annotation | | Functional annotation | | |
|---|---|---|---|---|---|---|---|---|---|---|---|---|
| | | | | | | | | Sequence components of *kabuli* genome | *Kabuli* gene accession IDs | NCBI-KOG | TFs | NCBI-nr database |
| CaPOPI_431 | Ca1 | 38597715 | ATAATTTTAATTTTAATTT/ATAATTTTAATTT | CATTTGCCTCCCTTGTTGTT | TGTAGTTGCTGCAAACGAGG | 60.0 | 551 | DRR | Ca_13956 | | | Vacuolar protein sorting-associated protein 62 |
| CaPOPI_432 | Ca1 | 38766832 | ACT/ACTCT | GAGCTTCTTCAAGTCGGTGG | GTTTGGAATACCCTGAGCCA | 60.0 | 222 | INTERGENIC | | | | |
| CaPOPI_433 | Ca1 | 38790509 | AC/A | AGAGCTCTGAAGTCAAGCGG | GAAATCCTTGTTGCCTTGGA | 59.9 | 163 | DRR | Ca_13953 | | | Vacuolar protein sorting-associated protein 62 |
| CaPOPI_434 | Ca1 | 38886459 | AAAATAAA/AAAA | GCATGTTGTATGCTTTCTTCCA | ATTTATCCATCCATGTCGCC | 60.1 | 863 | INTERGENIC | | | | |
| CaPOPI_435 | Ca1 | 39028142 | AATATATATATAT/AATATATATAT | TCATGGAAGGAGCAATCACA | TAAGGTTTAGGATGCCCCAA | 60.2 | 764 | INTRON | Ca_13946 | | | |
| CaPOPI_436 | Ca1 | 39038586 | GTATATATATATATATATATATATATATATATATA/GTATATATATATATATATATATATATATATATATATATATA | CGATTTTGAATTTGGGTCTCA | TTAAATAGGCTTCCAGGCCA | 59.9 | 708 | INTRON | Ca_13946 | | | |
| CaPOPI_437 | Ca1 | 39144961 | CACGTAC/CAC | CATCCTTTGCAGGATTGGTT | TTGTGCTTTGGAAAATGCAG | 59.9 | 494 | INTRON | Ca_13942 | | ARF | Plant lipid transfer protein/Par allergen |
| CaPOPI_438 | Ca1 | 39147028 | AT/ATT | AAAATGATCAGGGAAAATCATGT | ACAACTTCTCATGCTCGCAA | 58.7 | 339 | DRR | Ca_13942 | | ARF | Plant lipid transfer protein/Par allergen |
| CaPOPI_439 | Ca1 | 39261977 | A/AT | GGTTTGCTGTCGAGCCTTAG | AAACACAAGTCCAATCAAAAGG | 60.0 | 421 | INTERGENIC | | | | |
| CaPOPI_440 | Ca1 | 39384808 | T/TA | GGGCCAAGAAACAACAAAAA | TTCCACCAATCAATTAGGCA | 60.0 | 105 | INTERGENIC | | | | |
| CaPOPI_441 | Ca1 | 39444170 | TAAAA/TAA | AATGATGGAAAAGCCGAATG | GGAGGGACCTGAAGTCACAA | 59.9 | 838 | INTRON | Ca_13931 | Z | NAC | IQ motif, EF-hand binding site |
| CaPOPI_442 | Ca1 | 39598601 | TC/TCCC | TTTCGTTATTTAAGCTACAACGG | GCGAAGATGGTGCCAATAAT | 57.7 | 930 | INTERGENIC | | | | |
| CaPOPI_443 | Ca1 | 39609113 | AATAT/AAT | ATGGCCTTTCATTTTCAACG | TTGTCTCAAGCGTTCAATTTTT | 59.9 | 725 | INTERGENIC | | | | |
| CaPOPI_444 | Ca1 | 39675431 | CATATATATATATATAT/CATATATATATATATATATATATAT | CACCAAAGACAGGACCACCT | CCTAGGGTTTTTGGTTGGGT | 60.0 | 784 | DRR | Ca_13920 | | | Acid phosphatase/vanadium-dependent haloperoxidase- |
| CaPOPI_445 | Ca1 | 39871055 | TAAA/TAAAA | CATTGGCCACCTTTAGGAAA | TGCATTGCTTCAGTCATGGT | 59.9 | 329 | INTERGENIC | | | | |

| INDEL marker IDs | Chromosomes/ unanchored scaffolds | Physical positions (bp) | InDels (*Kabuli* reference genome-CDC Frontier/PI) | Forward primers (5'-3') | Reverse primers (5'-3') | Anneling temperature (0C) | Expected amplified product size (bp) | Structural annotation | | Functional annotation | | |
|---|---|---|---|---|---|---|---|---|---|---|---|---|
| | | | | | | | | Sequence components of *kabuli* genome | *Kabuli* gene accession IDs | NCBI-KOG | TFs | NCBI-nr database |
| CaPOPI_446 | Ca1 | 39976327 | CA/CATA | TTTAAGAGAAGCGTTTGAGCG | CTTTGACTACGTTTCCCCGA | 59.8 | 211 | INTERGENIC | | | | |
| CaPOPI_447 | Ca1 | 40225071 | ATTATTT/ATTATTTATTTATTT | GTTGGAACTGTCCATTGGCT | AAGTCCGTTTCAGGGTTGTG | 60.0 | 355 | INTERGENIC | | | | |
| CaPOPI_448 | Ca1 | 40380273 | CTATA/CTATATATA | CCTGGTCCCACTTTTACGAA | GAAAAAGCGCTGTAAATGGC | 60.0 | 487 | INTRON | Ca_13892 | | Trihelix | |
| CaPOPI_449 | Ca1 | 40391433 | ACCCCC/ACCCCCC | TCCTGAAGGGGATTCATCAG | GTTAGGCGATCGCGAATACT | 60.0 | 610 | INTERGENIC | | | | |
| CaPOPI_450 | Ca1 | 40424994 | C/CCAAAATATTATAA | TGCAAAACTTTGTTCATCGAG | GCATCATTCTCCTTCGAGACA | 59.0 | 748 | INTERGENIC | | | | |
| CaPOPI_451 | Ca1 | 40426155 | ATTTTTTTT/ATTTTTTTTTT | CTTTACTGCGTTGGACCACA | ATAAATTGGATTTGCACCGC | 59.8 | 544 | INTERGENIC | | | | |
| CaPOPI_452 | Ca1 | 40534315 | GAAA/GAAAA | CAATTTTGACCATGGAACCC | TGAATTGACCTCGCAGATTG | 60.0 | 730 | INTERGENIC | | | | |
| CaPOPI_453 | Ca1 | 40535465 | TTAATAATAATAATAATAATA/TTAATAATAATAATAATAATA | CATGCGTTCCTTGTGGTAGA | ATCACAGTTTGGCCAAGTCC | 59.7 | 599 | INTERGENIC | | | | |
| CaPOPI_454 | Ca1 | 40535882 | AATATATATATATATATATATATAT/AATATATATATATATATATATATATAT | GATGATGATGATGTCGTGCC | ATCACAGTTTGGCCAAGTCC | 59.9 | 311 | INTERGENIC | | | | |
| CaPOPI_455 | Ca1 | 40795125 | AATATATATATATATATATATATATAT/AATATATATATATATATATAT | TGAAATCAATTGAGGACATTCG | ACGTAGCGAGCCTCGAATTA | 59.9 | 547 | INTERGENIC | | | | |
| CaPOPI_456 | Ca1 | 40861492 | GTTTTTTTT/GTTTTTTTTTTTTT | ATTTCTAAACCCGCAAACCC | TCCACCCACGATACTGATGA | 60.2 | 941 | INTERGENIC | | | | |
| CaPOPI_457 | Ca1 | 40889769 | TTATATA/TTA | GCATCTCTTGATGCCCTTTT | AACATACGCCATACCCAAGC | 59.3 | 289 | INTERGENIC | | | | |
| CaPOPI_458 | Ca1 | 41058981 | CTTTTTTTTTTT/CTTTTTTTTTT | GCATGGGAAATGGATTCTCT | TGGCTCATACACAGTTTTCTGG | 58.9 | 679 | INTERGENIC | | | | |
| CaPOPI_459 | Ca1 | 41503912 | TCC/TC | TCGATTATGCGTAGGGAAGG | CTTGGTGGTTTTGAAACTCAAT | 60.1 | 540 | INTERGENIC | | | | |
| CaPOPI_460 | Ca1 | 41695402 | CTA/CTATA | TACTTCCATGCAAACCAGCA | CCAACTCTGAAATACAAAAGCCA | 60.3 | 486 | INTERGENIC | | | | |

| INDEL marker IDs | Chromosomes/ unanchored scaffolds | Physical positions (bp) | InDels (*Kabuli* reference genome-CDC Frontier/PI) | Forward primers (5'-3') | Reverse primers (5'-3') | Anneling temperature (0C) | Expected amplified product size (bp) | Structural annotation | | Functional annotation | | |
|---|---|---|---|---|---|---|---|---|---|---|---|---|
| | | | | | | | | Sequence components of *kabuli* genome | *Kabuli* gene accession IDs | NCBI-KOG | TFs | NCBI-nr database |
| CaPOPI_461 | Ca1 | 41704419 | CATAATAAT/CATAAT | GGGGAATTTTTCATTTT GTGG | TGTTTTGGTTTCAAGGG ACA | 60.4 | 311 | INTERGENIC | | | | |
| CaPOPI_462 | Ca1 | 41704503 | CTTTTTTT/CTTTTTT | GGGGAATTTTTCATTTT GTGG | TGTTTTGGTTTCAAGGG ACA | 60.4 | 311 | INTERGENIC | | | | |
| CaPOPI_463 | Ca1 | 41705653 | ATTTTTTTT/ATTTTTTT | CATCACCGTAACATGAC TTACCA | TCCCAATAATTTTTCCTT TTCG | 59.8 | 723 | INTERGENIC | | | | |
| CaPOPI_464 | Ca1 | 41718814 | T/TG | GGGTCGAATGTCGATTC TGT | TCATCTCTCAGCCCCAC TCT | 59.9 | 156 | INTERGENIC | | | | |
| CaPOPI_465 | Ca1 | 42432936 | AATATATATATATATATATATAT/AATATA TATATATATATAT | CAAATTCCTCTTGCTCCA CC | TGAAAGCTGATGAAAAT GGTC | 59.7 | 695 | INTERGENIC | | | | |
| CaPOPI_466 | Ca1 | 43119967 | AATATATATATATATATATATA/AATATAT ATATATATATA | TTTCCGGGAATAAATCC CTC | GAAGGAGAGTTCGCGTT GAC | 60.1 | 361 | INTERGENIC | | | | |
| CaPOPI_467 | Ca1 | 43335241 | AATATATATATATATATATATATATATAT ATATATATAT/AATATATATATATATATAT ATATATATATAT | CGTGTATATATGCGCAC CCA | CACAAACGGAAAAGTCG GAT | 60.4 | 788 | INTERGENIC | | | | |
| CaPOPI_468 | Ca1 | 43469250 | A/ATATATTATT | GAGGGTTTGCAGCTCTT GTC | AACGCATCGGTAAACAC TCC | 60.0 | 547 | INTERGENIC | | | | |
| CaPOPI_469 | Ca1 | 43638401 | A/ATATGTC | CGCTTCATGTGTGTTGC TTT | ACCCCTCCTAAGTGGGT TGA | 59.9 | 857 | INTERGENIC | | | | |
| CaPOPI_470 | Ca1 | 43867975 | AATATATATATATATATATATATATATA/A ATATATATATATATATATATATATATATA TATATA | TTGCTGGCATAAGTGAA TCG | CCGTGAATTTTCATGGA ATAAA | 59.8 | 711 | INTERGENIC | | | | |
| CaPOPI_471 | Ca1 | 44172001 | GTTTTT/GTTTTTT | ACCTATGCCCACTGATG AGG | GCAATTGCCAATGACGA CTA | 60.0 | 750 | INTERGENIC | | | | |
| CaPOPI_472 | Ca1 | 44247311 | GTTTTTTTTTT/GTTTTTTTTT | TTCTCCAGACTCTAGCC CGA | AGCTTCTCAGTAGGGGG CTC | 60.1 | 592 | INTERGENIC | | | | |
| CaPOPI_473 | Ca1 | 44351850 | TTATATATATATATA/TTATATATATAT ATATATA | TGAAATTCGGAAGAACC AGG | CGCATTCCCTTCACCTC TAA | 60.0 | 111 | INTERGENIC | | | | |
| CaPOPI_474 | Ca1 | 44467119 | GTT/GTTT | ATGCAGCTCAAAAACAG CCT | GGTTCACCCAAAGATGG CTA | 60.0 | 537 | INTERGENIC | | | | |
| CaPOPI_475 | Ca1 | 44650256 | T/TC | TCGTTTGCGTCATTGGA TTA | AGGGAGGACGAAAATGA CCT | 60.1 | 444 | INTERGENIC | | | | |

| INDEL marker IDs | Chromosomes/ unanchored scaffolds | Physical positions (bp) | InDels (*Kabuli* reference genome-CDC Frontier/PI) | Forward primers (5'-3') | Reverse primers (5'-3') | Anneling temperature (0C) | Expected amplified product size (bp) | Structural annotation | | Functional annotation | | |
|---|---|---|---|---|---|---|---|---|---|---|---|---|
| | | | | | | | | Sequence components of *kabuli* genome | *Kabuli* gene accession IDs | NCBI-KOG | TFs | NCBI-nr database |
| CaPOPI_476 | Ca1 | 44776081 | AATATATATATATATATATATATATATATATATATATATA/AATATATATATATATATATATATATATATATATATATATATATATATA | TGGAATACCATATATTTTACGTTTTCA | TGACGGATCCTTTCTTGACA | 59.1 | 795 | INTERGENIC | | | | |
| CaPOPI_477 | Ca1 | 44847079 | ATTTTTTTTTTT/ATATTTTTTTTTTTTTTT | GACATGCTCAGTAATTGCCC | AAAATCCAACAATCTCCCCC | 58.2 | 373 | INTERGENIC | | | | |
| CaPOPI_478 | Ca1 | 44847252 | ATCTGATGTTCTGATG/ATCTGATGTTCTGATGTTCTGATG | TGTTTTCTGCCTTTTTGCCT | AAAATCCAACAATCTCCCCC | 59.9 | 274 | INTERGENIC | | | | |
| CaPOPI_479 | Ca1 | 44847313 | TATAATAATAATAATAATAATA/TATAATAATAATAATAATA | TGTTTTCTGCCTTTTTGCCT | AAAATCCAACAATCTCCCCC | 59.9 | 274 | INTERGENIC | | | | |
| CaPOPI_480 | Ca1 | 44921329 | A/ATGATGCTAATCTT | CCTCAAACGATTTTCTGGGA | CTTTCCCAATGCGTGAAGAT | 60.0 | 478 | INTERGENIC | | | | |
| CaPOPI_481 | Ca1 | 45003074 | AA/AATTCCGAATCA | TCTTCCACATTGATTTTGTTGC | TGCTTATTCCCCAGATACCG | 60.0 | 988 | DRR | Ca_25714 | | | |
| CaPOPI_482 | Ca1 | 45033030 | ATTTTTTTT/ATTTTTTTTTT | TTTCCCCATGCAGAGTTAGG | TAAACCAGCCACTAAGGCGT | 60.1 | 894 | INTERGENIC | | | | |
| CaPOPI_483 | Ca1 | 45058076 | TAAAAAA/TAAAAAAA | AAACTAGATTATGGCCTGCG | TGCTCGCAATTAAAACATGC | 57.0 | 426 | INTERGENIC | | | | |
| CaPOPI_484 | Ca1 | 45104438 | TTAATAATAATAATAATAATAATAATAATAATAATAATAATAATAATAATAATAATAATAATAATAA/TTAATAATAATAATAATAATAATAATAATAATAATAATAATAATA | TTTCAGGCGATTTTCGTACC | AAGTGGGGAACGTTGACTTG | 60.1 | 744 | INTERGENIC | | | | |
| CaPOPI_485 | Ca1 | 45243785 | TA/TAGACA | TGTGCCAAGGTTTCAGAACA | CTTTTATTGCAAGACCCCGA | 60.3 | 605 | DRR | Ca_26020 | | | Uncharacterised protein family Ycf1 |
| CaPOPI_486 | Ca1 | 45246034 | C/CGTCTTGTT | CGATTTCGCTTTTAGCATCC | CAATTGCGATATTATGGGGG | 59.8 | 253 | INTERGENIC | | | | |
| CaPOPI_487 | Ca1 | 45339273 | TA/TAA | TGATTGGGAAATCGATTGGT | TTGATCCTTGCAGTCACTCG | 60.1 | 566 | DRR | Ca_24431 | | | |
| CaPOPI_488 | Ca1 | 45497100 | CTTTTTTTTTTT/CTTTTTTTTTTTTT | AGTATAAGTGGGGCCGTGTG | TGGATTGTGCCAACTTTTCA | 59.9 | 237 | INTERGENIC | | | | |
| CaPOPI_489 | Ca1 | 45609121 | C/CT | TCGCGTGAGAAAATAATCCC | CGAACCCTCGGTAAACAAAA | 60.0 | 295 | INTERGENIC | | | | |
| CaPOPI_490 | Ca1 | 45635524 | AATATATATATATATATATATATATATATAT/AATATATATATATATATATATATATATATATATATAT | ATGTTTTGGATTTTGGCTCG | GGAGAAGGAGGGAGAAATGG | 59.9 | 433 | INTERGENIC | | | | |

| INDEL marker IDs | Chromosomes/ unanchored scaffolds | Physical positions (bp) | InDels (*Kabuli* reference genome-CDC Frontier/PI) | Forward primers (5'-3') | Reverse primers (5'-3') | Anneling temperature (0C) | Expected amplified product size (bp) | Structural annotation | | Functional annotation | | |
|---|---|---|---|---|---|---|---|---|---|---|---|---|
| | | | | | | | | Sequence components of *kabuli* genome | *Kabuli* gene accession IDs | NCBI-KOG | TFs | NCBI-nr database |
| CaPOPI_491 | Ca1 | 45749239 | GAAAAAAAAAA/GAAAAAAAAA | TTGTGCGCTTGACTGATGTT | GTTTTGGCGTTGGTTTCACT | 60.5 | 857 | INTERGENIC | | | | |
| CaPOPI_492 | Ca1 | 45941271 | AATATATATATATA/AATATATATATATATA | GAAAGGAGATCACGAGCAGC | GTTGAATGGAACAGGCGAGT | 60.1 | 723 | INTERGENIC | | | | |
| CaPOPI_493 | Ca1 | 45941295 | GATATATATATATATATATATATAT/GATATATATATATATATATATATATAT | GAAAGGAGATCACGAGCAGC | CAAGTCGTGGGGAGCATTAT | 60.1 | 846 | INTERGENIC | | | | |
| CaPOPI_494 | Ca1 | 46023584 | CACTAGTTACTAGTTACTAGTTACTAGTTACT/CACTAGTTACTAGTTACTAGTTACT | TTTTCCACCAACAAACCTCA | AGTTGTGGAATCTGCCTGCT | 59.0 | 330 | INTERGENIC | | | | |
| CaPOPI_495 | Ca1 | 46088799 | CTATATATATATATATATATATATATATATAT/CTATATATATATATATATATATATATATATATAT | TAAGTCCGTAAGTGGTGCCC | GGGCTTTCCAAAGTGTTATCC | 60.0 | 586 | INTERGENIC | | | | |
| CaPOPI_496 | Ca1 | 46186559 | AGTGATTGGTTTGTTTGTGATTGGTTTGTTTG/AGTGATTGGTTTGTTTGTGATTGGTTTGTTTGTGATTGGTTTGTTTG | AAAGCACACGATTTTCACCC | TTCGGCATCTAGCACTTCCT | 60.0 | 775 | INTERGENIC | | | | |
| CaPOPI_497 | Ca1 | 46278771 | ATGGCCTGGCCTGGCCTGGCCTG/ATGGCCTGGCCTG | ACCTTCAGTCAGGGGCCTAT | AAGTCTGGTCTGGCCTGAAA | 60.0 | 538 | INTERGENIC | | | | |
| CaPOPI_498 | Ca1 | 46286614 | CTTTT/CTTT | CCAAGTTATGCTTGATCGCA | CGAGAACCATCCTGCATTTT | 59.8 | 820 | INTERGENIC | | | | |
| CaPOPI_499 | Ca1 | 46286661 | GTAGGGATA/GTA | CCAAGTTATGCTTGATCGCA | CGAGAACCATCCTGCATTTT | 59.8 | 820 | INTERGENIC | | | | |
| CaPOPI_500 | Ca1 | 46352138 | CACAAC/CACAACAAC | AGCGATGCATGTCTCAAGTG | TGCCCTTCTTCTTTTCCTAGTG | 60.0 | 396 | INTERGENIC | | | | |
| CaPOPI_501 | Ca1 | 46491737 | CAAG/CAAGAAG | GATGCATCTCACCAAGGGTT | CGGTGCAAACTCTACGTTCA | 59.9 | 420 | INTERGENIC | | | | |
| CaPOPI_502 | Ca1 | 46523548 | CAAAAAAAAAAAAAAAA/CAAAAAAAAAAAAA | GTGGACAAACCTGCATTCCT | ACCATGCACAGTTGCTGAGA | 60.0 | 675 | INTERGENIC | | | | |
| CaPOPI_503 | Ca1 | 46524349 | TAAAAAAAAA/TAAAAAAAAAAA | TGAAGCATGCACAAATCCTC | TTGATGTAGCAACGGCTGAG | 59.8 | 843 | INTERGENIC | | | | |
| CaPOPI_504 | Ca1 | 46525859 | GAAAAAA/GAAAAAAA | CCCACCTGGATACAAGGCTA | AGTAGAGGGAGTTCGGGTGC | 59.9 | 711 | INTERGENIC | | | | |
| CaPOPI_505 | Ca1 | 46526676 | TAAAA/TAAA | TTAAAATTCCGAGGCGAAAA | ACATGGTTGTGGTGGTGATG | 59.7 | 698 | INTERGENIC | | | | |

| INDEL marker IDs | Chromosomes/ unanchored scaffolds | Physical positions (bp) | InDels (*Kabuli* reference genome-CDC Frontier/PI) | Forward primers (5'-3') | Reverse primers (5'-3') | Anneling temperature (0C) | Expected amplified product size (bp) | Structural annotation | | Functional annotation | | |
|---|---|---|---|---|---|---|---|---|---|---|---|---|
| | | | | | | | | Sequence components of *kabuli* genome | *Kabuli* gene accession IDs | NCBI-KOG | TFs | NCBI-nr database |
| CaPOPI_506 | Ca1 | 46528558 | CT/CTT | TTAAAATTCCCTCCTCGGCT | AACTCTGGGAACCCTCCTGT | 60.0 | 398 | INTERGENIC | | | | |
| CaPOPI_507 | Ca1 | 46530950 | CAAATTGAAAATTGAAAATT/CAAATTGAAAATT | ATTTGAGAATGTTCGGGCAA | GTCTGCGGATAATTTCGCTT | 60.4 | 665 | DRR | Ca_21551 | M | | Pectinacetylesterase |
| CaPOPI_508 | Ca1 | 46531041 | T/TG | GCGAAAATAATGTTGATGCAGA | GTCTGCGGATAATTTCGCTT | 60.1 | 139 | DRR | Ca_21551 | M | | Pectinacetylesterase |
| CaPOPI_509 | Ca1 | 46539360 | CAAA/CAAAA | TCCCAAACAGCCTGATAACC | CTGATGACAAAAGGGGGAGA | 59.9 | 744 | INTERGENIC | | | | |
| CaPOPI_510 | Ca1 | 46598729 | GATATATATATAT/GATATATATATATAT | TGATTTGTTAGCTTGTGGCAA | TGGAAGCATGATCCTCAACA | 59.4 | 477 | INTERGENIC | | | | |
| CaPOPI_511 | Ca1 | 46831943 | CTTTT/CTTT | GTGGTTGGTTTGCAAGAGGT | AACTGGGTGAGAGCCTGAGA | 60.0 | 725 | INTRON | Ca_25116 | U | | |
| CaPOPI_512 | Ca1 | 46856347 | GCT/GCTGAACT | ATGCAATCCGATAAGCAACC | ACGATTTTAGAAGAGGCGCA | 59.9 | 980 | INTERGENIC | | | | |
| CaPOPI_513 | Ca1 | 46916401 | GATATATATATATATATATATA/GATATATATATATATATATATATA | CAGCAAGCACCAACCACTTA | TTGCCAGTTATTTTTGCAAGG | 59.9 | 363 | DRR | Ca_12949 | | | |
| CaPOPI_514 | Ca1 | 47016631 | ACTCTCTCTCTCTCT/ACTCTCTCTCTCTCTCT | ATTGCCTCTCAGTCGCGTTA | AAAATTCGACATCGTGAGGG | 60.9 | 531 | INTERGENIC | | | | |
| CaPOPI_515 | Ca1 | 47116478 | AATATATATATATATATATATATAT/AATATATATATATATATATATAT | ACACGCTTCCCTGTTGACAT | TAGGCCTGAACTGGGATGAC | 60.6 | 772 | INTRON | Ca_12935 | | ERF | Protein kinase, catalytic domain |
| CaPOPI_516 | Ca1 | 47455552 | TTAATAATAATAATAATAA/TTAATAATAATAATAA | TTGGGGTCAGTCAAACACAA | CGGTGAAATAGGTATGCCAAA | 60.0 | 600 | INTERGENIC | | | | |
| CaPOPI_517 | Ca1 | 47630703 | TAAAAAGAAAAAGAAAAAGAAA/TAAAAAGAAAAAGAAAAAGAAAAAGAAA | CGTGTCCGGCAAAGTAAAAT | GTGATGCACGCAGTAATTGG | 60.0 | 517 | INTERGENIC | | | | |
| CaPOPI_518 | Ca1 | 47630977 | AC/ACC | GGCCTTCCATCGTGAATAAA | TTTCGAATTCCAGAACTGCC | 59.9 | 916 | INTERGENIC | | | | |
| CaPOPI_519 | Ca1 | 47676057 | ATTTTTTTTTTT/ATTTTTTTTTTTT | AATCCCTCCCTGCCTAAAAA | ATGTGGTGGTCCAATCAACA | 59.9 | 619 | INTRON | Ca_12903 | | | |
| CaPOPI_520 | Ca1 | 47875198 | AT/ATT | GAAAAACAAGACCCGCGATA | TTCCTATTGCTCACCCCTTG | 60.1 | 466 | INTERGENIC | | | | |

| INDEL marker IDs | Chromosomes/ unanchored scaffolds | Physical positions (bp) | InDels (*Kabuli* reference genome-CDC Frontier/PI) | Forward primers (5'-3') | Reverse primers (5'-3') | Anneling temperature (0C) | Expected amplified product size (bp) | Structural annotation | | Functional annotation | | |
|---|---|---|---|---|---|---|---|---|---|---|---|---|
| | | | | | | | | Sequence components of *kabuli* genome | *Kabuli* gene accession IDs | NCBI-KOG | TFs | NCBI-nr database |
| CaPOPI_521 | Ca1 | 47916750 | ATT/ATTTCAGGTTT | GTTGGTGATGCCACTGAATG | GAGAGAAAGGCAAGTGTGGG | 60.0 | 979 | DRR | Ca_12885 | O | M-type | ATPase, AAA+ type, core |
| CaPOPI_522 | Ca1 | 47945221 | TCAAGCCA/TCA | AGGACAGTGCCAAAAGATGG | GTCATGTATGCATTGGGCAG | 60.1 | 226 | DRR | Ca_12882 | R | | Tetratricopeptide TPR-1 |
| CaPOPI_523 | Ca1 | 48064288 | TTATATATATATATATATATATATATA/TTATATATATATATATATATA | TGGGTGGATAGATGAGAGGC | TCTGAATAGGTTTGGTCGGC | 60.0 | 404 | INTERGENIC | | | | |
| CaPOPI_524 | Ca1 | 48091761 | TAAAAAAAA/TAAAAAAA | TCTGGAGATGGGACCTCTTG | CCAAATCGGAGGAAGTTGTT | 60.2 | 700 | INTERGENIC | | | | |
| CaPOPI_525 | Ca1 | 48114146 | TTATATATATATATATATATATATAT/TTATATATATATATATATATATAT | TGATTGTTGTTGGGCCACTA | TTTGTTCTCCATTTTGCTTTTG | 60.0 | 807 | INTERGENIC | | | | |
| CaPOPI_526 | Ca1 | 48172069 | AAATAATAATAATAATAATAATAATAATAATAATAATAATA/AAATAATAATAATAATAATAATAATAATAATAATAATAATAATAATAATA | TAAATGGCAGGTAAAGGCGA | AGACTTTGCTGCCACCTCAT | 60.6 | 315 | DRR | Ca_12862 | R | WRKY | Leucine-rich repeat |
| CaPOPI_527 | Ca1 | 48236817 | TAA/TA | TGGGTGTTGCCCATATATCA | TTTGGATCCATTTCATTTGATT | 59.6 | 626 | INTERGENIC | | | | |
| CaPOPI_528 | Ca2 | 42172 | TTATATATATATATATATATATATA/TTATATATATATATATA | TGGCAGTCTGTTTCACGTTT | CAAGGGACCAGATTCTGCAT | 59.3 | 553 | INTERGENIC | | | | |
| CaPOPI_529 | Ca2 | 55390 | GACACACACACACACACA/GACACACACACACACACA | GCACCGGGAGATACGTAAGA | TCACGGCTTCACAAAACAAG | 60.1 | 798 | INTERGENIC | | | | |
| CaPOPI_530 | Ca2 | 228183 | GTTTTTTTTTT/GTTTTTTTTT | TTCGAAACGTTGGATGAATG | GCAACCATCTGACCAAGTGA | 59.5 | 418 | INTERGENIC | | | | |
| CaPOPI_531 | Ca2 | 285679 | AAATAATAATAATAATAATAATAATAATAATAATAATAATACTAATAATAATAATAATAATAA/AAATAATAATAATAATAATAATAATAATAATAATAATAATAATACTAATAAT | AAACGACTCACACCCGAAAC | GCCATTGATTCATTTCTTCG | 60.0 | 614 | INTERGENIC | | | | |
| CaPOPI_532 | Ca2 | 422540 | G/GTCTTCCT | AGAGTTGGAGGGTGCAGCTA | CTTCGAATCCAACCTTGGTG | 60.0 | 829 | INTERGENIC | | | | |
| CaPOPI_533 | Ca2 | 469349 | TA/TAA | CCCAAGACAACCTTGCAGAT | ATGAAGTGCAAACCATGCTG | 60.1 | 674 | DRR | Ca_16986 | | | |
| CaPOPI_534 | Ca2 | 753916 | CTTTTTTTTTT/CTTTTTTTTT | TTGCCCATTAACTTTACCCG | ACTTGTGCATTTGCATTAGTTC | 59.8 | 555 | DRR | Ca_17000 | | ARF | |
| CaPOPI_535 | Ca2 | 833993 | TAATAAAATAAAATAAAATAAAATA/TAATAAAATAAAATA | TTGCATCATGGTTATGCGAC | CATAGCCTCGTACATGCAACA | 60.5 | 490 | INTERGENIC | | | | |

| INDEL marker IDs | Chromosomes/ unanchored scaffolds | Physical positions (bp) | InDels (*Kabuli* reference genome-CDC Frontier/PI) | Forward primers (5'-3') | Reverse primers (5'-3') | Anneling temperature (0C) | Expected amplified product size (bp) | Structural annotation | | Functional annotation | | |
|---|---|---|---|---|---|---|---|---|---|---|---|---|
| | | | | | | | | Sequence components of *kabuli* genome | *Kabuli* gene accession IDs | NCBI-KOG | TFs | NCBI-nr database |
| CaPOPI_536 | Ca2 | 904938 | GGTGT/GGTGTGT | TGAGCAGGTTGTTGCAATTC | CAGGGTTCTGTGGCATTTCT | 59.8 | 575 | INTERGENIC | | | | |
| CaPOPI_537 | Ca2 | 1233900 | C/CG | CGTTTTTGGAAGCCATTTGT | CTGCCATAAAGGCTGCAGAT | 60.0 | 830 | INTERGENIC | | | | |
| CaPOPI_538 | Ca2 | 1558409 | TCGGAAGNNNNNNNNNNCGGAAG/TCGGAAG | TCGAAATTTTAGGCCACGAC | TGAGGTTGTTGCTTTGACCA | 60.1 | 631 | INTERGENIC | | | | |
| CaPOPI_539 | Ca2 | 1640062 | ATTTTTTTTT/ATTTTTT | AGATCATGTGCAAAAAGGGG | AGTAACCGTGAGGGAACGTG | 59.9 | 509 | INTERGENIC | | | | |
| CaPOPI_540 | Ca2 | 1728113 | AATTATTATTATTATTATTATTATTATTATTATTATT/AATTATTATTATTATTATTATTATTATTATTATT | GAAAAGACACACATATTTGGCA | GTTTGTTGTGGTTGCAGTGG | 57.7 | 491 | INTERGENIC | | | | |
| CaPOPI_541 | Ca2 | 1835747 | TTATATATATATATATATATATATATAT/TTATATATATATATATATATATAT | CACAATGCACCATGTGACAA | TGGAACTGAAGCCATATCCC | 60.0 | 831 | INTRON | Ca_12601 | | | Raffinose synthase |
| CaPOPI_542 | Ca2 | 1879905 | CAAAAAAA/CAAAAAAAA | AACATCTCTAGCGTGTGCCA | CACACCCACTCCCTTGTCTT | 59.5 | 711 | DRR | Ca_12598 | YU | | Bacterial Ig-like, group 2 |
| CaPOPI_543 | Ca2 | 1919646 | TC/TCTCC | TTGAAGCGATGTGGTTGAAG | CCAAAACAACTATGCCCGTT | 59.8 | 339 | INTERGENIC | | | | |
| CaPOPI_544 | Ca2 | 2169056 | C/CG | AACCTCTCCAAGAGGATCGG | GGAGAGTGAAACGGATGGAA | 60.6 | 648 | INTERGENIC | | | | |
| CaPOPI_545 | Ca2 | 2169072 | TTCTCTCTC/TTCTCTCTCTC | AACCTCTCCAAGAGGATCGG | GGAGAGTGAAACGGATGGAA | 60.6 | 648 | INTERGENIC | | | | |
| CaPOPI_546 | Ca2 | 2235889 | TTATTA/TTATTACTATTA | TCTTGCACAACTCATTTCGG | AAAAAGCAAAGAAATTGTCAGAA | 59.8 | 330 | INTERGENIC | | | | |
| CaPOPI_547 | Ca2 | 2373367 | AATATATATATATATATATATATA/AATATATATATATATATATATATATATATA | AAAATTGTCAGGTTCACCGC | GGTGGTGATTTGGTGCTCTT | 60.0 | 896 | DRR | Ca_12541 | R | DBB | RAG1-activating protein-1-related |
| CaPOPI_548 | Ca2 | 2546989 | AT/ATCATAGTTT | CCCGTCGATCAAAAGAAAAA | TTGAATTGCTTTCCAGTGTCC | 60.0 | 705 | INTRON | Ca_10592 | R | | Haem peroxidase, animal |
| CaPOPI_549 | Ca2 | 2624920 | CGGCTGGCT/CGGCT | GGCCAAGTTGCTGAAGGATA | CGCTAAGAGGCCGTTATCAA | 60.2 | 610 | INTRON | Ca_10586 | | | Ribonuclease H domain |
| CaPOPI_550 | Ca2 | 2692293 | ATTTTTTTT/ATTTTTTT | AAAGGTGATGAAGCAGATTTTGA | TGGTGTCCTCTCTTCTCTAATCG | 60.1 | 478 | INTERGENIC | | | | |

| INDEL marker IDs | Chromosomes/ unanchored scaffolds | Physical positions (bp) | InDels (*Kabuli* reference genome-CDC Frontier/PI) | Forward primers (5'-3') | Reverse primers (5'-3') | Anneling temperature (0C) | Expected amplified product size (bp) | **Structural annotation** | | **Functional annotation** | | |
|---|---|---|---|---|---|---|---|---|---|---|---|---|
| | | | | | | | | **Sequence components of *kabuli* genome** | ***Kabuli* gene accession IDs** | **NCBI-KOG** | **TFs** | **NCBI-nr database** |
| CaPOPI_551 | Ca2 | 2749945 | CTTT/CTT | ATATGGATTCATGCCCCAAA | TTGCATTTTTCAGAGGGAGG | 60.0 | 580 | INTRON | Ca_10569 | GMO | E2F/DP | Nucleotidyl transferase |
| CaPOPI_552 | Ca2 | 2977761 | A/ACTT | CAACTTGCCAAACAGCTTCA | TGGTGCAAGGATAGTACGACA | 60.0 | 620 | INTERGENIC | | | | |
| CaPOPI_553 | Ca2 | 2978014 | CTAT/CTATAT | TTGCTGTAATTGTTGCACATGA | CCTCCCCAACTTGTAATTTCTG | 60.2 | 542 | INTERGENIC | | | | |
| CaPOPI_554 | Ca2 | 2984359 | CTTTTTTTTTTTT/CTTTTTTTTTT | GCTCACAACAGCAAAAACCA | TCTCTTTCTTAGACCGGAGCA | 59.9 | 443 | INTERGENIC | | | | |
| CaPOPI_555 | Ca2 | 2984403 | GAT/GATATAT | GCTCACAACAGCAAAAACCA | TCTCTTTCTTAGACCGGAGCA | 59.9 | 443 | INTERGENIC | | | | |
| CaPOPI_556 | Ca2 | 2985774 | ATGT/AT | GTGAAGCTGTATGGCCCAAT | AGATCTGGGTCGGGAAAAGT | 60.0 | 540 | INTERGENIC | | | | |
| CaPOPI_557 | Ca2 | 2987500 | TAAAAAAAAA/TAAAAAAAAAAA | GCCAAATGATTCGGTTGCTA | GCCGACCATGGAGTAATTGT | 61.0 | 700 | INTERGENIC | | | | |
| CaPOPI_558 | Ca2 | 2996172 | ATTT/ATT | TTCTAAGGACCAACGCAACA | GTATGATCGATTATGCGCGA | 59.3 | 434 | INTERGENIC | | | | |
| CaPOPI_559 | Ca2 | 3000644 | ATTTTTT/ATTTT | TGATTCAATTGTGAAGTTTTAATGTG | GGAGAAACATCACGTTGACC | 59.4 | 949 | INTERGENIC | | | | |
| CaPOPI_560 | Ca2 | 3001687 | CAAA/CAAAAA | TTCCAATTTTCGGCAACTTC | GCTGATGAAGGAGAGGAACG | 60.1 | 915 | INTERGENIC | | | | |
| CaPOPI_561 | Ca2 | 3009059 | ACCCCC/ACCCC | ATTCGCTTGCCTCTTTGGTA | CCCGAAACTTTCTGTGCATT | 59.8 | 658 | INTRON | Ca_10544 | G | | Glycosyl transferase, family 14 |
| CaPOPI_562 | Ca2 | 3011716 | G/GA | TGATTTGGGGTGTTCAGAAA | TCCGCACACCAACATTAAAA | 58.9 | 657 | INTERGENIC | | | | |
| CaPOPI_563 | Ca2 | 3016524 | GAAAAAAAA/GAAAAAAAAA | TGGGAGAGATAAAACGCTGC | TCTTTCACAACCTCAAAAGTGC | 60.4 | 353 | INTERGENIC | | | | |
| CaPOPI_564 | Ca2 | 3022835 | TTAA/TTAATAA | CCCGACTCGAAAGTTTCTCA | AAAATTGACACGGCTTCGTC | 60.4 | 544 | INTERGENIC | | | | |
| CaPOPI_565 | Ca2 | 3024878 | ATTTTTTTT/ATTTTTTTTTT | CTGGTTTTCAAACGCGCTAC | CGGGGTCGGTATTATTTTGA | 60.8 | 799 | INTERGENIC | | | | |

| INDEL marker IDs | Chromosomes/ unanchored scaffolds | Physical positions (bp) | InDels (*Kabuli* reference genome-CDC Frontier/PI) | Forward primers (5'-3') | Reverse primers (5'-3') | Anneling temperature (0C) | Expected amplified product size (bp) | Structural annotation | | Functional annotation | | |
|---|---|---|---|---|---|---|---|---|---|---|---|---|
| | | | | | | | | Sequence components of *kabuli* genome | *Kabuli* gene accession IDs | NCBI-KOG | TFs | NCBI-nr database |
| CaPOPI_566 | Ca2 | 3029449 | TTTAATAATTATT/TTTAATAATTATTAATAATTATT | ACCGCTCTATTAAGGGCGAG | TCCATCGCAATTGAATAATGA | 60.7 | 765 | INTERGENIC | | | | |
| CaPOPI_567 | Ca2 | 3030942 | CAAGA/C | TTTTCATTTTCTCCCTTCCCT | TCCATCGCAATTGAATAATGA | 59.1 | 592 | INTERGENIC | | | | |
| CaPOPI_568 | Ca2 | 3031259 | ATTTTT/ATTTTTT | TCAATTGCGATGGATAGAGAAA | AATTGGTGTACCGATTGCGT | 59.7 | 647 | INTERGENIC | | | | |
| CaPOPI_569 | Ca2 | 3033081 | TA/TAA | AACGCAACAAGCTCCAAAAG | CGTTAGGCCTTATCCTAGCC | 60.4 | 902 | INTERGENIC | | | | |
| CaPOPI_570 | Ca2 | 3038248 | GAAAAAAA/GAAAAAAAA | ACTTGCACTTTGGAGTGCCT | CCCTTGTGCCTTATGGTTTG | 59.9 | 784 | INTERGENIC | | | | |
| CaPOPI_571 | Ca2 | 3042033 | TTTTGTTTGCTT/TTT | TGCCAAATCATGCGTTTTTA | CTCTGATGCGAAGGTTGACA | 60.1 | 100 | INTRON | Ca_10543 | | | Conserved hypothetical protein CHP00275, flavoprotein HI0933- |
| CaPOPI_572 | Ca2 | 3047152 | CTTT/CTTTT | TGAAATTTAACGGGCCTTCA | GTGGAAAAACTGGACCCTCA | 60.4 | 643 | INTRON | Ca_10543 | | | Conserved hypothetical protein CHP00275, flavoprotein HI0933- |
| CaPOPI_573 | Ca2 | 3047767 | TGG/TGGG | TGAGGGTCCAGTTTTTCCAC | AACCAAGTTGGCCCAAAAA | 59.9 | 266 | DRR | Ca_10543 | | | Conserved hypothetical protein CHP00275, flavoprotein HI0933- |
| CaPOPI_574 | Ca2 | 3090922 | TA/T | GAGACTTTTGGGCATGGAAA | TGGTCAGTCAAATTCTCCGA | 60.1 | 368 | INTRON | Ca_10541 | T | HD-ZIP | Protein kinase, catalytic domain |
| CaPOPI_575 | Ca2 | 3096490 | ATTTT/ATTTTT | TGGTTTTCCTTAAACATGCAA | CTTTATCTTCATTTCGGCGG | 58.2 | 134 | DRR | Ca_10541 | T | HD-ZIP | Protein kinase, catalytic domain |
| CaPOPI_576 | Ca2 | 3098929 | TTTTCTT/TTT | CAAATGCTTCACGAAAGGGT | AGCCACAAAACCACAACACA | 60.1 | 361 | INTERGENIC | | | | |
| CaPOPI_577 | Ca2 | 3099476 | ATTT/ATTTT | GTTGTGGTTTTGTGGCTGTG | CCAATGCTTGACCCATCTTT | 60.0 | 649 | INTERGENIC | | | | |
| CaPOPI_578 | Ca2 | 3104148 | TAAAAAAA/TAAAAAAAA | GGAAACGCCGAGACATACAT | GCACAACCTTTTTGGCGTAT | 60.0 | 360 | DRR | Ca_10539 | P | WOX | Porin, eukaryotic type |
| CaPOPI_579 | Ca2 | 3113326 | AACTTATACTT/AACTTATACTTATACTT | ATTTGTTCTGGCCCACCTTT | AGCTTGATGCTGCAATTCCT | 60.7 | 535 | INTRON | Ca_10538 | | | Auxin efflux carrier |
| CaPOPI_580 | Ca2 | 3118907 | TAAAAA/TAAAAAAA | TGCACCAGAATGCCTTGTTA | TGGCCTTCACATTTCAAACA | 60.3 | 752 | INTRON | Ca_10537 | | AP2 | Pectate lyase/Amb allergen |

| INDEL marker IDs | Chromosomes/ unanchored scaffolds | Physical positions (bp) | InDels (*Kabuli* reference genome-CDC Frontier/PI) | Forward primers (5'-3') | Reverse primers (5'-3') | Anneling temperature (0C) | Expected amplified product size (bp) | Structural annotation | | Functional annotation | | |
|---|---|---|---|---|---|---|---|---|---|---|---|---|
| | | | | | | | | Sequence components of *kabuli* genome | *Kabuli* gene accession IDs | NCBI-KOG | TFs | NCBI-nr database |
| CaPOPI_581 | Ca2 | 3126083 | CTATATATAT/CTATATATATAT | AACGATTCCATGACTAATTTGAA | TTGAACGGTTTAAATGTGGCT | 57.7 | 518 | INTERGENIC | | | | |
| CaPOPI_582 | Ca2 | 3154111 | A/AT | TTCGTCCTGTCATCCAAAAT | TTTGACGGTGAAAAAGTGATATG | 58.0 | 975 | INTERGENIC | | | | |
| CaPOPI_583 | Ca2 | 3156648 | GTCT/GTCTCT | GGTCAGAACAAAGGGTTGGA | GTTTGTTCCCTTCTAGGCCC | 59.9 | 488 | INTERGENIC | | | | |
| CaPOPI_584 | Ca2 | 3158875 | GTATTATTATTATTATTAT/GTATTATTATTATTATTATTAT | CGCAAAAGTTGCATGAAGAA | TCTAAGAAATTTGCCCCAATG | 60.0 | 295 | INTERGENIC | | | | |
| CaPOPI_585 | Ca2 | 3159298 | ATTTTTTTT/ATTTTTTTTTT | CGCAAAAGTTGCATGAAGAA | TGCAGGACGGTCTAACAATG | 60.0 | 600 | INTERGENIC | | | | |
| CaPOPI_586 | Ca2 | 3162145 | CAAAAAA/CAAAAA | CTCTTGCTTCTCTCTCCCCC | GGCCTTCTGTGAGAAGCATT | 60.5 | 788 | INTRON | Ca_10531 | S | | Cysteamine dioxygenase |
| CaPOPI_587 | Ca2 | 3166543 | T/TA | CATGTTTTCAGCCCAACAAA | TTCCGCACATCAAACACAAT | 59.6 | 849 | INTERGENIC | | | | |
| CaPOPI_588 | Ca2 | 3169671 | ATTTTTTT/ATTTTTT | TTGTCGGTTTAAACTTGTGTGC | TTTTCCAAGGGATTTCAACTCT | 60.1 | 892 | INTERGENIC | | | | |
| CaPOPI_589 | Ca2 | 3184404 | AAAA/AAAATAAA | TGCATATCCTTGTGTTCCCA | CTTCCATGGGCTGTGAGAAT | 59.9 | 486 | INTRON | Ca_10528 | T | HD-ZIP | Protein kinase, catalytic domain |
| CaPOPI_590 | Ca2 | 3185239 | AAGA/AA | GATGTTGGAGGGCACCTAGA | GTGAAGGAGGGTTTGGAACA | 60.1 | 661 | INTRON | Ca_10528 | T | HD-ZIP | Protein kinase, catalytic domain |
| CaPOPI_591 | Ca2 | 3199849 | TT/TTATTATTAGT | GAGCATGTGTTGGTGAATGG | TCACCGGAGGTTAGATCTCG | 60.0 | 309 | INTERGENIC | | | | |
| CaPOPI_592 | Ca2 | 3200285 | TCA/T | CGAGATCTAACCTCCGGTGA | TTGATGCATGCTGCTACAAAT | 60.2 | 811 | DRR | Ca_10527 | K | HD-ZIP | Helix-turn-helix motif, lambda-like repressor |
| CaPOPI_593 | Ca2 | 3200442 | CAT/CATAT | GGAGTTGAATTAGCCTCTTTATCC | TTGATGCATGCTGCTACAAAT | 58.8 | 503 | DRR | Ca_10527 | K | HD-ZIP | Helix-turn-helix motif, lambda-like repressor |
| CaPOPI_594 | Ca2 | 3200504 | AA/AAAAAGATA | CATTTCCCGATTTTAGATTATGC | AATTGAAGGAAACATTTGCCA | 58.9 | 511 | DRR | Ca_10527 | K | HD-ZIP | Helix-turn-helix motif, lambda-like repressor |
| CaPOPI_595 | Ca2 | 3200632 | CAATT/CAATTAATT | AAGGAGTATCAACCAAAACAGAGG | AATTGAAGGAAACATTTGCCA | 59.9 | 423 | DRR | Ca_10527 | K | HD-ZIP | Helix-turn-helix motif, lambda-like repressor |

| INDEL marker IDs | Chromosomes/ unanchored scaffolds | Physical positions (bp) | InDels (*Kabuli* reference genome-CDC Frontier/PI) | Forward primers (5'-3') | Reverse primers (5'-3') | Anneling temperature (0C) | Expected amplified product size (bp) | Structural annotation | | Functional annotation | | |
|---|---|---|---|---|---|---|---|---|---|---|---|---|
| | | | | | | | | Sequence components of *kabuli* genome | *Kabuli* gene accession IDs | NCBI-KOG | TFs | NCBI-nr database |
| CaPOPI_596 | Ca2 | 3203039 | AT/A | TGGATTTCTGACACTGCCAC | ATGCTCTTCCCTGTCTCCAA | 59.7 | 348 | INTRON | Ca_10527 | K | HD-ZIP | Helix-turn-helix motif, lambda-like repressor |
| CaPOPI_597 | Ca2 | 3205849 | TC/T | ATTGTGGAGACATAACCGCC | ACATCGAGACGTACGGGAAA | 59.8 | 284 | INTERGENIC | | | | |
| CaPOPI_598 | Ca2 | 3630204 | CATATATATATATATATATATATAT/CATATATATATATATATATATAT | TGAAACAAGCATATGCGTCAA | TTTGCCTTCAATAATTGCACA | 60.3 | 158 | INTRON | Ca_10502 | P | ERF | SPX, N-terminal |
| CaPOPI_599 | Ca2 | 3723233 | TATTATCAATTATCAATTA/TATTATCAATTA | ATTTCGTTGCGTTTTGGACT | TGGTCAATTTTTGTTTGCCA | 59.6 | 234 | INTERGENIC | | | | |
| CaPOPI_600 | Ca2 | 3723695 | TAAAAAA/TAAAAAAA | TGGCAAACAAAAATTGACCA | TTCCTCAAATTGCAACACCA | 59.9 | 752 | INTERGENIC | | | | |
| CaPOPI_601 | Ca2 | 3733191 | TTAGAGACTAA/T | ACCTGCAACATGTGCGATAA | TGCATGAGATTGGAAATGGA | 60.1 | 349 | DRR | Ca_10489 | G | C3H | Alkaline phosphatase D-related |
| CaPOPI_602 | Ca2 | 3741294 | CT/CTTT | GGTGGAGAGGGATGATGAGA | GCAGAGCACCAAACACAAGA | 60.0 | 605 | DRR | Ca_10487 | | | Plastocyanin-like |
| CaPOPI_603 | Ca2 | 3743015 | A/AATACAAATGGTG | CACCACTGGATTTAGCCGTT | TCCTTTTCGCTGATGAAACA | 60.0 | 722 | INTRON | Ca_10487 | | | Plastocyanin-like |
| CaPOPI_604 | Ca2 | 3743829 | CTTTTTT/CTTTTT | TTGGCCAATACTCCAACCAT | CAGCAAGTCCCACGAAAGAT | 60.2 | 311 | INTRON | Ca_10487 | | | Plastocyanin-like |
| CaPOPI_605 | Ca2 | 3745143 | TAAAA/TAAA | GAAGCACTGGACCTCCCATA | TGACTTGGAATAGATTGGACCAT | 60.1 | 630 | INTERGENIC | | | | |
| CaPOPI_606 | Ca2 | 3746503 | TTA/TTAAACATA | TCCACATTGCACCAACACTT | TTGCTCCTCTGGTCCTCACT | 60.0 | 925 | INTERGENIC | | | | |
| CaPOPI_607 | Ca2 | 3754940 | GA/GATA | TCCTTCAACTGTGGAACCAA | GCTTCTTGTGTGGGCTTAGG | 59.1 | 751 | DRR | Ca_10486 | DL | | Hus1-like protein |
| CaPOPI_608 | Ca2 | 3761698 | TAA/TAAA | ACCCTCCATTGCTGTTTTTG | CTCGGAACATGCATAGAATGA | 60.0 | 262 | INTERGENIC | | | | |
| CaPOPI_609 | Ca2 | 3766913 | ATTTTTT/ATTTTTTT | AGAACCATGTGCTTCATCCC | CAACACAATTGAATGGCAGG | 59.9 | 478 | INTERGENIC | | | | |
| CaPOPI_610 | Ca2 | 3771310 | TTATATATATATATATA/TTATATATATATA | GCTCGACGGAATAGCATTTT | TGGCCAAGCATGATCTAACA | 59.3 | 612 | DRR | Ca_10483 | E | | Chorismate mutase, AroQ class, eukaryotic type |

| **INDEL marker IDs** | **Chromosomes/ unanchored scaffolds** | **Physical positions (bp)** | **InDels (*Kabuli* reference genome-CDC Frontier/PI)** | **Forward primers (5'-3')** | **Reverse primers (5'-3')** | **Anneling temperature (0C)** | **Expected amplified product size (bp)** | **Structural annotation** | | **Functional annotation** | | |
|---|---|---|---|---|---|---|---|---|---|---|---|---|
| | | | | | | | | **Sequence components of *kabuli* genome** | ***Kabuli* gene accession IDs** | **NCBI-KOG** | **TFs** | **NCBI-nr database** |
| CaPOPI_611 | Ca2 | 3771545 | AT/A | GCTCGACGGAATAGCATTTT | TGGCCAAGCATGATCTAACA | 59.3 | 612 | DRR | Ca_10483 | E | | Chorismate mutase, AroQ class, eukaryotic type |
| CaPOPI_612 | Ca2 | 3777372 | AATATA/AATATATA | AATTTTTGGGGGAAAAATGC | CTCTGATAGACCCTCCCACCT | 60.0 | 646 | INTERGENIC | | | | |
| CaPOPI_613 | Ca2 | 3782950 | TAAAAAAA/TAAAAAAAA | TGCCATGGGAGCTCTATGAT | CGCACTTGGAGTTGATGTGT | 60.6 | 554 | INTERGENIC | | | | |
| CaPOPI_614 | Ca2 | 3800163 | TA/TAA | TTCCTCATTTTTCTCTTGGTTTG | CCACACTGCGCTTCAGATAA | 59.6 | 377 | INTERGENIC | | | | |
| CaPOPI_615 | Ca2 | 3805996 | TAAAAA/TAAAAAA | GAACAAGAGCAGGCGTTAGG | CCACCCCTTGAATCAAAAGA | 60.0 | 567 | INTRON | Ca_10479 | | | |
| CaPOPI_616 | Ca2 | 3812277 | TATTAATTAATTAATTA/TATTAATTAATTA | TGTTCTGCAAAATCGCAAAG | TGGTGACATGACTAATTTTGGA | 60.0 | 414 | INTERGENIC | | | | |
| CaPOPI_617 | Ca2 | 3813617 | CAA/CAAA | TTGAAGCATACCTTGAGTGGTG | TTAATCTGGTTTTGGTGGGC | 60.2 | 570 | INTERGENIC | | | | |
| CaPOPI_618 | Ca2 | 3901844 | AC/ACTTCACGAGC | GTCGCTATCTCCCCAGTGAA | CATTCTGTGCCGCTATCTGA | 60.2 | 248 | DRR | Ca_10466 | GT | | UDP-glucose/GDP-mannose dehydrogenase, N-terminal |
| CaPOPI_619 | Ca2 | 4013903 | ATTTTTTT/ATTTTTTTT | CGGTCACAAGCATCTCATGT | TGATGCTTAAAAACTATGGGACAA | 59.7 | 669 | INTERGENIC | | | | |
| CaPOPI_620 | Ca2 | 4014195 | AATATATATATATATATATAT/AATATATATATATATATATATATATATAT | TGGAAATTATGAGTCCCTTGA | TGAATACCAACGACACACCG | 57.1 | 642 | INTERGENIC | | | | |
| CaPOPI_621 | Ca2 | 4060085 | AATATATATATATATATATATATATATATA/AATATATATATATATATATATATATA | GACCTTGCATGGAGTGTTGA | GGCATAGCCATGAAGAACTTG | 59.7 | 690 | INTERGENIC | | | | |
| CaPOPI_622 | Ca2 | 4116151 | GTTTTTTTTTT/GTTTTTTTTTTT | TTTTTATGACGCAGATGCCC | TGGATCCTTACATGGTCGGT | 61.0 | 665 | INTERGENIC | | | | |
| CaPOPI_623 | Ca2 | 4123258 | AATATATATATATA/AATATATATATATA | GCCCAATCATTTTCAAGGAA | CGTTCAACTCCCACAGGATT | 59.9 | 667 | INTERGENIC | | | | |
| CaPOPI_624 | Ca2 | 4204531 | GTATATATATATATATATATATATATA/GTATATATATATATATATATATA | GGGTTTGAACCGTCCCTACT | CTTTCTCCATCTCCTTCCCC | 60.2 | 633 | INTRON | Ca_21114 | | | Xklp2 targeting protein |
| CaPOPI_625 | Ca2 | 4285697 | TAAAA/TAAA | CCAGTCCCGAAATTAAACGA | ACTTGCGATAATCGATTGGG | 59.9 | 613 | INTERGENIC | | | | |

| INDEL marker IDs | Chromosomes/ unanchored scaffolds | Physical positions (bp) | InDels (*Kabuli* reference genome-CDC Frontier/PI) | Forward primers (5'-3') | Reverse primers (5'-3') | Anneling temperature (0C) | Expected amplified product size (bp) | Structural annotation | | Functional annotation | | |
|---|---|---|---|---|---|---|---|---|---|---|---|---|
| | | | | | | | | Sequence components of *kabuli* genome | *Kabuli* gene accession IDs | NCBI-KOG | TFs | NCBI-nr database |
| CaPOPI_626 | Ca2 | 4527479 | GAAGACAAAAAAAACATAAGACAAAAAAAACAT/GAAGACAAAAAAAACATAAGACAAAAAAAACATAAGACAAAAAAAACAT | GCAATTTCACCTCTTCTGCC | TGACGAAGCCCACAATAACA | 59.8 | 523 | INTERGENIC | | | | |
| CaPOPI_627 | Ca2 | 4700407 | ATTATTTATTTATTTATTTATTTATT/ATTATTTATTTATTTATTTATT | TCTCACGGTTGTGAGGACTG | CCTACCTCTGGAGTGTCCGT | 59.9 | 926 | INTERGENIC | | | | |
| CaPOPI_628 | Ca2 | 4812881 | ATTTTTTT/ATTTTTTTTT | AACCAGTTCCGTTCCAAGTG | TCCCCCAAACAGAATTTGAG | 60.0 | 731 | DRR | Ca_21090 | | MYB | Transferase |
| CaPOPI_629 | Ca2 | 4892794 | CATATATATATATATATATATATATATATATATAT/CATATATATATATATATATATATATATATATATATATATATATATATAT | GCGGACATAAGGAAGATGTGA | AGGTCTGCGAAGATGGAAAA | 60.1 | 606 | INTERGENIC | | | | |
| CaPOPI_630 | Ca2 | 4894086 | TA/TAGACAACTCAA | TTGAACTACAACCAGCGCAC | TTTTGTTCAATTTCTTGCTCCA | 59.9 | 493 | INTERGENIC | | | | |
| CaPOPI_631 | Ca2 | 4895677 | AA/AATATGA | CCAGGATAAAACAGCCCAGA | TGGATGCTTTCCCAACTTTC | 60.1 | 467 | INTERGENIC | | | | |
| CaPOPI_632 | Ca2 | 5096452 | AATATATATATATATATATATATAT/AATATATATATATATATATATATATAT | GTTTTGTGGTGCAGTTTGGA | TTTGAAAGAGTCCGAATTCAAATA | 59.6 | 570 | INTERGENIC | | | | |
| CaPOPI_633 | Ca2 | 5135372 | AATATATATATATATATATATATATATATATATATATATATATATAT/AATATATATATATATATATATATATATATATATATATATAT | TGATTTATTTCCACGCCTACG | TTGTTTCTCCCCAGTTTTGG | 60.0 | 952 | INTERGENIC | | | | |
| CaPOPI_634 | Ca2 | 5163788 | CAAAAAAAAA/CAAAAAAAAAA | TGCGAGAAAGTGAATTCCAG | GGAATGATGATGGGGTTTTG | 59.0 | 658 | DRR | Ca_14696 | | | |
| CaPOPI_635 | Ca2 | 5286155 | AATATATATATATATATATATATATATATATA/AATATATATATATATATATATATATATATATATATA | TTCATGCCTCCCATTTCTTC | CGCTCTGTCAACATGTCAGC | 60.0 | 752 | INTRON | Ca_14679 | T | | Protein phosphatase 2A, regulatory B subunit, B56 |
| CaPOPI_636 | Ca2 | 5566169 | GAAAAAAAAAAA/GAAAAAAAAAAAA | TGGGAGTATCCTTCACTGCC | TTCGGATTTTCCTTCCCTTT | 60.1 | 836 | INTERGENIC | | | | |
| CaPOPI_637 | Ca2 | 5626731 | ATTTTTTTTTTT/ATTTTTTTTTT | GAACCCAATATAGCCGCAAA | TTTGCTGCAACAACTCCAAG | 59.9 | 782 | INTRON | Ca_14650 | I | C3H | Alpha/beta hydrolase fold-1 |
| CaPOPI_638 | Ca2 | 5674299 | TCTTTACTTTACTT/TCTTTACTTTACTTTACTT | ATTAGTCCACTGTGCTGGGG | GCTTCCAAATTGCTTGTTCC | 60.0 | 815 | INTRON | Ca_14646 | O | FAR1 | ATPase, AAA+ type, core |
| CaPOPI_639 | Ca2 | 6317566 | GTATATATATATATATATATATATATATATAT/GTATATATATATATATATATATATATATAT | CACCAAATGATGACGACGAC | ATTCACGTCGTTCCTGTTCC | 60.0 | 324 | INTERGENIC | | | | |
| CaPOPI_640 | Ca2 | 6505178 | TA/TAA | CGTAATAACGGCGAGGAAAA | TGAGCAGGGGGAAGAGTAGA | 60.1 | 548 | INTERGENIC | | | | |

| INDEL marker IDs | Chromosomes/ unanchored scaffolds | Physical positions (bp) | InDels (*Kabuli* reference genome-CDC Frontier/PI) | Forward primers (5'-3') | Reverse primers (5'-3') | Anneling temperature (0C) | Expected amplified product size (bp) | Structural annotation | | Functional annotation | | |
|---|---|---|---|---|---|---|---|---|---|---|---|---|
| | | | | | | | | Sequence components of *kabuli* genome | *Kabuli* gene accession IDs | NCBI-KOG | TFs | NCBI-nr database |
| CaPOPI_641 | Ca2 | 6522206 | AT/ATT | GGAAGTGAAGGTCACGGTGT | TGAGCACGAATAATTGCAGC | 60.0 | 593 | DRR | Ca_20947 | | | |
| CaPOPI_642 | Ca2 | 6640316 | TAAAAAA/TAAAAAAA | ATGGCCACGAATCTGAAGAG | ACGGTTGTTTTGGCATTGAT | 60.2 | 890 | INTERGENIC | | | | |
| CaPOPI_643 | Ca2 | 6640452 | GAAAA/GAAAAA | AAAAAGGTCCTAGGGTGGGA | ACGGTTGTTTTGGCATTGAT | 59.8 | 473 | INTERGENIC | | | | |
| CaPOPI_644 | Ca2 | 6641737 | CATATAT/CATATATATAT | GTATTCAGGGGCGAAGGAAT | AATGCAAACGACAAAGGAGG | 60.3 | 500 | INTERGENIC | | | | |
| CaPOPI_645 | Ca2 | 6641916 | TG/T | GTTCTATGGGGACAGGGTCA | TATCAACTACCAGAGCGGGG | 59.8 | 476 | INTERGENIC | | | | |
| CaPOPI_646 | Ca2 | 6642515 | TTATATATATATATATA/TTATATATATATATATATA | CCCCGCTCTGGTAGTTGATA | ACCCACTTTGAGAAACGTGC | 60.1 | 479 | INTERGENIC | | | | |
| CaPOPI_647 | Ca2 | 6643765 | AT/ATT | ATGGAACAAGCAGCGAAGTT | GTTGATACGAGGGGGTTGAA | 59.9 | 537 | INTERGENIC | | | | |
| CaPOPI_648 | Ca2 | 6643843 | CTTTTTTTTTTT/CTTTTTTTT | ATGGAACAAGCAGCGAAGTT | GTTGATACGAGGGGGTTGAA | 59.9 | 537 | INTERGENIC | | | | |
| CaPOPI_649 | Ca2 | 6648530 | TAAAAAA/TAAAAAAAA | TTATCGATCGTCGGTGTCAA | TTCCATATGCACCTCCCATT | 60.1 | 612 | INTERGENIC | | | | |
| CaPOPI_650 | Ca2 | 6648980 | ATTTTTTTTTT/ATTTTTTTTTTT | AGCCTAATGGGAGGTGCATA | TATGAGGGGGTTGAATTGGA | 59.6 | 563 | INTERGENIC | | | | |
| CaPOPI_651 | Ca2 | 6815005 | ATTATTTATTTATTTATTTATTTA/ATTATTTATTTATTTATTTA | GAAATTTTAGGCGCAACAGC | TCCAATCCCATTGTCCACTT | 59.9 | 703 | INTERGENIC | | | | |
| CaPOPI_652 | Ca2 | 6831436 | AC/A | CCTGTGACATGTTGCCTTTG | AAATGGGCTGAGTTTGAACA | 60.2 | 360 | INTERGENIC | | | | |
| CaPOPI_653 | Ca2 | 6867609 | CAAAAAAAAAAA/CAAAAAAAAAA | TAACCACATTGCAAGCCAAA | CAATCAAAATGCAACAGACACA | 60.1 | 451 | INTERGENIC | | | | |
| CaPOPI_654 | Ca2 | 7144237 | A/AG | GGACCGGAATCATAATGCAA | TTTTTCCGTAAAAGTCCCCC | 60.7 | 743 | INTERGENIC | | | | |
| CaPOPI_655 | Ca2 | 7171869 | TTAT/TT | CGGCCATTAAATTTCTGACA | TTGCATTATGTTTCTGGCAAA | 58.6 | 320 | INTERGENIC | | | | |

| **INDEL marker IDs** | **Chromosomes/ unanchored scaffolds** | **Physical positions (bp)** | **InDels (*Kabuli* reference genome-CDC Frontier/PI)** | **Forward primers (5'-3')** | **Reverse primers (5'-3')** | **Anneling temperature (0C)** | **Expected amplified product size (bp)** | **Structural annotation** | | **Functional annotation** | | |
|---|---|---|---|---|---|---|---|---|---|---|---|---|
| | | | | | | | | **Sequence components of *kabuli* genome** | ***Kabuli* gene accession IDs** | **NCBI-KOG** | **TFs** | **NCBI-nr database** |
| CaPOPI_656 | Ca2 | 7171912 | TATTTAATTTAATTTAATT/TATTTAATTTAATT | CGGCCATTAAATTTCTGACA | TCATTTCATAACGAGTGACCCA | 58.6 | 584 | INTERGENIC | | | | |
| CaPOPI_657 | Ca2 | 7172121 | CTATATATATATAT/CTATATATATAT | TTTGCCAGAAACATAATGCAA | TCATTTCATAACGAGTGACCCA | 59.2 | 285 | INTERGENIC | | | | |
| CaPOPI_658 | Ca2 | 7172172 | ATTTTTTTTT/ATTTTTTTTTT | TTTGCCAGAAACATAATGCAA | TTTTGGAAAAATGAAGGACCA | 59.2 | 774 | INTERGENIC | | | | |
| CaPOPI_659 | Ca2 | 7173102 | GAAAAAA/GAAAAA | CTCAATCCAAACACACGCTT | AATGACCACCTCTTGTTGCC | 58.8 | 787 | INTERGENIC | | | | |
| CaPOPI_660 | Ca2 | 7276294 | CAAAA/CAAAAA | TCCACCACAATCGTTAAGCA | AGCGGAGATTTTACCGGATT | 60.1 | 676 | INTERGENIC | | | | |
| CaPOPI_661 | Ca2 | 7302005 | TTATATATATATATATATATA/TTATATATATATATATATATA | TGTAGCAAAACGTGCAGACC | GGATATTTTGCTCTCTCGCAA | 59.9 | 159 | INTERGENIC | | | | |
| CaPOPI_662 | Ca2 | 7535934 | TAAAAAAAAAA/TAAAAAAAAAAA | AACCGGACATTTTGTTGGAG | TGCACAAATTGGTGGAAAAA | 59.8 | 626 | INTERGENIC | | | | |
| CaPOPI_663 | Ca2 | 7540916 | TATAGTAATAAATAGTAATAAAT/TATAGTAATAAAT | AATATTCGAACCTCCCACCC | TTAATCGTCCAACTCCCACC | 60.0 | 890 | INTERGENIC | | | | |
| CaPOPI_664 | Ca2 | 7555681 | GTTTTTTTTTTT/GTTTTTTTTTT | AATTTTGCGTTCATCGGAGA | AATCACACACGACACCGAGA | 60.6 | 178 | INTERGENIC | | | | |
| CaPOPI_665 | Ca2 | 7617731 | CTAAAATAAAATAAAA/CTAAAATAAAATAAAATAAAATAAAA | CCAAGATCTCGTAGTGCAACA | TGGGTTTAGTCCGTTTTTCTTC | 58.9 | 468 | INTERGENIC | | | | |
| CaPOPI_666 | Ca2 | 7657147 | CTTTTT/CTTTTTT | TTATGTGGGGGATTTGGATG | TTCCACGTCCTTTTCATTCC | 60.4 | 705 | INTERGENIC | | | | |
| CaPOPI_667 | Ca2 | 7675590 | TAAAAAAA/TAAAAAAAA | ACTCCGGATTTGGAGAAAGG | AAAATGAGTTTACATTCACGGTACA | 60.4 | 882 | INTERGENIC | | | | |
| CaPOPI_668 | Ca2 | 7681881 | TAAAAA/TAAAA | GAAAAAGAGCCACGGTGGTA | GCGAGAAAAGATTTGTTGAGAA | 60.1 | 383 | INTRON | Ca_18121 | R | ERF | Multi antimicrobial extrusion protein |
| CaPOPI_669 | Ca2 | 7682063 | CTTTTTTT/CTTTTTT | GAAAAAGAGCCACGGTGGTA | TTTCCAGCTCCAAGCTCATT | 60.1 | 643 | INTRON | Ca_18121 | R | ERF | Multi antimicrobial extrusion protein |
| CaPOPI_670 | Ca2 | 7695766 | TG/T | GGCGGGACAATTATTTTGAA | CCATATAGGAAGTACGAGTACGTGA | 59.8 | 401 | INTERGENIC | | | | |

| INDEL marker IDs | Chromosomes/ unanchored scaffolds | Physical positions (bp) | InDels (*Kabuli* reference genome-CDC Frontier/PI) | Forward primers (5'-3') | Reverse primers (5'-3') | Anneling temperature (0C) | Expected amplified product size (bp) | Structural annotation | | Functional annotation | | |
|---|---|---|---|---|---|---|---|---|---|---|---|---|
| | | | | | | | | Sequence components of *kabuli* genome | *Kabuli* gene accession IDs | NCBI-KOG | TFs | NCBI-nr database |
| CaPOPI_671 | Ca2 | 7699107 | TAAAAAAA/TAAAAAAAA | CCAAAAATTGTGGAGATTTGG | GCTGATGATGCCTCCATTTT | 59.3 | 846 | INTERGENIC | | | | |
| CaPOPI_672 | Ca2 | 7699126 | TAAAAAAAA/TAAAAAAA | CCAAAAATTGTGGAGATTTGG | GCTGATGATGCCTCCATTTT | 59.3 | 846 | INTERGENIC | | | | |
| CaPOPI_673 | Ca2 | 7703919 | AAATAAAATAATAAAATA/AAATAAAATA | TCTCTTTTATTGGGGCATGG | AGATGTAAGGGGTGTGGAAAA | 59.9 | 910 | DRR | Ca_18123 | R | ERF | Multi antimicrobial extrusion protein |
| CaPOPI_674 | Ca2 | 7706488 | AAAATAATTAAATAATTAAAT/AAAATAATTAAATAATTAAATAATTAAATAATTAAAT | GCCTATTTTGCTTTTCCTGC | TCATCCACTTTCTTGCCTGT | 59.0 | 689 | INTERGENIC | | | | |
| CaPOPI_675 | Ca2 | 7709881 | TAAAAAAAA/TAAAAAAA | TGACACATGCAAAACACCCT | AGAAGCACGAAGATATGCACTT | 60.0 | 619 | INTERGENIC | | | | |
| CaPOPI_676 | Ca2 | 7716515 | TA/TACCTGA | ATGGGCTTTAAATGTGGCAG | ACCCCGTTTGGCTTCTATTT | 60.0 | 105 | DRR | Ca_18124 | | | |
| CaPOPI_677 | Ca2 | 7718496 | C/CT | CCTGCCACCACTCTTAGCTT | TGGGATGCTACCTCCTATGG | 59.5 | 511 | INTERGENIC | | | | |
| CaPOPI_678 | Ca2 | 7718955 | TGAAA/T | GAGCTTCATTGAAATCGGGA | TCATATAGGAGGTCGCACCC | 60.2 | 821 | INTERGENIC | | | | |
| CaPOPI_679 | Ca2 | 7720508 | TAAA/TAA | TAATCCGCCTAAATGGATGC | CTCCCATAGGATGAAGGGGT | 59.9 | 530 | INTERGENIC | | | | |
| CaPOPI_680 | Ca2 | 7720908 | AAT/A | TTCACCCCTTCATCCTATGG | TCCAAATGTTTGTGATTGTGC | 59.7 | 490 | INTERGENIC | | | | |
| CaPOPI_681 | Ca2 | 7724864 | TA/TAAAGAGATTGAGA | ACCAGAGACACTGCAGCAAA | AACAAGATTCTCGAATGCTCC | 59.6 | 464 | INTERGENIC | | | | |
| CaPOPI_682 | Ca2 | 7725038 | ATTTT/ATTT | ACCAGAGACACTGCAGCAAA | AGCCATTTGAGACTGTGCAA | 59.6 | 789 | INTERGENIC | | | | |
| CaPOPI_683 | Ca2 | 7731377 | GAAAAA/GAA | GTGGTTGTGATGTTGAACGC | CTCCCGCTAACTCCTCTTCC | 60.0 | 697 | INTERGENIC | | | | |
| CaPOPI_684 | Ca2 | 7743405 | CG/C | ATCCATCATGTTCATGCCCT | ATCTCCACCCGTTCTCTTGA | 60.2 | 579 | INTERGENIC | | | | |
| CaPOPI_685 | Ca2 | 7743661 | AATC/AATCGATC | TCAAGAGAACGGGTGGAGAT | GATTCAAAATGTGAGCGCAA | 59.7 | 530 | INTERGENIC | | | | |

| INDEL marker IDs | Chromosomes/ unanchored scaffolds | Physical positions (bp) | InDels (*Kabuli* reference genome-CDC Frontier/PI) | Forward primers (5'-3') | Reverse primers (5'-3') | Anneling temperature (0C) | Expected amplified product size (bp) | Structural annotation | | Functional annotation | | |
|---|---|---|---|---|---|---|---|---|---|---|---|---|
| | | | | | | | | Sequence components of *kabuli* genome | *Kabuli* gene accession IDs | NCBI-KOG | TFs | NCBI-nr database |
| CaPOPI_686 | Ca2 | 7747929 | TAAAAAAAA/TAAAAAAA | TGGTGAAATGCTGCAGAAAA | CTCAACTTGTCGATGCCTCA | 60.4 | 147 | INTERGENIC | | | | |
| CaPOPI_687 | Ca2 | 7753358 | C/CAAT | CGGAGGTTTGGTGTAAACTGA | AGCTTGCCATTGCAGCTACT | 60.0 | 447 | INTRON | Ca_18125 | | MYB_related | NPH3 |
| CaPOPI_688 | Ca2 | 7771490 | ATTTTTTTTT/ATTTTTTTTTTT | ACCATTTATTATTTTCGTGTTAATGA | GGTACCATGGATGATATTTAGCC | 57.3 | 188 | INTERGENIC | | | | |
| CaPOPI_689 | Ca2 | 7772671 | AAAAAAATAAAAA/AAAAAA | CAATTGGTGGCCGTTATTTT | TGCCCTAAGGGGAATAAGCTA | 59.7 | 750 | INTERGENIC | | | | |
| CaPOPI_690 | Ca2 | 7773617 | TTAAATAAAAT/TTAAATAAAATAAATAAAAT | GCTGAGCCCAAACAAGCTAA | CCACTGTGGAAAGGTAAATGC | 60.5 | 845 | INTERGENIC | | | | |
| CaPOPI_691 | Ca2 | 7778320 | ATT/ATTT | AGACCTCTTACTGTGCGCGT | TGCACTTTTGGTCCCTCAAT | 60.1 | 321 | INTERGENIC | | | | |
| CaPOPI_692 | Ca2 | 7780730 | ATTTTTTTTTTT/ATTTTTTTTTT | TTGTCTCCATCTCCAGCCTT | TGTTGTTTGAGTTGGGACACA | 59.8 | 995 | INTERGENIC | | | | |
| CaPOPI_693 | Ca2 | 7782164 | AG/A | TCATTTCAATCCCCCATCAT | TTTGATGCTATGCGAAGGAG | 60.0 | 388 | INTERGENIC | | | | |
| CaPOPI_694 | Ca2 | 7786133 | AC/A | GCTCCTTTGGTGTATCGCTC | CATTCCATTCCATGTTGCAC | 59.8 | 369 | INTERGENIC | | | | |
| CaPOPI_695 | Ca2 | 7788989 | TC/T | GCTCTTTTACGTTTGCCAGC | TGTTTTAGATTGTCTTCTATTTTACGG | 60.0 | 566 | INTERGENIC | | | | |
| CaPOPI_696 | Ca2 | 7790064 | CA/CATGTTA | AGAGTTGGCCAATTGGTTTG | GAATGTCAAGCATTGTGAAGGA | 60.0 | 467 | INTERGENIC | | | | |
| CaPOPI_697 | Ca2 | 7815016 | TAATAACAAATAACAAA/TAATAACAAATAACAAATAACAAA | TTTTCACCAACACCGTCAAA | AAATAATCGTGGCCTTGCAC | 60.0 | 901 | INTERGENIC | | | | |
| CaPOPI_698 | Ca2 | 7824116 | T/TG | TGTCTCGTGGATCTGGTTTG | TGACTTTGTGTCGGAATTTG | 59.7 | 258 | INTERGENIC | | | | |
| CaPOPI_699 | Ca2 | 7881274 | GTAT/GTATCGTTAT | GCCACTCCAACACCAATTTC | TGGAGACCACATGGAGATGA | 60.4 | 423 | INTERGENIC | | | | |
| CaPOPI_700 | Ca2 | 7881305 | TTAT/TT | GCCACTCCAACACCAATTTC | TGGAGACCACATGGAGATGA | 60.4 | 423 | DRR | Ca_18136 | | | |

| INDEL marker IDs | Chromosomes/ unanchored scaffolds | Physical positions (bp) | InDels (*Kabuli* reference genome-CDC Frontier/PI) | Forward primers (5'-3') | Reverse primers (5'-3') | Anneling temperature (0C) | Expected amplified product size (bp) | Structural annotation | | Functional annotation | | |
|---|---|---|---|---|---|---|---|---|---|---|---|---|
| | | | | | | | | Sequence components of *kabuli* genome | *Kabuli* gene accession IDs | NCBI-KOG | TFs | NCBI-nr database |
| CaPOPI_701 | Ca2 | 7881740 | ATATTCC/ATATTCCTATTCC | TCATCTCCATGTGGTCTCCA | TGCAAATGACCACCCACTTA | 60.0 | 642 | DRR | Ca_18136 | | | |
| CaPOPI_702 | Ca2 | 7887346 | AATAT/AATATAT | GTGCAGAGGAGCAAAAGGAC | TGGAATTTGAGACATGTCAACC | 60.0 | 889 | DRR | Ca_18137 | E | | 1-aminocyclopropane-1-carboxylate synthase |
| CaPOPI_703 | Ca2 | 7887518 | ATTTTTTT/ATTTTTTTT | AAGCAAAATACATCTCGAAGTTCT | TCCCAAGAAAAGCTTCAACC | 57.8 | 550 | DRR | Ca_18137 | E | | 1-aminocyclopropane-1-carboxylate synthase |
| CaPOPI_704 | Ca2 | 7887647 | CAACATA/C | AAGCAAAATACATCTCGAAGTTCT | TCCCAAGAAAAGCTTCAACC | 57.8 | 550 | DRR | Ca_18137 | E | | 1-aminocyclopropane-1-carboxylate synthase |
| CaPOPI_705 | Ca2 | 7916006 | A/AC | CTCATTTGCATCCGATTGGT | TATCGAAAGTTTCCGCGTTT | 60.9 | 525 | INTERGENIC | | | | |
| CaPOPI_706 | Ca2 | 7922625 | TAA/TA | GAAGCATCACTAGGTGGGGA | CAACCAAAACCATTTCAAAACT | 60.1 | 369 | DRR | Ca_18141 | | bHLH | Protein kinase, catalytic domain |
| CaPOPI_707 | Ca2 | 7933926 | ATT/AT | TTCCTTAAGCGTGCTTGTCA | CCAAAATAACCCACAATCGG | 59.6 | 539 | INTERGENIC | | | | |
| CaPOPI_708 | Ca2 | 7940136 | ATTTTTTT/ATTTTTTTT | TCTTGACCGTTAGATTGAACCA | CAAAATGGACAAAATTGGAGC | 59.6 | 714 | INTERGENIC | | | | |
| CaPOPI_709 | Ca2 | 8166930 | AAATAATAATAATAATAATAATAATAAT/AAATAATAATAATAATAATAATAATAATAATAAT | CCTAGGGATGCGACCATCTA | TCCTCCTCTTTTGGCACATT | 60.1 | 385 | INTERGENIC | | | | |
| CaPOPI_710 | Ca2 | 8332435 | GTTATTATTATTATTATTATTATTATTATTATTATTATTATT/GTTATTATTATTATTATTATTATTATTATTATTATTATTATT | CTACGGAGGATGGAACTGGA | TTTGGTGATCTGGGGAGAAG | 60.1 | 734 | INTRON | Ca_21468 | TU | | Phosphatidylinositol 3-/4-kinase, catalytic |
| CaPOPI_711 | Ca2 | 8343398 | AATATATATATATAT/AATATATATATATATAT | CCAGTGTTTGACCTCCAAGC | TTTGCAACAACGCGAAATAG | 60.7 | 743 | INTERGENIC | | | | |
| CaPOPI_712 | Ca2 | 8420270 | AACA/AACACA | CCAAGTTTGAAATGCCGATT | GCTGCATGATCTTATGCTCG | 59.9 | 451 | INTERGENIC | | | | |
| CaPOPI_713 | Ca2 | 8478800 | AATTT/ATT | CCCGTATGAATGAACGGAAC | CTACCCCCACATTTCAATCG | 60.2 | 519 | INTERGENIC | | | | |
| CaPOPI_714 | Ca2 | 8478910 | ACT/A | CCCGTATGAATGAACGGAAC | CTACCCCCACATTTCAATCG | 60.2 | 519 | INTERGENIC | | | | |
| CaPOPI_715 | Ca2 | 8708914 | ATTTTTTTT/ATTTTTTTTT | GCCCACACCGAAGAAATAAA | TTCATTTTGCTCCATCACTTTC | 59.9 | 714 | INTERGENIC | | | | |

| INDEL marker IDs | Chromosomes/ unanchored scaffolds | Physical positions (bp) | InDels (*Kabuli* reference genome-CDC Frontier/PI) | Forward primers (5'-3') | Reverse primers (5'-3') | Anneling temperature (0C) | Expected amplified product size (bp) | Structural annotation | | Functional annotation | | |
|---|---|---|---|---|---|---|---|---|---|---|---|---|
| | | | | | | | | Sequence components of *kabuli* genome | *Kabuli* gene accession IDs | NCBI-KOG | TFs | NCBI-nr database |
| CaPOPI_716 | Ca2 | 8777880 | ATTTTTTTTT/ATTTTTTTTTT | CATGTCCTCAGGTGGTTGTG | TTTGTCGTGGGATGAACTGA | 60.0 | 807 | INTERGENIC | | | | |
| CaPOPI_717 | Ca2 | 8820736 | ATTTTTTTTTTT/ATTTTTTTTTT | TTACCATGATGTCGGTGGTG | GTGAGCAAATGTTTCGGGAT | 60.2 | 690 | INTERGENIC | | | | |
| CaPOPI_718 | Ca2 | 9277232 | AGG/AG | ATCGTGCAATCAACCTGTCA | GTTTCATGCAAATTGGGTCC | 60.1 | 439 | DRR | Ca_20035 | | | Domain of unknown function DUF231, plant |
| CaPOPI_719 | Ca2 | 9287043 | ATTTTTTTTTT/ATTTTTTTTT | CTCTTTCTCCTCCCGAAACA | AAGGGTTTTGTTGAAAGGGG | 59.4 | 706 | INTERGENIC | | | | |
| CaPOPI_720 | Ca2 | 9408358 | CTT/CTTTCTATTATT | CATCAAAAACAGCAGCGAAA | TCGTGTCTTCTTTCCATCCA | 60.0 | 934 | INTERGENIC | | | | |
| CaPOPI_721 | Ca2 | 9485330 | TTTTTATTTTATTTTATTTT/TTTTTATTTTATTTT | TTATGCGCCACATCACATTT | CGCCGAAAAGGTTACACAAT | 60.0 | 604 | INTRON | Ca_20024 | T | M-type | NB-ARC |
| CaPOPI_722 | Ca2 | 9494071 | T/TA | GAACTGCAACAACAACGACAA | GGTCTCGAAATCCCTGATGA | 59.8 | 609 | INTERGENIC | | | | |
| CaPOPI_723 | Ca2 | 9500229 | CGTATGCTAGACCTGG/CGTATGCTAGACCTGGGTATGCTAGACCTGG | AGAGGTTGGGGGAGAAGAAA | GCAACAACAGCAGAGACAATG | 60.0 | 213 | INTERGENIC | | | | |
| CaPOPI_724 | Ca2 | 9650270 | CATATATATATATATATA/CATATATATATATATA | GAAGGGGTATGAACGAAGCA | TGAGTAGGGATGGAGGACCA | 60.1 | 590 | INTERGENIC | | | | |
| CaPOPI_725 | Ca2 | 9884034 | AATATATATAT/AATATATAT | TAAGTTGTTGTGGCATCGGA | CCGACCTAATCACTCATAAATATCG | 60.1 | 322 | INTERGENIC | | | | |
| CaPOPI_726 | Ca2 | 10013835 | ATTTTTTTTT/ATTTTTTTT | TACTCACCCAAATCCGCTTC | TAGACCCTCATCCTCATCCG | 60.1 | 393 | INTERGENIC | | | | |
| CaPOPI_727 | Ca2 | 10032701 | AATATATATATATATA/AATATATATATATA | TTCTTCAGAGAAAAGAGTGGTTTTT | CCGTGTTTACGCTCTGTTGA | 59.1 | 392 | INTERGENIC | | | | |
| CaPOPI_728 | Ca2 | 10187103 | AATATATATATATATATATATATA/AATATATATATATATATATATATATATATA | AAGGCGAGAACAAGCCATTA | CCTGAATATGCGGCGTTATC | 59.8 | 314 | INTERGENIC | | | | |
| CaPOPI_729 | Ca2 | 10498057 | CTTTTTTTTTTT/CTTTTTTTTTTTT | TGACCAAATCGGAAAGAAAAA | AGGGAATGAGAGCAATGACG | 59.6 | 454 | INTERGENIC | | | | |
| CaPOPI_730 | Ca2 | 10779124 | TTA/TTACATA | CACTTTTCTTATGCACCGCA | GCAGCGAGTAGGAGAAATGC | 59.9 | 663 | INTERGENIC | | | | |

| INDEL marker IDs | Chromosomes/ unanchored scaffolds | Physical positions (bp) | InDels (*Kabuli* reference genome-CDC Frontier/PI) | Forward primers (5'-3') | Reverse primers (5'-3') | Anneling temperature (0C) | Expected amplified product size (bp) | Structural annotation | | Functional annotation | | |
|---|---|---|---|---|---|---|---|---|---|---|---|---|
| | | | | | | | | Sequence components of *kabuli* genome | *Kabuli* gene accession IDs | NCBI-KOG | TFs | NCBI-nr database |
| CaPOPI_731 | Ca2 | 10853311 | ATTTTTTTTTT/ATTTTTTTTT | CAACTCAACCCCTAAACCGA | TGAGAAATTTTTCCGCTGCT | 60.0 | 261 | INTERGENIC | | | | |
| CaPOPI_732 | Ca2 | 10913431 | GAAAAAAAAAAA/GAAAAAAAAAA | TGCATAATCGATTGGCACAT | TCACTTACCGATTCCAGCCT | 59.9 | 838 | DRR | Ca_17557 | | | |
| CaPOPI_733 | Ca2 | 10928556 | ATTTTTTTT/ATTTTTTTTT | CATCCGTGGTCCCTTAACTT | GCTAAATTACATTCGTGGTCCC | 58.9 | 370 | INTERGENIC | | | | |
| CaPOPI_734 | Ca2 | 11226197 | GATATATATATATATATATATA/GATATATATATATATATATATATATATATA | CGCGATCAAAACAAAACGTA | CCAACATGGCATGAGTTGAC | 59.7 | 757 | INTERGENIC | | | | |
| CaPOPI_735 | Ca2 | 11240445 | ATTTTTT/ATTTTTTT | TCGACCGAATCAAACAATGA | ACGTTTCCTCACGTTTCCAC | 60.0 | 622 | INTERGENIC | | | | |
| CaPOPI_736 | Ca2 | 11625278 | AATATATATATATATATATATATATATATATA/AATATATATATATATATATATATATATATATATATATA | GTTTAGCGCTCCTCCATCAT | CTGCCCGTAAAAATCTTCCA | 59.3 | 839 | INTERGENIC | | | | |
| CaPOPI_737 | Ca2 | 11648268 | AATATATATATATATATATATA/AATATATATATATATATATATATATATA | TGAGTGCAAGCCTAGTGTGG | TGCACGGAAAACTGACTCTG | 60.0 | 521 | INTERGENIC | | | | |
| CaPOPI_738 | Ca2 | 11711787 | ATATTATTATTATTATTATTATTATTATTATTATTA/ATATTATTATTATTATTATTATTATTATTATTATTATTA | TTCAAAATTGCTGGTTTGGTC | TGAAAGAAGCGCGGTAAAAC | 60.0 | 596 | INTERGENIC | | | | |
| CaPOPI_739 | Ca2 | 12040246 | TTATATATATATATATATAT/TTATATATATATATAT | TGTCAATCCATTTATAATTTCTTTCAA | CAGCTTTTTGGTTGATGTGG | 59.3 | 490 | INTERGENIC | | | | |
| CaPOPI_740 | Ca2 | 12040519 | ATT/AT | CCACATCAACCAAAAAGCTG | GTGAGGTGGCATTCGAGAGT | 59.2 | 665 | INTERGENIC | | | | |
| CaPOPI_741 | Ca2 | 12048675 | AATAATTTATAATTTATA/AATAATTTATA | CAAACATGCTGATGTGGACC | CCATTGGTAAATGGCTAACAA | 60.0 | 278 | INTERGENIC | | | | |
| CaPOPI_742 | Ca2 | 12307967 | GAAA/GAAAA | TGTCTTTGGTCCCTGCAAAT | TCAAACCCCCATTTTTGTGT | 60.5 | 755 | INTERGENIC | | | | |
| CaPOPI_743 | Ca2 | 12360564 | TAAAAAAAAAAAA/TAAAAAAAAAAA | TCGCACTCACACAACAAAATC | CCTCTTCACTTTTTGGAAGGG | 59.8 | 155 | INTERGENIC | | | | |
| CaPOPI_744 | Ca2 | 12553140 | ATTTTTTTTTT/ATTTTTTTTTTTT | CCCCCTCCAACCTTTCATAC | ATCCAAATTCAATGCATCCC | 60.5 | 811 | INTERGENIC | | | | |
| CaPOPI_745 | Ca2 | 12707442 | GAATTTCAATTTCAAT/GAATTTCAATTTCAATTTCAAT | AGCAGCTGACCGAGCATATT | GGATGGGTGATTGAAAATCG | 60.0 | 902 | INTRON | Ca_18086 | R | | Methyltransferase type 11 |

| **INDEL marker IDs** | **Chromosomes/ unanchored scaffolds** | **Physical positions (bp)** | **InDels (*Kabuli* reference genome-CDC Frontier/PI)** | **Forward primers (5'-3')** | **Reverse primers (5'-3')** | **Anneling temperature (0C)** | **Expected amplified product size (bp)** | **Structural annotation** | | **Functional annotation** | | |
|---|---|---|---|---|---|---|---|---|---|---|---|---|
| | | | | | | | | **Sequence components of *kabuli* genome** | ***Kabuli* gene accession IDs** | **NCBI-KOG** | **TFs** | **NCBI-nr database** |
| CaPOPI_746 | Ca2 | 12933106 | A/AT | AGGTTGAAAGAAGCCCCACT | ATCGGTTGGTTGGTGAAATG | 60.1 | 834 | INTERGENIC | | | | |
| CaPOPI_747 | Ca2 | 12933573 | CACCAACCAACC/CACCAACC | TCATTGACAAGGAAAGAACATGA | CGAATTGGTTTTGGGATTTG | 59.6 | 413 | INTERGENIC | | | | |
| CaPOPI_748 | Ca2 | 12934202 | AAA/AAACAA | AGAAAGCAAGCAAATCCCAA | TTCCAACACCTCACGATTCA | 59.8 | 488 | INTERGENIC | | | | |
| CaPOPI_749 | Ca2 | 12937651 | ATTTT/ATTTTT | GATTGCTTTCCTCTATGCCG | GTTTTGGCGTTGGTTTCACT | 59.8 | 353 | INTERGENIC | | | | |
| CaPOPI_750 | Ca2 | 12939688 | AATATATA/AATATA | TTTTGGTGTTGAGGGTGTCA | TTAATCAAAGCGGCAAAAGAA | 60.0 | 564 | INTERGENIC | | | | |
| CaPOPI_751 | Ca2 | 12941200 | CAAAA/CAAA | TGTGCTTCTTCCTGATTTTGG | GGCCAAAGACGGATATGGTA | 60.2 | 888 | INTERGENIC | | | | |
| CaPOPI_752 | Ca2 | 12941800 | T/TA | CATATCCGTCTTTGGCCATT | CCTTGAACAATTTGGGCAGT | 59.8 | 643 | INTERGENIC | | | | |
| CaPOPI_753 | Ca2 | 12947970 | G/GT | TGCTTTTATGAGCATTACGGG | CGACTTCAAATCCCTTCGAG | 60.1 | 777 | INTERGENIC | | | | |
| CaPOPI_754 | Ca2 | 12952557 | ACATATCATATCA/ACATATCA | AAAGCATTTTATCCCGACCA | TTTTTCATCTCACTCTCTCCCA | 59.4 | 787 | INTERGENIC | | | | |
| CaPOPI_755 | Ca2 | 12952944 | TGTCATGATTAT/TGTCATGATTATAGTCATGATTAT | TGGGAGAGAGTGAGATGAAAAA | ACGGATGTCTAATCCGTTGG | 58.9 | 308 | INTERGENIC | | | | |
| CaPOPI_756 | Ca2 | 13247575 | ATTTTTTTT/ATTTTTTT | TGAAAAGCTAAATGCATGGAA | TCATGACCATGGACCCACTA | 58.4 | 937 | INTERGENIC | | | | |
| CaPOPI_757 | Ca2 | 13957739 | TGG/TG | ATTATCCCCACCCCCACTAC | TTTCAAAAACGGTGGTGTGA | 59.8 | 755 | INTERGENIC | | | | |
| CaPOPI_758 | Ca2 | 13959549 | ATTTTTTTTTT/ATTTTTTTTTTT | TGAAATTTGTGATTTAGTTGCTTAAAA | CATAGATTCCCCACCCAATG | 59.6 | 752 | INTERGENIC | | | | |
| CaPOPI_759 | Ca2 | 13983591 | ATTTTTTTTTT/ATTTTTTTTTTT | ACACGAGGCGGAGAAATAAA | TGCAATTGTGGTCCATTTGT | 59.7 | 893 | INTERGENIC | | | | |
| CaPOPI_760 | Ca2 | 14086143 | C/CT | GCTTCTGATGGTGCACTTGA | TGGCCTAAAGCAAGCTCCTA | 60.0 | 371 | INTERGENIC | | | | |

| INDEL marker IDs | Chromosomes/ unanchored scaffolds | Physical positions (bp) | InDels (*Kabuli* reference genome-CDC Frontier/PI) | Forward primers (5'-3') | Reverse primers (5'-3') | Anneling temperature (0C) | Expected amplified product size (bp) | Structural annotation | | Functional annotation | | |
|---|---|---|---|---|---|---|---|---|---|---|---|---|
| | | | | | | | | Sequence components of *kabuli* genome | *Kabuli* gene accession IDs | NCBI-KOG | TFs | NCBI-nr database |
| CaPOPI_761 | Ca2 | 14192522 | GAAAAAAAAAA/GAAAAAAAAAAA | TACAAATTGGATGCGGTTGA | CCCTCTGCAATGTCGGTAAT | 59.9 | 755 | INTERGENIC | | | | |
| CaPOPI_762 | Ca2 | 15479768 | ATTTTTTTTTTT/ATTTTTTTTTTTT | GGGGTTCTTCAAAATTGGGT | CAAACTTGGGGATGAAAACG | 60.0 | 461 | INTERGENIC | | | | |
| CaPOPI_763 | Ca2 | 15557649 | AATATATATATATATATATATATATATA/AATATATATATATATATATATATATATATA | GAAAGCAATTGGGCTACTGG | CCAAAAACGTCTTTTCAATGG | 59.7 | 251 | INTERGENIC | | | | |
| CaPOPI_764 | Ca2 | 15589036 | ATTT/ATTTT | ATACGCCTCAACAAAATGGC | CTGCTTTGCTTGATTCCGTT | 60.0 | 376 | INTERGENIC | | | | |
| CaPOPI_765 | Ca2 | 15691178 | TTATATATATATATATATATATATAT/TTATATATATATATATATATATATATAT | GACTGTCCAAATTTGTGGGG | TTTTTGGCATCAAGTTGTCTTTT | 60.2 | 581 | DRR | Ca_18540 | | | Alpha-1,4-glucan-protein synthase, UDP-forming |
| CaPOPI_766 | Ca2 | 17046294 | AATAAATT/AAAATTTATAAATT | CTATTCCATCCACGAATGCC | TCCATGGAGGATATTTATGTCG | 60.3 | 523 | DRR | Ca_24768 | | G2-like | Zinc finger, TTF-type |
| CaPOPI_767 | Ca2 | 17061321 | TAATATATA/TAATATATAAATATATA | TTTGGCTCGGAAGCTTTTTA | AGGAAGAGTGGAGTTGCGAA | 60.0 | 498 | INTERGENIC | | | | |
| CaPOPI_768 | Ca2 | 17061679 | T/TCCA | TTCGCAACTCCACTCTTCCT | CGGTAAAAATCTCTGCTCGC | 60.0 | 190 | INTERGENIC | | | | |
| CaPOPI_769 | Ca2 | 17551687 | G/GC | ATGTGGTTGATAAGGCCTCG | AGCAAATGCAATGCTCTGAA | 60.0 | 398 | CDS (FRAME SHIFT) | Ca_16000 | OE | | Peptidase S10, serine carboxypeptidase |
| CaPOPI_770 | Ca2 | 18225059 | GTTTTTTTTT/GTTTTTTTTTT | TTGTTTTGATGGATGATTTGGA | AAGCAAACATTTGGAATGGC | 60.2 | 460 | INTERGENIC | | | | |
| CaPOPI_771 | Ca2 | 18391802 | AATATATATATATATATATA/AATATATATATATATATA | CAAAGCGGGTAGCAAGGATA | GCCGCTCCTCTTCAAGTAAA | 60.2 | 524 | INTERGENIC | | | | |
| CaPOPI_772 | Ca2 | 18584340 | GAAAAAAAAAA/GAAAAAAAAAAA | ATGTACCTCCGCCTCATTTG | TCTCTGGCAACCGTTCTTCT | 60.0 | 257 | INTERGENIC | | | | |
| CaPOPI_773 | Ca2 | 18610007 | TAA/TAACAA | TTGATCCCTTATTTCCTGCG | TTATTTCACCGTCGTGTCCA | 60.0 | 492 | INTERGENIC | | | | |
| CaPOPI_774 | Ca2 | 18764616 | GAATAATAATAATAATAATAATA/GAATAATAATAATAATAATA | ATTTGCCTCCCTCAACCTTT | TGTCTTCATGGGTGCATTGT | 59.9 | 841 | INTERGENIC | | | | |
| CaPOPI_775 | Ca2 | 18833380 | TAAAAAAAAAAA/TAAAAAAAAAAAA | TCGAAAAATCCCTTGAAAAAGA | GGACCTATCAAGTGAGCTTGTG | 60.0 | 551 | INTERGENIC | | | | |

| INDEL marker IDs | Chromosomes/ unanchored scaffolds | Physical positions (bp) | InDels (*Kabuli* reference genome-CDC Frontier/PI) | Forward primers (5'-3') | Reverse primers (5'-3') | Anneling temperature (0C) | Expected amplified product size (bp) | Structural annotation | | Functional annotation | | |
|---|---|---|---|---|---|---|---|---|---|---|---|---|
| | | | | | | | | Sequence components of *kabuli* genome | *Kabuli* gene accession IDs | NCBI-KOG | TFs | NCBI-nr database |
| CaPOPI_776 | Ca2 | 19008979 | TAAAAAA/TAAAAAAAA | GGCTATATCTCCCATGCCAA | GCAAGAAACGAGGAAAGAAGG | 59.9 | 663 | INTRON | Ca_15954 | | bHLH | Terpene synthase-like |
| CaPOPI_777 | Ca2 | 19207562 | TAAAAAAA/TAAAAAAAA | ACAAACCAAAACCCAACCAA | TTTCACCGACCCAAACAAAT | 60.1 | 663 | INTERGENIC | | | | |
| CaPOPI_778 | Ca2 | 19219016 | ATTTT/ATTT | CCCGTGAGATCCTCCTAACA | TGAATCTACGGCTTTCTGGG | 60.1 | 498 | INTERGENIC | | | | |
| CaPOPI_779 | Ca2 | 19705772 | GAAAA/GAAAAA | TATTTTGGATTTTGGCTCGG | AGGAAGAGTGGAGTTGCGAA | 59.9 | 475 | INTERGENIC | | | | |
| CaPOPI_780 | Ca2 | 19791685 | C/CG | TAGGCCATTTTCGTCCTCAC | AATCCAATGACACAAACGCA | 60.1 | 392 | INTERGENIC | | | | |
| CaPOPI_781 | Ca2 | 20280019 | TAAAAAAAAAAA/TAAAAAAAAAA | GCAAGGTTTGACAGTGAGCA | CATCGACAACCAATCCACAG | 60.0 | 554 | INTERGENIC | | | | |
| CaPOPI_782 | Ca2 | 20353586 | T/TG | TCTCCCTCCTTCCTCCATTT | CATTAAGAGGTTCCCCCTCC | 60.0 | 844 | INTERGENIC | | | | |
| CaPOPI_783 | Ca2 | 20451589 | CAAAA/CA | ACCAAATTGACTCGGTGCTC | TGTGGGATGACCCTTTTTGT | 60.1 | 460 | INTERGENIC | | | | |
| CaPOPI_784 | Ca2 | 20647114 | AATATATATATATATATATAT/AATATATATATATATATATATAT | ATCACTCGCACAACAAACCA | TTGGAAGGAGCCCTTACCTT | 60.2 | 920 | INTERGENIC | | | | |
| CaPOPI_785 | Ca2 | 20970910 | ATTCCCT/A | AACAAAGGAACAAAATGGCG | AGGGACAAAGGGCAAGAAAT | 60.0 | 853 | INTERGENIC | | | | |
| CaPOPI_786 | Ca2 | 21088001 | TA/TAAAAGCATCA | AGAACGATGACACGTTGGGT | CTTCTGTCCAAAAAGCAGCC | 60.4 | 812 | INTERGENIC | | | | |
| CaPOPI_787 | Ca2 | 21312312 | GTTTTTT/GTTTTTTTT | TATCAGAGTTCTCCCCACCG | AGAAACCACAACGCAACACA | 60.1 | 356 | INTERGENIC | | | | |
| CaPOPI_788 | Ca2 | 21314611 | AG/AGG | CAAACCCTTTCAAGGTGCAT | GCACTGCCCCAAGAATTTTA | 60.0 | 443 | INTERGENIC | | | | |
| CaPOPI_789 | Ca2 | 21596317 | TTTATTATTATTATTATTATTATTATTATTATT/TTTATTATTATTATTATTATTATTATTATTATT | AATCCCTCCCTTCGTCACTT | TCCCTTTCTACAAGCGTTCA | 59.9 | 210 | INTERGENIC | | | | |
| CaPOPI_790 | Ca2 | 21672716 | ATT/AT | TGATGCCGTGCCATAAATAG | TAACCTTGCCTTTGGGTGAG | 59.5 | 595 | INTERGENIC | | | | |

| INDEL marker IDs | Chromosomes/ unanchored scaffolds | Physical positions (bp) | InDels (*Kabuli* reference genome-CDC Frontier/PI) | Forward primers (5'-3') | Reverse primers (5'-3') | Anneling temperature (0C) | Expected amplified product size (bp) | Structural annotation | | Functional annotation | | |
|---|---|---|---|---|---|---|---|---|---|---|---|---|
| | | | | | | | | Sequence components of *kabuli* genome | *Kabuli* gene accession IDs | NCBI-KOG | TFs | NCBI-nr database |
| CaPOPI_791 | Ca2 | 21783996 | ATTTTTT/ATTTTTTTT | ATCTCGTGGGTGGATCTGAC | GCTATGGGACCTATCAGGCA | 59.9 | 381 | DRR | Ca_23057 | | | |
| CaPOPI_792 | Ca2 | 21943275 | AGCGCG/AGCG | GCAGATTTGGAAGGTGTGGT | ACAATCAGCACGCAAGAATG | 60.0 | 461 | INTERGENIC | | | | |
| CaPOPI_793 | Ca2 | 22219214 | GTGGTGGTCG/GTGGTGGTCGTTGGTGGTCG | CAAAGCGGTCGAAGTAGAGG | GTTGTTCGGGGTTGTGAGTT | 60.0 | 391 | INTERGENIC | | | | |
| CaPOPI_794 | Ca2 | 22286038 | GAAGCGC/GAAGCGCACCCAAGCGC | GGGAGGCAACCCAATTTTAT | GCTTAGATCATCCGCTCCTG | 60.0 | 368 | INTERGENIC | | | | |
| CaPOPI_795 | Ca2 | 22650634 | TG/T | GAGAGGAGGGTTCCCAGAGT | GTGCATCCTCCATCTTTGGT | 59.7 | 550 | DRR | Ca_14991 | | | |
| CaPOPI_796 | Ca2 | 22846943 | ATTTTTTTTTT/ATTTTTTTTTTT | GGTTCCAAACTCTCGGTTGA | GCACGTGGCTAGGGTTATGT | 60.1 | 569 | DRR | Ca_14984 | | | Domain of unknown function DUF296 |
| CaPOPI_797 | Ca2 | 22890617 | TTATATATATATATATATATATAT/TTATATATATATATATATATATAT | GCCACCCTTGTGAGAGTTTC | CACGAGTCCGATAAGGGTGT | 59.7 | 554 | INTERGENIC | | | | |
| CaPOPI_798 | Ca2 | 23058191 | GTTTTTTTTTTT/GTTTTTTTTTTTT | GAAACTGTGTCACGAGGCAA | AGAGCCGTCCAGAAAAATCA | 59.9 | 121 | INTERGENIC | | | | |
| CaPOPI_799 | Ca2 | 23193466 | TATTTAATA/TTTTAATA | CCACAAATGCTCATCGTGAC | TTTGCAGCCACAAAAGACAG | 60.1 | 561 | INTERGENIC | | | | |
| CaPOPI_800 | Ca2 | 23408235 | AATATATATATATATATATATATATAT/AATATATATATATATATATATATATATAT | GGTAGGAATTGGGTGTGGTG | AGGAAGAGTGGAGTTGCGAA | 60.1 | 710 | INTERGENIC | | | | |
| CaPOPI_801 | Ca2 | 23472039 | CTTTATTTATTTATTTATTTATTTATTTATTTATTTAT/CTTTATTTATTTATTTATTTATTTATTTATTTATTTATTTAT | TTTTGCTAACCACACCCCTC | TGACCACGTGTTTTTGGGTA | 60.0 | 434 | INTRON | Ca_14958 | | | Rhamnogalacturonate lyase |
| CaPOPI_802 | Ca2 | 23509535 | AAAAACTTAAAACTTAAAACTTA/AAAAACTTAAAACTTA | CCCTCTTGAATTTTTGTTTTCC | CCCACACCCTCGACTAAAGA | 59.0 | 542 | INTERGENIC | | | | |
| CaPOPI_803 | Ca2 | 23792320 | GGAGTAAGAGAGTAA/GGAGTAAGAGAGTAAGAGAGTAA | TCAGAATCCCTGATCCCAAG | GCAACCAGGAGGCAAGTCTA | 60.0 | 490 | INTERGENIC | | | | |
| CaPOPI_804 | Ca2 | 23820380 | TTATATATATATATAT/TTATATATATATATATAT | GTGCTCTCGTCAAACGAACA | AAGCGCTGTAAATGGTGCAT | 60.0 | 716 | DRR | Ca_14945 | | | |
| CaPOPI_805 | Ca2 | 23851284 | GATATATATATATATATATATATATATA/GATATATATATATATATATATATATATATATA | TGCCTTCATCATGTGGTTGT | ATGGCAATTTCTCAGGATGG | 60.0 | 501 | INTERGENIC | | | | |

| INDEL marker IDs | Chromosomes/ unanchored scaffolds | Physical positions (bp) | InDels (*Kabuli* reference genome-CDC Frontier/PI) | Forward primers (5'-3') | Reverse primers (5'-3') | Anneling temperature (0C) | Expected amplified product size (bp) | Structural annotation | | Functional annotation | | |
|---|---|---|---|---|---|---|---|---|---|---|---|---|
| | | | | | | | | Sequence components of *kabuli* genome | *Kabuli* gene accession IDs | NCBI-KOG | TFs | NCBI-nr database |
| CaPOPI_806 | Ca2 | 23932608 | TAAATTAATGAATAAAAATTAAT/TAAATTAAT | AAGGCATCCAATCAAAGCAC | CTCAAGATTTTAGCGCCGTC | 60.1 | 460 | INTERGENIC | | | | |
| CaPOPI_807 | Ca2 | 23989802 | AAAAACAAAACAAAA/AAAAACAAAACAAAACAAAA | AATTCGTTGAGCCGGTCTAGT | AGCACGGGGAGTATGAGAAA | 60.1 | 421 | INTERGENIC | | | | |
| CaPOPI_808 | Ca2 | 23990182 | AAAAACAAAACAAAA/AAAAACAAAACAAAACAAAA | TCTCATACTCCCCGTGCTTT | ATCCCCTACACCAGATGCAC | 59.7 | 565 | INTERGENIC | | | | |
| CaPOPI_809 | Ca2 | 24102909 | GT/GTT | TGGGGGATCCAAGTTAAAAA | GAAAACTCTCCGGCATTCAA | 59.2 | 428 | CDS (FRAME SHIFT) | Ca_21670 | S | | Transmembrane receptor, eukaryota |
| CaPOPI_810 | Ca2 | 24252139 | TAA/TAAGAAA | GATCCTCAAATCGACCTCCA | TGTCACCATTGGTCTCCAAA | 60.0 | 465 | INTERGENIC | | | | |
| CaPOPI_811 | Ca2 | 24563119 | TCCCC/TCCC | CCATCTACTGCCCTTGGAAA | GATCCAGGCAAGGTCAATGT | 60.1 | 260 | INTERGENIC | | | | |
| CaPOPI_812 | Ca2 | 24943252 | CTTT/CTT | AACCCCTCCAAAGAACGACT | AGGGCCCCACTAACTCCTTA | 60.0 | 680 | INTERGENIC | | | | |
| CaPOPI_813 | Ca2 | 24948166 | CTTTTT/CTTTT | GGTGAGACTTTCGAGGGCTA | GGCATTGAACGTTTTCACAA | 59.4 | 295 | INTERGENIC | | | | |
| CaPOPI_814 | Ca2 | 25171520 | TCAACAA/TCAA | GGAAATGAGCTGAATGGGAA | CCAGAGGGGATGTTGAGAAA | 60.0 | 551 | INTRON | Ca_14250 | T | M-type | Disease resistance protein |
| CaPOPI_815 | Ca2 | 25241576 | GATATATATATATATA/GATATATATATATATATATA | TATTTTGGATTTTGGCTCGG | AAATGGAGGAAGGAGGGAGA | 59.9 | 433 | INTERGENIC | | | | |
| CaPOPI_816 | Ca2 | 25352886 | ATTTTTTTTTT/ATTTTTTTTTTT | AAAGAGACACATGACATAACTCCTC | ATGGGGGATGGACCTAGAAC | 57.9 | 685 | DRR | Ca_14268 | | | Histone acetylation protein 2 |
| CaPOPI_817 | Ca2 | 25427657 | TTATATATATATATATATATATATATATATAT/TTATATATATATATATATATATATATATATAT | TGTTGTTGAAGATGAAATATTGGC | TTTGGCGTAGTGAAGTTGACA | 60.2 | 723 | INTERGENIC | | | | |
| CaPOPI_818 | Ca2 | 25535050 | CTTTTTTTTT/CTTCTTTTTTTTTTTTTT | CCAGATTTGAACTGGGGAAA | AATCAAGTGGGTTCGAGTGG | 59.9 | 551 | DRR | Ca_14283 | R | MYB | Integrase, catalytic core |
| CaPOPI_819 | Ca2 | 25619256 | CTTTTTT/CTTTTTTT | TTGAGAGACCAAAGTGTCCAAA | AAGTGACTATTTCCTCGGTCCTT | 59.8 | 829 | INTERGENIC | | | | |
| CaPOPI_820 | Ca2 | 25767704 | GAAGTTAAGTT/GAAGTT | ATGAATTTTGGCATCCAACC | CGTGGACAAAGATCGTGATG | 59.6 | 654 | INTRON | Ca_14294 | G | NAC | Glycoside hydrolase, family 1 |

| INDEL marker IDs | Chromosomes/ unanchored scaffolds | Physical positions (bp) | InDels (*Kabuli* reference genome-CDC Frontier/PI) | Forward primers (5'-3') | Reverse primers (5'-3') | Anneling temperature (0C) | Expected amplified product size (bp) | Structural annotation | | Functional annotation | | |
|---|---|---|---|---|---|---|---|---|---|---|---|---|
| | | | | | | | | Sequence components of *kabuli* genome | *Kabuli* gene accession IDs | NCBI-KOG | TFs | NCBI-nr database |
| CaPOPI_821 | Ca2 | 26504489 | CAA/CAAAA | CCGACGTCCAGTCAGAATTT | CTCAATTGGACACCGTTCCT | 60.1 | 772 | INTERGENIC | | | | |
| CaPOPI_822 | Ca2 | 27153936 | TTATATATATATATATATATATAT/TTATATATATATATATATATAT | TCCAAACTAGGTGGCAACAA | TTGCCCACTCATTTCCTTTC | 59.2 | 478 | INTERGENIC | | | | |
| CaPOPI_823 | Ca2 | 27401550 | TAAAAAAAAAAAA/TAAAAAAAAAAAAA | GCCGTTGGGTTTGAGTTAGA | CCAAATCACCTCCAGTCACC | 60.1 | 678 | INTERGENIC | | | | |
| CaPOPI_824 | Ca2 | 27436271 | CAAA/CAA | TCCTACGTATCTTTTGATGCGA | CGTGCCCAATCTCTCAATCT | 59.7 | 382 | INTERGENIC | | | | |
| CaPOPI_825 | Ca2 | 27560889 | TTCCCTCCTATCCCTC/TTCCCTC | GGTGCATTTGGGAACCTTTA | CTGCAGCATCCCTACACTCA | 59.8 | 767 | INTERGENIC | | | | |
| CaPOPI_826 | Ca2 | 27614728 | AAATAATAATAATAATAATAATAATAATAATAATAATAATAATGCTAATAATAATAATAATAATTGTAATAATAATAATAATAATAACAATAATAATAATAATAA/AAATAATAAT | CGCGCGAGCCATAATCTAGT | CGACGCCAATCTGCTTATTT | 62.6 | 625 | INTERGENIC | | | | |
| CaPOPI_827 | Ca2 | 27692325 | TG/T | AGACAAAACGTTCAAGTTGTCG | TCTTTAATTGCATGCGCAAA | 59.3 | 762 | DRR | Ca_15671 | | ERF | Pathogenesis-related transcriptional factor/ERF, DNA-binding |
| CaPOPI_828 | Ca2 | 27804750 | TCC/TC | CAGGAAACTCTGCCTTTTCG | TAGCAGCTGTATGGTGCAGG | 60.0 | 137 | INTERGENIC | | | | |
| CaPOPI_829 | Ca2 | 28157957 | TTATATATATATATATATATATAT/TTATATATATATATATATATATATATATAT | GCATGGACATTCAAAAATGAGA | CCATGTCTAAGTGGCAGGGT | 59.9 | 583 | DRR | Ca_14385 | O | MYB_related | SANT domain, DNA binding |
| CaPOPI_830 | Ca2 | 28235432 | AATATATATATATATATATATA/AATATATATATATATATATATATA | TCCAAATAGCGACTTCCCAC | GCTGCATGTTCTTATGCTCG | 60.1 | 849 | INTERGENIC | | | | |
| CaPOPI_831 | Ca2 | 28465231 | CAAAAA/CAAAA | TCATCGCAGGTGTACCATGT | GGTATCAGAGCCCACCATGT | 60.0 | 908 | INTERGENIC | | | | |
| CaPOPI_832 | Ca2 | 28465336 | AAT/A | AAGAGAGGTCACTTTGGCGA | GCAATTCCCACAGGTATGCT | 60.0 | 886 | INTERGENIC | | | | |
| CaPOPI_833 | Ca2 | 28465620 | A/AT | AGCTCCATTGCAAAAACACC | GCAATTCCCACAGGTATGCT | 60.1 | 553 | INTERGENIC | | | | |
| CaPOPI_834 | Ca2 | 28569036 | TGG/TGGG | TCTCCAAATGATTGGAAGGC | GACTGGACGTAACCCACGTT | 60.0 | 913 | INTERGENIC | | | | |
| CaPOPI_835 | Ca2 | 28569281 | T/TC | CATCTGTAATGATGCGCCAC | GACTGGACGTAACCCACGTT | 60.1 | 503 | INTERGENIC | | | | |

| INDEL marker IDs | Chromosomes/ unanchored scaffolds | Physical positions (bp) | InDels (*Kabuli* reference genome-CDC Frontier/PI) | Forward primers (5'-3') | Reverse primers (5'-3') | Anneling temperature (0C) | Expected amplified product size (bp) | Structural annotation | | Functional annotation | | |
|---|---|---|---|---|---|---|---|---|---|---|---|---|
| | | | | | | | | Sequence components of *kabuli* genome | *Kabuli* gene accession IDs | NCBI-KOG | TFs | NCBI-nr database |
| CaPOPI_836 | Ca2 | 28617666 | TTTTATTTATTTATTTATT/TTTTATTTATTTATTTATTTATTTATT | ATCGACTCCCGAACTCAAAA | CCACGGTTCTTAGAGCCATTA | 59.7 | 621 | INTERGENIC | | | | |
| CaPOPI_837 | Ca2 | 29220253 | GATATATATATATATATATATATA/GATATATATATATATATATATATATATATATA | AGGCATGGTGTTGGAGTCAT | CTAGTTCGTAACCCGTGCGT | 60.4 | 253 | INTERGENIC | | | | |
| CaPOPI_838 | Ca2 | 29220546 | GATAATAATAATAATAATAATAATAATAATAAT/GATAATAATAATAATAATAATAATAATAAT | CACGTGTCAAACTTGCCAAT | GTAACCGTTGGAGCACCACT | 59.6 | 534 | INTERGENIC | | | | |
| CaPOPI_839 | Ca2 | 29238503 | A/AGTT | TGTGGAACACCAACGAATGT | ACCTCCCGCTAATGTCAATG | 59.9 | 746 | DRR | Ca_14317 | | | |
| CaPOPI_840 | Ca2 | 29238544 | AAATAATAATAATAATAATAATAATAATAATAATAATAATAATAATAATAA/AAATAATAATAATAATAATAATAATAATAATAATAATAA | TGTGGAACACCAACGAATGT | ACCTCCCGCTAATGTCAATG | 59.9 | 746 | DRR | Ca_14317 | | | |
| CaPOPI_841 | Ca2 | 29239999 | AAT/A | GGCTGCTGATCCTGTTCTTC | CTGACGGACGGACTCATTTT | 60.0 | 482 | INTERGENIC | | | | |
| CaPOPI_842 | Ca2 | 29240150 | TC/TCACC | CTTAGAGCATCTCCAACGGC | GAAGCATCGTTCCTTCCTGA | 60.0 | 134 | INTERGENIC | | | | |
| CaPOPI_843 | Ca2 | 29240273 | ATTTTTTTT/ATTTTTTT | CTTAGAGCATCTCCAACGGC | CAACGGTGAACTCAATATGGG | 60.0 | 671 | INTERGENIC | | | | |
| CaPOPI_844 | Ca2 | 29240382 | TCCC/TCC | CTTAGAGCATCTCCAACGGC | CCTTGCACTATCATTTTCGGA | 60.0 | 803 | INTERGENIC | | | | |
| CaPOPI_845 | Ca2 | 29252004 | GTT/GT | TCAAAGGTGCACTCTTGCTATT | TGGCTTCCGGAAAGACTAAA | 59.0 | 502 | INTERGENIC | | | | |
| CaPOPI_846 | Ca2 | 29253047 | TTTATTATTATTATTATTATTATTATTATTATTATTATTATTATTATTATTATTATT/TTTATTATTATTATTATTATTATTATTATTATTATTATTATTATTATTATTATTAT | GACACCCAAATATAGTGTCAACGA | CCCGGGTATAGGGTCTCACT | 60.2 | 583 | INTERGENIC | | | | |
| CaPOPI_847 | Ca2 | 29339791 | CAAA/CAAAA | CCACAGCCCTACCTGAACAT | CCCAAGGTATGCAGTGGATT | 60.0 | 502 | DRR | Ca_25183 | TZ | | Zinc finger, LIM-type |
| CaPOPI_848 | Ca2 | 29444244 | ATTTT/ATTTTT | AAATCCGACTGTCTAACCCG | TTTTCTCCTCCAATTTAGTCCAA | 59.1 | 826 | INTERGENIC | | | | |
| CaPOPI_849 | Ca2 | 29445927 | AACCCGAACCC/AACCCGAACCCACCCGAACCC | TGTCTAACCCGTCTAACCCG | CCCTACCTAGGGGTGGACAT | 60.0 | 138 | INTERGENIC | | | | |
| CaPOPI_850 | Ca2 | 29534597 | ATTTTTTTT/ATTTTTTT | AGAGTCGCTCCCAAGCATTA | GGAGTGGTTGACGTTGGATT | 60.0 | 472 | INTERGENIC | | | | |

| INDEL marker IDs | Chromosomes/ unanchored scaffolds | Physical positions (bp) | InDels (*Kabuli* reference genome-CDC Frontier/PI) | Forward primers (5'-3') | Reverse primers (5'-3') | Anneling temperature (0C) | Expected amplified product size (bp) | Structural annotation | | Functional annotation | | |
|---|---|---|---|---|---|---|---|---|---|---|---|---|
| | | | | | | | | Sequence components of *kabuli* genome | *Kabuli* gene accession IDs | NCBI-KOG | TFs | NCBI-nr database |
| CaPOPI_851 | Ca2 | 29608982 | TAAAAAAA/TAAAAAA | TTTGCCACAAACAAAAC GTG | TGAAATTTCCCTACGAT GCC | 60.6 | 504 | INTERGENIC | | | | |
| CaPOPI_852 | Ca2 | 29829940 | TT/TTTCGAAAAAAT | CTTTTCGTCGGAGTTTG TCC | TCGTCCTCTGGTTTTCT CGT | 59.7 | 476 | INTERGENIC | | | | |
| CaPOPI_853 | Ca2 | 29962779 | G/GC | AGCCAACGGACCACTAA CAC | TTCGCCATTCTTCTTCGT CT | 60.0 | 237 | INTERGENIC | | | | |
| CaPOPI_854 | Ca2 | 30095996 | TAAAAAAA/TAAAAAAAAA | CATTCCCGCTACATTTT GGT | GGAGAGAGGCCAATTTT TCA | 59.8 | 226 | INTERGENIC | | | | |
| CaPOPI_855 | Ca2 | 30096276 | TA/TAAA | CATTCCCGCTACATTTT GGT | GAAGCTGCTTGTGTGCC ATA | 59.8 | 876 | INTERGENIC | | | | |
| CaPOPI_856 | Ca2 | 30125989 | TAAAAAA/TAAAAAAA | TTCCACTTATGGTGAGT AATGATTTT | TTGTGAGCATGAGAAGT TGTTG | 59.3 | 641 | INTERGENIC | | | | |
| CaPOPI_857 | Ca2 | 30146454 | CAAAAAA/CAAAAAAAA | GCCCAAAACCAAATTTA GCA | AAGACCCAGAAACTTCC CGT | 59.9 | 452 | INTERGENIC | | | | |
| CaPOPI_858 | Ca2 | 30174020 | CTT/CTTT | TTATCCCCGCATATTGG ACT | AGTCCTTGGGACCGAAA AGT | 59.3 | 227 | INTERGENIC | | | | |
| CaPOPI_859 | Ca2 | 30243585 | CTT/CT | TTCCTACTCCCACATTTT AACGA | GCCTGTATCCAGCCATT TGT | 59.9 | 467 | INTERGENIC | | | | |
| CaPOPI_860 | Ca2 | 30341034 | TAA/TAAA | TGTCTTATCCTGAACTCA AACTCG | ATTTGGTCAACCACCCA AAA | 59.8 | 537 | INTERGENIC | | | | |
| CaPOPI_861 | Ca2 | 30342844 | ATTTTT/ATTT | TGCCTAATTTTGACTTCC CC | TCTCTAATCTGCTACGC ATCCA | 59.0 | 439 | INTERGENIC | | | | |
| CaPOPI_862 | Ca2 | 30342955 | TAAA/TAAAA | TGCCTAATTTTGACTTCC CC | TCTCTAATCTGCTACGC ATCCA | 59.0 | 439 | INTERGENIC | | | | |
| CaPOPI_863 | Ca2 | 30343145 | TAAA/TAA | TGGATGCGTAGCAGATT AGAGA | CGTCACTTGGTCACTTG GTG | 60.0 | 581 | INTERGENIC | | | | |
| CaPOPI_864 | Ca2 | 30343408 | TTAATAAA/T | TGGATGCGTAGCAGATT AGAGA | CGTCACTTGGTCACTTG GTG | 60.0 | 581 | INTERGENIC | | | | |
| CaPOPI_865 | Ca2 | 30343585 | CACCAAGTGACCAAGTGAC/CACCAAG TGAC | CAATCAATAATTTTGACG TCGG | CATTTCACGGGGCTCTT AAA | 59.4 | 658 | INTERGENIC | | | | |

| INDEL marker IDs | Chromosomes/ unanchored scaffolds | Physical positions (bp) | InDels (*Kabuli* reference genome-CDC Frontier/PI) | Forward primers (5'-3') | Reverse primers (5'-3') | Anneling temperature (0C) | Expected amplified product size (bp) | Structural annotation | | Functional annotation | | |
|---|---|---|---|---|---|---|---|---|---|---|---|---|
| | | | | | | | | Sequence components of *kabuli* genome | *Kabuli* gene accession IDs | NCBI-KOG | TFs | NCBI-nr database |
| CaPOPI_866 | Ca2 | 30345200 | TG/T | TAAGTTGGGCGAAATCATCC | TGGGGTGGGTGTTTAGAAAA | 59.9 | 524 | INTERGENIC | | | | |
| CaPOPI_867 | Ca2 | 30361475 | TATC/TATCGATC | TCCGCTAATAGTGGCAGATAAA | ACCAATTTGTGAAAGCTGGC | 58.9 | 617 | INTERGENIC | | | | |
| CaPOPI_868 | Ca2 | 30369374 | TAAAA/TAAAAA | TGTACTTCCTCCGATTCCAAA | TGAATGACGACGACGGTAAA | 59.6 | 632 | DRR | Ca_12515 | Q | | Multicopper oxidase, type 1 |
| CaPOPI_869 | Ca2 | 30373739 | TAAAAAAAAAA/TAAAAAAAAA | CGGGAATGAAAAGTGGAAGA | CCAGGGGCAAAACAAAGTAA | 60.0 | 518 | DRR | Ca_12514 | S | | Fcf2 pre-rRNA processing |
| CaPOPI_870 | Ca2 | 30375213 | TAAAAAAA/TAAAAAA | AATTGAATCAACCCCCTTCC | GGTTTGAAGCTAATGTCGTGC | 60.0 | 737 | DRR | Ca_12514 | S | | Fcf2 pre-rRNA processing |
| CaPOPI_871 | Ca2 | 30375265 | CT/C | AACCCCCTTCCTTTTGTGTC | TTGATTTTTGGGGTGGCTAA | 60.2 | 963 | DRR | Ca_12514 | S | | Fcf2 pre-rRNA processing |
| CaPOPI_872 | Ca2 | 30391104 | AT/ATT | TTGCATTCTCAAGGCTCAGA | TTTTGGGAGTGCTTGGTTTC | 59.7 | 461 | INTRON | Ca_12512 | R | | Leucine-rich repeat, cysteine-containing subtype |
| CaPOPI_873 | Ca2 | 30395203 | CATATATATA/CATATATATATA | CCCAACAACAACAAAAGCCT | AAACCAGCATGACAAAAGCA | 60.0 | 289 | INTERGENIC | | | | |
| CaPOPI_874 | Ca2 | 30433778 | TA/TAA | CGGTTGGCCCTTAACTGTAA | TCCTCAAACCCAAAATGGTC | 60.0 | 674 | INTERGENIC | | | | |
| CaPOPI_875 | Ca2 | 30447611 | A/AT | CTTTTGTCTTCTCGGCCTTG | CGTGCTCATCTCCATTGTTG | 60.0 | 891 | INTERGENIC | | | | |
| CaPOPI_876 | Ca2 | 30447804 | TTG/TTGTG | AACAAAAGGGGTTGGAAAGC | ATCTCCTCAGCATTGTCGCT | 60.3 | 809 | INTERGENIC | | | | |
| CaPOPI_877 | Ca2 | 30449164 | TAAAAAAA/TAAAAAAAA | TGTTGCAAAGAGAAAGAACTTCA | TGACGGTGGCAATCGATATT | 59.2 | 557 | DRR | Ca_12507 | | | |
| CaPOPI_878 | Ca2 | 30456719 | TAAAAAAAA/TAAAAAAA | TTCCTGATTCCGATCTCACC | CATACCTTGGGGATGCAAAG | 60.0 | 601 | DRR | Ca_12504 | | WOX | Homeobox |
| CaPOPI_879 | Ca2 | 30457303 | A/AT | TGCATCCCCAAGGTATGACT | CCACCATTAGATTTGCGGTT | 60.3 | 649 | DRR | Ca_12504 | | WOX | Homeobox |
| CaPOPI_880 | Ca2 | 30461396 | CTTTTTTT/CTTTTTTTTT | AAAAAGAAGTGGTTGTCCGC | AAAACGATGCTTATCCGTGAA | 59.2 | 929 | INTERGENIC | | | | |

| INDEL marker IDs | Chromosomes/ unanchored scaffolds | Physical positions (bp) | InDels (*Kabuli* reference genome-CDC Frontier/PI) | Forward primers (5'-3') | Reverse primers (5'-3') | Anneling temperature (0C) | Expected amplified product size (bp) | Structural annotation | | Functional annotation | | |
|---|---|---|---|---|---|---|---|---|---|---|---|---|
| | | | | | | | | Sequence components of *kabuli* genome | *Kabuli* gene accession IDs | NCBI-KOG | TFs | NCBI-nr database |
| CaPOPI_881 | Ca2 | 30480817 | TAAA/TAAAA | CTGCGTCCCATTTCAAGAAT | ATGTGTCTTTGGGAGCCAAC | 60.1 | 880 | INTRON | Ca_12502 | G | | Major intrinsic protein |
| CaPOPI_882 | Ca2 | 30481523 | TTTAAAAAAT/TTTAAAAAATATGTTAAAAAAT | GTTGGCTCCCAAAGACACAT | TCGATTTTCATTAAAGTGTTGGA | 60.0 | 631 | INTERGENIC | | | | |
| CaPOPI_883 | Ca2 | 30489361 | TTAT/TT | TGCAAAGTTTGATGGAGTGC | TAGAACCCCAATTTTGTCCG | 59.8 | 363 | INTERGENIC | | | | |
| CaPOPI_884 | Ca2 | 30490798 | AA/AACA | AGCCCAATGTGTAAGCCAAC | AAGCTTTTGCAAATGGGAGA | 60.0 | 410 | INTERGENIC | | | | |
| CaPOPI_885 | Ca2 | 30514265 | AAGA/AA | CACCAAATCACCCACAATCA | ATGGGCTGGTATATCATGGG | 60.2 | 972 | INTERGENIC | | | | |
| CaPOPI_886 | Ca2 | 30527508 | TAAAAAAAAAAAA/TAAAAAAAAAA | TTGAGATGCGTTGCAATAGG | CGTGCCACTTTCTTCCTTTC | 59.8 | 681 | INTERGENIC | | | | |
| CaPOPI_887 | Ca2 | 30553379 | GAAAA/GAAAAA | TTTTTATGAGGCGTGGAAGG | CCATGAACACGCCAAACATA | 60.1 | 874 | INTERGENIC | | | | |
| CaPOPI_888 | Ca2 | 30555764 | AAG/AAGACACGTGTAG | AAAACATAACCTTTTGCCCG | TCACTCCAAGCTTCCCAAAT | 59.0 | 257 | INTERGENIC | | | | |
| CaPOPI_889 | Ca2 | 30558600 | CTAT/CTATATAT | GATGCATACAAAAGTCACCCC | GGATTGATGAAGTGGTTGGG | 59.3 | 254 | INTERGENIC | | | | |
| CaPOPI_890 | Ca2 | 30568142 | AT/ATGT | CTTGTTTGTCGCAGTCGCTA | GGTCATACCAAGGCCAAGAA | 60.2 | 453 | INTRON | Ca_12495 | E | | Amino acid transporter, transmembrane |
| CaPOPI_891 | Ca2 | 30573602 | CGGG/CGGGG | TGGCTCATGACCCCTTTATC | GGCCCCTTTTCATTTTCATT | 59.9 | 709 | DRR | Ca_12495 | E | | Amino acid transporter, transmembrane |
| CaPOPI_892 | Ca2 | 30574328 | TAAAAA/TAAAAAAA | CCATTGAGGAGTGGAGAACTTT | CTTGTGTGTTCCCAATGCAA | 59.6 | 783 | INTERGENIC | | | | |
| CaPOPI_893 | Ca2 | 30575054 | AT/A | TGCAGCAACAATTATCCAGC | GACACACACCTCGTTTGCAT | 59.8 | 858 | INTERGENIC | | | | |
| CaPOPI_894 | Ca2 | 30622722 | A/ACCGTAATCC | CTTGGTCAGAATCACGGAGC | CACGTTGACCCTTTTTGTCA | 60.8 | 380 | INTRON | Ca_12490 | R | | Yippee-like protein |
| CaPOPI_895 | Ca2 | 30623473 | TAAA/TAAAAA | TGACAAAAAGGGTCAACGTG | CCGCAATCAATTTAGCGAAT | 59.6 | 686 | INTERGENIC | | | | |

| INDEL marker IDs | Chromosomes/ unanchored scaffolds | Physical positions (bp) | InDels (*Kabuli* reference genome-CDC Frontier/PI) | Forward primers (5'-3') | Reverse primers (5'-3') | Anneling temperature (0C) | Expected amplified product size (bp) | Structural annotation | | Functional annotation | | |
|---|---|---|---|---|---|---|---|---|---|---|---|---|
| | | | | | | | | Sequence components of *kabuli* genome | *Kabuli* gene accession IDs | NCBI-KOG | TFs | NCBI-nr database |
| CaPOPI_896 | Ca2 | 30623593 | TTATGTTTTAT/TTATGTTTTATATGTTTTAT | AGTAGAGCCCGACAAGATGC | CCGCAATCAATTTAGCGAAT | 59.5 | 483 | INTERGENIC | | | | |
| CaPOPI_897 | Ca2 | 30624454 | C/CT | TCATGAATGCAACGTGGTTT | TTTTGCTGTTTGTTTTACGTCAT | 60.0 | 646 | INTERGENIC | | | | |
| CaPOPI_898 | Ca2 | 30642500 | GAAAAAAAAA/GAAAAAAAAAAA | TGCAGTTGCCGACAATTTTAT | TCCTCGGTAACCAAACAAGC | 60.5 | 128 | INTERGENIC | | | | |
| CaPOPI_899 | Ca2 | 30645883 | TAAAAAAAAAA/TAAAAAAAAAAA | TGCTGGGTTGATGGATGTAA | TGCATGCATGGATCCCTAA | 59.9 | 816 | INTERGENIC | | | | |
| CaPOPI_900 | Ca2 | 30647034 | CAT/CATAT | TTGACGTACCCAACCATTGA | CCCTCACTTAAGCCCACAAA | 59.8 | 443 | INTERGENIC | | | | |
| CaPOPI_901 | Ca2 | 30665443 | ATTTTTTTTTT/ATTTTTTTT | TCATGGTGGACCGTAATCTG | CTACTGCCTTGGATTCCTTTT | 59.4 | 403 | INTRON | Ca_12489 | QI | bHLH | Cytochrome P450 |
| CaPOPI_902 | Ca2 | 30665911 | ATTTTTTTTTT/ATTTTTTTTTTTT | AAAAGGAATCCAAGGCAGTAG | TCCATTACCATCATACGCCA | 57.5 | 788 | INTRON | Ca_12489 | QI | bHLH | Cytochrome P450 |
| CaPOPI_903 | Ca2 | 30672058 | ATTTT/ATT | GGGCTGATCCAACACGTTAT | CCATGGGAAACGTTGGTAAC | 59.8 | 396 | DRR | Ca_12489 | QI | bHLH | Cytochrome P450 |
| CaPOPI_904 | Ca2 | 30672151 | TGGGG/TGGG | GGGCTGATCCAACACGTTAT | CCATGGGAAACGTTGGTAAC | 59.8 | 396 | DRR | Ca_12489 | QI | bHLH | Cytochrome P450 |
| CaPOPI_905 | Ca2 | 30675995 | A/AC | TTAAGTGGGTTGCGCTTTTT | AAGAACTTGGACGTTGGTGG | 59.8 | 536 | INTERGENIC | | | | |
| CaPOPI_906 | Ca2 | 30676386 | GAAAAAAAAAA/GAAAAAAAAAAA | ATCCACCAACGTCCAAGTTC | CCTTGGTATGACCCTAATTCCTT | 59.8 | 532 | INTERGENIC | | | | |
| CaPOPI_907 | Ca2 | 30676782 | ACTAGC/ACTAGCTAGC | GCTCCAGCAAAAATAGAAATGG | TCATCAATCAAACTACTTACGCAGA | 60.1 | 504 | INTERGENIC | | | | |
| CaPOPI_908 | Ca2 | 30690091 | CT/CTTT | GCGGTGTTTGTTTGGGTAGT | GGAGCACACAGATGCGAATA | 59.9 | 335 | INTERGENIC | | | | |
| CaPOPI_909 | Ca2 | 30690274 | A/AT | TTAGGGGAACGTGAAATTGG | CGGTCATACTGCACACACAA | 59.8 | 444 | INTERGENIC | | | | |
| CaPOPI_910 | Ca2 | 30690585 | AAATAATAATAATAATAA/AAATAATAATAATAATAATAATAA | TATTCGCATCTGTGTGCTCC | CGTGGTTGAGTAGATGTGCC | 59.8 | 670 | INTERGENIC | | | | |

| INDEL marker IDs | Chromosomes/ unanchored scaffolds | Physical positions (bp) | InDels (*Kabuli* reference genome-CDC Frontier/PI) | Forward primers (5'-3') | Reverse primers (5'-3') | Anneling temperature (0C) | Expected amplified product size (bp) | Structural annotation | | Functional annotation | | |
|---|---|---|---|---|---|---|---|---|---|---|---|---|
| | | | | | | | | Sequence components of *kabuli* genome | *Kabuli* gene accession IDs | NCBI-KOG | TFs | NCBI-nr database |
| CaPOPI_911 | Ca2 | 30695617 | CTTTTTTTTTT/CTTTTTTTTTTT | CCCATGCATATCACGTTTTG | ACGCAGGGCCATGATACTAC | 59.8 | 361 | DRR | Ca_12487 | KLB | YABBY | High mobility group, HMG1/HMG2 |
| CaPOPI_912 | Ca2 | 30701503 | T/TAATC | TTATCTGCCCTGGATTCTGC | TTGGAAGAGGCCAAATATGC | 60.2 | 753 | INTRON | Ca_12487 | KLB | YABBY | High mobility group, HMG1/HMG2 |
| CaPOPI_913 | Ca2 | 30702625 | GAAAAA/GAAAAAA | CTTCCGCCATCAATGACAC | AATCTCTTCAATCCCCCGTT | 60.1 | 566 | INTRON | Ca_12487 | KLB | YABBY | High mobility group, HMG1/HMG2 |
| CaPOPI_914 | Ca2 | 30702934 | ATTTTTTTTTT/ATTTTTTTTT | AACGGGGGATTGAAGAGATT | GCAAATTCTGCGGGTGTTAT | 59.8 | 508 | INTERGENIC | | | | |
| CaPOPI_915 | Ca2 | 30703181 | TAAAAAAA/TAAAAAA | AACGGGGGATTGAAGAGATT | CGCATACATACGGTGATTGC | 59.8 | 882 | INTERGENIC | | | | |
| CaPOPI_916 | Ca2 | 30704814 | CAA/CAAA | AAATTCAGTGCAACGCACAT | CCCACTTTGGATAACAATTCC | 59.2 | 445 | INTERGENIC | | | | |
| CaPOPI_917 | Ca2 | 30720315 | TATTA/T | TTGTGGCCGTTAATTTGTGA | GTTGATGTTATCAAATAAACAATTTCA | 60.0 | 344 | INTERGENIC | | | | |
| CaPOPI_918 | Ca2 | 30720521 | CAAAAAAAA/CAAAAAAA | TTGTGGCCGTTAATTTGTGA | CGAGTTTCTTTTTCCCGTCA | 60.0 | 787 | INTERGENIC | | | | |
| CaPOPI_919 | Ca2 | 30720601 | TAAAAA/TAAAAAA | TTGTGGCCGTTAATTTGTGA | TTGTTGTGGATGCGGTTAAA | 60.0 | 917 | INTERGENIC | | | | |
| CaPOPI_920 | Ca2 | 30732861 | A/AG | TCACAGAAGGGGTTTTGGTC | AAAATTGTGAACGGAGACCG | 59.9 | 394 | INTRON | Ca_12484 | T | RAV | C2 calcium-dependent membrane targeting |
| CaPOPI_921 | Ca2 | 30737308 | TAAAAAAAAA/TAAAAAAAAAAA | TTTTGCCATGGTCTTGGATT | TCAAGAAAATTGCCAACCCT | 60.3 | 618 | DRR | Ca_12484 | T | RAV | C2 calcium-dependent membrane targeting |
| CaPOPI_922 | Ca2 | 30737463 | TAAAAAA/TAAAAAAA | TTTTGCCATGGTCTTGGATT | TGGGCCCTCATGATATTGAT | 60.3 | 720 | DRR | Ca_12484 | T | RAV | C2 calcium-dependent membrane targeting |
| CaPOPI_923 | Ca2 | 30773455 | GAAAAAAAAA/GAAAAAAAAAA | TGACTCCATCCACTTATCCCA | CCCATGAGTTTATGTTGGGG | 60.3 | 158 | INTERGENIC | | | | |
| CaPOPI_924 | Ca2 | 30788694 | TTTTATTTATTTATT/TTTTATTTATTTATTTATT | TCTATCCTCACCACCTCAAGC | TGCATGTCAAGTTGGATTCAT | 59.3 | 774 | INTERGENIC | | | | |
| CaPOPI_925 | Ca2 | 30790808 | GAAAAAAAA/GAAAAAAAAA | CACTCAGGGGATGAATGTACG | CGAAGTTTGGACATGAGCTG | 60.4 | 480 | INTERGENIC | | | | |

| INDEL marker IDs | Chromosomes/ unanchored scaffolds | Physical positions (bp) | InDels (*Kabuli* reference genome-CDC Frontier/PI) | Forward primers (5'-3') | Reverse primers (5'-3') | Anneling temperature (0C) | Expected amplified product size (bp) | Structural annotation | | Functional annotation | | |
|---|---|---|---|---|---|---|---|---|---|---|---|---|
| | | | | | | | | Sequence components of *kabuli* genome | *Kabuli* gene accession IDs | NCBI-KOG | TFs | NCBI-nr database |
| CaPOPI_926 | Ca2 | 30794649 | TAAAAAAAAA/TAAAAAAAA | TGATTCTCGACTGAAAACATGAG | TTGCATTTCACTGGAGACGTT | 59.4 | 641 | INTERGENIC | | | | |
| CaPOPI_927 | Ca2 | 30796973 | CAAA/CAAAA | CGTGTCCCTGATATTTTGCC | GGGACAGTTTTGTTTGTGGC | 60.3 | 108 | INTERGENIC | | | | |
| CaPOPI_928 | Ca2 | 30799752 | T/TGAC | CCATGAAGTTGAAGGCAAGA | TCTCTGGTGGGAAGGTTCAG | 58.8 | 556 | DRR | Ca_12478 | R | MYB | Protein kinase, catalytic domain |
| CaPOPI_929 | Ca2 | 30846096 | GAAAA/GAAAAA | CCTTTGCGAACTTGAAGACC | TCCTCCCTCTTGCAGGTTTA | 59.9 | 815 | INTERGENIC | | | | |
| CaPOPI_930 | Ca2 | 30848508 | TTGATAAT/TTGATAATAATGATAAT | TTGAAGGACCAAAATCATGAAC | CATACAACGCAAGCTAGGCA | 58.9 | 533 | INTERGENIC | | | | |
| CaPOPI_931 | Ca2 | 30860320 | TAA/TAAA | TGAACAAAAAGGTTGGGTGA | TGCTCAACTTTGACCCATTG | 59.0 | 487 | INTERGENIC | | | | |
| CaPOPI_932 | Ca2 | 30860644 | CAAA/CA | TGGGTCAAAGTTGAGCATGA | CACCTTTGGAAAGAGCTGGA | 60.2 | 557 | INTERGENIC | | | | |
| CaPOPI_933 | Ca2 | 30872234 | CAT/CATAT | CAAAAGCACAACAAGCCAGA | AATTTAAAGAAAGGCCGGGA | 60.0 | 268 | INTRON | Ca_12473 | U | | Synaptobrevin |
| CaPOPI_934 | Ca2 | 30883523 | TGCG/TG | CACATCAACAGGGCAAAATG | GTGTGTGCAATCTCTGGCAT | 60.0 | 682 | INTRON | Ca_12473 | U | | Synaptobrevin |
| CaPOPI_935 | Ca2 | 30887613 | AT/A | TCAAGGGAGGATTACATGGG | AAAGGGAAAGTTGCATCACG | 59.7 | 826 | INTERGENIC | | | | |
| CaPOPI_936 | Ca2 | 30890128 | AAAATAAATAAATAAATAAA/AAAATAAATAAATAAA | GATGATGTAAATGGGAGCCAA | CATTTGTTGAATGTTGCCCA | 59.8 | 697 | INTERGENIC | | | | |
| CaPOPI_937 | Ca2 | 30890896 | ATTTTTT/ATTTTTTT | CTCTTTTCGAGTCTCACCGC | ATGCCATGACGTGAACAAAA | 60.1 | 566 | INTERGENIC | | | | |
| CaPOPI_938 | Ca2 | 30892153 | A/ATGTAC | AGGATGGGTTTCTGTGTTGC | CTAAGGGGCAAAAGCGTACA | 60.0 | 617 | INTRON | Ca_12472 | | | |
| CaPOPI_939 | Ca2 | 30901717 | TG/T | TCATTGGTGTTTACCGGGAT | TAGCCTTTTGCAATATGGGG | 60.1 | 886 | DRR | Ca_12470 | DO | | |
| CaPOPI_940 | Ca2 | 30945167 | GT/GTCT | GACCATGGAATCGGACAAGT | CTTCTTGCATCCCCTTGTGT | 59.8 | 684 | INTRON | Ca_12469 | U | bHLH | Clathrin, heavy chain/VPS, 7-fold repeat |

| INDEL marker IDs | Chromosomes/ unanchored scaffolds | Physical positions (bp) | InDels (*Kabuli* reference genome-CDC Frontier/PI) | Forward primers (5'-3') | Reverse primers (5'-3') | Anneling temperature (0C) | Expected amplified product size (bp) | Structural annotation | | Functional annotation | | |
|---|---|---|---|---|---|---|---|---|---|---|---|---|
| | | | | | | | | Sequence components of *kabuli* genome | *Kabuli* gene accession IDs | NCBI-KOG | TFs | NCBI-nr database |
| CaPOPI_941 | Ca2 | 30963140 | GT/G | TAAGCCCATTGAACTGCTCC | CACAATTGCCTACACCATCG | 60.2 | 780 | INTERGENIC | | | | |
| CaPOPI_942 | Ca2 | 30997225 | A/AT | TCACCCTCGTATCCACAACA | ATGTGGCCATTGACAAGTGA | 60.0 | 245 | DRR | Ca_12465 | | ERF | Pathogenesis-related transcriptional factor/ERF, DNA-binding |
| CaPOPI_943 | Ca2 | 31013894 | TAAAAAAAAA/TAAAAAAAAAAAA | GTGGTAGAGGGAAGGCACAA | TCATGGATTTGTGGTGTGCT | 60.1 | 615 | INTERGENIC | | | | |
| CaPOPI_944 | Ca2 | 31017880 | A/ATTG | TCCTTGATGCTAAGGTTGCC | GACCCATCTCCAATAGCCCT | 60.2 | 584 | DRR | Ca_12462 | R | G2-like | Bacterial surface antigen (D15) |
| CaPOPI_945 | Ca2 | 31041300 | GGAA/GGAAGAAGAA | TACATCGGCGGTTCCTTAAC | GGCTAAATTACATCTGTGGTCCTT | 60.0 | 553 | INTERGENIC | | | | |
| CaPOPI_946 | Ca2 | 31051512 | GTATATATATATATAT/GTATATATATATATATATATAT | CCAAACGGCCACTAATGTCT | CTGCATTTTCCATCCCAAGT | 60.0 | 614 | INTRON | Ca_12459 | D | | Rad21/Rec8-like protein, C-terminal, eukaryotic |
| CaPOPI_947 | Ca2 | 31085057 | T/TTAAA | CACCAACATAAACAATGTCGAA | ACCACAAATGGAGAAGGTGC | 58.5 | 942 | DRR | Ca_12455 | | | |
| CaPOPI_948 | Ca2 | 31105983 | TAAAAAA/TAAAAAAA | TGTGGATTATCACCACTCCAA | TTTTTCCCGCTTAAAATGTAGC | 58.8 | 557 | INTERGENIC | | | | |
| CaPOPI_949 | Ca2 | 31108593 | ATTTTTT/ATTTTTTT | TCCAACCAAGTGTCAATCCA | TGCTAGATTGGCCAGCATAA | 59.9 | 792 | INTERGENIC | | | | |
| CaPOPI_950 | Ca2 | 31118846 | ATATTATTATTATTATTAT/ATATTATTATTATTAT | AACGCCAAGTCAAGACGAGT | AAGTATGTTGCATTGCGATTTTT | 59.9 | 352 | INTERGENIC | | | | |
| CaPOPI_951 | Ca2 | 31125141 | TTCT/TT | TCTCTTCCGTCTGCCATTTT | CCACATTGGGTATTTTTGGG | 59.8 | 581 | INTERGENIC | | | | |
| CaPOPI_952 | Ca2 | 31131673 | GAATAATAA/GAATAATAATAA | TTTGGACAGCAAGAAGATGG | CATGCAAGAAATGCAAGGAA | 58.8 | 473 | INTERGENIC | | | | |
| CaPOPI_953 | Ca2 | 31133111 | CAA/CAAA | GGTGATCAAACACATCGTCG | TTAGGTGGAGAGGGTGAGGA | 60.0 | 807 | INTERGENIC | | | | |
| CaPOPI_954 | Ca2 | 31138425 | AA/AATA | TGATGAGACGTTGGTCAAACA | TTTCCTCCTTGTTCACCTCC | 60.1 | 464 | INTRON | Ca_12449 | | | Domain of unknown function DUF231, plant |
| CaPOPI_955 | Ca2 | 31146520 | CA/CACTAATTTTAGA | CTAGAAAGTCCGGCGTATCG | TTGGCCTGAAGAATGACCTC | 59.9 | 557 | INTERGENIC | | | | |

| INDEL marker IDs | Chromosomes/ unanchored scaffolds | Physical positions (bp) | InDels (*Kabuli* reference genome-CDC Frontier/PI) | Forward primers (5'-3') | Reverse primers (5'-3') | Anneling temperature (0C) | Expected amplified product size (bp) | Structural annotation | | Functional annotation | | |
|---|---|---|---|---|---|---|---|---|---|---|---|---|
| | | | | | | | | Sequence components of *kabuli* genome | *Kabuli* gene accession IDs | NCBI-KOG | TFs | NCBI-nr database |
| CaPOPI_956 | Ca2 | 31151939 | A/AT | AGCCAAAGTGAGAAAGGCAA | AAGGATCCAAGCCCACTTCT | 60.0 | 740 | INTERGENIC | | | | |
| CaPOPI_957 | Ca2 | 31158685 | GAAAA/GAAA | GGGAACCTGGATTACCACCT | TCCATTCAATGCTCACCGTA | 60.1 | 179 | INTRON | Ca_12447 | L | ERF | SNF2-related |
| CaPOPI_958 | Ca2 | 31160632 | ATTTTTTTTTTT/ATTTTTT | AGTTTGAGAGGAAAACACGCA | TTTTGAACGTCACTTTATGATCG | 59.9 | 426 | INTERGENIC | | | | |
| CaPOPI_959 | Ca2 | 31172482 | TATTTAAT/TAT | CCTTGAATAAAAATATGGGGCA | TCAATAACTTTTAACAGTCCGACAA | 60.0 | 526 | INTERGENIC | | | | |
| CaPOPI_960 | Ca2 | 31172925 | AATATATATATA/AATATATATATATA | TTGTCGGACTGTTAAAAGTTATTGA | TAGTGCGCGACATTTTATGC | 59.2 | 265 | INTERGENIC | | | | |
| CaPOPI_961 | Ca2 | 31177108 | ATT/ATTT | TCCAATTTGATATCGGTCTCTTG | AAACATAAAACAACGTGAGACAAA | 60.3 | 970 | INTERGENIC | | | | |
| CaPOPI_962 | Ca2 | 31178815 | CTTTTTTTTT/CTTTTTTT | TGTAACAAAAAGGGAGGAAAAA | ACGCAGATGCCAAAGTTGTA | 57.9 | 691 | INTERGENIC | | | | |
| CaPOPI_963 | Ca2 | 31180716 | TAA/TAAA | GGCCAATTGCTAAGTGGATG | GCTTTGATTCTTGCAATGTCC | 60.5 | 364 | INTERGENIC | | | | |
| CaPOPI_964 | Ca2 | 31189040 | GATCA/GATCATCA | CAATAGGGTTTGCATCCCAC | TTCATGGTTGAGAGCCACTG | 60.2 | 532 | INTRON | Ca_12445 | Q | | Multicopper oxidase, type 1 |
| CaPOPI_965 | Ca2 | 31232642 | GTTAATTAATTA/GTTAATTA | TGTCATCAAGATGCGTCAAAG | CCATTGTGATGTTGGTGGAA | 59.9 | 830 | INTERGENIC | | | | |
| CaPOPI_966 | Ca2 | 31233960 | AG/A | GGGTGGAAATCATGGTGAAC | TTGGTTGGAAGGAATGGAAG | 60.0 | 436 | INTRON | Ca_12440 | | | |
| CaPOPI_967 | Ca2 | 31240228 | TCAA/T | CTGCCCCCACTATTTCTCAA | GAAACACTTGGCATGTTAAGGA | 60.1 | 479 | INTERGENIC | | | | |
| CaPOPI_968 | Ca2 | 31244339 | AGG/AG | CAAGACTCAGGGTGGGTTGT | AAATCCATTGGGAAATGCAG | 60.0 | 547 | INTRON | Ca_12439 | | C3H | Biotin/lipoyl attachment |
| CaPOPI_969 | Ca2 | 31286944 | A/ATAATG | TGGTATATTTGCACGGACCA | AAATGCGAGTCTAAGAGGATGC | 59.8 | 330 | INTERGENIC | | | | |
| CaPOPI_970 | Ca2 | 31294019 | AG/AGAGG | AGACCGTCGGTTTCAATGAC | AAAAATCTTTAACAAGCGGGT | 60.0 | 494 | INTERGENIC | | | | |

| INDEL marker IDs | Chromosomes/ unanchored scaffolds | Physical positions (bp) | InDels (*Kabuli* reference genome-CDC Frontier/PI) | Forward primers (5'-3') | Reverse primers (5'-3') | Anneling temperature (0C) | Expected amplified product size (bp) | Structural annotation | | Functional annotation | | |
|---|---|---|---|---|---|---|---|---|---|---|---|---|
| | | | | | | | | Sequence components of *kabuli* genome | *Kabuli* gene accession IDs | NCBI-KOG | TFs | NCBI-nr database |
| CaPOPI_971 | Ca2 | 31299649 | TAAAAAAAAAA/TAAAAAAAA | GCTGCAATGGAAATGCA TAA | GCGAATTTCACAATCCC ACT | 59.7 | 972 | INTERGENIC | | | | |
| CaPOPI_972 | Ca2 | 31300005 | A/AG | CATTCCAATGTGCAACC TCT | GCGAATTTCACAATCCC ACT | 58.6 | 535 | INTERGENIC | | | | |
| CaPOPI_973 | Ca2 | 31317559 | GTT/GTTT | ATGGCAAACTTATGGCC AAG | GAGTGGGTTGGTTGTTT GGT | 60.0 | 831 | DRR | Ca_12431 | U | | NSF attachment protein |
| CaPOPI_974 | Ca2 | 31352669 | CTTTT/CTTT | CCCTTGCTGTGGATGGT AGT | TCCAAAATCTGCAAGAG GCT | 60.0 | 569 | INTERGENIC | | | | |
| CaPOPI_975 | Ca2 | 31355084 | AT/ATT | GCATGGAAAAATGAAGG CAT | GAATGCATTGCTCATTT GGA | 59.9 | 674 | INTERGENIC | | | | |
| CaPOPI_976 | Ca2 | 31365474 | CT/C | TTTACGGCAGAATTTGA CCA | TGGCTTTTTCCCTACTTC CA | 59.2 | 361 | INTERGENIC | | | | |
| CaPOPI_977 | Ca2 | 31367430 | TA/TAGGCAA | ATTGTAATGCGGTTCCG AAG | CTCCACACGTGCCATCA TAC | 60.0 | 485 | INTERGENIC | | | | |
| CaPOPI_978 | Ca2 | 31367840 | CTATA/CTA | GTATGATGGCACGTGTG GAG | CCTTCCCTTTCCCTTGA AAA | 60.0 | 399 | INTERGENIC | | | | |
| CaPOPI_979 | Ca2 | 31386815 | TAAA/TAA | CAGTCGAATCCGCCATA TCT | TTGGGAATCGCATTTTT CTC | 60.1 | 587 | INTRON | Ca_12422 | | MYB_rela ted | Plant peroxidase |
| CaPOPI_980 | Ca2 | 31388110 | TAAAA/TAAA | TCAGGGGACAATAACTT GGC | TCCATTTACCCACTAGA CCCA | 59.9 | 919 | DRR | Ca_12422 | | MYB_rela ted | Plant peroxidase |
| CaPOPI_981 | Ca2 | 31388227 | TAAAA/TAAAAA | CATCAGTCCCCTCACTG GAT | TCCATTTACCCACTAGA CCCA | 59.9 | 707 | DRR | Ca_12422 | | MYB_rela ted | Plant peroxidase |
| CaPOPI_982 | Ca2 | 31388368 | TTT/TTTTCTT | CATCAGTCCCCTCACTG GAT | CGAAACAGTTCCCGAAA GAA | 59.9 | 866 | DRR | Ca_12422 | | MYB_rela ted | Plant peroxidase |
| CaPOPI_983 | Ca2 | 31407170 | GAAAAAAA/GAAAAAAAA | TGGAACCCAGCCGTATA AAG | TCTTGCTCAAGGGTCAT TCA | 60.0 | 223 | INTRON | Ca_12421 | | ERF | Protein kinase, catalytic domain |
| CaPOPI_984 | Ca2 | 31419525 | T/TGG | GGCTTTCCATTTTGGGT TTT | ATTCCAATGTGGAAGGA CAA | 60.2 | 258 | INTERGENIC | | | | |
| CaPOPI_985 | Ca2 | 31439958 | GAAAAAAA/GAAAAAAAA | CAAATGTCAAGGTGAAA ATCCA | TGCTCCTTATGCCAACT CCT | 59.8 | 492 | INTERGENIC | | | | |

| INDEL marker IDs | Chromosomes/ unanchored scaffolds | Physical positions (bp) | InDels (*Kabuli* reference genome-CDC Frontier/PI) | Forward primers (5'-3') | Reverse primers (5'-3') | Anneling temperature (0C) | Expected amplified product size (bp) | Structural annotation | | Functional annotation | | |
|---|---|---|---|---|---|---|---|---|---|---|---|---|
| | | | | | | | | Sequence components of *kabuli* genome | *Kabuli* gene accession IDs | NCBI-KOG | TFs | NCBI-nr database |
| CaPOPI_986 | Ca2 | 31450111 | TCC/TC | TTCCCAATCGATCCTACGAG | ATTGTGGAGAATCTGCAGGG | 60.0 | 313 | INTERGENIC | | | | |
| CaPOPI_987 | Ca2 | 31456735 | ATTTTT/ATTTTTT | AGCGCGCAATATGTCTACCT | CCACTCTAGCTCGGTCCTTG | 59.9 | 300 | DRR | Ca_12418 | | Trihelix | |
| CaPOPI_988 | Ca2 | 31534378 | CG/CGG | AGGAAAGCAACGTGAACAGC | CCAAGACAGGCAAAGAAAGC | 60.4 | 519 | INTERGENIC | | | | |
| CaPOPI_989 | Ca2 | 31534854 | TT/TTCT | GCTTTCTTTGCCTGTCTTGG | ACTTTGTCCACTGCCAAACC | 60.0 | 331 | INTERGENIC | | | | |
| CaPOPI_990 | Ca2 | 31550288 | CTTTTT/CTTTTTT | ACAAATATCCCATCCCACGA | TGCACGTAAAATTGCCTAAAA | 60.0 | 721 | INTERGENIC | | | | |
| CaPOPI_991 | Ca2 | 31562756 | ACC/ACCC | CACTTAGGAATTCCCCACCA | AGCTTGAAACCCTTGACCCT | 59.8 | 476 | INTERGENIC | | | | |
| CaPOPI_992 | Ca2 | 31657254 | CTTTTTTTT/CTTTTTTTTT | GAACGTGGAAAACTTCCATCA | GTTGGGAGGGTTCACTTCTG | 60.0 | 570 | INTERGENIC | | | | |
| CaPOPI_993 | Ca2 | 31670858 | CGTCGCTGTCGCTGTCGCTGTCGCTG/ CGTCGCTGTCGCTG | ACCGCCACCATTGTAGTCTC | CCGAGCCATCAAAAGATGAT | 60.0 | 434 | CDS (large-effect mutations) | Ca_17848 | | | |
| CaPOPI_994 | Ca2 | 31678680 | TC/T | GGAGCGCTTTAAACACAAGC | TGGAAACCAAGGTCCAAGTC | 60.0 | 517 | INTERGENIC | | | | |
| CaPOPI_995 | Ca2 | 31694754 | A/AAT | GCTTTTCATAAGAAGCGTTGTAA | AATTGAGCTTGTTGATGTGGA | 57.9 | 305 | INTERGENIC | | | | |
| CaPOPI_996 | Ca2 | 31697056 | TTTT/TTTTTATTT | GTGTGCAGGTAAGGCTGACA | ATTTGGCATTGGGTTGGATA | 59.9 | 759 | INTERGENIC | | | | |
| CaPOPI_997 | Ca2 | 31713656 | TAAG/TAAGAAG | TCGGTTTCTCCTTCACCATC | TTGTCAAACACGCAATCCAT | 60.1 | 595 | INTERGENIC | | | | |
| CaPOPI_998 | Ca2 | 31746177 | AATAT/AATATAT | CGAGTTGTAAAATTTGGCTTCC | GGGGATGTGTATTAAAGGGGA | 60.0 | 914 | INTERGENIC | | | | |
| CaPOPI_999 | Ca2 | 31794099 | TAA/TAAA | TGTGTACGTGTGCATGGAAG | ATCCTTGGCCTTTTGGCTAT | 59.2 | 247 | INTRON | Ca_17840 | | | Glycine rich protein |
| CaPOPI_1000 | Ca2 | 31794610 | GAAAAA/GAAAAAAA | ATAGCCAAAAGGCCAAGGAT | CTGGTCTGTCACTTTCAGCG | 59.9 | 804 | INTERGENIC | | | | |

| INDEL marker IDs | Chromosomes/ unanchored scaffolds | Physical positions (bp) | InDels (*Kabuli* reference genome-CDC Frontier/PI) | Forward primers (5'-3') | Reverse primers (5'-3') | Anneling temperature (0C) | Expected amplified product size (bp) | Structural annotation | | Functional annotation | | |
|---|---|---|---|---|---|---|---|---|---|---|---|---|
| | | | | | | | | Sequence components of *kabuli* genome | *Kabuli* gene accession IDs | NCBI-KOG | TFs | NCBI-nr database |
| CaPOPI_1001 | Ca2 | 31799253 | TAAAAAA/TAAAAA | AAATGTCAACGTGGCGTACA | GCACGAGGATTGCTATGTGA | 60.0 | 832 | INTERGENIC | | | | |
| CaPOPI_1002 | Ca2 | 31814386 | CAAAAAAA/CAAAAAA | GGAGGCAACAACAAGGAGAG | CCATCTTTCAAGCCAACTCC | 59.8 | 685 | INTRON | Ca_17839 | | | IQ motif, EF-hand binding site |
| CaPOPI_1003 | Ca2 | 31828278 | ATTGTT/ATTGTTGTT | AAAAATCGCCATTGTTTTGG | AAAAATAGGGAATCCGGTGC | 59.8 | 510 | INTRON | Ca_17836 | | MYB_related | |
| CaPOPI_1004 | Ca2 | 31828392 | CTTTTTTT/CTTTTTT | AAAAATCGCCATTGTTTTGG | AAAAATAGGGAATCCGGTGC | 59.8 | 510 | INTRON | Ca_17836 | | MYB_related | |
| CaPOPI_1005 | Ca2 | 31838256 | GTGCGGTG/GTGCGGTGGAAATATGCGGTG | ACTTGACGATGGTTTTTGGC | CTAACCGCGAAACCAAACAT | 60.0 | 393 | DRR | Ca_17834 | | | |
| CaPOPI_1006 | Ca2 | 31853745 | ATTT/ATTATATTTT | CAACCGTAGCCCACCTAAAA | AATGCGCAAAGAAAGTTTGG | 60.0 | 610 | INTERGENIC | | | | |
| CaPOPI_1007 | Ca2 | 31853792 | AA/AAGA | CAACCGTAGCCCACCTAAAA | AATGCGCAAAGAAAGTTTGG | 60.0 | 610 | INTERGENIC | | | | |
| CaPOPI_1008 | Ca2 | 31861522 | ATTTT/ATTT | TTGCAAAATGCTTGAGAGAAAA | ATGGCCTATGCCAATGAAAC | 60.0 | 459 | INTERGENIC | | | | |
| CaPOPI_1009 | Ca2 | 31882952 | CTTTATTTATTTAT/CTTTATTTATTTATTTAT | TTTCTGGTGGGATCAGCTCT | GACATCCTGCGCCAGTTATT | 59.8 | 591 | INTRON | Ca_17830 | D | LBD | RZZ complex, subunit Zw10 |
| CaPOPI_1010 | Ca2 | 31883010 | AAGAG/AAG | TTTCTGGTGGGATCAGCTCT | GACATCCTGCGCCAGTTATT | 59.8 | 591 | INTRON | Ca_17830 | D | LBD | RZZ complex, subunit Zw10 |
| CaPOPI_1011 | Ca2 | 31892832 | TC/T | CATTTGGTCGGTCAAAAGAAA | TTCTACCCAAGAAAACATGCG | 60.0 | 473 | INTERGENIC | | | | |
| CaPOPI_1012 | Ca2 | 31905190 | ATTTT/ATTTTT | AATAAGGTGGTTTGGGGGAG | GGTATTCGTTGCGTCATGTG | 60.0 | 450 | INTRON | Ca_17827 | | C3H | |
| CaPOPI_1013 | Ca2 | 31905735 | CTACTATACT/CTACTATACTATACTATACT | TTCCAACGGTGAAAACAACA | CGATATAGGGTTGGTTTGCG | 60.0 | 207 | DRR | Ca_17826 | O | | Zinc finger, RING-type |
| CaPOPI_1014 | Ca2 | 31912393 | TTATCTA/TTATCTATCTA | TGAGAAATCTCCATCCCCAG | CGCATCCTTGACAACTCTGA | 60.0 | 650 | INTRON | Ca_17826 | O | | Zinc finger, RING-type |
| CaPOPI_1015 | Ca2 | 31920016 | AATTATAATCAATTAT/AATTATAATCAATTATTATAATCAATTAT | TTCAGAAGAAGGAAACAGCACA | GATCCACAAAATTGGTTTTTCA | 60.0 | 877 | INTERGENIC | | | | |

| INDEL marker IDs | Chromosomes/ unanchored scaffolds | Physical positions (bp) | InDels (*Kabuli* reference genome-CDC Frontier/PI) | Forward primers (5'-3') | Reverse primers (5'-3') | Anneling temperature (0C) | Expected amplified product size (bp) | Structural annotation | | Functional annotation | | |
|---|---|---|---|---|---|---|---|---|---|---|---|---|
| | | | | | | | | Sequence components of *kabuli* genome | *Kabuli* gene accession IDs | NCBI-KOG | TFs | NCBI-nr database |
| CaPOPI_1016 | Ca2 | 31921460 | TA/TAA | GGAAAGTCAAACTCTTAAATGGGA | TCTGTATCGCATGGTTGCTT | 59.9 | 533 | INTERGENIC | | | | |
| CaPOPI_1017 | Ca2 | 31925279 | GTTTTTTTTT/GTTTTTTTT | GACCAGCCTCCCTAACCTTC | TGTTGCCGATAGACCATGAA | 60.1 | 538 | INTERGENIC | | | | |
| CaPOPI_1018 | Ca2 | 31939044 | ATTTTTTTT/ATTTTTTT | ATCACATTGCAAAGCAACGA | TGTATTCACGGTTTGGGTTTT | 60.3 | 586 | DRR | Ca_17823 | R | C3H | Zinc finger, CCCH-type |
| CaPOPI_1019 | Ca2 | 31945989 | ATTTTTTTTT/ATTTTTTT | CACATGGAAGGCTAAAGGAA | CCACAAGTGTGGATGACAGG | 57.8 | 838 | INTERGENIC | | | | |
| CaPOPI_1020 | Ca2 | 32088009 | TTAATAATAATAATAATAATAATAATA/TTAATAATAATAATAATAATAATAATAATA | TCAGCAAGCAAGCATTATGG | ATGGGGAACGTTGACAGAAG | 60.0 | 605 | INTERGENIC | | | | |
| CaPOPI_1021 | Ca2 | 32127033 | TGC/TGCGC | TGCAAGTTGTGATGGGTTGT | TGTCTGTTCTGCACGGAGTC | 60.0 | 650 | INTRON | Ca_17812 | O | | Ubiquitin |
| CaPOPI_1022 | Ca2 | 32146442 | AT/ATT | ATTCCCACGCCACTTAGATG | CACTGTCACAGAGATAAGATGCAA | 60.0 | 807 | INTRON | Ca_17808 | | G2-like | Peptidase S8/S53, subtilisin/kexin/sedolisin |
| CaPOPI_1023 | Ca2 | 32161753 | TCCCC/TCCC | AGGCCATTAAGCATGACACC | TTCTTGGAACCCTGCATACC | 60.0 | 867 | INTERGENIC | | | | |
| CaPOPI_1024 | Ca2 | 32163428 | CATA/CATATATATATA | TCAAAGAGACATGATTTTGTCCA | GGTCAATCAGCTGTGAGCAA | 59.6 | 415 | INTERGENIC | | | | |
| CaPOPI_1025 | Ca2 | 32173774 | TC/T | ATGTTTTGATTGGGGCTTTG | TAAATCAGCCCTTTTGGTGG | 59.8 | 631 | DRR | Ca_17806 | | WRKY | Glycoside hydrolase, family 9 |
| CaPOPI_1026 | Ca2 | 32209131 | T/TG | GCAAAGATAGACACCACCCC | AACCATTCCCATGGAACAAA | 59.4 | 577 | INTERGENIC | | | | |
| CaPOPI_1027 | Ca2 | 32209334 | CTATATATA/CTATA | TCCATGGGAATGGTTTTGAT | TTGAAGAGGAAGTTTTGGCA | 60.0 | 342 | INTERGENIC | | | | |
| CaPOPI_1028 | Ca2 | 32209671 | TC/TCC | TCCATGGGAATGGTTTTGAT | TTTTAGTGTGCACCAATGTCAA | 60.0 | 899 | INTERGENIC | | | | |
| CaPOPI_1029 | Ca2 | 32210099 | CA/CAAATA | TCAGCTTCAATTATCTCCAACAA | TCGGCGACAAAAATCACATA | 58.9 | 644 | INTERGENIC | | | | |
| CaPOPI_1030 | Ca2 | 32210241 | TA/T | TGACATTGGTGCACACTAAAAA | CCACGTCATCTTCCTTTGGT | 59.1 | 505 | INTERGENIC | | | | |

| INDEL marker IDs | Chromosomes/ unanchored scaffolds | Physical positions (bp) | InDels (*Kabuli* reference genome-CDC Frontier/PI) | Forward primers (5'-3') | Reverse primers (5'-3') | Anneling temperature (0C) | Expected amplified product size (bp) | Structural annotation | | Functional annotation | | |
|---|---|---|---|---|---|---|---|---|---|---|---|---|
| | | | | | | | | Sequence components of *kabuli* genome | *Kabuli* gene accession IDs | NCBI-KOG | TFs | NCBI-nr database |
| CaPOPI_1031 | Ca2 | 32211970 | A/AG | ATGGAATTCTGAATGGTGGG | AAAACCACAAAATGGGGTCA | 59.6 | 763 | INTERGENIC | | | | |
| CaPOPI_1032 | Ca2 | 32213171 | GTGAT/GTGATGAT | CAACATGACAAGGACTTTCCAA | TTACCGGTATCGCAAGGAAC | 60.0 | 529 | INTERGENIC | | | | |
| CaPOPI_1033 | Ca2 | 32213424 | CAAA/CAAAA | AGGGATAGATAACGGCACGA | AAGGCCCTGCTAGAATGATG | 59.6 | 504 | INTERGENIC | | | | |
| CaPOPI_1034 | Ca2 | 32243132 | TCAA/TCAACAA | TCTGAGGGGATTCGTTATGC | TCCATAACTCTTGCCAACCC | 60.0 | 524 | CDS (large-effect mutations) | Ca_10292 | | bHLH | Protein kinase, catalytic domain |
| CaPOPI_1035 | Ca2 | 32282292 | CATTAGTGATT/CATTAGTGATTAGTGATT | ACCCTCCAGGGTGCTAAGAT | GCCAACATTTTCTGGTTGCT | 60.0 | 726 | INTRON | Ca_10287 | S | | |
| CaPOPI_1036 | Ca2 | 32293632 | AAGAAATTCAGAAATTCA/AAGAAATTCAGAAATTCAGAAATTCA | AAACCCTACGAAGTTTGGCA | TTGTCACCCGTCCAAATACA | 59.6 | 237 | INTERGENIC | | | | |
| CaPOPI_1037 | Ca2 | 32332565 | AAAATAAATAAATAAATA/AAAATAAATAAATAAATAAATA | CGCCAAAGTCATTGATTGAA | CATCTTGTCAGCCATAGCCA | 59.7 | 338 | INTERGENIC | | | | |
| CaPOPI_1038 | Ca2 | 32340350 | TGG/TG | TCCATTCAATGCTTTCAAATTC | TTGTGGTCATTTAAGGTGGTTG | 59.1 | 530 | INTERGENIC | | | | |
| CaPOPI_1039 | Ca2 | 32341023 | GAAT/GAATGTTAAAAT | CAACCACCTTAAATGACCACAA | AAAAACAATGTCATGCAGAGG | 59.8 | 682 | INTERGENIC | | | | |
| CaPOPI_1040 | Ca2 | 32353326 | G/GA | GAACGACAACAATTTCGCCT | CAATGGATTTCTTTGGCCTT | 60.1 | 513 | DRR | Ca_10283 | | | |
| CaPOPI_1041 | Ca2 | 32353407 | TGAAC/T | GAACGACAACAATTTCGCCT | CAATGGATTTCTTTGGCCTT | 60.1 | 513 | DRR | Ca_10283 | | | |
| CaPOPI_1042 | Ca2 | 32357617 | CTTT/CTTTT | TCCCAACTCTTCGAAATCGT | TTGGATGATAGACATTGAAATTTTG | 59.7 | 367 | INTERGENIC | | | | |
| CaPOPI_1043 | Ca2 | 32358261 | CAAAAAAA/CAAAAAA | TCAAAATTTCAATGTCTATCATCCA | GCGAGGATGAGAAGTTGTTGT | 59.7 | 658 | INTERGENIC | | | | |
| CaPOPI_1044 | Ca2 | 32362657 | TAAAAAAA/TAAAAAAAA | GCAAAAGCACAACCAAATCA | AATGACCACAATTTGCATTGA | 59.7 | 260 | INTERGENIC | | | | |
| CaPOPI_1045 | Ca2 | 32369826 | CT/CTCGT | TGGTTTCAAGGTCTTCCACA | GAAAAGTTAAAATCCAAAATAACATTC | 59.1 | 794 | INTERGENIC | | | | |

| INDEL marker IDs | Chromosomes/ unanchored scaffolds | Physical positions (bp) | InDels (*Kabuli* reference genome-CDC Frontier/PI) | Forward primers (5'-3') | Reverse primers (5'-3') | Anneling temperature (0C) | Expected amplified product size (bp) | Structural annotation | | Functional annotation | | |
|---|---|---|---|---|---|---|---|---|---|---|---|---|
| | | | | | | | | Sequence components of *kabuli* genome | *Kabuli* gene accession IDs | NCBI-KOG | TFs | NCBI-nr database |
| CaPOPI_1046 | Ca2 | 32379585 | AATTAATTTT/AATTAATTTTGATTAATTTT | TGTGCGAGTTTATTGCGGTA | CATTTGGTTCAAAATGTCATGG | 60.3 | 624 | INTERGENIC | | | | |
| CaPOPI_1047 | Ca2 | 32380306 | GTTTGAATTTAATTA/GTTTGAATTTAATTATTTGAATTTAATTA | AACTAACTTTTCGCAGCCCA | GGCAACTTGGCCAACACTAT | 59.9 | 587 | INTERGENIC | | | | |
| CaPOPI_1048 | Ca2 | 32393042 | ATTTTTTTT/ATTTTTTTTT | TGCCCATCCTTATGCTCTTC | ATCACGTGCAACCACAAGTC | 60.2 | 626 | INTERGENIC | | | | |
| CaPOPI_1049 | Ca2 | 32393117 | T/TC | TGCCCATCCTTATGCTCTTC | ATCACGTGCAACCACAAGTC | 60.2 | 626 | INTERGENIC | | | | |
| CaPOPI_1050 | Ca2 | 32393187 | AATAT/A | TGCCCATCCTTATGCTCTTC | ATCACGTGCAACCACAAGTC | 60.2 | 626 | INTERGENIC | | | | |
| CaPOPI_1051 | Ca2 | 32393441 | TGG/TGTGGG | TCGCACTTTTTCGTGTAGGA | GGGCTTGTTTGACGCATTAT | 59.5 | 588 | INTERGENIC | | | | |
| CaPOPI_1052 | Ca2 | 32393633 | TAACATAATAA/TA | GACTTGTGGTTGCACGTGAT | GGGCTTGTTTGACGCATTAT | 59.6 | 445 | INTERGENIC | | | | |
| CaPOPI_1053 | Ca2 | 32399194 | T/TATTAATGTCTAA | TCTTGCACGGGTGAGAACTA | CCTATTTGAAGAGTGCCCCA | 59.4 | 587 | INTERGENIC | | | | |
| CaPOPI_1054 | Ca2 | 32399302 | ATTTTTTTTT/ATTTTTTTTTT | AATTTGGGGCACTCTTCAAA | GCCTCTTCAAAAGTCGGTCA | 59.5 | 219 | INTERGENIC | | | | |
| CaPOPI_1055 | Ca2 | 32417284 | ATTTTTTTT/ATTTTTTTTT | AAATTTGCGTGGAAAGGTTG | CTCTGGTGGCATGTATCCAA | 60.0 | 207 | INTRON | Ca_10278 | | bHLH | Protein kinase, catalytic domain |
| CaPOPI_1056 | Ca2 | 32423881 | CAAAA/CAAAAA | TGAGGCATGATAATTGAGCG | TGTTGTCATCAATCACGCAG | 59.8 | 536 | DRR | Ca_10277 | | | Protein of unknown function DUF247, plant |
| CaPOPI_1057 | Ca2 | 32476919 | ATGT/AT | CATGGATGGTGCTTCAAATG | CAACTGCAATGTTCCATATTCG | 59.9 | 885 | DRR | Ca_10273 | R | C3H | Leucine-rich repeat |
| CaPOPI_1058 | Ca2 | 32480670 | T/TA | TCCACATGCTTCCAATCAAA | ACCACTCCAACCAGTCGTTT | 60.0 | 584 | INTERGENIC | | | | |
| CaPOPI_1059 | Ca2 | 32480992 | GTTT/GTT | CATGCTTCCAATCAAACACG | CAACGCCTCAAGTAGGGGTA | 60.1 | 740 | INTERGENIC | | | | |
| CaPOPI_1060 | Ca2 | 32481790 | ATATAT/A | GGGTGGAAATGCAGAAGAAA | AAGCTTTCGGTGATTGCTGT | 60.1 | 535 | INTERGENIC | | | | |

| INDEL marker IDs | Chromosomes/ unanchored scaffolds | Physical positions (bp) | InDels (*Kabuli* reference genome-CDC Frontier/PI) | Forward primers (5'-3') | Reverse primers (5'-3') | Anneling temperature (0C) | Expected amplified product size (bp) | Structural annotation | | Functional annotation | | |
|---|---|---|---|---|---|---|---|---|---|---|---|---|
| | | | | | | | | Sequence components of *kabuli* genome | *Kabuli* gene accession IDs | NCBI-KOG | TFs | NCBI-nr database |
| CaPOPI_1061 | Ca2 | 32642955 | ATTTTTTTTTT/ATTTTTTTTT | CAAAGTTGAGGAAAGGCCAG | GGAATCACCCATCCACATTC | 59.8 | 869 | DRR | Ca_10258 | | | |
| CaPOPI_1062 | Ca2 | 32713700 | TTTTTTTTT/TTTTTTTTTTT | AACGCATCGGTAAACACTCC | GAGGTACTACAGCTTGCCCC | 60.0 | 234 | INTERGENIC | | | | |
| CaPOPI_1063 | Ca2 | 32713895 | CAAAAAAA/CAAAACAAAAAAAA | AACGCATCGGTAAACACTCC | TGTTTTGCAGCAAGATGTCC | 60.0 | 695 | INTERGENIC | | | | |
| CaPOPI_1064 | Ca2 | 33257195 | ATTTTTTTTT/ATTTTTTTTTT | ATCGCATTTTGCCTTTGTTT | GCTGTCGTTTGCAGCATAAA | 59.6 | 801 | INTERGENIC | | | | |
| CaPOPI_1065 | Ca2 | 33306373 | TTATATATATATATATATA/TTATATATATATATA | GAATCCGTTTCCGAGTTTGA | TCAATGGAAATCAAAGTTGAAGG | 60.1 | 644 | INTERGENIC | | | | |
| CaPOPI_1066 | Ca2 | 33345639 | GTT/GT | CTCCATTTTGGGCAGGTTTA | GGATGATCTGTTTTTCGCGT | 59.9 | 573 | INTRON | Ca_10191 | T | FAR1 | Protein kinase, catalytic domain |
| CaPOPI_1067 | Ca2 | 33346809 | C/CA | ATGCACATGAACCGATTGAA | AAACAAGGTTGAATCACGCC | 59.9 | 718 | INTRON | Ca_10191 | T | FAR1 | Protein kinase, catalytic domain |
| CaPOPI_1068 | Ca2 | 33398441 | TACACAC/TACAC | AGAGCCTACAGCAAGCCAAA | AAATGCAGGGTCACAACACA | 60.2 | 205 | DRR | Ca_10186 | T | M-type | Disease resistance protein |
| CaPOPI_1069 | Ca2 | 33405227 | AATATATATATA/AATATATATA | AAAGAGACATGACTTTGTCCATCA | TTTCGCTACCCATCATACCC | 60.0 | 586 | INTERGENIC | | | | |
| CaPOPI_1070 | Ca2 | 33639777 | TAAAAAAAA/TAAAAAAA | CGACGGAATAGTTTGCTCTCT | CGGAAAACCGTAGCTTAATACG | 58.6 | 892 | INTERGENIC | | | | |
| CaPOPI_1071 | Ca2 | 33715359 | ANNNNNNNNNN/ANNNNNNNN | CAAAAGGCGTGCATGAATTA | CTTGCCTTATTTTTCCTGCG | 59.7 | 271 | DRR | Ca_10165 | K | G2-like | SNF2-related |
| CaPOPI_1072 | Ca2 | 33959397 | ATATTTATTTATTT/ATATTTATTT | GGCTAATTTTCGTTATATGGATGC | ACTAGTGATGGCCGAAATGG | 60.1 | 764 | INTERGENIC | | | | |
| CaPOPI_1073 | Ca2 | 33984310 | CTATATATATATATATATATATATA/CCATATATATATATATATATATATATATA | TGGCTATGATCACAATTGGC | CCCTTTGCAAGAGACTCCAA | 59.5 | 869 | INTERGENIC | | | | |
| CaPOPI_1074 | Ca2 | 34028835 | AC/A | AAAGGGGTTTTGGCTATGCT | CATGATCATAATTGCATGGAAAA | 60.0 | 229 | INTERGENIC | | | | |
| CaPOPI_1075 | Ca2 | 34353805 | GTTTTTTTT/GTTTTTTTTT | AGTTGAAGGAGTGCATGAGGA | TAGGAGGCATATGTGAGGGG | 59.9 | 633 | INTERGENIC | | | | |

| INDEL marker IDs | Chromosomes/ unanchored scaffolds | Physical positions (bp) | InDels (*Kabuli* reference genome-CDC Frontier/PI) | Forward primers (5'-3') | Reverse primers (5'-3') | Anneling temperature (0C) | Expected amplified product size (bp) | Structural annotation | | Functional annotation | | |
|---|---|---|---|---|---|---|---|---|---|---|---|---|
| | | | | | | | | Sequence components of *kabuli* genome | *Kabuli* gene accession IDs | NCBI-KOG | TFs | NCBI-nr database |
| CaPOPI_1076 | Ca2 | 34355206 | CTTGGGCT/CTTGGGCTTTGTTGGGCT | TCTTTGATGCAATGCCAGAG | ATCATTCGAGGTGCCATCAT | 59.9 | 975 | INTERGENIC | | | | |
| CaPOPI_1077 | Ca2 | 34355457 | TAAAAAA/TAAAAAAA | ATTCTTGGGCTGTGTTTTGG | GGGTGATGAGGACTGGTTGT | 60.0 | 725 | INTERGENIC | | | | |
| CaPOPI_1078 | Ca2 | 34515091 | TTTATTATTATTATTATTATTATTATTATTATTATTATTATTATTATTATTATTATTA/TTTATTATTATTATTATTATTATTATTATTATTATTATTATTATTATTATTATTATTA | TGCCAATGTAATGATTCAGGA | AGCGTAAGTTTCGAGGACCA | 59.0 | 766 | INTERGENIC | | | | |
| CaPOPI_1079 | Ca2 | 34654101 | GTATATATATATATATATATATATATAT/GTATATATATATATATATATATAT | AGGACGAGACAAAATGAGCC | TGGAAACCAAGGTCCAAGTC | 59.3 | 219 | INTERGENIC | | | | |
| CaPOPI_1080 | Ca2 | 34683764 | TC/TCC | GTTTTTCGCAACCACCTTTC | CGCTTTTTGCACTAACCTCC | 59.6 | 682 | INTERGENIC | | | | |
| CaPOPI_1081 | Ca2 | 34684386 | CAACGTTAAC/CAAC | ACAATACTGCGGCCTGTTTC | GATTCCGATCGTTTTTCACG | 60.1 | 830 | INTERGENIC | | | | |
| CaPOPI_1082 | Ca2 | 34684923 | ATTTTTTT/ATTTTTTTT | TCGTGAAAAACGATCGGAAT | TGCTTGATTTTAATGGATGCC | 60.4 | 550 | INTERGENIC | | | | |
| CaPOPI_1083 | Ca2 | 34685252 | CATAAATAAATAAATAAA/CATAAATAAA | TCGTGAAAAACGATCGGAAT | TGGAGAGAAACAAAATGGGG | 60.4 | 816 | INTERGENIC | | | | |
| CaPOPI_1084 | Ca2 | 34685921 | TAAAAAAA/TAAAAA | CCAAAACTGGGGAGAAACAA | TACGATAGGGGGATCTCACG | 59.9 | 503 | INTERGENIC | | | | |
| CaPOPI_1085 | Ca2 | 34983521 | AATATATATATATATATATATATATATATATATATATATA/AATATATATATATATATATATATATATATATATATATATATATA | CAAATGCATGCTCCCCTAAT | CTAAAAGCCCAAAGCTGCAC | 59.9 | 450 | INTERGENIC | | | | |
| CaPOPI_1086 | Ca2 | 35258590 | A/ATT | AAAAAGTTTTAACACGGAGGTTTTT | TGTTGTTGGATTGGACATGG | 59.8 | 487 | INTERGENIC | | | | |
| CaPOPI_1087 | Ca2 | 35288109 | ATTTTTT/ATTTTTTT | TGCTTTATAGTGCTATTATGTGCCA | CGTCATTAAAAGGGAAACCAA | 60.1 | 757 | INTERGENIC | | | | |
| CaPOPI_1088 | Ca2 | 35288455 | TT/TTAT | TTCGATGGTATGCAGTAATTGG | TTCCCATGAAAGAGAGGTGG | 59.9 | 433 | INTERGENIC | | | | |
| CaPOPI_1089 | Ca2 | 35289402 | ACCCCC/ACCCC | ACCCACCTCTTTTTCTTGGG | TGGTCCTCGAAATTTGTAATGA | 60.3 | 609 | INTERGENIC | | | | |
| CaPOPI_1090 | Ca2 | 35298866 | AA/AACA | TGGGGGCTTAGAGCTTTGTA | CGATGTTGTTGATCTGATATCGT | 59.8 | 527 | INTERGENIC | | | | |

| INDEL marker IDs | Chromosomes/ unanchored scaffolds | Physical positions (bp) | InDels (*Kabuli* reference genome-CDC Frontier/PI) | Forward primers (5'-3') | Reverse primers (5'-3') | Anneling temperature (0C) | Expected amplified product size (bp) | Structural annotation | | Functional annotation | | |
|---|---|---|---|---|---|---|---|---|---|---|---|---|
| | | | | | | | | Sequence components of *kabuli* genome | *Kabuli* gene accession IDs | NCBI-KOG | TFs | NCBI-nr database |
| CaPOPI_1091 | Ca2 | 35303488 | ATTTTTT/ATTTTTTT | TTCCGAATAAGGATTTCTTGG | ATGGTTTGGGGAAAAGAACC | 58.2 | 137 | INTERGENIC | | | | |
| CaPOPI_1092 | Ca2 | 35304698 | C/CG | GCTTGGAAACAGCCTCAGAC | CAGGGTGGTACTGCAATGTG | 60.0 | 536 | INTERGENIC | | | | |
| CaPOPI_1093 | Ca2 | 35329169 | AATATATATATATATATATATATATAT/AATATATATATATATATATATATAT | CAATTTTGGAAGACGATTTTGA | TGTCCCGAAAAAGTTCATCC | 59.1 | 330 | INTERGENIC | | | | |
| CaPOPI_1094 | Ca2 | 35944964 | CAAAA/CA | TCCAACCATACCTGAAAGGC | CTCGTGCGAACTTGTGTGTT | 59.9 | 313 | CDS (large-effect mutations) | Ca_09769 | | | Ferredoxin-dependent bilin reductase |
| CaPOPI_1095 | Ca2 | 36146623 | GAAAAAA/GAAAAA | AAACAGCAACAAAAATCCCAA | TTTGGAAATCAAGAGGTGGG | 59.5 | 201 | INTERGENIC | | | | |
| CaPOPI_1096 | Ca2 | 36146917 | TCCCCC/TCCCCCC | AGGGAAACCCACCTCTTGAT | AAATCCAAAGTGCTTGCGTT | 59.8 | 623 | INTERGENIC | | | | |
| CaPOPI_1097 | Ca2 | 36224008 | CTTTTTTTTTT/CTTTTTTTTTTT | TTTTCACCACGACACACAAA | TGCACGTTTATAAGGAATCGG | 58.6 | 354 | INTERGENIC | | | | |
| CaPOPI_1098 | Ca3 | 62942 | AATATATATATATATATATATATA/AATATATATATATATATATATATATATATA | TTCTCTCGAGGATAGTCCGA | AGTTAAACGAGGGGTTTGCC | 57.6 | 936 | INTERGENIC | | | | |
| CaPOPI_1099 | Ca3 | 135372 | CA/C | GTTGTAATGGGGCTTAGGCA | TTGGTCGTTTGAGCCTTTCT | 60.0 | 478 | INTERGENIC | | | | |
| CaPOPI_1100 | Ca3 | 213670 | AATATATATATATATATAT/AATATATATATATATATATATAT | TTCCTTTTCCAATTTGCAGG | TCACGTAGAATGCTATCACTTTGA | 60.0 | 544 | DRR | Ca_22935 | M | MYB_related | Glycosyl transferase, family 48 |
| CaPOPI_1101 | Ca3 | 451560 | ATAACGTGATCTTA/ATA | AGCAGAAAGGGAGAATGGGT | ACGGTGAAGCTCCGAAAATA | 60.1 | 734 | INTERGENIC | | | | |
| CaPOPI_1102 | Ca3 | 454494 | AGGG/AGG | GGATTGGAGTGTTCAGCCAT | TTGTCATTAGTTTCAATCTCACCAA | 59.9 | 460 | INTERGENIC | | | | |
| CaPOPI_1103 | Ca3 | 454633 | GATATATATATATAT/GATATATATATAT | GGATTGGAGTGTTCAGCCAT | TTGTCATTAGTTTCAATCTCACCAA | 59.9 | 460 | INTERGENIC | | | | |
| CaPOPI_1104 | Ca3 | 474304 | GTTTTTT/GTTTTTTT | ACGTTGCGAGCAGAGATTTT | TTGGCTTCTGAATTTGGTCC | 60.0 | 655 | INTERGENIC | | | | |
| CaPOPI_1105 | Ca3 | 571479 | GTT/GTTGATT | GTGAGTGGTGAGGGCCTAAT | TTGCATACCTCGTATCATCCC | 59.0 | 407 | INTERGENIC | | | | |

| INDEL marker IDs | Chromosomes/ unanchored scaffolds | Physical positions (bp) | InDels (*Kabuli* reference genome-CDC Frontier/PI) | Forward primers (5'-3') | Reverse primers (5'-3') | Anneling temperature (0C) | Expected amplified product size (bp) | Structural annotation | | Functional annotation | | |
|---|---|---|---|---|---|---|---|---|---|---|---|---|
| | | | | | | | | Sequence components of *kabuli* genome | *Kabuli* gene accession IDs | NCBI-KOG | TFs | NCBI-nr database |
| CaPOPI_1106 | Ca3 | 613347 | ATTTTTTTTTTT/ATTTTTTTTTT | TGGAGCTTTGTTTTGGACCT | AGTAAGTGTCGTCGTTGGGC | 59.7 | 409 | INTERGENIC | | | | |
| CaPOPI_1107 | Ca3 | 726429 | ATTTTTTT/ATTTTTTTT | TCCTGTATCTTACCAAACAAGGC | GTTTGTTGTCGGCTTACGGT | 59.6 | 715 | INTERGENIC | | | | |
| CaPOPI_1108 | Ca3 | 781722 | CAA/CAAAA | AAAACGCGCCACTAATGACT | AGCAAGAGTGCAAAGTCGGT | 59.8 | 546 | DRR | Ca_22643 | V | B3 | Protease inhibitor I4, serpin |
| CaPOPI_1109 | Ca3 | 794982 | GTTTTTTTTTTT/GTTTTTTTTTT | TAGAGGGAGGAGGTGCAAGA | GGTGATATAGACGGGGCAGA | 59.9 | 686 | INTERGENIC | | | | |
| CaPOPI_1110 | Ca3 | 939073 | GTT/GTTTT | ATTGGTTCTGCTCTGGGTTG | GGGACCACAGCACCTATGTT | 60.1 | 296 | INTERGENIC | | | | |
| CaPOPI_1111 | Ca3 | 943806 | ATCT/ATCTCT | GTGAATTCATCCCATGTCCC | AGCCCTGGTAAGCCTTTTGT | 60.0 | 515 | INTERGENIC | | | | |
| CaPOPI_1112 | Ca3 | 1149110 | TAAAAAAAAAAAA/TAAAAAAAAAAA | TGTGCAATCGTTTTAACCCA | TGAGGGGTAATTTGAACCGA | 60.0 | 272 | INTERGENIC | | | | |
| CaPOPI_1113 | Ca3 | 1149320 | ATTTTTTTTT/ATTTTTTTTTT | TGTGCAATCGTTTTAACCCA | CCCTTACCACAAAATCAACTCA | 60.0 | 719 | INTERGENIC | | | | |
| CaPOPI_1114 | Ca3 | 1288242 | GTTTTTTTTTTT/GTTTTTTTTTT | TGGTGAGATCAACGATACGC | TGGTAACAAATCTCCGAAGGA | 59.7 | 371 | INTRON | Ca_19421 | R | | |
| CaPOPI_1115 | Ca3 | 1371048 | CAAAAAAAAAAAA/CAAAAAAAAAAA | CGGATCTCAGTTCGTGGGTA | CAGAGAGAGCGAGAGGGAGA | 61.1 | 567 | INTERGENIC | | | | |
| CaPOPI_1116 | Ca3 | 1403982 | GAA/GAAGAAA | TCCCCCGTAATAGGTGCATA | TCTTTGTTTCTGCGAACCCT | 60.2 | 521 | INTERGENIC | | | | |
| CaPOPI_1117 | Ca3 | 1533357 | TCAAAA/TCAAAACTCCAAAA | ACCCAAAAGCTAACTTGCCA | GCATCAACAAAGCAGCAGAA | 59.7 | 533 | INTERGENIC | | | | |
| CaPOPI_1118 | Ca3 | 1534003 | AAACTGCAACTAACT/AAACTGCAACTAACTGCAACTAACT | TTCTGCTGCTTTGTTGATGC | GTGGATGGGTGTTTTCCATC | 60.1 | 774 | INTERGENIC | | | | |
| CaPOPI_1119 | Ca3 | 1573297 | TAAAAAAAAAAA/TAAAAAAAAAA | ATAAGACCAAAGAAGCGGCA | GCATGCAATTGGTTTGACAC | 59.8 | 607 | INTERGENIC | | | | |
| CaPOPI_1120 | Ca3 | 2065051 | AATTATTATTATTATTATTATTATTATTATTATTA/AATTATTATTATTATTATTATTATTATTATTATTA | CGACCATGTAGTGAGGCAAA | AATAATTGCCCTACACCAACAA | 59.7 | 685 | INTERGENIC | | | | |

| INDEL marker IDs | Chromosomes/ unanchored scaffolds | Physical positions (bp) | InDels (*Kabuli* reference genome-CDC Frontier/PI) | Forward primers (5'-3') | Reverse primers (5'-3') | Anneling temperature (0C) | Expected amplified product size (bp) | Structural annotation | | Functional annotation | | |
|---|---|---|---|---|---|---|---|---|---|---|---|---|
| | | | | | | | | Sequence components of *kabuli* genome | *Kabuli* gene accession IDs | NCBI-KOG | TFs | NCBI-nr database |
| CaPOPI_1121 | Ca3 | 2176981 | GATTATTATTATTATTATTATTATTATTATTATTATTATTATTATGATTATTATTATTA/GATTATTATTATTATTATTATTATTATTATTATTATTATTATGATTATTATTATTA | ACGGATTGTGTTTTGCTTCT | CCTCTTCTTCGGTGATCTTTTG | 57.3 | 906 | INTERGENIC | | | | |
| CaPOPI_1122 | Ca3 | 2273008 | CAAAAAAAAAAA/CAAAAAAAAAAAA | TAAAGGATCGGACCCACTTG | GGCGATCCAAGAGTTCAATC | 59.9 | 822 | INTERGENIC | | | | |
| CaPOPI_1123 | Ca3 | 2327052 | CAAAAAAAAAAAAAA/CAAAAAAAAAAAAA | GATACGGAACCATCGCACTT | TTGGTTTCGTTCTCCATTCA | 60.0 | 343 | DRR | Ca_19393 | K | MYB | SANT domain, DNA binding |
| CaPOPI_1124 | Ca3 | 2414966 | A/AC | TTAATGAGGGGCTGAATTGG | AAGCGTACCTGTGGGAATTG | 59.9 | 259 | INTERGENIC | | | | |
| CaPOPI_1125 | Ca3 | 2855993 | TAAA/TAA | CTTGGTTGGAATTGCATGTG | CGTTTTGGAAGGTAGGGTGT | 60.0 | 514 | INTERGENIC | | | | |
| CaPOPI_1126 | Ca3 | 2930472 | ATCTC/ATCTCTC | AGGCGATGGAGGATAAAACC | CGGAAACGAGTGAAACCAAT | 60.3 | 502 | INTRON | Ca_22158 | D | | Tetratricopeptide repeat-containing |
| CaPOPI_1127 | Ca3 | 2962592 | TTTATTATTATTATTATTATTATTATTATT/TTTATTATTATTATTATTATTATTATT | AGAGGCAAACAAGAACCGAA | AACTCGGTGCCAACAGTCTT | 59.9 | 596 | INTERGENIC | | | | |
| CaPOPI_1128 | Ca3 | 3022233 | A/ATTTTCC | AGTTTTGGTAATTGTTGGTTTTATTT | CTCTAAGCCCCACAGAGTCG | 57.8 | 328 | INTERGENIC | | | | |
| CaPOPI_1129 | Ca3 | 3054800 | TAAAAAAAAAA/TAAAAAAAAA | TCTACGTATCTCTCGGTGCG | TGTCTTCTTCGTTGTTGCCA | 59.1 | 639 | INTERGENIC | | | | |
| CaPOPI_1130 | Ca3 | 3185857 | TAAAAAAAAAA/TAAAAAAAA | CGCCTCTCTTTTTCTATGCG | TGTTGCTGTTGTTGCCTCTC | 60.1 | 468 | INTERGENIC | | | | |
| CaPOPI_1131 | Ca3 | 3235341 | AGCTTCAGTCGCT/AGCT | TGGAGTGAGCTGTGGTTGAG | TGGTCACCACTTGGTGTTTG | 60.0 | 840 | INTERGENIC | | | | |
| CaPOPI_1132 | Ca3 | 3235471 | TAAA/TAA | ATAGGCCAAGCAACCAAAGA | GACGATGGTGAAGATGACGA | 59.7 | 663 | INTERGENIC | | | | |
| CaPOPI_1133 | Ca3 | 3235549 | GCACACA/GCACACACA | ATAGGCCAAGCAACCAAAGA | GACGATGGTGAAGATGACGA | 59.7 | 663 | INTERGENIC | | | | |
| CaPOPI_1134 | Ca3 | 3272993 | TTATATATATATATATATATATATATATATATA/TTATATATATATATATATATATATATATATA | TTGTAGTATCCCGACAATGTAAAA | TGATTCAACAATGATCCCCA | 57.7 | 687 | INTERGENIC | | | | |
| CaPOPI_1135 | Ca3 | 3411224 | CATATATATATATATATATAT/CATATATATATATATATATATATATATATAT | TGAATTTTTAAATGATTTTCTGCAA | ATAGTCGGCAAAACCCTCCT | 59.1 | 785 | INTERGENIC | | | | |

| INDEL marker IDs | Chromosomes/ unanchored scaffolds | Physical positions (bp) | InDels (*Kabuli* reference genome-CDC Frontier/PI) | Forward primers (5'-3') | Reverse primers (5'-3') | Anneling temperature (0C) | Expected amplified product size (bp) | Structural annotation | | Functional annotation | | |
|---|---|---|---|---|---|---|---|---|---|---|---|---|
| | | | | | | | | Sequence components of *kabuli* genome | *Kabuli* gene accession IDs | NCBI-KOG | TFs | NCBI-nr database |
| CaPOPI_1136 | Ca3 | 3424341 | CAAAAAAAAA/CAAAAAAAAAA | TTGCTCATGCGGTCTATCAA | CGTTGACAAAGGGATCGAGT | 60.4 | 600 | INTERGENIC | | | | |
| CaPOPI_1137 | Ca3 | 3632559 | ATTTTTTTTTTTTTT/ATTTTTTTTTTTTT | TTTGAGTCTAACCCGAACCAA | GTGGGGTAGGCAGAGACAAA | 59.6 | 765 | INTERGENIC | | | | |
| CaPOPI_1138 | Ca3 | 3818047 | GT/GGAT | ATGCTCTGCACTTGCAGCTA | TTCATCGCCTCAAAGTGTTG | 59.9 | 707 | INTERGENIC | | | | |
| CaPOPI_1139 | Ca3 | 3847338 | CATATATATATAT/CATATATATATATATAT | AGGAAGAGTGGAGTTGCGAA | ATTTTGGTTCGGAGGCTTTT | 60.0 | 496 | INTERGENIC | | | | |
| CaPOPI_1140 | Ca3 | 3881329 | AAAAATATATAAAATATATAAAATA/AAAAATATATAAAATATATAAAATATATAAAATA | AGGTAGGCAAGTCTGAGGCA | CAAACGCATGACCAAGACAG | 60.0 | 773 | INTERGENIC | | | | |
| CaPOPI_1141 | Ca3 | 3962696 | AAAAATATTT/AAAAATATTTAATATAAAATATTT | AGTTCCATGGGCTTTTCCTT | AGACTGTGGCACACAATGGA | 59.9 | 552 | INTERGENIC | | | | |
| CaPOPI_1142 | Ca3 | 4042829 | GATATATATATATATATATATATAT/GATATATATATATATATATATATATAT | ACACGAAGAGCAGGATCGTT | AAACAAAAATAAAAACTCGGAGACA | 59.9 | 743 | DRR | Ca_21325 | QR | | |
| CaPOPI_1143 | Ca3 | 4073796 | CT/CTT | CTCAACATCCACCATGCAAT | TGCAAATTTCATCAAATGCATAA | 59.4 | 746 | INTERGENIC | | | | |
| CaPOPI_1144 | Ca3 | 4076583 | ATATTATTATTATTATTATTATTATTATTATTATTATTATTATTATTATT/ATATTATTATTATTATTATTATTATTATTATTATTATTATTATTATTATTATT | ATAGTCCTCACCCACGCATC | AAGCGTACCTGTGGGAATTG | 60.0 | 607 | INTERGENIC | | | | |
| CaPOPI_1145 | Ca3 | 4622794 | ATATTATTATTATTATTATTATTATTATTATTATTATTATTA/ATATTATTATTATTATTATTATTATTATTATTATTATTATTATTATTA | TTGTCAAGTTAGGGCACATTTTT | TCTTGAAGTGTGTGGCCATT | 59.9 | 627 | INTERGENIC | | | | |
| CaPOPI_1146 | Ca3 | 4689138 | TAAAAAA/TAAAAAAA | CAGCTTGCATTTGCTTTTGA | TTGCTCCTACGTATCTCCCG | 60.1 | 888 | INTERGENIC | | | | |
| CaPOPI_1147 | Ca3 | 4965548 | ATTTT/ATTTTT | CGGGGAATGTGATTGATAGG | GTTGGAGAGATCGGTGGAAA | 60.1 | 948 | INTERGENIC | | | | |
| CaPOPI_1148 | Ca3 | 4965627 | T/TGAG | TGCAAATCTGGAGCAGAGAA | GTTGGAGAGATCGGTGGAAA | 59.7 | 510 | INTERGENIC | | | | |
| CaPOPI_1149 | Ca3 | 4966098 | CTTTTTTTT/CTTTTTTT | TTTCCACCGATCTCTCCAAC | AAACCCGCTCCTTTCAAAAT | 60.1 | 514 | INTERGENIC | | | | |
| CaPOPI_1150 | Ca3 | 4975195 | AATATATATATATATA/AATATATATATA | CAAAGTTCAATACGCCAGCA | AGGTTTTGGCGTAACTCCCT | 59.9 | 455 | INTERGENIC | | | | |

| INDEL marker IDs | Chromosomes/ unanchored scaffolds | Physical positions (bp) | InDels (*Kabuli* reference genome-CDC Frontier/PI) | Forward primers (5'-3') | Reverse primers (5'-3') | Anneling temperature (0C) | Expected amplified product size (bp) | Structural annotation | | Functional annotation | | |
|---|---|---|---|---|---|---|---|---|---|---|---|---|
| | | | | | | | | Sequence components of *kabuli* genome | *Kabuli* gene accession IDs | NCBI-KOG | TFs | NCBI-nr database |
| CaPOPI_1151 | Ca3 | 5182639 | ATTTTTTTTT/ATTTTTTTTTT | TCTCTTTCTAAAACTAGGCAACCA | TTGAGGTGGCCAAAGCTACT | 58.7 | 492 | INTERGENIC | | | | |
| CaPOPI_1152 | Ca3 | 5239462 | AATATATATATATATATATATATATATAT/AATATATATATATATATATATATATATAT | TGAGATCAGTTTTTGTGGAGGA | CAGTCACCACCCTTGCTTTT | 59.7 | 482 | INTERGENIC | | | | |
| CaPOPI_1153 | Ca3 | 5270478 | TATCAATC/TATC | ACGAAACGAAAGCGAGAAAA | CGACCTCGAAATAATCGGAA | 60.0 | 521 | INTERGENIC | | | | |
| CaPOPI_1154 | Ca3 | 5286221 | ATTT/ATT | CACCCCTCCCAAAACATCT | TTGTGAGTGCATTTGATTTTGTT | 59.8 | 876 | INTERGENIC | | | | |
| CaPOPI_1155 | Ca3 | 5288032 | AATATATATATATATATATATATATAT/AATATATATATATATATATAT | AGAAGGTCGCATCTCCAAGA | TGGCACTTGTATTCACTCGC | 60.0 | 456 | INTERGENIC | | | | |
| CaPOPI_1156 | Ca3 | 5347423 | CAATAATAATAATAATAATAATAATAATAATAATAA/CAATAATAATAATAATAATAATAATAA | TTGCTCCTACGTATCTCCCG | TTGTGACAATTTGATGGTTCTAAA | 60.2 | 563 | INTERGENIC | | | | |
| CaPOPI_1157 | Ca3 | 5502243 | TG/T | TGCGTTTTTCTGCATTTCTG | ATTCTGCGCGACTATCAACC | 60.0 | 661 | INTERGENIC | | | | |
| CaPOPI_1158 | Ca3 | 5507746 | AATATATATATATATATATATATATATATATAT/AATATATATATATATATATATATATATATATATATATATATATATAT | TATTTTGGATTTTGGGCTCG | GGAGGGATAAGGTTTCTCGC | 59.9 | 427 | INTERGENIC | | | | |
| CaPOPI_1159 | Ca3 | 5520403 | GTATATATATATATATATATATATATA/GTATATATATATATATATATATATATA | CAAATGACGACGACGATGAC | GAATGATGACAATGCGGTTG | 60.1 | 503 | INTERGENIC | | | | |
| CaPOPI_1160 | Ca3 | 5689325 | ATTT/ATT | TTGGTCAATGTGGCTTCTCA | TGAACTCTGAGCGTGTGACC | 60.2 | 439 | INTERGENIC | | | | |
| CaPOPI_1161 | Ca3 | 6243942 | CTTATT/CTT | TGATGGACTTCGTGCTCTTG | CCTCAGGATAAAACAGCCCA | 60.0 | 298 | INTERGENIC | | | | |
| CaPOPI_1162 | Ca3 | 6289426 | CATATATATATAT/CATATATATATATATAT | CGGTTCAGAACTGCATTGAA | CCTTACAAGGCCCACCAATA | 59.8 | 457 | INTERGENIC | | | | |
| CaPOPI_1163 | Ca3 | 6386934 | TGG/TGGG | TGTACGAGAAGGTCGTGTGC | GCCCAACAGCAAGCTATTTC | 59.9 | 574 | INTRON | Ca_24834 | | Trihelix | |
| CaPOPI_1164 | Ca3 | 6587330 | T/TA | TTGAAGGGTTGCATGAATGA | GGAGGTCGAAAATGACCAAA | 60.0 | 741 | INTERGENIC | | | | |
| CaPOPI_1165 | Ca3 | 6664320 | TAAAAAA/TAAAAAAA | TGAAATTATGGGTGGGCATT | GCTGAAAAAGTGCCGAAGAA | 60.0 | 569 | INTERGENIC | | | | |

| INDEL marker IDs | Chromosomes/ unanchored scaffolds | Physical positions (bp) | InDels (*Kabuli* reference genome-CDC Frontier/PI) | Forward primers (5'-3') | Reverse primers (5'-3') | Anneling temperature (0C) | Expected amplified product size (bp) | Structural annotation | | Functional annotation | | |
|---|---|---|---|---|---|---|---|---|---|---|---|---|
| | | | | | | | | Sequence components of *kabuli* genome | *Kabuli* gene accession IDs | NCBI-KOG | TFs | NCBI-nr database |
| CaPOPI_1166 | Ca3 | 6672422 | TTTTATTTATTTATTTATTTATTTATTTAT/TTTTATTTATTTATTTATTTATTTATTTATTTTAT | GCGCCGCAATAGAATAGAAG | CAACAAAGGGAGGCAAATGT | 60.0 | 672 | INTRON | Ca_25772 | A | | |
| CaPOPI_1167 | Ca3 | 6772905 | GTTT/GTT | TCCCTTTGTCTTTTCGCACT | CACTGCCGTTCTTCACTTCA | 59.9 | 383 | DRR | Ca_16559 | R | NAC | Pentatricopeptide repeat |
| CaPOPI_1168 | Ca3 | 6774064 | T/TTAA | TTTGCTGGACTTTGAAGCCT | CCCGGTCCTAGAGAGAAAGA | 60.0 | 640 | DRR | Ca_16559 | R | NAC | Pentatricopeptide repeat |
| CaPOPI_1169 | Ca3 | 6788233 | AATTTATTAT/AAT | TTTTTCACTATTGTGTGAAATCAGT | TTTCCTACCATATTCCCGCT | 57.0 | 826 | DRR | Ca_16556 | R | NAC | Pentatricopeptide repeat |
| CaPOPI_1170 | Ca3 | 6821985 | TTATATATATATATATATATATATATA/TTATATATATATATATATATATATATATATATATA | GCGTGATCCCAAGCTACAAT | CATCTTTTAAGGTGCGGGTG | 60.1 | 139 | INTERGENIC | | | | |
| CaPOPI_1171 | Ca3 | 6838667 | AGCA/AGCAGGATCCAATGCAATGGCA | GGAACCCCACAATCATCATC | GCAGCCAATTTTGTTGCATA | 60.0 | 547 | CDS (large-effect mutations) | Ca_16550 | R | bHLH | WD40 repeat |
| CaPOPI_1172 | Ca3 | 7076805 | CG/CCCGAATGTG | TCTTCGATTCCATTCCAACC | TTGGGGGAGACGATATTCTG | 59.9 | 701 | INTERGENIC | | | | |
| CaPOPI_1173 | Ca3 | 7367436 | CGAAGAAGA/CGAAGA | TGATTGTTTGTCTGGTGGGA | CACTCTCTTCCTTGGCCTTG | 59.9 | 331 | INTERGENIC | | | | |
| CaPOPI_1174 | Ca3 | 7467677 | AACATTAGA/AACATTAGACATTAGA | CCACACCCCTCACACTTTCT | AGCAAAGGGGATGAAAAGGT | 60.0 | 461 | INTERGENIC | | | | |
| CaPOPI_1175 | Ca3 | 7580880 | TAAAAAAAAA/TAAAAAAAAAA | GTGGTTCCGGAAAAGATGAA | CCCCATCCATTTCCCTATCT | 59.9 | 373 | INTERGENIC | | | | |
| CaPOPI_1176 | Ca3 | 7792015 | TAT/TATTATTCTTTCCCTAAT | TAGGTAAAGGTGGTTTGCCG | TGAGGGATAGATGCAGGTCA | 60.0 | 611 | INTERGENIC | | | | |
| CaPOPI_1177 | Ca3 | 7880432 | TCTAG/T | AGGAAGAGTGGAGTTGCGAA | TATTTTGGATTTTGGCTCGG | 60.0 | 503 | INTERGENIC | | | | |
| CaPOPI_1178 | Ca3 | 7922530 | TTATATATATATATATATAT/TTATATATATATATATAT | AGGAAGAGTGGAGTTGCGAA | TATTTTGGATTTTGGCTCGG | 60.0 | 504 | INTERGENIC | | | | |
| CaPOPI_1179 | Ca3 | 7959750 | TTAATTTGATTATGTTAAGTAATTTGATTATGTTAA/TTAATTTGATTATGTTAAGTAATTTGATTATGTTAAGTAATTTGATTATGTTAA | TTGGCATAGGTCATGGAAGTT | TTTGCCAATGTTGCTAGCTG | 59.4 | 848 | INTERGENIC | | | | |
| CaPOPI_1180 | Ca3 | 8059631 | GTTT/GTT | TGTGCGACCATCTACTGCTC | GCAATCTCCTCAACTCGGTC | 60.0 | 813 | INTERGENIC | | | | |

| **INDEL marker IDs** | **Chromosomes/ unanchored scaffolds** | **Physical positions (bp)** | **InDels (*Kabuli* reference genome-CDC Frontier/PI)** | **Forward primers (5'-3')** | **Reverse primers (5'-3')** | **Anneling temperature (0C)** | **Expected amplified product size (bp)** | **Structural annotation** | | **Functional annotation** | | |
|---|---|---|---|---|---|---|---|---|---|---|---|---|
| | | | | | | | | **Sequence components of *kabuli* genome** | ***Kabuli* gene accession IDs** | **NCBI-KOG** | **TFs** | **NCBI-nr database** |
| CaPOPI_1181 | Ca3 | 8245494 | TCT/TCTTCTGCT | TGCGCCTCTTCTAAAATCGT | TATCGCAATCAGAGGAACCC | 60.0 | 670 | INTERGENIC | | | | |
| CaPOPI_1182 | Ca3 | 8393314 | GTATATATATATATATATAT/GTATATATATATATATATATATAT | GTTGGTTCCCTAGCACCGTA | TATTTTGGATTTTGGCTCGG | 60.0 | 602 | INTERGENIC | | | | |
| CaPOPI_1183 | Ca3 | 8523296 | ATTTTTTTTTT/ATTTTTTTT | CACATGACAAAATTGGTGGAA | CGTTAAGGCACATCTCACTCC | 59.3 | 508 | INTERGENIC | | | | |
| CaPOPI_1184 | Ca3 | 8754025 | GTATATATATATATATATATATATATATAT/GTATATATATATATATATATATATATATATAT | AAAATCCTTGGCCTTTCGAT | GATGAGGTATGTGCGTGTGG | 59.9 | 863 | INTRON | Ca_23720 | | B3 | Protein of unknown function DUF674 |
| CaPOPI_1185 | Ca3 | 8942193 | AT/ATT | TGGGGAAAAACCCTTACCTT | CACATGATTTCAATTGGCTTTG | 59.7 | 326 | INTERGENIC | | | | |
| CaPOPI_1186 | Ca3 | 9186815 | CTATATATATATATATATATATATA/CTATATATATATATATATATATATA | TCCACAAACACGATAACATTGA | TGTAACAAGCATTTGTCGGG | 58.9 | 693 | INTERGENIC | | | | |
| CaPOPI_1187 | Ca3 | 9189477 | AATATATATATATATATATATATATATATATAT/AATATATATATATATATATATATATATATATATATATAT | AAAGGTTTGGTTGGAGATACG | TTGGCTGCAAAATATCCCTC | 58.1 | 856 | INTERGENIC | | | | |
| CaPOPI_1188 | Ca3 | 9577976 | AGG/AGGG | TTGCGTTCAAAGACTCGATG | TGAATTGCAGAATCCCATGA | 60.0 | 575 | INTRON | Ca_20977 | | | |
| CaPOPI_1189 | Ca3 | 10159468 | T/TA | TGGGGCAAACAAAGCTATTC | CTAAGGGCGCTACAACAAGC | 60.1 | 107 | INTERGENIC | | | | |
| CaPOPI_1190 | Ca3 | 10312456 | TAAAA/TAAAAA | TGACACGCAATATAGAACCG | CATCTTGGTGTGTGAAAGCG | 57.3 | 598 | INTERGENIC | | | | |
| CaPOPI_1191 | Ca3 | 10350276 | CAAGAAG/CAAGAAGAAG | ATTGGCAAATCGGTTCAGTC | GCCAAACCCTTACGAGATCA | 59.9 | 459 | DRR | Ca_18818 | | | |
| CaPOPI_1192 | Ca3 | 10398321 | G/GATTGATCTTTCCC | CGGATTGCCAAGTACAAGGT | CAAACCACAACCAGCACAAC | 60.0 | 770 | DRR | Ca_18819 | J | | Ribosomal protein L10/acidic P0 |
| CaPOPI_1193 | Ca3 | 10684192 | CTATTATTATTATTATTATTATTATTATTATTATTA/CTATTATTATTATTATTATTATTATTATTA | TTTCATCCAATGCTTCAGGA | TTGTGGTTGCATCCCTAACA | 59.2 | 822 | DRR | Ca_18828 | O | | Glutaredoxin |
| CaPOPI_1194 | Ca3 | 10846962 | AATATATATATATATATATATATATATATA/AATATATATATATATATATATATATATATATATATA | CCAAATGACGACTTCCCACT | CAAGACGTCGTCAAAATCCC | 60.0 | 683 | INTERGENIC | | | | |
| CaPOPI_1195 | Ca3 | 10860554 | TG/T | GCACACTGGTGTTGGATTTG | TCAATGGTAACTTCCCCAGC | 60.0 | 851 | INTRON | Ca_19358 | G | | Acetylhexosaminidase, subunit a/b |

| **INDEL marker IDs** | **Chromosomes/ unanchored scaffolds** | **Physical positions (bp)** | **InDels (*Kabuli* reference genome-CDC Frontier/PI)** | **Forward primers (5'-3')** | **Reverse primers (5'-3')** | **Anneling temperature (0C)** | **Expected amplified product size (bp)** | **Structural annotation** | | **Functional annotation** | | |
|---|---|---|---|---|---|---|---|---|---|---|---|---|
| | | | | | | | | **Sequence components of *kabuli* genome** | ***Kabuli* gene accession IDs** | **NCBI-KOG** | **TFs** | **NCBI-nr database** |
| CaPOPI_1196 | Ca3 | 10923237 | T/TC | GCTGATTGAAATCGAGCACA | TTAAAACAAGCACCAAGGGC | 60.0 | 347 | INTERGENIC | | | | |
| CaPOPI_1197 | Ca3 | 11044475 | CAAAAAAAAAA/CAAAAAAAAA | GCATCCATCCAAACGACTTT | CATTACATCTTATTTGAGGGAGAGC | 59.9 | 286 | INTERGENIC | | | | |
| CaPOPI_1198 | Ca3 | 11182328 | GAAAAAAAAAA/GAAAAAAAAAAA | AGGTGCCCTTCTTTTTGGTT | CCATCGTCGACCTTGAGAAT | 60.0 | 527 | INTERGENIC | | | | |
| CaPOPI_1199 | Ca3 | 11273271 | ATTTTTTTTT/ATTTTTTTT | GCGCGCGGATAAAATAAATA | TCATGACGCAGCACTTTTTC | 60.0 | 738 | INTERGENIC | | | | |
| CaPOPI_1200 | Ca3 | 11497481 | ATTATTTATTTATTTATTTATTTATTTATGTATTTATGTATTTATGTATTTATTTATTCATTTATTTATTTATT/ATTATTTATTTATTTATTTATTTATTTATTTATGTATTTATG | GGGTTTGCACTTGAAGGTGT | CCTCCGAGCCAAAATACAAA | 60.0 | 617 | INTERGENIC | | | | |
| CaPOPI_1201 | Ca3 | 11587128 | CAAAA/CAAAAA | TTTGGGCTTACTTTCCATGC | GGTGTCAATTTCTTTGCGGT | 60.1 | 543 | DRR | Ca_19379 | | | Transposase, MuDR, plant |
| CaPOPI_1202 | Ca3 | 11765428 | ATTT/ATTTT | GATCAGATCTGGCAGCAACA | TGGCATACATCTAGGGGGAG | 60.0 | 574 | DRR | Ca_19390 | | | |
| CaPOPI_1203 | Ca3 | 11877050 | TAAAAAAAAA/TAAAAAAAA | TTCAAACCTTTCAATTTTGCTC | GCGATTTTGGTGAAATCCAT | 58.4 | 594 | INTERGENIC | | | | |
| CaPOPI_1204 | Ca3 | 11919605 | GAA/GA | CGGAAATTGAAATCGGAAAA | ATCGCGAAAGTAGTCTCCCA | 59.9 | 633 | INTERGENIC | | | | |
| CaPOPI_1205 | Ca3 | 12140676 | A/AT | TTCCTATTGCTCACCCCTTG | GAAAAACAAGACCCGCGATA | 60.1 | 525 | INTERGENIC | | | | |
| CaPOPI_1206 | Ca3 | 12697436 | GTTTTTATTTTTATTTTTATTTTTATTTTTATTTT/GTTTTTATTTTTATTTTTATTTTTATTTTTATTTTTATTTT | AGCTTGCCAACTTCTGGGTA | TGCAAAACGTGTCATCCATT | 59.9 | 670 | INTERGENIC | | | | |
| CaPOPI_1207 | Ca3 | 12715466 | T/TACACATTAAAG | TATTCCCGATTAATGCAGCC | GTCCCGAATGCAATGTCTCT | 59.9 | 667 | INTERGENIC | | | | |
| CaPOPI_1208 | Ca3 | 12747032 | TGGG/TGG | CCTACGGACGGGAATGTAGA | CGTTTTTCTAAGCGAGGGTG | 59.9 | 398 | INTERGENIC | | | | |
| CaPOPI_1209 | Ca3 | 12911294 | C/CCT | TATCGAGTGTTTCCGGTTCC | CAGGTTCACGTGAGCAAGAA | 59.9 | 337 | INTERGENIC | | | | |
| CaPOPI_1210 | Ca3 | 12969818 | TAAAAAAA/TAAAAAAAA | TTACACCAATTCCCCCAAGA | CATGCAAGGTTGGGCTAGAT | 60.2 | 400 | INTERGENIC | | | | |

| INDEL marker IDs | Chromosomes/ unanchored scaffolds | Physical positions (bp) | InDels (*Kabuli* reference genome-CDC Frontier/PI) | Forward primers (5'-3') | Reverse primers (5'-3') | Anneling temperature (0C) | Expected amplified product size (bp) | Structural annotation | | Functional annotation | | |
|---|---|---|---|---|---|---|---|---|---|---|---|---|
| | | | | | | | | Sequence components of *kabuli* genome | *Kabuli* gene accession IDs | NCBI-KOG | TFs | NCBI-nr database |
| CaPOPI_1211 | Ca3 | 13097036 | A/AT | CCTTCTTTACGGGACCCAAT | TCATCAGTATCGTGGGTGGA | 60.2 | 364 | INTERGENIC | | | | |
| CaPOPI_1212 | Ca3 | 13236644 | AATATATATATATATATATATATAT/AATATATATATATATATATATATAT | AAGACCTGTGGGATCATTGC | TTTTGTGATAGGTTCCTGATGC | 59.9 | 928 | INTERGENIC | | | | |
| CaPOPI_1213 | Ca3 | 13313491 | CATTATTATTATTATTATTATTATTATTATTATTAATATTATTATTAT/CATTATTATTATTATTATTATTATTATTAATATTATTATTAT | AACCCACCTCTTGATTTCCA | CGACAATTTTGTTGGTGACG | 59.4 | 637 | INTERGENIC | | | | |
| CaPOPI_1214 | Ca3 | 13796912 | CAAAAAAAAAAA/CAAAAAAAAAAAA | TATCATGCATTTGCGGTTGT | CCTGAGAGAGAAAGGGGCTT | 60.0 | 650 | INTERGENIC | | | | |
| CaPOPI_1215 | Ca3 | 13827207 | TTATATATATATATATATAT/TTATATATATATATATATATAT | CTACTCCCGACACTGAAGCC | TTTTCCAGGAAGGAATCGTG | 59.9 | 527 | DRR | Ca_25325 | | | |
| CaPOPI_1216 | Ca3 | 13836046 | TAAAAAAAAA/TAAAAAAAAAA | TGAGCTGATGGTGAAGTGATTT | TGGTTGAGCTTCAGACATGC | 59.7 | 530 | INTERGENIC | | | | |
| CaPOPI_1217 | Ca3 | 14653349 | A/AG | AGGAGCTTCTGCCATTGAAA | TAAAAGGAGCAAAGGCCAGA | 60.0 | 408 | INTRON | Ca_22505 | O | | Zinc finger, RING-type |
| CaPOPI_1218 | Ca3 | 14801451 | GATATATATATATATATATATAT/GATATATATATATATATATATATAT | ATGAGCCACAAGCTCCAAGT | TGCCATTTGGTGAACGATTA | 59.9 | 357 | INTERGENIC | | | | |
| CaPOPI_1219 | Ca3 | 14843003 | AATATATATATATATATATATATATATATA/AATATATATATATATATATATATATATATATATA | GAGAAAGAAGATATCGGATTCTCAA | AAAAATCAACGCAGTGGGAG | 59.3 | 356 | INTERGENIC | | | | |
| CaPOPI_1220 | Ca3 | 14874833 | GCC/GCCC | AGGTCATTTTCGTCCTCCCT | CAAACACACCTGTTGGATGC | 59.9 | 679 | INTERGENIC | | | | |
| CaPOPI_1221 | Ca3 | 14880871 | TG/TGTCG | TTTCGGTCATTTTCGTCCTC | ACACACCTATCGGATGCACA | 60.1 | 616 | INTERGENIC | | | | |
| CaPOPI_1222 | Ca3 | 14902851 | GAA/GAAA | CGGTGGGGAGAACTCTGATA | TTCACATGACATCATCCCGT | 60.1 | 474 | INTERGENIC | | | | |
| CaPOPI_1223 | Ca3 | 14919264 | GTTT/GTTTT | CGATTGTGTTTGTCACAGGG | GAAGCAAATCGATTGGGAAA | 60.0 | 303 | INTERGENIC | | | | |
| CaPOPI_1224 | Ca3 | 15039270 | TC/T | CAACACGATCATGACAAGCA | GCATTTTGCATTTGTCAACG | 59.2 | 677 | INTERGENIC | | | | |
| CaPOPI_1225 | Ca3 | 15047295 | TA/T | TACTTTGTGCCATGCTCTGC | ACACAAGCCAACACATCCAA | 60.0 | 117 | INTERGENIC | | | | |

| INDEL marker IDs | Chromosomes/ unanchored scaffolds | Physical positions (bp) | InDels (*Kabuli* reference genome-CDC Frontier/PI) | Forward primers (5'-3') | Reverse primers (5'-3') | Anneling temperature (0C) | Expected amplified product size (bp) | Structural annotation | | Functional annotation | | |
|---|---|---|---|---|---|---|---|---|---|---|---|---|
| | | | | | | | | Sequence components of *kabuli* genome | *Kabuli* gene accession IDs | NCBI-KOG | TFs | NCBI-nr database |
| CaPOPI_1226 | Ca3 | 15072147 | AATATATATATATATATATATATATATA/AATATATATATATATATATATATA | GAGTGGTGTTGGAGTCATGTTT | AGACATGTGTTTCCCTTCCG | 59.0 | 424 | DRR | Ca_17011 | | | |
| CaPOPI_1227 | Ca3 | 15232531 | TAAAAA/TAAAA | CTTTTACAGCGCTTTTTGGG | TTCGTAATGGACGGGATGAT | 59.9 | 245 | INTERGENIC | | | | |
| CaPOPI_1228 | Ca3 | 15318264 | TAAAAAAA/TAAAAAA | CGAAGGAGCTTTTCTTGTGG | CGCATTTTGCATTTGTCAAC | 60.0 | 651 | INTERGENIC | | | | |
| CaPOPI_1229 | Ca3 | 15388201 | CATATATATAT/CATATATAT | AAACACATGCGCTTTCATTG | AATCGATTGGCTGAAATCCA | 59.7 | 479 | INTERGENIC | | | | |
| CaPOPI_1230 | Ca3 | 15463471 | CATATATATATATATATATATA/CATATATATATATATATATA | TTTGTGGATTTGCATTGATGA | TGAATTCACCGTAAACGGAA | 59.9 | 783 | INTERGENIC | | | | |
| CaPOPI_1231 | Ca3 | 15503247 | C/CTAAAATATTAGGGAA | ATTGGGAAGTGGGGAAACTC | CGGGGTACATTGATAGCGTT | 60.2 | 800 | INTERGENIC | | | | |
| CaPOPI_1232 | Ca3 | 15524418 | GTTTTTT/GTTTTTTT | GACGTGTGTGTGGAAAGAGC | CGGCCAAAGTAATGGATTGT | 59.3 | 720 | INTERGENIC | | | | |
| CaPOPI_1233 | Ca3 | 15660475 | TA/TAA | AAGTGAGCTAACGGGGGTTT | TGGACAGAGCAAGTGAGGTG | 60.0 | 140 | INTERGENIC | | | | |
| CaPOPI_1234 | Ca3 | 16133157 | GTTTTATTTTATTTTATTT/GTTTTATTTTATTTTATTTTATTT | CGTCTCTTGAAGCGATGAAA | CATTGTTGGTCACTAGCAAATCA | 59.1 | 698 | INTERGENIC | | | | |
| CaPOPI_1235 | Ca3 | 16133384 | CAATAATAATAATAATAATAATAATAATAATAATAATA/CAATAATAATAATAATAATAATAATAATAATAATAATAATA | CGTCTCTTGAAGCGATGAAA | ATAAGCCGTCAATCTGCCAC | 59.1 | 807 | INTERGENIC | | | | |
| CaPOPI_1236 | Ca3 | 16133499 | TA/TACACAAAGAA | TTTGCCCCATTATAAACAAAA | ATAAGCCGTCAATCTGCCAC | 57.2 | 625 | INTERGENIC | | | | |
| CaPOPI_1237 | Ca3 | 16137267 | CATATATATATATATATA/CATATATATATATATATATA | TGGCACATCTAAAAATAGGAGGA | AGAAAGTCGCATCCCAGAGA | 60.0 | 544 | INTERGENIC | | | | |
| CaPOPI_1238 | Ca3 | 16138352 | TTATATATATATATATATATATATATA/TTATATATATATATATATATATATATATATATATA | GCCACAATCTGTCCCCAATA | TAGTGAAGGCATTGGCAGAA | 60.7 | 855 | INTERGENIC | | | | |
| CaPOPI_1239 | Ca3 | 16166714 | TTTTG/TTTTGCTTTG | TCAACCATCTGGGTTGGAAT | CCCCTAGCAAAGGAAACAAA | 60.2 | 674 | INTERGENIC | | | | |
| CaPOPI_1240 | Ca3 | 16168531 | AG/A | TGGAGGAGGATTTCCATCTG | TGCTTTCTTGCCCTGTTTTT | 60.0 | 547 | INTERGENIC | | | | |

| INDEL marker IDs | Chromosomes/ unanchored scaffolds | Physical positions (bp) | InDels (*Kabuli* reference genome-CDC Frontier/PI) | Forward primers (5'-3') | Reverse primers (5'-3') | Anneling temperature (0C) | Expected amplified product size (bp) | Structural annotation | | Functional annotation | | |
|---|---|---|---|---|---|---|---|---|---|---|---|---|
| | | | | | | | | Sequence components of *kabuli* genome | *Kabuli* gene accession IDs | NCBI-KOG | TFs | NCBI-nr database |
| CaPOPI_1241 | Ca3 | 16171083 | T/TG | TACAGCGCTTCATTCCACAG | CGTGCATGAGCAAAAGAAAA | 60.0 | 468 | INTERGENIC | | | | |
| CaPOPI_1242 | Ca3 | 16182804 | ATTTTTTTT/ATTTTTTTTTT | CCGGCATCACCTATCAGAGT | TCCGACCTCCTTTGAGAAGA | 60.1 | 583 | INTERGENIC | | | | |
| CaPOPI_1243 | Ca3 | 16190775 | CA/C | TCGCGGTTCTTGATGATGTA | AGCTCGACTTATCCTTCGCA | 60.2 | 423 | INTERGENIC | | | | |
| CaPOPI_1244 | Ca3 | 16195131 | AT/ATT | TGATAAAAGACTAACGATGCAAACA | TAAACCATCAATCTGCCACG | 60.1 | 655 | INTERGENIC | | | | |
| CaPOPI_1245 | Ca3 | 16195368 | CATATAT/CATATATAT | ACGTGGCAGATTGATGGTTT | TTTGATGTTGGCGTAGAGGA | 60.4 | 350 | INTERGENIC | | | | |
| CaPOPI_1246 | Ca3 | 16195709 | AC/A | ACGTGGCAGATTGATGGTTT | GAACGTACGGAAGAGAAGCG | 60.4 | 754 | INTERGENIC | | | | |
| CaPOPI_1247 | Ca3 | 16197261 | TCTTAA/T | CTGCATCGAATTAATGCAACA | AAGGGGGATCATGGAAAACT | 59.7 | 316 | DRR | Ca_18251 | | | |
| CaPOPI_1248 | Ca3 | 16212378 | A/AACATTC | ATGCACTTTGCCACTGTTGT | GAGGGGATGATGCTTCAAAA | 59.2 | 879 | INTERGENIC | | | | |
| CaPOPI_1249 | Ca3 | 16347875 | CAAAAAA/CAAAAAAA | GCGTTTTTATGGTTGCTGGT | TATGGAAGGTGAGACAGGGG | 60.0 | 690 | INTERGENIC | | | | |
| CaPOPI_1250 | Ca3 | 16423935 | AATATATATATATATATATATAT/AATATATATATATATATATATAT | TCCAGAAAATAAATCTTCTTCACG | CAAGGATGGGTTGATCGTCT | 58.9 | 702 | INTERGENIC | | | | |
| CaPOPI_1251 | Ca3 | 16518599 | A/AACC | CTTTGGAGCAAGCTGGGTAG | TTTTGCGATGTTTGATCGTT | 60.0 | 544 | INTERGENIC | | | | |
| CaPOPI_1252 | Ca3 | 16544552 | GTTATTATTATTATTATTATTATTATTATTATTATTATTATTA/GTTATTATTATTATTATTATTATTATTATTATTA | TGCTCGACATGTTTCTCCTT | CCACACAAACTGACAACTCCA | 58.4 | 605 | INTERGENIC | | | | |
| CaPOPI_1253 | Ca3 | 16778238 | GAAAAAAA/GAAAAAA | TCCTATGGTGGCTCATCTCC | TTTTGCCTCAACAATCTCCC | 60.0 | 359 | INTERGENIC | | | | |
| CaPOPI_1254 | Ca3 | 16820332 | AATATATATATATATATA/AATATATATATATATATA | AGAGGACATCGGTTAGCTGC | AAGAAAGAAGGGGTCCCAAA | 59.5 | 633 | INTERGENIC | | | | |
| CaPOPI_1255 | Ca3 | 16885390 | CTATATATATATATATATATA/CTATATATATATATATA | CATTTTCATCAAAGGTCGCA | CAAATTTGCCACTGTCTTGC | 59.7 | 244 | INTERGENIC | | | | |

| INDEL marker IDs | Chromosomes/ unanchored scaffolds | Physical positions (bp) | InDels (*Kabuli* reference genome-CDC Frontier/PI) | Forward primers (5'-3') | Reverse primers (5'-3') | Anneling temperature (0C) | Expected amplified product size (bp) | Structural annotation | | Functional annotation | | |
|---|---|---|---|---|---|---|---|---|---|---|---|---|
| | | | | | | | | Sequence components of *kabuli* genome | *Kabuli* gene accession IDs | NCBI-KOG | TFs | NCBI-nr database |
| CaPOPI_1256 | Ca3 | 16954725 | CATATATATATATATATATATATATA/CATATATATATATATATATATATATATA | TAAATGACCAAGCCGGAAAA | CGTTTGATCGATTGCAGTTC | 60.4 | 573 | INTERGENIC | | | | |
| CaPOPI_1257 | Ca3 | 17033772 | AAATAATAATAATAATAATAATAATAATAATAATAATAATAAT/AAATAATAATAATAATAATAATAATAATAATAATAATAATAATAATAATAAT | ATGCGATCATGTGGATGAGA | TACCCCTTGTTGGTCCTGAG | 60.0 | 840 | INTERGENIC | | | | |
| CaPOPI_1258 | Ca3 | 17039245 | CAAAAAA/CAAAAAAAA | TTCTAACTTTGCTGTCCCATTTT | GGGGTAAAACCAGGAGGAAG | 59.2 | 750 | INTERGENIC | | | | |
| CaPOPI_1259 | Ca3 | 17122676 | ATTTTTTTTTT/ATTTTTTTTTTT | AATGCACCACTTTATGCACG | CCAGTTTTCATTCAGGCACA | 59.6 | 358 | INTERGENIC | | | | |
| CaPOPI_1260 | Ca3 | 17201946 | TTATATATATATATATATATATATATATATATATAT/TTATATATATATATATATATATATATATAT | AAAATCAAACATGGTGGGGA | AAGGCATGATGTTGGAGTCA | 60.0 | 398 | DRR | Ca_24619 | S | | Domain of unknown function DUF862, eukaryotic |
| CaPOPI_1261 | Ca3 | 17217773 | TCC/TC | ACAATCGGACACCGTTTCAT | TCATGTGGCAACACTGTTCA | 60.2 | 253 | INTERGENIC | | | | |
| CaPOPI_1262 | Ca3 | 17491839 | AATATATATATATATA/AATATATATATATA | TGAATTCATGGCTAAAAAGCCT | GCAAAGATGATAATTCGGCAA | 60.1 | 654 | INTERGENIC | | | | |
| CaPOPI_1263 | Ca3 | 17579559 | TAAAA/TAAATAAAAA | TGCATTTTCACCAACATCGT | AATCACCGAGCTGAAATCAAA | 60.0 | 912 | INTERGENIC | | | | |
| CaPOPI_1264 | Ca3 | 17819480 | AATATATATATATATATATATATATATATATA/AATATATATATATATATATATATATA | AACGATCTGATTGTAATGTGTTTCTT | GTCGTGAACGGTGCTACATC | 59.4 | 884 | INTERGENIC | | | | |
| CaPOPI_1265 | Ca3 | 17940721 | GAAAAAAAAAAA/GAAAAAAAAAA | GGCTAAATTACATTTGTGGTCCC | GGCTAAATTATATTCGTGGTCCC | 60.8 | 382 | INTERGENIC | | | | |
| CaPOPI_1266 | Ca3 | 18015978 | TTATATATATATATATATATATATATATAT/TTATATATATATATATATATATATATATAT | TCAGCAATAGAAGCCAAATGC | CGAACTTTGCCGTACGTCTT | 60.4 | 470 | INTERGENIC | | | | |
| CaPOPI_1267 | Ca3 | 18241779 | CTATATATATATATA/CTATATATATATATATA | CTCTACCACGCGATTACGGT | GGAAGTGATGGGTGAAATGG | 60.2 | 390 | INTERGENIC | | | | |
| CaPOPI_1268 | Ca3 | 18268210 | GAAAAAAAAAA/GAAAAAAAAAAAA | AACGAAGAAGTTGAGGTGGC | CCGAATTGCTCTTCATTGGT | 59.3 | 940 | DRR | Ca_19613 | O | | PI31 proteasome regulator |
| CaPOPI_1269 | Ca3 | 18345308 | TAAAAAAA/TAAAAAAAA | GGTTTCCCTCCTAAGTTAAGGGT | GCGGTGCAATCAGTAAACCT | 60.1 | 271 | DRR | Ca_19619 | O | | Glutathione S-transferase, N-terminal |
| CaPOPI_1270 | Ca3 | 18647131 | ATTTTTTTTT/ATTTTTTTTTT | AATTGATTTAAGCCCAAAAATAGA | AGAAGTGGCAAGATGGATGG | 57.5 | 576 | DRR | Ca_19638 | Q | | Multicopper oxidase, type 1 |

| INDEL marker IDs | Chromosomes/ unanchored scaffolds | Physical positions (bp) | InDels (*Kabuli* reference genome-CDC Frontier/PI) | Forward primers (5'-3') | Reverse primers (5'-3') | Anneling temperature (0C) | Expected amplified product size (bp) | Structural annotation | | Functional annotation | | |
|---|---|---|---|---|---|---|---|---|---|---|---|---|
| | | | | | | | | Sequence components of *kabuli* genome | *Kabuli* gene accession IDs | NCBI-KOG | TFs | NCBI-nr database |
| CaPOPI_1271 | Ca3 | 18943181 | AAT/A | TGGAGTGTTGATGTCGTTGTC | AAATCGAAGATCGCAACAGT | 59.6 | 428 | INTERGENIC | | | | |
| CaPOPI_1272 | Ca3 | 19056367 | ATATTATTATTATTATTATTATTATTATTATTATTATTATTGTTATTATTATTATTATTATTATTAT/ATATTATTATTATTATTATTATTATTATTATTATTATTATTGTTATTAT | CAACACAACGATAATGGGCA | GACATTGGTTGGGGTTTGAC | 60.4 | 717 | INTERGENIC | | | | |
| CaPOPI_1273 | Ca3 | 19062819 | AATATATATATATATATATATATATATATATATATATATATATA/AATATATATATATATATATATATATATATATATATATATATA | CATGCTAAATAACATGAAGAATGC | GCAGGTTTGACAGTGAGCAA | 57.9 | 637 | INTERGENIC | | | | |
| CaPOPI_1274 | Ca3 | 19148213 | CATATATATATATATA/CATATATATATATATATA | GCACAAGACTTTACGTCCATCTC | TATTTTGTGTCGTGCGCATT | 60.2 | 691 | INTERGENIC | | | | |
| CaPOPI_1275 | Ca3 | 19163962 | GGTGTGTGTGTGT/GGTGTGTGTGT | AGTTAACCGACGAAACCCCT | TTAGCAATCAAACCAACGCA | 59.9 | 321 | INTERGENIC | | | | |
| CaPOPI_1276 | Ca3 | 19358625 | GTTTTTTTTTTTTT/GTTTTTTTT | AAGATTTGGTCCGTTATGCG | GTGGGAATGAACAAAGGACG | 60.0 | 517 | INTERGENIC | | | | |
| CaPOPI_1277 | Ca3 | 19359038 | TAAAA/TAAA | CGTCCTTTGTTCATTCCCAC | TGAGGGCCTTTAGAATCGTG | 60.4 | 670 | INTERGENIC | | | | |
| CaPOPI_1278 | Ca3 | 19359334 | CCAA/CA | AGGGAAATCAACGACAGAGC | TGAGGGCCTTTAGAATCGTG | 59.3 | 365 | INTERGENIC | | | | |
| CaPOPI_1279 | Ca3 | 19368426 | GTTTTTTTT/GTTTTTTTTT | GGCACTCAAAGTGTTGGGTT | TGGGTTAGCTTTTGGGATTG | 60.0 | 920 | DRR | Ca_19336 | | | Toll-Interleukin receptor |
| CaPOPI_1280 | Ca3 | 19368565 | GTTTTT/GTTTTTT | GGTGATGCATGATGATTTGC | TGGGTTAGCTTTTGGGATTG | 59.9 | 630 | DRR | Ca_19336 | | | Toll-Interleukin receptor |
| CaPOPI_1281 | Ca3 | 19369038 | CCAACAAC/CCAAC | CAATCCCAAAAGCTAACCCA | AAGAATCTGCGCCAACATCT | 59.9 | 173 | INTERGENIC | | | | |
| CaPOPI_1282 | Ca3 | 19371062 | TAAAAAAAA/TAAAAAAAAA | TTCATTGGATTGTTTGTGGG | GAATTCTGCGTTTTTCCTGC | 59.2 | 845 | INTERGENIC | | | | |
| CaPOPI_1283 | Ca3 | 19373696 | TAA/TA | GCTGAATGCGTGAAGAACAA | GGGTAGAAAACACGCGGAAT | 60.0 | 339 | INTERGENIC | | | | |
| CaPOPI_1284 | Ca3 | 19376077 | A/ATTT | TGACCTTGTGCATTGTTTTGA | GCAAACTCAACCTTGCCAAT | 60.1 | 333 | INTERGENIC | | | | |
| CaPOPI_1285 | Ca3 | 19383912 | CAAA/CAA | GTCAGGTCAGGCTGGAAAAA | TCGTCTTAATCCACTTGGTGC | 60.2 | 436 | DRR | Ca_19337 | | | |

| INDEL marker IDs | Chromosomes/ unanchored scaffolds | Physical positions (bp) | InDels (*Kabuli* reference genome-CDC Frontier/PI) | Forward primers (5'-3') | Reverse primers (5'-3') | Anneling temperature (0C) | Expected amplified product size (bp) | Structural annotation | | Functional annotation | | |
|---|---|---|---|---|---|---|---|---|---|---|---|---|
| | | | | | | | | Sequence components of *kabuli* genome | *Kabuli* gene accession IDs | NCBI-KOG | TFs | NCBI-nr database |
| CaPOPI_1286 | Ca3 | 19402891 | AGA/AGATGA | ACCCAATCCAAGTTTGTCCA | GAAGGCGACCTCAAAGACTG | 60.2 | 632 | INTERGENIC | | | | |
| CaPOPI_1287 | Ca3 | 19560633 | TAAAAAAAAA/TAAAAAAAAAA | TCAGTGCGATGGAGAAATCA | TGACATTTTAAACCTTGAGTTGC | 60.4 | 453 | INTERGENIC | | | | |
| CaPOPI_1288 | Ca3 | 19639547 | CT/CTTATAT | TGCAATCAAGAGAGGACACAA | CGAGAAATAGCTCACGAGGC | 59.4 | 521 | INTERGENIC | | | | |
| CaPOPI_1289 | Ca3 | 19969942 | GATCAACGCATC/GATCAACGCATCAACGCATC | CATAAGGATCGGGGAACAAA | AGGATCATCCATCGTCTTCG | 59.8 | 541 | INTERGENIC | | | | |
| CaPOPI_1290 | Ca3 | 20005707 | AATATATATATATATATA/AATATATATATATATA | CCACAACAGAACGATGTTGG | TTCCCAAAACTATTTATGCATGG | 60.0 | 312 | INTERGENIC | | | | |
| CaPOPI_1291 | Ca3 | 20011560 | GTTGACACTT/GTTGACACTTGACACTT | GCAATTGCAAGAGCGTGATA | TTTGGCATGCAATCAAACAT | 60.0 | 277 | DRR | Ca_09404 | | | |
| CaPOPI_1292 | Ca3 | 20445164 | TTATATATATATATATATATATATAT/TTATATATATATATATATATATAT | AGTGCCACATGTAACACCCC | GTTTTGGCGTTGGTTTCACT | 60.7 | 815 | INTERGENIC | | | | |
| CaPOPI_1293 | Ca3 | 20526351 | TTATATATATATATATATATATATATATAT/TTATATATATATATATATATATAT | TGCATGTTCTTACGCTCGTC | AATTCACAAGACGCCATTCC | 60.0 | 504 | INTERGENIC | | | | |
| CaPOPI_1294 | Ca3 | 20617847 | AATATATATATATATATATATATA/AATATATATATATATATATATATA | AATCGATTGGCTGAAATCCA | TCGAGTTTGAGCATGTGAGG | 60.4 | 809 | INTERGENIC | | | | |
| CaPOPI_1295 | Ca3 | 20750487 | CTTTATTTATTTTATTTATTTTATTTATTTTATTTATTTTATTTATTTTATTT/CTTTATTTATTTTATTTATTTTATTTATTTTATTTATTTTATTTATTTTATTT | TGTTATGTTGGGTTTGGGCT | TGCAGATGGGTTGAGTGTGT | 60.2 | 875 | INTERGENIC | | | | |
| CaPOPI_1296 | Ca3 | 20988635 | AATATATATATATATATATATATATATATATATATATATATAT/AATATATATATATATATATATATATATATATATATATATATATATATATAT | CAAGCTGAAGGACTTCCACA | ATTATGTGGCGAGTGACGGT | 59.0 | 477 | INTERGENIC | | | | |
| CaPOPI_1297 | Ca3 | 21097843 | ATTTTTTTTT/ATTTTTTTTTT | GGCATTTTGATTTGATTCGC | CAAATTTTGTGTTTCAAAAACCATT | 60.4 | 653 | INTERGENIC | | | | |
| CaPOPI_1298 | Ca3 | 21311568 | GAAAAAAAAA/GAAAAAAAAAA | CACGTCACATCGCAATTTTT | CATGTCATTTCAACCCACGA | 59.6 | 542 | INTERGENIC | | | | |
| CaPOPI_1299 | Ca3 | 21319728 | AACTTACTTA/AACTTA | TGCTGTTCTAGTGTTCTAATTGCTC | GCCTGCCAGACGTTTTACAT | 59.2 | 562 | INTERGENIC | | | | |
| CaPOPI_1300 | Ca3 | 21424576 | CTTTTTT/CTTTTT | ACGTAGTTGGGTCTTGGCAC | CAAATTCTGTCCCGTCTGGT | 60.0 | 385 | INTERGENIC | | | | |

| INDEL marker IDs | Chromosomes/ unanchored scaffolds | Physical positions (bp) | InDels (*Kabuli* reference genome-CDC Frontier/PI) | Forward primers (5'-3') | Reverse primers (5'-3') | Anneling temperature (0C) | Expected amplified product size (bp) | Structural annotation | | Functional annotation | | |
|---|---|---|---|---|---|---|---|---|---|---|---|---|
| | | | | | | | | Sequence components of *kabuli* genome | *Kabuli* gene accession IDs | NCBI-KOG | TFs | NCBI-nr database |
| CaPOPI_1301 | Ca3 | 21599415 | CAAAAAA/CAAAAAAA | GCAGCAGTTGTTTGCAGAAG | TCTCTTGCATGCCTAACACG | 59.8 | 941 | INTERGENIC | | | | |
| CaPOPI_1302 | Ca3 | 21635589 | GCATCATCA/GCATCA | AAGCATGCCAAACCAAAAAC | TGAACATCAAAGATCTGCGG | 60.0 | 521 | INTRON | Ca_09516 | | | |
| CaPOPI_1303 | Ca3 | 21897869 | ATATTATTATTATTATTATTATTATTA/ATATTATTATTATTATTATTATTA | GAGTTGAGTGTTGCCAGGGT | AGCTACCAAGCTGATGGTGAA | 60.2 | 296 | INTERGENIC | | | | |
| CaPOPI_1304 | Ca3 | 21989858 | TTATATATATATATATATATATATATATATAT/TTATATATATATATATATATATATATAT | TGAAGCAAACCAAATAAATCCA | GACCGGTGGTAGACCTTTGA | 59.5 | 592 | INTERGENIC | | | | |
| CaPOPI_1305 | Ca3 | 22042563 | CTTTTTT/CTTTTTTT | AGGAAAGTGCCATGTGAAAAA | TCGTAGGTCAAGTTCAGACCG | 59.6 | 764 | INTERGENIC | | | | |
| CaPOPI_1306 | Ca3 | 22377503 | TGTT/TGTTAGTT | TTCATTAACGCTTATTCCCTCA | TGCAACTCTAACAGCGACAA | 58.8 | 694 | INTERGENIC | | | | |
| CaPOPI_1307 | Ca3 | 22661182 | AATTATTATTATTATTATTATTATTATTATTATTATTATTAT/AATTATTATTATTATTATTATTATTATTATTATTATTAT | AGAGAAGGTGAGGGGGTGTT | AGCCTGATAGGTGGTGGAAA | 60.0 | 763 | INTRON | Ca_06245 | R | | SPX, N-terminal |
| CaPOPI_1308 | Ca3 | 22934054 | G/GCCGATACAATTTGT | CCACATTATGCCTTCGGTTT | GGTTTTCGCGATATTTTCCA | 59.8 | 210 | INTERGENIC | | | | |
| CaPOPI_1309 | Ca3 | 22936637 | TTAATAATAATAATAATAATAATAATAATAATAAAAATAAAAATAATAATAATAATAATAATATTAATATTAATAATAATAATAATAATAATAATAATAATAATAATAATAATAAT | ACCACAAATTTCGAGGACGA | TGAAAGATGTGGGTAGCAATAA | 60.5 | 778 | INTERGENIC | | | | |
| CaPOPI_1310 | Ca3 | 23016776 | TCCTACCT/TCCT | GCACACCTATGTGGTCTTGC | CCATTTTGTCGAAGTTTGGTT | 59.2 | 615 | DRR | Ca_06208 | QR | B3 | Oxoglutarate/iron-dependent oxygenase |
| CaPOPI_1311 | Ca3 | 23102761 | CTATATATAT/CTATATATATAT | CACCTTGTCACATTCACAGACA | CCTTGAAGGTTAACAATTTCGT | 59.6 | 543 | INTERGENIC | | | | |
| CaPOPI_1312 | Ca3 | 23112762 | TT/TTTCCTTGATACTTCGT | TGATGCCAATTTGGTTCTGA | TTCGAATCCGTCACAAATGA | 60.0 | 628 | INTERGENIC | | | | |
| CaPOPI_1313 | Ca3 | 23201611 | TAGGTCGATCAG/TAG | AAAAAGGAGAGTTCTCCGGC | ATCGAGCGAACACATTGTCA | 59.8 | 677 | INTERGENIC | | | | |
| CaPOPI_1314 | Ca3 | 23308159 | CTTTTTTTTTT/CTTTTTTTTTTT | TTGCCTTCATGCTTTCTCAA | CATGTTCCGATATGCCACTG | 59.5 | 461 | INTERGENIC | | | | |
| CaPOPI_1315 | Ca3 | 23424382 | TATTAAAAAAT/TATTAAAAAATTAAAAAAT | CATTGTCTGGACTACATTGTGTGA | GAAGGGATCAATTTCTACCCA | 60.0 | 674 | INTERGENIC | | | | |

| INDEL marker IDs | Chromosomes/ unanchored scaffolds | Physical positions (bp) | InDels (*Kabuli* reference genome-CDC Frontier/PI) | Forward primers (5'-3') | Reverse primers (5'-3') | Anneling temperature (0C) | Expected amplified product size (bp) | Structural annotation | | Functional annotation | | |
|---|---|---|---|---|---|---|---|---|---|---|---|---|
| | | | | | | | | Sequence components of *kabuli* genome | *Kabuli* gene accession IDs | NCBI-KOG | TFs | NCBI-nr database |
| CaPOPI_1316 | Ca3 | 23530917 | AATATATATATATATATATATATAT/AATATATATATATATATATATATATAT | ACCCATTAGCGTAGGAGTGC | AACGTAAATGCACTTGAACCG | 59.2 | 786 | INTERGENIC | | | | |
| CaPOPI_1317 | Ca3 | 23541848 | TTATATATATATATATATATATATATAT/TTATATATATATATATATATATATATATATATATATATATAT | ATAAGTTGGCGTTGCCTCTG | CGTCATATGGCGAAATAGATTG | 60.3 | 518 | INTERGENIC | | | | |
| CaPOPI_1318 | Ca3 | 23551096 | AACAATTTACAATTTACGATTTACAATTT/AACAATTTACAATTTACAATTTACGATTTACAATTT | TCCCATTTTGATTCCCTTCA | TCCCTAAATCGCTGAATCTGA | 60.2 | 363 | INTERGENIC | | | | |
| CaPOPI_1319 | Ca3 | 23830553 | ACTCTCTCTCTCTCTCTCTCTCTCTCTC/ACTCTCTCTCTCTCTCTCTCTCTCTCTCTCTC | TCACGAAATTGGTCCCCTTA | GGGACCAAAACTGGAGAGAA | 60.3 | 878 | INTERGENIC | | | | |
| CaPOPI_1320 | Ca3 | 24033300 | TTAATAATAATAATAATAATAATAATA/TTAATAATAATAATAATAATAATA | TCCAAGAAAAGTGTTGGAAATG | CCTTCCTACGCCTCAAATCA | 59.1 | 376 | INTERGENIC | | | | |
| CaPOPI_1321 | Ca3 | 24117383 | TTACATACATACATAC/TTACATACATAC | GAAGAGGTGCTTGGTGGAGA | TACACCAGAATTTGCTCCCC | 60.4 | 342 | DRR | Ca_06107 | O | | Calreticulin/calnexin |
| CaPOPI_1322 | Ca3 | 24193117 | ATTT/ATT | TAAGGTTCAGGTTATGGCCG | CGCCACTATGTCGGCTATTT | 60.0 | 695 | INTRON | Ca_06100 | J | | Aminoacyl-tRNA synthetase, class II (D/K/N) |
| CaPOPI_1323 | Ca3 | 24272959 | GAATAATAA/GAATAATAATAA | GCATGTCCGTTACCTCCACT | GCGATTTATTATTCAGAGTTATTAGCG | 60.0 | 646 | INTERGENIC | | | | |
| CaPOPI_1324 | Ca3 | 24701158 | ATTTTTTTTTT/ATTTTTTTTT | TTTGGCACATCTATTCAACTGC | CGCATTCTTGGGATGCTTAT | 60.1 | 136 | INTERGENIC | | | | |
| CaPOPI_1325 | Ca3 | 24984768 | CTTTTTTTTT/CTTTTTTTT | TATATGGGCTGCTCTCCCTG | TTTTGCTTTTGAAGCCAACC | 60.2 | 663 | INTRON | Ca_06031 | | SAP | |
| CaPOPI_1326 | Ca3 | 25585812 | TAAAAAAAA/TAAAAAAA | TGTTTCGTTTTTGTTCCCTCT | TCCATTGCAATCGTACTTAATGA | 58.7 | 735 | DRR | Ca_05976 | | B3 | Transcriptional factor B3 |
| CaPOPI_1327 | Ca3 | 25758872 | TAAAAAAAAA/TAAAAAAAA | GGATCCGGTGCTACTCCATA | ATTTGAGGTTGTGATTATGCAA | 59.9 | 713 | INTERGENIC | | | | |
| CaPOPI_1328 | Ca3 | 25888060 | AT/ATT | TGTGGAAGACATCCTTTACTGC | ACCTGACCTATTTCCACCCC | 59.2 | 551 | INTERGENIC | | | | |
| CaPOPI_1329 | Ca3 | 25888907 | GTTTTTTT/GTTTTTTTT | CAGGTCATGCCAGACTTTGA | TCTGACCCGACCTGGTCTAC | 59.8 | 578 | INTERGENIC | | | | |
| CaPOPI_1330 | Ca3 | 25889145 | ATTTTTTTT/ATTTTTTTTT | GTAGACCAGGTCGGGTCAGA | TGATATGTCACATTGTCGCAC | 60.1 | 403 | INTERGENIC | | | | |

| INDEL marker IDs | Chromosomes/ unanchored scaffolds | Physical positions (bp) | InDels (*Kabuli* reference genome-CDC Frontier/PI) | Forward primers (5'-3') | Reverse primers (5'-3') | Anneling temperature (0C) | Expected amplified product size (bp) | Structural annotation | | Functional annotation | | |
|---|---|---|---|---|---|---|---|---|---|---|---|---|
| | | | | | | | | Sequence components of ***kabuli*** **genome** | ***Kabuli*** **gene accession IDs** | **NCBI-KOG** | **TFs** | **NCBI-nr database** |
| CaPOPI_1331 | Ca3 | 25891751 | TAAA/TAAAA | CGGTTCCTAATTGAAAAGGC | CAACGCTTTGTTCTCTCAACA | 58.7 | 341 | INTERGENIC | | | | |
| CaPOPI_1332 | Ca3 | 26020458 | CATATATATATATATATATAT/CATATATATATATATATATATATAT | CCCACTAAAGGCCCATATCA | TTCAAATTCACGCCCCTAAC | 59.8 | 789 | INTERGENIC | | | | |
| CaPOPI_1333 | Ca3 | 26071275 | ATTTTT/ATTTTTTT | TGTGTTTGTTTGGTTTCATCTTG | TGTCAACGTGATTGGAGCAT | 59.9 | 563 | INTRON | Ca_08292 | | MYB_related | SAM dependent carboxyl methyltransferase |
| CaPOPI_1334 | Ca3 | 26071877 | TAAA/TAAAA | TGCTCCAATCACGTTGACAT | AGACGCTGCAACATCAAAGC | 60.1 | 397 | INTRON | Ca_08292 | | MYB_related | SAM dependent carboxyl methyltransferase |
| CaPOPI_1335 | Ca3 | 26114065 | AG/A | TTTATCCTGGGGACTTCGTG | AGGAAAATACACGGGCAAGA | 59.9 | 364 | INTRON | Ca_08292 | | MYB_related | SAM dependent carboxyl methyltransferase |
| CaPOPI_1336 | Ca3 | 26114282 | ATTATAGTTTTTATT/AT | TTTATCCTGGGGACTTCGTG | ATACACCGATGGAGGGCTTT | 59.9 | 838 | INTRON | Ca_08292 | | MYB_related | SAM dependent carboxyl methyltransferase |
| CaPOPI_1337 | Ca3 | 26115029 | AACG/A | TGGCATGGTACTCAACCAGA | TTCACATGAATGATTGTTGGGT | 60.1 | 545 | INTRON | Ca_08292 | | MYB_related | SAM dependent carboxyl methyltransferase |
| CaPOPI_1338 | Ca3 | 26245714 | C/CA | CGCCTGTACCGGTTGATACT | ACCCATTCCCAGTGTTTTCA | 60.0 | 678 | INTRON | Ca_08279 | QI | B3 | Cytochrome P450 |
| CaPOPI_1339 | Ca3 | 26349764 | GTTAACA/G | TCTGCATTGTCCAAAACCAA | TTATCCATGATTTGGGAGGG | 60.1 | 737 | INTERGENIC | | | | |
| CaPOPI_1340 | Ca3 | 26777304 | GTATATATATATATATAT/GTATATATATATATATAT | CACTCATCACAACGGGACAC | CAATGAATCGATGAAATGAATTG | 60.0 | 754 | DRR | Ca_08228 | P | | Cation/H+ exchanger |
| CaPOPI_1341 | Ca3 | 26872317 | CAGAGAGAGAGAGAGAGAGAGAGAG/CAGAGAGAGAGAGAGAGAGAGAGAGAG | CAATGTGCTCGATTTCTCCA | TTACATTCCTTGCCTCGTCA | 59.8 | 389 | INTERGENIC | | | | |
| CaPOPI_1342 | Ca3 | 26961684 | ATTTTTTTTTTTT/ATTTTTTTTTTT | CACGGTTGTGATGACTCGAC | GTCGCGTCAACGTTTGTTTA | 60.2 | 538 | INTERGENIC | | | | |
| CaPOPI_1343 | Ca3 | 26998517 | AATATATATATATATATATATATATATAT/AATATATATATATATATATATATATATATATATATATATAT | TGGGATGTTTCAAATTTCTGC | AAAATGGCATTGGTGTGGTT | 59.9 | 576 | INTERGENIC | | | | |
| CaPOPI_1344 | Ca3 | 27382854 | GTTTTTTTTTTTT/GTTTTTTTTTTT | GCGGTAAAAGAAGGCAAGTG | TGTGGGTACCTTAAACCCTAAAAA | 59.9 | 591 | INTERGENIC | | | | |
| CaPOPI_1345 | Ca3 | 27648871 | AATATATATATATATATATATATAT/AATATATATATATATATATATATATAT | AATCCCATGGCACTGAGAAC | AGCGTGATCACATTCACAGC | 59.9 | 670 | INTERGENIC | | | | |

| INDEL marker IDs | Chromosomes/ unanchored scaffolds | Physical positions (bp) | InDels (*Kabuli* reference genome-CDC Frontier/PI) | Forward primers (5'-3') | Reverse primers (5'-3') | Anneling temperature (0C) | Expected amplified product size (bp) | Structural annotation | | Functional annotation | | |
|---|---|---|---|---|---|---|---|---|---|---|---|---|
| | | | | | | | | Sequence components of *kabuli* genome | *Kabuli* gene accession IDs | NCBI-KOG | TFs | NCBI-nr database |
| CaPOPI_1346 | Ca3 | 27854154 | TGG/TG | TCGATAATGAGTGATTAGGTGGG | ACCTCACCCTCGAATGTCAC | 60.2 | 459 | INTERGENIC | | | | |
| CaPOPI_1347 | Ca3 | 27854804 | T/TA | GGATTGAGAGGGGAGAAAGG | TTTGAAAGCAAGAGCGTCAA | 60.0 | 516 | INTERGENIC | | | | |
| CaPOPI_1348 | Ca3 | 27856755 | TAAAAAAAAA/TAAAAAAAA | CGAAGATGAAGAGGCTTGAAA | CAACAACTTCTGATGCTCGC | 59.6 | 593 | INTERGENIC | | | | |
| CaPOPI_1349 | Ca3 | 27866552 | TTAATAATAATAATAATAATAATAATAATAATAATAATAATAATAGTAATAATAATAATA/TTAATAATAATAATAATAATAATAATAATAATAATAGTAATAATAATAATA | TCAGAATTTTGGTGAGTTGGC | GGGATTCGCTGGTTATCTCA | 60.1 | 552 | INTERGENIC | | | | |
| CaPOPI_1350 | Ca3 | 28113252 | ATTTTTTTT/ATTTTTTT | TCCTTCTCCTCTTCCTTCCA | TGTTTGTGCAATTGAATTATAGTTATG | 58.9 | 849 | INTERGENIC | | | | |
| CaPOPI_1351 | Ca3 | 28188438 | CAAAA/CAAAAA | ACCCATAACGCACTCGTTTC | TCCTCCATTTTTCGTTTTCG | 60.0 | 434 | INTERGENIC | | | | |
| CaPOPI_1352 | Ca3 | 28191077 | CTTTTTTTTT/CTTTTTTTTTT | TAAGATGCGTGGGTTTAGGG | GCTTGTGTTTAAAGCGCTCC | 60.0 | 659 | INTERGENIC | | | | |
| CaPOPI_1353 | Ca3 | 28293830 | TAAAAAAAAAA/TAAAAAAAAA | GATTACTGCTAGGGCCCAAAT | ATCCCACTTGCAACACTTCC | 59.5 | 147 | INTERGENIC | | | | |
| CaPOPI_1354 | Ca3 | 28294648 | AATATATATATATATATATAT/AATATATATATATATATATATATAT | TACCAGTACCAGGAGGCAGG | TGGTGGGTAATCCGATGAAT | 60.1 | 670 | INTERGENIC | | | | |
| CaPOPI_1355 | Ca3 | 28428889 | TTATATATATATATATATATA/TTATATATATATATATATATATA | AGGCAACTCGATTCAAGCAT | AAACAAACAAGCCCACCAAC | 59.8 | 923 | INTERGENIC | | | | |
| CaPOPI_1356 | Ca3 | 28514570 | ATATTATTATTATTATTATTATTATTATTATT/ATATTATTATTATTATTATTATTATTATT | CAAAGAAAATGGTGGGGATG | AAATTGGACTATGCCACCGA | 60.2 | 841 | INTERGENIC | | | | |
| CaPOPI_1357 | Ca3 | 28797139 | CAA/CAATTCGATAA | CAAGGAGCTGTTTTGCTTCA | GTAAAATGGCACGCGGTAGT | 59.2 | 609 | INTERGENIC | | | | |
| CaPOPI_1358 | Ca3 | 28797825 | A/AG | CATAGTCTGCGAAACCAGCA | GGTGGAATTGATCGGAGAGA | 60.0 | 454 | INTERGENIC | | | | |
| CaPOPI_1359 | Ca3 | 28799476 | G/GTC | TCTTGGACCTCACTGGGTTT | TGTCCTTGATCTTGTTTCCG | 59.5 | 836 | INTERGENIC | | | | |
| CaPOPI_1360 | Ca3 | 28799571 | AG/A | TCTTGGACCTCACTGGGTTT | TGTCCTTGATCTTGTTTCCG | 59.5 | 836 | INTERGENIC | | | | |

| INDEL marker IDs | Chromosomes/ unanchored scaffolds | Physical positions (bp) | InDels (*Kabuli* reference genome-CDC Frontier/PI) | Forward primers (5'-3') | Reverse primers (5'-3') | Anneling temperature (0C) | Expected amplified product size (bp) | Structural annotation | | Functional annotation | | |
|---|---|---|---|---|---|---|---|---|---|---|---|---|
| | | | | | | | | Sequence components of *kabuli* genome | *Kabuli* gene accession IDs | NCBI-KOG | TFs | NCBI-nr database |
| CaPOPI_1361 | Ca3 | 28859122 | CAAAAAAAAA/CAAAAAAAAAA | TTTCCCGACTTGGTCCTTTA | ATGACATTTCGCCAAACACA | 59.5 | 432 | INTERGENIC | | | | |
| CaPOPI_1362 | Ca3 | 28966806 | TTATATATATATATATATATATATAT/TTATATATATATATATATATATATATATAT | TCGATATTTAGAATTTACGAACGAGA | ATTCAAGAAAGTTCGCCGTG | 59.6 | 850 | INTERGENIC | | | | |
| CaPOPI_1363 | Ca3 | 28984656 | GTCTCTCT/GTCTCT | GAGAACGCAAGCTAACGGAC | GGATCAGAGAAAACGTGGGA | 60.0 | 326 | INTERGENIC | | | | |
| CaPOPI_1364 | Ca3 | 28994933 | AC/A | CAAATTAAGCAGAGCCCGTC | TTTTGGACAATGTCAACCCA | 59.8 | 165 | INTRON | Ca_07207 | KTDL | | GCN5-related N-acetyltransferase (GNAT) domain |
| CaPOPI_1365 | Ca3 | 28995118 | CAA/CAAA | CAAATTAAGCAGAGCCCGTC | CGGAATTTGAACAAAACGCT | 59.8 | 769 | INTRON | Ca_07207 | KTDL | | GCN5-related N-acetyltransferase (GNAT) domain |
| CaPOPI_1366 | Ca3 | 28999636 | CT/CTT | TCTGAATTTCTGTACCGCCC | GGGATAGGGCATGTGTGAGT | 60.1 | 536 | INTRON | Ca_07207 | KTDL | | GCN5-related N-acetyltransferase (GNAT) domain |
| CaPOPI_1367 | Ca3 | 29053872 | TTATATATATATATATATATA/TTATATATATATATATATATA | TCCTCCGTCTTTTCATTTGG | GGTTGGGGGAGTGTTAGCTT | 60.0 | 456 | INTERGENIC | | | | |
| CaPOPI_1368 | Ca3 | 29228366 | TTATATATATATATATATATAT/TTATATATATATATATAT | TCGGAGGCAGAGACAAGTTT | TTAATTGGGGGAAATCCACA | 60.0 | 364 | INTERGENIC | | | | |
| CaPOPI_1369 | Ca3 | 29334875 | TTATATATATATATATATATAT/TTATATATATATATATATATATAT | GGGTGGCTCATGAAGCTTAG | TCTCAACCGTTTTGGTGACA | 59.8 | 576 | INTERGENIC | | | | |
| CaPOPI_1370 | Ca3 | 29446926 | GCA/GCACA | CAGCAGGGGCATACGTTAAT | GATTCCCCTCCCTGATTCTC | 60.0 | 306 | INTERGENIC | | | | |
| CaPOPI_1371 | Ca3 | 29506460 | CGTT/C | TGCCCCTGTCATCAATGTTA | TCAATCCCCATCAATCATCA | 59.9 | 598 | DRR | Ca_07256 | T | FAR1 | Protein kinase, catalytic domain |
| CaPOPI_1372 | Ca3 | 29563741 | TATTCAAATAAATT/TATTCAAATAAATTCAAATAAATT | GCACATGCGGTCCTTTAACT | TTCCCCTCTCTCCCTCTCTC | 60.1 | 648 | INTERGENIC | | | | |
| CaPOPI_1373 | Ca3 | 29572508 | TTTA/TTTAGCCTAAAAAGTATTA | GGACCAATTCGGAAAAAGAA | TTGGCACCCCAAATTTTAAG | 59.0 | 359 | DRR | Ca_07262 | R | bHLH | Pentatricopeptide repeat |
| CaPOPI_1374 | Ca3 | 29748543 | CATTATTATTATTATTATTATTATTATTATTATTATTATTATTATTATTATTATTATTATTATTATTAT/CATTATTATTATTATTATTATTATTATTATTATTATTATTATTATTATTA | TGCACCAAGTCACTAACAAAAA | GGTGGCCCTCAACTTTCTTT | 58.4 | 404 | INTERGENIC | | | | |
| CaPOPI_1375 | Ca3 | 29752847 | TAAAAAAAAAA/TAAAAAAAAAAA | AATCGAGATGCCTTAGTGCG | CCTATTCCCACCCCCTTTTA | 60.4 | 868 | INTERGENIC | | | | |

| INDEL marker IDs | Chromosomes/ unanchored scaffolds | Physical positions (bp) | InDels (*Kabuli* reference genome-CDC Frontier/PI) | Forward primers (5'-3') | Reverse primers (5'-3') | Anneling temperature (0C) | Expected amplified product size (bp) | Structural annotation | | Functional annotation | | |
|---|---|---|---|---|---|---|---|---|---|---|---|---|
| | | | | | | | | Sequence components of *kabuli* genome | *Kabuli* gene accession IDs | NCBI-KOG | TFs | NCBI-nr database |
| CaPOPI_1376 | Ca3 | 29771272 | CTTTTTTTTTTT/CTTTTTTTTTT | GGATTGCATTATTGCCAAGTT | GCGAGACATTTTGGGTGAAT | 59.0 | 264 | INTERGENIC | | | | |
| CaPOPI_1377 | Ca3 | 29772076 | ATTTTTTTTTTTT/ATTTTTTTTTTTTT | CCAGCTCCACAACCATCTTT | GCAAATGTTCTGGTCGCATA | 60.1 | 391 | INTERGENIC | | | | |
| CaPOPI_1378 | Ca3 | 29779379 | TTATATATATATATATATAT/TTATATATATATATATATATAT | GAGGAAAGCTTGTTGCTTGG | CCGAATATAAATCATTTGTGGAAA | 60.0 | 323 | DRR | Ca_07285 | | | |
| CaPOPI_1379 | Ca3 | 29970428 | CATATAATATAATATAATAT/CATATAATATAATAT | GCAAATAACATCGAGGTTCAGG | AACGACTCAGATGAAATGCAA | 60.8 | 454 | INTERGENIC | | | | |
| CaPOPI_1380 | Ca3 | 30071057 | TAAAAAAAAAA/TAAAAAAAAA | TAAAAGCCAATGGAACTGGG | ATCGCTCTCCTCCTCAAACA | 59.9 | 247 | INTERGENIC | | | | |
| CaPOPI_1381 | Ca3 | 30100381 | ATATTATTATTATTATTATTATT/ATATTATTATTATTATT | TTGCACATTTACTCAACCGC | ATTCCAGAAATGCGACCTTG | 59.7 | 319 | INTERGENIC | | | | |
| CaPOPI_1382 | Ca3 | 30339041 | AAA/AAATAA | CCTCCTCCAACCATGAAATG | CCTGTGTGGTCAGAAAGGGT | 60.3 | 496 | INTERGENIC | | | | |
| CaPOPI_1383 | Ca3 | 30450479 | ATCTTCTTCTTCTTCTTCTTCT/ATCTTCTTCTTCTTCTTCT | TCATGGGCCACAGCATATAA | GTCGTGCCAGCACAAGAGTA | 59.9 | 366 | INTERGENIC | | | | |
| CaPOPI_1384 | Ca3 | 30602335 | TTGA/TA | GCCCTCAGATGAGAGTCAGG | AAATTAAACCCTCATCCCCG | 59.9 | 842 | INTERGENIC | | | | |
| CaPOPI_1385 | Ca3 | 30930504 | GTTTTTATTTTTATTTTTA/GTTTTTATTTTTA | GCATGGACCGTTTAAAATTG | TTTAGGTGGAAAATTCATTAGAAAA | 58.0 | 818 | INTERGENIC | | | | |
| CaPOPI_1386 | Ca3 | 31101603 | ATTTTTTTTTTT/ATTTTTTTTTTTT | ATGGTCCCAATGAAATCCAA | TGTGTTGTCTCTGGCCTTTG | 60.0 | 843 | INTRON | Ca_12207 | | | Zeta toxin, P-loop nucleoside triphosphate hydrolase |
| CaPOPI_1387 | Ca3 | 31290021 | T/TTGA | GGTTCAAACGTGTTCCCTTG | ACGCTCTCAAATCGACCATC | 60.4 | 929 | DRR | Ca_12225 | P | ERF | General substrate transporter |
| CaPOPI_1388 | Ca3 | 31293455 | TG/TGG | TGGGATGTTTGAGAAAAGCA | ATCCCATCCCACCAACAATA | 59.2 | 522 | INTERGENIC | | | | |
| CaPOPI_1389 | Ca3 | 31295937 | AAAAAA/AAAAAACAAAAA | GGACCATGGACTCCAGAAGA | TCGTCCTCCATCAGTTCGAT | 60.0 | 688 | INTRON | Ca_12226 | K | MYB | SANT domain, DNA binding |
| CaPOPI_1390 | Ca3 | 31296813 | CGTGTGT/CGTGT | TCGGTTTTAAGGGAAGCAAA | TGCTGCAATAGCTGACCATC | 59.7 | 806 | INTRON | Ca_12226 | K | MYB | SANT domain, DNA binding |

| INDEL marker IDs | Chromosomes/ unanchored scaffolds | Physical positions (bp) | InDels (*Kabuli* reference genome-CDC Frontier/PI) | Forward primers (5'-3') | Reverse primers (5'-3') | Anneling temperature (0C) | Expected amplified product size (bp) | Structural annotation | | Functional annotation | | |
|---|---|---|---|---|---|---|---|---|---|---|---|---|
| | | | | | | | | Sequence components of *kabuli* genome | *Kabuli* gene accession IDs | NCBI-KOG | TFs | NCBI-nr database |
| CaPOPI_1391 | Ca3 | 31298126 | GTTATTATTATTAT/GTTATTATTATTATTAT | GGACTTTTGGCACGATGTTT | TTTGCAACTTTTGACGGACA | 60.0 | 716 | DRR | Ca_12226 | K | MYB | SANT domain, DNA binding |
| CaPOPI_1392 | Ca3 | 31298291 | CT/CTTTT | TGAGAGGTCGATTTTGGAGG | TTTGCAACTTTTGACGGACA | 60.2 | 506 | DRR | Ca_12226 | K | MYB | SANT domain, DNA binding |
| CaPOPI_1393 | Ca3 | 31319681 | GAAAA/GAAAAA | CGTGGCTCCTCAAAAGTCTC | ATGCACAAGGCTTCCATACC | 60.0 | 599 | DRR | Ca_12232 | U | bHLH | VHS |
| CaPOPI_1394 | Ca3 | 31326387 | GTTTT/GTTT | CGGGTACAGCCGGTAAGTAA | GGGTGCCTTTAAAGTGTGGA | 60.0 | 639 | INTERGENIC | | | | |
| CaPOPI_1395 | Ca3 | 31328317 | ATTTTTT/ATTTTTTT | TCCAACTAACCGCAATTCAA | TCATGGCAAATTTAGGATGTTG | 59.2 | 447 | INTERGENIC | | | | |
| CaPOPI_1396 | Ca3 | 31336604 | T/TC | AGCAGGCCTGGACCTTTACT | GGAATGCCAACGAATGAAAA | 60.3 | 555 | INTERGENIC | | | | |
| CaPOPI_1397 | Ca3 | 31339236 | T/TC | CCAACCCATGTACCGACATT | AGAGACCGTTTCATGAGATTTG | 60.5 | 525 | INTERGENIC | | | | |
| CaPOPI_1398 | Ca3 | 31343075 | CAATAAAAATAAAAATAAAAAT/CAATAAAAATAAAAAT | TGGCAAGTTTTCACTTCCATT | GATCACGGGAGGATCGACTA | 59.6 | 792 | DRR | Ca_12234 | | | Domain of unknown function DUF640 |
| CaPOPI_1399 | Ca3 | 31353219 | CAAAAA/CAAAAAA | TCCCTAAAAGTTTCTTCATCCAA | GAATGACATATTGTGGCAGCA | 59.2 | 225 | INTERGENIC | | | | |
| CaPOPI_1400 | Ca3 | 31364505 | CTTTTTTTT/CTTTTTTTTT | ACCAATGTCTCGCAAGCTCT | GGTAAACATTTGCATCGTTGG | 60.0 | 458 | INTERGENIC | | | | |
| CaPOPI_1401 | Ca3 | 31366021 | A/ATT | CCCTCTTGTGCCTGTCTTTC | CAACGGGAATCTTCCGTCTA | 59.8 | 469 | INTERGENIC | | | | |
| CaPOPI_1402 | Ca3 | 31371532 | TATCAT/TATCATCAT | GGATCAGCCCAATTGGTAAA | TAGACCATAAGCCCCTGACG | 59.8 | 322 | INTERGENIC | | | | |
| CaPOPI_1403 | Ca3 | 31381715 | GATATA/GATA | TAAACCTTGGCTGGGTCAAC | TTGTTTATTTTGTTCGTCGTGTT | 60.0 | 403 | INTERGENIC | | | | |
| CaPOPI_1404 | Ca3 | 31381813 | AT/A | TAAACCTTGGCTGGGTCAAC | TTGCTACCATTGTAAAGCTTGTTT | 60.0 | 611 | INTERGENIC | | | | |
| CaPOPI_1405 | Ca3 | 31391982 | ATTTT/ATTT | CGTGCCCACATTATCAACAA | TCTTGCTCATGGTGCTTCTG | 60.4 | 793 | INTERGENIC | | | | |

| INDEL marker IDs | Chromosomes/ unanchored scaffolds | Physical positions (bp) | InDels (*Kabuli* reference genome-CDC Frontier/PI) | Forward primers (5'-3') | Reverse primers (5'-3') | Anneling temperature (0C) | Expected amplified product size (bp) | Structural annotation | | Functional annotation | | |
|---|---|---|---|---|---|---|---|---|---|---|---|---|
| | | | | | | | | Sequence components of *kabuli* genome | *Kabuli* gene accession IDs | NCBI-KOG | TFs | NCBI-nr database |
| CaPOPI_1406 | Ca3 | 31402077 | TAAAA/TAAAAA | TGTACACGTCGGTGAACAACT | TGTGGCGACAACTAACTTTGA | 59.1 | 634 | INTERGENIC | | | | |
| CaPOPI_1407 | Ca3 | 31413249 | AA/AATA | TGTTGCACGTTGGACAAGTT | GACTGCTACCAACCCAGCAT | 60.2 | 374 | DRR | Ca_12238 | A | | H/ACA ribonucleoprotein complex, subunit Nop10 |
| CaPOPI_1408 | Ca3 | 31426177 | C/CCCAACAAGTGT | TGAGTCCTCATCGGTCACAA | TCATCAATGACTAAAATTTGTTTTGTA | 60.2 | 573 | DRR | Ca_12239 | R | YABBY | Protein kinase, catalytic domain |
| CaPOPI_1409 | Ca3 | 31441706 | AT/ATGAAATT | AACAATTTTTGGAACCCCTAA | TTTGCAAGTGCAACTTCTGC | 57.5 | 669 | DRR | Ca_12242 | | | PAK-box/P21-Rho-binding |
| CaPOPI_1410 | Ca3 | 31446074 | CTTTTT/CTTTT | CATCCCAATTTGCATTGGTT | TGGAGGCTATTGGTACAGGG | 60.6 | 531 | DRR | Ca_12243 | G | | Glycolipid transfer protein domain |
| CaPOPI_1411 | Ca3 | 31471455 | CTTATTTATTTATTT/CTTATTTATTTATTTATTT | AAAACCCAAAAGCTGAATCG | CCCAAAGTTGGCATGAAGTT | 59.2 | 624 | INTERGENIC | | | | |
| CaPOPI_1412 | Ca3 | 31476304 | ATTTTT/ATTTT | TGGTTCAGATTTGTTTGGTGA | CAAACGTGAGTGAAACAACCA | 59.0 | 568 | INTERGENIC | | | | |
| CaPOPI_1413 | Ca3 | 31478334 | TCC/TCCC | TGGTGTGTGTTAGCCATAAAGG | GGGGCCTCTTCTTTTGTTTC | 59.9 | 614 | INTRON | Ca_12246 | | | Glucose-methanol-choline oxidoreductase, N-terminal |
| CaPOPI_1414 | Ca3 | 31495232 | CTTT/CTT | CCAAAATGGTGAGAGACTTCG | TTCGGTGCTTTGTCTCTTCC | 59.7 | 809 | INTERGENIC | | | | |
| CaPOPI_1415 | Ca3 | 31498407 | ATTT/ATTTT | CTAGCTCGGGTTTTCTGACG | TTGGCCACAGTTTAACCACA | 60.0 | 531 | DRR | Ca_12248 | T | | RHO protein GDP dissociation inhibitor |
| CaPOPI_1416 | Ca3 | 31519164 | C/CT | TACACGTGGCCTCAAACAAA | TCAAGCATGGTTCCAATCAG | 60.1 | 364 | INTERGENIC | | | | |
| CaPOPI_1417 | Ca3 | 31530560 | TTGGGT/TTGGGTGGGT | AACTCCTTCTGCACATTGGG | TGGGAAATAGATCGGTCTCG | 60.1 | 449 | INTRON | Ca_12252 | E | TALE | Oligopeptide transporter |
| CaPOPI_1418 | Ca3 | 31540731 | CTATATATATATATATATATATA/CTATATATATATATATATATATATA | CTGTTCCATTGTCCAACACG | CACATGGGTGACATCTGAGG | 60.0 | 566 | INTRON | Ca_12253 | LK | | BRCT |
| CaPOPI_1419 | Ca3 | 31560705 | A/AAG | TGTTCACTGCCAAACTCTGC | GCAGTGCACAACTGATGGTT | 60.0 | 585 | INTERGENIC | | | | |
| CaPOPI_1420 | Ca3 | 31563003 | CATA/CATATA | GAATGGTGTTCCTTTGTCCC | AATTTCACCCAAAGACACGG | 59.2 | 274 | INTERGENIC | | | | |

| **INDEL marker IDs** | **Chromosomes/ unanchored scaffolds** | **Physical positions (bp)** | **InDels (*Kabuli* reference genome-CDC Frontier/PI)** | **Forward primers (5'-3')** | **Reverse primers (5'-3')** | **Anneling temperature (0C)** | **Expected amplified product size (bp)** | **Structural annotation** | | **Functional annotation** | | |
|---|---|---|---|---|---|---|---|---|---|---|---|---|
| | | | | | | | | **Sequence components of *kabuli* genome** | ***Kabuli* gene accession IDs** | **NCBI-KOG** | **TFs** | **NCBI-nr database** |
| CaPOPI_1421 | Ca3 | 31566099 | CG/C | AAAGGGCATGGGGAAAATAG | TTTTCATGCATTTGAGACGC | 60.1 | 338 | DRR | Ca_12255 | | G2-like | Myb, DNA-binding |
| CaPOPI_1422 | Ca3 | 31595214 | CATTTATAAACAT/C | TTGATGTTATTTATTGGTGCAAAA | AGTAACGACAACCTCCGTGG | 58.5 | 623 | INTERGENIC | | | | |
| CaPOPI_1423 | Ca3 | 31602428 | GAGTGCTAGTGCTA/GAGTGCTAGTGCTAGTGCTA | GCACGAGCACAACTTCAAAA | AAAGAACCATCATCGCAGGT | 60.0 | 618 | INTERGENIC | | | | |
| CaPOPI_1424 | Ca3 | 31602911 | ATGTT/ATGTTGTT | ACCGAATATTCCATGCTCCA | CCAAGTGATGTGTCATGGCT | 60.3 | 565 | INTERGENIC | | | | |
| CaPOPI_1425 | Ca3 | 31607270 | ATTTTTTTT/ATTTTTTT | TACCCTAACGGTGCTCCATC | AAGATCATTCCCCAAAACCC | 60.0 | 567 | INTRON | Ca_12260 | | | Gnk2-homologous domain |
| CaPOPI_1426 | Ca3 | 31609545 | T/TTA | GACAAAGCCAGAACCTTTGC | ACCCAAGCAGGCATACAAAC | 59.9 | 538 | DRR | Ca_12260 | | | Gnk2-homologous domain |
| CaPOPI_1427 | Ca3 | 31611126 | GAAAAGTAAAAGTAAAA/GAAAAGTAAAA | TTTGGATTAGCCATAACATCCA | CTTCCACCTTCGTGGAAAGA | 59.3 | 617 | INTERGENIC | | | | |
| CaPOPI_1428 | Ca3 | 31629115 | ATTTTTT/ATTTTT | GTTTGTTTCGCAGGTTGGTT | ATTTTGGAGGGATTTGGAGG | 60.0 | 498 | INTRON | Ca_12262 | T | | RNA recognition motif domain |
| CaPOPI_1429 | Ca3 | 31755384 | CTATATATATATATATATATATAT/CTATATATATATATATATATATATAT | GCAGCTAAAGAAGATTGGCCT | TTTGCGATATGAACCAAATCA | 60.0 | 467 | INTERGENIC | | | | |
| CaPOPI_1430 | Ca3 | 31810634 | AAC/AACCTTTGAC | TGTGTGATTGCACAGGAACA | TTAGCGTGGGCAAATAGTCA | 59.7 | 253 | INTERGENIC | | | | |
| CaPOPI_1431 | Ca3 | 31852962 | TTTATTATTATTATTATTATTATTATTATTATTATTATTATTATTATTATTATTATT/TTTATTATTATTATTATTATTATTATTATTATTATTATTATTATTATTATTATTATTAT | TTTAGGGAAATGGGTCATGG | TCAATCTGTCTCGTTTTGATTGA | 59.6 | 576 | DRR | Ca_12289 | G | STAT | Ribokinase |
| CaPOPI_1432 | Ca3 | 31908295 | TAAAAAAAAAAAA/TAAAAAAAAAA | GAGGTGAAAATGGTGTAGATTAGAG | TCGTTCAACACCAAATGGAA | 57.5 | 863 | INTERGENIC | | | | |
| CaPOPI_1433 | Ca3 | 31912229 | GAAAA/GAAA | CTCGGATACTGCCGATTGAT | TGCCTGTTCTCTGTGATTGC | 60.1 | 958 | INTRON | Ca_12292 | | | Protein of unknown function DUF3741 |
| CaPOPI_1434 | Ca3 | 32399895 | CATATATATATATATATATATATATATATATATAT/CATATATATATATATATATATATATATATATATATATATAT | TTTCTTTCCGAAAATGGAGA | TGTGAATCCCGCTTTAACAA | 57.4 | 523 | INTERGENIC | | | | |
| CaPOPI_1435 | Ca3 | 32563568 | TTATATATATATATATATAT/TTATATATATATATATATATAT | CGAAAGAAAAAGAAAACATCGC | GAAGGTGGCTCGGCATATAA | 60.2 | 574 | INTERGENIC | | | | |

| INDEL marker IDs | Chromosomes/ unanchored scaffolds | Physical positions (bp) | InDels (*Kabuli* reference genome-CDC Frontier/PI) | Forward primers (5'-3') | Reverse primers (5'-3') | Anneling temperature (0C) | Expected amplified product size (bp) | Structural annotation | | Functional annotation | | |
|---|---|---|---|---|---|---|---|---|---|---|---|---|
| | | | | | | | | Sequence components of *kabuli* genome | *Kabuli* gene accession IDs | NCBI-KOG | TFs | NCBI-nr database |
| CaPOPI_1436 | Ca3 | 32604694 | CTTCTTGATTTATTTATTTCTTGAT/CTTCTTGATTTATTTATTTCTTGATTTATTTATTTCTTGAT | TCGGCACATCACAATCATTT | TTGGCTTGCTTCACACATTC | 59.9 | 677 | DRR | Ca_11988 | NI | | Lipin, N-terminal |
| CaPOPI_1437 | Ca3 | 32629755 | TAAAAAAAA/TAAAAAAA | TTTTTGCGGTCCTTTGAATTA | GCGGTATTCACTCCAAACTGA | 59.6 | 819 | INTERGENIC | | | | |
| CaPOPI_1438 | Ca3 | 33196113 | TAAAAAAA/TAAAAAA | CAGGGGCACAAATACATCAA | TTGCAATTCCTTGTGAACCA | 59.4 | 334 | INTERGENIC | | | | |
| CaPOPI_1439 | Ca3 | 33243720 | CTT/CT | GATGTGGCTGCAACAGCTTA | TGACAGACTCGTCGTTCAGG | 60.0 | 462 | CDS (FRAME SHIFT) | Ca_12059 | | | Glycosyl transferase, family 2 |
| CaPOPI_1440 | Ca3 | 33346296 | GATATATATATATATATATATATA/GATATATATATATATATATATATA | ATTTTGCCACATGATACGCA | GCTTCACACGGAAACAACAA | 60.0 | 531 | DRR | Ca_23675 | | | PWWP |
| CaPOPI_1441 | Ca3 | 33387153 | AATATATATATATATATATATATATATAT/AATATATATATATATATATATATATATATATATATATATAT | ATACCTACGGATTTGTGCCG | TTTGTTGTTGAAGATGGCAA | 59.8 | 553 | INTERGENIC | | | | |
| CaPOPI_1442 | Ca3 | 33531472 | CAAA/CAAAA | TTGCCTTTGTATCACTGCCA | CTTGCCACTTGGTTGGAAAT | 60.3 | 228 | INTRON | Ca_23669 | S | | |
| CaPOPI_1443 | Ca3 | 33545135 | C/CTATCAGTAAACATT | ACGTCATCAGTCGAAGTCCC | AGTTTGCGTAGGTTGTTGGG | 60.1 | 329 | INTERGENIC | | | | |
| CaPOPI_1444 | Ca3 | 33748722 | CTTTTTTTTTTT/CTTTTTTTTTTTT | AGGTGCCAAAATTGATGAGG | CAAGTTGCGATGAACACCTG | 59.9 | 399 | INTERGENIC | | | | |
| CaPOPI_1445 | Ca3 | 33748791 | TAGTTTCAG/TAGTTTCAGTTTCAG | AGGTGCCAAAATTGATGAGG | CCTAGAAGGTCGCATCTCCA | 59.9 | 417 | INTERGENIC | | | | |
| CaPOPI_1446 | Ca3 | 33828272 | TTTATTATTATTATTATTATTATTATTATTATTATTATTATTATTATTATT/TTTATTATTATTATTATTATTATTATTATTATTATTATTATTATTATTATTATT | AGAGGCAAACAAGAACCGAA | TGCATATAAGGAATTGACGCC | 59.9 | 318 | INTERGENIC | | | | |
| CaPOPI_1447 | Ca3 | 33859343 | TT/TTATTATTAT | CAATTGAATTCATTCTTCATACAGG | TTGGAAAATGTTGCAAGTTCA | 59.0 | 978 | INTERGENIC | | | | |
| CaPOPI_1448 | Ca3 | 33954528 | ATATTATTATTATTATTATTATTATTATTATTATTATTATTATTATTATTATT/ATATTATTATTATTATTATTATTATTATTATTATTATTATTATTATTATTATTA | GGCTTCAAAGCCAACATCAT | TCATACAAAAACCAAACGGG | 60.1 | 622 | INTERGENIC | | | | |
| CaPOPI_1449 | Ca3 | 33983043 | TA/TAGACACTGA | GGTGTTTTACGAATGGGGTG | GGGACTCATTTATGCCGATG | 60.1 | 353 | INTERGENIC | | | | |
| CaPOPI_1450 | Ca3 | 34032860 | CCAAAGC/CC | TAAATGTTGCGAACCTGCTG | CGTCGCGGTTATCACTTACA | 59.9 | 678 | INTERGENIC | | | | |

| **INDEL marker IDs** | **Chromosomes/ unanchored scaffolds** | **Physical positions (bp)** | **InDels (*Kabuli* reference genome-CDC Frontier/PI)** | **Forward primers (5'-3')** | **Reverse primers (5'-3')** | **Anneling temperature (0C)** | **Expected amplified product size (bp)** | **Structural annotation** | | **Functional annotation** | | |
|---|---|---|---|---|---|---|---|---|---|---|---|---|
| | | | | | | | | **Sequence components of *kabuli* genome** | ***Kabuli* gene accession IDs** | **NCBI-KOG** | **TFs** | **NCBI-nr database** |
| CaPOPI_1451 | Ca3 | 34161169 | GAAAA/GAAAAA | GAAGCAAATCGATTGGGAAA | CTGGGGTTCCACTGTGACTT | 60.0 | 239 | INTERGENIC | | | | |
| CaPOPI_1452 | Ca3 | 34365238 | T/TC | TGACCCAAAATGTGACTTGC | ATGCCAAGAATCCAACTCGT | 59.5 | 865 | INTERGENIC | | | | |
| CaPOPI_1453 | Ca3 | 34456382 | AGG/AG | TCCCCTGTTTGGTTGTTGAT | GTAGTGTTGGTTTGTGGGGG | 60.2 | 418 | INTERGENIC | | | | |
| CaPOPI_1454 | Ca3 | 34474422 | TAAAAAAAA/TAAAAAAA | AAAAATCAATTTATGCCGTTGT | TTATGCCGGATTGACACTCTT | 57.7 | 478 | INTERGENIC | | | | |
| CaPOPI_1455 | Ca3 | 34520648 | GAA/GAAA | AAAGACGAAAGGGAACACCA | GAAATTGGAAACAAAGTCCGA | 59.6 | 411 | INTERGENIC | | | | |
| CaPOPI_1456 | Ca3 | 34521509 | CTTTTTT/CTTTTTTT | ACCCATCTTGCGTTTTTCAG | TTAAATGGCGGCAAATTGAT | 60.1 | 310 | INTERGENIC | | | | |
| CaPOPI_1457 | Ca3 | 34565949 | AA/AAGA | TGCTTTAGTTTGGCTTTGGC | TGATAGGATTTGTTGGAAATCG | 60.4 | 876 | DRR | Ca_00727 | GC | HSF | UDP-glucuronosyl/UDP-glucosyltransferase |
| CaPOPI_1458 | Ca3 | 34568477 | CCTCCGA/CCTCCGACTCCGA | GTTTGTGCCCAGTCCAAAGT | ACAAACACAACGCCAAACAA | 60.0 | 549 | INTERGENIC | | | | |
| CaPOPI_1459 | Ca3 | 34582473 | TG/T | GGGGTGTTGAAGAATCGAGA | GGGAAATAGCGGTTTGTTCA | 60.1 | 614 | INTERGENIC | | | | |
| CaPOPI_1460 | Ca3 | 34605719 | CTTTTTAATTTTTTAATTTTTTAAT/CTTTTTAATTTTTTAAT | ATGTCTTTGGTTGGGCAGAG | GGGTTTGGAGGAGAGGAGAA | 60.1 | 404 | INTERGENIC | | | | |
| CaPOPI_1461 | Ca3 | 34608178 | ATTG/A | TGGAGAAACAAAATACGGGA | TTGGGTAGACATTTTCCCTTG | 58.1 | 575 | INTERGENIC | | | | |
| CaPOPI_1462 | Ca3 | 34619372 | CAAA/CAA | CGGCTCAGTGATCCTCTTTC | CCAAGGGAAAAGATAACCAACA | 60.0 | 334 | INTERGENIC | | | | |
| CaPOPI_1463 | Ca3 | 34623936 | ATTTTT/ATTTT | CAGATAATTTGGGCAAGAGAA | GGAGCCAAGCTCCAGTTATT | 57.0 | 716 | INTERGENIC | | | | |
| CaPOPI_1464 | Ca3 | 34626639 | C/CAAATGATT | TTTGCAAACCAAATTCTTCAAA | GATGGGTCCTATTGGTGGAA | 59.6 | 938 | DRR | Ca_00734 | FH | | Permease, cytosine/purines, uracil, thiamine, allantoin |
| CaPOPI_1465 | Ca3 | 34660669 | GAA/GAAA | TCTTGCACCAGCAATTTGAG | TAAACCCAATGGATAGCCGA | 60.0 | 824 | INTRON | Ca_00740 | AJ | NAC | RNA recognition motif domain |

| INDEL marker IDs | Chromosomes/ unanchored scaffolds | Physical positions (bp) | InDels (*Kabuli* reference genome-CDC Frontier/PI) | Forward primers (5'-3') | Reverse primers (5'-3') | Anneling temperature (0C) | Expected amplified product size (bp) | Structural annotation | | Functional annotation | | |
|---|---|---|---|---|---|---|---|---|---|---|---|---|
| | | | | | | | | Sequence components of *kabuli* genome | *Kabuli* gene accession IDs | NCBI-KOG | TFs | NCBI-nr database |
| CaPOPI_1466 | Ca3 | 34665269 | TGGGGG/TGGGG | CAAGAAGGTGGGAGGATTCA | TTGGTACAAGCCCAGACAAA | 60.0 | 593 | INTERGENIC | | | | |
| CaPOPI_1467 | Ca3 | 34674867 | TAAAAAA/TAAAAAAA | GGAGGAACTCAAACCCACAA | GTTTCGACTGGTGCAGATCA | 59.9 | 659 | INTERGENIC | | | | |
| CaPOPI_1468 | Ca3 | 34681160 | ATAAAATTAAAAT/ATAAAATTAAAATTAAAAT | AGGGGCCATGCTAATCTTCT | TTTGAAGGGAGAAATCCATCA | 60.1 | 552 | DRR | Ca_00743 | V | GRAS | Lipase, GDXG, active site |
| CaPOPI_1469 | Ca3 | 34681675 | GTTTTTTT/GTTTTTT | TGATGGATTTCTCCCTTCAAA | TTGGTTGTGGACCTGTGGTA | 59.5 | 475 | DRR | Ca_00743 | V | GRAS | Lipase, GDXG, active site |
| CaPOPI_1470 | Ca3 | 34685742 | TAAAAAAA/TAAAAAA | TTATGTCTCGTTTGGTGGACA | TTAGTGACATGCGTGGCTTC | 59.0 | 586 | INTERGENIC | | | | |
| CaPOPI_1471 | Ca3 | 34717035 | CAAAATTAAAATT/CAAAATTAAAATTAAAATT | AAAACATGTAGATCCATTTGAGTGA | AACTTTTTGGCTTTCCGGTT | 59.0 | 640 | INTERGENIC | | | | |
| CaPOPI_1472 | Ca3 | 34721625 | ACTATCATTCTATCA/ACTATCA | CAATCCGTTTAGAACTCCCG | TGTTGCACGTAGCCTAAGAAA | 59.6 | 625 | DRR | Ca_00746 | GMW | ERF | Exostosin-like |
| CaPOPI_1473 | Ca3 | 34731798 | TAAAAAAAA/TAAAAAAAAAA | TTTTCTGCCAAAATCATCCC | AAGAAAGAGTACCGTCGCCA | 59.9 | 671 | DRR | Ca_00748 | | | Protein of unknown function DUF81 |
| CaPOPI_1474 | Ca3 | 34732162 | TAAA/TAAAAA | TGGCGACGGTACTCTTTCTT | CACACTTTTAGGCCACCCAT | 59.9 | 389 | DRR | Ca_00748 | | | Protein of unknown function DUF81 |
| CaPOPI_1475 | Ca3 | 34737485 | CTTTTTTT/CTTTTTT | TGCTCTTCGGTTCGTTTCTT | ACCCAAGGATACAATGCCAA | 60.0 | 650 | DRR | Ca_00749 | J | | Ribosomal protein L24e |
| CaPOPI_1476 | Ca3 | 34739285 | TT/TTGGT | AGGTAGAGCCAGTGAGGTGG | TCTTTGGATGTGGTGGAACA | 59.3 | 841 | DRR | Ca_00749 | J | | Ribosomal protein L24e |
| CaPOPI_1477 | Ca3 | 34739482 | ATTTT/ATTT | AGGCAATTCAAAGTTAAAGGTG | TCTTTGGATGTGGTGGAACA | 57.5 | 508 | INTERGENIC | | | | |
| CaPOPI_1478 | Ca3 | 34746354 | GAT/GATCAT | TGGTGTATAACTAAGAGACACGTCAA | GGGGAGGAGGAAGAAGCTC | 59.2 | 810 | INTERGENIC | | | | |
| CaPOPI_1479 | Ca3 | 34749435 | TC/T | TGGGGGAGAGACGTCATAAT | TGTCCTCGACATTTCTCTCCA | 59.4 | 834 | INTERGENIC | | | | |
| CaPOPI_1480 | Ca3 | 34749745 | A/ACCTTAT | CGTCAATATTTGTTGCAGGC | TATGGGTGGTTCCATTCCAC | 59.2 | 332 | INTERGENIC | | | | |

| INDEL marker IDs | Chromosomes/ unanchored scaffolds | Physical positions (bp) | InDels (*Kabuli* reference genome-CDC Frontier/PI) | Forward primers (5'-3') | Reverse primers (5'-3') | Anneling temperature (0C) | Expected amplified product size (bp) | Structural annotation | | Functional annotation | | |
|---|---|---|---|---|---|---|---|---|---|---|---|---|
| | | | | | | | | Sequence components of *kabuli* genome | *Kabuli* gene accession IDs | NCBI-KOG | TFs | NCBI-nr database |
| CaPOPI_1481 | Ca3 | 34753443 | TAA/TAACAA | TCCAAAACCACAACAACCAA | CTAAAATGTCCCCCGTCTCA | 59.8 | 689 | INTERGENIC | | | | |
| CaPOPI_1482 | Ca3 | 34755544 | T/TA | TTTTTCCATTTTACCGGTCG | TTTTCAAGTGGCATCAACCA | 59.8 | 587 | DRR | Ca_00751 | | HD-ZIP | F-box domain, cyclin-like |
| CaPOPI_1483 | Ca3 | 34762413 | T/TA | GGTGCTCAAAATGGTCGAGT | GCTCCTGGTTTTGTCTTTGC | 60.1 | 360 | INTERGENIC | | | | |
| CaPOPI_1484 | Ca3 | 34790577 | CATATTAATATTAAT/CATATTAATATTAATATTAAT | CCTCACAGCTGCCCCTATTA | AAAACACACAGGGCATGATAA | 60.2 | 692 | INTERGENIC | | | | |
| CaPOPI_1485 | Ca3 | 34797791 | GAAAA/GAAAAA | TTGTCAAACATTGCAACAAGAA | CACACTGAAGGTCTCCGGTT | 59.3 | 902 | INTERGENIC | | | | |
| CaPOPI_1486 | Ca3 | 34797856 | GTTTTTTTT/GTTTTTT | TTGTCAAACATTGCAACAAGAA | CACACTGAAGGTCTCCGGTT | 59.3 | 902 | INTERGENIC | | | | |
| CaPOPI_1487 | Ca3 | 34818875 | AAATAATAATAAT/AAATAATAATAATAATAAT | TTGTTGTCGCCATCATTGTT | ACTCCCGCAGTCCCTATTCT | 60.0 | 455 | INTRON | Ca_00758 | | | Glycosyl transferase, family 2 |
| CaPOPI_1488 | Ca3 | 34836958 | TTTTTTTAAATTTTTTA/TTTTTTTA | GTGGTAATCCAACCGTCGAG | GAACCGATCCAACATGCTTT | 60.4 | 443 | INTRON | Ca_00761 | G | | Enolase |
| CaPOPI_1489 | Ca3 | 34840506 | GTTTTTTTTTT/GTTTTTTTT | GTTGGTGATGAAGGTGGCTT | ATCCGGTCCCCATAACTTTC | 60.0 | 457 | INTRON | Ca_00761 | G | | Enolase |
| CaPOPI_1490 | Ca3 | 34845314 | GCAAA/G | ATGGGTACACACGAAAAGCA | ACACCCCCGTACTTCAAACA | 59.1 | 563 | INTERGENIC | | | | |
| CaPOPI_1491 | Ca3 | 34851854 | TATAA/TATAATAA | ACAAACCCTTCACTTGGCAC | ACCGCAAACTCTATGTTGGG | 60.0 | 648 | INTRON | Ca_00763 | | C2H2 | Zinc finger, C2H2-type |
| CaPOPI_1492 | Ca3 | 34852048 | CTACTTTA/CTACTTTACTTTA | ACAAACCCTTCACTTGGCAC | ACCGCAAACTCTATGTTGGG | 60.0 | 648 | INTRON | Ca_00763 | | C2H2 | Zinc finger, C2H2-type |
| CaPOPI_1493 | Ca3 | 34866347 | CTATT/CTATTATT | TACCATGATGATGAAGCGGA | TCAACCGAATCAACAACCAA | 60.0 | 308 | DRR | Ca_00764 | A | C2H2 | RNA polymerase II, large subunit, CTD |
| CaPOPI_1494 | Ca3 | 34872921 | ATTTTTT/ATTTTTTT | CACAAATGCGATTAAGGGCT | CATGTTGAATGGAAGCACACA | 60.1 | 685 | INTERGENIC | | | | |
| CaPOPI_1495 | Ca3 | 34873766 | CAAAAAAA/CAAAAAA | AACACATTTTTGCAAGTGCG | GGCTTCTGAAAAACACAGCC | 59.8 | 728 | INTERGENIC | | | | |

| **INDEL marker IDs** | **Chromosomes/ unanchored scaffolds** | **Physical positions (bp)** | **InDels (*Kabuli* reference genome-CDC Frontier/PI)** | **Forward primers (5'-3')** | **Reverse primers (5'-3')** | **Anneling temperature (0C)** | **Expected amplified product size (bp)** | **Structural annotation** | | **Functional annotation** | | |
|---|---|---|---|---|---|---|---|---|---|---|---|---|
| | | | | | | | | **Sequence components of *kabuli* genome** | ***Kabuli* gene accession IDs** | **NCBI-KOG** | **TFs** | **NCBI-nr database** |
| CaPOPI_1496 | Ca3 | 34887394 | GT/GTT | TCTTCCACACAAAACCCTCC | ACGTGCATCACAAAACCAAA | 59.9 | 571 | INTRON | Ca_00767 | G | ERF | Fructose-bisphosphate aldolase, class-I |
| CaPOPI_1497 | Ca3 | 34897346 | TAAAAAAA/TAAAAAAAA | AGAACAAATTTTGGCGCAAC | AGTTTTGTCGGTCCATGAGC | 60.1 | 537 | INTERGENIC | | | | |
| CaPOPI_1498 | Ca3 | 34906102 | CTTTTTTT/CTTTTTTTTT | TGGATGGTGATTATCCGACTC | TCCAAGTCTTTCAATACAAAACACA | 59.8 | 605 | INTERGENIC | | | | |
| CaPOPI_1499 | Ca3 | 34910960 | GTTTTTT/GTTTTTTT | TCAAGGTCAAGTGTGGCAAA | CTTTGGTCGACGATGAGGAT | 60.3 | 819 | INTERGENIC | | | | |
| CaPOPI_1500 | Ca3 | 34913784 | GTATATATATATA/GTATATATATATATATA | ACCCGTTTCTTTGTTGTTGG | CTATCCAAGAAGCATCCCCA | 59.9 | 427 | DRR | Ca_00768 | | ERF | Pathogenesis-related transcriptional factor/ERF, DNA-binding |
| CaPOPI_1501 | Ca3 | 34913867 | AA/AACACTTGA | TGGGGATGCTTCTTGGATAG | AAAATTTCGAGGCCCATACC | 60.0 | 154 | DRR | Ca_00768 | | ERF | Pathogenesis-related transcriptional factor/ERF, DNA-binding |
| CaPOPI_1502 | Ca3 | 34914599 | T/TCGGG | TGGGAAGATGGAGGAAGAAA | GCCGGTTTCACACTTTTGAT | 59.6 | 387 | INTERGENIC | | | | |
| CaPOPI_1503 | Ca3 | 34916105 | GTTTT/GTTTTTT | TCGACCATCGTCATACTCCA | TCTCCCCTTCATATGACCCTT | 60.1 | 671 | INTERGENIC | | | | |
| CaPOPI_1504 | Ca3 | 34932929 | TAAAAAAA/TAAAAAAAA | ACTTGCTTTGCACCTCGTTT | TTGAAAGATGACTCTTCACCGA | 59.9 | 631 | INTERGENIC | | | | |
| CaPOPI_1505 | Ca3 | 34943202 | ATTT/ATTTT | TGCATTTTTGTGATTGTGTGTG | GCTGGCTCTTTGGGTATGAG | 60.5 | 454 | INTERGENIC | | | | |
| CaPOPI_1506 | Ca3 | 34943765 | ATTTTTTTTT/ATTTTTTTT | GTGTTTGCTTGCTTTGACCA | CGGAAACTACTCCATCGTGAA | 59.9 | 384 | DRR | Ca_00771 | | S1Fa-like | Golgin, RAB6-interacting |
| CaPOPI_1507 | Ca3 | 34953897 | TAAAAAA/TAAAAAAA | CCCGTGATTCCTCTACAACA | GAACTCGCTTGAGGACGAAG | 58.6 | 395 | INTERGENIC | | | | |
| CaPOPI_1508 | Ca3 | 34954280 | TC/T | CTTCGTCCTCAAGCGAGTTC | TGCATGCAGGAGAAGTATTTATT | 60.1 | 681 | INTERGENIC | | | | |
| CaPOPI_1509 | Ca3 | 34958868 | ACCCC/ACCC | GCAACACCATGATAACGTGC | GATGAGATGTTCGCCCTGTT | 60.0 | 220 | INTERGENIC | | | | |
| CaPOPI_1510 | Ca3 | 34964095 | GCC/GCCCC | GGTGACCATGAAGCTCGTTT | GCAATCAACAAACAAGTGTGC | 60.1 | 488 | INTERGENIC | | | | |

| INDEL marker IDs | Chromosomes/ unanchored scaffolds | Physical positions (bp) | InDels (*Kabuli* reference genome-CDC Frontier/PI) | Forward primers (5'-3') | Reverse primers (5'-3') | Anneling temperature (0C) | Expected amplified product size (bp) | Structural annotation | | Functional annotation | | |
|---|---|---|---|---|---|---|---|---|---|---|---|---|
| | | | | | | | | Sequence components of *kabuli* genome | *Kabuli* gene accession IDs | NCBI-KOG | TFs | NCBI-nr database |
| CaPOPI_1511 | Ca3 | 34967661 | GTATTTTTATTTGATATATTTT/GTATTTT | TGGTGGTTTTGGACTTGATG | ATCTGTAATTGGCAGGCAGG | 59.4 | 773 | INTERGENIC | | | | |
| CaPOPI_1512 | Ca3 | 34970200 | CTTTTTTTT/CTTTTTTT | AACACCAACAGATTCCCCAG | CAAGCTAAAATGGCTTCCCA | 59.8 | 234 | INTRON | Ca_00772 | | GRF | WRC |
| CaPOPI_1513 | Ca3 | 34972470 | TA/T | ATGAAGCTTTGGATTGTGGG | TCCACAGCGTACCAACAAAA | 59.9 | 491 | INTRON | Ca_00772 | | GRF | WRC |
| CaPOPI_1514 | Ca3 | 35019374 | A/AG | CCCCTGAAAATCGTATCACAA | GCTGAGTTTAATGACCCGGA | 59.8 | 765 | DRR | Ca_00776 | Q | E2F/DP | ABC transporter-like |
| CaPOPI_1515 | Ca3 | 35030161 | GC/GCTACTAAAGTTC | GATGTCCAACTCCGACGACT | GAAACCGGATTTTCCCACTT | 60.1 | 370 | INTERGENIC | | | | |
| CaPOPI_1516 | Ca3 | 35031349 | CAA/C | ACTCATCTGTTCCCACAGCC | ATTCATGATGGTCCCTCAGC | 60.1 | 584 | INTERGENIC | | | | |
| CaPOPI_1517 | Ca3 | 35033028 | GATATA/GATA | TTCAAACTAGTCACACCAACGG | TTTCATGTTGTTCCCCCAAT | 60.1 | 477 | DRR | Ca_00777 | R | | |
| CaPOPI_1518 | Ca3 | 35035644 | TA/TAA | GCTGAACTCCGGATTACCAA | TCATGCTGCTTCAAAGGTTG | 60.1 | 701 | INTRON | Ca_00777 | R | | |
| CaPOPI_1519 | Ca3 | 35040031 | GA/GAA | CATTCCAACCACACTTCCCT | GAACAACCACCATCTTTGGG | 59.8 | 521 | INTRON | Ca_00777 | R | | |
| CaPOPI_1520 | Ca3 | 35040555 | ATT/AAAAAAAAGTT | AGCAAGGCCATCTTTCTTCA | TCAAGCTCTGCATATGGGTG | 60.0 | 259 | INTRON | Ca_00777 | R | | |
| CaPOPI_1521 | Ca3 | 35061749 | A/AACTGT | TCAATACCCCAGCACCAAAT | TTCTGCCTTCCATTCAAACC | 60.2 | 710 | CDS (FRAME SHIFT) | Ca_00778 | Q | E2F/DP | ABC transporter-like |
| CaPOPI_1522 | Ca3 | 35077901 | ATTTTT/ATTTTTTT | GTCCATCACGACTTGGACCT | TGCAGTCCAGCATTGGTAAC | 60.0 | 281 | INTERGENIC | | | | |
| CaPOPI_1523 | Ca3 | 35086678 | ATTTTTT/ATTTTT | TCTGCTTCATGTGGCAAAAG | CATGTTGGAGACTGCGATTG | 60.0 | 487 | INTRON | Ca_00781 | | G2-like | Peptidase S8/S53, subtilisin/kexin/sedolisin |
| CaPOPI_1524 | Ca3 | 35086999 | TGTTGCAGTTGCAGTTG/TGTTGCAGTTGCAGTTGCAGTTG | TGTTGTGGTGGTGTTGTTCC | TCACTCATTTCGCGTTCTTG | 60.3 | 458 | CDS (large-effect mutations) | Ca_00781 | | G2-like | Peptidase S8/S53, subtilisin/kexin/sedolisin |
| CaPOPI_1525 | Ca3 | 35100602 | TAAAAAAA/TAAAAAAAA | TTATAAAGTTTGGGGCACCG | CAACAGCGGTACAATCATCG | 59.8 | 701 | INTERGENIC | | | | |

| **INDEL marker IDs** | **Chromosomes/ unanchored scaffolds** | **Physical positions (bp)** | **InDels (*Kabuli* reference genome-CDC Frontier/PI)** | **Forward primers (5'-3')** | **Reverse primers (5'-3')** | **Anneling temperature (0C)** | **Expected amplified product size (bp)** | **Structural annotation** | | **Functional annotation** | | |
|---|---|---|---|---|---|---|---|---|---|---|---|---|
| | | | | | | | | **Sequence components of *kabuli* genome** | ***Kabuli* gene accession IDs** | **NCBI-KOG** | **TFs** | **NCBI-nr database** |
| CaPOPI_1526 | Ca3 | 35101262 | TTAAAAAGTAAAA/TTAAAAAGTAAAAAGTAAAA | CGATGATTGTACCGCTGTTG | CGAAGGAAGTGTTATCCGAA | 60.1 | 885 | INTERGENIC | | | | |
| CaPOPI_1527 | Ca3 | 35106644 | AATATATATATATA/AATATATATATA | TGTCAAATGGGAAAGGGAAG | TTTGCTGCAATACATTTTCAGG | 59.9 | 608 | INTRON | Ca_00783 | S | | Protein of unknown function DUF914, eukaryotic |
| CaPOPI_1528 | Ca3 | 35117240 | CTTT/CTT | GGTGCACCATACAATAACAACAA | CATGGCATCGTAACAACGAC | 59.7 | 580 | DRR | Ca_00785 | G | C3H | Metallo-dependent phosphatase |
| CaPOPI_1529 | Ca3 | 35119008 | G/GCACTTTTA | AGTTGGACCCACTTTGTTGG | AAAATGGATGGGTCTGTTGC | 59.9 | 545 | INTRON | Ca_00785 | G | C3H | Metallo-dependent phosphatase |
| CaPOPI_1530 | Ca3 | 35136413 | CTAT/CTATATAT | ATACCACTAGCACGCACACG | TTTATGCATATCACATCAACAAAAA | 59.8 | 660 | INTERGENIC | | | | |
| CaPOPI_1531 | Ca3 | 35138675 | TAA/TAAA | AGGGCCAGATTCTCTTGGTT | GTCAGATGAATTCCCGCATC | 60.1 | 563 | INTRON | Ca_00787 | G | C3H | |
| CaPOPI_1532 | Ca3 | 35143342 | ATTT/ATTTT | CACATGCACTTTGGAGTTGG | TCTTCGTCTCCCTCCATTTC | 60.2 | 428 | DRR | Ca_00788 | G | C3H | Metallo-dependent phosphatase |
| CaPOPI_1533 | Ca3 | 35147082 | T/TAAAGA | GTCGGAGCCAAAAATGAAAA | TGTGTTTCTTTCCAGCAGAGAC | 60.1 | 317 | INTERGENIC | | | | |
| CaPOPI_1534 | Ca3 | 35166785 | TAGAAAG/TAG | TGGCATGTGGTTTGTCATTT | AATGTGACCAACTTCCTCGG | 59.8 | 859 | INTERGENIC | | | | |
| CaPOPI_1535 | Ca3 | 35187164 | GTCT/GTCTCT | GGGCACTAAAACAAAAGGCA | TCAAATCCTGTGGTTGTGGA | 60.1 | 588 | DRR | Ca_00791 | | bZIP | Basic-leucine zipper (bZIP) transcription factor |
| CaPOPI_1536 | Ca3 | 35204680 | AT/ATCT | TGGCCAAATCACAAACCTATT | AATACCAATTTGGCGAGTGC | 59.3 | 560 | INTERGENIC | | | | |
| CaPOPI_1537 | Ca3 | 35213093 | CTTTT/CTTT | GCCATCACATTCATGACAAAA | TGATATTGCAAGAAACCCCA | 59.4 | 758 | INTERGENIC | | | | |
| CaPOPI_1538 | Ca3 | 35215168 | TTTTTCCTTTCATTTTCCTTT/TTTTTCCTTTCATTTTCCTTTCATTTTCCTTT | GCGAAGAAAAAGAGGTCGTG | GTAATCGACAGCCTGCAACA | 60.0 | 316 | INTRON | Ca_00796 | C | | Ubiquinol-cytochrome C reductase, UQCRX/QCR9-like |
| CaPOPI_1539 | Ca3 | 35230228 | CAA/CAAA | ACTCGCCTAAATTCTGCACG | GAAACTCCACGCTGAAAAGC | 60.4 | 502 | INTERGENIC | | | | |
| CaPOPI_1540 | Ca3 | 35241980 | CTT/CTTAGTT | GGGACGCTCATACAATGGTT | CGCACAGTGAAGTGCAAAGT | 59.8 | 331 | INTRON | Ca_00801 | | | SOUL haem-binding protein |

| INDEL marker IDs | Chromosomes/ unanchored scaffolds | Physical positions (bp) | InDels (*Kabuli* reference genome-CDC Frontier/PI) | Forward primers (5'-3') | Reverse primers (5'-3') | Anneling temperature (0C) | Expected amplified product size (bp) | Structural annotation | | Functional annotation | | |
|---|---|---|---|---|---|---|---|---|---|---|---|---|
| | | | | | | | | Sequence components of *kabuli* genome | *Kabuli* gene accession IDs | NCBI-KOG | TFs | NCBI-nr database |
| CaPOPI_1541 | Ca3 | 35247904 | CATATATAT/CATATAT | CACTATCTTTGGGCGGTTGT | TTCTCTGACCGTGAAAGTGG | 60.0 | 382 | INTERGENIC | | | | |
| CaPOPI_1542 | Ca3 | 35248340 | AATTATATT/AATT | ACCACTTTCACGGTCAGAGA | TACAATTTGCACTCCACCCA | 58.3 | 870 | INTERGENIC | | | | |
| CaPOPI_1543 | Ca3 | 35260978 | TACT/TACTGTAACTGCACT | ATGGGAACCAATTTGTTGGA | GCCTTGTGTTTGGGCTATGT | 60.0 | 537 | DRR | Ca_00803 | O | | Glutaredoxin |
| CaPOPI_1544 | Ca3 | 35261808 | CGTCATAATGTCATAATGT/CGTCATAATGT | ACATAGCCCAAACACAAGGC | GAACAGTTTCACCCCCAAAA | 60.0 | 584 | DRR | Ca_00803 | O | | Glutaredoxin |
| CaPOPI_1545 | Ca3 | 35265195 | TC/TCC | TTGGAGTCAGGAAATATCCCA | GCAACGAGGATTGACCGTAT | 59.4 | 820 | INTERGENIC | | | | |
| CaPOPI_1546 | Ca3 | 35284220 | ACT/A | TCACGTTCAACGATTGGCTA | CCTCGCTGATGCTTGTTGTA | 60.3 | 151 | INTERGENIC | | | | |
| CaPOPI_1547 | Ca3 | 35291358 | GAAAAA/GAAAA | ATGCATGCAGATCAGACGAC | TGCACAAATGGGTTCTGTGT | 59.8 | 283 | INTRON | Ca_00808 | U | | Signal recognition particle, SRP54 subunit, GTPase |
| CaPOPI_1548 | Ca3 | 35299387 | TAAA/TAAAA | CCTCTGGCATCGAAAGAAAG | ATTGAAGGCCCTCTACAGCA | 59.9 | 639 | INTRON | Ca_00809 | T | | PAK-box/P21-Rho-binding |
| CaPOPI_1549 | Ca3 | 35300047 | CAAAAAAA/CAAAAAA | AAAAGGAATGTTTGGCCTGA | GGCTGGCCTTCTGATGTTAG | 59.5 | 773 | INTRON | Ca_00809 | T | | PAK-box/P21-Rho-binding |
| CaPOPI_1550 | Ca3 | 35303259 | AAAG/A | ACTAACCATTCGCTGCCATC | CACCACCACCTCATTGTTTG | 60.1 | 821 | INTERGENIC | | | | |
| CaPOPI_1551 | Ca3 | 35303338 | TAAAAAAAAAA/TAAAAAAAA | AGTAAGGAATTGGGGAAGCC | CACCACCACCTCATTGTTTG | 59.4 | 683 | INTERGENIC | | | | |
| CaPOPI_1552 | Ca3 | 35307396 | TAAA/TAA | CAATCATGCGATTTGCATTC | ATTTGGTTTTGCGATGAAGC | 60.0 | 636 | INTRON | Ca_00810 | A | | Like-Sm ribonucleoprotein (LSM) domain |
| CaPOPI_1553 | Ca3 | 35307977 | AT/ATT | GCTTCATCGCAAAACCAAAT | TATAATCGCAAAAATGCCGC | 60.1 | 212 | INTRON | Ca_00810 | A | | Like-Sm ribonucleoprotein (LSM) domain |
| CaPOPI_1554 | Ca3 | 35317314 | TATATAATATAATAT/TATATAATAT | TGACGTTGTTGGAGATGAAAA | TGTCGGTCCACTCTCTACCC | 59.2 | 590 | INTRON | Ca_00811 | | | Condensation domain |
| CaPOPI_1555 | Ca3 | 35329937 | TA/TAA | ATAATATGCGCCTGCTTGCT | TCCAAATCGGCTCGTAAAAC | 59.9 | 473 | INTRON | Ca_00812 | Z | MYB_related | Kinesin, motor domain |

| INDEL marker IDs | Chromosomes/ unanchored scaffolds | Physical positions (bp) | InDels (*Kabuli* reference genome-CDC Frontier/PI) | Forward primers (5'-3') | Reverse primers (5'-3') | Anneling temperature (0C) | Expected amplified product size (bp) | Structural annotation | | Functional annotation | | |
|---|---|---|---|---|---|---|---|---|---|---|---|---|
| | | | | | | | | Sequence components of *kabuli* genome | *Kabuli* gene accession IDs | NCBI-KOG | TFs | NCBI-nr database |
| CaPOPI_1556 | Ca3 | 35336928 | GT/GTT | TCTAAAACTCTCCGCCCTTG | ACCGAACCTTTTGGGTTGTA | 59.4 | 607 | INTRON | Ca_00813 | G | | Glycoside hydrolase, family 32 |
| CaPOPI_1557 | Ca3 | 35361414 | AAATAATAATAATAATAA/AAATAATAATAATAA | GGAAGAAGAAGCTACGCCAA | AGAACCAAACCAACCACTCG | 59.6 | 696 | INTERGENIC | | | | |
| CaPOPI_1558 | Ca3 | 35362095 | ATATTATT/ATATTATTATT | CGAGTGGTTGGTTTGGTTCT | TGCTTAATTTTCAACAAAAGTTGC | 60.0 | 789 | INTERGENIC | | | | |
| CaPOPI_1559 | Ca3 | 35365941 | ATTTTTTTTT/ATTTTTTTTTTT | AACAACGGGGTGTCACCTAC | TTGGTGGGTATCTTGTGAAGC | 59.7 | 681 | DRR | Ca_00817 | | | Protein of unknown function DUF584 |
| CaPOPI_1560 | Ca3 | 35368592 | TA/T | TCAACTCACCACCAATCCAA | GCCGATTCTGAATATGTGTAAGG | 59.9 | 617 | INTERGENIC | | | | |
| CaPOPI_1561 | Ca3 | 35370619 | GAAAAAAAAA/GAAAAAAAA | AAAATGTTTTACAGTCCCGTTG | CCAAATCTTTGGCACTCGAT | 58.1 | 388 | INTERGENIC | | | | |
| CaPOPI_1562 | Ca3 | 35390580 | CG/C | TAGATCGGACGGTCGAAATC | CACGCACCTAGAACTTGCAG | 60.0 | 704 | INTRON | Ca_00819 | O | | Peptidyl-prolyl cis-trans isomerase, cyclophilin-type |
| CaPOPI_1563 | Ca3 | 35393263 | CTTTTT/CTTTTTT | AAGGAGATGCATGCCCTATG | CACCATCCCATATTTGGACC | 60.1 | 715 | INTRON | Ca_00820 | | | Protein of unknown function DUF1022 |
| CaPOPI_1564 | Ca3 | 35395949 | AG/A | AAGTGCACCACCTCCAAACT | TGAAGACATGCAAATGTTTACCA | 59.6 | 575 | DRR | Ca_00820 | | | Protein of unknown function DUF1022 |
| CaPOPI_1565 | Ca3 | 35396014 | CAAAAA/CAAAA | AAGTGCACCACCTCCAAACT | ATCTGTGTCGACCGAAAAGG | 59.6 | 721 | DRR | Ca_00820 | | | Protein of unknown function DUF1022 |
| CaPOPI_1566 | Ca3 | 35397271 | TGTC/T | GATTCTCTTCACCGCTGCTC | ACAAACCGTTATTTTCCGCA | 60.1 | 663 | INTRON | Ca_00821 | KA | | Dcp1-like decapping |
| CaPOPI_1567 | Ca3 | 35403982 | ATCT/AT | TTCGCCTGCTCTCTTTTTGT | TTGGTCAAATCATTCAATCAGA | 60.1 | 730 | INTERGENIC | | | | |
| CaPOPI_1568 | Ca3 | 35436004 | ATTTTTTTTT/ATTTTTTTT | GAAATAACGTTCCGCGTGTT | CATCATTGCCAAGTGCAAGT | 60.0 | 454 | INTERGENIC | | | | |
| CaPOPI_1569 | Ca3 | 35440193 | T/TCA | AACTGTGCAGCATATCCATAAA | CAAGGGCGGGTCTAGTATGT | 57.4 | 410 | INTERGENIC | | | | |
| CaPOPI_1570 | Ca3 | 35468557 | TG/T | CGAACCATGCTTTGGAGATT | GCAGATGGTCCTTTTTCAGC | 60.1 | 472 | INTERGENIC | | | | |

| INDEL marker IDs | Chromosomes/ unanchored scaffolds | Physical positions (bp) | InDels (*Kabuli* reference genome-CDC Frontier/PI) | Forward primers (5'-3') | Reverse primers (5'-3') | Anneling temperature (0C) | Expected amplified product size (bp) | Structural annotation | | Functional annotation | | |
|---|---|---|---|---|---|---|---|---|---|---|---|---|
| | | | | | | | | Sequence components of *kabuli* genome | *Kabuli* gene accession IDs | NCBI-KOG | TFs | NCBI-nr database |
| CaPOPI_1571 | Ca3 | 35488328 | GAAA/GAA | CTCGCTGGAATTTGCTAACC | AGGACGTTGCTGCAAGATTT | 59.8 | 502 | INTRON | Ca_00832 | S | | TLDc |
| CaPOPI_1572 | Ca3 | 35505847 | TAAAA/TAAA | CCGTCCTCCAATATAACCCA | TGTTTGGAGGGAAAACAAGG | 59.6 | 334 | INTERGENIC | | | | |
| CaPOPI_1573 | Ca3 | 35528890 | ATTAT/ATTATTTTTAT | AACGTCGACCAGCCTACACT | ATCCACACAAAACTCGGCAT | 59.8 | 422 | INTERGENIC | | | | |
| CaPOPI_1574 | Ca3 | 35532624 | TG/T | CACATAATGTTGGCGTGGTC | ACCGTATGTGGACTCTCGCT | 59.8 | 797 | INTERGENIC | | | | |
| CaPOPI_1575 | Ca3 | 35558006 | TAA/TA | ATCCCGCGTAATTCCTCTCT | ATCCCCTAACATTTCCGACC | 60.1 | 403 | INTERGENIC | | | | |
| CaPOPI_1576 | Ca3 | 35562721 | A/AT | TGATCTATTTGGTGGATATGAGG | TGCAATAGGTAAAGCCACCT | 58.0 | 795 | INTERGENIC | | | | |
| CaPOPI_1577 | Ca3 | 35568618 | AACGTACGT/AACGT | TGTGGCATGCAAATGCTTAT | TCCTGTGTTGTTGTGTCCGT | 60.1 | 572 | DRR | Ca_00841 | | | NIF system FeS cluster assembly, NifU, C-terminal |
| CaPOPI_1578 | Ca3 | 35574829 | AG/A | TTCAAGATTGAAAATGCTTTGTG | AATCGCATCGGCTAAACAAT | 59.3 | 412 | INTERGENIC | | | | |
| CaPOPI_1579 | Ca3 | 35591231 | TAAAA/T | CACACCTTTGAAGATGGGTCT | TGTCTCCGGATAGATGACATTG | 59.1 | 287 | DRR | Ca_00843 | | bZIP | Late embryogenesis abundant protein, group 2 |
| CaPOPI_1580 | Ca3 | 35596803 | ATTTTTTTT/ATTTTTTTTT | GTCAACGCAAGCGAAAACTA | CGAAAAATCATCCGGAGAAA | 59.1 | 524 | DRR | Ca_00844 | | bZIP | Late embryogenesis abundant protein, group 2 |
| CaPOPI_1581 | Ca3 | 35600454 | TAA/TAAA | AATTTCACAGTCCCGTCACA | CAATGCGACTGGGTTTACCT | 59.0 | 334 | DRR | Ca_00845 | | | Late embryogenesis abundant protein, group 2 |
| CaPOPI_1582 | Ca3 | 35602083 | CATGTTTTAT/CAT | GGGTGCGTTAGTGCATTTTT | GCCATGCTTTAACGAAGAGG | 60.0 | 940 | INTERGENIC | | | | |
| CaPOPI_1583 | Ca3 | 35614019 | ATTTTTTTTT/ATTTTTTTTTT | TGATCAAGATTTGAATTTTTGTTTTC | ATCCCCGTGTGTTGTTGTTT | 59.8 | 256 | INTERGENIC | | | | |
| CaPOPI_1584 | Ca3 | 35618022 | GTTTTTTTT/GTTTTTTT | TGCGTACCTTAATCAGGAACAA | TTTACGCGGGCGTATTTTAC | 59.6 | 882 | INTERGENIC | | | | |
| CaPOPI_1585 | Ca3 | 35618119 | T/TC | TGTGAAACGAACAAAGCTTAAA | TTTACGCGGGCGTATTTTAC | 57.7 | 555 | INTERGENIC | | | | |

| INDEL marker IDs | Chromosomes/ unanchored scaffolds | Physical positions (bp) | InDels (*Kabuli* reference genome-CDC Frontier/PI) | Forward primers (5'-3') | Reverse primers (5'-3') | Anneling temperature (0C) | Expected amplified product size (bp) | Structural annotation | | Functional annotation | | |
|---|---|---|---|---|---|---|---|---|---|---|---|---|
| | | | | | | | | Sequence components of *kabuli* genome | *Kabuli* gene accession IDs | NCBI-KOG | TFs | NCBI-nr database |
| CaPOPI_1586 | Ca3 | 35626631 | ATTTTTT/ATTTTTTT | TTGAATCTGAATCACCGCTTT | CCAAAAGGAGTATGGATCAGTAA | 59.7 | 459 | DRR | Ca_00850 | | | |
| CaPOPI_1587 | Ca3 | 35635776 | CTTTT/CTTT | GTAATTTTGGCGCTGGTGTT | GCGAATGCATAGAGCCTACC | 60.0 | 635 | INTERGENIC | | | | |
| CaPOPI_1588 | Ca3 | 35644626 | TTCTC/TTCTCTCTC | CTCCCAAGAGTGAGAGCACC | CAGTTGGAGGTACCACCGTT | 60.0 | 269 | INTRON | Ca_00852 | S | | |
| CaPOPI_1589 | Ca3 | 35652779 | GTT/GTTTT | AAGTGTTTGGTGGTGGTGGT | CCACTGTCCAGTCTCTGCAA | 60.2 | 499 | INTRON | Ca_00853 | | | |
| CaPOPI_1590 | Ca3 | 35678085 | TTCT/TT | TTGAGCACGACAGAATCACC | ACAATGGCCCAACTCTGTTC | 59.8 | 441 | DRR | Ca_00855 | | | Glycosyl transferase, group 1 |
| CaPOPI_1591 | Ca3 | 35679181 | ATTTTTTTT/ATTTTTTT | TTCATTCTTTGGCTTGGGAC | CGTCATGGTCGTGGCTTTAT | 60.1 | 959 | DRR | Ca_00856 | R | SBP | Malate transporter, aliminium toerance |
| CaPOPI_1592 | Ca3 | 35697688 | TAAAAAAA/TAAAAAAAAA | TTTCATATCCACCCGTCAAA | TGAATTTGCCTAATTTGGTCC | 58.8 | 628 | INTERGENIC | | | | |
| CaPOPI_1593 | Ca3 | 35699071 | CTGTAATGTAAT/CTGTAAT | ACGAATCTCTCAACCATCGG | TGAGGCTCTCTTTGTGACTTTG | 60.1 | 711 | INTERGENIC | | | | |
| CaPOPI_1594 | Ca3 | 35699306 | TAAAAA/TAAAAAA | GCTTCCTGGTAGGACAAACAA | TGAGGCTCTCTTTGTGACTTTG | 59.2 | 455 | INTERGENIC | | | | |
| CaPOPI_1595 | Ca3 | 35703336 | CAAAAAA/CAAAAAAA | CGGATATGCGGACAAAAACT | TTAGCTTTGGGGTTTGATGG | 60.0 | 600 | INTERGENIC | | | | |
| CaPOPI_1596 | Ca3 | 35703988 | CA/CATAATAA | CCATCAAACCCCAAAGCTAA | CACTGTTTCTTTCACAACTCTCG | 59.9 | 942 | INTERGENIC | | | | |
| CaPOPI_1597 | Ca3 | 35706702 | AAAATTAAATT/AAAATT | TCGGCCACTATTTCTTCCAC | CGTTGTGCCTAAATTGGGAT | 60.1 | 985 | INTERGENIC | | | | |
| CaPOPI_1598 | Ca3 | 35725827 | TAAAAAA/TAAAAAAA | AAATCATAAGAACAAGGAGGATCG | GCACCCCTCCTTTAGAGTCC | 59.9 | 549 | INTERGENIC | | | | |
| CaPOPI_1599 | Ca3 | 35725900 | CTAAA/CTAAAAGTAAA | TGTGTGTGTCTACCAAAATACTCG | TTTCCACACTCTAATCGGGG | 59.1 | 743 | INTERGENIC | | | | |
| CaPOPI_1600 | Ca3 | 35726533 | TTATATA/TTATATATATA | CCCCGATTAGAGTGTGGAAA | GCACTTTCACATGGTGTGGT | 59.9 | 410 | INTERGENIC | | | | |

| INDEL marker IDs | Chromosomes/ unanchored scaffolds | Physical positions (bp) | InDels (*Kabuli* reference genome-CDC Frontier/PI) | Forward primers (5'-3') | Reverse primers (5'-3') | Anneling temperature (0C) | Expected amplified product size (bp) | Structural annotation | | Functional annotation | | |
|---|---|---|---|---|---|---|---|---|---|---|---|---|
| | | | | | | | | Sequence components of *kabuli* genome | *Kabuli* gene accession IDs | NCBI-KOG | TFs | NCBI-nr database |
| CaPOPI_1601 | Ca3 | 35730551 | CATT/CATTAATT | TTAAAAGGCCATGGGCA TAA | TGGTGGTGCTGGACAAT TAG | 60.3 | 125 | INTERGENIC | | | | |
| CaPOPI_1602 | Ca3 | 35733605 | CTTT/CTT | TCTCCCCTTTCCTTTCAT TT | AAAAACGTTAATTTGAGA CAGCAA | 57.7 | 360 | INTERGENIC | | | | |
| CaPOPI_1603 | Ca3 | 35767362 | AAAAA/AAAAAATAAAA | GGGTGCTTCCTCAATCT TCA | GACTTTGGAAGTCGGCA AAA | 60.2 | 798 | INTERGENIC | | | | |
| CaPOPI_1604 | Ca3 | 35768780 | T/TC | CCTTATCCCCGAAATTT GAA | GCGGATAGCAAATCGTT CAT | 58.9 | 271 | INTERGENIC | | | | |
| CaPOPI_1605 | Ca3 | 35768860 | TAAAAAA/TAAAAAAA | CCTTATCCCCGAAATTT GAA | GCGGATAGCAAATCGTT CAT | 58.9 | 271 | INTERGENIC | | | | |
| CaPOPI_1606 | Ca3 | 35770921 | GTAAT/GT | GCATGGTGCAGTGAAAG AGA | AATTGAGGTCCCGTCAG TTG | 60.0 | 448 | DRR | Ca_00863 | U | bZIP | Target SNARE coiled-coil domain |
| CaPOPI_1607 | Ca3 | 35776584 | TTATATAT/TTATATATAT | GGTGGTTTTGCACTTTT GCT | TCACATACTCGCCAGAC TGC | 60.2 | 712 | DRR | Ca_00865 | R | G2-like | RNA recognition motif domain |
| CaPOPI_1608 | Ca3 | 35779416 | CTTTTTTTTTTTTT/CTTTTTTTTTTT | GCAAATCCTCTGCTCCT GTC | TTCCATTTCACTCACCAC CA | 60.0 | 548 | DRR | Ca_00866 | K | NF-YA | CCAAT-binding transcription factor, subunit B |
| CaPOPI_1609 | Ca3 | 35783476 | ACTATTC/AC | TGGAAAGTCAACTCAAT GCG | CTTACATCGAGCCGACA CAA | 59.8 | 745 | INTRON | Ca_00866 | K | NF-YA | CCAAT-binding transcription factor, subunit B |
| CaPOPI_1610 | Ca3 | 35783609 | ATGT/ATGTGT | TGGAAAGTCAACTCAAT GCG | CTTACATCGAGCCGACA CAA | 59.8 | 745 | INTRON | Ca_00866 | K | NF-YA | CCAAT-binding transcription factor, subunit B |
| CaPOPI_1611 | Ca3 | 35784715 | TAAAAAA/TAAAAA | GGTGCTGCTCCTGAATT AGC | GGCTCCAATCTAATGGG GTT | 60.0 | 798 | INTERGENIC | | | | |
| CaPOPI_1612 | Ca3 | 35788163 | GATAAATATTTTATAAA/GATAAATATTT TATAAATATTTTATAAA | CCTTCACCTTAGGCCAT TGA | CACCTTGATTTTACCACA CTCG | 60.1 | 645 | INTERGENIC | | | | |
| CaPOPI_1613 | Ca3 | 35796430 | CTTTT/CTTT | GCAATCTTGAGCTGGAG ACC | GGCACAAGTCAAATGGT CAA | 60.0 | 909 | INTERGENIC | | | | |
| CaPOPI_1614 | Ca3 | 35799677 | ATTTTT/ATTTT | TAACTGCATTTGATTGC GGA | TGCTTTTGCAAGTGTTTT TGA | 60.2 | 610 | INTERGENIC | | | | |
| CaPOPI_1615 | Ca3 | 35801766 | TAA/TA | TTACCATGATTGAGGCA CCA | CCTTGTTTGGTTTTGGTT GG | 59.9 | 395 | INTERGENIC | | | | |

| INDEL marker IDs | Chromosomes/ unanchored scaffolds | Physical positions (bp) | InDels (*Kabuli* reference genome-CDC Frontier/PI) | Forward primers (5'-3') | Reverse primers (5'-3') | Anneling temperature (0C) | Expected amplified product size (bp) | Structural annotation | | Functional annotation | | |
|---|---|---|---|---|---|---|---|---|---|---|---|---|
| | | | | | | | | Sequence components of *kabuli* genome | *Kabuli* gene accession IDs | NCBI-KOG | TFs | NCBI-nr database |
| CaPOPI_1616 | Ca3 | 35809791 | CTTTTT/CTTTTTT | CCGGAAGGAAAATTCCAAAT | TGCGGTTTGTTTTGTGTGTT | 60.1 | 297 | INTRON | Ca_00868 | | | |
| CaPOPI_1617 | Ca3 | 35811115 | TTTTTATTTTATTTTATTTTATTTTATTTTA/TTTTTATTTTATTTTATTTTATTTTA | TTTTGGGTTTCTCACTTGCC | TGGTAGTAAATCACTCGCAGTTG | 60.1 | 455 | INTERGENIC | | | | |
| CaPOPI_1618 | Ca3 | 35811467 | CTATAT/CTAT | TGAACAAATAAAATTCAACTGCG | TCTGAATTTCTCCCGTGGAT | 59.2 | 450 | INTERGENIC | | | | |
| CaPOPI_1619 | Ca3 | 35833491 | TTA/T | TGTGAGAAAGCATCAAAACCT | CAAACTACGGTCGGTGTCCT | 57.5 | 238 | DRR | Ca_00871 | | | Dihydroneopterin aldolase |
| CaPOPI_1620 | Ca3 | 35852219 | AAGGAATTGACTAG/AAG | CCGGCCTGTCAGACCTAATA | TGGCCAACAAGACAATTTCA | 60.1 | 639 | INTERGENIC | | | | |
| CaPOPI_1621 | Ca3 | 35856021 | ATTTTTTTTTTTT/ATTTTTTTTTTTTTTT | CTTTGACCGAAGCATTAGGC | ACCCCAAAAAGGAGGTGTTC | 59.8 | 435 | INTERGENIC | | | | |
| CaPOPI_1622 | Ca3 | 35859927 | TGGG/TGGGG | GAAATCAATTGAAAGAAAAGCCA | TGTCAACAATGTTATCTTCACAAAAA | 59.6 | 808 | INTERGENIC | | | | |
| CaPOPI_1623 | Ca3 | 35859979 | ATCTTCTT/ATCTT | AAACGTCATAATTGCTTATGGG | TGGCTTTTGTTTGAAGATTTTG | 58.1 | 205 | INTERGENIC | | | | |
| CaPOPI_1624 | Ca3 | 35862589 | ACCC/ACC | TTCAACTGTTCTACGGCAACA | TGGGGTTTCCAAAAGTTCAG | 59.4 | 654 | INTERGENIC | | | | |
| CaPOPI_1625 | Ca3 | 35873093 | TC/T | TTCAATGGGAATGGGAATGT | GTCTCCGACACCGACACTTT | 60.0 | 663 | INTERGENIC | | | | |
| CaPOPI_1626 | Ca3 | 35887062 | CT/CTAATAATT | GCAACATGAAAGCACGTTTG | CGTTGGAACTTGGAACCATT | 60.3 | 507 | INTERGENIC | | | | |
| CaPOPI_1627 | Ca3 | 35894033 | AACCTTTTACC/AACC | CACACAAATTGGTTGGTGCT | TCAAAAGTTTGACTCCTAAACGG | 59.5 | 508 | INTERGENIC | | | | |
| CaPOPI_1628 | Ca3 | 35894525 | GTATATATATATATA/GTATATATATATATATATATATA | TGAAACCTTTTACCAGACTCCAA | GTTAATCAAAGGGCCGACAA | 60.0 | 952 | INTERGENIC | | | | |
| CaPOPI_1629 | Ca3 | 35895341 | CAAAAAA/CAAAAAAAA | TTTCCTTAGGACGCGAAGAA | CGGAAGTTCCAATGGAGAAA | 60.0 | 553 | INTERGENIC | | | | |
| CaPOPI_1630 | Ca3 | 35911655 | AG/A | GCATGCGACTCATTTACCAA | TCGAATTATTTTTGAGTTGGTTTG | 59.7 | 599 | INTERGENIC | | | | |

| INDEL marker IDs | Chromosomes/ unanchored scaffolds | Physical positions (bp) | InDels (*Kabuli* reference genome-CDC Frontier/PI) | Forward primers (5'-3') | Reverse primers (5'-3') | Anneling temperature (0C) | Expected amplified product size (bp) | Structural annotation | | Functional annotation | | |
|---|---|---|---|---|---|---|---|---|---|---|---|---|
| | | | | | | | | Sequence components of *kabuli* genome | *Kabuli* gene accession IDs | NCBI-KOG | TFs | NCBI-nr database |
| CaPOPI_1631 | Ca3 | 35917238 | AC/A | ATCTTAGCCCACTCAATCCG | GTGTCGTAAGCCTCCTCGAA | 59.2 | 522 | INTERGENIC | | | | |
| CaPOPI_1632 | Ca3 | 35928795 | ATTTTTTT/ATTTTTT | GATGGGCATCATTAGTGTTTGA | TTCAGTTGCACCTCAACGTC | 59.8 | 336 | DRR | Ca_00885 | | | |
| CaPOPI_1633 | Ca3 | 35947883 | CTTT/CT | TCCTGTCTAGCCGTAGTCCTG | TTGAAATTTTGACCGCATCA | 59.5 | 434 | DRR | Ca_00889 | J | | Ribosomal protein L35A |
| CaPOPI_1634 | Ca3 | 35960562 | AGTTTGATT/AGTTTGATTGTTTGATT | TCCGACTTGGTAAAACAGCA | TTTGGACGAAATAAATTTGAAAAA | 59.3 | 398 | DRR | Ca_00891 | V | GRAS | Alpha/beta hydrolase fold-3 |
| CaPOPI_1635 | Ca3 | 35960868 | TAA/TAAA | GGATGCTACATTTCTTGGTGG | TGAAGGAGGGAGAAACATGG | 59.4 | 835 | DRR | Ca_00891 | V | GRAS | Alpha/beta hydrolase fold-3 |
| CaPOPI_1636 | Ca3 | 35973469 | AC/A | TGCCAGTGATCATCGAAAAA | AGGGGCAAAGGTAACAAAGC | 60.2 | 374 | INTRON | Ca_00892 | C | | FAD-linked oxidase, C-terminal |
| CaPOPI_1637 | Ca3 | 35973692 | CG/C | TGCCAGTGATCATCGAAAAA | TTCTGTGAAGGATGCAGCAG | 60.2 | 778 | INTRON | Ca_00892 | C | | FAD-linked oxidase, C-terminal |
| CaPOPI_1638 | Ca3 | 35974404 | GAA/GGAAA | TAGCAACATCTGCTGCATCC | ATCACACGGGAAATCTGAGC | 60.0 | 721 | INTRON | Ca_00892 | C | | FAD-linked oxidase, C-terminal |
| CaPOPI_1639 | Ca3 | 35982476 | TGG/TG | TCGAAAAGAACTTTATCCGCA | TGAATTGCTGTAGCCTGTGG | 59.8 | 408 | DRR | Ca_00893 | | | Zinc finger, RING-type |
| CaPOPI_1640 | Ca3 | 35990406 | ACTG/A | TGAATTTACACCCTGCCCTC | GCTCGAGATGAAGGGACTTG | 59.9 | 331 | INTERGENIC | | | | |
| CaPOPI_1641 | Ca3 | 35994489 | CT/CTAAATATT | TGAATGATGTTTCATTCCATCA | GAATTCGGAACAAAACGTTCA | 58.8 | 311 | INTERGENIC | | | | |
| CaPOPI_1642 | Ca3 | 36019541 | AT/ATGT | ACCTTTGGATTCATGGTTGG | CCGATAGCCATTTGGACACT | 59.6 | 614 | INTERGENIC | | | | |
| CaPOPI_1643 | Ca3 | 36020478 | ATT/ATTT | AGGTGAAGGTTGCGTTAGTGA | TCAAATTGTGTGGGAGAAACA | 59.8 | 536 | INTERGENIC | | | | |
| CaPOPI_1644 | Ca3 | 36021252 | GAT/G | GTTTGCGGATTTGGTTGAGT | TATTTTAGGACCGCCTGACG | 60.0 | 442 | DRR | Ca_00901 | O | CAMTA | MATH |
| CaPOPI_1645 | Ca3 | 36032758 | ATTTTTT/ATTTTTTT | GGTCATTTTGGTTCAGCGTT | TCGATTGGATAGTGCCATCA | 60.0 | 705 | DRR | Ca_00902 | S | bZIP | Ubiquitin-associated/translation elongation factor EF1B, N-terminal |

| INDEL marker IDs | Chromosomes/ unanchored scaffolds | Physical positions (bp) | InDels (*Kabuli* reference genome-CDC Frontier/PI) | Forward primers (5'-3') | Reverse primers (5'-3') | Anneling temperature (0C) | Expected amplified product size (bp) | Structural annotation | | Functional annotation | | |
|---|---|---|---|---|---|---|---|---|---|---|---|---|
| | | | | | | | | Sequence components of *kabuli* genome | *Kabuli* gene accession IDs | NCBI-KOG | TFs | NCBI-nr database |
| CaPOPI_1646 | Ca3 | 36040732 | ATTTT/ATTT | CCACCACTGCAGAAGTGAGA | TCCGAACTGGAGAGTGTGAA | 60.0 | 461 | INTRON | Ca_00902 | S | bZIP | Ubiquitin-associated/translation elongation factor EF1B, N-terminal |
| CaPOPI_1647 | Ca3 | 36041204 | ATT/AT | TTCACACTCTCCAGTTCGGA | AAGTCGACGTTTGTGAAGAGTTC | 59.4 | 699 | INTERGENIC | | | | |
| CaPOPI_1648 | Ca3 | 36043188 | AC/ATC | TGGAATGAATTAAACCATCACG | GCCACAAAATCATGCTGAAA | 59.7 | 245 | INTERGENIC | | | | |
| CaPOPI_1649 | Ca3 | 36045650 | ATT/ATTT | TGTGATTGAAAAGATGTGTCAAAA | GGGGTACGGAGTGGAGGTAT | 59.5 | 622 | INTERGENIC | | | | |
| CaPOPI_1650 | Ca3 | 36076083 | ATAAAA/ATAAAAATTAAAA | TGTCAAAATTTGGATTGCCA | CCTTGTTGAAACAAATGCAAGA | 59.9 | 539 | INTERGENIC | | | | |
| CaPOPI_1651 | Ca3 | 36086627 | AC/ACC | ATTCCAACGGAAAACATCCA | CTCCCAAACAAAGCCTACGA | 60.2 | 605 | INTRON | Ca_00907 | I | | Phosphatidic acid phosphatase type 2/haloperoxidase |
| CaPOPI_1652 | Ca3 | 36089072 | GAAAAAAAAAAAA/GAAAAAAAAAAA | CACCTACTAAAAAGCGTGCTGA | TCCAAAACAAAACAAAGCCC | 59.6 | 146 | INTERGENIC | | | | |
| CaPOPI_1653 | Ca3 | 36107876 | ATTTT/ATTT | CAATCCCTTTAACTTCGCCA | GGTGGGTGACAATGATTTCC | 60.1 | 658 | INTERGENIC | | | | |
| CaPOPI_1654 | Ca3 | 36109027 | CTTTTT/CTTTTTT | TCCATGCCATCAAAATCAAA | TTTCGTGAAGGCATACTCCA | 59.9 | 316 | DRR | Ca_00909 | J | | Ribosomal protein L21e |
| CaPOPI_1655 | Ca3 | 36109369 | CTTG/CTTGGTTG | TGGAGTATGCCTTCACGAAA | TGCCGTGGTTAAGGGAATAG | 59.3 | 652 | DRR | Ca_00909 | J | | Ribosomal protein L21e |
| CaPOPI_1656 | Ca3 | 36120455 | TGGG/TGG | AAAGCACCTGCAAGAAGGAA | GGCTTGGTCTCTTCCAATCA | 60.0 | 479 | INTRON | Ca_00911 | D | | |
| CaPOPI_1657 | Ca3 | 36120818 | TA/TAA | TGATTGGAAGAGACCAAGCC | CAATGGTGCAACAAACTTGG | 60.2 | 680 | INTRON | Ca_00911 | D | | |
| CaPOPI_1658 | Ca3 | 36129655 | GATATA/GATA | TTGCTCGTTTGATTGGTGTG | TTTTTGTGATTTTACGTAAGGGTA | 60.7 | 589 | DRR | Ca_00912 | YU | | NPL4 |
| CaPOPI_1659 | Ca3 | 36129790 | T/TC | TTGCTCGTTTGATTGGTGTG | TTTTTGTGATTTTACGTAAGGGTA | 60.7 | 589 | DRR | Ca_00912 | YU | | NPL4 |
| CaPOPI_1660 | Ca3 | 36135574 | ATT/AT | CAAATTAATTGGTTTGGATTTGA | TTTAGCACCGTTCTGGTGTG | 58.0 | 873 | DRR | Ca_00913 | | M-type | Plant peroxidase |

| INDEL marker IDs | Chromosomes/ unanchored scaffolds | Physical positions (bp) | InDels (*Kabuli* reference genome-CDC Frontier/PI) | Forward primers (5'-3') | Reverse primers (5'-3') | Anneling temperature (0C) | Expected amplified product size (bp) | Structural annotation | | Functional annotation | | |
|---|---|---|---|---|---|---|---|---|---|---|---|---|
| | | | | | | | | Sequence components of *kabuli* genome | *Kabuli* gene accession IDs | NCBI-KOG | TFs | NCBI-nr database |
| CaPOPI_1661 | Ca3 | 36136545 | CA/CATACATACATA | GCTTTGACACGTTTGCTTCA | ATTGTGGGAAGTGCTTACGG | 60.0 | 368 | INTRON | Ca_00913 | | M-type | Plant peroxidase |
| CaPOPI_1662 | Ca3 | 36138195 | TAAAAAAAA/TAAAAAAAAA | AGCTAATGAGCGGAAGCAAA | AATCTCCAAATTGGTGGTGG | 60.1 | 558 | INTERGENIC | | | | |
| CaPOPI_1663 | Ca3 | 36148820 | TAAAA/TAAA | TTGCTTATGTCTCACGGCTG | CACCTTTGGAAGCAAACAGAG | 60.0 | 901 | INTERGENIC | | | | |
| CaPOPI_1664 | Ca3 | 36168799 | CTCATAAGTCA/CTCA | GCCTGCGAAAGACAAAGTTC | ATTTGCAGGAGCTGCTGTTT | 60.0 | 610 | INTRON | Ca_00918 | KTDL | | BRCT |
| CaPOPI_1665 | Ca3 | 36178743 | TAA/TAAA | TGGATAAGACCGGAGAATGC | TCCTTATAAAATTATCATGCACTCAA | 60.0 | 690 | DRR | Ca_00921 | Q | B3 | Pyridine nucleotide-disulphide oxidoreductase, class-II |
| CaPOPI_1666 | Ca3 | 36195467 | TTTTATTTATTTATTTATTTATTTATTTATTTATTT/TTTTATTTATTTATTTATTTATTT | TATTGCCGACGTGAGTCTGA | TTCCCTTTCTTTTCGTCTGC | 60.4 | 283 | DRR | Ca_00923 | | bHLH | KIP1-like |
| CaPOPI_1667 | Ca3 | 36203025 | CACTTAC/CAC | ATCGAAGCCGGTTAGGTTTT | TACATCTTAATTGGGCCGGA | 60.0 | 168 | INTERGENIC | | | | |
| CaPOPI_1668 | Ca3 | 36204918 | ATTT/ATTTATTAAGTTT | GTCGTTGGAGTCTTTGCGAT | TTCACCAAATGTTGACCGAA | 60.3 | 452 | DRR | Ca_00925 | | | rRNA processing |
| CaPOPI_1669 | Ca3 | 36207784 | GA/GAA | TGGGTTCATTAGCCACATCA | GTTCAAACTTCGTTGCCTCC | 59.9 | 237 | INTRON | Ca_00925 | | | rRNA processing |
| CaPOPI_1670 | Ca3 | 36208259 | CTT/CTTACATATAATTTTT | GAGGCAACGAAGTTTGAACC | GGTTTCGTCGTCGTTTGAGT | 59.7 | 675 | INTRON | Ca_00925 | | | rRNA processing |
| CaPOPI_1671 | Ca3 | 36218063 | CAAAAAAAA/CAAAAAAAAAA | TTGATGTCTCCCACAAGCTG | TAATCAGATGATCGCGCAAG | 59.8 | 480 | INTRON | Ca_00927 | | CO-like | Zinc finger, B-box |
| CaPOPI_1672 | Ca3 | 36219342 | GTTT/GTT | CATGTGGCGAAGAGAGTGAA | GTATTTCCTTTGGGCGTGAA | 60.0 | 531 | INTERGENIC | | | | |
| CaPOPI_1673 | Ca3 | 36219993 | TCA/T | TTCACGCCCAAAGGAAATAC | ACCGGTGATGACTCCAGTTC | 59.9 | 757 | DRR | Ca_00928 | | | Ubiquitin |
| CaPOPI_1674 | Ca3 | 36226234 | AATAGAT/AATAGATAGAT | TGGAGCCCATCGAAATAAAG | CCACATGGTGAAGAGCAGAA | 60.0 | 729 | INTERGENIC | | | | |
| CaPOPI_1675 | Ca3 | 36226833 | CGAGGAGG/CGAGG | CACGAAATCCGAAATGGAAC | TTGTTTGCTCTGATTTTGGAGA | 60.3 | 537 | INTERGENIC | | | | |

| INDEL marker IDs | Chromosomes/ unanchored scaffolds | Physical positions (bp) | InDels (*Kabuli* reference genome-CDC Frontier/PI) | Forward primers (5'-3') | Reverse primers (5'-3') | Anneling temperature (0C) | Expected amplified product size (bp) | Structural annotation | | Functional annotation | | |
|---|---|---|---|---|---|---|---|---|---|---|---|---|
| | | | | | | | | Sequence components of *kabuli* genome | *Kabuli* gene accession IDs | NCBI-KOG | TFs | NCBI-nr database |
| CaPOPI_1676 | Ca3 | 36230990 | T/TA | TCCAGTCCTTTCCTTTTGGA | TGAGTTAAAGGACCACATGCC | 59.6 | 353 | DRR | Ca_00929 | J | | Ribosomal protein S19/S15 |
| CaPOPI_1677 | Ca3 | 36241653 | CC/CCAC | TTCGCAGAAAAACTATGCCC | TCCGTATTCGAATTTGAGACAA | 60.2 | 808 | INTERGENIC | | | | |
| CaPOPI_1678 | Ca3 | 36250776 | AAATAATAATAATAATAATAA/AAATAATAATAATAATAA | GCAAAGCCAAACCGCTATAA | AACGCTAACGTTGGTTCCAC | 60.2 | 935 | DRR | Ca_00933 | O | | Glutaredoxin |
| CaPOPI_1679 | Ca3 | 36274277 | T/TA | TGGGTTTTCATTGGTTTGGT | AAGCCGACTTCAAGAAGGAC | 60.1 | 419 | DRR | Ca_00937 | J | Dof | Peptide chain release factor class I/class II |
| CaPOPI_1680 | Ca3 | 36283526 | ATTTTTTTTT/ATTTTTTTTTTTT | GCGGTCGTGTGACAGGTACT | TCTTATGCCGTTTTCCTTCG | 61.2 | 654 | DRR | Ca_00938 | R | | Domain of unknown function DUF255 |
| CaPOPI_1681 | Ca3 | 36286597 | ATTTTTTT/ATTTTTTTT | TCATTTTGGCAACTTACCACC | TGCCTGAATTTATTGGGAATG | 59.9 | 754 | INTERGENIC | | | | |
| CaPOPI_1682 | Ca3 | 36295270 | TTGTTTTCATGTTT/TTGTTT | TGTAGAGAACGGAAATGGGG | TAAGGTGGCGAGGATTGAAC | 59.9 | 593 | INTRON | Ca_00939 | | | Zinc finger, CW-type |
| CaPOPI_1683 | Ca3 | 36308195 | T/TC | TTTGCCACTTGATACAGCAGA | TTGAACCTAAACGATGACACCTT | 59.5 | 309 | INTERGENIC | | | | |
| CaPOPI_1684 | Ca3 | 36312854 | AAA/AAATTAAGTAA | CTATGCACGAGTTTGGGGTT | CAATCAAGAAGCGCATTGAA | 60.0 | 586 | DRR | Ca_00941 | D | TCP | Cullin, N-terminal |
| CaPOPI_1685 | Ca3 | 36320561 | CTTTTTTTT/CTTTTTTTTTTT | GTCTCAAGCAATCCTCAGCC | CAACGAGAGAGTGGATGGGT | 60.0 | 442 | INTRON | Ca_00941 | D | TCP | Cullin, N-terminal |
| CaPOPI_1686 | Ca3 | 36328591 | GAAA/GAA | CACGATGAAATCACTATGAAAAGC | CGTTCCAGACCTCCATTGTT | 60.0 | 440 | INTERGENIC | | | | |
| CaPOPI_1687 | Ca3 | 36352882 | AT/ATT | TCACCTCGAGCAAACAAGTG | AAGCACCATTTGTAGTTTTCATGT | 60.0 | 380 | INTERGENIC | | | | |
| CaPOPI_1688 | Ca3 | 36356666 | CAAAAAAAAAAAA/CAAAAAAAAAAA | ATTTAAAAGTCACGCGGAGC | TCCAGGTATGCGGTAGTTCC | 59.4 | 373 | DRR | Ca_00944 | | | |
| CaPOPI_1689 | Ca3 | 36371742 | ATTTTTTTT/ATTTTTTT | GGGTTTTCTAGTCCCCCAAG | TGTTTTTCATTATGTGCCCG | 59.8 | 483 | INTERGENIC | | | | |
| CaPOPI_1690 | Ca3 | 36381407 | ATTTTTT/ATTTTTTT | TTTTGAGAGCGCATTTTGTG | CCATGCTTCCCACGTCTAAT | 60.0 | 352 | INTERGENIC | | | | |

| INDEL marker IDs | Chromosomes/ unanchored scaffolds | Physical positions (bp) | InDels (*Kabuli* reference genome-CDC Frontier/PI) | Forward primers (5'-3') | Reverse primers (5'-3') | Anneling temperature (0C) | Expected amplified product size (bp) | Structural annotation | | Functional annotation | | |
|---|---|---|---|---|---|---|---|---|---|---|---|---|
| | | | | | | | | Sequence components of *kabuli* genome | *Kabuli* gene accession IDs | NCBI-KOG | TFs | NCBI-nr database |
| CaPOPI_1691 | Ca3 | 36396966 | AAAATT/AAAATTTCAAATT | CTCACATCTGATCGAAGGCA | ACTGTAACCTTGTGCTCCCG | 59.9 | 332 | INTRON | Ca_00950 | P | | Heavy metal transport/detoxification protein |
| CaPOPI_1692 | Ca3 | 36397163 | TTATATATAT/TTATATATATATAT | CTCACATCTGATCGAAGGCA | TGCAGCAGCAGAACCATTAC | 59.9 | 708 | INTRON | Ca_00950 | P | | Heavy metal transport/detoxification protein |
| CaPOPI_1693 | Ca3 | 36399143 | ATTTTTT/ATTTTTTT | ATAATTGGGCCACACATCGT | TGCAATGTGGAAATCAGAGAA | 60.1 | 208 | DRR | Ca_00950 | P | | Heavy metal transport/detoxification protein |
| CaPOPI_1694 | Ca3 | 36399524 | T/TG | ATAATTGGGCCACACATCGT | AACAAGCAAGCGCAACACTA | 60.1 | 712 | DRR | Ca_00950 | P | | Heavy metal transport/detoxification protein |
| CaPOPI_1695 | Ca3 | 36410249 | GAAAAAAAAAA/GAAAAAAAAAAAA | CGGAAATGTCATGTGTGGTC | ATGTTTGTCCACTTGCTCCC | 59.8 | 458 | DRR | Ca_00953 | | HSF | Rapid ALkalinization Factor |
| CaPOPI_1696 | Ca3 | 36410296 | GTTTTTTT/GTTTTTTTTT | ATGGTTGTTCATCGTCCCAT | ATGTTTGTCCACTTGCTCCC | 60.1 | 126 | DRR | Ca_00953 | | HSF | Rapid ALkalinization Factor |
| CaPOPI_1697 | Ca3 | 36418781 | GAAAA/GAAA | TCCTCCTTACCACATCGGTC | ACGTAGAAACAATGGCCGTC | 59.9 | 929 | INTRON | Ca_00954 | G | | Glycosyltransferase, ALG3 |
| CaPOPI_1698 | Ca3 | 36420292 | GGAGC/G | TGTTTTTCGCTCATCCATTG | TGGTAGAAATCACGATGCCA | 59.7 | 710 | INTERGENIC | | | | |
| CaPOPI_1699 | Ca3 | 36420971 | AATATATATATATAT/AATATATATAT | TGGCATCGTGATTTCTACCA | GGCCATGGTGATTGTAAAGG | 60.1 | 746 | INTERGENIC | | | | |
| CaPOPI_1700 | Ca3 | 36443050 | ATG/A | TTGCAATTGAAGTTCCCACA | GGCAAGCTCATTTCTGCTTC | 60.1 | 427 | INTRON | Ca_00959 | R | | WD40 repeat |
| CaPOPI_1701 | Ca3 | 36443105 | GCATCAT/GCAT | TTGCAATTGAAGTTCCCACA | GGCAAGCTCATTTCTGCTTC | 60.1 | 427 | INTRON | Ca_00959 | R | | WD40 repeat |
| CaPOPI_1702 | Ca3 | 36454087 | CGAT/CGATCATATTGGAT | TGATTCGGTTTGTGCTGTGT | CGTGACAGCCCATAGTTGAA | 60.2 | 931 | INTERGENIC | | | | |
| CaPOPI_1703 | Ca3 | 36455748 | AC/AGTTTTACTCTTAATACATC | TCAAATTACAACAAGGGCGA | CGTAAGGGAGCAAAGTGGTC | 59.2 | 792 | INTERGENIC | | | | |
| CaPOPI_1704 | Ca3 | 36458050 | AG/A | TGGGTCCCAAAAGTGGTATC | CAATTTGGCCACTTGAATGA | 59.6 | 772 | INTERGENIC | | | | |
| CaPOPI_1705 | Ca3 | 36458326 | ATTTTTT/ATTTTTTT | TTTGCAAAGTGGCGGTAGTT | TGTTTTGCAAGGTGAATTGG | 60.7 | 525 | INTERGENIC | | | | |

| INDEL marker IDs | Chromosomes/ unanchored scaffolds | Physical positions (bp) | InDels (*Kabuli* reference genome-CDC Frontier/PI) | Forward primers (5'-3') | Reverse primers (5'-3') | Anneling temperature (0C) | Expected amplified product size (bp) | Structural annotation | | Functional annotation | | |
|---|---|---|---|---|---|---|---|---|---|---|---|---|
| | | | | | | | | Sequence components of *kabuli* genome | *Kabuli* gene accession IDs | NCBI-KOG | TFs | NCBI-nr database |
| CaPOPI_1706 | Ca3 | 36490446 | T/TCAC | CAGGGTCACCTGCTCAAAAT | TCCAAGATCAGTTGACTGCG | 60.1 | 333 | DRR | Ca_00965 | | | Pectinesterase inhibitor |
| CaPOPI_1707 | Ca3 | 36525538 | AAT/AAAATCTTTAT | TCCAAGGACTAAAACGTCCAA | TCCAAAGAGACTCATCCCAAA | 59.6 | 519 | DRR | Ca_00969 | | | Late embryogenesis abundant protein, group 3 |
| CaPOPI_1708 | Ca3 | 36529799 | AAAGA/AA | CCCTATCCAACATTCATGGG | ACCAAACTGGTGGAACTTGC | 60.0 | 722 | DRR | Ca_00971 | U | | Exocyst complex subunit Sec15-like |
| CaPOPI_1709 | Ca3 | 36530182 | T/TC | GATTTAGCAGGAGTGCCGAG | TCGGATCGACTCTTTGGAAC | 60.0 | 686 | DRR | Ca_00971 | U | | Exocyst complex subunit Sec15-like |
| CaPOPI_1710 | Ca3 | 36596139 | GAAAAAA/GAAAAAAA | AAAGTTTTTGGCAAGTCGGA | AGAGCAAGGAAACCGAATCA | 59.7 | 596 | INTERGENIC | | | | |
| CaPOPI_1711 | Ca3 | 36597966 | TAAAAAAAA/TAAAAAAA | TAACCAACAACCGCATGAAA | TCAATTTCTTAAAAGAGGTGGCA | 60.0 | 242 | INTERGENIC | | | | |
| CaPOPI_1712 | Ca3 | 36608541 | T/TA | TGGCTCAAAGATAGGATGGG | CCCTTCCTCCATCAATCTCA | 60.0 | 315 | DRR | Ca_00981 | | | |
| CaPOPI_1713 | Ca3 | 36609637 | CATATAT/CATAT | ATTGAAATGCGGGTTCTCAG | TTGATCACAACGAGGAGCTG | 60.1 | 519 | DRR | Ca_00981 | | | |
| CaPOPI_1714 | Ca3 | 36614124 | TGTA/T | TTGCATTTCAAAAGTTTCGC | TGTGGCTCAACGAGAGATTT | 58.9 | 277 | INTERGENIC | | | | |
| CaPOPI_1715 | Ca3 | 36624385 | CAAAAAAAAAAA/CAAAAAAAA | TGATTTCCACTGCACCACAT | TGCAACACAAACTCCTTTGC | 60.0 | 517 | INTERGENIC | | | | |
| CaPOPI_1716 | Ca3 | 36624455 | ATTTTTTT/ATTTTTTTT | TGATTTCCACTGCACCACAT | TGCAACACAAACTCCTTTGC | 60.0 | 517 | INTERGENIC | | | | |
| CaPOPI_1717 | Ca3 | 36624904 | GTTT/GTTTCTTTTTT | TGCAAAGGAGTTTGTGTTGC | AACATAAAAGAACAGAGCGGAGA | 59.9 | 211 | INTERGENIC | | | | |
| CaPOPI_1718 | Ca3 | 36625899 | TTATTTATTTA/T | ATTTCACTGACTGTCGCCCT | CAACAGGAAGGGGATTAGAAAA | 59.7 | 356 | INTERGENIC | | | | |
| CaPOPI_1719 | Ca3 | 36636712 | CTTTTTT/CTTTTT | CGGATAACACTTGTTTGTGAGTTT | GTCTTTTCTTGATCCCGACG | 59.5 | 776 | INTERGENIC | | | | |
| CaPOPI_1720 | Ca3 | 36638153 | TG/T | TCGTCAACTCGCAACTCATC | AAAAAGCCAAACAATTCTAGAAACA | 60.0 | 487 | INTERGENIC | | | | |

| INDEL marker IDs | Chromosomes/ unanchored scaffolds | Physical positions (bp) | InDels (*Kabuli* reference genome-CDC Frontier/PI) | Forward primers (5'-3') | Reverse primers (5'-3') | Anneling temperature (0C) | Expected amplified product size (bp) | Structural annotation | | Functional annotation | | |
|---|---|---|---|---|---|---|---|---|---|---|---|---|
| | | | | | | | | Sequence components of *kabuli* genome | *Kabuli* gene accession IDs | NCBI-KOG | TFs | NCBI-nr database |
| CaPOPI_1721 | Ca3 | 36638232 | AATTTT/A | TCGTCAACTCGCAACTCATC | AAAAAGCCAAACAATTCTAGAAACA | 60.0 | 487 | INTERGENIC | | | | |
| CaPOPI_1722 | Ca3 | 36652791 | TGGG/TG | TCACAAAGAAAGCCAGCTCA | TCCAGAGCTTTTCATGGCTT | 59.7 | 451 | DRR | Ca_00986 | | bZIP | Cellulose synthase |
| CaPOPI_1723 | Ca3 | 36682594 | GTTTTTTTTT/GTTTTTTTTTT | TTCATCGATGTTCGGACAAA | CCAACTCGGTTCATTTACTCG | 60.0 | 536 | INTERGENIC | | | | |
| CaPOPI_1724 | Ca3 | 36688981 | TTCTTTATCTTTATCTTTATC/TTCTTTATCTTTATCTTTATCTTTATC | AGGGGAGACAACCATAGGCT | GTGATGTGATTGGCTGGTTG | 60.0 | 862 | DRR | Ca_00990 | O | C3H | Zinc finger, CCCH-type |
| CaPOPI_1725 | Ca3 | 36697642 | ATT/AT | CGACGCATATTTCAAATGACTT | TCTTTTGGCAACAGGTTGGT | 59.1 | 634 | INTERGENIC | | | | |
| CaPOPI_1726 | Ca3 | 36697708 | AC/A | CGACGCATATTTCAAATGACTT | AGTTGCATGCTTCTTTTGGC | 59.1 | 645 | INTERGENIC | | | | |
| CaPOPI_1727 | Ca3 | 36707043 | CT/C | CTCCTCCCAGTGGTAACAAA | CAGTGAAAAATTGAATCCCCA | 57.6 | 595 | INTERGENIC | | | | |
| CaPOPI_1728 | Ca3 | 36707318 | TA/TAAA | CAAAAACAGTTTGGGGATTCA | CATTTGACCTTACGTCCATCC | 59.8 | 502 | INTERGENIC | | | | |
| CaPOPI_1729 | Ca3 | 36707636 | TTTTATTTATT/TTTTATTTATTTATTTATT | CAAAAACAGTTTGGGGATTCA | GATGATGGAAGGGGATGAGA | 59.8 | 868 | INTERGENIC | | | | |
| CaPOPI_1730 | Ca3 | 36716843 | GC/G | TTTTATTTGAGGGGTGACCG | GATGGATTGGTCGTGAGGTT | 59.8 | 554 | DRR | Ca_00995 | O | MYB | Heat shock protein DnaJ, N-terminal |
| CaPOPI_1731 | Ca3 | 36735770 | AATAAATAAATATAAATAAATATAAATA/AATAAATAAATATAAATA | TACGTGGCCTATTAGGTGGC | GCATATGGTGATTGCTGACTCT | 60.0 | 648 | INTERGENIC | | | | |
| CaPOPI_1732 | Ca3 | 36735874 | TAAAAAAA/TAAAAAA | TACGTGGCCTATTAGGTGGC | CGGCGGAGTCAGGTAATAAA | 60.0 | 834 | INTERGENIC | | | | |
| CaPOPI_1733 | Ca3 | 36735945 | AT/ATT | TACGTGGCCTATTAGGTGGC | ATGGGTAACCGGGTGTATCA | 60.0 | 874 | INTERGENIC | | | | |
| CaPOPI_1734 | Ca3 | 36751775 | C/CCTATTAACAAGGAAA | TAACATCCATGGCCTCACAA | CGGTTTGTCTTTGGATAATGG | 59.9 | 835 | INTRON | Ca_00998 | | C2H2 | Glycoside hydrolase, family 3, N-terminal |
| CaPOPI_1735 | Ca3 | 36752520 | AAGTAGTAGTAGTAGTA/AAGTAGTAGTAGTAGTAGTA | ACGAATCAACTTGGCCTACG | AGGGCAATGTACAATGGAGG | 60.1 | 602 | INTRON | Ca_00998 | | C2H2 | Glycoside hydrolase, family 3, N-terminal |

| INDEL marker IDs | Chromosomes/ unanchored scaffolds | Physical positions (bp) | InDels (*Kabuli* reference genome-CDC Frontier/PI) | Forward primers (5'-3') | Reverse primers (5'-3') | Anneling temperature (0C) | Expected amplified product size (bp) | Structural annotation | | Functional annotation | | |
|---|---|---|---|---|---|---|---|---|---|---|---|---|
| | | | | | | | | Sequence components of *kabuli* genome | *Kabuli* gene accession IDs | NCBI-KOG | TFs | NCBI-nr database |
| CaPOPI_1736 | Ca3 | 36756795 | TAAAAAA/TAAAAAAA | AAGTGCTTGTTGCTGGAGTG | TCGTCGCATCTACAAACAAAA | 59.1 | 529 | INTERGENIC | | | | |
| CaPOPI_1737 | Ca3 | 36932559 | GAAAAAAAAAAAAA/GAAAAAAAAAAAA | GTTACGATTTTCATTGGCGA | CTTTGGTATGTTGGGCCTGT | 58.6 | 917 | DRR | Ca_01018 | | WRKY | DNA-binding WRKY |
| CaPOPI_1738 | Ca3 | 37017817 | AATATATATATATATATATATATATATATATATATA/AATATATATATATATATATATATATATATATATATATATATATATATATATA | CAGAAGGCCAGGAGAAGAAA | TGTGCGTGAAGTTTCATGGT | 59.5 | 707 | INTERGENIC | | | | |
| CaPOPI_1739 | Ca3 | 37030222 | AATATATATATATATATATATATATATA/AATATATATATATATATATATATATATATATA | GAAAAGCCCATTTTGTCTCG | AAAGGTTTGAAAATGGATGGTC | 59.7 | 593 | DRR | Ca_01031 | G | C3H | Protein kinase, catalytic domain |
| CaPOPI_1740 | Ca3 | 37150967 | ATTTTTTTT/ATTTTTTTTT | TTTTGCTTGCGTGAAAATTG | CCAAATGATAATCCATGGGC | 59.9 | 604 | INTERGENIC | | | | |
| CaPOPI_1741 | Ca3 | 37191129 | TAAAAAAAAAA/TAAAAAAAAA | TGGTTTCATTCATCTTTTGCAG | GCCCAAATCAACCCATTTTA | 60.1 | 445 | INTERGENIC | | | | |
| CaPOPI_1742 | Ca3 | 37428682 | T/TGGA | TTGAGGATCAGCTACACCCC | TGGAACAATTTTGGTGGTGA | 60.1 | 546 | INTERGENIC | | | | |
| CaPOPI_1743 | Ca3 | 37594905 | TTATATATATATATATATATATATA/TTATATATATATATATATATATATATA | GTCCGCCCTAAAAAGGTAGC | TCAAAACACCACATCAAAACG | 60.1 | 188 | INTERGENIC | | | | |
| CaPOPI_1744 | Ca3 | 37989883 | GTATATATATATATATATATA/GTATATATATATATATATATATATATATA | TGTCACGGGTGGACAAATTA | TAGGCGATTGTAGGGCTTTG | 59.8 | 749 | INTERGENIC | | | | |
| CaPOPI_1745 | Ca3 | 38129457 | CATATATATATATATA/CATATATATATATATATA | CAAAAGCCGGACCAAAATTA | GGGCATGGGCAATAATCTAA | 59.9 | 398 | INTERGENIC | | | | |
| CaPOPI_1746 | Ca3 | 38139076 | A/AC | TTGCATTAAACTTGAACGTTTTT | TCAGGCCAACCCAGTACATT | 58.0 | 416 | INTERGENIC | | | | |
| CaPOPI_1747 | Ca3 | 38141259 | TTA/T | GGAAAGTCATGATGTCCTTTTT | GTCATTTGTAAATGGCGGCT | 57.2 | 436 | INTERGENIC | | | | |
| CaPOPI_1748 | Ca3 | 38141514 | ATTTTTT/ATTTTTTT | CCGCCATTTACAAATGACACT | TCGTCAAAATTATTTAGCGCAT | 59.9 | 536 | INTERGENIC | | | | |
| CaPOPI_1749 | Ca3 | 38149993 | ATTA/ATTAACTTA | AGTTATTACATGCGGCAGGG | GCCAAACAGTGTTGTAATTCG | 60.0 | 752 | DRR | Ca_01158 | C | | Cystathionine beta-synthase, core |
| CaPOPI_1750 | Ca3 | 38154708 | CGGAGGA/CGGAGGAGGA | AGAGCGGAAAAGTTATCGCA | TTCATGTTGTCCCTTTTGACA | 60.0 | 477 | DRR | Ca_01160 | L | MYB_related | DNA-directed DNA polymerase, family B, exonuclease domain |

| INDEL marker IDs | Chromosomes/ unanchored scaffolds | Physical positions (bp) | InDels (*Kabuli* reference genome-CDC Frontier/PI) | Forward primers (5'-3') | Reverse primers (5'-3') | Anneling temperature (0C) | Expected amplified product size (bp) | Structural annotation | | Functional annotation | | |
|---|---|---|---|---|---|---|---|---|---|---|---|---|
| | | | | | | | | Sequence components of *kabuli* genome | *Kabuli* gene accession IDs | NCBI-KOG | TFs | NCBI-nr database |
| CaPOPI_1751 | Ca3 | 38164329 | TG/TGAGAGAGAG | TATTCCCCAATTCCTAGCCC | CAAGATGCTGGCACAGAAAA | 60.1 | 740 | INTRON | Ca_01160 | L | MYB_related | DNA-directed DNA polymerase, family B, exonuclease domain |
| CaPOPI_1752 | Ca3 | 38170265 | TAA/TA | CCCAGATCTGCCTAATGGAA | GATGGGTTTGGAACATCCAG | 60.0 | 594 | INTRON | Ca_01160 | L | MYB_related | DNA-directed DNA polymerase, family B, exonuclease domain |
| CaPOPI_1753 | Ca3 | 38174447 | A/AT | TTCACATCCACACTCATCGG | CTGGCACTGGCATTCCTAAT | 60.5 | 628 | DRR | Ca_01161 | | NF-YA | Lipoxygenase |
| CaPOPI_1754 | Ca3 | 38205894 | TAAAAAAAAA/TAAAAAAAA | ACAACACTCGGCCTATCACC | CTCCCCCTTCTCCAGGATAG | 60.0 | 768 | DRR | Ca_01163 | E | | Creatinase |
| CaPOPI_1755 | Ca3 | 38206711 | GAAAAAAAAAAA/GAAAAAAAAAAAAA | CTATCCTGGAGAAGGGGGAG | GCTATTGCATTGGAGTTGCTC | 60.0 | 706 | DRR | Ca_01163 | E | | Creatinase |
| CaPOPI_1756 | Ca3 | 38206767 | AAGAG/AAG | CTATCCTGGAGAAGGGGGAG | GCTATTGCATTGGAGTTGCTC | 60.0 | 706 | DRR | Ca_01163 | E | | Creatinase |
| CaPOPI_1757 | Ca3 | 38215897 | CTT/CTTCTCTTCATT | TGTTGCCTGGAAATGAGTTG | TGCTGCATTGAAATCGAGTC | 59.7 | 688 | DRR | Ca_01165 | R | | |
| CaPOPI_1758 | Ca3 | 38219148 | A/AC | CTACCGGCTACTTTCTTGCG | TATGCATGGTGAAAAAGCGA | 60.0 | 463 | DRR | Ca_01166 | Z | B3 | Kinesin, motor domain |
| CaPOPI_1759 | Ca3 | 38221107 | TCTCCTC/TCTC | CCACTGGTGGATTCAGAGGT | GAGAACTCATGTGCCGGAAT | 60.0 | 566 | CDS (large-effect mutations) | Ca_01166 | Z | B3 | Kinesin, motor domain |
| CaPOPI_1760 | Ca3 | 38228425 | A/AGTGTGAATAC | ATGATCCACTGAGATTCCGC | CCAGAGTGTGACGCTTTTGA | 60.0 | 472 | DRR | Ca_01168 | R | bHLH | Pentatricopeptide repeat |
| CaPOPI_1761 | Ca3 | 38240356 | TCCCC/TCCC | GTGGTGAGTTCAGTTCCGGT | ACCGTTAGAGGAGAGGAGCC | 60.0 | 475 | INTERGENIC | | | | |
| CaPOPI_1762 | Ca3 | 38241149 | A/AAT | GGCTCCTCTCCTCTAACGGT | GACAAGTGCAACAATTTTCATCA | 59.8 | 527 | INTERGENIC | | | | |
| CaPOPI_1763 | Ca3 | 38241297 | TTTATT/TTT | TTGATGAAAATTGTTGCACTTG | TTGCTAACACAAAAGTATTTGAATGTC | 58.7 | 189 | INTERGENIC | | | | |
| CaPOPI_1764 | Ca3 | 38690322 | AATATATATATATATATATATA/AATATATATATATATATATATATATATATA | GAAGCAGGGAGGAGGAAAAC | TGTCAATGAGGATTATGCCAA | 60.2 | 910 | DRR | Ca_01224 | K | Trihelix | SANT domain, DNA binding |
| CaPOPI_1765 | Ca3 | 38764047 | TAAAAATAAAA/TAAAAATAAAAAATAAAA | TGGGCTCCGAGAGTCTTATG | CGTGGATTAATCGCTACCGT | 60.4 | 253 | DRR | Ca_01234 | | bZIP | Basic-leucine zipper (bZIP) transcription factor |

| INDEL marker IDs | Chromosomes/ unanchored scaffolds | Physical positions (bp) | InDels (*Kabuli* reference genome-CDC Frontier/PI) | Forward primers (5'-3') | Reverse primers (5'-3') | Anneling temperature (0C) | Expected amplified product size (bp) | Structural annotation | | Functional annotation | | |
|---|---|---|---|---|---|---|---|---|---|---|---|---|
| | | | | | | | | Sequence components of *kabuli* genome | *Kabuli* gene accession IDs | NCBI-KOG | TFs | NCBI-nr database |
| CaPOPI_1766 | Ca3 | 38870959 | AAATAATAATAATAATAATAATAATAATAATAATAATAATAATAA/AAATAATAATAATAATAATAATAATAATAATAATAATAA | TTTGCATATGACATTGAAAGCA | TGAAACAGGCAATCTTTCCC | 59.2 | 292 | INTERGENIC | | | | |
| CaPOPI_1767 | Ca3 | 39465898 | GTTATTATTATTATTATTATTATTATTATTATTATTATTATTAT/GTTATTATTATTATTATTATTATTATTATTATTATTATTATTATTAT | GAAGTTCGCTTCCTCCAAAA | TTGGCAGTACCAACGAATGA | 59.4 | 358 | DRR | Ca_01313 | | | Acid phosphatase/vanadium-dependent haloperoxidase- |
| CaPOPI_1768 | Ca3 | 39571556 | GAAAAAAAAA/GAAAAAAAA | AATTTGTTTCGGCAATTCCA | GCAAAAGTGGCTGATGACAA | 60.3 | 553 | INTERGENIC | | | | |
| CaPOPI_1769 | Ca3 | 39611074 | T/TC | ACCGGACACGATATCAGGAG | TGCTTCAACAAAGGCAAACA | 60.0 | 474 | INTRON | Ca_01338 | | | Profilin/allergen |
| CaPOPI_1770 | Ca3 | 39663553 | TAAAAAAAAA/TAAAAAAAA | GTGGCCACGTATCGAGGTAG | TCCAAAAGAAAATCATGGGG | 60.5 | 516 | INTERGENIC | | | | |
| CaPOPI_1771 | Ca3 | 39763068 | CAGAGAGAGAGAGAGAGAGA/CAGAGAGAGAGAGAGAGA | GGTCGCACATTGTCATCATC | AAAGGTTTCGACCACGTCAC | 59.9 | 522 | INTRON | Ca_01363 | R | | O-methyltransferase, family 2 |
| CaPOPI_1772 | Ca3 | 39866735 | C/CT | ACAGTCTTTGGTTCGATGGG | CTGCCCCAAAAGACACAAAT | 60.0 | 288 | INTRON | Ca_01372 | | WRKY | Protein kinase, catalytic domain |
| CaPOPI_1773 | Ca3 | 39988476 | GTTTT/GTTT | AGAACAGAGCTGGCCTCAAA | ATTGCAAGAAAACCAGCACC | 60.1 | 148 | INTERGENIC | | | | |
| CaPOPI_1774 | Ca4 | 323717 | ATTT/AT | CTGGGAATTGGACTTAAAAGAA | TTGTTGGGTTATCGTTGTCG | 57.4 | 791 | INTERGENIC | | | | |
| CaPOPI_1775 | Ca4 | 978893 | TAAAAAAAAA/TAAAAAAAA | TTTTGAGGTGCTTTCCCATC | CAATCCCCCAAGCTTAACAA | 60.1 | 535 | INTERGENIC | | | | |
| CaPOPI_1776 | Ca4 | 1144979 | CATATATATATATATATATATATATATATA/CATATATATATATATATATATATATATA | AACGCATGCAATCTATTCCC | ATTGTTGGCGTCTTCCAAAC | 59.9 | 813 | INTERGENIC | | | | |
| CaPOPI_1777 | Ca4 | 1309061 | GAAAAAA/GAAAAAAA | AGCTACAAAATGGGCAATGG | CCACGGTACCGAAAAATGAT | 60.0 | 237 | INTERGENIC | | | | |
| CaPOPI_1778 | Ca4 | 1310245 | ATTTTTTTTTTT/ATTTTTTTTTT | TGAAATTGCTTGATTTTGCTTT | CGACCGATTTTATCCGTCTC | 58.9 | 825 | INTERGENIC | | | | |
| CaPOPI_1779 | Ca4 | 1371815 | TATAATAATAATAATAATAA/TATAATAATAATAATAATAA | ATGAAGTTCCCCATGAGTGC | AGGCAAAAGGGAAAGACCAC | 59.9 | 463 | INTERGENIC | | | | |
| CaPOPI_1780 | Ca4 | 1665800 | CATATATATATATATATA/CATATATATATATATATATATA | CATTGAAAGCGCGTTGATAA | GGTGAATTGATTTATGCTGGAA | 59.8 | 394 | INTERGENIC | | | | |

| INDEL marker IDs | Chromosomes/ unanchored scaffolds | Physical positions (bp) | InDels (*Kabuli* reference genome-CDC Frontier/PI) | Forward primers (5'-3') | Reverse primers (5'-3') | Anneling temperature (0C) | Expected amplified product size (bp) | Structural annotation | | Functional annotation | | |
|---|---|---|---|---|---|---|---|---|---|---|---|---|
| | | | | | | | | Sequence components of *kabuli* genome | *Kabuli* gene accession IDs | NCBI-KOG | TFs | NCBI-nr database |
| CaPOPI_1781 | Ca4 | 1677374 | TAAAAAAA/TAAAAA | TCCAAACTAGTCCGACGTGA | AGCTGCTGGAGGAGTTGGTA | 59.3 | 450 | INTRON | Ca_07795 | | | |
| CaPOPI_1782 | Ca4 | 1771183 | TATATATA/TATATATAACATATATA | GATCAATATCTCCATGCGGG | GTTGGGCACCGTCATAAAGT | 60.3 | 423 | INTERGENIC | | | | |
| CaPOPI_1783 | Ca4 | 1775612 | TT/TTTCAATTTTTGCT | GAGAAATGGAGAGGGAAGGG | TCGTCTGACACCACCAACAT | 60.0 | 718 | DRR | Ca_07800 | K | HSF | Heat shock factor (HSF)-type, DNA-binding |
| CaPOPI_1784 | Ca4 | 1782351 | TAA/TA | TGGTTTGGATTTGGAGCTGT | TAGATCCGGAATGAAGACCG | 60.5 | 361 | INTRON | Ca_07801 | T | | Protein kinase, catalytic domain |
| CaPOPI_1785 | Ca4 | 1791916 | AGTTAGTTTGTTA/AGTTAGTTTGTTAGTTTGTTA | CGATATTGTCAAATGGCGTG | CAATGATCACACCAAGGTCG | 60.0 | 741 | INTRON | Ca_07801 | T | | Protein kinase, catalytic domain |
| CaPOPI_1786 | Ca4 | 1831456 | TTA/TTATA | TCCAATAAGGTTGCCTAAGGA | GAGATTAAGCCGGAAGACCC | 58.7 | 256 | DRR | Ca_07804 | | | Domain of unknown function DUF828 |
| CaPOPI_1787 | Ca4 | 1848187 | TATAATAATAA/TATAATAATAATAA | AGAAGCAAAAGAGCCCTTCC | ATAGGAATATAGGCGCGGGT | 60.0 | 323 | INTERGENIC | | | | |
| CaPOPI_1788 | Ca4 | 1855105 | TTTAT/TTTATTAT | CACGACCCTTTCGATTGTTT | AAACCAGCTGCCAATTCTGT | 60.0 | 430 | INTERGENIC | | | | |
| CaPOPI_1789 | Ca4 | 1943645 | CATATATATATATATATATATATAT/CATATATATATATATATATATAT | GCACATGGTGTGAAACTGCT | TTGGGGAGATGGAAGAAAAA | 59.8 | 662 | INTERGENIC | | | | |
| CaPOPI_1790 | Ca4 | 2022623 | AAATAATAATAATAATAATAATAATAATAATAATAA/AAATAATAATAATAATAATAATAATAATAATAA | AACGTGTTTGAGTGCGAAAA | TCGAGATGCGATTATGTGGA | 59.4 | 819 | INTERGENIC | | | | |
| CaPOPI_1791 | Ca4 | 2032207 | TC/T | TGCTGAGTTCGAGGAAAGGT | CACATCTCTTGTTCGCTCCA | 60.0 | 517 | CDS (FRAME SHIFT) | Ca_07822 | D | | RNA-processing protein, HAT helix |
| CaPOPI_1792 | Ca4 | 2032547 | CTGTGTGTGT/CTGTGTGT | TGCTGAGTTCGAGGAAAGGT | CACATCTCTTGTTCGCTCCA | 60.0 | 517 | INTRON | Ca_07822 | D | | RNA-processing protein, HAT helix |
| CaPOPI_1793 | Ca4 | 2034424 | CTTTT/CTTTTT | ACCAGATACCGCACCAAAAG | TTCTGCATACTTGCTCCACG | 60.0 | 352 | INTRON | Ca_07822 | D | | RNA-processing protein, HAT helix |
| CaPOPI_1794 | Ca4 | 2035282 | GTTTTTT/GTTTTTTT | CGCGCCAGAAGTATGTGATA | TGGGCAGCTTAGACTGGACT | 59.9 | 275 | INTRON | Ca_07822 | D | | RNA-processing protein, HAT helix |
| CaPOPI_1795 | Ca4 | 2035697 | ATTT/AT | AGTCCAGTCTAAGCTGCCCA | TGGAATCATGCCATGCTAGA | 60.0 | 436 | DRR | Ca_07823 | R | MYB | Reverse transcriptase, RNA-dependent DNA polymerase |

| INDEL marker IDs | Chromosomes/ unanchored scaffolds | Physical positions (bp) | InDels (*Kabuli* reference genome-CDC Frontier/PI) | Forward primers (5'-3') | Reverse primers (5'-3') | Anneling temperature (0C) | Expected amplified product size (bp) | Structural annotation | | Functional annotation | | |
|---|---|---|---|---|---|---|---|---|---|---|---|---|
| | | | | | | | | Sequence components of *kabuli* genome | *Kabuli* gene accession IDs | NCBI-KOG | TFs | NCBI-nr database |
| CaPOPI_1796 | Ca4 | 2035798 | A/AGTGT | AGTCCAGTCTAAGCTGCCCA | TGGAATCATGCCATGCTAGA | 60.0 | 436 | DRR | Ca_07823 | R | MYB | Reverse transcriptase, RNA-dependent DNA polymerase |
| CaPOPI_1797 | Ca4 | 2139392 | TCCCCC/TCCCCCC | TATCTGGAACGACGACATGC | TCGTTCGCCATTAAAGAGGT | 59.7 | 558 | INTERGENIC | | | | |
| CaPOPI_1798 | Ca4 | 2445623 | AATATATATATATATATATATATATATATATATATA/AATATATATATATATATATATATATATATATATA | GGAGGTCACAACCACTCCAA | CGGACTTTCGACTTTCTTGC | 61.0 | 654 | INTERGENIC | | | | |
| CaPOPI_1799 | Ca4 | 2589224 | CAGTTGCAAGTTGCAAG/CAGTTGCAAGTTGCAAGTTGCAAG | AAGATCGATCAGTTCCACCG | ATTGGGAACCAACTGACGAG | 60.1 | 396 | INTERGENIC | | | | |
| CaPOPI_1800 | Ca4 | 2688293 | AT/A | AGCATGCTTCCACAAAGGAT | TTGGAGGTTGTGTTGTTGGA | 59.7 | 532 | DRR | Ca_12072 | | | Domain of unknown function DUF296 |
| CaPOPI_1801 | Ca4 | 2729716 | A/AGACATCAT | AGGGCTTGTAATGCCCTTTT | TTGGCTTCACAAGATACCCC | 60.0 | 654 | INTRON | Ca_12075 | Z | | Actin-like |
| CaPOPI_1802 | Ca4 | 2755654 | CAAAAAA/CAAAAAAA | AATTCGTTGCACAATTTGGTT | GCATGCTAAATGCCATCGTA | 59.4 | 801 | INTERGENIC | | | | |
| CaPOPI_1803 | Ca4 | 2819881 | TAAAAAA/TAAAAAAA | TTGTTACCTCAATCGTGGGG | TTCGCCCCTACAACTACCAC | 60.7 | 674 | INTERGENIC | | | | |
| CaPOPI_1804 | Ca4 | 2891639 | A/AG | TGTTGTGGTGCCACCTTTTA | TAATACACACGGCCAAGCAA | 60.0 | 823 | INTERGENIC | | | | |
| CaPOPI_1805 | Ca4 | 2894996 | GAAAAAAAAA/GAAAAAAAAAA | AAAAGAAACGGAGGAAGTGTCA | TAAATACGGTGATCGGCCTC | 60.1 | 127 | DRR | Ca_12086 | P | ERF | General substrate transporter |
| CaPOPI_1806 | Ca4 | 2897791 | ATATTTATTTATTTATTTATTTATTT/ATATTTATTTATTTATTTATTTATTTATTT | AAAAAGACATGCTTTCAATTTGG | AAGCCTTGTGGAGCAGTTGT | 59.5 | 948 | INTERGENIC | | | | |
| CaPOPI_1807 | Ca4 | 2929266 | CATATATATATATATATA/CATATATATATATATATATATA | AAATGGCAATGAAATCCCTG | TCACTCTATCAAATGTTCAAGCAA | 59.8 | 628 | INTERGENIC | | | | |
| CaPOPI_1808 | Ca4 | 3017713 | ATTATTTTAAATTTATTTTAAATTTATTTTAAATTTATTTTAAATTTATTTTAAATT/ATTATTTTAAATTTATTTTAAATTTATTTTAAATTTATTTTAAATTTATTTTAAATTTA | GACAAATTAGTGGGGCAACC | TTGTATTGTTGGTGAGGCCA | 59.3 | 902 | INTERGENIC | | | | |
| CaPOPI_1809 | Ca4 | 3069954 | TTTTATTTATTTATTTATTTAT/TTTTATTTATTTATTTATTTATTTAT | AAACCAAAACTGGCGAAAAA | TGAGAAAACATATTGGGAAGGC | 59.6 | 686 | DRR | Ca_12105 | | | |
| CaPOPI_1810 | Ca4 | 3081231 | TAAAAAAAAAAA/TAAAAAAAAAA | TCGCCCCTTAATTGTTTGTC | TGTCTAGGTGGCCCTGTGAT | 59.9 | 571 | INTERGENIC | | | | |

| **INDEL marker IDs** | **Chromosomes/ unanchored scaffolds** | **Physical positions (bp)** | **InDels (*Kabuli* reference genome-CDC Frontier/PI)** | **Forward primers (5'-3')** | **Reverse primers (5'-3')** | **Anneling temperature (0C)** | **Expected amplified product size (bp)** | **Structural annotation** | | **Functional annotation** | | |
|---|---|---|---|---|---|---|---|---|---|---|---|---|
| | | | | | | | | **Sequence components of *kabuli* genome** | ***Kabuli* gene accession IDs** | **NCBI-KOG** | **TFs** | **NCBI-nr database** |
| CaPOPI_1811 | Ca4 | 3832898 | ATTT/ATTTTTT | CGGAGGGTACGAACCTGTAA | ACTGTTGTGGAAATCTGGGC | 60.0 | 484 | INTERGENIC | | | | |
| CaPOPI_1812 | Ca4 | 3966806 | GAAAAAAAAAAA/GAAAAAAAAAA | CCGTCGCTAAACACACTCAA | TGCACCAACAATCCTTGCTA | 59.9 | 814 | INTERGENIC | | | | |
| CaPOPI_1813 | Ca4 | 3989437 | AATATATATATATATATATATATAT/AATATATATATATATATATATATATAT | TCCCGTTCCAAAACTTTTCA | TGATAGCACAAAATCGTGGA | 60.5 | 594 | DRR | Ca_03854 | G | | Major intrinsic protein |
| CaPOPI_1814 | Ca4 | 4030815 | CAAAAA/CAAAA | TGGATCCACTAATAACATGGGA | CCTTTAAGGGGATTGAAGGA | 59.2 | 516 | DRR | Ca_03849 | | Dof | Zinc finger, Dof-type |
| CaPOPI_1815 | Ca4 | 4031880 | GTTTTTTT/GTTTTTTTTT | GGGTTTGTTTTATTTGATTTTGA | GATCTGGAAAGGAGAAGGGC | 57.2 | 238 | INTERGENIC | | | | |
| CaPOPI_1816 | Ca4 | 4039327 | TACTC/T | TTTTGGTTTGGTAATTGTGGG | TGATGGAGAAATCCTCACCC | 59.6 | 456 | INTERGENIC | | | | |
| CaPOPI_1817 | Ca4 | 4044991 | A/AATTGGATC | GGGTGCATTTGTTGGAACTT | TTGGTACGTGTAGAACATCGTG | 59.8 | 676 | INTERGENIC | | | | |
| CaPOPI_1818 | Ca4 | 4045724 | ATTTTTTTTT/ATTTTTTT | TGTTGGGTTTGTTTCCTCCT | GCTTGATAATTTAAGGGCACCA | 59.4 | 515 | INTERGENIC | | | | |
| CaPOPI_1819 | Ca4 | 4086337 | TAAAAAAAA/TAAAAAAAAAAA | GGGGCGTGTCAAAGTTAAAA | ACCATCCGTATAAAGCACGC | 60.0 | 453 | INTERGENIC | | | | |
| CaPOPI_1820 | Ca4 | 4104993 | TTATAT/TTAT | GAAGTGGGAATGGGCTGTTA | AAGTTAGGGGGACACAACCC | 59.9 | 677 | DRR | Ca_03843 | T | BBR-BPC | Protein kinase, catalytic domain |
| CaPOPI_1821 | Ca4 | 4116752 | TA/TACA | ACATGTTGGAAATCCGGGTA | TCGGCTACTCAAACAAAGGTG | 60.1 | 516 | INTERGENIC | | | | |
| CaPOPI_1822 | Ca4 | 4121415 | ATT/AT | CTCATATGAACGGCCCATCT | TGTTTGTACGGACTCGACCA | 59.9 | 403 | INTERGENIC | | | | |
| CaPOPI_1823 | Ca4 | 4196538 | ATTTT/ATTT | ATTGGCCGGAAATTGTACTG | TAACCCAAGGCCATATCCTG | 59.8 | 841 | DRR | Ca_03838 | R | | Alpha/beta hydrolase fold-1 |
| CaPOPI_1824 | Ca4 | 4302695 | ATATTATTATTATTATTATTATTATTATTATTATTATTATTATTATTAATATTATTATTATTATTA/ATATTATTATTATTATTATTATTATTATTATTATTATTATTATTATTATT | GCATCTCCAAAATGCGATCT | TGAGTCCAAAACAAATGGTGTC | 60.2 | 730 | INTERGENIC | | | | |
| CaPOPI_1825 | Ca4 | 4471743 | TAAGAAAAATACAAG/TAAGAAAAATACAAGAAAAATACAAG | ACATTTGATCACGGTCCTCC | AGGCATTGATTCTTCGTTCG | 59.8 | 615 | INTRON | Ca_03810 | GOT | | Tetratricopeptide TPR-1 |

| INDEL marker IDs | Chromosomes/ unanchored scaffolds | Physical positions (bp) | InDels (*Kabuli* reference genome-CDC Frontier/PI) | Forward primers (5'-3') | Reverse primers (5'-3') | Anneling temperature (0C) | Expected amplified product size (bp) | Structural annotation | | Functional annotation | | |
|---|---|---|---|---|---|---|---|---|---|---|---|---|
| | | | | | | | | Sequence components of *kabuli* genome | *Kabuli* gene accession IDs | NCBI-KOG | TFs | NCBI-nr database |
| CaPOPI_1826 | Ca4 | 4478589 | TAAGTTAAAAGTTAAAAGTTAAAAGTTAAAA/TAAGTTAAAAGTTAAAAGTTAAAA | TGTAGACGCGCAGAGAGAGA | AATCATTTGCCTGGTGCTTT | 60.0 | 593 | INTERGENIC | | | | |
| CaPOPI_1827 | Ca4 | 4480054 | TT/TTATTTGAAT | TCCTCCATTTTTCCACGCTA | TTTCATCCTCCATGTGACCA | 60.6 | 409 | INTERGENIC | | | | |
| CaPOPI_1828 | Ca4 | 4480380 | TC/TCC | TGGTCACATGGAGGATGAAA | TTTAATACCTCGTATTTCCTATCAAGA | 59.9 | 609 | DRR | Ca_03809 | | S1Fa-like | Protein kinase, catalytic domain |
| CaPOPI_1829 | Ca4 | 4481135 | TAA/TAAA | TGAAATCAAATTTTCTCAAGGC | AAACGACATTACGTTATTTCTTCATT | 58.4 | 120 | DRR | Ca_03809 | | S1Fa-like | Protein kinase, catalytic domain |
| CaPOPI_1830 | Ca4 | 4481516 | T/TTC | TGAAATCAAATTTTCTCAAGGC | GTTCCGCCTCTTCTACCTCC | 58.4 | 760 | DRR | Ca_03809 | | S1Fa-like | Protein kinase, catalytic domain |
| CaPOPI_1831 | Ca4 | 4481679 | TAAAAAAAA/TAAAAAAAAA | TTTTGCTTTTGATTACTTTCTTTTT | GTTCCGCCTCTTCTACCTCC | 57.2 | 314 | DRR | Ca_03809 | | S1Fa-like | Protein kinase, catalytic domain |
| CaPOPI_1832 | Ca4 | 4487412 | AT/ATTAATAAAGT | TCAGACTCTCCCGAACGATT | CGTACGACGTCTTTCGTCAA | 59.8 | 281 | DRR | Ca_03808 | | | DNA-binding SAP |
| CaPOPI_1833 | Ca4 | 4488536 | TAAA/T | ACAGCACATTTCTGGTGGTG | TCTGCTAAACAGGGGATTGG | 59.6 | 450 | DRR | Ca_03808 | | | DNA-binding SAP |
| CaPOPI_1834 | Ca4 | 4502063 | C/CG | GCTGGATCCGTCGTTACAAT | CCATCTGAGAAAGCTAGCCG | 60.0 | 441 | DRR | Ca_03807 | | FAR1 | Lipase, GDSL |
| CaPOPI_1835 | Ca4 | 4503851 | TAAAAAAAAA/TAAAAAAAA | TTCTCCCTCCGTCTCACATC | TGGAGGCAATGATTTTGTCA | 60.2 | 421 | INTRON | Ca_03807 | | FAR1 | Lipase, GDSL |
| CaPOPI_1836 | Ca4 | 4508477 | T/TA | GTCTCTTGGTTTACCGTGCC | ATGAGTGGTTTGCCACATGA | 59.6 | 452 | INTERGENIC | | | | |
| CaPOPI_1837 | Ca4 | 4511887 | ATTTTT/ATTTTTT | CGTTGTTGGTGAAACTGCAT | CCGGCGAGAATTTTTCAATA | 59.6 | 700 | INTERGENIC | | | | |
| CaPOPI_1838 | Ca4 | 4517245 | AAGA/AA | AAAATGCGTTTCACTCGGAC | CCGTTTTTCGTCACAATTCA | 60.1 | 550 | INTERGENIC | | | | |
| CaPOPI_1839 | Ca4 | 4527520 | TTATATATATATATATATATATATATATATATATAT/TTATATATATATATATATATATATATATAT | AAGGTATGCTGTTTCAAGGGAA | AATGTGCATCTGCCACCATA | 60.0 | 892 | INTERGENIC | | | | |
| CaPOPI_1840 | Ca4 | 4537624 | TAATAAAATAAAATAAAAT/TAATAAAATAAAATAAAATAAAAT | TGCATCGCCCATATCAATAA | TATTGGACCGACCCTGAAAA | 59.9 | 511 | INTERGENIC | | | | |

| **INDEL marker IDs** | **Chromosomes/ unanchored scaffolds** | **Physical positions (bp)** | **InDels (*Kabuli* reference genome-CDC Frontier/PI)** | **Forward primers (5'-3')** | **Reverse primers (5'-3')** | **Anneling temperature (0C)** | **Expected amplified product size (bp)** | **Structural annotation** | | **Functional annotation** | | |
|---|---|---|---|---|---|---|---|---|---|---|---|---|
| | | | | | | | | **Sequence components of *kabuli* genome** | ***Kabuli* gene accession IDs** | **NCBI-KOG** | **TFs** | **NCBI-nr database** |
| CaPOPI_1841 | Ca4 | 4539713 | TTA/TTATA | TCGACCCAACCTATTTCCAC | GGTTAACCGTCGTAACACACTTT | 59.8 | 340 | INTERGENIC | | | | |
| CaPOPI_1842 | Ca4 | 4568058 | ATTTGATTTT/ATTTGATTTTTGATTTT | GTGGGGCATTACGATACCAC | GGGGAAGCCATATCACATCA | 60.1 | 636 | DRR | Ca_03802 | UZ | | Vps52/Sac2 |
| CaPOPI_1843 | Ca4 | 4574989 | AA/AAAGTA | TGCCACTAAATAGGGTTGGC | CGTTCTGAAAACGCGCTTAG | 60.0 | 460 | DRR | Ca_03801 | | | Domain of unknown function DUF296 |
| CaPOPI_1844 | Ca4 | 4576739 | GAAAAA/GAAAAAA | TCCATATGATTTGAACTCGATTTTT | GGTTGGTTTAACCGTTGGAA | 60.1 | 729 | INTERGENIC | | | | |
| CaPOPI_1845 | Ca4 | 4589864 | CTTT/CTT | TTTCGCTCAATGTGAAAAGC | AACGGAGGCAGTAATTGTCG | 59.0 | 322 | INTERGENIC | | | | |
| CaPOPI_1846 | Ca4 | 4784534 | ATTTTTTTTT/ATTTTTTTT | CCCTTCGGAAGACCACATAA | TTCATTTCTTTACCCCGTTCA | 59.9 | 657 | DRR | Ca_03778 | K | G2-like | SNF2-related |
| CaPOPI_1847 | Ca4 | 4826016 | AT/A | GGATTGATTGCAGTTCCAAAA | GGCAAAATGCCTTTAGACCA | 59.9 | 445 | DRR | Ca_03777 | O | FAR1 | Peptidase M41 |
| CaPOPI_1848 | Ca4 | 4840884 | AC/A | TTGGGCCTTTGTGGAGTAAG | TACCAAAGTGGTGATGGTGG | 60.1 | 496 | DRR | Ca_03775 | | | Transposase, Ptta/En/Spm, plant |
| CaPOPI_1849 | Ca4 | 4842046 | AAATAATAATAATAATAAT/AAATAATAATAAT | TGAGAAGAAGGGGCTTAGCA | CGAGGAGGTTATTGGCGTAA | 60.1 | 754 | INTERGENIC | | | | |
| CaPOPI_1850 | Ca4 | 4875484 | GC/G | AGCCTATGGCTTGGCCTATT | GCCTAAGCTTGACTTGTGGC | 60.1 | 687 | INTERGENIC | | | | |
| CaPOPI_1851 | Ca4 | 4891737 | AATTATTATTATTATTATT/AATTATTATTATTATTATTATT | TGCCTTGTATCGTTCCATCA | TTCCTGCTTTTTCTTTCCGA | 60.1 | 540 | INTRON | Ca_03769 | Z | NAC | IQ motif, EF-hand binding site |
| CaPOPI_1852 | Ca4 | 4897344 | ATTT/ATTTT | CTCTGCAGCCTTGATTGGTT | ATGGAGTATGGGGCTGTGAC | 60.4 | 278 | INTRON | Ca_03768 | K | | SSXT |
| CaPOPI_1853 | Ca4 | 4978107 | AAATAAATAA/AAA | GTCTAATCCCCCATTTTGGC | TCTTGTGGTCGTTGCTTGAG | 60.5 | 557 | INTRON | Ca_03758 | R | | Barwin |
| CaPOPI_1854 | Ca4 | 5081255 | GATTATTATTATTATTATTATTATTATTAT/GATTATTATTATTATTATTATTATTATTATTATTAT | CACCAGCAATAGCTTGCTCA | GAAAAACAGGGCTAGCAACG | 60.2 | 711 | CDS (large-effect mutations) | Ca_03748 | K | Trihelix | MYB-like |
| CaPOPI_1855 | Ca4 | 5300098 | TAAAAAAAAAA/TAAAAAAAAA | GGCTGGAGAAAGAATATGCG | AAAAGGAGTTTGGAAGGGGA | 59.8 | 598 | INTERGENIC | | | | |

| INDEL marker IDs | Chromosomes/ unanchored scaffolds | Physical positions (bp) | InDels (*Kabuli* reference genome-CDC Frontier/PI) | Forward primers (5'-3') | Reverse primers (5'-3') | Anneling temperature (0C) | Expected amplified product size (bp) | Structural annotation | | Functional annotation | | |
|---|---|---|---|---|---|---|---|---|---|---|---|---|
| | | | | | | | | Sequence components of *kabuli* genome | *Kabuli* gene accession IDs | NCBI-KOG | TFs | NCBI-nr database |
| CaPOPI_1856 | Ca4 | 5525661 | GAAAAAAAAAA/GAAAAAAAAA | TATTTAGCCAAAAGGGCAGC | TCCATGTGAATATGATGAACCAA | 59.3 | 320 | INTERGENIC | | | | |
| CaPOPI_1857 | Ca4 | 5570234 | CTTTTTTTTTT/CTTTTTTTTTTT | TGATGCATAAGTACAGGTGGG | AAGTTTTTGGCTTTAAATAGTGTCA | 58.5 | 581 | INTERGENIC | | | | |
| CaPOPI_1858 | Ca4 | 5655244 | TATA/TATAAATA | TGAACCCCCACCAAAATAAA | GCACCGCTCAAAATGAAATA | 60.0 | 928 | DRR | Ca_03683 | R | | F-box domain, cyclin-like |
| CaPOPI_1859 | Ca4 | 5707384 | AATATATATATATATATATAT/AATATATATATATATATATAT | AAGCTAAGCACCGGTAACGA | TTCACGTCTTGAAATTTTCCG | 59.9 | 711 | DRR | Ca_03679 | DKL | MYB_related | Transcription regulator cyclin |
| CaPOPI_1860 | Ca4 | 5961971 | T/TGTG | CCTCTAATATCGTCGCTCCAA | ATAATCGGTGCGGATTTCCT | 59.3 | 668 | INTERGENIC | | | | |
| CaPOPI_1861 | Ca4 | 6145049 | ATTTTTTTTTTTT/ATTTTTTTTTTTTT | ATCCCCTCAGAATCGGCTAT | TTCGTCTATGCATTTGTTCCTG | 59.9 | 565 | INTERGENIC | | | | |
| CaPOPI_1862 | Ca4 | 6186297 | GTTTTTTTTT/GTTTTTTTTTT | TGTGTTTTCTCAATTTGACGATG | GCCATTTGAACCAAATTAAGAG | 60.0 | 751 | INTERGENIC | | | | |
| CaPOPI_1863 | Ca4 | 6271908 | GTATATATATATATATATAT/GTATATATATATATATATATAT | AAAAATTACGGGATGCATAAAAA | TCCCCAAACAATGACAAAAA | 58.9 | 775 | INTERGENIC | | | | |
| CaPOPI_1864 | Ca4 | 6389441 | TT/TTCCTTATGTGGAAAT | GCCCCACCTTCAGTGTTAAA | ATGCTGGCAAATGATGTCAC | 60.0 | 728 | DRR | Ca_03602 | | ERF | X8 |
| CaPOPI_1865 | Ca4 | 6581955 | TAAAAAAAA/TAAAAAAA | GCACGTGTGTAATGGTGCTC | TCTTGCTCAACGAAAATCCC | 60.2 | 612 | INTERGENIC | | | | |
| CaPOPI_1866 | Ca4 | 6875620 | TGG/TG | GACAGTTTTGTGCAATCCGA | AACCTATACCCGCACAATGC | 59.7 | 859 | INTERGENIC | | | | |
| CaPOPI_1867 | Ca4 | 6907181 | TTATATATATATATATAT/TTATATATATATATAT | TTTGAAATGGCGACTTCCTT | CCAAATGGCAACTTCCAACT | 59.7 | 569 | INTERGENIC | | | | |
| CaPOPI_1868 | Ca4 | 7089091 | GA/GAACAAAAGAGATTCACA | CTTTGCATACCCCTCTCGAC | TTGGGAGGATTGGTTTCAAG | 59.7 | 644 | INTERGENIC | | | | |
| CaPOPI_1869 | Ca4 | 7127718 | GAAAAAAAAAA/GAAAAAAAAA | AATTTGGACAATACCGCGAA | AGGGAAGGGGAAGAAGATCA | 60.3 | 580 | INTERGENIC | | | | |
| CaPOPI_1870 | Ca4 | 7388463 | CAAAAAAAAAAA/CAAAAAAAAAA | TTTTCTCTAATCCCCGACTCA | TGTTGTTGTCAAACACCTCAAA | 58.8 | 565 | INTERGENIC | | | | |

| INDEL marker IDs | Chromosomes/ unanchored scaffolds | Physical positions (bp) | InDels (*Kabuli* reference genome-CDC Frontier/PI) | Forward primers (5'-3') | Reverse primers (5'-3') | Anneling temperature (0C) | Expected amplified product size (bp) | Structural annotation | | Functional annotation | | |
|---|---|---|---|---|---|---|---|---|---|---|---|---|
| | | | | | | | | Sequence components of *kabuli* genome | *Kabuli* gene accession IDs | NCBI-KOG | TFs | NCBI-nr database |
| CaPOPI_1871 | Ca4 | 7415395 | TAA/TA | TTTCTGTCCTCACCCTCACC | TGTAGGTTTGGACGGTAGGC | 60.1 | 535 | INTRON | Ca_03496 | F | YABBY | CTP synthase, N-terminal |
| CaPOPI_1872 | Ca4 | 7620418 | GATATATATATAT/GATATATATATATAT | CGATTTTTGTGAAGTGCGTC | TCTCATCGCCGACAAAGTAA | 59.3 | 837 | INTERGENIC | | | | |
| CaPOPI_1873 | Ca4 | 7710147 | AATATTATTATATATTA/AATATTATTATATATTATTATATATTA | TCGTTACCCCTCTCGTCATC | ACAGAATGCCTCATTGGTGG | 60.1 | 927 | DRR | Ca_03470 | R | Nin-like | Pentatricopeptide repeat |
| CaPOPI_1874 | Ca4 | 8039801 | ATAAAATTAAAAAACTAAAATTAAAAAACTAAAA/ATAAAATTAAAAAACTAAAATTAAAAAACTAAAATTAAAAAACTAAAA | TTCGAAAATTGGATTGCCA | TTTGTGCGAGATAGGTACGAAA | 60.0 | 267 | INTERGENIC | | | | |
| CaPOPI_1875 | Ca4 | 8115754 | CATATATATATATATATATATATATA/CATATATATATATATATATATATATATA | GCTGCTAGGGATGCTGTTTC | GAATATTGGCCAAGGCTTCA | 60.0 | 208 | INTRON | Ca_03421 | | M-type | Plant peroxidase |
| CaPOPI_1876 | Ca4 | 8169426 | GTCGATC/GTC | GACATCCGAAATGGGCTAAA | ATTTCCAAGGTCACGGTTTG | 59.9 | 907 | INTERGENIC | | | | |
| CaPOPI_1877 | Ca4 | 8169626 | TAAATAAAAA/TAAATAAAAAATAAAAA | TATGGCACCTCCAGTTGAAA | TGTTAAATCCTCCAGACGGC | 59.1 | 858 | INTERGENIC | | | | |
| CaPOPI_1878 | Ca4 | 8186328 | CTATATATATATATA/CTATATATATATA | TGCTTTTCATTTGCAGAATCC | TTTCACCTCCTGGTAGACGG | 60.2 | 626 | DRR | Ca_08308 | T | | Signal transduction histidine kinase, phosphotransfer (Hpt) domain |
| CaPOPI_1879 | Ca4 | 8187730 | TCC/TC | CTAAGCCGAGCAGAAGCAAT | TCCCTTTAATGCAAAGGTGG | 59.8 | 499 | INTRON | Ca_08308 | T | | Signal transduction histidine kinase, phosphotransfer (Hpt) domain |
| CaPOPI_1880 | Ca4 | 8193266 | TAAAAAAAAA/TAAAAAAAAAA | GCATCTCTCCAAGATGTCTGC | TGGGAATTGGGAGCTATCAG | 60.0 | 620 | DRR | Ca_08309 | R | | WD40 repeat |
| CaPOPI_1881 | Ca4 | 8206232 | GTATA/GTATACTATA | CAAGGGGTTGAAGTTGCATT | TTGAAGCCACCCTCTGATCT | 60.0 | 763 | INTERGENIC | | | | |
| CaPOPI_1882 | Ca4 | 8209835 | CTTTTTTTT/CTTTTTTT | AGACCAAGAGAGAGAGGCCC | GGGTTTGGTTTGTGCGTAGT | 60.0 | 306 | INTERGENIC | | | | |
| CaPOPI_1883 | Ca4 | 8216534 | GTT/GTTT | CGAATCCGCTTCGTGTTAAT | CGACCCGCTTAATATGTGGT | 60.1 | 507 | INTERGENIC | | | | |
| CaPOPI_1884 | Ca4 | 8216710 | T/TG | CGAATCCGCTTCGTGTTAAT | CGACCCGCTTAATATGTGGT | 60.1 | 507 | INTERGENIC | | | | |
| CaPOPI_1885 | Ca4 | 8222692 | A/AC | TTCTGTCTTTGTTGTTGCGG | TGCTAAGGATTATGCAGGGG | 59.9 | 483 | INTRON | Ca_08312 | | | Endonuclease/exonuclease/phosphatase |

| INDEL marker IDs | Chromosomes/ unanchored scaffolds | Physical positions (bp) | InDels (*Kabuli* reference genome-CDC Frontier/PI) | Forward primers (5'-3') | Reverse primers (5'-3') | Anneling temperature (0C) | Expected amplified product size (bp) | Structural annotation | | Functional annotation | | |
|---|---|---|---|---|---|---|---|---|---|---|---|---|
| | | | | | | | | Sequence components of *kabuli* genome | *Kabuli* gene accession IDs | NCBI-KOG | TFs | NCBI-nr database |
| CaPOPI_1886 | Ca4 | 8223032 | GAAAAAAAA/GAAAAAAA | CAACCTTTGGTGTTGGCTTT | ACGCGATAATCCAATCGAAC | 60.0 | 252 | INTRON | Ca_08312 | | | Endonuclease/exonuclease/phosphatase |
| CaPOPI_1887 | Ca4 | 8226617 | TG/T | TATATGTGTTGCTGTGGGGG | GTACACCAAGGCGATTCGAT | 59.3 | 969 | INTERGENIC | | | | |
| CaPOPI_1888 | Ca4 | 8281328 | CA/CCTAA | TTGGTGTCATTCGCCTTTTT | CAATCGCAAGGATAATGGCT | 60.5 | 711 | INTERGENIC | | | | |
| CaPOPI_1889 | Ca4 | 8293335 | AT/AATT | CAATGGTTTTGCTTTTGGCT | TTCTCGAATGCGAAGAGTCA | 60.1 | 492 | INTERGENIC | | | | |
| CaPOPI_1890 | Ca4 | 8295749 | ATTTTTATT/ATTTTTATTATTTTTTATT | TCAACCCTCATTCAACCACA | CCTGCGACAATCTGAAGGAT | 59.9 | 840 | INTERGENIC | | | | |
| CaPOPI_1891 | Ca4 | 8305998 | GAAA/GAA | CATCATGCAGTCGCCTCTAA | ACCACATCTGCCATCAATCA | 60.0 | 510 | INTRON | Ca_08319 | S | | Oxoglutarate/iron-dependent oxygenase |
| CaPOPI_1892 | Ca4 | 8308551 | TAA/TAAA | TAACAGGTGAGGGCATGTCA | TATGCAAACCCAATTGACCA | 60.1 | 321 | INTERGENIC | | | | |
| CaPOPI_1893 | Ca4 | 8309234 | TCAACACAA/TCAACACAACACAA | GAATTTAAGAGGAAGGCGATGA | TTCACCCTAATTCGTAGGCG | 59.7 | 450 | INTERGENIC | | | | |
| CaPOPI_1894 | Ca4 | 8310925 | CAAAAA/CAAAAAA | CATGTGTGGGACTCTTTGGA | CTCAACACGGTTCTGCTCTG | 59.5 | 764 | INTERGENIC | | | | |
| CaPOPI_1895 | Ca4 | 8315933 | CTT/CTTATT | GGACCCGTGATGCTGTTTAT | TCTCGGTTGATGTCATTATGCT | 59.8 | 586 | INTERGENIC | | | | |
| CaPOPI_1896 | Ca4 | 8316304 | GTTTTTTT/GTTTTTTTT | GCATAATGACATCAACCGAGAG | AAAAGAAATGGTTGTTTCTCGTTC | 59.6 | 235 | INTERGENIC | | | | |
| CaPOPI_1897 | Ca4 | 8316722 | ACTCT/ACT | GAACGAGAAACAACCATTTCTTTT | CACGAGTGACACGCCATTTA | 59.9 | 583 | INTERGENIC | | | | |
| CaPOPI_1898 | Ca4 | 8325707 | TA/T | ACGGGGCTGTTACTTTACCC | TTCTCAATGGGTGCGTACAA | 60.2 | 739 | INTERGENIC | | | | |
| CaPOPI_1899 | Ca4 | 8333443 | GCTTCTTC/GCTTC | TCAAAGGTGATCATGGATGG | ACCCTTGCCAAAACACTACG | 59.3 | 593 | INTERGENIC | | | | |
| CaPOPI_1900 | Ca4 | 8342865 | T/TTAAG | GAGCTTCAACCACAAGCACA | GCTTTTTGAGCAACTCGTCC | 60.0 | 691 | CDS (FRAME SHIFT) | Ca_08321 | | | E3 Ubiquitin ligase |

| INDEL marker IDs | Chromosomes/ unanchored scaffolds | Physical positions (bp) | InDels (*Kabuli* reference genome-CDC Frontier/PI) | Forward primers (5'-3') | Reverse primers (5'-3') | Anneling temperature (0C) | Expected amplified product size (bp) | Structural annotation | | Functional annotation | | |
|---|---|---|---|---|---|---|---|---|---|---|---|---|
| | | | | | | | | Sequence components of *kabuli* genome | *Kabuli* gene accession IDs | NCBI-KOG | TFs | NCBI-nr database |
| CaPOPI_1901 | Ca4 | 8372871 | T/TG | TCTTTTTCTGCCCCACTAGC | CAGCTGAGTTGTGAGGTCCA | 59.5 | 150 | INTERGENIC | | | | |
| CaPOPI_1902 | Ca4 | 8373181 | TACA/TACACA | GGCAAAATGGACCTCACAAC | GGAGAGTCGGTCTTTGTGGA | 60.4 | 706 | INTERGENIC | | | | |
| CaPOPI_1903 | Ca4 | 8373461 | AAC/AACCAC | TGAGCATAAAGTGGCATTGG | GTCGGTCTTTGTGGAGAGGA | 59.7 | 398 | INTERGENIC | | | | |
| CaPOPI_1904 | Ca4 | 8377254 | ATTTTTTTTTTT/ATTTTTTTTTTTTT | CCACATATATCTGCCCCACC | TGACTGTGTGCACTGAAACAA | 60.0 | 563 | DRR | Ca_08327 | KL | | BSD |
| CaPOPI_1905 | Ca4 | 8377411 | AT/ATATATATATT | TAGTGTACGTGTTTGGGGCA | GGGTGAGACTGAAGCTGGTC | 60.0 | 949 | DRR | Ca_08327 | KL | | BSD |
| CaPOPI_1906 | Ca4 | 8377585 | ATTT/ATT | TTCGTCCATTTGAATCCTGA | CCTGCAAGCACTTACGACAA | 59.0 | 891 | DRR | Ca_08327 | KL | | BSD |
| CaPOPI_1907 | Ca4 | 8381452 | TAAAAAAA/TAAAAAAAA | CAATCCGTTAAGGACCTGGA | GATGATTCCCTTGCTGGAAA | 59.9 | 388 | INTRON | Ca_08327 | KL | | BSD |
| CaPOPI_1908 | Ca4 | 8384923 | AGGGGGG/AGGGGG | GCCGCAATAGTGTTTTTGGT | GCGTATCGGTTGGAATTGTT | 60.0 | 807 | INTRON | Ca_08327 | KL | | BSD |
| CaPOPI_1909 | Ca4 | 8396183 | T/TG | TTTACAACACATTGCGCACC | CGTTTATTGACGGAAGGCAT | 60.6 | 681 | DRR | Ca_08329 | O | B3 | Chaperonin Cpn60 |
| CaPOPI_1910 | Ca4 | 8400811 | GAATTACTAAT/GAATTACTAATTACTAAT | CTTAAGCATCAAAGCCCGAG | CCTCAATTGCCACCAAAACT | 60.0 | 469 | INTRON | Ca_08329 | O | B3 | Chaperonin Cpn60 |
| CaPOPI_1911 | Ca4 | 8413764 | TA/T | AATGGTGAACAAGGAGCAGG | TCAAATATTAACTCCAAGCGCA | 60.1 | 714 | INTERGENIC | | | | |
| CaPOPI_1912 | Ca4 | 8422191 | GAAA/GAA | AAGACGGATGCATTTTGCTC | GACACATGCCCAACTGAATG | 60.2 | 841 | INTERGENIC | | | | |
| CaPOPI_1913 | Ca4 | 8441953 | GTTTGTA/GTTTGTATTTTGTA | AGCAGGAGAGCTGAGATTGG | TACTGTGTATCGGGGCAACA | 59.7 | 731 | INTRON | Ca_08335 | R | | Polycomb protein, VEFS-Box |
| CaPOPI_1914 | Ca4 | 8467235 | CCATCATCATCATC/CCATCATCATC | GGCAAGCATTCAATTTCCAT | GGTTGGGATGTTATGTTCGG | 59.9 | 449 | INTERGENIC | | | | |
| CaPOPI_1915 | Ca4 | 8475153 | ATTT/ATT | TGGACCGTACATTTTCGTGA | GTGGTAGCAGAAGGAGCAGG | 60.0 | 829 | INTRON | Ca_08339 | G | | Phosphofructokinase domain |

| INDEL marker IDs | Chromosomes/ unanchored scaffolds | Physical positions (bp) | InDels (*Kabuli* reference genome-CDC Frontier/PI) | Forward primers (5'-3') | Reverse primers (5'-3') | Anneling temperature (0C) | Expected amplified product size (bp) | Structural annotation | | Functional annotation | | |
|---|---|---|---|---|---|---|---|---|---|---|---|---|
| | | | | | | | | Sequence components of *kabuli* genome | *Kabuli* gene accession IDs | NCBI-KOG | TFs | NCBI-nr database |
| CaPOPI_1916 | Ca4 | 8478290 | A/AT | TTTAGCTTGCCAAAACA GCA | CCTTTCTTCGTACGTCT CGC | 59.6 | 607 | INTRON | Ca_08339 | G | | Phosphofructokinase domain |
| CaPOPI_1917 | Ca4 | 8489479 | CACATACA/CACA | CCCAAATCACACACATG AGC | AGAGTTGTTTCATCTCC AAACG | 60.0 | 415 | INTERGENIC | | | | |
| CaPOPI_1918 | Ca4 | 8497257 | TAAACTGAAGAAACT/TAAACTGAAGAA ACTGAAGAAACT | AGCGTGAGAGGAACCTG TGT | GACTGGCAACAGCAATT TGA | 59.9 | 564 | INTRON | Ca_08341 | | CPP | WD40 repeat |
| CaPOPI_1919 | Ca4 | 8509176 | CTTTT/CTT | GCCTTCTGGACAAAACG AAG | TCTGCAGGTGAAGCAAC ATC | 59.9 | 578 | INTRON | Ca_08343 | YU | | |
| CaPOPI_1920 | Ca4 | 8514326 | CTTTT/CTTTTT | TGTCGTGCGGTAAGTTT GAC | GAAGCAAAGCTAGGGGT GTG | 59.8 | 449 | DRR | Ca_08343 | YU | | |
| CaPOPI_1921 | Ca4 | 8521020 | CATA/CATATATA | AATTCAACCAAATAAATT CCCA | CACGCAAAATGAAAGGT TGA | 57.4 | 308 | INTERGENIC | | | | |
| CaPOPI_1922 | Ca4 | 8522151 | TG/T | TGTGATTGTTAGGATGA TGACG | CCAAAACTGGGGAGAAA CAA | 58.5 | 288 | INTERGENIC | | | | |
| CaPOPI_1923 | Ca4 | 8522296 | GT/GTT | GCAGTTTTGGTCCCCCT ATT | GCAGTTTTGGTCCCCCT ATT | 60.2 | 328 | INTERGENIC | | | | |
| CaPOPI_1924 | Ca4 | 8612629 | T/TTA | TTTCTTTCTGCTGTCGG CTT | CATGCCATCCACTATGT TGC | 60.1 | 393 | INTERGENIC | | | | |
| CaPOPI_1925 | Ca4 | 8613689 | TTTTTCT/TT | TGCATTTGGTTGAAAAT GAAA | TGGTGGGCCTACGAGTA AAG | 59.0 | 374 | INTERGENIC | | | | |
| CaPOPI_1926 | Ca4 | 8621480 | GAAAAAAAA/GAAAAAAAAA | GTGCATGACCCCTTCTT GTT | TGTCAGGCAACAGAAAA CCA | 60.0 | 889 | INTERGENIC | | | | |
| CaPOPI_1927 | Ca4 | 8689007 | A/AT | AGGACCCACAACATCCA CAT | ACCAATCCATGCACGCT ACT | 60.1 | 505 | INTERGENIC | | | | |
| CaPOPI_1928 | Ca4 | 8698891 | AAGAGTAGTAGAGTAGT/AAGAGTAGT | AAGCTGGCCACTGTAAG CAT | TCACAATCTCGACAACC CAA | 59.9 | 246 | INTRON | Ca_08365 | G | | Glycoside hydrolase, family 13, N-terminal |
| CaPOPI_1929 | Ca4 | 8704241 | G/GC | TTTGTACCCAACAGGGC ATT | ATGCATGCGTTGACAAT GTT | 60.2 | 569 | DRR | Ca_08366 | J | NAC | Ribosomal protein L6 |
| CaPOPI_1930 | Ca4 | 8705159 | GTT/GTTT | TCAATGGTGGAGCAAAT TCA | GGTTCCCTTCCCCAATT TAG | 60.0 | 399 | DRR | Ca_08366 | J | NAC | Ribosomal protein L6 |

| INDEL marker IDs | Chromosomes/ unanchored scaffolds | Physical positions (bp) | InDels (*Kabuli* reference genome-CDC Frontier/PI) | Forward primers (5'-3') | Reverse primers (5'-3') | Anneling temperature (0C) | Expected amplified product size (bp) | Structural annotation | | Functional annotation | | |
|---|---|---|---|---|---|---|---|---|---|---|---|---|
| | | | | | | | | Sequence components of *kabuli* genome | *Kabuli* gene accession IDs | NCBI-KOG | TFs | NCBI-nr database |
| CaPOPI_1931 | Ca4 | 8709656 | CAAAAAAA/CAAAAAAAA | AATTTGTGGGTGGGCATAAA | TGCATTGAGAGAATCACCAACT | 60.1 | 701 | INTERGENIC | | | | |
| CaPOPI_1932 | Ca4 | 8709676 | TG/T | AATTTGTGGGTGGGCATAAA | TGCATTGAGAGAATCACCAACT | 60.1 | 701 | INTERGENIC | | | | |
| CaPOPI_1933 | Ca4 | 8720139 | AAAGT/AAAGTTTAAGT | GGCTGATGGAGAATTCCAAA | ACCAAAGACCAGATTGGCAC | 60.0 | 860 | INTRON | Ca_08368 | Q | | Multicopper oxidase, type 1 |
| CaPOPI_1934 | Ca4 | 8730605 | CGTG/CTG | TTCACACCAACACCCTCAAA | ATTTTGGAAGAACAATCCGC | 60.0 | 517 | INTERGENIC | | | | |
| CaPOPI_1935 | Ca4 | 8730686 | TAA/TA | TTCACACCAACACCCTCAAA | ATTTTGGAAGAACAATCCGC | 60.0 | 517 | INTERGENIC | | | | |
| CaPOPI_1936 | Ca4 | 8733054 | TGGG/TGG | AAAAATCACCCAAAATTAACATCT | CACTTTCCTTGCTCCCTCTG | 57.2 | 867 | INTERGENIC | | | | |
| CaPOPI_1937 | Ca4 | 8748201 | AG/A | CATTTCAATCATTCACCCCC | TCACAACATGAGAATGGGACA | 60.0 | 218 | INTERGENIC | | | | |
| CaPOPI_1938 | Ca4 | 8753314 | TGCTGGAACGCTGGAAC/TGCTGGAAC | TTCTTTCCCAGTCATTTCGG | CCAAATGGAGCTTCCACCTA | 60.0 | 841 | INTRON | Ca_08371 | | NAC | No apical meristem (NAM) protein |
| CaPOPI_1939 | Ca4 | 8758495 | A/AG | TCACCATTCGTGAGTTGGAA | TGCTCATGTCTTCTAAAGATCCA | 60.1 | 762 | INTERGENIC | | | | |
| CaPOPI_1940 | Ca4 | 8836055 | TATTTTTAATTTT/TATTTTTAATTTTTAATTTT | TGACCTGTAGGGCCGTAATC | TCACCAAATCGAAGCTCAAA | 60.0 | 508 | INTERGENIC | | | | |
| CaPOPI_1941 | Ca4 | 8836902 | T/TG | AGCTTCGATTTGGTGAGGTG | TTGAGTTTCAATATGGTGATGTGA | 60.3 | 854 | INTERGENIC | | | | |
| CaPOPI_1942 | Ca4 | 8858467 | TTACTAA/TTACTAATATTATAATATACTAA | TTGGTTTCCAAGTTCCCATC | TTCAACTTCGAGCAAAAGGG | 59.8 | 602 | INTRON | Ca_08382 | | MIKC | |
| CaPOPI_1943 | Ca4 | 8861999 | CAAAAAAAA/CAAAAAAAAAAA | CGAGGGAGCATTATGCAACT | TCCTTCGCCTCTCAAGAAAA | 60.2 | 432 | DRR | Ca_08383 | C | | Globin, subset |
| CaPOPI_1944 | Ca4 | 8867611 | AT/A | AGCTCCCATACCAGGACCTT | GTAGCCACCATTTTCCTCCA | 60.0 | 374 | INTERGENIC | | | | |
| CaPOPI_1945 | Ca4 | 8890826 | CAGGTAG/CAG | TTTGAGGAGTGTGTGGTGGA | ACCCAACTCGCACTTTGAAT | 60.1 | 360 | INTERGENIC | | | | |

| INDEL marker IDs | Chromosomes/ unanchored scaffolds | Physical positions (bp) | InDels (*Kabuli* reference genome-CDC Frontier/PI) | Forward primers (5'-3') | Reverse primers (5'-3') | Anneling temperature (0C) | Expected amplified product size (bp) | Structural annotation | | Functional annotation | | |
|---|---|---|---|---|---|---|---|---|---|---|---|---|
| | | | | | | | | Sequence components of *kabuli* genome | *Kabuli* gene accession IDs | NCBI-KOG | TFs | NCBI-nr database |
| CaPOPI_1946 | Ca4 | 8896251 | CAAAAA/CAAAA | GTCGCTAAACTGCAAAAGGC | TTTGTCCGGATGAGTTATCAAA | 60.0 | 477 | INTERGENIC | | | | |
| CaPOPI_1947 | Ca4 | 8903771 | GAATTAAT/GAAT | GGATCTCATTGGTTCTATGGG | CGAGTGTGGTGGGTTTTCTT | 58.3 | 490 | INTERGENIC | | | | |
| CaPOPI_1948 | Ca4 | 8903859 | CATAT/CAT | GGATCTCATTGGTTCTATGGG | CGAGTGTGGTGGGTTTTCTT | 58.3 | 490 | INTERGENIC | | | | |
| CaPOPI_1949 | Ca4 | 9030140 | ATTTTTTTTTTT/ATTTTTTTTTTTT | TGGTGTTGGAGTCATGCTTT | AAATTTGCAATGACGGCTTC | 59.1 | 656 | INTERGENIC | | | | |
| CaPOPI_1950 | Ca4 | 9066423 | ACC/AC | GCAACCCGTAGGTGTAGGAA | TGTGCGTTGGTAGTCAATGTG | 60.0 | 546 | INTERGENIC | | | | |
| CaPOPI_1951 | Ca4 | 9079145 | ATTTTTTTTTTT/ATTTTTTTTTT | AGTTGGAAGTCGCCAAAGAA | ATGGTGCAGTTCTTGCCATA | 59.9 | 629 | INTERGENIC | | | | |
| CaPOPI_1952 | Ca4 | 9371255 | GAAAAAAAA/GAAAAAAAAA | GGAGCATATGCCTCGTTGTT | TGGAAATTCAACTTTTTGGACA | 60.1 | 549 | INTERGENIC | | | | |
| CaPOPI_1953 | Ca4 | 9388140 | C/CTTCAT | TCGTGTTGCGCCTAACAAT | AGTTTTTAATCCGTGCGTCG | 60.3 | 976 | INTERGENIC | | | | |
| CaPOPI_1954 | Ca4 | 9460620 | ATTTTTTTTT/ATTTTTTTTTT | TTGGTGGAATAAATGCCAAA | TTGCGTGTTTTAAGAATTCGG | 58.9 | 283 | INTERGENIC | | | | |
| CaPOPI_1955 | Ca4 | 9569592 | CTT/C | CAAATGTCGAATTTAAGTATTGCG | TTGTCCTTCTCCCATTGTCC | 59.9 | 355 | INTERGENIC | | | | |
| CaPOPI_1956 | Ca4 | 9630289 | ATTAGTTAGTT/ATTAGTTAGTTAGTT | CCTCTTCCAAACAAATCTTCG | CCAGCTCATCAAAGCCATTT | 58.8 | 550 | INTERGENIC | | | | |
| CaPOPI_1957 | Ca4 | 9632653 | TG/T | TGGATTGTCGTGATCTCAAA | CACACCCGTACTCAGCATTG | 57.6 | 630 | INTERGENIC | | | | |
| CaPOPI_1958 | Ca4 | 9653112 | TAAAAAAAAAA/TAAAAAAAAAAA | GGGTTGTACGATGACAGCAA | AAATCGTCGCAAATCAGTCC | 59.6 | 208 | INTERGENIC | | | | |
| CaPOPI_1959 | Ca4 | 9658674 | TAAAAAAA/TAAAA | TGCAATTTGATACCGAGCCT | TCCCCTTCTTTGAGGGATTT | 60.6 | 914 | INTERGENIC | | | | |
| CaPOPI_1960 | Ca4 | 9732872 | A/AC | CGGTGGTTGATAAAGTCGTG | GAATTCAAATGGGGAAATGC | 59.0 | 416 | INTERGENIC | | | | |

| INDEL marker IDs | Chromosomes/ unanchored scaffolds | Physical positions (bp) | InDels (*Kabuli* reference genome-CDC Frontier/PI) | Forward primers (5'-3') | Reverse primers (5'-3') | Anneling temperature (0C) | Expected amplified product size (bp) | Structural annotation | | Functional annotation | | |
|---|---|---|---|---|---|---|---|---|---|---|---|---|
| | | | | | | | | Sequence components of *kabuli* genome | *Kabuli* gene accession IDs | NCBI-KOG | TFs | NCBI-nr database |
| CaPOPI_1961 | Ca4 | 9764739 | GTTTTTTT/GTTTTTTTT | TCAGGCGGGTTTTATAGACA | CTCTGCTCACAACCCAACAA | 58.2 | 701 | INTERGENIC | | | | |
| CaPOPI_1962 | Ca4 | 9847676 | TAAAA/TAAA | GAGTTGCAGGGGATGTAGGA | CACAAAGAAAGGCCTGGAAG | 60.1 | 240 | INTRON | Ca_08462 | Z | | VHS |
| CaPOPI_1963 | Ca4 | 9857350 | TGTGCGAGTGCGAGTGCGAGT/TGTGCGAGTGCGAGT | TGAGACGTTTTTCACACCCA | CTGCAACAATGAATCTTGAAGT | 60.1 | 469 | INTERGENIC | | | | |
| CaPOPI_1964 | Ca4 | 9923455 | AATATATATATATATATATA/AATATATATATATATA | TGTGAACGTCGTCGTATGGT | CCGATAACAAAAGCTCTCGC | 60.0 | 892 | INTERGENIC | | | | |
| CaPOPI_1965 | Ca4 | 10033685 | TTATATATATATATATATAT/TTATATATATATATATATAT | CTAGTTCGTAACCCGTGCGT | AGGCATGGTGTTGGAGTCAT | 60.2 | 248 | INTERGENIC | | | | |
| CaPOPI_1966 | Ca4 | 10057169 | AAAATTCAAATTCAAATTCAAATTCAAA/AAAATTCAAATTCAAATTCAAA | AACGTGCATCGACACTTTGA | AACGGAAGAATACGCCCTTTA | 60.3 | 542 | INTERGENIC | | | | |
| CaPOPI_1967 | Ca4 | 10173137 | ATTTTTTTT/ATTTTTTTTT | CCCGTTATTTTTGACGAAACA | TGTGTTGAGTGGTGGCATTT | 59.9 | 791 | INTERGENIC | | | | |
| CaPOPI_1968 | Ca4 | 10233239 | GACAATTATTTGGTACAATTAT/GACAATTATTTGGTACAATTATTTGGTACAATTAT | TGTCGGTTGTTGCTTGAAGA | ATACAATCATTTTCCCGCCA | 60.4 | 403 | INTERGENIC | | | | |
| CaPOPI_1969 | Ca4 | 10460957 | CATATATATATATATATATATATAT/CATATATATATATATATATATATATATATAT | TTAATTCCAATGGCGGGTAG | TCACATTCAGCTTTTGGTCG | 59.8 | 428 | INTERGENIC | | | | |
| CaPOPI_1970 | Ca4 | 10624424 | TTAATAATAATAATAATAATA/TTAATAATAATAATAATA | GGATGTCCCAAAGGGTTTTT | GGATGATTCTGGAGCGAGAC | 60.0 | 303 | INTERGENIC | | | | |
| CaPOPI_1971 | Ca4 | 10657750 | T/TCTAACACTCTTTCC | ATACAAAAACATTTGGGCCG | TTCACATGAAGTGGCGAGAC | 59.7 | 417 | DRR | Ca_04301 | | | Tetratricopeptide TPR-1 |
| CaPOPI_1972 | Ca4 | 10939792 | G/GAGTGTTGGTC | GCAGCAAAAAGGGATAACCA | AAGAAACCTCGTGTGCCATC | 60.1 | 231 | DRR | Ca_04328 | GMW | NAC | Exostosin-like |
| CaPOPI_1973 | Ca4 | 11209323 | TAAAAAAA/TAAAAAA | TTAGTGTTGAATTGACGGCG | CAATGCCTAGAATCACGGGT | 59.7 | 960 | INTERGENIC | | | | |
| CaPOPI_1974 | Ca4 | 11220000 | AAATAATAATAA/AAATAATAATAATAA | ATTTCTTCGTGTACCGCACC | CTGATAGACCGACACACACACA | 60.0 | 465 | INTERGENIC | | | | |
| CaPOPI_1975 | Ca4 | 11285447 | C/CAA | TCCACATGCACCTGTGAACT | AGTAGTGCCTCAAACCACGG | 60.2 | 382 | DRR | Ca_04361 | GE | GATA | Domain of unknown function DUF250 |

| INDEL marker IDs | Chromosomes/ unanchored scaffolds | Physical positions (bp) | InDels (*Kabuli* reference genome-CDC Frontier/PI) | Forward primers (5'-3') | Reverse primers (5'-3') | Anneling temperature (0C) | Expected amplified product size (bp) | Structural annotation | | Functional annotation | | |
|---|---|---|---|---|---|---|---|---|---|---|---|---|
| | | | | | | | | Sequence components of *kabuli* genome | *Kabuli* gene accession IDs | NCBI-KOG | TFs | NCBI-nr database |
| CaPOPI_1976 | Ca4 | 11340703 | CG/C | TCATCACCGTCCAATTTCAA | CTCCTTGCCCAATTTGTTGT | 59.9 | 333 | INTERGENIC | | | | |
| CaPOPI_1977 | Ca4 | 11435815 | AAC/A | CGGAGGTTTCAGTTCAGCTC | GTGAATTGGTCGAGCGGTAT | 60.0 | 906 | DRR | Ca_04378 | S | | |
| CaPOPI_1978 | Ca4 | 11506583 | CTTA/CTTAATTA | CTCGACATCATGGTTCATGG | CGGATCTATGCATGGGATAA | 59.9 | 507 | INTERGENIC | | | | |
| CaPOPI_1979 | Ca4 | 11551467 | TTTTTTTTAATTTTTTTA/TTTTTTTTA | ATCCAAAAGCCCCCAATAAC | TCACTGTTGCATCTCCAAGC | 60.0 | 844 | INTERGENIC | | | | |
| CaPOPI_1980 | Ca4 | 11567821 | CA/CACGAATA | TTTCTTTTGCCCTCATTTCAA | TTCCACCTGATCATCATTTCA | 59.7 | 463 | INTERGENIC | | | | |
| CaPOPI_1981 | Ca4 | 11866901 | TATAATAATAATAATAA/TATAATAATAA | GAATAGACCAGGCCAGACCA | CTATTAGGCCTGACAAGGCG | 60.1 | 493 | INTERGENIC | | | | |
| CaPOPI_1982 | Ca4 | 12027795 | AAAAAAGAAATAAAAAGAAATAAA/AAAAAAGAAATAAA | GATGTCGTCATGGCTGTTTG | TCATTGCGTTCTTTAGTGCG | 60.1 | 510 | INTERGENIC | | | | |
| CaPOPI_1983 | Ca4 | 12313260 | G/GA | ATATTTTCGATGTGCAGGGC | TGAAGGAGGCTTCTAGGACG | 59.9 | 545 | DRR | Ca_04455 | J | | Ribosomal protein L36e |
| CaPOPI_1984 | Ca4 | 12334767 | C/CTATT | GTCCTCCAACTGACCCAAAA | AGGTTAGGTCGTCCGTCAAG | 59.9 | 377 | INTERGENIC | | | | |
| CaPOPI_1985 | Ca4 | 12490666 | TGTAGTAGTAGTAGTAGTAGTAGTAGTA/TGTAGTAGTAGTAGTAGTAGTAGTAGTAGTAGTAGTAGTAGTAGTA | GGAATATGGCAGCAGACGAT | GCCTGCATATGGCTAAGAGC | 60.1 | 734 | DRR | Ca_04472 | R | NAC | Pentatricopeptide repeat |
| CaPOPI_1986 | Ca4 | 12504806 | CAAA/CAAAAA | TGGCACCAAAGATACCATCA | TGCAACCGCAATATTTACCA | 59.9 | 328 | INTRON | Ca_04474 | | MIKC | |
| CaPOPI_1987 | Ca4 | 12505639 | GTCTTAAA/GTCTTAAATATCTTAAA | GCAAGGACAAGGGATGTAGG | TCAACCGCAATTGAGATTTTC | 59.6 | 677 | INTERGENIC | | | | |
| CaPOPI_1988 | Ca4 | 12506017 | ATTTTTTT/ATTTTTTTT | TCAATTGCGGTTGAATTATCA | AAGCTGCTCTCAACACCCAT | 59.0 | 485 | INTERGENIC | | | | |
| CaPOPI_1989 | Ca4 | 12518595 | ATTTTTTTT/ATTTTTTTTT | GGAGCCTTACACATGTGGCT | TCATTTGTGAGATTGTCACGTT | 60.1 | 364 | INTERGENIC | | | | |
| CaPOPI_1990 | Ca4 | 12528293 | AT/ATT | TTCCTTCGACCAACAAAACC | CATGGGACCCTAGCGTTAAA | 59.9 | 573 | DRR | Ca_04475 | C | bZIP | Cytochrome b561, eukaryote |

| INDEL marker IDs | Chromosomes/ unanchored scaffolds | Physical positions (bp) | InDels (*Kabuli* reference genome-CDC Frontier/PI) | Forward primers (5'-3') | Reverse primers (5'-3') | Anneling temperature (0C) | Expected amplified product size (bp) | Structural annotation | | Functional annotation | | |
|---|---|---|---|---|---|---|---|---|---|---|---|---|
| | | | | | | | | Sequence components of *kabuli* genome | *Kabuli* gene accession IDs | NCBI-KOG | TFs | NCBI-nr database |
| CaPOPI_1991 | Ca4 | 12529895 | TAA/TAAA | AACGTGTTCAAACTCGGAGC | ACAACACCTTATTCTGCGCC | 60.3 | 630 | INTERGENIC | | | | |
| CaPOPI_1992 | Ca4 | 12529954 | CTTTT/CTTTTT | AACGTGTTCAAACTCGGAGC | AGCTTAGCTGGGTGGGTTTT | 60.3 | 707 | INTERGENIC | | | | |
| CaPOPI_1993 | Ca4 | 12529978 | T/TC | AACGTGTTCAAACTCGGAGC | AGCTTAGCTGGGTGGGTTTT | 60.3 | 707 | INTERGENIC | | | | |
| CaPOPI_1994 | Ca4 | 12532996 | C/CA | ATGAATTTGATGAGCAGGGC | GTTCCAGTTGCCCTGTGTTT | 60.0 | 349 | DRR | Ca_04477 | K | MYB | SANT domain, DNA binding |
| CaPOPI_1995 | Ca4 | 12542560 | TAAAAAAA/TAAAAAAAA | TGCCAAATTGTGTTTGTCGT | TATGACGGGAATGCATTTGA | 60.0 | 631 | INTERGENIC | | | | |
| CaPOPI_1996 | Ca4 | 12566654 | AG/A | TCAGTCTTTCCATATTCTCTCCG | AAAACCCAGGAGGTCAGCTT | 59.7 | 619 | INTERGENIC | | | | |
| CaPOPI_1997 | Ca4 | 12567928 | AATATATATAT/AATATATAT | ATTCGGCAATTCTACCCCTT | GAGGTAGAGCTCGACGATGG | 59.8 | 446 | INTERGENIC | | | | |
| CaPOPI_1998 | Ca4 | 12571013 | GTATATA/GTATA | GGCTCACATCAGGAATGGAT | GGCTCTTGGTCATTGGATGT | 59.9 | 968 | DRR | Ca_04479 | E | | Homoserine kinase |
| CaPOPI_1999 | Ca4 | 12572622 | TAAAAAAAAA/TAAAAAAAAAA | TGGAAGAGCATGCAAAACAA | TGAGATCTTGAGGCATGGTG | 60.4 | 644 | INTERGENIC | | | | |
| CaPOPI_2000 | Ca4 | 12578358 | TAT/TATACCAGCAT | AGGCTTCTTAGGCATTGGGT | ATTCTTGTAATCCCGCGTTG | 60.1 | 346 | INTRON | Ca_04480 | L | | DNA mismatch repair protein MutS, C-terminal domain |
| CaPOPI_2001 | Ca4 | 12581069 | TAA/TA | TTTTAGTTGCCGCCTTTTTG | CTTCAGGAGGGTGATACCGA | 60.2 | 468 | INTRON | Ca_04480 | L | | DNA mismatch repair protein MutS, C-terminal domain |
| CaPOPI_2002 | Ca4 | 12589173 | ATTTTTTTTTT/ATTTTTTTTT | CTTTCGAGATCTGATGCGTG | ACAACGTCTTCCATTACGCC | 59.5 | 753 | INTERGENIC | | | | |
| CaPOPI_2003 | Ca4 | 12589473 | G/GA | TGTGGCATTTTGGTAATGCT | ACAACGTCTTCCATTACGCC | 59.0 | 200 | INTERGENIC | | | | |
| CaPOPI_2004 | Ca4 | 12592291 | ATA/ATACAAATACTTGATGTA | TGACTGCCGCTTTGTCATTA | TTTCGATTCAAACCCAAACC | 60.4 | 588 | DRR | Ca_04481 | R | | |
| CaPOPI_2005 | Ca4 | 12593132 | AT/ATGTTGT | TCACTGTAAGTCCATGCCTCC | GCCCTTGTCAAAACAAGTTGA | 60.1 | 454 | INTERGENIC | | | | |

| INDEL marker IDs | Chromosomes/ unanchored scaffolds | Physical positions (bp) | InDels (*Kabuli* reference genome-CDC Frontier/PI) | Forward primers (5'-3') | Reverse primers (5'-3') | Anneling temperature (0C) | Expected amplified product size (bp) | Structural annotation | | Functional annotation | | |
|---|---|---|---|---|---|---|---|---|---|---|---|---|
| | | | | | | | | Sequence components of *kabuli* genome | *Kabuli* gene accession IDs | NCBI-KOG | TFs | NCBI-nr database |
| CaPOPI_2006 | Ca4 | 12593182 | TAAAA/TAAA | TCACTGTAAGTCCATGCCTCC | GCCCTTGTCAAAACAAGTTGA | 60.1 | 454 | INTERGENIC | | | | |
| CaPOPI_2007 | Ca4 | 12597000 | AT/ATCTAATGGT | CACATGGTTCCAGGTTCCTT | CTTATGGCGTGGCCTTAAAC | 59.8 | 820 | DRR | Ca_04482 | R | | Cytochrome b5 |
| CaPOPI_2008 | Ca4 | 12604165 | CTTTTTTTTTTTTT/CTTTTTTTTTTT | CGATTTCACTTCCCCAACTT | AGCAGCAGAAAGCATGGACT | 59.0 | 209 | INTERGENIC | | | | |
| CaPOPI_2009 | Ca4 | 12644877 | CATATATATATATATAT/CATATATATATATATATATAT | TCACTTGTTAGTATGACCACAACA | TTTGAGGCCATTAGTTTGGG | 57.3 | 877 | INTERGENIC | | | | |
| CaPOPI_2010 | Ca4 | 12676299 | CATATGTGATATGTGATA/CATATGTGATATGTGATATGTGATA | GATCTCGGGTTAGATGGTCG | TCCACTCTCGCTCTCAATCC | 59.5 | 740 | INTERGENIC | | | | |
| CaPOPI_2011 | Ca4 | 12715012 | TA/TATAGAAAGA | AGAAGCGATGCAGACAACTTT | AGGGGCGAATATCGCTAACT | 59.1 | 373 | INTERGENIC | | | | |
| CaPOPI_2012 | Ca4 | 12724267 | GAAAAAAAAAAA/GAAAAAAAAAAAAA | CCAAGTGCAATGACGAGCTA | CAAGGATATGGATCCAATGTGA | 60.0 | 610 | INTERGENIC | | | | |
| CaPOPI_2013 | Ca4 | 12866217 | GGG/GGGAGCTTTATTTTTTGG | TAGACTCCACTAAGCCCCCA | CCTTCCAACGCAACATTTCT | 59.7 | 294 | DRR | Ca_04506 | T | FAR1 | Protein kinase, catalytic domain |
| CaPOPI_2014 | Ca4 | 12885868 | ATTTTTTTTTT/ATTTTTTTTT | AGGTCATTGGTGTGCAAGGT | TGAAGCTGACTGCACTTTGG | 60.4 | 666 | DRR | Ca_04508 | R | | Glucose-methanol-choline oxidoreductase, N-terminal |
| CaPOPI_2015 | Ca4 | 13028368 | CTTGTTG/CTTGTTGTTG | TGCACGCTCCACATTATTTC | CTTTTCTGGGGCAAATTGAA | 59.7 | 367 | INTRON | Ca_04525 | T | bHLH | Protein kinase, catalytic domain |
| CaPOPI_2016 | Ca4 | 13068436 | AATATATATATATATATATATATATAT/AATATATATATATATATATATATATATATATATATAT | GCTCAATTTTTCTTAGGGCG | TGTCGCAACAATTTGTCTCTT | 59.0 | 432 | INTERGENIC | | | | |
| CaPOPI_2017 | Ca4 | 13133292 | ATTTT/ATATATTTTTT | CAGCCGCCTCCAACTATTAC | CCATCCATGCAATTGAACAC | 59.7 | 408 | INTERGENIC | | | | |
| CaPOPI_2018 | Ca4 | 13325790 | ATTTTTT/ATTT | TTGATCATCCTATTTCAAAAGTTCA | ATTTTCGACTCCACACACGG | 59.1 | 640 | INTERGENIC | | | | |
| CaPOPI_2019 | Ca4 | 13433311 | TA/T | CGAGCAAACACAATGAATGG | TTGGTTGGTTCATTGGTTGA | 60.1 | 569 | INTERGENIC | | | | |
| CaPOPI_2020 | Ca4 | 13509527 | ATTTT/ATTTTATTAAAAAATTTATTTTT | CCTCTTCACGGTGCTCATTT | CCTCGTTTTAAGCCCAATCA | 60.3 | 158 | INTERGENIC | | | | |

| INDEL marker IDs | Chromosomes/ unanchored scaffolds | Physical positions (bp) | InDels (*Kabuli* reference genome-CDC Frontier/PI) | Forward primers (5'-3') | Reverse primers (5'-3') | Anneling temperature (0C) | Expected amplified product size (bp) | Structural annotation | | Functional annotation | | |
|---|---|---|---|---|---|---|---|---|---|---|---|---|
| | | | | | | | | Sequence components of *kabuli* genome | *Kabuli* gene accession IDs | NCBI-KOG | TFs | NCBI-nr database |
| CaPOPI_2021 | Ca4 | 13604124 | GA/G | TTCAGACCCCAGTTTCATCA | GACATGTATCGTGTGCGGTC | 59.1 | 631 | INTERGENIC | | | | |
| CaPOPI_2022 | Ca4 | 13617448 | TG/TGG | GGGCCTTTTCTTCAGCTTTT | TGCATGTTATCCGCAATCTC | 59.8 | 713 | DRR | Ca_04579 | | | Protein of unknown function DUF1677, plant |
| CaPOPI_2023 | Ca4 | 13643715 | GTT/GTTT | ATAGTTGTTGAAGGGGCACG | GGGTGAAGAATGTATGCCAAA | 60.0 | 798 | INTERGENIC | | | | |
| CaPOPI_2024 | Ca4 | 13657824 | ATATT/AT | TGCATGCATGAATAGTGTTTGA | TCTCCCAATGTGATTGCTCA | 60.1 | 395 | INTERGENIC | | | | |
| CaPOPI_2025 | Ca4 | 13666161 | ACTG/ACTGCTG | CCCATTTCACCTCTTTGGAA | TTCCCGCAACAAATAGATCC | 59.9 | 840 | CDS (large-effect mutations) | Ca_04586 | Q | B3 | Cytochrome P450 |
| CaPOPI_2026 | Ca4 | 13691259 | GTT/GT | CCATTTATTTCCGGCATTTC | AGCCGACCAAACACTATTGG | 59.3 | 271 | INTERGENIC | | | | |
| CaPOPI_2027 | Ca4 | 13692964 | TGTATCAGTAT/TGTAT | TGTATCAAGTTCGTCCACCG | TTGATTTACGCCTGTTTCCC | 59.6 | 247 | DRR | Ca_04590 | | | XS domain |
| CaPOPI_2028 | Ca4 | 13730284 | TTATATATATATATATATATATATATAT/TTATATATATATATATATATATATATAT | AACACATAACCACTTCGCCC | TGCAGCGAAATTATTCGAGA | 59.9 | 458 | INTERGENIC | | | | |
| CaPOPI_2029 | Ca4 | 13743876 | GAA/GAAGCATATGCAA | CTGGTTCATGCCCCTACAAT | TCAAAGCACTAGCCACATGC | 59.8 | 740 | DRR | Ca_04598 | R | MYB_related | Pentatricopeptide repeat |
| CaPOPI_2030 | Ca4 | 13757687 | ACTTGC/AC | TAACCTGTTGCCATGGTTCA | AAGATGGGGAGAGGGAAAGA | 60.0 | 649 | INTRON | Ca_04600 | | NF-YB | Aminotransferase, class IV |
| CaPOPI_2031 | Ca4 | 13776936 | GTATATATATATA/GTATATATATA | TGTGTCTGCCCATGAGTGTT | ATATGCGGTTTCATCCTTGC | 60.2 | 301 | INTERGENIC | | | | |
| CaPOPI_2032 | Ca4 | 13896280 | CGAAGAAGAAGAAGAA/CGAAGAAGAAGAA | TACACTGGCCCATCATCTTG | GCCTCGTTGGAATGATTTGT | 59.5 | 482 | INTERGENIC | | | | |
| CaPOPI_2033 | Ca4 | 13908536 | TC/T | CTCCTCCGATGTCTGGAAAA | CGCGGTTGCTTCAATTCTAT | 60.2 | 232 | INTERGENIC | | | | |
| CaPOPI_2034 | Ca4 | 13909254 | CA/CAA | CATCACGATTTGACGAGTTGA | AAAAACATGACTCCAAACACCA | 59.7 | 636 | INTERGENIC | | | | |
| CaPOPI_2035 | Ca4 | 14011652 | TAA/TAACAA | GCACCGACTTGCTGATGTAA | GCCTCATGCTCCAATTTTGT | 59.9 | 755 | INTERGENIC | | | | |

| INDEL marker IDs | Chromosomes/ unanchored scaffolds | Physical positions (bp) | InDels (*Kabuli* reference genome-CDC Frontier/PI) | Forward primers (5'-3') | Reverse primers (5'-3') | Anneling temperature (0C) | Expected amplified product size (bp) | Structural annotation | | Functional annotation | | |
|---|---|---|---|---|---|---|---|---|---|---|---|---|
| | | | | | | | | Sequence components of *kabuli* genome | *Kabuli* gene accession IDs | NCBI-KOG | TFs | NCBI-nr database |
| CaPOPI_2036 | Ca4 | 14196867 | CTATATATATATATATAT/CTATATATATATATAT | CACTCGTGTTTCCCAAGGTT | TTGCAAGTGCATCCAAATTC | 60.0 | 584 | INTERGENIC | | | | |
| CaPOPI_2037 | Ca4 | 14397300 | GATATATATATATATATATGTATATATATATA/GATATATATATATATATATATATATGTATATATATATA | TAAATGCATGCTCCCCTGAT | AACACATCCGGGATCCATTA | 60.4 | 273 | INTERGENIC | | | | |
| CaPOPI_2038 | Ca4 | 14405755 | ATCTCTCTCT/ATCTCTCT | ATGTGCATGCCAAGTTGTGT | ACCTATAAGCCCTTTGCGCT | 60.0 | 963 | DRR | Ca_05663 | E | MYB | Amino acid transporter, transmembrane |
| CaPOPI_2039 | Ca4 | 14406037 | GTCTTT/GT | TTGGTTCCACATTGTACCCC | TTGTTGCTGGAGTTGGATCA | 60.5 | 639 | DRR | Ca_05663 | E | MYB | Amino acid transporter, transmembrane |
| CaPOPI_2040 | Ca4 | 14406133 | CAAA/CAAAA | TTGGTTCCACATTGTACCCC | CCATTGGTTGGGATATGGAG | 60.5 | 892 | DRR | Ca_05663 | E | MYB | Amino acid transporter, transmembrane |
| CaPOPI_2041 | Ca4 | 14416755 | TTATATATATATA/TTATATATATATATA | TCATTTCAAACCCTTAAACTCCA | TTGCGGTCAATTGTATGAAGAC | 59.9 | 392 | INTERGENIC | | | | |
| CaPOPI_2042 | Ca4 | 14677950 | CAAAAAAA/CAAAAAAAA | TGTTGGGCAAAATAAATTGAGA | CAAACCTTACCACCGTCTTG | 59.5 | 424 | INTERGENIC | | | | |
| CaPOPI_2043 | Ca4 | 14967549 | GTAATAATAATAATAATAATAATAATAATAA/GTAATAATAATAATAATAATAATAATAATAATAATAA | CAAAGTGCTTTTGTACCCCC | CCGTTTGTTTTGGGACATCT | 59.5 | 899 | INTERGENIC | | | | |
| CaPOPI_2044 | Ca4 | 15012755 | AATATATATATATATA/AATATATATATATATATATA | TACTCCCAAATGGCGACTTC | GACATTTCGAACTTGAATTTCCT | 60.1 | 322 | INTERGENIC | | | | |
| CaPOPI_2045 | Ca4 | 15045995 | CTATATATATATATATATATATATATATATATA/CTATATATATATATATATATATATATATATATATATA | GAAAATTATATTCTTTCCCAAATGC | GGAGGAGAGAGACGAAGGCT | 58.5 | 402 | INTERGENIC | | | | |
| CaPOPI_2046 | Ca4 | 15048181 | TA/TAA | CAAAAGCTGTAACACCCCGT | AATCGATTGATTCAGCCCAG | 60.0 | 528 | INTERGENIC | | | | |
| CaPOPI_2047 | Ca4 | 15216075 | TAAAAAAAAAA/TAAAAAAAAAAA | TGAACCAAAAATCATCGCAA | AGGCTCTTGTTAAGCGACCA | 60.1 | 402 | INTERGENIC | | | | |
| CaPOPI_2048 | Ca4 | 15221737 | ATTTTTTT/ATTTTTTTT | AGGGTGCGATCAGGTAAATG | AACAAGTGGTGCGATTGTGA | 60.0 | 430 | INTERGENIC | | | | |
| CaPOPI_2049 | Ca4 | 15252487 | GTA/GTACATATA | TTTTTGAGTGAAACCAGTTTGG | CCAACCATGTTGGTGTGCTA | 59.2 | 533 | INTERGENIC | | | | |
| CaPOPI_2050 | Ca4 | 15272177 | AG/A | GTTTCGGCTAATGGGTTGAA | TTGCTTGTAATTTTGGGTTTTG | 59.9 | 878 | DRR | Ca_05572 | A | | Zinc finger, RING-CH-type |

| INDEL marker IDs | Chromosomes/ unanchored scaffolds | Physical positions (bp) | InDels (*Kabuli* reference genome-CDC Frontier/PI) | Forward primers (5'-3') | Reverse primers (5'-3') | Anneling temperature (0C) | Expected amplified product size (bp) | Structural annotation | | Functional annotation | | |
|---|---|---|---|---|---|---|---|---|---|---|---|---|
| | | | | | | | | Sequence components of *kabuli* genome | *Kabuli* gene accession IDs | NCBI-KOG | TFs | NCBI-nr database |
| CaPOPI_2051 | Ca4 | 15473254 | TTATATATATATATATATAT/TTATATATATATATATAT | TTGGACCGAAGAAATTGTCA | CTCCGTTAATCCTCATGCGT | 59.1 | 702 | INTERGENIC | | | | |
| CaPOPI_2052 | Ca4 | 15481101 | AATA/AA | TAGTAGGGCTCTTGCTCCCA | CCAATTGTACCGGGATCTTC | 60.0 | 933 | INTERGENIC | | | | |
| CaPOPI_2053 | Ca4 | 15643039 | T/TTACTGTAGGGA | TGCACCGACAAACTGAAGTC | ACGCGTGAGTTTGCTCACTA | 59.9 | 592 | INTERGENIC | | | | |
| CaPOPI_2054 | Ca4 | 15891944 | CTTT/CTTTT | CGACGTCAACACAAATTGC | AGATGCGCGGTGATTAGAGT | 58.7 | 802 | INTERGENIC | | | | |
| CaPOPI_2055 | Ca4 | 15892004 | AC/ACCGACTC | CGACGTCAACACAAATTGC | AGATGCGCGGTGATTAGAGT | 58.7 | 802 | INTERGENIC | | | | |
| CaPOPI_2056 | Ca4 | 15898293 | T/TA | CCTTCAAAATTTGTGAGTCCG | ATGGGAATTGGTGAATGGAA | 59.6 | 941 | INTERGENIC | | | | |
| CaPOPI_2057 | Ca4 | 15926036 | AAAGA/AAAGAAGA | GTTTCCCTTACGCCAGATGA | GCATTGCTGTATTGGTGCAT | 60.1 | 532 | DRR | Ca_05513 | R | | Beta-lactamase-like |
| CaPOPI_2058 | Ca4 | 15926090 | ACT/ACTCT | AGCAGCACCCACCAAATATC | TGCTGGTGTTCTAGTTGGCA | 60.0 | 674 | DRR | Ca_05513 | R | | Beta-lactamase-like |
| CaPOPI_2059 | Ca4 | 15939075 | AT/ATT | GTGCACCGGTAACGAAGTTT | TTCGTTGGAGGTTTGGATTC | 60.0 | 452 | INTERGENIC | | | | |
| CaPOPI_2060 | Ca4 | 15945430 | AAAA/AAAAATCAAA | AGTCCAGCCTCTGCACAAAT | GGAGCAAGATGGAACAATCAA | 59.9 | 212 | DRR | Ca_05512 | O | C2H2 | Heat shock protein DnaJ, N-terminal |
| CaPOPI_2061 | Ca4 | 15950510 | GTTATTTTTATT/GTTATTTTTATTATTATTTTTATT | AAAAGGGATTGTCTTCCCAGA | CAAAGGTAGCCCTGCTTCAG | 59.9 | 601 | INTERGENIC | | | | |
| CaPOPI_2062 | Ca4 | 15962571 | ACTT/ACTTCTCTT | CAGGAAATTGTGCATCATGG | TGGTGCATGACTGAATGGTT | 59.9 | 723 | INTERGENIC | | | | |
| CaPOPI_2063 | Ca4 | 15969051 | CAA/CA | TAAGGCGTTAACTTGCACCC | TGGTTGTGGCGATTACAAGA | 60.1 | 494 | DRR | Ca_05510 | J | NF-YA | Cysteinyl-tRNA synthetase, class Ia |
| CaPOPI_2064 | Ca4 | 15983115 | AATT/AATTATT | TGATGTTGATGATATGACGTGG | ATTGTTCTGCAAATGAGGGG | 59.3 | 729 | INTERGENIC | | | | |
| CaPOPI_2065 | Ca4 | 16000633 | TACA/TA | CGATGTGATTTTTGTAGTCAAGC | TATTTTCCGCACACCAATGA | 58.8 | 392 | INTERGENIC | | | | |

| INDEL marker IDs | Chromosomes/ unanchored scaffolds | Physical positions (bp) | InDels (*Kabuli* reference genome-CDC Frontier/PI) | Forward primers (5'-3') | Reverse primers (5'-3') | Anneling temperature (0C) | Expected amplified product size (bp) | Structural annotation | | Functional annotation | | |
|---|---|---|---|---|---|---|---|---|---|---|---|---|
| | | | | | | | | Sequence components of *kabuli* genome | *Kabuli* gene accession IDs | NCBI-KOG | TFs | NCBI-nr database |
| CaPOPI_2066 | Ca4 | 16027847 | A/AG | AATTTTGGCTTTTTGCGTTG | ATCATATCCATCAAACGGGC | 60.1 | 536 | INTERGENIC | | | | |
| CaPOPI_2067 | Ca4 | 16028817 | CGTGTGT/CGTGTGTGT | TGATTTTACACAAGGGTAATTTTG | CGCCGCATACTGTAATGGTA | 57.3 | 951 | INTERGENIC | | | | |
| CaPOPI_2068 | Ca4 | 16048595 | TG/T | GACCATTTTTGGTGATTGGG | AGGTACCACCAACCAAGTGC | 60.0 | 258 | INTRON | Ca_05504 | J | bHLH | Glutamyl/glutaminyl-tRNA synthetase, class Ic |
| CaPOPI_2069 | Ca4 | 16066680 | CAAAA/CAAA | TCTCGATTGAAGTGCATTGG | CATTAGAGTTCACTTGGCGG | 59.8 | 514 | INTERGENIC | | | | |
| CaPOPI_2070 | Ca4 | 16066890 | ATTTTT/ATTTT | AGAACCGATTTTGGTGATGC | GGGACCAGTGATTTAATTTAGCC | 59.9 | 705 | INTERGENIC | | | | |
| CaPOPI_2071 | Ca4 | 16080327 | CTTTTTTTTTTTTT/CTTTTTTTTTTT | TTCCTCACGTGATCCCTTTC | ACAATTGCTCCAACGGTCAT | 60.1 | 567 | INTERGENIC | | | | |
| CaPOPI_2072 | Ca4 | 16095440 | TA/TAA | AGCCCAAGAAAGAAAACGCT | AAGGCAAAAGTCGTCCAATC | 60.4 | 726 | INTERGENIC | | | | |
| CaPOPI_2073 | Ca4 | 16095927 | GATAATATAATATAATATA/GATAATATAATATAATATAATATA | TACAGAATCGCTCCCTCCAC | TACATGGTTCGGTTGGGTTT | 60.2 | 665 | INTERGENIC | | | | |
| CaPOPI_2074 | Ca4 | 16120236 | AATATATATATATATATATA/AATATATATATATATATA | TCGAAAGCATTCTTCTCCAAA | TGGTTTGACCTATTTATGGTTGG | 59.9 | 550 | INTERGENIC | | | | |
| CaPOPI_2075 | Ca4 | 16138106 | TTTATTATTATTATTATTATTATTATTATT/TTTATTATTATTATTATTATTATTATTATTATT | CAATGACATTACTTGGGCCTT | GGGAGAAGAAAAGAATGGGAA | 59.0 | 391 | INTERGENIC | | | | |
| CaPOPI_2076 | Ca4 | 16186698 | T/TC | TTGTTTACAAGAGATGTTTTGTCATTT | AAACCACGACTCCAAGCAAT | 59.5 | 372 | DRR | Ca_05493 | Z | | Domain of unknown function DUF292, eukaryotic |
| CaPOPI_2077 | Ca4 | 16329274 | A/AAGCATAC | TTGGACGATAGTACCGTATTCAA | CGAAGCAAATAGCAAGACTGC | 58.6 | 815 | INTERGENIC | | | | |
| CaPOPI_2078 | Ca4 | 16787893 | CTTTTTTTT/CTTTTTTT | CCCTTTAAAATGAGAGAATTGAAG | ATCGTCACGTGCAAACAATC | 57.7 | 628 | INTERGENIC | | | | |
| CaPOPI_2079 | Ca4 | 16827901 | AATATTGATTATA/AATATTGATTATATTGATTATA | ACGCCAACATTTGTGAGTGA | TCGGATTCACTTAGTTGCACA | 60.2 | 853 | INTERGENIC | | | | |
| CaPOPI_2080 | Ca4 | 16846897 | C/CTT | ACATGTGCCTTGGGACTAGC | GCGAAAAGTCGAAGATCCTG | 60.1 | 571 | INTRON | Ca_05437 | | | Amino acid-binding ACT |

| INDEL marker IDs | Chromosomes/ unanchored scaffolds | Physical positions (bp) | InDels (*Kabuli* reference genome-CDC Frontier/PI) | Forward primers (5'-3') | Reverse primers (5'-3') | Anneling temperature (0C) | Expected amplified product size (bp) | Structural annotation | | Functional annotation | | |
|---|---|---|---|---|---|---|---|---|---|---|---|---|
| | | | | | | | | Sequence components of *kabuli* genome | *Kabuli* gene accession IDs | NCBI-KOG | TFs | NCBI-nr database |
| CaPOPI_2081 | Ca4 | 16925907 | TAAAAAAAAAAAA/TAAAAAAAAAAA | TGCAAAACGTCGCTGTCTAC | TGGTCACTGGTCACTCTTGG | 60.1 | 566 | DRR | Ca_05430 | R | C2H2 | Zinc finger, C2H2-type |
| CaPOPI_2082 | Ca4 | 16933042 | TAAAAAAAAAA/TAAAAAAAAA | CCAAATAAACTACTTTTGTGAGACGA | CACACTCTGCCTCCACTGAA | 60.0 | 388 | INTERGENIC | | | | |
| CaPOPI_2083 | Ca4 | 16943222 | TATGAATGAAT/TATGAATGAATGAAT | CTCAAGCTCAGTCCCAGTCC | TGCGTTTTGTTTGGTGTAGC | 60.0 | 668 | INTERGENIC | | | | |
| CaPOPI_2084 | Ca4 | 16984107 | ATTTTTTTT/ATTTTTTT | TAACAAGGGTGCCATGTGTG | ATTGATTTGGCAATGGTGGT | 60.4 | 822 | INTRON | Ca_05426 | U | | Zinc finger, Sec23/Sec24-type |
| CaPOPI_2085 | Ca4 | 17017613 | ATTTTT/ATTTTTT | GGAAGTTGACTTGTGCCGAT | TTGTTCTGAAAGAAACGGGG | 60.1 | 527 | INTERGENIC | | | | |
| CaPOPI_2086 | Ca4 | 17029753 | ATTTTTTTTT/ATTTTTTTT | ACCATTTCCTCCTGTGTTGC | CAAAAATTTGAGGGTTCAAACA | 60.0 | 728 | INTERGENIC | | | | |
| CaPOPI_2087 | Ca4 | 17047766 | ATTTTTTTTTTT/ATTTTTTTTTT | GAACCCAGTGCACAACTCCT | AAGCGACCAGTTGAAGGAAA | 60.2 | 662 | DRR | Ca_05418 | DA | | mRNA splicing factor SYF2 |
| CaPOPI_2088 | Ca4 | 17047788 | ATTTTTTTTT/ATTTTTTTTTT | GAACCCAGTGCACAACTCCT | AAGCGACCAGTTGAAGGAAA | 60.2 | 662 | DRR | Ca_05418 | DA | | mRNA splicing factor SYF2 |
| CaPOPI_2089 | Ca4 | 17063810 | TAAAAAAAA/TAAAAAAAAAA | CAAAAGAAAAGCTTTATACGGTGA | TTCGGTTTATGTAGCAGCCC | 59.0 | 558 | INTERGENIC | | | | |
| CaPOPI_2090 | Ca4 | 17071722 | ATTTTTTTTT/ATTTTTTTTTT | GGGCTGCTACATAAACCGAA | TCCGACATGCCATTTTGTTA | 60.1 | 189 | INTERGENIC | | | | |
| CaPOPI_2091 | Ca4 | 17081756 | TTATA/TTATATA | TACCGGAAGATTCGGAAATG | CTGCAGGTAAGCATCCCAAT | 59.9 | 694 | INTRON | Ca_05415 | | | |
| CaPOPI_2092 | Ca4 | 17081772 | TAATA/T | TACCGGAAGATTCGGAAATG | CTGCAGGTAAGCATCCCAAT | 59.9 | 694 | INTRON | Ca_05415 | | | |
| CaPOPI_2093 | Ca4 | 17082915 | CTTTTT/CTTTTTT | TCAGCTCACTCTCGCTTTGA | TGATGGAAGAATAAGGGGTCC | 60.0 | 503 | DRR | Ca_05415 | | | |
| CaPOPI_2094 | Ca4 | 17090015 | ATATTTATTTATTTATTTATTT/ATATTTATTTATTTATTT | CCAACATTAAAACGGCCCTA | GCATTTTCACAATGTGGTGC | 59.8 | 698 | INTERGENIC | | | | |
| CaPOPI_2095 | Ca4 | 17101140 | TAACAAC/TAACAACAAC | TGGTTTGATTTATTGTGGTGC | CATTGGTTTTAACAAGTGTTGTTGT | 58.4 | 472 | DRR | Ca_05414 | | | Plastocyanin-like |

| INDEL marker IDs | Chromosomes/ unanchored scaffolds | Physical positions (bp) | InDels (*Kabuli* reference genome-CDC Frontier/PI) | Forward primers (5'-3') | Reverse primers (5'-3') | Anneling temperature (0C) | Expected amplified product size (bp) | Structural annotation | | Functional annotation | | |
|---|---|---|---|---|---|---|---|---|---|---|---|---|
| | | | | | | | | Sequence components of *kabuli* genome | *Kabuli* gene accession IDs | NCBI-KOG | TFs | NCBI-nr database |
| CaPOPI_2096 | Ca4 | 17101199 | A/AC | TGGTTTGATTTATTGTG GTGC | GTTCTTTGAAATTGCAC GAATA | 58.4 | 812 | DRR | Ca_05414 | | | Plastocyanin-like |
| CaPOPI_2097 | Ca4 | 17101268 | TAAAAAA/TAAAAAAA | TGGTTTGATTTATTGTG GTGC | TCGAGAGTTGGGTCATG GTC | 58.4 | 881 | DRR | Ca_05414 | | | Plastocyanin-like |
| CaPOPI_2098 | Ca4 | 17101583 | AG/A | TGATGTTAACTGACAAAT GCTGTTT | GTCGAGAGTTGGGTCAT GGT | 60.0 | 322 | DRR | Ca_05414 | | | Plastocyanin-like |
| CaPOPI_2099 | Ca4 | 17141313 | T/TC | TTTCAATCGTATCAAGTT TTTGC | AAACTGAAAGGCCTAAT TCCAA | 58.4 | 682 | DRR | Ca_05410 | CR | Nin-like | Monooxygenase, FAD-binding |
| CaPOPI_2100 | Ca4 | 17157766 | TTAAT/TTAATAAT | AGAACCTTGCTATGCGT CGT | CATGGTGTGGACAAGAT TGG | 59.9 | 501 | DRR | Ca_05407 | | | Translation initiation factor 3 |
| CaPOPI_2101 | Ca4 | 17167273 | T/TC | TTCCACTCTCCACCATTT CC | CACCAACAAGGATCGGA TTT | 59.9 | 429 | INTERGENIC | | | | |
| CaPOPI_2102 | Ca4 | 17193639 | ATTTT/ATTT | AAGCAGCAAGGATCACC AAT | CCCCATTAGTATTGCCG CTA | 59.7 | 532 | DRR | Ca_05405 | T | | Protein phosphatase 2C, manganese/magnesium aspartate binding |
| CaPOPI_2103 | Ca4 | 17196085 | TTCTCTCTCTCTCTCTCTCTCTCTCTCT /TTCTCTCTCTCTCTCTCTCTCTCTCT | CCTCAACCATGGCCTGT ACT | AGTTGCATTGGAGACAA GGG | 60.0 | 118 | INTERGENIC | | | | |
| CaPOPI_2104 | Ca4 | 17203357 | TAAGA/TAAGAAGA | TGTGCTCCATGTCTCTT TGC | CTCTTGGCTGGTAGCTT TGG | 60.0 | 611 | INTRON | Ca_05403 | | | Protein of unknown function DUF3353 |
| CaPOPI_2105 | Ca4 | 17206399 | ATTTTTT/ATTTTT | AACCGTTTGATGCGAAA AAT | ATCTATTGCCATCTGCC AGG | 59.5 | 925 | INTERGENIC | | | | |
| CaPOPI_2106 | Ca4 | 17206661 | T/TA | AATCAATCAACCAAGAA CGGA | ATCTATTGCCATCTGCC AGG | 59.4 | 598 | INTERGENIC | | | | |
| CaPOPI_2107 | Ca4 | 17206969 | TAAAAAAAA/TAAAAAAA | CCTGGCAGATGGCAATA GAT | GAAAATGGATCCGACGA AGA | 60.1 | 593 | INTERGENIC | | | | |
| CaPOPI_2108 | Ca4 | 17209722 | CT/CTT | CCATTCAGAAAAGGGCA AGA | AAGCCTCGGGTAAAATT GGT | 60.2 | 442 | INTRON | Ca_05402 | C | | ATPase, alpha/beta subunit, nucleotide-binding domain |
| CaPOPI_2109 | Ca4 | 17210535 | CTT/CT | TTCAGGGAGTTCCATCA ACC | TAGCCTTCTGAATTCCC CCT | 59.9 | 448 | INTRON | Ca_05402 | C | | ATPase, alpha/beta subunit, nucleotide-binding domain |
| CaPOPI_2110 | Ca4 | 17247653 | GAAAAAAAA/GAAAAAAAAAA | TTTATGTTTGGCCAACTC CC | TTTTTAGGGACGCAATT CAAA | 59.8 | 539 | INTERGENIC | | | | |

| INDEL marker IDs | Chromosomes/ unanchored scaffolds | Physical positions (bp) | InDels (*Kabuli* reference genome-CDC Frontier/PI) | Forward primers (5'-3') | Reverse primers (5'-3') | Anneling temperature (0C) | Expected amplified product size (bp) | Structural annotation | | Functional annotation | | |
|---|---|---|---|---|---|---|---|---|---|---|---|---|
| | | | | | | | | Sequence components of *kabuli* genome | *Kabuli* gene accession IDs | NCBI-KOG | TFs | NCBI-nr database |
| CaPOPI_2111 | Ca4 | 17247848 | TAAAAAAAAA/TAAAAAAAAAA | TTTATGTTTGGCCAACTCCC | TTTTTAGGGACGCAATTCAAA | 59.8 | 539 | INTERGENIC | | | | |
| CaPOPI_2112 | Ca4 | 17250847 | ATTTT/ATTT | GCCGAAAAACCAAAGTGTGT | CGTTGGATTCCTTTCCTCTC | 60.0 | 514 | INTERGENIC | | | | |
| CaPOPI_2113 | Ca4 | 17253834 | AAATAA/AAA | TGCAAATATCATTTCATGTTCTTG | TGATGAAGTGAGTCGCTTGG | 59.0 | 843 | INTERGENIC | | | | |
| CaPOPI_2114 | Ca4 | 17253939 | T/TA | TCAACATCTAGTTAGAGGTGCAAAA | TGATGAAGTGAGTCGCTTGG | 59.4 | 780 | INTERGENIC | | | | |
| CaPOPI_2115 | Ca4 | 17254750 | GTAATAA/GTAA | CAAATATTGTGCCAGCCAAA | TCTTAATCTTGACCAGCCCG | 59.6 | 898 | INTERGENIC | | | | |
| CaPOPI_2116 | Ca4 | 17257056 | ATTTT/ATTT | CCAACAAGCATTACATTGCG | ACGTTGACATTCCAGCATGA | 60.1 | 423 | INTERGENIC | | | | |
| CaPOPI_2117 | Ca4 | 17257974 | AATA/AATATA | ACATGGCAGGTTGGATTCAT | TCATGGGTACCCCACCTTAG | 60.2 | 605 | INTERGENIC | | | | |
| CaPOPI_2118 | Ca4 | 17284874 | GATA/GA | TTGAATATGAGCCTCCAAGAGA | CTTGTTGAGGCTTTGGGTGT | 58.9 | 794 | DRR | Ca_05393 | R | | Ankyrin repeat-containing domain |
| CaPOPI_2119 | Ca4 | 17285160 | ACT/A | CATTTCCTTTGAGTCATGAGAGG | CTTGTTGAGGCTTTGGGTGT | 60.1 | 310 | DRR | Ca_05393 | R | | Ankyrin repeat-containing domain |
| CaPOPI_2120 | Ca4 | 17286686 | CATTGGATTGGATTG/CATTGGATTG | TGCATTTCATGCCTTTGCTA | TGATTAGCCCGTATTTTGGG | 60.4 | 648 | INTRON | Ca_05393 | R | | Ankyrin repeat-containing domain |
| CaPOPI_2121 | Ca4 | 17290871 | GTTTAATT/GTTTAATTTTAATT | CCAAGTATGGCAGGTGGAAT | TCATGAGCTAGAATGGTTGTAAAAA | 59.8 | 615 | INTERGENIC | | | | |
| CaPOPI_2122 | Ca4 | 17297028 | TA/TAA | TTTGCCTCAGCTGTGTTGAC | TCATCAGCTGGACAACTTCG | 60.0 | 607 | INTRON | Ca_05392 | R | | Ankyrin repeat-containing domain |
| CaPOPI_2123 | Ca4 | 17313521 | TAAA/TAA | GCTAGACAGGTTTGCCTTCG | AAGGCTCCTGCAAGCATTTA | 60.0 | 966 | INTERGENIC | | | | |
| CaPOPI_2124 | Ca4 | 17314621 | ATTTTTT/ATTTTT | ACGCGATTGAGAACAAAAGG | CTTCCCCACTCACAGTCCAT | 60.2 | 604 | DRR | Ca_05390 | M | | Mechanosensitive ion channel MscS |
| CaPOPI_2125 | Ca4 | 17315192 | TAAA/TAAAA | ATGGACTGTGAGTGGGGAAG | TTGGTCCAACAGCTTGATGA | 60.0 | 716 | DRR | Ca_05390 | M | | Mechanosensitive ion channel MscS |

| INDEL marker IDs | Chromosomes/ unanchored scaffolds | Physical positions (bp) | InDels (*Kabuli* reference genome-CDC Frontier/PI) | Forward primers (5'-3') | Reverse primers (5'-3') | Anneling temperature (0C) | Expected amplified product size (bp) | Structural annotation | | Functional annotation | | |
|---|---|---|---|---|---|---|---|---|---|---|---|---|
| | | | | | | | | Sequence components of *kabuli* genome | *Kabuli* gene accession IDs | NCBI-KOG | TFs | NCBI-nr database |
| CaPOPI_2126 | Ca4 | 17318086 | A/AG | CTCAAAAGTGGGATAGG GCA | ACAGCGTGAAACAAGAT CCC | 60.1 | 429 | INTRON | Ca_05390 | M | | Mechanosensitive ion channel MscS |
| CaPOPI_2127 | Ca4 | 17329245 | TCACA/TCA | GGCATGATAAAGAGGGA GGA | TAACCTCTCACGTGCAA TCG | 59.1 | 386 | INTERGENIC | | | | |
| CaPOPI_2128 | Ca4 | 17354782 | TAAAAAAAA/TAAAAAAA | TATTGATTCGGTCAATTA TGACA | TGGATTAGGCCTTGAAG CAT | 57.1 | 728 | INTERGENIC | | | | |
| CaPOPI_2129 | Ca4 | 17360464 | TTATATATATATATATAT/TTATATATATA TATATATATATATATATAT | GTGTGCGATTGAGTTGA ACG | CTTGCTGGGCTTTTCAA GAC | 60.3 | 579 | INTRON | Ca_05388 | T | NAC | Protein kinase, catalytic domain |
| CaPOPI_2130 | Ca4 | 17365284 | TA/TAA | CCCCGGCTTACACATTC TTA | TATTGGTGTAACCGAAC GCA | 60.0 | 557 | INTRON | Ca_05388 | T | NAC | Protein kinase, catalytic domain |
| CaPOPI_2131 | Ca4 | 17367667 | AC/ACC | TAAATGTGATTTGCGAG CGA | ATGCATGGAGGTGATGA TGA | 60.4 | 284 | INTERGENIC | | | | |
| CaPOPI_2132 | Ca4 | 17376716 | ATTTTTTTTT/ATTTTTTTTTTT | CCCCCATCTTCAAATGT GTT | AGATGTAGCAACGCAAA CCA | 59.6 | 698 | DRR | Ca_05387 | R | | F-box domain, cyclin-like |
| CaPOPI_2133 | Ca4 | 17388357 | TAAAAAAAAAAAAA/TAAAAAAAAA | AAATCGCCATTTGGAAC TTG | CACGATCATGTGTTCCA CAA | 59.9 | 308 | INTERGENIC | | | | |
| CaPOPI_2134 | Ca4 | 17420097 | TA/TAA | TCGTTCAACCATTGCAT CAT | TCCGAATGAGTGACCAA AATC | 59.9 | 612 | INTERGENIC | | | | |
| CaPOPI_2135 | Ca4 | 17421248 | G/GTTTAA | CCCGTGAGTTGTTGGAA GTT | TCGATAATTTGAAACCG CCT | 60.0 | 569 | INTERGENIC | | | | |
| CaPOPI_2136 | Ca4 | 17423293 | TTATATA/TTA | GTGTGGGTTGGCTTTCA TCT | GAAACGCTATGATGCCG TAAA | 60.0 | 492 | INTERGENIC | | | | |
| CaPOPI_2137 | Ca4 | 17431572 | GC/G | TCTGTCATTCCTGCCATT TTT | TCGCAATGTAGCTCCCT CTT | 59.6 | 858 | INTRON | Ca_05384 | K | | Transcription factor jumonji/aspartyl beta-hydroxylase |
| CaPOPI_2138 | Ca4 | 17436350 | CTTTTT/CTTT | AAGATCCTGCCCCTCAA GAT | TGCTCCAACTGAGTGGT GAC | 60.0 | 459 | INTRON | Ca_05384 | K | | Transcription factor jumonji/aspartyl beta-hydroxylase |
| CaPOPI_2139 | Ca4 | 17448021 | TATTTTCATTTTC/TATTTTCATTTTCATT TTC | GAGTTGAGGAAACAATT ATGTCAAA | TTCTTTTTCTGCGATTTC TCG | 58.7 | 654 | INTERGENIC | | | | |
| CaPOPI_2140 | Ca4 | 17470871 | GTGGCTA/GTGGCTAATGGCTA | GTCCCACCAAAGAAAGC AAA | CACTTGAAGAGGCTCCA TCC | 60.1 | 343 | DRR | Ca_05380 | AB | | SKI-interacting protein SKIP, SNW domain |

| INDEL marker IDs | Chromosomes/ unanchored scaffolds | Physical positions (bp) | InDels (*Kabuli* reference genome-CDC Frontier/PI) | Forward primers (5'-3') | Reverse primers (5'-3') | Anneling temperature (0C) | Expected amplified product size (bp) | Structural annotation | | Functional annotation | | |
|---|---|---|---|---|---|---|---|---|---|---|---|---|
| | | | | | | | | Sequence components of *kabuli* genome | *Kabuli* gene accession IDs | NCBI-KOG | TFs | NCBI-nr database |
| CaPOPI_2141 | Ca4 | 17475440 | GT/GTTGTTATT | CGGGCAGTAGCATTAGATGG | GGATGAATGGGAGGTGGTTA | 60.6 | 210 | INTERGENIC | | | | |
| CaPOPI_2142 | Ca4 | 17484481 | ATTTTTTTTT/ATTTTTTTTTT | TCGCCTGATGCTTGTACTTG | AGGAGGCTTTTCCACAGGTT | 60.0 | 361 | INTRON | Ca_05379 | O | | Oligosaccharyl transferase, STT3 subunit |
| CaPOPI_2143 | Ca4 | 17532742 | TAAAA/TAAAAA | CCCATCTTGTTTGGTTCACA | CTGAAAGGGCATAAAGGCAA | 59.4 | 954 | INTERGENIC | | | | |
| CaPOPI_2144 | Ca4 | 17533392 | CTT/CTTT | CTTTTTGCTGGTGCCATTTT | GGCATTGTGCTGAGTGATTTT | 60.1 | 679 | INTERGENIC | | | | |
| CaPOPI_2145 | Ca4 | 17533529 | GT/G | CTTTTTGCTGGTGCCATTTT | TGCAAGAAGAAGGAAGCCAT | 60.1 | 713 | INTERGENIC | | | | |
| CaPOPI_2146 | Ca4 | 17534928 | TAAAAAAAAA/TAAAAAAAA | TTTTTGTGTGTGTGCGGTTT | TGTGTCAAAGATGGTATTTCAATG | 60.1 | 410 | INTERGENIC | | | | |
| CaPOPI_2147 | Ca4 | 17535175 | ACT/ACTCT | TTTTTGTGTGTGTGCGGTTT | GGCTGGAAGTGAGCTAGTGG | 60.1 | 811 | INTERGENIC | | | | |
| CaPOPI_2148 | Ca4 | 17545627 | TAAAAAAAA/TAAAAAAA | AGTTTAGGGGCAAGCACTGA | TGCATCGGAGATCACATCTT | 59.9 | 363 | INTERGENIC | | | | |
| CaPOPI_2149 | Ca4 | 17552174 | GTT/GTTT | AAGTGAGGCCACTGGAAAGA | ATGTGTTGGAACTTTTCCGC | 59.8 | 204 | INTERGENIC | | | | |
| CaPOPI_2150 | Ca4 | 17600797 | TC/T | AAGGGTGATGGACGAAAAAC | TTAAATGGTCGCATAGAGCAA | 58.9 | 682 | INTERGENIC | | | | |
| CaPOPI_2151 | Ca4 | 17600811 | TAAAAAA/TAAAAAAA | AAGGGTGATGGACGAAAAAC | TCCAACAACATCAAATTCATCC | 58.9 | 951 | INTERGENIC | | | | |
| CaPOPI_2152 | Ca4 | 17601510 | TC/T | GGATGAATTTGATGTTGTTGGA | CCGTGTCTATCCCCATTTTG | 59.7 | 515 | INTERGENIC | | | | |
| CaPOPI_2153 | Ca4 | 17617805 | GAAAAAAAA/GAAAAAAA | CATCTAGTGTTGGGGAATGTGA | ACGTTTCGCCCTACATCATT | 59.9 | 838 | INTERGENIC | | | | |
| CaPOPI_2154 | Ca4 | 17629803 | ATTTTTTTT/ATTTTTTTTTTT | CTTTTCCGCGAACACATTTT | GTACATACATGGACGCGACG | 60.1 | 587 | INTERGENIC | | | | |
| CaPOPI_2155 | Ca4 | 17636026 | C/CCAAGATATA | GGCCTCATCTCTAGCCAATG | CCAAGGAAGTTTCACCAGGA | 59.8 | 297 | INTRON | Ca_05367 | Z | WRKY | IQ motif, EF-hand binding site |

| INDEL marker IDs | Chromosomes/ unanchored scaffolds | Physical positions (bp) | InDels (*Kabuli* reference genome-CDC Frontier/PI) | Forward primers (5'-3') | Reverse primers (5'-3') | Anneling temperature (0C) | Expected amplified product size (bp) | Structural annotation | | Functional annotation | | |
|---|---|---|---|---|---|---|---|---|---|---|---|---|
| | | | | | | | | Sequence components of *kabuli* genome | *Kabuli* gene accession IDs | NCBI-KOG | TFs | NCBI-nr database |
| CaPOPI_2156 | Ca4 | 17637226 | CTATT/C | ACACAAGTTAGGTGGGGCAG | CTGCATCTGTAGTGAGGCCA | 60.0 | 346 | INTRON | Ca_05367 | Z | WRKY | IQ motif, EF-hand binding site |
| CaPOPI_2157 | Ca4 | 17637458 | GAC/G | ACACAAGTTAGGTGGGGCAG | GCATGGCTTGATGTGAGAGA | 60.0 | 475 | INTRON | Ca_05367 | Z | WRKY | IQ motif, EF-hand binding site |
| CaPOPI_2158 | Ca4 | 17640423 | CTTTTTTT/CTTTTTT | CAAGAAGAAGTGGTGGGGAA | GAACCCTTTCCATCGAGTCA | 60.1 | 524 | DRR | Ca_05367 | Z | WRKY | IQ motif, EF-hand binding site |
| CaPOPI_2159 | Ca4 | 17649020 | TTTTAAA/TTTTAAATTTAAA | CCAAAGAGCTGAAATGCACA | GGAAGCCGAAAAACTAAGGC | 60.0 | 399 | DRR | Ca_05365 | H | | Molybdopterin cofactor biosynthesis C (MoaC) domain |
| CaPOPI_2160 | Ca4 | 17656569 | GATA/GATATA | AATTTGGGGCGTTACAAACA | GTGCAAGATCCGCTACCAGT | 60.2 | 427 | INTERGENIC | | | | |
| CaPOPI_2161 | Ca4 | 17697196 | TAAAAAAAAA/TAAAAAAAAAA | GGAAATTTGGAAAGTTGGCA | ATAAGGTTCCCCCTGCAATC | 59.9 | 564 | INTERGENIC | | | | |
| CaPOPI_2162 | Ca4 | 17715218 | GAAAAAAAAAA/GAAAAAAAA | CTGTTCCTCACATCACGCAT | GGAGTCACCCTCCAAATTGA | 59.7 | 143 | INTERGENIC | | | | |
| CaPOPI_2163 | Ca4 | 17715291 | CTT/CT | TCAATTTGGAGGGTGACTCC | TACCAGCTTTGGTTTCTGCC | 59.9 | 250 | INTERGENIC | | | | |
| CaPOPI_2164 | Ca4 | 17715629 | AT/ATATATT | TCAATTTGGAGGGTGACTCC | GCGCTGATGCAATACATACAC | 59.9 | 532 | INTERGENIC | | | | |
| CaPOPI_2165 | Ca4 | 17717044 | TGG/TG | TCTGTCAAAGTCGGTGCAAG | ATAATGGCGCCACAGAAAAG | 60.0 | 821 | INTERGENIC | | | | |
| CaPOPI_2166 | Ca4 | 17720367 | CTGATCTTGATC/CTGATCTTGATCTTGATC | ACCCTCAGCATGGTCAAAAG | TAGTGGTGGCTCCCCTAATG | 60.1 | 480 | DRR | Ca_05352 | KL | bHLH | SNF2-related |
| CaPOPI_2167 | Ca4 | 17735379 | ATATT/AT | TTTTTAAAGGGCCCGATTG | TGATTGTCACGTCATCAAAATTC | 59.9 | 554 | INTERGENIC | | | | |
| CaPOPI_2168 | Ca4 | 17735574 | T/TA | TCACGATCGAAAAACCTATTTG | AAACATGACAAAAGTTGATGTTGAA | 59.1 | 916 | INTERGENIC | | | | |
| CaPOPI_2169 | Ca4 | 17740594 | GTTTTTTTT/GTTTTTTT | ATATGGCAAGGAACAACGGA | TACCTTGGAATTGGCTGGTC | 60.3 | 664 | INTERGENIC | | | | |
| CaPOPI_2170 | Ca4 | 17757375 | T/TC | TAAACCAACCCCACACCATT | ACAATGCCTTTTTGCCACTC | 59.9 | 252 | DRR | Ca_05349 | O | | Peptidase S9, prolyl oligopeptidase, catalytic domain |

| INDEL marker IDs | Chromosomes/ unanchored scaffolds | Physical positions (bp) | InDels (*Kabuli* reference genome-CDC Frontier/PI) | Forward primers (5'-3') | Reverse primers (5'-3') | Anneling temperature (0C) | Expected amplified product size (bp) | Structural annotation | | Functional annotation | | |
|---|---|---|---|---|---|---|---|---|---|---|---|---|
| | | | | | | | | Sequence components of *kabuli* genome | *Kabuli* gene accession IDs | NCBI-KOG | TFs | NCBI-nr database |
| CaPOPI_2171 | Ca4 | 17758892 | GTTTTTTTT/GTTTTTTT | TTTGATGCAAACAAGTTCCTG | CGATGAAGGAGTTCTCAGCC | 58.8 | 499 | DRR | Ca_05349 | O | | Peptidase S9, prolyl oligopeptidase, catalytic domain |
| CaPOPI_2172 | Ca4 | 17766593 | TAAAA/TAAA | ATTGCATTGTGCTCCACTTG | AGGGGTTGGTATAGTAGAAGGAA | 59.7 | 512 | DRR | Ca_05348 | | | |
| CaPOPI_2173 | Ca4 | 17769321 | GAACAAACAAACAAACAAACAA/GAACAAACAAACAAACAA | GAGCCCATTCCAAAAGATGA | TTCAGCCTCGACCAGTTTTT | 60.0 | 814 | INTRON | Ca_05348 | | | |
| CaPOPI_2174 | Ca4 | 17772365 | AACTT/AACTTACTT | TCTCATCCCGATTCCAAAAG | CGTAAGTTTCCCGTTCCAAA | 60.0 | 387 | INTRON | Ca_05347 | R | | D111/G-patch |
| CaPOPI_2175 | Ca4 | 17775890 | TAAA/TA | TGCATTCAGCAAAAAGTACCA | GGCACTGCCGTCAAAAGTAT | 59.4 | 525 | DRR | Ca_05347 | R | | D111/G-patch |
| CaPOPI_2176 | Ca4 | 17777647 | CATCTGAATCTGAA/CATCTGAA | CCTTAGGGGAGATTTGCATTC | TGCAGATAAATGGCCAAACA | 59.9 | 852 | INTERGENIC | | | | |
| CaPOPI_2177 | Ca4 | 17821019 | AAGA/AA | GGAACTGTCGTATTGTTCCCA | AGATGTGGGCATGTGTTTCA | 59.8 | 805 | INTERGENIC | | | | |
| CaPOPI_2178 | Ca4 | 17827610 | A/AT | TCAACTTCGGTGCTTGACAG | TTGGGGTACACGAACAATGA | 60.0 | 141 | INTERGENIC | | | | |
| CaPOPI_2179 | Ca4 | 17830486 | ATTTTTTTTT/ATTTTTTTTTT | TGGCTTGCATTCTCATTCAG | TTTGGATGCATAGGCCATTT | 59.9 | 296 | INTERGENIC | | | | |
| CaPOPI_2180 | Ca4 | 17847500 | CTCCTTTTCATC/CTCCTTTTCATCCTTTTCATC | TGAACGATGGTGGAAAGACA | GCAGAGCTCTTTGGCAGAGT | 60.1 | 391 | INTRON | Ca_18621 | J | | Methionyl-tRNA synthetase |
| CaPOPI_2181 | Ca4 | 17862487 | CTTT/CTT | TTTTCTTCGGATGGAAATGC | ACAAAAATGGCAATGCACAA | 60.0 | 571 | INTRON | Ca_18623 | | WOX | Homeobox |
| CaPOPI_2182 | Ca4 | 17862773 | AGT/AGTGT | TTTTCTTCGGATGGAAATGC | ACAAAAATGGCAATGCACAA | 60.0 | 571 | INTRON | Ca_18623 | | WOX | Homeobox |
| CaPOPI_2183 | Ca4 | 17863620 | CTTATTTGGAATTATTTGGAATTATT/CTTATTTGGAATTATT | TTTGATTGTGGCTTTTGCAG | ACGAGCTAAAGGATGGGTTG | 59.8 | 540 | DRR | Ca_18623 | | WOX | Homeobox |
| CaPOPI_2184 | Ca4 | 17942733 | CTATATATATATATAT/CTATATATATATATATAT | CATCGTCAGCACATCACAAA | CTTAGGAGGTCTTGCCCCTC | 59.2 | 447 | INTERGENIC | | | | |
| CaPOPI_2185 | Ca4 | 18033913 | CT/CTT | CTGAGTGACCTCCCTTCCAA | TCATTCCAGAAGGAGATGGG | 60.2 | 433 | INTRON | Ca_18645 | | | |

| INDEL marker IDs | Chromosomes/ unanchored scaffolds | Physical positions (bp) | InDels (*Kabuli* reference genome-CDC Frontier/PI) | Forward primers (5'-3') | Reverse primers (5'-3') | Anneling temperature (0C) | Expected amplified product size (bp) | Structural annotation | | Functional annotation | | |
|---|---|---|---|---|---|---|---|---|---|---|---|---|
| | | | | | | | | Sequence components of *kabuli* genome | *Kabuli* gene accession IDs | NCBI-KOG | TFs | NCBI-nr database |
| CaPOPI_2186 | Ca4 | 18121565 | TAAGAAG/TAAG | GCTGCACCATGAGATCTTGA | ATGGTGCTGGAAGTTGCTTT | 60.0 | 440 | INTRON | Ca_18652 | | | Chalcone isomerase, subgroup |
| CaPOPI_2187 | Ca4 | 18196212 | ATTT/ATTTAATCTTTTT | GTGGTCCAGATTCTCCCAAA | TGTCGCTTCAGCAAAAAGAA | 59.9 | 549 | INTERGENIC | | | | |
| CaPOPI_2188 | Ca4 | 18407838 | AATATATATATATATATATATATATA/AATATATATATATATATATATATATATA | TAATAGGAACCCACACCCCA | ATGCTCCGGTGAACAATCTC | 60.0 | 360 | INTERGENIC | | | | |
| CaPOPI_2189 | Ca4 | 18446227 | GTTTTTT/GTTTTTTT | TCTCACGGTTGTGAGGACTG | GGTGGAGGTGAAGGTAAACAA | 59.9 | 304 | INTERGENIC | | | | |
| CaPOPI_2190 | Ca4 | 18515805 | TATAAATAAATAAATAAATAAATAAAT/TATAAATAAATAAATAAATAAATAAATAAAT | TTTTTGAGATTGGTACGGGA | TCAGCGTTTGTTTGTTGACTT | 58.1 | 738 | INTERGENIC | | | | |
| CaPOPI_2191 | Ca4 | 18649901 | AATATATATATATATATATATATATATATATAT/AATATATATATATATATATATATATATATAT | CTCTTTGGTTCTACGGCTGC | TATGGCTTACGAGGGACTGG | 60.0 | 911 | INTERGENIC | | | | |
| CaPOPI_2192 | Ca4 | 18781017 | TAAAAAAAAAAA/TAAAAAAAAAA | GAGAAACAACGCAGGGAAAC | ATTATTGCCGCCAATTCAAA | 59.7 | 350 | INTERGENIC | | | | |
| CaPOPI_2193 | Ca4 | 18821207 | CATTATTATTATTATTATTATTATTATT/CATTATTATTATTATTATTATTATTATTATTATT | AAAGTCGCATTCTGGGGATA | ATGTGCCAACTTCGACAATG | 59.5 | 722 | INTERGENIC | | | | |
| CaPOPI_2194 | Ca4 | 18860078 | TTATATATATATATATATATATATAT/TTATATATATATATATATATATATATATATATAT | GAGAAACTCCGCTCTTGTGG | GAACTCCCCGTCCAACACTA | 60.0 | 700 | INTERGENIC | | | | |
| CaPOPI_2195 | Ca4 | 18993059 | GTTTTTTT/GTTTTTT | CGTTAAGAATGCGTTGCAGA | TCGCAACTTTTGTTGGTTTTT | 60.0 | 302 | INTERGENIC | | | | |
| CaPOPI_2196 | Ca4 | 19045820 | A/AT | GGGCTTGCTTGGTTTGAATA | TCAACTGCACGTGTTAAATGC | 60.1 | 825 | INTERGENIC | | | | |
| CaPOPI_2197 | Ca4 | 19046060 | CAAAAAAA/CAAAAAAAA | TGAAAATGAGATTAGAACCACCAA | TCTTCACCTGCATCGAAATG | 59.9 | 677 | INTERGENIC | | | | |
| CaPOPI_2198 | Ca4 | 19198535 | CTTTTTTTTTTT/CTTTTTTTTTTTTT | AGAGGCGGGTGAGAAATTGT | CGTGCTATTCGGCAATCATA | 61.0 | 707 | INTRON | Ca_14019 | O | | Peptidyl-prolyl cis-trans isomerase, cyclophilin-type |
| CaPOPI_2199 | Ca4 | 19294536 | TTATATATATATATATATATATATATAT/TTATATATATATATATATATATATATATATATATAT | GCATACTCTGTCCTTTCGTCC | GGCTGCCATCACTTCATCTC | 58.8 | 586 | INTERGENIC | | | | |
| CaPOPI_2200 | Ca4 | 19643688 | CTTTTT/CTTTTTT | GGTGCAGTTGTTTGATGCAC | ATGCGATTTACATGGTTGGC | 60.2 | 255 | INTERGENIC | | | | |

| **INDEL marker IDs** | **Chromosomes/ unanchored scaffolds** | **Physical positions (bp)** | **InDels (*Kabuli* reference genome-CDC Frontier/PI)** | **Forward primers (5'-3')** | **Reverse primers (5'-3')** | **Anneling temperature (0C)** | **Expected amplified product size (bp)** | **Structural annotation** | | **Functional annotation** | | |
|---|---|---|---|---|---|---|---|---|---|---|---|---|
| | | | | | | | | **Sequence components of *kabuli* genome** | ***Kabuli* gene accession IDs** | **NCBI-KOG** | **TFs** | **NCBI-nr database** |
| CaPOPI_2201 | Ca4 | 19678061 | CAA/CGGCTGGTACAAAA | ATTTTGGCAACGGCTGATAC | CCACAATCCTCCGTTGTCTT | 60.0 | 492 | INTERGENIC | | | | |
| CaPOPI_2202 | Ca4 | 19780699 | CTTGTAATTTGTAATTTGT/CTTGTAATTTGTAATTTGTAATTTGT | AGCAAACCACACAACCACAA | TTTGTGACCACCACTTCCAA | 60.0 | 484 | INTERGENIC | | | | |
| CaPOPI_2203 | Ca4 | 20425177 | TTTATTATTATTATTATTATTATTATTATTATTATTATTATTATTATT/TTTATTATTATTATTATTATTATTATTATTATTATTATTATTATTATTATT | AATGAAAAACCGTGCAAAGC | ATTGGTGAATGGCAATTGGT | 60.1 | 529 | INTERGENIC | | | | |
| CaPOPI_2204 | Ca4 | 20650435 | TTATATATATATATATATATATATAT/TTATATATATATATATATATATAT | CGTGTGAGAAAACCTCGACA | CACCCTTACAAGTCGCCATT | 59.9 | 733 | INTERGENIC | | | | |
| CaPOPI_2205 | Ca4 | 20709240 | ATTTTTTT/ATTTTTTTT | ACTTTGCCCATCAGTTTTGC | TCCGTTGGTGAAAGTTTGAA | 60.1 | 251 | INTERGENIC | | | | |
| CaPOPI_2206 | Ca4 | 20714850 | AAAAATGATTTTT/AAAAATGATTTTTTTAACAAAATGATTTTT | CATGTGGAAAGTAGGGGTGAA | ACAACGTCCGTCTGAGGTCT | 59.8 | 930 | INTERGENIC | | | | |
| CaPOPI_2207 | Ca4 | 20798832 | TTTTATTTATTTATTTATT/TTTTATTTATTTATT | TAGTGCTTTTGCATGCCTCA | TTTGTCCTGGGAAAATGCTC | 60.5 | 316 | INTERGENIC | | | | |
| CaPOPI_2208 | Ca4 | 20870966 | ATTTTTTTT/ATTTTTTT | ACTCATTGTGTTGGCTTTCG | GTGGATGAAGGGGGTAGGTT | 58.8 | 776 | DRR | Ca_19043 | | | Late embryogenesis abundant protein, group 4 |
| CaPOPI_2209 | Ca4 | 21028273 | AATATATATATATATATATAT/AATATATATATATATATAT | CCACCACTTGGTTCCAATCT | AGGCCATCTAGCTAAGCACAA | 59.8 | 380 | INTERGENIC | | | | |
| CaPOPI_2210 | Ca4 | 21060178 | GC/G | GGATGATAACTCCTTCAAAGCG | TGCTCATTTAATTTGACTGGGA | 60.1 | 711 | INTERGENIC | | | | |
| CaPOPI_2211 | Ca4 | 21071879 | T/TA | AATTGAGTGATCGAGTCGGC | ATGACGCGTGTCCAATTACA | 60.2 | 837 | INTERGENIC | | | | |
| CaPOPI_2212 | Ca4 | 21181744 | A/ATGG | GAGGGAGATGATCGTGGAAA | GCTTCTTGGAAAAAGCGTTG | 60.0 | 294 | INTERGENIC | | | | |
| CaPOPI_2213 | Ca4 | 21226048 | AAA/AAAAAGGAACCAA | CACGGCTCTACGAAGGAAAG | TGAACACCCCCAAGGTTAAG | 60.0 | 287 | INTERGENIC | | | | |
| CaPOPI_2214 | Ca4 | 21376315 | CATTATTATTATTATTATTATTATTATTATTATTATTATTATTATTATTA/CATTATTATTATTATTATTATTATTATTATTATTATTATTATTATTATTA | CCCCCTCTAAACCAACGTAA | CATTGGACTTGTGGCTTGTG | 58.9 | 575 | INTERGENIC | | | | |
| CaPOPI_2215 | Ca4 | 21426612 | ATTTTT/ATTTTTT | TGCAGATAAATGGCCAAACA | AGAACGGTTGCCAGAGAAGA | 60.1 | 370 | INTERGENIC | | | | |

| INDEL marker IDs | Chromosomes/ unanchored scaffolds | Physical positions (bp) | InDels (*Kabuli* reference genome-CDC Frontier/PI) | Forward primers (5'-3') | Reverse primers (5'-3') | Anneling temperature (0C) | Expected amplified product size (bp) | Structural annotation | | Functional annotation | | |
|---|---|---|---|---|---|---|---|---|---|---|---|---|
| | | | | | | | | Sequence components of *kabuli* genome | *Kabuli* gene accession IDs | NCBI-KOG | TFs | NCBI-nr database |
| CaPOPI_2216 | Ca4 | 21514634 | ATGT/ATGTGT | TTGGAATCATGAGTTGATCTCG | AGAAGAACCACGACAAAGGC | 60.1 | 656 | INTERGENIC | | | | |
| CaPOPI_2217 | Ca4 | 21529168 | CAAAA/CAA | TGGGGGATTGTATTCCAAAA | TATGGAATGGACCTCCTGCT | 60.0 | 499 | INTERGENIC | | | | |
| CaPOPI_2218 | Ca4 | 21529316 | T/TA | TGGGGGATTGTATTCCAAAA | TGAAATCGTCACCGTCTCAA | 60.0 | 886 | INTERGENIC | | | | |
| CaPOPI_2219 | Ca4 | 21657934 | TTATATATATATATATATATA/TTATATATATATATATATATATATA | AAATGTGAAAGCCACAACCC | CCATGTAGCATGGCAACAAC | 59.8 | 570 | INTERGENIC | | | | |
| CaPOPI_2220 | Ca4 | 21804779 | CTGT/CTGTTATGT | CCGCATCATAAACAGCCTTT | CTAAAGCTGGTGCTTTTGGC | 60.1 | 159 | INTERGENIC | | | | |
| CaPOPI_2221 | Ca4 | 21910161 | ATTTTTTTTTTT/ATTTTTTTTTT | TGCCCGTAGTTGTACCATGA | CGATGCAAACGTGGATTATG | 60.0 | 383 | INTERGENIC | | | | |
| CaPOPI_2222 | Ca4 | 22097668 | ATTTTTTTTTTTT/ATTTTTTTTTTT | TGAAGCACCTTTGATGAGGA | GACGTGATAACATGAAGGATGC | 59.4 | 850 | INTERGENIC | | | | |
| CaPOPI_2223 | Ca4 | 22134995 | CATAT/CAT | GCACCTATGGTGGCAAAAAC | TTTGATGCCCACATTGAAGA | 60.4 | 506 | DRR | Ca_20134 | J | | Brix domain |
| CaPOPI_2224 | Ca4 | 22309344 | ATTTTTTTTT/ATTTTTTTTTT | TGCTTTGTTCAATTGCTTGC | AGTGCCAAGTAAGGTTGCTGA | 60.0 | 566 | INTERGENIC | | | | |
| CaPOPI_2225 | Ca4 | 22324512 | TAAAAAAAAAAA/TAAAAAAAAAAAA | CGCTGATTCATCCTTTTCGT | TTGAACTCCTTTCTGGGTGG | 60.2 | 937 | INTERGENIC | | | | |
| CaPOPI_2226 | Ca4 | 22381273 | AATATATATATATATATA/AATATATATATATATATATA | CAGTGGCGAAATCTGCATTA | TGGACGTAGGTTGTTGTGGA | 59.8 | 702 | INTERGENIC | | | | |
| CaPOPI_2227 | Ca4 | 22441886 | CTA/CTATA | TCCAATGGTGGAAAATAAACG | TCAATCACAAATTTCAAGGAGG | 59.7 | 848 | INTERGENIC | | | | |
| CaPOPI_2228 | Ca4 | 22499567 | A/ACACGACGCTAAAATACCC | ATGCAATGCGTATATGCCAA | TACATGGTGACCGCGATAGA | 59.9 | 352 | INTERGENIC | | | | |
| CaPOPI_2229 | Ca4 | 22508434 | CATATATATATATATATATATATATAT/CATATATATATATATATATATATATATATATATATATATAT | TGGAAGGAAAACAATCGAGG | GCATTGCAATAATCCATCCA | 60.0 | 647 | INTERGENIC | | | | |
| CaPOPI_2230 | Ca4 | 22546535 | AATATATATATATA/AATATATATATATATA | CATTTTGGTTTCAAAGCCGT | CCAGGGGATGAGATCATAGG | 60.0 | 714 | INTERGENIC | | | | |

| INDEL marker IDs | Chromosomes/ unanchored scaffolds | Physical positions (bp) | InDels (*Kabuli* reference genome-CDC Frontier/PI) | Forward primers (5'-3') | Reverse primers (5'-3') | Anneling temperature (0C) | Expected amplified product size (bp) | Structural annotation | | Functional annotation | | |
|---|---|---|---|---|---|---|---|---|---|---|---|---|
| | | | | | | | | Sequence components of *kabuli* genome | *Kabuli* gene accession IDs | NCBI-KOG | TFs | NCBI-nr database |
| CaPOPI_2231 | Ca4 | 22601747 | TAAAAAAAAA/TAAAAAAAAAAA | GTCGCGTCTTCCATTCA TTT | GATTTTCCCTTGTCAACA ATCA | 60.1 | 229 | INTERGENIC | | | | |
| CaPOPI_2232 | Ca4 | 22656873 | TA/TAGAAAAGCAGA | GACACTCAAGGCTGAAG TAGAAAA | ACCTGACAACCCCAAAT TGA | 59.1 | 246 | INTERGENIC | | | | |
| CaPOPI_2233 | Ca4 | 22664879 | AA/AAGA | TCGCGTTCGATTTGTTC ATA | GGTCATTCTTATTGATTG ACCCA | 60.2 | 570 | INTERGENIC | | | | |
| CaPOPI_2234 | Ca4 | 22674188 | CAAAAAAAAAA/CAAAAAAAAA | AATGCATGTTGCACCAC AGT | GCTGAAAAGGAAGGGAA AGG | 60.0 | 292 | INTERGENIC | | | | |
| CaPOPI_2235 | Ca4 | 22675724 | TTT/TTTCTT | CACAGAAAACTGAAACG CGA | ATGGCAATTAGGGTGTG CTC | 60.0 | 325 | INTERGENIC | | | | |
| CaPOPI_2236 | Ca4 | 22703169 | ATTTTTT/ATTTTTTT | GTGCAAACAAGGAGCAA CAA | GCCTCCAATCCATGTCT GAT | 59.9 | 322 | INTERGENIC | | | | |
| CaPOPI_2237 | Ca4 | 22719677 | TATAAA/TAA | TTTTGGCACGTCTTCTC AACT | CATCGACGATTGTACCA AAAA | 59.9 | 548 | INTERGENIC | | | | |
| CaPOPI_2238 | Ca4 | 22723833 | TGGGGG/TGGGGGG | TCTAACACCGGCAACAT CAA | AGGCCACGAAAGTGAAG AGA | 60.1 | 527 | DRR | Ca_14476 | | | Protein of unknown function DUF593 |
| CaPOPI_2239 | Ca4 | 22727877 | TAAA/TAA | TTGGTTCCTTTATTAACT AATTTTTGG | GGGACAAACGTTTTTGC TATG | 59.3 | 809 | INTERGENIC | | | | |
| CaPOPI_2240 | Ca4 | 22735575 | AAAA/AAAATAAA | TTGAAGTTGTCGATCCC CTC | CATATTCAACGCAACAA CGC | 60.1 | 553 | DRR | Ca_14475 | T | FAR1 | Protein kinase, catalytic domain |
| CaPOPI_2241 | Ca4 | 22746108 | ACC/ACCC | GGAAGAAGCATGTGGGT TGT | TTGGCCTTGAGACCAGC TAT | 60.0 | 721 | INTERGENIC | | | | |
| CaPOPI_2242 | Ca4 | 22755158 | ATTTTTTT/ATTTTTTTT | CTATGTGCGTCGTCAGG AAA | CCAAAAACGGTTTCACC AGT | 59.9 | 595 | INTERGENIC | | | | |
| CaPOPI_2243 | Ca4 | 22765623 | CTT/CTTT | TGGGGACCTTCCCAATA TAC | TATGGCCGATGCTACAA CAA | 58.6 | 219 | INTERGENIC | | | | |
| CaPOPI_2244 | Ca4 | 22766072 | AATATA/AATATATATA | TAATTACTTTCCGCGGA TGC | GGTGTAGCCTTTGCTTG GAG | 60.1 | 424 | INTERGENIC | | | | |
| CaPOPI_2245 | Ca4 | 22766863 | T/TGG | GCATAATCAAAAGCATT GGC | ATCGATTCTCAGTTGGA CCG | 58.3 | 756 | INTERGENIC | | | | |

| INDEL marker IDs | Chromosomes/ unanchored scaffolds | Physical positions (bp) | InDels (*Kabuli* reference genome-CDC Frontier/PI) | Forward primers (5'-3') | Reverse primers (5'-3') | Anneling temperature (0C) | Expected amplified product size (bp) | Structural annotation | | Functional annotation | | |
|---|---|---|---|---|---|---|---|---|---|---|---|---|
| | | | | | | | | Sequence components of *kabuli* genome | *Kabuli* gene accession IDs | NCBI-KOG | TFs | NCBI-nr database |
| CaPOPI_2246 | Ca4 | 22767329 | AACAC/AAC | CCAACTGAGAATCGATGGGT | TCTCCTTTTCAAATTCCTCCAA | 59.9 | 274 | INTERGENIC | | | | |
| CaPOPI_2247 | Ca4 | 22792612 | ATTT/ATT | CCTTGAGTGAGTTTGACAGGAA | AAACCATAAAGCCCCAAACC | 59.4 | 577 | DRR | Ca_14472 | | | |
| CaPOPI_2248 | Ca4 | 22793993 | ATTT/ATT | CGAGTGGCAGAACTTCAAAA | GAATTATGCCGCAAGTACGA | 59.0 | 318 | DRR | Ca_14472 | | | |
| CaPOPI_2249 | Ca4 | 22799910 | AAAA/AAAAAAATTAAA | TCGGTGTGATTTGTGTGTGA | GTGCAATCATGCAATCAAGG | 59.5 | 672 | INTERGENIC | | | | |
| CaPOPI_2250 | Ca4 | 22824416 | CCGT/CCGTACGT | TCTATTCCCGGTGTTGAAGC | TGCACTCTAATGGGATTATTTTGA | 60.1 | 147 | INTERGENIC | | | | |
| CaPOPI_2251 | Ca4 | 22825034 | TAAAAAA/TTAAAAAAA | TCCTCTAAAACATTTGTGTTGTCA | TCACTCTCTTACTATGGAAGCCAA | 58.7 | 609 | INTERGENIC | | | | |
| CaPOPI_2252 | Ca4 | 22837601 | CAAAAA/CAAAA | TTGAAATGGCAAAGAGGGTC | ATGCATGGGTTGAAATACCC | 60.1 | 556 | DRR | Ca_14468 | | | |
| CaPOPI_2253 | Ca4 | 22837725 | AAATAATAA/AAATAA | TTGAAATGGCAAAGAGGGTC | ATGCATGGGTTGAAATACCC | 60.1 | 556 | DRR | Ca_14468 | | | |
| CaPOPI_2254 | Ca4 | 22848996 | TAAAAAAA/TAAAAAAAA | TTCATTATCTGTTCCCTCCCA | ACGACTGGCTAAGGTGCTGT | 59.4 | 546 | INTERGENIC | | | | |
| CaPOPI_2255 | Ca4 | 22864646 | TAGAGAGA/TAGAGA | TGAGACTGCAATTTGGCTTG | ACGACGATGTTCATTCCACA | 60.0 | 914 | DRR | Ca_14466 | S | | Protein of unknown function DUF408 |
| CaPOPI_2256 | Ca4 | 22867454 | GAAAAAAAAAA/GAAAAAAAAA | GATCTTCATCCCCTGCAGAC | CACCACCACCACAAAACACT | 59.6 | 292 | INTERGENIC | | | | |
| CaPOPI_2257 | Ca4 | 22884702 | CAAAA/CAAA | TTTGGTACCCAATTTTCCCA | GGTGAAAGCGTTTGATGGAT | 60.0 | 564 | INTERGENIC | | | | |
| CaPOPI_2258 | Ca4 | 22889620 | TATAAAAATAAAAATAAAA/TATAAAAATAAAAATAAAAATAAAA | GGGTCAGGATACTTACAACCCA | TCCACCCCCAAATTTTATCA | 60.1 | 717 | INTERGENIC | | | | |
| CaPOPI_2259 | Ca4 | 22896917 | GTTTTTTTTTTT/GTTTTTTTTT | CGATGCGCATGATAGAAAGA | AACGGAACCCCACTTAAAGG | 59.9 | 590 | INTERGENIC | | | | |
| CaPOPI_2260 | Ca4 | 22897920 | ATTTTTTTTT/ATTTTTTTTTT | GGTTGGCTTGTGATCTCCAT | TGCTTGTGGTCTGAAACCTG | 59.9 | 463 | INTERGENIC | | | | |

| **INDEL marker IDs** | **Chromosomes/ unanchored scaffolds** | **Physical positions (bp)** | **InDels (*Kabuli* reference genome-CDC Frontier/PI)** | **Forward primers (5'-3')** | **Reverse primers (5'-3')** | **Anneling temperature (0C)** | **Expected amplified product size (bp)** | **Structural annotation** | | **Functional annotation** | | |
|---|---|---|---|---|---|---|---|---|---|---|---|---|
| | | | | | | | | **Sequence components of *kabuli* genome** | ***Kabuli* gene accession IDs** | **NCBI-KOG** | **TFs** | **NCBI-nr database** |
| CaPOPI_2261 | Ca4 | 22898673 | CAAAA/CAA | AACCCCACTTAAAGGGCAAC | AGGGTGAAGAAGCTCACACAA | 60.2 | 684 | INTERGENIC | | | | |
| CaPOPI_2262 | Ca4 | 22901962 | CAAAA/CAAA | AGGGACTCAGTTTTCTGCGA | CAGACAAGCCTCTCGATTCC | 60.0 | 593 | INTERGENIC | | | | |
| CaPOPI_2263 | Ca4 | 22914105 | GTTTTTTTT/GTTTTTTTTTTT | AAAACCCTTTTGCTTCCGTT | TCCTACCAAAAACTTCATTCACAA | 60.0 | 458 | INTERGENIC | | | | |
| CaPOPI_2264 | Ca4 | 22914458 | CTTT/CT | ATTGCGCTTGCATCATCTTT | CCAAAGTTTCAACTTCGAGACA | 60.8 | 671 | INTERGENIC | | | | |
| CaPOPI_2265 | Ca4 | 22921547 | TAA/TAAAATAAATAAAAAA | TCGTCCGAACACAATCACAT | TGATCTCGTGCTTCTACGTGTT | 60.0 | 311 | INTERGENIC | | | | |
| CaPOPI_2266 | Ca4 | 22933272 | CAAATAAAAAATAAAAAAT/CAAATAAAAAAT | CTATAGTGACCGCACTCCGC | TTCCAAAATTCAGGAATCGC | 60.8 | 774 | INTRON | Ca_14461 | | | Heat shock protein DnaJ, N-terminal |
| CaPOPI_2267 | Ca4 | 22933918 | CTGAACAATT/CTGAACAATTGCGGTGAACAATT | AGAAACCCAAAAGGGGAAGA | TTTGAACCCAAGCTTCAACC | 59.9 | 750 | INTRON | Ca_14461 | | | Heat shock protein DnaJ, N-terminal |
| CaPOPI_2268 | Ca4 | 22973209 | TAAAAAAAAA/TAAAAAAAAAAA | ATTTTATTGGCATCGGCATT | TGCGAATGTGCTTTCTGTCT | 59.3 | 672 | DRR | Ca_14460 | | | |
| CaPOPI_2269 | Ca4 | 22974338 | A/AAT | ATTTTGGGCGAAATCCTTTT | GAACCACTCATCCTCCCAAA | 59.8 | 442 | DRR | Ca_14460 | | | |
| CaPOPI_2270 | Ca4 | 22981343 | ATTTTTTTT/ATTTTTTT | TCCGCCGTTCTAAATTCTTG | CAAGCCCTCTCCCTCTCTTC | 60.2 | 874 | INTERGENIC | | | | |
| CaPOPI_2271 | Ca4 | 22984796 | GTTTA/GTTTAAACTTTA | CAAATTCCCTTTAGTGCCACA | TGCTTATGTGTTCCATTTGCTC | 60.0 | 802 | INTERGENIC | | | | |
| CaPOPI_2272 | Ca4 | 23002548 | ATTATTTTATTTTATTTTATT/ATTATTTTATTTTATTTTATTTTATT | TCGATCGCTGATGTCAAACT | TGGGCTTGACCTATGTGTGT | 59.4 | 147 | INTERGENIC | | | | |
| CaPOPI_2273 | Ca4 | 23002623 | TTTACTATTACTATT/TTTACTATTACTATTACTATT | TTTATGGCGGCTGGTTTTAC | ACGATGCATTTGATTGATGTT | 60.0 | 546 | INTERGENIC | | | | |
| CaPOPI_2274 | Ca4 | 23018840 | AA/AATTA | CAAAATAAATCAAATGATTGAAGTGA | GGCCTGACGACACAGCTAAT | 58.6 | 651 | INTERGENIC | | | | |
| CaPOPI_2275 | Ca4 | 23020256 | TTAT/TT | TCATGACCAAGAAACAACGG | TCACATATCAAATGTTGGCACA | 59.5 | 354 | INTERGENIC | | | | |

| INDEL marker IDs | Chromosomes/ unanchored scaffolds | Physical positions (bp) | InDels (*Kabuli* reference genome-CDC Frontier/PI) | Forward primers (5'-3') | Reverse primers (5'-3') | Anneling temperature (0C) | Expected amplified product size (bp) | Structural annotation | | Functional annotation | | |
|---|---|---|---|---|---|---|---|---|---|---|---|---|
| | | | | | | | | Sequence components of *kabuli* genome | *Kabuli* gene accession IDs | NCBI-KOG | TFs | NCBI-nr database |
| CaPOPI_2276 | Ca4 | 23027444 | T/TC | CCATTGGGAATTAGGCATTG | AAGGTTGTGGATCGATTCGT | 60.1 | 868 | INTERGENIC | | | | |
| CaPOPI_2277 | Ca4 | 23055622 | TAAGAA/TAAGAAAGAA | TGAATTAAAGGATTGCATGAACA | CCGTTGAATGGAAAATCACC | 59.5 | 530 | DRR | Ca_14459 | | | |
| CaPOPI_2278 | Ca4 | 23056577 | TAAAAAAAAAA/TAAAAAAAAA | ATGAATAGCATGCCACGACA | TCATGTGGGCTCATTTGGTA | 60.1 | 771 | INTERGENIC | | | | |
| CaPOPI_2279 | Ca4 | 23060568 | ATTTTTTTTT/ATTTTTTTTTT | ATGGGCATTATGCAGTCACA | TTTGCCAATTTATCATGTTTGC | 60.0 | 766 | INTERGENIC | | | | |
| CaPOPI_2280 | Ca4 | 23067690 | GTTTTTTTTT/GTTTTTTTTTT | CGGAAATAACTGACCGCAAT | GGAATCGAAAGTCGTGGAAA | 60.0 | 373 | INTERGENIC | | | | |
| CaPOPI_2281 | Ca4 | 23077402 | TAAAAAAAA/TAAAAAAAAA | ACCAAAGAATCCATCGGTTG | TGAATTCATGTGTATTGTCGGA | 59.8 | 842 | INTERGENIC | | | | |
| CaPOPI_2282 | Ca4 | 23077586 | ATTTTTT/ATTTTTTT | AAGAAAAGGGTTGCGTTTGA | TGAATTCATGTGTATTGTCGGA | 59.7 | 644 | INTERGENIC | | | | |
| CaPOPI_2283 | Ca4 | 23078030 | GAAAAAA/GAAAAAAAA | TTTGAAGAACCAGAGTTTGAAGAA | TTCAATGCCCATAATCGTGA | 59.5 | 656 | INTERGENIC | | | | |
| CaPOPI_2284 | Ca4 | 23079340 | CAAA/CAAAA | AATTGCAAGTGGACTGGACC | CCGAGCCAAAATCCAAAATA | 60.0 | 787 | INTERGENIC | | | | |
| CaPOPI_2285 | Ca4 | 23134474 | ATTGTTGTTGT/ATTGTTGT | ATGCTGCGAAATTGTTGAAA | AAAAGCGTTGTAAATTGGATTGA | 59.3 | 342 | INTERGENIC | | | | |
| CaPOPI_2286 | Ca4 | 23135482 | GAAAAAA/GAAAAA | ATTCAGCAATGAGAGGTCCG | GATGATGCATGCCATTTGAG | 60.2 | 752 | INTERGENIC | | | | |
| CaPOPI_2287 | Ca4 | 23217567 | AATATATATATATATATATAT/AATATATATATATATATATAT | CTCTAGGGAAGCGACCTCCT | CCAACAACCATTGTGGGAGT | 60.0 | 633 | INTERGENIC | | | | |
| CaPOPI_2288 | Ca4 | 23525491 | GTTTTTTTTTT/GTTTTTTTTT | TGAGATTATATGCACGCGGA | TGAATCGCGAAAGTAGTCCC | 60.2 | 574 | INTERGENIC | | | | |
| CaPOPI_2289 | Ca4 | 23756363 | TTATATATATATATATATA/TTATATATATATATATATATA | TTGGAACGTAATTTGATGCG | CCCGTTGCTTCTGTATGCTT | 59.6 | 872 | INTERGENIC | | | | |
| CaPOPI_2290 | Ca4 | 23960230 | AAATAATAATAATAATAATAATAAT/AAATAATAATAATAATAATAATAATAAT | GTCTGTGATTTGGTGCAACG | AGGCCAATGAGGCAAGAGTA | 60.2 | 617 | INTERGENIC | | | | |

| INDEL marker IDs | Chromosomes/ unanchored scaffolds | Physical positions (bp) | InDels (*Kabuli* reference genome-CDC Frontier/PI) | Forward primers (5'-3') | Reverse primers (5'-3') | Anneling temperature (0C) | Expected amplified product size (bp) | Structural annotation | | Functional annotation | | |
|---|---|---|---|---|---|---|---|---|---|---|---|---|
| | | | | | | | | Sequence components of *kabuli* genome | *Kabuli* gene accession IDs | NCBI-KOG | TFs | NCBI-nr database |
| CaPOPI_2291 | Ca4 | 24100684 | AATATATATATATATATATATATATA/AATATATATATATATATATATATATA | AATGGTCCAAGGAAAAAGGG | ATCCCCAATTGGAACCTACC | 60.2 | 753 | INTERGENIC | | | | |
| CaPOPI_2292 | Ca4 | 24115337 | ATATAT/ATATATATCTATAT | CACACGTGCATGGATAGAGG | TTCACCGTGGATGAATTTGA | 60.1 | 341 | INTERGENIC | | | | |
| CaPOPI_2293 | Ca4 | 24140540 | AT/ATATT | TTATTTCACCGTCGTGTCCA | TTGTGTGTTGGCCGATTTTA | 60.0 | 234 | INTERGENIC | | | | |
| CaPOPI_2294 | Ca4 | 24273906 | TTATATATATATATATATATATATATA/TTATATATATATATATATATATATA | GGGGGAGTTGTTATTCGGTT | GAAAAGTGTTGTCGTGTGTGC | 60.1 | 646 | INTERGENIC | | | | |
| CaPOPI_2295 | Ca4 | 24359275 | TAAAAAAAA/TAAAAAAAAAA | GGGAGGGTGGGAGTAAATGT | TGTGGCTTTCCAATGTACGA | 60.1 | 571 | INTERGENIC | | | | |
| CaPOPI_2296 | Ca4 | 24368839 | TAAAAAAA/TAAAAAA | TTTTTCCGACTTGGTCCTTT | GGACTTTGGTTCTCTTGGCA | 58.7 | 381 | INTERGENIC | | | | |
| CaPOPI_2297 | Ca4 | 24427573 | GTT/GTTT | CTGGAAAATCCTCCTCCTCC | TTCGTCTCAACAATCTCCCC | 60.0 | 346 | INTERGENIC | | | | |
| CaPOPI_2298 | Ca4 | 24442934 | GCA/GCATTCA | TCGCCTTCAAAGAAAACCAC | GGCATGAAATCCATCACCTT | 60.2 | 568 | DRR | Ca_20866 | R | NAC | Pentatricopeptide repeat |
| CaPOPI_2299 | Ca4 | 24455402 | TTATATATATATATAT/TTATATATATATATATAT | AGCACCAAAGCCACCATTAC | AGCCTCTTTTTGGGACCACT | 60.0 | 236 | INTERGENIC | | | | |
| CaPOPI_2300 | Ca4 | 24478956 | CAA/CAAA | CGGTTCTACACAAGTTGCGA | TCGATTATGCGTAGGGAAGG | 59.9 | 746 | INTERGENIC | | | | |
| CaPOPI_2301 | Ca4 | 24479035 | ATTTTTTT/ATTTTTTTTTT | CGGTTCTACACAAGTTGCGA | TCGATTATGCGTAGGGAAGG | 59.9 | 746 | INTERGENIC | | | | |
| CaPOPI_2302 | Ca4 | 24510502 | CTGGAACATTGGAACATTG/CTGGAACATTG | CTGCAAAAGCCCTCACAAAT | TCCTCTTGCACACCACAAGA | 60.2 | 969 | INTERGENIC | | | | |
| CaPOPI_2303 | Ca4 | 24553735 | GATTAT/GAT | TGGTGCGATTGTGGAAAATA | GAGTAGATGTGCCAACAGCG | 59.9 | 361 | INTERGENIC | | | | |
| CaPOPI_2304 | Ca4 | 24828297 | ATTTTTTTTTT/ATTTTTTTTTTT | TGGAATTTCAGACTGCCTTG | GGGCACGAAGAAGCACTATC | 58.8 | 716 | INTERGENIC | | | | |
| CaPOPI_2305 | Ca4 | 24897433 | GAAAAAAAAA/GAAAAAAAAAA | TGCACAAACCTTTCAATGCT | TTTTTGAATTTTGAACGAAACA | 59.3 | 775 | INTERGENIC | | | | |

| INDEL marker IDs | Chromosomes/ unanchored scaffolds | Physical positions (bp) | InDels (*Kabuli* reference genome-CDC Frontier/PI) | Forward primers (5'-3') | Reverse primers (5'-3') | Anneling temperature (0C) | Expected amplified product size (bp) | Structural annotation | | Functional annotation | | |
|---|---|---|---|---|---|---|---|---|---|---|---|---|
| | | | | | | | | Sequence components of *kabuli* genome | *Kabuli* gene accession IDs | NCBI-KOG | TFs | NCBI-nr database |
| CaPOPI_2306 | Ca4 | 24905867 | ATTTTTTTTTTTTT/ATTTTTTTTTTTT | GCAATTTTGCAGAACATGGA | CCTTTCAGCCTTTTTGTTGG | 59.7 | 140 | INTERGENIC | | | | |
| CaPOPI_2307 | Ca4 | 24940280 | GAAAAAAAAAAA/GAAAAAAAAAAAA | TTGTTGTCGCAATGGAATGT | TGCTTGGTCACTTTGGCATA | 60.0 | 315 | INTERGENIC | | | | |
| CaPOPI_2308 | Ca4 | 25070539 | TTTTATTTATTTATTTATTTATTTATTT/TTTTATTTATTTATTTATTTATTT | TTTGCAAAAACCAATGAATACTT | GTTTTGAGCCCATTCACGAT | 57.8 | 596 | INTERGENIC | | | | |
| CaPOPI_2309 | Ca4 | 25071336 | CA/C | TGAGACTGCTTGGCTACCCT | ATCTGAATCATCCCAAGCCA | 60.0 | 488 | INTERGENIC | | | | |
| CaPOPI_2310 | Ca4 | 25097629 | CAAAAAA/CAAAAAAA | TCAATGATGGTGACTTGGGA | TACCCATTTGGTCTTGGGTC | 59.9 | 820 | INTERGENIC | | | | |
| CaPOPI_2311 | Ca4 | 25177814 | CAGAGAGA/CAGAGA | TCCTTGATCGGTTAAGTCGG | TAAAGGACCAAGTCGGGAAA | 60.1 | 373 | INTERGENIC | | | | |
| CaPOPI_2312 | Ca4 | 25186812 | TAAAAAAAAA/TAAAAAAAA | CCACAGGGTTAAGTTTCCGA | GCCAAACTCTCCTCCCTACC | 60.0 | 657 | INTRON | Ca_16562 | | | Coenzyme F420 hydrogenase/dehydrogenase beta subunit, N-terminal |
| CaPOPI_2313 | Ca4 | 25205443 | TAGATTTGGCACAAAAGATTTGG/TAGATTTGG | GATGGAGGATCAGCCACATT | ATGGTGGAATGCCTCAAAAG | 59.9 | 648 | INTERGENIC | | | | |
| CaPOPI_2314 | Ca4 | 25213751 | GAG/GAGATCATAG | TCATTCAACGTCATCGTATCAA | TGTGTTTGTATCAATATCGAAGGC | 59.0 | 562 | INTERGENIC | | | | |
| CaPOPI_2315 | Ca4 | 25228841 | AATATATATATATAT/AATATATATATATATATAT | ATGATTACCTCGGCATGGAG | AGCACGAGATCAAAGCACAA | 59.9 | 349 | INTRON | Ca_16565 | | | |
| CaPOPI_2316 | Ca4 | 25290328 | GTTTTTTTT/GTTTTTTT | TCACACCGTGAGATTTTCCA | GAGGCTCCAGATCGAAGTTG | 60.1 | 629 | INTERGENIC | | | | |
| CaPOPI_2317 | Ca4 | 25292705 | GTTAATTAA/GTTAA | GGCAGAAATTTGAACGTGGT | GGACAAAAGAAGAGCAAAAAGG | 60.0 | 227 | INTERGENIC | | | | |
| CaPOPI_2318 | Ca4 | 25352495 | GGTT/G | GGAAATGGTTCTTTGAAAGCC | AGCCTTACGCATGTATTGCC | 59.9 | 719 | INTERGENIC | | | | |
| CaPOPI_2319 | Ca4 | 25352610 | T/TA | GGAAATGGTTCTTTGAAAGCC | AGCCTTACGCATGTATTGCC | 59.9 | 719 | INTERGENIC | | | | |
| CaPOPI_2320 | Ca4 | 25353133 | GAAAAAAAA/GAAAAAAA | CGTCGCACTAAGACGATGAA | GGGTAAGGGTACGTTGAGTCC | 60.0 | 415 | INTERGENIC | | | | |

| INDEL marker IDs | Chromosomes/ unanchored scaffolds | Physical positions (bp) | InDels (*Kabuli* reference genome-CDC Frontier/PI) | Forward primers (5'-3') | Reverse primers (5'-3') | Anneling temperature (0C) | Expected amplified product size (bp) | Structural annotation | | Functional annotation | | |
|---|---|---|---|---|---|---|---|---|---|---|---|---|
| | | | | | | | | Sequence components of *kabuli* genome | *Kabuli* gene accession IDs | NCBI-KOG | TFs | NCBI-nr database |
| CaPOPI_2321 | Ca4 | 25358106 | TTAAATAAATAAATAAATAAATAA/TTAAATAAATAAATAAATAA | GGGACTACTTTCGCGATTCA | ATTACACGCTTGCAGACGAA | 60.2 | 842 | INTERGENIC | | | | |
| CaPOPI_2322 | Ca4 | 25365917 | ATTTT/ATT | AATTTTGTTGCCCCTCCAAT | TGCTATGGCACAAGTCTCCA | 60.5 | 547 | INTERGENIC | | | | |
| CaPOPI_2323 | Ca4 | 25367189 | A/AT | TTTATTGTAGGTGGAGGCGG | GGCCAAATTGATGCAAAGTT | 60.0 | 271 | INTERGENIC | | | | |
| CaPOPI_2324 | Ca4 | 25457334 | ATTTTTTT/ATTTTTTTT | CATTTTACTCATCGATCTTTGTGG | TGGACAAAATCATGTCTCTTTGA | 59.9 | 336 | DRR | Ca_16577 | | | |
| CaPOPI_2325 | Ca4 | 25466972 | TAAAAAAA/TAAAAAAAA | TATAGGGAGCGGAACACACC | TCTAACTCCAGGTAACGCCG | 60.0 | 840 | INTERGENIC | | | | |
| CaPOPI_2326 | Ca4 | 25468253 | ATTTTTTT/ATTTTTTTT | CAAAATACAAATGTGATGGAAAAA | TTTTTGTTTCCTTATTGTTTGACA | 58.0 | 648 | INTERGENIC | | | | |
| CaPOPI_2327 | Ca4 | 25470351 | CTTTTTTTTT/CTTTTTTTTTT | TGCAATTATCCAAAGATGTATGAAA | TCCTTTTGTCCGTCGTCTCT | 59.8 | 479 | INTERGENIC | | | | |
| CaPOPI_2328 | Ca4 | 25478172 | TAAAA/TAAAAA | TGTGATGCAGTTGCTCCATT | GACCTTGCTCTCTTGATGGG | 60.3 | 703 | INTERGENIC | | | | |
| CaPOPI_2329 | Ca4 | 25501013 | TAAAAAAAA/TAAAAAAAAA | GGGCATTGGTAACTCCAAAA | CCAATTGTGCTCTCCTCTCC | 59.8 | 909 | INTERGENIC | | | | |
| CaPOPI_2330 | Ca4 | 25512047 | CTTTATT/CTTTATTTTATT | GCCACTGAATTGTCGGAGAT | ACCCCCAATGAATGAAAACA | 60.1 | 324 | INTRON | Ca_16579 | S | | TMS membrane protein/tumour differentially expressed protein |
| CaPOPI_2331 | Ca4 | 25523236 | TAAAA/TAAA | TGTGGTGGTGATCCTTGAAA | GATTCTCGCATCTGCTAGGC | 59.9 | 786 | INTERGENIC | | | | |
| CaPOPI_2332 | Ca4 | 25527454 | TAAAAAAAAAAAAAA/TAAAAAAAAAAAAAAAAAA | TCATGTGTAATTTTTATCTCAAGGAA | GGCTTGATTGATTCACATCCT | 58.3 | 743 | DRR | Ca_16580 | | | Plant disease resistance response protein |
| CaPOPI_2333 | Ca4 | 25579370 | TAAAAAAAAAA/TAAAAAAAAA | AAATCGGCTCCTTTTAAAACAATA | TCTTCTTCGACGTTTTTGGC | 59.5 | 699 | INTERGENIC | | | | |
| CaPOPI_2334 | Ca4 | 25596032 | TTAT/TTATAT | AGTGATGTGGGCAACAATGA | TGCATTTTACAGCGCTTTTG | 60.0 | 515 | INTERGENIC | | | | |
| CaPOPI_2335 | Ca4 | 25623331 | CATTATTATTATTATTATTATTATTATTATTATTATTATTATTATTATTATTATTATTAT/CATTATTATTATTATTATTATTATTATTATTATTATTATTATTATTATTATTATTA | CACCGGAACCAAGCTTTTTA | GAGGAGGTTTGTTGGATGGA | 60.1 | 376 | INTERGENIC | | | | |

| INDEL marker IDs | Chromosomes/ unanchored scaffolds | Physical positions (bp) | InDels (*Kabuli* reference genome-CDC Frontier/PI) | Forward primers (5'-3') | Reverse primers (5'-3') | Anneling temperature (0C) | Expected amplified product size (bp) | Structural annotation | | Functional annotation | | |
|---|---|---|---|---|---|---|---|---|---|---|---|---|
| | | | | | | | | Sequence components of *kabuli* genome | *Kabuli* gene accession IDs | NCBI-KOG | TFs | NCBI-nr database |
| CaPOPI_2336 | Ca4 | 25651231 | CTTTTTTTTTT/CTTTTTTTTTTT | CGTTTGTCTGTGAAATGGGA | CAAGCGGTAAGCACTAGGGA | 59.5 | 825 | INTRON | Ca_16590 | G | | Glycosyl transferase, family 14 |
| CaPOPI_2337 | Ca4 | 25758636 | CTTTT/CTTTTTT | TGCACTTCAGTTAAATTGGGC | AGAACTTGCGTGGTGATTCC | 60.1 | 530 | INTERGENIC | | | | |
| CaPOPI_2338 | Ca4 | 25768326 | TAAAAAA/TAAAAAAA | ATGTTTCGTGCAATGGTCAA | CACATGGCTGAACGAGAAGA | 60.0 | 382 | INTERGENIC | | | | |
| CaPOPI_2339 | Ca4 | 25776089 | G/GAC | TGGAATGAGAGGGGAAGATG | TCCGAGACAGGTATTTTGGC | 60.0 | 366 | INTERGENIC | | | | |
| CaPOPI_2340 | Ca4 | 25783388 | CTTTT/CTTTTT | GCGTGATGCATGATCTAGGA | TCTTTTGATTCCGAGTTGGG | 59.8 | 658 | INTERGENIC | | | | |
| CaPOPI_2341 | Ca4 | 25790471 | CAAACGAAACGAAACGAAAC/CAAACGAAAC | CCTCAAATTCCAATTGTGCC | TCATCCTCACCCCCAGTTAG | 60.3 | 646 | INTERGENIC | | | | |
| CaPOPI_2342 | Ca4 | 25812288 | CT/C | ATGGTTGTTGCAAGTTTCCC | CCTTGAAGACATATGGTGGCA | 59.8 | 522 | INTRON | Ca_16599 | R | | Aminoglycoside phosphotransferase |
| CaPOPI_2343 | Ca4 | 25822023 | ATTTTTTTTTT/ATTTTTTTTT | ACGCAAGAGCAACATCTACG | TGAAGTCTCGGGTTCAAACA | 59.1 | 617 | INTERGENIC | | | | |
| CaPOPI_2344 | Ca4 | 25822369 | CAAAAAAA/CAAAAAA | TGTTTGAACCCGAGACTTCAC | CGTGGCATTTGAAAAAGCAT | 60.1 | 611 | INTERGENIC | | | | |
| CaPOPI_2345 | Ca4 | 25844453 | AATTATTATTATTATTATTATTATTATTAATGTTATTATTATTATTATTATTATTATT/AATTATTATTATTATTATTATTATTATTATTATTAATGTTATTATTATTATTATTATT | CACTTGGGTCACTGCTGAAG | GATGCTGCATGTGTTGATCC | 59.5 | 662 | DRR | Ca_16601 | R | | Ankyrin repeat |
| CaPOPI_2346 | Ca4 | 25847698 | T/TG | CATGCTACCAAAGCGATTGA | ATCGATTTCGCGAGTCTTGT | 59.8 | 370 | INTERGENIC | | | | |
| CaPOPI_2347 | Ca4 | 25884525 | TA/T | AGCAACCAAATCGAAACACC | GGGAAATTTTGGAACCAATG | 60.0 | 307 | INTERGENIC | | | | |
| CaPOPI_2348 | Ca4 | 25916811 | CTTTTTTTTTTT/CTTTTTTTTTTTTTT | ATGGTAACAGATATCGCCGC | ATTGTAGCATTGCCCAAAGG | 60.0 | 627 | DRR | Ca_16606 | I | | Thiolase |
| CaPOPI_2349 | Ca4 | 25924178 | ATTTTTT/ATTTTTTT | TGATGATGTCATTGATGTGTTTG | CTTGTAAGTTGTAGGCGGGC | 59.3 | 778 | INTERGENIC | | | | |
| CaPOPI_2350 | Ca4 | 25937535 | GC/GCTTTTGCTC | TCGACGTTGTCCATTTGTGT | ACACGAGCGTTTGAGCTTTT | 60.0 | 621 | INTERGENIC | | | | |

| INDEL marker IDs | Chromosomes/ unanchored scaffolds | Physical positions (bp) | InDels (*Kabuli* reference genome-CDC Frontier/PI) | Forward primers (5'-3') | Reverse primers (5'-3') | Anneling temperature (0C) | Expected amplified product size (bp) | Structural annotation | | Functional annotation | | |
|---|---|---|---|---|---|---|---|---|---|---|---|---|
| | | | | | | | | Sequence components of *kabuli* genome | *Kabuli* gene accession IDs | NCBI-KOG | TFs | NCBI-nr database |
| CaPOPI_2351 | Ca4 | 25954241 | A/ATTC | TTGCAAGAATCCATCCATCA | TTGAGCATTTCAGCAGAAGC | 60.0 | 493 | INTERGENIC | | | | |
| CaPOPI_2352 | Ca4 | 25956176 | ATTTTTTTTTTTTT/ATTTTTTTTTT | ATATTGGGACCGGTTAAGGG | TTTGGGTGTGTTGACTGCAT | 59.9 | 784 | INTERGENIC | | | | |
| CaPOPI_2353 | Ca4 | 25962811 | GTATATATAT/GTATATATATAT | TGGTTGGAAGCTCCAGAAGT | GAAAAGGTGAGGAATCGCTG | 59.8 | 275 | DRR | Ca_16607 | | B3 | |
| CaPOPI_2354 | Ca4 | 25962938 | CTT/CT | TGGTTGGAAGCTCCAGAAGT | GTGGTAAGTTGTGTGGCCCT | 59.8 | 577 | DRR | Ca_16607 | | B3 | |
| CaPOPI_2355 | Ca4 | 25963240 | GAAAAAAA/GAAAAAAAA | AATCCAAGCACCAAAAGCAC | GTGGTAAGTTGTGTGGCCCT | 60.1 | 353 | DRR | Ca_16607 | | B3 | |
| CaPOPI_2356 | Ca4 | 25963279 | TTTTATTT/TTTT | AATCCAAGCACCAAAAGCAC | GTGGTAAGTTGTGTGGCCCT | 60.1 | 353 | DRR | Ca_16607 | | B3 | |
| CaPOPI_2357 | Ca4 | 25965378 | ACCCCCC/ACCCCC | GGTGTGCAAGAGGACTGGAT | GGTCCAAGAATATGCCCAAA | 60.1 | 481 | INTERGENIC | | | | |
| CaPOPI_2358 | Ca4 | 25966069 | ATTT/ATTTT | GGCATGGACAACTTCACCTT | CGAAATCCTCAAAATCGGAA | 60.0 | 484 | INTERGENIC | | | | |
| CaPOPI_2359 | Ca4 | 25967972 | ATTTTTTTT/ATTTTTTTTT | TTGTACCCTTGTGCAACCAA | TGGTTGGATACTTGCAATGG | 60.0 | 180 | INTERGENIC | | | | |
| CaPOPI_2360 | Ca4 | 25968820 | GTTT/GTTTT | TTCTTCTGCCCAAAAATGCT | CCTCAAGCAGACCTTTGGAG | 59.8 | 412 | INTERGENIC | | | | |
| CaPOPI_2361 | Ca4 | 25969511 | ACC/AC | TCCCTTGCCTCTAGCCAGTA | GCAGATGTTCTCAACAGCCA | 60.0 | 350 | INTERGENIC | | | | |
| CaPOPI_2362 | Ca4 | 25970159 | AC/A | TGGTTGGGTCTTGGTTTGTT | CACCTGCTATGGATGCAATG | 60.2 | 709 | INTERGENIC | | | | |
| CaPOPI_2363 | Ca4 | 25975114 | TGGGG/TGGGGG | ACACATGTTGGCCAATGAGA | GAGTCCGATTTCCATGAGGA | 60.0 | 621 | CDS (FRAME SHIFT) | Ca_16608 | | | |
| CaPOPI_2364 | Ca4 | 25978445 | CT/CTTT | TGCCTCAAAGGCTTCATCTT | AGCACTCATCCTCGGGTAGA | 60.0 | 435 | INTERGENIC | | | | |
| CaPOPI_2365 | Ca4 | 25979689 | TG/TGG | GTCCTCGTCAGGAGGTTGAG | CCACTGTTGCTGGCAGATAA | 59.8 | 707 | INTERGENIC | | | | |

| INDEL marker IDs | Chromosomes/ unanchored scaffolds | Physical positions (bp) | InDels (*Kabuli* reference genome-CDC Frontier/PI) | Forward primers (5'-3') | Reverse primers (5'-3') | Anneling temperature (0C) | Expected amplified product size (bp) | Structural annotation | | Functional annotation | | |
|---|---|---|---|---|---|---|---|---|---|---|---|---|
| | | | | | | | | Sequence components of *kabuli* genome | *Kabuli* gene accession IDs | NCBI-KOG | TFs | NCBI-nr database |
| CaPOPI_2366 | Ca4 | 25983794 | A/AC | TGACAACCCCAGATTGTTGA | TGGGTCCATGTTCCAATTTT | 59.9 | 671 | INTERGENIC | | | | |
| CaPOPI_2367 | Ca4 | 25984075 | TA/TAA | CGATTTTTAACAGCGTGGGT | TGGGTCCATGTTCCAATTTT | 60.0 | 276 | INTERGENIC | | | | |
| CaPOPI_2368 | Ca4 | 25984442 | TGAGAG/TGAGAGAG | AGATCCTGTTGACACGGCTT | CGGTTGTTGTTGTGATTTGC | 59.7 | 524 | INTERGENIC | | | | |
| CaPOPI_2369 | Ca4 | 25987244 | AATATATATAT/AATATATATATATATATAT | TCTACTCTCGTCGCAGCTCA | TGCTTAGGCATTTACCCCAG | 60.0 | 250 | INTERGENIC | | | | |
| CaPOPI_2370 | Ca4 | 25987622 | TCC/TC | TTCAACTGGGGTAAATGCCT | TGTTCTAGTCCAGATCAAAACGA | 59.4 | 323 | INTERGENIC | | | | |
| CaPOPI_2371 | Ca4 | 25988978 | AAAGAAGAAGAAG/AAAGAAGAAG | AAGCTGGATGTGGACTTTGC | AGTTCCAATTGGCCTCCTTT | 60.3 | 404 | INTERGENIC | | | | |
| CaPOPI_2372 | Ca4 | 25989281 | TCCCC/TCCC | AAGCTGGATGTGGACTTTGC | AGTTCCAATTGGCCTCCTTT | 60.3 | 404 | INTERGENIC | | | | |
| CaPOPI_2373 | Ca4 | 25989322 | GTTTTT/GTTTTTT | GTACGCGATCTAGTCCCCAA | CCGGGGAGAAAGTTCTAGTG | 60.1 | 168 | INTERGENIC | | | | |
| CaPOPI_2374 | Ca4 | 25994447 | G/GT | ATCCACAACCGAGTTAGCGT | TACCCACACCCCTCAATCTT | 59.6 | 389 | INTERGENIC | | | | |
| CaPOPI_2375 | Ca4 | 25994721 | AC/A | TTAAGATTGAGGGGTGTGGG | CACCACTTCATTCGGGACTT | 59.8 | 516 | INTERGENIC | | | | |
| CaPOPI_2376 | Ca4 | 25995802 | ATTTT/ATTT | CCCTAAATCAAAGCAGCGTC | TGCACTGTTGTTGTTGCTGA | 59.8 | 470 | INTERGENIC | | | | |
| CaPOPI_2377 | Ca4 | 25996238 | GAAAAAAAAAAA/GAAAAAAAAA | CCCTAAATCAAAGCAGCGTC | AACGTTGGAGGAACAAAACG | 59.8 | 949 | INTERGENIC | | | | |
| CaPOPI_2378 | Ca4 | 25998410 | TTA/TTATGTA | TATTGTTGCCCTGCATGTGT | TGGAGGCCAATTTGTTTCTC | 60.0 | 531 | DRR | Ca_16609 | | B3 | |
| CaPOPI_2379 | Ca4 | 26011159 | AT/AACAGAACTCCT | GAAACCCCACAACAAAATGG | AGCATCATGGATTTCTTCCG | 60.1 | 438 | DRR | Ca_16611 | C | | Aldo/keto reductase |
| CaPOPI_2380 | Ca4 | 26015617 | AATAAATATA/AATA | TGCGAGGGCTAGCTTAAAAA | TGAGACAAAAGTTTGCGGTG | 60.1 | 635 | INTERGENIC | | | | |

| INDEL marker IDs | Chromosomes/ unanchored scaffolds | Physical positions (bp) | InDels (*Kabuli* reference genome-CDC Frontier/PI) | Forward primers (5'-3') | Reverse primers (5'-3') | Anneling temperature (0C) | Expected amplified product size (bp) | Structural annotation | | Functional annotation | | |
|---|---|---|---|---|---|---|---|---|---|---|---|---|
| | | | | | | | | Sequence components of *kabuli* genome | *Kabuli* gene accession IDs | NCBI-KOG | TFs | NCBI-nr database |
| CaPOPI_2381 | Ca4 | 26058753 | TAAA/TAAAA | AGGAACCAATCCTACCAGCC | TCTGGTCATGCATCCTTTGA | 60.3 | 433 | INTERGENIC | | | | |
| CaPOPI_2382 | Ca4 | 26110333 | TCCC/TCCCC | CCAACAATCTCCCCCTTTTT | GCATCTGGTTGCAGTTCTCA | 60.2 | 583 | INTERGENIC | | | | |
| CaPOPI_2383 | Ca4 | 26118906 | CTTTTTTTTTT/CTTTTTTTTT | CTGGATTCCAACAATCTCCC | TTCTTTGCTTTTGCGCTTTT | 59.3 | 851 | INTERGENIC | | | | |
| CaPOPI_2384 | Ca4 | 26176935 | GAAAAAAA/GAAAAAAAA | CACAATCAATCACGCCCTTA | TGTCATTTTTGGCGTAGTGC | 59.5 | 500 | INTERGENIC | | | | |
| CaPOPI_2385 | Ca4 | 26203930 | TTATATATATATATATAT/TTATATATATATATATATATATAT | TTGGAAATAAGGACAAAACACC | TCCAAATTTGCTGGAATCCT | 58.0 | 626 | INTERGENIC | | | | |
| CaPOPI_2386 | Ca4 | 26236726 | TAAAAAAAAAAAA/TAAAAAAAAAA | GTTACGATTGTGTTCGGCCT | TAATCTCACTGCGCACCTTG | 60.0 | 368 | INTERGENIC | | | | |
| CaPOPI_2387 | Ca4 | 26251759 | CTTTTTTTTT/CTTTTTTTTTT | TAAAACGCAATCGGAAGACC | GAGAAACTTTCCGCTGCTTG | 60.1 | 545 | INTERGENIC | | | | |
| CaPOPI_2388 | Ca4 | 26251857 | ATTTTTTTTTTT/ATTTTTTTTTTTTT | AGCTTTCATGTAACACCCCG | GCCCTCAGATGAGAGTCAGG | 60.0 | 453 | INTERGENIC | | | | |
| CaPOPI_2389 | Ca4 | 26276734 | ATAATTAA/ATAA | GGTGCTACAGTGTCGAAGCA | TGCATTTTACAGCGCTTTTG | 60.1 | 725 | INTERGENIC | | | | |
| CaPOPI_2390 | Ca4 | 26308021 | CTTTTTT/CTTTTT | ATAAAACATCATCGGGCGAC | TTCAGTGGCATTGAGACATTG | 59.8 | 405 | DRR | Ca_25307 | S | | |
| CaPOPI_2391 | Ca4 | 26326139 | GAAAAAAAA/GAAAAAAA | CTCCAACAGCATCAATTCCA | GATCCTGGGATTGTGAGCAT | 59.6 | 724 | DRR | Ca_25306 | R | | Alcohol dehydrogenase superfamily, zinc-containing |
| CaPOPI_2392 | Ca4 | 26363618 | T/TTTAC | GCCAAAATTTCCTTCGTTGA | ATTCCCCAAGAACATCACCA | 60.1 | 613 | INTERGENIC | | | | |
| CaPOPI_2393 | Ca4 | 26389354 | TTATATATATATAT/TTATATATATATATAT | AGGAAGAGTGGAGTTGCGAA | TCATATTCGGTAGCCGTTCC | 60.0 | 759 | INTERGENIC | | | | |
| CaPOPI_2394 | Ca4 | 26495554 | GAAAGTTGTTC/GAAAGTTGTTCAAAGTTGTTC | AACGATTTATCCTGGTCCCC | CCCGAATACAAACCCAAGTC | 60.0 | 844 | INTERGENIC | | | | |
| CaPOPI_2395 | Ca4 | 26529457 | ATCTCTCTCTCTCTC/ATCTCTCTCTCTCTCTC | ATAGCAGCAATTTCCATCCG | GTCCCACGAAGTAAGTCCCA | 60.1 | 286 | INTERGENIC | | | | |

| INDEL marker IDs | Chromosomes/ unanchored scaffolds | Physical positions (bp) | InDels (*Kabuli* reference genome-CDC Frontier/PI) | Forward primers (5'-3') | Reverse primers (5'-3') | Anneling temperature (0C) | Expected amplified product size (bp) | Structural annotation | | Functional annotation | | |
|---|---|---|---|---|---|---|---|---|---|---|---|---|
| | | | | | | | | Sequence components of *kabuli* genome | *Kabuli* gene accession IDs | NCBI-KOG | TFs | NCBI-nr database |
| CaPOPI_2396 | Ca4 | 26620467 | A/AGTAATT | CCGAAACCGAACAACAGATT | TGGGAACAAAACACAGCAAG | 60.0 | 819 | INTERGENIC | | | | |
| CaPOPI_2397 | Ca4 | 26624241 | C/CG | CCAAAAGTCCAACGATTGCT | TGTGCATGGTGCTTTTTGAT | 60.1 | 447 | INTERGENIC | | | | |
| CaPOPI_2398 | Ca4 | 26648267 | ATTTTTTTTT/ATTTTTTTT | TCGAACAGAAACCTGAGCCT | GATTCGGGAGTTGGTTGATG | 60.0 | 440 | INTRON | Ca_24999 | L | | Excinuclease ABC, C subunit, N-terminal |
| CaPOPI_2399 | Ca4 | 26664091 | TAAAAAAAAA/TAAAAAAAAAA | TGATACAATTGATTCGGCCA | TTTTTGTCACGCGAGCTCTA | 59.9 | 371 | INTERGENIC | | | | |
| CaPOPI_2400 | Ca4 | 26672920 | CTTTT/CTTT | ACGGATGTCGTAGGGTGCTA | AAGACCCGAATCAGCATTTG | 60.5 | 438 | INTERGENIC | | | | |
| CaPOPI_2401 | Ca4 | 26748572 | GAAAAAAAA/GAAAAAAAAA | GGGTGTGAGTTGAAATGACG | TTTGTCGCAACAACACCATT | 59.0 | 858 | INTERGENIC | | | | |
| CaPOPI_2402 | Ca4 | 26752184 | ATTTTTTTTTTT/ATTTTTTTTTT | TTGGGATCATAGAATCATTTGGT | TGATCACGGTCACTCCAAAG | 59.6 | 475 | INTERGENIC | | | | |
| CaPOPI_2403 | Ca4 | 26753050 | GAA/GAAA | AACACTCGCAAAATCTTCGG | ATTGTTTGAGTTGCCGCTCT | 60.2 | 623 | INTERGENIC | | | | |
| CaPOPI_2404 | Ca4 | 26763494 | TAAAAAAA/TAAAAAAAA | AAAAATACCGCGTCATTTCATT | TCGCTCAAAAACACAACGAG | 59.8 | 572 | INTERGENIC | | | | |
| CaPOPI_2405 | Ca4 | 26765991 | GAAAAAAAAA/GAAAAAAAA | AGTTTTGGCCCCTTTTGATT | AAAAACTCGCACACAACTGTTA | 59.8 | 508 | DRR | Ca_20437 | K | MYB | SANT domain, DNA binding |
| CaPOPI_2406 | Ca4 | 26787145 | CTTTTTTTTTT/CTTTTTTTTT | GCGACTATGTCCGTCAACTG | TCGAAGCATGAAAGAAAAGGA | 59.3 | 803 | INTERGENIC | | | | |
| CaPOPI_2407 | Ca4 | 26847079 | AAAAATAAAATAAAATAAAATA/AAAAATAAAATA | GGAGAACGTTAAGGCAGCAG | GTGGTGTAGGTTTCGATCCG | 60.0 | 689 | INTERGENIC | | | | |
| CaPOPI_2408 | Ca4 | 26847495 | A/AC | AGCTTCAAGTCGGATCGAAA | AAAAATCTTGTGGGAATAATTCAA | 60.0 | 216 | INTERGENIC | | | | |
| CaPOPI_2409 | Ca4 | 26876873 | ATTTTTTTTTTT/ATTTTTTTTTTTT | CCACCGTTGTGCCTCTTATT | GCCAGAGAATGATCGAGAGG | 60.0 | 439 | INTERGENIC | | | | |
| CaPOPI_2410 | Ca4 | 26898145 | CTTTTTTTTTT/CTTTTTTTTT | CGAAACTCAAAAACAACGGG | CGGCCCAATTCAAACTAAAA | 60.5 | 588 | INTERGENIC | | | | |

| **INDEL marker IDs** | **Chromosomes/ unanchored scaffolds** | **Physical positions (bp)** | **InDels (*Kabuli* reference genome-CDC Frontier/PI)** | **Forward primers (5'-3')** | **Reverse primers (5'-3')** | **Anneling temperature (0C)** | **Expected amplified product size (bp)** | **Structural annotation** | | **Functional annotation** | | |
|---|---|---|---|---|---|---|---|---|---|---|---|---|
| | | | | | | | | **Sequence components of *kabuli* genome** | ***Kabuli* gene accession IDs** | **NCBI-KOG** | **TFs** | **NCBI-nr database** |
| CaPOPI_2411 | Ca4 | 26903304 | T/TG | ACTCGATTGGTCCATGCTTC | ATGTTTTGGACGCACACAGA | 60.1 | 532 | INTERGENIC | | | | |
| CaPOPI_2412 | Ca4 | 26905366 | TAAAAAAAAAA/TAAAAAAAAA | TGAAAGCCCAAAACTAATCAAGA | ATTGCTCGGTCGGTTTAGTC | 60.1 | 471 | DRR | Ca_20443 | R | G2-like | RNA recognition motif domain |
| CaPOPI_2413 | Ca4 | 26991333 | ATTTTTTTTTT/ATTTTTTTTT | ACAAGGAAGCGCACCTCTAA | GTGCGACGAGCAAGAATGTA | 60.0 | 762 | INTERGENIC | | | | |
| CaPOPI_2414 | Ca4 | 27017251 | GTTTTTTTTT/GTTTTTTTTTT | CCTTTGCATCTCAGGGACAT | TGAGACCGGGTTTGAGTACC | 60.1 | 453 | INTERGENIC | | | | |
| CaPOPI_2415 | Ca4 | 27074666 | GAAAAAAAAA/GAAAAAAAAAA | ATTGGCATCAGAGCCAGTCT | TTTTCCAAGAGAGCGCATTT | 59.8 | 566 | INTERGENIC | | | | |
| CaPOPI_2416 | Ca4 | 27079794 | AAA/ACAA | AAACACTACCCGGCATCAAG | CAATTTTCTGCACTCTCGCA | 60.0 | 516 | INTERGENIC | | | | |
| CaPOPI_2417 | Ca4 | 27087434 | TA/TAAAA | TTATTGCGATGCCGACTATG | AATGATGTGGCTGGGTTAGC | 59.7 | 656 | INTERGENIC | | | | |
| CaPOPI_2418 | Ca4 | 27149358 | ATTTTTTTT/ATTTTTTT | CGATGATGCTACCAGAGGGT | CGGGGATTCCCATATCACTA | 60.1 | 611 | INTERGENIC | | | | |
| CaPOPI_2419 | Ca4 | 27168549 | CAAAAAA/CAAAAA | AGGGCCAAGGGACAATAAGT | TGGTTTGTGCAATGTCCCTA | 59.8 | 523 | INTERGENIC | | | | |
| CaPOPI_2420 | Ca4 | 27184150 | AAATAATAATAATAATA/AAATAATAATAATAATAATA | TTTCTTGTTGCCTTGGTGTG | TAGAAACCGGTTGCCAAGAA | 59.7 | 744 | INTERGENIC | | | | |
| CaPOPI_2421 | Ca4 | 27217904 | CAAAAAAAAAA/CAAAAAAAAAAA | TGTAGTGGCATGTTTGGCAT | TTTGGAGGACTTTTGGGTTG | 60.0 | 662 | INTERGENIC | | | | |
| CaPOPI_2422 | Ca4 | 27241081 | CTTTTTTTTTTT/CTTTTTTTTTTTTTT | AAGGACCAAGTCGGGAAAAA | AGGTTTTGATGTGTTTGACGG | 60.8 | 705 | INTRON | Ca_20460 | | | Protein of unknown function DUF3527 |
| CaPOPI_2423 | Ca4 | 27258927 | ATTTTTTTTTT/ATTTTTTTTT | AGCGCGGAGAGATAGTTGAA | TGGGTGACTTCATTTGGACA | 60.1 | 393 | INTERGENIC | | | | |
| CaPOPI_2424 | Ca4 | 27267123 | TAAAAA/TAAAA | CCCTCAACTCAACTGAGATCC | TGCATTGGTTTGAATTTGGA | 58.7 | 568 | DRR | Ca_20461 | R | | Sugar/inositol transporter |
| CaPOPI_2425 | Ca4 | 27283830 | GTTT/GTTTT | TATTGGTCGTCAAGCAGCAG | TTTCCCAATCGATTTGCTTC | 60.0 | 681 | DRR | Ca_20463 | | | |

| INDEL marker IDs | Chromosomes/ unanchored scaffolds | Physical positions (bp) | InDels (*Kabuli* reference genome-CDC Frontier/PI) | Forward primers (5'-3') | Reverse primers (5'-3') | Anneling temperature (0C) | Expected amplified product size (bp) | Structural annotation | | Functional annotation | | |
|---|---|---|---|---|---|---|---|---|---|---|---|---|
| | | | | | | | | Sequence components of *kabuli* genome | *Kabuli* gene accession IDs | NCBI-KOG | TFs | NCBI-nr database |
| CaPOPI_2426 | Ca4 | 27285679 | G/GAA | TATTTCGAGGGCGTACCAAC | CCAGCCTACCCCTTTTATCTG | 60.0 | 549 | INTERGENIC | | | | |
| CaPOPI_2427 | Ca4 | 27357133 | GAAAA/GAAA | TTGATTTGGTGCACATCCAT | TAAAACTTGTCCCCAACCCC | 59.8 | 561 | INTERGENIC | | | | |
| CaPOPI_2428 | Ca4 | 27367807 | CTTTT/CTTT | GCATCCTTGCTCACAACTCA | TAAAGGCCCAAAGAAGCAAA | 60.0 | 415 | INTERGENIC | | | | |
| CaPOPI_2429 | Ca4 | 27376069 | TAA/TAAA | ATCCTCTCCATTCCTTGCCT | TTGCTTTCACAGTCCAATGC | 60.0 | 304 | INTRON | Ca_21323 | | | |
| CaPOPI_2430 | Ca4 | 27379933 | CAAAAAAA/CAAAAAAAA | ATGGTACAAAGTGGCAAGGC | GAGAAAAGCAGGGTGAATGC | 60.0 | 748 | DRR | Ca_21323 | | | |
| CaPOPI_2431 | Ca4 | 27380010 | ATTT/ATT | ATGGTACAAAGTGGCAAGGC | GTGGACTGCAGTGGGTCTTT | 60.0 | 909 | DRR | Ca_21323 | | | |
| CaPOPI_2432 | Ca4 | 27405693 | TAAAA/TAAAAAA | GAGTTGGTATTTCATTTGTTCGG | GTGGTTAAGATGGACCACCG | 59.8 | 531 | INTERGENIC | | | | |
| CaPOPI_2433 | Ca4 | 27483001 | ATTTTTT/ATTTTTTT | GAAATCCAAGCCATTCTCCA | TGGTTGCGGAAATAAACACA | 60.0 | 485 | INTRON | Ca_21318 | | | |
| CaPOPI_2434 | Ca4 | 27491121 | ATTTTTTTT/ATTTTTTT | ATGCAGCACCTAGTTTTGGC | CATGTTCGTCGCTCTCATGT | 60.3 | 531 | INTERGENIC | | | | |
| CaPOPI_2435 | Ca4 | 27491848 | ATTTTTTT/ATTTTTT | TACGCGCTTTCTTTCATTCA | CGTCCCCTTCCTCAAACATA | 59.6 | 503 | INTERGENIC | | | | |
| CaPOPI_2436 | Ca4 | 27496407 | CTG/CTGTG | TCGGAAACGAATTGGGAATA | GGAGTTTGAGAAAGGGGTCC | 60.3 | 750 | INTERGENIC | | | | |
| CaPOPI_2437 | Ca4 | 27503266 | AG/AGG | GGGGTTCTCCTTGAACCACT | CCTGCCATTTTCTTCCAAAA | 60.3 | 343 | INTERGENIC | | | | |
| CaPOPI_2438 | Ca4 | 27512574 | CAAAAAAAAA/CAAAAAAAA | GTTGGCTGGGTGGAAATCTA | ATACGCGCTCCCTTAACTTG | 59.9 | 806 | INTERGENIC | | | | |
| CaPOPI_2439 | Ca4 | 27548804 | TAAAA/TAAA | TTCCCCATTCTTAACACCCA | TTCGCAACTCCACTCTTCCT | 60.2 | 672 | INTERGENIC | | | | |
| CaPOPI_2440 | Ca4 | 27574473 | ATTTTTTTTTTT/ATTTTTTTTT | CACCCTTACCTTGACCCCTT | GCTCGTATGCGTCAATTCAA | 60.2 | 571 | INTRON | Ca_21314 | | | Peptidase C48, SUMO/Sentrin/Ubl1 |

| INDEL marker IDs | Chromosomes/ unanchored scaffolds | Physical positions (bp) | InDels (*Kabuli* reference genome-CDC Frontier/PI) | Forward primers (5'-3') | Reverse primers (5'-3') | Anneling temperature (0C) | Expected amplified product size (bp) | Structural annotation | | Functional annotation | | |
|---|---|---|---|---|---|---|---|---|---|---|---|---|
| | | | | | | | | Sequence components of *kabuli* genome | *Kabuli* gene accession IDs | NCBI-KOG | TFs | NCBI-nr database |
| CaPOPI_2441 | Ca4 | 27580274 | GAAAAAAAAA/GAAAAAAAAAA | TGGGGTCCATGTACCACTTT | AAGAAATGTGCCTCAGGTGC | 60.1 | 305 | INTRON | Ca_21314 | | | Peptidase C48, SUMO/Sentrin/Ubl1 |
| CaPOPI_2442 | Ca4 | 27594072 | CTTTTTTT/CTTTTTTTTT | ATCAGAGGTTGCGCAAAAAT | TTTCCTTCCAAATTTACGCC | 59.7 | 668 | INTRON | Ca_21314 | | | Peptidase C48, SUMO/Sentrin/Ubl1 |
| CaPOPI_2443 | Ca4 | 27604352 | AAATGTTGATAAT/AAAT | ATTAGGAGTCTTGCTGCCCA | CCCTCCGAACTCTCAAACAG | 59.8 | 320 | INTRON | Ca_21314 | | | Peptidase C48, SUMO/Sentrin/Ubl1 |
| CaPOPI_2444 | Ca4 | 27637430 | TG/TGG | TTTGGAGTGGTGGTTCTCAA | CCAATGATTTTCATGCCACA | 59.1 | 482 | DRR | Ca_21310 | | | |
| CaPOPI_2445 | Ca4 | 27649411 | TTTTATTT/TTTT | AAAATAAGGCATGGTGTTGGA | TAGTTTGTAACCCGTGCGTC | 59.3 | 256 | INTERGENIC | | | | |
| CaPOPI_2446 | Ca4 | 27656062 | TAAAAAAAAAAAAA/TAAAAAAAAAAAAAAA | GCGAGCATGAGAAGTTGTTG | TTTAAAATAGACTCCCGATGTCA | 59.6 | 101 | INTERGENIC | | | | |
| CaPOPI_2447 | Ca4 | 27859134 | ATTTTTTTTT/ATTTTTTTTTT | ACCCTCCTTTTAGGTGTGCC | GGGAGATTTGGGGTTTTGAT | 60.4 | 569 | INTERGENIC | | | | |
| CaPOPI_2448 | Ca4 | 27865338 | AAATAATAATAATAATAATAATAATAATAATAATAATAATAA/AAATAATAATAATAATAATAATAATAATAATAATAA | TCCTAAAACAAATGCACCCA | TTAGGCTTGATATGCCGACC | 59.0 | 557 | INTERGENIC | | | | |
| CaPOPI_2449 | Ca4 | 27920147 | GTATATATATATATAT/GTATATATATATATATATAT | GCCCATCAAAACCCACATAC | CACAATCCTTTTCAACCGCT | 60.1 | 466 | INTERGENIC | | | | |
| CaPOPI_2450 | Ca4 | 27928820 | CACAAC/CACAACAAC | AGCGATGCATGTCTCAAGTG | AGCGCTTTGTGACCTCCTTA | 60.0 | 754 | INTERGENIC | | | | |
| CaPOPI_2451 | Ca4 | 27929931 | GTTTT/GTTTTT | CCTTCTCATTGCGAACCCTA | TGGTTGCACCACATTTGACT | 60.2 | 427 | INTERGENIC | | | | |
| CaPOPI_2452 | Ca4 | 27943723 | ATTTTTT/ATTTTT | CGATTGAATTAAGCCCAGGA | TCTCCATTTTCGTCCGTAGG | 60.0 | 403 | INTERGENIC | | | | |
| CaPOPI_2453 | Ca4 | 27951126 | CTT/CTTTCATTT | TGTGTAACCTCCCCCATGAT | AAAGGGCACCAAATACTCCA | 60.1 | 858 | INTERGENIC | | | | |
| CaPOPI_2454 | Ca4 | 28042488 | T/TG | GTCCTTTTCCTTTGGCCTTC | CCTGAGGATAGCCTCGTGAA | 60.1 | 159 | INTERGENIC | | | | |
| CaPOPI_2455 | Ca4 | 28093890 | GAA/G | GAGAGTGCTCCTTTGGGAAA | GGGAAAAATTGCTTGGTGAA | 59.4 | 300 | DRR | Ca_23175 | | | Crotonase, core |

| INDEL marker IDs | Chromosomes/ unanchored scaffolds | Physical positions (bp) | InDels (*Kabuli* reference genome-CDC Frontier/PI) | Forward primers (5'-3') | Reverse primers (5'-3') | Anneling temperature (0C) | Expected amplified product size (bp) | **Structural annotation** | | **Functional annotation** | | |
|---|---|---|---|---|---|---|---|---|---|---|---|---|
| | | | | | | | | **Sequence components of *kabuli* genome** | ***Kabuli* gene accession IDs** | **NCBI-KOG** | **TFs** | **NCBI-nr database** |
| CaPOPI_2456 | Ca4 | 28108572 | TAAAAAAAA/TAAAAAAAAA | GCAAATGTTGGCGAAGTACA | AGTGTGTGCTTGTGGGATCA | 59.7 | 301 | INTERGENIC | | | | |
| CaPOPI_2457 | Ca4 | 28109349 | ATTTTTT/ATTTTTTT | CGGGGGTAGGGTTTTAGAGA | GCTGCTTGGTTGTGCTATGA | 60.3 | 491 | INTERGENIC | | | | |
| CaPOPI_2458 | Ca4 | 28120543 | TAAAAAA/TAAAAAAA | CCAACTCCCTTCCCCTTTTA | CAATACGAGCACCTGGCTCT | 60.3 | 994 | INTERGENIC | | | | |
| CaPOPI_2459 | Ca4 | 28120712 | ATTTTTTTTTT/ATTTTTTTT | CATCAGTCAAACGCACAAAAA | CAATACGAGCACCTGGCTCT | 59.8 | 433 | INTERGENIC | | | | |
| CaPOPI_2460 | Ca4 | 28122657 | GAAAAAAAAAAAA/GAAAAAAAAAAA | CCCCGAGTTTGAGAAATTGA | CCTCCCGTCCTCTTTCTTTC | 60.0 | 393 | INTERGENIC | | | | |
| CaPOPI_2461 | Ca4 | 28147858 | AT/ATTTAATAATTT | GGGAAAAGCCCCAAACTAAG | TCCAACGTCGATTATGACCA | 59.9 | 424 | INTERGENIC | | | | |
| CaPOPI_2462 | Ca4 | 28159857 | TAAAAAAAAA/TAAAAAAAAAAA | TTTGGGGGTACAAGGGAAAT | TGCAACCTCATACTGGGAAA | 60.4 | 645 | INTERGENIC | | | | |
| CaPOPI_2463 | Ca4 | 28160415 | GAAAAAA/GAAAAAAA | CAAAATGCCCTTTGGCACTA | TGACACGTTGTTTGTTTTGACA | 61.0 | 457 | INTERGENIC | | | | |
| CaPOPI_2464 | Ca4 | 28179566 | AGGGG/AGGGGG | TGGGTAAAGTTCTGCCCACT | TAGCATGCTCGAAAACGAAA | 59.6 | 129 | INTERGENIC | | | | |
| CaPOPI_2465 | Ca4 | 28180097 | ATTTTTTTTT/ATTTTTTT | TCGAGCATGCTATAGTCCGA | ACCAATGTCCAACGGGAATA | 59.5 | 869 | INTERGENIC | | | | |
| CaPOPI_2466 | Ca4 | 28184807 | ATTT/ATTTT | GACCTTTGTTGACCTTGCGT | TGTTGAGTTGGAGATGAAGCA | 60.2 | 595 | INTERGENIC | | | | |
| CaPOPI_2467 | Ca4 | 28209971 | T/TA | ATTGATGGAGCGAATCGAAC | AATTCCTCTTTTGGTGGGCT | 60.0 | 891 | INTERGENIC | | | | |
| CaPOPI_2468 | Ca4 | 28254483 | CAACAA/CAACAAGACAAACAA | GGGGAACTCTCCAAGTCACA | CGTGGAAACCAACCTTGTCT | 60.1 | 703 | INTERGENIC | | | | |
| CaPOPI_2469 | Ca4 | 28274340 | CAAAAAAAAAA/CAAAAAAAAA | CCAGAAGCAGAAAAACCAGG | TTTGAAACTGTCGCGACTCA | 59.8 | 631 | INTERGENIC | | | | |
| CaPOPI_2470 | Ca4 | 28306771 | CAAAAAAAAA/CAAAAAAAAAAA | TGATGGTCCATGAACTGACAT | CGAGCCAAAATCCAAAACAT | 58.8 | 430 | INTERGENIC | | | | |

| INDEL marker IDs | Chromosomes/ unanchored scaffolds | Physical positions (bp) | InDels (*Kabuli* reference genome-CDC Frontier/PI) | Forward primers (5'-3') | Reverse primers (5'-3') | Anneling temperature (0C) | Expected amplified product size (bp) | Structural annotation | | Functional annotation | | |
|---|---|---|---|---|---|---|---|---|---|---|---|---|
| | | | | | | | | Sequence components of *kabuli* genome | *Kabuli* gene accession IDs | NCBI-KOG | TFs | NCBI-nr database |
| CaPOPI_2471 | Ca4 | 28321521 | CCGTACAGGCGTACA/CCGTACA | TCGAGTTGTGAAATTGAGCG | GCTGAAGAACCCGAGAAAGA | 60.0 | 202 | INTERGENIC | | | | |
| CaPOPI_2472 | Ca4 | 28331028 | CAAAAAAAA/CAAAAAAAAA | CTTGAAGGGACGGCTAGATG | TTGCCATAATGTTGAGAAGGG | 59.8 | 458 | INTERGENIC | | | | |
| CaPOPI_2473 | Ca4 | 28336605 | CAAAAAAAAAAAAA/CAAAAAAAAAAAAA | CAAAGCAGGGAGTTTCCATC | TGGTTTAAGTCCTCGGCTGT | 59.7 | 723 | INTERGENIC | | | | |
| CaPOPI_2474 | Ca4 | 28338433 | GTTT/GTT | TGACTCGGGCTGCAGATTAT | GCTTTGAGCCCCATGAAATA | 60.8 | 739 | INTERGENIC | | | | |
| CaPOPI_2475 | Ca4 | 28342637 | GCCC/GCCCC | TCACAAATCCTGGTGGTTGA | GGAAGAAAGCAATGGTGGAA | 59.9 | 359 | INTERGENIC | | | | |
| CaPOPI_2476 | Ca4 | 28346696 | A/AT | ACCACAGGTGAAAGAAACCG | TTTTCAACTCTATCCGTAGCCC | 60.0 | 340 | INTERGENIC | | | | |
| CaPOPI_2477 | Ca4 | 28372820 | ATTTT/ATTTTT | TAAGGGAAGGGGCTCTAGGA | CCCCTAAAATTTAAAAGGCCC | 60.2 | 389 | INTERGENIC | | | | |
| CaPOPI_2478 | Ca4 | 28380287 | ATTTT/ATTTTT | ACTTTTCTCATTTTTAAGTCATCAAAT | CTTCAAAGTTGCCGGTGATT | 57.3 | 919 | INTERGENIC | | | | |
| CaPOPI_2479 | Ca4 | 28390462 | TAA/TAAA | GTCATCAAAAGCCGACGAAT | GGCCCACAATTTTGAAGAGA | 60.1 | 786 | INTERGENIC | | | | |
| CaPOPI_2480 | Ca4 | 28401988 | TAATTAA/TAATTAATTAAATAAAATTAA | ACTTGGATGAAGGGAAAGGG | GCATTTCGCAAAAATTTGAA | 60.3 | 561 | INTERGENIC | | | | |
| CaPOPI_2481 | Ca4 | 28414876 | CAAT/CAATAAT | GCCCCATGTTTGTGCTTAAT | GGATTCAGCATCCGTCAAGT | 59.8 | 741 | INTERGENIC | | | | |
| CaPOPI_2482 | Ca4 | 28420944 | TAAAGTTAGAAGAA/TAA | GTGTGGGCGGAAAAATCTAA | CACAGTGAAAAACCGATGGA | 59.9 | 693 | INTERGENIC | | | | |
| CaPOPI_2483 | Ca4 | 28423269 | T/TA | TAGGTTTCCCCATGTGGTGT | AGCACTTGCCCCTTAGGAGT | 60.1 | 697 | INTERGENIC | | | | |
| CaPOPI_2484 | Ca4 | 28447880 | ATTTTTTTT/ATTTTTTTTT | TCTTCTGTGTGAGCGGATTG | GCTCAACCTTGAGACCAAGC | 60.0 | 252 | INTERGENIC | | | | |
| CaPOPI_2485 | Ca4 | 28457671 | TCTCTA/TCTCTATTTAAACTCTA | TACAAAGCACCTGACCCTCC | CCCAAATCAAAGCAAGCAAT | 60.1 | 765 | INTERGENIC | | | | |

| INDEL marker IDs | Chromosomes/ unanchored scaffolds | Physical positions (bp) | InDels (*Kabuli* reference genome-CDC Frontier/PI) | Forward primers (5'-3') | Reverse primers (5'-3') | Anneling temperature (0C) | Expected amplified product size (bp) | Structural annotation | | Functional annotation | | |
|---|---|---|---|---|---|---|---|---|---|---|---|---|
| | | | | | | | | Sequence components of *kabuli* genome | *Kabuli* gene accession IDs | NCBI-KOG | TFs | NCBI-nr database |
| CaPOPI_2486 | Ca4 | 28501292 | AAATATTTTATTATT/AAATATTTTATTATTAATATTTTATTATT | GCACGCACACACATATACCC | TTTGCAGGCACAAAAGACAG | 59.9 | 747 | INTERGENIC | | | | |
| CaPOPI_2487 | Ca4 | 28505491 | TAAAAAAAA/TAAAAAAAAA | GGATTTCACGGTGGTCTTTT | TCACACACCTCAAAACCCCT | 58.9 | 433 | INTERGENIC | | | | |
| CaPOPI_2488 | Ca4 | 28534748 | ATTTTTTTTTTT/ATTTTTTTTTT | TGGTCAACATGCCAAAGAAA | TTGTTTTTCCTCCAAACAAATG | 60.1 | 958 | INTERGENIC | | | | |
| CaPOPI_2489 | Ca4 | 28535714 | TTT/TTTATT | GCCAGCTCCAATTACAATGC | CATGTCTTTTGAATTTTGATCAGC | 60.6 | 816 | INTERGENIC | | | | |
| CaPOPI_2490 | Ca4 | 28581947 | TAAAAAAAAAAA/TAAAAAAAAAA | CATTTCCCCACACCAATTTC | AAGGATGATGATTGTGCATGT | 60.0 | 379 | INTERGENIC | | | | |
| CaPOPI_2491 | Ca4 | 28581991 | CAAAAAAAAAA/CAAAAAAAAAAA | CATTTCCCCACACCAATTTC | AAGGATGATGATTGTGCATGT | 60.0 | 379 | INTERGENIC | | | | |
| CaPOPI_2492 | Ca4 | 28623056 | A/AGAATTTGG | ATATGGTTGCGCACAAATGA | TGTCCGAAACATTAAACCCA | 60.0 | 704 | INTERGENIC | | | | |
| CaPOPI_2493 | Ca4 | 28714992 | GATAT/GAT | AGAGCCAACGCTCATAGGAA | TTAAAATTCCCTCCTCGGCT | 60.0 | 531 | INTERGENIC | | | | |
| CaPOPI_2494 | Ca4 | 28783547 | CTTTTTTTTT/CTTTTTTTTTT | TGGAAAGCTTGCTCCTTCAT | CAAGTGATGTTGCCTATTCTGG | 60.0 | 124 | INTERGENIC | | | | |
| CaPOPI_2495 | Ca4 | 28791900 | TCACA/T | TGCCCCTCATGGTTATCCTA | CACAGAGTTCGACACCGCTA | 60.3 | 580 | INTERGENIC | | | | |
| CaPOPI_2496 | Ca4 | 28793395 | TAA/TAAAAACAA | CCAAAATTACACCAACGGTTC | TTGTTCGCATGCTCATTCTC | 59.2 | 275 | INTERGENIC | | | | |
| CaPOPI_2497 | Ca4 | 28806476 | T/TA | TGTTTGTTTTTGCAAGCAGG | GCAAGAAGCCATCCACAAGT | 59.9 | 351 | INTERGENIC | | | | |
| CaPOPI_2498 | Ca4 | 28849010 | GAAAAAAAA/GAAAAAAAAA | AGAGGAAATGATGCCACAATG | AAACACTTGCGTAACCTGGC | 59.9 | 794 | INTERGENIC | | | | |
| CaPOPI_2499 | Ca4 | 28850439 | ATTATTTTTATTTT/ATTATTTTTATTTTTATTTT | GTCAGAGGCAGTGAGCCTTC | TTTTAGCTTTCCAACGCCTG | 60.1 | 500 | INTERGENIC | | | | |
| CaPOPI_2500 | Ca4 | 28856354 | ATTTTTTT/ATTTTTTTT | GCGCGAATATCTCAACCAAT | TCCACCAACAACAAGATCCA | 60.1 | 212 | INTERGENIC | | | | |

| INDEL marker IDs | Chromosomes/ unanchored scaffolds | Physical positions (bp) | InDels (*Kabuli* reference genome-CDC Frontier/PI) | Forward primers (5'-3') | Reverse primers (5'-3') | Anneling temperature (0C) | Expected amplified product size (bp) | Structural annotation | | Functional annotation | | |
|---|---|---|---|---|---|---|---|---|---|---|---|---|
| | | | | | | | | Sequence components of *kabuli* genome | *Kabuli* gene accession IDs | NCBI-KOG | TFs | NCBI-nr database |
| CaPOPI_2501 | Ca4 | 28887918 | TAAAAAAAAA/TAAAAAAAAAA | TGCCCTATTTTGTGTCCAGG | TGCGTTTGGCTGATGTTAAG | 60.9 | 573 | INTERGENIC | | | | |
| CaPOPI_2502 | Ca4 | 28910424 | ATTTTCTTTTTCTTTTTCTT/ATTTTCTTTTTCTT | CTGTTTCCTTCCAAACTGGG | TGTAGAGGAATGTCTCGCCC | 59.6 | 393 | INTERGENIC | | | | |
| CaPOPI_2503 | Ca4 | 28910579 | A/AC | CTGTTTCCTTCCAAACTGGG | TGTAGAGGAATGTCTCGCCC | 59.6 | 393 | INTERGENIC | | | | |
| CaPOPI_2504 | Ca4 | 28913641 | AAATAATAATAATAATAATAAT/AAATAATAATAATAATAAT | TACGTCGCTCCATGCAAATA | CAACGTCCTCCCATTTGTTT | 60.2 | 822 | INTERGENIC | | | | |
| CaPOPI_2505 | Ca4 | 28914437 | GA/GAAA | AAACAAATGGGAGGACGTTG | TGTTCCAGATATTTTGCCGA | 59.8 | 508 | INTERGENIC | | | | |
| CaPOPI_2506 | Ca4 | 28925061 | CAAAAAA/CAAAAAAAAA | GGGGTGGTTTTTAGCTTGGT | TAGCCCAGTTTGGAAGGAAA | 60.2 | 773 | INTERGENIC | | | | |
| CaPOPI_2507 | Ca4 | 28925610 | TAA/TA | TTTCCTTCCAAACTGGGCTA | AATGCGGTCTTCCCCTAAGT | 59.7 | 622 | INTERGENIC | | | | |
| CaPOPI_2508 | Ca4 | 28988768 | TAAAAAAAA/TAAAAAAAAA | AAAAGTCGACAGTTTTGGTTCA | ACTGGGTTGTCAATTGCCTC | 58.8 | 681 | INTERGENIC | | | | |
| CaPOPI_2509 | Ca4 | 28990312 | CAAAAA/CAAA | GTTTCGCAATCTGGGTTTGT | AACACGTGTAGCAAAGCACG | 60.0 | 770 | INTERGENIC | | | | |
| CaPOPI_2510 | Ca4 | 29037043 | GAAAA/GAAAAAAA | CCATTGAGCTACACTGTGCG | TTGGACAACGCTTCAAACTG | 60.5 | 434 | INTERGENIC | | | | |
| CaPOPI_2511 | Ca4 | 29049755 | TTA/TTAAAGCGAGAAATA | GGTTTTATAGGGCATGCTGG | TCCCAAGAAATCAATGCTCC | 59.4 | 749 | INTERGENIC | | | | |
| CaPOPI_2512 | Ca4 | 29064946 | TA/T | GTTTCCCGAAGGTTTTGGAT | TCATCTTGGATCACACTCAACC | 60.2 | 424 | INTERGENIC | | | | |
| CaPOPI_2513 | Ca4 | 29065135 | TAAAAAAAAA/TAAAAAAAA | GTTTCCCGAAGGTTTTGGAT | TCATCTTGGATCACACTCAACC | 60.2 | 424 | INTERGENIC | | | | |
| CaPOPI_2514 | Ca4 | 29085024 | CT/CTT | AAGGAGAAAAGTGGGGGAGA | ACGTATCCCCAGGTGCAATA | 60.0 | 602 | INTERGENIC | | | | |
| CaPOPI_2515 | Ca4 | 29110502 | ATTTTT/ATTTT | TGCAAACATGGATGGGAATA | CACGGTTTCTAACACCATTTGA | 59.7 | 617 | INTERGENIC | | | | |

| INDEL marker IDs | Chromosomes/ unanchored scaffolds | Physical positions (bp) | InDels (*Kabuli* reference genome-CDC Frontier/PI) | Forward primers (5'-3') | Reverse primers (5'-3') | Anneling temperature (0C) | Expected amplified product size (bp) | Structural annotation | | Functional annotation | | |
|---|---|---|---|---|---|---|---|---|---|---|---|---|
| | | | | | | | | Sequence components of *kabuli* genome | *Kabuli* gene accession IDs | NCBI-KOG | TFs | NCBI-nr database |
| CaPOPI_2516 | Ca4 | 29156658 | CATGCAAATGCAAA/CATGCAAA | AGTTGGTTTGGTGGTGAAGG | GCATGGTAAAGGCTGTTGGT | 59.9 | 841 | DRR | Ca_23711 | | | Domain of unknown function DUF296 |
| CaPOPI_2517 | Ca4 | 29159894 | CAT/CATAT | TGATTTTCAAAGAGAAACCCG | TTCCTGCGATGTCATTTGTT | 59.2 | 450 | INTERGENIC | | | | |
| CaPOPI_2518 | Ca4 | 29190573 | GA/G | GCCAGTCTTCTCAAACAGCC | TTTGACCAAGTGGAAAAGAAGA | 60.0 | 648 | INTERGENIC | | | | |
| CaPOPI_2519 | Ca4 | 29191237 | CTTTTTTTT/CTTTTTTTTT | CCAACTTGGTTCCAACAATCT | GGAGAAAAGTTAGGGGGTGC | 58.9 | 119 | INTERGENIC | | | | |
| CaPOPI_2520 | Ca4 | 29202977 | AATGATAG/A | TCACCCCGATGTCACCTATT | ACATGCCATTGAATTACGCA | 60.2 | 449 | INTERGENIC | | | | |
| CaPOPI_2521 | Ca4 | 29205093 | C/CCAAG | ACATATGGCTTCGCATGACA | TTAGGAGTGGCTTTGGATGG | 60.1 | 492 | INTERGENIC | | | | |
| CaPOPI_2522 | Ca4 | 29238761 | A/AATT | CCATGAGATCCACATAATCCG | TTGCAAGAATCCATCCATCA | 60.2 | 513 | INTERGENIC | | | | |
| CaPOPI_2523 | Ca4 | 29244811 | CAAAA/CAAA | CCGAGATGGTTTTGGAGAAA | GCTTCATAGCTTGCAAAGGG | 60.0 | 488 | INTERGENIC | | | | |
| CaPOPI_2524 | Ca4 | 29245084 | ATA/ATACTTA | GCCCTTTGCAAGCTATGAAG | TGGTGCAATTTGGTTGGTAA | 60.0 | 418 | INTERGENIC | | | | |
| CaPOPI_2525 | Ca4 | 29270177 | CTTTTTTTT/CTTTTTTT | TTTCCCGACTTGGTCCTTTA | TCGACTCCCCACCTAATGAC | 59.5 | 696 | INTERGENIC | | | | |
| CaPOPI_2526 | Ca4 | 29274800 | GTTTTTTTTTT/GTTTTTTTTTTT | TTGGCGTGATAATTACATGAGG | GCCTACCGGAAATGAATGAA | 59.9 | 864 | INTERGENIC | | | | |
| CaPOPI_2527 | Ca4 | 29278808 | GTTTTTTTTTT/GTTTTTTTTTTT | CCACCGGATTTATGAAGGAA | GGGCATGAAATATGAGACTGC | 59.8 | 415 | INTERGENIC | | | | |
| CaPOPI_2528 | Ca4 | 29292913 | ATTTTTTTTT/ATTTTTTTTTT | ACACGCTAGCCATTAGAGCC | TCCTTTCTTTCCAAGAGGTTCA | 59.5 | 306 | INTERGENIC | | | | |
| CaPOPI_2529 | Ca4 | 29310722 | ATTTTTTTTTT/ATTTTTTTTT | GTGGTGTCCATCTCGGTCTT | TAAGCCAGCAACTCCAGGAT | 60.0 | 658 | INTERGENIC | | | | |
| CaPOPI_2530 | Ca4 | 29321184 | ATTTTTTT/ATTTTTTTT | AACACATCAGCAAATGGCAA | TGGGTTCGGGTACAAGCTAC | 60.1 | 682 | INTERGENIC | | | | |

| INDEL marker IDs | Chromosomes/ unanchored scaffolds | Physical positions (bp) | InDels (*Kabuli* reference genome-CDC Frontier/PI) | Forward primers (5'-3') | Reverse primers (5'-3') | Anneling temperature (0C) | Expected amplified product size (bp) | Structural annotation | | Functional annotation | | |
|---|---|---|---|---|---|---|---|---|---|---|---|---|
| | | | | | | | | Sequence components of *kabuli* genome | *Kabuli* gene accession IDs | NCBI-KOG | TFs | NCBI-nr database |
| CaPOPI_2531 | Ca4 | 29330235 | ATTTT/ATTT | TTTTGTTCAGATTGCGCTTG | GAGGGGATCCATTTTCCCTA | 60.0 | 762 | INTERGENIC | | | | |
| CaPOPI_2532 | Ca4 | 29348534 | AGGGGGG/AGGGGGGG | GTTGTGCGGAAGCCATTTAT | GAGGACTGGACGTACCCTTG | 60.0 | 248 | INTERGENIC | | | | |
| CaPOPI_2533 | Ca4 | 29356210 | ATTTTTT/ATTTTT | GCGGGTTTCCTTTTATAGGC | TTTTCCAGCATGTGTGCATC | 59.9 | 311 | INTERGENIC | | | | |
| CaPOPI_2534 | Ca4 | 29356293 | CTTTTTTTTT/CTTTTTTTT | GCGGGTTTCCTTTTATAGGC | TTTTCCAGCATGTGTGCATC | 59.9 | 311 | INTERGENIC | | | | |
| CaPOPI_2535 | Ca4 | 29363363 | TAA/TA | CTCGCGGTATCTTCATGACT | AAAAACAGAGCAGGTACGAGTG | 57.9 | 313 | INTERGENIC | | | | |
| CaPOPI_2536 | Ca4 | 29368326 | TC/T | TCGATTGTTTAGAGGCTATACGTG | TCGCTTGTGAGAGAGGACAA | 59.7 | 511 | DRR | Ca_14154 | | | |
| CaPOPI_2537 | Ca4 | 29388732 | ATTTT/ATTT | GGCATCCCAGATCTGAAAAA | AAGTCGGTACAGCACCATCC | 60.0 | 931 | INTERGENIC | | | | |
| CaPOPI_2538 | Ca4 | 29418053 | GAATAAATAAATAAA/GAATAAATAAATAAATAAA | TTGCTCCTACGTATCTCCCG | AACTTCCACCGTACACCACC | 60.2 | 836 | INTERGENIC | | | | |
| CaPOPI_2539 | Ca4 | 29418531 | TT/TTAT | GGTGGTGTACGGTGGAAGTT | GTCAAGGCAGACCAGAAAGC | 59.7 | 383 | INTERGENIC | | | | |
| CaPOPI_2540 | Ca4 | 29419703 | A/AC | TGATTTTACATCTGCACCATGA | GGCATAAGCTTGCATTGTGA | 59.0 | 262 | INTERGENIC | | | | |
| CaPOPI_2541 | Ca4 | 29421150 | TAAAAAAAAAAAAAAAAA/TAAAAAAAA | TCGCCGTTGCTTTTCTACTT | AGAGAGCCAAACGGCAGATA | 60.0 | 728 | INTERGENIC | | | | |
| CaPOPI_2542 | Ca4 | 29426569 | ATTTTTTTTT/ATTTTTTTTTT | ATCCAGCGAATTTTTACCGA | TTTTGACACACACACACCCA | 59.5 | 781 | INTERGENIC | | | | |
| CaPOPI_2543 | Ca4 | 29498412 | ATTTTTTTT/ATTTTTTT | TTGGATTGATTTTTGGATTTGTC | AACTTGATTTGAATATTGCCATTG | 60.0 | 779 | INTERGENIC | | | | |
| CaPOPI_2544 | Ca4 | 29503488 | ATTTTTTTTTT/ATTTTTTTTTTTT | TGTATGTCCCTCCTCTTTGTCA | GGGGTACTCAACCCACAAAA | 59.6 | 735 | DRR | Ca_14160 | | | |
| CaPOPI_2545 | Ca4 | 29515377 | TAAAAAAAAA/TAAAAAAAA | CGATGAGAGATGGTTAGGGTG | GGGAAGAAGCTTGATTCACCT | 59.6 | 436 | INTERGENIC | | | | |

| INDEL marker IDs | Chromosomes/ unanchored scaffolds | Physical positions (bp) | InDels (*Kabuli* reference genome-CDC Frontier/PI) | Forward primers (5'-3') | Reverse primers (5'-3') | Anneling temperature (0C) | Expected amplified product size (bp) | Structural annotation | | Functional annotation | | |
|---|---|---|---|---|---|---|---|---|---|---|---|---|
| | | | | | | | | Sequence components of *kabuli* genome | *Kabuli* gene accession IDs | NCBI-KOG | TFs | NCBI-nr database |
| CaPOPI_2546 | Ca4 | 29544316 | GATAT/GAT | CCATCAAACCACAACAAACA | CCCAATTTCCTTCCGTTTTT | 57.8 | 706 | INTERGENIC | | | | |
| CaPOPI_2547 | Ca4 | 29545135 | AATTATTTATTATTTAT/AATTATTTATTATTTATTATTTAT | CATACGTGATACGCTGGGTG | GTGAGGGAGGACCAAAACAA | 60.0 | 659 | INTERGENIC | | | | |
| CaPOPI_2548 | Ca4 | 29547360 | CTTTTTTTT/CTTTTTTT | TCCCACACATCCAAAACAAA | TTGGTCCCTATAAGTTGGCG | 59.8 | 613 | INTERGENIC | | | | |
| CaPOPI_2549 | Ca4 | 29564628 | GTTTT/GTTTTTT | GAACGGTCCATGCAAAAGTT | AGTTTCCCCGATCCTTATGC | 60.0 | 535 | INTERGENIC | | | | |
| CaPOPI_2550 | Ca4 | 29566926 | GTTTTTTTTTTTT/GTTTTTTTTTT | CTCTTTTGCCCTGCGATTAG | TGAAACCAGATTTTGAGTCCG | 60.0 | 701 | INTERGENIC | | | | |
| CaPOPI_2551 | Ca4 | 29582409 | TAAAA/TAAA | AGCTGTATTGGGCCTCCTTT | GCCGAATAAAACCCTCACAA | 60.1 | 212 | INTERGENIC | | | | |
| CaPOPI_2552 | Ca4 | 29619182 | AG/A | TTGTCGTGGTGATTAATGAAAAA | GCCGACACCTTCAATCAAAT | 59.4 | 751 | INTERGENIC | | | | |
| CaPOPI_2553 | Ca4 | 29622506 | ATTTTTTT/ATTTTTTTTT | CCCCACGATCCTGAAATAAA | AACGATGATCGCAACAACAA | 59.8 | 281 | INTERGENIC | | | | |
| CaPOPI_2554 | Ca4 | 29626397 | CTTTTTTTTTTT/CTTTTTTTTT | ACCCCATTTCATCCTCATCA | CTTAGTGAGTGGAGAGCGCC | 60.1 | 398 | INTERGENIC | | | | |
| CaPOPI_2555 | Ca4 | 29626809 | CTTTTTTTT/CTTTTTTT | CTAGGAGAAGGGGGATTTGG | TGTGTTTGAATTCTTGATTGGG | 59.9 | 239 | INTERGENIC | | | | |
| CaPOPI_2556 | Ca4 | 29640045 | TAAAGA/TAAAGAAAGA | GCCTGTCTGCATTGTTTCCT | CGGACAGCAACAATTTCCTT | 60.3 | 753 | INTERGENIC | | | | |
| CaPOPI_2557 | Ca4 | 29658881 | TGAGGAG/TGAG | CTTTTGAGGCATTCCACCAT | ATTTTGTTGGGGTGCTCTTG | 59.9 | 176 | INTERGENIC | | | | |
| CaPOPI_2558 | Ca4 | 29681493 | G/GT | ATCCCCCACACGATGAATAA | CCAAAGAGCTGAGCGAAAAT | 60.0 | 404 | INTRON | Ca_14166 | Z | MYB_related | Kinesin, motor domain |
| CaPOPI_2559 | Ca4 | 29684929 | ATTTTTTTTT/ATTTTTTTTTT | GCCCTGGCCTAAGAATAACC | CAAGCCAACAGAACCTGGAT | 59.9 | 562 | INTRON | Ca_14166 | Z | MYB_related | Kinesin, motor domain |
| CaPOPI_2560 | Ca4 | 29699958 | TTG/TTGGATG | TGGCCTTATTTGCTTTTTGG | CCAACATCATGCTCTCCAAC | 60.1 | 446 | INTERGENIC | | | | |

| **INDEL marker IDs** | **Chromosomes/ unanchored scaffolds** | **Physical positions (bp)** | **InDels (*Kabuli* reference genome-CDC Frontier/PI)** | **Forward primers (5'-3')** | **Reverse primers (5'-3')** | **Anneling temperature (0C)** | **Expected amplified product size (bp)** | **Structural annotation** | | **Functional annotation** | | |
|---|---|---|---|---|---|---|---|---|---|---|---|---|
| | | | | | | | | **Sequence components of *kabuli* genome** | ***Kabuli* gene accession IDs** | **NCBI-KOG** | **TFs** | **NCBI-nr database** |
| CaPOPI_2561 | Ca4 | 29701374 | G/GTTTAAATTTC | AGATCTTTCCCCTCATGGCT | CCTTGACAAGAAGCAACACG | 60.0 | 474 | INTERGENIC | | | | |
| CaPOPI_2562 | Ca4 | 29702135 | CTTTTTTT/CTTTTTTTTT | AATTAGGTGTGGAGGGCAAA | TTTCATTTTTAAAACCCCCTCA | 59.4 | 421 | INTERGENIC | | | | |
| CaPOPI_2563 | Ca4 | 29717512 | GAAATAAATA/GAAATAAATAAATA | GTTGGCATTTCTCCACTCGT | GTGCCCAACAAACAAGGACT | 60.1 | 458 | INTERGENIC | | | | |
| CaPOPI_2564 | Ca4 | 29719863 | ATTTTTTT/ATTTTTTTT | GAGTTGCGCCCAAACTTTTA | AGGAACAGCAAGAACGGTGT | 60.2 | 883 | INTRON | Ca_14167 | | | Domain of unknown function DUF231, plant |
| CaPOPI_2565 | Ca4 | 29776445 | TAAAAAAAAA/TAAAAAAAAAA | TATTTTGGGCCTGGTGTTGT | TGGGCCACCCTATACCAATA | 60.2 | 729 | INTRON | Ca_14170 | K | HD-ZIP | Homeobox |
| CaPOPI_2566 | Ca4 | 29793706 | TAAAAAAAAAA/TAAAAAAAAAAA | AGATGTTGCTTCCCCAAGAA | CAAAAGAACCTTAGCTTCCCC | 59.7 | 718 | INTERGENIC | | | | |
| CaPOPI_2567 | Ca4 | 29821605 | CTTTTTTTT/CTTTTTTT | GACTCATCCAAGGTTGCCAT | TTTGATGGCTCAGAAAGCCT | 59.9 | 524 | INTERGENIC | | | | |
| CaPOPI_2568 | Ca4 | 29866382 | CAAA/CAA | TGGGGAGGAAGTTGACTCAG | CTTCGCACCTCTATCTTGCC | 60.2 | 465 | INTERGENIC | | | | |
| CaPOPI_2569 | Ca4 | 29900525 | TTATA/TTA | AACAAAGAAGAACACGGGGT | CTTTCCTGCGCATAATCGAC | 58.6 | 574 | INTERGENIC | | | | |
| CaPOPI_2570 | Ca4 | 29925448 | G/GC | ATCGGACAGAGTTGACCCTC | GTTTTCCAAATCGTGGCCTA | 59.1 | 686 | INTERGENIC | | | | |
| CaPOPI_2571 | Ca4 | 29932586 | TAAAAAAAAAA/TAAAAAAAAAAAA | TCTACGGCGTCTAGTGGACA | AGCCCGAAAGGATCTAAAGG | 59.5 | 487 | INTERGENIC | | | | |
| CaPOPI_2572 | Ca4 | 29933754 | ATTTTTTT/ATTTTTTTT | AGCCACACGATGTTGATTGA | TGATTAGGGAATCACCCGTT | 60.1 | 845 | INTERGENIC | | | | |
| CaPOPI_2573 | Ca4 | 29941572 | TAAAAAAA/TAAAAAA | CCGTGTAGGTATGATCTTTTGG | CCTTTCCTGTAAATCGGCAA | 58.5 | 324 | INTERGENIC | | | | |
| CaPOPI_2574 | Ca4 | 29987923 | GATGGTAT/GATGGTATGGTAT | GAGCAGAGCTGCCTAATGCT | GGCGTTGTTGTAGAGTGCAA | 59.9 | 419 | INTERGENIC | | | | |
| CaPOPI_2575 | Ca4 | 30045432 | CATATAT/CATATATAT | GCGTGCATAAGTGGTTGATG | ACTCACGGTGATGAGTGTGG | 60.1 | 345 | DRR | Ca_14181 | | S1Fa-like | Protein kinase, catalytic domain |

| INDEL marker IDs | Chromosomes/ unanchored scaffolds | Physical positions (bp) | InDels (*Kabuli* reference genome-CDC Frontier/PI) | Forward primers (5'-3') | Reverse primers (5'-3') | Anneling temperature (0C) | Expected amplified product size (bp) | Structural annotation | | Functional annotation | | |
|---|---|---|---|---|---|---|---|---|---|---|---|---|
| | | | | | | | | Sequence components of *kabuli* genome | *Kabuli* gene accession IDs | NCBI-KOG | TFs | NCBI-nr database |
| CaPOPI_2576 | Ca4 | 30046973 | GAAAAAAAAAA/GAAAAAAAAA | TGAATGAAATTTCTGGCAGG | CGTTTTGTTTGTGGCACATT | 58.7 | 280 | INTERGENIC | | | | |
| CaPOPI_2577 | Ca4 | 30075059 | CTAA/CTAATTGTTTTTATAA | TCGAAACCAACCACCTTTTC | GCAATTCGACAATGCACAAC | 59.9 | 547 | INTERGENIC | | | | |
| CaPOPI_2578 | Ca4 | 30077264 | ATTTTTT/ATTTTTTT | GCATATTTGGGAGCGCTTTA | TGGATGATTGTGACAAAATTGC | 60.2 | 508 | INTERGENIC | | | | |
| CaPOPI_2579 | Ca4 | 30082739 | AAAAACAAAACAG/AAAAACAAAACAGTGGAAAACAAAACAG | TGCGTTGTATCATGTCGGTT | TCTGGTTTGTTCCTCCGTTT | 60.0 | 389 | INTERGENIC | | | | |
| CaPOPI_2580 | Ca4 | 30086302 | TTTTCATG/TTTTCATGCAAACTTTCATG | TTGATGTGGACTTTCTTGCG | TTTTCGGCGTAAAACCTTTG | 59.8 | 238 | INTERGENIC | | | | |
| CaPOPI_2581 | Ca4 | 30086898 | ACTTTCT/ACT | ACGCCGAAAAACACTTTCAC | TCCATTCGACTGTCCTTCAA | 60.2 | 630 | INTERGENIC | | | | |
| CaPOPI_2582 | Ca4 | 30097465 | GAAA/GAA | GCAAAACGGGGACTAGAACA | CCAGGGGTTGCCTTAGATTT | 60.1 | 307 | INTERGENIC | | | | |
| CaPOPI_2583 | Ca4 | 30118694 | CATAGATA/CATAGATAGATA | CTCCTTCATTTCCTCCCTCC | AAGGTGGGTTAAAGAACCGC | 60.0 | 778 | INTERGENIC | | | | |
| CaPOPI_2584 | Ca4 | 30120661 | TAAAA/TAAA | TTTGACATCCATCTTATGCAGC | CCCTTGGGTGCGTTAGTTAT | 60.1 | 379 | INTERGENIC | | | | |
| CaPOPI_2585 | Ca4 | 30122560 | GAA/GAAAA | ATGTCAATGGGCCACTTAGC | GTGGGGCCATTGATGAGTAA | 60.0 | 962 | INTERGENIC | | | | |
| CaPOPI_2586 | Ca4 | 30134439 | CAAAAAA/CAAAA | TCCTTTTGCATTACTATCCAAGC | GGGGGTTCCATGTAAAACATT | 59.7 | 419 | INTERGENIC | | | | |
| CaPOPI_2587 | Ca4 | 30141789 | TTAT/TT | GAACAATGACCAAGTGAATTTTGA | TATGGGATGCGTTGAGATGA | 60.3 | 428 | INTERGENIC | | | | |
| CaPOPI_2588 | Ca4 | 30148542 | ATTTTTTTT/ATTTTTTTTT | GTTGTTGCTGTGAGTGCTCC | TTAGTCCCTTACGTCGTGGG | 59.5 | 302 | INTRON | Ca_14185 | | | Plastid lipid-associated protein/fibrillin conserved domain |
| CaPOPI_2589 | Ca4 | 30178438 | AAATTTCAATATTTAATTTCAATAT/AAATTTCAATATTTAATTTCAATATTTAATTTCAATAT | CAAGCAATTATGGTGCAACTG | AGTGAGGCAAATGGAAATGG | 59.2 | 669 | INTERGENIC | | | | |
| CaPOPI_2590 | Ca4 | 30184517 | TA/T | CGAGGGGCGTAAAAATCATA | GCGAGATTTGTTTCCGTTGT | 59.9 | 579 | INTERGENIC | | | | |

| INDEL marker IDs | Chromosomes/ unanchored scaffolds | Physical positions (bp) | InDels (*Kabuli* reference genome-CDC Frontier/PI) | Forward primers (5'-3') | Reverse primers (5'-3') | Anneling temperature (0C) | Expected amplified product size (bp) | Structural annotation | | Functional annotation | | |
|---|---|---|---|---|---|---|---|---|---|---|---|---|
| | | | | | | | | Sequence components of *kabuli* genome | *Kabuli* gene accession IDs | NCBI-KOG | TFs | NCBI-nr database |
| CaPOPI_2591 | Ca4 | 30185691 | TAAAAAAAA/TAAAAAAAAAA | CATCGCTAACTCAGTGCCAA | AATGCGTTACGGGTGAAATG | 60.0 | 876 | INTERGENIC | | | | |
| CaPOPI_2592 | Ca4 | 30210048 | ATATCACTTT/AT | GGTTTGCTTATGGGGCAGTA | CACCCCGAATGAACTTTAGAA | 60.0 | 631 | INTERGENIC | | | | |
| CaPOPI_2593 | Ca4 | 30211443 | T/TA | ACATCAAACAAACAGCGCAC | CCGCTAAAATTGCCCAATAA | 59.8 | 714 | INTERGENIC | | | | |
| CaPOPI_2594 | Ca4 | 30226816 | CAAAAAA/CAAAA | GCTTATGCAATGCACGAAAA | CATGGGTTGAAATGGAGACA | 59.8 | 528 | INTERGENIC | | | | |
| CaPOPI_2595 | Ca4 | 30330570 | CTATATATATATATATATATA/CTATATATATATATATATATATA | TTCGATGGCTAGATTACATACTGC | CCCCTCAACTCAAGAATGATG | 59.7 | 561 | INTERGENIC | | | | |
| CaPOPI_2596 | Ca4 | 30351459 | CTTTTTTT/CTTTTTTTT | TCTTTGAAACACATCACTTTCG | AAAATTTGATCGGTGGTGGA | 57.9 | 314 | INTERGENIC | | | | |
| CaPOPI_2597 | Ca4 | 30390669 | T/TA | CTACTGAAGCCCAACGAAGG | CTTTCAGATTCGTGGCCATT | 59.9 | 864 | INTERGENIC | | | | |
| CaPOPI_2598 | Ca4 | 30404870 | AATAACAT/AAT | TTAACTGTTGGTGCGCAGTC | CGTCACATTTAACACGCCAT | 59.9 | 798 | INTERGENIC | | | | |
| CaPOPI_2599 | Ca4 | 30415073 | GCATCATCATCATCA/GCATCATCATCATCATCA | TTGAACATATAGGGAGGCGG | CCATCCTTTTCAATGCGAGT | 59.9 | 423 | CDS (large-effect mutations) | Ca_14197 | | | |
| CaPOPI_2600 | Ca4 | 30423088 | TAAAAAAA/TAAAAAAAA | AGGGGCAAAAATACTGCTTG | CTTGATCGGCTCATGTGTTG | 59.2 | 938 | INTERGENIC | | | | |
| CaPOPI_2601 | Ca4 | 30429374 | TATTCATT/TATT | TGATCGCCATATCCTTAGCC | ATTTGGTTTGGCATTGTTGC | 60.0 | 811 | INTERGENIC | | | | |
| CaPOPI_2602 | Ca4 | 30468296 | AAT/AATAT | ATTGTGCATATTCCCTCCCA | CCTTAATCCACCAAAAGCCA | 60.2 | 747 | DRR | Ca_14199 | | G2-like | Myb, DNA-binding |
| CaPOPI_2603 | Ca4 | 30468822 | AAA/AAAGAA | TGGCTTTTGGTGGATTAAGG | GGTCTCACGACCAAGTCACA | 59.9 | 438 | DRR | Ca_14199 | | G2-like | Myb, DNA-binding |
| CaPOPI_2604 | Ca4 | 30477859 | CTTTTT/CTTTTTT | AAAATCAATCGGGGACATCA | GAATCAAACAAAGCCCCAAA | 60.1 | 648 | INTERGENIC | | | | |
| CaPOPI_2605 | Ca4 | 30501632 | AGT/A | TGGGGATTTATCCCTTCAAA | GCAAGTGTCAACTCACTCAATCA | 59.2 | 595 | INTERGENIC | | | | |

| INDEL marker IDs | Chromosomes/ unanchored scaffolds | Physical positions (bp) | InDels (*Kabuli* reference genome-CDC Frontier/PI) | Forward primers (5'-3') | Reverse primers (5'-3') | Anneling temperature (0C) | Expected amplified product size (bp) | Structural annotation | | Functional annotation | | |
|---|---|---|---|---|---|---|---|---|---|---|---|---|
| | | | | | | | | Sequence components of *kabuli* genome | *Kabuli* gene accession IDs | NCBI-KOG | TFs | NCBI-nr database |
| CaPOPI_2606 | Ca4 | 30535832 | TATAAATAAATAAATAAA/TATAAATAAATAAATAAATAAATAAATAAA | CCATTCAGAATTAGAAAATCACCA | CCAAATTTCCCAAGGGTTCT | 59.4 | 582 | INTERGENIC | | | | |
| CaPOPI_2607 | Ca4 | 30536932 | GTTTTTTTTT/GTTTTTTTTTTTTTT | ATCATTGAACGACCGGAAAC | CGGATACAATGGCAATCAAA | 59.8 | 431 | INTRON | Ca_14204 | | NAC | No apical meristem (NAM) protein |
| CaPOPI_2608 | Ca4 | 30537523 | CA/CATA | TTTGATTGCCATTGTATCCG | CAAAATGGAAATGCAAGAACAA | 59.4 | 803 | INTRON | Ca_14204 | | NAC | No apical meristem (NAM) protein |
| CaPOPI_2609 | Ca4 | 30538166 | AAAGAAG/AAAGAAGAAG | TTGTTCTTGCATTTCCATTTTG | CGCCATCTTCCTCTTCTTTG | 60.0 | 380 | CDS (large-effect mutations) | Ca_14204 | | NAC | No apical meristem (NAM) protein |
| CaPOPI_2610 | Ca4 | 30542137 | TAAAAAA/TAAAAAAA | TAAGGCATGATAAGGCCGAC | GTCATGTCACCCGTGATGAG | 60.1 | 299 | DRR | Ca_14205 | | | |
| CaPOPI_2611 | Ca4 | 30552355 | TG/T | TGTCGCATGCAACCTCTTAG | ACCTGTGTGTTCCCTTCCAC | 60.0 | 528 | INTERGENIC | | | | |
| CaPOPI_2612 | Ca4 | 30558963 | GC/G | CCCCTTTTGGCATCTATCAA | TCATCTCATTTTGCTTCCCT | 59.9 | 510 | INTERGENIC | | | | |
| CaPOPI_2613 | Ca4 | 30561689 | TAAAAA/TAAAA | GGAGGCACCATACTTTTCCA | AGAATGGCCACTTCAAGTCAA | 59.9 | 198 | INTERGENIC | | | | |
| CaPOPI_2614 | Ca4 | 30566057 | CGGG/CGG | TTTTTGCAATTGGGCATACA | ACTGGGTTTGGGTTACCCTC | 59.9 | 515 | INTERGENIC | | | | |
| CaPOPI_2615 | Ca4 | 30570798 | TAAAAAAA/TAAAAAAAA | TGCTTCTCAATGACTAACACCAA | TGTTGGTGTGACTTTTCCTTCT | 59.8 | 881 | INTERGENIC | | | | |
| CaPOPI_2616 | Ca4 | 30573766 | T/TC | CATTATGATCACAGACGATGGTG | TCCTCTTCGATCAAGCATCC | 60.3 | 259 | DRR | Ca_14206 | A | | D111/G-patch |
| CaPOPI_2617 | Ca4 | 30582286 | GAAAAAAAAAA/GAAAAAAAAAAAA | TTCCCTCCCTTCCTTTGAGT | TTTTCTGCAAGTTTGTCGTTG | 60.0 | 615 | INTERGENIC | | | | |
| CaPOPI_2618 | Ca4 | 30585901 | CTTTTTCTAATTTTTCTA/CTTTTTCTA | AAAGGTCAGTCATCCCAAACA | GAAACAGAAAACGGGAACCA | 59.4 | 381 | INTERGENIC | | | | |
| CaPOPI_2619 | Ca4 | 30619694 | CAAAAA/CAAAA | ACTCACTCTGCCCCTGTGTT | CCAACTTGGTTCCAACAAAAA | 59.8 | 566 | INTERGENIC | | | | |
| CaPOPI_2620 | Ca4 | 30653740 | AC/ACC | TGCCCATTGATGCTAAACAA | GAATGATGTCGCGGTAGGAT | 60.1 | 513 | INTERGENIC | | | | |

| INDEL marker IDs | Chromosomes/ unanchored scaffolds | Physical positions (bp) | InDels (*Kabuli* reference genome-CDC Frontier/PI) | Forward primers (5'-3') | Reverse primers (5'-3') | Anneling temperature (0C) | Expected amplified product size (bp) | Structural annotation | | Functional annotation | | |
|---|---|---|---|---|---|---|---|---|---|---|---|---|
| | | | | | | | | Sequence components of *kabuli* genome | *Kabuli* gene accession IDs | NCBI-KOG | TFs | NCBI-nr database |
| CaPOPI_2621 | Ca4 | 30685617 | TTA/TTATCAATA | GGGAAGAATGGATTTGGGAT | GGATGTTTCATTCCACCAAAG | 60.0 | 192 | INTERGENIC | | | | |
| CaPOPI_2622 | Ca4 | 30697175 | CTTTT/CTTT | AGATTCCATGGCACACACAA | CCCCAAGATTAACAAAGGCA | 60.0 | 647 | INTERGENIC | | | | |
| CaPOPI_2623 | Ca4 | 30741967 | TCAATATTGAACA/T | ATCCGGTTCCTTTTTGGTTC | TAAGGACCGGTGATCTGACA | 60.2 | 656 | INTERGENIC | | | | |
| CaPOPI_2624 | Ca4 | 30755137 | ATTTTTTT/ATTTTTT | CAGTAGGAAGTCACATTCCCAGA | GCAACTGAGCTGAGAGCGTA | 60.5 | 860 | INTERGENIC | | | | |
| CaPOPI_2625 | Ca4 | 30760524 | ATT/ATTT | TTTGGAAGCGGTCTCACTCT | AAACGAATACTGCAACGCAA | 60.0 | 217 | DRR | Ca_14211 | | ERF | C2 calcium-dependent membrane targeting |
| CaPOPI_2626 | Ca4 | 30790861 | TAAAAAAAAA/TAAAAAAAAAA | GAATTTTGGCCATCACCACT | TGTCTCGCACACCCATTTTA | 59.8 | 530 | INTERGENIC | | | | |
| CaPOPI_2627 | Ca4 | 30844654 | CTTTTTTTTTT/CTTTTTTTTT | CACCTGCTTTTGACCCATCT | GGCAATTGCTTTACGCTAGG | 60.1 | 959 | INTERGENIC | | | | |
| CaPOPI_2628 | Ca4 | 30866796 | A/AC | AAGTTGCCTCTGCCTTTGAA | ATGTTTGCAACCCGTTTCTC | 60.0 | 255 | INTERGENIC | | | | |
| CaPOPI_2629 | Ca4 | 30873864 | ACC/ACCC | CAAAGAGAGAAACTTGCCCG | GGAAAGCGAGAAGTTGAACG | 60.0 | 566 | INTERGENIC | | | | |
| CaPOPI_2630 | Ca4 | 30880709 | ATT/ATTTT | TGCAGAGTAAACCAGCAACG | TTGCAAGTTGTGACTGAGGC | 60.1 | 557 | INTERGENIC | | | | |
| CaPOPI_2631 | Ca4 | 30880741 | CAA/CAAA | TGCAGAGTAAACCAGCAACG | TTGCAAGTTGTGACTGAGGC | 60.1 | 557 | INTERGENIC | | | | |
| CaPOPI_2632 | Ca4 | 30887008 | TCTCCTC/TCTC | CCTGACCCTACAAGCCAATC | GTAGTCTCGCAAGGACTCCG | 59.6 | 218 | INTERGENIC | | | | |
| CaPOPI_2633 | Ca4 | 30928717 | TAAAAAAA/TAAAAAAAA | CAATGTCCATGTCGACCAAA | TCTGTCGCACCATCTCAGTC | 60.4 | 949 | INTERGENIC | | | | |
| CaPOPI_2634 | Ca4 | 30968151 | CTTTTTTTT/CTTTTTTT | CACATCCCTTGCCATTTCTT | CCCGCATTTGTAACCATCTT | 59.9 | 827 | INTERGENIC | | | | |
| CaPOPI_2635 | Ca4 | 31063154 | AATATATATATATATATATATAT/AATATATATATATATATATATAT | GAGGGGATGAATCATTAACTCG | GTAAGACCCCGCATTGTGTT | 59.8 | 499 | INTERGENIC | | | | |

| INDEL marker IDs | Chromosomes/ unanchored scaffolds | Physical positions (bp) | InDels (*Kabuli* reference genome-CDC Frontier/PI) | Forward primers (5'-3') | Reverse primers (5'-3') | Anneling temperature (0C) | Expected amplified product size (bp) | Structural annotation | | Functional annotation | | |
|---|---|---|---|---|---|---|---|---|---|---|---|---|
| | | | | | | | | Sequence components of *kabuli* genome | *Kabuli* gene accession IDs | NCBI-KOG | TFs | NCBI-nr database |
| CaPOPI_2636 | Ca4 | 31079661 | TGG/TG | GCTCGTGTCATTGCAGAAAA | CCAATTACCACAGATTCGGG | 60.0 | 337 | INTERGENIC | | | | |
| CaPOPI_2637 | Ca4 | 31106521 | CCTT/CCTTCTT | ATAAGAGTGTGGCGGTTTGG | TGGTTGAAATGGACGACAAA | 60.0 | 584 | INTERGENIC | | | | |
| CaPOPI_2638 | Ca4 | 31108602 | GGAAGAAGAA/GGAAGAAGAAGAA | GGATCAGGCCCTATTGTTGA | AGTTGGCTTTGCTTGATGCT | 59.9 | 476 | INTERGENIC | | | | |
| CaPOPI_2639 | Ca4 | 31110947 | TAAAAAA/TAAAAA | GCTAAAGCAATCACCAAGGC | GCGTTTGGTGACATAGCTGA | 59.9 | 412 | INTERGENIC | | | | |
| CaPOPI_2640 | Ca4 | 31137565 | GAAAAAA/GAAAAAAA | TTTCCGAATCGGGAGTATGA | GTTCCTGCCATCCAACCTTA | 60.4 | 113 | DRR | Ca_14223 | | bHLH | Helix-loop-helix DNA-binding domain |
| CaPOPI_2641 | Ca4 | 31140324 | TAAAA/TAAAAA | ACTTCCATTCGCTTCAGCAT | AAATGATTCTGGTGGGGACA | 59.8 | 536 | INTRON | Ca_14223 | | bHLH | Helix-loop-helix DNA-binding domain |
| CaPOPI_2642 | Ca4 | 31172943 | TA/T | TCGCCTAAGGTAAGGGCTTT | TTACACCAATTCCCCCAAGA | 60.2 | 421 | INTERGENIC | | | | |
| CaPOPI_2643 | Ca4 | 31182932 | T/TC | CGCATATTAGCATGGAGGGT | TCGCTCGAACCAGAGTTCTT | 59.9 | 317 | INTERGENIC | | | | |
| CaPOPI_2644 | Ca4 | 31192815 | TGG/TG | TTGAAGGAGAAGCCAGAGTCA | TGAGGCATGCAGAAACGTAG | 60.1 | 238 | INTERGENIC | | | | |
| CaPOPI_2645 | Ca4 | 31194912 | CTTTTTTTTTTT/CTTTTTTTTTT | TTCCATGTTTGTGTTGAAGCA | GCACATTGCAAGGGTATTGA | 60.1 | 822 | DRR | Ca_14224 | | S1Fa-like | Protein kinase, catalytic domain |
| CaPOPI_2646 | Ca4 | 31195163 | GAAAAAAAA/GAAAAAAAAA | TTTCCCGACTTGGTCCTTTA | TGCATGCAGATCTTGCTCTC | 59.5 | 643 | DRR | Ca_14224 | | S1Fa-like | Protein kinase, catalytic domain |
| CaPOPI_2647 | Ca4 | 31200114 | TA/TAGACCA | TTGTCTTAGGCAGCACTGGA | TGGTGAACGAAAGTGACGTG | 59.6 | 903 | INTERGENIC | | | | |
| CaPOPI_2648 | Ca4 | 31230420 | TTCTCTCTCTCT/TTCTCTCTCT | CTTTCCATCTCACTCCCTCG | TAGCCGACAGGGTCTCAGTT | 59.8 | 435 | INTERGENIC | | | | |
| CaPOPI_2649 | Ca4 | 31235705 | ATTTTTTTTT/ATTTTTTTTTT | CAATTGCGGTTTCCACTTTT | TTCATGCATTTTCAATGGGA | 60.0 | 434 | INTERGENIC | | | | |
| CaPOPI_2650 | Ca4 | 31236090 | ATATTATTATTATTATT/ATATTATTATTATT | CAATTGCGGTTTCCACTTTT | TTTTTACCGTGGGTGAATCC | 60.0 | 956 | INTERGENIC | | | | |

| INDEL marker IDs | Chromosomes/ unanchored scaffolds | Physical positions (bp) | InDels (*Kabuli* reference genome-CDC Frontier/PI) | Forward primers (5'-3') | Reverse primers (5'-3') | Anneling temperature (0C) | Expected amplified product size (bp) | Structural annotation | | Functional annotation | | |
|---|---|---|---|---|---|---|---|---|---|---|---|---|
| | | | | | | | | Sequence components of *kabuli* genome | *Kabuli* gene accession IDs | NCBI-KOG | TFs | NCBI-nr database |
| CaPOPI_2651 | Ca4 | 31258911 | GTT/GT | CAGGTCGTGCCAATTCTTTT | GGCTAGGATTTGCAACAGGT | 60.1 | 264 | INTERGENIC | | | | |
| CaPOPI_2652 | Ca4 | 31259145 | GATATATATATATATATATATATAT/GATATATCTATATATATATATATATATATAT | CAGGTCGTGCCAATTCTTTT | TTCATTGACGAATGTACCATCA | 60.1 | 404 | INTERGENIC | | | | |
| CaPOPI_2653 | Ca4 | 31274972 | TAAAAAAAAAA/TAAAAAAAA | CTATGGCACACACCAGCAAC | GATCAAGTAGGGCGGATTCA | 60.2 | 699 | INTERGENIC | | | | |
| CaPOPI_2654 | Ca4 | 31275360 | ATTTTTTTTT/ATTTTTTTT | TTCAATTAGGGTTTGGGTCG | ACAGTTTGTGCGTTGTTGGT | 59.8 | 631 | INTERGENIC | | | | |
| CaPOPI_2655 | Ca4 | 31279281 | ACCC/ACC | GCCACCTGTCCAACTTCCTA | ATACCCAAGAGGGTATGGGC | 60.1 | 454 | INTERGENIC | | | | |
| CaPOPI_2656 | Ca4 | 31284485 | ATT/ATTT | ATGGCTACTCCAATGCATCC | ACATGGCTCCCCCTTACTCT | 59.9 | 962 | INTERGENIC | | | | |
| CaPOPI_2657 | Ca4 | 31302749 | GAAAAAAAAAAA/GAAAAAAAAAA | CACCCCACACCAAACTCTCT | GTTAGGTTGGACGGGACCTC | 60.0 | 680 | INTRON | Ca_14231 | | bHLH | Helix-loop-helix DNA-binding domain |
| CaPOPI_2658 | Ca4 | 31303190 | CTTTTTTTTTTT/CTTTTTTTTTT | GAGGTCCCGTCCAACCTAAC | TCAGCACCGAGAGTGTCAAC | 60.7 | 488 | INTRON | Ca_14231 | | bHLH | Helix-loop-helix DNA-binding domain |
| CaPOPI_2659 | Ca4 | 31317473 | ATTTTTTTTT/ATTTTTTTT | TGAGGCGTCATGGTAGAACA | CAAATCGACATTGTTGCCAG | 60.3 | 707 | INTERGENIC | | | | |
| CaPOPI_2660 | Ca4 | 31317508 | TAAAAAAAA/TAAAAAAA | TTCGCATCAGCCATATTTTG | CAAATCGACATTGTTGCCAG | 59.7 | 509 | INTERGENIC | | | | |
| CaPOPI_2661 | Ca4 | 31322894 | GAAAAAAAA/GAAAAAAA | CTCCTAAGGGTGGCCTTTTC | GGGTTGTTCTTGGACCTTCA | 60.1 | 623 | INTERGENIC | | | | |
| CaPOPI_2662 | Ca4 | 31406944 | GTTTTTTTTT/GTTTTTTTTTT | CAAATTATACTCGCACAACTTGC | AAAGTGGACAAACACAAAACGA | 58.9 | 782 | INTERGENIC | | | | |
| CaPOPI_2663 | Ca4 | 31424757 | GTTTTTT/GTTTTTTT | AATTGGCGAAACCACTTTTT | TGCACTTGTTGGGGTTGATA | 58.6 | 501 | INTERGENIC | | | | |
| CaPOPI_2664 | Ca4 | 31445256 | ATT/ATTT | AGATCTATGCATCGGCAACC | AGGGTACTACAGCTGCCCCT | 60.1 | 338 | INTERGENIC | | | | |
| CaPOPI_2665 | Ca4 | 31452576 | CTTTTTTTT/CTTTTTTTTT | CCACTCGTGGTCCCTTAACT | TCGGCTAAATACCATTTGTGG | 59.1 | 566 | INTERGENIC | | | | |

| INDEL marker IDs | Chromosomes/ unanchored scaffolds | Physical positions (bp) | InDels (*Kabuli* reference genome-CDC Frontier/PI) | Forward primers (5'-3') | Reverse primers (5'-3') | Anneling temperature (0C) | Expected amplified product size (bp) | Structural annotation | | Functional annotation | | |
|---|---|---|---|---|---|---|---|---|---|---|---|---|
| | | | | | | | | Sequence components of *kabuli* genome | *Kabuli* gene accession IDs | NCBI-KOG | TFs | NCBI-nr database |
| CaPOPI_2666 | Ca4 | 31529637 | CTTTTTTTTTT/CTTTTTTTTT | CCAAATCCGATGAGGAAAGA | TTTAAGACCCGCGATTTCAG | 60.0 | 820 | INTERGENIC | | | | |
| CaPOPI_2667 | Ca4 | 31531525 | G/GGT | AGGGATTGCTTGCAAATGAC | AATTCCCAAACCAATAGCCC | 60.1 | 387 | INTERGENIC | | | | |
| CaPOPI_2668 | Ca4 | 31534458 | CAAAAAAA/CAAAAAAAAA | CAAGCCAACGTGTGTTTGTT | TGTGGGGGATCAAATTGTTT | 59.7 | 371 | INTERGENIC | | | | |
| CaPOPI_2669 | Ca4 | 31570022 | TGGGGGG/TGGGGGGG | TATTTTGGATTTTGGCTCGG | AGGAAGAGTGGAGTTGCGAA | 59.9 | 505 | INTERGENIC | | | | |
| CaPOPI_2670 | Ca4 | 31609144 | ATTTT/ATTT | TCGACGTTTATTTGATGGCA | TTGAGAAACTTAATTGTGGGCA | 60.1 | 651 | DRR | Ca_15441 | A | | |
| CaPOPI_2671 | Ca4 | 31611840 | GTTTTTTTTTT/GTTTTTTTTTTTT | CGTTTTCAGCTGAGCAAATTC | TTGATCCATTGGGATTGTGA | 60.0 | 392 | INTERGENIC | | | | |
| CaPOPI_2672 | Ca4 | 31611867 | AAT/AATAT | CGTTTTCAGCTGAGCAAATTC | TTGATCCATTGGGATTGTGA | 60.0 | 392 | INTERGENIC | | | | |
| CaPOPI_2673 | Ca4 | 31612473 | TAAAAAAA/TAAAAAAAA | TAAAATCCAAAACCGCAAGC | CACTTAAGCAAACGGGGTGT | 60.1 | 462 | INTERGENIC | | | | |
| CaPOPI_2674 | Ca4 | 31630531 | AGG/AG | AGTTGAAGGTGCAAACACGA | AATTTACTTGGAGGGTCGGG | 59.3 | 288 | INTERGENIC | | | | |
| CaPOPI_2675 | Ca4 | 31630588 | TCA/TA | AAGGTGCAAACACGAGAAGG | AATTTACTTGGAGGGTCGGG | 60.3 | 283 | INTERGENIC | | | | |
| CaPOPI_2676 | Ca4 | 31631888 | AAAA/AAAATAAA | CCAACTTGGTTCCAACATCTG | TGGCTAATCATAGCGAAACG | 60.4 | 832 | INTERGENIC | | | | |
| CaPOPI_2677 | Ca4 | 31633774 | AG/AGG | AAATATTGTTCAATCCGCCG | CTCCTCCAAGCCAAGTGAAG | 59.8 | 491 | INTERGENIC | | | | |
| CaPOPI_2678 | Ca4 | 31635242 | ATTTACCT/A | TCAATGTGAACGGGCATCTA | TGGTGCCTCCTACAATTTCC | 60.1 | 778 | INTERGENIC | | | | |
| CaPOPI_2679 | Ca4 | 31635868 | AAT/A | GGAAATTGTAGGAGGCACCA | GTTGCGCTACGAGATGTTGA | 59.9 | 695 | INTERGENIC | | | | |
| CaPOPI_2680 | Ca4 | 31639620 | GTTTT/GTTTTT | ATTTGGAGAATGATTTGGCG | GTGTTTGGTTCAAGGGGAGA | 59.9 | 412 | INTERGENIC | | | | |

| INDEL marker IDs | Chromosomes/ unanchored scaffolds | Physical positions (bp) | InDels (*Kabuli* reference genome-CDC Frontier/PI) | Forward primers (5'-3') | Reverse primers (5'-3') | Anneling temperature (0C) | Expected amplified product size (bp) | Structural annotation | | Functional annotation | | |
|---|---|---|---|---|---|---|---|---|---|---|---|---|
| | | | | | | | | Sequence components of *kabuli* genome | *Kabuli* gene accession IDs | NCBI-KOG | TFs | NCBI-nr database |
| CaPOPI_2681 | Ca4 | 31642398 | TAAAAAAAA/TAAAAAAAAA | TGTCACTGCACTGCCAC ATA | TTTCCGGCAAAGTTCTC ATAA | 59.9 | 482 | INTRON | Ca_15442 | U | EIL | SPX, N-terminal |
| CaPOPI_2682 | Ca4 | 31644451 | TA/T | AATGTCGGGAGTTTTCA TCG | GCCATCTTAGGCTTTCC CTT | 59.9 | 953 | INTRON | Ca_15442 | U | EIL | SPX, N-terminal |
| CaPOPI_2683 | Ca4 | 31646633 | AA/AACA | ATGGAGAAGAGGGGGA GTGT | ACCAGTCTTTTACGGGC CTT | 59.9 | 135 | INTRON | Ca_15442 | U | EIL | SPX, N-terminal |
| CaPOPI_2684 | Ca4 | 31647747 | ATT/AT | CACGTGTGAAGGTCGAG AGA | AAGCTACCTGATCAATG CGG | 60.0 | 863 | INTRON | Ca_15442 | U | EIL | SPX, N-terminal |
| CaPOPI_2685 | Ca4 | 31647770 | GTTTTTTTTT/GTTTTTTTT | CACGTGTGAAGGTCGAG AGA | AAGCTACCTGATCAATG CGG | 60.0 | 863 | INTRON | Ca_15442 | U | EIL | SPX, N-terminal |
| CaPOPI_2686 | Ca4 | 31649916 | AT/A | TGAGGCATTTGGAGACA ACA | ATTGACCGGTGGAAGAC ATT | 60.2 | 585 | INTRON | Ca_15442 | U | EIL | SPX, N-terminal |
| CaPOPI_2687 | Ca4 | 31649942 | CT/CTT | CGTGCCCTGCAGGTATT TAT | ATTGACCGGTGGAAGAC ATT | 60.0 | 454 | INTRON | Ca_15442 | U | EIL | SPX, N-terminal |
| CaPOPI_2688 | Ca4 | 31652250 | CTTTT/CTTTTT | CCATTACCCTTTCGTGA TGC | TGAAAGCAAAGCCACGT ATG | 60.3 | 796 | DRR | Ca_15442 | U | EIL | SPX, N-terminal |
| CaPOPI_2689 | Ca4 | 31653241 | GATAAA/GATAAAAAATAAA | TTCAAACGATGGCAACA AAA | TCATGGGATTAAGCCTC CAC | 60.1 | 268 | DRR | Ca_15442 | U | EIL | SPX, N-terminal |
| CaPOPI_2690 | Ca4 | 31718401 | AT/ATT | ATTCCTCTAATGTCGGG GCT | GCCACCCTTGTGAGAGT TTC | 59.9 | 501 | INTERGENIC | | | | |
| CaPOPI_2691 | Ca4 | 31757461 | CTTTTTTTTT/CTTTTTTTT | TATGCTAAACCAACCGC CTC | TTTTTCCTGACTTGGTC CTTT | 60.1 | 383 | DRR | Ca_15447 | R | bZIP | |
| CaPOPI_2692 | Ca4 | 31806633 | ATGTGACTAATTTT/ATGTGACTAATTTT GTGACTAATTTT | ACAAAAGCCTGGATAGC GAA | TCACATATACCCTTAAAA TCGCA | 59.8 | 410 | INTERGENIC | | | | |
| CaPOPI_2693 | Ca4 | 31816562 | ATTTTTTTTTTT/ATTTTTTTTTT | GGCTCCGATCAAAAGAA ACA | GCCCTCAGATGAGAGTC AGG | 60.2 | 545 | INTERGENIC | | | | |
| CaPOPI_2694 | Ca4 | 31837452 | ATTTTTTTT/ATTTTTTTTT | TGGAGACCATGGAAATG CTT | TTTTGTGAATAGTCCATG CTTCA | 60.5 | 456 | INTERGENIC | | | | |
| CaPOPI_2695 | Ca4 | 31859597 | TAAAAAAAAA/TAAAAAAAAAAA | GTTGGAGCCCCCACTTT ATT | TGCAAGCAGCATCAGAG AAC | 60.2 | 793 | INTERGENIC | | | | |

| INDEL marker IDs | Chromosomes/ unanchored scaffolds | Physical positions (bp) | InDels (*Kabuli* reference genome-CDC Frontier/PI) | Forward primers (5'-3') | Reverse primers (5'-3') | Anneling temperature (0C) | Expected amplified product size (bp) | Structural annotation | | Functional annotation | | |
|---|---|---|---|---|---|---|---|---|---|---|---|---|
| | | | | | | | | Sequence components of *kabuli* genome | *Kabuli* gene accession IDs | NCBI-KOG | TFs | NCBI-nr database |
| CaPOPI_2696 | Ca4 | 31892250 | CTTTTTTTT/CTTTTTTTTT | AACCGTTAACCACCGAAACA | TGCGGTCAACACACTTAGGA | 60.3 | 520 | INTERGENIC | | | | |
| CaPOPI_2697 | Ca4 | 31930111 | AATATATATATA/AATATATATATATATA | GAGGAGCATTGATGGAGGAA | TTTGGAAGACTTTTGGGCTC | 60.2 | 574 | INTERGENIC | | | | |
| CaPOPI_2698 | Ca4 | 31931258 | TAAAAAAAAA/TAAAAAAAA | TCAAACCCGAGAACAAGTGA | TAACAAGCGCGCAATGTATC | 59.3 | 595 | INTERGENIC | | | | |
| CaPOPI_2699 | Ca4 | 31937240 | TGG/TGGG | CTAAGGCCCTATGTGCAAGC | CGAGACTACTCGTGGGCTTC | 59.9 | 835 | CDS (FRAME SHIFT) | Ca_15455 | | Dof | |
| CaPOPI_2700 | Ca4 | 31967535 | T/TC | TACCCTTAATGGCTGGGATG | AAAAACACTTGCGATGACCC | 59.8 | 510 | INTRON | Ca_15456 | | | |
| CaPOPI_2701 | Ca4 | 31972811 | CT/CTT | TTTGTGAAATTGGTTCCCTG | GGTCCAGCAGCATAGGACAT | 58.4 | 523 | INTERGENIC | | | | |
| CaPOPI_2702 | Ca4 | 31974174 | AC/A | GCTGAAAAAGTGGGTGTGGT | GAGAGTGCTGTTGGCTGTGA | 60.0 | 655 | INTERGENIC | | | | |
| CaPOPI_2703 | Ca4 | 31989643 | CTTTTTTTT/CTTTTTTTTTTT | GAAAGATTCCTCCCACTCCC | TGCACATTGGAAAATTGGTG | 59.9 | 246 | INTERGENIC | | | | |
| CaPOPI_2704 | Ca4 | 31991301 | GTTTTTTTT/GTTTTTT | AGACAACGACTTTGGCGTTC | ATTGCTCCTTCAATCATGGG | 60.3 | 534 | INTERGENIC | | | | |
| CaPOPI_2705 | Ca4 | 31994404 | TAAAA/TAAAAA | ATCAGCGAGGATGCAGAACT | CATCGCTCAGTTTGGACTCA | 60.0 | 796 | DRR | Ca_15458 | | | |
| CaPOPI_2706 | Ca4 | 32008597 | TAAAAAA/TAAAAAAA | CAGTTTGCTTGACTTCGCAA | TTCGTACAATTCATACGCGG | 60.2 | 659 | INTERGENIC | | | | |
| CaPOPI_2707 | Ca4 | 32045859 | T/TA | TGGGGTTTTGATCCATTTGT | TGATGTCTTTCGATCCACCT | 60.0 | 607 | DRR | Ca_15459 | | | Domain of unknown function DUF828 |
| CaPOPI_2708 | Ca4 | 32047782 | G/GT | TGGGGTTTTGATCCATTTGT | AAAGCCCAAACCAACATTGA | 60.0 | 329 | INTERGENIC | | | | |
| CaPOPI_2709 | Ca4 | 32048513 | TAAAAAAAA/TAAAAAAAAA | CGATTTTGTCGGTCAATCTCT | TCACCGTAGGAAAGCTGGAT | 59.2 | 355 | INTERGENIC | | | | |
| CaPOPI_2710 | Ca4 | 32069410 | A/AC | CCAGTGGTGTCCCTCTTGAT | GAGACGGAATTTGGGACAGA | 60.0 | 767 | DRR | Ca_15461 | R | | Ankyrin repeat |

| INDEL marker IDs | Chromosomes/ unanchored scaffolds | Physical positions (bp) | InDels (*Kabuli* reference genome-CDC Frontier/PI) | Forward primers (5'-3') | Reverse primers (5'-3') | Anneling temperature (0C) | Expected amplified product size (bp) | Structural annotation | | Functional annotation | | |
|---|---|---|---|---|---|---|---|---|---|---|---|---|
| | | | | | | | | Sequence components of *kabuli* genome | *Kabuli* gene accession IDs | NCBI-KOG | TFs | NCBI-nr database |
| CaPOPI_2711 | Ca4 | 32101684 | TG/T | TCATTGCCTTGTCTGCAAAA | GCACTAACCCGACCCTAACA | 60.4 | 632 | INTRON | Ca_15462 | | | Transposase, Ptta/En/Spm, plant |
| CaPOPI_2712 | Ca4 | 32118643 | AAAT/A | GCCAAAGGTAACAAAGGCAA | TGATTTCTTCATTGCATCGG | 60.1 | 569 | INTERGENIC | | | | |
| CaPOPI_2713 | Ca4 | 32119212 | ATT/AT | TGTTTCGTTGTTGATAAGCATGT | TGTCCTCCTCCCTCCTTTTT | 59.6 | 721 | INTERGENIC | | | | |
| CaPOPI_2714 | Ca4 | 32121836 | AATA/AATATA | CCCCAAGCTTGTAAAGCAAA | AATGAAAGCGTTGATTGGGT | 60.2 | 588 | INTERGENIC | | | | |
| CaPOPI_2715 | Ca4 | 32122386 | CTTTTTTTTT/CTTTTTTTTTT | TGGGTTTTACCCAATCAACG | CACCAGCCAAGTTCTTCCTT | 60.6 | 359 | INTERGENIC | | | | |
| CaPOPI_2716 | Ca4 | 32143759 | GATATATA/GATATA | CGTTATCATTGCTTGAACGG | TTGAGCACTTACTGGTGGGA | 59.2 | 941 | INTERGENIC | | | | |
| CaPOPI_2717 | Ca4 | 32144375 | TAAAAAA/TAAAAAAA | TCCCACCAGTAAGTGCTCAA | GTCGTTGTTGGTCTGTGTGG | 59.3 | 308 | INTERGENIC | | | | |
| CaPOPI_2718 | Ca4 | 32156632 | TAAAAAA/TAAAAAAA | TTCACTTGGTTGCTTTGCAG | CTTAAACCATAATCCCCGCA | 60.0 | 692 | INTERGENIC | | | | |
| CaPOPI_2719 | Ca4 | 32159052 | TAAAA/TAAA | TCTCTTTTAATGGGCCATGC | ATGCATGGAATCCAGCTTTC | 60.0 | 688 | INTERGENIC | | | | |
| CaPOPI_2720 | Ca4 | 32159427 | ATTTTTTTTTT/ATTTTTTTTT | TTCCCTTGAAAGTGCATTGA | GCTTCTATGGGCAACGTACC | 59.2 | 224 | INTERGENIC | | | | |
| CaPOPI_2721 | Ca4 | 32173778 | GAAA/GAA | TGCAACACCGGAACTTCATA | TTTCAACCAATTGTTGCTCG | 60.1 | 299 | INTERGENIC | | | | |
| CaPOPI_2722 | Ca4 | 32179388 | T/TA | TTCTTGCGCTTTGTCTCTCA | TGGAAGTGAACTAAACCCCTTT | 59.9 | 744 | INTERGENIC | | | | |
| CaPOPI_2723 | Ca4 | 32179682 | CAATAATCAAACAAA/CAATAATCAAACAAATAATCAAACAAA | TTGCATACCTCATACCATCCC | TTGATGCATTACCTCCCACA | 59.7 | 731 | INTERGENIC | | | | |
| CaPOPI_2724 | Ca4 | 32180768 | CAA/CA | TAAGCCAATGAAGAATCGCC | TTTTCATCCCACCCCAAATA | 60.2 | 425 | INTERGENIC | | | | |
| CaPOPI_2725 | Ca4 | 32212035 | CTCA/CTCATTGATGCTTATGTCA | TGAATCAACAAATGAAAGCAGTG | TCCCGCTTCGATACAGAGAT | 60.2 | 448 | INTERGENIC | | | | |

| INDEL marker IDs | Chromosomes/ unanchored scaffolds | Physical positions (bp) | InDels (*Kabuli* reference genome-CDC Frontier/PI) | Forward primers (5'-3') | Reverse primers (5'-3') | Anneling temperature (0C) | Expected amplified product size (bp) | Structural annotation | | Functional annotation | | |
|---|---|---|---|---|---|---|---|---|---|---|---|---|
| | | | | | | | | Sequence components of *kabuli* genome | *Kabuli* gene accession IDs | NCBI-KOG | TFs | NCBI-nr database |
| CaPOPI_2726 | Ca4 | 32214848 | ATTTTTT/ATTTTTTT | CGCATGTGTATCCCTTTGTG | CGTTCTCGGAACCTTTCTTTC | 60.0 | 471 | INTRON | Ca_15467 | T | HB-other | Calcium-binding EF-hand |
| CaPOPI_2727 | Ca4 | 32215092 | AT/A | CGCATGTGTATCCCTTTGTG | ATGGGAATGGAACCATTGAA | 60.0 | 918 | INTRON | Ca_15467 | T | HB-other | Calcium-binding EF-hand |
| CaPOPI_2728 | Ca4 | 32222918 | ACTCT/ACT | TCCATCATCGTTCTCATTGG | TCGAACAAAAGGCAACACAA | 59.5 | 225 | INTERGENIC | | | | |
| CaPOPI_2729 | Ca4 | 32225876 | TTAT/TT | TCGTCGCATTGGTGTAATGT | TCCGTGAGGACACAAGTGAG | 60.0 | 506 | INTERGENIC | | | | |
| CaPOPI_2730 | Ca4 | 32227293 | AAAAA/AAAAATTAAAA | TCTTCACAACCCCTTGAACC | TTCTTCCAAACGAGCCTCTC | 59.9 | 868 | INTERGENIC | | | | |
| CaPOPI_2731 | Ca4 | 32239930 | TT/TTGGT | CAGGTAGAGTGCAGATTGCG | TAATGCTTGATGCCAAACCA | 59.6 | 366 | INTERGENIC | | | | |
| CaPOPI_2732 | Ca4 | 32264601 | TA/TAA | GATCAAATTCTCTATGCATGTGCT | CCAATTGAAGGGTAAGTGCC | 59.7 | 608 | INTERGENIC | | | | |
| CaPOPI_2733 | Ca4 | 32264980 | AAT/AATAT | GCACTTACCCTTCAATTGGG | AGGAGAGGTCCATTTCTCAGG | 59.4 | 262 | INTERGENIC | | | | |
| CaPOPI_2734 | Ca4 | 32301599 | TAAAAAAA/TAAAA | TGGATTGCAAAACTGGTTGA | CACGAGCTTACAAACCCACA | 60.1 | 174 | DRR | Ca_15472 | DO | MYB_related | WD40 repeat |
| CaPOPI_2735 | Ca4 | 32335179 | AAAGAAGAAGAAGAAGAAGAA/AAAGAAGAAGAAGAAGAA | AAAGTCCGTTTGTGAATCCG | TGGGCATTCAAATCAAACAA | 60.0 | 507 | INTERGENIC | | | | |
| CaPOPI_2736 | Ca4 | 32367636 | ATTTTTTTTTTTTT/ATTTTTTTTTTTT | TCCTCCTTGTGATCTTGGCT | CATTGCTCCCTCTTACTGCC | 59.8 | 429 | INTERGENIC | | | | |
| CaPOPI_2737 | Ca4 | 32369377 | TA/T | TTGGTTTCGTTTGCTTGTTG | AATAGTGCGCATGTGTGGTC | 59.7 | 822 | INTERGENIC | | | | |
| CaPOPI_2738 | Ca4 | 32395281 | TA/TAA | GGTTAACAAATCTCCTAAAGGCA | TTTTGTTAAAGAATTGCGACCTC | 58.7 | 523 | INTERGENIC | | | | |
| CaPOPI_2739 | Ca4 | 32396185 | TCCC/TCC | TCGTCATTTCTTTGTTTGGTTG | TCCAATTTTCCGTGTCATGT | 60.0 | 346 | INTERGENIC | | | | |
| CaPOPI_2740 | Ca4 | 32410630 | TA/TAA | TTATTCCACCCCACCTCAAA | CTCAGCTCATTGGTTTGGTG | 60.2 | 262 | INTERGENIC | | | | |

| INDEL marker IDs | Chromosomes/ unanchored scaffolds | Physical positions (bp) | InDels (*Kabuli* reference genome-CDC Frontier/PI) | Forward primers (5'-3') | Reverse primers (5'-3') | Anneling temperature (0C) | Expected amplified product size (bp) | Structural annotation | | Functional annotation | | |
|---|---|---|---|---|---|---|---|---|---|---|---|---|
| | | | | | | | | Sequence components of *kabuli* genome | *Kabuli* gene accession IDs | NCBI-KOG | TFs | NCBI-nr database |
| CaPOPI_2741 | Ca4 | 32431643 | TTAATAATAA/TTAATAA | ACCCAACCTCATCTGCATTC | TTTCAGAAAAACTACGGAACAAA | 59.9 | 362 | DRR | Ca_15476 | | | Armadillo |
| CaPOPI_2742 | Ca4 | 32460225 | TAAAAAAAAA/TAAAAAAAAAA | CGGAGATCCATAACACGGTC | TTTGAAAGAGAAATCCACCCA | 60.3 | 242 | INTERGENIC | | | | |
| CaPOPI_2743 | Ca4 | 32473583 | TTTTTTTATTTTTTATTTT/TTTTTTTATTTTTTATTTTTTTATTTT | TGATCTTAGCGATGGTTCCC | TGAGCGTTCGATTTTGTTTG | 60.0 | 687 | INTERGENIC | | | | |
| CaPOPI_2744 | Ca4 | 32484353 | CTTTTTT/CTTTTTTT | TTATCCTTTGCTGCCCAAAC | ATCATGTTGCCCCTAATCCA | 60.1 | 753 | INTERGENIC | | | | |
| CaPOPI_2745 | Ca4 | 32489291 | ATT/ATTGTT | CGACAAAGAGGTGGATGGTT | CCTCCATCAATAATAATAAAACCCC | 60.0 | 423 | INTERGENIC | | | | |
| CaPOPI_2746 | Ca4 | 32492742 | AAGA/AA | AGTGGATTGGGTGGAAAATG | GCCAATTTCAATTAAGCCGA | 59.6 | 296 | INTERGENIC | | | | |
| CaPOPI_2747 | Ca4 | 32494646 | GAAAAAAA/GAAAAAAAA | CAATTCACTTGAAACGTCTCTCAT | CACAAAATCGACAAAGACATGC | 59.7 | 386 | INTERGENIC | | | | |
| CaPOPI_2748 | Ca4 | 32494672 | TA/T | CAATTCACTTGAAACGTCTCTCAT | CACAAAATCGACAAAGACATGC | 59.7 | 386 | INTERGENIC | | | | |
| CaPOPI_2749 | Ca4 | 32498847 | AG/A | TAGCACGGACCCCAATTAAG | TGAACCCCCATCAAAGGATA | 60.0 | 897 | INTERGENIC | | | | |
| CaPOPI_2750 | Ca4 | 32512065 | AG/A | TTTCTCCCGTTATGTGTCGG | TGAGTGCTGGGAACTAACCC | 60.9 | 704 | INTERGENIC | | | | |
| CaPOPI_2751 | Ca4 | 32514320 | GACATTGACTTTTT/GACATTGACTTTTTTCAACATTGACTTTTT | ATTACTGGACCAGAGGCACG | AATGTCCATTCTGGGACGAG | 60.1 | 510 | INTERGENIC | | | | |
| CaPOPI_2752 | Ca4 | 32522290 | GAAAAAAAAA/GAAAAAAAAAA | ACTCATTCATTCCACAGCCC | TTGCTAACGGTAACACAACGA | 59.9 | 851 | INTERGENIC | | | | |
| CaPOPI_2753 | Ca4 | 32529368 | GATATATATATAT/GATATATATATATAT | GGGTGTGATTAATGGGTTCG | TCAGGGGTTGCATGGTATTT | 60.1 | 457 | INTERGENIC | | | | |
| CaPOPI_2754 | Ca4 | 32531473 | TAA/T | TAAAATGGATGCATCGAGCC | ACAAATCAACCAGTGTCGCA | 60.9 | 662 | INTERGENIC | | | | |
| CaPOPI_2755 | Ca4 | 32548891 | GT/GTTAT | CGAACCAAGTGAGGAAAGAGA | TGATTGGCATGAGTGCTGAC | 59.5 | 603 | INTERGENIC | | | | |

| INDEL marker IDs | Chromosomes/ unanchored scaffolds | Physical positions (bp) | InDels (*Kabuli* reference genome-CDC Frontier/PI) | Forward primers (5'-3') | Reverse primers (5'-3') | Anneling temperature (0C) | Expected amplified product size (bp) | Structural annotation | | Functional annotation | | |
|---|---|---|---|---|---|---|---|---|---|---|---|---|
| | | | | | | | | Sequence components of *kabuli* genome | *Kabuli* gene accession IDs | NCBI-KOG | TFs | NCBI-nr database |
| CaPOPI_2756 | Ca4 | 32549547 | GTATA/GTA | TTGATGTCTATTGATGCAACG | ACATTGTGCCATCCCTTGAT | 57.2 | 747 | INTERGENIC | | | | |
| CaPOPI_2757 | Ca4 | 32550360 | ATTT/ATTTT | TGAATTAGCTTGGCCGTTTT | GAGAAAATCAACCCAACGTCA | 59.7 | 530 | INTERGENIC | | | | |
| CaPOPI_2758 | Ca4 | 32555948 | AAAGAA/AAA | GTGTGTTTCCCCATACCACC | TGGGAATTTTGTTTGTGGGT | 60.0 | 449 | INTERGENIC | | | | |
| CaPOPI_2759 | Ca4 | 32559742 | AAAGAAAGAAGAAAGAAGA/AAAGAAAGAAGA | TTCATTTCCAGTCTGTGGCA | ACGGCCGTTATAGGTTTCCT | 60.2 | 669 | INTERGENIC | | | | |
| CaPOPI_2760 | Ca4 | 32562355 | CTTTTT/CTTTTTT | AAATGGAGGAGAAGGAGGGA | CGGTGAATCCTTTACGGAGA | 60.0 | 286 | INTERGENIC | | | | |
| CaPOPI_2761 | Ca4 | 32586827 | GAAAAAAAA/GAAAAAAAAA | TATTCTTTGCCGCTCCAATC | AAGGACCAAAACCAATTGTCTC | 60.2 | 750 | INTERGENIC | | | | |
| CaPOPI_2762 | Ca4 | 32622005 | T/TTAG | CATTGTTGCGCCTTAAATGA | GAATTTGAAATGCACCCGTT | 59.7 | 525 | DRR | Ca_15485 | O | | Peptidase S9, prolyl oligopeptidase, catalytic domain |
| CaPOPI_2763 | Ca4 | 32623000 | TAAAAAAAAAA/TAAAAAAAAAAAA | TCGATGTTGTTGGAGATGGA | TCCATCTTTTGGAGGAATGG | 60.0 | 566 | DRR | Ca_15485 | O | | Peptidase S9, prolyl oligopeptidase, catalytic domain |
| CaPOPI_2764 | Ca4 | 32634327 | TTTACTTACTTA/TTTACTTA | CAGACCCGTGTGAACACAAA | GCCTTTGGAGTTGAAGATTGA | 60.6 | 591 | INTERGENIC | | | | |
| CaPOPI_2765 | Ca4 | 32700117 | CTTTTTTT/CTTTTTTTT | TACGTGTCGCTTAGGCACTG | CGTCTTGTGCGGTGTATTTG | 60.1 | 461 | INTERGENIC | | | | |
| CaPOPI_2766 | Ca4 | 32886390 | AC/ACC | AATCATCCCATGTTGCCATT | CGAGATTTCACACCAAGGGT | 60.0 | 519 | INTERGENIC | | | | |
| CaPOPI_2767 | Ca4 | 33050402 | ATTTTTTTTTTT/ATTTTTTTTTT | TTCCTCACACGCAAATTGTC | AAAAGAATTTGGGGTCTTTCG | 59.7 | 531 | DRR | Ca_24017 | GC | HSF | UDP-glucuronosyl/UDP-glucosyltransferase |
| CaPOPI_2768 | Ca4 | 33054018 | TAA/TAAA | GGAATGGCAAACTTTCATGC | TTTGCATTACGGATTTGATGA | 60.5 | 520 | INTERGENIC | | | | |
| CaPOPI_2769 | Ca4 | 33054232 | TAAAAAAAA/TAAAAAAAAA | TCATCAAATCCGTAATGCAAA | GCACATGGGGTACCAGACTT | 59.0 | 445 | INTERGENIC | | | | |
| CaPOPI_2770 | Ca4 | 33064079 | ATTGTTGTTGTTGTTGTTGTTG/ATTGTTGTTGTTGTTGTTGTTGTTG | TCGGGCCACTATCTGGATTA | GTGTGCCGTCACAAACAATC | 60.4 | 810 | INTERGENIC | | | | |

| INDEL marker IDs | Chromosomes/ unanchored scaffolds | Physical positions (bp) | InDels (*Kabuli* reference genome-CDC Frontier/PI) | Forward primers (5'-3') | Reverse primers (5'-3') | Anneling temperature (0C) | Expected amplified product size (bp) | Structural annotation | | Functional annotation | | |
|---|---|---|---|---|---|---|---|---|---|---|---|---|
| | | | | | | | | Sequence components of *kabuli* genome | *Kabuli* gene accession IDs | NCBI-KOG | TFs | NCBI-nr database |
| CaPOPI_2771 | Ca4 | 33100738 | GAAAAAAAAAA/GAAAAAAAAAAAA | TTGGGTTGTGAGGAATGGAT | TCATCTGTTAGGTACCCCGC | 60.2 | 304 | INTERGENIC | | | | |
| CaPOPI_2772 | Ca4 | 33145619 | TAAAAAAA/TAAAAAA | AAACCGTCGTTCCAGTTGTC | AGGCAACAAACATAGAATCGC | 60.0 | 550 | INTERGENIC | | | | |
| CaPOPI_2773 | Ca4 | 33195041 | T/TAGAATTCGGTGA | TTGTCCGTGAGAGACTGCAC | GAATCCTGGGCTGAAATCAA | 60.0 | 595 | INTERGENIC | | | | |
| CaPOPI_2774 | Ca4 | 33296074 | TAAAAAAAAAA/TAAAAAAAAAAA | TGACCTCCCACTGCATACAA | AAGAGACCGCATACGTGGAT | 60.1 | 532 | INTERGENIC | | | | |
| CaPOPI_2775 | Ca4 | 33308913 | GAAAA/GAAA | TCATTTTCCCCTCGTTTCTG | TTGGCCAAAACCTTTTGATG | 60.0 | 183 | DRR | Ca_24021 | R | C2H2 | Zinc finger, C2H2-type |
| CaPOPI_2776 | Ca4 | 33314599 | AC/A | ATGTCCATAACGCATGCAGA | CATCATGCCACTTTGGATTG | 60.1 | 661 | INTERGENIC | | | | |
| CaPOPI_2777 | Ca4 | 33315743 | CTT/CTTT | ACATGAGTCTTTGTTGCCCC | TGGCACGTGATTTGTTTGAT | 60.0 | 477 | INTERGENIC | | | | |
| CaPOPI_2778 | Ca4 | 33321015 | AATATA/AATATATA | TATTGTTGCACCCCCAATTT | ATGTCTGCCCCATTTTCTTG | 60.1 | 229 | INTERGENIC | | | | |
| CaPOPI_2779 | Ca4 | 33327822 | TAAAAAAAAAA/TAAAAAAAAAAAA | CGAAAATTCGAATGGGTAGC | GGTCAAGTGTTTGCCGAAAT | 59.5 | 516 | INTERGENIC | | | | |
| CaPOPI_2780 | Ca4 | 33339958 | T/TA | TGGTGACAACAAGGTTTCCA | CCACATTTTCTCATCCACACA | 60.0 | 437 | INTERGENIC | | | | |
| CaPOPI_2781 | Ca4 | 33346959 | TT/TTAT | TTTTCGGTTTGTTCAAAGGC | GGAAAACATTGGGAAACCCT | 60.1 | 766 | INTERGENIC | | | | |
| CaPOPI_2782 | Ca4 | 33397164 | TTATATATATATATATATA/TTATATATATATATATATATATA | ATAGGCTTTGCACTTCCCCT | CCCCAACAAAATGGAGCTAA | 60.1 | 582 | DRR | Ca_24022 | | | Protein of unknown function DUF1950 |
| CaPOPI_2783 | Ca4 | 33397720 | ATTTTTTTTTTT/ATTTTTTTTTT | CTCCATTTTGTTGGGGCTTA | GCCAACAAACAACCATGCTA | 59.9 | 584 | DRR | Ca_24022 | | | Protein of unknown function DUF1950 |
| CaPOPI_2784 | Ca4 | 33431052 | GAAAAAAAAAA/GAAAAAAAAA | TTTATGCTCCGTTTGTGCTG | CACACACCACCTCTCCCTTT | 59.9 | 435 | INTERGENIC | | | | |
| CaPOPI_2785 | Ca4 | 33467450 | CAAAAAAAAAA/CAAAAAAAAAAA | TGATGTAAAAATGATGATGTGAAA | AATTTGAGTTGCAATTTGATGA | 57.1 | 558 | INTERGENIC | | | | |

| INDEL marker IDs | Chromosomes/ unanchored scaffolds | Physical positions (bp) | InDels (*Kabuli* reference genome-CDC Frontier/PI) | Forward primers (5'-3') | Reverse primers (5'-3') | Anneling temperature (0C) | Expected amplified product size (bp) | Structural annotation | | Functional annotation | | |
|---|---|---|---|---|---|---|---|---|---|---|---|---|
| | | | | | | | | Sequence components of *kabuli* genome | *Kabuli* gene accession IDs | NCBI-KOG | TFs | NCBI-nr database |
| CaPOPI_2786 | Ca4 | 33467899 | ATTTTTT/ATTTTTTT | TCATATCATCAAATTGCAACTCA | TGATGTTAAAAGCTACAGCAACAA | 58.2 | 455 | INTERGENIC | | | | |
| CaPOPI_2787 | Ca4 | 33615467 | ATT/ATTT | GGTCCTTGTTGAGTCCAACG | CTCAAAAACCTGAGCATGGC | 60.5 | 646 | INTERGENIC | | | | |
| CaPOPI_2788 | Ca4 | 33615658 | AG/A | GGTCCTTGTTGAGTCCAACG | AATGGAATAGCGGAGTGTGC | 60.5 | 898 | INTERGENIC | | | | |
| CaPOPI_2789 | Ca4 | 33627666 | TATTTAAAATTTAAAATTTAAAATTTAAAAT/TATTTAAAATTTAAAATTTAAAAT | AGTGACATGACGGAAGTAGCA | TTGGTTCCAGTGTGCATGTT | 58.4 | 432 | INTERGENIC | | | | |
| CaPOPI_2790 | Ca4 | 33633372 | GTTTTTTT/GTTTTTT | ACGAGGAAACATATCGTCCG | ATGTCCACACATTGGGTGTC | 60.0 | 726 | INTERGENIC | | | | |
| CaPOPI_2791 | Ca4 | 33714267 | T/TCTTAATC | GAACACGTTGTTAGACCGGC | TGACAAAATGGGGAGAAAGG | 60.6 | 713 | INTERGENIC | | | | |
| CaPOPI_2792 | Ca4 | 33714529 | ATT/ATTGTT | CGTTGTTAGACCGGCTCTTG | TGGGCTGACATAATCACCAA | 60.8 | 940 | INTERGENIC | | | | |
| CaPOPI_2793 | Ca4 | 33715942 | TAAAAAAAAAAAA/TAAAAAAAAAAAAAAAA | TTGGGTACGTTGGTGTAGCA | ATTGACTGGGCACCATGATT | 60.0 | 634 | INTERGENIC | | | | |
| CaPOPI_2794 | Ca4 | 33736905 | ATTTTTTTT/ATTTTTTTTT | GGCTTTTCAAAGTTCATCGG | TTGAAGGATTTGAATTCCCC | 59.7 | 789 | INTERGENIC | | | | |
| CaPOPI_2795 | Ca4 | 33738599 | GAAAAAAAAA/GAAAAAAAAAAAAA | ATTGTTGCGCACAAATTGAA | CACCACCAAATCACAAGACG | 60.1 | 734 | INTERGENIC | | | | |
| CaPOPI_2796 | Ca4 | 33743516 | CAAAAAA/CAAAAA | TGTAGCTGGTGTCCACGAAG | AATGCAAGAATCCACCTTGG | 59.9 | 435 | INTERGENIC | | | | |
| CaPOPI_2797 | Ca4 | 33784625 | GTAT/GTATAT | CCGGTCTCAATTTTCCTCAA | TGTACGCTAGATTTCACTTTTGGA | 60.0 | 440 | INTERGENIC | | | | |
| CaPOPI_2798 | Ca4 | 33795008 | TTATATATATATATAT/TTATATATATATAT | GTCGTGTCAATGCAACAACC | TCGACTAAGGACATTGGGCT | 60.0 | 514 | INTERGENIC | | | | |
| CaPOPI_2799 | Ca4 | 33807474 | TAAAAAAA/TAAAAAAAA | GTCATTTTGGCGGAACCTAA | TTGAGGTGGGATATGTTTGC | 59.9 | 694 | INTERGENIC | | | | |
| CaPOPI_2800 | Ca4 | 33813152 | TAA/TA | AGAGAAGTTGGCCGACAAAA | CTCCAATGCCCAACAAAAGT | 59.9 | 734 | INTERGENIC | | | | |

| INDEL marker IDs | Chromosomes/ unanchored scaffolds | Physical positions (bp) | InDels (*Kabuli* reference genome-CDC Frontier/PI) | Forward primers (5'-3') | Reverse primers (5'-3') | Anneling temperature (0C) | Expected amplified product size (bp) | Structural annotation | | Functional annotation | | |
|---|---|---|---|---|---|---|---|---|---|---|---|---|
| | | | | | | | | Sequence components of *kabuli* genome | *Kabuli* gene accession IDs | NCBI-KOG | TFs | NCBI-nr database |
| CaPOPI_2801 | Ca4 | 33833847 | TA/TAACTGCA | GCGTCATCGTCCCTACAAAT | TCCAACTCGCAAGGCTATCT | 60.0 | 375 | INTERGENIC | | | | |
| CaPOPI_2802 | Ca4 | 33835234 | TAAAAAAA/TAAAAAAAA | CCAATCAAATTGAGCATACCC | TGTGTTTGCTTTGCATGCTT | 59.3 | 529 | INTERGENIC | | | | |
| CaPOPI_2803 | Ca4 | 33857426 | AAAAATTAAAAT/AAAAATTAAAATTAAAAT | GTTTTGTGATGGGGGATGAC | CAAATTTGGCGTTGTTCCTT | 60.0 | 611 | INTERGENIC | | | | |
| CaPOPI_2804 | Ca4 | 33912297 | ATATCT/AT | AGAATCCAAATCGAAGCCCT | ACCCGCTCTAAGTCCAAAGC | 60.0 | 606 | INTERGENIC | | | | |
| CaPOPI_2805 | Ca4 | 33921285 | ATGCT/A | TGGGTGGTTGACACTTGAAA | CAGAGGAAAATGGGGTCAGA | 60.0 | 560 | INTRON | Ca_20365 | O | GATA | Peptidase C19, ubiquitin carboxyl-terminal hydrolase 2 |
| CaPOPI_2806 | Ca4 | 34026279 | TAAAAAAAAAA/TAAAAAAAAAAA | AAACAACCGCCATCCAACT | TTTCATGGACAAACAAGCCA | 60.4 | 213 | DRR | Ca_20364 | | | Transposase, Ptta/En/Spm, plant |
| CaPOPI_2807 | Ca4 | 34039084 | TAAAAAAAAAAA/TAAAAAAAAA | TACCAAAGTGTCGCCAACAA | AAAAAGGAAAAGCCAGCCTC | 60.1 | 742 | INTERGENIC | | | | |
| CaPOPI_2808 | Ca4 | 34169705 | CAATGA/CAATGATAATGA | TCAAAGTGGCTCACAAGTGC | TCGACTCAAGAAGGCAAGTG | 60.0 | 781 | INTERGENIC | | | | |
| CaPOPI_2809 | Ca4 | 34227066 | A/AG | ACGGCAAAAACCAGTGAAAT | TGAGGAAAATTGAGACCGGA | 59.5 | 327 | INTERGENIC | | | | |
| CaPOPI_2810 | Ca4 | 34240189 | CC/CCTGATGC | AGCGACCAGCTCTGATTTGT | GGGAGCTGCTTGATGAGAAG | 60.0 | 168 | DRR | Ca_20356 | L | | DNA mismatch repair protein MutS, C-terminal domain |
| CaPOPI_2811 | Ca4 | 34246830 | ATT/AT | CGTCTTCCGTACAAAGCCTC | GATTGGGGACCATTCCATTA | 59.9 | 320 | INTERGENIC | | | | |
| CaPOPI_2812 | Ca4 | 34254143 | GTTTTTTTTTTT/GTTTTTTTTTTTT | TTTTGCGACATTTGATCGTT | TGTTCTCGCACACCAAAAAG | 59.2 | 454 | INTERGENIC | | | | |
| CaPOPI_2813 | Ca4 | 34330740 | TAAAAAAAAAAA/TAAAAAAAAAAAA | TGCACACATCTACCCCTGAA | GGCATGGCAACTTCACTTTT | 60.1 | 502 | INTERGENIC | | | | |
| CaPOPI_2814 | Ca4 | 34375164 | ATTTTTTT/ATTTTTT | CAAATCTTCTTCAAATCTTGACCAT | CTATCGACCAAGCCACCATT | 59.9 | 768 | INTERGENIC | | | | |
| CaPOPI_2815 | Ca4 | 34382417 | GC/G | AAGGGGCTTTGAGGTAAGGA | TGGAAAATGGATGTTGACGA | 60.1 | 572 | INTERGENIC | | | | |

| INDEL marker IDs | Chromosomes/ unanchored scaffolds | Physical positions (bp) | InDels (*Kabuli* reference genome-CDC Frontier/PI) | Forward primers (5'-3') | Reverse primers (5'-3') | Anneling temperature (0C) | Expected amplified product size (bp) | Structural annotation | | Functional annotation | | |
|---|---|---|---|---|---|---|---|---|---|---|---|---|
| | | | | | | | | Sequence components of *kabuli* genome | *Kabuli* gene accession IDs | NCBI-KOG | TFs | NCBI-nr database |
| CaPOPI_2816 | Ca4 | 34388082 | CATATATATATATAT/CATATATATATATATATATAT | GTTGGTTCCCTAGCACCGTA | CTGTCAATTGAGTTGCGACG | 60.0 | 484 | INTERGENIC | | | | |
| CaPOPI_2817 | Ca4 | 34388181 | GAAA/GAAAA | GTTGGTTCCCTAGCACCGTA | CTGTCAATTGAGTTGCGACG | 60.0 | 484 | INTERGENIC | | | | |
| CaPOPI_2818 | Ca4 | 34473954 | CTTTTTTT/CTTTTTTTT | ACCCAAATCCAGTGTTCTGC | GGCCTTGTCGAATGATTGTT | 60.0 | 839 | INTERGENIC | | | | |
| CaPOPI_2819 | Ca4 | 34475378 | GAAAAAAA/GAAAAAAAA | CATTGTGGATGCAGTTTTGC | CGAAATAAGATGTCCACCGC | 60.1 | 535 | INTERGENIC | | | | |
| CaPOPI_2820 | Ca4 | 34475759 | GAAAA/GAAA | GCGGTGGACATCTTATTTCG | CGCATTGAAGCATAAACCCT | 60.5 | 446 | INTERGENIC | | | | |
| CaPOPI_2821 | Ca4 | 34482261 | GCCGCC/GCCGCCGTAAACCGCC | ACGCCATCTGCCATATGTTT | TTTGGGGTGATGGAAATGAT | 60.4 | 497 | INTRON | Ca_20350 | R | G2-like | Domain of unknown function DUF676, hydrolase-like |
| CaPOPI_2822 | Ca4 | 34518791 | ATTTTTTT/ATTTTTT | CAGCTTCCCACCAAGGTAAA | TCGATGTGAACAATAGGCCA | 60.1 | 463 | DRR | Ca_20349 | | ERF | Pathogenesis-related transcriptional factor/ERF, DNA-binding |
| CaPOPI_2823 | Ca4 | 34566424 | ATT/AT | CCCTGAGACTCACGAAGAGG | AAAATCCAACAATCTCCCCC | 60.0 | 612 | INTERGENIC | | | | |
| CaPOPI_2824 | Ca4 | 34599013 | AGGGG/AGGG | TGTGCATCGAAAAGGTGTGT | AGCCTACGGAGGACCAAAAT | 60.2 | 568 | INTERGENIC | | | | |
| CaPOPI_2825 | Ca4 | 35237197 | TTTATTATTATTATTATTATTATTATTA/TTTATTATTATTATTATTATTATTATTATTATTATTATTATTA | TCACTGCTCACACACCAACA | TTGAAATGGGGTTGAAGGAC | 59.9 | 276 | INTERGENIC | | | | |
| CaPOPI_2826 | Ca4 | 35295864 | TAA/TAAA | CACGATGAATTAAATAGCATCCA | ATTCACATGGTTGTTCGCAA | 59.0 | 239 | INTERGENIC | | | | |
| CaPOPI_2827 | Ca4 | 35319446 | ATTTTTTTTT/ATTTTTTTTTT | GGTCAGTTCATCCTAGGGCA | TTTTTGGTTGCCTAAGTGGG | 60.1 | 462 | INTERGENIC | | | | |
| CaPOPI_2828 | Ca4 | 35343662 | TCCACAACC/TCCACAACCACAACC | CCACAACATGGTGCAAACTC | AACCGGTTCAGGTTCTGTTG | 60.0 | 679 | INTRON | Ca_20018 | Z | NAC | IQ motif, EF-hand binding site |
| CaPOPI_2829 | Ca4 | 35369859 | GATTAA/GATTAATTAA | TTTAGTGCGCATGAATTTTTACA | TTTCCACAAGCTAACAAAAATGA | 59.7 | 494 | INTERGENIC | | | | |
| CaPOPI_2830 | Ca4 | 35432013 | TTAT/TTATAT | TGTTCAACATCCACGAACAA | TGCCATACTCATGTCGGAAA | 58.5 | 669 | INTERGENIC | | | | |

| INDEL marker IDs | Chromosomes/ unanchored scaffolds | Physical positions (bp) | InDels (*Kabuli* reference genome-CDC Frontier/PI) | Forward primers (5'-3') | Reverse primers (5'-3') | Anneling temperature (0C) | Expected amplified product size (bp) | Structural annotation | | Functional annotation | | |
|---|---|---|---|---|---|---|---|---|---|---|---|---|
| | | | | | | | | Sequence components of *kabuli* genome | *Kabuli* gene accession IDs | NCBI-KOG | TFs | NCBI-nr database |
| CaPOPI_2831 | Ca4 | 35432836 | TTATATATATATATATATATAT/TTATATATATATATATATATATATAT | TTTCCGACATGAGTATGGCA | TTTGGAAAGTAACACTGCACG | 60.1 | 785 | INTERGENIC | | | | |
| CaPOPI_2832 | Ca4 | 35457008 | TTGC/T | AATTGCAGAAAAATGCGGTC | TTGTACCAGATATTGGCCCG | 60.1 | 776 | INTERGENIC | | | | |
| CaPOPI_2833 | Ca4 | 35620689 | TGA/TGAGA | ACAATCCCGACACAAAGTCA | GAAGCGAGAGGTAAGCGATG | 59.0 | 499 | INTERGENIC | | | | |
| CaPOPI_2834 | Ca4 | 35639160 | ATT/ATTT | GGAAAAGATGGTATTTGCCG | CTTCCCTGCTGGCAACTTAG | 59.4 | 339 | INTERGENIC | | | | |
| CaPOPI_2835 | Ca4 | 35818039 | AAATAATAATAATAATA/AAATAATAATAATA | GTTCATAACGAAGATGACAATAACA | TGCAGTCAAACTTTAAAAGGAAG | 57.4 | 728 | INTERGENIC | | | | |
| CaPOPI_2836 | Ca4 | 35822348 | TGG/TG | GATCAAATCGAGGACCGAAA | CGAACAAAACGATGGGAAGT | 60.0 | 695 | INTERGENIC | | | | |
| CaPOPI_2837 | Ca4 | 35999977 | ATTTTTTTTTT/ATTTTTTTTTTT | GCTGGCAGCACAAATAAACA | TCGAACCAATGACCTCACAA | 59.9 | 439 | INTERGENIC | | | | |
| CaPOPI_2838 | Ca4 | 36079126 | TTGTG/TTGTGTG | ATTTTGATGCTCACGGTTCC | CGGAGATGATGGTTCAGGTT | 59.9 | 595 | INTERGENIC | | | | |
| CaPOPI_2839 | Ca4 | 36094744 | TAAAAAAAAAAA/TAAAAAAAAAA | CCTACCATTGCCTGACCTGT | TTCCGAGTTGTTTGAGAGGA | 60.0 | 427 | INTERGENIC | | | | |
| CaPOPI_2840 | Ca4 | 36245892 | TTATATATATATATATATATATATAT/TTATATATATATATATATATAT | TACATGCAAGCGAAATGGAA | AGGCATGGTGTTGGAGTCAT | 60.2 | 287 | DRR | Ca_14846 | O | | Zinc finger, RING-type |
| CaPOPI_2841 | Ca4 | 36256375 | ATTTTTTTTTT/ATTTTTTTTTTT | GATCATCGAGTTTCCCGTGT | AGTTCTGAGGGAGGCTCACA | 59.9 | 350 | INTERGENIC | | | | |
| CaPOPI_2842 | Ca4 | 36335313 | ATTTTTT/ATTTTTTT | TTGCGACAAGATCAAGCAAC | TCAACACCAAACACAAGGGA | 60.0 | 519 | INTERGENIC | | | | |
| CaPOPI_2843 | Ca4 | 36336918 | TAAA/TATAAAA | ACAACAAGGGTGACCTCCAC | TGCTTGTCTATTCTGCGTCAA | 59.9 | 736 | INTERGENIC | | | | |
| CaPOPI_2844 | Ca4 | 36337139 | GC/G | TGCAATGTCAATATGAACCAAA | CCACAACCACTCATCACGAC | 58.9 | 479 | INTERGENIC | | | | |
| CaPOPI_2845 | Ca4 | 36337338 | TT/TTATTAT | TGACGCAGAATAGACAAGCAA | CCACAACCACTCATCACGAC | 59.6 | 341 | INTERGENIC | | | | |

| INDEL marker IDs | Chromosomes/ unanchored scaffolds | Physical positions (bp) | InDels (*Kabuli* reference genome-CDC Frontier/PI) | Forward primers (5'-3') | Reverse primers (5'-3') | Anneling temperature (0C) | Expected amplified product size (bp) | Structural annotation | | Functional annotation | | |
|---|---|---|---|---|---|---|---|---|---|---|---|---|
| | | | | | | | | Sequence components of *kabuli* genome | *Kabuli* gene accession IDs | NCBI-KOG | TFs | NCBI-nr database |
| CaPOPI_2846 | Ca4 | 36472761 | TAAAAAAAAA/TAAAAAAAAAA | CGTAGGACAGAAACCGTGGTA | TATGTAGCCCGTGTCAAGCA | 60.0 | 679 | INTERGENIC | | | | |
| CaPOPI_2847 | Ca4 | 36529935 | TTATATATATATATATATATATATATATA/TTATATATATATATATATATATATATATA | TGGTGTAATTTGATTTCTTCCCT | TCTGGAAAAACTTGAACCACAA | 58.9 | 523 | INTERGENIC | | | | |
| CaPOPI_2848 | Ca4 | 36593028 | TAAAAAAAAAAA/TAAAAAAAAAAAA | CGCGGGGCATATTCTAGTTA | TGATTTGGCATTGTGTTTGC | 60.1 | 527 | INTERGENIC | | | | |
| CaPOPI_2849 | Ca4 | 36690853 | CTTTT/CTTT | TTGACAAGCGCTGTGAAATC | CAATGTGTATTCAACGACCGA | 60.0 | 538 | INTERGENIC | | | | |
| CaPOPI_2850 | Ca4 | 36952818 | TT/TTGAACT | GGGTTCATGTTTGAAGGGTG | ATGGGCCCTCTTTTGTCTTT | 60.2 | 846 | INTRON | Ca_14797 | R | C2H2 | Zinc finger, C2H2-type |
| CaPOPI_2851 | Ca4 | 36953596 | AATATATATAT/AATATATATATATAT | AAAAGAGGGCCCATGAGATT | TGTGCATTGGGAACAACACT | 59.9 | 693 | INTRON | Ca_14797 | R | C2H2 | Zinc finger, C2H2-type |
| CaPOPI_2852 | Ca4 | 36968194 | TAAAAAAAA/TAAAAAAAAA | GGCGAAAGTACCCAAACAAA | GACTTCTGCAAAGGACCTCG | 60.0 | 426 | INTERGENIC | | | | |
| CaPOPI_2853 | Ca4 | 36968275 | G/GTTAATAC | GGCGAAAGTACCCAAACAAA | GACTTCTGCAAAGGACCTCG | 60.0 | 426 | INTERGENIC | | | | |
| CaPOPI_2854 | Ca4 | 36969837 | CTTTTTTTTTT/CTTTTTTTTTTT | AGGTCATGCCATCAAAGACA | GAGCTGTGGAGAAGAAAAGCA | 59.1 | 145 | INTERGENIC | | | | |
| CaPOPI_2855 | Ca4 | 36972358 | CTTT/CTT | ACAACCAACAAAATGCGACA | AGCCTTGAGCGACCACTTTA | 60.0 | 484 | INTERGENIC | | | | |
| CaPOPI_2856 | Ca4 | 36972412 | GTT/GTTT | ACAACCAACAAAATGCGACA | TTGAAACATTCTCAGCCTTGTT | 60.0 | 564 | INTERGENIC | | | | |
| CaPOPI_2857 | Ca4 | 37034691 | ATTTTT/ATTTT | CTCCATTCCTTTCCACTCCA | AGGGAAAACAAGGGTTTTGG | 60.0 | 522 | INTERGENIC | | | | |
| CaPOPI_2858 | Ca4 | 37035676 | GTAATAATAATAATAA/GTAATAATAATAATAATAA | TCAAACCCAATGAAAGGCTC | TCCCTTTACCTTGCCTCATTT | 60.1 | 640 | INTERGENIC | | | | |
| CaPOPI_2859 | Ca4 | 37035741 | TCC/TC | CCCAATGAAAGGCTCACAAT | TCCCTTTACCTTGCCTCATTT | 59.9 | 635 | INTERGENIC | | | | |
| CaPOPI_2860 | Ca4 | 37038560 | TGAGA/TGAGAGA | TGGTTTATAACACCGCTTATATTCA | AGGATTCAACCACAAAGGACA | 59.0 | 242 | INTERGENIC | | | | |

| INDEL marker IDs | Chromosomes/ unanchored scaffolds | Physical positions (bp) | InDels (*Kabuli* reference genome-CDC Frontier/PI) | Forward primers (5'-3') | Reverse primers (5'-3') | Anneling temperature (0C) | Expected amplified product size (bp) | Structural annotation | | Functional annotation | | |
|---|---|---|---|---|---|---|---|---|---|---|---|---|
| | | | | | | | | Sequence components of *kabuli* genome | *Kabuli* gene accession IDs | NCBI-KOG | TFs | NCBI-nr database |
| CaPOPI_2861 | Ca4 | 37039540 | TAAAAAAAAAAA/TAAAAAAAA | TTTATTGACGAGCATCGTTCC | TGGCAAGTGCAACCAAAATA | 60.1 | 386 | INTERGENIC | | | | |
| CaPOPI_2862 | Ca4 | 37044142 | TAAAAAA/TAAAAAAA | TGATTGTTTTGATCCCCTCA | TCACGAAATTAGCCCTCCAA | 58.9 | 328 | INTERGENIC | | | | |
| CaPOPI_2863 | Ca4 | 37047512 | CAAA/CA | GAAGTGGGAAATGGGTTTGA | ATGATTGTGCCTCGTGAACA | 59.8 | 662 | INTERGENIC | | | | |
| CaPOPI_2864 | Ca4 | 37047776 | GC/GCC | ATACTCTGCACCACACACGG | ATGATTGTGCCTCGTGAACA | 59.6 | 241 | INTERGENIC | | | | |
| CaPOPI_2865 | Ca4 | 37055713 | A/AT | GCCGGACAGTGTATTGTGTTT | CCATATGCCACCATTTCCTT | 59.9 | 552 | INTERGENIC | | | | |
| CaPOPI_2866 | Ca4 | 37057959 | TA/T | TTTGTGCTCCATACACACATGA | CACCGTCTTCAAAGAATTCCA | 60.0 | 771 | INTERGENIC | | | | |
| CaPOPI_2867 | Ca4 | 37099831 | TAAAAAAA/TAAAAAA | TGAATAATTCGCGTACGGCT | TGTGAGGAGCTCTTTGTGGA | 60.6 | 589 | INTERGENIC | | | | |
| CaPOPI_2868 | Ca4 | 37117919 | AT/A | TACGGAAATCGGCTGGATAG | GCACGATAAATGCAACCCTT | 60.1 | 496 | INTERGENIC | | | | |
| CaPOPI_2869 | Ca4 | 37118197 | ATTTTTTT/ATTTTTT | AAGGGTTGCATTTATCGTGC | CCAGCTCGTTTGTTTGAAGA | 60.0 | 237 | INTERGENIC | | | | |
| CaPOPI_2870 | Ca4 | 37128543 | TAAAAA/TAAAA | TCGCACTTTTGGAGATTGAA | TGCATCCATCGTCAAATACAA | 59.4 | 462 | INTERGENIC | | | | |
| CaPOPI_2871 | Ca4 | 37130048 | ATTTTTTTTTT/ATTTTTTTTTTT | TTGCAGAAGGTTCCCAAATC | TGTTGGCCATCTTTTAGCTTT | 60.1 | 841 | INTERGENIC | | | | |
| CaPOPI_2872 | Ca4 | 37131217 | TC/T | TTGTGACCAAAGGTTGGAGG | AATTTGCGAGCATTACCTGC | 60.9 | 710 | INTERGENIC | | | | |
| CaPOPI_2873 | Ca4 | 37311820 | GATATATATATATATATATATATA/GATATATATATATATATATATATA | CGCTGTAAAATGCATGTGCT | TTGGGTGAACCATTAGGCAT | 59.9 | 528 | INTERGENIC | | | | |
| CaPOPI_2874 | Ca4 | 37443931 | CT/CTAT | CCGCATTTTCTTCATTAGGG | ACGTTAAGGCATGAGTTGGG | 59.5 | 364 | INTERGENIC | | | | |
| CaPOPI_2875 | Ca4 | 37519504 | CTATTTATTTATTTATTTATTT/CTATTTATTTATTTATTT | GAAAAAGGAGTTGCCACCAA | TGGGGATACGTAGGAGCAAG | 60.1 | 306 | INTERGENIC | | | | |

| INDEL marker IDs | Chromosomes/ unanchored scaffolds | Physical positions (bp) | InDels (*Kabuli* reference genome-CDC Frontier/PI) | Forward primers (5'-3') | Reverse primers (5'-3') | Anneling temperature (0C) | Expected amplified product size (bp) | Structural annotation | | Functional annotation | | |
|---|---|---|---|---|---|---|---|---|---|---|---|---|
| | | | | | | | | Sequence components of *kabuli* genome | *Kabuli* gene accession IDs | NCBI-KOG | TFs | NCBI-nr database |
| CaPOPI_2876 | Ca4 | 37822334 | TT/TTATAGAACTCTAAT | TCAACCAAAATCCCCCAATA | CCCAATTTCGATTTACCCCT | 60.0 | 329 | INTERGENIC | | | | |
| CaPOPI_2877 | Ca4 | 38080710 | GTATATATATATATATATATATATATATATATATATAT/GTATATATATATATATATATATATATATATATATATATAT | GCTTTTGAGGCGTAAAACCA | TTCCTCCAATAGAAATGCCG | 60.2 | 520 | INTERGENIC | | | | |
| CaPOPI_2878 | Ca4 | 38682630 | TTATATATATATATATATA/TTATATATATATATATATATA | ACGGTATCAATTAACACAGGAAA | GTGGGGGCTTGTTGAGTTAC | 57.6 | 707 | INTERGENIC | | | | |
| CaPOPI_2879 | Ca4 | 38884678 | CAAAAAAA/CAAAAAA | GGTGGTCATGGTGTAGTTTGG | TCACACGTCCTTTCAAAATCA | 60.1 | 575 | INTERGENIC | | | | |
| CaPOPI_2880 | Ca4 | 39020626 | TAAAAAAAAA/TAAAAAAAAAA | TGGCATGACACTTAGCTCAAA | CCCCAATGTATTGGAACCAC | 59.5 | 864 | INTERGENIC | | | | |
| CaPOPI_2881 | Ca4 | 39038460 | ACTTGCTTG/ACTTG | CCGGTCACATTCAATAGCCT | ATTGCGTTGTTTCTTCCAGG | 60.0 | 411 | DRR | Ca_13078 | A | | RNA recognition motif domain |
| CaPOPI_2882 | Ca4 | 39070213 | TAATAAATAA/TAATAA | TATTTTGGATTTTGGCTCGG | AAGGAAGCAACGAAAACGAA | 59.9 | 446 | INTERGENIC | | | | |
| CaPOPI_2883 | Ca4 | 39147993 | AATATATATATATATATATATATATAT/AATATATATATATATATATATATATATATATAT | GGAAGGAGCCCTTATCTTCG | ATGTCAATTGATTTGCGACG | 60.2 | 422 | INTERGENIC | | | | |
| CaPOPI_2884 | Ca4 | 39165742 | TAAAAATAAAAAATAAAAAA/TAAAAATAAAAAATAAAAAATAAAAAA | CGTATACCGCTGCTCATCAA | TGATGTGATTTGTGCCATGA | 59.9 | 259 | INTERGENIC | | | | |
| CaPOPI_2885 | Ca4 | 39689187 | AC/ACC | ATTACCGACATTGCAGAGGG | CTCCCATTCTGCCCTTGATA | 60.0 | 329 | INTERGENIC | | | | |
| CaPOPI_2886 | Ca4 | 39740716 | AG/A | TCCTAGGGCTCATGAGTTGC | GAGATGTGCAGTTTAATTCGTCC | 60.4 | 882 | INTERGENIC | | | | |
| CaPOPI_2887 | Ca4 | 40485498 | GAAAAAAAA/GAAAAAAA | TTGGCTCCATCAATTGAACA | GGTCCCTTGAGCACATTAGC | 60.0 | 625 | INTERGENIC | | | | |
| CaPOPI_2888 | Ca4 | 40498066 | AACATAACTAAATATTACA/AACATAACTAAATATTACATAACTAAATATTACA | CCACCAAACATCATCACAGC | TTGCATTCGAGTTGGATTTTT | 60.0 | 403 | DRR | Ca_14875 | | | Protein of unknown function DUF688 |
| CaPOPI_2889 | Ca4 | 40498650 | AAT/A | TCATTTGAAACATGGACCACA | GCTTGACAATCATGGGGTTT | 59.8 | 262 | DRR | Ca_14875 | | | Protein of unknown function DUF688 |
| CaPOPI_2890 | Ca4 | 40501876 | ATTTTTTTTT/ATTTTTTTT | ACCCATGGTACTTGGTCCAC | CATGAGTTATTGATGGTGCGA | 59.6 | 353 | INTERGENIC | | | | |

| INDEL marker IDs | Chromosomes/ unanchored scaffolds | Physical positions (bp) | InDels (*Kabuli* reference genome-CDC Frontier/PI) | Forward primers (5'-3') | Reverse primers (5'-3') | Anneling temperature (0C) | Expected amplified product size (bp) | Structural annotation | | Functional annotation | | |
|---|---|---|---|---|---|---|---|---|---|---|---|---|
| | | | | | | | | Sequence components of *kabuli* genome | *Kabuli* gene accession IDs | NCBI-KOG | TFs | NCBI-nr database |
| CaPOPI_2891 | Ca4 | 40508840 | GAAAAAAAA/GAAAAAAAAAA | GGGGAAAAAGAAAGGAAGGA | GTGGCTGCAAAAGCTAAAGG | 59.5 | 294 | DRR | Ca_14874 | | | IQ motif, EF-hand binding site |
| CaPOPI_2892 | Ca4 | 40514871 | AATA/AA | GGAGAACCAGCTTCCTCCTT | CGCTCTTGCTTCCTTTCACT | 59.8 | 278 | INTERGENIC | | | | |
| CaPOPI_2893 | Ca4 | 40521264 | TTACTACTA/TTACTA | AGATGCGACACAATTCCACA | CATTAAAATTTCAAAGATTTTGCG | 60.1 | 162 | INTERGENIC | | | | |
| CaPOPI_2894 | Ca4 | 40521734 | ATTTTTTTTTTT/ATTTTTTTTTTTT | TCATCGGGACAACTTTGTATG | CGGCTCATCTCAATCCTTGT | 58.5 | 655 | INTERGENIC | | | | |
| CaPOPI_2895 | Ca4 | 40522888 | AAATATATATA/AAATATATATATTTTTAGAATATATATA | GATGGGTTGGTTTGATTTGG | CGACAATTTTGGTCTCTCCA | 60.0 | 946 | INTERGENIC | | | | |
| CaPOPI_2896 | Ca4 | 40528670 | GTTTTTTTTT/GTTTTTTTT | AAATGCAACAGGAAGATGCC | TGGACTTGGAAGAGAAACGG | 60.1 | 558 | INTERGENIC | | | | |
| CaPOPI_2897 | Ca4 | 40529406 | CTTTTTTTTT/CTTTTTTTTTT | CCGTTTCTCTTCCAAGTCCA | CGCTAAGCACCAGTTTCCTC | 60.2 | 608 | INTERGENIC | | | | |
| CaPOPI_2898 | Ca4 | 40560588 | TAAAAAA/TAAAAAAA | TCATTCTCCCGATCTTCCAC | TTTCTGTGGACTCACGCATC | 60.0 | 636 | INTERGENIC | | | | |
| CaPOPI_2899 | Ca4 | 40561270 | TTTCAA/T | ACTGATGCGTGAGTCCACAG | GGATGTTGGCTCCCAAGTTA | 59.9 | 785 | INTERGENIC | | | | |
| CaPOPI_2900 | Ca4 | 40573928 | CAAAA/CAA | TTCGTGGAATATCACAAAAGTTC | TGGACAAAAACTCATCTGCATT | 58.2 | 895 | INTERGENIC | | | | |
| CaPOPI_2901 | Ca4 | 40574863 | A/AC | TGCAGATGAGTTTTTGTCCA | AAAATTAAGACAAAAGGAAGTTCAACA | 57.8 | 658 | INTERGENIC | | | | |
| CaPOPI_2902 | Ca4 | 40575649 | ATTTTTT/ATTTTT | TTTCTCATTTTCATGACTCCTATCA | TCACCAGTGACACGTTCGAT | 59.2 | 375 | INTERGENIC | | | | |
| CaPOPI_2903 | Ca4 | 40593121 | CAAGT/CAAGTAAGT | CATTCCCACCATCCTTCACT | GGCCTATCGAACCACACAGT | 59.8 | 932 | INTRON | Ca_14867 | R | BES1 | Zinc finger, RING-type |
| CaPOPI_2904 | Ca4 | 40593400 | GTT/GTTTT | AAGGATTTGCTTGCTGATGC | GGCCTATCGAACCACACAGT | 60.4 | 276 | INTRON | Ca_14867 | R | BES1 | Zinc finger, RING-type |
| CaPOPI_2905 | Ca4 | 40615032 | TAA/TA | GAGCCCAACGATGTTCAAAT | TGAACCCCACCAGCATTATT | 59.9 | 793 | INTERGENIC | | | | |

| INDEL marker IDs | Chromosomes/ unanchored scaffolds | Physical positions (bp) | InDels (*Kabuli* reference genome-CDC Frontier/PI) | Forward primers (5'-3') | Reverse primers (5'-3') | Anneling temperature (0C) | Expected amplified product size (bp) | Structural annotation | | Functional annotation | | |
|---|---|---|---|---|---|---|---|---|---|---|---|---|
| | | | | | | | | Sequence components of *kabuli* genome | *Kabuli* gene accession IDs | NCBI-KOG | TFs | NCBI-nr database |
| CaPOPI_2906 | Ca4 | 40620475 | A/AT | TATGTGAAATTGCCACCGAA | ACCTCATCGAGCCATTGATT | 59.9 | 509 | INTERGENIC | | | | |
| CaPOPI_2907 | Ca4 | 40622124 | TAAAAAAAAAAAAA/TAAAAAAAAAAAA | ACATACACACGTCACGGGAA | TTTGCAAATGAGATTTCCTAAAGA | 59.9 | 391 | INTERGENIC | | | | |
| CaPOPI_2908 | Ca4 | 40626965 | TAA/TAAA | GACGTGCTCACTGAAAGGGT | CATCGAGGCCCATTATGATT | 60.3 | 687 | INTERGENIC | | | | |
| CaPOPI_2909 | Ca4 | 40649020 | ATTTTTT/ATTTTTTTT | TGTGATTATCCGGTACCACAAC | TGTATTCAGGACCTCGCAGA | 59.6 | 207 | INTERGENIC | | | | |
| CaPOPI_2910 | Ca4 | 40700886 | AGAC/A | TCTTGATGTAACGTTTTGTTTCAGA | TGAATATTTGAGTTACGAATTTGATGA | 60.1 | 478 | INTERGENIC | | | | |
| CaPOPI_2911 | Ca4 | 40702737 | ACTCT/ACT | CCCCCATCATAAAATTGTGC | AAGATTAGTGGGCGACCCTT | 60.0 | 392 | INTERGENIC | | | | |
| CaPOPI_2912 | Ca4 | 40704105 | ATTTTTT/ATTTTT | GGATCTCCACTGCCAGGTTA | TTTCGTTTCCCATTCTACGC | 60.1 | 648 | INTERGENIC | | | | |
| CaPOPI_2913 | Ca4 | 40719941 | CTTTTTTTTTTT/CTTTTTTTTTT | TCCTCCCTTTTGTTTTGTGC | TCGTCATCACCCACACTCAT | 60.1 | 410 | INTRON | Ca_23911 | BK | FAR1 | PWWP |
| CaPOPI_2914 | Ca4 | 40728044 | TATTTGTAATT/TATTTGTAATTTGTAATT | AGCATACATTGGCACCACAA | CATGCATTTCATGGTAACCG | 60.0 | 502 | INTERGENIC | | | | |
| CaPOPI_2915 | Ca4 | 40729597 | T/TG | TTGTGAAATTGAAAAGGTTCCA | CAACCATGTGTGAGGTCCAT | 59.5 | 817 | INTERGENIC | | | | |
| CaPOPI_2916 | Ca4 | 40743968 | A/AAAC | TGTTGAAAAACACCGCAAGA | GCAATGAAGTGTGTGCCAAT | 60.3 | 575 | INTERGENIC | | | | |
| CaPOPI_2917 | Ca4 | 40753856 | GTATATATATATATA/GTATATATA | TTACGATATTGCCGCAACTG | GCCCTTTCTTTCAGGCTTCT | 59.7 | 444 | INTRON | Ca_23905 | J | | Diphthamide synthesis, DPH1/DHP2 |
| CaPOPI_2918 | Ca4 | 40761334 | A/ATATAT | GAACTTTGTTATTGCATTCGTTTT | TGGATACACCGTCCAAAGTG | 58.7 | 714 | INTERGENIC | | | | |
| CaPOPI_2919 | Ca4 | 40767434 | GTTTT/GTTTTT | CCAAGAAGTCCACGTTTGGT | AGGTACGTCCAGGCATCAAG | 60.0 | 644 | INTRON | Ca_23904 | Q | | Pyridine nucleotide-disulphide oxidoreductase, class-II |
| CaPOPI_2920 | Ca4 | 40773929 | TTAT/TTATAT | GTGTGCACCATAGGGAAGGT | ATCCTTGCACATCCCAAAAA | 59.9 | 405 | INTERGENIC | | | | |

| INDEL marker IDs | Chromosomes/ unanchored scaffolds | Physical positions (bp) | InDels (*Kabuli* reference genome-CDC Frontier/PI) | Forward primers (5'-3') | Reverse primers (5'-3') | Anneling temperature (0C) | Expected amplified product size (bp) | Structural annotation | | Functional annotation | | |
|---|---|---|---|---|---|---|---|---|---|---|---|---|
| | | | | | | | | Sequence components of *kabuli* genome | *Kabuli* gene accession IDs | NCBI-KOG | TFs | NCBI-nr database |
| CaPOPI_2921 | Ca4 | 40788340 | ATTTTTTTTT/ATTTTTTTT | GCATCGGAGCATGAAATGTA | TTCGCACACGATGAAGACTAA | 59.7 | 167 | INTERGENIC | | | | |
| CaPOPI_2922 | Ca4 | 40797521 | ATT/AT | CAAGGTCCCCAAACATCACT | ATGGACCATCGATTCTGGAG | 59.8 | 610 | INTERGENIC | | | | |
| CaPOPI_2923 | Ca4 | 40799151 | TTAGCATA/TTA | CCAGCAATCTAATGAACGCA | ATGTCACCAATGGGGTTTGT | 59.8 | 323 | DRR | Ca_23902 | R | | DNA-directed DNA polymerase, family A, palm domain |
| CaPOPI_2924 | Ca4 | 40799742 | TCCCCC/TCCCCCC | ACAAACCCCATTGGTGACAT | TGGCTTGTAAGGCTGTGTTG | 60.0 | 740 | DRR | Ca_23902 | R | | DNA-directed DNA polymerase, family A, palm domain |
| CaPOPI_2925 | Ca4 | 40801294 | AAAA/AAAATACCAGTAAA | AGCTTGCCTCTGAGCTCTTG | TTTATGGCATGGGACCAAAT | 60.0 | 736 | INTRON | Ca_23902 | R | | DNA-directed DNA polymerase, family A, palm domain |
| CaPOPI_2926 | Ca4 | 40802030 | A/AC | CATGAAGCCAAGAAGCAACA | TTGACACCTGTCACACATGC | 60.0 | 518 | INTRON | Ca_23902 | R | | DNA-directed DNA polymerase, family A, palm domain |
| CaPOPI_2927 | Ca4 | 40809750 | A/AC | TGCAAGAACAAATCCTTCCC | GCAGCTTTTGGCAGTTCTCT | 60.1 | 278 | INTRON | Ca_23902 | R | | DNA-directed DNA polymerase, family A, palm domain |
| CaPOPI_2928 | Ca4 | 40810384 | G/GAAAAAA | AAAAACGGCAGTAAAAGCCA | GGGACGAGGTACATGCAGAT | 59.8 | 298 | INTRON | Ca_23902 | R | | DNA-directed DNA polymerase, family A, palm domain |
| CaPOPI_2929 | Ca4 | 40836419 | TAAAAAAAA/TAAAAAAA | GTGTCGGTCACGACATTCAG | AGATTGAAAAGTTGCACCCG | 60.2 | 714 | INTERGENIC | | | | |
| CaPOPI_2930 | Ca4 | 40837444 | TAAAAAAAAA/TAAAAAAAA | GGAAGCAGATGGAAAAGGAA | TCAACTTTGAGGACCCAACA | 59.2 | 494 | INTERGENIC | | | | |
| CaPOPI_2931 | Ca4 | 40842793 | TC/T | CTGGATTGAGTTGAGGCCAT | TGGACCAAACCAAGCACATA | 60.1 | 568 | INTERGENIC | | | | |
| CaPOPI_2932 | Ca4 | 40844159 | TAAAAAA/TAAAAAAA | AACATTAATGCAACACGCCA | GACTTCCGTCCCTTACAGAGAA | 60.0 | 199 | INTERGENIC | | | | |
| CaPOPI_2933 | Ca4 | 40887107 | GAAAAAAAAA/GAAAAAAAA | TGAATCCAATCCATCCAACA | ACTATCGACCAACTGCCCAC | 59.7 | 705 | INTERGENIC | | | | |
| CaPOPI_2934 | Ca4 | 40891579 | AATATATA/AATA | GCACAAAAGCAGTGTAGCCA | CCCGAACCTCTCCACAATAA | 60.1 | 869 | INTRON | Ca_11020 | R | G2-like | C2 calcium-dependent membrane targeting |
| CaPOPI_2935 | Ca4 | 40891712 | TCC/TC | ATCGAATCCTCGTTGGAATG | CCCGAACCTCTCCACAATAA | 59.9 | 725 | INTRON | Ca_11020 | R | G2-like | C2 calcium-dependent membrane targeting |

| INDEL marker IDs | Chromosomes/ unanchored scaffolds | Physical positions (bp) | InDels (*Kabuli* reference genome-CDC Frontier/PI) | Forward primers (5'-3') | Reverse primers (5'-3') | Anneling temperature (0C) | Expected amplified product size (bp) | Structural annotation | | Functional annotation | | |
|---|---|---|---|---|---|---|---|---|---|---|---|---|
| | | | | | | | | Sequence components of *kabuli* genome | *Kabuli* gene accession IDs | NCBI-KOG | TFs | NCBI-nr database |
| CaPOPI_2936 | Ca4 | 40901246 | AAA/AAATAA | GGGTATATTAATTTAACAACTCCCTCC | TTAGGAGGTCGCATCTTCGT | 59.7 | 462 | INTERGENIC | | | | |
| CaPOPI_2937 | Ca4 | 40907158 | TATACAT/TATACATAATACAT | TGTCGTTGAAATCAAAGCAAT | ATCGTTTGGGTGAAGAATCG | 58.3 | 334 | INTERGENIC | | | | |
| CaPOPI_2938 | Ca4 | 40929080 | T/TC | ATGGTGGGGGTAAGCAATCT | AAGAAGCGCGTATTGGTGTT | 60.6 | 813 | INTRON | Ca_11015 | | bZIP | Basic-leucine zipper (bZIP) transcription factor |
| CaPOPI_2939 | Ca4 | 40931865 | TCCCC/TCCC | TGCTTGGAAAACAAGCAATTT | AACTCGTGGGAACGCACTAC | 59.8 | 779 | INTRON | Ca_11015 | | bZIP | Basic-leucine zipper (bZIP) transcription factor |
| CaPOPI_2940 | Ca4 | 40937505 | GTTTTTTTTT/GTTTTTTTTTT | TAATCCGATGGCAACATGAA | CGGTAGGAAATGATGAAACCA | 59.9 | 615 | INTERGENIC | | | | |
| CaPOPI_2941 | Ca4 | 40949429 | TT/TTAT | CCATGTGCACCATAAATTGC | CAGAATGTTCTTTCGGAATGG | 59.8 | 612 | INTRON | Ca_11013 | | | Protein of unknown function DUF599 |
| CaPOPI_2942 | Ca4 | 40967580 | TAAAAAAAAAA/TAAAAAAAAAAA | AATGAAAAACTTGTTGCGGC | GTTTGGAATCGGGTAACGAA | 60.1 | 748 | INTERGENIC | | | | |
| CaPOPI_2943 | Ca4 | 40968955 | TC/TCC | CACTGGAATGATTGCCAAAA | TTCTACCCAAGATCGGATGG | 59.5 | 398 | INTERGENIC | | | | |
| CaPOPI_2944 | Ca4 | 40996595 | GAAAAA/GAAAA | CGGTGTTAAAAATTGGGGTG | AAACGTGGAGGTGATTTTGC | 60.1 | 445 | DRR | Ca_11010 | | | Glycoside hydrolase, family 28 |
| CaPOPI_2945 | Ca4 | 40997097 | C/CT | GCAAAATCACCTCCACGTTT | AACGGTCGTCTCCATTCATC | 60.0 | 762 | DRR | Ca_11010 | | | Glycoside hydrolase, family 28 |
| CaPOPI_2946 | Ca4 | 41016384 | ATTTTTTT/ATTTTTTTT | AAACACGGGGCAACATAAAG | TGTTGGTGGTGAACGAGGTA | 59.9 | 631 | INTERGENIC | | | | |
| CaPOPI_2947 | Ca4 | 41027713 | AATATATATATATATATATATATATATATAT/AATATATATATATATATATATATATATATATATATATAT | TGAATCCCAAAATTTGAACCA | TTTGATCGCAAGGGTTTAGG | 60.2 | 637 | INTERGENIC | | | | |
| CaPOPI_2948 | Ca4 | 41029836 | AATATATATATATATA/AATATATATATATATA | TTTCTCTCCATCGACCCATC | GAACCTTTGGGAAATCAGCA | 60.0 | 760 | INTERGENIC | | | | |
| CaPOPI_2949 | Ca4 | 41159362 | ATTTTTTTTTTT/ATTTTTTTTTTTT | ATGCTTGGACCACCATTCTC | CGTGTGAAACCTTTGCTGAA | 59.9 | 891 | INTERGENIC | | | | |
| CaPOPI_2950 | Ca4 | 41272417 | GATTATTATTATTATTATTATTATTATTATTATT/GATTATTATTATTATTATTATTATTATTATT | GGTTCACATGGAACTCCAACA | AGCCTTTTGCTTCCTTCCTC | 60.8 | 737 | INTERGENIC | | | | |

| INDEL marker IDs | Chromosomes/ unanchored scaffolds | Physical positions (bp) | InDels (*Kabuli* reference genome-CDC Frontier/PI) | Forward primers (5'-3') | Reverse primers (5'-3') | Anneling temperature (0C) | Expected amplified product size (bp) | Structural annotation | | Functional annotation | | |
|---|---|---|---|---|---|---|---|---|---|---|---|---|
| | | | | | | | | Sequence components of *kabuli* genome | *Kabuli* gene accession IDs | NCBI-KOG | TFs | NCBI-nr database |
| CaPOPI_2951 | Ca4 | 41713312 | TAAAAA/TAAAAAA | CCATCAAAACATGCATCCAC | CATGACAACACAACCAACCA | 59.8 | 748 | INTERGENIC | | | | |
| CaPOPI_2952 | Ca4 | 42078586 | AAGGCATGAAA/AAGGCATGAAACACAGGCATGAAA | TCTTTTGAACACTCCCCCTG | CCAAAGGAAACCACGACAAT | 60.1 | 810 | INTERGENIC | | | | |
| CaPOPI_2953 | Ca4 | 42083362 | CA/CAA | TTTCAAAGGTTTGCATTGTGA | AAAATTAACCGTATGGCCGA | 59.2 | 327 | INTERGENIC | | | | |
| CaPOPI_2954 | Ca4 | 42120439 | TAAAAAAAAA/TAAAAAAAA | CACAAGCATTGGAGTCCCTT | AACTCGTGGGTTGGATTTTG | 60.1 | 495 | INTRON | Ca_10910 | | C2H2 | Glycoside hydrolase, family 28 |
| CaPOPI_2955 | Ca4 | 42120489 | TAAAA/TAAA | CACAAGCATTGGAGTCCCTT | AACTCGTGGGTTGGATTTTG | 60.1 | 495 | INTRON | Ca_10910 | | C2H2 | Glycoside hydrolase, family 28 |
| CaPOPI_2956 | Ca4 | 42374619 | ATTTTTTTT/ATTTTTTT | TTTGATGTGTTCACGGCACT | CGGCATGCATGATTATTTTG | 60.2 | 331 | INTERGENIC | | | | |
| CaPOPI_2957 | Ca4 | 42407053 | GTTT/GTTTT | GCATAAGGATCGGGGAAACT | AATGATCGTCTTCGGATTGG | 60.3 | 392 | INTERGENIC | | | | |
| CaPOPI_2958 | Ca4 | 42546801 | CAAAA/CAAAAA | GGTTGGTCAATATTAATTCCGC | CTAATATCATCGCCCCTTCG | 59.6 | 686 | INTERGENIC | | | | |
| CaPOPI_2959 | Ca4 | 42548370 | ATTTTTTTT/ATTTTTTTTT | CGTGCCTTTGTCGTCTCTTT | AGAGGCCCAATATCCTTGCT | 60.4 | 528 | INTERGENIC | | | | |
| CaPOPI_2960 | Ca4 | 42561342 | AATAGAGATAGAGATAGAGATAGAGA/AATAGAGATAGAGATAGAGA | CGGCAAAACAGAGACAATCA | TCTCACGGTTGTGAGGACTG | 59.8 | 310 | INTERGENIC | | | | |
| CaPOPI_2961 | Ca4 | 42633986 | TATAATAATAATAATAATAATAATAATAATAATA/TATAATAATAATAATAATAATAATAATAATAATAATAATA | TTGCTTTAGATGAATCAAATCCC | CGACAGGCTTAGGTTAGACCA | 59.5 | 362 | INTERGENIC | | | | |
| CaPOPI_2962 | Ca4 | 42693293 | TC/TCC | AAATGCTCGAACGAAAATGC | TCGAAGTTGGATGCTTTTGA | 60.2 | 503 | INTERGENIC | | | | |
| CaPOPI_2963 | Ca4 | 42945720 | CATATATATATAT/CATATATATATATAT | TGTCAATGTTACTTTATTTCAAAACTC | TTGGGTTATCTTCATTGCAAAA | 57.2 | 739 | INTERGENIC | | | | |
| CaPOPI_2964 | Ca4 | 43005724 | CTTTTTTTTTTT/CTTTTTTTTTT | TTGTAGTTGTATTCAATGACGAAGA | CAGAATTAATTTATTTGACAACTCCA | 58.0 | 628 | INTERGENIC | | | | |
| CaPOPI_2965 | Ca4 | 43222555 | TTATATATATATATATATATAT/TTATATATATATATATAT | AAAGATTTAAAGGGCAAAAAGTG | CTGGCAGCTGGGAAAGATT | 58.0 | 908 | INTERGENIC | | | | |

| INDEL marker IDs | Chromosomes/ unanchored scaffolds | Physical positions (bp) | InDels (*Kabuli* reference genome-CDC Frontier/PI) | Forward primers (5'-3') | Reverse primers (5'-3') | Anneling temperature (0C) | Expected amplified product size (bp) | Structural annotation | | Functional annotation | | |
|---|---|---|---|---|---|---|---|---|---|---|---|---|
| | | | | | | | | Sequence components of *kabuli* genome | *Kabuli* gene accession IDs | NCBI-KOG | TFs | NCBI-nr database |
| CaPOPI_2966 | Ca4 | 43278176 | GTTTTTTTT/GTTTTTTT | ACTGCGTCTCCATGGGTAAC | TTGGAATGGGCTTCTTTTTG | 60.0 | 592 | DRR | Ca_23681 | | | |
| CaPOPI_2967 | Ca4 | 43291624 | TAA/TAAAA | GAGCGATTACCGGTGTTGTT | GCCTTCAAATGTAACCCCCT | 60.0 | 946 | INTERGENIC | | | | |
| CaPOPI_2968 | Ca4 | 43301091 | ATAA/ATAATGTAA | ACCATAAAGGGAAACAGGGG | TCATGGTCTGCAATCTCGTC | 60.0 | 714 | INTERGENIC | | | | |
| CaPOPI_2969 | Ca4 | 43301725 | ATTTT/ATTT | GACGAGATTGCAGACCATGA | TGGGCCCTTAGTCTCTTTTT | 59.8 | 753 | INTERGENIC | | | | |
| CaPOPI_2970 | Ca4 | 43362642 | TG/T | AGAATCACACTGCCCCAAAC | TTGCAAGGGAATTTTTGTCA | 60.0 | 590 | DRR | Ca_23687 | | AP2 | |
| CaPOPI_2971 | Ca4 | 44040432 | TAAAAAAA/TAAAAAAAA | GATTGCGGAATTAGCCTTCTT | TGGTTAAGTGAAGCGCAAAC | 59.7 | 774 | INTERGENIC | | | | |
| CaPOPI_2972 | Ca4 | 44098644 | ATTTTTTT/ATTTTTTTT | TCACCTTCATCCATCGTCAA | TGTGGTGTGCAAGATGACTG | 60.0 | 408 | INTERGENIC | | | | |
| CaPOPI_2973 | Ca4 | 44134460 | AAC/AACACAC | TACCAGATTGCCCCACTTTC | GCCAAAACGGATGAAGTTGT | 59.9 | 554 | INTERGENIC | | | | |
| CaPOPI_2974 | Ca4 | 44428431 | CATATATATATATATATATAT/CATATATATATATATATAT | TGTTGCGGAAATGTTAGTTCA | TTCATCCATGCAATATTCAAAAA | 59.2 | 699 | INTERGENIC | | | | |
| CaPOPI_2975 | Ca4 | 44498939 | CTTT/CTT | ACCTTGGGCCTTAATTGAAA | CGGAACATCACTGTTCACAAA | 58.6 | 410 | INTERGENIC | | | | |
| CaPOPI_2976 | Ca4 | 44507569 | CTTTT/CTTT | AGCGGTTGTTCAAAATGACC | AAAGCAAGTCCGACCAAAAA | 60.0 | 421 | INTERGENIC | | | | |
| CaPOPI_2977 | Ca4 | 44507737 | TTTCTTCTT/TTTCTTCTTCTT | AGCGGTTGTTCAAAATGACC | AAAGCAAGTCCGACCAAAAA | 60.0 | 421 | INTERGENIC | | | | |
| CaPOPI_2978 | Ca4 | 44511339 | ATTTT/ATTTTT | GATGCAGGTCATGAAACCAA | GTCCTCAACGCTTCAAGAGG | 59.5 | 494 | INTERGENIC | | | | |
| CaPOPI_2979 | Ca4 | 44522624 | GAAAAAA/GAAAAAAA | GAAGTTGCTTGCTCCGATTC | CCCTAGCAAGTGCTAACCCA | 60.0 | 675 | DRR | Ca_09124 | | ERF | Pathogenesis-related transcriptional factor/ERF, DNA-binding |
| CaPOPI_2980 | Ca4 | 44523921 | ATTTTTTTTTTT/ATTTTTTTTTTTT | GCAATGTGCTCACTTTTTGTCT | AACGAAGGAGGAGGGAAGAA | 59.4 | 651 | DRR | Ca_09124 | | ERF | Pathogenesis-related transcriptional factor/ERF, DNA-binding |

| INDEL marker IDs | Chromosomes/ unanchored scaffolds | Physical positions (bp) | InDels (*Kabuli* reference genome-CDC Frontier/PI) | Forward primers (5'-3') | Reverse primers (5'-3') | Anneling temperature (0C) | Expected amplified product size (bp) | Structural annotation | | Functional annotation | | |
|---|---|---|---|---|---|---|---|---|---|---|---|---|
| | | | | | | | | Sequence components of *kabuli* genome | *Kabuli* gene accession IDs | NCBI-KOG | TFs | NCBI-nr database |
| CaPOPI_2981 | Ca4 | 44525230 | TAAAAAAAAAAAA/TAAAAAAAA | ATGACGTTGGTGATTGGACA | GCGCTAGCAAAAATAGCCAC | 59.8 | 884 | INTERGENIC | | | | |
| CaPOPI_2982 | Ca4 | 44526873 | ACTCCAAA/ACTCCAAATGCTCCTCCAAA | TTCCATTCTTTTTGCCAAGG | TTGGCTCAAGTGGGATAAGC | 60.0 | 397 | INTERGENIC | | | | |
| CaPOPI_2983 | Ca4 | 44533305 | TAAAAAAAA/TAAAAAAA | CAAAATGCCGTCACAGAATG | CCTTGGTTTCGATGCCTAAA | 60.1 | 368 | DRR | Ca_09125 | L | | Smr protein/MutS2 C-terminal |
| CaPOPI_2984 | Ca4 | 44559914 | TTATATATATAT/TTATATATATATAT | GCGGGGCATAAACTGATAGA | CATTTTCATATCACCAAATCACG | 60.1 | 465 | INTERGENIC | | | | |
| CaPOPI_2985 | Ca4 | 44694709 | TAGAGAG/TAGAG | GATTCGATCATCTGGGGAAA | AGGCTTCTGGTGCTGTTCTT | 59.8 | 539 | INTERGENIC | | | | |
| CaPOPI_2986 | Ca4 | 44773439 | ATTTTTT/ATTTTTTTT | TTGTTTCACTTTTTCCATTCCA | TAGGGTTCTTGATCCATCGC | 59.5 | 596 | INTERGENIC | | | | |
| CaPOPI_2987 | Ca4 | 44886483 | TTCT/TT | TAATGGAGGGCAATTTCGAG | AAAACACCCAACCCCCTAAT | 60.0 | 349 | INTERGENIC | | | | |
| CaPOPI_2988 | Ca4 | 44902733 | AAGAGAGAGAGAGAGAG/AAGAGAGAGAGAGAGAGAGAG | TGTGAAGCAACAAGGAACAGA | TCGATTCCCACATGACACTG | 59.5 | 133 | DRR | Ca_09152 | | C2H2 | Protein of unknown function DUF3511 |
| CaPOPI_2989 | Ca4 | 44953374 | TACACACACA/TACACA | TCCCTCCATCACCAAAACTC | ATGTGCAGTGACCAGAGCAG | 59.9 | 349 | INTRON | Ca_09159 | G | | Glycoside hydrolase, family 32 |
| CaPOPI_2990 | Ca4 | 44965984 | TTCAACTT/TTCAACTTAATTTCTCAACTT | ATCTTTCCCCTCCACACACA | TGGTCGCATTCGCATATAAA | 60.4 | 601 | INTERGENIC | | | | |
| CaPOPI_2991 | Ca4 | 44968120 | ATTCTTCT/ATATTATTACTTCTTCTTCTTCT | ATTCTCCCAATCCACCATCA | GCATGTTTAAGTGGGGCCTA | 60.1 | 184 | CDS (large-effect mutations) | Ca_09160 | R | C2H2 | Zinc finger, C2H2-type |
| CaPOPI_2992 | Ca4 | 44970459 | CAAA/CA | CATTGCACGAGCACAACTG | TGGAACGTAATTCCTCCATT | 60.0 | 661 | DRR | Ca_09160 | R | C2H2 | Zinc finger, C2H2-type |
| CaPOPI_2993 | Ca4 | 44987442 | TG/T | TCATGAAAACGTTTGGTTAATTG | AAAAGGGATACTGAATTTCATCCA | 58.9 | 373 | INTERGENIC | | | | |
| CaPOPI_2994 | Ca4 | 44987779 | GA/GAATA | ACTTTGGAACAAAAGGAGGAA | TGGTCAAGATGTGTTGACTTTG | 57.8 | 570 | INTERGENIC | | | | |
| CaPOPI_2995 | Ca4 | 44999560 | GTCTCTC/GTCTC | TTCATTTTCCCACCCCTACA | TCCCTACTCAAAGGGGTCAA | 60.2 | 630 | INTERGENIC | | | | |

| INDEL marker IDs | Chromosomes/ unanchored scaffolds | Physical positions (bp) | InDels (*Kabuli* reference genome-CDC Frontier/PI) | Forward primers (5'-3') | Reverse primers (5'-3') | Anneling temperature (0C) | Expected amplified product size (bp) | Structural annotation | | Functional annotation | | |
|---|---|---|---|---|---|---|---|---|---|---|---|---|
| | | | | | | | | Sequence components of *kabuli* genome | *Kabuli* gene accession IDs | NCBI-KOG | TFs | NCBI-nr database |
| CaPOPI_2996 | Ca4 | 45005102 | A/AAT | CTCCGTTCAAGCCTCTTGTC | GAAAGTGTCGAGCAACACGA | 60.0 | 225 | INTERGENIC | | | | |
| CaPOPI_2997 | Ca4 | 45068051 | CAA/CAAA | CGTGGACTTCCCGACTACAT | ACAATTTACTCCCTCCGCCT | 60.0 | 584 | DRR | Ca_09168 | T | WRKY | Disease resistance protein |
| CaPOPI_2998 | Ca4 | 45076892 | CG/CGG | GGTCCAACATTCCTCCTTGA | TCCACAAATAACAGGTTTTCCC | 59.9 | 606 | INTERGENIC | | | | |
| CaPOPI_2999 | Ca4 | 45350262 | TTA/TTATA | AGAATCGTTTTGTTGGGATCA | GTCCTTGGTAGCGAGTTGGA | 59.4 | 778 | INTERGENIC | | | | |
| CaPOPI_3000 | Ca4 | 45366808 | TT/TTAT | ACACCCCAATCCAAAATTGA | ATGCTCAAATGGGTAGCAGG | 60.0 | 771 | INTRON | Ca_09200 | K | MYB | SANT domain, DNA binding |
| CaPOPI_3001 | Ca4 | 45369151 | CTTTTTT/CTTTTT | TTGCATTCAATGTCTGCATTT | GGGCAGTCACCTCCACTAAA | 59.2 | 575 | INTERGENIC | | | | |
| CaPOPI_3002 | Ca4 | 45380400 | ACTACG/A | TTGCCGTTAATTCTTGGAGC | GTGGAGAAAACAACAACGGG | 60.2 | 774 | INTERGENIC | | | | |
| CaPOPI_3003 | Ca4 | 45397379 | TA/TAA | CACGTGAGTGTGACATGGTG | CAATGGCAAAAATATCCCAAA | 59.6 | 626 | INTERGENIC | | | | |
| CaPOPI_3004 | Ca4 | 45399331 | ATTTTTTTTTTTTTTTT/ATTTTTTTTTTT | ACCACTTCACCACAAGAGGG | ATTCCGCTTTGCTGTCAAAC | 60.0 | 445 | INTERGENIC | | | | |
| CaPOPI_3005 | Ca4 | 45399782 | AG/A | GTTTGACAGCAAAGCGGAAT | ACAGCTTGCAACTGATGTGG | 60.3 | 334 | INTERGENIC | | | | |
| CaPOPI_3006 | Ca4 | 45505990 | AATTATTATTATTATTATTATTATTATTATTATTATTATTATTATTATTATTATTA/AATTATTATTATTATTATTATTATTATTATTATTATTATTATTATTATTATTATTAT | GAATCACGCAGCATTCAAAA | CGGAGATTGCAAGTAAAAGAGTC | 59.8 | 402 | INTERGENIC | | | | |
| CaPOPI_3007 | Ca4 | 45567618 | TTATATATATATATATATAT/TTATATATATATATATAT | TCACAAATGGCAGTTTTGTCTT | CCAATCCTAGCTTCTGAAAATG | 59.7 | 635 | INTERGENIC | | | | |
| CaPOPI_3008 | Ca4 | 45626888 | ATTT/ATTTT | TATCAGCAGCACCAAATGGA | GCCTGATGTTTGTCAGGGTT | 60.2 | 286 | INTRON | Ca_09219 | Z | MYB_related | Kinesin, motor domain |
| CaPOPI_3009 | Ca4 | 45949230 | AATTATTATTATTATTATTATTATTATTATTATGATTATTATTATTAT/AATTATTATTATTATTATTATTATTATTATTATTATTATTATTATGATTATTATTATTAT | TCGAAAGAACCTCGACTCGT | TCAATATGAGGTCGCTTCCC | 60.0 | 508 | INTERGENIC | | | | |
| CaPOPI_3010 | Ca4 | 45959235 | TTTATTATTATTATTATTATTATTAT/TTTATTATTATTATTATTATTATTATTAT | GGCCTATGGCCTAGCCTACT | ACGGCCTACCATAGGCTTTT | 59.7 | 555 | INTERGENIC | | | | |

| INDEL marker IDs | Chromosomes/ unanchored scaffolds | Physical positions (bp) | InDels (*Kabuli* reference genome-CDC Frontier/PI) | Forward primers (5'-3') | Reverse primers (5'-3') | Anneling temperature (0C) | Expected amplified product size (bp) | Structural annotation | | Functional annotation | | |
|---|---|---|---|---|---|---|---|---|---|---|---|---|
| | | | | | | | | Sequence components of *kabuli* genome | *Kabuli* gene accession IDs | NCBI-KOG | TFs | NCBI-nr database |
| CaPOPI_3011 | Ca4 | 46001807 | GATAATAATAATAATAATAATAATAATAATAATAATAATA/GATAATAATAATAATAATAATAATAATAATAATA | TTGCTCCTACGTATCCCCAG | CTCAATCGTGGGTGACCTTT | 60.1 | 385 | INTERGENIC | | | | |
| CaPOPI_3012 | Ca4 | 46003281 | TTTATTATTATTATTATTATTATTATTATTATTATT/TTTATTATTATTATTATTATTATTATTATTATT | ATTCTCTCCCCCTGGTGTCT | TGTTTGGCCTAAAATTGGGT | 59.9 | 724 | INTERGENIC | | | | |
| CaPOPI_3013 | Ca4 | 46004838 | TAAAAAA/TAAAAAAA | TGATTTCAATGAGAGGATTTTGTT | GCTCGATTGATGTTAAGGGC | 59.0 | 425 | INTERGENIC | | | | |
| CaPOPI_3014 | Ca4 | 46301292 | TAAAAAAAAAA/TAAAAAAAAA | CGCTTTTGGTCTCTAATGATTT | ATATGTCGCAGTCTCCCACC | 57.6 | 184 | INTERGENIC | | | | |
| CaPOPI_3015 | Ca4 | 46384693 | T/TCCCTATAAAAA | GAGGAGGGTGGAGAATGGAT | GGGTGCCTGGTACAAAACTC | 60.3 | 708 | INTERGENIC | | | | |
| CaPOPI_3016 | Ca4 | 46386159 | ATT/ATTT | GAGGAGGATGTCCCTAAGCC | AGAATCCAATGCTCCACAGG | 60.0 | 779 | INTRON | Ca_19274 | | Dof | Aminotransferase-like, plant mobile domain |
| CaPOPI_3017 | Ca4 | 46496090 | TAAAAAAAAA/TAAAAAAAAAA | AAAGTGGGGTCAACTTTTTGAA | TGTCAAGGAGACCCACCACT | 59.9 | 481 | INTERGENIC | | | | |
| CaPOPI_3018 | Ca4 | 46674801 | A/AG | GGATGACTCGAAACGGTGTT | TTACCCAAAACCCCAAATCA | 60.0 | 667 | INTERGENIC | | | | |
| CaPOPI_3019 | Ca4 | 47188403 | TTATATATATATATATATATATATATAT/TTATATATATATATATATATATATATATAT | ACGTCCACTGATTGGTCACA | ACAATAGCGCAATGCTCGTT | 60.0 | 583 | INTERGENIC | | | | |
| CaPOPI_3020 | Ca4 | 47193005 | TTTAT/TTTATTAT | CAATGTGGGTGAATTTGCTG | GATTCTGGCAAATTCGTCGT | 60.0 | 287 | INTRON | Ca_10862 | | | Protein of unknown function DUF1645 |
| CaPOPI_3021 | Ca4 | 47238699 | ATTT/ATT | TTTTGATCGAATCTGACCCA | TCGGCCATTTTTCTTCAATC | 59.0 | 659 | INTERGENIC | | | | |
| CaPOPI_3022 | Ca4 | 47442661 | AATATATATATATATATATATATATATAT/AATATATATATATATATATATATATATATATAT | TGTGGGAGCAAAATCACACT | GACGATCAAATCGGAAGAAAA | 59.1 | 688 | DRR | Ca_10843 | U | | Inositol polyphosphate-related phosphatase |
| CaPOPI_3023 | Ca4 | 47705093 | C/CG | TTCGCATCCGTATCAAATCA | TCACACCCATGAAGATATGAGC | 60.0 | 269 | INTERGENIC | | | | |
| CaPOPI_3024 | Ca4 | 47755689 | CTTTTTTTT/CTTTTTTT | TTTTCCTTTTGGTGGATTGG | TGTGCAACTTTCTCACCCAG | 59.8 | 480 | INTERGENIC | | | | |
| CaPOPI_3025 | Ca4 | 47793461 | TATAAATAAATAAATAAATAAA/TATAAATAAATAAATAAA | TGTGCCAATGGATTGAGAAAT | CTGCAAGCACGTATGCCTTA | 60.3 | 396 | DRR | Ca_10815 | C | | Flavodoxin |

| **INDEL marker IDs** | **Chromosomes/ unanchored scaffolds** | **Physical positions (bp)** | **InDels (*Kabuli* reference genome-CDC Frontier/PI)** | **Forward primers (5'-3')** | **Reverse primers (5'-3')** | **Anneling temperature (0C)** | **Expected amplified product size (bp)** | **Structural annotation** | | **Functional annotation** | | |
|---|---|---|---|---|---|---|---|---|---|---|---|---|
| | | | | | | | | **Sequence components of *kabuli* genome** | ***Kabuli* gene accession IDs** | **NCBI-KOG** | **TFs** | **NCBI-nr database** |
| CaPOPI_3026 | Ca4 | 47848250 | TAAAAAAAAA/TAAAAAAAAAA | GACGGTGTTTGTAGCAGCAG | TTTCTTCCCCTTTTTGGGTT | 59.5 | 273 | INTERGENIC | | | | |
| CaPOPI_3027 | Ca4 | 47895561 | ATTT/ATTTT | AAAGGAGGCTAAATTCAAGAACC | AAAGAATGATCAAACGCAAGG | 59.2 | 317 | DRR | Ca_10803 | S | | |
| CaPOPI_3028 | Ca4 | 47961030 | TG/T | CAAATTTATAACGATTCTCTCACAAA | GCAAATGCATAGAGCAGCAA | 58.0 | 270 | DRR | Ca_10794 | | ARF | Transcriptional factor B3 |
| CaPOPI_3029 | Ca4 | 47961387 | TG/T | GCTGCTCTATGCATTTGCTG | GAGCATGGACGTTTGGATTT | 59.7 | 527 | DRR | Ca_10794 | | ARF | Transcriptional factor B3 |
| CaPOPI_3030 | Ca4 | 47966378 | CTTTTTTTTTT/CTTTTTTTT | AGCAAATTGGGAACCTTGTG | CGAGAAACCCATGAGGAAAA | 60.0 | 361 | DRR | Ca_10792 | | B3 | |
| CaPOPI_3031 | Ca4 | 47982618 | TTTTTATTTTATTTTATTTTATTTT/TTTTTATTTTATTTTATTTT | CCAAACTCCAATCAAATGGG | CAAATGAGAATTTTCTGGGCA | 60.2 | 483 | INTRON | Ca_10790 | U | ARF | Armadillo |
| CaPOPI_3032 | Ca4 | 47982672 | TC/TCC | CCAAACTCCAATCAAATGGG | CAAATGAGAATTTTCTGGGCA | 60.2 | 483 | INTRON | Ca_10790 | U | ARF | Armadillo |
| CaPOPI_3033 | Ca4 | 47983864 | CTATATAT/C | GCTTTGTTTCCATAAGCCCA | GGACCTCTTTCTACCCCTGG | 60.1 | 332 | INTRON | Ca_10790 | U | ARF | Armadillo |
| CaPOPI_3034 | Ca4 | 47985512 | G/GGTCAATATTGTTAA | ATGGGAAGGGAGCTTTTTGT | TTTGGATACTATTTGCGAAGTTT | 59.9 | 383 | INTRON | Ca_10790 | U | ARF | Armadillo |
| CaPOPI_3035 | Ca4 | 48019545 | AATATTTATTTTATATTTATTTTATATTTATTT/AATATTTATTTTATATTTATTT | AAGCAACCCCGTTATTTGTG | TTCGTACCATACCGGCAGAT | 59.9 | 588 | INTERGENIC | | | | |
| CaPOPI_3036 | Ca4 | 48116724 | AATATATATATATATA/AATATATATATATATATA | TTTGACGTTCGAAAAACCAC | ATGGAACAATCGGACACCAT | 58.6 | 433 | INTERGENIC | | | | |
| CaPOPI_3037 | Ca4 | 48196964 | AATATATATATATATAT/AATATATATATATATATAT | GGGTTTTTACCTAGACGGTGA | TCATGACATGGCGTGGTAGT | 58.1 | 709 | INTERGENIC | | | | |
| CaPOPI_3038 | Ca4 | 48204819 | T/TAA | AAAGAGGAAGAGGGCAAAGG | GCAAACACCGATAGAGAGGG | 59.8 | 425 | INTERGENIC | | | | |
| CaPOPI_3039 | Ca4 | 48207508 | AGATATAGT/A | CCGCCCCTTATCCTTATTGT | TTTCAAGCAGCTAAAACGCA | 60.2 | 568 | INTERGENIC | | | | |
| CaPOPI_3040 | Ca4 | 48214982 | GAAT/GAATTAAT | ATGCTTTCAAACTCATCGCC | CCATCGGATAGGTCAGAACC | 60.2 | 525 | INTERGENIC | | | | |

| INDEL marker IDs | Chromosomes/ unanchored scaffolds | Physical positions (bp) | InDels (*Kabuli* reference genome-CDC Frontier/PI) | Forward primers (5'-3') | Reverse primers (5'-3') | Anneling temperature (0C) | Expected amplified product size (bp) | Structural annotation | | Functional annotation | | |
|---|---|---|---|---|---|---|---|---|---|---|---|---|
| | | | | | | | | Sequence components of *kabuli* genome | *Kabuli* gene accession IDs | NCBI-KOG | TFs | NCBI-nr database |
| CaPOPI_3041 | Ca4 | 48227983 | TTCTCT/TTCTCTCT | AAATTCGATCTCGGCAGCTA | AGACATGGATTCGCCACTTC | 59.9 | 594 | INTERGENIC | | | | |
| CaPOPI_3042 | Ca4 | 48230328 | A/AC | TCCTTGAGGGACCAGGATAA | ATTGTGGATTTTGTGTCGCA | 59.5 | 431 | INTERGENIC | | | | |
| CaPOPI_3043 | Ca4 | 48243356 | CCTTCT/CCT | TGCATTTGTTGGACGCTTAG | AAATCGATAGTTTTGGCCCC | 59.9 | 601 | INTERGENIC | | | | |
| CaPOPI_3044 | Ca4 | 48260228 | GT/GTTTTAAGTT | TGTTGCCATTTTGTTTGCAT | ACCTGCACCACAAGAAAACC | 60.0 | 635 | INTRON | Ca_10761 | K | B3 | DNA-directed RNA polymerase, subunit 2, domain 6 |
| CaPOPI_3045 | Ca4 | 48278382 | CGAGAGA/CGAGA | ATGTTCCGGATTTGAACTCG | ACCAACGACGAGATAGGGTG | 59.9 | 604 | INTERGENIC | | | | |
| CaPOPI_3046 | Ca4 | 48314680 | ATTT/ATTTTT | GTAATGCGTTTGACCTGCAA | ATAGGCGCATCTCAATCACC | 59.7 | 649 | DRR | Ca_10756 | | BBR-BPC | GAGA binding-like |
| CaPOPI_3047 | Ca4 | 48314877 | TA/T | TGCGCCTATCTCTCTGTGTG | TTCGATCCATTTTACCACCC | 60.2 | 365 | DRR | Ca_10756 | | BBR-BPC | GAGA binding-like |
| CaPOPI_3048 | Ca4 | 48318482 | AATA/AA | CAACATATTTGCGGTTACCAA | CTCACCGTTTCACGTGATTG | 58.5 | 920 | INTERGENIC | | | | |
| CaPOPI_3049 | Ca4 | 48323095 | AACACACACACA/AACACACACACACA | GCAGCCAAACCAGCTTAGAC | AATTTGCAAAAATTCGGCTG | 60.0 | 515 | DRR | Ca_10754 | Q | GeBP | Cytochrome P450 |
| CaPOPI_3050 | Ca4 | 48330370 | AC/A | GCAAAGGAGCTTCTCAATGG | GCATCAGGAACAACCATCCT | 60.0 | 617 | INTRON | Ca_10753 | J | | Ribosomal protein L13 |
| CaPOPI_3051 | Ca4 | 48366728 | A/AGAG | CAAGGGCTGGATTGTTTTGT | TTGGCTTTACACTTGGTCCC | 60.0 | 673 | DRR | Ca_10750 | A | bHLH | Helicase, C-terminal |
| CaPOPI_3052 | Ca4 | 48398628 | GTATA/GTATATA | ATGGCCCAATTAAGCTTTGA | CTGTTCTTGCGTGACCTTCA | 59.5 | 565 | INTERGENIC | | | | |
| CaPOPI_3053 | Ca4 | 48414216 | CATAT/CAT | ATGAGCATAGCATGTGCCAG | GCATGGTTATCGGCTGATTC | 59.9 | 418 | INTERGENIC | | | | |
| CaPOPI_3054 | Ca4 | 48435268 | A/AG | TTGCAGTTGAATTTCGAAAGAA | TGATGTGATCCTAGGGTGAAGA | 59.9 | 610 | INTERGENIC | | | | |
| CaPOPI_3055 | Ca4 | 48443839 | AT/A | TGCCATAACTTTGAAAAATTGC | GAGTTTGAGAAGTGCAGGGC | 59.2 | 955 | INTERGENIC | | | | |

| INDEL marker IDs | Chromosomes/ unanchored scaffolds | Physical positions (bp) | InDels (*Kabuli* reference genome-CDC Frontier/PI) | Forward primers (5'-3') | Reverse primers (5'-3') | Anneling temperature (0C) | Expected amplified product size (bp) | Structural annotation | | Functional annotation | | |
|---|---|---|---|---|---|---|---|---|---|---|---|---|
| | | | | | | | | Sequence components of *kabuli* genome | *Kabuli* gene accession IDs | NCBI-KOG | TFs | NCBI-nr database |
| CaPOPI_3056 | Ca4 | 48454259 | CTTTT/CTTT | TTGCTTTTCCTACCACCACC | CAGCAGAGTCCAATTCAGCA | 60.0 | 376 | INTERGENIC | | | | |
| CaPOPI_3057 | Ca4 | 48465859 | GTTTTTTTTTTT/GTTTTTTTTTT | TGTGTGTCTCAGTGGTGGGT | GCCTTTCCTTGACAGTCTGC | 60.0 | 619 | INTRON | Ca_10746 | LA | | Zinc finger, CCHC-type |
| CaPOPI_3058 | Ca4 | 48467313 | TAG/TAGAG | CTATTTGCGTCTTTTCCCCA | ACGTGAACTTTTTGTTGCCC | 60.1 | 202 | INTRON | Ca_10746 | LA | | Zinc finger, CCHC-type |
| CaPOPI_3059 | Ca4 | 48522137 | TTTATTATTATTATTATTATTATT/TTTATTATTATTATTATTATTATT | CATTGCAATCGGCCTCTATC | TCCCACTTTCACTTTGGGTC | 60.6 | 657 | DRR | Ca_10740 | | C2H2 | Zinc finger, C2H2-type |
| CaPOPI_3060 | Ca4 | 48541135 | TAAAAAAAAAAAA/TAAAAAAAAA | AATTCCCACACGTACGCTTC | GATGTTCTTGGCTTGTGGCT | 60.0 | 723 | INTERGENIC | | | | |
| CaPOPI_3061 | Ca4 | 48575054 | TGGGG/TGGGGG | GCAACAATTGAATCAACCCC | CTTCATGAATTGCACCATGC | 60.2 | 705 | CDS (FRAME SHIFT) | Ca_23007 | P | | Heavy metal transport/detoxification protein |
| CaPOPI_3062 | Ca4 | 48576925 | AT/ATT | ATCTCACCCCAAGACAACCA | TCATTCCAGAAGGAGATGGG | 60.4 | 211 | INTRON | Ca_23007 | P | | Heavy metal transport/detoxification protein |
| CaPOPI_3063 | Ca4 | 48577228 | A/AC | AACACATCCGGGATCCATTA | TGATGACGAAGACGAAGACG | 60.0 | 303 | INTERGENIC | | | | |
| CaPOPI_3064 | Ca4 | 48651388 | AACTTTTAAGAGTACTTTTA/AACTTTTA | AAAACCACTCCAGACATCGG | CTTGCAATACCCGCAAAGTT | 60.0 | 682 | DRR | Ca_23016 | O | LBD | Heat shock protein Hsp90 |
| CaPOPI_3065 | Ca4 | 48683487 | TAAA/TAAAA | GTGTTTGTGCGATGATTTGG | TGGCATCATAAATTTGTGTGTGTA | 60.0 | 200 | INTERGENIC | | | | |
| CaPOPI_3066 | Ca4 | 48687183 | TTTACTTA/TTTACTTACTTA | TTCATAATTGCTGCACCCTG | ATGGAACCCAATGATCCTGA | 59.7 | 677 | INTRON | Ca_23019 | R | | C2 calcium-dependent membrane targeting |
| CaPOPI_3067 | Ca4 | 48766746 | TAAAA/TAAAAA | ATATCCAGCCGTGTTTGCAT | TTCTCCCCACCACTAACTCG | 60.4 | 261 | INTRON | Ca_23023 | | FAR1 | Lipase, GDSL |
| CaPOPI_3068 | Ca4 | 48772701 | AT/A | GGTTGGAAAACGGAAAAACA | AAGAAAATCTAAACGCCAAGG | 59.8 | 295 | DRR | Ca_23023 | | FAR1 | Lipase, GDSL |
| CaPOPI_3069 | Ca4 | 48781102 | TAT/TATGAT | TCGGCATTTTCATTGTCTCA | TGTGTACCGACCGATGTTTG | 60.2 | 190 | INTERGENIC | | | | |
| CaPOPI_3070 | Ca4 | 48793555 | TTCTCTCTCTCTCTCTCTC/TTCTCTCTCTCTCTCTCTC | ACGGAGAGTTTGGGTGTGAC | GTTATTCCACCGCGAGAAAA | 60.0 | 451 | INTERGENIC | | | | |

| INDEL marker IDs | Chromosomes/ unanchored scaffolds | Physical positions (bp) | InDels (*Kabuli* reference genome-CDC Frontier/PI) | Forward primers (5'-3') | Reverse primers (5'-3') | Anneling temperature (0C) | Expected amplified product size (bp) | Structural annotation | | Functional annotation | | |
|---|---|---|---|---|---|---|---|---|---|---|---|---|
| | | | | | | | | Sequence components of *kabuli* genome | *Kabuli* gene accession IDs | NCBI-KOG | TFs | NCBI-nr database |
| CaPOPI_3071 | Ca4 | 48809672 | GAAAAAAA/GAAAAAAAA | GCGAGCGAATTAAACTCACC | GGGAACATGACTTGGTTCGT | 59.9 | 190 | INTERGENIC | | | | |
| CaPOPI_3072 | Ca4 | 48831208 | GTATATA/GTATATATA | TATTTCCCAAGACCTCGTGC | ATGTGGTTGTTGTTGCCTGA | 60.1 | 255 | INTERGENIC | | | | |
| CaPOPI_3073 | Ca4 | 48846764 | ACATCATCATCATCA/ACATCATCATCA | GGTAAACGTGTGTTGCATGG | ACAGCAGTAGCCCTTTGAGC | 59.9 | 715 | INTERGENIC | | | | |
| CaPOPI_3074 | Ca4 | 48850057 | GA/GATCA | GCATCAATATGAATCACCAATCA | TGACGATTTTATTTACGCCAA | 59.7 | 857 | INTERGENIC | | | | |
| CaPOPI_3075 | Ca4 | 49073579 | GTT/GT | GTGACTTGGAGGGAGTGGAA | ATCCGCTAACCAATCACAGC | 60.1 | 453 | INTERGENIC | | | | |
| CaPOPI_3076 | Ca4 | 49074099 | ATTTTTTTTTT/ATTTTTTTTT | GCTGTGATTGGTTAGCGGAT | CGACGCGGGTTACACACTA | 60.1 | 694 | INTERGENIC | | | | |
| CaPOPI_3077 | Ca4 | 49135046 | ATATTATTATTATTATTATTATTATTATTATTATTATTATTATTATTATTATTATTATTATTATTATTATTATTAT/ATATTATTATTATTATTATTATTATTATTATTATTATTATTATTATTATTAT | TATAAAGCGGTTTGGTTCGG | ATCAACACAACATCCACCCA | 60.0 | 938 | INTERGENIC | | | | |
| CaPOPI_3078 | Ca5 | 115778 | TTCACCGGTCA/TTCA | AAAGATTGCAATTTGCCTGC | TTTTTAGGGGGCGATTGTATT | 60.2 | 511 | INTERGENIC | | | | |
| CaPOPI_3079 | Ca5 | 132937 | CAA/CAAA | CCAAAGACACAGCAAAGCAA | CACTTCCACCAGTTGCAAAA | 60.0 | 569 | INTERGENIC | | | | |
| CaPOPI_3080 | Ca5 | 133283 | AATATAT/AATAT | ACCTTTACATGGTGCCAAGC | GCGTTGTGAAAACAGGACAA | 60.0 | 433 | INTERGENIC | | | | |
| CaPOPI_3081 | Ca5 | 133428 | GAACA/GAACAATTTTTATAATAACA | ACCTTTACATGGTGCCAAGC | GCGTTGTGAAAACAGGACAA | 60.0 | 433 | INTERGENIC | | | | |
| CaPOPI_3082 | Ca5 | 146635 | GAAAAAAAA/GAAAAAAAAA | TGCATTTATTACTTCTTTACCAACA | CGAGGAAGGTAATACCATTTTGA | 57.2 | 712 | INTERGENIC | | | | |
| CaPOPI_3083 | Ca5 | 155645 | TAAAAAA/TAAAAAAA | GAGTGGTCACTCACGGGTTT | CCAATTTTCCCACTTGGTTG | 60.0 | 773 | INTERGENIC | | | | |
| CaPOPI_3084 | Ca5 | 158794 | TAAAAAA/TAAAAA | TGATGCGTCATGGACATAAA | TTTTAAAAATGTCCATTTACACCG | 58.5 | 498 | INTERGENIC | | | | |
| CaPOPI_3085 | Ca5 | 164138 | T/TGTAAATG | GGTAAAAGAGACTCGCGTGG | CTGATGTGCTCGTTGTTGCT | 59.9 | 695 | INTERGENIC | | | | |

| **INDEL marker IDs** | **Chromosomes/ unanchored scaffolds** | **Physical positions (bp)** | **InDels (*Kabuli* reference genome-CDC Frontier/PI)** | **Forward primers (5'-3')** | **Reverse primers (5'-3')** | **Anneling temperature (0C)** | **Expected amplified product size (bp)** | **Structural annotation** | | **Functional annotation** | | |
|---|---|---|---|---|---|---|---|---|---|---|---|---|
| | | | | | | | | **Sequence components of *kabuli* genome** | ***Kabuli* gene accession IDs** | **NCBI-KOG** | **TFs** | **NCBI-nr database** |
| CaPOPI_3086 | Ca5 | 180097 | ATTTTTTTTT/ATTTTTTTT | ATGACCAAGGAGGCACAGAC | GAATCAGAATGACGATTGTTTGA | 60.1 | 782 | INTERGENIC | | | | |
| CaPOPI_3087 | Ca5 | 188946 | CTT/CTTT | CCCCATTTTTACAATCCACG | CATGTATGAGCAGCCACACC | 60.0 | 366 | INTERGENIC | | | | |
| CaPOPI_3088 | Ca5 | 191792 | ACTATCTATCTATCTA/ACTATCTATCTATCTATCTA | TCACACTTGCACTTTGGAGC | CTTGGAATCGAAACCGTTGT | 60.0 | 680 | INTERGENIC | | | | |
| CaPOPI_3089 | Ca5 | 195971 | GATAT/GATATTTTGTTAAGTGTTATAT | ATCACGGAAAGCTTGTGTCC | TTTCACCTTCATCCTCTGGC | 60.1 | 866 | INTRON | Ca_18174 | L | | ATPase, AAA+ type, core |
| CaPOPI_3090 | Ca5 | 198698 | AA/AAGA | CCGGAGCACGTAAAGCTTAG | GTGGCATCCACAATGACAAA | 60.0 | 488 | DRR | Ca_18174 | L | | ATPase, AAA+ type, core |
| CaPOPI_3091 | Ca5 | 200719 | GTTTT/GTTT | CGATCACCTCCAACCAAAAT | AAGTGTTTCCGCATGGTAGG | 59.8 | 665 | DRR | Ca_18175 | | | 4-diphosphocytidyl-2C-methyl-D-erythritol synthase |
| CaPOPI_3092 | Ca5 | 210200 | ATTTTTTTTT/ATTTTTTTTTT | TTAAACACCCGTGTTGTGGA | AAATGGCTTAGGGAGGGAGA | 59.9 | 607 | DRR | Ca_18176 | | C2H2 | F-box domain, cyclin-like |
| CaPOPI_3093 | Ca5 | 210510 | ATT/ATTT | TCTCCCTCCCTAAGCCATTT | GAATTATCCGCTGCTTGCAT | 60.0 | 201 | DRR | Ca_18176 | | C2H2 | F-box domain, cyclin-like |
| CaPOPI_3094 | Ca5 | 217922 | CTT/CTTAGTTT | AAGGACATCCACAATGGAAA | ATCACAACACCACCACGCTA | 57.8 | 406 | INTERGENIC | | | | |
| CaPOPI_3095 | Ca5 | 218592 | TATAATAATAATAATAATA/TATAATAATAATAATAATAATA | TAGCGTGGTGGTGTTGTGAT | CCTTTCAAGGTCAGCCACAT | 60.0 | 739 | INTERGENIC | | | | |
| CaPOPI_3096 | Ca5 | 236073 | CGAAGAAAAT/CGAAGAAAATGAAGAAAATGAAGAAAAT | TTGAATTGACCGGAGGAATC | GGGATAGGGAGGGTTGTGTT | 59.9 | 680 | INTERGENIC | | | | |
| CaPOPI_3097 | Ca5 | 241183 | ATTTTTTT/ATTTTTTTT | AAAACACAACCCTTCAAGAGACA | GGGACGTCTTCGCATACATT | 60.1 | 129 | INTERGENIC | | | | |
| CaPOPI_3098 | Ca5 | 244348 | TA/TAATACAAA | TTTTGCGCGAGAATTGATAA | GCACTTACCACCGCTCTCTT | 59.4 | 918 | INTERGENIC | | | | |
| CaPOPI_3099 | Ca5 | 262311 | CTTTT/CTTT | TCGCTCGCACAAGTAAAATG | GGTCGATGCGTAGTTTCCAT | 60.0 | 859 | INTRON | Ca_18180 | A | | Tudor domain |
| CaPOPI_3100 | Ca5 | 276846 | T/TGCATCGGTTCA | TTGTTGCTTTTTCTTCGGCT | ACAGGCACTCCAGGATCAAC | 60.0 | 330 | INTRON | Ca_18181 | D | | Mitotic checkpoint |

| INDEL marker IDs | Chromosomes/ unanchored scaffolds | Physical positions (bp) | InDels (*Kabuli* reference genome-CDC Frontier/PI) | Forward primers (5'-3') | Reverse primers (5'-3') | Anneling temperature (0C) | Expected amplified product size (bp) | Structural annotation | | Functional annotation | | |
|---|---|---|---|---|---|---|---|---|---|---|---|---|
| | | | | | | | | Sequence components of *kabuli* genome | *Kabuli* gene accession IDs | NCBI-KOG | TFs | NCBI-nr database |
| CaPOPI_3101 | Ca5 | 280542 | AATTATTATTATT/AATTATTATTATTATT | CAAACAAGCTTTTTCACTTTCTCA | AGGACCTCCCTTTTTCTCCA | 60.0 | 636 | DRR | Ca_18183 | | ARF | Protein of unknown function DUF482 |
| CaPOPI_3102 | Ca5 | 291054 | TAAAAAAAAA/TAAAAAAAAAAA | TGCTTCATATGCTTGACCACA | TGGTGCTTCTTTGGCTCTTT | 60.3 | 476 | INTRON | Ca_18185 | R | B3 | Multi antimicrobial extrusion protein |
| CaPOPI_3103 | Ca5 | 291688 | ATTTTTT/ATTTTTTTT | AAAGAGCCAAAGAAGCACCA | AGCAAATGCGACTGTGATTG | 60.0 | 722 | DRR | Ca_18186 | | | |
| CaPOPI_3104 | Ca5 | 291943 | GCGCAACCGCAA/GCGCAACCGCAACCGCAA | TTCGTGGCTTAATCCCACTC | GTCTGGTGCTCTTCCCAATC | 60.1 | 517 | DRR | Ca_18186 | | | |
| CaPOPI_3105 | Ca5 | 292149 | A/AC | AAAGTGCCGAAGTTTGCAGT | TGCTTTCCCTTCAACAGTCA | 59.9 | 485 | DRR | Ca_18186 | | | |
| CaPOPI_3106 | Ca5 | 360986 | ATTTTTTTTTTT/ATTTTTTTTTT | TGTGAGTTTAAGGGAGGGGA | GCATCCAGGATCAGAGGTGT | 59.5 | 390 | DRR | Ca_18192 | P | | |
| CaPOPI_3107 | Ca5 | 378415 | T/TG | AGCTTTAGCTTGAGGGGGAG | TCGATTTAATCCGCAGAAGG | 60.0 | 510 | DRR | Ca_18193 | E | | Amino acid/polyamine transporter I |
| CaPOPI_3108 | Ca5 | 435121 | CA/CAAA | TGCAAATCAGGTGATCCAAA | CAGCAACCACAATTCAACTCA | 60.0 | 497 | DRR | Ca_18196 | | | |
| CaPOPI_3109 | Ca5 | 456196 | CTTTTTTTT/CTTTTTTTTTTT | TGCCTACAACCACACATGCT | CGTTTTCCGATTCATGCTTT | 60.2 | 685 | INTERGENIC | | | | |
| CaPOPI_3110 | Ca5 | 456689 | GGAAAACGAAAACG/GGAAAACG | TGTTGAGTTTTTGCACAGGC | TGAAAGGAGTCGGTTTTGTG | 59.9 | 257 | INTERGENIC | | | | |
| CaPOPI_3111 | Ca5 | 457302 | CG/CAAAGACCGTTGTG | CACAAGAAAGGGAATGACACTG | ACCACACAACTTTGGGCTTC | 59.6 | 643 | DRR | Ca_18199 | | | |
| CaPOPI_3112 | Ca5 | 622630 | G/GGCAACTCA | CATGCTTACCCTCTGGCCTA | ACAGCAGAATGGGGTAGTGG | 60.2 | 651 | INTERGENIC | | | | |
| CaPOPI_3113 | Ca5 | 927603 | TTAATAATAATAATAATAATAATAATAATAATAA/TTAATAATAATAATAATAATAATAATAATAATAATAATAA | GGTCTTTTGAAAATAATCTTAACGC | AAGGGGGAAGTTAAGCCAGA | 58.8 | 664 | INTERGENIC | | | | |
| CaPOPI_3114 | Ca5 | 1065182 | TAAAA/TAAAAA | AATGGCCACGAATCTGAAAG | ACCATTTTCAACTGCATCCC | 60.1 | 634 | INTERGENIC | | | | |
| CaPOPI_3115 | Ca5 | 1677289 | CTATATATATATATATATATATATATATAT/CTATATATATATATATATATATATATATATATAT | AAGCGCTGTAAAAGGTGCAT | ATGTGAATCCCACCAGGAAA | 59.9 | 794 | INTERGENIC | | | | |

| INDEL marker IDs | Chromosomes/ unanchored scaffolds | Physical positions (bp) | InDels (*Kabuli* reference genome-CDC Frontier/PI) | Forward primers (5'-3') | Reverse primers (5'-3') | Anneling temperature (0C) | Expected amplified product size (bp) | **Structural annotation** | | **Functional annotation** | | |
|---|---|---|---|---|---|---|---|---|---|---|---|---|
| | | | | | | | | **Sequence components of *kabuli* genome** | ***Kabuli* gene accession IDs** | **NCBI-KOG** | **TFs** | **NCBI-nr database** |
| CaPOPI_3116 | Ca5 | 1709760 | A/ATT | TGATGGGGGAAACATTTTGT | AAAAAGGGCATGTGAATGATCT | 60.0 | 247 | INTERGENIC | | | | |
| CaPOPI_3117 | Ca5 | 1970073 | ATTTTTTTT/ATTTTTTTTT | GATGAAATCGAGGACTGGGA | TTCGGACTAGACGAGGGCTA | 60.0 | 596 | INTERGENIC | | | | |
| CaPOPI_3118 | Ca5 | 2100697 | ATTTTTTT/ATTTTTT | TGGACACGACGGTGAAATAA | TTCGGTTCTTGTTTGCCTCT | 60.0 | 642 | INTERGENIC | | | | |
| CaPOPI_3119 | Ca5 | 2140832 | G/GC | GCCCGGATTGCAAGTATAAA | TTTGTGAGAGGGTGGGAGTC | 59.9 | 549 | INTRON | Ca_26505 | B | | Helicase, C-terminal |
| CaPOPI_3120 | Ca5 | 2295220 | GAA/GA | GCACCAGCATAAACTGCACA | GCAGGAGTTTTCCTTGCATT | 60.9 | 739 | INTERGENIC | | | | |
| CaPOPI_3121 | Ca5 | 2323461 | GAAAA/GAAAAA | GCGCCAGATTTCAAACACTA | ACCAGCAGACCAAAATGGAC | 58.9 | 262 | INTERGENIC | | | | |
| CaPOPI_3122 | Ca5 | 2327252 | TAA/TAAA | TCGAAATGAAATCGAGGACC | ACAGACGAGGGCTAGGGATT | 60.0 | 721 | INTERGENIC | | | | |
| CaPOPI_3123 | Ca5 | 2375435 | TTTA/T | ATGTTTTGGATTTTGGCTCG | AGGAAGAGTGGAGTTGCGAA | 59.9 | 571 | INTRON | Ca_18664 | | B3 | |
| CaPOPI_3124 | Ca5 | 2774211 | CTCT/CTCTTTCCCTTAATCT | ATCCGACATTTCTTTGGCAG | CAAAGCAAGGTGAAGTGCAA | 60.1 | 842 | INTERGENIC | | | | |
| CaPOPI_3125 | Ca5 | 3061873 | GATAATAATAATAATAATAATAATAATAATAATAATAATAATAATAATAATA/GATAATAATAATAATAATAATAATAATAATAATAATAATAATAATAATA | TTGCTCCTACGTATCCCCAG | TTATTTCACCGTCGTGTCCA | 60.1 | 332 | INTERGENIC | | | | |
| CaPOPI_3126 | Ca5 | 3106419 | CACA/CACATAACTACA | CAATTTTCGTGATGTGGCAG | TGAGTGATGCGAGTTGTTCG | 60.1 | 749 | INTERGENIC | | | | |
| CaPOPI_3127 | Ca5 | 3337230 | CATATATATATAT/CATATATATATATATAT | TCATCCCGAATCAGTTCACA | GGCATGATGCAAGGGATAGT | 60.0 | 291 | DRR | Ca_18697 | | | |
| CaPOPI_3128 | Ca5 | 3353130 | A/AC | GCGGTCACCTCCACATAGTT | TGTGTGGAGCCCATATGAAA | 60.0 | 659 | INTERGENIC | | | | |
| CaPOPI_3129 | Ca5 | 3742109 | TT/TTACATAT | ATTTACGAGCAGCGGAAAGA | TGGTCATGCAGAAACTCAGC | 60.0 | 605 | INTERGENIC | | | | |
| CaPOPI_3130 | Ca5 | 3742639 | TA/TAA | TGCTGAGTTTCTGCATGACC | CCCGTGATAGGCAGTACGTT | 60.0 | 597 | INTERGENIC | | | | |

| INDEL marker IDs | Chromosomes/ unanchored scaffolds | Physical positions (bp) | InDels (*Kabuli* reference genome-CDC Frontier/PI) | Forward primers (5'-3') | Reverse primers (5'-3') | Anneling temperature (0C) | Expected amplified product size (bp) | Structural annotation | | Functional annotation | | |
|---|---|---|---|---|---|---|---|---|---|---|---|---|
| | | | | | | | | Sequence components of *kabuli* genome | *Kabuli* gene accession IDs | NCBI-KOG | TFs | NCBI-nr database |
| CaPOPI_3131 | Ca5 | 3850375 | ATTTTTTTTTTT/ATTTTTTTTTTTT | TTTGAATCTTGAGGGTGGAAA | GAATGATATATTGATTGGGTGTGTG | 59.5 | 396 | INTERGENIC | | | | |
| CaPOPI_3132 | Ca5 | 3862372 | TAAA/TAA | TTCACATCCCCTCTAAACCAA | TCCCAATGACTCATCGTCAA | 59.4 | 349 | INTERGENIC | | | | |
| CaPOPI_3133 | Ca5 | 3874588 | AC/A | GACCTTGCTTTGGGGTTGTA | CACATCCCCTCTAAACCAACC | 60.0 | 409 | INTERGENIC | | | | |
| CaPOPI_3134 | Ca5 | 4035916 | CTTTTTTTTTTTT/CTTTTTTTTTTT | GCGGTGGGTTTGAAAAAGTA | ACACCAGTCAAACGCACAAA | 60.0 | 420 | INTERGENIC | | | | |
| CaPOPI_3135 | Ca5 | 4064742 | G/GC | TATGTTTGTTTTGCCGTCCA | AGGATGTACCATTCTTGCGG | 60.0 | 375 | INTERGENIC | | | | |
| CaPOPI_3136 | Ca5 | 4176359 | T/TG | TGCAATGGTCTCAATGTATTCAA | CCCGAAACCCTAAACCCTAA | 60.4 | 703 | INTERGENIC | | | | |
| CaPOPI_3137 | Ca5 | 4179599 | GATATATATATAT/GATATATATAT | CGGTTTAGGGATTGGGTTTT | TCGATTGAGTCTTTTGCACTT | 60.0 | 166 | INTERGENIC | | | | |
| CaPOPI_3138 | Ca5 | 4275629 | GC/G | TTAGGGTTTAGGGTTTCGGG | TGACTTTTGCACTAATTGGACG | 60.2 | 516 | INTERGENIC | | | | |
| CaPOPI_3139 | Ca5 | 4307171 | AGGTTTAGGGTTTAGGGTTT/AGGTTTAGGGTTTAGGGTTTAGGGTTT | AGGGTGTAAGGGTCTTGGTCT | CAAAAGCCAAAATGACCTCAA | 59.0 | 747 | INTERGENIC | | | | |
| CaPOPI_3140 | Ca5 | 4470545 | TGT/TGTCGCGTCGT | GATTGAACTTGGTGCGGATT | AATTCCAAACCAAACCCTCC | 59.9 | 557 | INTERGENIC | | | | |
| CaPOPI_3141 | Ca5 | 4968830 | AATATATATATATA/AATATATATATATATA | TTTCCAAAGTACAACGCATCA | TTGGAGAAACTTTTCCGCTG | 59.2 | 529 | INTERGENIC | | | | |
| CaPOPI_3142 | Ca5 | 5190851 | CTATATATATATATATATATATAT/CTATATATATATATATATATATATAT | AGCGCTATGTGACCCTTTTG | AATGTGAATCCCACCAGGAA | 60.3 | 555 | INTERGENIC | | | | |
| CaPOPI_3143 | Ca5 | 5318962 | TC/T | ACGTTGACCAATTCAGCTCC | CACTAGGGACCCTTTGGTGA | 60.1 | 720 | INTERGENIC | | | | |
| CaPOPI_3144 | Ca5 | 5602407 | CATATATATATATATATATATATATATAT/CATATATATATATATATATATATATATATATAT | GTTGGTTCCCTAGCACCGTA | CAATTGAGTTGCGACGAGAG | 60.0 | 552 | INTERGENIC | | | | |
| CaPOPI_3145 | Ca5 | 5836817 | TACCCTAAACC/TACCCTAAACCCTAAACCCTAAACC | GTATGAGGAGGATCTTGCGG | TCAAAATGCACCATAAACCG | 59.7 | 594 | INTERGENIC | | | | |

| INDEL marker IDs | Chromosomes/ unanchored scaffolds | Physical positions (bp) | InDels (*Kabuli* reference genome-CDC Frontier/PI) | Forward primers (5'-3') | Reverse primers (5'-3') | Anneling temperature (0C) | Expected amplified product size (bp) | Structural annotation | | Functional annotation | | |
|---|---|---|---|---|---|---|---|---|---|---|---|---|
| | | | | | | | | Sequence components of *kabuli* genome | *Kabuli* gene accession IDs | NCBI-KOG | TFs | NCBI-nr database |
| CaPOPI_3146 | Ca5 | 5837067 | ACCTAAACCCTAAACCCTAAACCCTAA/ ACCTAAACCCTAAACCCTAA | AAAGAAAAACACTCGCG CTG | AGCGTTTTGGGTTTAGG GTT | 60.6 | 825 | INTERGENIC | | | | |
| CaPOPI_3147 | Ca5 | 6199138 | TTTATTATTATTATTATTAT/TTTATTATT ATTATTATTATTATTATTAT | GCATTCCCGTATGCGAC TAT | GGCACATGTTATTTGTTT TGG | 60.0 | 511 | INTERGENIC | | | | |
| CaPOPI_3148 | Ca5 | 6200121 | CT/CTT | TTCGCAACTCCACTCTT CCT | GATCCATTTTCCCCCAA ATC | 60.0 | 342 | INTERGENIC | | | | |
| CaPOPI_3149 | Ca5 | 6369660 | TC/TCAC | GAGGAAACACGGTGAAT GGT | CGCTCACTAGTCGAACG AGA | 59.8 | 486 | INTERGENIC | | | | |
| CaPOPI_3150 | Ca5 | 6401833 | GAAAAAAA/GAAAAAA | CCTTTTGCACAAGCTTC ACA | TTATGCATGTTGGGTTT CCA | 60.0 | 551 | INTERGENIC | | | | |
| CaPOPI_3151 | Ca5 | 6532288 | AAAAAGAACAAAA/AAAAA | TGTCTTCAACTTCTGCG GTG | TTGCAACGTGTCATTGT CCT | 60.0 | 831 | INTERGENIC | | | | |
| CaPOPI_3152 | Ca5 | 6727251 | CGG/CGGG | TTTGGATTTTGACTCGG AGG | AGGAAGAGTGGAGTTGC GAA | 60.0 | 509 | INTERGENIC | | | | |
| CaPOPI_3153 | Ca5 | 6764095 | GTATATATATATATATATATATAT/GTAT ATATATATATATATATATATAT | TCAATTTGGGTTCAGGG TGT | ATGATGGGCCAGACGTT AAG | 60.2 | 841 | INTERGENIC | | | | |
| CaPOPI_3154 | Ca5 | 6765423 | GCAACA/GCAACAACA | CGAAGCTGCCAGGTAAA AAG | TGAGTCCATTCTCCAAA CCA | 60.0 | 652 | INTERGENIC | | | | |
| CaPOPI_3155 | Ca5 | 6772280 | CTTTTTTTTTTTT/CTTTTTTTTT | CCAATGGCTTCGACATT TCT | AAGTCACCCCTCGTTGT TTG | 60.1 | 726 | INTERGENIC | | | | |
| CaPOPI_3156 | Ca5 | 6772456 | GCACACA/GCACA | TGCACAACAAAAGCACA ACA | TGTACCATCATAGTGGC GGA | 59.9 | 512 | INTERGENIC | | | | |
| CaPOPI_3157 | Ca5 | 6773384 | AATATATATATATATAT/AATATATATATA TATATATATATATATAT | TATTTTGGATTTTGGCTC GG | AAACCGGTAAAATCTCC GCT | 59.9 | 681 | INTERGENIC | | | | |
| CaPOPI_3158 | Ca5 | 6833150 | TTTATTATTATTATTATTATTATTATT/TT TATTATTATTATTATTATTATTATTATT | TGCTAGGAACCGAGCCT TTA | TTGGCATCGTGATTTTAT GC | 60.0 | 924 | INTERGENIC | | | | |
| CaPOPI_3159 | Ca5 | 6833700 | ATATT/AT | GCATAAAATCACGATGC CAA | CGATTTTTCGTCTACATG TGC | 59.5 | 211 | INTERGENIC | | | | |
| CaPOPI_3160 | Ca5 | 6833833 | AATA/AATATA | AAATCACGATGCCAAAG AAAA | AAGAAAGTTGTAAATGT GTTTTATGGA | 59.6 | 616 | INTERGENIC | | | | |

| INDEL marker IDs | Chromosomes/ unanchored scaffolds | Physical positions (bp) | InDels (*Kabuli* reference genome-CDC Frontier/PI) | Forward primers (5'-3') | Reverse primers (5'-3') | Anneling temperature (0C) | Expected amplified product size (bp) | Structural annotation | | Functional annotation | | |
|---|---|---|---|---|---|---|---|---|---|---|---|---|
| | | | | | | | | Sequence components of *kabuli* genome | *Kabuli* gene accession IDs | NCBI-KOG | TFs | NCBI-nr database |
| CaPOPI_3161 | Ca5 | 6833924 | ATTT/ATT | AAATCACGATGCCAAAGAAAA | AAGAAAGTTGTAAATGTGTTTTATGGA | 59.6 | 616 | INTERGENIC | | | | |
| CaPOPI_3162 | Ca5 | 6834075 | ATATTATTATTATTATTATTATTATTATTATTATTATTATTATTATTATTATTA/ATATTATTATTATTATTATTATTATTATTATTATTATTATTATTATTATTA | GCACATGTAGACGAAAAATCG | ATGGCATGGTGATTTTATGC | 58.3 | 775 | INTERGENIC | | | | |
| CaPOPI_3163 | Ca5 | 6834263 | CT/C | GCTTAATCCATAAAACACATTTACAA | TTCTCATGCGCTTGACTAATG | 57.9 | 526 | INTERGENIC | | | | |
| CaPOPI_3164 | Ca5 | 6834706 | TAAAAAA/TAAAAAAA | TGCATAAAATCACCATGCCA | TTGTACGTTTCTTTTCTATGATTTGC | 60.9 | 360 | INTERGENIC | | | | |
| CaPOPI_3165 | Ca5 | 6837967 | TAAAAAAAAAAAA/TAAAAAAAAA | TCGAGAACAACTCGTCTCGTG | AATTTGCAGTGTCGCAAAAA | 61.6 | 254 | INTERGENIC | | | | |
| CaPOPI_3166 | Ca5 | 6872599 | ATTATTTATTTATT/ATTATTTATTTATTTATT | CGGCCATCACACAATGATAA | TCAGGATGTCACCACGAAGA | 60.3 | 454 | INTERGENIC | | | | |
| CaPOPI_3167 | Ca5 | 6907615 | A/AGTG | GGTTGTGTTATCCCTCGACA | AGGGTGCCACATTTACTCCA | 58.4 | 539 | INTERGENIC | | | | |
| CaPOPI_3168 | Ca5 | 6907876 | GA/GAATA | GGTTGTGTTATCCCTCGACA | AGGGTGCCACATTTACTCCA | 58.4 | 539 | INTERGENIC | | | | |
| CaPOPI_3169 | Ca5 | 6908168 | GTAAT/GTAATTAAT | GGAGTAAATGTGGCACCCTG | CCAACACTAGTCGCAAAGTCA | 60.4 | 588 | INTERGENIC | | | | |
| CaPOPI_3170 | Ca5 | 6909501 | TATATATAATTTAAATATATAATTTAAAT/TATATATAATTTAAAT | AATGATGGAGGGAGTGAAGC | AACTGTCCATTTGTGAGGGC | 59.1 | 405 | INTERGENIC | | | | |
| CaPOPI_3171 | Ca5 | 6910554 | A/AC | CTTTCAGGAACCAACAACCA | CCAAATTACCAAACGCCAAC | 58.6 | 328 | INTERGENIC | | | | |
| CaPOPI_3172 | Ca5 | 6964667 | CTAATA/CTAATAATA | CAAAATGCAGCAGCAATCAT | CCAACGGCAAATATTGTGGTA | 59.8 | 634 | INTERGENIC | | | | |
| CaPOPI_3173 | Ca5 | 7495764 | ATTTTT/ATTTTTT | AAGGGACAGGGGTAAAATGG | TTGTCATCTGGCAATCCTGA | 60.0 | 914 | INTRON | Ca_18748 | O | | Peptidase C13, legumain |
| CaPOPI_3174 | Ca5 | 7523624 | ATTT/ATT | TGGATGAAGTTTTTCGGTGG | CCTTACCGCTACCTCCATCA | 60.9 | 615 | INTRON | Ca_18747 | O | | Peptidase C13, legumain |
| CaPOPI_3175 | Ca5 | 7595427 | CAAAAAA/CAAAAAAAAA | TTTTTCGATTTTCATCCGGT | GGTGACCACGATCCATAATCA | 59.4 | 549 | INTERGENIC | | | | |

| INDEL marker IDs | Chromosomes/ unanchored scaffolds | Physical positions (bp) | InDels (*Kabuli* reference genome-CDC Frontier/PI) | Forward primers (5'-3') | Reverse primers (5'-3') | Anneling temperature (0C) | Expected amplified product size (bp) | Structural annotation | | Functional annotation | | |
|---|---|---|---|---|---|---|---|---|---|---|---|---|
| | | | | | | | | Sequence components of *kabuli* genome | *Kabuli* gene accession IDs | NCBI-KOG | TFs | NCBI-nr database |
| CaPOPI_3176 | Ca5 | 7605369 | T/TCAAAA | CGAAGATGGGAACCAACACT | TATTTGAGCCGAACCGAATC | 60.0 | 760 | INTERGENIC | | | | |
| CaPOPI_3177 | Ca5 | 7639450 | AAGTTAGTT/AAGTTAGTTAGTT | AACCAAGCCAACCTCAACAG | CCCTTTCATGCCAACAAAAT | 60.1 | 392 | DRR | Ca_18738 | | B3 | Transcriptional factor B3 |
| CaPOPI_3178 | Ca5 | 7661539 | CA/CATA | GATAGGTGGTGGGTGGGTC | GGAACCACTGCGTTGAAAAT | 60.0 | 720 | DRR | Ca_18734 | A | | Regulator of nonsense-mediated decay, UPF3 |
| CaPOPI_3179 | Ca5 | 7661611 | ATTTTTTT/ATTTTTT | CCCCAAAATCTTAAATCCGA | GGAACCACTGCGTTGAAAAT | 58.9 | 327 | DRR | Ca_18734 | A | | Regulator of nonsense-mediated decay, UPF3 |
| CaPOPI_3180 | Ca5 | 7662046 | TAGAAG/TAG | ATTTTCAACGCAGTGGTTCC | TTCGGTTTTGGGATGTGTTT | 60.0 | 671 | DRR | Ca_18734 | A | | Regulator of nonsense-mediated decay, UPF3 |
| CaPOPI_3181 | Ca5 | 7663256 | GTTT/GTT | TTGTTGCACTGACCGAGTGT | GAAATTTTACTCGCCGTGGA | 60.4 | 795 | DRR | Ca_18734 | A | | Regulator of nonsense-mediated decay, UPF3 |
| CaPOPI_3182 | Ca5 | 7666528 | TAAAAAAA/TAAAAAAAA | CGGGACCACATTAAAGGCTA | CCAACATCCCCAGCTAAAGA | 60.0 | 648 | INTRON | Ca_18734 | A | | Regulator of nonsense-mediated decay, UPF3 |
| CaPOPI_3183 | Ca5 | 7670175 | CAAG/CAAGTAAG | AAATTTGCGCAATGTTGTGA | AAAGGAAGCTGAGCAAGCTG | 60.1 | 921 | INTRON | Ca_18734 | A | | Regulator of nonsense-mediated decay, UPF3 |
| CaPOPI_3184 | Ca5 | 7674333 | CAAAAAAAA/CAAAAAA | GCAAAAGCCACACATCAGAA | TCAAAATTTGCCTCTTTCTCTTTC | 59.8 | 252 | INTRON | Ca_18733 | K | MIKC | Transcription factor, K-box |
| CaPOPI_3185 | Ca5 | 7680447 | AG/AGG | TGTTCCTCGTCAACTTCATCC | AATCAACCGCTTTAGCTTGC | 60.1 | 867 | INTRON | Ca_18733 | K | MIKC | Transcription factor, K-box |
| CaPOPI_3186 | Ca5 | 7682051 | CAAAAAAA/CAAAAAAAA | CCTCTCCCCATTTTTCAACA | TGGGATTCCAAATTTTTCCA | 59.9 | 856 | INTRON | Ca_18733 | K | MIKC | Transcription factor, K-box |
| CaPOPI_3187 | Ca5 | 7695472 | A/AGTTG | GATGCGACAAATGACCAATG | CAAAGGGTTCTTAACACATGGA | 59.9 | 239 | INTRON | Ca_18733 | K | MIKC | Transcription factor, K-box |
| CaPOPI_3188 | Ca5 | 7695635 | T/TG | GATGCGACAAATGACCAATG | GGCAAAATTGCAAAAGGAAA | 59.9 | 802 | INTRON | Ca_18733 | K | MIKC | Transcription factor, K-box |
| CaPOPI_3189 | Ca5 | 7696426 | TAA/TA | TGGTGGCAATGCTTGTTCTA | TCTCTTTGCCACTCAATCCA | 60.3 | 476 | INTRON | Ca_18733 | K | MIKC | Transcription factor, K-box |
| CaPOPI_3190 | Ca5 | 7696679 | AGTATGTA/AGTA | TGTCTTGTCCTAGCCGTCAA | ATGGAATTGGAAAGGTGGGT | 59.4 | 904 | INTRON | Ca_18733 | K | MIKC | Transcription factor, K-box |

| INDEL marker IDs | Chromosomes/ unanchored scaffolds | Physical positions (bp) | InDels (*Kabuli* reference genome-CDC Frontier/PI) | Forward primers (5'-3') | Reverse primers (5'-3') | Anneling temperature (0C) | Expected amplified product size (bp) | Structural annotation | | Functional annotation | | |
|---|---|---|---|---|---|---|---|---|---|---|---|---|
| | | | | | | | | Sequence components of *kabuli* genome | *Kabuli* gene accession IDs | NCBI-KOG | TFs | NCBI-nr database |
| CaPOPI_3191 | Ca5 | 7696740 | A/AAT | TGTCTTGTCCTAGCCGTCAA | ATGCACAAGTAGGGTCGAGG | 59.4 | 978 | INTRON | Ca_18733 | K | MIKC | Transcription factor, K-box |
| CaPOPI_3192 | Ca5 | 7707739 | A/ATCCGTCATATTGGG | CATGTGCTTGTGAGTTGGCT | TTGAGATTGTTGCCTTGCAG | 59.9 | 344 | INTRON | Ca_18733 | K | MIKC | Transcription factor, K-box |
| CaPOPI_3193 | Ca5 | 7786396 | GAAAAAAAA/GAAAAAAA | ACACAAATGGTACTGCCCAA | AAAGCATTGGGGTGATATGC | 58.9 | 596 | INTERGENIC | | | | |
| CaPOPI_3194 | Ca5 | 7828844 | TAAA/TAA | TGAAAACTCGAAAGAGCGGT | GCCGATGCGTAGATCGTAAT | 60.0 | 791 | DRR | Ca_18728 | | | |
| CaPOPI_3195 | Ca5 | 7846426 | TAAAATAAAAA/TAAAATAAAAATAAAAA | GGAGCCATTTCTGTTTGCAT | TTTTGTGGTGGTGCATGACT | 60.1 | 596 | DRR | Ca_18725 | A | MYB_related | RNA recognition motif domain |
| CaPOPI_3196 | Ca5 | 7858765 | TGGGGG/TGGGGGG | GGAAATTTCAGCAAGGTCCA | GGCTTTTGTATCCCCCATTT | 60.1 | 128 | INTERGENIC | | | | |
| CaPOPI_3197 | Ca5 | 7866386 | AAAATATA/AA | TCGAAAATATCCTTCATCGAAAA | TCAAAGGATTAGCCAAACAAAAA | 59.9 | 383 | DRR | Ca_18723 | | | Glutathione S-transferase, N-terminal |
| CaPOPI_3198 | Ca5 | 7886991 | ATTTTTTTTTTTT/ATTTTTTTTT | GTCCAGGTGTACAGTTCCCG | TCGCATTTTCTTAGCACTTTTG | 60.4 | 605 | INTERGENIC | | | | |
| CaPOPI_3199 | Ca5 | 7892187 | CTT/CT | AGTTGTCTTACATTGGCGGG | TGAAGCCTGCCCTAGTCAAT | 60.0 | 458 | INTRON | Ca_18722 | R | | Methyltransferase type 11 |
| CaPOPI_3200 | Ca5 | 7913105 | TG/TGG | CCTGCCGTCCTTGAAGAATA | CAGTGGATCACACAATTGGC | 60.2 | 180 | INTERGENIC | | | | |
| CaPOPI_3201 | Ca5 | 7915989 | C/CG | ATTAGGGACCGATTTCGTGA | GGCATGTCGGCAAAATTAAG | 59.4 | 551 | INTERGENIC | | | | |
| CaPOPI_3202 | Ca5 | 7918404 | GTTTTT/GTTTTTT | TGCATCATGCGAATTCATTT | AGAACACACATTTCTTTCTCGTT | 60.0 | 592 | INTERGENIC | | | | |
| CaPOPI_3203 | Ca5 | 7937565 | GT/GTT | ATCGTTTTGTTTCGGGACTG | ATGCCCATGCATGTTTGTAA | 60.0 | 404 | INTERGENIC | | | | |
| CaPOPI_3204 | Ca5 | 7957712 | CAAAAA/CAAAAAA | CTCTGCCTTGGAAGTGAAGG | GCAGAAACCTCACCCAATGT | 60.0 | 519 | INTERGENIC | | | | |
| CaPOPI_3205 | Ca5 | 7963920 | CAA/CAAAA | TTGGATTTTCTGGGCATCTC | CACATTGGAAAGACCGTCAA | 60.0 | 321 | INTERGENIC | | | | |

| INDEL marker IDs | Chromosomes/ unanchored scaffolds | Physical positions (bp) | InDels (*Kabuli* reference genome-CDC Frontier/PI) | Forward primers (5'-3') | Reverse primers (5'-3') | Anneling temperature (0C) | Expected amplified product size (bp) | Structural annotation | | Functional annotation | | |
|---|---|---|---|---|---|---|---|---|---|---|---|---|
| | | | | | | | | Sequence components of *kabuli* genome | *Kabuli* gene accession IDs | NCBI-KOG | TFs | NCBI-nr database |
| CaPOPI_3206 | Ca5 | 7966801 | GTTTTTTT/GTTTTTT | CCAAAAAGGTTGGTTTGAGC | TGGCATCAAATGCCTACAAA | 59.6 | 387 | INTERGENIC | | | | |
| CaPOPI_3207 | Ca5 | 7967489 | GTTTTTT/GTTTTTTT | TTGATGCCATTGTTGAAGGA | ACAGATCACCGTTGTTTCCC | 60.0 | 439 | INTERGENIC | | | | |
| CaPOPI_3208 | Ca5 | 7967914 | AATATATATATATATATA/AATATATATATATATATA | GGGAAACAACGGTGATCTGT | GGGGATATTTTTGTGTGCGT | 59.8 | 858 | INTERGENIC | | | | |
| CaPOPI_3209 | Ca5 | 7978058 | TTAATAATAATAATAATAATAATAATAATAATAAT/TTAATAATAATAATAATAATAAT | CTCCATGCTTTTGGGTGTTT | GGTTTTTGCAATGCCTGTTT | 60.0 | 454 | INTRON | Ca_18720 | E | | Amino acid transporter, transmembrane |
| CaPOPI_3210 | Ca5 | 7982148 | CC/CCTTC | CTTGTAGCCATTTTTGCCGT | GGACTTTTGTCGTCGGGTAA | 60.1 | 377 | INTERGENIC | | | | |
| CaPOPI_3211 | Ca5 | 8071914 | ATTTT/ATTTTT | TTTTCGTTTCAATAATTGTTTTCTT | AATTGATTCGTGTGTTGCCA | 57.6 | 658 | INTERGENIC | | | | |
| CaPOPI_3212 | Ca5 | 8088108 | CAAAAAAAA/CAAAAAAAAAA | CACTGTACATGCAGTCCTTGC | TAGGCTGGGATGGAAATCAG | 59.4 | 604 | DRR | Ca_18715 | C | | Glycerophosphoryl diester phosphodiesterase |
| CaPOPI_3213 | Ca5 | 8090658 | CTT/CTTGCTTT | GCATGTCGAATTTTTCCCAT | GGACGCCTTCGTCAGATAAG | 59.8 | 604 | INTERGENIC | | | | |
| CaPOPI_3214 | Ca5 | 8090888 | ATTTTTTTT/ATTTTTTT | TTTTCCCTCAATTCTTTTCAACA | GGACGCCTTCGTCAGATAAG | 60.0 | 291 | INTERGENIC | | | | |
| CaPOPI_3215 | Ca5 | 8097767 | GAAATTTTA/GA | TGTTTCTGACAGTTCTTTCCCA | TGGAAAGTCATGCTTTGTGG | 59.8 | 664 | INTERGENIC | | | | |
| CaPOPI_3216 | Ca5 | 8098608 | ATAGTACGTTA/ATA | TCGTCAATGTTTGTGCCATT | TTTCATGTTTGCAAGAAGAAGG | 60.0 | 378 | INTERGENIC | | | | |
| CaPOPI_3217 | Ca5 | 8100992 | AATAACAACCTTATAACAACCTT/AATAACAACCTT | TTTTCCCCCTAGTTGTGCAG | AATCTCCAGCCACTTAGGCA | 60.1 | 533 | INTERGENIC | | | | |
| CaPOPI_3218 | Ca5 | 8164282 | TAAAAAAA/TAAAAAAAA | CTACAAACGGAGAAGTCGGG | TTGACTGATGTTAAGTATGCACG | 59.7 | 816 | INTERGENIC | | | | |
| CaPOPI_3219 | Ca5 | 8199807 | TTATATATATATATATATATATATAT/TTATATATATATATATAT | TGCATGTTCTTACGCTCGTC | AGTTATCGGTCGCTGGTGTC | 60.0 | 704 | INTERGENIC | | | | |
| CaPOPI_3220 | Ca5 | 8241804 | ATTTT/ATTT | TGACAGGAGCTTCCCTGAGT | GTACGAACTTCCTCCCACGA | 60.0 | 678 | INTERGENIC | | | | |

| INDEL marker IDs | Chromosomes/ unanchored scaffolds | Physical positions (bp) | InDels (*Kabuli* reference genome-CDC Frontier/PI) | Forward primers (5'-3') | Reverse primers (5'-3') | Anneling temperature (0C) | Expected amplified product size (bp) | Structural annotation | | Functional annotation | | |
|---|---|---|---|---|---|---|---|---|---|---|---|---|
| | | | | | | | | Sequence components of *kabuli* genome | *Kabuli* gene accession IDs | NCBI-KOG | TFs | NCBI-nr database |
| CaPOPI_3221 | Ca5 | 8267659 | AT/ATT | TAATACACACACGCACACGC | GTTGCTTCCCCAATTGAGAC | 59.2 | 344 | INTERGENIC | | | | |
| CaPOPI_3222 | Ca5 | 8315904 | ATTTTTTTTTT/ATTTTTTTTT | TGTGTTCCGTGAGAATTGGA | TTGTGTTTGACTGACGTGGC | 60.1 | 700 | INTERGENIC | | | | |
| CaPOPI_3223 | Ca5 | 8489533 | G/GA | AGATATTGCAATGCCGGAAG | TTCGGTACACCCACATCTCA | 60.1 | 320 | INTERGENIC | | | | |
| CaPOPI_3224 | Ca5 | 8583752 | GATAATAATAATAATAATAATAATAATAATAATAATAATAATAATAATAA/GATAATAATAATAATAATAATAATAATAATAATAATAATAATAA | CGATGACCATTGTTGACACC | TCTTCTTGTGGCCGACTCTT | 59.8 | 556 | INTERGENIC | | | | |
| CaPOPI_3225 | Ca5 | 8721019 | TG/T | GCTATAGCGGTGCTATTCGG | TAAGCCTTGCAACGACCTCT | 59.9 | 720 | INTERGENIC | | | | |
| CaPOPI_3226 | Ca5 | 8729583 | AT/A | TCTCTTCCGCAATAGCCACT | TGGTGAAAGCATTTGATGGA | 60.0 | 653 | INTERGENIC | | | | |
| CaPOPI_3227 | Ca5 | 8768889 | GCATTTC/GCATTTCATTTC | GACAAGCAAGGTTCGAGTCC | GCCACACGTTTACTTCCGTT | 59.9 | 618 | INTERGENIC | | | | |
| CaPOPI_3228 | Ca5 | 8775868 | ATTTTT/ATTTT | AGATCTTCTCCGACACCGAA | ACCCCATAAGTTTTCCCACG | 59.8 | 625 | INTERGENIC | | | | |
| CaPOPI_3229 | Ca5 | 8782889 | TT/TTCTTATGT | ACCCTGGCTTTAATGGGTCT | TGCAAATGCAATGTTAAATGA | 59.8 | 538 | INTERGENIC | | | | |
| CaPOPI_3230 | Ca5 | 8787077 | ATT/AT | TTGAAGGAAAGGGAATGGAA | CAATCCCCTTAACCATGTGC | 59.5 | 384 | INTERGENIC | | | | |
| CaPOPI_3231 | Ca5 | 8794913 | TAAAAAA/TAAAAA | TGCCATTAGTTAAAGCCCAAA | CCGTTGGTGAAAGTGGAGAT | 59.6 | 475 | INTRON | Ca_20334 | J | | Ribosomal protein L5 |
| CaPOPI_3232 | Ca5 | 8823123 | ATTTGAAAG/ATTTGAAAGTTTGAAAG | TGGTTGATGTGAGTGGCTTC | CCAAATCAAAGGAGATCCGA | 59.7 | 573 | INTERGENIC | | | | |
| CaPOPI_3233 | Ca5 | 8823474 | C/CT | AAAAATACGCGCACACACAT | CCACAAAGGCTGCATACAAA | 59.1 | 512 | DRR | Ca_20336 | A | | Ribosomal RNA adenine methylase transferase |
| CaPOPI_3234 | Ca5 | 8832454 | AC/A | TTCATTGAGGTTCATTGCACA | GGTGGTTCCTCAACGAAAAA | 60.1 | 890 | INTERGENIC | | | | |
| CaPOPI_3235 | Ca5 | 8837420 | TG/TGG | GCAGGAAAAATGGGTTGAGA | CAAGGGGCTGCAAAAGTAAC | 60.1 | 326 | DRR | Ca_20337 | | | |

| INDEL marker IDs | Chromosomes/ unanchored scaffolds | Physical positions (bp) | InDels (*Kabuli* reference genome-CDC Frontier/PI) | Forward primers (5'-3') | Reverse primers (5'-3') | Anneling temperature (0C) | Expected amplified product size (bp) | Structural annotation | | Functional annotation | | |
|---|---|---|---|---|---|---|---|---|---|---|---|---|
| | | | | | | | | Sequence components of *kabuli* genome | *Kabuli* gene accession IDs | NCBI-KOG | TFs | NCBI-nr database |
| CaPOPI_3236 | Ca5 | 8877960 | AG/AGG | AAAGGAAAGATAAAGTGGAAGTGAGA | ATAGTGTTGCCCCCATTGAA | 60.1 | 441 | INTRON | Ca_20339 | | bZIP | Zinc finger, RING-type |
| CaPOPI_3237 | Ca5 | 8929713 | GA/GACA | AGTTTTGTGGCTAGGAGGCA | ATGCAAGGAGGCCATAAAAA | 59.9 | 782 | INTERGENIC | | | | |
| CaPOPI_3238 | Ca5 | 8930267 | GA/GAA | TTTTTATGGCCTCCTTGCAT | TGGGATTTCATTCCCATTTG | 59.5 | 721 | INTERGENIC | | | | |
| CaPOPI_3239 | Ca5 | 8934749 | TAAAAA/TAAAA | GGCAAGCGAAGGTAAGTGAG | AGACTGGTGAGCAAATTCGG | 60.0 | 427 | INTERGENIC | | | | |
| CaPOPI_3240 | Ca5 | 8937226 | AGT/AGTGT | CCACAAGCTAGGATACTGCCA | CGTAACCCAAGTCGCTAATCA | 60.3 | 420 | INTERGENIC | | | | |
| CaPOPI_3241 | Ca5 | 8952513 | TAAAAAA/TAAAAA | GCCAATCACCGCGTATAACT | TCTTGCTTCGCCCTTACCTA | 60.0 | 566 | INTERGENIC | | | | |
| CaPOPI_3242 | Ca5 | 8969612 | TAAAAAA/TAAAAAAA | TGCAATGATGGTTTCTCCAA | TTGCTTCGATCTTGAGGTCC | 60.0 | 562 | INTERGENIC | | | | |
| CaPOPI_3243 | Ca5 | 8970639 | AATATATATATATATA/AATATATATATATATATA | GATTTTAAGCGGCCAAACAA | CGGTCGCTAGTGTAAAAGGC | 60.1 | 425 | INTERGENIC | | | | |
| CaPOPI_3244 | Ca5 | 8975579 | CAAAAAAAAA/CAAAAAAAAAAA | TCATGAGGTGTTGCTCATCG | TTGACTTTCTCCCCGTTGAC | 60.8 | 110 | INTERGENIC | | | | |
| CaPOPI_3245 | Ca5 | 9002459 | TTATATATATAT/TTATATATATATAT | CCCAACCATGAAATGATGAA | GCAACAACCCAAAACCTCAT | 59.2 | 377 | INTERGENIC | | | | |
| CaPOPI_3246 | Ca5 | 9015474 | CT/C | TCCACACCTCATTGGTTGTC | TTGGTTGATGACGTTCAAGG | 59.4 | 548 | INTERGENIC | | | | |
| CaPOPI_3247 | Ca5 | 9015926 | AG/A | CCTTGAACGTCATCAACCAA | TCACGGCATAAAGAGGATGA | 59.5 | 413 | INTERGENIC | | | | |
| CaPOPI_3248 | Ca5 | 9032003 | ATAAGT/AT | TCTTCTTTGGAGGTTGGGTG | CAAAGCATCAAACTTCCCCT | 60.1 | 173 | DRR | Ca_19958 | R | | Sugar/inositol transporter |
| CaPOPI_3249 | Ca5 | 9075563 | T/TAC | CTCTGAAACCATGCAAGCAA | TGACGGCTGAATTGTCCATA | 60.0 | 676 | DRR | Ca_19962 | O | | Prohibitin |
| CaPOPI_3250 | Ca5 | 9112753 | TTT/TTTATT | CCACATTCATACACAAAGGGG | GGAAACTCGGGAGCCTTATC | 60.1 | 325 | DRR | Ca_19963 | IT | | Diacylglycerol kinase, catalytic domain |

| INDEL marker IDs | Chromosomes/ unanchored scaffolds | Physical positions (bp) | InDels (*Kabuli* reference genome-CDC Frontier/PI) | Forward primers (5'-3') | Reverse primers (5'-3') | Anneling temperature (0C) | Expected amplified product size (bp) | Structural annotation | | Functional annotation | | |
|---|---|---|---|---|---|---|---|---|---|---|---|---|
| | | | | | | | | Sequence components of *kabuli* genome | *Kabuli* gene accession IDs | NCBI-KOG | TFs | NCBI-nr database |
| CaPOPI_3251 | Ca5 | 9122149 | CCTCT/CCTCTCT | GCTGATGCACACCTTTCAGA | CAGAGAGTTGGTGTGCCTGA | 60.0 | 829 | INTERGENIC | | | | |
| CaPOPI_3252 | Ca5 | 9123724 | AT/A | TTTTAATTGATTCGCCCTCG | CCAATTGAATCCCTGCATTT | 60.0 | 673 | DRR | Ca_19964 | | C2H2 | Protein kinase, catalytic domain |
| CaPOPI_3253 | Ca5 | 9132512 | CTTTTT/CTTTTTT | ACTTGCGGATAAAACCACCA | TGGATTGGTGTTCAAGCATC | 60.4 | 704 | INTERGENIC | | | | |
| CaPOPI_3254 | Ca5 | 9133458 | AAT/AATTTCAT | ATTGGTTCAGTGGTTGGCTC | TTGTCAAATACTCCATTCAACCC | 60.0 | 399 | INTERGENIC | | | | |
| CaPOPI_3255 | Ca5 | 9140493 | AAAGAAGAAGAAGAAGAAGAAGAAGAA/AAAGAAGAAGAAGAAGAAGAAGAAGAAGAAGAA | CACTAGCACCGTAGCATGGA | AATTGCGGCGAGAGAAGATA | 59.9 | 378 | INTERGENIC | | | | |
| CaPOPI_3256 | Ca5 | 9148867 | GA/GAA | AATTCCTTGGCCTTGAGGTT | AAGTCACATCCCCCATCAAA | 59.9 | 859 | INTERGENIC | | | | |
| CaPOPI_3257 | Ca5 | 9163773 | AG/A | TCTGGTCATTTTCCTTGGTTG | CTCCAGCGATCTGTAAAGCC | 60.0 | 180 | INTERGENIC | | | | |
| CaPOPI_3258 | Ca5 | 9163864 | CT/CTT | TCTGGTCATTTTCCTTGGTTG | TCTCTTTAAAAAGTCGTTGATTTGG | 60.0 | 235 | INTERGENIC | | | | |
| CaPOPI_3259 | Ca5 | 9172890 | TT/TTATCT | GCCTCCAAACCTGAAACAAA | TAGCGACCACACGAAGATGT | 60.1 | 618 | INTERGENIC | | | | |
| CaPOPI_3260 | Ca5 | 9194215 | CATATAT/CATAT | GTGGCGCTCTAGGGAATACA | TTGGTTTCATGACCTGCATC | 60.2 | 456 | INTERGENIC | | | | |
| CaPOPI_3261 | Ca5 | 9231109 | AAT/A | ATTGATGACGACGTTGCTTT | TTTTGCAATTGTGGTCCATC | 58.2 | 634 | INTERGENIC | | | | |
| CaPOPI_3262 | Ca5 | 9233301 | CAAAA/CAAAAA | TTGTAATGCTTTGTAATGCTTGG | TTTTGGAGGTGTGTATTTTGGA | 59.2 | 538 | INTERGENIC | | | | |
| CaPOPI_3263 | Ca5 | 9308007 | GTTTTTTTTTTTTTTTT/GTTTTTTTTTT | TAACCCACGGAGGAATCAAG | ATTGGTGGTGTCATGGATGT | 59.9 | 720 | INTERGENIC | | | | |
| CaPOPI_3264 | Ca5 | 9348625 | TA/TACA | ATATCCCATGTCCGGTTTGA | CATTCGCTAACAATCTCCACA | 60.0 | 508 | INTERGENIC | | | | |
| CaPOPI_3265 | Ca5 | 9349749 | TA/TAA | CATTTGAGTTGTGTGTGGTTCA | TATGGTCCATTTGGGGTGAT | 59.5 | 846 | INTERGENIC | | | | |

| **INDEL marker IDs** | **Chromosomes/ unanchored scaffolds** | **Physical positions (bp)** | **InDels (*Kabuli* reference genome-CDC Frontier/PI)** | **Forward primers (5'-3')** | **Reverse primers (5'-3')** | **Anneling temperature (0C)** | **Expected amplified product size (bp)** | **Structural annotation** | | **Functional annotation** | | |
|---|---|---|---|---|---|---|---|---|---|---|---|---|
| | | | | | | | | **Sequence components of *kabuli* genome** | ***Kabuli* gene accession IDs** | **NCBI-KOG** | **TFs** | **NCBI-nr database** |
| CaPOPI_3266 | Ca5 | 9352151 | ATT/AT | TCAGAAAGCGCAAACTG ATG | TTTGCCACGTCTAATGC AAA | 60.1 | 720 | INTERGENIC | | | | |
| CaPOPI_3267 | Ca5 | 9360960 | CAAA/CAA | GAGGGACTGCAGGGTC ATTA | TAAACCCAGTGAAGGCA ACC | 60.1 | 421 | DRR | Ca_19977 | T | | |
| CaPOPI_3268 | Ca5 | 9361061 | CACAAA/CACAAACAAA | GAGGGACTGCAGGGTC ATTA | TAAACCCAGTGAAGGCA ACC | 60.1 | 421 | DRR | Ca_19977 | T | | |
| CaPOPI_3269 | Ca5 | 9361874 | TAAAAA/TAAAA | TTGCCCAAGCAATAACA TCA | GACTGCATTTGGAGCTG TGA | 60.1 | 303 | DRR | Ca_19977 | T | | |
| CaPOPI_3270 | Ca5 | 9363778 | TGG/TGGG | ACCACCTTGTGTGATGA CGA | TGACCTCAATCCCATTG ACA | 60.0 | 552 | INTERGENIC | | | | |
| CaPOPI_3271 | Ca5 | 9403421 | TA/TACCTTA | GGCACTTTTCACGTCCA TTT | ACAGCAGAATGGGGTAG TGG | 60.0 | 836 | INTERGENIC | | | | |
| CaPOPI_3272 | Ca5 | 9416660 | TA/TAA | CCATCTTAAATCATGGT GTTGG | TTCAACTCCTTTTGTTCC CG | 59.2 | 522 | INTERGENIC | | | | |
| CaPOPI_3273 | Ca5 | 9436009 | TAAAAAAA/TAAAAAAAA | TTTTCAATTCCTTTAAAA TTCGAGA | CCAAAATTTCCAAAGCT CCA | 59.6 | 726 | INTRON | Ca_19988 | | | Protein of unknown function DUF566 |
| CaPOPI_3274 | Ca5 | 9441399 | AATTTTATTTT/AATTTTATTTTATTTT | TGCGATGGAGAAATCAA AGC | CTAGTTTGGCTTGACGG GAA | 61.3 | 865 | INTERGENIC | | | | |
| CaPOPI_3275 | Ca5 | 9441477 | CAAAAAAAA/CAAAAAA | TCGTGTCTCAAAATTCAA GGAG | CTAGTTTGGCTTGACGG GAA | 59.4 | 675 | INTERGENIC | | | | |
| CaPOPI_3276 | Ca5 | 9467922 | T/TAGCCAATAAAC | CCTCTTTGGTTCCATGT GCT | AAGCGTACCTGTGGGAA TTG | 60.1 | 489 | INTERGENIC | | | | |
| CaPOPI_3277 | Ca5 | 9520123 | TAA/TA | TGAGACACATGACTCCA AGATTC | CAAATACATTTGCATCGA ATTACA | 59.2 | 484 | INTERGENIC | | | | |
| CaPOPI_3278 | Ca5 | 9520244 | CAAAAAAAAA/CAAAAAAA | TGAGACACATGACTCCA AGATTC | TCGCGTTGTTTAAAGTG TGG | 59.2 | 754 | INTERGENIC | | | | |
| CaPOPI_3279 | Ca5 | 9524095 | A/AC | GTCCCAATTGCCTTCTG AAA | ATCAAATGGCGGAAGCT ATG | 60.1 | 799 | INTERGENIC | | | | |
| CaPOPI_3280 | Ca5 | 9535247 | GTT/GTAAAAGTCTTTT | GGAAGTGTGGAGAAGTT GCC | GCTAGTCCTGCACCCCC TAT | 59.7 | 915 | INTERGENIC | | | | |

| INDEL marker IDs | Chromosomes/ unanchored scaffolds | Physical positions (bp) | InDels (*Kabuli* reference genome-CDC Frontier/PI) | Forward primers (5'-3') | Reverse primers (5'-3') | Anneling temperature (0C) | Expected amplified product size (bp) | Structural annotation | | Functional annotation | | |
|---|---|---|---|---|---|---|---|---|---|---|---|---|
| | | | | | | | | Sequence components of *kabuli* genome | *Kabuli* gene accession IDs | NCBI-KOG | TFs | NCBI-nr database |
| CaPOPI_3281 | Ca5 | 9560422 | ATTTTTT/ATTTTTTT | TGTTACAATTCCCATACCAAAAA | AGAGGGACGAAAACAGAGCA | 58.3 | 668 | INTERGENIC | | | | |
| CaPOPI_3282 | Ca5 | 9570925 | CTTT/CTTTAATTT | TGGTGAAACTGATCTCAAGCA | CTTAATCAACGTGCAGGCAA | 59.4 | 458 | INTERGENIC | | | | |
| CaPOPI_3283 | Ca5 | 9570971 | AATCATATAT/AAT | TGGTGAAACTGATCTCAAGCA | CTTAATCAACGTGCAGGCAA | 59.4 | 458 | INTERGENIC | | | | |
| CaPOPI_3284 | Ca5 | 9582075 | TT/TTAT | AGCTGACACCACACAACCAC | TCTTGAACCTGTCCCGAATC | 59.6 | 438 | DRR | Ca_23783 | | | Pectinesterase inhibitor |
| CaPOPI_3285 | Ca5 | 9583627 | TT/TTGACAAATCTGT | TTGGCTAGTAGGTGGGATGG | GGTATTTTGTGTTGCCATGTG | 59.9 | 260 | INTERGENIC | | | | |
| CaPOPI_3286 | Ca5 | 9615819 | TAAAAAA/TAAAAAAA | TTGCCTTTAACTGGTTTGGG | CGGGTATGGTTTGACTTTATCC | 60.0 | 149 | DRR | Ca_23788 | C | | Pyridine nucleotide-disulphide oxidoreductase, NAD-binding region |
| CaPOPI_3287 | Ca5 | 9620206 | ATTTT/ATTT | ACCACATGGACTGGAAAAGC | CTCTTCAGCATCAGCCATCA | 60.0 | 528 | INTERGENIC | | | | |
| CaPOPI_3288 | Ca5 | 9626102 | CCACA/CCA | AGTTTCCTCCCCGAAACCTA | TGGCAAATCGATTGACAAAA | 59.9 | 657 | INTERGENIC | | | | |
| CaPOPI_3289 | Ca5 | 9733773 | AA/AACA | TGTCACTTAAGGAACGAAAATGAA | CAAGGAAAGAATTGGATGGG | 60.0 | 295 | INTERGENIC | | | | |
| CaPOPI_3290 | Ca5 | 9767795 | ATTTTTTT/ATTTTTTTT | AATACCCAAAATGCCCCTTC | CTGAACTGGATTCACGCAAA | 60.0 | 490 | INTERGENIC | | | | |
| CaPOPI_3291 | Ca5 | 9768398 | TA/T | TTTGCGTGAATCCAGTTCAG | TTTGATACGAGTGGGTTTGC | 59.8 | 829 | INTERGENIC | | | | |
| CaPOPI_3292 | Ca5 | 9768596 | T/TTTCTAAATAAATGA | TTCTGAAAAATTGGTGCCAT | TTTGATACGAGTGGGTTTGC | 58.1 | 574 | INTERGENIC | | | | |
| CaPOPI_3293 | Ca5 | 9774862 | TAAAAAAA/TAAAAAA | TGTCTTCACAGCTCAATTGAAAA | TGAGTCTGGCTCAGTGTGCT | 59.9 | 755 | INTERGENIC | | | | |
| CaPOPI_3294 | Ca5 | 9775672 | CAAAAAA/CAAAAAAA | CAACCCTGCAATTCCAACTT | GAGTAATGGATCTCGTCCGC | 60.0 | 148 | INTERGENIC | | | | |
| CaPOPI_3295 | Ca5 | 9782845 | ACCC/ACC | AAACATGCTTAAGAAAATTCGG | CGCAAAAACACTAACCGACA | 57.5 | 689 | INTERGENIC | | | | |

| INDEL marker IDs | Chromosomes/ unanchored scaffolds | Physical positions (bp) | InDels (*Kabuli* reference genome-CDC Frontier/PI) | Forward primers (5'-3') | Reverse primers (5'-3') | Anneling temperature (0C) | Expected amplified product size (bp) | Structural annotation | | Functional annotation | | |
|---|---|---|---|---|---|---|---|---|---|---|---|---|
| | | | | | | | | Sequence components of *kabuli* genome | *Kabuli* gene accession IDs | NCBI-KOG | TFs | NCBI-nr database |
| CaPOPI_3296 | Ca5 | 9808086 | TT/TTCT | GTGACGAAGAATGCCAA CAA | GACCTCAAGTTTCACAA TGTGGT | 59.7 | 396 | INTERGENIC | | | | |
| CaPOPI_3297 | Ca5 | 9809593 | AATTCATTCATTCATTC/AATTCATTCAT TCATTCATTC | GCATTCAAATCTCCTTG GGA | CTGAGGGCTTCGAAGAA ATG | 60.0 | 542 | INTRON | Ca_20501 | O | | Peptidase M3A/M3B, thimet/oligopeptidase F |
| CaPOPI_3298 | Ca5 | 9819633 | GATA/GATAGTTCATTATA | CCAAGTGGCAAGTTTGG TTT | TCGGTGCAAGTTGAGTT CTG | 60.0 | 545 | INTRON | Ca_20501 | O | | Peptidase M3A/M3B, thimet/oligopeptidase F |
| CaPOPI_3299 | Ca5 | 9828911 | CTTTTTTTTT/CTTTTTTTT | ATGAACGCTCACACTTTT CG | TGGAGAAAACCATGGAA GGA | 58.9 | 539 | INTERGENIC | | | | |
| CaPOPI_3300 | Ca5 | 9829260 | CTTAATTA/CTTAATTAATTA | ATGAACGCTCACACTTTT CG | TGGAGAAAACCATGGAA GGA | 58.9 | 539 | INTERGENIC | | | | |
| CaPOPI_3301 | Ca5 | 9836320 | ATTTTTTTT/ATTTTTTTTT | CATGGTGAAACATGTGA TTCTG | CCTCTTCAAAGCTCAAC AACC | 58.9 | 700 | DRR | Ca_20502 | U | | Vacuolar sorting protein 9 |
| CaPOPI_3302 | Ca5 | 9836548 | T/TGTATTGTCAA | AGCATGTGGTTGTTGAG CTTT | CACACGAAATTTGAGGT GTCA | 59.8 | 408 | DRR | Ca_20502 | U | | Vacuolar sorting protein 9 |
| CaPOPI_3303 | Ca5 | 9859158 | GATA/GATAATA | TTTTGCAACGGAAGTTT GGT | ATAGCTCATCCATTGTC GGC | 60.5 | 717 | INTERGENIC | | | | |
| CaPOPI_3304 | Ca5 | 9883179 | TAA/TA | TCGTGGAAGGATAATCC ACA | CCTTGCCCCCTACATCT ACA | 58.9 | 477 | INTERGENIC | | | | |
| CaPOPI_3305 | Ca5 | 9884435 | ATTTTT/ATTTT | TGGTGGATTTGTGTTTG GAA | ATACATGCTTCGGCCCT ATG | 59.8 | 547 | INTERGENIC | | | | |
| CaPOPI_3306 | Ca5 | 9900286 | C/CA | CATTTTGCTTGCATCCAT TG | TGGAATAGAGTGTGATT GCTTTC | 60.1 | 172 | INTERGENIC | | | | |
| CaPOPI_3307 | Ca5 | 9905958 | AGA/AGACATTTTCTTGA | GTTCGGCGATGATAACA ACC | TTTGAGTCAGGGTGCTG GAT | 60.3 | 713 | DRR | Ca_20506 | | STAT | Protein kinase, catalytic domain |
| CaPOPI_3308 | Ca5 | 9917719 | GAAAAAA/GAAAAAAA | TGGACAGAATCAATGCT CCA | TGAGCTACGTCCAGTCC TCA | 60.2 | 677 | INTERGENIC | | | | |
| CaPOPI_3309 | Ca5 | 9919003 | TG/TGG | TGATTGGAGTGGGAATG GAT | TAAAGCCCTTGTGTGCC TCT | 60.1 | 395 | INTERGENIC | | | | |
| CaPOPI_3310 | Ca5 | 9953374 | CTTTTTTTT/CTTTTTTTTT | GTGACCTTACTCTTTGG CCG | AAATCACAAAATGGCTG GCT | 59.7 | 532 | INTERGENIC | | | | |

| INDEL marker IDs | Chromosomes/ unanchored scaffolds | Physical positions (bp) | InDels (*Kabuli* reference genome-CDC Frontier/PI) | Forward primers (5'-3') | Reverse primers (5'-3') | Anneling temperature (0C) | Expected amplified product size (bp) | Structural annotation | | Functional annotation | | |
|---|---|---|---|---|---|---|---|---|---|---|---|---|
| | | | | | | | | Sequence components of *kabuli* genome | *Kabuli* gene accession IDs | NCBI-KOG | TFs | NCBI-nr database |
| CaPOPI_3311 | Ca5 | 9955402 | GA/G | ACAAGGCCAGCTGTAAATGA | AAGTGGGGGAGGATCAACTT | 58.4 | 581 | INTERGENIC | | | | |
| CaPOPI_3312 | Ca5 | 9957931 | AAGGTT/AAGGTTTTATTGAGGTT | TTCTTGTGTGTGGTCTCCCA | TCGGCTATTTTCCCCTTCTT | 60.1 | 366 | INTRON | Ca_20508 | | WRKY | DNA-binding WRKY |
| CaPOPI_3313 | Ca5 | 9964705 | A/AT | GCCAAGGTAAAGAGAACGCA | TTGGCGCATAACAAAAATGA | 60.4 | 603 | INTRON | Ca_20509 | GC | HSF | UDP-glucuronosyl/UDP-glucosyltransferase |
| CaPOPI_3314 | Ca5 | 9965556 | CTT/CTTTT | TCTCAGGCGACCTGTTTTCT | TTTCCGGATCTCAAACGAAC | 60.0 | 385 | INTRON | Ca_20509 | GC | HSF | UDP-glucuronosyl/UDP-glucosyltransferase |
| CaPOPI_3315 | Ca5 | 9981523 | TA/T | AAATCCGTGATTCCAAGCTG | GCCGTGAGTCCTGATTCATT | 60.1 | 342 | DRR | Ca_20510 | GC | HSF | UDP-glucuronosyl/UDP-glucosyltransferase |
| CaPOPI_3316 | Ca5 | 9986447 | TAAAAAAA/TAAAAAAAA | TTCAAAATCAACCACGACCA | AAGGCTTGGGCCATTATACC | 59.9 | 589 | INTRON | Ca_20511 | P | | Cystathionine beta-synthase, core |
| CaPOPI_3317 | Ca5 | 10004188 | GTTGTTGTTATTGT/GTTGT | TCGCCATTTCCAACTACTCC | AATTTCCTCCTCCCACCAGT | 60.1 | 703 | INTERGENIC | | | | |
| CaPOPI_3318 | Ca5 | 10024689 | TAAA/TAAAA | GGGTCTTTAGGCCCTCTTGT | ACCTCGTGTGACTGATGATGA | 59.6 | 565 | INTERGENIC | | | | |
| CaPOPI_3319 | Ca5 | 10025602 | TAAAAAAAA/TAAAAAAA | TGAGTGCTCGACGACATTTT | AGACCTGTTGATTGTGTTTTGG | 59.4 | 700 | INTERGENIC | | | | |
| CaPOPI_3320 | Ca5 | 10054215 | ACTCT/ACTCTCT | TTGCCCAACTTGATGAAACA | GCCATAGCACCTCTGTAGGC | 60.1 | 531 | DRR | Ca_20515 | | | |
| CaPOPI_3321 | Ca5 | 10084501 | CTTGTTTTTGTTTTT/CTTGTTTTTGTTTTTGTTTTT | TTTTTGCCATTCCCATCATT | TCATGAAAATCATAAGCCACG | 60.1 | 603 | INTERGENIC | | | | |
| CaPOPI_3322 | Ca5 | 10131669 | TAAAAAAAAAAA/TAAAAAAAAAAAAAA | AATTAGACGACACGGGGTTG | CGTATCAATTTCTTTTCAAATGG | 59.9 | 635 | DRR | Ca_20519 | | | |
| CaPOPI_3323 | Ca5 | 10132207 | CTTTTTT/CTTTTT | TCCATTTGAAAAGAAATTGATACG | TCATCAACGGTCATTCAACC | 59.4 | 737 | DRR | Ca_20519 | | | |
| CaPOPI_3324 | Ca5 | 10159630 | TG/T | CCAACAGGGCAAACTGAAAT | GCACAATGGGCTTATTCGAT | 60.0 | 197 | INTERGENIC | | | | |
| CaPOPI_3325 | Ca5 | 10176653 | CA/C | CTCTCAAACGGTTGCATTGA | TGTTTCAGCTGTGATGCAAA | 59.8 | 571 | INTERGENIC | | | | |

| INDEL marker IDs | Chromosomes/ unanchored scaffolds | Physical positions (bp) | InDels (*Kabuli* reference genome-CDC Frontier/PI) | Forward primers (5'-3') | Reverse primers (5'-3') | Anneling temperature (0C) | Expected amplified product size (bp) | Structural annotation | | Functional annotation | | |
|---|---|---|---|---|---|---|---|---|---|---|---|---|
| | | | | | | | | Sequence components of *kabuli* genome | *Kabuli* gene accession IDs | NCBI-KOG | TFs | NCBI-nr database |
| CaPOPI_3326 | Ca5 | 10191175 | CTTAAATTAT/C | TCTTGTTCGTGTGTCAT AGTTCG | CATTTTTGCGTGTGTGT GCT | 60.2 | 407 | INTERGENIC | | | | |
| CaPOPI_3327 | Ca5 | 10223215 | CATAAAAA/C | AATGGGGGTACATGGGA AAT | TCAATTCCACTTGCAGC AATA | 60.1 | 547 | INTERGENIC | | | | |
| CaPOPI_3328 | Ca5 | 10289518 | A/AT | TTGTGACACTTTTTACCC GTG | GTTACGGTGTGCGGTTT TCT | 58.6 | 343 | INTERGENIC | | | | |
| CaPOPI_3329 | Ca5 | 10299950 | CAA/CAAAA | CCTTTTCAGCATTCCAAT TCA | GGTTAATTATTTTTAATG GGCGG | 60.1 | 356 | INTERGENIC | | | | |
| CaPOPI_3330 | Ca5 | 10300146 | GA/GATGATTAATAA | CCTTTTCAGCATTCCAAT TCA | GGTTAATTATTTTTAATG GGCGG | 60.1 | 356 | INTERGENIC | | | | |
| CaPOPI_3331 | Ca5 | 10331676 | ATTTTTTTT/ATTTTTTTTT | AATGCCAAAATCTCAAAC CG | GCCATGTTTGTCTTTGC ACT | 59.9 | 688 | DRR | Ca_21237 | O | E2F/DP | 14-3-3 protein |
| CaPOPI_3332 | Ca5 | 10348801 | ACC/ACCC | TGTGCCTGAAAATGATT GGA | CAGCTTCGTACACTTTG CCA | 60.0 | 615 | INTERGENIC | | | | |
| CaPOPI_3333 | Ca5 | 10389582 | CTACGTTA/CTA | TGGTTGCTTCATTGCTT GTG | GCGAAGGATATTCGACC AAG | 60.8 | 216 | INTERGENIC | | | | |
| CaPOPI_3334 | Ca5 | 10396682 | TAAAAAAAAAA/TAAAAAAAAA | TTGGAGGTCAAATCTGG GAG | AAAATTTTATGCCGCCA CAC | 60.0 | 310 | INTERGENIC | | | | |
| CaPOPI_3335 | Ca5 | 10409173 | TTGTGT/TTGTGTGT | TGGCTCTTGAAAAGAAA CGAA | TCAATGGTGCTTTTGTT GGA | 60.0 | 805 | DRR | Ca_21231 | | WRKY | Glycoside hydrolase, family 9 |
| CaPOPI_3336 | Ca5 | 10413807 | GTTTTTTTTTTTTT/GTTTTTTTTTTT | TTCTCATGCTCACTGGT TGC | TCGCATTTAACCATTCAC ACA | 60.0 | 569 | INTERGENIC | | | | |
| CaPOPI_3337 | Ca5 | 10414029 | TACCCAC/TAC | TTCTCATGCTCACTGGT TGC | TCGCATTTAACCATTCAC ACA | 60.0 | 569 | INTERGENIC | | | | |
| CaPOPI_3338 | Ca5 | 10457260 | CACAATACTC/CACAATACTCTACAATA CTC | GGGGCGAGATATTCAGA CAA | TGATTGTTCTAGTGGTC GCTCT | 60.0 | 108 | INTERGENIC | | | | |
| CaPOPI_3339 | Ca5 | 10497866 | AT/ATT | GCAAACGGGTTAATTGG AGA | GTCTTATGAGCCGAAGG TCG | 59.9 | 764 | INTERGENIC | | | | |
| CaPOPI_3340 | Ca5 | 10507484 | ATTTTTTTTT/ATTTTTTTTTT | TGCAAGTTAAAGAATAAT TTAGAGCG | GGAAACGATTGGATTCG AGA | 59.5 | 897 | INTERGENIC | | | | |

| INDEL marker IDs | Chromosomes/ unanchored scaffolds | Physical positions (bp) | InDels (*Kabuli* reference genome-CDC Frontier/PI) | Forward primers (5'-3') | Reverse primers (5'-3') | Anneling temperature (0C) | Expected amplified product size (bp) | Structural annotation | | Functional annotation | | |
|---|---|---|---|---|---|---|---|---|---|---|---|---|
| | | | | | | | | Sequence components of *kabuli* genome | *Kabuli* gene accession IDs | NCBI-KOG | TFs | NCBI-nr database |
| CaPOPI_3341 | Ca5 | 10515370 | A/AT | CGATTAAAGTCAAAGTTTCACGA | AGCAAACCAGGTTGTCCTGT | 58.5 | 560 | INTERGENIC | | | | |
| CaPOPI_3342 | Ca5 | 10516286 | TTTTTATTTTATTTT/TTTTTATTTTATTTTATTTT | ATTTGGGTTGAAGTGTTGCC | ATTCAATGCAACTTCCCTGC | 59.8 | 598 | INTERGENIC | | | | |
| CaPOPI_3343 | Ca5 | 10550017 | C/CATATTTTACCATTGAT | CTCCTCATATTGCAGGCGTT | CGGCTAAACAACAAGCCATT | 60.2 | 703 | DRR | Ca_21226 | | | |
| CaPOPI_3344 | Ca5 | 10559602 | AT/ATTTATCATTCATATGT | TGTCATTTCAAATTAGACCCAAAA | GCTTTTACGGAATGGCAAAA | 59.8 | 250 | INTERGENIC | | | | |
| CaPOPI_3345 | Ca5 | 10564601 | ATTTT/ATT | TCCAAATCCCAAGCTACCAA | GGGCCTCATTTCCCTTTAAC | 60.4 | 408 | INTRON | Ca_21225 | | | |
| CaPOPI_3346 | Ca5 | 10605600 | GT/GTT | AGTGAAACCAACGCCAAAAC | TCACTCAAAGGGCTACTGCC | 60.0 | 272 | DRR | Ca_21224 | | | |
| CaPOPI_3347 | Ca5 | 10625989 | TAAAAAAA/TAAAAAAAA | TGTAGGGGTACAGGGGAAAG | AACTTCCGTTGTTTTTGCCA | 58.9 | 165 | INTERGENIC | | | | |
| CaPOPI_3348 | Ca5 | 10679625 | TAAAAAAAA/TAAAAAAAAA | GGGATTGTTTTGAATATGAGGG | TCACACGATCATTTTGGCAT | 59.6 | 596 | INTERGENIC | | | | |
| CaPOPI_3349 | Ca5 | 10686981 | TAAAAAAAA/TAAAAAAAAAAA | GCTCCTTCTGCATCAACACA | TGCCCCACATTTTAGACCTC | 60.0 | 509 | INTERGENIC | | | | |
| CaPOPI_3350 | Ca5 | 10689102 | TAAAA/TAA | ATGGGCCACCTATTGAGACA | TGCCAAAGAAAAAGGTTTCG | 60.3 | 623 | INTERGENIC | | | | |
| CaPOPI_3351 | Ca5 | 10689622 | AGAAGCATG/AG | CGAAACCTTTTTCTTTGGCA | AACGGATTGTCCCTTCATTG | 60.2 | 418 | INTERGENIC | | | | |
| CaPOPI_3352 | Ca5 | 10693789 | CTTGTGATTGTTTTGTTGTGATTG/CTTGTGATTG | CCAATTTGAATTGTCCGTCC | TTGCAAAAGCTGGGTCAAGT | 60.2 | 406 | INTERGENIC | | | | |
| CaPOPI_3353 | Ca5 | 10995810 | AAATAATAATAATAATAATAAT/AAATAATAATAATAATAAT | ACTTCAGGTGGTCGATGTCC | GGGAGCGACCTCACACTAGA | 60.0 | 923 | INTERGENIC | | | | |
| CaPOPI_3354 | Ca5 | 11131180 | GT/GTT | CAAGTGCACAGCGGAATTTA | TCCGATTGGATTTTCAGGAC | 59.9 | 148 | INTERGENIC | | | | |
| CaPOPI_3355 | Ca5 | 11168411 | AATA/AA | TTCAAACGGAACTGATGCTTT | TTGGTTGGGGGAGTTATTGA | 59.7 | 954 | INTERGENIC | | | | |

| INDEL marker IDs | Chromosomes/ unanchored scaffolds | Physical positions (bp) | InDels (*Kabuli* reference genome-CDC Frontier/PI) | Forward primers (5'-3') | Reverse primers (5'-3') | Anneling temperature (0C) | Expected amplified product size (bp) | Structural annotation | | Functional annotation | | |
|---|---|---|---|---|---|---|---|---|---|---|---|---|
| | | | | | | | | Sequence components of *kabuli* genome | *Kabuli* gene accession IDs | NCBI-KOG | TFs | NCBI-nr database |
| CaPOPI_3356 | Ca5 | 11168801 | ACTATTAGTCTATTA/ACTATTA | TCGTTGAGGAAAAACAAACAAA | TTGGTTGGGGGAGTTATTGA | 59.6 | 557 | INTERGENIC | | | | |
| CaPOPI_3357 | Ca5 | 11168904 | GTCTCTCT/GTCTCT | TCAATAACTCCCCCAACCAA | TCACAATTGGGTTTGGATCA | 60.2 | 227 | INTERGENIC | | | | |
| CaPOPI_3358 | Ca5 | 11169035 | CG/C | TCAATAACTCCCCCAACCAA | TGACGCCGTGAAAAACATTA | 60.2 | 626 | INTERGENIC | | | | |
| CaPOPI_3359 | Ca5 | 11233017 | TAAAAA/TAAA | ATTGGACGTATCCCGAGTTG | GTTGTGGGATGAACTGACCC | 59.8 | 633 | INTERGENIC | | | | |
| CaPOPI_3360 | Ca5 | 11420785 | TTATTA/TTACTATTA | ATTAAGTGAATGGATCGCGG | AGTGTGGAAGGACGAGCAAG | 59.9 | 485 | INTERGENIC | | | | |
| CaPOPI_3361 | Ca5 | 11505972 | TTTCTTC/TTTC | GGACCTAAATTTTGCAACAACA | GAACGCGAGATATTGGGTGT | 59.0 | 199 | INTERGENIC | | | | |
| CaPOPI_3362 | Ca5 | 11628665 | CNNNNNNNNNN/CNN | TATCCCCGTCCATTAACCAA | GGGAGTGGTTGAGACCTTGA | 60.0 | 248 | INTERGENIC | | | | |
| CaPOPI_3363 | Ca5 | 11729570 | AAATAATAATAA/AAATAATAA | CACGGTCCCTAGAGCCAATA | CGGGAGATACGTAGGAGCAA | 60.1 | 672 | INTERGENIC | | | | |
| CaPOPI_3364 | Ca5 | 11878820 | CATATATATATATATATATATATATATAT/CATATATATATATATATATATATAT | TATTTTGGATTTTGGCTCGG | AGAGGGGTTTGTGTTTGACG | 59.9 | 599 | INTERGENIC | | | | |
| CaPOPI_3365 | Ca5 | 11879108 | TAA/TAAA | AAGATTTCCTCCCTCCTCCA | CTTTAACATGCGTTTCCGGT | 60.0 | 327 | INTERGENIC | | | | |
| CaPOPI_3366 | Ca5 | 11886997 | GATAATAATAATAATAATAATAA/GATAATAATAATAATAATAATAATAA | TGCCTTCGGTTTTCCTATTG | AATGTTTGGAATTCCCCCTC | 60.1 | 888 | INTERGENIC | | | | |
| CaPOPI_3367 | Ca5 | 11983861 | GTCT/GATCTTCT | CTGCTGTCAACTCCACTCCA | TCAAAATCCCCCAATAACCA | 60.0 | 713 | DRR | Ca_17106 | | | |
| CaPOPI_3368 | Ca5 | 12040897 | T/TTTTAAATGAAG | TCGATGATGTTAACTCGCAA | GCGGTGTCATTTTGTTTCAA | 57.9 | 636 | INTERGENIC | | | | |
| CaPOPI_3369 | Ca5 | 12448859 | ATTTT/ATTTTT | TTCTACCCGAAATTTGTGCC | GTCAGTCAGCCTCTCTTGCC | 59.9 | 361 | INTERGENIC | | | | |
| CaPOPI_3370 | Ca5 | 12619050 | AATATATATATATATATATATATA/AATATATATATATATATATATATATATATA | TCCTAACCTTTTGACTAATTTTTCTTC | TCAATCCTCTTAAGATATTTCCCTG | 58.9 | 685 | INTERGENIC | | | | |

| INDEL marker IDs | Chromosomes/ unanchored scaffolds | Physical positions (bp) | InDels (*Kabuli* reference genome-CDC Frontier/PI) | Forward primers (5'-3') | Reverse primers (5'-3') | Anneling temperature (0C) | Expected amplified product size (bp) | Structural annotation | | Functional annotation | | |
|---|---|---|---|---|---|---|---|---|---|---|---|---|
| | | | | | | | | Sequence components of *kabuli* genome | *Kabuli* gene accession IDs | NCBI-KOG | TFs | NCBI-nr database |
| CaPOPI_3371 | Ca5 | 12631984 | GCGTC/GC | GGTTATCACCGTGGTGCTCT | TGGCTTCTGGTTGGATTTTC | 60.0 | 648 | INTERGENIC | | | | |
| CaPOPI_3372 | Ca5 | 13636401 | A/AC | ATCGACAAACCAACATGCAA | GGGAAACTCACTTGCGGTTA | 60.0 | 735 | INTERGENIC | | | | |
| CaPOPI_3373 | Ca5 | 13636921 | CTGT/CT | TAACCGCAAGTGAGTTTCCC | GCGGTGGCTGAGTTTCTTAC | 60.1 | 387 | INTERGENIC | | | | |
| CaPOPI_3374 | Ca5 | 13714998 | TA/T | TGATGTGAGGTGACAGGCAT | GTAGCGTGGAGAGGAGCAAC | 60.1 | 374 | INTERGENIC | | | | |
| CaPOPI_3375 | Ca5 | 13825062 | ATATTATTATTATTATTATTATTATCATTATTATTATTAATATGATTATTATTATTATTATTATTATTATTATTATTATTATTATTATTATTATT/ATATTATTATTATTATTATTA | TGGTTTCATAACGTTTGTCCA | TGATTTGGTCATTTGGTATTCA | 58.9 | 823 | INTERGENIC | | | | |
| CaPOPI_3376 | Ca5 | 13855690 | TA/TAA | CCAGGTTTGCACTCAACTCA | GGGTGATGAGGACTCAGGAA | 59.9 | 573 | INTERGENIC | | | | |
| CaPOPI_3377 | Ca5 | 13858932 | A/AC | GATTCCCCATTCTTAACGCC | TCGCGAAGAGATAGAAGGGA | 60.6 | 554 | INTERGENIC | | | | |
| CaPOPI_3378 | Ca5 | 14022502 | ATTT/ATTTT | GGAAATGGATGTCTGCCAAT | TTGCAATCTCATCAACTCGG | 59.8 | 318 | INTERGENIC | | | | |
| CaPOPI_3379 | Ca5 | 14084083 | A/ACAAGGATG | TGTTGTTGTGATTGTGCCCT | TTAGCATGCACCTTGAAAGC | 60.0 | 385 | DRR | Ca_20825 | K | | Nascent polypeptide-associated complex NAC |
| CaPOPI_3380 | Ca5 | 14307259 | TTTATTATTATTATTATTATTATTATTATTATTATTATTATTATTATTATTATTAT/TTTATTATTATTATTATTATTATTATTATTATTATTATTATTATTATTATTATTATT | GCTCTCCAACAACAAATCAACA | CACTTGAAATTTTAGGCCGC | 60.2 | 798 | INTERGENIC | | | | |
| CaPOPI_3381 | Ca5 | 14330970 | ATTTTTTTTTT/ATTTTTTTTTTT | TGATTAAGAAGTTTTGATTTGCG | GGTTTGGACAATCTGCCTTG | 58.5 | 795 | INTERGENIC | | | | |
| CaPOPI_3382 | Ca5 | 14394852 | CTTTTT/CTTTT | TCTTCCACCCAGTTTTCACC | TTGAGGGCTATGTTTTTCGG | 59.9 | 337 | INTERGENIC | | | | |
| CaPOPI_3383 | Ca5 | 15176756 | AATATATATATATATATATATATATATAT/AATATATATATATATATATATATATATATAT | ACATTTAGGGCCACTTGGTG | GTTGGTTCCCTAGCACCGTA | 59.9 | 725 | INTERGENIC | | | | |
| CaPOPI_3384 | Ca5 | 15184248 | AGGGGGGG/AGGGGGGGG | TCTAACCCGGAATCCTTTGA | TGAGGATTGAACGTAACCCA | 59.5 | 300 | DRR | Ca_21631 | | | |
| CaPOPI_3385 | Ca5 | 15339602 | GAAAAAAAAAA/GAAAAAAAAA | AGGAGAGAAAAAGGGGGAAA | GGGAAACCCACCTCTTGATT | 59.1 | 211 | INTERGENIC | | | | |

| INDEL marker IDs | Chromosomes/ unanchored scaffolds | Physical positions (bp) | InDels (*Kabuli* reference genome-CDC Frontier/PI) | Forward primers (5'-3') | Reverse primers (5'-3') | Anneling temperature (0C) | Expected amplified product size (bp) | Structural annotation | | Functional annotation | | |
|---|---|---|---|---|---|---|---|---|---|---|---|---|
| | | | | | | | | Sequence components of *kabuli* genome | *Kabuli* gene accession IDs | NCBI-KOG | TFs | NCBI-nr database |
| CaPOPI_3386 | Ca5 | 15756736 | GACAC/GAC | GCGCGATTTACCAAAAATGT | TTTGAGTGGTGGGTTGTGAA | 60.0 | 431 | INTERGENIC | | | | |
| CaPOPI_3387 | Ca5 | 15773178 | GATTATTATTATTATTATTATTATTATTATTATTATTATTATTATT/GATTATTATTATTATTATTATTATTATTATTATTATTATTATTATTATTATT | GCCTTAAAATTTGGCAATGG | TCTTCGCAAATCAAAAGAATGA | 59.4 | 807 | INTERGENIC | | | | |
| CaPOPI_3388 | Ca5 | 15835478 | CAAAA/CAAA | TGCATGATTCCAGAATTCCA | AGCGGTGAGGAGAACTCTGA | 60.0 | 690 | INTERGENIC | | | | |
| CaPOPI_3389 | Ca5 | 15877436 | GC/G | GGGCTACATCCAGTCCTCAC | TTTGGCTGTAGAGGTTCCAAA | 59.5 | 474 | INTERGENIC | | | | |
| CaPOPI_3390 | Ca5 | 15918981 | TTTATTATTATTATTATTATTATTATTATTATTATTATTATTATTATTATT/TTTATTATTATTATTATTATTATTATTATTATTATTATTATTATTATTATTATTATT | CCTCCCCCATTTGTCTTCTT | GCCTGATGTTCGAATTTTTCA | 60.3 | 462 | INTERGENIC | | | | |
| CaPOPI_3391 | Ca5 | 15929348 | ACCC/ACC | TGATGACGAAGACGAAGACG | AACACATCCGGGATCCATTA | 60.0 | 416 | INTERGENIC | | | | |
| CaPOPI_3392 | Ca5 | 16319965 | TGGG/TGGGG | TTGTGTGCAGGATCCACATT | TTGACCATTGCACGAAACAT | 60.0 | 502 | INTERGENIC | | | | |
| CaPOPI_3393 | Ca5 | 16363340 | GTT/GTTT | AATCCTGGTGCACGATTTTC | ACATCGCCATTACCGAACTC | 59.9 | 445 | DRR | Ca_17921 | R | MYB_related | Pentatricopeptide repeat |
| CaPOPI_3394 | Ca5 | 16393546 | CTTTTTTTTTT/CTTTTTTTTT | CACAACTCCGCCATTACCTT | GTTGCAAGTTGCCGTGTCTA | 60.0 | 713 | INTERGENIC | | | | |
| CaPOPI_3395 | Ca5 | 16765808 | TAGA/TA | CGATCGTGCTACGTCTTCAA | TGGTGGGAATGTTGTGATTG | 60.0 | 677 | DRR | Ca_17941 | R | HSF | Pentatricopeptide repeat |
| CaPOPI_3396 | Ca5 | 16889364 | AATATATATATATATATATAT/AATATATATATATATATATATATAT | GGAGAAAATTCATTTGGCATT | CCTTTGCATGGGACAGTTTT | 58.0 | 985 | INTERGENIC | | | | |
| CaPOPI_3397 | Ca5 | 16958294 | AT/A | GGGAAGGATGGAATTTTGGT | GGTGGAGAGTGAGGTGGTGT | 60.0 | 684 | INTERGENIC | | | | |
| CaPOPI_3398 | Ca5 | 17494887 | CTGA/CTGACCTCCTATATGTGA | CTACGCCAAGAAACTGCTCC | GTACGCGTCCAGACATAGCA | 60.0 | 245 | INTERGENIC | | | | |
| CaPOPI_3399 | Ca5 | 17612584 | CATTCATAAAAATTCA/CATTCATAAAAATTCATAAAAATTCA | GTCTCTTGCTCTCCCACCAA | TTTATGTGGTGAATTGGCGA | 60.4 | 769 | INTERGENIC | | | | |
| CaPOPI_3400 | Ca5 | 17757862 | TC/TCC | AGAGTCTTTCCAAACGGCCT | TGTTGGAATCAAGTTGGTTTT | 60.2 | 815 | DRR | Ca_22474 | | | |

| INDEL marker IDs | Chromosomes/ unanchored scaffolds | Physical positions (bp) | InDels (*Kabuli* reference genome-CDC Frontier/PI) | Forward primers (5'-3') | Reverse primers (5'-3') | Anneling temperature (0C) | Expected amplified product size (bp) | Structural annotation | | Functional annotation | | |
|---|---|---|---|---|---|---|---|---|---|---|---|---|
| | | | | | | | | Sequence components of *kabuli* genome | *Kabuli* gene accession IDs | NCBI-KOG | TFs | NCBI-nr database |
| CaPOPI_3401 | Ca5 | 17760753 | TAATAAAAATAAAAATAAAAATAAAA/TAATAAAAATAAAAATAAAAATAAAAATAAAA | TGAAACCGCGTTTGATTGTA | GGTTGGGACCACTTCTCAAA | 60.1 | 168 | INTERGENIC | | | | |
| CaPOPI_3402 | Ca5 | 17876530 | CAA/CAAA | TTTCATCCATCACCGACAAA | CTCAACGAATGCCTGAAACA | 59.9 | 573 | INTERGENIC | | | | |
| CaPOPI_3403 | Ca5 | 17876693 | CACAAA/CAA | TGTTTCAGGCATTCGTTGAG | TCAATACCATTTTCACCGCA | 59.8 | 471 | INTERGENIC | | | | |
| CaPOPI_3404 | Ca5 | 18049660 | CATATATATATAT/CATATATATAT | GCACGTTGTAAAGAGTGGACTG | AAAAGGCCCACAAGAGTTGA | 59.8 | 694 | INTERGENIC | | | | |
| CaPOPI_3405 | Ca5 | 18136111 | CTTTTTTTTTTT/CTTTTTTTTTT | CGGGTCATTTTCTTTCTCCA | TTGTGGTCCTTTAAGATGGTTG | 60.0 | 327 | INTERGENIC | | | | |
| CaPOPI_3406 | Ca5 | 18176797 | TAAAAAAAAAA/TAAAAAAAAAAA | TGGGTTGAAAGTTGGATGTG | ATCCTGGTTGGGGAAGGTAG | 59.4 | 473 | INTERGENIC | | | | |
| CaPOPI_3407 | Ca5 | 18191704 | ATTTTTTTT/ATTTTTTTTT | TGAAATCCGCCTAAATCCTG | TAATTGGGGAACCAAAAAGC | 60.0 | 715 | INTERGENIC | | | | |
| CaPOPI_3408 | Ca5 | 18299239 | GTTTTTTTTT/GTTTTTTTTTT | ATCACAAATTTTCGCGACCT | CGGGATTGTGAAGAAGTGGT | 59.6 | 880 | INTERGENIC | | | | |
| CaPOPI_3409 | Ca5 | 18404984 | CATATATATATATATATATATATA/CATATATATATATATATATATATATATATA | ATTTTGGACAACAGGTTCGC | GCAAGAACATGTGAGGAGCA | 60.0 | 397 | INTERGENIC | | | | |
| CaPOPI_3410 | Ca5 | 18589511 | TTTATTATTATTATTATTATTATTATTATTATTATTATTATTATTATTATTATTATTATTATTATTATTAT/TTTATTATTATTATTATTATTATTATTATTATTATTATTATTATTATTATT | CACGTTCCCACACTCTCTCA | TCCATTTACGGCAAATTCAA | 59.9 | 829 | INTERGENIC | | | | |
| CaPOPI_3411 | Ca5 | 18683897 | AGCG/AGCGCGAGTCATCAGCTCGCG | ATGGCCGTTCTTAGTTGGTG | GTACAAAGGGCAGGGACGTA | 60.0 | 395 | INTRAGENIC | | | | |
| CaPOPI_3412 | Ca5 | 18944656 | CAAAA/CAA | TCCTGTCATGTGCTTTGAGC | CTAACCTCCCAGGACCCAAT | 60.0 | 131 | INTERGENIC | | | | |
| CaPOPI_3413 | Ca5 | 19351420 | ATTTTTTTT/ATTTTTTT | CAAGACCCGCACAAATACAA | AGGCAACACTTCTTGGTTGTC | 59.6 | 618 | INTERGENIC | | | | |
| CaPOPI_3414 | Ca5 | 19391673 | ATTTTTT/ATTTTTTT | CATGTTCGGCTGCACTTCTA | GATTTTGGGCAATTTTACGTC | 60.0 | 663 | INTERGENIC | | | | |
| CaPOPI_3415 | Ca5 | 19579350 | A/AC | ACGAACGTTGAAAATCCAGC | GTATCAAGTGCACAGCGGAA | 60.1 | 489 | INTERGENIC | | | | |

| INDEL marker IDs | Chromosomes/ unanchored scaffolds | Physical positions (bp) | InDels (*Kabuli* reference genome-CDC Frontier/PI) | Forward primers (5'-3') | Reverse primers (5'-3') | Anneling temperature (0C) | Expected amplified product size (bp) | Structural annotation | | Functional annotation | | |
|---|---|---|---|---|---|---|---|---|---|---|---|---|
| | | | | | | | | Sequence components of *kabuli* genome | *Kabuli* gene accession IDs | NCBI-KOG | TFs | NCBI-nr database |
| CaPOPI_3416 | Ca5 | 19591188 | CATATATATATATATATATATATATATATATAT/CATATATATATATATATATATATATATATATATATATATAT | CCACAGCTTCAACAAAAGCA | TGGATGATTTGATGTCACATAGG | 60.0 | 525 | INTERGENIC | | | | |
| CaPOPI_3417 | Ca5 | 19746235 | TAAAAAAA/TAAAAAAAA | ACCATATCGGCATAGTGCAT | CCGAGCCAAAATCCAAAATA | 57.9 | 259 | INTERGENIC | | | | |
| CaPOPI_3418 | Ca5 | 19815809 | CA/CATACAGTGA | TTTTGGGTTGGGATGACATT | ACCAGGAAAAGAAGAAGGGC | 60.0 | 378 | INTERGENIC | | | | |
| CaPOPI_3419 | Ca5 | 19899928 | AATATATATATATATATATATATATATATATATATATATAT/AATATATATATATATATATATATATATATATATATATATATATATATATATATAT | ATGACATGTCGTGCGATAAA | GGTTGCGTGATTGCCTTAGT | 57.6 | 483 | INTERGENIC | | | | |
| CaPOPI_3420 | Ca5 | 19922331 | A/AG | ATGTTGACTACGCGGGAGAC | CCCCTTTTTGCACATGATCT | 60.1 | 334 | INTERGENIC | | | | |
| CaPOPI_3421 | Ca5 | 20087836 | GAAGCCTATTTAAAA/GAAGCCTATTTAAAAGCCTATTTAAAA | AAACGAATTTTGAGGCCCTT | GGCTAGGCTTAGGCCATTTT | 59.9 | 697 | INTERGENIC | | | | |
| CaPOPI_3422 | Ca5 | 20430611 | AAAA/AAAATAAAAA | AGACAAAAGGGCCAACAAAA | AACACATATTAAATTGGGGCTCA | 59.6 | 193 | INTERGENIC | | | | |
| CaPOPI_3423 | Ca5 | 20455904 | TTTATTATTATTATTATTATTATTATTATTATTATTATTATTGTTATTATTGTTATTATTATTATTATTATTATTATTATTATTATTATT/TTTATTATTATTATTATTATTATTAT | TCACTTTTCCAGCCTCCACT | CCCGGTCAAACTCCAAATTA | 59.8 | 729 | DRR | Ca_18207 | | | |
| CaPOPI_3424 | Ca5 | 20547334 | AACCAACACCAACACCAACA/AACCAACACCAACACCAACACCAACA | AGGAGATGGAGCGTTGTTTG | TTGGTTAGCGTGCATCTGAG | 60.3 | 462 | INTERGENIC | | | | |
| CaPOPI_3425 | Ca5 | 20743462 | AA/AATA | AAAATAAAGCATGGCGTTGG | AGTTTGTAAACCGTGCGTCG | 60.0 | 258 | INTERGENIC | | | | |
| CaPOPI_3426 | Ca5 | 20759180 | CAATAATAATAATAATAATAATAATAATAATAATAATAAT/CAATAATAATAATAATAATAATAATAATAATAAT | AACAGGAGGTCGTTGTGACC | ATCAGGTTCCGTTGCAAGAC | 60.0 | 757 | INTERGENIC | | | | |
| CaPOPI_3427 | Ca5 | 21085449 | A/AT | TTAGGCGACCACTGTATCCC | TGAGCCACTTTGATCAGCAC | 60.0 | 758 | INTERGENIC | | | | |
| CaPOPI_3428 | Ca5 | 21106566 | TAGAA/TAGAAGAA | TGAAGGTGAGCAAGGAAAGAA | AAAAACCACAAGTGTGCTTAGG | 60.0 | 236 | INTERGENIC | | | | |
| CaPOPI_3429 | Ca5 | 21121401 | CACATA/CA | TGTCACCATTCAAGGAAGCA | TGCTACTAGGCCCTCAGCAT | 60.2 | 721 | INTERGENIC | | | | |
| CaPOPI_3430 | Ca5 | 21144099 | ATATTATTATTATTATTATTATTATTATTATTATTATTATT/ATATTATTATTATTATTATTATTATTATTATTATTATTATTATTATT | AATCGTTGGTGGCCTTAGTG | AGGGGAAAACCTTGCTCCTA | 60.0 | 484 | INTERGENIC | | | | |

| INDEL marker IDs | Chromosomes/ unanchored scaffolds | Physical positions (bp) | InDels (*Kabuli* reference genome-CDC Frontier/PI) | Forward primers (5'-3') | Reverse primers (5'-3') | Anneling temperature (0C) | Expected amplified product size (bp) | Structural annotation | | Functional annotation | | |
|---|---|---|---|---|---|---|---|---|---|---|---|---|
| | | | | | | | | Sequence components of *kabuli* genome | *Kabuli* gene accession IDs | NCBI-KOG | TFs | NCBI-nr database |
| CaPOPI_3431 | Ca5 | 21152020 | ATTTTTT/ATTTTTTT | AAATTGTGGGTTTCGCGTAG | GAGGTAGGTGGCAAAGGACA | 60.0 | 459 | INTERGENIC | | | | |
| CaPOPI_3432 | Ca5 | 21411216 | TAAAAAAAA/TAAAAAAAAAAA | CCAAAGTGGGTCAATAACGC | CTTCGACAGCCAGTTCATCA | 60.4 | 883 | INTERGENIC | | | | |
| CaPOPI_3433 | Ca5 | 21623515 | AAATTAATT/AAATT | AGGTCATTTTCATCCACCCA | ACACACCTATGGGATGCACA | 60.2 | 678 | INTERGENIC | | | | |
| CaPOPI_3434 | Ca5 | 21755455 | CATAG/CATAGTATAG | TGGCTTTGTTGATCTCATGC | ACCAATGCAAGCTGGAAGAA | 59.8 | 363 | INTERGENIC | | | | |
| CaPOPI_3435 | Ca5 | 21866481 | GA/GAA | TCCGATTGGATTTTCAGGAC | GCTTCGTTCTTCGTTCTTCG | 59.9 | 437 | INTERGENIC | | | | |
| CaPOPI_3436 | Ca5 | 21883868 | AATATATATATATATATATA/AATATATATATATATATATATA | GCTCCATTTGTGGTTCCAAT | CTATTTTGCTATGCCGCTCC | 59.8 | 405 | INTRON | Ca_24467 | | C2H2 | Glycoside hydrolase, family 28 |
| CaPOPI_3437 | Ca5 | 21899576 | TAAAAAAAAAAAA/TAAAAAAAAAAA | TGACCTGACCTGGCCTATTC | CCCAAACAAAGCCTTATGGA | 60.1 | 519 | INTERGENIC | | | | |
| CaPOPI_3438 | Ca5 | 21965060 | AG/AGG | ACGCTCAGAGTTCACTGGGT | GGCAAATCGATTGGACAAGT | 59.9 | 365 | INTERGENIC | | | | |
| CaPOPI_3439 | Ca5 | 22218019 | TAAATTAAAATTAAAATTAAA/TAAATTAAAATTAAAATTAAAATTAAAATTAAA | AAATCCTGCATTTGTGAGGG | TGCTTATTGTGGCTGCTTTG | 59.9 | 676 | INTERGENIC | | | | |
| CaPOPI_3440 | Ca5 | 22631833 | TGAGAGAGAGAGAGAGAGAGAGAGAGAGAGAGA/TGAGAGAGAGAGAGAGAGAGAGAGAGAGAGAGAGAGA | TGGAGAATTGGCTGGAGTTC | GCTTAAACTCATGGCGAAGC | 60.2 | 749 | INTRON | Ca_20079 | | | Bromodomain |
| CaPOPI_3441 | Ca5 | 22918447 | T/TG | TTATCGGCCTTATCCACTGC | GGATTCCACCAAATTCAGGA | 60.1 | 721 | INTERGENIC | | | | |
| CaPOPI_3442 | Ca5 | 22918526 | AT/ATT | TTATCGGCCTTATCCACTGC | TGAAAATCCATGAACCGTGA | 60.1 | 915 | INTERGENIC | | | | |
| CaPOPI_3443 | Ca5 | 22919631 | AG/A | GATTTGGTGGAATCTCCGAA | GCCACCGACTACTTCTCCCT | 59.9 | 336 | DRR | Ca_21962 | | | |
| CaPOPI_3444 | Ca5 | 22919722 | GTT/GT | GATTTGGTGGAATCTCCGAA | GCCACCGACTACTTCTCCCT | 59.9 | 336 | DRR | Ca_21962 | | | |
| CaPOPI_3445 | Ca5 | 23051252 | ATTTTTTTTT/ATTTTTTTTTT | TGGGACTAAAACATAGCGCC | ACGGATGAGTGACGTGACAA | 60.1 | 118 | DRR | Ca_21972 | U | | Target SNARE coiled-coil domain |

| INDEL marker IDs | Chromosomes/ unanchored scaffolds | Physical positions (bp) | InDels (*Kabuli* reference genome-CDC Frontier/PI) | Forward primers (5'-3') | Reverse primers (5'-3') | Anneling temperature (0C) | Expected amplified product size (bp) | Structural annotation | | Functional annotation | | |
|---|---|---|---|---|---|---|---|---|---|---|---|---|
| | | | | | | | | Sequence components of *kabuli* genome | *Kabuli* gene accession IDs | NCBI-KOG | TFs | NCBI-nr database |
| CaPOPI_3446 | Ca5 | 23096429 | TGTG/TGTGAGTG | GAATGTGCCGCCTATTGTTT | TGTCCTCCCTTAATTGCACA | 60.0 | 662 | INTERGENIC | | | | |
| CaPOPI_3447 | Ca5 | 23111759 | ACCCCC/ACCCCCC | TCTCACGGTTGTGAGGACTG | TAGAGAAGTGTGCACCGTGG | 59.9 | 600 | INTERGENIC | | | | |
| CaPOPI_3448 | Ca5 | 23663097 | TAATGAATGAATGAA/TAATGAATGAA | GAGGGTTTTGGGTCAGTGAA | AGTTTTCAAGGGTAGCGGGT | 59.9 | 789 | INTERGENIC | | | | |
| CaPOPI_3449 | Ca5 | 23664574 | TT/TTCCAACT | CAAGAAGGAACGAGGTGCTC | TAGCCTAGAGCAGGGGTGAA | 60.0 | 626 | INTERGENIC | | | | |
| CaPOPI_3450 | Ca5 | 23672060 | TC/TCGCCC | TGAGTAAGGGGGCTGAAATG | TGAGAGACGGTGGTTTACCC | 60.1 | 822 | CDS (FRAME SHIFT) | Ca_17683 | | | |
| CaPOPI_3451 | Ca5 | 23770283 | ATTTTT/ATTTTTT | TTGGCCTTCCTTTTACATGC | CCATCAAGCGTCTGTTGAGA | 60.1 | 882 | INTERGENIC | | | | |
| CaPOPI_3452 | Ca5 | 23812349 | TAAAAAAAAAAA/TAAAAAAAAAAAAAAAAA | ATTGCTAGCCACATTTTGGG | CGCTATGATAGGATGGTCCAA | 60.0 | 664 | INTERGENIC | | | | |
| CaPOPI_3453 | Ca5 | 23990846 | GCCC/GCC | TTCAGCTGGTTGCACCATAG | AGTTGGAAGTCGCCATTTTG | 59.9 | 261 | INTERGENIC | | | | |
| CaPOPI_3454 | Ca5 | 24097297 | AAGCTTTGGATAC/A | CAGAGCCAACACTACAGGCA | ATTTGAAGGGCCATGTGAAG | 60.0 | 329 | CDS (large-effect mutations) | Ca_17656 | S | | Protein of unknown function DUF3437 |
| CaPOPI_3455 | Ca5 | 24602434 | GTAAATAAATAAATAAATAAATAAATAA/GTAAATAAATAAATAAATAAATAAATAAATAAATAA | TCACATCAGTTAGGCCACCA | GTTTCGAACACCGGACCTTA | 60.1 | 235 | INTRON | Ca_15595 | G | bHLH | Protein kinase, catalytic domain |
| CaPOPI_3456 | Ca5 | 24881793 | TAAAAAAAAAAAA/TAAAAAAAAAAA | CCGAAAGAATAGCTGGTGGA | TTCAAAAATCTGACGGAGGG | 60.2 | 681 | INTERGENIC | | | | |
| CaPOPI_3457 | Ca5 | 24932738 | ATTTTTTTT/ATTTTTTT | TGAAGAAACTTTTCCGCTGC | TGGAAATTTTACGGTGCAGA | 60.5 | 710 | INTERGENIC | | | | |
| CaPOPI_3458 | Ca5 | 24982766 | GTATATATATATATATATATAT/GTATATATATATATATATATATATATAT | CTTGGCCAGAAAATTGGAAA | CGTTGAATACGCCTAACGGT | 60.0 | 676 | INTERGENIC | | | | |
| CaPOPI_3459 | Ca5 | 25050486 | AATATATATATATATATATATATATATATATATATATAT/AATATATATATATATATATATATATATATATATATATATATATATATAT | AGTGGCGGAGCTTGATAGAA | GGGTCCAAGTTGAACTAAACCA | 60.0 | 677 | INTERGENIC | | | | |
| CaPOPI_3460 | Ca5 | 25186556 | GAAAAAAAA/GAAAAAAAAA | TCAGAGGTACATTCGCCAGA | GTCAATTTGATAGGCCCCCT | 59.4 | 686 | DRR | Ca_15627 | L | | Smr protein/MutS2 C-terminal |

| INDEL marker IDs | Chromosomes/ unanchored scaffolds | Physical positions (bp) | InDels (*Kabuli* reference genome-CDC Frontier/PI) | Forward primers (5'-3') | Reverse primers (5'-3') | Anneling temperature (0C) | Expected amplified product size (bp) | Structural annotation | | Functional annotation | | |
|---|---|---|---|---|---|---|---|---|---|---|---|---|
| | | | | | | | | Sequence components of *kabuli* genome | *Kabuli* gene accession IDs | NCBI-KOG | TFs | NCBI-nr database |
| CaPOPI_3461 | Ca5 | 25202952 | ATTTTTTTTTTTTT/ATTTTTTTTTTTT | AACCAGGGTCTATTCCATGC | TTGGTAATGCGTTGATGGTG | 58.9 | 790 | DRR | Ca_15629 | O | | Peptidyl-prolyl cis-trans isomerase, cyclophilin-type |
| CaPOPI_3462 | Ca5 | 25250090 | ATCTT/AT | TGCTCTGACTCATTCAACCG | TCTTTAGCTAGACGCCCTGC | 60.0 | 638 | INTERGENIC | | | | |
| CaPOPI_3463 | Ca5 | 25288223 | AGAAACTTGTGATTGAGGAAAC/AGAAAC | TTGCTTGAAGACATGAACGC | TGGATGTCAAGCAGTCTTGC | 60.0 | 493 | INTERGENIC | | | | |
| CaPOPI_3464 | Ca5 | 25600693 | A/AT | CGTGCACTTTCACGACAAAT | TTGCTGTACTTGGTTGCTCG | 59.8 | 801 | INTRON | Ca_09030 | E | | Asparagine synthase |
| CaPOPI_3465 | Ca5 | 25661861 | TAAAAAAAAA/TAAAAAAAAAA | GGAGTGACAATGCCCTCAAT | TCCGCTTGGGACAATTTTAG | 59.9 | 432 | INTERGENIC | | | | |
| CaPOPI_3466 | Ca5 | 25726766 | AG/A | TCAAAGCGCGAGGAAATAGT | ATTGTTGCCCAATGGAGAAG | 60.0 | 315 | INTERGENIC | | | | |
| CaPOPI_3467 | Ca5 | 25785107 | CATA/CA | TGCCCGTGCTAATAATTTTG | AAGCACGGAAACAACGATTT | 58.7 | 580 | INTERGENIC | | | | |
| CaPOPI_3468 | Ca5 | 25803856 | TAAAAAAAAAAAAA/TAAAAAAAAAAA | ATCCGATAAATCGCATTGTG | CCCACAAGGTCCGAGAATTA | 58.5 | 203 | DRR | Ca_09020 | | | Peptidase C48, SUMO/Sentrin/Ubl1 |
| CaPOPI_3469 | Ca5 | 25803970 | TGGGGG/TGGGG | GGGGCGTTACATGTGGTATC | TGTAGGGCACATTGGGTTTT | 60.1 | 510 | DRR | Ca_09020 | | | Peptidase C48, SUMO/Sentrin/Ubl1 |
| CaPOPI_3470 | Ca5 | 25836482 | AG/A | AAGGCAACAACAAACAAGCC | ATGCCATTCAGCACATTTGA | 60.2 | 344 | INTERGENIC | | | | |
| CaPOPI_3471 | Ca5 | 25922321 | TTTATTATTATTATTATTATTATTATTATTATTATTATTATTATTATTA/TTTATTATTATTATTATTATTATTATTATTATTATTATTATTATTA | AGGCGTTTGTGTATGGAAGG | ATTGGACAAAAGGAGGACCC | 60.0 | 580 | INTERGENIC | | | | |
| CaPOPI_3472 | Ca5 | 26816643 | AATATATATATATATATATA/AATATATATATATATATATATA | CGCACAAAAATCATGTGGAC | GGACACCCCAAATTTCTCAA | 60.0 | 896 | INTERGENIC | | | | |
| CaPOPI_3473 | Ca5 | 26852877 | TGAA/TGAACGTCGTCGATGTTAGGAA | CCATTGCTTGCTCGCTAAAT | CCTTCAAAGAATTTGGTTGGG | 60.4 | 218 | INTERGENIC | | | | |
| CaPOPI_3474 | Ca5 | 26889418 | TAAAAAAAAAA/TAAAAAAAAA | TCATCGCAGGTGTACCATGT | TCCCTTTCGCAGACAAGAAT | 60.0 | 712 | INTERGENIC | | | | |
| CaPOPI_3475 | Ca5 | 26904550 | GTTTTTTTTTT/GTTTTTTTTT | CGTGGTAGACCTTCGGTGAT | TCCTTCACGAGTAAAATGTCTCA | 60.0 | 786 | INTERGENIC | | | | |

| INDEL marker IDs | Chromosomes/ unanchored scaffolds | Physical positions (bp) | InDels (*Kabuli* reference genome-CDC Frontier/PI) | Forward primers (5'-3') | Reverse primers (5'-3') | Anneling temperature (0C) | Expected amplified product size (bp) | Structural annotation | | Functional annotation | | |
|---|---|---|---|---|---|---|---|---|---|---|---|---|
| | | | | | | | | Sequence components of *kabuli* genome | *Kabuli* gene accession IDs | NCBI-KOG | TFs | NCBI-nr database |
| CaPOPI_3476 | Ca5 | 26995171 | TAG/TAGCACAAGCACTAAG | GGAGCGGGAATATGACTGAA | GCGGCAGAAGAACCTATTTG | 60.0 | 576 | DRR | Ca_08931 | R | MYB | |
| CaPOPI_3477 | Ca5 | 26996806 | A/AAAAGCAAAATTA | TCGTTTTGGGTGAATTGTGA | TGCCTCCTCGTTCCTTTTTA | 59.9 | 410 | INTERGENIC | | | | |
| CaPOPI_3478 | Ca5 | 27000467 | AA/AAGA | TTGGCACTGAATAGTGCTCG | TCGAAGCTGTGCTAATGGTG | 60.0 | 361 | INTERGENIC | | | | |
| CaPOPI_3479 | Ca5 | 27000752 | CTTTTTTTTTTTTTTT/CTTTTTTTTTTTTT | TTGGCACTGAATAGTGCTCG | CGTCCCGAATAGACGAACAT | 60.0 | 698 | INTERGENIC | | | | |
| CaPOPI_3480 | Ca5 | 27000834 | ATCTCT/ATCT | TTGGCACTGAATAGTGCTCG | CTCAGCGGGTAGAGTATCGC | 60.0 | 940 | INTERGENIC | | | | |
| CaPOPI_3481 | Ca5 | 27099226 | GAA/GAAA | TGGCAGACAAACCAAACAAA | TGGTTTTGCCATGAGACAGA | 60.1 | 406 | INTERGENIC | | | | |
| CaPOPI_3482 | Ca5 | 27364017 | AAAAAAAAATAAAA/AAAAAAAAATAAAAAAAATAAAA | CAAGAAGAGTCCATTGGGGA | GGTAAGACCACTCGATCAATCA | 60.0 | 748 | INTERGENIC | | | | |
| CaPOPI_3483 | Ca5 | 27389155 | A/AAAAAAT | CACGAGCTTCAAGAGAGCCT | ATGAGATCGTTGTTGGGAGC | 59.9 | 507 | INTERGENIC | | | | |
| CaPOPI_3484 | Ca5 | 27393057 | CAGTCATA/CAGTCATAGAGTAGTCATA | TGGTGCTGATGAATTTTCACTT | TGCAATTTCCCTTACGCTTT | 59.6 | 639 | INTERGENIC | | | | |
| CaPOPI_3485 | Ca5 | 27458409 | ATTATATATAAAATTTTATATATAAAATTTTAT/ATTATATATAAAATTTTATATATAAAATTTTATATATAAAATTTTAT | CAATTGGTCGGCAGAGTTTT | CCCACAACCATGCAATAGAA | 60.1 | 531 | INTERGENIC | | | | |
| CaPOPI_3486 | Ca5 | 27460399 | GAAAAAAAAAA/GAAAAAAAAA | GCACATGTGTTGGCAACTTT | AAAGCTCACCAAATGCCACT | 59.6 | 456 | INTERGENIC | | | | |
| CaPOPI_3487 | Ca5 | 27472549 | AG/A | GCATTATGCCGTCATCATTTT | TTCGCATTCAAAATTGTTGTT | 59.8 | 491 | INTERGENIC | | | | |
| CaPOPI_3488 | Ca5 | 27477754 | ATTTTTTTATTTTTTTTTTATTTTTTTTT/ATTTTTTTATTTTTTTTTTATTTTTTTTTTATTTTTTTT | CCGCCCTCCTTTTCTAGTCT | AAAAACCCAAAATATGCCCC | 59.8 | 678 | INTRON | Ca_08905 | | bHLH | Protein kinase, catalytic domain |
| CaPOPI_3489 | Ca5 | 27603143 | GATATATATATATATAT/GATATATATATATAT | CTTGCCTTCATGCGAATTTT | GATTTCAAAGATGCAGGGGA | 60.2 | 555 | DRR | Ca_08896 | | | NAD(P)H-quinone oxidoreductase, subunit N |
| CaPOPI_3490 | Ca5 | 27675113 | GATATATATATATATATATATATA/GATATATATATATATATATATATATATA | TTGCCACTTGATACAGCAAAA | ATCGGTGGGAATAAGAGCCT | 59.4 | 891 | INTERGENIC | | | | |

| INDEL marker IDs | Chromosomes/ unanchored scaffolds | Physical positions (bp) | InDels (*Kabuli* reference genome-CDC Frontier/PI) | Forward primers (5'-3') | Reverse primers (5'-3') | Anneling temperature (0C) | Expected amplified product size (bp) | Structural annotation | | Functional annotation | | |
|---|---|---|---|---|---|---|---|---|---|---|---|---|
| | | | | | | | | Sequence components of *kabuli* genome | *Kabuli* gene accession IDs | NCBI-KOG | TFs | NCBI-nr database |
| CaPOPI_3491 | Ca5 | 27724401 | GATATATATATATATATATA/GATATATATATATATATA | ACATCATTGGCATTGGGTTT | TGCAAAACTACAACGATTCTCC | 60.1 | 678 | INTERGENIC | | | | |
| CaPOPI_3492 | Ca5 | 27880873 | GAAAAAAAAAAA/GAAAAAAAAAA | GGCAGCTTCTGTTTTGTCAAC | CATTTCTCCTCCCAAAGGGT | 59.9 | 786 | INTERGENIC | | | | |
| CaPOPI_3493 | Ca5 | 27945125 | TAATAATAA/TAATAATAAAAATAATAA | TGGCTACATCCAGTCCCTTC | TTGGGCAAATTTAGTTCTTGTTT | 60.1 | 579 | INTERGENIC | | | | |
| CaPOPI_3494 | Ca5 | 28012407 | AATATATATATATATAT/AATATATATATATAT | TTGAAGGAAGATGAATCGTTGA | ATGCGGATACTCTCTGCCAC | 59.7 | 622 | DRR | Ca_13417 | S | | GINS complex, subunit Psf3 |
| CaPOPI_3495 | Ca5 | 28068856 | CTATATATATATATATATATA/CTATATATATATATATATATATA | GATTCGTCGGTTCACTCGTT | TGATGATGAATGTTGCACGA | 60.1 | 139 | INTERGENIC | | | | |
| CaPOPI_3496 | Ca5 | 28131912 | ATTT/ATT | TGCCATATTCAGGAGTGTGC | ATGTACATTGCATGAGGCCA | 59.7 | 850 | INTERGENIC | | | | |
| CaPOPI_3497 | Ca5 | 28188039 | T/TC | CAAGAAAAGTAACCATCGTAATGA | TGTGGATTCATGATCCTTGTG | 57.5 | 494 | INTERGENIC | | | | |
| CaPOPI_3498 | Ca5 | 28234704 | AATATATATATATATATATAT/AATATATATATATATATATAT | TTTCAAGCTTTTGATGCACG | TTTCTTTTCCAGTTCCACCG | 60.0 | 751 | INTERGENIC | | | | |
| CaPOPI_3499 | Ca5 | 28342481 | TTCAAAATCAAAATCAAAATCAAAATCA/TTCAAAATCAAAATCAAAATCA | CTCCTTAAGTGTACGGCCCA | GGACTATTTTCGCGAATGAGTAA | 60.1 | 329 | INTERGENIC | | | | |
| CaPOPI_3500 | Ca5 | 28413401 | A/AT | TTCTCCTTGAAGCTGTCGGT | ATTGCCAACATTTCCGGTAG | 60.0 | 252 | INTERGENIC | | | | |
| CaPOPI_3501 | Ca5 | 28588926 | TGGG/TGG | CAACTGCTCCTGTTCTCCAA | CTAGGAGCATGTCGCTCTCC | 59.0 | 412 | INTERGENIC | | | | |
| CaPOPI_3502 | Ca5 | 28864108 | AATATATATATATATATATATAT/AATATATATATATATATAT | AAAAGGGACGATACTTGCCA | TCCTCTCAAAAGCCCTCAAA | 59.6 | 787 | DRR | Ca_13346 | | NF-YC | Histone chaperone domain CHZ |
| CaPOPI_3503 | Ca5 | 28865362 | TTCT/TT | TTTAGTGGTGCAAGTGCATGT | CGAAAGCTAGCCATGAGGTT | 59.3 | 104 | INTERGENIC | | | | |
| CaPOPI_3504 | Ca5 | 28974386 | TAAAAAAAAAA/TAAAAAAAAAAA | GCCATAATCGAGAGGAATGG | ATTAATCCCCGTCAAGACCC | 59.5 | 527 | INTERGENIC | | | | |
| CaPOPI_3505 | Ca5 | 29247345 | ATTTTTTTTTT/ATTTTTTTTTTT | CGTGGAAAACTTCCCTCAAA | TGACGAGCGTAAGAACATGC | 60.1 | 853 | INTERGENIC | | | | |

| INDEL marker IDs | Chromosomes/ unanchored scaffolds | Physical positions (bp) | InDels (*Kabuli* reference genome-CDC Frontier/PI) | Forward primers (5'-3') | Reverse primers (5'-3') | Anneling temperature (0C) | Expected amplified product size (bp) | Structural annotation | | Functional annotation | | |
|---|---|---|---|---|---|---|---|---|---|---|---|---|
| | | | | | | | | Sequence components of *kabuli* genome | *Kabuli* gene accession IDs | NCBI-KOG | TFs | NCBI-nr database |
| CaPOPI_3506 | Ca5 | 29418887 | TTATATATATATATATA/TTATATATATATATATATA | CCCAAGCTAGAAGTGTCACGA | TTGCATGGGCATAGTGAGAG | 60.4 | 671 | DRR | Ca_16633 | G | | Trehalose-phosphatase |
| CaPOPI_3507 | Ca5 | 29601698 | ATTATTTATTTATTTAT/ATTATTTATTTATTTATTTATTTAT | ATCTTTGGTTGGTTGGTTGC | TGCATATCCATTGAGTTTCACA | 59.8 | 607 | INTERGENIC | | | | |
| CaPOPI_3508 | Ca5 | 29665140 | ATCACATTTCACATTTCACATTT/ATCACATTTCACATTTCACATTTCACATTT | TGGAAAGCGATTTATCCTGG | AGAGATCTTCTTGCGGTGGA | 60.0 | 579 | INTERGENIC | | | | |
| CaPOPI_3509 | Ca5 | 29778782 | TAAAAAAAAAA/TAAAAAAAAAAA | TTCGATTCACCCGAAGAAAT | GTGGCGTGAAACAACAAATG | 59.5 | 329 | INTERGENIC | | | | |
| CaPOPI_3510 | Ca5 | 29827143 | TAAAAAAAAAAA/TAAAAAAAAAA | ATCTGGTTAACGGATGCGAG | TTGGGACTGACAGAAAGAAAAA | 60.1 | 669 | INTERGENIC | | | | |
| CaPOPI_3511 | Ca5 | 29881935 | CT/CTCTAT | ACATGCTTGGAGAGTTGCCT | TCCATGCAATTCGTTTTTGA | 59.9 | 425 | INTRON | Ca_16670 | K | | Bromodomain |
| CaPOPI_3512 | Ca5 | 29933300 | TAAAAAAAAAAA/TAAAAAAAAAA | TGTTTCAAGCATGCATTTAATTTT | TCAGCGTACACTTTTCGACG | 59.9 | 758 | INTERGENIC | | | | |
| CaPOPI_3513 | Ca5 | 29933558 | AATATATATATATATATATATATATATAT/AATATATATATATATATATATATATATATATATATATATATAT | GACCTTTTCAAAGGATGCATTA | TCAGCGTACACTTTTCGACG | 58.3 | 369 | INTERGENIC | | | | |
| CaPOPI_3514 | Ca5 | 29933647 | GATATATATATATA/GATATATATATATATA | GACCTTTTCAAAGGATGCATTA | CGTGATGACAGACCAACACC | 58.3 | 713 | INTERGENIC | | | | |
| CaPOPI_3515 | Ca5 | 29938246 | ATT/ATTT | GCAAAAACAGCGACACTGAA | TTGTGTGGGAAAGGTGACAA | 60.0 | 764 | DRR | Ca_16674 | | | |
| CaPOPI_3516 | Ca5 | 29978017 | CTATTATTATTATTATTATTATTATTATTATTATTATTA/CTATTATTATTATTATTATTATTATTATTATTATTA | CATCCATTCTCCAAACACCT | TGTGATATGGATGAGATGAAGCA | 57.4 | 806 | INTERGENIC | | | | |
| CaPOPI_3517 | Ca5 | 30080567 | CAAAAAAAAAA/CAAAAAAAAA | AGATTTGGTGATTGGGCAAG | CATGCTAAATTGATTTTTCAGTCTTC | 59.9 | 326 | INTERGENIC | | | | |
| CaPOPI_3518 | Ca5 | 30107423 | TG/TGG | CAATTTGGGTCAATGTGCAA | GCCAACCTAGTCTCAGAAGCA | 60.4 | 475 | INTERGENIC | | | | |
| CaPOPI_3519 | Ca5 | 30114886 | TTATATATATATATATATATATATATATATA/TTATATATATATATATATATATATA | CGGGTGGCTTAGTGAGAAGT | AAACTCTTTCCGCTCACCTTT | 59.4 | 325 | INTERGENIC | | | | |
| CaPOPI_3520 | Ca5 | 30126893 | ATTTTTTTTT/ATTTTTTTTTT | GTGGCTTCCAAAGAGAAACG | TTCGGTACACAATTTTCCCC | 59.9 | 612 | INTERGENIC | | | | |

| INDEL marker IDs | Chromosomes/ unanchored scaffolds | Physical positions (bp) | InDels (*Kabuli* reference genome-CDC Frontier/PI) | Forward primers (5'-3') | Reverse primers (5'-3') | Anneling temperature (0C) | Expected amplified product size (bp) | Structural annotation | | Functional annotation | | |
|---|---|---|---|---|---|---|---|---|---|---|---|---|
| | | | | | | | | Sequence components of *kabuli* genome | *Kabuli* gene accession IDs | NCBI-KOG | TFs | NCBI-nr database |
| CaPOPI_3521 | Ca5 | 30128968 | GATTATAATTAAAAATA/GATTATAATTAAAAATAATTATAATTAAAAATA | TGTTTCTCTCTCCGCTCACA | CCGCCGACTATACCTGTTTT | 59.7 | 681 | INTERGENIC | | | | |
| CaPOPI_3522 | Ca5 | 30131050 | AATATATATATATATATATATATA/AATATATATATATATATATATA | CAAATTTCGAGCAACAATGC | AACACAAATGTTAAATTGCGAAGA | 59.3 | 571 | INTERGENIC | | | | |
| CaPOPI_3523 | Ca5 | 30135601 | CAT/CATAT | CTGGTCCTCCTTCTGTCGAG | ATCCCATCAAAATAGCTGCG | 60.0 | 426 | DRR | Ca_04690 | J | E2F/DP | Alanyl-tRNA synthetase, class IIc |
| CaPOPI_3524 | Ca5 | 30355494 | CATATATATATATATATATATA/CATATATATATATATATATATATA | CCTTTGGTTCAAGTAATGGCA | TGCACACACCAATTCCAAGT | 60.0 | 531 | INTERGENIC | | | | |
| CaPOPI_3525 | Ca5 | 30712358 | G/GGATAGAAA | TATTGGATTGGGCCAAACAT | TTCGTTGGTTTTGGGTTCTC | 60.0 | 534 | INTERGENIC | | | | |
| CaPOPI_3526 | Ca5 | 30835479 | TTATATATAT/TTATATATATAT | TCGGCGATGTAAAATTCTCG | TGATAGACAAGCCACATGCC | 61.1 | 495 | INTERGENIC | | | | |
| CaPOPI_3527 | Ca5 | 30858994 | TTT/TTTATT | ATGAAGGCTGCGTTCTGACT | GGCCCTTGGAAGTCACATAA | 60.0 | 214 | DRR | Ca_04781 | | NF-YB | Protein kinase, catalytic domain |
| CaPOPI_3528 | Ca5 | 30860511 | CAA/CAAA | ATAGCAACACTCCATCCGCT | TTAGCAACTGATCGCCACTG | 59.7 | 580 | INTERGENIC | | | | |
| CaPOPI_3529 | Ca5 | 30861801 | TTAAT/TTAATGTAAT | TTACCTACTCCGAATGCCCA | TGATGGTGGTGAGGATCAAA | 60.5 | 658 | INTERGENIC | | | | |
| CaPOPI_3530 | Ca5 | 30864475 | TAA/TAAA | TTCTGCAAGACGGTTCTGTG | CGGATGTCTACCCTCGTGTT | 60.0 | 260 | INTRON | Ca_04782 | T | bHLH | Protein kinase, catalytic domain |
| CaPOPI_3531 | Ca5 | 30868972 | AT/A | ACCATTCATTTTCTGCAGCC | ATTTCCATGTCCAACAAGGC | 60.1 | 526 | DRR | Ca_04782 | T | bHLH | Protein kinase, catalytic domain |
| CaPOPI_3532 | Ca5 | 30875873 | ATT/ATTT | GTCGTCCAGCGGTTAGGATA | AAGAGAATTAGAGAAGAGTGCGG | 60.1 | 318 | DRR | Ca_04783 | | BES1 | Glycoside hydrolase, family 17 |
| CaPOPI_3533 | Ca5 | 30886397 | CTT/CTTT | GTGACATATTGAGGGTGGGG | CTCCCCAACGTAGAACCAAA | 60.1 | 791 | INTRON | Ca_04785 | R | | WD40 repeat |
| CaPOPI_3534 | Ca5 | 30900578 | AAAA/AAAAATAAA | AGTGTCACGTGAGAGTGCCA | TCAAAGGAGGCAAATGGTAAA | 60.5 | 570 | DRR | Ca_04787 | T | MYB_related | Protein kinase, catalytic domain |
| CaPOPI_3535 | Ca5 | 30948697 | CAA/CAAA | ATATTGTTGGTAGCGTCGGC | TGTGCGGAGACATCTTATGC | 60.0 | 601 | INTERGENIC | | | | |

| INDEL marker IDs | Chromosomes/ unanchored scaffolds | Physical positions (bp) | InDels (*Kabuli* reference genome-CDC Frontier/PI) | Forward primers (5'-3') | Reverse primers (5'-3') | Anneling temperature (0C) | Expected amplified product size (bp) | Structural annotation | | Functional annotation | | |
|---|---|---|---|---|---|---|---|---|---|---|---|---|
| | | | | | | | | Sequence components of *kabuli* genome | *Kabuli* gene accession IDs | NCBI-KOG | TFs | NCBI-nr database |
| CaPOPI_3536 | Ca5 | 30959016 | CTATAT/CTAT | GGGGCATACTGCTACGATGT | TTCGCTACACCTTGACCACA | 60.0 | 639 | INTERGENIC | | | | |
| CaPOPI_3537 | Ca5 | 30959641 | AT/ATGATACT | TGTGGTCAAGGTGTAGCGAA | TGCCATGTATTCTGAGCGTC | 60.3 | 425 | INTERGENIC | | | | |
| CaPOPI_3538 | Ca5 | 30961731 | A/AC | GCCTGTGCTATCTTGGCTTC | AGGATTGCTCCAGCAAAGAA | 60.0 | 493 | INTRON | Ca_04795 | R | | Sugar/inositol transporter |
| CaPOPI_3539 | Ca5 | 30986745 | GATAAAT/GATAAATTTGTCATTAATAAAT | AAAAAGACTGCAACAAGAAAAACT | TCTCATAACTGCATCCGCTG | 57.9 | 957 | INTERGENIC | | | | |
| CaPOPI_3540 | Ca5 | 30997360 | GA/GAA | TTGGTGCCAAATTGGTGTAA | CTCTCGACTCTCAAATTCCGA | 59.8 | 419 | DRR | Ca_04797 | K | | ARID/BRIGHT DNA-binding domain |
| CaPOPI_3541 | Ca5 | 31001905 | AAT/AATATAT | CGACTCACGACAGAGGATGA | CGAAGAGGAAATTCTTGTTTGTTT | 60.0 | 836 | INTERGENIC | | | | |
| CaPOPI_3542 | Ca5 | 31038384 | ATACTACT/ATACT | TCAAAACACAATCCTTCCTTCA | TGGAATAATGACGGTGACGA | 59.6 | 625 | INTERGENIC | | | | |
| CaPOPI_3543 | Ca5 | 31045566 | AT/ATT | AACTCACTTCCACAATGCCC | CACTTGCAAATCAACCCCTT | 60.0 | 231 | INTRON | Ca_04803 | O | | Peptidase, cysteine peptidase active site |
| CaPOPI_3544 | Ca5 | 31052451 | TAAAAAAAAAA/TAAAAAAAAA | TGGATCTTGCCATGTGGATA | TCTGAAACCTGGTGGGAGAC | 59.9 | 581 | INTERGENIC | | | | |
| CaPOPI_3545 | Ca5 | 31065540 | TATAA/TATAAATAA | GTGCATCCTTGTTTTCGGTT | GTGATGACTTCAAGGGGCAT | 60.0 | 938 | INTRON | Ca_04805 | R | | Sugar/inositol transporter |
| CaPOPI_3546 | Ca5 | 31072835 | A/AC | GTTTTCATGCATTGCTCCCT | TTCAACCCAATGCACTTCAA | 60.1 | 622 | INTERGENIC | | | | |
| CaPOPI_3547 | Ca5 | 31084271 | TAAC/TAACTCAAC | GATTCGGGGCAAACAAGTAA | ATTTCACCCCAACCCTTAGC | 59.9 | 524 | DRR | Ca_04808 | | | |
| CaPOPI_3548 | Ca5 | 31094152 | AATATATA/AATATA | TTTCATGCAATTCAAAAATCG | CTGCCCCAATGTCTAATGCT | 58.7 | 438 | DRR | Ca_04810 | O | | Peptidyl-prolyl cis-trans isomerase, FKBP-type, domain |
| CaPOPI_3549 | Ca5 | 31110488 | AGGATAATG/AG | GGTGAGTCAACGGCATTTTT | CCGGGTGCAAGTTCAGTAAA | 60.0 | 279 | INTERGENIC | | | | |
| CaPOPI_3550 | Ca5 | 31121983 | TCAACTCCAACTCCAA/TCAACTCCAACTCCAACTCCAA | CTGCTGCCAAACTTCTCCTC | CCGTTTGTGCAGTGTTCATC | 60.1 | 614 | INTRON | Ca_04814 | | NF-YC | Mlo-related protein |

| INDEL marker IDs | Chromosomes/ unanchored scaffolds | Physical positions (bp) | InDels (*Kabuli* reference genome-CDC Frontier/PI) | Forward primers (5'-3') | Reverse primers (5'-3') | Anneling temperature (0C) | Expected amplified product size (bp) | Structural annotation | | Functional annotation | | |
|---|---|---|---|---|---|---|---|---|---|---|---|---|
| | | | | | | | | Sequence components of *kabuli* genome | *Kabuli* gene accession IDs | NCBI-KOG | TFs | NCBI-nr database |
| CaPOPI_3551 | Ca5 | 31188289 | TAAAA/TAAA | AAAGTGTTCGAGGCGAGAAA | GCATGAGTTCATTGGTGGTG | 60.0 | 479 | INTERGENIC | | | | |
| CaPOPI_3552 | Ca5 | 31193802 | T/TTC | TTAATTCCAATGCGGAAAGC | CCTTTCCAATGCACCAAACT | 60.0 | 367 | DRR | Ca_04825 | R | WOX | High mobility group, HMG1/HMG2 |
| CaPOPI_3553 | Ca5 | 31197193 | TA/T | TTGGCTAAATACCTCTCGTTCC | GTCGGTTGAGCTAGGGCTTA | 59.6 | 488 | INTERGENIC | | | | |
| CaPOPI_3554 | Ca5 | 31229749 | GATATATA/GATATATATA | TCGGCTCCTTTTCATTTACG | CTACCGATTCGCAATCCAAT | 60.2 | 272 | INTERGENIC | | | | |
| CaPOPI_3555 | Ca5 | 31241531 | CTTTTTTTT/CTTTTTTTTTT | CGATGAACGCAAACTCAAGA | TAATCAACCAAAACCCCCAA | 60.0 | 689 | INTRON | Ca_04830 | R | | Bromodomain |
| CaPOPI_3556 | Ca5 | 31255829 | TG/T | AGACGGCAACAAATACTGGC | TGTCTATTTTGGTGTGCGTAAAA | 60.1 | 673 | INTERGENIC | | | | |
| CaPOPI_3557 | Ca5 | 31256076 | TA/TAA | TTTTTGTTGTAACGATGGCTAA | CGCTCAAACACACGTTCATT | 57.5 | 672 | INTERGENIC | | | | |
| CaPOPI_3558 | Ca5 | 31264749 | TTATATATATATAT/TTATATATATATATAT | CTGGAGCTGGTGTGTCATTG | GGCCATTTGCAAAAACTTGT | 60.3 | 374 | DRR | Ca_04832 | T | WRKY | Protein kinase, catalytic domain |
| CaPOPI_3559 | Ca5 | 31311316 | CTTTT/CTTTTT | TTGGCACATATGGGGTTTTT | CCAACGCATAAGTCATGTGG | 60.1 | 730 | DRR | Ca_04834 | | Nin-like | Phox/Bem1p |
| CaPOPI_3560 | Ca5 | 31333742 | CAA/CA | GCGTTTGGGGTGACTTACAT | TGGTTCGTTCGTGTGTTGAT | 59.9 | 780 | INTRON | Ca_04835 | P | | Catalase |
| CaPOPI_3561 | Ca5 | 31334829 | TATTATAAAAATT/TATT | AATGTCCCCAACCAATTTGA | ATGGGTGATAAATACCTGTTTTG | 60.0 | 774 | INTERGENIC | | | | |
| CaPOPI_3562 | Ca5 | 31375160 | ATTT/ATT | TTGTCCGTTTGTTACGTGGA | TCCAAAGCCTGAAGCTCAAT | 60.0 | 429 | INTERGENIC | | | | |
| CaPOPI_3563 | Ca5 | 31412254 | AATTA/AA | CACGTGTCCATCAAAATTGC | TGCAAGCGAATCAAACAAAA | 60.0 | 721 | INTERGENIC | | | | |
| CaPOPI_3564 | Ca5 | 31435124 | CTTTTT/CTTTTTT | TTGAAATCCGATTGGTGACA | ATTTGGTGTCCCTTGTCTCG | 59.9 | 538 | INTERGENIC | | | | |
| CaPOPI_3565 | Ca5 | 31481808 | TG/T | GATGGTTTGGTTGTTTTGGG | ACTACAGTCCACCGGCCTTA | 60.1 | 507 | INTERGENIC | | | | |

| **INDEL marker IDs** | **Chromosomes/ unanchored scaffolds** | **Physical positions (bp)** | **InDels (*Kabuli* reference genome-CDC Frontier/PI)** | **Forward primers (5'-3')** | **Reverse primers (5'-3')** | **Anneling temperature (0C)** | **Expected amplified product size (bp)** | **Structural annotation** | | **Functional annotation** | | |
|---|---|---|---|---|---|---|---|---|---|---|---|---|
| | | | | | | | | **Sequence components of *kabuli* genome** | ***Kabuli* gene accession IDs** | **NCBI-KOG** | **TFs** | **NCBI-nr database** |
| CaPOPI_3566 | Ca5 | 31526667 | CTTTTTT/CTTTTTTT | GGATGTTTATTGGTCATTTTTCG | TTGTGAAATGCACGAGAGAA | 59.6 | 254 | INTERGENIC | | | | |
| CaPOPI_3567 | Ca5 | 31532842 | CTT/CTTT | AAGCAAAAGGGAGGGATGAT | CCTGAAAACTCAACCCAAGG | 59.9 | 577 | INTERGENIC | | | | |
| CaPOPI_3568 | Ca5 | 31539844 | GTTTT/GTT | TGTTTGAAGGACCCAATGAA | ACCGGAGAGGGCATAAAAGT | 58.9 | 146 | INTERGENIC | | | | |
| CaPOPI_3569 | Ca5 | 31585638 | TTTAAATATTAAATATTAAAT/TTTAAATATTAAATATTAAATATTAAAT | CACAGAATTTGGTGGCATTG | TGATCATGCGTGGATTTTGT | 60.0 | 745 | DRR | Ca_04861 | R | | Tetracycline resistance protein, TetA |
| CaPOPI_3570 | Ca5 | 31609524 | T/TGTATATGAA | GCAGGCTGCCTATTTGAGTC | ATGTAGCAAACTTGCGGGTC | 60.0 | 627 | DRR | Ca_04863 | | | |
| CaPOPI_3571 | Ca5 | 31611802 | TGGG/TGG | TTCTTACCTGGGCTGAATGG | GCAGGCTGTTTCACAACTGA | 60.1 | 689 | INTERGENIC | | | | |
| CaPOPI_3572 | Ca5 | 31621509 | CTT/CTGTATATCTACTTTT | ATCCGAGTTTGCGTATGTCC | GGGACGTGGAGATTGTTGAT | 60.0 | 635 | INTERGENIC | | | | |
| CaPOPI_3573 | Ca5 | 31694006 | GGAATGAATGAATGAATGA/GGAATGAATGAATGAATGAATGAATGA | CCAAGCATGCTCATCAGAAA | TGAGAATGGGATCAGGGAAG | 59.9 | 484 | INTRON | Ca_04872 | | G2-like | Peptidase S8/S53, subtilisin/kexin/sedolisin |
| CaPOPI_3574 | Ca5 | 31710056 | CA/CAGA | GCATTGAGTCAAACAGCCAA | TTTTGCTTGAAGCCAGGTCT | 59.8 | 353 | DRR | Ca_04874 | | | |
| CaPOPI_3575 | Ca5 | 31716024 | AAA/AAATAA | CAATGAAACCCTGCAACCTT | CACGAGAATCTCCCCTACCA | 60.0 | 717 | INTRON | Ca_04875 | | FAR1 | Lipase, GDSL |
| CaPOPI_3576 | Ca5 | 31732127 | CTGT/CT | TGCTAGCTTCTTCCCACGTT | AAAACGAACAAAAATAACATTCG | 60.0 | 306 | INTERGENIC | | | | |
| CaPOPI_3577 | Ca5 | 31754975 | TTAT/TT | TTTGGTGCGAGTACACTTCC | TGAGGATTTGGTTGAGAGGTG | 58.8 | 261 | INTERGENIC | | | | |
| CaPOPI_3578 | Ca5 | 31755077 | CTTT/CT | TACACTTCCGTGCACCGTTA | TGAGGATTTGGTTGAGAGGTG | 60.2 | 250 | INTERGENIC | | | | |
| CaPOPI_3579 | Ca5 | 31759396 | G/GC | CCACAAGCAGCACAAAGTGT | GGATTAGCGCGTCTGACTTC | 59.9 | 490 | DRR | Ca_04880 | O | MYB_related | SANT domain, DNA binding |
| CaPOPI_3580 | Ca5 | 31772153 | AATTAATATTAATAT/AATTAATAT | CTTGTGCCCACACACATTTT | TCCATTGTTGTTGTCGTCGT | 59.5 | 498 | INTERGENIC | | | | |

| INDEL marker IDs | Chromosomes/ unanchored scaffolds | Physical positions (bp) | InDels (*Kabuli* reference genome-CDC Frontier/PI) | Forward primers (5'-3') | Reverse primers (5'-3') | Anneling temperature (0C) | Expected amplified product size (bp) | Structural annotation | | Functional annotation | | |
|---|---|---|---|---|---|---|---|---|---|---|---|---|
| | | | | | | | | Sequence components of *kabuli* genome | *Kabuli* gene accession IDs | NCBI-KOG | TFs | NCBI-nr database |
| CaPOPI_3581 | Ca5 | 31787653 | CA/C | GAACCCCAAACACGCTTCTA | GCTTTAACGGCGCATATGTT | 60.1 | 626 | DRR | Ca_04883 | | | |
| CaPOPI_3582 | Ca5 | 31805131 | GAAAAAA/GAAAAAAA | TGCAGTGGATAGATAACGGG | TCTTTTGGCAGTGTCAGCAC | 58.6 | 919 | INTERGENIC | | | | |
| CaPOPI_3583 | Ca5 | 31807859 | TCC/TC | AATCATACACGGTGGGTCGT | TTGCATTGTTCACATGCTCA | 60.1 | 224 | INTERGENIC | | | | |
| CaPOPI_3584 | Ca5 | 31809508 | TAAAAAAAAA/TAAAAAAAA | TCTGTTCTCGACGTTGTTCG | CAAAAGAACATGGACCACGA | 60.0 | 637 | INTRON | Ca_04886 | Z | | Tubulin |
| CaPOPI_3585 | Ca5 | 31835201 | TAAAAAAAAAA/TAAAAAAA | AGAGTGCATCCTATGCGACC | CCAACAAAAGAGGGGGAGTT | 60.2 | 211 | INTERGENIC | | | | |
| CaPOPI_3586 | Ca5 | 31844171 | CGG/CG | CAAGAACAGGGCCGTTAAGA | CGGTAGCCTAATGAACTCGC | 60.2 | 371 | INTERGENIC | | | | |
| CaPOPI_3587 | Ca5 | 31869532 | TAA/TA | GCTGGAGGCACTTCAAGAGT | TTGGTCATTGAAATAATTGGGTT | 59.6 | 788 | DRR | Ca_04893 | | | |
| CaPOPI_3588 | Ca5 | 31883810 | AAAATTA/AAAATTATAAATTA | TGTGGTGTTGTTAAATCAGGATG | TGATTGCGGGATTTTATGGT | 59.8 | 780 | INTERGENIC | | | | |
| CaPOPI_3589 | Ca5 | 31886554 | AATA/AA | AGGCTTCAAGGTCAGACCAG | AATGGCTGGGCAGTAAAACA | 59.5 | 235 | INTERGENIC | | | | |
| CaPOPI_3590 | Ca5 | 31887987 | ATTTTTTTTT/ATTTTTTTTTTTT | GTGAGGTGTGACTGGTGCAT | TTCAAGGACATGAATTTTGCC | 59.6 | 722 | INTERGENIC | | | | |
| CaPOPI_3591 | Ca5 | 31932559 | AAACAACA/AAACAACAACA | CAAATGGGAGGAAAAACCAA | TCATCAAAAGGGAAACCTGC | 59.8 | 284 | DRR | Ca_04898 | | | Protein of unknown function DUF869, plant |
| CaPOPI_3592 | Ca5 | 31941021 | AATATATATATATATATATATATATATATA/AATATATATATATATATATATATATATATATATA | CGTAAACAAGGCCCAAACAC | GCCCCCTTCAATAAATCCAT | 60.4 | 568 | INTERGENIC | | | | |
| CaPOPI_3593 | Ca5 | 31967141 | AATATATATATAT/AATATATATATATATAT | ACGGGTCATTTTAAACACGC | TTCGCCACCAAGTCATGATA | 59.9 | 550 | DRR | Ca_04900 | | | Glyoxal oxidase, N-terminal |
| CaPOPI_3594 | Ca5 | 31970233 | CATATATATA/CATATATATATA | CGCTGTCACTAGGGAATGTGT | CTGTTTTTACCACCCAAGCG | 60.2 | 435 | INTERGENIC | | | | |
| CaPOPI_3595 | Ca5 | 31974245 | GTTTTTTTTTTT/GTTTTTTTT | GGCTAAGTTTTCTTTGGGGG | GCACTCCATGAATCAAAGCA | 59.9 | 696 | INTERGENIC | | | | |

| INDEL marker IDs | Chromosomes/ unanchored scaffolds | Physical positions (bp) | InDels (*Kabuli* reference genome-CDC Frontier/PI) | Forward primers (5'-3') | Reverse primers (5'-3') | Anneling temperature (0C) | Expected amplified product size (bp) | Structural annotation | | Functional annotation | | |
|---|---|---|---|---|---|---|---|---|---|---|---|---|
| | | | | | | | | Sequence components of *kabuli* genome | *Kabuli* gene accession IDs | NCBI-KOG | TFs | NCBI-nr database |
| CaPOPI_3596 | Ca5 | 31999312 | GA/G | AACGGTTATTTTCCGGCTTT | TTACAACAGCACGTGCAACC | 59.8 | 433 | DRR | Ca_04903 | | S1Fa-like | Protein kinase, catalytic domain |
| CaPOPI_3597 | Ca5 | 32021738 | CAAA/CA | CTTGGGAAGGTCACAACGAT | TACTGTCAGCATGGCACCTC | 60.0 | 659 | INTRON | Ca_04906 | S | | Protein of unknown function DUF726 |
| CaPOPI_3598 | Ca5 | 32041461 | CTT/CT | GAAGTAGCGGTCATGCCAAT | ATAGTGGAGGAGCTGGAGCA | 60.1 | 338 | DRR | Ca_04909 | K | MIKC | Transcription factor, MADS-box |
| CaPOPI_3599 | Ca5 | 32044892 | T/TC | CTGTTTTTCACCGCCAAAAT | TCTTCCCTAATCACGCATCC | 60.0 | 763 | INTERGENIC | | | | |
| CaPOPI_3600 | Ca5 | 32062989 | ATATTATTATTATTATT/ATATTATTATTATT | GAGGGAGAGCTCATGTGGAA | TTCGTGCATGCAAATTGAGT | 60.3 | 376 | INTERGENIC | | | | |
| CaPOPI_3601 | Ca5 | 32068203 | TT/TTGATAAATGAAT | TGAAAAATGGAATCCCACTAGC | GAGCATTTAGTGGAGCGGAG | 60.3 | 683 | INTERGENIC | | | | |
| CaPOPI_3602 | Ca5 | 32087284 | TAA/TAAA | GGATGTCACATTTTGGGACC | TTGAGTTATACCATGCTTCTCAACA | 60.0 | 553 | DRR | Ca_04911 | K | LBD | Transcription initiation factor IIF, beta subunit |
| CaPOPI_3603 | Ca5 | 32087748 | TAAAAAA/TAAAAAAAAAAA | TTCTAAATGTTGAGAAGCATGG | TGTGTGTGGTGGGTCAATCT | 57.1 | 630 | DRR | Ca_04911 | K | LBD | Transcription initiation factor IIF, beta subunit |
| CaPOPI_3604 | Ca5 | 32096296 | AGAT/AGATGAT | TTGTGGATACCAAAAGAATCTCAA | CACACCACCTCCTCCTGTTT | 59.9 | 648 | INTERGENIC | | | | |
| CaPOPI_3605 | Ca5 | 32100080 | CATAT/CATATATAT | GTGGCATCAGGAATAGCGTT | CACCTAAAGGTCCCAACCAA | 60.1 | 480 | INTRON | Ca_04912 | | ERF | Drug/metabolite transporter |
| CaPOPI_3606 | Ca5 | 32149202 | TCAC/TCACAC | AAATTCCATTTTCGGGAACC | GGTGGGAATCCTTGGAAAAT | 60.0 | 466 | DRR | Ca_04917 | G | C2H2 | Tetrapyrrole methylase |
| CaPOPI_3607 | Ca5 | 32163070 | CCTTCTT/CCTTCTTCTT | CACGTGCCTTTTCTCACTCA | GTTGATCTTTACGGCCTCCA | 60.0 | 552 | INTRON | Ca_04917 | G | C2H2 | Tetrapyrrole methylase |
| CaPOPI_3608 | Ca5 | 32196357 | TC/TCC | AGCATTTCCAAAGGGGACTC | CCAAAACGGTGGACTGGTTA | 60.4 | 779 | INTRON | Ca_04919 | K | TALE | Homeobox |
| CaPOPI_3609 | Ca5 | 32201348 | A/AC | ATGGAGTTGGCGTTTCTGTT | TTCTCCTGCCTCTCTCCAAA | 59.6 | 772 | INTRON | Ca_04920 | M | | Glycosyl transferase, group 1 |
| CaPOPI_3610 | Ca5 | 32226752 | ACC/ACCC | TTGGGCGACGTTACTTTTAGA | GGAGTGGGTTATTCTTTTGGC | 59.8 | 189 | DRR | Ca_04923 | T | WRKY | Protein kinase, catalytic domain |

| INDEL marker IDs | Chromosomes/ unanchored scaffolds | Physical positions (bp) | InDels (*Kabuli* reference genome-CDC Frontier/PI) | Forward primers (5'-3') | Reverse primers (5'-3') | Anneling temperature (0C) | Expected amplified product size (bp) | Structural annotation | | Functional annotation | | |
|---|---|---|---|---|---|---|---|---|---|---|---|---|
| | | | | | | | | Sequence components of *kabuli* genome | *Kabuli* gene accession IDs | NCBI-KOG | TFs | NCBI-nr database |
| CaPOPI_3611 | Ca5 | 32241464 | GTAATAATAATAATA/GTAATAATAATAATAATAATA | ATCATGGCCAAAAGAGGTTG | TCGGTTTGGTTTGGGTTAAA | 59.9 | 724 | INTERGENIC | | | | |
| CaPOPI_3612 | Ca5 | 32324347 | GCTCTCTCTCTCTCTCTC/GCTCTCTCTCTCTCTC | TGATGCCACGTGAGAAAGAG | CTGTTCAAGATGCAGGCAAA | 60.0 | 899 | DRR | Ca_04930 | I | bHLH | Cellular retinaldehyde binding/alpha-tocopherol transport |
| CaPOPI_3613 | Ca5 | 32349699 | TAAAAA/TAAAAAA | AGGGGCCATGTGTAAGTACG | CATTAACACCGATGAGGGCT | 59.9 | 566 | DRR | Ca_04933 | | | |
| CaPOPI_3614 | Ca5 | 32435142 | TC/T | CCATCGCAAGGCTGTAAAAT | ACATGTCAAGTGGGGGTGTT | 60.1 | 873 | INTERGENIC | | | | |
| CaPOPI_3615 | Ca5 | 32540371 | TA/T | TGGTTGCTTGTTGGTTTCTG | GTGAAAGAGCGTTAGAGCGG | 59.7 | 706 | INTERGENIC | | | | |
| CaPOPI_3616 | Ca5 | 32649599 | ATTTTTTTTTTT/ATTTTTTTTTT | TCGGACAAGTTAGCAAAAACAA | CCGTATTTTTGAATATCAGTGGC | 59.8 | 561 | INTERGENIC | | | | |
| CaPOPI_3617 | Ca5 | 32745764 | TAAA/TAAAA | AGGCATCACATTGAGGGACT | CCTCGGTGACCCTGAATAAA | 59.5 | 148 | INTERGENIC | | | | |
| CaPOPI_3618 | Ca5 | 32848829 | GAAAAAAAAAA/GAAAAAAAAAAA | AACATTTACCAGTCCAGCGG | TCACCGTATGCAAAGACGAG | 60.0 | 848 | INTERGENIC | | | | |
| CaPOPI_3619 | Ca5 | 32912336 | AATATATATATATATATATATATATAT/AATATATATATATATATATATATATATATATATATATAT | ACCCCCTCACCTCCTTATTG | TTTTCTTGCGTCACTTGTTGA | 60.2 | 499 | INTERGENIC | | | | |
| CaPOPI_3620 | Ca5 | 33146690 | TAAAAAAAAA/TAAAAAAAAAA | TACCCGAACAACTTTGACCC | TGCTTTCGTGATTCCAACAA | 59.8 | 731 | DRR | Ca_05009 | D | YABBY | Protein kinase, catalytic domain |
| CaPOPI_3621 | Ca5 | 33444959 | GATATA/GATATATA | TTATGGAGGCCACATTCACA | GAAATTAAGGCCTCTTGATCTTTTT | 59.9 | 961 | DRR | Ca_01912 | O | MYB_related | SANT domain, DNA binding |
| CaPOPI_3622 | Ca5 | 33456475 | TTATGTTAAATATGTTAA/TTATGTTAAATATGTTAAATATGTTAA | GATGAAGTGCTGATCAGAGGC | ACCAGGAAACATTGCCAGAC | 60.0 | 789 | DRR | Ca_01911 | O | MYB_related | SANT domain, DNA binding |
| CaPOPI_3623 | Ca5 | 33457018 | TTTTATTTATTTATTTA/TTTTATTTATTTATTTATTTA | GTCTGGCAATGTTTCCTGGT | GGTGGTGTATGTATATCTTTACGGG | 60.0 | 430 | DRR | Ca_01911 | O | MYB_related | SANT domain, DNA binding |
| CaPOPI_3624 | Ca5 | 33457459 | ATT/ATTT | TCTTCAAATTAAAATTCCACCCA | GTTGAAATTTTGGGTGTCGG | 59.7 | 750 | DRR | Ca_01911 | O | MYB_related | SANT domain, DNA binding |
| CaPOPI_3625 | Ca5 | 33457924 | ATTTTTTT/ATTTTTTTT | CCGACACCCAAAATTTCAAC | GCCCTCAGATGAGAGTCAGG | 60.2 | 680 | DRR | Ca_01911 | O | MYB_related | SANT domain, DNA binding |

| INDEL marker IDs | Chromosomes/ unanchored scaffolds | Physical positions (bp) | InDels (*Kabuli* reference genome-CDC Frontier/PI) | Forward primers (5'-3') | Reverse primers (5'-3') | Anneling temperature (0C) | Expected amplified product size (bp) | Structural annotation | | Functional annotation | | |
|---|---|---|---|---|---|---|---|---|---|---|---|---|
| | | | | | | | | Sequence components of *kabuli* genome | *Kabuli* gene accession IDs | NCBI-KOG | TFs | NCBI-nr database |
| CaPOPI_3626 | Ca5 | 33600042 | CT/C | TAACCATTCCACAGCCACAA | CTTAGAGGTCGCTTCAACGG | 60.0 | 680 | DRR | Ca_01896 | | | |
| CaPOPI_3627 | Ca5 | 33687861 | TTATATATATATATAT/TTATATATATATAT | GACTTCCTAAGGAGGGTGGC | ATTTCCACGGCAAATTCGTA | 60.1 | 381 | DRR | Ca_01887 | | | |
| CaPOPI_3628 | Ca5 | 34072036 | CCCAAAGCAGAT/C | CATTGGTGTTGTCTGTTCGG | ACCGTTCCGTTTGTTCTGAC | 60.0 | 262 | INTERGENIC | | | | |
| CaPOPI_3629 | Ca5 | 34164848 | C/CA | CTTCCAGGGGATGCAAGATA | CGTAGGCAATAAGACACGCA | 60.0 | 377 | DRR | Ca_01832 | | NF-YB | FAS1 domain |
| CaPOPI_3630 | Ca5 | 34629428 | A/ACTTAAAAAGTTG | TTAGGTTGAGGCAGTTCGCT | TTTGGGTTTCGACCATAAAAA | 60.0 | 587 | INTERGENIC | | | | |
| CaPOPI_3631 | Ca5 | 34698063 | GATAATAATAATAATAATAATAATAATAATAATAA/GATAATAATAATAATAATAATAATAATAATAATAA | ATGGCTTTTCCTACCTTCGG | ACCAAAACAAAAACCAACGG | 60.4 | 560 | INTERGENIC | | | | |
| CaPOPI_3632 | Ca5 | 34871969 | GA/GAAATGAA | TGACGTTGCATTCACCAAAT | AGGGAGGACGAAAATGACCT | 60.0 | 609 | INTERGENIC | | | | |
| CaPOPI_3633 | Ca5 | 35072594 | GATATATATATATATATATATAT/GATATATATATATATATATATATATATATAT | GGTAGAGGCACATGGGAAAA | CTTGAACTCTCACATGCATGAAC | 59.9 | 760 | INTERGENIC | | | | |
| CaPOPI_3634 | Ca5 | 35104483 | TTATATATATATATATATAT/TTATATATATATATATATATATATAT | GCTTCACCCACCTTCCACTA | TCTTGTTTTAGAGTGTTGCAAATGA | 60.1 | 833 | INTERGENIC | | | | |
| CaPOPI_3635 | Ca5 | 35190711 | GAAAA/GAAAAA | TGTCACTTTTTGTTTCGTCCT | TGAAATGTTGCCCACTGTGT | 57.4 | 454 | DRR | Ca_01716 | O | | Proteasome component (PCI) domain |
| CaPOPI_3636 | Ca5 | 35231327 | ATTTTTTTT/ATTTTTTTTT | CACGAACCATAGCATGGGA | ATTGGATCGGGCCTAGTGTA | 60.5 | 834 | INTERGENIC | | | | |
| CaPOPI_3637 | Ca5 | 35328160 | ATTTTTTTTT/ATTTTTTTTTT | ACCACGTGACCCCTTAACTT | TGATGAAATTACGTTATATTGAGGATG | 58.4 | 204 | INTERGENIC | | | | |
| CaPOPI_3638 | Ca5 | 35599782 | TTATATATATATATATATATATA/TTATATATATATATATATA | ATTTCCAACTGTTTGGGCAG | TACTAGATCGCGTCCCACG | 60.0 | 665 | INTERGENIC | | | | |
| CaPOPI_3639 | Ca5 | 35607633 | CAATAATAATAATAATAATAATAATAATAAAAATAATAATAATAATAA/CAATAATAATAATAATAATAATAATAATAATAATAAAAATAATAATAATAATAA | TCGTTTACAGCATCGACCAA | GAACAATCTTTGCACGTCCC | 60.3 | 531 | INTERGENIC | | | | |
| CaPOPI_3640 | Ca5 | 35734976 | CNNNNNNNNNN/CNNN | TTTTGGGCAACTTGGTTAGG | TCAATATAAACGGCGAAGGG | 60.0 | 570 | INTERGENIC | | | | |

| INDEL marker IDs | Chromosomes/ unanchored scaffolds | Physical positions (bp) | InDels (*Kabuli* reference genome-CDC Frontier/PI) | Forward primers (5'-3') | Reverse primers (5'-3') | Anneling temperature (0C) | Expected amplified product size (bp) | Structural annotation | | Functional annotation | | |
|---|---|---|---|---|---|---|---|---|---|---|---|---|
| | | | | | | | | Sequence components of *kabuli* genome | *Kabuli* gene accession IDs | NCBI-KOG | TFs | NCBI-nr database |
| CaPOPI_3641 | Ca5 | 36120649 | GA/GATAA | GCTTTCAGGCCAGACTATGC | AGGGGTAGGAATAGGCCAGA | 60.0 | 118 | INTERGENIC | | | | |
| CaPOPI_3642 | Ca5 | 36176137 | AATATATATATAT/AATATATATATATATAT | TCATTGACATTTTGTCCCCA | TTGGTTGGAAAGGTTTGTCA | 59.8 | 617 | DRR | Ca_01608 | | | |
| CaPOPI_3643 | Ca5 | 36212540 | GTT/GTTAGATT | TTTCATTGTTTCCGTCACCA | TTGAGTCAATGCAAGAGTCCTT | 59.9 | 874 | DRR | Ca_01603 | I | B3 | Lecithin:cholesterol acyltransferase |
| CaPOPI_3644 | Ca5 | 36214320 | G/GC | TACCAACCCAACAATGCAGA | TGGTGATGGTGATGGAACTG | 60.0 | 475 | DRR | Ca_01603 | I | B3 | Lecithin:cholesterol acyltransferase |
| CaPOPI_3645 | Ca5 | 36308359 | TTTATT/TTTATTGGTATTATTATTATT | GTTTGTGGCGGATTTTGTTT | CCGATACAACGGAATTGTCC | 59.8 | 423 | DRR | Ca_01593 | Z | | Interferon-related developmental regulator, C-terminal |
| CaPOPI_3646 | Ca5 | 36583877 | CATATATATATATATATAT/CATATATATATATATATATATAT | TCACTTTTAGGATCCGTTTGA | CACAATGGACCATGCACTTC | 57.4 | 809 | INTERGENIC | | | | |
| CaPOPI_3647 | Ca5 | 36742914 | CTTTTTTTTTTT/CTTTTTTTTTT | CAAATGTTGGGCTTGGAGTC | ATTTCTTTGGGGATTTTGGG | 60.5 | 351 | INTERGENIC | | | | |
| CaPOPI_3648 | Ca5 | 36897567 | TTATATATATATATATATATATATATAT/TTATATATATATATATATATATATATATAT | AGTCCCCATCTCAACTCGTG | TGATTTGGACGGAGAAAACC | 60.1 | 383 | INTERGENIC | | | | |
| CaPOPI_3649 | Ca5 | 37015456 | TTATATATATATATA/TTATATATATATATATA | TTGCATTTTGGTCTCTCTCTCA | TGTCAGCATCATGCACTTCA | 60.0 | 534 | INTERGENIC | | | | |
| CaPOPI_3650 | Ca5 | 37229002 | CCACACACACACACACACACA/CCACACACACACACACA | GAGCTGCTGAGCTGGACTTT | GCAACTGACTTGTTGTGCGT | 59.9 | 281 | INTERGENIC | | | | |
| CaPOPI_3651 | Ca5 | 37799581 | CTTTTT/CTTTTTT | GCACCGTGATTTTGATGATG | ATCAAGTCAGTGCTGCATCG | 59.9 | 594 | DRR | Ca_01443 | | | VQ |
| CaPOPI_3652 | Ca5 | 38005397 | CTCT/C | TGAGAGACCTTGATTGATACAAATG | TCAATTTGGTTCCAACGTCAT | 59.6 | 540 | INTERGENIC | | | | |
| CaPOPI_3653 | Ca5 | 38016624 | TAAGAAA/TAA | ATCCTCGAGAAGGAAGGAGC | CCTTTTCCCTCCAATGTCAA | 59.9 | 276 | INTRON | Ca_01419 | A | | Pseudouridine synthase, RsuA and RluB/C/D/E/F |
| CaPOPI_3654 | Ca5 | 38019468 | ATTTTTTTT/ATTTTTTT | TTTTTCCAGCTTTGGACCAG | CAAACGTAACCATTGTGCCA | 60.2 | 644 | DRR | Ca_01419 | A | | Pseudouridine synthase, RsuA and RluB/C/D/E/F |
| CaPOPI_3655 | Ca5 | 38021365 | AAAGCA/AAAGCAAGCA | TCTTGCATTTTCCTCAGCCT | ATTTTGGCAGCTGTCTTTGG | 60.0 | 507 | INTERGENIC | | | | |

| INDEL marker IDs | Chromosomes/ unanchored scaffolds | Physical positions (bp) | InDels (*Kabuli* reference genome-CDC Frontier/PI) | Forward primers (5'-3') | Reverse primers (5'-3') | Anneling temperature (0C) | Expected amplified product size (bp) | Structural annotation | | Functional annotation | | |
|---|---|---|---|---|---|---|---|---|---|---|---|---|
| | | | | | | | | Sequence components of *kabuli* genome | *Kabuli* gene accession IDs | NCBI-KOG | TFs | NCBI-nr database |
| CaPOPI_3656 | Ca5 | 38023980 | AACACAC/AACAC | CCGCAGTAAGAGTTTGACCA | TTCGGAGGAATGGTTGTTTC | 58.9 | 548 | INTERGENIC | | | | |
| CaPOPI_3657 | Ca5 | 38032027 | ATCTCTCTCTC/ATCTCTCTCTCTC | CAGTGGTGCGAGAAAACAGA | TTCCATAATGTCCACGCAAA | 60.0 | 429 | INTERGENIC | | | | |
| CaPOPI_3658 | Ca5 | 38034605 | CG/CGG | CTGCTATTGCGATCCCTCTC | TTTTGAACGCAAGAAAACAGAA | 59.9 | 800 | INTERGENIC | | | | |
| CaPOPI_3659 | Ca5 | 38034841 | TTTA/TTTAATTA | TCGTCCGATTTATACCGACA | CGACGAGGTATATGCCAACA | 59.0 | 856 | INTERGENIC | | | | |
| CaPOPI_3660 | Ca5 | 38042134 | T/TA | TTGTCATAGGTTATACGGTAGGTCTC | GGGGACCAAAACCACAATTA | 59.0 | 265 | INTERGENIC | | | | |
| CaPOPI_3661 | Ca5 | 38043572 | TAAAA/TAAA | AGGAGGCTAATTGAGGGAGC | TGTGGGTGCATGGTAGAAAA | 59.8 | 519 | INTERGENIC | | | | |
| CaPOPI_3662 | Ca5 | 38045991 | ATT/ATTTTCTT | TGACATTTTCAAATGCCCAG | TGAAATGCGAAAGAGGTGAA | 59.5 | 652 | INTERGENIC | | | | |
| CaPOPI_3663 | Ca5 | 38052063 | CATATGAATATATGAAT/CATATGAAT | CAACAAAATTTCCCAAAAATTGA | TTTTTGGCAGTTATCGACATT | 60.1 | 822 | INTERGENIC | | | | |
| CaPOPI_3664 | Ca5 | 38067693 | CTATATA/CTATATATA | GAGCACAATAATTTTCGTCAACA | GCGACAGATTCAACTCACAGA | 59.2 | 593 | DRR | Ca_01413 | | | |
| CaPOPI_3665 | Ca5 | 38068190 | GAAAAGAAAAAGA/GAAAAGAAAAAGAAAAAGA | GCAATGTGCACAATCCAAAC | CCCTTACCTGCTAAGCGAAA | 60.0 | 580 | DRR | Ca_01413 | | | |
| CaPOPI_3666 | Ca5 | 38075218 | GTTTTTTTTTTTT/GTTTTTTTTTT | ATTTTGGAAGCTGTTGGTGG | TGATCCACTCCCCTCTTGTC | 60.0 | 457 | DRR | Ca_01412 | O | MYB_related | Ubiquitin-associated/translation elongation factor EF1B, N-terminal |
| CaPOPI_3667 | Ca5 | 38075506 | TCC/TC | GACAAGAGGGGAGTGGATCA | CACAACGGCAAGACAGACAT | 60.0 | 441 | DRR | Ca_01412 | O | MYB_related | Ubiquitin-associated/translation elongation factor EF1B, N-terminal |
| CaPOPI_3668 | Ca5 | 38102985 | TA/T | GCTGGTGGCATACTTTGGTT | GTCCATGACAGCAGCAAAAA | 60.0 | 122 | CDS (FRAME SHIFT) | Ca_01408 | P | | |
| CaPOPI_3669 | Ca5 | 38107026 | AAAACAA/AAA | GGGCTTTCTTGAAGGGTTTC | TGTGCCTAATGAAGTTGCCA | 60.1 | 564 | INTERGENIC | | | | |
| CaPOPI_3670 | Ca5 | 38110715 | CATATATATATATA/CATATATATATA | TATACGCAGCCCACAATCAC | GATTGTCTGCTGGATTGGGT | 59.6 | 426 | DRR | Ca_01407 | S | | |

| INDEL marker IDs | Chromosomes/ unanchored scaffolds | Physical positions (bp) | InDels (*Kabuli* reference genome-CDC Frontier/PI) | Forward primers (5'-3') | Reverse primers (5'-3') | Anneling temperature (0C) | Expected amplified product size (bp) | Structural annotation | | Functional annotation | | |
|---|---|---|---|---|---|---|---|---|---|---|---|---|
| | | | | | | | | Sequence components of *kabuli* genome | *Kabuli* gene accession IDs | NCBI-KOG | TFs | NCBI-nr database |
| CaPOPI_3671 | Ca5 | 38117183 | ATTTTTTT/ATTTTTTTT | AATTTTGAATGTTCAAGTGCATTA | AACAATTCTACAAGTTCGGAAGTG | 57.8 | 570 | INTERGENIC | | | | |
| CaPOPI_3672 | Ca5 | 38121467 | AA/AAAACAATA | ATTGGATCTAGTGCGATGCC | GGTGATAGGTGGACGGCTTA | 60.1 | 198 | INTERGENIC | | | | |
| CaPOPI_3673 | Ca5 | 38137873 | TC/T | GGCTAATGGCTCAGTTCGAC | TTTCATGGGTGCAAACAAAA | 59.8 | 622 | INTERGENIC | | | | |
| CaPOPI_3674 | Ca5 | 38401552 | TTCTCTCTCTCTCTCTC/TTCTCTCTCTCTCTCTCTC | TTGTCTCCAGTTTTGGTCCC | GTTTAGAGGCAACACAGCCC | 59.9 | 482 | INTERGENIC | | | | |
| CaPOPI_3675 | Ca5 | 38407056 | CT/C | TCTCAAAGTCTCAATGGTCCAA | TTGAAGTCAAATTTCGCCAG | 59.7 | 658 | INTERGENIC | | | | |
| CaPOPI_3676 | Ca5 | 38476895 | C/CTTTGCGAAAT | GAGGACCAAAACATGCCACT | CCGTGCACAACACAATTTTC | 60.0 | 665 | INTERGENIC | | | | |
| CaPOPI_3677 | Ca5 | 38719512 | ATTTTTTTTTTT/ATTTTTTTTTT | TTTGGCTGGTTATGCAATCA | GGATGGACAGAGAGCCAGAG | 60.1 | 558 | INTERGENIC | | | | |
| CaPOPI_3678 | Ca5 | 38755750 | TGGGG/TGGGGG | GAAGTTGAGCGCAATGTTGA | TTAAGACGGGTGATGGAAGC | 60.0 | 528 | INTERGENIC | | | | |
| CaPOPI_3679 | Ca5 | 38755876 | TA/TATGA | GAAGTTGAGCGCAATGTTGA | TTAAGACGGGTGATGGAAGC | 60.0 | 528 | INTERGENIC | | | | |
| CaPOPI_3680 | Ca5 | 38755924 | TAAAA/TAAA | GTCTGCCACATCACCCTTTT | CGGATCAACTTTTCTTTGTGG | 60.0 | 661 | INTERGENIC | | | | |
| CaPOPI_3681 | Ca5 | 38854400 | TATTATTCATT/TATTATTCATTATTCATT | TCCCTAATTTAAAGTTTTGGGCT | CGTGTTGCATTTAGGTGGTG | 59.5 | 464 | INTERGENIC | | | | |
| CaPOPI_3682 | Ca5 | 38902709 | TAAAAAA/TAAAAAAA | GAAGGGGTGAATGCTGACAT | ATGACCCCCTTCACTTTGTG | 59.9 | 729 | INTERGENIC | | | | |
| CaPOPI_3683 | Ca5 | 39482180 | AATATATATATATATATATATATATATATAT/AATATATATATATATATATATATATATATATATATAT | TGGTCGGAGAAGAGGAGCTA | AATGGATGATAGCGAGTGGTT | 60.1 | 522 | INTERGENIC | | | | |
| CaPOPI_3684 | Ca5 | 39515362 | TAAAAAAAAA/TAAAAAAAA | TGGTCATCTCCCTAAATGGC | CATGAGCAAATGAACCATCG | 59.9 | 491 | DRR | Ca_07487 | H | | Domain of unknown function DUF89 |
| CaPOPI_3685 | Ca5 | 39557215 | GATATATATATATATATATAT/GATATATATATATATATATATAT | GCAAAAAGGGGCACAATAAA | AATGTCAATCCTCCATTGCC | 59.9 | 357 | INTRON | Ca_07489 | TBLD | | Phosphatidylinositol 3-/4-kinase, catalytic |

| INDEL marker IDs | Chromosomes/ unanchored scaffolds | Physical positions (bp) | InDels (*Kabuli* reference genome-CDC Frontier/PI) | Forward primers (5'-3') | Reverse primers (5'-3') | Anneling temperature (0C) | Expected amplified product size (bp) | Structural annotation | | Functional annotation | | |
|---|---|---|---|---|---|---|---|---|---|---|---|---|
| | | | | | | | | Sequence components of *kabuli* genome | *Kabuli* gene accession IDs | NCBI-KOG | TFs | NCBI-nr database |
| CaPOPI_3686 | Ca5 | 39872057 | GATATATATATATATATATATATATATA/GATATATATATATATATATATATATATATA | AGGAAAACATTGGTGGGACA | ATCAAACAACCGAAAGTGCC | 60.2 | 656 | DRR | Ca_07512 | F | | Xanthine/uracil/vitamin C permease |
| CaPOPI_3687 | Ca5 | 39927528 | TTATATATATATATATATAT/TTATATATATATATATAT | ATAGGGGCAACTAGGGGCTA | AAACATGGACAAGAGACCGC | 59.9 | 360 | INTERGENIC | | | | |
| CaPOPI_3688 | Ca5 | 39996193 | ATTTTTTTTT/ATTTTTTTTTT | GCATCCCAAGAATACAACCG | AACAAACCTCTTCCGCAATG | 60.3 | 413 | INTERGENIC | | | | |
| CaPOPI_3689 | Ca5 | 40119931 | GAAAAAAAAAAA/GAAAAAAAAAAAA | AAATTGTTGGGAAATTGGCA | GGGAAAAGCGCTTCTAAGGT | 60.2 | 757 | INTERGENIC | | | | |
| CaPOPI_3690 | Ca5 | 40191220 | AAATAATAATAATAATAATAATAATAATAATAATAATAATAATAAT/AAATAATAATAATAATAATAATAATAATAATAATAATAATAATAATAATAATAATAAT | TTCATGAGTGGGTGGTAGTTTG | TTGAATTTTTGTTTGGCTTGA | 59.9 | 257 | INTERGENIC | | | | |
| CaPOPI_3691 | Ca5 | 40223853 | ATTGTTTGTT/ATTGTT | GTCTCTCCCAAACCACCTCA | CCTTCATTGGTTGTCGTGTG | 60.1 | 451 | INTRON | Ca_07547 | R | | RNA recognition motif domain |
| CaPOPI_3692 | Ca5 | 40392907 | GTATATATATATATATATATATATATATATATAT/GTATATATATATATATATATATATATATAT | GCATTATCACGAGCAACACG | TCAATGTGGTTCGCAAAAGA | 60.3 | 192 | INTERGENIC | | | | |
| CaPOPI_3693 | Ca5 | 40471737 | CTTTTTTTTTTT/CTTTTTTTTTT | TGATTTTCCCTGTGCATCAA | AATGTGGCAAAGGATTCCAG | 60.0 | 757 | DRR | Ca_07567 | | | Double-stranded RNA-binding |
| CaPOPI_3694 | Ca5 | 40493832 | CTATATATATATATATATATATATA/CTATATATATATATATATATATA | TGCACAAAATATCGGGAGAA | GGGACCACGGGAACTATTTT | 59.1 | 779 | DRR | Ca_07570 | | C3H | Leucine-rich repeat |
| CaPOPI_3695 | Ca5 | 40750478 | TGGGG/TGGG | ATGTTATCAACCCTCGCTCG | TCAATTCCTTCTTTTTAATAACGTG | 60.1 | 510 | INTERGENIC | | | | |
| CaPOPI_3696 | Ca5 | 40968327 | TAAAAAAAAA/TAAAAAAAA | CAAAATTTTCAATCAAAACCCA | TACATTCCCGTGGTCCCTTA | 58.9 | 393 | INTERGENIC | | | | |
| CaPOPI_3697 | Ca5 | 40970000 | AATATATATATATATATATATATATAT/AATATATATATATATATATATATATAT | TGCATTTAATCAGAGACGTAAAGG | TAAAGGGGCACATTCAAAGC | 59.7 | 246 | INTERGENIC | | | | |
| CaPOPI_3698 | Ca5 | 40983709 | AATATATATATATATATATATATATATATATAT/AATATATATATATATATATATAT | TTGTCAGCACATCACAACCA | CAAAGAGTCGTCACGTTGACTT | 59.7 | 603 | INTERGENIC | | | | |
| CaPOPI_3699 | Ca5 | 41063761 | CTTTTTTT/CTTTT | TTTGCAGCCACAAAAGACAG | GCCCTCACAAACGTTGATTT | 60.0 | 122 | INTERGENIC | | | | |
| CaPOPI_3700 | Ca5 | 41124249 | TCAACA/TCAACAACA | ATATTGTTCAGCACCCGAGG | CAGACGACGAAATGAAGCAA | 60.0 | 361 | INTERGENIC | | | | |

| INDEL marker IDs | Chromosomes/ unanchored scaffolds | Physical positions (bp) | InDels (*Kabuli* reference genome-CDC Frontier/PI) | Forward primers (5'-3') | Reverse primers (5'-3') | Anneling temperature (0C) | Expected amplified product size (bp) | Structural annotation | | Functional annotation | | |
|---|---|---|---|---|---|---|---|---|---|---|---|---|
| | | | | | | | | Sequence components of *kabuli* genome | *Kabuli* gene accession IDs | NCBI-KOG | TFs | NCBI-nr database |
| CaPOPI_3701 | Ca5 | 41179556 | AA/AAAGAGTGTTGAAAAGA | GACTCAATGCAGATGGGGTT | GTCCCATACCTGCAAAAGGA | 59.9 | 596 | INTRON | Ca_07636 | | | |
| CaPOPI_3702 | Ca5 | 41226745 | CAAAAAAAAAA/CAAAAAAAAAAAA | GCCCAAAATCAAAAACTCCA | TGTGTCGTAAAAAGATGTCTAAGCA | 59.9 | 611 | INTERGENIC | | | | |
| CaPOPI_3703 | Ca5 | 41250178 | TTATATATATATATATATATAT/TTATATATATATATATATAT | CAGTTGAGACTGGGCAGACA | GCATCCCATCTTGAGTTGCT | 60.0 | 640 | DRR | Ca_07641 | | CO-like | Zinc finger, B-box |
| CaPOPI_3704 | Ca5 | 41455567 | AATATATATATATATATATATATATATA/AATATATATATATATATATATATATA | TTGCCACTTGATACAGCAAAA | TGCATGTTGAAACACCGACT | 59.4 | 757 | INTERGENIC | | | | |
| CaPOPI_3705 | Ca5 | 41459766 | CTATTATTATTATTATTATT/CTATTATTATTATTATTATTATT | GGACGTAGAAAATTAATGACATGG | TAGGGAGGGCTTTGTAAGCA | 58.9 | 479 | INTERGENIC | | | | |
| CaPOPI_3706 | Ca5 | 41467556 | TA/TAA | AAAGGATGCAGTATGGGTTTTT | TCACTTGGGTGGAAAAATGAG | 58.9 | 453 | INTERGENIC | | | | |
| CaPOPI_3707 | Ca5 | 41467725 | CTT/CTTT | CTCATTTTTCCACCCAAGTGA | AGCGACAAGATGAAAGCTCC | 60.0 | 529 | INTERGENIC | | | | |
| CaPOPI_3708 | Ca5 | 41477742 | CAA/CAAA | TGAGGCAAGCATGTGTAAGC | AATGTGCATGAGAGGCTGTG | 60.0 | 223 | DRR | Ca_19232 | B | MYB_related | SANT domain, DNA binding |
| CaPOPI_3709 | Ca5 | 41603707 | GTATATATATATATATATATATATAT/GTATATATATATATATATATATATATATATAT | ACGTCCATTTTCCATGTGGT | TTGCCATTTCCATCTTCCTC | 60.1 | 444 | INTERGENIC | | | | |
| CaPOPI_3710 | Ca5 | 41614182 | AATATATATATATATATATAT/AATATATATATATATATATATATAT | TGTGGATTCATGATCCTTGTG | TTTAACGTCAATGCCAACTTT | 59.4 | 916 | INTERGENIC | | | | |
| CaPOPI_3711 | Ca5 | 41678103 | TCT/TCTTTCCCTTAAAGGGACT | CAGTGGGTCCTCTTCCACAT | CCCGAACTCCTGTTACTCCA | 60.0 | 551 | INTRON | Ca_19215 | J | C3H | Argonaute/Dicer protein, PAZ |
| CaPOPI_3712 | Ca5 | 41795988 | ATT/AT | TGGAATGCTACACAAGCTGC | TTGAGGTGCATCGATCAAAA | 60.0 | 317 | INTERGENIC | | | | |
| CaPOPI_3713 | Ca5 | 41854653 | AATATATATATATATATATATATATA/AATATATATATATATATATATATA | TTACGGCTACCACCGAAAAC | CTCCCACTCAAAAGTTTGTTCA | 60.0 | 488 | INTERGENIC | | | | |
| CaPOPI_3714 | Ca5 | 41858516 | TTATATATATATATATATATATATATATATAT/TTATATATATATATATATATATATATATATATATATAT | AGCAATGAACAACGCATCAA | AAGGCATCATCATTTGAGAAAGA | 60.3 | 614 | INTERGENIC | | | | |
| CaPOPI_3715 | Ca5 | 42231500 | CC/CCGAGAACGTAC | TCAAAATCCACACCCCACTT | GCCATCTCCAATAGCACCAT | 60.2 | 888 | INTERGENIC | | | | |

| INDEL marker IDs | Chromosomes/ unanchored scaffolds | Physical positions (bp) | InDels (*Kabuli* reference genome-CDC Frontier/PI) | Forward primers (5'-3') | Reverse primers (5'-3') | Anneling temperature (0C) | Expected amplified product size (bp) | Structural annotation | | Functional annotation | | |
|---|---|---|---|---|---|---|---|---|---|---|---|---|
| | | | | | | | | Sequence components of *kabuli* genome | *Kabuli* gene accession IDs | NCBI-KOG | TFs | NCBI-nr database |
| CaPOPI_3716 | Ca5 | 42425779 | CATGTTTTATGTTTTA/CATGTTTTA | TCAATGGAATGGAGTCGTCA | CGACAATTTCATTATCCGTCC | 60.0 | 749 | INTERGENIC | | | | |
| CaPOPI_3717 | Ca5 | 42427468 | TTATATATATATATATATATATATATATAT/TTATATATATATATATATATATATATAT | CAACTGATCGGAGTGGGATT | AAATCCTTAAGATCGGCGGT | 59.9 | 618 | DRR | Ca_11375 | | | Proteinase inhibitor I25, cystatin, conserved region |
| CaPOPI_3718 | Ca5 | 42975249 | CTTATTATTATTATTATTATTATTATTATTATT/CTTATTATTATTATTATTATTATTATTATT | TCTGATGAGTTGCTTGTGCC | GGCCGCAATTATTGTTTTGT | 60.0 | 528 | INTERGENIC | | | | |
| CaPOPI_3719 | Ca5 | 43073124 | CA/CAA | ACACGGCTCAAGCACTTTTT | GACACCATGTCCAACATGTCA | 59.9 | 665 | INTRON | Ca_11300 | OU | WRKY | Membrane insertion protein, OxaA/YidC |
| CaPOPI_3720 | Ca5 | 43722797 | TAAAAAAAA/TAAAAAAA | CATTGTCCAATGGCATGTTT | CGAGTGTCTGTTAACCGCTG | 59.3 | 822 | DRR | Ca_12680 | P | MIKC | Sulphate transporter/antisigma-factor antagonist STAS |
| CaPOPI_3721 | Ca5 | 43921147 | CTATA/CTATATA | TGGGACCAAACATGTGAAGA | TCTAATTGTTTTGATTTATCCCTCA | 59.9 | 583 | INTERGENIC | | | | |
| CaPOPI_3722 | Ca5 | 44596809 | ATT/ATTT | TTGGCACATGCATTTTTGTT | GACCAAATCTTGTTGTTGCAGA | 60.0 | 582 | INTERGENIC | | | | |
| CaPOPI_3723 | Ca5 | 44597112 | CTATA/CTATATATA | GGGGTTAGTGCAAGCCAAAT | GTTCGCACGTGATGAACAAT | 61.2 | 862 | INTERGENIC | | | | |
| CaPOPI_3724 | Ca5 | 45086826 | TA/TAA | CAAACGCAAAATTAAACACACAA | AATACAGCTGGGTTTGTGGC | 60.0 | 367 | INTERGENIC | | | | |
| CaPOPI_3725 | Ca5 | 45098220 | ATTTTTT/ATTTTTTT | ATGTTCGAAACGCTGATTCC | TTGCCAAGCCTTTTGAAACT | 60.1 | 121 | INTERGENIC | | | | |
| CaPOPI_3726 | Ca5 | 45449782 | GAAAAAAAAAAA/GAAAAAAAAAA | TCCATATTCGGTAGCCGTTC | TGATGCACCCAAAAATACGA | 59.9 | 579 | INTERGENIC | | | | |
| CaPOPI_3727 | Ca5 | 45511101 | TA/TAA | CAAGGTTTCTCTTTCGCTGC | CTCAAATTGGTCCCCTGAAA | 60.1 | 539 | INTRON | Ca_03998 | T | | Protein phosphatase 2C, manganese/magnesium aspartate binding |
| CaPOPI_3728 | Ca5 | 45989618 | TAAAAAAAAAA/TAAAAAAAAA | TTGCTCCTCTGGTCCTCACT | GTTGGCTTTCGTACCCTCAA | 60.0 | 325 | INTRON | Ca_04045 | | SAP | Aminotransferase-like, plant mobile domain |
| CaPOPI_3729 | Ca5 | 46056306 | GTTATTATTATTATTATTATTATTATTATTATTATTATTATTATTATT/GTTATTATTATTATTATTATTATTATTATTATTATTATTATTATT | TTGTTTGGTCCACATTCCTG | GGAGCATATCAATGGAAAATCA | 59.4 | 779 | INTERGENIC | | | | |
| CaPOPI_3730 | Ca5 | 46070562 | AATATATATATATATATATA/AATATATATATATATA | TGGCAAAATTCATACGTGGA | AAAACCGTTCCAACATCCAT | 59.9 | 515 | INTERGENIC | | | | |

| INDEL marker IDs | Chromosomes/ unanchored scaffolds | Physical positions (bp) | InDels (*Kabuli* reference genome-CDC Frontier/PI) | Forward primers (5'-3') | Reverse primers (5'-3') | Anneling temperature (0C) | Expected amplified product size (bp) | Structural annotation | | Functional annotation | | |
|---|---|---|---|---|---|---|---|---|---|---|---|---|
| | | | | | | | | Sequence components of *kabuli* genome | *Kabuli* gene accession IDs | NCBI-KOG | TFs | NCBI-nr database |
| CaPOPI_3731 | Ca5 | 46132848 | TTTATTATTATTATTATTATTATTATTATTATTATTATT/TTTATTATTATTATTATTATTATTATTATTATTATTATT | TGTACCGTCACATTGCACCT | TACTGTATCAACGCCGCAAG | 60.0 | 771 | INTERGENIC | | | | |
| CaPOPI_3732 | Ca5 | 46208612 | AAT/AATAT | ATGCAGCACAAAATGCAAGT | TTCGAATCTTATTTTGACAATTTTT | 59.4 | 502 | INTERGENIC | | | | |
| CaPOPI_3733 | Ca5 | 46212141 | AATGATG/AATG | AAAAATCAATCCTTTCTCCAATG | AGTTTCAAAAACGGTGGTGG | 58.6 | 107 | INTERGENIC | | | | |
| CaPOPI_3734 | Ca5 | 46231130 | ATTTTTTTT/ATTTTTTT | GGAAGCCAAGCATTCAAGAG | TGGAAAAAGAATGAAATTGCTTA | 60.0 | 849 | INTERGENIC | | | | |
| CaPOPI_3735 | Ca5 | 46271953 | TTTTAATT/TTTTAATTTAATT | CCATCAAATGTTGGTCACTCC | ATTCATCTCGCCTTTGCTGT | 60.2 | 352 | INTERGENIC | | | | |
| CaPOPI_3736 | Ca5 | 46425096 | ATT/ATTT | GCGAGAATGGACTCCAGAAA | TCTCGTATTGACGGAATGACA | 60.3 | 323 | DRR | Ca_04100 | | WRKY | DNA-binding WRKY |
| CaPOPI_3737 | Ca5 | 46429100 | GAAAAAAAAAAA/GAAAAAAAAAA | TGCTCACGTTCTTTTGTTGG | TTGGTCTTGCTTCTTTCAAATG | 59.9 | 501 | INTERGENIC | | | | |
| CaPOPI_3738 | Ca5 | 46490687 | CTTTTTTTTTT/CTTTTTTTTT | CTCTGAACCCCTCTCACCAC | CCAACTCCTTGGCAGGTTTA | 59.7 | 481 | INTERGENIC | | | | |
| CaPOPI_3739 | Ca5 | 46637208 | TGAG/TGAGAG | TGGACTTAGTGGGCCTATGG | TATGGGCATGAGCTGTCAAA | 59.9 | 361 | DRR | Ca_04123 | V | | Dual specificity phosphatase, catalytic domain |
| CaPOPI_3740 | Ca5 | 46654455 | AATATATATATATATATATATATATATAT/AATATATATATATATATATATATATATATAT | ATGCTCTTGGAATCGTGGTC | CATCGTTTTCTCGTTCATGC | 60.1 | 781 | INTERGENIC | | | | |
| CaPOPI_3741 | Ca5 | 46661039 | AATATATATATATATATATATAT/AATATATATATATATATATATAT | TGGAATTCAAGGGACCAAAA | ACTTGCGAGATAATTTAGTAGGGA | 60.3 | 863 | INTERGENIC | | | | |
| CaPOPI_3742 | Ca5 | 46886435 | T/TTAACTATACTCAAAC | CAGCCATGCTCGTATGCTAA | AGAACCCCATCCAAACAATG | 60.0 | 444 | INTERGENIC | | | | |
| CaPOPI_3743 | Ca5 | 47118403 | GTATTATTATTATTATTATTATTATT/GTATTATTATTATTATTATTATT | ATTCGAATAATGGGTGGCAA | TTGGGTCAAGTAGGAGGTCG | 60.2 | 515 | INTERGENIC | | | | |
| CaPOPI_3744 | Ca5 | 47138535 | CTATTATTATTATTATTATTATTATTA/CTATTATTATTATTATTATTATTATTATTATTA | TGAATCTTTCCGCATTTGTG | CCTGAAAGACTTAGACGGCG | 59.7 | 651 | INTERGENIC | | | | |
| CaPOPI_3745 | Ca5 | 47166855 | GAAAAAAAAA/GAAAAAAAA | CGTGGAATCCATCGAATTTT | GTGGCACCTTTTTGCTCAAT | 59.8 | 117 | DRR | Ca_04173 | J | | Ribosomal protein S2 |

| INDEL marker IDs | Chromosomes/ unanchored scaffolds | Physical positions (bp) | InDels (*Kabuli* reference genome-CDC Frontier/PI) | Forward primers (5'-3') | Reverse primers (5'-3') | Anneling temperature (0C) | Expected amplified product size (bp) | Structural annotation | | Functional annotation | | |
|---|---|---|---|---|---|---|---|---|---|---|---|---|
| | | | | | | | | Sequence components of *kabuli* genome | *Kabuli* gene accession IDs | NCBI-KOG | TFs | NCBI-nr database |
| CaPOPI_3746 | Ca5 | 47226447 | TTTATATTATATTATATTATAT/TTTATATTATATTATATTATATTATAT | ACCGTTACCTTTTGTGGCTG | GAAGCTTTCACGTACGGAGC | 60.0 | 449 | INTERGENIC | | | | |
| CaPOPI_3747 | Ca5 | 47512183 | GTTTTTTTTTT/GTTTTTTTTTTT | TGGCATATTCCCATCCCATA | TGTGGGTATAAATGAGGGGC | 60.9 | 404 | INTERGENIC | | | | |
| CaPOPI_3748 | Ca5 | 47553707 | GAA/GAAA | ATGATGAATGTCCGACAACG | CAAAAGACAACCAAGGGCAT | 59.4 | 744 | INTERGENIC | | | | |
| CaPOPI_3749 | Ca5 | 47588093 | AT/A | TGTGGTTAGAATCCTGGCTCA | TTCGAATTCAGGACAAGACG | 60.6 | 385 | INTERGENIC | | | | |
| CaPOPI_3750 | Ca5 | 47838081 | AATATATATATATATATATATATA/AATATATATATATATATATATATATATA | TACCTCATTGTAGGCCCAGC | TAGTTCGGGGCAAATGGTAG | 60.1 | 168 | INTERGENIC | | | | |
| CaPOPI_3751 | Ca5 | 47945436 | AATATATATATATATATATATATATATA/AATATATATATATATATATATATATATATATATATATA | ACATCAAAGGCGGATCCATA | TCCTCGCAACACACACATTT | 60.3 | 392 | INTERGENIC | | | | |
| CaPOPI_3752 | Ca5 | 48059653 | TTCTC/TTC | CGAAACTCCACTGCAGACAA | GCATATTTGCAAGCACCAAA | 60.0 | 804 | DRR | Ca_04266 | R | ERF | Pentatricopeptide repeat |
| CaPOPI_3753 | Ca5 | 48121332 | TTTTATTTATTTATTTATTTATTTATTTATTTAT/TTTTATTTATTTATTTATTTATTTATTTAT | ATTTTATGGCCAACCCAACA | ACTCGGCTTCCATTGATGAG | 60.1 | 178 | INTERGENIC | | | | |
| CaPOPI_3754 | Ca5 | 48164734 | AGG/AG | GGTTGCAATTATGGGAAATGTT | AACTCTAAATCCCCGCAAGG | 60.0 | 485 | INTERGENIC | | | | |
| CaPOPI_3755 | Ca6 | 112870 | TG/T | AACTCCTTGGGACCGTGATT | ACCTTGCAACTGGAGCAAAT | 60.7 | 242 | INTERGENIC | | | | |
| CaPOPI_3756 | Ca6 | 233726 | CTATTTATTTATTTATTTATTTATTTATTTATTTATTTATTTATTTATTTAT/CTATTTATTTATTTATTTATTTATTTATTTATTTATTTATTTATTTATTTATTTATTTATT | CGGATCCCACCACTTGATAA | TTGATCGTTGGGTTGATGAAT | 60.7 | 640 | INTERGENIC | | | | |
| CaPOPI_3757 | Ca6 | 472874 | AT/A | TTTTCCTTCTCCCTTTGCAC | AGCAAGAAACAAACGACGCT | 59.3 | 719 | DRR | Ca_13267 | | G2-like | Myb, DNA-binding |
| CaPOPI_3758 | Ca6 | 582030 | AATATATATATATATATA/AATATATATATATATATATA | AAAATCGAAGATCTTTCACAAAA | TGGCCTAATCTTTGTGGGAC | 57.2 | 264 | DRR | Ca_13279 | O | SRS | Peptidase A1 |
| CaPOPI_3759 | Ca6 | 1239992 | CATATATATA/CATATATATATA | CGAGACCAAAGGTTTCTTGC | GACATGGCAATTTAGCTCAACA | 59.9 | 684 | INTERGENIC | | | | |
| CaPOPI_3760 | Ca6 | 1343859 | GAAAAA/GAAAAAA | CAACGAGTTTGATTTCTATTCTAACG | TTGGGTAGCCAAGGTTTCAC | 59.7 | 721 | INTERGENIC | | | | |

| INDEL marker IDs | Chromosomes/ unanchored scaffolds | Physical positions (bp) | InDels (*Kabuli* reference genome-CDC Frontier/PI) | Forward primers (5'-3') | Reverse primers (5'-3') | Anneling temperature (0C) | Expected amplified product size (bp) | Structural annotation | | Functional annotation | | |
|---|---|---|---|---|---|---|---|---|---|---|---|---|
| | | | | | | | | Sequence components of *kabuli* genome | *Kabuli* gene accession IDs | NCBI-KOG | TFs | NCBI-nr database |
| CaPOPI_3761 | Ca6 | 1350878 | A/AGTCTGT | ACACATCAGCTTTTTGGCTG | AGCCCACTCACCTTCTTCCT | 58.9 | 428 | INTERGENIC | | | | |
| CaPOPI_3762 | Ca6 | 1353833 | CAA/CAAA | TGTTTAAAGGGGTGGCACTT | ACGGAAGGATCCAGTAGGCT | 59.5 | 513 | INTERGENIC | | | | |
| CaPOPI_3763 | Ca6 | 1354322 | GAA/GAACCAA | AATTTGCATAGGCGGTGAAG | GGTGTTACAGCTTTCATGAACAAA | 60.1 | 420 | INTERGENIC | | | | |
| CaPOPI_3764 | Ca6 | 1468628 | TAAAAAAAAAAA/TAAAAAAAAAAAA | AAGAATGGAGCTCAAGGGGT | GCCTCCAAAAAGTAATCCCC | 60.1 | 405 | INTERGENIC | | | | |
| CaPOPI_3765 | Ca6 | 1491943 | CT/CTT | TGGTTTTCGTACCGGACTCT | CAATGTCATCAAATAGCGGC | 59.6 | 772 | INTRON | Ca_27033 | H | | Folylpolyglutamate synthetase |
| CaPOPI_3766 | Ca6 | 1492206 | TAAGAAGAAAAG/TAAGAAGAAAAGAAGAAAAG | CCGTGCATATGACAGAAATGA | ATGCACCGTTGAAACAACAA | 59.6 | 510 | INTRON | Ca_27033 | H | | Folylpolyglutamate synthetase |
| CaPOPI_3767 | Ca6 | 1492346 | ATAGC/ATAGCGCCGCTGTAGC | CCGTGCATATGACAGAAATGA | ATGCACCGTTGAAACAACAA | 59.6 | 510 | INTRON | Ca_27033 | H | | Folylpolyglutamate synthetase |
| CaPOPI_3768 | Ca6 | 1492728 | AT/ATT | TTGTTGTTTCAACGGTGCAT | ATGGTTGGGGGATAAAAAGG | 60.0 | 651 | INTRON | Ca_27033 | H | | Folylpolyglutamate synthetase |
| CaPOPI_3769 | Ca6 | 1542114 | ATT/ATTT | TGCACTTTGTGCACTCCTTA | GTAATGCTGCGTGCAAAACA | 58.1 | 334 | INTERGENIC | | | | |
| CaPOPI_3770 | Ca6 | 1556325 | TAAA/TAAAAAAAAAAAAA | ATTGTTCTCCGTTGTCAGGG | GTGATCGACATACTTCGGCA | 60.0 | 864 | INTERGENIC | | | | |
| CaPOPI_3771 | Ca6 | 1556869 | TAAAAA/TAAAAAA | CAATTTGGTTGGGCGTAAAC | TTGCTTTATACTCATATTTTGGCCT | 60.2 | 528 | INTERGENIC | | | | |
| CaPOPI_3772 | Ca6 | 1557117 | TAAAAAAAAA/TAAAAAAAAAA | GCTTTTCTTTCATATTGCCGTT | TTTTGAGCGGAAAGAGTCGT | 59.6 | 674 | INTERGENIC | | | | |
| CaPOPI_3773 | Ca6 | 1559285 | GTTA/GTTATTA | CAGAGCAGTGTGACAGCAAGA | TCAAAAATTTCAAACAAAATCCA | 60.4 | 456 | INTERGENIC | | | | |
| CaPOPI_3774 | Ca6 | 1559495 | GAACAAA/GA | GGATTTTGTTTGAAATTTTTGATG | TATTTTATTCCCACCGCTGC | 58.9 | 445 | INTERGENIC | | | | |
| CaPOPI_3775 | Ca6 | 1559581 | ATTTTTTTTTTTT/ATTTTTTTTTT | TGAAGTTAAATTAAAGGATCGCA | TATTTTATTCCCACCGCTGC | 58.0 | 283 | INTERGENIC | | | | |

| INDEL marker IDs | Chromosomes/ unanchored scaffolds | Physical positions (bp) | InDels (*Kabuli* reference genome-CDC Frontier/PI) | Forward primers (5'-3') | Reverse primers (5'-3') | Anneling temperature (0C) | Expected amplified product size (bp) | Structural annotation | | Functional annotation | | |
|---|---|---|---|---|---|---|---|---|---|---|---|---|
| | | | | | | | | Sequence components of *kabuli* genome | *Kabuli* gene accession IDs | NCBI-KOG | TFs | NCBI-nr database |
| CaPOPI_3776 | Ca6 | 1560016 | TTT/TTTAATT | AATATTACTGCAGCGGTGGG | GGACCACAAGAAATCCTCCA | 60.0 | 719 | INTERGENIC | | | | |
| CaPOPI_3777 | Ca6 | 1682722 | GTGCATCTGTTT/G | GAGGCATTGTCTTTGGTGGT | GCAAATGTTTGGAATGGCTT | 60.0 | 656 | INTERGENIC | | | | |
| CaPOPI_3778 | Ca6 | 1683094 | TATTAAC/TATTAACCATTGATTAAC | AATAAGTCCATCCGGCTCCT | TTGTGGATGGCGTTTGATAA | 59.9 | 276 | DRR | Ca_10299 | | HB-other | Rop nucleotide exchanger, PRONE |
| CaPOPI_3779 | Ca6 | 1683226 | CAA/CA | AATAAGTCCATCCGGCTCCT | TTGTGGATGGCGTTTGATAA | 59.9 | 276 | DRR | Ca_10299 | | HB-other | Rop nucleotide exchanger, PRONE |
| CaPOPI_3780 | Ca6 | 1683327 | AC/A | TTATCAAACGCCATCCACAA | CACTTGCGTGTGGTAGTGCT | 59.9 | 504 | DRR | Ca_10299 | | HB-other | Rop nucleotide exchanger, PRONE |
| CaPOPI_3781 | Ca6 | 1728817 | GTTTTTTTTT/GTTTTTTTTTT | ATGGCTCCTTGATTTCGATG | TCAAAAAGGACAATTAAGCCA | 60.0 | 539 | INTERGENIC | | | | |
| CaPOPI_3782 | Ca6 | 1788325 | CTTTTTTTTTTT/CTTTTTTTTTT | AGGGTGTAAGGCATGGATGA | CCATCAATCAAAGCAAACCA | 60.3 | 330 | INTERGENIC | | | | |
| CaPOPI_3783 | Ca6 | 1820872 | TTG/TTGGTG | CCCACGGGTAACAACTCAAT | CGGGTTGGTTTGATTCAAGT | 59.7 | 707 | DRR | Ca_10309 | | | Acid phosphatase (Class B) |
| CaPOPI_3784 | Ca6 | 1824130 | GT/GTT | TTGCAGAATGCATGGGAATA | TCCAAACTCTCCGACCAAAC | 60.0 | 779 | INTERGENIC | | | | |
| CaPOPI_3785 | Ca6 | 1824314 | CA/CTCACAAAA | TTGCAGAATGCATGGGAATA | TCCAAACTCTCCGACCAAAC | 60.0 | 779 | INTERGENIC | | | | |
| CaPOPI_3786 | Ca6 | 1825931 | C/CT | TCAAATCACATCACCGTTTG | AGCTCCCAATTTTGCACTTG | 57.9 | 508 | INTERGENIC | | | | |
| CaPOPI_3787 | Ca6 | 1832442 | A/AC | AAAGGTACGTGAGGTGTGCC | CGTAATTAATGATGGTGGAGTGG | 60.0 | 614 | DRR | Ca_10310 | G | | Fructose-1,6-bisphosphatase class 1/Sedoheputulose-1,7-bisphosphatase |
| CaPOPI_3788 | Ca6 | 1846516 | GCC/GC | CCATAATCTGGAAGCTGGGA | AATCTTGCATGTGGCCTTTC | 60.0 | 746 | DRR | Ca_10312 | R | | Leucine-rich repeat, cysteine-containing subtype |
| CaPOPI_3789 | Ca6 | 1846574 | CAAAAAAA/CAAAAAA | CCATAATCTGGAAGCTGGGA | AATCTTGCATGTGGCCTTTC | 60.0 | 746 | DRR | Ca_10312 | R | | Leucine-rich repeat, cysteine-containing subtype |
| CaPOPI_3790 | Ca6 | 1854353 | ATTTTTTTTT/ATTTTTTTT | GCATTTTTGTCCTCTTTGTCG | AGAGGACGCGTACACTTCAAA | 59.7 | 288 | INTERGENIC | | | | |

| INDEL marker IDs | Chromosomes/ unanchored scaffolds | Physical positions (bp) | InDels (*Kabuli* reference genome-CDC Frontier/PI) | Forward primers (5'-3') | Reverse primers (5'-3') | Anneling temperature (0C) | Expected amplified product size (bp) | Structural annotation | | Functional annotation | | |
|---|---|---|---|---|---|---|---|---|---|---|---|---|
| | | | | | | | | Sequence components of *kabuli* genome | *Kabuli* gene accession IDs | NCBI-KOG | TFs | NCBI-nr database |
| CaPOPI_3791 | Ca6 | 1976018 | AAT/AATAGAT | GAACTAGATGCCGCAAAAGC | GTACGTGGTCCTTCCCTGAA | 60.0 | 560 | DRR | Ca_10322 | | | |
| CaPOPI_3792 | Ca6 | 1976736 | TC/TCCC | TTATTCTCCATTGGGCATCC | TCCTGCGGGTAATGATAAGC | 59.7 | 963 | DRR | Ca_10322 | | | |
| CaPOPI_3793 | Ca6 | 1977351 | TTT/TTTTTT | GCTTATCATTACCCGCAGGA | TAGCATAGTCCAAGCGGACC | 60.1 | 465 | DRR | Ca_10322 | | | |
| CaPOPI_3794 | Ca6 | 1978360 | A/ATCG | CGTGGAGCATTCCGAGTATT | GGAGTTAGGGCGTGTGATGT | 60.1 | 575 | INTRON | Ca_10322 | | | |
| CaPOPI_3795 | Ca6 | 1978522 | C/CCTGAGG | ACATCACACGCCCTAACTCC | CTTAGCCTTGCAAATGAGGC | 60.0 | 197 | INTRON | Ca_10322 | | | |
| CaPOPI_3796 | Ca6 | 2189256 | CGG/CG | GTGTTGCGTGTTCGAGTCTT | ATTGAAATCATCCGTGCCTC | 58.9 | 388 | INTERGENIC | | | | |
| CaPOPI_3797 | Ca6 | 2478363 | TAAAAAAAAAAAAAA/TAAAAAAAAAAAAA | TGCAATGTGGAACGAAGAAA | CATCAATTCATCAACTTGTGGAC | 60.2 | 736 | INTERGENIC | | | | |
| CaPOPI_3798 | Ca6 | 2518566 | TG/TGG | TAGTCACGCTTGGTCACTCG | AATGCATTTGCCTGACTCAA | 60.0 | 952 | DRR | Ca_10384 | | | Protein of unknown function DUF599 |
| CaPOPI_3799 | Ca6 | 2518811 | TA/TAA | AATCTGGCTCAAAATGCAGC | AATGCATTTGCCTGACTCAA | 60.4 | 723 | DRR | Ca_10384 | | | Protein of unknown function DUF599 |
| CaPOPI_3800 | Ca6 | 2528953 | AATAT/AAT | TGGGTCAATGTTAAGAGGGC | CGTCAACGTCGTGCATTTAC | 59.9 | 515 | INTERGENIC | | | | |
| CaPOPI_3801 | Ca6 | 2533591 | AT/ATT | CTCTTATGTCGGTCGCCAAT | AACTTTGTGGAAGCTGTGGG | 60.1 | 533 | INTERGENIC | | | | |
| CaPOPI_3802 | Ca6 | 2553132 | GCACACACACACACACACA/GCACACACACACACACACACACA | TTGCTTGCAATGCACATTTT | TGGTCTAACTTTCGGCACAA | 60.3 | 647 | INTERGENIC | | | | |
| CaPOPI_3803 | Ca6 | 2586276 | GTTTTTTTTTTT/GTTTTTTTTTTTT | GGTGCCACACATACATTGGA | TAATGCGCGGATCTTATTCC | 60.2 | 490 | INTERGENIC | | | | |
| CaPOPI_3804 | Ca6 | 2674985 | ATGTT/AT | GAAACTTCGTGCCTTTTTGC | ACGATAGTAGATGTGGCGGC | 59.9 | 152 | INTRON | Ca_10401 | V | | Protein of unknown function DUF1712, fungi |
| CaPOPI_3805 | Ca6 | 2960468 | CATATATATATATATATAT/CATATATATATATATATATAT | AAGAAAGGGGTATTGGTGGC | CACAAATTTGACAATAAACTTTGACA | 60.2 | 698 | INTRON | Ca_10438 | T | bHLH | Protein kinase, catalytic domain |

| **INDEL marker IDs** | **Chromosomes/ unanchored scaffolds** | **Physical positions (bp)** | **InDels (*Kabuli* reference genome-CDC Frontier/PI)** | **Forward primers (5'-3')** | **Reverse primers (5'-3')** | **Anneling temperature (0C)** | **Expected amplified product size (bp)** | **Structural annotation** | | **Functional annotation** | | |
|---|---|---|---|---|---|---|---|---|---|---|---|---|
| | | | | | | | | **Sequence components of *kabuli* genome** | ***Kabuli* gene accession IDs** | **NCBI-KOG** | **TFs** | **NCBI-nr database** |
| CaPOPI_3806 | Ca6 | 3176026 | AGG/AGGG | GCAATGGAAGGTGAAAATGG | GCATAGGAGCGTGGAAATTG | 60.3 | 522 | INTERGENIC | | | | |
| CaPOPI_3807 | Ca6 | 3232392 | ATTTATTTTTATTT/ATTTATTTTTATTTTTATTT | ACGTGGCTCCTTATACCACG | TTTTTGAGCCTTTTTCACCAA | 60.0 | 563 | INTERGENIC | | | | |
| CaPOPI_3808 | Ca6 | 3255826 | AATATATATATATATAT/AATATATATATATATAT | TGGCTTGTTTGATTTAGGACA | ATTCGAATGATCAGTTGCCC | 58.3 | 547 | INTERGENIC | | | | |
| CaPOPI_3809 | Ca6 | 3330798 | AATTATTATTATTATTATTATTATTATTATTATT/AATTATTATTATTATTATTATTATTATTATTATTATT | TCAATTGATTTTCGGTATCCTTC | GACCTCCCAAAGGCATTTTT | 59.3 | 406 | INTERGENIC | | | | |
| CaPOPI_3810 | Ca6 | 3351662 | GATATAT/GATATATATAT | CGAAAATGGAAAACACCACC | TCGAGTTGCGTAACTGACATAG | 60.2 | 687 | INTERGENIC | | | | |
| CaPOPI_3811 | Ca6 | 3351745 | CATATTATATATA/CATA | GGTTGATTTAGGCTTCACGG | TCGAGTTGCGTAACTGACATAG | 59.6 | 580 | INTERGENIC | | | | |
| CaPOPI_3812 | Ca6 | 3352912 | AATTA/AA | AAAGTGGGTGGCAATTAACG | TTATCCAATTCCGTTGCACA | 59.9 | 247 | INTERGENIC | | | | |
| CaPOPI_3813 | Ca6 | 3355103 | GCTATTGATCAATCTATTGAT/GCTATTGAT | TGAATAGATGTGCGCGAGAC | TCCACGGGAGTGAGAGAGAT | 60.0 | 401 | INTERGENIC | | | | |
| CaPOPI_3814 | Ca6 | 3355875 | CTTT/CTTTT | AATTTGGCCCTATTGGCTTT | AGAAACGGAATGAGTCACGG | 59.8 | 709 | INTERGENIC | | | | |
| CaPOPI_3815 | Ca6 | 3375286 | AATATATATATATATATATAT/AATATATATATATATATATATAT | GATCAGTTGACCCGCGTAGT | TTGATGCCACCTATTTCTCG | 60.1 | 789 | INTERGENIC | | | | |
| CaPOPI_3816 | Ca6 | 3377584 | CTTTTT/CTT | ATGCGGTGATGGTGATATTG | TATGGCGAATATGGATGGGT | 59.2 | 745 | INTERGENIC | | | | |
| CaPOPI_3817 | Ca6 | 3382708 | AA/AAGGGTTGGA | ATCACCGACCACTCACCTTC | TCAATTTTTCTTCGTTGACGG | 60.0 | 787 | INTERGENIC | | | | |
| CaPOPI_3818 | Ca6 | 3382789 | CTTTTT/CTTT | ATCACCGACCACTCACCTTC | GTTCAATCAGTGCAGCGTGT | 60.0 | 810 | INTERGENIC | | | | |
| CaPOPI_3819 | Ca6 | 3382946 | CAAAAA/CAAAA | TAACCACCAACATTCTGCCA | TGTTCCGTCCCACTCATACA | 60.0 | 927 | INTERGENIC | | | | |
| CaPOPI_3820 | Ca6 | 3387853 | AGTACGTA/AGTA | GAGCCAACAGGATAAGAGCG | GAAAATTGAGTTCCATGACGTTT | 60.0 | 477 | INTERGENIC | | | | |

| INDEL marker IDs | Chromosomes/ unanchored scaffolds | Physical positions (bp) | InDels (*Kabuli* reference genome-CDC Frontier/PI) | Forward primers (5'-3') | Reverse primers (5'-3') | Anneling temperature (0C) | Expected amplified product size (bp) | Structural annotation | | Functional annotation | | |
|---|---|---|---|---|---|---|---|---|---|---|---|---|
| | | | | | | | | Sequence components of *kabuli* genome | *Kabuli* gene accession IDs | NCBI-KOG | TFs | NCBI-nr database |
| CaPOPI_3821 | Ca6 | 3387937 | A/AG | GAGCCAACAGGATAAGAGCG | GAAAATTGAGTTCCATGACGTTT | 60.0 | 477 | INTERGENIC | | | | |
| CaPOPI_3822 | Ca6 | 3388065 | GATATATATAT/GATATAT | GAGCCAACAGGATAAGAGCG | TTGTCTTTCACTTCTTTTTCCG | 60.0 | 841 | INTERGENIC | | | | |
| CaPOPI_3823 | Ca6 | 3420698 | TTATATATATATATATATATATATATATAT/TTATATATATATATATATATATATAT | TAGCCGACAACATTGGAACA | TGAACTCGCACATTTGAGGA | 60.1 | 386 | INTERGENIC | | | | |
| CaPOPI_3824 | Ca6 | 3519049 | GTTTTTTTTTTT/GTTTTTTTTTT | AAGACTGCCCTGACCAGAGA | GTGGGGGACTAAGCTAAGGG | 60.0 | 334 | INTERGENIC | | | | |
| CaPOPI_3825 | Ca6 | 3520685 | CTATATATATATATAT/CTATATATATATAT | GTTTCGCGATTTCTCGTGTT | CACGCATTTGACTCGAAAAC | 60.3 | 249 | INTERGENIC | | | | |
| CaPOPI_3826 | Ca6 | 3742066 | ATATTATTATTATTATTATTATTATTATTATTATTA/ATATTATTATTATTATTATTATTATTATTATTATTATTATTATTATTATTA | TGAAAGTTTAGCGGCAATCA | AATTGTTGGCACGCTTTTTC | 59.4 | 609 | INTERGENIC | | | | |
| CaPOPI_3827 | Ca6 | 3756968 | TAAAAAAAAA/TAAAAAAAAAA | TCCGTTACCACTCACACCAA | ACCCCTCAAATCTGTTGTGC | 60.0 | 640 | INTRON | Ca_05928 | | bZIP | Ribosomal protein S4/S9, N-terminal |
| CaPOPI_3828 | Ca6 | 3780129 | CTTTTTT/CTTTTTTTT | GCGTTGTCAAAATTACAAATTCA | TTATTTGAATCCCCCAAAACA | 59.1 | 693 | INTERGENIC | | | | |
| CaPOPI_3829 | Ca6 | 3920180 | GTTGGAAATCTTTT/GTTGGAAATCTTTTGGAAATCTTTT | TGGCATCCAACAATTGTCAT | TTGTGCAACCAATCACTTGAA | 59.8 | 899 | DRR | Ca_05908 | R | | Inositol oxygenase |
| CaPOPI_3830 | Ca6 | 4290626 | ACATCATCATCATCAT/ACATCATCATCATCATCAT | CATTCCAGACATTGACGGTG | ACCTGGAAACATTTTGCTCG | 60.0 | 684 | CDS (large-effect mutations) | Ca_05873 | | C2H2 | Protein kinase, catalytic domain |
| CaPOPI_3831 | Ca6 | 4363547 | AT/ATAGGT | TTGCCTCTTCATTCTTTTGC | TAAAAACCGGTTCGGTTCAG | 58.1 | 338 | INTERGENIC | | | | |
| CaPOPI_3832 | Ca6 | 4432554 | ATTTTTTTTTT/ATTTTTTTTT | TTTCCTTTGCCAGAGAGACG | AAGTGTCTTTGGGGACGTTG | 60.5 | 424 | INTERGENIC | | | | |
| CaPOPI_3833 | Ca6 | 4621073 | ATTTTTTTTTTT/ATTTTTTTTTTTT | CCAACGCTACAACCCTTCAT | GGAGGAAACAAATTGATCCAAA | 60.0 | 761 | INTERGENIC | | | | |
| CaPOPI_3834 | Ca6 | 4649762 | AATAAGT/AATAAGTATAAGT | GGTCGAACCACTTGAGGTGT | AATCCACTCCCACTCCACTG | 60.0 | 389 | INTERGENIC | | | | |
| CaPOPI_3835 | Ca6 | 4673406 | ATTTTTTTTTT/ATTTTTTTTTTT | TTTTTACTCGGGGTGGAATAA | CGCCAAAGAGAAAAACAAATG | 58.5 | 585 | INTERGENIC | | | | |

| INDEL marker IDs | Chromosomes/ unanchored scaffolds | Physical positions (bp) | InDels (*Kabuli* reference genome-CDC Frontier/PI) | Forward primers (5'-3') | Reverse primers (5'-3') | Anneling temperature (0C) | Expected amplified product size (bp) | Structural annotation | | Functional annotation | | |
|---|---|---|---|---|---|---|---|---|---|---|---|---|
| | | | | | | | | Sequence components of *kabuli* genome | *Kabuli* gene accession IDs | NCBI-KOG | TFs | NCBI-nr database |
| CaPOPI_3836 | Ca6 | 4731176 | ATTTTTTTTTT/ATTTTTTTTT | GACACGTGCAAACCACTAGC | AAGCAACGATGATAAAATACGACA | 59.4 | 611 | INTERGENIC | | | | |
| CaPOPI_3837 | Ca6 | 4801698 | TGG/TGGG | ATAACCGACACAGACGAGGC | GTTGTTGCTTCTGCTCCTCC | 60.1 | 278 | INTERGENIC | | | | |
| CaPOPI_3838 | Ca6 | 4836563 | GATACATAAT/GATACATAATACATAAT | TGGCACGATAGTTCACCAAA | TGGTGCAAATAAATTATGAGTTGG | 60.1 | 524 | DRR | Ca_05811 | R | | High mobility group, HMG1/HMG2 |
| CaPOPI_3839 | Ca6 | 5295925 | TTTATTATTTATTA/TTTATTATTTATTATTATTTATTA | TTGACATTTCAAACCGGTCTAT | TGGAAAAGACTCCTGAATATGGA | 58.5 | 359 | INTERGENIC | | | | |
| CaPOPI_3840 | Ca6 | 5853411 | GTATTATTATTATTA/GTATTATTATTATTATTATTA | TCGAACATAACACCTCGCAG | TCCGGTTACATTCCTTCTTTTG | 59.9 | 467 | INTERGENIC | | | | |
| CaPOPI_3841 | Ca6 | 5971753 | GATATATATATATATATATAT/GATATATATATATATATATATATATATATAT | AGAGAGACGATTGTCAAGGCA | GCTTGTTTCACAAGTCATTTCAA | 60.0 | 737 | INTERGENIC | | | | |
| CaPOPI_3842 | Ca6 | 6163420 | ATTTTTTTTT/ATTTTTTTTTT | CCGTCCCTTAGCAAAATTCA | AAGCAACTATGGCAAATGGG | 60.1 | 443 | DRR | Ca_05680 | T | G2-like | Arf GTPase activating protein |
| CaPOPI_3843 | Ca6 | 6319723 | CTTTTTT/CTTTTTTT | CATGATGGAAAACATGGGTG | CAGTGGCGTTGGTTAGCATA | 59.6 | 540 | INTERGENIC | | | | |
| CaPOPI_3844 | Ca6 | 6409588 | TTATATATATATATATAT/TTATATATATATATATATATATAT | TCCACGGCCTCTTGTGTTAT | AGAGCAAGCAAGTTGGTGGT | 60.5 | 581 | INTERGENIC | | | | |
| CaPOPI_3845 | Ca6 | 6411336 | AAT/AATAT | ATCTCGTGGTGCAACAATCA | TTTGGGAAGGGTCAAGAAAA | 60.1 | 628 | INTERGENIC | | | | |
| CaPOPI_3846 | Ca6 | 6411391 | AATTTATTTATTTATTTAT/AATTTATTTATTTATTTATTTATTTAT | ATCTCGTGGTGCAACAATCA | TTTGGGAAGGGTCAAGAAAA | 60.1 | 628 | INTERGENIC | | | | |
| CaPOPI_3847 | Ca6 | 6447647 | TTATATATATATATATATAT/TTATATATATATATATATATATAT | TAATTCGTGCGTCGTATGGA | TTTTCATCAATAACATTGAGCG | 60.1 | 219 | INTERGENIC | | | | |
| CaPOPI_3848 | Ca6 | 6583135 | ATTTTTTTTTTT/ATTTTTTTTTT | TACGCGGATACGGGTTATGT | AGGACAAATGTGGGAATTGG | 60.2 | 421 | INTERGENIC | | | | |
| CaPOPI_3849 | Ca6 | 6747248 | TAA/TAAA | ACATGATAACCAATGCGGGT | GGGGGTTGAAACGAGGTATT | 60.1 | 509 | DRR | Ca_16935 | | NF-YA | Plant self-incompatibility S1 |
| CaPOPI_3850 | Ca6 | 6862212 | ACC/AGAATGAGTTATCC | TGATGATGAGAGGCGAGACA | GGTGGAGCTGTTTATTGGGA | 60.5 | 198 | INTERGENIC | | | | |

| INDEL marker IDs | Chromosomes/ unanchored scaffolds | Physical positions (bp) | InDels (*Kabuli* reference genome-CDC Frontier/PI) | Forward primers (5'-3') | Reverse primers (5'-3') | Anneling temperature (0C) | Expected amplified product size (bp) | Structural annotation | | Functional annotation | | |
|---|---|---|---|---|---|---|---|---|---|---|---|---|
| | | | | | | | | Sequence components of *kabuli* genome | *Kabuli* gene accession IDs | NCBI-KOG | TFs | NCBI-nr database |
| CaPOPI_3851 | Ca6 | 6918472 | ATGGGTAGGGT/ATGGGTAGGGTTTGGGTAGGGT | TTGTTCGCCATTGATTTTGA | TGATACCCAAGAGGGTACGG | 60.1 | 333 | INTERGENIC | | | | |
| CaPOPI_3852 | Ca6 | 6954085 | ATAACTAACT/ATAACT | ACAGCTTCGAACTTGCACCT | GCATTCAGTGGTCCAATGTG | 60.1 | 482 | INTRON | Ca_09688 | | NF-YB | Protein of unknown function DUF248, methyltransferase putative |
| CaPOPI_3853 | Ca6 | 7254332 | AATATATATATATATATATATATAT/AATATATATATATATATATAT | TCACAGTCAAGCACGTCTCC | AGGGTTGGGTTGTGTTCAAA | 60.0 | 708 | INTERGENIC | | | | |
| CaPOPI_3854 | Ca6 | 7373908 | TTATATATATATATATATATATAT/TTATATATATATATATATATATAT | GGAAAGGCTTCACAAAGCTG | TAGCCCCTCCTGCTCATAGA | 60.0 | 696 | DRR | Ca_09643 | Q | | Alcohol dehydrogenase superfamily, zinc-containing |
| CaPOPI_3855 | Ca6 | 7548995 | ATTTTTTTT/ATTTTTTTTT | ATTTCTCAACAGGGTGGTGC | GATCGCAAGGGATGATGAAT | 60.0 | 667 | INTERGENIC | | | | |
| CaPOPI_3856 | Ca6 | 7555065 | GGTCTA/GGTCTAGTCTA | GGCATCAACCCCCTTTTAAT | TATCCAAGCTGGGAATGGAG | 60.0 | 746 | INTRON | Ca_09627 | S | | |
| CaPOPI_3857 | Ca6 | 7568051 | GACTAA/GACTAACTAA | TGGGGGAAACTTTGCTACAC | TTTTGACCCATGAGGAGACC | 60.0 | 615 | INTRON | Ca_09627 | S | | |
| CaPOPI_3858 | Ca6 | 7572118 | CTTTTTTTT/CTTTTTTT | CTTTGCTAAGGGCGTTTGAG | CCCATGCAAAAGATTTGGTT | 60.0 | 536 | INTRON | Ca_09627 | S | | |
| CaPOPI_3859 | Ca6 | 7587896 | TTT/TTTAATT | AGTTGCGATTGTCCGAAGTT | GATGAGACTTTTCCCTAGCCTT | 59.7 | 485 | INTERGENIC | | | | |
| CaPOPI_3860 | Ca6 | 7588189 | TT/TTGCAT | AAGGCTAGGGAAAAGTCTCATC | GCTCTCAGTTGAATAGCACGG | 58.1 | 504 | INTERGENIC | | | | |
| CaPOPI_3861 | Ca6 | 7592871 | GAAGAAA/GAAGAAAGAAA | AGCACGAAAGTTTCCACCAG | AGAGACGGTATCCGGTGTTG | 60.3 | 646 | INTRON | Ca_09625 | | ERF | Glycoside hydrolase, family 17 |
| CaPOPI_3862 | Ca6 | 7593280 | CAAA/CAAAAA | CCTTCTTGCTGGCATTTAGC | TGGCTTAAAGAGATCGGTGC | 60.0 | 477 | INTERGENIC | | | | |
| CaPOPI_3863 | Ca6 | 7606703 | ATT/AT | TTTCGTGGCATGGAATACAA | CCAAAAATTTGCCAGCAGTT | 59.9 | 597 | INTRON | Ca_09624 | R | | Domain of unknown function DUF699, exodeoxyribonuclease V alpha chain |
| CaPOPI_3864 | Ca6 | 7632106 | A/AACT | CGCTTTTGTGTTCAGTTGTGA | GGAAAAATGTGAAATCCAAAAA | 59.9 | 930 | INTERGENIC | | | | |
| CaPOPI_3865 | Ca6 | 7643356 | ATTTTTT/ATTTTT | TTGCATTCCATAAACACGGA | TGATCAGATAGATATGTTGACCGA | 59.9 | 695 | INTERGENIC | | | | |

| **INDEL marker IDs** | **Chromosomes/ unanchored scaffolds** | **Physical positions (bp)** | **InDels (*Kabuli* reference genome-CDC Frontier/PI)** | **Forward primers (5'-3')** | **Reverse primers (5'-3')** | **Anneling temperature (0C)** | **Expected amplified product size (bp)** | **Structural annotation** | | **Functional annotation** | | |
|---|---|---|---|---|---|---|---|---|---|---|---|---|
| | | | | | | | | **Sequence components of *kabuli* genome** | ***Kabuli* gene accession IDs** | **NCBI-KOG** | **TFs** | **NCBI-nr database** |
| CaPOPI_3866 | Ca6 | 7644118 | ATTTTTTTTTT/ATTTTTTTTT | CCAGAGGTTGAATCAATGAGA | GAGGAAGCACGAACGAAGTC | 57.7 | 674 | INTERGENIC | | | | |
| CaPOPI_3867 | Ca6 | 7658418 | TTAAAAATAAAAAT/TTAAAAATAAAAATAAAAAT | AGATCAGCCGCAGAATTTGT | CACCCGTGAAACTGTTGATG | 59.8 | 606 | DRR | Ca_09616 | | | |
| CaPOPI_3868 | Ca6 | 7685952 | AATATATATATATATATATATATAT/AATATATATATATATATATAT | CAGCGCCATAGTTGAGCATA | GGGTGGTGTTGGAGTCATTT | 60.0 | 591 | INTERGENIC | | | | |
| CaPOPI_3869 | Ca6 | 7861860 | AACTTCAGGCA/AA | GGAGGCGTTGACAATAGTGAT | TATTTGGCGTCGTCTGTCTG | 59.1 | 616 | INTERGENIC | | | | |
| CaPOPI_3870 | Ca6 | 7912887 | GTT/GTTTTT | CCTTGAAGCTTCTCCCATCA | CTCCCTCTCTGTGGTTTTGG | 60.3 | 896 | INTRON | Ca_09584 | U | M-type | Inositol polyphosphate-related phosphatase |
| CaPOPI_3871 | Ca6 | 7961982 | A/AAT | CGTTGCAGCTGCTTTGTTTA | TTGGGGAAGAAAGTTGGAGA | 60.2 | 841 | INTERGENIC | | | | |
| CaPOPI_3872 | Ca6 | 8032854 | AATATATATATATATATATATATATAT/AATATATATATATATATATATATATATATAT | TTGCAATACTTTATCCTAGAGCCC | TGGTGAGGGCATCAATTTTT | 60.0 | 929 | INTERGENIC | | | | |
| CaPOPI_3873 | Ca6 | 8085419 | ATA/ATACAGTTTA | TTTCCCCCGTAGACACTGAC | TGATAAAAAGACGGAATCTTTCAA | 60.0 | 565 | DRR | Ca_09563 | C | | Mitochondrial carrier protein |
| CaPOPI_3874 | Ca6 | 8313713 | GTTTTTTTTTT/GTTTTTTTTTTT | TTTGACAATCTCATGTTTTGCAG | GGGTCAAAAAGTGTGCAGGT | 60.2 | 730 | INTERGENIC | | | | |
| CaPOPI_3875 | Ca6 | 8557817 | TGAAGAAGAAGAAGAAGAAGAAGAAGAAGAAGAAGAAGAAG/TGAAGAAGAAGAAGAAGAAGAAGAAGAAGAAGAAGAAGAAG | TTTGGAGACCAATGGTGACA | CTCCGTTTGTAGCGCAAAAT | 59.9 | 783 | INTERGENIC | | | | |
| CaPOPI_3876 | Ca6 | 8796967 | A/AG | GCCAAACAATAGTTGAAACCAA | AACGTTACGCATGAACCTCC | 59.0 | 588 | INTRON | Ca_16754 | E | | Amino acid transporter, transmembrane |
| CaPOPI_3877 | Ca6 | 8904663 | GATATATATATATATATATATATATATATATATAT/GATATATATATATATATATATATATATATATAT | TGAATTGCTTCGAACCTGTG | TGCATTTCAAGCTCTCATGC | 59.8 | 465 | INTERGENIC | | | | |
| CaPOPI_3878 | Ca6 | 9130006 | ATGATTTGATTTGATTT/ATGATTTGATTTGATTTGATTTGATTT | ATTGTGATCGGAGATCGAGG | AAATGTGGTGGAGGCTTGTC | 60.0 | 400 | INTRON | Ca_08684 | O | Nin-like | Rab GDI protein |
| CaPOPI_3879 | Ca6 | 9203559 | G/GTTATATT | TCGTGAAATAGTGGACGGTG | TGAACATCAAAATGCCCAAA | 59.6 | 763 | DRR | Ca_08676 | OT | | Peptidase, cysteine peptidase active site |
| CaPOPI_3880 | Ca6 | 9257795 | TAAAAAAAAAAA/TAAAAAAAAAA | GCATCCTCGGAAAATTTCAA | CACGACAAGTCTCGTGGCTA | 60.0 | 352 | INTERGENIC | | | | |

| INDEL marker IDs | Chromosomes/ unanchored scaffolds | Physical positions (bp) | InDels (*Kabuli* reference genome-CDC Frontier/PI) | Forward primers (5'-3') | Reverse primers (5'-3') | Anneling temperature (0C) | Expected amplified product size (bp) | Structural annotation | | Functional annotation | | |
|---|---|---|---|---|---|---|---|---|---|---|---|---|
| | | | | | | | | Sequence components of *kabuli* genome | *Kabuli* gene accession IDs | NCBI-KOG | TFs | NCBI-nr database |
| CaPOPI_3881 | Ca6 | 9403873 | AATATATATATATATA/AATATATATATATATATA | TTTTGTCGCAATGATAGTTTGA | TAGCGTTCCGAATCTTTCGT | 58.4 | 891 | INTERGENIC | | | | |
| CaPOPI_3882 | Ca6 | 9472058 | CAAAAAAAAAAA/CAAAAAAAAAAAA | TTCATTTGGTTCTCCCTTCG | TGTTTAGTACTTTTCCCAATGCC | 60.0 | 498 | INTERGENIC | | | | |
| CaPOPI_3883 | Ca6 | 9606829 | TAAAAAAAAAAAAA/TAAAAAAAAAAAAAAA | CGCTACATGAACTTTGTCACTCA | AAAGGTGATGGTCGAAGGTG | 60.4 | 567 | INTERGENIC | | | | |
| CaPOPI_3884 | Ca6 | 9884658 | AT/ATGT | GCCTTTGAGCGGTAATGAAA | TCGGAAATTTGGAGTCAAGC | 60.2 | 716 | INTERGENIC | | | | |
| CaPOPI_3885 | Ca6 | 9890136 | CTTTTTTTTTTT/CTTTTTTTT | AGTCTTCCACGTTGGTGTCC | CCTTTCTTCAGCCAAACCAA | 60.0 | 381 | INTRON | Ca_08601 | | FAR1 | Protein kinase, catalytic domain |
| CaPOPI_3886 | Ca6 | 9893250 | TAA/TAAA | CCGAATCATGGAAGGAGAGA | AACCGCGAAACTTCATTCAC | 60.2 | 774 | INTERGENIC | | | | |
| CaPOPI_3887 | Ca6 | 9894814 | T/TA | AAATTTCGAAACGCGAGAGA | GGATCTTGCAACGTGGACTT | 60.0 | 391 | DRR | Ca_08600 | O | | Peptidase, cysteine peptidase active site |
| CaPOPI_3888 | Ca6 | 9900963 | CATGAT/CAT | ATGTTGCCAAGGCAGAGAGT | TCGTCGAGGCATCACAACTA | 59.9 | 928 | INTERGENIC | | | | |
| CaPOPI_3889 | Ca6 | 9903379 | ATTT/ATTTT | TTCGATGTTGCCTAGTCCCT | GTCGTCGAGGCATCAAAACT | 59.7 | 230 | INTERGENIC | | | | |
| CaPOPI_3890 | Ca6 | 9925899 | CATTATTATTATTATTATT/CATTATTATTATTATT | CCAATTTTGGTGTTGCTCCT | GGTGAGGACACTTCAATGGG | 60.0 | 448 | INTERGENIC | | | | |
| CaPOPI_3891 | Ca6 | 9935576 | CGAGAAAAACAGAGAAAAA/CGAGAAAAACAGAGAAAAACAGAGAAAAA | TTCATTTCCGTTGCAAAATG | AATGAAGAATTTGAGCGCGT | 59.5 | 381 | INTERGENIC | | | | |
| CaPOPI_3892 | Ca6 | 9957991 | TAAAAAAAAA/TAAAAAAA | TTAGAGTTAATTTGATGGACAGTGA | AAAATTAAAAAGTGCTAAGAGCAAA | 57.1 | 525 | INTERGENIC | | | | |
| CaPOPI_3893 | Ca6 | 9961842 | CAAAAAAA/CAAAAAA | ACGTTTTCGAATTGGCTCAT | AGAAATCATTCGTCCGTTCC | 59.6 | 366 | DRR | Ca_08594 | O | | Preprotein translocase Sec, Sec61-beta subunit |
| CaPOPI_3894 | Ca6 | 9987106 | CA/CAA | TTGACATCAAAATTCGACAACA | TCACCACACAATCAAACCCA | 59.1 | 337 | INTERGENIC | | | | |
| CaPOPI_3895 | Ca6 | 10002215 | ATT/ATTT | CCTAATGTAAATGGAGGGGG | CAAGAGGTTCATTTGACCGA | 58.3 | 291 | INTERGENIC | | | | |

| **INDEL marker IDs** | **Chromosomes/ unanchored scaffolds** | **Physical positions (bp)** | **InDels (*Kabuli* reference genome-CDC Frontier/PI)** | **Forward primers (5'-3')** | **Reverse primers (5'-3')** | **Anneling temperature (0C)** | **Expected amplified product size (bp)** | **Structural annotation** | | **Functional annotation** | | |
|---|---|---|---|---|---|---|---|---|---|---|---|---|
| | | | | | | | | **Sequence components of *kabuli* genome** | ***Kabuli* gene accession IDs** | **NCBI-KOG** | **TFs** | **NCBI-nr database** |
| CaPOPI_3896 | Ca6 | 10002372 | CAATTTATTAAATTT/CAATTT | TCGGTCAAATGAACCTCTTG | TCGAATACTGTTTTGAGAACCTTT | 58.7 | 328 | INTERGENIC | | | | |
| CaPOPI_3897 | Ca6 | 10012800 | A/AT | ACCATATCCCAAGCAAGCAC | CTATGCCTGGGGCATGATTA | 60.0 | 792 | DRR | Ca_08591 | R | G2-like | Domain of unknown function DUF676, hydrolase-like |
| CaPOPI_3898 | Ca6 | 10046135 | GTTTTTTT/GTTTTTTTT | CAGCCGTTGGTTCAATAGGT | TCATGCATGTTTTTATCAATTTTGT | 60.0 | 722 | INTERGENIC | | | | |
| CaPOPI_3899 | Ca6 | 10050244 | TTTTATTTATTTATTTATTTATT/TTTTATTTATTTATTTATTTATTTATTTATT | GCAAATCGACAACGAATCCT | TTTTAGGGACGAAAATGATGG | 60.1 | 560 | INTERGENIC | | | | |
| CaPOPI_3900 | Ca6 | 10050426 | AAT/ACGGTTGAT | GCAAATCGACAACGAATCCT | TTTTAGGGACGAAAATGATGG | 60.1 | 560 | INTERGENIC | | | | |
| CaPOPI_3901 | Ca6 | 10061419 | A/AT | TTGAAATTGGTAACGAATGAGAAA | CATCTAACAACAAACACCGTCA | 59.9 | 674 | INTERGENIC | | | | |
| CaPOPI_3902 | Ca6 | 10061549 | ATTTTTTT/ATTTTTTTT | TTGTGACAAATATGTATCGTGTAATTG | TCCGTAAAGATGTTGTTGATGC | 59.2 | 860 | INTERGENIC | | | | |
| CaPOPI_3903 | Ca6 | 10107731 | CTTTTTTTTT/CTTTTTTTT | GCGCAAGGTTGCATAAATCT | ACTCGTGCTAGCGACATTGA | 60.2 | 766 | INTERGENIC | | | | |
| CaPOPI_3904 | Ca6 | 10107926 | CACAATAA/CA | TGCACATCAACCTTAAATTCCA | GTTTCCGATTGCCAGGTTTA | 60.4 | 745 | INTERGENIC | | | | |
| CaPOPI_3905 | Ca6 | 10123378 | AAGTA/AA | TTTCTTTCCGTCACAAGCTG | TTTTTGTGATTTCGCAGACG | 59.0 | 175 | INTERGENIC | | | | |
| CaPOPI_3906 | Ca6 | 10135848 | TTTTTTT/TTTTTTTATTTTTT | TTCCAAGGATCCCATTACCA | TATTTTGTCGGTGTGACCCA | 60.1 | 440 | INTERGENIC | | | | |
| CaPOPI_3907 | Ca6 | 10163284 | ATTTTTTT/ATTTTTT | CCACAACAAAGGACGAGACA | CATGGACACAAATTGCAAGG | 59.7 | 827 | INTERGENIC | | | | |
| CaPOPI_3908 | Ca6 | 10178382 | TC/T | TATCTAACGTGCCCCCTCAC | ACAGGGGAAACTAGACCCGT | 60.0 | 635 | INTRON | Ca_08573 | | ERF | Glycoside hydrolase, family 17 |
| CaPOPI_3909 | Ca6 | 10178910 | TA/T | ACGGGTCTAGTTTCCCCTGT | TTAATGCCTAGATCCTGCGG | 59.9 | 166 | INTRON | Ca_08573 | | ERF | Glycoside hydrolase, family 17 |
| CaPOPI_3910 | Ca6 | 10180510 | CTCTTTCTTTC/CTCTTTC | GAGACAAACTGAAGGCCGAG | GGTTGGTCTCGAATAAGGCA | 60.0 | 561 | DRR | Ca_08573 | | ERF | Glycoside hydrolase, family 17 |

| INDEL marker IDs | Chromosomes/ unanchored scaffolds | Physical positions (bp) | InDels (*Kabuli* reference genome-CDC Frontier/PI) | Forward primers (5'-3') | Reverse primers (5'-3') | Anneling temperature (0C) | Expected amplified product size (bp) | Structural annotation | | Functional annotation | | |
|---|---|---|---|---|---|---|---|---|---|---|---|---|
| | | | | | | | | Sequence components of *kabuli* genome | *Kabuli* gene accession IDs | NCBI-KOG | TFs | NCBI-nr database |
| CaPOPI_3911 | Ca6 | 10185405 | TAAA/TAAAA | TTTTTGACGAGATTGGCAATTA | AGGCCACAACTTGTCATTTG | 59.6 | 528 | INTERGENIC | | | | |
| CaPOPI_3912 | Ca6 | 10190857 | C/CT | AGGGTATTTTCACGCAGGTG | CGAACGCACACACTTTTCAT | 60.0 | 433 | INTRON | Ca_08570 | I | | AMP-dependent synthetase/ligase |
| CaPOPI_3913 | Ca6 | 10208472 | ATTTTT/ATTTT | TGATCGCATTAACCTTTGACTG | AGAAAATCCCTCTTCCCCAC | 60.1 | 656 | INTERGENIC | | | | |
| CaPOPI_3914 | Ca6 | 10209877 | ACTCTCTCT/ACTCTCT | AGGGAGAGTCGTATTCGGGT | CAGGGTTTTGGGTTTGAAGA | 60.0 | 137 | INTERGENIC | | | | |
| CaPOPI_3915 | Ca6 | 10224803 | TC/TCC | CCCCGCAGTTTGTTTTGTAT | GATGCTATTGTGGGCATGTG | 59.9 | 641 | DRR | Ca_08566 | T | FAR1 | Metallo-dependent phosphatase |
| CaPOPI_3916 | Ca6 | 10250923 | CAAAA/CAAAAA | TTATAAGCACTCGCGTTCCA | AAATCCGCGATTGGTTTCTA | 59.5 | 409 | DRR | Ca_08565 | R | bZIP | |
| CaPOPI_3917 | Ca6 | 10251637 | TTTCAATAATT/TTTCAATAATTCAATAATT | GGATTGCAGGAAGATACCCA | TCGTAAACGGTTTCTTTGGC | 59.9 | 640 | INTERGENIC | | | | |
| CaPOPI_3918 | Ca6 | 10251944 | CTCATCATCATCATC/CTCATCATCATCATCATC | CATATAGGGTGGGGTTGGTG | ATCCAAAACGACGACGAATC | 59.9 | 344 | INTERGENIC | | | | |
| CaPOPI_3919 | Ca6 | 10259432 | GTTTTTTT/GTTTTTTTTTT | ATTCCGTAGTCGTCGTCACC | AAGGCAGCGTTGAAGGTAGA | 60.0 | 505 | INTERGENIC | | | | |
| CaPOPI_3920 | Ca6 | 10265158 | ACATGTCTAACTACTTCA/ACATGTCTAACTACTTCATGTCTAACTACTTCA | TTGATTTGTGCATTGGTGCT | TCCCTTGATTGTTTCTTTTGG | 60.1 | 199 | DRR | Ca_08562 | S | | YL1 nuclear, C-terminal |
| CaPOPI_3921 | Ca6 | 10272224 | G/GGCGTATAT | TAGGGTGCTAGCGGATTTTG | AAGGCACCCATTAGTTGACG | 60.2 | 550 | DRR | Ca_08561 | | | Plant lipid transfer protein/seed storage/trypsin-alpha amylase inhibitor |
| CaPOPI_3922 | Ca6 | 10275563 | CTTT/CTTTT | AGGTCTAGGCCAAATCAGAAA | ATGTGCCATGTCGTAGGCTT | 57.9 | 497 | DRR | Ca_08560 | R | | |
| CaPOPI_3923 | Ca6 | 10316984 | C/CG | TGCAGAACAACAAACATGGC | ATTGCATCCGATTGTTCTCC | 60.7 | 613 | INTERGENIC | | | | |
| CaPOPI_3924 | Ca6 | 10318872 | CTTTTTTTTTTTTT/CTTTTTTTTTTTT | TTAACCACCCGGTTCTCAAG | TGACATGGTTGGCTGTGAAT | 60.0 | 724 | INTERGENIC | | | | |
| CaPOPI_3925 | Ca6 | 10377538 | ATA/ATATTATTATTA | CTCGATTTGGGCGAAAATAA | TTGGCGCATCAATTTTTGTA | 60.0 | 549 | INTERGENIC | | | | |

| INDEL marker IDs | Chromosomes/ unanchored scaffolds | Physical positions (bp) | InDels (*Kabuli* reference genome-CDC Frontier/PI) | Forward primers (5'-3') | Reverse primers (5'-3') | Anneling temperature (0C) | Expected amplified product size (bp) | Structural annotation | | Functional annotation | | |
|---|---|---|---|---|---|---|---|---|---|---|---|---|
| | | | | | | | | Sequence components of *kabuli* genome | *Kabuli* gene accession IDs | NCBI-KOG | TFs | NCBI-nr database |
| CaPOPI_3926 | Ca6 | 10377821 | ATGT/AT | CTCGATTTGGGCGAAAATAA | TTGGCGCATCAATTTTTGTA | 60.0 | 549 | INTERGENIC | | | | |
| CaPOPI_3927 | Ca6 | 10395343 | TAAAAAAAAAAAAA/TAAAAAAAAAAA | GTGTGTGGGTTTTTGGCTTT | TTTGCAGCAAGGCAACATAG | 59.9 | 565 | INTRON | Ca_08553 | R | MYB_related | Small ubiquitin-related modifier, SUMO |
| CaPOPI_3928 | Ca6 | 10412258 | GAAAAA/GAAAAAA | AGGGCCAAACTTGGATCTTT | TTTTCACAAGCTCACAGGGA | 59.9 | 374 | INTERGENIC | | | | |
| CaPOPI_3929 | Ca6 | 10417732 | TGG/T | CTCACTTGTTGGGTTTCCTCT | CCCCCTCGAACAACAAAATA | 58.3 | 369 | INTERGENIC | | | | |
| CaPOPI_3930 | Ca6 | 10438851 | CA/C | TCTCTTGGAACCTCTCGACAA | ATTGGTCCTCTACCCCCATT | 60.0 | 302 | DRR | Ca_08552 | R | | Rab-GAP/TBC domain |
| CaPOPI_3931 | Ca6 | 10457521 | TA/TCTTTTTCTAAGTCA | TTCCTTCGGTCTTGTCTGCT | ATTTCGGATCATGCAACACA | 60.0 | 551 | INTRON | Ca_08551 | | | |
| CaPOPI_3932 | Ca6 | 10458622 | TGGATG/T | CAGAGTTGGAGTGGTGCAAA | TTTTCAGTTTCGCCATGGAT | 59.9 | 567 | INTRON | Ca_08551 | | | |
| CaPOPI_3933 | Ca6 | 10472712 | TAAAAAAA/TAAAAAAAAA | AACCAGATCAGATTGTCGCC | TGGGATGAATTTGGATCATTT | 60.1 | 852 | INTERGENIC | | | | |
| CaPOPI_3934 | Ca6 | 10476151 | TA/T | GCCCATTTATGGGGGTATTT | GCTCTGTGGGAACTGAGGAG | 59.8 | 595 | INTERGENIC | | | | |
| CaPOPI_3935 | Ca6 | 10482318 | CA/C | CAAGTGGGAAAATGGCAAGT | ATTGGATTGAGTTGGGCTTG | 60.0 | 535 | INTRON | Ca_08548 | E | CAMTA | Spermine synthase |
| CaPOPI_3936 | Ca6 | 10486008 | ATTT/ATTTTT | AATCAAGTATTTGTCCGGCG | TCTGTGGAAGCAAGATGCAC | 60.0 | 650 | DRR | Ca_08548 | E | CAMTA | Spermine synthase |
| CaPOPI_3937 | Ca6 | 10497132 | CAAATAAATAAATAAATAAATAAA/CAAATAAATAAATAAATAAA | ACCACCTTTGAGACAAACCG | GTTTGCAGTGGTTTCCCTGT | 60.0 | 291 | INTRON | Ca_08547 | G | C2H2 | D-galactoside/L-rhamnose binding SUEL lectin domain |
| CaPOPI_3938 | Ca6 | 10514411 | CAAAAAAAAA/CAAAAAAAAAAA | TTCGCAATCTGTGTTTTTCG | CAAGAATTCGTATCGTCTAAATGC | 59.8 | 642 | INTERGENIC | | | | |
| CaPOPI_3939 | Ca6 | 10559368 | T/TG | CGCCCTAGGTGCTTGTTTTA | TGGATGGATGTTTGCAATGT | 60.3 | 417 | INTERGENIC | | | | |
| CaPOPI_3940 | Ca6 | 10564033 | AATATAT/AAT | GAAAATCCCAATCCAAATTTCA | GCAGCACACAGCATAGGAAA | 60.0 | 567 | INTERGENIC | | | | |

| INDEL marker IDs | Chromosomes/ unanchored scaffolds | Physical positions (bp) | InDels (*Kabuli* reference genome-CDC Frontier/PI) | Forward primers (5'-3') | Reverse primers (5'-3') | Anneling temperature (0C) | Expected amplified product size (bp) | Structural annotation | | Functional annotation | | |
|---|---|---|---|---|---|---|---|---|---|---|---|---|
| | | | | | | | | Sequence components of *kabuli* genome | *Kabuli* gene accession IDs | NCBI-KOG | TFs | NCBI-nr database |
| CaPOPI_3941 | Ca6 | 10564108 | ATTTTTTTTT/ATTTTTT | GAAAATCCCAATCCAAATTTCA | GCAGCACACAGCATAGGAAA | 60.0 | 567 | INTERGENIC | | | | |
| CaPOPI_3942 | Ca6 | 10564399 | CTT/CTTT | TTTCCTATGCTGTGTGCTGC | CTTTTCACCCTTGATCGGAA | 60.0 | 611 | INTERGENIC | | | | |
| CaPOPI_3943 | Ca6 | 10576490 | CTTTTT/CTTTTTT | TTGCCAAAGGTTAATGCACA | ATGGTGGTTGCTAGGTTTGC | 60.1 | 888 | INTERGENIC | | | | |
| CaPOPI_3944 | Ca6 | 10587047 | A/AC | CACCCAGTCGCTCACAATTA | CACGACCTTCCTTTGGTTGT | 59.7 | 824 | INTERGENIC | | | | |
| CaPOPI_3945 | Ca6 | 10604574 | TTAT/TTATTTATTTATCTAT | GCAAAGGCTTGTTGGTGTTT | AAAGCGAGATCTGAACCGAA | 60.2 | 264 | INTRON | Ca_08537 | H | | Adenosylhomocysteinase |
| CaPOPI_3946 | Ca6 | 10608315 | TG/TGG | ACCCGCTTAATTGTGGCTAA | ACATTACCCGCATCCACCTA | 59.6 | 383 | INTERGENIC | | | | |
| CaPOPI_3947 | Ca6 | 10611247 | TAATAAATAAATAAATAAA/TAATAAATAAATAAATAAATAAA | TCTGACATGACAAAATGGTGG | TCGGAATTGGTCCGAAATTA | 59.4 | 409 | DRR | Ca_08536 | K | HSF | Heat shock factor (HSF)-type, DNA-binding |
| CaPOPI_3948 | Ca6 | 10642500 | C/CCTATAGA | CGATTTGACAGCAAAAGCAA | GACAACCTTCGTTGCCCTAA | 60.0 | 437 | INTERGENIC | | | | |
| CaPOPI_3949 | Ca6 | 10649918 | GTTTGTTTTGTTTTGTTTT/GTTTGTTTTGTTTTGTTTTGTTTT | GACGGTTATGGAGCCTTCAA | TTCGATATCTTGTGCCTCCC | 60.1 | 363 | DRR | Ca_08531 | I | | AMP-dependent synthetase/ligase |
| CaPOPI_3950 | Ca6 | 10657533 | C/CTT | ACATCTCTCATTGCCCATCC | ATCAGGTGCTTTGCTGCTTT | 59.9 | 769 | INTRON | Ca_08531 | I | | AMP-dependent synthetase/ligase |
| CaPOPI_3951 | Ca6 | 10660162 | TTAT/TT | CTAGTGAGCATTGCGAGCAG | CTTGTGAGGCAGTCCAATCA | 59.9 | 372 | INTRON | Ca_08531 | I | | AMP-dependent synthetase/ligase |
| CaPOPI_3952 | Ca6 | 10664790 | TC/T | TGCTTGGTACCACAAAGACAAA | AAAATTTCGTTTCAAAAATCTTGT | 60.6 | 767 | INTERGENIC | | | | |
| CaPOPI_3953 | Ca6 | 10665216 | ATTTTTTT/ATTTTTTTT | CAAAAACAAGATTTTTGAAACGAA | TCAAAAGTTTCGAGCAATACGA | 59.6 | 398 | INTERGENIC | | | | |
| CaPOPI_3954 | Ca6 | 10677110 | TCCCC/TCCCCC | TTCAACACGTGCATGGAAAT | GCTAACACATGATTTCCGCA | 60.0 | 581 | INTRON | Ca_08530 | | bHLH | Aspartate/glutamate/uridylate kinase |
| CaPOPI_3955 | Ca6 | 10686252 | TACAC/TAC | CCAGCTTCTTGTTGTGTGGA | CAGAGGCAACAAGCTCAGTG | 59.9 | 609 | INTRON | Ca_08529 | | G2-like | Peptidase S8/S53, subtilisin/kexin/sedolisin |

| INDEL marker IDs | Chromosomes/ unanchored scaffolds | Physical positions (bp) | InDels (*Kabuli* reference genome-CDC Frontier/PI) | Forward primers (5'-3') | Reverse primers (5'-3') | Anneling temperature (0C) | Expected amplified product size (bp) | Structural annotation | | Functional annotation | | |
|---|---|---|---|---|---|---|---|---|---|---|---|---|
| | | | | | | | | Sequence components of *kabuli* genome | *Kabuli* gene accession IDs | NCBI-KOG | TFs | NCBI-nr database |
| CaPOPI_3956 | Ca6 | 11315858 | ATTT/ATTTT | GTTTGTTGTGGTTGCAGTGG | GCTGATTGGTTGCCTCTCTC | 60.0 | 399 | INTERGENIC | | | | |
| CaPOPI_3957 | Ca6 | 11344092 | CTTTTTTTTTT/CTTTTTTTTTTT | TTGACATGTTTTATTGGTGTGAA | CGTGGATGTAGGCAAGAGGT | 58.0 | 652 | INTERGENIC | | | | |
| CaPOPI_3958 | Ca6 | 11367342 | CAAAAAAAAAA/CAAAAAAAAA | TAGATCCACCTTCAAACCCG | AAAAGCGGTGACAGATGGAC | 59.9 | 667 | INTRON | Ca_23422 | K | ARR-B | Signal transduction response regulator, receiver domain |
| CaPOPI_3959 | Ca6 | 11464290 | TAAAA/TAA | AGGAAAAGGAGGAGGATGGA | GTGCCAAAACAAATGACGTG | 60.0 | 638 | INTERGENIC | | | | |
| CaPOPI_3960 | Ca6 | 11546492 | GTAAAA/G | TCGGAAAGGTCATTCAAAGC | TTTGTGTCCAAAGCATTCTCC | 60.2 | 472 | INTERGENIC | | | | |
| CaPOPI_3961 | Ca6 | 11569101 | T/TAAGTAA | CAAAAGGGGGTATTTTGGTTC | CGTTCAATTTCCTGCCAAAT | 59.6 | 499 | INTERGENIC | | | | |
| CaPOPI_3962 | Ca6 | 11617529 | GA/GAA | TTGGGAGTTCATTTGCATCA | TTTGTGAAGATATTTGTTTCCTCG | 60.0 | 905 | INTERGENIC | | | | |
| CaPOPI_3963 | Ca6 | 11617707 | AATATATATATATATATATATATATATAT/AATATATATATATATATATATATATATATATATATAT | GAGGCATCGTTTCACAATGA | AGGTTTGCGAGGACTGTGTT | 59.7 | 809 | INTERGENIC | | | | |
| CaPOPI_3964 | Ca6 | 11630320 | AAGTAGT/AAGT | TGCATATGTGTGGGGGAATA | GCTTGATACTTGGCAAAGCC | 59.6 | 621 | INTERGENIC | | | | |
| CaPOPI_3965 | Ca6 | 11650123 | TTAA/T | GGAAGGGTCCACGCATAGTA | GCTTTTGTGGTGGCACTTTT | 60.0 | 827 | DRR | Ca_25059 | J | | Ribosomal protein S7 |
| CaPOPI_3966 | Ca6 | 11655132 | TAAAACTAAAAACTAAAAACTAAAAACTA/TAAAACTAAAAACTAAAAACTA | GCATGGATGAAATCAAAAGC | TTGGGATGATCCAACAACAA | 58.2 | 333 | DRR | Ca_25060 | | FAR1 | |
| CaPOPI_3967 | Ca6 | 11655226 | AATAACTTTTTA/AA | GCATGGATGAAATCAAAAGC | TTGGGATGATCCAACAACAA | 58.2 | 333 | DRR | Ca_25060 | | FAR1 | |
| CaPOPI_3968 | Ca6 | 11680950 | C/CA | TAATCCATGGCGAACACAAA | TCCAAAGTTTGCAGCATTCA | 59.9 | 581 | INTERGENIC | | | | |
| CaPOPI_3969 | Ca6 | 11681178 | TTGTGTGTGTGTGTGT/TTGTGTGTGTGTGTGTGTGTGT | ATCAATTGCCATTTGCATCA | TAGCATTTTTGAGGGGGAAA | 59.9 | 692 | INTERGENIC | | | | |
| CaPOPI_3970 | Ca6 | 11688551 | GAAAA/GAAAAA | TCTCCTCCTCCTCCTTCTCC | TGTTGCCCAAAACCATCATA | 59.9 | 557 | INTERGENIC | | | | |

| **INDEL marker IDs** | **Chromosomes/ unanchored scaffolds** | **Physical positions (bp)** | **InDels (*Kabuli* reference genome-CDC Frontier/PI)** | **Forward primers (5'-3')** | **Reverse primers (5'-3')** | **Anneling temperature (0C)** | **Expected amplified product size (bp)** | **Structural annotation** | | **Functional annotation** | | |
|---|---|---|---|---|---|---|---|---|---|---|---|---|
| | | | | | | | | **Sequence components of *kabuli* genome** | ***Kabuli* gene accession IDs** | **NCBI-KOG** | **TFs** | **NCBI-nr database** |
| CaPOPI_3971 | Ca6 | 11691624 | T/TA | CTTTGCAGCAAGTCATTGGA | CCAAAACTGGGGAGAAACAA | 60.0 | 871 | INTERGENIC | | | | |
| CaPOPI_3972 | Ca6 | 11722419 | TATATT/TATATTAATATT | CCTTCGCCGTATATGATTCC | AAGCAAAGGTCCTGAGCAAA | 59.4 | 185 | INTERGENIC | | | | |
| CaPOPI_3973 | Ca6 | 11737556 | CTATATATATATATAT/CTATATATATATATATATATAT | GCAAGCGCTATGTGACCTTT | AATGTGAATCCCACCAGGAA | 60.4 | 770 | INTERGENIC | | | | |
| CaPOPI_3974 | Ca6 | 11742023 | ATTTTTTT/ATTTTTTTT | GCGCTTCAATTTACAACGCT | TTTTCGCACGAAATCAATCA | 60.4 | 614 | INTERGENIC | | | | |
| CaPOPI_3975 | Ca6 | 11785408 | ATTTT/ATTT | TGCAAACGCACATATACACG | AATTTGAGGCGTGTGGGTAG | 59.2 | 711 | INTERGENIC | | | | |
| CaPOPI_3976 | Ca6 | 11792161 | AAT/A | TGTTGGACGGACAATTGAAA | CAAAATGCCATAAAATCGGC | 59.9 | 408 | INTERGENIC | | | | |
| CaPOPI_3977 | Ca6 | 11806357 | CATATATATATATATATAT/CATATATATATATATATATAT | TCACCTAGGAATTCCGCATC | CGAGGTTGGAGTTGGATCAC | 60.0 | 711 | INTERGENIC | | | | |
| CaPOPI_3978 | Ca6 | 12253200 | AATATATATATATATATATATATATATATATATATATATAT/AATATATATATATATATATATATATATATATATATATATATATATATATATATAT | CTCCGTTTATCTCCGTTGGA | CGTGATGATTGATGCATTTCA | 60.1 | 746 | INTERGENIC | | | | |
| CaPOPI_3979 | Ca6 | 12254420 | AATATATATATATATATATATATATATATATA/AATATATATATATATATATATATATATATATATATA | CCTGTGGTTGGTTGTAGCCT | CTGGAGGTCACCTTCGACAT | 60.0 | 703 | INTERGENIC | | | | |
| CaPOPI_3980 | Ca6 | 12335628 | TGAAG/TG | GGAAGGGTGTTGGTGTGTTT | TTGGTCACTAAGGGGGTGAG | 59.7 | 968 | DRR | Ca_24849 | | | Protein of unknown function DUF688 |
| CaPOPI_3981 | Ca6 | 12543039 | TTATATATATATATATATATATATATATATATATATATATATATA/TTATATATATATATATATATATATATATATATATATATATATATATATATATA | TTTCGACATAGGTTGTTAAGCTTTT | GAAACCAGTGAACTTCTCCCC | 59.7 | 590 | INTERGENIC | | | | |
| CaPOPI_3982 | Ca6 | 12663993 | TAAA/TAAAA | ATTGCCTTGCACACACGTAA | CGGCTCGAACGGTACATATT | 60.2 | 636 | INTERGENIC | | | | |
| CaPOPI_3983 | Ca6 | 12787897 | AATATATATATATATATATATAT/AATATATATATATATATATATATAT | ATCAAACAAATGTTGCGAGC | CAAACCCCTATTTTTGTGATTGA | 58.8 | 749 | INTERGENIC | | | | |
| CaPOPI_3984 | Ca6 | 12853638 | TAAAA/TAAA | CTCATCCATCACACCAAAGC | CTCAGATTCTGCGTCCATCA | 59.1 | 875 | INTRON | Ca_05058 | T | TALE | Protein kinase, catalytic domain |
| CaPOPI_3985 | Ca6 | 12856182 | TC/TCAAGTTCGAC | TCCAATGCTGGATTAGGCTC | GCATTTATGAACATAACGCCAA | 60.2 | 512 | DRR | Ca_05058 | T | TALE | Protein kinase, catalytic domain |

| INDEL marker IDs | Chromosomes/ unanchored scaffolds | Physical positions (bp) | InDels (*Kabuli* reference genome-CDC Frontier/PI) | Forward primers (5'-3') | Reverse primers (5'-3') | Anneling temperature (0C) | Expected amplified product size (bp) | Structural annotation | | Functional annotation | | |
|---|---|---|---|---|---|---|---|---|---|---|---|---|
| | | | | | | | | Sequence components of *kabuli* genome | *Kabuli* gene accession IDs | NCBI-KOG | TFs | NCBI-nr database |
| CaPOPI_3986 | Ca6 | 12865294 | ATTGT/ATTGTATATTTATTTTTTGT | CAAAATGGACACTTAAGGGGA | CTTCCACCCATTATGCCACT | 58.9 | 948 | DRR | Ca_05059 | O | MYB_related | Ubiquitin-conjugating enzyme, E2 |
| CaPOPI_3987 | Ca6 | 12877302 | CG/CGCTTTTTTTG | GTTCGTAAAGGACGGGATGA | CTTTTACAGCGCTTTCTGGG | 59.9 | 235 | INTERGENIC | | | | |
| CaPOPI_3988 | Ca6 | 12879345 | TAAAAAAAAAA/TAAAAAAAAA | TAAAGGACCAAGTCGGGAAA | ATCCAAAGGAAAGCATCGTG | 59.5 | 599 | INTERGENIC | | | | |
| CaPOPI_3989 | Ca6 | 12891974 | GAAAAAAAAAA/GAAAAAAAAA | GGTAAAAGCCCACTTGGTGA | CCCTGTATTTGCTCTCCGAA | 60.0 | 552 | INTERGENIC | | | | |
| CaPOPI_3990 | Ca6 | 12899744 | TAAAAAAAAAA/TAAAAAAAAA | TTGGATGTATACGGCGAACA | CAATCCACCTGAATGAAATCC | 60.0 | 881 | INTERGENIC | | | | |
| CaPOPI_3991 | Ca6 | 12909901 | AT/ATT | TGGACAGGTGAAGACCAACA | TTCCTTCCTCCAATCAATGC | 60.1 | 696 | DRR | Ca_05064 | T | bHLH | Protein kinase, catalytic domain |
| CaPOPI_3992 | Ca6 | 12920553 | TAAAAAAA/TAAAAAAAA | GGAAGGAAGGAGGTTCTTCAA | TGCACTGGTTTATTTTGAGGG | 59.7 | 585 | INTERGENIC | | | | |
| CaPOPI_3993 | Ca6 | 12922857 | A/AC | TTTTACCGCGGAAACAAATC | CAGAAACAGCAGAGACAGCG | 59.9 | 686 | INTERGENIC | | | | |
| CaPOPI_3994 | Ca6 | 12934565 | ATTTTTTTT/ATTTTTTT | CGGTTTGAAGATGAGGTTCAC | TATTCAAGCAACGGCTGGT | 59.6 | 691 | INTERGENIC | | | | |
| CaPOPI_3995 | Ca6 | 12934614 | GAAAAAAAA/GAAAAAAAAAA | CGGTTTGAAGATGAGGTTCAC | ATTATTCAAGCAACGGCTGG | 59.6 | 693 | INTERGENIC | | | | |
| CaPOPI_3996 | Ca6 | 12935007 | TA/T | GCAAACAATCTAGAGGGAGGAA | GGGGGCACAAAGACAACTAA | 59.7 | 362 | INTERGENIC | | | | |
| CaPOPI_3997 | Ca6 | 12940701 | TAAAAAAA/TAAAAAA | TAGCCTCTCCTCCCAACCTT | ATTCATGGATTGGCATTGGT | 60.2 | 665 | DRR | Ca_05066 | | | Protein of unknown function DUF1423, plant |
| CaPOPI_3998 | Ca6 | 12950692 | AATATATATAT/AATATATATATATAT | ATCATCGAAGAAGCATTGGG | TCAATTGAGCGAGTGTAGCG | 60.0 | 633 | INTERGENIC | | | | |
| CaPOPI_3999 | Ca6 | 12954436 | TAAAAA/TAAAA | TGAAATGATGAGGGGGAGAC | CCCTTCATCCCTTACCCCTA | 59.9 | 716 | INTERGENIC | | | | |
| CaPOPI_4000 | Ca6 | 12957093 | TAAAAAAAAA/TAAAAAAAA | TGAATCCCGAAATTGACCAT | AAAAGCAAACAGGTGAACCG | 60.1 | 404 | INTERGENIC | | | | |

| INDEL marker IDs | Chromosomes/ unanchored scaffolds | Physical positions (bp) | InDels (*Kabuli* reference genome-CDC Frontier/PI) | Forward primers (5'-3') | Reverse primers (5'-3') | Anneling temperature (0C) | Expected amplified product size (bp) | **Structural annotation** | | **Functional annotation** | | |
|---|---|---|---|---|---|---|---|---|---|---|---|---|
| | | | | | | | | **Sequence components of *kabuli* genome** | ***Kabuli* gene accession IDs** | **NCBI-KOG** | **TFs** | **NCBI-nr database** |
| CaPOPI_4001 | Ca6 | 12957392 | ATTTT/ATTT | TGAATCCCGAAATTGACCAT | AACTTTTGGCACTTAAAAGGGA | 60.1 | 919 | INTERGENIC | | | | |
| CaPOPI_4002 | Ca6 | 12961933 | GCA/GCATGTCATAGAATTTTAACA | TGGGCTTCCAAGTATTGACC | CAGGGGATACGACCTCTCAA | 59.9 | 693 | DRR | Ca_05068 | | MYB_related | Glycoside hydrolase, family 16 |
| CaPOPI_4003 | Ca6 | 12963634 | CAAAAAA/CAAAAAAA | TTTATTTTCCCCTTTCGGCT | AAAGAGAACCATGCCACGAG | 59.9 | 612 | INTERGENIC | | | | |
| CaPOPI_4004 | Ca6 | 12965366 | ATTTTTTTTTTT/ATTTTTTTTT | TTTCAACTGGACACTATTCTCTTCA | ATGATTGGATGAAAGAGCGG | 59.4 | 341 | INTERGENIC | | | | |
| CaPOPI_4005 | Ca6 | 12965600 | CAAAAAA/CAAAAA | CCGCTCTTTCATCCAATCAT | ACATTTTTGCCCTCATTTCG | 60.0 | 657 | INTERGENIC | | | | |
| CaPOPI_4006 | Ca6 | 12972982 | GTTTTTTTTTTT/GTTTTTTTTTT | AGTTGGTGAGAGGAAGCGAA | GTCCAACCCAGTGGAGAAAA | 60.0 | 487 | INTRON | Ca_05069 | S | | Oxoglutarate/iron-dependent oxygenase |
| CaPOPI_4007 | Ca6 | 12981169 | T/TA | ATTGAAAATGCACCACGACA | GGAATTTTGGTGGGGAGAAT | 60.0 | 417 | INTRON | Ca_05071 | O | E2F/DP | Metallo-dependent phosphatase |
| CaPOPI_4008 | Ca6 | 13007668 | AT/A | TTGAACCTCATTGTGGCAAA | ATAAGTTTTGCCTCCCCACC | 60.1 | 116 | DRR | Ca_05074 | U | M-type | Inositol polyphosphate-related phosphatase |
| CaPOPI_4009 | Ca6 | 13019215 | GAAAAAAAA/GAAAAAAAAA | TAGGCCTATGCCATCAAACC | ACGTGACACCACGCAAAGTA | 59.9 | 627 | INTERGENIC | | | | |
| CaPOPI_4010 | Ca6 | 13292268 | AATATATATATATATATAT/AATATATATATATATATAT | AAATGATGACGTGGGATGTTT | TGCGATTTCATCCACTACATTC | 59.2 | 686 | INTERGENIC | | | | |
| CaPOPI_4011 | Ca6 | 13330838 | A/ATTATTATTATTATAT | TTTCAATTTGGCTTGATGGA | GCAACTCACAACCACCTCAA | 59.1 | 567 | DRR | Ca_05104 | | | Zinc finger, RING-type |
| CaPOPI_4012 | Ca6 | 13394015 | GATATATATATATATATATATATATA/GATATATATATATATATATATATA | TTGCCACTTGATACAGCAAAA | TGCAACTTGCGTCTAGGATG | 59.4 | 771 | DRR | Ca_05112 | Q | B3 | Cytochrome P450 |
| CaPOPI_4013 | Ca6 | 13422865 | CAAAAAAAAAA/CAAAAAAAAAAA | CATCGTCGTCAACCAGTGTT | TGTGAGCCTGCTCATTGTTC | 59.6 | 550 | INTERGENIC | | | | |
| CaPOPI_4014 | Ca6 | 13428719 | CTATTTATTTATTTATTT/CTATTTATTTATTTATTTATTTATTTATTT | GTCTGCGAAAAAGGAGTTGC | TTTCCCTATTGCACCTGGAG | 60.0 | 329 | INTERGENIC | | | | |
| CaPOPI_4015 | Ca6 | 13481060 | GAAAAAAAAAAA/GAAAAAAAAAA | AAAGGTGCTGCCTGTCCTTA | GGAGTTGGAACTCGGAACAA | 59.9 | 483 | INTRON | Ca_05119 | S | | Mitochondrial matrix Mmp37 |

| INDEL marker IDs | Chromosomes/ unanchored scaffolds | Physical positions (bp) | InDels (*Kabuli* reference genome-CDC Frontier/PI) | Forward primers (5'-3') | Reverse primers (5'-3') | Anneling temperature (0C) | Expected amplified product size (bp) | Structural annotation | | Functional annotation | | |
|---|---|---|---|---|---|---|---|---|---|---|---|---|
| | | | | | | | | Sequence components of *kabuli* genome | *Kabuli* gene accession IDs | NCBI-KOG | TFs | NCBI-nr database |
| CaPOPI_4016 | Ca6 | 13541161 | TTATATATATATATATATATATATATATATATAT/TTATATATATATATATATATATATATATATAT | TTTTGTGTTTTCCAAGATGGG | TCCTCATCACGCAGTATAGCC | 59.8 | 298 | INTERGENIC | | | | |
| CaPOPI_4017 | Ca6 | 13838996 | CCT/CCTTCT | TGTGTGATTGGTGTTGAGCA | GCAACAACGGGTGAAAATCT | 59.7 | 563 | INTERGENIC | | | | |
| CaPOPI_4018 | Ca6 | 14084331 | C/CA | AGGATGTGCACTGTTGTTGC | ACTAGATGGTCGCATCCTGG | 59.8 | 453 | INTERGENIC | | | | |
| CaPOPI_4019 | Ca6 | 14206895 | CCATCATCAATC/CCATCATCAATCATCATCAATC | TGCAACCAGAACCAATGTGT | TGACCATACCTCACCAACCC | 60.0 | 930 | INTERGENIC | | | | |
| CaPOPI_4020 | Ca6 | 14217212 | CAGAGAAGAGAAGAGAAGAGAAGAGA/CAGAGAAGAGAAGAGAAGAGAAGAGAAGAGA | AGGGGTGTTGGGAAAATAGG | GTTGCTTTCCGGATAAAACG | 60.0 | 439 | INTERGENIC | | | | |
| CaPOPI_4021 | Ca6 | 14246280 | ATATTATTATTATTATTATTATTATTATTAT/ATATTATTATTATTATTATTATTAT | GCATGGAGCAATGGAAGATT | TGGTTTTTGAAATTAAACGCA | 60.0 | 849 | DRR | Ca_05192 | QR | | Oxoglutarate/iron-dependent oxygenase |
| CaPOPI_4022 | Ca6 | 14338064 | TTATATATATATATATATATATATATATATATATAT/TTATATATATATATATATATATATATATATATATATATAT | AAAGCTTGCTTCGAGAAAAA | CTCCCACTCCCACCTACAAG | 57.1 | 740 | INTERGENIC | | | | |
| CaPOPI_4023 | Ca6 | 14553627 | TTATATATATAT/TTATATATAT | TGTTCATCAAGACCTGCACTG | CAAAAATTTGTGAGAGACCAAAA | 59.9 | 232 | INTERGENIC | | | | |
| CaPOPI_4024 | Ca6 | 14696179 | GTTATTATTATTATTATTATTATTATTATTATTATTATTAT/GTTATTATTATTATTATTATTATTATTATTATTATTAT | TGCATTTATGCCAGGAGACA | CAAACCCTCCTTTGTCCTCTC | 60.2 | 556 | INTERGENIC | | | | |
| CaPOPI_4025 | Ca6 | 15411815 | TC/TCC | TTTTGTGATAGGTTCCTGATGC | ATACCTACGGATTTTCGCCG | 59.1 | 811 | INTERGENIC | | | | |
| CaPOPI_4026 | Ca6 | 15555599 | AATATATA/AATATA | TGTGGGCACATTAGTAGGCA | TCATCGTCGTTGCTTGAGTC | 60.1 | 640 | INTERGENIC | | | | |
| CaPOPI_4027 | Ca6 | 15559125 | CTTT/CTT | TCACCAAACAAAAACTGGGG | CTACATTCCGGTTAGGGCAA | 60.8 | 480 | INTERGENIC | | | | |
| CaPOPI_4028 | Ca6 | 15577471 | TTCATTATCATTATCATTATCATTATCATTATCATTAT/TTCATTATCATTATCATTATCATTATCATTATCATTAT | ACATCCAGTCCTGCAAATCC | TGAACTCTGAGCGTGTGACC | 59.9 | 852 | INTERGENIC | | | | |
| CaPOPI_4029 | Ca6 | 15630356 | T/TG | CCTCATTTGTGGCCAACTTT | CAACGTTGTGATTGTGAGGG | 60.0 | 187 | DRR | Ca_05342 | | | |
| CaPOPI_4030 | Ca6 | 15727094 | ATTTATTTTATTTT/ATTTATTTTATTTTATTTT | TCCCCCGTCTAACTAGGGTT | CCACTTGTGTCCTAACAATTCAA | 59.8 | 268 | INTERGENIC | | | | |

| INDEL marker IDs | Chromosomes/ unanchored scaffolds | Physical positions (bp) | InDels (*Kabuli* reference genome-CDC Frontier/PI) | Forward primers (5'-3') | Reverse primers (5'-3') | Anneling temperature (0C) | Expected amplified product size (bp) | Structural annotation | | Functional annotation | | |
|---|---|---|---|---|---|---|---|---|---|---|---|---|
| | | | | | | | | Sequence components of *kabuli* genome | *Kabuli* gene accession IDs | NCBI-KOG | TFs | NCBI-nr database |
| CaPOPI_4031 | Ca6 | 15727514 | ATTTATTTTATTTTATTTTATTT/ATTTATTTTATTTTATTTTATTTTATTT | TTAGTTTGGGCTTGGGTTTT | TTGCTCCTACGTATCTCCCG | 58.6 | 911 | INTERGENIC | | | | |
| CaPOPI_4032 | Ca6 | 15728024 | AA/AAATA | CGGGAGATACGTAGGAGCAA | TCGATTATGCGTAGGGAAGG | 60.2 | 248 | INTERGENIC | | | | |
| CaPOPI_4033 | Ca6 | 15730699 | TAAA/TAA | ATTTGAACCTGGCAGCAAAA | GGTTTGAAAAAGGGGTGATG | 60.6 | 175 | INTERGENIC | | | | |
| CaPOPI_4034 | Ca6 | 15731057 | TAAAAA/TAA | ATTTGAACCTGGCAGCAAAA | GGGACAAAAGGCTAGTTTGG | 60.6 | 909 | INTERGENIC | | | | |
| CaPOPI_4035 | Ca6 | 15732116 | TAAAAAA/TAAAAAAA | AGAGTGTTTAGCGGCTCAATG | TCAACACCCCCTCTTAGGTG | 59.5 | 472 | INTERGENIC | | | | |
| CaPOPI_4036 | Ca6 | 15877864 | GTATA/GTA | TGAGAACTAAATGCATGCGG | GGTCTTCAACGGAGATGGAA | 59.8 | 216 | DRR | Ca_22023 | R | | Sugar/inositol transporter |
| CaPOPI_4037 | Ca6 | 15999975 | A/AG | AAGTGTGGGCATTTCCTTTG | ATGGACCTCTGTATTCGCCA | 60.0 | 137 | INTERGENIC | | | | |
| CaPOPI_4038 | Ca6 | 16012701 | TA/TAA | TAAGAGTTTGGTTCCGGTGG | TGTGCAGATAAATGGCCAAA | 60.0 | 397 | INTERGENIC | | | | |
| CaPOPI_4039 | Ca6 | 16286125 | CTTACTTTTT/CTTACTTTTTACTTTTT | TGTTGGGATATTTCCTTTGGA | CTTGAAAGAGCCGCAAGAAG | 59.3 | 576 | INTERGENIC | | | | |
| CaPOPI_4040 | Ca6 | 16322518 | GTT/GTTT | AGAAGGGAAAAAGAGAGGCG | TCAAAGGAAAATCCCTGACG | 60.0 | 160 | DRR | Ca_19667 | FP | bHLH | Inositol monophosphatase |
| CaPOPI_4041 | Ca6 | 16413106 | GTTATTATTATTATTATTATTAT/GTTATTATTATTATTATTAT | AGAGAGGAGATGCCAAATGC | GGGGCAAGGGGTATGATATT | 59.4 | 177 | INTERGENIC | | | | |
| CaPOPI_4042 | Ca6 | 16476384 | AGAGAA/AGAGAAATGACGAGAA | CTTGATGTTTCCGATGTGGA | TCTCTAAGGGGACACCATCG | 59.5 | 671 | INTERGENIC | | | | |
| CaPOPI_4043 | Ca6 | 16547931 | GA/GAGAGCTTA | TTCCTTCCTGCTCGTGCTAT | TTCTTTGGCTGAAATGGTCC | 60.0 | 640 | INTRON | Ca_06284 | A | | PSP, proline-rich |
| CaPOPI_4044 | Ca6 | 16548981 | CAT/CATAT | CTTCCAAGTCACCCCAGTGT | GGTCTTTAGTGGCACGGTGT | 60.0 | 460 | INTRON | Ca_06284 | A | | |
| CaPOPI_4045 | Ca6 | 16723215 | C/CA | GCGAGGATTAGGGTTTGTGA | GTGGCCAGGTGAGAGAAGAG | 60.1 | 208 | INTERGENIC | | | | |

| INDEL marker IDs | Chromosomes/ unanchored scaffolds | Physical positions (bp) | InDels (*Kabuli* reference genome-CDC Frontier/PI) | Forward primers (5'-3') | Reverse primers (5'-3') | Anneling temperature (0C) | Expected amplified product size (bp) | Structural annotation | | Functional annotation | | |
|---|---|---|---|---|---|---|---|---|---|---|---|---|
| | | | | | | | | Sequence components of *kabuli* genome | *Kabuli* gene accession IDs | NCBI-KOG | TFs | NCBI-nr database |
| CaPOPI_4046 | Ca6 | 16866680 | TATAATAATAATAATAATAATAATAATAATAATAATAA/TATAATAATAATAATAATAATAATAATAATAA | TGTCTACCGGTCCATACAAAAA | CACATTACAGATGATCCTTGCAT | 59.4 | 816 | INTERGENIC | | | | |
| CaPOPI_4047 | Ca6 | 17086650 | GTATATATATATATATATATATATA/GTATATATATATATATATATATATATATATATATA | AACGGTGAACAAATGCACAA | ACAAGCCACATTCCCCATAC | 60.0 | 520 | INTERGENIC | | | | |
| CaPOPI_4048 | Ca6 | 17100171 | AATATATATATATATATATAT/AATATATATATATATATATATAT | CTCGAAACCAGCAAACCATT | TTTTCTTTGTTGTCATCGACATTT | 60.1 | 562 | INTERGENIC | | | | |
| CaPOPI_4049 | Ca6 | 17249239 | CTTATTATTATTATTATTATTATTA/CTTATTATTATTATTATTATTATTATTATTA | CCCTTTGACCATCAATCACA | GCACAAATGCAAACGAGAGA | 59.3 | 257 | INTERGENIC | | | | |
| CaPOPI_4050 | Ca6 | 17312806 | CT/C | ACCACAATCAGTTTGCCTCC | TTCCCGCTCAAATTAGGATG | 60.0 | 720 | INTERGENIC | | | | |
| CaPOPI_4051 | Ca6 | 17914426 | CAA/CAAA | ATCTTTTGGTGGAAATTGCG | CGCGAAAACAGCGTTATGTA | 59.9 | 744 | INTERGENIC | | | | |
| CaPOPI_4052 | Ca6 | 18382715 | TAAAAAAAAA/TAAAAAAA | GTGGCAACCTTCTCTTCGAC | GGCTACCTCGCGTGTAACAT | 59.9 | 664 | DRR | Ca_06453 | | | |
| CaPOPI_4053 | Ca6 | 18526506 | CATATATATATATATATATATATATATATATATA/CATATATATATATATATATATATATATATATA | ATTTATGGTTTCTTGGCCCC | TGATTTTTGTTTTGTGGGCA | 60.0 | 719 | INTERGENIC | | | | |
| CaPOPI_4054 | Ca6 | 18790835 | TCCGTTGAG/T | TGCATGGTACGCCAAATAAA | AGCGGGTGGTTGCTAAAGTA | 60.0 | 869 | INTERGENIC | | | | |
| CaPOPI_4055 | Ca6 | 18932008 | ATTTTTTTTTTT/ATTTTTTTTTT | GCAATTTCCAGGCTATTCCA | GGTTCTGGTGAGACGAAGGA | 60.0 | 754 | DRR | Ca_06500 | U | | WD40 repeat |
| CaPOPI_4056 | Ca6 | 19172700 | TTTTTAAATTAATTTTTTATTTTAA/TTTTTAAATTAATTTTTTATTTTAAATTAATTTTTTATTTTAA | GTGGCACAGTAGACCCCATT | CGTGACATTAACCTTGGCCT | 59.9 | 712 | DRR | Ca_06524 | R | | Ribokinase |
| CaPOPI_4057 | Ca6 | 19355099 | CTTTTTTTTT/CTTTTTTTT | TTGTTCATGAAGTCTTTGGCA | CCGTGATCATATTTTCAAACCA | 59.3 | 400 | INTERGENIC | | | | |
| CaPOPI_4058 | Ca6 | 19591689 | CATATATATATATATATATATATATATAT/CATATATATATATATATATATATATATATATAT | TGGAACACACATTTGTCATTGAT | TGTGCATATCTGCAGTTTCATTT | 60.1 | 709 | INTERGENIC | | | | |
| CaPOPI_4059 | Ca6 | 19680352 | TTATATATATATATATATATAT/TTATATATATATATATATATATAT | GGGAAAATGAACATGTCACAGA | CTTTGATAAGTGGCGGTGGT | 59.8 | 654 | INTERGENIC | | | | |
| CaPOPI_4060 | Ca6 | 20531282 | CTTTTTTTTTT/CTTTTTTTTTTT | TTGGTGGAACTCTTGAAAATGA | CCTTTTCAGCAAAAACTCCG | 59.6 | 630 | INTERGENIC | | | | |

| INDEL marker IDs | Chromosomes/ unanchored scaffolds | Physical positions (bp) | InDels (*Kabuli* reference genome-CDC Frontier/PI) | Forward primers (5'-3') | Reverse primers (5'-3') | Anneling temperature (0C) | Expected amplified product size (bp) | Structural annotation | | Functional annotation | | |
|---|---|---|---|---|---|---|---|---|---|---|---|---|
| | | | | | | | | Sequence components of *kabuli* genome | *Kabuli* gene accession IDs | NCBI-KOG | TFs | NCBI-nr database |
| CaPOPI_4061 | Ca6 | 20851411 | TTTCTTCTTCTT/TTTCTTCTTCTTCTT | ACACAAAACTGCCGATCCAT | CTTGAAAACGAATTGGGACC | 60.4 | 335 | INTERGENIC | | | | |
| CaPOPI_4062 | Ca6 | 20852278 | TAAAA/TAA | TGCAATTTCAAGGGACAAGA | CAAGGATGATAGGCGATGTG | 59.2 | 731 | INTERGENIC | | | | |
| CaPOPI_4063 | Ca6 | 20853701 | GA/GAATAAGCA | TCCTGAGGAACATTCTCCGT | AAGCAACAAAAGAACATCACACA | 59.7 | 327 | INTERGENIC | | | | |
| CaPOPI_4064 | Ca6 | 20920548 | CATATT/CATATTATATT | TTCCTGGAACCTGCAAAATC | AAAGACCAGCACCATCAACC | 60.1 | 353 | INTRON | Ca_16338 | U | | Domain of unknown function DUF1692 |
| CaPOPI_4065 | Ca6 | 21021586 | CTATTTATTTATTTATTTATTTATTT/CTATTTATTTATTTATTTATTT | CACCGTCGTGTTCATCTGTC | TTATTTCACCGTCGTGTCCA | 60.2 | 458 | INTERGENIC | | | | |
| CaPOPI_4066 | Ca6 | 21139884 | ATTTTTTTT/ATTTTTTTTT | CAGAAGGATTGCTCGTGTGA | TGCATAGGCAAAAATGATTGA | 60.0 | 528 | INTERGENIC | | | | |
| CaPOPI_4067 | Ca6 | 21141563 | GTTTTTT/GTTTTT | TCCGATAGGAAGAGCCAATG | ACGTTTATTTTTCGGACCCC | 60.2 | 697 | INTERGENIC | | | | |
| CaPOPI_4068 | Ca6 | 21146689 | AT/A | CACACCTTTTGGATGCACAC | TGGTCATTTTCGTCATCCCT | 60.0 | 379 | INTERGENIC | | | | |
| CaPOPI_4069 | Ca6 | 21353236 | GTTTTTTTTTT/GTTTTTTTTT | TTATCGGTGACGGAGCTAGG | TGCATTATTTCTCAGCGGGT | 60.2 | 548 | INTERGENIC | | | | |
| CaPOPI_4070 | Ca6 | 21399662 | AAATAATAATAATAATAATAA/AAATAATAATAATAATAA | TCCCTAGTTGGAGGTCGATG | GTCCACCTAAATGCCCTTGA | 60.1 | 447 | INTERGENIC | | | | |
| CaPOPI_4071 | Ca6 | 21401312 | C/CT | AGCTCAGTCCTCACAACCGT | TTAGATCGGACAAGAAACCGA | 59.9 | 344 | INTERGENIC | | | | |
| CaPOPI_4072 | Ca6 | 21401897 | ATATTACACTATTA/ATA | TCGGTTTCTTGTCCGATCTAA | ATTCATGGATGCGGGTATGT | 59.7 | 721 | DRR | Ca_19861 | E | | Serine hydroxymethyltransferase |
| CaPOPI_4073 | Ca6 | 21408015 | ATTTTTTTT/ATTTTTTT | AGGCCTTTTATTTCGTTGCC | TTGAATCGCAAAACAACCAA | 60.4 | 897 | DRR | Ca_19860 | | | Methyltransferase type 11 |
| CaPOPI_4074 | Ca6 | 21485595 | GTTTTTTT/GTTTTTTTT | TGCGGGCTAGCTCACTTAAT | TCAAAACAAATAGGCAGGGG | 60.0 | 115 | INTERGENIC | | | | |
| CaPOPI_4075 | Ca6 | 21605985 | CTATATATATATATATATA/CTATATATATATATATATA | GATTTTCGCAGTGAACGGAT | TCCCCTTCTATTTCCCTTGAA | 60.1 | 437 | INTERGENIC | | | | |

| INDEL marker IDs | Chromosomes/ unanchored scaffolds | Physical positions (bp) | InDels (*Kabuli* reference genome-CDC Frontier/PI) | Forward primers (5'-3') | Reverse primers (5'-3') | Anneling temperature (0C) | Expected amplified product size (bp) | Structural annotation | | Functional annotation | | |
|---|---|---|---|---|---|---|---|---|---|---|---|---|
| | | | | | | | | Sequence components of *kabuli* genome | *Kabuli* gene accession IDs | NCBI-KOG | TFs | NCBI-nr database |
| CaPOPI_4076 | Ca6 | 22099449 | ATTTTTTTTT/ATTTTTTTTTTTT | TGTGTTGCTTCTTGGCTTTG | ACGTGTGGGTTTCAGGTCTC | 60.0 | 620 | INTERGENIC | | | | |
| CaPOPI_4077 | Ca6 | 22915019 | AATATATATATATATAT/AATATATATATATATATAT | CCTAACCAACAAACCTCCGA | TGAAAAATCGAACATTGGCA | 60.0 | 369 | INTERGENIC | | | | |
| CaPOPI_4078 | Ca6 | 23042823 | CATATATATATATAT/CATATATATATAT | CAACGATCTGCACCACACAT | GGTGCAGCAAGTGAGCAATA | 60.6 | 665 | INTRON | Ca_11143 | | ERF | Glycoside hydrolase, family 17 |
| CaPOPI_4079 | Ca6 | 23042859 | TCAATCCAATCCAATCCAAT/TCAATCCAATCCAATCCAATCCAAT | CAACGATCTGCACCACACAT | GGTGCAGCAAGTGAGCAATA | 60.6 | 665 | INTRON | Ca_11143 | | ERF | Glycoside hydrolase, family 17 |
| CaPOPI_4080 | Ca6 | 23042981 | TAAA/TAA | CAACGATCTGCACCACACAT | GGTGCAGCAAGTGAGCAATA | 60.6 | 665 | INTRON | Ca_11143 | | ERF | Glycoside hydrolase, family 17 |
| CaPOPI_4081 | Ca6 | 23064332 | CCATGTGTGTTATTC/CC | TGGGTTGATTTTCCCACATT | TGGAGTTATAGAGACCATTCCG | 60.0 | 391 | INTERGENIC | | | | |
| CaPOPI_4082 | Ca6 | 23064745 | CA/C | TGGGTTGATTTTCCCACATT | TCTCGAGCAAATCACAAGTTAAA | 60.0 | 571 | INTERGENIC | | | | |
| CaPOPI_4083 | Ca6 | 23186768 | ATTTTTTTTTTTTT/ATTTTTTTTTTTT | GGTTCACCAATGGACCCTTA | CAAATTTTTCAAACAAAACCCA | 59.6 | 762 | INTERGENIC | | | | |
| CaPOPI_4084 | Ca6 | 23231829 | ATTTTTTTT/ATTTTTTTTT | AAGCAACAAACATCATGCCA | GAGGCCAAAACGAAACAAAA | 60.1 | 584 | INTERGENIC | | | | |
| CaPOPI_4085 | Ca6 | 23238479 | GTTTTTTT/GTTTTTT | TCAACGGATTTCCACCCTTA | TCTTGTCTCCACCCGTTAAAA | 60.3 | 368 | INTERGENIC | | | | |
| CaPOPI_4086 | Ca6 | 23642077 | TC/TTTCCC | GATTACCATGGTTGGGATCG | CAATTTCGGTGCTTGTTCCT | 60.0 | 137 | INTRON | Ca_11091 | R | | |
| CaPOPI_4087 | Ca6 | 23741801 | GTATATATATATATATATATAT/GTATATATATATATATATAT | TAGTGCTCTGCATTTGTCCG | TGAAGGACGATGAATTCCTAAAA | 60.0 | 768 | INTERGENIC | | | | |
| CaPOPI_4088 | Ca6 | 23755333 | TAC/T | GGAGGAGCAGAAGCAACAAC | CTGTGGGATCAATTGGCTCT | 60.0 | 256 | INTERGENIC | | | | |
| CaPOPI_4089 | Ca6 | 23873936 | CAT/CATCCTTTAAT | GAGCATAGTTCCTTTTCACGG | AATTTTGGTCCCCAAACAGA | 58.8 | 441 | INTERGENIC | | | | |
| CaPOPI_4090 | Ca6 | 24092648 | CTATTATTATTATTATTATTATTATTATTATTATTATTATTATTATTATT/CTATTATTATTATTATTATTATTATTATTATTATTATTATT | TTGCTCTTTTTGGTTGCTCA | CCAACTATTTGTCCCCTTTGA | 59.6 | 345 | INTERGENIC | | | | |

| INDEL marker IDs | Chromosomes/ unanchored scaffolds | Physical positions (bp) | InDels (*Kabuli* reference genome-CDC Frontier/PI) | Forward primers (5'-3') | Reverse primers (5'-3') | Anneling temperature (0C) | Expected amplified product size (bp) | Structural annotation | | Functional annotation | | |
|---|---|---|---|---|---|---|---|---|---|---|---|---|
| | | | | | | | | Sequence components of *kabuli* genome | *Kabuli* gene accession IDs | NCBI-KOG | TFs | NCBI-nr database |
| CaPOPI_4091 | Ca6 | 24232847 | TTATATATATATATATATAT/TTATATATATATATATAT | TGAGGTGCTGTCCAAATTGT | ATAAATTCCGCAACCGATCA | 59.1 | 521 | INTERGENIC | | | | |
| CaPOPI_4092 | Ca6 | 24296638 | CATATATATATATATATATATA/CATATATATATATATATATATATA | GAGAAGGAGGGAAAAATGGC | ATGTTTTGGATTTTGGCTCG | 60.0 | 421 | INTERGENIC | | | | |
| CaPOPI_4093 | Ca6 | 24459206 | TTATATATATATATATATATATAT/TTATATATATATATATATATAT | ATCGAGCGGTGGAGTGAATA | TTTGGTATACATGGTCGGTAAGG | 60.6 | 831 | INTERGENIC | | | | |
| CaPOPI_4094 | Ca6 | 24637049 | TTTCTTCTTCTTCTTCTTCTTCTTCT/TTTCTTCTTCTTCTTCTTCTTCTTCT | TGTTTTTGGTGAAACGTGGA | AGGATGAATGTGGTGTTGAAGA | 60.0 | 376 | INTERGENIC | | | | |
| CaPOPI_4095 | Ca6 | 24647579 | ATTTTTTTTT/ATTTTTTTTTT | GTTCTGGATGTGTTTGGCCT | ATTCAACTGCATGTCGCATC | 60.0 | 485 | INTERGENIC | | | | |
| CaPOPI_4096 | Ca6 | 24730447 | GTAATAATAATAATAATAATAATAATAA/GTAATAATAATAATAATAATAATAATAATAA | TGAGGACTGGACGTAGCCTT | ACCCCAATTTTCAACCATCA | 59.9 | 498 | INTERGENIC | | | | |
| CaPOPI_4097 | Ca6 | 25002657 | AATATATATATATATATAT/AATATATATATATATATATAT | CTACATATGCAAAAGCGGCA | GGATGTGTTTTCCCCTTCAA | 59.9 | 400 | INTERGENIC | | | | |
| CaPOPI_4098 | Ca6 | 25594919 | AATATATATATATATATATATATATATATATATATA/AATATATATATATATATATATATATATATATA | TATTTTGGATTTTGGCTCGG | CGAAAATGAAAACGGGAGAA | 59.9 | 293 | INTERGENIC | | | | |
| CaPOPI_4099 | Ca6 | 25682308 | GTTTAGGTTTTAGGTTTTAGG/GTTTAGGTTTTAGGTTTTAGGTTTTAGG | TGGGTCTTGGGCTGTTTTAG | CCCTTAACCCTTAACCCCAA | 60.1 | 838 | INTERGENIC | | | | |
| CaPOPI_4100 | Ca6 | 25762575 | TTATATATATATATATATATAT/TTATATATATATATATAT | TCTCTTCGCGAAACGAATCT | TTGGTATTGAACTCAGGGGC | 60.1 | 576 | INTERGENIC | | | | |
| CaPOPI_4101 | Ca6 | 25959668 | TACATCAAA/TACATCAAATATATGACATCAAA | TGTCATCCCAAACACGTCAT | TTTTCCCCATGCAAGGTTAG | 59.8 | 339 | DRR | Ca_25499 | R | C2H2 | Pentatricopeptide repeat |
| CaPOPI_4102 | Ca6 | 26092614 | GATATATATATATATATATATATAT/GATATATATATATATATATATATATATAT | ATGTTTTGGATTTTGGCTCG | AATTCCTTCCCACACAACCA | 59.9 | 716 | INTERGENIC | | | | |
| CaPOPI_4103 | Ca6 | 26277672 | T/TG | TTAGGTGCGCTTGACTGATG | GTTTTGGCGTTGGTTTCACT | 60.0 | 558 | INTERGENIC | | | | |
| CaPOPI_4104 | Ca6 | 26590044 | GATAATAATAATAATAATAATA/GATAATAATAATAATAATA | CCTTTGTGAGGGCTAAAAGC | TTGTTGTTGACCAAGCTAGGAA | 59.0 | 731 | INTERGENIC | | | | |
| CaPOPI_4105 | Ca6 | 26764661 | TAGAGAGAGAGAGAGAGAGAGAGA/TAGAGAGAGAGAGAGAGAGAGA | CCATAGGATGAAATGCTGGC | GCAGCCATGAACCTTCAACT | 60.4 | 436 | INTERGENIC | | | | |

| INDEL marker IDs | Chromosomes/ unanchored scaffolds | Physical positions (bp) | InDels (*Kabuli* reference genome-CDC Frontier/PI) | Forward primers (5'-3') | Reverse primers (5'-3') | Anneling temperature (0C) | Expected amplified product size (bp) | Structural annotation | | Functional annotation | | |
|---|---|---|---|---|---|---|---|---|---|---|---|---|
| | | | | | | | | Sequence components of *kabuli* genome | *Kabuli* gene accession IDs | NCBI-KOG | TFs | NCBI-nr database |
| CaPOPI_4106 | Ca6 | 26867680 | CC/CCCTAAATATAAC | TGCAGAATCGTTCAAACCAA | CAAGTGGCCTCGAACTCAAT | 60.2 | 720 | INTERGENIC | | | | |
| CaPOPI_4107 | Ca6 | 27289106 | GATATATAT/GATATATATAT | CAATTCCCACAGGTACGCTT | AATTTCAAGGATTGGCGAAG | 60.0 | 612 | INTERGENIC | | | | |
| CaPOPI_4108 | Ca6 | 27332756 | AAGAGAGAGAGAGAGAGAGAGAGAGAGAGAGAGA/AAGAGAGAGAGAGAGAGAGAGAGAGAGAGAGAGA | CACACAGGGGCATATCCTTT | GCAGCTGAGTAGTGTGCGAG | 59.8 | 648 | INTRON | Ca_14617 | K | | RNA recognition motif domain |
| CaPOPI_4109 | Ca6 | 27628955 | GTATATATATATATATA/GTATATATATATATATATATA | TTTTGTTTGTCTCATTTTGTTTTT | ACGGGACATATTGTTTTTACTGA | 57.1 | 388 | INTERGENIC | | | | |
| CaPOPI_4110 | Ca6 | 27681598 | CTTTTTTTTTTTT/CTTTTTTTTTTT | TTGTGCTGAGAAGTCGACAAA | TCAATTTTAATCACTGCACAATCA | 59.6 | 518 | INTERGENIC | | | | |
| CaPOPI_4111 | Ca6 | 27808764 | GATATATATATATATATAT/GATATATATATATATATATAT | CCTCAAAGGGAAAGGGAAAG | CGTTCTTTGTTCTGGTACGG | 60.0 | 632 | INTERGENIC | | | | |
| CaPOPI_4112 | Ca6 | 27929163 | TG/TGGTGTCGG | GGACAAACAAAATGAGGGGA | GCAGAACACTGGATTTGGGT | 59.8 | 198 | INTERGENIC | | | | |
| CaPOPI_4113 | Ca6 | 28065874 | ATTTTTTTTT/ATTTTTTTTTT | TTGCAAGAAGCAAGGAGCTA | CCCAAACACTTCAATCAGCA | 58.9 | 456 | INTERGENIC | | | | |
| CaPOPI_4114 | Ca6 | 28086166 | C/CTT | TGGTAACCCAGCGTATTTGC | GTGTGATGATTGTGCTTGCC | 60.9 | 162 | INTERGENIC | | | | |
| CaPOPI_4115 | Ca6 | 28098136 | ATTTTTTTTTTT/ATTTTTTTTTT | TTTGGATTGTGTCACTGTAGCA | TGCGTCTTACAAATTTCAATCA | 59.2 | 318 | INTERGENIC | | | | |
| CaPOPI_4116 | Ca6 | 28159515 | TAAAAAAAAA/TAAAAAAAA | AAAAACTATGCACAAACTGACG | ATGGCGGCTAAGAAGACAGA | 57.1 | 456 | INTERGENIC | | | | |
| CaPOPI_4117 | Ca6 | 28190654 | A/ATAAAAG | AGGTCTCATACCAGAGATGCG | TGTGGTTGAGTAAATGTGCCA | 59.3 | 580 | INTERGENIC | | | | |
| CaPOPI_4118 | Ca6 | 28190835 | ATATTATTATTATTATT/ATATTATTATTATTATTATT | AGGTCTCATACCAGAGATGCG | AAGTTAAGGGACCGCAAATG | 59.3 | 871 | INTERGENIC | | | | |
| CaPOPI_4119 | Ca6 | 28193559 | AGGG/AGG | AAGGACTAAGTCGGGAAGAAAAA | GATGGTTTTTGTCCATTGAATTT | 59.7 | 131 | INTERGENIC | | | | |
| CaPOPI_4120 | Ca6 | 28301391 | CA/C | ATAAACCGGTGACCATTGGA | TGAACACGTCAAACACGACA | 60.1 | 551 | INTERGENIC | | | | |

| INDEL marker IDs | Chromosomes/ unanchored scaffolds | Physical positions (bp) | InDels (*Kabuli* reference genome-CDC Frontier/PI) | Forward primers (5'-3') | Reverse primers (5'-3') | Anneling temperature (0C) | Expected amplified product size (bp) | Structural annotation | | Functional annotation | | |
|---|---|---|---|---|---|---|---|---|---|---|---|---|
| | | | | | | | | Sequence components of *kabuli* genome | *Kabuli* gene accession IDs | NCBI-KOG | TFs | NCBI-nr database |
| CaPOPI_4121 | Ca6 | 28326755 | AT/ATT | TTTGGGCTTGAAGATGAAAA | TTTAAAAGCAAGGGTGGTGG | 58.3 | 336 | DRR | Ca_25968 | A | | Ribonuclease T2 |
| CaPOPI_4122 | Ca6 | 28327723 | CATATATATATATAT/CATATATATATAT | CAAGAGCATCGTGTCAAGGA | GTCCTGGCGTGACAAGAAAT | 60.0 | 424 | DRR | Ca_25968 | A | | Ribonuclease T2 |
| CaPOPI_4123 | Ca6 | 28332319 | ATTTT/ATTTTT | TTTCAAAAAGTCCCAATCGC | GGTTTGGAGAAGAAGACGAAA | 60.1 | 751 | INTERGENIC | | | | |
| CaPOPI_4124 | Ca6 | 28332579 | A/AG | GTCGGAGCTCTGCCATAAAA | GGTTTGGAGAAGAAGACGAAA | 60.4 | 599 | INTERGENIC | | | | |
| CaPOPI_4125 | Ca6 | 28332651 | C/CT | GTCGGAGCTCTGCCATAAAA | GGTTTGGAGAAGAAGACGAAA | 60.4 | 599 | INTERGENIC | | | | |
| CaPOPI_4126 | Ca6 | 28332758 | TAAA/TAAAA | TTTCGTCTTCTTCTCCAAACC | CCCAACATATTACCTGTTACCCA | 58.4 | 502 | INTERGENIC | | | | |
| CaPOPI_4127 | Ca6 | 28332811 | A/AG | TTTCGTCTTCTTCTCCAAACC | CCCAACATATTACCTGTTACCCA | 58.4 | 502 | INTERGENIC | | | | |
| CaPOPI_4128 | Ca6 | 28332973 | A/AT | TTTCGTCTTCTTCTCCAAACC | CGTGCAAAGTCGAAGCGTAT | 58.4 | 669 | INTERGENIC | | | | |
| CaPOPI_4129 | Ca6 | 28336699 | ATTTT/ATTT | TCTCCCATTTTTACCATGCC | TTTGGCTTTGGTTGATTCCT | 59.8 | 980 | INTERGENIC | | | | |
| CaPOPI_4130 | Ca6 | 28443583 | CTCTTTTGTTGTTA/CTCTTTTGTTGTTATATCTTTTGTTGTTA | AAAAACACCAAAAGCCCCTC | CATAAAATGCCATTGATCCCT | 60.3 | 499 | INTERGENIC | | | | |
| CaPOPI_4131 | Ca6 | 28444744 | GATAATAATAATAATAATAA/GATAATAATAATAATAATAATAATAA | TCCCAAACAGCCTAACAACC | CAAACGCAATCTGAACAAAA | 60.0 | 509 | INTERGENIC | | | | |
| CaPOPI_4132 | Ca6 | 28503466 | ATAAT/ATAATTAAT | TTTGTGCCTCCATTTCCTTC | TTTTTAACTAATTGATCACCGCT | 60.1 | 497 | DRR | Ca_25961 | | | |
| CaPOPI_4133 | Ca6 | 28513162 | TA/TCTTCAACTTTCCCTACCA | CCCACACCTTCTTACCAGGA | GGGACCCAAATTGTCGATAG | 60.0 | 492 | INTERGENIC | | | | |
| CaPOPI_4134 | Ca6 | 28513941 | ANNNNNNNNNN/ANNNNN | TGAAGAAAATTTAAAGGGACGAA | GTGTTGCATCTCATTGGTGG | 59.2 | 569 | INTERGENIC | | | | |
| CaPOPI_4135 | Ca6 | 28515482 | GAAAAAAA/GAAAAAAAA | GGCATAGGTGAGATGACCGT | GCTCCTTATCAAACCATTTGC | 60.0 | 507 | INTERGENIC | | | | |

| INDEL marker IDs | Chromosomes/ unanchored scaffolds | Physical positions (bp) | InDels (*Kabuli* reference genome-CDC Frontier/PI) | Forward primers (5'-3') | Reverse primers (5'-3') | Anneling temperature (0C) | Expected amplified product size (bp) | Structural annotation | | Functional annotation | | |
|---|---|---|---|---|---|---|---|---|---|---|---|---|
| | | | | | | | | Sequence components of *kabuli* genome | *Kabuli* gene accession IDs | NCBI-KOG | TFs | NCBI-nr database |
| CaPOPI_4136 | Ca6 | 28520924 | CAA/CAATTAAA | TTAGAAACAGGGACCCCTCC | TTGTCTGTGAACCGAAGCTG | 60.3 | 577 | INTERGENIC | | | | |
| CaPOPI_4137 | Ca6 | 28521027 | A/ACATAG | TCTCATTCTTGCCTTGCCTT | TTGTCTGTGAACCGAAGCTG | 60.0 | 172 | INTERGENIC | | | | |
| CaPOPI_4138 | Ca6 | 28521076 | AACTAT/A | TCTCATTCTTGCCTTGCCTT | TTGTCTGTGAACCGAAGCTG | 60.0 | 172 | INTERGENIC | | | | |
| CaPOPI_4139 | Ca6 | 28537234 | ATT/AT | ATTCCTCAAAGCCCTCACCT | TTCCACTTCCTTTTCCACCA | 60.1 | 436 | INTERGENIC | | | | |
| CaPOPI_4140 | Ca6 | 28610431 | CT/C | AGGATTTGGTTCAAGGGTCC | TGTCCTGACTCTTGGCAGTG | 60.2 | 643 | DRR | Ca_17488 | K | | |
| CaPOPI_4141 | Ca6 | 28832556 | AAAATAAATAAATAAATAAATAAATAAATAAATA/AAAATAAATAAATAAATAAATAAATA | GCTTTCTTGTATCAAGTGGCAA | TGCAATTGTGGTTTCTGTCTG | 59.4 | 648 | INTERGENIC | | | | |
| CaPOPI_4142 | Ca6 | 29148934 | GTTATTATTATTATTATTATTATTATT/GTTATTATTATTATTATTATTATTATTATT | TTGTGTCGTGTTGCTGTTCA | TCGTGTTTCAAAATTCAAGGA | 59.9 | 871 | INTERGENIC | | | | |
| CaPOPI_4143 | Ca6 | 29174756 | AATATATATATATA/AATATATATATATATA | ACCAGCTCCTTTTTGTGACC | CAGCCCCCAATACTCCTACA | 59.2 | 595 | INTERGENIC | | | | |
| CaPOPI_4144 | Ca6 | 29231459 | AAAGTCTCAAGTCTCAAGTCTCAA/AAAGTCTCAAGTCTCAAGTCTCAAGTCTCAA | ACTCCCACACACCTCAAAGG | TTTCAAGTCTAGTCGAAGTTCATCA | 60.0 | 834 | INTERGENIC | | | | |
| CaPOPI_4145 | Ca6 | 29640665 | AATATATATATATATATATATATATAT/AATATATATATATATATATATATAT | CACACATCGCGAGTAATGATG | AACTTCTCGAGGACGCAAAA | 60.2 | 541 | INTERGENIC | | | | |
| CaPOPI_4146 | Ca6 | 29833274 | A/AG | GCCGAATAAAACCCTCACAA | CAAAGCAACCACAAAAAGCA | 59.9 | 561 | INTERGENIC | | | | |
| CaPOPI_4147 | Ca6 | 30102387 | ATTTTTTTTTTTT/ATTTTTTTTTTT | TTGGGAGATGCATGTATTGG | TTAGACGTAACCCACCTTGGA | 59.4 | 596 | INTERGENIC | | | | |
| CaPOPI_4148 | Ca6 | 30588229 | AATCGC/A | GGACGTCTACAACATTGGGG | CCACATGAGTGTGTTGAGGG | 60.2 | 948 | DRR | Ca_16461 | | SAP | Aminotransferase-like, plant mobile domain |
| CaPOPI_4149 | Ca6 | 30816682 | GTTTTTTTTT/GTTTTTTTT | ATAAACCTTCGGAGGGGAAA | ACCCCTCTCAAAGCACAAGA | 59.8 | 518 | INTERGENIC | | | | |
| CaPOPI_4150 | Ca6 | 30901162 | ACT/ACTCT | CACTCCAAAAGGCACTGGTT | CACAGATGATGGGCAGAAGA | 60.1 | 341 | INTERGENIC | | | | |

| INDEL marker IDs | Chromosomes/ unanchored scaffolds | Physical positions (bp) | InDels (*Kabuli* reference genome-CDC Frontier/PI) | Forward primers (5'-3') | Reverse primers (5'-3') | Anneling temperature (0C) | Expected amplified product size (bp) | Structural annotation | | Functional annotation | | |
|---|---|---|---|---|---|---|---|---|---|---|---|---|
| | | | | | | | | Sequence components of *kabuli* genome | *Kabuli* gene accession IDs | NCBI-KOG | TFs | NCBI-nr database |
| CaPOPI_4151 | Ca6 | 31097180 | ATTT/ATTTTT | TCCCAATTGAGTGACATCTTTC | CTTATAGCCGAATGGCCAAA | 59.0 | 587 | INTERGENIC | | | | |
| CaPOPI_4152 | Ca6 | 31099504 | TA/T | TGGGATGATGTTGGGAAAGT | TTGCAAAGAAAACCTCGCTT | 60.2 | 129 | INTERGENIC | | | | |
| CaPOPI_4153 | Ca6 | 31132134 | TAAA/TA | CACTCCAGGTTCCATTCCTC | GCTGGTGGCTAATTATTCCATT | 59.5 | 708 | INTERGENIC | | | | |
| CaPOPI_4154 | Ca6 | 31335349 | TTATATATATATATATATATATATATATATATAT/TTATATATATATATATATATATATATAT | TCGGTTCATAAAACGAGTTAAAA | GAAGCTTCTATGGTCCCGTG | 58.0 | 392 | INTERGENIC | | | | |
| CaPOPI_4155 | Ca6 | 31413696 | CAAAAAA/CAAAAA | GAACGGTGATGACAGCTTGA | GGCCTCCTTGTACCTCCTCT | 59.8 | 491 | INTERGENIC | | | | |
| CaPOPI_4156 | Ca6 | 31769480 | A/AT | CGGGAGATACGTAGGAGCAA | TCGATTATGCGTAGGGAAGG | 60.2 | 250 | DRR | Ca_15194 | | | |
| CaPOPI_4157 | Ca6 | 31950641 | ATTTTTTTT/ATTTTTTT | CCACTTCAGCGTGCAATCTA | TTCTTGGGTAGAAAATGCGG | 60.0 | 612 | INTERGENIC | | | | |
| CaPOPI_4158 | Ca6 | 32372827 | CAA/CA | GAATACGCGGCTGATTCATT | CGCCCCGTTTTAACTCATTA | 60.1 | 613 | INTERGENIC | | | | |
| CaPOPI_4159 | Ca6 | 32391786 | AATATATATATATATA/AATATATATATATATATA | CGCTATGCCCCATTAGATGT | TTTGATTGACTCATTGCGTG | 59.9 | 294 | INTERGENIC | | | | |
| CaPOPI_4160 | Ca6 | 32519592 | AG/A | CGCATGCCACTCACTCTAAA | CGCTGCCACTTTCTACATCA | 60.0 | 611 | INTERGENIC | | | | |
| CaPOPI_4161 | Ca6 | 32521477 | GACATGATAC/GAC | TTGCCAACGTGTCAATCACT | TTTTCCCTACTATTTTGCAGCC | 60.2 | 259 | INTERGENIC | | | | |
| CaPOPI_4162 | Ca6 | 32521795 | ATTTTGTTTGTTTT/ATTTT | TTGCCAACGTGTCAATCACT | TGCTAGGACCAACGATCATGT | 60.2 | 902 | INTERGENIC | | | | |
| CaPOPI_4163 | Ca6 | 32522263 | CA/CAA | TTGTTCATTTTTGGTCGTTCA | CCTTGGCGGATATAGGGAAT | 59.0 | 347 | INTERGENIC | | | | |
| CaPOPI_4164 | Ca6 | 32540181 | TATATAT/TATATACATATAT | GTTTATCAATGGACGCGGAC | TTCCCCTAAAAACGGTTGTG | 60.3 | 341 | INTERGENIC | | | | |
| CaPOPI_4165 | Ca6 | 32540257 | TACATAA/TACATAAGAAACATAA | GTTTATCAATGGACGCGGAC | TTCCCCTAAAAACGGTTGTG | 60.3 | 341 | INTERGENIC | | | | |

| INDEL marker IDs | Chromosomes/ unanchored scaffolds | Physical positions (bp) | InDels (*Kabuli* reference genome-CDC Frontier/PI) | Forward primers (5'-3') | Reverse primers (5'-3') | Anneling temperature (0C) | Expected amplified product size (bp) | Structural annotation | | Functional annotation | | |
|---|---|---|---|---|---|---|---|---|---|---|---|---|
| | | | | | | | | Sequence components of *kabuli* genome | *Kabuli* gene accession IDs | NCBI-KOG | TFs | NCBI-nr database |
| CaPOPI_4166 | Ca6 | 32540480 | GTTTTTTTT/GTTTTTTT | CACAACCGTTTTTAGGGGAA | GAGGATTGCTATGGACAACCA | 59.8 | 214 | INTERGENIC | | | | |
| CaPOPI_4167 | Ca6 | 32617100 | AAA/AAATAA | GAACGGTCCATGCAAAAGTT | CCCCCGATCCTTATACGACT | 60.0 | 554 | INTERGENIC | | | | |
| CaPOPI_4168 | Ca6 | 32630124 | TAAA/TAAAA | TTTGAGGGAGGGAATTAGGG | TAGTGATTGGAAAGGACGGC | 60.3 | 583 | INTERGENIC | | | | |
| CaPOPI_4169 | Ca6 | 32759448 | AATATATATATATATATATATATAT/AATATATATATATATATATATATAT | AATTTTGAAGTGGCCGTGAC | TCGACCTTCATTTCATACATTTTT | 60.0 | 701 | INTERGENIC | | | | |
| CaPOPI_4170 | Ca6 | 32839696 | TTATATATATATATATATATATATATATATATATATATATA/TTATATATATATATATATATATATATATATATATATATA | CCATATTAAACCGTGCACACA | CAAACGCTGTTCCACAAGAA | 59.4 | 863 | INTERGENIC | | | | |
| CaPOPI_4171 | Ca6 | 32932244 | TTATATATATATATATATATATATATATA/TTATATATATATATATATATATATATATATATATATA | GCAAATGGCGATTTCCTTTA | GTAATGTTGGGCTTCGGAGA | 60.0 | 713 | DRR | Ca_22231 | | ARF | Transferase |
| CaPOPI_4172 | Ca6 | 32971894 | CTTTTTTTTTTT/CTTTTTTTTTT | TTAACCTACCAATTCGAATACCA | TTAATGCATCTTGATGGTGG | 57.7 | 257 | INTERGENIC | | | | |
| CaPOPI_4173 | Ca6 | 32974504 | AAT/AATGAT | TCGAGATGTGTTTGGGTGAA | TTTTACGATGGACAACCGTG | 60.1 | 940 | INTERGENIC | | | | |
| CaPOPI_4174 | Ca6 | 33094621 | ATTT/ATT | TCAAAATGTAGCGGGTTTCC | AAGCCTTCCTCAAAATCCAA | 59.9 | 398 | INTERGENIC | | | | |
| CaPOPI_4175 | Ca6 | 33094845 | CAAAAA/CAAAAAA | TCAAAATGTAGCGGGTTTCC | AAGTTGGACAAAATTGGGGT | 59.9 | 950 | INTERGENIC | | | | |
| CaPOPI_4176 | Ca6 | 33229235 | CTT/CTTT | GCTTTTATTCCAAATTATGGCG | AAATTTTAGACCGCGACACG | 59.8 | 758 | INTERGENIC | | | | |
| CaPOPI_4177 | Ca6 | 33229272 | GAAAA/GAAA | TGTTGACGGTGTGTTTTTAAGG | AAATTTTAGACCGCGACACG | 59.9 | 477 | INTERGENIC | | | | |
| CaPOPI_4178 | Ca6 | 33229296 | TG/TGG | CGGTGTGTTTTTAAGGATTGG | AAATTTTAGACCGCGACACG | 59.4 | 471 | INTERGENIC | | | | |
| CaPOPI_4179 | Ca6 | 33229369 | ATTTTTTTTTTTTTT/AATGTTTTTTTTTTTTTT | CGGTGTGTTTTTAAGGATTGG | AAATTTTAGACCGCGACACG | 59.4 | 471 | INTERGENIC | | | | |
| CaPOPI_4180 | Ca6 | 33229523 | AAAAACGAAAA/AAAAA | CGGTGTGTTTTTAAGGATTGG | GCGGATATCGTAGGGTGCTA | 59.4 | 644 | INTERGENIC | | | | |

| INDEL marker IDs | Chromosomes/ unanchored scaffolds | Physical positions (bp) | InDels (*Kabuli* reference genome-CDC Frontier/PI) | Forward primers (5'-3') | Reverse primers (5'-3') | Anneling temperature (0C) | Expected amplified product size (bp) | Structural annotation | | Functional annotation | | |
|---|---|---|---|---|---|---|---|---|---|---|---|---|
| | | | | | | | | Sequence components of *kabuli* genome | *Kabuli* gene accession IDs | NCBI-KOG | TFs | NCBI-nr database |
| CaPOPI_4181 | Ca6 | 33229803 | TAAAAAAAA/TAAAAAA | AAGCAAGCCAAAAACGAAAA | GCGGATATCGTAGGGTGCTA | 59.9 | 382 | INTERGENIC | | | | |
| CaPOPI_4182 | Ca6 | 33231576 | AT/ATT | TGGTGTATTGGGTGCTGATG | GACCCATCGATTTGAAGCAT | 60.4 | 975 | INTERGENIC | | | | |
| CaPOPI_4183 | Ca6 | 33233620 | G/GAA | CCGCCCACTAAAAAGTTGAA | AATCGCCATAACTTGAAATGC | 60.1 | 935 | INTERGENIC | | | | |
| CaPOPI_4184 | Ca6 | 33240261 | A/AAAGAG | GTTTGGTATTGGGCTCCCTT | CACAATTCCTCCCTCTCCAA | 60.2 | 314 | INTERGENIC | | | | |
| CaPOPI_4185 | Ca6 | 33240380 | A/AG | GTTTGGTATTGGGCTCCCTT | CGAAACTCACCCCTTGTTGT | 60.2 | 750 | INTERGENIC | | | | |
| CaPOPI_4186 | Ca6 | 33240742 | AA/AATATA | TTGGAGAGGGAGGAATTGTG | CGAAACTCACCCCTTGTTGT | 60.0 | 456 | INTERGENIC | | | | |
| CaPOPI_4187 | Ca6 | 33256778 | ATATTATTATTATTATTATTATTATTATTATTATTATTATTATTATTATTA/ATATTATTATTATTATTATTATTATTATTATTATTATTA | TGGCTATTTATGGCACGACA | TAATGTGGAAACCCCCTCAA | 60.1 | 596 | INTERGENIC | | | | |
| CaPOPI_4188 | Ca6 | 33337104 | ATTT/ATT | CCAATTTTTGATTCCCTCCA | TTAGGCTCCTGCTTCATGCT | 59.7 | 527 | INTERGENIC | | | | |
| CaPOPI_4189 | Ca6 | 33340808 | GTTTTTTT/GTTTTTTTT | TTCAATGTCATACACAGGCCA | ATGATAACGCATGAATGGCA | 60.0 | 841 | INTERGENIC | | | | |
| CaPOPI_4190 | Ca6 | 33488777 | GATATATATATATATATAT/GATATATATATATATATATAT | TTACCCAACCCCAATTTTCA | AAGTTTTGGGAAAGGGGAAA | 60.0 | 641 | INTERGENIC | | | | |
| CaPOPI_4191 | Ca6 | 33780561 | ATTTT/ATTTTT | TGGGAGCTTCAGGAATCACT | TGCATGTTTGATAATGCGGT | 59.8 | 250 | DRR | Ca_22060 | G | C3H | Metallo-dependent phosphatase |
| CaPOPI_4192 | Ca6 | 34222252 | CATATATATATATATAT/CATATATATATATATAT | TGCCTTTTGTGTTTCCCTTC | AGTTTAATGTCGTGACCGGC | 60.1 | 496 | INTERGENIC | | | | |
| CaPOPI_4193 | Ca6 | 34570118 | TTTTTTG/T | TGGTCGACAAGCAACTGAAG | AACAATCTCCCCCTTTTTGG | 60.0 | 583 | DRR | Ca_15816 | | | |
| CaPOPI_4194 | Ca6 | 34728984 | TTATATATATATATATATAT/TTATATATATATATATATATATAT | TAGGCACATCAAGTCAACCG | TCAGACCATCCGATAGAGGC | 59.7 | 570 | INTERGENIC | | | | |
| CaPOPI_4195 | Ca6 | 34775621 | T/TCAATTCAC | AAATGAACTTGTCACGGGCT | TCCCAACATTAGCTGATCCC | 59.6 | 702 | INTRON | Ca_15807 | R | | |

| INDEL marker IDs | Chromosomes/ unanchored scaffolds | Physical positions (bp) | InDels (*Kabuli* reference genome-CDC Frontier/PI) | Forward primers (5'-3') | Reverse primers (5'-3') | Anneling temperature (0C) | Expected amplified product size (bp) | Structural annotation | | Functional annotation | | |
|---|---|---|---|---|---|---|---|---|---|---|---|---|
| | | | | | | | | Sequence components of *kabuli* genome | *Kabuli* gene accession IDs | NCBI-KOG | TFs | NCBI-nr database |
| CaPOPI_4196 | Ca6 | 34859064 | GATATATATATATATATATATATATA/GATATATATATATATATATATATATATA | GAAAGAGTTTTGAGCCACGG | AAGTCCAATCTCGGTCAAACTT | 59.9 | 625 | INTERGENIC | | | | |
| CaPOPI_4197 | Ca6 | 34936983 | AATATATATATATATATATA/AATATATATATATATA | TCATCTTGCACTTGGCTTTG | TCGCGTCGAAAACTAGATCA | 60.0 | 708 | INTERGENIC | | | | |
| CaPOPI_4198 | Ca6 | 35161798 | TATAAATATAA/TGAATATAAAATAAATATAA | AGGAAGAGTGGAGTTGCGAA | CGGTTGAGAACCAAGGATGT | 60.0 | 649 | INTERGENIC | | | | |
| CaPOPI_4199 | Ca6 | 35399356 | GTTT/GAATTT | TTGAAGCTCAGATGGCACAC | CTCCCCCTTTTTGGCATAAT | 60.0 | 718 | INTERGENIC | | | | |
| CaPOPI_4200 | Ca6 | 35528115 | AACA/AACACA | AAGACCCATCTCCAGCACAC | CACCCATCGGATAATGCTTC | 60.1 | 783 | INTERGENIC | | | | |
| CaPOPI_4201 | Ca6 | 35616799 | C/CCTT | GTTTCTGAGGCAAAATCCCA | TGCATTTGTCTTCCCATTGA | 60.1 | 537 | INTERGENIC | | | | |
| CaPOPI_4202 | Ca6 | 35719325 | AATAGAATACTTTATTAT/AATAGAATACTTTATTATAGAATACTTTATTAT | TGCATCGTTTTTCATCTTTCA | GCGGCTAATTTCAACCAAAT | 59.3 | 630 | INTRON | Ca_15773 | | | |
| CaPOPI_4203 | Ca6 | 35801189 | AAATAATAATAATAATAATAATAA/AAATAATAATAATAATAATAA | CTTCCATAAGCCGCATTGTT | TTGAACAATAAAAATTAATCGCAAA | 60.1 | 548 | INTERGENIC | | | | |
| CaPOPI_4204 | Ca6 | 35802672 | TTTCT/TT | GAGCCTTAGAGTTGGGAGCA | ACCCAACTTGTCGAACCAAC | 59.6 | 778 | INTERGENIC | | | | |
| CaPOPI_4205 | Ca6 | 35810337 | C/CT | TCATCGATTGCAACTTGCTT | CCCTAAAATTTTGTGGGCTT | 59.4 | 672 | INTERGENIC | | | | |
| CaPOPI_4206 | Ca6 | 36029920 | CTTT/CTTTT | TTTGGAACCAAAAACTTCTTCAA | GGAGAAAAAGAATTAAAATCCTCTAAA | 60.0 | 520 | INTERGENIC | | | | |
| CaPOPI_4207 | Ca6 | 36039668 | CATAA/C | CAAGTCGTCGCTTCCAAAGT | AATTGCGGCGAGAGAAGATA | 60.4 | 394 | INTERGENIC | | | | |
| CaPOPI_4208 | Ca6 | 36237842 | CATTATTATTATTATTATTATTATTATTATTATTATTATTATTATTATTATTATTAT/CATTATTATTATTATTATTATTATTATTATTATTATTATTATTATTATTATTATTA | TCTACCCCCAATCCCTCTCT | ATTTGCCCTGATGTTTTTGG | 59.9 | 573 | INTERGENIC | | | | |
| CaPOPI_4209 | Ca6 | 36281940 | CATATATATATATATATATA/CATATATATATATATATA | AGTGACCATCTCCCAAGGTG | ATTTTAACGGATGTCGTGGG | 60.0 | 646 | INTERGENIC | | | | |
| CaPOPI_4210 | Ca6 | 36452738 | AATATATATATATATATA/AATATATATATATATATATA | CAATCCCATTTCCCATTTTG | AATGGGCGCTGTTAAATGTC | 60.0 | 778 | INTERGENIC | | | | |

| INDEL marker IDs | Chromosomes/ unanchored scaffolds | Physical positions (bp) | InDels (*Kabuli* reference genome-CDC Frontier/PI) | Forward primers (5'-3') | Reverse primers (5'-3') | Anneling temperature (0C) | Expected amplified product size (bp) | **Structural annotation** | | **Functional annotation** | | |
|---|---|---|---|---|---|---|---|---|---|---|---|---|
| | | | | | | | | **Sequence components of *kabuli* genome** | ***Kabuli* gene accession IDs** | **NCBI-KOG** | **TFs** | **NCBI-nr database** |
| CaPOPI_4211 | Ca6 | 36722414 | ACCC/ACC | CTTAGGTTTGCACTCAGCCC | TCGCGGATCTGCTCTTAAAT | 59.9 | 685 | INTERGENIC | | | | |
| CaPOPI_4212 | Ca6 | 36722630 | TA/TAA | TTGCTCCTACGTATCCCCAG | TCGCGGATCTGCTCTTAAAT | 60.1 | 295 | INTERGENIC | | | | |
| CaPOPI_4213 | Ca6 | 36880735 | AATATATATATATATATAT/AATATATATATATATAT | GGGCCAAGCCCATATTTTAT | TTGTTTGCAAATACTCCCCT | 60.0 | 177 | INTERGENIC | | | | |
| CaPOPI_4214 | Ca6 | 36898169 | AATATATATATAT/AATATATATATATAT | AAGAGAGGTCACTTTGGCGA | CAATTCCCACAGGTACGCTT | 60.0 | 692 | INTERGENIC | | | | |
| CaPOPI_4215 | Ca6 | 36951265 | TTTATTATTATTATTATTATTATTATTATTATTATTAT/TTTATTATTATTATTATTATTATTATTATTATTATTATTATTAT | TGGGCAGAATTGATAAATCG | CTCCCTCTCATCTGTTCAAAAA | 58.6 | 578 | INTERGENIC | | | | |
| CaPOPI_4216 | Ca6 | 37640608 | CAAAATTTAAAAATTTAAAAATTTAAAA/CAAAATTTAAAAATTTAAAAATTTAAAAATTTAAAA | TTTGGCGTAGTGTGACTTGC | CTCGTGAGGGGAAGCATAAG | 59.9 | 308 | INTERGENIC | | | | |
| CaPOPI_4217 | Ca6 | 37714381 | CATATATATATATATATA/CATATATATATATATATATA | TCGATCTCACCAGCAACAAC | TGGTGTCATGACCAAAATGG | 59.8 | 352 | INTERGENIC | | | | |
| CaPOPI_4218 | Ca6 | 37788611 | GTATATATATATATATATAT/GTATATATATATATATAT | AATGGAGGAAGGAGGGAGAA | TTTGGATTTTGACTCGGAGG | 60.0 | 435 | INTERGENIC | | | | |
| CaPOPI_4219 | Ca6 | 37849692 | CAAAA/CAAAAA | TCGAAACCCATGACGTTGTA | GAATCCTGGGCTGAAATCAA | 60.0 | 547 | INTERGENIC | | | | |
| CaPOPI_4220 | Ca6 | 37890277 | AATATATATATAT/AATATATATATATATAT | TCACGGCGAATATGAAATGA | TGCTATGCGTTTGGATCTTG | 60.0 | 652 | INTERGENIC | | | | |
| CaPOPI_4221 | Ca6 | 37902107 | TT/TTGATAT | GTTTTGACAGGCCCGAATAA | ATGGTGGTTCGGTCACATTT | 59.9 | 462 | INTERGENIC | | | | |
| CaPOPI_4222 | Ca6 | 37981858 | TAAA/TAA | TGCAATGACCTTGAAATTCG | CTTTTAGCCTTCATCGCCAG | 59.7 | 817 | INTERGENIC | | | | |
| CaPOPI_4223 | Ca6 | 37982172 | AATGAT/AAT | GGTACACTTTCCCATCAGCAA | CTTTTAGCCTTCATCGCCAG | 60.0 | 468 | INTERGENIC | | | | |
| CaPOPI_4224 | Ca6 | 38201434 | GTTTTTTT/GTTTTTTTT | GGTTCGTAATGGATGGGATG | AAGGCCTTTTACAACGCTCA | 60.0 | 609 | INTERGENIC | | | | |
| CaPOPI_4225 | Ca6 | 38317090 | ACC/ACCAAAATGCC | CAATCACAACCTTTGCCCTT | GTGGGAAGTCGCCATTTAGA | 60.0 | 531 | INTERGENIC | | | | |

| INDEL marker IDs | Chromosomes/ unanchored scaffolds | Physical positions (bp) | InDels (*Kabuli* reference genome-CDC Frontier/PI) | Forward primers (5'-3') | Reverse primers (5'-3') | Anneling temperature (0C) | Expected amplified product size (bp) | Structural annotation | | Functional annotation | | |
|---|---|---|---|---|---|---|---|---|---|---|---|---|
| | | | | | | | | Sequence components of *kabuli* genome | *Kabuli* gene accession IDs | NCBI-KOG | TFs | NCBI-nr database |
| CaPOPI_4226 | Ca6 | 38822653 | T/TCGTTTACATTGTTCA | TGAGAAAGGGGAAAGGGAAT | TGAGGTTGTTGCTTTGACCA | 59.9 | 737 | INTERGENIC | | | | |
| CaPOPI_4227 | Ca6 | 39182715 | GAAAAAAAAA/GAAAAAAAAAA | TGGACGAAGTCAATGGTCAA | TCAATGGAATTTTTCGAGTCAA | 60.1 | 843 | DRR | Ca_15911 | P | ERF | SPX, N-terminal |
| CaPOPI_4228 | Ca6 | 39781733 | CATATATATATATATATATATATATATA/ CATATATATATATATATATATATATATATA | TCTCCGCATAAGCTTTCGAT | TGCTTCGACACAGTAGCACC | 59.9 | 478 | DRR | Ca_15927 | | | |
| CaPOPI_4229 | Ca6 | 40508760 | GAAAA/GAAAAA | AATTGACGTCGAACCCTGTC | CGTAGCGAAGTCGGATGAAT | 60.0 | 364 | INTERGENIC | | | | |
| CaPOPI_4230 | Ca6 | 41139265 | AA/AAACAGGA | TTCGGGCTTAACCAAAAATG | AGCTGCAAGTGCAGAGCATA | 59.9 | 534 | INTERGENIC | | | | |
| CaPOPI_4231 | Ca6 | 41432949 | GAATTAAATTAA/GAATTAAATTAAATTAA | TCGAATTTTGAGACGGAAAGA | GGTTCGAGTGTGTGCCATAA | 59.8 | 684 | INTERGENIC | | | | |
| CaPOPI_4232 | Ca6 | 41493875 | TTATATATATATATATATATAT/TTATATATATATATATATATATAT | GGATCCAAATCTTCTCGTTAAA | AGTGGACGGTCTCAACTTGG | 57.4 | 993 | INTERGENIC | | | | |
| CaPOPI_4233 | Ca6 | 41520820 | AATATATATATATATATATAT/AATATATATATATATATATATAT | GCATTTTGTTTGAAGATTTTGATG | ATTCAATCACCCAAACCGAA | 59.9 | 414 | INTERGENIC | | | | |
| CaPOPI_4234 | Ca6 | 41574227 | CATAATAATAATAATAAT/CATAATAATAATAATAATAAT | TGATTAATCGCAAATTGTCCA | TTTTTGGGGTCGTTTTATGC | 59.0 | 471 | INTERGENIC | | | | |
| CaPOPI_4235 | Ca6 | 41574282 | TTTATTATTATTATTATT/TTTATTATTATTATTATTATT | TGATTAATCGCAAATTGTCCA | TTTTTGGGGTCGTTTTATGC | 59.0 | 471 | INTERGENIC | | | | |
| CaPOPI_4236 | Ca6 | 41654561 | GAAAAAAAAAAA/GAAAAAAAAA | CCTTTTGTGAAAGAGGCAGC | CACGGGGAAAGTTGTTTTGT | 60.0 | 623 | INTERGENIC | | | | |
| CaPOPI_4237 | Ca6 | 41659483 | CAAAA/CAAAAA | CCATCAGAAACTGGGATGGT | TGGCCCTTCATTTTTCAATC | 59.8 | 495 | INTERGENIC | | | | |
| CaPOPI_4238 | Ca6 | 41669872 | ATT/ATTT | TGTAGAATCCACCAATGTCCA | ACCAATCATGTTTCGTGGAA | 58.8 | 651 | INTERGENIC | | | | |
| CaPOPI_4239 | Ca6 | 41875891 | ATTTTT/ATTTTTT | GGATCTATGATTTCCCTCTTGC | AGTCCCCATGTAATTGTCACG | 59.1 | 568 | INTERGENIC | | | | |
| CaPOPI_4240 | Ca6 | 41957220 | CTATATATATAT/CTATATATAT | TCCCCAACTTTTGAATTTGC | TGCTTCAGTCAATGGGACAG | 59.9 | 877 | DRR | Ca_21949 | O | | Armadillo |

| INDEL marker IDs | Chromosomes/ unanchored scaffolds | Physical positions (bp) | InDels (*Kabuli* reference genome-CDC Frontier/PI) | Forward primers (5'-3') | Reverse primers (5'-3') | Anneling temperature (0C) | Expected amplified product size (bp) | Structural annotation | | Functional annotation | | |
|---|---|---|---|---|---|---|---|---|---|---|---|---|
| | | | | | | | | Sequence components of *kabuli* genome | *Kabuli* gene accession IDs | NCBI-KOG | TFs | NCBI-nr database |
| CaPOPI_4241 | Ca6 | 42309198 | AATATATATATATATATATA/AATATATATATATATATATATA | AGGAAGCCCAAGTTCCAAAT | CCAAGAGAGGTGCAACCAAT | 59.9 | 569 | INTERGENIC | | | | |
| CaPOPI_4242 | Ca6 | 42398678 | ATTTTTTT/ATTTTTT | GGTTTGGGAGAGAAAAAGGAA | TGGTTAAAACTCATTGGTCCC | 59.6 | 900 | INTERGENIC | | | | |
| CaPOPI_4243 | Ca6 | 42869037 | TAA/TAAA | TTGTCGTGATTATTAGGGGACA | TTGTCAAAACATATGCCGTTTC | 59.3 | 263 | INTERGENIC | | | | |
| CaPOPI_4244 | Ca6 | 42907383 | A/AAGAGGG | ACTGGCTGACCACTCTCGTT | CAGCGCTTATTTGGGAAAAA | 59.9 | 840 | INTERGENIC | | | | |
| CaPOPI_4245 | Ca6 | 43017644 | ACATCAT/ACATCATCAT | GGAAGGATTTTGAAGAAACCA | GCCGTGCCTTTTAAATGTGT | 58.1 | 529 | INTERGENIC | | | | |
| CaPOPI_4246 | Ca6 | 43043105 | CA/C | TGATTTGGTCGCAACAGCTA | AAAAATTGTGCCAAAGTTATGTGA | 60.4 | 556 | INTERGENIC | | | | |
| CaPOPI_4247 | Ca6 | 43064353 | A/AACTG | CGCCACAACTCCTACTCCTT | GAGGTGCGACAACAGATGAA | 59.4 | 785 | INTERGENIC | | | | |
| CaPOPI_4248 | Ca6 | 43116900 | AATATATATATATATATATATATATATATATA/AATATATATATATATATATATATATATATATATATA | GTTTGGGGTTACTCATGGGA | ATTCCCAAACATTGGCAAAA | 59.6 | 195 | INTERGENIC | | | | |
| CaPOPI_4249 | Ca6 | 43138332 | GAAAA/GAAA | GTTCCCTTTGGAATGCTTGA | TCTTTGGGCCAGTGAAAATC | 60.1 | 741 | INTERGENIC | | | | |
| CaPOPI_4250 | Ca6 | 43144384 | TTTCTTCTTCTTCTTCTTCTTCTTCT/TTTCTTCTTCTTCTTCTTCTTCTTCTTCT | TCATGTGGACTAAATGGGCA | ACAGCTCAAGCGAAACCCTA | 59.9 | 399 | INTERGENIC | | | | |
| CaPOPI_4251 | Ca6 | 43144709 | TTTATTATTATTATTATTATTATTATTATTATTATTATTATAATTATAATTATTATTATTATTATTATTATTATTATTATTATTATT/TTTATTATTATTATTATTATTATTAT | TAGGGTTTCGCTTGAGCTGT | TCCGGATCCTTCAGATTCAC | 60.0 | 656 | INTERGENIC | | | | |
| CaPOPI_4252 | Ca6 | 43901335 | GCCC/GCCCCC | GACCTTTTGAGGGTTCCCAT | ATTGACAACCTTCCCACTGC | 60.2 | 260 | INTERGENIC | | | | |
| CaPOPI_4253 | Ca6 | 43909185 | TTGAAG/TTGAAGCAAGTACAACTCATGAAG | TGATTGGGTTTGGGTTCATT | ACATAGATGCCGCCTTCATT | 60.0 | 445 | INTERGENIC | | | | |
| CaPOPI_4254 | Ca6 | 44072205 | TTATATATATATATATATATATATATAT/TTATATATATATATATATATATATATAT | GACATTTCGAACTTGAATTTCCT | TTTGGAATTGCGACTTCCTT | 58.7 | 363 | INTERGENIC | | | | |
| CaPOPI_4255 | Ca6 | 44266489 | AATATATATATATATATATATATATATATA/AATATATATATATATATATATATA | CCGCATTATAATTCCGCTGT | GTGCCGAGAAAATGTGGATT | 60.0 | 819 | INTERGENIC | | | | |

| INDEL marker IDs | Chromosomes/ unanchored scaffolds | Physical positions (bp) | InDels (*Kabuli* reference genome-CDC Frontier/PI) | Forward primers (5'-3') | Reverse primers (5'-3') | Anneling temperature (0C) | Expected amplified product size (bp) | Structural annotation | | Functional annotation | | |
|---|---|---|---|---|---|---|---|---|---|---|---|---|
| | | | | | | | | Sequence components of *kabuli* genome | *Kabuli* gene accession IDs | NCBI-KOG | TFs | NCBI-nr database |
| CaPOPI_4256 | Ca6 | 44386840 | GAT/G | ACCCCAGTGCCTTTTCTCTT | GCGTCCAGAGGAAGAATCAG | 60.1 | 577 | INTERGENIC | | | | |
| CaPOPI_4257 | Ca6 | 44418513 | CCTCTCTCTCTCTCTCTCT/CCTCTCTCTCTCTCTCTCT | ATAGGTGGCCATTGTTCCAA | TGTGGAGTGAAAGCATGAGG | 60.2 | 543 | INTRON | Ca_24238 | | MYB_related | Toll-Interleukin receptor |
| CaPOPI_4258 | Ca6 | 45018203 | AA/AAGTAACA | TCAAGACGGGAATTGGAAAG | TTGCAAAGGATGTATTGGCA | 60.0 | 433 | INTERGENIC | | | | |
| CaPOPI_4259 | Ca6 | 45329060 | T/TTTAAAA | TTTTTGCTATTTTATTCTCGTGG | TCGATTATGCGTAGGGAAGG | 57.6 | 762 | INTERGENIC | | | | |
| CaPOPI_4260 | Ca6 | 45469975 | TGTTCAGTTCAGTTCAGTTCA/TGTTCAGTTCAGTTCA | TGCGAAAAATGGGTTTGAGT | ATGTGAATCCCACCAGGAAA | 60.5 | 637 | INTERGENIC | | | | |
| CaPOPI_4261 | Ca6 | 45484176 | GTTTTTTTTTTTT/GTTTTTTTTTTTTTT | CACCAAAAATTGTACCACTTGC | GAGGCAACGACTCAAACCTC | 59.4 | 864 | INTERGENIC | | | | |
| CaPOPI_4262 | Ca6 | 45922563 | GTTTTTTTTTTTTT/GTTTTTTTTTTT | GTGGCCACTCACCAAAAGAT | TGAACAAAACCACCAAACCA | 60.0 | 564 | INTERGENIC | | | | |
| CaPOPI_4263 | Ca6 | 46031146 | G/GGA | GGAAATGGATGTCTGCCAAT | AGCCTCGTAGGTTCGACTGA | 59.8 | 551 | INTERGENIC | | | | |
| CaPOPI_4264 | Ca6 | 46128884 | AAATAATAATAATAATAATAATAATAATAATAATAATAATAATAAT/AAATAATAATAATAATAATAATAATAATAATAATAATAATAATAAT | ATGCCAGCTTGGACAATGTT | TGGAGATGCGACCGTCTACT | 60.5 | 741 | INTERGENIC | | | | |
| CaPOPI_4265 | Ca6 | 46391549 | ATTTTTTTT/ATTTTTTTTTT | GAACATGAAAAGGGTCACCG | GGAGGAATCTTTTGTTGCCA | 60.4 | 564 | INTERGENIC | | | | |
| CaPOPI_4266 | Ca6 | 46517053 | T/TAAATAATAATAATAA | TCTTCGTCGTCAACATGAGC | AGCAACGGAAAGGATACACG | 60.0 | 574 | INTERGENIC | | | | |
| CaPOPI_4267 | Ca6 | 46772986 | A/ATT | GATTATTCTGCCTCCCGTCA | AAACGCAATCCTTGAGTTCG | 60.0 | 633 | INTERGENIC | | | | |
| CaPOPI_4268 | Ca6 | 46804307 | AATATATATATATATATATATATATATAT/AATATATATATATATATATATATAT | TAAACCAAGTTCCCCGTCAG | CAATGGCTAAGAGCAAAGGG | 60.0 | 799 | INTERGENIC | | | | |
| CaPOPI_4269 | Ca6 | 46855085 | AATATATATATATATATATA/AATATATATATATATATA | CTGTATCACGTGGACAACGG | CGATCAGGCCGGATATTAGA | 60.0 | 699 | INTERGENIC | | | | |
| CaPOPI_4270 | Ca6 | 46877609 | TTTATTATTATTATTATTATTATTATTA/TTTATTATTATTATTATTATTATTA | TGGATGGATTGGTTGAATGA | TGAAGGAGGGACCAAAACTG | 59.7 | 198 | INTERGENIC | | | | |

| INDEL marker IDs | Chromosomes/ unanchored scaffolds | Physical positions (bp) | InDels (*Kabuli* reference genome-CDC Frontier/PI) | Forward primers (5'-3') | Reverse primers (5'-3') | Anneling temperature (0C) | Expected amplified product size (bp) | Structural annotation | | Functional annotation | | |
|---|---|---|---|---|---|---|---|---|---|---|---|---|
| | | | | | | | | Sequence components of *kabuli* genome | *Kabuli* gene accession IDs | NCBI-KOG | TFs | NCBI-nr database |
| CaPOPI_4271 | Ca6 | 47001052 | A/AG | GAAGCGGTACTTCCCATTGA | GGTTACAATGTGGGGACGAG | 60.1 | 417 | INTERGENIC | | | | |
| CaPOPI_4272 | Ca6 | 47040606 | TTTATTATTATTATTATTATTATTATTATTATTATTATTATTAT/TTTATTATTATTATTATTATTATTATTATTATTATTATTATTAT | TATCATCACCTCACCCCCAT | TCCAGTCAATGTGCAAAAACA | 60.0 | 550 | INTERGENIC | | | | |
| CaPOPI_4273 | Ca6 | 47149113 | TAAAA/TAAAAA | AAACGGGAACTCCAGAATGA | TTTCCACCCAGAAAGCAATC | 59.5 | 617 | INTERGENIC | | | | |
| CaPOPI_4274 | Ca6 | 47312521 | GTT/GTTT | TATCTGTGGTCGTGTGGGAA | TCACGGGTCAACACGATTTA | 60.0 | 822 | INTERGENIC | | | | |
| CaPOPI_4275 | Ca6 | 47393714 | AAATATCAATA/AAATATCAATATCAATA | TTATCTGCCGCTACCCAAAG | TCTTGGAGCTTTTGGCTTGT | 60.2 | 755 | INTERGENIC | | | | |
| CaPOPI_4276 | Ca6 | 47697947 | GTTCTGATTAATTTATTTTCTG/GTTCTGATTAATTTATTTTCTGATTAATTTATTTTCTG | TATTTTGGGGTTATCGAGCG | TTGGTTTATTTGGGTTGGGA | 59.9 | 801 | INTERGENIC | | | | |
| CaPOPI_4277 | Ca6 | 47816277 | AATATATATATATATATATATATATATA/AATATATATATATATATATATATATA | TGGGAATAGGCCAGACTTTG | TTCCGAGTTGTTTGAGAGGA | 60.1 | 748 | INTERGENIC | | | | |
| CaPOPI_4278 | Ca6 | 48011269 | GATATATATATATAT/GATATATATATATATAT | TTTGTTGCGTAAATGTTGTTGA | TAGCTGTTGCGACCAAATCA | 59.2 | 665 | INTERGENIC | | | | |
| CaPOPI_4279 | Ca6 | 48260819 | GAAAATTAATAAAATA/GAAAATTAATAAAATAAAATTAATAAAATA | GTGGATGGTGGGGAATAGTG | CTGAGGTCCCAAGCTACGTC | 60.1 | 504 | INTERGENIC | | | | |
| CaPOPI_4280 | Ca6 | 48271447 | TTTATTATTATTATTATTATTATTATTATTATTATTATTATTATTATTATTAT/TTTATTATTATTATTATTATTATTATTATTATTATTATTATTATTAT | TGACTAATTGGTCGCCCTTC | GGATGCGATCATGTGAAGAA | 60.1 | 685 | INTERGENIC | | | | |
| CaPOPI_4281 | Ca6 | 48271722 | CATATATATATATATATATATATATAT/CATATATATATATATATATATAT | TGGACCAAAGCAGTGATGAG | TGACATTTTCAGGAGGCACA | 59.8 | 804 | INTERGENIC | | | | |
| CaPOPI_4282 | Ca6 | 48271982 | GTATATATATATATATATATATATAT/GTATATATATATATATATATAT | GAGGATGCGACCTCCTACTG | TGACATTTTCAGGAGGCACA | 59.8 | 337 | INTERGENIC | | | | |
| CaPOPI_4283 | Ca6 | 48279188 | T/TA | TCAAGAACGTTTCGACCACA | GCAACTCGCGGGTTAGTATC | 60.3 | 562 | INTERGENIC | | | | |
| CaPOPI_4284 | Ca6 | 48279626 | ATTTTTTT/ATTTTTT | AACCAGATACTAACCCGCGA | TCTGGAAGTTTCTTGACCGTG | 59.6 | 620 | INTERGENIC | | | | |
| CaPOPI_4285 | Ca6 | 48280097 | GAAAAAAA/GAAAAAAAA | GACACGGTCAAGAAACTTCCA | TGCGTTGACTCTTCAATACACA | 60.1 | 376 | INTERGENIC | | | | |

| INDEL marker IDs | Chromosomes/ unanchored scaffolds | Physical positions (bp) | InDels (*Kabuli* reference genome-CDC Frontier/PI) | Forward primers (5'-3') | Reverse primers (5'-3') | Anneling temperature (0C) | Expected amplified product size (bp) | **Structural annotation** | | **Functional annotation** | | |
|---|---|---|---|---|---|---|---|---|---|---|---|---|
| | | | | | | | | **Sequence components of *kabuli* genome** | ***Kabuli* gene accession IDs** | **NCBI-KOG** | **TFs** | **NCBI-nr database** |
| CaPOPI_4286 | Ca6 | 48280225 | GTAT/GT | GACACGGTCAAGAAACTTCCA | TGTGGCACATTCCAAATTTAT | 60.1 | 601 | INTERGENIC | | | | |
| CaPOPI_4287 | Ca6 | 48280286 | ATTTTTTTTTTTTTTT/ATTTTTTTTTTTT | GACACGGTCAAGAAACTTCCA | TGTGGCACATTCCAAATTTAT | 60.1 | 601 | INTERGENIC | | | | |
| CaPOPI_4288 | Ca6 | 48280766 | TCTA/TCTAAAACTA | TGTATTGATGAGTCGTTGAATGAT | GGAGACGTTAAAGTCCCACA | 58.5 | 902 | INTERGENIC | | | | |
| CaPOPI_4289 | Ca6 | 48281397 | TAAAAAAAA/TGAAAAAAAAAA | GGGACTTTAACGTCTCCAATACA | CTTGAGAACGATCTGCCACA | 59.4 | 473 | INTERGENIC | | | | |
| CaPOPI_4290 | Ca6 | 48281570 | TA/TAATTAAA | TAGGGCAATTGTGCACATGA | CTCCAGGTAACGGTTGTGGT | 61.1 | 335 | INTERGENIC | | | | |
| CaPOPI_4291 | Ca6 | 48316872 | T/TAGCC | AGGAGGTGTCACCGTCAATC | TGTTTAGGGTACAATGCCGA | 60.0 | 164 | DRR | Ca_24607 | Q | | Multicopper oxidase, type 1 |
| CaPOPI_4292 | Ca6 | 48788599 | CATATAT/CATATATATAT | TACAATTGATTGGCGGCATA | ATTGTGGTGGAGGTTGTGGT | 59.9 | 738 | INTERGENIC | | | | |
| CaPOPI_4293 | Ca6 | 48905266 | CTAAATAAATAAATAAATAA/CTAAATAAATAAATAAATAAATAAATAA | CACGGTCCCTAGAGCCAATA | ATCGACTCCCGAACTCAAAA | 60.1 | 666 | INTERGENIC | | | | |
| CaPOPI_4294 | Ca6 | 49178234 | TTCTCTCTCTCTCTCTCTCTCTCTCT/TTCTCTCTCTCTCTCTCTCTCT | CATAACCCCTTTTTGTGCGT | CTTTGGACGACCTTGGAAGA | 59.9 | 280 | INTERGENIC | | | | |
| CaPOPI_4295 | Ca6 | 49218883 | TAAAAAA/TAAAAAAAA | CCCCTTCATATGCTTCCAGA | GTGCTTGCTTTTACTTCGCC | 60.0 | 446 | INTERGENIC | | | | |
| CaPOPI_4296 | Ca6 | 49733827 | TCC/TC | TTGCAATCTCATCAACTCGG | GTAAAATGAAGCGCTTTCGC | 59.8 | 810 | INTERGENIC | | | | |
| CaPOPI_4297 | Ca6 | 50818138 | AAAAATTTTATA/AA | CACATACTTCACATTTTGCATTGA | TGTAAAACCCTTACCGTGTGTC | 59.9 | 682 | INTERGENIC | | | | |
| CaPOPI_4298 | Ca6 | 51012538 | TTAATAATAATAATAATAATAATAAT/TTAATAATAATAATAATAATAAT | TGGCACATCTATTCAACCACA | CATGCGATGCAAACATGTAA | 60.0 | 540 | INTERGENIC | | | | |
| CaPOPI_4299 | Ca6 | 51337603 | AG/AGG | TGATGGGGTGCAAATTATGA | CAGGGACCAAGAAATTCAGG | 59.7 | 877 | INTRON | Ca_19171 | R | | Tetratricopeptide repeat-containing |
| CaPOPI_4300 | Ca6 | 51429120 | AATATATATATATATATATATATATA/AATATATATATATATATATATATATATATA | CATGACCCCAATTTTTCAGG | TGGACAACGAATCTAACGGA | 60.2 | 943 | INTERGENIC | | | | |

| INDEL marker IDs | Chromosomes/ unanchored scaffolds | Physical positions (bp) | InDels (*Kabuli* reference genome-CDC Frontier/PI) | Forward primers (5'-3') | Reverse primers (5'-3') | Anneling temperature (0C) | Expected amplified product size (bp) | Structural annotation | | Functional annotation | | |
|---|---|---|---|---|---|---|---|---|---|---|---|---|
| | | | | | | | | Sequence components of *kabuli* genome | *Kabuli* gene accession IDs | NCBI-KOG | TFs | NCBI-nr database |
| CaPOPI_4301 | Ca6 | 51482572 | TAAAAAAAA/TAAAAAAAAA | TGCACAATTGTTTATGATGTTTTT | ACACTCCCAAAATCGTCCAG | 58.5 | 552 | INTERGENIC | | | | |
| CaPOPI_4302 | Ca6 | 51672087 | AT/AATATATAACCT | ATGGAATGAATCCACACGGT | TCTTCCACCCAGTTTTGACC | 60.1 | 360 | INTERGENIC | | | | |
| CaPOPI_4303 | Ca6 | 51675507 | A/AG | GGAAGGGATTTTTGTGGTGA | TTCGTAATGGACGGGATGAT | 59.8 | 596 | INTERGENIC | | | | |
| CaPOPI_4304 | Ca6 | 51771487 | AATAAATATAAATATA/AATAAATATA | CGACCCACATGTTGAAGTTG | TCCCCCTTTGTCTTCTTGTG | 60.0 | 685 | INTERGENIC | | | | |
| CaPOPI_4305 | Ca6 | 51776432 | AATATATATATATATATATATATATATAT/AATATATATATATATATATATATAT | ATTTTTCGTTGAAACGCACC | GGCCTATAGCCTGGCCTACT | 60.0 | 481 | INTERGENIC | | | | |
| CaPOPI_4306 | Ca6 | 51959426 | CAA/CAAAA | TAATGGTTTTCACGCCAACA | ACCGGAAACGCATGTTAAAG | 60.0 | 586 | INTERGENIC | | | | |
| CaPOPI_4307 | Ca6 | 52074906 | TTTATTAAGCGATTTATTATTCAGCGATTTATTATTA/TTTATTCAGCGATTTATTATTA | TCCTGAAGATGAGATGCGTG | GGCTATCGCTTATGCAGGAC | 59.9 | 797 | DRR | Ca_17514 | | SAP | Aminotransferase-like, plant mobile domain |
| CaPOPI_4308 | Ca6 | 52075058 | GTTATTATTATT/GTTATTATTATTATT | GCGTGTCTTATTGGAGAGAGC | GGCTATCGCTTATGCAGGAC | 59.1 | 782 | DRR | Ca_17514 | | SAP | Aminotransferase-like, plant mobile domain |
| CaPOPI_4309 | Ca6 | 52104833 | A/AC | CAGAAAAAGGTGCAACCACA | TCCTCCAACCACCATTTCAT | 59.7 | 793 | INTERGENIC | | | | |
| CaPOPI_4310 | Ca6 | 52477923 | TTTATTATTATTATTATTATTATTATTATTATTATTATTATTATTATTATT/TTTATTATTATTATTATTATTATTATTATTATTATTATTATTATTATTATT | TGCGATTGAATAGATGTGCC | ACTTTGGAGGAACGACTTGG | 59.7 | 733 | INTERGENIC | | | | |
| CaPOPI_4311 | Ca6 | 52486383 | GTTTTTTTTTTTTTTTTT/GTTTTTTTTTT | GTTCCATGCTGCCTTTTCAT | AGAGGAAGCAGCCACTGTGT | 60.1 | 271 | INTERGENIC | | | | |
| CaPOPI_4312 | Ca6 | 52486696 | ATTTTTTTTTTT/ATTTTTTTT | AGGCCATCACATTCCTTTTG | CACTTTGGGGACGTCAAGTT | 59.9 | 449 | INTERGENIC | | | | |
| CaPOPI_4313 | Ca6 | 52522417 | CTTTTTTTTT/CTTTTTTTT | TGCAAAATCAATTTCAATCAGG | GGGCCTTTTAGGTCCATACC | 59.9 | 480 | INTERGENIC | | | | |
| CaPOPI_4314 | Ca6 | 52529525 | TAAAAAAAAAAA/TAAAAAAAAAAAAAA | CCCCAGGTGCAATAGAGAAA | TCGCCTACTCACGGATTTTT | 60.1 | 278 | INTERGENIC | | | | |
| CaPOPI_4315 | Ca6 | 52669647 | TTTATTATTATTATTATTATTATTATT/TTTATTATTATTATTATTATTATT | TGATGATAATCGGTGTCCCA | AGTTGGAGGTCACTAGCCCA | 59.7 | 372 | INTERGENIC | | | | |

| INDEL marker IDs | Chromosomes/ unanchored scaffolds | Physical positions (bp) | InDels (*Kabuli* reference genome-CDC Frontier/PI) | Forward primers (5'-3') | Reverse primers (5'-3') | Anneling temperature (0C) | Expected amplified product size (bp) | **Structural annotation** | | **Functional annotation** | | |
|---|---|---|---|---|---|---|---|---|---|---|---|---|
| | | | | | | | | **Sequence components of *kabuli* genome** | ***Kabuli* gene accession IDs** | **NCBI-KOG** | **TFs** | **NCBI-nr database** |
| CaPOPI_4316 | Ca6 | 52676255 | GATATATATATATATATATATATAT/GATATATATATATATATATATATAT | ATGGGTATAAGTACCCGCGA | TGTGTCCATCCCTAAAACGA | 59.3 | 223 | INTERGENIC | | | | |
| CaPOPI_4317 | Ca6 | 52848807 | ACTCGCT/A | CTGCCAAATACTGCGACTCA | TCAATTTTGCTTGTAGGATGGA | 60.0 | 381 | INTERGENIC | | | | |
| CaPOPI_4318 | Ca6 | 52850074 | ACTAATTTCTAATT/ACTAATTTCTAATTTCTAATT | TCTGATTCGTTAATGACAACCTTT | CGTGGGAGCAATTATGTCCT | 59.1 | 218 | INTERGENIC | | | | |
| CaPOPI_4319 | Ca6 | 52850184 | TA/T | AGGACATAATTGCTCCCACG | TCCATCTCCAACAACATCAAA | 60.0 | 305 | INTERGENIC | | | | |
| CaPOPI_4320 | Ca6 | 52862596 | T/TTATAA | ACATGAAGGCTTGGGTTGAC | GAGGAGCATGATAGCAAGGC | 60.0 | 193 | INTERGENIC | | | | |
| CaPOPI_4321 | Ca6 | 52889876 | ATT/ATTT | TAAAGGACCAAGTCGGGAAA | TGCTCATGGAGTTAGAAGCAA | 59.5 | 471 | INTERGENIC | | | | |
| CaPOPI_4322 | Ca6 | 52890257 | TT/TTTGT | TTGCTTCTAACTCCATGAGCA | TTATTTGGGTGCATGAGCAA | 58.7 | 413 | INTERGENIC | | | | |
| CaPOPI_4323 | Ca6 | 53305336 | ATTTTTTTT/ATTTTTTTTT | CAAGATTTCGTGGTGCAAGA | TTGAGGACAAAAGCTGCTGA | 59.8 | 660 | INTRON | Ca_22925 | G | | Major intrinsic protein |
| CaPOPI_4324 | Ca6 | 53311252 | AT/ATT | GATGGTGGCCATTGAAGAGT | CAACAGTGTATGCACCTGCC | 59.9 | 721 | INTERGENIC | | | | |
| CaPOPI_4325 | Ca6 | 53312302 | AAAAATAAAATAAAATAAAATAAAA/AAAAATAAAATAAAATAAAATAAAATAAAA | ATAGCAATTGGTGCATCCCT | ATGACCAAGGCATTGAGGAC | 59.4 | 761 | INTERGENIC | | | | |
| CaPOPI_4326 | Ca6 | 53413835 | ATT/ATTT | CGTCATTTGGGGGACTAAAA | GAGTTCGGGAGTAGGGAAGG | 59.8 | 978 | INTERGENIC | | | | |
| CaPOPI_4327 | Ca6 | 53680637 | CATATATATATATATATATATATATAT/CATATATATATATATATATATATATATAT | TCTATGGCACTTGGAGCCTT | AATTTGCATGTCTTTTGCCC | 59.8 | 667 | INTRON | Ca_18901 | | | |
| CaPOPI_4328 | Ca6 | 53780329 | GATTAATTAATTAATTAATTAATTAATTAATTAATTAATTAATT/GATTAATTAATTAATTAATTAATTAATTAATTAATTAATT | CACACCCTCTCAAGGTGGAT | TCGAATGGTTGATAAAATTAACG | 60.0 | 723 | INTERGENIC | | | | |
| CaPOPI_4329 | Ca6 | 53799025 | ATTT/ATTTT | GGCCCAATATTTGAAACCCT | CAACTTGCAAAGACCCATCA | 60.0 | 704 | INTERGENIC | | | | |
| CaPOPI_4330 | Ca6 | 54068748 | CCATCAT/CCAT | GTAAGTGAAAGCGGGAGTGC | ACCACCAACTTGCTTGCTCT | 59.9 | 527 | INTERGENIC | | | | |

| INDEL marker IDs | Chromosomes/ unanchored scaffolds | Physical positions (bp) | InDels (*Kabuli* reference genome-CDC Frontier/PI) | Forward primers (5'-3') | Reverse primers (5'-3') | Anneling temperature (0C) | Expected amplified product size (bp) | Structural annotation | | Functional annotation | | |
|---|---|---|---|---|---|---|---|---|---|---|---|---|
| | | | | | | | | Sequence components of *kabuli* genome | *Kabuli* gene accession IDs | NCBI-KOG | TFs | NCBI-nr database |
| CaPOPI_4331 | Ca6 | 54068881 | TAATATAAAAA/TAATATAAAAATATAAAAA | GATCCCAACCATCATTGTCC | ACCACCAACTTGCTTGCTCT | 60.0 | 258 | INTERGENIC | | | | |
| CaPOPI_4332 | Ca6 | 54068968 | GA/GATA | GATCCCAACCATCATTGTCC | ACCACCAACTTGCTTGCTCT | 60.0 | 258 | INTERGENIC | | | | |
| CaPOPI_4333 | Ca6 | 54069131 | ATTTT/ATTTTTT | GATCCCAACCATCATTGTCC | AATTACCACGGCCTCTTGTG | 60.0 | 824 | INTERGENIC | | | | |
| CaPOPI_4334 | Ca6 | 54070088 | ATT/ATTT | GCAATTGTCGTGATTTTATTTTTG | TTTTGGTGGGTTCCACTTTT | 59.8 | 379 | INTERGENIC | | | | |
| CaPOPI_4335 | Ca6 | 54070236 | TAAAA/TAA | TTTCTTTCCTCATGCTTTAGAGG | TTTTGGTGGGTTCCACTTTT | 59.1 | 123 | INTERGENIC | | | | |
| CaPOPI_4336 | Ca6 | 54143384 | T/TAA | CCAAGAGATATACGGGGCAA | TGAAAGTCTGATCCGATCTAATAGT | 59.9 | 623 | INTERGENIC | | | | |
| CaPOPI_4337 | Ca6 | 54143633 | AATATATA/AATATATATA | CCAAGAGATATACGGGGCAA | CGACAAAACAAACCGCATAA | 59.9 | 907 | INTERGENIC | | | | |
| CaPOPI_4338 | Ca6 | 54158111 | CTTTT/CTTT | ATTCGAAACCATCGGAACTG | TTGGTGTTGCAAAATGAAAA | 59.9 | 588 | INTERGENIC | | | | |
| CaPOPI_4339 | Ca6 | 54158243 | ATTTTGAATTTTT/ATTTT | ATTCGAAACCATCGGAACTG | TTGGTGTTGCAAAATGAAAA | 59.9 | 588 | INTERGENIC | | | | |
| CaPOPI_4340 | Ca6 | 54160975 | AAATAT/AAATATTAATAT | TTGATTTGACAATTTTCATTGCTT | TCAGTTTTCCGGTTTCTTGC | 59.9 | 703 | INTERGENIC | | | | |
| CaPOPI_4341 | Ca6 | 54192734 | ATTTTTTTT/ATTTTTTTTT | GGACGGCGAAAATAAAACAA | TTGAAAATGGCTAAGGCAAAA | 59.9 | 642 | INTERGENIC | | | | |
| CaPOPI_4342 | Ca6 | 54339604 | GAGGATCA/GA | ATCATGCGTTTCTCACATGC | TACGGGGTTGGGGATAATTT | 59.7 | 216 | DRR | Ca_16289 | O | WRKY | Peptidase M41 |
| CaPOPI_4343 | Ca6 | 54339840 | CAAAAA/CAAAA | TAAATTATCCCCAACCCCGT | CTGATGAGCTTTCGGTGCTT | 60.3 | 205 | INTERGENIC | | | | |
| CaPOPI_4344 | Ca6 | 54339929 | TC/T | AAATTATCCCCAACCCCGTA | TCATTGGACTCTCATCCTCTCA | 60.3 | 628 | INTERGENIC | | | | |
| CaPOPI_4345 | Ca6 | 54341513 | T/TCTCTC | GATGTTGGGTCGCTTTCAAT | AAAACCAACACTGCCAAAGG | 59.9 | 688 | INTERGENIC | | | | |

| **INDEL marker IDs** | **Chromosomes/ unanchored scaffolds** | **Physical positions (bp)** | **InDels (*Kabuli* reference genome-CDC Frontier/PI)** | **Forward primers (5'-3')** | **Reverse primers (5'-3')** | **Anneling temperature (0C)** | **Expected amplified product size (bp)** | **Structural annotation** | | **Functional annotation** | | |
|---|---|---|---|---|---|---|---|---|---|---|---|---|
| | | | | | | | | **Sequence components of *kabuli* genome** | ***Kabuli* gene accession IDs** | **NCBI-KOG** | **TFs** | **NCBI-nr database** |
| CaPOPI_4346 | Ca6 | 54344134 | ATTTTTTT/ATTTTTTTT | AAGTCACAATGGAAAACGGC | AGGAACTGTAGCAGCCTCCA | 60.0 | 564 | INTRON | Ca_16290 | R | | |
| CaPOPI_4347 | Ca6 | 54712565 | AAAACTAA/AAAACTAAACTAA | GCATACCCTTGGTAAATTGCTC | TGCTAGAAATAGAATTTGGCACA | 59.9 | 352 | INTERGENIC | | | | |
| CaPOPI_4348 | Ca6 | 54952283 | TTATATATATATATATATATATATATATAT/TTATATATATATATATATATATATATAT | CCATTGTAACACCCCAAATTTTA | TTTCCTCCATTTCTTCGTGG | 59.9 | 937 | INTERGENIC | | | | |
| CaPOPI_4349 | Ca6 | 55143076 | A/AAAGAGTGTAAGGTAAT | ATAAAGCCTCGCGATGAAGA | CACCCAATACGGGTTACGTC | 59.9 | 466 | INTERGENIC | | | | |
| CaPOPI_4350 | Ca6 | 55490894 | T/TTAATAC | TGAAGAGACGCACATTGGAG | TGTCGGTTACTAAACATGATGGA | 60.0 | 542 | INTERGENIC | | | | |
| CaPOPI_4351 | Ca6 | 55540809 | TAAAAAAAAA/TAAAAAAAAAA | TGTTTGCAGAACAAGCAGAGA | ACTTCCTCTCACTACGCCGA | 59.8 | 548 | INTERGENIC | | | | |
| CaPOPI_4352 | Ca6 | 55769150 | C/CT | TCGATTATGCGTAGGGAAGG | TTTTTCCTTTGTAGGCCCAA | 60.1 | 943 | INTERGENIC | | | | |
| CaPOPI_4353 | Ca6 | 55771133 | GTC/GTCTTC | GGAAACGTGTGAACGGAAGT | GGTAACCAATCCGAAGACGA | 60.0 | 575 | INTERGENIC | | | | |
| CaPOPI_4354 | Ca6 | 55771190 | TAT/TATCGTCTTCGGAT | TTACCGATGCATTGATCGAC | GGTAACCAATCCGAAGACGA | 59.5 | 143 | INTERGENIC | | | | |
| CaPOPI_4355 | Ca6 | 55781099 | GTTTTT/GTTTT | AGAAGGCTACCACTACGCGA | ATCGACTCCCGAACTCAAAA | 60.0 | 500 | INTERGENIC | | | | |
| CaPOPI_4356 | Ca6 | 55876495 | TTAATAATAATAATAATAATAATAATAATAATAATAATAATA/TTAATAATAATAATAATAATAATAATAATAATAATAATAATAATAATA | ACATCTGCCCACATTGATGA | CTTTCAAAGTCAGCGGGAAC | 59.9 | 567 | INTERGENIC | | | | |
| CaPOPI_4357 | Ca6 | 56087656 | CTTT/CTTTT | TTGTGAGCATGAGAAGTTGTTG | TGAAAAATTATTGAAAAAGTTACTTCC | 59.0 | 737 | INTERGENIC | | | | |
| CaPOPI_4358 | Ca6 | 56191156 | CAAATAAATAAATAAATAAATAAATAAA/CAAATAAATAAATAAATAAATAAA | TCGATTATGCGTAGGGAAGG | TCCTTGAGTTTTGGGACACA | 60.1 | 482 | INTERGENIC | | | | |
| CaPOPI_4359 | Ca6 | 56504001 | ATTTTT/ATTTT | GTTGGCGCAAACTCTTCTTC | TGCAACTTTCAGGTCCCTTT | 60.0 | 583 | INTERGENIC | | | | |
| CaPOPI_4360 | Ca6 | 56504140 | AAAAGAAA/AAAA | AAATGCAAAGTCGGATGGTC | TGCGAAGAAGAATGATCACG | 59.9 | 896 | INTERGENIC | | | | |

| INDEL marker IDs | Chromosomes/ unanchored scaffolds | Physical positions (bp) | InDels (*Kabuli* reference genome-CDC Frontier/PI) | Forward primers (5'-3') | Reverse primers (5'-3') | Anneling temperature (0C) | Expected amplified product size (bp) | Structural annotation | | Functional annotation | | |
|---|---|---|---|---|---|---|---|---|---|---|---|---|
| | | | | | | | | Sequence components of *kabuli* genome | *Kabuli* gene accession IDs | NCBI-KOG | TFs | NCBI-nr database |
| CaPOPI_4361 | Ca6 | 56504753 | ATTTTTTTTTTTTT/ATTTTTTTTTT | CGTGATCATTCTTCTTCGCA | TCGGTCACCAAGTTACCACA | 59.9 | 614 | INTERGENIC | | | | |
| CaPOPI_4362 | Ca6 | 56505598 | AC/A | TGGTAACTTGGTGACCGACA | AATTTTTGCAGAAGAAAGAAAAAG | 60.0 | 485 | INTERGENIC | | | | |
| CaPOPI_4363 | Ca6 | 56507452 | TATCA/TA | CGTGATCATTCTTCTTCGCA | ATCCAAACATTGGCCATTTC | 59.9 | 685 | INTERGENIC | | | | |
| CaPOPI_4364 | Ca6 | 56507723 | GTTTTTTTTTTTTTT/GTTTTTTTTTTTTTTTTT | CGTGATCATTCTTCTTCGCA | CTCCCAATTTGCAGTGTCCT | 59.9 | 966 | INTERGENIC | | | | |
| CaPOPI_4365 | Ca6 | 56507798 | CA/CAA | AAATTCAAGTCTCGACGCAAA | CTCCCAATTTGCAGTGTCCT | 59.9 | 511 | INTERGENIC | | | | |
| CaPOPI_4366 | Ca6 | 56727392 | TAA/TAAA | AGGCAAAACATGTTCCCTCA | AATGTTAGTGCACCGTGTCG | 60.5 | 706 | INTERGENIC | | | | |
| CaPOPI_4367 | Ca6 | 56775193 | TTTATTATTATTATTATTATTATTATTA/TTTATTATTATTATTATTATTATTATTATTA | CGTGTGCGAACTTGCTTAAA | GAATCCCTGGTTCACAGCAT | 60.1 | 279 | INTERGENIC | | | | |
| CaPOPI_4368 | Ca6 | 57055878 | ATTTTTTTT/ATTTTTTTTT | TCCAAATGAAGCAAGATCAAAA | GCGGAAAGAAACAAATCTCAA | 59.7 | 600 | INTERGENIC | | | | |
| CaPOPI_4369 | Ca6 | 57058024 | TAAAAAAA/TAAAAAAAA | AGGTTCACGGTTAAATTTGGA | CAGATCGAATCGGATTTTGAA | 58.5 | 351 | INTERGENIC | | | | |
| CaPOPI_4370 | Ca6 | 57132721 | CAAAAAAAAA/CAAAAAAAAAA | ACAAGATCGGAGGGAGGAGT | TTTTGTGGTTTTGGTTCGTG | 60.1 | 580 | INTRON | Ca_17439 | | | Glycosyl-phosphatidyl inositol-anchored, plant |
| CaPOPI_4371 | Ca6 | 58084151 | TAAAAAAAA/TAAAAAAAAA | GGCTACATCGCCATCATCTT | TCAAGTCGATCCCATCAAATC | 60.1 | 770 | INTERGENIC | | | | |
| CaPOPI_4372 | Ca6 | 58373671 | GATATATATATATATATATATATA/GATATATATATATATATATATATATATA | TCACGCAAAAGATTCATAAAAGA | AGTTTGTAAGCTGTGCGTCG | 58.9 | 500 | INTERGENIC | | | | |
| CaPOPI_4373 | Ca6 | 58523123 | TTATATATATATATATATATATA/TTATATATATATATATATATATATATATA | TTTTGGAAACAAATCTAACGGAA | GACACGAGCCTCACCAAAGT | 59.9 | 238 | INTERGENIC | | | | |
| CaPOPI_4374 | Ca6 | 58597676 | CG/C | CGATCATGCTGCTTGTTTTG | TCGTTCATCTTGAATGTGACAA | 60.4 | 737 | DRR | Ca_15402 | | | Pectinesterase inhibitor |
| CaPOPI_4375 | Ca6 | 58834846 | GTTTTTTTTTT/GTTTTTTTTTTT | TGAAACGTCAACTTTTCGGA | AGGACTTCACCATCAATTCCA | 59.3 | 292 | INTERGENIC | | | | |

| INDEL marker IDs | Chromosomes/ unanchored scaffolds | Physical positions (bp) | InDels (*Kabuli* reference genome-CDC Frontier/PI) | Forward primers (5'-3') | Reverse primers (5'-3') | Anneling temperature (0C) | Expected amplified product size (bp) | Structural annotation | | Functional annotation | | |
|---|---|---|---|---|---|---|---|---|---|---|---|---|
| | | | | | | | | Sequence components of *kabuli* genome | *Kabuli* gene accession IDs | NCBI-KOG | TFs | NCBI-nr database |
| CaPOPI_4376 | Ca6 | 58966264 | AATATATATATATATATATAT/AATATATATATATATATATATAT | TCAGCCTAAGCAGATGCAAA | CAATTCCCACAGGTACGCTT | 59.7 | 414 | INTERGENIC | | | | |
| CaPOPI_4377 | Ca6 | 59059948 | CT/CTTT | ATAGGACGCAAGAAAAGCCA | TCCACAACAACCTACGTCCA | 59.8 | 263 | INTERGENIC | | | | |
| CaPOPI_4378 | Ca6 | 59093194 | ATTTTTTTTTTT/ATTTTTTTTTT | GGAGGCAACCCAACTCTATG | TCGCATAATTGGAACGTCAA | 59.6 | 271 | INTERGENIC | | | | |
| CaPOPI_4379 | Ca6 | 59184042 | TGAAGAAGAAGAAGAAGAAGAAGAA/TGAAGAAGAAGAAGAAGAAGAAGAAGAAGAAGAAGAA | CAAAACTGCCTAATCCCCAA | CGTTTGAGTGGGATGAGGTT | 59.9 | 157 | INTERGENIC | | | | |
| CaPOPI_4380 | Ca6 | 59193085 | CTTTTTTTTT/CTTTTTTTTTT | ATGGGGTTTACCCATTTGAA | ACATGATGGCAAGACCAAGA | 59.0 | 511 | INTERGENIC | | | | |
| CaPOPI_4381 | Ca6 | 59273276 | TA/TAAA | TGAAGCTTGTGCCAAAGGTA | GGTGGAGAATGAAACGGAGA | 59.5 | 754 | INTERGENIC | | | | |
| CaPOPI_4382 | Ca6 | 59273311 | TA/T | TTTTTGGGCTGAAGCCATAC | GGTGGAGAATGAAACGGAGA | 60.1 | 359 | INTERGENIC | | | | |
| CaPOPI_4383 | Ca6 | 59349983 | AATATATATATATATATATATATATATATA/AATATATATATATATATATATATATATATA | TTTCCATTGCACTCACTCCA | CCTATCATGGCCTGACCTGT | 60.2 | 777 | INTERGENIC | | | | |
| CaPOPI_4384 | Ca6 | 59422588 | TTATATATATATATATATATATAT/TTATATATATATATATATATATATATAT | CGGTCAAACTTCATTGTTGC | AGCGAGTCTGCTCCTAAACG | 59.2 | 702 | INTERGENIC | | | | |
| CaPOPI_4385 | Ca7 | 180428 | T/TGAGGCAA | TCAACAGCAGCTTTGCATTC | TTTCGCAGCTGGTATGTGAG | 60.1 | 280 | INTERGENIC | | | | |
| CaPOPI_4386 | Ca7 | 357521 | CTTT/CTTTT | TATTGGTTCCTTATGGGGCA | AGCTCTGGAGACCTCCTTCC | 60.1 | 581 | INTERGENIC | | | | |
| CaPOPI_4387 | Ca7 | 509621 | ATTTTTTTT/ATTTTTTTTT | AGCATCAGCTGCTGGTTTTT | GCACCAAACCAAGCCTATGT | 60.0 | 646 | INTERGENIC | | | | |
| CaPOPI_4388 | Ca7 | 540192 | C/CAT | TGAGGCAATTTTGGTGACAA | ATACCCTCATGCCACATGGT | 60.1 | 421 | INTERGENIC | | | | |
| CaPOPI_4389 | Ca7 | 586986 | AA/AATTA | TTCCATTTCCTCCACAAAGC | CAACCGCATTGTCATCATTC | 60.1 | 531 | DRR | Ca_24415 | | | |
| CaPOPI_4390 | Ca7 | 760518 | AACACACACA/AACACACA | TCTAGCGACGGGTCTGATCT | CAAATGCCAACAAGCGTTTA | 60.0 | 365 | DRR | Ca_03403 | R | | Short-chain dehydrogenase/reductase SDR |

| INDEL marker IDs | Chromosomes/ unanchored scaffolds | Physical positions (bp) | InDels (*Kabuli* reference genome-CDC Frontier/PI) | Forward primers (5'-3') | Reverse primers (5'-3') | Anneling temperature (0C) | Expected amplified product size (bp) | Structural annotation | | Functional annotation | | |
|---|---|---|---|---|---|---|---|---|---|---|---|---|
| | | | | | | | | Sequence components of *kabuli* genome | *Kabuli* gene accession IDs | NCBI-KOG | TFs | NCBI-nr database |
| CaPOPI_4391 | Ca7 | 760856 | GTTGAAGATTG/GTTGAAGATTGAAGATTG | TAAACGCTTGTTGGCATTTG | AGTTCGCAGGCTCTGTTGTT | 59.7 | 445 | DRR | Ca_03403 | R | | Short-chain dehydrogenase/reductase SDR |
| CaPOPI_4392 | Ca7 | 819442 | ATTAG/A | TTCACGAAATTTGTTCCCCT | TGTACATTTCCTAAAAACACATTGA | 59.4 | 784 | INTERGENIC | | | | |
| CaPOPI_4393 | Ca7 | 1018745 | C/CT | TTGCAAACATGATAGCTGCC | TTGTTGGCATTGTTGTGGTT | 59.8 | 467 | INTERGENIC | | | | |
| CaPOPI_4394 | Ca7 | 1018777 | ATTGCAT/ATTGCATATTTGCAT | TTGTTCCCGTAAAAGATCGC | TTGTTGGCATTGTTGTGGTT | 60.1 | 233 | INTERGENIC | | | | |
| CaPOPI_4395 | Ca7 | 1038892 | TTATATATATATATATATATATATATATATAT/TTATATATATATATATATATATATATATATATATAT | GTACGAGATGACGACGCCTT | ATGAATGCAGTTACGTGGCA | 60.3 | 717 | INTERGENIC | | | | |
| CaPOPI_4396 | Ca7 | 1176487 | TTATATATATATATAT/TTATATATATATAT | GGGATGGGTGGATTTCTTCT | TGGTCGGGACTTCAAGAAAC | 60.1 | 542 | DRR | Ca_03355 | R | bHLH | Short-chain dehydrogenase/reductase SDR |
| CaPOPI_4397 | Ca7 | 1219494 | TG/T | GGAGGGATGAATTTGGTTGA | CATCCACATGTATGCTTTCCA | 59.7 | 442 | INTERGENIC | | | | |
| CaPOPI_4398 | Ca7 | 1245279 | A/AATTTCAT | TGCACAAAACACACAACCCT | TCGCTCCTGTGTCAGTTTTG | 60.0 | 468 | INTERGENIC | | | | |
| CaPOPI_4399 | Ca7 | 1307924 | A/AGAT | CAAAAATAAGGCATAGTGTTGGA | TAGTTTGTAACCCCGTGCGT | 58.3 | 260 | INTERGENIC | | | | |
| CaPOPI_4400 | Ca7 | 1807047 | CAAAAAAAAA/CAAAAAAAAAA | TGTTGTCACACCAACTCTTGC | GCTTGGTCCCATTTGAGAGA | 59.8 | 846 | DRR | Ca_03288 | | | Zinc finger, RING-type |
| CaPOPI_4401 | Ca7 | 1919879 | TTATATATATATATATATAT/TTATATATATATATATATATATAT | TGAATTTTGGGGTCAACCAT | ACACAGCCCCAAAAGTTGTC | 60.0 | 435 | DRR | Ca_03281 | | | BNR repeat |
| CaPOPI_4402 | Ca7 | 2047396 | TTATATATATATATATATATATATAT/TTATATATATATATATATATATATATATAT | ATGGGACTAGGCAACACGTC | TTTCAACCGTAAATCATACTTGG | 60.0 | 630 | DRR | Ca_03268 | GC | HSF | UDP-glucuronosyl/UDP-glucosyltransferase |
| CaPOPI_4403 | Ca7 | 2168224 | CATA/CATATA | TTCTGAGATTGGCAAAAGGG | CGCGATCGTTCACTGATATG | 60.2 | 820 | INTRON | Ca_03253 | P | | Porin, eukaryotic type |
| CaPOPI_4404 | Ca7 | 2169830 | AAT/A | CAGGGCCTTACTGCTACAGG | CAACGGAAAACATCGTTCAA | 59.9 | 397 | CDS (FRAME SHIFT) | Ca_03253 | P | | Porin, eukaryotic type |
| CaPOPI_4405 | Ca7 | 2169903 | T/TA | CAGGGCCTTACTGCTACAGG | CAACGGAAAACATCGTTCAA | 59.9 | 397 | INTRON | Ca_03253 | P | | Porin, eukaryotic type |

| INDEL marker IDs | Chromosomes/ unanchored scaffolds | Physical positions (bp) | InDels (*Kabuli* reference genome-CDC Frontier/PI) | Forward primers (5'-3') | Reverse primers (5'-3') | Anneling temperature (0C) | Expected amplified product size (bp) | Structural annotation | | Functional annotation | | |
|---|---|---|---|---|---|---|---|---|---|---|---|---|
| | | | | | | | | Sequence components of *kabuli* genome | *Kabuli* gene accession IDs | NCBI-KOG | TFs | NCBI-nr database |
| CaPOPI_4406 | Ca7 | 2380600 | ATTTAATTTGTTTAA/ATTTAATTTGTTTAATTTGTTTAA | CCACCATTTTTGATGACGTG | TGGAAAAACCACAAATATTACATTG | 59.8 | 593 | INTERGENIC | | | | |
| CaPOPI_4407 | Ca7 | 2432614 | TTATATATATATATATATATAT/TTATATATATATATATATATAT | CAAATGGATTGGTGAGACGA | GGCATGAGTGAGTATGGCCT | 59.5 | 640 | INTERGENIC | | | | |
| CaPOPI_4408 | Ca7 | 2525335 | CAATACAATTGATTAATA/CAATACAATTGATTAATACAATTGATTAATA | TGGTGGAAAAATAGCATTTGG | CCTCTCCCGAATGACTCAAG | 59.8 | 868 | INTERGENIC | | | | |
| CaPOPI_4409 | Ca7 | 2639799 | CTCATCATCATCATCATCATC/CTCATCATCATCATCATCATCATC | TTCAAGACAAAGAGGGGGTG | CAATGCAGCAATGAGAGCAT | 60.1 | 382 | CDS (large-effect mutations) | Ca_03214 | O | | |
| CaPOPI_4410 | Ca7 | 2940930 | AAATAATAATAATAATAATAATAATAATAATAATAATAATAATA/AAATAATAATAATAATAATAATAATAATAATAATAATAATAATAATAATAATA | TCTTCAGACCAAAAATGGGC | TGCAAGTTTTCCTGTCCAAA | 60.1 | 581 | DRR | Ca_03181 | | FAR1 | |
| CaPOPI_4411 | Ca7 | 2974132 | CTATA/CTA | ATTACCTCCCCTCTCCTCCA | AAACAGTGAACGGAACCAGG | 59.9 | 465 | DRR | Ca_03176 | R | | WD40 repeat |
| CaPOPI_4412 | Ca7 | 3243289 | AGTTATTTTGATTTTGTTATTTTGATTTTGT/AGTTATTTTGATTTTGT | TGAGTTTGGATATCGTTTTTACAC | CATTTTTGTCTAGCCACGCA | 57.4 | 350 | INTERGENIC | | | | |
| CaPOPI_4413 | Ca7 | 3259173 | TCCC/TCCCC | TAGAAAGGAATTGGCGGTGA | TCATTTTCATTTAGAAGCATGAGC | 60.6 | 767 | INTERGENIC | | | | |
| CaPOPI_4414 | Ca7 | 3317609 | GTTT/GTTTT | CCACGGGTTCTGAAGTTGTT | TGGGAAGTTGGTCTGTGTGA | 60.0 | 599 | INTERGENIC | | | | |
| CaPOPI_4415 | Ca7 | 3523561 | CAAAAAA/CAAAAAAA | TTGGATGGGAAGACCTTCTG | TCATTGCAAGAAGATCACGC | 60.0 | 405 | INTRON | Ca_03116 | I | | Synaptojanin, N-terminal |
| CaPOPI_4416 | Ca7 | 3528666 | CTATAT/CTATATAT | GGGGAACACATCAAAACCAC | TTATTTTTCACTAATTTTGATCTCTCA | 60.1 | 778 | INTERGENIC | | | | |
| CaPOPI_4417 | Ca7 | 3540216 | GTTTTTT/GTTTTTTT | TCAACAATGAAGCAACACATCA | CATTGCGCAAGAGAATACGA | 60.2 | 634 | INTERGENIC | | | | |
| CaPOPI_4418 | Ca7 | 3552289 | TAAA/TAAAA | CCCTGCTTCAATTTATTCTCTCA | TGTTCGTTGGCACAGATGTT | 59.7 | 586 | INTERGENIC | | | | |
| CaPOPI_4419 | Ca7 | 3613493 | CTTT/CTTTT | GCAAGTGCTCCCATTTCATT | ACTTGGCAATGCAAGAGTCC | 60.1 | 297 | INTERGENIC | | | | |
| CaPOPI_4420 | Ca7 | 3617634 | TAAA/TAA | CCGCTACTTTTTCGAACACTTC | TCAAACCACCCCCAAAAATA | 60.3 | 626 | INTERGENIC | | | | |

| INDEL marker IDs | Chromosomes/ unanchored scaffolds | Physical positions (bp) | InDels (*Kabuli* reference genome-CDC Frontier/PI) | Forward primers (5'-3') | Reverse primers (5'-3') | Anneling temperature (0C) | Expected amplified product size (bp) | Structural annotation | | Functional annotation | | |
|---|---|---|---|---|---|---|---|---|---|---|---|---|
| | | | | | | | | Sequence components of *kabuli* genome | *Kabuli* gene accession IDs | NCBI-KOG | TFs | NCBI-nr database |
| CaPOPI_4421 | Ca7 | 3629960 | TTTATTA/TTTA | ATTGCCATGTGGGTCAGAGT | TGGCAACATAATGTCCATGC | 60.4 | 390 | INTRON | Ca_03106 | E | | Saccharopine dehydrogenase / Homospermidine synthase |
| CaPOPI_4422 | Ca7 | 3670558 | A/AT | AAGGCAATAGGTGATGGTGG | TCACTATTCACACGCGGAAG | 59.8 | 717 | DRR | Ca_03102 | TU | | Zinc finger, FYVE-type |
| CaPOPI_4423 | Ca7 | 3672046 | AT/A | ACATAGGTCGGCATACTCGG | TACAGTTTCAGCCTGGGTCC | 60.0 | 330 | DRR | Ca_03102 | TU | | Zinc finger, FYVE-type |
| CaPOPI_4424 | Ca7 | 3676987 | ACTC/ACTCTC | TTGAGTGGCATTTCAGCAAG | TCCTTCTCCAAAATCGAAGG | 60.0 | 492 | INTERGENIC | | | | |
| CaPOPI_4425 | Ca7 | 3683696 | TA/T | CCATGAAATCACCTTAAATGACA | CCGACAAGGCGACAAGTAAT | 58.9 | 451 | DRR | Ca_03101 | | | Tify |
| CaPOPI_4426 | Ca7 | 3689798 | TAGAGAG/TAGAG | ATGGTAGGGGGAAGAGGAGT | CTTTTCGTTTGGCCAATGTT | 58.9 | 381 | DRR | Ca_03100 | S | | Uncharacterised protein family UPF0136, Transmembrane |
| CaPOPI_4427 | Ca7 | 3691358 | TAAA/TAA | GCACAAAAAGGAATGCATCA | AAGAAGGATCGGACGGAGAT | 59.7 | 963 | INTRON | Ca_03100 | S | | Uncharacterised protein family UPF0136, Transmembrane |
| CaPOPI_4428 | Ca7 | 3905558 | TG/T | ACTCTGTGTTTGAGGTGGGG | TGAGGGATGAAGAGGACAAAA | 60.0 | 468 | INTERGENIC | | | | |
| CaPOPI_4429 | Ca7 | 3934957 | AATATATATATATATATATATATAT/AATATATATATATATATATATATATAT | TTCCATATTTGGGTGCCATT | TGTGCATCTTTTTCCAATGC | 60.0 | 547 | INTERGENIC | | | | |
| CaPOPI_4430 | Ca7 | 4001096 | CATATATATATATATATATA/CATATATATATATATATATATA | AAAATTAAGGGTTGCCCCC | TTCAAAACAGTACGTGCGGA | 60.0 | 922 | INTERGENIC | | | | |
| CaPOPI_4431 | Ca7 | 4233766 | TTCTCTCTCTCTCTCT/TTCTCTCTCTCTCTCTCT | TATGTTCTGCCACAAGCAGC | CGCCACTCAAAAACATGCTA | 60.0 | 740 | INTERGENIC | | | | |
| CaPOPI_4432 | Ca7 | 4329744 | AATATATATATATATATATA/AATATATATATATATATATATA | TTCATGAAGTGGATGTGGTGA | AAACAAAAAGTTGCTTTTACGAA | 60.0 | 561 | INTERGENIC | | | | |
| CaPOPI_4433 | Ca7 | 4351119 | TAAAAAAAAAAAA/TAAAAAAAAAAAAA | AGCGCTCAACCATAAATTCG | TGTGGAGGTCGGTGTTTGTA | 60.2 | 223 | DRR | Ca_03023 | T | HB-other | Calcium-binding EF-hand |
| CaPOPI_4434 | Ca7 | 4369825 | ATTTTTTTT/ATTTTTTT | GATCGCGGTTGACGAGTATT | TGGTCCTCTAAATTTTGCCA | 60.1 | 912 | INTERGENIC | | | | |
| CaPOPI_4435 | Ca7 | 4385687 | GAAAAAA/GAAAAAAA | TTTAATGGGGCCTAAGGGAG | GTGAGAGAGTGCCCCCAATA | 60.3 | 776 | INTERGENIC | | | | |

| INDEL marker IDs | Chromosomes/ unanchored scaffolds | Physical positions (bp) | InDels (*Kabuli* reference genome-CDC Frontier/PI) | Forward primers (5'-3') | Reverse primers (5'-3') | Anneling temperature (0C) | Expected amplified product size (bp) | Structural annotation | | Functional annotation | | |
|---|---|---|---|---|---|---|---|---|---|---|---|---|
| | | | | | | | | Sequence components of *kabuli* genome | *Kabuli* gene accession IDs | NCBI-KOG | TFs | NCBI-nr database |
| CaPOPI_4436 | Ca7 | 4418125 | A/ATC | CAAGTCAGCACCGACAAAGA | ATAAAAGGGTGACGTGGCAG | 60.0 | 592 | INTERGENIC | | | | |
| CaPOPI_4437 | Ca7 | 4674504 | AATATATATATATATATATATATATAT/AATATATATATATATATATATAT | GCCCAATTTTACCCACACAT | GCCACCTATGTGCTATTTGC | 59.6 | 679 | INTERGENIC | | | | |
| CaPOPI_4438 | Ca7 | 4805132 | ATTTTTT/ATT | AAATTTGTCGCATCTCAATCG | TGCACCTCCACAATTGTACC | 60.1 | 371 | INTERGENIC | | | | |
| CaPOPI_4439 | Ca7 | 4867122 | AATATATATATATATATATATATATATATATTTATATATATATAT/AATATATATATATATATATATATATATATATATTTATATATATATAT | AGGTGGTGAGCGTTTTTGAA | TTGATGATGTGAAGGAATGACAG | 60.7 | 670 | INTERGENIC | | | | |
| CaPOPI_4440 | Ca7 | 4999972 | GAAAAAAAAAAA/GAAAAAAAAAA | CCTCAGATGAGAGCCAGGAC | TTGGTTGTAACACCCCGTTT | 59.9 | 453 | INTERGENIC | | | | |
| CaPOPI_4441 | Ca7 | 5014340 | CAA/CAAA | AATACCACCACCGACCACAT | AATGAGCTCCAACGGTCATC | 60.0 | 787 | INTERGENIC | | | | |
| CaPOPI_4442 | Ca7 | 5024464 | ATTTTTT/ATTTTTTT | CCCCGCATGTATTATTGGAG | ACCCACATCCGTTTCATAGC | 60.2 | 294 | INTERGENIC | | | | |
| CaPOPI_4443 | Ca7 | 5033957 | AATATATATATATATATATAT/AATATATATATATATATATATATATATATATAT | TGGATCGTGTCTTGATCATTG | AATAAACCGTTGCAAGTGGG | 59.5 | 592 | INTERGENIC | | | | |
| CaPOPI_4444 | Ca7 | 5034485 | ATTTTTTTTT/ATTTTTTTTTT | CCCACTTGCAACGGTTTATT | TGAGGTAAGCTTTGCATCATGT | 59.9 | 837 | INTERGENIC | | | | |
| CaPOPI_4445 | Ca7 | 5140984 | GTTTTTTTTTT/GTTTTTTTTTTT | CCCCTTCTATTCAGCTTCCC | TTGAGCCAATTGACCTACCC | 60.0 | 612 | INTERGENIC | | | | |
| CaPOPI_4446 | Ca7 | 5342023 | CTTTTTTTT/CTTTTTTT | GGCATAACCGCGAAACTTTA | CATGAAATCAAACAACACCACA | 60.1 | 506 | INTERGENIC | | | | |
| CaPOPI_4447 | Ca7 | 5343899 | CTTTTTTTTTT/CTTTTTTTTTTT | TGAGGTGATGTGTCCATTGAG | TGCAGAGAACAAGCACATTG | 59.5 | 477 | INTERGENIC | | | | |
| CaPOPI_4448 | Ca7 | 5398943 | TAATAA/TAATAACAATAA | CTTTTCCTTGTGCTGCATGA | TCGCTTCACCTCCATCTTCT | 60.0 | 348 | INTRON | Ca_06830 | R | | Kelch repeat type 1 |
| CaPOPI_4449 | Ca7 | 5399126 | CAAAA/CAAAAA | CTTTTCCTTGTGCTGCATGA | AGGTGAGTGGGCTATGTTGG | 60.0 | 819 | INTRON | Ca_06830 | R | | Kelch repeat type 1 |
| CaPOPI_4450 | Ca7 | 5405233 | AC/A | TGGCCTCTCTGAAACCATCT | TCCCTTTCCAGAATTGGATG | 59.8 | 905 | INTERGENIC | | | | |

| INDEL marker IDs | Chromosomes/ unanchored scaffolds | Physical positions (bp) | InDels (*Kabuli* reference genome-CDC Frontier/PI) | Forward primers (5'-3') | Reverse primers (5'-3') | Anneling temperature (0C) | Expected amplified product size (bp) | Structural annotation | | Functional annotation | | |
|---|---|---|---|---|---|---|---|---|---|---|---|---|
| | | | | | | | | Sequence components of *kabuli* genome | *Kabuli* gene accession IDs | NCBI-KOG | TFs | NCBI-nr database |
| CaPOPI_4451 | Ca7 | 5434245 | ATTTTTTT/ATTTTTTTT | CCAATTATGCCATTGGAATTT | TCAAATTCAAAATCACAACGCT | 58.7 | 685 | INTERGENIC | | | | |
| CaPOPI_4452 | Ca7 | 5434750 | AG/A | GCGTTGTGATTTTGAATTTGA | CATGGTCCACTTCGAGGAAT | 58.7 | 524 | DRR | Ca_06825 | | | |
| CaPOPI_4453 | Ca7 | 5434792 | CTTTT/CTTT | GAAAGAGGATCAACCCGACA | CATGGTCCACTTCGAGGAAT | 60.1 | 466 | DRR | Ca_06825 | | | |
| CaPOPI_4454 | Ca7 | 5436041 | GTTTTTTTTTT/GTTTTTTTTTTT | TGTTTAGATGTGGTGGTGCTG | CAAGCAGGTATGTGAGGGGT | 59.6 | 721 | DRR | Ca_06825 | | | |
| CaPOPI_4455 | Ca7 | 5443791 | TATAAATAAATAAATAA/TATAAATAAATAA | TATTTGCGAGCGTGTCTTCA | TTTTGATGCTTTTTCCACCA | 60.5 | 516 | INTERGENIC | | | | |
| CaPOPI_4456 | Ca7 | 5443871 | TAAAA/TAAAAA | TATTTGCGAGCGTGTCTTCA | TTTTGATGCTTTTTCCACCA | 60.5 | 516 | INTERGENIC | | | | |
| CaPOPI_4457 | Ca7 | 5446544 | ATATTTATTTATTTATTTATTTATTTATTTATT/ATATTTATTTATTTATTTATTTATT | CCCCTTTCGGAATTTCATTT | CACAAAGCCTTCTTTCCTGC | 60.1 | 527 | INTRON | Ca_06824 | | | |
| CaPOPI_4458 | Ca7 | 5459177 | ATTTTTTTTTT/ATTTTTTTTTTT | GAAAAAGGTTGCAATCCGAA | TCAAGGTGTTTGGGTTTGTG | 60.1 | 817 | INTERGENIC | | | | |
| CaPOPI_4459 | Ca7 | 5465549 | TAACCGGAA/TAA | AGTCCTAATGCGGCTGTGAT | ATGTGGGAATTGTGGCATTT | 59.7 | 207 | DRR | Ca_06821 | | | Uncharacterised protein family UPF0497, trans-membrane plant |
| CaPOPI_4460 | Ca7 | 5470006 | TTATATATATATAT/TTATATATATAT | GGGATCTATTAAAAACCGAACCA | TGGAATTGTCCACCACAACA | 60.4 | 632 | INTERGENIC | | | | |
| CaPOPI_4461 | Ca7 | 5482693 | TATT/TATTTTATAATT | TTCGTGTAGTTGATTCAAACCAT | AACAAAAGGCAATAAATGAACTGA | 58.6 | 576 | INTERGENIC | | | | |
| CaPOPI_4462 | Ca7 | 5482708 | ATTTT/ATTT | TTCGTGTAGTTGATTCAAACCAT | AACAAAAGGCAATAAATGAACTGA | 58.6 | 576 | INTERGENIC | | | | |
| CaPOPI_4463 | Ca7 | 5524631 | ATACAATTTTTACAATTT/ATACAATTTTTACAATTTTTACAATTT | CTTGGTTCACACGGTTCCTT | CAAAACGCGAATTGCTATGA | 60.0 | 840 | DRR | Ca_06814 | | TCP | Heat shock protein DnaJ, N-terminal |
| CaPOPI_4464 | Ca7 | 5540728 | TC/T | TCAATGTTGGAGTTTGTGCAT | CGCATATCAAACATTGCAGC | 59.0 | 569 | DRR | Ca_06810 | R | G2-like | Sugar/inositol transporter |
| CaPOPI_4465 | Ca7 | 5540919 | T/TTATTA | TCAATGTTGGAGTTTGTGCAT | GCCAATTGCCTAGCAGTAGC | 59.0 | 645 | DRR | Ca_06810 | R | G2-like | Sugar/inositol transporter |

| INDEL marker IDs | Chromosomes/ unanchored scaffolds | Physical positions (bp) | InDels (*Kabuli* reference genome-CDC Frontier/PI) | Forward primers (5'-3') | Reverse primers (5'-3') | Anneling temperature (0C) | Expected amplified product size (bp) | Structural annotation | | Functional annotation | | |
|---|---|---|---|---|---|---|---|---|---|---|---|---|
| | | | | | | | | Sequence components of *kabuli* genome | *Kabuli* gene accession IDs | NCBI-KOG | TFs | NCBI-nr database |
| CaPOPI_4466 | Ca7 | 5572652 | ATTTTTTTT/ATTTTTTTTTT | AAAAATGGCGTTCATCAAATG | TTTGCCAATTTTTAGGGACC | 59.8 | 502 | INTERGENIC | | | | |
| CaPOPI_4467 | Ca7 | 5572728 | GTTTAATTTTAATTTTAATTTTA/GTTTAATTTTAATTTTA | ATGGGTCCCTAAAAATTGGC | GCTTGTTACTTATATTCAGAGGGCA | 60.0 | 559 | INTERGENIC | | | | |
| CaPOPI_4468 | Ca7 | 5575534 | ATTTTTTTT/ATTTTTTT | GGTGAAGGAGAAATGCAAGAA | TTATTAACGTGGGTGTGGGC | 59.3 | 556 | INTERGENIC | | | | |
| CaPOPI_4469 | Ca7 | 5578208 | AG/AGAATGAATG | ATGGGAAATTGTGAATGGGA | TGCCCGTTAGTCAATGTTTG | 60.0 | 435 | DRR | Ca_06805 | | | |
| CaPOPI_4470 | Ca7 | 5582721 | AATATATATATATATATATATATATATAT/AATATATATATATATATATATATATATATATATATAT | TGCCCACTCATTTGGCTATT | AAGACGGTCTGGTAACGACG | 60.5 | 494 | INTERGENIC | | | | |
| CaPOPI_4471 | Ca7 | 5585752 | TAAAAAAAAAA/TAAAAAAAAAAA | ATTCACACGACCAAAAACCT | TGATGAGATGTATTCGATGCAA | 57.0 | 496 | DRR | Ca_06804 | | | Protein of unknown function DUF679 |
| CaPOPI_4472 | Ca7 | 5633058 | T/TAAG | GCAAGCCAAACCACAAAAAT | ACAGGCGCTTCCTTCTTGTA | 60.0 | 386 | DRR | Ca_06796 | GC | HSF | UDP-glucuronosyl/UDP-glucosyltransferase |
| CaPOPI_4473 | Ca7 | 5648526 | TAAAAAAAA/TAAAAAAA | TGTGCACCAAACAAACATCA | TGGGGGATGACATGTACCTT | 59.6 | 448 | INTERGENIC | | | | |
| CaPOPI_4474 | Ca7 | 5649774 | CTTTTTTTTT/CTTTTTTTT | AAGGGAATGCATTGTCTCTCA | TTTGTTATGTGTGGCTTGTAGTGA | 59.7 | 359 | INTERGENIC | | | | |
| CaPOPI_4475 | Ca7 | 5650363 | AAATAATAATAATA/AAATAATAATAATAATA | TCGTTCCCTCTGAAATGAAA | TCGAGTGAACGTGGAAGATG | 58.3 | 239 | INTERGENIC | | | | |
| CaPOPI_4476 | Ca7 | 5668000 | TAAAAAAAAAAA/TAAAAAAAA | TCATACTCGTATTCGTGCCG | CAGGGTCCTTGTCGTGATTT | 59.7 | 764 | INTERGENIC | | | | |
| CaPOPI_4477 | Ca7 | 5684395 | TTAT/TTATAT | CGTTCAAGCTTGTCGTGAAA | GAGATGTTCAATGGCCTGGT | 60.0 | 716 | DRR | Ca_06789 | K | | RNA polymerase I specific transcription initiation factor RRN3 |
| CaPOPI_4478 | Ca7 | 5694761 | TAAAAAA/TAAAAAAA | GGAATTCTGTTTGCAAGGGA | AACCCATCACTTGCTGATCC | 60.1 | 627 | INTERGENIC | | | | |
| CaPOPI_4479 | Ca7 | 5698697 | ATT/ATTTT | AAGTCGATTACGATCCACGC | CCCCCTTAAAAGACAATTCAAA | 60.1 | 430 | INTERGENIC | | | | |
| CaPOPI_4480 | Ca7 | 5702622 | GTATATATA/GTATATA | TATGCCACGCCATAAGATGA | GGCCCAAACAAGAAGTTATCC | 60.1 | 726 | INTERGENIC | | | | |

| INDEL marker IDs | Chromosomes/ unanchored scaffolds | Physical positions (bp) | InDels (*Kabuli* reference genome-CDC Frontier/PI) | Forward primers (5'-3') | Reverse primers (5'-3') | Anneling temperature (0C) | Expected amplified product size (bp) | Structural annotation | | Functional annotation | | |
|---|---|---|---|---|---|---|---|---|---|---|---|---|
| | | | | | | | | Sequence components of *kabuli* genome | *Kabuli* gene accession IDs | NCBI-KOG | TFs | NCBI-nr database |
| CaPOPI_4481 | Ca7 | 5778254 | AAACAA/AAACAACAA | CAAATCCATCCACTCTTCACAA | CTGCAAAAACCCTCTTCTCG | 60.0 | 910 | INTERGENIC | | | | |
| CaPOPI_4482 | Ca7 | 5779009 | ATTTTTTTT/ATTTTTTTTT | GCTGAAGCCAGTTTCTGGAC | CACACCAAACCAAGCAACAC | 60.0 | 458 | INTRON | Ca_06780 | F | | Ribonucleotide reductase |
| CaPOPI_4483 | Ca7 | 5785096 | GTCATCATCA/GTCATCA | TTTGCAACTTGTGTCACCTCT | CGCTCTCTATCTCTCCGCAC | 58.4 | 789 | INTERGENIC | | | | |
| CaPOPI_4484 | Ca7 | 5785418 | GTCT/GT | TGCGGAGAGATAGAGAGCGT | GGGTGATTTGATTGTTCTTGC | 60.3 | 206 | INTERGENIC | | | | |
| CaPOPI_4485 | Ca7 | 5799102 | TTATAAATATAAA/TTATAAATATAAATATAAA | ATGACATGTCTCGAGCTCCC | TTGGCCCTCTTTTCTCTCAA | 60.2 | 312 | DRR | Ca_06778 | R | | RNA recognition motif domain |
| CaPOPI_4486 | Ca7 | 5800832 | TTTCTT/TTT | AAAAGCGGGCCATTCTAGTT | TTCTAGCATTGTGGCTGTGC | 60.1 | 375 | DRR | Ca_06778 | R | | RNA recognition motif domain |
| CaPOPI_4487 | Ca7 | 5841878 | CTT/CT | TGCAACTTTGAAGGTGCTTG | CAACAAAGCCACAAAAGCAA | 60.0 | 731 | DRR | Ca_06775 | | | |
| CaPOPI_4488 | Ca7 | 5843643 | AT/ATT | GGTGAGTTTTTCTCTCCCCC | TTCATCGTCTGAAGTCACGG | 59.9 | 501 | INTERGENIC | | | | |
| CaPOPI_4489 | Ca7 | 5858608 | TACTAA/TA | TGTTTAGTCATGCGGTGATTG | CCCGTCAAAGGGTATTTGAA | 59.6 | 846 | INTERGENIC | | | | |
| CaPOPI_4490 | Ca7 | 5870900 | GAAAAAA/GAAAAAAAA | TTGTGGCTAATATTTCGGTCAA | CACAACTCTCCAGAGCCAAA | 59.5 | 485 | INTERGENIC | | | | |
| CaPOPI_4491 | Ca7 | 5871833 | TAAAAAA/TAAAAA | AATTGTGGTTCTTCATGCCC | TTCGTATTGTTATTGCGGGA | 59.8 | 487 | INTERGENIC | | | | |
| CaPOPI_4492 | Ca7 | 5874667 | CAAA/CAA | AAGCTGATGTGACAAGCCAA | TTATAAACATGGGCGAACCC | 59.4 | 391 | INTERGENIC | | | | |
| CaPOPI_4493 | Ca7 | 5884631 | ATTTTTT/ATTTTTTTT | TCTTGTTTGCCTGTCTCGTG | ATGCGAAGGAGCAATTATGG | 60.0 | 832 | INTERGENIC | | | | |
| CaPOPI_4494 | Ca7 | 5889222 | CTTT/CTT | ATTGGCAAGAGTGTTACGGG | GTGTGACTTTGGCCACCTTA | 60.0 | 520 | DRR | Ca_06769 | O | | Peptidase A1 |
| CaPOPI_4495 | Ca7 | 5890273 | TTATATATATAT/TTATATATATATAT | TGTTCATATACACATGCATCGC | TGAGAGTGAGATGGTGGTCA | 59.5 | 127 | DRR | Ca_06769 | O | | Peptidase A1 |

| INDEL marker IDs | Chromosomes/ unanchored scaffolds | Physical positions (bp) | InDels (*Kabuli* reference genome-CDC Frontier/PI) | Forward primers (5'-3') | Reverse primers (5'-3') | Anneling temperature (0C) | Expected amplified product size (bp) | Structural annotation | | Functional annotation | | |
|---|---|---|---|---|---|---|---|---|---|---|---|---|
| | | | | | | | | Sequence components of *kabuli* genome | *Kabuli* gene accession IDs | NCBI-KOG | TFs | NCBI-nr database |
| CaPOPI_4496 | Ca7 | 5893840 | CAAAAAAAA/CAAAAAAAAA | AAATGAAAGCAGGCCTCAAA | CGGTGTTGTCCGTTTTTCTT | 59.8 | 411 | INTERGENIC | | | | |
| CaPOPI_4497 | Ca7 | 5894005 | GTTTTTTTTTT/GTTTTTTTTTTT | AAGAAAAACGGACAACACCG | TGTTGTGCATGACTTGAAGG | 60.0 | 375 | INTERGENIC | | | | |
| CaPOPI_4498 | Ca7 | 5894787 | TTTTAAAA/TTTTAAAAATTTAAAA | CGGTTCAATCAGGTTGACATT | TGGCCCCAAAATTGATACAT | 59.8 | 421 | INTERGENIC | | | | |
| CaPOPI_4499 | Ca7 | 5895013 | CAAAAAAAAA/CAAAAA | CGGTTCAATCAGGTTGACATT | TGGCCCCAAAATTGATACAT | 59.8 | 421 | INTERGENIC | | | | |
| CaPOPI_4500 | Ca7 | 5916457 | TATCATCATCATCATCATCATCA/TATCATCATCATCATCATCATCA | CATGCAATCATGTATCCCCA | CTACCTGCACCCCCTATTGA | 60.2 | 475 | CDS (large-effect mutations) | Ca_06766 | G | G2-like | Glycoside hydrolase, family 18, catalytic domain |
| CaPOPI_4501 | Ca7 | 5934199 | GTATATATATATA/GTATATATA | TTGAAAACAATTTTGCAGGC | CACGAGCGCTTTAAAAATCC | 58.8 | 903 | DRR | Ca_06764 | P | GRAS | ATPase, P-type, H+ transporting proton pump |
| CaPOPI_4502 | Ca7 | 5955426 | CA/C | TTCCCAATCACTTTGGCTTC | ATTGTGGCACTAGTGGAGGG | 60.1 | 947 | INTRON | Ca_06761 | A | AP2 | Ribonuclease III |
| CaPOPI_4503 | Ca7 | 5975269 | TTATATATATAT/TTATATATAT | AGAGCAGAATGGAACACGCT | TGCAATTTTAATTTTCATATTTTCG | 60.0 | 472 | INTERGENIC | | | | |
| CaPOPI_4504 | Ca7 | 5977012 | GTATATAT/GTATAT | TGAAACCACATTGCTCACCT | CCTCCACTTTTGAGCACCAT | 59.1 | 481 | INTERGENIC | | | | |
| CaPOPI_4505 | Ca7 | 5978931 | TTAGATACTAG/TTAG | TGCCAAAATGTTTGGTTGTG | CTGGGGACTCGATTGAGAAA | 60.4 | 843 | DRR | Ca_06760 | | | Lipid-binding START |
| CaPOPI_4506 | Ca7 | 5989176 | GATACA/GA | TGCTCTAAACTGAAGGGGGA | GTTTCGTGGACAAGACGGAT | 59.8 | 311 | INTRON | Ca_06760 | | | Lipid-binding START |
| CaPOPI_4507 | Ca7 | 5992633 | GC/G | AACGCAATCCATGACAAACA | GTATGCTCTGTTTCCCCCAA | 60.0 | 497 | INTRON | Ca_06760 | | | Lipid-binding START |
| CaPOPI_4508 | Ca7 | 5995821 | ATGT/AT | GACCGTCAAGGGGTTGTTTA | AGTTTATTGGCGCACCAGAG | 59.8 | 916 | INTERGENIC | | | | |
| CaPOPI_4509 | Ca7 | 6008667 | ATTTT/ATTT | CCATCCCGATTTGTTATTGG | CCCTTCGGTCATCAACGTAT | 60.0 | 486 | DRR | Ca_06759 | | | |
| CaPOPI_4510 | Ca7 | 6016711 | ATTTTTTTTT/ATTTTTTTT | GCCTTTTCTTTTTCAAGCCC | CATTTTGACAAGTGCATAGTTTGA | 60.2 | 306 | INTERGENIC | | | | |

| INDEL marker IDs | Chromosomes/ unanchored scaffolds | Physical positions (bp) | InDels (*Kabuli* reference genome-CDC Frontier/PI) | Forward primers (5'-3') | Reverse primers (5'-3') | Anneling temperature (0C) | Expected amplified product size (bp) | Structural annotation | | Functional annotation | | |
|---|---|---|---|---|---|---|---|---|---|---|---|---|
| | | | | | | | | Sequence components of *kabuli* genome | *Kabuli* gene accession IDs | NCBI-KOG | TFs | NCBI-nr database |
| CaPOPI_4511 | Ca7 | 6018232 | CTAA/C | GAGCCAACTTGTTGTCTACGG | CGAGGTGACACACATTTTCG | 59.8 | 271 | INTERGENIC | | | | |
| CaPOPI_4512 | Ca7 | 6020433 | TAAAAAAA/TAAAAAAAA | AATCCGTTGAGATTGGGATG | TTTTTGTTCCATTGGTGTTTATTTT | 59.8 | 677 | DRR | Ca_06757 | R | | Sugar/inositol transporter |
| CaPOPI_4513 | Ca7 | 6020761 | TCA/TA | AAACAAAGGAAAGGACAAAAATG | TGACGTTTAGTTTTTCTGTTTTATTG | 58.6 | 826 | DRR | Ca_06757 | R | | Sugar/inositol transporter |
| CaPOPI_4514 | Ca7 | 6021501 | CTTCTTT/CTT | CAATAAAACAGAAAAACTAAACGTCA | TTTTGTTCCAATTTCACGGA | 58.1 | 786 | DRR | Ca_06757 | R | | Sugar/inositol transporter |
| CaPOPI_4515 | Ca7 | 6021592 | GTTTTTTTTTT/GTTTTTTTTTTT | TCTTGCAAAAACACGTTTCAA | TTTTGTTCCAATTTCACGGA | 59.4 | 366 | DRR | Ca_06757 | R | | Sugar/inositol transporter |
| CaPOPI_4516 | Ca7 | 6021693 | ATTTTTTTTTT/ATTTTTTTTT | TCTTGCAAAAACACGTTTCAA | TTTTGTTCCAATTTCACGGA | 59.4 | 366 | DRR | Ca_06757 | R | | Sugar/inositol transporter |
| CaPOPI_4517 | Ca7 | 6024790 | GAAAAAAA/GAAAAAAAA | TCTCGATAGAAAACGTGGAAA | TTTCGACCCCTCTCTTTTAGG | 57.5 | 918 | INTRON | Ca_06757 | R | | Sugar/inositol transporter |
| CaPOPI_4518 | Ca7 | 6033948 | ATTTTTT/ATTTTTTT | ACTTCAGCATCAAGCCAGGT | AAAATTTTTGCAAGTTGGTTGTT | 59.9 | 291 | DRR | Ca_06755 | A | | D111/G-patch |
| CaPOPI_4519 | Ca7 | 6044320 | A/AATT | TCACCCACTTTGGTTGCATA | CAATTAACTGAAGTGGCCAAA | 60.0 | 339 | INTERGENIC | | | | |
| CaPOPI_4520 | Ca7 | 6055513 | TA/TTTAGTATGA | TTTGTGTCTTTCCAGGTCCC | AGAAGGTCGCATCCAGACAC | 59.9 | 507 | INTERGENIC | | | | |
| CaPOPI_4521 | Ca7 | 6056347 | GTATACACTATACA/GTATACA | CCATCGATTTAAAAATGAGCA | TATGGGGGCGGATAATTACA | 57.8 | 517 | INTERGENIC | | | | |
| CaPOPI_4522 | Ca7 | 6060869 | ACT/ACTCT | CCTTGGTCTGTCCCACTTGT | GAACACGGACATGTTGTTGC | 60.0 | 454 | INTRON | Ca_06754 | K | TALE | Homeobox |
| CaPOPI_4523 | Ca7 | 6062336 | GTTAT/GTTATATTAT | CAAAGGAAAAGGAATTGGCA | TGCTTTCACCTGCACTGTTC | 60.0 | 188 | DRR | Ca_06753 | | | |
| CaPOPI_4524 | Ca7 | 6081820 | CTTTTTTTTTTTT/CTTTTTTTT | GATTTCTCGGGTTTTGTGGA | AACGAGAGTCTAACGCCTTCC | 59.9 | 825 | INTERGENIC | | | | |
| CaPOPI_4525 | Ca7 | 6083464 | AATATA/AATATATA | GGACCGTTTTGCTGTCAAAT | CACATTCCTGGCTTCCAACT | 60.0 | 548 | INTERGENIC | | | | |

| INDEL marker IDs | Chromosomes/ unanchored scaffolds | Physical positions (bp) | InDels (*Kabuli* reference genome-CDC Frontier/PI) | Forward primers (5'-3') | Reverse primers (5'-3') | Anneling temperature (0C) | Expected amplified product size (bp) | Structural annotation | | Functional annotation | | |
|---|---|---|---|---|---|---|---|---|---|---|---|---|
| | | | | | | | | Sequence components of *kabuli* genome | *Kabuli* gene accession IDs | NCBI-KOG | TFs | NCBI-nr database |
| CaPOPI_4526 | Ca7 | 6097018 | AG/A | CTCAGCAGCACAAATCCAAA | GCACAACATCACCATATCGC | 60.0 | 472 | DRR | Ca_06748 | | | |
| CaPOPI_4527 | Ca7 | 6100952 | CTTTTTTTTT/CTTTTTTTT | TATTGCATGTTTGACGGCAT | CGGACTCCCCCTTTTTATTC | 60.0 | 558 | INTRON | Ca_06748 | | | |
| CaPOPI_4528 | Ca7 | 6104896 | AG/A | TTGAACTCCCCAAGGAAACA | TCGGTTGAATTTTGAATCATCA | 60.5 | 315 | INTERGENIC | | | | |
| CaPOPI_4529 | Ca7 | 6106310 | TTAT/TT | ACATCCTGGACCTGCTCATC | ATTCGAACGCGAATTTTCAG | 60.1 | 546 | DRR | Ca_06747 | | | |
| CaPOPI_4530 | Ca7 | 6107406 | AAAAATTTAAAATTT/AAAAATTTAAAATTTAAAATTT | TGTTCCACTTTCACCATTTTCA | TGGGTGTTTTATTTTTCAGATTACTG | 60.4 | 204 | DRR | Ca_06747 | | | |
| CaPOPI_4531 | Ca7 | 6107658 | TG/T | TGTTCCACTTTCACCATTTCA | GAGCAGTGTGGGGTTTTAGG | 60.4 | 703 | DRR | Ca_06747 | | | |
| CaPOPI_4532 | Ca7 | 6122129 | GTTTTTT/GTTTTTTT | TGGGTTCGAGCCTAATTTTG | CCGATTCATCCAAAGCCTTA | 60.1 | 435 | INTERGENIC | | | | |
| CaPOPI_4533 | Ca7 | 6125384 | TAAAATTC/TAAAATTCAAAATTC | TCAACTTCCACACTTGCTTAAA | TCAACTATCATCCCTTGGTCTTT | 57.6 | 902 | INTERGENIC | | | | |
| CaPOPI_4534 | Ca7 | 6126281 | TAAAAA/TAAAA | TTTCTGCCTAGAAAAGTTCCAT | TGTAGCCACGTAAGGAAACCT | 57.2 | 246 | DRR | Ca_06744 | O | | Ubiquitin |
| CaPOPI_4535 | Ca7 | 6162985 | TTT/TTTATT | CTACCCCACTTGCCATGATT | TCACAAACGCGGTTGATTTA | 59.8 | 624 | INTERGENIC | | | | |
| CaPOPI_4536 | Ca7 | 6195527 | GTATATATATATATATATAT/GTATATATATATATATATATAT | GATCTAACGGCTGAGCGAAC | CAGAAGCTCCAAGAACTGCC | 60.0 | 156 | DRR | Ca_06738 | | | |
| CaPOPI_4537 | Ca7 | 6314366 | GTTTTTTTTT/GTTTTTTTTTT | TGACGTCAAAGATCCCAAAA | TGCTCCCTTAATTTGCATGA | 59.1 | 567 | INTERGENIC | | | | |
| CaPOPI_4538 | Ca7 | 6347731 | ATAATTA/ATAATTAATTA | TGTTAATCACCCCGGAAAAA | GCTGCATTTCAGAAGCATCA | 60.2 | 521 | DRR | Ca_06721 | R | NAC | Pentatricopeptide repeat |
| CaPOPI_4539 | Ca7 | 6481476 | ATTTT/ATTT | TCCTCGCTGTTGACAATCAC | TTGTTAAGTGGTGGGAAGGC | 59.8 | 454 | INTERGENIC | | | | |
| CaPOPI_4540 | Ca7 | 6686273 | TAAAAAAAA/TAAAAAAA | TGCCATAAATGTCAAGATGTGA | CCAATTTTGTGAAGGAGGGA | 59.0 | 887 | INTERGENIC | | | | |

| INDEL marker IDs | Chromosomes/ unanchored scaffolds | Physical positions (bp) | InDels (*Kabuli* reference genome-CDC Frontier/PI) | Forward primers (5'-3') | Reverse primers (5'-3') | Anneling temperature (0C) | Expected amplified product size (bp) | Structural annotation | | Functional annotation | | |
|---|---|---|---|---|---|---|---|---|---|---|---|---|
| | | | | | | | | Sequence components of *kabuli* genome | *Kabuli* gene accession IDs | NCBI-KOG | TFs | NCBI-nr database |
| CaPOPI_4541 | Ca7 | 6842219 | GA/GAAA | CCAATTCGGGGGATAAAGTT | TGGGATGACAAAACGAGTCA | 60.0 | 124 | INTERGENIC | | | | |
| CaPOPI_4542 | Ca7 | 6854971 | ATTTTTTTTTT/ATTTTTTTTTTT | TATGAGGAGTGTGCTGCTGG | ACTCGCGGACTTAACTTCCA | 60.0 | 348 | INTRON | Ca_06677 | T | YABBY | Protein kinase, catalytic domain |
| CaPOPI_4543 | Ca7 | 6912264 | TTATATATATATATATATATATATAT/TTATATATATATATATATATATAT | TGCACTTGCACATGTTGAATAA | AATCCCTGCAATTGTGGAAG | 60.2 | 560 | INTERGENIC | | | | |
| CaPOPI_4544 | Ca7 | 7243214 | CTATATATATATATATATA/CTATATATATATATATATATA | CGTCCGTGAAAGGTTTCCTA | TGACCGACTATACAATCTTACATGC | 60.1 | 669 | INTERGENIC | | | | |
| CaPOPI_4545 | Ca7 | 7284443 | AATATATATATATATATATATATATATATATATA/AATATATATATATATATATATATATATATATATATATATATATA | TTGAATTCGGAGTTTTCCTTTT | CCTCACTTCATTGCTGCTCA | 59.1 | 992 | INTERGENIC | | | | |
| CaPOPI_4546 | Ca7 | 7332178 | TC/T | TCGACAGTTTTGGTCACTGTCT | TCTCATCTCCTCCAATCTCTCA | 59.8 | 592 | INTERGENIC | | | | |
| CaPOPI_4547 | Ca7 | 7332238 | CATATAATTATAT/CATATAATTATATAATTATAT | TCGACAGTTTTGGTCACTGTCT | TCTCATCTCCTCCAATCTCTCA | 59.8 | 592 | INTERGENIC | | | | |
| CaPOPI_4548 | Ca7 | 7335750 | TAAAAAAAAAAAA/TAAAAAAAAA | GTGTTTGGGATTGCGTTCTT | CTCCCGCAAATTGAAAATGT | 60.0 | 583 | INTERGENIC | | | | |
| CaPOPI_4549 | Ca7 | 7380960 | AAT/AATTAT | GGTGAACCCATTGCAATTTT | TCAGGCCAAGAAAGGAGAAA | 59.7 | 766 | INTERGENIC | | | | |
| CaPOPI_4550 | Ca7 | 7385094 | GGT/GGTGT | ATTCATTTCAAGGTTTGCGG | CAAATGAGCAGCTCGGTGTA | 59.9 | 750 | INTRON | Ca_06620 | S | | Protein of unknown function DUF92, TMEM19 |
| CaPOPI_4551 | Ca7 | 7404264 | TAAAAAAA/TAAAAAAAAAA | GGTGGGCTCAGAAAACAGAG | TCCCAGGTATTGCATTGTGA | 59.8 | 603 | DRR | Ca_06618 | | | Acyl-ACP thioesterase |
| CaPOPI_4552 | Ca7 | 7411561 | TAAA/TAA | AGTTACCCGATGTGCATGGT | TGCAAACTTTGGTGTGGGTA | 60.3 | 631 | INTERGENIC | | | | |
| CaPOPI_4553 | Ca7 | 7411632 | TAAGTCA/TA | AGTTACCCGATGTGCATGGT | TGCAAACTTTGGTGTGGGTA | 60.3 | 631 | INTERGENIC | | | | |
| CaPOPI_4554 | Ca7 | 7411899 | TAAAAAAAA/TAAAAAA | AGTTACCCGATGTGCATGGT | TGCAAACTTTGGTGTGGGTA | 60.3 | 631 | INTERGENIC | | | | |
| CaPOPI_4555 | Ca7 | 7440742 | GGAG/GGAGAG | ATCATCCACGACCCAATCAT | CACAAACAGAATGTGCACCC | 60.0 | 562 | INTRON | Ca_06616 | | bHLH | Protein of unknown function DUF936, plant |

| INDEL marker IDs | Chromosomes/ unanchored scaffolds | Physical positions (bp) | InDels (*Kabuli* reference genome-CDC Frontier/PI) | Forward primers (5'-3') | Reverse primers (5'-3') | Anneling temperature (0C) | Expected amplified product size (bp) | Structural annotation | | Functional annotation | | |
|---|---|---|---|---|---|---|---|---|---|---|---|---|
| | | | | | | | | Sequence components of *kabuli* genome | *Kabuli* gene accession IDs | NCBI-KOG | TFs | NCBI-nr database |
| CaPOPI_4556 | Ca7 | 7458735 | A/AT | TTTTGCAATTTGACCGTC TTT | GACCCACATAGTGAGGG GTG | 59.6 | 693 | INTERGENIC | | | | |
| CaPOPI_4557 | Ca7 | 7459713 | TACA/T | TTTTTGTCTTAACTGGG CCG | TGGAGGTCTCCTCGTTC ACT | 60.1 | 541 | INTERGENIC | | | | |
| CaPOPI_4558 | Ca7 | 7462087 | TTTACTTACTT/TTTACTTACTTACTT | TTGAAGCTGCTGCTGAA AGA | CAGATGTCCAACACCGA CAC | 60.0 | 323 | INTRON | Ca_06613 | B | NAC | Histone deacetylase superfamily |
| CaPOPI_4559 | Ca7 | 7491994 | AAAGAA/AAAGAAGAA | GCAAAGCTTGCTTTTTG TCC | ATAGAGGGCGCATGTCA CTT | 60.0 | 411 | INTRON | Ca_06610 | L | GeBP | DNA mismatch repair protein MutS, C-terminal domain |
| CaPOPI_4560 | Ca7 | 7510196 | TC/TCC | TTGGAAGGGAAAAAGCA AGA | TGCATTTTATTCTGCACC TGA | 59.8 | 555 | DRR | Ca_06609 | | TCP | Transcription factor, TCP |
| CaPOPI_4561 | Ca7 | 7543253 | AG/A | ATTAATATGCCAACTGG CCG | ACCACTACCCAATTCTT GCG | 59.8 | 596 | INTRON | Ca_06607 | K | C2H2 | Transcription factor TFIIE, alpha subunit |
| CaPOPI_4562 | Ca7 | 7547228 | CTGAACATGAACAT/CTGAACAT | TGACGATTGTTGTGAAA GGG | TTCATCAACCTTCCTCCC AG | 59.5 | 746 | INTERGENIC | | | | |
| CaPOPI_4563 | Ca7 | 7550729 | ATTTTTT/ATTTTT | CCGTGAATTTGGGTTCA ACT | CTCGCGTAATCCCACGT ATT | 59.8 | 602 | INTERGENIC | | | | |
| CaPOPI_4564 | Ca7 | 7597807 | A/AT | TCCTTTATGATCAGCCC ACC | CGTTCAACGGAGCAATT TTT | 59.9 | 356 | DRR | Ca_06600 | A | | RNA helicase, ATP-dependent, DEAD-box, conserved site |
| CaPOPI_4565 | Ca7 | 7626364 | CTTTTT/CTTTTTT | AGTCAGCTGCAAACCCA AGT | GGGAAGGTCATGCCAAC TTA | 59.9 | 819 | DRR | Ca_06598 | Z | MYB_related | Kinesin, motor domain |
| CaPOPI_4566 | Ca7 | 7668650 | ATTT/ATTTT | CATTTTCCATCCAATTTT TGAG | CCACTTTCTCATTTTTGT TTGG | 58.5 | 501 | DRR | Ca_06594 | R | | ATPase, P-type, K/Mg/Cd/Cu/Zn/Na/Ca/Na/H-transporter |
| CaPOPI_4567 | Ca7 | 7671749 | AA/AATA | TGCACCATTTATGTGAG GCT | TTCCAAATTCACATGCAT CC | 59.2 | 571 | DRR | Ca_06593 | J | MYB_related | Translation elongation factor IF5A |
| CaPOPI_4568 | Ca7 | 7683130 | TAA/TAAA | TTCGATAAACCGCTAAT GCAC | GTACTTGGCTGCTTTGG AGC | 60.1 | 847 | INTERGENIC | | | | |
| CaPOPI_4569 | Ca7 | 7688075 | TGGG/TGG | TTGTCATGGATGATGGT GCT | CCTCCATGGGTGAAAGT TTG | 59.9 | 664 | INTERGENIC | | | | |
| CaPOPI_4570 | Ca7 | 7689690 | GTTTTTTTTTT/GTTTTTTTT | TCAGAAACATCATCAAAC GCA | TGGTTTCAAAACAAAAAT GCAG | 60.3 | 952 | INTERGENIC | | | | |

| INDEL marker IDs | Chromosomes/ unanchored scaffolds | Physical positions (bp) | InDels (*Kabuli* reference genome-CDC Frontier/PI) | Forward primers (5'-3') | Reverse primers (5'-3') | Anneling temperature (0C) | Expected amplified product size (bp) | Structural annotation | | Functional annotation | | |
|---|---|---|---|---|---|---|---|---|---|---|---|---|
| | | | | | | | | Sequence components of *kabuli* genome | *Kabuli* gene accession IDs | NCBI-KOG | TFs | NCBI-nr database |
| CaPOPI_4571 | Ca7 | 7694767 | AATAGATAGATA/AATAGATA | TCTCATTGATGACCCAAAGG | CACAATCACAAAGTGGGACG | 58.5 | 722 | INTERGENIC | | | | |
| CaPOPI_4572 | Ca7 | 7701750 | CA/CAA | CCGATTCTATTTGTTGAAGGC | TAACCCCTCCAACGCTACAC | 58.7 | 492 | INTERGENIC | | | | |
| CaPOPI_4573 | Ca7 | 7703445 | AA/AATATGGATTAGTAGACA | TCCAAAAGGTTCTTGCATCC | AACATGGAGAATGAAACCGC | 60.1 | 641 | INTRON | Ca_06588 | | | Protein of unknown function DUF3353 |
| CaPOPI_4574 | Ca7 | 7724983 | GTTT/GTT | TCAACAAAGCTTAATTGGCG | ACACTGCCGAAAGTTTTGCT | 59.0 | 724 | DRR | Ca_06585 | J | | Phenylalanyl-tRNA synthetase |
| CaPOPI_4575 | Ca7 | 7738038 | TC/T | GATTCCATTGGGATTGGTTG | CACGTTTTAGAGATGTCCATTTTG | 60.0 | 505 | INTERGENIC | | | | |
| CaPOPI_4576 | Ca7 | 7738595 | GTATATATATATATATATATATATA/GTATATATATATATATATATATA | CAAAATGGACATCTCTAAAACGTG | TCTCTCACCCAATCACCAAA | 59.9 | 583 | INTERGENIC | | | | |
| CaPOPI_4577 | Ca7 | 7753007 | GTTTTTT/GTTTTTTTT | GGAGAATCGCCAACCAGATA | GGGGAAAACAATGCAGGTAA | 60.0 | 542 | CDS (FRAME SHIFT) | Ca_13243 | KL | G2-like | SNF2-related |
| CaPOPI_4578 | Ca7 | 7850911 | CAAAAAAAAAAAAAAA/CAAAAAAAAA | TCGATGATATTTTTGTTCGCTT | TGGAGCTTGCAGCTCATACA | 58.8 | 440 | INTERGENIC | | | | |
| CaPOPI_4579 | Ca7 | 7861934 | ATTT/ATTTT | TCGCTTGGTGTTTGTATAGGTG | TTCTAATGCTTCGGCAATCC | 60.1 | 452 | INTERGENIC | | | | |
| CaPOPI_4580 | Ca7 | 7875761 | ATT/ATTT | CTTTCCACCAAGGATAGGCA | GGCTTTCTGCCACTCATCTC | 60.1 | 820 | INTRON | Ca_13230 | | MIKC | GDP-fucose protein O-fucosyltransferase |
| CaPOPI_4581 | Ca7 | 7880271 | A/ACTGTTAG | TGGATATTCCACACAGCATTAAG | TTGGAACTTGTCTCAACCGA | 59.0 | 727 | INTERGENIC | | | | |
| CaPOPI_4582 | Ca7 | 7881505 | CTTTTT/CTTTT | ACCTTCTGGATATGCCATGC | GAGATGGGATCTCCAACATCA | 59.9 | 189 | INTERGENIC | | | | |
| CaPOPI_4583 | Ca7 | 7888043 | A/AC | TAACTGAGACCCAGGAGGGA | TTCAACACAGCCCATAACCA | 59.6 | 645 | DRR | Ca_13229 | | | BSD |
| CaPOPI_4584 | Ca7 | 7910843 | ATA/ATACTTA | AACACACGTTCACGCAACTC | AAGCATGTTTTACCTTTTCACACA | 59.8 | 477 | INTERGENIC | | | | |
| CaPOPI_4585 | Ca7 | 7944979 | TATGAAATGAAAT/TATGAAAT | ATTTAATGGCTTCCACACGC | CGTACTCAACAGAGTTGGATCG | 60.0 | 640 | DRR | Ca_13222 | K | MIKC | Transcription factor, MADS-box |

| INDEL marker IDs | Chromosomes/ unanchored scaffolds | Physical positions (bp) | InDels (*Kabuli* reference genome-CDC Frontier/PI) | Forward primers (5'-3') | Reverse primers (5'-3') | Anneling temperature (0C) | Expected amplified product size (bp) | Structural annotation | | Functional annotation | | |
|---|---|---|---|---|---|---|---|---|---|---|---|---|
| | | | | | | | | Sequence components of *kabuli* genome | *Kabuli* gene accession IDs | NCBI-KOG | TFs | NCBI-nr database |
| CaPOPI_4586 | Ca7 | 7950061 | CATTGCAATTGCAATTGCA/CATTGCAATTGCAATTGCAATTGCA | GCCACAGCCTTTTTCTCTTG | TGCTGAAGTTGCTCTCATCG | 60.0 | 442 | INTRON | Ca_13222 | K | MIKC | Transcription factor, MADS-box |
| CaPOPI_4587 | Ca7 | 7952171 | AGGGG/AGGG | TATCCAAATTGCGTCGGATT | ACTCGGATGTCTCAATGCAA | 60.3 | 307 | INTERGENIC | | | | |
| CaPOPI_4588 | Ca7 | 7954760 | AAATAATAATAATAATAATAATAAT/AAATAATAATAATAATAAT | TGCAATTTTGGATCTCCCAT | TGGAAAAATCAAGGTGTCCAT | 60.3 | 422 | INTERGENIC | | | | |
| CaPOPI_4589 | Ca7 | 7958075 | CAATGGTAAT/CAATGGTAATGGTAAT | CGAGCATCAAAGGTGAGGAT | ATTGGGGAGGATTCTTTGGA | 60.2 | 321 | INTERGENIC | | | | |
| CaPOPI_4590 | Ca7 | 7962835 | A/ATTCT | TGGAATAAACTCTGTGCCCC | CGATGACGACAACATAACGG | 59.9 | 283 | INTERGENIC | | | | |
| CaPOPI_4591 | Ca7 | 7962974 | AACAGAGTTTGTTTAG/A | TGGAATAAACTCTGTGCCCC | GACGGCGACAAAAACAAAAG | 59.9 | 365 | INTERGENIC | | | | |
| CaPOPI_4592 | Ca7 | 8010935 | TAAAAAAA/TAAAAAA | AGGCCACAAATGCAGCTTAG | CAAGTCAGCTTTTTCCAGGC | 60.4 | 586 | DRR | Ca_13217 | R | NF-YA | Kelch repeat type 1 |
| CaPOPI_4593 | Ca7 | 8016813 | TAATAAATAAATAAATAAATAAAT/TAATAAATAAATAAATAAATAAATAAAT | TGACCTCTGCAACCTTTTCC | GGTGGACGTGCTACTTGTGA | 60.2 | 200 | INTERGENIC | | | | |
| CaPOPI_4594 | Ca7 | 8019951 | ACTACAT/ACTACATAGTCTACAT | TAATGTATAGGGGGAGGGGG | CTGTCGTCATCACATCTTGCT | 59.9 | 305 | INTERGENIC | | | | |
| CaPOPI_4595 | Ca7 | 8023022 | TAAA/TATAAAA | GGAACCACGAGAGAAGGAGA | GGGTGAGATCTTGCAGTGGT | 59.4 | 468 | INTERGENIC | | | | |
| CaPOPI_4596 | Ca7 | 8028807 | AT/ATT | TCAAAGGTTTCTCCCACCAC | GGTGGCTTCTTGTTGGTGTT | 59.9 | 608 | DRR | Ca_13215 | | | |
| CaPOPI_4597 | Ca7 | 8032032 | GAAAAAAAAA/GAAAAAAA | CAAGAAATTCCGATCACTCCA | CACAACACACGATGACTCCC | 60.1 | 639 | INTERGENIC | | | | |
| CaPOPI_4598 | Ca7 | 8082220 | TC/T | CGATTCAATCTTGATTCCGT | CGTGCAATTTTTCCTATTGGT | 57.6 | 491 | INTERGENIC | | | | |
| CaPOPI_4599 | Ca7 | 8107965 | G/GTT | CACAAAATTTTAGGGGGCCT | AGAAAGGGGGATCAAGGAAA | 60.2 | 679 | INTERGENIC | | | | |
| CaPOPI_4600 | Ca7 | 8147843 | CT/C | ATGTACCGACTCTTCCGCTG | GTTGTGGGATGAATTGACCA | 60.3 | 752 | INTERGENIC | | | | |

| INDEL marker IDs | Chromosomes/ unanchored scaffolds | Physical positions (bp) | InDels (*Kabuli* reference genome-CDC Frontier/PI) | Forward primers (5'-3') | Reverse primers (5'-3') | Anneling temperature (0C) | Expected amplified product size (bp) | Structural annotation | | Functional annotation | | |
|---|---|---|---|---|---|---|---|---|---|---|---|---|
| | | | | | | | | Sequence components of *kabuli* genome | *Kabuli* gene accession IDs | NCBI-KOG | TFs | NCBI-nr database |
| CaPOPI_4601 | Ca7 | 8158726 | ATTATTTTTATT/ATTATTTTTATTTTTATT | TTCGACCACTACTGCCATCA | TTTCTCCTCCGTTCATGAGG | 60.3 | 677 | INTERGENIC | | | | |
| CaPOPI_4602 | Ca7 | 8160639 | CATAT/CATATAT | TCCCTCCTTCCCCCTATCTA | GTGGATCATTACCATGGCCT | 59.9 | 443 | INTERGENIC | | | | |
| CaPOPI_4603 | Ca7 | 8168245 | TCC/TCCC | GAAAGAGAGATGGTGGGTGC | ATCCTTCAGCAACGATCCAC | 59.7 | 568 | DRR | Ca_13197 | S | | Uncharacterised protein family, transmembrane-40 |
| CaPOPI_4604 | Ca7 | 8215292 | AG/AGG | CACTAACGCCAACCATGAGA | AAAAGTTACAAAGCGGAGATGC | 59.7 | 345 | DRR | Ca_13193 | | | |
| CaPOPI_4605 | Ca7 | 8216237 | TGG/TGGG | TTCTTGAACCTTGGGCATTC | CAATTGTTGGGGAGGAAATG | 60.1 | 766 | DRR | Ca_13193 | | | |
| CaPOPI_4606 | Ca7 | 8241298 | CATATATATATATATA/CATATATATATATATATATATATATA | ATGCATAAATGACCTTGGCA | ATGGTCCATGTGTTAGGGGA | 59.0 | 298 | INTERGENIC | | | | |
| CaPOPI_4607 | Ca7 | 8246509 | CTAGAAAAT/CTAGAAAATAGAAAAT | TGATTTGGGGGTATCACGTT | AAAAGCCTCTTGCTATGCCA | 60.1 | 634 | INTERGENIC | | | | |
| CaPOPI_4608 | Ca7 | 8250120 | TATATTGATATTG/TATATTGATATTGATATTG | GCAGCTGGTGTTGTTTCTGA | TGCAAGAACTGAAGTGGGTG | 60.0 | 546 | DRR | Ca_13190 | R | C2H2 | Zinc finger, C2H2-type |
| CaPOPI_4609 | Ca7 | 8303689 | CTTTTTTTTTTT/CTTTTTTTTT | TTTTGGACCACTTTGCTGTG | ACGAGGCTTGTGACCTCATT | 59.7 | 326 | DRR | Ca_13182 | | FAR1 | Protein kinase, catalytic domain |
| CaPOPI_4610 | Ca7 | 8310630 | ATTTTT/ATTTTTTTT | TTGGTGGTGGAAAGTGACAA | TGAACACCCACAACACAAGG | 60.0 | 562 | DRR | Ca_13181 | | | Protein of unknown function DUF827, plant |
| CaPOPI_4611 | Ca7 | 8311161 | AT/ATGT | CCTTGTGTTGTGGGTGTTCA | TCATCCCCGAAAACTACACC | 60.5 | 670 | DRR | Ca_13181 | | | Protein of unknown function DUF827, plant |
| CaPOPI_4612 | Ca7 | 8315825 | CAATCATTT/CAATCATTTACTAATCATTT | GCCATATCACCTTTGATCAAGTC | TGCCATGACACAAACAACAC | 59.9 | 142 | INTERGENIC | | | | |
| CaPOPI_4613 | Ca7 | 8363466 | CCTTT/CCTTTCTTT | GCAACCACAGACACAGAACG | TCAAATGTGGTGTAGGGGGT | 60.4 | 599 | INTERGENIC | | | | |
| CaPOPI_4614 | Ca7 | 8366630 | G/GTAT | AAGCAGTGATCCAACTCCTCA | TGGAAATTATAAACCGATTAAGGTG | 59.9 | 210 | INTERGENIC | | | | |
| CaPOPI_4615 | Ca7 | 8384302 | CTAATAATAATAATAATAATAATAATAATAATAATAATAATAATA/CTAATAATAATAATAATAATAATAATAATAATAATAATA | AAGAAATGCAGGCAATCACC | AAAAGGCTTCGCGTGAGTTA | 60.1 | 386 | INTRON | Ca_13172 | | | Actin-binding FH2/DRF autoregulatory |

| INDEL marker IDs | Chromosomes/ unanchored scaffolds | Physical positions (bp) | InDels (*Kabuli* reference genome-CDC Frontier/PI) | Forward primers (5'-3') | Reverse primers (5'-3') | Anneling temperature (0C) | Expected amplified product size (bp) | Structural annotation | | Functional annotation | | |
|---|---|---|---|---|---|---|---|---|---|---|---|---|
| | | | | | | | | Sequence components of *kabuli* genome | *Kabuli* gene accession IDs | NCBI-KOG | TFs | NCBI-nr database |
| CaPOPI_4616 | Ca7 | 8409838 | CGGG/CGGGG | TGCAGCCTTTTCACCTTCTT | CGGTGCACAACTTATCTTGC | 60.0 | 122 | INTERGENIC | | | | |
| CaPOPI_4617 | Ca7 | 8437623 | CT/CTTT | TTTGACTACTTGCGTGGCAG | ATGTTGCCTGGGTTGCTATC | 60.1 | 534 | DRR | Ca_13163 | O | TCP | Zinc finger, RING-type |
| CaPOPI_4618 | Ca7 | 8438347 | AGGGG/AGGGGG | GATAGCAACCCAGGCAACAT | ACATCGTGATGCAATGGAAA | 60.0 | 510 | DRR | Ca_13163 | O | TCP | Zinc finger, RING-type |
| CaPOPI_4619 | Ca7 | 8440281 | T/TG | TTTAGCTCCCAGGCTTTTCA | CATCAAAGCAGGAGCTGACA | 60.0 | 608 | INTRON | Ca_13162 | G | MYB | Ribulose-phosphate 3-epimerase |
| CaPOPI_4620 | Ca7 | 8442048 | GGTTA/GGTTATTAGTGAGTTA | TTGGCGATTTCTGTGGTGTA | TTTGAAGCACAAGATGGCTG | 60.1 | 597 | INTRON | Ca_13162 | G | MYB | Ribulose-phosphate 3-epimerase |
| CaPOPI_4621 | Ca7 | 8445186 | TAAAAAAAAAAAAAA/TAAAAAAAAAAA | GCATGCCATTGGTTTAAGTTT | GGACTAAAACGTTGCCTGAAA | 59.0 | 443 | INTERGENIC | | | | |
| CaPOPI_4622 | Ca7 | 8466786 | TAA/TAATAAA | GTTTTTATTGCAACCCGTGG | CTATCCTTCGCCGACAACAT | 60.2 | 186 | INTERGENIC | | | | |
| CaPOPI_4623 | Ca7 | 8467239 | GC/G | ATGTTGTCGGCGAAGGATAG | TCCGTCCTAAATTGTATGCCA | 60.1 | 918 | INTERGENIC | | | | |
| CaPOPI_4624 | Ca7 | 8473510 | CATTATTTTCTTGTGTA/CATTATTTTCTTGTGTATTATTTTCTTGTGTA | CGAAGTTGTGGTACAACACATGA | CCCACCACAACATAGACCCT | 60.9 | 409 | INTERGENIC | | | | |
| CaPOPI_4625 | Ca7 | 8477670 | GA/GAA | CATCAGTTGCTTTTGGAGCA | CGATTTGTCGATGGGAATCT | 60.0 | 624 | INTERGENIC | | | | |
| CaPOPI_4626 | Ca7 | 8492749 | TAA/TA | GACTCTCTGCGGGAGATTTG | TTCCGGCTGATGATCTTTTC | 60.0 | 690 | INTERGENIC | | | | |
| CaPOPI_4627 | Ca7 | 8492888 | TAAA/TAA | TACCGTGCATCCTTTTCTCC | TTCCGGCTGATGATCTTTTC | 60.1 | 455 | INTERGENIC | | | | |
| CaPOPI_4628 | Ca7 | 8522491 | CAA/CAAA | GGATTCGTTTTTCCCCAAAT | TTCGCAACTCCACTCTTCCT | 60.0 | 343 | INTERGENIC | | | | |
| CaPOPI_4629 | Ca7 | 8529147 | TAAAAAAA/TAAAAAAAAA | CACAAACCAAAACAACCTGTG | AATGAGTCATCCCTTTTGCG | 59.0 | 459 | DRR | Ca_13150 | | | Protein of unknown function DUF3223 |
| CaPOPI_4630 | Ca7 | 8529891 | TATAAA/TA | CATGAATTCAGGATTTTCCCA | TTCCCACCCCAATTTTGTTA | 59.7 | 489 | DRR | Ca_13150 | | | Protein of unknown function DUF3223 |

| INDEL marker IDs | Chromosomes/ unanchored scaffolds | Physical positions (bp) | InDels (*Kabuli* reference genome-CDC Frontier/PI) | Forward primers (5'-3') | Reverse primers (5'-3') | Anneling temperature (0C) | Expected amplified product size (bp) | Structural annotation | | Functional annotation | | |
|---|---|---|---|---|---|---|---|---|---|---|---|---|
| | | | | | | | | Sequence components of *kabuli* genome | *Kabuli* gene accession IDs | NCBI-KOG | TFs | NCBI-nr database |
| CaPOPI_4631 | Ca7 | 8597223 | CTG/CTGTG | AGTGACAAGCGGTGGAA ATC | GGTAGGACCAAGAACAC AACC | 60.1 | 931 | INTERGENIC | | | | |
| CaPOPI_4632 | Ca7 | 8601211 | TC/T | AGCGGTTTTTGCGAGAG ATA | CACCTCACACCTAACCC TCA | 60.0 | 769 | INTERGENIC | | | | |
| CaPOPI_4633 | Ca7 | 8619938 | TAG/TAGAG | TTCCATGGTTTTCTCCCA AG | TCTAATAAATGGCAACG GGC | 59.9 | 218 | INTERGENIC | | | | |
| CaPOPI_4634 | Ca7 | 8633845 | TA/TAA | TTATGTTTGTGGTGGTT GCG | AAATGTCGGTGGAATGA AGC | 60.4 | 618 | DRR | Ca_15354 | DZ | C2H2 | Calponin homology domain |
| CaPOPI_4635 | Ca7 | 8634841 | ATTTT/ATTT | TACAAAGGGAGAATGGG CAG | GCACCCTCCTATGGTCG TAA | 60.1 | 536 | DRR | Ca_15354 | DZ | C2H2 | Calponin homology domain |
| CaPOPI_4636 | Ca7 | 8662282 | AGTGTG/AGTG | TGAGAATAGTTTTACGG TAGTTTTGG | CATTGGCAACACTTCCT CCT | 59.2 | 641 | DRR | Ca_15350 | R | bZIP | Kelch repeat type 1 |
| CaPOPI_4637 | Ca7 | 8662645 | AATA/AA | CTTCTGCAGATGTTTTG GCA | TGCATTGACACGTGGTT TTT | 60.0 | 374 | DRR | Ca_15350 | R | bZIP | Kelch repeat type 1 |
| CaPOPI_4638 | Ca7 | 8666289 | ATTTTTTTCTTT/ATTT | TCACTCCAACAACCAAT GACA | CAACAATCAACAAGGCT CCA | 60.0 | 566 | INTERGENIC | | | | |
| CaPOPI_4639 | Ca7 | 8670801 | AA/AAAAGA | CACATGCGGACCCTTAA CTT | GCCCAATTGGTCGCTTA ATA | 60.0 | 798 | INTERGENIC | | | | |
| CaPOPI_4640 | Ca7 | 8673839 | CC/CCGTTAAAATGAAC | TCGAGCTTTGGAGTTAA GGTTT | TGAAATCGCCCCAAATT AAA | 59.4 | 442 | INTERGENIC | | | | |
| CaPOPI_4641 | Ca7 | 8678324 | CTTATTTTATTTTATTTT/CTTATTTTATT TTATTTTATTTT | AAAAAGGGTTGGTTGGT TCC | TGTCCCCTTCTGATCTT GGA | 60.1 | 679 | INTRON | Ca_15349 | T | WRKY | Protein kinase, catalytic domain |
| CaPOPI_4642 | Ca7 | 8681581 | AT/ATT | ATAATTGCGTGGCTTGC ATT | ATTGCTTTGGAGACACA GGG | 60.5 | 488 | DRR | Ca_15348 | G | | Aldose 1-epimerase |
| CaPOPI_4643 | Ca7 | 8709902 | CAAAA/CAA | ATCAATCATGCGTCAAA ACG | AAACACAAGCAGGGTTG GTC | 59.5 | 392 | INTERGENIC | | | | |
| CaPOPI_4644 | Ca7 | 8737215 | TAAATAAAAATAAAAATAA/TAAATAAAA ATAAAAATAAAAATAA | TTTTGACACTCTAGCCTT TGTG | TGGGATTTTGTTGTGCT TTTT | 57.2 | 575 | INTERGENIC | | | | |
| CaPOPI_4645 | Ca7 | 8761317 | CTTT/CTT | GGGCAATGCTTTTAAAG GTG | ACGCATGCACATTGATT TGT | 59.6 | 818 | INTRON | Ca_15340 | | HD-ZIP | Pectate lyase/Amb allergen |

| **INDEL marker IDs** | **Chromosomes/ unanchored scaffolds** | **Physical positions (bp)** | **InDels (*Kabuli* reference genome-CDC Frontier/PI)** | **Forward primers (5'-3')** | **Reverse primers (5'-3')** | **Anneling temperature (0C)** | **Expected amplified product size (bp)** | **Structural annotation** | | **Functional annotation** | | |
|---|---|---|---|---|---|---|---|---|---|---|---|---|
| | | | | | | | | **Sequence components of *kabuli* genome** | ***Kabuli* gene accession IDs** | **NCBI-KOG** | **TFs** | **NCBI-nr database** |
| CaPOPI_4646 | Ca7 | 8762041 | GTTTTTTTT/GTTTTTTTTTT | ACAAATCAATGTGCATGCGT | TCTCACATTGCACAAATCTCG | 60.0 | 484 | INTERGENIC | | | | |
| CaPOPI_4647 | Ca7 | 8792391 | TTATAT/TTAT | TTTTGTTTGCACTTCCTTGAAA | CGGCAGACGCTTCTTAATTT | 59.8 | 772 | INTERGENIC | | | | |
| CaPOPI_4648 | Ca7 | 8793998 | TAAAAAAAA/TAAAAAAAAAA | TCATTGGTTATTGGTTGGGAA | GAAGGAACCAAAGGGAGACC | 60.0 | 823 | INTERGENIC | | | | |
| CaPOPI_4649 | Ca7 | 8839299 | C/CAA | CCAATTGCCCCAAAATAAAA | GTTTCCCCTTTGAGTCCGAT | 59.6 | 450 | DRR | Ca_15328 | E | BBR-BPC | Oligopeptide transporter |
| CaPOPI_4650 | Ca7 | 8841846 | TATAAATAAATAAATAAA/TATAAATAAATAAATAAATAAATAAA | TTCATTTTTGCCTCAACACG | AATGGGTGATTGCATGCTG | 59.7 | 399 | DRR | Ca_15327 | P | GRAS | ATPase, P-type, H+ transporting proton pump |
| CaPOPI_4651 | Ca7 | 8859288 | TAAA/TAA | CCCTATTGATGTCCGTGTCA | GGTACGATTGCCTTTTCCAA | 59.4 | 191 | INTERGENIC | | | | |
| CaPOPI_4652 | Ca7 | 8875146 | TTT/TTTAAATT | CATTATACACCCTGGACAAATGG | TTAATGAAAATTTGGTCCGTG | 60.4 | 304 | INTERGENIC | | | | |
| CaPOPI_4653 | Ca7 | 8898762 | GAGAAGAAGA/GAGAAGA | TGCCACGTCATTGAAAATGT | CTGCGTCGAAATTTAGGGAA | 60.0 | 859 | INTRON | Ca_15325 | | C2H2 | Domain of unknown function DUF629 |
| CaPOPI_4654 | Ca7 | 8907832 | TATCAT/TAT | TCATGACAATGAGTCAATTTGC | GACTCTCGCCAGAATCATGTC | 58.6 | 656 | INTERGENIC | | | | |
| CaPOPI_4655 | Ca7 | 8955893 | CTTTT/CTTT | CATACCAGTGGGAGGCAGTT | TTGCTTCCCCATTAAGCATC | 60.0 | 636 | INTRON | Ca_15319 | | | |
| CaPOPI_4656 | Ca7 | 8967220 | AAATTAATTAATTA/AAATTAATTA | TTATTTGGCTCACACCCTCA | ACAACAGGCTGAGCCAAGTC | 59.1 | 539 | INTERGENIC | | | | |
| CaPOPI_4657 | Ca7 | 8973095 | TA/T | GGCGGTGTTGTTAGCAATTT | TGTGGAACAAGTTCCCAAGG | 60.0 | 626 | INTERGENIC | | | | |
| CaPOPI_4658 | Ca7 | 8974976 | ATTTTTT/ATTTT | TAGAAGCCCAAAGGCTCAAA | GGTACAGAACGTGGTGCAAA | 60.0 | 918 | INTERGENIC | | | | |
| CaPOPI_4659 | Ca7 | 8994610 | CTTTTTTT/CTTTTTTTTTT | CTGCTGCGTTTCTGATTGAA | AACGAAAAAGACCGCAACAT | 60.1 | 471 | INTERGENIC | | | | |
| CaPOPI_4660 | Ca7 | 9020687 | AC/A | ATTACTTTGAAGCCCTGGCA | TGGCACACATGACAACTTGA | 59.7 | 489 | INTRON | Ca_15313 | U | bHLH | Exportin-1/Importin-beta-like |

| INDEL marker IDs | Chromosomes/ unanchored scaffolds | Physical positions (bp) | InDels (*Kabuli* reference genome-CDC Frontier/PI) | Forward primers (5'-3') | Reverse primers (5'-3') | Anneling temperature (0C) | Expected amplified product size (bp) | Structural annotation | | Functional annotation | | |
|---|---|---|---|---|---|---|---|---|---|---|---|---|
| | | | | | | | | Sequence components of *kabuli* genome | *Kabuli* gene accession IDs | NCBI-KOG | TFs | NCBI-nr database |
| CaPOPI_4661 | Ca7 | 9023557 | CTT/CTTT | GACAGAGGGGGAGCTAATCC | AGAACCGCCTTCCCAGTTAT | 60.0 | 621 | INTRON | Ca_15313 | U | bHLH | Exportin-1/Importin-beta-like |
| CaPOPI_4662 | Ca7 | 9030127 | GAAATATAAATATAAAT/GAAATATAAATATAAATATAAAT | CACGATAGCGTTGGAGCTAA | TTGCACCTAAGTTTTGTTCCG | 59.1 | 755 | DRR | Ca_15313 | U | bHLH | Exportin-1/Importin-beta-like |
| CaPOPI_4663 | Ca7 | 9031079 | CTTTTTT/CTTTTTTT | ACCCTCCATTTTCTCCCACT | ACTGCGCCATGAATGATTTC | 59.8 | 703 | DRR | Ca_15313 | U | bHLH | Exportin-1/Importin-beta-like |
| CaPOPI_4664 | Ca7 | 9032345 | ATTCTATCTTT/ATT | ACCACCTGAATTGCTTTTGG | TCTCGCACCTTGACAGTTTG | 60.0 | 936 | INTRON | Ca_15312 | R | | Tubby, C-terminal |
| CaPOPI_4665 | Ca7 | 9034671 | CAAA/CAAAA | TTTCATGGTGGTGGTACTGG | TTCGCAAAGGTGTCAAACTG | 59.3 | 463 | DRR | Ca_15312 | R | | Tubby, C-terminal |
| CaPOPI_4666 | Ca7 | 9037363 | GTTT/GTT | CCACAAAGGAGTGGGACCTA | GTGGTGTCATTGGCCCTAGT | 60.0 | 295 | INTERGENIC | | | | |
| CaPOPI_4667 | Ca7 | 9044478 | GTT/GTTTT | AAGTTGCAGGGGTTTCTTGA | TGAATTTATTGCGATTTAATGAATG | 59.7 | 247 | INTERGENIC | | | | |
| CaPOPI_4668 | Ca7 | 9051626 | A/AT | TGAGAGGATCTCCTTCCAACTT | TTCAAAAATTCGGTCCCATT | 59.3 | 120 | INTERGENIC | | | | |
| CaPOPI_4669 | Ca7 | 9064807 | TGA/T | CAGGAACGTGAACCAAACAA | AATGCTACGTCCAAACCTGG | 59.6 | 785 | DRR | Ca_15308 | R | AP2 | Smr protein/MutS2 C-terminal |
| CaPOPI_4670 | Ca7 | 9075277 | TA/T | AGGCACGGTCTTTCCTTTTT | ATTGCTAGGGGTGTTGATGG | 60.1 | 355 | INTERGENIC | | | | |
| CaPOPI_4671 | Ca7 | 9084742 | TTAT/TT | TGAGATCATGAAAATGCCCA | GGAGTTGATTTGATTTGCGG | 60.0 | 975 | INTERGENIC | | | | |
| CaPOPI_4672 | Ca7 | 9101816 | AAGA/AA | CAAGTGGTCATTCAAAAGCAAA | TTTAAGTGGACCACGCCATT | 60.1 | 549 | INTERGENIC | | | | |
| CaPOPI_4673 | Ca7 | 9109063 | AG/AGG | TGGACCTATTGGCCTACGTT | TCGTTCAAAAAGGAAACATCG | 59.5 | 904 | INTERGENIC | | | | |
| CaPOPI_4674 | Ca7 | 9129170 | GAGCTA/GAGCTAGCTA | TTGTGAGGCGGAGAGAGTTT | AGCATCCGACAATGTCACAA | 60.0 | 480 | DRR | Ca_15303 | K | ARR-B | Signal transduction response regulator, receiver domain |
| CaPOPI_4675 | Ca7 | 9134590 | CATAAAAAA/CATAAAAAAATAAAAAA | TTTTCTGGGAGATGGATAAAAA | ACATTGCGAGTCCAAACACA | 57.7 | 503 | INTERGENIC | | | | |

| INDEL marker IDs | Chromosomes/ unanchored scaffolds | Physical positions (bp) | InDels (*Kabuli* reference genome-CDC Frontier/PI) | Forward primers (5'-3') | Reverse primers (5'-3') | Anneling temperature (0C) | Expected amplified product size (bp) | Structural annotation | | Functional annotation | | |
|---|---|---|---|---|---|---|---|---|---|---|---|---|
| | | | | | | | | Sequence components of *kabuli* genome | *Kabuli* gene accession IDs | NCBI-KOG | TFs | NCBI-nr database |
| CaPOPI_4676 | Ca7 | 9180941 | TA/TAGA | ATGATTTCCACGAGCCAAAC | TGCAACATTTCCTGATGAGC | 59.9 | 322 | INTERGENIC | | | | |
| CaPOPI_4677 | Ca7 | 9181192 | AGT/AGTCGT | ATGATTTCCACGAGCCAAAC | ACGACATGCCAATGTCTCAA | 59.9 | 641 | INTERGENIC | | | | |
| CaPOPI_4678 | Ca7 | 9181858 | CT/C | TATTACCCAACCAAGCCAGC | TGAACTTTGATTGCCGCTAA | 60.0 | 554 | INTERGENIC | | | | |
| CaPOPI_4679 | Ca7 | 9183560 | CCACA/CCA | CGATTGCAAATATGCCCTTT | GTGTTGGCATTGTCTTCCCT | 59.9 | 337 | INTERGENIC | | | | |
| CaPOPI_4680 | Ca7 | 9210664 | CTTTTTTTT/CTTTTTTTTT | GTGTGCCACATATCCACAGC | GGACCAAATTGAGGAACCAA | 60.0 | 539 | INTERGENIC | | | | |
| CaPOPI_4681 | Ca7 | 9232233 | GAAAAA/GAAAA | GAAACGTTGGTGGACATTCA | ATGCCATATTCTCGAGTCGG | 59.4 | 546 | INTRON | Ca_17335 | | | rRNA small subunit methyltransferase G |
| CaPOPI_4682 | Ca7 | 9255165 | CACTTACTTACTTACTTAC/CACTTACTTAC | AATTTGCAACCAATTACCGC | ATCCACCAACAACAGCAACA | 59.8 | 220 | INTERGENIC | | | | |
| CaPOPI_4683 | Ca7 | 9259824 | AATTTTTTGA/AATTTTTTGATTTTTTGA | AGTGGGAGAGCAGCTGAGAG | ATCGGTGCAAGACACATTGA | 59.9 | 352 | INTERGENIC | | | | |
| CaPOPI_4684 | Ca7 | 9261954 | ATGA/ATGACGATACTGA | GGGAAAGTCTTACTGTGTGCG | TGGACCAAATTGATGAACCA | 59.8 | 808 | INTERGENIC | | | | |
| CaPOPI_4685 | Ca7 | 9276938 | TAA/TCGAATCGAAA | GGAGCCTGATAATTCGGTGA | GCAATTCGATTTTCTGCGAT | 60.0 | 241 | INTERGENIC | | | | |
| CaPOPI_4686 | Ca7 | 9312827 | A/AC | TTTTGGCCTGGGTCATTTAG | AGACGGAAGCAACACAACAA | 59.9 | 520 | DRR | Ca_17329 | | C2H2 | HhH-GPD domain |
| CaPOPI_4687 | Ca7 | 9367031 | CGATTAAAATTTAT/C | TCAGCTTATTGTGCGAGACG | CACACAACAAGTCCCACAGG | 60.2 | 391 | INTRON | Ca_17324 | | | Gibberellin regulated protein |
| CaPOPI_4688 | Ca7 | 9386132 | G/GC | TCCTCCAGTGGAACTCATGTC | TTTTTCTCATGGCCCATTGT | 60.1 | 509 | DRR | Ca_17321 | | WRKY | |
| CaPOPI_4689 | Ca7 | 9401104 | AT/ATTT | AATAGGGTCCGGATGTTTCC | TTGAACGGAGAAAACTCGCT | 60.0 | 345 | INTERGENIC | | | | |
| CaPOPI_4690 | Ca7 | 9406189 | AAATAATAATAATAATAATAA/AAATAATAATAATAATAA | CGCTCAATACTCTTCAAAGTTCA | TTCCCTTGTGCATTTCAGTG | 58.7 | 545 | INTERGENIC | | | | |

| INDEL marker IDs | Chromosomes/ unanchored scaffolds | Physical positions (bp) | InDels (*Kabuli* reference genome-CDC Frontier/PI) | Forward primers (5'-3') | Reverse primers (5'-3') | Anneling temperature (0C) | Expected amplified product size (bp) | Structural annotation | | Functional annotation | | |
|---|---|---|---|---|---|---|---|---|---|---|---|---|
| | | | | | | | | Sequence components of *kabuli* genome | *Kabuli* gene accession IDs | NCBI-KOG | TFs | NCBI-nr database |
| CaPOPI_4691 | Ca7 | 9421127 | TGGG/TGG | ATTGTGATGAAATGGGGACA | GTGGGTGGGATGCATAATTC | 58.6 | 569 | INTERGENIC | | | | |
| CaPOPI_4692 | Ca7 | 9421758 | A/AT | CTTCGCAAAAATGCGAAACT | GCTATCGCGCTATTCCAAAC | 60.4 | 486 | INTERGENIC | | | | |
| CaPOPI_4693 | Ca7 | 9422013 | AATAT/AATATAT | CTTCGCAAAAATGCGAAACT | CATGATGATGCAGAAAACGG | 60.4 | 573 | INTERGENIC | | | | |
| CaPOPI_4694 | Ca7 | 9440061 | TTACT/TTACTACT | GGGTGTTGATTTCCCTTGATT | ATTTCCAGTTGGAGTGGTCG | 60.0 | 867 | DRR | Ca_17315 | O | TCP | Zinc finger, RING-type |
| CaPOPI_4695 | Ca7 | 9452345 | CTTCA/CTTCAATTCA | GCTTCACATTAGGCCTCTGC | CCACGAGTAAGCTAAAGCCG | 60.0 | 129 | CDS (FRAME SHIFT) | Ca_17312 | | | Ribonuclease III |
| CaPOPI_4696 | Ca7 | 9459566 | ATT/ATTT | TGGCGATAATGTTTGCATGT | AGAGCAAACGGGAAAGTGTG | 60.0 | 131 | DRR | Ca_17311 | | | |
| CaPOPI_4697 | Ca7 | 9465063 | TAAAA/TAAA | TGAGTGGGATTGGTAGGGAG | GCAACCATGACACGAAATTG | 59.9 | 917 | DRR | Ca_17310 | C | bHLH | Aldehyde dehydrogenase domain |
| CaPOPI_4698 | Ca7 | 9468329 | TTA/T | TTGGAGCAATGTTCCCAAAT | TTTGAGGAGTGGCCTCAAGT | 60.3 | 642 | INTERGENIC | | | | |
| CaPOPI_4699 | Ca7 | 9494173 | AACA/AACACA | TACGCAGCCTACTGTTGTGC | TTGTTGCTGACCACGCTATC | 60.1 | 594 | INTERGENIC | | | | |
| CaPOPI_4700 | Ca7 | 9495205 | TTTATTAT/TTTAT | TCTCGCTATCCACTTTTGGG | CCCCAGAGAACAACTTTCCA | 60.2 | 801 | INTERGENIC | | | | |
| CaPOPI_4701 | Ca7 | 9542781 | ATTT/ATT | ATTTTTGGCCTCGTTTTCCT | TTGAAGTTGGTGAATGATGGA | 59.9 | 759 | INTERGENIC | | | | |
| CaPOPI_4702 | Ca7 | 9559628 | A/AAAC | CCAAATTGTAAACCGTAAAATGAA | TTTTATCCAATCTTCCGCGT | 59.3 | 420 | INTERGENIC | | | | |
| CaPOPI_4703 | Ca7 | 9586429 | AA/AAGGA | ACATGCGCATTTGTGTGTTT | GAAGGTTTTTCCGAAGAGGG | 60.0 | 439 | INTERGENIC | | | | |
| CaPOPI_4704 | Ca7 | 9593254 | TA/TAA | CTCCTTGTTGGCAAATGGTT | GGTGTACCGGTTGTTATGCC | 60.0 | 586 | INTERGENIC | | | | |
| CaPOPI_4705 | Ca7 | 9624778 | GA/G | GAACAGCCAGTGCTTCTTCC | TGGGTCATGTTGGCAGTTTA | 60.0 | 580 | INTRON | Ca_17293 | | | UvrB/UvrC protein |

| INDEL marker IDs | Chromosomes/ unanchored scaffolds | Physical positions (bp) | InDels (*Kabuli* reference genome-CDC Frontier/PI) | Forward primers (5'-3') | Reverse primers (5'-3') | Anneling temperature (0C) | Expected amplified product size (bp) | Structural annotation | | Functional annotation | | |
|---|---|---|---|---|---|---|---|---|---|---|---|---|
| | | | | | | | | Sequence components of *kabuli* genome | *Kabuli* gene accession IDs | NCBI-KOG | TFs | NCBI-nr database |
| CaPOPI_4706 | Ca7 | 9639157 | T/TTA | ATTTACCCAATTTCTGGCCC | AAATGGTGGGTAGCCTTCCT | 60.0 | 123 | INTRON | Ca_17292 | A | | Mpp10 protein |
| CaPOPI_4707 | Ca7 | 9648140 | CA/CAA | CAAGTTCGTGCTTGCATCAT | TTCTACGGGTCGTATTTGCC | 59.9 | 694 | INTERGENIC | | | | |
| CaPOPI_4708 | Ca7 | 9655480 | TA/TAA | GAGTTGGACTTGATGCACGA | ACATAGGCAAAGCTAGGCGA | 59.8 | 508 | INTRON | Ca_17290 | | TALE | Protein kinase, catalytic domain |
| CaPOPI_4709 | Ca7 | 9665800 | TA/T | CTGGGGTGGCAACATCTTAT | TTGCATAGCTCACAACTGGC | 59.8 | 223 | INTRON | Ca_17290 | | TALE | Protein kinase, catalytic domain |
| CaPOPI_4710 | Ca7 | 9672464 | TAAA/TAA | TAATGGAATGTGCGTGTGGT | ACTTGTGTCGCCATTTTCCT | 59.8 | 294 | INTERGENIC | | | | |
| CaPOPI_4711 | Ca7 | 9676622 | CAA/CAAA | CCGTTAGGAAACTTGTGGGA | GGGACTGAGGTCTTAAGCAAA | 60.0 | 113 | INTERGENIC | | | | |
| CaPOPI_4712 | Ca7 | 9699894 | TA/T | AACTCTATCCCCACACGCAC | CAAACACGTCGGATTCGTAA | 60.0 | 476 | INTERGENIC | | | | |
| CaPOPI_4713 | Ca7 | 9701171 | GAAAA/GAAA | CCCAAACACACACACACAAA | ATGCAATTGTTCGACGTGAC | 58.9 | 817 | INTERGENIC | | | | |
| CaPOPI_4714 | Ca7 | 9705402 | GAAAAAA/GAAAAAAA | TGGAGACATACAGTTAAGAAAACAA | CACATGTGCTGATTGCCATA | 57.2 | 240 | INTERGENIC | | | | |
| CaPOPI_4715 | Ca7 | 9710850 | ACCCC/ACCC | TGAACCAAATTGGAAACCCT | GAATTGTGCACGATGGATTG | 59.3 | 595 | INTERGENIC | | | | |
| CaPOPI_4716 | Ca7 | 9711096 | TG/TGG | CTGAACCAAATTGGAAACCC | ATAGGCATGCAATGATGCAA | 59.3 | 667 | INTERGENIC | | | | |
| CaPOPI_4717 | Ca7 | 9774600 | GGTT/G | GAAGTCCACTTTTCAAATGACG | GGAAGTCCACTTTTTAAATGACG | 58.7 | 299 | INTERGENIC | | | | |
| CaPOPI_4718 | Ca7 | 9814381 | CTA/CTATA | TGCTTTATGGTTGCGTTGAG | TGACAAATTGTGATTGGTTGG | 59.9 | 109 | DRR | Ca_12746 | S | | Cwf15/Cwc15 cell cycle control protein |
| CaPOPI_4719 | Ca7 | 9814956 | TAAAAA/TAAAA | GAACGCAACCTTTAAAAACGA | TGACTGTTCCAACACGGAAG | 59.3 | 252 | DRR | Ca_12746 | S | | Cwf15/Cwc15 cell cycle control protein |
| CaPOPI_4720 | Ca7 | 9821392 | CCTCT/CCTCTCT | ATTGCTTCCGTTGATGTTCC | CATCCCCACAAACCCATAAG | 59.9 | 402 | INTRON | Ca_12747 | G | | Glycosyl transferase, family 8 |

| INDEL marker IDs | Chromosomes/ unanchored scaffolds | Physical positions (bp) | InDels (*Kabuli* reference genome-CDC Frontier/PI) | Forward primers (5'-3') | Reverse primers (5'-3') | Anneling temperature (0C) | Expected amplified product size (bp) | Structural annotation | | Functional annotation | | |
|---|---|---|---|---|---|---|---|---|---|---|---|---|
| | | | | | | | | Sequence components of *kabuli* genome | *Kabuli* gene accession IDs | NCBI-KOG | TFs | NCBI-nr database |
| CaPOPI_4721 | Ca7 | 9843413 | C/CCTGATTA | TGACAAGGGTGTTCAACTGC | TGGCTTTGGAGGACTGAATC | 59.7 | 532 | INTERGENIC | | | | |
| CaPOPI_4722 | Ca7 | 9843838 | AA/AATAATA | TTGAAATTTCCCGAAGATGC | CAACTGCCTCCTATTTTTATTTTGA | 60.0 | 304 | INTERGENIC | | | | |
| CaPOPI_4723 | Ca7 | 9843850 | AT/A | TTGAAATTTCCCGAAGATGC | CAACTGCCTCCTATTTTTATTTTGA | 60.0 | 304 | INTERGENIC | | | | |
| CaPOPI_4724 | Ca7 | 9844158 | TTTT/TTTTATTT | TTGAAATTTCCCGAAGATGC | GCAACGAGGCCTTAGAACTG | 60.0 | 787 | INTERGENIC | | | | |
| CaPOPI_4725 | Ca7 | 9844646 | ATGTT/ATGTTGTT | GCAGTTCTAAGGCCTCGTTG | CCCATCCACCTACGAACATC | 60.0 | 672 | DRR | Ca_12750 | JO | | Ribosomal protein S30 |
| CaPOPI_4726 | Ca7 | 9868243 | TAA/TAAA | CATTCCTTCCTCCACCAAAA | ACGCGTATACCAACTTTGCC | 59.9 | 568 | INTERGENIC | | | | |
| CaPOPI_4727 | Ca7 | 9927302 | CTT/CT | TATCACTAACCGCACACCCA | TGGTTGAAAATAGTACAAGCCG | 60.0 | 525 | INTERGENIC | | | | |
| CaPOPI_4728 | Ca7 | 9941741 | TCC/TC | ACGAAGATGGAGCGAAAAGA | AAAGTGAGAAGAATGTCGGTCA | 60.0 | 688 | INTERGENIC | | | | |
| CaPOPI_4729 | Ca7 | 9951203 | GAAAAAA/GAAAAAAAA | AATTGCTTGTCTCCGATTGC | TCTTTTGGCATGTGTATTGGA | 60.2 | 865 | INTERGENIC | | | | |
| CaPOPI_4730 | Ca7 | 9960561 | TTCTGAAAA/T | GGTTTGAAGCACGGGAAATA | GGTTGGTTGAGTGGTTCTTTG | 59.9 | 359 | INTRON | Ca_12761 | GMW | ERF | Exostosin-like |
| CaPOPI_4731 | Ca7 | 9967617 | ATTTTTTTT/ATTTTTTTTTT | TCAATGTAATGTTGGTTCGCA | GAATGCGCCAATACGTTTCT | 60.0 | 576 | DRR | Ca_12763 | | MYB | |
| CaPOPI_4732 | Ca7 | 9968032 | TTAAATATAAAT/TTAAATATAAATATAAAT | GCGGGAGTTTCCTCAACTAA | GTGCTGCTTCTCTGGGTTTC | 59.3 | 822 | DRR | Ca_12763 | | MYB | |
| CaPOPI_4733 | Ca7 | 9975017 | TGG/TG | ACTTTCCATCACCAACGAGG | CGGGGTTAAGCCATCAACTA | 60.0 | 688 | INTERGENIC | | | | |
| CaPOPI_4734 | Ca7 | 9992899 | ACCCCC/ACCCC | GATTTGTCGGATGCTTTGGT | GGGGTTTGTTTGAAATTTTTG | 59.9 | 406 | INTERGENIC | | | | |
| CaPOPI_4735 | Ca7 | 10007202 | TG/T | CGCAACATCTCTTTACACCG | AAACCATAACACTCGCAGCC | 59.3 | 853 | INTERGENIC | | | | |

| INDEL marker IDs | Chromosomes/ unanchored scaffolds | Physical positions (bp) | InDels (*Kabuli* reference genome-CDC Frontier/PI) | Forward primers (5'-3') | Reverse primers (5'-3') | Anneling temperature (0C) | Expected amplified product size (bp) | Structural annotation | | Functional annotation | | |
|---|---|---|---|---|---|---|---|---|---|---|---|---|
| | | | | | | | | Sequence components of *kabuli* genome | *Kabuli* gene accession IDs | NCBI-KOG | TFs | NCBI-nr database |
| CaPOPI_4736 | Ca7 | 10016031 | CAAA/CAAAA | TAAAAACGCGTACCGAATCC | GTCGGCTTTTCAAGCAACTC | 60.0 | 588 | DRR | Ca_12767 | | MYB_related | SANT domain, DNA binding |
| CaPOPI_4737 | Ca7 | 10058702 | A/AC | TCGCTTTCAAAAATGAGCAA | TTATAGGGCCAGCTTGGTTG | 59.6 | 519 | INTERGENIC | | | | |
| CaPOPI_4738 | Ca7 | 10081345 | GGT/GGTATGT | TGATCATGTGCTCCAACACTC | CCACGGCTGCATACATACAC | 59.7 | 820 | INTERGENIC | | | | |
| CaPOPI_4739 | Ca7 | 10111639 | TGG/TG | AAATGATTTTCATGGTAATGGAAT | GAAGCAACTGTATCGTGGGG | 57.8 | 494 | INTERGENIC | | | | |
| CaPOPI_4740 | Ca7 | 10112519 | GAGAAAGAAAAA/GAGAAAGAAAAATAAGAAAGAAAAA | GGCCGACCCAAGAATTAGAT | TGTTGCTTTTTCCCAAATCA | 60.3 | 923 | INTERGENIC | | | | |
| CaPOPI_4741 | Ca7 | 10119011 | TTTT/TTTTCTTT | TTTGGCCCGACTTATCTTTG | TAAATCAGGCGGTTTTGGTC | 60.1 | 516 | INTERGENIC | | | | |
| CaPOPI_4742 | Ca7 | 10129752 | TTC/T | AAACACATGCATTCCCTCAA | GAACATGAGAGGCCCACAAT | 59.0 | 387 | INTERGENIC | | | | |
| CaPOPI_4743 | Ca7 | 10138374 | AAA/AAAGTTGAAACAA | CTACCCCTTCTTTTGTGCCA | TCCACCAACTACTTTCACCG | 60.1 | 551 | DRR | Ca_12775 | | ERF | Pathogenesis-related transcriptional factor/ERF, DNA-binding |
| CaPOPI_4744 | Ca7 | 10192729 | TTTTTTTCATATA/TTTTTTTCATATAATGTTTTTTCATATA | CCCATTCCGAATACATTGCT | GAGTTGGGTGCACGATTTTT | 59.8 | 624 | INTERGENIC | | | | |
| CaPOPI_4745 | Ca7 | 10194004 | ATAATTAATT/ATAATT | TTGCTCGATAGAGTTAAATTCCG | CATTTGCCAAGTTTTCATTTTT | 59.8 | 672 | INTERGENIC | | | | |
| CaPOPI_4746 | Ca7 | 10204089 | CTTTTT/CTTTTTT | GATGGGATGACTTTTGTTTGC | GGTTTGTTCCCGTGAGTGTT | 59.4 | 475 | INTERGENIC | | | | |
| CaPOPI_4747 | Ca7 | 10208171 | GAAA/GAAAA | GCATGATGTGGGAGAGGAGT | CAAACTCTCCAAAAGGCAGC | 60.1 | 482 | DRR | Ca_12782 | TU | bHLH | Ras GTPase |
| CaPOPI_4748 | Ca7 | 10213506 | AAAAT/A | ACCGGTATCAGAGATCGTCG | TGGATTTCACATAAATTCCGTC | 60.1 | 639 | INTERGENIC | | | | |
| CaPOPI_4749 | Ca7 | 10214018 | AATGTATATATATTATGTATATATATTAT/AATGTATATATATTAT | TCTCACCTAAAGTAAAGTGACGGA | AAACAAGTGCTTTCGTTGGAA | 59.4 | 741 | INTERGENIC | | | | |
| CaPOPI_4750 | Ca7 | 10224561 | AT/ATGTT | GCAGAAATTCCCGCAGTAAA | GCAGCATGCAAAAACTAGCA | 60.2 | 851 | INTRON | Ca_12784 | U | B3 | Major sperm protein |

| INDEL marker IDs | Chromosomes/ unanchored scaffolds | Physical positions (bp) | InDels (*Kabuli* reference genome-CDC Frontier/PI) | Forward primers (5'-3') | Reverse primers (5'-3') | Anneling temperature (0C) | Expected amplified product size (bp) | Structural annotation | | Functional annotation | | |
|---|---|---|---|---|---|---|---|---|---|---|---|---|
| | | | | | | | | Sequence components of *kabuli* genome | *Kabuli* gene accession IDs | NCBI-KOG | TFs | NCBI-nr database |
| CaPOPI_4751 | Ca7 | 10247426 | AT/ATATTT | TTCAATCACCTTAACTTTTGATCG | TTTTAAGAAATTATGTACGATGTCCAA | 59.6 | 579 | INTERGENIC | | | | |
| CaPOPI_4752 | Ca7 | 10260220 | TTTTATTTATTTATTT/TTTTATTTATTTATTTATTT | GATGATCAATCCTAACATGCGA | AAGTCCCCGCACCTCTACTT | 59.9 | 486 | INTERGENIC | | | | |
| CaPOPI_4753 | Ca7 | 10289954 | TA/TAA | TGACACGGTACAAGGTGGAA | ATGGAATGTGTTAGCCCTGC | 60.0 | 870 | INTERGENIC | | | | |
| CaPOPI_4754 | Ca7 | 10313209 | TAATTTCTAAATTTCTAAATT/TAATTTCTAAATT | TTTCCATGCATTGATCTCCA | TCCCTAAGATGCATTCGTCC | 60.0 | 620 | INTRON | Ca_12794 | A | | tRNA/rRNA methyltransferase, SpoU |
| CaPOPI_4755 | Ca7 | 10339253 | AAACAAAA/AAACAAAAAGAACAAAA | GAAGCATCCCCATTGAAAGA | GGTGCATTTTGTCACCTCCT | 60.0 | 549 | INTRON | Ca_12794 | A | | tRNA/rRNA methyltransferase, SpoU |
| CaPOPI_4756 | Ca7 | 10343265 | TAAAAAA/TAAAAAAA | AAGCGCCTTCTTTTTAGTTTAACA | TGATCAAATGGAAGGACAAAAA | 59.9 | 132 | INTERGENIC | | | | |
| CaPOPI_4757 | Ca7 | 10351925 | CATATATATAT/CATATATATATAT | TTGTGTGGTATCAAGCCCAA | AAGCTAAAGCCTCCTCGGTC | 60.0 | 663 | DRR | Ca_12795 | | | Cupin 2, conserved barrel |
| CaPOPI_4758 | Ca7 | 10380474 | TAAAAAA/TAAAAAAA | TGATTCTGGATGTCGTTTTGG | GCAGAACTTGGCTGGTTTGA | 60.9 | 646 | INTERGENIC | | | | |
| CaPOPI_4759 | Ca7 | 10393062 | ATTT/ATTTT | TCCTCCATGTCAACCCTCTC | CGTTGCCCTCTTTTGGTATC | 60.0 | 653 | INTERGENIC | | | | |
| CaPOPI_4760 | Ca7 | 10395585 | GGA/G | TGGGTTTGACGAAAGGAGAG | CTGGATCGTGGGACTCCTAA | 60.2 | 559 | INTERGENIC | | | | |
| CaPOPI_4761 | Ca7 | 10396287 | TAAA/TAAAA | TGACAAATAAGCTAACGGTGC | TCGAGGTCAATTTTACCATCAAT | 58.0 | 413 | INTERGENIC | | | | |
| CaPOPI_4762 | Ca7 | 10405924 | TC/TCC | ATTGCTGGTTGGCAAGGTAG | CCATAATCACATTGCGGGAT | 60.1 | 669 | INTRON | Ca_12799 | T | | Recoverin |
| CaPOPI_4763 | Ca7 | 10415974 | T/TTATA | GACATTGACATATGGAGGCG | TGCTTCTGCCCTTTTCTGTT | 59.0 | 743 | INTERGENIC | | | | |
| CaPOPI_4764 | Ca7 | 10423937 | ATTT/ATTTT | TTCATTCGATGCTTGTGACC | GAGGTGAAGCCTCCATGTGT | 59.7 | 607 | INTERGENIC | | | | |
| CaPOPI_4765 | Ca7 | 10434123 | AGTTTG/A | TACAACTTTTGCAATGGGCA | ACCCTCTCCCTAGATGGGAA | 60.1 | 783 | INTERGENIC | | | | |

| **INDEL marker IDs** | **Chromosomes/ unanchored scaffolds** | **Physical positions (bp)** | **InDels (*Kabuli* reference genome-CDC Frontier/PI)** | **Forward primers (5'-3')** | **Reverse primers (5'-3')** | **Anneling temperature (0C)** | **Expected amplified product size (bp)** | **Structural annotation** | | **Functional annotation** | | |
|---|---|---|---|---|---|---|---|---|---|---|---|---|
| | | | | | | | | **Sequence components of *kabuli* genome** | ***Kabuli* gene accession IDs** | **NCBI-KOG** | **TFs** | **NCBI-nr database** |
| CaPOPI_4766 | Ca7 | 10447170 | GTA/GTATA | AGGATTTATTGTGCCCGTCA | TGCCTTTGGAAGTGTTGGTT | 60.3 | 679 | INTERGENIC | | | | |
| CaPOPI_4767 | Ca7 | 10449940 | AA/AAGA | TTGACTTCACAGGTTCAAACAA | CTCCCTCGTGAATGGTGATT | 58.3 | 664 | INTERGENIC | | | | |
| CaPOPI_4768 | Ca7 | 10459620 | TA/T | GATAATGGACCCGCGACTTA | CGCCATTTGAATGACGTATG | 59.9 | 680 | DRR | Ca_12806 | T | WRKY | Protein kinase, catalytic domain |
| CaPOPI_4769 | Ca7 | 10464523 | CAAA/CAAAA | ATTCGGAACAACTGCAATCC | GCTGAAGCTGCTCTTGTGTG | 59.9 | 879 | DRR | Ca_12807 | | | |
| CaPOPI_4770 | Ca7 | 10471583 | GAA/GAATCGCTTTTTAAAA | ATCTCGGATGGGTTTCTTCA | TTTCATCTCTTCCACTCAACACA | 59.5 | 228 | INTERGENIC | | | | |
| CaPOPI_4771 | Ca7 | 10473226 | TA/TAA | GGTAAACCTCCACCGAAACA | TCCCCAAGTCTTTGATTTGC | 59.8 | 648 | DRR | Ca_12809 | R | | Endoribonuclease XendoU |
| CaPOPI_4772 | Ca7 | 10482051 | TTCTCTCT/TTCTCT | CAAGAGGTAGCAGGCACACA | TTATGGCTCCGGGTAGACAG | 60.0 | 383 | INTRON | Ca_12810 | | SRS | Protein of unknown function DUF702 |
| CaPOPI_4773 | Ca7 | 10490031 | TC/T | AGAGTTTAAAGTTAATTTTTACGGTTG | TATGCCTCCAAATAGGCCAG | 57.1 | 687 | INTERGENIC | | | | |
| CaPOPI_4774 | Ca7 | 10515194 | ATTTTTTT/ATTTTTTTT | TGAAGGGTTATCAAGAAATTTGG | TTGCTCCCTTAAAATGACCG | 59.4 | 626 | INTERGENIC | | | | |
| CaPOPI_4775 | Ca7 | 10517966 | TTATATATATATATAT/TTATATATATATAT | AGCATTGTCATGCCTAGAACC | ACAGGTAGGTCAGGGTGCAG | 59.2 | 382 | INTERGENIC | | | | |
| CaPOPI_4776 | Ca7 | 10552099 | CAA/CAAA | GCAGCTTCACCCCAAAAATA | AAACGGGGTTGTTCATCAAA | 60.1 | 139 | INTERGENIC | | | | |
| CaPOPI_4777 | Ca7 | 10557993 | A/ACAC | GCGGCTAAATCAGGTCCATA | AAGAAAAAGGCGACAGGACA | 60.1 | 685 | DRR | Ca_12813 | O | | Peptidase S16, Lon protease, C-terminal |
| CaPOPI_4778 | Ca7 | 10568493 | TTATATATATATATATATATATATATATAT/TTATATATATATATATATATATATAT | ACGATGGAGGCCAAAGTATG | AAAATAAGGCATGGTGTTGGA | 60.0 | 487 | INTERGENIC | | | | |
| CaPOPI_4779 | Ca7 | 10572777 | TCTAC/T | TCAAAATGTGACCAGGTGAAAC | CCTGGCCCAACTTGTCATAC | 59.9 | 650 | INTERGENIC | | | | |
| CaPOPI_4780 | Ca7 | 10579803 | ATT/AT | TGACATTTATGCCTCACTAGCC | AATGCTTGTGGGTCTGTGGT | 59.3 | 489 | INTERGENIC | | | | |

| INDEL marker IDs | Chromosomes/ unanchored scaffolds | Physical positions (bp) | InDels (*Kabuli* reference genome-CDC Frontier/PI) | Forward primers (5'-3') | Reverse primers (5'-3') | Anneling temperature (0C) | Expected amplified product size (bp) | Structural annotation | | Functional annotation | | |
|---|---|---|---|---|---|---|---|---|---|---|---|---|
| | | | | | | | | Sequence components of *kabuli* genome | *Kabuli* gene accession IDs | NCBI-KOG | TFs | NCBI-nr database |
| CaPOPI_4781 | Ca7 | 10591717 | TAAA/TAAAA | TCTGCTCCAATTAAATGGTGTTT | CACCGTTCCTTCCTGACAGT | 59.9 | 764 | INTERGENIC | | | | |
| CaPOPI_4782 | Ca7 | 10607434 | GAAA/GAA | TATATGGAGACCCGTGCCTT | CTCCATTGCTGTTGCTTTGA | 59.4 | 526 | INTRON | Ca_12815 | O | | Peptidase S16, Lon protease, C-terminal |
| CaPOPI_4783 | Ca7 | 10613236 | CTTT/CTT | TCCCTCTGAGCTGTATGCCT | GGCAACAAGGTCATCCTCAT | 60.0 | 649 | INTRON | Ca_12815 | O | | Peptidase S16, Lon protease, C-terminal |
| CaPOPI_4784 | Ca7 | 10620555 | CATG/CATGATG | GCAACTGCATCATCTTGCTC | TACCCCTGATTTCCGTCAAA | 59.6 | 604 | DRR | Ca_12815 | O | | Peptidase S16, Lon protease, C-terminal |
| CaPOPI_4785 | Ca7 | 10631442 | TTGCAACATGCAACAT/TTGCAACAT | AGAGAGATATGCCTGGCGAA | CTTCAGCAAGGGGAAAACAA | 59.9 | 136 | INTRON | Ca_12818 | U | Trihelix | Ras GTPase |
| CaPOPI_4786 | Ca7 | 10672753 | TTAT/TT | TGGTGTTGAGATGGGAATGA | GGATACAAATGTTGGCCCAT | 59.9 | 583 | DRR | Ca_12824 | | NF-YB | Protein of unknown function DUF248, methyltransferase putative |
| CaPOPI_4787 | Ca7 | 10672818 | TCC/TC | TGGTGTTGAGATGGGAATGA | GGATACAAATGTTGGCCCAT | 59.9 | 583 | DRR | Ca_12824 | | NF-YB | Protein of unknown function DUF248, methyltransferase putative |
| CaPOPI_4788 | Ca7 | 10676593 | TGATCGAT/TGATCGATCGAT | AAAACCGGAGCAACAATTAAGA | ACCCGGGAAAAGAGTTTTGT | 60.0 | 557 | INTERGENIC | | | | |
| CaPOPI_4789 | Ca7 | 10680712 | ATTTTTT/ATTTTTTT | TGGTGGAGGAAGAGGACGTA | TTCATGGTTCACACATTGGC | 60.6 | 497 | INTERGENIC | | | | |
| CaPOPI_4790 | Ca7 | 10681107 | GTAT/GTATATAT | TGGTGGAGGAAGAGGACGTA | TTCCCTCGGTCACAAACATT | 60.6 | 801 | INTERGENIC | | | | |
| CaPOPI_4791 | Ca7 | 10694557 | TAAAA/TAA | TGTGGATTTCAACATCGCAA | TGCCAAAATTTAAAGGACGAA | 61.1 | 582 | DRR | Ca_12826 | K | MYB | SANT domain, DNA binding |
| CaPOPI_4792 | Ca7 | 10718905 | T/TC | ATTGTTCGGGCTGCAAATAG | AGGAATCTTTTGGGGAAGGA | 60.1 | 399 | INTRON | Ca_12827 | | | Tetraspanin |
| CaPOPI_4793 | Ca7 | 10731505 | AAG/AAGAG | CAGAACCTGTCGGGATTCAT | TCGGCAACTTCAATTTCCTC | 59.9 | 417 | DRR | Ca_12828 | U | M-type | Inositol polyphosphate-related phosphatase |
| CaPOPI_4794 | Ca7 | 10732290 | A/ATT | GCAGTGAAAGAAGAAATCTCCG | AGAAGTACCCGCGAAGATGA | 60.4 | 482 | INTERGENIC | | | | |
| CaPOPI_4795 | Ca7 | 10741982 | ATCAATTT/AT | TTTGAAAGATGAATTCGGGG | AAAATACATCCCGCGATCCT | 59.9 | 855 | DRR | Ca_12829 | FE | | Phosphoribosyltransferase |

| INDEL marker IDs | Chromosomes/ unanchored scaffolds | Physical positions (bp) | InDels (*Kabuli* reference genome-CDC Frontier/PI) | Forward primers (5'-3') | Reverse primers (5'-3') | Anneling temperature (0C) | Expected amplified product size (bp) | Structural annotation | | Functional annotation | | |
|---|---|---|---|---|---|---|---|---|---|---|---|---|
| | | | | | | | | Sequence components of *kabuli* genome | *Kabuli* gene accession IDs | NCBI-KOG | TFs | NCBI-nr database |
| CaPOPI_4796 | Ca7 | 10838599 | GAAAAA/GAAAA | TGGAAGGACGAGGAAAGTTG | CTTGAAGTGCTCCTTGAGACAA | 60.2 | 453 | INTERGENIC | | | | |
| CaPOPI_4797 | Ca7 | 10839999 | CTTTTT/CTTTT | CACATTTGCCCATGAAATTG | ACAATTTGGAAACTTTGCCG | 59.8 | 395 | INTERGENIC | | | | |
| CaPOPI_4798 | Ca7 | 10896204 | GTTT/GTTTT | TCCGATTTATGCTTTGTTCG | TTTAGGCTAATTGATCCGCTG | 58.8 | 121 | INTERGENIC | | | | |
| CaPOPI_4799 | Ca7 | 10899870 | ATCT/AT | TTCCAGTGTCCATCCATTCA | ATATGGAGCTGGTGGTGGAG | 59.9 | 594 | INTERGENIC | | | | |
| CaPOPI_4800 | Ca7 | 10904696 | AT/A | CAGGTGCATCCTCTCCAAAT | TGCAGCATCGACACTTTAGG | 60.1 | 333 | DRR | Ca_12847 | | | Ferredoxin |
| CaPOPI_4801 | Ca7 | 10905837 | ATTT/ATT | TGCACGGCCAACAAATATAA | ACTCCAGCTAGCCCAAGTCA | 60.0 | 383 | INTRON | Ca_12847 | | | Ferredoxin |
| CaPOPI_4802 | Ca7 | 10910160 | GATATATATATATAT/GATATATATAT | AGCGCAAAACATTGACACAT | TTCCTTGCTTCTTGCAATTTT | 59.2 | 779 | INTERGENIC | | | | |
| CaPOPI_4803 | Ca7 | 10925493 | CATATTTATCCAATATTTA/CATATTTA | TTGCATGATTGATTGTTGGG | CCGGCATAGAGATCGAAGAC | 60.3 | 517 | INTERGENIC | | | | |
| CaPOPI_4804 | Ca7 | 10937736 | TCAACACAACACA/TCAACACA | TGCCACGTCAACTGATGTTT | CAAAGATCTCGCAGCAATGA | 60.2 | 681 | INTERGENIC | | | | |
| CaPOPI_4805 | Ca7 | 10938162 | TAA/TAAAA | AACACAACACACGGGTAGCA | TATTCCATCCCTCCGACAAC | 60.1 | 661 | INTERGENIC | | | | |
| CaPOPI_4806 | Ca7 | 11034555 | GTGTA/GTGTATGTA | TGTCAATGTCAACCCATGCT | TCAAAAGGCCACCTCTTCAT | 60.0 | 270 | INTERGENIC | | | | |
| CaPOPI_4807 | Ca7 | 11065887 | AC/ACTTAAACTTC | ACCGTGGCTAGACAAAGTGG | TTGGGGTTGAGACACTGACA | 60.2 | 768 | INTRON | Ca_09379 | | bHLH | Helix-loop-helix DNA-binding domain |
| CaPOPI_4808 | Ca7 | 11070487 | AATATATATATATATATATATAT/AATATATATATATATATAT | GGCCTGCCAATTCTGTTTAT | GTTCGCATTCTGGGGATCTA | 59.0 | 290 | INTERGENIC | | | | |
| CaPOPI_4809 | Ca7 | 11074298 | TAAATTAAAAATTAAA/TAAATTAAA | TACCGATTGCCATCCTTAGC | TTTTCAAGTAGGGTACCTCAATTTTT | 60.1 | 831 | INTERGENIC | | | | |
| CaPOPI_4810 | Ca7 | 11075792 | ATATTACTATTA/ATATTA | ATGCAAAAGTCGGTCATCCT | GATTTGGAAATGAGTGCGGT | 59.6 | 544 | INTERGENIC | | | | |

| INDEL marker IDs | Chromosomes/ unanchored scaffolds | Physical positions (bp) | InDels (*Kabuli* reference genome-CDC Frontier/PI) | Forward primers (5'-3') | Reverse primers (5'-3') | Anneling temperature (0C) | Expected amplified product size (bp) | Structural annotation | | Functional annotation | | |
|---|---|---|---|---|---|---|---|---|---|---|---|---|
| | | | | | | | | Sequence components of *kabuli* genome | *Kabuli* gene accession IDs | NCBI-KOG | TFs | NCBI-nr database |
| CaPOPI_4811 | Ca7 | 11079201 | CAAA/CAAAA | AAAGAAGGGGTTGAGAGGGA | GAGCAGAAAGGTGGAAGTGC | 60.0 | 553 | DRR | Ca_09378 | | bHLH | Helix-loop-helix DNA-binding domain |
| CaPOPI_4812 | Ca7 | 11092070 | CATAT/CATATAT | CGTCCGGTCAAAATGAAGTT | CCGAACCCAATTTCCCTAAT | 60.0 | 497 | INTERGENIC | | | | |
| CaPOPI_4813 | Ca7 | 11118723 | CAAA/CAAAA | TGCATCCAAGAACACCGTAA | AATGTCGTTTTGGTTCCAGC | 60.1 | 774 | INTERGENIC | | | | |
| CaPOPI_4814 | Ca7 | 11120511 | TA/TAATTTAAA | CCACTCACTGCTTGCTTATCC | TCGGGAAATGTTGTTTGTCA | 59.9 | 913 | INTERGENIC | | | | |
| CaPOPI_4815 | Ca7 | 11122293 | CTTTTT/CTTTTTT | GTAGCCCACTCAATTGGCAT | TTTTTGCAATCCAGCTTCCT | 60.0 | 634 | INTERGENIC | | | | |
| CaPOPI_4816 | Ca7 | 11154403 | AACCGCA/AACCGCACCGCA | ATCGACCGAAAATCAATCCA | AAAGAGTGAGCCGACCATTG | 60.3 | 317 | INTERGENIC | | | | |
| CaPOPI_4817 | Ca7 | 11166262 | ATTTTTTTTTT/ATTTTTTTTTTTTT | TTCCAATTCAATGGCATGAG | CATTGACACGCCAAACTGTC | 59.5 | 830 | INTERGENIC | | | | |
| CaPOPI_4818 | Ca7 | 11180939 | TCCGG/T | TTCCAAACCGAGATGAATCC | CCCACTCATTTACACAACATCC | 59.9 | 659 | INTERGENIC | | | | |
| CaPOPI_4819 | Ca7 | 11205713 | AA/AAATA | ACCATGTCACTCCCCATTGT | TTCCATTTGTTTCTCTGCCC | 60.1 | 395 | INTERGENIC | | | | |
| CaPOPI_4820 | Ca7 | 11224360 | TGATCTTAA/TGATCTTAAGATCTTAA | AACCAAAGTGTGTGCAGCAG | AGGGTGGCCTCCAGTAGTTT | 59.9 | 534 | INTRON | Ca_09369 | | | |
| CaPOPI_4821 | Ca7 | 11229799 | TATCAATGTTTCTAAT/TAT | TCCGCACAAAAGACAGACAA | CCTGGTGCAGGTTTGATCTT | 60.4 | 473 | INTERGENIC | | | | |
| CaPOPI_4822 | Ca7 | 11230451 | GAGAAA/GA | CAGAGCCGGAACACAAAGAT | AGCCCTCCCCATGTTTTAAT | 60.3 | 953 | INTERGENIC | | | | |
| CaPOPI_4823 | Ca7 | 11246939 | GAAAAAA/GAAAAAAA | ATTTACGCCGCTGATTTCAA | AATTGCATACCACCGGAAAA | 60.6 | 747 | INTERGENIC | | | | |
| CaPOPI_4824 | Ca7 | 11268569 | CATATATATATAT/CATATATATATATAT | TTTTAACTCGTCCGGGTCAC | TCTCCCAAATTCTTACCCCC | 60.0 | 486 | DRR | Ca_09366 | | | Methionine biosynthesis MetW |
| CaPOPI_4825 | Ca7 | 11272809 | TAAA/TAAATAAAA | ACGTCTCGTGGAACTTGCTT | ACGGAGTTCGTAGTGCGTTT | 59.9 | 516 | INTERGENIC | | | | |

| INDEL marker IDs | Chromosomes/ unanchored scaffolds | Physical positions (bp) | InDels (*Kabuli* reference genome-CDC Frontier/PI) | Forward primers (5'-3') | Reverse primers (5'-3') | Anneling temperature (0C) | Expected amplified product size (bp) | Structural annotation | | Functional annotation | | |
|---|---|---|---|---|---|---|---|---|---|---|---|---|
| | | | | | | | | Sequence components of *kabuli* genome | *Kabuli* gene accession IDs | NCBI-KOG | TFs | NCBI-nr database |
| CaPOPI_4826 | Ca7 | 11286406 | GAAATAAATAAATAAATAAATAAA/GAAATAAATAAATAAATAAATAAATAAA | GTAGCAATCCCCACCAAGAA | TCATGCAAGCAAGAAGATGG | 59.9 | 460 | INTRON | Ca_09365 | | AP2 | Pathogenesis-related transcriptional factor/ERF, DNA-binding |
| CaPOPI_4827 | Ca7 | 11313507 | CTTTTTT/CTTTTT | TGGACAAATATTGCATCATGG | CCTTCACCTGTTTAAGGAAAATATAA | 59.2 | 593 | INTERGENIC | | | | |
| CaPOPI_4828 | Ca7 | 11343583 | ATTCACTTCACTTC/ATTCACTTC | TGTTCCATACACCCCACCTT | TGCAGTTCAACATTTGTTTCC | 60.1 | 531 | DRR | Ca_09360 | | | VQ |
| CaPOPI_4829 | Ca7 | 11345461 | CTTTTTTTTTTTT/CTTTTTTTT | CCACTGGTGCTTTCTTCCAT | GATGCTCCAAATTCTTCCCA | 60.1 | 401 | INTERGENIC | | | | |
| CaPOPI_4830 | Ca7 | 11375539 | TA/T | CAGATTTGTTCGGACTGGGT | GGTCGACACATCCTCACCTT | 60.0 | 759 | INTERGENIC | | | | |
| CaPOPI_4831 | Ca7 | 11397404 | GTTTTTTTTT/GTTTTTTTTTTT | TCAAGGTTGCGTTTTTCCTT | CAACGTTAAGAATCGATGGGA | 59.7 | 670 | DRR | Ca_09356 | C | | NADH dehydrogenase ubiquinone Fe-S protein 4, mitochondrial |
| CaPOPI_4832 | Ca7 | 11413494 | TA/TAA | AAGTCCCCTGCAAATACCAA | GGAAGCATGGAAGGAAATGA | 59.4 | 598 | DRR | Ca_09355 | | MYB_related | SANT domain, DNA binding |
| CaPOPI_4833 | Ca7 | 11428616 | GAAAAA/GAAAA | CCATGCAACTTCGACTTCAA | GGTGGTCAATGACACAGTGC | 59.8 | 658 | DRR | Ca_09352 | | GRAS | Transcription factor GRAS |
| CaPOPI_4834 | Ca7 | 11430402 | CTTGTTGTTGTTGTTGTTG/CTTGTTGTTGTTGTTG | TCTTGGGGAGGTGAGAAAGA | CTCAACTCCACCACCACCTT | 59.8 | 308 | CDS (large-effect mutations) | Ca_09352 | | GRAS | Transcription factor GRAS |
| CaPOPI_4835 | Ca7 | 11438197 | ATTT/ATT | CTGTGCGTCATTTCTTTCCA | TTGGCAATGTTCCCAAGAAT | 59.8 | 802 | INTERGENIC | | | | |
| CaPOPI_4836 | Ca7 | 11441772 | ACTCT/ACT | GTGGAGGGCATTCACATTTT | TTCATGTTTAATGCGAAGTGAGA | 59.8 | 265 | INTERGENIC | | | | |
| CaPOPI_4837 | Ca7 | 11481982 | ATT/AT | GGAGGGACGATGTTACTCCA | TCCACCAATTCGGTTGAATC | 59.9 | 257 | INTERGENIC | | | | |
| CaPOPI_4838 | Ca7 | 11487806 | T/TG | TTGATGTTGAAGGGGGAAAG | GTCATTGGATATGGCCTCGT | 59.9 | 631 | INTERGENIC | | | | |
| CaPOPI_4839 | Ca7 | 11494762 | CCTT/CCTTCTT | AGGAGCAGTTTGTGAGGTGC | GAAAGGTGACTTCCTCACGG | 60.5 | 223 | DRR | Ca_09346 | | | GCN5-related N-acetyltransferase (GNAT) domain |
| CaPOPI_4840 | Ca7 | 11511673 | C/CCCGCGAAACTT | TGACCCTAAGAACCCACCAG | GGATGGGGTAAACATTGTCC | 60.0 | 514 | INTERGENIC | | | | |

| INDEL marker IDs | Chromosomes/ unanchored scaffolds | Physical positions (bp) | InDels (*Kabuli* reference genome-CDC Frontier/PI) | Forward primers (5'-3') | Reverse primers (5'-3') | Anneling temperature (0C) | Expected amplified product size (bp) | Structural annotation | | Functional annotation | | |
|---|---|---|---|---|---|---|---|---|---|---|---|---|
| | | | | | | | | Sequence components of *kabuli* genome | *Kabuli* gene accession IDs | NCBI-KOG | TFs | NCBI-nr database |
| CaPOPI_4841 | Ca7 | 11517081 | CTATA/CTA | GGTGCAGTACACGACCTCAA | GGATGAGGGAACAATGTGCT | 59.8 | 487 | INTERGENIC | | | | |
| CaPOPI_4842 | Ca7 | 11518406 | TTTAAAGACACATATTAAAGA/TTTAAAGA | CCTCAAGTGTGGCGTAGACA | ACGTGGCATGTGTTGTGAAT | 59.9 | 796 | INTERGENIC | | | | |
| CaPOPI_4843 | Ca7 | 11542682 | ATTTTTTTTT/ATTTTTTT | AACTGCCATGGAGCTAGGAA | TTTTTGGTTTGAGTATTATGCGA | 59.8 | 737 | INTERGENIC | | | | |
| CaPOPI_4844 | Ca7 | 11543117 | ATGTGTGTGT/ATGTGTGTGTGT | TCGCATAATACTCAAACCAAAAA | TTCAATCTCCGAGTCCGTTC | 58.7 | 432 | INTERGENIC | | | | |
| CaPOPI_4845 | Ca7 | 11545129 | CTTAA/CTTAAGAAAGTAAGGCATTAA | CCAAACGTTTATAAGCATGCAA | TGTGAAAGGGAAGGAGATGG | 60.0 | 311 | INTERGENIC | | | | |
| CaPOPI_4846 | Ca7 | 11580871 | GTTTTT/GTTTTTT | TGGTGGGCCACAGTTATGTA | GCATGTCCAAAATCATTCCC | 59.8 | 694 | INTERGENIC | | | | |
| CaPOPI_4847 | Ca7 | 11585943 | CTTTT/CTTTTT | CTTAACCCACACGTGCCTTT | ATGCGAATTGAAGACGGAAC | 60.0 | 212 | INTERGENIC | | | | |
| CaPOPI_4848 | Ca7 | 11600742 | CAT/CATAT | AGCTTCGCCGATGAATAGAA | ATCGACCATCTTCATGCTCA | 59.9 | 734 | INTERGENIC | | | | |
| CaPOPI_4849 | Ca7 | 11624553 | TATTAATTAAT/TATTAAT | AGACTCCTTTTGGGGTTTCC | GCAAGACATTGACAAAACTTTCA | 59.4 | 303 | INTERGENIC | | | | |
| CaPOPI_4850 | Ca7 | 11678977 | TTTTATT/TTTTATTTATTTATT | CCCAGCTCATTTCAAGAGAA | AATTGCGTGAGAGCTTTTGG | 58.0 | 732 | INTERGENIC | | | | |
| CaPOPI_4851 | Ca7 | 11691921 | TA/TAA | GTGGAAAAGGAAGTCGCAAG | GTTGGGGATTTGGGGTTTTA | 59.9 | 654 | INTERGENIC | | | | |
| CaPOPI_4852 | Ca7 | 11692218 | AA/AATA | GGATGCACATTAGAGCGACA | GCTGAAGACACGTATGCGAA | 59.8 | 759 | INTERGENIC | | | | |
| CaPOPI_4853 | Ca7 | 11714431 | ATTTT/ATTT | AACGATGAGATGGCAAAAGG | GGCTTTGGTGCTAAGTCCAG | 60.1 | 598 | INTERGENIC | | | | |
| CaPOPI_4854 | Ca7 | 11729720 | CAAAAAA/CAAAAAAA | AACAAATCTTGGCCATGGAG | GGCGTGTGCTTGGTAAGAGT | 59.9 | 711 | INTRON | Ca_09324 | O | NAC | Peptidase A1 |
| CaPOPI_4855 | Ca7 | 11732346 | TC/TCC | TTTTCAGGCCAGACCAAACT | GGTTCTTGATCCATGCCAGT | 59.7 | 324 | DRR | Ca_09323 | | ERF | Frigida-like |

| INDEL marker IDs | Chromosomes/ unanchored scaffolds | Physical positions (bp) | InDels (*Kabuli* reference genome-CDC Frontier/PI) | Forward primers (5'-3') | Reverse primers (5'-3') | Anneling temperature (0C) | Expected amplified product size (bp) | Structural annotation | | Functional annotation | | |
|---|---|---|---|---|---|---|---|---|---|---|---|---|
| | | | | | | | | Sequence components of *kabuli* genome | *Kabuli* gene accession IDs | NCBI-KOG | TFs | NCBI-nr database |
| CaPOPI_4856 | Ca7 | 11751654 | CTTT/CTT | CATGTTGACCACCTCCAATG | AGCCAAATTGGTTTCCACAT | 59.8 | 428 | DRR | Ca_09321 | | C2H2 | Zinc finger, C2H2-type |
| CaPOPI_4857 | Ca7 | 11756608 | ATAT/ATATAATTAT | CTTTGGGACAGTCTGGTGGT | CAAGACGGACTAGTGGGATGA | 60.0 | 476 | INTERGENIC | | | | |
| CaPOPI_4858 | Ca7 | 11757918 | ATTTTT/ATTATTTTTT | TTTCCCACAAATGCTGTTCTT | CTTCCGATTCTCCGTGTGAT | 59.6 | 588 | INTERGENIC | | | | |
| CaPOPI_4859 | Ca7 | 11757957 | A/AG | TTTCCCACAAATGCTGTTCTT | CTTCCGATTCTCCGTGTGAT | 59.6 | 588 | INTERGENIC | | | | |
| CaPOPI_4860 | Ca7 | 11834363 | GTATATATAT/GTATATATATAT | TGTTGCTGACGGACATTGAT | CATCCCCAATTTTCTTTGACA | 60.1 | 542 | INTERGENIC | | | | |
| CaPOPI_4861 | Ca7 | 11884796 | GAT/G | AAGGGAAAGACCTCCCTTCA | AGGGCTGCATGAACAAATTC | 60.0 | 566 | INTRON | Ca_09310 | P | C3H | ATPase, P-type, K/Mg/Cd/Cu/Zn/Na/Ca/Na/H-transporter |
| CaPOPI_4862 | Ca7 | 11892192 | TATTACTA/TATTACTAATATATAAATTACTA | ATTAGGGAGAAACGGAGGGA | TTTTGTGCCCTAACACTTAGCA | 59.9 | 441 | INTERGENIC | | | | |
| CaPOPI_4863 | Ca7 | 11902508 | TAAAAAA/TAAAAA | CCATTGCACAAGTCCTCAAA | CCATGCGGATTGAAAAACTT | 59.7 | 757 | INTERGENIC | | | | |
| CaPOPI_4864 | Ca7 | 11911166 | AAGA/AAGAGA | CAAATACAATGGCAGCTGGA | TGCAGGAGCATATGACCAAC | 59.7 | 305 | INTERGENIC | | | | |
| CaPOPI_4865 | Ca7 | 11960847 | ATAT/ATGTATGGATGTAT | TGATGGCACATCATTGTCCT | AACCCCATCCCCAAAATAAG | 59.9 | 221 | INTERGENIC | | | | |
| CaPOPI_4866 | Ca7 | 11961138 | CAAA/CAA | AGAGCCCCACTGGGTTACTT | ATTGAATAGGGAAGGGGTGG | 60.0 | 645 | INTERGENIC | | | | |
| CaPOPI_4867 | Ca7 | 11979039 | AAATAATAATAATAATAAT/AAATAATAATAAT | TCACTTGCCCAAATCATTCA | CATGCCTAGGATGAAGGAAGA | 60.0 | 558 | INTERGENIC | | | | |
| CaPOPI_4868 | Ca7 | 11990830 | TATAAAATAAA/TATAAAATAAAATAAA | TGATCGCATGAATAAAATGGTT | TCACTTGCCATGTGTACGCT | 59.3 | 193 | INTERGENIC | | | | |
| CaPOPI_4869 | Ca7 | 11990906 | CTA/CTATATA | TGATCGCATGAATAAAATGGTT | TCACTTGCCATGTGTACGCT | 59.3 | 193 | INTERGENIC | | | | |
| CaPOPI_4870 | Ca7 | 12006713 | AT/ATGT | CCCAATCAGTGGCAGAACTT | TGGCTCTACAATGGATGCAG | 60.1 | 720 | INTERGENIC | | | | |

| INDEL marker IDs | Chromosomes/ unanchored scaffolds | Physical positions (bp) | InDels (*Kabuli* reference genome-CDC Frontier/PI) | Forward primers (5'-3') | Reverse primers (5'-3') | Anneling temperature (0C) | Expected amplified product size (bp) | Structural annotation: Sequence components of *kabuli* genome | Structural annotation: *Kabuli* gene accession IDs | Functional annotation: NCBI-KOG | Functional annotation: TFs | Functional annotation: NCBI-nr database |
|---|---|---|---|---|---|---|---|---|---|---|---|---|
| CaPOPI_4871 | Ca7 | 12024461 | AATATATATATATA/AATATATATATATATATA | TGGTTCACAAATCAGATGGG | GATGCATCCCGATGAAAAA | 59.3 | 260 | DRR | Ca_09302 | | WRKY | DNA-binding WRKY |
| CaPOPI_4872 | Ca7 | 12024656 | TGAAGAAGAAGAAGAAGAA/TGAAGAAGAAGAAGAA | TGGTTCACAAATCAGATGGG | CACACTAATAAGTGCTCTAAGGACCA | 59.3 | 746 | INTERGENIC | | | | |
| CaPOPI_4873 | Ca7 | 12092590 | GAAAAAAAGATAAA/GAAAAAAAGATAAAAAAAAAGATAAA | AAATTGGAGTTGCCAAACAGA | TCGTCTTAAAATCCACAAACCC | 59.6 | 757 | INTERGENIC | | | | |
| CaPOPI_4874 | Ca7 | 12106813 | T/TATACCCAAAAAA | ATTGGGAGGAGATTGTGTGC | ATTGGAGATCAGCATTTGCC | 59.9 | 567 | INTERGENIC | | | | |
| CaPOPI_4875 | Ca7 | 12125579 | C/CG | AGATGGAGGCAGAGGGAAAT | AAGAAATGCAGGTTGGATGG | 60.0 | 689 | DRR | Ca_09295 | | AP2 | Pathogenesis-related transcriptional factor/ERF, DNA-binding |
| CaPOPI_4876 | Ca7 | 12138547 | TG/T | TGAGTTGTGTCAAAGGCAGAA | TGAAGCAAATTCGTCAATCAA | 59.5 | 710 | INTERGENIC | | | | |
| CaPOPI_4877 | Ca7 | 12143576 | ATTTTTTTT/ATTTTTTTTTT | AAACCAAATATTTTCGGCCC | TTTGTTTACAAGGTATTCTTGCCA | 60.0 | 454 | INTERGENIC | | | | |
| CaPOPI_4878 | Ca7 | 12145921 | TTTATT/TTT | TTTCAATCCACACAGAAACGA | TCTTTGGGTCAAAACTTGGC | 59.2 | 646 | INTERGENIC | | | | |
| CaPOPI_4879 | Ca7 | 12210192 | TTGTGTGTGTGTGTGT/TTGTGTGTGTGTGTGTGT | TGAAAATATGTGCTCCCGTCT | ACGAAGGGCAACAACAAAC | 59.6 | 823 | INTRON | Ca_09287 | Q | | O-methyltransferase, family 3 |
| CaPOPI_4880 | Ca7 | 12212766 | ATTTTTT/ATTTTTTT | GGGCAATTTGGAAATCAAGA | TCGCTTTTGATGATGACACC | 59.9 | 719 | INTERGENIC | | | | |
| CaPOPI_4881 | Ca7 | 12212967 | TTAAACCA/T | ATCACCACATCGTCCCTAAA | CAAAGGCTTGGACAAAGAGC | 57.9 | 754 | INTERGENIC | | | | |
| CaPOPI_4882 | Ca7 | 12227697 | ATT/AT | TCTTGTAGTCAACGAATCTTCATCA | GTCAACGAGAATTGTTGCCA | 60.2 | 431 | INTERGENIC | | | | |
| CaPOPI_4883 | Ca7 | 12234527 | AA/AATA | AGACGACGAACAACACAACG | ATTTTCGATTTCCGAGGACC | 59.8 | 353 | INTERGENIC | | | | |
| CaPOPI_4884 | Ca7 | 12266841 | CATT/CATTGAAAAGTATATT | GTTGCAACATCGGAGGTTTT | AAAAAGCGGGGGATTTAGAA | 60.0 | 508 | INTERGENIC | | | | |
| CaPOPI_4885 | Ca7 | 12280015 | TAAAA/TAAAAA | TGGTTTTAATGTGTTGCGGA | TTTGGGATAATTTCACCCTTTG | 60.0 | 501 | INTERGENIC | | | | |

| INDEL marker IDs | Chromosomes/ unanchored scaffolds | Physical positions (bp) | InDels (*Kabuli* reference genome-CDC Frontier/PI) | Forward primers (5'-3') | Reverse primers (5'-3') | Anneling temperature (0C) | Expected amplified product size (bp) | Structural annotation | | Functional annotation | | |
|---|---|---|---|---|---|---|---|---|---|---|---|---|
| | | | | | | | | Sequence components of *kabuli* genome | *Kabuli* gene accession IDs | NCBI-KOG | TFs | NCBI-nr database |
| CaPOPI_4886 | Ca7 | 12283477 | C/CGACACATAATT | ATAGCCCCTCGTACAAACCC | GGGTCTATCTCGAATTGGCA | 60.2 | 943 | INTERGENIC | | | | |
| CaPOPI_4887 | Ca7 | 12285646 | TTCTC/T | TTGAAACCTCATTTGGGAGG | CTTCCGTTTGTGGTGGTCTT | 59.9 | 269 | INTERGENIC | | | | |
| CaPOPI_4888 | Ca7 | 12291131 | TCC/TCCC | TAAAGCCAACAACCACACCA | GGCCCAATTATTTGCCTCTT | 60.0 | 190 | INTERGENIC | | | | |
| CaPOPI_4889 | Ca7 | 12300332 | GTAAATTCCCTA/GTA | TTTTCATCCATCGGTCATCA | TGGAAGGCCTTTTTATAGCAAC | 59.9 | 665 | INTERGENIC | | | | |
| CaPOPI_4890 | Ca7 | 12321093 | GTT/GT | GAGCGAAAATGAAACGAAGC | GATGGGTTAGCCAGCTTTCA | 60.0 | 854 | INTRON | Ca_09281 | R | G2-like | RNA recognition motif domain |
| CaPOPI_4891 | Ca7 | 12332691 | TCACAC/TCAC | CATGGGAGATCGATTTGGAC | CCTGCAACAAGTACCCGAAT | 60.3 | 659 | INTERGENIC | | | | |
| CaPOPI_4892 | Ca7 | 12339660 | CAAA/CAAAA | AAATGTGGGTTGAGGGTCAG | GTGTCGTGTCTGGCTGAGAA | 59.8 | 280 | DRR | Ca_09278 | | | |
| CaPOPI_4893 | Ca7 | 12347854 | CATATATATAT/CATATATATATATAT | AATCATGCAACTACACCACCA | AACGTCGTTCCAGACACTCC | 58.9 | 434 | INTERGENIC | | | | |
| CaPOPI_4894 | Ca7 | 12348225 | TA/T | TTTTGAAAAGGACGGGATTG | GCCAAATGGTTTGTTGCTCT | 59.9 | 534 | INTERGENIC | | | | |
| CaPOPI_4895 | Ca7 | 12361782 | TAA/TAAA | GACAAAAATGATGCGGTCAA | GAAAAACTTAGGCAACCCCC | 59.5 | 300 | INTERGENIC | | | | |
| CaPOPI_4896 | Ca7 | 12422509 | GATGTATA/GATGTATATGTATA | TTCAAAAGCAAAAATTATTTCCAA | TGTTGATGTGGTTTGCCATT | 59.1 | 464 | INTERGENIC | | | | |
| CaPOPI_4897 | Ca7 | 12432563 | GTTT/GTTTT | GCCATAGTTGGTGTTGGCTT | CCAAAGCCTTTTCTGAAGCA | 60.0 | 819 | INTRON | Ca_09272 | | | |
| CaPOPI_4898 | Ca7 | 12477809 | ATTT/ATTTT | AATAGGTTGCAGTTGGTCGG | CGCAACACATAAGACATGGG | 60.0 | 849 | INTRON | Ca_09269 | Z | | CTLH, C-terminal LisH motif |
| CaPOPI_4899 | Ca7 | 12483012 | T/TAAGGAG | GCGCAAACTGCAAAGTCATA | CCTTTTCGGACAGGAAATCA | 60.0 | 723 | DRR | Ca_09268 | R | bHLH | Pentatricopeptide repeat |
| CaPOPI_4900 | Ca7 | 12489580 | CG/C | CATGCTTCTCAGGTCCCATT | TTCTCCTTCAACGCACACTG | 60.1 | 748 | INTERGENIC | | | | |

| INDEL marker IDs | Chromosomes/ unanchored scaffolds | Physical positions (bp) | InDels (*Kabuli* reference genome-CDC Frontier/PI) | Forward primers (5'-3') | Reverse primers (5'-3') | Anneling temperature (0C) | Expected amplified product size (bp) | Structural annotation | | Functional annotation | | |
|---|---|---|---|---|---|---|---|---|---|---|---|---|
| | | | | | | | | Sequence components of *kabuli* genome | *Kabuli* gene accession IDs | NCBI-KOG | TFs | NCBI-nr database |
| CaPOPI_4901 | Ca7 | 12495945 | TA/T | GATTTCCAATCCCATGTTGC | AAAATGGAAGTGGCATGAGG | 60.1 | 961 | INTERGENIC | | | | |
| CaPOPI_4902 | Ca7 | 12506632 | TTATCATATCATATCATATCA/TTATCATATCATATCA | AAAGAGCATGTTAGCCCACG | GGACTGTGACCAGGGTCTGT | 60.3 | 686 | INTERGENIC | | | | |
| CaPOPI_4903 | Ca7 | 12520999 | GAAA/GAAAA | AGCGCTCAAAAATTGCATCT | TGTGTGCGGTCATTAATTGG | 60.0 | 377 | DRR | Ca_09264 | | | |
| CaPOPI_4904 | Ca7 | 12524095 | TG/T | TGAGATTGGAGGGATGAAGG | ATGCAAAAATTCATCGCAAA | 60.0 | 334 | INTERGENIC | | | | |
| CaPOPI_4905 | Ca7 | 12550642 | TAATTAT/TAATTATGCAAGAATTAT | TTTTAGGCCCACAACTCCTG | ATTTGACCATCACCCCAAAA | 60.1 | 419 | INTERGENIC | | | | |
| CaPOPI_4906 | Ca7 | 12583983 | TC/T | GCCATTCCAAATGACGACTT | GGAGCGTGTGGTTTATGTCA | 59.9 | 204 | INTERGENIC | | | | |
| CaPOPI_4907 | Ca7 | 12585103 | TATTTAATTTAAT/TATTTAAT | ACCCGTGCACATCAATTAAA | TCACCATACGTTTGTGTACGG | 58.9 | 762 | INTERGENIC | | | | |
| CaPOPI_4908 | Ca7 | 12608444 | GGTGTG/GGTG | ATGCATTGAAGGAAGGGAAA | TCCAAAATGTATGCCACCAA | 59.5 | 311 | DRR | Ca_09260 | | SBP | Domain of unknown function DUF547 |
| CaPOPI_4909 | Ca7 | 12609027 | TTATAT/TTAT | TTGGTGGCATACATTTTGGA | ACAAGCATGATAGAGGCAAAA | 59.8 | 780 | DRR | Ca_09260 | | SBP | Domain of unknown function DUF547 |
| CaPOPI_4910 | Ca7 | 12645517 | TAAATTAAAAATTAAAAA/TAAATTAAAAATTAAAAAATTAAAAA | TGGACCAAAGCAGAACACAG | GGAGGCAGTCATTAGTTGGG | 59.9 | 500 | INTERGENIC | | | | |
| CaPOPI_4911 | Ca7 | 12653885 | TTTAATTAAT/TTTAAT | AAGTGTGTTGAAGATGAAGCGA | ATGCACTTGCACTGCCATAA | 59.9 | 400 | INTERGENIC | | | | |
| CaPOPI_4912 | Ca7 | 12663192 | AGGGG/AGGGGG | AACTTTCCCCATCCTCCAAC | GGAGCTAGCAGAAGCAGCAT | 60.2 | 906 | DRR | Ca_09255 | T | FAR1 | Protein kinase, catalytic domain |
| CaPOPI_4913 | Ca7 | 12689465 | AATGA/AA | GCACGTTGCAAGTCAGAAGA | GTTGTGGTTCGTCATGGTCA | 60.2 | 496 | INTRON | Ca_09253 | G | | Phosphoglycerate/bisphosphoglycerate mutase, active site |
| CaPOPI_4914 | Ca7 | 12712927 | AAAAGAA/AAAAGAAAGAGGAACAAAGAA | CAGGGGTTTTAGGATTTGGG | CTGTGTGTTGCTGGGTCCTA | 60.5 | 610 | INTERGENIC | | | | |
| CaPOPI_4915 | Ca7 | 12724633 | AATATATATAT/AATATATATATAT | GTGCATTTTCCCATGTCCTT | ACGAAATACGATGAAAGCCG | 59.8 | 571 | INTERGENIC | | | | |

| INDEL marker IDs | Chromosomes/ unanchored scaffolds | Physical positions (bp) | InDels (*Kabuli* reference genome-CDC Frontier/PI) | Forward primers (5'-3') | Reverse primers (5'-3') | Anneling temperature (0C) | Expected amplified product size (bp) | Structural annotation | | Functional annotation | | |
|---|---|---|---|---|---|---|---|---|---|---|---|---|
| | | | | | | | | Sequence components of *kabuli* genome | *Kabuli* gene accession IDs | NCBI-KOG | TFs | NCBI-nr database |
| CaPOPI_4916 | Ca7 | 12730150 | TGAAGAG/TG | CGCAGCTGTCATTTTCTTCA | TTCGACGCAGATACTTGCAC | 60.1 | 426 | INTRON | Ca_09247 | | LBD | Lateral organ boundaries, LOB |
| CaPOPI_4917 | Ca7 | 12745134 | AAACTT/AAACTTGAATTATATAACTT | TTTGGAACACAACAAACTCCA | GTAGGGTTAAAGGGGACCGA | 59.1 | 206 | INTERGENIC | | | | |
| CaPOPI_4918 | Ca7 | 12762980 | ATATTATT/ATATT | CGTTGTGTTTTGCAATGACC | GTTCACCATTGGACCGAGTT | 60.0 | 516 | DRR | Ca_09243 | H | | Tetrapyrrole methylase |
| CaPOPI_4919 | Ca7 | 12773412 | GT/GTT | TTGGACTTGAGAAAGGTGGG | TTGTGTTGACTGGTTTGGGA | 60.1 | 747 | INTRON | Ca_09242 | | LBD | Disease resistance protein |
| CaPOPI_4920 | Ca7 | 12808768 | AATATATATATATATATATATATAT/AATATATATATATATATATAT | AATCATGAGCCCAACGTTTC | GGCGGCATATACCGTCATAA | 59.9 | 874 | DRR | Ca_09239 | Q | | Multicopper oxidase, type 1 |
| CaPOPI_4921 | Ca7 | 12810410 | A/AC | GGTAGATCGGCTCAAGAACG | AAAGTGTTTCCACAAACGGC | 59.8 | 391 | DRR | Ca_09238 | | Dof | Zinc finger, Dof-type |
| CaPOPI_4922 | Ca7 | 12810847 | CATATATATATATATATATATATA/CATATATATATATATATATATATATATA | GCCGTTTGTGGAAACACTTT | ATGCTGCAAGTTTCGATTGT | 60.0 | 509 | DRR | Ca_09238 | | Dof | Zinc finger, Dof-type |
| CaPOPI_4923 | Ca7 | 12844900 | CTTATTTTTATTTTTATTTTTATTTTTATTTTTATTTTTATTTTTATTTTTA/CTTATTTTTATTTTTATTTTTATTTTTATTTTTATTTTTATTTTTATTTTTA | TGCTCTTCAAAAGTAATTGTTCC | AGATCGACTGCTCCCAAATG | 57.6 | 458 | INTRON | Ca_09236 | | | Alpha/beta hydrolase fold-1 |
| CaPOPI_4924 | Ca7 | 12848385 | C/CTAG | AGGTTCCCAACGGGAATATC | TGCTCGAATCTTATGTCCCC | 60.0 | 367 | DRR | Ca_09235 | | | |
| CaPOPI_4925 | Ca7 | 12850002 | AATTTACAACAT/A | CGTATCTGCAGCTCAGCAAG | ACTCTGACTTGTCCGGTTGG | 59.9 | 622 | INTRON | Ca_09235 | | | |
| CaPOPI_4926 | Ca7 | 12853842 | GTTT/GTTTT | CCAAACGTGGAAGTGGAACT | GCTTGGTCGACGAATCTCTC | 60.0 | 589 | INTRON | Ca_09235 | | | |
| CaPOPI_4927 | Ca7 | 12858631 | CT/CTAT | AATGAAGTAGGGTGGCGATG | TGAGAGAGAGAGAAAGTAGCACCA | 60.0 | 487 | INTERGENIC | | | | |
| CaPOPI_4928 | Ca7 | 12865284 | TTAA/T | TGACACTCATTGTGTCACGG | CGGACAGCATTGTTTGTTTG | 59.1 | 177 | INTERGENIC | | | | |
| CaPOPI_4929 | Ca7 | 12897407 | ACCCC/ACCCCC | TGATGTTTTTCCATCCAGCA | ACCTCACAAATGCACTCGAA | 60.0 | 707 | DRR | Ca_09230 | O | | Peptidase S9, prolyl oligopeptidase, catalytic domain |
| CaPOPI_4930 | Ca7 | 12921443 | T/TAG | TCAGAGTTCTCTCCACCGCT | AGGGAGGTGTTGGTGTTGAG | 60.1 | 526 | DRR | Ca_09228 | | | |

| INDEL marker IDs | Chromosomes/ unanchored scaffolds | Physical positions (bp) | InDels (*Kabuli* reference genome-CDC Frontier/PI) | Forward primers (5'-3') | Reverse primers (5'-3') | Anneling temperature (0C) | Expected amplified product size (bp) | Structural annotation | | Functional annotation | | |
|---|---|---|---|---|---|---|---|---|---|---|---|---|
| | | | | | | | | Sequence components of *kabuli* genome | *Kabuli* gene accession IDs | NCBI-KOG | TFs | NCBI-nr database |
| CaPOPI_4931 | Ca7 | 12923877 | AC/A | GAAGTGTAGCCTTTCAGGCG | CCTGGACTCGACAAAAGGAA | 60.0 | 556 | INTERGENIC | | | | |
| CaPOPI_4932 | Ca7 | 13032221 | TG/T | GGCAGTACAAGCATCAAGCA | AACGCTATCACACACCACGA | 60.0 | 480 | INTERGENIC | | | | |
| CaPOPI_4933 | Ca7 | 13032927 | GAAAAA/GAAAA | TTGAATGCAACCTTGGTGTC | GTTTGGAGACCTGTGCCCTA | 59.5 | 602 | INTERGENIC | | | | |
| CaPOPI_4934 | Ca7 | 13033158 | AAAA/AAAAGAAA | TAGGGCACAGGTCTCCAAAC | GAATATGTCGAGTCGCGCTT | 60.1 | 145 | INTERGENIC | | | | |
| CaPOPI_4935 | Ca7 | 13037732 | TA/TAA | AAATGTGCTGATCGGGTTTC | GGCCGATACGATAATGGGTA | 59.9 | 715 | INTERGENIC | | | | |
| CaPOPI_4936 | Ca7 | 13060580 | TCGTGACC/TCGTGACCGTGACC | CCCTTCACGTGAGGATTGTT | CCAGTTTTGCCACCCTACAT | 60.0 | 767 | DRR | Ca_09222 | | MYB_related | BTB/POZ-like |
| CaPOPI_4937 | Ca7 | 13066626 | CAGTTAGTTAGTTAGTT/CAGTTAGTTAGTT | GGAAAGGTGCACACAAGTGA | GAGACCGAATCCGACTTGAA | 59.7 | 442 | INTERGENIC | | | | |
| CaPOPI_4938 | Ca7 | 13071654 | GAAGTAAG/GAAG | ACTCGCGATTCATCAACCTT | TTTGTGGCTAGAGGCTATGACA | 59.7 | 445 | DRR | Ca_09221 | | | Cold acclimation WCOR413 |
| CaPOPI_4939 | Ca7 | 13178303 | TCAT/TCATATCAAACAT | GGATCTGCTGCTGTTTCACA | AAAACACTGGACCCACCATC | 60.0 | 338 | INTRON | Ca_16019 | | | Uncharacterised protein family UPF0497, trans-membrane plant |
| CaPOPI_4940 | Ca7 | 13188851 | TTAAATAAA/TTAAATAAATAAA | CTGCAGCAAAAACAGGACAA | TGCATCGTACCGTCGTGTAT | 60.0 | 424 | INTERGENIC | | | | |
| CaPOPI_4941 | Ca7 | 13193883 | CG/CGG | TTCGACATACTAAATTAGCCTCACA | TCTTAAACCGAGACGAACAAAA | 59.3 | 412 | INTERGENIC | | | | |
| CaPOPI_4942 | Ca7 | 13283430 | TAAAAAAA/TAAAAAAAAA | ACATCAGGCTGTCTGGGAAT | CCATATTCATCCATCGCCAC | 59.5 | 747 | INTERGENIC | | | | |
| CaPOPI_4943 | Ca7 | 13285824 | AAAAACAAT/AAAAACAATTCTCAAAACAAT | AACGACGGTCTTGTAAAGCAA | CACAAGACCCGAAAAAGGAA | 59.8 | 628 | INTERGENIC | | | | |
| CaPOPI_4944 | Ca7 | 13299994 | GC/G | CCCTAAGATTTTGAGCAGCG | GAAGGTGGGCTTGACTATGC | 60.0 | 213 | INTERGENIC | | | | |
| CaPOPI_4945 | Ca7 | 13306728 | AAGAGAGA/AAGAGA | TGTGTTTGGATAAGAGGGAAAAA | TTTCTGGACCATTGCAGTTATG | 59.9 | 185 | INTERGENIC | | | | |

| INDEL marker IDs | Chromosomes/ unanchored scaffolds | Physical positions (bp) | InDels (*Kabuli* reference genome-CDC Frontier/PI) | Forward primers (5'-3') | Reverse primers (5'-3') | Anneling temperature (0C) | Expected amplified product size (bp) | Structural annotation | | Functional annotation | | |
|---|---|---|---|---|---|---|---|---|---|---|---|---|
| | | | | | | | | Sequence components of *kabuli* genome | *Kabuli* gene accession IDs | NCBI-KOG | TFs | NCBI-nr database |
| CaPOPI_4946 | Ca7 | 13313904 | TAAAA/TAAAAA | ACCCTGGCATTAATGAACCA | TCGCATTTGATTCTTTTGGA | 60.2 | 694 | INTRON | Ca_16026 | K | TALE | Homeobox |
| CaPOPI_4947 | Ca7 | 13321942 | TTT/TTTGTT | CTTTGCTCTTCACCACCTCC | GTTGGTTCCCAAAGGACGTA | 59.8 | 774 | INTERGENIC | | | | |
| CaPOPI_4948 | Ca7 | 13381675 | GTTTTT/GTTTTTT | AAGCCATCAAGCGAACCTTA | CTCTAGACACGCGGTGAACA | 59.8 | 310 | DRR | Ca_16030 | | HD-ZIP | Pectate lyase/Amb allergen |
| CaPOPI_4949 | Ca7 | 13456910 | GA/G | ATTGGACAACCGCGTAAAAT | CCCATGAAATCACAATAAAATCC | 59.3 | 630 | INTERGENIC | | | | |
| CaPOPI_4950 | Ca7 | 13467995 | TA/TAAA | TGCACTTGGAAAATACACCG | TTCTTATTCTCCCACCGACG | 59.6 | 545 | DRR | Ca_16035 | I | | GNS1/SUR4 membrane protein |
| CaPOPI_4951 | Ca7 | 13468281 | TA/TAATGA | AAACAAGCGCGATGTTTTTC | TTCTTATTCTCCCACCGACG | 60.3 | 268 | DRR | Ca_16035 | I | | GNS1/SUR4 membrane protein |
| CaPOPI_4952 | Ca7 | 13479302 | AGA/AGAGGA | AATGCACATACCAGCATCCA | TAGGGCCAATGAAAATGAGG | 60.0 | 505 | INTERGENIC | | | | |
| CaPOPI_4953 | Ca7 | 13495702 | ATAA/ATAAGTAA | TGGACATTTTCGTAAAAGGGA | CTCACTGAGGTTGGGTTGGT | 59.4 | 581 | INTERGENIC | | | | |
| CaPOPI_4954 | Ca7 | 13497952 | CTATATA/CTATATATA | TTGGCATTTCGGGTTCTATC | GCCTACTAGACGCCACCATC | 59.9 | 552 | INTERGENIC | | | | |
| CaPOPI_4955 | Ca7 | 13500441 | TAAAAAAA/TAAAAAAAAA | GGGACAAGCATTGCTCACTT | TGTCATTTCTGGCGTAGTGTG | 60.3 | 994 | INTERGENIC | | | | |
| CaPOPI_4956 | Ca7 | 13522224 | TTATATATATATATATAT/TTATATATATATATATATAT | AAGGGTTTGTGGTTTTGCAG | ACCACCACACTCCCTCTCAC | 60.0 | 314 | INTERGENIC | | | | |
| CaPOPI_4957 | Ca7 | 13599607 | TCAATTAATA/T | TGTGGTGTATGGAGGTTTTCC | ATGTGGTGCTGCGAATAACA | 59.7 | 127 | INTERGENIC | | | | |
| CaPOPI_4958 | Ca7 | 13622541 | GCCCC/GCCC | TTTCTTCATCCGCGTTATCC | CCGGGATGTTTCTCTTGGTA | 60.0 | 661 | INTERGENIC | | | | |
| CaPOPI_4959 | Ca7 | 13655144 | TAAAAAAA/TAAAAAA | CGAGACATTGAGGTAACACGG | TTGGCATCGTTGTGTTGATT | 60.6 | 597 | DRR | Ca_16047 | DR | | Leucine-rich repeat |
| CaPOPI_4960 | Ca7 | 13656688 | GTAATACT/GTAATACTAATACT | GGAAGAGGTGGCAGAGACAG | ATGATCCATCAGCGTCATCA | 60.0 | 688 | INTERGENIC | | | | |

| INDEL marker IDs | Chromosomes/ unanchored scaffolds | Physical positions (bp) | InDels (*Kabuli* reference genome-CDC Frontier/PI) | Forward primers (5'-3') | Reverse primers (5'-3') | Anneling temperature (0C) | Expected amplified product size (bp) | Structural annotation | | Functional annotation | | |
|---|---|---|---|---|---|---|---|---|---|---|---|---|
| | | | | | | | | Sequence components of *kabuli* genome | *Kabuli* gene accession IDs | NCBI-KOG | TFs | NCBI-nr database |
| CaPOPI_4961 | Ca7 | 13676108 | ATTTT/ATTT | AACTTTCAGGAAGCAGCCAA | TTTTGCCCGTACATTTGTGA | 60.0 | 651 | INTRON | Ca_16049 | | | Protein of unknown function DUF81 |
| CaPOPI_4962 | Ca7 | 13684363 | AAGA/AA | TAAGCATTTCCATTGTGGCA | TGAGTTTGTTGTTTCTTCAAATTCT | 60.1 | 485 | INTERGENIC | | | | |
| CaPOPI_4963 | Ca7 | 13684569 | TA/T | AGGTTGGCAAACTTGAGGAT | CGAGGTGCCATAACAAATGA | 58.6 | 776 | INTERGENIC | | | | |
| CaPOPI_4964 | Ca7 | 13686933 | ATTATTATTTATTATTTATTATTTA/ATTATTATTTATTATTTATTATTTATTATTTA | CGAAGTTCGAACCTTGAAGC | TTAATCTGCGGGGATTTTTG | 60.0 | 363 | INTERGENIC | | | | |
| CaPOPI_4965 | Ca7 | 13690598 | CT/CTGTATCGGAGTT | GGCGGAGAGATGAGTTTTCA | TGTGTATCGTTGTCCCTTCG | 60.3 | 890 | INTERGENIC | | | | |
| CaPOPI_4966 | Ca7 | 13800047 | TTATATATATATATATATATATATATATATA/TTATATATATATATATATATATATATA | ATGGTGGAGGTGGATGGTAG | TTGACTCAATTTATTCGTTATGCAA | 59.7 | 649 | INTERGENIC | | | | |
| CaPOPI_4967 | Ca7 | 13909902 | AATATATATATATATATA/AATATATATATATATATATA | TCGTCATTTGAAACAGCGAC | GAAAACTTCGGCAATGTGGT | 59.8 | 611 | INTERGENIC | | | | |
| CaPOPI_4968 | Ca7 | 14026857 | GAAAAA/GAAAA | TACAAATTGGATGCGGTTGA | TCTTCCACCCAGTTTTCACC | 59.9 | 233 | INTERGENIC | | | | |
| CaPOPI_4969 | Ca7 | 14075293 | CATATATATATATATAT/CATATATATATATAT | TTTGTTTAGCCACAACTTTTGA | CGTGCTCGGGATTTATTTGT | 57.6 | 862 | INTERGENIC | | | | |
| CaPOPI_4970 | Ca7 | 14157143 | T/TTCC | TGTTTAGATCAACAATTCCCCA | CAAAGGCAGCTGTCAACAAA | 59.3 | 522 | INTERGENIC | | | | |
| CaPOPI_4971 | Ca7 | 14317932 | A/AT | CGAAAAGCGACAAAGAAAGG | GCTTCGGAGTAGTCACTGCC | 60.0 | 938 | INTERGENIC | | | | |
| CaPOPI_4972 | Ca7 | 14352764 | CAATAAATAAATAAATAAATAAATAAATAAATAAATAAATAAA/CAATAAATAAATAAATAAATAAATAAATAAATAAA | GCCTCTTCCTTCTTTTGCCT | TTTATTCATGCCCCGAAGAC | 60.0 | 165 | INTERGENIC | | | | |
| CaPOPI_4973 | Ca7 | 14379603 | ACC/AC | TTCCCCATTCTTAACGCTCA | GGTAAGGGCTCCTTCCAAAC | 60.6 | 452 | INTERGENIC | | | | |
| CaPOPI_4974 | Ca7 | 14780927 | ATTTT/ATTTTT | AAATGAAATTTCCGGGAAGG | AGGGATGGAAAACACACGAC | 60.1 | 473 | DRR | Ca_09968 | O | bHLH | Heat shock protein DnaJ, N-terminal |
| CaPOPI_4975 | Ca7 | 14803293 | CTATTATTATTATTATTATTATTATTATTATTATTATTATTAT/CTATTATTATTATTATTATTATTATTATTATTATTATTATTAT | CGACAGTTGATTGCGTATGG | ACGTAGGAGCAAGATCGCAT | 60.1 | 196 | INTERGENIC | | | | |

| INDEL marker IDs | Chromosomes/ unanchored scaffolds | Physical positions (bp) | InDels (*Kabuli* reference genome-CDC Frontier/PI) | Forward primers (5'-3') | Reverse primers (5'-3') | Anneling temperature (0C) | Expected amplified product size (bp) | Structural annotation | | Functional annotation | | |
|---|---|---|---|---|---|---|---|---|---|---|---|---|
| | | | | | | | | Sequence components of *kabuli* genome | *Kabuli* gene accession IDs | NCBI-KOG | TFs | NCBI-nr database |
| CaPOPI_4976 | Ca7 | 14831868 | AATATATATATATATATATATATATATATATATATA/AATATATATATATATATATATATATATATATATATATATATATATATA | GGCCTATGGCCTGACCTACT | TGCAACAACCTTCCCATAAA | 60.5 | 796 | INTERGENIC | | | | |
| CaPOPI_4977 | Ca7 | 14896597 | CTTTTTTTTT/CTTTTTTTTTT | TCTTTTGAGGGGTAAACGGA | AAGCATCCGCTTTTTAACCA | 59.5 | 351 | DRR | Ca_09961 | P | | Rhodanese-like |
| CaPOPI_4978 | Ca7 | 14992578 | TTATATATATATATATATATATAT/TTATATATATATATATATATAT | GACTTGCACAACGAAGCAAA | GCCCTATTTAATGCAGGCAA | 60.0 | 345 | INTRON | Ca_09954 | A | | RNA helicase, ATP-dependent, DEAD-box, conserved site |
| CaPOPI_4979 | Ca7 | 15094001 | AATATATATATATATATAT/AATATATATATATATAT | GGGACTGGGGTCATTTAGGT | AGCGACATCCAACTGCGTAT | 60.1 | 675 | INTERGENIC | | | | |
| CaPOPI_4980 | Ca7 | 15202855 | ATTTTTT/ATTTTTTT | TCGTGATTCCCTAGTCCACC | CAATCTCGCGCTCAAATGTA | 59.9 | 692 | INTERGENIC | | | | |
| CaPOPI_4981 | Ca7 | 15268881 | AAATAATAATAATAATAATAATAATAATAATAATAATAATAATAATAATAATA/AAATAATAATAATAATAATAATAATAATAATAATAATAATAATAATAATAATA | CAAATCAGCAAACACTTTACCC | TAGTGCCTGAAGCCTTGGTT | 58.7 | 549 | INTERGENIC | | | | |
| CaPOPI_4982 | Ca7 | 15278671 | GAAAAAAAAAAA/GAAAAAAAAAA | CATTTCAAGTTGGGGCAGTT | TGGATTGCGTAGATCGAACA | 60.0 | 617 | INTERGENIC | | | | |
| CaPOPI_4983 | Ca7 | 15392479 | AAATAATAATAATAATAATAATAATAATAATAATAATAAT/AAATAATAATAATAATAATAATAATAATAATAATAATAATAAT | CTCGTGTCTCAAAATCCAAGG | TCACGTGAGGCAATATCAAG | 59.7 | 911 | INTERGENIC | | | | |
| CaPOPI_4984 | Ca7 | 15394134 | ATATTATTATTATTATTATTATTATTATTATTA/ATATTATTATTATTATTATTATTATTATTATTATTA | GAGGGTTTGCAGCTCTTGTC | TTGCACTCCACTGTTTTTCG | 60.0 | 341 | INTERGENIC | | | | |
| CaPOPI_4985 | Ca7 | 15467222 | TTTATTATTATTATTATTATTATTATTATTATTATTATTATTAT/TTTATTATTATTATTATTATTATTATTATTATTATTATTATTATTATTATTAT | TGGGAAACGGTGCTAAACTT | TCCATTTACGGCAAATTCAA | 59.6 | 716 | INTERGENIC | | | | |
| CaPOPI_4986 | Ca7 | 15518207 | CATATATATATATATATATATATATATATATAT/CATATATATATATATATATATATATATATATATATAT | TTCTCATTGTGGCTGCTTTG | AAATCAATATGTTTTACATGTGTTTGA | 60.0 | 581 | DRR | Ca_09915 | | | |
| CaPOPI_4987 | Ca7 | 15626341 | TTATATATATATATATATATATATAT/TTATATATATATATATATATAT | ATGAGTGCGTTTGTTTCACG | CATGATGCTCTCCACATACCA | 59.8 | 417 | DRR | Ca_09907 | OR | | Ubiquitin-associated/translation elongation factor EF1B, N-terminal |
| CaPOPI_4988 | Ca7 | 15667150 | CTTTTTTTTTT/CTTTTTTTTTTT | TTTCAAGCTCCATTTTCTTCG | CGGAAACATTTGTAAATCAATCA | 59.5 | 177 | INTERGENIC | | | | |
| CaPOPI_4989 | Ca7 | 15688440 | AATATATATATATATATATATATATATAT/AATATATATATATATATATATATATATATATATAT | TCATGGAGAACATTCTTCGTTTT | TTTCCGTCTTGATGCAATGT | 60.0 | 865 | INTERGENIC | | | | |
| CaPOPI_4990 | Ca7 | 15841363 | GC/GTGATC | CTTTCGGAAGTTCAGGCAAC | GGTGCTGTCTCGGATGAAAT | 59.9 | 415 | INTERGENIC | | | | |

| INDEL marker IDs | Chromosomes/ unanchored scaffolds | Physical positions (bp) | InDels (*Kabuli* reference genome-CDC Frontier/PI) | Forward primers (5'-3') | Reverse primers (5'-3') | Anneling temperature (0C) | Expected amplified product size (bp) | Structural annotation | | Functional annotation | | |
|---|---|---|---|---|---|---|---|---|---|---|---|---|
| | | | | | | | | Sequence components of *kabuli* genome | *Kabuli* gene accession IDs | NCBI-KOG | TFs | NCBI-nr database |
| CaPOPI_4991 | Ca7 | 15902473 | TTATATATATATATATAT/TTATATATATATATATAT | TTGATAGGTGTTTGGAGCCC | GGACAACAAAGCGAGCCTAA | 59.9 | 593 | INTERGENIC | | | | |
| CaPOPI_4992 | Ca7 | 16001405 | AATTATTATTATTATTATTATTATTATTAT/AATTATTATTATTATTATTATTATTATTAT | AAAACACAACGTGCAAACGA | GGGTCACCAAGCACAGTTTT | 60.2 | 679 | INTERGENIC | | | | |
| CaPOPI_4993 | Ca7 | 16122158 | AAATAATAATAATAATAATAATAATA/AAATAATAATAATAATAATAATA | TCATTAGAAATTTCGTTTGGTCA | GGGTGAAATTGGTTTACAACG | 58.7 | 620 | INTERGENIC | | | | |
| CaPOPI_4994 | Ca7 | 16240640 | TCAACAACAA/TCAACAACAACAA | AACCCAAAGTTAGGGATCCAA | CACTTCACGTAGAGGACGCA | 59.7 | 306 | INTERGENIC | | | | |
| CaPOPI_4995 | Ca7 | 16354763 | ATTTTTTTTTTTT/ATTTTTTTTTTT | TATACCGGTTTTCCATCCCA | TCAAACTCATACCAATTCAAATCAA | 60.0 | 902 | INTERGENIC | | | | |
| CaPOPI_4996 | Ca7 | 16405742 | ATTTTTTTTTTT/ATTTTTTTTTT | TTGACTGCAATGCTTCGTTC | TGTAACTCCGGGATTAAAAGATG | 60.0 | 661 | INTERGENIC | | | | |
| CaPOPI_4997 | Ca7 | 16414718 | TGTCAATAAAAT/TGTCAATAAAATATTATAGTCAATAAAAT | GTGGTTCTTTTGTTGCCGTT | GATAGCTGACGTTTGCGACA | 60.0 | 607 | INTRON | Ca_09848 | | | Glucose-methanol-choline oxidoreductase, N-terminal |
| CaPOPI_4998 | Ca7 | 16453107 | CTATATATATATATATATATATATATAT/CTATATATATATATATATATATATATATATATATAT | TTGGGTTCGTGGTTTTCAAT | GCCGCATTTCAAATTCACTT | 60.2 | 259 | INTERGENIC | | | | |
| CaPOPI_4999 | Ca7 | 16548666 | TG/T | TCCCTTCTACTTGGTGGTGG | GTGATTCATGCTCCAAGGCT | 60.0 | 624 | DRR | Ca_20555 | R | | Sugar/inositol transporter |
| CaPOPI_5000 | Ca7 | 16610320 | TAAAAAAA/TAAAAAAAA | CCACATCTCGTCTCCCTCAT | TTGCAGGTCATGTGGCTAAC | 60.1 | 247 | INTERGENIC | | | | |
| CaPOPI_5001 | Ca7 | 16941588 | TAAA/TAAAA | TGCCGTAGTATTCACCACCA | TGCCATGAAATTCTGCCATA | 60.0 | 852 | INTERGENIC | | | | |
| CaPOPI_5002 | Ca7 | 17133790 | ATTTTTTT/ATTTTTT | GACACTACCCGGCATCAAGT | CCAACCCAAATAAATGAAAATCA | 60.0 | 530 | INTERGENIC | | | | |
| CaPOPI_5003 | Ca7 | 17139267 | GATTATTATTATTATTATTATTATTATTATTATTATTATTATTATT/GATTATTATTATTATTATTATTATTATTATTATTATTATT | AAAAAGCGCTGTTGTGTCCT | CCAAGTGAACAGTACGTGCC | 59.9 | 517 | INTERGENIC | | | | |
| CaPOPI_5004 | Ca7 | 17172621 | AATCATAT/AATCATATCATAT | GGCTAAACCTTGTCTTTGGC | AGACTCTGCTTGACCGGAAA | 58.8 | 541 | INTERGENIC | | | | |
| CaPOPI_5005 | Ca7 | 17374590 | CTTTTTTTTTTTT/CTTTTTTTTT | GCATCTGTAAAACAAGACCCG | ACATCGATAAGAGGCAGCGT | 59.6 | 525 | INTERGENIC | | | | |

| INDEL marker IDs | Chromosomes/ unanchored scaffolds | Physical positions (bp) | InDels (*Kabuli* reference genome-CDC Frontier/PI) | Forward primers (5'-3') | Reverse primers (5'-3') | Anneling temperature (0C) | Expected amplified product size (bp) | Structural annotation | | Functional annotation | | |
|---|---|---|---|---|---|---|---|---|---|---|---|---|
| | | | | | | | | Sequence components of *kabuli* genome | *Kabuli* gene accession IDs | NCBI-KOG | TFs | NCBI-nr database |
| CaPOPI_5006 | Ca7 | 17922655 | TGGGGGG/TGGGGG | AAGTCGAGTGCTTGAGCCAT | TCTTTGCGTGCGTGCTATAC | 60.0 | 606 | DRR | Ca_15831 | | bHLH | Protein kinase, catalytic domain |
| CaPOPI_5007 | Ca7 | 18121209 | TAAAA/TAAAAA | TGAAAAGCAATCCCAAATGT | AATGGGCATTCGTAAATCGT | 58.1 | 429 | INTERGENIC | | | | |
| CaPOPI_5008 | Ca7 | 18417161 | A/AG | TGGTGAAACTGCACTTCTGC | TCGGGGTGTTACACTTCACA | 60.0 | 593 | DRR | Ca_22130 | | | |
| CaPOPI_5009 | Ca7 | 18587623 | AAAGGCGGAAGGCGG/AAAGGCGG | CGATGATGACTGATTGGGGT | TGTTTGTGTTTTGAGGCGTT | 60.7 | 374 | INTERGENIC | | | | |
| CaPOPI_5010 | Ca7 | 18587996 | CTTTTTTTTT/CTTTTTTTT | GAGTGTACAAAGGCGGAAGG | GGGTGTTACAAGGAGTCTTGG | 59.7 | 865 | INTERGENIC | | | | |
| CaPOPI_5011 | Ca7 | 18588675 | TTATAT/TTATATAT | CCAAGACTCCTTGTAACACCC | GCACATATATACACGTAAAATCGCA | 58.6 | 299 | INTERGENIC | | | | |
| CaPOPI_5012 | Ca7 | 18588937 | ACTATATAT/AATATAT | CCAAGACTCCTTGTAACACCC | TTCTCCGTTTTCGTAAGACCA | 58.6 | 792 | INTERGENIC | | | | |
| CaPOPI_5013 | Ca7 | 18918117 | C/CG | TGAGTGATTCGGTGGGTTTT | CAACAACTTCTCATGCTCGC | 60.4 | 374 | INTERGENIC | | | | |
| CaPOPI_5014 | Ca7 | 18918765 | TAA/TAAA | TGTTTGTGCAATTGAATTATAGTTATG | CCCACATCAGCCAAAAAGAT | 58.6 | 971 | INTERGENIC | | | | |
| CaPOPI_5015 | Ca7 | 18928675 | TAAAAAAAAAAAA/TAAAAAAAAAAAAA | AGCTCACAGAAAAACACGGG | TGTCCCGTGCGATTTTTAAT | 60.3 | 392 | INTERGENIC | | | | |
| CaPOPI_5016 | Ca7 | 19290788 | TTATATATATATATATA/TTATATATATATATATATA | CTGCCATGTTCAGTTGCCTA | TCAACTTGGTGTGACGAGGA | 59.9 | 475 | INTERGENIC | | | | |
| CaPOPI_5017 | Ca7 | 19359942 | TA/TATGCCATTGA | ACGAGTGCGGGTATCAATGT | GAGAAATGGAGGCAATGGAA | 60.4 | 456 | INTERGENIC | | | | |
| CaPOPI_5018 | Ca7 | 19393876 | CAAAAAAAAAAAAA/CAAAAAAAAAAA | TGGCGAAAGCCATCTTAACT | TTAGTGGCGCATTTTATCCC | 59.8 | 385 | DRR | Ca_12340 | | bHLH | Protein kinase, catalytic domain |
| CaPOPI_5019 | Ca7 | 19589201 | TTATATATATATATATATATATATAT/TTATATATATATATATATATAT | GGTCGTCCAAATAGTACCCG | AGGAAGCCCAAGTTCCAAAT | 59.3 | 828 | INTERGENIC | | | | |
| CaPOPI_5020 | Ca7 | 19614333 | TTATATATATATATATA/TTATATATATATATATATA | TCTGCTAAAGAGCTGGAGGG | CCAATTTGATACGCACCCTT | 59.7 | 176 | INTERGENIC | | | | |

| INDEL marker IDs | Chromosomes/ unanchored scaffolds | Physical positions (bp) | InDels (*Kabuli* reference genome-CDC Frontier/PI) | Forward primers (5'-3') | Reverse primers (5'-3') | Anneling temperature (0C) | Expected amplified product size (bp) | Structural annotation | | Functional annotation | | |
|---|---|---|---|---|---|---|---|---|---|---|---|---|
| | | | | | | | | Sequence components of *kabuli* genome | *Kabuli* gene accession IDs | NCBI-KOG | TFs | NCBI-nr database |
| CaPOPI_5021 | Ca7 | 19644529 | AATATATATATATATATAT/AATATATATATATATATATAT | ATGTTTTGGATTTTGGCTCG | AGGAAGAGTGGAGTTGCGAA | 59.9 | 521 | INTERGENIC | | | | |
| CaPOPI_5022 | Ca7 | 19731743 | ATTTTTTTT/ATTTTTTTTTTT | TCAAAACCTCCAAACGAACC | TTTGCATAGGAGCAAAGCCT | 59.9 | 628 | INTERGENIC | | | | |
| CaPOPI_5023 | Ca7 | 19831369 | ACTCTCTCTCTCTCTCTCTCTCTCTCTCTCTCTC/ACTCTCTCTCTCTCTCTCTCTCTCTCTCTCTCTCTCTCTCTCTC | AACCCATTCCCTCCTTTCAC | TCCTCATCATCAGGTCCTCC | 60.2 | 351 | INTERGENIC | | | | |
| CaPOPI_5024 | Ca7 | 19857139 | AAATAATAATAATAATAATAATAATAATAATAATAA/AAATAATAATAATAATAATAATAATAATAATAATAA | CCATATGTCATAGTGCGGTCA | AGAGGCAAACAAGAACCGAA | 59.4 | 314 | INTERGENIC | | | | |
| CaPOPI_5025 | Ca7 | 19860378 | GT/G | CCTCAATATGTTGGGACATGC | TGATTCTCAGGGAGGGTGTC | 60.2 | 464 | INTERGENIC | | | | |
| CaPOPI_5026 | Ca7 | 19965866 | A/AC | CTCACGGTTGTGAGGACTGA | TTTGCTTTGATCTAGGCGGT | 59.9 | 566 | INTERGENIC | | | | |
| CaPOPI_5027 | Ca7 | 19968336 | TG/TGCGTAGGG | TCAAATTTGGAGTTCGGGAG | CCCAATACCCTAAACCCTAACC | 60.0 | 405 | INTERGENIC | | | | |
| CaPOPI_5028 | Ca7 | 19977838 | TG/TGG | ACCCAATTACTGGAGATGCG | AGGAAGAGTGGAGTTGCGAA | 60.0 | 693 | INTERGENIC | | | | |
| CaPOPI_5029 | Ca7 | 20036621 | T/TC | TTTGGAAAGAGGGCATTTTG | TTGTGTCCTTCCCTTCCATC | 60.0 | 337 | INTERGENIC | | | | |
| CaPOPI_5030 | Ca7 | 20060914 | AATATATATATATATATATAT/AATATATATATATATATATATAT | ATATTGGCTCGGAGGCTTTT | GTTGGTTCCCTAGCACCGTA | 60.1 | 640 | INTERGENIC | | | | |
| CaPOPI_5031 | Ca7 | 20217805 | TAT/TATATATTAAT | GCTTTGTGGAGGAAATGGAA | CGATGAGATCGACCCGTACT | 60.1 | 558 | INTERGENIC | | | | |
| CaPOPI_5032 | Ca7 | 20237932 | TAAAAAAAAAAAAA/TAAAAAAAAAAAA | GCATATTGGAGATGTGTTTTGC | CACAACCGCCACTGTCTCTA | 59.5 | 539 | INTERGENIC | | | | |
| CaPOPI_5033 | Ca7 | 20238379 | AATATATATATATATATATATATATATATATAT/AATATATATATATATATATATATATATATAT | TAGAGACAGTGGCGGTTGTG | TCGATTTCCCAATCGATTTTA | 59.9 | 852 | INTERGENIC | | | | |
| CaPOPI_5034 | Ca7 | 20252580 | GTAGAATA/GTAGAATAGAATA | ACCTGTCAGCTAAGAGCCCA | CACATGTGTGAACCTCCTGG | 60.0 | 641 | DRR | Ca_12383 | | | Protein of unknown function DUF220 |
| CaPOPI_5035 | Ca7 | 20480020 | AATTATAAAATTATTATAAAATTATTATAAAATTATT/AATTATAAAATTATTATAAAATTATTATAAAATTATTATAAAATTATT | TTAAAACAGACCCCCGAATG | TCATTACTCACCATAAGCGGAA | 59.8 | 577 | INTERGENIC | | | | |

| INDEL marker IDs | Chromosomes/ unanchored scaffolds | Physical positions (bp) | InDels (*Kabuli* reference genome-CDC Frontier/PI) | Forward primers (5'-3') | Reverse primers (5'-3') | Anneling temperature (0C) | Expected amplified product size (bp) | Structural annotation | | Functional annotation | | |
|---|---|---|---|---|---|---|---|---|---|---|---|---|
| | | | | | | | | Sequence components of *kabuli* genome | *Kabuli* gene accession IDs | NCBI-KOG | TFs | NCBI-nr database |
| CaPOPI_5036 | Ca7 | 20495725 | CACAACAACAAC/CACAACAAC | TCCCATTAAAATTGTAGCGTGTT | GTATGATTCGAATGCGGCTT | 59.8 | 843 | DRR | Ca_12400 | | AP2 | Pathogenesis-related transcriptional factor/ERF, DNA-binding |
| CaPOPI_5037 | Ca7 | 20501449 | AA/AAATA | TAATGCACCTTCCGTGTCAA | GACTCCTTCGGTATCCTTGG | 60.1 | 245 | INTERGENIC | | | | |
| CaPOPI_5038 | Ca7 | 20501733 | TGTA/TGTAGTA | TAATGCACCTTCCGTGTCAA | TTATAGGAAAAAGAACGAAGGAAA | 60.1 | 636 | INTERGENIC | | | | |
| CaPOPI_5039 | Ca7 | 20502385 | TAAA/TAAAA | TTCCGATTTACTTCCTCTAAAATGA | GCTTCCTCCTATTCATGCGT | 59.6 | 456 | INTERGENIC | | | | |
| CaPOPI_5040 | Ca7 | 20502470 | CAAAAAA/CAAAAAAA | TTCCGATTTACTTCCTCTAAAATGA | GCTTCCTCCTATTCATGCGT | 59.6 | 456 | INTERGENIC | | | | |
| CaPOPI_5041 | Ca7 | 20503526 | T/TCAC | TTGAAGGTTTTTGGGAATATGG | ATGAAGGAGTGGAGCGAGAA | 60.0 | 141 | INTERGENIC | | | | |
| CaPOPI_5042 | Ca7 | 20503738 | GTTTTTTT/GTTTTTT | TTCTCGCTCCACTCCTTCAT | CGAAACTAAAAGAGGGAGGCT | 60.0 | 571 | INTERGENIC | | | | |
| CaPOPI_5043 | Ca7 | 20505830 | TAAAAAAA/TAAAAAAAA | GGCATGGAAAATTCTGAAAAA | AAGAAGCGACGAAGAGAATTTG | 59.0 | 732 | INTERGENIC | | | | |
| CaPOPI_5044 | Ca7 | 20505889 | ATA/ATAGAAATTA | GGCATGGAAAATTCTGAAAAA | TTCATTTCTCGATTGAGGGC | 59.0 | 803 | INTERGENIC | | | | |
| CaPOPI_5045 | Ca7 | 20508181 | TTATT/TTATTATATT | TCAAAAGCAGTCGTTCTTGG | AAGGTTGTCCCTCCAGCTTT | 59.0 | 558 | INTERGENIC | | | | |
| CaPOPI_5046 | Ca7 | 20510109 | ATTTT/ATTT | GAACAATGCAAGAGCCACAA | AATGCACTGGCTTCTCGTTT | 59.8 | 575 | INTERGENIC | | | | |
| CaPOPI_5047 | Ca7 | 20510427 | TTT/TTTATTAGTGTATTAAAATT | TGCATTGCTCCCTAGACACA | GGCACTGCTTTATGCACTCA | 60.4 | 503 | INTERGENIC | | | | |
| CaPOPI_5048 | Ca7 | 20517567 | GAAAAAAAAAA/GAAAAAAAAAAA | GAACTGACCCAACAAACCGT | ACCACCTCGAAATGGACATC | 59.9 | 277 | INTERGENIC | | | | |
| CaPOPI_5049 | Ca7 | 20518314 | TTATATATATATATA/TTATATATATATATATA | TGCAAAGCAGCTCTTGTACG | CAGTTTACCCACCTAGCCCA | 60.3 | 588 | INTERGENIC | | | | |
| CaPOPI_5050 | Ca7 | 20530900 | AACG/AACGACG | GCAAACTCCTAGTTGCTGAGG | CGAGTGTCACATAGATGGCAA | 59.1 | 890 | INTERGENIC | | | | |

| INDEL marker IDs | Chromosomes/ unanchored scaffolds | Physical positions (bp) | InDels (*Kabuli* reference genome-CDC Frontier/PI) | Forward primers (5'-3') | Reverse primers (5'-3') | Anneling temperature (0C) | Expected amplified product size (bp) | Structural annotation | | Functional annotation | | |
|---|---|---|---|---|---|---|---|---|---|---|---|---|
| | | | | | | | | Sequence components of *kabuli* genome | *Kabuli* gene accession IDs | NCBI-KOG | TFs | NCBI-nr database |
| CaPOPI_5051 | Ca7 | 20538907 | TAACCAAA/TAA | GGCCATTGGATCTAGCAAAA | GCCATTCATCAAGGAAGGAA | 60.0 | 635 | DRR | Ca_12404 | F | | Xanthine/uracil/vitamin C permease |
| CaPOPI_5052 | Ca7 | 20554410 | AA/AACCTA | CATCTGACCTGGACTTCGGT | TTTTCTGAAGGGTTGTTGCC | 60.1 | 682 | INTRON | Ca_12405 | K | | SGT1 |
| CaPOPI_5053 | Ca7 | 20556895 | TCACAC/TCAC | TACTTTGCCGCCTCTGCTAT | CAAACGCGCAGATCAGTAAA | 60.0 | 589 | DRR | Ca_12405 | K | | SGT1 |
| CaPOPI_5054 | Ca7 | 20560193 | CAAAAAAAA/CAAAAAAA | TGCATTTGAAATTTGCAGGA | TCAACCCCAATACACCCCTA | 60.2 | 602 | DRR | Ca_12406 | | M-type | KIP1-like |
| CaPOPI_5055 | Ca7 | 20573093 | GCAAGACAA/GCAAGACAAGACAA | AATGCAAGCAACCAAAAACC | ATAGACCTGGCTGCAAGGAA | 60.0 | 617 | INTRON | Ca_12407 | | | |
| CaPOPI_5056 | Ca7 | 20600846 | T/TC | CGTGAATGTTTATGGGGGAC | TTTTTCAGCAATTGGCAACA | 60.1 | 544 | DRR | Ca_12410 | | bHLH | |
| CaPOPI_5057 | Ca7 | 20604250 | CTAATATAATATAATA/CTAATATAATATAATATAATA | ACCATTTCCCCTTTCGATTT | TGCTTTGTTCAATTGCTTGC | 59.6 | 589 | DRR | Ca_12412 | YU | | |
| CaPOPI_5058 | Ca7 | 20617780 | C/CAAT | GGCGAGGGATGGAATTTTAG | GAATTGGGACTTCTGCCGTA | 60.8 | 777 | INTERGENIC | | | | |
| CaPOPI_5059 | Ca7 | 21099731 | T/TGCGCAA | GAGAAATCAAAATTGCCCCA | AGTGCCTCCTGTTCCTGAGA | 59.9 | 500 | INTERGENIC | | | | |
| CaPOPI_5060 | Ca7 | 21115383 | TAAAAAAAAAAA/TAAAAAAAAA | CATCACTGAGGTTTTGGCTG | CATCCATCACCTGTCAATGC | 59.3 | 386 | INTERGENIC | | | | |
| CaPOPI_5061 | Ca7 | 21130339 | AAGAGAGAGAGAGAGAGAGAGAGAGAGA/AAGAGAGAGAGAGAGAGAGAGA | CACCGATGGGCTGATATTTT | TCTCCCCCTTTTTGATGATG | 59.8 | 430 | INTERGENIC | | | | |
| CaPOPI_5062 | Ca7 | 21171384 | ATTTTTTTT/ATTTTTTTTT | GTTCGCCGTTTTATGTCGTT | TGTGGCTGTAAACCTGCAAA | 60.0 | 581 | INTERGENIC | | | | |
| CaPOPI_5063 | Ca7 | 21182517 | GTTTTTTTTTTTT/GTTTTTTTTTTTTT | AACCCGAGACCTAGCAGTTG | AGAAGCTTGAGTAACATTGCCA | 59.4 | 628 | INTERGENIC | | | | |
| CaPOPI_5064 | Ca7 | 21184582 | TTATATATATATATATATA/TTATATATATATATATATA | GGTGGGAACTGCTGGTAAAA | AACTGCATCCTGTGCTCCTT | 60.0 | 592 | INTERGENIC | | | | |
| CaPOPI_5065 | Ca7 | 21213067 | TAAAAA/TAAAA | AGGCATAAGCAAGGCAGAGA | GGCTGTGAAAAACCAGAACG | 60.1 | 366 | INTERGENIC | | | | |

| **INDEL marker IDs** | **Chromosomes/ unanchored scaffolds** | **Physical positions (bp)** | **InDels (*Kabuli* reference genome-CDC Frontier/PI)** | **Forward primers (5'-3')** | **Reverse primers (5'-3')** | **Anneling temperature (0C)** | **Expected amplified product size (bp)** | **Structural annotation** | | **Functional annotation** | | |
|---|---|---|---|---|---|---|---|---|---|---|---|---|
| | | | | | | | | **Sequence components of *kabuli* genome** | ***Kabuli* gene accession IDs** | **NCBI-KOG** | **TFs** | **NCBI-nr database** |
| CaPOPI_5066 | Ca7 | 21598915 | TTATATATATATATATAT/TTATATATATATATAT | TTTCATGGTTCGCATCACAT | AACCCCATGTGTGAAATGCT | 59.9 | 242 | INTERGENIC | | | | |
| CaPOPI_5067 | Ca7 | 21670841 | A/AAGAT | TGACAAATCTTTGCAACCCA | ATTGCTCATTTCATGCCCTC | 60.1 | 217 | INTERGENIC | | | | |
| CaPOPI_5068 | Ca7 | 21675444 | AAAATAA/AAA | TTTGATGCGTGAAGAGGTTG | TGGACTGTGACTGCACCATT | 59.8 | 510 | INTERGENIC | | | | |
| CaPOPI_5069 | Ca7 | 21685577 | TT/TTAT | TTTATAAATTGGCCCCATCG | TAGGGGGACCAAGAAACTGG | 59.6 | 501 | INTERGENIC | | | | |
| CaPOPI_5070 | Ca7 | 21728387 | CAAAA/CAAA | ATCTCTGCGCAATATCGCTT | AGCCGACTTTTCCTACCACA | 60.0 | 674 | INTERGENIC | | | | |
| CaPOPI_5071 | Ca7 | 21729097 | TAAAAA/TAAAA | TGCTCAAAAGGGGAAGAAAA | GCCACGTGGTTGAAAGAAGT | 59.8 | 240 | INTERGENIC | | | | |
| CaPOPI_5072 | Ca7 | 21729303 | T/TC | TTGCTCAAAAGGGGAAGAAA | TTGTTATTTTCTTTATGCCGTTG | 59.8 | 426 | INTERGENIC | | | | |
| CaPOPI_5073 | Ca7 | 21743270 | CAA/CA | TTGCCCCTCTAAATTCTTGG | TCCCTCGTATTTAGCCCTCA | 59.2 | 686 | DRR | Ca_14521 | | | Transposase, Ptta/En/Spm, plant |
| CaPOPI_5074 | Ca7 | 21743627 | ATTTTTTT/ATTTTTTTT | TGAGGGCTAAATACGAGGGA | GGGATTGAGCGCCTATACAA | 59.7 | 461 | DRR | Ca_14521 | | | Transposase, Ptta/En/Spm, plant |
| CaPOPI_5075 | Ca7 | 21743947 | AA/AAATA | CCTGGTGTGCATTGAAAAGA | AATTGCGGTTTTGGTTCAAG | 59.7 | 410 | DRR | Ca_14521 | | | Transposase, Ptta/En/Spm, plant |
| CaPOPI_5076 | Ca7 | 21745626 | TTTTATATTTAT/TTTTAT | ATAGGGCCCATTTTACAGCG | CCAGTTGCCCTGTCCATACT | 61.2 | 455 | INTRON | Ca_14522 | O | MYB | SANT domain, DNA binding |
| CaPOPI_5077 | Ca7 | 21838175 | TAAT/TAATAAAT | CCCTGCCTCAATTGTTTTGT | AGCATATATTGTTCCCGCGT | 60.0 | 300 | INTERGENIC | | | | |
| CaPOPI_5078 | Ca7 | 22014557 | GTGT/GTGTTGT | AACTTGCAGCAAAGAACCGT | CACTACCCCGTACTTCGCAT | 59.9 | 419 | INTERGENIC | | | | |
| CaPOPI_5079 | Ca7 | 22028394 | G/GA | TCATCAAATCCGTAACGCAA | TCCATGTTTTGCTCTATTGGC | 60.1 | 271 | DRR | Ca_14536 | R | MYB | Integrase, catalytic core |
| CaPOPI_5080 | Ca7 | 22036269 | GCTC/G | GCTCAGCCTTGAAATCGAAC | TTCATCATCATCTTCAAACAAAA | 60.0 | 627 | INTERGENIC | | | | |

| INDEL marker IDs | Chromosomes/ unanchored scaffolds | Physical positions (bp) | InDels (*Kabuli* reference genome-CDC Frontier/PI) | Forward primers (5'-3') | Reverse primers (5'-3') | Anneling temperature (0C) | Expected amplified product size (bp) | Structural annotation | | Functional annotation | | |
|---|---|---|---|---|---|---|---|---|---|---|---|---|
| | | | | | | | | Sequence components of *kabuli* genome | *Kabuli* gene accession IDs | NCBI-KOG | TFs | NCBI-nr database |
| CaPOPI_5081 | Ca7 | 22038011 | C/CT | CTCCAAATTTCCAGCCAAAA | ACACGGACGGGTCTTGTATC | 60.0 | 822 | INTERGENIC | | | | |
| CaPOPI_5082 | Ca7 | 22043317 | T/TG | TGGTTCAAATGCTGTAATCTTCTC | ATTCGCTCAATACACGGGTC | 59.7 | 884 | INTERGENIC | | | | |
| CaPOPI_5083 | Ca7 | 22045478 | ATTTTTTTT/ATTTTTTT | TGTGGTGTTCCCAAAAGACA | CAAAGCGAAGTGCAAAATGA | 60.0 | 246 | INTERGENIC | | | | |
| CaPOPI_5084 | Ca7 | 22046891 | CATAATAATAATAATAATAATAATAATAATAATAATAATAATAAT/CATAATAATAATAATAATAATAATAATAATAATAATAATAAT | ATAATTTGCCTTTGCGGTTG | TTCTGTGAGCAAGGCTGATG | 60.0 | 319 | INTERGENIC | | | | |
| CaPOPI_5085 | Ca7 | 22046957 | CTATATATATATATATATATATATAT/CTATATATATATATATATATATAT | ATAATTTGCCTTTGCGGTTG | TTCTGTGAGCAAGGCTGATG | 60.0 | 319 | INTERGENIC | | | | |
| CaPOPI_5086 | Ca7 | 22046996 | TGCGCGC/TGC | ATAATTTGCCTTTGCGGTTG | ACCTGGTCGTAGATGCCACT | 60.0 | 414 | INTERGENIC | | | | |
| CaPOPI_5087 | Ca7 | 22049141 | TTATATATATATATAT/TTATATATATATATATATATAT | ATTTTGGAAATTCCCGCTCT | TTGGTTGAGGCTAAAATGGTG | 59.9 | 521 | INTERGENIC | | | | |
| CaPOPI_5088 | Ca7 | 22336659 | CGTGT/CGT | CCTGGAAAATTCGTTGATGG | TTAGTTGCAGCCGTCTTCG | 60.3 | 592 | INTRON | Ca_14549 | K | | Transcription initiation factor TFIID component TAF4 |
| CaPOPI_5089 | Ca7 | 22380060 | CATATATATATATATATATATATA/CATATATATATATATATATA | GCGAATTGCGATTAATGTGA | GCCACTTGTTTGTTGTTGATG | 59.7 | 510 | INTERGENIC | | | | |
| CaPOPI_5090 | Ca7 | 22440146 | TAATATA/TAATATAAAATATA | GAAGTTGTAATCGGAGGCCA | GGCAGAGATGGAGGTGATGT | 60.1 | 542 | INTERGENIC | | | | |
| CaPOPI_5091 | Ca7 | 22519524 | TTAATAATAATAATAATAATAATAATAATAATAA/TTAATAATAATAATAATAATAATAATAATAA | ATGATCTTCCTTCTCGGCCT | CCGTTTCTTCCTCTCCCTCT | 60.2 | 257 | INTERGENIC | | | | |
| CaPOPI_5092 | Ca7 | 22587400 | TTATATATATATATATATATATATATATAT/TTATATATATATATATATATATATATATATATAT | AAGGAACAAAACGTCATTTTCAA | AGTCAGACGCTACTCGGCAT | 59.9 | 519 | INTERGENIC | | | | |
| CaPOPI_5093 | Ca7 | 22658192 | GTATATATATATATATATATATATAT/GTATATATATATATATATATATAT | TACACCTGAGTCCGCTACCC | TCCCAAGTTTCAAATGCTGA | 60.1 | 494 | INTERGENIC | | | | |
| CaPOPI_5094 | Ca7 | 22678046 | AG/AGG | ACCAGTCAACAAATGCCCTC | CCACCAACTTTAGCTCCGAA | 60.0 | 314 | INTERGENIC | | | | |
| CaPOPI_5095 | Ca7 | 22745128 | G/GGTAGTTAGTGAT | GGAGTTGGTCGCTACACCAT | CGAGGCATTCCTTCACAAAT | 60.0 | 634 | INTRON | Ca_19530 | R | | Haloacid dehalogenase-like hydrolase |

| INDEL marker IDs | Chromosomes/ unanchored scaffolds | Physical positions (bp) | InDels (*Kabuli* reference genome-CDC Frontier/PI) | Forward primers (5'-3') | Reverse primers (5'-3') | Anneling temperature (0C) | Expected amplified product size (bp) | Structural annotation | | Functional annotation | | |
|---|---|---|---|---|---|---|---|---|---|---|---|---|
| | | | | | | | | Sequence components of *kabuli* genome | *Kabuli* gene accession IDs | NCBI-KOG | TFs | NCBI-nr database |
| CaPOPI_5096 | Ca7 | 22886024 | G/GCTAC | GCCCTTCTATGCTTGTCAGC | TTGGGAAGGTATGAAGGCAG | 60.0 | 728 | CDS (FRAME SHIFT) | Ca_19525 | S | ARF | G-protein, beta subunit |
| CaPOPI_5097 | Ca7 | 22890324 | A/AT | GTATCACACCCCTGCAACCT | TGTGGTGTTGGGTACTTCAGG | 59.9 | 705 | DRR | Ca_19524 | S | | WD40 repeat |
| CaPOPI_5098 | Ca7 | 22950306 | TTATATATATATATATATATAT/TTATATATATATATATAT | ACCCAAGAGAGGTGCGACTA | AATGGCGACTTACCAAAGGA | 59.9 | 832 | INTERGENIC | | | | |
| CaPOPI_5099 | Ca7 | 23038315 | TCCCCCC/TCCCCCCCCC | CTAATTTAGAGGGGGAGCGG | TGATGTTGTTGCCTCTCTGC | 60.0 | 825 | INTERGENIC | | | | |
| CaPOPI_5100 | Ca7 | 23667025 | AAATAATAATAATAATAATA/AAATAATAATAATAATAATA | ACACTGATTTCCCAAAACGC | TTTCCTTCCCTTCTCATTGC | 60.0 | 728 | DRR | Ca_26877 | G | FAR1 | NAD-dependent epimerase/dehydratase |
| CaPOPI_5101 | Ca7 | 23688628 | TTAATAATAATAATAATAATAATAATA/TTAATAATAATAATAATAATAATAATAATAATAATA | CCACAAAGACAATCGAGCAA | CGTGCAATCTGCTTGGAGTA | 59.8 | 308 | INTERGENIC | | | | |
| CaPOPI_5102 | Ca7 | 23780060 | TTATATATATATATAT/TTATATATATATAT | CGCTCGTCTATGCGATTGTA | TAAAATCTCCGCTCGCAACT | 60.0 | 946 | INTERGENIC | | | | |
| CaPOPI_5103 | Ca7 | 23797195 | AATATATATATATATATATATATATATATA/AATATATATATATATATATATATATATATATATA | AAGAAATCGCGAGTCACAGG | TTAATAGAACGCGCCAAAGG | 60.4 | 652 | INTERGENIC | | | | |
| CaPOPI_5104 | Ca7 | 23907440 | AATATATATAT/AATATATATATATAT | TTGGAGAATTCAAAAATAAGGCA | CGTGACAAAGCACGAAAGAA | 60.0 | 513 | INTERGENIC | | | | |
| CaPOPI_5105 | Ca7 | 23984802 | AGTTGTTG/AGTTG | AAGCTGGCATGATGAGGACT | GGGGAATTGAGCCTAAAAGC | 59.8 | 359 | INTERGENIC | | | | |
| CaPOPI_5106 | Ca7 | 24096310 | TCCCCC/TCCCCCC | CTTGCCACCACTAATGCTCA | ACAACCGATTTCAAGCCATC | 59.9 | 818 | DRR | Ca_19914 | | | |
| CaPOPI_5107 | Ca7 | 24260186 | ATTTTTT/ATTTTTTT | TCAGCTTTTTGGCTGATGTG | TTTCTTTCAATTTATGTCAACCCA | 60.0 | 667 | INTERGENIC | | | | |
| CaPOPI_5108 | Ca7 | 24260251 | CTTTTTT/CTTTTTTT | AGAATGAGGTGACATTCGGG | TCTCCTTCAATCCTCCTCTCA | 59.9 | 978 | INTERGENIC | | | | |
| CaPOPI_5109 | Ca7 | 24347972 | TAAAA/TAAAAA | CTCTCATCTGAGGGCAAAGC | CGATTGAATTAAGCCCAGGA | 60.1 | 356 | INTERGENIC | | | | |
| CaPOPI_5110 | Ca7 | 24348355 | TAA/TAAA | TCCTGGGCTTAATTCAATCG | CGTTCCTGAGGGAATTGTGT | 60.0 | 378 | INTERGENIC | | | | |

| INDEL marker IDs | Chromosomes/ unanchored scaffolds | Physical positions (bp) | InDels (*Kabuli* reference genome-CDC Frontier/PI) | Forward primers (5'-3') | Reverse primers (5'-3') | Anneling temperature (0C) | Expected amplified product size (bp) | Structural annotation | | Functional annotation | | |
|---|---|---|---|---|---|---|---|---|---|---|---|---|
| | | | | | | | | Sequence components of *kabuli* genome | *Kabuli* gene accession IDs | NCBI-KOG | TFs | NCBI-nr database |
| CaPOPI_5111 | Ca7 | 24421160 | AATATATATATATATATATATATA/AATATATATATATATATA | AGGAAGCCCAAGTTCCAAAT | ACAGCCAACAACCACATCAA | 59.9 | 469 | INTERGENIC | | | | |
| CaPOPI_5112 | Ca7 | 24450388 | TTATATATATATATATATATAT/TTATATATATATATATATATAT | TTGTTAGTTTGACGAGGGCA | TGGGTGCTATTTGCAATCAA | 59.3 | 380 | INTERGENIC | | | | |
| CaPOPI_5113 | Ca7 | 24485265 | C/CAACTTT | GTCCCACCCTAGGAGCTTTC | CGGACCAAAAATTAAAGCGA | 60.1 | 986 | INTERGENIC | | | | |
| CaPOPI_5114 | Ca7 | 24495058 | TTTATTATTATTATTATTATTATTATTATTATTATTATTATTATTATTATTATTATT/TTTATTATTATTATTATTATTATTATTATTATTATTATTATTATTATT | TCCGTCGTAGCTTTTTGACA | TCTGAATCCGACATAGTGCG | 59.5 | 625 | INTERGENIC | | | | |
| CaPOPI_5115 | Ca7 | 24660866 | CTTTT/CT | ACGTTCTCCATTTTCGTTGC | AATCCGCTCCTTAGGGAAAA | 60.1 | 838 | INTERGENIC | | | | |
| CaPOPI_5116 | Ca7 | 24876128 | TAAA/TA | GCTTTAGAGGCAACAACCGA | CTCCAAGAACCAACTCCTGC | 60.4 | 686 | INTERGENIC | | | | |
| CaPOPI_5117 | Ca7 | 25111194 | AATATATATATATATA/AATATATATATATATATA | GGGATTGAGAGGAGAGGGTC | ATTTAAATGTCGTGACCGGC | 60.0 | 489 | INTERGENIC | | | | |
| CaPOPI_5118 | Ca7 | 25240070 | CTATATATATATATATATAT/CTATATATATATAT | GTTGTAGCCGGTCCAAGGTA | GTTTAAGCCCCTTTGAGCCT | 60.0 | 406 | INTERGENIC | | | | |
| CaPOPI_5119 | Ca7 | 25613830 | TAAAAAAAAA/TAAAAAAAA | TCGATGGCTACCTCAAATCC | GGTGGTCCTCTGCTCTTACG | 60.0 | 512 | INTERGENIC | | | | |
| CaPOPI_5120 | Ca7 | 25626892 | CGG/CGGG | TTGGCCAAGTCCCTGTTTAC | CCCGTGATAGGCAGTACGTT | 60.0 | 420 | INTERGENIC | | | | |
| CaPOPI_5121 | Ca7 | 25834002 | GTTTTTTTT/GTTTTTTTTT | TGCTCTTAGTGGTGACCGTG | CATCGTCATCGTTGCAATTC | 59.9 | 461 | INTERGENIC | | | | |
| CaPOPI_5122 | Ca7 | 26035041 | TAAAAAAAA/TAAAAAAAAA | TAAAGAGATTCGCAGCGGTT | CATGTTTTCGAAGCTCACCA | 60.0 | 407 | INTERGENIC | | | | |
| CaPOPI_5123 | Ca7 | 26056806 | CTTTTTT/CTTTTTTTTT | GTTGCCACTTGGCGTAAAAT | GGCCGATTTACTGCTCACAT | 60.0 | 393 | INTERGENIC | | | | |
| CaPOPI_5124 | Ca7 | 26160184 | AG/A | GTGGCTACCTTCTTGGGTGA | TTTCATCGCCGAAACCTATC | 60.1 | 141 | INTERGENIC | | | | |
| CaPOPI_5125 | Ca7 | 26179604 | AT/ATT | TCAGGGATGAGTTTCACTTCG | TTGGGAAGTTGGGAGACTTG | 60.2 | 625 | INTERGENIC | | | | |

| INDEL marker IDs | Chromosomes/ unanchored scaffolds | Physical positions (bp) | InDels (*Kabuli* reference genome-CDC Frontier/PI) | Forward primers (5'-3') | Reverse primers (5'-3') | Anneling temperature (0C) | Expected amplified product size (bp) | Structural annotation | | Functional annotation | | |
|---|---|---|---|---|---|---|---|---|---|---|---|---|
| | | | | | | | | Sequence components of *kabuli* genome | *Kabuli* gene accession IDs | NCBI-KOG | TFs | NCBI-nr database |
| CaPOPI_5126 | Ca7 | 26221720 | AATATATATATATATATATATAT/AATATATATATATATAT | CTTGGGAATTGGAGAGCAAG | GGGTTTCTTTCCTTGAAGCC | 59.8 | 671 | INTERGENIC | | | | |
| CaPOPI_5127 | Ca7 | 26228410 | GAAAAAAAAAA/GAAAAAAAAAAAA | ATGGTGCAAAATGGTGACAA | GAAAGTCGGTGCATGGAAGT | 59.8 | 304 | INTERGENIC | | | | |
| CaPOPI_5128 | Ca7 | 26233274 | TAAAAAAA/TAAAAAAAA | TACGCCATCCAAAAATGTGA | CCCTCATCCGAAAAGATGAA | 59.9 | 945 | INTERGENIC | | | | |
| CaPOPI_5129 | Ca7 | 26235537 | TTATATATATATA/TTATATATATATATATATA | TGCGACCTCAGTTTCTCAAA | GGGGTCATAGCATCCCCTAT | 59.6 | 157 | INTERGENIC | | | | |
| CaPOPI_5130 | Ca7 | 26240583 | G/GTAACATATT | GTTTCCCACATCCAGAGGAA | GAGATCAACGTGAATTGGTCA | 59.9 | 330 | INTRON | Ca_23643 | | HD-ZIP | Lipid-binding START |
| CaPOPI_5131 | Ca7 | 26326487 | GTATATATATATATATATATATATATATA/GTATATATATATATATATATATATA | GGTGGTAGCTGGTAGGGTGA | TGACAACTACGAGGTTGCTTG | 60.0 | 657 | INTERGENIC | | | | |
| CaPOPI_5132 | Ca7 | 26662956 | CAA/CA | TCCAAGCTTCCTTCAATGGT | CTCAAAAGCGCTCCAATTTC | 59.7 | 542 | INTERGENIC | | | | |
| CaPOPI_5133 | Ca7 | 26694675 | ACC/ACCCCCCC | CTCGGCCATCCGAGATATAA | GCTCACTAACTCTGCCTGGG | 60.0 | 881 | CDS (FRAME SHIFT) | Ca_16250 | | | |
| CaPOPI_5134 | Ca7 | 26699922 | A/AACTAG | AAACTCCAGCCCGGTAAACT | AACCCATTTGAGGTGACTGC | 60.0 | 465 | DRR | Ca_16249 | | | |
| CaPOPI_5135 | Ca7 | 26701106 | A/ACTAGGAATTACTCCC | CAATAGGGCGAGTCGAATGT | AAGTCCAGTAGCCGAAAGCA | 60.1 | 838 | DRR | Ca_16249 | | | |
| CaPOPI_5136 | Ca7 | 26701534 | C/CG | AAGCTTCATCCTGGTCATGG | AAGTCCAGTAGCCGAAAGCA | 60.1 | 381 | DRR | Ca_16249 | | | |
| CaPOPI_5137 | Ca7 | 26906603 | ATTTTT/ATTTT | TGGAGTTCCAACACGCACTA | CAAGGTAATTTGCATTGAAGGA | 60.3 | 112 | INTERGENIC | | | | |
| CaPOPI_5138 | Ca7 | 27115436 | GATATATATATATATAT/GATATATATAT | CAATTCCCACAGGTACGCTT | CAAACCCTAATTTTTCAAACACG | 60.0 | 347 | INTERGENIC | | | | |
| CaPOPI_5139 | Ca7 | 27346895 | TTATATATATATATATATATATATAT/TTATATATATATATATATATATATATATAT | TGCTTGATTGCTTGAGTTCC | CGCGCAGATTGTCAAATAGA | 59.0 | 641 | INTERGENIC | | | | |
| CaPOPI_5140 | Ca7 | 27520803 | AATATATATATATAT/AATATATATATATATATAT | GCCGCAAGGAATTTAACTCA | GGAGAAAGAGGGAATTTGGC | 60.2 | 618 | INTERGENIC | | | | |

| INDEL marker IDs | Chromosomes/ unanchored scaffolds | Physical positions (bp) | InDels (*Kabuli* reference genome-CDC Frontier/PI) | Forward primers (5'-3') | Reverse primers (5'-3') | Anneling temperature (0C) | Expected amplified product size (bp) | Structural annotation | | Functional annotation | | |
|---|---|---|---|---|---|---|---|---|---|---|---|---|
| | | | | | | | | Sequence components of *kabuli* genome | *Kabuli* gene accession IDs | NCBI-KOG | TFs | NCBI-nr database |
| CaPOPI_5141 | Ca7 | 27521328 | CAAA/CAA | GCCAAATTCCCTCTTTCTCC | CCTTTCCACACAACCACAGA | 60.0 | 393 | INTERGENIC | | | | |
| CaPOPI_5142 | Ca7 | 27521551 | TC/T | GGTAAGGGCTCCTTCCAAAC | GATTCCCCCTCCTTAACGTC | 59.9 | 576 | INTERGENIC | | | | |
| CaPOPI_5143 | Ca7 | 27935917 | AGGGG/AGG | GGCTGTTAGCCCAGTTTTTG | GTTGGTTCCCTAGCACCGTA | 59.7 | 664 | INTERGENIC | | | | |
| CaPOPI_5144 | Ca7 | 28056896 | TAAAAAAAA/TAAAAAAA | TCCAAAAAGATATGGCCGAC | GTATTAGCTGGTGCGCTGGT | 59.9 | 406 | INTERGENIC | | | | |
| CaPOPI_5145 | Ca7 | 28190197 | AGTTTATTTGG/AGTTTATTTGGTTTATTTGG | TTGGTTTATTTGGGTTGGGA | ACCAGGAAAAGAAGAAGGGC | 60.0 | 519 | INTERGENIC | | | | |
| CaPOPI_5146 | Ca7 | 28335175 | AAAATATA/AA | GGTACTGTTCACTGCGCAAA | TCAACCAAAATCCCCCAATA | 59.9 | 236 | INTRON | Ca_18921 | | | GOLD |
| CaPOPI_5147 | Ca7 | 28355456 | AATATATATATATATATATA/AATATATATATATATATA | GCCAACTCATCAAATCGTGA | ATGGAACAATCGGACACCAT | 59.7 | 521 | INTERGENIC | | | | |
| CaPOPI_5148 | Ca7 | 28400317 | AATATATATAT/AATATATATATATAT | CTGACCAAAATTCCAAAAGACA | TCGGTTTTGAAACCAAAATG | 59.1 | 637 | INTERGENIC | | | | |
| CaPOPI_5149 | Ca7 | 28454557 | GTT/GTTACTT | TGTCCGTCAATCCACTTTTT | CCCAAACCATTCCATTTTTG | 58.0 | 411 | INTERGENIC | | | | |
| CaPOPI_5150 | Ca7 | 28561460 | CCAC/CCACAC | CACTGCTTTAGCAACCACCA | TTCTAGAAGCGGCGTTGTTT | 59.9 | 678 | DRR | Ca_18943 | L | C3H | Domain of unknown function DUF223 |
| CaPOPI_5151 | Ca7 | 28702659 | ATCCTCC/ATCC | TCCATTAACCCTCTTGAGGAAA | TCAGGATGAAGAAGGGGAGA | 59.9 | 531 | INTERGENIC | | | | |
| CaPOPI_5152 | Ca7 | 28707261 | ATTT/AT | TGATGGTGGATATGCGAAAA | TTGGTTTTTGATGGTGGGAT | 59.9 | 314 | INTERGENIC | | | | |
| CaPOPI_5153 | Ca7 | 28707412 | GAAATAACAATAAAAACAAATAACAATAAAA/GAAATAACAATAAAAACAAATAACAATAAAAACAAATAACAATAAAA | ATCCCACCATCAAAAACCAA | GATATGTTCCGAGTGCCGTT | 60.0 | 491 | INTERGENIC | | | | |
| CaPOPI_5154 | Ca7 | 28914738 | TAAAAAA/TAAAAAAA | TGAACCCAAAACACAAACACA | AACATTTCGTGCCTTTTCGT | 59.9 | 827 | DRR | Ca_11811 | QR | B3 | Oxoglutarate/iron-dependent oxygenase |
| CaPOPI_5155 | Ca7 | 28918944 | GTGCT/GT | TGCCGTCAAAATTGTAGCAG | TCCCCATCCATTTCATTTGT | 59.9 | 505 | INTERGENIC | | | | |

| **INDEL marker IDs** | **Chromosomes/ unanchored scaffolds** | **Physical positions (bp)** | **InDels (*Kabuli* reference genome-CDC Frontier/PI)** | **Forward primers (5'-3')** | **Reverse primers (5'-3')** | **Anneling temperature (0C)** | **Expected amplified product size (bp)** | **Structural annotation** | | **Functional annotation** | | |
|---|---|---|---|---|---|---|---|---|---|---|---|---|
| | | | | | | | | **Sequence components of *kabuli* genome** | ***Kabuli* gene accession IDs** | **NCBI-KOG** | **TFs** | **NCBI-nr database** |
| CaPOPI_5156 | Ca7 | 28925485 | AAGTAGTAG/AAGTAG | TCATCAAAAGACCGAAAGCA | ACCACCACTTAGAGAGGCCA | 59.4 | 410 | INTERGENIC | | | | |
| CaPOPI_5157 | Ca7 | 28935886 | GTTATATCA/G | TGGAACCAGTCCCTTAGACG | TCCATCATGGCAAGTTTTTCT | 60.1 | 797 | INTERGENIC | | | | |
| CaPOPI_5158 | Ca7 | 28936338 | TAAAAAAA/TAA | TTCGGAATTCAACCGATAAA | TAAAAGAGGGTGCAAGGTCC | 58.1 | 866 | INTERGENIC | | | | |
| CaPOPI_5159 | Ca7 | 28937372 | ATT/ATTTT | AACCTCATGTTCCCCTTTCC | TGGGTCATGGCCATCTATTT | 60.2 | 702 | INTERGENIC | | | | |
| CaPOPI_5160 | Ca7 | 28941166 | TAT/TATATACAAT | TCAACCACATGTGAAGATGGA | TTGGCTCCCTACATTTTTCTTC | 60.0 | 752 | INTERGENIC | | | | |
| CaPOPI_5161 | Ca7 | 28942484 | AATTA/AA | AGCTTGTGAAGGTTTGGTGG | CAAGAGCCGGTGAATGAAGT | 60.1 | 559 | INTERGENIC | | | | |
| CaPOPI_5162 | Ca7 | 28949507 | TTATA/TTATATA | CCACCATTGGCATTCTTTCT | GCTGCTCAAATTCGACAACA | 59.9 | 599 | INTERGENIC | | | | |
| CaPOPI_5163 | Ca7 | 28979318 | TA/TATATTCAACA | TTCTGGCCCGTTAGCATTAC | CTCAGCCTTCCCACCAAATA | 60.1 | 453 | DRR | Ca_11805 | C | | Malic oxidoreductase |
| CaPOPI_5164 | Ca7 | 28999036 | CAAAAAAAA/CAAAAAAAAAA | CGTGGACAAAGATCGTGATG | ATTCCCCGTGGTCTTGAATA | 60.1 | 447 | INTERGENIC | | | | |
| CaPOPI_5165 | Ca7 | 29001394 | TA/TATAAATATATTTATGA | TTGACTTGCATTCCATCGTC | ATGCTTCCTCCCGAAAATCT | 59.7 | 569 | INTERGENIC | | | | |
| CaPOPI_5166 | Ca7 | 29001560 | ATTTTT/ATTTT | ACTTGCATTCCATCGTCCAT | ATGCTTCCTCCCGAAAATCT | 60.3 | 566 | INTERGENIC | | | | |
| CaPOPI_5167 | Ca7 | 29004077 | ATGA/ATGACACTGA | CGTCGGTGGTCAGGTAAAAT | TCACAATCCCTGCAATTCAA | 59.9 | 681 | INTRON | Ca_11804 | UR | HB-other | Dynamin central domain |
| CaPOPI_5168 | Ca7 | 29006131 | AATATA/AATA | TTTCCTCATCCTCCTGGTTG | TGAGGGATCAACTTCCCTTG | 60.0 | 752 | INTRON | Ca_11804 | UR | HB-other | Dynamin central domain |
| CaPOPI_5169 | Ca7 | 29010477 | CAAAAAA/CAAAAA | TTTTCATCGGGTCACAGTCA | TGGATATGACCCGTGCTTTT | 60.1 | 738 | DRR | Ca_11804 | UR | HB-other | Dynamin central domain |
| CaPOPI_5170 | Ca7 | 29012088 | TAA/TAAA | TTTGCCCCTCAAGATTGTTC | ATTGGTGGCTCATGACCTGT | 60.1 | 102 | INTRON | Ca_11803 | F | | Adenylate kinase |

| INDEL marker IDs | Chromosomes/ unanchored scaffolds | Physical positions (bp) | InDels (*Kabuli* reference genome-CDC Frontier/PI) | Forward primers (5'-3') | Reverse primers (5'-3') | Anneling temperature (0C) | Expected amplified product size (bp) | Structural annotation | | Functional annotation | | |
|---|---|---|---|---|---|---|---|---|---|---|---|---|
| | | | | | | | | Sequence components of *kabuli* genome | *Kabuli* gene accession IDs | NCBI-KOG | TFs | NCBI-nr database |
| CaPOPI_5171 | Ca7 | 29014917 | TCCC/T | TTTGGCATACACTCCCCTTC | GGGAGCTGATGAGTGTGGTT | 59.9 | 225 | INTERGENIC | | | | |
| CaPOPI_5172 | Ca7 | 29020483 | TATAAA/TATAAATAAA | TGGTATAAGCATTCGGTAGACG | CAACGCTAAGTTTGTAAGCAACA | 59.2 | 982 | INTERGENIC | | | | |
| CaPOPI_5173 | Ca7 | 29021071 | TTA/TTATA | TTAGCCAAAATTGTGGTCCC | TCATGACGTAGTCATCGCGT | 59.8 | 209 | INTERGENIC | | | | |
| CaPOPI_5174 | Ca7 | 29021112 | TAAAAAAAAAAAA/TAAAAAAA | GTCCGCAACTGGAACCTTTA | TCATGACGTAGTCATCGCGT | 60.1 | 148 | INTERGENIC | | | | |
| CaPOPI_5175 | Ca7 | 29022459 | TAAAAAAAA/TAAAAAAA | TTAGAACAATGCGACGTGAA | TCACCCGAAACATCTCCTTC | 57.9 | 261 | INTERGENIC | | | | |
| CaPOPI_5176 | Ca7 | 29022601 | AGG/AG | TTAGAACAATGCGACGTGAA | TCACCCGAAACATCTCCTTC | 57.9 | 261 | INTERGENIC | | | | |
| CaPOPI_5177 | Ca7 | 29023514 | AAGA/AA | GGACCACGTGTGTATTTAGCC | TCACCAGCAATGATCTGAGC | 59.4 | 482 | INTERGENIC | | | | |
| CaPOPI_5178 | Ca7 | 29067406 | ATTAAAATTTA/ATTAAAATTTAAAATTTA | AGCTTTTGGCTGACATTGTG | AAAATACTGCGTGGCCTTTG | 58.9 | 522 | INTERGENIC | | | | |
| CaPOPI_5179 | Ca7 | 29069731 | AG/A | AGTCTTTCCCTTCCCTCCAA | CATACACCACCAAATCCACG | 60.0 | 369 | DRR | Ca_11799 | | | Uncharacterised protein, Wax2 C-terminal |
| CaPOPI_5180 | Ca7 | 29069848 | AGG/AG | AGTCTTTCCCTTCCCTCCAA | CAACATGGCTTCCAACCTCT | 60.0 | 875 | DRR | Ca_11799 | | | Uncharacterised protein, Wax2 C-terminal |
| CaPOPI_5181 | Ca7 | 29070532 | TATTAGATACCAT/TAT | TCATTACCCTCCTTGGCAAC | AAACACCGCAAGGATTGTTC | 59.9 | 265 | INTRON | Ca_11799 | | | Uncharacterised protein, Wax2 C-terminal |
| CaPOPI_5182 | Ca7 | 29085797 | TAAAAAA/TAAAAAAAA | CCAAATTATCCTCTCTTTTTCCC | GAATTGGACCGAATGCAAGT | 59.4 | 656 | INTERGENIC | | | | |
| CaPOPI_5183 | Ca7 | 29090138 | GTATA/GTA | TGTCCTGTTTGTGAAACTTGC | ATCAAGTCAAACTCACCACAAA | 58.8 | 745 | INTERGENIC | | | | |
| CaPOPI_5184 | Ca7 | 29095794 | ATTTTTTTTTT/ATTTTTTTT | CATTGAATCTATTTGTCCGCAA | CTTTGGTAGGACAGCAAAAGC | 60.0 | 390 | INTERGENIC | | | | |
| CaPOPI_5185 | Ca7 | 29104256 | AC/ACTC | TTTTGATTTCAGGCCTCCAC | TCGTATTGATGCAGCTCCTG | 60.1 | 573 | INTRON | Ca_11798 | Q | | ABC transporter, transmembrane domain |

| INDEL marker IDs | Chromosomes/ unanchored scaffolds | Physical positions (bp) | InDels (*Kabuli* reference genome-CDC Frontier/PI) | Forward primers (5'-3') | Reverse primers (5'-3') | Anneling temperature (0C) | Expected amplified product size (bp) | Structural annotation | | Functional annotation | | |
|---|---|---|---|---|---|---|---|---|---|---|---|---|
| | | | | | | | | Sequence components of *kabuli* genome | *Kabuli* gene accession IDs | NCBI-KOG | TFs | NCBI-nr database |
| CaPOPI_5186 | Ca7 | 29118994 | CATTTAATTTAATTTAATTT/CATTTAATTTAATTTAATTTAATTT | TTTAACTTCTCCGCGGATTC | CTCCCAAACGCTCTCGTAAG | 59.3 | 490 | INTRON | Ca_11796 | FE | | Phosphoribosyltransferase |
| CaPOPI_5187 | Ca7 | 29121824 | GATAAATAAATAAATAA/GATAAATAAATAAATAAATAA | TTAGTGCCAACAAATGCGAG | TTGCCACGTCATTGAAAGAG | 59.9 | 492 | INTRON | Ca_11795 | S | | NADH dehydrogenase [ubiquinone] 1 alpha subcomplex assembly factor 3 |
| CaPOPI_5188 | Ca7 | 29127420 | CT/CTTT | GCAGTGTCCAGACCCTCATT | CACAACCACAAAGATGGCAC | 60.1 | 460 | INTRON | Ca_11794 | R | | tRNA (guanine-N-7) methyltransferase |
| CaPOPI_5189 | Ca7 | 29128437 | TC/T | TCTATAATTCCCGTGTTGCG | ACCTGCAATTTTGGATGAGC | 58.7 | 599 | DRR | Ca_11794 | R | | tRNA (guanine-N-7) methyltransferase |
| CaPOPI_5190 | Ca7 | 29133168 | CAAA/CAAAAAA | AGAGCAGCTTTTTCCCCATT | GAAGAAAAGTATTGCACGTTTGAA | 60.2 | 720 | DRR | Ca_11793 | | | |
| CaPOPI_5191 | Ca7 | 29135633 | ATTAATTTTAATTTTAATT/ATTAATTTTAATTTTAATTTTAATT | CGACGATTTCGACAATCAAA | AAATGCAAACCCAACTCACC | 59.7 | 955 | INTERGENIC | | | | |
| CaPOPI_5192 | Ca7 | 29164762 | CAAT/CAATAAAAT | ATGGTCATGTCAAATGGACG | GGAAAAAGTGGATTGGGGTT | 59.2 | 610 | INTERGENIC | | | | |
| CaPOPI_5193 | Ca7 | 29172192 | GAAAAAAAAA/GAAAAAAA | CACCACCACTACCCCATTTC | TGCTTGTTCCTCACTTGTGC | 60.1 | 628 | INTERGENIC | | | | |
| CaPOPI_5194 | Ca7 | 29177568 | CAAAAA/CAAA | GCTTATTCCTGGTCCCCATT | TGATACAAAGCAACCCAGCA | 60.2 | 381 | INTRON | Ca_11788 | | | EF-HAND 2 |
| CaPOPI_5195 | Ca7 | 29179462 | CTTT/CTT | CTGAAGGTGTCCCTGGTGTT | GCGGCTACCAAACTTGACTC | 60.0 | 489 | DRR | Ca_11788 | | | EF-HAND 2 |
| CaPOPI_5196 | Ca7 | 29191236 | GAAA/GAA | TACCGGAAAACTTCACGGAC | GAACCAATGAGCATGAGCAA | 60.0 | 611 | DRR | Ca_11786 | J | | Glutamyl/glutaminyl-tRNA synthetase, class Ic |
| CaPOPI_5197 | Ca7 | 29196375 | CTT/CTTT | CTTCTCGGAAACTGACAGGG | CCACATGAGCGTGAACAATC | 59.8 | 359 | DRR | Ca_11785 | | | |
| CaPOPI_5198 | Ca7 | 29208189 | CTTT/CTT | AAACTTTCACGACCCTCCCT | CACCGGGTTACAAATTCTGC | 60.0 | 307 | INTERGENIC | | | | |
| CaPOPI_5199 | Ca7 | 29219777 | CT/CTCACGAT | TGAAGTTGTAGGGCTTGCCT | TGCTTAGAGCCTTTTGGCAT | 59.9 | 588 | CDS (large-effect mutations) | Ca_11784 | | | Protein of unknown function DUF3550/UPF0682 |
| CaPOPI_5200 | Ca7 | 29221906 | CTTTTT/CTT | TATCGCCAATCTCCCATCTC | CTCGATGACAGTGGCAAAAA | 60.0 | 429 | DRR | Ca_11783 | C | | NADH:ubiquinone oxidoreductase, 17.2kDa subunit |

| INDEL marker IDs | Chromosomes/ unanchored scaffolds | Physical positions (bp) | InDels (*Kabuli* reference genome-CDC Frontier/PI) | Forward primers (5'-3') | Reverse primers (5'-3') | Anneling temperature (0C) | Expected amplified product size (bp) | Structural annotation | | Functional annotation | | |
|---|---|---|---|---|---|---|---|---|---|---|---|---|
| | | | | | | | | Sequence components of *kabuli* genome | *Kabuli* gene accession IDs | NCBI-KOG | TFs | NCBI-nr database |
| CaPOPI_5201 | Ca7 | 29223450 | CA/CAA | AGGCCTCAAGAAGGTTGTGA | ATTTCATGGGGTGGAAAACA | 59.8 | 414 | DRR | Ca_11783 | C | | NADH:ubiquinone oxidoreductase, 17.2kDa subunit |
| CaPOPI_5202 | Ca7 | 29226958 | AATTGATA/AATTGATATTGATA | TTCCATCTGGAAGGCATCTC | TCCTGTTTGAGGGGGTGTAA | 60.2 | 577 | INTRON | Ca_11783 | C | | NADH:ubiquinone oxidoreductase, 17.2kDa subunit |
| CaPOPI_5203 | Ca7 | 29240073 | CAAA/CAAAAAA | GCAGAGGTCCGAGAGAAATG | GCATCCACTACCGCTGAAAT | 60.0 | 583 | INTERGENIC | | | | |
| CaPOPI_5204 | Ca7 | 29265546 | T/TA | ATTGTACCTTCGCGAACACC | TAGCAATGTCACGAAATGCC | 60.0 | 707 | INTRON | Ca_11779 | | C2H2 | Glycoside hydrolase, family 28 |
| CaPOPI_5205 | Ca7 | 29280548 | ATATTATTATTATTATTATTATTATTATTATT/ATATTATTATTATTATTATTATTATTATT | TTGTTTGAAAAACCAATATGACA | TTGCAACTACCAAAATAAACAATTTC | 57.2 | 875 | INTERGENIC | | | | |
| CaPOPI_5206 | Ca7 | 29296655 | AATTATTATTATTAT/AATTATTATTATTATTATTAT | TGCATGTCTTCTAAGGGTATTTTTC | AGGGAGCTGGTAGGGGATTA | 59.9 | 837 | INTERGENIC | | | | |
| CaPOPI_5207 | Ca7 | 29322502 | TATTTTGAT/TATTTTGATTTTGAT | AAACGCTCTTAAAAACATTCTTCC | CTCGTACAATGGCCCAGATT | 59.3 | 852 | INTERGENIC | | | | |
| CaPOPI_5208 | Ca7 | 29324634 | TTT/TTTATT | TGGCAAATGCCTACAATTCA | TTTTTCCCCATATTTTTCGTTG | 60.1 | 865 | INTERGENIC | | | | |
| CaPOPI_5209 | Ca7 | 29326662 | CAAAAA/CAAA | AAGTCAACGGAATCTCATCCA | GCAAAGCGATTAATGACAGG | 59.5 | 978 | INTERGENIC | | | | |
| CaPOPI_5210 | Ca7 | 29334299 | ACATCATCA/ACATCATCATCATCA | AATCCCCACCTGGATACCTC | GCTCGCTACGTACGACAACA | 60.0 | 609 | INTERGENIC | | | | |
| CaPOPI_5211 | Ca7 | 29344358 | TTA/TTAGAGTATTTATA | TTATCATCATGTAACCATGTGTCTT | CACGCTCCCACTAATCTTACA | 57.5 | 227 | INTERGENIC | | | | |
| CaPOPI_5212 | Ca7 | 29346372 | G/GC | CAAAAGGGAAGATGCAATTTTT | TTTGCGGGATGAATTCTTTT | 59.5 | 206 | INTERGENIC | | | | |
| CaPOPI_5213 | Ca7 | 29347288 | CAAT/CAATTAAT | TTCAAATCCACGATTAGGGTG | CATCAGCTATGCCATCTCCA | 59.8 | 829 | INTERGENIC | | | | |
| CaPOPI_5214 | Ca7 | 29357883 | TTTATT/TTT | ATGAATGATTGCGTCCATGA | GGGACCACCGGTGTTATTTA | 59.9 | 517 | INTERGENIC | | | | |
| CaPOPI_5215 | Ca7 | 29388652 | GTTTTT/GTTTT | AATAATTCTTCGGCATGCAA | ACATCAGTCAAGCGCACAAA | 58.3 | 832 | INTERGENIC | | | | |

| INDEL marker IDs | Chromosomes/ unanchored scaffolds | Physical positions (bp) | InDels (*Kabuli* reference genome-CDC Frontier/PI) | Forward primers (5'-3') | Reverse primers (5'-3') | Anneling temperature (0C) | Expected amplified product size (bp) | Structural annotation | | Functional annotation | | |
|---|---|---|---|---|---|---|---|---|---|---|---|---|
| | | | | | | | | Sequence components of *kabuli* genome | *Kabuli* gene accession IDs | NCBI-KOG | TFs | NCBI-nr database |
| CaPOPI_5216 | Ca7 | 29392132 | TAAAAAAA/TAAAAAAAA | TCAATTGAGGACATTCGTCG | CGATTCGTATAATTTCACCCA | 59.6 | 542 | INTERGENIC | | | | |
| CaPOPI_5217 | Ca7 | 29410137 | GTCT/GT | TTGTAGGGCGGATCTGGTTA | GGGCATTCCTACATGCAGAT | 60.5 | 541 | INTERGENIC | | | | |
| CaPOPI_5218 | Ca7 | 29416537 | ATATAAATTATAAATTATAAATTAT/ATATAAATTATAAATTATAAATTATAAATTAT | AAAATCATTACTCACCATAAGTGGAA | TGCTCTCCTCCTTCAACAACT | 59.3 | 436 | INTERGENIC | | | | |
| CaPOPI_5219 | Ca7 | 29418151 | GT/G | CATGTACACGTCACATCGGA | TTGTAGGCTGGACATCATGC | 59.0 | 329 | INTERGENIC | | | | |
| CaPOPI_5220 | Ca7 | 29446111 | CATT/CATTGATT | GTCTCCGCTCAATCAGAAGG | TCATTCCCAACATTATTTCGTG | 60.0 | 763 | DRR | Ca_11775 | R | | Alpha/beta hydrolase fold-1 |
| CaPOPI_5221 | Ca7 | 29446201 | ATTTTTTTTTT/ATTTTTTTTTTT | CGTTGCGCATGTGTAATTTG | TCATTCCCAACATTATTTCGTG | 61.1 | 639 | DRR | Ca_11775 | R | | Alpha/beta hydrolase fold-1 |
| CaPOPI_5222 | Ca7 | 29458658 | GCA/GCAATCA | CACTGTGGTATGACCGGAAG | GCCCTTTGATCATGCAACTT | 59.0 | 460 | INTRON | Ca_11773 | S | | Autophagy-related protein 3 |
| CaPOPI_5223 | Ca7 | 29465694 | GCTC/GCTCTC | AATCATTTTCACCCACCTGC | GGGATATGGTCCTCGAGTCA | 59.8 | 807 | DRR | Ca_11772 | | ERF | Frigida-like |
| CaPOPI_5224 | Ca7 | 29489393 | C/CT | TCGACCGGTACTGATTCAAA | AAATGATAAGCCTCGTTGCG | 59.1 | 280 | INTERGENIC | | | | |
| CaPOPI_5225 | Ca7 | 29496210 | TG/T | TTTTTCTTTCAAATGCAAGCC | TGTGCTTCTGAGCTTCATGG | 59.4 | 300 | INTERGENIC | | | | |
| CaPOPI_5226 | Ca7 | 29535944 | CTT/CT | AGGTTGATCTGGACTGGTGG | TGGTTCACCCAATTCAGGTT | 60.0 | 109 | INTERGENIC | | | | |
| CaPOPI_5227 | Ca7 | 29537259 | T/TG | TTTGCACCACACCTTGGTTA | GAACAAAAAGGAAAGGGGAAA | 60.0 | 528 | INTERGENIC | | | | |
| CaPOPI_5228 | Ca7 | 29550695 | AT/A | AAGCATCCCAATTTCGTCTG | AGAACTTGAGGCGACCAGAA | 60.1 | 466 | DRR | Ca_11767 | | SBP | Profilin/allergen |
| CaPOPI_5229 | Ca7 | 29552593 | TATTAATTA/TATTA | TTTGCACCACACCTTGGTTA | GCTTTTGAAGGGGATTATGTAGG | 60.0 | 586 | INTERGENIC | | | | |
| CaPOPI_5230 | Ca7 | 29572407 | GT/GTTT | CGTCCAAGACCATTCAACCT | CGCTCCATTCGCTTTCTTAC | 60.0 | 324 | INTERGENIC | | | | |

| INDEL marker IDs | Chromosomes/ unanchored scaffolds | Physical positions (bp) | InDels (*Kabuli* reference genome-CDC Frontier/PI) | Forward primers (5'-3') | Reverse primers (5'-3') | Anneling temperature (0C) | Expected amplified product size (bp) | Structural annotation | | Functional annotation | | |
|---|---|---|---|---|---|---|---|---|---|---|---|---|
| | | | | | | | | Sequence components of *kabuli* genome | *Kabuli* gene accession IDs | NCBI-KOG | TFs | NCBI-nr database |
| CaPOPI_5231 | Ca7 | 29580185 | TAAAAAA/TAAAAAAA | GTTCACACACGTAGGCCAAA | TCATGTGCGTTTGACTGATG | 59.6 | 537 | INTERGENIC | | | | |
| CaPOPI_5232 | Ca7 | 29582004 | AA/AATAAATA | GCAATGTGATTTCATTCGTGA | AGCCCAATCTTTTGGTTTCA | 59.5 | 297 | INTERGENIC | | | | |
| CaPOPI_5233 | Ca7 | 29582896 | ATTTTTTTT/ATTTTTTTTT | ATGGCTAAATAACACCCGCA | TAACCAAGGTGTGGTGCAAA | 60.3 | 377 | INTERGENIC | | | | |
| CaPOPI_5234 | Ca7 | 29584512 | ATTTTTT/ATTTTTTTT | GGTATGAAGGTGCAATGGCT | TGAAGCTCAATTTCATTGGAGA | 60.0 | 771 | INTERGENIC | | | | |
| CaPOPI_5235 | Ca7 | 29595027 | AAAGAAGAAGAAGAAGAAGAAGAA/AAAGAAGAAGAAGAAGAAGAA | TCCAAGTGGTGAAAATGTGTG | CAAACCTTGGCAAAGGAGAA | 59.5 | 254 | INTERGENIC | | | | |
| CaPOPI_5236 | Ca7 | 29609973 | TAAAAAAAAAAA/TAAAAAAAAA | AATCACATTCAACTGCCGTG | TGAGTTAAGCCCCGCTAGAA | 59.6 | 702 | DRR | Ca_11764 | | | F-box domain, cyclin-like |
| CaPOPI_5237 | Ca7 | 29610103 | ATTTTTT/ATTTTTTTT | AACAGAAAGAAGGCCGTTTG | TGAGTTAAGCCCCGCTAGAA | 59.4 | 134 | DRR | Ca_11764 | | | F-box domain, cyclin-like |
| CaPOPI_5238 | Ca7 | 29615212 | ATTTT/ATTTTT | TGCCAATTCCACAATGAAAA | AGTGAAACCAACGCCAAAAC | 59.9 | 474 | INTERGENIC | | | | |
| CaPOPI_5239 | Ca7 | 29615433 | AA/AATCGATCTAACTCGA | TGCCAATTCCACAATGAAAA | AGTGAAACCAACGCCAAAAC | 59.9 | 474 | INTERGENIC | | | | |
| CaPOPI_5240 | Ca7 | 29615700 | TA/TAA | GGCGTTGGTTTCACTCAAAT | ATGAAGCTTACGGCCATCAC | 60.0 | 277 | INTERGENIC | | | | |
| CaPOPI_5241 | Ca7 | 29616903 | ATTTTTTTTT/ATTTTTTTTTT | GGGGTGTTACACTTGAGGCA | TCGGAGAAAAATAGGAGCAAA | 61.0 | 551 | INTERGENIC | | | | |
| CaPOPI_5242 | Ca7 | 29617184 | TTTTTTTATTTTTTATTT/TTTTTTTATTTTTTATTTTTTATTT | TTTGCTCCTATTTTTCTCCGA | GTAAGTCATGCCATAGGCCC | 59.0 | 378 | INTERGENIC | | | | |
| CaPOPI_5243 | Ca7 | 29645650 | ATA/ATATAAATCTTA | GCCTCATCTACGATTACGTCG | CTTGCAAAGCATTTGGATCA | 59.7 | 693 | INTERGENIC | | | | |
| CaPOPI_5244 | Ca7 | 29645891 | CAA/CAAA | GAAAGCGAGAAAGAGGGAAAA | CTTGCAAAGCATTTGGATCA | 60.0 | 483 | INTERGENIC | | | | |
| CaPOPI_5245 | Ca7 | 29672845 | AA/AATA | TTTCTCAATTACGGGAGCATC | TCATCAAATTCGTAACGCAAA | 59.2 | 834 | DRR | Ca_11757 | P | MIKC | Heavy metal transport/detoxification protein |

| INDEL marker IDs | Chromosomes/ unanchored scaffolds | Physical positions (bp) | InDels (*Kabuli* reference genome-CDC Frontier/PI) | Forward primers (5'-3') | Reverse primers (5'-3') | Anneling temperature (0C) | Expected amplified product size (bp) | Structural annotation | | Functional annotation | | |
|---|---|---|---|---|---|---|---|---|---|---|---|---|
| | | | | | | | | Sequence components of *kabuli* genome | *Kabuli* gene accession IDs | NCBI-KOG | TFs | NCBI-nr database |
| CaPOPI_5246 | Ca7 | 29673545 | TTA/TTAAATA | AAAGGACCAAGTCGGAAAAA | GGCAATAAGGGCAAAGAAGA | 58.7 | 341 | DRR | Ca_11757 | P | MIKC | Heavy metal transport/detoxification protein |
| CaPOPI_5247 | Ca7 | 29736728 | ATTT/ATT | ATGCGGCCAACTTGTTAAAG | TGAATCATCGAATCCAAATCA | 60.1 | 209 | INTERGENIC | | | | |
| CaPOPI_5248 | Ca7 | 29745710 | A/AG | TTTGTTTACAAGAGTATCTTTGCCA | TTACACCCCACAAGCAACAA | 59.3 | 632 | INTERGENIC | | | | |
| CaPOPI_5249 | Ca7 | 29755622 | GAAAAGAGAAAAAGAGAAAAAGA/GAAAAGAGAAAAAGA | TGGATTATTGACCCGATTGC | TCCAGAAACTTTGGTTTGGG | 60.7 | 760 | INTRON | Ca_11749 | U | | Zinc finger, Sec23/Sec24-type |
| CaPOPI_5250 | Ca7 | 29770911 | ACCC/ACCCC | GCAACAGAGCCGTCCTAAAG | GGGGGAGACAGAGAAACCTC | 60.0 | 732 | INTRON | Ca_11747 | OE | WRKY | Peptidase S10, serine carboxypeptidase |
| CaPOPI_5251 | Ca7 | 29773203 | TAAAAAAAAAAA/TAAAAAA | GCGTGTGTATCAGACGCAGT | TTGTGTTTTGGGGGTAGGAA | 59.9 | 870 | INTRON | Ca_11747 | OE | WRKY | Peptidase S10, serine carboxypeptidase |
| CaPOPI_5252 | Ca7 | 29785311 | CTTGACTTTT/CTTGACTTTTGACTTTT | TTTCCCCATGCAGAGTTAGG | TCAAACCACAGATCACCAGC | 60.1 | 727 | INTERGENIC | | | | |
| CaPOPI_5253 | Ca7 | 29800193 | GTCTCTCTCTCTCTCTCTCTCTCTCTCTCTCTC/GTCTCTCTCTCTCTCTCTCTCTCTCTCTCTCTCTCTC | TTATTGGAGGGATGAGGGGT | GCAGGAAAGTGAGAAGACGG | 60.5 | 508 | INTRON | Ca_11746 | Z | | Spc97/Spc98 |
| CaPOPI_5254 | Ca7 | 29805110 | CAGGAGG/CAGG | AATTGCACACACACACACCC | CAATTTCTGGAGCCAAAAGC | 60.3 | 511 | INTRON | Ca_11745 | Z | | Calponin homology domain |
| CaPOPI_5255 | Ca7 | 29805558 | TTAATAATA/T | GCTTTTGGCTCCAGAAATTG | AGTGGAAGGATCCCCAAATC | 59.8 | 253 | INTRON | Ca_11745 | Z | | Calponin homology domain |
| CaPOPI_5256 | Ca7 | 29815328 | AT/ATTT | TTCAGGTGATGTGGAAGCAA | AAAGCTAATGTGCCCCAAAA | 60.2 | 451 | INTRON | Ca_11744 | | | Spc97/Spc98 |
| CaPOPI_5257 | Ca7 | 29843656 | AT/ATCTT | GAGTGTTTCGACCTTTTGGC | GTTGGCTTATGTGCAGGGAT | 59.7 | 575 | INTRON | Ca_11739 | | | Lipase, class 3 |
| CaPOPI_5258 | Ca7 | 29852813 | TTA/T | AATCCCTGTATTTGATGCGG | TTGCATACGCCATCGTAAAA | 59.8 | 317 | INTERGENIC | | | | |
| CaPOPI_5259 | Ca7 | 29856343 | TT/TTATAGT | CTTTGGAACATGTTAGTGGCA | TTCACAAACGACCGTTGAAA | 58.7 | 249 | DRR | Ca_11738 | T | | DOMON domain |
| CaPOPI_5260 | Ca7 | 29865664 | CATATA/CATA | ATTTTTCATCATGCTTTTGTCA | TGCAGTTCTGTTTGGATTGC | 57.3 | 629 | INTERGENIC | | | | |

| INDEL marker IDs | Chromosomes/ unanchored scaffolds | Physical positions (bp) | InDels (*Kabuli* reference genome-CDC Frontier/PI) | Forward primers (5'-3') | Reverse primers (5'-3') | Anneling temperature (0C) | Expected amplified product size (bp) | Structural annotation | | Functional annotation | | |
|---|---|---|---|---|---|---|---|---|---|---|---|---|
| | | | | | | | | Sequence components of *kabuli* genome | *Kabuli* gene accession IDs | NCBI-KOG | TFs | NCBI-nr database |
| CaPOPI_5261 | Ca7 | 29866869 | TAAAAAAAAA/TAAAAAAAAAAAAA | AGGTGAAACTTCCGAAATGC | AAAATAGGGCCTCACCTGCT | 59.2 | 548 | DRR | Ca_11737 | R | | Protein kinase, catalytic domain |
| CaPOPI_5262 | Ca7 | 29969276 | ATTTT/ATTTTT | ATAGGCACAACAACTTGCCC | AGTCACGTTCATCGAGAGACAA | 60.0 | 681 | DRR | Ca_11731 | | | |
| CaPOPI_5263 | Ca7 | 29969403 | CAT/C | CGTCGTAGAGCCATTGATGA | TGGCTATGAAACCCGGATAC | 59.8 | 729 | DRR | Ca_11731 | | | |
| CaPOPI_5264 | Ca7 | 29969461 | TG/T | CGTCGTAGAGCCATTGATGA | GGGAGGATGGACAATGCTTA | 59.8 | 871 | DRR | Ca_11731 | | | |
| CaPOPI_5265 | Ca7 | 29972956 | TAAAAAA/TAAAAAAA | ATGGATCCCAGTATCCCACA | TAACAAGCTTGGAATGCACG | 60.0 | 269 | INTERGENIC | | | | |
| CaPOPI_5266 | Ca7 | 29976341 | CAA/CA | CCCCAATTGTTCATGTTTCC | TGGCTCAACTTGCACAAAAC | 60.0 | 288 | DRR | Ca_11730 | KR | | Mitochodrial transcription termination factor-related |
| CaPOPI_5267 | Ca7 | 29979176 | A/AGAGTT | ATTACGCAACGAGGGACAAC | TATGCACATGCTTTCACCGT | 60.0 | 445 | INTERGENIC | | | | |
| CaPOPI_5268 | Ca7 | 29979856 | TTT/TTTTATT | CAAATGAAAAGAAGAGACATTCCA | TTGAGAACCAAACAACTTTCCTT | 59.6 | 888 | INTERGENIC | | | | |
| CaPOPI_5269 | Ca7 | 29980723 | CAAAAA/CAAAA | CACATTTTGTGTCCAATCTGTTG | ATCCTGTCCATCAAACGAGC | 60.3 | 756 | INTERGENIC | | | | |
| CaPOPI_5270 | Ca7 | 29980870 | T/TC | CAACCGCAATCTTAACAAAAA | ATCCTGTCCATCAAACGAGC | 57.9 | 402 | INTERGENIC | | | | |
| CaPOPI_5271 | Ca7 | 29982388 | ATTT/ATT | TCTCCACCCAATTTTTCGAC | GTGCGAATTCAGGTTCGATT | 59.9 | 397 | DRR | Ca_11729 | | | Protein of unknown function DUF506, plant |
| CaPOPI_5272 | Ca7 | 29983649 | TAAAAAAAA/TAAAAAAA | TCAATGGAGCTCAATCTTCAAA | TTGGATTGGGTCGATTTTGT | 59.8 | 619 | DRR | Ca_11729 | | | Protein of unknown function DUF506, plant |
| CaPOPI_5273 | Ca7 | 29986240 | A/AT | AAATGAACGGCACCAACATT | CCAATTCCAAATGCCAAATC | 60.2 | 536 | INTERGENIC | | | | |
| CaPOPI_5274 | Ca7 | 29994584 | TTATA/TTA | CTCCGCAATTGTATGCAGAA | CGTCGGTGCTGTAATGTGTC | 59.8 | 315 | INTERGENIC | | | | |
| CaPOPI_5275 | Ca7 | 30002970 | ATTTTTTT/ATTTTTT | CGAAATTACCGGTTTGCCTA | CGTTGTCGGGTTCACATAGA | 60.0 | 495 | INTERGENIC | | | | |

| INDEL marker IDs | Chromosomes/ unanchored scaffolds | Physical positions (bp) | InDels (*Kabuli* reference genome-CDC Frontier/PI) | Forward primers (5'-3') | Reverse primers (5'-3') | Anneling temperature (0C) | Expected amplified product size (bp) | Structural annotation | | Functional annotation | | |
|---|---|---|---|---|---|---|---|---|---|---|---|---|
| | | | | | | | | Sequence components of *kabuli* genome | *Kabuli* gene accession IDs | NCBI-KOG | TFs | NCBI-nr database |
| CaPOPI_5276 | Ca7 | 30007344 | AAACAAC/AAACAACAAC | CCTTTTCTTGAACATCCCTCC | TTGATCCGTTGACTTTTCCC | 59.9 | 385 | INTERGENIC | | | | |
| CaPOPI_5277 | Ca7 | 30007406 | GAAAAA/GAAAA | CCTTTTCTTGAACATCCCTCC | TTGATCCGTTGACTTTTCCC | 59.9 | 385 | INTERGENIC | | | | |
| CaPOPI_5278 | Ca7 | 30009218 | ATTATTTAT/ATTATTTATTTAT | AATTGTGGTTGTGGTTCGGT | GATGGTGCTGGGGAATCTAA | 60.1 | 881 | INTRON | Ca_11728 | | bHLH | Helix-loop-helix DNA-binding domain |
| CaPOPI_5279 | Ca7 | 30009540 | T/TTTG | ACGGAGGGAGAAAATCAGTG | GATGGTGCTGGGGAATCTAA | 59.1 | 580 | INTRON | Ca_11728 | | bHLH | Helix-loop-helix DNA-binding domain |
| CaPOPI_5280 | Ca7 | 30015362 | CTTTTTTT/CTTTTTTTTT | CCAAAATTAGAAAAGCTTTGAACG | CCCCAAGCAAAAGAAACAAA | 60.5 | 562 | INTERGENIC | | | | |
| CaPOPI_5281 | Ca7 | 30026336 | AATGATG/AATG | GAGCTGAGGAAGGGTTGATG | TCTTTGCCCAACTAACCTGG | 59.8 | 507 | INTRON | Ca_11727 | | | Glycosyl transferase, family 2 |
| CaPOPI_5282 | Ca7 | 30041676 | CA/C | ATGCGTGAAGAAGAGGGATG | AATCGGACAAGGGACACAAA | 60.2 | 428 | INTERGENIC | | | | |
| CaPOPI_5283 | Ca7 | 30048460 | GAAAAAAAAAAAAAAA/GAAAAAAAAAAA | TGTGTGTGTGTGTAAATGGCA | AAGAGCGGGGACGTATTTTT | 59.5 | 585 | DRR | Ca_11723 | O | GeBP | Peptidyl-prolyl cis-trans isomerase, FKBP-type, domain |
| CaPOPI_5284 | Ca7 | 30053126 | TAA/TA | AAAGAGAGAACGGACATAATCCA | TCGCATATTTTTCTTTTCAGTCC | 59.1 | 550 | INTERGENIC | | | | |
| CaPOPI_5285 | Ca7 | 30053648 | CAAAGT/C | GGACTGAAAAGAAAAATATGCGA | GTGCTCTCTTTTGCCGATTC | 59.6 | 774 | INTERGENIC | | | | |
| CaPOPI_5286 | Ca7 | 30072638 | GATAATTACAAA/GATAATTACAAATAATTACAAA | TGAGATTTGGAACTAACGGTGA | AAAGGATCAAATCGGAGAAAA | 59.6 | 349 | INTERGENIC | | | | |
| CaPOPI_5287 | Ca7 | 30073842 | AGTGT/AGTGTGT | CGGACAACGAGTGTTATTTAGC | GCCAATGGGTGCTGATTATT | 58.8 | 944 | DRR | Ca_11719 | | NAC | Uncharacterised protein domain XH |
| CaPOPI_5288 | Ca7 | 30091306 | ATTTT/ATTT | ACATTCCACGGTCTAGCTGC | GATGAATTTCCAGGCCTCAC | 60.3 | 888 | INTERGENIC | | | | |
| CaPOPI_5289 | Ca7 | 30099390 | TA/TAA | TGGGCACATGTCACAAAACT | TGAGGTATGGGAAGGGTGAG | 60.0 | 840 | INTERGENIC | | | | |
| CaPOPI_5290 | Ca7 | 30107818 | TA/TAACCAGTTTATAA | GAAGGCTGCAACTCCTTGAC | AACGTGAAACCCACCAAAAC | 60.0 | 764 | INTRON | Ca_11716 | T | WRKY | Protein kinase, catalytic domain |

| INDEL marker IDs | Chromosomes/ unanchored scaffolds | Physical positions (bp) | InDels (*Kabuli* reference genome-CDC Frontier/PI) | Forward primers (5'-3') | Reverse primers (5'-3') | Anneling temperature (0C) | Expected amplified product size (bp) | Structural annotation | | Functional annotation | | |
|---|---|---|---|---|---|---|---|---|---|---|---|---|
| | | | | | | | | Sequence components of *kabuli* genome | *Kabuli* gene accession IDs | NCBI-KOG | TFs | NCBI-nr database |
| CaPOPI_5291 | Ca7 | 30129775 | AATGAT/AATGATGATGAT | CCACGTGAGTTACCATGCTG | AATGCATTCAATTCGGAACC | 60.2 | 203 | INTRON | Ca_11715 | U | | Citron-like |
| CaPOPI_5292 | Ca7 | 30137991 | AAAAGC/AAAAGCTCTTAATTAAAGC | TGGCTATTTTTGTGTTGCCA | TTCCCTCTGCCAACAATTTC | 60.1 | 424 | DRR | Ca_11715 | U | | Citron-like |
| CaPOPI_5293 | Ca7 | 30140331 | TAAA/TAA | CTTTGGCCAGAAGGGACATA | TTTATGCTGGTGGGATGGAT | 60.1 | 522 | INTRON | Ca_11714 | H | | GTP cyclohydrolase I/Nitrile oxidoreductase |
| CaPOPI_5294 | Ca7 | 30154601 | ATCTTCTTC/ATCTTC | TGGGTTGTTAACATAATTTTGAATG | CTTAACCCACCAACCACCAG | 59.1 | 293 | INTERGENIC | | | | |
| CaPOPI_5295 | Ca7 | 30157722 | CG/C | CCGAGATGAATGGGAAGAAA | AACACCTAGGAGGAGAAGAAAACA | 60.0 | 354 | INTERGENIC | | | | |
| CaPOPI_5296 | Ca7 | 30157868 | GA/GAA | CCGAGATGAATGGGAAGAAA | GCTCCTGTTCAATAGAGTGCAT | 60.0 | 716 | INTERGENIC | | | | |
| CaPOPI_5297 | Ca7 | 30166196 | CAA/CTTAAA | TGAGCCCATAATGTGCAGAA | CTTTTTGAAAGGGCATCAGC | 60.2 | 520 | DRR | Ca_11711 | Q | B3 | Cytochrome P450 |
| CaPOPI_5298 | Ca7 | 30173467 | ATTTTTTTTT/ATTTTTTTTTT | TGGTCAATTCACCTGCAAAA | TTCCTTGTGAACCAATTCGC | 60.1 | 542 | INTERGENIC | | | | |
| CaPOPI_5299 | Ca7 | 30192100 | A/ATT | CATCGTTGGTTGTTTGGTTG | TGCAAAAATGACTCGGACAA | 59.9 | 279 | INTRON | Ca_11709 | U | | ER lumen protein retaining receptor |
| CaPOPI_5300 | Ca7 | 30192636 | ATAAGCATT/AT | CCTTCAGCAATTTTGGCAGT | TCCCCATGGTTCAAAGTTGT | 60.2 | 594 | INTRON | Ca_11709 | U | | ER lumen protein retaining receptor |
| CaPOPI_5301 | Ca7 | 30208302 | CGA/C | TTGGACGATGTGGATTGAAA | CCATCTCAGCATGTGTTTGG | 59.9 | 642 | INTRON | Ca_11706 | | | Mitochondrial inner membrane translocase complex, subunit Tim17/22 |
| CaPOPI_5302 | Ca7 | 30208770 | ATTT/ATT | CCAAACACATGCTGAGATGG | CTTCCTTTGCACGACCGTAT | 60.1 | 707 | INTRON | Ca_11706 | | | Mitochondrial inner membrane translocase complex, subunit Tim17/22 |
| CaPOPI_5303 | Ca7 | 30221695 | T/TGAA | CTTGTTGATGCAATGTTGGC | TTTGGCCACACAAAAACAAA | 60.1 | 538 | INTERGENIC | | | | |
| CaPOPI_5304 | Ca7 | 30228676 | TTTTAA/TTTTAACTTTAA | AGACATGTCGCATCATGGAA | TGCTTTGAACTTTGGAAACG | 60.1 | 408 | INTERGENIC | | | | |
| CaPOPI_5305 | Ca7 | 30235069 | TTTCTTCTTCTTCTTCT/TTTCTTCTTCTTCT | TGCGTGTTCGAGTTGTAAGG | AACGTGCCAAAAACCACTTC | 59.9 | 606 | INTERGENIC | | | | |

| INDEL marker IDs | Chromosomes/ unanchored scaffolds | Physical positions (bp) | InDels (*Kabuli* reference genome-CDC Frontier/PI) | Forward primers (5'-3') | Reverse primers (5'-3') | Anneling temperature (0C) | Expected amplified product size (bp) | Structural annotation | | Functional annotation | | |
|---|---|---|---|---|---|---|---|---|---|---|---|---|
| | | | | | | | | Sequence components of *kabuli* genome | *Kabuli* gene accession IDs | NCBI-KOG | TFs | NCBI-nr database |
| CaPOPI_5306 | Ca7 | 30240282 | ACGT/ACGTGATCGT | TAAATGCGGCAATGTCAAAA | TCCATTTCGTGAGTAAAACATCA | 60.1 | 219 | INTERGENIC | | | | |
| CaPOPI_5307 | Ca7 | 30269288 | AAAT/A | ATTTAATTCGGCTTTGCGTG | AAAACGGGTGGCACAATAGA | 60.1 | 620 | INTERGENIC | | | | |
| CaPOPI_5308 | Ca7 | 30276513 | AAAT/AAATAAT | TTTCCATCATCAATTCCTTTTG | GAGGTATGGTGCAACCTGCT | 58.9 | 686 | INTERGENIC | | | | |
| CaPOPI_5309 | Ca7 | 30278558 | ATT/ATTT | CCCGTGGTTATTGCATTTTC | AATACCAGATTTGGGCGTTG | 60.2 | 529 | DRR | Ca_11700 | C | | Ribosomal protein/NADH dehydrogenase domain |
| CaPOPI_5310 | Ca7 | 30279217 | T/TCTAGGCC | CAACGCCCAAATCTGGTATT | TGCTAATCAGCATTTTCCCC | 59.8 | 517 | INTRON | Ca_11700 | C | | Ribosomal protein/NADH dehydrogenase domain |
| CaPOPI_5311 | Ca7 | 30280166 | GAAA/GAAAA | GATGTCCTCTCCATGCCATT | ATGTGAAGCATAGGCTTGGG | 59.9 | 300 | INTERGENIC | | | | |
| CaPOPI_5312 | Ca7 | 30294894 | A/AT | GTCGTGGTCCTACTCCGAAA | AAAGTCGCATCCTCTCAAAAA | 60.1 | 320 | INTRON | Ca_11698 | QI | bHLH | Cytochrome P450 |
| CaPOPI_5313 | Ca7 | 30298307 | GATATATATA/GATATATA | GCTTGGTGTCCCAAACAAGT | TGGGACCAAAACAGAAGAGAA | 60.0 | 583 | INTRON | Ca_11698 | QI | bHLH | Cytochrome P450 |
| CaPOPI_5314 | Ca7 | 30298620 | CTATATATATATA/CTATATATATA | GCTTGGTGTCCCAAACAAGT | CTTTCATGCATGCCGATTTA | 60.0 | 923 | INTRON | Ca_11698 | QI | bHLH | Cytochrome P450 |
| CaPOPI_5315 | Ca7 | 30303300 | AACACACACACA/AACACACACA | GAACCGATGGTTACACTCCAA | CGTCATCGGATTGAAGGAAT | 59.8 | 666 | INTERGENIC | | | | |
| CaPOPI_5316 | Ca7 | 30308260 | GAAAAAAAAA/GAAAAAAA | ACCCATGTCGTCATCCATTT | ATCATGGCTCCTATGGTTGG | 60.1 | 602 | INTRON | Ca_11697 | QI | B3 | Cytochrome P450 |
| CaPOPI_5317 | Ca7 | 30309875 | GTTACTTACTT/GTTACTT | CAAAAAGACAAGGGAGAAAATGA | TGGGATGGCCTATTATTGGA | 59.6 | 125 | CDS (FRAME SHIFT) | Ca_11697 | QI | B3 | Cytochrome P450 |
| CaPOPI_5318 | Ca7 | 30317892 | T/TTG | CATTGCACGAAAATGTGGTC | TTGCCTTTTCAATTTTTCGG | 60.0 | 633 | INTRON | Ca_11696 | QI | B3 | Cytochrome P450 |
| CaPOPI_5319 | Ca7 | 30359172 | CT/CTTT | CGGATAAGCACGCTCTTAGG | GAGATTGAAGGGTTTTGCCA | 60.0 | 349 | DRR | Ca_11693 | O | C2H2 | PUG domain |
| CaPOPI_5320 | Ca7 | 30365181 | CAAAA/CAA | TTTTCTTCTGCGATTCGCTT | CGGTCACATTGGAACACAAG | 60.1 | 610 | INTRON | Ca_11693 | O | C2H2 | PUG domain |

| INDEL marker IDs | Chromosomes/ unanchored scaffolds | Physical positions (bp) | InDels (*Kabuli* reference genome-CDC Frontier/PI) | Forward primers (5'-3') | Reverse primers (5'-3') | Anneling temperature (0C) | Expected amplified product size (bp) | Structural annotation | | Functional annotation | | |
|---|---|---|---|---|---|---|---|---|---|---|---|---|
| | | | | | | | | Sequence components of *kabuli* genome | *Kabuli* gene accession IDs | NCBI-KOG | TFs | NCBI-nr database |
| CaPOPI_5321 | Ca7 | 30365537 | GAAAAAAA/GAAAAAAAA | CTTGTGTTCCAATGTGACCG | GCAATACCAGCTTAGAGGAAGC | 60.0 | 299 | INTRON | Ca_11693 | O | C2H2 | PUG domain |
| CaPOPI_5322 | Ca7 | 30366631 | CTCTTCT/CTCTTCTTCT | TCACTTTTGGAACAGCCATTT | AAAAATCCTGTGCGATTCCA | 59.6 | 867 | INTERGENIC | | | | |
| CaPOPI_5323 | Ca7 | 30394719 | TATA/T | TCATCTTCGATGCAAATCCA | TGTCGTCGGATTTAAATGTTTTT | 60.2 | 375 | INTRON | Ca_11690 | | MYB_related | Glycoside hydrolase, family 16 |
| CaPOPI_5324 | Ca7 | 30394911 | TAAAAAAA/TAAAAAA | TCATCTTCGATGCAAATCCA | TTTTCTCACCAATCAAGCTGTC | 60.2 | 783 | INTRON | Ca_11690 | | MYB_related | Glycoside hydrolase, family 16 |
| CaPOPI_5325 | Ca7 | 30395077 | AAGAGTTTA/AA | AAAGGTGCTCGCATCTATGTA | TTTTCTCACCAATCAAGCTGTC | 57.6 | 540 | INTRON | Ca_11690 | | MYB_related | Glycoside hydrolase, family 16 |
| CaPOPI_5326 | Ca7 | 30395813 | CTATATATATAT/CTATATATATATAT | TCGATTCTCTCCCATGAAATC | AGGGAACGAAATTCTGCTCA | 59.1 | 556 | INTRON | Ca_11690 | | MYB_related | Glycoside hydrolase, family 16 |
| CaPOPI_5327 | Ca7 | 30415401 | ATG/A | GGAGTCATTCTCTCCCATGAA | GTCCTCGTCCCTCATGTTGT | 59.1 | 683 | INTERGENIC | | | | |
| CaPOPI_5328 | Ca7 | 30420041 | AATGTATCTA/AA | ATGCAGGGATTAGGCAGTTG | CCGATTCTATCATCGGCAAT | 60.1 | 667 | INTERGENIC | | | | |
| CaPOPI_5329 | Ca7 | 30662891 | TAAAAAAAA/TAAAAAAA | TTGGGGTAGTAAAACCTCAAGAGT | GGTCTGGGTGTCAAACCTGT | 59.5 | 275 | INTERGENIC | | | | |
| CaPOPI_5330 | Ca7 | 30668720 | ATTTTAATTATAATAATTTTTAATTATAAT/ATTTTAATTATAATAATTTTTAATTATAATAATTTTTAATTATAAT | TTGACACCAACGGAACGTAA | CAAGGTAGCATCCCCCACTA | 60.0 | 905 | INTERGENIC | | | | |
| CaPOPI_5331 | Ca7 | 30709199 | T/TA | GCTGCAATTTCGTAAGCCAT | TTTGGAGGCTCAATTTTGCT | 60.2 | 724 | INTERGENIC | | | | |
| CaPOPI_5332 | Ca7 | 30744719 | GAAAATTAAAAAAA/GAAAATTAAAAAAAATTAAAAAAA | ACTCCATGAATCACGAAGGC | CCCACACTCATATTCCCACTC | 60.1 | 668 | INTERGENIC | | | | |
| CaPOPI_5333 | Ca7 | 30746774 | TAAAAAA/TAAAAA | GTTTTGCACCCAATCAAACA | ATCGTGTTCGTAGGGGAGTT | 59.4 | 588 | INTERGENIC | | | | |
| CaPOPI_5334 | Ca7 | 30760339 | AA/AACA | TCAGCCAGAATTCCATTTCC | CGCGTATGAGTTGTTGTCGT | 60.0 | 399 | INTERGENIC | | | | |
| CaPOPI_5335 | Ca7 | 30763146 | C/CAG | CTTAGCCAGAGATTGAGCCG | CCATTGCCAAAGGACAGATT | 60.1 | 237 | INTRON | Ca_23324 | | HD-ZIP | Homeobox |

| INDEL marker IDs | Chromosomes/ unanchored scaffolds | Physical positions (bp) | InDels (*Kabuli* reference genome-CDC Frontier/PI) | Forward primers (5'-3') | Reverse primers (5'-3') | Anneling temperature (0C) | Expected amplified product size (bp) | Structural annotation | | Functional annotation | | |
|---|---|---|---|---|---|---|---|---|---|---|---|---|
| | | | | | | | | Sequence components of *kabuli* genome | *Kabuli* gene accession IDs | NCBI-KOG | TFs | NCBI-nr database |
| CaPOPI_5336 | Ca7 | 30770470 | GAA/GAAA | GAAAAAGTTGGGCCGACATA | CCATGGCTGATAATGGTGTG | 59.9 | 488 | INTERGENIC | | | | |
| CaPOPI_5337 | Ca7 | 30778209 | CTATAAATAGAAT/CTATAAATAGAATTATAAATAGAAT | CAACATCAATTCACATCTCTTCG | CAACCAAGTCTTACGATGCAAA | 59.6 | 178 | INTERGENIC | | | | |
| CaPOPI_5338 | Ca7 | 30795636 | A/AAAC | AAAGCTGAGAAGCAATCCAAA | CATGGCGTCTCACCTACCTT | 59.1 | 354 | INTERGENIC | | | | |
| CaPOPI_5339 | Ca7 | 30805803 | AAGAAATAGAAATAGAAATAGAAA/AAGAAATAGAAATAGAAA | CCTCGCTTTGCTACTCTGGT | CTGCGGCAGACTGTAAACAA | 59.6 | 771 | INTRON | Ca_23321 | O | | Peptidase M16, N-terminal |
| CaPOPI_5340 | Ca7 | 30806501 | TG/T | GCTCGTCATGGGGAGTAAAA | GAAATACCCCGCCAGTGTTA | 60.1 | 673 | INTRON | Ca_23321 | O | | Peptidase M16, N-terminal |
| CaPOPI_5341 | Ca7 | 30807395 | T/TA | TAACACTGGCGGGGTATTTC | CTCGTCAAGACGGACATGAA | 59.8 | 720 | INTRON | Ca_23321 | O | | Peptidase M16, N-terminal |
| CaPOPI_5342 | Ca7 | 30824955 | TTTGGGT/TT | CCAAAATTTAAGCGCCAAAC | TGACTCGGATTCTTTTCCAGA | 59.6 | 577 | INTRON | Ca_23321 | O | | Peptidase M16, N-terminal |
| CaPOPI_5343 | Ca7 | 30840351 | TAAAAAAAAA/TAAAAAAAAAAA | TTCAGACGCTAACAATTATATGCAA | CAAATTTGGGTCACCAATCA | 60.1 | 554 | DRR | Ca_23320 | | | |
| CaPOPI_5344 | Ca7 | 30858655 | TCATAAACATA/T | TTGGGTGCAAAATTGAAAAA | CGTTGTTCCAACATACAGGTTA | 59.0 | 860 | DRR | Ca_23319 | T | HB-other | Protein kinase, catalytic domain |
| CaPOPI_5345 | Ca7 | 30869621 | CA/CAA | CAAACCGATGTGTCATTCCA | CCAATTCTCACCGGATTTGT | 60.4 | 492 | INTERGENIC | | | | |
| CaPOPI_5346 | Ca7 | 30870269 | GTTGAATACACTTGAATACACTTGAATACACTTGAA/GTTGAATACACTTGAATACACTTGAA | TTAAGATTTGGTTGGGCTGC | GAACCAGACCCAGCAGTTGT | 60.1 | 307 | INTERGENIC | | | | |
| CaPOPI_5347 | Ca7 | 30874290 | TAA/TAAA | TTGTCTCAACAATCTCCCCC | ACCACCTCACTGCCCTACAC | 59.9 | 734 | DRR | Ca_23317 | | | |
| CaPOPI_5348 | Ca7 | 30930614 | TCC/TC | CCACTCAAGGCCAGTGATTT | ATGAGCAGCACCAACATCAA | 60.1 | 222 | DRR | Ca_23314 | | | |
| CaPOPI_5349 | Ca7 | 30967683 | ATTTT/ATTTTT | CCCCATGATCTTCCCTTTTT | GCAACAAACCTATGGCAGGT | 60.1 | 355 | DRR | Ca_23312 | | | |
| CaPOPI_5350 | Ca7 | 31087270 | TAAAAAAA/TAAAAAAAA | ACAGGGAGCTCCATTTCCTT | TGCAATGCAATGTCCATTTT | 60.1 | 190 | INTERGENIC | | | | |

| **INDEL marker IDs** | **Chromosomes/ unanchored scaffolds** | **Physical positions (bp)** | **InDels (*Kabuli* reference genome-CDC Frontier/PI)** | **Forward primers (5'-3')** | **Reverse primers (5'-3')** | **Anneling temperature (0C)** | **Expected amplified product size (bp)** | **Structural annotation** | | **Functional annotation** | | |
|---|---|---|---|---|---|---|---|---|---|---|---|---|
| | | | | | | | | **Sequence components of *kabuli* genome** | ***Kabuli* gene accession IDs** | **NCBI-KOG** | **TFs** | **NCBI-nr database** |
| CaPOPI_5351 | Ca7 | 31112471 | TGG/TG | GCAAAGGCACTCATTCCATT | GCAAAAACACGAGAAGGAGG | 60.1 | 493 | INTERGENIC | | | | |
| CaPOPI_5352 | Ca7 | 31133335 | GTTTT/GTTTTT | TTCTCAATCATTACTCCGACCA | TCACCGATCACTTACGTTGC | 59.6 | 562 | INTERGENIC | | | | |
| CaPOPI_5353 | Ca7 | 31146442 | TTATATATAT/TTATAT | TAGGGATTGAGCTGGCTCTG | TAGAAGACCTTGACCGTGCC | 60.5 | 765 | INTERGENIC | | | | |
| CaPOPI_5354 | Ca7 | 31152594 | CA/CAAA | TGAACACTTAGACCGTGTTTGG | CAGCGTAATGCCGACCTATT | 60.1 | 540 | INTERGENIC | | | | |
| CaPOPI_5355 | Ca7 | 31154886 | ATTTTTTT/ATTTTTTTT | CTGCGATAATGACGATGACG | TGTAACTTCTTGACTTTGTCCTTTTG | 60.2 | 627 | INTERGENIC | | | | |
| CaPOPI_5356 | Ca7 | 31167770 | TATAGA/TA | TCGAGATGTCATCGCAAATAA | TTTTGTTCTCCCTCACTCCG | 59.3 | 316 | DRR | Ca_10142 | Q | E2F/DP | ABC transporter-like |
| CaPOPI_5357 | Ca7 | 31175781 | ATT/ATTT | TTGCACCATAAAAGTGCAGC | CTCACCCCACCATAGCATTT | 59.9 | 592 | DRR | Ca_10141 | C | | Malic oxidoreductase |
| CaPOPI_5358 | Ca7 | 31181804 | T/TCAAAAA | TCACACACAAAATTGAAGATGGA | CCATTGCTTGCATTGATTTG | 60.4 | 600 | INTERGENIC | | | | |
| CaPOPI_5359 | Ca7 | 31188755 | TAAAAA/TAAAA | TTAACCAATGACACTGGAAGG | CCCTCAAATCATCTTCAAATCA | 57.6 | 848 | INTERGENIC | | | | |
| CaPOPI_5360 | Ca7 | 31188947 | ATTTTT/ATTTT | TTGCACGTTGAATTAAAAACTCTT | TCACGCTTTCTTTCGTTCCT | 59.3 | 501 | INTERGENIC | | | | |
| CaPOPI_5361 | Ca7 | 31196149 | ATTTT/ATTT | CAACCAAGATTTTGATGAGTCG | CACGAGGGTAGTTTTGTCTTTC | 59.6 | 622 | DRR | Ca_10138 | | | |
| CaPOPI_5362 | Ca7 | 31199747 | TGG/TG | TCAAGCGATTTCACGACAAG | TGGTCGTGTTGCGTTTAAGA | 60.0 | 406 | DRR | Ca_10137 | | bHLH | Protein kinase, catalytic domain |
| CaPOPI_5363 | Ca7 | 31202147 | TA/TAA | CATGCTCATGGAATCAGGTG | ATTTGGCGTTGTCCTTTTTG | 60.1 | 370 | INTRON | Ca_10137 | | bHLH | Protein kinase, catalytic domain |
| CaPOPI_5364 | Ca7 | 31204267 | TAAATTAAACAAAT/TAAAT | TCCTAAGCCACCAATGATCC | CTGGTGATCCTTTTCCTCCA | 59.9 | 267 | INTRON | Ca_10137 | | bHLH | Protein kinase, catalytic domain |
| CaPOPI_5365 | Ca7 | 31205971 | CAAAAAAAAAAAAA/CAAAAAAAAAA | ACCTGGCAACACATCAAACA | TGCAAGTCGCTTTCTCCTTT | 60.0 | 785 | INTERGENIC | | | | |

| INDEL marker IDs | Chromosomes/ unanchored scaffolds | Physical positions (bp) | InDels (*Kabuli* reference genome-CDC Frontier/PI) | Forward primers (5'-3') | Reverse primers (5'-3') | Anneling temperature (0C) | Expected amplified product size (bp) | Structural annotation | | Functional annotation | | |
|---|---|---|---|---|---|---|---|---|---|---|---|---|
| | | | | | | | | Sequence components of *kabuli* genome | *Kabuli* gene accession IDs | NCBI-KOG | TFs | NCBI-nr database |
| CaPOPI_5366 | Ca7 | 31210939 | CTC/CTCAATC | CCTTTCTCAGCCCATATCCA | TCAACAATGCAAACGAAGGA | 60.0 | 306 | DRR | Ca_10136 | | ARF | AUX/IAA protein |
| CaPOPI_5367 | Ca7 | 31213637 | TAAAAAAA/TAAAAAA | GTTTGCATGCACCACAATTC | AAAGAAGCCACGAAAAGGGT | 60.0 | 356 | INTRON | Ca_10136 | | ARF | AUX/IAA protein |
| CaPOPI_5368 | Ca7 | 31215378 | GAAAAAAAAA/GAAAAAAAAAA | GTTGTGCCGCTTAAGTTGGT | ATGGGACCACTTGGTGGTAA | 60.2 | 854 | DRR | Ca_10135 | G | | Hexokinase |
| CaPOPI_5369 | Ca7 | 31215551 | AT/ATT | GCACTGGATTTGGAAATTGG | ATGGGACCACTTGGTGGTAA | 60.3 | 496 | DRR | Ca_10135 | G | | Hexokinase |
| CaPOPI_5370 | Ca7 | 31231997 | CATATATATATATATATATAT/CATATATATAT | TCGTTGAAACCATACGACCA | TCCTTGTAAATTTGATTACCCCA | 60.0 | 909 | INTERGENIC | | | | |
| CaPOPI_5371 | Ca7 | 31252978 | GTTATTTATTTATTTA/GTTATTTATTTATTTATTTA | TCCCATTGACTGCTCCTTTT | TTGCAACACTTTCTGGAGCA | 59.7 | 567 | INTERGENIC | | | | |
| CaPOPI_5372 | Ca7 | 31257051 | TAAAA/TAAAAA | ACAATGAGGCTGAGGGAGAG | GCCTTCACAAAGTAGCTGGC | 59.4 | 178 | DRR | Ca_10132 | | | IQ motif, EF-hand binding site |
| CaPOPI_5373 | Ca7 | 31277211 | TAAAAAAA/TAAAAAAAA | GGTGCATTTGAGTTTGCTGA | CCACAAGTTTCTGGGGAAAA | 59.8 | 336 | INTERGENIC | | | | |
| CaPOPI_5374 | Ca7 | 31294686 | AA/AAACA | ACGAAAACCAATCCAATCCA | TGAATCTGTGAACCGCATGT | 60.2 | 753 | DRR | Ca_10130 | O | | Peptidase M16, zinc-binding site |
| CaPOPI_5375 | Ca7 | 31300454 | TTAAA/TTAAATAAA | CAGGCCATAAACTCGATGGT | CAACATTTAATGGATGGGGG | 60.0 | 592 | DRR | Ca_10129 | | C2H2 | ATPase, AAA+ type, core |
| CaPOPI_5376 | Ca7 | 31302590 | CAATGTCAGA/CAATGTCAGAGTAGAAAAATGTCAGA | CATGTTCTTGGAGACGCAGA | GCTGTTTCAAATGCCAGTGA | 60.0 | 691 | INTRON | Ca_10129 | | C2H2 | ATPase, AAA+ type, core |
| CaPOPI_5377 | Ca7 | 31313382 | GTTT/GTT | ATTTCCTTTGAAGCACACGG | ACCCTCTGTAGCACCCACAC | 60.1 | 799 | INTRON | Ca_10128 | | | |
| CaPOPI_5378 | Ca7 | 31330956 | AT/ATT | TTGTTCTCCTTTGCCACCTT | TGTGATGATGTCATGGGGAC | 59.7 | 156 | INTRON | Ca_10126 | R | | Inositol oxygenase |
| CaPOPI_5379 | Ca7 | 31335294 | CAAG/CAAAGGAAG | TTTCTTTCCGTTTCCAATGC | ACAGCCTGTGCACTGTTTTG | 60.1 | 605 | INTERGENIC | | | | |
| CaPOPI_5380 | Ca7 | 31352192 | CAAAA/CAA | GCGAGCAAATTCTGAACCAT | TGTTAGGGTCAGTTCTGCCA | 60.2 | 245 | DRR | Ca_10122 | U | | Vacuolar protein sorting-associated protein 26 |

| **INDEL marker IDs** | **Chromosomes/ unanchored scaffolds** | **Physical positions (bp)** | **InDels (*Kabuli* reference genome-CDC Frontier/PI)** | **Forward primers (5'-3')** | **Reverse primers (5'-3')** | **Anneling temperature (0C)** | **Expected amplified product size (bp)** | **Structural annotation** | | **Functional annotation** | | |
|---|---|---|---|---|---|---|---|---|---|---|---|---|
| | | | | | | | | **Sequence components of *kabuli* genome** | ***Kabuli* gene accession IDs** | **NCBI-KOG** | **TFs** | **NCBI-nr database** |
| CaPOPI_5381 | Ca7 | 31357954 | CCTACTA/CCTA | TTAAAGGACTGCCGGAGAAA | ATGGAATCTGATGGAAAGCG | 59.8 | 183 | INTERGENIC | | | | |
| CaPOPI_5382 | Ca7 | 31361859 | TA/TAGTTCAA | TGGTTATAATCAAAATCGGTGG | TCTTCCTTAATTCGCGTCGT | 58.7 | 426 | INTERGENIC | | | | |
| CaPOPI_5383 | Ca7 | 31374467 | CAAAA/CAAAAGAAAA | TTTGGACTCGCTCTACATGG | GCTGATGACCCAAAACCATT | 58.9 | 524 | INTERGENIC | | | | |
| CaPOPI_5384 | Ca7 | 31384635 | TAA/TAAA | GATTGGTGAAAAATGGGGTG | TGGCAATATCAATCCACCTAGT | 60.0 | 629 | INTERGENIC | | | | |
| CaPOPI_5385 | Ca7 | 31393273 | TAAAAAAAAAAAA/TAAAAAAAAA | CATGGTGAGGGTGATTTGTG | TTTGAGTTGCAATTTGATGACA | 59.8 | 717 | INTERGENIC | | | | |
| CaPOPI_5386 | Ca7 | 31745904 | TCCCC/TCCC | TGCATGGAATGGTGAGTGAT | ATTGATGGGTTTACTTGGCG | 59.9 | 219 | INTERGENIC | | | | |
| CaPOPI_5387 | Ca7 | 31808923 | TTCTCTCTCTCTCTCT/TTCTCTCTCTCTCTCTCT | AACTGTACCCGAGTGATGCC | GATACAAGGGGTTTCTGCCA | 60.0 | 304 | INTRON | Ca_10083 | R | G2-like | Root hair defective 3 GTP-binding |
| CaPOPI_5388 | Ca7 | 31862312 | ATATTATTATTATTATTATTATTATTATTATTATTATTATTATTATTATTATTATTATTA/ATATTATTATTATTATTATTATTATTATTATTATTATTATTATTATTATTATTATT | CAAATACAATAACACCTGTCTCCG | ACTTTTCCCACCCAACTTCA | 59.8 | 498 | INTERGENIC | | | | |
| CaPOPI_5389 | Ca7 | 31895626 | AAAGAAGAAGAAGAAGAA/AAAGAAGAAGAAGAAGAAGAA | TGAGTTTGTGGTGGAACCAA | TGCAACGTCAGTGTTTAGCC | 60.0 | 816 | INTERGENIC | | | | |
| CaPOPI_5390 | Ca7 | 32114393 | A/AAC | ATATTCAAGGCAAAGGTGCG | CGAGACTACTCGTGGGCTTC | 60.1 | 505 | INTERGENIC | | | | |
| CaPOPI_5391 | Ca7 | 32479028 | AATATATATATATATATATATATAT/AATATATATATATATATATATATATATAT | AGGGAGTTTAGTGGTGGGCT | CGGATGCATGAAAGTGAAAA | 60.0 | 636 | INTERGENIC | | | | |
| CaPOPI_5392 | Ca7 | 32601479 | A/AGGAATGCT | CAGCAACAGCGCAAAGATTA | GGATGACAAAGAAAGGCCAA | 60.2 | 543 | INTRON | Ca_10025 | TU | | Calcium-binding EF-hand |
| CaPOPI_5393 | Ca7 | 32601813 | T/TG | CAGCAACAGCGCAAAGATTA | GCAAGTGATTGCAAAGCAAA | 60.2 | 652 | INTRON | Ca_10025 | TU | | Calcium-binding EF-hand |
| CaPOPI_5394 | Ca7 | 32614218 | AAAAAATTATAAA/AAAA | AAATAAAATCTTTCGCCCCC | GGAGGGATTGCAGTGACAGT | 59.3 | 525 | INTERGENIC | | | | |
| CaPOPI_5395 | Ca7 | 32649545 | T/TC | TTGCAGGATGCACAGAAAAG | AGGTGCATCCCCTGTAACTG | 60.0 | 415 | INTERGENIC | | | | |

| INDEL marker IDs | Chromosomes/ unanchored scaffolds | Physical positions (bp) | InDels (*Kabuli* reference genome-CDC Frontier/PI) | Forward primers (5'-3') | Reverse primers (5'-3') | Anneling temperature (0C) | Expected amplified product size (bp) | Structural annotation | | Functional annotation | | |
|---|---|---|---|---|---|---|---|---|---|---|---|---|
| | | | | | | | | Sequence components of *kabuli* genome | *Kabuli* gene accession IDs | NCBI-KOG | TFs | NCBI-nr database |
| CaPOPI_5396 | Ca7 | 32650059 | TAATAATGTAAAAAA/T | ATGGGACTGTGGGAGAGAGA | GAAAAAGGCTTCAAACATCAAA | 59.6 | 398 | INTERGENIC | | | | |
| CaPOPI_5397 | Ca7 | 32653182 | G/GA | TTTTCAAATTTCCGGTTTGC | CCAATTCCTGTGGAAATGCT | 59.9 | 582 | INTERGENIC | | | | |
| CaPOPI_5398 | Ca7 | 32654894 | AAATAATAATAATAATAAT/AAATAATAATAATAATAATAATAAT | AACGAAGTGAGACGTGAGCA | TGATGGCTATGGTGGAACAA | 59.6 | 618 | INTERGENIC | | | | |
| CaPOPI_5399 | Ca7 | 32655163 | TTAATA/TTA | AACGAAGTGAGACGTGAGCA | TGATGGCTATGGTGGAACAA | 59.6 | 618 | INTERGENIC | | | | |
| CaPOPI_5400 | Ca7 | 32655249 | TATTAATTAA/TATTAA | TGTTGTTCCAAATTTTACCTGAA | TACAGACCGGGCTCAAAATC | 58.6 | 935 | INTERGENIC | | | | |
| CaPOPI_5401 | Ca7 | 33055618 | CTGTG/CTG | TCCTCAAGGTGAATCTGACAAA | TTATCATGCGCTGAAAAACG | 59.7 | 345 | INTERGENIC | | | | |
| CaPOPI_5402 | Ca7 | 33061943 | TA/T | CGATCAATGACTGTCCAACG | TCCCTTTAGAATTGACGCTTTT | 60.1 | 252 | INTERGENIC | | | | |
| CaPOPI_5403 | Ca7 | 33068212 | ATTT/ATT | CTTCTTCGTCATCCATTGGC | AAGCCTATCACTCATCCAATCAA | 60.6 | 624 | INTERGENIC | | | | |
| CaPOPI_5404 | Ca7 | 33069173 | ATTTT/ATTTTT | TGACATGCATGATTCAACTGG | TTTGAATTGGTCCCTGATTTT | 60.5 | 617 | INTERGENIC | | | | |
| CaPOPI_5405 | Ca7 | 33102819 | ATT/AT | AACAGTCCCGCATTGGATAG | CAGCACTGTCCAAATTGTGC | 60.0 | 447 | INTERGENIC | | | | |
| CaPOPI_5406 | Ca7 | 33211070 | TAA/TAAA | ACCTGCCATCTTTTGAGCAT | AATTTCCGTCGGGTCTTTTT | 59.7 | 622 | INTERGENIC | | | | |
| CaPOPI_5407 | Ca7 | 33237657 | GATATAT/GATATATATAT | TTTGGCCTTATCACCCAAAC | CAACTTGGTTCAACGCAAAA | 59.8 | 171 | INTERGENIC | | | | |
| CaPOPI_5408 | Ca7 | 33247703 | ATCTCT/ATCTCTCT | TGTAACGCGTAGGGTAAGGG | TAAGCATCCGCTTCTTCAGG | 60.0 | 136 | INTERGENIC | | | | |
| CaPOPI_5409 | Ca7 | 33251332 | AAATAAT/AAAT | TGTTGATAAATGATAATTGGTTGTTG | GACTATCGCTCCCCTAAGCC | 59.2 | 836 | DRR | Ca_16152 | A | | Like-Sm ribonucleoprotein (LSM) domain |
| CaPOPI_5410 | Ca7 | 33275253 | TAAAAA/TAAAAAAA | TTTAATGTATTGGATCACTCCTCC | CTGGCTCTCCTCACATGACA | 58.5 | 393 | INTERGENIC | | | | |

| INDEL marker IDs | Chromosomes/ unanchored scaffolds | Physical positions (bp) | InDels (*Kabuli* reference genome-CDC Frontier/PI) | Forward primers (5'-3') | Reverse primers (5'-3') | Anneling temperature (0C) | Expected amplified product size (bp) | Structural annotation | | Functional annotation | | |
|---|---|---|---|---|---|---|---|---|---|---|---|---|
| | | | | | | | | Sequence components of *kabuli* genome | *Kabuli* gene accession IDs | NCBI-KOG | TFs | NCBI-nr database |
| CaPOPI_5411 | Ca7 | 33288649 | ATTT/ATTTT | TCCCCTTCTCGTATTTG CAC | TTTTGGACCGAATATGA TTATGC | 60.1 | 442 | INTERGENIC | | | | |
| CaPOPI_5412 | Ca7 | 33295433 | GTTAATTA/GTTA | CGACCACGAGTTTAATC GAA | AAAAGGAACAACCGTCG TCA | 58.8 | 510 | INTERGENIC | | | | |
| CaPOPI_5413 | Ca7 | 33298775 | AAG/AAGAG | TGGAAAACCCCTGCATT AAG | CACCCAACCATTGATGT TCA | 59.9 | 571 | INTRON | Ca_16157 | | | Tify |
| CaPOPI_5414 | Ca7 | 33329178 | GTTTTTT/GTTTTTTT | TCACCTTCTGACCTTGA ATGAA | TGGTGGGCTATGAACAA ACA | 59.7 | 619 | INTERGENIC | | | | |
| CaPOPI_5415 | Ca7 | 33343840 | TA/T | TGGTGACTGGTGAGTTC CAA | CGTCGATTTCAAGCACT CAA | 60.1 | 519 | DRR | Ca_16160 | | | |
| CaPOPI_5416 | Ca7 | 33350171 | TG/T | TGTGGCTGAAGTCAATG AGC | AATTTGTCCTCAACAACC CG | 60.0 | 547 | INTRON | Ca_16161 | YU | | Nuclear pore protein 84/107 |
| CaPOPI_5417 | Ca7 | 33358560 | CTTGT/CTTGTTGT | AACAAGGGCAAGTTGGT TTG | GGGTTTTTCCCCTTCAC AAT | 60.0 | 397 | INTRON | Ca_16161 | YU | | Nuclear pore protein 84/107 |
| CaPOPI_5418 | Ca7 | 33412820 | ACATATCGAATT/ACATATCGAATTTCC ATATCGAATT | TTTCTTGCTTTCAACTCG CA | CCAGTTTCTCAATCCCTT GG | 59.7 | 548 | INTRON | Ca_16164 | | C3H | Zinc finger, CCCH-type |
| CaPOPI_5419 | Ca7 | 33413065 | CA/CAAA | AATTTGACAACCTTGGC ACC | ATGGTGGAGGGGAGAA AAAG | 59.8 | 508 | INTRON | Ca_16164 | | C3H | Zinc finger, CCCH-type |
| CaPOPI_5420 | Ca7 | 33472559 | ATTTTT/ATTTT | GCTGCAAAGGATTGAGG ATT | TGTCTCGTCATCGTTTT CCA | 59.3 | 532 | INTERGENIC | | | | |
| CaPOPI_5421 | Ca7 | 33473339 | TAAAAAA/TAAAAA | TTAAAGTCAGTGGGTAG CTCAAA | TTGCCAAAATTATGCATG TGA | 58.1 | 185 | INTERGENIC | | | | |
| CaPOPI_5422 | Ca7 | 33473368 | TAAAAAAAA/TAAAAAAA | TTAAAGTCAGTGGGTAG CTCAAA | TTGCCAAAATTATGCATG TGA | 58.1 | 185 | INTERGENIC | | | | |
| CaPOPI_5423 | Ca7 | 33519406 | ATTTTTTTT/ATTTTTTTTT | CGCCGCCATTTTATTAT CAC | TTTGCCTTTTTGATGGAA CA | 60.3 | 802 | INTERGENIC | | | | |
| CaPOPI_5424 | Ca7 | 33524828 | A/AAATGTC | CCGAGAACACGAGATGG ATT | ACCAGGTCTTGCTGAGC ACT | 60.1 | 316 | INTRON | Ca_16172 | S | M-type | NADH:ubiquinone oxidoreductase intermediate-associated protein 30 |
| CaPOPI_5425 | Ca7 | 33528371 | ATTTTTTT/ATTTTTT | GAATATTTGGTGGAGAT CGCA | AATGGAGCGGAATTTTC CTT | 59.9 | 482 | INTERGENIC | | | | |

| INDEL marker IDs | Chromosomes/ unanchored scaffolds | Physical positions (bp) | InDels (*Kabuli* reference genome-CDC Frontier/PI) | Forward primers (5'-3') | Reverse primers (5'-3') | Anneling temperature (0C) | Expected amplified product size (bp) | Structural annotation | | Functional annotation | | |
|---|---|---|---|---|---|---|---|---|---|---|---|---|
| | | | | | | | | Sequence components of *kabuli* genome | *Kabuli* gene accession IDs | NCBI-KOG | TFs | NCBI-nr database |
| CaPOPI_5426 | Ca7 | 33532784 | T/TG | GCCTAGCTCAGAAGACAAGTCC | CGATGCTTCATTATAAATCAACTCTT | 59.7 | 534 | INTERGENIC | | | | |
| CaPOPI_5427 | Ca7 | 33539636 | GTTTT/GTTT | AAATTGGACGGTGCTAATCC | CACGAAAGGTTTCATCACAAA | 58.9 | 243 | INTERGENIC | | | | |
| CaPOPI_5428 | Ca7 | 33558611 | TTTG/TTTGTTAGTTTTTG | TTCAAGGACATTGCCAATTTAT | TGCATTTTTCCTTCTTCCACTT | 58.5 | 492 | INTERGENIC | | | | |
| CaPOPI_5429 | Ca7 | 33559679 | CTTTTT/CTTTTTT | GCTCATTGCAAGAAGAGGATG | TCTCAAAGTGACAATGGTGTGA | 60.0 | 417 | DRR | Ca_16175 | QI | B3 | Cytochrome P450 |
| CaPOPI_5430 | Ca7 | 33561063 | GTACTATTCATAC/GTACTATTCATACTATTCATAC | CGTGTACTCCCCTTTCCCTT | GGCCATTTTGTGCAGATTTT | 60.4 | 431 | DRR | Ca_16175 | QI | B3 | Cytochrome P450 |
| CaPOPI_5431 | Ca7 | 33561157 | TATCATCATCAT/TATCATCAT | CGTGTACTCCCCTTTCCCTT | GGCCATTTTGTGCAGATTTT | 60.4 | 431 | DRR | Ca_16175 | QI | B3 | Cytochrome P450 |
| CaPOPI_5432 | Ca7 | 33576122 | GTTTTTTTTTT/GTTTTTTTTT | TAATTTAACGAACGGCCGAG | TTGGGTGACTCATGGTGAGA | 60.1 | 410 | DRR | Ca_16178 | I | | Synaptojanin, N-terminal |
| CaPOPI_5433 | Ca7 | 33597364 | CTAATTAATT/CTAATT | GATGTTAACGCACCTCCACC | CACACTTCGATCTCCGATCA | 60.4 | 483 | INTERGENIC | | | | |
| CaPOPI_5434 | Ca7 | 33605853 | TATAATTAAATAATAAT/TATAATTAAATAATAATTAAATAATAAT | CCACACACCCTCATAGCCTT | TTTAAACCCCGTCAACACCT | 60.0 | 665 | INTERGENIC | | | | |
| CaPOPI_5435 | Ca7 | 33606263 | TT/TTCT | TTCGAAGACAGGTGTTGACG | AAGTGTTTCATTTGTTTGTGATTTTT | 59.9 | 529 | INTERGENIC | | | | |
| CaPOPI_5436 | Ca7 | 33619410 | TTTCCTTCCTT/TTTCCTT | TCGTGGCTCATTCCACATTA | GGGTTGTGTCCCAAAAGCTA | 60.1 | 431 | DRR | Ca_16179 | J | | Ribosomal protein/NADH dehydrogenase domain |
| CaPOPI_5437 | Ca7 | 33638562 | AATGAAT/AATGAATATGAAT | TCGCAAGAGTTTGGACCTTT | TTCTAAAAACGGAAAGCTATTTTGA | 59.9 | 643 | INTERGENIC | | | | |
| CaPOPI_5438 | Ca7 | 33638744 | A/AC | TCGCAAGAGTTTGGACCTTT | GCACATGGTTTCGAATGTTG | 59.9 | 882 | INTERGENIC | | | | |
| CaPOPI_5439 | Ca7 | 33640082 | TAA/TAAA | CCAAATCGTCGGTGTGAATA | CCTTTGTTTGCTGGGATCAT | 59.4 | 614 | DRR | Ca_16180 | | ERF | Pathogenesis-related transcriptional factor/ERF, DNA-binding |
| CaPOPI_5440 | Ca7 | 33640133 | CAAA/CAAAAATAAAA | CCAAATCGTCGGTGTGAATA | CCTTTGTTTGCTGGGATCAT | 59.4 | 614 | DRR | Ca_16180 | | ERF | Pathogenesis-related transcriptional factor/ERF, DNA-binding |

| INDEL marker IDs | Chromosomes/ unanchored scaffolds | Physical positions (bp) | InDels (*Kabuli* reference genome-CDC Frontier/PI) | Forward primers (5'-3') | Reverse primers (5'-3') | Anneling temperature (0C) | Expected amplified product size (bp) | Structural annotation | | Functional annotation | | |
|---|---|---|---|---|---|---|---|---|---|---|---|---|
| | | | | | | | | Sequence components of *kabuli* genome | *Kabuli* gene accession IDs | NCBI-KOG | TFs | NCBI-nr database |
| CaPOPI_5441 | Ca7 | 33644014 | TTTTT/TTTTTCTTTT | GTGCCCCACAACTTCTGTTT | TGTTTTTATCCAATCCAACATCA | 60.0 | 489 | INTERGENIC | | | | |
| CaPOPI_5442 | Ca7 | 33644686 | TTTA/TTTATTA | TTTTCTCAATCAAGAGGCCA | CAATATGACATGACGGCTGG | 58.4 | 581 | INTERGENIC | | | | |
| CaPOPI_5443 | Ca7 | 33645082 | CTT/CTTT | TTGACATTGCGCTTCTCAAG | CAATATGACATGACGGCTGG | 60.1 | 202 | INTERGENIC | | | | |
| CaPOPI_5444 | Ca7 | 33662614 | ATTTTTTT/ATTTTTTTTT | AAGGCACTCAAACAAATGGG | GGGAACTAATTAATGTGAACATTGTC | 60.0 | 528 | INTERGENIC | | | | |
| CaPOPI_5445 | Ca7 | 33662685 | TTATATATAT/TTATATAT | AAGGCACTCAAACAAATGGG | TGAATTTGGATTTGTCAATGATG | 60.0 | 662 | INTERGENIC | | | | |
| CaPOPI_5446 | Ca7 | 33662861 | CAAAAAAA/CAAAAAAAA | AAGGCACTCAAACAAATGGG | TGAATTTGGATTTGTCAATGATG | 60.0 | 662 | INTERGENIC | | | | |
| CaPOPI_5447 | Ca7 | 33662912 | ATTTTTTTTTTT/ATTTTTTTTTT | AAGGCACTCAAACAAATGGG | TGAATTTGGATTTGTCAATGATG | 60.0 | 662 | INTERGENIC | | | | |
| CaPOPI_5448 | Ca7 | 33663454 | TA/TAA | GGTCCAACAAGTCACTCATGC | AGTCAACTCAACAGCTCATGGA | 60.6 | 137 | INTERGENIC | | | | |
| CaPOPI_5449 | Ca7 | 33672380 | CATTA/CATTAATTA | CTCTGTGTTGGGCCAAGATT | TCACAGGAGACACGTAGATGAAA | 60.1 | 347 | DRR | Ca_16181 | | G2-like | Peptidase S8/S53, subtilisin/kexin/sedolisin |
| CaPOPI_5450 | Ca7 | 33683185 | GCAAGTCA/GCA | GGGCATTTCAGGAATATTGTTT | GATTGAGCCGAAAGTGAAGC | 59.2 | 919 | INTERGENIC | | | | |
| CaPOPI_5451 | Ca7 | 33713974 | ATTTTT/ATTTT | CTATGGGCCGACCTTGATTA | CCAATTGGTCCCTCAGAAAA | 59.9 | 699 | INTERGENIC | | | | |
| CaPOPI_5452 | Ca7 | 33726416 | T/TC | TAAAATTGCCGTGACGATGA | TCGATTCAGATTGAAGGCTTTT | 60.1 | 366 | INTERGENIC | | | | |
| CaPOPI_5453 | Ca7 | 33766122 | ATTT/ATTTT | AATATTTGGCTCCACCACCA | GGGTTACAAACTTCCTGCCA | 60.2 | 564 | INTRON | Ca_16185 | | | Initiation factor 2B-related |
| CaPOPI_5454 | Ca7 | 33768691 | TAAA/TAAAA | TCATGTGGGGAAGAAATTCA | AAAAAGACAAAACTACCCTTGTGA | 58.9 | 577 | INTERGENIC | | | | |
| CaPOPI_5455 | Ca7 | 33770972 | ATTTTTTTTT/ATTTTTTTTTT | TTTCTTTCTTCCCGATTTAGTCC | TGGCTCGTTGGGCTAGATTA | 60.0 | 639 | INTERGENIC | | | | |

| INDEL marker IDs | Chromosomes/ unanchored scaffolds | Physical positions (bp) | InDels (*Kabuli* reference genome-CDC Frontier/PI) | Forward primers (5'-3') | Reverse primers (5'-3') | Anneling temperature (0C) | Expected amplified product size (bp) | Structural annotation | | Functional annotation | | |
|---|---|---|---|---|---|---|---|---|---|---|---|---|
| | | | | | | | | Sequence components of *kabuli* genome | *Kabuli* gene accession IDs | NCBI-KOG | TFs | NCBI-nr database |
| CaPOPI_5456 | Ca7 | 33774070 | CAAA/CA | TTATGGCCCGCTAAAATGAC | TGCTTGCACGAGTTTGGATA | 59.9 | 776 | INTERGENIC | | | | |
| CaPOPI_5457 | Ca7 | 33781250 | AA/AATA | TTGAGGTGACTAAAGCTCCGA | TCGAACCACACAGAATTCCA | 60.0 | 379 | INTERGENIC | | | | |
| CaPOPI_5458 | Ca7 | 33781676 | ATT/ATTATTT | ATGGAATTCTGTGTGGTTCG | TCGTAAATACCAGCATCATCAA | 58.4 | 508 | INTERGENIC | | | | |
| CaPOPI_5459 | Ca7 | 33795392 | GAAAAAAAAAA/GAAAAAAAAA | TGGCCCTTCATCATATCCTT | TAAACGAAGCTATGCCCCAC | 59.3 | 441 | INTRON | Ca_16187 | | | |
| CaPOPI_5460 | Ca7 | 33806939 | AAAAAATTAAAAATTAAAA/AAAAAATTAAAAATTAAAAATTAAAA | AGGCATAGGCACAGGAAAGA | TTCGATAGTCGCACAACCAC | 59.8 | 842 | INTERGENIC | | | | |
| CaPOPI_5461 | Ca7 | 33810289 | ATT/ATTT | AACTCAACGCCAAACCAGAC | CCATGCTTGGATTTTTGCTT | 60.2 | 232 | INTRON | Ca_16189 | | C3H | Remorin, C-terminal |
| CaPOPI_5462 | Ca7 | 33812293 | TCACA/TCACACA | TTGGATGAAAGAGACAAGCAAA | GTATCCATGAATTGGGGGTG | 59.9 | 687 | DRR | Ca_16189 | | C3H | Remorin, C-terminal |
| CaPOPI_5463 | Ca7 | 33813164 | TAA/TA | TAAAAGAATTGCATTGGCCC | GGAAGAGTGATCCTCAAACCC | 59.9 | 471 | INTERGENIC | | | | |
| CaPOPI_5464 | Ca7 | 33814827 | TAT/TATTATAGTTTTTAACCAT | TGTTTGATGGCGTTAATGTGA | TCTTTAATCTTTGCAAATCAATGG | 60.0 | 422 | DRR | Ca_16190 | E | | 1-aminocyclopropane-1-carboxylate synthase |
| CaPOPI_5465 | Ca7 | 33815491 | GT/GTT | TTTGTTTTCATGCATGTGTCC | TCTGCATGGCAGTGAATAGC | 59.4 | 162 | DRR | Ca_16190 | E | | 1-aminocyclopropane-1-carboxylate synthase |
| CaPOPI_5466 | Ca7 | 33815769 | TAAA/TA | ATGGCTATTCACTGCCATGC | ATCATCCTTGGTCCCGAATA | 61.0 | 434 | DRR | Ca_16190 | E | | 1-aminocyclopropane-1-carboxylate synthase |
| CaPOPI_5467 | Ca7 | 33843751 | TTTCTATTTTCTCCTCT/TT | TAGAGCATGGCATTTCACCA | GGCCTTTAAATTGGGCTTTC | 60.2 | 229 | DRR | Ca_16192 | | NAC | Pectinesterase, catalytic |
| CaPOPI_5468 | Ca7 | 33855265 | GTTTTT/GTTTT | TCCAGAAGATGGTGAGAGTGG | ACTTGATTGCACATGGATCG | 60.2 | 578 | INTERGENIC | | | | |
| CaPOPI_5469 | Ca7 | 33855439 | CAA/CA | TCCAGAAGATGGTGAGAGTGG | ACTTGATTGCACATGGATCG | 60.2 | 578 | INTERGENIC | | | | |
| CaPOPI_5470 | Ca7 | 33855544 | CT/C | TGCAACATTTTACTCAATCCG | ACTTGATTGCACATGGATCG | 58.7 | 137 | INTERGENIC | | | | |

| **INDEL marker IDs** | **Chromosomes/ unanchored scaffolds** | **Physical positions (bp)** | **InDels (*Kabuli* reference genome-CDC Frontier/PI)** | **Forward primers (5'-3')** | **Reverse primers (5'-3')** | **Anneling temperature (0C)** | **Expected amplified product size (bp)** | **Structural annotation** | | **Functional annotation** | | |
|---|---|---|---|---|---|---|---|---|---|---|---|---|
| | | | | | | | | **Sequence components of *kabuli* genome** | ***Kabuli* gene accession IDs** | **NCBI-KOG** | **TFs** | **NCBI-nr database** |
| CaPOPI_5471 | Ca7 | 33855578 | ATTTTTTT/ATTTTTT | CGATCCATGTGCAATCAAGT | TCCAACAAGCGATTCATTCTT | 59.5 | 497 | INTERGENIC | | | | |
| CaPOPI_5472 | Ca7 | 33856959 | GAAAAAAAA/GAAAAAA | TCCCATCATAAATCTTGTGCC | ACTTTTGCTCTGGCTTCCAA | 59.8 | 161 | INTERGENIC | | | | |
| CaPOPI_5473 | Ca7 | 33859459 | AAG/A | AAAATGCGAAGATGTTTACGG | CTTCACCCTCTCGTAGCACC | 59.1 | 768 | INTERGENIC | | | | |
| CaPOPI_5474 | Ca7 | 33872304 | T/TG | TCATGCCACTTTAATGCCTG | TGCCAACCTTAAACCACAGA | 59.7 | 507 | INTERGENIC | | | | |
| CaPOPI_5475 | Ca7 | 33880924 | TAAAAAAA/TAAAAAAAAAA | TTTTTGTAATGATTGCTTGAATTT | CCGTTTCACTTTACCTTCGC | 57.1 | 711 | DRR | Ca_16194 | O | | Ubiquitin-conjugating enzyme, E2 |
| CaPOPI_5476 | Ca7 | 34109983 | GAAA/GAAAA | TCGGGACCCACTTATTTGAC | AAAATTGTGTGGTGGTGGGT | 59.8 | 497 | INTERGENIC | | | | |
| CaPOPI_5477 | Ca7 | 34136390 | T/TA | TGCCCATTAATTCTTGCCTC | AGGCTGATTCTCCCTTGGAT | 60.0 | 458 | INTRON | Ca_21006 | K | ERF | DNA-directed RNA polymerase, subunit 2, domain 6 |
| CaPOPI_5478 | Ca7 | 34138307 | GTTTTTTTTT/GTTTTTTTTTT | CAAGATGCACAGGAGTGGAA | GGACAAAGCATTCCCCACTA | 59.8 | 451 | INTRON | Ca_21006 | K | ERF | DNA-directed RNA polymerase, subunit 2, domain 6 |
| CaPOPI_5479 | Ca7 | 34173121 | TAAAAAAAA/TAAAAAA | TCGGCAACCTAATTCAAACC | AATGCGTAACATTTGACCCC | 59.9 | 609 | INTERGENIC | | | | |
| CaPOPI_5480 | Ca7 | 34173546 | GT/GTATCT | TGGGGTCAAATGTTACGCAT | TTTGATCTTCTTCGGTCGCT | 61.1 | 499 | INTERGENIC | | | | |
| CaPOPI_5481 | Ca7 | 34174102 | CTTTTTTTT/CTTTTTTTTTT | AGCGACCGAAGAAGATCAAA | GCTTGCATTTTGGGTGAGAT | 60.0 | 509 | INTERGENIC | | | | |
| CaPOPI_5482 | Ca7 | 34176709 | TTTTATTT/TTTT | AGCGTTGAAATTATGGACCG | TCGTTTGTGTGCAATTGTTTC | 60.0 | 682 | INTERGENIC | | | | |
| CaPOPI_5483 | Ca7 | 34187425 | GAA/GAAA | AGGTCTGGATTCGTGGTTTG | ACATCCGTGCAAGGAAAAAG | 60.0 | 646 | INTRON | Ca_21004 | S | | |
| CaPOPI_5484 | Ca7 | 34189872 | AATAT/AAT | CATTCAGGCATTTGTTTCCC | AAAAACTAGGGCTATTTGGAGC | 60.3 | 594 | DRR | Ca_21004 | S | | |
| CaPOPI_5485 | Ca7 | 34213991 | TCAGAATTACAGAATTACAG/TCAGAATTACAG | TGCGCGATCTCTAATCAATG | CGGCAACCTTACCTCAACTC | 59.9 | 763 | INTERGENIC | | | | |

| INDEL marker IDs | Chromosomes/ unanchored scaffolds | Physical positions (bp) | InDels (*Kabuli* reference genome-CDC Frontier/PI) | Forward primers (5'-3') | Reverse primers (5'-3') | Anneling temperature (0C) | Expected amplified product size (bp) | Structural annotation | | Functional annotation | | |
|---|---|---|---|---|---|---|---|---|---|---|---|---|
| | | | | | | | | Sequence components of *kabuli* genome | *Kabuli* gene accession IDs | NCBI-KOG | TFs | NCBI-nr database |
| CaPOPI_5486 | Ca7 | 34238786 | TAAA/TAA | ACACAAATTTAATATGGATTGAGTACA | CAAAGCAAAACAAAGTGCCA | 57.3 | 426 | INTERGENIC | | | | |
| CaPOPI_5487 | Ca7 | 34241485 | CTTTT/CTT | TGGTTGTTCCATCACATGTCT | CAAAGCTACCACACCTTACTTGC | 58.8 | 536 | INTERGENIC | | | | |
| CaPOPI_5488 | Ca7 | 34251183 | AAT/A | AACCATGGGAACCAATTGAA | TGGCCCAATGGATCATTTAT | 60.0 | 748 | INTERGENIC | | | | |
| CaPOPI_5489 | Ca7 | 34256619 | TCA/TCACA | CGGAGTGGACTCTAACCCAA | TTAGGTGATTCTATGCGCCC | 60.1 | 532 | DRR | Ca_20996 | S | | Basic helix-loop-helix, Nulp1-type |
| CaPOPI_5490 | Ca7 | 34274334 | AAATTATTTTCAA/AAA | AAAACCACCGAGTCATCAGC | AGCCCGTTTGCTACCTTTTT | 60.1 | 555 | DRR | Ca_20991 | | M-type | Chlorophyll A-B binding protein, plant |
| CaPOPI_5491 | Ca7 | 34274392 | TAAAA/TAAA | ACCACCGAGTCATCAGCTCT | AGCCCGTTTGCTACCTTTTT | 59.9 | 552 | DRR | Ca_20991 | | M-type | Chlorophyll A-B binding protein, plant |
| CaPOPI_5492 | Ca7 | 34276465 | ATTTCAC/A | ACACTTTGTTGTGGCAGCAG | CGTTGCCACAGTGATTTTGT | 59.9 | 493 | INTRON | Ca_20991 | | M-type | Chlorophyll A-B binding protein, plant |
| CaPOPI_5493 | Ca7 | 34277307 | T/TG | CAATGGCAGAAGAAGCACAA | TTTGTGGTGCCAGTGTGAAT | 60.0 | 335 | INTERGENIC | | | | |
| CaPOPI_5494 | Ca7 | 34278471 | T/TAAATAC | TTTGACCCATCAAATCATCAAA | CCTTCGCCATTTAATTTTGG | 60.2 | 102 | INTERGENIC | | | | |
| CaPOPI_5495 | Ca7 | 34283992 | GAAAAAAAAA/GAAAAAAAA | CATAACCCATATGCGAGCAA | TCCATGAATTTGTCAACCGA | 59.5 | 662 | DRR | Ca_20990 | A | | Brix domain |
| CaPOPI_5496 | Ca7 | 34286263 | TAAAAAAAA/TAAAAAA | ACGTCAGACATTGGATGTGC | TTGTTTTCAAGGAGTTGCGA | 59.6 | 666 | INTERGENIC | | | | |
| CaPOPI_5497 | Ca7 | 34286533 | AAG/AAGAG | CCTCTAAAAATGGAAAATGACCA | GTCTCTGCCGCTGAACTCTT | 59.4 | 793 | INTERGENIC | | | | |
| CaPOPI_5498 | Ca7 | 34295146 | AATTTGT/AATTTGTTATCTATTTGT | CCAACTGGCCTAGGATGAAA | TGGGAGAAAAACAAGCAAGG | 60.1 | 579 | INTERGENIC | | | | |
| CaPOPI_5499 | Ca7 | 34305236 | AATCATAACCATCATAACCATCATA/AATCATAACCATCATA | ACCGATGCATTGATGAAGGT | CAATGGTGTGCTTGTAACCG | 60.3 | 806 | INTERGENIC | | | | |
| CaPOPI_5500 | Ca7 | 34305420 | CT/CTT | TGCAACTCTAGATGTGAGCCA | CAATGGTGTGCTTGTAACCG | 59.6 | 727 | INTERGENIC | | | | |

| INDEL marker IDs | Chromosomes/ unanchored scaffolds | Physical positions (bp) | InDels (*Kabuli* reference genome-CDC Frontier/PI) | Forward primers (5'-3') | Reverse primers (5'-3') | Anneling temperature (0C) | Expected amplified product size (bp) | Structural annotation | | Functional annotation | | |
|---|---|---|---|---|---|---|---|---|---|---|---|---|
| | | | | | | | | Sequence components of *kabuli* genome | *Kabuli* gene accession IDs | NCBI-KOG | TFs | NCBI-nr database |
| CaPOPI_5501 | Ca7 | 34312118 | AAATTAAT/AAAT | TTGCTAATTGCTAATTGCTTTG | TGGAGTGAGTGTGAGAAAGGAG | 57.4 | 388 | INTERGENIC | | | | |
| CaPOPI_5502 | Ca7 | 34312433 | GATAAATA/GATA | CTCCTTTCTCACACTCACTCCA | TTGGTGGACTTCCCAACTTC | 59.5 | 515 | INTERGENIC | | | | |
| CaPOPI_5503 | Ca7 | 34312678 | ATTTTTTTTT/ATTTTTTTT | CTCCTTTCTCACACTCACTCCA | TTGGTGGACTTCCCAACTTC | 59.5 | 515 | INTERGENIC | | | | |
| CaPOPI_5504 | Ca7 | 34314534 | ATTTTTTT/ATTTTTT | AAAGATGGCCTCAACCTATTCA | TTGACAATTGTTAGAAGTGATTCCA | 60.0 | 453 | INTERGENIC | | | | |
| CaPOPI_5505 | Ca7 | 34318595 | CATTATTACAGATTT/CATTATTACAGATTTATTATTACAGATTT | TCAATGACTGTGACAAAATCCA | GCTAGCAGCCACCTTCTTTG | 59.0 | 503 | INTERGENIC | | | | |
| CaPOPI_5506 | Ca7 | 34319877 | CTTGTTGTTGTTGTTGTTGTTGTTGT/CTTGTTGTTGTTGTTGTTGTTGT | CCACACTCCATGTTCACACC | AGCACCACCATCAATGTCAA | 59.8 | 246 | INTERGENIC | | | | |
| CaPOPI_5507 | Ca7 | 34321138 | GAAAAAAA/GAAAAAA | AAATTGGGGAGGGAAAAATG | TGAAGGCACCGACTCTTACA | 60.0 | 247 | INTERGENIC | | | | |
| CaPOPI_5508 | Ca7 | 34323694 | TAAAA/TAAA | TATGAGCGGGTTGGATTTGT | ATGGGTACGGGTAATCCACA | 60.3 | 402 | INTERGENIC | | | | |
| CaPOPI_5509 | Ca7 | 34328524 | TCC/TCCC | AGGGAGGCACTTCGGAGTAT | TCAATTGTCACAAACATCCTGA | 60.1 | 433 | INTERGENIC | | | | |
| CaPOPI_5510 | Ca7 | 34336122 | T/TTTA | TATTTGACCCGTGAGACGTG | CAACCACAATTCAATTTATGCAA | 59.6 | 539 | INTERGENIC | | | | |
| CaPOPI_5511 | Ca7 | 34337758 | TAAAA/TAAA | TGCAAACACAACTGCAAAAAG | TGCAACATCCATTGATGACC | 59.9 | 755 | INTERGENIC | | | | |
| CaPOPI_5512 | Ca7 | 34342253 | TTTTT/TTTTTATTTT | AATGATAGGTGGACGTTCAAA | GGGTAACCTCGCCACAACTA | 57.1 | 725 | INTERGENIC | | | | |
| CaPOPI_5513 | Ca7 | 34350011 | AC/ACC | TTGAGTGTTGGATGGCTTCA | GGAGATTCGTCGCTTCTTTG | 60.2 | 439 | INTRON | Ca_20986 | C | | Inorganic pyrophosphatase |
| CaPOPI_5514 | Ca7 | 34383642 | TTCT/TT | TTAATTCAGTCGATGCCACG | TCTGGAAAATACACGTTGAATACAT | 59.7 | 841 | INTERGENIC | | | | |
| CaPOPI_5515 | Ca7 | 34391793 | CTTTTTTTTT/CTTTTTTTTTTTT | GGCGAGCATGAAATACCAAT | CAATTGATGCAGGCACACTAA | 59.9 | 256 | INTERGENIC | | | | |

| INDEL marker IDs | Chromosomes/ unanchored scaffolds | Physical positions (bp) | InDels (*Kabuli* reference genome-CDC Frontier/PI) | Forward primers (5'-3') | Reverse primers (5'-3') | Anneling temperature (0C) | Expected amplified product size (bp) | Structural annotation | | Functional annotation | | |
|---|---|---|---|---|---|---|---|---|---|---|---|---|
| | | | | | | | | Sequence components of *kabuli* genome | *Kabuli* gene accession IDs | NCBI-KOG | TFs | NCBI-nr database |
| CaPOPI_5516 | Ca7 | 34408338 | GTTTT/GTTT | TTCCCCGGTGACATAACAGT | CACTGCATAAGCCTGCAAAA | 60.2 | 758 | INTERGENIC | | | | |
| CaPOPI_5517 | Ca7 | 34509865 | AGAGAGAGAGAGAG/AGAGAGAGAGAGAGGGAGAGAGAGAGAG | TTTGTGGTTCTTCCTCGACC | GGGTGGATATCAAATGACCAA | 60.1 | 173 | INTERGENIC | | | | |
| CaPOPI_5518 | Ca7 | 34903171 | AATATATATATATATATATATATA/AATATATATATATATATATATATATA | AAAGGCTCGAGGCATTTTTA | TGTCTGCATTTTAAGCAACGA | 59.0 | 790 | DRR | Ca_17624 | | ARF | Transcriptional factor B3 |
| CaPOPI_5519 | Ca7 | 35020588 | GATATATATATATATATATATATATA/GATATATATATATATATATATATATATATATATATA | ATACTCGCGGTTTGCTTTTG | TACATGCATTGCTGGCATTA | 60.3 | 476 | INTERGENIC | | | | |
| CaPOPI_5520 | Ca7 | 35121887 | GAAAAAAAA/GAAAAAAAAA | AAAAATAATTGGCACAAACCAA | GGGCAATTTTCTGTCTCCAA | 58.5 | 851 | INTERGENIC | | | | |
| CaPOPI_5521 | Ca7 | 35131579 | T/TA | TCGTGGTCGCATTGAACTTA | GAATCAAAATGGAGCGGAAA | 60.3 | 376 | INTERGENIC | | | | |
| CaPOPI_5522 | Ca7 | 35138981 | ATTTTTTTTT/ATTTTTTTT | ATGGGTGCGTTTCTTTTCAG | TCGCTCTCATCAATCACATCA | 60.1 | 171 | INTERGENIC | | | | |
| CaPOPI_5523 | Ca7 | 35148641 | ATCTCT/ATCT | GTTGCCAAATGTTAGCCGTT | GTCTTTGTCAGGGGAGTGGA | 60.0 | 221 | INTERGENIC | | | | |
| CaPOPI_5524 | Ca7 | 35150109 | TTTTATTTATTTATTTATTTATTT/TTTTATTTATTTATTTATTTATTTATTT | GGGGAAACTTTTGGGGAGTA | CACCTTCGCCAATTTCATCT | 60.2 | 820 | INTERGENIC | | | | |
| CaPOPI_5525 | Ca7 | 35154545 | ATTT/ATTTT | TCCATGTCAGATTCGGATGA | CAATGTCCATTTCAGCATGG | 60.0 | 749 | INTERGENIC | | | | |
| CaPOPI_5526 | Ca7 | 35154835 | TTATATATATATAT/TTATATATATAT | CAAAACGATAGAAAGATAATGCAAGA | CAATGTCCATTTCAGCATGG | 60.0 | 374 | INTERGENIC | | | | |
| CaPOPI_5527 | Ca7 | 35166389 | CATGT/CATGTTATTTTTATGT | ATCAAGGTGGGGATTGTTGA | TTGTAGTTTTTCACGTGGTGC | 60.2 | 781 | INTERGENIC | | | | |
| CaPOPI_5528 | Ca7 | 35166415 | TC/T | ATCAAGGTGGGGATTGTTGA | TTGTAGTTTTTCACGTGGTGC | 60.2 | 781 | INTERGENIC | | | | |
| CaPOPI_5529 | Ca7 | 35166612 | GAAAAAAA/GAAAAAA | TTCAATTTTGTTGTTGTTGGAAA | TTGTAGTTTTTCACGTGGTGC | 59.4 | 571 | INTERGENIC | | | | |
| CaPOPI_5530 | Ca7 | 35175468 | CC/CCTTC | TACTTTGCGTGTTCTCGTCG | CCCATGAAGGAAAAATAGCG | 60.1 | 657 | INTERGENIC | | | | |

| INDEL marker IDs | Chromosomes/ unanchored scaffolds | Physical positions (bp) | InDels (*Kabuli* reference genome-CDC Frontier/PI) | Forward primers (5'-3') | Reverse primers (5'-3') | Anneling temperature (0C) | Expected amplified product size (bp) | Structural annotation | | Functional annotation | | |
|---|---|---|---|---|---|---|---|---|---|---|---|---|
| | | | | | | | | Sequence components of *kabuli* genome | *Kabuli* gene accession IDs | NCBI-KOG | TFs | NCBI-nr database |
| CaPOPI_5531 | Ca7 | 35175572 | AAATA/AA | GGCCATGGGAACTAAAACTG | CCCATGAAGGAAAAATAGCG | 59.4 | 239 | INTERGENIC | | | | |
| CaPOPI_5532 | Ca7 | 35177660 | AATA/ATAAAAATAAATATA | GCCAACCGACTCCATCTTTA | TTTAGCAGTTTTTCAATTTCCAA | 60.1 | 690 | INTERGENIC | | | | |
| CaPOPI_5533 | Ca7 | 35180198 | AAAGAACAAGAACAAGA/AAAGAACAAGAACAAGAACAAGA | GGGTTTTAGGTTGGGGTGAT | TGTAACACGGTCCAATCCAA | 59.9 | 203 | CDS (large-effect mutations) | Ca_17639 | | bHLH | Helix-loop-helix DNA-binding domain |
| CaPOPI_5534 | Ca7 | 35206628 | GT/GTAT | CGGACATCTGTTTGTCCTTG | TCCCATTTTATTTGTCCCCA | 59.1 | 334 | INTERGENIC | | | | |
| CaPOPI_5535 | Ca7 | 35206915 | CATATA/CATA | TTGGGGACAAATAAAATGGG | AAACGTGTTTCGTATTTTTGATG | 59.5 | 548 | INTERGENIC | | | | |
| CaPOPI_5536 | Ca7 | 35213604 | CTTTTTT/CTTTT | TTGGAGCAGTACCAACTGACA | GCAATGTGTCAAAGGCTCAA | 59.4 | 681 | INTERGENIC | | | | |
| CaPOPI_5537 | Ca7 | 35217866 | ATTTTTT/ATTTTTTT | AAGGCTAGGATTCTCGGGAT | ATGCGGGATGTTCTTTTCAT | 59.2 | 369 | INTERGENIC | | | | |
| CaPOPI_5538 | Ca7 | 35227195 | GA/GACA | AAAGCGAACGCCATTTTAGA | TCAGAGCAACTGCACTTCAA | 59.9 | 809 | INTERGENIC | | | | |
| CaPOPI_5539 | Ca7 | 35230535 | C/CAT | TCAAAAATGGAAATGGGAAGA | TGGGTGAGGCTTTGCTACTT | 59.4 | 320 | DRR | Ca_17641 | | | |
| CaPOPI_5540 | Ca7 | 35230825 | ATTTTTTTTT/ATTTTTTTT | AAGTAGCAAAGCCTCACCCA | CGGATAATTTCAAATGGATCACT | 59.9 | 401 | INTERGENIC | | | | |
| CaPOPI_5541 | Ca7 | 35275008 | AGGTGGTG/AGGTG | CAACCTTGGTGTTTGGCTTT | CTTCTTCATTCGCCTGGAAC | 60.0 | 262 | DRR | Ca_17646 | R | | |
| CaPOPI_5542 | Ca7 | 35283537 | T/TCCACGATGAA | GCCCAATTGGTTCAGTCAGT | CATCCTGAGGGCAGTGATTT | 60.0 | 770 | INTRON | Ca_17646 | R | | |
| CaPOPI_5543 | Ca7 | 35289806 | CAAAAAA/CAAAAA | ATTGGGAAAACCTTTGGTCA | GAAGGCAGTTTGGGGACATA | 59.3 | 315 | INTRON | Ca_17646 | R | | |
| CaPOPI_5544 | Ca7 | 35290233 | TC/TCC | TATGTCCCCAAACTGCCTTC | CCCAATTTTGCAATTTTCTGT | 59.9 | 442 | INTRON | Ca_17646 | R | | |
| CaPOPI_5545 | Ca7 | 35292570 | GT/GTT | TGAACCAAAATTCTATATGGGGA | GACCCCTCTTTGTAACGCAA | 59.6 | 692 | INTERGENIC | | | | |

| INDEL marker IDs | Chromosomes/ unanchored scaffolds | Physical positions (bp) | InDels (*Kabuli* reference genome-CDC Frontier/PI) | Forward primers (5'-3') | Reverse primers (5'-3') | Anneling temperature (0C) | Expected amplified product size (bp) | Structural annotation | | Functional annotation | | |
|---|---|---|---|---|---|---|---|---|---|---|---|---|
| | | | | | | | | Sequence components of *kabuli* genome | *Kabuli* gene accession IDs | NCBI-KOG | TFs | NCBI-nr database |
| CaPOPI_5546 | Ca7 | 35292736 | ATTTTTTTT/ATTTTTTTTT | TGAACCAAAATTCTATATGGGGA | GACCCCTCTTTGTAACGCAA | 59.6 | 692 | INTERGENIC | | | | |
| CaPOPI_5547 | Ca7 | 35292837 | AGG/AGGG | TGCAGGATTTAGTGGTGGTG | GACCCCTCTTTGTAACGCAA | 59.6 | 154 | INTERGENIC | | | | |
| CaPOPI_5548 | Ca7 | 35313643 | TC/T | TTCGATAAAGCAAAAGCTCCA | AACTGAATATGGGCCAGTCG | 60.0 | 805 | INTERGENIC | | | | |
| CaPOPI_5549 | Ca7 | 35317473 | ATTTT/ATTTTT | CGATGCACACTCATTTTTGC | TGGTTCATTTGCAATGGTTG | 60.3 | 535 | INTERGENIC | | | | |
| CaPOPI_5550 | Ca7 | 35330032 | TTCT/TTCTCTCT | TCACGTGCACTATGCCTCTC | TCGGTGATGGGTATTTGACA | 60.0 | 421 | INTRON | Ca_17647 | BK | FAR1 | PWWP |
| CaPOPI_5551 | Ca7 | 35333033 | C/CT | TGCAAAGCACCCAATTGTAG | CTTTTCCATGCCACGGTACT | 59.7 | 974 | DRR | Ca_17647 | BK | FAR1 | PWWP |
| CaPOPI_5552 | Ca7 | 35337950 | C/CT | CATGATTGCTTTCCATGCAG | GGCTAGGGATGAACTACCCC | 60.2 | 345 | INTRON | Ca_17648 | | TCP | Zinc finger, RING-type |
| CaPOPI_5553 | Ca7 | 35347410 | T/TTATTTTA | AGTTTCCACATGTGATAAATTGC | CCTCCTATCCAACCCATGAA | 58.1 | 749 | INTERGENIC | | | | |
| CaPOPI_5554 | Ca7 | 35348488 | GAAAAAA/GAAAAA | GTGGGTGGTACAAGGTGGAG | AAGCAAATAACAAGGTGTGCC | 60.3 | 669 | INTERGENIC | | | | |
| CaPOPI_5555 | Ca7 | 35364737 | AGATTAGGA/AGA | CGCATTCCTCTCGAAAAGTC | TTGGAGAAAGATCTCCGGTT | 60.0 | 439 | INTERGENIC | | | | |
| CaPOPI_5556 | Ca7 | 35366366 | CA/CAA | GCAGTGCACGGGTCTAAAAT | AGTAGTTGGGTGGACATGCG | 60.1 | 877 | INTERGENIC | | | | |
| CaPOPI_5557 | Ca7 | 35379781 | ATTT/ATT | TCCTCATCACCTGCACAATC | TCCTCTACCCACCAAACCAA | 59.6 | 445 | INTERGENIC | | | | |
| CaPOPI_5558 | Ca7 | 35379825 | CTTTTTTT/CTTTTTTTT | TCCTCATCACCTGCACAATC | TCCTCTACCCACCAAACCAA | 59.6 | 445 | INTERGENIC | | | | |
| CaPOPI_5559 | Ca7 | 35379938 | G/GTGAGTT | TCCTCATCACCTGCACAATC | TCCTCTACCCACCAAACCAA | 59.6 | 445 | INTERGENIC | | | | |
| CaPOPI_5560 | Ca7 | 35385155 | GT/GTT | TGCGAAATGTGAGAAAATGAA | AAGTCAAGATGGCTTGGTGG | 59.3 | 225 | INTERGENIC | | | | |

| INDEL marker IDs | Chromosomes/ unanchored scaffolds | Physical positions (bp) | InDels (*Kabuli* reference genome-CDC Frontier/PI) | Forward primers (5'-3') | Reverse primers (5'-3') | Anneling temperature (0C) | Expected amplified product size (bp) | Structural annotation | | Functional annotation | | |
|---|---|---|---|---|---|---|---|---|---|---|---|---|
| | | | | | | | | Sequence components of *kabuli* genome | *Kabuli* gene accession IDs | NCBI-KOG | TFs | NCBI-nr database |
| CaPOPI_5561 | Ca7 | 35386085 | CAAAAAAAAAAAAAA/CAAAAAAAAAA | TTGTTTCAAAGCTCCAGCAA | CATCGACCAAAAGGAGTTTGA | 59.6 | 505 | INTERGENIC | | | | |
| CaPOPI_5562 | Ca7 | 35389419 | TAAAAA/TAAAAAA | TTCGGTTCTTGTCTGCCTCT | GCCGCGACCATTAATTCTAA | 60.0 | 796 | INTERGENIC | | | | |
| CaPOPI_5563 | Ca7 | 35442260 | AG/A | TATGCTGTCCACCCATCAAA | CAAGCGCATGCATTTTCTAA | 59.9 | 628 | INTERGENIC | | | | |
| CaPOPI_5564 | Ca7 | 35535446 | CTTTTGTTTTGTTTTGTTTTGT/CTTTTGTTTTGTTTTGTTTTGTTTTGT | CAGGCATGTAGAGCATTGGA | AGTTTGGCTCGTGATGGTTC | 59.8 | 528 | DRR | Ca_17760 | | | Protein of unknown function DUF2358 |
| CaPOPI_5565 | Ca7 | 35684957 | ATTTTTT/ATTTTT | CCCCCTCTTAGGACCAGTGT | TATCGCACTCCGACAACAAA | 60.4 | 592 | DRR | Ca_17775 | O | ERF | Glutathione S-transferase, N-terminal |
| CaPOPI_5566 | Ca7 | 35794267 | CTTTT/CTTT | ATGTAGACGATTGCTTCCCG | GCAACAAACTTTGCACTCCA | 60.1 | 700 | INTERGENIC | | | | |
| CaPOPI_5567 | Ca7 | 35921881 | CTTTTTTTTTT/CTTTTTTTTTTT | TGCAATAAGATGAAAAACACCAA | GCATTGGAGCTGAAAAGGAG | 59.5 | 332 | INTRON | Ca_17797 | A | | Ribonuclease T2 |
| CaPOPI_5568 | Ca7 | 36117059 | CTTTA/CTTTTCTTTATTTTA | TGGATTGGGCTTTCCAATAA | TGCCTTAACGTACCCAAACA | 60.3 | 435 | DRR | Ca_13721 | G | Trihelix | Protein of unknown function DUF604 |
| CaPOPI_5569 | Ca7 | 36244087 | GTCTCTCTCTCTCTCTCTCTCTCTCT/GTCTCTCTCTCTCTCTCTCTCTCTCTCT | TCCATGAAACCAAAGCATGA | TGAATTTTCGGCAATCACAA | 60.0 | 681 | INTERGENIC | | | | |
| CaPOPI_5570 | Ca7 | 36353585 | TTAATTAAATAAT/TTAATTAAATAATTAAATAAT | CACCGATGTGAAATCTCTATTTTT | GCGTTTCAGCCAACTTGTGT | 58.6 | 996 | INTERGENIC | | | | |
| CaPOPI_5571 | Ca7 | 36358822 | AAGAGAGAGAGAGAGAGAGAGAGA/AAGAGAGAGAGAGAGAGAGA | AAAACCCCAACTCAACCGTA | TCGCGCTTTCTTTCTCTCTC | 59.3 | 587 | INTERGENIC | | | | |
| CaPOPI_5572 | Ca7 | 36475569 | GA/GAA | GAAAATGTGAGTTTAATTCCCCA | TGGCCTATGTGCTTCTATTGG | 59.3 | 235 | INTERGENIC | | | | |
| CaPOPI_5573 | Ca7 | 36636517 | AC/A | ACATAAGCCAAACAACCCCA | TGGCAACTTTGCTCGTCATA | 60.2 | 722 | INTERGENIC | | | | |
| CaPOPI_5574 | Ca7 | 36803710 | TTT/TTTTATT | AAGTGATCATTTGATGCACAGG | TGACATGTGCAAATTGAAAGG | 60.0 | 720 | INTERGENIC | | | | |
| CaPOPI_5575 | Ca7 | 36871436 | AAGAGAGAGAGAGAGAGAGAGAGAGA/AAGAGAGAGAGAGAGAGAGAGAGAGAGA | TGAGCTGAAACAACATGCGT | TGTCGGGAACCATTTCCTTA | 60.5 | 893 | DRR | Ca_13774 | R | | Lipopolysaccharide-modifying protein |

| INDEL marker IDs | Chromosomes/ unanchored scaffolds | Physical positions (bp) | InDels (*Kabuli* reference genome-CDC Frontier/PI) | Forward primers (5'-3') | Reverse primers (5'-3') | Anneling temperature (0C) | Expected amplified product size (bp) | Structural annotation | | Functional annotation | | |
|---|---|---|---|---|---|---|---|---|---|---|---|---|
| | | | | | | | | Sequence components of *kabuli* genome | *Kabuli* gene accession IDs | NCBI-KOG | TFs | NCBI-nr database |
| CaPOPI_5576 | Ca7 | 36884880 | TTTCTTCTTCTTCTTCTTC/TTTCTTCTTCTTCTTC | TTGGGGTAAGTTGGCTCTTG | TTCTACACGTTTGCAGCAGG | 60.1 | 570 | DRR | Ca_13775 | I | | |
| CaPOPI_5577 | Ca7 | 37205527 | A/AAT | TTGAAATAATTGCGTTGCCA | TGTGCCGATGATGATGTTCT | 60.1 | 666 | INTERGENIC | | | | |
| CaPOPI_5578 | Ca7 | 37282057 | GAATAAATAAATAAAT/GAATAAATAATAAAATAAATAAATAAAT | GCGAGGGAATTATAGCCACA | TCGTGTAGCCAATAGCTTTGTT | 60.1 | 374 | INTERGENIC | | | | |
| CaPOPI_5579 | Ca7 | 37568106 | CT/C | TGTGTTGCAAGTGTGCTTGA | CCAAGTCTCATCTGGAAGCC | 60.1 | 259 | INTERGENIC | | | | |
| CaPOPI_5580 | Ca7 | 37569442 | TCTGATGCTGATGCTG/TCTGATGCTG | CCAACTGAGAAGCTTTTGGC | GATGCCTCCAACATGTTCCT | 60.0 | 698 | INTERGENIC | | | | |
| CaPOPI_5581 | Ca7 | 37595168 | ATGTGTGTGTGTGTGTGTGT/ATGTGTGTGTGTGTGT | CGTTTTGCCTCCTTTCAGTC | TGGAACCCGGTAGATACTTTG | 59.9 | 805 | INTERGENIC | | | | |
| CaPOPI_5582 | Ca7 | 37622315 | TAAAAAAAA/TAAAAAAAAA | TACCCCACGGTAGCTCTTTG | TTCGATCATCACCCTCAACA | 60.1 | 722 | INTERGENIC | | | | |
| CaPOPI_5583 | Ca7 | 37711486 | TG/T | TACCCTGTGGATGGGAATGT | TTGTCGATCCGAAATGTGAA | 60.1 | 870 | DRR | Ca_22800 | | | |
| CaPOPI_5584 | Ca7 | 37752611 | ATTTTTTT/ATTTTTTTT | TCGACAAAATGCAATGTCGTA | CTGGTTCCCCTTATGACCAA | 60.1 | 860 | INTERGENIC | | | | |
| CaPOPI_5585 | Ca7 | 37928727 | GATATATATATATATATATATATATATA/GATATATATATATATATATATATA | ATTGTGTCCGCAGGAAAATC | GTGTGTGTTCCACTGCCATC | 59.9 | 329 | INTERGENIC | | | | |
| CaPOPI_5586 | Ca7 | 37964071 | T/TTTCAA | CCACCATGCAAGGAGTTTTT | GCACGTTGTAATGAACGGTG | 60.0 | 470 | DRR | Ca_16386 | | HD-ZIP | Homeobox |
| CaPOPI_5587 | Ca7 | 38073484 | GATAATAATAATAATAATAATAATAATAATAATAATA/GATAATAATAATAATAATAATAATAATAATAATAATAATA | TGGAGAGAAACAAAATCGGG | TCTGTCTCGCTCACATACCG | 60.0 | 424 | INTERGENIC | | | | |
| CaPOPI_5588 | Ca7 | 38331525 | AAGAGAGAGAG/AAGAGAGAGAGAG | TGTGCAACACCAAAACCCTA | CACTATCTGCTGCACCCTCC | 60.0 | 174 | INTERGENIC | | | | |
| CaPOPI_5589 | Ca7 | 38383093 | ATTTTT/ATTTTTT | TTTCGACGACGATAAAACAATG | ATTTTGTTGGTGATGTGGCA | 60.0 | 170 | DRR | Ca_16412 | | | |
| CaPOPI_5590 | Ca7 | 38449321 | ATA/ATATAATTA | GCGACTTTCAACGTGGTTTT | CCGATTTCCCAAAGGTTTTT | 60.2 | 501 | INTERGENIC | | | | |

| INDEL marker IDs | Chromosomes/ unanchored scaffolds | Physical positions (bp) | InDels (*Kabuli* reference genome-CDC Frontier/PI) | Forward primers (5'-3') | Reverse primers (5'-3') | Anneling temperature (0C) | Expected amplified product size (bp) | Structural annotation | | Functional annotation | | |
|---|---|---|---|---|---|---|---|---|---|---|---|---|
| | | | | | | | | Sequence components of *kabuli* genome | *Kabuli* gene accession IDs | NCBI-KOG | TFs | NCBI-nr database |
| CaPOPI_5591 | Ca7 | 38457330 | CATATATATATATA/CATATATATATATATA | TCATGATGAGGCAATTTGGA | TTGGTTTATTTGGGTTGGGA | 60.0 | 385 | INTERGENIC | | | | |
| CaPOPI_5592 | Ca7 | 38539687 | AATATATATATATATATATATATATATA/AATATATATATATATATATATATATATATA | TGGCCATAAAATATGCCTGA | AGACCGAGTTATAGTTGTGTTCG | 59.0 | 635 | INTERGENIC | | | | |
| CaPOPI_5593 | Ca7 | 38579414 | TTATATATATATATATATATATATATATATATATATATATAT/TTATATATATATATATATATATATATATATATATATATATATATATAT | AGCCATGACTTCATAGTGCATC | CATTAGATGAAAGACAAAGTCCCA | 59.2 | 859 | INTERGENIC | | | | |
| CaPOPI_5594 | Ca7 | 38613431 | CA/CATTAAGAA | TTGAGTGGGCTCTATACCCG | CAAAACTGCCGGTACTGGAT | 60.1 | 796 | INTERGENIC | | | | |
| CaPOPI_5595 | Ca7 | 38626397 | GATAATAATAATAATAATAATAATAATAATAATAATAATA/GATAATAATAATAATAATAATAATAATAATAATAATAATAATAATA | CCAAGCCCAATAAAACCCTT | GGACTCACTCCCCAAATTGA | 60.2 | 131 | INTERGENIC | | | | |
| CaPOPI_5596 | Ca7 | 38669981 | TTTTATTTATTTATTTATT/TTTTATTTATTTATTTATTTATT | CCTGCCGGTTTATTTTGTTC | GCGATCGAATGGCAATATCT | 59.5 | 657 | INTERGENIC | | | | |
| CaPOPI_5597 | Ca7 | 38743655 | CTATTTATTTATTTATTTATTTATTTATTT/CTATTTATTTATTTATTTATTTATTTATTTATTTATTT | CTTAGGTTTGCACTCAGCCC | CTCAATCGTGGGTGACCTTT | 59.9 | 932 | INTERGENIC | | | | |
| CaPOPI_5598 | Ca7 | 38744999 | TTTATTATTATTATTATTATTATTATTATTATTATTATTATTATTATTATTATTATTATTATT/TTTATTATTATTATTATTATTATTATTATTATTATTATTATTATTATTATTATTAT | TCTTCTTGTGGCCGACTCTT | TTGCAAAACCAAAAAGGGAA | 60.0 | 765 | INTERGENIC | | | | |
| CaPOPI_5599 | Ca7 | 39149719 | AT/ATT | GCACTAAGTCAGGGGCTCAG | TGGAACCCATTCACACAAGA | 60.0 | 577 | INTERGENIC | | | | |
| CaPOPI_5600 | Ca7 | 39282936 | GATATATATATATATATATATATAT/GATATATATATATATATATATATATATATATAT | TTCAATGTGAGCAAAGTCGAT | TCCCCCAAATTCAATATCCA | 58.4 | 391 | INTERGENIC | | | | |
| CaPOPI_5601 | Ca7 | 39409758 | TTATATATATATATATA/TTATATATATATATATATA | TCCAAACTCAAACCAAATTCAA | GAAAACAAATCCCCCTCCAT | 59.5 | 485 | INTERGENIC | | | | |
| CaPOPI_5602 | Ca7 | 39525063 | ATTTTTTTTTT/ATTTTTTTTTTT | AACGACCAACTTGGGAAAAA | GGAGTGGGTCACCGAGAATA | 59.4 | 520 | INTERGENIC | | | | |
| CaPOPI_5603 | Ca7 | 39830592 | AGGGGGGG/AGGGGGG | CCTGCTCAAAGCACACGTTA | TAATCATCCTCGGGTTGAGG | 60.1 | 636 | DRR | Ca_25100 | | | |
| CaPOPI_5604 | Ca7 | 39852561 | AAAAATGTTAAAA/AAAAATGTTAAAATGTTAAAA | TTAATCCAACCATCACACAAAA | CAAGCGCAGGTGTATGAAAA | 57.9 | 749 | INTERGENIC | | | | |
| CaPOPI_5605 | Ca7 | 39987914 | ATTTT/ATTTTT | CAAATAATGCACCACTCCCA | CAAGTCTCGCCCACTAAACC | 59.4 | 141 | INTERGENIC | | | | |

| INDEL marker IDs | Chromosomes/ unanchored scaffolds | Physical positions (bp) | InDels (*Kabuli* reference genome-CDC Frontier/PI) | Forward primers (5'-3') | Reverse primers (5'-3') | Anneling temperature (0C) | Expected amplified product size (bp) | Structural annotation | | Functional annotation | | |
|---|---|---|---|---|---|---|---|---|---|---|---|---|
| | | | | | | | | Sequence components of *kabuli* genome | *Kabuli* gene accession IDs | NCBI-KOG | TFs | NCBI-nr database |
| CaPOPI_5606 | Ca7 | 40086967 | A/ATTTGCTACC | GGTGAAAACTGGGTGGAAGA | ATACAATTGTGCCTGCTCCC | 59.9 | 880 | INTERGENIC | | | | |
| CaPOPI_5607 | Ca7 | 40196153 | AATATATATATATATATA/AATATATATATATATATATA | TGAATGCCTACATATGGCGA | TTTGACCTTTATGGCGGTTC | 60.1 | 741 | INTERGENIC | | | | |
| CaPOPI_5608 | Ca7 | 40418637 | TATAATAATAATAATAAT/TATAATAATAATAATAAT | CGGATTCACAAACGGACTTT | GCATTCGATTATGCCGATCT | 60.0 | 888 | INTERGENIC | | | | |
| CaPOPI_5609 | Ca7 | 40430153 | AAGTAAGTA/AAGTAAGTAGTAAGTA | GTTGGTTCAAAAGCAACCGT | ACAAGGATACGTGCAGGGAC | 60.0 | 493 | CDS (FRAME SHIFT) | Ca_20258 | | Trihelix | Transposase, Ptta/En/Spm, plant |
| CaPOPI_5610 | Ca7 | 40591240 | AAATAATAATAATAATAATAATAATAATAATAATAATAATA/AAATAATAATAATAATAATAATAATAATAATAATAATAATAATAATAATAATA | TTGTTGGAATTGCATAGAATTGA | CACCGACCTCAAATTCAACA | 59.5 | 852 | INTERGENIC | | | | |
| CaPOPI_5611 | Ca7 | 40985025 | TAAAA/TAAAAA | GCAACTGCCTTAAAAATGGAA | GGAACGTGCGTACATTGTTG | 59.2 | 303 | INTERGENIC | | | | |
| CaPOPI_5612 | Ca7 | 41008241 | CAAAAAAAAAAAAA/CAAAAAAAAAAA | GCCAATCAGGACACATCTCA | TCAGAACATTCCATCGGTGA | 59.6 | 563 | INTERGENIC | | | | |
| CaPOPI_5613 | Ca7 | 41009960 | ATTTTTTTT/ATTTTTTTTTT | CATGACCTCGGTTTTCAATAA | CCCTCACATTTTTAAGGCACA | 57.6 | 814 | INTERGENIC | | | | |
| CaPOPI_5614 | Ca7 | 41010142 | CAA/CAAAA | TGCAATGAATGAAAGTATGCTACC | GAAAAAGCGCTGTAAATGGC | 60.4 | 815 | INTERGENIC | | | | |
| CaPOPI_5615 | Ca7 | 41170096 | T/TA | TAAGGTTGTCTGGGGAGGTG | CGGCGAATTTGTTTTCTTTT | 60.0 | 667 | INTERGENIC | | | | |
| CaPOPI_5616 | Ca7 | 41226170 | AATATATATATATATATATA/AATATATATATATATATATATA | TTCATCAATGGCACAAGAAAA | TCCTTGGGGATTTTGTGAAA | 59.1 | 500 | INTERGENIC | | | | |
| CaPOPI_5617 | Ca7 | 41250872 | AT/ATTATT | GTGATATTGGGGGTCGAATG | AAGGGTCCTCTTGAAGCGTT | 60.0 | 680 | INTERGENIC | | | | |
| CaPOPI_5618 | Ca7 | 41251256 | AG/A | CCAATAGTTTAGTGGGCCAAA | AAGGGTCCTCTTGAAGCGTT | 59.0 | 213 | INTERGENIC | | | | |
| CaPOPI_5619 | Ca7 | 41252327 | TTAT/TT | TGCAATGCAATGTCCATTTT | TTTTTCCTCATGGTGCAGTG | 59.9 | 324 | INTERGENIC | | | | |
| CaPOPI_5620 | Ca7 | 41283416 | A/AAG | GGTAAACCAATAGCAGCGGA | GAAAAACAGCCCCAATTCAA | 60.1 | 566 | INTERGENIC | | | | |

| INDEL marker IDs | Chromosomes/ unanchored scaffolds | Physical positions (bp) | InDels (*Kabuli* reference genome-CDC Frontier/PI) | Forward primers (5'-3') | Reverse primers (5'-3') | Anneling temperature (0C) | Expected amplified product size (bp) | Structural annotation | | Functional annotation | | |
|---|---|---|---|---|---|---|---|---|---|---|---|---|
| | | | | | | | | Sequence components of *kabuli* genome | *Kabuli* gene accession IDs | NCBI-KOG | TFs | NCBI-nr database |
| CaPOPI_5621 | Ca7 | 41429174 | TAA/TA | TGAGACCGGGTTTGAGTACC | CTGCTGCTCTTGCATCTCAG | 60.0 | 385 | INTERGENIC | | | | |
| CaPOPI_5622 | Ca7 | 41484126 | GTATATATATATATATATATAT/GTATATATATATATATAT | AGATTCGCAGGCAGTTGAGT | CGAGAGAGTGTCACGCATGT | 60.0 | 701 | INTERGENIC | | | | |
| CaPOPI_5623 | Ca7 | 41564191 | CTATATATATATATATATATATATAT/CTATATATATATATATATATATATATATAT | TGCACTCACTCCAACACACA | CCTATCATGGTTTGGCCTGT | 59.9 | 762 | INTERGENIC | | | | |
| CaPOPI_5624 | Ca7 | 42085502 | ATTTTTTTTTTT/ATTTTTTTTTT | TGCTCAACCTCAACGAAATG | GGACATTTGAAGGGCAAAAA | 59.8 | 227 | INTERGENIC | | | | |
| CaPOPI_5625 | Ca7 | 42364254 | AATATATATATATATATATATATATATATATAT/AATATATATATATATATATATATATATATATAT | CATCTCGGGTTGCTGAAAAT | TTTGAAAACCCCTAACACCG | 60.1 | 363 | INTERGENIC | | | | |
| CaPOPI_5626 | Ca7 | 42487577 | AATATATATATATATATATATATATAT/AATATATATATATATATATATATATATATAT | TGATTGATTGGGTGCTCTGT | TAAGGCCCATGTTCAAGGTT | 59.1 | 489 | INTERGENIC | | | | |
| CaPOPI_5627 | Ca7 | 42568760 | AT/A | GCAAGGTTTGCACGTCACTA | AAATTTGTCTTCGCATTTCCA | 59.9 | 293 | DRR | Ca_20212 | K | | Protein of unknown function DUF667 |
| CaPOPI_5628 | Ca7 | 42582021 | TTATATATATATATATATATATATATATATATAT/TTATATATATATATATATATATATATATATAT | TCAATCGGAAGGAGAAGGTG | TGCCCACTTATACAGCAAAGG | 60.2 | 740 | DRR | Ca_20211 | | | |
| CaPOPI_5629 | Ca7 | 42603074 | GTATATATATATATATAT/GTATATATATATAT | GGGCAACCGTTCTTTCTACA | AAATTTCAGCGATCAAACGA | 60.1 | 741 | INTERGENIC | | | | |
| CaPOPI_5630 | Ca7 | 42738535 | TAAAAAAA/TAAAAAAAA | TCCCGCTACACTTTGAGGAC | TCAAAAGCAAGTGTTGCAGG | 60.3 | 592 | INTERGENIC | | | | |
| CaPOPI_5631 | Ca7 | 42757617 | T/TGGTTTGAAGTTTGC | TTTGCTCTGAAAGCCCAAAT | AGTTCCTGCAACTCCGAAGA | 59.8 | 527 | INTRON | Ca_20201 | | | Domain of unknown function DUF239, plant |
| CaPOPI_5632 | Ca7 | 42834810 | AATATATATATATATATATATATATATA/AATATATATATATATATATATA | TTTCTTCGCGTTTGACTAATG | GACATAAGAATGGAGGCGGA | 58.1 | 691 | INTERGENIC | | | | |
| CaPOPI_5633 | Ca7 | 43011755 | TTATATATATATATATAT/TTATATATATATATAT | TGCCAATTTTTCAAAGGTTTTT | GCCCAAGTTCGAAATGTTGT | 59.9 | 351 | INTERGENIC | | | | |
| CaPOPI_5634 | Ca7 | 43375115 | CAAAA/CAAA | CCAATATCTCGTGTTCCCGT | CATTTCAAGTTGGGGCAGTT | 59.8 | 335 | INTERGENIC | | | | |
| CaPOPI_5635 | Ca7 | 43487665 | GTT/GT | AGCTTTTCATTCCACACCGA | CGCACTGCAGATGCAATTTA | 60.6 | 636 | INTERGENIC | | | | |

| INDEL marker IDs | Chromosomes/ unanchored scaffolds | Physical positions (bp) | InDels (*Kabuli* reference genome-CDC Frontier/PI) | Forward primers (5'-3') | Reverse primers (5'-3') | Anneling temperature (0C) | Expected amplified product size (bp) | Structural annotation | | Functional annotation | | |
|---|---|---|---|---|---|---|---|---|---|---|---|---|
| | | | | | | | | Sequence components of *kabuli* genome | *Kabuli* gene accession IDs | NCBI-KOG | TFs | NCBI-nr database |
| CaPOPI_5636 | Ca7 | 43554492 | GA/GATA | CCGTAAATCCCCATGTTTTG | CAAGGTCTTCGAGGTGATCC | 60.0 | 759 | INTRON | Ca_25034 | QI | bHLH | Cytochrome P450 |
| CaPOPI_5637 | Ca7 | 43606803 | TATAAATAAATAAATAAA/TATAAATAAATAAATAAATAAA | TTTTGCCATGTCTCAGCTTG | TCTCCTCATTCCCAAAGTGG | 60.0 | 814 | INTERGENIC | | | | |
| CaPOPI_5638 | Ca7 | 43621219 | GTATATATATATATATATATATA/GTATATATATATATATATATA | TGCATGTAAAACTGGTGGGA | AGCTGAACGCCTGAACATCT | 60.0 | 412 | INTERGENIC | | | | |
| CaPOPI_5639 | Ca7 | 43811202 | CAAA/CAA | GGGTCTGGGATCAACTCTGA | TTGAACTGAATGCAAGCCAC | 60.0 | 630 | INTRON | Ca_21355 | S | | GINS complex, subunit Psf2 |
| CaPOPI_5640 | Ca7 | 44001613 | T/TG | TGAAACTGCCATTGGACAAA | CAATTTCTTTGCGTGTGGTG | 60.1 | 476 | INTERGENIC | | | | |
| CaPOPI_5641 | Ca7 | 44083169 | T/TTC | ATGCTCTGCACTTGCAGCTA | GACGAAATTCTTTTAAGAGGCG | 59.9 | 734 | INTERGENIC | | | | |
| CaPOPI_5642 | Ca7 | 44150096 | T/TTTA | AGTCTTACCAGCGCCACACT | TCCCAACTACAACGAGGACC | 59.9 | 567 | DRR | Ca_21366 | O | | Prohibitin |
| CaPOPI_5643 | Ca7 | 44372091 | GAATAATAATAATAATAATAATAATAATA/GAATAATAATAATAATAATAATAATAATAATA | CGGAGAGTTGATTTCACACAA | CGTCAAAGACGTTCTTCAATCA | 58.8 | 498 | DRR | Ca_21371 | | | Plant lipid transfer protein/Par allergen |
| CaPOPI_5644 | Ca7 | 44396434 | ACC/ACCC | ATGCATGTGAAGTAAGGCCC | ACTCAGCCTCTCTCCAACCA | 60.0 | 458 | INTERGENIC | | | | |
| CaPOPI_5645 | Ca7 | 44474070 | AT/ATT | TAGCAACAAAAATGGGGGAG | TGGTAGCGTACCGTTTGACA | 59.9 | 634 | INTERGENIC | | | | |
| CaPOPI_5646 | Ca7 | 44488959 | ATATTATTATTATTATTATTATTATTATTATTATTATT/ATATTATTATTATTATTATTATTATTATTATTATTATTATTATT | GAGGGGAGGGGAGTATTCTG | GATTGTGCGAAGAGGAGGAG | 59.9 | 588 | INTERGENIC | | | | |
| CaPOPI_5647 | Ca7 | 44822805 | GATATATATATATATATATAT/GATATATATATATATATATATATAT | CTTTCGGGTTGTACTGAGGC | TTGGTCGTTTGAGCCTTTCT | 59.7 | 529 | INTERGENIC | | | | |
| CaPOPI_5648 | Ca7 | 44825811 | TAAAAA/TAAAAAAA | TAGGGCACAAGTCTCCCAAG | GCAGTGCTGAATTTTGGTGA | 60.2 | 385 | INTERGENIC | | | | |
| CaPOPI_5649 | Ca7 | 45088200 | GCTTCTTCTTCTTCTTCTTCTTCTTCTTCTTC/GCTTCTTCTTCTTCTTCTTCTTCTTCTTCTTCTTC | AACCAAGACCGGCTTTATCA | TTTGGAGACCAATGGTGACA | 59.6 | 900 | INTERGENIC | | | | |
| CaPOPI_5650 | Ca7 | 45206871 | GTATATATATATATA/GTATATATATATATATA | AGGAGGTTTGTGTTTGACGG | TTTGGATTTTGACTCGGAGG | 60.0 | 617 | INTERGENIC | | | | |

| INDEL marker IDs | Chromosomes/ unanchored scaffolds | Physical positions (bp) | InDels (*Kabuli* reference genome-CDC Frontier/PI) | Forward primers (5'-3') | Reverse primers (5'-3') | Anneling temperature (0C) | Expected amplified product size (bp) | Structural annotation | | Functional annotation | | |
|---|---|---|---|---|---|---|---|---|---|---|---|---|
| | | | | | | | | Sequence components of *kabuli* genome | *Kabuli* gene accession IDs | NCBI-KOG | TFs | NCBI-nr database |
| CaPOPI_5651 | Ca7 | 45219689 | GTAATAATAATAATAATAATAATAATAATAATAATAATAATAATAA/GTAATAATAATAATAATAATAATAATAATAATAATAATAATAATAATAATAATAATAA | TGCCACAAGGTGTTTATTGC | TTTCTCTTTTGGTGGTTGCC | 59.6 | 769 | INTERGENIC | | | | |
| CaPOPI_5652 | Ca7 | 45241482 | AATATATATATATATATATATATATATATAT/AATATATATATATATATATATATATATATATATATAT | TTTACCCCAACACGTAAGCC | CAGATCGGCGCTACACTACA | 59.9 | 554 | INTERGENIC | | | | |
| CaPOPI_5653 | Ca7 | 45247821 | GAAA/GAAAA | CAAAGACCTAGCGGAACTCG | AACCTGCTCAACTTGCGACT | 60.0 | 767 | INTRON | Ca_15725 | I | bHLH | Alpha/beta hydrolase fold-1 |
| CaPOPI_5654 | Ca7 | 45829437 | TA/TAGTAGGGGTTTTCAAA | TCGTGAGTTTGAGGGGAACT | CGAAAAATCCTTTCGCCATA | 59.7 | 536 | INTERGENIC | | | | |
| CaPOPI_5655 | Ca7 | 46075915 | CTATATATATATATATATA/CTATATATATATATATATA | AAACAATCCAGACCAGTGGC | TCAAGCCAAACCCTTATCTTTT | 60.0 | 543 | INTERGENIC | | | | |
| CaPOPI_5656 | Ca7 | 46332309 | GAACACAA/GAACACAATCAACACAA | AACGTACTGCCTATCACGGG | TTCGCAACTCCACTCTTCCT | 60.0 | 649 | INTERGENIC | | | | |
| CaPOPI_5657 | Ca7 | 46560466 | AATATATATATATATATATA/AACATATATATATATATATATATATA | GGAGGCATTGTTTCCTCAAA | AGGAAGAGTGGAGTTGCGAA | 60.1 | 658 | INTERGENIC | | | | |
| CaPOPI_5658 | Ca7 | 46868801 | TATAATAATAATAATAATAATAATAATAA/TATAATAATAATAATAATAATAATAATAATAATAA | GTACGAAGGTTTGATCCCGA | AACCCACCGTGAAAACAAAC | 59.9 | 276 | INTERGENIC | | | | |
| CaPOPI_5659 | Ca7 | 47130515 | CAT/CATAT | TCGTGTCCCAAAATGTGAAA | TGAAATGGGGCAGGTTTAAG | 59.9 | 189 | DRR | Ca_19486 | | | |
| CaPOPI_5660 | Ca7 | 47359557 | ATTTTT/AT | TGTGCCATGAGGTTCTTTTG | TGCTACGTACGAACCTTCCC | 59.7 | 867 | INTERGENIC | | | | |
| CaPOPI_5661 | Ca7 | 47396377 | ATATTATTATTATTATTATTATTATTATTATTATTATT/ATATTATTATTATTATTATTATTATTATTATTATTATTATTATT | AAAAATTAACGGCATAGAGTTTTCA | GGAAATGAAAGGGGACATCA | 59.5 | 483 | INTERGENIC | | | | |
| CaPOPI_5662 | Ca7 | 48210281 | TTTCATTCATTCATTCATTCATTC/TTTCATTCATTCATTCATTC | TCTTGGTAGCGAGCACATCC | CAAGGGATTCCAGAAACGAA | 61.3 | 171 | INTERGENIC | | | | |
| CaPOPI_5663 | Ca7 | 48234648 | AT/ATT | TTGCAAAAGTCAAACCCACA | ATACTTCTCGCGCAGAGTCC | 60.1 | 660 | INTERGENIC | | | | |
| CaPOPI_5664 | Ca7 | 48311764 | GAAA/GAA | TTCAGATCTGGGATGCCTCT | GGTACGTCCAGTCCTCTTGC | 59.8 | 402 | INTERGENIC | | | | |
| CaPOPI_5665 | Ca7 | 48312616 | ATC/ATATCTC | CGAAGCACAAGGAGATGACA | TTTCCGAGTGCTGTCAAATG | 60.0 | 382 | INTERGENIC | | | | |

| INDEL marker IDs | Chromosomes/ unanchored scaffolds | Physical positions (bp) | InDels (*Kabuli* reference genome-CDC Frontier/PI) | Forward primers (5'-3') | Reverse primers (5'-3') | Anneling temperature (0C) | Expected amplified product size (bp) | Structural annotation | | Functional annotation | | |
|---|---|---|---|---|---|---|---|---|---|---|---|---|
| | | | | | | | | Sequence components of *kabuli* genome | *Kabuli* gene accession IDs | NCBI-KOG | TFs | NCBI-nr database |
| CaPOPI_5666 | Ca7 | 48390576 | CTTTATTTATTTATTTATTTATTTATT/CTTTATTTATTTATTTATTTATTTATTTATT | TGTCAATGGCAACCATGTCT | TTGCTCCTACGTATTTCCCG | 60.0 | 938 | INTERGENIC | | | | |
| CaPOPI_5667 | Ca7 | 48392684 | TAAAAAAAAA/TAAAAAAAAAA | TGATTCCCCAACATCCAACT | ATGATATTGGAGTTTGCGCC | 60.2 | 165 | INTERGENIC | | | | |
| CaPOPI_5668 | Ca7 | 48703820 | CAAAAAAAA/CAAAAAAAAAA | TCAGTCAAACGCACACAAAA | TTCGGTTTGAGGGAAGTCAC | 58.9 | 496 | DRR | Ca_26166 | | | Domain of unknown function DUF239, plant |
| CaPOPI_5669 | Ca7 | 48775188 | ATTTTTT/ATTTTTTT | CAGATGCAAAAGATGCGAAA | CCAAGTTCCCCAGAGTCAAA | 60.0 | 191 | INTERGENIC | | | | |
| CaPOPI_5670 | Ca7 | 48835104 | A/AC | TGCCACCCAAACTATCCAAT | TGTTGAGCTCCCATTTTTCC | 60.2 | 743 | INTERGENIC | | | | |
| CaPOPI_5671 | Ca7 | 48845811 | TAAAAAAAAAA/TAAAAAAAAA | GACTTGACGGATATTTCACGC | TACATCCCTAGGATGCGACC | 59.6 | 421 | INTERGENIC | | | | |
| CaPOPI_5672 | Ca7 | 48879159 | TTATATATATATATATATAT/TTATATATATATATATATATAT | AGTTGATTGGATTCGGTTCG | AGCAAATTCGATCACCCAAC | 59.9 | 408 | INTERGENIC | | | | |
| CaPOPI_5673 | Ca8 | 4957 | AAATAATAATAATAATAATAATAATAATAATAATAATAATAATAA/AAATAATAATAATAATAATAATAATAATAATAATAATAATAATAATAA | TTGGTTTCAAGAAGGATGGC | CGGGTTGAGTTGTTGTTCCT | 60.1 | 738 | INTERGENIC | | | | |
| CaPOPI_5674 | Ca8 | 13774 | CAAAAA/CAAAA | CGAGGCACAACATCATCATC | TGGTGCTTCAATGAACTTGG | 60.1 | 616 | INTERGENIC | | | | |
| CaPOPI_5675 | Ca8 | 104807 | TTATATATATATATATATATAT/TTATATATATATATATATAT | AACCACTAAATCGCATTGCTC | CGGGAAAGAAAAAGATAAAGGA | 59.2 | 278 | INTERGENIC | | | | |
| CaPOPI_5676 | Ca8 | 419332 | TAAAAAAAAA/TAAAAAAAAAA | AAGGTGACAACAGGCCACAT | ATCGATTTTTGTCCGTTGAA | 60.4 | 410 | INTERGENIC | | | | |
| CaPOPI_5677 | Ca8 | 465058 | TAAAAAAAA/TAAAAAAA | CCCAAACTCAAACGTTCACC | ACATGGGACATGTGCAAAAA | 60.4 | 863 | INTERGENIC | | | | |
| CaPOPI_5678 | Ca8 | 610757 | CAAAAAAAAA/CAAAAAAAA | TTTTTCCGACTTGGTCATTT | TTTGCGTTACGAATTTGATGA | 57.2 | 178 | DRR | Ca_11875 | O | | Heat shock protein Hsp70 |
| CaPOPI_5679 | Ca8 | 728618 | GTATATATATATATATATATATATATAT/GTATATATATATATATATATATATATATAT | TGAGGGGCATCTAGAAGGAA | GCAAGTTAAAACCATCTTTCGG | 59.8 | 504 | INTERGENIC | | | | |
| CaPOPI_5680 | Ca8 | 763423 | TTATATATATATATAT/TTATATATATATATATAT | AAACTGCATTTGTAAATGACGAC | CGTTGGACTTGTGAACGAGA | 58.2 | 729 | INTERGENIC | | | | |

| INDEL marker IDs | Chromosomes/ unanchored scaffolds | Physical positions (bp) | InDels (*Kabuli* reference genome-CDC Frontier/PI) | Forward primers (5'-3') | Reverse primers (5'-3') | Anneling temperature (0C) | Expected amplified product size (bp) | Structural annotation | | Functional annotation | | |
|---|---|---|---|---|---|---|---|---|---|---|---|---|
| | | | | | | | | Sequence components of *kabuli* genome | *Kabuli* gene accession IDs | NCBI-KOG | TFs | NCBI-nr database |
| CaPOPI_5681 | Ca8 | 807972 | AAGAGAGAGAGAGAGAGAGAGAGAGAGAGAGA/AAGAGAGAGAGAGAGAGAGAGAGAGAGAGA | CCCTTTCACGCCAATAAAGA | AATCCTTAACCCTCCCTCCA | 60.1 | 565 | INTERGENIC | | | | |
| CaPOPI_5682 | Ca8 | 813049 | ACGCG/ACG | ACTTTCACCAGAAACCGGAA | AGAAGTCAAGGCGAACGAAA | 59.6 | 372 | INTERGENIC | | | | |
| CaPOPI_5683 | Ca8 | 1050452 | GTT/GTTT | AAAGCCGAGAAAATAGGGGA | TTGAGGCTGAATTTGAAGGG | 60.0 | 273 | DRR | Ca_15081 | | | PWWP |
| CaPOPI_5684 | Ca8 | 1079907 | AC/A | ATTAGGTAGCCATGAGCCCC | TTCTCAGATTTGGCATGCTG | 60.3 | 638 | INTRON | Ca_15076 | CR | NAC | NADH:flavin oxidoreductase/NADH oxidase, N-terminal |
| CaPOPI_5685 | Ca8 | 1080030 | TAAAAAAAAAAAA/TAAAAAAAAAA | ACATGATTTGCAAAAAGCCC | AAGGTATCCAAACACAGCGG | 59.9 | 457 | INTRON | Ca_15076 | CR | NAC | NADH:flavin oxidoreductase/NADH oxidase, N-terminal |
| CaPOPI_5686 | Ca8 | 1184414 | GAAAAAAAAAA/GAAAAAAAAA | TTCGTACCGTTCTCTCCTCAA | TTGTACACAACTCCCTTGCG | 59.9 | 563 | INTERGENIC | | | | |
| CaPOPI_5687 | Ca8 | 1342981 | CAAAA/CAAAAA | CCTCTAGGAAACCATCAACATCA | CCTTGCCACCTCATCTCATT | 60.4 | 423 | INTERGENIC | | | | |
| CaPOPI_5688 | Ca8 | 1343227 | AAAAGAAAGAAAGAAA/AAAAGAAAGAAAGAAAGAAA | TGAGAAATTAAGCTTGTGGGG | CCTTGCCACCTCATCTCATT | 59.2 | 206 | INTERGENIC | | | | |
| CaPOPI_5689 | Ca8 | 1779502 | AATATATATATATATATATATATATATAT/AATATATATATATATATATATAT | TTGTCAACTCTCCATGTGTGG | TGAGTTGTTGGGTGTGGAGA | 59.6 | 536 | INTERGENIC | | | | |
| CaPOPI_5690 | Ca8 | 1993541 | A/AGGGAATAATAAT | ATTTCATCCCCAACCGTGTA | GGACTCCAAAGGAAATGCAA | 60.1 | 666 | DRR | Ca_02403 | R | | Glucose-methanol-choline oxidoreductase, N-terminal |
| CaPOPI_5691 | Ca8 | 2033135 | TC/TCC | ACGCTCGATTTCCGAGAGTA | CACTGTGTGGGTTGTTCGAC | 60.0 | 828 | INTERGENIC | | | | |
| CaPOPI_5692 | Ca8 | 2037724 | ATTTTTTTTTCTTTTTTTTTCTT/ATTTTTTTTTCTTTTTTTTTCTTTTTTTTTCTT | TTTTGAGGACTTTCGGGTTG | CATTTGCTAGCCCTTTGAGC | 60.1 | 448 | INTERGENIC | | | | |
| CaPOPI_5693 | Ca8 | 2172364 | GT/GTT | TGGGAGCTACCACGCTTTAC | GCTGTGATCATCGTTTCGTG | 60.3 | 830 | DRR | Ca_02379 | R | | |
| CaPOPI_5694 | Ca8 | 2755315 | GT/GTGAAGAGCGGTAGATAAT | GGTTGCCCTACAACACTGCT | TACTCCGCATCAAATGGACT | 60.2 | 602 | INTERGENIC | | | | |
| CaPOPI_5695 | Ca8 | 3128241 | GTTCGAAAGTTTCGAAAGTTTCGAAAGTTT/GTTCGAAAGTTTCGAAAGTTT | CAGTTGTCCACCCTCCACTT | CACTTTAACTTCCCACCCCC | 60.0 | 856 | DRR | Ca_02267 | E | | Peptidase M20 |

| **INDEL marker IDs** | **Chromosomes/ unanchored scaffolds** | **Physical positions (bp)** | **InDels (*Kabuli* reference genome-CDC Frontier/PI)** | **Forward primers (5'-3')** | **Reverse primers (5'-3')** | **Anneling temperature (0C)** | **Expected amplified product size (bp)** | **Structural annotation** | | **Functional annotation** | | |
|---|---|---|---|---|---|---|---|---|---|---|---|---|
| | | | | | | | | **Sequence components of *kabuli* genome** | ***Kabuli* gene accession IDs** | **NCBI-KOG** | **TFs** | **NCBI-nr database** |
| CaPOPI_5696 | Ca8 | 3313290 | TTATATATATATATATATATAT/TTATATATATATATATATATATAT | TGGCGTATTGTCATTTCCAA | AAAATTCTGTTTGGGGGACC | 59.9 | 674 | INTERGENIC | | | | |
| CaPOPI_5697 | Ca8 | 3556737 | AATATATATATATATATATATATATATATATATA/AATATATATATATATATATATATATATATATATA | GTGAGGGCGGTTATTTCAGA | TGGACAATTCCTCTAAGCTCG | 60.1 | 803 | INTERGENIC | | | | |
| CaPOPI_5698 | Ca8 | 4049374 | AG/AGG | TTGCAGTGACAGTGAGGAGG | GAAAAGCAAAGCTCGTGAGG | 60.0 | 416 | INTRON | Ca_02166 | K | GRAS | SET domain |
| CaPOPI_5699 | Ca8 | 4534293 | CTTTTTTTTTT/CTTTTTTTTTTT | CAGAGGGAGGAATTGTGGAA | GCAGAAAAGAATCACACGCA | 60.0 | 607 | INTERGENIC | | | | |
| CaPOPI_5700 | Ca8 | 4630639 | GATATATATATATATATATATATATA/GATATATATATATATATATATA | TGAAGCATGAACGGGTAGAA | ATGGGTACCGGTCAAAAACC | 59.3 | 265 | INTERGENIC | | | | |
| CaPOPI_5701 | Ca8 | 4841766 | AATATATATATATATATAT/AATATATATATATATATAT | TGCAACTCAGCTATGCATCC | GAGAATGGCAATGAGGTGGT | 60.0 | 719 | INTERGENIC | | | | |
| CaPOPI_5702 | Ca8 | 4937020 | ATTTTTTTT/ATTTTTTT | AGGTCATTCCATTCACCGTC | ATGCTGGTTGTCCTCCTGTC | 59.8 | 845 | INTRON | Ca_02063 | | | Protein of unknown function DUF620 |
| CaPOPI_5703 | Ca8 | 5016373 | TAAAAAA/TAAAAAAA | TTGTCCGTTATAACATTAAACACTTT | GAAAAGACAACCCCGCATTA | 57.5 | 279 | INTERGENIC | | | | |
| CaPOPI_5704 | Ca8 | 5069926 | GTTTT/GTTT | AGGTTTCACATCGGAGGTTG | TGGAAGTAGCCATCTTTGGG | 60.0 | 454 | INTERGENIC | | | | |
| CaPOPI_5705 | Ca8 | 5203286 | TATAAAAAATAAAAAATAAAAAATAAAA/TATAAAAAATAAAAAATAAAAAATAAAAAATAAAA | TGTAGAGAAAGTGATTGACCACC | AATTTGGCATGGGAGACTTG | 58.2 | 643 | DRR | Ca_02032 | O | FAR1 | ATPase, AAA+ type, core |
| CaPOPI_5706 | Ca8 | 5269336 | GG/GGGTCAGG | ATCCAGAATGGAGCAACTGG | CCACGAGATTCCCTGGTAAA | 60.1 | 478 | INTRON | Ca_02025 | R | | Cytidylyltransferase |
| CaPOPI_5707 | Ca8 | 5667391 | TTCTCTCTCTCTCTCTCTCTC/TTCTCTCTCTCTCTCTCTC | TTCACCACCAAAACACCTGA | AGATCCCATGGTTCGATACG | 60.0 | 423 | INTERGENIC | | | | |
| CaPOPI_5708 | Ca8 | 5862916 | CTT/CTTT | GGCCTTGTAACATTTGAGGG | GCTCGCCTTTGCTATGAGTC | 59.4 | 643 | INTERGENIC | | | | |
| CaPOPI_5709 | Ca8 | 6036137 | CAAAAAAAAAA/CAAAAAAAAA | CACACACATAAATGAAACGAACG | CATGCTTTGACCATCAGGTG | 60.3 | 576 | DRR | Ca_01945 | O | NAC | SKP1 component |
| CaPOPI_5710 | Ca8 | 6056579 | TTATATATATATATAT/TTATATATATATAT | TATTCAGATCCCTTGCCCAG | TGCACGGCAATTACAACAAT | 60.0 | 493 | INTERGENIC | | | | |

| INDEL marker IDs | Chromosomes/ unanchored scaffolds | Physical positions (bp) | InDels (*Kabuli* reference genome-CDC Frontier/PI) | Forward primers (5'-3') | Reverse primers (5'-3') | Anneling temperature (0C) | Expected amplified product size (bp) | Structural annotation | | Functional annotation | | |
|---|---|---|---|---|---|---|---|---|---|---|---|---|
| | | | | | | | | Sequence components of *kabuli* genome | *Kabuli* gene accession IDs | NCBI-KOG | TFs | NCBI-nr database |
| CaPOPI_5711 | Ca8 | 6100294 | TAAAAAAAAAAAAAAA/TAAAAAAAAAAAAA | TGCAAAAACCTTAAACGTATGG | TTAGATGCAAACCCAAAGGG | 59.1 | 792 | INTERGENIC | | | | |
| CaPOPI_5712 | Ca8 | 6253206 | TTATATATATATATATATATAT/TTATATATATATATATATAT | ACCCGTTATGAAACGAGTGC | CAGTAATCGGAGAAAACCCG | 60.0 | 602 | INTERGENIC | | | | |
| CaPOPI_5713 | Ca8 | 6307106 | TATACATATAATACAT/TATACAT | GCAATTCAACGAAAATGCAA | CCACTAAGTCATTTGCAAGGC | 59.7 | 942 | INTERGENIC | | | | |
| CaPOPI_5714 | Ca8 | 6492029 | A/ATCAACC | TCTGTTTTGCCCAACCTTTC | ATCTCAACGATCCAACGGTC | 60.1 | 455 | INTRON | Ca_10739 | D | | |
| CaPOPI_5715 | Ca8 | 6492546 | CAAAA/CAAA | CGTAAGCAACCCACCAATCT | TTGTCAACAACTCAGCGTAAAAA | 60.0 | 142 | INTERGENIC | | | | |
| CaPOPI_5716 | Ca8 | 6492603 | ATTTTTTTTTTTTT/ATTTTTTTTTTT | CGTAAGCAACCCACCAATCT | TTGTCAACAACTCAGCGTAAAAA | 60.0 | 142 | INTERGENIC | | | | |
| CaPOPI_5717 | Ca8 | 6492758 | ATTTTTTT/ATTTTTTTT | CGTAAGCAACCCACCAATCT | TGGACATCATACGTTATCAATCG | 60.0 | 672 | INTERGENIC | | | | |
| CaPOPI_5718 | Ca8 | 6493315 | TAAAAAAAA/TAAAAAAAAAAAA | TTTCAAAAGTGAAATTTGGTTGAA | GAGTCAGAAAACAAACGCTCTT | 59.9 | 570 | INTERGENIC | | | | |
| CaPOPI_5719 | Ca8 | 6850537 | GTTTT/GTTTTTT | AAAGCAAGACGCGTCAGAAT | CAAGAGGATATCAAAGCAACAA | 60.0 | 925 | INTRON | Ca_10703 | O | | |
| CaPOPI_5720 | Ca8 | 7065316 | AATATATATATATATATATATATAT/AATATATATATATATATATATATATAT | TGGACCACAATTTAAGTGCAA | GGTTGAGTGGAAGGATGGAA | 59.1 | 448 | INTERGENIC | | | | |
| CaPOPI_5721 | Ca8 | 7211763 | AATATATATATATAT/AATATATATATATATAT | GTTGTTTCCGTTTGGACCAC | TTGGCATTTGGCCTATTCTC | 60.3 | 350 | INTERGENIC | | | | |
| CaPOPI_5722 | Ca8 | 7288711 | TTATATATATATATATATATATATATATATATATAT/TTATATATATATATATATATATATATATATATATATATATATATAT | TACACCTGAGTCCGCTACCC | CAAACGACGACTTCCCAAAT | 60.1 | 633 | INTERGENIC | | | | |
| CaPOPI_5723 | Ca8 | 7323946 | GA/GAACAA | CAGTAATTTCGCCATCCACC | ACGTGAAAGCAGTTGAAGGA | 60.3 | 911 | INTERGENIC | | | | |
| CaPOPI_5724 | Ca8 | 7741922 | A/AG | TGTGCTCCAATGTAACTGGC | GAGCATAGGAGCTGGCTTGA | 59.7 | 511 | DRR | Ca_10611 | J | | Ribosomal protein L35A |
| CaPOPI_5725 | Ca8 | 7777713 | CACATAACATA/CACATA | TCCAGTGGCACCATAACAAA | CTCCCAAGAACCCTTCTTCC | 60.0 | 744 | INTRON | Ca_10607 | Z | MYB_related | Kinesin, motor domain |

| INDEL marker IDs | Chromosomes/ unanchored scaffolds | Physical positions (bp) | InDels (*Kabuli* reference genome-CDC Frontier/PI) | Forward primers (5'-3') | Reverse primers (5'-3') | Anneling temperature (0C) | Expected amplified product size (bp) | Structural annotation | | Functional annotation | | |
|---|---|---|---|---|---|---|---|---|---|---|---|---|
| | | | | | | | | Sequence components of *kabuli* genome | *Kabuli* gene accession IDs | NCBI-KOG | TFs | NCBI-nr database |
| CaPOPI_5726 | Ca8 | 7896482 | TC/T | TTTGATATGTTGCTTCC GCA | CGATGCAGCCTCTTAGG TGT | 60.2 | 689 | INTERGENIC | | | | |
| CaPOPI_5727 | Ca8 | 7910743 | CAAAAAAAAAAA/CAAAAAAAAAA | ATGATTGGGCTCGTTTG TTC | CCAAAATCAACTGCAAC CCT | 59.9 | 390 | INTERGENIC | | | | |
| CaPOPI_5728 | Ca8 | 7913547 | A/AT | CCGATATGTCAGAAAAC ATCACA | GGCCTTCAACTTCAACT TGCT | 59.9 | 685 | INTERGENIC | | | | |
| CaPOPI_5729 | Ca8 | 7927288 | CAAA/CAAAA | AGGACCGTGCAAATGTT TCT | TGGCCTACTTATGGTCT GGC | 59.6 | 349 | DRR | Ca_11431 | J | | Protein synthesis factor, GTP-binding |
| CaPOPI_5730 | Ca8 | 7927539 | GTATTGCTTTTAT/GTATTGCTTTTATTG CTTTTAT | GCCAGACCATAAGTAGG CCA | GCAACTGGATTGCTGGA TTT | 60.1 | 509 | DRR | Ca_11430 | J | | Translation elongation factor EFTu/EF1A, C-terminal |
| CaPOPI_5731 | Ca8 | 7927639 | ATT/AT | GCCAGACCATAAGTAGG CCA | GCAACTGGATTGCTGGA TTT | 60.1 | 509 | DRR | Ca_11430 | J | | Translation elongation factor EFTu/EF1A, C-terminal |
| CaPOPI_5732 | Ca8 | 7948182 | TCA/TCACA | GCCGCAAAGAAGAGAAG AGA | TGCCCAAGTCACTAATG CTG | 59.8 | 443 | INTERGENIC | | | | |
| CaPOPI_5733 | Ca8 | 7948249 | ACTCTCT/ACTCT | GCCGCAAAGAAGAGAAG AGA | TGCCCAAGTCACTAATG CTG | 59.8 | 443 | INTERGENIC | | | | |
| CaPOPI_5734 | Ca8 | 7950561 | CAAAAAA/CAAAAA | GTCTTTGGCCTCCAATC AAA | TGGAGTTGGGCCAATAA TTT | 60.1 | 359 | INTERGENIC | | | | |
| CaPOPI_5735 | Ca8 | 7951970 | TAAAAAAAAA/TAAAAAAAA | GCAACCATAAAAGAGCA AGTTGA | AGAGCAACACCATTTTG AAGG | 60.6 | 377 | INTERGENIC | | | | |
| CaPOPI_5736 | Ca8 | 7952607 | GAAAAAA/GAAAAAAAA | CCTTCAAAATGGTGTTG CTCT | GTCATTTTGCCGATCAC CTT | 59.2 | 676 | INTERGENIC | | | | |
| CaPOPI_5737 | Ca8 | 7953093 | T/TC | AAGGTGATCGGCAAAAT GAC | CAGCCCTTCAGTGTCAT GAGT | 59.9 | 609 | INTERGENIC | | | | |
| CaPOPI_5738 | Ca8 | 7953809 | TACCTCAA/TACCTCAAACCTCAA | ACCTGCGACACGTGAAC ATA | GAAAACGAGGCGTAAAC GAG | 60.2 | 360 | INTERGENIC | | | | |
| CaPOPI_5739 | Ca8 | 7954393 | TAA/TA | CTCGTTTACGCCTCGTT TTC | CCGTAGCATGAGAGTGA CCA | 59.9 | 561 | INTERGENIC | | | | |
| CaPOPI_5740 | Ca8 | 7954496 | CATATA/CATATATA | TTTCGTCGTATAAAGAC GGAGAG | CCGTAGCATGAGAGTGA CCA | 59.8 | 545 | INTERGENIC | | | | |

| INDEL marker IDs | Chromosomes/ unanchored scaffolds | Physical positions (bp) | InDels (*Kabuli* reference genome-CDC Frontier/PI) | Forward primers (5'-3') | Reverse primers (5'-3') | Anneling temperature (0C) | Expected amplified product size (bp) | Structural annotation | | Functional annotation | | |
|---|---|---|---|---|---|---|---|---|---|---|---|---|
| | | | | | | | | Sequence components of *kabuli* genome | *Kabuli* gene accession IDs | NCBI-KOG | TFs | NCBI-nr database |
| CaPOPI_5741 | Ca8 | 7955526 | TCTC/T | CATGCAAGAGAAGGGAAAGAA | GTGTGGGAGAGAAAGCATCC | 59.4 | 296 | INTERGENIC | | | | |
| CaPOPI_5742 | Ca8 | 7955567 | TTCTCTCTCTCT/TTCTCTCTCTCTCTCT | CATGCAAGAGAAGGGAAAGAA | GTGTGGGAGAGAAAGCATCC | 59.4 | 296 | INTERGENIC | | | | |
| CaPOPI_5743 | Ca8 | 7956165 | GTTATATAATATTA/GTTATATAATATTATTATATAATATTA | TTGATTTGTCTAGAAGCAGCACA | CACTTCTTGGCTGAGTTGGA | 60.1 | 476 | DRR | Ca_11432 | | G2-like | Myb, DNA-binding |
| CaPOPI_5744 | Ca8 | 8003394 | CAAAAAAAA/CAAAAAAAAA | TCTCTCAATTCATGCTATGGAAAA | ACCCGCATGTGATCATGTTT | 60.1 | 703 | INTERGENIC | | | | |
| CaPOPI_5745 | Ca8 | 8041385 | TAGAGAGAGAGAGAGAGAGAGAGA/TAGAGAGAGAGAGAGAGAGA | CGGCGCGAATTTAGAGTTAG | GTTTCTGCCACTCTCTTCGG | 60.0 | 212 | INTRON | Ca_11441 | U | M-type | SCAMP |
| CaPOPI_5746 | Ca8 | 8075837 | ATACT/ATACTTACT | TTGAGCCAAAAGCCTCCTTA | TTTTCAACAAAATGTGCGGA | 60.0 | 824 | INTERGENIC | | | | |
| CaPOPI_5747 | Ca8 | 8084688 | CATA/CA | TCACCCTACAACATGACCGA | ATGGATGCAACACCAAAATG | 60.0 | 977 | INTERGENIC | | | | |
| CaPOPI_5748 | Ca8 | 8240879 | TC/TCC | GTCGCCGCTACGAGATAAAC | TTTGTGCTGATGGTGATGGT | 59.9 | 729 | INTERGENIC | | | | |
| CaPOPI_5749 | Ca8 | 8272916 | TTAT/TT | TGGCACAGAAACTTCGATTG | TTACCGCCTCTTCCTTTTCA | 59.8 | 727 | INTERGENIC | | | | |
| CaPOPI_5750 | Ca8 | 8273148 | GT/GTGAT | GTCACCAGTCACCAGTCACG | TTACCGCCTCTTCCTTTTCA | 60.2 | 550 | INTERGENIC | | | | |
| CaPOPI_5751 | Ca8 | 8312325 | AAATAGGAATGTTTA/AAATAGGAATGTTTAATAGGAATGTTTA | CGGCGATCATTAAGGAGAAA | ATTTTGAACAAGCGGACCTG | 60.2 | 561 | INTERGENIC | | | | |
| CaPOPI_5752 | Ca8 | 8427403 | TAAAA/TAAAAA | TGCCATGCAAGTGTTTCAAT | GTCCACGATGTTGACGTGAC | 60.1 | 456 | INTERGENIC | | | | |
| CaPOPI_5753 | Ca8 | 8623323 | ATTTTTTTT/ATTTTTTTTT | GGGGTGATTATGGAGCAAGA | GGTCACGTGTGTTCCAAGAA | 59.9 | 963 | INTERGENIC | | | | |
| CaPOPI_5754 | Ca8 | 8624257 | ATTAAAAGC/ATTAAAAGCTCAACTTTAAAAGC | CTTTGATTGGGGTTTTGGAA | TGGTGACAATTATTTTTCCCGT | 59.8 | 664 | INTERGENIC | | | | |
| CaPOPI_5755 | Ca8 | 8624418 | CAAAAATTTAGAAAAAT/CAAAAATTTAGAAAAATTTAGAAAAAT | TCCTTTCAATGGACTGGTGA | GTCCCGAGCATGACGTTTAT | 59.1 | 888 | INTERGENIC | | | | |

| INDEL marker IDs | Chromosomes/ unanchored scaffolds | Physical positions (bp) | InDels (*Kabuli* reference genome-CDC Frontier/PI) | Forward primers (5'-3') | Reverse primers (5'-3') | Anneling temperature (0C) | Expected amplified product size (bp) | Structural annotation | | Functional annotation | | |
|---|---|---|---|---|---|---|---|---|---|---|---|---|
| | | | | | | | | Sequence components of *kabuli* genome | *Kabuli* gene accession IDs | NCBI-KOG | TFs | NCBI-nr database |
| CaPOPI_5756 | Ca8 | 8771102 | AATTAATTTA/AATTAATTTATTAATTTA | TCATAGAAATTTGGAGGCGG | GTGCTGTTATTTGCACCCCT | 60.0 | 742 | INTERGENIC | | | | |
| CaPOPI_5757 | Ca8 | 8845260 | ATTTTTTTTTTT/ATTTTTTTTTT | TTTGCTCCTCCTCTTTCTGC | TCGTGTTATCCAACCCACTCT | 59.7 | 697 | INTERGENIC | | | | |
| CaPOPI_5758 | Ca8 | 8848853 | TAAAAAAAA/TAAAAAAAAA | GTTGGGTCTAGTGGGGTTTG | TGTACTTTTTAGGTGCCGGG | 59.3 | 589 | INTERGENIC | | | | |
| CaPOPI_5759 | Ca8 | 8848887 | TAAAAAAAA/TAAAAAAA | GTTGGGTCTAGTGGGGTTTG | TGTACTTTTTAGGTGCCGGG | 59.3 | 589 | INTERGENIC | | | | |
| CaPOPI_5760 | Ca8 | 8857167 | ATTTTTTT/ATTTTTT | GCCCAATTTGTTTCTTGGAA | ACTTGGCCAATCTTGTTTCG | 59.9 | 929 | INTERGENIC | | | | |
| CaPOPI_5761 | Ca8 | 8858935 | TAA/TA | TTATTGGACCATCCGCATTT | TTTTGGGTTACCTTGGCTTG | 60.2 | 750 | INTERGENIC | | | | |
| CaPOPI_5762 | Ca8 | 8860779 | TAA/TA | TCTGGGACACAACCAAAACA | CAATTGCATGTGATTCAGCC | 60.0 | 517 | INTERGENIC | | | | |
| CaPOPI_5763 | Ca8 | 8862222 | TAAAAAAAAA/TAAAAAAA | AGGTTTCGAACCATGCATTT | TGCGAGTCACATGAATTTCG | 59.4 | 753 | INTERGENIC | | | | |
| CaPOPI_5764 | Ca8 | 8868822 | TATTTATTAATTTATT/TATTTATT | TGGTGGTTCAAAGGTAAGGG | AAAACGACGGTGTGACATATTTT | 59.8 | 830 | INTERGENIC | | | | |
| CaPOPI_5765 | Ca8 | 8869164 | TAAAAAAA/TAAAAAA | TGAGATGCTCTCCATCTCCAT | AAAACGACGGTGTGACATATTTT | 59.8 | 575 | INTERGENIC | | | | |
| CaPOPI_5766 | Ca8 | 8871176 | T/TA | AGGACAAGCTCCACCAACAT | TTACTTGATGCCCCCACTTC | 59.6 | 271 | INTERGENIC | | | | |
| CaPOPI_5767 | Ca8 | 8873400 | TTA/TTATTATTCATA | TTTCTTGTCGTGGCTTACCA | TGCATCACGTATATGACCCATT | 59.3 | 797 | INTERGENIC | | | | |
| CaPOPI_5768 | Ca8 | 8881428 | CA/CAA | CCAAAGTCAACTGGTCAAAGG | TCGTGAACTTGCTGAATTGG | 59.6 | 790 | INTERGENIC | | | | |
| CaPOPI_5769 | Ca8 | 8882090 | T/TTCCAAAC | ATCATTCCACGGATTTTCCA | TGAAAACAAAACAAGAAACAGAAA | 60.1 | 108 | DRR | Ca_11519 | | | |
| CaPOPI_5770 | Ca8 | 8885048 | ATTTTTT/ATTTTTTT | GTGTGGAAGCACGAGACAGA | AAAGGAACATTGTTGGACCG | 60.0 | 723 | DRR | Ca_11520 | C | | Mitochondrial substrate/solute carrier |

| INDEL marker IDs | Chromosomes/ unanchored scaffolds | Physical positions (bp) | InDels (*Kabuli* reference genome-CDC Frontier/PI) | Forward primers (5'-3') | Reverse primers (5'-3') | Anneling temperature (0C) | Expected amplified product size (bp) | Structural annotation | | Functional annotation | | |
|---|---|---|---|---|---|---|---|---|---|---|---|---|
| | | | | | | | | Sequence components of *kabuli* genome | *Kabuli* gene accession IDs | NCBI-KOG | TFs | NCBI-nr database |
| CaPOPI_5771 | Ca8 | 8886584 | ACA/ACATATAAAAATCTGTTCA | CATTTGCACCCTACAACACG | AAGGTTTTGGTCCTGCAATG | 60.0 | 536 | DRR | Ca_11520 | C | | Mitochondrial substrate/solute carrier |
| CaPOPI_5772 | Ca8 | 8901973 | TCTCTCTCTCAC/TC | TTGCAATGGTTCAAGCTACG | TCATTTATCACCTACGGCCC | 59.9 | 393 | INTRON | Ca_11521 | G | | Major facilitator superfamily MFS-1 |
| CaPOPI_5773 | Ca8 | 8907150 | ATT/AT | ATTCTTCGTCCAAACATGGC | GGAGGGGGAAAACTTTGGTA | 59.9 | 866 | INTERGENIC | | | | |
| CaPOPI_5774 | Ca8 | 8910515 | GAAAAAAAAA/GAAAAAAAA | TTTCATCAATGGCAAGGTCA | CGATTGTTCTGGTTGTCCCT | 60.0 | 530 | DRR | Ca_11522 | | | Starch synthase, catalytic domain |
| CaPOPI_5775 | Ca8 | 8924623 | GTTTTTTTT/GTTTTTTTTT | TAAGCGTAAATAGGCGCCAG | GGGAGGAAAAGAGGAGGTTG | 60.4 | 188 | INTRON | Ca_11523 | | | Chromatin SPT2 |
| CaPOPI_5776 | Ca8 | 8938717 | G/GATA | GGTGTTGAGGGTGCAGATTT | GCAAGACATTCACGTTGCAC | 60.0 | 751 | INTERGENIC | | | | |
| CaPOPI_5777 | Ca8 | 8940349 | TAAAAAAAA/TAAAAAAA | TGCATTTTAACCTTGTTACATCG | TACCGGAGAAAGCCACAAAC | 59.1 | 770 | INTERGENIC | | | | |
| CaPOPI_5778 | Ca8 | 8954679 | TAAAAAAAAA/TAAAAAAAA | ATCCATCGAGCAAGTCGTTC | TTGTCCTCCTTGTACTTCCTCC | 60.2 | 425 | INTERGENIC | | | | |
| CaPOPI_5779 | Ca8 | 8964218 | AGG/AGGG | GGCTGGCCTCTACAACTGAG | AGGGCCACAACACTCCCTAT | 60.0 | 221 | INTERGENIC | | | | |
| CaPOPI_5780 | Ca8 | 8964234 | A/AT | GGCTGGCCTCTACAACTGAG | AGGGCCACAACACTCCCTAT | 60.0 | 221 | INTERGENIC | | | | |
| CaPOPI_5781 | Ca8 | 8969277 | TAAAAAA/TAAAAAAA | GCAACATTGGCAAAAAGTGA | CACATGGCCGTGGGTAAAT | 59.7 | 534 | INTERGENIC | | | | |
| CaPOPI_5782 | Ca8 | 8994344 | A/AGG | TTCACTTCAACGGTTGTGGA | CGAAAATTCAAAATGAAGGAACA | 60.1 | 665 | INTERGENIC | | | | |
| CaPOPI_5783 | Ca8 | 8995565 | CCTCT/CCTCTCT | GTTGCAAAGTCAACGGGTTT | TGAAAAATTGAAATGTTGATTCATCT | 60.0 | 330 | INTERGENIC | | | | |
| CaPOPI_5784 | Ca8 | 8998721 | TCC/TC | TTAACGACGAAAATGAATTAAAAA | AAGTATGCATTTGTTGCCTTCA | 57.2 | 546 | INTERGENIC | | | | |
| CaPOPI_5785 | Ca8 | 9004500 | TA/TAA | CAATTTTGCACTTGATAGCCAA | GTGCTGCGTAGTGACCGTTA | 60.1 | 233 | INTERGENIC | | | | |

| INDEL marker IDs | Chromosomes/ unanchored scaffolds | Physical positions (bp) | InDels (*Kabuli* reference genome-CDC Frontier/PI) | Forward primers (5'-3') | Reverse primers (5'-3') | Anneling temperature (0C) | Expected amplified product size (bp) | Structural annotation | | Functional annotation | | |
|---|---|---|---|---|---|---|---|---|---|---|---|---|
| | | | | | | | | Sequence components of *kabuli* genome | *Kabuli* gene accession IDs | NCBI-KOG | TFs | NCBI-nr database |
| CaPOPI_5786 | Ca8 | 9005048 | GTTTTTTTT/GTTTTTTT | TAACGGTCACTACGCAGCAC | CGGTGGCCACATTACATACA | 59.9 | 809 | INTERGENIC | | | | |
| CaPOPI_5787 | Ca8 | 9057136 | TAAAAAAA/TAAAAAAAA | GCGGAGACGCAACTACTGTT | TGGCTTTGTGAACTCCAAGA | 60.5 | 599 | DRR | Ca_11528 | | | |
| CaPOPI_5788 | Ca8 | 9081856 | ATT/AT | TGGCACCACCTCATATTTCA | CCTTTTCCACCTTGCATCAT | 59.9 | 322 | INTERGENIC | | | | |
| CaPOPI_5789 | Ca8 | 9084472 | TACTTCA/TA | GGGCCAAAAAGTTTCACAAA | TCCATTTCCCCTAATTTCCC | 60.0 | 661 | INTERGENIC | | | | |
| CaPOPI_5790 | Ca8 | 9087628 | ATATTATTATTATT/ATATTATTATTATTATT | TTGGACCCCATCTTACTTGG | TGTAGCATCCCATTTTCCACT | 59.8 | 741 | INTERGENIC | | | | |
| CaPOPI_5791 | Ca8 | 9088156 | TAAAAAA/TAAAAA | AGTGGAAAATGGGATGCTACA | CACAAATTTCAACGACAAATCG | 59.4 | 534 | INTERGENIC | | | | |
| CaPOPI_5792 | Ca8 | 9088516 | CTTT/CTT | CGATTTGTCGTTGAAATTTGTG | CCATACAAAACACAAGGTGTCG | 60.4 | 479 | INTERGENIC | | | | |
| CaPOPI_5793 | Ca8 | 9089194 | TAATAAAATAAAATAAAATAAAA/TAATAAAATAAAATAAAA | CGCGTTCCCAAATGTTTAAT | CCCTAATCTTCACCAACCGA | 59.8 | 513 | INTERGENIC | | | | |
| CaPOPI_5794 | Ca8 | 9101623 | CT/CTTT | CAAGGTTCTCCAGAAACCCA | ACACGTGTGCGCTCAAAATA | 60.1 | 671 | DRR | Ca_11533 | | C2H2 | Glycoside hydrolase, family 3, N-terminal |
| CaPOPI_5795 | Ca8 | 9129065 | TAG/TAGAAG | TCCATGGACCTTCACTCCTC | GCCTTCTTCACCTTGTGCTC | 60.0 | 619 | CDS (large-effect mutations) | Ca_11536 | Q | Nin-like | ABC transporter-like |
| CaPOPI_5796 | Ca8 | 9131797 | AAGA/AA | TTCGCTTCAGATTTCCCTTT | TCAACTCTTTCAACCACTGGC | 58.9 | 447 | DRR | Ca_11536 | Q | Nin-like | ABC transporter-like |
| CaPOPI_5797 | Ca8 | 9137514 | GTTTT/GTTTTT | TTTTGGGGTATCATTGAGCC | TCGTTTGGGAGATTTCGAGT | 59.8 | 536 | INTERGENIC | | | | |
| CaPOPI_5798 | Ca8 | 9149110 | CTTTTTTT/CTTTTTT | GCAAAAAGTGAAGGATTCGC | TGCATGCAGTAAAAAGTATCCA | 59.8 | 557 | INTERGENIC | | | | |
| CaPOPI_5799 | Ca8 | 9152450 | ATT/ATTT | CAGCCTGTTCAAAAATGCAA | GCATTTGAGGATGCTGGATT | 59.8 | 257 | INTRON | Ca_11539 | O | | Peptidase M3A/M3B, thimet/oligopeptidase F |
| CaPOPI_5800 | Ca8 | 9153262 | CA/CAAGTA | CAGAAACTCTGCGGTCAACA | TGCTGCCAGAACTTATCGTG | 60.0 | 431 | INTRON | Ca_11539 | O | | Peptidase M3A/M3B, thimet/oligopeptidase F |

| INDEL marker IDs | Chromosomes/ unanchored scaffolds | Physical positions (bp) | InDels (*Kabuli* reference genome-CDC Frontier/PI) | Forward primers (5'-3') | Reverse primers (5'-3') | Anneling temperature (0C) | Expected amplified product size (bp) | Structural annotation | | Functional annotation | | |
|---|---|---|---|---|---|---|---|---|---|---|---|---|
| | | | | | | | | Sequence components of *kabuli* genome | *Kabuli* gene accession IDs | NCBI-KOG | TFs | NCBI-nr database |
| CaPOPI_5801 | Ca8 | 9153702 | AAAGAAG/AAAG | CAGCACGATAAGTTCTGGCA | GGGGAATAGAGTGGGATGCT | 60.0 | 420 | INTRON | Ca_11539 | O | | Peptidase M3A/M3B, thimet/oligopeptidase F |
| CaPOPI_5802 | Ca8 | 9199467 | ATT/AT | AGCTCCCAAGGACAGATTCA | GCAAAGATATGATTGGGCGT | 59.8 | 692 | INTERGENIC | | | | |
| CaPOPI_5803 | Ca8 | 9201647 | CA/CAA | GTCTCGATCGCAAGTCACAA | GATATATACGGCTGACGGCG | 60.0 | 300 | INTERGENIC | | | | |
| CaPOPI_5804 | Ca8 | 9215036 | GTTTTTTT/GTTTTTTTT | CATCCTCTGGTTGGACGACT | TTTTTCCCCCGACACATAAG | 60.1 | 837 | INTERGENIC | | | | |
| CaPOPI_5805 | Ca8 | 9220067 | C/CTA | AAAATTAGGGGGAACACAGGA | GTATGTGTGGTGCCATTTGC | 59.7 | 909 | DRR | Ca_11542 | | | Legume lectin, beta chain |
| CaPOPI_5806 | Ca8 | 9274060 | AA/AATA | AACAACAGAGCCTGCGAACT | TCCATGTTTCCGGTTTTCAT | 60.1 | 486 | INTRON | Ca_11549 | | NAC | Protein kinase, catalytic domain |
| CaPOPI_5807 | Ca8 | 9327905 | GTCTC/GTC | GCATGGAAGTATGCCTCCTC | AATTGAGAACCATTTTGCCG | 59.7 | 698 | DRR | Ca_11554 | Z | B3 | Kinesin, motor domain |
| CaPOPI_5808 | Ca8 | 9355298 | TTCT/TT | GAAGAGCTAACAGTTGGCGG | GGGAATTGGCAAGTAGTGGA | 60.0 | 431 | INTRON | Ca_11556 | AR | | K Homology |
| CaPOPI_5809 | Ca8 | 9357060 | TATTA/T | CTTTGTTCGGTTGTTCCGTT | CATCATCCACGGGTGACATA | 60.0 | 620 | INTERGENIC | | | | |
| CaPOPI_5810 | Ca8 | 9364522 | ATT/ATTT | TTGTCGGTGAGATTGTGTGAA | AAAGATTGTGATTTTGGCGG | 60.1 | 802 | INTERGENIC | | | | |
| CaPOPI_5811 | Ca8 | 9953316 | TTGTGT/TTGTGTGT | AGCAGCAACAACCGTCTTTT | AAAAATCGATCGGGTGCATA | 59.9 | 312 | DRR | Ca_18394 | | | Protein of unknown function DUF1442 |
| CaPOPI_5812 | Ca8 | 9960024 | CATAATAATAATAAT/CATAATAATAATAATAATAATAAT | CCATTGGGATGTTTGCTTTT | GCTCATGTGGTGGTGTTTTG | 59.8 | 280 | INTRON | Ca_18395 | | | Stress up-regulated Nod 19 |
| CaPOPI_5813 | Ca8 | 10161190 | GAAAAAAAAAA/GAAAAAAAAA | GCAGATGTGCTCATTGCTTT | GCTCCCAGCCCAACTATTTT | 59.0 | 554 | INTERGENIC | | | | |
| CaPOPI_5814 | Ca8 | 10357104 | ATTTT/ATTTTT | AATGTTACCTTGCAGGACCG | TGAGTGACCGAGCTGACAAC | 60.0 | 763 | INTERGENIC | | | | |
| CaPOPI_5815 | Ca8 | 10366674 | CTT/CT | CAAGAAGACGCCGATGAAAT | GTCGAACGTTCTCCGTTTGT | 60.2 | 571 | INTERGENIC | | | | |

| INDEL marker IDs | Chromosomes/ unanchored scaffolds | Physical positions (bp) | InDels (*Kabuli* reference genome-CDC Frontier/PI) | Forward primers (5'-3') | Reverse primers (5'-3') | Anneling temperature (0C) | Expected amplified product size (bp) | Structural annotation | | Functional annotation | | |
|---|---|---|---|---|---|---|---|---|---|---|---|---|
| | | | | | | | | Sequence components of *kabuli* genome | *Kabuli* gene accession IDs | NCBI-KOG | TFs | NCBI-nr database |
| CaPOPI_5816 | Ca8 | 10484577 | GCCC/GCC | TGAGGAGTATCCATGTGGCA | GAGTTCGGAAGTGAGCCAAG | 60.1 | 939 | INTERGENIC | | | | |
| CaPOPI_5817 | Ca8 | 10509590 | GA/GAA | GCCATGTCAACCGAAGAGAT | ATGCCAAGCGTTTCACTTTT | 60.1 | 596 | DRR | Ca_18435 | | | Stress up-regulated Nod 19 |
| CaPOPI_5818 | Ca8 | 10590161 | ATTTTTT/ATTTTTTT | AACGGAGGAGGAACAAACAC | TTAGAAGGCCTTGCATACGA | 59.0 | 212 | INTERGENIC | | | | |
| CaPOPI_5819 | Ca8 | 10591114 | AAA/AAAGAA | TCCCGAGCATATCAACATCA | TGTGGGAGTTTGTCTTGCAG | 60.0 | 555 | INTERGENIC | | | | |
| CaPOPI_5820 | Ca8 | 10612849 | CTTTT/CTTT | AGTGGGAGTTTCGTCCATTG | AGCACATGGAACCAAAGAGG | 60.0 | 299 | INTERGENIC | | | | |
| CaPOPI_5821 | Ca8 | 10684139 | TAAAA/TAAA | AAAAAGAAAGGAACGGTCATAAA | AAAGTCCACTCTGCAATATGATCT | 58.3 | 403 | INTERGENIC | | | | |
| CaPOPI_5822 | Ca8 | 10686546 | ATTTT/ATTT | TGCTATCGGTACCAATTTTGC | TCCGTTGTCTCATTCGATCA | 60.0 | 628 | INTERGENIC | | | | |
| CaPOPI_5823 | Ca8 | 10701972 | ATGTT/AT | TCAACGGTTATACGGCAACA | TTTTATCTATGAAAAATGCCTCACA | 60.0 | 373 | INTERGENIC | | | | |
| CaPOPI_5824 | Ca8 | 10727907 | GAAAAA/GAAAA | GGTAGGCATTGGAGCATTGT | CATCTTTTGCAGCATGGCTA | 60.0 | 581 | INTERGENIC | | | | |
| CaPOPI_5825 | Ca8 | 10934374 | AT/ATT | TCCGATTCATCCTCTTGGTC | AATGAAAAAGCGGGGAAACT | 60.0 | 368 | DRR | Ca_19747 | R | NAC | Pentatricopeptide repeat |
| CaPOPI_5826 | Ca8 | 10973766 | TATA/TATAAATAAATAAATAAATA | CGGACGTATGCATCAAATTC | GGACTCGCTCGATGTACGTT | 59.0 | 387 | INTERGENIC | | | | |
| CaPOPI_5827 | Ca8 | 11019474 | TTATATATATATATATATATATATAT/TTATATATATATATATATATATATATAT | CGTCGCACGGATACAAAATA | AAAGAGTGGTGCAGGAGTCG | 59.6 | 234 | INTERGENIC | | | | |
| CaPOPI_5828 | Ca8 | 11070760 | AG/A | GCTTCTAGCTGGTGCTGCTT | AGGGAATCAATTGTAGGGGG | 59.9 | 900 | INTERGENIC | | | | |
| CaPOPI_5829 | Ca8 | 11113946 | GA/GAATA | CCAGAAAACGAAAACCAAACA | TTTGATTTTGAGGCAGAGCA | 60.0 | 480 | INTERGENIC | | | | |
| CaPOPI_5830 | Ca8 | 11144283 | A/ATTTAATC | TTGCCATGCCTACTTAAGGG | AATGACATTGAATTCCCCGA | 60.1 | 292 | INTERGENIC | | | | |

| INDEL marker IDs | Chromosomes/ unanchored scaffolds | Physical positions (bp) | InDels (*Kabuli* reference genome-CDC Frontier/PI) | Forward primers (5'-3') | Reverse primers (5'-3') | Anneling temperature (0C) | Expected amplified product size (bp) | Structural annotation | | Functional annotation | | |
|---|---|---|---|---|---|---|---|---|---|---|---|---|
| | | | | | | | | Sequence components of *kabuli* genome | *Kabuli* gene accession IDs | NCBI-KOG | TFs | NCBI-nr database |
| CaPOPI_5831 | Ca8 | 11145618 | TTTATTA/TTTATTATTA | TCTTTCTGGTTGCAAATTGTTTT | CATGCTTTAAAGAGCCAATGC | 60.0 | 758 | INTERGENIC | | | | |
| CaPOPI_5832 | Ca8 | 11145831 | TCC/TC | TCTTTCTGGTTGCAAATTGTTTT | AAATTGAAGCAAACGGAACC | 60.0 | 980 | INTERGENIC | | | | |
| CaPOPI_5833 | Ca8 | 11146284 | TGTGTCTTAGGT/TGT | TGTGATTGAGGCACTATCAAGC | CCGGGTGAAATGGTTATTTG | 60.3 | 280 | INTERGENIC | | | | |
| CaPOPI_5834 | Ca8 | 11146309 | ATTTTTTTTTTT/ATTTTTTTTT | TGGTTTGCTGGAATTGTGTG | CCGGGTGAAATGGTTATTTG | 60.6 | 167 | INTERGENIC | | | | |
| CaPOPI_5835 | Ca8 | 11146772 | TAAA/TAAAA | TAACCTGTGCGCTATTCGTG | TGCAAATAATTCCTCATCATTTTT | 59.9 | 329 | INTERGENIC | | | | |
| CaPOPI_5836 | Ca8 | 11151543 | GAAAAAAA/GAAAAAAAAA | TCCTATTGCCGCTATGTTGA | TTGAGCATCGTGTTGGAGAG | 59.3 | 805 | INTERGENIC | | | | |
| CaPOPI_5837 | Ca8 | 11193555 | ATTTTTTTT/AATTTTTTTTTT | TAGCCGTAAATGTTTTGGGG | ATTGTGATCACCTTCGCACA | 59.8 | 523 | INTERGENIC | | | | |
| CaPOPI_5838 | Ca8 | 11206549 | GT/GTCTATTTAT | GGTTTTTCACTTTCGGTGGA | TTTGCCTGACCTGACATTTTT | 59.9 | 464 | INTERGENIC | | | | |
| CaPOPI_5839 | Ca8 | 11212615 | TT/TTTTATCCCTCT | TTCTCTCCTCGATTTGCTCC | TTCGCACGTGTATTTTTGGA | 59.5 | 715 | INTERGENIC | | | | |
| CaPOPI_5840 | Ca8 | 11356172 | GT/GTT | TTGCGTGATTTGTGTTCGTT | TCACCTATCACGGGTCAACA | 60.2 | 771 | INTRON | Ca_16822 | G | NAC | Glycoside hydrolase, family 1 |
| CaPOPI_5841 | Ca8 | 11536143 | CATTATTATTATTATTATT/CATTATTATTATTATTATTATT | TCTTACACTGCAGCAATAAAAATCA | TGGTTGATGCCCAAGTACAA | 60.2 | 576 | DRR | Ca_16812 | R | MYB | Integrase, catalytic core |
| CaPOPI_5842 | Ca8 | 11545234 | CTAATAATAATAATAATAATAATAATAAT/CTAATAATAATAATAATAATAATAATAATAAT | CTGGTCATTACCCTGCACCT | TTGTTTCTGTCCATTTCCCC | 60.0 | 125 | CDS (large-effect mutations) | Ca_16811 | | | Auxin efflux carrier |
| CaPOPI_5843 | Ca8 | 11875776 | ATCTCTCTCTCTCTCTCTCTCTCTCTCTCTCTCTC/ATCTCTCTCTCTCTCTCTCTCTCTCTCTCTCTCTCTCTC | GTCAACACTCTTGGCCACCT | TTTAGGCCCAAATCATCAGC | 60.2 | 483 | INTERGENIC | | | | |
| CaPOPI_5844 | Ca8 | 11946471 | CTTTTTTTT/CTTTTTTTTT | AAATGCGAACTTACATGGCA | TTCCCCATGAATCTGCTCTC | 59.2 | 785 | INTERGENIC | | | | |
| CaPOPI_5845 | Ca8 | 11955442 | T/TG | GGCCGATTTACTGCTCACAT | GTTGCCACTTGGCGTAAAAT | 60.1 | 430 | INTERGENIC | | | | |

| INDEL marker IDs | Chromosomes/ unanchored scaffolds | Physical positions (bp) | InDels (*Kabuli* reference genome-CDC Frontier/PI) | Forward primers (5'-3') | Reverse primers (5'-3') | Anneling temperature (0C) | Expected amplified product size (bp) | Structural annotation | | Functional annotation | | |
|---|---|---|---|---|---|---|---|---|---|---|---|---|
| | | | | | | | | Sequence components of *kabuli* genome | *Kabuli* gene accession IDs | NCBI-KOG | TFs | NCBI-nr database |
| CaPOPI_5846 | Ca8 | 11955768 | CTAATAATAA/CTAATAA | CTAATGCCCCCAACTCACAT | TTGTTTGGTGAAGGCTCTTG | 59.8 | 307 | INTERGENIC | | | | |
| CaPOPI_5847 | Ca8 | 11975713 | CT/C | TATACTCGTGCACTACGCGG | GGATGGAGACAATGATGAGGA | 59.9 | 135 | INTERGENIC | | | | |
| CaPOPI_5848 | Ca8 | 11975949 | AATTTATGTGTAGATGTATTTAT/AATTTATGTGTAGATGTATTTATGTGTAGATGTATTTAT | TATACTCGTGCACTACGCGG | TTGTTGGGGTGAACTTAATCG | 59.9 | 663 | INTERGENIC | | | | |
| CaPOPI_5849 | Ca8 | 11976112 | AATAT/AAT | TATACTCGTGCACTACGCGG | TTGTTGGGGTGAACTTAATCG | 59.9 | 663 | INTERGENIC | | | | |
| CaPOPI_5850 | Ca8 | 11976190 | TA/T | CACTACGCGGGTTAGCATCT | TTGTTGGGGTGAACTTAATCG | 60.3 | 653 | INTERGENIC | | | | |
| CaPOPI_5851 | Ca8 | 12016131 | ACCCC/ACCCCC | CAACTCTCGTCGCAACTCAA | ATGCAACTTGGGAATTGGAG | 60.2 | 362 | INTRON | Ca_23846 | T | | Apoptosis inhibitory 5 |
| CaPOPI_5852 | Ca8 | 12046116 | GTTTTTTTT/GTTTTTTTTT | GTAGTTCGATCCTGCATGGG | TGGATAACTTGCATTTGGGC | 60.5 | 327 | INTERGENIC | | | | |
| CaPOPI_5853 | Ca8 | 12048312 | TA/TTATGGATCCTACA | TTCCCCCTAAAACAGAACCA | TGGAAGAAGGTGGGAAAGAA | 59.4 | 269 | INTERGENIC | | | | |
| CaPOPI_5854 | Ca8 | 12048375 | ACC/ACCC | TTCCCCCTAAAACAGAACCA | GTTGTTGCTGCCCTTTGTTT | 59.4 | 723 | INTERGENIC | | | | |
| CaPOPI_5855 | Ca8 | 12072367 | GAAAAAAAAAA/GAAAAAAAAAAA | TGGAGAGGTTGTGATGTCCC | ACGGGCATCTTGATCTTCTG | 60.9 | 782 | INTERGENIC | | | | |
| CaPOPI_5856 | Ca8 | 12979016 | C/CG | ATATGTTGGGGGTGATGGAA | GGGGTCGGACATTTACTAGC | 59.9 | 921 | INTERGENIC | | | | |
| CaPOPI_5857 | Ca8 | 12979079 | TTTA/TTTAATTA | CGTGCAGTTTAGGGAAGGAT | GGGGTCGGACATTTACTAGC | 59.2 | 873 | INTERGENIC | | | | |
| CaPOPI_5858 | Ca8 | 12980984 | TC/T | ATCCACTGCAAAACTCCACC | GAAAAATGATGCAAGAGACAAGAA | 60.0 | 659 | INTERGENIC | | | | |
| CaPOPI_5859 | Ca8 | 12983474 | ATTTTT/ATTTT | TGCAGATGCACGAATCTCTT | TGCACTAATTTATCGGGACCA | 59.6 | 496 | INTERGENIC | | | | |
| CaPOPI_5860 | Ca8 | 12983910 | TAA/TA | TTGGTCCCGATAAATTAGTGC | GCCCAAGTTAAGACAACCCA | 59.0 | 442 | INTERGENIC | | | | |

| INDEL marker IDs | Chromosomes/ unanchored scaffolds | Physical positions (bp) | InDels (*Kabuli* reference genome-CDC Frontier/PI) | Forward primers (5'-3') | Reverse primers (5'-3') | Anneling temperature (0C) | Expected amplified product size (bp) | Structural annotation | | Functional annotation | | |
|---|---|---|---|---|---|---|---|---|---|---|---|---|
| | | | | | | | | Sequence components of *kabuli* genome | *Kabuli* gene accession IDs | NCBI-KOG | TFs | NCBI-nr database |
| CaPOPI_5861 | Ca8 | 12984097 | CGTGTGT/CGTGT | AGTGTTAACCGGGATCAAACA | GCCCAAGTTAAGACAACCCA | 59.3 | 301 | INTERGENIC | | | | |
| CaPOPI_5862 | Ca8 | 12990010 | CTA/CTATA | TGGCTAATAAATTGTGCCCAT | TCATGTCCCCTCCTTGAATC | 59.3 | 552 | INTERGENIC | | | | |
| CaPOPI_5863 | Ca8 | 13297997 | TA/TAA | AAATTGATTGGATGGTGGGA | AAATGCAGAGGCACCACTCT | 60.0 | 442 | INTERGENIC | | | | |
| CaPOPI_5864 | Ca8 | 13476730 | AA/AAGA | GATGTTCCAGCTCTCTTGGC | TGGGAAATCGATTAAGCCTG | 60.0 | 504 | DRR | Ca_20118 | | | Protein of unknown function DUF819 |
| CaPOPI_5865 | Ca8 | 13776463 | GTT/GT | TACGGTGCTAGGGAACCAAC | TAATGGTTTTCACGCCAACA | 60.0 | 534 | INTERGENIC | | | | |
| CaPOPI_5866 | Ca8 | 13940610 | AG/A | ATTGGGTCAGTTGGTTTTGC | TTAGTCGCCTCACTCCATCA | 59.8 | 924 | INTERGENIC | | | | |
| CaPOPI_5867 | Ca8 | 13993907 | GTTTTT/GTTTT | CTCCTCAACCTCAATCCCAA | TCACCGGTCAATCAATTAAAAA | 60.0 | 228 | DRR | Ca_22739 | K | | CASP, C-terminal |
| CaPOPI_5868 | Ca8 | 14042124 | ATTTTTTTT/ATTTTTTT | TTCGGTCGTTTGTAATGGTG | CTCAACCAACCACCCACTCT | 59.4 | 932 | INTERGENIC | | | | |
| CaPOPI_5869 | Ca8 | 14064085 | CATATATATATATATATA/CATATATATATATATA | TGTGTGTGTGTGTCCAGAGG | GGTATCCATGGGAGCTTTGA | 59.1 | 804 | INTERGENIC | | | | |
| CaPOPI_5870 | Ca8 | 14064157 | C/CTTCATAT | TTAACCCTATGGGGGTTGATT | GGTATCCATGGGAGCTTTGA | 59.4 | 467 | INTERGENIC | | | | |
| CaPOPI_5871 | Ca8 | 14073516 | A/AT | TTCCTTCTCAACCAACAGGC | GAAGTTGATGAACCCGTCGT | 60.2 | 578 | INTERGENIC | | | | |
| CaPOPI_5872 | Ca8 | 14074282 | CCGCG/CCG | AAACAAAATCCACCGCACTC | TAGGTTCCAAAGGTGCCAAC | 60.0 | 606 | CDS (FRAME SHIFT) | Ca_22744 | U | | Clathrin/coatomer adaptor, adaptin-like, N-terminal |
| CaPOPI_5873 | Ca8 | 14076848 | CTT/CTTGCTATTTTT | CCCAGGCCTATAGTGGTGAA | ACCGGGGATGACATATCAAA | 59.9 | 491 | CDS (large-effect mutations) | Ca_22744 | U | | Clathrin/coatomer adaptor, adaptin-like, N-terminal |
| CaPOPI_5874 | Ca8 | 14077330 | TAGAGAAAAGAAGAGAAA/TAGAGAAA | TTGATATGTCATCCCCGGTT | TTCTTTCCCACCCTTCTTCC | 60.0 | 696 | DRR | Ca_22744 | U | | Clathrin/coatomer adaptor, adaptin-like, N-terminal |
| CaPOPI_5875 | Ca8 | 14077398 | GA/GAAA | TTGATATGTCATCCCCGGTT | AGCTGCATCTTCCACGATTT | 60.0 | 940 | DRR | Ca_22744 | U | | Clathrin/coatomer adaptor, adaptin-like, N-terminal |

| **INDEL marker IDs** | **Chromosomes/ unanchored scaffolds** | **Physical positions (bp)** | **InDels (*Kabuli* reference genome-CDC Frontier/PI)** | **Forward primers (5'-3')** | **Reverse primers (5'-3')** | **Anneling temperature (0C)** | **Expected amplified product size (bp)** | **Structural annotation** | | **Functional annotation** | | |
|---|---|---|---|---|---|---|---|---|---|---|---|---|
| | | | | | | | | **Sequence components of *kabuli* genome** | ***Kabuli* gene accession IDs** | **NCBI-KOG** | **TFs** | **NCBI-nr database** |
| CaPOPI_5876 | Ca8 | 14113684 | TAAAAAA/TAAAAA | AACACGCCTCACACCACATA | CCAATTCACAAAATCGGTCC | 60.0 | 447 | INTERGENIC | | | | |
| CaPOPI_5877 | Ca8 | 14121923 | GAAAAAAAA/GAAAAAAAAA | TGGTCTTTGATTCCTGACGA | GCTTTTGTTGAGTCCACTTGC | 59.2 | 690 | INTRON | Ca_13048 | | GRAS | Drug/metabolite transporter |
| CaPOPI_5878 | Ca8 | 14127063 | A/AC | TCCAACTGAAACACAGCACAG | TGGCCTTCTCTGGAGATTTG | 59.9 | 154 | INTRON | Ca_13047 | T | B3 | Protein phosphatase 2C-like |
| CaPOPI_5879 | Ca8 | 14130339 | CATATA/CATA | TTGTCGTTCATTGCTTTTTCA | TTGGACAACGAGCTCACAAC | 59.3 | 240 | INTERGENIC | | | | |
| CaPOPI_5880 | Ca8 | 14159846 | AATATA/AATATATA | AAATGCAAACATCAATGCCA | GAAAATTAAGAGGGAGGGAGGA | 59.9 | 290 | INTERGENIC | | | | |
| CaPOPI_5881 | Ca8 | 14165620 | AAATTAA/AAATTAATTAA | TTTTTCCGACTTGGTCCTTT | TGGGGGTATTGACAGGTGTT | 58.7 | 752 | INTERGENIC | | | | |
| CaPOPI_5882 | Ca8 | 14218237 | A/AT | TCTCCACAAATGCAAGGAAA | AAGCCTGGCCCAACTAATTT | 59.2 | 465 | INTERGENIC | | | | |
| CaPOPI_5883 | Ca8 | 14231465 | T/TATA | TGCGTTTATTTCTTGGTGGTT | AATGGTACTCCCTCCATCCC | 59.5 | 701 | INTERGENIC | | | | |
| CaPOPI_5884 | Ca8 | 14238334 | AT/A | AGGGTTGGAATCCATATGTCA | TGGCTCGTGAAGAAAAACAA | 59.1 | 713 | INTERGENIC | | | | |
| CaPOPI_5885 | Ca8 | 14245101 | TAAAAAAA/TAAAAAAAA | AAAGGGATGGACACCATTGA | AGCAGAGTGAAAACAGGGGA | 60.2 | 786 | INTERGENIC | | | | |
| CaPOPI_5886 | Ca8 | 14250642 | GAAAAAAAAA/GAAAAAAA | TGTCGTTTTGGTGCAACATT | TCTTCAACACTTGCTCGCAG | 60.0 | 642 | INTERGENIC | | | | |
| CaPOPI_5887 | Ca8 | 14261406 | ATTTTTTTTT/ATTTTTTTTTT | TGGAGATTGTGGATTTGCAG | GATTCACCATCCCATTTGATTT | 59.6 | 592 | INTERGENIC | | | | |
| CaPOPI_5888 | Ca8 | 14264326 | GATATATATA/GATATATA | GTTGGTTGGATTGATTTGGG | TGAAACCCACGCACATTAAA | 60.0 | 406 | INTERGENIC | | | | |
| CaPOPI_5889 | Ca8 | 14287691 | CTTTATCTTTTTATCTTTTTAT/CTTTATCTTTTTAT | AGTGGTTTTGTTTTGGTGGG | GGTTGGCAAAGACTGTGGTT | 59.7 | 467 | DRR | Ca_13035 | | | |
| CaPOPI_5890 | Ca8 | 14302503 | AG/A | CCTAACAACCTCACAACCCC | CGTCTTTGACCAAATTGAAAAA | 59.3 | 842 | INTERGENIC | | | | |

| INDEL marker IDs | Chromosomes/ unanchored scaffolds | Physical positions (bp) | InDels (*Kabuli* reference genome-CDC Frontier/PI) | Forward primers (5'-3') | Reverse primers (5'-3') | Anneling temperature (0C) | Expected amplified product size (bp) | Structural annotation | | Functional annotation | | |
|---|---|---|---|---|---|---|---|---|---|---|---|---|
| | | | | | | | | Sequence components of *kabuli* genome | *Kabuli* gene accession IDs | NCBI-KOG | TFs | NCBI-nr database |
| CaPOPI_5891 | Ca8 | 14306794 | ATT/AT | TATTGAACTTGGGGAGGCTG | CCCTTTAAATGTGGCAAAAA | 60.1 | 839 | INTERGENIC | | | | |
| CaPOPI_5892 | Ca8 | 14307818 | CTTTT/CTTTTT | CATGGGCAGCAACAAAGTAA | GAGGACCAATGAAGGCAAAA | 59.7 | 819 | INTERGENIC | | | | |
| CaPOPI_5893 | Ca8 | 14345244 | GAAAATGAAAATGAAA/GAAAATGAAAATGAAAATGAAAATGAAA | TTTCCAACTCCCAGCTCAAC | GCAAGGTCGTGGAGAAACAT | 60.2 | 726 | INTRON | Ca_13030 | T | bHLH | Protein kinase, catalytic domain |
| CaPOPI_5894 | Ca8 | 14355037 | CTTT/CT | TTTGAGGGTTAGGTGGTTCG | TGGGAAAATAGTCTCCTTCATGT | 60.0 | 369 | INTERGENIC | | | | |
| CaPOPI_5895 | Ca8 | 14362355 | ATATTATTATTATT/ATATTATTATT | ATCTTTCGTCGCAATTCACC | TGACAGAGATTAGGATTGAAAGGA | 60.1 | 506 | INTERGENIC | | | | |
| CaPOPI_5896 | Ca8 | 14363844 | CTATATA/CTATA | GTTGCCACTTGACCTTGGAT | TGGACTTCATCCAAAAAGGG | 60.0 | 351 | INTERGENIC | | | | |
| CaPOPI_5897 | Ca8 | 14370547 | TAA/TAAAAA | GGCCAATCAATTTTTGATGC | TGGGAGATTGGAGCATGATT | 60.3 | 590 | INTERGENIC | | | | |
| CaPOPI_5898 | Ca8 | 14370711 | A/AAAT | GGCCAATCAATTTTTGATGC | TGGGAGATTGGAGCATGATT | 60.3 | 590 | INTERGENIC | | | | |
| CaPOPI_5899 | Ca8 | 14373850 | AGAAAATGAAAATGAAAA/AGAAAATGAAAATGAAAATGAAAATGAAAA | GGAAATTTGGCGTGTGACTT | GTTTCGTTGTTGTGGCAATG | 60.0 | 373 | INTERGENIC | | | | |
| CaPOPI_5900 | Ca8 | 14404073 | GTTTTTTTTTT/GTTTTTTTTT | AGATGTGTGATTGGGCCTTC | CCACTCCCTTTGTAGCATCA | 59.9 | 597 | INTERGENIC | | | | |
| CaPOPI_5901 | Ca8 | 14408424 | AT/A | GTTTTGCCACCCCTACACAC | GTCCCGTGAAGACTTTCAGC | 60.3 | 684 | INTERGENIC | | | | |
| CaPOPI_5902 | Ca8 | 14516248 | GNNNNNNNNNN/GNNNNNNN | GGCAAAACATTCATCCGTTT | ATCTTTGGCTTCTCCTGCAA | 59.8 | 679 | INTERGENIC | | | | |
| CaPOPI_5903 | Ca8 | 14568462 | TATATAT/TATATATATAGATATAGATATAT | TTGCACCATGTTTGTTATTCAA | GGCAAAGGGTTTTTGCAATC | 59.0 | 198 | INTERGENIC | | | | |
| CaPOPI_5904 | Ca8 | 14580122 | GAAAAAAAAAA/GAAAAAAAAA | TCCAAATCCAAGACCTCCAC | TTTGAATGATGCAGCTGAGG | 59.9 | 499 | INTRON | Ca_13009 | Z | MYB_related | Kinesin, motor domain |
| CaPOPI_5905 | Ca8 | 14603641 | GCA/GCACTATACCA | AATGCCGTTAAGTTGATGGG | CGTATTGCGGAACAACAATG | 59.8 | 268 | DRR | Ca_13007 | C | | NADH-ubiquinone oxidoreductase, subunit 10 |

| INDEL marker IDs | Chromosomes/ unanchored scaffolds | Physical positions (bp) | InDels (*Kabuli* reference genome-CDC Frontier/PI) | Forward primers (5'-3') | Reverse primers (5'-3') | Anneling temperature (0C) | Expected amplified product size (bp) | Structural annotation | | Functional annotation | | |
|---|---|---|---|---|---|---|---|---|---|---|---|---|
| | | | | | | | | Sequence components of *kabuli* genome | *Kabuli* gene accession IDs | NCBI-KOG | TFs | NCBI-nr database |
| CaPOPI_5906 | Ca8 | 14603917 | TA/T | CATTGTTGTTCCGCAATACG | TAAAACTTCCCTGCACGAGG | 60.0 | 310 | DRR | Ca_13007 | C | | NADH-ubiquinone oxidoreductase, subunit 10 |
| CaPOPI_5907 | Ca8 | 14613573 | ATTTTTTT/ATTTTTT | CGTGGTGACATCAAACTTGG | CCAATCCCTCAGACCAGAAA | 60.0 | 144 | INTRON | Ca_13006 | K | C3H | Endonuclease/exonuclease/phosphatase |
| CaPOPI_5908 | Ca8 | 14617610 | CATTTAT/CATTTATATTTAT | GCTCCAACCGTAAAACCAAA | TGCAGGGAGAAGAGGGAGTA | 60.0 | 670 | INTERGENIC | | | | |
| CaPOPI_5909 | Ca8 | 14623218 | CTTTTTTTTTTTTT/CTTTTTTTTTTT | CAGCAGCAACTTTTGGATGA | TGCTGCTGCTATTCCTTTGA | 60.0 | 531 | INTRON | Ca_13005 | U | | SNARE-complex protein Syntaxin-18 N-terminal |
| CaPOPI_5910 | Ca8 | 14625324 | TC/T | GCGTCAACACTTCCAACACA | TAAGGACTGGACGTAACCCG | 60.8 | 682 | DRR | Ca_13005 | U | | SNARE-complex protein Syntaxin-18 N-terminal |
| CaPOPI_5911 | Ca8 | 14645402 | A/AAATAATTATAAAT | TTTGAGAATCAATGTCATATCCG | GGACCCTAAGCATTCTGTGC | 59.0 | 568 | INTERGENIC | | | | |
| CaPOPI_5912 | Ca8 | 14647157 | GTTT/GTTTT | TTGCTTGTCCATGACGTAGG | TCTGCAACGAAGATTAAACCAA | 59.7 | 297 | INTERGENIC | | | | |
| CaPOPI_5913 | Ca8 | 14659373 | AT/ATT | AATTTGGAAGCACATCGACC | CACACATTTCTTGGCCCTCT | 59.9 | 401 | INTERGENIC | | | | |
| CaPOPI_5914 | Ca8 | 14659986 | TAAAAAAAAAAA/TAAAAAAAAAAAAA | AGAGGGCCAAGAAATGTGTG | GCATGCATAACAATTTTGTGTGA | 60.1 | 551 | INTERGENIC | | | | |
| CaPOPI_5915 | Ca8 | 14662169 | GAA/G | CTTCCTTTGGCCACCTAACA | CATCATAACATTTAGCCCCTCA | 60.1 | 150 | INTERGENIC | | | | |
| CaPOPI_5916 | Ca8 | 14662220 | GTTTTTTTTTTTT/GTTTTTTTTTTT | CTTCCTTTGGCCACCTAACA | TTTAAATGCACTTTTGATTCTCC | 60.1 | 617 | INTERGENIC | | | | |
| CaPOPI_5917 | Ca8 | 14671445 | TAAAAAAAAA/TAAAAAAAA | CCTTCCCGATTTGATCCTTT | TCGACAACTTACATTTCATCATCC | 60.3 | 831 | INTERGENIC | | | | |
| CaPOPI_5918 | Ca8 | 14688273 | AAACA/AA | GATTTAATTTGTTTGGGGATTTTT | TGCGTGATCGATGCATATAGA | 58.7 | 446 | INTERGENIC | | | | |
| CaPOPI_5919 | Ca8 | 14707819 | AGT/A | TCATGGCTATGCATGGATGT | ACCGCATCTGCAATAGGTTC | 59.9 | 407 | INTRON | Ca_12998 | R | SBP | Malate transporter, aliminium toerance |
| CaPOPI_5920 | Ca8 | 14722667 | TA/TAGA | TTCATTGCACCCACACTTTC | TCGGAAACAAATGGTGGATT | 59.5 | 578 | INTRON | Ca_12995 | O | NAC | Peptidase A1 |

| INDEL marker IDs | Chromosomes/ unanchored scaffolds | Physical positions (bp) | InDels (*Kabuli* reference genome-CDC Frontier/PI) | Forward primers (5'-3') | Reverse primers (5'-3') | Anneling temperature (0C) | Expected amplified product size (bp) | Structural annotation | | Functional annotation | | |
|---|---|---|---|---|---|---|---|---|---|---|---|---|
| | | | | | | | | Sequence components of *kabuli* genome | *Kabuli* gene accession IDs | NCBI-KOG | TFs | NCBI-nr database |
| CaPOPI_5921 | Ca8 | 14733700 | ATTTTTTTTTT/ATTTTTTTTT | TCTGAACGAAGTTGGAGCAA | GGTATGTTTGTTTTGAAGGGG | 59.6 | 787 | INTRON | Ca_12995 | O | NAC | Peptidase A1 |
| CaPOPI_5922 | Ca8 | 14742163 | T/TGA | TGGTGGTTGATCATACGAGAG | GCCAAGAAATAGCCACTTGC | 58.6 | 644 | INTRON | Ca_12995 | O | NAC | Peptidase A1 |
| CaPOPI_5923 | Ca8 | 14746760 | ATTCA/ATTCATTATTTTCA | TGGATCTCATCCTCATCCCT | TTGAAGTGCTTTTGGTGCTG | 59.4 | 573 | INTRON | Ca_12994 | | | Uncharacterised protein family Cys-rich |
| CaPOPI_5924 | Ca8 | 14751128 | TC/T | TGATGCGTTGATAGTGTTGG | GGCACTTGGTTGGTCTTCAT | 58.1 | 619 | INTRON | Ca_12994 | | | Uncharacterised protein family Cys-rich |
| CaPOPI_5925 | Ca8 | 14764445 | TGG/TG | GGCATCATAGTGTGAGACCAA | ACGCGCCAGAAATATAAACG | 58.6 | 609 | INTERGENIC | | | | |
| CaPOPI_5926 | Ca8 | 14770790 | ACC/AC | TAAGGAGTTGGGTGCTTGCT | TTTGGCAAACACTTGTAAAATGA | 59.9 | 461 | INTERGENIC | | | | |
| CaPOPI_5927 | Ca8 | 14778020 | AACTAT/AACTATAATACTAT | TTGGCATTATATGTGGCACC | TGACTCACGATCAGTTTGGC | 59.3 | 750 | DRR | Ca_12990 | | | Adaptor protein ClpS, core |
| CaPOPI_5928 | Ca8 | 14780556 | TAAAAAAAA/TAAAAAAAAA | CTCCAACAAACCACCACCTT | GTCCATGACGTGTCACCGTA | 59.9 | 531 | INTRON | Ca_12990 | | | Adaptor protein ClpS, core |
| CaPOPI_5929 | Ca8 | 14781231 | T/TG | ATCGTTGGTCCTTGTTTTGG | AAATGGCAGGACACATTGTT | 59.8 | 608 | INTERGENIC | | | | |
| CaPOPI_5930 | Ca8 | 14783369 | ATTTTT/ATTTT | GCAACAATTGTGCAGATGAA | AGGCCTTCAGAAAAGCAACA | 58.3 | 216 | INTERGENIC | | | | |
| CaPOPI_5931 | Ca8 | 14808806 | TG/T | TTCCCAACCCAAAGTACCTC | TTTGATATTGTTGTTGGCGA | 58.9 | 553 | INTERGENIC | | | | |
| CaPOPI_5932 | Ca8 | 14815482 | GTTT/GTTTTT | CTTGGAGAAGCACCAAGGAG | GTGCCCACACAAATGACAAT | 60.0 | 568 | INTERGENIC | | | | |
| CaPOPI_5933 | Ca8 | 14820833 | TTATATATATATA/TTATATATATATATA | ACATCCGTGATGGTCACACA | GGGAATGGTGAAATCAATGC | 60.9 | 530 | DRR | Ca_12987 | D | bHLH | Mrp, conserved site |
| CaPOPI_5934 | Ca8 | 14921456 | TAAAAAAAA/TAAAAAAA | CCATGGTTGAGTTGACCAAA | ATGTGTGGAGGAGTTGGGAG | 59.4 | 626 | INTERGENIC | | | | |
| CaPOPI_5935 | Ca8 | 14924927 | GAAAAAAAAAAA/GAAAAAAAAAA | AGCCACCATGACGAAAAGTC | GTCCTCGTCAACATTCGACA | 60.1 | 484 | DRR | Ca_12980 | P | MIKC | Sulphate transporter/antisigma-factor antagonist STAS |

| INDEL marker IDs | Chromosomes/ unanchored scaffolds | Physical positions (bp) | InDels (*Kabuli* reference genome-CDC Frontier/PI) | Forward primers (5'-3') | Reverse primers (5'-3') | Anneling temperature (0C) | Expected amplified product size (bp) | Structural annotation | | Functional annotation | | |
|---|---|---|---|---|---|---|---|---|---|---|---|---|
| | | | | | | | | Sequence components of *kabuli* genome | *Kabuli* gene accession IDs | NCBI-KOG | TFs | NCBI-nr database |
| CaPOPI_5936 | Ca8 | 14932583 | GAAAAAAAAAA/GAAAAAAAAA | CACCATTCTCCTTCAACCAAA | AACCACGCGACTCATACACA | 60.0 | 716 | INTRON | Ca_12980 | P | MIKC | Sulphate transporter/antisigma-factor antagonist STAS |
| CaPOPI_5937 | Ca8 | 14984060 | T/TGCTGCTAATCC | GCTCGAATGGACCTTGAAAC | AATATTTGGTCCCGTCATGG | 59.7 | 235 | DRR | Ca_12975 | K | | Helix-turn-helix type 3 |
| CaPOPI_5938 | Ca8 | 15048107 | TAAAAAAAAAA/TAAAAAAAAAAA | ATGGCGGATATTCAAACCAA | TTGTTCAGATCCCTCTCTTGC | 60.2 | 627 | INTERGENIC | | | | |
| CaPOPI_5939 | Ca8 | 15162330 | TTAATAATAATAATAATAATAA/TTAATAATAATAATAATAA | TTCGTCGTGTAGGTCCAGTG | GGGAAACCCACCTCTTGATT | 59.7 | 757 | INTERGENIC | | | | |
| CaPOPI_5940 | Ca8 | 15315884 | TCC/TC | ATTCCATCTCATGCTCCCTG | CAAACTGCAAGACTGCCAAA | 60.0 | 345 | INTERGENIC | | | | |
| CaPOPI_5941 | Ca8 | 15360832 | GTTTTTTTTTTT/GTTTTTTTTTT | TGATCGACTGCCAAACACAC | ATGCCACATCATACCATTCG | 60.7 | 684 | INTERGENIC | | | | |
| CaPOPI_5942 | Ca8 | 15435370 | TTTAATTAATTAATTAATTAATTAATTAATTAAT/TTTAATTAATTAATTAATTAATTAATTAATTAATTAAT | AATGGGGAAACAAGCTGATG | TGGGGCAATGAAGAAAGCTA | 59.9 | 549 | INTRON | Ca_17897 | | bHLH | |
| CaPOPI_5943 | Ca8 | 15439013 | CTTTTTTTTTT/CTTTTTTTTT | GCTAAATTACATTCGTGGTCCC | TTTCATCAAATCCGTAACACAAA | 59.7 | 155 | INTERGENIC | | | | |
| CaPOPI_5944 | Ca8 | 15558716 | GAAAAAAAAA/GAAAAAAAAAA | TTCCTTTGTCACTGCTTCCA | CATGCTTTCAAAAGATGCCA | 59.4 | 490 | INTERGENIC | | | | |
| CaPOPI_5945 | Ca8 | 15577896 | CCA/CCATCA | TTTTCAGCAAACCCCAAAAG | TAGAGGCTTGTCATTCGGCT | 60.1 | 665 | CDS (large-effect mutations) | Ca_17886 | T | EIL | Protein phosphatase 2C, manganese/magnesium aspartate binding |
| CaPOPI_5946 | Ca8 | 15580121 | A/AATTTAATTT | TCCTCAATTTTGAGCACACG | TGGACACCACACTTCAAATCA | 59.8 | 657 | INTERGENIC | | | | |
| CaPOPI_5947 | Ca8 | 15642015 | ATTTTTTTTT/ATTTTTTTTTT | GGGGTGGGATGACAAATAGA | TGAGCCCTCAAAACCCTAAA | 59.6 | 656 | DRR | Ca_17880 | | | Polyketide cyclase/dehydrase |
| CaPOPI_5948 | Ca8 | 15685177 | AGG/AG | GCCCAACAACAGAACCAAGT | GACTCCAAAACCCTGTGCAT | 60.0 | 420 | INTERGENIC | | | | |
| CaPOPI_5949 | Ca8 | 15685323 | TAAAAAAA/TAAAAAAAA | GCCCAACAACAGAACCAAGT | GACTCCAAAACCCTGTGCAT | 60.0 | 420 | INTERGENIC | | | | |
| CaPOPI_5950 | Ca8 | 15685626 | T/TA | ATGCACAGGGTTTTGGAGTC | ACTTGGTTCTGTTGTTGGGC | 60.0 | 416 | INTERGENIC | | | | |

| INDEL marker IDs | Chromosomes/ unanchored scaffolds | Physical positions (bp) | InDels (*Kabuli* reference genome-CDC Frontier/PI) | Forward primers (5'-3') | Reverse primers (5'-3') | Anneling temperature (0C) | Expected amplified product size (bp) | Structural annotation | | Functional annotation | | |
|---|---|---|---|---|---|---|---|---|---|---|---|---|
| | | | | | | | | Sequence components of ***kabuli*** genome | ***Kabuli*** gene accession IDs | NCBI-KOG | TFs | NCBI-nr database |
| CaPOPI_5951 | Ca8 | 15685828 | TAAAAAAAA/TAAAAAAAAAA | ATGCACAGGGTTTTGGAGTC | ACTTGGTTCTGTTGTTGGGC | 60.0 | 416 | INTERGENIC | | | | |
| CaPOPI_5952 | Ca8 | 15685888 | A/AG | ATGCACAGGGTTTTGGAGTC | ACTTGGTTCTGTTGTTGGGC | 60.0 | 416 | INTERGENIC | | | | |
| CaPOPI_5953 | Ca8 | 15694311 | GTATATATATATATATATATATATATATATATA/GTATATATATATATATATATATATATATATA | CCGTCTCTTTCACATGGGTT | AAAGGCTCGGGTCAGTCATA | 60.0 | 438 | INTRON | Ca_17876 | A | | RNA-dependent RNA polymerase, eukaryotic-type |
| CaPOPI_5954 | Ca8 | 15788537 | TAAAAAAAAAA/TAAAAAAAAAAA | TCGGTTCCTTTCAACTTTGG | ATCAATGCTTCCACCCTTTG | 60.1 | 588 | INTERGENIC | | | | |
| CaPOPI_5955 | Ca8 | 15850345 | AATA/AATATGTGTTATA | TTATTGTTTCCCTCTTGCGG | TCTTCAAATTACTCCCTCCATCA | 60.1 | 527 | INTERGENIC | | | | |
| CaPOPI_5956 | Ca8 | 15891448 | GTTTTTTTTTT/GTTTTTTTTTTTTTT | TGCAAGAAAGTTGTGTTGGG | GTTCATGCACCTTCTTGGGT | 59.7 | 385 | INTERGENIC | | | | |
| CaPOPI_5957 | Ca8 | 15892786 | AAAGA/AAAGAAGA | AGCAGTTGAACTTGCCTCGT | CGATGATTCATTCCCAAACC | 60.1 | 513 | INTERGENIC | | | | |
| CaPOPI_5958 | Ca8 | 15918262 | A/ATTAATTCTC | AAACGTCAGAAAACATCGGG | TGCCCCCAGACATAAAGTTC | 60.0 | 243 | INTERGENIC | | | | |
| CaPOPI_5959 | Ca8 | 15918552 | TAAAAAAAAAA/TAAAAAAAAAAA | AAACGTCAGAAAACATCGGG | GAGTCAACTTTGCTGAGGGC | 60.0 | 717 | INTERGENIC | | | | |
| CaPOPI_5960 | Ca8 | 16066420 | AATATATATATATATATATAT/AATATATATATATATATAT | TATTTTGGGATTTTGGCTCG | AGGAAGAGTGGAGTTGCGAA | 59.9 | 512 | INTERGENIC | | | | |
| CaPOPI_5961 | Ca8 | 16315020 | TAAAA/TAAA | AATGCAAAGCAACAAGGTTTC | TTGTATGCATGTGTGATGTGTG | 59.3 | 504 | INTERGENIC | | | | |
| CaPOPI_5962 | Ca8 | 16315069 | CTGATGAT/CT | AATGCAAAGCAACAAGGTTTC | GCACATTGAACCCCATACAA | 59.3 | 605 | INTERGENIC | | | | |
| CaPOPI_5963 | Ca8 | 16315111 | GAT/G | AATGCAAAGCAACAAGGTTTC | GCACATTGAACCCCATACAA | 59.3 | 605 | INTERGENIC | | | | |
| CaPOPI_5964 | Ca8 | 16316035 | CAAAA/CAAAAAA | GGCATCACCCCAAGACATAG | CCCTTGATTTTTCAATCACTCC | 60.3 | 352 | INTERGENIC | | | | |
| CaPOPI_5965 | Ca8 | 16360078 | T/TA | GAAAAATGAAGGAGGTGGCA | GGAAAGAATGTTACGCCTGC | 60.1 | 159 | INTRON | Ca_15558 | I | | Prenyltransferase/squalene oxidase |

| INDEL marker IDs | Chromosomes/ unanchored scaffolds | Physical positions (bp) | InDels (*Kabuli* reference genome-CDC Frontier/PI) | Forward primers (5'-3') | Reverse primers (5'-3') | Anneling temperature (0C) | Expected amplified product size (bp) | Structural annotation | | Functional annotation | | |
|---|---|---|---|---|---|---|---|---|---|---|---|---|
| | | | | | | | | Sequence components of *kabuli* genome | *Kabuli* gene accession IDs | NCBI-KOG | TFs | NCBI-nr database |
| CaPOPI_5966 | scaffold10 | 68796 | GATAATAATAATAATAATAATAATAATAATAATAA/GATAATAATAATAATAATAATAATAATAATAATAA | AAAGCAAACCAATGGGCATA | ATGAATGGAAGACGTGACGA | 60.3 | 657 | INTERGENIC | | | | |
| CaPOPI_5967 | scaffold1003 | 13636 | GAAGTAAAG/GAAG | TGCCACTATCGGGCTAATTT | ATTTTCGCATCCAATTACCG | 59.6 | 885 | DRR | Ca_27802 | QR | B3 | Oxoglutarate/iron-dependent oxygenase |
| CaPOPI_5968 | scaffold1003 | 41332 | G/GAGCGGT | GGGATTTGAAGGTGCTGAAA | TAGTGGCCACAACCAACTCA | 60.1 | 452 | INTERGENIC | | | | |
| CaPOPI_5969 | scaffold1003 | 41466 | AAAGA/AAAGAAGA | GGGATTTGAAGGTGCTGAAA | TAGTGGCCACAACCAACTCA | 60.1 | 452 | INTERGENIC | | | | |
| CaPOPI_5970 | scaffold1006 | 109456 | T/TC | TTGATTTCAGCCCAGGATTC | TTTACAAGCAGCGGGAAAGT | 60.0 | 493 | INTERGENIC | | | | |
| CaPOPI_5971 | scaffold1006 | 212542 | AATATATATATATATATAT/AATATATATATATATATAT | TTTAAGGAGGGAGCGAGTTG | TTCTATGTGCACCGCTTAGG | 59.4 | 477 | INTERGENIC | | | | |
| CaPOPI_5972 | scaffold1006 | 403767 | GTTATTATTATTATTATTATTATTATTATT/GTTATTATTATTATTATTATTATTATT | CGCGAGACAACTTTCCCTAA | AATGGTGTTTTCTGGTTGCC | 60.4 | 520 | INTERGENIC | | | | |
| CaPOPI_5973 | scaffold1006 | 520865 | ATTTTTT/ATTTTTTT | TGGAGTGTGCCAAAAAGTTG | CAACCTCCCTTTGTTTTGCT | 59.7 | 567 | DRR | Ca_18036 | M | HB-other | Sucrose synthase |
| CaPOPI_5974 | scaffold1006 | 624934 | CT/CTTAAAAGAATT | TCCATTGCATAAAAGTTTTGTTT | TCATTTCTTGCGTCTCCCTT | 57.8 | 123 | INTERGENIC | | | | |
| CaPOPI_5975 | scaffold1006 | 655490 | GTTTTTTTT/GTTTTTTTTT | CTTTTGGTTGTGCAATGGTG | ATGCCTTCATCAACTTTGGG | 60.0 | 615 | INTERGENIC | | | | |
| CaPOPI_5976 | scaffold1008 | 12678 | TC/T | TCTTGGTTGTAACCCTTGGC | GGGCTGATGCCATAAACACT | 60.0 | 727 | INTERGENIC | | | | |
| CaPOPI_5977 | scaffold1008 | 20458 | T/TATA | TGTTTGACTGATGTTAAGTGTGC | GGGGTGCAGATTTCTGATGT | 57.9 | 562 | INTERGENIC | | | | |
| CaPOPI_5978 | scaffold1010 | 21084 | ATGC/ATGCTGC | TGTAACCCGTGTTGGATGAA | ACCGGTTTGTGATTGTCCTC | 59.8 | 729 | INTERGENIC | | | | |
| CaPOPI_5979 | scaffold1013 | 39772 | AATATATATATATATATATATATATATATAT/AATATATATATATATATATATATATATATAT | ACGAAACAGGGAGGTTTGTG | CGGTGAATCCTTTACGGAGA | 60.0 | 624 | INTERGENIC | | | | |
| CaPOPI_5980 | scaffold10158 | 16048 | TC/TCC | CTGTGGGGGCTATTCTGAAA | AAACGATCAAATCCAACCCA | 60.1 | 487 | INTERGENIC | | | | |

| INDEL marker IDs | Chromosomes/ unanchored scaffolds | Physical positions (bp) | InDels (*Kabuli* reference genome-CDC Frontier/PI) | Forward primers (5'-3') | Reverse primers (5'-3') | Anneling temperature (0C) | Expected amplified product size (bp) | Structural annotation | | Functional annotation | | |
|---|---|---|---|---|---|---|---|---|---|---|---|---|
| | | | | | | | | Sequence components of *kabuli* genome | *Kabuli* gene accession IDs | NCBI-KOG | TFs | NCBI-nr database |
| CaPOPI_5981 | scaffold1017 | 10794 | AT/A | GTTTGGACATTTCAGGCGAT | GTTTCGGGTTCCACGTTAGA | 59.9 | 725 | INTERGENIC | | | | |
| CaPOPI_5982 | scaffold10180 | 748 | AT/ATT | GGTGAGTTTGTGTCGTTGGA | AGGGAGGACGAAGATGACCT | 59.6 | 448 | INTERGENIC | | | | |
| CaPOPI_5983 | scaffold10227 | 6464 | AATATATATATATATATATATATATATATATATATA/AATATATATATATATATATATA | GGGAACGTGAAGAACTCAGC | TGCAACAACCACAACCACTT | 59.9 | 518 | INTRON | Ca_28086 | | bHLH | Helix-loop-helix DNA-binding domain |
| CaPOPI_5984 | scaffold1027 | 152594 | CAA/CA | TGAGTGCGACGGATTGTAAA | CTCAGCCCCTGACTTAATGC | 60.3 | 692 | INTERGENIC | | | | |
| CaPOPI_5985 | scaffold10285 | 986 | CTAGGAAAAAA/CTAGGAAAAAATTAGGAAAAAA | CGAAAACTCAGAAAGGCGAC | GCCTTATGTGGAAAGACACCA | 60.0 | 513 | INTERGENIC | | | | |
| CaPOPI_5986 | scaffold1038 | 720 | TGG/TG | CGAAAATCGCCCAGAAAATA | TCCTGGTCCTCGATTTCATC | 60.0 | 842 | INTERGENIC | | | | |
| CaPOPI_5987 | scaffold1039 | 3089 | ATTTT/ATTT | CGAAGTTCCTTTCAGCCAAC | CCCATCCTTCTTCTTGACCA | 59.9 | 358 | INTERGENIC | | | | |
| CaPOPI_5988 | scaffold1039 | 32679 | T/TGTTG | TTCCCTAGATCCTTCGCAGA | TGGATGGTAATCCAACGTGA | 59.9 | 623 | INTERGENIC | | | | |
| CaPOPI_5989 | scaffold1039 | 41739 | AATATATATATATATATATATA/AATATATATATATATATATA | CTTGGGAACTGGAGAGCAAG | ACCTTCACGTTCGATTACGG | 60.0 | 809 | INTERGENIC | | | | |
| CaPOPI_5990 | scaffold1039 | 48817 | ATTTTTTTTTTT/ATTTTTTTT | CGACATGCATGCCTTATTTG | CATGAGGAAACAATTGTGCAG | 60.1 | 571 | INTERGENIC | | | | |
| CaPOPI_5991 | scaffold1039 | 59288 | CAAAAAAAAAAAA/CAAAAAAAAAAA | GCAATCTCCTCAACTCGGTC | TACTGTGCGGCCATCTACTG | 59.8 | 610 | INTERGENIC | | | | |
| CaPOPI_5992 | scaffold10412 | 1146 | GCTC/GC | TGATTCCATTGGGGTTTCAT | ATTGTTTTCTCGAGGTTGCG | 60.0 | 611 | INTERGENIC | | | | |
| CaPOPI_5993 | scaffold10415 | 1018 | AT/ATT | GAGGAAGATGCCACCAGAAA | TTTGTATCCCACAGAAGCCT | 60.2 | 774 | INTERGENIC | | | | |
| CaPOPI_5994 | scaffold10415 | 1060 | TTGTG/TTGTGTG | GAGGAAGATGCCACCAGAAA | TTTGTATCCCACAGAAGCCT | 60.2 | 774 | INTERGENIC | | | | |
| CaPOPI_5995 | scaffold10419 | 1064 | TAAAAAA/TAAAAAAA | TTTTGGCTGATGTGGATGAA | GTTTGGGTTTGCTTGTTTGG | 60.0 | 537 | INTERGENIC | | | | |

| INDEL marker IDs | Chromosomes/ unanchored scaffolds | Physical positions (bp) | InDels (*Kabuli* reference genome-CDC Frontier/PI) | Forward primers (5'-3') | Reverse primers (5'-3') | Anneling temperature (0C) | Expected amplified product size (bp) | Structural annotation | | Functional annotation | | |
|---|---|---|---|---|---|---|---|---|---|---|---|---|
| | | | | | | | | Sequence components of *kabuli* genome | *Kabuli* gene accession IDs | NCBI-KOG | TFs | NCBI-nr database |
| CaPOPI_5996 | scaffold1043 | 1901 | GTATATATATATATATATATATATATATATATATAT/GTATATATATATATATATATATATATATATATATATATAT | CGAAGCAAAAGAAAGAACCG | TGGATTACGGGTGAAATGGT | 60.0 | 321 | INTERGENIC | | | | |
| CaPOPI_5997 | scaffold1045 | 12241 | CAAA/CAA | TGTGCATCAAATAGGTGCGT | GCGGACGAAAATGACCTAAA | 60.1 | 650 | INTERGENIC | | | | |
| CaPOPI_5998 | scaffold1046 | 11369 | GA/G | AGCCTAGGGAGGACGAATGT | TACCAATCCTGTGCATCCAA | 60.1 | 464 | INTERGENIC | | | | |
| CaPOPI_5999 | scaffold1046 | 17706 | ACC/AC | CATTGGATGCACAAGATTGG | AGGTCATTTTCGTCCTCCCT | 59.9 | 871 | INTERGENIC | | | | |
| CaPOPI_6000 | scaffold1046 | 20332 | TAAAAAAAA/TAAAAAAA | GCGCAAACAAACCTATGTGA | TCAGGTCATTTTCGTCCTCC | 59.7 | 788 | INTERGENIC | | | | |
| CaPOPI_6001 | scaffold1046 | 21076 | GAAA/GAAAA | GGAGGACGAAAATGACCTGA | ACCGATCTTGTGCATCCAAT | 60.1 | 671 | INTERGENIC | | | | |
| CaPOPI_6002 | scaffold1047 | 43221 | ATTTT/ATTTTT | AAGTTTAATGGGTTTGGCCC | GGCAATTTTGTTTGGTCGTT | 60.1 | 771 | INTERGENIC | | | | |
| CaPOPI_6003 | scaffold1047 | 59966 | TTATAT/TTATATATAT | GGGTCTTCAAGTCCATTCCA | TGCCCATATTGAAGCTTTCC | 59.9 | 266 | CDS (FRAME SHIFT) | Ca_17176 | G | | Glycoside hydrolase, family 32 |
| CaPOPI_6004 | scaffold1047 | 72978 | CAAGAAG/CAAG | TCCCCAGCCACTACTTATGC | ACCAACATTGATCAGTGGCA | 60.1 | 409 | DRR | Ca_17177 | Z | Dof | Actin-like |
| CaPOPI_6005 | scaffold1047 | 93852 | ATT/AT | GGCTCCTTTGGTGGTAGTGA | TCATCATCCTATTGCAGCCA | 60.1 | 484 | INTRON | Ca_17178 | | bZIP | RNA recognition motif domain |
| CaPOPI_6006 | scaffold1047 | 132182 | TTTGC/T | TTTCTTTTGGGCCCTCCTAT | AGTTTCCTTTGTCGCCCTTT | 59.9 | 705 | INTERGENIC | | | | |
| CaPOPI_6007 | scaffold1047 | 282094 | TTATATATATATATATATATAT/TTATATATATATATATATATATAT | GTCACGGGAGACAATCCACT | TTGAGGGGTGGGAGTTAAAA | 60.0 | 280 | INTERGENIC | | | | |
| CaPOPI_6008 | scaffold1047 | 313122 | GAAAAAAAAA/GAAAAAAAAAA | AAAATTCCACACGTCCGAAG | TCAAAAGATCGCAGTTGTGTG | 60.0 | 354 | INTRON | Ca_17199 | I | | Patatin/Phospholipase A2-related |
| CaPOPI_6009 | scaffold1047 | 419626 | C/CAAACATG | CCGATGAAAATTCACCCATC | CCATTGATAAAGCGTGTACGG | 60.1 | 327 | INTERGENIC | | | | |
| CaPOPI_6010 | scaffold1047 | 461378 | TTATATATATATATATATATATA/TTATATATATATATATATATATATATA | TATATGCTTGCACGAGGCTG | ACTCCAAAAGCACCGACAAT | 60.0 | 514 | INTERGENIC | | | | |

| INDEL marker IDs | Chromosomes/ unanchored scaffolds | Physical positions (bp) | InDels (*Kabuli* reference genome-CDC Frontier/PI) | Forward primers (5'-3') | Reverse primers (5'-3') | Anneling temperature (0C) | Expected amplified product size (bp) | Structural annotation | | Functional annotation | | |
|---|---|---|---|---|---|---|---|---|---|---|---|---|
| | | | | | | | | Sequence components of *kabuli* genome | *Kabuli* gene accession IDs | NCBI-KOG | TFs | NCBI-nr database |
| CaPOPI_6011 | scaffold1047 | 527453 | GTTTTTTTTT/GTTTTTTTT | ATCAGAGGCAAAGTGCCAAT | TACCCAGGCTTTTCTCATGG | 59.7 | 378 | INTERGENIC | | | | |
| CaPOPI_6012 | scaffold1047 | 595067 | GTT/GTTT | AGGAACGCCTGGAGAAAGAT | CGCACCTAAACCTCCTTTCA | 60.2 | 507 | INTRON | Ca_17227 | Z | | Actin-like |
| CaPOPI_6013 | scaffold1049 | 75199 | ACC/AC | ACGTTCCTGAGGGAATTGTG | GTAAAGGGTCACCAACCGAA | 60.0 | 828 | INTERGENIC | | | | |
| CaPOPI_6014 | scaffold1049 | 293719 | TCC/TCCC | CGTTTTTCAAAGCGAGGGTA | GCCCTCAGATGAGAGTCAGG | 60.2 | 439 | INTERGENIC | | | | |
| CaPOPI_6015 | scaffold1052 | 11395 | GTATATAT/GTATATATAT | GGTAACCAATCCGAAGACGA | GCCCTCAGATGAGAGTCAGG | 59.9 | 518 | INTERGENIC | | | | |
| CaPOPI_6016 | scaffold1052 | 16411 | ATT/AT | CCGTTGTTTCAACTTCCACC | GCAGCAGCCATTCATACAGA | 60.4 | 323 | INTERGENIC | | | | |
| CaPOPI_6017 | scaffold1052 | 16426 | T/TG | CCGTTGTTTCAACTTCCACC | GCAGCAGCCATTCATACAGA | 60.4 | 323 | INTERGENIC | | | | |
| CaPOPI_6018 | scaffold1052 | 20929 | TAAAAAAAAA/TAAAAAAAAAAA | TGTACCACCACAAGTCCATCA | TGCAATTTTGCATGGTTGTT | 59.9 | 238 | INTERGENIC | | | | |
| CaPOPI_6019 | scaffold1052 | 48422 | TAAAAAAAAAAAA/TAAAAAAAAAA | TGTGTTGCGTTGTGGATTCT | CCCAACACACTCGCTATTCA | 60.2 | 686 | INTERGENIC | | | | |
| CaPOPI_6020 | scaffold1052 | 51298 | AGTG/AGTGTG | GGGAGAATTGTAACACCCCA | AACCAAACACACAAGGAAATATGA | 59.6 | 337 | INTERGENIC | | | | |
| CaPOPI_6021 | scaffold1052 | 147202 | TGGGG/TGGGGG | AACCAACCCGTTTTTCATCA | TCATCAACCTCCATCATCTTCA | 60.2 | 506 | INTRON | Ca_27109 | J | | Protein synthesis factor, GTP-binding |
| CaPOPI_6022 | scaffold1052 | 186937 | TC/TCC | ATTCCCTGATGTTCACTCGG | ATGCCACTACAAAAGTGGGC | 59.9 | 588 | INTERGENIC | | | | |
| CaPOPI_6023 | scaffold1055 | 16854 | TAAAAAA/TAAAAA | CACCAGTCCCCTAAACCAAG | TCCCTTGAAAGTGTTGATGG | 59.4 | 379 | INTERGENIC | | | | |
| CaPOPI_6024 | scaffold1055 | 56180 | GGGAGGAG/GGGAGGAGGAG | GCACCGTGAGTAAATTCGGT | CTTGGGCTGCTAAGAATTGC | 60.0 | 577 | INTERGENIC | | | | |
| CaPOPI_6025 | scaffold1055 | 56306 | CATATATATATATATATAT/CATATATATATATATATATATAT | GCACCGTGAGTAAATTCGGT | CTTGGGCTGCTAAGAATTGC | 60.0 | 577 | INTERGENIC | | | | |

| INDEL marker IDs | Chromosomes/ unanchored scaffolds | Physical positions (bp) | InDels (*Kabuli* reference genome-CDC Frontier/PI) | Forward primers (5'-3') | Reverse primers (5'-3') | Anneling temperature (0C) | Expected amplified product size (bp) | Structural annotation | | Functional annotation | | |
|---|---|---|---|---|---|---|---|---|---|---|---|---|
| | | | | | | | | Sequence components of *kabuli* genome | *Kabuli* gene accession IDs | NCBI-KOG | TFs | NCBI-nr database |
| CaPOPI_6026 | scaffold1055 | 157848 | TTATATATATATATATATATATATATATATATATATA/TTATATATATATATATATATATA | CGGAACTCATTGTTCATCCA | TCATAAACGGAGAAGTCGGG | 59.5 | 625 | INTERGENIC | | | | |
| CaPOPI_6027 | scaffold1055 | 206130 | ATATTATTATTATTATTATTATTATTATTATTATTATTATTATTATTATTATTATT/ATTATATTATTATTATTATTATTATTATTATTATTATTATTATTATTATTATTA | TTTATTGCCTGCCCAAACTC | GGAACCAGCAACTGCAAAAT | 60.1 | 608 | INTERGENIC | | | | |
| CaPOPI_6028 | scaffold1055 | 339319 | ATTTTT/ATTT | TTTAAGGTGTGGGACCAAGC | GGAAACAATAAACCGAGGCA | 60.0 | 540 | INTERGENIC | | | | |
| CaPOPI_6029 | scaffold1055 | 340051 | GAAAAAAA/GAAAAAAAA | TGTTGTTCGGCATACGACAT | TGCACATACATAATGGACCACA | 60.0 | 516 | INTERGENIC | | | | |
| CaPOPI_6030 | scaffold1055 | 340491 | T/TATC | TGCAATAAAGCTTTTGAAGTGG | ACATACTCCCTCACTCCCCC | 59.4 | 413 | INTERGENIC | | | | |
| CaPOPI_6031 | scaffold1055 | 340641 | ATGGGGGAGTG/ATG | TGCAATAAAGCTTTTGAAGTGG | AAGTTGTGTCACCATTGCCA | 59.4 | 813 | INTERGENIC | | | | |
| CaPOPI_6032 | scaffold1056 | 72718 | GAA/GAAA | TTCTCCGCATATTTGAATTCCT | TTCGCATGACCAAACAAATG | 59.9 | 206 | INTERGENIC | | | | |
| CaPOPI_6033 | scaffold1056 | 116197 | ACCGAGCCGAGCCGA/ACCGAGCCGAGCCGAGCCGAGCCGA | ACCTTGTGGATGGCAGTTTC | TGGCATTGATGATGGCTTTA | 60.0 | 342 | INTERGENIC | | | | |
| CaPOPI_6034 | scaffold1056 | 135454 | TG/T | GCTACGGTGTTAGGGAACCA | CTATCACGGGTCTCCACGAT | 60.0 | 879 | INTERGENIC | | | | |
| CaPOPI_6035 | scaffold1056 | 142810 | TAAAAAAAAAA/TAAAAAAAAAAA | TTGCTCCTACGTATCCCCAG | TGGCCACAAGAAGACAATGA | 60.1 | 705 | INTERGENIC | | | | |
| CaPOPI_6036 | scaffold1056 | 142961 | ATTTTTTTTTT/ATTTTTTTTTTT | CCGACGGCGGTAATAATAAG | TGGCCACAAGAAGACAATGA | 59.5 | 404 | INTERGENIC | | | | |
| CaPOPI_6037 | scaffold1056 | 148159 | TTAGATAGA/TTAGA | TGAAAGAATGTGAAAAGACATGG | AAATGGTATTGGGACGAACG | 59.1 | 493 | INTERGENIC | | | | |
| CaPOPI_6038 | scaffold1060 | 591 | TAAAAA/TAAAA | GTGATAAAGGCGTCAACGGT | CCAAGGCAGACAAAGAAAGC | 60.0 | 410 | INTRON | Ca_21773 | | ERF | Pathogenesis-related transcriptional factor/ERF, DNA-binding |
| CaPOPI_6039 | scaffold1060 | 10684 | TAGAGAGAGAGAGAGAGAGAGAGA/TAGAGAGAGAGAGAGAGAGAGAGAGA | CTCCAAAGGGACAAACATGA | AGTTGCACTGGGGTTAATGG | 58.5 | 738 | INTRON | Ca_21773 | | ERF | |
| CaPOPI_6040 | scaffold1060 | 51771 | CTTTTTTTTTT/CTTTTTTTTTTT | CACATGAAGCCAAGGGAAGT | GGAATCACACCATGTCACCA | 60.1 | 758 | INTERGENIC | | | | |

| INDEL marker IDs | Chromosomes/ unanchored scaffolds | Physical positions (bp) | InDels (*Kabuli* reference genome-CDC Frontier/PI) | Forward primers (5'-3') | Reverse primers (5'-3') | Anneling temperature (0C) | Expected amplified product size (bp) | Structural annotation | | Functional annotation | | |
|---|---|---|---|---|---|---|---|---|---|---|---|---|
| | | | | | | | | Sequence components of *kabuli* genome | *Kabuli* gene accession IDs | NCBI-KOG | TFs | NCBI-nr database |
| CaPOPI_6041 | scaffold1060 | 389867 | CAT/CATAT | ACCAGGATCCTCCGAACTCT | CAAACGCAAAATTAAACACACAA | 60.1 | 414 | INTRON | Ca_21793 | | | Transposase, Ptta/En/Spm, plant |
| CaPOPI_6042 | scaffold1061 | 27664 | CAAA/CAA | GCCAGATCTTTCGATTCAGC | GGAACATGGCCTCAGTTGAT | 59.9 | 302 | INTERGENIC | | | | |
| CaPOPI_6043 | scaffold1061 | 32882 | T/TC | ACTAACCATCGTTTGGCCTG | GCAATTTTCGTACCCCTTGA | 60.0 | 227 | INTERGENIC | | | | |
| CaPOPI_6044 | scaffold1061 | 49764 | TAAA/TAA | CCTTGTCACACAGTTGGCTG | TCTTGGTTGTAACCCTTGGC | 60.3 | 150 | INTERGENIC | | | | |
| CaPOPI_6045 | scaffold1061 | 76996 | CGG/CG | TATTTGGACCAGAGGATGGC | CCCAAACAGTAGGACCAGGA | 59.9 | 109 | INTERGENIC | | | | |
| CaPOPI_6046 | scaffold1067 | 190632 | GTTT/GTTTTT | TCGCGTAGTGGTAGCCTTCT | TCACAAGGGAGGCTCTATGG | 60.0 | 182 | INTERGENIC | | | | |
| CaPOPI_6047 | scaffold1067 | 202690 | AC/ACC | GCAGCCCATTTACGTTTTGT | CGAATTCTCTTCCTCCTCCC | 60.0 | 595 | DRR | Ca_24425 | | | |
| CaPOPI_6048 | scaffold1067 | 203101 | ATTTTTTTT/AATTTTTTTTT | TGAAACTGGGAGGAGGAAGA | CAGTGCGATGGAGAAATCAA | 59.8 | 418 | DRR | Ca_24425 | | | |
| CaPOPI_6049 | scaffold1067 | 214347 | A/AGCAGTGCGG | GATAGGAGCATTCGGTGGAA | TCAAAGCGAAAACCCATTTC | 60.0 | 658 | INTERGENIC | | | | |
| CaPOPI_6050 | scaffold1067 | 229129 | CTTTT/CTTTTT | ACATGGTTCGGAAACGTAGG | TCCATGCAAGAAGAGCTCAA | 59.9 | 341 | INTERGENIC | | | | |
| CaPOPI_6051 | scaffold1067 | 242965 | CAT/CATAT | TTTCTTCGCTAGCCGTTGTT | TCTTGTGTTGGGTTAAGGGC | 60.0 | 941 | INTERGENIC | | | | |
| CaPOPI_6052 | scaffold1067 | 243320 | TATCCAAAT/TATCCAAATCCAAAT | TTATTGTTGTTGCTTGGGCA | TCTTGTGTTGGGTTAAGGGC | 60.1 | 447 | INTERGENIC | | | | |
| CaPOPI_6053 | scaffold1067 | 243540 | AGGGTTGGG/AGGG | GCCCTTAACCCAACACAAGA | CTCCCGTTGTGGAACTGATT | 60.0 | 594 | INTERGENIC | | | | |
| CaPOPI_6054 | scaffold1067 | 243805 | CAAGCCT/CAAGCCTAAAGCCT | GCCCTTAACCCAACACAAGA | AGGCGACCCTATACCGAAGT | 60.0 | 643 | INTERGENIC | | | | |
| CaPOPI_6055 | scaffold1067 | 263503 | AA/AGCTAGTCAGCA | CAGTGAGTAGCAAGCAAGCG | CCCAGCCTAGTGACATCCAT | 60.0 | 445 | INTERGENIC | | | | |

| INDEL marker IDs | Chromosomes/ unanchored scaffolds | Physical positions (bp) | InDels (*Kabuli* reference genome-CDC Frontier/PI) | Forward primers (5'-3') | Reverse primers (5'-3') | Anneling temperature (0C) | Expected amplified product size (bp) | **Structural annotation** | | **Functional annotation** | | |
|---|---|---|---|---|---|---|---|---|---|---|---|---|
| | | | | | | | | **Sequence components of *kabuli* genome** | ***Kabuli* gene accession IDs** | **NCBI-KOG** | **TFs** | **NCBI-nr database** |
| CaPOPI_6056 | scaffold1067 | 266043 | CTTTTTTTT/CTTTTTTT | ATCCAGAATGGAGCAACTGG | CGGAGAAATCCGTTTTGCTA | 60.1 | 575 | INTERGENIC | | | | |
| CaPOPI_6057 | scaffold1081 | 39164 | T/TC | TTCAAATTCACCACCATTTCA | GGCCGTTGTTACTGCAAAAT | 58.9 | 774 | INTERGENIC | | | | |
| CaPOPI_6058 | scaffold1081 | 86270 | GAAAAAAA/GTAAAAAAAA | TTATTCGGGCCTGTCAAAAC | CATGCGTTCCTTGTGGTAGA | 59.9 | 251 | INTERGENIC | | | | |
| CaPOPI_6059 | scaffold1081 | 88052 | TT/TTGT | TGAGCATGGCTAAGTTGCAC | AAGGGGAAGGGCATAATGTT | 60.0 | 522 | INTERGENIC | | | | |
| CaPOPI_6060 | scaffold1082 | 21212 | ACCTAAACCCTAAACCCTAA/ACCTAAACCCTAA | TTGGACGAACTGCAATGAAT | GCGTTTCGGGTTTATGGTTA | 59.1 | 687 | INTERGENIC | | | | |
| CaPOPI_6061 | scaffold1083 | 74707 | AATATATATATATATATATATATATATATATATAT/AATATATATATATATATATATATATATAT | ATCTCGGGTGCTGAAAATTG | GGTTCCCTAGCACCATAGCA | 60.1 | 546 | INTERGENIC | | | | |
| CaPOPI_6062 | scaffold1083 | 122050 | C/CT | AAGCCTAGGGAGGACGAAAA | AACGAATCATGTGCATCCAA | 60.2 | 772 | INTERGENIC | | | | |
| CaPOPI_6063 | scaffold1087 | 12601 | CTT/CTTT | GCTTAGGCAGCCACAAAATC | CGGTTATTTGATATCACGGGTT | 59.9 | 732 | INTERGENIC | | | | |
| CaPOPI_6064 | scaffold1087 | 13529 | TTTTATTTATTTATTTATT/TTTTATTTATTTATT | TTCCGCATAACACTAAGTGGC | CCATGCTAATGGATGCTGTG | 60.1 | 521 | INTERGENIC | | | | |
| CaPOPI_6065 | scaffold1087 | 14399 | CGAGAGA/CGAGA | TGGGTTGTACCGTTAATTGG | ACCCCAAATATTGCCATGAA | 58.3 | 810 | INTERGENIC | | | | |
| CaPOPI_6066 | scaffold1087 | 23029 | CTTTTTTTTTT/CTTTTTTTT | TGCACCACGAGATCTTGAAA | CGTTGATACATAACAAAAATACAAACA | 60.4 | 597 | INTERGENIC | | | | |
| CaPOPI_6067 | scaffold1087 | 23220 | T/TG | TGTGGGATATCAAGTTTTGGA | AAAAATGCACGGGACAAATA | 57.9 | 704 | INTERGENIC | | | | |
| CaPOPI_6068 | scaffold1092 | 65384 | GTTTTTTTT/GTTTTTTT | AACCAATCTTGTGCATCCAA | AACCCTAGGGAGGACGAAAA | 59.0 | 778 | INTERGENIC | | | | |
| CaPOPI_6069 | scaffold1092 | 108212 | AGGG/AGG | TCCTCCCCAGGCTTATTCTT | TTGCCTAAAAACCCGAATTG | 60.0 | 544 | INTERGENIC | | | | |
| CaPOPI_6070 | scaffold1092 | 121415 | AT/A | AGGTCATTTTCGTCCTCCCT | AATCCAATGGCACAAACACA | 59.9 | 648 | INTERGENIC | | | | |

| INDEL marker IDs | Chromosomes/ unanchored scaffolds | Physical positions (bp) | InDels (*Kabuli* reference genome-CDC Frontier/PI) | Forward primers (5'-3') | Reverse primers (5'-3') | Anneling temperature (0C) | Expected amplified product size (bp) | Structural annotation | | Functional annotation | | |
|---|---|---|---|---|---|---|---|---|---|---|---|---|
| | | | | | | | | Sequence components of *kabuli* genome | *Kabuli* gene accession IDs | NCBI-KOG | TFs | NCBI-nr database |
| CaPOPI_6071 | scaffold1092 | 146775 | GTTT/GTT | CTTCCTCCCTCGGCTTATTC | AGGGAGGACGAAAATGACCT | 60.2 | 365 | INTERGENIC | | | | |
| CaPOPI_6072 | scaffold1101 | 3795 | TCC/TC | TTGGCTCATTGCTTGAGTTG | AGCCAAGGGAGGATGAAAAT | 60.0 | 214 | INTERGENIC | | | | |
| CaPOPI_6073 | scaffold1102 | 7455 | CAAAA/CAAAAA | AGGGAGGACGAAAATGACCT | AACCAATGTTGTGCATCCAA | 59.9 | 470 | INTERGENIC | | | | |
| CaPOPI_6074 | scaffold1109 | 14028 | ATTTTTTTTTTTTT/ATTTTTTTT | TTTGAAAACCTCACCAAGGG | TAAACTGTCAGGGGCGAAGA | 59.9 | 416 | INTERGENIC | | | | |
| CaPOPI_6075 | scaffold1109 | 14081 | GTT/GTTAATT | TTTGAAAACCTCACCAAGGG | TATAAACTGTCAGGGGCGAA | 59.9 | 418 | INTERGENIC | | | | |
| CaPOPI_6076 | scaffold1109 | 109368 | GCAGAAAATAAATTAATCAGAAAATAAATTAATCAGAA/GCAGAAAATAAATTAATCAGAAAATAAATTAATCAGAAAATAAATTAATCAGAA | TTGGTTTATTTGGGTTGGGA | AATAGGGTGGGATGCAAAGA | 60.0 | 753 | INTERGENIC | | | | |
| CaPOPI_6077 | scaffold1109 | 114315 | ATTT/ATT | TCCCTACTCGTAATCGACGC | ACTCAGCCCGGGTATAGAGC | 60.2 | 392 | INTERGENIC | | | | |
| CaPOPI_6078 | scaffold111 | 98560 | GATATATATATATATATATATATATATATATATATA/GATATATATATATATATATATATATATATATATATATA | CGACTGTCGGATATCTGCTC | TCGATGCACTTTGGTCTGAA | 58.4 | 509 | INTERGENIC | | | | |
| CaPOPI_6079 | scaffold111 | 128540 | ATTTTTTTTTTT/ATTTTTTTTTTTT | AACGGGAATGGACTTTACGA | TATGACAATCCCCGACAACA | 59.4 | 507 | INTERGENIC | | | | |
| CaPOPI_6080 | scaffold1115 | 3807 | ATATATTTA/ATA | TTCTTCATTAATGTCAATCCATTTTT | GCGAGCATGAGAAGTTGTTG | 59.3 | 340 | INTERGENIC | | | | |
| CaPOPI_6081 | scaffold1116 | 16701 | TG/T | GGCTTGTGATCTTCACGACA | CCTCAGATGAGAGCCAGGAC | 59.8 | 673 | INTERGENIC | | | | |
| CaPOPI_6082 | scaffold1116 | 30008 | AATTATTATTATTATTATTATTATTATTATTATTATTATTATTATTATTATTATT/AATTATTATTATTATTATTATTATTATTATTATTATTATTATTATTATTATTATT | AAGGTGCCAACACGATAAGG | CAAGTGGATTGGCCCTTTTA | 60.0 | 775 | INTERGENIC | | | | |
| CaPOPI_6083 | scaffold1116 | 42811 | TC/TCC | GATGAAACCGAGGACCAAGA | GTGTTTTCGGACCAGACGAT | 60.1 | 599 | INTERGENIC | | | | |
| CaPOPI_6084 | scaffold1116 | 94918 | TATAATAATAATA/TATAATAATAATAATA | TCTTCGTTATGCAGTCAAACCT | ACCCAACAAAAATGGACCAA | 58.9 | 728 | INTERGENIC | | | | |
| CaPOPI_6085 | scaffold1120 | 11138 | C/CA | TTCATCCAAAGAATCTGGGC | TGCAAGAGGAGTGCCTTTCT | 60.0 | 486 | URR | Ca_27900 | | | |

| INDEL marker IDs | Chromosomes/ unanchored scaffolds | Physical positions (bp) | InDels (*Kabuli* reference genome-CDC Frontier/PI) | Forward primers (5'-3') | Reverse primers (5'-3') | Anneling temperature (0C) | Expected amplified product size (bp) | Structural annotation | | Functional annotation | | |
|---|---|---|---|---|---|---|---|---|---|---|---|---|
| | | | | | | | | Sequence components of *kabuli* genome | *Kabuli* gene accession IDs | NCBI-KOG | TFs | NCBI-nr database |
| CaPOPI_6086 | scaffold1128 | 45001 | CAAAAAAAAAAA/CAAAAAAAAAAAA | ATGGGAAGGAACAACAATGC | CACAAAATTAAACCCAAATTGACA | 59.8 | 453 | INTERGENIC | | | | |
| CaPOPI_6087 | scaffold1128 | 46039 | ATTTTTTTTTTT/ATTTTTTTTTT | CGAATGCAAGTTCGTGGTAA | TGTCAATTGGGCAGACACAT | 59.7 | 510 | INTERGENIC | | | | |
| CaPOPI_6088 | scaffold1128 | 151757 | CTTTTTTTTTTTTTT/CTTTTTTTTTTTT | TTTGACTCCCCACTTATGGC | TGTTTCGCCTCTTTGGAGTC | 59.9 | 597 | INTERGENIC | | | | |
| CaPOPI_6089 | scaffold1128 | 198535 | CAAAAAAAAAAAA/CAAAAAAAAAAA | TAGGGGACTGTCTCGGATTG | GGATCCTCACTTGCAAGACC | 60.1 | 653 | INTERGENIC | | | | |
| CaPOPI_6090 | scaffold1128 | 348170 | TAAAAAAAAAAA/TAAAAAAAAAAAAA | GGTATGGTCCCGATTTGAAG | GCCTAGCCTGACCTATTCCC | 59.2 | 678 | INTERGENIC | | | | |
| CaPOPI_6091 | scaffold1128 | 413406 | TAA/TA | GACCTCCCAAAGGCATTTTT | CCGGTCATCCAACAGAAAGT | 60.3 | 600 | DRR | Ca_23739 | | | |
| CaPOPI_6092 | scaffold1128 | 483765 | GTCAAAACTAAACTAAAAT/GTCAAAACTAAACTAAAATCAAAACTAAACTAAAAT | CAGGATCCTTCCGTATTGGA | TTCTTTCCGGCATGATTTTC | 59.9 | 639 | INTERGENIC | | | | |
| CaPOPI_6093 | scaffold1135 | 9704 | GTTTGTGCT/GT | TTTCGGTTGGGTTTGCTTAC | TACCCGTGCTCAACATCAAG | 60.0 | 704 | INTERGENIC | | | | |
| CaPOPI_6094 | scaffold1135 | 14111 | TTAAATAAATAAATAA/TTAAATAAATAAATAAATAA | TCTGGCACATGAACTTCAGC | CACTCGACAATTGGGAGGTT | 60.0 | 532 | INTRON | Ca_26843 | | SAP | Aminotransferase-like, plant mobile domain |
| CaPOPI_6095 | scaffold1135 | 67023 | TAAAAAAAAAAA/TAAAAAAAAAA | TTTTGCCATGTGTATGAATTG | TCAAGTATGAATCACGGCTGA | 57.0 | 773 | INTERGENIC | | | | |
| CaPOPI_6096 | scaffold1135 | 72246 | ACTT/ACTTCTT | AAACCGAAAACCGAAAACCT | CAATGTAGTTTGTCTAAGAAGTGCAA | 59.9 | 667 | INTERGENIC | | | | |
| CaPOPI_6097 | scaffold1135 | 74034 | AAACCCT/AAACCCTTAACCCT | CAAACCCTAAACCCAAACCC | AGGTCATTTTGGTCTTTGGTG | 60.4 | 358 | INTERGENIC | | | | |
| CaPOPI_6098 | scaffold1139 | 1257 | CT/CTTT | TCTCAAACGGAGCGGTAATC | AAACTCCACATGGATGGGTC | 60.2 | 388 | INTERGENIC | | | | |
| CaPOPI_6099 | scaffold1139 | 1393 | CT/CTTT | TCATCCAGTGTGGGGTACAA | TTGTGTGTGTTGGAGATGAGC | 59.8 | 313 | INTERGENIC | | | | |
| CaPOPI_6100 | scaffold1139 | 1605 | TAACAACAA/TAACAACAACAA | TCATCCAGTGTGGGGTACAA | TTTGAGAATTTGGGATGGAAA | 59.8 | 785 | INTERGENIC | | | | |

| INDEL marker IDs | Chromosomes/ unanchored scaffolds | Physical positions (bp) | InDels (*Kabuli* reference genome-CDC Frontier/PI) | Forward primers (5'-3') | Reverse primers (5'-3') | Anneling temperature (0C) | Expected amplified product size (bp) | Structural annotation | | Functional annotation | | |
|---|---|---|---|---|---|---|---|---|---|---|---|---|
| | | | | | | | | Sequence components of *kabuli* genome | *Kabuli* gene accession IDs | NCBI-KOG | TFs | NCBI-nr database |
| CaPOPI_6101 | scaffold1142 | 4296 | T/TGA | AGGTCATTTTCGTCCTCCCT | TTGCATGCACAAGATTGGTT | 59.9 | 268 | INTERGENIC | | | | |
| CaPOPI_6102 | scaffold1145 | 3948 | ACTTCT/ACTTCTTCT | AATCCTCAAAGCCCTCACCT | TTTTGGAACCTTCGTCCTTT | 60.1 | 750 | INTERGENIC | | | | |
| CaPOPI_6103 | scaffold1145 | 7358 | TAAAAA/TAAAAAAA | TGAATTTTCCAAGTATTGCATCC | GTTAAGGGACCGCAAGAACA | 60.2 | 614 | INTERGENIC | | | | |
| CaPOPI_6104 | scaffold1145 | 15770 | ATATTATTATTATTATTATTATTA/ATATTATTATTATTATTA | TGGCACAACATCATCATCATT | GCGCATTCTTCATCTTCTCC | 59.8 | 496 | INTERGENIC | | | | |
| CaPOPI_6105 | scaffold1145 | 16689 | AAAGTAAAATAAGTAAAA/AAAGTAAAATAAGTAAAATAAGTAAAA | GGCCAAATGCCAAATCTCTA | TAGGAAAAGGGAAACCCACC | 60.0 | 683 | INTERGENIC | | | | |
| CaPOPI_6106 | scaffold1145 | 67865 | AT/A | AAGCGTACCTGTGGGAATTG | TTTACCGCGCTGAAAAGACT | 60.0 | 921 | INTERGENIC | | | | |
| CaPOPI_6107 | scaffold115 | 52073 | GA/GACTA | CAATTAACACAAACGCCCCT | TTGGCCTCCCTAGGCTTATT | 59.9 | 577 | INTERGENIC | | | | |
| CaPOPI_6108 | scaffold1151 | 18791 | CTTT/CTTTT | AGCCGTCATGACTTTTGTCC | ACGGACCCTTTGTTGAGACA | 60.1 | 392 | INTERGENIC | | | | |
| CaPOPI_6109 | scaffold1153 | 18767 | GAAAAAAAAA/GAAAAAAAA | TTGGAGACGAAAACATGAGTCT | AGTTCATCAACACCACATCAGTG | 58.8 | 472 | INTERGENIC | | | | |
| CaPOPI_6110 | scaffold1159 | 27402 | TG/TGG | AGGGAGGACGAAAATGACCT | CCTTCCTTGGCTTGTTCTTG | 59.9 | 345 | INTERGENIC | | | | |
| CaPOPI_6111 | scaffold118 | 104154 | AATATATATATATATATATATATATATA/AATATATATATATATATATATATATATATATATA | CTCGAGCAGGAGGATCAAAC | TCCGGTACCGCAAGTAAATC | 60.0 | 861 | INTERGENIC | | | | |
| CaPOPI_6112 | scaffold118 | 173557 | TTATATATATATATATA/TTATATATATATATATA | TGTCATGCTAATATCCGCCA | TGGATGGATTTCTAGTTGCCTT | 60.1 | 489 | INTERGENIC | | | | |
| CaPOPI_6113 | scaffold1180 | 2599 | ATT/ATTTT | GAGAGGTCATGGCACCCTTA | GAGCAACACATTGCATGTCC | 60.1 | 166 | INTERGENIC | | | | |
| CaPOPI_6114 | scaffold1180 | 2913 | A/AC | GAGAGGTCATGGCACCCTTA | TCTTGCCCAACGTTTTACAT | 60.1 | 513 | INTERGENIC | | | | |
| CaPOPI_6115 | scaffold1180 | 3744 | GA/G | GTGCACGAAAGTGTGGCTTA | ACTTTACCATTGCCTTCCCC | 59.9 | 766 | INTERGENIC | | | | |

| INDEL marker IDs | Chromosomes/ unanchored scaffolds | Physical positions (bp) | InDels (*Kabuli* reference genome-CDC Frontier/PI) | Forward primers (5'-3') | Reverse primers (5'-3') | Anneling temperature (0C) | Expected amplified product size (bp) | Structural annotation | | Functional annotation | | |
|---|---|---|---|---|---|---|---|---|---|---|---|---|
| | | | | | | | | Sequence components of *kabuli* genome | *Kabuli* gene accession IDs | NCBI-KOG | TFs | NCBI-nr database |
| CaPOPI_6116 | scaffold1180 | 36749 | CTATTTATTTATTTATTTATTTATTTATTT/CTATTTATTTATTTATTTATTTATTT | TACGGTTCGATCTCATGCAA | TGCCTCTTCCCCTGTTTCTA | 60.2 | 419 | DRR | Ca_25737 | | G2-like | Peptidase S8/S53, subtilisin/kexin/sedolisin |
| CaPOPI_6117 | scaffold1180 | 82368 | GAAAAAAAAA/GAAAAAAAAAAA | AATCCCATTCAGGTAACCGT | CATGCTATAAATTTTCAGCTGGTTT | 58.2 | 742 | INTERGENIC | | | | |
| CaPOPI_6118 | scaffold1180 | 102429 | TAAAAAAA/TAAAAAA | ACAAAATCGGCCAGTGAATC | TCTCTTGCCACAACGGATAA | 59.9 | 663 | INTERGENIC | | | | |
| CaPOPI_6119 | scaffold1180 | 135503 | TTATAT/TTATATAT | TTGTGTGCGTTTGACTGACA | AAGCGGAGAAGTCGGAGATT | 59.9 | 789 | INTERGENIC | | | | |
| CaPOPI_6120 | scaffold1180 | 206096 | AATATATATATATATATA/AATATATATATATATATATA | CCATGTGGTTCTTTTGCATTT | AACTTCGCCATTTGTCATCC | 59.9 | 548 | INTERGENIC | | | | |
| CaPOPI_6121 | scaffold1180 | 208476 | TA/T | TAAGAGGGGCTTGTTACCCC | CCAACCTAATATTCATGCCACA | 60.3 | 216 | INTERGENIC | | | | |
| CaPOPI_6122 | scaffold1180 | 209213 | ACTAT/ACTATTCTAT | TTGATTTTTGACATGTTGTGCTT | TTGAGGGGTGGCAAGTTTAG | 59.5 | 607 | INTERGENIC | | | | |
| CaPOPI_6123 | scaffold1180 | 216830 | TTATATATATATATATATATATAT/TTATATATATATATATATATAT | AAGCGTCTTCTTTTGGGGAT | CATATGCACGTAAAATCGCCT | 60.1 | 345 | INTERGENIC | | | | |
| CaPOPI_6124 | scaffold1180 | 241101 | ATTTTTTTTTTTTTT/ATTTTTTTTTTT | AAAACAAGAAGGGGCAACCT | TAAATCAGAGGATGCGGGTC | 60.0 | 760 | INTERGENIC | | | | |
| CaPOPI_6125 | scaffold1180 | 247801 | TATTCATTCATTCATTCATT/TATTCATTCATTCATTCATTCATT | TGAAAACCTGAAAGGGCAAC | CTTCAATATGCGGGTCGTTT | 60.1 | 413 | INTERGENIC | | | | |
| CaPOPI_6126 | scaffold1181 | 6020 | AACAAATAC/AAC | AAACCCTAAATCCCAAACCC | TTTAGGTCATTTTGGCCTTTTT | 59.0 | 465 | INTERGENIC | | | | |
| CaPOPI_6127 | scaffold1181 | 6839 | GAAAAAAAAA/GAAAAAAAA | GACCCTAGACCCTTAACCCTT | TCAATTTTTGTCGTTGTCCA | 57.7 | 176 | INTERGENIC | | | | |
| CaPOPI_6128 | scaffold1183 | 131590 | AATATATATATATATATATATATATATATAT/AATATATATATATATATATATATATATATATATAT | GAAATGGGTTTCGCGTAGAG | TTTGGCTCGAAGGCTTTTTA | 59.7 | 428 | INTERGENIC | | | | |
| CaPOPI_6129 | scaffold1183 | 142757 | TAAAAAAAAAAA/TAAAAAAAAAA | TCAAATCCATTGGTCCCTTT | AAAGATGCATTTGCCAGGAC | 59.2 | 916 | INTERGENIC | | | | |
| CaPOPI_6130 | scaffold1183 | 239750 | CTTTTTTTTTTT/CTTTTTTTTTTTT | CCCTTCCGTGAGGTTACAAA | CCGGTGGATATGATGAGCTT | 60.0 | 403 | INTERGENIC | | | | |

| INDEL marker IDs | Chromosomes/ unanchored scaffolds | Physical positions (bp) | InDels (*Kabuli* reference genome-CDC Frontier/PI) | Forward primers (5'-3') | Reverse primers (5'-3') | Anneling temperature (0C) | Expected amplified product size (bp) | Structural annotation | | Functional annotation | | |
|---|---|---|---|---|---|---|---|---|---|---|---|---|
| | | | | | | | | Sequence components of *kabuli* genome | *Kabuli* gene accession IDs | NCBI-KOG | TFs | NCBI-nr database |
| CaPOPI_6131 | scaffold1185 | 47365 | AAATAATAATAATAATAATAATAATAATA/AAATAATAATAATAATAATAATAATA | TTCCTTATAGGCATGATTTCTTGA | TCGTTTTGGTGTTGGTTTCA | 59.2 | 809 | INTERGENIC | | | | |
| CaPOPI_6132 | scaffold1185 | 67322 | GATATATATATAT/GATATATATAT | CAATTCCCACAGGTACGCTT | GACGCGAAAACAACATTTCA | 60.0 | 683 | INTERGENIC | | | | |
| CaPOPI_6133 | scaffold1185 | 67468 | T/TAGGGTGA | CAATTCCCACAGGTACGCTT | GACGCGAAAACAACATTTCA | 60.0 | 683 | INTERGENIC | | | | |
| CaPOPI_6134 | scaffold119 | 16292 | AACA/AACACACA | AGGAAGAGTGGAGTTGCGAA | TATTTTGGATTTTGGCTCGG | 60.0 | 513 | INTERGENIC | | | | |
| CaPOPI_6135 | scaffold119 | 16337 | T/TAA | AGGAAGAGTGGAGTTGCGAA | TATTTTGGATTTTGGCTCGG | 60.0 | 513 | INTERGENIC | | | | |
| CaPOPI_6136 | scaffold119 | 35751 | A/AG | ACTCATATGCAAAATGCCCC | TTATGCTGGAGATGGGAAGG | 59.8 | 444 | INTERGENIC | | | | |
| CaPOPI_6137 | scaffold1193 | 7288 | TCAA/TCAATTTAAGCAA | AATGCTTTCCAGAAAACCGA | TGATCTGATGGCTGACTTTCA | 59.7 | 367 | INTERGENIC | | | | |
| CaPOPI_6138 | scaffold1193 | 51363 | CTATTATTATTATTATTATTATTATTATTATTATTA/CTATTATTATTATTATTATTATTATTATTATTATTATTATTATTA | ATACACGCAAAATCGCATGA | TGCCAAACTACCTTCCCATC | 60.1 | 351 | INTERGENIC | | | | |
| CaPOPI_6139 | scaffold1195 | 21554 | CTTATTATTATTATTATTATTATTATT/CTTATTATTATTATTATTATTATT | AAGGGTGCAACCGTATTGAG | GAAAGGCTCAAACGACCAAA | 60.0 | 594 | INTERGENIC | | | | |
| CaPOPI_6140 | scaffold1196 | 44249 | TTATATATATATATATATATATAT/TTATATATATATATATATATATATATATATAT | ACCCAATAGAGGTGCAACCA | CCATTTGGAATTGCGACTTT | 60.4 | 593 | INTERGENIC | | | | |
| CaPOPI_6141 | scaffold1196 | 112009 | AGGGGGGG/AGGGGGG | GTTCTCCCTTCCATCGTTCA | TGTTAGGTGTTTTGGTGGCA | 60.1 | 525 | INTERGENIC | | | | |
| CaPOPI_6142 | scaffold1196 | 182114 | CTTTTTTTT/CTTTTTTTTT | GGGGGAGGATGAAGATGAAT | TTGTGTGCTCTAGGAACCGA | 60.1 | 650 | INTERGENIC | | | | |
| CaPOPI_6143 | scaffold1196 | 184515 | TTTATTATTATTATTATTATTATTATTATTATTATTATTATTATTATTATTATT/TTTATTATTATTATTATTATTATTATTATTATTATTATTATTATTATTATTATTATTAT | AGAGGCAAACAAGAACCGAA | TGCTGTCGTAGCGGAATACA | 59.9 | 376 | INTERGENIC | | | | |
| CaPOPI_6144 | scaffold1197 | 5120 | GT/GTT | TCGCAGCTCAATTGGTAGTG | GAGAGCTACCGTGCAACACA | 60.0 | 486 | INTERGENIC | | | | |
| CaPOPI_6145 | scaffold1197 | 75749 | TTTATTATTATTATTATTATTATTATTAT/TTTATTATTATTATTATTATTATTATTATTAT | TAACCCAAAAATCCCAACCA | TGGATCGCATAACTCCATGA | 60.0 | 396 | INTERGENIC | | | | |

| INDEL marker IDs | Chromosomes/ unanchored scaffolds | Physical positions (bp) | InDels (*Kabuli* reference genome-CDC Frontier/PI) | Forward primers (5'-3') | Reverse primers (5'-3') | Anneling temperature (0C) | Expected amplified product size (bp) | Structural annotation | | Functional annotation | | |
|---|---|---|---|---|---|---|---|---|---|---|---|---|
| | | | | | | | | Sequence components of *kabuli* genome | *Kabuli* gene accession IDs | NCBI-KOG | TFs | NCBI-nr database |
| CaPOPI_6146 | scaffold1197 | 87034 | GATATATATATATATATATATATATATAT/GATATATATATATATATATATATAT | CCGATCAACCCGTTAAAATC | ATCTTCAGGGTGGAGGCTCT | 59.3 | 310 | INTERGENIC | | | | |
| CaPOPI_6147 | scaffold1197 | 209617 | ATTTT/ATTT | GCCCTCAGATGAGAGTCAGG | AATGTGAACTCGCCCAAGAC | 59.9 | 514 | INTERGENIC | | | | |
| CaPOPI_6148 | scaffold1197 | 244953 | TAAAAAAA/TAAAAAAAA | CACTTTTTGTTTCATCGCCA | ACTGTGATCCGGTGATTTGA | 59.7 | 574 | INTERGENIC | | | | |
| CaPOPI_6149 | scaffold1197 | 285281 | ATTTTTTTTTT/ATTTTTTTTT | AGTTTGCCATTCCAACCATC | TCCCACAACAAAATTCGTCA | 59.8 | 467 | INTERGENIC | | | | |
| CaPOPI_6150 | scaffold1197 | 540966 | ACCC/ACC | TAACTGGGCGTTAAGAACGG | TTGAGGGACATATTTTGGGC | 60.1 | 901 | INTERGENIC | | | | |
| CaPOPI_6151 | scaffold12 | 154021 | AATATATATATATATATATATATATATATATATATAT/AATATATATATATATATATATATATATATATATATAT | TCGAGTTTGCAAAGGCAAC | ATGCCAATTTGCCAAGCTAA | 60.0 | 350 | INTERGENIC | | | | |
| CaPOPI_6152 | scaffold12 | 271141 | TACA/TA | TGATGATTTTGATGCGGAAA | CCGTGTAAACTTCGGACAGAG | 60.0 | 854 | INTERGENIC | | | | |
| CaPOPI_6153 | scaffold12 | 352195 | GAAA/GAAAAA | GTTGTCCGGCGTATATTGCT | TCATCAAAGGCATGCAAATC | 60.0 | 572 | INTERGENIC | | | | |
| CaPOPI_6154 | scaffold1202 | 6534 | TAAAAAAA/TAAAAAA | AGTGCCCTCGTCAAACAAAC | TTACAGCGCGTGTTGAAAAG | 60.2 | 824 | DRR | Ca_24658 | | | |
| CaPOPI_6155 | scaffold1202 | 25847 | CTTTTTCTTTTTTTTTC/CTTTTTCTTTTTTTTTCTTTTTTTTTC | TTGTCCACAGTGGCATATTGA | TGGCAGATATGGGGGTGTAT | 60.0 | 737 | INTERGENIC | | | | |
| CaPOPI_6156 | scaffold1202 | 82238 | AATATATATATATATA/AATATATATATATATATA | AACACATCCGGGTTCCATTA | TTTGATCCATTTGCCCTAGC | 60.1 | 739 | INTERGENIC | | | | |
| CaPOPI_6157 | scaffold1202 | 83479 | GTATATATATATATATAT/GTATATATATATATAT | ACGGTGTAGGCAAGATGGAC | AGGATGCATGGGATGAAAAG | 60.0 | 458 | INTERGENIC | | | | |
| CaPOPI_6158 | scaffold1202 | 177457 | TAT/TATCATTTAGAGGAAT | CAGGCCTGGGTAATCTTTGA | CACACTTGGGTGTCCCTCTT | 60.1 | 397 | DRR | Ca_24666 | | | |
| CaPOPI_6159 | scaffold1202 | 178836 | CCG/CCGTAAGGCG | TCTGACATGTGTGCGAGTCA | GCCACACCGTGCTACCTATT | 60.0 | 406 | CDS (FRAME SHIFT) | Ca_24666 | | | |
| CaPOPI_6160 | scaffold1202 | 179607 | AG/AGG | AATAGGTAGCACGGTGTGGC | TCCCTTGCCTACATTGTTCC | 60.0 | 814 | INTRON | Ca_24666 | | | |

| INDEL marker IDs | Chromosomes/ unanchored scaffolds | Physical positions (bp) | InDels (*Kabuli* reference genome-CDC Frontier/PI) | Forward primers (5'-3') | Reverse primers (5'-3') | Anneling temperature (0C) | Expected amplified product size (bp) | Structural annotation | | Functional annotation | | |
|---|---|---|---|---|---|---|---|---|---|---|---|---|
| | | | | | | | | Sequence components of *kabuli* genome | *Kabuli* gene accession IDs | NCBI-KOG | TFs | NCBI-nr database |
| CaPOPI_6161 | scaffold1202 | 184065 | C/CATGATTGGTT | CATCGAGTCTTTGAACGCAA | TCACAAATGGCAACGTGTTT | 60.0 | 319 | DRR | Ca_24667 | | | Transposase, Ptta/En/Spm, plant |
| CaPOPI_6162 | scaffold1206 | 920 | A/AC | TATCACCTATCAGAGCGGGG | GAGTTATTCGGGTGGGGAAT | 60.1 | 546 | INTERGENIC | | | | |
| CaPOPI_6163 | scaffold1215 | 12684 | TAAAAAAAAA/TAAAAAAAAAAAA | TCTCTCCCGATTTGGTCATT | TTTGCTCCTCTTCTTCTGGC | 59.5 | 588 | INTERGENIC | | | | |
| CaPOPI_6164 | scaffold1215 | 12961 | GAAAAAAAA/GAAAAAAA | GCCAGAAGAAGAGGAGCAAA | AAACATTCAATCACCAAGGGA | 59.7 | 470 | INTERGENIC | | | | |
| CaPOPI_6165 | scaffold1215 | 13648 | TAAAAAA/TAAAAA | TCGTGGGACTAAAACTGAGGA | CGCACATATGAATGATCCCA | 59.7 | 576 | INTERGENIC | | | | |
| CaPOPI_6166 | scaffold1215 | 14063 | GGGATCAT/G | AATTGAAGGAAGATGGAAAACA | ACAAACTGGAGGGCAACAAC | 57.7 | 822 | INTERGENIC | | | | |
| CaPOPI_6167 | scaffold1215 | 41563 | GATATATATATATATATATATATA/GATATATATATATATATATA | CCCTTCATTTCCTTCGCATA | GAAGCAGGCTTCGATGGATA | 60.0 | 415 | INTRON | Ca_28164 | | G2-like | Peptidase S8/S53, subtilisin/kexin/sedolisin |
| CaPOPI_6168 | scaffold1215 | 44629 | TAAAAA/TAAAA | AAACATCTCCTTTGTGTGTGTGA | AATCTTTCGCTCAACAATGCT | 59.6 | 391 | URR | Ca_28164 | | G2-like | Peptidase S8/S53, subtilisin/kexin/sedolisin |
| CaPOPI_6169 | scaffold1215 | 45663 | ATT/ATTT | TGGCTTTTGTATTTCAGAGTTGG | AGTGGGACCATGATTTTTGAA | 60.5 | 492 | INTERGENIC | | | | |
| CaPOPI_6170 | scaffold1215 | 45939 | ATTTT/ATTT | TTTCAAAAATCATGGTCCCAC | GGGGTGGTGTCTATCTTTGC | 59.7 | 337 | INTERGENIC | | | | |
| CaPOPI_6171 | scaffold1219 | 7175 | AT/ATT | GGAAACCTCACCCTACCACA | TGGGTCGTTCTTTTTGGAGT | 59.8 | 925 | INTERGENIC | | | | |
| CaPOPI_6172 | scaffold1219 | 7693 | CAA/CAAA | ACTCCAAAAAGAACGACCCA | GGACATTCCTCACCTGCAAC | 59.6 | 104 | INTERGENIC | | | | |
| CaPOPI_6173 | scaffold1219 | 40006 | GTATATATATATATATAT/GTATATATATATATAT | TGGTTTAATGTTGGCAAATGG | ATCCAGTTGGTGAGGAAAAA | 60.6 | 317 | INTERGENIC | | | | |
| CaPOPI_6174 | scaffold1221 | 73998 | AATATATATATATATATATATATATATATATATATAT/AATATATATATATATATATATATATATATATATATATATATAT | ACGGCACCGTGAGTAAATTC | GAAACTACCGAGCAAATGGC | 60.0 | 840 | INTERGENIC | | | | |
| CaPOPI_6175 | scaffold123 | 361645 | TCA/TCAAAGAACTCTTTCCCAACA | ATCCACCCACCCTTTCTTTC | CAGAGCAACCAGGAACAACA | 60.2 | 293 | CDS (large-effect mutations) | Ca_24703 | QR | B3 | Oxoglutarate/iron-dependent oxygenase |

| INDEL marker IDs | Chromosomes/ unanchored scaffolds | Physical positions (bp) | InDels (*Kabuli* reference genome-CDC Frontier/PI) | Forward primers (5'-3') | Reverse primers (5'-3') | Anneling temperature (0C) | Expected amplified product size (bp) | Structural annotation | | Functional annotation | | |
|---|---|---|---|---|---|---|---|---|---|---|---|---|
| | | | | | | | | Sequence components of *kabuli* genome | *Kabuli* gene accession IDs | NCBI-KOG | TFs | NCBI-nr database |
| CaPOPI_6176 | scaffold1238 | 155840 | CATATATATATATATAT/CATATATATATATATAT | CCAGTAAAATCTTCGCTCGC | TTGGTATTGAACTCGGAGGC | 60.0 | 682 | INTERGENIC | | | | |
| CaPOPI_6177 | scaffold1239 | 4656 | T/TC | TCGAGTGGTACGAAAATCCC | TCCCAGTCCTCGATTTCATC | 59.9 | 539 | INTERGENIC | | | | |
| CaPOPI_6178 | scaffold1240 | 70748 | CA/CAA | TTCCTTCATCACCCCTTACG | TTGAGGATATACACGGGCAA | 59.9 | 668 | INTERGENIC | | | | |
| CaPOPI_6179 | scaffold1240 | 74630 | CTATTATTATTATTATTATTATTATTATTATTATTATT/CTATTATTATTATTATTATTATTATTATTATTATTATTATTATT | GACGCATATGAGCAGGCTTT | TCTGTTGTCGTACACGGGTC | 60.4 | 858 | INTERGENIC | | | | |
| CaPOPI_6180 | scaffold1240 | 105707 | A/AG | ACATGGAACCCATCCAAAGA | GCCCTCAGATGAGAGTCAGG | 60.2 | 268 | INTERGENIC | | | | |
| CaPOPI_6181 | scaffold1242 | 2626 | AATATATATATATATATA/AATATATATATATATATA | TGGTGTTGGAGTCATGTTTTTC | TTCTCAACTGACGCATGAGG | 59.9 | 388 | INTERGENIC | | | | |
| CaPOPI_6182 | scaffold1252 | 98018 | CA/CAA | TTTGCCAATCGATTATCGTG | TGCACAAGTCAGTAGCACCC | 59.5 | 561 | INTERGENIC | | | | |
| CaPOPI_6183 | scaffold1252 | 112875 | CATATATATATATATATATAT/CATATATATATATATATATATATATATATATATAT | TTGGAAGGAGCCCTTACCTT | TGAAAAGGGGTAGGCAACAC | 60.1 | 787 | INTERGENIC | | | | |
| CaPOPI_6184 | scaffold1256 | 617 | ATTTTTTTTTTT/ATTTTTTTTTT | TCCAACACCCTCACAAATCA | TTGGACTACGGGTTAGTGGG | 59.9 | 914 | INTERGENIC | | | | |
| CaPOPI_6185 | scaffold1259 | 75294 | AA/ATGACAAAGGGAAAGAGGA | TCCACAGAACCTGCAGACAG | GCTGGATAAGCATTGCCATT | 60.0 | 709 | INTRON | Ca_27885 | I | | Phospholipase/carboxylesterase |
| CaPOPI_6186 | scaffold127 | 61054 | AG/AGG | TATGCTTCCACCTTCCCATC | GACTAAGGAAGAGCATGGCG | 59.9 | 475 | INTERGENIC | | | | |
| CaPOPI_6187 | scaffold127 | 152263 | TCTAATCGATTGC/TCTAATCGATTGCCTAATCGATTGC | TCGACTTGTCAATCGCTTTG | CTCTCTGTCCTCGAAATCCG | 60.0 | 836 | INTERGENIC | | | | |
| CaPOPI_6188 | scaffold127 | 256092 | TC/T | TTTCAGGCAATTTTCGAACC | ATTATGTGAAAGCGCAAGGC | 60.1 | 797 | INTERGENIC | | | | |
| CaPOPI_6189 | scaffold127 | 270423 | AATTTTCATTTTCATTT/AATTTTCATTT | GCCCACATATCTTGTTGCCT | TTCAAGCACATACTGCACCAG | 60.0 | 687 | INTRON | Ca_26800 | | | |
| CaPOPI_6190 | scaffold1272 | 21606 | TC/TCC | TTCCTTTCCACACAACCACA | CCATCTTCTTCCTCTGTCGC | 60.0 | 362 | INTERGENIC | | | | |

| INDEL marker IDs | Chromosomes/ unanchored scaffolds | Physical positions (bp) | InDels (*Kabuli* reference genome-CDC Frontier/PI) | Forward primers (5'-3') | Reverse primers (5'-3') | Anneling temperature (0C) | Expected amplified product size (bp) | Structural annotation | | Functional annotation | | |
|---|---|---|---|---|---|---|---|---|---|---|---|---|
| | | | | | | | | Sequence components of *kabuli* genome | *Kabuli* gene accession IDs | NCBI-KOG | TFs | NCBI-nr database |
| CaPOPI_6191 | scaffold1272 | 125862 | CAAA/CAA | CTCTCATCTGAGGGCAAAGC | ATTTGGCAACACAGGGACTC | 60.1 | 387 | DRR | Ca_26855 | | Trihelix | |
| CaPOPI_6192 | scaffold1274 | 6685 | T/TGTG | TCATTTTCGTCCTTCCTTGG | ACACACCTATTGGACGCACA | 60.0 | 272 | INTERGENIC | | | | |
| CaPOPI_6193 | scaffold1275 | 6195 | CAA/CAAGAGATAA | TGTTGCCTCGTAATTCAACG | CAGGGACTAAAACTGTTTGCTG | 59.7 | 556 | INTERGENIC | | | | |
| CaPOPI_6194 | scaffold1276 | 1178 | CTTTTT/CTTTTTT | TCAGCCATGACTTGAGGTTG | CTCACTTTGGTCCCATGCTT | 59.8 | 361 | INTERGENIC | | | | |
| CaPOPI_6195 | scaffold1276 | 1671 | TT/TTATAT | AAGCATGGGACCAAAGTGAG | TTTCCCAATCGATTTGCTTC | 60.1 | 942 | INTERGENIC | | | | |
| CaPOPI_6196 | scaffold1278 | 29766 | CAAAAA/CAAAA | GAGAATTCCGGGAAGGAGAC | TTTCTTCTTCTCTCAGGCCG | 60.0 | 320 | INTERGENIC | | | | |
| CaPOPI_6197 | scaffold1278 | 45110 | CATATATATATATATATATATAT/CATATATATATATATATAT | TATTTGCTCGCAACACAACC | GTTTAAGCCCCTTTGAGCCT | 59.7 | 253 | INTERGENIC | | | | |
| CaPOPI_6198 | scaffold1278 | 60991 | GTATATATATATATATATATATAT/GTATATATATATATATATATATAT | TTAAAGAGATTCGCAGCGGT | TTTCAGGTTATCGATTGGGG | 60.0 | 359 | INTERGENIC | | | | |
| CaPOPI_6199 | scaffold1278 | 63103 | GAAAAA/GAAAA | ACGAAGGGTGTAGGACGATG | ATTTGTTTGCCGATCGCTAC | 60.0 | 611 | INTERGENIC | | | | |
| CaPOPI_6200 | scaffold1278 | 63247 | TAAAAAAAA/TAAAAAAA | ACGAAGGGTGTAGGACGATG | ATTTGTTTGCCGATCGCTAC | 60.0 | 611 | INTERGENIC | | | | |
| CaPOPI_6201 | scaffold1278 | 63301 | ATT/ATTT | ACGAAGGGTGTAGGACGATG | ATTTGTTTGCCGATCGCTAC | 60.0 | 611 | INTERGENIC | | | | |
| CaPOPI_6202 | scaffold1278 | 63474 | TTATATATATATATATATATATATAT/TTAGTTATATATATATATATATATATATATAT | CCTTTGATCCGCTTTCTGAG | TGCCATTATTACTGCTACCCA | 59.9 | 598 | INTERGENIC | | | | |
| CaPOPI_6203 | scaffold1278 | 65072 | TTTATTATTATTATTATTATTATTATTATTATTA/TTTATTATTATTATTATTATTATTATTATTATTATTA | TGGGTAGCAGTAATAATGGCA | TGAGCAAGATGTGTTTTGTGG | 58.2 | 341 | INTERGENIC | | | | |
| CaPOPI_6204 | scaffold1278 | 107077 | CCT/CCTACTATCACT | GCGATTATGGCACCAAAGTT | ACCGTCAAACACAAACCTCC | 60.0 | 114 | INTERGENIC | | | | |
| CaPOPI_6205 | scaffold128 | 43105 | AATATATATATATATATATATATATATATA/AATATATATATATATATATATATATATA | CCTGATTTTCTCCGTCCAAA | ACTCGCAGCAAACATCCTCT | 60.0 | 691 | INTERGENIC | | | | |

| INDEL marker IDs | Chromosomes/ unanchored scaffolds | Physical positions (bp) | InDels (*Kabuli* reference genome-CDC Frontier/PI) | Forward primers (5'-3') | Reverse primers (5'-3') | Anneling temperature (0C) | Expected amplified product size (bp) | Structural annotation | | Functional annotation | | |
|---|---|---|---|---|---|---|---|---|---|---|---|---|
| | | | | | | | | Sequence components of *kabuli* genome | *Kabuli* gene accession IDs | NCBI-KOG | TFs | NCBI-nr database |
| CaPOPI_6206 | scaffold128 | 437260 | ATTTTTTT/ATTTTTT | TGTGCGTATATACACGTAAAATCGT | GCACTTTCTGGAAACCTTGC | 59.8 | 526 | INTERGENIC | | | | |
| CaPOPI_6207 | scaffold128 | 547816 | CAAA/CAA | CAACGCAACATAACACCCAG | GGAAAGTTGCTCACCACCAT | 60.0 | 361 | INTERGENIC | | | | |
| CaPOPI_6208 | scaffold128 | 588046 | TAA/TAAA | AGTACTGAGCCCCCTGGTTT | ATGATGTTGTAGCGATTGCG | 60.0 | 308 | INTERGENIC | | | | |
| CaPOPI_6209 | scaffold128 | 732668 | A/ATAAAC | GCTCAAAGCACATGACAGGA | CATTTTGAACCAAAGCAGCA | 60.0 | 479 | INTERGENIC | | | | |
| CaPOPI_6210 | scaffold128 | 747239 | TCCC/TCC | GGTGTGTTTGTGACGTTGGA | GGAGGACGAAAATGATCGAA | 60.5 | 646 | INTERGENIC | | | | |
| CaPOPI_6211 | scaffold128 | 747253 | G/GT | GGTGTGTTTGTGACGTTGGA | GGAGGACGAAAATGATCGAA | 60.5 | 646 | INTERGENIC | | | | |
| CaPOPI_6212 | scaffold1281 | 46557 | T/TTTGC | CAATTCGACATGCATTTGCT | AATTTCATGCACCGTCAATTT | 59.7 | 650 | DRR | Ca_21505 | K | | Bromodomain |
| CaPOPI_6213 | scaffold1281 | 376337 | GTTTTTTTTTT/GTTTTTTTT | TGTTTAGGCGCTAGTTTGGC | TCGATTATGCGTAGGGAAGG | 60.4 | 665 | INTERGENIC | | | | |
| CaPOPI_6214 | scaffold1281 | 381985 | CAATAAATAAATAAATAAATAA/CAATAAATAAATAAATAAATAAATAA | GGTAGGGTCCCCCACTCTTA | AGGATCCGAATTAATGGCAG | 60.2 | 845 | INTERGENIC | | | | |
| CaPOPI_6215 | scaffold1281 | 440047 | CTTTTTTTTT/CTTTTTTTT | TTGGTTGGTAGAGTTGTGCG | CTCACCGCTGCTTCAATTTT | 59.8 | 190 | INTERGENIC | | | | |
| CaPOPI_6216 | scaffold1281 | 469460 | GAAAAAAAAAAAA/GAAAAAAAAAAA | TCTCTGGCAACCGTTCTTCT | ACCTCGTTGAGACACGCTTT | 60.0 | 566 | INTERGENIC | | | | |
| CaPOPI_6217 | scaffold1281 | 471713 | AATATATATATATA/AATATATATATATATA | GAGCATTGTGACAAAATTGAGC | CGGGAATTCTTGATGAATCG | 59.8 | 541 | INTERGENIC | | | | |
| CaPOPI_6218 | scaffold1281 | 472602 | CATA/CATATATA | CAAATGGAGGTTATTGCATTG | AGCAGTGCTTCAACTCATGC | 58.0 | 569 | INTERGENIC | | | | |
| CaPOPI_6219 | scaffold1281 | 473339 | GATATATATATATATATATA/GATATATATATATATATA | ACGCATGGATATTGCAGTCA | TCCGATTGTTGACCAGTTTTT | 60.1 | 783 | INTERGENIC | | | | |
| CaPOPI_6220 | scaffold1281 | 492209 | TAAAAA/TAAAA | GGAATGGAAAACACATTCAACA | GAATGCTTGAGAAACTTTGGG | 59.7 | 499 | INTERGENIC | | | | |

| INDEL marker IDs | Chromosomes/ unanchored scaffolds | Physical positions (bp) | InDels (*Kabuli* reference genome-CDC Frontier/PI) | Forward primers (5'-3') | Reverse primers (5'-3') | Anneling temperature (0C) | Expected amplified product size (bp) | Structural annotation | | Functional annotation | | |
|---|---|---|---|---|---|---|---|---|---|---|---|---|
| | | | | | | | | Sequence components of *kabuli* genome | *Kabuli* gene accession IDs | NCBI-KOG | TFs | NCBI-nr database |
| CaPOPI_6221 | scaffold1281 | 504417 | GAAA/GAAAA | TGCATGTTTGTCTGCAATGA | CAGGCATTTTGCCTCAATTT | 59.8 | 525 | INTERGENIC | | | | |
| CaPOPI_6222 | scaffold1283 | 64512 | AT/A | AGGTCATTTTCGTCCTCCCT | ATTTTGGGAATTTCCGCTCT | 59.9 | 560 | INTERGENIC | | | | |
| CaPOPI_6223 | scaffold1292 | 7985 | CAAAAAAAAA/CAAAAAAA | TTGCTGTTACCCAATCCTTG | TTTGGGGATTTAGGGTGTCA | 58.6 | 575 | INTERGENIC | | | | |
| CaPOPI_6224 | scaffold1294 | 29929 | CT/C | CCAAAGGAGGACGAAAATGA | CTTGTGCATCCAATAGGGGT | 60.0 | 389 | INTERGENIC | | | | |
| CaPOPI_6225 | scaffold1294 | 33981 | AT/A | GGAGGACGAAAATGACCAAA | TGTGCATCCAATAAGCGTGT | 59.9 | 562 | INTERGENIC | | | | |
| CaPOPI_6226 | scaffold1307 | 6228 | A/AG | ACACACCTATTGGACGCACA | AGGTCAATTTCGTCCTCCCT | 60.0 | 377 | INTERGENIC | | | | |
| CaPOPI_6227 | scaffold1308 | 11986 | TACAACAACAAC/TACAACAACAACAAC | CCAAAGGAGGACGAAAATGA | TGCATAATCGATTGGCACAT | 60.0 | 718 | INTERGENIC | | | | |
| CaPOPI_6228 | scaffold1308 | 16327 | ACCC/ACC | AGGGAGGACGAAAATGACCT | CCAATTGGGGTTTTTAACCA | 59.9 | 488 | INTERGENIC | | | | |
| CaPOPI_6229 | scaffold1309 | 49935 | CATATATATATATATA/CATATATATATATATATATA | TGGGTGCTATTTGCAATCAA | TTTTGCTTCGACACTGTTGC | 60.1 | 245 | INTRON | Ca_26762 | | Trihelix | Transposase, Ptta/En/Spm, plant |
| CaPOPI_6230 | scaffold1309 | 58470 | CTTTT/CTTTTT | TGGGTAACGTAACCTACGGC | ATCCGGTAAAAATCTCCGCT | 59.9 | 951 | DRR | Ca_26763 | | | |
| CaPOPI_6231 | scaffold1309 | 88698 | TAAAAAAAA/TAAAAAAAAA | AGCAATTGCAGCTTGTGATG | AAAGCGTTGTGGAATCGAAC | 60.0 | 931 | INTRON | Ca_26765 | | Nin-like | |
| CaPOPI_6232 | scaffold1312 | 70692 | GATATATATATATATATATATATATATATAT/GATATATATATATATATATATATAT | CTTACGCAGTGGCTGGTACA | AACTCACCCTTCGTGGTTTG | 59.9 | 714 | INTERGENIC | | | | |
| CaPOPI_6233 | scaffold1312 | 78520 | TA/TAA | GCATTAAATGTGGAACAAGCA | TTACCTGTGTGGGACAACGA | 58.7 | 526 | INTERGENIC | | | | |
| CaPOPI_6234 | scaffold1312 | 78648 | TAAA/TAAAA | GCATTAAATGTGGAACAAGCA | TTACCTGTGTGGGACAACGA | 58.7 | 526 | INTERGENIC | | | | |
| CaPOPI_6235 | scaffold1312 | 78828 | GAAAAAA/GAAAAAAA | GCATTAAATGTGGAACAAGCA | TTACCTGTGTGGGACAACGA | 58.7 | 526 | INTERGENIC | | | | |

| INDEL marker IDs | Chromosomes/ unanchored scaffolds | Physical positions (bp) | InDels (*Kabuli* reference genome-CDC Frontier/PI) | Forward primers (5'-3') | Reverse primers (5'-3') | Anneling temperature (0C) | Expected amplified product size (bp) | Structural annotation | | Functional annotation | | |
|---|---|---|---|---|---|---|---|---|---|---|---|---|
| | | | | | | | | Sequence components of *kabuli* genome | *Kabuli* gene accession IDs | NCBI-KOG | TFs | NCBI-nr database |
| CaPOPI_6236 | scaffold1312 | 78884 | TAAGAAGAAGAAGAAGAA/TAAGAAGAAGAAGAA | ATTTGACCTCGACACAACGG | GCCGCTTGATCGTTAATTGT | 61.0 | 425 | INTERGENIC | | | | |
| CaPOPI_6237 | scaffold1312 | 78931 | CG/C | TCGACACAACGGACAAGAGT | GCCGCTTGATCGTTAATTGT | 59.3 | 417 | INTERGENIC | | | | |
| CaPOPI_6238 | scaffold1312 | 80040 | GAAAAAAAAA/GAAAAAAAAAA | GCATTGGCCTTTTATTCCAA | TGGCCATGTTACAACCTGAA | 59.9 | 639 | INTERGENIC | | | | |
| CaPOPI_6239 | scaffold1312 | 80554 | ATTT/ATTTTTT | GCCAAGGTAAGGGTGAGACA | ATGAATTGTCCCAATCTCGG | 60.1 | 322 | INTERGENIC | | | | |
| CaPOPI_6240 | scaffold1312 | 81111 | TAA/TAAA | CCGAGATTGGGACAATTCAT | TTGGAAAAGCCCACCTACAC | 59.8 | 661 | INTERGENIC | | | | |
| CaPOPI_6241 | scaffold1312 | 82340 | CAAA/CAAAAA | GTGGGCCAAAAGAAACTTGA | AACTGAGATTGATCCGGTGG | 60.1 | 455 | INTERGENIC | | | | |
| CaPOPI_6242 | scaffold1312 | 83212 | T/TC | TCTAGCGGATGCATGATGAG | ATACTTGAACGGTTGGCTGG | 59.9 | 461 | INTERGENIC | | | | |
| CaPOPI_6243 | scaffold1312 | 89732 | ATTTTTTT/ATTTTTTTT | TTCATTACGCCATTGACACTTT | ACAATGTGTGCCCAACAAAA | 59.5 | 457 | INTERGENIC | | | | |
| CaPOPI_6244 | scaffold1313 | 102532 | AATATA/AATA | GCTGTCTTTTCTGCTTCGCT | CACCAAATGCGTTGTAGCAC | 59.9 | 396 | INTERGENIC | | | | |
| CaPOPI_6245 | scaffold1313 | 155204 | GTCAAACTCTAGCGAGATCAA/GTCAAACTCTAGCGAGATCAAACTCTAGCGAGATCAA | AGCGTCACAGCCTATCCATC | TGCATAATCGATTGGCACAT | 60.2 | 385 | INTERGENIC | | | | |
| CaPOPI_6246 | scaffold1313 | 180679 | ATTTTTTTTTT/ATTTTTTTTT | TCAGGGTTAGGTCATCAGGG | AGGGAGGCAAAATTCGCTAT | 59.9 | 554 | INTERGENIC | | | | |
| CaPOPI_6247 | scaffold1315 | 121793 | TG/TGG | TACGTGTTAACGTGGTCGGA | TCCATTGATCCGCCTTCTAC | 60.0 | 312 | INTERGENIC | | | | |
| CaPOPI_6248 | scaffold1315 | 172506 | TTATATATATATATATAT/TTATATATATATATATAT | TTTCCCTTTTTGTTCGGATG | CATGGTTTGAAAAGTGGTTCG | 59.9 | 528 | URR | Ca_26616 | R | | Sugar/inositol transporter |
| CaPOPI_6249 | scaffold1316 | 4181 | TA/TAA | GTAAAGGGTCACCAACCGAA | TTGTCCGTGAGAGACTGCAC | 59.8 | 813 | INTERGENIC | | | | |
| CaPOPI_6250 | scaffold1316 | 13036 | AATTTATTTATTTATTTATTTATT/AATTTATTTATTTATTTATTTATTTATT | ACATGCACAGAGAATGCCAA | GTGCCAGTCGATTGCCTAAT | 60.3 | 631 | DRR | Ca_28021 | | | |

| INDEL marker IDs | Chromosomes/ unanchored scaffolds | Physical positions (bp) | InDels (*Kabuli* reference genome-CDC Frontier/PI) | Forward primers (5'-3') | Reverse primers (5'-3') | Anneling temperature (0C) | Expected amplified product size (bp) | Structural annotation | | Functional annotation | | |
|---|---|---|---|---|---|---|---|---|---|---|---|---|
| | | | | | | | | Sequence components of *kabuli* genome | *Kabuli* gene accession IDs | NCBI-KOG | TFs | NCBI-nr database |
| CaPOPI_6251 | scaffold1317 | 46249 | AC/A | CATGGGTGAAGACTGTGGTG | ACCACTGTTGCATGCCTAAA | 60.0 | 134 | INTERGENIC | | | | |
| CaPOPI_6252 | scaffold1317 | 59870 | AAAGAG/AAAGAGAAAGCAAGAG | AAAATCAGCCAAATCCAACG | TTGGAAAATCTCATTAAGCCAGA | 59.9 | 930 | INTERGENIC | | | | |
| CaPOPI_6253 | scaffold1317 | 63682 | AAGAGAGAGAGGGAGAGAGAGAGAGAGAGA/AAGAGAGAGAGAGGGAGAGAGAGAGAGAGAGA | TTTCAAGCTCAAACTTCCCC | CATGTGTTTGGATGAAGGGTT | 59.3 | 364 | INTERGENIC | | | | |
| CaPOPI_6254 | scaffold1317 | 69339 | AC/A | TGGGCTTATAGGATTGGTCG | TGCGAAAGTCACATCCACAT | 59.9 | 668 | INTERGENIC | | | | |
| CaPOPI_6255 | scaffold132 | 44427 | TA/TAGAA | CATCGTCACGTCTTCCATTC | TGTTTACACACTTTAAGATGAAGAGTT | 59.1 | 427 | INTERGENIC | | | | |
| CaPOPI_6256 | scaffold1323 | 6638 | ATTT/ATTTT | GGCCACTGACTTGCAGTGTA | TTGTGCATCCAATACGTGTG | 59.9 | 717 | INTERGENIC | | | | |
| CaPOPI_6257 | scaffold1324 | 8519 | ATTTT/ATTT | ATTTGTTGCATTGTCGGTCA | CTGCCTCACGATTTCACTCA | 60.0 | 899 | INTERGENIC | | | | |
| CaPOPI_6258 | scaffold1324 | 49560 | AT/ATT | ATTTTTCGGTTTGGAAGCAA | CGATCAAGATTTCGTTCCTG | 59.6 | 602 | DRR | Ca_19753 | P | | Heavy metal transport/detoxification protein |
| CaPOPI_6259 | scaffold1324 | 53855 | GATATATATATATATATATATATA/GATATATATATATATATATA | TAGCTTCGCAAGCTCAACAA | ACGCTTTTCCACAAAGCACT | 59.9 | 655 | INTERGENIC | | | | |
| CaPOPI_6260 | scaffold1324 | 108053 | AATATATATATATATATATATATATA/AATATATATATATATATATATATA | TAGACGCATGAACACCTTGC | ACCCGATTTTTACCCGTACC | 59.9 | 249 | INTERGENIC | | | | |
| CaPOPI_6261 | scaffold1324 | 215253 | ATCTTCTTCTTCTT/ATCTTCTTCTTCTTCTT | CCAAATTCAAATTGCAAGACC | CAATCGCCTTTTTATTGGGA | 59.4 | 219 | INTERGENIC | | | | |
| CaPOPI_6262 | scaffold1324 | 227334 | TG/T | TTGGTGACATTTAGCATTTCTCA | GTCCTTTGTGTCGTGGGTCT | 59.6 | 511 | INTERGENIC | | | | |
| CaPOPI_6263 | scaffold1324 | 235091 | CGTTGT/CGT | TCTTGGTTGTAACCCTTGGC | TTTCGACAAAAGGTTCTCCG | 60.0 | 431 | INTERGENIC | | | | |
| CaPOPI_6264 | scaffold1324 | 236638 | GAAAAA/GAAAA | ATGCACCTTGAAAGGGTTTG | TTCGAGGAGGGTTTTGATTG | 60.0 | 597 | INTERGENIC | | | | |
| CaPOPI_6265 | scaffold1324 | 244504 | CATATATATATATATATATATATATATATATATAT/CACACACACATATATATATATATATATATATATATATATATATAT | TTGTTTTCTAACCCGCCAAG | CGATTGAGTTGCAACGAGAG | 60.1 | 370 | INTERGENIC | | | | |

| INDEL marker IDs | Chromosomes/ unanchored scaffolds | Physical positions (bp) | InDels (*Kabuli* reference genome-CDC Frontier/PI) | Forward primers (5'-3') | Reverse primers (5'-3') | Anneling temperature (0C) | Expected amplified product size (bp) | Structural annotation | | Functional annotation | | |
|---|---|---|---|---|---|---|---|---|---|---|---|---|
| | | | | | | | | Sequence components of *kabuli* genome | *Kabuli* gene accession IDs | NCBI-KOG | TFs | NCBI-nr database |
| CaPOPI_6266 | scaffold1324 | 252648 | T/TTCTTTCAAC | CCTATAGGCCCACTAAGCCC | TTGCAAGCAAACTTCACGAT | 59.9 | 587 | INTERGENIC | | | | |
| CaPOPI_6267 | scaffold1324 | 252816 | CGTGAAGT/CGT | TGTTAACATGATCAGGCCCA | ACATAGCGCCACAACATGAA | 59.9 | 789 | INTERGENIC | | | | |
| CaPOPI_6268 | scaffold1324 | 253776 | ATTTTTTTTTTTT/ATTTTTTTTTT | GAGTGCTCCTTGCACATGAA | ATGAAGACCAAGCCATCCAC | 60.0 | 514 | INTERGENIC | | | | |
| CaPOPI_6269 | scaffold1324 | 255649 | CTTTTTTTTTTTT/CTTTTTTTTT | CCTTGGAGGCAGTGATTTGT | GAGCTACCCCAATGGTTGAA | 60.1 | 613 | INTERGENIC | | | | |
| CaPOPI_6270 | scaffold1324 | 255844 | GAAAAAA/GAAAAAAA | CCTTGGAGGCAGTGATTTGT | GAGCTACCCCAATGGTTGAA | 60.1 | 613 | INTERGENIC | | | | |
| CaPOPI_6271 | scaffold1324 | 255890 | ATTTTT/ATTTT | CCTTGGAGGCAGTGATTTGT | GAGCTACCCCAATGGTTGAA | 60.1 | 613 | INTERGENIC | | | | |
| CaPOPI_6272 | scaffold1324 | 256337 | CAAAA/CAAA | TTCAACCATTGGGGTAGCTC | TTCACTTGAATATTTTGTGGCA | 59.9 | 474 | INTERGENIC | | | | |
| CaPOPI_6273 | scaffold1324 | 266713 | AATCTATTATCTATTATCTATTAT/AATCTATTATCTATTAT | TGTATGCATCAACAATGTCAAGA | TCAGAGGATGTGGCATAGAGG | 59.1 | 160 | INTERGENIC | | | | |
| CaPOPI_6274 | scaffold1324 | 266796 | CA/CAA | TCCTCTATGCCACATCCTCTG | CACAAATAGTTGTACATTGGTTGC | 60.2 | 238 | INTERGENIC | | | | |
| CaPOPI_6275 | scaffold1324 | 267973 | A/AT | CCAAGGCACACACAAATGAA | AGAGAATAACCCGCACGTTG | 60.6 | 344 | INTERGENIC | | | | |
| CaPOPI_6276 | scaffold1324 | 268436 | A/AGTTCG | CAACGTGCGGGTTATTCTCT | GGCACGTGAAGTCCACTTTT | 60.1 | 358 | INTERGENIC | | | | |
| CaPOPI_6277 | scaffold1324 | 270833 | GTT/GT | ACAAAATCGGCCACTGAATC | TCCCGTTCATCTCTCTCACC | 59.9 | 500 | INTERGENIC | | | | |
| CaPOPI_6278 | scaffold1324 | 272116 | TGGGG/TGGG | CCCCGCTCTGATAGGTGATA | TGTTTGTCATGCGAGCATTT | 60.1 | 767 | INTERGENIC | | | | |
| CaPOPI_6279 | scaffold1324 | 504029 | AATATATATATATATATATATAT/AATATATATATATATATATAT | TCCATTCGAGTTTCTCCACC | CATCCGCCTCGGAAGTAATA | 60.1 | 249 | INTERGENIC | | | | |
| CaPOPI_6280 | scaffold1324 | 514795 | TTATATATATATATATATATATATA/TTATATATATATATATATATATATATATA | ATCAGCGAGGATGCAGAACT | GATGACATGGATGTGTTCGAG | 60.0 | 557 | INTERGENIC | | | | |

| INDEL marker IDs | Chromosomes/ unanchored scaffolds | Physical positions (bp) | InDels (*Kabuli* reference genome-CDC Frontier/PI) | Forward primers (5'-3') | Reverse primers (5'-3') | Anneling temperature (0C) | Expected amplified product size (bp) | Structural annotation | | Functional annotation | | |
|---|---|---|---|---|---|---|---|---|---|---|---|---|
| | | | | | | | | Sequence components of *kabuli* genome | *Kabuli* gene accession IDs | NCBI-KOG | TFs | NCBI-nr database |
| CaPOPI_6281 | scaffold1324 | 516063 | ATTTTTTTTTTT/ATTTTTTTTTT | AAAACACGCTAGGCTCCAAT | TGGTCATGTGGTTTTTGCAT | 58.9 | 642 | INTERGENIC | | | | |
| CaPOPI_6282 | scaffold1324 | 520260 | AATTATTATTATTATTATTATTATTATTATTATTATT/AATTATTATTATTATTATTATTATTATTATTATTATT | TTCTTTTCGCTATTTTATTTTCGTG | TCTTACGTATCTCCCGGTGC | 60.0 | 734 | INTERGENIC | | | | |
| CaPOPI_6283 | scaffold1324 | 642408 | ATTTTTT/ATTTTTTT | CCTTGCCCTTTTATTTCGGT | TCCTAGAACATGCATCACGC | 60.3 | 496 | INTERGENIC | | | | |
| CaPOPI_6284 | scaffold1324 | 698355 | GTTTTATTTTTATTTTT/GTTTTATTTTTATTTTTATTTTT | TTTCACATACGCCGCATAGA | TAAGCAGTCGGTTTGCAATG | 60.2 | 311 | DRR | Ca_19785 | T | | Peptidase S54, rhomboid |
| CaPOPI_6285 | scaffold1328 | 11774 | C/CCT | GGTTTCGAGGAAACTGGACA | AGGGAGGACGAAAATGACCT | 60.1 | 958 | INTERGENIC | | | | |
| CaPOPI_6286 | scaffold1331 | 50640 | GT/GTT | AATGGGCGCTGTTAAATGTC | TGGTGTCGTTGGCACTCTAC | 60.0 | 411 | INTERGENIC | | | | |
| CaPOPI_6287 | scaffold1331 | 50773 | AAT/AATAGGAT | AATGGGCGCTGTTAAATGTC | TGGTGTCGTTGGCACTCTAC | 60.0 | 411 | INTERGENIC | | | | |
| CaPOPI_6288 | scaffold1331 | 83953 | AG/AGG | TCCTTTCACTATAAACTCTTCCGC | AGCGGTTCGTAGGATATTGA | 60.1 | 471 | INTERGENIC | | | | |
| CaPOPI_6289 | scaffold1332 | 500 | TAAAAAAAA/TAAAAAAAAA | CCAAAATTTGAAGTCGGGAG | ACACGGGTCGTACAACTTGA | 59.5 | 443 | INTERGENIC | | | | |
| CaPOPI_6290 | scaffold1332 | 8212 | CTTTTTTTT/CTTTTTTTTTT | ACGAACTCAACTACAACTCTTGG | TCAGACACTACCCGACATCAA | 57.6 | 624 | INTERGENIC | | | | |
| CaPOPI_6291 | scaffold1332 | 8473 | ATTTTTTT/ATTTTTTTT | CACAATTTCTTTTCTATGTGTGGA | AACCCGTCACACAGGAAGTC | 58.2 | 894 | INTERGENIC | | | | |
| CaPOPI_6292 | scaffold1332 | 9986 | ATGTTGTTGT/ATGTTGT | CCAATTCTCCGGTACTCCAA | GACGATTTCTCCACCCTTGA | 59.9 | 808 | INTERGENIC | | | | |
| CaPOPI_6293 | scaffold1335 | 46272 | TC/T | TTCGTGCAACTGACGCTAAC | CCATTTGGATTTAAAAGAAGCC | 60.1 | 345 | DRR | Ca_28106 | S | | |
| CaPOPI_6294 | scaffold1337 | 40902 | ATTT/ATTTTCAACTTTT | TAGCATGCAAATCAGGTTGC | CCCGACAAAAATAGACCGAA | 59.8 | 425 | INTERGENIC | | | | |
| CaPOPI_6295 | scaffold1337 | 46228 | AAAACCCTAAACCCTAAA/AAAACCCTAAACCCTAAACCCTAAA | AAACCCCAAACCTTAAACCC | TTGGGACATTTCGGTTTTTG | 59.1 | 353 | INTERGENIC | | | | |

| INDEL marker IDs | Chromosomes/ unanchored scaffolds | Physical positions (bp) | InDels (*Kabuli* reference genome-CDC Frontier/PI) | Forward primers (5'-3') | Reverse primers (5'-3') | Anneling temperature (0C) | Expected amplified product size (bp) | Structural annotation | | Functional annotation | | |
|---|---|---|---|---|---|---|---|---|---|---|---|---|
| | | | | | | | | Sequence components of *kabuli* genome | *Kabuli* gene accession IDs | NCBI-KOG | TFs | NCBI-nr database |
| CaPOPI_6296 | scaffold134 | 60134 | AATATATATATATATATATATATAT/AATATATATATATATATATATATAT | TGCACATTGGAGTTTGAGATG | TCTAAGGCCTAACCAACGGA | 59.7 | 541 | INTRON | Ca_22578 | O | C2H2 | Peptidase A1 |
| CaPOPI_6297 | scaffold134 | 276694 | GAA/GA | GAGCAGCACATTTTTGCTCA | TTGATTTGGGGTTGTACCCA | 60.1 | 567 | DRR | Ca_22585 | | ERF | Pathogenesis-related transcriptional factor/ERF, DNA-binding |
| CaPOPI_6298 | scaffold134 | 357959 | CAGCAGGAGCAGGA/CAGCAGGA | GGAGATCAACAGCAGCAACA | CTTCCTGAACAAACCCCAAA | 60.0 | 805 | CDS (large-effect mutations) | Ca_22591 | | | |
| CaPOPI_6299 | scaffold135 | 113512 | A/AG | GCTTATTGGGAGAAGCGTTG | GCCCTCAGATGAGAGTCAGG | 59.8 | 514 | INTERGENIC | | | | |
| CaPOPI_6300 | scaffold1351 | 275138 | TTTTATTTATTTATTT/TTTTATTT | TCATCGCAGGTGTACCATGT | GAACAAGTGGGTGAAGGCAT | 60.0 | 544 | INTERGENIC | | | | |
| CaPOPI_6301 | scaffold1358 | 778 | AC/A | TCCACAAGAAAACCTCCATTG | AGTGAAACCAACGCCAAAAC | 60.0 | 522 | INTERGENIC | | | | |
| CaPOPI_6302 | scaffold1359 | 74591 | ATTTTTTTT/ATTTTTTTTT | TGGGTGGGAGTAATTTTTCG | TTAAAGAGATTCGCTGCGGT | 59.8 | 297 | DRR | Ca_26683 | KR | G2-like | Mitochodrial transcription termination factor-related |
| CaPOPI_6303 | scaffold136 | 12993 | CTTTTTTTTT/CTTTTTTTTTT | TTTGGAAGTCACCGGAAAAC | CACGATACGAACCATTTCCC | 59.9 | 165 | INTERGENIC | | | | |
| CaPOPI_6304 | scaffold1360 | 17330 | AAATAAT/AAAT | CGCCGTAAATTGAAACTATGC | AACCGCTGCGAATCTCTTTA | 59.6 | 729 | INTERGENIC | | | | |
| CaPOPI_6305 | scaffold1365 | 59505 | G/GTTTGAAAACAAAGGAT | ACGTGGTGAAATTCTTTCGG | ATTTTCACCAAACATCCCCA | 60.0 | 480 | INTERGENIC | | | | |
| CaPOPI_6306 | scaffold1365 | 74375 | CATG/CATGATG | TATTGTGAAGAGGCGTGTGC | TGTTGGGATTTGGTTGTGAA | 59.9 | 351 | INTERGENIC | | | | |
| CaPOPI_6307 | scaffold1371 | 80775 | TTATATATATATATATATATATAT/TTATATATATATATATATATATAT | TGTCGCACGGATACAATGAT | AGGCATGGTGTTGGAGTCAT | 60.0 | 234 | INTERGENIC | | | | |
| CaPOPI_6308 | scaffold1376 | 39940 | TNNNNNNNNNNNNN/TN | CCTGAATCGGTCAACCTTGT | GGGTTTTGCATCTTGCATCT | 60.0 | 612 | INTERGENIC | | | | |
| CaPOPI_6309 | scaffold1380 | 5644 | ATTTTTTTT/ATTTTTTTTT | TTTTAGCGGATGTCGTAGGG | TCACAAGGGGGACTCTATGG | 60.1 | 719 | INTERGENIC | | | | |
| CaPOPI_6310 | scaffold1380 | 9721 | CTGTGTGT/CTGTGT | CGTTGACAACTGAACCAGGA | GCACATTCACTGCCAAGAGA | 59.7 | 429 | INTERGENIC | | | | |

| INDEL marker IDs | Chromosomes/ unanchored scaffolds | Physical positions (bp) | InDels (*Kabuli* reference genome-CDC Frontier/PI) | Forward primers (5'-3') | Reverse primers (5'-3') | Anneling temperature (0C) | Expected amplified product size (bp) | Structural annotation | | Functional annotation | | |
|---|---|---|---|---|---|---|---|---|---|---|---|---|
| | | | | | | | | Sequence components of *kabuli* genome | *Kabuli* gene accession IDs | NCBI-KOG | TFs | NCBI-nr database |
| CaPOPI_6311 | scaffold1382 | 29087 | TAAA/TAAAAA | TGCTTTTGTCGTATAATTGAGCA | TGATGGACCAGTAGACCACCT | 59.8 | 317 | INTERGENIC | | | | |
| CaPOPI_6312 | scaffold1382 | 183210 | GTTTTTTT/GTTTTTT | CAAAAGGTTTGAAAGGCATCA | GGATATGGCACAAGCGAACT | 60.1 | 343 | INTERGENIC | | | | |
| CaPOPI_6313 | scaffold1382 | 225615 | GTTTTT/GTTTTTT | AGGTCATTTTCGTCCTCCCT | CCCCCTAGGGTGTAAATGGT | 59.9 | 805 | INTERGENIC | | | | |
| CaPOPI_6314 | scaffold1382 | 339331 | CCTT/CCTTCTT | TCAACCCACTTTCACCATCA | CAGGTCCTGAATTGCCGTAT | 59.9 | 712 | INTERGENIC | | | | |
| CaPOPI_6315 | scaffold1386 | 8928 | ATTTTTTT/ATTTTTTTT | GTGGTGCGCTACATTGGATT | GTGCAAACGACACCTGAAGA | 60.9 | 558 | INTERGENIC | | | | |
| CaPOPI_6316 | scaffold1386 | 9543 | TAAAAAAAA/TAAAAAAAAA | TCCATTGAATTGTTTGTGCAT | ATAAATTACACCCGCGCAAG | 58.9 | 421 | INTERGENIC | | | | |
| CaPOPI_6317 | scaffold1386 | 13609 | CAAAA/CAAAAA | TTTGTGGTAATTTATGGTTGCTACA | TTTCCGTTTCCTTTGTTTGC | 59.7 | 424 | INTERGENIC | | | | |
| CaPOPI_6318 | scaffold1386 | 59518 | TAAAAAAA/TAAAAAA | GCAAACAGGACCATTACGCT | GAGAGGATTGAGGGAGATTTGA | 60.1 | 909 | INTERGENIC | | | | |
| CaPOPI_6319 | scaffold140 | 2406 | CATATATATATATATATATATATATATATATAT/CATATATATATATATATATATATAT | CTTGTAAAATCTCCGCTCGC | TTGGTATTGAACTCGGAGGC | 60.0 | 665 | INTERGENIC | | | | |
| CaPOPI_6320 | scaffold140 | 7449 | ATT/AT | TGTCAATTTTGGCGTAGTGG | AACTGTCATTTTCGGTTGGC | 59.6 | 362 | INTERGENIC | | | | |
| CaPOPI_6321 | scaffold140 | 25774 | AATTT/A | TCCTAGAACATGCATCACGC | CTCAAGTCGACAAGCCTTCC | 59.8 | 666 | INTERGENIC | | | | |
| CaPOPI_6322 | scaffold1401 | 26858 | CATATATATATATATATATAT/CATATATATATATATAT | CATTGCAGGTGCTATGGTTG | TTCTCTTACGAAGCGGAGGA | 60.1 | 648 | INTERGENIC | | | | |
| CaPOPI_6323 | scaffold1408 | 24952 | GTTTTTT/GTTTTT | TGTGCGTCCAATAGGTGTGT | GACACGAATAAGCCAAGGGA | 60.0 | 478 | INTERGENIC | | | | |
| CaPOPI_6324 | scaffold1408 | 26856 | TC/T | AACCAATGTTGTGCATCCAA | AGGGAGGACGAAAATGACCT | 59.8 | 571 | INTERGENIC | | | | |
| CaPOPI_6325 | scaffold1408 | 92293 | GTTTTT/GTTTTTT | AGGTCATTTTCGTCCTCCCT | TAATTGGGCAAGAATGAGCC | 59.9 | 640 | INTERGENIC | | | | |

| INDEL marker IDs | Chromosomes/ unanchored scaffolds | Physical positions (bp) | InDels (*Kabuli* reference genome-CDC Frontier/PI) | Forward primers (5'-3') | Reverse primers (5'-3') | Anneling temperature (0C) | Expected amplified product size (bp) | Structural annotation | | Functional annotation | | |
|---|---|---|---|---|---|---|---|---|---|---|---|---|
| | | | | | | | | Sequence components of *kabuli* genome | *Kabuli* gene accession IDs | NCBI-KOG | TFs | NCBI-nr database |
| CaPOPI_6326 | scaffold1408 | 94374 | G/GT | AGGTCATTTTCGTCCTCCCT | TGGGTGCACAAGATTGGTTA | 59.9 | 467 | INTERGENIC | | | | |
| CaPOPI_6327 | scaffold1409 | 9222 | AAATAATAATAATAATAA/AAATAATAATAATAATAATAATAATAA | GCTTAAACCCATTGGTCCCT | GGAAGATGTGACGATGCCTT | 60.2 | 516 | INTERGENIC | | | | |
| CaPOPI_6328 | scaffold1409 | 12185 | TCC/TCCC | TGTGCATTCAATAGGCGTGT | CCAAGGGAGGATGAAAATGA | 60.1 | 259 | INTERGENIC | | | | |
| CaPOPI_6329 | scaffold1414 | 38236 | GTTATTATTAT/GTTATTAT | TGGTCCTAGATTTCATCGGG | CGTTATTAAGTACAACTACAAAACGAA | 59.9 | 871 | INTERGENIC | | | | |
| CaPOPI_6330 | scaffold1414 | 75044 | AATATATATATATATATATATATATA/AATATATATATATATATATATATATATATA | ATCGGTTGCTACGAAAATCG | GCTCCTTCCATATCCCTTCC | 60.1 | 737 | INTERGENIC | | | | |
| CaPOPI_6331 | scaffold1414 | 190587 | GAA/GAAA | TCGGATCACGGTCTGATACA | GAGGCAAATGGGACAAGGTA | 60.1 | 611 | CDS (FRAME SHIFT) | Ca_25542 | R | | Zinc finger, CCHC-type |
| CaPOPI_6332 | scaffold1417 | 23009 | CTTATTTATTTATTTATTTATTTATTTATTTATTTATT/CTTATTTATTTATTTATTTATTTATTTATTTATT | GCCCATAGGAACAAAATGGA | AACCTTCCCCTTTCTTTGGA | 59.8 | 323 | INTERGENIC | | | | |
| CaPOPI_6333 | scaffold1417 | 78392 | AATATATATATATATATATATATATA/AATATATATATATATATATATATATATATATA | TCTTATATGGACTAATCCCCCAA | TTGTGTTTGGGCTTTCATCA | 58.8 | 436 | INTERGENIC | | | | |
| CaPOPI_6334 | scaffold1417 | 149554 | AATATATATATATATATATATATAT/AATATATATATATATATATATATATAT | ATGTTTTGGATTTTGGCTCG | AGGAAGAGTGGAGTTGCGAA | 59.9 | 522 | INTERGENIC | | | | |
| CaPOPI_6335 | scaffold1418 | 5209 | TATAATAATAATAATAATAATAATAATAATAATAATAAT/TATAATAATAATAATAATAATAATAATAATAATAATAAT | CACAATTTCACGATTTTCACG | CGGCTAGGTTGACCCTTACA | 59.1 | 819 | INTERGENIC | | | | |
| CaPOPI_6336 | scaffold1418 | 13866 | AAGAGAGAGAGAGAGAGAGAGAGAGAGAGAGAGA/AAGAGAGAGAGAGAGAGAGAGAGAGAGAGAGAGA | ACTGCCGCATATCATTCTCC | AGAAGTTGGCAGTTGCGTTT | 60.1 | 605 | DRR | Ca_25260 | R | bHLH | Pentatricopeptide repeat |
| CaPOPI_6337 | scaffold1418 | 18174 | AC/A | GCATTGTATCCATCCCCATC | ACCCACGTTTAAAGGTTCCC | 60.0 | 797 | INTERGENIC | | | | |
| CaPOPI_6338 | scaffold1418 | 18332 | A/ATAT | TGAGAACATGGCCACCAATA | ACCCACGTTTAAAGGTTCCC | 59.9 | 601 | INTERGENIC | | | | |
| CaPOPI_6339 | scaffold1418 | 21418 | AG/A | GCCATAATTGCTTCTGCAGTTT | TCGCGGTGTGGTAAGTCATA | 60.6 | 235 | INTRON | Ca_25261 | QR | B3 | Oxoglutarate/iron-dependent oxygenase |
| CaPOPI_6340 | scaffold1418 | 31681 | G/GT | GTCGCAATTTTTGTCCCCTA | TTTCTTTCCGATTTGGTTCTTT | 59.9 | 733 | DRR | Ca_25262 | S | | Domain of unknown function DUF21 |

| INDEL marker IDs | Chromosomes/ unanchored scaffolds | Physical positions (bp) | InDels (*Kabuli* reference genome-CDC Frontier/PI) | Forward primers (5'-3') | Reverse primers (5'-3') | Anneling temperature (0C) | Expected amplified product size (bp) | Structural annotation | | Functional annotation | | |
|---|---|---|---|---|---|---|---|---|---|---|---|---|
| | | | | | | | | Sequence components of *kabuli* genome | *Kabuli* gene accession IDs | NCBI-KOG | TFs | NCBI-nr database |
| CaPOPI_6341 | scaffold1418 | 38513 | CTTTTATTTTTTATTT/CTTTTATTTTTTATTTTTTATTT | CTTTCCCTAAAGCCCAATCC | TTGCTCCCTTTACTCATCTGC | 59.9 | 811 | INTRON | Ca_25262 | S | | Domain of unknown function DUF21 |
| CaPOPI_6342 | scaffold1418 | 106529 | AATATATATATATATATATATATATATA/AATATATATATATATATATATATATA | AATATCGCTCGCTGACACCT | AGCAGCAGGACCTTCACAAT | 59.9 | 762 | INTERGENIC | | | | |
| CaPOPI_6343 | scaffold1419 | 10604 | ATTTTTTTTTTTT/ATTTTTTTTT | CCTCTTGCTGCAAACACAAA | AACTTTTTCCGCTGCTTGAA | 60.0 | 294 | INTERGENIC | | | | |
| CaPOPI_6344 | scaffold1419 | 22458 | AT/A | TCATGCGCATCAAATACACA | TTTCAAAATTCGTCGTTGGA | 59.7 | 265 | INTERGENIC | | | | |
| CaPOPI_6345 | scaffold1419 | 30683 | T/TG | TCCATCTCCAACAACATCCA | TAATTGCTCTTGCGGTCCTT | 59.9 | 306 | INTERGENIC | | | | |
| CaPOPI_6346 | scaffold1419 | 33257 | TTATA/TTA | TGCATTTTCTTGAAATTGATGG | AAAGGTCGTGAGCAGAGGAA | 59.9 | 595 | INTERGENIC | | | | |
| CaPOPI_6347 | scaffold1419 | 49527 | TGTAAACC/TGTAAACCGTAAACC | AACCCTTATCGCAGAACCCT | GGTATTGGGTTTGGGGTTTC | 60.0 | 609 | INTERGENIC | | | | |
| CaPOPI_6348 | scaffold1422 | 687 | TT/TTATGTCAT | AGGTCATTTTCGTCCTCCCT | CAAATGAACTTATTGGATGCACA | 59.9 | 274 | INTERGENIC | | | | |
| CaPOPI_6349 | scaffold1424 | 15042 | TC/T | CGACTCACGTTGAGGAGACA | CCCCCTTTTTGCATAAGTGA | 60.0 | 668 | INTERGENIC | | | | |
| CaPOPI_6350 | scaffold1424 | 48624 | A/AT | TAGGTCATTGTCGTCCTCCC | TGGATGCACAAGATCGGTTA | 59.9 | 272 | INTERGENIC | | | | |
| CaPOPI_6351 | scaffold1426 | 5555 | AG/A | CCTCAGATGAGAGCCAGGAC | ACACCCCGTTTTTCAAAGTG | 59.9 | 434 | INTERGENIC | | | | |
| CaPOPI_6352 | scaffold1426 | 5828 | T/TTC | AACGGGGTGTTACAATGAGC | CTGGTTTGACAATGCGTTTG | 59.9 | 623 | INTERGENIC | | | | |
| CaPOPI_6353 | scaffold1426 | 6734 | TCATCAC/TCATCACCATCAC | TTGATACTATGCCACCACGC | GCATTTTGCTTGTGGACTCA | 59.6 | 635 | INTERGENIC | | | | |
| CaPOPI_6354 | scaffold1426 | 7221 | TAAAAAA/TAAAAAAA | TGAGTCCACAAGCAAAATGC | GGTTCCTCTTGGTTTTGGGT | 59.8 | 494 | INTERGENIC | | | | |
| CaPOPI_6355 | scaffold143 | 67526 | TA/TAA | GGATTGTTGGTTCATTTGGG | TTAGGAAATCGATTGGGGTG | 60.0 | 495 | INTERGENIC | | | | |

| INDEL marker IDs | Chromosomes/ unanchored scaffolds | Physical positions (bp) | InDels (*Kabuli* reference genome-CDC Frontier/PI) | Forward primers (5'-3') | Reverse primers (5'-3') | Anneling temperature (0C) | Expected amplified product size (bp) | Structural annotation | | Functional annotation | | |
|---|---|---|---|---|---|---|---|---|---|---|---|---|
| | | | | | | | | Sequence components of *kabuli* genome | *Kabuli* gene accession IDs | NCBI-KOG | TFs | NCBI-nr database |
| CaPOPI_6356 | scaffold143 | 68109 | CATT/CATTTTAATT | CACCCCAATCGATTTCCTAA | ACATCTGTTCCCTCCGTGAC | 59.8 | 472 | INTERGENIC | | | | |
| CaPOPI_6357 | scaffold1430 | 1269 | ATTTTT/ATTTT | TCATGTGCATTTCTACCACCA | TGATTCGAGTCAAACCACCA | 60.0 | 315 | INTERGENIC | | | | |
| CaPOPI_6358 | scaffold1432 | 2456 | TAAAAAAAA/TAAAAAAA | CCATGACATGACCTTAGGGG | CAATGTAATTTGCATCATAAAGGC | 60.2 | 234 | INTERGENIC | | | | |
| CaPOPI_6359 | scaffold1432 | 59960 | TTATATATATATATATATATATATATAT/TTATATATATATATATATATATATATATAT | TGCTTCAATGGTTCAATCAAA | GAGGTGCATTAGAGATCACGG | 59.1 | 705 | INTERGENIC | | | | |
| CaPOPI_6360 | scaffold1432 | 80163 | ATTT/ATTTT | TCTTTGGATGGGTTCCATGT | TGAAAATGTTGTTGGTAGTGGC | 60.2 | 522 | INTERGENIC | | | | |
| CaPOPI_6361 | scaffold1432 | 80490 | CTTTTTTTT/CTTTTTTT | GCCACTACCAACAACATTTTCA | ATTTGTGTTTGTGGGGGAAA | 59.9 | 713 | INTERGENIC | | | | |
| CaPOPI_6362 | scaffold1432 | 80541 | AAATAATAATAATAATAATAATAAT/AAATAATAATAATAATAATAATAATAAT | GCCACTACCAACAACATTTTCA | ATTTGTGTTTGTGGGGGAAA | 59.9 | 713 | INTERGENIC | | | | |
| CaPOPI_6363 | scaffold1433 | 21153 | A/AG | TTCCGGGTCAGATTTCAAAC | GGTCCTCGATTTCATCTGGA | 59.9 | 417 | INTERGENIC | | | | |
| CaPOPI_6364 | scaffold1433 | 21769 | AGCATTATTTTC/A | TCCAGATGAAATCGAGGACC | TCATATGTACGTTTCGGGCA | 60.0 | 498 | INTERGENIC | | | | |
| CaPOPI_6365 | scaffold1433 | 21950 | TAAAAAA/TAAAAA | GATGAAATGGAGGACCGAGA | TCATATGTACGTTTCGGGCA | 60.0 | 332 | INTERGENIC | | | | |
| CaPOPI_6366 | scaffold1433 | 33630 | CAAAA/CAAAAA | AGGCGATTTTCGTACCACAC | CGTGTCATTTATGTCGGGTG | 60.0 | 112 | INTERGENIC | | | | |
| CaPOPI_6367 | scaffold1439 | 105314 | GAAAAGAAAAAAAGAAA/GAAAAGAAAAAAAGAAAAAAAGAAA | AGACCACCATCAGAAGCCAC | TGAGAGGCACGAACGAAAAT | 60.1 | 643 | INTERGENIC | | | | |
| CaPOPI_6368 | scaffold1446 | 21277 | TATATA/TATATACATATA | GGCGTTGGAGTCATGTTTTT | TTCTCAACTGACGCATGAGG | 60.0 | 362 | INTERGENIC | | | | |
| CaPOPI_6369 | scaffold1448 | 11427 | CAAA/CAA | AATCCAATGACGCAAACACA | AGGTCATTTTCGTCCTCCCT | 60.0 | 792 | INTERGENIC | | | | |
| CaPOPI_6370 | scaffold1448 | 34387 | CAAAAAAAAAA/CAAAAAAAAAAA | CACACATTCACCTAACCCCC | TTCAAAGTTGTGTTGTCTGCG | 60.1 | 867 | INTERGENIC | | | | |

| **INDEL marker IDs** | **Chromosomes/ unanchored scaffolds** | **Physical positions (bp)** | **InDels (*Kabuli* reference genome-CDC Frontier/PI)** | **Forward primers (5'-3')** | **Reverse primers (5'-3')** | **Anneling temperature (0C)** | **Expected amplified product size (bp)** | **Structural annotation** | | **Functional annotation** | | |
|---|---|---|---|---|---|---|---|---|---|---|---|---|
| | | | | | | | | **Sequence components of *kabuli* genome** | ***Kabuli* gene accession IDs** | **NCBI-KOG** | **TFs** | **NCBI-nr database** |
| CaPOPI_6371 | scaffold1448 | 99427 | GAATTTAATATAAATTAATTTAATATAAATT/GAATTTAATATAAATTAATTTAATATAAATTAATTTAATATAAATT | CCAGGAAATGACCCCCTATT | TTTATTTTAGCGCGGTGTCC | 60.0 | 774 | DRR | Ca_26688 | | | |
| CaPOPI_6372 | scaffold1448 | 133221 | GTTTTTTTTTTTT/GTTTTTTTTTT | AACCCACCCTCGAAAGAGTT | CGTCGGATATTGGAGGAAAA | 60.0 | 170 | INTERGENIC | | | | |
| CaPOPI_6373 | scaffold1449 | 3971 | AATATATATATATATATATATATATATAT/AATATATATATATATATATATATATAT | AGGAAGAGTGGAGTTGCGAA | CTGTAAACTGAAGCGCCCTC | 60.0 | 456 | INTERGENIC | | | | |
| CaPOPI_6374 | scaffold1452 | 1227 | GTATATATATATATATATATATAT/GTATATATATATATATATATATATAT | CGAAATCTTTGGGTTTTGGA | ACCTAAATTGGGGAGCTCGT | 59.9 | 618 | INTERGENIC | | | | |
| CaPOPI_6375 | scaffold1452 | 29608 | C/CAG | GAAGCCTTTTCAGCAGGTTG | TAGTGTGCCACCACTCCAAA | 60.0 | 648 | URR | Ca_27551 | R | ZF-HD | Pentatricopeptide repeat |
| CaPOPI_6376 | scaffold1452 | 29738 | ATTT/ATTTT | GAAGCCTTTTCAGCAGGTTG | TAGTGTGCCACCACTCCAAA | 60.0 | 648 | URR | Ca_27551 | R | ZF-HD | Pentatricopeptide repeat |
| CaPOPI_6377 | scaffold1452 | 30210 | TAAA/TAAAA | TGCCCAATTACCACAATCAA | ACCAACCCTTCATTTCCCTC | 59.8 | 465 | INTERGENIC | | | | |
| CaPOPI_6378 | scaffold1452 | 30615 | AGC/AAAAATCTTGC | TCCATTTCATGAACCCGATT | TGTACATTGTTTCCGTCCGA | 60.1 | 662 | INTERGENIC | | | | |
| CaPOPI_6379 | scaffold1455 | 12569 | TCACCTCCACCTCCACCTCCACCTCC/TCACCTCCACCTCCACCTCC | TTATAGCCGTTGGAAGTGGG | TCAGGAAGGAAGAAGGCAGA | 60.0 | 594 | INTERGENIC | | | | |
| CaPOPI_6380 | scaffold1455 | 19091 | CG/C | TATTGGGTGCACCGTTTACA | TTAGGTGGTACGGAAATCGC | 59.8 | 438 | DRR | Ca_27725 | | | |
| CaPOPI_6381 | scaffold1457 | 19983 | G/GA | TGGCTTTCAAATTCCACACA | GCGAGCAGAGATTTTTACCG | 60.1 | 468 | INTERGENIC | | | | |
| CaPOPI_6382 | scaffold146 | 9554 | GTTCTT/GTT | GGTGTGCAAGAGGACTGGAT | AAAAACGAGCCTAGCGTGAA | 60.1 | 508 | INTERGENIC | | | | |
| CaPOPI_6383 | scaffold146 | 71228 | ATATTATTATTATTATT/ATATTATTATTATT | TGAAATAAAACCATAAAACGACGA | CATTTTAATGTTTGACTGCATAATGA | 59.8 | 450 | INTERGENIC | | | | |
| CaPOPI_6384 | scaffold1462 | 12436 | TGG/TGGG | TCAGCCAAAAAGCTGATGTG | TTGTCCATGAAAGGATGATATTT | 60.0 | 380 | INTERGENIC | | | | |
| CaPOPI_6385 | scaffold1462 | 14707 | CAAA/CAAAA | TTTCGTCAAATAAATACAAATACCATT | ATCAGTGGTCCCGTGAGAAG | 58.5 | 360 | INTERGENIC | | | | |

| INDEL marker IDs | Chromosomes/ unanchored scaffolds | Physical positions (bp) | InDels (*Kabuli* reference genome-CDC Frontier/PI) | Forward primers (5'-3') | Reverse primers (5'-3') | Anneling temperature (0C) | Expected amplified product size (bp) | Structural annotation | | Functional annotation | | |
|---|---|---|---|---|---|---|---|---|---|---|---|---|
| | | | | | | | | Sequence components of *kabuli* genome | *Kabuli* gene accession IDs | NCBI-KOG | TFs | NCBI-nr database |
| CaPOPI_6386 | scaffold1465 | 3149 | AATATATATATATATATATAT/AATATATATATATATATATATATAT | AAGGCTCAACCTTACGTGGT | TTTTTAGCAATGGCACAACATT | 58.7 | 558 | INTERGENIC | | | | |
| CaPOPI_6387 | scaffold1465 | 11699 | CTTT/CTTTT | CGAGGCATTTAGCTAGCCTG | CACCCCTTACCTCGACTGAA | 60.1 | 647 | INTERGENIC | | | | |
| CaPOPI_6388 | scaffold1465 | 20135 | TTA/TTAGATA | ATAGATCCCCTCAGCCCAAC | TTTCGGTAGTCCCGTTCATC | 60.3 | 604 | INTERGENIC | | | | |
| CaPOPI_6389 | scaffold1465 | 22512 | ATTT/ATTTT | TTGCTTTGTCTTGGCAAGTG | AACCTCCCATCGAGAAGGAT | 60.0 | 735 | INTERGENIC | | | | |
| CaPOPI_6390 | scaffold1465 | 36700 | CAAAAAAAAAAAAA/CAAAAAAAAA | CTTTCGTTTCGCCTCGTTAG | CCGAAACAAACATGTCAAAAA | 60.0 | 393 | INTERGENIC | | | | |
| CaPOPI_6391 | scaffold1466 | 216791 | CTCAACCTTCAACCTT/CTCAACCTT | GTCACCCAAGCCAAACTTGT | CTTCCCCCTATCCCTACCAA | 60.0 | 810 | INTERGENIC | | | | |
| CaPOPI_6392 | scaffold1467 | 11911 | AATATATATATATATATATATATATAT/AATATATATATATATATATATATATATAT | TCGCAGCTGTGATGCTAAGT | ATGGGAGGTCTTGTGCAGTT | 59.8 | 359 | DRR | Ca_26807 | Q | GeBP | Cytochrome P450 |
| CaPOPI_6393 | scaffold1467 | 16133 | GTC/GTCAATC | ACTTTCAAACGGTCAATCCG | ACATTATGTGAGGGCGCTTC | 60.0 | 596 | INTERGENIC | | | | |
| CaPOPI_6394 | scaffold1467 | 55724 | TTTATTATTATTATTATTATTATTATTATT/TTTATTATTATTATTATTATTATTATT | TGCGATCAATATACCCCACA | CCGGAGATGCAACCATCTAT | 59.8 | 812 | INTERGENIC | | | | |
| CaPOPI_6395 | scaffold1483 | 6428 | TAAAAAAAAAAAAA/TAAAAAAAA | TAACTTGCATGCCCATTGTC | CAAAAGACACACATATTTGGCA | 59.5 | 573 | INTERGENIC | | | | |
| CaPOPI_6396 | scaffold1483 | 6873 | ATGT/ATGTGT | GCACCACGTGCGATAACA | TGTGTGAGCGTTATTGGTTTG | 60.3 | 984 | INTERGENIC | | | | |
| CaPOPI_6397 | scaffold1483 | 44049 | GAAAAAA/GAAAAA | CATCTTCAGGGGCATGATCT | TGATGGATTGGTAAAGCGTG | 60.0 | 211 | INTRON | Ca_26253 | R | | Aldo/keto reductase |
| CaPOPI_6398 | scaffold1483 | 57089 | GTGAGATTGAGATTGAGATTG/GTGAGATTGAGATTGAGATTGAGATTG | ACCCAACAAGCAACATCTCC | CATCTTCTCCATTTCTCCGC | 60.0 | 751 | INTERGENIC | | | | |
| CaPOPI_6399 | scaffold1483 | 72991 | G/GT | TCAGAATGGCGGTTCCTTAG | CATTAGTGATTGTGTGCCCG | 60.2 | 815 | URR | Ca_26256 | R | NAC | Pentatricopeptide repeat |
| CaPOPI_6400 | scaffold1488 | 9466 | CAAAAAAAAAAA/CAAAAAAAAA | TCCATTAATGCGTTGAGACTTTT | TTAGGGTTTAGGGTTTGGGG | 60.0 | 238 | INTERGENIC | | | | |

| INDEL marker IDs | Chromosomes/ unanchored scaffolds | Physical positions (bp) | InDels (*Kabuli* reference genome-CDC Frontier/PI) | Forward primers (5'-3') | Reverse primers (5'-3') | Anneling temperature (0C) | Expected amplified product size (bp) | Structural annotation | | Functional annotation | | |
|---|---|---|---|---|---|---|---|---|---|---|---|---|
| | | | | | | | | Sequence components of *kabuli* genome | *Kabuli* gene accession IDs | NCBI-KOG | TFs | NCBI-nr database |
| CaPOPI_6401 | scaffold1490 | 1256 | GAAAAA/GAAAAAA | CAAACACACCTGTTGGATGG | AGGTCATTTTCGTCCTCCCT | 59.8 | 281 | INTERGENIC | | | | |
| CaPOPI_6402 | scaffold1493 | 65515 | AC/A | AGGCAATTTGGGATTCCTCT | GGCTGCTTTGAGTTCCTTTTT | 59.9 | 372 | INTERGENIC | | | | |
| CaPOPI_6403 | scaffold1496 | 126659 | AATATATATATATATATATA/AATATATATATATATATA | TCGGGGACGAAATTCTTTTA | ACGCTTCATTTTACTGCGCT | 59.5 | 490 | INTERGENIC | | | | |
| CaPOPI_6404 | scaffold1496 | 233940 | ATTTTTT/ATTTTTTT | CATTGCCAATCATTATCCCC | TGACAACGACATCAAAGGGA | 60.0 | 620 | INTERGENIC | | | | |
| CaPOPI_6405 | scaffold1496 | 236426 | AATATATATATATATATA/AATATATATATATATA | TTCAACCTCGAATTTCAGCA | AGCGGTGGAGAGAACTCTGA | 59.4 | 764 | INTERGENIC | | | | |
| CaPOPI_6406 | scaffold1496 | 292609 | GTATA/GTA | GGCTCCATGAGTTGATTGGT | GATGTTTCCGTCGTCAAGGT | 59.9 | 460 | INTERGENIC | | | | |
| CaPOPI_6407 | scaffold1496 | 307581 | T/TA | TGGGTGCACAAGATTGGTTA | AGGTCATTTTTGCCCTCCTT | 60.0 | 467 | INTERGENIC | | | | |
| CaPOPI_6408 | scaffold1499 | 931 | TAAAAA/TAAAA | TTCTTATTCACCTGGTTCCTTTT | TTGAAGGATACATTACGAGATTCA | 58.3 | 734 | INTERGENIC | | | | |
| CaPOPI_6409 | scaffold1499 | 9817 | CTTTTTTTTT/CTTTTTTTTTT | TGCATTTTTCTGCACCTCTG | TGGTTGAGGCACTAGCCTTT | 60.0 | 214 | INTERGENIC | | | | |
| CaPOPI_6410 | scaffold1499 | 17023 | TG/TGG | GAGGTTGGTGCTTGGACAAT | AATTTTCAGCCCCCAATACC | 60.0 | 676 | INTERGENIC | | | | |
| CaPOPI_6411 | scaffold1499 | 48638 | AAAATAAATAAATAAATAAA/AAAATAAATAAATAAA | CCGGGTGTAAGGTTCCACTA | TTTGCAAAAACCAATGAATACTT | 59.8 | 284 | INTERGENIC | | | | |
| CaPOPI_6412 | scaffold1499 | 62316 | ATTTT/ATTT | ATGATTGGCTTGGTAGTGCC | ATGCCCCCAAGCTTCTTATT | 60.0 | 573 | INTERGENIC | | | | |
| CaPOPI_6413 | scaffold1499 | 74379 | ATTTTTTTTTTT/ATTTTTTTTTTTT | CTGCTCGTTGCACCATTAGA | TTTTCTGTTTGGTCCTTGGG | 60.0 | 802 | INTERGENIC | | | | |
| CaPOPI_6414 | scaffold1499 | 85779 | CAAAAAAAAAA/CAAAAAAAAAAA | TGGATCAACTAGCACCATCG | CCACCAAAACCCAAAGGTTA | 59.7 | 606 | INTERGENIC | | | | |
| CaPOPI_6415 | scaffold1499 | 112672 | CTTTTGTCAATT/CTTTTGTCAATTTTGTCAATT | CGGTTCACGTTTTTGTTACG | ACCTCCTTTCTCGTGTTTGC | 59.1 | 391 | INTERGENIC | | | | |

| **INDEL marker IDs** | **Chromosomes/ unanchored scaffolds** | **Physical positions (bp)** | **InDels (*Kabuli* reference genome-CDC Frontier/PI)** | **Forward primers (5'-3')** | **Reverse primers (5'-3')** | **Anneling temperature (0C)** | **Expected amplified product size (bp)** | **Structural annotation** | | **Functional annotation** | | |
|---|---|---|---|---|---|---|---|---|---|---|---|---|
| | | | | | | | | **Sequence components of *kabuli* genome** | ***Kabuli* gene accession IDs** | **NCBI-KOG** | **TFs** | **NCBI-nr database** |
| CaPOPI_6416 | scaffold1499 | 129736 | GTTTTTTTT/GTTTTTTTTT | CAAGTGAACGTTACAAAAACGTG | CCCTGAGTCATTCACACCCT | 59.6 | 318 | INTERGENIC | | | | |
| CaPOPI_6417 | scaffold15 | 144204 | CAA/CAAA | AGGTTTGAGGATCGGGGTAT | CAAGATTTGGGCAATCTGCT | 59.7 | 757 | INTERGENIC | | | | |
| CaPOPI_6418 | scaffold150 | 4518 | ATT/ATTT | TGGACAAGAATTAGCCCAGG | AGGTCATTTTCGTCCTCCCT | 60.1 | 436 | INTERGENIC | | | | |
| CaPOPI_6419 | scaffold1504 | 55137 | ATTTTTTTTTTT/ATTTTTTTTTT | GGAACAAGTTCCCATGAAGG | AATCCAACTTCATTCGCCAT | 59.4 | 375 | INTERGENIC | | | | |
| CaPOPI_6420 | scaffold1504 | 129111 | TTATATATATATATATATATATATATA/TTATATATATATATATATATATATATA | TGGAATGGCGACTTCCTAAA | ACTGGCCATACTCGAAAACG | 60.6 | 206 | INTRON | Ca_24092 | | Trihelix | |
| CaPOPI_6421 | scaffold1504 | 200103 | CATATATATATATATATATATA/CATATATATATATATATATATATA | TCTCCGCATAAGCTTTCGAT | CCGTACCGTTTCTGGGATTA | 59.9 | 685 | INTERGENIC | | | | |
| CaPOPI_6422 | scaffold1504 | 290680 | CATATATATATATATATATATATATATATAT/CATATATATATATATATATATATATATATATATAT | CACGTTAGCACTTTCTGGCA | AGCAAATATCCACGAGACAAA | 60.1 | 736 | INTERGENIC | | | | |
| CaPOPI_6423 | scaffold1506 | 37155 | GTATTATTATTATTATTATTATTATTATTA/GTATTATTATTATTATTATTATTATTA | TTTGTGCCTTGAAATTGCTG | ATGAAGACCAGAGGAGCGAA | 59.8 | 559 | INTERGENIC | | | | |
| CaPOPI_6424 | scaffold1508 | 60830 | T/TACA | TCACGGGTCAACACGATTTA | AGTACATGGCGACCGGATAG | 60.0 | 247 | INTERGENIC | | | | |
| CaPOPI_6425 | scaffold1508 | 85452 | TTATATATATAT/TTATATATATATAT | GTTGGTTCCCTAGCACCGTA | CACCCTGGGATTCTTGCTAC | 60.0 | 850 | INTERGENIC | | | | |
| CaPOPI_6426 | scaffold151 | 814 | ATTTTTTTTT/ATTTTTTTTTT | GGAGACTCCCATACATTGGC | TGCTATCCCACTTTGAGCCT | 59.4 | 332 | INTERGENIC | | | | |
| CaPOPI_6427 | scaffold151 | 1987 | GAAAA/GAAAAA | GGACATTTGTGAACAAGGCA | TTGGAGCTTATGTGTTCCTCAA | 59.5 | 732 | INTERGENIC | | | | |
| CaPOPI_6428 | scaffold151 | 4221 | AGG/AG | TATAAGCCGAAAATGGCAGG | ATGGCGTTTGGATTGATGAT | 60.1 | 520 | INTERGENIC | | | | |
| CaPOPI_6429 | scaffold151 | 4988 | G/GT | ATCATCAATCCAAACGCCAT | TCGAGAGCACAATCGAAGTTT | 60.2 | 385 | INTERGENIC | | | | |
| CaPOPI_6430 | scaffold151 | 7801 | CT/CGAACATTAAAAATTT | GGTGAAAACTGGGTGGAAGA | CCCTCTGCAATGTCGGTAAT | 59.9 | 277 | INTERGENIC | | | | |

| INDEL marker IDs | Chromosomes/ unanchored scaffolds | Physical positions (bp) | InDels (*Kabuli* reference genome-CDC Frontier/PI) | Forward primers (5'-3') | Reverse primers (5'-3') | Anneling temperature (0C) | Expected amplified product size (bp) | Structural annotation | | Functional annotation | | |
|---|---|---|---|---|---|---|---|---|---|---|---|---|
| | | | | | | | | Sequence components of *kabuli* genome | *Kabuli* gene accession IDs | NCBI-KOG | TFs | NCBI-nr database |
| CaPOPI_6431 | scaffold151 | 35903 | AAT/AATAT | ACACACGCATAACACACGAG | TTGGAGTGCATGTGACCATT | 58.2 | 459 | INTERGENIC | | | | |
| CaPOPI_6432 | scaffold151 | 57230 | A/ATTAGAGACCTAC | ATGCAATGCGTATATGCCAA | TTCGTGTGGATTTTGTGGAA | 59.9 | 577 | INTERGENIC | | | | |
| CaPOPI_6433 | scaffold151 | 68819 | ATATTATTATTATTATTATTATTATTATTATTATTATTATT/ATATTATTATTATTATTATTATTATTATTATTATTATTATTATT | AAATGAATGGAAGACGCGAT | AAAATTCTCTTGAATCCCCATT | 59.5 | 851 | INTERGENIC | | | | |
| CaPOPI_6434 | scaffold151 | 221306 | TCCCC/TCCCCC | TTCAAAGGAGATGGTTTGCC | ATGACAATTCCAATGCACCC | 60.1 | 738 | INTERGENIC | | | | |
| CaPOPI_6435 | scaffold151 | 294117 | ATTTTTTTTT/ATTTTTTTTTT | CATTTCTTTTTCTCGCGCAT | CCAAGTTCCCCAGAGTCAAA | 60.3 | 656 | INTERGENIC | | | | |
| CaPOPI_6436 | scaffold1511 | 36830 | ATTTTTTTTT/ATTTTTTTT | CCGAGTTGGATTGGGTATTG | TGGTCAACTTGTTTCCTCCA | 60.2 | 447 | INTERGENIC | | | | |
| CaPOPI_6437 | scaffold1511 | 110106 | TAA/TAAA | TTAATGCAGGGGTTTGTGCT | ATTGAAGCCCACTATGGCTG | 60.5 | 396 | INTERGENIC | | | | |
| CaPOPI_6438 | scaffold1511 | 135714 | GAAAAAAAAA/GAAAAAAAAAA | AGGGGCTAGCAAAGGGTAAA | GGGAGGCAACCCAATTTTAT | 60.1 | 875 | INTERGENIC | | | | |
| CaPOPI_6439 | scaffold1511 | 136948 | CAAATAAAATAAAA/CAAATAAAATAAAATAAAA | TCAAGATTTGCCCCTCAATC | AAACGCAATCCTTGAGTTCG | 60.0 | 357 | INTERGENIC | | | | |
| CaPOPI_6440 | scaffold1511 | 167354 | T/TA | GGCCTGGCCTACTTAAGGTC | GCATGTTCCTTGGCCATTAT | 60.1 | 759 | INTERGENIC | | | | |
| CaPOPI_6441 | scaffold1521 | 1157 | CA/C | TCATGTGAACAATGCAACCA | AACTAGGCGCTATACGGGGT | 59.5 | 394 | DRR | Ca_26976 | | | |
| CaPOPI_6442 | scaffold1521 | 10780 | CA/CATCGA | TCATGTGAACAATGCAACCA | CCACAGCTTGTCTGTCGGTA | 59.5 | 296 | DRR | Ca_26977 | | bHLH | Protein kinase, catalytic domain |
| CaPOPI_6443 | scaffold1521 | 15206 | T/TTTGAAATAAATTAT | GGGGTTAGGGCTCATACGAT | GTTGCAGCGTGTTGTTCTGT | 60.2 | 821 | INTERGENIC | | | | |
| CaPOPI_6444 | scaffold1521 | 15603 | GAA/GAAA | TCGTGGTTCAATAATTGGCA | CAAAATAACGATTCGGGCTC | 59.9 | 228 | INTERGENIC | | | | |
| CaPOPI_6445 | scaffold1521 | 15896 | TAAAAAAA/TAAAAAAAAA | TCGTGGTTCAATAATTGGCA | AAATTAGGCATACAGAAATGAAGGA | 59.9 | 403 | INTERGENIC | | | | |

| INDEL marker IDs | Chromosomes/ unanchored scaffolds | Physical positions (bp) | InDels (*Kabuli* reference genome-CDC Frontier/PI) | Forward primers (5'-3') | Reverse primers (5'-3') | Anneling temperature (0C) | Expected amplified product size (bp) | Structural annotation | | Functional annotation | | |
|---|---|---|---|---|---|---|---|---|---|---|---|---|
| | | | | | | | | Sequence components of *kabuli* genome | *Kabuli* gene accession IDs | NCBI-KOG | TFs | NCBI-nr database |
| CaPOPI_6446 | scaffold153 | 28056 | GAAA/GAA | AATGGCCACGAATCTGAAAG | TGTTTGAAATATGACGCGGA | 60.1 | 518 | DRR | Ca_27484 | | | |
| CaPOPI_6447 | scaffold1530 | 16153 | ACCCCC/ACCCCCC | TCTCACGGTTGTGAGGACTG | ACCGTTCTTGGTTTCTGACC | 59.9 | 746 | INTERGENIC | | | | |
| CaPOPI_6448 | scaffold1530 | 17519 | TA/T | GTGCAGTTGAATGAGCCAGA | TTGGAACCCTTCTCCAACAC | 60.0 | 508 | INTERGENIC | | | | |
| CaPOPI_6449 | scaffold1530 | 19122 | AA/AAGA | CATCGCCATTCATCTGTTTG | CTTGCTTCCATGAATCAACAA | 60.1 | 623 | INTERGENIC | | | | |
| CaPOPI_6450 | scaffold1530 | 21107 | TC/TCC | TCTAAAAGATTTTGAACCCCAAA | TCCAAGGGTGTTAGAAACGG | 59.1 | 812 | INTERGENIC | | | | |
| CaPOPI_6451 | scaffold1530 | 23128 | AATATATATATATATATATA/AATATATATATATATATATATATATATATATATA | TCTCCATTGCACACCCAATA | GGTGCTGAAATCGGAAAATC | 59.9 | 189 | INTERGENIC | | | | |
| CaPOPI_6452 | scaffold1530 | 24452 | AC/A | AGAAGGAGAATGTCACGATGG | GACGTGTGCTGAACTCTCCA | 59.2 | 261 | INTERGENIC | | | | |
| CaPOPI_6453 | scaffold1530 | 24697 | CTTTTTTTT/CTTTTTTTTTT | TGGAGAGTTCAGCACACGTC | CCTCCGGTACCATAAACCCT | 60.0 | 249 | INTERGENIC | | | | |
| CaPOPI_6454 | scaffold1530 | 25494 | TAT/TATGTCAAAT | CTTTTTCCGTTTTCAGCCAC | TTTACCCCTCGGTAAAACCTC | 59.7 | 607 | INTERGENIC | | | | |
| CaPOPI_6455 | scaffold1530 | 25850 | TTTTTTTATTTTTTATTT/TTTTTTTATTT | TCGATCTTGAGCGAATTTGA | CAATACCAATTTCACGAAAATTCA | 59.5 | 482 | INTERGENIC | | | | |
| CaPOPI_6456 | scaffold1530 | 26727 | CT/CTT | TGCTTTTGGTAAAGGTGCTT | ATTCGCAAACCAAACTCGAT | 57.6 | 742 | INTERGENIC | | | | |
| CaPOPI_6457 | scaffold1530 | 27039 | T/TCA | CGTTTTCGACATCAAGATGGTA | TCGCAAACCAAACTCGATAA | 60.0 | 134 | INTERGENIC | | | | |
| CaPOPI_6458 | scaffold1530 | 27186 | CTTATTTAT/CTTAT | TCGAGTTTGGTTTGCGAATA | CGATTGCTCATAAAATCGCTC | 59.3 | 538 | INTERGENIC | | | | |
| CaPOPI_6459 | scaffold1530 | 27815 | ATTTTTTTT/ATTTTTTTTT | TTTGAGAGGGCCTTTGGTAA | TCGACAGGTTTGTTTGTTCG | 59.7 | 405 | INTERGENIC | | | | |
| CaPOPI_6460 | scaffold1530 | 27996 | CCTATGGTG/C | TTTGAGAGGGCCTTTGGTAA | TCGACAGGTTTGTTTGTTCG | 59.7 | 405 | INTERGENIC | | | | |

| INDEL marker IDs | Chromosomes/ unanchored scaffolds | Physical positions (bp) | InDels (*Kabuli* reference genome-CDC Frontier/PI) | Forward primers (5'-3') | Reverse primers (5'-3') | Anneling temperature (0C) | Expected amplified product size (bp) | Structural annotation | | Functional annotation | | |
|---|---|---|---|---|---|---|---|---|---|---|---|---|
| | | | | | | | | Sequence components of *kabuli* genome | *Kabuli* gene accession IDs | NCBI-KOG | TFs | NCBI-nr database |
| CaPOPI_6461 | scaffold1530 | 28367 | CAATTAA/CAA | GGCCCTATGGTGTTATGGTG | TTCGAAGCCATTATGTTTGATG | 60.1 | 817 | INTERGENIC | | | | |
| CaPOPI_6462 | scaffold1530 | 28720 | ATTTTTTTTTTTT/ATTTTTTTTT | CAAATCTGAGCACACACGAGA | ATAGTGACATCGGGTTGGGA | 60.0 | 510 | INTERGENIC | | | | |
| CaPOPI_6463 | scaffold1531 | 24929 | AT/ATTCT | TCAGGATTTTGGGTTTAGGG | TTTTGTAGTTGTACTCAATAACAAGGA | 58.9 | 482 | INTERGENIC | | | | |
| CaPOPI_6464 | scaffold1531 | 27008 | AC/A | CCTGAAAAGTCCCGATGAAA | CTAACGCAGAACCTGCAACA | 60.0 | 376 | INTERGENIC | | | | |
| CaPOPI_6465 | scaffold1531 | 29443 | AG/AGGG | TCCAGATGAAATCGAGGACC | GATTTTCGTTCCACCCTTCA | 60.0 | 621 | INTERGENIC | | | | |
| CaPOPI_6466 | scaffold1532 | 69027 | TAAAAATAAAAAATAAAAA/TAAAAATAAAAAATAAAAAATAAAAA | ACCCAATCTTGGGGGATAAA | TCTTTGCTCACAACACAGCC | 60.4 | 220 | INTERGENIC | | | | |
| CaPOPI_6467 | scaffold1533 | 39114 | CTATATATATATATATATATATATATATAT/CTATATATATATATATATATATATAT | TCCGCGAGAGAGTTATAGCC | CACGAAACCACAATTTCTGC | 59.6 | 790 | INTERGENIC | | | | |
| CaPOPI_6468 | scaffold1533 | 40798 | ATATTATTATTATTATTATTATTATTATTATTATTATTATTATTATTATTATTA/ATATTATTATTATTATTATTATTATTATTATTATTATTATTATTATTATTA | GGCACGACATCATCATCATC | TTTTCACAAGGCTCAACTAATTACA | 59.9 | 575 | INTERGENIC | | | | |
| CaPOPI_6469 | scaffold1544 | 14620 | CTTT/CTT | CCACTGGATGAATCTCGGTT | TAGAAGGAACGGAGGGGTTT | 59.9 | 794 | INTERGENIC | | | | |
| CaPOPI_6470 | scaffold1545 | 95527 | ATCTACACATGT/AT | AAGGTTCACCGTGTTTTTGG | CATCAAAAGTTGCATCACCG | 59.9 | 874 | INTERGENIC | | | | |
| CaPOPI_6471 | scaffold1548 | 1087 | GCTCTCTCTCTC/GCTCTCTCTC | AAATGGCTTGGTTATGGCTG | TGTAATGCCGCAAATTCAAA | 60.0 | 432 | DRR | Ca_27706 | QI | | |
| CaPOPI_6472 | scaffold1550 | 44368 | TAAAA/TAAAAA | TGGACTTTTCGAGCGACTTT | AGTTATGACGTAGAACTATAAAACGGT | 60.0 | 381 | INTERGENIC | | | | |
| CaPOPI_6473 | scaffold1550 | 56017 | TAAAAA/TAAA | AGGCGATTTTCGTACCACAC | TCCAGATGAAATCGAGGACC | 60.0 | 322 | INTERGENIC | | | | |
| CaPOPI_6474 | scaffold1552 | 1897 | AGG/AGGG | ACCAAGAATGTCCCTGCAAC | TCTTGTGGAAGGAAGCTCGT | 60.0 | 223 | INTERGENIC | | | | |
| CaPOPI_6475 | scaffold1552 | 2208 | CCCGACGCTAA/CCCGACGCTAATCCGACGCTAA | ACGAGCTTCCTTCCACAAGA | GGGCTGTTGTTCGTCAGAAT | 60.0 | 736 | INTERGENIC | | | | |

| INDEL marker IDs | Chromosomes/ unanchored scaffolds | Physical positions (bp) | InDels (*Kabuli* reference genome-CDC Frontier/PI) | Forward primers (5'-3') | Reverse primers (5'-3') | Anneling temperature (0C) | Expected amplified product size (bp) | **Structural annotation** | | **Functional annotation** | | |
|---|---|---|---|---|---|---|---|---|---|---|---|---|
| | | | | | | | | **Sequence components of *kabuli* genome** | ***Kabuli* gene accession IDs** | **NCBI-KOG** | **TFs** | **NCBI-nr database** |
| CaPOPI_6476 | scaffold1559 | 551 | GTTTTTTT/GTTTTTTTT | AGGTGTTTTTCGTCCTCCCT | TTCGATGCACATGATTGGTT | 60.0 | 473 | INTERGENIC | | | | |
| CaPOPI_6477 | scaffold1569 | 32526 | ACC/ACCC | CAGGGCCTTAAACCATGAAA | GGTTTGGGGTTTGGGATTTA | 59.9 | 601 | INTERGENIC | | | | |
| CaPOPI_6478 | scaffold1569 | 32980 | TAA/TA | ATAAACCGAACCGCAAAACA | GGTTTTATGGTTTAACGGCCT | 60.4 | 714 | INTERGENIC | | | | |
| CaPOPI_6479 | scaffold1569 | 33746 | T/TC | AAACCCTAAACCCTAACCCC | ATGGTTTAAGGGCCTTGGTA | 58.2 | 612 | INTERGENIC | | | | |
| CaPOPI_6480 | scaffold157 | 127896 | A/AGG | TCCTGTAGATTTTCCTGCGG | GTGCAGATTTTCATGCGAGA | 60.2 | 276 | INTERGENIC | | | | |
| CaPOPI_6481 | scaffold157 | 212151 | AATATATATATATATATATATA/AATATATATATATATATATATATATA | CTTAGGGTGAGGGAAGGGAG | CATCTAAGGGCAAAACGCTC | 60.1 | 542 | INTRON | Ca_21071 | C | GATA | Mitochondrial substrate/solute carrier |
| CaPOPI_6482 | scaffold157 | 248333 | CAA/CA | TTGTATTGCCTCCATCTCAAAA | TGGAAGTTGTGGATGCAATG | 59.6 | 483 | INTERGENIC | | | | |
| CaPOPI_6483 | scaffold157 | 254639 | GTTT/GTTTTT | TGGGATGAAAAGGGAGTTTG | TTGACTCTGATGAACGTGCC | 59.9 | 605 | INTERGENIC | | | | |
| CaPOPI_6484 | scaffold157 | 255873 | GAT/GATAT | TTTGGTCTTGCCTTTGATGA | TGCGGTTAAGGTTGGTTCTC | 59.2 | 888 | INTERGENIC | | | | |
| CaPOPI_6485 | scaffold157 | 289273 | T/TATC | GCACATTCAGTGCCAAGAGA | ATTGAGGGCATTGTCACTCC | 60.0 | 492 | INTERGENIC | | | | |
| CaPOPI_6486 | scaffold157 | 308958 | A/AC | ATGTGGACATGGCACAAGAA | TTTATCTTTCGGATCACCGC | 60.0 | 677 | INTERGENIC | | | | |
| CaPOPI_6487 | scaffold157 | 335191 | GAAAAAAA/GAAAAAAAA | TTCGAAGTTTCGGTAGGGTG | GTCGAATGAAACCCCAACAC | 60.1 | 543 | INTERGENIC | | | | |
| CaPOPI_6488 | scaffold157 | 349077 | TA/T | TGCCAATTCCCCAAACTTTA | GCGGGACATAACACCTAACG | 60.3 | 369 | INTERGENIC | | | | |
| CaPOPI_6489 | scaffold157 | 355168 | GAAA/GAAAA | CTGTTGAGGTCGGGAAACAG | CTTCACTTTTGCTGATGGCA | 60.7 | 575 | INTRON | Ca_21079 | | SBP | DEP domain |
| CaPOPI_6490 | scaffold157 | 358753 | ATTTTT/ATTTT | ATTTAGATCGGCGGTTGAGA | AACGTAGCTTTTGTGCGCTA | 59.7 | 216 | DRR | Ca_21079 | | SBP | DEP domain |

| INDEL marker IDs | Chromosomes/ unanchored scaffolds | Physical positions (bp) | InDels (*Kabuli* reference genome-CDC Frontier/PI) | Forward primers (5'-3') | Reverse primers (5'-3') | Anneling temperature (0C) | Expected amplified product size (bp) | Structural annotation | | Functional annotation | | |
|---|---|---|---|---|---|---|---|---|---|---|---|---|
| | | | | | | | | Sequence components of *kabuli* genome | *Kabuli* gene accession IDs | NCBI-KOG | TFs | NCBI-nr database |
| CaPOPI_6491 | scaffold1578 | 76566 | CATATATATAT/CATATATATATAT | TCATTGTTTGTGTTTGGCGT | CTTGTTTTCTCCATGGTCGC | 60.0 | 816 | INTERGENIC | | | | |
| CaPOPI_6492 | scaffold1578 | 76623 | AGG/AG | TCATTGTTTGTGTTTGGCGT | CTTGTTTTCTCCATGGTCGC | 60.0 | 816 | INTERGENIC | | | | |
| CaPOPI_6493 | scaffold1578 | 76801 | ATCTCT/ATCTCTCT | CTGATAGTGACACCGGGTTG | CTTGTTTTCTCCATGGTCGC | 59.0 | 664 | INTERGENIC | | | | |
| CaPOPI_6494 | scaffold1578 | 77306 | T/TG | GCGACCATGGAGAAAACAAG | TTGGTTGAATCCCACACAAAT | 60.6 | 563 | INTERGENIC | | | | |
| CaPOPI_6495 | scaffold1578 | 79075 | CT/C | CCAGACCTCATAGATCCCCA | CATGTAAAATCGCCGAAATC | 59.9 | 467 | INTERGENIC | | | | |
| CaPOPI_6496 | scaffold1579 | 35194 | CAAAAAA/CAAAA | CATTTTTAATTGACAACTCAATTTTCT | TGAAATAAAACCATAAAACGAAGA | 58.3 | 240 | INTERGENIC | | | | |
| CaPOPI_6497 | scaffold1579 | 64472 | A/AT | GAGCGAATTGAGAAACGAGG | TTTTCAAATAAAGTAACATTGACAACA | 60.0 | 927 | INTERGENIC | | | | |
| CaPOPI_6498 | scaffold158 | 131634 | T/TAAC | GTGACTGGTGACTGGGGACT | TTATGCCTGCTGTTCTGCAC | 60.0 | 227 | INTRAGENIC | | | | |
| CaPOPI_6499 | scaffold158 | 132271 | TAAACGTGAGGGACAAAAC/TAAACGTGAGGGACAAAACGTGAGGGACAAAAC | GTGCAGAACAGCAGGCATAA | CGTGGCGTGACTACATTGAG | 60.0 | 753 | INTERGENIC | | | | |
| CaPOPI_6500 | scaffold1580 | 61955 | AAA/AAATAA | AAGAACGCGATAACCAAAGC | TTGGACCGATGGATCATTTT | 59.4 | 894 | INTERGENIC | | | | |
| CaPOPI_6501 | scaffold1580 | 69955 | ATTTTT/ATTTT | ATCACCAGCCACTTAGGCAA | TCCCTTATGACAATTCATGGTG | 60.7 | 639 | INTERGENIC | | | | |
| CaPOPI_6502 | scaffold1580 | 99509 | AAATAATAATAATAATAATAATAATAATAATAA/AAATAATAATAATAATAATAATAATAATAA | TACCCAGAAGTGGAGATCGG | ATCATCAATGTCGTGCCTCA | 60.1 | 709 | INTERGENIC | | | | |
| CaPOPI_6503 | scaffold1580 | 251123 | CNNNNNNNNNN/C | TGTTCTCTTAGGGGGTGGTG | GGCCATCTTTTAAACCCCTT | 60.0 | 569 | CDS (FRAME SHIFT) | Ca_20799 | B | | DEK, C-terminal |
| CaPOPI_6504 | scaffold1580 | 472285 | TC/T | TGATGCAAGAAGCCACATCT | CTTGAAAGCCATTGGAGAGC | 59.4 | 677 | INTERGENIC | | | | |
| CaPOPI_6505 | scaffold1580 | 472375 | CATA/CATATA | TTTTGGTCAGGGGAAGGTTT | CTTGAAAGCCATTGGAGAGC | 60.7 | 578 | INTERGENIC | | | | |

| **INDEL marker IDs** | **Chromosomes/ unanchored scaffolds** | **Physical positions (bp)** | **InDels (*Kabuli* reference genome-CDC Frontier/PI)** | **Forward primers (5'-3')** | **Reverse primers (5'-3')** | **Anneling temperature (0C)** | **Expected amplified product size (bp)** | **Structural annotation** | | **Functional annotation** | | |
|---|---|---|---|---|---|---|---|---|---|---|---|---|
| | | | | | | | | **Sequence components of *kabuli* genome** | ***Kabuli* gene accession IDs** | **NCBI-KOG** | **TFs** | **NCBI-nr database** |
| CaPOPI_6506 | scaffold1580 | 472426 | CT/CTT | TTTTGGTCAGGGGAAGGTTT | CTTGAAAGCCATTGGAGAGC | 60.7 | 578 | INTERGENIC | | | | |
| CaPOPI_6507 | scaffold1586 | 1838 | AC/ACC | AGGGAGGACGAAAATGACCT | TGACCAATTTTGAGCATCCA | 59.9 | 471 | INTERGENIC | | | | |
| CaPOPI_6508 | scaffold1607 | 47435 | CAA/CA | TCACCGATGTTGGATTTTGA | CGATTATCGCGATCTTGGTT | 59.9 | 806 | INTERGENIC | | | | |
| CaPOPI_6509 | scaffold1607 | 61142 | ACTCTCTCTCTCTCTCTCTCTCT/ACTCTCTCTCTCTCTCTCTCTCT | AGACCAGCAAGAAGCATTGG | CCTTAAAGCAACCGTCCAAA | 60.4 | 473 | INTERGENIC | | | | |
| CaPOPI_6510 | scaffold1607 | 117990 | CAA/CAAA | TGGGATGTTAAACTCCATACCC | ATATTGGAAACCCGTGTGGA | 59.9 | 347 | INTERGENIC | | | | |
| CaPOPI_6511 | scaffold1609 | 2922 | A/AAGTTCATTT | TGTGTGTTTGTGTCATCGGA | ATCCTTGGGAGGACGAAAAT | 59.5 | 546 | INTERGENIC | | | | |
| CaPOPI_6512 | scaffold1613 | 98233 | TAT/TATAAAGGTAAGGAT | GACGCGTAGTTTCGAGGATG | TGGCAAGGAGCAACTTTCTT | 60.8 | 651 | INTERGENIC | | | | |
| CaPOPI_6513 | scaffold1613 | 98434 | A/AG | AAGAAAGTTGCTCCTTGCCA | AGGAGCACTTACCTCCGTTG | 60.0 | 133 | INTERGENIC | | | | |
| CaPOPI_6514 | scaffold1613 | 106669 | C/CGATATCATAGCCAAG | ACAATTTACGCCACCGAAAG | AGAAGCGACCCCATAGGTTT | 60.0 | 549 | INTERGENIC | | | | |
| CaPOPI_6515 | scaffold1614 | 8293 | ATATATATATATATAT/ATATATATATATATATGTATATATATATATAT | CTTCTGATGCCCCGATTAAA | AAGATGGAGGAAATGCGATG | 60.0 | 436 | INTERGENIC | | | | |
| CaPOPI_6516 | scaffold1614 | 131511 | AATATATATATAT/AATATATATATATATAT | CCGCGAGTAATTCCGATAAA | ATCAGCCTAAGGGGGAAGAA | 60.1 | 875 | INTERGENIC | | | | |
| CaPOPI_6517 | scaffold1614 | 188751 | GTTTTTTTTT/GTTTTTTTTTTT | TGAAAGCCAATTCCACTTCC | ACTGGAAGGACAGGCTGATG | 60.1 | 703 | INTERGENIC | | | | |
| CaPOPI_6518 | scaffold1626 | 121557 | TTTATTATTATTATTATTATTATT/TTTATTATTATTATTATTATTATTATT | AGAGGCAAACAAGAACCGAA | AGCCGAGAAAATAACTCGCA | 59.9 | 311 | INTERGENIC | | | | |
| CaPOPI_6519 | scaffold1630 | 6306 | T/TA | GTACCACCCGACAAAAATGG | TCCAGATGAAATCGAGGACC | 60.1 | 530 | INTERGENIC | | | | |
| CaPOPI_6520 | scaffold1631 | 32889 | TGGTTTAAAAA/T | GTCCACTTCCGTTTCGGTAA | GTCGCTTCAAAAGTTGGCTC | 60.0 | 625 | INTERGENIC | | | | |

| INDEL marker IDs | Chromosomes/ unanchored scaffolds | Physical positions (bp) | InDels (*Kabuli* reference genome-CDC Frontier/PI) | Forward primers (5'-3') | Reverse primers (5'-3') | Anneling temperature (0C) | Expected amplified product size (bp) | Structural annotation | | Functional annotation | | |
|---|---|---|---|---|---|---|---|---|---|---|---|---|
| | | | | | | | | Sequence components of *kabuli* genome | *Kabuli* gene accession IDs | NCBI-KOG | TFs | NCBI-nr database |
| CaPOPI_6521 | scaffold1632 | 26463 | AAGTAG/AAG | ACGTTTGTTTGTCGACGGTT | ATCCAATCCACTTCTTTCCG | 60.5 | 554 | DRR | Ca_26993 | | | Domain of unknown function DUF239, plant |
| CaPOPI_6522 | scaffold1635 | 74246 | TCCC/TCCCC | GGTAAACGTGTGTTGCATGG | GGAGTACCATAGGCCAACGA | 59.9 | 258 | INTERGENIC | | | | |
| CaPOPI_6523 | scaffold1643 | 107792 | TTATATATATATATATATATATAT/TTATATATATATATATATAT | GGCGATTTTACATTTATACGTGAG | ACATATGGGTCAAACCCGTC | 59.0 | 402 | INTERGENIC | | | | |
| CaPOPI_6524 | scaffold1643 | 109664 | CTATATATATATATATATATATATATATATATATATAT/CTATATATATATATATATATATATATATATATATATATATATAT | TTATGCTTTGTGTCATGGGC | CCAACCCAAATTGAACAGGT | 59.5 | 550 | INTERGENIC | | | | |
| CaPOPI_6525 | scaffold1643 | 147473 | AAATAATAATAATAATAATAATAA/AAATAATAATAATAATAATAATAA | ATGGTGGTGCAAACTCAACA | CAAGCGTAGTGCAAGTTGTGA | 60.0 | 677 | INTERGENIC | | | | |
| CaPOPI_6526 | scaffold1643 | 152357 | GGGAAATAGAGGAAA/GGGAAA | AGGAGGATGACTCAGTGACCA | TTTGAATTTCATCCCTGCAA | 59.7 | 680 | INTERGENIC | | | | |
| CaPOPI_6527 | scaffold1643 | 163768 | AATATATATATATATATATATATATATA/AATATATATATATATATATATATA | ATTTCCAGCATGCCAATTTC | CTTCCAGAAACTAGCCGCAC | 59.9 | 719 | INTERGENIC | | | | |
| CaPOPI_6528 | scaffold1643 | 166873 | CAAAAAAAA/CAAAAAAA | GCAACTAGGGGTGTTCAGGA | ATGCACACTTGTTGCACCTT | 60.1 | 610 | INTERGENIC | | | | |
| CaPOPI_6529 | scaffold1643 | 167442 | AC/A | GCCGTTTACAGTGCTTTTTCA | AAGCAGCGCGTTTTTAGAAG | 60.3 | 123 | INTERGENIC | | | | |
| CaPOPI_6530 | scaffold1643 | 169454 | CAAAAAAAAAAA/CAAAAAAAAA | GTCATTTTTGGCGTAGTGGG | TTTCCGGTCAACACTCATCA | 60.4 | 133 | INTERGENIC | | | | |
| CaPOPI_6531 | scaffold1643 | 171022 | TTTA/TTTAATTA | TAACCCCATCAAATTTGGGA | TCTTGAAGGAGCGGGTAGAA | 60.0 | 563 | INTERGENIC | | | | |
| CaPOPI_6532 | scaffold1643 | 180974 | ATGATGAATCTTGATGAATCTTGATGAAT/ATGATGAATCTTGATGAAT | CCCACGCTTCTTTAGCAGTC | TTTCACTTTCCCGCTTCTGT | 60.0 | 859 | INTERGENIC | | | | |
| CaPOPI_6533 | scaffold1643 | 192850 | TATAAATA/TATA | GAGGGGGAGAAAGCTGAGAG | TGCTGACATGATTGTTGATGTG | 60.5 | 535 | INTERGENIC | | | | |
| CaPOPI_6534 | scaffold1643 | 192933 | CG/C | GAGGGGGAGAAAGCTGAGAG | TAGCATGCTGGAAATTGCTG | 60.5 | 604 | INTERGENIC | | | | |
| CaPOPI_6535 | scaffold1643 | 204405 | TCC/TC | TGCGAAATCGCAGTTGTTAC | ACAAATGGAGGGACGATGAA | 59.9 | 712 | INTERGENIC | | | | |

| INDEL marker IDs | Chromosomes/ unanchored scaffolds | Physical positions (bp) | InDels (*Kabuli* reference genome-CDC Frontier/PI) | Forward primers (5'-3') | Reverse primers (5'-3') | Anneling temperature (0C) | Expected amplified product size (bp) | Structural annotation | | Functional annotation | | |
|---|---|---|---|---|---|---|---|---|---|---|---|---|
| | | | | | | | | Sequence components of *kabuli* genome | *Kabuli* gene accession IDs | NCBI-KOG | TFs | NCBI-nr database |
| CaPOPI_6536 | scaffold1643 | 207973 | TAAA/TAAAA | CGAATATTGCTTTTCAAAACACC | GGCGAAAACAAGATTTCCAT | 59.9 | 327 | INTERGENIC | | | | |
| CaPOPI_6537 | scaffold1643 | 210197 | ATTTTTT/ATTTTTTT | AATACAGCGAAAAAGCTCGC | TCGAAAAGAAATCGAGACCG | 59.6 | 710 | INTERGENIC | | | | |
| CaPOPI_6538 | scaffold1643 | 210540 | ATTTTTTTT/ATTTTTTTTTT | CACACAATCACAAATTTTTCTCAA | AAAGAAATCGAGACCGTTGC | 59.1 | 419 | INTERGENIC | | | | |
| CaPOPI_6539 | scaffold1643 | 211018 | TGTA/T | TTCTCTCCACCGCTAACTCG | ATGGTGGGGGTATGAATGAA | 60.5 | 611 | INTERGENIC | | | | |
| CaPOPI_6540 | scaffold1643 | 211096 | TTCT/TT | ACGGTCTCGATTTCTTTTCG | ATGGTGGGGGTATGAATGAA | 59.3 | 585 | INTERGENIC | | | | |
| CaPOPI_6541 | scaffold1643 | 213932 | TAAAAAAAA/TATAAAAAAAAA | CGGATTTCGTCTCTTGAAGC | CATTGGTGCAGTTGTTCGTC | 60.0 | 662 | INTERGENIC | | | | |
| CaPOPI_6542 | scaffold1651 | 5134 | GAAAAAAA/GAAAAAAAAAA | ATGGCACAAGTCTCCCAACT | GGGGCCACTTAGGGTTGTAT | 59.6 | 276 | INTERGENIC | | | | |
| CaPOPI_6543 | scaffold1651 | 9845 | TAAAAAAAAAA/TAAAAAAAAA | AACGGAGGAAGAACATGTGG | TCACACAAGCATTCCCAAAA | 60.0 | 364 | DRR | Ca_27410 | | | |
| CaPOPI_6544 | scaffold1651 | 11557 | GAAAAAAAAAA/GAAAAAAAAA | TTCTAAGTTTGGGGTGGTGG | ATCATGCTCCAAGGTTGCTT | 59.8 | 426 | INTERGENIC | | | | |
| CaPOPI_6545 | scaffold1651 | 12361 | GTTTTTTT/GTTTTTTTT | AAATTGTTGCTTGGGGACAA | TGACGTCATGACCTGCATTT | 60.3 | 882 | INTERGENIC | | | | |
| CaPOPI_6546 | scaffold1651 | 13370 | TATTAATTAATTAATT/TATTAATTAATTAATTAATT | CCTTGAGTTCGACACTCGGT | TGGTGACCGTGTAATTGTTG | 60.3 | 337 | INTERGENIC | | | | |
| CaPOPI_6547 | scaffold1651 | 13586 | CAAAAAAA/CAAAAAAAA | CCTTGAGTTCGACACTCGGT | CAGGATGTGAACCGTAAATCTTC | 60.3 | 547 | INTERGENIC | | | | |
| CaPOPI_6548 | scaffold1651 | 14250 | ATATTTTAAGGTATTTTAAGGTAT/ATATTTTAAGGTAT | AAAAATGGAGAGATCGAAAGTG | TTGATGCTAGGGGGATTTTG | 57.5 | 553 | INTERGENIC | | | | |
| CaPOPI_6549 | scaffold1651 | 16309 | CATA/CATATA | TGCCAAGCATTGTTTCTCAC | TCCCTTTTACATCCCTTCCC | 59.8 | 497 | INTERGENIC | | | | |
| CaPOPI_6550 | scaffold1651 | 16500 | CA/CAAA | GGGAAGGGATGTAAAAGGGA | ACGTCGTGATTTGTGCTTCA | 60.1 | 231 | INTERGENIC | | | | |

| INDEL marker IDs | Chromosomes/ unanchored scaffolds | Physical positions (bp) | InDels (*Kabuli* reference genome-CDC Frontier/PI) | Forward primers (5'-3') | Reverse primers (5'-3') | Anneling temperature (0C) | Expected amplified product size (bp) | Structural annotation | | Functional annotation | | |
|---|---|---|---|---|---|---|---|---|---|---|---|---|
| | | | | | | | | Sequence components of *kabuli* genome | *Kabuli* gene accession IDs | NCBI-KOG | TFs | NCBI-nr database |
| CaPOPI_6551 | scaffold1651 | 16843 | GACC/GACCACC | GGGAAGGGATGTAAAAGGGA | AAAATCTGGCCTGGACCTG | 60.1 | 510 | INTERGENIC | | | | |
| CaPOPI_6552 | scaffold1651 | 16898 | TAAAA/TAAA | TAAGGCATGGTATGGGGAAA | CTAAGCCTGAGTTTGGCCTG | 60.1 | 921 | INTERGENIC | | | | |
| CaPOPI_6553 | scaffold1651 | 17729 | GATAT/GATATAT | TCTCTTGGGTGATTTTTCGG | ACACCCAAACTCTTGTTGGC | 60.0 | 561 | INTERGENIC | | | | |
| CaPOPI_6554 | scaffold1651 | 18006 | A/AG | TGATTGGGGGCATACATTTT | ACACCCAAACTCTTGTTGGC | 60.0 | 171 | INTERGENIC | | | | |
| CaPOPI_6555 | scaffold1651 | 18574 | CA/C | CTGGATTGGAATTGTCAGCA | TCACGCACACTCCATCTCTC | 59.6 | 679 | INTERGENIC | | | | |
| CaPOPI_6556 | scaffold1651 | 18823 | AG/A | GAGAGATGGAGTGTGCGTGA | AACCCAGAACTCGAGCTGAA | 60.0 | 461 | INTERGENIC | | | | |
| CaPOPI_6557 | scaffold1651 | 22832 | CA/CAAAA | GTCCAGCAAAAGTCGTGTGA | TTGAAGGCATTTCTCCATCC | 59.9 | 675 | INTERGENIC | | | | |
| CaPOPI_6558 | scaffold1659 | 96268 | GTTT/GTTTT | TTGCTCACTACCACGCTACG | CACCAAAGCACATCCATTCA | 60.1 | 382 | INTERGENIC | | | | |
| CaPOPI_6559 | scaffold1659 | 269627 | AATATATATATATATATATAT/AATATATATATATATATATATAT | CACCGTAGTGTGGTGGTGAG | CGGTGAATCCTTTACGGAGA | 60.1 | 531 | INTERGENIC | | | | |
| CaPOPI_6560 | scaffold1661 | 70196 | A/AGT | CAGCTTATGCGGAGACAAGA | AACATGGACAACAACGACGA | 59.2 | 303 | INTRON | Ca_27588 | | | |
| CaPOPI_6561 | scaffold1661 | 91058 | AATATATATATATATAT/AATATATATATATATATAT | GTTGGATCCCTAGCACCGTA | AAAAATGAAAAGGGGAGCGT | 60.0 | 905 | INTERGENIC | | | | |
| CaPOPI_6562 | scaffold1664 | 9660 | GAAAAAAAAAAAAA/GAAAAAAAAAAAA | TGGTCACCACGTGTCATTTT | ATGATTGCTTCGGATTACGG | 59.9 | 584 | INTERGENIC | | | | |
| CaPOPI_6563 | scaffold1664 | 59110 | AAAAGAGAAAGAGAAAGAGAAAGAGA/AAAAGAGAAAGAGAAAGAGA | TTGTTTGGACCAACATTCCA | TGAATTGGGTCGGTGAATTT | 59.8 | 294 | INTERGENIC | | | | |
| CaPOPI_6564 | scaffold167 | 12696 | AT/ATCAAATATGTTTGT | TTTTTGTTTGTGCCATTGGA | AGGGAGGACGAAAATGACCT | 59.9 | 465 | INTERGENIC | | | | |
| CaPOPI_6565 | scaffold167 | 29049 | CTTT/CTT | TTACCGGCTATTCATGCCTC | TCCCTTAATTTTCCTGCCCT | 60.1 | 508 | INTERGENIC | | | | |

| INDEL marker IDs | Chromosomes/ unanchored scaffolds | Physical positions (bp) | InDels (*Kabuli* reference genome-CDC Frontier/PI) | Forward primers (5'-3') | Reverse primers (5'-3') | Anneling temperature (0C) | Expected amplified product size (bp) | Structural annotation | | Functional annotation | | |
|---|---|---|---|---|---|---|---|---|---|---|---|---|
| | | | | | | | | Sequence components of *kabuli* genome | *Kabuli* gene accession IDs | NCBI-KOG | TFs | NCBI-nr database |
| CaPOPI_6566 | scaffold167 | 342564 | CATATATATATATATA/CATATATATATATATATA | GCGCAAATTGCCAGATAGAT | CGACATTGTAGCACCCCTTT | 60.2 | 508 | DRR | Ca_24157 | | | |
| CaPOPI_6567 | scaffold167 | 353330 | A/AGTTCTCCCATC | CATGCAGTTTCAAATGGTGC | TGCTAAGTTGATGCAGCAGG | 60.1 | 472 | INTERGENIC | | | | |
| CaPOPI_6568 | scaffold1679 | 2481 | CGGGTTTAGGGTTTAGGGTTTAGGGTT/CGGGTTTAGGGTTTAGGGTT | TTACGGTTTTGCGTTTAGGG | AACCAAAATCCGACACCCTT | 60.0 | 503 | INTERGENIC | | | | |
| CaPOPI_6569 | scaffold1681 | 934 | ATTTT/ATTTTT | GAGATTTTCGTACCACCCGA | TCCAGATGAAATCGAGGACC | 59.9 | 797 | INTERGENIC | | | | |
| CaPOPI_6570 | scaffold1682 | 8324 | TTGTGT/TTGT | AGGTCGTTTTCGTCCTTCCT | CACCCAATGACACCAATGAA | 60.1 | 693 | INTERGENIC | | | | |
| CaPOPI_6571 | scaffold1684 | 69381 | TAA/TA | CGGGTTGTACAAAAATCGCT | CAGCGATGGCTAGTGTTTGA | 60.0 | 458 | INTERGENIC | | | | |
| CaPOPI_6572 | scaffold1687 | 165898 | ATAGTA/ATA | TACCCACACTCATGCTCCAA | GGTTGTAGAGAAAACATCCAGACA | 60.1 | 428 | INTERGENIC | | | | |
| CaPOPI_6573 | scaffold1696 | 4415 | ATTTTTTTTTT/ATTTTTTTTT | ATAACCCGAACCTCAAACCC | TGTTTGAAATATGACGCCGA | 60.1 | 645 | INTERGENIC | | | | |
| CaPOPI_6574 | scaffold1696 | 5390 | TGTTTCGGGTTTCGGGTTTCG/TGTTTCGGGTTTCG | TTGAAGTACGAAAATCGCCC | TTCGTTATGCGGTCAAACCT | 60.1 | 813 | INTERGENIC | | | | |
| CaPOPI_6575 | scaffold1701 | 101096 | TT/TTCT | AGGTCATTTTCGTCCTCCCT | GCCCTCAGATGAGAGTCAGG | 59.9 | 515 | INTERGENIC | | | | |
| CaPOPI_6576 | scaffold1703 | 131019 | TGAAGAAGAAGAAGAAGAAGAAG/TGAAGAAGAAGAAGAAGAAGAAGAAG | CCAAAACGATTTCCCTTCAA | TTTCCAAAAGTGACCGAACC | 59.9 | 234 | INTERGENIC | | | | |
| CaPOPI_6577 | scaffold1703 | 243731 | TTATATATATATATATATATA/TTATATATATATATATATATATA | GAGAAAGGAGGGAATTTGGC | TATTTTGGATTTTGGCTCGG | 60.0 | 296 | INTERGENIC | | | | |
| CaPOPI_6578 | scaffold1704 | 9054 | ATTTTTTT/ATTTTTT | TGTGACATCTCTGCCTCCTG | AACATCAGTCAAGCACACAAAAA | 60.0 | 356 | INTERGENIC | | | | |
| CaPOPI_6579 | scaffold1704 | 11771 | AATATA/AATATATA | AACGATCAAGAGTCGCAAAA | ACCCTAAACCCTAAAGCCCA | 58.5 | 347 | INTERGENIC | | | | |
| CaPOPI_6580 | scaffold1708 | 11531 | AATATAATTTATT/AATATAATTTATTATATAATTTATT | TCACCTTCTTCGTTGTTTGC | GCGCATAAGTCACAAGTCCA | 58.9 | 734 | INTERGENIC | | | | |

| INDEL marker IDs | Chromosomes/ unanchored scaffolds | Physical positions (bp) | InDels (*Kabuli* reference genome-CDC Frontier/PI) | Forward primers (5'-3') | Reverse primers (5'-3') | Anneling temperature (0C) | Expected amplified product size (bp) | Structural annotation | | Functional annotation | | |
|---|---|---|---|---|---|---|---|---|---|---|---|---|
| | | | | | | | | Sequence components of *kabuli* genome | *Kabuli* gene accession IDs | NCBI-KOG | TFs | NCBI-nr database |
| CaPOPI_6581 | scaffold1708 | 11960 | ATTTTTT/ATTTTTTT | TGAGTTGCTTGTCAGCCTTG | TGGTACAACCACAAATAGGTAAGG | 60.2 | 444 | INTERGENIC | | | | |
| CaPOPI_6582 | scaffold1708 | 14932 | GTTT/GT | GCCATTTGGGTGGTTGATAG | CTGCTTGAGGACAAGCAACA | 60.2 | 901 | INTERGENIC | | | | |
| CaPOPI_6583 | scaffold1708 | 40978 | ATTTTTTTTTT/ATTTTTTTTT | GACACCCTAAACGCCAAAAA | GCGTTTGCGAAATCGTAGTT | 60.0 | 395 | INTERGENIC | | | | |
| CaPOPI_6584 | scaffold1708 | 49501 | AATATATATATATA/AATATATATATA | TTCCAACAATGACGATCTGC | TTTGAACGAAATTGAAAACCG | 59.7 | 659 | INTERGENIC | | | | |
| CaPOPI_6585 | scaffold1708 | 68435 | TCC/TCCC | GAAAAAGCGCTGTAAATGGC | CATTCGAGACGTGAGCAAAA | 59.9 | 510 | DRR | Ca_27642 | | | RNA polymerase Rpb1, domain 5 |
| CaPOPI_6586 | scaffold1708 | 87295 | AG/A | TGACTGTTTGCGTGTTCTCC | ATCCCATGGTGGGAATTGTA | 59.9 | 336 | INTERGENIC | | | | |
| CaPOPI_6587 | scaffold1708 | 88337 | AATATATATATATA/AATATATATATATATATATA | TTTGCCACTGACTTGCTTTG | GAGAAAGGAGGGAATTTGGC | 60.0 | 567 | INTERGENIC | | | | |
| CaPOPI_6588 | scaffold1708 | 88410 | GAAGGAAAAGGAAAAGGAAAAG/GAAGGAAAAGGAAAAGGAAAAGGAAAAG | TTTGCCACTGACTTGCTTTG | GAGAAAGGAGGGAATTTGGC | 60.0 | 567 | INTERGENIC | | | | |
| CaPOPI_6589 | scaffold171 | 130237 | GAA/GAAAA | CATGCGTGTCTAGGCATTTG | TGAATTCATGGCCAGCATAA | 60.3 | 341 | INTERGENIC | | | | |
| CaPOPI_6590 | scaffold171 | 150122 | CAATAATAATAATAATAAT/CAATAATAATAATAAT | CGAGTTGAGGTTCTTTCGGT | GGCACAGTGGGATATTGCTT | 59.3 | 770 | INTERGENIC | | | | |
| CaPOPI_6591 | scaffold171 | 190541 | GAA/GAAA | CTCTGATCAGCTCCATTCCC | CTAGGGCTCCTCCTTCGACT | 59.8 | 160 | CDS (FRAME SHIFT) | Ca_25949 | | | Transposase, Ptta/En/Spm, plant |
| CaPOPI_6592 | scaffold171 | 224792 | TAAA/TAAAA | AAAATCGATCCCCTATCACAT | TAGCAGCTGGAGGAACATGA | 57.4 | 348 | INTERGENIC | | | | |
| CaPOPI_6593 | scaffold1710 | 13152 | CATTATTATTATTATTATTATTATTATTATTATT/CATTATTATTATTATTATTATTATTATTATTATTATTATT | GGTCGCATCCTTGTACTGTG | AACAACTTCCTCGGATTTGG | 59.2 | 540 | INTERGENIC | | | | |
| CaPOPI_6594 | scaffold1710 | 43434 | AT/ATT | TCGCTTTTTGTGTTGAGCAG | ATGGCTCTCTCCCGGTAAAT | 60.2 | 813 | INTRON | Ca_25722 | | | |
| CaPOPI_6595 | scaffold1710 | 177159 | AAGAGAGAGAGAGAGAGAGAGAGAGAGAGAG/AAGAGAGAGAGAGAGAGAGAGAGAGAG | TCCAAACAGCTTCTAACCGC | GCCGTCGTTTCAACCACTAT | 60.4 | 271 | INTRON | Ca_25728 | O | | Abortive infection protein |

| INDEL marker IDs | Chromosomes/ unanchored scaffolds | Physical positions (bp) | InDels (*Kabuli* reference genome-CDC Frontier/PI) | Forward primers (5'-3') | Reverse primers (5'-3') | Anneling temperature (0C) | Expected amplified product size (bp) | Structural annotation | | Functional annotation | | |
|---|---|---|---|---|---|---|---|---|---|---|---|---|
| | | | | | | | | Sequence components of *kabuli* genome | *Kabuli* gene accession IDs | NCBI-KOG | TFs | NCBI-nr database |
| CaPOPI_6596 | scaffold1722 | 65936 | A/AT | CGTAGCGATAAAACCTGGGA | CGGGCCAGAATACTTACAGC | 60.1 | 802 | INTRON | Ca_26630 | G | | Ribulose-phosphate 3-epimerase |
| CaPOPI_6597 | scaffold1722 | 132942 | TGG/TG | ATGGTGGATACTTGGGTGGA | CCCTGTAAGAAACGTGCCAT | 60.1 | 206 | INTERGENIC | | | | |
| CaPOPI_6598 | scaffold1723 | 1064 | ACTCTC/ACTC | CCTCATAGGTTCGACTGGGA | GCGCCCTCACATAATGTTTT | 60.1 | 467 | INTERGENIC | | | | |
| CaPOPI_6599 | scaffold1725 | 28680 | TTT/TTTATT | GATTCAGTGGCCGATTTTGT | TTGGACGAAAAAGGAGGAAA | 59.9 | 917 | INTERGENIC | | | | |
| CaPOPI_6600 | scaffold1730 | 64331 | ACATATGATTTTTCATAT/ACATAT | TTGGATGTTTTCGCTTACCA | CAAGTTAGTGGTGAACCGGG | 59.2 | 882 | INTERGENIC | | | | |
| CaPOPI_6601 | scaffold1730 | 87897 | T/TTA | TTGAGGCGTCAAGCTAATGA | CGAGCAACCCCAAACTTAGA | 59.6 | 707 | INTERGENIC | | | | |
| CaPOPI_6602 | scaffold1730 | 100035 | TAAAAAAAAAA/TAAAAAAAAAAA | ACGGGACGGCTATCAGTATG | GGCGTAATTGCACATTCCAT | 60.0 | 612 | INTERGENIC | | | | |
| CaPOPI_6603 | scaffold1731 | 39857 | AATATATATATATATATATAT/AATATATATATATATATAT | ATGTTTTGGATTTTGGCTCG | GCACCGTGAGTAAATTCGGT | 59.9 | 704 | INTERGENIC | | | | |
| CaPOPI_6604 | scaffold1731 | 49034 | CATATATATATATATATATATA/CATATATATATATATATA | ATGGGAAGCAACAACGATTC | CCGTACCGTTTCTGGGATAA | 59.9 | 784 | DRR | Ca_27041 | IR | | ABC transporter-like |
| CaPOPI_6605 | scaffold1731 | 53952 | AATATATATATATATAT/AATATATATATATATATAT | TTTGGATATTGGTTCGGAGG | AGGAGGTTTGTGTTTGACGG | 59.8 | 514 | INTERGENIC | | | | |
| CaPOPI_6606 | scaffold1731 | 65474 | TTTATTATTATTATTATTAT/TTTATTATTATTATTATTATTAT | TAATCGGGGACTCAACATCC | CCCTTTGGATGCGAAATAAA | 59.8 | 279 | INTERGENIC | | | | |
| CaPOPI_6607 | scaffold1731 | 85329 | CATATATATATATATATATA/CATATATATATATATATATATA | TCTCCGCATAAGCTTTCGAT | GCTTCGACACTGTAGCACCTC | 59.9 | 478 | DRR | Ca_27043 | | | |
| CaPOPI_6608 | scaffold1731 | 86422 | AATATATATATATATATATATAT/AATATATATATATATATATAT | TTTTGGGAATTCCTGCTCTG | GTGACACCCTAAACCCCAAA | 60.2 | 299 | DRR | Ca_27043 | | | |
| CaPOPI_6609 | scaffold1736 | 8297 | TG/T | GTGAGATGAACGGGAATGCT | ACAACGAGGGGTGAGTATCG | 60.1 | 935 | INTERGENIC | | | | |
| CaPOPI_6610 | scaffold1736 | 8593 | GTA/GTATA | TGGCATAGTATCAGGCCTCC | ACAACGAGGGGTGAGTATCG | 60.1 | 237 | INTERGENIC | | | | |

| INDEL marker IDs | Chromosomes/ unanchored scaffolds | Physical positions (bp) | InDels (*Kabuli* reference genome-CDC Frontier/PI) | Forward primers (5'-3') | Reverse primers (5'-3') | Anneling temperature (0C) | Expected amplified product size (bp) | Structural annotation | | Functional annotation | | |
|---|---|---|---|---|---|---|---|---|---|---|---|---|
| | | | | | | | | Sequence components of *kabuli* genome | *Kabuli* gene accession IDs | NCBI-KOG | TFs | NCBI-nr database |
| CaPOPI_6611 | scaffold1736 | 21426 | GTTTTTTTTTT/GTTTTTTTTTTT | CTTTTGCAGGGTGTTGGTTT | TCTCGAGGCATCACATTCAG | 60.0 | 790 | INTERGENIC | | | | |
| CaPOPI_6612 | scaffold174 | 19539 | GAAAAAAAAAAA/GAAAAAAAAAA | CGATGATGGTTGAAGTGGTG | GGGTGGCTGTGAACTTTTGT | 60.0 | 225 | INTERGENIC | | | | |
| CaPOPI_6613 | scaffold174 | 82773 | AAGAG/AAG | AACCGTCAAGGGTGTGAAAG | ACTCTTGTTGAAGCATGGGG | 60.0 | 506 | INTRON | Ca_21709 | | | PDZ/DHR/GLGF |
| CaPOPI_6614 | scaffold174 | 111558 | TAAA/TAAAA | CCAATCAAACATCATTCATGC | ATTGGTGGTTGCCTAAGTGG | 58.8 | 556 | INTERGENIC | | | | |
| CaPOPI_6615 | scaffold174 | 111791 | TGGGG/TGGG | CCGGATATAACCAGCCACAC | ATTGGTGGTTGCCTAAGTGG | 60.2 | 289 | INTERGENIC | | | | |
| CaPOPI_6616 | scaffold174 | 136798 | TG/T | GAAGGTAGACGTTGGGGACA | CTGTGGACGACCTTTTGGAT | 60.0 | 585 | INTERGENIC | | | | |
| CaPOPI_6617 | scaffold174 | 140361 | TAAAAAAAAAAAAAA/TAAAAAAAAAAAA | CGGGTGAATGTGGCTTTATT | TTTCTTGTTTTGGACCCCTG | 59.8 | 395 | INTERGENIC | | | | |
| CaPOPI_6618 | scaffold174 | 159321 | ATTTTTTTT/ATTTTTTTTTTT | CTAACGAGTCTGCAACGCAA | TGAAAGGTAATAATCGGTGCG | 60.2 | 319 | INTERGENIC | | | | |
| CaPOPI_6619 | scaffold174 | 169758 | CG/C | GTCAATCACAAGGAAGGGGA | TCGAAAGTTGGTGTGAAAAGC | 59.9 | 326 | INTERGENIC | | | | |
| CaPOPI_6620 | scaffold174 | 192838 | GATA/GATAAAACATCTTTTCATA | GCGCCGTTTATATAGCCAGT | AAATCCACACTAACATTGAAAACTT | 59.3 | 355 | INTERGENIC | | | | |
| CaPOPI_6621 | scaffold174 | 206548 | GCTCTCTCTCT/GCTCTCTCT | ACCAAAGCAAAAGCTCCTCA | TTATTCACGAGGTCCTTGGC | 60.0 | 455 | INTERGENIC | | | | |
| CaPOPI_6622 | scaffold174 | 225469 | CAAAAAAAA/CAAAAAAAAA | GTGATTTGCACTTCAAGCGA | CCGAGGATGATGGTCAATTT | 60.0 | 653 | INTERGENIC | | | | |
| CaPOPI_6623 | scaffold174 | 279541 | ATTTTTTT/ATTTTTTTT | AATGTAGCAGCAAACCACCC | TTGATGCATTGTCTTCTCGC | 60.0 | 602 | INTERGENIC | | | | |
| CaPOPI_6624 | scaffold174 | 327909 | ATTTTTTTTTTT/ATTTTTTTTTTTTT | TGGCAACCACAAGGAGTGTA | TCAAAAGGGAAAACAATTGACA | 60.2 | 661 | INTERGENIC | | | | |
| CaPOPI_6625 | scaffold174 | 351626 | GAAAAAAAAAAA/GAAAAAAAAAA | CCTCGATCACTTCGTCTACCA | AGAGCAAGGCCTCACAAAAA | 60.3 | 478 | INTERGENIC | | | | |

| INDEL marker IDs | Chromosomes/ unanchored scaffolds | Physical positions (bp) | InDels (*Kabuli* reference genome-CDC Frontier/PI) | Forward primers (5'-3') | Reverse primers (5'-3') | Anneling temperature (0C) | Expected amplified product size (bp) | Structural annotation | | Functional annotation | | |
|---|---|---|---|---|---|---|---|---|---|---|---|---|
| | | | | | | | | Sequence components of *kabuli* genome | *Kabuli* gene accession IDs | NCBI-KOG | TFs | NCBI-nr database |
| CaPOPI_6626 | scaffold174 | 359071 | GAAAAAAAAAAA/GAAAAAAAAAA | TCTGTCAATTGAGCTGCGAC | CGTCCATGAAAAGAGGGAAA | 60.1 | 446 | INTERGENIC | | | | |
| CaPOPI_6627 | scaffold174 | 373713 | TAAAAAAAAAA/TAAAAAAAAA | AGTTGATTCGCTCATGGGTT | TCCACAAAGCACACATACATCA | 59.6 | 307 | INTERGENIC | | | | |
| CaPOPI_6628 | scaffold174 | 376719 | ATTTTTTTTTT/ATTTTTTTTTTT | TCGAGTCCCAACACATGGTA | GCAGAGGTCATGGGATTCAT | 60.0 | 503 | INTERGENIC | | | | |
| CaPOPI_6629 | scaffold174 | 490723 | ATTTTTT/ATTTTTTT | GCCGTTGCCTTATTTTGTGT | TTTTTCTTCAGCTTGATGTTGAT | 60.0 | 574 | INTERGENIC | | | | |
| CaPOPI_6630 | scaffold1740 | 2698 | TAA/TA | CGATTTGGCCATACAGGAAT | CCCATCTACATACCCATCCG | 59.8 | 498 | INTERGENIC | | | | |
| CaPOPI_6631 | scaffold1750 | 124578 | A/AGTGCGAAAT | TTACGATCACCAGCCATGAA | TCCAAAGAGACCACTCTTCTCA | 60.1 | 730 | INTERGENIC | | | | |
| CaPOPI_6632 | scaffold1756 | 5242 | CGGTA/C | GGTGAGGTCTTTTGCCTCAA | TTGGATGAAGGCCATAAAGG | 60.2 | 665 | INTERGENIC | | | | |
| CaPOPI_6633 | scaffold1756 | 43727 | CATATATATATATATATATATATATAT/CATATATATATATATATATATATATATATATATATAT | GTTGGTTCCCTAGCACCGTA | GGCTGTTAGCCCAGTTTTTG | 60.0 | 658 | INTERGENIC | | | | |
| CaPOPI_6634 | scaffold1757 | 1167 | CTT/CTTATT | AAGCGTACCTGTGGGAATTG | TTAATGAGGGGCTGAATTGG | 60.0 | 259 | INTERGENIC | | | | |
| CaPOPI_6635 | scaffold1757 | 47237 | ATTTTTTTTTT/ATTTTTTTTT | ATGCTACTGGACAACCCGAC | TGTCAGCTTAAATGTATTTTTGTTTTT | 60.0 | 594 | INTERGENIC | | | | |
| CaPOPI_6636 | scaffold1761 | 69178 | CTT/CTTAGTT | GAGATTGGCGTGGTTTTTGT | GAGGGAAAAGCCCAGATTTC | 60.0 | 951 | INTERGENIC | | | | |
| CaPOPI_6637 | scaffold1768 | 12788 | T/TC | GGTCCTCGATTTCATCTGGA | GTACGAAAATCGCCCAAAAA | 60.0 | 320 | INTERGENIC | | | | |
| CaPOPI_6638 | scaffold1777 | 47085 | T/TAAAACTTG | CTCAACAGGGGCAACTCATT | AGAGGCGAAACAAAGTGCAT | 60.1 | 698 | INTERGENIC | | | | |
| CaPOPI_6639 | scaffold1777 | 282839 | CCAGATGCA/CCA | TGCATCAGTATCTGCCTTCG | TTCTTTCAAGAGGTCGCAGC | 60.0 | 329 | DRR | Ca_23266 | L | ERF | SNF2-related |
| CaPOPI_6640 | scaffold1777 | 347891 | ATTTTT/ATTTTTT | CCCAAAACTCACGGGATAAA | ATTTGGGCCCATCATAAACA | 59.8 | 729 | INTERGENIC | | | | |

| INDEL marker IDs | Chromosomes/ unanchored scaffolds | Physical positions (bp) | InDels (*Kabuli* reference genome-CDC Frontier/PI) | Forward primers (5'-3') | Reverse primers (5'-3') | Anneling temperature (0C) | Expected amplified product size (bp) | Structural annotation | | Functional annotation | | |
|---|---|---|---|---|---|---|---|---|---|---|---|---|
| | | | | | | | | Sequence components of *kabuli* genome | *Kabuli* gene accession IDs | NCBI-KOG | TFs | NCBI-nr database |
| CaPOPI_6641 | scaffold1777 | 513940 | C/CT | ATGCTGTCTTGTGCTGGATG | CGCTTTTATTTCGAATTGCTG | 59.9 | 205 | DRR | Ca_23271 | BK | MYB_related | SET domain |
| CaPOPI_6642 | scaffold1778 | 1269 | C/CG | ACTTCCGCGCTAGATTTCAA | TCCTGGTCCTCGATTTCATC | 60.0 | 282 | INTERGENIC | | | | |
| CaPOPI_6643 | scaffold1779 | 131414 | CAAAAAAAAAA/CAAAAAAAAAAAA | TTGTTTCTCCCCAGTTTTGG | TTCCATATCACACGGAACCA | 59.9 | 275 | INTERGENIC | | | | |
| CaPOPI_6644 | scaffold1779 | 131497 | ATTTTTTTT/ATTTTTTT | TTGTTTCTCCCCAGTTTTGG | TTCCATATCACACGGAACCA | 59.9 | 275 | INTERGENIC | | | | |
| CaPOPI_6645 | scaffold1779 | 132071 | ATTTGTTGTTGTT/AT | ATGGAAAAGTGCTGCAAAAA | TTTCGCCCTCAATTTCAAAC | 58.4 | 688 | INTERGENIC | | | | |
| CaPOPI_6646 | scaffold178 | 605 | AACACAC/AACAC | CAATAGGCCAAGGAAGGACA | ACATTGAGGCCATTTTCGTC | 60.1 | 236 | INTERGENIC | | | | |
| CaPOPI_6647 | scaffold1802 | 20448 | CG/C | ATTTTCTCCGCTTCCGATTT | AAGGAAGCGCACTAGCAGAG | 60.0 | 816 | INTERGENIC | | | | |
| CaPOPI_6648 | scaffold1810 | 98576 | GGTTGT/GGTTGTTGT | TGACGATACGAAACGATGGA | AGCGATGCATGTCTCAAGTG | 60.1 | 430 | URR | Ca_26004 | | | |
| CaPOPI_6649 | scaffold1810 | 103255 | GC/GCC | TTGCTAGTTGCTGATGTGGC | GGTGTCATCGGGAGTCTGTT | 60.0 | 300 | INTERGENIC | | | | |
| CaPOPI_6650 | scaffold1810 | 103852 | GAAAA/GAAAAA | AACAGACTCCCGATGACACC | AACATTTATGACGATATTTATGAGTGA | 60.0 | 438 | INTERGENIC | | | | |
| CaPOPI_6651 | scaffold1815 | 5752 | A/ATAC | TGTCCATCACAGGCTACATGA | TGTGGGCAAAATCTTGTGGT | 60.1 | 434 | INTERGENIC | | | | |
| CaPOPI_6652 | scaffold1816 | 53607 | ATATATAATTTATATAATTTATATAATTT/ATATATAATTTATATAATTT | TGGTGAGGAAATGAGCAACA | ACAAAATGAAGGTGCGGAAG | 60.2 | 679 | INTERGENIC | | | | |
| CaPOPI_6653 | scaffold1822 | 17416 | GTT/GTTT | AAGCACCAATTACCGAGGTG | TTGTATTTGGCGTGTCCGTA | 60.0 | 720 | INTERGENIC | | | | |
| CaPOPI_6654 | scaffold1825 | 148166 | ATTTTTTTTTT/ATTTTTTTTT | GTTTTGGCGTTGGTTTCACT | TACGTGTATATTCGCGCACC | 60.0 | 904 | INTERGENIC | | | | |
| CaPOPI_6655 | scaffold1830 | 4465 | CTATA/CTA | CCAAGGGAAGACGAAAATGA | TGCATCCAACAGGTGTGTTT | 60.0 | 665 | INTERGENIC | | | | |

| INDEL marker IDs | Chromosomes/ unanchored scaffolds | Physical positions (bp) | InDels (*Kabuli* reference genome-CDC Frontier/PI) | Forward primers (5'-3') | Reverse primers (5'-3') | Anneling temperature (0C) | Expected amplified product size (bp) | Structural annotation | | Functional annotation | | |
|---|---|---|---|---|---|---|---|---|---|---|---|---|
| | | | | | | | | Sequence components of *kabuli* genome | *Kabuli* gene accession IDs | NCBI-KOG | TFs | NCBI-nr database |
| CaPOPI_6656 | scaffold1835 | 8173 | CAA/CA | TCATTGCTCCTTTGAGTTGC | ATTTGCCACAGATACCGCTC | 59.0 | 453 | INTERGENIC | | | | |
| CaPOPI_6657 | scaffold1835 | 8463 | TAAAAAAAA/TAAAAAAAAA | GAGCGGTATCTGTGGCAAAT | CGGTTTGTGGCTTTCCTAAA | 60.1 | 486 | INTERGENIC | | | | |
| CaPOPI_6658 | scaffold1835 | 10086 | GCC/GCCC | TGGTTAACCTTGCACCACAA | GGATGCTTGAATTATGCCGT | 60.0 | 672 | INTERGENIC | | | | |
| CaPOPI_6659 | scaffold1835 | 11243 | GAACAACAAC/GAACAAC | AAACGCATCCCGTAAATCAG | CGATTGCCCTAGAAAATGGA | 60.0 | 610 | INTERGENIC | | | | |
| CaPOPI_6660 | scaffold1844 | 2095 | AAA/AAAATAA | AACATGGGACGAGCTGTTTC | GGGATATTCAATAAGTGAAATTCAAA | 60.1 | 923 | INTERGENIC | | | | |
| CaPOPI_6661 | scaffold1844 | 2214 | T/TC | TGCAATTATGATCATGCTTTATATG | GGGATATTCAATAAGTGAAATTCAAA | 58.2 | 732 | INTERGENIC | | | | |
| CaPOPI_6662 | scaffold1844 | 2526 | CA/CAA | GACCTTTGAATTTATCTCTCTTTGAA | TTACAACATGTCAGGCCGAA | 58.5 | 373 | INTERGENIC | | | | |
| CaPOPI_6663 | scaffold1844 | 40976 | TAAAAA/TAAAA | CACTCGCATTTGCATAGTTCA | ATTTGGTGCTTTTGTGGAGG | 59.9 | 578 | DRR | Ca_24135 | | | Epsin domain, N-terminal |
| CaPOPI_6664 | scaffold1844 | 101225 | CTTTTTTTTTT/CTTTTTTTTTTTT | CCTTCGCGAAACTCACTGAT | TTGGAATTTCTTCGCAGCTT | 60.4 | 116 | INTERGENIC | | | | |
| CaPOPI_6665 | scaffold1844 | 123387 | GTT/GTTT | TCGCGTAGTGGTAGCCTTCT | TCACAAGGGAGGCTCTATGG | 60.0 | 181 | INTERGENIC | | | | |
| CaPOPI_6666 | scaffold1844 | 136314 | CTTTTTTT/CTTTTTTTTTT | CCACCATTGAATCAAGTCCA | GACTCCATACAGGGCCGTC | 59.3 | 354 | INTERGENIC | | | | |
| CaPOPI_6667 | scaffold1844 | 146939 | TT/TTAGACTTGT | CAATCAAAATTGGTCTAGCCG | TCAAAATGGGCCAGTGTTTT | 59.6 | 274 | INTERGENIC | | | | |
| CaPOPI_6668 | scaffold1844 | 155952 | GAAAAAAAA/GAAAAAAA | TCAATCGGTTCCTCTCACAA | TTAAATGCGTTCGACAGATCA | 59.2 | 857 | DRR | Ca_24144 | | MYB_related | Glycoside hydrolase, family 16 |
| CaPOPI_6669 | scaffold1844 | 165772 | TATATCTC/TATATCTCTTAATATCTC | CCCCCTCTTAGGCGTACTTC | CTTGTTCGGGTACCTCGTGT | 60.1 | 340 | INTERGENIC | | | | |
| CaPOPI_6670 | scaffold1844 | 166678 | TC/T | GGCTAGACGAGTTCGACGAC | CTAGATGGTCGCATTCTGGG | 60.0 | 418 | DRR | Ca_24145 | | | |

| INDEL marker IDs | Chromosomes/ unanchored scaffolds | Physical positions (bp) | InDels (*Kabuli* reference genome-CDC Frontier/PI) | Forward primers (5'-3') | Reverse primers (5'-3') | Anneling temperature (0C) | Expected amplified product size (bp) | Structural annotation | | Functional annotation | | |
|---|---|---|---|---|---|---|---|---|---|---|---|---|
| | | | | | | | | Sequence components of *kabuli* genome | *Kabuli* gene accession IDs | NCBI-KOG | TFs | NCBI-nr database |
| CaPOPI_6671 | scaffold1844 | 167253 | CAAAAAA/CAAAA | CCCCAATTTGCGATCAATAA | TCAAAACTAAGAAACTCGCCAA | 60.6 | 694 | DRR | Ca_24145 | | | |
| CaPOPI_6672 | scaffold1844 | 167398 | GTATATAT/GTATATATAT | CCCCAATTTGCGATCAATAA | TCAAAACTAAGAAACTCGCCAA | 60.6 | 694 | DRR | Ca_24145 | | | |
| CaPOPI_6673 | scaffold1845 | 47422 | GA/G | GACCCAACCAGATTTCGGTA | TTAGGTTAGCATGGCCCAAC | 59.8 | 505 | INTERGENIC | | | | |
| CaPOPI_6674 | scaffold1845 | 71268 | AATGTA/AA | CAAAGCGATGATCGAACAAA | TGGATTAAAAGTTCGGGTCG | 59.8 | 285 | INTERGENIC | | | | |
| CaPOPI_6675 | scaffold1852 | 76175 | ATTTTTTTT/ATTTTTTTTT | GGTTGGTCTTCCAACGCTAA | GGTGTGTTTGTTACAACTTTTTGG | 60.1 | 358 | INTERGENIC | | | | |
| CaPOPI_6676 | scaffold1852 | 94390 | AT/ATGCTCAACCATT | GGTCCAACATCGACGCTAAT | CGAGCACACTCCATGGTATC | 60.0 | 234 | INTERGENIC | | | | |
| CaPOPI_6677 | scaffold1852 | 94526 | ATTTTTT/ATTTTT | GGTCCAACATCGACGCTAAT | TCGGATGTTACACACCTCACA | 60.0 | 515 | INTERGENIC | | | | |
| CaPOPI_6678 | scaffold1852 | 94771 | GGTGTGT/GGTGT | GGTCCAACATCGACGCTAAT | TTTCAGTCATTTGATTTCGGA | 60.0 | 531 | INTERGENIC | | | | |
| CaPOPI_6679 | scaffold1852 | 94935 | ATTGTT/ATT | TACCATGGAGTGTGCTCGAA | TTTCAGAACACAAATCTGCATGT | 60.3 | 765 | INTERGENIC | | | | |
| CaPOPI_6680 | scaffold1852 | 103209 | ATTTTTTTTTTT/ATTTTTTTTTTTTTTT | TTGAGGACCAATGAGTGTCG | GTTAAATTCGGGGTCGAGGT | 59.7 | 553 | INTERGENIC | | | | |
| CaPOPI_6681 | scaffold1864 | 5989 | ATAGT/AT | TTGCCCATTCTCCAAGTTTC | CGGATATAAGCATGGCATCA | 60.1 | 659 | INTERGENIC | | | | |
| CaPOPI_6682 | scaffold1864 | 14836 | TAAAAAAA/TAAAAAA | CGCTCTGTTAAGTCGGTTCA | CATCCACGGAGCAAAAACTT | 59.1 | 251 | INTERGENIC | | | | |
| CaPOPI_6683 | scaffold1866 | 62792 | AAATAAA/AAATAAATAGAATAAA | TTGCTCCTACGTATCTCCCG | TTAAGTTGGTTTGGGCTTGG | 60.2 | 855 | INTRON | Ca_26041 | R | | Uncharacterised protein family, ATP binding |
| CaPOPI_6684 | scaffold1866 | 113475 | AAATTATTTTACAATTATTT/AAATTATTT | TTAAAACAGACCTCGCCACC | GAGGAGAAGTCCACGTCAGC | 60.1 | 595 | INTERGENIC | | | | |
| CaPOPI_6685 | scaffold1866 | 116123 | GAA/GAAA | TTCCACTTATGGTGAGTAATGATG | GCGAGCATGAGAAGTTGTTG | 58.1 | 632 | INTERGENIC | | | | |

| INDEL marker IDs | Chromosomes/ unanchored scaffolds | Physical positions (bp) | InDels (*Kabuli* reference genome-CDC Frontier/PI) | Forward primers (5'-3') | Reverse primers (5'-3') | Anneling temperature (0C) | Expected amplified product size (bp) | Structural annotation | | Functional annotation | | |
|---|---|---|---|---|---|---|---|---|---|---|---|---|
| | | | | | | | | Sequence components of *kabuli* genome | *Kabuli* gene accession IDs | NCBI-KOG | TFs | NCBI-nr database |
| CaPOPI_6686 | scaffold1867 | 226694 | ATTT/ATTTT | ATCGATTTTGTCCGTTTTGC | GGAGGGTTTTGGTTTGGAAT | 59.9 | 473 | INTERGENIC | | | | |
| CaPOPI_6687 | scaffold1867 | 228749 | CGGGG/CGGGGG | TTGGAAGGAGCCCTTACCTT | TTCCCAACTCCTTCCTCCTT | 60.1 | 255 | INTERGENIC | | | | |
| CaPOPI_6688 | scaffold1869 | 19075 | AG/A | CTCGATTTCATCTGGCCATT | CGAAAGTCGCTCCAAAAGTC | 60.0 | 524 | INTERGENIC | | | | |
| CaPOPI_6689 | scaffold1869 | 20055 | CAA/CAAA | TTTCAGGCGATTTTCGTACC | ATTGTGTTTCGGGTTTCGAG | 60.1 | 825 | INTERGENIC | | | | |
| CaPOPI_6690 | scaffold1870 | 21211 | TAAAA/TAAA | ATGGACTGAAATCGAGTGGC | CATATAACAAAATTGAGTTGCAAAA | 60.1 | 333 | INTERGENIC | | | | |
| CaPOPI_6691 | scaffold1878 | 7439 | T/TTA | TGGCCTTCGATCTTTGAAGT | CATTGGTTTGACCAACCTTGT | 59.8 | 693 | INTERGENIC | | | | |
| CaPOPI_6692 | scaffold1878 | 40480 | CTCA/CTCAAGGATTTTGATTTCA | GCTTGGAAACAGCCTCAGAC | ACATTCAAGCGCAGGTCTCT | 60.0 | 612 | INTERGENIC | | | | |
| CaPOPI_6693 | scaffold1878 | 45211 | TAAAAAAAAAA/TAAAAAAAAAAA | GCCCTCAGATGAGAGTCAGG | GGAGGGTAGGCTTATTTGGC | 59.9 | 780 | INTERGENIC | | | | |
| CaPOPI_6694 | scaffold1878 | 56174 | TCTTCAAATC/TCTTCAAATCTACTTCAAATC | CCTTGACCCTACAAGCCAAG | TCATACCTGGCTGATGTTGTG | 59.7 | 896 | INTERGENIC | | | | |
| CaPOPI_6695 | scaffold1878 | 73171 | ATTTTTTTT/ATTTTTTTTT | GGTTGGGTCCTGGTTTGTTA | CCTCTGGATCCAACACAGGT | 59.7 | 626 | INTERGENIC | | | | |
| CaPOPI_6696 | scaffold1878 | 77007 | TAAAAAAAAAAA/TAAAAAAAAAA | TTGCGAGAGCGGTCTTAAAT | CCTCAAGATCTCGTGTGCAA | 60.0 | 358 | INTERGENIC | | | | |
| CaPOPI_6697 | scaffold1885 | 40622 | TAACC/T | CCATTGTGGTCGTTTAGGCT | CCCAAATGACATCAAAACCC | 60.0 | 572 | INTERGENIC | | | | |
| CaPOPI_6698 | scaffold1885 | 47510 | A/AG | GTAGCTTCGGCTACACCGAC | ATTCCGTTGTGCACTTGATG | 59.9 | 664 | INTERGENIC | | | | |
| CaPOPI_6699 | scaffold1886 | 17788 | TTAT/TT | GAGAGAGGCATTGCACAACA | CGAAATTTCTTTAACGAGGGG | 60.0 | 537 | INTERGENIC | | | | |
| CaPOPI_6700 | scaffold1886 | 17993 | TA/TAA | GAGAGAGGCATTGCACAACA | CGAAATTTCTTTAACGAGGGG | 60.0 | 537 | INTERGENIC | | | | |

| INDEL marker IDs | Chromosomes/ unanchored scaffolds | Physical positions (bp) | InDels (*Kabuli* reference genome-CDC Frontier/PI) | Forward primers (5'-3') | Reverse primers (5'-3') | Anneling temperature (0C) | Expected amplified product size (bp) | Structural annotation | | Functional annotation | | |
|---|---|---|---|---|---|---|---|---|---|---|---|---|
| | | | | | | | | Sequence components of *kabuli* genome | *Kabuli* gene accession IDs | NCBI-KOG | TFs | NCBI-nr database |
| CaPOPI_6701 | scaffold1899 | 8615 | TTTATTATTATTATTA/TTTATTATTATTA | CCCCACTCCCCGTACTTATT | TGGTGGATGTGAACGACACT | 60.1 | 484 | INTERGENIC | | | | |
| CaPOPI_6702 | scaffold1905 | 1617 | CTG/CTCTGATG | ACTGATGTCAAGAGGGCCAG | AGAGGGTGGCAATGGTAATG | 60.3 | 251 | INTERGENIC | | | | |
| CaPOPI_6703 | scaffold1911 | 12969 | CAGAAGA/CAGA | GTGCCAGTCGATTGCCTAAT | TGCAATTCAAGCTCACTTGG | 60.1 | 993 | INTERGENIC | | | | |
| CaPOPI_6704 | scaffold1918 | 17379 | TAAAAAAA/TAAAAAAAA | GCGGATGCTATTTAACCGAG | TAATCGGTTGCACCGAAAAG | 59.7 | 107 | INTERGENIC | | | | |
| CaPOPI_6705 | scaffold1918 | 23098 | GATATAT/GAT | TGTTGTTCAAAATGGGCTTG | GCAGCCAACAAAAGAACAGA | 59.6 | 712 | INTERGENIC | | | | |
| CaPOPI_6706 | scaffold1918 | 31828 | GTTT/GTT | TTACACTTGCTTTTTCGGGG | TGTGCGTATTGGTGCGTATT | 60.1 | 584 | INTERGENIC | | | | |
| CaPOPI_6707 | scaffold1918 | 78141 | AGCGCGCGCGC/AGCGCGCGCGCGC | TATTTGCGCCACATCGTTTA | TCACGGTTGTGTGAGATGCT | 60.1 | 482 | INTERGENIC | | | | |
| CaPOPI_6708 | scaffold1918 | 187805 | TAAAAAAA/TAAAAAA | GTTCGAGTTTCTCCGTCTGC | AAAACTTGAGTGTGGGGACG | 60.0 | 309 | INTERGENIC | | | | |
| CaPOPI_6709 | scaffold1918 | 244709 | TCACATACA/TCA | GCATGATTGGTCGATGTGAG | CAAAGGAAAGTGGCAGGAAA | 60.1 | 498 | INTERGENIC | | | | |
| CaPOPI_6710 | scaffold1923 | 10722 | ATTTTTTTTTT/ATTTTTTTTT | GGCCCCCAATATTTTGGTAA | ATTATTGGCCCCAACTCACA | 60.7 | 383 | INTERGENIC | | | | |
| CaPOPI_6711 | scaffold1928 | 13480 | ATATATATATGTATATAT/ATATATAT | ACCAGGGAGGAAGGAAATGT | TTGAGTTGCAACGAGAGTGG | 59.8 | 307 | INTERGENIC | | | | |
| CaPOPI_6712 | scaffold1928 | 14034 | TCC/TC | GGGTTCCAAATCACCTTGAA | CTTATCGGATTGCATTGCCT | 59.8 | 222 | INTERGENIC | | | | |
| CaPOPI_6713 | scaffold193 | 64250 | GTT/GT | AACCAAACACCAATTCAGCC | TGCAAGGGTGTGTTTCATGT | 59.8 | 628 | INTERGENIC | | | | |
| CaPOPI_6714 | scaffold193 | 79630 | TAAAAAAA/TAAAAAA | AAAATAAATGCGCCGTATGC | AGATCATGTGCAAAAAGGGG | 60.0 | 477 | INTERGENIC | | | | |
| CaPOPI_6715 | scaffold193 | 79687 | TAATAA/TAATAATAAAATAA | AAAATAAATGCGCCGTATGC | AGATCATGTGCAAAAAGGGG | 60.0 | 477 | INTERGENIC | | | | |

| INDEL marker IDs | Chromosomes/ unanchored scaffolds | Physical positions (bp) | InDels (*Kabuli* reference genome-CDC Frontier/PI) | Forward primers (5'-3') | Reverse primers (5'-3') | Anneling temperature (0C) | Expected amplified product size (bp) | Structural annotation | | Functional annotation | | |
|---|---|---|---|---|---|---|---|---|---|---|---|---|
| | | | | | | | | Sequence components of *kabuli* genome | *Kabuli* gene accession IDs | NCBI-KOG | TFs | NCBI-nr database |
| CaPOPI_6716 | scaffold193 | 208565 | TTA/TTATA | CGTTCCTGAGGGAATTGTGT | AGTACTGAGCCCCCTGGTTT | 60.0 | 485 | INTERGENIC | | | | |
| CaPOPI_6717 | scaffold193 | 255384 | GAA/GA | GTCTCCTCGAGCTTGGTTTG | CGCTTGATTCGATCTTCTCC | 60.0 | 776 | DRR | Ca_25167 | R | MYB | Integrase, catalytic core |
| CaPOPI_6718 | scaffold193 | 403373 | CATATATATATATATATATATATATATATATATATA/CATATATATATATATATATATATATATATATATATATATATA | GTTGGTTCCCTAGCACCGTA | ACCCAATTACTGGAGATGCG | 60.0 | 817 | INTERGENIC | | | | |
| CaPOPI_6719 | scaffold1930 | 40318 | GTTT/GTT | ATTAGCTCGAAAAGTGCCGA | GTTTGAAATCTGGCGAGGAA | 60.0 | 265 | INTERGENIC | | | | |
| CaPOPI_6720 | scaffold1943 | 2994 | TAAAAAAAAA/TAAAAAAAA | TTTGTAGCCTGCTCGGATTT | TCCATCGAGACTCATTTATAAGCA | 59.8 | 807 | INTERGENIC | | | | |
| CaPOPI_6721 | scaffold1943 | 10178 | GTTTTTTTTT/GTTTTTTTTTT | TTGCTTTGGTTTGATGTGGA | TCACCCCTTTTTGAACGAAG | 60.1 | 528 | INTERGENIC | | | | |
| CaPOPI_6722 | scaffold1943 | 60462 | ATTTTTTTTTT/ATTTTTTTTTTTT | AAATTGGATTCAACAACCATCC | CCATCCACTTGCATTGAAAA | 59.9 | 458 | INTERGENIC | | | | |
| CaPOPI_6723 | scaffold1943 | 104145 | CTTTTTTTTT/CTTTTTTTTTTT | TAAAAACCGGTTCGGTTCAG | AGTCTGAAATGACGTTGGGA | 60.0 | 685 | INTERGENIC | | | | |
| CaPOPI_6724 | scaffold1943 | 121284 | A/ATTCT | TTGGATCAAATTGCTTGGAAC | TCGACTTGTCAATCGCTTTG | 59.9 | 388 | INTERGENIC | | | | |
| CaPOPI_6725 | scaffold1943 | 243638 | TACACACA/TACACACACA | TGGTGGCATAACTAAGTGTCTCA | AAAGGAACCAAGTTTTGGACAG | 59.7 | 181 | INTRON | Ca_21926 | R | | Metallo-dependent phosphatase |
| CaPOPI_6726 | scaffold1943 | 375682 | TAAAAAAAAAA/TAAAAAAAAA | TGTGCGGTATTCGAACAAAA | AGCACGATGCAAGTTACGAG | 60.1 | 174 | INTERGENIC | | | | |
| CaPOPI_6727 | scaffold1943 | 393620 | ATC/ATCTTC | TGGTTCTCATGATACGTGTGG | GCGCAAACACAAACAACAAA | 59.4 | 635 | INTERGENIC | | | | |
| CaPOPI_6728 | scaffold1945 | 1161 | ATTT/ATT | TTCTAGCTGGCTTTTCTCGC | TGCACAAGTCAGTAGCACCC | 59.9 | 871 | INTERGENIC | | | | |
| CaPOPI_6729 | scaffold1945 | 4916 | GTTT/GTT | CGTCAAACACAAACCTCCCT | TGGTTTTCACGCCAACATTA | 60.0 | 659 | INTERGENIC | | | | |
| CaPOPI_6730 | scaffold1945 | 4979 | ATTT/ATT | CGTCAAACACAAACCTCCCT | TGGTTTTCACGCCAACATTA | 60.0 | 659 | INTERGENIC | | | | |

| INDEL marker IDs | Chromosomes/ unanchored scaffolds | Physical positions (bp) | InDels (*Kabuli* reference genome-CDC Frontier/PI) | Forward primers (5'-3') | Reverse primers (5'-3') | Anneling temperature (0C) | Expected amplified product size (bp) | Structural annotation | | Functional annotation | | |
|---|---|---|---|---|---|---|---|---|---|---|---|---|
| | | | | | | | | Sequence components of *kabuli* genome | *Kabuli* gene accession IDs | NCBI-KOG | TFs | NCBI-nr database |
| CaPOPI_6731 | scaffold1946 | 1221 | TTATATATATATATATAT/TTATATATATATATATATATAT | ACATTAAAATTCGGGGGAGG | CACACTCTCACCCGGCTAAT | 60.0 | 388 | INTERGENIC | | | | |
| CaPOPI_6732 | scaffold1946 | 40975 | TGAAAATTTAAG/TGAAAATTTAAGAAAATTTAAG | AATATACGGTTGTCGCAGGC | GCGGAGAATTCAATTGGTTG | 60.0 | 582 | INTERGENIC | | | | |
| CaPOPI_6733 | scaffold1954 | 1371 | TA/TAGTAA | TCCTATATTCGCCCCACTTG | GTGGCATGGGAGTCTGTTTT | 59.9 | 433 | INTERGENIC | | | | |
| CaPOPI_6734 | scaffold1956 | 63720 | GTAAAACCATAAAACCATAAAACCATAAAACCATAAAAC/GTAAAACCATAAAACCATAAAACCATAAAAC | TTCGTTATGCAGTCAAATTTCTAA | TCTGACGAAGAACCCGAAAC | 58.1 | 667 | INTERGENIC | | | | |
| CaPOPI_6735 | scaffold1956 | 63893 | ATATTATTATTATTATTATTATTATT/ATATTATTATTATTATTATTATTATTATTATT | TTCGTTATGCAGTCAAATTTCTAA | AAAATGGACCAACGTCCGTA | 58.1 | 719 | INTERGENIC | | | | |
| CaPOPI_6736 | scaffold1956 | 85954 | GGTTTAG/GGTTTAGGGTTTAGTGTTTAG | TAGGCGATTTTCGTACCACC | ATAACCCGAACCTCAAACCC | 60.0 | 282 | INTERGENIC | | | | |
| CaPOPI_6737 | scaffold1959 | 47247 | GC/GCC | TCCTCCTCAAGTTGGAGCAT | ATATTGCATTGCAGTGGTGG | 59.8 | 458 | DRR | Ca_26462 | K | G2-like | Signal transduction response regulator, receiver domain |
| CaPOPI_6738 | scaffold1959 | 67541 | GT/G | CCTACGGATGACCTCTCTCG | TAACGGTGAGTTGACGACGA | 59.8 | 400 | INTERGENIC | | | | |
| CaPOPI_6739 | scaffold1959 | 95637 | CAAAAAAAAAA/CAAAAAAAAAAAA | AAATTCCAACAATGTCCCGA | CGGATCAAATTAGTGCCGTT | 60.2 | 354 | DRR | Ca_26466 | | | |
| CaPOPI_6740 | scaffold1962 | 124223 | T/TATTATTATA | TTATCACGATTTTGGTGGCA | TCCCCCAAACCAAAATATCA | 59.9 | 498 | INTERGENIC | | | | |
| CaPOPI_6741 | scaffold1962 | 159146 | AAATATAAGCAGAAAATTAA/AAATATAAGCAGAAAATTAATATAAGCAGAAAATTAA | CTTTTTACAGCGCTTGTGGG | AAGCCGTGTAATCTGAACCG | 60.8 | 557 | INTERGENIC | | | | |
| CaPOPI_6742 | scaffold1962 | 170714 | GTTTTTTTTT/GTTTTTTTT | TCAAGGACCCAAAGCACATT | TACTCCCCCTTTGAGCTTGA | 60.5 | 423 | INTERGENIC | | | | |
| CaPOPI_6743 | scaffold1964 | 11011 | A/ACTC | ATGTTCCTCGCCAATCATTC | TTCCAAAAGAGGAATGGTGC | 59.9 | 771 | DRR | Ca_25209 | G | | Histidine phosphatase superfamily, clade-1 |
| CaPOPI_6744 | scaffold1964 | 11658 | AACACA/AACA | GCACCATTCCTCTTTTGGAA | GATGGACCACCTTCTCTCCA | 60.1 | 553 | CDS (FRAME SHIFT) | Ca_25209 | G | | Histidine phosphatase superfamily, clade-1 |
| CaPOPI_6745 | scaffold1964 | 11803 | TAGAATTATGCAG/TAGAATTATGCAGAATTATGCAG | GCACCATTCCTCTTTTGGAA | GATGGACCACCTTCTCTCCA | 60.1 | 553 | CDS (FRAME SHIFT) | Ca_25209 | G | | Histidine phosphatase superfamily, clade-1 |

| INDEL marker IDs | Chromosomes/ unanchored scaffolds | Physical positions (bp) | InDels (*Kabuli* reference genome-CDC Frontier/PI) | Forward primers (5'-3') | Reverse primers (5'-3') | Anneling temperature (0C) | Expected amplified product size (bp) | Structural annotation | | Functional annotation | | |
|---|---|---|---|---|---|---|---|---|---|---|---|---|
| | | | | | | | | Sequence components of *kabuli* genome | *Kabuli* gene accession IDs | NCBI-KOG | TFs | NCBI-nr database |
| CaPOPI_6746 | scaffold1964 | 20327 | TTTCTT/TTT | TGTTGTTTCCAACCACTCCA | TTGGATCGAAATTTGGGTGT | 60.0 | 861 | INTERGENIC | | | | |
| CaPOPI_6747 | scaffold1964 | 21448 | CAAAATAAAAATAA/CAAAATAA | GGGTCAGTTCATCCCATGAC | TATGCATCTTTGTTGACGCC | 60.2 | 292 | INTERGENIC | | | | |
| CaPOPI_6748 | scaffold1964 | 172119 | ATTGG/A | TTGTTTTGTTAGAAACGCCG | ACCCACATAGCGCAAGATTC | 58.9 | 313 | INTERGENIC | | | | |
| CaPOPI_6749 | scaffold198 | 70179 | ATTTTTTTTTTT/ATTTTTTTTTT | TCAAAATCCCCCAATAACCA | GCTGTCGAGTTCCCAACAAT | 60.0 | 302 | INTERGENIC | | | | |
| CaPOPI_6750 | scaffold198 | 267630 | A/AG | TCCTAGAACATGCATCACGC | AGCCACATTCCAAGCAAAAC | 59.8 | 473 | DRR | Ca_20909 | | | |
| CaPOPI_6751 | scaffold198 | 274491 | ATTTTTTTTTTT/ATTTTTTTTTT | TGGATTGTTCATCCCCTTTT | TGTGTGTTTCGTTCAACGGT | 59.2 | 293 | INTERGENIC | | | | |
| CaPOPI_6752 | scaffold198 | 501505 | CA/CAA | TTGGCCAAGTCCCTGTTTAC | CGTTCCTGAGGGAATTGTGT | 60.0 | 498 | INTERGENIC | | | | |
| CaPOPI_6753 | scaffold198 | 626672 | TAAAAAAAAA/TAAAAAAAA | TGGACACCTTCAATTATGCG | CGCAATTCTTGAGTTCGACA | 59.5 | 404 | INTERGENIC | | | | |
| CaPOPI_6754 | scaffold198 | 637595 | TTATTTTATATATTTTATATATTTT/TTATTTTATATATTTT | TGACCGCAACCAGTTCAATA | CCAACTCACAAATACATCGCC | 60.1 | 386 | INTERGENIC | | | | |
| CaPOPI_6755 | scaffold1981 | 100589 | AAATTTATAATTTATAA/AAATTTATAA | ATTTCCCACATCACCCAAAA | TCATTACTCACACCATAAGTGGAA | 60.0 | 436 | INTERGENIC | | | | |
| CaPOPI_6756 | scaffold1981 | 117409 | TTTATTATTATTATTATTA/TTTATTATTATTATTATTATTA | TTCATTAATTGTCCAATTGCTAAGT | AGGTGCGATCACGTAAAACC | 58.3 | 534 | INTERGENIC | | | | |
| CaPOPI_6757 | scaffold1981 | 118118 | ACTCTCTC/ACTCTC | GGTTTTACGTGATCGCACCT | ATCGGAATTTTTGTGCCTTG | 60.0 | 783 | INTERGENIC | | | | |
| CaPOPI_6758 | scaffold1981 | 183798 | TACTTATAAAACTTATAAAA/TACTTATAAAA | AACAACTTCTCATGTTCGCAA | CACTTCATTCCGCTCCATTT | 58.4 | 746 | INTERGENIC | | | | |
| CaPOPI_6759 | scaffold1981 | 239519 | AATATATATATATATATATATATATATATA/AATATATATATATATATATATATATATATATATATATATA | AAATTTGCGTTTAAATAACGGG | TGGATCCTCCCTTTTGTACG | 59.3 | 779 | INTERGENIC | | | | |
| CaPOPI_6760 | scaffold1981 | 287943 | TTTATTATTATTATTATTATTATTATTATTATTATTATTA/TTTATTATTATTATTATTATTATTATTATTATTATTATTATTATTATTATTA | CTTTCAAATAAAACCCGCCA | TTTAGGCATTGTTGCTGCTG | 59.9 | 179 | INTERGENIC | | | | |

| INDEL marker IDs | Chromosomes/ unanchored scaffolds | Physical positions (bp) | InDels (*Kabuli* reference genome-CDC Frontier/PI) | Forward primers (5'-3') | Reverse primers (5'-3') | Anneling temperature (0C) | Expected amplified product size (bp) | Structural annotation | | Functional annotation | | |
|---|---|---|---|---|---|---|---|---|---|---|---|---|
| | | | | | | | | Sequence components of *kabuli* genome | *Kabuli* gene accession IDs | NCBI-KOG | TFs | NCBI-nr database |
| CaPOPI_6761 | scaffold1981 | 317439 | TAAA/TAAAA | TCTCATTTGCTCCCGACTCT | TTGTCTCAACAATCTCCCCC | 60.0 | 501 | DRR | Ca_21265 | | | |
| CaPOPI_6762 | scaffold1981 | 345037 | ATTTTTTTTTT/ATTTTTTTTT | ACACTTCCAATGCCAAGGTC | TGGCTTGTGGTTGTGAAATC | 60.0 | 655 | INTRON | Ca_21268 | | | Polyketide cyclase/dehydrase |
| CaPOPI_6763 | scaffold1981 | 355117 | TCCC/TCCCC | AAACCTCGAATTTTGTTGAATC | GGGGCCTTTCAAAATATCGT | 57.3 | 502 | INTERGENIC | | | | |
| CaPOPI_6764 | scaffold1981 | 355366 | A/AC | ACGATATTTTGAAAGGCCCC | CGCGGATCCAATATCTCACT | 60.2 | 426 | INTERGENIC | | | | |
| CaPOPI_6765 | scaffold1981 | 357850 | GTTTT/GTTTTT | TCAAGTGTTCTATTTTGTTGGCA | CCTTTTTGCAATTGGTTGGT | 59.7 | 559 | INTERGENIC | | | | |
| CaPOPI_6766 | scaffold1981 | 358146 | TAAAAAAA/TAAAAAA | ACCAACCAATTGCAAAAAGG | TAAACTTGTTGTCCGGAGGC | 59.8 | 553 | INTERGENIC | | | | |
| CaPOPI_6767 | scaffold1981 | 366756 | GTTTTTTAAATTTTTTAAATTTTTTT/GTTTTTTAAATTTTTT | TCGGACTACCTGGCTATTCAA | GCACCATGAACACCCCTACT | 59.7 | 784 | INTERGENIC | | | | |
| CaPOPI_6768 | scaffold1981 | 370536 | AAATAATAATAATAATAATAATA/AAATAATAATAATA | GGTGTTTGCTTTTAATAGGCTCA | TCACCCACTCTACTCTTTTTGTTT | 59.7 | 934 | INTERGENIC | | | | |
| CaPOPI_6769 | scaffold1981 | 371440 | TAAAAAAA/TATAAAAAAAAAA | TTTCGTGGTCCTAAATTGCC | AAAAGGGACATGGAACAACAA | 59.9 | 698 | INTERGENIC | | | | |
| CaPOPI_6770 | scaffold1981 | 421474 | A/AT | GCGCGAGTATGGGCTTATTA | ATTGATTGGATGAGGGACCA | 60.2 | 714 | INTERGENIC | | | | |
| CaPOPI_6771 | scaffold1981 | 421693 | TAG/T | TCATTGGATCAATAAGCCTCA | ATTGATTGGATGAGGGACCA | 58.2 | 206 | INTERGENIC | | | | |
| CaPOPI_6772 | scaffold1983 | 23052 | GTT/GTTT | AGCAGGTCTGTCCAATCAGG | CATCAGCCCCTCTAAACCAA | 60.3 | 852 | INTERGENIC | | | | |
| CaPOPI_6773 | scaffold1983 | 63035 | TAAA/TA | GCGGGTGTAGTGACATGTTG | TGTGGCCGTAAACATTCAAA | 60.0 | 415 | INTERGENIC | | | | |
| CaPOPI_6774 | scaffold1984 | 106927 | ATG/ATGTTG | GCAAGGAATTCCCCCATTAT | TGTGACCGATTTTGTGACTGA | 60.0 | 825 | INTERGENIC | | | | |
| CaPOPI_6775 | scaffold1984 | 284025 | CAAAA/CAAA | GCTCTCATCTGAGGGCAAAG | ATTTGGGAAGTTACAGGGGC | 60.1 | 562 | INTERGENIC | | | | |

| INDEL marker IDs | Chromosomes/ unanchored scaffolds | Physical positions (bp) | InDels (*Kabuli* reference genome-CDC Frontier/PI) | Forward primers (5'-3') | Reverse primers (5'-3') | Anneling temperature (0C) | Expected amplified product size (bp) | Structural annotation | | Functional annotation | | |
|---|---|---|---|---|---|---|---|---|---|---|---|---|
| | | | | | | | | Sequence components of *kabuli* genome | *Kabuli* gene accession IDs | NCBI-KOG | TFs | NCBI-nr database |
| CaPOPI_6776 | scaffold1984 | 323626 | T/TCCG | GCTTGTCCGCGTAACTCTTC | ACGATTTTAGAAGAGGCGCA | 60.0 | 601 | INTERGENIC | | | | |
| CaPOPI_6777 | scaffold1984 | 386637 | GTT/GT | CGGTAAAAAGTGTGCGGATT | TGAATGGAAGTGGGACAACA | 60.0 | 491 | INTERGENIC | | | | |
| CaPOPI_6778 | scaffold1985 | 19601 | TCTTTTCCTTTTCCTTTTCCTT/TCTTTTCCTTTTCCTTTTCCTTTTCCTT | AGGAAGAGTGGAGTTGCGAA | TATTTTGGATTTTGGCTCGG | 60.0 | 511 | INTERGENIC | | | | |
| CaPOPI_6779 | scaffold1988 | 7769 | TACACACAATCAAA/TACACACACAATCAAA | ACCATACAAGTTCCCGCATC | GAAGGAGCAGTAAGCCATCG | 59.8 | 725 | INTERGENIC | | | | |
| CaPOPI_6780 | scaffold1990 | 8359 | TTGAAATGAAAT/TTGAAAT | ACGTCTTTCTTTGTGGGTCG | TTAAGCAACAGGCCCAATTC | 60.1 | 196 | INTERGENIC | | | | |
| CaPOPI_6781 | scaffold1996 | 1333 | TG/TGG | CGGATGAATCATTGGGAAAT | AGTGAAACCAACGCCAAAAC | 59.6 | 325 | INTERGENIC | | | | |
| CaPOPI_6782 | scaffold1996 | 5730 | GAAAAAAAA/GAAAAAAAAAA | TGTTAGGAGGATCTCACGGG | ATTGTTGGAAAATTGGCTGC | 60.1 | 823 | INTERGENIC | | | | |
| CaPOPI_6783 | scaffold1999 | 8100 | GAAAA/GAAAAA | TGGATGCACGAGATTGGTTA | TGCATCCAATAGGCGTTTTT | 60.1 | 938 | INTERGENIC | | | | |
| CaPOPI_6784 | scaffold1999 | 24246 | TAAAAA/TAAAA | GCCTAGGGAGGACGAAAATC | AACCAATGTTGTGCATCCAA | 60.0 | 375 | INTERGENIC | | | | |
| CaPOPI_6785 | scaffold1999 | 27916 | CAAA/CAAAA | AGGGAGGACGAAAATGACCT | CTTGGGCATCCAATAGGTGT | 59.9 | 763 | INTERGENIC | | | | |
| CaPOPI_6786 | scaffold1999 | 29603 | GACA/GACACA | AGGGAGGACGAAAATGACCT | AACCAATCTTGTGCATCCAA | 59.9 | 670 | INTERGENIC | | | | |
| CaPOPI_6787 | scaffold1999 | 29835 | TAAAAAAA/TAAAAAA | AATCCAACGACAACGGAACT | CCGATGTTGTGCATCCAATA | 59.5 | 541 | INTERGENIC | | | | |
| CaPOPI_6788 | scaffold1999 | 30095 | AT/A | ATCCAACGACAACGGAACTT | AACCAATGTTGTGCATCCAA | 59.5 | 939 | INTERGENIC | | | | |
| CaPOPI_6789 | scaffold2 | 18928 | A/AT | GTCAGGCATTCCCCTCTACA | TTCGGTTGGTGACCCTTTAC | 60.1 | 118 | INTERGENIC | | | | |
| CaPOPI_6790 | scaffold2 | 69919 | GC/G | TTCGCAACTCCACTCTTCCT | CAACGCAACATAACACCCAG | 60.0 | 477 | INTERGENIC | | | | |

| INDEL marker IDs | Chromosomes/ unanchored scaffolds | Physical positions (bp) | InDels (*Kabuli* reference genome-CDC Frontier/PI) | Forward primers (5'-3') | Reverse primers (5'-3') | Anneling temperature (0C) | Expected amplified product size (bp) | Structural annotation | | Functional annotation | | |
|---|---|---|---|---|---|---|---|---|---|---|---|---|
| | | | | | | | | Sequence components of *kabuli* genome | *Kabuli* gene accession IDs | NCBI-KOG | TFs | NCBI-nr database |
| CaPOPI_6791 | scaffold2 | 515747 | TTATATATATATATATATATAT/TTATATATATATATATATATATAT | TGGACTGGCATGATTGGATA | ACCACTCAAAGTGTTTGGGC | 59.9 | 833 | INTERGENIC | | | | |
| CaPOPI_6792 | scaffold2000 | 1544 | GAAAAAAA/GAAAAAAAA | GCCTAGGGATGACGAAAATG | TGTGTTTGCGTCATTGGATT | 59.5 | 566 | INTERGENIC | | | | |
| CaPOPI_6793 | scaffold2000 | 14213 | CA/C | AAGCCTAGGGAGGACGAAAA | TGCCACTGTGACTTATCCAATC | 60.2 | 509 | INTERGENIC | | | | |
| CaPOPI_6794 | scaffold2000 | 22359 | AATTGGCCT/A | GCTGTGCACTTGATGCTGAT | TGTGTCGTTCGGTGAACATT | 60.0 | 465 | INTERGENIC | | | | |
| CaPOPI_6795 | scaffold2003 | 11976 | T/TG | TCATTGGCTTATTCTTGCCC | TAGGGACGACGAAAATGACC | 60.0 | 504 | INTERGENIC | | | | |
| CaPOPI_6796 | scaffold2003 | 13286 | ATT/AT | CAATCTTGTGCATCCAATCG | AGGGAGGACGAAAATGACCT | 60.1 | 567 | INTERGENIC | | | | |
| CaPOPI_6797 | scaffold2003 | 32381 | ATTTTTT/ATTTTTTT | CTGTGGAGTTCTAGGGGCTG | TGGCATAAGAGAGTGGCTCA | 59.9 | 479 | INTERGENIC | | | | |
| CaPOPI_6798 | scaffold2003 | 33197 | GTT/GTTT | AATTGCGTTTCAAACCTTCG | AAACTGGCCAACAGAGGATG | 60.1 | 344 | INTERGENIC | | | | |
| CaPOPI_6799 | scaffold2005 | 14756 | TAAAAAAA/TAAAAAAAA | AAAATCCAACAATCTCCCCC | CGGTCTCAACCTCTCCCATA | 60.0 | 515 | DRR | Ca_28003 | | | |
| CaPOPI_6800 | scaffold2005 | 32399 | ATTTTTTTTTT/ATTTTTTTTTTT | CCAGACCAGACTTCGAAAGG | GACTGTCTGTCAGGGGCCTA | 59.8 | 549 | INTERGENIC | | | | |
| CaPOPI_6801 | scaffold2010 | 3634 | AT/ATT | AATCGATTGATTCAGCCCAG | TGATTTCCAACGGTCATCAA | 60.0 | 869 | DRR | Ca_27976 | | | |
| CaPOPI_6802 | scaffold2010 | 6749 | T/TC | TTCCCTTTCGTTGTGTATCG | GGGCACCGATGTTACATAAA | 58.6 | 857 | URR | Ca_27976 | | | |
| CaPOPI_6803 | scaffold2010 | 7925 | ATTTTTTTTT/ATTTTTTTTTT | AAAGTGCCTTCTTCTTGGCA | ATGTTTTCTTTTGGGTTGCG | 60.0 | 564 | URR | Ca_27976 | | | |
| CaPOPI_6804 | scaffold2018 | 4354 | TGGAG/TG | CATGGTTTTGTTTGTCGTGC | TGTTTGCTTTAAGCGATGTCA | 60.0 | 462 | INTERGENIC | | | | |
| CaPOPI_6805 | scaffold2018 | 16998 | CAATAATAATAATAATAATAATAATAATAATAATAATAATAATAATAATAATAAT/CAATAATAATAATAATAATAATAATAATAATAATAATAATAATAATAATAATAATAA | AAGGCTCACTTGCAAAGACG | GGTATTGCTGCAGTTTTTGG | 60.6 | 445 | INTERGENIC | | | | |

| INDEL marker IDs | Chromosomes/ unanchored scaffolds | Physical positions (bp) | InDels (*Kabuli* reference genome-CDC Frontier/PI) | Forward primers (5'-3') | Reverse primers (5'-3') | Anneling temperature (0C) | Expected amplified product size (bp) | Structural annotation | | Functional annotation | | |
|---|---|---|---|---|---|---|---|---|---|---|---|---|
| | | | | | | | | Sequence components of *kabuli* genome | *Kabuli* gene accession IDs | NCBI-KOG | TFs | NCBI-nr database |
| CaPOPI_6806 | scaffold202 | 17670 | ATTTTTT/ATTTTTTT | TCACAAGGGTAGTTTTGGGTTT | CTGCCAGCTCACCTCCTATC | 59.8 | 685 | INTERGENIC | | | | |
| CaPOPI_6807 | scaffold202 | 17816 | AAA/AAATAA | TCACAAGGGTAGTTTTGGGTTT | CTGCCAGCTCACCTCCTATC | 59.8 | 685 | INTERGENIC | | | | |
| CaPOPI_6808 | scaffold202 | 19978 | GT/G | GGAATTTTGCAGAAAACGGA | CTGTCCGCCTAATTTTCGAG | 60.1 | 778 | INTERGENIC | | | | |
| CaPOPI_6809 | scaffold202 | 20536 | CTA/C | TGCATCCAACTTTGGTGTGT | ACACCGTTGTTTATGCACGA | 60.0 | 373 | INTERGENIC | | | | |
| CaPOPI_6810 | scaffold202 | 22635 | CG/C | GGATTACATTCGCTTCTCCG | GCAAACAATGCGAAACAGAA | 59.7 | 804 | INTERGENIC | | | | |
| CaPOPI_6811 | scaffold202 | 22768 | AT/A | TTGCAATCATGAGGCAAGTC | GCAAACAATGCGAAACAGAA | 59.8 | 235 | INTERGENIC | | | | |
| CaPOPI_6812 | scaffold202 | 36674 | TAAA/TAA | AAATCGGTGTCAACGGTCTC | TTCACCTGTCAATTGGGTTG | 60.0 | 559 | INTERGENIC | | | | |
| CaPOPI_6813 | scaffold202 | 37860 | TAAA/TAA | ATTGGAGGCTGGTTTTGTTG | ATCGTGGGGATGAATTTGAA | 60.0 | 539 | INTERGENIC | | | | |
| CaPOPI_6814 | scaffold202 | 37967 | T/TC | ATTGGAGGCTGGTTTTGTTG | ATCGTGGGGATGAATTTGAA | 60.0 | 539 | INTERGENIC | | | | |
| CaPOPI_6815 | scaffold202 | 39185 | TATG/TATGATG | GCACCGGAAAATACGTAGGA | ATTATCTTGTCGCGGCCTAA | 60.0 | 359 | INTERGENIC | | | | |
| CaPOPI_6816 | scaffold202 | 39293 | CTTTT/CTTTTT | GCACCGGAAAATACGTAGGA | ATTATCTTGTCGCGGCCTAA | 60.0 | 359 | INTERGENIC | | | | |
| CaPOPI_6817 | scaffold202 | 61873 | GTAAAATGAAATAAAATGA/GTAAAATGAAATAAAATGAAATAAAATGA | CAGCCCTTGAATTCGATCAT | GCTGTTTGGCTCAACCATTT | 60.0 | 331 | INTRON | Ca_27125 | | | |
| CaPOPI_6818 | scaffold202 | 93888 | A/ACAAGATC | GTGCAGTGAAAGGGTTCCAT | AATTTGATATCGTGGCGGAG | 60.0 | 881 | INTRON | Ca_27126 | U | | Vacuolar protein sorting-associated protein |
| CaPOPI_6819 | scaffold202 | 99484 | TAAAGTTTATATTTTAAAAGTTTATATTTTAA/TAAAGTTTATATTTTAA | CCTCTTTCTCAATTGCAGCC | ATGTTTGGGAGAGCCATTTG | 60.0 | 792 | DRR | Ca_27127 | A | | Like-Sm ribonucleoprotein (LSM) domain |
| CaPOPI_6820 | scaffold202 | 100589 | A/AG | CATCAAATCACGAATGGCAG | GTTAGGGGAGATGGGGTGAT | 60.1 | 543 | DRR | Ca_27126 | U | | Vacuolar protein sorting-associated protein |

| INDEL marker IDs | Chromosomes/ unanchored scaffolds | Physical positions (bp) | InDels (*Kabuli* reference genome-CDC Frontier/PI) | Forward primers (5'-3') | Reverse primers (5'-3') | Anneling temperature (0C) | Expected amplified product size (bp) | Structural annotation | | Functional annotation | | |
|---|---|---|---|---|---|---|---|---|---|---|---|---|
| | | | | | | | | Sequence components of *kabuli* genome | *Kabuli* gene accession IDs | NCBI-KOG | TFs | NCBI-nr database |
| CaPOPI_6821 | scaffold202 | 103994 | ATTTTTTTTTT/ATTTTTTTTTTTTT | AAATTGCTTATTTAGTAGTGTCAATCG | AACTCAGCAAAACCCAAAACA | 58.5 | 409 | URR | Ca_27127 | A | | Like-Sm ribonucleoprotein (LSM) domain |
| CaPOPI_6822 | scaffold202 | 104373 | TAAAAAAAAAAA/TAAAAAAAAA | TGTTTTGGGTTTTGCTGAGTT | TGGATGGATTGGATGAATGA | 59.6 | 180 | URR | Ca_27127 | A | | Like-Sm ribonucleoprotein (LSM) domain |
| CaPOPI_6823 | scaffold202 | 104727 | TTA/T | TCATTCATCCAATCCATCCA | TCGAGTGTCGATCTCAAGGA | 59.7 | 489 | INTERGENIC | | | | |
| CaPOPI_6824 | scaffold2020 | 7500 | AATA/AA | CTTGCTAGAGCTTGGCGATT | ACATTTCTTTGGCGGTTTTG | 59.8 | 569 | INTERGENIC | | | | |
| CaPOPI_6825 | scaffold2024 | 1945 | CAAA/CAAAA | GAATGTTAGGGTTTCCGGGT | GATGAAATCGAGGACTGGGA | 60.1 | 338 | INTERGENIC | | | | |
| CaPOPI_6826 | scaffold2027 | 955 | GAAAAAAAA/GAAAAAAAAA | AGAAAGGCTCAAACGACCAA | ACAGAGGAAGCTGCCACCTA | 59.9 | 672 | INTERGENIC | | | | |
| CaPOPI_6827 | scaffold2030 | 4596 | ATT/ATTTT | AGGGTTTAGGATTTCGCGTT | CATATCAAAAACCCAAATGACC | 60.0 | 243 | INTERGENIC | | | | |
| CaPOPI_6828 | scaffold2033 | 183883 | CGAAGAAGAAG/CGAAGAAGAAGAAG | CTTGGGGTGGCAAGTACAGT | TCGAATACATGAATTAACAAAAGGA | 60.0 | 371 | DRR | Ca_26143 | | bHLH | Lipase, GDSL |
| CaPOPI_6829 | scaffold2036 | 83593 | TAATAAATAAATAAATAAATAAATAAATAAATAAATAAATAAA/TAATAAATAAATAAATAAATAAATAAATAAATAAATAAATAAATAAA | TGAATTGGAGGTCATGGTGA | TCCAACGAATTGATGTCCAA | 59.9 | 551 | INTERGENIC | | | | |
| CaPOPI_6830 | scaffold2036 | 151113 | AATATATATATATATATATA/AATATATATATATATATATATA | TATTTTGGATTTTGGCTCGG | AGGAAGAGTGGAGTTGCGAA | 59.9 | 394 | INTERGENIC | | | | |
| CaPOPI_6831 | scaffold2036 | 238131 | TGTAGTA/TGTA | TTCAAAAAGCGCGGTAAATC | AGAATCCACCCACACAAAGC | 60.2 | 821 | DRR | Ca_26067 | | ARF | Transferase |
| CaPOPI_6832 | scaffold2039 | 15621 | C/CTAGTT | GCAAGAGGAAAAGACGAACG | CGTTTTGCTTTTTGTTGGGT | 60.0 | 516 | INTERGENIC | | | | |
| CaPOPI_6833 | scaffold2039 | 52513 | A/ACTTTCTCT | TGCAAAAACCAATGTGGAAA | AGGCCAAACTCGTGCTTAGA | 59.9 | 575 | INTERGENIC | | | | |
| CaPOPI_6834 | scaffold2039 | 83114 | TAAAAAAAAA/TAAAAAAAAAAA | CCACTCACTCGAATCTCGAAC | GACTCAAAAGCTGGGCACTC | 59.9 | 661 | INTERGENIC | | | | |
| CaPOPI_6835 | scaffold2039 | 128633 | ATTTTTTTTTTTTT/ATTTTTTTTTTT | TTGTCCCCTGAATGATTCGT | TTTGTGGCTGTCAAAGCAG | 60.3 | 613 | INTERGENIC | | | | |

| INDEL marker IDs | Chromosomes/ unanchored scaffolds | Physical positions (bp) | InDels (*Kabuli* reference genome-CDC Frontier/PI) | Forward primers (5'-3') | Reverse primers (5'-3') | Anneling temperature (0C) | Expected amplified product size (bp) | Structural annotation | | Functional annotation | | |
|---|---|---|---|---|---|---|---|---|---|---|---|---|
| | | | | | | | | Sequence components of *kabuli* genome | *Kabuli* gene accession IDs | NCBI-KOG | TFs | NCBI-nr database |
| CaPOPI_6836 | scaffold2040 | 102148 | A/AC | TGGTCCTCAACGAACAACAA | TTGCAAAAGTGAGTGCCTTG | 60.1 | 386 | INTERGENIC | | | | |
| CaPOPI_6837 | scaffold2040 | 154810 | AATATATATATATATATATAT/AATATATATATATATATAT | ATTTTGGTTCGGAGGCTTTT | TCTCTCACGCCGTTTTTCTT | 59.9 | 539 | INTERGENIC | | | | |
| CaPOPI_6838 | scaffold2040 | 159577 | CA/CAGGTA | TGAAGAGGGGAATCCATCTG | TTAACAACTCCTGGTTCGGG | 60.0 | 372 | INTERGENIC | | | | |
| CaPOPI_6839 | scaffold205 | 40693 | C/CAAG | CTACTCTCAATTTTCCGCCG | GCACAATCAGAAGCGCATTA | 59.8 | 761 | DRR | Ca_25491 | | | |
| CaPOPI_6840 | scaffold205 | 46882 | GAGGA/GAGGAAGGA | GAGAGGATTTGTGTTCCCGA | GGAATGCTCAAAGGTTGCAT | 60.1 | 235 | INTERGENIC | | | | |
| CaPOPI_6841 | scaffold205 | 75804 | G/GA | TGAAGAGGGGAATCCATCTG | ACTCCAAACGGCATAACCAG | 60.0 | 745 | INTERGENIC | | | | |
| CaPOPI_6842 | scaffold2051 | 10215 | ATTTTTT/ATTTT | CCTTTCCATTTCTTTGAGGG | GGCACCCAACTAGGTTTGAA | 58.6 | 818 | DRR | Ca_25399 | R | | Sugar/inositol transporter |
| CaPOPI_6843 | scaffold206 | 162025 | GTTTTT/GTTTT | CTTGGATTCATTGATCCGCT | GTTTGGTTTTCTTTGGGGGT | 60.0 | 423 | INTERGENIC | | | | |
| CaPOPI_6844 | scaffold206 | 176077 | T/TATAG | CTCTCGCTATGAAAAACGGG | TGGGTTCAATTGGCATTTTT | 59.8 | 759 | INTERGENIC | | | | |
| CaPOPI_6845 | scaffold206 | 190834 | CATATATATATATATATAT/CATATATATATATATATATAT | ATCCCTAACGCCGTTTTTCT | ATATTGGCTCGGAGGCTTTT | 60.0 | 480 | INTERGENIC | | | | |
| CaPOPI_6846 | scaffold207 | 6894 | AC/A | GCAAACTCGACAGGAAAAGC | TTGGTGAAGATGTCAGCCAG | 60.0 | 370 | INTERGENIC | | | | |
| CaPOPI_6847 | scaffold208 | 95031 | ATATTATTATTATTATTATTACTATTATTATTAT/ATATTATTATTATTATTATTATTATTATTACTATTATTATTAT | CATTGGATGCACCATTTCAG | TCAATCACCACAAACATCCC | 59.9 | 720 | INTERGENIC | | | | |
| CaPOPI_6848 | scaffold208 | 131475 | GTT/GT | TGCAAAAACCACTTTCAAGG | TTTGGACCAATTTTGTGGCT | 58.8 | 620 | INTERGENIC | | | | |
| CaPOPI_6849 | scaffold208 | 174233 | GAAAA/GAAA | CCTTGGAGTATTCTCGGCAG | TGCTGATCCGTGAGAGATTG | 59.8 | 894 | INTERGENIC | | | | |
| CaPOPI_6850 | scaffold210 | 31686 | CAA/CAAA | TTTTCTCGCAAAACATGCAG | TTCGCAACTCCACTCTTCCT | 60.0 | 712 | INTERGENIC | | | | |

| INDEL marker IDs | Chromosomes/ unanchored scaffolds | Physical positions (bp) | InDels (*Kabuli* reference genome-CDC Frontier/PI) | Forward primers (5'-3') | Reverse primers (5'-3') | Anneling temperature (0C) | Expected amplified product size (bp) | Structural annotation | | Functional annotation | | |
|---|---|---|---|---|---|---|---|---|---|---|---|---|
| | | | | | | | | Sequence components of *kabuli* genome | *Kabuli* gene accession IDs | NCBI-KOG | TFs | NCBI-nr database |
| CaPOPI_6851 | scaffold210 | 32630 | TAAAA/TAAAAA | AGGAAGAGTGGAGTTGCGAA | TATTTTGGATTTTGGCTCGG | 60.0 | 512 | INTERGENIC | | | | |
| CaPOPI_6852 | scaffold210 | 411319 | GTTTTTTT/GTTTTTTTTT | CCACAACTCCTCACATCCCT | TCACAGTTGGTAGTGGCACC | 60.0 | 239 | INTERGENIC | | | | |
| CaPOPI_6853 | scaffold210 | 532061 | CATATATAT/CATATAT | AAGCGCTGTAATATGCCCAC | CTGGGCAAGGGATCTGAATA | 60.1 | 639 | DRR | Ca_21912 | | Trihelix | Peptidase C48, SUMO/Sentrin/Ubl1 |
| CaPOPI_6854 | scaffold210 | 577121 | AGAAATCGATTTGG/AGAAATCGATTTGGAAATCGATTTGG | TTGCCAAGTGTGGATGTTGT | GGGTACGAACCGGTAGGATT | 60.0 | 947 | INTERGENIC | | | | |
| CaPOPI_6855 | scaffold2103 | 124174 | GTTTTC/GTTTTCATTTTC | AACCCAATCACTGGAGATGC | AGGAAGAGTGGAGTTGCGAA | 59.9 | 540 | INTERGENIC | | | | |
| CaPOPI_6856 | scaffold2103 | 145039 | GTATATATATATATATATATAT/GTATATATATATATATATAT | GCCAGAACACCGACCATACT | CCAAGTGGTACAACTGGTGTCT | 60.0 | 581 | INTERGENIC | | | | |
| CaPOPI_6857 | scaffold212 | 125792 | TA/TTCA | TGTCCAATCTTAGGGGTCAAA | AATTTGCCAATTCAAGGCAG | 59.4 | 641 | INTERGENIC | | | | |
| CaPOPI_6858 | scaffold212 | 180804 | T/TA | CGAAGATTCGAAGGCAAAAG | CCGTTCTTGGTTTTCACACA | 60.0 | 722 | INTERGENIC | | | | |
| CaPOPI_6859 | scaffold2126 | 3526 | GATATATATATATA/GATATATATATATATA | CCATGAAAAGAAGAAGGGCA | GCCTTTTACTGCACTTTGGC | 60.2 | 472 | INTERGENIC | | | | |
| CaPOPI_6860 | scaffold2134 | 5442 | TTA/T | GAAAAAGGAACGAAGGGAGG | AACGGAACGAGCGAGTCTTA | 60.0 | 566 | INTERGENIC | | | | |
| CaPOPI_6861 | scaffold2152 | 34103 | CTTTTT/CTTTT | GGGGTGCTCAACTTGTTTGT | TGATCAAATTCCCCCACACT | 60.0 | 589 | INTERGENIC | | | | |
| CaPOPI_6862 | scaffold2152 | 76304 | AATTATTATTATTATTATTATTATTATTATTATTATTGTTATTATTATTAT/AATTATTATTATTATTATTATTATTATTATTATTATTGTTATTATTATTAT | TTCATGGGAACACAAGCAAG | GAAGGAAAGGGTCAAGGCTC | 59.7 | 258 | INTERGENIC | | | | |
| CaPOPI_6863 | scaffold2152 | 76522 | AATTATTATTATTATTATTATTATTATTAT/AATTATTATTATTATTATTATTATTATTATTAT | GAGCCTTGACCCTTTCCTTC | TGGGGGTATAAACCATCAAAA | 60.2 | 596 | INTERGENIC | | | | |
| CaPOPI_6864 | scaffold2152 | 108576 | CT/CTATATTTCATATCTT | TTTCAGCTTCATCGCATTCA | GAGATTGCCAAGAACAAGGC | 60.5 | 448 | INTERGENIC | | | | |
| CaPOPI_6865 | scaffold2153 | 19763 | CTATTTATTTATTTATTT/CTATTTATTTATTTATTTATTT | TGGAAAACACAAGAAAGGGG | TGGGGATACGTAGGAGCAAG | 59.9 | 606 | INTERGENIC | | | | |

| INDEL marker IDs | Chromosomes/ unanchored scaffolds | Physical positions (bp) | InDels (*Kabuli* reference genome-CDC Frontier/PI) | Forward primers (5'-3') | Reverse primers (5'-3') | Anneling temperature (0C) | Expected amplified product size (bp) | Structural annotation | | Functional annotation | | |
|---|---|---|---|---|---|---|---|---|---|---|---|---|
| | | | | | | | | Sequence components of *kabuli* genome | *Kabuli* gene accession IDs | NCBI-KOG | TFs | NCBI-nr database |
| CaPOPI_6866 | scaffold2158 | 81348 | AATATATATATATATATATATATATATATTTATATATATAT/AATATATATATATATATATATATATATTTATATATATAT | GGATTTTGGCTAGGAGGCTT | AAATGGAGGAGAAGGAGGGA | 59.7 | 438 | INTERGENIC | | | | |
| CaPOPI_6867 | scaffold2158 | 133975 | ACCCCCCCCCCCC/ACCCCCCCCCC | GTAGCAAGAATCCCAGGGTG | AATGGCCACTTCGAGTCAAC | 59.6 | 317 | DRR | Ca_24818 | | | |
| CaPOPI_6868 | scaffold2158 | 178374 | TTACTAC/TTACTACTAC | TATCTCAACCCCCACCCATA | ATCAACAGCCAGACCCATTC | 60.0 | 829 | INTERGENIC | | | | |
| CaPOPI_6869 | scaffold2161 | 5214 | GGGGT/GGGGTGGGT | TACCCTTCTGGGTTGCTTCA | AAGCCTCCAATGCTTCTTCA | 60.6 | 154 | INTRON | Ca_26956 | H | | Folylpolyglutamate synthetase |
| CaPOPI_6870 | scaffold2161 | 14357 | TAAAAAAAA/TAAAAAA | TTTCAGTATCGTTGCGTGGA | ACGCACCAAACACATTGAGA | 60.3 | 575 | INTERGENIC | | | | |
| CaPOPI_6871 | scaffold2161 | 32357 | GAAAAAAAAAAA/GAAAAAAAAAAAA | GAGGGATGAGAAGGTGACGA | TTTTTGTGCCGTAGTCCCTC | 60.2 | 744 | INTERGENIC | | | | |
| CaPOPI_6872 | scaffold2161 | 184512 | CATTATTATTATTATTATTATTATTATTATTATTATTGTTATTATTATTATTATTATTATTATAATTATTATTATT/CATTATTATTATTATTATTATTATTATTATTATTATTATT | TGCATTGGTATATGGAGGCT | GGCCTGCCAGCTTATCATAG | 58.1 | 841 | INTERGENIC | | | | |
| CaPOPI_6873 | scaffold2164 | 67799 | TTGTG/TTG | TAAACCCCATTACAACCCGA | TCCTAGAACATGCATCACGC | 60.0 | 109 | INTERGENIC | | | | |
| CaPOPI_6874 | scaffold2166 | 41038 | AG/AGG | GCTCGAAACTGGTGGCTACT | TCCACAACAACCTACGTCCA | 59.5 | 245 | INTERGENIC | | | | |
| CaPOPI_6875 | scaffold2166 | 49190 | CTTTTTTTTTT/CTTTTTTTTT | TGTTGGTTCAAAATGGGGTT | TCCATGGTGCCTTTGTATCA | 60.1 | 872 | INTERGENIC | | | | |
| CaPOPI_6876 | scaffold2166 | 85838 | AATATATATATATATATATATAT/AATATATATATATATATATATATAT | TTTCAAAGGATTCACTGCCC | GCACCATAATGCCCCTGTAT | 60.1 | 536 | INTERGENIC | | | | |
| CaPOPI_6877 | scaffold2170 | 4201 | ATTTGGCTTCGTGGTCTTTTGGCT/ATTTGGCT | GCTATCGGTCACTGGTTGGT | CGAGGACCAGCTTTGGTAAG | 60.0 | 372 | INTERGENIC | | | | |
| CaPOPI_6878 | scaffold2170 | 5002 | AACCTTACCTTACCTTACCT/AACCTTACCTTACCT | GCAAATCAAGGTTGCAGTCA | ACACTCTAATGGGGACAGCG | 59.8 | 222 | INTRON | Ca_26928 | YU | | |
| CaPOPI_6879 | scaffold2170 | 5357 | C/CTATTGGG | CGCTGTCCCCATTAGAGTGT | GTTTGCCAATTAGGGCACAT | 60.1 | 387 | INTRON | Ca_26928 | YU | | |
| CaPOPI_6880 | scaffold2170 | 12258 | CTTTTT/CTTTT | TGCTTTGTTCAATTGCTTGC | GGAGTCCCGGAAAATTTCAT | 60.0 | 531 | INTRON | Ca_26929 | O | | |

| INDEL marker IDs | Chromosomes/ unanchored scaffolds | Physical positions (bp) | InDels (*Kabuli* reference genome-CDC Frontier/PI) | Forward primers (5'-3') | Reverse primers (5'-3') | Anneling temperature (0C) | Expected amplified product size (bp) | Structural annotation | | Functional annotation | | |
|---|---|---|---|---|---|---|---|---|---|---|---|---|
| | | | | | | | | Sequence components of *kabuli* genome | *Kabuli* gene accession IDs | NCBI-KOG | TFs | NCBI-nr database |
| CaPOPI_6881 | scaffold2170 | 12676 | ATATGTAT/ATATGTATGTAT | GAAATTTTCCGGGACTCCAT | AGCTCTTTCCACTCCAAGCA | 60.1 | 618 | INTRON | Ca_26929 | O | | |
| CaPOPI_6882 | scaffold2170 | 14347 | AC/ACC | GCTCATTCTTGTCCCCAAAA | GCCTCTGCTAATTGGTCTCG | 60.1 | 583 | INTRON | Ca_26929 | O | | |
| CaPOPI_6883 | scaffold2173 | 19315 | TAAAAAAAAA/TAAAAAAAA | GGACAAAAATGACCCAAACG | TTTTGTCCTCCCTTGGCTTA | 60.2 | 513 | INTERGENIC | | | | |
| CaPOPI_6884 | scaffold2173 | 19619 | TAAAAAA/TAAAAAAA | GGATGACACAAACGCACCTA | ATTTTTGTCCTCCCTTGGCT | 59.6 | 385 | INTERGENIC | | | | |
| CaPOPI_6885 | scaffold2173 | 19824 | A/AG | CCAAGGGAGGACAAAAATGA | TTTTCGTCCTTCACAGGCTT | 59.9 | 515 | INTERGENIC | | | | |
| CaPOPI_6886 | scaffold2173 | 56248 | ATT/AT | CCTTGGGAAGACGAAAATGA | CCATCCAATACGTGTGCTTG | 60.0 | 363 | INTERGENIC | | | | |
| CaPOPI_6887 | scaffold2173 | 56515 | GACACCAACACACCAA/GACACCAA | CCTTGGGAAGACGAAAATGA | TCCAATCTGGTCCATCCAAT | 60.0 | 673 | INTERGENIC | | | | |
| CaPOPI_6888 | scaffold2177 | 10444 | CATTAATT/CATT | CCGTGGAATTGATGGAACTT | CTGCCACTTCGAGAACAACA | 59.8 | 331 | INTRON | Ca_27883 | | | Glycoside hydrolase, family 28 |
| CaPOPI_6889 | scaffold2179 | 20401 | CTTTT/CTTT | GGGACACGTGTTGTCCTTTT | AATGTCAAGGATGGCCTCAC | 59.9 | 601 | INTERGENIC | | | | |
| CaPOPI_6890 | scaffold2179 | 20793 | TCCCCC/TCCCC | ATCAGCGAGGATGCAGAACT | CATCGCTCAGTTTGGACTCA | 60.0 | 789 | INTERGENIC | | | | |
| CaPOPI_6891 | scaffold2179 | 35934 | TG/TGCG | GAATTTGAACCTCCAAGCCA | ATAGCGGAGAAGCCCATACA | 60.1 | 404 | INTERGENIC | | | | |
| CaPOPI_6892 | scaffold2179 | 35973 | TC/TCC | GAATTTGAACCTCCAAGCCA | ATAGCGGAGAAGCCCATACA | 60.1 | 404 | INTERGENIC | | | | |
| CaPOPI_6893 | scaffold2179 | 36066 | ATAG/A | GAATTTGAACCTCCAAGCCA | TGCATTCCACATACAAGGGA | 60.1 | 582 | INTERGENIC | | | | |
| CaPOPI_6894 | scaffold2183 | 19955 | TATTAATTAATT/TATTAATT | TAGGGCGATTTTCGTACCAC | TTTTCGCATATTTATTTGACAACTC | 60.0 | 468 | INTERGENIC | | | | |
| CaPOPI_6895 | scaffold2183 | 67523 | GT/G | AGTCGCTCGAAAAGTCCAAA | TCCTGGTCCTCGATTTCATC | 60.0 | 528 | INTERGENIC | | | | |

| INDEL marker IDs | Chromosomes/ unanchored scaffolds | Physical positions (bp) | InDels (*Kabuli* reference genome-CDC Frontier/PI) | Forward primers (5'-3') | Reverse primers (5'-3') | Anneling temperature (0C) | Expected amplified product size (bp) | Structural annotation | | Functional annotation | | |
|---|---|---|---|---|---|---|---|---|---|---|---|---|
| | | | | | | | | Sequence components of *kabuli* genome | *Kabuli* gene accession IDs | NCBI-KOG | TFs | NCBI-nr database |
| CaPOPI_6896 | scaffold2183 | 69871 | ATTTTT/ATTTTTT | GATGAAATCGAGGACTGGGA | TGAAATCTAGCGCAGACCCT | 60.0 | 245 | INTERGENIC | | | | |
| CaPOPI_6897 | scaffold2183 | 70044 | AGGGG/AGGGGG | AGGGTCTGCGCTAGATTTCA | AGGGTTTCTCGGTCCTTGAT | 60.0 | 127 | INTERGENIC | | | | |
| CaPOPI_6898 | scaffold2192 | 2178 | GA/GAGAA | CATGGAGTTGAAATGGCAAA | CGTTGTTAAAAATCGGGGTG | 59.5 | 589 | INTERGENIC | | | | |
| CaPOPI_6899 | scaffold2193 | 31576 | AA/ACAAACACA | GCCAATAGAGGATGGAAGCA | TATGCCAAAGCCATGTTTCA | 60.2 | 437 | INTERGENIC | | | | |
| CaPOPI_6900 | scaffold2193 | 161568 | ATTTTTTTTT/ATTTTTTTTTT | CCGTTTCGAGTCTCAAGGAG | GCTTCCATGTTGCTCCTCTC | 60.0 | 679 | INTERGENIC | | | | |
| CaPOPI_6901 | scaffold22 | 24507 | AAT/A | ATGAGGCTCAAAATGCCATC | GCCAACTTTTCCAATGCCTA | 60.0 | 727 | INTERGENIC | | | | |
| CaPOPI_6902 | scaffold22 | 34524 | GTTTTT/GTTTTTTT | GCAGCATCGCAGATCATAGA | CGAATTGGGTTTTCGTTCTC | 60.1 | 181 | INTERGENIC | | | | |
| CaPOPI_6903 | scaffold2205 | 49159 | ATT/ATTT | TTTTTAACCAATCTTCGGCG | AGGGAGGACGAAAATGACCT | 60.1 | 272 | INTERGENIC | | | | |
| CaPOPI_6904 | scaffold2208 | 20005 | CA/CATA | CAAACACACCTGTTGGATGC | CCCTAGGCTTATTCTTGCCC | 60.0 | 362 | INTERGENIC | | | | |
| CaPOPI_6905 | scaffold221 | 61534 | CT/CTAAGTGTTATGT | CCCGTGATAGGCAGTACGTT | CGATTTCCCTGCATGTTTTT | 60.0 | 352 | INTERGENIC | | | | |
| CaPOPI_6906 | scaffold2210 | 15184 | CG/CGGATTTG | TCCAGATGAAATCGAGGACC | AAATCCGACACGTAACTCGG | 60.0 | 531 | INTERGENIC | | | | |
| CaPOPI_6907 | scaffold2210 | 36171 | AATTATATTA/AATTA | TCTTCGTTATGCAGTCAAACCT | TGTCAAGAAAACACCATGAATTG | 58.9 | 612 | INTERGENIC | | | | |
| CaPOPI_6908 | scaffold2216 | 16725 | AATATATATATATATATAT/AATATATATATATATAT | CTATCGGCTCGACCAATGAT | TTTTGGCTGGGTATAGGCAC | 60.1 | 451 | INTERGENIC | | | | |
| CaPOPI_6909 | scaffold222 | 40304 | CTTTTTTTTTT/CTTTTTTTTT | GGTTGTTTCCCTAACCAGCA | GGTATCTGGGGAACAGATTTCTC | 60.0 | 279 | INTERGENIC | | | | |
| CaPOPI_6910 | scaffold2222 | 1209 | G/GGATGCACAA | TGGATGCACAAGGTTGGTTA | AGGTCATTTTCGTCCTCCCT | 60.0 | 559 | INTERGENIC | | | | |

| INDEL marker IDs | Chromosomes/ unanchored scaffolds | Physical positions (bp) | InDels (*Kabuli* reference genome-CDC Frontier/PI) | Forward primers (5'-3') | Reverse primers (5'-3') | Anneling temperature (0C) | Expected amplified product size (bp) | Structural annotation | | Functional annotation | | |
|---|---|---|---|---|---|---|---|---|---|---|---|---|
| | | | | | | | | Sequence components of *kabuli* genome | *Kabuli* gene accession IDs | NCBI-KOG | TFs | NCBI-nr database |
| CaPOPI_6911 | scaffold2227 | 7998 | TAAAAAAAAAA/TAAAAAAAAA | TTTGACCAAAACTCATTTGCAC | GGTCCTCGATTTCATCTGGA | 60.0 | 418 | INTERGENIC | | | | |
| CaPOPI_6912 | scaffold2227 | 27932 | CCCCAATCGATTG/CCCCAATCGATTGCCCAATCGATTG | TCAGCCCAGGATTTTGTTTC | CCCGTGAGATCCTCCTAACA | 60.1 | 537 | INTERGENIC | | | | |
| CaPOPI_6913 | scaffold2227 | 31662 | AATATATATATATATATATATA/AATATATATATATATATATA | CTTGGGAACTGGAGAGCAAG | AATGGAGGAAGGAGGGAGAA | 60.0 | 541 | INTERGENIC | | | | |
| CaPOPI_6914 | scaffold223 | 82674 | GTTTTTT/GTTTTTTT | AATCTTCCCATGGATGTTTCA | TCAGCAAAGTCAAGAAGGCA | 59.2 | 541 | INTERGENIC | | | | |
| CaPOPI_6915 | scaffold223 | 95879 | AGCGACTACT/A | TTGGTCTCCTTGTGTTGTGC | TGGAGACCCCTTGATGAAAC | 59.7 | 502 | INTERGENIC | | | | |
| CaPOPI_6916 | scaffold223 | 177357 | TACT/TACTAACAACT | GACCAGCAGCAAACTCTTCC | AGATGTAAGCTCCGATGCGT | 60.0 | 971 | INTERGENIC | | | | |
| CaPOPI_6917 | scaffold2235 | 878 | GTTTT/GTTTTT | AGGTCATTTTCGTCCTCCCT | TTGGATGCACAAGATTGGTT | 59.9 | 267 | INTERGENIC | | | | |
| CaPOPI_6918 | scaffold2249 | 1002 | CT/CTT | TATTCCCACCCACGATCATT | CAACCAAGCCATTTTCCATT | 60.0 | 871 | INTERGENIC | | | | |
| CaPOPI_6919 | scaffold2249 | 12435 | CCAA/CCAACAA | CAGCTGAATACCAAGCGTGA | TGTCACCAAAATCTCGTCCA | 60.0 | 772 | INTERGENIC | | | | |
| CaPOPI_6920 | scaffold2269 | 43256 | A/ACGATTC | TCATCCCAAGGGAAAATGAA | CTTTATTGGTCCCCCACCTT | 60.2 | 308 | INTERGENIC | | | | |
| CaPOPI_6921 | scaffold2269 | 45593 | T/TAA | CTTTGAGAAGCAGGCCATTC | CGGTTGCGTGAGGTTTTTAT | 60.0 | 562 | DRR | Ca_27076 | | | |
| CaPOPI_6922 | scaffold2269 | 121644 | TAAAAAAAAAAA/TAAAAAAAAAAAA | TTTGATGAAGTTGATGCCCA | TGACTTGTGTTCTTCACGGC | 60.0 | 645 | DRR | Ca_27079 | R | G2-like | RNA recognition motif domain |
| CaPOPI_6923 | scaffold227 | 135497 | TCC/TCCC | CTTGGATGTGGTAGCCGTTT | GGTCTTGTGGTGGCTTTTGT | 60.0 | 651 | INTRON | Ca_25190 | | | |
| CaPOPI_6924 | scaffold227 | 140947 | TTA/TTAAAAGTA | ACCCTTTTGTTCCACACGAG | TACGGATGTTGACGCAATGT | 60.0 | 511 | INTRON | Ca_25190 | | | |
| CaPOPI_6925 | scaffold227 | 144419 | C/CACAATAG | CCTCAAACTTCCATGGCCTA | CAACTGGAATTTTCGGGAGA | 60.1 | 641 | DRR | Ca_25191 | | | |

| INDEL marker IDs | Chromosomes/ unanchored scaffolds | Physical positions (bp) | InDels (*Kabuli* reference genome-CDC Frontier/PI) | Forward primers (5'-3') | Reverse primers (5'-3') | Anneling temperature (0C) | Expected amplified product size (bp) | Structural annotation | | Functional annotation | | |
|---|---|---|---|---|---|---|---|---|---|---|---|---|
| | | | | | | | | Sequence components of *kabuli* genome | *Kabuli* gene accession IDs | NCBI-KOG | TFs | NCBI-nr database |
| CaPOPI_6926 | scaffold227 | 194790 | TAAAAAAAAA/TAAAAAAAA | GCCGAAACTCAATCACACCT | TGCCAGTTTGTCGGATAAGTT | 60.1 | 429 | INTERGENIC | | | | |
| CaPOPI_6927 | scaffold227 | 195682 | CAA/CAAA | AGACACAAACCCCGCAATAG | GGATGCCACCATTCAAGTTT | 60.0 | 338 | INTERGENIC | | | | |
| CaPOPI_6928 | scaffold227 | 196111 | TAAAAAAA/TAAAAAAAA | AAACTTGAATGGTGGCATCC | AGTAATGAATGGGCGAGTGG | 59.8 | 595 | INTERGENIC | | | | |
| CaPOPI_6929 | scaffold227 | 196280 | TAAAA/TAAAAA | AAACTTGAATGGTGGCATCC | AGTAATGAATGGGCGAGTGG | 59.8 | 595 | INTERGENIC | | | | |
| CaPOPI_6930 | scaffold227 | 197474 | TAAAAAAAA/TAAAAAAAAAA | CCTAATGGCGGAAATTGTGT | TAATCAGACCGTGTGGGTGA | 59.8 | 626 | INTERGENIC | | | | |
| CaPOPI_6931 | scaffold227 | 325938 | CTATATATATATATATATATATAT/CTATATATATATATATATATATATATATATAT | CAATTCTTGATTGGCATGCTT | GCTAATTTTTCGCGTCTTCG | 60.1 | 327 | INTERGENIC | | | | |
| CaPOPI_6932 | scaffold2273 | 24986 | GAAAAA/GAAAA | AAAATCCAACAATCTCCCCC | CCCTGCTACTCCTGCTTTTG | 60.0 | 590 | DRR | Ca_28242 | | | |
| CaPOPI_6933 | scaffold2275 | 15734 | AATATATATATATATATATATATAT/AATATATATATATATATATATAT | AGTGTATAACCCGTGCGTCG | TTGGAGTCATGCTTTTCATCA | 61.0 | 229 | INTERGENIC | | | | |
| CaPOPI_6934 | scaffold228 | 136549 | A/AC | GATGAAATCGAGGACAGGGA | TGCGTCTTTCTCAAGTGCAT | 60.0 | 445 | INTERGENIC | | | | |
| CaPOPI_6935 | scaffold228 | 176693 | ATATTATTATTATTAT/ATATTATTATTAT | GGTTTCGAGGAAACTGGACA | TTGTTTATTTGACAACTCAATTGTTCT | 60.1 | 794 | INTERGENIC | | | | |
| CaPOPI_6936 | scaffold2289 | 12871 | ATTT/ATTTT | TGTGTTTGTGCCATTGGATT | AGGGAGGATGAAAACGACCT | 59.8 | 475 | INTERGENIC | | | | |
| CaPOPI_6937 | scaffold229 | 13642 | A/AG | TTGGATGCCTAAGACTGCCT | AGGTCATTTTCGTCCTCCCT | 59.8 | 168 | INTERGENIC | | | | |
| CaPOPI_6938 | scaffold229 | 81575 | AAGG/AAGGAGG | TAGGGAGGACGAAAATGACG | AACCATTCATGTGCATCCAA | 60.1 | 865 | INTERGENIC | | | | |
| CaPOPI_6939 | scaffold2301 | 3312 | C/CT | CCTAGGGTGGACGAAAATCA | ATCCTCCCGTGGCTTATTCT | 59.9 | 232 | INTERGENIC | | | | |
| CaPOPI_6940 | scaffold2301 | 22791 | CTT/CT | CAAACACACCTGTTGGATGC | CCTCGCTTGGCTTATTCTTG | 60.0 | 466 | INTERGENIC | | | | |

| INDEL marker IDs | Chromosomes/ unanchored scaffolds | Physical positions (bp) | InDels (*Kabuli* reference genome-CDC Frontier/PI) | Forward primers (5'-3') | Reverse primers (5'-3') | Anneling temperature (0C) | Expected amplified product size (bp) | Structural annotation | | Functional annotation | | |
|---|---|---|---|---|---|---|---|---|---|---|---|---|
| | | | | | | | | Sequence components of *kabuli* genome | *Kabuli* gene accession IDs | NCBI-KOG | TFs | NCBI-nr database |
| CaPOPI_6941 | scaffold2301 | 53889 | CAAATGAACTTATTGGAT/C | ACACAAATGCACCTGTTGGA | TCATTTTCGTCCTCCTTTGG | 60.0 | 554 | INTERGENIC | | | | |
| CaPOPI_6942 | scaffold2301 | 54382 | ATG/ATGTG | CCAAAGGAGGACGAAAATGA | GCGTGTTTGTGACATTGGAG | 60.0 | 349 | INTERGENIC | | | | |
| CaPOPI_6943 | scaffold2301 | 54476 | C/CA | TGGATGCACATGATTGGTTT | AGGTCATTTTCGTCCTCCCT | 59.8 | 316 | INTERGENIC | | | | |
| CaPOPI_6944 | scaffold2301 | 56350 | TT/TTTAT | GAGGATACCGGGAATAAGGG | TGACAAAAACTAAGGGAGGTTG | 59.6 | 351 | INTERGENIC | | | | |
| CaPOPI_6945 | scaffold2325 | 58485 | TAA/TA | AGGTCGTTTTCGTCCTCCTT | GCATGCCCAAGATTGGTAAA | 60.1 | 268 | INTERGENIC | | | | |
| CaPOPI_6946 | scaffold2328 | 13583 | GTTTT/GTTTTT | TGTGTTTGTGCCATTGGAGT | AGGGAGGACGAAAATGACCT | 60.0 | 245 | INTERGENIC | | | | |
| CaPOPI_6947 | scaffold2345 | 549 | C/CA | CCCGAAAAGTCCAGATGAAA | TTCGGACTAGACGAGGGCTA | 60.0 | 453 | INTERGENIC | | | | |
| CaPOPI_6948 | scaffold2346 | 28393 | GA/GAA | TGGGCGATTTTTGTACTTCC | CCCGAAAAGTCCAGATGAAA | 59.9 | 395 | INTERGENIC | | | | |
| CaPOPI_6949 | scaffold235 | 57295 | TAAAAAA/TAAAAAAA | GCCAAAACGACCACAAAGTT | GGGAGTAGAGCTGATCAGAGGA | 60.0 | 779 | INTERGENIC | | | | |
| CaPOPI_6950 | scaffold235 | 178169 | CATATATATATATATATATATATATAT/CATATATATATATATATATATATATATATATATAT | TGGAAGTTGCACCAGACAAA | AAAGGACCAAATCAGGAAAAA | 60.3 | 930 | INTERGENIC | | | | |
| CaPOPI_6951 | scaffold2356 | 103676 | AATTATTATTATTATTATTATTATTATTATTATTATTATTATTATTATTATTATTAT/AATTATTATTATTATTATTATTATTATTATTATTATTATTATTATTATTATTATTA | GGAACTCACATGAATTAGCAGAA | GTTTTTCACCTTTGCTTGGC | 58.4 | 299 | INTERGENIC | | | | |
| CaPOPI_6952 | scaffold2356 | 138194 | TT/TTCT | CCTTGAACTGTTGTCTGGCA | CCAACAACATCAAATTCATCG | 59.9 | 687 | INTRON | Ca_24344 | Q | | Alcohol dehydrogenase superfamily, zinc-containing |
| CaPOPI_6953 | scaffold2356 | 138866 | TG/TGAG | ACCCAATTCCTTCATGTCCA | AGATCCACCAAAGGCAACAG | 60.2 | 213 | INTRON | Ca_24344 | Q | | Alcohol dehydrogenase superfamily, zinc-containing |
| CaPOPI_6954 | scaffold2356 | 144020 | CAAAAAAAAAAA/CAAAAAAAAAA | GGATAACAGCGATGGGAAGA | ATCCCTCATTTGCAATCCAC | 60.0 | 590 | DRR | Ca_24345 | A | | Ribonuclease T2 |
| CaPOPI_6955 | scaffold2358 | 1353 | ATTTTT/ATTTT | GATGAAATCGAGGACTGGGA | CTTTTCGGGCGATTTTCATA | 60.0 | 479 | INTERGENIC | | | | |

| INDEL marker IDs | Chromosomes/ unanchored scaffolds | Physical positions (bp) | InDels (*Kabuli* reference genome-CDC Frontier/PI) | Forward primers (5'-3') | Reverse primers (5'-3') | Anneling temperature (0C) | Expected amplified product size (bp) | Structural annotation | | Functional annotation | | |
|---|---|---|---|---|---|---|---|---|---|---|---|---|
| | | | | | | | | Sequence components of *kabuli* genome | *Kabuli* gene accession IDs | NCBI-KOG | TFs | NCBI-nr database |
| CaPOPI_6956 | scaffold236 | 45554 | GTT/GTTT | TGGACTTTTCGAGCGACTTT | CTTCGTTTTGTGTTTCGGGT | 60.0 | 511 | INTERGENIC | | | | |
| CaPOPI_6957 | scaffold236 | 60071 | TCC/TC | GGTTCCGCGTTAGATTTCAA | GCGGAACCTGAAACACATTT | 60.1 | 820 | INTERGENIC | | | | |
| CaPOPI_6958 | scaffold2368 | 53512 | TGGG/TGGGG | AACCCTGGAGATGGGAAAGT | CTAGCCATTAGCTTCGACCG | 59.8 | 607 | DRR | Ca_26720 | P | | Zinc/iron permease |
| CaPOPI_6959 | scaffold2368 | 54204 | GTT/GTTT | CATCGGTCAAACACCATCTG | GCTTTGCAGAACATGCGTAA | 60.0 | 490 | DRR | Ca_26720 | P | | Zinc/iron permease |
| CaPOPI_6960 | scaffold2368 | 54372 | CAGATA/CAGATAGATA | CATCGGTCAAACACCATCTG | TTTGGCGTACATGATGCAAT | 60.0 | 800 | CDS (FRAME SHIFT) | Ca_26720 | P | | Zinc/iron permease |
| CaPOPI_6961 | scaffold2368 | 59799 | TC/TCTCC | CTCCAAATCCCAATCTCCAA | CTCACTGTCAAGGTGCCGTA | 59.9 | 725 | URR | Ca_26721 | | MYB | |
| CaPOPI_6962 | scaffold2371 | 73153 | TAAAAAAAAAA/TAAAAAAAA | TGTGTCGGAGAATCTTCCCT | ATCGACGATAAGTCATCGGG | 59.7 | 950 | INTERGENIC | | | | |
| CaPOPI_6963 | scaffold2371 | 73366 | ATTTTTATTTT/ATTTTTATTTTTATTTTTTATTTT | CAAGAAAAAGTTTTGACTTCCGA | ATCCACGCCACATAAACCAT | 59.8 | 919 | INTERGENIC | | | | |
| CaPOPI_6964 | scaffold2371 | 73819 | GTT/GT | ATGGTTTATGTGGCGTGGAT | TGAATGAAGCGAAAATGTGC | 60.1 | 321 | INTERGENIC | | | | |
| CaPOPI_6965 | scaffold2371 | 150056 | TGGG/TGG | GCATTCGGGGGTATGTTTTA | CATCTTCCTTCCACAATTTCTTT | 59.7 | 970 | INTERGENIC | | | | |
| CaPOPI_6966 | scaffold2371 | 287452 | CTTTTTTTTTT/CTTTTTTTTT | CGACTCGTGTCCTAAAACTCAA | TTTCATGCCATAAGTCATTGTG | 59.4 | 884 | INTERGENIC | | | | |
| CaPOPI_6967 | scaffold2371 | 323810 | CAGAGAGAGAGAGAGAGAGAGAGAGAGAGAGAGA/CAGAGAGAGAGAGAGAGAGAGAGAGAGAGAGAGA | GAAAACACCAAGAAGGGCAA | ACGAAACTCATCGGAAAACG | 60.1 | 672 | INTERGENIC | | | | |
| CaPOPI_6968 | scaffold2373 | 4581 | TGGTGAGGGTGAGGGTGAGGG/TGGTGAGGGTGAGGGTGAGGGTGAGGG | AGGATGTGGGTGAGGTTGAG | TCACCAGCAGCAGAAACATC | 60.0 | 463 | INTERGENIC | | | | |
| CaPOPI_6969 | scaffold2373 | 39709 | TG/T | GGCAGTGGAGGAGAACACAT | TCAAACAGATGCTAAAATGCAAA | 60.1 | 269 | INTERGENIC | | | | |
| CaPOPI_6970 | scaffold2373 | 40568 | CATTATTATTATTATTATTATTATTATTATTATTATTATTATTATTATTA/CATTATTATTATTATTATTATTATTATTATTATTATTATTATTA | TGCATATTTAGAAACGTCAACGA | TTCAGGTCAAACGGTTTTCA | 59.7 | 339 | INTERGENIC | | | | |

| INDEL marker IDs | Chromosomes/ unanchored scaffolds | Physical positions (bp) | InDels (*Kabuli* reference genome-CDC Frontier/PI) | Forward primers (5'-3') | Reverse primers (5'-3') | Anneling temperature (0C) | Expected amplified product size (bp) | **Structural annotation** | | **Functional annotation** | | |
|---|---|---|---|---|---|---|---|---|---|---|---|---|
| | | | | | | | | **Sequence components of *kabuli* genome** | ***Kabuli* gene accession IDs** | **NCBI-KOG** | **TFs** | **NCBI-nr database** |
| CaPOPI_6971 | scaffold2373 | 53283 | TTATATATATATATATATATAT/TTATATATATATATATATATATAT | GTCGCACGGAAATAACGATT | AAGGCATGGTATTGGAGTCA | 60.0 | 234 | INTERGENIC | | | | |
| CaPOPI_6972 | scaffold2373 | 69117 | TCA/TCACA | GCGAAGTCTCCATCCTTTCTT | AAAAATGCGCACGACAGATT | 59.8 | 254 | INTRON | Ca_27075 | | | Transposase, Ptta/En/Spm, plant |
| CaPOPI_6973 | scaffold2373 | 88920 | TT/TTTTGTATAAT | TGTGGATCTGTTTTGATCGC | TCCACACAAGCCAACTGAAA | 59.7 | 110 | INTRON | Ca_27075 | | | Transposase, Ptta/En/Spm, plant |
| CaPOPI_6974 | scaffold2373 | 97344 | TAAAAAAAAAA/TAAAAAAAAAAA | AATTGTTGGATCTGCCCTTG | GAGATTGAACACGCCACTTCT | 59.9 | 888 | DRR | Ca_27075 | | | Transposase, Ptta/En/Spm, plant |
| CaPOPI_6975 | scaffold2378 | 17251 | AGGTGAATGGTGAATGGTGAA/AGGTGAATGGTGAATGGTGAATGGTGAA | CATCACGTGTGTGTATTACTTTATTTG | AAAACTTTTCCGCTGCTTGA | 59.0 | 357 | INTERGENIC | | | | |
| CaPOPI_6976 | scaffold2385 | 5093 | TCC/TC | GAGTGAATTGTAACAGCCCAAA | GGACTTGCGGTGAAGAAGAA | 59.1 | 720 | INTERGENIC | | | | |
| CaPOPI_6977 | scaffold2385 | 5335 | TT/TTTCAAT | GAGTGAATTGTAACAGCCCAAA | TCATTTCGACGCTTTCACAC | 59.1 | 968 | INTERGENIC | | | | |
| CaPOPI_6978 | scaffold239 | 109266 | CA/C | GGTTTCAACCCCAATGCTTA | TCGACTTGTCAATCGCTTTG | 59.8 | 808 | INTERGENIC | | | | |
| CaPOPI_6979 | scaffold2392 | 10820 | TTTCTTCTTCTTCTTCT/TTTCTTCTTCTTCTTCTTCT | GGTAGGCCTTGGGCTAGTGT | TTGGAAGGAGCCCTTACCTT | 60.5 | 729 | INTERGENIC | | | | |
| CaPOPI_6980 | scaffold2392 | 18107 | AT/A | TTGCTCCACGAACATCTCTG | CTGAGTTGCATTGATCTCGC | 60.0 | 870 | INTERGENIC | | | | |
| CaPOPI_6981 | scaffold2392 | 20138 | CATATAT/CATAT | CGCATCGAGACGAGAAAACT | GCGCGACCCCTTATATTACA | 60.5 | 871 | INTERGENIC | | | | |
| CaPOPI_6982 | scaffold2392 | 61104 | AAGAGAGAGAGAGAGAGAGAGAGAGAGAGAGAGAGAGA/AAGAGAGAGAGAGAGAGAGAGAGAGAGAGAGAGAGAGAGA | CATCTGCGGTTTTCTTCACA | CCGTTACACGGTTTTGTTCA | 59.8 | 180 | INTERGENIC | | | | |
| CaPOPI_6983 | scaffold2392 | 321808 | ATTT/ATT | GGAAAACTAGGCCAAAACCC | CATGCACACCCCATGTTAAA | 59.8 | 692 | INTERGENIC | | | | |
| CaPOPI_6984 | scaffold2392 | 326211 | AATATTT/A | TCATTCCCCACCTACACAAG | GTGCATGTGGCTGAAAAACA | 58.4 | 552 | INTERGENIC | | | | |
| CaPOPI_6985 | scaffold2392 | 354336 | AAACAA/AAA | TGTTGCGTTTTCATTCTTTCA | TACCAAATATCCCAGGCCAA | 59.3 | 859 | DRR | Ca_25138 | KL | | Xeroderma pigmentosum group D protein |

| INDEL marker IDs | Chromosomes/ unanchored scaffolds | Physical positions (bp) | InDels (*Kabuli* reference genome-CDC Frontier/PI) | Forward primers (5'-3') | Reverse primers (5'-3') | Anneling temperature (0C) | Expected amplified product size (bp) | Structural annotation | | Functional annotation | | |
|---|---|---|---|---|---|---|---|---|---|---|---|---|
| | | | | | | | | Sequence components of *kabuli* genome | *Kabuli* gene accession IDs | NCBI-KOG | TFs | NCBI-nr database |
| CaPOPI_6986 | scaffold2392 | 355121 | GTTTTTTTTTTTT/GTTTTTTTTTT | TGCACCCAATCTGCAAATAA | TGGGCTAGTTAGCTTTTGCC | 60.1 | 433 | INTERGENIC | | | | |
| CaPOPI_6987 | scaffold2392 | 355152 | ATTT/ATTTT | TGCACCCAATCTGCAAATAA | TGGGCTAGTTAGCTTTTGCC | 60.1 | 433 | INTERGENIC | | | | |
| CaPOPI_6988 | scaffold2392 | 355244 | AAATTATATT/AAATTATATTTTAATTATATT | TGCACCCAATCTGCAAATAA | TGGGCTAGTTAGCTTTTGCC | 60.1 | 433 | INTERGENIC | | | | |
| CaPOPI_6989 | scaffold240 | 38776 | TTATATATATATATATATATAT/TTATATATATATATATATATATAT | TATTTTGGATTTTGGCTCGG | TAGGAGAAACGGAGGGGTTT | 59.9 | 575 | INTERGENIC | | | | |
| CaPOPI_6990 | scaffold240 | 73293 | TA/TAA | CAAGGTGTCGATGTTGGTTG | CTGGTGGCTACTTTCTTGGG | 60.0 | 472 | INTERGENIC | | | | |
| CaPOPI_6991 | scaffold240 | 150038 | GTATATATATATATATATATATAT/GTATATATATATATATATATAT | GGAGGCTTTGAAATTAGGGC | CTCCAGCGCACAACATCTAA | 60.0 | 783 | INTERGENIC | | | | |
| CaPOPI_6992 | scaffold240 | 151385 | TAAAAAAA/TAAAAAAAA | AGATGCATGTGATGAAACCAA | TACTCCCATTTCCTCCCCTT | 59.0 | 727 | INTERGENIC | | | | |
| CaPOPI_6993 | scaffold240 | 156860 | AAACAACA/AAACA | GACAATCGCAGGAAGTGGTT | GCATTGCACCAAAACACAAC | 60.1 | 699 | INTERGENIC | | | | |
| CaPOPI_6994 | scaffold2408 | 4043 | TTC/T | TGTTCTCATGAGGCAATATGCT | CAAAGAATTCACTTGATACATGGC | 59.7 | 594 | INTERGENIC | | | | |
| CaPOPI_6995 | scaffold2415 | 126110 | AGG/AG | CGTTTTTCAAAGCGAGGGTA | TGGAGAAACTCTTCCGCTGT | 60.2 | 128 | INTERGENIC | | | | |
| CaPOPI_6996 | scaffold2415 | 137080 | AATATATATATATATATATATATATATA/AATATATATATATATATATATATATA | ATCGTTGGAAGTAGGGACCA | CCACCAAACAACAATTCCCT | 59.4 | 484 | INTERGENIC | | | | |
| CaPOPI_6997 | scaffold242 | 53694 | TC/TCAC | TGCTTTGCGAGAAGATGTTG | ATATGCCAAATGGGAGGTCA | 60.1 | 459 | DRR | Ca_22259 | R | C2H2 | |
| CaPOPI_6998 | scaffold242 | 123686 | TTCAGAAAAACTCAGAAAAACTC/TTCAGAAAAACTC | GCATCATGAGTTTTGTCCCA | CAATGAAGGTGACACGGTTG | 59.5 | 367 | INTERGENIC | | | | |
| CaPOPI_6999 | scaffold242 | 225770 | CAAAAAAAA/CAAAAAAA | TGTGTCAGAAGGATCTTGCG | CCTCTTTGATTGAGGACCCA | 60.0 | 667 | INTERGENIC | | | | |
| CaPOPI_7000 | scaffold242 | 238083 | TATGCA/TA | TGCCAAATGAAGTTGGACAC | CTCCAATATTTGACCGCCAC | 59.5 | 391 | INTERGENIC | | | | |

| INDEL marker IDs | Chromosomes/ unanchored scaffolds | Physical positions (bp) | InDels (*Kabuli* reference genome-CDC Frontier/PI) | Forward primers (5'-3') | Reverse primers (5'-3') | Anneling temperature (0C) | Expected amplified product size (bp) | Structural annotation | | Functional annotation | | |
|---|---|---|---|---|---|---|---|---|---|---|---|---|
| | | | | | | | | Sequence components of *kabuli* genome | *Kabuli* gene accession IDs | NCBI-KOG | TFs | NCBI-nr database |
| CaPOPI_7001 | scaffold242 | 260122 | T/TTG | TTCACCAACTTTGCACTCCA | AAAGAAAGCCGCTTAGAGGG | 60.3 | 620 | INTERGENIC | | | | |
| CaPOPI_7002 | scaffold242 | 316249 | TAAAAAAAA/TAAAAAAAAA | CAGATGGCTGCTGTTCTCAA | GAAGGACATCGTGGAAGGAA | 60.1 | 614 | INTERGENIC | | | | |
| CaPOPI_7003 | scaffold242 | 393693 | CAAAA/CAAAAA | TTAGAGGGGCTAGGAGGGAA | TCGCCTTTGCAAACACATAC | 60.2 | 487 | INTERGENIC | | | | |
| CaPOPI_7004 | scaffold242 | 393744 | ATTTTTTTTTT/ATTTTTTTTT | TTAGAGGGGCTAGGAGGGAA | TCGCCTTTGCAAACACATAC | 60.2 | 487 | INTERGENIC | | | | |
| CaPOPI_7005 | scaffold242 | 416343 | GAAAAA/GAAAA | CCAGGAGATGAGAGGTCCAA | TGGCTTTCAACCATTTTTCC | 60.2 | 435 | INTERGENIC | | | | |
| CaPOPI_7006 | scaffold242 | 430857 | CAAAAAAAAAA/CAAAAAAAAA | CCATACCAACTACTTGTTTCCCA | GCTATCCCACTTTTGGGGTT | 60.1 | 802 | INTERGENIC | | | | |
| CaPOPI_7007 | scaffold242 | 458674 | TAAAAAAAA/TAAAAAAAAA | GGCAACAAGCATATTGCTCA | TTGGATTCATCATTGCCTCA | 59.8 | 294 | INTERGENIC | | | | |
| CaPOPI_7008 | scaffold242 | 494583 | ATTTTTTTT/ATTTTTTTTT | GTTGGACACCCAAACACCTC | AATTAGGGGGCATAGGGATG | 60.3 | 794 | INTERGENIC | | | | |
| CaPOPI_7009 | scaffold242 | 494657 | TAAAA/TAAA | GTTGGACACCCAAACACCTC | AATTAGGGGGCATAGGGATG | 60.3 | 794 | INTERGENIC | | | | |
| CaPOPI_7010 | scaffold242 | 509048 | TAA/TAAA | AAAGGCCTGAAAGGGAAAAA | TGGGCTAACAGAAAATCGCT | 60.0 | 679 | INTERGENIC | | | | |
| CaPOPI_7011 | scaffold242 | 509072 | GTATTATTATTATTATTATT/GTATTATTATTATTATTATTATTATT | AAAGGCCTGAAAGGGAAAAA | TGGGCTAACAGAAAATCGCT | 60.0 | 679 | INTERGENIC | | | | |
| CaPOPI_7012 | scaffold242 | 523949 | TC/TCC | CAAGGTGTGAGATCAACCGA | TCATGACTTTTGACCGACGA | 59.7 | 417 | INTERGENIC | | | | |
| CaPOPI_7013 | scaffold242 | 593752 | ACCC/ACC | GGTTGTTTGAGCAAACTGCAT | TTTGATTTGTTTGATGAGCCA | 60.2 | 634 | INTERGENIC | | | | |
| CaPOPI_7014 | scaffold242 | 643894 | ATTTTTT/ATTTTTTT | TGAAATGGTTCCTTGCATTC | ATGCCTTCCTTGTTTGATGG | 58.5 | 318 | INTERGENIC | | | | |
| CaPOPI_7015 | scaffold242 | 692807 | GAAA/GAAAA | TTGTCTTGCTTGCATCCTTG | TGTGTAAAGATTGGCGGTTG | 60.0 | 399 | DRR | Ca_22275 | F | | Guanylate kinase |

| INDEL marker IDs | Chromosomes/ unanchored scaffolds | Physical positions (bp) | InDels (*Kabuli* reference genome-CDC Frontier/PI) | Forward primers (5'-3') | Reverse primers (5'-3') | Anneling temperature (0C) | Expected amplified product size (bp) | Structural annotation | | Functional annotation | | |
|---|---|---|---|---|---|---|---|---|---|---|---|---|
| | | | | | | | | Sequence components of *kabuli* genome | *Kabuli* gene accession IDs | NCBI-KOG | TFs | NCBI-nr database |
| CaPOPI_7016 | scaffold242 | 696950 | TTATATATATATATAT/TTATATATATATATATAT | CTGGCCATGAGGAATGAGTT | AACTACGCCTTGGTGGATTG | 60.1 | 412 | INTERGENIC | | | | |
| CaPOPI_7017 | scaffold242 | 710240 | TTTATTATTA/TTTATTA | ACTTGTTTCACCACATGCCA | CTTCACGTGAGCCAAACTCA | 60.0 | 510 | INTERGENIC | | | | |
| CaPOPI_7018 | scaffold242 | 721932 | ATT/ATTGTT | TGGTGACTCGTTCATATACCCA | AAAGACGGTGCCAAAAATGT | 60.2 | 882 | INTERGENIC | | | | |
| CaPOPI_7019 | scaffold242 | 722081 | GAAAAA/GAAAA | CACAAGATCTTTAAGTTCATATTTTCA | TTTCTCCATCCAAATTCCCA | 57.3 | 951 | INTERGENIC | | | | |
| CaPOPI_7020 | scaffold242 | 735562 | A/AC | CGAACTTGAGAATTCACCGC | GTGGAAAAGGGGATTGGATT | 60.8 | 371 | INTERGENIC | | | | |
| CaPOPI_7021 | scaffold242 | 771258 | TAAAAAAAAA/TAAAAAAAAAA | CGAGATGGTGCAGCTGAATA | CACACGAATACGATTCCACG | 60.0 | 984 | INTERGENIC | | | | |
| CaPOPI_7022 | scaffold242 | 771316 | TAAAAAAAAAA/TAAAAAAAAA | TGTGCTTCATGGGTCAAGTC | CACACGAATACGATTCCACG | 59.7 | 469 | INTERGENIC | | | | |
| CaPOPI_7023 | scaffold242 | 804134 | CTTT/CTTTT | TGAAATGGAAATGCTACCATCA | TGGTCAGAAACAACGCAAGA | 60.3 | 710 | INTERGENIC | | | | |
| CaPOPI_7024 | scaffold242 | 804233 | CTTTTTTTTT/CTTTTTTTTTT | TGAAATGGAAATGCTACCATCA | TGGTCAGAAACAACGCAAGA | 60.3 | 710 | INTERGENIC | | | | |
| CaPOPI_7025 | scaffold2433 | 2492 | ATT/AT | TATGATCCCGCTTGACACAA | TTTGGGAAAATCCACAAACA | 60.1 | 369 | URR | Ca_27860 | | | |
| CaPOPI_7026 | scaffold2434 | 76263 | TGG/TG | TTCTTCGTTTTGTAGTTGTACTCAATG | GGCAAACAAGAACCGAAAAC | 60.0 | 638 | INTERGENIC | | | | |
| CaPOPI_7027 | scaffold2434 | 76563 | TAAAA/TAAAAA | TTCGGTTCTTGTTTGCCTCT | CCTTTATCTTCTTGTGGCCG | 59.9 | 382 | INTERGENIC | | | | |
| CaPOPI_7028 | scaffold2446 | 7253 | A/AC | TTCCTCTGCGAAAATAAACCTC | TGCCCTTGTTTTCAGTTCAA | 59.7 | 394 | INTERGENIC | | | | |
| CaPOPI_7029 | scaffold249 | 73354 | AA/AATTGA | CAGCCCAAAATAACTGCCAT | CTTTGTTGGTGCGTGTGTTC | 60.0 | 422 | INTERGENIC | | | | |
| CaPOPI_7030 | scaffold25 | 35858 | ATTTT/ATTT | AGGTCATTTTCGTCCTCCCT | TGGATGCACAAGATCGGTTA | 59.9 | 168 | INTERGENIC | | | | |

| INDEL marker IDs | Chromosomes/ unanchored scaffolds | Physical positions (bp) | InDels (*Kabuli* reference genome-CDC Frontier/PI) | Forward primers (5'-3') | Reverse primers (5'-3') | Anneling temperature (0C) | Expected amplified product size (bp) | Structural annotation | | Functional annotation | | |
|---|---|---|---|---|---|---|---|---|---|---|---|---|
| | | | | | | | | Sequence components of *kabuli* genome | *Kabuli* gene accession IDs | NCBI-KOG | TFs | NCBI-nr database |
| CaPOPI_7031 | scaffold25 | 51854 | AC/A | TCCGTCCTCCCTAGGCTTAT | GGACCAAAATGACCCAAATG | 60.1 | 388 | INTERGENIC | | | | |
| CaPOPI_7032 | scaffold25 | 72914 | ATTTT/ATTTTT | TGAACCAATCTTGTGCATCC | AGGGAGGACGAAAATGACCT | 59.5 | 672 | INTERGENIC | | | | |
| CaPOPI_7033 | scaffold2501 | 7458 | TG/T | AACCGATCTTGTCGATCCAA | AGGGAGGACGAAAATGACCT | 60.5 | 373 | INTERGENIC | | | | |
| CaPOPI_7034 | scaffold2505 | 1462 | TCCC/TCCCC | ATTCCAAACCAAAACCCTCC | TTGCTCACTACCACGCTACG | 60.0 | 884 | INTERGENIC | | | | |
| CaPOPI_7035 | scaffold2505 | 1793 | TG/TGGAAGG | CGTTTCCACACAACCACAAA | TTGCTCACTACCACGCTACG | 60.4 | 112 | INTERGENIC | | | | |
| CaPOPI_7036 | scaffold2511 | 1442 | TTGTG/TTGTGTG | AAGCTTCAATGGCCAAAAGA | CCTGACAAGTTTCCTTGGGA | 59.8 | 246 | INTERGENIC | | | | |
| CaPOPI_7037 | scaffold2511 | 7402 | CT/C | TGTTGAAGAAGGTTTTGGGG | CCAATAACGATCAAGCAATCG | 59.9 | 359 | INTERGENIC | | | | |
| CaPOPI_7038 | scaffold2522 | 24043 | ATTT/ATTTT | AGGTGTGTTTGTGCATTGGA | AGGGAGGACGAAAATGACCT | 60.0 | 248 | INTERGENIC | | | | |
| CaPOPI_7039 | scaffold2528 | 19502 | GTTTTT/GTTTT | GTCCTCCGTTGGCTTATTCA | GGACGAAAATGACCAAAAGG | 60.1 | 488 | INTERGENIC | | | | |
| CaPOPI_7040 | scaffold2542 | 2207 | TAGGAG/TAG | TCATTTGGACTTTTCGGGAG | TCAATTTGGGTTCAGGGTGT | 60.0 | 816 | INTERGENIC | | | | |
| CaPOPI_7041 | scaffold2557 | 49964 | AA/AATTA | CTTGGGCAAACACCTCCTAA | TGCATTGGTATATGGAGGCT | 60.1 | 907 | INTERGENIC | | | | |
| CaPOPI_7042 | scaffold2557 | 101354 | CATATATATATATA/CATATATATATATATA | TCCCTCCAAACAAACACACA | CGGTGAATCCTTTACGGAGA | 60.0 | 130 | INTERGENIC | | | | |
| CaPOPI_7043 | scaffold2557 | 102326 | CT/CGCTCTTTTTTGT | ATGGCGAACAAATCACACAA | TTGTAGCAATCATGTGGGGA | 60.0 | 644 | INTERGENIC | | | | |
| CaPOPI_7044 | scaffold2557 | 112937 | CGG/CGGTTGG | TTTTAGCTTTCCATCGCCTG | CTCAGAGGGTCCAAATTCCA | 60.3 | 717 | INTERGENIC | | | | |
| CaPOPI_7045 | scaffold2560 | 60629 | T/TA | GGAGAACATTATCCGTTTTCCA | TCAAATTTCATCCTCACCCA | 60.2 | 536 | INTERGENIC | | | | |

| INDEL marker IDs | Chromosomes/ unanchored scaffolds | Physical positions (bp) | InDels (*Kabuli* reference genome-CDC Frontier/PI) | Forward primers (5'-3') | Reverse primers (5'-3') | Anneling temperature (0C) | Expected amplified product size (bp) | Structural annotation | | Functional annotation | | |
|---|---|---|---|---|---|---|---|---|---|---|---|---|
| | | | | | | | | Sequence components of *kabuli* genome | *Kabuli* gene accession IDs | NCBI-KOG | TFs | NCBI-nr database |
| CaPOPI_7046 | scaffold2560 | 61168 | ACACGCACGCAC/ACACGCACGCACGCACGCAC | GGGTGAGGATGAAATTTGAAGA | ATTCGGTAGCACATTCGTCC | 60.3 | 864 | INTERGENIC | | | | |
| CaPOPI_7047 | scaffold2560 | 61734 | TC/T | GGACGAATGTGCTACCGAAT | GCACCCGCAAAGAATAACAT | 60.0 | 121 | INTERGENIC | | | | |
| CaPOPI_7048 | scaffold2569 | 65788 | GATATATATATATATATATATATAT/GATATATATATATATATATATATATAT | GCCGCGGTCTATTTGTGTAT | CCCTGTCCAGATTGAGTGGT | 60.0 | 646 | INTERGENIC | | | | |
| CaPOPI_7049 | scaffold2575 | 196132 | C/CA | ACTTTTCACCACATCCCTCG | ATCCATTCCCCACATTGAAA | 60.0 | 347 | INTERGENIC | | | | |
| CaPOPI_7050 | scaffold2575 | 245742 | ATTTTTTTT/ATTTTTTTTT | CGCAATAAATTTTTGACGCC | TACCCATCAGGGCAGAACTC | 60.4 | 535 | INTERGENIC | | | | |
| CaPOPI_7051 | scaffold2575 | 278772 | TGAAGAAG/TGAAG | TGGTGGCAAATGATGAAGAA | CAGGGACTACATCCAACAAAAA | 60.0 | 769 | INTERGENIC | | | | |
| CaPOPI_7052 | scaffold2575 | 279175 | CTAT/CTATTAT | TTTGATTGACTTAGCACCACG | TAAAAGATGATGTGTTGGTGTGT | 58.8 | 557 | INTERGENIC | | | | |
| CaPOPI_7053 | scaffold2575 | 279306 | TAA/TA | TTTTTGTTGGATGTAGTCCCTG | CATGAACATTTAAGAGTTTGCATTT | 59.0 | 583 | INTERGENIC | | | | |
| CaPOPI_7054 | scaffold2575 | 280082 | C/CATTGATA | TCCCAAGGTATCATTGTCTCTTT | TCGGAGTTTTGTGTGAGTGTG | 59.0 | 316 | INTERGENIC | | | | |
| CaPOPI_7055 | scaffold2575 | 280195 | TTA/T | CACCTTTGTCCCTGAGCTGT | TCGGAGTTTTGTGTGAGTGTG | 60.3 | 178 | INTERGENIC | | | | |
| CaPOPI_7056 | scaffold2575 | 282023 | CGAGAG/CGAG | GAACCCGCCTAAACATTTCA | TTCAATTGTCCATAAGCAGTCA | 59.9 | 401 | INTERGENIC | | | | |
| CaPOPI_7057 | scaffold26 | 145340 | CAAACAAAAACAAA/CAAACAAA | GAGGAGAAGGGAGAAATGGG | GGCTCGGAGGTATTTTAGCC | 60.0 | 287 | INTERGENIC | | | | |
| CaPOPI_7058 | scaffold26 | 506230 | GTT/GTTT | ATTGGCGTGAAAACCGTTAG | TGACGGGTCTGCACAATTTA | 60.0 | 538 | INTERGENIC | | | | |
| CaPOPI_7059 | scaffold260 | 287454 | AATATATATATATATATATATATATATAT/AATATATATATATATATATATATATATATATAT | CGTGAGACAGGGAGAGGAAG | CGGTGAATCCTTTACGGAGA | 60.0 | 281 | INTERGENIC | | | | |
| CaPOPI_7060 | scaffold260 | 341696 | AT/ATGT | AGGAAGAGTGGAGTTGCGAA | GCATTCAGCAGCAAAAACAA | 60.0 | 670 | INTERGENIC | | | | |

| INDEL marker IDs | Chromosomes/ unanchored scaffolds | Physical positions (bp) | InDels (*Kabuli* reference genome-CDC Frontier/PI) | Forward primers (5'-3') | Reverse primers (5'-3') | Anneling temperature (0C) | Expected amplified product size (bp) | Structural annotation | | Functional annotation | | |
|---|---|---|---|---|---|---|---|---|---|---|---|---|
| | | | | | | | | Sequence components of *kabuli* genome | *Kabuli* gene accession IDs | NCBI-KOG | TFs | NCBI-nr database |
| CaPOPI_7061 | scaffold260 | 356606 | GTTT/GTTTT | ATCGATTGATTTCAGCCCAG | TTCTCATCTCCATTCCCGTC | 60.0 | 154 | INTERGENIC | | | | |
| CaPOPI_7062 | scaffold260 | 413792 | A/AG | TATTTTGGATTTTGGCTCGG | AAAATCTCTGCTCGCGATGT | 59.9 | 668 | INTERGENIC | | | | |
| CaPOPI_7063 | scaffold2603 | 6501 | CCA/CCACA | AGCAGCTTCAAGCAAAGCTC | TGAACCAGTCCAATTCAACAGT | 60.0 | 485 | INTERGENIC | | | | |
| CaPOPI_7064 | scaffold2603 | 9101 | TA/TAA | GGCCAACCAAAAAGTTTGAA | ATTGAGTGAGTTTGGCCCAC | 60.0 | 552 | INTERGENIC | | | | |
| CaPOPI_7065 | scaffold2603 | 19509 | ATTTTTTTTTT/ATTTTTTTTTTT | TTGCGTGTTGATCTATGGGA | TCACCTTAGGGTTGAAATGGA | 60.1 | 884 | INTERGENIC | | | | |
| CaPOPI_7066 | scaffold2603 | 67369 | GTT/G | AATCCGCAACGGAGTTTATG | TGAACGAGCTGAGAGAAGCA | 60.0 | 617 | INTERGENIC | | | | |
| CaPOPI_7067 | scaffold2617 | 66007 | AATATATATATATATATATATATATATATATAT/AATATATATATATATATATATATATATATATATATAT | GACCCAACCATGTGGAGTTT | GGTGAGTGATCGCATCCTTT | 59.7 | 594 | INTERGENIC | | | | |
| CaPOPI_7068 | scaffold2617 | 195587 | GAATATATTAAATA/GAATATATTAAATATATTAAATA | GACCGCGTAGATGTGGAACT | GAATGCCCTACTTTTTGGCA | 60.1 | 634 | DRR | Ca_21875 | | | Aminotransferase-like, plant mobile domain |
| CaPOPI_7069 | scaffold2617 | 234200 | AATATATATATATATATATATATATAT/AATATATATATATATATATATAT | TTTGGGAAACTGAACCAACA | AAAATTGAAATTATCAAGAAAGTTGTG | 59.0 | 322 | DRR | Ca_21877 | | | |
| CaPOPI_7070 | scaffold2617 | 320997 | TAAAA/TAAA | TGGGGTATTCCTTTACGTCTT | TGAAAGAGGGGAAAAGGAGC | 57.6 | 296 | DRR | Ca_21883 | | WRKY | DNA-binding WRKY |
| CaPOPI_7071 | scaffold2617 | 321825 | TAAAAA/TAAAAAAAAAA | TTGGTCATGCGCACATAATA | GATGGCTGTGTCGGAGACTT | 58.6 | 529 | DRR | Ca_21883 | | WRKY | DNA-binding WRKY |
| CaPOPI_7072 | scaffold2617 | 324497 | TGTG/T | AAACAAGTTGGTGGTGGAGC | AAAGTTAATTCAAATGCACTGTCA | 60.0 | 290 | INTERGENIC | | | | |
| CaPOPI_7073 | scaffold2617 | 402483 | GAA/GAAA | GTTGCAAGGGTGGAGTGATT | AACAATTGTGAAACGTCTCCG | 60.0 | 640 | INTERGENIC | | | | |
| CaPOPI_7074 | scaffold2617 | 402519 | TG/T | GTTGCAAGGGTGGAGTGATT | AACAATTGTGAAACGTCTCCG | 60.0 | 640 | INTERGENIC | | | | |
| CaPOPI_7075 | scaffold263 | 177694 | A/AC | TCTGATGCAGTGTTTGGCTC | TTTTCTGGTCTGCCTTGGTC | 60.0 | 329 | INTERGENIC | | | | |

| INDEL marker IDs | Chromosomes/ unanchored scaffolds | Physical positions (bp) | InDels (*Kabuli* reference genome-CDC Frontier/PI) | Forward primers (5'-3') | Reverse primers (5'-3') | Anneling temperature (0C) | Expected amplified product size (bp) | Structural annotation | | Functional annotation | | |
|---|---|---|---|---|---|---|---|---|---|---|---|---|
| | | | | | | | | Sequence components of *kabuli* genome | *Kabuli* gene accession IDs | NCBI-KOG | TFs | NCBI-nr database |
| CaPOPI_7076 | scaffold2651 | 4275 | TT/TTGT | CTTGTGCATCCAATAGGGGT | TCTTCATCCAATGCCACAAA | 59.8 | 640 | INTERGENIC | | | | |
| CaPOPI_7077 | scaffold2660 | 12451 | TAAAA/TAAAAA | CCTAGGGACGACGAAAATGA | TGCATCCAACAGGTGTGTTT | 60.1 | 462 | INTERGENIC | | | | |
| CaPOPI_7078 | scaffold2662 | 1313 | T/TC | TGCACTTGCAACACATCTCA | TGGCTATTGATTATCCCATTTTG | 60.0 | 866 | INTERGENIC | | | | |
| CaPOPI_7079 | scaffold2662 | 10211 | AAATAATAATAATAATAATAATAATAATAAT/AAATAATAATAATAATAATAATAATAATAATAAT | TTGCTCCTACGTATCTCCCG | TTCTTTTCGCTATTTTATTTTCGTG | 60.2 | 699 | INTERGENIC | | | | |
| CaPOPI_7080 | scaffold2662 | 26227 | TT/TTCCT | AACACCATGAAACGCTCCTC | CGAAGCCGAGAAAATGACTC | 60.1 | 589 | INTERGENIC | | | | |
| CaPOPI_7081 | scaffold2662 | 31062 | TAA/TA | AGAGGATGCGGATCAATGTC | GGACAACCTGGGAAAAGTGA | 60.0 | 898 | INTERGENIC | | | | |
| CaPOPI_7082 | scaffold2669 | 16328 | AATATTGATATTGAT/AATATTGAT | TTTTAGGCGATTCTACGTGC | TTGAGGGGTGGCAAGTTTAG | 58.1 | 641 | INTERGENIC | | | | |
| CaPOPI_7083 | scaffold2692 | 13450 | GGTTAAGTT/GGTT | TTCAGAAAAACCGCCATACC | CCCTCTAAAGCCCCAGGTTA | 59.9 | 730 | INTERGENIC | | | | |
| CaPOPI_7084 | scaffold2692 | 14114 | TATAATAATAATAATAATAATAATAATAATAATAATAATAATAATAATAATAATA/TATAATAATAATAATAATAATAATAATAATAATAATAATAATAATA | TAACCTGGGGCTTTAGAGGG | TTTGACAGGTCAATTTCAAACC | 60.4 | 654 | INTERGENIC | | | | |
| CaPOPI_7085 | scaffold2692 | 14565 | ATTTTTTT/ATTTTTTTT | ACCTGTCAAAAGTTTTCAAGCA | TCCTTCACCAACCAAACAAA | 58.9 | 420 | INTERGENIC | | | | |
| CaPOPI_7086 | scaffold2692 | 14759 | ATTTTTTT/ATTTTTT | ACCTGTCAAAAGTTTTCAAGCA | TTTCCTTCACCAACCAAACA | 58.9 | 422 | INTERGENIC | | | | |
| CaPOPI_7087 | scaffold271 | 179145 | ATTTTTTTT/ATTTTTTTTT | GAGGTGCAGAAAAATGCAGC | TTGCTCCTACGTATCTCCCG | 60.9 | 592 | INTERGENIC | | | | |
| CaPOPI_7088 | scaffold271 | 196964 | ATTTTTTTTTT/ATTTTTTTTT | TATCATTTAGGGTGAGGGCG | AGAAGTGGAAAAGGGTGCCT | 59.9 | 605 | INTERGENIC | | | | |
| CaPOPI_7089 | scaffold271 | 205973 | GAA/GA | GCACCGTGAGTAAATTCGGT | TATTTTGGATTTTGGCTCGG | 60.0 | 692 | DRR | Ca_25334 | | | |
| CaPOPI_7090 | scaffold271 | 244115 | TTATATATATATATATATATATATATAT/TTATATATATATATATATATATATATATATAT | GAGAAAGGAGGGAAATTGGC | CATCGCCCACCGTAGTAAGT | 60.0 | 577 | INTERGENIC | | | | |

| INDEL marker IDs | Chromosomes/ unanchored scaffolds | Physical positions (bp) | InDels (*Kabuli* reference genome-CDC Frontier/PI) | Forward primers (5'-3') | Reverse primers (5'-3') | Anneling temperature (0C) | Expected amplified product size (bp) | Structural annotation | | Functional annotation | | |
|---|---|---|---|---|---|---|---|---|---|---|---|---|
| | | | | | | | | Sequence components of *kabuli* genome | *Kabuli* gene accession IDs | NCBI-KOG | TFs | NCBI-nr database |
| CaPOPI_7091 | scaffold2722 | 70279 | TAAAAAAAA/TAAAAAAAAA | TTTTGACCAATCCATCAGTTTTT | TGCAGTTGAGTAGATATGCCAAA | 59.7 | 883 | INTERGENIC | | | | |
| CaPOPI_7092 | scaffold2722 | 77542 | ATTTTTTTTT/ATTTTTTTT | GGAAGGAGAACGCCTAAGGT | AAAAATGGATCTCACGGTGC | 59.7 | 946 | URR | Ca_27216 | | C2H2 | Glycoside hydrolase, family 28 |
| CaPOPI_7093 | scaffold2726 | 4506 | G/GA | CAAACACCCCTATTGGATGC | AGGTCATTTTCGTCCTCCCT | 60.2 | 380 | INTERGENIC | | | | |
| CaPOPI_7094 | scaffold2729 | 5336 | AAAA/AAAACAAA | CAAATGCCACACATTTAACGA | AATTGTGTTTCGAGTTCCGC | 59.5 | 787 | INTERGENIC | | | | |
| CaPOPI_7095 | scaffold273 | 49066 | TAAAAAAAAAA/TAAAAAAAA | TTTCAGGCGAAAATCGTACC | ATAACCCGAACCTCAAACCC | 60.1 | 547 | INTERGENIC | | | | |
| CaPOPI_7096 | scaffold273 | 190574 | CAAAAAAAAAA/CAAAAAAAAA | GAAGAAGGTCCCAAGGGTTC | CGCTTCTTCCCCTTCTCTTT | 59.9 | 757 | DRR | Ca_26226 | | | |
| CaPOPI_7097 | scaffold2734 | 606 | TGCAAG/TGCAAGCAAG | TTTCTTTTTCCCTCCAAGCA | GGGGGAGAGAACAAATCCAT | 59.8 | 550 | INTERGENIC | | | | |
| CaPOPI_7098 | scaffold2736 | 3361 | ATTTTTTTT/ATTTTTTTTTT | TGGACAAAAGGCGTTGTAAA | GACTGGGCATTTTCCTTGAA | 59.2 | 340 | INTERGENIC | | | | |
| CaPOPI_7099 | scaffold2736 | 3536 | GAAAAAA/GAAAAA | TTCAAGGAAAATGCCCAGTC | TGTTTGCGACAACACAACAA | 60.1 | 385 | INTERGENIC | | | | |
| CaPOPI_7100 | scaffold2737 | 9370 | AAATAATAATAATAA/AAATAATAATAATAATAA | TCTTCGTTTTACGGTTTTATTTCA | AACATATAACAAAATTGAGTTGTCAAA | 59.2 | 419 | INTERGENIC | | | | |
| CaPOPI_7101 | scaffold2738 | 4048 | ATTT/ATTTT | GAGAGCTTTCGTTTCGATGG | GAGCCACGAGGCATGATATT | 60.0 | 775 | INTERGENIC | | | | |
| CaPOPI_7102 | scaffold2738 | 27122 | GTTTTTT/GTTTTTTTTT | TACCCTGTGGATGGGAATGT | CAAAAGATGTGTAACGGCCC | 60.1 | 441 | INTERGENIC | | | | |
| CaPOPI_7103 | scaffold2738 | 27897 | ATATGAAACTTAATGTTATGAAACTTAA/ATATGAAACTTAA | GGAGAAGGCATGGATGTTGT | TTGCATCGTACCACTTTGGA | 59.9 | 519 | INTERGENIC | | | | |
| CaPOPI_7104 | scaffold2744 | 48855 | T/TCG | ACCCAAATGCATCAGACTCC | TGTGTGTTTGTTTGGAGGGA | 59.9 | 565 | INTERGENIC | | | | |
| CaPOPI_7105 | scaffold2748 | 27802 | TCA/TCACA | TCTCGTTTAGATCGTCCGCT | TGGAATTCTTCTCCAATGCC | 60.0 | 551 | INTRON | Ca_28188 | | | |

| INDEL marker IDs | Chromosomes/ unanchored scaffolds | Physical positions (bp) | InDels (*Kabuli* reference genome-CDC Frontier/PI) | Forward primers (5'-3') | Reverse primers (5'-3') | Anneling temperature (0C) | Expected amplified product size (bp) | Structural annotation | | Functional annotation | | |
|---|---|---|---|---|---|---|---|---|---|---|---|---|
| | | | | | | | | Sequence components of *kabuli* genome | *Kabuli* gene accession IDs | NCBI-KOG | TFs | NCBI-nr database |
| CaPOPI_7106 | scaffold275 | 67453 | AG/A | CGTACTTGCATCCATGAAACA | CATCGCCGTAGTCAAACTCA | 59.6 | 563 | INTERGENIC | | | | |
| CaPOPI_7107 | scaffold275 | 162362 | TG/TGG | CGAAGCACAAGGAGATGACA | GGTTTGGGCAATCTGCTTTA | 60.0 | 762 | INTERGENIC | | | | |
| CaPOPI_7108 | scaffold275 | 187416 | CTT/C | CAGCAGCAGGAAAACATCAA | TCTTATCCTTGCGGGAATTG | 60.0 | 393 | INTERGENIC | | | | |
| CaPOPI_7109 | scaffold275 | 377072 | AATATATATATATATATATATATATAT/AATATATATATATATATATATATATATATATAT | TCAAGATACCCAGTGCACCA | AGCGACATGTGAACAACAGC | 60.1 | 551 | INTERGENIC | | | | |
| CaPOPI_7110 | scaffold2763 | 9760 | C/CCTCACG | ATGCCAGTCGTTATTCCCAG | GAGATGCCATTGAAGCCATT | 60.0 | 400 | CDS (large-effect mutations) | Ca_24393 | R | | |
| CaPOPI_7111 | scaffold2763 | 81370 | T/TG | AACCAACTGGTCCTGACTGG | GAATGGAGAAGGTCGTGCAT | 60.0 | 536 | DRR | Ca_24399 | | | |
| CaPOPI_7112 | scaffold2766 | 1362 | GTATATATATATATATATATATATATATAT/GTGTATATATATATATATATATATATATATATATAT | TTTGGAATTGTGATTATGTGTGTG | CAAACCCATCGGTCTCCTTA | 59.7 | 421 | INTERGENIC | | | | |
| CaPOPI_7113 | scaffold2766 | 6783 | AATATATATATATATATATATATATATATATATATATATAT/AATATATATATATATATATATATATATATATATATATATATATATATATAT | CGGCAGTAGGGATAACCAAA | TGTTGAAGATGAAATCTTGGCA | 60.0 | 652 | INTERGENIC | | | | |
| CaPOPI_7114 | scaffold277 | 4447 | TACTGA/TA | TAAAGGAGCATCCGTGGAAT | TGCATGTATCATAGACCGCC | 59.5 | 472 | INTERGENIC | | | | |
| CaPOPI_7115 | scaffold277 | 4542 | AATATATATATATATATATA/AATATATATATATATATA | TAAAGGAGCATCCGTGGAAT | TGCATGTATCATAGACCGCC | 59.5 | 472 | INTERGENIC | | | | |
| CaPOPI_7116 | scaffold277 | 4861 | AATATATAT/AATATAT | TGGAGAAACCTTGTGAACCA | GAAACGGAAAGGGGAACAAT | 59.1 | 316 | INTERGENIC | | | | |
| CaPOPI_7117 | scaffold277 | 205504 | TTATATATATATATATATATAT/TGATATATATATATATATATATAT | GGTTTAGGTGTGGGAGTGGA | TGATACAAGGTTCAACCGCA | 59.8 | 444 | DRR | Ca_25752 | | | |
| CaPOPI_7118 | scaffold277 | 252454 | CTCTT/CT | CCCATTGCATTGCCTTCTAT | TAAAGGACCATGTCGGGAAG | 59.9 | 329 | INTERGENIC | | | | |
| CaPOPI_7119 | scaffold2770 | 650 | AT/ATT | AATCGATTGATTCAGCCCAG | TTTACAGGCAGCGGAAAAGT | 60.0 | 682 | INTERGENIC | | | | |
| CaPOPI_7120 | scaffold2777 | 4052 | GTTAAC/G | CATTGATTAAGAACGCAAATGG | AGTTGGTCCCAAAAGTGCAA | 59.5 | 199 | INTERGENIC | | | | |

| INDEL marker IDs | Chromosomes/ unanchored scaffolds | Physical positions (bp) | InDels (*Kabuli* reference genome-CDC Frontier/PI) | Forward primers (5'-3') | Reverse primers (5'-3') | Anneling temperature (0C) | Expected amplified product size (bp) | Structural annotation | | Functional annotation | | |
|---|---|---|---|---|---|---|---|---|---|---|---|---|
| | | | | | | | | Sequence components of *kabuli* genome | *Kabuli* gene accession IDs | NCBI-KOG | TFs | NCBI-nr database |
| CaPOPI_7121 | scaffold2782 | 6224 | AGG/AGGG | CTTTTCGGGCGATTTTCATA | GATGAAATCGAGGACTGGGA | 60.0 | 483 | INTERGENIC | | | | |
| CaPOPI_7122 | scaffold2782 | 27408 | GAAAAAAAA/GAAAAAAAAAA | CTCGAAACCCGAAACACAAT | CGAATTTCGTTGTCAACTCAT | 60.0 | 574 | INTERGENIC | | | | |
| CaPOPI_7123 | scaffold2792 | 14181 | GTTTTT/GTTTT | AGGTTCATTTGGGTCATTGG | AACCTTGGGAGGACGAAAAT | 59.6 | 973 | INTERGENIC | | | | |
| CaPOPI_7124 | scaffold280 | 15504 | ACTAAAACCTAAAACCTAAA/ACTAAAACCTAAAACCTAAAACCTAAA | AAACCTGAACCGCAAAACAC | TCGTCCAATAAGAACAAAAGTCAA | 60.0 | 150 | INTERGENIC | | | | |
| CaPOPI_7125 | scaffold280 | 52649 | CTCAAT/CTCAATTGATTTATCAAT | TGGTCATGCAGAAACTCAGC | ACTCTCATCTGAGGGCGAAG | 60.0 | 330 | INTERGENIC | | | | |
| CaPOPI_7126 | scaffold280 | 83786 | CAAAACCTAAAACCTAAAACCTAAAAC/ CAAAACCTAAAACCTAAAACCTAAAACCTAAAAC | CCAAAAACCAAAAACCAAAAA | TCAAGCTATTAGTGCAAAAGAGTCA | 58.9 | 532 | INTERGENIC | | | | |
| CaPOPI_7127 | scaffold280 | 114213 | AG/AGCTTTGG | AAGGCTACTCACTCGCGGTA | ATGAGTTCTTTGCATTGGGC | 60.0 | 575 | INTERGENIC | | | | |
| CaPOPI_7128 | scaffold280 | 128741 | CAA/CAAA | ATTCCAAACCAAAACCCTCC | TGATTTTGGGGGAAAATGAA | 60.0 | 943 | INTERGENIC | | | | |
| CaPOPI_7129 | scaffold280 | 266426 | TAAC/T | TCTTCGTTATGCAGTCAAACCT | CGGAACCCGAAACAAAGTTA | 58.9 | 598 | INTERGENIC | | | | |
| CaPOPI_7130 | scaffold281 | 1263 | CAA/CAATTAA | TGTTCGACACTCAGTCTTACGG | TGCATGCCCTTTGATTTACA | 60.3 | 287 | INTERGENIC | | | | |
| CaPOPI_7131 | scaffold281 | 1388 | GAAAAAAAAAA/GAAAAAAAAA | TGTTCGACACTCAGTCTTACGG | TGCATGCCCTTTGATTTACA | 60.3 | 287 | INTERGENIC | | | | |
| CaPOPI_7132 | scaffold281 | 1467 | GGCATGCATG/GGCATG | TGTTCGACACTCAGTCTTACGG | ATCGGAAAAGTGACATTGGG | 60.3 | 515 | INTERGENIC | | | | |
| CaPOPI_7133 | scaffold281 | 2224 | CT/C | TGGGCAGATTTGTAACACGA | GCGAAACCCGATTCTATTGT | 60.1 | 593 | INTERGENIC | | | | |
| CaPOPI_7134 | scaffold281 | 3002 | TAAAAA/TAAAAAA | ACAATAGAATCGGGTTTCGC | AGGACTATTTTTGGTCTCCCA | 59.0 | 623 | INTERGENIC | | | | |
| CaPOPI_7135 | scaffold281 | 3090 | AATATATATATATAT/AATATATATAT | ACAATAGAATCGGGTTTCGC | AGGACTATTTTTGGTCTCCCA | 59.0 | 623 | INTERGENIC | | | | |

| INDEL marker IDs | Chromosomes/ unanchored scaffolds | Physical positions (bp) | InDels (*Kabuli* reference genome-CDC Frontier/PI) | Forward primers (5'-3') | Reverse primers (5'-3') | Anneling temperature (0C) | Expected amplified product size (bp) | Structural annotation | | Functional annotation | | |
|---|---|---|---|---|---|---|---|---|---|---|---|---|
| | | | | | | | | Sequence components of *kabuli* genome | *Kabuli* gene accession IDs | NCBI-KOG | TFs | NCBI-nr database |
| CaPOPI_7136 | scaffold281 | 3414 | GAAAAGAAAA/GAAAAGAAAAAGAAAA | TGGGAGACCAAAAATAGTCCT | CGTTGACCATTTTAAGCTTGG | 57.7 | 534 | INTERGENIC | | | | |
| CaPOPI_7137 | scaffold281 | 3735 | A/AATT | TGGGAGACCAAAAATAGTCCT | CAAGAGGGGTTGCAAGTGTT | 57.7 | 648 | INTERGENIC | | | | |
| CaPOPI_7138 | scaffold281 | 4125 | TGG/TG | AACACTTGCAACCCCTCTTG | ATGATGATTTTCTCCCCGTG | 60.1 | 595 | INTERGENIC | | | | |
| CaPOPI_7139 | scaffold281 | 4227 | AG/AGGAATTATG | AACACTTGCAACCCCTCTTG | ATGATGATTTTCTCCCCGTG | 60.1 | 595 | INTERGENIC | | | | |
| CaPOPI_7140 | scaffold2812 | 19841 | CT/C | GACGCTTCTTCCTCCTGCTA | TGAAGATAAAGCAATCCCCG | 59.7 | 761 | INTRON | Ca_26641 | | TALE | Serine/threonine-protein kinase domain |
| CaPOPI_7141 | scaffold2815 | 71444 | T/TGCG | CGGTGCTAGTGGACCAATTT | CCCGCCTGACAGTTAATCAT | 60.0 | 788 | INTERGENIC | | | | |
| CaPOPI_7142 | scaffold284 | 4461 | CTC/CTCCTCCTCCTCCTCATC | TACGATGATGAACAGTCGGC | TTGGTTTCAAGACCTGCATCT | 59.7 | 424 | INTERGENIC | | | | |
| CaPOPI_7143 | scaffold284 | 14484 | ATTTT/ATTTTT | TTTGCCTCATTGCTTTTTCC | CCCCTTTCTCCTAGAATCGC | 60.2 | 294 | INTERGENIC | | | | |
| CaPOPI_7144 | scaffold284 | 15317 | ATTTTTTTTTCTGCGTTTTTTTTTC/ATTTTTTTTTC | CCAGAATCGGACTTGTTTCG | TTCTTGTTGTGGAGTGTGGC | 60.6 | 515 | INTERGENIC | | | | |
| CaPOPI_7145 | scaffold284 | 40796 | TA/T | TCCTGCACTTGAATGGACAG | TTGGGTTTTTCTTTCAACGG | 59.8 | 693 | INTERGENIC | | | | |
| CaPOPI_7146 | scaffold284 | 205698 | AATATATATATATATAT/AATATATATATATATATAT | GGTCATGAGAAGGGGAGTGA | AAATTGGTCCACTAGCACCG | 60.0 | 480 | INTERGENIC | | | | |
| CaPOPI_7147 | scaffold284 | 428305 | CTATTATTATTATTATT/CTATTATTATTATTATT | TGTTGTTTACACACTTTAAGATGAAGA | GTCGCGTCTTCCATTCATTT | 58.5 | 361 | INTERGENIC | | | | |
| CaPOPI_7148 | scaffold284 | 483743 | ATTTTTTTTTTT/ATTTTTTTTTT | CAATCCGAAAGTCCTCCAAA | GCTTGAGGCACAAATTGACA | 60.0 | 847 | INTERGENIC | | | | |
| CaPOPI_7149 | scaffold284 | 516497 | AATATATATATATATATATATATATATATATATATAT/AATATATATATATATATATATATATATATAT | CCTCCAAATAGCCCCAAAAT | GTTCCTTTGGCAAGTCAGGA | 60.1 | 720 | DRR | Ca_20600 | C | C3H | Oxidoreductase, molybdopterin-binding domain |
| CaPOPI_7150 | scaffold284 | 680277 | CATATATATATATATATATATATA/CATATATATATATATATATATA | CCCACATGAAAAACTCCACC | TAAGGGCTGAACCAAAATCG | 60.2 | 779 | INTERGENIC | | | | |

| INDEL marker IDs | Chromosomes/ unanchored scaffolds | Physical positions (bp) | InDels (*Kabuli* reference genome-CDC Frontier/PI) | Forward primers (5'-3') | Reverse primers (5'-3') | Anneling temperature (0C) | Expected amplified product size (bp) | Structural annotation | | Functional annotation | | |
|---|---|---|---|---|---|---|---|---|---|---|---|---|
| | | | | | | | | Sequence components of *kabuli* genome | *Kabuli* gene accession IDs | NCBI-KOG | TFs | NCBI-nr database |
| CaPOPI_7151 | scaffold2845 | 27323 | CA/CAA | CCCTGATGGAAAATCAATGC | TCCAGTCCCCCTAGTCAGAA | 60.3 | 858 | INTERGENIC | | | | |
| CaPOPI_7152 | scaffold2848 | 2441 | GCTCCTCCT/GCTCCT | AAGGCAGCAGACCTCAAAAA | GCACACGACCTTCTCGTACA | 60.0 | 449 | CDS (large-effect mutations) | Ca_27862 | | | Peptidase C48, SUMO/Sentrin/Ubl1 |
| CaPOPI_7153 | scaffold2871 | 30418 | AGT/AGTGT | GGTTTTGGATTTGGGCTACA | GGGTTATGGCTTTGATCATGT | 59.8 | 521 | INTERGENIC | | | | |
| CaPOPI_7154 | scaffold2871 | 32080 | TG/TGG | CCCTGACCAAAACCGTAAAA | GTGCCATTTCATCCTTGCTT | 59.8 | 916 | INTERGENIC | | | | |
| CaPOPI_7155 | scaffold2871 | 33759 | GTTTTATTTTATTTTAT/GTTTTATTTTAT | TACCAATTGCGTTAGCGATG | TCGTTCTCCTTCGTCGTCTT | 59.7 | 487 | INTERGENIC | | | | |
| CaPOPI_7156 | scaffold2893 | 10683 | AGG/AGGG | GCCAGATCTTTCGATTCAGC | CCTTCAATGGAACCGCATAC | 59.9 | 255 | INTERGENIC | | | | |
| CaPOPI_7157 | scaffold2896 | 30850 | GAAAAAAAAAAA/GAAAAAAAAAA | CTGGCACAACAAGCACAACT | AGAGACAGAGGTGGGAAGCA | 59.9 | 521 | INTERGENIC | | | | |
| CaPOPI_7158 | scaffold29 | 101084 | AGG/AGGG | CGCGGAACCTGAAATACAAT | TCCAGATGAAATCGAGGACC | 60.0 | 401 | INTERGENIC | | | | |
| CaPOPI_7159 | scaffold290 | 112115 | A/ACTAAC | CATGGGAAAATCGTTGAGGT | TTGGGTCCTAGCTTTCTCCA | 59.8 | 839 | INTERGENIC | | | | |
| CaPOPI_7160 | scaffold290 | 122806 | AG/AAGCTTTTGCGGG | ACATTTGTGGCGGTCTTAGG | ACAAAGGGAGACGTTTGTGG | 60.0 | 243 | INTERGENIC | | | | |
| CaPOPI_7161 | scaffold290 | 124623 | CTTTTTTTTTT/CTTTTTTTTTTT | AATTTTGGTTGACTTTGCCG | GAGGCTTTTTGCATGGAGAG | 60.0 | 526 | INTERGENIC | | | | |
| CaPOPI_7162 | scaffold290 | 178205 | TAAAAAAAAAAAA/TAAAAAAAAAA | CACATCGAAGCAATCGTCAT | TCAGAACTGAACTTTGCCCA | 59.7 | 767 | INTRON | Ca_19830 | F | | Xanthine/uracil/vitamin C permease |
| CaPOPI_7163 | scaffold290 | 189028 | TAAAAAAAAAA/TAAAAAAAAAAA | TCAGGAGGATGACGGGTTAC | TTGATCATGGGGGTGATTTT | 59.9 | 624 | INTRON | Ca_19830 | F | | Xanthine/uracil/vitamin C permease |
| CaPOPI_7164 | scaffold290 | 204899 | GTACACAATAT/GT | ACCTTGGTCCGATGACAAAC | TTTCCCAATTGAAAGGATTGA | 59.8 | 656 | INTRON | Ca_19830 | F | | Xanthine/uracil/vitamin C permease |
| CaPOPI_7165 | scaffold290 | 206705 | GAT/GATCAATTAAAT | TCCCTCTTTTCAAGTCAACTCA | TCGCTCTAGGCTAATCCAACT | 59.0 | 318 | INTRON | Ca_19830 | F | | Xanthine/uracil/vitamin C permease |

| INDEL marker IDs | Chromosomes/ unanchored scaffolds | Physical positions (bp) | InDels (*Kabuli* reference genome-CDC Frontier/PI) | Forward primers (5'-3') | Reverse primers (5'-3') | Anneling temperature (0C) | Expected amplified product size (bp) | Structural annotation | | Functional annotation | | |
|---|---|---|---|---|---|---|---|---|---|---|---|---|
| | | | | | | | | Sequence components of *kabuli* genome | *Kabuli* gene accession IDs | NCBI-KOG | TFs | NCBI-nr database |
| CaPOPI_7166 | scaffold290 | 231729 | AATATATATATATATATA/AATATATATATATATATATA | TTTCACACCTCAATGCGAAG | ACCGAATGGCAGATTCAAAC | 59.8 | 789 | INTERGENIC | | | | |
| CaPOPI_7167 | scaffold290 | 233617 | A/AC | GGACGACCTAGCCTATTCCC | TGATCCGTTACAAAATGCCA | 59.9 | 430 | INTERGENIC | | | | |
| CaPOPI_7168 | scaffold290 | 280894 | TAATTATTG/TAATTATTGAATTATTG | GGTTCGCATATTTGCCTTGT | TTGGCACATCTACTTCACTGC | 60.0 | 222 | INTERGENIC | | | | |
| CaPOPI_7169 | scaffold290 | 285456 | AG/AGG | TTGGTCGTTTGAGCCTTTCT | GGGGCTTCAGTAAGGGTAGG | 59.9 | 436 | INTERGENIC | | | | |
| CaPOPI_7170 | scaffold290 | 293241 | ATTATTTATTTATTTATTTATTTATT/ATTATTTATTTATTTATTTATTTATTTATT | TTGCTCTTAATTTTTCCTCGAA | GTGAGTCTGCGACCACTTGA | 58.2 | 602 | INTERGENIC | | | | |
| CaPOPI_7171 | scaffold290 | 377065 | ATT/AT | TGCTTCAAGCTGCAATCTCT | CGCCTTGGTTACCCTTCAT | 58.9 | 789 | INTERGENIC | | | | |
| CaPOPI_7172 | scaffold290 | 400824 | TAAAAAAAAAA/TAAAAAAAAAAAA | TTGTGTTGCATTGTGGATTCT | CACAGCCCCTCTAAACCAAC | 59.0 | 650 | INTERGENIC | | | | |
| CaPOPI_7173 | scaffold290 | 418174 | GT/GTT | GAAGAGGCTAAACGGGGAAG | ATGCAAAGGTTGGTCATTCC | 60.2 | 409 | INTERGENIC | | | | |
| CaPOPI_7174 | scaffold290 | 429778 | ATTTTTTTTT/ATTTTTTTT | ACTCCGATGCACTGTTACCC | TAATGTGTCGTGTCTTCGCC | 60.0 | 751 | INTERGENIC | | | | |
| CaPOPI_7175 | scaffold290 | 434027 | CTTTTTTTT/CTTTTTTTTT | GGGACCATGCTTTTTGCTAT | ACAAGGCAAATATGCGAACC | 59.0 | 243 | INTERGENIC | | | | |
| CaPOPI_7176 | scaffold290 | 434453 | ATTATTTTTATTTTTATT/ATTATTTTTATTTTTATTTTTATT | GGTTCGCATATTTGCCTTGT | TGCGGTAGAGTAAATGTGACAAA | 60.0 | 570 | INTERGENIC | | | | |
| CaPOPI_7177 | scaffold290 | 452067 | GAAA/GAA | GAAAAATTCACCACCGATTAGG | TACAATCAGGATGCGTCCAA | 59.7 | 632 | INTERGENIC | | | | |
| CaPOPI_7178 | scaffold290 | 533287 | TAAAAAAAA/TAAAAAAA | CGATGATCATTCCACTTACCA | ACATTGGGTTTGGTTATGCC | 58.4 | 839 | INTERGENIC | | | | |
| CaPOPI_7179 | scaffold290 | 571667 | ATTTTT/ATTTTTT | ATTCCCCATTCTTAATGCCC | GGTAAGGGCTCCTTCCAAAC | 60.0 | 529 | INTERGENIC | | | | |
| CaPOPI_7180 | scaffold290 | 572854 | AATATATATATATATATAT/AATATATATATATATATATATAT | TCACCAAGCAAACCCACATA | CATGCAACCAAATCGACAAC | 60.0 | 554 | INTERGENIC | | | | |

| INDEL marker IDs | Chromosomes/ unanchored scaffolds | Physical positions (bp) | InDels (*Kabuli* reference genome-CDC Frontier/PI) | Forward primers (5'-3') | Reverse primers (5'-3') | Anneling temperature (0C) | Expected amplified product size (bp) | Structural annotation | | Functional annotation | | |
|---|---|---|---|---|---|---|---|---|---|---|---|---|
| | | | | | | | | Sequence components of *kabuli* genome | *Kabuli* gene accession IDs | NCBI-KOG | TFs | NCBI-nr database |
| CaPOPI_7181 | scaffold290 | 591539 | AATATATATATATATATATATATATATAT/AATATATATATATATATATATATATATATATATATATAT | AACAAGTCGTCGAGGCGTAT | AGGAAGAGTGGAGTTGCGAA | 59.8 | 620 | INTERGENIC | | | | |
| CaPOPI_7182 | scaffold290 | 605386 | GATATATATATATATATATATATATATATATATATA/GATATATATATATATATATATATATATA | TACGAACCCTCACAACCACA | GAGCGCTGTAAAAGACCTCC | 60.0 | 663 | INTERGENIC | | | | |
| CaPOPI_7183 | scaffold290 | 674413 | GAATTGAAAATTGAAA/GAATTGAAA | AACTCGAATCGTTTGGCAAG | AATTTTTCGACGCTTGATCG | 60.2 | 309 | INTERGENIC | | | | |
| CaPOPI_7184 | scaffold290 | 720833 | CTTTTTTTTTT/CTTTTTTTTT | AACTCCAACAACCCGTTGAG | ATGGGCCAATTACCCAACTT | 60.0 | 341 | INTERGENIC | | | | |
| CaPOPI_7185 | scaffold290 | 721068 | TAAAAAAA/TAAAAAAAA | AACTCCAACAACCCGTTGAG | TCTCCTAGTGCGATGGAGAAA | 60.0 | 936 | INTERGENIC | | | | |
| CaPOPI_7186 | scaffold290 | 867556 | AGGCCTACG/AGGCCTACGCCTGGCCTACG | TGCATTTTGAGTCTTGCTCG | GCCGAAGAAAAAGCCTATGA | 60.1 | 132 | INTERGENIC | | | | |
| CaPOPI_7187 | scaffold290 | 877992 | CTTTTTTTTTTT/CTTTTTTTTTT | TTTGACATGAGGCGTGTGTT | TCCGAAGACTTCCGCTACAT | 60.2 | 426 | INTERGENIC | | | | |
| CaPOPI_7188 | scaffold290 | 879217 | ATTTTTTTTTT/ATTTTTTTTT | ACACCCAATATCTCGCGTTC | GAAACTTTTTCCGCTGCTTG | 60.0 | 350 | INTERGENIC | | | | |
| CaPOPI_7189 | scaffold2900 | 7979 | ATT/AT | TATGAAAATCGCCCGAAAAG | GGTCCTCGATTTCATCTGGA | 60.0 | 355 | INTERGENIC | | | | |
| CaPOPI_7190 | scaffold2907 | 21102 | AAT/AATGTATGTTGTAT | GGTCTCAGCCCCTTCCTTAG | AAAATCCAACAATCTCCCCC | 60.2 | 562 | DRR | Ca_27009 | | | |
| CaPOPI_7191 | scaffold2907 | 30453 | T/TA | TATTCAGATCCCTTGCCCAG | TGGGTGCTATTTGCAATCAA | 60.0 | 635 | INTRON | Ca_27010 | | Trihelix | |
| CaPOPI_7192 | scaffold2924 | 26942 | T/TG | TGCAGGTAGCACTCCAAATG | TGGAGAAACCTCGCTCTGAT | 59.9 | 652 | INTERGENIC | | | | |
| CaPOPI_7193 | scaffold2928 | 10516 | ATGTTGTT/ATGTT | TGATTTCAATCCCCAATGCT | ACTTGCGCAATTTTCATTCC | 60.3 | 584 | DRR | Ca_27959 | | | |
| CaPOPI_7194 | scaffold2928 | 22766 | ATTTT/ATTTTT | AACCAATGATGTGCATCCAA | AGGGAGGACGAAAATGACCT | 59.8 | 471 | INTERGENIC | | | | |
| CaPOPI_7195 | scaffold2928 | 23701 | TTGT/TT | AGACCTCGCTTGGCTGATTA | ACGGAGGAGGAAAATGACCT | 60.0 | 210 | INTERGENIC | | | | |

| INDEL marker IDs | Chromosomes/ unanchored scaffolds | Physical positions (bp) | InDels (*Kabuli* reference genome-CDC Frontier/PI) | Forward primers (5'-3') | Reverse primers (5'-3') | Anneling temperature (0C) | Expected amplified product size (bp) | Structural annotation | | Functional annotation | | |
|---|---|---|---|---|---|---|---|---|---|---|---|---|
| | | | | | | | | Sequence components of *kabuli* genome | *Kabuli* gene accession IDs | NCBI-KOG | TFs | NCBI-nr database |
| CaPOPI_7196 | scaffold2932 | 2304 | GTTTT/GTTTTT | ATTGTCGTCCTCCCTAGGCT | ACACCTATTGGATGCCCAAG | 60.1 | 670 | INTERGENIC | | | | |
| CaPOPI_7197 | scaffold2950 | 515 | CA/CATTA | TGGAATAATCCACCGGGTAA | ACAACGACAACGACAACGTG | 60.0 | 782 | INTERGENIC | | | | |
| CaPOPI_7198 | scaffold2950 | 671 | CTTTTTTTTTTT/CTTTTTTTTT | TAACGGTCTCCAAATCCCTG | TTTGTTGGGTTTGCCTTACC | 59.9 | 846 | INTERGENIC | | | | |
| CaPOPI_7199 | scaffold2950 | 1436 | ATTTT/ATTTTT | GTCCTCTGAAACCTCCCACA | TTTTGGGTCGTGACACTTGA | 60.1 | 337 | INTERGENIC | | | | |
| CaPOPI_7200 | scaffold2950 | 6843 | A/AG | CCAAATTACAACCGACCCAC | AAAGGCGCTCCTCTATCACA | 60.1 | 650 | INTERGENIC | | | | |
| CaPOPI_7201 | scaffold2950 | 6903 | AAA/AAATAA | CCAAATTACAACCGACCCAC | AAAGGCGCTCCTCTATCACA | 60.1 | 650 | INTERGENIC | | | | |
| CaPOPI_7202 | scaffold2950 | 7749 | ATTTT/ATTTTT | CACGTTTTAAAGGGGTTTGC | GGTAAAGCCCATTCTGACCA | 59.5 | 845 | INTRON | Ca_27517 | L | FAR1 | BRCT |
| CaPOPI_7203 | scaffold2950 | 8204 | T/TG | TGGTCAGAATGGGCTTTACC | TTCAGAAGGGCCAGAAGAAA | 59.9 | 506 | INTRON | Ca_27517 | L | FAR1 | BRCT |
| CaPOPI_7204 | scaffold2950 | 10165 | AGG/AGGG | AGTTGTTTGGATACACCGGC | AGTGCTGTGGCTGGACCTAT | 59.9 | 662 | INTRON | Ca_27517 | L | FAR1 | BRCT |
| CaPOPI_7205 | scaffold2950 | 10696 | G/GT | TGATGATATCAGGCAACGGA | TGTCCCCACAGGTAGCTTTC | 60.0 | 588 | INTRON | Ca_27517 | L | FAR1 | BRCT |
| CaPOPI_7206 | scaffold2955 | 6116 | TTTTGAGAAA/TTTTGAGAAATTTGAGAAA | ACAAGTTGATGGGACTTGGC | GTCATCTGACCCCAACGACT | 60.0 | 572 | INTERGENIC | | | | |
| CaPOPI_7207 | scaffold2955 | 9708 | T/TCTAC | ACTATGCAAAGGTGCAAGGC | CACCATTTTCCGTCCACTCT | 60.3 | 374 | INTERGENIC | | | | |
| CaPOPI_7208 | scaffold2955 | 10080 | CT/CTT | AGAGTGGACGGAAAATGGTG | AAACTTGGTGCCCTGACTTG | 60.0 | 621 | INTERGENIC | | | | |
| CaPOPI_7209 | scaffold2955 | 11390 | ATTTT/ATTTTT | TAGAATAAGGCTCGACCGGA | TCCAAGCCCTTTCTCTTTCA | 59.8 | 402 | INTERGENIC | | | | |
| CaPOPI_7210 | scaffold2955 | 11713 | GTTTT/GTT | TGAAAGAGAAAGGGCTTGGA | AACTCGTTTGTTCATTGGTGA | 59.9 | 649 | INTERGENIC | | | | |

| INDEL marker IDs | Chromosomes/ unanchored scaffolds | Physical positions (bp) | InDels (*Kabuli* reference genome-CDC Frontier/PI) | Forward primers (5'-3') | Reverse primers (5'-3') | Anneling temperature (0C) | Expected amplified product size (bp) | Structural annotation | | Functional annotation | | |
|---|---|---|---|---|---|---|---|---|---|---|---|---|
| | | | | | | | | Sequence components of *kabuli* genome | *Kabuli* gene accession IDs | NCBI-KOG | TFs | NCBI-nr database |
| CaPOPI_7211 | scaffold296 | 11327 | TCC/TCCC | TGGACGTAGTCCGAGTTGGT | CTTGTGCTTCGGTTACAGCA | 60.6 | 517 | INTERGENIC | | | | |
| CaPOPI_7212 | scaffold296 | 16183 | TAAAAA/TAAAAAA | GCAGGGATCCAAAAGTTCAA | GTTTGGCAGTGAGAAAAGGC | 60.1 | 456 | INTERGENIC | | | | |
| CaPOPI_7213 | scaffold296 | 78708 | TTATA/TTA | TATGGACCGGAAATGGATGT | GACATTGCATTTTGGACACG | 60.0 | 606 | INTERGENIC | | | | |
| CaPOPI_7214 | scaffold296 | 84993 | TAAAAAAAA/TAAAAAAA | TCTCCGCATAAGCTTTCGAT | TGATTGCAAATAGCACCCAA | 59.9 | 241 | INTRON | Ca_20533 | | | |
| CaPOPI_7215 | scaffold296 | 257003 | ATTTTTTT/ATTTTTTTT | CAAATGTCGTCCAAAAAGCA | CTCAATAGGAGTTCGCAGGC | 59.7 | 433 | INTERGENIC | | | | |
| CaPOPI_7216 | scaffold296 | 259048 | A/ATT | AAGGTCAAGCAGTGGCAGAT | CATCTTCAAAGGGGCATGAT | 59.9 | 741 | INTERGENIC | | | | |
| CaPOPI_7217 | scaffold296 | 261410 | AAGAGAGAG/AAGAGAGAGAG | CATTGTGGGAGCGTAAGACA | TCGCAACTCTCATGTGTATGG | 59.7 | 388 | INTERGENIC | | | | |
| CaPOPI_7218 | scaffold296 | 261628 | TACACACACAC/TACACACACACAC | CATTGTGGGAGCGTAAGACA | TCGCAACTCTCATGTGTATGG | 59.7 | 388 | INTERGENIC | | | | |
| CaPOPI_7219 | scaffold296 | 261904 | CAAAAA/CAAAA | CCATACACATGAGAGTTGCGA | TTGACTCAGCCTTAAATTTTTATTGA | 59.7 | 534 | DRR | Ca_20542 | | | |
| CaPOPI_7220 | scaffold296 | 262823 | ATATTATTATTATTATTATTA/ATATTATTATTATTATTA | CACTTTTTAACCGGAGGAATG | GCAGTGGATAAGGCCGATAA | 58.6 | 140 | DRR | Ca_20542 | | | |
| CaPOPI_7221 | scaffold296 | 265325 | AACTACTA/AACTA | TCAAGCCATAAATGATGCCA | ATCTGCCACTGCTTGACCTT | 60.0 | 257 | INTERGENIC | | | | |
| CaPOPI_7222 | scaffold296 | 335726 | TTATATATATATATATATATATAT/TTATATATATATATATATAT | TGATCTTCGGGGCACTACTC | ATCGTTCCCCAAAGTGACAG | 60.2 | 445 | INTERGENIC | | | | |
| CaPOPI_7223 | scaffold296 | 406805 | GTTATTATTATTATTATTATTATTATTATTATTATTATTATTATTAATATTATTATTAT/GTTATTATTATTATTATTATTATTATTATTATTATTATTATTATTATTATTATTATT | AGTTATTTACGACGCACGGG | GGCACGACATCATCATCATC | 60.0 | 547 | INTERGENIC | | | | |
| CaPOPI_7224 | scaffold298 | 2633 | AAT/ATAT | CATCCACCCCTCTAAACCAA | TCGATTGATAGACCTTGCATTT | 59.8 | 416 | INTERGENIC | | | | |
| CaPOPI_7225 | scaffold298 | 8123 | TGA/T | AAATGAAAAGTGGCGAGGAC | AGTTACCCATTTGTGGCAGC | 59.2 | 740 | INTERGENIC | | | | |

| INDEL marker IDs | Chromosomes/ unanchored scaffolds | Physical positions (bp) | InDels (*Kabuli* reference genome-CDC Frontier/PI) | Forward primers (5'-3') | Reverse primers (5'-3') | Anneling temperature (0C) | Expected amplified product size (bp) | Structural annotation | | Functional annotation | | |
|---|---|---|---|---|---|---|---|---|---|---|---|---|
| | | | | | | | | Sequence components of *kabuli* genome | *Kabuli* gene accession IDs | NCBI-KOG | TFs | NCBI-nr database |
| CaPOPI_7226 | scaffold298 | 76407 | ATTTTTTTTTT/ATTTTTTTTT | GTTCAAACAACACGCTCACG | GATTGGACTTGTGGCTTGCT | 60.3 | 569 | INTERGENIC | | | | |
| CaPOPI_7227 | scaffold298 | 208119 | GTTTTTTTT/GTTTTTTT | AGTCGGAGGTTCCTAGCACA | ATTGTTGGGCTTGTCAGTCC | 59.9 | 471 | INTERGENIC | | | | |
| CaPOPI_7228 | scaffold2981 | 12753 | ATTTTTTT/ATTTTTTTT | TGTTATAGGTTTCGCCCCAG | CTTTTCGGGCGATTTTCATA | 60.0 | 840 | INTERGENIC | | | | |
| CaPOPI_7229 | scaffold299 | 27346 | GATAATAATAATAATAATAATAATAATAA/GATAATAATAATAATAATAATAATAA | CTACCGCGATCAAATACGGT | CCCTTTTGTCTTCTTGTGGC | 60.0 | 977 | INTERGENIC | | | | |
| CaPOPI_7230 | scaffold299 | 63392 | AATATATATATATATATATATATATATATA/AATATATATATATATATATATATATATA | AAATATCTTCTCTCGCCGCA | CTGAGATGCAGCGTTTTTCA | 59.9 | 528 | INTERGENIC | | | | |
| CaPOPI_7231 | scaffold299 | 213491 | AGG/AGGG | ACGGGAGCAAAAGTCTCTGA | CCAAAAGAAAATGCCCAAGA | 60.0 | 705 | INTERGENIC | | | | |
| CaPOPI_7232 | scaffold3015 | 68656 | A/AACCGGCACAGCC | TTCGCAGACAAAACTCATGC | AGCATTATGGGCCATCTGAG | 60.0 | 283 | CDS (large-effect mutations) | Ca_27318 | | | |
| CaPOPI_7233 | scaffold3015 | 69170 | AG/A | GGCCCATAATGCTCAAGTGT | TCATAGGCATGGGATTTGAA | 60.0 | 472 | DRR | Ca_27318 | | | |
| CaPOPI_7234 | scaffold3015 | 73264 | TGGCATAG/TG | TTTGTGTTTGCGATGACGAT | TAAGAGCGGAGTTTCTCCCA | 60.1 | 792 | INTERGENIC | | | | |
| CaPOPI_7235 | scaffold3015 | 73454 | TTTTGACTTTGACTTT/TTTTGACTTTGACTTTGACTTT | GTCTCGCGTTGAGTTAAGGG | TAAGAGCGGAGTTTCTCCCA | 59.9 | 489 | INTERGENIC | | | | |
| CaPOPI_7236 | scaffold3015 | 73940 | TG/T | TGGGAGAAACTCCGCTCTTA | TGATGGCTCCAAGGTGTATG | 59.9 | 538 | INTERGENIC | | | | |
| CaPOPI_7237 | scaffold3015 | 74020 | AAAATCATA/AA | TGGGAGAAACTCCGCTCTTA | TGATGGCTCCAAGGTGTATG | 59.9 | 538 | INTERGENIC | | | | |
| CaPOPI_7238 | scaffold3015 | 74169 | TTT/TTTCTT | TTTTGAAATTTAGGGTGTCACG | TGATGGCTCCAAGGTGTATG | 59.0 | 221 | INTERGENIC | | | | |
| CaPOPI_7239 | scaffold3019 | 18112 | CAAA/CA | ACCCGCAACTGGTAGACATC | AATTGTTTGGCCTCAAATGG | 60.0 | 597 | INTRON | Ca_28069 | | | XS domain |
| CaPOPI_7240 | scaffold3019 | 18761 | GAAAAAAAAAAAAAA/GAAAAAAAAAA | ACCCCAACCAACAAAGACAG | CAAGCCTGGAGCTATTGAGG | 59.9 | 369 | INTRON | Ca_28069 | | | XS domain |

| INDEL marker IDs | Chromosomes/ unanchored scaffolds | Physical positions (bp) | InDels (*Kabuli* reference genome-CDC Frontier/PI) | Forward primers (5'-3') | Reverse primers (5'-3') | Anneling temperature (0C) | Expected amplified product size (bp) | Structural annotation | | Functional annotation | | |
|---|---|---|---|---|---|---|---|---|---|---|---|---|
| | | | | | | | | Sequence components of *kabuli* genome | *Kabuli* gene accession IDs | NCBI-KOG | TFs | NCBI-nr database |
| CaPOPI_7241 | scaffold3019 | 20892 | AT/AATGGTT | TGCATGAGTTGTTAAGGCGA | TATGCAAAACGGGACTTCCT | 60.4 | 582 | URR | Ca_28069 | | | |
| CaPOPI_7242 | scaffold303 | 16790 | ATTTTTTTTT/ATTTTTTTTTT | TTTTTCTCTGCCCAAGGCTA | ACCATGCAAAGACATCCACA | 60.0 | 467 | INTERGENIC | | | | |
| CaPOPI_7243 | scaffold303 | 162542 | AAGCGCTGT/AAGCGCTGTGGAAACGAGCGCTGT | GCCATTTACAGCGCTTTTTC | TGAAATTATGGGTGGGCATT | 59.9 | 357 | DRR | Ca_26661 | | Trihelix | |
| CaPOPI_7244 | scaffold3032 | 11288 | GCCCCC/GCCCCCC | AGGTCATTTTCGTCCTCCCT | TATCCAATGACCAAAACGCA | 59.9 | 487 | INTERGENIC | | | | |
| CaPOPI_7245 | scaffold3038 | 10393 | TG/T | TGTGCATCCAAGAGGTGTGT | GGCGAGAATGACCAAAATGT | 60.2 | 756 | INTERGENIC | | | | |
| CaPOPI_7246 | scaffold3038 | 17812 | C/CTTAAGT | AGGTCATTTTCGTCCTCCCT | TCGACTTGTCAATCGCTTTG | 59.9 | 645 | INTERGENIC | | | | |
| CaPOPI_7247 | scaffold3046 | 14446 | AGGG/AGG | TTGGATGAACATTTCGGTCA | AAGCCTAGGGAGGACGAAAA | 59.9 | 240 | INTERGENIC | | | | |
| CaPOPI_7248 | scaffold305 | 168595 | TTAATAATAATAATAATAATAATAATAATAAT/TTAATAATAATAATAATAATAATAAT | AAGACACAAGGCAATGCAAA | GGTGGTGGAGGGTTACCTTT | 59.3 | 540 | INTRON | Ca_25903 | | | Phospholipid/glycerol acyltransferase |
| CaPOPI_7249 | scaffold3051 | 4826 | AATA/AA | GGAGGAACCCTCCTTGAAAA | ACAAACAAGTTGAGCACCCC | 60.4 | 648 | INTERGENIC | | | | |
| CaPOPI_7250 | scaffold306 | 128535 | TA/T | ACCGAAAGGAGAACACATGG | TGCAAATGAAAGTTTAAAGACCA | 60.0 | 240 | INTERGENIC | | | | |
| CaPOPI_7251 | scaffold306 | 128902 | AACA/AA | AAGGAGAACACATGGACTTGC | TGAGGCTTCAAGGAATTTGG | 59.2 | 961 | INTERGENIC | | | | |
| CaPOPI_7252 | scaffold306 | 130271 | GAAAAAAAAAA/GAAAAAAAA | TGCAAATTTCATCAAATCCA | CGTAGCCTTTGTTTGTGCTG | 57.5 | 415 | INTERGENIC | | | | |
| CaPOPI_7253 | scaffold306 | 166393 | TAAATCGATTG/TAAATCGATTGGGAAATCGATTG | TGCACAAGTCAGTAGCACCC | GCCAATCGATTACCCTCAAA | 59.9 | 138 | INTERGENIC | | | | |
| CaPOPI_7254 | scaffold306 | 196935 | T/TA | CAAGCGTTGGAGTTTTGGTT | TTTATTTGTGGGGGTTCTCG | 60.1 | 859 | INTERGENIC | | | | |
| CaPOPI_7255 | scaffold306 | 477894 | TAAAAAAAAAA/TAAAAAAAAA | CGTCATTTGCTTCTTCCCAT | TGGGACTTAAAAGCGTCGTT | 60.1 | 880 | INTRON | Ca_22723 | C | NAC | Cytochrome b561, eukaryote |

| **INDEL marker IDs** | **Chromosomes/ unanchored scaffolds** | **Physical positions (bp)** | **InDels (*Kabuli* reference genome-CDC Frontier/PI)** | **Forward primers (5'-3')** | **Reverse primers (5'-3')** | **Anneling temperature (0C)** | **Expected amplified product size (bp)** | **Structural annotation** | | **Functional annotation** | | |
|---|---|---|---|---|---|---|---|---|---|---|---|---|
| | | | | | | | | **Sequence components of *kabuli* genome** | ***Kabuli* gene accession IDs** | **NCBI-KOG** | **TFs** | **NCBI-nr database** |
| CaPOPI_7256 | scaffold3092 | 1124 | TAAAAAAA/TAAAAAAAA | GAGGTATTTTACAGCGCTCTTTC | TTGGGTCTGAAGAATGGCTC | 58.7 | 117 | INTERGENIC | | | | |
| CaPOPI_7257 | scaffold3093 | 19732 | GATATATATATATAT/GATATATATATATATAT | GAACCCTCACAATCACACGA | GAGCGCTGTAAAACACCTCC | 59.5 | 863 | INTERGENIC | | | | |
| CaPOPI_7258 | scaffold311 | 26152 | TA/TAA | TCAACCAAGTGCATCCAAAA | GGAGGCCGAAAATGACATAA | 60.1 | 564 | INTERGENIC | | | | |
| CaPOPI_7259 | scaffold311 | 91768 | CAAAAA/CAAAA | TGGGCAAGAAAAAGCCTATG | AGGTCATTTTCGTCCTCCCT | 60.2 | 836 | INTERGENIC | | | | |
| CaPOPI_7260 | scaffold311 | 131360 | A/ACAT | GAAGCTCAGATGGCACACAA | CAATCTCCCCCTTGAGTTGA | 60.0 | 716 | DRR | Ca_26332 | | | |
| CaPOPI_7261 | scaffold3115 | 46887 | TC/T | TGTCTACATTTCTCTTTTTCATCTTGA | TTTCTAAAATCGTTTCTTGTCCC | 59.7 | 600 | INTERGENIC | | | | |
| CaPOPI_7262 | scaffold3115 | 55767 | TATACATACATACATACATACATA/TATACATACATACATACATACATACATA | TGCATGTCCCTCAAAGGTAA | TCATTTGCTTACAAAGCCCC | 59.1 | 830 | INTERGENIC | | | | |
| CaPOPI_7263 | scaffold3115 | 62578 | ATTTTTTTTTTTT/ATTTTTTTTTTTTTT | CTTGGTGGTCCACTATGTGC | GGAGGTCAAAATACTAACTGCGA | 59.0 | 594 | INTERGENIC | | | | |
| CaPOPI_7264 | scaffold3115 | 62639 | CTTTTTTT/CTTTTTTTT | CTTGGTGGTCCACTATGTGC | TTCATAGGAGGCTGCCATTC | 59.0 | 632 | INTERGENIC | | | | |
| CaPOPI_7265 | scaffold3115 | 62933 | TTTATTATTATTATTATTATTATTATTATTTTTATTATTATTATTATTATAATTATTATTATTA/TTTATTATTATTATTATTATTATTATTTTTATTATTATTATTATTATAATTA | CTTGGTGGTCCACTATGTGC | TTCATAGGAGGCTGCCATTC | 59.0 | 632 | INTERGENIC | | | | |
| CaPOPI_7266 | scaffold3115 | 63986 | AATATATATATATATATATATATATATATA/AATATATATATATATATATATATATATATATATATA | TGAAGCATATGCCGTGAGAG | GCGTCAATGAACTTTACATGC | 60.0 | 559 | INTERGENIC | | | | |
| CaPOPI_7267 | scaffold3115 | 87750 | T/TAAGA | GGGGACTTGAATTTCTGCAA | ATGCCTGCATTTTTGAGGTC | 60.1 | 220 | INTERGENIC | | | | |
| CaPOPI_7268 | scaffold3127 | 8863 | A/AT | AATCCAATGGCACAAACACA | GGACATTTTCGTCCTGCCTA | 59.8 | 488 | INTERGENIC | | | | |
| CaPOPI_7269 | scaffold3134 | 12154 | ACATC/A | TAAACGAGGTAAACCGGTGC | GAAAGTCGCATGTGCAACAC | 60.0 | 437 | DRR | Ca_27104 | O | C2H2 | Peptidase A1 |
| CaPOPI_7270 | scaffold3134 | 13610 | A/AAG | TTGATTCGTGCAAATCAGGA | CCAAGAGAAAGGCAGTCGAG | 60.2 | 289 | DRR | Ca_27104 | O | C2H2 | Peptidase A1 |

| INDEL marker IDs | Chromosomes/ unanchored scaffolds | Physical positions (bp) | InDels (*Kabuli* reference genome-CDC Frontier/PI) | Forward primers (5'-3') | Reverse primers (5'-3') | Anneling temperature (0C) | Expected amplified product size (bp) | Structural annotation | | Functional annotation | | |
|---|---|---|---|---|---|---|---|---|---|---|---|---|
| | | | | | | | | Sequence components of *kabuli* genome | *Kabuli* gene accession IDs | NCBI-KOG | TFs | NCBI-nr database |
| CaPOPI_7271 | scaffold3134 | 15211 | TAAA/TAAAAA | CCTGGTTGAGTCTGGATAGCA | GCATTGGCGTATCTTGTTCA | 60.3 | 874 | INTRON | Ca_27104 | O | C2H2 | Peptidase A1 |
| CaPOPI_7272 | scaffold3134 | 109334 | AATATATATATATATATATATAT/AATATATATATATATAT | AGGAAGAGTGGAGTTGCGAA | TTAATGTGTTGCGGGTGAAA | 60.0 | 342 | INTERGENIC | | | | |
| CaPOPI_7273 | scaffold3135 | 4161 | TCC/TC | TCCAGATGAAATCGAGGACC | CCCGAAAAGTCCAGATGAAA | 60.0 | 882 | INTERGENIC | | | | |
| CaPOPI_7274 | scaffold314 | 79817 | GAA/GA | GAAGGCCCTCAAAATCATCA | CACAGCACCAGGTTATCCCT | 60.0 | 615 | INTERGENIC | | | | |
| CaPOPI_7275 | scaffold314 | 451895 | TTTCCACCATT/TTTCCACCATTCTTCCACCATT | CAAAGGCTTCAGCACACAAA | CGAGGAGGGTCTGTTGGATA | 60.0 | 678 | INTERGENIC | | | | |
| CaPOPI_7276 | scaffold314 | 543872 | C/CTA | AACCAAGGGAGGACGAAAAT | CTTGGGCATCCAATAGGTGT | 59.8 | 268 | INTERGENIC | | | | |
| CaPOPI_7277 | scaffold3149 | 9644 | TAG/TAGGAG | TTTCATCTGGACTTTTCGGG | ATCGTCGATGGGTACGAAAG | 60.0 | 452 | INTERGENIC | | | | |
| CaPOPI_7278 | scaffold3155 | 24057 | GAAAAAAAA/GAAAAAAA | TGCATATTTTGAAACGAATGACA | TGCAACCATCGGTTAAAATG | 60.4 | 505 | INTRON | Ca_27114 | R | NF-YB | |
| CaPOPI_7279 | scaffold3160 | 9833 | TGG/TG | AACCAGTCACTCAAGCGTCC | TGGCTGGTGATTTGTGTGAT | 60.3 | 165 | INTERGENIC | | | | |
| CaPOPI_7280 | scaffold3160 | 10499 | AG/AGG | TGGCTTTTTAGTTAGAGCACCA | GCAAACAAAGGAAACGGAAA | 59.1 | 629 | INTERGENIC | | | | |
| CaPOPI_7281 | scaffold3164 | 2274 | ACC/AC | CGTATTTCGGGGAAGAGTCA | TCGAGATTGTGTGCAAGGAG | 60.1 | 614 | INTERGENIC | | | | |
| CaPOPI_7282 | scaffold317 | 8959 | TTATATATATATATATATATATATATAT/TTATATATATATATATATATATATATATATATAT | AATGGAGGAAGGAGGGAGAA | GACCAAGTCCAACCTTGCAT | 60.0 | 492 | INTERGENIC | | | | |
| CaPOPI_7283 | scaffold317 | 30584 | CAAAAAATAAAAAAAAA/CAAAAAATAAAAAAAAAATAAAAAAAAA | CTCGCTCGGTTACCCTCATA | AGTTCGATTCAAAGACCCCA | 60.2 | 682 | DRR | Ca_25635 | D | | Cyclin |
| CaPOPI_7284 | scaffold317 | 203820 | AATATATATATATATATATA/AATATATATATATATATATATATA | GAAATTTTGACGGAAACGGA | AACGACTGTAACACGCCAAA | 59.9 | 974 | INTERGENIC | | | | |
| CaPOPI_7285 | scaffold3170 | 61619 | TTATATATATATATAT/TTATATATATATATATAT | AGCATTAGCAATCCTCCGAA | ACGAGAAAGCTGGACAGGAA | 59.8 | 484 | INTERGENIC | | | | |

| INDEL marker IDs | Chromosomes/ unanchored scaffolds | Physical positions (bp) | InDels (*Kabuli* reference genome-CDC Frontier/PI) | Forward primers (5'-3') | Reverse primers (5'-3') | Anneling temperature (0C) | Expected amplified product size (bp) | Structural annotation | | Functional annotation | | |
|---|---|---|---|---|---|---|---|---|---|---|---|---|
| | | | | | | | | Sequence components of *kabuli* genome | *Kabuli* gene accession IDs | NCBI-KOG | TFs | NCBI-nr database |
| CaPOPI_7286 | scaffold3170 | 205155 | T/TTTG | TATGTCGCATGATTGAGGGA | TCTTTCAACTCCACAACCCC | 60.0 | 443 | INTERGENIC | | | | |
| CaPOPI_7287 | scaffold3170 | 216441 | CA/CAA | CGGGTTACGTATGCAGGTCT | TTGCTTCATTCTCATTGGCA | 60.0 | 515 | INTERGENIC | | | | |
| CaPOPI_7288 | scaffold3171 | 2398 | C/CTA | TGATGGAGGATTGAGGAAGG | GTTGACAAATGCAAAATGCG | 60.0 | 431 | INTERGENIC | | | | |
| CaPOPI_7289 | scaffold3171 | 2647 | ATTTTTT/ATTTT | TGATGGAGGATTGAGGAAGG | GTTGACAAATGCAAAATGCG | 60.0 | 431 | INTERGENIC | | | | |
| CaPOPI_7290 | scaffold3175 | 11875 | CT/C | CGAAAATCGCTCGAAAAGTC | GGTCCTCGATTTCATCTGGA | 60.0 | 613 | INTERGENIC | | | | |
| CaPOPI_7291 | scaffold319 | 3243 | AATATATATATATATATATATATATATATATAT/AATATATATATATATATATATATATATATATATATATATAT | GAGAATTCCGGGAAGGAGAC | GTTGGTTCCCTAGCACCGTA | 60.0 | 790 | INTERGENIC | | | | |
| CaPOPI_7292 | scaffold3191 | 26393 | ATTTTTT/ATTTTT | TTCGTAATGGACGGGATGAT | GGGTTTTGCATGTTGAGCTT | 60.2 | 492 | DRR | Ca_27210 | | M-type | |
| CaPOPI_7293 | scaffold3209 | 943 | TC/TTTTTCC | GGTCCTCGATTTCATCTGGA | TTTGTCGAGGGGTACGAAAC | 60.0 | 620 | INTERGENIC | | | | |
| CaPOPI_7294 | scaffold321 | 38512 | A/AT | CCAAGGAGGACGAAAATGAC | ACCAATCTTGTGCATCCCAT | 59.5 | 768 | INTERGENIC | | | | |
| CaPOPI_7295 | scaffold3212 | 725 | CTT/CTTTT | CGAGTCGTGTCTTGATAATGGA | CCCATTAGTGGTGATGCTCC | 60.1 | 125 | INTERGENIC | | | | |
| CaPOPI_7296 | scaffold3212 | 1519 | GAAAAAAAA/GAAAAAAAAAA | AATGACATTTGTCCCCGTAGA | ATCGGTGGCATGTCCTTACT | 59.3 | 741 | INTERGENIC | | | | |
| CaPOPI_7297 | scaffold3212 | 1828 | TAATCTAA/TA | GCAGAAATTAAAATGAGTGGTGTG | TGGAAGGTGGAGGTTCAGAG | 59.9 | 715 | INTERGENIC | | | | |
| CaPOPI_7298 | scaffold3212 | 2564 | A/AC | TGAACCTCCACCTTCCATTC | CAAACAAATGAGTGGCGATG | 59.9 | 439 | INTERGENIC | | | | |
| CaPOPI_7299 | scaffold3212 | 6334 | ATTTTTTTT/ATTTTTTT | GAGCACATGATCTCGCAGAA | CACTGCTGAAGCGGAATACA | 60.1 | 213 | INTERGENIC | | | | |
| CaPOPI_7300 | scaffold3217 | 4699 | TTA/TTACTATA | CGCTTTGCCCTTAGTTTGAG | ATTCCCCATCAATTTGGACA | 60.0 | 298 | INTERGENIC | | | | |

| INDEL marker IDs | Chromosomes/ unanchored scaffolds | Physical positions (bp) | InDels (*Kabuli* reference genome-CDC Frontier/PI) | Forward primers (5'-3') | Reverse primers (5'-3') | Anneling temperature (0C) | Expected amplified product size (bp) | Structural annotation | | Functional annotation | | |
|---|---|---|---|---|---|---|---|---|---|---|---|---|
| | | | | | | | | Sequence components of *kabuli* genome | *Kabuli* gene accession IDs | NCBI-KOG | TFs | NCBI-nr database |
| CaPOPI_7301 | scaffold322 | 4062 | CT/CCTGTAGAAGACTGGT | TGCAAGAGCATATGGCAAAG | AATAAGCGACCATTGTTCCG | 60.0 | 227 | INTERGENIC | | | | |
| CaPOPI_7302 | scaffold322 | 32031 | ATT/AT | AGGTCATTTCCTTCGTCCCT | GGACGAAAACGACCTAAACG | 59.9 | 216 | INTERGENIC | | | | |
| CaPOPI_7303 | scaffold322 | 55500 | TCTACT/TCT | AAGTGCATATCAGCAGCGTG | ACCAGGTATTGAATGCAGCC | 60.0 | 163 | INTERGENIC | | | | |
| CaPOPI_7304 | scaffold322 | 77924 | GTTGATATTTTG/GTTGATATTTTGATATTTTG | TTAGGAATCGAACCCGTGTC | TCCTTGAAGACAAGCAACCA | 59.9 | 346 | INTERGENIC | | | | |
| CaPOPI_7305 | scaffold3225 | 20595 | AT/AGT | TTTGATGCCCCGATTAAAAA | GGTACCGCGAGTAATTCCAA | 60.3 | 646 | INTERGENIC | | | | |
| CaPOPI_7306 | scaffold3228 | 1705 | GAA/GAAA | TTGGTGAAGGAGGAGGATTG | CTCACCCTTGAATGCCACTT | 60.0 | 149 | INTERGENIC | | | | |
| CaPOPI_7307 | scaffold3228 | 1858 | CATA/CATATA | TCAGCTTTTTGGCTGATGTG | CTTGTGGATTGGGTCTTGTG | 60.0 | 247 | INTERGENIC | | | | |
| CaPOPI_7308 | scaffold3228 | 47967 | C/CAA | TGACAAAAGGGGGAGATTGA | TGCGTCTTTCTCAAGTGCAT | 60.4 | 414 | DRR | Ca_27761 | | | |
| CaPOPI_7309 | scaffold3228 | 48150 | GAATCGATTGATAAATCGATTG/GAATCGATTG | ACCAAAGTTGGAGGATGCAC | TTTCCATCAATCGATTTGCC | 60.0 | 596 | DRR | Ca_27761 | | | |
| CaPOPI_7310 | scaffold3229 | 5378 | TA/TATTGCA | AGCTGAAATTCCACAAGGCA | GATAATGGCCGCATGAAAAG | 60.8 | 366 | INTERGENIC | | | | |
| CaPOPI_7311 | scaffold3229 | 6703 | GAAAAAAAA/GAAAAAAAAAA | CAACGATTTAGCCATTGTTGA | TTGATGCCTCTTGGCTCTTT | 58.7 | 398 | INTERGENIC | | | | |
| CaPOPI_7312 | scaffold3229 | 8934 | GATATATATATATATATATATATATATAT/GATATATATATATATATATATATATATATAT | TGAAGCACAATGTTGGTTTTG | TGTAAAGTGCAACACCCGTC | 59.6 | 692 | INTERGENIC | | | | |
| CaPOPI_7313 | scaffold3234 | 4828 | A/AATCC | TCACGGGTCAACACGATTTA | CCACGAGATGATTCCATGTG | 60.0 | 285 | INTERGENIC | | | | |
| CaPOPI_7314 | scaffold324 | 86326 | TTATATATATATATATAT/TTATATATATATATATAT | ATCCCAATAAAGTGCCATCG | ATGCCCACCCATAATTTTCA | 59.8 | 826 | DRR | Ca_24915 | | NAC | Peptidase C48, SUMO/Sentrin/Ubl1 |
| CaPOPI_7315 | scaffold324 | 147338 | GGGT/G | TCCAGATGAAATCGAGGACC | TTTTCGGGCGATTTTTGTAG | 60.0 | 488 | INTERGENIC | | | | |

| INDEL marker IDs | Chromosomes/ unanchored scaffolds | Physical positions (bp) | InDels (*Kabuli* reference genome-CDC Frontier/PI) | Forward primers (5'-3') | Reverse primers (5'-3') | Anneling temperature (0C) | Expected amplified product size (bp) | Structural annotation | | Functional annotation | | |
|---|---|---|---|---|---|---|---|---|---|---|---|---|
| | | | | | | | | Sequence components of *kabuli* genome | *Kabuli* gene accession IDs | NCBI-KOG | TFs | NCBI-nr database |
| CaPOPI_7316 | scaffold3243 | 20346 | AATATATATATATATATATATATATATATATATATATAT/AATATATATATATATATATATATATATATATATATATATAT | TCTGGTGTGGGGGATGTTAT | CACGAATCGCATTTCATTTG | 60.1 | 421 | INTERGENIC | | | | |
| CaPOPI_7317 | scaffold3258 | 34096 | GTTTTTTTTTTTT/GTTTTTTTTTTT | GGGTTTGCTTACCGAACTCA | GGTTTCCACGGTTTGTCTGT | 60.1 | 270 | URR | Ca_27898 | | | Transposase, Ptta/En/Spm, plant |
| CaPOPI_7318 | scaffold3258 | 34164 | TAAAAAAAAAA/TAAAAAAAAA | GGGTTTGCTTACCGAACTCA | TACGGAAAATTACCCACGGA | 60.1 | 375 | URR | Ca_27898 | | | Transposase, Ptta/En/Spm, plant |
| CaPOPI_7319 | scaffold3258 | 34821 | TAAAAAAAAA/TAAAAAAAA | TGCAGAATATTGTCTTCATGCTT | GTGACACGTCGGAAGGAAAT | 58.9 | 837 | URR | Ca_27898 | | | Transposase, Ptta/En/Spm, plant |
| CaPOPI_7320 | scaffold3258 | 41416 | AT/A | TTTTTGTGTTTGAATACACGGTT | TTTTTCCTCGAGTGCAGCTT | 58.5 | 588 | INTERGENIC | | | | |
| CaPOPI_7321 | scaffold3258 | 46908 | GAC/GACCGGCTACGTAC | ATGGCCCCATTATTGAGGTT | TGCACGAGTCTAATGAGGGA | 60.4 | 696 | INTERGENIC | | | | |
| CaPOPI_7322 | scaffold3258 | 48852 | TTATGT/TT | ATGACACGGCTGAATGTCAA | ACGCACGTATGTTGTCCAAA | 60.1 | 374 | INTERGENIC | | | | |
| CaPOPI_7323 | scaffold3258 | 52201 | TA/T | TTTTTGGGTCCCTATCAACTAA | TCCAACAATAATTTAAAACCTTACAA | 57.7 | 500 | INTERGENIC | | | | |
| CaPOPI_7324 | scaffold3258 | 66747 | TACA/TA | GAAAGCGCTGTAAAAGGTGC | TGATTTGCTGGGAAATGACA | 60.0 | 504 | INTERGENIC | | | | |
| CaPOPI_7325 | scaffold3258 | 74031 | ATTTTTTT/ATTTTTT | AACTTCCACACCTGTCTCCG | AATTCAAAAGAATGTCGGCG | 60.2 | 726 | INTERGENIC | | | | |
| CaPOPI_7326 | scaffold3258 | 74657 | CTTTTTTTTTTT/CTTTTTTTTTT | AATGCAATCGTCAGGACACA | TATGGACCGGAAATGGATGT | 60.1 | 630 | INTERGENIC | | | | |
| CaPOPI_7327 | scaffold3258 | 77642 | AATTATTATTATTATTATTATTATTATTAT/AATTATTATTATTATTATTATTATTAT | TGCATGCATGAAAGGAAAAT | TCCCTCTTTAGCAAAAATAATTGG | 59.1 | 507 | INTERGENIC | | | | |
| CaPOPI_7328 | scaffold3258 | 81448 | GTTTTTTTTT/GTTTTTTTTTT | CCATATTGAATGCACATGGTC | AAGGTCCAACTCCAAACGAA | 58.7 | 517 | INTERGENIC | | | | |
| CaPOPI_7329 | scaffold330 | 10108 | CGTTGTT/CGTT | CCAATGACTCGTCGTCGATA | GCCATAGTGAATGAGGGGAA | 59.7 | 435 | INTERGENIC | | | | |
| CaPOPI_7330 | scaffold330 | 10431 | TAAAAAAAAA/TAAAAAAAAAAA | CCAATGACTCGTCGTCGATA | GCCATAGTGAATGAGGGGAA | 59.7 | 435 | INTERGENIC | | | | |

| INDEL marker IDs | Chromosomes/ unanchored scaffolds | Physical positions (bp) | InDels (*Kabuli* reference genome-CDC Frontier/PI) | Forward primers (5'-3') | Reverse primers (5'-3') | Anneling temperature (0C) | Expected amplified product size (bp) | Structural annotation | | Functional annotation | | |
|---|---|---|---|---|---|---|---|---|---|---|---|---|
| | | | | | | | | Sequence components of *kabuli* genome | *Kabuli* gene accession IDs | NCBI-KOG | TFs | NCBI-nr database |
| CaPOPI_7331 | scaffold330 | 10729 | AC/A | TTCCCCTCATTCACTATGGC | GTGCTCGAAATTTTGTCTCG | 59.9 | 561 | INTERGENIC | | | | |
| CaPOPI_7332 | scaffold331 | 202046 | C/CGA | ATTCCAAACCAAAACCCTCC | GTGATTTGGTGCGAATTGTG | 60.0 | 581 | INTERGENIC | | | | |
| CaPOPI_7333 | scaffold331 | 207791 | CC/CCTCTTCTCCAGATCAC | GCTGTCATGATTCGCTTCAA | GAACAACGCTGCAAAAGTCA | 60.0 | 273 | CDS (large-effect mutations) | Ca_25648 | | | |
| CaPOPI_7334 | scaffold3314 | 5786 | TTTTTTTCTTTTTTCTTTTTT/TTTTTTTCTTTTTTCTTTTTTCTTTTTT | AAGGTGAATCGTTCGAGAGAA | GCAAATCAATTTTTCTGGGG | 58.9 | 587 | INTERGENIC | | | | |
| CaPOPI_7335 | scaffold3317 | 732 | TA/TAA | TACCAACTGCGGATGCATAA | TTTCGCTACCCATCATACCC | 60.1 | 717 | INTERGENIC | | | | |
| CaPOPI_7336 | scaffold3317 | 3339 | TGACGAC/TGAC | GCATCTGTGTAGCCATCCAA | TAGGTCATTTTCGTCCTCCG | 59.7 | 738 | INTERGENIC | | | | |
| CaPOPI_7337 | scaffold3321 | 35578 | TGG/TG | GGAAAGGGATCATCAGCAAA | TGTGCAGATCCACACACTCA | 60.0 | 410 | DRR | Ca_25707 | U | FAR1 | Zinc finger, RING-type |
| CaPOPI_7338 | scaffold3321 | 59785 | GAAGCA/GAAGCAAGCA | GATTGTCGAAAGGGTGGAGA | AACCTCTGCAAATACCAGCG | 60.1 | 586 | DRR | Ca_25712 | GC | HSF | UDP-glucuronosyl/UDP-glucosyltransferase |
| CaPOPI_7339 | scaffold3321 | 61130 | C/CTTT | AAAATGTCACGCCACCAAAT | GGACCGCATATGTAATTTAGCC | 60.2 | 507 | DRR | Ca_25712 | GC | HSF | UDP-glucuronosyl/UDP-glucosyltransferase |
| CaPOPI_7340 | scaffold3321 | 69689 | TAA/TA | TCTTGCAGGAGCTTGGATTT | TGTGCCGACAATTAACCAAA | 60.0 | 681 | INTERGENIC | | | | |
| CaPOPI_7341 | scaffold3321 | 69753 | AAAAAGAGAAAAGAGA/AAAAAGAGA | TCTTGCAGGAGCTTGGATTT | TGTGCCGACAATTAACCAAA | 60.0 | 681 | INTERGENIC | | | | |
| CaPOPI_7342 | scaffold3322 | 4199 | TAAAAAAA/TAAAAAAAAA | GCCACAAAATTGGTCCAAAA | CGTGAGGCTGTTGGGATATT | 60.7 | 544 | INTERGENIC | | | | |
| CaPOPI_7343 | scaffold333 | 8945 | TCC/TC | GATGAAATCGAGGACCAGGA | GACTTTTCGAGCGATTTTCG | 60.0 | 299 | INTERGENIC | | | | |
| CaPOPI_7344 | scaffold333 | 91813 | GAACAACAAC/GAACAAC | GATGAAATCGAGGACCAGGA | GTGCCAGTCGATTGCCTAAT | 60.0 | 519 | INTERGENIC | | | | |
| CaPOPI_7345 | scaffold3336 | 8274 | GATTATTATTATTATTATTATTATTATTATTATTATTATTATTATTATTATTATTATT/GATTATTATTATTATTATTATTATTATTATTATTATTATTATTATTATTATTATTATTATT | CGGCCGTTTCTAACATCATT | TTGCTTTTGAAACCAATTTAGGA | 60.0 | 569 | INTERGENIC | | | | |

| INDEL marker IDs | Chromosomes/ unanchored scaffolds | Physical positions (bp) | InDels (*Kabuli* reference genome-CDC Frontier/PI) | Forward primers (5'-3') | Reverse primers (5'-3') | Anneling temperature (0C) | Expected amplified product size (bp) | Structural annotation | | Functional annotation | | |
|---|---|---|---|---|---|---|---|---|---|---|---|---|
| | | | | | | | | Sequence components of *kabuli* genome | *Kabuli* gene accession IDs | NCBI-KOG | TFs | NCBI-nr database |
| CaPOPI_7346 | scaffold3336 | 39173 | TAAAAAAAA/TAAAAAAA | TGAGATTGAATTGATATTGTGTTTGA | TCTCTTTCTCCTGCCCTTCA | 59.8 | 502 | INTERGENIC | | | | |
| CaPOPI_7347 | scaffold335 | 45267 | GATATATATATATATATATA/GATATATATATATATATATATATA | ACAAACCACGTACTCCCTCG | GCCCATTGCCATGTCTAAAT | 60.0 | 553 | INTERGENIC | | | | |
| CaPOPI_7348 | scaffold335 | 88156 | GTTATTATTATTATTATTATTATTATTATTATT/GTTATTATTATTATTATTATTATTATTATTATTATT | TTGTCTTCTTGTGGCCGACT | TTTTACAGGCGATTTGGAGC | 60.8 | 785 | INTERGENIC | | | | |
| CaPOPI_7349 | scaffold335 | 208290 | AATATATATATAT/AATATATATATATATATATATATAT | CGAAACAGGGAGGTTTGTGT | TATTCTGGATTTTGGCTCGG | 60.0 | 576 | INTERGENIC | | | | |
| CaPOPI_7350 | scaffold335 | 233856 | GTTTTTTTTTT/GTTTTTTTTTTT | ATGCCGTGTTATATTTCCGC | CCATTCTTCATGTGTGGTGC | 59.8 | 409 | INTERGENIC | | | | |
| CaPOPI_7351 | scaffold3354 | 665 | T/TA | CAACACAATTGAGCTGTCAAA | TTCGTAAGTACAACTTCAAAACGAA | 57.4 | 585 | INTERGENIC | | | | |
| CaPOPI_7352 | scaffold336 | 292805 | A/AT | TTCATCTTTTATCTTCATTCGGC | GCGCTGTTGTATCCTCCATT | 59.6 | 640 | INTERGENIC | | | | |
| CaPOPI_7353 | scaffold336 | 360914 | A/ATTATGG | TGCACATTTTACAGCGCTTC | CTGGGCAAGGGATCTGAATA | 60.0 | 666 | INTERGENIC | | | | |
| CaPOPI_7354 | scaffold336 | 441916 | T/TC | TCCCTAGTTCGAATCTGCGT | CCTCCATCGAATTTCTCCAA | 59.8 | 663 | INTERGENIC | | | | |
| CaPOPI_7355 | scaffold336 | 546259 | A/AG | TTTCGCCGATAAATTTCCTG | GGGCATCACTCAAGTCCTGT | 60.0 | 642 | INTERGENIC | | | | |
| CaPOPI_7356 | scaffold336 | 624730 | AG/AGTCACTCTCATGTTCCTG | TTAACCCAAGATTTCACGGC | CCCGACAAAGGGTAGTTGAA | 59.9 | 617 | DRR | Ca_21292 | | | |
| CaPOPI_7357 | scaffold336 | 633921 | A/AC | GAGAAAGGCAAGACCAGTGC | TTGCAATTCAACTGATGGGA | 60.0 | 365 | INTERGENIC | | | | |
| CaPOPI_7358 | scaffold336 | 825176 | TAAAAAAAAAA/TAAAAAAAAAAA | TTCTTATCCTCAACGTCCCG | TCTTTTAGGACGCAAGTTGTGA | 60.1 | 714 | INTERGENIC | | | | |
| CaPOPI_7359 | scaffold3364 | 43644 | T/TG | AGCCAAGGGAGGATGAAAAT | CGTCCACCCTTTGATTATCC | 59.9 | 214 | INTERGENIC | | | | |
| CaPOPI_7360 | scaffold3369 | 18856 | T/TA | TTCAACGTGACCTGCTCATC | ATGGGTCCAGGTCATCTCAG | 59.8 | 671 | INTERGENIC | | | | |

| INDEL marker IDs | Chromosomes/ unanchored scaffolds | Physical positions (bp) | InDels (*Kabuli* reference genome-CDC Frontier/PI) | Forward primers (5'-3') | Reverse primers (5'-3') | Anneling temperature (0C) | Expected amplified product size (bp) | Structural annotation | | Functional annotation | | |
|---|---|---|---|---|---|---|---|---|---|---|---|---|
| | | | | | | | | Sequence components of *kabuli* genome | *Kabuli* gene accession IDs | NCBI-KOG | TFs | NCBI-nr database |
| CaPOPI_7361 | scaffold3369 | 22053 | TATAATAATAATAATAATAATAATAATAATAATA/TATAATAATAATAATAATAATAATAATAATAATAATA | TCGCCACCGATTTTATTTTT | AATTAATGGTTGTCGCTGCC | 59.4 | 674 | INTERGENIC | | | | |
| CaPOPI_7362 | scaffold3371 | 7999 | AAAATAAATAAATAAATAAATAAATAAATA/AAAATAAATAAATAAATAAATAAATAAATAAATAAATA | CCTGGGGATATGTAGGAGCA | CATTATTGTTGTTGCGTCCG | 59.9 | 348 | INTERGENIC | | | | |
| CaPOPI_7363 | scaffold3371 | 33866 | AC/A | CGAGCCAAAATCCCAAAATA | ATGGTTGATTGGTTGCGAAT | 59.9 | 256 | INTERGENIC | | | | |
| CaPOPI_7364 | scaffold338 | 76520 | GTATATATATATATATAT/GTATATATATATATATATAT | CTTGGCCATAAAATCGGAAA | AGGATGCATGGGATGAAAAG | 59.9 | 661 | INTERGENIC | | | | |
| CaPOPI_7365 | scaffold3397 | 17802 | TATTGCAAATTGCA/TATTGCA | GATCAGATCTGGCAGCAACA | GAGGATGGAAATTGCTGCAT | 60.0 | 366 | DRR | Ca_26271 | | | |
| CaPOPI_7366 | scaffold340 | 51387 | A/ACT | TCCATCAATTTCATCCACCA | CCTTTGCCTCTAGCGATTTG | 59.7 | 587 | INTRON | Ca_24956 | DL | | ATPase, AAA+ type, core |
| CaPOPI_7367 | scaffold340 | 66713 | ATTTTTTTT/ATTTTTTTTT | AACACACGCACCAACAAAGA | TGAATATGCGTCGACTGAGC | 60.2 | 559 | INTERGENIC | | | | |
| CaPOPI_7368 | scaffold340 | 100873 | TTCTC/TTC | AACGTGGGTTACGTCCAGTC | AAATGCAGAGCACCTCCAAC | 59.9 | 530 | INTRON | Ca_24959 | U | | Longin |
| CaPOPI_7369 | scaffold340 | 119249 | GAAAAAAA/GAAAAAAAA | GGTTGCGTAGTGGTAGCCAT | CTCCGAGCCAATATCCAAAA | 60.0 | 623 | INTERGENIC | | | | |
| CaPOPI_7370 | scaffold340 | 135811 | ATTTTTTTT/ATTTTTTTTT | TTTGACGCTTTAACTATCCCG | AACTTTGTCCATTTTATTGGCTT | 59.3 | 530 | INTERGENIC | | | | |
| CaPOPI_7371 | scaffold340 | 161360 | ATTTTTTT/ATTTTTTTT | GCGGATATCGTAGGGTGCTA | TACATCCAGAAGGCTACCGC | 60.1 | 539 | INTERGENIC | | | | |
| CaPOPI_7372 | scaffold340 | 205523 | TAA/TA | CCCCAAAAAGTTGGAGAACA | TTCTAACCTTGCAACCGGAG | 59.9 | 398 | INTERGENIC | | | | |
| CaPOPI_7373 | scaffold340 | 210507 | GA/GAA | AACGGAGAATGTTCACCAGG | TGCAGAATCGTGGAGATTGA | 60.0 | 383 | INTERGENIC | | | | |
| CaPOPI_7374 | scaffold340 | 225242 | TAAAAAAAA/TAAAAAAA | TCACCTTGGAAAAGTCTGCC | TATCCCCCAAGATTTGGTCA | 60.2 | 683 | INTERGENIC | | | | |
| CaPOPI_7375 | scaffold340 | 261525 | GTTTTTTTTT/GTTTTTTTTTTT | TTTCCAAGAGTTTCAACCCG | TGTTTAGGCTGCTGATGGTG | 60.1 | 387 | INTERGENIC | | | | |

| INDEL marker IDs | Chromosomes/ unanchored scaffolds | Physical positions (bp) | InDels (*Kabuli* reference genome-CDC Frontier/PI) | Forward primers (5'-3') | Reverse primers (5'-3') | Anneling temperature (0C) | Expected amplified product size (bp) | Structural annotation | | Functional annotation | | |
|---|---|---|---|---|---|---|---|---|---|---|---|---|
| | | | | | | | | Sequence components of *kabuli* genome | *Kabuli* gene accession IDs | NCBI-KOG | TFs | NCBI-nr database |
| CaPOPI_7376 | scaffold340 | 261564 | ATTTTT/ATTTTTT | TTTCCAAGAGTTTCAACCCG | TGTTTAGGCTGCTGATGGTG | 60.1 | 387 | INTERGENIC | | | | |
| CaPOPI_7377 | scaffold340 | 287997 | ATTATTTATTTATTTATTTATTT/ATTATTTATTTATTTATTT | GTCACAGAGGGAACGGATGT | TTGGAAAAGCCCACCTACAC | 60.0 | 524 | INTERGENIC | | | | |
| CaPOPI_7378 | scaffold340 | 318031 | CAAAAAAAAAAAAAA/CAAAAAAAAAAAA | ATGTACCTCCGCCTCATTTG | CTTTTACAGCGCTCGATTCC | 60.0 | 175 | INTERGENIC | | | | |
| CaPOPI_7379 | scaffold341 | 135211 | TATATATATAGAAA/TA | AATGGAGTTGCGACGAGAGT | TCTCCGTAAAGGATTCACCG | 59.9 | 540 | INTERGENIC | | | | |
| CaPOPI_7380 | scaffold341 | 135303 | GAAAA/GAAAAA | AATGGAGTTGCGACGAGAGT | TCTCCGTAAAGGATTCACCG | 59.9 | 540 | INTERGENIC | | | | |
| CaPOPI_7381 | scaffold342 | 164779 | TTATATATATATATATAT/TTATATATATATATAT | TTCCAACATGCTTGAAACTCA | GGCCGAAATTTCATTCAACA | 59.3 | 703 | INTERGENIC | | | | |
| CaPOPI_7382 | scaffold342 | 249187 | ATTTTTTTTT/ATTTTTTTT | CAACGGTGAAGCCCATAACT | TGGGTGTTAAAATTCTCCTCTTG | 60.0 | 841 | INTERGENIC | | | | |
| CaPOPI_7383 | scaffold342 | 310549 | CATATATAT/CATAT | TTGTGACATTTCATCAAACACA | CAACCGACGTTGAAGAACAA | 57.5 | 933 | INTERGENIC | | | | |
| CaPOPI_7384 | scaffold342 | 340574 | TC/T | TGTTCCAGCATACCAAATGC | AGCAAAATCCGAGGAGGAAT | 59.5 | 557 | INTERGENIC | | | | |
| CaPOPI_7385 | scaffold342 | 370625 | TTATCAT/TT | TGGGGTCCTTTCTCTTCTCC | TATGCCCATTGAGTTGGACA | 60.6 | 387 | INTERGENIC | | | | |
| CaPOPI_7386 | scaffold3432 | 23969 | CAATAAT/CAAT | CCTGAATCCGAGACAAGACG | TCATGGGTTGTATGGAAGCA | 60.8 | 487 | INTERGENIC | | | | |
| CaPOPI_7387 | scaffold3434 | 7567 | GAAAAAA/GAAAAAAA | CGACATTCGTCATGGTTTTG | CATAGCAATCCAACAACAAGAAA | 60.0 | 876 | INTERGENIC | | | | |
| CaPOPI_7388 | scaffold3434 | 11167 | AGGGG/AGGG | CTTTCTCCATTTCCACCCAA | TCACTCAAAGGGCTACTGCC | 59.9 | 532 | INTERGENIC | | | | |
| CaPOPI_7389 | scaffold3436 | 9281 | GAGACTTAT/G | GGCCATGAATTCAACAACAA | TTGCCAAATACAAAAAGAAATGAA | 59.4 | 570 | INTERGENIC | | | | |
| CaPOPI_7390 | scaffold3436 | 9959 | TAATA/TTAATAATATACAAATA | TGGAGTTCGGGCTAAGTACAG | CGTGCTTGCAGAAAAACAAA | 59.4 | 309 | INTERGENIC | | | | |

| INDEL marker IDs | Chromosomes/ unanchored scaffolds | Physical positions (bp) | InDels (*Kabuli* reference genome-CDC Frontier/PI) | Forward primers (5'-3') | Reverse primers (5'-3') | Anneling temperature (0C) | Expected amplified product size (bp) | Structural annotation | | Functional annotation | | |
|---|---|---|---|---|---|---|---|---|---|---|---|---|
| | | | | | | | | Sequence components of *kabuli* genome | *Kabuli* gene accession IDs | NCBI-KOG | TFs | NCBI-nr database |
| CaPOPI_7391 | scaffold3453 | 1173 | CA/CAAA | ATATGCCTCTACCGTGCAGC | TCGTGAAAGCAAACAAAGGA | 60.3 | 507 | INTERGENIC | | | | |
| CaPOPI_7392 | scaffold3472 | 6763 | GTATATATATATATATATATATATATATA/GTATATATATATATATATATATATATA | CAAGCATGATCCAGGGAGAT | CTCGGCGAGTTTGTGTGTAA | 60.0 | 471 | INTERGENIC | | | | |
| CaPOPI_7393 | scaffold3472 | 7148 | TATTAAATAATT/TATTAAATAATTAAATAATT | CAGGCTAGCTAAATGCCTCG | ACCATGGTTGTTGCAAGTGA | 60.1 | 720 | INTERGENIC | | | | |
| CaPOPI_7394 | scaffold3472 | 7670 | CA/C | AAACCCAAAAATAGTCCGCC | CCGTAACACATGCTTTCCCT | 60.2 | 361 | INTERGENIC | | | | |
| CaPOPI_7395 | scaffold3472 | 9063 | CAA/CAAA | GCTCCATGTGCTTCACACAC | GTGACACGTGACAATCCCAC | 60.3 | 107 | INTERGENIC | | | | |
| CaPOPI_7396 | scaffold3472 | 9396 | TC/TCC | TTTTGACAGAGAAACCCGCT | CTGTGAGAGGCGAAGATGGT | 59.9 | 223 | INTERGENIC | | | | |
| CaPOPI_7397 | scaffold3472 | 71251 | CTACAGCGCTT/CTACAGCGCTTGTTTTACAGCGCTT | GAAAGCGCTTCCGTTTACAG | GACATTTAACAGCGCCCATT | 60.0 | 159 | INTERGENIC | | | | |
| CaPOPI_7398 | scaffold3475 | 6139 | AG/A | GGCCTGGAGACCCTTATAGC | ACCACTCGACGAAAATGGAC | 60.1 | 616 | INTERGENIC | | | | |
| CaPOPI_7399 | scaffold3475 | 6197 | AT/ATT | GGCCTGGAGACCCTTATAGC | ACCACTCGACGAAAATGGAC | 60.1 | 616 | INTERGENIC | | | | |
| CaPOPI_7400 | scaffold3475 | 31282 | TG/TGG | GCTATTTTCGTACCACCCGA | GCCCAAAAAGTCCAGATGAA | 60.0 | 326 | INTERGENIC | | | | |
| CaPOPI_7401 | scaffold3489 | 2052 | TAATAAAAAATAAAAAATAAAAAATAAAAA/TAATAAAAAATAAAAAATAAAAAA | TCGTTCTGTTGGACCCTTTC | AGTGGAAAATATGCCGTTGC | 60.1 | 521 | INTERGENIC | | | | |
| CaPOPI_7402 | scaffold3489 | 2940 | ATTTTTTTTT/ATTTTTTTT | GCACATTCCTAAGGGGCATA | AGTGCAAATGAGTTTTGGTCA | 59.9 | 373 | INTERGENIC | | | | |
| CaPOPI_7403 | scaffold349 | 70134 | GTATATATATATATATATATATAT/GTATATATATATATATATATATAT | TTCGGAAGTCCATCGGTATC | AATGCATGCTCCCCTAATTG | 59.9 | 643 | INTERGENIC | | | | |
| CaPOPI_7404 | scaffold349 | 177787 | GTTATTGAATT/GTTATTGAATTCCTTTATTGAATT | GCGATAATCCCCGTAAGGAT | TAGGTGCAGGTCATGAAACC | 60.1 | 652 | INTERGENIC | | | | |
| CaPOPI_7405 | scaffold349 | 335267 | ATT/ATTT | TTGGTCAATGTGGCTTCTCA | CTGAGCTTGTGACCAAATCG | 60.2 | 531 | INTERGENIC | | | | |

| INDEL marker IDs | Chromosomes/ unanchored scaffolds | Physical positions (bp) | InDels (*Kabuli* reference genome-CDC Frontier/PI) | Forward primers (5'-3') | Reverse primers (5'-3') | Anneling temperature (0C) | Expected amplified product size (bp) | Structural annotation | | Functional annotation | | |
|---|---|---|---|---|---|---|---|---|---|---|---|---|
| | | | | | | | | Sequence components of *kabuli* genome | *Kabuli* gene accession IDs | NCBI-KOG | TFs | NCBI-nr database |
| CaPOPI_7406 | scaffold350 | 67917 | CTTTT/CTTT | CAACATGCGGGTCTTTTTCT | TGATTTGAATGGTCGTGTCC | 60.1 | 267 | CDS (FRAME SHIFT) | Ca_26734 | | | |
| CaPOPI_7407 | scaffold350 | 165550 | ATGTGTG/ATGTGTGTG | CAATTCCATGGCTCCAAAGT | CAATGCCTCCCCATAACAAT | 59.9 | 670 | INTERGENIC | | | | |
| CaPOPI_7408 | scaffold3500 | 6141 | C/CTGTA | GCGTTCGATTCCACATCTTT | TCATTATGCGTGTTCCGCTA | 60.1 | 434 | INTERGENIC | | | | |
| CaPOPI_7409 | scaffold3500 | 30406 | GTATATATATATATATATATATATATATATATAT/GTATATATATATATATATATATATATATATATAT | TTTAACGTGTCGCGTCTTTG | GGATGAATACATTGGGCTGG | 59.9 | 602 | INTERGENIC | | | | |
| CaPOPI_7410 | scaffold3514 | 19067 | ATTTTTTTTTT/ATTTTTTTTT | TGACACCCTTAATTAACCCCA | TTAATTGTGAGCGTGTGGGA | 59.2 | 533 | INTERGENIC | | | | |
| CaPOPI_7411 | scaffold3514 | 40711 | GAAA/GAAAA | TGACCAAATCGGAATTGATTTT | CATTTCCGAATCTTCCCGTA | 60.5 | 723 | INTERGENIC | | | | |
| CaPOPI_7412 | scaffold3514 | 41395 | C/CCGTTGCA | TACGGGAAGATTCGGAAATG | TGATTGCCACGTCATTGTTT | 59.9 | 721 | INTRON | Ca_26926 | | FAR1 | Lipase, GDSL |
| CaPOPI_7413 | scaffold352 | 33761 | TA/T | TTTCATTTCACTCTTCCCCG | ATTTATCATAGGGGCGGCTC | 60.0 | 885 | INTERGENIC | | | | |
| CaPOPI_7414 | scaffold352 | 93620 | GAA/GAAA | TTCCCTCTCTTGCTCAAAGG | GACTGCCATCACAGAAGCAA | 59.5 | 466 | INTERGENIC | | | | |
| CaPOPI_7415 | scaffold352 | 141916 | AT/ATT | TTGCCTTGGATTCTTTGACC | CGCAAGACAGAAAACAACCA | 60.1 | 417 | INTERGENIC | | | | |
| CaPOPI_7416 | scaffold352 | 178010 | ATTT/ATT | AACGCATCTTTTTACCCACG | GGCCTGAATCTCATCCAAGA | 60.0 | 568 | INTERGENIC | | | | |
| CaPOPI_7417 | scaffold352 | 178511 | ATGAGTTGAGTTGAGTT/ATGAGTTGAGTT | TCGCAATGCAACATCTCTTT | GCTTGTGAGTGTGTGTGCCT | 59.4 | 823 | INTERGENIC | | | | |
| CaPOPI_7418 | scaffold352 | 192510 | ATTTTTTTTTTT/ATTTTTTTTTTTTT | TGTGTAAGGCTTATGTGGCG | GCAAGGGAGAGCAAGTTTTG | 59.8 | 762 | INTERGENIC | | | | |
| CaPOPI_7419 | scaffold3520 | 11867 | CTGTGTGTGTGTGTGTGTGTG/CTGTGTGTGTGTGTGTGTGTGTG | TTGGCAATCAACTCCCTCTT | TCCTCGTTTTATCATCACATCA | 59.7 | 488 | INTERGENIC | | | | |
| CaPOPI_7420 | scaffold3529 | 3004 | GGGA/GGGAGGA | TGTTCATCCAATGACCCAAA | CATTTTCATCCTCCCTACGC | 59.8 | 389 | INTERGENIC | | | | |

| INDEL marker IDs | Chromosomes/ unanchored scaffolds | Physical positions (bp) | InDels (*Kabuli* reference genome-CDC Frontier/PI) | Forward primers (5'-3') | Reverse primers (5'-3') | Anneling temperature (0C) | Expected amplified product size (bp) | Structural annotation | | Functional annotation | | |
|---|---|---|---|---|---|---|---|---|---|---|---|---|
| | | | | | | | | Sequence components of *kabuli* genome | *Kabuli* gene accession IDs | NCBI-KOG | TFs | NCBI-nr database |
| CaPOPI_7421 | scaffold3539 | 5454 | TAAAA/TAAAAA | GGAGGGCGAAAATGATGTAA | CTTGCGCATCCAACAAGTTA | 59.9 | 861 | INTERGENIC | | | | |
| CaPOPI_7422 | scaffold3542 | 507 | AGGGGGGG/AGGGGGG | GAAGAGTTACGCGGACAAGC | CCGAGCCAAAATCCAAAATA | 60.0 | 566 | INTERGENIC | | | | |
| CaPOPI_7423 | scaffold3542 | 22091 | G/GAT | ATTGCAACTGCCGGAAATAC | GGGCTCACAGCTAGTTTTCG | 60.0 | 874 | INTERGENIC | | | | |
| CaPOPI_7424 | scaffold3542 | 24843 | TTAG/TGATTAGCTGGGTAG | GGTGGGTTCCATCGTTTCTA | CAAGACAGCCAATCCGAAAT | 59.8 | 530 | INTERGENIC | | | | |
| CaPOPI_7425 | scaffold355 | 1384 | GTTTTT/GTTTT | GGGGCGTTACATGTGGTATC | CAGATTCACGAAGGTCACACA | 60.1 | 314 | INTERGENIC | | | | |
| CaPOPI_7426 | scaffold3562 | 1038 | ATTT/ATT | AGGGAGGACGAAAATGACCT | CCAATTGGTGTGTTTGCATC | 59.9 | 556 | INTERGENIC | | | | |
| CaPOPI_7427 | scaffold3594 | 965 | GAGCTAAAAAGCTAAAAAGCT/GAGCTAAAAAGCT | CGTTCAAAACGAGATCGTCA | TACAATGCCGAGCAAATCAA | 59.8 | 575 | INTERGENIC | | | | |
| CaPOPI_7428 | scaffold36 | 138770 | GAAAAA/GAAAA | CCAATCATTGTCCAAGAGAGC | TTGGGCTGATGCAATAAACA | 59.7 | 607 | INTERGENIC | | | | |
| CaPOPI_7429 | scaffold36 | 294954 | AATATATATATATA/AATATATATATATATA | CTTGGGCTTTACCCATGAAA | TGTCGTGTGCTTCAAAAAGG | 59.9 | 598 | INTERGENIC | | | | |
| CaPOPI_7430 | scaffold3604 | 961 | AAATCGATT/AAATCGATTAAGTAATCGATT | AATTGCACAAGTCAGTGGCA | TATCGCGGGTCTTGTTTTTC | 60.3 | 591 | INTERGENIC | | | | |
| CaPOPI_7431 | scaffold362 | 29056 | A/AATTCGT | AAATAATTTTGGGGCGAAGC | TCCAATGGCAAATGATTCAA | 60.3 | 625 | INTERGENIC | | | | |
| CaPOPI_7432 | scaffold362 | 148917 | T/TG | ATTTCTCCATGCTCAATCCG | TTGTTGGTTATCCAGGCACA | 60.0 | 255 | INTERGENIC | | | | |
| CaPOPI_7433 | scaffold362 | 166434 | TGA/T | CCCGGTATTGTTTGTGCTCT | CACATCAGCCACATTTGACC | 60.0 | 774 | DRR | Ca_24991 | C | | Aldehyde dehydrogenase NAD(P)-dependent |
| CaPOPI_7434 | scaffold362 | 246595 | AATATATATATATATATATATATA/AATATATATATATATATATATATATA | CGACTGCCGGTATCAATTTT | TCATATTTCCTTGTGTGTTTGGTT | 60.0 | 648 | INTERGENIC | | | | |
| CaPOPI_7435 | scaffold362 | 267543 | A/AC | TATCTTGTTCCGACCCAACC | CTTGAATTCCATGCGTCCTT | 59.8 | 698 | INTERGENIC | | | | |

| INDEL marker IDs | Chromosomes/ unanchored scaffolds | Physical positions (bp) | InDels (*Kabuli* reference genome-CDC Frontier/PI) | Forward primers (5'-3') | Reverse primers (5'-3') | Anneling temperature (0C) | Expected amplified product size (bp) | Structural annotation | | Functional annotation | | |
|---|---|---|---|---|---|---|---|---|---|---|---|---|
| | | | | | | | | Sequence components of *kabuli* genome | *Kabuli* gene accession IDs | NCBI-KOG | TFs | NCBI-nr database |
| CaPOPI_7436 | scaffold363 | 83340 | CTGT/CT | AGGTTCCTCTACGCCGTTCT | CTATCCATTTGTGACGGCCT | 60.3 | 502 | INTERGENIC | | | | |
| CaPOPI_7437 | scaffold3643 | 22491 | AG/A | AGCCTAGGGTGGACGAAAAT | CCATCCAATACGTGTGCTTG | 60.0 | 164 | INTERGENIC | | | | |
| CaPOPI_7438 | scaffold3643 | 22623 | TAAAAAAAA/TAAAAAAAAAA | CAAGCACACGTATTGGATGG | TTTGTGCGTGACATTGGATT | 60.0 | 111 | INTERGENIC | | | | |
| CaPOPI_7439 | scaffold3659 | 2503 | AT/ATT | GCCATTAATTATGTTAACGTTTGG | CAGAACATTTAACGTGAGCAAGA | 58.8 | 665 | INTERGENIC | | | | |
| CaPOPI_7440 | scaffold3663 | 4120 | T/TG | TTTTATTGTTTTACTACATAACGACGA | AAGTTTGACTGCATAACAATGACA | 57.7 | 631 | INTERGENIC | | | | |
| CaPOPI_7441 | scaffold3663 | 4430 | TAAA/TAAAA | TGTCATTGTTATGCAGTCAAACTT | TCTTCGTTTTATGGTTTTATTTCA | 58.7 | 280 | INTERGENIC | | | | |
| CaPOPI_7442 | scaffold3666 | 3244 | GATATATATATATAT/GATATATATATAT | ACAAATGATGCTTGCTCGTG | TCAAATTGGTCCTGTCCTCA | 59.9 | 534 | INTERGENIC | | | | |
| CaPOPI_7443 | scaffold3666 | 3853 | TTATATATATAT/TTATATATATATAT | CCAATTTGAATTGTCCGTCC | TATCGATCACCAGTGCCAAA | 60.2 | 909 | INTERGENIC | | | | |
| CaPOPI_7444 | scaffold367 | 6329 | AGGGG/AGGG | GACACCCTAGGCCAAATGAA | TAGATTGGGTGGATTCGCTC | 59.9 | 556 | INTERGENIC | | | | |
| CaPOPI_7445 | scaffold367 | 15516 | GTTCTTCTT/GTTCTT | CCTCATCATGTATGCCATCG | TTGGAGAAACTTTTCCGCTG | 59.9 | 449 | DRR | Ca_27355 | | | |
| CaPOPI_7446 | scaffold3673 | 1634 | ATTTT/AT | TATTTTGGATTTTGGCTCGG | AAATGGAGGAAGGAGGGAGA | 59.9 | 431 | INTERGENIC | | | | |
| CaPOPI_7447 | scaffold3689 | 16240 | AATT/AATTATT | GACATGCCAACACCAGACAC | TAAATCATGGGGGTTCCTCA | 60.0 | 201 | INTERGENIC | | | | |
| CaPOPI_7448 | scaffold369 | 73666 | GTATATATATATATATATATATATATAT/GTATATATATATATATATATATAT | CGGTGAATCCTTTACGGAGA | AATGGAGGAAGGAGGGAGAA | 60.1 | 297 | INTERGENIC | | | | |
| CaPOPI_7449 | scaffold370 | 37851 | GAA/GA | TTATTTAGTACCACTACAAAACGAAGA | AGTCCAGATGAAAGCGAGGA | 57.0 | 395 | INTERGENIC | | | | |
| CaPOPI_7450 | scaffold370 | 75163 | AC/A | GATTTTCGTACCCCTCGTCA | AGTCGCTCGAAAAGTCCAAA | 59.9 | 533 | INTERGENIC | | | | |

| **INDEL marker IDs** | **Chromosomes/ unanchored scaffolds** | **Physical positions (bp)** | **InDels (*Kabuli* reference genome-CDC Frontier/PI)** | **Forward primers (5'-3')** | **Reverse primers (5'-3')** | **Anneling temperature (0C)** | **Expected amplified product size (bp)** | **Structural annotation** | | **Functional annotation** | | |
|---|---|---|---|---|---|---|---|---|---|---|---|---|
| | | | | | | | | **Sequence components of *kabuli* genome** | ***Kabuli* gene accession IDs** | **NCBI-KOG** | **TFs** | **NCBI-nr database** |
| CaPOPI_7451 | scaffold370 | 89771 | GAAA/GAAAA | GGTTCGAAAATTGCCTGAAA | CCCGAAACGCAGTTACAAAT | 60.1 | 416 | INTERGENIC | | | | |
| CaPOPI_7452 | scaffold370 | 101037 | CAA/CA | TCCTGGTCCTCGATTTCATC | CGGGTTCAAACACTAGCCAT | 60.0 | 434 | INTERGENIC | | | | |
| CaPOPI_7453 | scaffold3705 | 4075 | T/TGGGCGAAAACC | CAATAACCCTTCAATGGCTCA | GCTTTGCTTCCCTTTCCTCT | 59.9 | 393 | INTERGENIC | | | | |
| CaPOPI_7454 | scaffold3724 | 53525 | TAAAAA/TAAA | AAGCACTTGCTCCCAATGAC | ACGAGTTGCTGGTTGACCTT | 60.3 | 372 | DRR | Ca_27522 | A | HD-ZIP | Queuine/other tRNA-ribosyltransferase |
| CaPOPI_7455 | scaffold373 | 20809 | TAAAA/TAAAAA | TCCAACGACACAAATGAACG | AGGTCATTTTCGTCCTCCCT | 60.6 | 290 | INTERGENIC | | | | |
| CaPOPI_7456 | scaffold373 | 25857 | ACTTC/ACTTCTTC | AGCCACGAATTTAAGCTCCA | AGGGACAAAGGGCAAGAAAT | 59.8 | 559 | INTERGENIC | | | | |
| CaPOPI_7457 | scaffold374 | 13002 | AC/ACC | CAAATTCAACGAGGTCACACA | ATTTGGAGCGTTACATTGGG | 59.6 | 184 | INTERGENIC | | | | |
| CaPOPI_7458 | scaffold374 | 14684 | CAA/CA | AGTCACTCATACCAACGGGG | TTTTGGGGGAATTGGTAAAA | 59.8 | 633 | INTERGENIC | | | | |
| CaPOPI_7459 | scaffold374 | 142006 | TAAA/TAA | AATTCCTTCCCACACAACCA | AGCGGAGATTTTACCGGAAT | 60.2 | 128 | INTERGENIC | | | | |
| CaPOPI_7460 | scaffold374 | 149528 | CTT/CTTTT | AAGGGTGGAAGAGGAGGAAA | TGATGTTTGAGATGCAAAAGC | 60.0 | 473 | INTERGENIC | | | | |
| CaPOPI_7461 | scaffold374 | 154680 | TAAAAAAAAAAAAAA/TAAAAAAAAA | GATTTGATGTTGGGACCGTT | TCAAATGCTTCAACTTGTGCTT | 59.7 | 477 | INTERGENIC | | | | |
| CaPOPI_7462 | scaffold374 | 158468 | ACACGATCAGCACGA/ACACGA | CCTATCACAGGTCAGCACGA | GGTGTGGATGGAATTTGGAG | 59.9 | 531 | INTERGENIC | | | | |
| CaPOPI_7463 | scaffold374 | 205813 | CAT/CATCCTGAT | AGCTGTTTCTGCTAGGCTGG | TTTATTTACGCGAATGCCCT | 59.8 | 941 | DRR | Ca_26214 | O | | K Homology |
| CaPOPI_7464 | scaffold374 | 205921 | ATTTTTTTTTT/ATTTTTTTTTTTT | CAAAAGTTGCGATTCTTCTGG | TTTATTTACGCGAATGCCCT | 59.9 | 483 | DRR | Ca_26214 | O | | K Homology |
| CaPOPI_7465 | scaffold3742 | 3802 | TTAT/TTATAT | CGATAGACTCAAGTTGGACCTG | GTGTCTGCTTGTTTGCCTGA | 58.9 | 632 | INTERGENIC | | | | |

| INDEL marker IDs | Chromosomes/ unanchored scaffolds | Physical positions (bp) | InDels (*Kabuli* reference genome-CDC Frontier/PI) | Forward primers (5'-3') | Reverse primers (5'-3') | Anneling temperature (0C) | Expected amplified product size (bp) | Structural annotation | | Functional annotation | | |
|---|---|---|---|---|---|---|---|---|---|---|---|---|
| | | | | | | | | Sequence components of *kabuli* genome | *Kabuli* gene accession IDs | NCBI-KOG | TFs | NCBI-nr database |
| CaPOPI_7466 | scaffold3742 | 11903 | ACC/AC | TTATAGCCGTTGGAAGTGGG | TTCAGCCTCGAATCCAGAGT | 60.0 | 804 | CDS (FRAME SHIFT) | Ca_27449 | | | |
| CaPOPI_7467 | scaffold377 | 75306 | C/CAT | TCACGGGTCGACACAATTTA | AACTGGACGTTGAGAATGGG | 60.0 | 403 | INTERGENIC | | | | |
| CaPOPI_7468 | scaffold3784 | 2352 | AATATATATATATATATATA/AATATATATATATATATA | TAAGACGAGGGAGGACGAAA | GAAACGAACACCGGAGAAAA | 59.8 | 764 | INTERGENIC | | | | |
| CaPOPI_7469 | scaffold3789 | 5837 | CAAAAAAAAAA/CAAAAAAAAAAAAA | TGCTCCATCAATCAAATCCA | TTACGTGGTGGTGAAGGTGA | 60.0 | 280 | INTERGENIC | | | | |
| CaPOPI_7470 | scaffold379 | 131210 | GAAAAAAAAAAA/GAAAAAAAAAA | TTATTGTGGGCATTGGGATT | GTGCCATTGAATTGGGAAAT | 60.0 | 590 | INTERGENIC | | | | |
| CaPOPI_7471 | scaffold379 | 132536 | TAAAAAAA/TAAAAAAAA | GTAATGAAGTGGCTGCACGA | GTTTTTGTTCAATGGCGGTC | 59.9 | 407 | INTERGENIC | | | | |
| CaPOPI_7472 | scaffold379 | 194523 | AATATATATATATATATATATA/AATATATATATATATATATA | CCGAACTTTACTGGGCCTAA | ACCGTTTTGGTGTTTCTTGC | 59.2 | 602 | INTERGENIC | | | | |
| CaPOPI_7473 | scaffold3794 | 17881 | ATTTTTTTTT/ATTTTTTTTTT | ATTGATCAGGTCGCATCTCC | GCAACGAAAGAAAGAGTGGC | 60.0 | 508 | INTERGENIC | | | | |
| CaPOPI_7474 | scaffold3794 | 37384 | CAAAAAAA/CAAAAAA | TGTCGACGAGTAAAAGTGCAA | TTTTGACCCCACATGTTTCA | 59.5 | 391 | DRR | Ca_26695 | GMW | | |
| CaPOPI_7475 | scaffold3794 | 59832 | GAAAAAAAAAAA/GAAAAAAAAAA | TCGATTATGCGTAGGGAAGG | TTTTCTGAGTTGTTAGGCCACA | 60.1 | 605 | INTERGENIC | | | | |
| CaPOPI_7476 | scaffold3794 | 61760 | TTTAAATT/TTTAAATTAAATT | GTTTAGGCCGCCACAAAATA | GGGACTTCTGTTGTCGGGTA | 60.0 | 703 | INTERGENIC | | | | |
| CaPOPI_7477 | scaffold3794 | 71924 | A/AT | TTGATCAAAGGTGTGTCCCA | CCCCATTGACCAAAATCATC | 59.9 | 440 | URR | Ca_26696 | GMW | NAC | Exostosin-like |
| CaPOPI_7478 | scaffold3794 | 79952 | TAAAAAAAAA/TAAAAAAAAAA | AAGTTTGCAAGAACCTTCCAT | CATATATGGTGGGGATTGGC | 57.9 | 616 | INTERGENIC | | | | |
| CaPOPI_7479 | scaffold3794 | 82476 | CCAC/CC | CAAAGGGACCACACCTCTTT | TTGGCCCCTTTAGTGTATCG | 59.0 | 554 | INTERGENIC | | | | |
| CaPOPI_7480 | scaffold38 | 25353 | TAAAAAAAAAA/TAAAAAAAAAAA | TGGATGTGTTGCATTGTCTT | CAAACTTGGTTTAAATGTTTGGC | 57.5 | 568 | INTERGENIC | | | | |

| INDEL marker IDs | Chromosomes/ unanchored scaffolds | Physical positions (bp) | InDels (*Kabuli* reference genome-CDC Frontier/PI) | Forward primers (5'-3') | Reverse primers (5'-3') | Anneling temperature (0C) | Expected amplified product size (bp) | Structural annotation | | Functional annotation | | |
|---|---|---|---|---|---|---|---|---|---|---|---|---|
| | | | | | | | | Sequence components of *kabuli* genome | *Kabuli* gene accession IDs | NCBI-KOG | TFs | NCBI-nr database |
| CaPOPI_7481 | scaffold38 | 135300 | GTTTTTT/GTTTTTTT | TGGTTGAAATTAAAGCCAAGG | TCACATCAGGAAAAATGGCA | 59.1 | 565 | INTERGENIC | | | | |
| CaPOPI_7482 | scaffold38 | 138674 | TA/TAGCAAA | CGTCGCAACTCAATTGACAG | TTTTTCTCGGTGTGTAGGGG | 60.4 | 950 | INTERGENIC | | | | |
| CaPOPI_7483 | scaffold38 | 281889 | TAAAAAAAAAA/TAAAAAAAAAAA | ATCACCATTGTGGCATTGAC | TTGCAATAATGCGAACCAAA | 59.2 | 464 | INTERGENIC | | | | |
| CaPOPI_7484 | scaffold38 | 285083 | CTTTTTTTT/CTTTTTTTTT | TTCATAATTTTTCTTGTTGAGCCA | TCTGTGTCCCACGTCATTTG | 60.0 | 399 | INTERGENIC | | | | |
| CaPOPI_7485 | scaffold38 | 334106 | CACTCAAGACATACTCAA/CACTCAAGACATACTCAAGACATACTCAA | GAAACTCATTCTGCCTTCCG | CACACACCGAATCAATTTGC | 59.8 | 699 | INTERGENIC | | | | |
| CaPOPI_7486 | scaffold38 | 350628 | GTTTTTTTT/GTTTTTTTTT | ACCAAGACAACCTCTCCCCT | TGGGTACCCTTCTCCTTCCT | 60.0 | 199 | INTERGENIC | | | | |
| CaPOPI_7487 | scaffold38 | 350706 | C/CAT | ACCAAGACAACCTCTCCCCT | TGGGTACCCTTCTCCTTCCT | 60.0 | 199 | INTERGENIC | | | | |
| CaPOPI_7488 | scaffold38 | 412147 | ATTT/ATT | GGGAGTGGAGCGATTGATTA | ATTTGTGAGGGTCAAGGACG | 60.0 | 567 | INTERGENIC | | | | |
| CaPOPI_7489 | scaffold38 | 413791 | ATTTT/ATTTTT | CTTCGCTGCAAAATTTCCTC | TTGATGTCGGGTAGTGTCTGA | 60.0 | 755 | INTERGENIC | | | | |
| CaPOPI_7490 | scaffold38 | 474073 | TAAAAAAAAAAAAA/TAAAAAAAAAAA | TGTGTCAGAAGGATCTTGCG | TGAGATTCGCCAGTTAACCC | 60.0 | 652 | INTERGENIC | | | | |
| CaPOPI_7491 | scaffold381 | 6830 | AAG/A | TGCAAAAGGGTCACTTGATTT | CCCCAAACCCTAAACCCTAA | 59.6 | 355 | INTERGENIC | | | | |
| CaPOPI_7492 | scaffold3811 | 19567 | GAAAAAAAAAAA/GAAAAAAAAAA | GCACATCCATGCCATGTTTA | TGGATGCGTCCATCATTGTA | 60.4 | 754 | INTERGENIC | | | | |
| CaPOPI_7493 | scaffold3811 | 24750 | CTATATATATATATATATATATATAT/CTATATATATATATATATATATAT | TACTATGCAACCATCCCGGT | TGAAACAAGAAACCCGAACC | 60.2 | 663 | INTERGENIC | | | | |
| CaPOPI_7494 | scaffold3823 | 3884 | CAAACAATAA/CAA | TGGCAAATCGATTGACAGAA | GTCCTGAAAATCCAAACGGA | 60.2 | 780 | DRR | Ca_27909 | | | |
| CaPOPI_7495 | scaffold3824 | 21914 | TACACAC/TACAC | ACGGTTGAGAATTGAGTGGC | ACAAAAACCACATTCGCACA | 60.1 | 572 | INTERGENIC | | | | |

| INDEL marker IDs | Chromosomes/ unanchored scaffolds | Physical positions (bp) | InDels (*Kabuli* reference genome-CDC Frontier/PI) | Forward primers (5'-3') | Reverse primers (5'-3') | Anneling temperature (0C) | Expected amplified product size (bp) | Structural annotation | | Functional annotation | | |
|---|---|---|---|---|---|---|---|---|---|---|---|---|
| | | | | | | | | Sequence components of *kabuli* genome | *Kabuli* gene accession IDs | NCBI-KOG | TFs | NCBI-nr database |
| CaPOPI_7496 | scaffold3837 | 2371 | ATTTTTTTTTTTTT/ATTTTTTTTTTT | CTTCTTGGTGGCATGGTTTT | TACCCTGTGGATGGGAATGT | 60.0 | 591 | INTERGENIC | | | | |
| CaPOPI_7497 | scaffold3837 | 5661 | AT/ATT | GCCTCCGAGTCAATACCAAA | ATTGAGGGTAGGGGAGCAGT | 60.1 | 415 | INTERGENIC | | | | |
| CaPOPI_7498 | scaffold3837 | 5910 | ACCC/ACC | ACCTCGATCTGGAAATCACG | ATTGAGGGTAGGGGAGCAGT | 60.1 | 467 | CDS (FRAME SHIFT) | Ca_27570 | | | |
| CaPOPI_7499 | scaffold387 | 16023 | CAT/C | CACACCCCTTGTCAACACTG | TTAAGCTGAGAGCCTTCCCA | 60.0 | 439 | INTERGENIC | | | | |
| CaPOPI_7500 | scaffold387 | 16971 | TT/TTGT | CTCAACAAGGACGCCAATTT | GGATTGATTACGCTTGTTGGA | 60.1 | 299 | INTERGENIC | | | | |
| CaPOPI_7501 | scaffold387 | 19261 | TATAATAGAT/TAT | CCTTGAGGAACAACAAAACCA | TTTTGGGGTTTAGGGTGTCA | 60.0 | 757 | INTERGENIC | | | | |
| CaPOPI_7502 | scaffold387 | 22975 | AATC/AATCATC | CGCAATCTGAACAAATGACAA | TCCACTAACCACCTTGACCC | 59.7 | 338 | INTERGENIC | | | | |
| CaPOPI_7503 | scaffold387 | 23485 | ATTTTT/ATTTTTTT | GGGTCAAGGTGGTTAGTGGA | TCCTCTGCCTTTCATGTGTG | 59.8 | 496 | INTERGENIC | | | | |
| CaPOPI_7504 | scaffold387 | 23589 | ATGTGTGTG/ATGTGTGTGTG | GGGTCAAGGTGGTTAGTGGA | TCCTCTGCCTTTCATGTGTG | 59.8 | 496 | INTERGENIC | | | | |
| CaPOPI_7505 | scaffold387 | 160969 | TTA/TTACTA | AGCTCCAAATCCAGAGCAAA | GGGATATGATCTTCTACCCCA | 60.0 | 835 | INTERGENIC | | | | |
| CaPOPI_7506 | scaffold39 | 39921 | TTATATATATATATATATAT/TTATATATATATATATATAT | CGGCCATCTACGTTGTCTTT | AACAAACTCCTTCGCTCCAA | 60.1 | 316 | INTERGENIC | | | | |
| CaPOPI_7507 | scaffold3917 | 4350 | ATTT/ATTTT | TGAGGCGTATCATGTGTTGA | CAACTTTGGTGAGGCCATTT | 58.6 | 662 | INTERGENIC | | | | |
| CaPOPI_7508 | scaffold3918 | 4292 | TAAAAAAAA/TAAAAAAAAAA | ATGCCTTCGTCCTCTCCTTT | ACATTGAAACTGCCCATTCC | 60.2 | 612 | INTERGENIC | | | | |
| CaPOPI_7509 | scaffold3918 | 4537 | CTTAATT/CTT | ATGCCTTCGTCCTCTCCTTT | CGAGTGAAGGCATGGGTATT | 60.2 | 914 | INTERGENIC | | | | |
| CaPOPI_7510 | scaffold3923 | 3230 | GA/GAA | TGCTGCAACTTCTACATCCG | AGAAAGCGTGAAAACTCCGA | 60.0 | 695 | INTERGENIC | | | | |

| INDEL marker IDs | Chromosomes/ unanchored scaffolds | Physical positions (bp) | InDels (*Kabuli* reference genome-CDC Frontier/PI) | Forward primers (5'-3') | Reverse primers (5'-3') | Anneling temperature (0C) | Expected amplified product size (bp) | Structural annotation | | Functional annotation | | |
|---|---|---|---|---|---|---|---|---|---|---|---|---|
| | | | | | | | | Sequence components of *kabuli* genome | *Kabuli* gene accession IDs | NCBI-KOG | TFs | NCBI-nr database |
| CaPOPI_7511 | scaffold3927 | 1574 | ATTTTT/ATTTTTTT | GAATCCTGGGCTGAAATCAA | CGTGGTAATCGCGTAGGAAT | 60.0 | 342 | INTERGENIC | | | | |
| CaPOPI_7512 | scaffold3939 | 3327 | GATAATAATAATAATAATAATAA/GATAATAATAATAATAATAATAA | TCCAGATCAAATCGAGGACC | AGAGGCAAACAAGAACCGAA | 60.0 | 450 | INTERGENIC | | | | |
| CaPOPI_7513 | scaffold395 | 7068 | T/TCTGA | TTTACAGCGCTTCTCCCAAT | TCCCAACCCAAATAAACCAA | 59.8 | 667 | INTERGENIC | | | | |
| CaPOPI_7514 | scaffold395 | 42393 | TA/TATTTTGAA | TGACACTTGTAACACCCCCA | ATGGCAAATAATTGGTGGGA | 59.8 | 537 | INTERGENIC | | | | |
| CaPOPI_7515 | scaffold395 | 101675 | ATATTATTATTATTATTATTATTATTATTATTATTA/ATATTATTATTATTATTATTATTATTATTATTA | ACGCGACAATCGAGGTAAAC | TACGTACGGCAGTAGCCCTT | 60.1 | 787 | INTRON | Ca_24514 | K | G2-like | Signal transduction response regulator, receiver domain |
| CaPOPI_7516 | scaffold3950 | 7394 | GTATATATATATA/GTATATATATATATATA | CCAGATGTCGTTTCTCCGAT | TTGTATGACATCTCGCCACC | 60.1 | 665 | INTERGENIC | | | | |
| CaPOPI_7517 | scaffold3950 | 7697 | C/CTT | TTTCTCTTTTGGTGGTTGCC | TCCACCAATATGCAAAAGCA | 60.1 | 854 | INTERGENIC | | | | |
| CaPOPI_7518 | scaffold3950 | 7767 | TAAAAA/TAAAA | GGTGGCGAGATGTCATACAA | TCCACCAATATGCAAAAGCA | 59.5 | 366 | INTERGENIC | | | | |
| CaPOPI_7519 | scaffold3950 | 8174 | TAAAAAAA/TAAAAAAAA | TGCTTTTGCATATTGGTGGA | AAAACATGGCCTTCAACTGG | 60.1 | 475 | INTERGENIC | | | | |
| CaPOPI_7520 | scaffold3950 | 8477 | TAAAAAAAAAA/TAAAAAAAA | TGCTTTTGCATATTGGTGGA | AAAACATGGCCTTCAACTGG | 60.1 | 475 | INTERGENIC | | | | |
| CaPOPI_7521 | scaffold3950 | 8667 | AAATAATAATAATAA/AAATAATAATAA | CCAGTTGAAGGCCATGTTTT | TTTTATGGAAGCATGTGGCA | 60.0 | 528 | INTERGENIC | | | | |
| CaPOPI_7522 | scaffold3950 | 12638 | T/TTCAGCA | CAAGAGCCTAAGGATGCTGC | AGCATGAAACGGTGGAGAAT | 60.1 | 919 | INTERGENIC | | | | |
| CaPOPI_7523 | scaffold3951 | 1843 | CAA/CA | GAGTCACAGTGGCACTCCAG | TCGACTTGTCAATCGCTTTG | 59.4 | 226 | INTERGENIC | | | | |
| CaPOPI_7524 | scaffold396 | 371269 | TAAAAAAAA/TAAAAAAA | GTTTTGACTGGCCCAAAGAG | GAGCGCTGTAAAAGACCACC | 59.7 | 588 | INTERGENIC | | | | |
| CaPOPI_7525 | scaffold396 | 372299 | CATATATATATATATATATATATA/CATATATATATATATATATATA | TGTATTTCTGCGCATTCTCG | TTGTTAGTTTGACGAGGGCA | 60.0 | 357 | INTERGENIC | | | | |

| INDEL marker IDs | Chromosomes/ unanchored scaffolds | Physical positions (bp) | InDels (*Kabuli* reference genome-CDC Frontier/PI) | Forward primers (5'-3') | Reverse primers (5'-3') | Anneling temperature (0C) | Expected amplified product size (bp) | Structural annotation | | Functional annotation | | |
|---|---|---|---|---|---|---|---|---|---|---|---|---|
| | | | | | | | | Sequence components of *kabuli* genome | *Kabuli* gene accession IDs | NCBI-KOG | TFs | NCBI-nr database |
| CaPOPI_7526 | scaffold396 | 577559 | GAA/GA | TCCCAACCCAAATAAACCAA | AAGCTCAACATGCAAAACCC | 60.0 | 538 | INTERGENIC | | | | |
| CaPOPI_7527 | scaffold396 | 577641 | ACCC/ACCCC | TCCCAACCCAAATAAACCAA | AAGCTCAACATGCAAAACCC | 60.0 | 538 | INTERGENIC | | | | |
| CaPOPI_7528 | scaffold396 | 588477 | AAATAATAATAATAATAATAATAATAATAATAATAATAATAATAATAA/AAATAATAATAATAATAATAATAATAATAATAATAATAATAATAATAA | AGGCGATTTTCATCCTCCTT | AGAGGCAAACAAGAACCGAA | 60.0 | 513 | INTERGENIC | | | | |
| CaPOPI_7529 | scaffold397 | 23446 | A/AT | TGGAGGCGGCTACTAGTGTT | ATTGAATTCGCCATAAACGG | 59.9 | 411 | INTERGENIC | | | | |
| CaPOPI_7530 | scaffold397 | 167388 | AATATATATATATATATATATATAT/AATATATATATATATATATATATATATAT | GATTTGCTTTGCGTGACAGA | CGGTGAATCCTTTACGGAGA | 60.0 | 279 | INTERGENIC | | | | |
| CaPOPI_7531 | scaffold398 | 17888 | CTTTTTTTTTTTT/CTTTTTTTTTT | AAACGCAATCCTTGAGTTCG | AAATGTTGCAAATCCTCCCA | 60.2 | 362 | INTERGENIC | | | | |
| CaPOPI_7532 | scaffold398 | 17995 | ATAAATCAACTAA/ATAA | AAACGCAATCCTTGAGTTCG | TCGATCAATTGAAAATCCATCA | 60.2 | 601 | INTERGENIC | | | | |
| CaPOPI_7533 | scaffold398 | 33224 | CTTTTTTTTT/CTTTTTTTT | AGTGATCCAACTCCAACATCG | TCGAATAAATCAATCCAAAATCAA | 60.0 | 561 | INTERGENIC | | | | |
| CaPOPI_7534 | scaffold398 | 33315 | AG/AGG | AGTGATCCAACTCCAACATCG | TCGAATAAATCAATCCAAAATCAA | 60.0 | 561 | INTERGENIC | | | | |
| CaPOPI_7535 | scaffold398 | 33778 | AATAT/AATATAT | TTTGATTTTGGATTGATTTATTCG | CATGGCCACTCAAAGCACTA | 59.3 | 494 | INTERGENIC | | | | |
| CaPOPI_7536 | scaffold398 | 33851 | A/ATTTTT | TTTGATTTTGGATTGATTTATTCG | CATGGCCACTCAAAGCACTA | 59.3 | 494 | INTERGENIC | | | | |
| CaPOPI_7537 | scaffold398 | 45043 | T/TGTA | CCCTACGACGTCCGTTAAAA | TCGCTCGTTGCACCATATTA | 60.0 | 554 | INTERGENIC | | | | |
| CaPOPI_7538 | scaffold398 | 133623 | AAAATAAATAAATAAATAAA/AAAATAAATAAATAAATAAATAAATAAA | TTGACCTATGTCCCCTCTCG | CGCACACTTAGCAAATGCAG | 60.1 | 269 | INTERGENIC | | | | |
| CaPOPI_7539 | scaffold398 | 160107 | CAAA/CAA | CAACTTTCCGAAACTCTGGG | TTTCCGCAAGATGTGATTGA | 59.7 | 250 | DRR | Ca_22283 | | C2H2 | Zinc finger, TRAF-type |
| CaPOPI_7540 | scaffold398 | 213400 | TAAAAAAAAAAAA/TAAAAAAAAAA | CATGCCTCTAAAGCCCTTGT | TGAAGGAAACGACACATGGA | 59.3 | 729 | INTERGENIC | | | | |

| INDEL marker IDs | Chromosomes/ unanchored scaffolds | Physical positions (bp) | InDels (*Kabuli* reference genome-CDC Frontier/PI) | Forward primers (5'-3') | Reverse primers (5'-3') | Anneling temperature (0C) | Expected amplified product size (bp) | Structural annotation | | Functional annotation | | |
|---|---|---|---|---|---|---|---|---|---|---|---|---|
| | | | | | | | | Sequence components of *kabuli* genome | *Kabuli* gene accession IDs | NCBI-KOG | TFs | NCBI-nr database |
| CaPOPI_7541 | scaffold398 | 252607 | CAAAAAAAA/CAAAAAAAAA | TGCTCAGACCACCAAATGAA | CAACTTCAAACCGACAAACG | 60.2 | 299 | INTERGENIC | | | | |
| CaPOPI_7542 | scaffold398 | 260710 | AC/ACC | TTGTCCTTCAACCACGTTCA | CACAAAGCAATTCCAAACCA | 60.1 | 379 | DRR | Ca_22288 | I | | Male sterility |
| CaPOPI_7543 | scaffold398 | 261122 | CTATATATATATATATATATATATA/CTATATATATATATATATATATA | ATGGACTCTCCCTCTCACGA | TCTGCATGTCAGCCTACCAG | 59.8 | 589 | DRR | Ca_22288 | I | | Male sterility |
| CaPOPI_7544 | scaffold398 | 269493 | GTTTTTTTTTT/GTTTTTTTT | TACTGGAGTGATGCAGGCAA | CAAAAGTCCAATAAAATGGAGCA | 60.4 | 330 | INTERGENIC | | | | |
| CaPOPI_7545 | scaffold398 | 270063 | CAAAAAAA/CAAAAAAAAAA | TGCTCCATTTTATTGGACTTTTG | TGTTGCAAAGATCGAACTGC | 60.3 | 579 | INTERGENIC | | | | |
| CaPOPI_7546 | scaffold398 | 270743 | TAAAAAAAAAA/TAAAAAAAAAAA | GTAGTGGTTGAAGTGGCGGT | CTACTCCATCGGGTCTCCAA | 60.0 | 462 | INTERGENIC | | | | |
| CaPOPI_7547 | scaffold398 | 288064 | AGGG/AGG | AATTCTCAGGCCAGCTCAAA | TGAAGCTTACCAATTCCCCA | 60.0 | 444 | INTERGENIC | | | | |
| CaPOPI_7548 | scaffold398 | 289050 | CAAAAA/CAAAAAAA | TGATGAGCTAGGAACCTCCG | GCAGTTTAAGTCTGCTCCCG | 60.4 | 592 | INTERGENIC | | | | |
| CaPOPI_7549 | scaffold398 | 290361 | ATTTTT/ATTTTTT | AAAACGGATTTTACGGTCCC | AGAGCACCCCCATACCCTAT | 60.1 | 598 | INTERGENIC | | | | |
| CaPOPI_7550 | scaffold398 | 290707 | CAAAAAA/CAAAAAAA | AAAACGGATTTTACGGTCCC | GTTGGTCCTTTTGGACCTCA | 60.1 | 990 | INTERGENIC | | | | |
| CaPOPI_7551 | scaffold398 | 319085 | TG/T | TTGCATGAAAACATGGCAGT | GTTGTTGATGTGGAGGGCTT | 60.1 | 337 | INTERGENIC | | | | |
| CaPOPI_7552 | scaffold398 | 339301 | TAAA/TAA | GGATGCTGGAATTTTGAAGG | CGCAGTAATATGTGATGGGTTT | 59.5 | 713 | DRR | Ca_22294 | K | C3H | Endonuclease/exonuclease/phosphatase |
| CaPOPI_7553 | scaffold398 | 339502 | T/TG | GGATGCTGGAATTTTGAAGG | TTTTACTTCCGCCAGCTTGT | 59.5 | 810 | DRR | Ca_22294 | K | C3H | Endonuclease/exonuclease/phosphatase |
| CaPOPI_7554 | scaffold398 | 344928 | CCAATTTGCAATTTGCAATTT/CCAATTTGCAATTT | CGAACTCCTAAAAACGCGAG | TGTTTTCCAGTGGTGTTTCTTG | 60.0 | 434 | INTRON | Ca_22294 | K | C3H | Endonuclease/exonuclease/phosphatase |
| CaPOPI_7555 | scaffold398 | 348013 | TAAAAAAAA/TAAAAAAAAA | AAATTATGAGGCGCAGTTGG | TCCGGTTGGAATTGCTCTTA | 60.1 | 348 | INTERGENIC | | | | |

| INDEL marker IDs | Chromosomes/ unanchored scaffolds | Physical positions (bp) | InDels (*Kabuli* reference genome-CDC Frontier/PI) | Forward primers (5'-3') | Reverse primers (5'-3') | Anneling temperature (0C) | Expected amplified product size (bp) | Structural annotation | | Functional annotation | | |
|---|---|---|---|---|---|---|---|---|---|---|---|---|
| | | | | | | | | Sequence components of *kabuli* genome | *Kabuli* gene accession IDs | NCBI-KOG | TFs | NCBI-nr database |
| CaPOPI_7556 | scaffold398 | 372482 | TTGTGTG/TTGTGTGTG | TGGGAGTTTGTGAGGTATTTCC | GCCAAAGAAGAAGTGAACCG | 60.2 | 772 | DRR | Ca_22296 | J | | Protein synthesis factor, GTP-binding |
| CaPOPI_7557 | scaffold398 | 379143 | AGAA/AGAAATTGAA | TCTGAGGAAGAGGAGGACGA | AGCGTGGCAGATTTTGAAAG | 60.1 | 778 | INTERGENIC | | | | |
| CaPOPI_7558 | scaffold398 | 443064 | ATTTTTTTTTT/ATTTTTTTTT | TTCCGATGGAGCTATTCTGG | TTTTCTTGGGTAGCAATCGG | 60.2 | 609 | INTERGENIC | | | | |
| CaPOPI_7559 | scaffold398 | 454951 | GTTTTTTTT/GTTTTTTT | GGTGGAGAATGAAACGGAGA | GATCTCTTTGGGCCAATCAA | 60.1 | 775 | INTERGENIC | | | | |
| CaPOPI_7560 | scaffold398 | 459574 | CAAAAAAAAAAA/CAAAAAAAAAA | TCGGGTTGTAATGGGGTTTA | TTGGTCGTTTGAGCCTTTCT | 60.0 | 477 | INTERGENIC | | | | |
| CaPOPI_7561 | scaffold398 | 466018 | ACCCCC/ACCCC | TGCATGCAGACAAGAGTCAA | TTGGAAAAGCCCACCTACAC | 59.1 | 468 | INTERGENIC | | | | |
| CaPOPI_7562 | scaffold398 | 479794 | CTTTTTTTT/CTTTTTTT | GCTGGCCTTATCCATTTGAA | TGAAATCAGTGCAGGGAGTG | 60.0 | 580 | INTERGENIC | | | | |
| CaPOPI_7563 | scaffold398 | 485833 | ATTTTTTTTT/ATTTTTTTT | TGGTTGTGCACCTCAAACAT | TCTCTCCCTCCTACCCTTCC | 60.0 | 870 | INTERGENIC | | | | |
| CaPOPI_7564 | scaffold398 | 492512 | ATTTTTTTTTTT/ATTTTTTTTTT | AGCTTCTTGAATAGCTGCGG | CCATTGATTCGACCATTGTG | 59.8 | 740 | INTERGENIC | | | | |
| CaPOPI_7565 | scaffold398 | 495732 | ATTTTTTTTTT/ATTTTTTTTT | TCAACAAACCAAGAACGATGA | GGGAAGGACTGGATGTAGCA | 59.2 | 636 | INTERGENIC | | | | |
| CaPOPI_7566 | scaffold40 | 41230 | CTTT/CTTTT | TGGGAGTCATGGATGAGTGA | CCGAGCCAAAATCCAAAATA | 60.0 | 341 | INTERGENIC | | | | |
| CaPOPI_7567 | scaffold40 | 41514 | GATATATATATATATATATA/GATATATATATATATA | TATTTTGGATTTTGGCTCGG | GAGGAAAGAGGGAGAATGGG | 59.9 | 432 | INTERGENIC | | | | |
| CaPOPI_7568 | scaffold40 | 56808 | CATA/CATATA | ATTTGCACGGAAAGTCATCC | CCTCCACCTCCTCCTCCTAC | 59.9 | 810 | INTERGENIC | | | | |
| CaPOPI_7569 | scaffold40 | 123508 | TA/T | TTTGGTCCCCAAACACCTTA | TTAGTTCACGTGTCCCCCTC | 60.2 | 959 | INTERGENIC | | | | |
| CaPOPI_7570 | scaffold40 | 466805 | ATTTTT/ATTTT | GCCTGAAATATGCCGAGGTA | TCGGCTCTCCATGTCAGATA | 60.1 | 420 | INTERGENIC | | | | |

| INDEL marker IDs | Chromosomes/ unanchored scaffolds | Physical positions (bp) | InDels (*Kabuli* reference genome-CDC Frontier/PI) | Forward primers (5'-3') | Reverse primers (5'-3') | Anneling temperature (0C) | Expected amplified product size (bp) | Structural annotation | | Functional annotation | | |
|---|---|---|---|---|---|---|---|---|---|---|---|---|
| | | | | | | | | Sequence components of *kabuli* genome | *Kabuli* gene accession IDs | NCBI-KOG | TFs | NCBI-nr database |
| CaPOPI_7571 | scaffold40 | 922923 | TAAAAAAAAA/TAAAAAAAAAA | GATTTGCATTAGATATTTGTGGTGA | CATTCACATGGTCGTTTTGG | 59.3 | 851 | INTERGENIC | | | | |
| CaPOPI_7572 | scaffold40 | 973137 | TTGTGTAACAAATCAT/TTGTGTAACAAATCATGTGTAACAAATCAT | GCCAAGAAGGGTGATTTTCA | CATATGGGTTTGGCACCTCT | 60.1 | 314 | DRR | Ca_18995 | S | | |
| CaPOPI_7573 | scaffold400 | 44195 | GTTTTTTTTT/GTTTTTTTT | CGATGACAAGCAATCAATGG | CCAAAGATCTCTACCCCTACCA | 60.1 | 123 | INTERGENIC | | | | |
| CaPOPI_7574 | scaffold4011 | 17091 | CAAAAAAA/CAAAAAA | TTTTCTTCCACAGGGGATTG | TGGCGGTTTATCAGCTCTCT | 59.9 | 269 | INTERGENIC | | | | |
| CaPOPI_7575 | scaffold4011 | 114159 | AAATAACACACAATAAC/AAATAACACACAATAACACACAATAAC | TCTGTGTGCCATCTCTCGAC | TAGCTCGGCCAAACTTTGTT | 60.0 | 481 | INTERGENIC | | | | |
| CaPOPI_7576 | scaffold4011 | 143675 | GAAGGTTGTAAGGTTGT/GAAGGTTGT | TCTGGTGGTGGTCAGGTGTA | TAATTTGGTGTTGTGCGAGC | 60.0 | 612 | INTERGENIC | | | | |
| CaPOPI_7577 | scaffold4028 | 27326 | T/TG | GGTTTGACTGCATAACGGAGA | TTGAATACACCTACAAAACGAAGA | 60.1 | 552 | INTERGENIC | | | | |
| CaPOPI_7578 | scaffold4038 | 835 | AATATA/AATATATA | GCCCTCAGATGAGAGTCAGG | AGGGTTACAGTCTACCCGCA | 59.9 | 780 | INTERGENIC | | | | |
| CaPOPI_7579 | scaffold4038 | 1082 | TG/T | TTGGAGAAACTTTTCCGCTG | CGAACTGGGCTCAGAGAAAC | 60.4 | 632 | INTERGENIC | | | | |
| CaPOPI_7580 | scaffold404 | 161683 | T/TC | CGTGAGATCCCCCTATCGTA | TTGATTTCAGCCCAGGATTC | 59.9 | 334 | INTERGENIC | | | | |
| CaPOPI_7581 | scaffold404 | 180224 | CAA/CA | AGTTGGACACTTGCGCTTCT | TCGCATCATTTTACCGCATA | 60.1 | 856 | INTERGENIC | | | | |
| CaPOPI_7582 | scaffold404 | 202624 | AATATATATATATATATATATATATATATATATATATATA/AATATATATATATATATATATATATATATATATA | TATTTTGGATTTTGGCTCGG | AAATGGAGGAGAAGGAGGGA | 59.9 | 417 | INTERGENIC | | | | |
| CaPOPI_7583 | scaffold404 | 237330 | TTTATTATTATTATTATTATTATTATTATTATTATTATTATT/TTTATTATTATTATTATTATTATTATTATTATTATTATT | AGAGGCAAACAAGAACCGAA | GCAACCAAATTGAAACACCA | 59.9 | 581 | INTERGENIC | | | | |
| CaPOPI_7584 | scaffold4041 | 994 | CT/CTTT | TCATTCTAATTCCTGCCTGATG | GTCCAAACCCAGCACAATCT | 59.2 | 494 | INTERGENIC | | | | |
| CaPOPI_7585 | scaffold4041 | 15702 | T/TG | AGGAATTGGATCGGGAGATT | AAGGAAATGAGGAAAATGGGA | 59.7 | 626 | INTERGENIC | | | | |

| INDEL marker IDs | Chromosomes/ unanchored scaffolds | Physical positions (bp) | InDels (*Kabuli* reference genome-CDC Frontier/PI) | Forward primers (5'-3') | Reverse primers (5'-3') | Anneling temperature (0C) | Expected amplified product size (bp) | Structural annotation | | Functional annotation | | |
|---|---|---|---|---|---|---|---|---|---|---|---|---|
| | | | | | | | | Sequence components of *kabuli* genome | *Kabuli* gene accession IDs | NCBI-KOG | TFs | NCBI-nr database |
| CaPOPI_7586 | scaffold4041 | 15753 | TAAAAAAAAA/TAAAAAAAAAAAAA | AGGAATTGGATCGGGAGATT | AAGGAAATGAGGAAAATGGGA | 59.7 | 626 | INTERGENIC | | | | |
| CaPOPI_7587 | scaffold4041 | 15867 | AT/ATT | GGGTTGGGATATGTTAGCAAAA | GAATGCGAAAGCCACAATTT | 60.1 | 851 | INTERGENIC | | | | |
| CaPOPI_7588 | scaffold4041 | 15956 | TAAAAAAAA/TAAAAAAA | TCCCATTTTCCTCATTTCCTT | GAATGCGAAAGCCACAATTT | 59.8 | 493 | INTERGENIC | | | | |
| CaPOPI_7589 | scaffold4041 | 16171 | AAA/AAATTAA | GGCACGTCCCTATTTTTGAA | GAATGCGAAAGCCACAATTT | 59.9 | 345 | INTERGENIC | | | | |
| CaPOPI_7590 | scaffold4045 | 1604 | GTTT/GTTTT | CACAACGCCAAGATGCTAGA | CCCCCTTTTTGCATAAGTGA | 60.0 | 779 | INTERGENIC | | | | |
| CaPOPI_7591 | scaffold4064 | 835 | AT/ATTAGT | ATGCTCTGCACTTGCAGCTA | TGACTTGACTCTTGTCCGCA | 59.9 | 581 | INTERGENIC | | | | |
| CaPOPI_7592 | scaffold4064 | 1366 | TTAT/TT | AAAAGCATGCAGTGCAACAC | TCCCGTCTGAAAATTATGGC | 59.9 | 367 | INTERGENIC | | | | |
| CaPOPI_7593 | scaffold4069 | 1182 | AATATATATATATATATATA/AATATATATATATATA | AACCGACACTCAACCCACTC | TTTGAAGGAACCCTGCTTTG | 60.0 | 260 | INTERGENIC | | | | |
| CaPOPI_7594 | scaffold408 | 3361 | T/TGTTGA | CAAGGAGGAGCAAGATCAGG | GGGAAAGCGGACGTGTATTA | 59.9 | 631 | INTERGENIC | | | | |
| CaPOPI_7595 | scaffold408 | 3569 | TCCCC/TCCCCC | TAATACACGTCCGCTTTCCC | CGAACCTGCTGTCCAAAAAT | 60.0 | 394 | INTERGENIC | | | | |
| CaPOPI_7596 | scaffold408 | 130988 | T/TG | GTGGTGATATTTGGGAAGCC | TCGTGGTTTTGGTGTTGGTA | 59.2 | 662 | INTERGENIC | | | | |
| CaPOPI_7597 | scaffold408 | 131029 | AT/AAGT | GTGGTGATATTTGGGAAGCC | TCGTGGTTTTGGTGTTGGTA | 59.2 | 662 | INTERGENIC | | | | |
| CaPOPI_7598 | scaffold4088 | 5270 | AT/A | ATCAACTTGCAAGGCAGCTT | TACCATTCAATTGGCGAACA | 60.0 | 454 | INTERGENIC | | | | |
| CaPOPI_7599 | scaffold4088 | 17489 | TTCT/TT | TAGGTGATGGAGATCTCGGC | TTGTCACTGTGCGCATCATA | 60.2 | 592 | INTERGENIC | | | | |
| CaPOPI_7600 | scaffold41 | 114200 | ATTTTTTTTT/ATTTTTTTTTTTT | ACCAGTCCCCTAAACCAACA | TTGATTGAAACATTGATTTCCG | 59.3 | 660 | INTERGENIC | | | | |

| INDEL marker IDs | Chromosomes/ unanchored scaffolds | Physical positions (bp) | InDels (*Kabuli* reference genome-CDC Frontier/PI) | Forward primers (5'-3') | Reverse primers (5'-3') | Anneling temperature (0C) | Expected amplified product size (bp) | Structural annotation | | Functional annotation | | |
|---|---|---|---|---|---|---|---|---|---|---|---|---|
| | | | | | | | | Sequence components of *kabuli* genome | *Kabuli* gene accession IDs | NCBI-KOG | TFs | NCBI-nr database |
| CaPOPI_7601 | scaffold41 | 122306 | ATT/ATTT | TCGCTACGGCTCTAGTG GTT | AAGTCCTGTAGCCGAAA GCA | 60.0 | 294 | INTERGENIC | | | | |
| CaPOPI_7602 | scaffold41 | 122382 | C/CG | TCGCTACGGCTCTAGTG GTT | AAGTCCTGTAGCCGAAA GCA | 60.0 | 294 | INTERGENIC | | | | |
| CaPOPI_7603 | scaffold41 | 122436 | GC/G | TGCTTTCGGCTACAGGA CTT | CGGGGAGTTGAAAATAA GCA | 60.0 | 282 | INTERGENIC | | | | |
| CaPOPI_7604 | scaffold4107 | 9634 | CT/CTAAATT | CCTAAAACCTAAAACCAA AAACC | TCGTCCAATAAGAACAA AAGTCAA | 57.4 | 127 | INTERGENIC | | | | |
| CaPOPI_7605 | scaffold4108 | 8749 | CTT/CT | CAAAACCGGAAACGCTT AAC | TTTTGGGCTTCGAGTTT AGG | 59.6 | 294 | INTERGENIC | | | | |
| CaPOPI_7606 | scaffold411 | 193597 | CAATAATAATAATAATAATAATAATAATA ATAATA/CAATAATAATAATAATAATAAT AATAATAATA | TGTTCGGCTTATTGCAG TTG | AGAGGAGGTGATGGTGA TGG | 59.9 | 482 | INTERGENIC | | | | |
| CaPOPI_7607 | scaffold411 | 251051 | TTTATTATTATTATTATTATTATTATTAT TA/TTTATTATTATTATTATTATTATTATT ATTATTA | CCCTTCTCCTTCCGTAG AGC | TTGACACGGTCTGCGTA GTT | 60.3 | 371 | INTERGENIC | | | | |
| CaPOPI_7608 | scaffold411 | 298521 | A/AGATTG | CTTGCATGGCAATTATC CAA | TTGGGCTGATGCAATAA ACA | 59.5 | 469 | INTERGENIC | | | | |
| CaPOPI_7609 | scaffold411 | 334354 | GAA/GA | GAGGTTTCACAGTGCAG CAA | TTCTTAGCCATGCTGGT GTG | 60.0 | 649 | INTERGENIC | | | | |
| CaPOPI_7610 | scaffold411 | 339806 | T/TA | GACAATGTCCCCACTTG AGAA | CACGGTTGTGATGACTG GAC | 60.0 | 671 | INTERGENIC | | | | |
| CaPOPI_7611 | scaffold411 | 340187 | TAAAAA/TAAAA | ATTGGTGTTGTGCAGGT GAA | CACGGTTGTGATGACTG GAC | 60.0 | 109 | INTERGENIC | | | | |
| CaPOPI_7612 | scaffold411 | 343473 | A/AGTG | TCATTTTCATGAAGACCA AACA | TCATTTCGACCATCAATC CA | 58.1 | 462 | INTERGENIC | | | | |
| CaPOPI_7613 | scaffold411 | 436040 | CATA/CATATA | ACCCGAATACCTCAAGG GTC | TCGTTTATTCGGATTCG CTC | 60.2 | 922 | INTERGENIC | | | | |
| CaPOPI_7614 | scaffold414 | 21975 | GAAAAA/GAAAA | TTCCTATTGCTCACCCC TTG | GAAAAACAAGACCCGCG ATA | 60.1 | 516 | DRR | Ca_27849 | | | |
| CaPOPI_7615 | scaffold4155 | 7060 | AATATATAT/AATATATATAT | CGGTTCAACGTAACCGA CTT | TTGGGCAACAATCGATG TAA | 60.0 | 402 | INTERGENIC | | | | |

| INDEL marker IDs | Chromosomes/ unanchored scaffolds | Physical positions (bp) | InDels (*Kabuli* reference genome-CDC Frontier/PI) | Forward primers (5'-3') | Reverse primers (5'-3') | Anneling temperature (0C) | Expected amplified product size (bp) | Structural annotation | | Functional annotation | | |
|---|---|---|---|---|---|---|---|---|---|---|---|---|
| | | | | | | | | Sequence components of *kabuli* genome | *Kabuli* gene accession IDs | NCBI-KOG | TFs | NCBI-nr database |
| CaPOPI_7616 | scaffold418 | 8392 | T/TC | TAATCTTCAGCAGTGGTGGC | TTTTCTTTTCGATTTGATCCG | 58.9 | 680 | INTERGENIC | | | | |
| CaPOPI_7617 | scaffold418 | 154196 | AA/AATA | ACTCCTCAAAACTCGGCAAA | TTGAACTTGCATTCGTTCCA | 59.9 | 719 | DRR | Ca_24681 | | | |
| CaPOPI_7618 | scaffold418 | 157125 | TCC/TC | TCGGTTCTCAATCTAACCATTTG | TTTTATGGGTTTTGGTTGAAAA | 60.4 | 660 | INTERGENIC | | | | |
| CaPOPI_7619 | scaffold419 | 148627 | CA/CAA | AACAAGCAAGCCATTCAAGC | GGTGAGGATCTGGTGTGGTT | 60.4 | 657 | INTERGENIC | | | | |
| CaPOPI_7620 | scaffold419 | 335182 | TAAAAAAAAAAA/TAAAAAAAAAAAA | GGGCATTTTTCAACAAGGTG | ATGTTCCAACAATCTCCCCA | 60.3 | 457 | INTERGENIC | | | | |
| CaPOPI_7621 | scaffold4192 | 3302 | C/CGATTGGTAAATCGA | AGGGAGGACGAAAATGACCT | CGATTGCCTCAAACTTGGAT | 59.9 | 724 | INTERGENIC | | | | |
| CaPOPI_7622 | scaffold4192 | 3668 | T/TC | ATCCAAGTTTGAGGCAATCG | CGATTTTCGCAGGTCTTGTT | 60.1 | 484 | INTERGENIC | | | | |
| CaPOPI_7623 | scaffold4197 | 16348 | GAAA/GAA | AATGGCCACGAATCTGAAAG | GTGCGGAAGCCATGAGTAAT | 60.1 | 483 | INTERGENIC | | | | |
| CaPOPI_7624 | scaffold4197 | 38400 | AATATA/AATA | GGCCTGGGTTATGGGTTAAT | AAATTGAGTTTCAAAACCACCAA | 59.9 | 830 | DRR | Ca_27296 | | | |
| CaPOPI_7625 | scaffold4197 | 56545 | GAAA/GAAAA | TCCAGATGAAATCGAGGACC | TATTTTCGGACCAGACGAGG | 60.0 | 606 | INTERGENIC | | | | |
| CaPOPI_7626 | scaffold420 | 69655 | CAA/CAAA | ATGGCGAAGAATTATGGTGC | CATGTACCGCCCATCTCTTT | 59.9 | 265 | INTERGENIC | | | | |
| CaPOPI_7627 | scaffold420 | 86760 | GATCAT/GAT | TGTTGCTAGGCAGAGTGTGG | AGAAAGGTCCAACCGGCTAT | 60.0 | 415 | DRR | Ca_22197 | | | |
| CaPOPI_7628 | scaffold420 | 109389 | TTTATTATTATTATTATTATTATTATTATTATTATTATTATTATTATTATTATTATT/TTTATTATTATTATTATTATTATTATTATTATTATTATTATTATTATT | CCCAAATCCCTAACATGTGG | GTTGTCCATTGTTTTGCGTG | 60.0 | 520 | INTERGENIC | | | | |
| CaPOPI_7629 | scaffold420 | 156465 | TTATATATATATATAT/TTATATATATATATATAT | TATGTCCCATTCACACTCGG | CTGGATTTTGCACTCTGCCT | 59.4 | 411 | INTERGENIC | | | | |
| CaPOPI_7630 | scaffold420 | 265273 | GAAAAAAAA/GAAAAAAAAA | CGTGATCGTGTTTGGTTGTC | GGGAATGGCAGCAAATAGAA | 60.0 | 587 | INTERGENIC | | | | |

| INDEL marker IDs | Chromosomes/ unanchored scaffolds | Physical positions (bp) | InDels (*Kabuli* reference genome-CDC Frontier/PI) | Forward primers (5'-3') | Reverse primers (5'-3') | Anneling temperature (0C) | Expected amplified product size (bp) | Structural annotation | | Functional annotation | | |
|---|---|---|---|---|---|---|---|---|---|---|---|---|
| | | | | | | | | Sequence components of *kabuli* genome | *Kabuli* gene accession IDs | NCBI-KOG | TFs | NCBI-nr database |
| CaPOPI_7631 | scaffold4221 | 569 | AAAGAA/AAA | ATTGATTTTGAAATCGCCCA | CAGTTTCCTGCCAGCCTTAG | 60.3 | 441 | INTERGENIC | | | | |
| CaPOPI_7632 | scaffold4221 | 691 | A/AG | CTAAGGCTGGCAGGAAACTG | CGCTTCTTTGGTCATGCAA | 60.0 | 443 | INTERGENIC | | | | |
| CaPOPI_7633 | scaffold4221 | 7762 | TAAAAA/TAAAA | TGCCAGAACTGATTTTCTGCT | GAGCGCTGTAAAACACCTCC | 60.0 | 540 | INTERGENIC | | | | |
| CaPOPI_7634 | scaffold4221 | 54414 | GTTTTTTTTTTTTT/GTTTTTTTTTT | GAGCCCGAATCGTTATTTTG | TTGTGGTATACCCGACAAAGAA | 59.5 | 695 | INTERGENIC | | | | |
| CaPOPI_7635 | scaffold4221 | 54516 | A/ATTTAGGG | GAGCCCGAATCGTTATTTTG | TTGTGGTATACCCGACAAAGAA | 59.5 | 695 | INTERGENIC | | | | |
| CaPOPI_7636 | scaffold424 | 29738 | T/TA | ACTCGACAAAAATGGAACGG | CTTTTGGCCTATTAGCGTCG | 60.0 | 496 | DRR | Ca_27938 | L | bHLH | RNA-binding, CRM domain |
| CaPOPI_7637 | scaffold4243 | 1042 | CGG/CG | GGTACGGAAATTGCCTGAAA | GGTCCTCGATTTCATCTGGA | 59.9 | 204 | INTERGENIC | | | | |
| CaPOPI_7638 | scaffold4243 | 14917 | CT/CTT | TGCTTGATTGCTTGAGTTCC | GATGAAATCGAGGACTGGGA | 59.0 | 537 | INTERGENIC | | | | |
| CaPOPI_7639 | scaffold4243 | 16781 | CTTTTTT/CTTTTT | TTTCATCTGGACTTTTCGGG | TCGAGGATAGGGAGACCCTT | 60.0 | 964 | INTERGENIC | | | | |
| CaPOPI_7640 | scaffold4244 | 559 | TAA/TAAA | CCCAGTTCCCAAGAGTCAAA | GGAACGCATTTCTCGTGTTT | 60.1 | 122 | INTERGENIC | | | | |
| CaPOPI_7641 | scaffold429 | 33115 | TAAAAAA/TAAAAAAA | AATTAGCGTGTTGATTCGGG | AATCCTGGTGCACGATTTTC | 60.0 | 720 | INTERGENIC | | | | |
| CaPOPI_7642 | scaffold4319 | 1139 | ATTT/AT | CGATTTCCCTCACGACAAAT | TTTTGGACAGCTACAAGTGGTT | 59.9 | 582 | INTERGENIC | | | | |
| CaPOPI_7643 | scaffold4319 | 1245 | TAAAAAA/TAAAA | CGATTTCCCTCACGACAAAT | TTTTGGACAGCTACAAGTGGTT | 59.9 | 582 | INTERGENIC | | | | |
| CaPOPI_7644 | scaffold433 | 8383 | GAAAAAAAAA/GAAAAAAAAAAA | ACATGTAAGGCAAGGCCAAC | GCCAGTCACCCCAACTTTTA | 60.0 | 377 | INTERGENIC | | | | |
| CaPOPI_7645 | scaffold433 | 18841 | TAAAAAAAAAA/TAAAAAAAAA | CGGCGATAGAAAGCCTACTG | ATGCAAGGTGGGTGCTAAAG | 60.0 | 371 | INTERGENIC | | | | |

| INDEL marker IDs | Chromosomes/ unanchored scaffolds | Physical positions (bp) | InDels (*Kabuli* reference genome-CDC Frontier/PI) | Forward primers (5'-3') | Reverse primers (5'-3') | Anneling temperature (0C) | Expected amplified product size (bp) | Structural annotation | | Functional annotation | | |
|---|---|---|---|---|---|---|---|---|---|---|---|---|
| | | | | | | | | Sequence components of *kabuli* genome | *Kabuli* gene accession IDs | NCBI-KOG | TFs | NCBI-nr database |
| CaPOPI_7646 | scaffold433 | 47351 | AAAAA/AAAAACAAAA | GAGGCGTCAAGCTAATGACC | AAGCTCAAATGGGCTAGCAA | 59.8 | 530 | INTERGENIC | | | | |
| CaPOPI_7647 | scaffold433 | 74147 | TTATATATATATA/TTATATATATATATATATA | GCAGCCGTTCTCTTTTGTTC | ACGTTGTCGGACATTCATCA | 60.0 | 709 | INTERGENIC | | | | |
| CaPOPI_7648 | scaffold433 | 86247 | GTTT/GTT | GCCCTCAGATGAGAGTCAGG | AGAATTTACGAGCAGCGGAA | 59.9 | 287 | INTERGENIC | | | | |
| CaPOPI_7649 | scaffold433 | 98245 | CTAACTTAACTTA/CTAACTTA | CAAGATCCAGTTTGTCCCGT | CGCATGGAGGAGAAAGGATA | 60.0 | 266 | INTERGENIC | | | | |
| CaPOPI_7650 | scaffold433 | 144718 | GCC/GCCC | TGGCTAATCGCCAGTTTTCT | AATGATCGTCTTCGGATTGC | 59.8 | 854 | INTERGENIC | | | | |
| CaPOPI_7651 | scaffold433 | 146170 | TAAAAAAAAA/TAAAAAAAA | GTTGCCAAAGCAGGGTAAAA | TGAAACGGATTTTCAACCAA | 60.1 | 434 | INTERGENIC | | | | |
| CaPOPI_7652 | scaffold433 | 146841 | AATTATTATTATTATTATTATTATTATT/AATTATTATTATTATTATTATT | TGGTGACAATTTGATGGTTCT | CAGTGCGATGGAGAAATCAA | 57.9 | 523 | INTERGENIC | | | | |
| CaPOPI_7653 | scaffold433 | 147086 | ATTTTTTTTT/ATTTTTTTTTT | TGGTGACAATTTGATGGTTCT | TCGATTATGCGTAGGGAAGG | 57.9 | 770 | INTERGENIC | | | | |
| CaPOPI_7654 | scaffold433 | 147750 | TTTTTATTTTAT/TTTTTATTTTATTTTAT | TTTTAGCGGATGTCGTAGGG | TCACAAGGGAGGCTTTATGG | 60.1 | 717 | INTERGENIC | | | | |
| CaPOPI_7655 | scaffold433 | 157672 | GATATATA/GATATA | GCATTTCAAGATGGGCAGTT | AATGATCGTCTTCGGATTGG | 60.1 | 714 | INTERGENIC | | | | |
| CaPOPI_7656 | scaffold433 | 165941 | A/AG | AAAATTGAAACATCCGACGC | ACTTGTCGCTGGACGAATCT | 59.9 | 512 | DRR | Ca_26821 | | | |
| CaPOPI_7657 | scaffold4331 | 1786 | ATT/AAATCTCCTT | CCTTGTGTGTCTTGGGTTGA | AGAAGCAATGGCTCGAAGAA | 59.6 | 528 | INTERGENIC | | | | |
| CaPOPI_7658 | scaffold4349 | 1831 | GTTTT/GTTT | AGGTCATTTTCGTCCTCCCT | CAAACACACCTGTTGGATGC | 59.9 | 182 | INTERGENIC | | | | |
| CaPOPI_7659 | scaffold436 | 67143 | A/AC | CAAACACACCTGTTGGATGC | AGGTCATTTTCGTCCTCCCT | 60.0 | 478 | INTERGENIC | | | | |
| CaPOPI_7660 | scaffold436 | 68438 | A/AT | GGCCGGATGAAAATGAACTA | ATTTTCGTCCTCCAAAGGCT | 59.9 | 513 | INTERGENIC | | | | |

| INDEL marker IDs | Chromosomes/ unanchored scaffolds | Physical positions (bp) | InDels (*Kabuli* reference genome-CDC Frontier/PI) | Forward primers (5'-3') | Reverse primers (5'-3') | Anneling temperature (0C) | Expected amplified product size (bp) | Structural annotation | | Functional annotation | | |
|---|---|---|---|---|---|---|---|---|---|---|---|---|
| | | | | | | | | Sequence components of *kabuli* genome | *Kabuli* gene accession IDs | NCBI-KOG | TFs | NCBI-nr database |
| CaPOPI_7661 | scaffold4366 | 635 | A/AG | ATGCACACGATTGGTGAAAA | TCATTTTCGTCCTCCAAAGG | 60.0 | 757 | INTERGENIC | | | | |
| CaPOPI_7662 | scaffold4366 | 1890 | G/GC | GGACATGAATAGGGCAATGG | GGTCATTTTTGTCCACGCTT | 60.2 | 532 | INTERGENIC | | | | |
| CaPOPI_7663 | scaffold4367 | 11881 | CTAAAAATTAAAAATTAA/CTAAAAATTAA | TGGATGCACAAGATGGGTTA | ATTTTCATCCTCCCAAGGCT | 59.9 | 351 | INTERGENIC | | | | |
| CaPOPI_7664 | scaffold437 | 2817 | ACCC/ACC | CCTGCTTTTCCAAACCATGT | TGGCACAGTTCCAAGATCAA | 60.0 | 507 | INTERGENIC | | | | |
| CaPOPI_7665 | scaffold437 | 11434 | TATGG/TATGGGTGAAATGG | ACAATCCCCCAAATGTCAAA | GTTGGTTCCCTAGCACCGTA | 60.0 | 841 | INTERGENIC | | | | |
| CaPOPI_7666 | scaffold437 | 45718 | CAATATTAAAAAATATTAAA/CAATATTAAAAAATATTAAAATATTAAAAAATATTAAA | TCGAAGAGGACCATTCATCA | AGGTCGCACTCCAAATATCG | 59.2 | 777 | INTERGENIC | | | | |
| CaPOPI_7667 | scaffold4374 | 728 | GCCTAAACCCTAAACCCTAA/GCCTAAACCCTAA | ATAAACCCGAACCGCAAAAC | AGGGTTTAGGGTTTAGGCCA | 61.1 | 848 | INTERGENIC | | | | |
| CaPOPI_7668 | scaffold4374 | 1159 | CTAAACC/CTAAACCATAAACC | TAAACCCCAAATGACCGAAA | AGGGGTTAGGGGTTAGGGTT | 60.2 | 444 | INTERGENIC | | | | |
| CaPOPI_7669 | scaffold4377 | 6572 | AA/AAGA | TCCAGATGAAATCGAGGACC | AGGCGATTTTCGTACCACAC | 60.0 | 319 | INTERGENIC | | | | |
| CaPOPI_7670 | scaffold4387 | 5507 | ATAGTATA/ATAGTATAGCTAGTATA | GCACAAAAACCTTATCCCCA | AAATGCTCGCCTCTTTTTGA | 59.8 | 311 | INTERGENIC | | | | |
| CaPOPI_7671 | scaffold4397 | 932 | TAAAAAAA/TAAAAAA | GTCAAGGCAGACCAGAAAGC | GAACGACGGTGATGCCTACT | 60.0 | 168 | INTERGENIC | | | | |
| CaPOPI_7672 | scaffold44 | 290551 | TTTA/TTTACAATTA | AACCAAAGAGGCTTTCGTCA | GGAGCAGTTTCTTGGCGTAG | 59.9 | 705 | DRR | Ca_25417 | | | |
| CaPOPI_7673 | scaffold440 | 30262 | CAAAA/CAAA | ACCAAAATAAGCCCAGGGAG | TTGCCATTGCATGAACATTT | 60.3 | 354 | INTERGENIC | | | | |
| CaPOPI_7674 | scaffold442 | 20594 | TGGGGGGGG/TGGGGGGG | TGGACGCACAAGATTGGTTA | AGGTCATTTTCGTCCTCCCT | 60.1 | 370 | INTERGENIC | | | | |
| CaPOPI_7675 | scaffold446 | 219694 | GGT/GGTGT | TCCCAGTCCTCGATTTCATC | CGGGTTATGCGTCAGATTTT | 60.0 | 699 | INTERGENIC | | | | |

| INDEL marker IDs | Chromosomes/ unanchored scaffolds | Physical positions (bp) | InDels (*Kabuli* reference genome-CDC Frontier/PI) | Forward primers (5'-3') | Reverse primers (5'-3') | Anneling temperature (0C) | Expected amplified product size (bp) | Structural annotation | | Functional annotation | | |
|---|---|---|---|---|---|---|---|---|---|---|---|---|
| | | | | | | | | Sequence components of *kabuli* genome | *Kabuli* gene accession IDs | NCBI-KOG | TFs | NCBI-nr database |
| CaPOPI_7676 | scaffold446 | 224556 | AAATAATAATAATAATAATAATAATAATAATAATAATAATAATAATA/AAATAATAATAATAATAATAATAATAATAATAATAATAATAATAATAATAATA | TTCATCTGGTCTTTTTGGGC | CATTGAGTACAACTACAAAACGACG | 60.1 | 771 | INTERGENIC | | | | |
| CaPOPI_7677 | scaffold446 | 235656 | TAA/TA | TCTGACAACTCAATTTTGCTCTATG | GTTGTACTTCATAACGAACACAATACT | 59.8 | 521 | INTERGENIC | | | | |
| CaPOPI_7678 | scaffold450 | 9894 | CTTTTTTTTTT/CTTTTTTTTT | TCATTGCTTTGGGTGATTGA | TGGTAACGTGGACGATTTGA | 60.0 | 597 | INTERGENIC | | | | |
| CaPOPI_7679 | scaffold450 | 255127 | GAAAAAAAAAA/GAAAAAAAAAAA | ACGACCCATATTGAGCAAGG | AGTTACGTGGCATGACCCTC | 60.0 | 528 | INTRON | Ca_23798 | | | |
| CaPOPI_7680 | scaffold450 | 299447 | ATTTTTTTTTTT/ATTTTTTTTTT | TGCCACTACTAGGCACTAGCC | TCGCATTTGATTATTTCACACA | 59.6 | 659 | INTERGENIC | | | | |
| CaPOPI_7681 | scaffold450 | 322861 | TA/T | ACACGTAACATGCAGGTCCA | TTGTTCTCATTAAGTGTTGGATTGA | 60.0 | 283 | INTERGENIC | | | | |
| CaPOPI_7682 | scaffold4508 | 4751 | TAAAAAAAAAAA/TAAAAAAAAAA | CCGGAAAACTTCATTCCAAA | AGAAAACCGAATTTGCCCTT | 59.9 | 810 | INTERGENIC | | | | |
| CaPOPI_7683 | scaffold4508 | 36727 | TAA/TA | TTGTGTTTGTCACAGGCCAT | TCAGGAACAACAACTCAACCC | 60.0 | 371 | INTERGENIC | | | | |
| CaPOPI_7684 | scaffold451 | 10137 | ATATTATTATTATTATTATTATTATTATTATTATTATTATTATTATTATTATTATTATTATTATTATTATT/ATATTATTATTATTATTATTATTATTATTATTATTATTATTATTATTA | TGTTTCAACACGTCTTTTCTTTTT | TGGTGTCTGTGGCAACACTT | 59.3 | 574 | INTERGENIC | | | | |
| CaPOPI_7685 | scaffold451 | 17388 | AAGTA/AA | TGCTCAAGGATGCACTCAAC | GCCATTGATGAGTACGCTGA | 60.0 | 671 | CDS (large-effect mutations) | Ca_22599 | | C3H | |
| CaPOPI_7686 | scaffold451 | 30168 | TA/TAA | GCAGCCACAAATAAGGTGGT | AACCAAAGCCTGAGCTTGAA | 60.0 | 810 | INTERGENIC | | | | |
| CaPOPI_7687 | scaffold451 | 37858 | TTATATATATATATATAT/TTATATATATATATAT | ACAGCGAACAACCACATCAA | TAAATGCCGATTTCCCAAAG | 60.2 | 569 | INTERGENIC | | | | |
| CaPOPI_7688 | scaffold451 | 324149 | G/GAAAAACCCTTATCT | TGAATTCAACGCAAATGATCT | TCAACAAAACCCTAGCCACC | 58.2 | 162 | INTERGENIC | | | | |
| CaPOPI_7689 | scaffold4511 | 11167 | CATATA/CATA | TGAGTGGTAGGAAAATCGCC | TTTCATTTGGACTTTTCGGG | 60.1 | 844 | INTERGENIC | | | | |
| CaPOPI_7690 | scaffold4511 | 22911 | CAAAAA/CAAAA | CTCCCCCTGAGTTTTGGAAT | GACGACAATCACCCGAAGAT | 60.3 | 753 | DRR | Ca_28219 | | Trihelix | Peptidase C48, SUMO/Sentrin/Ubl1 |

| **INDEL marker IDs** | **Chromosomes/ unanchored scaffolds** | **Physical positions (bp)** | **InDels (*Kabuli* reference genome-CDC Frontier/PI)** | **Forward primers (5'-3')** | **Reverse primers (5'-3')** | **Anneling temperature (0C)** | **Expected amplified product size (bp)** | **Structural annotation** | | **Functional annotation** | | |
|---|---|---|---|---|---|---|---|---|---|---|---|---|
| | | | | | | | | **Sequence components of *kabuli* genome** | ***Kabuli* gene accession IDs** | **NCBI-KOG** | **TFs** | **NCBI-nr database** |
| CaPOPI_7691 | scaffold4511 | 22953 | TAAAA/TAAAAA | CTCCCCCTGAGTTTTGGAAT | GACGACAATCACCCGAAGAT | 60.3 | 753 | DRR | Ca_28219 | | Trihelix | Peptidase C48, SUMO/Sentrin/Ubl1 |
| CaPOPI_7692 | scaffold4511 | 38356 | GTTTTTTTTTT/GTTTTTTTTTTTT | AATGCCCACCCATAATTTCA | CTGGGCAAGGGATCTGAATA | 60.0 | 642 | INTRON | Ca_28219 | | Trihelix | Peptidase C48, SUMO/Sentrin/Ubl1 |
| CaPOPI_7693 | scaffold4515 | 721 | TG/T | GTGTTTGTGTCGTTGGATGC | ACCTATTGGATGCACAAGCC | 60.0 | 677 | INTERGENIC | | | | |
| CaPOPI_7694 | scaffold453 | 76859 | GAAAAAAAA/GAAAAAAAAAA | TTCATTCATGTGTGCTTTGGA | CGGCAATGCAAGTTTGACTA | 60.1 | 478 | INTRON | Ca_21728 | | MYB_related | Toll-Interleukin receptor |
| CaPOPI_7695 | scaffold453 | 80166 | T/TCATG | AATTTAAACCGGCCAAAACC | TCACTCATAAATATCCTCATGGACA | 60.1 | 578 | INTRON | Ca_21728 | | MYB_related | Toll-Interleukin receptor |
| CaPOPI_7696 | scaffold453 | 281880 | CATATATATATATATAT/CATATATATATATATATAT | GTTGGTTCCCTAGCACCGTA | AACGTGGTATCAGCCCTTTG | 60.0 | 777 | INTERGENIC | | | | |
| CaPOPI_7697 | scaffold453 | 497792 | CAATAATAATAATAATA/CAATAATAATAATAATAATA | GCCGTGATTTGGAAAAAGAA | TGTCAGTTTTGAGCCCAATG | 60.1 | 479 | URR | Ca_21748 | | | SAC3/GANP/Nin1/mts3/eIF-3 p25 |
| CaPOPI_7698 | scaffold453 | 498751 | GATAT/GATATAT | GCCCCTCAAAGATAACCCAT | GACCATGATTTCTTACTAGCCGTT | 60.2 | 838 | URR | Ca_21748 | | | SAC3/GANP/Nin1/mts3/eIF-3 p25 |
| CaPOPI_7699 | scaffold453 | 503657 | TAAA/TAA | GAACATTGCAGAAACGCAGA | CTATTGACGCCTTGCGTGTA | 60.0 | 728 | INTERGENIC | | | | |
| CaPOPI_7700 | scaffold4580 | 704 | CATATA/CATATATATA | TTGGTTTATTTGGGTTGGGA | CCATGAAAAGAAGAAGGGCA | 60.0 | 717 | INTERGENIC | | | | |
| CaPOPI_7701 | scaffold4582 | 7612 | A/AG | TCCAGATGAAATCGAGGACC | CGAAATTTGACACGGAACCT | 60.0 | 258 | INTERGENIC | | | | |
| CaPOPI_7702 | scaffold46 | 6079 | CTTTT/CTTTTT | GGGCCGAGAGTCCCTTATAG | GGTCCTCGATTTCATCTGGA | 60.1 | 304 | INTERGENIC | | | | |
| CaPOPI_7703 | scaffold461 | 26285 | CAAAAA/CAAAAAAAA | CGATTCCCACATCGATTCTT | TGCTCAAGACACGTGAAAGG | 59.9 | 207 | INTERGENIC | | | | |
| CaPOPI_7704 | scaffold4643 | 53525 | CAAA/CAAAA | ATGCAATGCGTATATGCCAA | GCCTGTGATAGGCGGTACAT | 59.9 | 185 | INTERGENIC | | | | |
| CaPOPI_7705 | scaffold466 | 12138 | AATATATA/AATATATATA | ATTCAGTTCCCTTGCAGCAT | TCTCCGCATAAGCTTTCGAT | 59.7 | 661 | INTERGENIC | | | | |

| INDEL marker IDs | Chromosomes/ unanchored scaffolds | Physical positions (bp) | InDels (*Kabuli* reference genome-CDC Frontier/PI) | Forward primers (5'-3') | Reverse primers (5'-3') | Anneling temperature (0C) | Expected amplified product size (bp) | **Structural annotation** | | **Functional annotation** | | |
|---|---|---|---|---|---|---|---|---|---|---|---|---|
| | | | | | | | | **Sequence components of *kabuli* genome** | ***Kabuli* gene accession IDs** | **NCBI-KOG** | **TFs** | **NCBI-nr database** |
| CaPOPI_7706 | scaffold466 | 168483 | TTATTAAAAAATATTAAAAAATATTAAAAA/TTATTAAAAAATATTAAAAAATATTAAAAAATATTAAAAA | AGCAAAAAGTGTCCGAGAGC | AGTCACCAGTCACCAGTCCC | 59.6 | 655 | INTRON | Ca_25699 | | | |
| CaPOPI_7707 | scaffold466 | 169717 | A/ATGCAAAAGATTCTA | CTTTCGTGCCCTTGATTTGT | CGTGGCCTATCGATCCTTTA | 60.1 | 740 | INTRON | Ca_25699 | | | |
| CaPOPI_7708 | scaffold466 | 170442 | CA/CACGGA | ACCCTTTTGTTCCACACGAG | AAGCTGACTTCGGTTGGCTA | 60.0 | 270 | INTRON | Ca_25699 | | | |
| CaPOPI_7709 | scaffold466 | 254290 | TCTTCTCCACTT/TCTT | GGATTCCGCGTAAGATTTCA | ACTAGGTTTTGGGTTCCGCT | 60.0 | 465 | INTERGENIC | | | | |
| CaPOPI_7710 | scaffold4696 | 3564 | T/TA | CAAACACACCTGTTGGATGC | AGGTCATTTTCGTCCTCCCT | 60.0 | 180 | INTERGENIC | | | | |
| CaPOPI_7711 | scaffold470 | 169493 | TAAAAAAAAAAAAA/TAAAAAAAAAAAA | TCAAGAGGAAGCGAAAGGAA | GCCCCCTATTTTTGCAATTT | 60.1 | 277 | INTERGENIC | | | | |
| CaPOPI_7712 | scaffold470 | 318535 | TTATATATATATATATATATATATATAT/TTATATATATATATATATATATATATATATAT | AATTAGGGGATCGAGGGAAA | AGGTGGCAACCTTCTCATTG | 59.7 | 472 | INTERGENIC | | | | |
| CaPOPI_7713 | scaffold470 | 336358 | TTATGTAT/TTAT | GTCATCCTAGCCATCGTCGT | GTGTCCCAAAACTCAAGGGA | 60.1 | 876 | INTERGENIC | | | | |
| CaPOPI_7714 | scaffold470 | 365605 | CTTTTTTTTTTTTT/CTTTTTTTTTTT | AGAAGCAAGCTAGCTCGTGG | ATTGGAGCACATTCAGGCTT | 59.9 | 815 | INTERGENIC | | | | |
| CaPOPI_7715 | scaffold470 | 370786 | TAAAAAAA/TAAAAAA | CATGCCAGTTGCTACATGCT | GCTTGGACTCAGTGGCTAGG | 59.9 | 949 | INTERGENIC | | | | |
| CaPOPI_7716 | scaffold470 | 372719 | GAAA/GAAAA | ATTTTATGCTGCGACATCCC | GCAAATCCCACACTTGGAGT | 59.9 | 265 | INTERGENIC | | | | |
| CaPOPI_7717 | scaffold470 | 375954 | AATATATATATATATATATATATATATATATATATATATAT/ATATATATATATATATATATATATATATATATATATATAT | AGGGAATCGAGGGAAAAATG | AGGTGGCAACCTTCTCATTG | 60.3 | 463 | INTERGENIC | | | | |
| CaPOPI_7718 | scaffold4708 | 20906 | CTTT/CTTTT | TGTTTGAAATCAGATGCGGA | AATTGTGTTTCGGGTTCTGC | 60.2 | 526 | INTERGENIC | | | | |
| CaPOPI_7719 | scaffold4711 | 5708 | GTTTTTTTT/GTTTTTTT | AGGTCATTTTCGTCCTCCCT | TTGGATGCACAAGATTGGTT | 59.9 | 271 | INTERGENIC | | | | |
| CaPOPI_7720 | scaffold4711 | 6211 | GTTTTTTT/GTTTTTTTT | TTTTCGTCCTCCCTAGGCTT | AGGAACTTATTGGATGCCCA | 60.2 | 470 | INTERGENIC | | | | |

| INDEL marker IDs | Chromosomes/ unanchored scaffolds | Physical positions (bp) | InDels (*Kabuli* reference genome-CDC Frontier/PI) | Forward primers (5'-3') | Reverse primers (5'-3') | Anneling temperature (0C) | Expected amplified product size (bp) | Structural annotation | | Functional annotation | | |
|---|---|---|---|---|---|---|---|---|---|---|---|---|
| | | | | | | | | Sequence components of *kabuli* genome | *Kabuli* gene accession IDs | NCBI-KOG | TFs | NCBI-nr database |
| CaPOPI_7721 | scaffold4711 | 9037 | CA/CAA | AACCAATCTTGTGCATCCAA | AAGACGAGGGACGACAAAAA | 59.0 | 379 | INTERGENIC | | | | |
| CaPOPI_7722 | scaffold4713 | 1946 | TG/TGG | TCATCCAATGACACAAACGC | AGGTCATTTTCGTCCTCCCT | 60.5 | 193 | INTERGENIC | | | | |
| CaPOPI_7723 | scaffold4733 | 507 | AT/ATT | ACAGTGAACGTGCACCCATA | CTGGTTATGCCGTTTGGAGT | 60.0 | 697 | INTERGENIC | | | | |
| CaPOPI_7724 | scaffold475 | 141188 | A/AAAATGGGG | GAGGAGCGACCAGTTGTTTC | GAAACTAATGCGACCGGTGT | 59.9 | 884 | INTERGENIC | | | | |
| CaPOPI_7725 | scaffold475 | 163127 | C/CCT | TTCAGGCAATCCAATCTTCC | TGGGTCCAAAGGACAGGTAG | 60.0 | 503 | INTERGENIC | | | | |
| CaPOPI_7726 | scaffold475 | 174713 | GTTTTTTT/GTTTTTTTT | AAGGTTGACTGCATAACGAAGA | CAACACCATTTGACAACACCA | 58.9 | 730 | INTERGENIC | | | | |
| CaPOPI_7727 | scaffold477 | 10236 | CAAAAAAAA/CAAAAAAA | TTGGAAAAGCCCACCTACAC | TGCAGTGCAATGTCCATTTT | 60.0 | 208 | INTERGENIC | | | | |
| CaPOPI_7728 | scaffold477 | 11283 | C/CA | TGTCATCTTGTTTGCCTTGG | TGATGAAACTCTGAACTCGTGG | 59.7 | 114 | INTERGENIC | | | | |
| CaPOPI_7729 | scaffold477 | 20714 | TA/TGA | CCCCCTAATGCTCAATTTCA | TGCAACAAGAGGCAAAACAC | 59.9 | 288 | INTERGENIC | | | | |
| CaPOPI_7730 | scaffold477 | 28410 | TAAAAA/TAAA | GTAGGAGGATCTCAAGGGGC | CCAAAATTAGGAAGCACCCA | 60.0 | 567 | INTERGENIC | | | | |
| CaPOPI_7731 | scaffold477 | 46653 | GAAAAAA/GAAAAAAA | ATGCAACTGTCGTTTTAGCA | TTAGAGCGCCTTCTGATGGT | 57.0 | 405 | INTERGENIC | | | | |
| CaPOPI_7732 | scaffold477 | 49382 | GCTCTCACTCTCACTCTC/GCTCTCACTCTCACTCTCACTCTC | CCCGCGAATAACTCCAATAA | TACCCACACCACTGACCAGA | 59.9 | 583 | INTERGENIC | | | | |
| CaPOPI_7733 | scaffold4773 | 12822 | ATCT/AT | CTTGTGAAAAACGCGCTGTA | TAGCTTGTCGCGTTGATTTG | 60.1 | 612 | INTERGENIC | | | | |
| CaPOPI_7734 | scaffold4773 | 12850 | TA/T | CTTGTGAAAAACGCGCTGTA | TAGCTTGTCGCGTTGATTTG | 60.1 | 612 | INTERGENIC | | | | |
| CaPOPI_7735 | scaffold4773 | 13699 | TTTTATTTATTTATTTATTTATTTATT/TTTTATTTATTTATTTATT | TATGCCACATGCCAACCTAC | TAGGCCGACTGCTCTTATGG | 59.4 | 247 | INTERGENIC | | | | |

| INDEL marker IDs | Chromosomes/ unanchored scaffolds | Physical positions (bp) | InDels (*Kabuli* reference genome-CDC Frontier/PI) | Forward primers (5'-3') | Reverse primers (5'-3') | Anneling temperature (0C) | Expected amplified product size (bp) | Structural annotation | | Functional annotation | | |
|---|---|---|---|---|---|---|---|---|---|---|---|---|
| | | | | | | | | Sequence components of *kabuli* genome | *Kabuli* gene accession IDs | NCBI-KOG | TFs | NCBI-nr database |
| CaPOPI_7736 | scaffold4773 | 14894 | CAAAA/CAAAAA | CCGCAGTCCCTTTGTTTTTA | CTCCACGGTTCTGTCATCAG | 60.1 | 684 | INTERGENIC | | | | |
| CaPOPI_7737 | scaffold4773 | 16885 | TNNNNNNNNNN/TNNNNNNNNN | TGCGGCTATTCATAACATGG | GCATATCTGAGGCTGCCAAT | 59.5 | 600 | INTERGENIC | | | | |
| CaPOPI_7738 | scaffold4773 | 18351 | GAAA/GAAAA | TGGGGGATTGTTGGAAAATA | GGGACAGAAAGAAAGGGGAG | 60.0 | 313 | INTERGENIC | | | | |
| CaPOPI_7739 | scaffold4773 | 19083 | A/ATTCACAGCATTGT | TTTAAGCGCCATATTGAGGG | GTGGTGTTGGCTGCAACTTA | 60.1 | 448 | INTERGENIC | | | | |
| CaPOPI_7740 | scaffold4773 | 19668 | TAAA/TAA | TAAGTTGCAGCCAACACCAC | GGGTTTTCCCCATTTAGCAT | 59.8 | 700 | INTERGENIC | | | | |
| CaPOPI_7741 | scaffold4773 | 19970 | ATT/ATTT | ATGCTAAATGGGGAAAACCC | CACGAAAGGTTAGGAGCAAGA | 60.0 | 106 | INTERGENIC | | | | |
| CaPOPI_7742 | scaffold4773 | 29645 | CAAAAAAAAAAAAA/CAAAAAAAAAAA | GGCATGCTGCGATTAATTTT | TTGTTACTCGGTGGACGACA | 60.1 | 282 | INTERGENIC | | | | |
| CaPOPI_7743 | scaffold4773 | 30145 | AATATATATATATATATATATATATATATATATATATATAT/AATATATATATATATATATATATATATATATATATATATATAT | GCGTTTACATGCAATCGTGT | TGATTTTGGTTTTGTTCCTTGTT | 59.6 | 910 | INTERGENIC | | | | |
| CaPOPI_7744 | scaffold4773 | 33470 | GAAAA/GAAAAA | TGAATAGTGGTGGTGAGTTTTCA | TGTGGAGAAGAGGGAGCAAT | 59.5 | 405 | INTERGENIC | | | | |
| CaPOPI_7745 | scaffold4773 | 36112 | T/TCCTGC | CGCAAATGGGTAAAACCCTA | AGCCTTCGAGATACGGTTGA | 59.8 | 663 | INTERGENIC | | | | |
| CaPOPI_7746 | scaffold4773 | 36184 | GATATATATATATATAT/GATATATATATATATATAT | CGCAAATGGGTAAAACCCTA | AGCCTTCGAGATACGGTTGA | 59.8 | 663 | INTERGENIC | | | | |
| CaPOPI_7747 | scaffold4773 | 52490 | ACTTCTTTGG/ACTTCTTTGGCTTCTTTGG | CCAAGGTAGGCGTTGGATAA | CACGTGATGTTTGGGCATAG | 60.0 | 408 | INTERGENIC | | | | |
| CaPOPI_7748 | scaffold4773 | 55151 | CCTTCTT/CCTT | GCTGCTGGTTCGTTTTCTTC | CTGTTGGCTTCCACTTCCAT | 60.0 | 307 | INTERGENIC | | | | |
| CaPOPI_7749 | scaffold4777 | 29741 | CAAAAAAAAAA/CAAAAAAAAAAA | TGGCTTGTGTTTCTCTCCCT | TGTTGTTCTACATGCGCTGA | 59.8 | 663 | INTERGENIC | | | | |
| CaPOPI_7750 | scaffold4777 | 118728 | CTTTTTTT/CTTTTTTTTT | AACGGTTTGGTTTGATCTGC | ACGTCGTCAAACAGATGCAC | 60.0 | 231 | INTRON | Ca_26711 | R | | WD40 repeat |

| INDEL marker IDs | Chromosomes/ unanchored scaffolds | Physical positions (bp) | InDels (*Kabuli* reference genome-CDC Frontier/PI) | Forward primers (5'-3') | Reverse primers (5'-3') | Anneling temperature (0C) | Expected amplified product size (bp) | Structural annotation | | Functional annotation | | |
|---|---|---|---|---|---|---|---|---|---|---|---|---|
| | | | | | | | | Sequence components of *kabuli* genome | *Kabuli* gene accession IDs | NCBI-KOG | TFs | NCBI-nr database |
| CaPOPI_7751 | scaffold4784 | 684 | TG/TGATGGCTAG | TCCTGGTCCTCGATTTCATC | GGGCCGAGAGACCCTTATAG | 60.0 | 339 | INTERGENIC | | | | |
| CaPOPI_7752 | scaffold479 | 7594 | TTA/TTAGGCATA | TGTGTTTGTGGCATTGGATT | AGGGAGGACGAAAATGACCT | 59.8 | 329 | INTERGENIC | | | | |
| CaPOPI_7753 | scaffold479 | 125417 | CTTTT/CTTTTT | ACAAACCAATCGATTTCCCA | CGGAGGACGAAAATGACCTA | 60.2 | 180 | INTERGENIC | | | | |
| CaPOPI_7754 | scaffold48 | 11811 | AAATAATAATAATAATAATAATA/AAATAATAATAATAATAATAATAATA | GCTAACTGACTTGCATTGCG | ATTCATCAGTTGCGCAAAAA | 59.6 | 758 | INTERGENIC | | | | |
| CaPOPI_7755 | scaffold48 | 15048 | AAATAATAATAATAATAATAATAATAATAATAATAATAATAATAATAATAATAATAATAATAA/AAATAATAATAATAATAATAATAATAATAATAATAATAATAATAATAAT | TCGGTTCATCATGTTGGAAAT | TCTTTTGATGTGGGTTAGTGAGAA | 60.2 | 677 | INTERGENIC | | | | |
| CaPOPI_7756 | scaffold48 | 140670 | C/CTTTCTTCCCT | TTTGCCCTTTTTGATTGGTC | GCAAATTGGACAGCAGATGA | 59.9 | 467 | INTERGENIC | | | | |
| CaPOPI_7757 | scaffold480 | 43150 | TCCC/TCCCC | AACGTACTGCCTATCACGGG | TGTTCATCGTATTTGGCGTG | 60.0 | 836 | INTERGENIC | | | | |
| CaPOPI_7758 | scaffold480 | 69498 | TTTTATTTATTTATTTATTT/TTTTATTTATTTATTTATTTATTT | AGAGGTGCGCACTATGGACT | CTGGAAGTGGCAGTGAATGA | 59.9 | 695 | INTERGENIC | | | | |
| CaPOPI_7759 | scaffold4804 | 893 | TAAAAAAAA/TAAAAAAA | AGCCTAGGGGTGACGAAAAT | GTCCTCCCCAGGCTTAATTC | 60.0 | 516 | INTERGENIC | | | | |
| CaPOPI_7760 | scaffold4804 | 1921 | TAAG/TAAGCCAAG | TTGGATGCACAACATTGGTT | AGGTCATTTTCGTCCTCCCT | 59.8 | 561 | INTERGENIC | | | | |
| CaPOPI_7761 | scaffold4804 | 8867 | CTT/CT | TGGATGCACCAGATTGGTTA | AGGTCATTTTCGTCCTCCCT | 59.9 | 570 | INTERGENIC | | | | |
| CaPOPI_7762 | scaffold4822 | 847 | A/AGAATACT | CAGCGGATCCCCAGATATAC | AGGTCATTTTCGTCCTCCCT | 59.4 | 545 | INTERGENIC | | | | |
| CaPOPI_7763 | scaffold4829 | 6279 | TCCC/TCCCC | GACCCGCATTTTCCTCATTA | GGAGAACGTTAAGGCAGCAG | 59.9 | 507 | INTERGENIC | | | | |
| CaPOPI_7764 | scaffold4829 | 6512 | AGGGG/AGGG | CTGCTGCCTTAACGTTCTCC | CCAAAAATCAGCCCGATCTA | 60.0 | 262 | INTERGENIC | | | | |
| CaPOPI_7765 | scaffold4829 | 8205 | TAAAA/TAAA | CAATAGCATGCAGTTGGAGG | GGAGACTCCCATACATTGGC | 59.3 | 500 | INTERGENIC | | | | |

| INDEL marker IDs | Chromosomes/ unanchored scaffolds | Physical positions (bp) | InDels (*Kabuli* reference genome-CDC Frontier/PI) | Forward primers (5'-3') | Reverse primers (5'-3') | Anneling temperature (0C) | Expected amplified product size (bp) | Structural annotation | | Functional annotation | | |
|---|---|---|---|---|---|---|---|---|---|---|---|---|
| | | | | | | | | Sequence components of *kabuli* genome | *Kabuli* gene accession IDs | NCBI-KOG | TFs | NCBI-nr database |
| CaPOPI_7766 | scaffold4833 | 740 | TTTTTTGTTTTT/TTTTTT | TGGGAATGTAGTGGATGAACC | TCCCGAACACCTATGCTTTT | 59.7 | 529 | INTERGENIC | | | | |
| CaPOPI_7767 | scaffold4833 | 3774 | T/TA | CCAAGCCAGTCAAACACAAA | GGGCTCGCTATGTACAGCA | 59.7 | 839 | INTERGENIC | | | | |
| CaPOPI_7768 | scaffold4833 | 3926 | CATATATATAT/CATATATATATAT | CAATTCAAATTGGCCCTGTC | GGGCTCGCTATGTACAGCA | 60.3 | 477 | INTERGENIC | | | | |
| CaPOPI_7769 | scaffold484 | 11805 | CAATATCAAATATCAAATATCAAATATCAAATATCAAATA/CAATATCAAATATCAAATATCAAATATCAAATA | CGAATACCTCCAAGGTCCAA | TCGTTTATTCGGATTCGCTC | 59.9 | 814 | INTERGENIC | | | | |
| CaPOPI_7770 | scaffold484 | 136302 | TCCCC/TCCC | ACCGAGGTGCCACTTATCAC | ATAGTGGTGACTGGATGGGC | 60.0 | 229 | INTERGENIC | | | | |
| CaPOPI_7771 | scaffold484 | 435397 | CATATATATATAT/CATATATATAT | TTGCTATGAAGGGACCAAGG | TCAAGTGGGGAGTGGAGAAC | 60.1 | 252 | INTERGENIC | | | | |
| CaPOPI_7772 | scaffold484 | 575607 | AGA/AGATTGA | TCCGATGACTTTGTGAAGCA | AAGATGATGGGAGGCAAACA | 60.4 | 559 | INTERGENIC | | | | |
| CaPOPI_7773 | scaffold484 | 656687 | AC/A | TATCACCTATCAGAGCGGGG | TGATTCCGGGGAAGTACAAC | 60.1 | 472 | INTERGENIC | | | | |
| CaPOPI_7774 | scaffold484 | 793859 | CACACTA/CACACTAACACTA | GGAAGGTGTGTGGGAAGTGT | ATATTTGCCTCCTCCCCTGT | 59.9 | 631 | INTERGENIC | | | | |
| CaPOPI_7775 | scaffold485 | 131454 | TCC/TC | TCAATCCACACCACTGGGTA | TGTTGGTTTATGGGGGAAGA | 59.8 | 475 | INTERGENIC | | | | |
| CaPOPI_7776 | scaffold4851 | 2996 | G/GCGATTTTC | GGTCCTCGATTTCATCTGGA | GGGCCGAGAGACCCTTATAG | 60.0 | 130 | INTERGENIC | | | | |
| CaPOPI_7777 | scaffold4851 | 3617 | TAAAAAAA/TAAAAAAAA | CGCGGAACCTGAAACACTAT | CCGATGCTAAAAATGGCAGT | 60.1 | 616 | INTERGENIC | | | | |
| CaPOPI_7778 | scaffold487 | 87751 | T/TC | ACATTGAGGTCATTTTCGCC | AGCCTAGGGAGGATGCAAAT | 59.9 | 229 | INTERGENIC | | | | |
| CaPOPI_7779 | scaffold4872 | 661 | G/GTTCTATCA | TGGGCACATGCTTCTCAGTA | TACTGGAACGGACCACACAA | 60.4 | 498 | INTERGENIC | | | | |
| CaPOPI_7780 | scaffold4902 | 30200 | AAATTCCAAAA/AAATTCCAAAAATTAATTCCAAAA | TCACCATGTGCTACTTCATTCA | GTGACGGTGTTCGTTGCTAA | 59.2 | 503 | INTERGENIC | | | | |

| INDEL marker IDs | Chromosomes/ unanchored scaffolds | Physical positions (bp) | InDels (*Kabuli* reference genome-CDC Frontier/PI) | Forward primers (5'-3') | Reverse primers (5'-3') | Anneling temperature (0C) | Expected amplified product size (bp) | **Structural annotation** | | **Functional annotation** | | |
|---|---|---|---|---|---|---|---|---|---|---|---|---|
| | | | | | | | | **Sequence components of *kabuli* genome** | ***Kabuli* gene accession IDs** | **NCBI-KOG** | **TFs** | **NCBI-nr database** |
| CaPOPI_7781 | scaffold4902 | 30281 | ATTTTTT/ATTTTTTT | TCACCATGTGCTACTTCATTCA | GACACCCAAAATCTCAGGGA | 59.2 | 779 | INTERGENIC | | | | |
| CaPOPI_7782 | scaffold4902 | 65969 | ATTTTTTTTT/ATTTTTTTTTT | TGAAAAACGCAACGAAGATG | CTTTTGCCAATTAACCCGAA | 59.8 | 746 | INTERGENIC | | | | |
| CaPOPI_7783 | scaffold4902 | 72921 | CTT/CT | GGGGTTACATTAACGGGGTT | CACCTCATAACGTGTTGGGC | 59.8 | 613 | INTERGENIC | | | | |
| CaPOPI_7784 | scaffold4902 | 73019 | AA/AACTGAAATTTA | GGGGTTACATTAACGGGGTT | CACCTCATAACGTGTTGGGC | 59.8 | 613 | INTERGENIC | | | | |
| CaPOPI_7785 | scaffold4902 | 73796 | CTTTTTTTTT/CTTTTTTTT | GTCGGCCCAACACGTTAT | AGCGGCCTCTCTATCAAACA | 59.4 | 532 | INTERGENIC | | | | |
| CaPOPI_7786 | scaffold4902 | 73891 | CGG/CGGGG | TTTGATAGAGAGGCCGCTTG | ACTAAGCGTGGAAAACGGTG | 60.5 | 372 | INTERGENIC | | | | |
| CaPOPI_7787 | scaffold4902 | 75211 | ATTTTTTTTTT/ATTTTTTTT | GGTTGTGGCGTTTTCTTCAT | AAAACTTGTTTACATGGACAATGC | 60.0 | 458 | INTERGENIC | | | | |
| CaPOPI_7788 | scaffold4902 | 82598 | TAA/TAAA | TTGTCACACTACAAGGTTGCAT | CCAATCCATTTCCATCAAGC | 58.2 | 635 | INTERGENIC | | | | |
| CaPOPI_7789 | scaffold4902 | 83305 | CTTT/CTTTTT | GCTTGATGGAAATGGATTGG | AAAAACGCAACCAACATCAA | 60.3 | 609 | INTERGENIC | | | | |
| CaPOPI_7790 | scaffold4920 | 19062 | CA/C | CAGAAAATCGATTGCCCAGT | AGTCCAACTAGCAAATGCCG | 60.1 | 497 | INTERGENIC | | | | |
| CaPOPI_7791 | scaffold4923 | 11425 | AATATATATATATATATATATATA/AATATATATATATATATATATA | TTGGATTTTGGCTCATAGGC | CGCGTTAACAGAGGAAGAGG | 60.0 | 392 | INTERGENIC | | | | |
| CaPOPI_7792 | scaffold493 | 25789 | GT/GTCATAGGTCTTTCCCTATT | ACAATCGTGCCTAAATTGCC | TTGGACTGCAAATGAAAGGA | 60.0 | 495 | INTERGENIC | | | | |
| CaPOPI_7793 | scaffold493 | 149227 | CTTTTTTTT/CTTTTTTTTT | AAGCCTATTACAGCGCTCTTTG | GGTCTTTTAAAGCGCTTGTTG | 60.1 | 930 | INTERGENIC | | | | |
| CaPOPI_7794 | scaffold493 | 297027 | AT/ATATATATATGTGT | ATGTTTTGGATTTTGGCTCG | AAATTGGTCCACTAGCACCG | 59.9 | 643 | INTERGENIC | | | | |
| CaPOPI_7795 | scaffold4943 | 1324 | A/AC | GAAGTCGGGTGGTACGAAAA | CGCGGAACCAAAAACATAGT | 60.0 | 731 | INTERGENIC | | | | |

| **INDEL marker IDs** | **Chromosomes/ unanchored scaffolds** | **Physical positions (bp)** | **InDels (*Kabuli* reference genome-CDC Frontier/PI)** | **Forward primers (5'-3')** | **Reverse primers (5'-3')** | **Anneling temperature (0C)** | **Expected amplified product size (bp)** | **Structural annotation** | | **Functional annotation** | | |
|---|---|---|---|---|---|---|---|---|---|---|---|---|
| | | | | | | | | **Sequence components of *kabuli* genome** | ***Kabuli* gene accession IDs** | **NCBI-KOG** | **TFs** | **NCBI-nr database** |
| CaPOPI_7796 | scaffold495 | 33497 | TAAAAAAAAAAAAAAA/TAAAAAAAAAAAAA | AACTCTTTGGCTTTCTGCCA | GGGGAAATTCGCTTTGATTT | 60.0 | 480 | INTERGENIC | | | | |
| CaPOPI_7797 | scaffold495 | 126369 | GAAAAAAAAAA/GAAAAAAAAAAA | TACATTCGCGGTCCCTTAAC | AGCCAAATTTTAGGGAGGCA | 60.0 | 859 | INTERGENIC | | | | |
| CaPOPI_7798 | scaffold496 | 54994 | CAAA/CAAAAGTTGAAAA | GGAATAAGGAAAAGTCGCCC | TCAAGTTGCCACTGACTTGC | 59.9 | 637 | INTERGENIC | | | | |
| CaPOPI_7799 | scaffold496 | 121429 | CT/C | TCCTGGTCCTCGATTTCATC | AATCGCCTGAAAAGACGAGA | 60.0 | 379 | INTERGENIC | | | | |
| CaPOPI_7800 | scaffold496 | 211993 | GTATATATATATAT/GTATATATATAT | CCTTCATGAACCGCAATACA | TCTCCGCATAAGCTTTCGAT | 59.5 | 651 | INTRON | Ca_27203 | | | |
| CaPOPI_7801 | scaffold497 | 69293 | ATTTATTTT/ATTTATTTTATTTT | GGTAGTCAAGCCTGAGGCAG | TCTTGTATGTTTGATTAATGTTAGGTG | 60.0 | 585 | INTERGENIC | | | | |
| CaPOPI_7802 | scaffold497 | 69995 | GTTTTTTTTTTTTT/GTTTTTTTTT | TGGTGCTTTTGGAAAAAGTG | ATTTGGTTTGGTTGCATTCG | 58.8 | 446 | INTERGENIC | | | | |
| CaPOPI_7803 | scaffold497 | 70043 | CAATAAATAAATAAATAAAT/CAATAAATAAAT | TGGTGCTTTTGGAAAAAGTG | ATTTGGTTTGGTTGCATTCG | 58.8 | 446 | INTERGENIC | | | | |
| CaPOPI_7804 | scaffold4978 | 33825 | AGG/AG | CACAATTGGGGGAACAAGAG | CGAACCGACGTCGCTAATAA | 60.3 | 399 | INTERGENIC | | | | |
| CaPOPI_7805 | scaffold498 | 206145 | GTTTTTTTT/GTTTTTTT | CACCGCCTCTGTTCCATATT | GAGATTTAGAACCATCAAATTGTCA | 60.0 | 829 | INTERGENIC | | | | |
| CaPOPI_7806 | scaffold498 | 214228 | CATAATAAAACTAATAATAAAACTAATAAT/CATAATAAAACTAATAATAAAACTAATAATAAAACTAATAAT | TTTCATGGTCATGTTTTGGC | GAAAGGCCAATGTCCAGAAA | 59.4 | 781 | INTERGENIC | | | | |
| CaPOPI_7807 | scaffold498 | 214273 | TACACACACACACAC/TACACACACACAC | TTTCATGGTCATGTTTTGGC | GAAAGGCCAATGTCCAGAAA | 59.4 | 781 | INTERGENIC | | | | |
| CaPOPI_7808 | scaffold498 | 214558 | CTTTTT/CTTTTTT | CACACACACGTAAGAAGGCTG | TTGGACAGAATCAAGGCTCC | 59.4 | 544 | INTERGENIC | | | | |
| CaPOPI_7809 | scaffold4983 | 27276 | ATTT/ATT | AAGGTGGTACGAAAATCCCC | GCACGTGGGAAAATGAGATT | 60.1 | 893 | INTERGENIC | | | | |
| CaPOPI_7810 | scaffold50 | 6994 | AGGG/AGG | ACCTGTGTAGCCGACAAACC | AATCCCGAACCTCCTCAAGT | 60.0 | 769 | INTERGENIC | | | | |

| INDEL marker IDs | Chromosomes/ unanchored scaffolds | Physical positions (bp) | InDels (*Kabuli* reference genome-CDC Frontier/PI) | Forward primers (5'-3') | Reverse primers (5'-3') | Anneling temperature (0C) | Expected amplified product size (bp) | Structural annotation | | Functional annotation | | |
|---|---|---|---|---|---|---|---|---|---|---|---|---|
| | | | | | | | | Sequence components of *kabuli* genome | *Kabuli* gene accession IDs | NCBI-KOG | TFs | NCBI-nr database |
| CaPOPI_7811 | scaffold50 | 86754 | TTTATTATTATTATTATTATTATTATTATTATTATTATT/TTTATTATTATTATTATTATTATTATTATTATTATTATT | CTTCGGGCTCCGATTTTTA | TCTGAAATTCCCAAACCAGG | 60.2 | 857 | INTERGENIC | | | | |
| CaPOPI_7812 | scaffold50 | 191851 | GAAAAAAAAAA/GAAAAAAAAA | GTTCTTCCGCAGAAGTCAGG | GAGATCTCGGTGCTACTGCC | 60.0 | 325 | INTERGENIC | | | | |
| CaPOPI_7813 | scaffold50 | 192238 | A/AGTGATAGAAATT | CTGCCCATTAGCCCTTCATA | CTGAATGCAAGAGAGAGGGG | 60.1 | 291 | INTERGENIC | | | | |
| CaPOPI_7814 | scaffold501 | 66521 | TCC/TCCC | ATTTTTGGACTCCCTTGGCT | AATCCAATGCCACAAACACA | 59.9 | 686 | INTERGENIC | | | | |
| CaPOPI_7815 | scaffold501 | 87430 | CATATATATATATATAT/CATATATATATATATATAT | TGGGTGTGCCTATATACACGTAA | TTCTTTGCCACACACCATGT | 59.3 | 519 | INTERGENIC | | | | |
| CaPOPI_7816 | scaffold5030 | 906 | AAATAATAAT/AAATAAT | TGGGGATACGTAGGAGCAAG | TGTTGGTCCATTTTTGACGA | 60.1 | 432 | INTERGENIC | | | | |
| CaPOPI_7817 | scaffold506 | 18074 | TAAA/TAAAA | CTATGGTAGAGCGGTTTCGC | TTCTCCCGAGTTTTGGTCTC | 59.9 | 487 | INTERGENIC | | | | |
| CaPOPI_7818 | scaffold506 | 18975 | TA/TAA | TCTCGATACCATCGTGGACA | TCCCATTTGATTGCTGAACA | 60.1 | 489 | INTERGENIC | | | | |
| CaPOPI_7819 | scaffold506 | 33758 | TTCT/TT | TGACCAACTCTTTTGTGCCA | AGCCAACCTCTGTCTCATGG | 60.3 | 667 | INTERGENIC | | | | |
| CaPOPI_7820 | scaffold506 | 39512 | GAA/GA | AAACCTTTGAGAAATGCCCA | AATGGAGTTCGGAGATTGGA | 59.5 | 563 | INTERGENIC | | | | |
| CaPOPI_7821 | scaffold5083 | 2034 | AG/A | AACCCAAAACGCTAAACCCT | TCCGTCCAATAAGAGCAAATG | 59.9 | 559 | INTERGENIC | | | | |
| CaPOPI_7822 | scaffold5086 | 3516 | ACC/AC | TAGGGAGGACGAAAATGACG | CCAATCGTGTGCATCCAATA | 60.1 | 469 | INTERGENIC | | | | |
| CaPOPI_7823 | scaffold5089 | 3720 | TAAAA/TAAAAA | AGCCTAGGGAGGACGAAAAA | AAACAATCGTGTGCATCCAA | 60.2 | 541 | INTERGENIC | | | | |
| CaPOPI_7824 | scaffold509 | 266782 | AG/A | GGTGGTAGCTGGTAGGGTGA | TGCACTTGACCTTTCTTCCC | 60.0 | 935 | INTERGENIC | | | | |
| CaPOPI_7825 | scaffold5094 | 2305 | TAA/TA | TGGCACATGAATAGTTCCACA | GCCACCTCATTCAAATATTGC | 60.0 | 669 | INTERGENIC | | | | |

| INDEL marker IDs | Chromosomes/ unanchored scaffolds | Physical positions (bp) | InDels (*Kabuli* reference genome-CDC Frontier/PI) | Forward primers (5'-3') | Reverse primers (5'-3') | Anneling temperature (0C) | Expected amplified product size (bp) | Structural annotation | | Functional annotation | | |
|---|---|---|---|---|---|---|---|---|---|---|---|---|
| | | | | | | | | Sequence components of *kabuli* genome | *Kabuli* gene accession IDs | NCBI-KOG | TFs | NCBI-nr database |
| CaPOPI_7826 | scaffold510 | 85609 | TAACA/TA | TTGTCAAATAAAGTAACAACAAAACG | TTGCAACAATTGTACCCGAA | 58.7 | 995 | INTERGENIC | | | | |
| CaPOPI_7827 | scaffold510 | 86131 | TAAA/TAAAA | TTTTGGTCCCCCAAACATAA | AAAAGTCTCTCCCGCTCCTC | 60.0 | 514 | INTERGENIC | | | | |
| CaPOPI_7828 | scaffold511 | 101350 | AATATATATATATATATATATATATATAT/AATATATATATATATATATATATATATATATAT | CGGTGAATCCTTTACGGAGA | GGAGGAGAAGGAGGGATTTG | 60.1 | 306 | INTERGENIC | | | | |
| CaPOPI_7829 | scaffold511 | 101474 | GAAAAAAAAA/GAAAAAAAA | CGGTGAATCCTTTACGGAGA | GGAGGAGAAGGAGGGATTTG | 60.1 | 306 | INTERGENIC | | | | |
| CaPOPI_7830 | scaffold511 | 193038 | AATATATATATATATATATA/AATATATATATATATATATATA | TTTCAGGTTATCGACTGGGG | AACAAGAAAGCGCAAGCAAT | 59.9 | 729 | INTERGENIC | | | | |
| CaPOPI_7831 | scaffold511 | 194529 | AAA/AAACAA | AATCTGCTTCCGTTAGCTGC | TGCACAATAAACCTGCTTGC | 59.6 | 667 | INTERGENIC | | | | |
| CaPOPI_7832 | scaffold512 | 111039 | GAAAAAAAA/GAAAAAAA | GGTCCTCGATTTCATCTGGA | GACCGGGAGACCCTTATAGC | 60.0 | 168 | INTERGENIC | | | | |
| CaPOPI_7833 | scaffold5120 | 14840 | TTATATATATATATATATATATATATATAT/TTATATATATATATATATATATATATATAT | TATCGTGCCTGTTGAGAACG | CTAATTAGCGTCGGAGTCGG | 59.9 | 527 | INTERGENIC | | | | |
| CaPOPI_7834 | scaffold5120 | 15547 | TAGAGTACGAGA/TAGA | CCGAAGTAATAGTGGGGCAA | GGACCAGGTTCTGCATCAAT | 60.0 | 214 | INTERGENIC | | | | |
| CaPOPI_7835 | scaffold515 | 42759 | C/CT | GGTCCTCGATTTCATCTGGA | TTTCATCTGGACTTTTCGGG | 60.0 | 652 | INTERGENIC | | | | |
| CaPOPI_7836 | scaffold515 | 43016 | GAAAAA/GAAAA | CAAGAGTCTCCTGGTCCTCG | CTCCCGAAAAGTCCAAATGA | 60.0 | 912 | INTERGENIC | | | | |
| CaPOPI_7837 | scaffold515 | 216518 | ATTTTTTTTTTT/ATTTTTTTTTT | TAATAAAATGAGGCGGTGGC | TTAGCATCGTCATTTTCTTCAA | 59.9 | 902 | INTERGENIC | | | | |
| CaPOPI_7838 | scaffold515 | 235422 | CTATATATA/CTATATATATA | TTGCTCCAAATTGATCACGA | AACCGATGCTAATCCGACAC | 60.2 | 818 | INTERGENIC | | | | |
| CaPOPI_7839 | scaffold515 | 266354 | TAG/TAGAG | CCACCCAACAAAAATGGAAC | CATAGAACAAAATTGAGTTGTGAGA | 60.1 | 635 | INTERGENIC | | | | |
| CaPOPI_7840 | scaffold5152 | 20235 | CTTTTTTTTTT/CTTTTTTTTTTT | CCAAGGGGAGCAATTATGAA | TGATGCAATTGTGTGAGTCATT | 59.9 | 366 | INTERGENIC | | | | |

| INDEL marker IDs | Chromosomes/ unanchored scaffolds | Physical positions (bp) | InDels (*Kabuli* reference genome-CDC Frontier/PI) | Forward primers (5'-3') | Reverse primers (5'-3') | Anneling temperature (0C) | Expected amplified product size (bp) | Structural annotation | | Functional annotation | | |
|---|---|---|---|---|---|---|---|---|---|---|---|---|
| | | | | | | | | Sequence components of *kabuli* genome | *Kabuli* gene accession IDs | NCBI-KOG | TFs | NCBI-nr database |
| CaPOPI_7841 | scaffold5158 | 6384 | TAA/TA | CCAAACAAATCTATTCCCCATC | TGATTGTTCTGGACGCATCT | 59.6 | 463 | INTERGENIC | | | | |
| CaPOPI_7842 | scaffold5158 | 17908 | A/AT | CAATTGTGGGCGAATTTCTT | TACCCCCAAAATGGAAATGA | 59.9 | 203 | INTERGENIC | | | | |
| CaPOPI_7843 | scaffold5165 | 1609 | CTTTTTTTTTT/CTTTTTTTTTTTTT | TTTCAGTTAATTTCGTGGTTCC | CAAAATTTTCAAACAAAACGCA | 58.2 | 412 | INTERGENIC | | | | |
| CaPOPI_7844 | scaffold5186 | 6668 | CAAAAAA/CAAAAA | ATTTAGGTCATTTCCGCCCT | TGGATGCACAAGATGGGTTA | 59.8 | 363 | INTERGENIC | | | | |
| CaPOPI_7845 | scaffold5186 | 8653 | GA/G | GAGTGTTGGGGTCGTTGAAT | CCTAGGGAGGCTAAAATGGC | 59.8 | 270 | INTERGENIC | | | | |
| CaPOPI_7846 | scaffold5186 | 27510 | AATATATATATATATATATATATATATATATAT/AATATATATATATATATATATATATAT | TCGTATGCAACCACTAAGCG | AAGGAGGAAGGAGTTGGGAA | 59.9 | 823 | INTERGENIC | | | | |
| CaPOPI_7847 | scaffold520 | 127701 | T/TCATCGC | ATCGAGGATCGAGAAACCCT | GGTCCTCGATTTCATCTGGA | 60.0 | 166 | INTERGENIC | | | | |
| CaPOPI_7848 | scaffold5211 | 3755 | TAAAAAAAAA/TAAAAAAAA | GTTCTCGAAGCAGAAAACGG | CTTTGGTCAAGTCTGGCACA | 60.0 | 702 | INTERGENIC | | | | |
| CaPOPI_7849 | scaffold5251 | 1190 | CTCTTCTT/CTCTT | TGGCCACCATTAGCATTACA | AGCGCAGTGGTGAAAAGAAT | 60.0 | 383 | INTERGENIC | | | | |
| CaPOPI_7850 | scaffold526 | 72192 | GTCTTCC/G | ACCATCCTTGTGCATCCAAT | AGGGAGGACGAAAATGACCT | 60.2 | 170 | INTERGENIC | | | | |
| CaPOPI_7851 | scaffold527 | 4805 | AT/ATTACTT | TCGTTGGTGCATTATTTCCA | TTGCAAGAAGGGGTTTTGAC | 59.9 | 437 | INTERGENIC | | | | |
| CaPOPI_7852 | scaffold527 | 52781 | TTATATATATATATAT/TTATATATATATATATAT | CTTAATGCCCTCCTTGGACA | AGGAAGAGTGGAGTTGCGAA | 60.1 | 605 | INTERGENIC | | | | |
| CaPOPI_7853 | scaffold527 | 108544 | ATATTATTATTATTATTATTATTATTATTATTATTATTATTATTATTATTATTATTAT/ATATTATTATTATTATTATTATTATTATTATTATTATTATTATTATTATTATTATTAT | GGTGGAGTATTCTGCCGAGA | ATGGGTGTTTGTGGTGGATT | 60.2 | 578 | INTERGENIC | | | | |
| CaPOPI_7854 | scaffold528 | 69096 | AG/A | TGGATGCTTTCTATGATGTGC | TATCGATCACTGGTGCCAAA | 58.7 | 868 | INTERGENIC | | | | |
| CaPOPI_7855 | scaffold528 | 183983 | CAAAAAA/CAAAAA | TGAACTCAAAATTCGATTTGTCT | TGAAAAACATTTGCCTGAACA | 57.9 | 651 | INTERGENIC | | | | |

| INDEL marker IDs | Chromosomes/ unanchored scaffolds | Physical positions (bp) | InDels (*Kabuli* reference genome-CDC Frontier/PI) | Forward primers (5'-3') | Reverse primers (5'-3') | Anneling temperature (0C) | Expected amplified product size (bp) | Structural annotation | | Functional annotation | | |
|---|---|---|---|---|---|---|---|---|---|---|---|---|
| | | | | | | | | **Sequence components of *kabuli* genome** | ***Kabuli* gene accession IDs** | **NCBI-KOG** | **TFs** | **NCBI-nr database** |
| CaPOPI_7856 | scaffold528 | 215682 | CAA/CAAATAA | TTTCAATTTATGTCAATCGATTTTT | TGCGAGAATGAGAAGTTGTTG | 58.2 | 572 | INTERGENIC | | | | |
| CaPOPI_7857 | scaffold528 | 241367 | ATTTT/ATTTTT | TGAGGCGTTACAAGAGGTGA | ATGTGCTCCCTCCAACAATC | 59.4 | 443 | INTERGENIC | | | | |
| CaPOPI_7858 | scaffold528 | 312579 | A/AAAT | TTGAGGGAAGGCATAATTGC | TGGAATAGTGTGGGGGAAAA | 60.0 | 294 | INTERGENIC | | | | |
| CaPOPI_7859 | scaffold53 | 1354 | T/TG | ACCACAATGTAACACCCCGT | CGATTGAATTAAGCCCAGGA | 60.0 | 788 | INTERGENIC | | | | |
| CaPOPI_7860 | scaffold53 | 137170 | CTT/CTTTT | GGACTGGTGGCTCAAAGGTA | TCGACCGGGAGTTTTGATTA | 60.1 | 671 | INTERGENIC | | | | |
| CaPOPI_7861 | scaffold530 | 10667 | CG/C | ACCAAATTTGTCGAACCAGC | TTGAGTGTGAGTGTCCAGGG | 60.0 | 342 | INTRON | Ca_27188 | J | | Ribosomal protein L14b/L23e |
| CaPOPI_7862 | scaffold5306 | 9364 | GAAAAAAAAAA/GAAAAAAAAAAA | TAGATGGGTGTGACGGTGAA | TGAGGGAGCACTATTGGGAG | 60.0 | 590 | INTERGENIC | | | | |
| CaPOPI_7863 | scaffold531 | 11911 | A/AT | ATTCCATTTGGCATGCTTTC | TTTGGGTACAAAATCAGAAAATTG | 59.9 | 892 | INTRON | Ca_22388 | | | Domain of unknown function DUF231, plant |
| CaPOPI_7864 | scaffold531 | 83931 | CCGG/CCGGCCGGTTCGG | GAGAACGCAAGCTAACGGAC | ACCTTGTGGATGGCAGTTTC | 60.0 | 180 | DRR | Ca_22395 | | | |
| CaPOPI_7865 | scaffold535 | 94648 | GATATATATAT/GATATATATATAT | AATGGAGGAGAAGGGAGGAA | TGAAAAGGGGTAGGCAACAC | 60.0 | 563 | INTERGENIC | | | | |
| CaPOPI_7866 | scaffold5420 | 685 | ACC/ACCC | AAGGGAGGATGGAAATGACC | ATTTTTGTCCTCCTTGGGCT | 60.1 | 613 | INTERGENIC | | | | |
| CaPOPI_7867 | scaffold543 | 69059 | TGGGG/TGGGGG | TCGCTACTGAAGATGGGCTT | TTGATAACTTGAAAACGAAATGAAA | 60.0 | 363 | CDS (FRAME SHIFT) | Ca_24780 | | | |
| CaPOPI_7868 | scaffold543 | 229568 | ATTTTT/ATTTT | TTTGCCCCCAACTCTACATC | ATGGGCACATGCTATCATCA | 59.9 | 755 | DRR | Ca_24786 | | | |
| CaPOPI_7869 | scaffold544 | 41662 | CT/CTT | GTACCCTGTGGATGGGAATG | TGGATGCACTCGATTGGTAA | 60.1 | 606 | INTERGENIC | | | | |
| CaPOPI_7870 | scaffold544 | 58853 | ATTT/ATTTT | GCATTCGATTGGTAAAACCC | AGGTCATTTTCGTCCTCCCT | 59.3 | 261 | INTERGENIC | | | | |

| INDEL marker IDs | Chromosomes/ unanchored scaffolds | Physical positions (bp) | InDels (*Kabuli* reference genome-CDC Frontier/PI) | Forward primers (5'-3') | Reverse primers (5'-3') | Anneling temperature (0C) | Expected amplified product size (bp) | Structural annotation | | Functional annotation | | |
|---|---|---|---|---|---|---|---|---|---|---|---|---|
| | | | | | | | | Sequence components of *kabuli* genome | *Kabuli* gene accession IDs | NCBI-KOG | TFs | NCBI-nr database |
| CaPOPI_7871 | scaffold544 | 82660 | AGGG/AGG | AATCCAATGACCCCAATGAA | TCATTCTCATCCTCCCAAGG | 60.0 | 190 | INTERGENIC | | | | |
| CaPOPI_7872 | scaffold544 | 147706 | CAAAAA/CAAAA | CCCCCTTTTTGCATAAGTGA | GAGGAGACATCTGCTGAGGG | 59.9 | 582 | DRR | Ca_26540 | | | |
| CaPOPI_7873 | scaffold544 | 342056 | GTTTTTTTTT/GTTTTTTTTTT | CGGGGTGTTACACTCATCCT | CGACATCCGTCAAACACAAC | 59.8 | 846 | INTERGENIC | | | | |
| CaPOPI_7874 | scaffold545 | 116816 | ATATTATTATTATTATTATTATTATTATTATTATTATTATT/ATATTATTATTATTATTATTATTATTATTATTATTATTATTATTATTATTATT | TTGCGGCCTACATTCTTAGG | TCACATTCATTTCCAAAGCG | 60.2 | 775 | INTERGENIC | | | | |
| CaPOPI_7875 | scaffold545 | 193109 | CAAAAA/CAAAAAA | AATCTCCCCCTGAGTTTTGG | ATCTTTGGACACCGTCGTTC | 60.3 | 643 | INTERGENIC | | | | |
| CaPOPI_7876 | scaffold546 | 67935 | AG/A | TTTGGACCAATTTTGTGGCT | TGCAAAAACCACTTTCAAGG | 60.3 | 652 | INTERGENIC | | | | |
| CaPOPI_7877 | scaffold547 | 96984 | CAAAA/CAAAAA | CACGATGGCTAGTGCTTGAA | CGATCGGTATGAACATTGACC | 60.0 | 276 | INTERGENIC | | | | |
| CaPOPI_7878 | scaffold547 | 155914 | ATCAAA/ATCAAAGTTCAAA | TCGATCTGTACGAACATTGACC | CGGAACCCGAAACACTTTTA | 60.0 | 295 | INTERGENIC | | | | |
| CaPOPI_7879 | scaffold547 | 156143 | CAAA/CAAAA | TTCGGTCTATTTTTGTCGGG | CGGAACCCGAAACACTTTTA | 59.9 | 219 | INTERGENIC | | | | |
| CaPOPI_7880 | scaffold548 | 114800 | ACCCC/ACCCCC | AATCGACATTCGACCCACTC | CCCCGCTCTGATAGGTGATA | 59.9 | 384 | INTERGENIC | | | | |
| CaPOPI_7881 | scaffold548 | 212010 | G/GA | GGAACACTTCGGTGTGGACT | CATGTCCCGAAAAGAGCAAT | 60.0 | 563 | DRR | Ca_21489 | | | |
| CaPOPI_7882 | scaffold548 | 238316 | AATATATATATATATATATATATATATATATATATA/AATATATATATATATATATATATATATATATATATATATATATA | AACAAACTCCTTCGCTCCAA | GTGCTTGCTAAACACAAGCG | 59.9 | 470 | INTERGENIC | | | | |
| CaPOPI_7883 | scaffold548 | 523039 | GTTATTATTATTATTATTATTATTATTATTATTATTATTATTATTATTATTATT/GTTATTATTATTATTATTATTATTATTATTATTATTATTATTATTATTATTATTAT | ATTTTTGCCTAGGTTGCCCT | CTAAATGAAGAGCCGCAAGG | 60.0 | 170 | INTERGENIC | | | | |
| CaPOPI_7884 | scaffold548 | 593356 | CATATATATATATATATATATATATATATATA/CATATATATATATATATATATATATATATATATATATATATA | CTAGCACCTTAGCGTGGAGG | AAAACGTGGTTTGGGAAGTG | 60.0 | 481 | INTERGENIC | | | | |
| CaPOPI_7885 | scaffold548 | 612534 | A/AT | TATTTTGGATTTTGGCTCGG | AGGAAGAGTGGAGTTGCGAA | 59.9 | 416 | INTERGENIC | | | | |

| INDEL marker IDs | Chromosomes/ unanchored scaffolds | Physical positions (bp) | InDels (*Kabuli* reference genome-CDC Frontier/PI) | Forward primers (5'-3') | Reverse primers (5'-3') | Anneling temperature (0C) | Expected amplified product size (bp) | Structural annotation | | Functional annotation | | |
|---|---|---|---|---|---|---|---|---|---|---|---|---|
| | | | | | | | | Sequence components of *kabuli* genome | *Kabuli* gene accession IDs | NCBI-KOG | TFs | NCBI-nr database |
| CaPOPI_7886 | scaffold549 | 19237 | ATTTTTTTT/ATTTTTTT | AATTGGCCACGTCAAAGAAC | TGACGAAATCATTCGTTTGG | 60.0 | 531 | INTERGENIC | | | | |
| CaPOPI_7887 | scaffold549 | 41362 | CTATATATATATATATATATATATATAT/CTATATATATATATATATATATATATATAT | TGTCGTGCCATAAATATCCG | CAAGCATGATCCAGGGAGAT | 59.4 | 522 | INTERGENIC | | | | |
| CaPOPI_7888 | scaffold549 | 44384 | CATATATATAT/CATATATATATATATATAT | TCAATGCAGAAAACTCAACCT | AGGTGGTGTTGGTGAAGGAG | 57.5 | 518 | INTERGENIC | | | | |
| CaPOPI_7889 | scaffold549 | 61007 | GTATATATATATATATA/GTATATATATATATA | TGCCAATTCGAAGATCAAAA | CACCCCTTACCTCGACTGAA | 59.2 | 570 | INTERGENIC | | | | |
| CaPOPI_7890 | scaffold5504 | 1323 | T/TTA | ACCAATCAAGTGCATCCCAT | TAAGCCTAAGGAGGACGCAA | 60.2 | 175 | INTERGENIC | | | | |
| CaPOPI_7891 | scaffold5514 | 3914 | TG/TGG | AGGAAGAGTGGAGTTGCGAA | CTTGGGAGTTGGAGAGCAAG | 60.0 | 489 | INTERGENIC | | | | |
| CaPOPI_7892 | scaffold553 | 2669 | CTT/CT | TTGGTTCACTCCCTGGATTC | AACATCAGTCAAGCGCACAA | 59.9 | 484 | INTERGENIC | | | | |
| CaPOPI_7893 | scaffold553 | 2745 | TAAAAAAA/TAAAAAAAA | TTGGTTCACTCCCTGGATTC | AACATCAGTCAAGCGCACAA | 59.9 | 484 | INTERGENIC | | | | |
| CaPOPI_7894 | scaffold553 | 325514 | CT/CTAT | ATCGGAAGTTGGACAGGTTG | TGGCAAATCAGCAGAGACAC | 60.0 | 317 | INTRON | Ca_19934 | L | | |
| CaPOPI_7895 | scaffold553 | 871030 | TTATATATATATATATATATATATATATATATATATAT/TTATATATATATATATATATATATATATATATATATAT | TCGTCACTCTTAAACCGCCT | AACCCATCGCAAAAGAGAAA | 59.9 | 652 | INTERGENIC | | | | |
| CaPOPI_7896 | scaffold5551 | 550 | CTTTTTTT/CTTTTTTTT | GATACGTTGGAGCATCGACA | CACGAAACACCCTCTTCCAT | 59.7 | 230 | INTERGENIC | | | | |
| CaPOPI_7897 | scaffold556 | 1805 | G/GC | TCATTTTCGTTCTCCCAAGG | TGGATGCACAAGATGGGTTA | 60.0 | 664 | INTERGENIC | | | | |
| CaPOPI_7898 | scaffold5580 | 1246 | GAAAAA/GAAAAAA | CGAGGATCGATTGTCCCTTA | GGTCCTCGATTTCATCTGGA | 60.0 | 824 | INTERGENIC | | | | |
| CaPOPI_7899 | scaffold5598 | 9627 | TAAAAAAAAAAAAAA/TAAAAAAAAAAAAAAAA | TTTCCAACAAGTGAGGGGTC | AAAAACCCCATCCAACAACA | 59.9 | 472 | INTERGENIC | | | | |
| CaPOPI_7900 | scaffold5598 | 13569 | ATTTTTTTTTTTTT/ATTTTTTTTTTT | CGGCATTGGATCATGGTTAG | TTTCGACTGCCTAGCGATTT | 61.2 | 549 | INTERGENIC | | | | |

| INDEL marker IDs | Chromosomes/ unanchored scaffolds | Physical positions (bp) | InDels (*Kabuli* reference genome-CDC Frontier/PI) | Forward primers (5'-3') | Reverse primers (5'-3') | Anneling temperature (0C) | Expected amplified product size (bp) | Structural annotation | | Functional annotation | | |
|---|---|---|---|---|---|---|---|---|---|---|---|---|
| | | | | | | | | Sequence components of *kabuli* genome | *Kabuli* gene accession IDs | NCBI-KOG | TFs | NCBI-nr database |
| CaPOPI_7901 | scaffold560 | 30079 | TATAATAAATAATAAATAA/TATAATAAATAATAAATAATAAATAA | GCATTCTCGTTCATCTCACG | CGAATCTTGATTTCGGTAACCT | 59.4 | 906 | INTERGENIC | | | | |
| CaPOPI_7902 | scaffold560 | 156538 | ATTTTTTTT/ATTTTTTTTTTT | AGGTGGTGTTGGTGAAGGAG | TCAATGCAGAAAACTCAACCT | 60.0 | 522 | INTERGENIC | | | | |
| CaPOPI_7903 | scaffold560 | 160741 | ATT/AT | TGTTCATCATGAGGAGAGGG | TGGTCCAAATGAATGAGTCTTG | 58.6 | 360 | INTERGENIC | | | | |
| CaPOPI_7904 | scaffold5602 | 4886 | TG/TGG | ACAAAGGAATGAGTGGCACC | TCAAGTCTCGCACAACAACC | 60.0 | 347 | INTERGENIC | | | | |
| CaPOPI_7905 | scaffold561 | 25431 | CTCTTTCT/CTCT | TTGATTCATCAAACACCGGA | AAATTGGGTTCTGCCAATCA | 59.9 | 399 | DRR | Ca_26215 | | MYB_related | |
| CaPOPI_7906 | scaffold564 | 2634 | ATTTTT/ATTTT | TCGAATGCTCGACTTTACCC | GCGGAATAACATGGTTGCTT | 60.2 | 641 | INTERGENIC | | | | |
| CaPOPI_7907 | scaffold564 | 5212 | G/GAAC | TGGGATGCAAACAATTCTCA | TTGTCAATTTGGGAGTGCAA | 60.0 | 560 | INTERGENIC | | | | |
| CaPOPI_7908 | scaffold564 | 8317 | TAAAAAA/TAAAAAAA | CAACATCCAAATTTTGTTATAGAGC | CGACATGCATGCCTTATTTG | 58.3 | 143 | INTERGENIC | | | | |
| CaPOPI_7909 | scaffold564 | 13897 | T/TG | GAGCACATTGGAGGACACTG | CGGAGATAAAAACAATTGGCA | 59.3 | 659 | INTERGENIC | | | | |
| CaPOPI_7910 | scaffold564 | 15097 | TTAT/TTATAT | TGGAGAAATGCTAAATCCCG | GATGGCAGGTAAAAGCGAAA | 60.0 | 599 | INTERGENIC | | | | |
| CaPOPI_7911 | scaffold564 | 15227 | ATATCTATTATCTATTAT/ATATCTATTAT | TGGAGAAATGCTAAATCCCG | AGATGGCAGGTAAAAGCGAA | 60.0 | 600 | INTERGENIC | | | | |
| CaPOPI_7912 | scaffold564 | 25971 | TA/TAGTTTATTAATAGTTAA | GTATCTGAGCCTCTGCCAGC | AATTGGTGTGTCTGCGTCAA | 60.1 | 555 | INTERGENIC | | | | |
| CaPOPI_7913 | scaffold564 | 26112 | A/AG | GTATCTGAGCCTCTGCCAGC | AATTGGTGTGTCTGCGTCAA | 60.1 | 555 | INTERGENIC | | | | |
| CaPOPI_7914 | scaffold571 | 18167 | TTACATCTCATA/TTA | CCTCACGGTTTCAATTGCTT | CTTAATGCACAGCCTACCCC | 60.1 | 670 | INTRON | Ca_27520 | T | NAC | Serine-threonine/tyrosine-protein kinase |
| CaPOPI_7915 | scaffold571 | 41234 | GAAAA/GAAA | GAGGGCAAAGATGACCTGAA | AGGTTGTTTTCGTCCTCCCT | 60.2 | 693 | INTERGENIC | | | | |

| INDEL marker IDs | Chromosomes/ unanchored scaffolds | Physical positions (bp) | InDels (*Kabuli* reference genome-CDC Frontier/PI) | Forward primers (5'-3') | Reverse primers (5'-3') | Anneling temperature (0C) | Expected amplified product size (bp) | Structural annotation | | Functional annotation | | |
|---|---|---|---|---|---|---|---|---|---|---|---|---|
| | | | | | | | | Sequence components of *kabuli* genome | *Kabuli* gene accession IDs | NCBI-KOG | TFs | NCBI-nr database |
| CaPOPI_7916 | scaffold5730 | 659 | ATTTTTTTTTTTTTTTTT/ATTTTTTTTT | ACAAAGGCAACCTTTTGTGG | CTGCCCACCGTTGTTATTCT | 60.0 | 866 | INTERGENIC | | | | |
| CaPOPI_7917 | scaffold575 | 65125 | G/GT | TTGAAGTTAAGGGGTGCTGC | TCCATCCAACCTTGTGGATT | 60.2 | 531 | DRR | Ca_27003 | | | |
| CaPOPI_7918 | scaffold575 | 66804 | CTTTTTTTT/CTTTTTTT | AACCGGATGTTGGGATGTAA | CCCATTCACTCCCACTCTGT | 60.1 | 348 | INTERGENIC | | | | |
| CaPOPI_7919 | scaffold575 | 67025 | ATTTAGGGTA/ATTTAGGGTATGGTTTAGGGTA | ACAGAGTGGGAGTGAATGGG | CCTAAGCCTGACCCTACCCT | 60.0 | 397 | INTERGENIC | | | | |
| CaPOPI_7920 | scaffold575 | 67830 | TGGTTTAGGGTTTAGGGTT/TGGTTTAGGGTT | GGGTTAGGGTAGGGTCAGGA | AGGCTGGAATCGGTAAGTGA | 60.2 | 357 | INTERGENIC | | | | |
| CaPOPI_7921 | scaffold575 | 82549 | TAAAAAA/TAAAAAAA | GCAATAACGAAATGGCTGTG | CCCTAACTCCCTAAACCCCA | 59.2 | 101 | INTERGENIC | | | | |
| CaPOPI_7922 | scaffold575 | 89948 | TAAAAAAA/TAAAAAAAAA | GGATTGGCCCCAAATAGAAT | CCCTTGTCATTGCTGCTGTA | 60.0 | 722 | INTERGENIC | | | | |
| CaPOPI_7923 | scaffold575 | 90216 | G/GA | CCCAAATTGCAATAACCAGG | TACCGTTTCCCACACTCACA | 60.2 | 637 | INTERGENIC | | | | |
| CaPOPI_7924 | scaffold575 | 91034 | AATATATATATATATATATATATAT/AATATATATATATATATATATAT | TGTGAGTGTGGGAAACGGTA | GGCAATGTGCATAAACAAAAA | 60.0 | 426 | INTERGENIC | | | | |
| CaPOPI_7925 | scaffold575 | 98810 | TC/T | TAAGCCAGGGCTTTCTTTCA | CCTCCACTCCAAACCTACCA | 60.0 | 404 | INTERGENIC | | | | |
| CaPOPI_7926 | scaffold575 | 99188 | CT/CTGT | TGGTAGGTTTGGAGTGGAGG | GGCTAAGTCTGAAAGTGGCG | 60.0 | 263 | INTERGENIC | | | | |
| CaPOPI_7927 | scaffold575 | 103253 | AGGGG/AGGGGG | AAACAACGAGTATGGGCGAC | AGCTTGCTTTGAAAGGGACA | 60.0 | 113 | INTERGENIC | | | | |
| CaPOPI_7928 | scaffold575 | 103640 | ACCCCCC/ACCCC | AAACAACGAGTATGGGCGAC | CGCAATTAGCCTCTTTCGTC | 60.0 | 707 | INTERGENIC | | | | |
| CaPOPI_7929 | scaffold575 | 103819 | AT/ATGCCTTATT | TCTTCTCGCTGACTTGGGAT | CGCAATTAGCCTCTTTCGTC | 60.0 | 205 | INTERGENIC | | | | |
| CaPOPI_7930 | scaffold575 | 110510 | GT/GCTT | CTTTCTGGTCAAGGCCTCAG | CTAGACCTGCGGAAGACAGG | 60.0 | 446 | INTERGENIC | | | | |

| INDEL marker IDs | Chromosomes/ unanchored scaffolds | Physical positions (bp) | InDels (*Kabuli* reference genome-CDC Frontier/PI) | Forward primers (5'-3') | Reverse primers (5'-3') | Anneling temperature (0C) | Expected amplified product size (bp) | Structural annotation | | Functional annotation | | |
|---|---|---|---|---|---|---|---|---|---|---|---|---|
| | | | | | | | | Sequence components of *kabuli* genome | *Kabuli* gene accession IDs | NCBI-KOG | TFs | NCBI-nr database |
| CaPOPI_7931 | scaffold575 | 111632 | TA/TAAACAAATAA | GTTCCTCGCTGCTTATCGAC | TTTAACAACCGTGGGCTAGG | 60.0 | 229 | INTERGENIC | | | | |
| CaPOPI_7932 | scaffold575 | 113273 | ATT/AT | TTCGGCAACATTCCGTATTT | GCCTGACCTATGGCCTATCA | 60.3 | 283 | INTERGENIC | | | | |
| CaPOPI_7933 | scaffold575 | 113513 | ATCTC/ATCTCTC | TGATAGGCCATAGGTCAGGC | GCTAAGTGGTCTTTCCGCTG | 60.1 | 440 | INTERGENIC | | | | |
| CaPOPI_7934 | scaffold575 | 113710 | GAAAAAA/GAAAAAAA | TGATAGGCCATAGGTCAGGC | GCTAAGTGGTCTTTCCGCTG | 60.1 | 440 | INTERGENIC | | | | |
| CaPOPI_7935 | scaffold5761 | 842 | CTTTT/CTTTTT | GGTCCTCGATTTCATCTGGA | AACGCCTTAAAAGCCCAAAT | 60.0 | 684 | INTERGENIC | | | | |
| CaPOPI_7936 | scaffold5761 | 1101 | C/CG | TCCTGGTCCTCGATTTCATC | AACGCCTTAAAAGCCCAAAT | 60.0 | 362 | INTERGENIC | | | | |
| CaPOPI_7937 | scaffold5777 | 16517 | CTT/CTTT | TTGCAATTGGCACACAAAAT | TGAGTTGCTTCAAAGTGATGTG | 60.0 | 742 | INTERGENIC | | | | |
| CaPOPI_7938 | scaffold5777 | 16560 | TAATAAATAAATAAATA/TAATAAATAAATAAATAAATA | TTGCAATTGGCACACAAAAT | TGAGTTGCTTCAAAGTGATGTG | 60.0 | 742 | INTERGENIC | | | | |
| CaPOPI_7939 | scaffold578 | 11617 | CTTTTTTTT/CTTTTTT | TTCATTTGATCTTTTCGGGG | CCTCGAGAAAAATGGACCAA | 59.9 | 513 | INTERGENIC | | | | |
| CaPOPI_7940 | scaffold578 | 38009 | C/CGGTCTT | GACTTTTCGAGCGATTTTCG | ATCGAGGATGAGGAGACCCT | 60.0 | 312 | INTERGENIC | | | | |
| CaPOPI_7941 | scaffold5780 | 2877 | AC/A | GGAATCCGGTTTCTTTCCAT | GACCATTATTCACACGGTGG | 60.1 | 765 | INTERGENIC | | | | |
| CaPOPI_7942 | scaffold5780 | 5394 | CAAAAAAA/CAAAAAAAAAA | CGTGTTCTCCCCGTGTATTT | ATGTGACACCCTAAACCCCA | 59.9 | 488 | INTERGENIC | | | | |
| CaPOPI_7943 | scaffold5780 | 5464 | ATT/AT | CGTGTTCTCCCCGTGTATTT | ATGTGACACCCTAAACCCCA | 59.9 | 488 | INTERGENIC | | | | |
| CaPOPI_7944 | scaffold5780 | 5895 | ATT/ATTT | TGGGGTTTAGGGTGTCACAT | AGGCTTTTCAGGCCCTATCT | 60.1 | 659 | INTERGENIC | | | | |
| CaPOPI_7945 | scaffold5780 | 8742 | CTTTTT/CTTTT | AAGTTCCGCATTAGGCCTTT | GTTTGGGTCAAGGCTATGGA | 60.1 | 444 | INTERGENIC | | | | |

| INDEL marker IDs | Chromosomes/ unanchored scaffolds | Physical positions (bp) | InDels (*Kabuli* reference genome-CDC Frontier/PI) | Forward primers (5'-3') | Reverse primers (5'-3') | Anneling temperature (0C) | Expected amplified product size (bp) | Structural annotation | | Functional annotation | | |
|---|---|---|---|---|---|---|---|---|---|---|---|---|
| | | | | | | | | Sequence components of *kabuli* genome | *Kabuli* gene accession IDs | NCBI-KOG | TFs | NCBI-nr database |
| CaPOPI_7946 | scaffold5780 | 9297 | GAAAC/GAAACGTCAATAAAAAC | TCCATAGCCTTGACCCAAAC | CCCACTGCTTGCAATAATGA | 59.9 | 404 | INTERGENIC | | | | |
| CaPOPI_7947 | scaffold5781 | 12542 | A/AT | CAAGGCTGTTTCAGCATTCA | TTGTCCGTGAGAGACTGCAC | 60.0 | 574 | INTERGENIC | | | | |
| CaPOPI_7948 | scaffold5792 | 19474 | TTATATATATATATATATATATATATAT/TTATATATATATATATATATATAT | CCCTTTTCTCACCATCGAAA | AAAATCTCCTTGACACCCCC | 60.0 | 424 | INTERGENIC | | | | |
| CaPOPI_7949 | scaffold5812 | 30560 | G/GC | GTGCTCCATGTTTTCCCAGT | CCGGGATCCTTAGGGTATGT | 60.0 | 515 | INTRON | Ca_27755 | | | |
| CaPOPI_7950 | scaffold5823 | 938 | GAA/GA | ACACACCTATTGGACGCACA | AGGTCATTTTCGTCCTCCCT | 60.0 | 278 | INTERGENIC | | | | |
| CaPOPI_7951 | scaffold583 | 4145 | CTTTTTTT/CTTTTTT | TGAATGAAACCAATGCCAAA | TGATTGCAAATAGCACCCAA | 59.9 | 152 | INTERGENIC | | | | |
| CaPOPI_7952 | scaffold583 | 129627 | TAAAAA/TAAAA | GCTTACGGTGAAGGAGAACG | CAACACATATTTTAAACACATGCAA | 59.9 | 770 | INTERGENIC | | | | |
| CaPOPI_7953 | scaffold583 | 164184 | G/GA | CTCTCATCTGAGGGCAAAGC | ATTAAGTCAGGGGCTCCGTT | 60.1 | 389 | INTERGENIC | | | | |
| CaPOPI_7954 | scaffold584 | 233927 | AGCCGAGG/AG | ATTGGATGCATACGGTTGGT | AGGTCATTTTCGTCCTCCCT | 60.1 | 271 | INTERGENIC | | | | |
| CaPOPI_7955 | scaffold5867 | 874 | TA/T | TCCAGATGAATTCGAGGACC | CTGAACGTTTTGGGCAATTT | 60.0 | 431 | INTERGENIC | | | | |
| CaPOPI_7956 | scaffold587 | 18087 | T/TCA | GTCCTTGCGCTTATGCTTGT | TGCTTGATTGCTTGAGATCC | 60.4 | 347 | DRR | Ca_27621 | | | |
| CaPOPI_7957 | scaffold587 | 23045 | AC/A | CCCGAAAAGTCCAGATGAAA | GCGATTTCCGTACCACCTAA | 60.0 | 329 | INTERGENIC | | | | |
| CaPOPI_7958 | scaffold587 | 50853 | TAAAAA/TAAAAAA | AGGGGAATTTTCGTACCACC | ATCGAAGACCAGGAGACCCT | 60.1 | 638 | INTERGENIC | | | | |
| CaPOPI_7959 | scaffold587 | 51041 | CAAAAAAAAAAA/CAAAAAAAA | AGGGGAATTTTCGTACCACC | ATCGAAGACCAGGAGACCCT | 60.1 | 638 | INTERGENIC | | | | |
| CaPOPI_7960 | scaffold587 | 53018 | CAAAAA/CAAAA | TACACGAAACCCGAAACACA | CGAGGACCAGGAGACCATTA | 60.0 | 749 | INTERGENIC | | | | |

| INDEL marker IDs | Chromosomes/ unanchored scaffolds | Physical positions (bp) | InDels (*Kabuli* reference genome-CDC Frontier/PI) | Forward primers (5'-3') | Reverse primers (5'-3') | Anneling temperature (0C) | Expected amplified product size (bp) | Structural annotation | | Functional annotation | | |
|---|---|---|---|---|---|---|---|---|---|---|---|---|
| | | | | | | | | Sequence components of *kabuli* genome | *Kabuli* gene accession IDs | NCBI-KOG | TFs | NCBI-nr database |
| CaPOPI_7961 | scaffold587 | 54888 | AATATATATATATATATA/AATATATATATATATA | CAAGACATGCGAAAGTCGAA | TCCAGCTTCCTCAAAGCCTA | 60.0 | 484 | INTERGENIC | | | | |
| CaPOPI_7962 | scaffold587 | 56115 | GAA/GAAA | AACTGCCAAAGAGATGTGGG | TGCAGTTCCATCTCATGCTC | 60.1 | 338 | INTERGENIC | | | | |
| CaPOPI_7963 | scaffold587 | 70502 | CAAA/CAAAA | GATGAAATCCAGGACCGAGA | TTCGAACGATTTTCGTACCC | 60.0 | 732 | INTERGENIC | | | | |
| CaPOPI_7964 | scaffold589 | 178513 | ACCCC/ACCC | TCAGAAGGCTTCACTCGGAT | GTTTGGGGTTACCAATGCAC | 60.0 | 693 | INTERGENIC | | | | |
| CaPOPI_7965 | scaffold589 | 309093 | TTAAAAAA/T | TTTGAAAAACGGGGTGCTAC | GTTTTGGCGTTGGTTTCACT | 60.0 | 886 | INTERGENIC | | | | |
| CaPOPI_7966 | scaffold5891 | 5025 | TTATATAT/TTATAT | GCCAATCGATTATCGCAAGT | CCCTGTGACAAACACAATCG | 60.1 | 481 | DRR | Ca_27904 | | | |
| CaPOPI_7967 | scaffold590 | 3079 | AT/ATT | AATAAAAGACCAAATCGGAAAAA | TGACAACCATTTATATTTGCCAC | 58.2 | 393 | INTERGENIC | | | | |
| CaPOPI_7968 | scaffold590 | 3166 | TTATATATATATATATA/TTATATATATATATA | AATAAAAGACCAAATCGGAAAAA | TCAACCAAAGTTGGAGGATG | 58.2 | 578 | INTERGENIC | | | | |
| CaPOPI_7969 | scaffold590 | 19258 | TT/TTACCATAGT | TCCTAAGAAAGCTACCCCACC | TGGCAAAGCACAAGACACTC | 59.6 | 398 | INTERGENIC | | | | |
| CaPOPI_7970 | scaffold590 | 19474 | AG/AGG | CTATCCATGCATGTGCAACAC | TGGCAAAGCACAAGACACTC | 60.0 | 175 | INTERGENIC | | | | |
| CaPOPI_7971 | scaffold590 | 96189 | TTATATATATATAT/TTATATATATATATAT | AGGAAGAGTGGAGTTGCGAA | TATTTTGGATTTTGGCTCGG | 60.0 | 385 | INTERGENIC | | | | |
| CaPOPI_7972 | scaffold5901 | 5039 | CAT/CATATAT | AATCGTGGCACTTTTGCTCT | TTCAGGATTGTTGGGGAATC | 59.9 | 493 | INTERGENIC | | | | |
| CaPOPI_7973 | scaffold5904 | 17347 | TG/T | TCCGTCGAATAACTTGTGGA | ACACCTTCTTTTGCCCTTGA | 59.1 | 958 | INTERGENIC | | | | |
| CaPOPI_7974 | scaffold5904 | 23362 | CTATATATATATAT/CTATATATATATATATAT | AATGGAAGGAACCGAGTCAG | GGTCCAAGATCCATCCATGT | 59.1 | 854 | INTERGENIC | | | | |
| CaPOPI_7975 | scaffold5916 | 1178 | CAAAA/CAAAAA | TGACACCATTCTCTAGGCCC | AGGGAGGACGAAAATGACCT | 60.1 | 310 | INTERGENIC | | | | |

| INDEL marker IDs | Chromosomes/ unanchored scaffolds | Physical positions (bp) | InDels (*Kabuli* reference genome-CDC Frontier/PI) | Forward primers (5'-3') | Reverse primers (5'-3') | Anneling temperature (0C) | Expected amplified product size (bp) | Structural annotation | | Functional annotation | | |
|---|---|---|---|---|---|---|---|---|---|---|---|---|
| | | | | | | | | Sequence components of *kabuli* genome | *Kabuli* gene accession IDs | NCBI-KOG | TFs | NCBI-nr database |
| CaPOPI_7976 | scaffold592 | 18365 | GTTTTTTT/GTTTTTTTT | TAACCAATCTTGTGCGTCCA | AGGGAGGACGAAAATGACCT | 60.1 | 873 | INTERGENIC | | | | |
| CaPOPI_7977 | scaffold592 | 21132 | TT/TTAT | TTTTGGTCCTCCCTAGGCTT | GGGCGAAAATGACCTAAATG | 60.1 | 807 | INTERGENIC | | | | |
| CaPOPI_7978 | scaffold5920 | 7700 | AATATATATATATATATATATATATATAT/AATATATATATATATATATATATATATATATAT | CAAACCGACTAATCACTCCTAAA | CAGTTGATCAAAAGACCGGA | 57.5 | 514 | INTERGENIC | | | | |
| CaPOPI_7979 | scaffold5928 | 4085 | CTATATATATATATA/CTATATATATATATATA | AGCAAAAGGAAGCAACGAAA | ACACACCTATCGGATGCACA | 60.0 | 598 | INTERGENIC | | | | |
| CaPOPI_7980 | scaffold593 | 86436 | ATTTTTTT/ATTTTTT | TTGTTGGGTTATCGTTGTCG | GTGCAATGTTGCATGAGCTT | 59.4 | 575 | INTERGENIC | | | | |
| CaPOPI_7981 | scaffold593 | 181350 | AATATATATATATATATATAT/AATATATATATATATATAT | GCCCCAACCTGAATTAGTGA | ATTTGTGGGGCACAAGACTC | 59.9 | 871 | INTERGENIC | | | | |
| CaPOPI_7982 | scaffold593 | 191195 | CTTTT/CTTTTT | AGCCAAGTCGATTGACGAAT | CAGACTTTCCAGCGTCACAA | 59.7 | 738 | INTERGENIC | | | | |
| CaPOPI_7983 | scaffold593 | 209468 | TC/T | GCAAACCTTTGCAAAAAGAAA | GGAATAAAATATGCATCCGAGC | 59.4 | 153 | DRR | Ca_26412 | | C2H2 | Glycoside hydrolase, family 28 |
| CaPOPI_7984 | scaffold593 | 209698 | CT/CTTT | GCAAACCTTTGCAAAAAGAAA | GGAATGGAAATAATTGAAAAGTTTG | 59.4 | 847 | DRR | Ca_26412 | | C2H2 | Glycoside hydrolase, family 28 |
| CaPOPI_7985 | scaffold593 | 211628 | TT/TTGTGAT | TACGTACGGCAGTAGCCCTT | ATTTCCCCGGAATCATTAGG | 59.8 | 673 | URR | Ca_26412 | | C2H2 | Glycoside hydrolase, family 28 |
| CaPOPI_7986 | scaffold5954 | 1414 | A/AG | TGGATGCACAAGGTTGGTTA | AGGTCATTTTCGTCCTCCCT | 60.0 | 367 | INTERGENIC | | | | |
| CaPOPI_7987 | scaffold5977 | 4624 | GAA/GAATTTTTGGAAA | CGTGGTAATCGCGTAGGAAT | GGACTTCTGCTGAGCCAATC | 60.0 | 166 | INTERGENIC | | | | |
| CaPOPI_7988 | scaffold5977 | 7940 | GTTTT/GTTTTT | CCGCAATGAGACCAAGTTTT | TTTGCAATCCAACAACCAGA | 60.1 | 555 | INTERGENIC | | | | |
| CaPOPI_7989 | scaffold5977 | 14707 | GTTTTTTTTT/GTTTTTTTTTT | TGAGAATGATGCAAATGGGA | TGGTCAACCTTTTGTGGATG | 60.0 | 640 | INTERGENIC | | | | |
| CaPOPI_7990 | scaffold598 | 126131 | GAT/GATCAT | CCATCATCGATATTCATGCG | TTCCAAACTGAAGCAAAGAACA | 59.9 | 170 | INTERGENIC | | | | |

| INDEL marker IDs | Chromosomes/ unanchored scaffolds | Physical positions (bp) | InDels (*Kabuli* reference genome-CDC Frontier/PI) | Forward primers (5'-3') | Reverse primers (5'-3') | Anneling temperature (0C) | Expected amplified product size (bp) | Structural annotation | | Functional annotation | | |
|---|---|---|---|---|---|---|---|---|---|---|---|---|
| | | | | | | | | Sequence components of *kabuli* genome | *Kabuli* gene accession IDs | NCBI-KOG | TFs | NCBI-nr database |
| CaPOPI_7991 | scaffold598 | 131012 | GA/GAA | GAGGCTCCTCCTCTATCGGT | TCCTTAATGACACCCCCAAA | 59.8 | 727 | INTERGENIC | | | | |
| CaPOPI_7992 | scaffold598 | 165238 | ATTTTTTTTTTT/ATTTTTTTTTT | TGAAGGAGGAGGATCCTGAA | TGAGAGGGGTTCAAGAGGTG | 59.7 | 696 | DRR | Ca_21754 | | | |
| CaPOPI_7993 | scaffold598 | 232941 | CAAA/CAA | ACATCCATTTCCGGTCCATA | GCCCTCAGATGAGAGTCAGG | 60.0 | 582 | INTERGENIC | | | | |
| CaPOPI_7994 | scaffold598 | 311554 | GTTTTTTTTTT/GTTTTTTTTTTT | ACGAATGCCCACACTAAAGG | TTCATTGTGTGCTTTTCACCA | 60.0 | 408 | INTERGENIC | | | | |
| CaPOPI_7995 | scaffold598 | 318052 | GAAAAA/GAAAA | TCGGGAAAAAGAATACGTGG | AGATTGTGGCGCAGAAGACT | 59.9 | 116 | INTERGENIC | | | | |
| CaPOPI_7996 | scaffold598 | 392260 | CATATATATATATATATAT/CATATATATATATATAT | ATTCCCCCTTCATCTTGGAC | TTGTTTGCAAATGCATGCTC | 60.1 | 345 | INTERGENIC | | | | |
| CaPOPI_7997 | scaffold598 | 478255 | AATATATATATATATATA/AATATATATATATATA | GTTGGTTCCCTAGCACCGTA | TTTGGCATGAAGTGGAAACA | 60.0 | 753 | INTERGENIC | | | | |
| CaPOPI_7998 | scaffold598 | 563403 | CAAAAAA/CAAAAA | TTTTGCAATGTGACACACGA | AACGCATCAATCCTACGACC | 59.7 | 897 | INTERGENIC | | | | |
| CaPOPI_7999 | scaffold598 | 568507 | CTCAAAATCAAAATCAAAATCAAAATCAAAATC/CTCAAAATCAAAATCAAAATCAAAATCAAAATCAAAATC | CAGCCACTACATTGGCCTTT | AGGATTCTCCAAGTTGGCCT | 60.1 | 499 | INTERGENIC | | | | |
| CaPOPI_8000 | scaffold599 | 91366 | GTATATATATATAT/GTATATATATATATATATATAT | TAAAGAGATTCGCAGCGGTT | ACTTGCGAGAGCGCAGTTAT | 60.0 | 372 | INTERGENIC | | | | |
| CaPOPI_8001 | scaffold599 | 123496 | GAAAAAA/GAAAAAAA | AACTCGCCAAGCGAAAATAA | CTCGCAAGTCACAGTCGAAA | 59.9 | 952 | INTERGENIC | | | | |
| CaPOPI_8002 | scaffold599 | 187793 | CCT/C | TTGGTATTGAACTCGGAGGC | TTCCTTTCCACACAACCACA | 60.1 | 690 | INTERGENIC | | | | |
| CaPOPI_8003 | scaffold599 | 282543 | CTTTTTTTTTTTT/CTTTTTTTTTTT | AATTTTTGGCGTCATGGTTC | TCCATTGTGCCACATCAACT | 59.8 | 392 | INTERGENIC | | | | |
| CaPOPI_8004 | scaffold599 | 284681 | GAAAAAAAA/GAAAAAAA | AACGAACTCTACCGGGCTTT | GGCCTGAAAGCATACCAAAA | 60.1 | 634 | INTERGENIC | | | | |
| CaPOPI_8005 | scaffold599 | 286707 | AT/A | TTGAAATTACCGCTGGAGATG | CACTTGCGTATCACTGCGTT | 60.1 | 485 | INTERGENIC | | | | |

| **INDEL marker IDs** | **Chromosomes/ unanchored scaffolds** | **Physical positions (bp)** | **InDels (*Kabuli* reference genome-CDC Frontier/PI)** | **Forward primers (5'-3')** | **Reverse primers (5'-3')** | **Anneling temperature (0C)** | **Expected amplified product size (bp)** | **Structural annotation** | | **Functional annotation** | | |
|---|---|---|---|---|---|---|---|---|---|---|---|---|
| | | | | | | | | **Sequence components of *kabuli* genome** | ***Kabuli* gene accession IDs** | **NCBI-KOG** | **TFs** | **NCBI-nr database** |
| CaPOPI_8006 | scaffold599 | 291369 | GACTT/GACTTACTT | TTGTTTGTTTTGCAATCCTCC | TTTCCAACCGGTGAGTTTTC | 60.0 | 597 | INTERGENIC | | | | |
| CaPOPI_8007 | scaffold599 | 307088 | CTTATTATTATTATTATTATTATTATTATT/CTTATTATTATTATTATTATTATTATTATTATTATTATTATTATT | TCTTCCCATTCCTTGTCTGG | GGCGTAGCCCAAATATTCAA | 60.0 | 120 | INTERGENIC | | | | |
| CaPOPI_8008 | scaffold599 | 307530 | AATATATATATATATATATATATATATATATAT/AATATATATATATATATATATATAT | TCTTCCCATTCCTTGTCTGG | TGTGTGCTTCCTATGATGTTCT | 60.0 | 855 | INTERGENIC | | | | |
| CaPOPI_8009 | scaffold599 | 307947 | AT/A | AGAACATCATAGGAAGCACACA | CAACATTAAAAGAACGGCGA | 57.4 | 317 | INTERGENIC | | | | |
| CaPOPI_8010 | scaffold599 | 312899 | CTTTTTTTTTTT/CTTTTTTTTTTTTTT | CGTTGTCGTCGGTAGGATTT | TTCAAAAGTTATTCGGTGGAAA | 60.0 | 521 | INTERGENIC | | | | |
| CaPOPI_8011 | scaffold599 | 322634 | GTTTTTTTTTT/GTTTTTTTTTTTT | TTGATAAACCGCCATGACAA | TTTTGCTTTGCCGTATTTTT | 59.9 | 563 | INTERGENIC | | | | |
| CaPOPI_8012 | scaffold599 | 323301 | TTATATATATATATATATATATATATATA/TTATATATATATATATATATATATA | AAAAATACGGCAAAGCAAAA | TTGAGGTTGAGAGTCTTTTCCA | 57.5 | 732 | INTERGENIC | | | | |
| CaPOPI_8013 | scaffold599 | 329422 | CAAAAAAAAA/CAAAAAAAA | CACGTAAAATCGCCTAAAATCT | CCGATAGTAAAACGACATCCTG | 57.6 | 562 | INTERGENIC | | | | |
| CaPOPI_8014 | scaffold599 | 330437 | AAATATTTCAAATT/AAATATTTCAAATTAATATTTCAAATT | AAAATTCTGTTTGGGGGACC | TTCGAGTATTGCTTGCCTTG | 60.0 | 599 | INTERGENIC | | | | |
| CaPOPI_8015 | scaffold599 | 335351 | CTTTTTTTTTT/CTTTTTTTTTTTT | GCCAACCATGTAAATCGCAT | TCAATCATCAATTTTTGTGTGTTTC | 60.7 | 327 | INTERGENIC | | | | |
| CaPOPI_8016 | scaffold599 | 338561 | CTATATATATATATATATATATATATATA/CTATATATATATATATATATATATA | ATTGGGTGGGGATACAATGA | GATCTTGAAAAATTGATGGTGC | 59.9 | 307 | INTERGENIC | | | | |
| CaPOPI_8017 | scaffold599 | 347393 | CTTTTTTT/CTTTTTT | CCTCTTTTCTTCGCATTTCG | AACAACAGCCCCAATGTAGC | 60.0 | 504 | INTERGENIC | | | | |
| CaPOPI_8018 | scaffold599 | 358992 | CAAAAAAAAAA/CAAAAAAAAAAAA | GGGGTTTTTAGAAAACTCGTCA | TTTTGCCTTGCCATATCTTTTT | 59.5 | 349 | INTERGENIC | | | | |
| CaPOPI_8019 | scaffold599 | 366068 | ATTTTTTTTT/ATTTTTTTTTT | AGAGCGCAATCATTTCAGGT | CTTTTGGAAAGAAGCCCTCC | 59.8 | 613 | INTERGENIC | | | | |
| CaPOPI_8020 | scaffold599 | 369580 | T/TCA | AGGGCACTTTGGAAAACCTT | TCATTTAAAGGCCTCAATACGAT | 60.0 | 492 | INTERGENIC | | | | |

| INDEL marker IDs | Chromosomes/ unanchored scaffolds | Physical positions (bp) | InDels (*Kabuli* reference genome-CDC Frontier/PI) | Forward primers (5'-3') | Reverse primers (5'-3') | Anneling temperature (0C) | Expected amplified product size (bp) | Structural annotation | | Functional annotation | | |
|---|---|---|---|---|---|---|---|---|---|---|---|---|
| | | | | | | | | Sequence components of *kabuli* genome | *Kabuli* gene accession IDs | NCBI-KOG | TFs | NCBI-nr database |
| CaPOPI_8021 | scaffold599 | 371527 | TAAAAAAA/TAAAAAA | CAGCGTTGTTGTTGTTGTTTTT | TTTCGAATGAACATGGATGA | 60.1 | 531 | INTERGENIC | | | | |
| CaPOPI_8022 | scaffold599 | 373801 | TAAAAAAAAAAAA/TAAAAAAAAAAAAAAAA | CCCCCTAAGTTCATTCCACA | GGCTTGTCCGTCGGTAAATA | 59.8 | 567 | DRR | Ca_25347 | | | Transposase, Ptta/En/Spm, plant |
| CaPOPI_8023 | scaffold599 | 391375 | GTTTTTTTTTTT/GTTTTTTTTTTTT | TGCGGTGAAGAAGAACAGTG | CACATCAAAGAGGAAATGCC | 60.0 | 666 | INTERGENIC | | | | |
| CaPOPI_8024 | scaffold599 | 410067 | GTTTT/GTTT | ATCGATTTCCCAATCGGTTA | TATACCCTATAGCCACGGCG | 59.2 | 609 | INTERGENIC | | | | |
| CaPOPI_8025 | scaffold5992 | 921 | TTCTCCTCTCCTCTCC/TTCTCCTCTCCTCTCCTCTCC | TTGGAGAAACTTTTCCGCTG | TCATCTCACCCCTTACCTCG | 60.4 | 686 | INTERGENIC | | | | |
| CaPOPI_8026 | scaffold5992 | 1551 | G/GA | CGAGGTAAGGGGTGAGATGA | TCCCCGAATCACTAGGTTTG | 60.1 | 544 | INTERGENIC | | | | |
| CaPOPI_8027 | scaffold5993 | 18562 | GTTTTTTT/GTTTTTT | TTGATGTACGAAAAGTTTTAGCAAG | GGGGGCACTTATTTATCACTT | 59.0 | 267 | INTERGENIC | | | | |
| CaPOPI_8028 | scaffold5993 | 18598 | CTTATATTTTTATATTTTTATATTTT/CTTATATTTTTATATTTT | TTGATGTACGAAAAGTTTTAGCAAG | GGGGGCACTTATTTATCACTT | 59.0 | 267 | INTERGENIC | | | | |
| CaPOPI_8029 | scaffold601 | 49572 | CTTTTTTT/CTTTTTT | GGGGACATTTCGAGAGTCAA | TCACAAGGGAGGCTCTATGG | 60.1 | 519 | INTERGENIC | | | | |
| CaPOPI_8030 | scaffold604 | 14590 | TAATAAATAAATAAATAAATAAATAA/TAATAAATAAATAAATAAATAA | TGGGCTTCAACTTTTTGGAC | CGATTTTTGATTGCATGCTT | 60.1 | 536 | INTERGENIC | | | | |
| CaPOPI_8031 | scaffold604 | 40273 | CTTTTTTT/CTTTTTTTT | CCAACTCGCTGAGTAGGAGG | ACCGAATTGGATTGGTGAAA | 60.0 | 575 | DRR | Ca_27531 | | | |
| CaPOPI_8032 | scaffold604 | 70429 | CTTTTTTT/CTTTTTTTT | ACGCATTTATTGGATGCACA | TGAAACGTTTGGAGAGTGGA | 60.0 | 776 | INTERGENIC | | | | |
| CaPOPI_8033 | scaffold6066 | 2831 | CCT/C | CTGCAAAGCAACCATCTCAA | CCTCAGATGAGAGCCAGGAC | 60.0 | 797 | INTERGENIC | | | | |
| CaPOPI_8034 | scaffold6113 | 1438 | GAAAA/GAAAAA | ACAAGCCTATCGTTTGGCAC | GATGCAACATTGGAGCATGA | 60.1 | 589 | INTERGENIC | | | | |
| CaPOPI_8035 | scaffold612 | 28576 | GAAAAAA/GAAAAAAA | AAAAAGGGAGGGTCACATGAT | TCACCAATATCTTAACCACCAAAA | 59.7 | 701 | INTERGENIC | | | | |

| INDEL marker IDs | Chromosomes/ unanchored scaffolds | Physical positions (bp) | InDels (*Kabuli* reference genome-CDC Frontier/PI) | Forward primers (5'-3') | Reverse primers (5'-3') | Anneling temperature (0C) | Expected amplified product size (bp) | Structural annotation | | Functional annotation | | |
|---|---|---|---|---|---|---|---|---|---|---|---|---|
| | | | | | | | | Sequence components of *kabuli* genome | *Kabuli* gene accession IDs | NCBI-KOG | TFs | NCBI-nr database |
| CaPOPI_8036 | scaffold612 | 30300 | TAAA/TAA | TGCCACAAATTTCGAGGACT | ACACGTTAAATCGCATAAAATCT | 60.6 | 321 | INTERGENIC | | | | |
| CaPOPI_8037 | scaffold6120 | 2075 | CTTTTTT/CTTTTT | AATCAAAGTCGGTCTGGTGG | AGCAAGCTCTGCTTGACACA | 60.0 | 797 | INTERGENIC | | | | |
| CaPOPI_8038 | scaffold6120 | 3713 | GAGATA/GA | TCTGAAAGCCGTGAAAGACC | CGTGACAACCACCAACTCTTT | 60.4 | 929 | INTERGENIC | | | | |
| CaPOPI_8039 | scaffold6125 | 1553 | TAAAGA/TA | ATGCGATCCTCTCGGTTCTA | GAGACCGAAGAGTATTGGGAGA | 59.8 | 838 | INTERGENIC | | | | |
| CaPOPI_8040 | scaffold613 | 78619 | CNNNNNNNNNN/CNNNNNNNN | ATTCGAAATTCGGTTTGTGC | TAAAAGACGACGGAGTTGGC | 59.9 | 763 | INTERGENIC | | | | |
| CaPOPI_8041 | scaffold6154 | 548 | CCGCCTCA/CCGCCTCAGGCGCCTCA | AACCGCTCCAATGGTGTTAG | GAAGATGATGTGCGCTTGAA | 60.0 | 221 | INTERGENIC | | | | |
| CaPOPI_8042 | scaffold6177 | 3708 | AG/A | TTTTTGTCATCATCGGACCA | AAATCCGTTTGGATTTGCAG | 59.9 | 613 | INTERGENIC | | | | |
| CaPOPI_8043 | scaffold6179 | 967 | TAAAAAAAA/TAAAAAAA | TGGGTGCACAAGATTGGTTA | AGGTCATTTTCGTCCTCCCT | 60.0 | 468 | INTERGENIC | | | | |
| CaPOPI_8044 | scaffold618 | 24350 | TA/TAA | GAACACGTTTGTGTTGGTCG | TTCAAATTGATGATGTTCCCA | 60.0 | 299 | INTERGENIC | | | | |
| CaPOPI_8045 | scaffold618 | 197211 | AATATATATATATATATATA/AATATATATATATATATATA | TGTTGAGCACCTAGGGACATT | AACATCAGTCAAGCGCACAC | 59.6 | 758 | INTERGENIC | | | | |
| CaPOPI_8046 | scaffold6185 | 2912 | GA/G | GCAAAGTCGCATTATGGTATGA | AGCCCTCACATTAATTCGCA | 60.0 | 740 | DRR | Ca_27924 | | | Lipase, GDSL |
| CaPOPI_8047 | scaffold6185 | 6573 | CAAAAAAAAAAAA/CAAAAAAAAAAAAAAA | TGCACATTTTACAGCGCTTC | AATCTTTTAAACTCCATGGTGAAA | 60.0 | 785 | URR | Ca_27924 | | | Lipase, GDSL |
| CaPOPI_8048 | scaffold62 | 150253 | AGTTGTCCAAT/A | AACATTTAAGAAAATTGAGTTGTCAAA | GTGTGTTGGGCTGTTTAGGG | 58.7 | 405 | INTERGENIC | | | | |
| CaPOPI_8049 | scaffold62 | 338714 | ATT/ATTT | CCATCGTCAAGTCCGAAAAT | CCAAAACTCGAAACCCAAAA | 59.9 | 565 | INTERGENIC | | | | |
| CaPOPI_8050 | scaffold622 | 10235 | TAAAAAA/TAAAAAAA | ATAGGCCTAGGGAGGACGAA | TTTAGCCAACTTGTGCATCG | 60.1 | 381 | INTERGENIC | | | | |

| INDEL marker IDs | Chromosomes/ unanchored scaffolds | Physical positions (bp) | InDels (*Kabuli* reference genome-CDC Frontier/PI) | Forward primers (5'-3') | Reverse primers (5'-3') | Anneling temperature (0C) | Expected amplified product size (bp) | Structural annotation | | Functional annotation | | |
|---|---|---|---|---|---|---|---|---|---|---|---|---|
| | | | | | | | | Sequence components of *kabuli* genome | *Kabuli* gene accession IDs | NCBI-KOG | TFs | NCBI-nr database |
| CaPOPI_8051 | scaffold622 | 30884 | A/AC | TGGGTGCACAAGATTGGTTA | AGGGGTGTTTGAGTCATTGG | 60.0 | 832 | INTERGENIC | | | | |
| CaPOPI_8052 | scaffold622 | 46025 | A/AC | CACTCCTATGGGATGCACAA | AGGTCATTTTCGTCCTCCCT | 59.5 | 373 | INTERGENIC | | | | |
| CaPOPI_8053 | scaffold622 | 61725 | GACACACACACACAC/GACACACACACAC | CAAACCCACCTATTGGATGC | GGTCATTTTCGTCCTCGCTA | 60.2 | 310 | INTERGENIC | | | | |
| CaPOPI_8054 | scaffold626 | 94461 | CATATATATATAT/CATATATATAT | AGTGTTGTCAATGTTACTTTATTTCAA | GGGTTTGAGGTTCGGGTTAT | 57.6 | 695 | INTERGENIC | | | | |
| CaPOPI_8055 | scaffold626 | 172733 | C/CAATTA | GTGAGCAAAATGCTGGGAAT | GTTGTTGAGTTGTTGGGGCT | 60.1 | 393 | INTERGENIC | | | | |
| CaPOPI_8056 | scaffold626 | 219233 | CAAA/CAAAA | GGATTTAGGGTTTTGGCCTT | GATGAAATCGAGGACTGGGA | 59.3 | 654 | INTERGENIC | | | | |
| CaPOPI_8057 | scaffold626 | 225194 | T/TG | TACAGATGTTCCAGCCCTCC | GAACCTGTACCTCCAGCCAA | 60.1 | 501 | INTERGENIC | | | | |
| CaPOPI_8058 | scaffold626 | 236880 | AGGG/AGG | CCCATCCACAAAAATATGCC | GATGAAATCGAGGACCAGGA | 60.0 | 302 | INTERGENIC | | | | |
| CaPOPI_8059 | scaffold626 | 242253 | A/AAAAATGATCGTTTTC | CCGATGCGTAGATTGACAGA | GGTAACCAATCCGAAGACGA | 59.8 | 177 | INTERGENIC | | | | |
| CaPOPI_8060 | scaffold626 | 258123 | ATTTTT/ATTTTTT | AACTGTGTTTCGGGTTCTGG | CTTTTCGGGCGATTTTCATA | 60.0 | 432 | INTERGENIC | | | | |
| CaPOPI_8061 | scaffold626 | 268228 | CAA/CAAA | TAACCCAAACCTCAAACCCA | TTCATCTGGACTTTTTGGGC | 60.2 | 760 | INTERGENIC | | | | |
| CaPOPI_8062 | scaffold626 | 334424 | C/CGCT | TGCATAGGCCTCTTGTCCTT | AACCTGTGTTTCAGGTTCCG | 59.8 | 728 | INTERGENIC | | | | |
| CaPOPI_8063 | scaffold626 | 452443 | CAAA/CAAAA | GGTCCTCGATTTCATCTGGA | CGAAAATCGCTCGAAAAGTC | 60.0 | 336 | INTERGENIC | | | | |
| CaPOPI_8064 | scaffold626 | 464649 | GT/G | CAGTGTTTCGGATTCTGGGT | TTCAACTGGACGTTTAGGGC | 60.0 | 769 | INTERGENIC | | | | |
| CaPOPI_8065 | scaffold626 | 504911 | AG/A | CTATGTTTTTGGTTCCGCGT | GTTTCGTACCCCTCGACAAA | 60.0 | 930 | INTERGENIC | | | | |

| INDEL marker IDs | Chromosomes/ unanchored scaffolds | Physical positions (bp) | InDels (*Kabuli* reference genome-CDC Frontier/PI) | Forward primers (5'-3') | Reverse primers (5'-3') | Anneling temperature (0C) | Expected amplified product size (bp) | Structural annotation | | Functional annotation | | |
|---|---|---|---|---|---|---|---|---|---|---|---|---|
| | | | | | | | | Sequence components of *kabuli* genome | *Kabuli* gene accession IDs | NCBI-KOG | TFs | NCBI-nr database |
| CaPOPI_8066 | scaffold626 | 512589 | ATTTA/ATTTAGGGTTTA | CCCGACAAAAACGGACTAAA | ATAACCCGAACCTCAAACCC | 60.0 | 359 | INTERGENIC | | | | |
| CaPOPI_8067 | scaffold626 | 515112 | TTAT/TTATAT | CGTTATAAAGTAAAACTACATTGCGAA | AAATTTAGGAGACGAATGCTTTTT | 59.2 | 848 | INTERGENIC | | | | |
| CaPOPI_8068 | scaffold626 | 520360 | CTTT/CTTTT | AAGGTCTCCTGGTCCTTGGT | CACGAAACCCGAAACAAAGT | 60.0 | 515 | INTERGENIC | | | | |
| CaPOPI_8069 | scaffold626 | 522371 | CTTT/CTT | TTTGTCGAGGGGTACGAAAC | GGTCCTCGATTTCATCTGGA | 60.0 | 691 | INTERGENIC | | | | |
| CaPOPI_8070 | scaffold626 | 524724 | ATTTTTTTTTTT/ATTTTTTTT | AAATCCCCCGAAAATACCAG | TCCAGATCAAATCGAGGACC | 60.0 | 670 | INTERGENIC | | | | |
| CaPOPI_8071 | scaffold626 | 553162 | AT/A | TCCAGATGAAATCGAGGACC | TTTCATTTGGGCTTTTCAGG | 60.0 | 325 | INTERGENIC | | | | |
| CaPOPI_8072 | scaffold626 | 554690 | TAAGAAGAAGAA/TAAGAAGAA | GGTCCTCGATTTCATCTGGA | CCATCGTCTGGTTCGAAAAT | 60.0 | 415 | INTERGENIC | | | | |
| CaPOPI_8073 | scaffold626 | 631432 | ATAT/A | TTGATTTGACCACCCATTTTT | CAGCCCAACACACTAAACCC | 59.2 | 242 | INTERGENIC | | | | |
| CaPOPI_8074 | scaffold63 | 6585 | GTT/GTTT | CCCCAGCTTTGTTGTTTCAT | CCAATTTTGAATGAAGGCGT | 60.0 | 600 | INTERGENIC | | | | |
| CaPOPI_8075 | scaffold63 | 8644 | TAAA/TAA | ACCCCATCCTTGTTGTTTCA | TATGTTTCGAGCCGGCTATC | 60.2 | 425 | INTERGENIC | | | | |
| CaPOPI_8076 | scaffold63 | 59319 | TAA/TAATAAA | CGAGGTAAGGGGTGAGATGA | AGGTTTGTCAGACCCTCCCT | 60.1 | 481 | INTERGENIC | | | | |
| CaPOPI_8077 | scaffold637 | 107635 | ATT/ATTT | GCCTTGAGGAATCGGTATCA | GTGTGAATGCAACCTTCCCT | 60.0 | 313 | INTERGENIC | | | | |
| CaPOPI_8078 | scaffold6382 | 1419 | CTCCCTATGCTT/CT | AGGTCATTTTCGTCCTCCCT | CAAACACACGTTTTGGATGC | 59.9 | 181 | INTERGENIC | | | | |
| CaPOPI_8079 | scaffold639 | 11325 | T/TC | TGGTGCAATTTACTTTCTCGG | TTCGGTTCTTGTTTGCCTTT | 60.1 | 783 | INTERGENIC | | | | |
| CaPOPI_8080 | scaffold639 | 103317 | AATATATATATATATAT/AATATATATATATATATAT | TTCGACTCCCTTCCCTTTTT | CATTCTTTTATGCGTTTGGC | 60.0 | 551 | INTERGENIC | | | | |

| INDEL marker IDs | Chromosomes/ unanchored scaffolds | Physical positions (bp) | InDels (*Kabuli* reference genome-CDC Frontier/PI) | Forward primers (5'-3') | Reverse primers (5'-3') | Anneling temperature (0C) | Expected amplified product size (bp) | Structural annotation | | Functional annotation | | |
|---|---|---|---|---|---|---|---|---|---|---|---|---|
| | | | | | | | | Sequence components of *kabuli* genome | *Kabuli* gene accession IDs | NCBI-KOG | TFs | NCBI-nr database |
| CaPOPI_8081 | scaffold642 | 60062 | AATATATATATATATATATATATATATATA/AATATATATATATATATATA | TATTTTGGGATCTTGGCTCG | CGTAGCGTGGTAGTGAGCAA | 60.0 | 619 | INTERGENIC | | | | |
| CaPOPI_8082 | scaffold642 | 78334 | TTATATATATATATATATATA/TTATATATATATATATATATA | CTGAGATGCAACGTTTTCCA | AGCAAAAGGAAGCAAACGAA | 59.8 | 334 | INTERGENIC | | | | |
| CaPOPI_8083 | scaffold642 | 79362 | CGTC/CGTCGTGTAGGTC | TTGGTGTGGTTTTTGTGGAA | CACATATCACGGGTCAGCAC | 59.8 | 375 | INTERGENIC | | | | |
| CaPOPI_8084 | scaffold642 | 171116 | CC/CCGCTCTTCCAAGTTTTC | GGCTCTGTTGGTCAGCTTTC | AAAATTGGGCTCGCTAGGTT | 60.0 | 157 | INTERGENIC | | | | |
| CaPOPI_8085 | scaffold642 | 287766 | TTTTATTTATTTATTTATTTATT/TTTTATTTATTTATTTATTTATTTATT | AAAAATGGGAGAAGGGGTTG | ATTTGGAACTTGGGCTTCCT | 60.2 | 366 | INTERGENIC | | | | |
| CaPOPI_8086 | scaffold642 | 500946 | GTTTTTTTTTTT/GTTTTTTTTTTTT | TGCAAACAACCCGATAACAA | CGAAGACATGCTCGTCTTAAA | 60.0 | 432 | INTERGENIC | | | | |
| CaPOPI_8087 | scaffold6429 | 4894 | CCTA/C | GGGTGGACGAAAATGAGCTA | TGTGTCATTGGATTCGCATT | 60.1 | 537 | INTERGENIC | | | | |
| CaPOPI_8088 | scaffold645 | 73896 | AGGGG/AGGGGG | ACATCTTGCTTGGGTTTTGG | CCCCCTCAGAATATGCTTCA | 60.0 | 229 | INTERGENIC | | | | |
| CaPOPI_8089 | scaffold645 | 254689 | AATATATATATATATATATATATATATATATATAT/AATATATATATATATATATATATATATATATATAT | CACCGTAGTGTGGTGGTGAG | ATTTTGGCTCGGAGGTTTTT | 60.1 | 659 | INTRON | Ca_25158 | I | Trihelix | Phospholipid/glycerol acyltransferase |
| CaPOPI_8090 | scaffold6458 | 24160 | T/TAATAAACAGA | AAGAGGCCTACACCGCTACA | AGATGTTTGTCCTGCTGCAA | 59.9 | 530 | INTRON | Ca_27555 | T | C3H | NUDIX hydrolase domain |
| CaPOPI_8091 | scaffold647 | 200535 | A/AT | CAAGCTGTCGGTTGAAGACA | AACAATCTCCCCCTTTTTGG | 60.0 | 603 | DRR | Ca_27495 | | | |
| CaPOPI_8092 | scaffold6480 | 530 | ATTTT/ATTT | TAATCGATCATCCCAGACCC | TACAATTGGGCTCCTTCACC | 59.7 | 900 | INTERGENIC | | | | |
| CaPOPI_8093 | scaffold6480 | 672 | CTTTTTTTTTTTT/CTTTTTTTT | AGAAACATTTGCACGGTCCT | TACAATTGGGCTCCTTCACC | 59.6 | 612 | INTERGENIC | | | | |
| CaPOPI_8094 | scaffold6568 | 659 | TAAATTAAAAAAAATTAAAAAAAA/TAAATTAAAAAAAA | CACGCACGGGTTACACACTA | ACAACTTCATCCGTTTTGGC | 60.6 | 270 | INTERGENIC | | | | |
| CaPOPI_8095 | scaffold661 | 266987 | GAAAAAAA/GAAAAAAAA | TTTTTCTTTCGATTCGGTCG | TCCATTCATATGATTCCATCCA | 60.2 | 749 | INTERGENIC | | | | |

| INDEL marker IDs | Chromosomes/ unanchored scaffolds | Physical positions (bp) | InDels (*Kabuli* reference genome-CDC Frontier/PI) | Forward primers (5'-3') | Reverse primers (5'-3') | Anneling temperature (0C) | Expected amplified product size (bp) | Structural annotation | | Functional annotation | | |
|---|---|---|---|---|---|---|---|---|---|---|---|---|
| | | | | | | | | Sequence components of *kabuli* genome | *Kabuli* gene accession IDs | NCBI-KOG | TFs | NCBI-nr database |
| CaPOPI_8096 | scaffold661 | 474773 | GTATATATATATATATATATATAT/GTATATATATATATATATATATATAT | CGTAGCGTGGTAGTGAGCAA | GTTTTGGATTTTGGCTTGGA | 60.1 | 668 | INTERGENIC | | | | |
| CaPOPI_8097 | scaffold661 | 559302 | ATTTTTT/ATTTTTTT | GGTGCATTTGAAGAAAGGGA | AAATGTGCCACAAATGCAAG | 60.1 | 625 | INTERGENIC | | | | |
| CaPOPI_8098 | scaffold6694 | 1671 | TAAAAAAAA/TAAAAAAAAA | CCCACACTACGACCACGTTA | CGTAACTCGAATGAACGGAAA | 59.5 | 671 | INTERGENIC | | | | |
| CaPOPI_8099 | scaffold670 | 197831 | T/TG | TGGCCAATGATCCAAGAGAT | CCGACAAAAATGGACCAAAA | 60.4 | 823 | INTERGENIC | | | | |
| CaPOPI_8100 | scaffold670 | 197970 | AAAT/AAATAAT | TGGCCAATGATCCAAGAGAT | CCGACAAAAATGGACCAAAA | 60.4 | 823 | INTERGENIC | | | | |
| CaPOPI_8101 | scaffold672 | 34143 | AATATATATATATATATATATATATATATATATATAT/AATATATATATATATATATATATATATATATATATATATATAT | ATCAGTCAAGCCCACATTAAA | AAACACGGTTCCTAGAGCCA | 57.2 | 511 | INTERGENIC | | | | |
| CaPOPI_8102 | scaffold6720 | 536 | TAAA/TAAATAAAA | CGATACTCACCCCTCGTTGT | ATGTGACACCCTAAACCCCA | 60.0 | 338 | INTERGENIC | | | | |
| CaPOPI_8103 | scaffold6720 | 5246 | CA/CAA | TTTTCGTCCTCCCTAGGCTT | CAACTATTGGATGCGCAAGA | 60.2 | 470 | INTERGENIC | | | | |
| CaPOPI_8104 | scaffold674 | 84428 | GC/G | TAAGAAAGCGAGGGTGATGC | GAAAGCTGAAGTTGGTTCGC | 60.4 | 668 | INTERGENIC | | | | |
| CaPOPI_8105 | scaffold674 | 226567 | TAAAAAAAA/TAAAAAAAAA | GCTCGGGGTATAGGTTCCTC | CACCGGGAGATACGTAGGAG | 59.9 | 652 | INTERGENIC | | | | |
| CaPOPI_8106 | scaffold674 | 232088 | GAAAAAAAAAA/GAAAAAAAAA | GTAACCCCAAGGATCCAACA | AGATGTGTATCGAGCGTCGT | 59.6 | 110 | INTERGENIC | | | | |
| CaPOPI_8107 | scaffold674 | 248531 | GAT/GATAT | ACTTTTATGTGCGTTTGACTGA | TCCAACTCCCTTCCCTTTTT | 57.5 | 534 | INTERGENIC | | | | |
| CaPOPI_8108 | scaffold674 | 263650 | AT/A | GCTTCCCCTTTTTCTCTCAA | AGATCGCTGTCGTCTCTGGT | 58.5 | 559 | INTERGENIC | | | | |
| CaPOPI_8109 | scaffold674 | 312426 | ACC/AC | TTCCCCAACTTGGTCCTTTA | CAACTCCACAAAGGGTCGTT | 59.4 | 410 | INTERGENIC | | | | |
| CaPOPI_8110 | scaffold674 | 343997 | AAATTTTTAATTTTTAATTTT/AAATTTTTAATTTTTAATTTTTAATTTT | TGGACCAACTTGTGTAAGATTTTT | GCTTTATTGCTTTTGGCAGC | 59.0 | 622 | INTERGENIC | | | | |

| INDEL marker IDs | Chromosomes/ unanchored scaffolds | Physical positions (bp) | InDels (*Kabuli* reference genome-CDC Frontier/PI) | Forward primers (5'-3') | Reverse primers (5'-3') | Anneling temperature (0C) | Expected amplified product size (bp) | Structural annotation | | Functional annotation | | |
|---|---|---|---|---|---|---|---|---|---|---|---|---|
| | | | | | | | | Sequence components of *kabuli* genome | *Kabuli* gene accession IDs | NCBI-KOG | TFs | NCBI-nr database |
| CaPOPI_8111 | scaffold674 | 394555 | A/AT | CGGCTACGTCACTCCTTCAT | TGCAAAAATTCCATACGGTG | 60.3 | 410 | INTERGENIC | | | | |
| CaPOPI_8112 | scaffold674 | 394638 | ATTTTTTTTT/ATTTTTTTTTT | CGGCTACGTCACTCCTTCAT | TGCAAAAATTCCATACGGTG | 60.3 | 410 | INTERGENIC | | | | |
| CaPOPI_8113 | scaffold674 | 401473 | TAAAA/TAAA | TGCATTAGTCGATGTCCCAA | AATCGATTTCGCGTTTGTTC | 60.1 | 134 | INTERGENIC | | | | |
| CaPOPI_8114 | scaffold674 | 437989 | TAACAACAACAACAACAACAA/TAACAACAACAACAACAACAACAA | GCATAAATCGTGCTCGACAA | CAGATTCAACCAAAGCAGCA | 59.8 | 333 | INTERGENIC | | | | |
| CaPOPI_8115 | scaffold674 | 439330 | A/AG | AAGACCTTGCCTGTCTCGAA | TGCATCTGTTACCACCATCG | 60.0 | 284 | INTERGENIC | | | | |
| CaPOPI_8116 | scaffold674 | 441706 | GATATATATATATATATATA/GATATATATATATATATATATA | ATGTGAATCCCACCAGGAAA | GAAAGAGCGCTGCAAAAGAC | 60.2 | 533 | INTERGENIC | | | | |
| CaPOPI_8117 | scaffold674 | 468778 | ATT/ATTT | GCTAAGAGCATCTCCAACGG | TTTAGAGCATCTCCAACGGG | 60.0 | 590 | INTERGENIC | | | | |
| CaPOPI_8118 | scaffold674 | 468848 | TAAAAAAAAA/TAAAAAAAAAA | GCTAAGAGCATCTCCAACGG | TTTAGAGCATCTCCAACGGG | 60.0 | 590 | INTERGENIC | | | | |
| CaPOPI_8119 | scaffold674 | 518331 | ATTTTTTTTTT/ATTTTTTTTTTT | TTTTGACAAACCTGCGAAAC | TTTTAAAGAGGGACTGACCACAA | 58.8 | 386 | INTERGENIC | | | | |
| CaPOPI_8120 | scaffold674 | 570233 | TATATATA/TATATATAAATATATA | CGGGGTGTTACACAACTGAG | CGCATCGTCACAATTCATTT | 59.1 | 601 | INTERGENIC | | | | |
| CaPOPI_8121 | scaffold674 | 617218 | C/CTCATTTGTTTT | GACAGCTCAACCCTTTCCAA | CACCCAATCCAAGCTACGTT | 60.2 | 534 | INTERGENIC | | | | |
| CaPOPI_8122 | scaffold674 | 705834 | CT/CTT | TTGAGCTCCCCTTTTTCTGA | GTCAAAGGTGACCCTTCTGC | 59.9 | 718 | INTERGENIC | | | | |
| CaPOPI_8123 | scaffold674 | 711627 | CAAAAAAAAAA/CAAAAAAAAA | TCCACCCTTTGATGCTTTTC | TGCTTTCGGTGTCACTTTTG | 60.1 | 669 | INTERGENIC | | | | |
| CaPOPI_8124 | scaffold674 | 833729 | ATTTTTTTT/ATTTTTTTTT | GCTTCAGGTAGGAGAAGGCA | TGCATATTCCAAGTGACTTTTTG | 59.6 | 665 | INTERGENIC | | | | |
| CaPOPI_8125 | scaffold674 | 868010 | GA/GACCTAAATATTAA | CCAAGGGAAGTCCAAAATGA | CATTTTCGTCACCCCTCAAC | 59.9 | 589 | INTERGENIC | | | | |

| **INDEL marker IDs** | **Chromosomes/ unanchored scaffolds** | **Physical positions (bp)** | **InDels (*Kabuli* reference genome-CDC Frontier/PI)** | **Forward primers (5'-3')** | **Reverse primers (5'-3')** | **Anneling temperature (0C)** | **Expected amplified product size (bp)** | **Structural annotation** | | **Functional annotation** | | |
|---|---|---|---|---|---|---|---|---|---|---|---|---|
| | | | | | | | | **Sequence components of *kabuli* genome** | ***Kabuli* gene accession IDs** | **NCBI-KOG** | **TFs** | **NCBI-nr database** |
| CaPOPI_8126 | scaffold675 | 81863 | CTTTTT/CTTTTTT | TATCAGAGTTCTCCCCACCG | AAACATGACGAGGGCATTTA | 60.1 | 895 | INTERGENIC | | | | |
| CaPOPI_8127 | scaffold675 | 81930 | TT/TTGAAAAT | TATCAGAGTTCTCCCCACCG | AAACATGACGAGGGCATTTA | 60.1 | 895 | INTERGENIC | | | | |
| CaPOPI_8128 | scaffold675 | 82372 | T/TC | TAAATGCCCTCGTCATGTTT | CCCCTTTCCACGGCTATTAT | 58.1 | 444 | INTERGENIC | | | | |
| CaPOPI_8129 | scaffold676 | 84081 | CTTTT/CTTT | CACGAAACCCGAAACACTTT | GCCATCGTCTAATCCGAAAA | 60.0 | 146 | INTERGENIC | | | | |
| CaPOPI_8130 | scaffold676 | 84534 | GCAC/GCACAAAACAC | TTTTCGGATTAGACGATGGC | GATGAAATCGAGGACCAGGA | 60.0 | 796 | INTERGENIC | | | | |
| CaPOPI_8131 | scaffold676 | 104156 | GAAAAAAAA/GAAAAAAA | TTTCATCTAGGCATTTCGGG | TCCAGATGAAATCGAGGACC | 60.0 | 475 | INTERGENIC | | | | |
| CaPOPI_8132 | scaffold676 | 104639 | ACC/ACCC | GGTCCTCGATTTCATCTGGA | GACACTCGCTCGAAAAGTCC | 60.0 | 686 | INTERGENIC | | | | |
| CaPOPI_8133 | scaffold6760 | 618 | TC/T | TGTTTTCAAAAGGGGGTCAG | GGGCTGAGATGGACACATTT | 59.9 | 621 | INTERGENIC | | | | |
| CaPOPI_8134 | scaffold6760 | 921 | TT/TTCT | TGTTTTCAAAAGGGGGTCAG | GGGCTGAGATGGACACATTT | 59.9 | 621 | INTERGENIC | | | | |
| CaPOPI_8135 | scaffold6760 | 1141 | ATTTTTT/ATTTTT | AAATGTGTCCATCTCAGCCC | TCAAAAACTCAAGGGGCAAT | 59.9 | 545 | INTERGENIC | | | | |
| CaPOPI_8136 | scaffold678 | 12833 | TTAT/TT | CGCCGCCCAATAAAGTATTA | TTTGGTGGAGAATTCCAAGG | 59.9 | 276 | INTERGENIC | | | | |
| CaPOPI_8137 | scaffold678 | 14639 | CG/C | GCTACCATGGGCTTGAAAAA | GTGGTACATGAGCGGAGGAT | 60.1 | 772 | DRR | Ca_26300 | | | |
| CaPOPI_8138 | scaffold678 | 39763 | TAGTTATTAAGTTATT/TAGTTATT | AGGAGTTTGAGCATCGCATT | ATCCCACCCCTAAAACCAAG | 59.8 | 470 | INTERGENIC | | | | |
| CaPOPI_8139 | scaffold678 | 40865 | CTATTATTATTATTATTATTATTATTAT/CTATTATTATTATTATTATTATTATTATTATTAT | GAACAGCTTTGAGGGCAATC | TTTGAATTTGGTCTTTTGAAAGTG | 59.8 | 647 | INTERGENIC | | | | |
| CaPOPI_8140 | scaffold678 | 40998 | TAAAAAAAAA/TAAAAAAAAAAA | GAACAGCTTTGAGGGCAATC | CGATCACCACGTGACACTTC | 59.8 | 849 | INTERGENIC | | | | |

| INDEL marker IDs | Chromosomes/ unanchored scaffolds | Physical positions (bp) | InDels (*Kabuli* reference genome-CDC Frontier/PI) | Forward primers (5'-3') | Reverse primers (5'-3') | Anneling temperature (0C) | Expected amplified product size (bp) | Structural annotation | | Functional annotation | | |
|---|---|---|---|---|---|---|---|---|---|---|---|---|
| | | | | | | | | Sequence components of *kabuli* genome | *Kabuli* gene accession IDs | NCBI-KOG | TFs | NCBI-nr database |
| CaPOPI_8141 | scaffold678 | 41072 | T/TG | TCATTTTGGCTTTTGTTGAAA | CGATCACCACGTGACACTTC | 58.3 | 753 | INTERGENIC | | | | |
| CaPOPI_8142 | scaffold678 | 42464 | TAAAAAA/TAAAAAAA | GCGCACAATAGATGGGTTTA | TTTTCCAACGAGTCCACCTC | 58.7 | 816 | INTERGENIC | | | | |
| CaPOPI_8143 | scaffold678 | 73978 | A/AG | TGGTTGATGAAGACGCGATA | CCGGAACCAAACCCTTATTT | 60.2 | 549 | INTERGENIC | | | | |
| CaPOPI_8144 | scaffold6782 | 544 | AC/A | ACTCACCCCACATCCAACAT | ATGGTACCGCAAAGAACGTC | 60.1 | 337 | INTERGENIC | | | | |
| CaPOPI_8145 | scaffold6782 | 758 | ATTTTTTTT/ATTTTTTTTT | GACGTTCTTTGCGGTACCAT | TCAAACGCTCACACACACAA | 60.0 | 292 | INTERGENIC | | | | |
| CaPOPI_8146 | scaffold6782 | 1068 | GA/GAGAAA | GACGTTCTTTGCGGTACCAT | CAAATGTCAACCACACATTTTG | 60.0 | 428 | INTERGENIC | | | | |
| CaPOPI_8147 | scaffold6782 | 1165 | C/CA | GACGTTCTTTGCGGTACCAT | TTTAAATTGCTGCTAAGATTGGT | 60.0 | 469 | INTERGENIC | | | | |
| CaPOPI_8148 | scaffold6790 | 910 | TATAATAATAATAATAATAATAATAATAATAATAATAATAATAATAATAATAATAATAATAATAA/TATAATAATAATAATAATAATAATAATAATAATAATAATAATAATAA | TAGTTATGCTGAGGGGACGG | TGCAAGTACATTCCCGATCA | 60.1 | 714 | INTERGENIC | | | | |
| CaPOPI_8149 | scaffold6790 | 1414 | C/CA | TCCTTGACCCCAATTCGTAG | TTTTGGGGTTTAGGGTGTCA | 59.9 | 356 | INTERGENIC | | | | |
| CaPOPI_8150 | scaffold6790 | 2258 | ACC/ACCC | TTCAAATTGGCCTTGTCCTC | CATAGACCAACGAGGGGAGA | 60.1 | 317 | INTERGENIC | | | | |
| CaPOPI_8151 | scaffold682 | 196858 | TTTTATTTATTTATTTATTTATTT/TTTTATTTATTTATTTATTTATTTATTT | TCTTTTAAGTGGACATCCGGT | GCGTTACAGTTGGTCATGGA | 58.6 | 840 | INTERGENIC | | | | |
| CaPOPI_8152 | scaffold682 | 239323 | AGATGATG/AGATG | CAACTCCCCCTGCAAACTTA | TGCGTGACAGGAACAAGAAG | 60.1 | 417 | DRR | Ca_23188 | R | ERF | Integrase, catalytic core |
| CaPOPI_8153 | scaffold682 | 302365 | A/AGAGAGAT | GCTGCAAAAGTCAACGTCAA | CACCCTTGAACTTTGGAGGA | 60.0 | 177 | INTERGENIC | | | | |
| CaPOPI_8154 | scaffold682 | 375189 | ATTT/ATTTT | GTGTTGCCTACCCCTTTTCA | GGTTTGATGTAGCTGTGCGA | 60.0 | 430 | INTERGENIC | | | | |
| CaPOPI_8155 | scaffold682 | 506392 | GATATATATATATATATATATATATATATATAT/GATATATATATATATATATATATATATATATATATATATAT | AGGGAGGTTTGTGTTTGACG | TTTAATGTGTTGCGGGTGAA | 60.0 | 408 | INTERGENIC | | | | |

| INDEL marker IDs | Chromosomes/ unanchored scaffolds | Physical positions (bp) | InDels (*Kabuli* reference genome-CDC Frontier/PI) | Forward primers (5'-3') | Reverse primers (5'-3') | Anneling temperature (0C) | Expected amplified product size (bp) | Structural annotation | | Functional annotation | | |
|---|---|---|---|---|---|---|---|---|---|---|---|---|
| | | | | | | | | Sequence components of *kabuli* genome | *Kabuli* gene accession IDs | NCBI-KOG | TFs | NCBI-nr database |
| CaPOPI_8156 | scaffold682 | 539867 | CT/CTTT | TCGCCATTGTACTTGCTCTG | CCATGCCTCCAATCAAAAGT | 60.0 | 886 | INTERGENIC | | | | |
| CaPOPI_8157 | scaffold682 | 540419 | TGAG/TGAGAG | ACTTTTGATTGGAGGCATGG | TAATATGGGGAGAGGGGAGG | 59.9 | 665 | INTERGENIC | | | | |
| CaPOPI_8158 | scaffold682 | 540642 | T/TC | ACTTTTGATTGGAGGCATGG | GAAGCAAAACCAGGGAATGA | 59.9 | 886 | INTERGENIC | | | | |
| CaPOPI_8159 | scaffold682 | 540845 | A/AT | AAGGACTGGTTCACCAGCAG | GAAGCAAAACCAGGGAATGA | 60.3 | 437 | INTERGENIC | | | | |
| CaPOPI_8160 | scaffold682 | 579426 | GTTATTATTATTATTATTATTATTATTATTATTATTATTATTATTATTATT/GTTATTATTATTATTATTATTATTATTATTATTATTATTATTATT | CGGAACAATGACGTCTCAAA | GGAGGAGTGGAGGGGTTTAG | 59.7 | 352 | URR | Ca_23199 | I | G2-like | AMP-dependent synthetase/ligase |
| CaPOPI_8161 | scaffold683 | 20681 | TAA/TAAA | GAAGGATGAAAATGGGGGTT | TTGGAAAGGCTGAAATCTGG | 60.0 | 670 | INTERGENIC | | | | |
| CaPOPI_8162 | scaffold683 | 25444 | T/TCAAGA | TGGCAAATCGATTGACAAAG | TGTTGCTCCTCACGAATCTG | 59.7 | 533 | INTERGENIC | | | | |
| CaPOPI_8163 | scaffold6846 | 522 | T/TC | TGCATTGGTATATGGGGGTT | CGATCGACAGGAAGAGGAAG | 59.9 | 538 | INTERGENIC | | | | |
| CaPOPI_8164 | scaffold686 | 108971 | GATATATATATATATATATATATA/GATATATATATATATATATATATA | GGGAGAAGGAGGAAGAATGG | CTTGGGAACTGGAGAGCAAG | 60.0 | 564 | INTERGENIC | | | | |
| CaPOPI_8165 | scaffold686 | 134728 | ATATTATTATTATTATTATTATTATTATTATT/ATATTATTATTATTATTATTATTATT | TGAGAAATGCACTCTTCCCAT | CATCCGCCTCTAAAGTGAGC | 59.7 | 506 | INTERGENIC | | | | |
| CaPOPI_8166 | scaffold686 | 140005 | TT/TTAT | AGGCTTATGATTCCCCAACA | TCGACCAACCATGTCTTTCA | 59.4 | 262 | INTERGENIC | | | | |
| CaPOPI_8167 | scaffold686 | 162105 | ATTTTTTTTTTTT/ATTTTTTTTTTTTTT | AGCTTAACAAAGCGCCGATA | CCCTTTACATGTGCATCATCA | 60.0 | 563 | INTERGENIC | | | | |
| CaPOPI_8168 | scaffold686 | 182712 | CTA/CTAATA | ATGGGGTTCAAACCATGCTA | GCAAGGACTTTTGCTTTTGC | 60.2 | 696 | INTERGENIC | | | | |
| CaPOPI_8169 | scaffold686 | 183469 | AT/ATT | TGGATTAAATCATTCCCCCA | ATCCCTGTTGATTTGCTTCG | 60.0 | 617 | INTERGENIC | | | | |
| CaPOPI_8170 | scaffold686 | 184091 | ACCC/ACC | AGGCCACGATACTATGCCAC | TAGGCCAACGAGAGGAGAAA | 60.0 | 259 | INTERGENIC | | | | |

| INDEL marker IDs | Chromosomes/ unanchored scaffolds | Physical positions (bp) | InDels (*Kabuli* reference genome-CDC Frontier/PI) | Forward primers (5'-3') | Reverse primers (5'-3') | Anneling temperature (0C) | Expected amplified product size (bp) | Structural annotation | | Functional annotation | | |
|---|---|---|---|---|---|---|---|---|---|---|---|---|
| | | | | | | | | Sequence components of *kabuli* genome | *Kabuli* gene accession IDs | NCBI-KOG | TFs | NCBI-nr database |
| CaPOPI_8171 | scaffold6873 | 1457 | T/TA | TACCCAATCTTGTGCAACCA | CCTAGGGTCGACGAAAATGA | 60.0 | 575 | INTERGENIC | | | | |
| CaPOPI_8172 | scaffold688 | 2385 | GAAT/GAT | AGGTCATTTCCGTCCTTCCT | GACACGAACACACCTCATGG | 59.9 | 388 | INTERGENIC | | | | |
| CaPOPI_8173 | scaffold6903 | 5314 | A/AT | GCACACCACAACAAAATGGA | GCATCCTCTGATTGTTTGGG | 60.4 | 458 | INTRON | Ca_28223 | | | Domain of unknown function DUF239, plant |
| CaPOPI_8174 | scaffold6903 | 17324 | CT/CTTCGATGT | CCCACATTATAAATCGCCTCA | TCCGATATGCATCACTCTGC | 59.8 | 470 | CDS (FRAME SHIFT) | Ca_28223 | | | |
| CaPOPI_8175 | scaffold692 | 50884 | GTATATATATATATA/GTATATATATATATATA | TCAAGTATGGACACGCCAAA | TTAGCTGCGGTGAATCCTTT | 60.1 | 605 | INTERGENIC | | | | |
| CaPOPI_8176 | scaffold696 | 39655 | T/TC | TCGTATGAAAAGTCAATGCCTC | TGTTCTGGTGCCACATCATT | 59.2 | 133 | INTERGENIC | | | | |
| CaPOPI_8177 | scaffold696 | 43108 | C/CT | GATGTGGCATCACAAAATGC | GCGTGAAGATCACCAAGGTT | 59.9 | 840 | INTERGENIC | | | | |
| CaPOPI_8178 | scaffold696 | 52341 | CATATAT/CATATATAT | AACGGAAGTCAAAAGAGGCA | TGACTGAGGCAAGATGGTTG | 59.9 | 692 | INTRON | Ca_25809 | | bHLH | Terpene synthase-like |
| CaPOPI_8179 | scaffold6980 | 22077 | AATATATATATA/AATATATATATATA | GCCAATTCTATCCCTCACCTC | CCGATGTTTGAGGGGTATTT | 59.9 | 859 | INTERGENIC | | | | |
| CaPOPI_8180 | scaffold7 | 215103 | A/AC | TTGATCCTTGCAGTCACTCG | TGGCACTCCGATTAACATGA | 60.0 | 805 | INTERGENIC | | | | |
| CaPOPI_8181 | scaffold702 | 49940 | ATATTATTA/ATATTA | GTTTTCGAATCAACGAGGGA | TTTGGCTCGAAGGCTTTTTA | 60.1 | 452 | INTERGENIC | | | | |
| CaPOPI_8182 | scaffold702 | 55303 | CAAAAAAAAAAA/CAAAAAAAAAAAA | GCATCTGCACATGAAAAACAA | CAAACCCAATTTGATCCGAC | 59.7 | 865 | INTERGENIC | | | | |
| CaPOPI_8183 | scaffold702 | 99680 | ATTTTGCATAGTTTTTGCATAGT/ATTTTGCATAGT | CGCTCGATAACCCCAAAATA | ATTACCGACATTGCAGAGGG | 59.9 | 577 | INTERGENIC | | | | |
| CaPOPI_8184 | scaffold702 | 114373 | AGGGG/AGGGGG | TACATTTGGAAGTGGGGACG | GATGGTTGAAAAAGCTTGGC | 60.7 | 596 | DRR | Ca_25203 | | | |
| CaPOPI_8185 | scaffold702 | 156460 | TAAAAAAAA/TAAAAAAAAA | TGGACAAAATTGGAATCCTACA | GTGACACGTCGGAAGGAAAT | 59.3 | 525 | INTERGENIC | | | | |

| INDEL marker IDs | Chromosomes/ unanchored scaffolds | Physical positions (bp) | InDels (*Kabuli* reference genome-CDC Frontier/PI) | Forward primers (5'-3') | Reverse primers (5'-3') | Anneling temperature (0C) | Expected amplified product size (bp) | Structural annotation | | Functional annotation | | |
|---|---|---|---|---|---|---|---|---|---|---|---|---|
| | | | | | | | | Sequence components of *kabuli* genome | *Kabuli* gene accession IDs | NCBI-KOG | TFs | NCBI-nr database |
| CaPOPI_8186 | scaffold702 | 183682 | ATTTTT/ATTTTTT | TGCCTTAAGGTTTTGGGTTG | GACGACAACTAGGGATGGGA | 60.0 | 941 | INTERGENIC | | | | |
| CaPOPI_8187 | scaffold702 | 216004 | GA/G | CTGTTTGGGCGTCGAATTAT | TATTTTCTCCCCTCGTTGGT | 60.0 | 630 | INTERGENIC | | | | |
| CaPOPI_8188 | scaffold702 | 223066 | TTTATTATTATTATTATTATTATTATTATTATTATTATTATTATTAT/TTTATTATTATTATTATTATTATTATTATTATTATTATTATTATTATTATTAT | GGACGAAATCAGGAAGAAAAA | CCTGAGTGTGGCCTACGATT | 58.3 | 556 | INTERGENIC | | | | |
| CaPOPI_8189 | scaffold702 | 246690 | CAATTAACAAATTAACAAATTAA/CAATTAACAAATTAA | ACAGGCTTCAAAGGCAAAAA | TCAACCACCCCACTGTACCT | 59.9 | 515 | INTERGENIC | | | | |
| CaPOPI_8190 | scaffold702 | 273004 | AG/A | ATTGGTTAAAAACCCCCGAT | AGGTCATTTTCGTCCTCCCT | 59.4 | 454 | INTERGENIC | | | | |
| CaPOPI_8191 | scaffold702 | 275210 | TAAAAAA/TAAAAA | GGACAAGAATATGCCTTGGG | AGGTCATTTTCGTCCTCCCT | 59.4 | 336 | INTERGENIC | | | | |
| CaPOPI_8192 | scaffold7038 | 1268 | TTATATATATATATATATATATATATAT/TTATATATATATATATATATATATATATATAT | GGTGCTTGTTAAGTTGTGCCT | CCAACTCACCTTTCTTTACCG | 59.3 | 517 | INTERGENIC | | | | |
| CaPOPI_8193 | scaffold705 | 17000 | TAAA/TAAAA | AGGGTCTTTCGATCCTCGAT | AGTTCCGCGCTAGATTTCAA | 60.0 | 556 | INTERGENIC | | | | |
| CaPOPI_8194 | scaffold705 | 32855 | CAAAAAA/CAAAAAAA | CATGGAACCCGAAAGACAGT | TCAACACTAGCCGTCGTCTG | 60.0 | 481 | INTERGENIC | | | | |
| CaPOPI_8195 | scaffold705 | 45256 | AGGG/AGGGG | TTTCATCTGGTCTTTTCGGG | CGATCCATTTTTGTCGTGTG | 60.0 | 215 | INTERGENIC | | | | |
| CaPOPI_8196 | scaffold707 | 25303 | ATTTTTTT/ATTTTTTTT | AGTTTTCGTGGAGACATGGG | CAAATCTCACGTCGCTCAAA | 60.0 | 555 | INTERGENIC | | | | |
| CaPOPI_8197 | scaffold707 | 61601 | CTTTTTTT/CTTTTTT | CCAGGACCAAAATTGTTGAA | GGTTCAAATTCGAGATCTCCTG | 58.4 | 206 | INTERGENIC | | | | |
| CaPOPI_8198 | scaffold707 | 69823 | TT/TTAAAAAAT | TGACGGCTCCAATAGAATCA | CGAACTCTCGACCCCTAAGA | 59.2 | 869 | INTERGENIC | | | | |
| CaPOPI_8199 | scaffold707 | 86080 | CACTCTAGAGTTTCA/CACTCTAGAGTTTCAACTCTAGAGTTTCA | GGAGGAGCAGAAGCAACAAC | GGAGCATCAATGGAAAGGTC | 60.0 | 715 | INTERGENIC | | | | |
| CaPOPI_8200 | scaffold707 | 90383 | ATTTTTTTT/ATTTTTTT | TTGAGGGACTTTTGTCGTCC | TGATGGAAAAGACACCCACA | 60.1 | 576 | INTERGENIC | | | | |

| INDEL marker IDs | Chromosomes/ unanchored scaffolds | Physical positions (bp) | InDels (*Kabuli* reference genome-CDC Frontier/PI) | Forward primers (5'-3') | Reverse primers (5'-3') | Anneling temperature (0C) | Expected amplified product size (bp) | **Structural annotation** | | **Functional annotation** | | |
|---|---|---|---|---|---|---|---|---|---|---|---|---|
| | | | | | | | | **Sequence components of *kabuli* genome** | ***Kabuli* gene accession IDs** | **NCBI-KOG** | **TFs** | **NCBI-nr database** |
| CaPOPI_8201 | scaffold7081 | 2451 | A/AC | TTAATGAGGGGCTGAATTGG | AAGCGTACCTGTGGGAATTG | 59.9 | 259 | INTERGENIC | | | | |
| CaPOPI_8202 | scaffold7081 | 2734 | ATTTT/ATTTTT | CAATTCCCACAGGTACGCTT | TGGGGGTTAAAGGGATTAGG | 60.0 | 465 | INTERGENIC | | | | |
| CaPOPI_8203 | scaffold709 | 4010 | GTTTTT/GTTTTTTT | ACTCAAATGATGTTGGCGCT | CATGAGGAAACAATTGTGCAG | 60.7 | 788 | INTERGENIC | | | | |
| CaPOPI_8204 | scaffold71 | 29022 | AATTATTATTATTATTATTATTATTATTATTATTATTATTATTATTATTAT/AATTATTATTATTATTATTATTATTATTATTATTATTATTATTATTATTAT | GGTCGCATCCTAGACTTGTGA | TCAAGGGTTTGCTACTTGGG | 60.3 | 555 | INTRON | Ca_27236 | | | Domain of unknown function DUF239, plant |
| CaPOPI_8205 | scaffold7107 | 12825 | AA/AAAGA | GGGGACTAAAATTGGGGAGA | TGCAGGCAATCAATGAAAGA | 60.1 | 459 | INTERGENIC | | | | |
| CaPOPI_8206 | scaffold7107 | 14640 | AA/AATTA | TAATAGAAGGTCGCATCCCG | TGTGTCAAACGAAATGATGTG | 60.1 | 368 | INTERGENIC | | | | |
| CaPOPI_8207 | scaffold7107 | 15792 | CAAAATCATCTAAAAAATCATC/CAAAATCATC | TCACTCGGTTGCATCACATT | GGAGGTTCTAGCCCCTATAGCTT | 60.1 | 323 | INTERGENIC | | | | |
| CaPOPI_8208 | scaffold7107 | 16409 | CTATATATATATATATATA/CTATATATATATATA | GGGGCTAGAACCTCCTACAA | CCATTTGCATGATTCCACAC | 58.3 | 946 | INTERGENIC | | | | |
| CaPOPI_8209 | scaffold7107 | 16449 | TTTCAT/TTTCATCTTCAT | TCCAATGCTTACAATCCTCTCTC | CCATTTGCATGATTCCACAC | 59.7 | 508 | INTERGENIC | | | | |
| CaPOPI_8210 | scaffold711 | 184195 | TTA/T | TCCCAAGCAGAATTTTGGAC | TAAGGGGTCAGTTCATCCCA | 60.1 | 477 | INTERGENIC | | | | |
| CaPOPI_8211 | scaffold711 | 198349 | AATATATATATATATATATATA/AATATATATATATATATATA | CAAAAGATGGGTCGTGTCTTG | TTGATCTGACATGTGACACAACTT | 60.5 | 711 | INTERGENIC | | | | |
| CaPOPI_8212 | scaffold711 | 204653 | GATATATATATATATATA/GATATATATATATATA | AATGTGAATCCCACCAGGAA | TTTACAGCGCTTGTCCTCAA | 60.2 | 796 | INTERGENIC | | | | |
| CaPOPI_8213 | scaffold716 | 112534 | GTTTTT/GTTTT | CCTCTTGACTCTACAGCCCG | GTTGCCAAGTCCGCAATAAT | 60.0 | 277 | INTERGENIC | | | | |
| CaPOPI_8214 | scaffold716 | 135403 | AATTATTATTATTATTATTATTATTATTATTATTATTATTATTATTAT/AATTATTATTATTATTATTATTATTATTATTATTATTATTAT | TGATCATATAAAAACAAATGCTCACA | TGTGCAAGATTCCTGTTCCA | 59.8 | 410 | INTRON | Ca_24965 | | bHLH | Protein kinase, catalytic domain |
| CaPOPI_8215 | scaffold716 | 163516 | C/CA | TCCCAACCCAAATAAACCAA | TTTACAGCGCTTCTCCCAAT | 60.0 | 744 | INTERGENIC | | | | |

| INDEL marker IDs | Chromosomes/ unanchored scaffolds | Physical positions (bp) | InDels (*Kabuli* reference genome-CDC Frontier/PI) | Forward primers (5'-3') | Reverse primers (5'-3') | Anneling temperature (0C) | Expected amplified product size (bp) | Structural annotation | | Functional annotation | | |
|---|---|---|---|---|---|---|---|---|---|---|---|---|
| | | | | | | | | Sequence components of *kabuli* genome | *Kabuli* gene accession IDs | NCBI-KOG | TFs | NCBI-nr database |
| CaPOPI_8216 | scaffold716 | 189823 | TAAA/TAAAA | AAGCTTGTGCAAAGGGAAAA | TGGTTTTGATGGGACCAAAT | 59.9 | 113 | INTERGENIC | | | | |
| CaPOPI_8217 | scaffold719 | 123762 | TAAAA/TAAA | AGGTCATTTTCGTCCTCCCT | TTCATCCAATGGCACAAAGA | 59.9 | 695 | INTERGENIC | | | | |
| CaPOPI_8218 | scaffold72 | 23400 | AATATATATATATATATATATATA/AATATATATATATATATATA | TCAAATGTTTACCGCAACGA | CCGCCACAAATGTTTTCTTT | 60.1 | 686 | DRR | Ca_27654 | | | Transposase, Ptta/En/Spm, plant |
| CaPOPI_8219 | scaffold720 | 372071 | T/TTA | ACCAACATTTCAGGCTCACC | TAGAGCGCCAAACCCTAATG | 60.0 | 486 | INTERGENIC | | | | |
| CaPOPI_8220 | scaffold721 | 191166 | GA/GAA | CGGAGGACGAAAATGACCTA | TGTGCATCCCATAGGTGTGT | 60.1 | 473 | INTERGENIC | | | | |
| CaPOPI_8221 | scaffold7210 | 7073 | GA/G | TTTTCGTCCTCCCTAGGCTT | TTGGATGCACATGATTGGTT | 60.2 | 364 | INTERGENIC | | | | |
| CaPOPI_8222 | scaffold724 | 100088 | ATTTTTTTT/ATTTTTTTTTT | ACAGGAGGGTGATCGAGAGA | TGCATACTTGTGTGGCATTTT | 59.8 | 727 | DRR | Ca_25051 | Q | NAC | Short-chain dehydrogenase/reductase SDR |
| CaPOPI_8223 | scaffold724 | 100193 | TAAAAAAAA/TAAAAAAA | CTGTAAGCGGAGAAAATTGG | TTGCATACTTGTGTGGCATTT | 57.5 | 319 | DRR | Ca_25051 | Q | NAC | Short-chain dehydrogenase/reductase SDR |
| CaPOPI_8224 | scaffold724 | 100328 | TAAAAAAA/TAAAAAA | CTGTAAGCGGAGAAAATTGG | AATGGTAGTCCCTCTGGCCT | 57.5 | 690 | DRR | Ca_25051 | Q | NAC | Short-chain dehydrogenase/reductase SDR |
| CaPOPI_8225 | scaffold724 | 100591 | AATTTAACATAT/A | AAATGCCACACAAGTATGCAA | AATGGTAGTCCCTCTGGCCT | 59.1 | 392 | DRR | Ca_25051 | Q | NAC | Short-chain dehydrogenase/reductase SDR |
| CaPOPI_8226 | scaffold724 | 126741 | CTTTTT/CTTTT | TCGCACTTCAATATGCTTCG | AAAGGCCATGCAAGAAGAGA | 60.0 | 851 | URR | Ca_25052 | Q | NAC | Short-chain dehydrogenase/reductase SDR |
| CaPOPI_8227 | scaffold7279 | 558 | AA/AAAGGGTGTTGGTCGA | GGAATCGAGAGCTCCAAGTG | TCTCAACGCCCAGTTAGCTT | 60.0 | 523 | INTERGENIC | | | | |
| CaPOPI_8228 | scaffold7280 | 1708 | AC/ACC | GTTTCGTACCCCTCGACAAA | TCCAGATGAAATCGAGGACC | 60.0 | 311 | INTERGENIC | | | | |
| CaPOPI_8229 | scaffold7293 | 13170 | AG/A | TGCCTCTGGATTGGTTGAAT | AATCTATCCACCCGGAAACC | 60.5 | 471 | INTERGENIC | | | | |
| CaPOPI_8230 | scaffold7293 | 15472 | TT/TTAGAAAAGTGATAGT | TTCATGCCAGCAACTCTTTG | TGCTTTGCTATGCACCAGTC | 60.0 | 626 | INTERGENIC | | | | |

| INDEL marker IDs | Chromosomes/ unanchored scaffolds | Physical positions (bp) | InDels (*Kabuli* reference genome-CDC Frontier/PI) | Forward primers (5'-3') | Reverse primers (5'-3') | Anneling temperature (0C) | Expected amplified product size (bp) | Structural annotation | | Functional annotation | | |
|---|---|---|---|---|---|---|---|---|---|---|---|---|
| | | | | | | | | Sequence components of *kabuli* genome | *Kabuli* gene accession IDs | NCBI-KOG | TFs | NCBI-nr database |
| CaPOPI_8231 | scaffold7293 | 20198 | TC/T | TTGCTGCCTTCTCCTGTTTT | ATGATGCACTTTGGGCCTAC | 60.0 | 661 | INTERGENIC | | | | |
| CaPOPI_8232 | scaffold7299 | 551 | AATATATATATATATATAAATATATATATA/AATATATATATATATATATATAAATATATATATA | CACCAAAACTTAATTTCCCCTTT | TGATTCCTATGCATCGTATTTTG | 59.7 | 459 | INTERGENIC | | | | |
| CaPOPI_8233 | scaffold731 | 94332 | ATTTTTTTTTT/ATTTTTTTT | GAGCGCTGTAAAAGACCTCC | TGAAGATGGTTTGCAATGGA | 59.1 | 188 | DRR | Ca_23810 | | B3 | |
| CaPOPI_8234 | scaffold731 | 315903 | GATGA/GATGAATGA | AACTCGAAGTGCCATGCTTT | CAATACGCAATGGTGGTGAG | 59.9 | 660 | DRR | Ca_23814 | J | | Ribosomal protein S11 |
| CaPOPI_8235 | scaffold731 | 322781 | T/TCAAATTCATTAAAA | ATCCCCGATTCCTCTCAAGT | AATTCCTACTGGATGCACGC | 59.9 | 479 | INTERGENIC | | | | |
| CaPOPI_8236 | scaffold731 | 323192 | ATT/AT | GCAGGAATCCTCGTACCTCA | CTGCGAATTGCGTGAAAGTA | 60.2 | 296 | INTERGENIC | | | | |
| CaPOPI_8237 | scaffold731 | 324478 | AT/AAATAGT | TGTTTGGAAGACCGAAAACC | CAACCATCCCGTTTGGTATC | 59.9 | 257 | INTERGENIC | | | | |
| CaPOPI_8238 | scaffold731 | 342154 | CAAAAAAAA/CAAAAA | AAATGGAGGAGAAGGAGGGA | ACCCAATTACTGGAGATGCG | 60.0 | 574 | INTERGENIC | | | | |
| CaPOPI_8239 | scaffold731 | 354972 | T/TATAATCATAC | ATAACCTTAGCGCATTCCCA | CCTCCCGTAAGATCCAACAA | 59.6 | 308 | INTERGENIC | | | | |
| CaPOPI_8240 | scaffold731 | 359790 | T/TAGCAAGTGC | ATTTCTGGGAGGCAACTCCT | AAGACGCGTAAGCGGATCTA | 60.1 | 298 | DRR | Ca_23818 | | | Cytochrome f |
| CaPOPI_8241 | scaffold731 | 361613 | TGG/TGGG | TGTGCTATTGCTCAAATCCG | AGCAGTCGTTTCCAGCTGTT | 59.8 | 640 | DRR | Ca_23818 | | | Cytochrome f |
| CaPOPI_8242 | scaffold731 | 366076 | GAA/GAAA | TCTCGCGGATTTTCATAACC | CCATCCATTTCCGTATTGCT | 60.0 | 100 | INTERGENIC | | | | |
| CaPOPI_8243 | scaffold731 | 374028 | AA/AAGGGAGATAGTGCA | TCCTTGGGGTGATCTCGTAG | ATGGACGGTAGTTGGAGTCG | 60.1 | 222 | INTRON | Ca_23820 | | | |
| CaPOPI_8244 | scaffold731 | 390482 | C/CAA | TATCATAGGCCGTAGGCCAG | TTATTCGTCCAATTCCCTCG | 60.1 | 482 | INTRON | Ca_23820 | | | |
| CaPOPI_8245 | scaffold731 | 392660 | TT/TTTTTGCTAATCTAAT | GAACGACGGAACCTGTGAAT | AGGAAGCCCAAGTTCCAAAT | 60.0 | 665 | INTRON | Ca_23820 | | | |

| INDEL marker IDs | Chromosomes/ unanchored scaffolds | Physical positions (bp) | InDels (*Kabuli* reference genome-CDC Frontier/PI) | Forward primers (5'-3') | Reverse primers (5'-3') | Anneling temperature (0C) | Expected amplified product size (bp) | Structural annotation | | Functional annotation | | |
|---|---|---|---|---|---|---|---|---|---|---|---|---|
| | | | | | | | | Sequence components of *kabuli* genome | *Kabuli* gene accession IDs | NCBI-KOG | TFs | NCBI-nr database |
| CaPOPI_8246 | scaffold731 | 392845 | TTATATATATATATATATATATATATATATAT/TTATATATATATATATATAT | GAACGACGGAACCTGTGAAT | AGGAAGCCCAAGTTCCAAAT | 60.0 | 665 | INTRON | Ca_23820 | | | |
| CaPOPI_8247 | scaffold7330 | 2275 | AG/A | TTGGTCGACAAGCAACTGAG | AAAATCCAACAATCTCCCCC | 60.0 | 699 | DRR | Ca_28207 | | | |
| CaPOPI_8248 | scaffold735 | 3461 | CTTTTTTTTTT/CTTTTTTTTT | TTCTGAAATATGCCCTTCCG | AAGTGATGGCGAGGAACAAC | 60.0 | 563 | INTERGENIC | | | | |
| CaPOPI_8249 | scaffold735 | 192011 | CTTTTTTTTT/CTTTTTTTTTT | AACTCAACCCAACATTGAACA | TGGTGATGTGGCAATCATTT | 58.0 | 824 | INTERGENIC | | | | |
| CaPOPI_8250 | scaffold7364 | 2154 | CAT/C | TCAATTTGGGTTTAGGGTGC | TGTAGTGGCATGTTTGGCAT | 59.8 | 497 | INTERGENIC | | | | |
| CaPOPI_8251 | scaffold7364 | 2278 | GTTTTTTTTTTT/GTTTTTTTTT | ATGCCAAACATGCCACTACA | TGCCACCTTGACTAATGCAC | 60.0 | 378 | INTERGENIC | | | | |
| CaPOPI_8252 | scaffold7377 | 545 | CT/CTT | TTTCTTCCCAACCTTCATCG | GCCGTTGGAACTTCATCATT | 60.0 | 816 | INTERGENIC | | | | |
| CaPOPI_8253 | scaffold738 | 2137 | T/TC | TGAATATCGCCCGAAAAGTC | TTTCATAGCAGACGAGGGCT | 60.0 | 292 | INTERGENIC | | | | |
| CaPOPI_8254 | scaffold738 | 5098 | ATTTTTT/ATTTTT | TCGGGTGGTACTTAAATCGC | ATCTGACGTGGAAACCGAAC | 60.0 | 274 | INTERGENIC | | | | |
| CaPOPI_8255 | scaffold738 | 10670 | GAAAA/GAAAAA | GTACCACCCCACAAAAATGG | CATCAGGACTTTTGGGGCTA | 59.9 | 637 | INTERGENIC | | | | |
| CaPOPI_8256 | scaffold743 | 14244 | AACACACA/AACACA | TGCTTGATTGCTTGAGTTCC | GTACCCTGATTGCCCTCTGA | 59.0 | 321 | INTERGENIC | | | | |
| CaPOPI_8257 | scaffold7448 | 21480 | GAAA/GAAAA | AGGATCTCCCGATTCTCGAT | TTCCGGGTCAGATTTCAAAC | 60.0 | 287 | INTERGENIC | | | | |
| CaPOPI_8258 | scaffold7477 | 2293 | TAAA/TAA | TTGGTGGATTTGAGACCACA | ACTTCCCTTTGCACATTTGG | 59.9 | 652 | INTERGENIC | | | | |
| CaPOPI_8259 | scaffold7477 | 6564 | TTTG/TTTGTTG | CCTGCAGAAAGCAAAACACA | ACCTTGGGAAGGAGGAGAAA | 60.0 | 590 | INTERGENIC | | | | |
| CaPOPI_8260 | scaffold749 | 1624 | CTATATATATATATATATATATATATAT/CTATATATATATATATATATAT | CTTGGGAACTGGAGAGCAAG | GAGGAGGGAGGGATTTTGAG | 60.0 | 536 | INTERGENIC | | | | |

| **INDEL marker IDs** | **Chromosomes/ unanchored scaffolds** | **Physical positions (bp)** | **InDels (*Kabuli* reference genome-CDC Frontier/PI)** | **Forward primers (5'-3')** | **Reverse primers (5'-3')** | **Anneling temperature (0C)** | **Expected amplified product size (bp)** | **Structural annotation** | | **Functional annotation** | | |
|---|---|---|---|---|---|---|---|---|---|---|---|---|
| | | | | | | | | **Sequence components of *kabuli* genome** | ***Kabuli* gene accession IDs** | **NCBI-KOG** | **TFs** | **NCBI-nr database** |
| CaPOPI_8261 | scaffold750 | 31851 | GAAAAAAAAA/GAAAAAA | TGAGTTTCTATGCAAGGGCA | TTCATGAAGACACCGGAACA | 59.4 | 273 | INTERGENIC | | | | |
| CaPOPI_8262 | scaffold750 | 32434 | AGG/AG | GCTTGTCCCCAAACAACAAT | TTGGGATGGTGATTCTCACA | 59.8 | 627 | INTERGENIC | | | | |
| CaPOPI_8263 | scaffold750 | 38979 | AT/A | CACTGATAACGCGACTAATTTGA | ACCGCCACAAATGATAAAGC | 59.3 | 748 | INTERGENIC | | | | |
| CaPOPI_8264 | scaffold750 | 58677 | TAA/TAAA | CGTAGCATCAGCAACCTTCA | TGCTTTTGGGTGTAACCATTT | 60.0 | 634 | INTERGENIC | | | | |
| CaPOPI_8265 | scaffold750 | 59023 | ATTGTTGTTGTT/ATTGTTGTTGTTGTT | CGTAGCATCAGCAACCTTCA | TCCCTCTTTTGTTTTTCCCA | 60.0 | 989 | INTERGENIC | | | | |
| CaPOPI_8266 | scaffold750 | 59256 | TATGTTCTGCTAATGT/TATGT | TGGTTACACCCAAAAGCAAA | TCCCTCTTTTGTTTTTCCCA | 59.1 | 373 | INTERGENIC | | | | |
| CaPOPI_8267 | scaffold750 | 248395 | CAAAA/CAAA | AGCTCGTCCAACGAAAGAAA | TCGACTTGTCAATCGCTTTG | 60.0 | 645 | INTERGENIC | | | | |
| CaPOPI_8268 | scaffold750 | 318580 | ATATAACTTAACTTATAACTTAACTTATAACTTAA/ATATAACTTAACTTATAACTTAACTTATAACTTAACTTATAACTTAA | AAGCGCTGTAAAAGGTGCAT | AGCATTCTGCAAGGGATCTG | 59.9 | 436 | DRR | Ca_25943 | | | |
| CaPOPI_8269 | scaffold750 | 360259 | GA/G | TGTTTGTCTTGCTGCTTTGC | AAAATCCAACAATCTCCCCC | 60.2 | 626 | DRR | Ca_25944 | | | |
| CaPOPI_8270 | scaffold7502 | 560 | TTATATATATATATATATATATATATATAT/TTATATATATATATATATATAT | GTTGGTTCCCTAGCACCGTA | TATCCAATCTTGTGCGTCCA | 60.0 | 806 | INTERGENIC | | | | |
| CaPOPI_8271 | scaffold752 | 3867 | CAAAAAAAAAA/CAAAAAAAAA | TGGATGACAATGGGTAGGGT | TATAGTACCCATTCCGCCCC | 60.1 | 645 | INTERGENIC | | | | |
| CaPOPI_8272 | scaffold752 | 41940 | ATTTTTTTTTT/ATTTTTTTTTTT | AGCACACATGACACAAAGCA | TGAATTGTGAGGGTGTTGGA | 58.9 | 569 | INTERGENIC | | | | |
| CaPOPI_8273 | scaffold752 | 131814 | GTATATATATATATATAT/GTATATATATATATATAT | GTTGGTTCCCTAGCACCGTA | ATTGCCAGAGGTGTTGGAGT | 60.0 | 754 | INTERGENIC | | | | |
| CaPOPI_8274 | scaffold752 | 505163 | TTTTATTTTATTTATTTTAT/TTTTATTTTATTTATTTTATTTATTTTAT | AGCCATTGCAGTCTTCGTCT | TCGTGTCTCAAAACTCAAGGAA | 60.0 | 854 | INTERGENIC | | | | |
| CaPOPI_8275 | scaffold758 | 13400 | AATATATATATATATATATATATATA/AATATATATATATATATATATA | CTCGTCCAATTCCCCTTTTT | CGATTTTACGTGAATATACGAACAC | 60.3 | 603 | INTERGENIC | | | | |

| INDEL marker IDs | Chromosomes/ unanchored scaffolds | Physical positions (bp) | InDels (*Kabuli* reference genome-CDC Frontier/PI) | Forward primers (5'-3') | Reverse primers (5'-3') | Anneling temperature (0C) | Expected amplified product size (bp) | Structural annotation | | Functional annotation | | |
|---|---|---|---|---|---|---|---|---|---|---|---|---|
| | | | | | | | | Sequence components of *kabuli* genome | *Kabuli* gene accession IDs | NCBI-KOG | TFs | NCBI-nr database |
| CaPOPI_8276 | scaffold758 | 26328 | TTATATATATATATATATATATATATATA TA/TTATATATATATATATATATATATATATA TATATATATATATATATA | TTTGGCTCGAAGGCTTT TTA | GTTGGTTCCCTAGCACC GTA | 60.0 | 600 | INTERGENIC | | | | |
| CaPOPI_8277 | scaffold7650 | 9735 | T/TG | TATGGCATTGTGGTGCA CTT | GGATTAAAATTGGAATT GTTCAAGA | 60.0 | 482 | URR | Ca_27809 | O | | SKP1 component |
| CaPOPI_8278 | scaffold7650 | 9922 | CA/CATA | CCGCAATTATATCTGCG ACA | AGTTCAAAAATACGCGG GTG | 59.7 | 857 | URR | Ca_27809 | O | | SKP1 component |
| CaPOPI_8279 | scaffold7657 | 660 | CACAACAA/CACAA | AGGGAGGACGAAAATGA CCT | GGTGTGTTTGTGTCGTT GGA | 59.9 | 412 | INTERGENIC | | | | |
| CaPOPI_8280 | scaffold766 | 179582 | GTTTTTT/GTTTTT | TGGGTGGATTTTTCTGA AGC | TTTTATGGGGCCAACAA CTC | 60.1 | 426 | DRR | Ca_24688 | | | Zinc finger, GRF-type |
| CaPOPI_8281 | scaffold766 | 183062 | TAAAAAAAAAA/TAAAAAAAAAAA | AAAAGAAAACGGAGGAG GGA | GCAACCTTTATTGATTG CTATGC | 60.0 | 592 | INTERGENIC | | | | |
| CaPOPI_8282 | scaffold768 | 560 | T/TG | CCATGAGAGTCGAAATC GGT | TCCCACAGATCCACATT CAA | 60.1 | 188 | INTERGENIC | | | | |
| CaPOPI_8283 | scaffold769 | 1084 | TTT/TTTCAATT | AAATGAACCAATTTAATG AGCTACC | CCCTCCCAAAACACCCT TAT | 59.0 | 479 | INTERGENIC | | | | |
| CaPOPI_8284 | scaffold769 | 2786 | TGGCAGCAG/TG | ACGTACACGTCAGCGTT TTG | CCCAATTCTCAATCCCA CTT | 59.8 | 147 | INTERGENIC | | | | |
| CaPOPI_8285 | scaffold769 | 3178 | ACTCTCT/ACTCT | ACGTACACGTCAGCGTT TTG | AGCTGGATGATCACCAT GATA | 59.8 | 975 | INTERGENIC | | | | |
| CaPOPI_8286 | scaffold769 | 3493 | CTAA/CTAATAA | TCCAAGAAAACAGTTTC CGA | TGAATGGTTTTATTGAG CTGGA | 58.3 | 588 | INTERGENIC | | | | |
| CaPOPI_8287 | scaffold769 | 5414 | AATATATATATATATATATATATATATAT ATAT/AATATATATATATATATATAT | TCACTCGCGTTAATTTTG ATG | GCGAGTGAATACATGTG CCA | 58.8 | 518 | INTERGENIC | | | | |
| CaPOPI_8288 | scaffold769 | 5924 | AATATATATATATATAT/AATATATATATA TATATAT | TGGCACATGTATTCACT CGC | TGTTGTTATTTGTGGCG GAA | 60.7 | 489 | INTERGENIC | | | | |
| CaPOPI_8289 | scaffold769 | 17748 | AATATATATATATATATATATATATAT/AA TATATATATATATATATATATATATATAT | TTGCTCCTTTTTGCAATT AAA | TTGTGTGGCAGCTTCTA ACG | 57.7 | 545 | INTERGENIC | | | | |
| CaPOPI_8290 | scaffold769 | 27694 | T/TAA | CTGGGCAAGGGATCTGA ATA | TCACAATCAGTGCACAC AACA | 60.0 | 311 | INTERGENIC | | | | |

| INDEL marker IDs | Chromosomes/ unanchored scaffolds | Physical positions (bp) | InDels (*Kabuli* reference genome-CDC Frontier/PI) | Forward primers (5'-3') | Reverse primers (5'-3') | Anneling temperature (0C) | Expected amplified product size (bp) | Structural annotation | | Functional annotation | | |
|---|---|---|---|---|---|---|---|---|---|---|---|---|
| | | | | | | | | Sequence components of *kabuli* genome | *Kabuli* gene accession IDs | NCBI-KOG | TFs | NCBI-nr database |
| CaPOPI_8291 | scaffold769 | 29353 | AGC/AGCCCACTAATATGC | GCACTCTCATTCATCCCACA | CCACATGTCATTTAACCCTGC | 59.6 | 370 | INTERGENIC | | | | |
| CaPOPI_8292 | scaffold7697 | 589 | G/GA | CTAGCCCTCGTCTGTTCCAG | AATCCGTATTTTCGCACCAG | 60.0 | 521 | INTERGENIC | | | | |
| CaPOPI_8293 | scaffold7698 | 946 | AAA/AAATAA | CCGGTGGATATGATGAGCTT | GGCTTTTGAATCACCGAAAA | 59.9 | 650 | INTERGENIC | | | | |
| CaPOPI_8294 | scaffold772 | 11892 | TAAA/TA | GGCACACTCCCGTAAAATTC | TTTTTCGCACACCAATGAGA | 59.4 | 539 | INTERGENIC | | | | |
| CaPOPI_8295 | scaffold772 | 64471 | CAAAAAAAAAAA/CAAAAAAAAAAAA | TTTCTCAACCACCCACTCCT | AGGGAGAGAGTGACACGCAT | 59.5 | 734 | INTERGENIC | | | | |
| CaPOPI_8296 | scaffold772 | 213642 | AGG/AGGG | ACTTGAGGGCGAAGAACAAA | TTGGATGACCTACGACTCCC | 59.9 | 573 | INTERGENIC | | | | |
| CaPOPI_8297 | scaffold772 | 251341 | CATATATATATATATATATATATAT/CATATATATATATATATATATATATAT | GCTCCACCCATGAAGACATT | TGGTTCCCTAACACCGTAGC | 59.9 | 768 | INTERGENIC | | | | |
| CaPOPI_8298 | scaffold774 | 3829 | GAAAATGACC/GAAAATGACCAAAATGACC | TGGATGCACAAGATTGGCTA | AGGTCATTTTCGTCCTCCCT | 60.2 | 468 | INTERGENIC | | | | |
| CaPOPI_8299 | scaffold775 | 110824 | TAAA/TAA | GCCGAATCGATTTTGTCAGT | GGAGGGTTTTGGTTTGGAAT | 60.1 | 521 | INTERGENIC | | | | |
| CaPOPI_8300 | scaffold775 | 388602 | CATATATATATATATATAT/CATATATATATATATATATAT | CTTCGCGAAATAAAGCTTGG | TTGGTATTGAACTCGGAGGC | 60.0 | 567 | INTERGENIC | | | | |
| CaPOPI_8301 | scaffold775 | 446063 | TTATATATATATAT/TTATATATATATATAT | TTTTGCAAGATCTGCCACTG | TGGAAGGATCCGGAACTATG | 60.0 | 867 | INTERGENIC | | | | |
| CaPOPI_8302 | scaffold775 | 449733 | CTTATCAATTGTGTTT/CTTATCAATTGTGTTTATCAATTGTGTTT | CTCTCTTCTGCGCATCAACT | TTGACCATGACACATGCCTT | 58.3 | 459 | DRR | Ca_23414 | | NAC | Pectinesterase, catalytic |
| CaPOPI_8303 | scaffold775 | 450377 | TG/TGG | AAGGCATGTGTCATGGTCAA | TTTACGGGAACGAGGAGAGA | 60.0 | 738 | DRR | Ca_23414 | | NAC | Pectinesterase, catalytic |
| CaPOPI_8304 | scaffold776 | 224423 | T/TTCCCTA | TGGGATACGTAGGAGCAAGG | TGTCCGTGAAAAATGAGTCG | 60.1 | 310 | INTERGENIC | | | | |
| CaPOPI_8305 | scaffold7765 | 977 | ATTTTTTTTTT/ATTTTTTTTTTTTT | AGATGGCTGCAGCTCAAAGT | TTTCCAAGAGGTTTTGGGTC | 60.2 | 464 | INTERGENIC | | | | |

| INDEL marker IDs | Chromosomes/ unanchored scaffolds | Physical positions (bp) | InDels (*Kabuli* reference genome-CDC Frontier/PI) | Forward primers (5'-3') | Reverse primers (5'-3') | Anneling temperature (0C) | Expected amplified product size (bp) | Structural annotation | | Functional annotation | | |
|---|---|---|---|---|---|---|---|---|---|---|---|---|
| | | | | | | | | Sequence components of *kabuli* genome | *Kabuli* gene accession IDs | NCBI-KOG | TFs | NCBI-nr database |
| CaPOPI_8306 | scaffold787 | 91954 | C/CA | AGGTCATTTTCGACCTCCCT | TAGAACAAGCCTCGGGAAGA | 59.9 | 331 | INTERGENIC | | | | |
| CaPOPI_8307 | scaffold787 | 149556 | ATTTTT/ATTTT | CGGGATGGTGGCATAATATC | AGCAGGGTTTCTCCCAAACT | 60.0 | 343 | INTERGENIC | | | | |
| CaPOPI_8308 | scaffold787 | 154027 | TAAAAAAAAA/TAAAAAAAAAAAAA | AATCCAATGACACAAACGCA | TCAGTCAAGCGCACATAAAA | 60.0 | 415 | INTERGENIC | | | | |
| CaPOPI_8309 | scaffold787 | 289233 | TAAAAAAAA/TAAAAAAAAA | CAAGCTATTCTTTGCCGACAT | CGACATTCAAACATGACCCTT | 59.4 | 621 | INTERGENIC | | | | |
| CaPOPI_8310 | scaffold787 | 334329 | ATTTTTTTTT/ATTTTTTTTTT | CTTCCTCTGTGCCACATCCT | TTCATTCGTGTCTGCTTTGG | 60.3 | 598 | INTERGENIC | | | | |
| CaPOPI_8311 | scaffold787 | 348087 | TTATATATATATAT/TTATATATATAT | GAGGGGATGGTGAGAGTGAA | GCAGACATCACATGGAAGGA | 60.0 | 595 | INTERGENIC | | | | |
| CaPOPI_8312 | scaffold787 | 429004 | G/GGA | TCATTTCAATGCCCTCTTCC | TCTTTGAGGGATTCGTTTGG | 60.0 | 316 | INTRON | Ca_25848 | R | NAC | RNA recognition motif domain |
| CaPOPI_8313 | scaffold787 | 437054 | CAAAAAAAAAAAAA/CAAAAAAAAAAAAAAA | TCAATGGCTCATCAAACCAA | TTTTGTGCATCGATTGTGGT | 60.0 | 397 | INTERGENIC | | | | |
| CaPOPI_8314 | scaffold787 | 460684 | CAT/CATAT | TATGGGAAGGAAATGTGGGA | TCACTCGCAACGAGAAGTTG | 60.1 | 601 | INTERGENIC | | | | |
| CaPOPI_8315 | scaffold787 | 472975 | T/TTA | ACGCTGTTGGTCCATGTAAA | TGAACAAAATCTAAAGCGTAAAAGG | 59.1 | 428 | INTERGENIC | | | | |
| CaPOPI_8316 | scaffold7890 | 680 | TG/TGG | TTTCTCCCCTTTCTTTCCGT | GAAGGGACTGCGGCATAATA | 60.0 | 162 | INTERGENIC | | | | |
| CaPOPI_8317 | scaffold7891 | 3768 | CAAA/CAAAA | TCTGGACTTTTCGAGCGATT | AGTGCATTTTAGTCGGGTGG | 60.0 | 379 | INTERGENIC | | | | |
| CaPOPI_8318 | scaffold7902 | 684 | CTT/CTTT | GGGTAGCATGGCAAGTTGTT | CGAGTAAAGCGTTTTGACTAACG | 60.0 | 363 | INTERGENIC | | | | |
| CaPOPI_8319 | scaffold7923 | 2123 | AAGCTCA/AA | TCGGGGTCTTTAGTCGTCAT | GCGAGGAAGCAAAAGCTAGA | 59.6 | 796 | DRR | Ca_27506 | O | | |
| CaPOPI_8320 | scaffold7923 | 22242 | CAAAAAAAAAA/CAAAAAAAAA | TTTCCCGACTTGGTCCTTTA | TTTGCGTTACGAATTTGATGA | 59.5 | 177 | DRR | Ca_27507 | QI | bHLH | Cytochrome P450 |

| INDEL marker IDs | Chromosomes/ unanchored scaffolds | Physical positions (bp) | InDels (*Kabuli* reference genome-CDC Frontier/PI) | Forward primers (5'-3') | Reverse primers (5'-3') | Anneling temperature (0C) | Expected amplified product size (bp) | Structural annotation | | Functional annotation | | |
|---|---|---|---|---|---|---|---|---|---|---|---|---|
| | | | | | | | | Sequence components of *kabuli* genome | *Kabuli* gene accession IDs | NCBI-KOG | TFs | NCBI-nr database |
| CaPOPI_8321 | scaffold7923 | 22388 | GAGAAGAA/GAGAA | TTTCCCGACTTGGTCCTTTA | GGAGGGAGAACATCTGTTGG | 59.5 | 741 | DRR | Ca_27507 | QI | bHLH | Cytochrome P450 |
| CaPOPI_8322 | scaffold7923 | 25395 | ATC/A | AAAATATGCCATTCGATCAAGA | AAATGGCTTGGTTATGGCTG | 58.6 | 200 | URR | Ca_27507 | QI | bHLH | Cytochrome P450 |
| CaPOPI_8323 | scaffold793 | 49023 | CATAATAATAATAATAATAATAATAATAA/CATAATAATAATAATAATAATAATAATAATAA | TGTATGAGCAACGGATGTTGA | GCAAGGCGCGAGTTAGTATC | 60.1 | 351 | INTERGENIC | | | | |
| CaPOPI_8324 | scaffold793 | 127009 | T/TA | TGGTGTCATCCAAGAAAATGA | TTCCAGCATAATCCGAGTCC | 59.0 | 577 | INTERGENIC | | | | |
| CaPOPI_8325 | scaffold793 | 135694 | TG/TGGG | CCAAACACACCATTTATGCG | CACTAGTGAATGGAAGGGGC | 59.8 | 254 | INTERGENIC | | | | |
| CaPOPI_8326 | scaffold793 | 248408 | T/TA | TGCTCATGCATTTTCATGCT | GAGTCCCTGTGTTGCCAAAT | 60.4 | 410 | INTERGENIC | | | | |
| CaPOPI_8327 | scaffold794 | 138143 | GA/G | CTGTCAAACCGGGTTCTGTT | TGCTTGCAGTGCTTCATCTT | 60.0 | 378 | INTERGENIC | | | | |
| CaPOPI_8328 | scaffold796 | 7462 | GTTTTTTT/GTTTTTTTT | TCCTCCCTTGGCTTATCCTT | GGAGGACGAAAATGACCTGA | 60.0 | 607 | INTERGENIC | | | | |
| CaPOPI_8329 | scaffold796 | 26479 | GTT/GTTT | TCATTTTCGTCCTCCTTTGG | GCCTATTGGATGCACAGGAT | 60.0 | 470 | INTERGENIC | | | | |
| CaPOPI_8330 | scaffold797 | 15287 | CAAAAAA/CAAAAAAAA | GGTGATTTTTATGATGTGCTAGTTTT | CCCTTTCAATTCCCGTTTTT | 59.0 | 689 | INTERGENIC | | | | |
| CaPOPI_8331 | scaffold798 | 16927 | T/TC | ATGCAATGCGTATATGCCAA | TAAATCGTGTTGACCCGTGA | 59.9 | 233 | INTERGENIC | | | | |
| CaPOPI_8332 | scaffold80 | 25932 | TAAAAAAAAAA/TAAAAAAAAAAA | AATGTAGTCCCAACCCGATG | CGATACTCACCCCTCGTTGT | 59.7 | 210 | INTERGENIC | | | | |
| CaPOPI_8333 | scaffold80 | 56806 | GCC/GC | ATGCCTCCTTCTTCTCCCTT | GGAAATTCATGGTTTCACGA | 59.3 | 544 | INTERGENIC | | | | |
| CaPOPI_8334 | scaffold80 | 301960 | GATATATATATATATATATATATA/GATATATATATATATATATATA | GCTTTGTGGAGGAAATGGAA | TTGTCTCTCGACGAACGATG | 60.1 | 745 | INTERGENIC | | | | |
| CaPOPI_8335 | scaffold801 | 4383 | CCTC/CC | ATGGTGGTGAGCAACTTTCC | TATTTTGGATTTTGGCTCGG | 60.0 | 626 | INTERGENIC | | | | |

| INDEL marker IDs | Chromosomes/ unanchored scaffolds | Physical positions (bp) | InDels (*Kabuli* reference genome-CDC Frontier/PI) | Forward primers (5'-3') | Reverse primers (5'-3') | Anneling temperature (0C) | Expected amplified product size (bp) | Structural annotation | | Functional annotation | | |
|---|---|---|---|---|---|---|---|---|---|---|---|---|
| | | | | | | | | Sequence components of *kabuli* genome | *Kabuli* gene accession IDs | NCBI-KOG | TFs | NCBI-nr database |
| CaPOPI_8336 | scaffold801 | 106576 | AGG/AG | CCGTGTTCGTGAAATCAATG | GGTGAGTTGGGCATGCTTAT | 60.0 | 348 | INTRON | Ca_26177 | | | WD40 repeat |
| CaPOPI_8337 | scaffold801 | 118356 | TG/TGAGTCGTGTAG | ATGGGCCACCGTGTTAGTAG | CAATTCTCGCACGACTCAAA | 59.9 | 507 | INTERGENIC | | | | |
| CaPOPI_8338 | scaffold801 | 152306 | ATTTTTTTTTTT/ATTTTTTTTTT | TCCCCATTCTCTTCTTCACC | TGGTGGTCTTGTTGGTTTCA | 59.1 | 778 | INTERGENIC | | | | |
| CaPOPI_8339 | scaffold801 | 161086 | TAAAAAAAAAAA/TAAAAAAAAAAAA | CAGGATGCGACCATGTAGTG | TTTGTGTTCCGACATCTCCA | 60.1 | 244 | INTERGENIC | | | | |
| CaPOPI_8340 | scaffold801 | 189547 | CTAAATTGATA/CTAAATTGATAAATTGATA | CAGGAAAAAGAAGGAACCGA | TGCAAACACTTTCCAATGTCA | 59.3 | 637 | INTERGENIC | | | | |
| CaPOPI_8341 | scaffold801 | 192338 | ATTTTTTTTT/ATTTTTTTTTT | TGTGCAGATAAATGGCCAAA | GAGCTGTGTCTTGGCAATCA | 60.1 | 792 | INTERGENIC | | | | |
| CaPOPI_8342 | scaffold801 | 202304 | TAAAAAAAAA/TAAAAAAAA | TGCTTCTTTTGCACGTTTTG | AGACGTCTTTCTTTTGCGGA | 60.0 | 837 | INTERGENIC | | | | |
| CaPOPI_8343 | scaffold801 | 260992 | CATAT/CAT | TCGTCCGTAAAAGATACCGC | GGAGGACCAAAATTTGAGCA | 60.1 | 661 | INTERGENIC | | | | |
| CaPOPI_8344 | scaffold806 | 37447 | AATATATATATATATATATA/AATATATATATATATATATATA | TTGGTATTGAACTCGGAGGC | CGAGGCAGAGGAAGAAAATG | 60.1 | 359 | INTERGENIC | | | | |
| CaPOPI_8345 | scaffold81 | 91419 | A/AC | TGAGGAGAAACAAAATGGGG | ATGGAAGGGTCGTTGACATT | 59.9 | 458 | INTERGENIC | | | | |
| CaPOPI_8346 | scaffold811 | 56559 | GATATATATATATATATATATATAT/GATATATATATATATATATATATATAT | TTTAACGTGTCGCGTCTTTG | GAATAAAATGCACCGGCACT | 59.9 | 748 | INTERGENIC | | | | |
| CaPOPI_8347 | scaffold811 | 159571 | TTATATATATATATATATATATATATAT/TTATATATATATATATATATATATAT | TTGCAACATGCTATTTCCCA | TGGGTGCTATTTGCAATCAA | 60.1 | 613 | INTRON | Ca_27462 | | | |
| CaPOPI_8348 | scaffold811 | 234762 | GTAATAATAATAATAATAATAATAAAAATAATAATAATAATAATA/GTAATAATAATAATAATAATAATAATAAAAATAATAATAATAATAATA | GGTGTGTCTGTGTGTTTGCC | GGTCCTCGATTTCATCTGGA | 60.1 | 683 | INTERGENIC | | | | |
| CaPOPI_8349 | scaffold811 | 238697 | CTATATATATATATATATATATA/CTATATATATATATATATATATATA | GGTGTGTCTGTGTGTTTGCC | CCGTGCTAATCTCTGCTTCC | 60.1 | 447 | INTERGENIC | | | | |
| CaPOPI_8350 | scaffold812 | 215247 | TGG/TG | AGAAGGAACAACGAGAACGC | TGTCCTTCTCATTGGGAACC | 59.5 | 178 | INTERGENIC | | | | |

| INDEL marker IDs | Chromosomes/ unanchored scaffolds | Physical positions (bp) | InDels (*Kabuli* reference genome-CDC Frontier/PI) | Forward primers (5'-3') | Reverse primers (5'-3') | Anneling temperature (0C) | Expected amplified product size (bp) | Structural annotation | | Functional annotation | | |
|---|---|---|---|---|---|---|---|---|---|---|---|---|
| | | | | | | | | Sequence components of *kabuli* genome | *Kabuli* gene accession IDs | NCBI-KOG | TFs | NCBI-nr database |
| CaPOPI_8351 | scaffold812 | 216839 | TAAAAAAAAA/TAAAAAAAA | GCATTTGATCGTCCCTCACT | GGACAAAGCACACCAGGAAT | 60.1 | 447 | INTERGENIC | | | | |
| CaPOPI_8352 | scaffold812 | 228611 | TAAAAAAAAA/TAAAAAAAA | CAAGTGCATTCCCCAAAGAT | CGCATGAATATTGTCAACGG | 59.9 | 529 | INTERGENIC | | | | |
| CaPOPI_8353 | scaffold812 | 245558 | GAAAAAAAAA/GAAAAAAAAAA | CAAAGTCTTCACACACCGACA | TGTTGACTCCAGTCACCGAG | 59.8 | 736 | INTERGENIC | | | | |
| CaPOPI_8354 | scaffold812 | 285596 | TAAAAAAAA/TAAAAAAAAA | GATGTTCTCGGCAGTTGACA | CCGGTACTTAATATGCGACTCA | 59.8 | 365 | INTERGENIC | | | | |
| CaPOPI_8355 | scaffold812 | 311738 | AATTATTATTATTATTATTATTATTATTATTATT/AATTATTATTATTATTATTATTATTATTATTATTATTATTATT | TTGTTTGTTTGGGCACGTTA | GGATGCGACCATCTACTGCT | 60.0 | 460 | INTERGENIC | | | | |
| CaPOPI_8356 | scaffold812 | 320576 | ATTTTTTTTTTTT/ATTTTTTTTTTTTTTT | AAAACGTTGGAGCATTTTGC | AGGGAAATGGAACTCAACCC | 60.1 | 442 | INTERGENIC | | | | |
| CaPOPI_8357 | scaffold812 | 350314 | GAAAAAAAA/GAAAAAAAAA | ACACCACGACGACGTACAAA | AATGGTTCTCACTATCGCCG | 60.1 | 239 | INTERGENIC | | | | |
| CaPOPI_8358 | scaffold812 | 354262 | TTTCTTCTTCTTCTTCTTCTTCTTCTTCT/TTTCTTCTTCTTCTTCTTCTTCTTCTTCTTCTTCT | GCTACGGCTAGTGCCTTCAC | GTCAAAAGCAACGATCAGCA | 60.0 | 514 | INTERGENIC | | | | |
| CaPOPI_8359 | scaffold812 | 378934 | TAAAAAAAAAA/TAAAAAAAAA | TTGGAAGGAGCCCTTACCTT | TGAATTGTGGTTTCGAGCTT | 60.1 | 716 | INTERGENIC | | | | |
| CaPOPI_8360 | scaffold812 | 407862 | ATTTATCATTTATGTTTTAT/ATTTATCATTTATGTTTTATCATTTATGTTTTAT | TTGTGCAAAGACATGGGAAA | TCAACTGTTGCACCTTCGAG | 60.1 | 527 | INTERGENIC | | | | |
| CaPOPI_8361 | scaffold812 | 487308 | GAACAAATAACAACA/GAACA | CTTCACCATGCTCAGTCCAA | CCCAAATCAGTCGGTTCTGT | 59.8 | 840 | INTERGENIC | | | | |
| CaPOPI_8362 | scaffold812 | 526031 | TGG/TG | TCGACTTGTCAATCGCTTTG | CACTCCAGTGCTGAGGGAAT | 60.0 | 215 | INTERGENIC | | | | |
| CaPOPI_8363 | scaffold820 | 15078 | A/AG | TCATCATGGGCTTACGACCT | GGCCAAACCCATTAAAACCT | 60.5 | 343 | INTERGENIC | | | | |
| CaPOPI_8364 | scaffold823 | 52739 | TATAATAACATATAATAA/TATAATAACATATAATAATAACATATAATAA | CCTTTTAAAGTTTGACTGCATAACAA | TGAAATAAAACCATAAAACGAAGA | 60.0 | 830 | INTERGENIC | | | | |
| CaPOPI_8365 | scaffold824 | 6128 | CA/C | CACCCCAGTTTTGAGGGTAA | CGATTGTGTTTGTCACAGGG | 59.8 | 191 | INTERGENIC | | | | |

| INDEL marker IDs | Chromosomes/ unanchored scaffolds | Physical positions (bp) | InDels (*Kabuli* reference genome-CDC Frontier/PI) | Forward primers (5'-3') | Reverse primers (5'-3') | Anneling temperature (0C) | Expected amplified product size (bp) | Structural annotation | | Functional annotation | | |
|---|---|---|---|---|---|---|---|---|---|---|---|---|
| | | | | | | | | Sequence components of *kabuli* genome | *Kabuli* gene accession IDs | NCBI-KOG | TFs | NCBI-nr database |
| CaPOPI_8366 | scaffold829 | 10973 | T/TA | TTTATTTGACAACTCGTTTTTGTT | GAGCGAATTGAGAAACGAGG | 57.1 | 799 | INTERGENIC | | | | |
| CaPOPI_8367 | scaffold829 | 12450 | GT/G | AATCGAATTCACAAGCGACC | GAACCAAGGGCCAAAAATCT | 60.1 | 153 | INTERGENIC | | | | |
| CaPOPI_8368 | scaffold8301 | 5927 | AGTTAGTTTTTTTATTGTTAGTTTTTT/AGTTAGTTTTTT | ATGTCGCGTCTTCATCAACC | ACAATTTATCCGTGTCGCGT | 61.1 | 546 | INTERGENIC | | | | |
| CaPOPI_8369 | scaffold831 | 50051 | A/AAG | TGACCAAAATGCTCATCCAA | AGGTCATTTTCGTCCTCCCT | 60.0 | 285 | INTERGENIC | | | | |
| CaPOPI_8370 | scaffold8314 | 868 | CAAAAAAAAAAAAA/CAAAAAAAAAAAA | TGAATGGTTGCATGGTTATCA | GTGGGAAGTGGTAGAGTGCG | 59.8 | 415 | INTERGENIC | | | | |
| CaPOPI_8371 | scaffold8314 | 1258 | CTTTTTTTT/CTTTTTTT | AATTTAGCTCATTGCTGCCG | TACCTCCTTTGGAAGCATGG | 60.4 | 353 | INTERGENIC | | | | |
| CaPOPI_8372 | scaffold8314 | 2052 | G/GC | TCAATTAGGCCCTTCACCAC | GTCCTCTGAAACCTCCCACA | 59.9 | 915 | INTERGENIC | | | | |
| CaPOPI_8373 | scaffold8315 | 17783 | GTATATATATATATATATAT/GTATATATATATATATATATATATATAT | AGGGTCTCCAAGTCCTCGAT | TTTTAGGGCTAGTTGTACACATGAA | 60.1 | 377 | INTERGENIC | | | | |
| CaPOPI_8374 | scaffold8315 | 18988 | T/TTTA | AAAAATTTATATTGCGTCAATTTGG | GTGCTGGCCATAGGAAATGT | 59.6 | 565 | INTERGENIC | | | | |
| CaPOPI_8375 | scaffold8315 | 20409 | ATT/AGTTTTT | TTTCCAACTCGGGAGTAATTG | TGGAAAGAATGTTTAAGGTTTTGA | 59.1 | 777 | INTERGENIC | | | | |
| CaPOPI_8376 | scaffold8315 | 20462 | GTTT/GTT | AAAAGGATATTCAACTCTCCAAAAA | CGATTTTGCTACACAAAATTTCC | 58.7 | 759 | INTERGENIC | | | | |
| CaPOPI_8377 | scaffold8393 | 1553 | ATTTT/ATTTTTT | CCGGTTGCACAACATTCTTA | TCTTTCTTATGGTTCTGTAAAGCTGA | 59.6 | 378 | INTERGENIC | | | | |
| CaPOPI_8378 | scaffold8412 | 961 | TAAAAAAAA/TAAAAAAAAA | TTTTCACATTTCAAAAGCATGA | TCACATCTTCCTTATGTCCGC | 58.3 | 342 | INTERGENIC | | | | |
| CaPOPI_8379 | scaffold842 | 20647 | TA/TAA | TGATTCCTGTAACACCCCAA | AACCAAACACACAAGGAAATATGA | 58.8 | 332 | INTERGENIC | | | | |
| CaPOPI_8380 | scaffold842 | 30005 | CAAAAA/CAAAAAAAAA | GATACTATGCCACCATCCCG | TGGTCATGCAGAAACTCAGC | 60.2 | 848 | INTERGENIC | | | | |

| INDEL marker IDs | Chromosomes/ unanchored scaffolds | Physical positions (bp) | InDels (*Kabuli* reference genome-CDC Frontier/PI) | Forward primers (5'-3') | Reverse primers (5'-3') | Anneling temperature (0C) | Expected amplified product size (bp) | Structural annotation | | Functional annotation | | |
|---|---|---|---|---|---|---|---|---|---|---|---|---|
| | | | | | | | | Sequence components of *kabuli* genome | *Kabuli* gene accession IDs | NCBI-KOG | TFs | NCBI-nr database |
| CaPOPI_8381 | scaffold8442 | 1501 | CA/C | AACCAAACTGGTGCATCACA | AGCCTAGGGTGGACGAAAAT | 60.0 | 676 | INTERGENIC | | | | |
| CaPOPI_8382 | scaffold845 | 2172 | TTA/TTCTTTAGTA | CAACATTTTGGGTGCATTTG | TGGTTATTTTGCATGTAGCGA | 59.8 | 657 | INTERGENIC | | | | |
| CaPOPI_8383 | scaffold845 | 2505 | ATTTTTT/ATTTTTTT | TCGCTACATGCAAAATAACCA | TCACGTCACTTTTTCAAATGG | 59.2 | 388 | INTERGENIC | | | | |
| CaPOPI_8384 | scaffold845 | 3348 | TAAAAAA/TAAAAA | ACAAATGAATGTGGGTGGGT | TGATCATCTTTTGTGGAGGTTC | 60.0 | 701 | INTERGENIC | | | | |
| CaPOPI_8385 | scaffold845 | 3378 | TAAA/TAA | ACAAATGAATGTGGGTGGGT | TGATCATCTTTTGTGGAGGTTC | 60.0 | 701 | INTERGENIC | | | | |
| CaPOPI_8386 | scaffold845 | 23562 | CT/C | TCGATCAACCTAGATTGGCA | TCTGAACACTATCGTATCATGTTTCTT | 59.2 | 655 | INTERGENIC | | | | |
| CaPOPI_8387 | scaffold845 | 31907 | CTATAACAA/C | TGTGGTTTGGTTGGATGATG | AGAGAATGTGGCCTTCACAA | 60.2 | 653 | INTERGENIC | | | | |
| CaPOPI_8388 | scaffold845 | 33752 | CATCAAATCAAAT/CATCAAAT | AAAAACCTATCATGCTGCCC | ACGCCCATTTCGTAGATGTC | 59.0 | 299 | INTERGENIC | | | | |
| CaPOPI_8389 | scaffold845 | 39704 | GAAAAAA/GAAAAAAA | TGAAACGTTGAAAATGGCAC | TTGTTATCGCAGGGGAAATC | 59.6 | 251 | INTERGENIC | | | | |
| CaPOPI_8390 | scaffold845 | 61188 | CTGTT/CT | TTCGAAAGTTTTGCATTTCG | TGGAGAAGACCATATTGCCC | 58.9 | 405 | INTERGENIC | | | | |
| CaPOPI_8391 | scaffold845 | 69832 | A/AT | TTGTTTTCAGGTTGGGGTTC | CCTACGGACTTGTCGTGGAT | 59.8 | 511 | DRR | Ca_22414 | R | | RNA recognition motif domain |
| CaPOPI_8392 | scaffold845 | 81876 | GAAAAAAAAA/GAAAAAAAA | GGTTTTTGGTTGGAATGTGG | AAAAGGGAGGGGCAATCTAA | 60.1 | 382 | INTERGENIC | | | | |
| CaPOPI_8393 | scaffold845 | 102274 | TTAAGTAAG/TTAAG | ACTCTAAGTGCCCTCCCGTT | TTGACGGTCAGGTGAGACAG | 60.1 | 233 | DRR | Ca_22415 | R | | |
| CaPOPI_8394 | scaffold845 | 102446 | CAA/CAAA | CTGACCGTCAAAAGCACTGA | ACGCATAATACCTGAACGGC | 60.0 | 480 | DRR | Ca_22415 | R | | |
| CaPOPI_8395 | scaffold845 | 111245 | ACGAAT/A | GGCTCGGTAGGTATCCGTAA | TTATATTAGTGCGGGCGTGG | 59.1 | 166 | INTERGENIC | | | | |

| INDEL marker IDs | Chromosomes/ unanchored scaffolds | Physical positions (bp) | InDels (*Kabuli* reference genome-CDC Frontier/PI) | Forward primers (5'-3') | Reverse primers (5'-3') | Anneling temperature (0C) | Expected amplified product size (bp) | Structural annotation | | Functional annotation | | |
|---|---|---|---|---|---|---|---|---|---|---|---|---|
| | | | | | | | | Sequence components of *kabuli* genome | *Kabuli* gene accession IDs | NCBI-KOG | TFs | NCBI-nr database |
| CaPOPI_8396 | scaffold845 | 132751 | CAAATAAAT/CAAATAAATAAAT | AGTGTTGTTTGGGTTGGGAG | TCGATTTGACATTTGCGTGT | 59.9 | 526 | INTERGENIC | | | | |
| CaPOPI_8397 | scaffold845 | 132810 | GA/GAAATTCTAA | AGTGTTGTTTGGGTTGGGAG | TCGATTTGACATTTGCGTGT | 59.9 | 526 | INTERGENIC | | | | |
| CaPOPI_8398 | scaffold845 | 133191 | CAAA/CAA | CCACCAAAAACCAACCAACT | GGCCTAACCTGGAAGCCTAT | 59.7 | 730 | INTERGENIC | | | | |
| CaPOPI_8399 | scaffold845 | 134750 | CAA/CAAAA | CCGTTTGGGCCCTTAGTTAT | TCGTGTCCATTGCTATTTTCA | 60.2 | 546 | DRR | Ca_22417 | U | | Zinc finger, RING-type |
| CaPOPI_8400 | scaffold845 | 141061 | TAA/TAAA | CCGGCATAATCTGACAACCT | GTGCTTTGTGGGGATGATCT | 60.0 | 453 | INTRON | Ca_22417 | U | | Zinc finger, RING-type |
| CaPOPI_8401 | scaffold845 | 152531 | GTACTTAC/GTACTTACTTAC | CACGTGCAAGTAAAAGCCAA | TGCTGCACCATAATTCCAAA | 59.9 | 537 | INTERGENIC | | | | |
| CaPOPI_8402 | scaffold845 | 170625 | ATTT/ATTTT | ATTTGGTGCGCATGATAAGA | AAAAATGAGGTGGGTTAGAAATTA | 59.1 | 777 | INTERGENIC | | | | |
| CaPOPI_8403 | scaffold845 | 171362 | ACA/ACAATGTCA | TCAATCACACCCGTTCAAAT | TGATTGCCGACAATACCAAG | 58.8 | 159 | INTERGENIC | | | | |
| CaPOPI_8404 | scaffold845 | 174410 | CA/C | GCATAGATGCAGTGGAAGCA | ATTACCTGCAGGAGCCCTTT | 60.0 | 848 | INTERGENIC | | | | |
| CaPOPI_8405 | scaffold845 | 194654 | GGTGTGATTTTCTGT/GGTGTGATTTTCTGTGTGATTTTCTGT | TGAATTTTGAATGCCCATGA | CATTCGCCACACGTCAATAC | 59.9 | 318 | INTERGENIC | | | | |
| CaPOPI_8406 | scaffold845 | 331956 | TG/TGG | TTGCTTGGAAAGGAAAATCG | GGGGCCAAATAATGTTGCTA | 60.2 | 802 | INTERGENIC | | | | |
| CaPOPI_8407 | scaffold845 | 357903 | GTTTTTTTTTTT/GTTTTTTTTTT | TGGAATTCAATACCCCCAAA | CCCGGCCAATTTTATTTCTA | 60.0 | 304 | DRR | Ca_22428 | R | G2-like | RNA recognition motif domain |
| CaPOPI_8408 | scaffold847 | 117106 | TCAATCGATTGGGCAATCGATTG/TCAATCGATTG | CCCGTGAGATCCTCCTAACA | CTCGTGTTCGTTCGTGAGAA | 60.1 | 642 | INTERGENIC | | | | |
| CaPOPI_8409 | scaffold848 | 72755 | ATTT/ATT | ACAACATGCACTCGTGGGTA | CGTTTGTCGCGATATTGAAA | 60.0 | 608 | INTERGENIC | | | | |
| CaPOPI_8410 | scaffold848 | 226354 | GTCT/GT | TTTTGGCGTAGTGTGTGCTC | CAGCAAACGTTCTCCGTTTT | 59.9 | 678 | INTERGENIC | | | | |

| INDEL marker IDs | Chromosomes/ unanchored scaffolds | Physical positions (bp) | InDels (*Kabuli* reference genome-CDC Frontier/PI) | Forward primers (5'-3') | Reverse primers (5'-3') | Anneling temperature (0C) | Expected amplified product size (bp) | Structural annotation | | Functional annotation | | |
|---|---|---|---|---|---|---|---|---|---|---|---|---|
| | | | | | | | | Sequence components of *kabuli* genome | *Kabuli* gene accession IDs | NCBI-KOG | TFs | NCBI-nr database |
| CaPOPI_8411 | scaffold8493 | 9496 | A/AT | CGACCATGATTTAACGACCA | GTTTTCAAGAGGTGGCCTTG | 59.4 | 360 | INTERGENIC | | | | |
| CaPOPI_8412 | scaffold855 | 8963 | AATATATATATATATATATATA/AATATATATATATATATATATA | TGGACTCACTACGTACGGCA | TTTTTCTTCTATCCCCATGTTATTC | 60.3 | 521 | INTERGENIC | | | | |
| CaPOPI_8413 | scaffold855 | 30812 | TTATATATATATATATATATATA/TTATATATATATATATATATATATA | AGGCCGTGATGTTTTTGAAC | GCATCATATGTGTGGGCAAG | 60.0 | 268 | INTERGENIC | | | | |
| CaPOPI_8414 | scaffold855 | 31194 | ACCC/ACC | CTCGTACGCTTTTGGTGTGA | GTAGGGGGTAGAAAACCCCA | 59.9 | 556 | INTERGENIC | | | | |
| CaPOPI_8415 | scaffold855 | 31470 | TTAATATCC/T | AATTGGATACCCCTTTTGGC | GTAGGGGGTAGAAAACCCCA | 60.0 | 384 | INTERGENIC | | | | |
| CaPOPI_8416 | scaffold855 | 31510 | TTAT/TTATATAT | AATTGGATACCCCTTTTGGC | GTAGGGGGTAGAAAACCCCA | 60.0 | 384 | INTERGENIC | | | | |
| CaPOPI_8417 | scaffold8565 | 6875 | GTTTTTTTT/GTTTTTTTTTT | TGACCAAAATCCATCCCTTC | TTTACAGCGCTTGTTCCAAA | 59.7 | 517 | INTERGENIC | | | | |
| CaPOPI_8418 | scaffold864 | 221220 | GGAGAGAGAGAGAGAGAGAGAGAGAGAGA/GGAGAGAGAGAGAGAGAGAGAGAGAGAGAGAGA | TAACCCAAAAATCCCAACCA | GGGTCACGTCCTTGTCTACC | 60.0 | 377 | INTERGENIC | | | | |
| CaPOPI_8419 | scaffold864 | 247808 | TGGGG/TGGGGG | TTCTGCCCTCATGGATTTTC | CATGGCTCGAGTGAAACAGA | 60.0 | 349 | CDS (FRAME SHIFT) | Ca_24213 | | | |
| CaPOPI_8420 | scaffold864 | 319669 | CAA/CA | TTCACTTGTTGACCAATCGC | TGCGTGAAACAAGACGAAAC | 59.7 | 727 | INTERGENIC | | | | |
| CaPOPI_8421 | scaffold8646 | 2745 | TAAAAAA/TAAAAAAA | AGTCTACGACGAAGCGTTCC | TCCATAGGAGGTCGATGGTC | 59.5 | 532 | INTERGENIC | | | | |
| CaPOPI_8422 | scaffold866 | 36146 | CA/CAGA | GAACAACAAGTGGGATGCCT | TGTGAAAAATGAACACCCCA | 60.0 | 360 | INTERGENIC | | | | |
| CaPOPI_8423 | scaffold866 | 36277 | ACCC/ACC | GAACAACAAGTGGGATGCCT | TGTGAAAAATGAACACCCCA | 60.0 | 360 | INTERGENIC | | | | |
| CaPOPI_8424 | scaffold866 | 142956 | CTT/CT | GGATCCCCAATCGATAACCT | CTGTTGAGCCTTAGGGCAAG | 60.0 | 450 | INTERGENIC | | | | |
| CaPOPI_8425 | scaffold868 | 30710 | TGG/TG | AGGGAGGACGAAAATGACCT | AGGGTTTTTGCCCAATCTTT | 59.9 | 180 | INTERGENIC | | | | |

| INDEL marker IDs | Chromosomes/ unanchored scaffolds | Physical positions (bp) | InDels (*Kabuli* reference genome-CDC Frontier/PI) | Forward primers (5'-3') | Reverse primers (5'-3') | Anneling temperature (0C) | Expected amplified product size (bp) | Structural annotation | | Functional annotation | | |
|---|---|---|---|---|---|---|---|---|---|---|---|---|
| | | | | | | | | Sequence components of *kabuli* genome | *Kabuli* gene accession IDs | NCBI-KOG | TFs | NCBI-nr database |
| CaPOPI_8426 | scaffold869 | 6196 | TAAAATAAAAATA/TAAAATA | CACCCATGGCAAAAAGAAAT | TTGCTGAACCATTTTCCTCC | 59.8 | 389 | INTERGENIC | | | | |
| CaPOPI_8427 | scaffold869 | 39678 | TTATATATATATATATAT/TTATATATATATATAT | GTAAAATGAAGCGCTTTCGC | GCATTCTTCACTGCAAACTCA | 60.0 | 658 | INTERGENIC | | | | |
| CaPOPI_8428 | scaffold869 | 135112 | ATTTT/ATTTTT | TTAGTCATGGTCGCTCCGAT | TAACGGGATTTAAGGTGGGG | 60.6 | 360 | INTERGENIC | | | | |
| CaPOPI_8429 | scaffold87 | 361359 | GAAAAAAAAAAA/GAAAAAAAAAA | GTTCAGGAACGAGAGATGGG | AGGACTGAGCCTGGCTTACA | 59.7 | 803 | INTERGENIC | | | | |
| CaPOPI_8430 | scaffold87 | 468940 | TT/TTCGGAT | GAGAATGGCAGCCCAAAATA | TCGGTATTTGCCATGAGACA | 60.0 | 533 | INTERGENIC | | | | |
| CaPOPI_8431 | scaffold87 | 585278 | TTTTTTTATTTTT/TTTTTT | TATGCATGCAGGCAAGAGTC | TTGGAAAAGCCCACCTACAC | 60.0 | 570 | INTERGENIC | | | | |
| CaPOPI_8432 | scaffold87 | 594427 | CAGATA/CA | TGACCGATTTGGTGACTGAA | TTGGTGATCATGATGCTGGT | 60.1 | 555 | INTERGENIC | | | | |
| CaPOPI_8433 | scaffold87 | 597245 | TAAAAAAAAAAA/TAAAAAAAAA | TAAGCCAAATAACCCCCTCC | TCTCCCCATCCTCTCATCAC | 60.1 | 573 | INTERGENIC | | | | |
| CaPOPI_8434 | scaffold870 | 39934 | TTTATTATTATTATTATTATTATTATTATTATTATTATTATTATTAT/TTTATTATTATTATTATTATTATTATTATTATTATTATTATTATTATTATTAT | AGAGGCAAACAAGAACCGAA | GGTGAGCCACCTTCATTTGT | 59.9 | 517 | INTERGENIC | | | | |
| CaPOPI_8435 | scaffold870 | 82369 | GTTTTTT/GTTTTTTT | TGGTGGTGCAATTCACATCT | TTTCACCGTCATGCTTTTTG | 60.0 | 540 | INTERGENIC | | | | |
| CaPOPI_8436 | scaffold870 | 82445 | GT/GTT | TGGTGGTGCAATTCACATCT | TTTCACCGTCATGCTTTTTG | 60.0 | 540 | INTERGENIC | | | | |
| CaPOPI_8437 | scaffold873 | 18311 | GAAAAAAA/GAAAAAA | TGTTGGGTCCACAAAGAGAG | TGTTGTTCTCCTCTGACACAATG | 58.7 | 402 | INTERGENIC | | | | |
| CaPOPI_8438 | scaffold873 | 199356 | CTTATTATTATTATTATTATTATT/CTTATTATTATTATTATTATT | CCCTTACTTGGACCAAAGTTACC | CTGCGAACTGGACAAATGAA | 60.1 | 585 | DRR | Ca_23485 | | | Clp, N-terminal |
| CaPOPI_8439 | scaffold873 | 269556 | GATATATATATATATATATATATATATATATATATATAT/GATATATATATATATATATATATATATATATATATATATATATATAT | TTTTGCAATGAAACCCATCA | TGTGGCAAGCGTTTATTCTG | 59.9 | 614 | INTERGENIC | | | | |
| CaPOPI_8440 | scaffold873 | 309839 | TAAAAAAA/TAAAAAAAA | TTCATGGTGTTTTCTTGGCA | ATGGGGAAAATCGCTTACCT | 60.1 | 691 | INTERGENIC | | | | |

| INDEL marker IDs | Chromosomes/ unanchored scaffolds | Physical positions (bp) | InDels (*Kabuli* reference genome-CDC Frontier/PI) | Forward primers (5'-3') | Reverse primers (5'-3') | Anneling temperature (0C) | Expected amplified product size (bp) | Structural annotation | | Functional annotation | | |
|---|---|---|---|---|---|---|---|---|---|---|---|---|
| | | | | | | | | Sequence components of *kabuli* genome | *Kabuli* gene accession IDs | NCBI-KOG | TFs | NCBI-nr database |
| CaPOPI_8441 | scaffold8734 | 801 | TAA/TAAA | TTGGGCGAAAATGGATAAAG | CCCCGCTCTGATAGGTGATA | 59.9 | 802 | INTERGENIC | | | | |
| CaPOPI_8442 | scaffold8734 | 1079 | TAAA/TAAAA | CTGTGACACCCTAAATCCCA | GATCTATGAGGACAGGGCCA | 58.4 | 798 | INTERGENIC | | | | |
| CaPOPI_8443 | scaffold876 | 3909 | CCTGTCGCG/C | CACGAGTGCCGATATGTGTC | TAGTGGCGATTCCTTTTTCG | 60.1 | 531 | INTERGENIC | | | | |
| CaPOPI_8444 | scaffold876 | 4050 | TACGACGACG/TACGACG | AAATCCAACATTGCTCTGCC | CTATTGCACCTGGGGATACG | 60.1 | 388 | INTERGENIC | | | | |
| CaPOPI_8445 | scaffold876 | 34017 | AC/A | ACGATCCACACCAAAACACA | TACGGTGCTAGGGAACCAAC | 59.9 | 449 | INTERGENIC | | | | |
| CaPOPI_8446 | scaffold876 | 63698 | CAA/CAAA | GCTCGTCGAACTTTTTCAGG | TCATCAGTATCGTGGGTGGA | 60.0 | 602 | INTERGENIC | | | | |
| CaPOPI_8447 | scaffold876 | 163249 | ATTTTTTTTTTT/ATTTTTTTTTT | AAATAGGCCGACCTCTCGTT | TGAGGGAGAAAAACAAGCGT | 60.1 | 479 | DRR | Ca_24591 | K | ARR-B | Signal transduction response regulator, receiver domain |
| CaPOPI_8448 | scaffold876 | 622726 | TAA/TAAA | TCAGGAACAACAATCCAATCC | TTACCCTCAAAACTGGGGTG | 59.8 | 331 | INTERGENIC | | | | |
| CaPOPI_8449 | scaffold877 | 10844 | TAAAAA/TAAAA | GCAGCAGTTGCAGCAGTTTA | TGATTTAGCGTCGGTGTCAG | 60.3 | 601 | DRR | Ca_28083 | F | | Orotidine 5'-phosphate decarboxylase domain |
| CaPOPI_8450 | scaffold88 | 20403 | ATTTTTTTTTTTT/ATTTTTTTTTTT | AATGGATTGCTCTGTTTGGC | TCCCTCTATGCTTTGTCCCT | 60.1 | 728 | INTERGENIC | | | | |
| CaPOPI_8451 | scaffold88 | 63256 | TAAAAAAAAA/TAAAAAAAAAA | AAATCCTTTTGGGGTGAAGC | TTTTTGAAAACTGCCAGGTG | 60.3 | 655 | INTERGENIC | | | | |
| CaPOPI_8452 | scaffold882 | 70530 | CAAAAAA/CAAAAA | CACATCAACAGCAGCCTTGT | TGATGTTGCCTCTGTTTTGC | 59.9 | 385 | INTERGENIC | | | | |
| CaPOPI_8453 | scaffold882 | 314543 | CTTTTTTTTTTT/CTTTTTTTTTT | TCTGGAATTGCTTTGACGTG | TGCATTGTCACGTGTTCTTG | 59.8 | 813 | INTERGENIC | | | | |
| CaPOPI_8454 | scaffold882 | 326048 | GTATTATTATTATTATTAT/GTATTATTATTATTATTAT | TTTCGAAGAAAGCGATAACAA | GAGGAAAAACAAATGGACAAGC | 57.7 | 757 | INTERGENIC | | | | |
| CaPOPI_8455 | scaffold882 | 344839 | GAAAAAAAAA/GAAAAAAAAAA | TTTGTGTTTGACTGGCTTGG | GACACCCTAAACCCCGAAAT | 59.7 | 416 | DRR | Ca_23984 | | Trihelix | |

| INDEL marker IDs | Chromosomes/ unanchored scaffolds | Physical positions (bp) | InDels (*Kabuli* reference genome-CDC Frontier/PI) | Forward primers (5'-3') | Reverse primers (5'-3') | Anneling temperature (0C) | Expected amplified product size (bp) | Structural annotation | | Functional annotation | | |
|---|---|---|---|---|---|---|---|---|---|---|---|---|
| | | | | | | | | Sequence components of *kabuli* genome | *Kabuli* gene accession IDs | NCBI-KOG | TFs | NCBI-nr database |
| CaPOPI_8456 | scaffold882 | 436721 | GAAAA/GAAA | CGTACCAAAGGGCAATGAAT | GCTCTGCATCCCATCAAAAT | 59.8 | 323 | INTERGENIC | | | | |
| CaPOPI_8457 | scaffold882 | 446893 | GTTT/GTTTT | TGGTTGGTTTTTGTGGTGAA | TCACGGGCAAACACAATTTA | 59.8 | 644 | INTERGENIC | | | | |
| CaPOPI_8458 | scaffold882 | 513030 | AATATATATATATATATATATATATATATA/AATATATATATATATATATATA | ATTCTTGATTCGCCTCCCTT | CACCGTAGTGTGGTGGTGAG | 60.0 | 680 | URR | Ca_23986 | | | |
| CaPOPI_8459 | scaffold882 | 639280 | CTTTTTTTTTT/CTTTTTTTTT | AGGAATGACCAAAACGTTCG | CACTTGTATTATTGAAACACAAAGGT | 60.0 | 881 | INTERGENIC | | | | |
| CaPOPI_8460 | scaffold8825 | 968 | TAAAAA/TAAAAAA | AATCGATTGGCATCAACACA | CCAGTCAAGCATTCCCATCT | 59.9 | 475 | INTERGENIC | | | | |
| CaPOPI_8461 | scaffold8836 | 981 | GAAATAAAAA/GAAA | CAACACAACAAGAACGAGCAA | TCCAAGTAGGTCTGCCCAAT | 59.9 | 288 | INTERGENIC | | | | |
| CaPOPI_8462 | scaffold886 | 151276 | AG/AGCTG | AGGGATGCTTGTAGTGGTTGA | TTTGACGTGCATCCAATGTT | 59.6 | 162 | DRR | Ca_26671 | | | |
| CaPOPI_8463 | scaffold886 | 151448 | TA/T | GACAACATTGGATGCACGTC | TCGTGGTATGCTTGACCTGT | 60.0 | 259 | DRR | Ca_26671 | | | |
| CaPOPI_8464 | scaffold886 | 164957 | AGG/AG | GCTAATTGGCGGCACTTCTA | CGAGTTAGTGAATCATCCCCTC | 60.4 | 614 | INTERGENIC | | | | |
| CaPOPI_8465 | scaffold886 | 172563 | AATATATATATATATATATATATAT/AATATATATATATATATATATATATAT | AAGCCGACAATTTGTTTTTCT | TCATTCCTGATTTGGGGGTA | 57.9 | 382 | INTERGENIC | | | | |
| CaPOPI_8466 | scaffold8866 | 2222 | CTAAAATTAAAAT/CTAAAAT | TCTGATACGCCTCATAGCCC | AGGGCAAAAGTCTCAAAGGA | 60.2 | 519 | INTERGENIC | | | | |
| CaPOPI_8467 | scaffold89 | 23867 | ACAT/ACATCAT | CATGGTAGCTCCCGACATTT | ATTTCCAAACGATTCATCCG | 60.0 | 712 | INTERGENIC | | | | |
| CaPOPI_8468 | scaffold89 | 30029 | AATATATATATATATATATATA/AATATATATATATATATATA | GGATTGAACCTCCTATGCGA | TTCCGAGTTGTTTGAGAGGA | 60.0 | 716 | INTERGENIC | | | | |
| CaPOPI_8469 | scaffold890 | 1120 | ATATTATTATTATTATTCTTATTATTATT/ATATTATTATTATTATTATTATTCTTATTATTATT | TTCGGGCAAATCATTACACA | TGGCTGAAATACGCCACATA | 59.9 | 359 | INTERGENIC | | | | |
| CaPOPI_8470 | scaffold8913 | 2269 | TG/T | CAATGGATGGGGTAGGATTG | AGCGACAGTGAACGATGTTG | 60.0 | 366 | INTERGENIC | | | | |

| INDEL marker IDs | Chromosomes/ unanchored scaffolds | Physical positions (bp) | InDels (*Kabuli* reference genome-CDC Frontier/PI) | Forward primers (5'-3') | Reverse primers (5'-3') | Anneling temperature (0C) | Expected amplified product size (bp) | Structural annotation | | Functional annotation | | |
|---|---|---|---|---|---|---|---|---|---|---|---|---|
| | | | | | | | | Sequence components of *kabuli* genome | *Kabuli* gene accession IDs | NCBI-KOG | TFs | NCBI-nr database |
| CaPOPI_8471 | scaffold8913 | 2342 | ATTTTTT/ATTTT | CAATGGATGGGGTAGGATTG | GCAGGAGATTTAGATGGAGGG | 60.0 | 878 | INTERGENIC | | | | |
| CaPOPI_8472 | scaffold892 | 26717 | ATT/ATTT | TCAAGCTTCCTCTCCACACA | AATTAATATCCACGCAACGC | 59.5 | 255 | INTERGENIC | | | | |
| CaPOPI_8473 | scaffold892 | 29801 | CAAAAAA/CAAAAA | GAAGCCATGAAAACCTTCCA | ATGCAAACATTTCCCACCTC | 60.1 | 392 | INTERGENIC | | | | |
| CaPOPI_8474 | scaffold892 | 33546 | TTTCTT/TTTCTTCTT | TCCCAATTTGGTTTTGGTGT | TGGAATCTTCACCCTGACAA | 60.1 | 713 | DRR | Ca_27455 | R | | Sugar/inositol transporter |
| CaPOPI_8475 | scaffold892 | 33776 | TC/T | TCAAAAGACACCAAATGGAAAA | TGGAATCTTCACCCTGACAA | 59.5 | 251 | DRR | Ca_27455 | R | | Sugar/inositol transporter |
| CaPOPI_8476 | scaffold892 | 38411 | AATATATATATATATATAT/AATATATATATATATATATAT | GTAAATGGCAGGTAGAGGCG | ACCACTAACTCGGTGCCAAC | 59.7 | 826 | INTERGENIC | | | | |
| CaPOPI_8477 | scaffold892 | 42751 | TGGGG/TGGGGG | GACGATGAAAAATATGCGGG | TGTTGATTGGCGAACACAAT | 60.3 | 387 | DRR | Ca_27456 | | | |
| CaPOPI_8478 | scaffold892 | 44352 | TAAAAAAAA/TAAAAAAA | CTGAGTTGCGACGAGAGTTG | TTGGAAGGAGCCCTTACCTT | 59.8 | 465 | INTERGENIC | | | | |
| CaPOPI_8479 | scaffold892 | 56842 | TAAAAAAAAA/TAAAAAAAA | CCCCCTTTAGTGGAAAGTTG | TTGGTATAGGCGATTTTGGG | 58.6 | 471 | INTERGENIC | | | | |
| CaPOPI_8480 | scaffold8920 | 817 | A/AAT | CATCAGGGTGTCTTGGTCCT | GGTACGAAAATCGCCTGAAA | 60.0 | 699 | INTERGENIC | | | | |
| CaPOPI_8481 | scaffold893 | 6971 | CATATATATATATATATAT/CATATATATATATATAT | CTTTAACATGCGTTTCCGGT | TCAAGTCGACAAGCCTTCCT | 60.0 | 819 | DRR | Ca_27923 | | B3 | |
| CaPOPI_8482 | scaffold893 | 10885 | A/AG | GAGATTGTGGCCAATTCGAT | CTCTCACCCAAGAAGATCGC | 59.9 | 395 | INTERGENIC | | | | |
| CaPOPI_8483 | scaffold895 | 103999 | TTTATTATTATTATTATTATTATTATTATT/TTTATTATTATTATTATTATTATTATTATTATTATT | AGAGGCAAACAAGAACCGAA | GAAGGCTATGGTTGATGGGA | 59.9 | 479 | INTERGENIC | | | | |
| CaPOPI_8484 | scaffold896 | 47144 | TAAA/TAAAA | AGGGAGGACGAAAATGACCT | TGTGCATCAAATAGGCGTGT | 59.9 | 259 | INTERGENIC | | | | |
| CaPOPI_8485 | scaffold898 | 27647 | TA/T | TGTAATTGTATTCAATGGCAAAGA | CGTTATTAAGTACAACAATAAAACGAA | 58.6 | 469 | INTERGENIC | | | | |

| INDEL marker IDs | Chromosomes/ unanchored scaffolds | Physical positions (bp) | InDels (*Kabuli* reference genome-CDC Frontier/PI) | Forward primers (5'-3') | Reverse primers (5'-3') | Anneling temperature (0C) | Expected amplified product size (bp) | Structural annotation | | Functional annotation | | |
|---|---|---|---|---|---|---|---|---|---|---|---|---|
| | | | | | | | | Sequence components of *kabuli* genome | *Kabuli* gene accession IDs | NCBI-KOG | TFs | NCBI-nr database |
| CaPOPI_8486 | scaffold9 | 34748 | CACAACAA/CACAA | GGACAAACTACCCCTCCTTTTC | TCTTCCCCCATAAACCAACA | 60.2 | 317 | INTRON | Ca_27384 | | WOX | |
| CaPOPI_8487 | scaffold900 | 12737 | GCAC/GCACTAGTACAC | CCATTTTTGTCCTCTGCCAT | AAGGCCAATGCCACATAAAG | 59.9 | 604 | INTERGENIC | | | | |
| CaPOPI_8488 | scaffold901 | 229595 | TTATATATATATATATA/TTATATATATATATATA | CCATTTCAGCCGTTGATTTT | ACCTTGCTCCCTTACCCACT | 59.9 | 703 | INTERGENIC | | | | |
| CaPOPI_8489 | scaffold9059 | 539 | ATT/ATTT | TTAGGGCTCATACGATTCGG | GGAAAATTAGCCACGGATCA | 60.1 | 443 | INTERGENIC | | | | |
| CaPOPI_8490 | scaffold909 | 6733 | AATATA/AATA | TTTCATCTGAACCTTTCGGG | TAGGGTGGTACGAAAATCGC | 60.0 | 365 | INTERGENIC | | | | |
| CaPOPI_8491 | scaffold909 | 21317 | T/TA | CCCGACAAAAATAGACCGAA | TGCTTGATTGCTTGAGATCC | 59.9 | 429 | INTERGENIC | | | | |
| CaPOPI_8492 | scaffold913 | 140774 | CAT/CATAT | TTGAGTTATGGCTAATGAAGGTCA | AGCGAGCCGCTCAATTAGTA | 60.0 | 269 | INTERGENIC | | | | |
| CaPOPI_8493 | scaffold913 | 161492 | GTCTATCTAT/GTCTAT | ATGTGAAACGATGGTCGAAA | ATAAGCCAAGGGCCAACTCT | 59.0 | 464 | DRR | Ca_23205 | | HD-ZIP | Homeobox |
| CaPOPI_8494 | scaffold913 | 251896 | C/CA | AGCGTGAAAGGCAAGTGAGT | CCCATGCAAGGTATGCTTTT | 60.1 | 441 | INTERGENIC | | | | |
| CaPOPI_8495 | scaffold913 | 252010 | AGTGTG/AGTG | CCAAAAGTAGCGTGAAAGGC | ATGGGATTGACTTGCTCTGG | 59.9 | 947 | INTERGENIC | | | | |
| CaPOPI_8496 | scaffold913 | 252195 | T/TG | ATGCGTCCCAAGATTCTGTT | ATGGGATTGACTTGCTCTGG | 59.6 | 668 | INTERGENIC | | | | |
| CaPOPI_8497 | scaffold913 | 282492 | ATTTT/ATT | TTGGATTTCATACCCACACATC | AAAATCCGTTGGAAATTTTGG | 59.6 | 466 | INTERGENIC | | | | |
| CaPOPI_8498 | scaffold916 | 3519 | AATATATATATATATATAT/AATATATATATATATAT | ACATCTCATGCCATTGTGGA | GAGACAGTCAGAAGGCCACC | 59.9 | 520 | DRR | Ca_21034 | G | E2F/DP | Major facilitator superfamily MFS-1 |
| CaPOPI_8499 | scaffold916 | 4344 | GAAAAA/GAAAAAAAA | TGTTGACGAATGTCCATGAAA | CCTCCAAAACCCAACTACCA | 60.0 | 181 | INTERGENIC | | | | |
| CaPOPI_8500 | scaffold916 | 4805 | T/TACAATA | TGGTAGTTGGGTTTTGGAGG | TTCATGACATGCATCCATCC | 59.8 | 907 | INTERGENIC | | | | |

| INDEL marker IDs | Chromosomes/ unanchored scaffolds | Physical positions (bp) | InDels (*Kabuli* reference genome-CDC Frontier/PI) | Forward primers (5'-3') | Reverse primers (5'-3') | Anneling temperature (0C) | Expected amplified product size (bp) | Structural annotation | | Functional annotation | | |
|---|---|---|---|---|---|---|---|---|---|---|---|---|
| | | | | | | | | Sequence components of *kabuli* genome | *Kabuli* gene accession IDs | NCBI-KOG | TFs | NCBI-nr database |
| CaPOPI_8501 | scaffold916 | 4868 | GTTTTTTTT/GTTTTTTTTTT | TGGTAGTTGGGTTTTGGAGG | TTCATGACATGCATCCATCC | 59.8 | 907 | INTERGENIC | | | | |
| CaPOPI_8502 | scaffold916 | 5303 | A/AT | TGTCTCTTTTACGCCGCAAT | CTAGGCATTGCAACAGGGAT | 60.8 | 738 | INTERGENIC | | | | |
| CaPOPI_8503 | scaffold916 | 7772 | AG/A | TGTCCTTCTGTTCAATCAAATACC | TGGTGGTGCATAGTTGGTGT | 59.4 | 636 | INTERGENIC | | | | |
| CaPOPI_8504 | scaffold916 | 8185 | TT/TTCGATTAAT | ACACCAACTATGCACCACCA | ATAGGCCCCAATTAACCCAC | 59.9 | 376 | INTERGENIC | | | | |
| CaPOPI_8505 | scaffold916 | 8308 | ATT/AT | ACACCAACTATGCACCACCA | ATAGGCCCCAATTAACCCAC | 59.9 | 376 | INTERGENIC | | | | |
| CaPOPI_8506 | scaffold916 | 9278 | CTTT/CTTTT | TCACATAGGGATTGCCAACA | TCCCACCACATTCAATCCTT | 59.9 | 769 | INTERGENIC | | | | |
| CaPOPI_8507 | scaffold916 | 9496 | TACTA/T | CCCATATGGTTTGGGAATCA | TCCCACCACATTCAATCCTT | 60.4 | 257 | INTERGENIC | | | | |
| CaPOPI_8508 | scaffold916 | 9949 | AAG/A | AGGATTGAATGTGGTGGGAA | GCAGAAAAATAAGGGCGATG | 60.2 | 354 | INTERGENIC | | | | |
| CaPOPI_8509 | scaffold916 | 10023 | ACC/ACCC | AGGATTGAATGTGGTGGGAA | GCAGAAAAATAAGGGCGATG | 60.2 | 354 | INTERGENIC | | | | |
| CaPOPI_8510 | scaffold916 | 10051 | CCCT/C | AGGATTGAATGTGGTGGGAA | TAAACAATTTGGGGGTCCTG | 60.2 | 443 | INTERGENIC | | | | |
| CaPOPI_8511 | scaffold916 | 10102 | CTTTT/CTTT | CATGATAGACCATCGCCCTT | TAAACAATTTGGGGGTCCTG | 59.9 | 119 | INTERGENIC | | | | |
| CaPOPI_8512 | scaffold916 | 134889 | TTT/TTATT | AAATGTGGTCTCGGTTTTGC | TTGTTTTGTGAGTTTGAATGATGTT | 60.0 | 770 | INTERGENIC | | | | |
| CaPOPI_8513 | scaffold916 | 363659 | TAAAATTAAAAATTAAAAATTAAAAA/TAAAATTAAAAATTAAAAATTAAAAATTAAAAA | CCACCCTCTACCATGCAACT | TGTTTTCCATGTGTGCCACT | 60.0 | 569 | INTERGENIC | | | | |
| CaPOPI_8514 | scaffold916 | 429531 | ATTTTTTTTT/ATTTTTTTT | TGCGTTCAATTTAAAGGTGTTG | TCGCCTCAAAACATCAAGAA | 60.0 | 321 | INTERGENIC | | | | |
| CaPOPI_8515 | scaffold918 | 49291 | CAAA/CAAAA | TAGCGAGGACGAAAATGACC | CGTCCTCCCTGGGTTTATTC | 60.2 | 820 | INTERGENIC | | | | |

| INDEL marker IDs | Chromosomes/ unanchored scaffolds | Physical positions (bp) | InDels (*Kabuli* reference genome-CDC Frontier/PI) | Forward primers (5'-3') | Reverse primers (5'-3') | Anneling temperature (0C) | Expected amplified product size (bp) | Structural annotation | | Functional annotation | | |
|---|---|---|---|---|---|---|---|---|---|---|---|---|
| | | | | | | | | Sequence components of *kabuli* genome | *Kabuli* gene accession IDs | NCBI-KOG | TFs | NCBI-nr database |
| CaPOPI_8516 | scaffold919 | 73303 | GCTTCTTCTTCTTC/GCTTCTTCTTCTTCTTC | CACCCTCGCTTAGAAAAACG | TTTGGAGACCAATGGTGACA | 59.9 | 878 | INTERGENIC | | | | |
| CaPOPI_8517 | scaffold919 | 83832 | AAAAAAAATAAAAAAA/AAAAAAAATAAAAAAATAAAAAAA | GTTTGAAGTTGTCCGCGTCT | GACACTTGCGCCCCTATAAA | 60.3 | 479 | INTERGENIC | | | | |
| CaPOPI_8518 | scaffold92 | 45246 | ATATTATTATTATTATTATT/ATATTATTATTATTATTATTATTATTATT | TGCAATAAAACCATAATACGAAGA | TGTTTGACTGCATAACGAAGA | 57.9 | 438 | INTERGENIC | | | | |
| CaPOPI_8519 | scaffold922 | 47157 | ATTGTT/ATT | AGAGGAGGTGATGGTGATGG | TTTTGGTAATTGTTGGTTTTATTCAA | 59.9 | 357 | INTERGENIC | | | | |
| CaPOPI_8520 | scaffold924 | 202228 | TTATATATATATATAT/TTATATATATATAT | AGGAAGAGTGGAGTTGCGAA | CAGGTTTGATGCAACCATTG | 60.0 | 689 | INTERGENIC | | | | |
| CaPOPI_8521 | scaffold9240 | 617 | ATTTT/ATTTTT | TTTAGGTCATTTTCGTCGCC | CCTTTTGGACGCACAAGATT | 60.1 | 476 | INTERGENIC | | | | |
| CaPOPI_8522 | scaffold9247 | 528 | GATATATATATATATATATATAT/GATATATATATATATATAT | CAACCATTCAGCCTTGGTTT | CGTTTGTCGAATATCTGCTCA | 60.0 | 562 | INTERGENIC | | | | |
| CaPOPI_8523 | scaffold9281 | 4465 | CAAAA/CAAAAA | TGATCAAAGCAACAAAGCTCA | ATGTGCAGTCTGAGCTACCG | 59.6 | 316 | INTERGENIC | | | | |
| CaPOPI_8524 | scaffold929 | 17042 | TA/TAA | GCAGGAGCAACAACAGATGA | AGGCTTAATTGCACTTTTGG | 60.0 | 444 | INTERGENIC | | | | |
| CaPOPI_8525 | scaffold929 | 20949 | AA/AAACA | GATGAAATCGAGGACCAGGA | GATGAAATCGAGGACCAGGA | 60.0 | 133 | INTERGENIC | | | | |
| CaPOPI_8526 | scaffold93 | 6623 | TAAA/TAAAGGAAAA | GGATCGTAATCCGTGGGTAA | GAGAGAGCGATCATCAAGGC | 59.6 | 415 | INTERGENIC | | | | |
| CaPOPI_8527 | scaffold93 | 116733 | CATATATATATATATAT/CATATATATATATATAT | AGGAAGAGTGGAGTTGCGAA | TTATGATTTTGGCTCGGAGG | 60.0 | 505 | INTERGENIC | | | | |
| CaPOPI_8528 | scaffold93 | 141155 | CAA/CAAA | TCGATTGGCTTAAAACCAGG | AGAATTTACGAGCAGCGGAA | 60.1 | 617 | INTERGENIC | | | | |
| CaPOPI_8529 | scaffold93 | 338023 | TCCCC/TCCC | AAACTTGCAAGCCATTCCAG | TTTGAGAAATATGCTGGGGC | 60.2 | 876 | INTRON | Ca_23073 | L | GRAS | |
| CaPOPI_8530 | scaffold9303 | 1186 | CT/CTT | AAGACCACATCATTGTGTCACC | TGTTTGTTCGCGTCTTAATTTT | 59.8 | 439 | INTERGENIC | | | | |

| INDEL marker IDs | Chromosomes/ unanchored scaffolds | Physical positions (bp) | InDels (*Kabuli* reference genome-CDC Frontier/PI) | Forward primers (5'-3') | Reverse primers (5'-3') | Anneling temperature (0C) | Expected amplified product size (bp) | Structural annotation | | Functional annotation | | |
|---|---|---|---|---|---|---|---|---|---|---|---|---|
| | | | | | | | | Sequence components of *kabuli* genome | *Kabuli* gene accession IDs | NCBI-KOG | TFs | NCBI-nr database |
| CaPOPI_8531 | scaffold9303 | 1378 | TAAAAA/TAAAAAA | AAGACCACATCATTGTGTCACC | CAATGGAGGTTTTCTTGTGGA | 59.8 | 713 | INTERGENIC | | | | |
| CaPOPI_8532 | scaffold931 | 100930 | A/AC | TTTGAGCCCCCTTCAATATG | GATTCTCCTTGGGGATGGAT | 59.9 | 616 | INTERGENIC | | | | |
| CaPOPI_8533 | scaffold931 | 116282 | GTTTTTTTTTTTTTTT/GTTTTTTTTTTTT | TCAGTTGCAATTTCCTTCCC | TAAAACCGATTTGGGAGTCG | 60.1 | 519 | INTERGENIC | | | | |
| CaPOPI_8534 | scaffold931 | 116416 | ATTT/ATTTCCCTAGATGCTCTTT | CGACTCCCAAATCGGTTTTA | TTGGGCTGATGCAATAAACA | 59.9 | 260 | INTERGENIC | | | | |
| CaPOPI_8535 | scaffold931 | 116537 | ATT/ATTT | CGACTCCCAAATCGGTTTTA | TTGGGCTGATGCAATAAACA | 59.9 | 260 | INTERGENIC | | | | |
| CaPOPI_8536 | scaffold931 | 117076 | CAAA/CAA | TGCCACAACCAATCTTCATC | AGACAGTGGGTGTTCCAAGC | 59.5 | 391 | INTERGENIC | | | | |
| CaPOPI_8537 | scaffold931 | 118275 | GAAAA/GAAAAA | TGCTCTGACTCATTCAACCG | CACCAACAATTGGCATCAAG | 60.0 | 725 | INTERGENIC | | | | |
| CaPOPI_8538 | scaffold931 | 180349 | GATATATATATATATATA/GATATATATATATATA | TCGTCCCCATGTAATTGTCA | TGGATACCCAAAAACCATTCA | 59.8 | 281 | INTERGENIC | | | | |
| CaPOPI_8539 | scaffold931 | 181299 | G/GTT | GTAGAGTGCCAACGACACCA | TGAACTATTGATTGGATCGTGC | 59.8 | 665 | INTERGENIC | | | | |
| CaPOPI_8540 | scaffold931 | 246666 | TTATATATATATATATATATATATAT/TTATATATATATATATATATAT | GGGTGTCACATCACCCTTTT | GCGGAAGACTTTATAGTGAAAGGA | 59.7 | 191 | INTERGENIC | | | | |
| CaPOPI_8541 | scaffold931 | 250258 | CATATATATATATA/CATATATATATATATATA | GCCACCAATTCCAGACACTT | TTGATTGGATCGTGCAGAGA | 60.0 | 791 | INTERGENIC | | | | |
| CaPOPI_8542 | scaffold931 | 251946 | GAAAAAAAA/GAAAAAAAAAA | TTGACACCCTAAACCCCAAA | AATGGGCGCTGTTAAATGTC | 60.2 | 177 | INTERGENIC | | | | |
| CaPOPI_8543 | scaffold9314 | 650 | C/CATTAT | TAAGGTTGTCTGGGGAGGTG | GTTTGGGATCGGAATTGAAA | 60.0 | 463 | INTERGENIC | | | | |
| CaPOPI_8544 | scaffold933 | 3249 | TA/TAA | TTGTCTCTTGGACATTCCCC | TTTTCCAGTGCACCTTGTGA | 59.9 | 548 | INTERGENIC | | | | |
| CaPOPI_8545 | scaffold933 | 4208 | GTTTTTTTTT/GTTTTTTTTTTT | TCAAACCCAGTGGTCTCCTT | AGTCCGATGAAGGGTCACAG | 59.5 | 654 | INTERGENIC | | | | |

| INDEL marker IDs | Chromosomes/ unanchored scaffolds | Physical positions (bp) | InDels (*Kabuli* reference genome-CDC Frontier/PI) | Forward primers (5'-3') | Reverse primers (5'-3') | Anneling temperature (0C) | Expected amplified product size (bp) | Structural annotation | | Functional annotation | | |
|---|---|---|---|---|---|---|---|---|---|---|---|---|
| | | | | | | | | Sequence components of *kabuli* genome | *Kabuli* gene accession IDs | NCBI-KOG | TFs | NCBI-nr database |
| CaPOPI_8546 | scaffold9371 | 631 | TTTTT/TTTTTGTTTT | GCGGCTGAAAGAGAGAGAGA | AAAGATTTCCGGTACACACCA | 60.0 | 687 | INTERGENIC | | | | |
| CaPOPI_8547 | scaffold939 | 1093 | ATTT/ATT | GTGACGTTGGGGGAGTTAAA | TCGAGCCTTATGGCAAGATT | 59.8 | 317 | INTERGENIC | | | | |
| CaPOPI_8548 | scaffold9411 | 2770 | A/AG | TCAATAGACCGATATGGGGAA | GAAGCATCTCCAACAGCTCA | 59.2 | 838 | INTERGENIC | | | | |
| CaPOPI_8549 | scaffold9411 | 2893 | TAAC/T | GGATATGTCAGAAAAGGAACGG | GAAGCATCTCCAACAGCTCA | 59.8 | 393 | INTERGENIC | | | | |
| CaPOPI_8550 | scaffold943 | 141649 | AATATATATATATATATATATATAT/AATATATATATATATATATAT | AAACCGGTAAAATCTCCGCT | TATTTTGGATTTTGGCTCGG | 60.0 | 703 | INTERGENIC | | | | |
| CaPOPI_8551 | scaffold943 | 163051 | ATTT/ATT | TGGCAAATCGATTGAAACAA | GTGCCAGTCGATTGCCTAAT | 60.1 | 516 | INTERGENIC | | | | |
| CaPOPI_8552 | scaffold943 | 267949 | CAC/CACGGAATGAATTAATGAAC | AATCACGGTTTGTGGAAAGC | CGTCCAGTCCCGAAATTAAA | 60.0 | 544 | INTERGENIC | | | | |
| CaPOPI_8553 | scaffold944 | 672 | ATTTT/ATTT | TTGTAAGTTGAGTTGCGACGA | TGTGGTCGCAGGTTTTATGA | 59.5 | 308 | INTERGENIC | | | | |
| CaPOPI_8554 | scaffold944 | 2389 | CAAAAAAA/CAAAAAA | GAACGGGAAATATTCACGTCAT | TCGGACCACACTCCTAATCC | 60.1 | 495 | INTERGENIC | | | | |
| CaPOPI_8555 | scaffold944 | 2557 | TG/T | GGATTAGGAGTGTGGTCCGA | CAACTGGGTACCACTAAGCCA | 59.9 | 450 | INTERGENIC | | | | |
| CaPOPI_8556 | scaffold944 | 3122 | GAAAAAAAAA/GAAAAAAAA | TGGCTTAGTGGTACCCAGTTG | TGGCGCTTATATTCAAAGCA | 60.0 | 643 | INTERGENIC | | | | |
| CaPOPI_8557 | scaffold944 | 3226 | GTTTTTTT/GTTTTTTTT | TGGCTTAGTGGTACCCAGTTG | TGGCGCTTATATTCAAAGCA | 60.0 | 643 | INTERGENIC | | | | |
| CaPOPI_8558 | scaffold9444 | 2167 | AAGTTAAACTGAATGAGTTAAACTGAATGA/AAGTTAAACTGAATGA | TTTAAGTCAAGGTTTAGAAAGAAACA | TTGAGTAAGAGAAGGAAAGCAAAAA | 57.1 | 572 | INTERGENIC | | | | |
| CaPOPI_8559 | scaffold945 | 2482 | CATATTCTGTAATAAGTAATATT/CATATT | AAATCCGTTTGGATTTGCAG | TCCCAGAAAATCGTTTGAGG | 59.9 | 428 | INTERGENIC | | | | |
| CaPOPI_8560 | scaffold948 | 113626 | ATT/ATTT | TTTGTGGCTTGAATTGGATG | CATGCTCACTCCCACTGCTA | 59.5 | 174 | INTERGENIC | | | | |

| INDEL marker IDs | Chromosomes/ unanchored scaffolds | Physical positions (bp) | InDels (*Kabuli* reference genome-CDC Frontier/PI) | Forward primers (5'-3') | Reverse primers (5'-3') | Anneling temperature (0C) | Expected amplified product size (bp) | Structural annotation | | Functional annotation | | |
|---|---|---|---|---|---|---|---|---|---|---|---|---|
| | | | | | | | | Sequence components of *kabuli* genome | *Kabuli* gene accession IDs | NCBI-KOG | TFs | NCBI-nr database |
| CaPOPI_8561 | scaffold948 | 136923 | TAAAAAAAAAAAA/TAAAAAAAAAAA | TGCTCAAATAGGAATGGCATC | GGCATGCACGTTTACAACAC | 60.1 | 484 | INTERGENIC | | | | |
| CaPOPI_8562 | scaffold949 | 3822 | CAAAAAA/CAAAAAAA | AAGAGGGGCGTGAACTTTTT | GCTTCCATACCTGTTTTGGG | 60.1 | 646 | CDS (FRAME SHIFT) | Ca_25481 | | | Photosystem antenna protein-like |
| CaPOPI_8563 | scaffold949 | 12574 | GTTTTTTTTT/GTTTTTTTTTTTT | TTGGGCCATTGGATAAATGT | ATGCACACGATTGGTTGAAA | 60.0 | 589 | INTERGENIC | | | | |
| CaPOPI_8564 | scaffold949 | 26559 | GTT/GT | AGGTCATTTTCGTCCTCCCT | TCCAATGACACCAACACACC | 59.9 | 692 | INTERGENIC | | | | |
| CaPOPI_8565 | scaffold953 | 17853 | T/TG | AAGGATAGGTACGGGTTGGG | AAATTGAAATACATGCGCCC | 60.1 | 381 | INTERGENIC | | | | |
| CaPOPI_8566 | scaffold953 | 37096 | TAAA/TAA | TGCAAGAACCGCTCCTCTAT | GCGTGTGGATGCAAAATATG | 60.0 | 305 | INTERGENIC | | | | |
| CaPOPI_8567 | scaffold953 | 39457 | CTTTTTTTTT/CTTTTTTTT | CATGTTGTGACAATTTGGTGG | CGTGAGTTGGTGTTGATTCG | 59.7 | 812 | INTERGENIC | | | | |
| CaPOPI_8568 | scaffold953 | 44175 | ATTTTTTTT/ATTTTTTTTT | GCATGCATGTTGAGGTCATT | TGGATGGGGTAGATGTGTGTT | 59.5 | 494 | INTERGENIC | | | | |
| CaPOPI_8569 | scaffold953 | 44845 | CTTTTTT/CTTTTTTT | CAGGGGGAGACTACAACTGC | GGCGGATCTTGACATGTTTT | 59.7 | 272 | INTERGENIC | | | | |
| CaPOPI_8570 | scaffold953 | 69667 | GA/GATA | TGGATAAGGTTTCCCACGAG | TCGTTGAGAGCGTGTTTGTC | 59.9 | 650 | INTERGENIC | | | | |
| CaPOPI_8571 | scaffold953 | 75360 | GAAAAAAAAAAA/GAAAAAAAAAAAA | TCAATGGGATTCCTTCTATGC | CACCCTCGCTTAGAAAAACG | 59.0 | 762 | INTERGENIC | | | | |
| CaPOPI_8572 | scaffold953 | 135204 | CAGAAGAAG/CAGAAG | TTGCAAATACATCCCCCAAT | TGGCGTCATGTCAAGAGAAG | 60.0 | 403 | INTRON | Ca_26911 | | MYB | Protein of unknown function DUF3741 |
| CaPOPI_8573 | scaffold953 | 142447 | ATTTTTTT/ATTTTTT | GCACGTGCACCAGAAAAATA | GCCTCCGAGTTCAATACCAA | 59.7 | 505 | INTERGENIC | | | | |
| CaPOPI_8574 | scaffold954 | 3708 | GA/G | AGGTCATTTTCGTCCTCCCT | TGGATGCACGAGATTGGTTA | 59.9 | 568 | INTERGENIC | | | | |
| CaPOPI_8575 | scaffold956 | 19709 | G/GCTTCTT | TCCAGATGAAATCGAGGACC | GGATTAGCCTTTCGAGTCCC | 60.0 | 381 | INTERGENIC | | | | |

| INDEL marker IDs | Chromosomes/ unanchored scaffolds | Physical positions (bp) | InDels (*Kabuli* reference genome-CDC Frontier/PI) | Forward primers (5'-3') | Reverse primers (5'-3') | Anneling temperature (0C) | Expected amplified product size (bp) | Structural annotation | | Functional annotation | | |
|---|---|---|---|---|---|---|---|---|---|---|---|---|
| | | | | | | | | Sequence components of *kabuli* genome | *Kabuli* gene accession IDs | NCBI-KOG | TFs | NCBI-nr database |
| CaPOPI_8576 | scaffold959 | 57749 | GAAAAAAAAAA/GAAAAAAAA | AAGGCGTGCATATCCCATAC | TGTTCGTCAAAATGTCCCAA | 59.8 | 553 | INTRON | Ca_27234 | | Trihelix | Peptidase C48, SUMO/Sentrin/Ubl1 |
| CaPOPI_8577 | scaffold96 | 192974 | ATTATTTATTTATTTATTTATTTATTTATTTATTTATTTATTTATTTATT/ATTATTTATTTATTTATTTATTTATTTATTTATTTATTTATTTATTTATTTATT | CTAGTGGGTACCCGCAAAAA | AGTCGGGAAGACAATGATCG | 60.0 | 388 | INTERGENIC | | | | |
| CaPOPI_8578 | scaffold960 | 20793 | ATTTT/ATTT | TACGGTGCTAGGGAACCAAC | CAAGGCTGTTTCAGCATTCA | 60.0 | 845 | INTERGENIC | | | | |
| CaPOPI_8579 | scaffold962 | 157050 | AATATATATATATATATATATATATATATATATATATATA/AATATATATATATATATATATATATATATATATATATATATATATATA | AATCGATTGGCTGAAATCCA | TAAACACATGCGCTTCCAAA | 60.4 | 564 | INTERGENIC | | | | |
| CaPOPI_8580 | scaffold962 | 214783 | TT/TTCAT | GGATGCTTTCAATGTTGTGC | ATACGGCGTTTTAATTTGCG | 59.1 | 687 | INTERGENIC | | | | |
| CaPOPI_8581 | scaffold963 | 17319 | AATATATATATATATATATATATATATATATATATATAT/AATATATATATATATATATATATATATAT | CCGGGGATAATGGCTTAAAT | GGGGTATGCATTTGTTGGAC | 60.0 | 429 | INTERGENIC | | | | |
| CaPOPI_8582 | scaffold964 | 79837 | TATTAATAA/TATTAATAATAAATTAATAA | TTGAAGGTGCAATGCCTTTA | GCATGAACAGCAACGTCAAC | 59.3 | 469 | INTERGENIC | | | | |
| CaPOPI_8583 | scaffold9757 | 838 | TAAAA/TAAAAA | TGTTAATCCAACGGCACAAA | TCCCCTGGCTTATTGTTGTC | 60.0 | 279 | INTERGENIC | | | | |
| CaPOPI_8584 | scaffold977 | 6872 | GTGATGATG/GTGATGATGATGATG | CGTATTTCCCAACGATCCAT | ACGAGGTCGTACCAACCAAC | 59.6 | 637 | INTERGENIC | | | | |
| CaPOPI_8585 | scaffold98 | 168374 | CAAAAAAAAAAA/CAAAAAAAAAA | CGTCAAAAGAGGATTGATACCAC | CGTCTTGTTATGCCATTGTTG | 59.9 | 436 | INTERGENIC | | | | |
| CaPOPI_8586 | scaffold98 | 241916 | AATATATATATATATATATATATATAT/AATATATATATATATATATATATATATAT | ATTTGGGGTTTAAGGGTTGG | AACAAACAAAGAAAAGCAAGGC | 59.9 | 663 | INTERGENIC | | | | |
| CaPOPI_8587 | scaffold98 | 379604 | AAGTTTTA/AA | TTGCGATGGTAAGAAGGGTT | ATTCTTGGAGCCGATCAATG | 59.6 | 349 | DRR | Ca_21386 | OE | WRKY | Peptidase S10, serine carboxypeptidase |
| CaPOPI_8588 | scaffold98 | 474976 | TAA/TA | GAGGAAGGGATTTTGGTGGT | GCAAAACCCACGTAAGTGCT | 60.2 | 400 | INTERGENIC | | | | |
| CaPOPI_8589 | scaffold981 | 9701 | GAAA/GAAATCCTAAA | TCCCAAATCACTTAACCCGT | TGGGGTTTAGGGTTTCACAA | 59.3 | 555 | INTERGENIC | | | | |
| CaPOPI_8590 | scaffold981 | 10301 | GA/GAAA | ACCCGAAACCTAAAACCCTT | GGTTTAGGCGTTAGGGTTGA | 58.9 | 300 | INTERGENIC | | | | |

| INDEL marker IDs | Chromosomes/ unanchored scaffolds | Physical positions (bp) | InDels (*Kabuli* reference genome-CDC Frontier/PI) | Forward primers (5'-3') | Reverse primers (5'-3') | Anneling temperature (0C) | Expected amplified product size (bp) | Structural annotation | | Functional annotation | | |
|---|---|---|---|---|---|---|---|---|---|---|---|---|
| | | | | | | | | Sequence components of *kabuli* genome | *Kabuli* gene accession IDs | NCBI-KOG | TFs | NCBI-nr database |
| CaPOPI_8591 | scaffold981 | 10911 | CGAAACCTGAAACCTGAAACCTGAAACCT/CGAAACCTGAAACCTGAAACCT | AACCCGAGAACCAAAATCCT | GTTTCGGGTTTACGGTTTCA | 59.8 | 326 | INTERGENIC | | | | |
| CaPOPI_8592 | scaffold981 | 84130 | AT/ATCTTTTCCTT | GATGGAGAAGCACCGGATTA | CTTGCTAACCCACAAAAGCC | 60.0 | 110 | INTRON | Ca_27591 | U | | Endonuclease/exonuclease/phosphatase |
| CaPOPI_8593 | scaffold981 | 84325 | AATATATATATA/AATATATATA | GAAGCAGTCCCATGTGGTTT | TCACCTTTCACCATTCACCA | 60.0 | 981 | INTRON | Ca_27591 | U | | Endonuclease/exonuclease/phosphatase |
| CaPOPI_8594 | scaffold9813 | 1199 | GAAA/GAAAA | AAAATCGCGCGAAAAGTCTA | TCCTGGTCCTCGATTTCATC | 60.0 | 772 | INTERGENIC | | | | |
| CaPOPI_8595 | scaffold982 | 874 | ATT/ATTT | TTGGATGGAGTGCCACAATA | AACTTGGGGTTGAAGATGAAGA | 59.9 | 164 | INTERGENIC | | | | |
| CaPOPI_8596 | scaffold982 | 1298 | CTTTTTTTT/CTTTTTTT | TTGGATGGAGTGCCACAATA | TTTCCCGACTTGGTCCTTTA | 59.9 | 727 | INTERGENIC | | | | |
| CaPOPI_8597 | scaffold982 | 1547 | GAAAAAAAAAAA/GAAAAAAAAA | TTGGAGAGGAAGATGAAAAACC | GGCTAATATATCCGTGGTCCC | 59.6 | 461 | INTERGENIC | | | | |
| CaPOPI_8598 | scaffold982 | 14935 | CT/CTAAACTCTCGTAACAGT | GTCCATAGTCTTGCCGGTGT | GCGATGAGTTGGATGGAAGT | 60.0 | 632 | URR | Ca_28025 | | G2-like | Peptidase S8/S53, subtilisin/kexin/sedolisin |
| CaPOPI_8599 | scaffold983 | 71344 | ATT/ATTT | TATGATGGATCCTCCTTCGC | TCAACCCTAAGGCGAATGTC | 60.0 | 377 | INTERGENIC | | | | |
| CaPOPI_8600 | scaffold983 | 88220 | T/TG | CACCCACAAGGAGGAAGAAC | CATGGATCAAACAGGCTTCA | 59.5 | 517 | INTERGENIC | | | | |
| CaPOPI_8601 | scaffold983 | 88456 | AGTG/AG | TTTATTCGCATGTTGTTGCC | CATGGATCAAACAGGCTTCA | 59.6 | 274 | INTERGENIC | | | | |
| CaPOPI_8602 | scaffold983 | 88622 | A/ATTGTTG | TTGGTCCTACCAGACCAAGG | TCCGACAATTTGTGCACTTC | 60.0 | 458 | INTERGENIC | | | | |
| CaPOPI_8603 | scaffold983 | 142002 | AG/ACATAAAGG | GGTTGTTACAAAGCCAATTGAA | ACGGGTCTGCACGATTTACT | 59.0 | 905 | INTERGENIC | | | | |
| CaPOPI_8604 | scaffold983 | 169332 | GTTT/GTT | AAATCCGTTTGGATTTGCAG | TTTCAACAGCGTCAAGGATG | 59.9 | 535 | INTERGENIC | | | | |
| CaPOPI_8605 | scaffold983 | 255175 | GTTTT/GTTT | TTACCGGATTTACTCACGGC | ACCCGTCAAATACGACGAAC | 60.0 | 319 | INTERGENIC | | | | |

| INDEL marker IDs | Chromosomes/ unanchored scaffolds | Physical positions (bp) | InDels (*Kabuli* reference genome-CDC Frontier/PI) | Forward primers (5'-3') | Reverse primers (5'-3') | Anneling temperature (0C) | Expected amplified product size (bp) | Structural annotation | | Functional annotation | | |
|---|---|---|---|---|---|---|---|---|---|---|---|---|
| | | | | | | | | Sequence components of *kabuli* genome | *Kabuli* gene accession IDs | NCBI-KOG | TFs | NCBI-nr database |
| CaPOPI_8606 | scaffold983 | 267429 | GTTTTTTTTTT/GTTTTTTTTTTT | CGGTTTTGTCCATAAGCGAT | ACATTGCCAAGTTCGTAGGG | 60.0 | 371 | INTERGENIC | | | | |
| CaPOPI_8607 | scaffold984 | 143777 | CTTAATAATGTAATTAATAATGTAAT/CTTAATAATGTAATTAATAATGTAATTAATAATGTAAT | AGCGATTTTTGAACCACCTG | AACAATCTCCCCCTTTTTGG | 60.1 | 539 | INTERGENIC | | | | |
| CaPOPI_8608 | scaffold99 | 78741 | GATATATATATA/GATATATATATATATATATATATA | TATTTTGGATTTTGGCTCGG | AAATGGAGGAAGGAGGGAGA | 59.9 | 611 | INTERGENIC | | | | |
| CaPOPI_8609 | scaffold992 | 36461 | CATATATATATATATAT/CATATATATATATATATAT | AAATTGGTCCACTAGCACCG | TGCGTTACGTGTGAAATGGT | 60.0 | 475 | INTERGENIC | | | | |
| CaPOPI_8610 | scaffold992 | 36900 | ATT/AT | ACCATTTCACACGTAACGCA | GCCAGGTGCACTAAGGTTGT | 60.0 | 598 | INTERGENIC | | | | |
| CaPOPI_8611 | scaffold992 | 37820 | ACTC/ACTCTTTCTCTC | GCACCTCATAAAACGACGGT | AAAGAGCCAAGAGGCATCAA | 60.0 | 349 | INTERGENIC | | | | |
| CaPOPI_8612 | scaffold992 | 40484 | CA/CACCGA | TCAATGCCAAAACATGCAAT | TTCGGATGTCCCCTTAAACA | 59.9 | 513 | INTERGENIC | | | | |
| CaPOPI_8613 | scaffold992 | 42011 | CT/C | CAACCTTAACGCACCGTACC | TTTGTTCAATTTCATGCTCCC | 60.4 | 563 | INTERGENIC | | | | |
| CaPOPI_8614 | scaffold992 | 42112 | GAAAAAA/GAAAAAAA | TCGATAAAAGCAACGGATTG | AGGGAGAAAGACAGATGAGTGC | 58.8 | 676 | INTERGENIC | | | | |
| CaPOPI_8615 | scaffold992 | 42301 | CTCT/CTTTCT | GGGAGCATGAAATTGAACAAA | TCTTTTGCGTCTGAATTTGG | 59.9 | 708 | INTERGENIC | | | | |
| CaPOPI_8616 | scaffold992 | 46266 | GTTTTTT/GTTTTTTT | GCTTGGGGACAAGCAATAGA | CCCAGTTCCCAAGAGTCAAA | 60.2 | 393 | INTERGENIC | | | | |
| CaPOPI_8617 | scaffold996 | 37798 | GTATATATATATATATAT/GTATATATATATATAT | CGCTGTTTGAATCAATGGTG | GAATCAAGTGGGGAGGGAAT | 60.1 | 323 | INTERGENIC | | | | |
| CaPOPI_8618 | scaffold996 | 44276 | TAAGAAGA/TAAGA | CGGTCTTTCCGTGAGTAATGA | TGAATAAACTGCGCGTATGTG | 60.1 | 561 | INTERGENIC | | | | |
| CaPOPI_8619 | scaffold996 | 87555 | GTTTTTTT/GTTTTTTTTTTTT | TGGCGTACTGAGAGCTGCTA | TGTGCACCGGAAAACTTGTA | 59.9 | 435 | DRR | Ca_25583 | | | |
| CaPOPI_8620 | scaffold996 | 113638 | AAATAATAATAATAATAATAATAATAATAATAATAAT/AAATAATAATAATAATAATAATAATAATAATAATAAT | CAGTTAACGTCTTTGAGTGCG | GGGGATGCGACCATCTACTA | 58.6 | 689 | INTERGENIC | | | | |

| **INDEL marker IDs** | **Chromosomes/ unanchored scaffolds** | **Physical positions (bp)** | **InDels (*Kabuli* reference genome-CDC Frontier/PI)** | **Forward primers (5'-3')** | **Reverse primers (5'-3')** | **Anneling temperature (0C)** | **Expected amplified product size (bp)** | **Structural annotation** | | **Functional annotation** | | |
|---|---|---|---|---|---|---|---|---|---|---|---|---|
| | | | | | | | | **Sequence components of *kabuli* genome** | ***Kabuli* gene accession IDs** | **NCBI-KOG** | **TFs** | **NCBI-nr database** |
| CaPOPI_8621 | scaffold996 | 203068 | TTATATATATATATATATATATATATATATATATA/TTATATATATATATATATATATATATATATATATATATATA | TGGTGCGTTAAGCATGAAAC | TGTGTCCTGTTTGGGAACAA | 59.7 | 398 | INTERGENIC | | | | |
| CaPOPI_8622 | scaffold999 | 77364 | A/AT | AGGAAGAGTGGAGTTGCGAA | GGATCAAGGCAGTGGATTGT | 60.0 | 854 | DRR | Ca_28108 | | | Photosynthetic reaction centre, L/M |
| CaPOPI_8623 | scaffold999 | 123867 | CT/C | CCAAATGTTATGATCAGTATGCC | TGCGAAGAAGCTCACTTGAA | 58.4 | 734 | INTERGENIC | | | | |
| CaPOPI_8624 | scaffold999 | 130386 | TG/TGG | TGTAAACCTGCTTGCACACC | CTGCACAACAATCCCACAAC | 59.8 | 160 | INTERGENIC | | | | |
