# Supplementary material for: A High-Resolution InDel (Insertion–Deletion) Markers-Anchored Consensus Genetic Map Identifies Major QTLs Governing Pod Number and Seed Yield in Chickpea
Source: Front Plant Sci. 2016 Sep 16;7:1362. doi: 10.3389/fpls.2016.01362 (PMC5025440; doi:10.3389/fpls.2016.01362)
Supplement: Supplementary file 6 [file Table6.PDF]

Table S6. InDel markers (5263) exhibiting differentiation between high (Pusa 256 and HPNB) and low (ILWC 46 and LPNB) pod number-containing parental accessions and homozygous bulks of a mapping population (Pusa 256 x ILWC 46) with reference to *kabuli* (CDC Frontier) genome

INDEL marker IDs	Chromosomes /unanchored scaffolds	Physical positions (bp)	InDels (*Kabuli* reference genome-CDC Frontier/PI)	Forward primers (5'-3')	Reverse primers (5'-3')	Anneling temperature (0C)	Expected amplified product size (bp)	Structural annotation		Functional annotation		
								Sequence components of *kabuli* genome	*Kabuli* gene accession IDs	NCBI-KOG	TFs	NCBI-nr database
CaPOPII_5263	C11177592	7683	CAAA/CAA	CAGGCACACTGCACAAAGTT	TCCCTCGGATTATGTGAAGC	59.9	533	INTRON	Ca_27746	O	bHLH	Heat shock protein DnaJ, N-terminal
CaPOPII_1	Ca1	18088	TA/TAA	CCGCTTGTTTTTCTTTGCAG	TTGATTGCTTGTTGCTCCAG	60.9	543	INTERGENIC				
CaPOPII_2	Ca1	19168	GCTCTCT/GCTCT	CAGAACCGAGGAATTAAGCG	AGGCTTGAAGCGTGGTAATG	59.8	637	INTERGENIC				
CaPOPII_3	Ca1	186939	TAA/TAAA	CTCTTGGGGGATCTTCATCA	GCATGCCATGTAGTGCATTT	60.0	329	DRR	Ca_00014			Protein of unknown function DUF707
CaPOPII_4	Ca1	343407	CATA/CA	ATCAACTTCAGGTTGGGTGG	CCAAAGATGAGCCCAAGAAA	59.8	704	INTERGENIC				
CaPOPII_5	Ca1	345766	GAA/GA	TCAACTTTGAGGTCTCCGGT	TCACTTCCGTTTCTGCCTCT	59.7	182	INTRON	Ca_00035	O		NIF system FeS cluster assembly, NifU, C-terminal
CaPOPII_6	Ca1	374767	TCA/TCACA	AACACGGCAGTGGTATCTCC	CATCATCAAGGCCATCCTCT	60.0	722	INTRON	Ca_00038	S		Protein of unknown function DUF803
CaPOPII_7	Ca1	403989	CT/CTCGTATTGACAGT	TTGCACCTTATGCTTCACCA	CAATGGGAGCGCAGTTTTAT	60.3	228	INTERGENIC				
CaPOPII_8	Ca1	406876	C/CCCTCTTT	TGACACAAAATCAGGCCAAA	ACACATGGCAAAATTTCACG	60.1	629	INTRON	Ca_00043			Glycosyl-phosphatidyl inositol-anchored, plant
CaPOPII_9	Ca1	417847	CCAAAGCAA/CCAA	TAAGGGTAAGGCCACCAAAG	AACCGTTATACGGCAACGAC	59.1	594	INTERGENIC				
CaPOPII_10	Ca1	1397435	CA/C	TGGAAACAGCAACGTGGTAG	CAAAGGGTTGGATTTTCCAC	59.8	555	INTERGENIC				
CaPOPII_11	Ca1	1744972	CA/CACAGTTCCACTTTA	ACCCTGTGGCACAATACCAT	AGCACCGATCAAATCACACA	60.1	820	INTRON	Ca_00219		TALE	Protein kinase, catalytic domain
CaPOPII_12	Ca1	1746242	ACAAAT/A	AATGCAAAAAGCCGACATCT	GTGGGAGTCGTGAAACTCGT	59.7	281	INTRON	Ca_00219		TALE	Protein kinase, catalytic domain
CaPOPII_13	Ca1	1777834	CTCT/CTCTATCT	CGTCACAAAGCATCACAGGT	ATTGCACAACGGCTACAACA	59.8	906	INTERGENIC				
CaPOPII_14	Ca1	1785166	AG/A	TCTACTTGCGGCAACTTGTG	CATCATTCCAATCAAGCACG	60.1	319	DRR	Ca_00224	S		Zinc finger, RING-type
CaPOPII_15	Ca1	1785215	A/AAATG	TCTACTTGCGGCAACTTGTG	CATCATTCCAATCAAGCACG	60.1	319	DRR	Ca_00224	S		Zinc finger, RING-type

INDEL marker IDs	Chromosomes /unanchored scaffolds	Physical positions (bp)	InDels (*Kabuli* reference genome-CDC Frontier/PI)	Forward primers (5'-3')	Reverse primers (5'-3')	Anneling temperature (0C)	Expected amplified product size (bp)	Structural annotation		Functional annotation		
								Sequence components of *kabuli* genome	*Kabuli* gene accession IDs	NCBI-KOG	TFs	NCBI-nr database
CaPOPII_16	Ca1	1801730	ACACACAACGAGTC/ACACACAACGAGTCACACAACGAGTC	CAGGGAAAATTCCAAGCTGA	TGGACAAGAGCATCTGCAAC	60.2	565	CDS (large-effect mutations)	Ca_00228	F	MYB_related	Delayed-early response protein/equilibrative nucleoside transporter
CaPOPII_17	Ca1	1822026	TC/T	ATGTTTGGGGCTATCGACTG	GAGGAATTTTGCTCTCGTGC	60.0	449	INTERGENIC				
CaPOPII_18	Ca1	1841034	TTTCTTCT/TTTCT	TCAAGGTGTTGATTCGGTAGG	TTTTGCAACCCGGTCTATTC	60.0	595	INTERGENIC				
CaPOPII_19	Ca1	1841424	A/AT	GAATAGACCGGGTTGCAAAA	CCTCTTTCCGGACAACACAT	59.9	302	INTERGENIC				
CaPOPII_20	Ca1	1852604	TA/TAA	ACACGGCGTTCATTTTCTTC	CAAACCATGGGAGAGAGAGC	60.1	843	INTERGENIC				
CaPOPII_21	Ca1	1876420	AT/ATACGTAGTTCGGTTGAGT	CGGGTCTTCAAAATGGAATG	CAAAGGAAACGTGCTTCCAG	60.3	861	INTERGENIC				
CaPOPII_22	Ca1	1930262	ATTTTTTTT/ATTTTTTTTTT	GGCAACATTTTACAAAAGTTAAGG	AGTTTGATTAATGTCGGGCG	58.3	318	INTERGENIC				
CaPOPII_23	Ca1	1971572	AA/AAGAAATGCA	ATGTGCATCACCTCTGACGA	GCTGCCATGGAGGTAGTCAT	60.3	807	INTERGENIC				
CaPOPII_24	Ca1	1972044	ACTTCTTCT/ACTTCTTCTTCT	GCCCTCAGCCTCACTTTATG	AGGGAGGCTACGAACGATTT	59.8	356	INTERGENIC				
CaPOPII_25	Ca1	1987558	ATAGTAG/ATAG	GTGGCATAGGCTCCAATCAT	GGAATGGTGCTGGACATTTT	59.9	922	DRR	Ca_00248	T		Protein of unknown function DUF300
CaPOPII_26	Ca1	1989852	AAA/AAATAA	TTGGGCTTATTGGGTCACTC	TATCCAATCCCAACCCAAAA	59.9	115	INTERGENIC				
CaPOPII_27	Ca1	2004325	AC/ACCATC	TACCTGGTGTCATTGCTTGG	CATATCCTCATGGCAGTCCC	59.6	625	INTRON	Ca_00252		TCP	S1/P1 nuclease
CaPOPII_28	Ca1	2004592	T/TA	TCAGCAGTTGCTACCTCCCT	CGCCAAGAACAATAATGCCT	60.0	447	INTRON	Ca_00252		TCP	S1/P1 nuclease
CaPOPII_29	Ca1	2015582	TTGT/TT	CTCTAGAACCCCCAACTCCC	CTCCAATGTTGGGAGACTACG	59.9	499	DRR	Ca_00253	E		Amino acid transporter, transmembrane
CaPOPII_30	Ca1	2058843	CAAAA/CAA	GAGAAAGGGGAGGAGGATTG	AGACTCTGCAGCCACCTCAT	60.0	357	DRR	Ca_00261			Protein of unknown function DUF707
CaPOPII_31	Ca1	2073961	CTTTT/CTTT	AGAGGCACGAGATTTCCAGA	AAATATCCCCGCAATAAGGG	60.0	336	CDS (FRAME SHIFT)	Ca_00263	T	M-type	Disease resistance protein
CaPOPII_32	Ca1	2085412	AAGTTTTAT/AAGTTTTATAGAGTTTTAT	TTTTTGGGCTCACACAATTT	CAATTGTCCAAAACTCTTATCCAA	58.1	723	INTERGENIC				

**INDEL marker IDs**	**Chromosomes /unanchored scaffolds**	**Physical positions (bp)**	**InDels (*Kabuli* reference genome-CDC Frontier/PI)**	**Forward primers (5'-3')**	**Reverse primers (5'-3')**	**Anneling temperature (0C)**	**Expected amplified product size (bp)**	**Structural annotation**		**Functional annotation**		
								**Sequence components of *kabuli* genome**	***Kabuli* gene accession IDs**	**NCBI-KOG**	**TFs**	**NCBI-nr database**
CaPOPII_33	Ca1	2085895	GAAAAATAAAAA/GAAAAATAAAAATAAAAA	TGTTTTTGGATAAGAGTTTTGGA	TCCTCAATCGCGTTCTTTCT	57.9	626	INTERGENIC				
CaPOPII_34	Ca1	2113146	TAAAAAAAAAA/TAAAAAAAA	TGTGGACACGTTAAGTTGTGG	TGATCTCGCCCTATTATTCAAAA	59.5	409	INTERGENIC				
CaPOPII_35	Ca1	2115049	TAAAAAAAAA/TAAAAAAAAAA	TGGACTAAAATTGCTTTTTGGAA	TTAGAAAGAGCCACACGCAA	60.0	897	INTERGENIC				
CaPOPII_36	Ca1	2131678	TAT/TATTGAT	AGGAATGCATCGAATCCAAG	CCACAAGTGCCATTCAGCTA	60.0	516	INTERGENIC				
CaPOPII_37	Ca1	2250936	GA/GAA	TGAATATCCAGAGATCGCCC	CATTGCCCTTTCACCCTTTA	60.0	858	INTRON	Ca_00281	O		Zinc finger, RING-type
CaPOPII_38	Ca1	2313379	GTTTT/GTT	GCCAAAGGCATTCATTGTTT	TGTCACTAAAAGGGTTCCCG	59.9	528	INTRON	Ca_00290			Clathrin light chain
CaPOPII_39	Ca1	2315915	AACGTTACG/AACG	ACCGTCGGAGACAAAAACAC	AGCAAACAAGCAAAGCCAAC	60.0	412	INTERGENIC				
CaPOPII_40	Ca1	2317093	TG/T	TTGTGGTTGGTGGTGATGTC	CCTGGGTACATCATAACATTTTTC	60.3	664	INTERGENIC				
CaPOPII_41	Ca1	2324632	ATTTTTTTT/ATTTTTTTTTTT	TTAAGCTGAGCACTTCCAAGA	GAATCCGAAACCACACGAAT	57.9	523	DRR	Ca_00291	V	NAC	NAD-dependent epimerase/dehydratase
CaPOPII_42	Ca1	2332780	CTT/CT	CAAAAATGGCACATGCAAAG	TTCACCAATTCCGTGTTTCA	60.1	399	INTERGENIC				
CaPOPII_43	Ca1	2370923	CA/CAA	CTATGGAAAATGCTCCCGAA	CCGAAACTCACAATGAACCA	60.0	507	INTRON	Ca_00301	DR		Ribosomal RNA methyltransferase RrmJ/FtsJ
CaPOPII_44	Ca1	2411747	T/TA	TGTGTCTTGTTGGGGAAACA	TGACGCTTTTGAAGCAGTTG	60.0	547	INTERGENIC				
CaPOPII_45	Ca1	2412541	TTTATTA/TTTA	AGGACAATAGGACGAGGCAA	AGTCACAGAGCATGCACCAC	59.7	436	INTERGENIC				
CaPOPII_46	Ca1	2416502	GA/G	CAAAATCAGTCGCATGTTGG	TAGCCTTTTTGTTGGTTGGC	60.1	248	INTERGENIC				
CaPOPII_47	Ca1	2653521	T/TC	GGTGGGTGACCCTCACTTTA	CATTCCAGTTAGTTGAACGGGT	59.8	525	INTERGENIC				
CaPOPII_48	Ca1	2656475	A/AG	TTGCACAATTTCTGATTGGC	GATCATTGCATCCCCATTCT	59.7	569	INTERGENIC				
CaPOPII_49	Ca1	2661346	TAAAA/TAAAAA	CGCCGTTTTTCTTCAAACTC	TGGTGTTCATTTTGATTCAGGA	59.9	840	INTERGENIC				

INDEL marker IDs	Chromosomes /unanchored scaffolds	Physical positions (bp)	InDels (*Kabuli* reference genome-CDC Frontier/PI)	Forward primers (5'-3')	Reverse primers (5'-3')	Anneling temperature (0C)	Expected amplified product size (bp)	Structural annotation		Functional annotation		
								Sequence components of *kabuli* genome	*Kabuli* gene accession IDs	NCBI-KOG	TFs	NCBI-nr database
CaPOPII_50	Ca1	2663715	TGAAAAGAAAA/TGAAAA	GTTGCCATGCCTATAACGGT	TGAAGACCATGGACACCAAC	59.9	547	INTERGENIC				
CaPOPII_51	Ca1	2668175	AC/A	AATGCATCACGTGGGATTTT	CGATTGCACCCGTAGTTGTA	60.2	206	INTERGENIC				
CaPOPII_52	Ca1	2669284	TAAAAAAAAAAAAA/TAAAAAAAAAA	CCCCAAACTTTGAAGCAACT	TTTGTGCTGCTATGTGCCTC	59.2	344	DRR	Ca_00327		ERF	Pathogenesis-related transcriptional factor/ERF, DNA-binding
CaPOPII_53	Ca1	2674516	CTAGATTGGAT/CTAGATTGGATTAGATTGGAT	TGTCGGAATATGGGAAGCTC	GCCGTTGTTTTCAGATCCAT	60.0	934	INTERGENIC				
CaPOPII_54	Ca1	2693080	TAAAAAAAA/TAAAAAAAAA	CAACCAGTAATAATGCGCCA	CGTGACAGAGGACGCTTGTA	59.6	468	DRR	Ca_00330			
CaPOPII_55	Ca1	2725497	TAA/TA	GTGTAACCTCACGGGAAGGA	GGGTCAATTTGAAACAACGG	60.0	660	INTERGENIC				
CaPOPII_56	Ca1	2745007	GTTTTTTTTTT/GTTTTTTTTT	GAACGAACGGTGTGATGATG	GGGCCCACAATAACACAAAC	60.0	513	INTERGENIC				
CaPOPII_57	Ca1	2765671	GTTTTTTTTT/GTTTTTTT	TAAAAAGGGCCGATATGCAC	CATGCACGATTGTGGTAATTG	59.9	564	INTERGENIC				
CaPOPII_58	Ca1	2766077	CTTATGTT/CTTATGTTATGTT	AAAACAATTGATTGGGCTCC	CCAATCATACGCAGGTGAAA	58.9	561	INTERGENIC				
CaPOPII_59	Ca1	2766176	A/AG	AAAACAATTGATTGGGCTCC	CCAATCATACGCAGGTGAAA	58.9	561	INTERGENIC				
CaPOPII_60	Ca1	2766346	AATATA/AATATATA	TTTCACCTGCGTATGATTGG	TGACCTCTTATGCTTCTCGAAAT	59.5	501	INTERGENIC				
CaPOPII_61	Ca1	2766600	CTTTTTTTT/CTTTTTTT	TTTCACCTGCGTATGATTGG	TTGAAAGTTGAAACGTGGGA	59.5	779	INTERGENIC				
CaPOPII_62	Ca1	2797230	A/AC	TGTCTTAAAATCTGTGCATTCAA	AAATAGTTGACAAACCGCCG	57.5	581	INTERGENIC				
CaPOPII_63	Ca1	2802455	ATTTTT/ATTTT	GGGTTACAGACCGACCTTGA	GCGGATTTGTCCATCTTTGT	60.0	765	DRR	Ca_00344		NAC	No apical meristem (NAM) protein
CaPOPII_64	Ca1	2803396	GTGATGATG/GTGATG	ACAACGGAATTGGTCATGGT	TTCCTAATTCCAATCCCATGT	60.1	360	INTERGENIC				
CaPOPII_65	Ca1	2831499	GTTTTTTTTTTTTTT/GTTTTTTTTTTTT	CCTCTGACCACATGGGACTT	TCAAAAACAAAAGCGCACTG	60.0	625	DRR	Ca_00347		ARF	Transcriptional factor B3
CaPOPII_66	Ca1	2840718	GTTTTTTTTTT/GTTTTTTTTT	GCTACATGTAACGCAGCGAA	ATTTAAGATGGCGATGTGCC	60.0	512	INTERGENIC				

INDEL marker IDs	Chromosomes /unanchored scaffolds	Physical positions (bp)	InDels (*Kabuli* reference genome-CDC Frontier/PI)	Forward primers (5'-3')	Reverse primers (5'-3')	Anneling temperature (0C)	Expected amplified product size (bp)	Structural annotation		Functional annotation		
								Sequence components of *kabuli* genome	*Kabuli* gene accession IDs	NCBI-KOG	TFs	NCBI-nr database
CaPOPII_67	Ca1	2845108	TAAAAA/TAAA	CCACCTTTCAAAGACGAAACA	GCTTGATTTCAAAATCTCCTCC	60.1	686	INTERGENIC				
CaPOPII_68	Ca1	2845159	TAAAAAAAA/TAAAAAAA	CCACCTTTCAAAGACGAAACA	GCTTGATTTCAAAATCTCCTCC	60.1	686	INTERGENIC				
CaPOPII_69	Ca1	2867984	AAT/AATGAT	ATGAGAAAGGGGAAGAGGGA	AAGGGGTGAACAGTGAGGTG	60.0	646	INTERGENIC				
CaPOPII_70	Ca1	2875002	CTTTTTTTTTT/CTTTTTTTTT	TGCCAAAGGTAATTTTTCCG	GCCCGCAAAAGTTAAAAGAA	59.9	149	DRR	Ca_00349			
CaPOPII_71	Ca1	2884045	TAAA/TAA	GAGCTTCTCCACCTCACGTC	GTGGCATTCAGGTGAGGAAT	60.0	412	DRR	Ca_00351	T	M-type	Disease resistance protein
CaPOPII_72	Ca1	2884405	AGAGACATT/A	ATTCCTCACCTGAATGCCAC	TGAAAGCATCATGCACACAA	59.9	511	DRR	Ca_00351	T	M-type	Disease resistance protein
CaPOPII_73	Ca1	2884853	GTTTT/GTTT	TCTCCGTCGTGTTGTGTTGT	ATTGCGGAGTTAACAATGGC	60.2	743	DRR	Ca_00351	T	M-type	Disease resistance protein
CaPOPII_74	Ca1	2885377	ATTTTTTT/ATTTTTTTT	GCCATTGTTAACTCCGCAAT	CATTTGGAGAGGGCATAGGA	60.0	360	DRR	Ca_00351	T	M-type	Disease resistance protein
CaPOPII_75	Ca1	2898163	CAAAAAAAAAAA/CAAAAAAAAA	TGGTGTTTACCACTCCGTCA	TTAAATCACAGTGGCGGTCA	60.0	368	INTRON	Ca_00354		bHLH	Protein kinase, catalytic domain
CaPOPII_76	Ca1	2900742	AAC/A	CTGCAGGGATCTGAGTGACA	GGAATGGTTTGGGGAATTTT	60.0	637	INTRON	Ca_00354		bHLH	Protein kinase, catalytic domain
CaPOPII_77	Ca1	2912876	GGTAC/GGTACGTAC	TGTCATCCCTAGCAACACGA	TGTAATGCATTTCCCCTTGTC	60.3	192	INTERGENIC				
CaPOPII_78	Ca1	2919131	CAAAAAAAAAAA/CAAAAAAAAAA	GTATCCCATCCCCTGCAATA	TTCCATGTTCCCTTTGCTTC	59.6	202	INTERGENIC				
CaPOPII_79	Ca1	2921722	CAATA/CA	CAACTGGGCCAAATGCTTAT	TGATGATCACACGAGGGAGA	60.0	570	INTERGENIC				
CaPOPII_80	Ca1	2922949	CTTTTTTTTTTTT/CTTTTTTTTTTT	TTTGTGGATCGTATTGCGAA	AGCATATGGTCCCCTTTTCA	60.1	947	INTERGENIC				
CaPOPII_81	Ca1	2923053	TATGAA/TATGAATGAATGAA	CGCGAAATCTTATTTTCATGG	TGATGCGATTGTAGTCCCAA	59.6	606	INTERGENIC				
CaPOPII_82	Ca1	2923274	A/ATATTAAAATAAT	CGCGAAATCTTATTTTCATGG	TGATGCGATTGTAGTCCCAA	59.6	606	INTERGENIC				
CaPOPII_83	Ca1	2923894	TAAAAAAAAA/TAAAAAAAA	TTGGGACTACAATCGCATCA	ATTGACCCGATTTGCTGAAC	60.1	718	INTERGENIC				

INDEL marker IDs	Chromosomes /unanchored scaffolds	Physical positions (bp)	InDels (*Kabuli* reference genome-CDC Frontier/PI)	Forward primers (5'-3')	Reverse primers (5'-3')	Anneling temperature (0C)	Expected amplified product size (bp)	Structural annotation		Functional annotation		
								Sequence components of *kabuli* genome	*Kabuli* gene accession IDs	NCBI-KOG	TFs	NCBI-nr database
CaPOPII_84	Ca1	2923992	TTA/T	TTGGGACTACAATCGCATCA	ATTGACCCGATTTGCTGAAC	60.1	718	INTERGENIC				
CaPOPII_85	Ca1	2956674	TTATATATAT/TTATAT	CCCATCTCAAAGGAAGTTGC	CCACTGCTGCGCTATTATGA	59.7	641	INTERGENIC				
CaPOPII_86	Ca1	2966305	T/TTC	CCACGTCTGTAAATTCGCTTT	GCTCGCCACTCTCTTTCTCT	59.3	785	INTERGENIC				
CaPOPII_87	Ca1	3018574	CAATTAATTAATT/CAATTAATT	AGCCAACAGCCCAACATAAA	AAGGTTCATGTCCAAATCGC	60.5	242	INTERGENIC				
CaPOPII_88	Ca1	3020354	AGG/AGGG	CAAGCAACATGCTGAAGGAA	ATGTCCTTAGTGTCACGGGG	60.0	808	INTERGENIC				
CaPOPII_89	Ca1	3030441	AATATATAT/AATATAT	AATAGTGAGGGGTGGAGGCT	TGATGGATGAGACAAAATGCC	60.0	415	DRR	Ca_00367			
CaPOPII_90	Ca1	3037452	AT/ATTTAAAGTATTTGTT	ACACCAGCCGTTGAATAAGG	ATAACGGTGGAGCCTGTTTG	60.0	916	INTRON	Ca_00370		ARF	Transferase
CaPOPII_91	Ca1	3074826	A/AT	CACGAGGTTTGACCTCTTCC	CGTGTGAACATTCATACACCTCA	59.7	578	INTRON	Ca_00375		bHLH	Lipase, GDSL
CaPOPII_92	Ca1	3075028	A/AT	CACGAGGTTTGACCTCTTCC	CGATCGTCGCATTTTTCATA	59.7	841	INTRON	Ca_00375		bHLH	Lipase, GDSL
CaPOPII_93	Ca1	3089236	ATTT/ATT	GCAAAACGTCTTATCGGGAA	TGCAAACTATTTGACATTGACG	60.1	591	INTERGENIC				
CaPOPII_94	Ca1	3089287	TTGTAAT/TT	GCAAAACGTCTTATCGGGAA	TGCAAACTATTTGACATTGACG	60.1	591	DRR	Ca_00378	I		Lipase, class 3
CaPOPII_95	Ca1	3125906	GAAAAA/GAAAAAA	GAAATCCACACCATCGAACC	TGATTCATGTGCCTTACCCA	60.2	595	INTRON	Ca_00382			Domain of unknown function DUF231, plant
CaPOPII_96	Ca1	3125978	CAA/CAAA	GAAATCCACACCATCGAACC	TGATTCATGTGCCTTACCCA	60.2	595	INTRON	Ca_00382			Domain of unknown function DUF231, plant
CaPOPII_97	Ca1	3133490	AATTATATATTATA/AATTATA	CCACATTTTGCCACTTTTCA	TCGCCTTTTAAACCAACCAG	59.6	853	INTERGENIC				
CaPOPII_98	Ca1	3138107	A/ATT	ATCGGGGTGGACAAAACATA	GGGCTTTAAATTGAGTGAGGC	60.1	683	INTERGENIC				
CaPOPII_99	Ca1	3148374	TAAAAAA/TAAAAAAA	AATACCCCCATGACCCATCT	GTGGACACCAGATACGCCTT	60.3	579	INTRON	Ca_00385			LPPG:FO 2-phospho-L-lactate transferase CofD/UPF0052
CaPOPII_100	Ca1	3287281	TAAAAAAA/TAAAAAAAA	TGCTGAAAGAGTTGTCGGTG	AGAGGGACCACAAAGTCAAA	60.0	516	INTRON	Ca_00398		LBD	Protein kinase, catalytic domain

INDEL marker IDs	Chromosomes /unanchored scaffolds	Physical positions (bp)	InDels (*Kabuli* reference genome-CDC Frontier/PI)	Forward primers (5'-3')	Reverse primers (5'-3')	Anneling temperature (0C)	Expected amplified product size (bp)	Structural annotation		Functional annotation		
								Sequence components of *kabuli* genome	*Kabuli* gene accession IDs	NCBI-KOG	TFs	NCBI-nr database
CaPOPII_101	Ca1	3764450	GATATATATATATATATATATATA/GATATATATATATATATATATATATA	TTTTGGAGGTGTGATTAATGTATTTT	TGGTTGATGTGTCGAGTGTCT	59.2	515	INTERGENIC				
CaPOPII_102	Ca1	3783626	TATTTGCATTTG/TATTTG	TTCACAATTACCACAAAGATCG	GCGACTATCCAGGAAATCCA	57.7	433	INTERGENIC				
CaPOPII_103	Ca1	4171296	T/TCATTTTTTAGCTTA	CAACAAAACGCGCTAATGAC	CGAGATTTTGAGGCATGTTG	59.4	522	INTERGENIC				
CaPOPII_104	Ca1	4718653	CATAT/CAT	TTTGACACGACCAACCAAAA	TGGACACAGCAAGTTAAGCG	60.0	781	INTRON	Ca_00555	T	MYB_related	Protein kinase, catalytic domain
CaPOPII_105	Ca1	4720443	AT/A	TCAGAGTTCATTGCAAACGG	GGGATTCCCATTTTCCATCT	59.8	691	INTRON	Ca_00555	T	MYB_related	Protein kinase, catalytic domain
CaPOPII_106	Ca1	4720541	ATTTTTTTTTT/ATTTTTTTT	GCTTTACAATGCACCTGTTGA	GGGATTCCCATTTTCCATCT	58.8	359	INTRON	Ca_00555	T	MYB_related	Protein kinase, catalytic domain
CaPOPII_107	Ca1	4742142	TAAA/TAA	GACCGCGTAGATGTGGAACT	GGATGAGACCTCCTACTTGCC	60.1	677	INTERGENIC				
CaPOPII_108	Ca1	4900100	TATTCAAT/TATTCAATTCAAT	TCTTTGCAGCATGCTTTTTG	TTTCTGAAGCGACCAATTCC	60.1	561	DRR	Ca_00575			Protein of unknown function DUF679
CaPOPII_109	Ca1	5139845	TTTCTTCTTCTTCTTCTTCTTCTTCTTCTTCTTCTT/TTTCTTCTTCTTCTTCTTCTTCTTCTTCTTCTT	ACGTCTGAATCGGATTTTGC	CCCAGAAGAAGATGGAGCTG	60.1	306	INTERGENIC				
CaPOPII_110	Ca1	5180264	CTAATAATAATAATAATAATAATAATAATAATAATAAT/CTAATAATAATAATAATAATAATAATAATAATAATAATAAT	ATGCATGGAGCTATAAGCCG	TTCAAAGTTGGAATAACCCTTCTC	60.2	684	INTERGENIC				
CaPOPII_111	Ca1	5273611	TTATATATATATATATATATATATATAT/TTATATATATATATATATATATATATATATATATAT	TGCATTAATTTTGAAAACGTCAA	TGTTTGCCACGGTTGTTAAA	59.5	593	INTERGENIC				
CaPOPII_112	Ca1	5290057	ACC/AC	AGAAACGGTCACCTTCCCTT	CTCATTGCTTCCCAAACCAT	60.0	811	INTRON	Ca_00616	P		Heavy metal transport/detoxification protein
CaPOPII_113	Ca1	5351086	CAAA/CAA	TGTGGCAGACAGCCAACTAA	TTTGGCAATCGAAAATTGTG	60.4	607	INTERGENIC				
CaPOPII_114	Ca1	5446706	GTTTTT/GTTTTTTT	TGACGCAGTCTCATAGTCGG	GCAATTGTCAAGCACCATGT	60.0	336	DRR	Ca_00630			
CaPOPII_115	Ca1	5455204	TTGTT/TTGTTAATGTT	TGGTTGAACTGGGACCATTA	GGCATCAACAACAGCAAGAA	58.8	300	INTERGENIC				
CaPOPII_116	Ca1	5803481	TTAAGATAAGAT/TTAAGAT	GCTCATGAGTTCCCTACCCA	GTCAGGGGCTCGTGTATGAT	60.1	560	INTRON	Ca_00671	O	FAR1	ATPase, AAA+ type, core
CaPOPII_117	Ca1	5804081	AT/ATT	AATAACACGGGCACAAGAGG	AGGTCCACCAGGTTTGTCAG	60.0	457	INTRON	Ca_00671	O	FAR1	

INDEL marker IDs	Chromosomes /unanchored scaffolds	Physical positions (bp)	InDels (*Kabuli* reference genome-CDC Frontier/PI)	Forward primers (5'-3')	Reverse primers (5'-3')	Anneling temperature (0C)	Expected amplified product size (bp)	Structural annotation		Functional annotation		
								Sequence components of *kabuli* genome	*Kabuli* gene accession IDs	NCBI-KOG	TFs	NCBI-nr database
CaPOPII_118	Ca1	5998961	TATAATAATAATAATAATAATAATAATAATAATAATAATAATAATAATAATAATAATA/TATAATAATAATAATAATAATAATAATAATAATAATAATAATAATA	ATAGCGCACCGTCTCTCTGT	ATTTTGTGGCACATGAAGTCT	60.0	872	DRR	Ca_00687			Protein of unknown function DUF506, plant
CaPOPII_119	Ca1	6129000	CTTTTT/CTTT	CGTCATGTATCTTGGTCGGA	TGTTTAACACATTAACATGACCCA	59.5	556	INTERGENIC				
CaPOPII_120	Ca1	6138299	AT/ATT	GGCACGATGTTCTTGATTTTT	CCGATTGTTGATCGAGGTTT	59.1	846	INTERGENIC				
CaPOPII_121	Ca1	6148078	CAA/CA	TTGCTATTCCCTTTCCCCTT	CTCTCATCTCTGGCCTTTGG	59.9	558	DRR	Ca_00699	S		Mo25-like
CaPOPII_122	Ca1	6158400	TAAAA/TAAAAA	GGGGAGCATGAATGAGACAT	AAACCGCGACAATTTAATCTC	59.9	387	INTERGENIC				
CaPOPII_123	Ca1	6203281	GTTTTTTTTT/GTTTTTTTTTT	CTCCTTGAGTTTTGAGACACGA	TCGATTATGCGTAGGGAAGG	59.5	453	INTERGENIC				
CaPOPII_124	Ca1	6205233	CAAAAAAAAAAA/CAAAAAAAAAAAA	GGGTATTGGATAAAAATTTCAAAAA	TGAAAATTAAGGGACGGGTTT	58.7	604	DRR	Ca_07880	T		DOMON domain
CaPOPII_125	Ca1	6267078	GAAA/GA	TTTCTTGCACTGGTTTGTGC	TGGGTCCCTCATTGTACTCC	59.9	560	INTERGENIC				
CaPOPII_126	Ca1	6332779	CT/CTT	TTTTTCTGCCTGTTCTGCCT	TCATGATCGCGAAGTTGAAA	60.0	746	INTRON	Ca_07893			Bromo adjacent homology (BAH) domain
CaPOPII_127	Ca1	6374330	AAAGAAGAAGAAGAA/AAAGAAGAAGAA	CAAAATGATGGACACCCTTG	TCCATCTTGGACCAATCTCA	58.8	291	INTERGENIC				
CaPOPII_128	Ca1	6376706	TTGGATAAATTGTGGATAAATTGTG/TTGGATAAATTGTG	GCTCGTCCAAATATTTACGCA	CCCAGCAGTGAAGCAAACTT	60.1	379	INTERGENIC				
CaPOPII_129	Ca1	6538753	AT/ATT	CAAGGAAAGGGAATGGCATA	AATTGAAGGCTGAAACGTGG	59.9	787	DRR	Ca_07919	R	bZIP	Protein kinase, catalytic domain
CaPOPII_130	Ca1	6972063	CTATTATTATTATTATTATTATTATTATTATTATTATTATTATTATTATTATTATTATTATTATTATTATTATTAT/CTATTATTATTATTATTATTATTATTATTATTAT	GTTTTCGTTCTCTCCTCCCC	CCGGTGCATTTTAACCTTGT	60.1	578	INTERGENIC				
CaPOPII_131	Ca1	7215414	AA/AACA	AACCCCCAACTACAAGGTCC	CAATTTGACAAGCCCCCTAA	60.1	639	INTERGENIC				
CaPOPII_132	Ca1	7274145	AATATATATATATATATATATATATATA/AATATATATATATATATATATATATATA	TGAATGAACTTTTATCCTGCCA	CAGCGTGAACGACTTCAAAA	59.6	539	INTERGENIC				
CaPOPII_133	Ca1	7630412	G/GACAAAA	TCGTTGCGAAGATAGTGTGG	AGCATGAGTTGCACAAGTGG	59.9	768	CDS (large-effect mutations)	Ca_08016		HD-ZIP	Homeobox
CaPOPII_134	Ca1	7642434	TA/TAA	TGCCACACAAAAATCTTGGA	TAACCTACGCTTGTGGGGTC	60.1	219	INTERGENIC				

INDEL marker IDs	Chromosomes /unanchored scaffolds	Physical positions (bp)	InDels (*Kabuli* reference genome-CDC Frontier/PI)	Forward primers (5'-3')	Reverse primers (5'-3')	Anneling temperature (0C)	Expected amplified product size (bp)	Structural annotation		Functional annotation		
								Sequence components of *kabuli* genome	*Kabuli* gene accession IDs	NCBI-KOG	TFs	NCBI-nr database
CaPOPII_135	Ca1	7726229	CAAAAAAAA/CAAAAAAAAA	TTTAGTGCCCGAGAGGAAGA	TTTGAGTCCCAACCAAAGGA	59.9	394	INTERGENIC				
CaPOPII_136	Ca1	7750166	ATTT/ATTTT	TTGCAATAGGCGTTGAAGTTT	AGGTCAAGGGTTGGGCTACT	59.8	649	INTERGENIC				
CaPOPII_137	Ca1	7750284	AAAGAA/AAA	TTGACAATAGAATCGATCAAAAACA	AGGTCAAGGGTTGGGCTACT	59.9	312	INTERGENIC				
CaPOPII_138	Ca1	7810052	TCTAA/TCTAACTAA	CTTTGTGGGCAAACCAAACT	GGGTGGTTGAAACTTCAGGA	60.0	521	DRR	Ca_08031		FAR1	Protein kinase, catalytic domain
CaPOPII_139	Ca1	7914166	T/TG	TGCATAGGTTGTTGTGGGAA	GACGAGAGGTTGGAGACTCG	60.0	939	INTERGENIC				
CaPOPII_140	Ca1	7929432	T/TTA	ATATCCGCAAGCTGAATCGT	GGTTTGAACCCTCCACAAGA	59.7	677	INTERGENIC				
CaPOPII_141	Ca1	7933130	TG/TGG	TCCTGCATAACACCATTGGA	CCTTCACAAACTTGAAGGACC	59.9	883	INTERGENIC				
CaPOPII_142	Ca1	7988388	ATT/ATTTT	TCAATCCAAACAAGTCCAGAA	AATGCATGCTTTGGTTCCTC	58.2	761	DRR	Ca_08049		WRKY	DNA-binding WRKY
CaPOPII_143	Ca1	7992471	TGTAGTA/TGTAGTAGTA	GTGCAACACCAGCTGAAGAA	CAACATCATGATCAATGGCA	60.0	310	CDS (large-effect mutations)	Ca_08049		WRKY	DNA-binding WRKY
CaPOPII_144	Ca1	7995701	ATTAAATGAGTGTTAAAT/ATTAAAT	GCGTAAGTTATGCATGCCAAT	GGTGCAAGGGAAGAAAATGA	60.0	902	INTERGENIC				
CaPOPII_145	Ca1	7995766	CAAAAAAA/CAAAAAAAA	GCCAATTGCGTGTAGCTGT	GGTGCAAGGGAAGAAAATGA	59.9	887	INTERGENIC				
CaPOPII_146	Ca1	8003639	AC/A	TTGGTCATCTCCAAAGGTCC	GGACACAAAATATAATTGAGCCA	59.9	631	INTERGENIC				
CaPOPII_147	Ca1	8004049	AAT/AATATATAT	GGCTCAATTATATTTTGTGTCCC	TCATGATATTATGCACCGGG	58.8	551	INTERGENIC				
CaPOPII_148	Ca1	8018022	G/GAAACAT	TGTTTGGTTCGATGGTTTGA	TGTATTGGGCCAGTTTCACA	59.9	544	INTERGENIC				
CaPOPII_149	Ca1	8018283	GAAA/GAA	TGTTTGGTTCGATGGTTTGA	TGTATTGGGCCAGTTTCACA	59.9	544	INTERGENIC				
CaPOPII_150	Ca1	8020277	ATTT/ATTTGTTT	TCGCGGTGAATATCACAAGA	GTCGATTCAAGTTGGCGAAT	60.2	756	INTERGENIC				
CaPOPII_151	Ca1	8031692	TTA/TTATA	ATCTTCTTTTTGTGGGGCCT	CTTGGCAGATCTCGAGTATGAA	59.9	397	INTERGENIC				

INDEL marker IDs	Chromosomes /unanchored scaffolds	Physical positions (bp)	InDels (*Kabuli* reference genome-CDC Frontier/PI)	Forward primers (5'-3')	Reverse primers (5'-3')	Anneling temperature (0C)	Expected amplified product size (bp)	Structural annotation		Functional annotation		
								Sequence components of *kabuli* genome	*Kabuli* gene accession IDs	NCBI-KOG	TFs	NCBI-nr database
CaPOPII_152	Ca1	8031727	CG/C	ATCTTCTTTTTGTGGGGCCT	CTTGGCAGATCTCGAGTATGAA	59.9	397	INTERGENIC				
CaPOPII_153	Ca1	8033455	CTTTTTTTTT/CTTTTTTTTTTTT	TCACACCTTCATTCCCTTCC	CCCAGGAGAAGAAAATCGTG	59.9	766	INTERGENIC				
CaPOPII_154	Ca1	8041307	T/TG	CGGCGGCAATTGTTATAAGT	CAATTTCAAGCAAAATCGCA	60.0	890	INTERGENIC				
CaPOPII_155	Ca1	8041662	TAAC/TAACATTAAAC	CCCTTCATTGGAGATGTTCC	TTGGGGACACTAAGAGGTGG	59.3	866	INTERGENIC				
CaPOPII_156	Ca1	8063349	CTGGTTGGT/CTGGT	GCAGTATGGCAAGAGCAACA	TCATCACATTAAGGCCAGCA	60.0	486	INTRON	Ca_08053	O	MYB_related	Heat shock protein Hsp70
CaPOPII_157	Ca1	8130436	ATTTTT/ATTTT	TTACTCACCAGGAACCGTCC	GTTGCCGGGTAAGTTTTCAA	60.0	557	INTRON	Ca_08059	P		Bicarbonate transporter, eukaryotic
CaPOPII_158	Ca1	8430467	TT/TTATTATTAT	AGGTCATGGGCTGGATGTAG	ACCCTTTCACTGCGCTCTTA	60.0	612	INTERGENIC				
CaPOPII_159	Ca1	9130103	TTATATATATATATATATATAT/TTATATATATATATATATAT	GGGTCAAAATTCGTTGACCAT	TACGGGTCAAGACAGGTTCG	61.0	550	INTERGENIC				
CaPOPII_160	Ca1	9223996	TAAAAAAAAAA/TAAAAAAAAAAA	AATGGATTCTTCCGCTCAAA	ACAGAACGGTGCGTTGTAAA	59.6	489	INTERGENIC				
CaPOPII_161	Ca1	9871519	ATT/A	CTTAGTCCACGTGCAAACGA	AAAGTGCGGGCCAATATCTA	59.9	600	INTERGENIC				
CaPOPII_162	Ca1	10057638	CATATATATATATATATAT/CATATATATATATAT	TGATGGGAGCAATGAATGAA	TCAAATTCCGACTTGAGGGA	60.0	542	INTERGENIC				
CaPOPII_163	Ca1	10428835	GTTTTT/GTTTTTT	TCAAACAAAGCCTAAAACATCA	TCATGCCTTCTTTCACTTATTCA	57.5	361	INTERGENIC				
CaPOPII_164	Ca1	11195317	TTCTCTCTCTCTCTCTCTCTCTC/TTCTCTCTCTCTCTCTCTC	AGGTGGTAAAGACACGTGGG	TGAACTCGTCACGAACCTTG	59.9	338	DRR	Ca_02638			
CaPOPII_165	Ca1	11540175	AATATATATATATATATATATATATATATATATATATATAT/AATATATATATATATATATATATATATATATATATATATATATAT	GCGAAGGAAAGAAGTTCACG	CATTTAAAAGTTCAAAGGCCTGA	60.0	728	DRR	Ca_02595	KB		Methyl-CpG DNA binding
CaPOPII_166	Ca1	11735185	GTATTATTATTATTATT/GTATTATTATTATTATTATTATT	TGACAGTCAGAGTGCGATCC	TCTCTGGCAACCGTTCTTCT	60.0	441	INTERGENIC				
CaPOPII_167	Ca1	11788825	AATATATATATATATA/AATATATATATATATATA	TGGGAATAGGTCAGACCAGG	CCAAGTTGTTTGAGAGAATTTGTTT	59.9	696	INTERGENIC				
CaPOPII_168	Ca1	12110141	ATTTTTTTTTTTTTT/ATTTTTTTTTT	TGAATTGGGCCAAGACATTT	TGGCAGACCACAGATAACCA	60.3	943	INTERGENIC				

INDEL marker IDs	Chromosomes /unanchored scaffolds	Physical positions (bp)	InDels (*Kabuli* reference genome-CDC Frontier/PI)	Forward primers (5'-3')	Reverse primers (5'-3')	Anneling temperature (0C)	Expected amplified product size (bp)	Structural annotation		Functional annotation		
								Sequence components of *kabuli* genome	*Kabuli* gene accession IDs	NCBI-KOG	TFs	NCBI-nr database
CaPOPII_169	Ca1	12112319	C/CTTGG	GCGAAATTATTTGACGGAGTTT	CATTTCAGAAGTGAAGAGCAACA	59.5	874	INTERGENIC				
CaPOPII_170	Ca1	12420239	CAAAAAAAAA/CAAAAAAAAAA	AAATGCAATCTTGGTTTCCA	TTTTGGTAATGTGCCCATGATA	58.1	595	INTERGENIC				
CaPOPII_171	Ca1	12544202	TTATATATATATATAT/TTATATATATATAT	TCGGTGTCTCCCTTCATTTC	TCATTCAAGGATGCAACTGG	60.1	656	INTERGENIC				
CaPOPII_172	Ca1	12644727	TGTA/TGTACGTA	TTGATTTTCAATTGGGAGCC	TGCCAGGATGTAACCCTTTC	59.9	807	INTERGENIC				
CaPOPII_173	Ca1	12644922	AC/A	ACCCAACCGCAAAAACAATA	TGCCAGGATGTAACCCTTTC	60.2	440	INTERGENIC				
CaPOPII_174	Ca1	12690899	TTAAATAAATAAATAAATAA/TTAAATAAATAAATAAATAAATAA	TGCTTCATACCAACGTTCCA	CAGAGTTTTAAACGTATGACGGTG	60.1	516	INTERGENIC				
CaPOPII_175	Ca1	12721902	CATATATATATATATATATATATAT/CATATATATATATATATATATAT	CGCATGATTCAAACAAATGG	CCAACTCAGGTTCAGCAAGA	59.9	746	DRR	Ca_02480			
CaPOPII_176	Ca1	12776177	T/TC	ATCTCCCCGTCAGATATCCC	GACAATAACCACCCAGTGCC	60.1	225	INTERGENIC				
CaPOPII_177	Ca1	12947634	ATAAATTTAAA/ATAAATTTAAAATTTAAATTTAAA	AACTTTGTGGTCGTTTTGGC	TGCTTATTGTGGCTGCTTTG	60.0	647	INTERGENIC				
CaPOPII_178	Ca1	12956423	CAAAAAAA/CAAAAA	TTGTCGGATACCTGCTCAGT	TTGCAAATTCGGACAAACAA	58.3	455	INTERGENIC				
CaPOPII_179	Ca1	13007507	TTAT/TTATATAT	CACGGGTCCAGATGGTTAGT	CCAAGCATCTTGGTGCATAA	59.8	322	INTERGENIC				
CaPOPII_180	Ca1	13487363	TAAAAAAAAAAAA/TAAAAAAAAAAAAA	GATGATTGCAGCAAGTTGGA	TCGTGTTGGCTAATTCAACG	59.8	558	INTERGENIC				
CaPOPII_181	Ca1	13679372	CTTTT/CTTT	AGGTGCTCTCCTCTCCACAG	TTTGGCATGGATAGGGGTTA	59.6	429	DRR	Ca_14133		ERF	Pathogenesis-related transcriptional factor/ERF, DNA-binding
CaPOPII_182	Ca1	13697453	TAAAAAAA/TAAAAAAAA	TCTGAAAACAATTTCCCCTTG	CTAGACCATGGCCGTTCAAT	59.1	865	DRR	Ca_14135		bHLH	Terpene synthase-like
CaPOPII_183	Ca1	13701840	AGAATGTATAT/A	TGCAAAACCAAGTCTGTCCA	ACCTTGCCCCTGAAGTAGGT	60.3	632	INTRON	Ca_14135		bHLH	Terpene synthase-like
CaPOPII_184	Ca1	13939746	TA/TACTTTATTAAAAAA	TCAGGTGTCTCATCCACTGC	TGGATTTTCAATGCCAGACA	59.8	755	DRR	Ca_07137			
CaPOPII_185	Ca1	14309917	G/GAA	AACCATTGGAAGGCAAAATG	TCTTCCACCTCTTTCGCATC	59.8	106	INTERGENIC				

INDEL marker IDs	Chromosomes /unanchored scaffolds	Physical positions (bp)	InDels (*Kabuli* reference genome-CDC Frontier/PI)	Forward primers (5'-3')	Reverse primers (5'-3')	Anneling temperature (0C)	Expected amplified product size (bp)	Structural annotation		Functional annotation		
								Sequence components of *kabuli* genome	*Kabuli* gene accession IDs	NCBI-KOG	TFs	NCBI-nr database
CaPOPII_186	Ca1	14426009	CATATATATATATATATATATATATATAT/CATATATATATATATATATATATATATATATATAT	CAGCCAAGACCCAATTTCAT	ACTCTCCCAAAAAGGCGATT	59.9	432	INTERGENIC				
CaPOPII_187	Ca1	14891282	AATATATATATATATATATATATATAT/AATATATATATATATATATAT	TCACAAGATAGTTTTGCCTTTTT	CAAAATCATCGGAACGATTACA	57.2	360	INTERGENIC				
CaPOPII_188	Ca1	14995819	TTGTTATGTTAT/TTGTTATGTTATGTTAT	CAGCGCTTTTCCCAAATAAG	CCAAAAAGGGTGGAGATTGA	59.8	359	INTERGENIC				
CaPOPII_189	Ca1	14995847	ACATGCATGCA/ACATGCA	CAGCGCTTTTCCCAAATAAG	CCAAAAAGGGTGGAGATTGA	59.8	359	INTERGENIC				
CaPOPII_190	Ca1	15037301	CATATATATATATA/CATATATATATATATATA	GTCACACGAGCATGGAAAGT	TAACCTGGCCAGTCACCATT	58.7	578	INTERGENIC				
CaPOPII_191	Ca1	16206852	ATATTAT/ATAT	GAGTCTGTACTCGTAAGCCCCT	ATTTTGACAATGGCCACACA	58.9	697	INTERGENIC				
CaPOPII_192	Ca1	16629521	TTTAATTAATTAATTAATT/TTTAATTAATTAATTAATTAATTAATT	AACGTTTGAGGATTCCGTTG	GAAACTCCAGCTCCAAAACG	60.0	370	INTERGENIC				
CaPOPII_193	Ca1	16757631	AATATATATATATATATATATATATATA/AATATATATATATATATATATATATATATATATATATATATA	GATTCAAGACGAGACGCAAA	AAAAATGTTTTTCAATAAGCAAATC	59.0	562	INTERGENIC				
CaPOPII_194	Ca1	17436816	A/AC	GACGAAAAGATCACCCTCACA	CATGTGCCACAGGATCATTC	60.1	512	INTERGENIC				
CaPOPII_195	Ca1	17634013	TTATATATATATATATAT/TTATATATATATATATAT	TGATTGGATGTGGGTGTGAG	TTTCAAAGGCAACAACCCTC	60.4	529	INTERGENIC				
CaPOPII_196	Ca1	18181716	AATATATATATATATATATATATATATA/AATATATATATATATATATATATATATATATATA	CGAATACTCATGGTTTTTGATCTG	ACCCAACGTAGCACGTTACC	59.9	626	INTERGENIC				
CaPOPII_197	Ca1	18299880	TTCCTATCCTATCC/TTCCTATCC	TGGATCACTCACCTCTCTTCC	TGGAAGTGGTGGTTTTGTTG	59.2	185	INTERGENIC				
CaPOPII_198	Ca1	18300620	GTTTTTTTTT/GTTTTTTT	TTTCCATGAGTTATATTTGTCACGA	TGGCCTTCAATTTTCTTGCT	59.8	134	INTERGENIC				
CaPOPII_199	Ca1	18300800	ATTT/ATT	AGCAAGAAAATTGAAGGCCA	GGAAGCTTTAAAGGGAGCAAA	59.8	161	INTERGENIC				
CaPOPII_200	Ca1	18328227	GTCTATCT/GTCT	TTAAAACCACAAAATGGGGC	GAGGCAAAGCTTCCAAATCA	59.7	475	INTERGENIC				
CaPOPII_201	Ca1	18364053	GAAA/GAAAA	CCCGTAGCAAGACTTGAAGC	TTGGAGAAACTTTTCCGCTG	60.0	627	INTERGENIC				
CaPOPII_202	Ca1	18868658	CGG/CGGGAATGG	AATCGATTGATTCAGCCCAG	CGTTTTTCAAAGCGAGGGTA	60.0	707	INTERGENIC				

**INDEL marker IDs**	**Chromosomes /unanchored scaffolds**	**Physical positions (bp)**	**InDels (*Kabuli* reference genome-CDC Frontier/PI)**	**Forward primers (5'-3')**	**Reverse primers (5'-3')**	**Anneling temperature (0C)**	**Expected amplified product size (bp)**	**Structural annotation**		**Functional annotation**		
								**Sequence components of *kabuli* genome**	***Kabuli* gene accession IDs**	**NCBI-KOG**	**TFs**	**NCBI-nr database**
CaPOPII_203	Ca1	19422873	CTTATTTATTT/CTTATTT	GCCCATAACCTAGCCCATTT	TCGTGTCCCAAAACTCAATG	60.2	713	INTERGENIC				
CaPOPII_204	Ca1	19423044	TAAAAAAA/TAAAAAA	TCTTTGAGTTGTTAGGATACAAGTGG	TCGATTATGCGTAGGGAAGG	60.0	728	INTERGENIC				
CaPOPII_205	Ca1	19431535	TCA/T	TCTGATGCAGTGTTTGGCTC	GTGATAAAGGCGTCAACGGT	60.0	688	INTERGENIC				
CaPOPII_206	Ca1	19498678	TTTTGATCCTTTGAT/TTTTGAT	GCCCGAGTTAACAACAAACG	ATCGATTTCCTCAGCACTGG	60.5	374	INTERGENIC				
CaPOPII_207	Ca1	21746056	AC/ACACATATCAGCTTTC	CTATTGCCCCCTCCTCCTAC	TACCGATACCTCCAAGTGCC	59.9	585	INTRON	Ca_22116	S		Uncharacterised protein family FPL
CaPOPII_208	Ca1	21938049	CACTGAGTCCAGA/CACTGAGTCCAGACTGAGTCCAGA	TGTCAAAGGCCAAGATTCCT	CCTCACATCAAGAGCAGCAA	59.7	526	INTRON	Ca_22106		MIKC	GDP-fucose protein O-fucosyltransferase
CaPOPII_209	Ca1	22029011	TTATATATATATATATATATATATATATA/TTATATATATATATATATATA	CGAATCTTCACCCTCTTTGG	GCCGGATCCAAAATCAAAAT	59.7	839	INTERGENIC				
CaPOPII_210	Ca1	22131328	AATATATAT/AATATATATAT	GTGTGGACATGCCTAAAACG	GTTGCAATTGAGGTGCTGAA	59.1	403	INTERGENIC				
CaPOPII_211	Ca1	22131513	GAAAAA/GAAAA	GTGTGGACATGCCTAAAACG	GTTGCAATTGAGGTGCTGAA	59.1	403	INTERGENIC				
CaPOPII_212	Ca1	22204858	TTATATATATATATATATATAT/TTATATATATATATATAT	TGACATTTCGAACTTGAGTTTCC	CACAAGTCGCCATTTGGAAT	60.5	532	DRR	Ca_20635		TALE	Protein kinase, catalytic domain
CaPOPII_213	Ca1	22621219	TAAAAAAAAAA/TAAAAAAAAA	CATTTTTCCTCTGCACAACG	GCCGTCTTTTAGAAACATCGT	59.3	409	INTERGENIC				
CaPOPII_214	Ca1	22885854	TTA/TTATA	TGATTTGGGGTTTTGGGTAA	GGGTGAGTTCTTTGAGGGTG	60.0	531	INTERGENIC				
CaPOPII_215	Ca1	22886112	GAA/GAAA	TGATTTGGGGTTTTGGGTAA	GGGTGAGTTCTTTGAGGGTG	60.0	531	INTERGENIC				
CaPOPII_216	Ca1	23565928	AA/AACATCATATTA	GCGGTTAAGCCAATTTTGAG	TGGTGTAACGAGCATCAACAA	59.7	450	INTERGENIC				
CaPOPII_217	Ca1	23784964	ATTTTTTTT/ATTTTTT	GTCAACCAATTGACCCCAAC	TGCCAAGAAAACACCATGAA	60.1	663	INTERGENIC				
CaPOPII_218	Ca1	23838777	ATGTGTGTGTGT/ATGTGTGT	AATGCCCACCCATAATTTCA	TGTACGAGAAGGTCGTGTGC	60.0	593	INTERGENIC				
CaPOPII_219	Ca1	23966814	GA/GAA	AACTGTGGAGGAATCCATGC	GCTTCAAGTCTTGCTACGGG	59.9	490	INTERGENIC				

INDEL marker IDs	Chromosomes /unanchored scaffolds	Physical positions (bp)	InDels (*Kabuli* reference genome-CDC Frontier/PI)	Forward primers (5'-3')	Reverse primers (5'-3')	Anneling temperature (0C)	Expected amplified product size (bp)	Structural annotation		Functional annotation		
								Sequence components of *kabuli* genome	*Kabuli* gene accession IDs	NCBI-KOG	TFs	NCBI-nr database
CaPOPII_220	Ca1	24030518	CG/CGTGTTG	CCAATTCAAAAGCTCCCAAA	CAGATCCTCCTTTCCCAACA	60.0	424	DRR	Ca_19291	OK		E3 ubiquitin ligase, domain of unknown function DUF908
CaPOPII_221	Ca1	24801961	CTTTTTTTTT/CTTTTTTTTTT	TTTCAATGATGTTGGGTTGC	CAGCATATCCCCAACAAAAGA	59.4	998	INTERGENIC				
CaPOPII_222	Ca1	25296432	CTTT/CTTTT	GTTGGTGGCTGGACTCAGAT	TTCCCTAGTTGTTTGGGTGC	60.1	817	INTERGENIC				
CaPOPII_223	Ca1	25382154	GAA/GAAA	GAATCACTAGGCTTGCCAGG	GGGGTGAGAGAACGTTTGAA	59.8	714	INTERGENIC				
CaPOPII_224	Ca1	25437512	AATATATATATATATATATATATATATATATATATATATATA/AATATATATATATATATATATATATATATATATATATATATATA	GACTTTGATGCAAGTCACCG	CTCCCATCAGTTTAATCGGC	59.3	875	INTERGENIC				
CaPOPII_225	Ca1	25509778	AATATATATATATATATATATATATATATATATATCTATATATATAT/AATATATATATATATATATATATATATATATATATATATATATATATATATATATATCTATATATAT	ATTTTGGCTCGGAGGTTTTT	GTTTTCGAATCAACGAGGGA	59.9	550	INTERGENIC				
CaPOPII_226	Ca1	26377123	ATTTTTTT/ATTTTTTTT	TGTTCTAGATGGGGTTTGGC	GCCACTATCCAACAAATGGC	59.9	655	INTERGENIC				
CaPOPII_227	Ca1	26447765	ATATTATTATTATTATTATTATTATTATT/ATATTATTATTATTATTATTATTATTATTATTATT	AAATAGGGCCACACCAAAAA	TTAAATCGGAGGTATGGGGA	59.3	320	INTERGENIC				
CaPOPII_228	Ca1	26447923	CACATATCTG/C	AAATAGGGCCACACCAAAAA	GTAATGGGTATGGGTACGGG	59.3	420	INTERGENIC				
CaPOPII_229	Ca1	26473008	CTAAATT/CTAAATTCTGTAAATT	CCATTGACCCTTGCAAACTT	CTGCAGGAAGCTAAAATCCG	60.0	368	DRR	Ca_18608	R	NAC	Transcription factor jumonji/aspartyl beta-hydroxylase
CaPOPII_230	Ca1	26683611	TG/T	CAATTGTGTAAAACGTGCGG	TTGCATTTGGTTTGACTCCA	60.0	538	INTERGENIC				
CaPOPII_231	Ca1	26725325	CTATATATATATATATATATAT/CTATATATATATATATATAT	AATTAGCAGCGGAAGGAAAG	GCATCACAGTTAAATCATCGC	58.6	352	INTERGENIC				
CaPOPII_232	Ca1	26958642	TT/TTTGAT	TGCTCCATTTGATTGCTGAC	CCGACCAATAGACTTTTGTTGA	59.8	663	INTERGENIC				
CaPOPII_233	Ca1	27143610	AATATATATATATATATATATATATATA/AATATATATATATATATATATATATATATATATATATA	GTTCAATCAATACTCGGCGG	GGCATCTTTGATCAATGGTCTT	60.5	681	INTERGENIC				
CaPOPII_234	Ca1	27204677	C/CT	GCCGAAGTACCAATTACCGA	TACGGTGCTAGGGAACCAAC	60.0	705	INTERGENIC				
CaPOPII_235	Ca1	27232314	AATATATATATATATATATATATATAT/AATATATATATATATATATATATATATATATATAT	CTTGGGAACTGGAGAGCAAG	AGGGAGGTTTGTGTTTGACG	60.0	682	INTERGENIC				
CaPOPII_236	Ca1	27597440	ATT/AT	AAAATCCAACAATCTCCCCC	CCCTGAGACTCACGAAGAGG	60.0	559	DRR	Ca_25069			

INDEL marker IDs	Chromosomes /unanchored scaffolds	Physical positions (bp)	InDels (*Kabuli* reference genome-CDC Frontier/PI)	Forward primers (5'-3')	Reverse primers (5'-3')	Anneling temperature (0C)	Expected amplified product size (bp)	Structural annotation		Functional annotation		
								Sequence components of *kabuli* genome	*Kabuli* gene accession IDs	NCBI-KOG	TFs	NCBI-nr database
CaPOPII_237	Ca1	28339292	GCTCTCTCTCTCTCTCTCTCTCTCTCTCTCTCTCTCTCTCTCT/GCTCTCTCTCTCTCTCTCTCTCTCTCTCTCTCTCTCTCTCT	CGCCTTTCTGTTTTCCACAT	TGGTGTGCTTGGTGATGAAT	60.1	305	INTERGENIC				
CaPOPII_238	Ca1	28458887	AAATAATAATAATAATAATAATAATA/AAATAATAATAATAATAATAATAATAATAATA	CGTCGTTTGTTTTGACACATC	CGTTGTTTGTTTCGACACATCT	59.1	612	INTERGENIC				
CaPOPII_239	Ca1	28550132	ATTTTTTTTTT/ATTTTTTT	TGGAAGCACGCTTAAGGACT	TTTCCCAATCGATTTGCTTC	60.0	664	INTERGENIC				
CaPOPII_240	Ca1	28711868	TAGAGAGAGAGAGAGAGAGAG/TAGAGAGAGAGAGAGAGAG	TGTTGGGTTGACAAAAACCA	TTAATGGGGAAGCACAAAGC	59.8	532	INTERGENIC				
CaPOPII_241	Ca1	28713213	TTA/TTAGAAAGAGTGTA	TTCATTTCTCTGATTCCCCG	TCCGCATTATAACCTCTGCC	60.0	425	INTERGENIC				
CaPOPII_242	Ca1	28906702	TAAAAAAA/TAAAAAA	TATGGAGAGCAGCGACTCAA	TGTTGGGTTGCACAACATTC	59.7	184	INTERGENIC				
CaPOPII_243	Ca1	28910299	CATT/CATTAATT	GAAAATGATCGGTGGAGGAA	CTTGCGTGTTGCACGAGTAT	59.9	620	DRR	Ca_18486	R		Sugar/inositol transporter
CaPOPII_244	Ca1	28910417	CTTTTTTT/CTTTTTTTTTTT	GAGTCATTGGTGCCTTGGTT	CTTGCGTGTTGCACGAGTAT	60.0	385	DRR	Ca_18486	R		Sugar/inositol transporter
CaPOPII_245	Ca1	28910492	GATTAA/GATTAATTAA	GAGTCATTGGTGCCTTGGTT	CTTGCGTGTTGCACGAGTAT	60.0	385	DRR	Ca_18486	R		Sugar/inositol transporter
CaPOPII_246	Ca1	28930406	T/TA	TCAAAGACATGGTGCCTTCA	TTGGTGCCAATTTATGTATTTCA	60.2	605	INTERGENIC				
CaPOPII_247	Ca1	28941503	CAA/CAAAA	TGGGGTTTAGGGTGTCACAT	CTTTTGTAAAGGCAGCTCCG	60.1	207	INTERGENIC				
CaPOPII_248	Ca1	28941872	GAAAAAAA/GAAAAAAAAAA	TGGGGTTTAGGGTGTCACAT	TCAATTTGGATTTCTTGGTGG	60.1	847	INTERGENIC				
CaPOPII_249	Ca1	28943560	GG/GGTG	ACTTGGGACAAATCACATGG	TGATCACCAGCTTTCCAATG	58.3	252	INTERGENIC				
CaPOPII_250	Ca1	28944045	TAAAAAA/TAAAAA	TTGCCTTTAATGGACCAAGG	CAATATGAAGTCTGGTGCCAA	59.9	345	INTERGENIC				
CaPOPII_251	Ca1	28944496	TTATATATAT/TTATATAT	TTGGCACCAGACTTCATATTG	AAATGTACTTTGCGGCACAT	58.6	775	INTERGENIC				
CaPOPII_252	Ca1	29124803	ATTT/ATTTT	AGTGGCATTGGGCAAATTAC	ACCAAATATACGCGTCTGCC	59.8	350	INTERGENIC				
CaPOPII_253	Ca1	29386975	AATATATATATATATATATATATATATAT/AATATATATATATATATATATATATATATATATATATAT	TCGTTTCCCAGAGAAGCAAC	AGAAAATGCGGGTTATGTCG	60.4	743	DRR	Ca_22561	R	HB-other	

INDEL marker IDs	Chromosomes /unanchored scaffolds	Physical positions (bp)	InDels (*Kabuli* reference genome-CDC Frontier/PI)	Forward primers (5'-3')	Reverse primers (5'-3')	Anneling temperature (0C)	Expected amplified product size (bp)	Structural annotation		Functional annotation		
								Sequence components of *kabuli* genome	*Kabuli* gene accession IDs	NCBI-KOG	TFs	NCBI-nr database
CaPOPII_254	Ca1	29402165	TG/TGTAG	GGCCGTAAACCAGACTCAAA	GGGGTAAGTTTCCTTGACCA	60.1	627	INTERGENIC				
CaPOPII_255	Ca1	29422590	TT/TTATGAT	AGTGGATCGGGTGAAACAAG	GTCGCGTCGAAAACTAGCTC	60.0	783	DRR	Ca_22560			
CaPOPII_256	Ca1	29524255	ATTTTTTTTTTT/ATTTTTTTTTTTTT	GGCTTCTTTCCAAAATGCAG	CACCATATTCCCCCAAAACA	59.8	669	INTERGENIC				
CaPOPII_257	Ca1	29636835	AA/AAGA	TCACAAATCTCAACCCTCCC	ATCAGTTGGAGCCTGGTTGT	59.9	497	INTRON	Ca_24382	AR		K Homology, type 1
CaPOPII_258	Ca1	29637072	GAAAAAAAAA/GAAAAAAAAAAA	ACAACCAGGCTCCAACTGAT	ATGCTGCCAGCCTACAAAAT	59.6	503	INTRON	Ca_24382	AR		K Homology, type 1
CaPOPII_259	Ca1	29791416	GAAAA/GAAAAA	TCCGATAAAATCTCCGTTCG	AAAAACACGTTCCCCTCCTT	60.0	323	INTERGENIC				
CaPOPII_260	Ca1	29818737	ACTA/ACTAAATTACCGAGGTGCTA	CTCGGAATTCCAAACCAAAA	ATCGATGGCTCAATTGGTTC	59.9	359	INTERGENIC				
CaPOPII_261	Ca1	29825526	ATTTTT/ATTT	CAAGACTTAAGGCTCCCCCT	CCCATTTGCCTAACCAAGAA	59.7	500	INTERGENIC				
CaPOPII_262	Ca1	29842423	AC/A	TCATCAAAGGCATGCAAATC	GACCAAAACACAACGCAATG	59.6	359	INTERGENIC				
CaPOPII_263	Ca1	29843804	G/GA	ATGGATCAAGAGAAGGGGCT	CCTATGCGAAGTGGTGGTTT	60.0	535	INTERGENIC				
CaPOPII_264	Ca1	29844165	TTTATT/TTT	ATGGATCAAGAGAAGGGGCT	CCTATGCGAAGTGGTGGTTT	60.0	535	INTERGENIC				
CaPOPII_265	Ca1	29846530	CTTTT/CTTTTT	TGGATTGGGGATCATTCATT	TCAACATTTGGAAGCACAAGA	60.0	897	DRR	Ca_24388			
CaPOPII_266	Ca1	29846941	TAAAAAAAAAA/TAAAAAAAA	TGTTGCCAACTGAAAATCGT	ATACAGACACCGAATGGGGA	59.2	828	DRR	Ca_24388			
CaPOPII_267	Ca1	29847994	TAAAAAA/TAAAAAAAA	TCTTTTCATCAGGCCCAATC	GGAAAGTGAGGGAACCAATG	60.0	228	INTRON	Ca_24388			
CaPOPII_268	Ca1	29848026	ATGT/AT	TCTTTTCATCAGGCCCAATC	GGAAAGTGAGGGAACCAATG	60.0	228	INTRON	Ca_24388			
CaPOPII_269	Ca1	29853119	TAA/TA	TCCACGAGATTTTCCTTTGG	TGTCATCATTCTCTGCTGGC	60.0	357	INTERGENIC				
CaPOPII_270	Ca1	29853730	GTGGAAGTCT/GTGGAAGTCTGGAAGTCT	TTGCATTCCTAGGTTCAGCC	TATCGCTCGACCTCTGCTTT	60.2	602	INTERGENIC				

INDEL marker IDs	Chromosomes /unanchored scaffolds	Physical positions (bp)	InDels (*Kabuli* reference genome-CDC Frontier/PI)	Forward primers (5'-3')	Reverse primers (5'-3')	Anneling temperature (0C)	Expected amplified product size (bp)	Structural annotation		Functional annotation		
								Sequence components of *kabuli* genome	*Kabuli* gene accession IDs	NCBI-KOG	TFs	NCBI-nr database
CaPOPII_271	Ca1	29856647	CTTT/CTTTT	GCATTCCCTGCTCAAGACTC	AGACAAGGGAGAGTCCCACA	60.0	812	INTERGENIC				
CaPOPII_272	Ca1	29994387	GTTTT/GTTTTT	TCCAGCTTCCTCGAAACCTA	AACAAGACCTGCGAAAATCG	59.9	480	INTERGENIC				
CaPOPII_273	Ca1	30243449	AGG/AG	CCAAACCAAATAAAACATTCCAA	GAACTCTGCCTTCCAGCAAC	60.0	463	INTERGENIC				
CaPOPII_274	Ca1	30287511	GTTTT/GTTT	CCATCAAACATCGTCATGGA	ACTCAGATTGGTCTGTGGGG	60.3	305	INTERGENIC				
CaPOPII_275	Ca1	30352443	CACA/CACAACA	TCATCTTGGGAGGTCTCGAT	TTGTGGTCTAGGCGTCAAAA	59.6	673	INTERGENIC				
CaPOPII_276	Ca1	30398618	GTTTTTTTTTT/GTTTTTTTTTTTT	TTAGGCCCAGTACAGTTCGG	GGGGTGCTCAACTTGTTTGT	60.1	901	INTERGENIC				
CaPOPII_277	Ca1	30475415	A/AC	ACGGCAAGACCCTAAAACCT	AAACTTCTACTCGGGCCCAT	60.0	906	INTERGENIC				
CaPOPII_278	Ca1	30475519	ATTT/ATTTT	AGGATGCATGGGATGAAAAG	AAACTTCTACTCGGGCCCAT	59.9	766	INTERGENIC				
CaPOPII_279	Ca1	30682856	CATATATATATATATATATATAT/CATATATATATATATATATAT	GTTGGTTCCCTAGCACCGTA	TCCGCCTAGGAGATAGCAGA	60.0	672	INTERGENIC				
CaPOPII_280	Ca1	30755262	CAAAAAAAAAAA/CAAAAAAAAAA	AAGAGAGGTCACTTTGGCGA	TGACTGAGACAGCTCGATGG	60.0	489	INTERGENIC				
CaPOPII_281	Ca1	30770184	TAAA/TAAAA	AGTGGCTTGGAGGAGTTGGT	CGTTCGGCTTTCTTCTTCTG	61.1	535	INTERGENIC				
CaPOPII_282	Ca1	31072943	TAAAAAAA/TAAAAAA	CGCTCAGCTCGGGTATAGAG	CAAACCGGATGTGACAGTTG	60.1	615	INTERGENIC				
CaPOPII_283	Ca1	31117965	AATATA/AATA	GGGACATCAGAATGCTCACA	TGCGACACACGAATTACACA	59.6	536	INTERGENIC				
CaPOPII_284	Ca1	31163466	T/TAGA	ACTTTTGCGGAGAAGCACAT	CGTTCGTCTTTTCCTCTTGC	59.9	360	INTERGENIC				
CaPOPII_285	Ca1	31477691	CAAAA/CAAAAA	AAGGGAAACGGGTACCAAAC	ACCCGTAAACCCCAAATCTC	60.1	629	INTERGENIC				
CaPOPII_286	Ca1	31498685	AGG/AG	TTGGAGCTTCAGCATTTGTG	CCTCTTTTCTTCGCATTTCG	60.0	747	DRR	Ca_21863		M-type	
CaPOPII_287	Ca1	31592114	CT/CTTT	TCAGCTGCAGTTCCAACATC	GCACAAGTCCTTGAGGAAGC	60.0	375	INTRON	Ca_21868	F		Orotidine 5'-phosphate decarboxylase domain

INDEL marker IDs	Chromosomes /unanchored scaffolds	Physical positions (bp)	InDels (*Kabuli* reference genome-CDC Frontier/PI)	Forward primers (5'-3')	Reverse primers (5'-3')	Anneling temperature (0C)	Expected amplified product size (bp)	Structural annotation		Functional annotation		
								Sequence components of *kabuli* genome	*Kabuli* gene accession IDs	NCBI-KOG	TFs	NCBI-nr database
CaPOPII_288	Ca1	31592347	GAA/GAAAA	CCTGTGTAGCCAGCTTCCTC	TAGCCTCATTCACTCTCGCA	60.0	618	INTRON	Ca_21868	F		Orotidine 5'-phosphate decarboxylase domain
CaPOPII_289	Ca1	31602245	TTATATATATATATATATATATATA/TTATATATATATATATATA	TTTCGTGTGCGTCAAATCAT	AGAGGCACACGGGTCTAATG	60.1	756	INTERGENIC				
CaPOPII_290	Ca1	31768486	TT/TTTTTTAT	ACACATTATATTAAATGCGCGA	TGCCAAACGATAGGTTGTTG	57.4	646	INTERGENIC				
CaPOPII_291	Ca1	31783811	TTAATAATAATAATAATAATAATAATAATAATAAT/TTAATAATAATAATAATAATAATAATAATAAT	ATACTGCCTTCGATCCGTTG	CGGGAAAAAGGGTGGTAAAT	60.1	477	INTERGENIC				
CaPOPII_292	Ca1	31995500	CTTT/CTTTT	TGCTTTGAAGACCATGTGGA	AAAAGGATTCATGCGTGTCC	60.2	354	INTERGENIC				
CaPOPII_293	Ca1	32037334	CTTTTT/CTTTT	TCCAGTCATGTGGCTTCTCA	CTGAGCTTGTGACCAAATCG	60.4	559	INTERGENIC				
CaPOPII_294	Ca1	32078246	TA/TAAAAA	CTCGGTTCGAACAGGAGAAG	TATGTCAACAACCCCCTGGT	60.0	702	INTRON	Ca_26405			Uncharacterised protein family Ycf68
CaPOPII_295	Ca1	32147050	T/TA	CGGCCTCCAAATCTTTAACA	TTGTCATGAAAGTCTCGCCT	60.1	670	INTERGENIC				
CaPOPII_296	Ca1	32153452	TTTATTTTAT/TTTATTTTATATTATTTTAT	GTTTTGGCGTTGGTTTCACT	TGGGTTTGACCCTATGTGTG	60.0	353	INTERGENIC				
CaPOPII_297	Ca1	32244195	A/AG	TGAATAGAGGGAAACGGGTG	CAGTGACCTTGTGGAGAGCA	59.9	640	INTERGENIC				
CaPOPII_298	Ca1	32430396	TG/T	TCGTTTGAGCCTTTCGAACT	GCAATCACAATGCAGCAAAC	60.0	473	INTERGENIC				
CaPOPII_299	Ca1	32906329	TTATATATATATATATAT/TTATATATATATAT	TCATTAAATCAAAGGCACACAAA	TCGTATGTCATAGCGGTGCT	59.5	488	INTERGENIC				
CaPOPII_300	Ca1	33018948	CTT/CT	TTCGCAACTCCACTCTTCCT	CACATCATCACAACATGCCA	60.0	481	INTERGENIC				
CaPOPII_301	Ca1	33034774	AATATATATATATATA/AATATATATATA	ACCCCAACTTGTAAAGGCAA	TCACGTTGATTGAGGGATGA	59.5	618	INTERGENIC				
CaPOPII_302	Ca1	33102523	TTT/TTTATT	AAGGCTAAATCACATTCGTGG	TCACTTCCGTCCATGTCAAA	59.1	835	INTERGENIC				
CaPOPII_303	Ca1	33187249	GTAT/GTATAT	TAAAGGTTCCCTCGCATCTG	CACCACAGAAGAACTTGGCA	60.2	530	INTRON	Ca_19440	A		Helicase, C-terminal
CaPOPII_304	Ca1	33247147	TA/T	GTGTTGGACCGGTTCTTGAT	GTCTGATGACAAAAGGGGGA	59.8	643	INTERGENIC				

INDEL marker IDs	Chromosomes /unanchored scaffolds	Physical positions (bp)	InDels (*Kabuli* reference genome-CDC Frontier/PI)	Forward primers (5'-3')	Reverse primers (5'-3')	Anneling temperature (0C)	Expected amplified product size (bp)	Structural annotation		Functional annotation		
								Sequence components of *kabuli* genome	*Kabuli* gene accession IDs	NCBI-KOG	TFs	NCBI-nr database
CaPOPII_305	Ca1	33286471	ATTT/ATTTT	ATTATCAGAGAGTCCCCGGC	CAGATTGGCACCTATGAGCA	60.4	879	INTERGENIC				
CaPOPII_306	Ca1	33336930	GAAAAA/GAAAA	CAATGGCAACAAGGTCAATG	TCACATCTGTTGGTAACGGG	60.0	856	INTRON	Ca_19441		MIKC	Transcription factor, K-box
CaPOPII_307	Ca1	33365935	CTTTTTTTTTT/CTTTTTTTTTTTTT	CCACGTGTGGTCCCTTAACT	TTTTTCTCCCGATTTGGTCA	59.9	485	INTRON	Ca_19441		MIKC	Transcription factor, K-box
CaPOPII_308	Ca1	33407768	TA/TAA	TGGCCATCAATTAATCCACTC	GATCTGCAACTGGGGGTAAA	59.8	230	INTERGENIC				
CaPOPII_309	Ca1	33653494	TTTATTATTATTATTATTATTA/TTTATTATTATTATTATTATTATTA	TGGGCAAAATGTTAAGAACACA	AGAACAAATGCATGATTCTTGG	60.4	573	INTERGENIC				
CaPOPII_310	Ca1	33658726	ATTT/ATT	ATGTGAATCCCACCAGGAAA	TTGCACAGCCAAGTTCGTAG	60.2	778	INTERGENIC				
CaPOPII_311	Ca1	33777775	ATTT/ATT	CATGCTCAATACACGGATGG	AACGTTCTTGAGTGGCTTGG	59.9	870	INTERGENIC				
CaPOPII_312	Ca1	33781062	CTTTTTTT/CTTTTTTTTT	AATTTCCCAATCACCCACAA	TCCACAATGTCTCCCAACAA	60.0	517	CDS (FRAME SHIFT)	Ca_19460			Transposase, MuDR, plant
CaPOPII_313	Ca1	34160007	C/CAT	TGGAGACACTTGCCCTCAAT	TTTTATGTCAGGGCGAAACC	60.7	501	INTERGENIC				
CaPOPII_314	Ca1	34169923	GAA/GAAA	CCATTTCCTTTGTCCTCGTG	AAGCCTTGGAAGGAGAAAGC	60.5	538	INTERGENIC				
CaPOPII_315	Ca1	34250780	CTT/CTTCATTTTTATTTT	TTGGAGTCGAAGTGAAACCC	ACATTCAGGTGGTGGTGGAT	60.1	561	INTERGENIC				
CaPOPII_316	Ca1	34263760	TA/TTGCACA	ATCCAAAATGGGATGGATGA	GTTCGGAACATGCTGTCCTT	60.0	703	INTRON	Ca_25475	M	MYB_related	Glycosyl transferase, family 48
CaPOPII_317	Ca1	34332058	CATATAT/CATATATAT	AATTGGAAGCAGGGGTTTTT	TGAACCCGTTATAAATATTTTTCCT	59.8	356	INTERGENIC				
CaPOPII_318	Ca1	34362589	GATAAATA/GA	GGAGGAAACAAGCGTACGAA	TAAGGAGGCCTTTTACAGCG	60.2	859	INTERGENIC				
CaPOPII_319	Ca1	34386272	GTTT/GTTTT	AATCTGGAGGGCGGTAAGTT	ATATCGGTCAAACGCACACA	60.0	413	INTERGENIC				
CaPOPII_320	Ca1	34469736	AT/ATT	CACATAAAGGCATCATCCCC	ATTAAAGGGGCAACCGATTC	60.2	553	INTERGENIC				
CaPOPII_321	Ca1	34768259	AAAATAAATA/AAAATAAATAAATA	ATCGCCAGTGTCAATTTTCC	TGCGATTTTACGTGTATATGTGAG	59.9	677	INTERGENIC				

INDEL marker IDs	Chromosomes /unanchored scaffolds	Physical positions (bp)	InDels (*Kabuli* reference genome-CDC Frontier/PI)	Forward primers (5'-3')	Reverse primers (5'-3')	Anneling temperature (0C)	Expected amplified product size (bp)	Structural annotation		Functional annotation		
								Sequence components of *kabuli* genome	*Kabuli* gene accession IDs	NCBI-KOG	TFs	NCBI-nr database
CaPOPII_322	Ca1	34810237	GTTTTT/GTTTT	ATTGCCTCGCTTGAAGAATG	TTTGGATGAGCAACAAGCAG	60.4	598	INTERGENIC				
CaPOPII_323	Ca1	34814277	T/TG	GCGTGAACAAAAATCAAGCA	TCGAAGACGTCTCGTTGTTG	59.9	452	INTERGENIC				
CaPOPII_324	Ca1	34853862	CAAAAAAAAAAA/CAAAAAAAAA	AGTTTGGCTAGATGGGCCTT	TAATTCAAACCGCACTGCAA	60.1	256	INTERGENIC				
CaPOPII_325	Ca1	34865996	TAATAAATAAATAAA/TAATAAATAAATAAATAAA	TTGTTCGAGAATTTGGAGGG	TGAGGTGGTGACCAATTGAA	60.0	462	DRR	Ca_21411		MYB_related	SANT domain, DNA binding
CaPOPII_326	Ca1	34913480	AAT/AATAT	TTGTGTTCACCGAGGTTGTC	CAACAACCAACTTCGTGCAT	59.6	572	INTERGENIC				
CaPOPII_327	Ca1	34971020	CAAAAAAA/CAAAAA	AAATACCCCTTTTTCCCACG	TGAAAACGCTCAATGCCATA	60.0	689	INTERGENIC				
CaPOPII_328	Ca1	35044743	AG/A	GTACCGAATAGCCCGCATAA	CCAGTCCATATCGGGTCAGT	60.0	654	INTERGENIC				
CaPOPII_329	Ca1	35072166	CTA/CTATATA	AGTTGGCTCACTAGCACCGT	CACAAGTGGATGAACAACCG	59.9	696	INTERGENIC				
CaPOPII_330	Ca1	35240205	ATAT/ATATTAT	GTAGCATTTTTGGCCTCCCT	GTCGAGAAGAAGGCTTGCAT	60.5	782	DRR	Ca_26097	QI	bHLH	Cytochrome P450
CaPOPII_331	Ca1	35260458	ATT/ATTT	ATGGCTCATACTGGGATTGC	AAGACTGGGTTTGCCTGCTA	59.9	592	INTERGENIC				
CaPOPII_332	Ca1	35261821	ATT/AT	TGGATTGTCATGGTCTGGAA	AATGGTCTTGAATTTTGCCG	59.9	640	INTERGENIC				
CaPOPII_333	Ca1	35265473	TAAAAAAA/TAAAAAA	TTCCCTGAATCGAGTGAACC	TTTTCCAATAACTCTACCGCGT	60.1	674	INTERGENIC				
CaPOPII_334	Ca1	35281444	ATTTTTTTT/ATTTTTTT	ACAAAGGCCTGCCTAGATCA	TTTTTGATGTCTCTCTGTTCAAAT	59.8	591	INTRON	Ca_26099	QI	bHLH	Cytochrome P450
CaPOPII_335	Ca1	35281570	TC/T	ATGACACGTCATTCGTTTCG	GGTTCGAGACATCCATAATTCA	59.6	733	INTRON	Ca_26099	QI	bHLH	Cytochrome P450
CaPOPII_336	Ca1	35281940	TA/TAA	AAACGTGATCTATACAGTTTCCAAAA	TTCACATCTGGAAACAACGC	59.4	529	INTRON	Ca_26099	QI	bHLH	Cytochrome P450
CaPOPII_337	Ca1	35283797	TGGGGG/TGGGG	GGGTGTGTTTGATAGGAGGG	CTCCCCTTCCATCTCCTCTT	59.3	139	INTERGENIC				
CaPOPII_338	Ca1	35284017	ATTT/ATT	GAGATGGAAGGGGAGGAAAG	GCACAAAAGAAAAATACAAGTCCA	60.0	273	INTERGENIC				

INDEL marker IDs	Chromosomes /unanchored scaffolds	Physical positions (bp)	InDels (*Kabuli* reference genome-CDC Frontier/PI)	Forward primers (5'-3')	Reverse primers (5'-3')	Anneling temperature (0C)	Expected amplified product size (bp)	Structural annotation		Functional annotation		
								Sequence components of *kabuli* genome	*Kabuli* gene accession IDs	NCBI-KOG	TFs	NCBI-nr database
CaPOPII_339	Ca1	35284253	TT/TTGT	GAGATGGAAGGGGAGGAAAG	TCTGATCAAATATGTGGCAGAAA	60.0	657	INTERGENIC				
CaPOPII_340	Ca1	35284860	CAAAAA/CAA	TGAAGGGAGTGCTATTTGGT	TCACCTTTTGACTTCCTCATGTT	57.3	108	INTERGENIC				
CaPOPII_341	Ca1	35285638	TATT/TATTAATT	GACCTTATGCTCGTGCAATGT	GCCAGAGTGCAATAGGAAGG	60.2	419	INTERGENIC				
CaPOPII_342	Ca1	35290921	ATTT/ATTTT	CACACCTAATGCCTGTCACG	TGATGGACCAAACAAGTCCA	60.2	760	INTERGENIC				
CaPOPII_343	Ca1	35310736	ATTTTTTTTT/ATTTTTTTT	TTGATGCAACCTGTGACTCC	TTGGATCCATCCCCTAGATG	59.7	572	INTERGENIC				
CaPOPII_344	Ca1	35337257	CTTTTTT/CTTTTTTT	TACATGCACACACTCCACCA	TGGGAGCCTCAATTGCTTAG	59.6	290	INTERGENIC				
CaPOPII_345	Ca1	35353052	G/GTTGA	TCAGCAGCAAATAGGCTTCA	AGCCCATTCAGATTTTGTGG	59.7	352	INTERGENIC				
CaPOPII_346	Ca1	35353239	GAA/GAAA	CCAACAACTTCCTTTCGAGC	AGCCCATTCAGATTTTGTGG	59.9	147	INTERGENIC				
CaPOPII_347	Ca1	35537314	T/TG	ACATGGAACCCATCCAAAGA	AATCGATTGATTCAGCCCAG	60.2	475	INTERGENIC				
CaPOPII_348	Ca1	35553773	GTATCCTA/GTA	GTCGAGGAGATTGCAAAAGC	TTTGAATCGTCTTCACTGCG	60.0	469	INTERGENIC				
CaPOPII_349	Ca1	35569645	TAAAAAA/TAAAAA	TGAGTGCACCTTTTACAGCG	TTGCACGCTTAGTATGCACC	60.1	256	DRR	Ca_24107			
CaPOPII_350	Ca1	35977319	TA/TAA	AACTCGTTGGAACTTCCCTCT	GCAAGCAGCCTTGTTGACTA	59.2	540	INTERGENIC				
CaPOPII_351	Ca1	36044248	CGG/CG	CGATTTCCCTGCATGTTTTT	GCCTGTGATAGGCGGTACAT	59.9	540	INTERGENIC				
CaPOPII_352	Ca1	36724754	CTTTTTTTT/CTTTTTTTTTT	TTGCACCACACTTCTTCACC	CTTATTTCGACGCTTTTCGC	59.7	279	INTERGENIC				
CaPOPII_353	Ca1	36869128	CA/CAAA	TCCACAACAACCTACGTCCA	CCCAGAAATCGATTAGGCAA	60.0	555	INTERGENIC				
CaPOPII_354	Ca1	37078918	A/AAATTAATG	TGTGACACCCTAAACCCTAAGAA	TTGATTGCTTGCGTGTTCTC	59.9	499	INTERGENIC				
CaPOPII_355	Ca1	37122520	AGG/AG	TGCAATCATTGGTGCTTAGG	AAAAAGCGCCGTAAAATGAG	59.7	710	INTERGENIC				

INDEL marker IDs	Chromosomes /unanchored scaffolds	Physical positions (bp)	InDels (*Kabuli* reference genome-CDC Frontier/PI)	Forward primers (5'-3')	Reverse primers (5'-3')	Anneling temperature (0C)	Expected amplified product size (bp)	Structural annotation		Functional annotation		
								Sequence components of *kabuli* genome	*Kabuli* gene accession IDs	NCBI-KOG	TFs	NCBI-nr database
CaPOPII_356	Ca1	37122699	GTTT/GTT	GGAGGAGCGCAACTTAATCA	AAAAAGCGCCGTAAAATGAG	60.4	372	INTERGENIC				
CaPOPII_357	Ca1	37122810	TACAAC/TACAACAAC	GGAGGAGCGCAACTTAATCA	AAAAAGCGCCGTAAAATGAG	60.4	372	INTERGENIC				
CaPOPII_358	Ca1	37128515	ATTTTTTT/ATTTT	CAATTCCCACAGGTACGCTT	TTGGTCTCATCAATGATTGTAAAAA	60.0	568	INTERGENIC				
CaPOPII_359	Ca1	37197766	GTTT/GTT	TGAGAGGAAAGAAGACAACCATC	TTTTCTTTTTAAAGCGGCGA	59.7	605	DRR	Ca_21831	L	C3H	NUDIX hydrolase domain
CaPOPII_360	Ca1	37243078	AAATTAT/AAATTATAGAATTAT	TCATCCACATCAGCCAAAAA	TTTGTGCATTTTAATAATTGGAAG	60.0	490	INTERGENIC				
CaPOPII_361	Ca1	37412282	CCATG/CCATGCATG	CGTGGATGATGCTTTTCTCA	TGCTTCTCATCTGCTTGGTG	59.8	580	DRR	Ca_21842	R	GRAS	Pentatricopeptide repeat
CaPOPII_362	Ca1	37420494	ATTTTTTTTT/ATTTTTTT	GGGAAAAGGAAACCCAAAAA	TAGTGGAGTCCCCGTCAAGT	60.1	630	INTERGENIC				
CaPOPII_363	Ca1	37420598	ATTTT/ATTT	GGGAAAAGGAAACCCAAAAA	TGAGTGAGGGAGACAAAGGG	60.1	950	INTERGENIC				
CaPOPII_364	Ca1	37421082	CCTCACTCACTCACTCACTCAC/CCTCACTCACTCACTCAC	ACTTGACGGGGACTCCACTA	ACATGTTCCTTGTGCTCGTG	59.6	637	INTERGENIC				
CaPOPII_365	Ca1	37424329	CATATATATATATATATATATAT/CATATATATATATATATAT	AAAAAGTATTGAACAAACACGCT	TGAAATCCAAATTAAATTGCTGAA	57.2	549	INTERGENIC				
CaPOPII_366	Ca1	37424983	AC/A	GCAATTTAATTTGGATTTCATGG	TCACATCCATCCACGGTACA	59.6	761	INTERGENIC				
CaPOPII_367	Ca1	37425070	ATTTTTTTTTT/ATTTTTTTT	GCAATTTAATTTGGATTTCATGG	TCACATCCATCCACGGTACA	59.6	761	INTERGENIC				
CaPOPII_368	Ca1	37425153	G/GAT	GCAATTTAATTTGGATTTCATGG	TCACATCCATCCACGGTACA	59.6	761	INTERGENIC				
CaPOPII_369	Ca1	37425439	TG/T	TGTACCGTGGATGGATGTGA	TGACACGTATCAATTTAGGGAGAA	60.8	222	INTERGENIC				
CaPOPII_370	Ca1	37425620	ACG/A	TGTACCGTGGATGGATGTGA	TATGCATCCTCACGGTTTTG	60.8	675	INTERGENIC				
CaPOPII_371	Ca1	37433380	CAA/CAACGTAAA	AATAAATTAGTGCAGCAAACCAA	GGCAAAGAGAGGAAAGTGGA	58.0	756	INTERGENIC				
CaPOPII_372	Ca1	37489932	CAAAAA/CAAAA	ATTGTCCAGTTTCCTCACCG	CGATTGGTAAATCGATTGGG	60.0	594	INTERGENIC				

INDEL marker IDs	Chromosomes /unanchored scaffolds	Physical positions (bp)	InDels (*Kabuli* reference genome-CDC Frontier/PI)	Forward primers (5'-3')	Reverse primers (5'-3')	Anneling temperature (0C)	Expected amplified product size (bp)	Structural annotation		Functional annotation		
								Sequence components of *kabuli* genome	*Kabuli* gene accession IDs	NCBI-KOG	TFs	NCBI-nr database
CaPOPII_373	Ca1	37489959	AT/ATT	ATTGTCCAGTTTCCTCACCG	CGATTGGTAAATCGATTGGG	60.0	594	INTERGENIC				
CaPOPII_374	Ca1	37490066	A/AATGTAT	ATTGTCCAGTTTCCTCACCG	TGAACTCTGAGCGTGTGACC	60.0	775	INTERGENIC				
CaPOPII_375	Ca1	37693180	AATTAT/AAT	TGTGGAACATTCTCTGCGTC	TTCCCGAGAGATTTGTCTTCA	59.8	593	DRR	Ca_23533		G2-like	Myb, DNA-binding
CaPOPII_376	Ca1	37714757	ATTT/ATTTT	TCGCTCACATATAAGGTTGCAT	TTCGCCCATATTTCCTTTTG	59.6	640	INTERGENIC				
CaPOPII_377	Ca1	37721826	AAAA/AAAATAAA	TCCATAGCACCTCTTGATTTTG	CGTTGATGGCATCTTGATTG	59.2	420	DRR	Ca_23534			Protein of unknown function DUF620
CaPOPII_378	Ca1	37724987	A/ATT	ACCCTGTTGCTGCCAAATAC	GCTCCAATTCCCTCAGATCA	60.0	664	INTRON	Ca_23534			Protein of unknown function DUF620
CaPOPII_379	Ca1	37759391	GT/G	CCATCTTGATCCTTTGCCTC	TCAAACGGTCAAATTGGATG	59.6	268	INTERGENIC				
CaPOPII_380	Ca1	37798793	TATTA/TATTATTAATATCTGATTA	TGTGGAACTTGATTTTGGTGA	TATCGCCTCATGGAATCACA	59.0	583	INTRON	Ca_23537		NAC	No apical meristem (NAM) protein
CaPOPII_381	Ca1	37800107	TA/TATTTAAA	TGGGTCCTAAACCGCAATAC	CAAAAATGATGCAACCATCG	59.8	371	INTERGENIC				
CaPOPII_382	Ca1	37836904	CAGTTAGTTAGTTAGTTAGTTAGTTA/CAGTTAGTTAGTTAGTTAGTTAGTTAGTTAGTTA	ATGGATGTAGCGATTACGCC	CCAATACTCCACCTGCGATT	60.0	442	INTERGENIC				
CaPOPII_383	Ca1	37852569	TAAAAAAA/TAAAAAA	TGTGGTATGTGCGGTTCCTA	TTTGATGGGTTCATGTTCCA	60.0	814	INTERGENIC				
CaPOPII_384	Ca1	37875883	TTAT/TT	GCTGGGACTTATTTCCGTGA	TGCCAGGTGACAAAATCTCA	60.1	201	INTRON	Ca_23539	KT		Zinc finger, PHD-type
CaPOPII_385	Ca1	37887479	GTTTTTTTTTT/GTTTTTTTTTTTT	GATTGCAAAATCCGGAAAAA	TGGGCACTATTGTTTGGACA	59.9	530	INTRON	Ca_23539	KT		Zinc finger, PHD-type
CaPOPII_386	Ca1	37913622	AATATATATATATA/AATATATATATATATA	ACGTTCGCCACCTATCTCAT	CCTTATGCGGTAATCCCTGA	59.6	563	INTERGENIC				
CaPOPII_387	Ca1	37917967	ATATGACAGTTA/ATA	TCCACAAGTTATTCGACGAAAC	TTCCATTGATCTACGTTAATGTTTG	59.1	558	INTERGENIC				
CaPOPII_388	Ca1	37927331	TAAA/TAAATTGTATCAAA	ATTTTGATCGCGGAAGTTTG	TCAATCAACCAGATTTTGCATT	60.1	553	INTERGENIC				
CaPOPII_389	Ca1	37927862	G/GTA	TGCAAAATCTGGTTGATTGAG	TCCCAGATCAAAATTGGGTT	58.8	745	INTERGENIC				

INDEL marker IDs	Chromosomes /unanchored scaffolds	Physical positions (bp)	InDels (*Kabuli* reference genome-CDC Frontier/PI)	Forward primers (5'-3')	Reverse primers (5'-3')	Anneling temperature (0C)	Expected amplified product size (bp)	Structural annotation		Functional annotation		
								Sequence components of *kabuli* genome	*Kabuli* gene accession IDs	NCBI-KOG	TFs	NCBI-nr database
CaPOPII_390	Ca1	37929646	TAAAAAAAA/TAAAAAAAAAA	TGTGACGGTTAATATG GTGGTC	TTGACTTGTTTTGCGG ATTG	59.6	817	INTERGENIC				
CaPOPII_391	Ca1	37929753	ATTTTTTTTT/ATTTTTTTTTTT	TGTGACGGTTAATATG GTGGTC	TTGACTTGTTTTGCGG ATTG	59.6	817	INTERGENIC				
CaPOPII_392	Ca1	37954337	GTTTTT/GTTT	TCTTTTCTCCGATCCCA TTG	CGACTCCCGAACTCAA AATC	60.0	868	INTERGENIC				
CaPOPII_393	Ca1	37993665	CAAAAAA/CAAAAA	GTGAGCCCTTTACCAAT CCA	CAATTCCCACAGGTAC GCTT	59.9	654	INTERGENIC				
CaPOPII_394	Ca1	38070666	T/TA	AACCATTGGGGGAGAA TAGAA	TTGTTAGAACCATTGG GGGA	59.7	362	INTERGENIC				
CaPOPII_395	Ca1	38070895	ATGTGTGT/ATGTGT	CTCCCCCAATGGTTCTA ACA	CACTGGAATCCGTTTC TTTTG	59.8	587	INTERGENIC				
CaPOPII_396	Ca1	38071534	TACACACAC/TACACAC	CTCCTCCTCATTCACCC AAC	ACACACCCACTCTCCTT TGC	59.5	189	INTERGENIC				
CaPOPII_397	Ca1	38130303	C/CG	AAAGGGGGAGAACCAT CTGT	TGCATTTGCTTGACTTG GAG	59.8	680	INTERGENIC				
CaPOPII_398	Ca1	38131605	C/CT	CGTAACTCGTGTTGGG TGAA	GAAGCAAGAGGCAACT CCAC	59.6	379	INTERGENIC				
CaPOPII_399	Ca1	38147657	TC/TCC	TGTTGACACATCACATC CTTAACA	TTTCATGTCTCATTTCC CCA	60.3	858	INTERGENIC				
CaPOPII_400	Ca1	38148096	CAAAAAAAAAA/CAAAAAAAA	TGGGGAAATGAGACAT GAAA	ACGTTGCGCAGGTGTA ATTT	58.9	567	INTERGENIC				
CaPOPII_401	Ca1	38278656	C/CG	TCCAGCATTGCCTTTTC TTT	GCATGGGTGAGAATGG AGTT	59.8	687	DRR	Ca_13968	O		ATPase, AAA+ type, core
CaPOPII_402	Ca1	38303577	TT/TTGT	GGTCGTGCTGCAAGGA TTAT	GTGTGGTACTCATGCG ATGC	60.1	873	INTERGENIC				
CaPOPII_403	Ca1	38494341	TAAGAAGAAGAAGAAGAAGAAGAA/TAAGAAGAAGAAGAAGAAGAAGAA	ATCCGTCTTCAAGTTGT GGC	ACGCCCCTTGAGTGAG ATTA	60.1	462	INTERGENIC				
CaPOPII_404	Ca1	38597934	CAAAAA/CAAAAAA	CATTTGCCTCCCTTGTT GTT	TGTAGTTGCTGCAAAC GAGG	60.0	551	DRR	Ca_13956			Vacuolar protein sorting-associated protein 62
CaPOPII_405	Ca1	38613531	GAAAAAA/GAAAAA	CCTTCCTTCATGGTGAT TTGA	TCCCTGATCACACTTCC TCTG	59.9	610	INTERGENIC				
CaPOPII_406	Ca1	38636457	AAT/AATAT	AATATCCCAACAGCCTC ACG	TTGATTGAAACATTGAT TTCCG	60.0	710	INTERGENIC				

INDEL marker IDs	Chromosomes /unanchored scaffolds	Physical positions (bp)	InDels (*Kabuli* reference genome-CDC Frontier/PI)	Forward primers (5'-3')	Reverse primers (5'-3')	Anneling temperature (0C)	Expected amplified product size (bp)	Structural annotation		Functional annotation		
								Sequence components of *kabuli* genome	*Kabuli* gene accession IDs	NCBI-KOG	TFs	NCBI-nr database
CaPOPII_407	Ca1	38657360	TAAAAAAA/TAAAAAA	CCATCAGTGATAACTTGTCCCA	CACAGGACAAGTGCAATCTCA	59.9	451	INTERGENIC				
CaPOPII_408	Ca1	38671088	CTTGTGATT/CTTGTGATTATTTTGTTGTGATT	TGTTTGGGTCCTTGATGGTT	TTAGAGCGGAGCTTCTTCCA	60.2	825	INTERGENIC				
CaPOPII_409	Ca1	38771127	GTTATTATTATTAT/GTTATTATTATTATTATTAT	CCTTGTGGGAAGAAGCTTGA	ATATTGAGCGTCGTAACCCG	60.4	657	INTERGENIC				
CaPOPII_410	Ca1	38875592	TCA/T	ACCGAATCCAGTTGAACGAC	GGTCAATCCATCCATAGCCA	60.0	516	INTERGENIC				
CaPOPII_411	Ca1	38882967	GTTAGGA/GTTAGGACTTAGGA	ATTAGGAACCCTGGCATGTG	GGCAGCATTTGGTGAACTTT	59.8	464	DRR	Ca_13949	G		Glycoside hydrolase, family 20
CaPOPII_412	Ca1	38886569	TAAAAAAAA/TAAAAAAAAAAA	GCAACACTAGAACAACAGACGG	ATTTATCCATCCATGTCGCC	59.8	786	INTERGENIC				
CaPOPII_413	Ca1	39041564	TAAA/TAA	GAAAAAGCGCTGTTGTAGGG	TGGCCGCACAATATGTAGAG	59.9	435	INTRON	Ca_13946			
CaPOPII_414	Ca1	39141870	AATATAATATATAATATATAATAT/AATATAATATATAATAT	CAGCAAAACAACGCACATTC	AACAAGTGGGTGACCTGCAT	60.3	644	INTERGENIC				
CaPOPII_415	Ca1	39261977	A/AT	GGTTTGCTGTCGAGCCTTAG	AAACACAAGTCCAATCAAAAGG	60.0	421	INTERGENIC				
CaPOPII_416	Ca1	39319575	TTTATTTATTTGCTTATTTATTT/TTTATTTATTTGCTTATTTATTTGCTTATTTATTT	TTTGCCTCTTCTCCTCCAGA	CCACCAAATACAAATAAATTACTTACG	60.1	970	INTERGENIC				
CaPOPII_417	Ca1	39430506	ATT/ATTTT	TGCATCTCCCTCAAGCTTTT	TGGAAAATTTTGGGGTTCAA	60.0	976	INTERGENIC				
CaPOPII_418	Ca1	39431958	CAAA/CAA	TCAAGTGGAAGGGGGTAATG	ACCCCCAATCTGCATAAAAA	59.8	902	INTERGENIC				
CaPOPII_419	Ca1	39432122	CTTTTTTTT/CTTTTTTTTTTTTT	CAATTGCACAAATCATTGTTGA	CAGGTGCATTATTCGGTGTG	59.5	870	INTERGENIC				
CaPOPII_420	Ca1	39433392	CTTTTTTT/CTTTTTT	TATCCATACCACCCTGCCAT	CTCAGGATCCTCAACCCAAA	60.0	378	INTRON	Ca_13931	Z	NAC	IQ motif, EF-hand binding site
CaPOPII_421	Ca1	39442792	A/ACT	TGAATGGCATGCAAGAAAAG	GCCAGTGTTCGTGCTTCATA	59.8	631	INTRON	Ca_13931	Z	NAC	IQ motif, EF-hand binding site
CaPOPII_422	Ca1	39446823	GATT/G	GTTGTAAGCTTGCAGGAGGG	TTTTCGCCCCAATAAAGTTG	59.9	713	DRR	Ca_13931	Z	NAC	IQ motif, EF-hand binding site
CaPOPII_423	Ca1	39447104	TTAATG/TTAATGTTGATCTAATG	CAACTTTATTGGGGCGAAAA	CCCTCGATTAATTCTCTTGAGGT	59.9	253	DRR	Ca_13931	Z	NAC	IQ motif, EF-hand binding site

**INDEL marker IDs**	**Chromosomes /unanchored scaffolds**	**Physical positions (bp)**	**InDels (*Kabuli* reference genome-CDC Frontier/PI)**	**Forward primers (5'-3')**	**Reverse primers (5'-3')**	**Anneling temperature (0C)**	**Expected amplified product size (bp)**	**Structural annotation**		**Functional annotation**		
								**Sequence components of *kabuli* genome**	***Kabuli* gene accession IDs**	**NCBI-KOG**	**TFs**	**NCBI-nr database**
CaPOPII_424	Ca1	39450720	TAAAAAA/TAAAAAAA	CCTGTGCCTTTGCTTTATTTG	TTGCATAAATGGGAGGGAAG	59.8	705	INTERGENIC				
CaPOPII_425	Ca1	39463854	AAATTTAAT/AAAT	ATATTGAGAGGTTGCGTGGG	ACCATCTCGATGCAACACAA	60.0	556	INTERGENIC				
CaPOPII_426	Ca1	39508256	GAAAAAAAAA/GAAAAAAAAAAA	ACCTTTAGACTTGGGGCACA	CGGTTACCATTGTAAAAACAATCA	59.6	254	INTERGENIC				
CaPOPII_427	Ca1	39511343	CA/CAA	CGACGAAAGTGATGTGCATT	TGGATTTCACAATCATGGGTT	59.7	136	INTERGENIC				
CaPOPII_428	Ca1	39608275	AGGG/AGGGG	GGTCGACACATGGAGAGTTG	TTATTACCAAACTGCCCCCA	59.1	637	INTERGENIC				
CaPOPII_429	Ca1	39652228	ACCC/ACCCC	GGAGTGACAATGCCCTCAAT	CGGGAGATACGTAGGAGCAA	59.9	774	INTERGENIC				
CaPOPII_430	Ca1	39673797	ATTTTT/ATTTTTT	GCCTCAAAAGCGAAATTTGT	TTGGAACACATAAAAATTAATAATCCA	59.3	759	DRR	Ca_13921			
CaPOPII_431	Ca1	39682708	GCT/G	TCAATTGGCATTTTGCACAT	AAATTCAGCACTGCACCAAA	59.9	802	INTERGENIC				
CaPOPII_432	Ca1	39743942	TAAAA/TAAAAA	TTCCATTTCCTCCACAAAGC	TGCTACAACCGCATTGTCAT	60.1	563	DRR	Ca_13915			
CaPOPII_433	Ca1	39782012	TA/T	TTTTCCTAGACCATCTGCGG	CGATCAAATATTTTTCACCGAA	60.2	678	INTERGENIC				
CaPOPII_434	Ca1	39844169	ATT/ATCACTTT	AATTAATATTCGCGCAACGC	AATGGATGCATCAAATGGGT	60.1	879	INTRON	Ca_13909			
CaPOPII_435	Ca1	39847433	AAATA/AAATAATA	GAGGAAATTATTCCCGCAAA	TTTGGACTTCGTCGTTTTGA	59.0	735	INTERGENIC				
CaPOPII_436	Ca1	39955215	GTA/GTATA	CAAATGGATGAAGAGGAAAAGG	AACGACTTGCAATTGTCCCT	59.9	435	INTERGENIC				
CaPOPII_437	Ca1	40081687	AATTTATATAT/AAT	TTTCCACCATTGGATGATGA	TTGCCTGAAAAAGAGATGTCAA	59.7	475	INTERGENIC				
CaPOPII_438	Ca1	40147055	CGTTTCAAATTAAGT/CGT	ACGAAACAATCATTTGAACGTA	AGCGTTGCCAAAAATAATGC	57.3	532	INTERGENIC				
CaPOPII_439	Ca1	40298582	TAAAAAAAAAAA/TAAAAAAAAA	TTTTAAAGATGCTGAAACAAACAAA	AGCCGTGTTGATTTTCGCTA	59.3	251	INTERGENIC				
CaPOPII_440	Ca1	40326561	GTT/GTTT	CCGGCAAATATGACGAACTT	AAACCAAGAACAAAATGGCG	60.0	540	INTERGENIC				

INDEL marker IDs	Chromosomes /unanchored scaffolds	Physical positions (bp)	InDels (*Kabuli* reference genome-CDC Frontier/PI)	Forward primers (5'-3')	Reverse primers (5'-3')	Anneling temperature (0C)	Expected amplified product size (bp)	Structural annotation		Functional annotation		
								Sequence components of *kabuli* genome	*Kabuli* gene accession IDs	NCBI-KOG	TFs	NCBI-nr database
CaPOPII_441	Ca1	40355583	TAAAA/TAAAAA	GGAATTCTCAGGTTTTCGCA	TTCATGGTTGCTTTAAGGGG	60.2	616	INTRON	Ca_13893	S		Tetratricopeptide, MLP1/MLP2-like
CaPOPII_442	Ca1	40398387	CT/C	TGAAAAATACTTTTGGGCCG	AAAACGCCATGGATTGTCAT	59.9	858	INTERGENIC				
CaPOPII_443	Ca1	40408466	CTTATTTATTTATTTA/CTTATTTATTTATTTATTTA	TCACAAACAATTGCGAAAACA	CCACCTCCATGCCTCTAAAA	60.1	657	INTERGENIC				
CaPOPII_444	Ca1	40408801	ATT/ATTGAGCCAACTT	TTTTAGAGGCATGGAGGTGG	TTTTGCAAATGTTGGGTTCA	60.1	343	INTERGENIC				
CaPOPII_445	Ca1	40424994	C/CCAAAATATTATAA	TGCAAAACTTTGTTCATCGAG	GCATCATTCTCCTTCGAGACA	59.0	748	INTERGENIC				
CaPOPII_446	Ca1	40425232	AAA/AAATAA	CATTTTTAAGCTTTTGTTGCCA	GCATCATTCTCCTTCGAGACA	59.3	513	INTERGENIC				
CaPOPII_447	Ca1	40425317	GTCTC/GTCTCTC	CATTTTTAAGCTTTTGTTGCCA	TTTCATTAAGGTGAATCGTTCAGA	59.3	608	INTERGENIC				
CaPOPII_448	Ca1	40425540	ATTTTTTTT/ATTTTTTTTT	TGTCTCGAAGGAGAATGATGC	AAACCGGTTCAAAAATGCAC	60.4	600	INTERGENIC				
CaPOPII_449	Ca1	40535882	AATATATATATATATATATATAT/AATATATATATATATATATATATATAT	GATGATGATGATGTCGTGCC	ATCACAGTTTGGCCAAGTCC	59.9	311	INTERGENIC				
CaPOPII_450	Ca1	40735911	AATATATATATATATATATATATATATATATATATATATATATAT/AATATATATATATATATATATATATATATATAT	AAATGCAAAATTTTAAGCGTGA	CCATTGGAAAGTGTGTAGTGC	58.8	533	INTERGENIC				
CaPOPII_451	Ca1	40889769	TTATATA/TTA	GCATCTCTTGATGCCCTTTT	AACATACGCCATACCCAAGC	59.3	289	INTERGENIC				
CaPOPII_452	Ca1	40890559	ATTTTT/ATTTTTT	CGGATTTGCAAGTGTTCGTA	TGGGGTTTAGGATATCACATCA	59.7	707	INTERGENIC				
CaPOPII_453	Ca1	40890662	CAAA/CA	CAAGAAACAAATTGCAATGGA	TCAAGTTTGGGAGGAACCAC	58.7	704	INTERGENIC				
CaPOPII_454	Ca1	41695402	CTA/CTATA	TACTTCCATGCAAACCAGCA	CCAACTCTGAAATACAAAAGCCA	60.3	486	INTERGENIC				
CaPOPII_455	Ca1	41704419	CATAATAAT/CATAAT	GGGGAATTTTTCATTTTGTGG	TGTTTTGGTTTCAAGGGACA	60.4	311	INTERGENIC				
CaPOPII_456	Ca1	41704503	CTTTTTTT/CTTTTTT	GGGGAATTTTTCATTTTGTGG	TGTTTTGGTTTCAAGGGACA	60.4	311	INTERGENIC				
CaPOPII_457	Ca1	41718814	T/TG	GGGTCGAATGTCGATTCTGT	TCATCTCTCAGCCCCACTCT	59.9	156	INTERGENIC				

INDEL marker IDs	Chromosomes /unanchored scaffolds	Physical positions (bp)	InDels (*Kabuli* reference genome-CDC Frontier/PI)	Forward primers (5'-3')	Reverse primers (5'-3')	Anneling temperature (0C)	Expected amplified product size (bp)	Structural annotation		Functional annotation		
								Sequence components of *kabuli* genome	*Kabuli* gene accession IDs	NCBI-KOG	TFs	NCBI-nr database
CaPOPII_458	Ca1	41998443	CG/CGG	CCATTTTTCTCACGTGCTTTC	ATCCCTCTATTGGTGGGTCC	59.7	339	INTERGENIC				
CaPOPII_459	Ca1	42053807	CGTTGTTGTTGTTGTTGTTGTTGTTGT/CGTTGTTGTTGTTGTTGTTGTTGT	GGGATCTTTTTGGCTGATGA	CTCATCCTCGCCATTGTTTT	60.0	452	INTERGENIC				
CaPOPII_460	Ca1	43550360	ATTT/ATT	TGCAGAGTAATTCGGTTTGTTTT	GGCATTAAGCCTCAAATCCA	60.1	458	INTERGENIC				
CaPOPII_461	Ca1	43595794	AAAGAAGAAGAAGAAGAAGAAGAAGAAGAA/AAAGAAGAAGAAGAAGAAGAAGAAGAAGAA	GGAAGCCTGGAATGACACAT	TTCAATTCGGATTTTGACCC	59.9	555	INTERGENIC				
CaPOPII_462	Ca1	43687147	TAA/TA	TGTGGCTAAGATTAGAATCCCAT	TTGATGCAAACGTAATTCATAAAGA	59.0	586	INTERGENIC				
CaPOPII_463	Ca1	44247311	GTTTTTTTTTT/GTTTTTTTTT	TTCTCCAGACTCTAGCCCGA	AGCTTCTCAGTAGGGGGCTC	60.1	592	INTERGENIC				
CaPOPII_464	Ca1	44368738	GATAATAATAATAATAATAATAATAATAATAATAATAATAATAATAATAA/GATAATAATAATAATAATAATAATAATAATAATAATAATAA	TTGTAACTCCCCGAATTGTTG	AGAGGCAAACAAGAACCGAA	59.8	534	INTERGENIC				
CaPOPII_465	Ca1	44467151	GTTT/GTTTT	ATGCAGCTCAAAAACAGCCT	GGTTCACCCAAAGATGGCTA	60.0	537	INTERGENIC				
CaPOPII_466	Ca1	44650256	T/TC	TCGTTTGCGTCATTGGATTA	AGGGAGGACGAAAATGACCT	60.1	444	INTERGENIC				
CaPOPII_467	Ca1	44745225	A/AC	TCCGGATATAGTCCTGCCAC	TCCTTGTGAACGACTTGGTG	59.9	632	INTERGENIC				
CaPOPII_468	Ca1	44776081	AATATATATATATATATATATATATATATATATATATATATA/AATATATATATATATATATATATATATATATATATATATATATATATATA	TGGAATACCATATATTTTACGTTTTCA	TGACGGATCCTTTCTTGACA	59.1	795	INTERGENIC				
CaPOPII_469	Ca1	44847313	TATAATAATAATAATAATAATA/TATAATAATAATAATA	TGTTTTCTGCCTTTTTGCCT	AAAATCCAACAATCTCCCCC	59.9	274	INTERGENIC				
CaPOPII_470	Ca1	45057162	CATAAA/CATAAATAAA	TTGCTTGAATAGGCAATGGA	ACGTCGACGACATCAAAGTG	59.3	645	INTERGENIC				
CaPOPII_471	Ca1	45057964	CAAA/CAA	AAACTAGATTATGGCCTGCG	TGCTCGCAATTAAAACATGC	57.0	426	INTERGENIC				
CaPOPII_472	Ca1	45124635	GTATATATATATATATATATATATA/GTATATATATATATATATATATATATA	TCCGAGACACAAGACCAACA	TTACGAACACAATTGTTGCCA	60.3	836	INTERGENIC				
CaPOPII_473	Ca1	45238172	T/TGACA	ATTCTTTTTGGAGCGCAGAA	CCAATTAGGTTTACGGCGAA	60.0	401	INTERGENIC				
CaPOPII_474	Ca1	45243775	T/TATGCACTTCTTTTCTC	TGTGCCAAGGTTTCAGAACA	CTTTTATTGCAAGACCCCGA	60.3	605	DRR	Ca_26020			Uncharacterised protein family Ycf1

INDEL marker IDs	Chromosomes /unanchored scaffolds	Physical positions (bp)	InDels (*Kabuli* reference genome-CDC Frontier/PI)	Forward primers (5'-3')	Reverse primers (5'-3')	Anneling temperature (0C)	Expected amplified product size (bp)	**Structural annotation**		**Functional annotation**		
								**Sequence components of *kabuli* genome**	***Kabuli* gene accession IDs**	**NCBI-KOG**	**TFs**	**NCBI-nr database**
CaPOPII_475	Ca1	45244574	TC/TCATAAAGCTCAC	TCGGGGTCTTGCAATA AAAG	GACAATGGGAATTCGC CTAA	60.1	532	CDS (FRAME SHIFT)	Ca_26020			Uncharacterised protein family Ycf1
CaPOPII_476	Ca1	45246034	C/CGTCTTGTT	CGATTTCGCTTTTAGCA TCC	CAATTGCGATATTATGG GGG	59.8	253	INTERGENIC				
CaPOPII_477	Ca1	45251376	C/CCTATGA	CTAGCTCTGGGTTCGA GTGG	ATCCGACTAGTTCCGG GTTC	60.0	247	INTERGENIC				
CaPOPII_478	Ca1	45392704	ATTT/ATTTT	GGGGGCTCTTCATTTTT CTC	CACAGTTTCATCGCTC CTCA	60.0	476	INTERGENIC				
CaPOPII_479	Ca1	45443277	GTAATAATAATAATAATAATAATAATA ATAATAATAATAATAATAATAATAATA ATAATAGTAATAGTAATAATAATAAT AATAATAATAACAATAATAATAATAAT	CCCACATTCATGACGT GTTC	CTTAGAGGTGGGGAAC GTGA	59.8	741	INTERGENIC				
CaPOPII_480	Ca1	45609121	C/CT	TCGCGTGAGAAAATAA TCCC	CGAACCCTCGGTAAAC AAAA	60.0	295	INTERGENIC				
CaPOPII_481	Ca1	46088799	CTATATATATATATATATATATATATA TATAT/CTATATATATATATATATATAT ATATATATATATAT	TAAGTCCGTAAGTGGT GCCC	GGGCTTTCCAAAGTGT TATCC	60.0	586	INTERGENIC				
CaPOPII_482	Ca1	46178255	AATTTATAATTTAT/AAT	TCAAACATGCTCTGAAT GCC	AACCAATACATAACATC ACAGTGC	59.8	570	INTERGENIC				
CaPOPII_483	Ca1	46268212	TA/T	AAACGCAATCCTTGAGT TCG	CATGACTTGGATTGGT CGTG	60.2	226	INTERGENIC				
CaPOPII_484	Ca1	46278771	ATGGCCTGGCCTGGCCTGGCCTG/A TGGCCTGGCCTG	ACCTTCAGTCAGGGGC CTAT	AAGTCTGGTCTGGCCT GAAA	60.0	538	INTERGENIC				
CaPOPII_485	Ca1	46286614	CTTTT/CTTT	CCAAGTTATGCTTGATC GCA	CGAGAACCATCCTGCA TTTT	59.8	820	INTERGENIC				
CaPOPII_486	Ca1	46286661	GTAGGGATA/GTA	CCAAGTTATGCTTGATC GCA	CGAGAACCATCCTGCA TTTT	59.8	820	INTERGENIC				
CaPOPII_487	Ca1	46352138	CACAAC/CACAACAAC	AGCGATGCATGTCTCA AGTG	TGCCCTTCTTCTTTTCC TAGTG	60.0	396	INTERGENIC				
CaPOPII_488	Ca1	46491737	CAAG/CAAGAAG	GATGCATCTCACCAAG GGTT	CGGTGCAAACTCTACG TTCA	59.9	420	INTERGENIC				
CaPOPII_489	Ca1	46523548	CAAAAAAAAAAAAAAAA/CAAAAAAAA AAAAA	GTGGACAAACCTGCAT TCCT	ACCATGCACAGTTGCT GAGA	60.0	675	INTERGENIC				
CaPOPII_490	Ca1	46526481	CAAAAAAA/CAAAAAAAA	TTAAAATTCCGAGGCG AAAA	ACATGGTTGTGGTGGT GATG	59.7	698	INTERGENIC				
CaPOPII_491	Ca1	46528558	CT/CTT	TTAAAATTCCCTCCTCG GCT	AACTCTGGGAACCCTC CTGT	60.0	398	INTERGENIC				

INDEL marker IDs	Chromosomes /unanchored scaffolds	Physical positions (bp)	InDels (*Kabuli* reference genome-CDC Frontier/PI)	Forward primers (5'-3')	Reverse primers (5'-3')	Anneling temperature (0C)	Expected amplified product size (bp)	Structural annotation		Functional annotation		
								Sequence components of *kabuli* genome	*Kabuli* gene accession IDs	NCBI-KOG	TFs	NCBI-nr database
CaPOPII_492	Ca1	46528735	GTGACAGCCTGACAG/GTGACAG	TTAAAATTCCCTCCTCGGCT	GGGGAAAACATCCCGTTATT	60.0	865	INTERGENIC				
CaPOPII_493	Ca1	46531041	T/TG	GCGAAAATAATGTTGATGCAGA	GTCTGCGGATAATTTCGCTT	60.1	139	DRR	Ca_21551	M		Pectinacetylesterase
CaPOPII_494	Ca1	46539303	TTCAAATCTTCATCAAATCTTC/TTCAAATCTTC	TCAAGCCAATCTTCCCAAAC	TCTGATGACAAAAGGGGGAG	60.1	757	INTERGENIC				
CaPOPII_495	Ca1	46539360	CAAA/CAAAA	TCCCAAACAGCCTGATAACC	CTGATGACAAAAGGGGGAGA	59.9	744	INTERGENIC				
CaPOPII_496	Ca1	46633354	GTATATATATATATATATATATAT/GTATATATATATATATATATAT	CGACATGGCTCTGATACCAA	TCAGACCGCCTTTACCAATC	59.7	615	INTERGENIC				
CaPOPII_497	Ca1	46662012	AAT/AATAT	GCATTTACGTCGATATTTATGAGTG	TCAGCCAAAAAGCTGATGTG	59.1	617	INTERGENIC				
CaPOPII_498	Ca1	46831943	CTTTT/CTTT	GTGGTTGGTTTGCAAGAGGT	AACTGGGTGAGAGCCTGAGA	60.0	725	INTRON	Ca_25116	U		
CaPOPII_499	Ca1	46867363	CTTTT/CTTT	GCCCTCAGATGAGAGTCAGG	CAAACCTTTCATGCGGGTAT	59.9	836	DRR	Ca_25118			
CaPOPII_500	Ca1	46896717	AATATATATATATATATATATA/AATATATATATATATATATATATATA	CACGATGCATTGGTGTAGGT	TCGTTCAATAACCTTTTTCTTGG	59.4	555	INTERGENIC				
CaPOPII_501	Ca1	47161229	TAGAG/TAG	TCTGCAATGAAAGCAAACACA	AGTTTTGGCTCCCTATCGGT	60.4	829	INTERGENIC				
CaPOPII_502	Ca1	47337298	C/CAAATATATACT	ATTGGGCATAGGAAGCACTG	AGTTGACCACGGACCCATAG	60.1	533	DRR	Ca_12919			
CaPOPII_503	Ca1	47630703	TAAAAAGAAAAAGAAAAAGAAA/TAAAAAGAAAAAGAAAAAGAAAAAGAAA	CGTGTCCGGCAAAGTAAAAT	GTGATGCACGCAGTAATTGG	60.0	517	INTERGENIC				
CaPOPII_504	Ca1	47730180	GATATATATATATATATATATA/GATATATATATATATATATATA	TGCGTATCTTAGGAATGCGA	GGCTAGACGAGTTCGACGAC	59.4	321	INTERGENIC				
CaPOPII_505	Ca1	48055075	GTATATATATATATATATATATATATATATATA/GTATATATATATATATATATATATATATATATATATATA	TGACACTCGCATCTTTGTGA	GGCCATATCCGAAAACATTG	59.0	432	INTERGENIC				
CaPOPII_506	Ca2	37373	TTATATATATATATATATATATATAT/TTATATATATATATATATAT	TCATGGTCTGGGTTTGAACT	TGAATCGCGAAATTAGTCCC	58.0	708	INTERGENIC				
CaPOPII_507	Ca2	42172	TTATATATATATATATATATATATATA/TTATATATATATATATATATATATATATA	TGGCAGTCTGTTTCACGTTT	CAAGGGACCAGATTCTGCAT	59.3	553	INTERGENIC				
CaPOPII_508	Ca2	416248	TTATATATATATATATATATATATATATATAT/TTATATATATATATATATATATATAT	ATAAATGAAGGGGTGGGAGG	GTTTCCCATAAACAGCACCC	60.0	882	INTERGENIC				

INDEL marker IDs	Chromosomes /unanchored scaffolds	Physical positions (bp)	InDels (*Kabuli* reference genome-CDC Frontier/PI)	Forward primers (5'-3')	Reverse primers (5'-3')	Anneling temperature (0C)	Expected amplified product size (bp)	Structural annotation		Functional annotation		
								Sequence components of *kabuli* genome	*Kabuli* gene accession IDs	NCBI-KOG	TFs	NCBI-nr database
CaPOPII_509	Ca2	453297	TTATATATATATATATATATAT/TTATATATATATATATATATAT	TTCAAACCAAATGATCCAATGA	ATAAATGTTGGGTGGCCAAA	60.2	685	DRR	Ca_16985	L		Ubiquitin-associated/translation elongation factor EF1B, N-terminal
CaPOPII_510	Ca2	753916	CTTTTTTTTTT/CTTTTTTTTT	TTGCCCATTAACTTTACCCG	ACTTGTGCATTTGCATTAGTTC	59.8	555	DRR	Ca_17000		ARF	
CaPOPII_511	Ca2	981417	ACTCTCTCTCTCTCTCTCTCTCTCTCTCTCTCT/ACTCTCTCTCTCTCTCTCTCTCTCTCTCTCTCTCTCTCTCTCTCT	ACATAATTCCCAACCGACCA	CCAAAGGGGTGAGTTTTTGA	60.1	328	INTERGENIC				
CaPOPII_512	Ca2	1033751	CATTTATTA/C	TGTGTGATTTTCAATAGTCACCG	AAGTTAAGGGACCACGGGAT	59.9	845	INTERGENIC				
CaPOPII_513	Ca2	1640062	ATTTTTTTTT/ATTTTTT	AGATCATGTGCAAAAAGGGG	AGTAACCGTGAGGGAACGTG	59.9	509	INTERGENIC				
CaPOPII_514	Ca2	1842322	AG/AGGCAGG	CGGCTTCTGACACACAAAGA	CTAAATGCAGAAAAAGCCGC	60.0	395	INTRON	Ca_12600		SBP	Glycosyl transferase, family 14
CaPOPII_515	Ca2	1853896	AATATATATATATATATATATATAT/AATATATATATATATATATATATATATAT	TGAGTCCGCTTCCCAATTAC	TTGAGCACGACTTTTTGCAC	60.1	536	INTERGENIC				
CaPOPII_516	Ca2	2169056	C/CG	AACCTCTCCAAGAGGATCGG	GGAGAGTGAAACGGATGGAA	60.6	648	INTERGENIC				
CaPOPII_517	Ca2	2174282	ATTTTTTTTTTTTT/ATTTTTTTTTTT	TGACATCCATCTTATGTGGGTC	GGTCTACTCATGTTGAAAGACGC	59.7	674	INTERGENIC				
CaPOPII_518	Ca2	2388005	GTTTTTTTTT/GTTTTTTTTTT	GCCAACAACGTTACGAGCTA	CTTGCTAAATGCCCCACTTC	59.0	581	INTERGENIC				
CaPOPII_519	Ca2	2692293	ATTTTTTTT/ATTTTTTT	AAAGGTGATGAAGCAGATTTTGA	TGGTGTCCTCTCTTCTCTAATCG	60.1	478	INTERGENIC				
CaPOPII_520	Ca2	2726175	TTATATATATATATATATATATAT/TTATATATATATATATATATAT	CGTTTCGGGTTTCGTAAAAA	TTGTTGACAATTCGATCCAAAG	60.0	302	DRR	Ca_10571	Q	B3	Cytochrome P450
CaPOPII_521	Ca2	2975267	GAAAAAAAA/GAAAAAAA	TTCACGACTTGGAAAGGACC	TGATCGCCATTGATTGAGAA	60.1	750	INTERGENIC				
CaPOPII_522	Ca2	2996172	ATTT/ATT	TTCTAAGGACCAACGCAACA	GTATGATCGATTATGCGCGA	59.3	434	INTERGENIC				
CaPOPII_523	Ca2	2996631	CAAAAAAAAA/CAAAAAAAA	CTCGAGTCGGTGTTGTGATG	TTCTTCACGATCATCCATGC	60.3	633	INTERGENIC				
CaPOPII_524	Ca2	3009059	ACCCCC/ACCCC	ATTCGCTTGCCTCTTTGGTA	CCCGAAACTTTCTGTGCATT	59.8	658	INTRON	Ca_10544	G		Glycosyl transferase, family 14
CaPOPII_525	Ca2	3031259	ATTTTT/ATTTTTT	TCAATTGCGATGGATAGAGAAA	AATTGGTGTACCGATTGCGT	59.7	647	INTERGENIC				

INDEL marker IDs	Chromosomes /unanchored scaffolds	Physical positions (bp)	InDels (*Kabuli* reference genome-CDC Frontier/PI)	Forward primers (5'-3')	Reverse primers (5'-3')	Anneling temperature (0C)	Expected amplified product size (bp)	Structural annotation		Functional annotation		
								Sequence components of *kabuli* genome	*Kabuli* gene accession IDs	NCBI-KOG	TFs	NCBI-nr database
CaPOPII_526	Ca2	3031554	C/CCAAAATATCT	CATCTTGTCCATCAAACGGA	GGTGTACCGATTGCGTATCC	59.5	279	INTERGENIC				
CaPOPII_527	Ca2	3042033	TTTTGTTTGCTT/TTT	TGCCAAATCATGCGTTTTTA	CTCTGATGCGAAGGTTGACA	60.1	100	INTRON	Ca_10543			Conserved hypothetical protein CHP00275, flavoprotein HI0933-like
CaPOPII_528	Ca2	3045119	AA/AAGTGAGTCTA	CCTGATGTGCATGTGGAAAG	CGTTCCAACACATAGCTCCA	60.1	553	INTRON	Ca_10543			Conserved hypothetical protein CHP00275, flavoprotein HI0933-like
CaPOPII_529	Ca2	3049734	AGAT/A	CGATCTCATATGTGGGGACC	TGCAATCGAAAGAAGTGTGG	60.2	536	INTERGENIC				
CaPOPII_530	Ca2	3312282	TGAAGAAGAAGAAGAAGAAGAAGAA/TGAAGAAGAAGAAGAAGAAGAAGAAGAA	AATTGAGCCTTTTGCCATTG	TCCCTCCCTTCACTCTCCTT	60.1	215	INTERGENIC				
CaPOPII_531	Ca2	3857450	TTTTATTTATTTATTTATTTATTTATTT/TTTTATTTATTTATTTATTTATTTATTTATTTATTTATTTATTT	GGGAAATTGTGGTGTGAGGT	TTCGTGCAACGCTATAATTCC	59.7	513	INTERGENIC				
CaPOPII_532	Ca2	3901844	AC/ACTTCACGAGC	GTCGCTATCTCCCCAGTGAA	CATTCTGTGCCGCTATCTGA	60.2	248	DRR	Ca_10466	GT		UDP-glucose/GDP-mannose dehydrogenase, N-terminal
CaPOPII_533	Ca2	3949908	TAAAAAAAAAAAA/TAAAAAAAAAAAAA	TCAAAGAATAGAGGTATTGTCTCCG	CTAAAGCCTGCCCATTTCTG	60.0	795	INTERGENIC				
CaPOPII_534	Ca2	3955516	TATAATAATAATAATAATAATAA/TATAATAATAATAATAATAATAATAATAA	TTTCCGATTTCGAGGTGTTC	TGAATAGTGGAAAACACAACAATG	60.1	385	INTERGENIC				
CaPOPII_535	Ca2	4266265	A/ATTAATCCGAAAG	TGGACGTTTAACGGTTGACA	CCCAGTAGAAAAGAAGGGGC	60.0	705	INTERGENIC				
CaPOPII_536	Ca2	4285697	TAAAA/TAAA	CCAGTCCCGAAATTAAACGA	ACTTGCGATAATCGATTGGG	59.9	613	INTERGENIC				
CaPOPII_537	Ca2	4502043	ANNNNNNNNNN/ANNNNNN	GCTTTGGGTGAACAAATGCT	AGGCACCCTTCAACAAAATG	60.1	258	INTERGENIC				
CaPOPII_538	Ca2	4639205	TAAAAAAAAAAA/TAAAAAAAAAA	AACACTCGTACCTTTATTTCTGGG	ATGCAGCAATTTCCATCCTC	59.8	950	INTERGENIC				
CaPOPII_539	Ca2	4812881	ATTTTTTT/ATTTTTTTTT	AACCAGTTCCGTTCCAAGTG	TCCCCCAAACAGAATTTGAG	60.0	731	DRR	Ca_21090		MYB	Transferase
CaPOPII_540	Ca2	4891048	TTTTATTTATTTATTTATTTATTTATTT/TTTTATTTATTTATTTATTTATTTATTTATTT	AACGGTGTGAAAGGGAAATG	TGAATTGAATTTGGAAGGGG	59.8	515	INTERGENIC				
CaPOPII_541	Ca2	4891335	AAATTGAAG/A	AACGGTGTGAAAGGGAAATG	TGAATTGAATTTGGAAGGGG	59.8	515	INTERGENIC				
CaPOPII_542	Ca2	4892794	CATATATATATATATATATATATATATATATATAT/CATATATATATATATATATATATATATATATATATATATATATAT	GCGGACATAAGGAAGATGTGA	AGGTCTGCGAAGATGGAAAA	60.1	606	INTERGENIC				

INDEL marker IDs	Chromosomes /unanchored scaffolds	Physical positions (bp)	InDels (*Kabuli* reference genome-CDC Frontier/PI)	Forward primers (5'-3')	Reverse primers (5'-3')	Anneling temperature (0C)	Expected amplified product size (bp)	Structural annotation		Functional annotation		
								Sequence components of *kabuli* genome	*Kabuli* gene accession IDs	NCBI-KOG	TFs	NCBI-nr database
CaPOPII_543	Ca2	4894086	TA/TAGACAACTCAA	TTGAACTACAACCAGCGCAC	TTTTGTTCAATTTCTTGCTCCA	59.9	493	INTERGENIC				
CaPOPII_544	Ca2	4894836	ATGATT/AT	AAAGGGAAGAAATTTCGGGA	AACCGGACGATCATTTGAAG	59.9	498	INTERGENIC				
CaPOPII_545	Ca2	4915954	AAATAATAATAATAATAATAATAATAATAATAATAATAATAATAATAA/AAATCATAATAATAATAATAATAATAATAATAATAATAATAATAATAATA	TTATCGGACATTTTCCCGAG	CTAGGTCGCCCTTTCAGGTT	59.9	504	INTERGENIC				
CaPOPII_546	Ca2	5139260	AATATATATATATATATATATATATATATATATAT/AATATATATATATATATATATATATATATATATATATAT	GTAATTGACGCGTGTGATGC	GATAAAATGCAGAGGCGGAG	60.2	537	INTERGENIC				
CaPOPII_547	Ca2	5595728	AATATATATATATATATATATA/AATATATATATATATATATATATA	TTGCCACTTGATACAGCAAAA	CGCGTATTTATCGTGTGGAA	59.4	652	INTERGENIC				
CaPOPII_548	Ca2	6047361	AATTATTATTATTATTATTATTATTATTATTATTAT/AATTATTATTATTATTATTATTATTATTATTATTATTATTATTATTATTATTATTAT	ACCTTCCACACAAGCCAAAC	AGGCCTTTATGTGGCCTTTT	60.0	456	INTRON	Ca_19707	K	MYB	Transcription factor TFIIB-related
CaPOPII_549	Ca2	6056245	TATAAATAAATAAATAAATAAATAAATAAATAAATAAATAAATAAATAAA/TATAAATAAATAAATAAATAAATAAATAAATAAATAAATAAATAAATAA	ATCCCAGTTTTGGTTGTTGG	TCGTGGTCCAGAAAACATGA	59.7	489	INTRON	Ca_19707	K	MYB	Transcription factor TFIIB-related
CaPOPII_550	Ca2	6505178	TA/TAA	CGTAATAACGGCGAGGAAAA	TGAGCAGGGGGAAGAGTAGA	60.1	548	INTERGENIC				
CaPOPII_551	Ca2	6513260	AAATAATAATAATAATAATAATAATAATAATAAT/AAATAATAATAATAATAATAATAATAAT	ATGGTCCAAGGAAATTGTGC	AGTCGTCGAGGCCAAATCTA	59.8	265	INTERGENIC				
CaPOPII_552	Ca2	6640316	TAAAAAA/TAAAAAAA	ATGGCCACGAATCTGAAGAG	ACGGTTGTTTTGGCATTGAT	60.2	890	INTERGENIC				
CaPOPII_553	Ca2	6641737	CATATAT/CATATATATAT	GTATTCAGGGGCGAAGGAAT	AATGCAAACGACAAAGGAGG	60.3	500	INTERGENIC				
CaPOPII_554	Ca2	6641916	TG/T	GTTCTATGGGGACAGGGTCA	TATCAACTACCAGAGCGGGG	59.8	476	INTERGENIC				
CaPOPII_555	Ca2	6642487	ATTTT/ATTTTTTTTT	CCCCGCTCTGGTAGTTGATA	ACCCACTTTGAGAAACGTGC	60.1	479	INTERGENIC				
CaPOPII_556	Ca2	6643843	CTTTTTTTTTTT/CTTTTTTTT	ATGGAACAAGCAGCGAAGTT	GTTGATACGAGGGGGTTGAA	59.9	537	INTERGENIC				
CaPOPII_557	Ca2	6648530	TAAAAAA/TAAAAAAAA	TTATCGATCGTCGGTGTCAA	TTCCATATGCACCTCCCATT	60.1	612	INTERGENIC				
CaPOPII_558	Ca2	6657720	AATG/AATGATG	AAGAATTGTTGTCCCCCTCC	GGAACCAAAACAAAAGGCAA	60.2	635	INTERGENIC				
CaPOPII_559	Ca2	7043443	AATATATATATATATATATATATAT/AATATATATATATATATATATATATATATAT	ATGGGTCGTGTTCGATTGTT	AACCAACTATTGACCCCAAAAA	60.2	267	INTERGENIC				

INDEL marker IDs	Chromosomes /unanchored scaffolds	Physical positions (bp)	InDels (*Kabuli* reference genome-CDC Frontier/PI)	Forward primers (5'-3')	Reverse primers (5'-3')	Anneling temperature (0C)	Expected amplified product size (bp)	Structural annotation		Functional annotation		
								Sequence components of *kabuli* genome	*Kabuli* gene accession IDs	NCBI-KOG	TFs	NCBI-nr database
CaPOPII_560	Ca2	7171869	TTAT/TT	CGGCCATTAAATTTCTGACA	TTGCATTATGTTTCTGGCAAA	58.6	320	INTERGENIC				
CaPOPII_561	Ca2	7171912	TATTTAATTTAATTTAATT/TATTTAATTTAATT	CGGCCATTAAATTTCTGACA	TCATTTCATAACGAGTGACCCA	58.6	584	INTERGENIC				
CaPOPII_562	Ca2	7172121	CTATATATATATAT/CTATATATATAT	TTTGCCAGAAACATAATGCAA	TCATTTCATAACGAGTGACCCA	59.2	285	INTERGENIC				
CaPOPII_563	Ca2	7172172	ATTTTTTTTT/ATTTTTTTTTT	TTTGCCAGAAACATAATGCAA	TTTTGGAAAAATGAAGGACCA	59.2	774	INTERGENIC				
CaPOPII_564	Ca2	7276294	CAAAA/CAAAAA	TCCACCACAATCGTTAAGCA	AGCGGAGATTTTACCGGATT	60.1	676	INTERGENIC				
CaPOPII_565	Ca2	7528105	CAAAAAAAA/CAAAAAAA	AGAGGCCAACCTCTGTCTCA	TCGTCAGGAGGTTGATGATG	60.0	313	INTERGENIC				
CaPOPII_566	Ca2	7557453	TTATATATATATATATAT/TTATATATATATATATATAT	CGCACACACGAATATTCACC	ACGACAACATGGTGGAACAC	60.0	465	INTERGENIC				
CaPOPII_567	Ca2	7617731	CTAAAATAAAATAAAA/CTAAAATAAAATAAAATAAAATAAAA	CCAAGATCTCGTAGTGCAACA	TGGGTTTAGTCCGTTTTTCTTC	58.9	468	INTERGENIC				
CaPOPII_568	Ca2	7657147	CTTTTT/CTTTTTT	TTATGTGGGGGATTTGGATG	TTCCACGTCCTTTTCATTCC	60.4	705	INTERGENIC				
CaPOPII_569	Ca2	7669241	TAA/TAAA	TGTTCGCCTCTTCATCAGTG	TGTGTTTTGGGTTCGACTAGG	60.0	475	INTERGENIC				
CaPOPII_570	Ca2	7714112	TG/TTGG	CTCACTCGATCGATGGTTCA	TGACAATGCTGGCTGTTAGC	59.8	588	INTERGENIC				
CaPOPII_571	Ca2	7724864	TA/TAAAGAGATTGAGA	ACCAGAGACACTGCAGCAAA	AACAAGATTCTCGAATGCTCC	59.6	464	INTERGENIC				
CaPOPII_572	Ca2	7744794	CATGACATGAATGA/CATGA	CATTTTGATTGGGAATGTGC	CAACAACAGTCCAAAGTAGGCA	58.8	585	INTERGENIC				
CaPOPII_573	Ca2	7745257	CAAAA/CAAAAAA	TGGCATGACATGAATGAAGAA	GAGCAATTTGTGTGGACCCT	60.1	760	INTERGENIC				
CaPOPII_574	Ca2	7749962	TT/TTAT	GGAGCTGCAACCATCTTCTC	TGGATTTGGGCTGAATTAGG	60.0	321	INTERGENIC				
CaPOPII_575	Ca2	7753009	A/AC	AAGTGGCGAAGCTTGTTGAT	AGCTTGCCATTGCAGCTACT	59.9	797	INTRON	Ca_18125		MYB_related	NPH3
CaPOPII_576	Ca2	7756359	CAAA/CAAAA	AACCAGCATGTGTTGTTGGA	TGTGGTGGGGGAATTAAAAA	60.0	399	INTERGENIC				

INDEL marker IDs	Chromosomes /unanchored scaffolds	Physical positions (bp)	InDels (*Kabuli* reference genome-CDC Frontier/PI)	Forward primers (5'-3')	Reverse primers (5'-3')	Anneling temperature (0C)	Expected amplified product size (bp)	Structural annotation		Functional annotation		
								Sequence components of *kabuli* genome	*Kabuli* gene accession IDs	NCBI-KOG	TFs	NCBI-nr database
CaPOPII_577	Ca2	7772575	AGG/AGGG	TTAAGGGACCACGGGTGTTA	TCCCACCTCTCAATTTTTCAA	60.2	898	INTERGENIC				
CaPOPII_578	Ca2	7773617	TTAAATAAAAT/TTAAATAAAATAAATAAAAT	GCTGAGCCCAAACAAGCTAA	CCACTGTGGAAAGGTAAATGC	60.5	845	INTERGENIC				
CaPOPII_579	Ca2	7780730	ATTTTTTTTTTT/ATTTTTTTTTT	TTGTCTCCATCTCCAGCCTT	TGTTGTTTGAGTTGGGACACA	59.8	995	INTERGENIC				
CaPOPII_580	Ca2	7793328	GAAAAA/GAAAA	ATATCCCCTCCAACGAGGAA	TTTATTGCGGTTTCCCTTCA	60.7	877	INTERGENIC				
CaPOPII_581	Ca2	7811963	CAAAAAAA/CAAAAAAAA	ACTAACTCGGTGGGGAACAA	TCCCTTGTTCTTGCTGTGAA	59.4	330	INTERGENIC				
CaPOPII_582	Ca2	7818010	GATA/GATATTCTAATA	TGGACATAGAAAAACCAAATGGA	ATGTGGAAGTTGAACCCCAA	60.6	598	DRR	Ca_18128	QR	B3	Isopenicillin N synthase
CaPOPII_583	Ca2	7913902	GAAAAAAAAAA/GAAAAAAAAA	TCAAATCTACCTCTAACGATTCACA	AAGGACAGTTGTCCCAATGC	59.2	326	DRR	Ca_18139	FE		Phosphoribosyltransferase
CaPOPII_584	Ca2	7926426	CTTT/CTT	CTTGGCGAAGTGAAGGGATA	GAGGCTTGGTTCCCTAATCC	60.2	471	INTRON	Ca_18142	U		Clathrin adaptor, mu subunit
CaPOPII_585	Ca2	7933051	AC/A	CCCCAAAAAGTGCTTCAAAA	TGACAAGCACGCTTAAGGAA	60.1	694	INTERGENIC				
CaPOPII_586	Ca2	7938788	AA/AACA	TGATCAAATAATTGAAACAAGCG	GCCGTATTGGGAGAAGATGA	59.2	589	INTRON	Ca_18144			Allinase, C-terminal
CaPOPII_587	Ca2	7940111	ATTTTTTT/ATTTTTT	TCTTGACCGTTAGATTGAACCA	CAAAATGGACAAAATTGGAGC	59.6	714	INTERGENIC				
CaPOPII_588	Ca2	7940136	ATTTTTTT/ATTTTTTTT	TCTTGACCGTTAGATTGAACCA	CAAAATGGACAAAATTGGAGC	59.6	714	INTERGENIC				
CaPOPII_589	Ca2	8308316	TAAAAAAAA/TAAAAAAA	AATTGGGACCATTTGATAGGC	GCCTCTACCATCTTTGACAGC	60.0	171	INTERGENIC				
CaPOPII_590	Ca2	8328118	TATTTATTTATGCATTT/TATTTATTTATGCATTTATTTATGCATTT	AGGTTCAATTTGGTGGCAAG	TTGAATTGTTATGCCCGTGA	60.0	570	INTRON	Ca_21469		C2H2	Glycoside hydrolase, family 28
CaPOPII_591	Ca2	8374636	AATATATATATATATATATATAT/AATATATATATATATATAT	AAAGGGACAATGAAGGCAAG	TTGAAAATGTGATGTTTCTTCCA	59.2	636	INTERGENIC				
CaPOPII_592	Ca2	8420270	AACA/AACACA	CCAAGTTTGAAATGCCGATT	GCTGCATGATCTTATGCTCG	59.9	451	INTERGENIC				
CaPOPII_593	Ca2	8478800	AATTT/A	CCCGTATGAATGAACGGAAC	CTACCCCCACATTTCAATCG	60.2	519	INTERGENIC				

INDEL marker IDs	Chromosomes /unanchored scaffolds	Physical positions (bp)	InDels (*Kabuli* reference genome-CDC Frontier/PI)	Forward primers (5'-3')	Reverse primers (5'-3')	Anneling temperature (0C)	Expected amplified product size (bp)	Structural annotation		Functional annotation		
								Sequence components of *kabuli* genome	*Kabuli* gene accession IDs	NCBI-KOG	TFs	NCBI-nr database
CaPOPII_594	Ca2	8478910	ACT/A	CCCGTATGAATGAACGGAAC	CTACCCCCACATTTCAATCG	60.2	519	INTERGENIC				
CaPOPII_595	Ca2	9494071	T/TA	GAACTGCAACAACAACGACAA	GGTCTCGAAATCCCTGATGA	59.8	609	INTERGENIC				
CaPOPII_596	Ca2	9500229	CGTATGCTAGACCTGG/CGTATGCTAGACCTGGGTATGCTAGACCTGG	AGAGGTTGGGGGAGAAGAAA	GCAACAACAGCAGAGACAATG	60.0	213	INTERGENIC				
CaPOPII_597	Ca2	9690407	TAAAAAAAAAAAAA/TAAAAAAAAAAAA	TGATGCAAATATCTACCGCAA	TTCAAGGGACGTAAATGCAA	59.2	558	INTERGENIC				
CaPOPII_598	Ca2	9694226	TTATATATATATATATATATATATA/TTATATATATATATATATATA	TGTGCGTTGTAAAGGAGTGC	CGTCGGAATCCAAAAGTCAT	59.9	504	INTERGENIC				
CaPOPII_599	Ca2	10081600	CAAAAAAAAAAAAAAA/CAAAAAAAAAAAAAA	TTTTGATTGATTGATGATGGG	TGCAAATTCAACCTCACGAA	58.3	552	INTERGENIC				
CaPOPII_600	Ca2	10779124	TTA/TTACATA	CACTTTTCTTATGCACCGCA	GCAGCGAGTAGGAGAAATGC	59.9	663	INTERGENIC				
CaPOPII_601	Ca2	10948001	ATTA/ATTACACTGACGTTA	TGATTCTTGATCATGTTGTTTTCA	CCATCCTCACTCCAAAGACAA	59.5	702	INTERGENIC				
CaPOPII_602	Ca2	11085781	AATATATATATATATATATATATATATATATATATTTATATATATATAT/AATATATATATATATATATATATATATATTTATATATATATAT	TTTGGTTTTGAACTCGGAGG	CACCGTAGTGTGGTGGTGAG	60.1	660	INTERGENIC				
CaPOPII_603	Ca2	12048675	AATAATTTATAATTTATA/AATAATTTATA	CAAACATGCTGATGTGGACC	CCATTGGTAAATGGCTAACAA	60.0	278	INTERGENIC				
CaPOPII_604	Ca2	12475607	TTGTATTTGATGTAT/TTGTAT	TCAAAGAGAAATGACTTTGTCCA	TCACTTTGCTTTGTGTGTCTCA	58.9	852	INTERGENIC				
CaPOPII_605	Ca2	12567970	ATTTTTTTTTTT/ATTTTTTTTTTTT	GGAAGTTGCCTTTCCCTTTC	TCGCAAGGATCTACACACTGA	60.1	743	INTERGENIC				
CaPOPII_606	Ca2	12634541	CTTTTTTTT/CTTTTTTT	GTTCAAAGCAGCTGCAACAA	CCACAACTACCCTTGGCATT	60.2	236	INTERGENIC				
CaPOPII_607	Ca2	12933106	A/AT	AGGTTGAAAGAAGCCCCACT	ATCGGTTGGTTGGTGAAATG	60.1	834	INTERGENIC				
CaPOPII_608	Ca2	12933573	CACCAACCAACC/CACCAACC	TCATTGACAAGGAAAGAACATGA	CGAATTGGTTTTGGGATTTG	59.6	413	INTERGENIC				
CaPOPII_609	Ca2	12934202	AAA/AAACAA	AGAAAGCAAGCAAATCCCAA	TTCCAACACCTCACGATTCA	59.8	488	INTERGENIC				
CaPOPII_610	Ca2	12937651	ATTTT/ATTTTT	GATTGCTTTCCTCTATGCCG	GTTTTGGCGTTGGTTTCACT	59.8	353	INTERGENIC				

INDEL marker IDs	Chromosomes /unanchored scaffolds	Physical positions (bp)	InDels (*Kabuli* reference genome-CDC Frontier/PI)	Forward primers (5'-3')	Reverse primers (5'-3')	Anneling temperature (0C)	Expected amplified product size (bp)	Structural annotation		Functional annotation		
								Sequence components of *kabuli* genome	*Kabuli* gene accession IDs	NCBI-KOG	TFs	NCBI-nr database
CaPOPII_611	Ca2	12939688	AATATATA/AATATA	TTTTGGTGTTGAGGGTGTCA	TTAATCAAAGCGGCAAAAGAA	60.0	564	INTERGENIC				
CaPOPII_612	Ca2	12941200	CAAAA/CAAA	TGTGCTTCTTCCTGATTTTGG	GGCCAAAGACGGATATGGTA	60.2	888	INTERGENIC				
CaPOPII_613	Ca2	12941800	T/TA	CATATCCGTCTTTGGCCATT	CCTTGAACAATTTGGGCAGT	59.8	643	INTERGENIC				
CaPOPII_614	Ca2	12947970	G/GT	TGCTTTTATGAGCATTACGGG	CGACTTCAAATCCCTTCGAG	60.1	777	INTERGENIC				
CaPOPII_615	Ca2	12952557	ACATATCATATCA/ACATATCA	AAAGCATTTTATCCCGACCA	TTTTTCATCTCACTCTCTCCCA	59.4	787	INTERGENIC				
CaPOPII_616	Ca2	12952944	TGTCATGATTAT/TGTCATGATTATAGTCATGATTAT	TGGGAGAGAGTGAGATGAAAAA	ACGGATGTCTAATCCGTTGG	58.9	308	INTERGENIC				
CaPOPII_617	Ca2	12960292	GT/GTTAT	TTCATACATCCAATCGTGGTTT	CCACAAGAAGCAACACAAGC	59.2	527	INTERGENIC				
CaPOPII_618	Ca2	13639596	TAATTAAATAAAAT/TAAT	AAGCCAGCCGCATGTAAATA	TTCACTTCCTCTGCATCTTTCA	60.6	788	INTERGENIC				
CaPOPII_619	Ca2	13794561	CTTTTTTT/CTTTTTT	GATTCGGTGGAGAATCCAAA	CACCGGTGGTAACGAGAACT	59.9	834	INTRON	Ca_11639		SAP	Remorin, C-terminal
CaPOPII_620	Ca2	15313880	ATATTATTATTATTATTATTATTATTATTATTATTATTATTATTATTATTATTATTATTATTATTATT/ATATTATTATTATTATTATTATTATTATTATTATTATT	GCAAAAAGCAGCGCAATAAT	ACGAATCTAACGGTGGTTGC	60.4	360	INTERGENIC				
CaPOPII_621	Ca2	15589036	ATTT/ATTTT	ATACGCCTCAACAAAATGGC	CTGCTTTGCTTGATTCCGTT	60.0	376	INTERGENIC				
CaPOPII_622	Ca2	15598813	TA/TAA	GGAGGCGCATTAGATATGGA	GCCTCGTGAGTTGCATCATA	60.0	518	INTERGENIC				
CaPOPII_623	Ca2	15812866	TAAAAAAAAA/TAAAAAAAAAA	CATTGGCGGAGCTTTACAAT	CCATGGCCACTTCAAGATTT	60.1	298	INTERGENIC				
CaPOPII_624	Ca2	15920758	GTATATATATATATATAT/GTATATATATATATAT	TTCGGAAGTCCATCGGTATC	GCAAATGCTCCCCTAATTGA	59.9	546	INTERGENIC				
CaPOPII_625	Ca2	16496636	GTCAAAC/GTCAAACTAAAAGGACTATTCAAAC	TATGGACTTTGTGGTGGCAA	TTGGCACGTACTCCCATACA	60.0	441	INTERGENIC				
CaPOPII_626	Ca2	16514436	AC/A	TGTGCTGGAAAGGCACATAG	GTCTCCCCCACTACAGTCCA	59.9	812	INTERGENIC				
CaPOPII_627	Ca2	17061321	TAATATATA/TAATATATAAATATATA	TTTGGCTCGGAAGCTTTTTA	AGGAAGAGTGGAGTTGCGAA	60.0	498	INTERGENIC				

INDEL marker IDs	Chromosomes /unanchored scaffolds	Physical positions (bp)	InDels (*Kabuli* reference genome-CDC Frontier/PI)	Forward primers (5'-3')	Reverse primers (5'-3')	Anneling temperature (0C)	Expected amplified product size (bp)	Structural annotation		Functional annotation		
								Sequence components of *kabuli* genome	*Kabuli* gene accession IDs	NCBI-KOG	TFs	NCBI-nr database
CaPOPII_628	Ca2	17551687	G/GC	ATGTGGTTGATAAGGCCTCG	AGCAAATGCAATGCTCTGAA	60.0	398	CDS (FRAME SHIFT)	Ca_16000	OE		Peptidase S10, serine carboxypeptidase
CaPOPII_629	Ca2	18833380	TAAAAAAAAAAA/TAAAAAAAAAAAA	TCGAAAAATCCCTTGAAAAAGA	GGACCTATCAAGTGAGCTTGTG	60.0	551	INTERGENIC				
CaPOPII_630	Ca2	19219016	ATTTT/ATTT	CCCGTGAGATCCTCCTAACA	TGAATCTACGGCTTTCTGGG	60.1	498	INTERGENIC				
CaPOPII_631	Ca2	19387244	TTATATATATATATATAT/TTATATATATATATATAT	CGACATTGTAGCACCCCTTT	TTTAGATCCCTTGCACGACC	60.0	191	INTERGENIC				
CaPOPII_632	Ca2	19791685	C/CG	TAGGCCATTTTCGTCCTCAC	AATCCAATGACACAAACGCA	60.1	392	INTERGENIC				
CaPOPII_633	Ca2	20175480	GATATATATATATATATATA/GATATATATATATATATATATATA	TTTCAGAGTGTCTGTGGTTAGCA	GATCTGAGGTGTCATGGGCT	60.0	669	INTERGENIC				
CaPOPII_634	Ca2	20348649	AATATATATATATATATATAT/AATATATATATATATATATAT	TTCGTGGTTTACAAAATTCAATACA	GGTCGAAATTGGTGTTGGTC	59.7	664	INTERGENIC				
CaPOPII_635	Ca2	20474785	GAAAA/GAAAAA	ATACTGGCTCGGAGGCTTTT	CGTAGCGTGGTAGTGAGCAA	60.2	614	INTERGENIC				
CaPOPII_636	Ca2	20715795	T/TCATCA	GACATTGCATTTTGGACACG	AAATGGATTTCAGCCAATCG	60.0	539	INTERGENIC				
CaPOPII_637	Ca2	21314611	AG/AGG	CAAACCCTTTCAAGGTGCAT	GCACTGCCCCAAGAATTTTA	60.0	443	INTERGENIC				
CaPOPII_638	Ca2	21695844	GTTATTATTATTATTATTATTATTATTATTAATATTATTATTATTATTA/GTTATTATTATTATTATTATTATTATTATTATTAATATTATTATTATTATTA	GATGAAATTCAGGCTCGCTC	TTGACAAGGGAAAACCTTGC	59.9	444	INTERGENIC				
CaPOPII_639	Ca2	21717164	TA/T	GGTAGAGGCAGGCCATATCA	GATTTGTGAGGGATTTTTCAGC	60.1	841	INTERGENIC				
CaPOPII_640	Ca2	22468520	CAAAAAAAAAA/CAAAAAAAAAAA	TGGCCATGTGCATTGTTTAT	AGGCATCAACGCCTAAATTG	59.8	674	INTERGENIC				
CaPOPII_641	Ca2	22517081	ATTTTTTT/ATTTTTTTT	TGTGCAATTTCTCCACCAAA	CAGTTAACAGCCACCTTCCA	60.1	149	INTRON	Ca_14997	G		Phosphoglucose isomerase (PGI)
CaPOPII_642	Ca2	23050911	AATG/AATGGTCTATTGGCTTATG	TATGGGGATCGGAAATCAAA	ACAATCGAAATTTTAGGCCG	60.1	907	INTERGENIC				
CaPOPII_643	Ca2	23193466	TA/T	CCACAAATGCTCATCGTGAC	TTTGCAGCCACAAAAGACAG	60.1	561	INTERGENIC				
CaPOPII_644	Ca2	23509535	AAAAACTTAAAACTTAAAACTTA/AAAAACTTAAAACTTA	CCCTCTTGAATTTTTGTTTTCC	CCCACACCCTCGACTAAAGA	59.0	542	INTERGENIC				

INDEL marker IDs	Chromosomes /unanchored scaffolds	Physical positions (bp)	InDels (*Kabuli* reference genome-CDC Frontier/PI)	Forward primers (5'-3')	Reverse primers (5'-3')	Anneling temperature (0C)	Expected amplified product size (bp)	Structural annotation		Functional annotation		
								Sequence components of *kabuli* genome	*Kabuli* gene accession IDs	NCBI-KOG	TFs	NCBI-nr database
CaPOPII_645	Ca2	23932608	TAAATTAATGAATAAAAATTAAT/TAAATTAAT	AAGGCATCCAATCAAAGCAC	CTCAAGATTTTAGCGCCGTC	60.1	460	INTERGENIC				
CaPOPII_646	Ca2	23989802	AAAAACAAAACAAAA/AAAAACAAAACAAAACAAAA	AATTCGTTGAGCCGGTCTAGT	AGCACGGGGAGTATGAGAAA	60.1	421	INTERGENIC				
CaPOPII_647	Ca2	23990182	AAAAACAAAACAAAA/AAAAACAAAACAAAACAAAA	TCTCATACTCCCCGTGCTTT	ATCCCCTACACCAGATGCAC	59.7	565	INTERGENIC				
CaPOPII_648	Ca2	24251584	ACCCCC/ACCCCCC	GATTGCTTTCTGGGTGGAAA	TGATCAACCCTTGGTGTGAA	60.1	652	INTERGENIC				
CaPOPII_649	Ca2	24252139	TAA/TAAGAAA	GATCCTCAAATCGACCTCCA	TGTCACCATTGGTCTCCAAA	60.0	465	INTERGENIC				
CaPOPII_650	Ca2	24393200	G/GAAGATA	TCCTAGAACATGCATCACGC	TACCCTTTGCTAGCCCCTTT	59.8	505	INTERGENIC				
CaPOPII_651	Ca2	24943252	CTTT/CTT	AACCCCTCCAAAGAACGACT	AGGGCCCCACTAACTCCTTA	60.0	680	INTERGENIC				
CaPOPII_652	Ca2	25527401	GATAATAATAATAATAATAATAATAATAATAATAA/GATAATAATAATAATAATAATAATAATAATAATAA	ACCCTTCAATTTCGACATGC	CCAATTGGAATTTGCAACAA	59.9	598	INTERGENIC				
CaPOPII_653	Ca2	25554310	TTGTGTGTGTGTGT/TTGTGTGTGTGT	ATGCGGCAGGTATGAGAACA	ATATCAACGCCCTTTTGCAT	61.6	473	INTERGENIC				
CaPOPII_654	Ca2	26811414	TAAAAAAA/TAAAAAAAA	GGGCTTGTTTGATTGTGTTTT	GGCTCCATTCTTTGCTCTTG	59.0	584	INTERGENIC				
CaPOPII_655	Ca2	27776478	GTTTTTTTTTT/GTTTTTTTTTTT	CAATGAACTTGGTCAACCCC	GCCCATGTCTGCATAGTAACAA	60.2	407	INTERGENIC				
CaPOPII_656	Ca2	27776538	TAAAAAAA/TAAAAAA	CAATGAACTTGGTCAACCCC	GCCCATGTCTGCATAGTAACAA	60.2	407	INTERGENIC				
CaPOPII_657	Ca2	28037628	AATATATATATATATATATATATATATATAT/AATATATATATATATATATATATATATAT	CTAGTGTTGCACGGGTCAAA	CATAACGAATTCTCCCACCG	59.8	768	INTERGENIC				
CaPOPII_658	Ca2	28262473	GAA/GAAAA	CTGAGACACGGCGACAATAA	ATCGGAGCAATTCTGCATTC	59.9	305	INTERGENIC				
CaPOPII_659	Ca2	28365177	AATATATATATATATATATATAT/AATATATATATATATATATATAT	TCGGTCTCCTCATTTGATCC	GGGCCACATGTAATGCTTCT	60.0	768	INTERGENIC				
CaPOPII_660	Ca2	28465231	CAAAAA/CAAAA	TCATCGCAGGTGTACCATGT	GGTATCAGAGCCCACCATGT	60.0	908	INTERGENIC				
CaPOPII_661	Ca2	28465620	A/AT	AGCTCCATTGCAAAAACACC	GCAATTCCCACAGGTATGCT	60.1	553	INTERGENIC				

INDEL marker IDs	Chromosomes /unanchored scaffolds	Physical positions (bp)	InDels (*Kabuli* reference genome-CDC Frontier/PI)	Forward primers (5'-3')	Reverse primers (5'-3')	Anneling temperature (0C)	Expected amplified product size (bp)	Structural annotation		Functional annotation		
								Sequence components of *kabuli* genome	*Kabuli* gene accession IDs	NCBI-KOG	TFs	NCBI-nr database
CaPOPII_662	Ca2	29072040	ATCTGCAGACTTT/ATCTGCAGACTTTCTGCAGACTTT	TTGCTGGCAGAACAATCAAG	TGTGGGAAGCACCATGTTTA	60.0	546	DRR	Ca_14332			Protein of unknown function DUF707
CaPOPII_663	Ca2	29072081	TTATATATATATATATATATATATATATATA/TTATATATATATATATATATATATATATATA	TTGCTGGCAGAACAATCAAG	TGTGGGAAGCACCATGTTTA	60.0	546	DRR	Ca_14332			Protein of unknown function DUF707
CaPOPII_664	Ca2	29184413	GAAAAAAAAA/GAAAAAAAAAA	TGGTGGGTGATTGCTGATTA	AAGATTGGAACTTGGGCCTT	59.9	435	INTERGENIC				
CaPOPII_665	Ca2	29195958	CATATATATATATATATATA/CATATATATATATATATATATA	TTGTTTCGGAGGGTTGACTC	CCACCGATTGAAACATACCA	60.1	612	DRR	Ca_14321	PT	C3H	Cyclic nucleotide-binding domain
CaPOPII_666	Ca2	29220253	GATATATATATATATATATATATA/GATATATATATATATATATATATATA	AGGCATGGTGTTGGAGTCAT	CTAGTTCGTAACCCGTGCGT	60.4	253	INTERGENIC				
CaPOPII_667	Ca2	29240150	TC/TCACC	CTTAGAGCATCTCCAACGGC	GAAGCATCGTTCCTTCCTGA	60.0	134	INTERGENIC				
CaPOPII_668	Ca2	29240382	TCCC/TCC	CTTAGAGCATCTCCAACGGC	CCTTGCACTATCATTTTCGGA	60.0	803	INTERGENIC				
CaPOPII_669	Ca2	29252004	GTT/GT	TCAAAGGTGCACTCTTGCTATT	TGGCTTCCGGAAAGACTAAA	59.0	502	INTERGENIC				
CaPOPII_670	Ca2	29356033	CTTTTTTTTTTT/CTTTTTTTTTT	TGAAGTCAACCCGTCAACTG	AGGTGCAATTCCATCTACCG	59.7	638	INTERGENIC				
CaPOPII_671	Ca2	29417303	TA/TAA	CCCAGAAATCGATTAGGCAA	TCAACGTTCGTTTTGGTTCA	60.0	522	INTERGENIC				
CaPOPII_672	Ca2	29444244	ATTTT/ATTTTT	AAATCCGACTGTCTAACCCG	TTTTCTCCTCCAATTTAGTCCAA	59.1	826	INTERGENIC				
CaPOPII_673	Ca2	29445927	AACCCGAACCC/AACCCGAACCCACCCGAACCC	TGTCTAACCCGTCTAACCCG	CCCTACCTAGGGGTGGACAT	60.0	138	INTERGENIC				
CaPOPII_674	Ca2	29829940	TT/TTTCGAAAAAAT	CTTTTCGTCGGAGTTTGTCC	TCGTCCTCTGGTTTTCTCGT	59.7	476	INTERGENIC				
CaPOPII_675	Ca2	29847360	CTTTTTTTTTTTT/CTTTTTTTTTTT	AACGTTCTCCGTTTTTCAGC	GTGCGTCATGCAAATATGGT	59.4	838	INTERGENIC				
CaPOPII_676	Ca2	30050486	CAGAAGAAGAAGAAGAAGAAGAAGAA/CAGAAGAAGAAGAAGAAGAAGAAGAAGAA	AATCGGAGAAATGAAGCCCT	TTGAAAATGAAGGTGGGGTC	60.0	157	INTERGENIC				
CaPOPII_677	Ca2	30095996	TAAAAAAA/TAAAAAAAAA	CATTCCCGCTACATTTTGGT	GGAGAGAGGCCAATTTTTCA	59.8	226	INTERGENIC				
CaPOPII_678	Ca2	30096276	TA/TAAA	CATTCCCGCTACATTTTGGT	GAAGCTGCTTGTGTGCCATA	59.8	876	INTERGENIC				

INDEL marker IDs	Chromosomes /unanchored scaffolds	Physical positions (bp)	InDels (*Kabuli* reference genome-CDC Frontier/PI)	Forward primers (5'-3')	Reverse primers (5'-3')	Anneling temperature (0C)	Expected amplified product size (bp)	Structural annotation		Functional annotation		
								Sequence components of *kabuli* genome	*Kabuli* gene accession IDs	NCBI-KOG	TFs	NCBI-nr database
CaPOPII_679	Ca2	30096869	CATATATATATATATATATATATATATATATATATAT/CATATATATATATATATATATATATATATATATATATATATATATATATAT	CATTGCAGGTGCTATGGTTG	TTCTCTTATGAAGCGGAGGA	60.1	759	INTERGENIC				
CaPOPII_680	Ca2	30341034	TAA/TAAA	TGTCTTATCCTGAACTCAAACTCG	ATTTGGTCAACCACCCAAAA	59.8	537	INTERGENIC				
CaPOPII_681	Ca2	30352364	ATTTTTTTT/ATTTTTT	TAAAAAGTGGTTCGGTTCGG	TTATTTTTCCCTTTTGGCCC	60.0	826	INTERGENIC				
CaPOPII_682	Ca2	30369374	TAAAA/TAAAAA	TGTACTTCCTCCGATTCCAAA	TGAATGACGACGACGGTAAA	59.6	632	DRR	Ca_12515	Q		Multicopper oxidase, type 1
CaPOPII_683	Ca2	30395203	CATATATATA/CATATATATATA	CCCAACAACAACAAAAGCCT	AAACCAGCATGACAAAAGCA	60.0	289	INTERGENIC				
CaPOPII_684	Ca2	30399984	CATATATATATATATATAT/CATATATATATATATAT	AGGTGGGCCCATTACTTGAT	AGTGGAGGACCACTTCCTCA	60.6	836	INTERGENIC				
CaPOPII_685	Ca2	30401507	TT/TTGT	GCATCACAAAGGGTTTTTGG	TCGATGCGAATACAACTAAATCA	60.3	610	INTERGENIC				
CaPOPII_686	Ca2	30402988	ATT/AT	TACCGGATTGGTAACGTGGT	GCGTTTGATGCATATTTTCG	60.1	419	DRR	Ca_12511			
CaPOPII_687	Ca2	30403193	ATTGAGTT/ATTGAGTTGAGTT	AAAGTTGCACCAAGAGGTGG	TCTCTGGCCTTCGTTAGTCAA	60.1	989	DRR	Ca_12511			
CaPOPII_688	Ca2	30415849	CTTTTTT/CTTTTT	GCAACAACCCGTTGAAGATT	GATGGGATATGGACACCGTC	60.0	472	INTRON	Ca_12510			Signal peptidase complex subunit 2
CaPOPII_689	Ca2	30433806	CTTTT/CTTTTT	CGGTTGGCCCTTAACTGTAA	TCCTCAAACCCAAAATGGTC	60.0	674	INTERGENIC				
CaPOPII_690	Ca2	30445916	TAAAAAA/TAAAAAAA	CAAAAATATATCGATCGCAACAA	AATTGAAAGCTGCATTGGCT	59.0	406	INTERGENIC				
CaPOPII_691	Ca2	30447122	CATATATATATATAT/CATATATATATATATATATATATATAT	TGGTGCACACAATACAATCAAA	CAAGGCCGAGAAGACAAAAG	59.9	225	INTERGENIC				
CaPOPII_692	Ca2	30447611	A/AT	CTTTTGTCTTCTCGGCCTTG	CGTGCTCATCTCCATTGTTG	60.0	891	INTERGENIC				
CaPOPII_693	Ca2	30447653	AC/A	CTTTTGTCTTCTCGGCCTTG	ATCTCCTCAGCATTGTCGCT	60.0	943	INTERGENIC				
CaPOPII_694	Ca2	30449164	TAAAAAAA/TAAAAAAAA	TGTTGCAAAGAGAAAGAACTTCA	TGACGGTGGCAATCGATATT	59.2	557	DRR	Ca_12507			
CaPOPII_695	Ca2	30461229	AG/A	TCCCATTCAATCAAGACGTG	TTTTTGTCTCACATTGATTGTCTTT	59.5	666	INTERGENIC				

INDEL marker IDs	Chromosomes /unanchored scaffolds	Physical positions (bp)	InDels (*Kabuli* reference genome-CDC Frontier/PI)	Forward primers (5'-3')	Reverse primers (5'-3')	Anneling temperature (0C)	Expected amplified product size (bp)	Structural annotation		Functional annotation		
								Sequence components of *kabuli* genome	*Kabuli* gene accession IDs	NCBI-KOG	TFs	NCBI-nr database
CaPOPII_696	Ca2	30463432	TAAAAAAAAAA/TAAAAAAAA	ATTCTCAACCCTTGGTGTGC	AGTGTTTGTTTGGGAGGTGG	60.0	670	INTERGENIC				
CaPOPII_697	Ca2	30464150	CTT/CTTT	CCACCTCCCAAACAAACACT	GGCAAGTTGCTTTTACCAAAAC	59.9	602	INTERGENIC				
CaPOPII_698	Ca2	30481983	ATATTATTT/ATATTATTTAAATTATTATTT	TCAATGCATGGAAAATAATCTCT	TGGTCCTCATCCATCAAACA	57.8	401	INTERGENIC				
CaPOPII_699	Ca2	30484802	A/ATT	ACATGAGACCGTCAATGCAA	CGAAAAATCAAAATAACATCGC	60.1	386	INTERGENIC				
CaPOPII_700	Ca2	30494831	GAAAAAAAAA/GAAAAAAAAAA	TGAGAACGCCCATGTCAATA	TCTCTGGCAACCGTTCTTCT	60.1	404	INTERGENIC				
CaPOPII_701	Ca2	30510130	ATTTTTTTTTTTTTTT/ATTTTTTTTTTTTT	AGACCCCACGGTTAACATGA	AGCACCTTACAAGGGGGAGT	60.2	700	DRR	Ca_12500			
CaPOPII_702	Ca2	30520425	GAAAAAAAAA/GAAAAAAAAAAA	TTGACGTAGGGTCTTGGGTC	ATGTCCTGCTTCCATTCCAC	60.0	745	INTERGENIC				
CaPOPII_703	Ca2	30526988	GAAAA/GAAA	CTCGTGAAGGATTAGGTCGG	TTGCAACGCATCTCAATCTC	59.7	353	INTERGENIC				
CaPOPII_704	Ca2	30553379	GAAAA/GAAAAA	TTTTTATGAGGCGTGGAAGG	CCATGAACACGCCAAACATA	60.1	874	INTERGENIC				
CaPOPII_705	Ca2	30553679	TAAAAAAA/TAAAAAA	TGCACTTTATCACTTGGTAATCG	TGTTTGCGAGCTTCAATGAC	59.2	561	INTERGENIC				
CaPOPII_706	Ca2	30559073	AG/A	TGCATACAAAAGTCACCCCA	AAAAATTATGGGCTTTGGGC	60.0	982	INTERGENIC				
CaPOPII_707	Ca2	30566132	AT/ATT	CAGAGACAAAAGAGGGAACCA	TTGTGTTGTTGTTGTGTGCG	59.3	884	INTERGENIC				
CaPOPII_708	Ca2	30567133	TAAAAAAAA/TAAAAAAA	AAATTACCGCAACAATTGGC	GAAGCGCAACTAAACCAAGC	59.8	539	INTERGENIC				
CaPOPII_709	Ca2	30569240	AT/ATT	AGTTTTGCAGCTTTTGCCTC	CCGTCCAATTTTCTCTTGGA	59.6	609	INTRON	Ca_12495	E		Amino acid transporter, transmembrane
CaPOPII_710	Ca2	30578105	AC/A	GCCCCTGCACTAGTTTGAAG	GGCTTGTTAGTCTGCTTGCC	59.9	583	DRR	Ca_12493			Peptidase M50
CaPOPII_711	Ca2	30622722	A/ACCGTAATCC	CTTGGTCAGAATCACGGAGC	CACGTTGACCCTTTTTGTCA	60.8	380	INTRON	Ca_12490	R		Yippee-like protein
CaPOPII_712	Ca2	30665443	ATTTTTTTTTT/ATTTTTTTT	TCATGGTGGACCGTAATCTG	CTACTGCCTTGGATTCCTTTT	59.4	403	INTRON	Ca_12489	QI	bHLH	Cytochrome P450

INDEL marker IDs	Chromosomes /unanchored scaffolds	Physical positions (bp)	InDels (*Kabuli* reference genome-CDC Frontier/PI)	Forward primers (5'-3')	Reverse primers (5'-3')	Anneling temperature (0C)	Expected amplified product size (bp)	Structural annotation		Functional annotation		
								Sequence components of *kabuli* genome	*Kabuli* gene accession IDs	NCBI-KOG	TFs	NCBI-nr database
CaPOPII_713	Ca2	30671540	TAAAAAA/TAAAAA	TTCGATTGATGGTTAAGAGACA	ATAACGTGTTGGATCAGCCC	57.4	697	DRR	Ca_12489	QI	bHLH	Cytochrome P450
CaPOPII_714	Ca2	30676605	GAAAAA/GAAAA	GCTCCAGCAAAAATAGAAATGG	TCATCAATCAAACTACTTACGCAGA	60.1	504	INTERGENIC				
CaPOPII_715	Ca2	30676782	ACTAGC/ACTAGCTAGC	GCTCCAGCAAAAATAGAAATGG	TCATCAATCAAACTACTTACGCAGA	60.1	504	INTERGENIC				
CaPOPII_716	Ca2	30704638	TA/TAA	AAATTCAGTGCAACGCACAT	CCCACTTTGGATAACAATTCC	59.2	445	INTERGENIC				
CaPOPII_717	Ca2	30742309	CAGAGAGAG/CAGAGAGAGAGAGAG	CGTTGCGGTAACAAACAATG	TTGGAGAAAAGGCAGGCTTA	60.0	349	INTERGENIC				
CaPOPII_718	Ca2	30742679	TAAAAAA/TAAAAAAAA	TGTTTGCTTCATTGGCAGAG	AAATTTGATGTAGAATATAACCCAAAA	60.0	674	INTERGENIC				
CaPOPII_719	Ca2	30771512	TAAAAA/TAAAA	GACCACCTTTGTGGACCAGT	AATTTGATGGTTATGGGCGA	59.9	713	INTRON	Ca_12479	K	ERF	NLI interacting factor
CaPOPII_720	Ca2	30794400	CTTTTTTT/CTTTTTT	GAGTTGAATGCAGCTGACCA	TGCATTTCACTGGAGACGTT	60.0	741	INTERGENIC				
CaPOPII_721	Ca2	30805003	T/TA	TGCTGAATTGGATAGAGGGG	TTTTTGGTTTGTGTACATTTGTTTT	60.0	838	INTERGENIC				
CaPOPII_722	Ca2	30806790	T/TA	GAAGTCAACATGTGCATGGG	TTTAAAATTATGCGACGGCA	60.0	606	INTERGENIC				
CaPOPII_723	Ca2	30840723	TC/T	GCATGCGAACATGAATTTTG	GGCCACTCATGTTGTCTCCT	60.1	788	DRR	Ca_12475			
CaPOPII_724	Ca2	30844156	TTTAA/TTTAATTAA	TCCACACTCTTGTAGGTTGCAT	CAGAATCAGCACGCAAACAT	59.7	854	INTERGENIC				
CaPOPII_725	Ca2	30849743	ATT/ATTT	TCAACTTAGATATGCCGTCCG	TGGGTTGGTTTGAATCATCA	60.1	410	INTERGENIC				
CaPOPII_726	Ca2	30861518	GAAA/GAAAA	CCAAAGGTGGGTGAAGAAGA	CAGGCCCAATTCACAAAGTT	60.1	554	INTERGENIC				
CaPOPII_727	Ca2	30865173	GAAAAAAAA/GAAAAAAA	GCTGAGGGATTTCGAAAGTAA	ATAATACCCCTCAGGGTGGC	58.5	576	INTERGENIC				
CaPOPII_728	Ca2	30890128	AAAATAAATAAATAAATAAA/AAAATAAATAAATAAA	GATGATGTAAATGGGAGCCAA	CATTTGTTGAATGTTGCCCA	59.8	697	INTERGENIC				
CaPOPII_729	Ca2	30890182	AAA/AAAGAA	GATGATGTAAATGGGAGCCAA	GGAATTGATCCACAACTCGG	59.8	958	INTERGENIC				

INDEL marker IDs	Chromosomes /unanchored scaffolds	Physical positions (bp)	InDels (*Kabuli* reference genome-CDC Frontier/PI)	Forward primers (5'-3')	Reverse primers (5'-3')	Anneling temperature (0C)	Expected amplified product size (bp)	Structural annotation		Functional annotation		
								Sequence components of *kabuli* genome	*Kabuli* gene accession IDs	NCBI-KOG	TFs	NCBI-nr database
CaPOPII_730	Ca2	30897629	TAAAAAAAA/TAAAAAAA	CCAACAGATCAAGGGGAATG	ATCGAGATCTCACCGTGGAA	60.3	548	INTERGENIC				
CaPOPII_731	Ca2	30915268	A/ACAATCACTAAG	GAAGTGAAGTGGTTCTTTGCG	CAAGACCTTGTGGCTCATCA	59.9	783	INTRON	Ca_12470	DO		
CaPOPII_732	Ca2	30916172	TCC/TCCC	TGAATTGCCACAGGTCTTGA	TAAAGAGTGGCACCCCTCAC	60.2	411	INTRON	Ca_12470	DO		
CaPOPII_733	Ca2	30933751	TC/T	TCGAAACGTGTGGTGATGAT	ACAATTCCACGCAATGTGAG	60.0	895	INTERGENIC				
CaPOPII_734	Ca2	30936877	TAAAAAAA/TAAAAAAAA	TTGCACACAAGCGTTAGAGC	ATCAACCGCACACAATTCAA	60.2	393	INTERGENIC				
CaPOPII_735	Ca2	30968038	CAAA/CAA	GTGTGCGAAAGGTTGGTTTT	CGAACCTACCCGATCAAGAA	60.0	168	INTERGENIC				
CaPOPII_736	Ca2	30968401	CAAAAAAAA/CAAAAAAAAA	TTCTTGATCGGGTAGGTTCG	ATTTTGCTCCCGTCTCCTTT	60.1	507	INTERGENIC				
CaPOPII_737	Ca2	31240228	TCAA/T	CTGCCCCCACTATTTCTCAA	GAAACACTTGGCATGTTAAGGA	60.1	479	INTERGENIC				
CaPOPII_738	Ca2	31323113	GATATATATATATATATATA/GATATATATATATATATATA	TGTTCACCACCAAACTGGAA	TTCATTTTAAAGGCATCGTGA	60.0	901	INTERGENIC				
CaPOPII_739	Ca2	31846061	TTTATTATTATTATTATTATTATTATTATTATTATT/TTTATTATTATTATTATTATTATTATTATTATTATT	GATTGGAGCACCATCCTCTC	TTCGAGACAAAACGTTCAACC	59.6	805	INTERGENIC				
CaPOPII_740	Ca2	31858178	TTTAATATT/TTT	TGTGTTTCAGAGCTGCTAGTTGA	ATGAAAGGCATCCCAAAACA	60.2	368	INTERGENIC				
CaPOPII_741	Ca2	31882952	CTTTATTTATTTAT/CTTTATTTATTTATTTAT	TTTCTGGTGGGATCAGCTCT	GACATCCTGCGCCAGTTATT	59.8	591	INTRON	Ca_17830	D	LBD	RZZ complex, subunit Zw10
CaPOPII_742	Ca2	31883010	AAGAG/AAG	TTTCTGGTGGGATCAGCTCT	GACATCCTGCGCCAGTTATT	59.8	591	INTRON	Ca_17830	D	LBD	RZZ complex, subunit Zw10
CaPOPII_743	Ca2	31883642	TAA/TAAA	AATAACTGGCGCAGGATGTC	TAAAGATGGCACCAGGAAGG	60.1	632	INTRON	Ca_17830	D	LBD	RZZ complex, subunit Zw10
CaPOPII_744	Ca2	31939517	ACT/ACTCT	TCTTAAAACCCAAACCGTGAA	GAGCTTTTGGGGGATAGAGG	59.5	490	DRR	Ca_17823	R	C3H	Zinc finger, CCCH-type
CaPOPII_745	Ca2	31963928	GTACTT/GTACTTACTT	CCTCGAGAAACCCAGCTAGA	GCATTCATGAGTTTGCCCTT	59.6	187	INTERGENIC				
CaPOPII_746	Ca2	31964191	AT/ATT	AAGGGCAAACTCATGAATGC	CAAGGAAGGAGGAAAAGGAGA	60.1	609	INTERGENIC				

INDEL marker IDs	Chromosomes /unanchored scaffolds	Physical positions (bp)	InDels (*Kabuli* reference genome-CDC Frontier/PI)	Forward primers (5'-3')	Reverse primers (5'-3')	Anneling temperature (0C)	Expected amplified product size (bp)	Structural annotation		Functional annotation		
								Sequence components of *kabuli* genome	*Kabuli* gene accession IDs	NCBI-KOG	TFs	NCBI-nr database
CaPOPII_747	Ca2	31964265	TG/T	AAGGGCAAACTCATGAATGC	GGTGTGACAAAGGAAACGTG	60.1	674	INTERGENIC				
CaPOPII_748	Ca2	31981326	AT/ATTT	ACGTTGTGTGGACAATGAGG	TCCGGAGGGTTTTTCTACCT	59.4	353	INTERGENIC				
CaPOPII_749	Ca2	31983759	ATTTTTTTTT/ATTTTTTTTTTT	GACACTTGTTGCGTATGGGA	CAATGGAGATTCCAACCTGT	59.6	555	INTERGENIC				
CaPOPII_750	Ca2	32032083	ATATTTTTATTTTT/ATATTTTTATTTTTATTTTT	TGACAACGCATACGTGGATT	AAATAACACGAAATTCTAGCCCA	60.0	312	INTERGENIC				
CaPOPII_751	Ca2	32048288	AC/A	CCAAATCGAACTCTCTCCTCA	CGTGATGCACCGATAAATTG	59.4	634	DRR	Ca_17817	R		
CaPOPII_752	Ca2	32048344	CTTTTTT/CTTTTTTT	CCAAATCGAACTCTCTCCTCA	CGTGATGCACCGATAAATTG	59.4	634	DRR	Ca_17817	R		
CaPOPII_753	Ca2	32069395	TTATATATATATATAT/TTATATATATATATATATAT	ACCCCGTTGATCATAAGTCG	AAAATCCTGGCAGTCATCGT	59.8	922	DRR	Ca_17815	U		
CaPOPII_754	Ca2	32070956	TAAAAAAAAAAAA/TAAAAAAAAAAAAAA	TTTTTCCGACTTGGTCCTTT	GGAGCTGAAACTAACGCAGC	58.7	814	INTERGENIC				
CaPOPII_755	Ca2	32110176	AAAACAAA/AAAA	GCACCCTTCAAGAAGCAAAC	TTGGTCCATCGAATCACTTG	59.9	221	INTERGENIC				
CaPOPII_756	Ca2	32127033	TGC/TGCGC	TGCAAGTTGTGATGGGTTGT	TGTCTGTTCTGCACGGAGTC	60.0	650	INTRON	Ca_17812	O		Ubiquitin
CaPOPII_757	Ca2	32130215	ATTGAA/ATTGAATTTGAATTTGAA	CCGGGTCAGTTTCCTTCTTT	ACTCCACCAACTTCCAATGC	60.5	388	INTRON	Ca_17811	J		Phenylalanyl-tRNA synthetase
CaPOPII_758	Ca2	32145004	TA/TACA	GGCCTTCAAAATTCAATGAGA	TGGATACCTAGCACCTTAGCAA	59.1	598	INTRON	Ca_17808		G2-like	Peptidase S8/S53, subtilisin/kexin/sedolisin
CaPOPII_759	Ca2	32152633	T/TA	GAGTTGGGTAGGGTAGGGGA	TTTCCCCTTTTGCTTGTCAT	60.2	393	INTERGENIC				
CaPOPII_760	Ca2	32173774	TC/T	ATGTTTTGATTGGGGCTTTG	TAAATCAGCCCTTTTGGTGG	59.8	631	DRR	Ca_17806		WRKY	Glycoside hydrolase, family 9
CaPOPII_761	Ca2	32209131	T/TG	GCAAAGATAGACACCACCCC	AACCATTCCCATGGAACAAA	59.4	577	INTERGENIC				
CaPOPII_762	Ca2	32209334	CTATATATA/CTATA	TCCATGGGAATGGTTTTGAT	TTGAAGAGGAAGTTTTGGCA	60.0	342	INTERGENIC				
CaPOPII_763	Ca2	32209671	TC/TCC	TCCATGGGAATGGTTTTGAT	TTTTAGTGTGCACCAATGTCAA	60.0	899	INTERGENIC				

INDEL marker IDs	Chromosomes /unanchored scaffolds	Physical positions (bp)	InDels (*Kabuli* reference genome-CDC Frontier/PI)	Forward primers (5'-3')	Reverse primers (5'-3')	Anneling temperature (0C)	Expected amplified product size (bp)	Structural annotation		Functional annotation		
								Sequence components of *kabuli* genome	*Kabuli* gene accession IDs	NCBI-KOG	TFs	NCBI-nr database
CaPOPII_764	Ca2	32210099	CA/CAAATA	TCAGCTTCAATTATCTCCAACAA	TCGGCGACAAAAATCACATA	58.9	644	INTERGENIC				
CaPOPII_765	Ca2	32213171	GTGAT/GTGATGAT	CAACATGACAAGGACTTTCCAA	TTACCGGTATCGCAAGGAAC	60.0	529	INTERGENIC				
CaPOPII_766	Ca2	32213424	CAAA/CAAAA	AGGGATAGATAACGGCACGA	AAGGCCCTGCTAGAATGATG	59.6	504	INTERGENIC				
CaPOPII_767	Ca2	32218161	GTTTTTTTTTTT/GTTTTTTTTTT	ATTGTGCCGGAGCAACTACT	TGTTACGGCTGTGAATCTGG	59.8	138	INTERGENIC				
CaPOPII_768	Ca2	32245950	TA/TAGA	AACCGACAAAAGATTGCATGT	GCAGTTGCACAAGGTGGTAA	59.5	586	INTERGENIC				
CaPOPII_769	Ca2	32260990	GAGAA/GA	TGTTGTTTCCTCCAAACCCT	TTTTACCTTCTTTTCCGGGG	59.4	157	INTERGENIC				
CaPOPII_770	Ca2	32261025	AAAGAGAAGAGA/AAAGAGAAGAGAAGAGA	CCCCGGAAAAGAAGGTAAAA	CCTCTCTGTCTCCACCTGCT	60.3	126	INTERGENIC				
CaPOPII_771	Ca2	32293814	CT/CTTTGT	TGTATTTGGACGGGTGACAA	TACACCCACGAAACGTGAAA	59.8	571	INTERGENIC				
CaPOPII_772	Ca2	32347455	ATTTTTTT/ATTTT	ACAAGCTGTTCCCTCCAGAA	TGAGGTTCTTGCACAACTGC	59.8	678	INTRON	Ca_10283			
CaPOPII_773	Ca2	32356323	CAAA/CAAAA	GCTAGTTGCTGTGAATGGCA	ACGTGTGGATGATTGGATGA	60.0	711	INTERGENIC				
CaPOPII_774	Ca2	32366421	ATCAAAATTCAAAATT/ATCAAAATT	TCGTGACTTTGTCCATCATTTC	GCTTGGCAACATTCTTCACA	60.0	739	INTERGENIC				
CaPOPII_775	Ca2	32370223	AAAAAATTAATGAAAAATTAATGAAAAATTAA/AAAAAATTAATGAAAAATTAA	TCAAGCAAGTCACAACATTTCA	TGCTATTTTAATTGTGAGCATGAGA	59.4	637	INTERGENIC				
CaPOPII_776	Ca2	32378634	GTTTTT/GTTTT	GTGGAAGGTCGGTGGTCTTA	ACCACACCCAATCCTTTCAA	60.0	144	INTERGENIC				
CaPOPII_777	Ca2	32379585	AATTAATTTT/AATTAATTTTGATTAATTTT	TGTGCGAGTTTATTGCGGTA	CATTTGGTTCAAAATGTCATGG	60.3	624	INTERGENIC				
CaPOPII_778	Ca2	32393117	T/TC	TGCCCATCCTTATGCTCTTC	ATCACGTGCAACCACAAGTC	60.2	626	INTERGENIC				
CaPOPII_779	Ca2	32393187	AATAT/A	TGCCCATCCTTATGCTCTTC	ATCACGTGCAACCACAAGTC	60.2	626	INTERGENIC				
CaPOPII_780	Ca2	32393441	TGG/TGGG	TCGCACTTTTTCGTGTAGGA	GGGCTTGTTTGACGCATTAT	59.5	588	INTERGENIC				

INDEL marker IDs	Chromosomes /unanchored scaffolds	Physical positions (bp)	InDels (*Kabuli* reference genome-CDC Frontier/PI)	Forward primers (5'-3')	Reverse primers (5'-3')	Anneling temperature (0C)	Expected amplified product size (bp)	Structural annotation		Functional annotation		
								Sequence components of *kabuli* genome	*Kabuli* gene accession IDs	NCBI-KOG	TFs	NCBI-nr database
CaPOPII_781	Ca2	32393633	TAACATAATAA/TA	GACTTGTGGTTGCACGTGAT	GGGCTTGTTTGACGCATTAT	59.6	445	INTERGENIC				
CaPOPII_782	Ca2	32409946	ATTTTTT/ATTTT	TGGAGAGTTGAGAAGCCAGG	TACTCGAGGCCTCTCGCTAC	60.5	850	INTRON	Ca_10279		bHLH	Protein kinase, catalytic domain
CaPOPII_783	Ca2	32436418	CG/C	TCACATGGTGGCATCCTAGA	CGAGTCATCCATGCTCTGTT	60.1	302	DRR	Ca_10275	O	C2H2	Ubiquitin fusion degradation protein UFD1
CaPOPII_784	Ca2	32453109	ATTTTTTT/ATTTTTTTTT	TCTTCACACAATCCCACCAA	CTCAAGTCGACAAGCCTTCC	59.9	659	INTERGENIC				
CaPOPII_785	Ca2	32477117	TTTGTT/TTTGTTGTT	CTGCAATGATAATTGGGGCT	CAACTGCAATGTTCCATATTCG	59.9	555	DRR	Ca_10273	R	C3H	Leucine-rich repeat
CaPOPII_786	Ca2	32510389	TGGGGGGGGGG/TGGGGGGGGG	TACTGGCTGATTTGCGACAG	CATATTTGACAAGGACCGGG	60.0	880	INTRON	Ca_10271	O	GATA	Peptidase C19, ubiquitin carboxyl-terminal hydrolase 2
CaPOPII_787	Ca2	32520939	ATTTTTTT/ATTTTTT	AACATGGAAGATCGGTTGGA	GATCAATGCGAGTTTGTTGG	60.3	318	INTERGENIC				
CaPOPII_788	Ca2	32713890	AAAAACAAAA/AAAAACAAAACAAAA	AACGCATCGGTAAACACTCC	TGTTTTGCAGCAAGATGTCC	60.0	695	INTERGENIC				
CaPOPII_789	Ca2	32934778	TTAAATAAATAAATAAATAAA/TTAAATAAATAAATAAATAAATAAA	ACAGCGACACGAAGGACTCT	GGAACAAGTTTCCGTGAAGG	60.1	396	INTRON	Ca_10230	OJ	MYB_related	Protein of unknown function DM15
CaPOPII_790	Ca2	33257195	ATTTTTTTTT/ATTTTTTTTTT	ATCGCATTTTGCCTTTGTTT	GCTGTCGTTTGCAGCATAAA	59.6	801	INTERGENIC				
CaPOPII_791	Ca2	33278516	ATGTTGTTGTTGTTGTT/ATGTTGTTGTTGTTGTTGTT	AGGGGTCACATGGTTTTGAA	AGGTGGTTGCTGATAGGTGG	60.2	335	INTERGENIC				
CaPOPII_792	Ca2	33405227	AATATATATATA/AATATATATA	AAAGAGACATGACTTTGTCCATCA	TTTCGCTACCCATCATACCC	60.0	586	INTERGENIC				
CaPOPII_793	Ca2	33984310	CTATATATATATATATATATATATA/CCATATATATATATATATATATATATATA	TGGCTATGATCACAATTGGC	CCCTTTGCAAGAGACTCCAA	59.5	869	INTERGENIC				
CaPOPII_794	Ca2	33984629	CAAAA/CAAA	AACAAAGCAGAAATATCCTGTCG	AGCATAGCCAAAACCCCTTT	59.7	389	INTERGENIC				
CaPOPII_795	Ca2	33986265	TT/TTATAT	AACTTTGGATCTACACGCGA	ACTCCAAGATCATCATCGGC	58.4	623	INTERGENIC				
CaPOPII_796	Ca2	34028835	AC/A	AAAGGGGTTTTGGCTATGCT	CATGATCATAATTGCATGGAAAA	60.0	229	INTERGENIC				
CaPOPII_797	Ca2	34087701	CATATATATATATATATAT/CATATATATATATATATATATAT	ATGGTGGTGAGCAACTTTCC	AACAAACAGCCATTCCAAGG	60.0	707	INTERGENIC				

INDEL marker IDs	Chromosomes /unanchored scaffolds	Physical positions (bp)	InDels (*Kabuli* reference genome-CDC Frontier/PI)	Forward primers (5'-3')	Reverse primers (5'-3')	Anneling temperature (0C)	Expected amplified product size (bp)	Structural annotation		Functional annotation		
								Sequence components of *kabuli* genome	*Kabuli* gene accession IDs	NCBI-KOG	TFs	NCBI-nr database
CaPOPII_798	Ca2	34272124	AATATATATATATATATATATATATATA/AATATATATATATATATATATATATA	GGCACCTCATATGCAACCTT	TGGCACCAAACTTACACCAA	60.0	652	DRR	Ca_15251		WRKY	DNA-binding WRKY
CaPOPII_799	Ca2	34353539	CATATATATATATATATATATATATATATAT/CATATATATATATATATATATATATATATATAT	CATGAGCTTGAGTCGCAAAA	TAGGAGGCATATGTGAGGGG	60.1	760	INTERGENIC				
CaPOPII_800	Ca2	34353805	GTTTTTTTT/GTTTTTTTTT	AGTTGAAGGAGTGCATGAGGA	TAGGAGGCATATGTGAGGGG	59.9	633	INTERGENIC				
CaPOPII_801	Ca2	34355206	CTTGGGCT/CTTGGGCTTTGTTGGGCT	TCTTTGATGCAATGCCAGAG	ATCATTCGAGGTGCCATCAT	59.9	975	INTERGENIC				
CaPOPII_802	Ca2	34355457	TAAAAAA/TAAAAAAA	ATTCTTGGGCTGTGTTTTGG	GGGTGATGAGGACTGGTTGT	60.0	725	INTERGENIC				
CaPOPII_803	Ca2	34357732	CATATATATATATATATATATATATATATAT/CATATATATATATATATATATATATATATATATATATATATATAT	CATGAGCTTGAGTCGCAAAA	GCAACAAGATGGGGACAAGT	60.1	654	INTERGENIC				
CaPOPII_804	Ca2	34450150	TTATATATATATATATATATATATATATAT/TTATATATATATATATATATATAT	AGCTAGCTCAGAATGCAGCC	CCCACAAAACTTGCTTCGTT	59.9	324	INTERGENIC				
CaPOPII_805	Ca2	34562775	TCTTTA/TCTTTACTTTA	ATAGCGTGCAACGATCCTCT	CATAGCGCCCATAAAAGGAA	59.9	515	INTERGENIC				
CaPOPII_806	Ca2	34683764	TC/TCC	GTTTTTCGCAACCACCTTTC	CGCTTTTTGCACTAACCTCC	59.6	682	INTERGENIC				
CaPOPII_807	Ca2	34684386	CAACGTTAAC/CAAC	ACAATACTGCGGCCTGTTTC	GATTCCGATCGTTTTTCACG	60.1	830	INTERGENIC				
CaPOPII_808	Ca2	34684591	TTATATATATATATATATATATATATATATA/TTATATATATATATATATATATATATATATA	TGGAGTTTTGTGTGATGGACA	GATTCCGATCGTTTTTCACG	60.0	758	INTERGENIC				
CaPOPII_809	Ca2	34684688	CATCTT/C	CGTACGTAGCAAATGCAACAA	ATTCCGATCGTTTTTCACGA	59.8	229	INTERGENIC				
CaPOPII_810	Ca2	34684923	ATTTTTTT/ATTTTTTTT	TCGTGAAAAACGATCGGAAT	TGCTTGATTTTAATGGATGCC	60.4	550	INTERGENIC				
CaPOPII_811	Ca2	34685695	ATTTTTT/ATTTTTTT	CCCCATTTTGTTTCTCTCCA	TTGTTTCTCCCCAGTTTTGG	59.9	234	INTERGENIC				
CaPOPII_812	Ca2	34983521	AATATATATATATATATATATATATATATATATATATATATA/AATATATATATATATATATATATATATATATATA	CAAATGCATGCTCCCCTAAT	CTAAAAGCCCAAAGCTGCAC	59.9	450	INTERGENIC				
CaPOPII_813	Ca2	35258590	A/ATT	AAAAAGTTTTAACACGGAGGTTTTT	TGTTGTTGGATTGGACATGG	59.8	487	INTERGENIC				
CaPOPII_814	Ca2	35288109	ATTTTTT/ATTTTTTT	TGCTTTATAGTGCTATTATGTGCCA	CGTCATTAAAAGGGAAACCAA	60.1	757	INTERGENIC				

INDEL marker IDs	Chromosomes /unanchored scaffolds	Physical positions (bp)	InDels (*Kabuli* reference genome-CDC Frontier/PI)	Forward primers (5'-3')	Reverse primers (5'-3')	Anneling temperature (0C)	Expected amplified product size (bp)	Structural annotation		Functional annotation		
								Sequence components of *kabuli* genome	*Kabuli* gene accession IDs	NCBI-KOG	TFs	NCBI-nr database
CaPOPII_815	Ca2	35303488	ATTTTTT/ATTTTTTT	TTCCGAATAAGGATTTCTTGG	ATGGTTTGGGGAAAAGAACC	58.2	137	INTERGENIC				
CaPOPII_816	Ca2	35304698	C/CG	GCTTGGAAACAGCCTCAGAC	CAGGGTGGTACTGCAATGTG	60.0	536	INTERGENIC				
CaPOPII_817	Ca2	35329169	AATATATATATATATATATATATATAT/AATATATATATATATATATATAT	CAATTTTGGAAGACGATTTTGA	TGTCCCGAAAAAGTTCATCC	59.1	330	INTERGENIC				
CaPOPII_818	Ca2	35425064	GAAAAAAAAAA/GAAAAAAAAAAA	TGTCGAAATGATTAGCAAAAGAA	ACATTTTCCCTCCCTTGGTC	58.9	257	INTERGENIC				
CaPOPII_819	Ca2	35581740	AATATATATATATATATATATATATATA/AATATATATATATATATATATATATATA	TTGCCACTTGATACAGCAAAA	TTGAAAGGATAACATGGCCG	59.4	690	INTERGENIC				
CaPOPII_820	Ca2	35789864	AATTTGATTT/AATTTGATTTGATTT	TGCCTGCGAGATCTTAATCA	CCAAATCGAACCCAAATCAC	59.5	365	INTERGENIC				
CaPOPII_821	Ca2	35815027	ATTTTTTTT/ATTTTTTT	TAAGACCATGCATCTCACGC	CGTGCATGGATCAAACTAGG	59.8	650	INTERGENIC				
CaPOPII_822	Ca2	35834317	TTATATATATATATATATATATATAT/TTATATATATATATATATATATAT	CAAACCGTATTAGCCTCACCA	GGTTGAAGATGTTCGTCGGT	60.0	675	INTRON	Ca_09758	O	MYB	Heme A synthase/Protoheme IX farnesyltransferase
CaPOPII_823	Ca2	35846531	ATT/ATTT	TTATTTGTTCGGTGGCAACA	TGATCGGAATGCGAATAACA	60.0	455	DRR	Ca_09760	J		Ribosomal protein L47, mitochondrial
CaPOPII_824	Ca2	35853696	AAATAA/AAA	GTTGCCCAGCTTGTTCTTTC	GGGAGAGTGGGTGTTTATGC	59.9	404	INTERGENIC				
CaPOPII_825	Ca2	35892945	CAAA/CAA	CTCCAAATGGCAACCAAAAT	TTTAGGGGTGGACAAAATTCA	59.8	510	INTERGENIC				
CaPOPII_826	Ca2	35893811	ATTTTTTT/ATTTTTTTT	GGTTAGCTTGGCCCAAAAGT	GGGTAGAGTGGAGGAAAGGG	60.5	628	INTERGENIC				
CaPOPII_827	Ca2	35938206	AAGA/AA	TGCATATATTGCTGCAGGTTG	ATCAATGAAGTTTGGCCTGC	59.7	453	INTRON	Ca_09768		SAP	
CaPOPII_828	Ca2	35944964	CAAAA/CA	TCCAACCATACCTGAAAGGC	CTCGTGCGAACTTGTGTGTT	59.9	313	CDS (large-effect mutations)	Ca_09769			Ferredoxin-dependent bilin reductase
CaPOPII_829	Ca2	35945318	CCAA/C	AACATCATACTTTTGCGGGC	GCCTGAAATATGCCGAGGTA	60.0	417	INTRON	Ca_09769			Ferredoxin-dependent bilin reductase
CaPOPII_830	Ca2	35961921	TAAAA/TAAA	AGACGCTCATCACTCAATTTCA	TTCGACAATATGGCCAATGA	59.9	251	INTERGENIC				
CaPOPII_831	Ca2	35962691	ATTTTTTT/ATTTTTTTT	TGAGTCATGAGATCGACGAAA	TTCAAATTTAACGGTTGGATCA	59.4	367	INTERGENIC				

INDEL marker IDs	Chromosomes /unanchored scaffolds	Physical positions (bp)	InDels (*Kabuli* reference genome-CDC Frontier/PI)	Forward primers (5'-3')	Reverse primers (5'-3')	Anneling temperature (0C)	Expected amplified product size (bp)	Structural annotation		Functional annotation		
								Sequence components of *kabuli* genome	*Kabuli* gene accession IDs	NCBI-KOG	TFs	NCBI-nr database
CaPOPII_832	Ca2	35962726	GAAAA/GAAAAA	TGAGTCATGAGATCGACGAAA	TTCAAATTTAACGGTTGGATCA	59.4	367	INTERGENIC				
CaPOPII_833	Ca2	35965829	CAAAAA/CA	ATTTTTCTGCTAGCGGGACC	ACATTCCATGGGCTGCTTAG	60.6	260	DRR	Ca_09771	J	NAC	Ribosomal protein S26e
CaPOPII_834	Ca2	35971845	TAT/TATATCACAT	GCGGAGCAGAACCCTTATTA	TGACTTTTTATTTTTCCGCACTC	59.3	955	INTERGENIC				
CaPOPII_835	Ca2	35987956	ATGTTGT/ATGT	GGATTGGCCTCCCATAATTT	GCGTTTTCAAGGTTGGATGT	60.0	851	DRR	Ca_09772	T	LBD	Disease resistance protein
CaPOPII_836	Ca2	35995557	TAAAAAAAA/TAAAAAAAAAAAA	CGTGTGAGTTTCGTCGCTAA	TGGTTTGGGTGGTTCATTTT	60.1	499	INTERGENIC				
CaPOPII_837	Ca2	35996261	TTCTCT/TTCT	CCATTCGTCAACGGCATAAT	AAAATCAACGACGATGAATAAGAA	60.7	330	INTERGENIC				
CaPOPII_838	Ca2	35999918	CAAAAA/CAAAAAA	GGAATCAAAGCGGAATTCAA	AAGGAGCATGGTCCAATCAC	60.0	221	INTRON	Ca_09774		FAR1	
CaPOPII_839	Ca2	36065466	TTCTCTCTCTCTCTCTCT/TTCTCTCTCTCTCTCTCTCT	TGGGCACTCTCTTCCACTCT	GAACCACCAAACCAAACACC	60.0	500	INTERGENIC				
CaPOPII_840	Ca2	36146623	GAAAAAA/GAAAAA	AAACAGCAACAAAAATCCCAA	TTTGGAAATCAAGAGGTGGG	59.5	201	INTERGENIC				
CaPOPII_841	Ca2	36146925	TTTATTATTATTAT/TTTATTATTATTATTAT	AGGGAAACCCACCTCTTGAT	AAATCCAAAGTGCTTGCGTT	59.8	623	INTERGENIC				
CaPOPII_842	Ca2	36372982	GATTATTATTATTATTAATATTATTATTA/GATTATTATTATTATTATTATTATTAATATTATTATTA	TGAGCTGAAATTGTTCGTCG	CCAATCCTCGTTGTCAAGGT	60.0	274	CDS (large-effect mutations)	Ca_09826		HD-ZIP	Protein kinase, catalytic domain
CaPOPII_843	Ca2	36401773	TATAATAATAATAATA/TATAATAATAATA	GGATAAGGATCTGCATCGGA	ATCCATTTTTGTGGAACCAA	60.0	434	DRR	Ca_09829	R		Multi antimicrobial extrusion protein
CaPOPII_844	Ca3	173424	TAAAAAAAAAAAAA/TAAAAAAAAAAAA	GAGAGCGACAACACTGTCAAA	GTGGGAGGCGTGTTGTTATT	59.1	646	INTERGENIC				
CaPOPII_845	Ca3	414861	T/TG	CTATGCAACCACAGCTTCCA	GCAGATTCCGGTTCAAAAGA	59.9	438	INTRON	Ca_22928	O		Peptidase C13, legumain
CaPOPII_846	Ca3	454494	AGGG/AGG	GGATTGGAGTGTTCAGCCAT	TTGTCATTAGTTTCAATCTCACCAA	59.9	460	INTERGENIC				
CaPOPII_847	Ca3	454633	GATATATATATATAT/GATATATATATAT	GGATTGGAGTGTTCAGCCAT	TTGTCATTAGTTTCAATCTCACCAA	59.9	460	INTERGENIC				
CaPOPII_848	Ca3	939073	GTT/GTTTT	ATTGGTTCTGCTCTGGGTTG	GGGACCACAGCACCTATGTT	60.1	296	INTERGENIC				

INDEL marker IDs	Chromosomes /unanchored scaffolds	Physical positions (bp)	InDels (*Kabuli* reference genome-CDC Frontier/PI)	Forward primers (5'-3')	Reverse primers (5'-3')	Anneling temperature (0C)	Expected amplified product size (bp)	Structural annotation		Functional annotation		
								Sequence components of *kabuli* genome	*Kabuli* gene accession IDs	NCBI-KOG	TFs	NCBI-nr database
CaPOPII_849	Ca3	1038019	AAT/AATAATCAT	GGAGCAAAATGTGTTTTATGGA	GGAAACGGTCTTCCCTTTTT	59.0	173	INTERGENIC				
CaPOPII_850	Ca3	1488532	TTATATATATATATATATATATATA/TTATATATATATATATATATA	TCCAAACACCTTCACCATGA	CTGATCAGTGCTGTGGCATT	59.9	507	INTERGENIC				
CaPOPII_851	Ca3	1533357	TCAAAA/TCAAAACTCCAAAA	ACCCAAAAGCTAACTTGCCA	GCATCAACAAAGCAGCAGAA	59.7	533	INTERGENIC				
CaPOPII_852	Ca3	1534003	AAACTGCAACTAACT/AAACTGCAACTAACTGCAACTAACT	TTCTGCTGCTTTGTTGATGC	GTGGATGGGTGTTTTCCATC	60.1	774	INTERGENIC				
CaPOPII_853	Ca3	1955332	CAAA/CAA	TCATGGTTCTTTTCGAAGTGG	CACCCACTCATCCTTCACAA	60.1	930	INTERGENIC				
CaPOPII_854	Ca3	1964709	A/AT	TCTCTTTTCTCGAACCGCAT	AAACGCAATCCTTGAGTTCG	60.0	317	INTERGENIC				
CaPOPII_855	Ca3	2855993	TAAA/TAA	CTTGGTTGGAATTGCATGTG	CGTTTTGGAAGGTAGGGTGT	60.0	514	INTERGENIC				
CaPOPII_856	Ca3	3185857	TAAAAAAAAAA/TAAAAAAAA	CGCCTCTCTTTTTCTATGCG	TGTTGCTGTTGTTGCCTCTC	60.1	468	INTERGENIC				
CaPOPII_857	Ca3	3235341	AGCTTCAGTCGCT/AGCT	TGGAGTGAGCTGTGGTTGAG	TGGTCACCACTTGGTGTTTG	60.0	840	INTERGENIC				
CaPOPII_858	Ca3	3235471	TAAA/TAA	ATAGGCCAAGCAACCAAAGA	GACGATGGTGAAGATGACGA	59.7	663	INTERGENIC				
CaPOPII_859	Ca3	3524015	AATATATATATATAT/AATATATATATATATATATAT	GTTTGCAGGAAGAGGCAAAG	GCCAGCAGCCTTCAAGATAG	60.0	712	DRR	Ca_21350	J		Ribosomal protein 60S
CaPOPII_860	Ca3	3818047	G/GGA	ATGCTCTGCACTTGCAGCTA	TTCATCGCCTCAAAGTGTTG	59.9	707	INTERGENIC				
CaPOPII_861	Ca3	3900290	TTTTATTTATTTATTTATTTATTT/TTTTATTTATTTATTTATTT	CTGCAAGTGGTGTGTTTGCT	AATTACTCACGGCCCAAAAA	59.9	621	INTRON	Ca_21332	E		Amino acid transporter, transmembrane
CaPOPII_862	Ca3	3963300	CAAAAAAAAA/CAAAAAAAAAAAAA	TCCATTGTGTGCCACAGTCT	TCTAGTCCGCGACTCAACCT	60.2	505	INTERGENIC				
CaPOPII_863	Ca3	4017573	TAA/TAAA	ACCACAGATCACCAGTCGCT	ACGTCCAAGGTAAGGGCTTT	60.7	683	INTERGENIC				
CaPOPII_864	Ca3	4073796	CT/CTT	CTCAACATCCACCATGCAAT	TGCAAATTTCATCAAATGCATAA	59.4	746	INTERGENIC				
CaPOPII_865	Ca3	4132392	TTATATATATATATATATAT/TTATATATATATATATATATATATAT	CGTGTGAGAAAACCTCGACA	GAAGGCCAACTTCCAAATGA	59.9	738	INTERGENIC				

INDEL marker IDs	Chromosomes /unanchored scaffolds	Physical positions (bp)	InDels (*Kabuli* reference genome-CDC Frontier/PI)	Forward primers (5'-3')	Reverse primers (5'-3')	Anneling temperature (0C)	Expected amplified product size (bp)	Structural annotation		Functional annotation		
								Sequence components of *kabuli* genome	*Kabuli* gene accession IDs	NCBI-KOG	TFs	NCBI-nr database
CaPOPII_866	Ca3	4137886	TTATATATATATATATATATATATATATATATATATA/TTATATATATATATATATATATATATATATATATATATATATATATATATA	ATGTTCTCCTCCCCTTTTCA	GTTCCGACGAAAACGACATT	58.6	783	INTERGENIC				
CaPOPII_867	Ca3	4152638	CTTTTTTTTTT/CTTTTTTTTT	TGATGCAAAGTGTGCTGTGA	TTTTTGGTTGGGTGGGTAAA	60.0	675	INTERGENIC				
CaPOPII_868	Ca3	4689138	TAAAAAA/TAAAAAAA	CAGCTTGCATTTGCTTTTGA	TTGCTCCTACGTATCTCCCG	60.1	888	INTERGENIC				
CaPOPII_869	Ca3	4965548	ATTTT/ATTTTT	CGGGGAATGTGATTGATAGG	GTTGGAGAGATCGGTGGAAA	60.1	948	INTERGENIC				
CaPOPII_870	Ca3	4966098	CTTTTTTTT/CTTTTTTT	TTTCCACCGATCTCTCCAAC	AAACCCGCTCCTTTCAAAAT	60.1	514	INTERGENIC				
CaPOPII_871	Ca3	4975195	AATATATATATATATA/AACATATATATATATATATA	CAAAGTTCAATACGCCAGCA	AGGTTTTGGCGTAACTCCCT	59.9	455	INTERGENIC				
CaPOPII_872	Ca3	5269970	CAAACAATAA/CAA	GATTGGCACGTCGATTAACA	TTCTCGCTTTCGTTTCGTTT	59.5	563	INTERGENIC				
CaPOPII_873	Ca3	5270123	GAA/GA	ACGAAACGAAAGCGAGAAAA	CGACCTCGAAATAATCGGAA	60.0	521	INTERGENIC				
CaPOPII_874	Ca3	5270478	TATCAATC/TATC	ACGAAACGAAAGCGAGAAAA	CGACCTCGAAATAATCGGAA	60.0	521	INTERGENIC				
CaPOPII_875	Ca3	5286221	ATTT/ATT	CACCCCTCCCAAAACATCT	TTGTGAGTGCATTTGATTTTGTT	59.8	876	INTERGENIC				
CaPOPII_876	Ca3	5288032	AATATATATATATATATATATATAT/AATATATATATATATATATAT	AGAAGGTCGCATCTCCAAGA	TGGCACTTGTATTCACTCGC	60.0	456	INTERGENIC				
CaPOPII_877	Ca3	5347423	CAATAATAATAATAATAATAATAATAATAATAATAA/CAATAATAATAATAATAATAATAATAA	TTGCTCCTACGTATCTCCCG	TTGTGACAATTTGATGGTTCTAAA	60.2	563	INTERGENIC				
CaPOPII_878	Ca3	5502243	TG/T	TGCGTTTTTCTGCATTTCTG	ATTCTGCGCGACTATCAACC	60.0	661	INTERGENIC				
CaPOPII_879	Ca3	5520403	GTATATATATATATATATATATATATA/GTATATATATATATATATATATATA	CAAATGACGACGACGATGAC	GAATGATGACAATGCGGTTG	60.1	503	INTERGENIC				
CaPOPII_880	Ca3	5689325	ATTT/ATT	TTGGTCAATGTGGCTTCTCA	TGAACTCTGAGCGTGTGACC	60.2	439	INTERGENIC				
CaPOPII_881	Ca3	5698499	TTATAT/TTATATAT	GAGGATTTGGGGGAGAAGAG	AGAAGGGAGGAGATGGAAGC	60.0	397	INTERGENIC				
CaPOPII_882	Ca3	5805263	GT/GTT	CAATTGGTCGAAAGACGGAT	TTCGAATGAACATAATTGAACTTT	59.9	164	INTERGENIC				

INDEL marker IDs	Chromosomes /unanchored scaffolds	Physical positions (bp)	InDels (*Kabuli* reference genome-CDC Frontier/PI)	Forward primers (5'-3')	Reverse primers (5'-3')	Anneling temperature (0C)	Expected amplified product size (bp)	Structural annotation		Functional annotation		
								Sequence components of *kabuli* genome	*Kabuli* gene accession IDs	NCBI-KOG	TFs	NCBI-nr database
CaPOPII_883	Ca3	6345015	CAAAAAAAAAAA/CAAAAAAAAAA	CAGCTGTTCCGATGTCTTCA	GCTCATGGGATGTCAACCTT	60.0	979	DRR	Ca_24832		B3	
CaPOPII_884	Ca3	6352791	AATATATATATATATATATAT/AATATATATATATATATAT	TTTTTATGTTGGGCCCATGT	ATGCGCTTGACAAGAATGTG	60.1	655	INTERGENIC				
CaPOPII_885	Ca3	6386934	TGG/TGGG	TGTACGAGAAGGTCGTGTGC	GCCCAACAGCAAGCTATTTC	59.9	574	INTRON	Ca_24834		Trihelix	
CaPOPII_886	Ca3	6548012	CT/CTT	TTATGCCGTTTGGAGTTTCC	TTCAATTGCCTTGTGGCATA	59.9	615	INTERGENIC				
CaPOPII_887	Ca3	6772905	GTTT/GTT	TCCCTTTGTCTTTTCGCACT	CACTGCCGTTCTTCACTTCA	59.9	383	DRR	Ca_16559	R	NAC	Pentatricopeptide repeat
CaPOPII_888	Ca3	6774064	T/TTAA	TTTGCTGGACTTTGAAGCCT	CCCGGTCCTAGAGAGAAAGA	60.0	640	DRR	Ca_16559	R	NAC	Pentatricopeptide repeat
CaPOPII_889	Ca3	6790470	T/TA	GGCTGGCTACTTAGGGATCA	GACCTCGTCCAGCTTTTCAG	59.3	582	INTERGENIC				
CaPOPII_890	Ca3	6838667	AGCA/AGCAGGATCCAATGCAATGGCA	GGAACCCCACAATCATCATC	GCAGCCAATTTTGTTGCATA	60.0	547	CDS (large-effect mutations)	Ca_16550	R	bHLH	WD40 repeat
CaPOPII_891	Ca3	7312652	TAAAAAAAAA/TAAAAAAAAAA	CGCAAGATTTTACCTACCTGTG	TGATCAAGCTTCCAGTCCAA	58.8	720	DRR	Ca_16529		LFY	F-box domain, cyclin-like
CaPOPII_892	Ca3	7377309	TAAAAAA/TAAAAAAA	CATATTCGCATTCCCAAACC	ACCTGCAAACCCAACTCAAC	60.2	306	INTERGENIC				
CaPOPII_893	Ca3	7773838	AATATATATATATATAT/AATATATATATATAT	CATGCGAGTTGTAACACCCT	CGTGCATACTTAATATCAGTCAAGTG	58.7	365	INTERGENIC				
CaPOPII_894	Ca3	7959750	TTAATTTGATTATGTTAAGTAATTTGATTATGTTAA/TTAATTTGATTATGTTAAGTAATTTGATTATGTTAAGTAATTTGATTATGTTAA	TTGGCATAGGTCATGGAAGTT	TTTGCCAATGTTGCTAGCTG	59.4	848	INTERGENIC				
CaPOPII_895	Ca3	8245494	TCT/TCTTCTGCT	TGCGCCTCTTCTAAAATCGT	TATCGCAATCAGAGGAACCC	60.0	670	INTERGENIC				
CaPOPII_896	Ca3	8393314	GTATATATATATATATATAT/GTATATATATATATATATATATAT	GTTGGTTCCCTAGCACCGTA	TATTTTGGATTTTGGCTCGG	60.0	602	INTERGENIC				
CaPOPII_897	Ca3	8523296	ATTTTTTTTTT/ATTTTTTTT	CACATGACAAAATTGGTGGAA	CGTTAAGGCACATCTCACTCC	59.3	508	INTERGENIC				
CaPOPII_898	Ca3	8602266	AATATATATATATA/AATATATATATATATA	GCTCAACAGAGACCACCACA	CGTATTCGTGTGTGAAAGCG	59.9	835	INTERGENIC				
CaPOPII_899	Ca3	8775439	GTATATATATATATATATATATATATATATATAT/GTATATATATATATATATATATATATATATATATATATAT	AGTCGCATCTTCGTCGTTTC	GGCATCGGGAGTCTGTTTTA	60.4	537	INTERGENIC				

INDEL marker IDs	Chromosomes /unanchored scaffolds	Physical positions (bp)	InDels (*Kabuli* reference genome-CDC Frontier/PI)	Forward primers (5'-3')	Reverse primers (5'-3')	Anneling temperature (0C)	Expected amplified product size (bp)	Structural annotation		Functional annotation		
								Sequence components of *kabuli* genome	*Kabuli* gene accession IDs	NCBI-KOG	TFs	NCBI-nr database
CaPOPII_900	Ca3	8942193	AT/ATT	TGGGGAAAAACCCTTACCTT	CACATGATTTCAATTGGCTTTG	59.7	326	INTERGENIC				
CaPOPII_901	Ca3	9186815	CTATATATATATATATATATATA/CTATATATATATATATATATATATA	TCCACAAACACGATAACATTGA	TGTAACAAGCATTTGTCGGG	58.9	693	INTERGENIC				
CaPOPII_902	Ca3	9289555	TTATATATATATATATATATATAT/TTATATATATATATATATATATATATAT	GAACCAAGTTGCATGGGAAT	CCTAGGGACTTCGTGGTTGA	59.8	651	INTERGENIC				
CaPOPII_903	Ca3	9427257	TTTA/T	CATGCGTTCCTTGTGGTAGA	GGCCTAAGATCATAGCAATTCAA	59.7	165	INTERGENIC				
CaPOPII_904	Ca3	9500843	CAATCGATTTCC/CAATCGATTTCCAAATCGATTTCC	CCCGTGAGATCCTCCTAACA	CATGCAGGAATTCAGCAAAA	60.1	498	INTERGENIC				
CaPOPII_905	Ca3	9651257	CTTTTTT/CTTTTT	CTCCAAACTGGGGTGTCACT	CGGTCTCAACCTCTCCCATA	60.0	882	DRR	Ca_20979			
CaPOPII_906	Ca3	9953160	GAAAAAA/GAAAAAAA	TGCCACGTTTCTATTTCCCT	ATCACGCCATAAGCACCTTC	59.6	511	INTERGENIC				
CaPOPII_907	Ca3	10159468	T/TA	TGGGGCAAACAAAGCTATTC	CTAAGGGCGCTACAACAAGC	60.1	107	INTERGENIC				
CaPOPII_908	Ca3	10159672	AGGGGGGG/AGGGGGGGG	CACACCAACCCTTTCGTTCT	TGCTCCCACACACACAAACT	60.0	277	INTERGENIC				
CaPOPII_909	Ca3	10398321	G/GATTGATCTTTCCC	CGGATTGCCAAGTACAAGGT	CAAACCACAACCAGCACAAC	60.0	770	DRR	Ca_18819	J		Ribosomal protein L10/acidic P0
CaPOPII_910	Ca3	10846962	AATATATATATATATATATATATATAT ATA/AATATATATATATATATATATATATATATATATA	CCAAATGACGACTTCCCACT	CAAGACGTCGTCAAAATCCC	60.0	683	INTERGENIC				
CaPOPII_911	Ca3	10923237	T/TCC	GCTGATTGAAATCGAGCACA	TTAAAACAAGCACCAAGGGC	60.0	347	INTERGENIC				
CaPOPII_912	Ca3	11462251	ATTTTTTTTT/ATTTTTTTTTT	ATGATTCCCCAACATCCAAA	CACGAGTCCGATAAGGGTGT	60.0	149	INTERGENIC				
CaPOPII_913	Ca3	11519916	A/ACAGACTTATACTGTTT	CCGAGCCAAAATCCAAAATA	AATTTCAAACGCAAACCAGG	59.9	198	INTERGENIC				
CaPOPII_914	Ca3	11537812	CTTTTTTT/CTTTTTTTT	GATGCAACATCTTTTTAATCTTTGA	CCTACGTATCTCCTGGTGCG	58.8	688	INTERGENIC				
CaPOPII_915	Ca3	11919676	CNNNNNNNNNNNNNNNNNNN/CNNNNNNNNN	CGGAAATTGAAATCGGAAAA	ATCGCGAAAGTAGTCTCCCA	59.9	633	INTERGENIC				
CaPOPII_916	Ca3	12715466	T/TACACATTAAAG	TATTCCCGATTAATGCAGCC	GTCCCGAATGCAATGTCTCT	59.9	667	INTERGENIC				

INDEL marker IDs	Chromosomes /unanchored scaffolds	Physical positions (bp)	InDels (*Kabuli* reference genome-CDC Frontier/PI)	Forward primers (5'-3')	Reverse primers (5'-3')	Anneling temperature (0C)	Expected amplified product size (bp)	Structural annotation		Functional annotation		
								Sequence components of *kabuli* genome	*Kabuli* gene accession IDs	NCBI-KOG	TFs	NCBI-nr database
CaPOPII_917	Ca3	12969818	TAAAAAAA/TAAAAAAAA	TTACACCAATTCCCCCAAGA	CATGCAAGGTTGGGCTAGAT	60.2	400	INTERGENIC				
CaPOPII_918	Ca3	13096897	TCT/TCTGGAACT	TCTGGTCGACTTCTGCCTTT	TCATCAGTATCGTGGGTGGA	60.0	775	INTERGENIC				
CaPOPII_919	Ca3	13097036	A/AT	CCTTCTTTACGGGACCCAAT	TCATCAGTATCGTGGGTGGA	60.2	364	INTERGENIC				
CaPOPII_920	Ca3	13169603	ATT/ATTTTTTTT	TAGCTGGCAGGGTCTCAGTT	CGAAAGAAAGTTTCCCCTCC	60.0	419	INTERGENIC				
CaPOPII_921	Ca3	13185454	AT/ATTT	GCACACGACCTTCTCGTACA	AATCTGGATCGGGGAAAATC	59.9	302	CDS (FRAME SHIFT)	Ca_22687		Trihelix	
CaPOPII_922	Ca3	13647076	AATATATATATATATATATAT/AATATATATATATATATATATAT	TTGCACCTTATTTGATGCGT	TCTCTCTCTCATGCCACCCT	59.2	183	INTERGENIC				
CaPOPII_923	Ca3	14653349	A/AG	AGGAGCTTCTGCCATTGAAA	TAAAAGGAGCAAAGGCCAGA	60.0	408	INTRON	Ca_22505	O		Zinc finger, RING-type
CaPOPII_924	Ca3	14843003	AATATATATATATATATATATATATATATA/AATATATATATATATATATATATATATATATATA	GAGAAAGAAGATATCGGATTCTCAA	AAAAATCAACGCAGTGGGAG	59.3	356	INTERGENIC				
CaPOPII_925	Ca3	14874833	GCC/GCCC	AGGTCATTTTCGTCCTCCCT	CAAACACACCTGTTGGATGC	59.9	679	INTERGENIC				
CaPOPII_926	Ca3	14880871	TG/TGTCG	TTTCGGTCATTTTCGTCCTC	ACACACCTATCGGATGCACA	60.1	616	INTERGENIC				
CaPOPII_927	Ca3	14902851	GAA/GAAA	CGGTGGGGAGAACTCTGATA	TTCACATGACATCATCCCGT	60.1	474	INTERGENIC				
CaPOPII_928	Ca3	14919264	GTTT/GTTTT	CGATTGTGTTTGTCACAGGG	GAAGCAAATCGATTGGGAAA	60.0	303	INTERGENIC				
CaPOPII_929	Ca3	15039270	TC/T	CAACACGATCATGACAAGCA	GCATTTTGCATTTGTCAACG	59.2	677	INTERGENIC				
CaPOPII_930	Ca3	15072147	AATATATATATATATATATATATATAT A/AATATATATATATATATATATATATATA	GAGTGGTGTTGGAGTCATGTTT	AGACATGTGTTTCCCTTCCG	59.0	424	DRR	Ca_17011			
CaPOPII_931	Ca3	15396841	CATATATATATATATATATATATATATATATATATATATATATA/CATATATATATATATATATATATATATATATATATATATATATATATATA	TCAATGCGGTCTCTGAAGTG	CACATGCTAATCGACACCGT	60.0	412	DRR	Ca_17029		Trihelix	
CaPOPII_932	Ca3	15658525	TC/TCATTCC	AAAGCATTCCCACTACGGTG	TTGTCACAGCCTAAAACCCC	60.0	343	DRR	Ca_17043			
CaPOPII_933	Ca3	15660475	TA/TAA	AAGTGAGCTAACGGGGGTTT	TGGACAGAGCAAGTGAGGTG	60.0	140	INTERGENIC				

INDEL marker IDs	Chromosomes /unanchored scaffolds	Physical positions (bp)	InDels (*Kabuli* reference genome-CDC Frontier/PI)	Forward primers (5'-3')	Reverse primers (5'-3')	Anneling temperature (0C)	Expected amplified product size (bp)	Structural annotation		Functional annotation		
								Sequence components of *kabuli* genome	*Kabuli* gene accession IDs	NCBI-KOG	TFs	NCBI-nr database
CaPOPII_934	Ca3	15844609	C/CTAGG	ATGGGTAGTAAGCCCCGTCT	TCTTTCCAAAGGATGGATGG	59.8	831	INTERGENIC				
CaPOPII_935	Ca3	16094863	CTTTTTT/CTTTTTTT	TCTCCGCATAAGCTTTCGAT	TGATTGCAAATAGCACCCAA	59.9	248	DRR	Ca_17065			
CaPOPII_936	Ca3	16108101	TAAAAAAAAAAA/TAAAAAAAAA	AAAACAGGGCAAGAAAGCAA	AACAGCAAAAGAGAAGGCGA	59.9	292	INTERGENIC				
CaPOPII_937	Ca3	16133157	GTTTTATTTTATTTTATTT/GTTTTATTTTATTTTATTTTATTT	CGTCTCTTGAAGCGATGAAA	CATTGTTGGTCACTAGCAAATCA	59.1	698	INTERGENIC				
CaPOPII_938	Ca3	16133384	CAATAATAATAATAATAATAATAATAATAATAATAATA/CAATAATAATAATAATAATAATAATAATAATAATAATAATAATA	CGTCTCTTGAAGCGATGAAA	ATAAGCCGTCAATCTGCCAC	59.1	807	INTERGENIC				
CaPOPII_939	Ca3	16133499	TA/TACACAAAGAA	TTTGCCCCATTATAAACAAAA	ATAAGCCGTCAATCTGCCAC	57.2	625	INTERGENIC				
CaPOPII_940	Ca3	16137267	CATATATATATATATATA/CATATATATATATATATATATA	TGGCACATCTAAAAATAGGAGGA	AGAAAGTCGCATCCCAGAGA	60.0	544	INTERGENIC				
CaPOPII_941	Ca3	16166714	TTTTG/TTTTGCTTTG	TCAACCATCTGGGTTGGAAT	CCCCTAGCAAAGGAAACAAA	60.2	674	INTERGENIC				
CaPOPII_942	Ca3	16171083	T/TG	TACAGCGCTTCATTCCACAG	CGTGCATGAGCAAAAGAAAA	60.0	468	INTERGENIC				
CaPOPII_943	Ca3	16182804	ATTTTTTTT/ATTTTTTTTTT	CCGGCATCACCTATCAGAGT	TCCGACCTCCTTTGAGAAGA	60.1	583	INTERGENIC				
CaPOPII_944	Ca3	16190775	CA/C	TCGCGGTTCTTGATGATGTA	AGCTCGACTTATCCTTCGCA	60.2	423	INTERGENIC				
CaPOPII_945	Ca3	16195131	AT/ATT	TGATAAAAGACTAACGATGCAAACA	TAAACCATCAATCTGCCACG	60.1	655	INTERGENIC				
CaPOPII_946	Ca3	16195709	AC/A	ACGTGGCAGATTGATGGTTT	GAACGTACGGAAGAGAAGCG	60.4	754	INTERGENIC				
CaPOPII_947	Ca3	16212096	TAA/TAAA	AATGCACTTTGCCACTGTTG	CCAAGATTTTGCCAGATCAA	59.8	550	INTERGENIC				
CaPOPII_948	Ca3	16212378	A/AACATTC	ATGCACTTTGCCACTGTTGT	GAGGGGATGATGCTTCAAAA	59.2	879	INTERGENIC				
CaPOPII_949	Ca3	16471674	CA/CAA	GTGAGGACTGGACGTAACCC	TCCTTGCGGAGTTGTTCTTT	59.4	505	INTERGENIC				
CaPOPII_950	Ca3	16476513	TNNNNNNNNNN/TNNN	GGCTCACATGTGGAGGCTAT	CCAATGCCATACCATAGAGGA	60.1	344	INTERGENIC				

INDEL marker IDs	Chromosomes /unanchored scaffolds	Physical positions (bp)	InDels (*Kabuli* reference genome-CDC Frontier/PI)	Forward primers (5'-3')	Reverse primers (5'-3')	Anneling temperature (0C)	Expected amplified product size (bp)	Structural annotation		Functional annotation		
								Sequence components of *kabuli* genome	*Kabuli* gene accession IDs	NCBI-KOG	TFs	NCBI-nr database
CaPOPII_951	Ca3	16544552	GTTATTATTATTATTATTATTATTATT ATTATTATTATTATTA/GTTATTATTAT TATTATTATTATTATTATTATTATTA	TGCTCGACATGTTTCTC CTT	CCACACAAACTGACAA CTCCA	58.4	605	INTERGENIC				
CaPOPII_952	Ca3	16793468	TTATA/TTATATA	TAATCAAATGCCCCCAA TGT	TTGAGTTCAAGCGTGA GTGG	60.0	851	INTERGENIC				
CaPOPII_953	Ca3	17039245	CAAAAAA/CAAAAAAAA	TTCTAACTTTGCTGTCC CATTTT	GGGGTAAAACCAGGAG GAAG	59.2	750	INTERGENIC				
CaPOPII_954	Ca3	17039468	C/CA	TGTTTTGAGGACAAATG TTTTGA	GGGGTAAAACCAGGAG GAAG	59.5	301	INTERGENIC				
CaPOPII_955	Ca3	17135834	TCTC/TCTCTTTCCCTC	CAACGCGCTTTTAGAT GAGA	AAAGCTTTGAAGCAGG GTCA	59.2	655	INTERGENIC				
CaPOPII_956	Ca3	17187813	CT/CTTT	AAACAAATAAGCCTGG CGTG	GGAGAGACCAAGCTCT CGAA	60.1	462	INTRON	Ca_24618	S	B3	Protein of unknown function DUF647
CaPOPII_957	Ca3	17491839	AATATATATATATATA/AATATATATAT ATA	TGAATTCATGGCTAAAA AGCCT	GCAAAGATGATAATTC GGCAA	60.1	654	INTERGENIC				
CaPOPII_958	Ca3	17725458	CATATATATATATATATAT/CATAT ATATATATATATATATAT	AGAAGAAGGAGGATTC GCGT	GCGATTTGGGAGAGAA ACAG	60.3	572	INTERGENIC				
CaPOPII_959	Ca3	18943181	AAT/A	TGGAGTGTTGATGTCG TTGTC	AAATCGAAGATCGCAA CAGT	59.6	428	INTERGENIC				
CaPOPII_960	Ca3	19089322	T/TATC	CACCGTTGACCAGTTT GATG	CACGGGAAACTCGATG ATCT	60.0	713	INTERGENIC				
CaPOPII_961	Ca3	19359038	TAAAA/TAAA	CGTCCTTTGTTCATTCC CAC	TGAGGGCCTTTAGAAT CGTG	60.4	670	INTERGENIC				
CaPOPII_962	Ca3	19359334	CCA/C	AGGGAAATCAACGACA GAGC	TGAGGGCCTTTAGAAT CGTG	59.3	365	INTERGENIC				
CaPOPII_963	Ca3	19362553	TAAAAA/TAAAA	CCAAAATGATGTTGCA GGGT	TCATCATCATTGAAAAT CCGTT	60.8	361	INTERGENIC				
CaPOPII_964	Ca3	19366983	ATTTTTTTTT/ATTTTTTTTTT	AGCTACTCGTCATCCTC CGA	TTAAAAGTGAACACAC GCCG	60.0	807	DRR	Ca_19336			Toll-Interleukin receptor
CaPOPII_965	Ca3	19368565	GTTTTT/GTTTTTT	GGTGATGCATGATGAT TTGC	TGGGTTAGCTTTTGGG ATTG	59.9	630	DRR	Ca_19336			Toll-Interleukin receptor
CaPOPII_966	Ca3	19373696	TAA/TA	GCTGAATGCGTGAAGA ACAA	GGGTAGAAAACACGCG GAAT	60.0	339	INTERGENIC				
CaPOPII_967	Ca3	19375049	AAAAGAAA/AAAAGAAAGAAA	TCTCCGTTCCATTTACA CGA	AACTCCAAATCCCTGCT CAA	59.1	365	INTERGENIC				

INDEL marker IDs	Chromosomes /unanchored scaffolds	Physical positions (bp)	InDels (*Kabuli* reference genome-CDC Frontier/PI)	Forward primers (5'-3')	Reverse primers (5'-3')	Anneling temperature (0C)	Expected amplified product size (bp)	Structural annotation		Functional annotation		
								Sequence components of *kabuli* genome	*Kabuli* gene accession IDs	NCBI-KOG	TFs	NCBI-nr database
CaPOPII_968	Ca3	19375109	GAGAAGAAGAAGAAGAAGA/GAGAGAGAGAGAGAAGAAGAAGAAGAAGAAGA	TCTCCGTTCCATTTACACGA	AACTCCAAATCCCTGCTCAA	59.1	365	INTERGENIC				
CaPOPII_969	Ca3	19376077	A/ATTT	TGACCTTGTGCATTGTTTTGA	GCAAACTCAACCTTGCCAAT	60.1	333	INTERGENIC				
CaPOPII_970	Ca3	19382845	TTT/TTTATT	TAGGGGCATATGGTCTCGTC	ACCACATGATGCTCTCCACA	59.9	782	INTERGENIC				
CaPOPII_971	Ca3	19383912	CAAA/CAA	GTCAGGTCAGGCTGGAAAAA	TCGTCTTAATCCACTTGGTGC	60.2	436	DRR	Ca_19337			
CaPOPII_972	Ca3	19403411	T/TTCG	AGGGTAATCCGCCAGTCTTT	GCAACATTCGTCCTCTCCAT	60.0	674	INTERGENIC				
CaPOPII_973	Ca3	19960902	TAAAAAAAAAAA/TAAAAAAAAAA	AGCTTTGATTTCTCCATCGC	GGTATTAGCACCCTACGGCA	59.4	243	INTERGENIC				
CaPOPII_974	Ca3	20052639	AATTATTATTATTATTATTATTATTATTATTATTATTATTAT/AATTATTATTATTATTATTATTATTATTAT	ATCGTGTGCAAAGGAAGGAT	TGTGCTCAAATAAATAACGTGTCTC	59.6	856	INTERGENIC				
CaPOPII_975	Ca3	20315844	ATTTTTT/ATTTTTTT	CGCAAACACACTTGAACTGC	AAAGCATGTCACCAAATAAGCA	60.5	184	INTERGENIC				
CaPOPII_976	Ca3	20339565	TAA/TAAA	GGCAAAATGAATTTCCCTCA	AAACGCAATCCTTGAGTTCG	59.9	417	INTERGENIC				
CaPOPII_977	Ca3	20424638	CAAAAAAAAA/CAAAAAAAA	TACATTGGTTTGTGCGATGC	TGACTATGGGAGGTCAGGAAA	60.5	636	INTERGENIC				
CaPOPII_978	Ca3	21097843	ATTTTTTTTT/ATTTTTTTTTT	GGCATTTTGATTTGATTCGC	CAAATTTTGTGTTTCAAAAACCATT	60.4	653	INTERGENIC				
CaPOPII_979	Ca3	21192862	AATATATATATATATATATATATATATATATATAT/AATATATATATATATATATATATATATATATAT	TGTCTTTCCTCGTTTTTCAATG	TTTGTAGTTCACCAGCAGGG	59.2	414	INTERGENIC				
CaPOPII_980	Ca3	21311568	GAAAAAAAAA/GAAAAAAAAAA	CACGTCACATCGCAATTTTT	CATGTCATTTCAACCCACGA	59.6	542	INTERGENIC				
CaPOPII_981	Ca3	21635589	GCATCATCA/GCATCA	AAGCATGCCAAACCAAAAAC	TGAACATCAAAGATCTGCGG	60.0	521	INTRON	Ca_09516			
CaPOPII_982	Ca3	21696134	CTATTATTATTATTATTATTATTATTATTATTATTATTATTATT/CTATTATTATTATTATTATTATTATTATTATTATTATTATTATT	TTTTTCAACAACTCTCTCCGA	GCAAATGCAGACCAGAATCA	57.6	532	INTERGENIC				
CaPOPII_983	Ca3	21897869	ATATTATTATTATTATTATTATTATTA/ATATTATTATTATTATTATTATTA	GAGTTGAGTGTTGCCAGGGT	AGCTACCAAGCTGATGGTGAA	60.2	296	INTERGENIC				
CaPOPII_984	Ca3	22114232	ATTTTTTTTT/ATTTTTTTT	AAAAGTTGACGTGGCATTGA	ACTCGTATTTTACCCGTGCG	59.2	579	INTERGENIC				

INDEL marker IDs	Chromosomes /unanchored scaffolds	Physical positions (bp)	InDels (*Kabuli* reference genome-CDC Frontier/PI)	Forward primers (5'-3')	Reverse primers (5'-3')	Anneling temperature (0C)	Expected amplified product size (bp)	Structural annotation		Functional annotation		
								Sequence components of *kabuli* genome	*Kabuli* gene accession IDs	NCBI-KOG	TFs	NCBI-nr database
CaPOPII_985	Ca3	22438235	GAAAAAAAAAA/GAAAAAAAAAAA	TTCCAAACAAAGCCTTTTGC	TAGCAACGGAACAAACCACA	60.2	690	INTRON	Ca_06267		HD-ZIP	Homeobox
CaPOPII_986	Ca3	22487747	TACAGTGA/TA	GTAATGCCCCGTGAGTAGGA	CCCAATTGATTCCAAAAGAAA	60.0	778	INTERGENIC				
CaPOPII_987	Ca3	23014028	CAAAAAAAAAAAAAAA/CAAAAAAAAAAAAA	GGATGTTGCACAATCAAGGA	GGCAGTGGTAGTGAAGCACA	59.5	370	INTERGENIC				
CaPOPII_988	Ca3	23016776	TCCTACCT/TCCT	GCACACCTATGTGGTCTTGC	CCATTTTGTCGAAGTTTGGTT	59.2	615	DRR	Ca_06208	QR	B3	Oxoglutarate/iron-dependent oxygenase
CaPOPII_989	Ca3	23063614	TTATATATATATATATATATATATA/TTATATATATATATATATATATATATA	GTAACTTGCACCGACCCAAT	ATGACGGTGTTTGGAGACAA	59.9	599	INTERGENIC				
CaPOPII_990	Ca3	23253882	GAAAAAAAAAAA/GAAAAAAAAAA	TGGGTTGCGATCGTGTATTA	TCAGTCAGTTGCAACATAGGAGA	60.0	327	INTERGENIC				
CaPOPII_991	Ca3	23876448	AATATATATATATATAT/AATATATATATATATATATATAT	CATAATCCCATCGCTCTCGT	TCGGTTAACTGGCCAAACTC	60.1	568	DRR	Ca_06137			Cupin 1
CaPOPII_992	Ca3	23881356	TTATATATATATATATAT/TTATATATATATATATATATATAT	ACAGCCAACAACCACATCAA	CGTAAGTGTGACCGACGCTA	60.0	864	INTERGENIC				
CaPOPII_993	Ca3	24272959	GAATAATAA/GAATAATAATAA	GCATGTCCGTTACCTCCACT	GCGATTTATTATTCAGAGTTATTAGCG	60.0	646	INTERGENIC				
CaPOPII_994	Ca3	24439636	ATTTTTTTTTT/ATTTTTTTTT	ACTTTGCAAGCTTTTGCGAT	AACAATGTATGCACAGGCAAG	60.0	503	INTERGENIC				
CaPOPII_995	Ca3	24550785	AATATATATATATATATATAT/AATATATATATATATATATATATATAT	TCGTGCATATCAATTAAACTTAGGA	CCGGTTCAATGATGTGACCT	59.2	664	INTERGENIC				
CaPOPII_996	Ca3	24554701	ATGTG/ATG	GGAGTATTGTGAACTCCACACG	AGATTTTGATTGATTGGCGG	59.5	764	INTERGENIC				
CaPOPII_997	Ca3	24731209	ATTTTTTTTTTTT/ATTTTTTTTTTTTT	GGGGTTTCACTGGTTGAAGA	GCTTTGACCCCAGAATTCAA	59.9	467	INTERGENIC				
CaPOPII_998	Ca3	24791040	GAAAAAA/GAAAAA	GGGACCACGAATGTAATTTAGC	TGCGAGCATAAGAAGTTGTTG	59.7	740	INTERGENIC				
CaPOPII_999	Ca3	24796858	AA/AACATTATCACAAACTA	AAAACCAAATGCTTCACATGC	GGCAATGACTTGTCCCTGTT	60.0	784	INTERGENIC				
CaPOPII_1000	Ca3	24956986	TAAAAAAAAAA/TAAAAAAAAAAA	TAAAGGACCAAGTCGGGAAA	CGAGAAAAATGATTATCCCTCAA	59.5	315	DRR	Ca_06033		Trihelix	
CaPOPII_1001	Ca3	24969495	CATATATATATATATATATATATATATATATATA/CATATATATATATATATATATATATATATATATATA	TTGAAAATGTGATTCGATTGC	TTGAGGATTATTGCCTTGGG	58.6	469	DRR	Ca_06032		ERF	Pathogenesis-related transcriptional factor/ERF, DNA-binding

INDEL marker IDs	Chromosomes /unanchored scaffolds	Physical positions (bp)	InDels (*Kabuli* reference genome-CDC Frontier/PI)	Forward primers (5'-3')	Reverse primers (5'-3')	Anneling temperature (0C)	Expected amplified product size (bp)	Structural annotation		Functional annotation		
								Sequence components of *kabuli* genome	*Kabuli* gene accession IDs	NCBI-KOG	TFs	NCBI-nr database
CaPOPII_1002	Ca3	25195854	AAGAATACTCTATGTT/AAGAATACTCTATGTTAGAATACTCTATGTT	TGCCCTAATTAAGGGAATAAATG	GTGGTTTGGGCTACTTTCCA	58.5	267	INTERGENIC				
CaPOPII_1003	Ca3	25314505	GAAAAAAAAAAA/GAAAAAAAAAAAA	CCCTCGAATTTGTTTTGGAA	TGGAAAATGATTGTTTTTGGG	59.9	711	INTERGENIC				
CaPOPII_1004	Ca3	25519863	AATATATATATATATATATATATATATATAT/AATATATATATATATATATATATATATAT	TCAGTCAACCGCACATAAAAG	TCATCTATTCAAAAAGTCTCACCG	58.8	541	INTERGENIC				
CaPOPII_1005	Ca3	25585812	TAAAAAAAA/TAAAAAAA	TGTTTCGTTTTTGTTCCCTCT	TCCATTGCAATCGTACTTAATGA	58.7	735	DRR	Ca_05976		B3	Transcriptional factor B3
CaPOPII_1006	Ca3	25872845	ACTAAATTGGTCGCTAAAT/ACTAAAT	TGTGTACAAGATTGTGAAGGCA	CACGTAAGATCGCCAAAATC	59.2	680	INTERGENIC				
CaPOPII_1007	Ca3	25888907	GTTTTTTT/GTTTTTTTT	CAGGTCATGCCAGACTTTGA	TCTGACCCGACCTGGTCTAC	59.8	578	INTERGENIC				
CaPOPII_1008	Ca3	25889145	ATTTTTTTT/ATTTTTTTTT	GTAGACCAGGTCGGGTCAGA	TGATATGTCACATTGTCGCAC	60.1	403	INTERGENIC				
CaPOPII_1009	Ca3	25950181	AGATGGGGATGGGGATG/AGATGGGGATG	ACTTGCACCCCATCAGTTTC	TCCCCATCTTCATCTTCGTC	60.0	410	INTERGENIC				
CaPOPII_1010	Ca3	26071877	TAAA/TAAAA	TGCTCCAATCACGTTGACAT	AGACGCTGCAACATCAAAGC	60.1	397	INTRON	Ca_08292		MYB_related	SAM dependent carboxyl methyltransferase
CaPOPII_1011	Ca3	26114282	ATTATAGTTTTTATT/AT	TTTATCCTGGGGACTTCGTG	ATACACCGATGGAGGGCTTT	59.9	838	INTRON	Ca_08292		MYB_related	SAM dependent carboxyl methyltransferase
CaPOPII_1012	Ca3	26114943	GTTTT/GTTT	TGGCATGGTACTCAACCAGA	TTCACATGAATGATTGTTGGGT	60.1	545	INTRON	Ca_08292		MYB_related	SAM dependent carboxyl methyltransferase
CaPOPII_1013	Ca3	26115029	AACG/A	TGGCATGGTACTCAACCAGA	TTCACATGAATGATTGTTGGGT	60.1	545	INTRON	Ca_08292		MYB_related	SAM dependent carboxyl methyltransferase
CaPOPII_1014	Ca3	26159882	CATATATAT/CATATAT	TTGACCAGACCGAGATCACA	GGGGCATTATGGACTTGAAA	60.2	779	INTERGENIC				
CaPOPII_1015	Ca3	26724618	TCC/T	AGGAGTGTTTTTGGGGTTTG	GCCTCTGATGGTGTCAACTG	58.9	569	DRR	Ca_08235	J		Ribosomal protein S10
CaPOPII_1016	Ca3	26872317	CAGAGAGAGAGAGAGAGAGAGAGAG/CAGAGAGAGAGAGAGAGAGAGAGAGAGAG	CAATGTGCTCGATTTCTCCA	TTACATTCCTTGCCTCGTCA	59.8	389	INTERGENIC				
CaPOPII_1017	Ca3	27073479	TTTT/TTTTTTATTT	ATCATTTTCTTCAATGCGGA	ATTGGCGTCAACCTTGTTTC	58.2	727	DRR	Ca_08197	E		Amino acid/polyamine transporter I
CaPOPII_1018	Ca3	27378702	CAA/CAAA	GTCGGGGACTAGTGGACTCA	CGGGGAAGTTCTAAGGTGCT	60.1	388	INTERGENIC				

INDEL marker IDs	Chromosomes /unanchored scaffolds	Physical positions (bp)	InDels (*Kabuli* reference genome-CDC Frontier/PI)	Forward primers (5'-3')	Reverse primers (5'-3')	Anneling temperature (0C)	Expected amplified product size (bp)	Structural annotation		Functional annotation		
								Sequence components of *kabuli* genome	*Kabuli* gene accession IDs	NCBI-KOG	TFs	NCBI-nr database
CaPOPII_1019	Ca3	27854154	TGG/TG	TCGATAATGAGTGATTAGGTGGG	ACCTCACCCTCGAATGTCAC	60.2	459	INTERGENIC				
CaPOPII_1020	Ca3	27854804	T/TA	GGATTGAGAGGGGAGAAAGG	TTTGAAAGCAAGAGCGTCAA	60.0	516	INTERGENIC				
CaPOPII_1021	Ca3	27856755	TAAAAAAAAA/TAAAAAAAA	CGAAGATGAAGAGGCTTGAAA	CAACAACTTCTGATGCTCGC	59.6	593	INTERGENIC				
CaPOPII_1022	Ca3	28113252	ATTTTTTTT/ATTTTTTT	TCCTTCTCCTCTTCCTTCCA	TGTTTGTGCAATTGAATTATAGTTATG	58.9	849	INTERGENIC				
CaPOPII_1023	Ca3	28188438	CAAAA/CAAAAA	ACCCATAACGCACTCGTTTC	TCCTCCATTTTTCGTTTTCG	60.0	434	INTERGENIC				
CaPOPII_1024	Ca3	28262509	GTTTTTTTT/GTTTTTTTTTT	TACTTTTACCCCCATTGCCA	AAACACCCATCCCACGTATG	60.2	414	INTERGENIC				
CaPOPII_1025	Ca3	28797825	A/AG	CATAGTCTGCGAAACCAGCA	GGTGGAATTGATCGGAGAGA	60.0	454	INTERGENIC				
CaPOPII_1026	Ca3	28799402	AATATA/AATA	TCTAGCAAGTTCGCGCAATA	TTTGCTGCTGCAATTTAACAA	59.7	795	INTERGENIC				
CaPOPII_1027	Ca3	28994933	AC/A	CAAATTAAGCAGAGCCCGTC	TTTTGGACAATGTCAACCCA	59.8	165	INTRON	Ca_07207	KTDL		GCN5-related N-acetyltransferase (GNAT) domain
CaPOPII_1028	Ca3	28995118	CAA/CAAA	CAAATTAAGCAGAGCCCGTC	CGGAATTTGAACAAAACGCT	59.8	769	INTRON	Ca_07207	KTDL		GCN5-related N-acetyltransferase (GNAT) domain
CaPOPII_1029	Ca3	28996658	ATT/ATTT	AGGGCTTTGGTCGTGTATTG	AACAATTGCGGGTGTAGAGG	60.0	861	INTRON	Ca_07207	KTDL		GCN5-related N-acetyltransferase (GNAT) domain
CaPOPII_1030	Ca3	28999636	CT/CTT	TCTGAATTTCTGTACCGCCC	GGGATAGGGCATGTGTGAGT	60.1	536	INTRON	Ca_07207	KTDL		GCN5-related N-acetyltransferase (GNAT) domain
CaPOPII_1031	Ca3	29174780	TTATATATATATATAT/TTATATATATATATATATAT	ATCCACCCCACATTCAAATC	GGAGAGAAATACGCGTGGAG	59.5	439	INTERGENIC				
CaPOPII_1032	Ca3	29263411	TAAAAAAAA/TAAAAAAA	TTCACAAATGGACCGTGAAA	TTGCAACTTTACGAAACCCC	59.9	537	DRR	Ca_07235	IR	Nin-like	ABC transporter-like
CaPOPII_1033	Ca3	29263688	A/AAAGGT	TTGAAGATCTCTGTGGCGAA	CTTCTCTCCCCAGCTTGTTG	59.5	762	DRR	Ca_07235	IR	Nin-like	ABC transporter-like
CaPOPII_1034	Ca3	29486404	TAAAAAAAAA/TAAAAAAAAAA	TCACCCCAAACCAAAAGAAG	GAATTAAGACGTGGCCGGTA	59.9	592	INTRON	Ca_07254		C2H2	Glycoside hydrolase, family 28
CaPOPII_1035	Ca3	29512683	AATATATATATATATATATAT/AATATATATATATATATAT	TGCAATGAATGGAATCTTGG	TCCAAAACAACAAGGACACG	59.5	380	INTERGENIC				

INDEL marker IDs	Chromosomes /unanchored scaffolds	Physical positions (bp)	InDels (*Kabuli* reference genome-CDC Frontier/PI)	Forward primers (5'-3')	Reverse primers (5'-3')	Anneling temperature (0C)	Expected amplified product size (bp)	Structural annotation		Functional annotation		
								Sequence components of *kabuli* genome	*Kabuli* gene accession IDs	NCBI-KOG	TFs	NCBI-nr database
CaPOPII_1036	Ca3	29809142	ATAT/AATTAT	GCTCATGCCTCCTTCAAAAA	AAGCAGCCATAATGACAGGG	60.3	856	INTERGENIC				
CaPOPII_1037	Ca3	29829081	CAAAAA/CAAAA	TTCATTCATCGCATTGAGGA	CTTTGAATCCTTGCCCAAAA	60.2	337	INTERGENIC				
CaPOPII_1038	Ca3	29849306	AATTATTATTATTATTATTATTATTATTATTATTATTATTATTATTATTATTATTATT/AATTATTATTATTATTATTATTATTATTATTATTATTATTATTATTATTATT	TGGCAATCATTTGCTTTTTG	AGAGGCATTGACTTTGGTGG	59.7	228	INTERGENIC				
CaPOPII_1039	Ca3	30049696	CATATATATATATATATATATATATAT/CATATATATATATATATATATATATATATAT	TCCCCTTTTCCTCATCCTCT	TGCACTTTGTTCCCTCAACA	60.0	546	INTRON	Ca_07320			WRC
CaPOPII_1040	Ca3	30899185	G/GACA	CTTTAGTGGGGAGCCATGAA	GAAATTCGCCATTTTCTGGA	60.1	520	CDS (large-effect mutations)	Ca_12186	O		Peptidyl-prolyl cis-trans isomerase, cyclophilin-type
CaPOPII_1041	Ca3	30930504	GTTTTTATTTTTATTTTTA/GTTTTTATTTTTA	GCATGGACCGTTTAAAATTG	TTTAGGTGGAAAATTCATTAGAAAA	58.0	818	INTERGENIC				
CaPOPII_1042	Ca3	31266098	TC/T	TGCGTGCGTCTTTGATTAAC	TAGCCCAAGTCAACCGAATC	59.9	446	INTERGENIC				
CaPOPII_1043	Ca3	31290388	AG/A	TTTAAAGAAGGGAGGTCGCTC	TTGAAGCAGTGTTGTGGGAA	59.8	632	DRR	Ca_12225	P	ERF	General substrate transporter
CaPOPII_1044	Ca3	31324543	TAG/TAGTAATGTAATAAAG	CCTGGGTCCAATCCTTACCT	TCAGCTTATTAGGGCATGGG	60.2	660	INTRON	Ca_12232	U	bHLH	VHS
CaPOPII_1045	Ca3	31325736	ATTT/ATTTT	ATTAATTGGGCTGACGTGGA	GTCCACATTAGGGACACGCT	60.3	426	INTERGENIC				
CaPOPII_1046	Ca3	31338630	ATTT/ATT	TATCGTTCGTTGCTCTCGTG	GGCCGAAAGTTATTACGTGG	60.0	870	INTERGENIC				
CaPOPII_1047	Ca3	31339236	T/TC	CCAACCCATGTACCGACATT	AGAGACCGTTTCATGAGATTTG	60.5	525	INTERGENIC				
CaPOPII_1048	Ca3	31345376	AAATGGAAT/AAAT	ATGGAAAGCCAGCTGAAGAA	CTATGTTGTGGGAAGGTGGG	60.0	592	INTERGENIC				
CaPOPII_1049	Ca3	31350700	TCATG/TCATGCATG	TCTTAGCGTAGATTCGCCGT	AACATCTGGGCTAATCGGTG	60.0	280	INTERGENIC				
CaPOPII_1050	Ca3	31375853	TAATAAATAAATAA/TAATAAATAAATAAATAA	TGGTTGTCAGAGCAGGTTCA	CATATGTGAACCATGGCAGC	60.4	334	INTERGENIC				
CaPOPII_1051	Ca3	31415275	T/TG	GTAGCTAACCCAACCGGTGA	GGGCCCGTTATATATTGGCT	60.0	565	INTERGENIC				
CaPOPII_1052	Ca3	31448937	ATTT/ATTTAGAAAGTTT	TGGCCACCACTAACACAAAA	ACAGGTGATCCTCTTTCCCC	60.0	757	INTERGENIC				

INDEL marker IDs	Chromosomes /unanchored scaffolds	Physical positions (bp)	InDels (*Kabuli* reference genome-CDC Frontier/PI)	Forward primers (5'-3')	Reverse primers (5'-3')	Anneling temperature (0C)	Expected amplified product size (bp)	Structural annotation		Functional annotation		
								Sequence components of *kabuli* genome	*Kabuli* gene accession IDs	NCBI-KOG	TFs	NCBI-nr database
CaPOPII_1053	Ca3	31488477	CCTCT/CCT	AGGCCCTTATGACGTTGATG	ACACATTCATTCGCCAAACA	60.0	496	INTRON	Ca_12247	T		Tetratricopeptide repeat-containing
CaPOPII_1054	Ca3	31499806	CATATCAATATCAATATCAATATCAATAT/CATATCAATATCAATATCAATAT	GCGGCAAATAACATGTGAAA	TCCTAGACTTGTTGGGGCAC	59.6	565	DRR	Ca_12248	T		RHO protein GDP dissociation inhibitor
CaPOPII_1055	Ca3	31519164	C/CT	TACACGTGGCCTCAAACAAA	TCAAGCATGGTTCCAATCAG	60.1	364	INTERGENIC				
CaPOPII_1056	Ca3	31560705	A/AAG	TGTTCACTGCCAAACTCTGC	GCAGTGCACAACTGATGGTT	60.0	585	INTERGENIC				
CaPOPII_1057	Ca3	31588307	T/TC	CTTCCAAATACCTCCCCCAT	CCGTGGCCTTCTCTACTCTG	60.0	346	DRR	Ca_12258	BK	G2-like	SET domain
CaPOPII_1058	Ca3	31604401	TCTTACTTA/TCTTA	GATGAGCAAAGGGCATGATT	CAAATCAGCAGATTCCAGCA	60.0	393	INTERGENIC				
CaPOPII_1059	Ca3	31631967	AATAT/AAT	ATTTCAGCAGTCAACCCCAG	TCGAATTCCACAAATGCGTA	60.1	738	INTRON	Ca_12262	T		RNA recognition motif domain
CaPOPII_1060	Ca3	31895120	CTAATATCTTAA/CTAATATCTTAATATCTTAA	GCCCATCAAACTCTGTGGAT	ATCGGGTGTCAATTGTCGAT	59.9	771	INTERGENIC				
CaPOPII_1061	Ca3	31918341	TAATCTGTGAAAT/TAATCTGTGAAATCTGTGAAAT	TGGTCCAAAAACCACTCACA	TGAATCACCATTTGCCAGAA	60.0	761	INTERGENIC				
CaPOPII_1062	Ca3	32379633	TTAATAATAATAATAA/TTAATAATAATAATAATAA	TGCATTTTGCATTTTGTTGTT	TGCGGATTGTATTTTGGGTT	59.1	347	INTRON	Ca_11961		FAR1	Aromatic acid exporter
CaPOPII_1063	Ca3	32458289	CAAAAA/CAAA	CCTCCTCACGCAATTTTTGT	TCAACAGCGTCTTGTGGAAG	60.1	692	INTRON	Ca_11969	J		Proteasome component (PCI) domain
CaPOPII_1064	Ca3	32486522	AAATAATAATAATAATAATAATAATAATAATAATAAT/AAATAATAATAATAATAATAATAATAATAATAAT	AACCACTTTTAAATCGATGCG	TGCAGTAGTTTGCTGCCATC	59.1	498	DRR	Ca_11974			
CaPOPII_1065	Ca3	32563568	TTATATATATATATATATAT/TTATATATATATATATATATAT	CGAAAGAAAAAGAAAACATCGC	GAAGGTGGCTCGGCATATAA	60.2	574	INTERGENIC				
CaPOPII_1066	Ca3	32616318	GTTTTTTTTTTTTT/GTTTTTTTTTTT	TGTGGTTGTTGTGGGCTTAG	CGAATGTGAGGTTTGGGACT	59.6	512	INTERGENIC				
CaPOPII_1067	Ca3	32671018	CATATATATATATATATATATATATA/CATATATATATATATATATATATATATATATATA	TGGTTGGAGAGCAAAGAAGG	TTAGTGGATTGCTTCCCTGG	60.4	655	DRR	Ca_11996			Conserved hypothetical protein CHP00245
CaPOPII_1068	Ca3	32795871	ATTTTTT/ATTTTTTT	TTGATGGGTCTGGATGTTTT	TTGAAATATCCAAAAACTGTTACAAA	57.8	297	INTERGENIC				
CaPOPII_1069	Ca3	32853814	CTTAATATGA/CTTAATATGATTAATATGA	GAAGGAATCATCATGTGGCA	TTGCTCAAAACCAAGCTTCA	59.5	891	INTRON	Ca_12021	O		Glycosyl transferase, family 43

INDEL marker IDs	Chromosomes /unanchored scaffolds	Physical positions (bp)	InDels (*Kabuli* reference genome-CDC Frontier/PI)	Forward primers (5'-3')	Reverse primers (5'-3')	Anneling temperature (0C)	Expected amplified product size (bp)	Structural annotation		Functional annotation		
								Sequence components of *kabuli* genome	*Kabuli* gene accession IDs	NCBI-KOG	TFs	NCBI-nr database
CaPOPII_1070	Ca3	33099785	TAAAAAAA/TAAAAAAAA	GCTTTTTCACTCTCGGTTGC	ATATGAGTTACGGGATGCGG	60.0	683	INTERGENIC				
CaPOPII_1071	Ca3	33579150	AAAGNNNNNNNNNNNAAG/AAAG	TGTGGGAGCTGAAAGTGTTG	TTCCTTGCCATGTCACCATA	59.9	175	INTERGENIC				
CaPOPII_1072	Ca3	33693334	AATATATATATATATATATATAT/AATATATATATATATATAT	CTCACCATAAACCACCCACG	CGTCCCGTCCAAATCTTCTA	61.2	464	INTERGENIC				
CaPOPII_1073	Ca3	33828272	TTTATTATTATTATTATTATTATTATTATTATTATTATTATTATTATTATT/TTTATTATTATTATTATTATTATTATTATTATTATTATTATTATTATT	AGAGGCAAACAAGAACCGAA	TGCATATAAGGAATTGACGCC	59.9	318	INTERGENIC				
CaPOPII_1074	Ca3	33877257	AATATATATATATATAT/AATATATATATATATATAT	CTCACTTCCGCTCATGTCAA	ATATGGCAAGGATGGGAAAA	60.0	287	INTERGENIC				
CaPOPII_1075	Ca3	33954378	TTATATATATATATATATATAT/TTATATATATATATATATATATAT	GGCTTCAAAGCCAACATCAT	TTTGTGCTGCCAACATTTTC	60.1	440	INTERGENIC				
CaPOPII_1076	Ca3	33954528	ATATTATTATTATTATTATTATTATTATTATTATTATTATTATTATTATTATTATT/ATATTATTATTATTATTATTATTATTATTATTATTATTATTATTATT	GGCTTCAAAGCCAACATCAT	TCATACAAAAACCAAACGGG	60.1	622	INTERGENIC				
CaPOPII_1077	Ca3	34264150	TAAAAAAAAA/TAAAAAAAA	AATGGAAAGAGCATTGGGTG	GCAAACAAACAAAAACACATTGA	59.9	709	INTERGENIC				
CaPOPII_1078	Ca3	34307680	ATTTTTTTTTT/ATTTTTTTTTTT	TGAATTTGCAATTGGTCGAA	TGTCTTTGGTCTTTGCAATTATTA	60.1	607	INTERGENIC				
CaPOPII_1079	Ca3	34448761	GATATATATATATATATATATATA/GATATATATATATATATATATATATATA	TTTCCACACATGATCCAAAAA	GTGGAGAAAGAGTGGCCTTG	58.9	543	INTERGENIC				
CaPOPII_1080	Ca3	34474422	TAAAAAAAA/TAAAAAAA	AAAAATCAATTTATGCCGTTGT	TTATGCCGGATTGACACTCTT	57.7	478	INTERGENIC				
CaPOPII_1081	Ca3	34578013	AGAGG/AGAGGAGG	CCAAACCGCAAGATTGATTT	TTTCCCAATATTCTGCCTCG	59.9	480	INTERGENIC				
CaPOPII_1082	Ca3	34813314	GTTTTTTTTTTT/GTTTTTTTTTTTTT	AAAAATGCAAAAACACCATGAA	AAGTCGCCATTTGAAACTCG	59.4	340	INTERGENIC				
CaPOPII_1083	Ca3	34879718	ATTTTTTT/ATTTTTT	GGTAAAAGGTGTAACCGAAGAAA	GCAATTAGATGGGTCACGCT	58.7	447	INTERGENIC				
CaPOPII_1084	Ca3	35016413	ATTTTTTTTTT/ATTTTTTTTT	AGGCTTAAGAGAGGGGTCCA	CCCTAAAATTTTGTGGGCTT	60.2	342	INTERGENIC				
CaPOPII_1085	Ca3	35264037	GAAAAAAAA/GAAAAAAA	GAATCCAAGGTTGGTTGGTG	ATGCTTAGGCATGCTGGATT	60.2	566	INTERGENIC				
CaPOPII_1086	Ca3	35834316	GATATATATATATATATAT/GATATATATATATATATATAT	GCTCCCCCTTCATGTGACTA	CCTTTTCGCCATCTTTGTGT	60.1	595	INTERGENIC				

INDEL marker IDs	Chromosomes /unanchored scaffolds	Physical positions (bp)	InDels (*Kabuli* reference genome-CDC Frontier/PI)	Forward primers (5'-3')	Reverse primers (5'-3')	Anneling temperature (0C)	Expected amplified product size (bp)	Structural annotation		Functional annotation		
								Sequence components of *kabuli* genome	*Kabuli* gene accession IDs	NCBI-KOG	TFs	NCBI-nr database
CaPOPII_1087	Ca3	35863496	A/AG	CGTGAAGATATTGTTGCTTTGG	CCTTTCTACGACCAATTCCG	59.6	383	INTERGENIC				
CaPOPII_1088	Ca3	35878677	TAAAAAAAAAA/TAAAAAAAAA	TCCCTTCCCTTTGTTGTGTC	TTTTATGTTTCGCGGGGATA	59.9	753	INTERGENIC				
CaPOPII_1089	Ca3	35886280	GAAAAAAAA/GAAAAAAA	CATGATGTGGTCGAAAAGGA	TGCAAGCAATGATGAAAACTC	59.5	209	INTERGENIC				
CaPOPII_1090	Ca3	35950711	ATTTA/ATTTAAAATTACATGCAATTTTA	CATGCGATGGAACTGAATTG	GTAAAGTTGGCACAGGGGAA	60.1	479	INTERGENIC				
CaPOPII_1091	Ca3	35959327	GAAAAAAA/GAAAAAAAA	TGTTGACCAATAAGCAAAATTGA	TGACACACAACATACAAGCCA	59.5	360	DRR	Ca_00891	V	GRAS	Alpha/beta hydrolase fold-3
CaPOPII_1092	Ca3	35959933	GTTTTTTT/GTTTTTTTT	TCTGAACAAATGATCGGGTG	ACATGCAATTTCCATCCACA	59.5	164	DRR	Ca_00891	V	GRAS	Alpha/beta hydrolase fold-3
CaPOPII_1093	Ca3	35959985	CAAAAAAAAAAAAAAA/CAAAAAAAAAAAAA	TCTGAACAAATGATCGGGTG	ACATGCAATTTCCATCCACA	59.5	164	DRR	Ca_00891	V	GRAS	Alpha/beta hydrolase fold-3
CaPOPII_1094	Ca3	35993475	CATATA/CATA	TCACGGCTTTACACATGAGC	TTGATTCGAACCCAAAATCT	59.9	515	DRR	Ca_00894			Sterile alpha motif domain
CaPOPII_1095	Ca3	36000193	AATATATATA/AATATATATATA	TCCAAATCATCCGCACATAA	TCGGTAAATAAGTTTTTGGACACA	59.9	662	INTERGENIC				
CaPOPII_1096	Ca3	36019725	GAAA/GAA	ACCTTTGGATTCATGGTTGG	CCGATAGCCATTTGGACACT	59.6	614	INTERGENIC				
CaPOPII_1097	Ca3	36022347	AC/ACC	AACATTTACAAACATAAACGACGAA	TGGAGATGGAAATGGAGAGG	59.0	524	DRR	Ca_00901	O	CAMTA	MATH
CaPOPII_1098	Ca3	36022379	TCC/TC	AACATTTACAAACATAAACGACGAA	TGGAGATGGAAATGGAGAGG	59.0	524	DRR	Ca_00901	O	CAMTA	MATH
CaPOPII_1099	Ca3	36032000	TAATTTGAAATTTGAAATT/TAATTTGAAATT	TGGTAAGGACAAAAAGAGTTCCA	AACGCTGAACCAAAATGACC	60.0	801	INTERGENIC				
CaPOPII_1100	Ca3	36035545	GTTTTTTTTT/GTTTTTTTT	GTTCCCAACTTCCCATTTCC	TTACCTGTTTGGCATGGACA	60.5	559	INTRON	Ca_00902	S	bZIP	Ubiquitin-associated/translation elongation factor EF1B, N-terminal
CaPOPII_1101	Ca3	36039531	ACAATAGCA/ACAATAGCAATAGCA	CCTCTCTAGCTCAGAGCCCA	TACGCCGATAGTGCAACAAA	59.8	423	INTRON	Ca_00902	S	bZIP	Ubiquitin-associated/translation elongation factor EF1B, N-terminal
CaPOPII_1102	Ca3	36040095	TAAAAAAAAAA/TAAAAAAAAA	TGTTGCACTATCGGCGTAAA	TTTAAAACAGGCAGGGTTGC	60.3	782	INTRON	Ca_00902	S	bZIP	Ubiquitin-associated/translation elongation factor EF1B, N-terminal
CaPOPII_1103	Ca3	36041204	ATT/AT	TTCACACTCTCCAGTTCGGA	AAGTCGACGTTTGTGAAGAGTTC	59.4	699	INTERGENIC				

INDEL marker IDs	Chromosomes /unanchored scaffolds	Physical positions (bp)	InDels (*Kabuli* reference genome-CDC Frontier/PI)	Forward primers (5'-3')	Reverse primers (5'-3')	Anneling temperature (0C)	Expected amplified product size (bp)	Structural annotation		Functional annotation		
								Sequence components of *kabuli* genome	*Kabuli* gene accession IDs	NCBI-KOG	TFs	NCBI-nr database
CaPOPII_1104	Ca3	36042558	ATTTTTT/ATTTTTTT	CTCCAGTTTTGGACCCTCAA	TTTCCAAAATAAACTCGACGC	60.1	430	INTERGENIC				
CaPOPII_1105	Ca3	36052612	GTCACTTTCACTTT/GTCACTTT	CATCAATGCACGACCCATAC	TAAGTGAGCGCATCCATTTG	59.8	639	INTERGENIC				
CaPOPII_1106	Ca3	36076512	AGAATT/A	GCATTTGTTTCAACAAGGTAAGG	TTTGGAGGACAAAAACTGGG	59.9	395	INTERGENIC				
CaPOPII_1107	Ca3	36105355	CTTTTTT/CTTTTT	GAGTGTGGGAAGGATCGAAA	CCTGGAGAAGGCTGTCAAAG	60.1	372	INTERGENIC				
CaPOPII_1108	Ca3	36105726	TC/T	CTTTGACAGCCTTCTCCAGG	AGGCTCATAAAACGCCTCAA	60.0	440	INTERGENIC				
CaPOPII_1109	Ca3	36116816	CT/CTCATTAT	CGAAAAACCGCCATAAACAT	TGGCTCAATCATTCATCTGC	59.8	337	DRR	Ca_00911	D		
CaPOPII_1110	Ca3	36119763	ACTT/ACTTCTT	GCATATTCATCACCCCCATC	TCCTTCTTGCAGGTGCTTTT	60.0	376	CDS (large-effect mutations)	Ca_00911	D		
CaPOPII_1111	Ca3	36129194	GTTAAATTA/GTTAAATTAAATTA	GCAAAAAGCACCTCGACTTT	CACACCAATCAAACGAGCAA	59.5	696	DRR	Ca_00912	YU		NPL4
CaPOPII_1112	Ca3	36139586	CT/CTGT	ATGCAAATTACCCAAGACGC	TTATACCGGCTTCGTTGACC	60.0	580	INTERGENIC				
CaPOPII_1113	Ca3	36140094	CAA/CAAA	GGTCAACGAAGCCGGTATAA	CGAAAAATAACACCAATGCAAA	60.0	551	INTERGENIC				
CaPOPII_1114	Ca3	36163828	GTTTTTTTTTTT/GTTTTTTTTTT	AATCGTTTGAAAGACGTCGAA	TCAAACGGACCTCTCCATAAA	59.7	573	INTERGENIC				
CaPOPII_1115	Ca3	36188427	TAA/TAAA	GCATAGGACTAGGCCCAAAA	GGGAAGCGTCACACTAAAGC	59.2	521	DRR	Ca_00922	Q	B3	Pyridine nucleotide-disulphide oxidoreductase, class-II
CaPOPII_1116	Ca3	36197056	TTGTGT/TTGT	GTGGCGCAAAAAGAAAAGAG	TGCAGTAACTTCAACGGGTCT	60.0	756	INTRON	Ca_00923		bHLH	KIP1-like
CaPOPII_1117	Ca3	36204205	ATTT/ATTTT	ACATGCACAAATCCCTCACA	GGTAGCAAAGCAATTTCGGA	60.0	435	DRR	Ca_00925			rRNA processing
CaPOPII_1118	Ca3	36204911	ATATTAAATTT/ATATTAAATTTATTAAATTT	GTCGTTGGAGTCTTTGCGAT	TTCACCAAATGTTGACCGAA	60.3	452	DRR	Ca_00925			rRNA processing
CaPOPII_1119	Ca3	36205967	GT/GTCT	GCATGGCATCAACAAGAATG	CATCAGAAGTCTGTTGCCGA	60.1	467	DRR	Ca_00925			rRNA processing
CaPOPII_1120	Ca3	36212061	ATTTTTTTTTT/ATTTTTTTTT	TGGAGATGGAAAGTGGGAAG	CCAGAAATTTTAACGAACGAGG	60.0	326	INTRON	Ca_00926		LSD	Uncharacterised protein family UPF0497, trans-membrane plant

INDEL marker IDs	Chromosomes /unanchored scaffolds	Physical positions (bp)	InDels (*Kabuli* reference genome-CDC Frontier/PI)	Forward primers (5'-3')	Reverse primers (5'-3')	Anneling temperature (0C)	Expected amplified product size (bp)	Structural annotation		Functional annotation		
								Sequence components of *kabuli* genome	*Kabuli* gene accession IDs	NCBI-KOG	TFs	NCBI-nr database
CaPOPII_1121	Ca3	36215526	TA/TAA	TTGATTGCCGACAGTCTTTG	CGCAATTTTGAATGTCATCATC	59.8	622	DRR	Ca_00927		CO-like	Zinc finger, B-box
CaPOPII_1122	Ca3	36215688	ATTTTT/ATTTT	TGCTGTAACGATGATGCCAC	CAATGTGGATGCCTTTGTTG	60.7	981	DRR	Ca_00927		CO-like	Zinc finger, B-box
CaPOPII_1123	Ca3	36224870	AAGAGAGAGAGAGAGAGAGAG/AAGAGAGAGAGAGAGAGAGAGAGAGAG	ACCTGTGTGACTCTGAGGGG	CCCAGTAACCGCTCTCTCTG	60.2	721	INTERGENIC				
CaPOPII_1124	Ca3	36240433	TAAAAAAAAA/TAAAAAAAA	TTGTTTTGAATTTGGGCCAG	TTCCAAAATCTCACCATTCATT	60.8	859	INTERGENIC				
CaPOPII_1125	Ca3	36242951	GTTTTTTT/GTTTTTTTT	TGCTGCAAAAGACAATTTCG	AAGTGGTGGTGCTGTTTTCC	60.0	907	INTERGENIC				
CaPOPII_1126	Ca3	36256005	CTAAAGATA/CTAAAGATATAAAGATA	CCCTCCCTTGTTGACGAATA	ATTGGCGAAGAAATGCAATC	59.9	705	DRR	Ca_00935			
CaPOPII_1127	Ca3	36268678	ATTTTTTTTTT/ATTTTTTTTTTTT	ATCGGTATCGGAGCTTGATG	CATGGTTTTGAAAAATGGGG	60.1	967	INTRON	Ca_00937	J	Dof	Peptide chain release factor class I/class II
CaPOPII_1128	Ca3	36324828	T/TG	TCCATGAAACATTCCCCATT	CGCACTAAAACGACGAACAA	60.0	454	INTERGENIC				
CaPOPII_1129	Ca3	36327195	ATTTTTTTTT/ATTTTTTTTTT	CACTTTCTCCTTGCAACCAA	TGGAGAAACAATGCAAATGG	58.9	512	INTERGENIC				
CaPOPII_1130	Ca3	36336902	TAAA/TAA	CCCATCTCCAACTCCTTCAA	GGTGCTAAGTTTTGCCGTGT	60.0	818	INTRON	Ca_00942		HB-other	Homeobox
CaPOPII_1131	Ca3	36340133	A/AGTAGAGATATAATTG	TAGCCCGTCACAATTTAGGG	CTCTTTCTTTCCCTCCGCTC	60.0	910	DRR	Ca_00942		HB-other	Homeobox
CaPOPII_1132	Ca3	36342426	GAAAAAA/GAAAAAAA	AGTATTGAAGCATCACCGCC	TCGAGGCATTTGTCTGAGAG	60.1	195	INTERGENIC				
CaPOPII_1133	Ca3	36349322	TTCTC/TTCTCTC	TGACATTTTCCAAGGTCGAA	GTTGACGGTCGATTGAAACA	59.1	594	INTERGENIC				
CaPOPII_1134	Ca3	36361613	GACA/GA	ACTCATCTCCAACACGAGGG	TTGCTGCTGATTTTCTCGTC	60.1	769	INTERGENIC				
CaPOPII_1135	Ca3	36371828	GT/GTTAT	GGGTTTTCTAGTCCCCCAAG	TGTTTTTCATTATGTGCCCG	59.8	483	INTERGENIC				
CaPOPII_1136	Ca3	36381118	T/TTTTTGAAAGGGCA	AAAAAGCGCGGGTTAATTTT	CCATGCTTCCCACGTCTAAT	60.0	544	INTERGENIC				
CaPOPII_1137	Ca3	36403564	TCA/T	TGAGGTGCTAACACCGAAGA	AGTGACAACCCCTCAATTCG	59.4	223	DRR	Ca_00951	H		Tetrahydrofolate dehydrogenase/cyclohydrolase

INDEL marker IDs	Chromosomes /unanchored scaffolds	Physical positions (bp)	InDels (*Kabuli* reference genome-CDC Frontier/PI)	Forward primers (5'-3')	Reverse primers (5'-3')	Anneling temperature (0C)	Expected amplified product size (bp)	Structural annotation		Functional annotation		
								Sequence components of *kabuli* genome	*Kabuli* gene accession IDs	NCBI-KOG	TFs	NCBI-nr database
CaPOPII_1138	Ca3	36414069	TTT/TTTATT	GATGCGCTATCCAACCTCAC	GCCGCCCACATATCATTTAT	60.6	659	INTRON	Ca_00954	G		Glycosyltransferase, ALG3
CaPOPII_1139	Ca3	36415214	AAAGAAG/AAAG	GAGTCAACCTACCGGACCAA	TGGGAATGACCTTTGCTTTC	60.0	466	INTRON	Ca_00954	G		Glycosyltransferase, ALG3
CaPOPII_1140	Ca3	36420971	AATATATATATATAT/AATATATATAT	TGGCATCGTGATTTCTACCA	GGCCATGGTGATTGTAAAGG	60.1	746	INTERGENIC				
CaPOPII_1141	Ca3	36426774	TAGATGAAGATGAAGATGAAGATGA/TAGATGAAGATGAAGATGAAGATGAAGATGA	TCAAAGGAAGCTTAGCCCAA	GCAAACGCTTCTCTGGTTTC	60.0	861	CDS (large-effect mutations)	Ca_00956	GM	WRKY	Galactosyl transferase
CaPOPII_1142	Ca3	36432148	TA/TAA	CTTCTTGCAGGGAACGAAAG	AGTGCTCCAGCAACGAGAAT	60.0	378	INTRON	Ca_00957	H		Thiamin pyrophosphokinase, catalytic domain
CaPOPII_1143	Ca3	36447139	CAT/CATAT	GGATGAGTGCAATTTGGGAT	AATGAATGGCTGCGCTAGTT	59.8	473	DRR	Ca_00960	O		CS-like domain
CaPOPII_1144	Ca3	36448772	CAAAAAAAA/CAAAAAAA	TTCCTCCTAAGCCTCCCATT	CAATGTAGGAGTGCGAAGCA	60.0	489	INTRON	Ca_00960	O		CS-like domain
CaPOPII_1145	Ca3	36481853	TA/TAA	TCCTCGAGATCCAAAAGCAT	GTCCAATTCCCCCTTTCATT	59.8	272	INTERGENIC				
CaPOPII_1146	Ca3	36668334	GTTTT/GTTTTT	TGGGTAAAAGCTCGAGTTCC	CAGTGGTGGAAAAAGGCAAT	59.3	499	INTERGENIC				
CaPOPII_1147	Ca3	36829016	TAAAAAAAA/TAAAAAAA	CGTTCTTTGAATGAGTGACCAA	AGTGCAAAGAGGTTGCAAGG	60.1	321	INTERGENIC				
CaPOPII_1148	Ca3	37000758	TTAACTAACTAACTAACTAAC/TTAACTAACTAACTAAC	TGTGTGCTAACCAAATGCAAA	TGAATTGGATGCATCATCGT	60.2	664	INTRON	Ca_01028			Nitrogen regulatory protein PII
CaPOPII_1149	Ca3	37017817	AATATATATATATATATATATATATATATATATATA/AATATATATATATATATATATATATATA	CAGAAGGCCAGGAGAAGAAA	TGTGCGTGAAGTTTCATGGT	59.5	707	INTERGENIC				
CaPOPII_1150	Ca3	37078968	CT/C	GGAAAGTAGCTGCTGTCGCT	AATGGAGTGCCAGACAAACC	59.8	556	INTRON	Ca_01034	R		WD40 repeat
CaPOPII_1151	Ca3	37192959	T/TCA	TCAAATTTCATGTGCGCTGT	TAATAGTCCGGCCCACTTTG	60.3	478	INTERGENIC				
CaPOPII_1152	Ca3	37211475	GAACAACAACA/GAACAACAACAACAACAACA	TGGTCAAATTCTTGGAGCAA	TGATCACACCCCAAGTTTTG	59.2	336	CDS (large-effect mutations)	Ca_01053		bHLH	Helix-loop-helix DNA-binding domain
CaPOPII_1153	Ca3	37396352	CAATTGGGAATTGG/CAATTGGGAATTGGGAATTGG	CCTTTGCCTTTCTGAGATGG	GCGGTGAATATCCAAAGGAC	59.8	481	INTERGENIC				
CaPOPII_1154	Ca3	37719535	TGG/TGGG	TTGGGTCAAGAATTCATCTCG	CCCAAAATGTGGCTTGAAAT	60.1	581	INTERGENIC				

INDEL marker IDs	Chromosomes /unanchored scaffolds	Physical positions (bp)	InDels (*Kabuli* reference genome-CDC Frontier/PI)	Forward primers (5'-3')	Reverse primers (5'-3')	Anneling temperature (0C)	Expected amplified product size (bp)	Structural annotation		Functional annotation		
								Sequence components of *kabuli* genome	*Kabuli* gene accession IDs	NCBI-KOG	TFs	NCBI-nr database
CaPOPII_1155	Ca3	37756750	ATTTTTTTTT/ATTTTTTTTTT	AATAGGCCAGGTCAGGCTTT	GGATTCGTTGATGATGGCTT	60.1	656	INTERGENIC				
CaPOPII_1156	Ca3	37828643	CCAGAC/CC	GGGAATAGGCTAGACCGTCC	GCTTGGCCTACGACCTATCA	59.9	512	INTERGENIC				
CaPOPII_1157	Ca3	37875029	GT/GTT	ATGGCGTGACTGAACATGAA	AAGGGCAAGATCCCAAAAAC	60.1	672	INTERGENIC				
CaPOPII_1158	Ca3	37991623	TAAAAAAAAA/TAAAAAAAAAA	TAGATTCCCCACCAGTCCAG	TGGTACGCGACAATTTTTGTT	59.9	793	DRR	Ca_01140			Dormancyauxin associated
CaPOPII_1159	Ca3	38073514	CAAAAAAAAAAAAA/CAAAAAAAAAAAAAA	CTTCTGAATGGGTTTGGTGG	ATGGACTCAATTGGGAGTGG	60.3	439	DRR	Ca_01151	DL		Rad9
CaPOPII_1160	Ca3	38156627	ACTCT/ACT	CTGCAAGAGGGGTAAAACCA	ATGTTCTGAAAATGAGGCGG	60.1	560	INTRON	Ca_01160	L	MYB_related	DNA-directed DNA polymerase, family B, exonuclease domain
CaPOPII_1161	Ca3	38170925	CAAT/CAATCAACGCCATAAT	GAACAAATGCAGTGGCTGAA	TGGCGAGGAAGCTTGTAGAT	59.8	327	INTRON	Ca_01160	L	MYB_related	DNA-directed DNA polymerase, family B, exonuclease domain
CaPOPII_1162	Ca3	38174096	AGG/AG	CGAAACACGTTACCATCATCC	CCGATGAGTGTGGATGTGAA	60.2	410	DRR	Ca_01161		NF-YA	Lipoxygenase
CaPOPII_1163	Ca3	38174447	A/AT	TTCACATCCACACTCATCGG	CTGGCACTGGCATTCCTAAT	60.5	628	DRR	Ca_01161		NF-YA	Lipoxygenase
CaPOPII_1164	Ca3	38182839	AGTGTGTGTGTGTGTGT/AGTGTGTGTGTGT	AAAGCGTCTTTCATTAATAGTGTGTG	GGTTTTGCCAACATCTGCTT	60.0	319	INTERGENIC				
CaPOPII_1165	Ca3	38203857	CT/CTT	AGGAACTGGGCATGGTGTAG	TATTGGATTCCTCCAAAGCG	60.0	870	INTRON	Ca_01163	E		Creatinase
CaPOPII_1166	Ca3	38215897	CTT/CTTCTCTTCATT	TGTTGCCTGGAAATGAGTTG	TGCTGCATTGAAATCGAGTC	59.7	688	DRR	Ca_01165	R		
CaPOPII_1167	Ca3	38333156	TTATATATATATATATATATATAT/TTATATATATATATATATAT	CCTGCCTTGCACCTATCAGT	TAGGTGTGATTGCCCTTTTT	60.3	683	DRR	Ca_01182	R		Nicotinamide N-methyltransferase, putative
CaPOPII_1168	Ca3	38900161	GACAACA/GACA	GCCATTCCATTAAAGGGTCA	TCTGCCTAATCAAACATGCC	59.8	891	INTERGENIC				
CaPOPII_1169	Ca3	39027474	CGGGG/CGGGGG	CATATGCCACATACGCAAGG	CAAATGTTAATGGCGGTGTG	60.0	586	INTRON	Ca_01265	YU		
CaPOPII_1170	Ca3	39518552	AAATAATAATAATAATAATAATAATAATAATAATA/AAATAATAATAATAATAATAATAATAATAATAATAATAATA	ATTCCATGCAAACCACCATT	ACCACTGTAGCATTTTGGGG	60.1	423	INTRON	Ca_01323		NAC	
CaPOPII_1171	Ca3	39706199	TTATATATATATATATA/TTATATATATATATA	TGCTCAAACCAAATATGGCA	TTGGGCAAAACTCCATCTTC	60.1	770	INTERGENIC				

INDEL marker IDs	Chromosomes /unanchored scaffolds	Physical positions (bp)	InDels (*Kabuli* reference genome-CDC Frontier/PI)	Forward primers (5'-3')	Reverse primers (5'-3')	Anneling temperature (0C)	Expected amplified product size (bp)	Structural annotation		Functional annotation		
								Sequence components of *kabuli* genome	*Kabuli* gene accession IDs	NCBI-KOG	TFs	NCBI-nr database
CaPOPII_1172	Ca3	39963962	AGATCAGGATC/AGATC	TTGCAGCCAACACTGAACTC	CGTTCTTCGCCTTTCTCAAC	60.0	578	CDS (large-effect mutations)	Ca_01380			
CaPOPII_1173	Ca4	80132	G/GA	CCGTTTGGATTGTCAGGACT	TCGTTGAAAGCTGGAGGTCT	60.0	382	INTERGENIC				
CaPOPII_1174	Ca4	526483	TGAGAGAGAGAGAGAGAGAGAGAGAGAGAG/TGAGAGAGAGAGAGAGAGAGAGAGAGAG	GGGATTTTGAAAAACGCTCA	AAACGGTGTGGTCCAAAAAG	60.1	524	INTERGENIC				
CaPOPII_1175	Ca4	730137	TTATATATATATATATATATAT/TTATATATATATATATATAT	GTCAACCTTGGTGCTTGGTT	AAGTATTTCCGCCTGTCACG	60.0	775	DRR	Ca_07702	G		Ribokinase
CaPOPII_1176	Ca4	1169667	TTTATTATTATTATTATTATTATTATTATTATTATTATTATTATTAT/TTTATTATTATTATTTTTATTATTATTATTATTATTATTATTATTATTATTATTATTATT	TTTTAGCCCCGTTTTGTTCA	TAAGGTGTCCGACATCAGATT	60.5	701	DRR	Ca_07748	OU		
CaPOPII_1177	Ca4	1237884	AATATATATATATATATATATATATA/AATATATATATATATATATA	GTGGTCTGGAACATCGTCCT	TTGCAGTTGCAATATCGTTG	60.0	863	INTERGENIC				
CaPOPII_1178	Ca4	1677374	TAAAAAAA/TAAAAA	TCCAAACTAGTCCGACGTGA	AGCTGCTGGAGGAGTTGGTA	59.3	450	INTRON	Ca_07795			
CaPOPII_1179	Ca4	1764559	TAAAAAAAAAA/TAAAAAAAAAAA	GCCAACTCATGTCTTTGTCG	ATTCTCGTGAGTGGGGTTTG	59.3	758	INTERGENIC				
CaPOPII_1180	Ca4	1777960	CATAAAAAT/CATAAAAATAAAAAT	AGGGGACGTCTCCCATTTAT	AGATAGGACCCCCACCATTC	59.7	705	INTRON	Ca_07800	K	HSF	Heat shock factor (HSF)-type, DNA-binding
CaPOPII_1181	Ca4	1786291	GTTAGTTAGTT/GTTAGTTAGTTTGTTATTAGTTAGTT	AAATCGTGTCAACCGCTACC	ATCGTCGTCTCTCACCATCC	60.0	870	INTRON	Ca_07801	T		Protein kinase, catalytic domain
CaPOPII_1182	Ca4	1791824	GA/G	CGATATTGTCAAATGGCGTG	TTTTGGATTCCTCCCTCCTT	60.0	644	INTRON	Ca_07801	T		Protein kinase, catalytic domain
CaPOPII_1183	Ca4	1801951	TAAAAAAAAAA/TAAAAAAAAAAA	CGTAGCAAGTGCCACGTAGA	ATTTTACGACCTCGCACCAC	60.1	813	INTRON	Ca_07801	T		Protein kinase, catalytic domain
CaPOPII_1184	Ca4	1861881	GTTTATTTATTTATTTATTTATT/GTTTATTTATTTATTTATTTATTTATT	TGAGGACAAAAGATCCCCTG	AGGGTCCACAGAAAATCGTG	60.0	870	INTERGENIC				
CaPOPII_1185	Ca4	1907150	TATAAAAAATAAAAA/TATAAAAAATAAAAAATAAAAA	AAAGAGTTTAAAGGCGGGGA	GCGGTCATATTCCGAATGAA	60.1	437	INTERGENIC				
CaPOPII_1186	Ca4	1958352	TAAAAAA/TAAAAA	AACAAGCCCAATTTTGCAGT	TGGAGATTGTTCACCCATCA	59.6	463	INTERGENIC				
CaPOPII_1187	Ca4	2030157	GAAAAAAA/GAAAAAAAA	TGAAAGTGCCAGAAAACCCT	CGAATGTTTATGGTTGGGGT	59.7	354	INTERGENIC				
CaPOPII_1188	Ca4	2032207	TC/T	TGCTGAGTTCGAGGAAAGGT	CACATCTCTTGTTCGCTCCA	60.0	517	CDS (FRAME SHIFT)	Ca_07822	D		RNA-processing protein, HAT helix

INDEL marker IDs	Chromosomes /unanchored scaffolds	Physical positions (bp)	InDels (*Kabuli* reference genome-CDC Frontier/PI)	Forward primers (5'-3')	Reverse primers (5'-3')	Anneling temperature (0C)	Expected amplified product size (bp)	Structural annotation		Functional annotation		
								Sequence components of *kabuli* genome	*Kabuli* gene accession IDs	NCBI-KOG	TFs	NCBI-nr database
CaPOPII_1189	Ca4	2032547	CTGTGTGTGT/CTGTGTGT	TGCTGAGTTCGAGGAAAGGT	CACATCTCTTGTTCGCTCCA	60.0	517	INTRON	Ca_07822	D		RNA-processing protein, HAT helix
CaPOPII_1190	Ca4	2034663	TACA/TACACA	ACCAGATACCGCACCAAAAG	AGTTGGCAAGTTTGTCAGGC	60.0	570	INTRON	Ca_07822	D		
CaPOPII_1191	Ca4	2035282	GTTTTTT/GTTTTTTT	CGCGCCAGAAGTATGTGATA	TGGGCAGCTTAGACTGGACT	59.9	275	INTRON	Ca_07822	D		
CaPOPII_1192	Ca4	2035697	ATTT/AT	AGTCCAGTCTAAGCTGCCCA	TGGAATCATGCCATGCTAGA	60.0	436	DRR	Ca_07823	R	MYB	Reverse transcriptase, RNA-dependent DNA polymerase
CaPOPII_1193	Ca4	2035798	A/AGTGT	AGTCCAGTCTAAGCTGCCCA	TGGAATCATGCCATGCTAGA	60.0	436	DRR	Ca_07823	R	MYB	Reverse transcriptase, RNA-dependent DNA polymerase
CaPOPII_1194	Ca4	2233798	CT/C	TTCATTCTCGGGGAACTAGC	TTGTTATTTAGTCTTGCTTGCCA	59.3	264	INTERGENIC				
CaPOPII_1195	Ca4	2609181	T/TGTGCTATA	GCTTCTGCTAGGCATCCATC	ATGCATGTGCTGATGCTTGT	59.9	907	DRR	Ca_12062			
CaPOPII_1196	Ca4	3017713	ATTATTTTAAATTTATTTTAAATTTATTTTAAATTTATTTTAAATTTATTTTAAATT/ATTATTTTAAATTTATTTTAAATTTATTTTAAATTTATTTTAAATTTATTTT	GACAAATTAGTGGGGCAACC	TTGTATTGTTGGTGAGGCCA	59.3	902	INTERGENIC				
CaPOPII_1197	Ca4	3355845	CGA/CGATAAGGA	GTTGGGTATTGGTGTCCCAC	GAGGGTGATCAGCTTTCTCG	60.0	589	INTERGENIC				
CaPOPII_1198	Ca4	3356082	A/AGCCAAGC	TCACCGGAAAGTAACTCGCT	ATCGATATTCTCCGCACCTG	59.9	622	INTERGENIC				
CaPOPII_1199	Ca4	3683925	ATTTTTTTTT/ATTTTTTTTTT	CGACTGACCACATGGATTGT	CGAGTCATCTTATTGTTTGCCA	59.4	734	INTERGENIC				
CaPOPII_1200	Ca4	3912089	CTATATATATATATATAT/CTATATATATATATATATATATATATAT	TCCCTTAATTTTGTTGTTGTTGAA	GTTTGCAAGTACGTGGTGGA	59.8	457	INTRON	Ca_03861			Protein of unknown function DUF599
CaPOPII_1201	Ca4	4008216	TTATATA/TTATA	GCTTCCTAAGAAACCTCATTCAA	TGGTTTTAATTTAGAGTTGATCCAAG	58.9	796	INTERGENIC				
CaPOPII_1202	Ca4	4008273	TTTTAAAATATATTTAAAATA/TTTTAAAATATATTTAAAATATATTTAAAATA	GCTTCCTAAGAAACCTCATTCAA	TGGTTTTAATTTAGAGTTGATCCAAG	58.9	796	INTERGENIC				
CaPOPII_1203	Ca4	4013283	GTA/GTAAAGTGTGATGCTATA	TTGCCAACAAATTAGTGTTTGTG	CTTTGCCAAGCAAATCAACA	60.0	508	INTERGENIC				
CaPOPII_1204	Ca4	4029055	GATCATCATCATCAT/GATCATCATCATCATCATCAT	ACATTCCTGTTGGTGGTGGT	ACATTCCAGTCCAAAACCCA	60.1	612	CDS (large-effect mutations)	Ca_03849		Dof	Zinc finger, Dof-type
CaPOPII_1205	Ca4	4037177	GTTTTTTTTTTTT/GTTTTTTTTT	AAAGTGAAAGCCACTCCCAA	AATGTTCGCCACCACTTTTC	59.7	369	INTERGENIC				

INDEL marker IDs	Chromosomes /unanchored scaffolds	Physical positions (bp)	InDels (*Kabuli* reference genome-CDC Frontier/PI)	Forward primers (5'-3')	Reverse primers (5'-3')	Anneling temperature (0C)	Expected amplified product size (bp)	Structural annotation		Functional annotation		
								Sequence components of *kabuli* genome	*Kabuli* gene accession IDs	NCBI-KOG	TFs	NCBI-nr database
CaPOPII_1206	Ca4	4041384	A/AC	GGGTTTTGGTTGAAATTTTTG	TAATGACGACCAGCGAATGA	58.4	540	INTERGENIC				
CaPOPII_1207	Ca4	4068418	ATTTTTTTTTTT/ATTTTTTTTT	ATAGATGGTCGCATTCTGGG	TCATCAAATCCGTAACGCAA	59.9	698	INTERGENIC				
CaPOPII_1208	Ca4	4162107	AATTAAATT/AATTAAATTTATTAAATT	AGGGAGAGAGGCATGAGACA	ATGTGCGACATGAAAACCAA	59.9	735	INTERGENIC				
CaPOPII_1209	Ca4	4162204	GTTTTTTTT/GTTTTTTTTTT	AGGGAGAGAGGCATGAGACA	ATGTGCGACATGAAAACCAA	59.9	735	INTERGENIC				
CaPOPII_1210	Ca4	4173930	GAAAAAA/GAAAAAAA	GGACACCCCAATAACAAGGA	TCTTGACATAAAACTATTTTTCAGCG	59.6	699	INTERGENIC				
CaPOPII_1211	Ca4	4196538	ATTTT/ATTT	ATTGGCCGGAAATTGTACTG	TAACCCAAGGCCATATCCTG	59.8	841	DRR	Ca_03838	R		Alpha/beta hydrolase fold-1
CaPOPII_1212	Ca4	4242385	TTATATATATATATATATATATATATATAT/TTATATATATATATATATATATATATATATATATATAT	GAACCATTGAGAGGCCGTT	TCGGTTTCCGAGTAGTGGAC	60.1	359	INTERGENIC				
CaPOPII_1213	Ca4	4269269	TTATATATATATATATATATATAT/TTATATATATATATATATATATATATAT	ACACGGTGATCGTCAGTGAA	ACACGTGACACTTACCTAGCAA	60.2	596	INTERGENIC				
CaPOPII_1214	Ca4	4273619	ATTTTTTTTT/ATTTTTTTT	GCGCCCGTTGAAAATAATAA	ACACGTGTGGACGTGATTTG	59.9	408	INTERGENIC				
CaPOPII_1215	Ca4	4487086	ATA/ACAGAAATATATTA	GGCCAAATGAAGGACTCAAA	CGTACGACGTCTTTCGTCAA	60.1	932	DRR	Ca_03808			DNA-binding SAP
CaPOPII_1216	Ca4	4487412	AT/ATTAATAAAGT	TCAGACTCTCCCGAACGATT	CGTACGACGTCTTTCGTCAA	59.8	281	DRR	Ca_03808			DNA-binding SAP
CaPOPII_1217	Ca4	4489505	TTATA/TTA	AGTGGTCTGCAACATAGGGG	GAAATGCAAAATGCCTTGGT	60.0	698	INTRON	Ca_03808			DNA-binding SAP
CaPOPII_1218	Ca4	4507561	AAT/AAATAT	TTTGGGGACCAAAACTGAAG	TCCGAGCAACCATACTTTCA	59.9	698	INTERGENIC				
CaPOPII_1219	Ca4	4517051	ATTT/ATTTT	AAAATGCGTTTCACTCGGAC	GCACACTTGTGCTTGGAAGA	60.1	304	INTERGENIC				
CaPOPII_1220	Ca4	4517104	GATGAATTTATTAAT/GATGAATTTATTAATGAATTTATTAAT	AAAATGCGTTTCACTCGGAC	GCACACTTGTGCTTGGAAGA	60.1	304	INTERGENIC				
CaPOPII_1221	Ca4	4521076	AATATATATATATATATATA/AATATATATATATATA	CCACAGAAAATGTTGGGCTT	TCGTAAAATAGTTGGCCGGA	60.0	568	INTERGENIC				
CaPOPII_1222	Ca4	4522341	ATTTTTTTTTTT/ATTTTTTTTTT	TTTGTCCTGTGTTGTTTTATGTTT	GGGCCGAAAATTTGTCATT	57.7	276	INTERGENIC				

INDEL marker IDs	Chromosomes /unanchored scaffolds	Physical positions (bp)	InDels (*Kabuli* reference genome-CDC Frontier/PI)	Forward primers (5'-3')	Reverse primers (5'-3')	Anneling temperature (0C)	Expected amplified product size (bp)	Structural annotation		Functional annotation		
								Sequence components of *kabuli* genome	*Kabuli* gene accession IDs	NCBI-KOG	TFs	NCBI-nr database
CaPOPII_1223	Ca4	4527820	GAAAAAAAA/GAAAAAAA	TTTTCACTCGATACGGCTTTT	AATGTGCATCTGCCACCATA	58.9	163	INTERGENIC				
CaPOPII_1224	Ca4	4549797	TTTC/TTTCTTC	GGGCAATTTGGAAGTTACGA	TCTGCGCTATAAGCTGGGTT	59.9	383	INTRON	Ca_03803			Exosome complex, component CSL4
CaPOPII_1225	Ca4	4590029	C/CAT	TTTCGCTCAATGTGAAAAGC	CCACGATAACGATGTGATGC	59.0	749	INTERGENIC				
CaPOPII_1226	Ca4	4593540	CTAATTAATTAA/CTAATTAA	TTGAGGAATGTAAGGATGTCCA	TTAATGGCGGCTACGTTTTC	59.4	928	DRR	Ca_03798			Domain of unknown function DUF296
CaPOPII_1227	Ca4	4594832	GAAA/GAAAAATAAA	TATGAGTGGGGATGATGGGT	CCTTGTTTCCCGCACATAAT	60.0	403	INTERGENIC				
CaPOPII_1228	Ca4	4769266	TTATATATATATATATATAT/TTATATATATATATATATATAT	ATACCGATGCAGTACCCGAG	TGTTTGCCAACCATATGTGAA	60.0	375	INTERGENIC				
CaPOPII_1229	Ca4	4830250	A/ATTATTATAGTTAT	CAACATGGGCAACAAGACAC	CCCTTTCCCCTTCATCATTT	60.0	273	INTERGENIC				
CaPOPII_1230	Ca4	4851248	TAA/TA	GGAATGCATGGTATCCAACC	CAGCACTTCTCCCTTTTCCTT	60.0	678	INTERGENIC				
CaPOPII_1231	Ca4	4858835	G/GGT	TTCAGGCCAATTTTACGAGG	ATGTTCATTTTCTTCGCGCT	60.1	753	INTRON	Ca_03773			TENA/THI-4 protein/Coenzyme PQQ biosynthesis protein C domain
CaPOPII_1232	Ca4	4859197	GTCT/GT	TCCAGTTTTGGTTGCTGAGA	TAGGCATCACCGTTGTGTGT	59.4	737	INTRON	Ca_03773			TENA/THI-4 protein/Coenzyme PQQ biosynthesis protein C domain
CaPOPII_1233	Ca4	4891737	AATTATTATTATTATTATT/AATTATTATTATTATTATTATT	TGCCTTGTATCGTTCCATCA	TTCCTGCTTTTTCTTTCCGA	60.1	540	INTRON	Ca_03769	Z	NAC	IQ motif, EF-hand binding site
CaPOPII_1234	Ca4	4891996	GAAAAAAAA/GAAAAAAAAA	TCGGAAAGAAAAAGCAGGAA	GGCAAATTGCTACTGAAGGG	59.9	265	INTRON	Ca_03769	Z	NAC	IQ motif, EF-hand binding site
CaPOPII_1235	Ca4	4908883	T/TA	CAAACTTTTTCCCACGCAAT	TCATCATTGTGCCGTCATTT	60.0	318	DRR	Ca_03767	F		Phosphoribosylaminoimidazole carboxylase, core
CaPOPII_1236	Ca4	5028595	GTTTTTTTTTT/GTTTTTTTTTTT	TGATAAAAGGGGGAAAATCC	CGCATCCCCAGATCTAACAT	57.8	163	INTERGENIC				
CaPOPII_1237	Ca4	6209314	TTATATATATATATATATATA/TTATATATATATATATATA	AAACTGTTAGACTTTGCAGGATGA	TCGCCCCTTAAATTTGACAC	59.4	205	INTERGENIC				
CaPOPII_1238	Ca4	6324755	ATTAATAATTTAATAATTTAATAATT/ATTAATAATTTAATAATT	TGGGTGGAACATGACAGAGA	ATTTTGCCATTTGGGATTGA	60.1	588	INTERGENIC				
CaPOPII_1239	Ca4	6542618	TAAAAAAAAAA/TAAAAAAAAA	CCACTTTGGTCTGCCATTCT	GCTCACGCGGTTAAAGACTC	60.1	706	INTERGENIC				

INDEL marker IDs	Chromosomes /unanchored scaffolds	Physical positions (bp)	InDels (*Kabuli* reference genome-CDC Frontier/PI)	Forward primers (5'-3')	Reverse primers (5'-3')	Anneling temperature (0C)	Expected amplified product size (bp)	Structural annotation		Functional annotation		
								Sequence components of *kabuli* genome	*Kabuli* gene accession IDs	NCBI-KOG	TFs	NCBI-nr database
CaPOPII_1240	Ca4	6907181	TTATATATATATATATAT/TTATATATATATATAT	TTTGAAATGGCGACTTCCTT	CCAAATGGCAACTTCCAACT	59.7	569	INTERGENIC				
CaPOPII_1241	Ca4	7089091	GA/GAACAAAAGAGATTCACA	CTTTGCATACCCCTCTCGAC	TTGGGAGGATTGGTTTCAAG	59.7	644	INTERGENIC				
CaPOPII_1242	Ca4	7132134	TAAAAAAAAAAAAA/TAAAAAAAAAAAA	CACACCAGGGGAGACTCACT	GTCTCCCTACCCCCACATTT	60.2	595	INTERGENIC				
CaPOPII_1243	Ca4	7360660	TTTTATTTATTTATTTATTTATTTATTTATTTA/TTTTATTTATTTATTTATTTATTTATTTA	ACTACGAAAACGGTCATCCG	CAAGCATGCTCTATCTCTTTACG	60.0	380	INTERGENIC				
CaPOPII_1244	Ca4	7825515	TAAAAAAAAAAA/TAAAAAAAAAAAA	TGCACAAAAATAAAAACATTTCAA	AAAGGATGGAAGCCCATTTT	58.7	959	DRR	Ca_03457	G	NAC	Glycoside hydrolase, family 1
CaPOPII_1245	Ca4	7937193	TAAAAAAAAAAA/TAAAAAAAAAA	GCTCCGGTCAAACACAATTT	GAAAATTGAAAGGCATGGGA	60.0	403	INTERGENIC				
CaPOPII_1246	Ca4	8003681	GTATATATATATATATATATATATAT/GTATATATATATATATATATATATATATAT	AATGTGTCCTCGCCGTTAAA	TAGGGGGATGATGTGGAAAA	60.5	565	INTERGENIC				
CaPOPII_1247	Ca4	8167017	CAAAA/CAAA	GGGTGGGTTTAGAGGACCAT	TTTCTTGAATGGGCAAAACC	60.1	259	DRR	Ca_08305	V	GRAS	Lipase, GDXG, active site
CaPOPII_1248	Ca4	8167238	ATCTT/ATCTTCTT	GGGTGGGTTTAGAGGACCAT	TGAAGCTTTTTGCTACAAGGAA	60.1	737	DRR	Ca_08305	V	GRAS	Lipase, GDXG, active site
CaPOPII_1249	Ca4	8167347	ATATTATTTTTA/ATATTATTTTTATTATTTTTA	GGGTGGGTTTAGAGGACCAT	TGAAGCTTTTTGCTACAAGGAA	60.1	737	DRR	Ca_08305	V	GRAS	Lipase, GDXG, active site
CaPOPII_1250	Ca4	8169626	TAAATAAAAA/TAAATAAAAAATAAAAA	TATGGCACCTCCAGTTGAAA	TGTTAAATCCTCCAGACGGC	59.1	858	INTERGENIC				
CaPOPII_1251	Ca4	8170540	TAAAAAA/TAAAAA	TCAACCCCTTTGTAGTTTCACA	CTGCCAACTCCAAAAGATACC	59.5	713	INTERGENIC				
CaPOPII_1252	Ca4	8172016	T/TC	CTAGTGTTCGGCAACAAACG	GCCTCAATTGGAGTTTGCTC	59.4	204	INTERGENIC				
CaPOPII_1253	Ca4	8173963	TTAT/TTATAT	GAAGCTCGCTTTATCCAACG	TCCACGGAATTTTCTGCTTC	60.0	147	INTERGENIC				
CaPOPII_1254	Ca4	8193266	TAAAAAAAAA/TAAAAAAAAAA	GCATCTCTCCAAGATGTCTGC	TGGGAATTGGGAGCTATCAG	60.0	620	DRR	Ca_08309	R		WD40 repeat
CaPOPII_1255	Ca4	8210483	GATAT/GATATAT	GGCCAGGAAATCAACTTCAA	ATGAATTGCATGCATGGTGA	60.1	439	INTERGENIC				
CaPOPII_1256	Ca4	8210730	TCC/TC	GGGGATACTTGCCTCAATCA	TGATGCTATTCATTTTGCGG	59.9	814	INTERGENIC				

INDEL marker IDs	Chromosomes /unanchored scaffolds	Physical positions (bp)	InDels (*Kabuli* reference genome-CDC Frontier/PI)	Forward primers (5'-3')	Reverse primers (5'-3')	Anneling temperature (0C)	Expected amplified product size (bp)	Structural annotation		Functional annotation		
								Sequence components of *kabuli* genome	*Kabuli* gene accession IDs	NCBI-KOG	TFs	NCBI-nr database
CaPOPII_1257	Ca4	8211793	AG/A	CCAATATGTGATGGTTGTTTGG	CTACAATGGAATGAGGCGCT	60.0	473	INTERGENIC				
CaPOPII_1258	Ca4	8217219	AG/A	ACCACATATTAAGCGGGTCG	ATGTTGGTGTTGGTGGAGGT	59.8	781	INTERGENIC				
CaPOPII_1259	Ca4	8237892	TAAAAAAAAAA/TAAAAAAAAA	TCCAATCCTCAACCTCGTTC	CTTGCGTGTTCCCAATTTTT	60.1	364	INTERGENIC				
CaPOPII_1260	Ca4	8239012	CTTTT/CTTT	TTCTCGATCCACAAAAACAAAA	CAACCTCCTCAAATTGGACA	59.6	636	INTERGENIC				
CaPOPII_1261	Ca4	8297213	TAAAAAAA/TAAAAAAAA	GGGTTTTGTTTGAAAAATTTGAT	TGGTGCTAACATGTGGCTAA	58.4	676	INTERGENIC				
CaPOPII_1262	Ca4	8307784	ATTT/ATT	TACACCCATTGACGTGGCTA	TCAATCATGTCTGCATACCG	60.0	694	INTERGENIC				
CaPOPII_1263	Ca4	8309234	TCAACACAA/TCAACACAACACAA	GAATTTAAGAGGAAGGCGATGA	TTCACCCTAATTCGTAGGCG	59.7	450	INTERGENIC				
CaPOPII_1264	Ca4	8309498	A/ACC	CATCCCAGGATCAACACTCA	TTCACCCTAATTCGTAGGCG	59.5	272	INTERGENIC				
CaPOPII_1265	Ca4	8311844	TG/T	TTACTCCCTCCTTCCCATGA	ATGGATGGGGGATAAAAAGG	59.5	433	INTERGENIC				
CaPOPII_1266	Ca4	8313629	ATTTTTTTTTTTT/ATTTTTTTTTTT	CTGGTGGACTGGTTGTTGTG	GGCAGACAGGAGAGGGAGTA	60.0	563	INTERGENIC				
CaPOPII_1267	Ca4	8315933	CTT/CTTATT	GGACCCGTGATGCTGTTTAT	TCTCGGTTGATGTCATTATGCT	59.8	586	INTERGENIC				
CaPOPII_1268	Ca4	8316304	GTTTTTTT/GTTTTTTTT	GCATAATGACATCAACCGAGAG	AAAAGAAATGGTTGTTTCTCGTTC	59.6	235	INTERGENIC				
CaPOPII_1269	Ca4	8320140	C/CG	CTTTGCCCCCATCCATATAA	AGGCTGAAGACTTCGACGAG	59.6	358	INTERGENIC				
CaPOPII_1270	Ca4	8333830	ATTTTTTTTTT/ATTTTTTTTTTT	ACGTAGTGTTTTGGCAAGGG	TCACACATCACCGCAAACTT	60.0	342	INTERGENIC				
CaPOPII_1271	Ca4	8342083	AATCATCAT/AATCAT	TGATCAAATCCCCAAGAAGC	CCAATCTCTTCTGCTCCCCT	60.0	951	INTRON	Ca_08321			E3 Ubiquitin ligase
CaPOPII_1272	Ca4	8381452	TAAAAAAA/TAAAAAAAA	CAATCCGTTAAGGACCTGGA	GATGATTCCCTTGCTGGAAA	59.9	388	INTRON	Ca_08327	KL		BSD
CaPOPII_1273	Ca4	8409923	AAGAG/AAG	CGCATCAATACGACGAACAA	ACCACCACTTTGTAGCCACC	60.7	509	INTERGENIC				

**INDEL marker IDs**	**Chromosomes /unanchored scaffolds**	**Physical positions (bp)**	**InDels (*Kabuli* reference genome-CDC Frontier/PI)**	**Forward primers (5'-3')**	**Reverse primers (5'-3')**	**Anneling temperature (0C)**	**Expected amplified product size (bp)**	**Structural annotation**		**Functional annotation**		
								**Sequence components of *kabuli* genome**	***Kabuli* gene accession IDs**	**NCBI-KOG**	**TFs**	**NCBI-nr database**
CaPOPII_1274	Ca4	8441953	GTTTGTA/GTTTGTATTTTGTA	AGCAGGAGAGCTGAGATTGG	TACTGTGTATCGGGGCAACA	59.7	731	INTRON	Ca_08335	R		Polycomb protein, VEFS-Box
CaPOPII_1275	Ca4	8458330	A/ATC	GAGTTCTTTGAAGGGCTCACA	TCCCCTACCCAAAATTGGAA	59.5	569	INTERGENIC				
CaPOPII_1276	Ca4	8476596	GAAAAAA/GAAAAAAA	ACCTGCAGATGGAAGCTTGT	TGCAGTGTGTGCGTGATAGA	59.9	459	INTRON	Ca_08339	G		Phosphofructokinase domain
CaPOPII_1277	Ca4	8486971	AT/ATT	CAACTGCACATGCACACAAG	ACAGCAGGGCTGAATATTGG	59.9	733	INTRON	Ca_08340	R		WD40 repeat
CaPOPII_1278	Ca4	8490694	TTATATATATATATA/TTATATATATATATATATATA	TATGCCTTAGCGGTTTTTGG	GGTCCTTTGATCCCACGTTA	60.1	498	INTERGENIC				
CaPOPII_1279	Ca4	8491035	AAGA/AA	TAACGTGGGATCAAAGGACC	ATATGAAACCACCAACCCCA	59.8	385	INTERGENIC				
CaPOPII_1280	Ca4	8497257	TAAACTGAAGAAACT/TAAACTGAAGAAACTGAAGAAACT	AGCGTGAGAGGAACCTGTGT	GACTGGCAACAGCAATTTGA	59.9	564	INTRON	Ca_08341		CPP	WD40 repeat
CaPOPII_1281	Ca4	8514977	G/GT	CACACCCCTAGCTTTGCTTC	CACGAACGCCTGTGAACTTA	59.9	547	DRR	Ca_08343	YU		
CaPOPII_1282	Ca4	8616583	GATGT/GATGTATGT	GCGAGAATACAATTTCCAGCA	ACTCGATCGTGGAAGCAAGT	60.2	324	DRR	Ca_08355	O		
CaPOPII_1283	Ca4	8636225	GTT/GTTT	GGGAAACCAAGGAGTGTGAA	GCATGCGATCGTAATTCCTT	59.9	771	INTERGENIC				
CaPOPII_1284	Ca4	8689670	A/ATCTC	TGCAAATTTGGTGAAAACAGA	TGTCACATTTGCAGAAGAAAAA	59.2	300	INTERGENIC				
CaPOPII_1285	Ca4	8690768	TAAAAAAA/TAAAAAAAAA	GTTTTAATGCAAGCCATGTAGA	GGTGTTTTGATGCGAAGTGA	57.1	397	INTERGENIC				
CaPOPII_1286	Ca4	8698891	AAGAGTAGTAGAGTAGT/AAGAGTAGT	AAGCTGGCCACTGTAAGCAT	TCACAATCTCGACAACCCAA	59.9	246	INTRON	Ca_08365	G		Glycoside hydrolase, family 13, N-terminal
CaPOPII_1287	Ca4	8727347	TAAAAAAAAAAAA/TAAAAAAAAA	CGGGTCTCTTTGACTCTTCG	TCATCTTATTTTGGGCATTCA	60.0	563	INTERGENIC				
CaPOPII_1288	Ca4	8728353	TTTATT/TTTATTATT	AATCCAACGCTCTTTCAATCA	GGAACCAAACCAGCATGAAT	59.7	343	INTERGENIC				
CaPOPII_1289	Ca4	8757082	GATGGATATGGATA/GATGGATA	TGAGTTTTTGGCCAATTCAA	GCAGGTGCAGAAAGATTACCA	59.1	605	DRR	Ca_08371		NAC	No apical meristem (NAM) protein
CaPOPII_1290	Ca4	8758399	TAA/TA	CATAGCAGCTTCGATGCAAA	TCACAATTTGACAACCACGG	60.1	297	INTERGENIC				

INDEL marker IDs	Chromosomes /unanchored scaffolds	Physical positions (bp)	InDels (*Kabuli* reference genome-CDC Frontier/PI)	Forward primers (5'-3')	Reverse primers (5'-3')	Anneling temperature (0C)	Expected amplified product size (bp)	Structural annotation		Functional annotation		
								Sequence components of *kabuli* genome	*Kabuli* gene accession IDs	NCBI-KOG	TFs	NCBI-nr database
CaPOPII_1291	Ca4	8774335	CT/C	GACGATTGCGAATTTGAGGT	TGATTTATGTTTGTGGCGGA	60.1	295	DRR	Ca_08373	C		Lactate/malate dehydrogenase, N-terminal
CaPOPII_1292	Ca4	8775167	ATT/ATTT	TGTTCCCTGCAATTCACATC	ACGGGTCTCGATCAATGTGT	59.5	360	DRR	Ca_08373	C		Lactate/malate dehydrogenase, N-terminal
CaPOPII_1293	Ca4	8816656	AT/A	ATCGTTGTCGTAGATTGGGC	TTGGATCAAAATCTGAAGCG	60.0	789	INTERGENIC				
CaPOPII_1294	Ca4	8864736	TGGGG/TGG	TTTTTGGCTTATGGGGTGAG	TCAGCGTATTGCGAAACAAC	59.9	912	INTERGENIC				
CaPOPII_1295	Ca4	8871044	CATAAAAT/CATAAAATTTATAAAAT	AAAACATCGGAGTCCACGTC	AATTTCATTGCGGCAAATTC	60.0	699	INTERGENIC				
CaPOPII_1296	Ca4	8876702	A/AAGGAAT	ACTTCCCATGCCTTTGTGTC	TTGGTGGTGTTGATGCTGTT	60.0	393	INTRON	Ca_08385	U	B3	WD40 repeat
CaPOPII_1297	Ca4	8891981	CAAA/CAA	ATACACCACAACGCGTCAAA	AAGCATGGTATTCGGGAGTG	60.0	204	DRR	Ca_08386			
CaPOPII_1298	Ca4	8894416	CTATATATATATAT/CTATATATATATATATATAT	TGATATCCGGTTTGGGTGTT	CTCCCGTTATCCGAAACAGA	60.1	569	INTERGENIC				
CaPOPII_1299	Ca4	8897355	TA/T	GATCCACTCAGGGTCGTGTT	ACTTTTGCATCTGGATGGCT	60.0	461	INTERGENIC				
CaPOPII_1300	Ca4	8946430	TGAGAGAGAGAGAGA/TGAGAGAGAGAGAGAGA	ACAGCAACCGAAACTGCTTT	GGGGAGCATTGGTCACTAAA	59.9	432	INTERGENIC				
CaPOPII_1301	Ca4	9028821	TTATATATATATATATATAT/TTATATATATATATATATAT	ATGGAACAATCGGACACCAT	CTGTCAACTCACTCATCAAATCG	60.1	422	INTERGENIC				
CaPOPII_1302	Ca4	9066423	ACC/AC	GCAACCCGTAGGTGTAGGAA	TGTGCGTTGGTAGTCAATGTG	60.0	546	INTERGENIC				
CaPOPII_1303	Ca4	9229798	TGGG/TGGGG	AAGGCTCAACATGCAAAACC	TGCCAATTCGATTGGAATAA	60.1	522	INTERGENIC				
CaPOPII_1304	Ca4	9371255	GAAAAAAAA/GAAAAAAAAA	GGAGCATATGCCTCGTTGTT	TGGAAATTCAACTTTTTGGACA	60.1	549	INTERGENIC				
CaPOPII_1305	Ca4	9625757	ATATTATTATTATTATTATTATTATTATTATT/ATATTATTATTATTATTATTATTATTATTATTATT	ATGTGCGAGAAAGGGAGAGA	CACTTCGGTCATTCTCTTCAAA	60.0	543	INTERGENIC				
CaPOPII_1306	Ca4	9630152	GAA/GA	CCTCTTCCAAACAAATCTTCG	TCAAAATATGGCATGTGGGA	58.8	455	INTERGENIC				
CaPOPII_1307	Ca4	9630289	ATTAGTTAGTT/ATTAGTTAGTTAGTT	CCTCTTCCAAACAAATCTTCG	CCAGCTCATCAAAGCCATTT	58.8	550	INTERGENIC				

INDEL marker IDs	Chromosomes /unanchored scaffolds	Physical positions (bp)	InDels (*Kabuli* reference genome-CDC Frontier/PI)	Forward primers (5'-3')	Reverse primers (5'-3')	Anneling temperature (0C)	Expected amplified product size (bp)	Structural annotation		Functional annotation		
								Sequence components of *kabuli* genome	*Kabuli* gene accession IDs	NCBI-KOG	TFs	NCBI-nr database
CaPOPII_1308	Ca4	9632653	TG/T	TGGATTGTCGTGATCTCAAA	CACACCCGTACTCAGCATTG	57.6	630	INTERGENIC				
CaPOPII_1309	Ca4	9633059	TAAAAAAAAAAA/TAAAAAAAAA	CAATGCTGAGTACGGGTGTG	AACACAAGGAGGCCATGTTC	60.2	503	INTERGENIC				
CaPOPII_1310	Ca4	9704009	AAGTTAGGAGTTAGGAGTTAGGAGTTAGGAGTTAGG/AAGTTAGGAGTTAGGAGTTAGGAGTTAGGAGTTAGGAGTTAGG	TTCCAAAAGCAAACACACCA	CTCTTTCTTGACCTGCCCTG	60.1	745	DRR	Ca_08447	Q	Nin-like	ABC transporter-like
CaPOPII_1311	Ca4	9967472	ATTTTT/ATTTTTT	CCCCTTTTATGAAATGGTTGAA	TGAGACTGAAATCGGGTCTTTT	60.0	945	INTERGENIC				
CaPOPII_1312	Ca4	10033685	TTATATATATATATATATAT/TTATATATATATATATATATAT	CTAGTTCGTAACCCGTGCGT	AGGCATGGTGTTGGAGTCAT	60.2	248	INTERGENIC				
CaPOPII_1313	Ca4	10173137	ATTTTTTTT/ATTTTTTTTT	CCCGTTATTTTTGACGAAACA	TGTGTTGAGTGGTGGCATTT	59.9	791	INTERGENIC				
CaPOPII_1314	Ca4	10233239	GACAATTATTTGGTACAATTAT/GACAATTATTTGGTACAATTATTTGGTACAATTAT	TGTCGGTTGTTGCTTGAAGA	ATACAATCATTTTCCCGCCA	60.4	403	INTERGENIC				
CaPOPII_1315	Ca4	10241994	TAAAAAAAAAAA/TAAAAAAAAAAAA	CCTGCACCATTTCTCTTGGT	TTTTCTGGTAACTCGGACCC	60.1	649	INTERGENIC				
CaPOPII_1316	Ca4	10247608	CAATATTGTAAAATA/CAATATTGTAAAATAATATTGTAAAATA	GAGTTAGGGTTGGTGAGGCA	TTTTTCACGTCGCATAAATCT	60.1	236	INTERGENIC				
CaPOPII_1317	Ca4	10253630	GAAAAAAAA/GAAAAAAA	TGTGTTTGAATGGGCAAAAA	CTTCATTTAAACGCCCCAAA	59.9	312	INTERGENIC				
CaPOPII_1318	Ca4	10261671	ATTTTTT/ATTTTT	TGGATTTTGGACTTGGTCTTG	AACAACACGTGATTTGAATTGC	60.0	654	INTERGENIC				
CaPOPII_1319	Ca4	10456572	ATTTTTTTT/ATTTTTTT	ATCGATCAAAACCCGTGAAG	CGTTGTTGGTAAGGGAATCG	59.9	454	INTERGENIC				
CaPOPII_1320	Ca4	10612304	TTTATTATTATTATTATTATTATTATTATTATTATTATTATTATT/TTTATTATTATTATTATTATTATTATTATTATTATTATTATTATT	AATCACGCAAACAAGTGTCG	AGCGACATAAATGGCTGCTA	59.8	408	INTERGENIC				
CaPOPII_1321	Ca4	10784287	TAAAAAAAAAAA/TAAAAAAAAAA	TGGGCCGAATTTTGTGATAG	CGCACAATTGATACATCAACAC	60.8	486	DRR	Ca_04314	U		Vps51/Vps67
CaPOPII_1322	Ca4	11211716	AC/ACTCCC	GGAACATGGAATGTTGGAGG	GCAAAGTGCATGAAAAGCAA	60.2	500	INTRON	Ca_04353	U	M-type	Inositol polyphosphate-related phosphatase
CaPOPII_1323	Ca4	11216218	TAAAAAAAAA/TAAAAAAA	GCATAACCAATGCTCTTGGAA	TCAAACCAAGGATGATGCAA	60.1	853	DRR	Ca_04353	U	M-type	Inositol polyphosphate-related phosphatase
CaPOPII_1324	Ca4	11229115	ATATTATTA/ATATTA	CCATGTAAGTGAGTGAAATCGG	GTGCACTTAGGCTTGCATGA	59.5	702	INTRON	Ca_04355	G		Major intrinsic protein

INDEL marker IDs	Chromosomes /unanchored scaffolds	Physical positions (bp)	InDels (*Kabuli* reference genome-CDC Frontier/PI)	Forward primers (5'-3')	Reverse primers (5'-3')	Anneling temperature (0C)	Expected amplified product size (bp)	Structural annotation		Functional annotation		
								Sequence components of *kabuli* genome	*Kabuli* gene accession IDs	NCBI-KOG	TFs	NCBI-nr database
CaPOPII_1325	Ca4	11268024	AAAGAA/AAA	TAGTGCCCTCCAAAAGGATG	GCGTTGAGGTGTCAACTGTG	60.1	733	INTERGENIC				
CaPOPII_1326	Ca4	11270494	CATATAT/CATAT	TGTTGACAAACACAAAGACCG	ATCCACCATCAATGCCATCT	59.6	645	INTERGENIC				
CaPOPII_1327	Ca4	11281622	TG/T	TTTAAGGCATGTGAGTTCAGC	TGCAAAATCAATACATGGTTCG	58.0	641	DRR	Ca_04359			
CaPOPII_1328	Ca4	11298187	CTTTTTTTTTTTT/CTTTTTTTTT	AAATGCTCCAAAAACCAACG	ATTAAAATGCCAGCAGTGGG	60.0	471	INTERGENIC				
CaPOPII_1329	Ca4	11300403	ATATTATT/ATATT	GTGTTACTTCCGCCGTTGAT	TGTCAAGTTGTTTCATGCTGC	60.0	185	INTERGENIC				
CaPOPII_1330	Ca4	11311023	CATTATTATTATTATTATTAT/CATTATTATTATTATTATTATTAT	ATGTTTATGTGCCGTCCCTC	GGCCAACCATCAACCATATC	59.8	434	CDS (large-effect mutations)	Ca_04364	D	WRKY	Protein kinase, catalytic domain
CaPOPII_1331	Ca4	11311504	A/ACAC	TGATGGTTGGCCTAAATGGT	TTGCTGAAGACTGATGCCAC	60.2	463	DRR	Ca_04363			
CaPOPII_1332	Ca4	11317243	ATCTCTCTCTCTCTCTC/ATCTCTCTCTCTCTC	CCTTCCACTGCCAAAACATT	CAATTGGTCGGTTTCTGGTT	60.0	430	DRR	Ca_04364	D	WRKY	Protein kinase, catalytic domain
CaPOPII_1333	Ca4	11318155	ATTTTTTTTT/ATTTTTTT	ATTCTCATGGCAAGGGATTG	TGCGTAATGGACCAATTCAA	59.9	909	INTERGENIC				
CaPOPII_1334	Ca4	11322920	AAGCTCCTCTTC/A	TCGATGGCATGATGATTGTT	AAGAGGATTGGAGATAGGAGAGC	59.9	371	INTERGENIC				
CaPOPII_1335	Ca4	11338339	AAGA/AAGAGA	ACGGATAAAATGCCCATTGA	ATTGGTGTGAGTGCTGTGGA	60.2	821	INTERGENIC				
CaPOPII_1336	Ca4	11359837	ACCC/ACC	TGTTGCGGTTGTATGGTGTC	TGACATTGGGGGATTCATTT	60.4	455	INTERGENIC				
CaPOPII_1337	Ca4	11368276	CG/C	TGATGAATTTTCATTCACCAATC	TCCCAAGGTCAATTAGGCAG	58.8	116	INTERGENIC				
CaPOPII_1338	Ca4	11370815	TAAA/TAAAAA	GAGATGGTGAAATCCAACGC	AGGGAGGGGATGAGAGAAAA	60.5	641	INTERGENIC				
CaPOPII_1339	Ca4	11494600	ATAAATTTAAATTTAAATTTA/ATAAATTTAAATTTA	CAGGTTTTCTCTCTCGCACC	CAACACAACCCGTGAACATC	60.0	398	INTERGENIC				
CaPOPII_1340	Ca4	11539392	TA/TATGA	TGCGGTTTTCACAAAATGAC	CAACGGTTCATGATCCAATCT	59.6	192	INTERGENIC				
CaPOPII_1341	Ca4	11549653	TAA/TAAAA	AAATTCGTAAAGGTCCACCG	CCAACGGTGTGCATAATGAG	58.9	729	INTERGENIC				

INDEL marker IDs	Chromosomes /unanchored scaffolds	Physical positions (bp)	InDels (*Kabuli* reference genome-CDC Frontier/PI)	Forward primers (5'-3')	Reverse primers (5'-3')	Anneling temperature (0C)	Expected amplified product size (bp)	Structural annotation		Functional annotation		
								Sequence components of *kabuli* genome	*Kabuli* gene accession IDs	NCBI-KOG	TFs	NCBI-nr database
CaPOPII_1342	Ca4	11555971	CATATATATAT/CATATAT	CATTATTGGATTCGTTGGGG	GGGTGATAGATTCTTGATGCAAT	60.0	362	DRR	Ca_04385	K	Trihelix	SANT domain, DNA binding
CaPOPII_1343	Ca4	11769762	TA/T	TCCATAGTGGAGATGCGACA	GGAACATGCGAATCACAAAA	60.2	666	INTERGENIC				
CaPOPII_1344	Ca4	11818956	CAAA/CAAAA	CCAAAACATGAAAGCAACAAAA	TAACATTGATGGCAGAGGCA	60.0	170	INTERGENIC				
CaPOPII_1345	Ca4	12090377	TTATATATATATATATATATATATAT/TTATATATATATATATATATATATATATAT	ATTTGGTTCTGCGTTTGACC	ACTAGGTTGGAGCATGAGGC	60.0	567	INTERGENIC				
CaPOPII_1346	Ca4	12127716	AAATAATAATAATAATAATAATAATAAT/AAATAATAATAATAATAATAATAAT	CATTCGGTGGACCTTTTACG	TGAAGAGACGAAGCTGCAAA	60.4	343	INTERGENIC				
CaPOPII_1347	Ca4	12225286	G/GAGTCTC	TCATGGAGGCAAAGAAGGAG	TGACATGTGGATCGTGGTTT	60.3	724	DRR	Ca_04444			Protein of unknown function DUF677
CaPOPII_1348	Ca4	12270457	TCAGAC/TCAGACCAGAC	GGTCGTCGGTAGGGGTTTAT	GTGGAGAGTATGGGTGGGAA	60.1	552	INTRON	Ca_04451		C2H2	Zinc finger, C2H2-type
CaPOPII_1349	Ca4	12393377	TAAAAAAAAAAAAAAAA/TAAAAAAAAAAAAAA	TGTGCTGTTGTGATTAGGGG	AGCGTTCAAGGTGCAAAAGT	59.6	659	DRR	Ca_04463	QI	bHLH	Cytochrome P450
CaPOPII_1350	Ca4	12493651	ATTTTTT/ATTTTTTTT	ATTCCTCGTCCGTTTTCAGA	TTTTTCTTCCTGATTTGGTCC	59.7	293	DRR	Ca_04473			
CaPOPII_1351	Ca4	12520618	AATATATATATATAT/AATATATATATATATAT	TCAAAGCTGCATTTTGTTGC	TTCGTATGCTATGCACGCTC	60.0	441	INTERGENIC				
CaPOPII_1352	Ca4	12528995	CTTTT/CTTT	TAACGCTAGGGTCCCATGTC	TCGTTTTGATCATTTATCGTTTTT	60.0	720	INTERGENIC				
CaPOPII_1353	Ca4	12529201	TAAAAAAAAAA/TAAAAAAAA	CATGGCTCCAAGAAGCTTAAA	TTCGTTTTGATCATTTATCGTTTT	59.5	527	INTERGENIC				
CaPOPII_1354	Ca4	12535193	GAAAAAAAA/GAAAAAA	CGAAGCCTGCAACTTTTACC	CCAAACATGCAGGTTCCTCT	59.9	231	DRR	Ca_04476			Remorin, C-terminal
CaPOPII_1355	Ca4	12571013	GTATATA/GTATA	GGCTCACATCAGGAATGGAT	GGCTCTTGGTCATTGGATGT	59.9	968	DRR	Ca_04479	E		Homoserine kinase
CaPOPII_1356	Ca4	12571781	ACCCC/ACCCCC	ACATCCAATGACCAAGAGCC	CCACTTTCGGAACAATTTTGA	59.9	394	INTERGENIC				
CaPOPII_1357	Ca4	12577562	T/TC	TCTGTCAGCCAATGCACTTC	CAGGAATGCCAAAACCAAAT	60.0	409	INTRON	Ca_04480	L		DNA mismatch repair protein MutS, C-terminal domain
CaPOPII_1358	Ca4	12582939	TTAATATAA/TTAA	CAAGCTTGTACCTGGGGTGT	CCGAAAAAGCATCACTGGTT	60.0	503	INTRON	Ca_04480	L		DNA mismatch repair protein MutS, C-terminal domain

INDEL marker IDs	Chromosomes /unanchored scaffolds	Physical positions (bp)	InDels (*Kabuli* reference genome-CDC Frontier/PI)	Forward primers (5'-3')	Reverse primers (5'-3')	Anneling temperature (0C)	Expected amplified product size (bp)	Structural annotation		Functional annotation		
								Sequence components of *kabuli* genome	*Kabuli* gene accession IDs	NCBI-KOG	TFs	NCBI-nr database
CaPOPII_1359	Ca4	13433311	TA/T	CGAGCAAACACAATGAATGG	TTGGTTGGTTCATTGGTTGA	60.1	569	INTERGENIC				
CaPOPII_1360	Ca4	13509527	ATTTT/ATTTTATTAAAAAATTTATTTTT	CCTCTTCACGGTGCTCATTT	CCTCGTTTTAAGCCCAATCA	60.3	158	INTERGENIC				
CaPOPII_1361	Ca4	13546142	TAAA/TAA	GAGATTCACGAAGACCGAGG	GCCAATGCTAATCTGCAACA	59.8	665	DRR	Ca_04571	T		
CaPOPII_1362	Ca4	13579909	ATTT/AT	TTCCCAATTTTTCTTTCAATTTT	GCACTCACCAAGCAATAGCA	58.2	666	INTERGENIC				
CaPOPII_1363	Ca4	13608282	ATTTTTTTT/ATTTTTTTTTT	TCTTTGTCCAAAGGCCAATC	CACATTTTGCAAGGTTCGTG	60.1	795	INTERGENIC				
CaPOPII_1364	Ca4	13617448	TG/TGG	GGGCCTTTTCTTCAGCTTTT	TGCATGTTATCCGCAATCTC	59.8	713	DRR	Ca_04579			Protein of unknown function DUF1677, plant
CaPOPII_1365	Ca4	13627308	A/AT	TTGGCGTGAAAGACAACATT	TGTTGGGTGAGTTTTCCACA	59.2	938	INTERGENIC				
CaPOPII_1366	Ca4	13630632	AA/AAGAGTCTA	TATGCAGGCCCAAACTCTCT	TTTTCACAATCAATGCCCAA	59.8	370	INTERGENIC				
CaPOPII_1367	Ca4	13631062	GCC/GC	TTGGGCATTGATTGTGAAAA	TCGCAACTCGTATAGGACCA	59.9	271	INTERGENIC				
CaPOPII_1368	Ca4	13643836	ATTTTTT/ATTTTTTT	ATAGTTGTTGAAGGGGCACG	GGGTGAAGAATGTATGCCAAA	60.0	798	INTERGENIC				
CaPOPII_1369	Ca4	13657394	CAAAAAAA/CAAAAAAAA	TCGTTTTTCAACCATCAGTCA	TCTCCCAATGTGATTGCTCA	59.2	823	INTERGENIC				
CaPOPII_1370	Ca4	13671186	TCAA/T	CGTCCATTGTGGTTATGCTC	TTCCTAAAAAGTTAAACCGGCA	59.0	685	INTERGENIC				
CaPOPII_1371	Ca4	13678246	C/CT	CCGACGAATTTCCTTCAAAA	TGTTCGAGTCGGTGAGACAG	60.0	583	DRR	Ca_04588	E		Cysteine synthase/cystathionine beta-synthase P-phosphate-binding site
CaPOPII_1372	Ca4	13687679	CAGTTTAGTTTAGTTTAGTTTA/CAGTTTAGTTTAGTTTA	TGGACTCGGTATCAACCACA	GACGAGCTGCGGTATTCTTC	60.0	248	INTRON	Ca_04589			Frigida-like
CaPOPII_1373	Ca4	13708395	CAAA/CAA	TTGGAGGACCAAAACTGGAG	GGCTTGGACAATTTTGTGTG	60.1	795	INTRON	Ca_04592	R		
CaPOPII_1374	Ca4	13749552	AA/AAGA	TTGACTTCTCCTTGGTTGCC	TTTTTGTTGACATGAGGCCA	60.2	758	INTRON	Ca_04599	R	MIKC	Protein kinase, catalytic domain
CaPOPII_1375	Ca4	13789143	CAA/CAAA	ATCCACCACTCAAAGTTGGC	TTGGTACGAAACATCCATCAA	60.0	517	DRR	Ca_04603	Q		Copper amine oxidase

INDEL marker IDs	Chromosomes /unanchored scaffolds	Physical positions (bp)	InDels (*Kabuli* reference genome-CDC Frontier/PI)	Forward primers (5'-3')	Reverse primers (5'-3')	Anneling temperature (0C)	Expected amplified product size (bp)	Structural annotation		Functional annotation		
								Sequence components of *kabuli* genome	*Kabuli* gene accession IDs	NCBI-KOG	TFs	NCBI-nr database
CaPOPII_1376	Ca4	13792182	T/TA	GGCAACATAGATCCTCTCACC	GCTCACTCCGTCAGGTTAGG	58.6	719	INTRON	Ca_04604	Q		Copper amine oxidase
CaPOPII_1377	Ca4	13826117	ATTTTT/ATTTTTT	AGGTGGTTGTTGCACTTTGA	CAAATTTGTTCCGCTTCACA	59.2	474	INTERGENIC				
CaPOPII_1378	Ca4	13829467	AT/ATT	TGAGAATTTGCATGCCTCAC	AGCGAAGTGCTAGCAACACA	59.8	671	INTERGENIC				
CaPOPII_1379	Ca4	13854087	TAAAAAAAAA/TAAAAAAA	CGATCACTGACCATTATATGCAC	CCTTCTTTCCCACAATGTTGA	59.4	611	INTERGENIC				
CaPOPII_1380	Ca4	13865867	ACAC/A	TGACAAACGGATCCTCAAAG	GTCTCCAGCAAGTGTCGTGA	58.7	548	INTRON	Ca_04610	R		Sugar/inositol transporter
CaPOPII_1381	Ca4	13908536	TC/T	CTCCTCCGATGTCTGGAAAA	CGCGGTTGCTTCAATTCTAT	60.2	232	INTERGENIC				
CaPOPII_1382	Ca4	13909254	CA/CAA	CATCACGATTTGACGAGTTGA	AAAAACATGACTCCAAACACCA	59.7	636	INTERGENIC				
CaPOPII_1383	Ca4	14397300	GATATATATATATATATATATGTATATATATATATA/GATATATATATATATATATATATATATATGTATATATATATATA	TAAATGCATGCTCCCCTGAT	AACACATCCGGGATCCATTA	60.4	273	INTERGENIC				
CaPOPII_1384	Ca4	14405624	ATGTTGTT/ATGTTGTTGTT	CGGTGGTTGCTCTCTCTTTC	GGGGTACAATGTGGAACCAA	60.0	440	DRR	Ca_05663	E	MYB	Amino acid transporter, transmembrane
CaPOPII_1385	Ca4	14405755	ATCTCTCTCT/ATCTCTCT	ATGTGCATGCCAAGTTGTGT	ACCTATAAGCCCTTTGCGCT	60.0	963	DRR	Ca_05663	E	MYB	Amino acid transporter, transmembrane
CaPOPII_1386	Ca4	14406133	CAAA/CAAAA	TTGGTTCCACATTGTACCCC	CCATTGGTTGGGATATGGAG	60.5	892	DRR	Ca_05663	E	MYB	Amino acid transporter, transmembrane
CaPOPII_1387	Ca4	14677950	CAAAAAAA/CAAAAAAAA	TGTTGGGCAAAATAAATTGAGA	CAAACCTTACCACCGTCTTG	59.5	424	INTERGENIC				
CaPOPII_1388	Ca4	15048181	TA/TAA	CAAAAGCTGTAACACCCCGT	AATCGATTGATTCAGCCCAG	60.0	528	INTERGENIC				
CaPOPII_1389	Ca4	15465052	T/TG	GCAACTCATTCGTCCCATTT	GGAAACGAAAAATTTGCAGC	59.9	712	INTERGENIC				
CaPOPII_1390	Ca4	16067505	CAAAAAAAA/CAAAAAAA	GGCTAAATTAAATCACTGGTCCC	TACATCTGCGGCCCTTTAAC	60.1	572	INTERGENIC				
CaPOPII_1391	Ca4	16146480	CT/C	AAAGAAGCAGAGGAAGCGTG	GCAAACGTATGATGGGGACT	59.8	337	DRR	Ca_05497	I	bHLH	Alpha/beta hydrolase fold-1
CaPOPII_1392	Ca4	16238878	TTATATATATATATATATATATATA/TTATATATATATATATATATATA	TGTTGTTGCGTTATTCCAGC	CACCCATCGTTTCCCTCTTA	59.7	555	INTERGENIC				

INDEL marker IDs	Chromosomes /unanchored scaffolds	Physical positions (bp)	InDels (*Kabuli* reference genome-CDC Frontier/PI)	Forward primers (5'-3')	Reverse primers (5'-3')	Anneling temperature (0C)	Expected amplified product size (bp)	Structural annotation		Functional annotation		
								Sequence components of *kabuli* genome	*Kabuli* gene accession IDs	NCBI-KOG	TFs	NCBI-nr database
CaPOPII_1393	Ca4	16481759	CA/CAAA	TCGAGACACCAACACCGTAA	AATTCGTCAAACCCACAACC	60.2	732	INTERGENIC				
CaPOPII_1394	Ca4	16793556	AG/AGG	ACACTTGCTATGAAACGGGG	ACGCTTTGCAAGGTCTGAAT	60.0	590	INTERGENIC				
CaPOPII_1395	Ca4	16826129	A/AAATT	TTTTATCCACCAAAAATTCAAAAA	TGATTTTTCAGGAGGCCACT	58.9	521	DRR	Ca_05439			
CaPOPII_1396	Ca4	16943222	TATGAATGAAT/TATGAATGAATGAAT	CTCAAGCTCAGTCCCAGTCC	TGCGTTTTGTTTGGTGTAGC	60.0	668	INTERGENIC				
CaPOPII_1397	Ca4	16960625	GTT/GTTT	GCGAATTGCAGACTGAAACA	CTGGAAAATCCTCCTCCTCC	60.0	925	DRR	Ca_05428			
CaPOPII_1398	Ca4	17010005	AAAAAATAATATC/AAAAAATAATATCATTTAAAAATAATATC	ACGGCCTAGCTTATTCCCAT	GTATGCAAATGGGTGCTGTG	60.0	690	DRR	Ca_05423			
CaPOPII_1399	Ca4	17010261	GAGACA/GAGACAGACA	GTTAGCCTCACAGCACCCAT	GCTTCTAACTCGGCAAGTGG	60.1	342	DRR	Ca_05422		G2-like	Myb, DNA-binding
CaPOPII_1400	Ca4	17030182	TAA/TAAAA	AAGATTTTGTGTTTGAACCCTCA	TCTCGCCTTTTGTATCGTCA	59.9	426	DRR	Ca_05420	R	bHLH	Pentatricopeptide repeat
CaPOPII_1401	Ca4	17081756	TTATA/TTATATA	TACCGGAAGATTCGGAAATG	CTGCAGGTAAGCATCCCAAT	59.9	694	INTRON	Ca_05415			
CaPOPII_1402	Ca4	17082004	TGAT/TGATCGAT	TTTTGACAATGGGCACTCAA	CTGCAGGTAAGCATCCCAAT	60.1	295	DRR	Ca_05415			
CaPOPII_1403	Ca4	17096036	CTTTCAA/CTTTCAAGTTTCAA	CTGCAATTGTCAAGCTCCAA	TCCATATGTGCCCATTGCTA	60.0	276	INTERGENIC				
CaPOPII_1404	Ca4	17146270	AGGGG/AGGG	ATTCCAGCTCCCACAATCAC	GGCTCTTGATGGGTTGTGTT	59.9	442	DRR	Ca_05409	K		
CaPOPII_1405	Ca4	17196085	TTCTCTCTCTCTCTCTCTCTCTCTCTCT/TTCTCTCTCTCTCTCTCTCTCTCTCTCTCTCTCT	CCTCAACCATGGCCTGTACT	AGTTGCATTGGAGACAAGGG	60.0	118	INTERGENIC				
CaPOPII_1406	Ca4	17214261	GCTATC/G	GCTTTTTAGGCTGGGGAATC	AATAATTTGGTCAGGCGTGC	60.0	194	DRR	Ca_05402	C		ATPase, alpha/beta subunit, nucleotide-binding domain
CaPOPII_1407	Ca4	17214540	A/ATATAG	GCTTTTTAGGCTGGGGAATC	TTGGTCAATGCGGAAGTGTA	60.0	627	DRR	Ca_05402	C		ATPase, alpha/beta subunit, nucleotide-binding domain
CaPOPII_1408	Ca4	17214599	AC/ACTTC	GCTTTTTAGGCTGGGGAATC	TTGGTCAATGCGGAAGTGTA	60.0	627	DRR	Ca_05402	C		ATPase, alpha/beta subunit, nucleotide-binding domain
CaPOPII_1409	Ca4	17238317	CTTTATTT/CTTTATTTATTT	AAAAATGGGGTGGATGAACA	TTCCAATGTTGCAGAAGCAG	60.0	439	DRR	Ca_05400	YU		HEAT

INDEL marker IDs	Chromosomes /unanchored scaffolds	Physical positions (bp)	InDels (*Kabuli* reference genome-CDC Frontier/PI)	Forward primers (5'-3')	Reverse primers (5'-3')	Anneling temperature (0C)	Expected amplified product size (bp)	Structural annotation		Functional annotation		
								Sequence components of *kabuli* genome	*Kabuli* gene accession IDs	NCBI-KOG	TFs	NCBI-nr database
CaPOPII_1410	Ca4	17243538	AT/A	TTCACACATGCATTGTTTACCA	TGCAAAACTGCTCTACCCAA	59.9	422	INTERGENIC				
CaPOPII_1411	Ca4	17247653	GAAAAAAAA/GAAAAAAAAAA	TTTATGTTTGGCCAACTCCC	TTTTTAGGGACGCAATTCAAA	59.8	539	INTERGENIC				
CaPOPII_1412	Ca4	17259358	TAAA/TAAAA	ATGGGTGCATTTGTTTCAGA	GCCTGAGTTTGGCATACGAT	59.0	338	INTERGENIC				
CaPOPII_1413	Ca4	17268685	TGG/TGGG	CATCCCATCCAGGACCTTTA	CTGGTTCCCGGTTGAACTTA	59.7	308	DRR	Ca_05398	R		Major facilitator superfamily MFS-1
CaPOPII_1414	Ca4	17286686	CATTGGATTGGATTG/CATTGGATTG	TGCATTTCATGCCTTTGCTA	TGATTAGCCCGTATTTTGGG	60.4	648	INTRON	Ca_05393	R		Ankyrin repeat-containing domain
CaPOPII_1415	Ca4	17297039	TAAAAAA/TAAAAAAA	TTTGCCTCAGCTGTGTTGAC	TCATCAGCTGGACAACTTCG	60.0	607	INTRON	Ca_05392	R		Ankyrin repeat-containing domain
CaPOPII_1416	Ca4	17313148	TC/T	AGAGGATGCAGCTATGCGTT	CAACATTTTGGGAGGGAAGA	60.0	170	INTERGENIC				
CaPOPII_1417	Ca4	17313521	TAAA/TAA	GCTAGACAGGTTTGCCTTCG	AAGGCTCCTGCAAGCATTTA	60.0	966	INTERGENIC				
CaPOPII_1418	Ca4	17314848	CAA/CAAACCGAACTAGAA	TTGTAAAAACTGCATCAATCGAA	CTTCCCCACTCACAGTCCAT	59.7	488	DRR	Ca_05390	M		Mechanosensitive ion channel MscS
CaPOPII_1419	Ca4	17318086	A/AG	CTCAAAAGTGGGATAGGGCA	ACAGCGTGAAACAAGATCCC	60.1	429	INTRON	Ca_05390	M		Mechanosensitive ion channel MscS
CaPOPII_1420	Ca4	17318109	AA/AATATTA	CTCAAAAGTGGGATAGGGCA	ACAGCGTGAAACAAGATCCC	60.1	429	INTRON	Ca_05390	M		Mechanosensitive ion channel MscS
CaPOPII_1421	Ca4	17321954	TA/TATATATATATAAA	CTGCCGACCTGAATACCAAT	TGCCTTTAATGAAACACACCA	60.0	541	INTERGENIC				
CaPOPII_1422	Ca4	17328665	GAA/GAAAA	ACACGTGCAATATCGGTGAA	TCCTCCCTCTTTATCATGCC	60.0	750	INTERGENIC				
CaPOPII_1423	Ca4	17339892	ATTT/ATT	TTTTGGGGAACACAACCTTT	GACATTTCTCATAGGGGCGA	59.3	297	INTERGENIC				
CaPOPII_1424	Ca4	17367149	AATAT/AAT	GCAGCTAAAGGAGAGTGCCA	AGACAGTTCGCTCGCAAATC	60.7	506	INTERGENIC				
CaPOPII_1425	Ca4	17379518	TG/T	AACAAATGGTGCATCACGAA	TCTTCATTGGTCTCGTGCTT	60.0	740	INTRON	Ca_05387	R		F-box domain, cyclin-like
CaPOPII_1426	Ca4	17431572	GC/G	TCTGTCATTCCTGCCATTTTT	TCGCAATGTAGCTCCCTCTT	59.6	858	INTRON	Ca_05384	K		Transcription factor jumonji/aspartyl beta-hydroxylase

INDEL marker IDs	Chromosomes /unanchored scaffolds	Physical positions (bp)	InDels (*Kabuli* reference genome-CDC Frontier/PI)	Forward primers (5'-3')	Reverse primers (5'-3')	Anneling temperature (0C)	Expected amplified product size (bp)	Structural annotation		Functional annotation		
								Sequence components of *kabuli* genome	*Kabuli* gene accession IDs	NCBI-KOG	TFs	NCBI-nr database
CaPOPII_1427	Ca4	17464214	CTATATAT/CTATATATATAT	ACCACTCACCAACCTTCCAG	AACCCGGAGACCCAATTTAG	60.0	635	INTRON	Ca_05381	O	GRAS	Zinc finger, RING-type
CaPOPII_1428	Ca4	17464247	CA/CGAGGA	ACCACTCACCAACCTTCCAG	AACCCGGAGACCCAATTTAG	60.0	635	INTRON	Ca_05381	O	GRAS	Zinc finger, RING-type
CaPOPII_1429	Ca4	17475440	GT/GTTGTTATT	CGGGCAGTAGCATTAGATGG	GGATGAATGGGAGGTGGTTA	60.6	210	INTERGENIC				
CaPOPII_1430	Ca4	17480298	ATATTATTATTATTATTAT/ATATTATTATTAT	TTGTTGGCCGTCATATGCTA	GACCAATTACAAGCGAGGGA	60.1	617	INTRON	Ca_05379	O		Oligosaccharyl transferase, STT3 subunit
CaPOPII_1431	Ca4	17527467	AGT/AGTGT	CAAAGTTAAGAAGGCAGGCG	CAAAATTGGTGGGATTTTGG	60.0	578	INTRON	Ca_05374	R	NAC	Pentatricopeptide repeat
CaPOPII_1432	Ca4	17533077	CAGTG/CAGTGCATAAGTG	TGGTGAGTTGTACCGTGCTC	AAAATGGCACCAGCAAAAAG	59.8	358	INTERGENIC				
CaPOPII_1433	Ca4	17534710	ATATTT/ATT	CTGGATCCCCTCGGAAGTAT	TAGAAACCGCACACACACAA	60.3	317	INTERGENIC				
CaPOPII_1434	Ca4	17600797	TC/T	AAGGGTGATGGACGAAAAAC	TTAAATGGTCGCATAGAGCAA	58.9	682	INTERGENIC				
CaPOPII_1435	Ca4	17600811	TAAAAAA/TAAAAAAA	AAGGGTGATGGACGAAAAAC	TCCAACAACATCAAATTCATCC	58.9	951	INTERGENIC				
CaPOPII_1436	Ca4	17601888	TAAA/TAAAA	CAAAATGGGGATAGACACGG	TTTCAAATGAACGTTTGCTACA	60.2	440	INTERGENIC				
CaPOPII_1437	Ca4	17602358	GT/GTT	TTTGAATGTGTATTGTAGCAAACG	CCTATTCTCGAGAGCAACCG	59.2	322	INTERGENIC				
CaPOPII_1438	Ca4	17632165	TTTTTGCTT/TTTTTGCTTCAATTTTGCTT	ACACAGATCCCATCCCATGT	ATTTACGGCCACCAAAACTG	60.1	575	INTRON	Ca_05367	Z	WRKY	IQ motif, EF-hand binding site
CaPOPII_1439	Ca4	17654597	TCACACAC/TCACACACAC	TCCAATTGACACCACTGCTC	TTTTCAACATTCTAAAAATGGGC	59.7	542	DRR	Ca_05364	S		Protein of unknown function DUF829, TMEM53
CaPOPII_1440	Ca4	17672684	GATA/GA	TGTAGTCCCTCTGTCCCAAAA	CGGAAGATACCAAGTCGCAT	59.6	433	DRR	Ca_05360			GRAM
CaPOPII_1441	Ca4	17738882	AC/A	TTCTTTCTCTTGGTGTTGCG	TTGCTGTATCAAGTGGCAAAA	59.0	745	INTERGENIC				
CaPOPII_1442	Ca4	17766884	A/AAAT	TGCACCAGACATATTTTCGC	AAAAGCCACACAGGATGGAC	59.7	873	DRR	Ca_05348			
CaPOPII_1443	Ca4	17786473	TTTTTGT/TT	CGCGCCAACTTTACTTCTTC	TCCAAAATTTAGTGGCAGCA	60.0	621	INTERGENIC				

INDEL marker IDs	Chromosomes /unanchored scaffolds	Physical positions (bp)	InDels (*Kabuli* reference genome-CDC Frontier/PI)	Forward primers (5'-3')	Reverse primers (5'-3')	Anneling temperature (0C)	Expected amplified product size (bp)	Structural annotation		Functional annotation		
								Sequence components of *kabuli* genome	*Kabuli* gene accession IDs	NCBI-KOG	TFs	NCBI-nr database
CaPOPII_1444	Ca4	17823791	TAAAAAAAAAAAA/TAAAAAAAAAA	TCTCATGTCTTGCAAAATTCTTCT	TTTGTGATTGATATTTGCCGTT	59.4	354	INTERGENIC				
CaPOPII_1445	Ca4	17825306	TAAAA/TAAA	AATCCATCAAACTCGGTTGC	TGCTATTTGGTTGGCCTTTT	59.9	301	INTERGENIC				
CaPOPII_1446	Ca4	17825598	ACC/ACCC	AATCCATCAAACTCGGTTGC	GGGCCCCGTAAGTATTCAGT	59.9	679	INTERGENIC				
CaPOPII_1447	Ca4	17825906	ATTTTTTT/ATTTTTT	AAAAGGCCAACCAAATAGCA	CCTCAATTCTCGATTTCCCA	59.6	555	INTERGENIC				
CaPOPII_1448	Ca4	17825981	ATTTTTTTT/ATTTTTTT	GGGGCCCCATAATCATATTT	CCTCAATTCTCGATTTCCCA	59.7	164	INTERGENIC				
CaPOPII_1449	Ca4	17849398	AGGG/AGGGG	AGTGTAAGTGCCCAAGGTGG	CCGTTCGTTAAGTGCGAAAT	60.0	156	INTRON	Ca_18621	J		Methionyl-tRNA synthetase
CaPOPII_1450	Ca4	17865546	TAAAAAA/TAAAAAAA	GAGGTGTTTTATTGCCTCGG	TGTTAATCCCAATTCAATATCCC	59.6	133	INTERGENIC				
CaPOPII_1451	Ca4	17873043	TAAA/TA	CATTGAAATGCATGGACCAA	TTTTCTCCATGCACCCTACC	60.3	885	INTERGENIC				
CaPOPII_1452	Ca4	17942733	CTATATATATATATAT/CTATATATATATATATAT	CATCGTCAGCACATCACAAA	CTTAGGAGGTCTTGCCCCTC	59.2	447	INTERGENIC				
CaPOPII_1453	Ca4	17959700	C/CATTCCCTT	AGTGGCATGTGGGGATTAAA	TTTGGTGGAAATGGCATGTA	60.2	168	INTERGENIC				
CaPOPII_1454	Ca4	18041622	CTTTTTTT/CTTTTTTTT	CCTGTTTTCCTCCACCCTTT	ATGGGGACTTGGGGAGTAAC	60.3	660	INTERGENIC				
CaPOPII_1455	Ca4	18061059	TATAATAATAATAATAATAATAATAA/TATAATAATAATAATAATAATAATAATAATAA	GAAGCGACCACCTGAAGAAC	CAGAACAAATGACGTGTCCC	59.9	536	INTERGENIC				
CaPOPII_1456	Ca4	18091373	TCATCAC/T	CCACGGATGTGATTTAGCCT	GAAGCACCGTTCCTTCTTGA	60.0	401	INTERGENIC				
CaPOPII_1457	Ca4	18121565	TAAGAAG/TAAG	GCTGCACCATGAGATCTTGA	ATGGTGCTGGAAGTTGCTTT	60.0	440	INTRON	Ca_18652			Chalcone isomerase, subgroup
CaPOPII_1458	Ca4	18196212	ATTT/ATTTAATCTTTTT	GTGGTCCAGATTCTCCCAAA	TGTCGCTTCAGCAAAAAGAA	59.9	549	INTERGENIC				
CaPOPII_1459	Ca4	18388650	ATATTA/ATA	AGTTATCGATCACGGATGCC	CGTGTATATTCGCGCACTCA	59.9	514	INTERGENIC				
CaPOPII_1460	Ca4	18554841	TTATATATATATATATATATATATATATAT/TTATATATATATATATATATATATATAT	TGGACCATATGTGTTGGGTC	CCCGGATAATTGTTCACTTTG	59.1	809	INTERGENIC				

INDEL marker IDs	Chromosomes /unanchored scaffolds	Physical positions (bp)	InDels (*Kabuli* reference genome-CDC Frontier/PI)	Forward primers (5'-3')	Reverse primers (5'-3')	Anneling temperature (0C)	Expected amplified product size (bp)	Structural annotation		Functional annotation		
								Sequence components of *kabuli* genome	*Kabuli* gene accession IDs	NCBI-KOG	TFs	NCBI-nr database
CaPOPII_1461	Ca4	18703975	GACACA/GACA	CCACATTGACATAAACATCTTCTCA	TCAACAACGTTGCTCTTTTTG	60.3	173	INTERGENIC				
CaPOPII_1462	Ca4	18883677	AATATATATATATATATATATATATATATA/AATATATATATATATATATATATATATATATATATATATA	AAAATGTAGGGTGGACGGTG	TATTCCCCCAATGAAGGACA	59.7	750	DRR	Ca_13985	E	WRKY	Pyruvate carboxyltransferase
CaPOPII_1463	Ca4	19045820	A/AT	GGGCTTGCTTGGTTTGAATA	TCAACTGCACGTGTTAAATGC	60.1	825	INTERGENIC				
CaPOPII_1464	Ca4	19081618	CAATAATAATAATAATAATAATAA/CAATAATAATAATAATAATAATAATAA	CGACACTTCAACACAAAATTCA	TGTTGAAAACAACATGTTCCC	58.7	893	INTERGENIC				
CaPOPII_1465	Ca4	19228202	TATG/TATGATCGTGATG	GCAATCTGGAAAGATGGGAA	AAATTCGCCCTGTGTTTTTG	60.0	288	CDS (large-effect mutations)	Ca_14021	T	WRKY	Protein kinase, catalytic domain
CaPOPII_1466	Ca4	19342121	TAAAAAAAA/TAAAAAAAAA	TTCCGAAAATTCACATGCAC	GAATTTGGATTATTCGATTCTCTGA	59.5	388	INTERGENIC				
CaPOPII_1467	Ca4	19369951	CAAAAA/CAAAAAA	CCCATGTTTAAGGGTGGTTG	AATTTTTCACGACCTTCTTCAA	60.1	561	INTERGENIC				
CaPOPII_1468	Ca4	19397426	GAAAA/GAAA	GCCAACATATGCTTGATGGA	TTGAACTTTCTCCGTTTGGG	59.5	667	INTERGENIC				
CaPOPII_1469	Ca4	19643688	CTTTTT/CTTTTTT	GGTGCAGTTGTTTGATGCAC	ATGCGATTTACATGGTTGGC	60.2	255	INTERGENIC				
CaPOPII_1470	Ca4	19678061	C/CGGCTGGTACAA	ATTTTGGCAACGGCTGATAC	CCACAATCCTCCGTTGTCTT	60.0	492	INTERGENIC				
CaPOPII_1471	Ca4	19789343	A/AG	CTAGGGGATTGCATTCCAAA	TTTTCCTGCCAAACACCTTC	59.9	177	INTERGENIC				
CaPOPII_1472	Ca4	19886492	CATTTCTTATTTCTT/CATTTCTT	GACCCCCTGACAAAGACAGA	AGAGGGTTGTGCACTTGAGC	60.1	415	INTERGENIC				
CaPOPII_1473	Ca4	20051846	GTAT/GTATGTGATTAGTGACTAT	CACGAAGCGTATTGACTCCA	AAATTTGAAGGCGCTTTTTG	59.9	432	INTERGENIC				
CaPOPII_1474	Ca4	20615648	ATTTTTTTTTTTTTT/ATTTTTTTTTTTTT	CCATTGTCGTTGTGTTTTGC	TGAATAATCTGCACGTCCCA	60.0	460	INTERGENIC				
CaPOPII_1475	Ca4	20709240	ATTTTTTT/ATTTTTTTT	ACTTTGCCCATCAGTTTTGC	TCCGTTGGTGAAAGTTTGAA	60.1	251	INTERGENIC				
CaPOPII_1476	Ca4	20773849	CTTATTATTATTAT/CTTATTATTATTATTAT	TCTCGGCTGAAGGAACAGAT	AGCGGAACAACGACAAGATT	60.0	524	INTRON	Ca_17170		E2F/DP	
CaPOPII_1477	Ca4	20798832	TTTTATTTATTTATTTATT/TTTTATTTATTTATT	TAGTGCTTTTGCATGCCTCA	TTTGTCCTGGGAAAATGCTC	60.5	316	INTERGENIC				

INDEL marker IDs	Chromosomes /unanchored scaffolds	Physical positions (bp)	InDels (*Kabuli* reference genome-CDC Frontier/PI)	Forward primers (5'-3')	Reverse primers (5'-3')	Anneling temperature (0C)	Expected amplified product size (bp)	Structural annotation		Functional annotation		
								Sequence components of *kabuli* genome	*Kabuli* gene accession IDs	NCBI-KOG	TFs	NCBI-nr database
CaPOPII_1478	Ca4	21082826	AAATAATAATAATAATAATAATAA/AAATAATAATAATAATAATAA	GTGCGTGCTTTGGGATTTTA	CGAACTGAAGAACGATTAGGTG	61.0	332	INTERGENIC				
CaPOPII_1479	Ca4	21261688	AATATATATATATATATATATATATATATATATA/AATATATATATATATATATATATATATATATATATATATATATATATA	GAAAAATTTGCTGAGCTGGTG	TGCGACAGAATCTTAATGTGC	59.9	485	INTERGENIC				
CaPOPII_1480	Ca4	21529168	CAAAA/CAA	TGGGGGATTGTATTCCAAAA	TATGGAATGGACCTCCTGCT	60.0	499	INTERGENIC				
CaPOPII_1481	Ca4	21622605	TAATGTTAAAA/TAATGTTAAAAAAATGTTAAAA	CGTGTATATACGCACACCCAA	GTTTTGGCGTTGGTTTCACT	59.4	805	INTERGENIC				
CaPOPII_1482	Ca4	21728459	TAA/TAATAATAATAAAAA	CCATTTCGTTGCTCCATCTT	TGGTTTTTGTTGTTTGGTTTCT	60.1	267	INTERGENIC				
CaPOPII_1483	Ca4	21804779	CTGT/CTGTTATGT	CCGCATCATAAACAGCCTTT	CTAAAGCTGGTGCTTTTGGC	60.1	159	INTERGENIC				
CaPOPII_1484	Ca4	21933188	GAAAAAAAAAAA/GAAAAAAAAAA	GTTGGGCAATGGATCTGAAT	TGCTCACCGTTTGATCTTTG	59.8	862	DRR	Ca_20146			Peptidase C48, SUMO/Sentrin/Ubl1
CaPOPII_1485	Ca4	22134995	CATAT/CAT	GCACCTATGGTGGCAAAAAC	TTTGATGCCCACATTGAAGA	60.4	506	DRR	Ca_20134	J		Brix domain
CaPOPII_1486	Ca4	22441886	CTA/CTATA	TCCAATGGTGGAAAATAAACG	TCAATCACAAATTTCAAGGAGG	59.7	848	INTERGENIC				
CaPOPII_1487	Ca4	22546535	AATATATATATATA/AATATATATATATATA	CATTTTGGTTTCAAAGCCGT	CCAGGGGATGAGATCATAGG	60.0	714	INTERGENIC				
CaPOPII_1488	Ca4	22649924	A/AT	CCGTCCAAATCAAATGAAAAC	CATTTACCATGTGCTTTTTATGC	59.3	491	INTERGENIC				
CaPOPII_1489	Ca4	22719475	A/AT	ACACGTCATTTGTTTTGGCA	TGTTGCAATAGAAGGTCGCA	60.0	390	INTERGENIC				
CaPOPII_1490	Ca4	22728327	ATTTTTTTTT/ATTTTTTTT	GCAAAAACGTTTGTCCCTTG	CCATCGTAGTTGACAAATGGC	60.5	315	INTERGENIC				
CaPOPII_1491	Ca4	22728505	GTTG/GTTGTTTTTG	GCAAAAACGTTTGTCCCTTG	TCAGTGTTGTTCAGCACGTT	60.5	459	INTERGENIC				
CaPOPII_1492	Ca4	22782656	TTATAAATATACAT/TTATAAATATACATATAAATATACAT	AAGAGCAGGACAAATTAATGAGA	TTTGGCATTGATGAATTGGA	57.2	733	INTERGENIC				
CaPOPII_1493	Ca4	22792153	GAAAAAAAAA/GAAAAAAAAAA	CTCATTTTCATTCACCGGCT	ACGAAGCATTAGGCATGGAT	60.1	931	DRR	Ca_14472			
CaPOPII_1494	Ca4	22808161	A/AC	CATGGTTTTCGCTTACCGTT	TTGGGTGATGTGGCAAAATA	60.0	559	DRR	Ca_14470	H		Fumarate reductase/succinate dehydrogenase flavoprotein, N-terminal

INDEL marker IDs	Chromosomes /unanchored scaffolds	Physical positions (bp)	InDels (*Kabuli* reference genome-CDC Frontier/PI)	Forward primers (5'-3')	Reverse primers (5'-3')	Anneling temperature (0C)	Expected amplified product size (bp)	Structural annotation		Functional annotation		
								Sequence components of *kabuli* genome	*Kabuli* gene accession IDs	NCBI-KOG	TFs	NCBI-nr database
CaPOPII_1495	Ca4	22818473	GTATATATATAT/GTATATATATATAT	CAATTGCATGTTGAAATGGTG	TGGCCATATTGTTTTTGCAT	59.8	821	INTERGENIC				
CaPOPII_1496	Ca4	22825336	TTT/TTTGTT	TGGCTTCCATAGTAAGAGAGTGAA	AAATAACGTGTCACGGGGAT	59.5	546	INTERGENIC				
CaPOPII_1497	Ca4	22867161	ATTTT/ATTTTT	TCACCACTGACCAACCCTTT	CACCACCACCACAAAACACT	60.4	485	INTERGENIC				
CaPOPII_1498	Ca4	22867454	GAAAAAAAAAA/GAAAAAAAAA	GATCTTCATCCCCTGCAGAC	CACCACCACCACAAAACACT	59.6	292	INTERGENIC				
CaPOPII_1499	Ca4	22889580	AC/A	GGGTCAGGATACTTACAACCCA	TCCACCCCCAAATTTTATCA	60.1	717	INTERGENIC				
CaPOPII_1500	Ca4	23002623	TTTACTATTACTATT/TTTACTATTACTATTACTATT	TTTATGGCGGCTGGTTTTAC	ACGATGCATTTGATTGATGTT	60.0	546	INTERGENIC				
CaPOPII_1501	Ca4	23014862	CATATATA/CATATATATA	GAACATGTGCGTGCTACGTT	GCTGTTCAAAGGGCTAGACG	59.8	756	INTERGENIC				
CaPOPII_1502	Ca4	23076109	CTTT/CTTTT	TGGCATTGCAAGAAGAAACA	CCTCTTTCAACACCTTTGGC	60.4	760	INTERGENIC				
CaPOPII_1503	Ca4	23145296	TAAAAAAA/TAAAAAAAA	TGCATGTTTCATGGTGATCC	CACCAATTACAGTGGCATCAA	60.3	680	INTERGENIC				
CaPOPII_1504	Ca4	23145659	ATTTTTTT/ATTTTTT	TGGGAACTTAGGATTCCGTTC	CAAATTTGAATCCGCCTACTG	60.3	587	INTERGENIC				
CaPOPII_1505	Ca4	23272983	ATTTTTTTTT/ATTTTTTTT	ACCACATCGGAAGAAACGAC	GGACGAAGTAGAACGGACCA	60.0	596	INTERGENIC				
CaPOPII_1506	Ca4	23721197	TAAAACA/TAAAACAAAACA	ATTTGTGTGCATTTGCCTGA	TGGGCAATCGACTGACTAAA	60.1	353	INTERGENIC				
CaPOPII_1507	Ca4	24362931	TAA/TA	GGTTTAGATGGGGGATCGTT	CCCCGAGTTGGTCCTTTATT	60.0	947	INTERGENIC				
CaPOPII_1508	Ca4	24425317	GTTT/GTTTT	TGGGGTGTTTCATAGCATCA	ACCGTTTAATTGGCGAACAG	59.9	267	INTERGENIC				
CaPOPII_1509	Ca4	24427573	GTT/GTTT	CTGGAAAATCCTCCTCCTCC	TTCGTCTCAACAATCTCCCC	60.0	346	INTERGENIC				
CaPOPII_1510	Ca4	24438141	CAAAAAA/CAAAAA	CCGCTCTGGAAATCATTCAT	AAGACTCGGGATAATGGCCT	60.0	551	DRR	Ca_20867			
CaPOPII_1511	Ca4	24478956	CAA/CAAA	CGGTTCTACACAAGTTGCGA	TCGATTATGCGTAGGGAAGG	59.9	746	INTERGENIC				

INDEL marker IDs	Chromosomes /unanchored scaffolds	Physical positions (bp)	InDels (*Kabuli* reference genome-CDC Frontier/PI)	Forward primers (5'-3')	Reverse primers (5'-3')	Anneling temperature (0C)	Expected amplified product size (bp)	Structural annotation		Functional annotation		
								Sequence components of *kabuli* genome	*Kabuli* gene accession IDs	NCBI-KOG	TFs	NCBI-nr database
CaPOPII_1512	Ca4	24479035	ATTTTTTT/ATTTTTTTTTT	CGGTTCTACACAAGTTGCGA	TCGATTATGCGTAGGGAAGG	59.9	746	INTERGENIC				
CaPOPII_1513	Ca4	24488859	TAAA/TAAAA	TTGGAACTCATGCAAATGGA	CGATACTCACCCCTCGTTGT	60.0	557	INTERGENIC				
CaPOPII_1514	Ca4	24607917	GATGAATGAATGAATGAATGAATGAATGAATGA/GATGAATGAATGAATGAATGAATGAATGA	CTTCAGGGCCGAAAATAACA	CGAAAACATCCCTCCTTTCA	60.1	404	INTRON	Ca_20857			Brain/reproductive organ-expressed protein
CaPOPII_1515	Ca4	24677817	ATTTTTTTTT/ATTTTTTTTTT	CACGAAAACCTTAAACCCCA	TCTTAAAAGCGATGTAAAGGGC	59.8	526	INTERGENIC				
CaPOPII_1516	Ca4	24748886	GAAAAAAAAA/GAAAAAAAA	CACGACCACATCTGTGAGAAA	TTCCAAAATTTCTTCAACACTCAA	59.7	629	INTERGENIC				
CaPOPII_1517	Ca4	24828297	ATTTTTTTTTT/ATTTTTTTTTTT	TGGAATTTCAGACTGCCTTG	GGGCACGAAGAAGCACTATC	58.8	716	INTERGENIC				
CaPOPII_1518	Ca4	25036917	T/TA	ATCCAAGTTGAGCAAGCATT	CATTGAGACTTTGCCACGAA	57.4	605	INTERGENIC				
CaPOPII_1519	Ca4	25046968	TGCTGAGCTGA/TGCTGA	ATGTTTCGTGCAATGGTCAA	AAGGTCAAGGGCCAAAAACT	60.0	601	INTERGENIC				
CaPOPII_1520	Ca4	25071336	CA/C	TGAGACTGCTTGGCTACCCT	ATCTGAATCATCCCAAGCCA	60.0	488	INTERGENIC				
CaPOPII_1521	Ca4	25088043	CCT/CCTCCTTCTTCT	AACCTTCGCAAGAACCCTTC	ATCAATCTCGTTTTCCTGGG	60.6	219	INTERGENIC				
CaPOPII_1522	Ca4	25146347	GAAAAAAAA/GAAAAAAA	TCATCCTTGCCTCGATTAGG	GGAAATGCAAATCAAAACCAA	60.2	566	INTERGENIC				
CaPOPII_1523	Ca4	25168771	AA/AATA	GACCAAGGCAAGCAAGAAAG	GTGGAAGTTGAAACGACGGT	60.0	178	INTERGENIC				
CaPOPII_1524	Ca4	25252210	GTT/GT	AAGGGGGCCCTAATAAGGTT	GATGTGCCTGGTTGGAAAGT	60.0	855	DRR	Ca_16567	CD		GRIM-19
CaPOPII_1525	Ca4	25258427	GAAAAAAAAA/GAAAAAAAAAA	TTTGTGCGCAAACAACAGA	TGTGGGGAGCAAAAGTGTTT	60.0	626	INTERGENIC				
CaPOPII_1526	Ca4	25280052	CTTT/CTT	CTCACTTTTGTTCCCCCAAA	TGGTGCAAGGTCTAAAGGAGA	59.9	292	INTERGENIC				
CaPOPII_1527	Ca4	25352610	T/TA	GGAAATGGTTCTTTGAAAGCC	AGCCTTACGCATGTATTGCC	59.9	719	INTERGENIC				
CaPOPII_1528	Ca4	25457334	ATTTTTTT/ATTTTTTTT	CATTTTACTCATCGATCTTTGTGG	TGGACAAAATCATGTCTCTTTGA	59.9	336	DRR	Ca_16577			

INDEL marker IDs	Chromosomes /unanchored scaffolds	Physical positions (bp)	InDels (*Kabuli* reference genome-CDC Frontier/PI)	Forward primers (5'-3')	Reverse primers (5'-3')	Anneling temperature (0C)	Expected amplified product size (bp)	Structural annotation		Functional annotation		
								Sequence components of *kabuli* genome	*Kabuli* gene accession IDs	NCBI-KOG	TFs	NCBI-nr database
CaPOPII_1529	Ca4	25457701	AATATATATATATATATATATATATAT A/AATATATATATATATATATATATATA TATATA	TCAAAGAGACATGATTT TGTCCA	TCACTTTGCTTTGTGTG TCTCA	59.6	524	DRR	Ca_16577			
CaPOPII_1530	Ca4	25543812	TTTTCTTCTTTCTTCTTTC/TTTTCTTC TTTC	AGGACTTTCGACCACC ACAC	TACCCTTTGCTAGCCC CTTT	60.0	415	INTERGENIC				
CaPOPII_1531	Ca4	25596032	TTAT/TTATAT	AGTGATGTGGGCAACA ATGA	TGCATTTTACAGCGCTT TTG	60.0	515	INTERGENIC				
CaPOPII_1532	Ca4	25596215	ATGTGTGTGTGTGT/ATGTGTGTGTG T	TTCTCACGAAAGCGCT GTAA	GGGCAAGGGGATCTGA ATA	59.8	585	DRR	Ca_16586		Trihelix	
CaPOPII_1533	Ca4	25630012	TG/T	AGTAGACTTGCCTCCC GGTT	CCTTGTTGTGGAGTGT GGTG	60.1	788	INTERGENIC				
CaPOPII_1534	Ca4	25694964	CAAAAAAAAA/CAAAAAAAAAAAA	CCACTTCGCTTAGGGA TCAA	TATGGGCATTCTTCTTT GGG	60.2	675	INTERGENIC				
CaPOPII_1535	Ca4	25754372	AAAAATAAAATAAAATAAA/AAAAATA AAATAAA	AATGGGCGCTGTTAAA TGTC	TCATTTAAACCACTGCG TCAA	60.0	750	INTERGENIC				
CaPOPII_1536	Ca4	25760041	ATTTTTTT/ATTTTTT	CACAACGGAATTTGGG TTTC	TTGAGCTTGCAACTTG ATCACT	60.2	565	INTERGENIC				
CaPOPII_1537	Ca4	25774052	TAAAAAAAAAAAA/TAAAAAAAAAAA	TGTCAAATCCAACGCAA AGA	TCGCAACCAAACAAAG CATA	60.2	661	INTERGENIC				
CaPOPII_1538	Ca4	25779217	CTTAATTAATTAATTAATTAATTA/CTT AATTAATTAATTAATTA	ATACCGAGATGGACAC CACC	TAACCCTTCAACATCTG GGC	59.7	367	DRR	Ca_16597	R	NAC	Pentatricopeptide repeat
CaPOPII_1539	Ca4	25822369	CAAAAAAA/CAAAAAA	TGTTTGAACCCGAGAC TTCAC	CGTGGCATTTGAAAAA GCAT	60.1	611	INTERGENIC				
CaPOPII_1540	Ca4	25834950	AAGACGTTCCGTA/AA	TCGCTGCTTATTCATGT TCA	ACCACCAGTGAGGATT TTGC	58.0	336	INTERGENIC				
CaPOPII_1541	Ca4	25847698	T/TG	CATGCTACCAAAGCGA TTGA	ATCGATTTCGCGAGTC TTGT	59.8	370	INTERGENIC				
CaPOPII_1542	Ca4	25869082	ATTTTT/ATTTT	TATTTGTCGATTTGCGT GGA	TTATAAGTGCGGTTAG CGGG	60.1	693	INTERGENIC				
CaPOPII_1543	Ca4	25916811	CTTTTTTTTTTT/CTTTTTTTTTTTTT	ATGGTAACAGATATCG CCGC	ATTGTAGCATTGCCCA AAGG	60.0	627	DRR	Ca_16606	I		Thiolase
CaPOPII_1544	Ca4	25941584	A/AG	CGATCGTTGTTGGTAT GTCG	AGCTGACCAAATGCGT AAGG	60.0	244	INTERGENIC				
CaPOPII_1545	Ca4	25958010	TTATATATATAT/TTATATATATATAT	TCGGGGAAATCAATTC GTTA	TGGAAGTCATCGACCC ATTT	60.3	673	INTERGENIC				

INDEL marker IDs	Chromosomes /unanchored scaffolds	Physical positions (bp)	InDels (*Kabuli* reference genome-CDC Frontier/PI)	Forward primers (5'-3')	Reverse primers (5'-3')	Anneling temperature (0C)	Expected amplified product size (bp)	Structural annotation		Functional annotation		
								Sequence components of *kabuli* genome	*Kabuli* gene accession IDs	NCBI-KOG	TFs	NCBI-nr database
CaPOPII_1546	Ca4	25959074	GCC/GC	TGAAGATGAAGAAGAGGGCAA	TGTGGCATAAGGAGGAGTTTC	59.9	376	INTERGENIC				
CaPOPII_1547	Ca4	25962811	GTATATATAT/GTATATATATAT	TGGTTGGAAGCTCCAGAAGT	GAAAAGGTGAGGAATCGCTG	59.8	275	DRR	Ca_16607		B3	
CaPOPII_1548	Ca4	25967972	ATTTTTTTT/ATTTTTTTTT	TTGTACCCTTGTGCAACCAA	TGGTTGGATACTTGCAATGG	60.0	180	INTERGENIC				
CaPOPII_1549	Ca4	25968820	GTTT/GTTTT	TTCTTCTGCCCAAAAATGCT	CCTCAAGCAGACCTTTGGAG	59.8	412	INTERGENIC				
CaPOPII_1550	Ca4	25969511	ACC/AC	TCCCTTGCCTCTAGCCAGTA	GCAGATGTTCTCAACAGCCA	60.0	350	INTERGENIC				
CaPOPII_1551	Ca4	25970159	AC/A	TGGTTGGGTCTTGGTTTGTT	CACCTGCTATGGATGCAATG	60.2	709	INTERGENIC				
CaPOPII_1552	Ca4	25971278	GATTGAAGAA/GA	GCTTCGCCAAGCTTACAATC	CCAACGATCATCAAAGGGAT	60.0	268	INTERGENIC				
CaPOPII_1553	Ca4	25975114	TGGGG/TGGGGG	ACACATGTTGGCCAATGAGA	GAGTCCGATTTCCATGAGGA	60.0	621	CDS (FRAME SHIFT)	Ca_16608			
CaPOPII_1554	Ca4	25978445	CT/CTTT	TGCCTCAAAGGCTTCATCTT	AGCACTCATCCTCGGGTAGA	60.0	435	INTERGENIC				
CaPOPII_1555	Ca4	25979689	TG/TGG	GTCCTCGTCAGGAGGTTGAG	CCACTGTTGCTGGCAGATAA	59.8	707	INTERGENIC				
CaPOPII_1556	Ca4	25982514	CAATAATA/CAATAATAATA	GCGCAAAAACAAGAAGGAAG	GGTCCAGGTTGTTTGGAGAA	60.0	290	INTERGENIC				
CaPOPII_1557	Ca4	25984442	TGAGAG/TGAGAGAG	AGATCCTGTTGACACGGCTT	CGGTTGTTGTTGTGATTTGC	59.7	524	INTERGENIC				
CaPOPII_1558	Ca4	25987622	TCC/TC	TTCAACTGGGGTAAATGCCT	TGTTCTAGTCCAGATCAAAACGA	59.4	323	INTERGENIC				
CaPOPII_1559	Ca4	25988978	AAAGAAGAAGAAG/AAAGAAGAAG	AAGCTGGATGTGGACTTTGC	AGTTCCAATTGGCCTCCTTT	60.3	404	INTERGENIC				
CaPOPII_1560	Ca4	25989281	TCCCC/TCCC	AAGCTGGATGTGGACTTTGC	AGTTCCAATTGGCCTCCTTT	60.3	404	INTERGENIC				
CaPOPII_1561	Ca4	25989322	GTTTTT/GTTTTTT	GTACGCGATCTAGTCCCCAA	CCGGGGAGAAAGTTCTAGTG	60.1	168	INTERGENIC				
CaPOPII_1562	Ca4	25994543	GTTTTTT/GTTTTTTT	TTAAGATTGAGGGGTGTGGG	CACCACTTCATTCGGGACTT	59.8	516	INTERGENIC				

INDEL marker IDs	Chromosomes /unanchored scaffolds	Physical positions (bp)	InDels (*Kabuli* reference genome-CDC Frontier/PI)	Forward primers (5'-3')	Reverse primers (5'-3')	Anneling temperature (0C)	Expected amplified product size (bp)	Structural annotation		Functional annotation		
								Sequence components of *kabuli* genome	*Kabuli* gene accession IDs	NCBI-KOG	TFs	NCBI-nr database
CaPOPII_1563	Ca4	25994721	AC/A	TTAAGATTGAGGGGTGTGGG	CACCACTTCATTCGGGACTT	59.8	516	INTERGENIC				
CaPOPII_1564	Ca4	25995802	ATTTT/ATTT	CCCTAAATCAAAGCAGCGTC	TGCACTGTTGTTGTTGCTGA	59.8	470	INTERGENIC				
CaPOPII_1565	Ca4	26018351	TAAAAAAAA/TAAAAAAAAA	AGAACATGGTGGATGCACAG	TCCAAGAGCTTGGTCTGACA	59.6	373	INTERGENIC				
CaPOPII_1566	Ca4	26173966	CATAT/CAT	AGAGTGCATGTGGGATAGGG	TTCCATGATGCTCTTTTGGG	60.0	563	DRR	Ca_16612		HD-ZIP	Pectate lyase/Amb allergen
CaPOPII_1567	Ca4	26259040	ATTTTTTTTTT/ATTTTTTTTT	TTCTCGAATCAATTCCGTCC	CTCAGAGGCCGACCTATACA	60.0	888	INTERGENIC				
CaPOPII_1568	Ca4	26301785	GAAAAAAAAA/GAAAAAAAAAA	ACCACTACGCCACACATCAA	GTGACAGTACTGGGGGAAGC	60.0	428	INTERGENIC				
CaPOPII_1569	Ca4	26313656	ATTTTTTTTTTT/ATTTTTTTTTT	TGAGGTATGCCCAAAGAAGG	TTAGGCCGTAGTCAGGCTGT	60.1	837	INTERGENIC				
CaPOPII_1570	Ca4	26398945	AATTTAATTT/AATTTAATTTATCTATTTAATTT	GTGGCGAATTGATTTGTCCT	CGACGTAATCTGGGTTGGTT	59.9	413	INTERGENIC				
CaPOPII_1571	Ca4	26411287	GTTT/GTTTT	TGTTCTGCTTAAGGGGCCTA	GATCAAGAGGTCACCGGTTC	59.8	841	INTERGENIC				
CaPOPII_1572	Ca4	26418036	TCACACACACACACACA/TCACACACACACACACACA	CCAAAATTCAAACACCACCC	AACCCTGATTGACGGTGAAG	60.1	542	DRR	Ca_25299			
CaPOPII_1573	Ca4	26456492	T/TG	GCGAGCATGAGAAGTTGTTG	AATTCAATGGGAAAAACCACA	59.6	713	INTERGENIC				
CaPOPII_1574	Ca4	26483173	TAAAAAAA/TAAAAAA	TTTTGAAAACCAATGCACCA	CGCTATATCACAGCTTGGCA	59.9	413	INTRON	Ca_25006	O	C2H2	Peptidase A1
CaPOPII_1575	Ca4	26492251	ACTC/AC	CAAATGCACATGAGAACCGT	AATGATCCGGAACCATTTCA	59.6	519	INTERGENIC				
CaPOPII_1576	Ca4	26495554	GAAAGTTGTTC/GAAAGTTGTTCAAAGTTGTTC	AACGATTTATCCTGGTCCCC	CCCGAATACAAACCCAAGTC	60.0	844	INTERGENIC				
CaPOPII_1577	Ca4	26525402	GTTTTTTTTTT/GTTTTTTTTTTTT	TGTAGCTACCGCATTGATGG	GCTGCAAGGGATGATTTGAT	59.7	436	INTERGENIC				
CaPOPII_1578	Ca4	26547528	AT/A	TCCAAAACTTGTATATCCTCAAGC	TTTGTGCTTCTGCAACAAGG	58.8	815	INTERGENIC				
CaPOPII_1579	Ca4	26552272	TAAAAAAA/TAAAAAA	TCCTGGTTCACTCAATGTGG	CTCAAGTTGAAGGCCCAAAG	59.5	818	INTERGENIC				

INDEL marker IDs	Chromosomes /unanchored scaffolds	Physical positions (bp)	InDels (*Kabuli* reference genome-CDC Frontier/PI)	Forward primers (5'-3')	Reverse primers (5'-3')	Anneling temperature (0C)	Expected amplified product size (bp)	Structural annotation		Functional annotation		
								Sequence components of *kabuli* genome	*Kabuli* gene accession IDs	NCBI-KOG	TFs	NCBI-nr database
CaPOPII_1580	Ca4	26620833	GTT/GT	AAAGAAGGAAGGGTCTTGCC	TCAAAATCGAACCAAATCACA	59.7	593	INTERGENIC				
CaPOPII_1581	Ca4	26662668	ATTTTTTTTTT/ATTTTTTTTT	GTGACGTGGGGGAGTTAAGA	CAACGACCTTAACATTTGGTG	60.0	799	INTERGENIC				
CaPOPII_1582	Ca4	26753796	GT/GATTTAT	AGAGCGGCAACTCAAACAAT	TGCAGGGGACATCTCTTACC	59.9	613	INTERGENIC				
CaPOPII_1583	Ca4	26775300	TAAAAAAAAAAA/TAAAAAAAAAAAA	ACGGCGCTAAACCAACATAG	TTGTGTGCTTAACCTTTGCG	60.2	320	INTERGENIC				
CaPOPII_1584	Ca4	26847495	A/AC	AGCTTCAAGTCGGATCGAAA	AAAAATCTTGTGGGAATAATTCAA	60.0	216	INTERGENIC				
CaPOPII_1585	Ca4	26903304	T/TG	ACTCGATTGGTCCATGCTTC	ATGTTTTGGACGCACACAGA	60.1	532	INTERGENIC				
CaPOPII_1586	Ca4	26970810	TAAAAAAAA/TAAAAAAA	GAAAAGCTGAAACTGTATTTTGGAA	AACAGTGCCGATGAATAGTTTT	60.1	306	DRR	Ca_20452	A		Ribosomal protein L4/L1e
CaPOPII_1587	Ca4	27017251	GTTTTTTTTT/GTTTTTTTTTT	CCTTTGCATCTCAGGGACAT	TGAGACCGGGTTTGAGTACC	60.1	453	INTERGENIC				
CaPOPII_1588	Ca4	27233281	ATAATTCACAAGGTAATTAATTCACAAGGTA/ATAATTCACAAGGTAATTAATTCACAAGGTAATTAATTCACAAGGTA	CCAACGCTATTGGGTTTCAG	GCAGGAGAGAGAAAACGTGG	60.5	891	INTRON	Ca_20460			Protein of unknown function DUF3527
CaPOPII_1589	Ca4	27260077	GAAA/GAAAA	GCGTCCAAGTCTTTGAGGAA	TTTGGGGTTGCATAATTTGA	60.4	498	INTERGENIC				
CaPOPII_1590	Ca4	27267123	TAAAAA/TAAAA	CCCTCAACTCAACTGAGATCC	TGCATTGGTTTGAATTTGGA	58.7	568	DRR	Ca_20461	R		Sugar/inositol transporter
CaPOPII_1591	Ca4	27283830	GTTT/GTTTT	TATTGGTCGTCAAGCAGCAG	TTTCCCAATCGATTTGCTTC	60.0	681	DRR	Ca_20463			
CaPOPII_1592	Ca4	27376069	TAA/TAAA	ATCCTCTCCATTCCTTGCCT	TTGCTTTCACAGTCCAATGC	60.0	304	INTRON	Ca_21323			
CaPOPII_1593	Ca4	27378491	TAAAAAAAAAA/TAAAAAAAAA	GGATCCGTCTCCTGCATAAA	TTTGCACAGGTTGTTTGTTG	60.0	585	DRR	Ca_21323			
CaPOPII_1594	Ca4	27508639	T/TC	TCCAATGGAGATCAATGCAA	AACATAATGCCATGCCCAAT	60.0	233	INTERGENIC				
CaPOPII_1595	Ca4	27513599	ATTTTTTT/ATTTTTTTT	TGATGGGGTTTTGTGTGAGA	ATGCATTACACGACCGCATA	59.9	612	INTERGENIC				
CaPOPII_1596	Ca4	27537181	AGTGTGTGTGTGT/AGTGTGTGTGTGTGT	TTTCATCCTCTCTCACGGCT	TCTTCATGGCAACACTTCCA	60.0	478	INTERGENIC				

INDEL marker IDs	Chromosomes /unanchored scaffolds	Physical positions (bp)	InDels (*Kabuli* reference genome-CDC Frontier/PI)	Forward primers (5'-3')	Reverse primers (5'-3')	Anneling temperature (0C)	Expected amplified product size (bp)	Structural annotation		Functional annotation		
								Sequence components of *kabuli* genome	*Kabuli* gene accession IDs	NCBI-KOG	TFs	NCBI-nr database
CaPOPII_1597	Ca4	27539521	TAA/TAACATAGTGAA	CTTGTGAAAGCCTCAAAGCC	TTGATTTCGTGAGTTCATTTCAA	60.0	776	INTERGENIC				
CaPOPII_1598	Ca4	27539986	ATA/ATAGATTA	TTGAAATGAACTCACGAAATCAA	CACCCTCGCTTAGAAAAACG	59.6	542	INTERGENIC				
CaPOPII_1599	Ca4	27548804	TAAAA/TAAA	TTCCCCATTCTTAACACCCA	TTCGCAACTCCACTCTTCCT	60.2	672	INTERGENIC				
CaPOPII_1600	Ca4	27746278	CTTTTTTTTTTT/CTTTTTTTT	AGTTTTCTGTGGGTTCACCG	TGCCACATCACCCTAAATCA	60.0	546	INTERGENIC				
CaPOPII_1601	Ca4	27796696	AATATATATATATATATATATAT/AATATATATATATATATATAT	GGCAGAAGTGAAACAATGATGA	TAATAAAGGTGGTGGTCGGC	60.1	731	INTERGENIC				
CaPOPII_1602	Ca4	27878476	ATTTTTTTTTTTTT/ATTTTTTTTTTT	ATCCTCACCTGCAAGACCAT	TGTCTCGAATTACCCCGAAG	59.5	640	INTERGENIC				
CaPOPII_1603	Ca4	27902426	T/TC	CTGATTTGGCTCTGATGGCT	ATCATAGATCGCGAGCATCC	60.4	686	INTERGENIC				
CaPOPII_1604	Ca4	27928820	CACAAC/CACAACAAC	AGCGATGCATGTCTCAAGTG	AGCGCTTTGTGACCTCCTTA	60.0	754	INTERGENIC				
CaPOPII_1605	Ca4	28020575	TG/T	GGGAGCAAAAGCTTTGAATG	AATGAGACCTACGTCACGGG	59.8	427	INTERGENIC				
CaPOPII_1606	Ca4	28026871	TAAGAA/TAAGAAGAA	GACATCACACCAATTCGACG	TTTTGAAGGAGATGGGGTTG	60.0	885	INTERGENIC				
CaPOPII_1607	Ca4	28131693	TT/TTCGGTGGCT	TAGCAAGAACACCATCGCAA	TTAACCCTCAATTGCCGTTC	60.4	463	INTERGENIC				
CaPOPII_1608	Ca4	28148545	ATACTTACT/ATACT	TGGTCATAATCGACGTTGGA	CTGCAAGCAGCAGAAGAACA	59.9	956	INTERGENIC				
CaPOPII_1609	Ca4	28153242	CAA/C	TTGGCAGTGGTTCATTTTCA	AAAAGCTTGCGAACATCACC	60.1	365	INTERGENIC				
CaPOPII_1610	Ca4	28169092	GC/G	TTTTTGCCCATTCAAACACA	AAATGCGTCAGCCCAGTAAG	59.9	829	INTERGENIC				
CaPOPII_1611	Ca4	28209971	T/TA	ATTGATGGAGCGAATCGAAC	AATTCCTCTTTTGGTGGGCT	60.0	891	INTERGENIC				
CaPOPII_1612	Ca4	28213499	CTTTTTTTT/CTTTTTTTTT	ACTGACCTGACCTAGGCGTG	GTTGCAAAATTGGTGTCTGC	60.3	646	INTERGENIC				
CaPOPII_1613	Ca4	28254483	CAACAA/CAACAAGACAAACAA	GGGGAACTCTCCAAGTCACA	CGTGGAAACCAACCTTGTCT	60.1	703	INTERGENIC				

INDEL marker IDs	Chromosomes /unanchored scaffolds	Physical positions (bp)	InDels (*Kabuli* reference genome-CDC Frontier/PI)	Forward primers (5'-3')	Reverse primers (5'-3')	Anneling temperature (0C)	Expected amplified product size (bp)	Structural annotation		Functional annotation		
								Sequence components of *kabuli* genome	*Kabuli* gene accession IDs	NCBI-KOG	TFs	NCBI-nr database
CaPOPII_1614	Ca4	28281450	ATTTTTTTTTTT/ATTTTTTTTTTTTT	AATCGAACGACTGAGAACGG	GTTCTTTTGGCCACTTGCAT	60.3	261	INTRON	Ca_23166	TR	FAR1	Metallo-dependent phosphatase
CaPOPII_1615	Ca4	28281644	CTAT/CTATATAT	ATGCAAGTGGCCAAAAGAAC	ACCAAGAAATGCCTTCTGGA	60.1	405	INTRON	Ca_23166	TR	FAR1	Metallo-dependent phosphatase
CaPOPII_1616	Ca4	28281945	CTTTTTTT/CTTTTTT	ATGCAAGTGGCCAAAAGAAC	GCTGCCACATGAATACAACC	60.1	673	INTRON	Ca_23166	TR	FAR1	Metallo-dependent phosphatase
CaPOPII_1617	Ca4	28306538	CTTTTTTTT/CTTTTTTTTT	GGGTTTGTCTTTTGCGAGAA	AATTGTCCCATCCCTTACCC	60.2	772	INTERGENIC				
CaPOPII_1618	Ca4	28322339	A/ACCGT	TGCGTACACGAAAAAGCAAC	TGTTGTTGCCGACTGAGAAG	59.9	333	INTERGENIC				
CaPOPII_1619	Ca4	28338890	CAAAAAAAAA/CAAAAAAAA	TATTTCATGGGGCTCAAAGC	TAACCGCCTATTTTCTTGCG	60.0	555	INTERGENIC				
CaPOPII_1620	Ca4	28346696	A/AT	ACCACAGGTGAAAGAAACCG	TTTTCAACTCTATCCGTAGCCC	60.0	340	INTERGENIC				
CaPOPII_1621	Ca4	28390462	TAA/TAAA	GTCATCAAAAGCCGACGAAT	GGCCCACAATTTTGAAGAGA	60.1	786	INTERGENIC				
CaPOPII_1622	Ca4	28407567	CAAAAAAAA/CAAAAAAA	TGGTCATAAAGGGAACCAGG	GGAGGGGGTGAATTAGGTGT	59.8	146	INTERGENIC				
CaPOPII_1623	Ca4	28422388	TA/TAA	TGAAGGAAACTTTGATGCCA	TTGGGTGATGGTAGTTTCAATG	59.2	534	INTERGENIC				
CaPOPII_1624	Ca4	28536428	ATTTTTTTTTTT/ATTTTTTTTTTTTT	TTGTAACACCCCGTTTTTCG	CCTCAGATGAGAGCCAGGAC	60.8	433	INTERGENIC				
CaPOPII_1625	Ca4	28556813	TAAAAAAAAA/TAAAAAAAAAA	GCAAAAATTCATTCAAAAATTCG	TGGTGGTTGGGTGAAGAAGT	59.9	295	INTERGENIC				
CaPOPII_1626	Ca4	28714992	GATAT/GAT	AGAGCCAACGCTCATAGGAA	TTAAAATTCCCTCCTCGGCT	60.0	531	INTERGENIC				
CaPOPII_1627	Ca4	28793395	TAA/TAAAAACAA	CCAAAATTACACCAACGGTTC	TTGTTCGCATGCTCATTCTC	59.2	275	INTERGENIC				
CaPOPII_1628	Ca4	28832888	TGGTGTATA/T	CAGTTGTGCACTTTGAATGTTG	AAAATCACATGGAGGAACAAAAA	59.3	665	INTERGENIC				
CaPOPII_1629	Ca4	28949557	CAAAAAAAA/CAAAAAAA	TTTCATCATTTCTGGAGGCA	GTGAGGATGAGCTGACACGA	59.2	528	DRR	Ca_23705	J		Nop14-like protein
CaPOPII_1630	Ca4	28996068	CTTTTTTTTTTTT/CTTTTTTTTTT	TAACAGCATTGCATTGGGAA	TTCCAACACTAAAGGGGTGC	60.1	243	DRR	Ca_23706		NF-YB	Protein of unknown function DUF248, methyltransferase putative

INDEL marker IDs	Chromosomes /unanchored scaffolds	Physical positions (bp)	InDels (*Kabuli* reference genome-CDC Frontier/PI)	Forward primers (5'-3')	Reverse primers (5'-3')	Anneling temperature (0C)	Expected amplified product size (bp)	Structural annotation		Functional annotation		
								Sequence components of *kabuli* genome	*Kabuli* gene accession IDs	NCBI-KOG	TFs	NCBI-nr database
CaPOPII_1631	Ca4	29008246	GTTTT/GTTT	CCTACAATCCCAAGACAAGGA	ATTAATGCCCATGAAGGCAC	59.0	252	INTERGENIC				
CaPOPII_1632	Ca4	29100812	GGTAGTA/GGTA	TCAAGAAATAGTGGGGGCAG	TGGAGAAACTCTTCCGCTGT	60.1	840	INTERGENIC				
CaPOPII_1633	Ca4	29158154	T/TG	TTTGGGGAGACTGAAGATGG	GCTCACGTTTTTGTAATGTCCA	60.0	713	INTERGENIC				
CaPOPII_1634	Ca4	29191237	CTTTTTTTT/CTTTTTTTTT	CCAACTTGGTTCCAACAATCT	GGAGAAAAGTTAGGGGGTGC	58.9	119	INTERGENIC				
CaPOPII_1635	Ca4	29211121	AT/A	GATGTTTAAGGTCTGTTTGGCA	AACTTCAGCCGTCATTGGTT	59.1	641	DRR	Ca_14151			
CaPOPII_1636	Ca4	29222326	ATTTTTTT/ATTTTTTTT	TGCACCTCTCTATGTGCGTG	TTCGTTCCGGACAAATCTTC	61.0	342	INTERGENIC				
CaPOPII_1637	Ca4	29245578	T/TA	TTACCAACCAAATTGCACCA	CCCTAATGGGCCAAATTACA	59.8	583	INTERGENIC				
CaPOPII_1638	Ca4	29262137	AAT/AATTAT	TTCAAAGGTGCATTGTCGAT	CACTGCGACGAAACTTACCA	59.1	619	INTERGENIC				
CaPOPII_1639	Ca4	29315886	CTTTTTTTTT/CTTTTTTTTTT	CGTGGCAACCCAAGACTTAT	TCAATGTTCAAGAATCGGTCC	60.0	733	INTERGENIC				
CaPOPII_1640	Ca4	29379238	CAAAAAAA/CAAAAAA	ATGCAAGTGGGCCTAGACAG	CACTTCGTTTTGGAATCGGT	60.3	630	INTERGENIC				
CaPOPII_1641	Ca4	29418531	TT/TTAT	GGTGGTGTACGGTGGAAGTT	GTCAAGGCAGACCAGAAAGC	59.7	383	INTERGENIC				
CaPOPII_1642	Ca4	29501722	TGG/TGGG	ATGGAGGTGCAGGGAAGAAT	TGAGTGGAAAATGGCATCAA	60.9	790	INTERGENIC				
CaPOPII_1643	Ca4	29582409	TAAAA/TAAA	AGCTGTATTGGGCCTCCTTT	GCCGAATAAAACCCTCACAA	60.1	212	INTERGENIC				
CaPOPII_1644	Ca4	29646991	GGATCG/GG	AGGATTCGGTCTGTGTTTGG	TCAGGACTGCAAATAACCCA	60.0	555	DRR	Ca_14165	S		Activator of Hsp90 ATPase homologue 1-like
CaPOPII_1645	Ca4	29712689	CAAA/CAA	TTTGCTTACTCAAACCCCCA	AGTTCGGTAGTGGCTTCCCT	60.5	837	INTERGENIC				
CaPOPII_1646	Ca4	29811970	AGGCGCGC/AGGCGCGCAGGGCGCGC	GCGTTTTTATGGTTGCTGGT	ACTTTCATGAAGACACCGGG	60.0	152	INTERGENIC				
CaPOPII_1647	Ca4	29933754	ATTTTTTT/ATTTTTTTT	AGCCACACGATGTTGATTGA	TGATTAGGGAATCACCCGTT	60.1	845	INTERGENIC				

INDEL marker IDs	Chromosomes /unanchored scaffolds	Physical positions (bp)	InDels (*Kabuli* reference genome-CDC Frontier/PI)	Forward primers (5'-3')	Reverse primers (5'-3')	Anneling temperature (0C)	Expected amplified product size (bp)	Structural annotation		Functional annotation		
								Sequence components of *kabuli* genome	*Kabuli* gene accession IDs	NCBI-KOG	TFs	NCBI-nr database
CaPOPII_1648	Ca4	29939743	CAA/CA	TCCTTTTCGAGACTCAGGGA	GCACCACCTGTTTGATTTCC	59.9	718	INTERGENIC				
CaPOPII_1649	Ca4	29986497	GTGCGAATACATGCG/GTGCG	AAAAAGGATATAAATACCCGCAA	AAAAATCTTCTCCACAAGATGAA	58.1	449	INTERGENIC				
CaPOPII_1650	Ca4	30049126	G/GAA	TGAGGGCAAACAACTATTCAA	TTTGACCAATGTGCAATTCAA	58.3	771	INTERGENIC				
CaPOPII_1651	Ca4	30049460	C/CTAGT	GAGTGGGAGAGAAATGTTTAGTGAA	TGACAAATCCCCTTTTCCAA	60.1	233	INTERGENIC				
CaPOPII_1652	Ca4	30076786	GTTTTTTTT/GTTTTTTTTTT	GCACTTCCGCTAAAAGCATC	AAGCGCTCCCAAATATGCTA	60.0	432	INTERGENIC				
CaPOPII_1653	Ca4	30077287	CTTTTTTTT/CTTTTTTTTTT	GCATATTTGGGAGCGCTTTA	TGGATGATTGTGACAAAATTGC	60.2	508	INTERGENIC				
CaPOPII_1654	Ca4	30084699	GTTTTTTTTT/GTTTTTTTT	CGAAATTTTGTAGCCGGAAA	AAGTTGCATTGTGGTGGTCA	60.1	277	INTERGENIC				
CaPOPII_1655	Ca4	30086898	ACTTTCT/ACT	ACGCCGAAAAACACTTTCAC	TCCATTCGACTGTCCTTCAA	60.2	630	INTERGENIC				
CaPOPII_1656	Ca4	30102122	CTCTTCCAAT/CTCTTCCAATTCTTCCAAT	TGATTCTTCAATTGCCACCA	ACGAGAGACAAGTGAGGGGA	60.0	580	INTERGENIC				
CaPOPII_1657	Ca4	30116632	ATTTTTTTTTT/ATTTTTTTT	GATCACGCCAGTTGTTGGTA	TGTCGATTGCGAAGAGTTGT	59.6	896	INTERGENIC				
CaPOPII_1658	Ca4	30122538	ATT/AT	ATGTCAATGGGCCACTTAGC	TTGTTTGTTGTGCTTTGACCA	60.0	764	INTERGENIC				
CaPOPII_1659	Ca4	30138209	CAAAAAAAA/CAAAAAAA	GCATGCAAACATGGCTCTAA	CAAATTTGGTTGGGTCTTGG	59.8	553	INTERGENIC				
CaPOPII_1660	Ca4	30172265	TC/T	CTCAACAGCCTTATGTGCGA	TGCGATGGAATAATGTGTCG	60.0	752	INTERGENIC				
CaPOPII_1661	Ca4	30184775	TG/T	ACAACGGAAACAAATCTCGC	GGGGGTTAGGAGTAAGAAAGGA	60.1	116	INTERGENIC				
CaPOPII_1662	Ca4	30188259	TAA/TA	CTCCAAAACATGGTATGCCC	CACCATCAGCAGCCTAAACA	60.2	690	INTRON	Ca_14186		SAP	Aminotransferase-like, plant mobile domain
CaPOPII_1663	Ca4	30206461	TAAAAAAAAAAA/TAAAAAAAAA	TCCCTCGAGCTATAGGATGC	ATGCAATGGTTTATTCCCCA	59.4	643	DRR	Ca_14187			VQ
CaPOPII_1664	Ca4	30211443	T/TA	ACATCAAACAAACAGCGCAC	CCGCTAAAATTGCCCAATAA	59.8	714	INTERGENIC				

INDEL marker IDs	Chromosomes /unanchored scaffolds	Physical positions (bp)	InDels (*Kabuli* reference genome-CDC Frontier/PI)	Forward primers (5'-3')	Reverse primers (5'-3')	Anneling temperature (0C)	Expected amplified product size (bp)	Structural annotation		Functional annotation		
								Sequence components of *kabuli* genome	*Kabuli* gene accession IDs	NCBI-KOG	TFs	NCBI-nr database
CaPOPII_1665	Ca4	30327391	ATTTTTTT/ATTTTTTTT	CTCACGACTAAGGGTGGGAA	TTTTTCCTTCCTTGGGCTCT	60.1	497	INTERGENIC				
CaPOPII_1666	Ca4	30351459	CTTTTTTT/CTTTTTTTT	TCTTTGAAACACATCACTTTCG	AAAATTTGATCGGTGGTGGA	57.9	314	INTERGENIC				
CaPOPII_1667	Ca4	30404870	AATAACAT/AAT	TTAACTGTTGGTGCGCAGTC	CGTCACATTTAACACGCCAT	59.9	798	INTERGENIC				
CaPOPII_1668	Ca4	30408624	TC/T	TGCCTACGTCGTGGGATTAT	TCATCAAATCCGTAACGCAA	60.4	361	INTERGENIC				
CaPOPII_1669	Ca4	30408872	AA/AAAAGA	TTGCGTTACGGATTTGATGA	AGACTTTCGACCGACGCTAA	60.1	485	INTERGENIC				
CaPOPII_1670	Ca4	30535832	TATAAATAAATAAATAAA/TATAAATAAATAAATAAATAAATAAATAAA	CCATTCAGAATTAGAAAATCACCA	CCAAATTTCCCAAGGGTTCT	59.4	582	INTERGENIC				
CaPOPII_1671	Ca4	30537523	CA/CATA	TTTGATTGCCATTGTATCCG	CAAAATGGAAATGCAAGAACAA	59.4	803	INTRON	Ca_14204		NAC	No apical meristem (NAM) protein
CaPOPII_1672	Ca4	30538166	AAAGAAG/AAAGAAGAAG	TTGTTCTTGCATTTCCATTTTG	CGCCATCTTCCTCTTCTTTG	60.0	380	CDS (large-effect mutations)	Ca_14204		NAC	No apical meristem (NAM) protein
CaPOPII_1673	Ca4	30604376	CT/CTT	AATGCCCCACTTTTAATCCC	CGGAGGCTACCGATACCATA	60.0	185	INTRON	Ca_14208	T		Protein of unknown function DUF773
CaPOPII_1674	Ca4	30621604	ATTTTTTTTTTT/ATTTTTTTTTTTT	TGGGCTTCAATTTAGTCCTTG	CTGATGACAAAAGGGGGAGA	59.2	516	INTERGENIC				
CaPOPII_1675	Ca4	30687261	TA/TATCA	TCATCATTCATAGCTTGGGCT	AAATTTCCCAAGCAAAAGGG	59.7	393	INTERGENIC				
CaPOPII_1676	Ca4	30775212	CTTTTTTTTTT/CTTTTTTTTT	TGAACTCGTAACATAAGCCCAA	TGGGAATGCTTTCTTATGGG	59.6	931	INTERGENIC				
CaPOPII_1677	Ca4	30824879	AT/A	ACCGAGTGTCGAACTCAAGG	ACCAGCAACCATAAAAACGC	60.3	823	INTERGENIC				
CaPOPII_1678	Ca4	30928717	TAAAAAAA/TAAAAAAAA	CAATGTCCATGTCGACCAAA	TCTGTCGCACCATCTCAGTC	60.4	949	INTERGENIC				
CaPOPII_1679	Ca4	30955630	TAAAAAAAAA/TAAAAAAAA	CAAAGCCATGTTTCTTTCCA	TGAGCAAATGAGGCAAAATG	58.7	579	INTERGENIC				
CaPOPII_1680	Ca4	31101573	TGTCATTCCC/T	AATCGGACAAGAAACCGATCT	AGTTGTGACCCTCCGATACG	60.0	260	INTERGENIC				
CaPOPII_1681	Ca4	31172943	TA/T	TCGCCTAAGGTAAGGGCTTT	TTACACCAATTCCCCCAAGA	60.2	421	INTERGENIC				

INDEL marker IDs	Chromosomes /unanchored scaffolds	Physical positions (bp)	InDels (*Kabuli* reference genome-CDC Frontier/PI)	Forward primers (5'-3')	Reverse primers (5'-3')	Anneling temperature (0C)	Expected amplified product size (bp)	Structural annotation		Functional annotation		
								Sequence components of *kabuli* genome	*Kabuli* gene accession IDs	NCBI-KOG	TFs	NCBI-nr database
CaPOPII_1682	Ca4	31192815	TGG/TG	TTGAAGGAGAAGCCAGAGTCA	TGAGGCATGCAGAAACGTAG	60.1	238	INTERGENIC				
CaPOPII_1683	Ca4	31248634	ACC/AC	TGGGTCATTTCTCATTATGTCG	TGCTATGCCTTTTGATCGC	59.8	645	DRR	Ca_14228		Trihelix	
CaPOPII_1684	Ca4	31303190	CTTTTTTTTTTT/CTTTTTTTTTT	GAGGTCCCGTCCAACCTAAC	TCAGCACCGAGAGTGTCAAC	60.7	488	INTRON	Ca_14231		bHLH	Helix-loop-helix DNA-binding domain
CaPOPII_1685	Ca4	31316566	GAAA/GAA	CATTGTTTTGCTGTTGTGGG	CCTCACGAGCTGCTTTAACC	60.0	664	INTERGENIC				
CaPOPII_1686	Ca4	31445256	ATT/ATTT	AGATCTATGCATCGGCAACC	AGGGTACTACAGCTGCCCCT	60.1	338	INTERGENIC				
CaPOPII_1687	Ca4	31523716	AAATAATAATAATAATAATAATAATAATAATAATAATAATAATAATAATAA/AAATAATAATAATAATAATAATAATAATAATAATAATAATAATAATAATAATA	GAAACTCTCACAAGGGTGGC	TGGCTTTAGGAACCGTGTTT	59.7	832	INTERGENIC				
CaPOPII_1688	Ca4	31611840	GTTTTTTTTTT/GTTTTTTTTTTTT	CGTTTTCAGCTGAGCAAATTC	TTGATCCATTGGGATTGTGA	60.0	392	INTERGENIC				
CaPOPII_1689	Ca4	31611867	AAT/AATAT	CGTTTTCAGCTGAGCAAATTC	TTGATCCATTGGGATTGTGA	60.0	392	INTERGENIC				
CaPOPII_1690	Ca4	31636819	CAA/CAAA	TCTCAACATCTCGTAGCGCA	TTCATGATTCATTGGGTCATCT	60.7	771	INTERGENIC				
CaPOPII_1691	Ca4	31649942	CT/CTT	CGTGCCCTGCAGGTATTTAT	ATTGACCGGTGGAAGACATT	60.0	454	INTRON	Ca_15442	U	EIL	SPX, N-terminal
CaPOPII_1692	Ca4	31806633	ATGTGACTAATTTT/ATGTGACTAATTTTGTGACTAATTTT	ACAAAAGCCTGGATAGCGAA	TCACATATACCCTTAAAATCGCA	59.8	410	INTERGENIC				
CaPOPII_1693	Ca4	31822803	ATTTTTTT/ATTTTTTTT	CACAGATTTCTCGAGGCACA	TCTAAAGCTCATGTCCCGCT	60.0	841	INTERGENIC				
CaPOPII_1694	Ca4	31859597	TAAAAAAAAA/TAAAAAAAAAAA	GTTGGAGCCCCCACTTTATT	TGCAAGCAGCATCAGAGAAC	60.2	793	INTERGENIC				
CaPOPII_1695	Ca4	31978850	CAA/CAAA	ACATTCTTATGTCGGGGCAG	ACAGCCTTTTCTCCATCCCT	60.0	605	DRR	Ca_15457	S		Protein of unknown function DUF2363
CaPOPII_1696	Ca4	32016200	TGAGATACC/TGAGATACCGAGATACC	TCTAAATGTGGGTCGTGCAA	GAAGGATAAACATTGGGGCA	60.1	279	INTERGENIC				
CaPOPII_1697	Ca4	32038234	G/GA	ACAATGCCCGACTTTACTGG	ACTATGAGGTTTTGGTGGCG	60.0	812	INTERGENIC				
CaPOPII_1698	Ca4	32051935	ATTTTTTTTTTT/ATTTTTTTTT	TTGAAGATGGTGAATGTGGAA	TATGACCCGTTGGATTCGAT	59.0	797	INTERGENIC				

INDEL marker IDs	Chromosomes /unanchored scaffolds	Physical positions (bp)	InDels (*Kabuli* reference genome-CDC Frontier/PI)	Forward primers (5'-3')	Reverse primers (5'-3')	Anneling temperature (0C)	Expected amplified product size (bp)	Structural annotation		Functional annotation		
								Sequence components of *kabuli* genome	*Kabuli* gene accession IDs	NCBI-KOG	TFs	NCBI-nr database
CaPOPII_1699	Ca4	32057189	AATATAT/AATAT	CGCTGCTCTTCCTTGTTCTC	AAAGCGAAGCGAAGCAATAA	60.3	294	INTERGENIC				
CaPOPII_1700	Ca4	32057442	TAAAAAAA/TAAAAAAAA	TATTGCTTCGCTTCGCTTTT	GGGAAACTGCCCCTAATTCT	60.1	451	INTERGENIC				
CaPOPII_1701	Ca4	32121497	TAAAAAAAA/TAAAAAAAAAA	TGGCATACGACCAACTCAAA	TTTGCTTTACAAGCTTGGGG	60.1	440	INTERGENIC				
CaPOPII_1702	Ca4	32137832	CAAAAAAAA/CAAAAAAAAAA	CGACAGCACATTAGATAAACCG	ATGGATGATGGGCAGTAAGC	59.7	806	INTERGENIC				
CaPOPII_1703	Ca4	32149191	CTTTTTTT/CTTTTTT	CGGTGAAGGGACCACACTAT	TGCCCATAACTGCACAACAT	59.8	404	INTERGENIC				
CaPOPII_1704	Ca4	32167743	A/AG	GCAGCATATGATCGCAAAAA	AAAGTCCCACTTGATGGTGG	59.8	565	INTERGENIC				
CaPOPII_1705	Ca4	32179682	CAATAATCAAACAAA/CAATAATCAAACAAATAATCAAACAAA	TTGCATACCTCATACCATCCC	TTGATGCATTACCTCCCACA	59.7	731	INTERGENIC				
CaPOPII_1706	Ca4	32216199	ATTTTTTTT/ATTTTTTT	ATAAAGGTCAACGCACGCTT	GAAATCAACGGATCTCCATCA	59.8	384	INTERGENIC				
CaPOPII_1707	Ca4	32227293	AAAAA/AAAAATTAAAA	TCTTCACAACCCCTTGAACC	TTCTTCCAAACGAGCCTCTC	59.9	868	INTERGENIC				
CaPOPII_1708	Ca4	32241433	ATT/A	ACAATTGTTGCACCACGAGA	ATCGCAAAAGTAGTCCCCCT	60.2	148	INTERGENIC				
CaPOPII_1709	Ca4	32242240	TATTAAATTAAA/TATTAAA	TGCTAATGCTTTTCAAGGGC	GCGATGCACAGGTAAACAAA	60.3	482	INTERGENIC				
CaPOPII_1710	Ca4	32256317	A/AC	AAACAAAACTCATCCTTCCCAA	CGTGAGTCTCGTTGAAGCAA	59.9	336	INTERGENIC				
CaPOPII_1711	Ca4	32264980	AAT/AATAT	GCACTTACCCTTCAATTGGG	AGGAGAGGTCCATTTCTCAGG	59.4	262	INTERGENIC				
CaPOPII_1712	Ca4	32297893	CTGAAT/CTGAATGAAT	GCGGTGCGATATTCTTTGTT	TGGAAAATGTTGGAGCATGA	60.1	471	INTERGENIC				
CaPOPII_1713	Ca4	32335179	AAAGAAGAAGAAGAAGAAGAA/AAAGAAGAAGAAGAAGAA	AAAGTCCGTTTGTGAATCCG	TGGGCATTCAAATCAAACAA	60.0	507	INTERGENIC				
CaPOPII_1714	Ca4	32511208	GAAAAAAAAA/GAAAAAAAAAA	ACTGTTTTGCCCAGAAATGC	TGATGCATGTCAGAAAGGGA	60.1	475	INTERGENIC				
CaPOPII_1715	Ca4	32513097	TGGGGGG/TGGGGG	GATTTGCATTGGCCTCTTGT	ACAGTGGTTGCCATTTCCTC	60.1	479	INTERGENIC				

INDEL marker IDs	Chromosomes /unanchored scaffolds	Physical positions (bp)	InDels (*Kabuli* reference genome-CDC Frontier/PI)	Forward primers (5'-3')	Reverse primers (5'-3')	Anneling temperature (0C)	Expected amplified product size (bp)	Structural annotation		Functional annotation		
								Sequence components of *kabuli* genome	*Kabuli* gene accession IDs	NCBI-KOG	TFs	NCBI-nr database
CaPOPII_1716	Ca4	32525507	CAAAA/CAAAAA	GTCATGCATATGTTGGACGC	AAGTTTTCTTCCCGCTCACA	60.0	460	INTERGENIC				
CaPOPII_1717	Ca4	32531473	TAA/T	TAAAATGGATGCATCGAGCC	ACAAATCAACCAGTGTCGCA	60.9	662	INTERGENIC				
CaPOPII_1718	Ca4	32547296	AA/AACATTTATCAATTA	TGCGAGATTTTGAGTTCAACC	TGCTTATCTTGAACGGGACC	60.2	325	INTERGENIC				
CaPOPII_1719	Ca4	32562355	CTTTTT/CTTTTTT	AAATGGAGGAGAAGGAGGGA	CGGTGAATCCTTTACGGAGA	60.0	286	INTERGENIC				
CaPOPII_1720	Ca4	32663516	TTTATTATTATTATTATTATTATT/TTTATTATTATTATTATT	TGCGTTTCACTCTTCCTTCC	TGTCAAGCCCAAAAACAAAA	60.4	257	INTERGENIC				
CaPOPII_1721	Ca4	32886390	AC/ACC	AATCATCCCATGTTGCCATT	CGAGATTTCACACCAAGGGT	60.0	519	INTERGENIC				
CaPOPII_1722	Ca4	33256637	ATTTTTTTTT/ATTTTTTTT	CATTGATCATGGTTCTTGCC	GGCTTGTGAAAACAAATGGAA	58.9	329	INTERGENIC				
CaPOPII_1723	Ca4	33308913	GAAAA/GAAA	TCATTTTCCCCTCGTTTCTG	TTGGCCAAAACCTTTTGATG	60.0	183	DRR	Ca_24021	R	C2H2	Zinc finger, C2H2-type
CaPOPII_1724	Ca4	33327000	CA/C	AATATTCCCGTCGAATGTGC	TTTATTGCAACATCGCCTTTT	59.8	662	INTERGENIC				
CaPOPII_1725	Ca4	33346959	TT/TTAT	TTTTCGGTTTGTTCAAAGGC	GGAAAACATTGGGAAACCCT	60.1	766	INTERGENIC				
CaPOPII_1726	Ca4	33348179	ATT/AT	AGGTTGAGCGATTGGAGAAA	TCCTAAAAGACTGCCCGCTA	59.8	378	INTERGENIC				
CaPOPII_1727	Ca4	33413394	ATTTTTTTTTT/ATTTTTTTTT	TAGAGCGGGGCTCACTTAAA	GCGGAGATTGCCATATCACT	60.0	447	INTERGENIC				
CaPOPII_1728	Ca4	33615467	ATT/ATTT	GGTCCTTGTTGAGTCCAACG	CTCAAAAACCTGAGCATGGC	60.5	646	INTERGENIC				
CaPOPII_1729	Ca4	33617796	AAAAGAA/AAA	CCCCGATTTGGTCCTTTATT	TGCTAGTTGTTGCGACCAAA	60.0	765	INTERGENIC				
CaPOPII_1730	Ca4	33619321	TAAAAAAAA/TAAAAA	GAACATGCTGAAAACGGGAT	CCAATTCCAAGGTCATCTGG	59.9	448	INTERGENIC				
CaPOPII_1731	Ca4	33633372	GTTTTTTT/GTTTTTT	ACGAGGAAACATATCGTCCG	ATGTCCACACATTGGGTGTC	60.0	726	INTERGENIC				
CaPOPII_1732	Ca4	33714267	T/TCTTAATC	GAACACGTTGTTAGACCGGC	TGACAAAATGGGGAGAAAGG	60.6	713	INTERGENIC				

INDEL marker IDs	Chromosomes /unanchored scaffolds	Physical positions (bp)	InDels (*Kabuli* reference genome-CDC Frontier/PI)	Forward primers (5'-3')	Reverse primers (5'-3')	Anneling temperature (0C)	Expected amplified product size (bp)	Structural annotation		Functional annotation		
								Sequence components of *kabuli* genome	*Kabuli* gene accession IDs	NCBI-KOG	TFs	NCBI-nr database
CaPOPII_1733	Ca4	33714529	ATT/ATTGTT	CGTTGTTAGACCGGCTCTTG	TGGGCTGACATAATCACCAA	60.8	940	INTERGENIC				
CaPOPII_1734	Ca4	33717821	TAAAAAAAAAAAAAAAAAA/TAAAAAAAAAAA	CCAAAAACCCCTCTTTTTCC	CCACCAAAATTTGTCCAACC	59.8	730	INTERGENIC				
CaPOPII_1735	Ca4	33733792	AATATTTGTTTATATT/AATATTTGTTTATATTTGTTTATATT	CGTTATTCCCATATGAGAGGC	TGTGATGTTGGACGTGTGTG	58.5	280	INTERGENIC				
CaPOPII_1736	Ca4	33835105	CAAAAAAA/CAAAAAAAAA	TTTTCACCAAATCACTTGAAAGAA	TCGAGCCTTATTGTGGATGA	60.0	654	INTERGENIC				
CaPOPII_1737	Ca4	33835153	AAGCT/A	TTTTCACCAAATCACTTGAAAGAA	TCGAGCCTTATTGTGGATGA	60.0	654	INTERGENIC				
CaPOPII_1738	Ca4	33836766	GAAAAAAAAA/GAAAAAAAAAAA	GTTTTGTGATGGGGGATGAC	CCATTGAAAAACGAACGACC	60.0	205	INTERGENIC				
CaPOPII_1739	Ca4	33884406	GTTTTTTTTT/GTTTTTTTTTT	AGGCTAGTGCATCATCCAGG	GTACCCCAATTTTTCCCGTT	60.2	303	INTERGENIC				
CaPOPII_1740	Ca4	33924249	ATAGT/ATAGTAGT	CCGTCATTACATGTTGCTGG	TCGAGAGTTGCTCCTGGTTT	60.0	729	INTRON	Ca_20365	O	GATA	Peptidase C19, ubiquitin carboxyl-terminal hydrolase 2
CaPOPII_1741	Ca4	33953368	CAT/CATATCATAT	TATTTTGGGATTGTGGGCTC	AGATTAGGGCCTCAGGAACC	59.8	523	INTERGENIC				
CaPOPII_1742	Ca4	33972054	AAACAACA/AAACAACAACA	TGAGTTCCAAAATCGGGAAA	AATTGGCTAAGCAAGCTCCA	60.4	500	INTERGENIC				
CaPOPII_1743	Ca4	34169705	CAATGA/CAATGATAATGA	TCAAAGTGGCTCACAAGTGC	TCGACTCAAGAAGGCAAGTG	60.0	781	INTERGENIC				
CaPOPII_1744	Ca4	34262481	TAAAAAAAAA/TAAAAAAAAAA	GACCACCTACGCCACACTTT	TGTTCCAATTCCTCGTGTCA	60.0	807	INTERGENIC				
CaPOPII_1745	Ca4	34388181	GAAA/GAAAA	GTTGGTTCCCTAGCACCGTA	CTGTCAATTGAGTTGCGACG	60.0	484	INTERGENIC				
CaPOPII_1746	Ca4	34458862	CAAAA/CAAAAA	CCAAACGTTCTCCCACAACT	GAGGATGCCAAAATGCCTAA	60.0	744	DRR	Ca_20351			
CaPOPII_1747	Ca4	34468802	GTTTTTTT/GTTTTTTTT	GGGAAGCTTGTTTGCTTCTG	TAAGAGTGAGTGGGTTGGGC	60.0	353	INTERGENIC				
CaPOPII_1748	Ca4	34471336	ATTATTTATTTATTTATTTAT/ATTATTTATTTATTTATTTATTTAT	AACGTGACATTTTCGGAAGC	TCAAGTTTTCCGATATAGCCGT	60.1	577	INTERGENIC				
CaPOPII_1749	Ca4	34492879	TAAAAAAAAA/TAAAAAAAAAA	CTGATCAGTCGTTGGGAGGT	TGAGTTGATCGTTTCCACCA	60.1	538	DRR	Ca_20350	R	G2-like	Domain of unknown function DUF676, hydrolase-like

INDEL marker IDs	Chromosomes /unanchored scaffolds	Physical positions (bp)	InDels (*Kabuli* reference genome-CDC Frontier/PI)	Forward primers (5'-3')	Reverse primers (5'-3')	Anneling temperature (0C)	Expected amplified product size (bp)	Structural annotation		Functional annotation		
								Sequence components of *kabuli* genome	*Kabuli* gene accession IDs	NCBI-KOG	TFs	NCBI-nr database
CaPOPII_1750	Ca4	34566424	ATT/AT	CCCTGAGACTCACGAAGAGG	AAAATCCAACAATCTCCCCC	60.0	612	INTERGENIC				
CaPOPII_1751	Ca4	35345914	GTTTT/GTTTTTT	TTTGTGAAGGGATTTGGCTC	ATGCCTCTCTATGCAGCGTT	60.1	763	INTRON	Ca_20018	Z	NAC	IQ motif, EF-hand binding site
CaPOPII_1752	Ca4	35432013	TTAT/TTATAT	TGTTCAACATCCACGAACAA	TGCCATACTCATGTCGGAAA	58.5	669	INTERGENIC				
CaPOPII_1753	Ca4	35432836	TTATATATATATATATATATAT/TTATATATATATATATATATAT	TTTCCGACATGAGTATGGCA	TTTGGAAAGTAACACTGCACG	60.1	785	INTERGENIC				
CaPOPII_1754	Ca4	35433291	TTATATATATATATATATATATATAT/TTATATATATATATATATATATATATATATATATAT	CACGTGCAGTGTTACTTTCCA	TGACGAATTTCAAAAACCTGC	59.8	491	INTERGENIC				
CaPOPII_1755	Ca4	35457008	TTGC/T	AATTGCAGAAAAATGCGGTC	TTGTACCAGATATTGGCCCG	60.1	776	INTERGENIC				
CaPOPII_1756	Ca4	35822348	TGG/TG	GATCAAATCGAGGACCGAAA	CGAACAAAACGATGGGAAGT	60.0	695	INTERGENIC				
CaPOPII_1757	Ca4	35975112	TTTATTATTATTATTATTATTATTATTATTATTATTATTATTAT/TTTATTATTATTATTATTATTATTATTATTATTATTATTATTATTAT	AGCCAAAAGTGGGTAACGTG	TTTTGTGTAACGCCCAGTCA	60.0	193	INTERGENIC				
CaPOPII_1758	Ca4	36079126	TTGTG/TTGTGTG	ATTTTGATGCTCACGGTTCC	CGGAGATGATGGTTCAGGTT	59.9	595	INTERGENIC				
CaPOPII_1759	Ca4	36138277	ATT/AT	ACCATGAAAGACATGGGAGC	CGGAGTACCCGACCAAACTA	59.9	436	INTERGENIC				
CaPOPII_1760	Ca4	36336918	TAAA/TATAAAA	ACAACAAGGGTGACCTCCAC	TGCTTGTCTATTCTGCGTCAA	59.9	736	INTERGENIC				
CaPOPII_1761	Ca4	36337139	GC/G	TGCAATGTCAATATGAACCAAA	CCACAACCACTCATCACGAC	58.9	479	INTERGENIC				
CaPOPII_1762	Ca4	36390176	TATAATAATAA/TATAATAATAATAA	TATGCACGCTCTCTCTCCCT	AGGAGGAACCAAAACCGTCT	60.1	611	INTERGENIC				
CaPOPII_1763	Ca4	36455905	A/AC	GGTGGAGAGCTATTTGAGCG	AAGCAAAACTTCAGGAGCGA	60.0	668	INTRON	Ca_14832	T	YABBY	Protein kinase, catalytic domain
CaPOPII_1764	Ca4	36474350	ACATAATTCA/ACATAATTCATAATTCA	CAAACCACATCACCCTCCTT	GCTACACCGACCACCTGAAT	59.8	585	INTERGENIC				
CaPOPII_1765	Ca4	36502639	TCCCC/TC	GTGGCAAAATGGATCGAACT	GCCGTCTAGTGTTTCTTTAATGACT	59.9	551	INTERGENIC				
CaPOPII_1766	Ca4	36565480	T/TAAACCCC	CACGCTTTCTCCCAAAACAT	GGCACTGAGATCAACATGAGG	60.1	603	DRR	Ca_14822			

INDEL marker IDs	Chromosomes /unanchored scaffolds	Physical positions (bp)	InDels (*Kabuli* reference genome-CDC Frontier/PI)	Forward primers (5'-3')	Reverse primers (5'-3')	Anneling temperature (0C)	Expected amplified product size (bp)	Structural annotation		Functional annotation		
								Sequence components of *kabuli* genome	*Kabuli* gene accession IDs	NCBI-KOG	TFs	NCBI-nr database
CaPOPII_1767	Ca4	36622112	AGTGTTACGTGTTACGTGTT/AGTGTTACGTGTT	ACAAGTCAAAACCGCCTGAC	ATGCTGCCATCAGTAGGTCC	60.2	427	INTRON	Ca_14818	I	WRKY	AMP-dependent synthetase/ligase
CaPOPII_1768	Ca4	36638657	GAAAA/GAAAAA	CTGGGATTATGGGGTCATTG	ATTCAGCTTAGTGGCAAGGC	60.0	446	DRR	Ca_14816	H		FAD dependent oxidoreductase
CaPOPII_1769	Ca4	36644531	ACTCTC/ACTC	CAACACCAAATTGCCAAGTG	AGCTGCCTCAGTTCCACTGT	60.0	870	INTRON	Ca_14816	H		FAD dependent oxidoreductase
CaPOPII_1770	Ca4	36952734	ATTTTTTT/ATTTTTT	AACTCATGTGGTTTGTGGCA	ATGGGCCCTCTTTTGTCTTT	60.0	896	INTRON	Ca_14797	R	C2H2	Zinc finger, C2H2-type
CaPOPII_1771	Ca4	36960688	ATATCTTTGTTATTAT/ATATCTTTGTTATTATCTTTGTTATTAT	TTGATTTTGATGCAGGTGGA	CAAATACCTGTTGCCGAGGT	60.0	818	INTERGENIC				
CaPOPII_1772	Ca4	36968132	ATTTTTTTT/ATTTTTTTTT	GGCGAAAGTACCCAAACAAA	GACTTCTGCAAAGGACCTCG	60.0	426	INTERGENIC				
CaPOPII_1773	Ca4	36972358	CTTT/CTT	ACAACCAACAAAATGCGACA	AGCCTTGAGCGACCACTTTA	60.0	484	INTERGENIC				
CaPOPII_1774	Ca4	36972540	AATATATATATATA/AATATATATATATATATATATA	TAAAGTGGTCGCTCAAGGCT	TCTACATTCAAATTGGGGGC	60.0	608	INTERGENIC				
CaPOPII_1775	Ca4	37040426	TAAAAAAA/TAAAAAAAA	GCGCTACAGAGAGCCTTCAC	GGTCATAACAAAGGGCCAGA	60.3	648	INTERGENIC				
CaPOPII_1776	Ca4	37121983	ATTTTTTT/ATTTTTTTT	TCAACATGACCTCCTTATGCTTT	TCCAAACACTCCAAACACCA	60.0	407	INTERGENIC				
CaPOPII_1777	Ca4	37128859	G/GC	TTTGACGATGGATGCATAAGA	TTTAGGTTGGCTTTGGATGC	59.1	593	INTERGENIC				
CaPOPII_1778	Ca4	37131327	TAA/TAATAGTTTAAAAA	CAACTCAAACCACCTTCATTCA	AATTTGCGAGCATTACCTGC	60.0	633	INTERGENIC				
CaPOPII_1779	Ca4	37221016	A/AG	TGGTTGTCGACTTGTCTTGC	ATGCGGTAGCTTTTGATTCG	59.9	468	INTERGENIC				
CaPOPII_1780	Ca4	37249911	GAAAAA/GAAAAAA	TCGTCGATGAGATCAAATATGC	GGCACAAAAGAGCTGGTCAT	60.1	251	INTERGENIC				
CaPOPII_1781	Ca4	37283555	GT/GTT	TGAATGAAATATTGGATACGTTTATGA	GCAGGGCATTTGTCTCTAGC	59.9	530	INTERGENIC				
CaPOPII_1782	Ca4	37303497	ATTTTTT/ATTTT	CAGTCGAAATACCAGCCCAT	TTTCATTGCGGGACATACAA	60.0	113	INTRON	Ca_15145	J		Ribosomal protein S5
CaPOPII_1783	Ca4	37306375	C/CCT	TCAATGAAGTCCCACGTGAA	TGCAGTCGAAACCAAAAGTG	60.1	381	INTERGENIC				

INDEL marker IDs	Chromosomes /unanchored scaffolds	Physical positions (bp)	InDels (*Kabuli* reference genome-CDC Frontier/PI)	Forward primers (5'-3')	Reverse primers (5'-3')	Anneling temperature (0C)	Expected amplified product size (bp)	Structural annotation		Functional annotation		
								Sequence components of *kabuli* genome	*Kabuli* gene accession IDs	NCBI-KOG	TFs	NCBI-nr database
CaPOPII_1784	Ca4	37331887	CAA/CAAAA	TGACCGACGACTACAATTTCA	AATGGATCACAAAAATCATGTGT	59.2	782	INTERGENIC				
CaPOPII_1785	Ca4	37346206	TTTTATTTATTTATTT/TTTTATTTATTTATTTATTT	GCCCCAACGTGAACATTATT	TCAATAATGCTGCCATCCAA	59.7	208	INTERGENIC				
CaPOPII_1786	Ca4	37362171	GTTGT/GTTGTTTGT	CCATTGGTTTTTGCTTCGTT	CCACGCCTGGAGTTTATTGT	60.0	947	INTRON	Ca_15140	S		HEAT
CaPOPII_1787	Ca4	37366698	AAA/AAACCAA	TTACCCTCTGTTGCTTTGCC	ACCATGGACACCGTTTGTTT	60.2	430	DRR	Ca_15139	R	NF-YA	Ankyrin repeat
CaPOPII_1788	Ca4	37403650	CTTTT/CTTTTT	CTTATCCAAAAGCGAGGCAC	TGAGTTGGACTACAAGCCATCA	59.8	597	INTERGENIC				
CaPOPII_1789	Ca4	37443931	CT/CTAT	CCGCATTTTCTTCATTAGGG	ACGTTAAGGCATGAGTTGGG	59.5	364	INTERGENIC				
CaPOPII_1790	Ca4	37483206	TAAAAAAAAAAAAAA/TAAAAAAAAAAAAAAAA	AAATTGGGTGATGTGGTGGT	CCTAGAAGATGAGGGTGCCA	60.0	424	INTERGENIC				
CaPOPII_1791	Ca4	37608001	ACTCTCTCTCTCTCTC/ACTCTCTCTCTCTCTCTC	TTGGATTCCTGACACACACA	TAAAAATTTGGAGGGAGGGG	58.4	568	INTERGENIC				
CaPOPII_1792	Ca4	37646806	C/CAA	GCTCTGGTGGGTGATTTGTT	ATGTTCCGTTGGAAAGCATC	60.0	641	INTRON	Ca_15117	T	LBD	Disease resistance protein
CaPOPII_1793	Ca4	37671559	CAA/CA	TTTCCACCCAGAAAGCAATC	GCAAAACAGAGGCAACATCA	60.1	250	INTERGENIC				
CaPOPII_1794	Ca4	37688621	ATCTTCTTC/ATCTTC	GGATTTCCTGTGAGCTGCAT	AGAATTGGAATGCCTGAACG	60.2	492	INTRON	Ca_15113	S		Armadillo
CaPOPII_1795	Ca4	37696707	TA/TATAGACATAA	AATGTTTTTGGTAATCCCGGT	AACATTGGTGGTGCAAGACA	59.5	607	INTERGENIC				
CaPOPII_1796	Ca4	37701400	ATTTTTTTTTT/ATTTTTTTTTTT	TGAACACCGCATTAATTGGA	GGGTTTTTGTTCGAAGATTTTG	59.9	262	DRR	Ca_15111	F		ATP-grasp fold
CaPOPII_1797	Ca4	37703958	C/CAAG	TAGTGGGGAAGGTCTGTTGG	TGTTGCTGCGTCTTGAAATC	60.0	733	INTERGENIC				
CaPOPII_1798	Ca4	37704228	T/TG	AGGAGGGATCAAACCGAACT	TGTTGCTGCGTCTTGAAATC	59.9	472	INTERGENIC				
CaPOPII_1799	Ca4	37705943	GA/GATAA	GCCGGGATGTTCTTTTTGTA	TTTACTGAATGCCATGCTGC	59.9	679	DRR	Ca_15110	C	MYB	Cytochrome c oxidase subunit II C-terminal
CaPOPII_1800	Ca4	37706158	G/GA	GCCGGGATGTTCTTTTTGTA	GAGGGGAGGCCTTCTCATAC	59.9	818	DRR	Ca_15110	C	MYB	Cytochrome c oxidase subunit II C-terminal

INDEL marker IDs	Chromosomes /unanchored scaffolds	Physical positions (bp)	InDels (*Kabuli* reference genome-CDC Frontier/PI)	Forward primers (5'-3')	Reverse primers (5'-3')	Anneling temperature (0C)	Expected amplified product size (bp)	Structural annotation		Functional annotation		
								Sequence components of *kabuli* genome	*Kabuli* gene accession IDs	NCBI-KOG	TFs	NCBI-nr database
CaPOPII_1801	Ca4	37707068	C/CTGGCAAGGAGCTACT	TGAGGCCTTAGCTTCGTGAT	CACAAGGACCTGACGGTTTT	60.0	555	INTERGENIC				
CaPOPII_1802	Ca4	37707688	T/TTC	AAAACCGTCAGGTCCTTGTG	TGAGCGTTTTCTCGGTCTTT	60.0	386	INTERGENIC				
CaPOPII_1803	Ca4	37746382	GTTTTT/GTTTT	TTGCAATAGGCATAGGACCC	TTGTTTCTCCCCAGTTTTGG	59.9	448	INTERGENIC				
CaPOPII_1804	Ca4	37860521	CTTA/CTTAATTA	TGGTCCCTTGTTGCCTTTAG	AATGGACGGCTATGAGTTGC	60.1	739	INTRON	Ca_15089			Auxin efflux carrier
CaPOPII_1805	Ca4	37871651	ATTTTTT/ATTTTT	ACCCTACCCATTGTCAACCA	TGGATGGTTATGATTTGCTGA	60.1	668	INTERGENIC				
CaPOPII_1806	Ca4	37909050	GTTTTTTT/GTTTTTTTTTT	TGCATTTTAGGTCCATGATGA	ATTTGACCACCGAAAGATGC	59.0	190	INTERGENIC				
CaPOPII_1807	Ca4	37977728	CGTGGTGGT/CGTGGT	GTTCCCAACTTGTGTGCCTT	TGCATTTCATCACAGACATTGA	60.0	543	INTERGENIC				
CaPOPII_1808	Ca4	38021208	TA/TAAAAA	AAGATTGATTTTCATGGATTGACA	TTGGTGGATGTGAGGAAACA	59.7	584	INTRON	Ca_13143	E		Amino acid/polyamine transporter I
CaPOPII_1809	Ca4	38085487	AT/A	TCCCCTTTCTTACGAATCCC	CACTTGGAAGAGGGCAACAT	60.3	476	INTERGENIC				
CaPOPII_1810	Ca4	38237743	CTTTTTTTTT/CTTTTTTTTTTTTTT	CTCCATAGCCACAACAAGCA	TCCACAGGTTGCAATCACAT	59.9	604	INTRON	Ca_13131			Lipase, GDSL
CaPOPII_1811	Ca4	38240819	ACCC/ACC	CTGGTGAAACTCCGTATCCAA	TTGGATGTGCACGATGTTTT	60.0	258	INTRON	Ca_13131			Lipase, GDSL
CaPOPII_1812	Ca4	38367094	T/TAGATTGGAA	TCGTTTAGCGGTGAAACTCA	CAAGCTCTTGCCATGTTTGA	59.5	158	DRR	Ca_13120	S		C2 calcium-dependent membrane targeting
CaPOPII_1813	Ca4	38368647	A/ATTCTACTTTCAATT	TATGGTTGGATTTTCCGCTC	TTGTCTTCCCCGTTTAGTGG	59.9	751	INTERGENIC				
CaPOPII_1814	Ca4	38377327	TTT/TTTGAATT	TGATTGTGATGCACCGTAAAA	AGCGAAAGTTCTTGGAGGGT	60.0	470	INTERGENIC				
CaPOPII_1815	Ca4	38457252	CTGTGT/CTGT	CGATCACCAAGTCCCCTAAA	CCACTCCCTCCAATCTTCAA	59.9	344	INTERGENIC				
CaPOPII_1816	Ca4	38479454	CAAAA/CAAA	GGTGGGATCCATTTGAGTTG	CCGAAGGCTTTCTTCAACAG	60.2	676	INTERGENIC				
CaPOPII_1817	Ca4	38480745	TGG/TG	GTGGAGCTTCTTTGGGTGAC	ATCACCACCGAAAAACTTGG	59.7	844	INTERGENIC				

INDEL marker IDs	Chromosomes /unanchored scaffolds	Physical positions (bp)	InDels (*Kabuli* reference genome-CDC Frontier/PI)	Forward primers (5'-3')	Reverse primers (5'-3')	Anneling temperature (0C)	Expected amplified product size (bp)	Structural annotation		Functional annotation		
								Sequence components of *kabuli* genome	*Kabuli* gene accession IDs	NCBI-KOG	TFs	NCBI-nr database
CaPOPII_1818	Ca4	38482887	CAAAAA/CAAAA	CAAATGAGAGCGTTAAGGCAG	TGTCCTTCAATTTCATTTCCAA	60.0	501	INTERGENIC				
CaPOPII_1819	Ca4	38482961	A/AC	TTGGAAATGAAATTGAAGGACA	TTGTTGATTTCTAACCTTGCTCA	59.4	193	INTERGENIC				
CaPOPII_1820	Ca4	38483484	AATA/AATATATA	TGAGCAAGGTTAGAAATCAACAA	ACCATTGGTTTCCTTTGCAC	58.9	470	INTERGENIC				
CaPOPII_1821	Ca4	38484127	TTTATT/TTT	TCATCTTTGCTGCTTCTGGA	CTACCTCATGCTTGGCATCC	59.7	299	INTRON	Ca_13111	K	MIKC	Transcription factor, MADS-box
CaPOPII_1822	Ca4	38484269	ATTG/ATTGTTG	TCATCTTTGCTGCTTCTGGA	TGCCAGATCTGTTCATGGAT	59.7	523	INTRON	Ca_13111	K	MIKC	Transcription factor, MADS-box
CaPOPII_1823	Ca4	38484870	CATATA/CATA	AATGATGGCATGCAAGTTGA	GCCTGTTTCTCCCGGATAGT	60.1	268	INTRON	Ca_13111	K	MIKC	Transcription factor, MADS-box
CaPOPII_1824	Ca4	38567221	GAATAAAA/GAA	GACAAAAATGAGAAAGACCTGGA	AAATGCTGCCGAAGAAGCTA	59.6	824	INTERGENIC				
CaPOPII_1825	Ca4	38568495	ATATT/ATATTATT	CGAAATTGACAAAATCACAATCA	TGTCCATGAAGAAAGAGCCC	59.9	340	INTERGENIC				
CaPOPII_1826	Ca4	38618852	CATAA/CA	GCCGACAGAGAAAATCCAAA	ATTGGGGAAGGCCTTGATAA	60.2	532	INTERGENIC				
CaPOPII_1827	Ca4	38686412	AA/AAGGTGGAAGA	AGGTCAAGCACCCACTTCAG	TTCACAATCTTCGGGTCCTC	60.3	323	INTERGENIC				
CaPOPII_1828	Ca4	38705713	ATTTTTTT/ATTTTTTTTT	TTGGGTACAACTCCGTCACA	TCAAAATCTTCCTTCAATGCAA	60.0	715	INTERGENIC				
CaPOPII_1829	Ca4	38744817	CAAAAA/CAAAA	GAAGCACCATCGACCACTTT	TGGTGGCACTAGGTGTATTGA	60.1	194	INTERGENIC				
CaPOPII_1830	Ca4	38758073	GTTTTT/GTTT	TAGACAGGTTGGTCCCTCCC	ACCGTACAATCAAACCGCTC	61.3	840	INTRON	Ca_13097			Auxin efflux carrier
CaPOPII_1831	Ca4	38816584	AATTTATATTTA/AATTTATATTTATATTTA	ATGCCTTTACATGCACAAAAT	GCAAATATTGTCTGCCAAAAGTG	57.2	724	DRR	Ca_13093	K	NF-YB	Transcription factor, CBFA/NFYB, DNA topoisomerase
CaPOPII_1832	Ca4	38949892	GT/G	TCAAATCAGTATGCGAAGCG	ACCCACACAACACACATGCT	60.0	360	INTERGENIC				
CaPOPII_1833	Ca4	38955357	GATAAAATA/GATA	GAGTTTTGGGGTGACATGCT	CATCCTGCACAATGCTGTTC	60.0	251	INTERGENIC				
CaPOPII_1834	Ca4	38965155	TATAATAA/TATAA	AGCCATGCCAGATTATTTGC	CAGTGCGATCCATCTCTGAA	60.1	221	INTRON	Ca_13082	O	MYB_related	Ubiquitin-conjugating enzyme, E2

INDEL marker IDs	Chromosomes /unanchored scaffolds	Physical positions (bp)	InDels (*Kabuli* reference genome-CDC Frontier/PI)	Forward primers (5'-3')	Reverse primers (5'-3')	Anneling temperature (0C)	Expected amplified product size (bp)	Structural annotation		Functional annotation		
								**Sequence components of *kabuli* genome**	***Kabuli* gene accession IDs**	**NCBI-KOG**	**TFs**	**NCBI-nr database**
CaPOPII_1835	Ca4	38970599	CCTT/C	AGCGTACGAGAAGGAGTCCA	ACCAAAATGGCAATGAGGAC	60.0	510	INTERGENIC				
CaPOPII_1836	Ca4	38994620	ATGTATT/ATGTATTGTATT	GCATACCCTTTTCCGGTCTT	GCATGCTCTAATCCATTCCAA	60.3	769	INTERGENIC				
CaPOPII_1837	Ca4	38997590	TCAA/T	ACCCACCACTGATTCCATGT	TGTCTTCAACTAGCACTCGGG	60.1	480	INTERGENIC				
CaPOPII_1838	Ca4	39038460	ACTTGCTTG/ACTTG	CCGGTCACATTCAATAGCCT	ATTGCGTTGTTTCTTCCAGG	60.0	411	DRR	Ca_13078	A		RNA recognition motif domain
CaPOPII_1839	Ca4	39165742	TAAAAATAAAAAATAAAAAA/TAAAAATAAAAAATAAAAAATAAAAAA	CGTATACCGCTGCTCATCAA	TGATGTGATTTGTGCCATGA	59.9	259	INTERGENIC				
CaPOPII_1840	Ca4	39257138	ATTTTTTTTTT/ATTTTTTTTTTT	TTTGCGTTACGAATTTGATGA	TGAATTTTCCCGACTTGGTC	59.2	181	INTERGENIC				
CaPOPII_1841	Ca4	39689187	AC/ACC	ATTACCGACATTGCAGAGGG	CTCCCATTCTGCCCTTGATA	60.0	329	INTERGENIC				
CaPOPII_1842	Ca4	40111947	GAATAATAATAATAATAATAATAATAATA/GAATAATAATAATAATAATAATAATA	TGCAAGTTGCTATCCCATTG	TTGCAATACGGATCACAAAAA	59.7	753	INTERGENIC				
CaPOPII_1843	Ca4	40348298	AATATATATATATATATATATATATATATATATATA/AATATATATATATATATATATATATATATATATATATATATA	CCATCCTTCGAATTTGGCTA	TGGTTAAAGGCCAGGATTTC	60.0	668	DRR	Ca_14889	PET	NAC	Ionotropic glutamate receptor
CaPOPII_1844	Ca4	40479668	TCCC/TCC	CTCCCTAACCCTTCTCCCTG	CGGATATTGGCTGGCTTGTA	60.1	622	INTRON	Ca_14877	I		Alpha/beta hydrolase fold-1
CaPOPII_1845	Ca4	40491458	AAG/A	TCCGTTAGATTGCTGAATGC	CATCTCGAATGGCTTCCAAT	58.9	628	INTRON	Ca_14876		HD-ZIP	
CaPOPII_1846	Ca4	40499622	ATTTTT/ATTTT	GGCCAAATTCATCCAACAAA	GGGGTGGGTTGAAGAAAAAT	60.7	501	INTRON	Ca_14875			Protein of unknown function DUF688
CaPOPII_1847	Ca4	40506274	CAGTATAATAGTA/CAGTATAATAGTATAATAGTA	GTGGCTGTTTATGGATTGGG	TGATCCCCTTCATTCCTTCA	60.2	239	INTERGENIC				
CaPOPII_1848	Ca4	40584112	ATATCGT/AT	TGATATGCGGTAGCCATCAA	GACCCAAATCTGGCTTCAAA	60.1	547	INTRON	Ca_14868	K		Zinc finger, RING-type
CaPOPII_1849	Ca4	40649020	ATTTTTT/ATTTTTTTT	TGTGATTATCCGGTACCACAAC	TGTATTCAGGACCTCGCAGA	59.6	207	INTERGENIC				
CaPOPII_1850	Ca4	40763607	CAAAAA/CAAAA	GCTCTGCTTGATTGAAAATGC	CCAATGCATGAAATTTGTTCTT	60.0	696	INTERGENIC				
CaPOPII_1851	Ca4	40819629	TCAACAACAACAA/TCAACAACAACAACAA	GGCCTGGAACACTTTCCATA	CAAGGGGGTGAAAAATCAGA	59.9	407	INTRON	Ca_23900	R		DNA/RNA helicase, DEAD/DEAH box type, N-terminal

**INDEL marker IDs**	**Chromosomes /unanchored scaffolds**	**Physical positions (bp)**	**InDels (*Kabuli* reference genome-CDC Frontier/PI)**	**Forward primers (5'-3')**	**Reverse primers (5'-3')**	**Anneling temperature (0C)**	**Expected amplified product size (bp)**	**Structural annotation**		**Functional annotation**		
								**Sequence components of *kabuli* genome**	***Kabuli* gene accession IDs**	**NCBI-KOG**	**TFs**	**NCBI-nr database**
CaPOPII_1852	Ca4	40842793	TC/T	CTGGATTGAGTTGAGGCCAT	TGGACCAAACCAAGCACATA	60.1	568	INTERGENIC				
CaPOPII_1853	Ca4	40880303	GA/GAA	GGAAGTTTCCAAAGCACGAG	AAGTTCCATTGCTTGCTGGT	59.9	411	INTERGENIC				
CaPOPII_1854	Ca4	40893508	AG/A	TGAACTCGGCTAGGAGGAAA	CGCAAGTGTTGGACAATGAT	59.9	703	DRR	Ca_11020	R	G2-like	C2 calcium-dependent membrane targeting
CaPOPII_1855	Ca4	41015733	TAAAAAA/TAAAAAAA	CAAATTTTGATGATCTCACACGA	GCCCCATAAAAAGATGCTCA	60.0	504	INTERGENIC				
CaPOPII_1856	Ca4	41027713	AATATATATATATATATATATATATATATAT/AATATATATATATATATATATATATATATATATATATAT	TGAATCCCAAAATTTGAACCA	TTTGATCGCAAGGGTTTAGG	60.2	637	INTERGENIC				
CaPOPII_1857	Ca4	41172721	AC/A	TCAAGTTAAAGGTGGAGCGG	TTCCTCCCTTTCTTTGACAAC	60.2	761	INTERGENIC				
CaPOPII_1858	Ca4	41268887	AATATATATATATATATATA/AATATATATATATATATA	AAACCTCGTCAATTGGCTCA	CAATTGGTGGAACAATCGTG	60.6	299	INTERGENIC				
CaPOPII_1859	Ca4	41306366	CTTTTTTTTTT/CTTTTTTTTTTTT	GGTAGCATCCAACCATCTAGACA	GGGGATGTGAACACTTAAGCA	60.4	157	INTERGENIC				
CaPOPII_1860	Ca4	41581170	GA/GATCA	ACAAAAACTCCCAATGGTCG	TAGCCAACCACACATGGAAA	59.8	737	INTERGENIC				
CaPOPII_1861	Ca4	41806206	CTA/CTAATA	TGCCTTGAATCTCTTCTTGGA	TGGAAAACCATAATTGAGCG	59.9	905	INTRON	Ca_10934	QR	B3	Oxoglutarate/iron-dependent oxygenase
CaPOPII_1862	Ca4	41806539	ATTTT/ATT	TGGTGCTTGTAGTTGATGAGG	TTTGGGTCAGGACAAGAAGG	58.8	949	INTRON	Ca_10934	QR	B3	Oxoglutarate/iron-dependent oxygenase
CaPOPII_1863	Ca4	41806627	ATT/AT	AACACATCCAAATCACATGCT	ATTTGGAGCCCACTTGTTTG	57.9	964	INTRON	Ca_10934	QR	B3	Oxoglutarate/iron-dependent oxygenase
CaPOPII_1864	Ca4	41823980	CATCAGA/CA	CATCAAGACCATTTCCAGCA	CAATTGTTCGTGTTGCGTCT	59.6	114	INTERGENIC				
CaPOPII_1865	Ca4	41832968	AATATATATATATA/AATATATATATA	TTTCCATTGCACTCACTCCA	CCTATCATGGCCTGACCTGT	60.2	758	INTERGENIC				
CaPOPII_1866	Ca4	42120439	TAAAAAAAAA/TAAAAAAAA	CACAAGCATTGGAGTCCCTT	AACTCGTGGGTTGGATTTTG	60.1	495	INTRON	Ca_10910		C2H2	Glycoside hydrolase, family 28
CaPOPII_1867	Ca4	42120574	C/CG	CACAAGCATTGGAGTCCCTT	AACTCGTGGGTTGGATTTTG	60.1	495	INTRON	Ca_10910		C2H2	Glycoside hydrolase, family 28
CaPOPII_1868	Ca4	42517427	AATATATATATATATATATATATATATATATATATA/AATATATATATATATATATATATATA	ATAATGTTCAGCACCCGAGG	GAGCTGTCCACGACACTCAA	60.0	466	INTRON	Ca_10897		Dof	

INDEL marker IDs	Chromosomes /unanchored scaffolds	Physical positions (bp)	InDels (*Kabuli* reference genome-CDC Frontier/PI)	Forward primers (5'-3')	Reverse primers (5'-3')	Anneling temperature (0C)	Expected amplified product size (bp)	Structural annotation		Functional annotation		
								Sequence components of *kabuli* genome	*Kabuli* gene accession IDs	NCBI-KOG	TFs	NCBI-nr database
CaPOPII_1869	Ca4	42548370	ATTTTTTTT/ATTTTTTTTT	CGTGCCTTTGTCGTCTCTTT	AGAGGCCCAATATCCTTGCT	60.4	528	INTERGENIC				
CaPOPII_1870	Ca4	42574723	AGGG/AGGGG	TCGTCATCACACAAGGTGGT	CAGGTTGTAAGCAAAAGCCC	60.0	227	INTERGENIC				
CaPOPII_1871	Ca4	43339918	ATT/ATTT	TCATTCTCTTCATCGAAGATTTCA	TGGAGAATAATTAACAGAGCAGCA	60.2	565	INTERGENIC				
CaPOPII_1872	Ca4	43450204	A/AT	TTCGTGTCAGCAAGACCAAG	AGGGAGATTCTTGGAGAGAGC	60.0	194	DRR	Ca_23690			
CaPOPII_1873	Ca4	43518348	AATTTTTTTATTTTTTTATTTTT/AATTTTTTTATTTTT	GTGAGTCATGGGACGGTTCT	CTAGTTGGCCCATGGAATGT	60.0	749	INTRON	Ca_23694		bHLH	Helix-loop-helix DNA-binding domain
CaPOPII_1874	Ca4	43564232	CTTTTTTT/CTTTTTT	TCAAACAAAATTGGGTTACAACA	TTTGGTAATCACAAAAATCCAAAA	59.3	664	INTERGENIC				
CaPOPII_1875	Ca4	44344897	ATTTTTTTTTTT/ATTTTTTTTTT	TTGATTATTGATTACCCACGGA	TACCCATTGATCCGATTCGT	59.2	510	INTERGENIC				
CaPOPII_1876	Ca4	44428431	CATATATATATATATATATAT/CATATATATATATATATATATAT	TGTTGCGGAAATGTTAGTTCA	TTCATCCATGCAATATTCAAAAA	59.2	699	INTERGENIC				
CaPOPII_1877	Ca4	44522624	GAAAAAA/GAAAAAAA	GAAGTTGCTTGCTCCGATTC	CCCTAGCAAGTGCTAACCCA	60.0	675	DRR	Ca_09124		ERF	Pathogenesis-related transcriptional factor/ERF, DNA-binding
CaPOPII_1878	Ca4	44522868	A/AG	ACAATCCCTTCGTTCCCTCT	CCCTAGCAAGTGCTAACCCA	59.9	516	DRR	Ca_09124		ERF	Pathogenesis-related transcriptional factor/ERF, DNA-binding
CaPOPII_1879	Ca4	44525079	AGTATTTTTCTTA/AGTATTTTTCTTATGTATTTTTCTTA	ATGACGTTGGTGATTGGACA	TGGAGTAATCCCTAATCATTTCA	59.8	357	INTERGENIC				
CaPOPII_1880	Ca4	44540601	TAAAA/TAAAAA	AACAATGGGGTCTATCGTGTG	GGACCCATGGTCAAGAAAGA	59.7	678	INTERGENIC				
CaPOPII_1881	Ca4	44540632	TAAA/TAAAA	AACAATGGGGTCTATCGTGTG	GATTGGAACCATCGAGGAGA	59.7	896	INTERGENIC				
CaPOPII_1882	Ca4	44545143	AATAT/AAT	TTTTCAGCCCATTCCTTTTG	TAATATGCAAGTGGAGGCCC	60.0	910	INTERGENIC				
CaPOPII_1883	Ca4	44555024	T/TGAGATGGAGA	TTTCTCAAGGATCAAAATGGG	TGACCTGACCTATTCCCACC	59.0	629	INTERGENIC				
CaPOPII_1884	Ca4	44561281	ATTTTTTT/ATTTTTTTT	TCAAATCAAAGAATCAACAACAAAA	TTCGGTTAATTTTGCCAGGA	59.9	561	INTERGENIC				
CaPOPII_1885	Ca4	44563494	CTTCTTTT/CTT	GGCAAATCACATGCTAGGCT	TCGTCCAATCTTGTGGTTCA	60.2	513	INTERGENIC				

INDEL marker IDs	Chromosomes /unanchored scaffolds	Physical positions (bp)	InDels (*Kabuli* reference genome-CDC Frontier/PI)	Forward primers (5'-3')	Reverse primers (5'-3')	Anneling temperature (0C)	Expected amplified product size (bp)	Structural annotation		Functional annotation		
								Sequence components of *kabuli* genome	*Kabuli* gene accession IDs	NCBI-KOG	TFs	NCBI-nr database
CaPOPII_1886	Ca4	44578743	GAAAA/GAAAAA	TCATGAAGGAAAGCCAAACC	ATGGCTTGCAAGGTCATAGG	60.1	845	INTRON	Ca_09129	T	WRKY	Protein kinase, catalytic domain
CaPOPII_1887	Ca4	44585709	GAATTAAATATAAATTAAATATA/GAATTAAATATAAATTAAATATAAATTAAATATA	TTTCTATTTGGGGCCTAGCA	TCTCATTGTCACCGTTGCAT	59.7	587	INTERGENIC				
CaPOPII_1888	Ca4	44601700	TAAA/TAA	TGCAGGGATGAAATTCAAAAA	AGGACACGGATTCATTTGGA	60.4	380	INTERGENIC				
CaPOPII_1889	Ca4	44613140	T/TA	CCATCGTTGAAACGTTGTGAT	TGCGAGTTTCTTTATCATTTCC	60.8	428	INTERGENIC				
CaPOPII_1890	Ca4	44622646	CT/CTGTCAGTTT	TGTTGAGTCGCCGTTAAATG	ACGCAAAGCACACACAAGAG	59.7	701	INTERGENIC				
CaPOPII_1891	Ca4	44670060	AC/ACC	TGCCTAAGACCCCAAATCAA	GCCACTTCTGGAATCACCAT	60.4	682	DRR	Ca_09140	E		Spermine synthase
CaPOPII_1892	Ca4	44675409	CGATTGATTGATTG/CGATTGATTG	TGCTTCAGCACTCTGCATCT	GTTTTGGAAAGGTTTGCAGC	59.9	519	INTRON	Ca_09140	E		Spermine synthase
CaPOPII_1893	Ca4	44679413	CATA/CATATATA	AATCCTCTATGGATGCCGTG	TTGTGGGATTAAAAGTCGAAAAA	59.9	707	INTERGENIC				
CaPOPII_1894	Ca4	44679485	TCATG/TCATGCATG	AATCCTCTATGGATGCCGTG	TTGTGGGATTAAAAGTCGAAAAA	59.9	707	INTERGENIC				
CaPOPII_1895	Ca4	44694688	TAGA/TAGAGA	GATTCGATCATCTGGGGAAA	AGGCTTCTGGTGCTGTTCTT	59.8	539	INTERGENIC				
CaPOPII_1896	Ca4	44706474	CAAAAAAAAA/CAAAAAAAA	ACTACTCCTCCCCTGGGAAA	GGTACTAGAGTTGCGGTGGC	59.9	811	INTRON	Ca_09141	O		Pex, N-terminal
CaPOPII_1897	Ca4	44706655	GAAA/GAA	AATAAACAAAGCACGCCTGG	GGTACTAGAGTTGCGGTGGC	60.1	576	INTRON	Ca_09141	O		Pex, N-terminal
CaPOPII_1898	Ca4	44710559	GAAAAAA/GAAAAAAA	AGGAGTGGATGAGCCAAAAA	GGTGCTAAGTTCAAACACACAAA	59.7	822	INTERGENIC				
CaPOPII_1899	Ca4	44738779	TT/TTAT	GGTTGCCTCTTTTACATGGG	TCATGCATGGTTGGAAAAGA	59.4	649	INTERGENIC				
CaPOPII_1900	Ca4	44747851	ATTTTTTT/ATTTTTTTTT	TGGACTTGTGAAGCAAGCCT	CTTTCCCCGTGTGCTTACAT	61.0	580	INTERGENIC				
CaPOPII_1901	Ca4	44771286	CT/C	ATCGGATGTAAAATTCCCCC	TTGCTTCAGTTGGTGCATTC	59.9	411	INTERGENIC				
CaPOPII_1902	Ca4	44787142	CTGT/CTGTGT	TGGCTTCATTTTCCATGTCA	ATTCCAAGAATAGCCGCCTT	60.0	246	INTRON	Ca_09144		S1Fa-like	Protein kinase, catalytic domain

INDEL marker IDs	Chromosomes /unanchored scaffolds	Physical positions (bp)	InDels (*Kabuli* reference genome-CDC Frontier/PI)	Forward primers (5'-3')	Reverse primers (5'-3')	Anneling temperature (0C)	Expected amplified product size (bp)	Structural annotation		Functional annotation		
								Sequence components of *kabuli* genome	*Kabuli* gene accession IDs	NCBI-KOG	TFs	NCBI-nr database
CaPOPII_1903	Ca4	44863007	GAAAAAAA/GAAAAAA	TGAGCATGCAAAGTCCAATC	CAACCCTCCCAGTTTCACAT	59.8	285	INTERGENIC				
CaPOPII_1904	Ca4	44865492	TA/TAGA	TGACCATTGTGTGAAAATGAGA	TGGTCGTAAAGTGCATTGGA	59.0	685	INTERGENIC				
CaPOPII_1905	Ca4	44901004	GT/GTGTAT	AGCTTCAAGTGGCTGAAGGA	ATGAACACGGGCGTATCTTC	60.1	527	DRR	Ca_09152		C2H2	Protein of unknown function DUF3511
CaPOPII_1906	Ca4	44917486	AATATATATATA/AATATATATATATA	TTCCACCTTCAATGCAACAA	TTTGGAATTGGTCCAAGAGC	60.1	899	DRR	Ca_09155	Q		Cytochrome P450
CaPOPII_1907	Ca4	44922267	CTTTTTT/CTTTTTTT	TGATTTGAGGGAGTTGGAGTG	CCCATAATGTCCCGATTCTG	60.1	676	INTERGENIC				
CaPOPII_1908	Ca4	44927225	TTT/TTTATT	CGGGAGATACGTAGGAGCAA	GCTTGTGCATATCCCAACCT	60.2	438	INTERGENIC				
CaPOPII_1909	Ca4	44949173	AA/AAATTA	TGACGTGTCCCGTAACCTTT	ATAAGCCCATGTTGCGACTC	60.4	565	INTERGENIC				
CaPOPII_1910	Ca4	45122277	TAA/TAAA	GCGAGCGTATTGGTATTGGT	ATGTGACAAATTGGGGGAAA	60.0	551	DRR	Ca_09172	T		
CaPOPII_1911	Ca4	45161056	T/TG	GGATTGTGGAATTGGATTGG	CAAGACACGTGCACACACAG	60.0	496	INTRON	Ca_09179	P		Cation/H+ exchanger
CaPOPII_1912	Ca4	45165137	A/AC	TTGTTCTTCCTCCCCCTTCT	TTCAGCCCGCTAGCTATGAT	60.0	669	DRR	Ca_09179	P		Cation/H+ exchanger
CaPOPII_1913	Ca4	45182355	AATATATATATATATATATATAT/AATATATATATATATATAT	TCATTGTCTTGGTCTTCCCC	GTGGTATTTAGTAAAATGAATGTGTCA	59.9	428	INTERGENIC				
CaPOPII_1914	Ca4	45194041	CAATA/CA	GAGCGAGTCAAAACCGAGTC	GCAAGCAATCAAGTAAAAATGG	60.0	597	INTERGENIC				
CaPOPII_1915	Ca4	45205185	TC/T	GGAAATGGGAGGAGGGAATA	TCGTGTTTTGAATAAACAGGAAAA	60.1	300	INTERGENIC				
CaPOPII_1916	Ca4	45205721	ACC/AC	CGTATTTTGGCTTAAATATTTCCAA	CCTCCAACCCACTGATCCTA	59.7	451	INTERGENIC				
CaPOPII_1917	Ca4	45210841	CAAA/CA	TGGATCTTTTGTTGTCCCCT	ATGCTAAGCCACCAACCAAC	59.4	680	DRR	Ca_09183	O		SAC3/GANP/Nin1/mts3/eIF-3 p25
CaPOPII_1918	Ca4	45221189	GTTTTT/GTTTT	GCACTCAAAGAGCACCATCA	CTGGCTCCGAAATCTATTGG	60.0	505	DRR	Ca_09184	A	C2H2	K Homology
CaPOPII_1919	Ca4	45222131	TA/TAA	CCACCTTGTTCCCACTCCTA	TCCAATGAATTTGGAGAGCC	60.0	334	DRR	Ca_09184	A	C2H2	K Homology

INDEL marker IDs	Chromosomes /unanchored scaffolds	Physical positions (bp)	InDels (*Kabuli* reference genome-CDC Frontier/PI)	Forward primers (5'-3')	Reverse primers (5'-3')	Anneling temperature (0C)	Expected amplified product size (bp)	Structural annotation		Functional annotation		
								Sequence components of *kabuli* genome	*Kabuli* gene accession IDs	NCBI-KOG	TFs	NCBI-nr database
CaPOPII_1920	Ca4	45223304	ATTACT/ATGAATTACTTACT	TGAAGAGGGGAAATATGCAA	GAAAACGCTTTTATCATCATCCA	58.2	442	INTERGENIC				
CaPOPII_1921	Ca4	45226893	TTC/TTCGTC	ATTAGTTCCTGGCCGGTCTC	GGAGAGAGGACCCACAAACA	60.5	868	DRR	Ca_09185	S		Protein of unknown function DUF647
CaPOPII_1922	Ca4	45236013	CATGTGCGAACAATG/CATGTGCGAACAATGTGCGAACAATG	CAAACCTGAAAAGGGCACAT	TAACGATGGGATTGGTCCAT	60.0	461	INTRON	Ca_09187	A		Phosphorylated adapter RNA export protein, RNA-binding domain
CaPOPII_1923	Ca4	45247621	TA/TAA	GTTGGAAGCCATGGTGAACT	TTTTGGTTTTCAAGTATTGGCA	60.0	430	INTERGENIC				
CaPOPII_1924	Ca4	45267760	GT/GTAATCGTAATT	ATTGATGGCAAGAAAGGTGG	TTTCCAGGCATATAGGGTGC	59.9	758	INTRON	Ca_09190			Protein of unknown function DUF1639
CaPOPII_1925	Ca4	45274571	T/TG	TCGAACGACAATCCACACAT	CGTGGGGGAAATTTTTCTTT	60.0	518	DRR	Ca_09191	O		Ubiquitin-conjugating enzyme, E2
CaPOPII_1926	Ca4	45289876	GAAAAAAAAAA/GAAAAAAAA	AAGCGAGCATCAGAGAAAGC	CTTGATGGCAAAACCCAAGT	59.9	269	INTERGENIC				
CaPOPII_1927	Ca4	45335482	ATTTT/ATTT	GTTCCGATCACATGGCTTCT	TGAGACGCTTTCGTCACAAC	60.1	406	INTRON	Ca_09198	L		3'-5' exonuclease
CaPOPII_1928	Ca4	45341608	AAGAGAATCGGA/AAGAGAATCGGAGAGAATCGGA	GCTGGACAAGTCTGCCAAG	CTAGTGTGCAGTGGTGCCAT	59.6	874	DRR	Ca_09198	L		3'-5' exonuclease
CaPOPII_1929	Ca4	45385545	ACT/ATTATATATCT	CACCCATAATACTTTATGAGATGGAA	CACAAATTAAGGGAAGGCGA	59.6	363	INTERGENIC				
CaPOPII_1930	Ca4	45401996	TAAAAAAAAA/TAAAAAAAA	TTGATAGTGGCTGGCACAAA	GGGGTTTTGGTTTTGGTTTT	60.3	480	INTERGENIC				
CaPOPII_1931	Ca4	45414589	CTT/CTTT	TGCATAAATGCACGATGAAAA	GTTCAAACCGGCAATGTCTT	60.1	740	INTERGENIC				
CaPOPII_1932	Ca4	45645932	GT/G	AACTTGATGCCGAGTAGCGT	GCCGAATAAAACCCTCACAA	59.9	713	INTERGENIC				
CaPOPII_1933	Ca4	46001807	GATAATAATAATAATAATAATAATAATAATAATAATAATAATA/GATAATAATAATAATAATAATAATAATAATAATAATAATA	TTGCTCCTACGTATCCCCAG	CTCAATCGTGGGTGACCTTT	60.1	385	INTERGENIC				
CaPOPII_1934	Ca4	46004838	TAAAAAA/TAAAAAAA	TGATTTCAATGAGAGGATTTTGTT	GCTCGATTGATGTTAAGGGC	59.0	425	INTERGENIC				
CaPOPII_1935	Ca4	46493029	AATATATATATATATATATATATATA/AATATATATATATATATATATA	GGGAAAGTGATATTGGGGGT	CAAAGCTCCTTCAATTGTCTTG	59.9	407	INTERGENIC				
CaPOPII_1936	Ca4	46508599	AATATATATATATATATATATATA/AATATATATATATATATA	AAAGGGGGAGTATTGTTGGG	CAAAGCTCTTTCATAATTGTCTTGA	60.0	492	INTERGENIC				

INDEL marker IDs	Chromosomes /unanchored scaffolds	Physical positions (bp)	InDels (*Kabuli* reference genome-CDC Frontier/PI)	Forward primers (5'-3')	Reverse primers (5'-3')	Anneling temperature (0C)	Expected amplified product size (bp)	Structural annotation		Functional annotation		
								Sequence components of *kabuli* genome	*Kabuli* gene accession IDs	NCBI-KOG	TFs	NCBI-nr database
CaPOPII_1937	Ca4	46674801	A/AG	GGATGACTCGAAACGGTGTT	TTACCCAAAACCCCAAATCA	60.0	667	INTERGENIC				
CaPOPII_1938	Ca4	46711628	GTTATAG/GTTATAGACTTATAG	TCATTTACTCGTGCATGAAAGTT	ATGCTTTTTCACCATCCTCG	58.8	685	INTERGENIC				
CaPOPII_1939	Ca4	46716677	TAAA/TAA	AGATTGACGAGGAAGCTGGA	CTCTGCTTGCAGGGTTAAGG	60.0	947	INTRON	Ca_19246	S		PGAP1-like
CaPOPII_1940	Ca4	46730464	CTTTTT/CTTTTTT	CATTAGTCCACGTTGCTTCG	TAACAGACCCGTGCAATGAG	59.3	271	INTRON	Ca_19245	P		Haem oxygenase
CaPOPII_1941	Ca4	46732510	G/GCA	CGGTATAGCCTGGGAAATGA	GATGGAAGGAATGCCAAAGA	59.9	766	DRR	Ca_19245	P		Haem oxygenase
CaPOPII_1942	Ca4	47206262	A/ATAT	TCACCATTCAATCATTTTTGG	CCCGAGAAAGATTTTACGCA	58.3	550	INTERGENIC				
CaPOPII_1943	Ca4	47211846	TG/TGG	TTGAAATGCAACCTTGTCCA	AAGCCCCTGCTTTGATTTCT	60.1	774	DRR	Ca_10861		LBD	Disease resistance protein
CaPOPII_1944	Ca4	47238699	ATTT/ATT	TTTTGATCGAATCTGACCCA	TCGGCCATTTTTCTTCAATC	59.0	659	INTERGENIC				
CaPOPII_1945	Ca4	47242175	AAATAATAATAATAATAATAATAA/AAATAATAATAATAATAATAA	CGCTCCTAATTCCCACACAT	TTTCTCGCATGCATTCTCAC	60.0	835	INTERGENIC				
CaPOPII_1946	Ca4	47457546	CATTATTATTATTATTATTA/CATTATTATTATTATTATTATTATTA	GGTTGGGATGAAAACTGGAA	CCTGGAAAACATTAATTCCCC	59.8	543	INTERGENIC				
CaPOPII_1947	Ca4	47685878	AATATATATATATATA/AATATATATATATATATATATATATA	AAATTAAAGTTTCAAACCACGG	CCGACGAAATAAGTCCCTCA	57.3	667	INTERGENIC				
CaPOPII_1948	Ca4	47982672	TC/TCC	CCAAACTCCAATCAAATGGG	CAAATGAGAATTTTCTGGGCA	60.2	483	INTRON	Ca_10790	U	ARF	Armadillo
CaPOPII_1949	Ca4	47991117	GTTTTTT/GTTTTTTTT	ATTAGCCACGACGATGGTTC	TGGTGTACATGTAGGCGTGC	60.0	564	DRR	Ca_10789		B3	Transcriptional factor B3
CaPOPII_1950	Ca4	48074355	TG/T	GGTCGACATGACATCACTAAATG	AGTGTCAAAGGGGGAGGAAG	59.4	601	INTERGENIC				
CaPOPII_1951	Ca4	48202910	AAATAA/AAA	TCGTCCTTACACCGGAAAAC	CAAGTTTCGGTTGACTATACACG	60.0	638	INTERGENIC				
CaPOPII_1952	Ca4	48206793	CAT/CATAT	TATTTGGCCAACAGGAAAGG	AGAGCTGATTTGGTTCCTCG	59.9	893	INTERGENIC				
CaPOPII_1953	Ca4	48208929	TC/T	AAGTTTTTGAATACCGCAAAAT	AAGCTGCGATGGGACAATAC	57.0	490	INTERGENIC				

INDEL marker IDs	Chromosomes /unanchored scaffolds	Physical positions (bp)	InDels (*Kabuli* reference genome-CDC Frontier/PI)	Forward primers (5'-3')	Reverse primers (5'-3')	Anneling temperature (0C)	Expected amplified product size (bp)	Structural annotation		Functional annotation		
								Sequence components of *kabuli* genome	*Kabuli* gene accession IDs	NCBI-KOG	TFs	NCBI-nr database
CaPOPII_1954	Ca4	48218216	TATTA/TATTAATTA	CAACTTGAATTTGGTTTGGCT	TCTGTCGGCAGATAACCCTT	59.1	885	INTERGENIC				
CaPOPII_1955	Ca4	48247972	TA/TACAA	AAAATTGCCCTGTTTCCTTG	TCCAGTAGCAAAATGGCACA	59.1	195	INTRON	Ca_10763			Mediator complex, subunit Med11
CaPOPII_1956	Ca4	48314680	ATTT/ATTTTT	GTAATGCGTTTGACCTGCAA	ATAGGCGCATCTCAATCACC	59.7	649	DRR	Ca_10756		BBR-BPC	GAGA binding-like
CaPOPII_1957	Ca4	48351278	TAATTCCAATTCCAATT/TAATTCCAATTCCAATTCCAATT	GACACGTGATGTCTCTGTAATGG	AATGTATGTATCGAGAGCGCC	59.5	651	INTERGENIC				
CaPOPII_1958	Ca4	48404970	GTTTAATTT/GTTTAATTTTAATTT	TTGCATGACTGCTTTTGGAC	TTGTCATTGTTGCGGTTTGT	59.8	514	INTERGENIC				
CaPOPII_1959	Ca4	48453751	A/AT	GTTCCACCACCGCAAATATC	GGTGGTGGTAGGAAAAGCAA	60.2	742	INTERGENIC				
CaPOPII_1960	Ca4	48518535	AATAT/AATATAT	TGGTGGTTGACCACAAAAGA	ATCATCTTTGGCAGTAGCGG	60.0	927	INTRON	Ca_10740		C2H2	Zinc finger, C2H2-type
CaPOPII_1961	Ca4	48576925	AT/ATT	ATCTCACCCCAAGACAACCA	TCATTCCAGAAGGAGATGGG	60.4	211	INTRON	Ca_23007	P		Heavy metal transport/detoxification protein
CaPOPII_1962	Ca4	48577228	A/AC	AACACATCCGGGATCCATTA	TGATGACGAAGACGAAGACG	60.0	303	INTERGENIC				
CaPOPII_1963	Ca4	48670468	GATA/GATAAAATCTTATA	CTGTATTGTTAGTCGGCGCA	CCGACCCAACACGTTATGA	59.9	620	INTERGENIC				
CaPOPII_1964	Ca4	48708876	TTTTATTT/TTTT	CGCCACTTGTATTTCAAGCA	GCATCAGCTCTTCCAGCTTC	59.9	517	DRR	Ca_23020	Q	Nin-like	ABC transporter, transmembrane domain
CaPOPII_1965	Ca4	48716729	AATA/AATACATGGTTACATA	GACCAGAAAGCCCAATTGAA	CCGATTCGTGGTCTGTTTTT	60.1	576	INTRON	Ca_23021	U		Vacuolar protein sorting-associated protein 35
CaPOPII_1966	Ca4	48794099	TAA/TA	TTACGGTCGAAAATGAAGGG	TCGGGTAGCACAATCTTCAA	59.9	640	INTERGENIC				
CaPOPII_1967	Ca4	48837715	ATT/ATTT	TGTGTAACGGGTAAGAAATACGG	CAAAATACATCATCACGCGG	60.2	630	INTERGENIC				
CaPOPII_1968	Ca5	64660	CAAAAAA/CAAAA	TGGATCATCAAAGATCAACGG	ATTGAAAGTTGCGGGGACTA	60.8	438	INTERGENIC				
CaPOPII_1969	Ca5	132937	CAA/CAAA	CCAAAGACACAGCAAAGCAA	CACTTCCACCAGTTGCAAAA	60.0	569	INTERGENIC				
CaPOPII_1970	Ca5	133428	GAACA/GAACAATTTTTATAATAACA	ACCTTTACATGGTGCCAAGC	GCGTTGTGAAAACAGGACAA	60.0	433	INTERGENIC				

INDEL marker IDs	Chromosomes /unanchored scaffolds	Physical positions (bp)	InDels (*Kabuli* reference genome-CDC Frontier/PI)	Forward primers (5'-3')	Reverse primers (5'-3')	Anneling temperature (0C)	Expected amplified product size (bp)	Structural annotation		Functional annotation		
								Sequence components of *kabuli* genome	*Kabuli* gene accession IDs	NCBI-KOG	TFs	NCBI-nr database
CaPOPII_1971	Ca5	133648	GAAAAA/GAAAA	TTGTCCTGTTTTCACAACGC	TCAACAATTTGTGGGCTTCA	59.7	574	INTERGENIC				
CaPOPII_1972	Ca5	133972	CAAAAAA/CAAAAA	TTGTCCTGTTTTCACAACGC	TCAACAATTTGTGGGCTTCA	59.7	574	INTERGENIC				
CaPOPII_1973	Ca5	158794	TAAAAAA/TAAAAA	TGATGCGTCATGGACATAAA	TTTTAAAAATGTCCATTTACACCG	58.5	498	INTERGENIC				
CaPOPII_1974	Ca5	191704	CGGA/C	TCACACTTGCACTTTGGAGC	TTGTTGGGATCCCTGAAGAG	60.0	484	INTERGENIC				
CaPOPII_1975	Ca5	197674	CA/CATA	TGAATTTTTCTGTTTGGGGC	GAACCCGATTCATAACCCCT	59.9	309	INTRON	Ca_18174	L		ATPase, AAA+ type, core
CaPOPII_1976	Ca5	202535	TAAAAAAA/TAAAAAA	TTGGACTTGAAGTTGACATTGC	ACGAGTTGGATTGGGTTGAC	60.2	269	INTRON	Ca_18175			4-diphosphocytidyl-2C-methyl-D-erythritol synthase
CaPOPII_1977	Ca5	209466	AAAAAACTTAAAAACTTAAAA/AAAAAACTTAAAAACTTAAAAACTTAAAA	AATTGAAGCATCCCATAAACG	TTAGACCATCCACAACACGG	59.0	698	DRR	Ca_18176		C2H2	F-box domain, cyclin-like
CaPOPII_1978	Ca5	209690	ATTTTTTT/ATTTTTTTT	AAAACATTCAACCAACCACCA	TTAGACCATCCACAACACGG	60.1	320	DRR	Ca_18176		C2H2	F-box domain, cyclin-like
CaPOPII_1979	Ca5	209767	GAAAAAAAA/GAAAAAAAAAA	AAAACATTCAACCAACCACCA	TTAGACCATCCACAACACGG	60.1	320	DRR	Ca_18176		C2H2	F-box domain, cyclin-like
CaPOPII_1980	Ca5	209793	AAATTAAT/AAATTAATTAAT	AAAACATTCAACCAACCACCA	TTAGACCATCCACAACACGG	60.1	320	DRR	Ca_18176		C2H2	F-box domain, cyclin-like
CaPOPII_1981	Ca5	210200	ATTTTTTTTT/ATTTTTTTTTT	TTAAACACCCGTGTTGTGGA	AAATGGCTTAGGGAGGGAGA	59.9	607	DRR	Ca_18176		C2H2	F-box domain, cyclin-like
CaPOPII_1982	Ca5	210510	ATT/ATTT	TCTCCCTCCCTAAGCCATTT	GAATTATCCGCTGCTTGCAT	60.0	201	DRR	Ca_18176		C2H2	F-box domain, cyclin-like
CaPOPII_1983	Ca5	218592	TATAATAATAATAATAATA/TATAATAATAATAATAATAATA	TAGCGTGGTGGTGTTGTGAT	CCTTTCAAGGTCAGCCACAT	60.0	739	INTERGENIC				
CaPOPII_1984	Ca5	222234	TT/TTAAT	ACCGCTTTACAGAACAACCG	GGTGAGGACAAAACATGGCT	60.2	538	INTERGENIC				
CaPOPII_1985	Ca5	236073	CGAAGAAAAT/CGAAGAAAATGAAGAAAATGAAGAAAAT	TTGAATTGACCGGAGGAATC	GGGATAGGGAGGGTTGTGTT	59.9	680	INTERGENIC				
CaPOPII_1986	Ca5	237297	CTGTCAT/CTGTCATGTCAT	TCATTGCAATACCGGGAAAT	TGAATTTGGATAGGTGCAAAAA	60.2	233	INTERGENIC				
CaPOPII_1987	Ca5	238011	GTTTTTT/GTTTTT	CATGCACGATTCACACATGA	GTGTCGTCCAACCTCTCCAT	60.1	486	INTERGENIC				

INDEL marker IDs	Chromosomes /unanchored scaffolds	Physical positions (bp)	InDels (*Kabuli* reference genome-CDC Frontier/PI)	Forward primers (5'-3')	Reverse primers (5'-3')	Anneling temperature (0C)	Expected amplified product size (bp)	Structural annotation		Functional annotation		
								Sequence components of *kabuli* genome	*Kabuli* gene accession IDs	NCBI-KOG	TFs	NCBI-nr database
CaPOPII_1988	Ca5	239546	AATA/AATATA	CTCAACGCTGCCACAATAGA	TGGATTCCCAAACACTAGCC	60.0	473	INTERGENIC				
CaPOPII_1989	Ca5	240662	T/TCAATCCA	CCTTTAACCCAACACACCGT	TTGGTGCCTACAACATATTTTCC	59.8	268	INTERGENIC				
CaPOPII_1990	Ca5	260923	CATAT/CATATAT	CTTCGGCCTAAAGATTTTGC	ATTGCGGTTGTTCGGTTATC	59.0	358	DRR	Ca_18180	A		Tudor domain
CaPOPII_1991	Ca5	261320	TCAC/TC	GATAACCGAACAACCGCAAT	CGGAGCTTCGGTAGGACATA	59.8	754	DRR	Ca_18179	R		HEAT
CaPOPII_1992	Ca5	289898	ATT/ATTT	AAAGTGCCGAAGTTTGCAGT	TTTTCCCTTCAACAGTCATGG	59.9	351	INTRON	Ca_18185	R	B3	Multi antimicrobial extrusion protein
CaPOPII_1993	Ca5	291054	TAAAAAAAAA/TAAAAAAAAAAA	TGCTTCATATGCTTGACCACA	TGGTGCTTCTTTGGCTCTTT	60.3	476	INTRON	Ca_18185	R	B3	Multi antimicrobial extrusion protein
CaPOPII_1994	Ca5	291688	ATTTTTT/ATTTTTTTT	AAAGAGCCAAAGAAGCACCA	AGCAAATGCGACTGTGATTG	60.0	722	DRR	Ca_18186			
CaPOPII_1995	Ca5	291943	GCGCAACCGCAA/GCGCAACCGCAACCGCAA	TTCGTGGCTTAATCCCACTC	GTCTGGTGCTCTTCCCAATC	60.1	517	DRR	Ca_18186			
CaPOPII_1996	Ca5	292149	A/AC	AAAGTGCCGAAGTTTGCAGT	TGCTTTCCCTTCAACAGTCA	59.9	485	DRR	Ca_18186			
CaPOPII_1997	Ca5	317066	TAAA/TAAAA	GAGCATTGATCCGGATTGTAA	TTTGACGATGCAACGGTTTA	59.9	544	INTERGENIC				
CaPOPII_1998	Ca5	327512	ATTTTTTTTTT/ATTTTTTTTTTT	GCTTCTGCATGGTTTTGTCA	AGGATCATCCATTGGAGCAG	59.8	466	INTRON	Ca_18189	G		Glucosylceramidase
CaPOPII_1999	Ca5	360986	ATTTTTTTTTTT/ATTTTTTTTTT	TGTGAGTTTAAGGGAGGGGA	GCATCCAGGATCAGAGGTGT	59.5	390	DRR	Ca_18192	P		
CaPOPII_2000	Ca5	445610	GCCC/GCCCC	ACAGTGGCTTCAATTCCCAC	CCAGCCAAATAGCTGGAATG	60.0	336	INTERGENIC				
CaPOPII_2001	Ca5	460106	ATTT/ATTTT	ACACATACCCAAAGGGATGC	CCCCAGCAGTCGGTAAAATA	59.7	541	INTRON	Ca_18199			
CaPOPII_2002	Ca5	471069	GAAATTAAATTAAA/GAAATTAAATTAAATTAAA	GTGACACCGTCTCGTTGGTA	AACTGGGTGAGAGCCTGAGA	59.6	314	INTRON	Ca_18200	U		Inositol polyphosphate-related phosphatase
CaPOPII_2003	Ca5	479066	TAAAAAAAAAA/TAAAAAAAAAAA	CCTGTTCTGTAGTGGGAGCC	TCCATGTTACTTCTCCGCCT	59.7	508	DRR	Ca_18200	U		Inositol polyphosphate-related phosphatase
CaPOPII_2004	Ca5	479350	CATATATATATATATA/CATATATATATATATATA	TTGTCACACAATAAGGCGGA	TCATCACGATCTGAATCCCA	60.1	674	DRR	Ca_18200	U		Inositol polyphosphate-related phosphatase

INDEL marker IDs	Chromosomes /unanchored scaffolds	Physical positions (bp)	InDels (*Kabuli* reference genome-CDC Frontier/PI)	Forward primers (5'-3')	Reverse primers (5'-3')	Anneling temperature (0C)	Expected amplified product size (bp)	Structural annotation		Functional annotation		
								Sequence components of *kabuli* genome	*Kabuli* gene accession IDs	NCBI-KOG	TFs	NCBI-nr database
CaPOPII_2005	Ca5	482095	GC/G	TTGCATCTGTCAGCGAACTC	TTTTTCGGGGGTCAAATGTA	60.1	800	DRR	Ca_18201			Tetratricopeptide repeat-containing
CaPOPII_2006	Ca5	497099	GAAAA/GAAA	TTGAGTGCAGCTGGAGAAGA	TCACTCAAAAACCAAGCACG	59.9	110	INTERGENIC				
CaPOPII_2007	Ca5	927603	TTAATAATAATAATAATAATAATAATAATAATAA/TTAATAATAATAATAATAATAATAATAATAATAATAA	GGTCTTTTGAAAATAATCTTAACGC	AAGGGGGAAGTTAAGCCAGA	58.8	664	INTERGENIC				
CaPOPII_2008	Ca5	1230643	TT/TTCCGT	ACGAAATCGAAGAGAAGCGA	TCCAGGACACAAACAATCCA	60.1	455	INTERGENIC				
CaPOPII_2009	Ca5	1377883	TCC/TC	GACTCTTTCCATAGCGTGCC	CACCTTGTAGCCAACGGTTT	59.8	720	INTERGENIC				
CaPOPII_2010	Ca5	1586054	TAAAA/TAAA	CTGTTTTCTACCCCCACCCT	AGCGTTGTAAAAGATGCGCT	60.2	727	INTERGENIC				
CaPOPII_2011	Ca5	1678181	TTATATATATAT/TTATATATATATATAT	CGGTGAATCCTTTACGGAGA	AGGAAGAGTGGAGTTGCGAA	60.1	384	INTERGENIC				
CaPOPII_2012	Ca5	1794712	AATTATTATTATTATTATTATTATTATTATTATTATTATTATTATTATTATT/AATTATTATTATTATTATTATTATTATTATTATTATTATTATT	GAGAAGGCGAGAAGGAAGGT	GCAAGGTTTAGGTGGGTCAA	60.0	605	INTERGENIC				
CaPOPII_2013	Ca5	1903216	GA/GAA	TGCATAGGCCTCTTGTCCTT	AGTTCCGCGCTAGATTTCAA	59.8	553	INTERGENIC				
CaPOPII_2014	Ca5	1995521	ATGTT/ATGTTGTT	TCTTCGTTTTGCAGTCAAACTT	TCTTCGTTTTATGGTTTTATTTCA	59.1	279	INTERGENIC				
CaPOPII_2015	Ca5	2323461	GAAAA/GAAAAA	GCGCCAGATTTCAAACACTA	ACCAGCAGACCAAAATGGAC	58.9	262	INTERGENIC				
CaPOPII_2016	Ca5	2327252	TAA/TAAA	TCGAAATGAAATCGAGGACC	ACAGACGAGGGCTAGGGATT	60.0	721	INTERGENIC				
CaPOPII_2017	Ca5	2418306	ATTTTTTTTTTTTTT/ATTTTTTTTTTTTT	GCTCTGCATTTTCCTTGAGC	TGAGAAATTTTTCCGCTGCT	60.1	328	INTRON	Ca_18664		B3	
CaPOPII_2018	Ca5	2628560	AT/ATATATT	TTGTTTCTCCCCAGTTTTGG	TTTTAGCATTGACCGCTCCT	59.9	357	INTERGENIC				
CaPOPII_2019	Ca5	3115746	CAAAAAAAA/CAAAAAAA	GCAACATGGGATGATTCAGA	TCCAAGTCGTTCAGCTTGTG	59.5	628	INTERGENIC				
CaPOPII_2020	Ca5	3742109	TT/TTACATAT	ATTTACGAGCAGCGGAAAGA	TGGTCATGCAGAAACTCAGC	60.0	605	INTERGENIC				
CaPOPII_2021	Ca5	3865777	CTTTTTTT/CTTTTTTTT	ATGTGACACCCTAAACCCCA	TTGGTTTAGAGGGGATGTGAA	60.1	605	INTERGENIC				

INDEL marker IDs	Chromosomes /unanchored scaffolds	Physical positions (bp)	InDels (*Kabuli* reference genome-CDC Frontier/PI)	Forward primers (5'-3')	Reverse primers (5'-3')	Anneling temperature (0C)	Expected amplified product size (bp)	Structural annotation		Functional annotation		
								Sequence components of *kabuli* genome	*Kabuli* gene accession IDs	NCBI-KOG	TFs	NCBI-nr database
CaPOPII_2022	Ca5	4064742	G/GC	TATGTTTGTTTTGCCGTCCA	AGGATGTACCATTCTTGCGG	60.0	375	INTERGENIC				
CaPOPII_2023	Ca5	4179599	GATATATATATAT/GATATATATAT	CGGTTTAGGGATTGGGTTTT	TCGATTGAGTCTTTTGCACTT	60.0	166	INTERGENIC				
CaPOPII_2024	Ca5	4180242	AGGTTTAGGGTTTAGGGTTT/AGGTTTAGGGTTTAGGGTTTAGGGTTT	GGGTTTGGGGTTTAAGGTTT	TCCATTTCGTCTTTTGCACTT	59.1	321	INTERGENIC				
CaPOPII_2025	Ca5	4273567	CTATATATATATATATAT/CTATATATATATATATAT	TTGGAAGGAGCCCTTACCTT	CGATTGAGGACCAAGGATGT	60.1	908	INTERGENIC				
CaPOPII_2026	Ca5	4857245	GAAAA/GAAAAA	CCCACATTTCTTTGGCAGTT	TGTGCCTAAACTTGCTGGTG	60.0	501	INTERGENIC				
CaPOPII_2027	Ca5	4970490	T/TG	ATTCCTACGCGATTACCACG	CCCGTGATAGGCAGTACGTT	60.0	528	INTERGENIC				
CaPOPII_2028	Ca5	5190851	CTATATATATATATATATATATAT/CTATATATATATATATATATATATAT	AGCGCTATGTGACCCTTTTG	AATGTGAATCCCACCAGGAA	60.3	555	INTERGENIC				
CaPOPII_2029	Ca5	5318962	TC/T	ACGTTGACCAATTCAGCTCC	CACTAGGGACCCTTTGGTGA	60.1	720	INTERGENIC				
CaPOPII_2030	Ca5	5492827	TTTATTATTATTATTATTATTATTATTATTATTATTATTATTATTATTATTATTATTATTATTATTATTATTATTATTAT/TTTATTATTATTATTATTATTATTATTATT	TATCCCCTCGATTTGCTTCA	TTTTGCCTCAAAAAGTTTCACA	60.5	603	INTERGENIC				
CaPOPII_2031	Ca5	5705191	GA/G	AATCGATTGGCTTTGATTCG	CTCTCATCTGAGGGCAAAGC	60.0	412	INTERGENIC				
CaPOPII_2032	Ca5	5798731	TTATATATATATATATATATATA/TTATATATATATATATATATATATATATATA	CTTCGACACTGTAGCACCCA	ATATTTCTGCGCATTCTCGC	59.9	217	INTERGENIC				
CaPOPII_2033	Ca5	5836817	TACCCTAAACC/TACCCTAAACCCTAAACCCTAAACC	GTATGAGGAGGATCTTGCGG	TCAAAATGCACCATAAACCG	59.7	594	INTERGENIC				
CaPOPII_2034	Ca5	5837067	ACCTAAACCCTAAACCCTAAACCCTAA/ACCTAAACCCTAAACCCTAA	AAAGAAAAACACTCGCGCTG	AGCGTTTTGGGTTTAGGGTT	60.6	825	INTERGENIC				
CaPOPII_2035	Ca5	5888717	AATATATATATATATATATATATATATATA/AATATATATATATATATATATATATATATATATATATATATA	TCTTGAGCTACTGGGGCTTG	CAAGTTTGAAATGGAGGGGA	60.5	882	INTERGENIC				
CaPOPII_2036	Ca5	5912049	TAAATATACATCAAATATA/TAAATATACATCAAATATACATCAAATATA	TTTTTGTTGTCTCACTCGATCA	CAGTGTTAATTGGCACGAAA	58.4	276	INTERGENIC				
CaPOPII_2037	Ca5	6200121	CT/CTT	TTCGCAACTCCACTCTTCCT	GATCCATTTTCCCCCAAATC	60.0	342	INTERGENIC				
CaPOPII_2038	Ca5	6369660	TC/TCAC	GAGGAAACACGGTGAATGGT	CGCTCACTAGTCGAACGAGA	59.8	486	INTERGENIC				

INDEL marker IDs	Chromosomes /unanchored scaffolds	Physical positions (bp)	InDels (*Kabuli* reference genome-CDC Frontier/PI)	Forward primers (5'-3')	Reverse primers (5'-3')	Anneling temperature (0C)	Expected amplified product size (bp)	Structural annotation		Functional annotation		
								Sequence components of *kabuli* genome	*Kabuli* gene accession IDs	NCBI-KOG	TFs	NCBI-nr database
CaPOPII_2039	Ca5	6510151	CTTTTTTTTT/CTTTTTTTTTT	TCGCGTTCATGATTTTGTGT	CGGAGTGATAATTGGTGCAA	60.1	816	INTERGENIC				
CaPOPII_2040	Ca5	6532288	AAAAAGAACAAAA/AAAAA	TGTCTTCAACTTCTGCGGTG	TTGCAACGTGTCATTGTCCT	60.0	831	INTERGENIC				
CaPOPII_2041	Ca5	6663718	CAA/CAAA	AGGGAGGACGAAAATGACCT	TAACCAATCTTGTGCACCCA	59.9	171	INTERGENIC				
CaPOPII_2042	Ca5	6763101	AGGG/AGGGG	CAAATGTCGGGAGCTACCAT	GGATCCCCAATCGATAACCT	60.0	437	INTERGENIC				
CaPOPII_2043	Ca5	6763849	AATATATATATATATATATA/AATATATATATATATATA	TGGGTGGGAGTAATTCTTCG	GAGTTGCGGCGAGAGTAGAT	59.9	484	INTERGENIC				
CaPOPII_2044	Ca5	6765484	CATATATATATATATATATATATAT/CATATATATATATATATATATATATATAT	CGAAGCTGCCAGGTAAAAAG	TGAGTCCATTCTCCAAACCA	60.0	652	INTERGENIC				
CaPOPII_2045	Ca5	6772456	GCACACA/GCACA	TGCACAACAAAAGCACAACA	TGTACCATCATAGTGGCGGA	59.9	512	INTERGENIC				
CaPOPII_2046	Ca5	6833700	ATATT/AT	GCATAAAATCACGATGCCAA	CGATTTTTCGTCTACATGTGC	59.5	211	INTERGENIC				
CaPOPII_2047	Ca5	6833833	AATA/AATATA	AAATCACGATGCCAAAGAAAA	AAGAAAGTTGTAAATGTGTTTTATGGA	59.6	616	INTERGENIC				
CaPOPII_2048	Ca5	6833924	ATTT/ATT	AAATCACGATGCCAAAGAAAA	AAGAAAGTTGTAAATGTGTTTTATGGA	59.6	616	INTERGENIC				
CaPOPII_2049	Ca5	6834263	CT/C	GCTTAATCCATAAAACACATTTACAA	TTCTCATGCGCTTGACTAATG	57.9	526	INTERGENIC				
CaPOPII_2050	Ca5	6834706	TAAAAAA/TAAAAAAA	TGCATAAAATCACCATGCCA	TTGTACGTTTCTTTTCTATGATTTGC	60.9	360	INTERGENIC				
CaPOPII_2051	Ca5	6834978	ATATTATTATTATTATTATTATT/ATATTATTATTATTATTATTATTATT	TAGTCAAGCGCATGAGAAAA	GGCATGATGATTTTATGCATATTT	57.3	748	INTERGENIC				
CaPOPII_2052	Ca5	6837967	TAAAAAAAAAAAA/TAAAAAAAAA	TCGAGAACAACTCGTCTCGTG	AATTTGCAGTGTCGCAAAAA	61.6	254	INTERGENIC				
CaPOPII_2053	Ca5	6872599	ATTATTTATTTATT/ATTATTTATTTATTTATT	CGGCCATCACACAATGATAA	TCAGGATGTCACCACGAAGA	60.3	454	INTERGENIC				
CaPOPII_2054	Ca5	6907615	A/AGTG	GGTTGTGTTATCCCTCGACA	AGGGTGCCACATTTACTCCA	58.4	539	INTERGENIC				
CaPOPII_2055	Ca5	6909501	TATATATAATTTAAATATATAATTTAAAT/TATATATAATTTAAAT	AATGATGGAGGGAGTGAAGC	AACTGTCCATTTGTGAGGGC	59.1	405	INTERGENIC				

INDEL marker IDs	Chromosomes /unanchored scaffolds	Physical positions (bp)	InDels (*Kabuli* reference genome-CDC Frontier/PI)	Forward primers (5'-3')	Reverse primers (5'-3')	Anneling temperature (0C)	Expected amplified product size (bp)	Structural annotation		Functional annotation		
								Sequence components of *kabuli* genome	*Kabuli* gene accession IDs	NCBI-KOG	TFs	NCBI-nr database
CaPOPII_2056	Ca5	6909628	ATTTTTTT/ATTTTTT	AATGATGGAGGGAGTGAAGC	AACTGTCCATTTGTGAGGGC	59.1	405	INTERGENIC				
CaPOPII_2057	Ca5	6909656	TAAA/TAA	AATGATGGAGGGAGTGAAGC	AACTGTCCATTTGTGAGGGC	59.1	405	INTERGENIC				
CaPOPII_2058	Ca5	6909949	CAAAGA/CA	CCTAAGCCCTCACAAATGGA	TAAAGGGAGAAAAGCAGCCA	60.1	304	INTERGENIC				
CaPOPII_2059	Ca5	7107813	G/GA	TGTCTCGAGTTCTATCCCGAA	TCACACACTTTCTTCCAACCTTT	59.8	569	INTERGENIC				
CaPOPII_2060	Ca5	7941049	GA/GAA	TCTCATTTCCACTTAAACTTTCG	TAAGCAAGCCATTGCAAAAA	57.6	459	INTERGENIC				
CaPOPII_2061	Ca5	8314333	ATT/ATTT	TGGGGTTTAGGGTGTCACAT	TTCCATCATCAATTCTGCCA	60.1	375	INTERGENIC				
CaPOPII_2062	Ca5	8596558	GATAATAATAATAATAATAATAATAATAATAATAATAATAATAATAA/GATAATAATAATAATAATAATAATAATAATAATAATAATAATAA	CGAAGCCGAGAAAATGACTC	AGAGGCAAACAAGAACCGAA	60.0	343	INTERGENIC				
CaPOPII_2063	Ca5	8735941	AATATATATATATATATATATATATAT/AATATATATATATATATATATATAT	TCAAACTCTGCTGGCACATC	AAGCGACCTCCTACTGGGTT	60.0	646	INTERGENIC				
CaPOPII_2064	Ca5	8824148	A/ATGT	GGAGCTGAGGTTGAGGTTGA	CAATATCGGCAAATTGAAGTGA	60.4	476	DRR	Ca_20336	A		Ribosomal RNA adenine methylase transferase
CaPOPII_2065	Ca5	8998961	A/AAT	TGCAATAATGGCATTTCCAA	TGAGCTTGAATGGCAAGATG	59.9	556	INTERGENIC				
CaPOPII_2066	Ca5	8999793	CA/CAAA	CTCGATCAATGACCAGAGCA	TCACTTCTTCCCCCATTTTG	59.9	470	INTERGENIC				
CaPOPII_2067	Ca5	9264601	ATTTTTTTTTTT/ATTTTTTTTTTTT	TTCCAGAAAATTGCATTTTGAA	ACCTTCCACAAGATCGTCAA	59.6	756	INTERGENIC				
CaPOPII_2068	Ca5	9441399	AATTTTATTTT/AATTTTATTTTATTTT	TGCGATGGAGAAATCAAAGC	CTAGTTTGGCTTGACGGGAA	61.3	865	INTERGENIC				
CaPOPII_2069	Ca5	9708216	AATATATATATATATATATATATATA/AATATATATATATATATATATATATATATATATA	GGCAACGTATGTGTGTTTGG	AGAGTTGTCAATGGATCGGA	59.9	539	INTERGENIC				
CaPOPII_2070	Ca5	10420287	GTTTTTTTTT/GTTTTTTTT	GGTACAATTGTTGCACCACG	TTTGGGAGTAGGCCTTCCTT	59.9	702	INTERGENIC				
CaPOPII_2071	Ca5	10420664	AATATATATATATATATATA/AATATATATATATATATATATA	AAGGAAGGCCTACTCCCAAA	TACCAAGGCGTCGTCAAAAT	60.1	597	INTERGENIC				
CaPOPII_2072	Ca5	10572522	AATATATATATATATATATATATAT/AATATATATATATATATAT	CGTGGACAAAGATCGTGATG	TCATTGTTAAAGAGCGGTGC	60.1	382	INTERGENIC				

INDEL marker IDs	Chromosomes /unanchored scaffolds	Physical positions (bp)	InDels (*Kabuli* reference genome-CDC Frontier/PI)	Forward primers (5'-3')	Reverse primers (5'-3')	Anneling temperature (0C)	Expected amplified product size (bp)	Structural annotation		Functional annotation		
								Sequence components of *kabuli* genome	*Kabuli* gene accession IDs	NCBI-KOG	TFs	NCBI-nr database
CaPOPII_2073	Ca5	10634338	AC/ACC	AATTTTTCAGTCCCGTGTCG	ATCCCTCAAAGGGCTACTGC	60.0	229	INTERGENIC				
CaPOPII_2074	Ca5	10686981	TAAAAAAAA/TAAAAAAAAAAA	GCTCCTTCTGCATCAACACA	TGCCCCACATTTTAGACCTC	60.0	509	INTERGENIC				
CaPOPII_2075	Ca5	10687869	TAAAAAAAA/TAAAAAAAAA	CGAGGAACGACAAGGGTAAA	TGAGTTGAGTTAAAAACCAGCAGA	60.1	673	INTERGENIC				
CaPOPII_2076	Ca5	10688557	AGTTGTT/AGTT	TCCATCAAACTGATCCTAAGGG	TGGGGTTTAGATGATGGCTC	60.3	179	INTERGENIC				
CaPOPII_2077	Ca5	10689102	TAAAA/TAA	ATGGGCCACCTATTGAGACA	TGCCAAAGAAAAAGGTTTCG	60.3	623	INTERGENIC				
CaPOPII_2078	Ca5	10689622	AGAAGCATG/AG	CGAAACCTTTTTCTTTGGCA	AACGGATTGTCCCTTCATTG	60.2	418	INTERGENIC				
CaPOPII_2079	Ca5	10693789	CTTGTGATTGTTTTGTTGTGATTG/CTTGTGATTG	CCAATTTGAATTGTCCGTCC	TTGCAAAAGCTGGGTCAAGT	60.2	406	INTERGENIC				
CaPOPII_2080	Ca5	11138840	GT/G	TCGACTTGTCAATCGCTTTG	TCGCATTAACTACGAACGCA	60.0	676	INTERGENIC				
CaPOPII_2081	Ca5	11168411	AATA/AA	TTCAAACGGAACTGATGCTTT	TTGGTTGGGGGAGTTATTGA	59.7	954	INTERGENIC				
CaPOPII_2082	Ca5	11168904	GTCTCTCT/GTCTCT	TCAATAACTCCCCCAACCAA	TCACAATTGGGTTTGGATCA	60.2	227	INTERGENIC				
CaPOPII_2083	Ca5	11169035	CG/C	TCAATAACTCCCCCAACCAA	TGACGCCGTGAAAAACATTA	60.2	626	INTERGENIC				
CaPOPII_2084	Ca5	11169613	C/CG	TGAAGCAGTTGTTTGGGTGA	GAGCCCAAATCCAAAATTCA	60.3	225	CDS (FRAME SHIFT)	Ca_23744	A		Ribonuclease CAF1
CaPOPII_2085	Ca5	11414411	A/AACCT	TCTGAATCGGAGTACCCCAC	AATGGCTTTCCTTCGTGATG	59.9	770	INTERGENIC				
CaPOPII_2086	Ca5	11729570	AAATAATAATAA/AAATAATAA	CACGGTCCCTAGAGCCAATA	CGGGAGATACGTAGGAGCAA	60.1	672	INTERGENIC				
CaPOPII_2087	Ca5	11879108	TAA/TAAA	AAGATTTCCTCCCTCCTCCA	CTTTAACATGCGTTTCCGGT	60.0	327	INTERGENIC				
CaPOPII_2088	Ca5	11983861	G/GATCTTCGATCCT	CTGCTGTCAACTCCACTCCA	TCAAAATCCCCCAATAACCA	60.0	713	DRR	Ca_17106			
CaPOPII_2089	Ca5	12095777	CAAAAAAAAA/CAAAAAA	CTCTTTAAGACGTTTCGCGG	TGCTCGCCTAATTTTCGAGT	60.0	461	INTERGENIC				

INDEL marker IDs	Chromosomes /unanchored scaffolds	Physical positions (bp)	InDels (*Kabuli* reference genome-CDC Frontier/PI)	Forward primers (5'-3')	Reverse primers (5'-3')	Anneling temperature (0C)	Expected amplified product size (bp)	Structural annotation		Functional annotation		
								Sequence components of *kabuli* genome	*Kabuli* gene accession IDs	NCBI-KOG	TFs	NCBI-nr database
CaPOPII_2090	Ca5	13624288	ATTTT/ATTTTTT	TGAAAATGTTGTTGGTAGTGGC	CGACGGTGCCAATTTTCTAT	59.9	948	INTERGENIC				
CaPOPII_2091	Ca5	13636401	A/AC	ATCGACAAACCAACATGCAA	GGGAAACTCACTTGCGGTTA	60.0	735	INTERGENIC				
CaPOPII_2092	Ca5	13636921	CTGT/CT	TAACCGCAAGTGAGTTTCCC	GCGGTGGCTGAGTTTCTTAC	60.1	387	INTERGENIC				
CaPOPII_2093	Ca5	13970765	T/TG	TATTTGGACAAACGAAGGGC	CCCCTTTTGCTTGTTGGTTA	59.9	282	INTERGENIC				
CaPOPII_2094	Ca5	13971864	AATATATATATATATATATATATATATA/AATATATATATATATATATATATATATATATATA	ATGGAGACTTTCTGCAGCGT	TCCTCCGGCTGCTAAATAAA	60.0	691	INTERGENIC				
CaPOPII_2095	Ca5	14022502	ATTT/ATTTT	GGAAATGGATGTCTGCCAAT	TTGCAATCTCATCAACTCGG	59.8	318	INTERGENIC				
CaPOPII_2096	Ca5	14300751	G/GA	TTTGCGTTACGAATTTGATGA	AAATGAACGAATTTGCACTCG	59.2	595	INTERGENIC				
CaPOPII_2097	Ca5	14394852	CTTTTT/CTTTT	TCTTCCACCCAGTTTTCACC	TTGAGGGCTATGTTTTTCGG	59.9	337	INTERGENIC				
CaPOPII_2098	Ca5	15756736	GACAC/GAC	GCGCGATTTACCAAAAATGT	TTTGAGTGGTGGGTTGTGAA	60.0	431	INTERGENIC				
CaPOPII_2099	Ca5	15929247	AACCAC/AAC	TGATGACGAAGACGAAGACG	AACACATCCGGGATCCATTA	60.0	416	INTERGENIC				
CaPOPII_2100	Ca5	16319965	TGGG/TGGGG	TTGTGTGCAGGATCCACATT	TTGACCATTGCACGAAACAT	60.0	502	INTERGENIC				
CaPOPII_2101	Ca5	16715406	GAAAAAAAAA/GAAAAAAAAAA	TTGCGTTACGGATTTGATGA	GCTAAATTACATTCGTGGTCCC	60.1	240	INTERGENIC				
CaPOPII_2102	Ca5	16765808	TAGA/TA	CGATCGTGCTACGTCTTCAA	TGGTGGGAATGTTGTGATTG	60.0	677	DRR	Ca_17941	R	HSF	Pentatricopeptide repeat
CaPOPII_2103	Ca5	16948578	CATATATATATATATATAT/CATATATATATATATATATAT	GTTTTCGAATCAACGAGGGA	GCTCAGAGGCTTTTTAGCCA	60.1	509	INTERGENIC				
CaPOPII_2104	Ca5	17048541	AATATATATATATATATATATAT/AATATATATATATATATATATATAT	GGTCAGCATTTACCCCATGA	TTTCTCCACCTTCCTTGGTG	60.7	534	INTERGENIC				
CaPOPII_2105	Ca5	17244450	AG/A	GTACCCTCCTCCTCGGTCTC	TTGTGTTTCAACAACTCCCAA	60.1	729	INTERGENIC				
CaPOPII_2106	Ca5	17314539	TAAAAAAAAAA/TAAAAAAAAAAA	TCGATTATGCGTAGGGAAGG	TTGCGACAACTTGGTGATTC	60.1	824	INTERGENIC				

INDEL marker IDs	Chromosomes /unanchored scaffolds	Physical positions (bp)	InDels (*Kabuli* reference genome-CDC Frontier/PI)	Forward primers (5'-3')	Reverse primers (5'-3')	Anneling temperature (0C)	Expected amplified product size (bp)	Structural annotation		Functional annotation		
								Sequence components of *kabuli* genome	*Kabuli* gene accession IDs	NCBI-KOG	TFs	NCBI-nr database
CaPOPII_2107	Ca5	17448710	CACTATACAA/CACTATACAACTATACAA	TGTGGATGTTCAAGGGACAA	ACAAATGGCGTATGCCTCTC	59.9	864	INTERGENIC				
CaPOPII_2108	Ca5	17494887	CTGA/CTGACCTCCTATATGTGA	CTACGCCAAGAAACTGCTCC	GTACGCGTCCAGACATAGCA	60.0	245	INTERGENIC				
CaPOPII_2109	Ca5	17526738	T/TG	GATTGGGGGAAGATGAGGAT	TTTGCGTTACGAATTTGATGA	60.1	465	INTERGENIC				
CaPOPII_2110	Ca5	17733319	A/ATCGGTTCAGC	CGCGATGTCGTCTAAAGTGA	GAGGGGAGGGTTTTTGCTAC	60.0	583	INTERGENIC				
CaPOPII_2111	Ca5	17876530	CAA/CAAA	TTTCATCCATCACCGACAAA	CTCAACGAATGCCTGAAACA	59.9	573	INTERGENIC				
CaPOPII_2112	Ca5	17876974	ATTTTT/ATTTTTTT	TGTTTCAGGCATTCGTTGAG	TCAATACCATTTTCACCGCA	59.8	471	INTERGENIC				
CaPOPII_2113	Ca5	18049660	CATATATATATAT/CATATATATAT	GCACGTTGTAAAGAGTGGACTG	AAAAGGCCCACAAGAGTTGA	59.8	694	INTERGENIC				
CaPOPII_2114	Ca5	18179865	CAAAAAAAAAAAA/CAAAAAAAAAAA	GGCAAAATATCTTTCATTCCG	TCTGAACAGTGTTGCCACAGA	58.6	352	INTERGENIC				
CaPOPII_2115	Ca5	18683897	AGCG/AGCGCGAGTCATCAGCTCGCG	ATGGCCGTTCTTAGTTGGTG	GTACAAAGGGCAGGGACGTA	60.0	395	INTRAGENIC				
CaPOPII_2116	Ca5	18944656	CAAAA/CAA	TCCTGTCATGTGCTTTGAGC	CTAACCTCCCAGGACCCAAT	60.0	131	INTERGENIC				
CaPOPII_2117	Ca5	18962064	AAATAATAATAATAATAATAATAATAATAATAATAATAATAA/AAATAATAATAATAATAATAATAATAATAATAATAATAA	GCCCTCTCCTCGTGTTACAA	CCGAAAATAGCGATGAGAGC	60.3	394	INTERGENIC				
CaPOPII_2118	Ca5	20107894	ATTTTTTTTTT/ATTTTTTTTTTT	TTTGAAGCCATTGAGTCGTG	CTCACCCACCTTCACCATTT	59.8	207	INTERGENIC				
CaPOPII_2119	Ca5	20438947	AT/A	AGTTTGGAACCAAGCCACTG	TAAGCCGTGGGAACGTTAAG	60.1	343	INTERGENIC				
CaPOPII_2120	Ca5	20547334	AACCAACACCAACACCAACA/AACCAACACCAACACCAACACCAACA	AGGAGATGGAGCGTTGTTTG	TTGGTTAGCGTGCATCTGAG	60.3	462	INTERGENIC				
CaPOPII_2121	Ca5	20781552	GATATATATATATATATATATATATAT/GATATATATATATATATATATATATATATAT	GCTGAGGTGGGAAACATTTG	CCAAATCAGAACCAAAGGCA	60.5	598	DRR	Ca_18223		ARF	Transferase
CaPOPII_2122	Ca5	21085449	A/AT	TTAGGCGACCACTGTATCCC	TGAGCCACTTTGATCAGCAC	60.0	758	INTERGENIC				
CaPOPII_2123	Ca5	21598205	TA/TAA	TTGGTGTGGATTGTCGATTG	GGGGAAATCGATTGAATGAA	60.4	953	INTERGENIC				

INDEL marker IDs	Chromosomes /unanchored scaffolds	Physical positions (bp)	InDels (*Kabuli* reference genome-CDC Frontier/PI)	Forward primers (5'-3')	Reverse primers (5'-3')	Anneling temperature (0C)	Expected amplified product size (bp)	Structural annotation		Functional annotation		
								Sequence components of *kabuli* genome	*Kabuli* gene accession IDs	NCBI-KOG	TFs	NCBI-nr database
CaPOPII_2124	Ca5	21623515	AAATTAATT/AAATT	AGGTCATTTTCATCCACCCA	ACACACCTATGGGATGCACA	60.2	678	INTERGENIC				
CaPOPII_2125	Ca5	21899576	TAAAAAAAAAAAA/TAAAAAAAAAAA	TGACCTGACCTGGCCTATTC	CCCAAACAAAGCCTTATGGA	60.1	519	INTERGENIC				
CaPOPII_2126	Ca5	21965060	AG/AGG	ACGCTCAGAGTTCACTGGGT	GGCAAATCGATTGGACAAGT	59.9	365	INTERGENIC				
CaPOPII_2127	Ca5	22389657	AT/ATT	TGCCTTTGCTAAGTCCCACT	TAAATAGCACCTGCGGTTCC	59.9	432	INTERGENIC				
CaPOPII_2128	Ca5	22659444	AATATATATATATATATATATATATATATA/AATATATATATATATATATATATATATATATATATATA	CCAAGTTTGAAATGCCGATT	GCTGCATGTTCTTATGCTCG	59.9	485	INTERGENIC				
CaPOPII_2129	Ca5	22918526	AT/ATT	TTATCGGCCTTATCCACTGC	TGAAAATCCATGAACCGTGA	60.1	915	INTERGENIC				
CaPOPII_2130	Ca5	22919631	AG/A	GATTTGGTGGAATCTCCGAA	GCCACCGACTACTTCTCCCT	59.9	336	DRR	Ca_21962			
CaPOPII_2131	Ca5	22919722	GTT/GT	GATTTGGTGGAATCTCCGAA	GCCACCGACTACTTCTCCCT	59.9	336	DRR	Ca_21962			
CaPOPII_2132	Ca5	23275268	TAAAAAAAAA/TAAAAAAAAAA	AAGCCTAATTTCAGCCAGCA	TGATAGCCTCCAACGACAAA	60.0	482	INTERGENIC				
CaPOPII_2133	Ca5	23643411	ATTTTTTT/ATTTTTTTT	AAGCAATCAACGCATTCACA	TTGACTCTCGAAATGTCCCC	60.3	462	INTERGENIC				
CaPOPII_2134	Ca5	23663124	C/CGA	GAGGGTTTTGGGTCAGTGAA	AGTTTTCAAGGGTAGCGGGT	59.9	789	INTERGENIC				
CaPOPII_2135	Ca5	23664574	TT/TTCCAACT	CAAGAAGGAACGAGGTGCTC	TAGCCTAGAGCAGGGGTGAA	60.0	626	INTERGENIC				
CaPOPII_2136	Ca5	23672060	TC/TCGCCC	TGAGTAAGGGGGCTGAAATG	TGAGAGACGGTGGTTTACCC	60.1	822	CDS (FRAME SHIFT)	Ca_17683			
CaPOPII_2137	Ca5	23675051	GT/G	TCCATGCACATTGCTCTCTC	TGGGTTATTTTCATGGGCTT	60.0	514	INTERGENIC				
CaPOPII_2138	Ca5	23995501	CATATATATATATATATATATA/CATATATATATATATATATA	TTGCGAGCATGAGAAGTTGT	AAAAACCACCTAATCGCTCA	59.6	714	INTERGENIC				
CaPOPII_2139	Ca5	24054306	CTATTATTATTATTATTATTATTATTATTATTATTAT/CTATTATTATTATTATTATTATTATTATTATTATTATTAT	TCAATAGAAGGTCGCATCCC	TCGCACATCTACCTAAACGC	60.0	759	INTERGENIC				
CaPOPII_2140	Ca5	24097297	AAGCTTTGGATAC/A	CAGAGCCAACACTACAGGCA	ATTTGAAGGGCCATGTGAAG	60.0	329	CDS (large-effect mutations)	Ca_17656	S		Protein of unknown function DUF3437

INDEL marker IDs	Chromosomes /unanchored scaffolds	Physical positions (bp)	InDels (*Kabuli* reference genome-CDC Frontier/PI)	Forward primers (5'-3')	Reverse primers (5'-3')	Anneling temperature (0C)	Expected amplified product size (bp)	Structural annotation		Functional annotation		
								Sequence components of *kabuli* genome	*Kabuli* gene accession IDs	NCBI-KOG	TFs	NCBI-nr database
CaPOPII_2141	Ca5	24297832	AATATATATATATATA/AATATATATATATATATA	CCCAAGTTTTAAATGGGCAA	GGAGGCCACCTTCAACATTA	59.8	556	INTERGENIC				
CaPOPII_2142	Ca5	24625027	GT/G	GGTCCAAACAATGGCAAATC	ACCCACAACTCCGTATGTCA	60.2	503	INTERGENIC				
CaPOPII_2143	Ca5	24701450	CCATACATACATACATACATACATACATACATACATACATACATACATACATACA/CCATACATACATACATACATACATACATACATACATACATACATACATACATA	CGTGTCCGAGTTAACAGCAA	TGTGTGTGGGTTATCACGCT	59.9	621	INTERGENIC				
CaPOPII_2144	Ca5	25027142	TAAAAAAAAAAAAAA/TAAAAAAAAAA	AAATGGAATAACTTTGAAAATGGAA	CTCCATCGCACACTTTCTCA	59.2	602	INTERGENIC				
CaPOPII_2145	Ca5	25232481	T/TC	CCACCCTGATTTTTGGAGAA	AAAATCCAACAATCTCCCCC	59.9	790	INTERGENIC				
CaPOPII_2146	Ca5	25544088	TAAAAAAAAAA/TAAAAAAAAAAA	TTCACAACCATTCAATTCCAA	TGGTTATGCATGTTCGTCGT	58.9	175	INTERGENIC				
CaPOPII_2147	Ca5	25574427	CATATATATATATATATATATATATAT/CATATATATATATATATATATATATATATAT	TTTTTGCAATCCACTCTTGAA	GATTGTTACACAATTCTTCACGC	58.4	301	INTERGENIC				
CaPOPII_2148	Ca5	25600693	A/AT	CGTGCACTTTCACGACAAAT	TTGCTGTACTTGGTTGCTCG	59.8	801	INTRON	Ca_09030	E		Asparagine synthase
CaPOPII_2149	Ca5	25802252	ATTTTT/ATTTTTTT	TGATGGACTGGAATTGTCAAAG	TGCCAAGAAAACACCATGAA	60.0	846	INTERGENIC				
CaPOPII_2150	Ca5	25803856	TAAAAAAAAAAAAA/TAAAAAAAAAAA	ATCCGATAAATCGCATTGTG	CCCACAAGGTCCGAGAATTA	58.5	203	DRR	Ca_09020			Peptidase C48, SUMO/Sentrin/Ubl1
CaPOPII_2151	Ca5	26280416	CTTTTTTTTTT/CTTTTTTTTTTT	GGGGAAAAAGGTACAAGCCT	AGCGACCTCCTACTTGGTGA	59.5	251	INTERGENIC				
CaPOPII_2152	Ca5	26559939	AATATATATATATATAT/AATATATATATATATATAT	TTTTTGGGTTGAGGACAAGG	GCACTATCCTATCAAATGGTCTCTC	59.9	145	DRR	Ca_08966		bHLH	
CaPOPII_2153	Ca5	26989148	AG/A	CCAAAGCAAGCCAGAAAGAC	GCGAGGTGGAACCACACTAT	60.0	609	INTERGENIC				
CaPOPII_2154	Ca5	26994998	TC/TCC	GGAGCGGGAATATGACTGAA	GGGAGAGGTTGGGAGCTTAC	60.0	492	DRR	Ca_08931	R	MYB	
CaPOPII_2155	Ca5	26995171	TAG/TAGCACAAGCACTAAG	GGAGCGGGAATATGACTGAA	GCGGCAGAAGAACCTATTTG	60.0	576	DRR	Ca_08931	R	MYB	
CaPOPII_2156	Ca5	26996169	TG/TGATCG	ATGTTGGGAACTTGCACTCC	AACTATGTCCTGTGGACCGC	60.0	458	DRR	Ca_08931	R	MYB	
CaPOPII_2157	Ca5	26996806	A/AAAAGCAAAATTA	TCGTTTTGGGTGAATTGTGA	TGCCTCCTCGTTCCTTTTTA	59.9	410	INTERGENIC				

INDEL marker IDs	Chromosomes /unanchored scaffolds	Physical positions (bp)	InDels (*Kabuli* reference genome-CDC Frontier/PI)	Forward primers (5'-3')	Reverse primers (5'-3')	Anneling temperature (0C)	Expected amplified product size (bp)	Structural annotation		Functional annotation		
								Sequence components of *kabuli* genome	*Kabuli* gene accession IDs	NCBI-KOG	TFs	NCBI-nr database
CaPOPII_2158	Ca5	27000752	CTTTTTTTTTTTTTTT/CTTTTTTTTTTTTT	TTGGCACTGAATAGTGCTCG	CGTCCCGAATAGACGAACAT	60.0	698	INTERGENIC				
CaPOPII_2159	Ca5	27000834	ATCTCT/ATCT	TTGGCACTGAATAGTGCTCG	CTCAGCGGGTAGAGTATCGC	60.0	940	INTERGENIC				
CaPOPII_2160	Ca5	27472549	AG/A	GCATTATGCCGTCATCATTTT	TTCGCATTCAAAATTGTTGTT	59.8	491	INTERGENIC				
CaPOPII_2161	Ca5	27718024	TAAAAAAA/TAAAAAAAA	TGGCAACAGAAACCAAACAA	TTCTTTTAGCCCGTTGCTGT	60.1	573	INTERGENIC				
CaPOPII_2162	Ca5	27776620	ATTTTTTTTTT/ATTTTTTTTTTT	TCCAAACTTGGGTTGGTTTC	CATGAGCATGCAAGATGGAT	59.8	603	INTERGENIC				
CaPOPII_2163	Ca5	27953689	TA/T	AACAACAGCCCCAATGTAGC	ACGCTTTTCAAACAAACGCT	60.0	794	INTERGENIC				
CaPOPII_2164	Ca5	28192036	CATATATATATATATATATATAT/CATATATATATATATATATATATAT	CCACATGCTCATTGTGTGACT	CCATTCTCCTTCACCCAGAC	59.6	653	INTERGENIC				
CaPOPII_2165	Ca5	28305464	GTGTATATATG/GTGTATATATGTATATATG	AATATGTGGCGATTAATCTATTGTT	TGGTTGATCATTTCGTTAGTACG	57.3	960	INTERGENIC				
CaPOPII_2166	Ca5	28370910	TTATATATATATATATATATATATATATATAT/TTATATATATATATATATATATATATATAT	CTAGTTCGTAACCCGTGCGT	AGGCATGGTGTTGGAGTCAT	60.2	260	INTERGENIC				
CaPOPII_2167	Ca5	28399764	GT/GTACTAGCATCCT	AGAGTCGCCAGCTGAACAAT	TGATTTCTCCATAGCACCGA	60.0	502	INTERGENIC				
CaPOPII_2168	Ca5	28413401	AATATATA/ATATATATA	TTCTCCTTGAAGCTGTCGGT	ATTGCCAACATTTCCGGTAG	60.0	252	INTERGENIC				
CaPOPII_2169	Ca5	28463884	TAAAAAAAAA/TAAAAAAAAAA	TGTTTCCTTTCGACACATTGA	TGTTGAGCCCAAAATACAAGG	59.2	590	INTERGENIC				
CaPOPII_2170	Ca5	28588926	TGGG/TGG	CAACTGCTCCTGTTCTCCAA	CTAGGAGCATGTCGCTCTCC	59.0	412	INTERGENIC				
CaPOPII_2171	Ca5	28977874	CAATGCAGAA/CAATGCAGAATAATGCAGAA	TGCAGAACCAGAACAAGCAC	TCTGGATTTTGATGTGCTGC	60.0	137	INTERGENIC				
CaPOPII_2172	Ca5	29384323	CTTTTTTTTTTT/CTTTTTTTTT	CTTGGACAATTGAGGCATTTC	ATAGACAAACGTCCGCCTTC	59.6	545	INTERGENIC				
CaPOPII_2173	Ca5	29418196	CAAAAAAAAA/CAAAAAAAA	TGAAAAACACGGAGTCAAACA	CGTGACACTTCTAGCTTGGGA	59.2	512	INTERGENIC				
CaPOPII_2174	Ca5	29663443	AATATATATATATATATA/AATATATATATATATATATA	CTCCTCCACTAAAAATAATTCCAA	GGGGAGGAGCTTCCTATATCC	57.9	756	INTERGENIC				

INDEL marker IDs	Chromosomes /unanchored scaffolds	Physical positions (bp)	InDels (*Kabuli* reference genome-CDC Frontier/PI)	Forward primers (5'-3')	Reverse primers (5'-3')	Anneling temperature (0C)	Expected amplified product size (bp)	Structural annotation		Functional annotation		
								Sequence components of *kabuli* genome	*Kabuli* gene accession IDs	NCBI-KOG	TFs	NCBI-nr database
CaPOPII_2175	Ca5	29664249	GAAAAAAAAAAA/GAAAAAAAAAA	ATTCATAAACACATGGCCCC	TAGGGTTAGGTGAGGGGCTT	59.5	255	INTERGENIC				
CaPOPII_2176	Ca5	29933647	GATATATATATATA/GATATATATATATATA	GACCTTTTCAAAGGATGCATTA	CGTGATGACAGACCAACACC	58.3	713	INTERGENIC				
CaPOPII_2177	Ca5	29938246	ATT/ATTT	GCAAAAACAGCGACACTGAA	TTGTGTGGGAAAGGTGACAA	60.0	764	DRR	Ca_16674			
CaPOPII_2178	Ca5	30121835	TTATATATATATATATATATATATATAT/TTATATATATATATATATATATATATATATATATATATAT	CATGCGTGAATTTGATCGTC	AAACGCGTCACTTTACCTTCTC	60.1	224	INTERGENIC				
CaPOPII_2179	Ca5	30126893	ATTTTTTTTT/ATTTTTTTTTT	GTGGCTTCCAAAGAGAAACG	TTCGGTACACAATTTTCCCC	59.9	612	INTERGENIC				
CaPOPII_2180	Ca5	30128968	GATTATAATTAAAAATA/GATTATAATTAAAAATAATTATAATTAAAAATA	TGTTTCTCTCTCCGCTCACA	CCGCCGACTATACCTGTTTT	59.7	681	INTERGENIC				
CaPOPII_2181	Ca5	30131050	AATATATATATATATATATATATA/AATATATATATATATATATATATATATATATA	CAAATTTCGAGCAACAATGC	AACACAAATGTTAAATTGCGAAGA	59.3	571	INTERGENIC				
CaPOPII_2182	Ca5	30284983	ATATTATTATTATTATTATTATTATTATTATTATTATTGTTATTGTTATTATTATTATTAT/ATATTATTATTATTATTATTATTATTATTATTATTATTATTATTATT	TGTTGTCACATGCTTTGGGT	TTGTGCACATTTTATTCTTCAAA	60.0	651	INTERGENIC				
CaPOPII_2183	Ca5	30612967	TAAAAAAAAA/TAAAAAAAAAA	CGGCATATCATCACCCTTTT	ACCCCCGAAAAGTGCTAGT	59.8	378	INTERGENIC				
CaPOPII_2184	Ca5	31133950	A/AC	GAGGAGATTGGGGAGCAACT	CTCATTGGCCATGAAAGGAT	60.6	539	DRR	Ca_04815			
CaPOPII_2185	Ca5	31136023	AAAAT/A	TCACAATTTCCCAACCCAAT	AATTTCTACCAATGCGTCCG	60.0	796	INTRON	Ca_04816	G	Trihelix	Domain of unknown function CP12
CaPOPII_2186	Ca5	31183021	ATTT/ATTTTTT	CCAAACACTCAAACCGACTC	AAAGACCAAATTGGACACTTCA	58.2	817	DRR	Ca_04823		WOX	Homeobox
CaPOPII_2187	Ca5	31237915	TAAAAAAA/TAAAAAAAA	ACACAAATTCGCCAAAATCC	CATCATGCCTCAAGATCACG	59.8	768	INTERGENIC				
CaPOPII_2188	Ca5	31621509	CT/CTGTATATCTACTTT	ATCCGAGTTTGCGTATGTCC	GGGACGTGGAGATTGTTGAT	60.0	635	INTERGENIC				
CaPOPII_2189	Ca5	31821908	ATTTTTTTTTTT/ATTTTTTTTTT	TGGGTGGTATCCAAGAAAGC	TGCTCTTTGCGACCCTTACT	59.9	631	INTERGENIC				
CaPOPII_2190	Ca5	32299791	ATTTTTTTTTT/ATTTTTTTTTTT	CAAGCAAAACCCTCAAAAGC	GGCGGATCAACTCTTTGTGT	59.9	364	INTERGENIC				
CaPOPII_2191	Ca5	32388967	CTATTATTTTATTATTTTATTA/CTATTATTTTATTATTTTATTATTTTATTA	AATTGCGATAAGCAATTGGG	TAGGATTACACGGGGCAGAC	59.9	821	DRR	Ca_04938		bZIP	

INDEL marker IDs	Chromosomes /unanchored scaffolds	Physical positions (bp)	InDels (*Kabuli* reference genome-CDC Frontier/PI)	Forward primers (5'-3')	Reverse primers (5'-3')	Anneling temperature (0C)	Expected amplified product size (bp)	Structural annotation		Functional annotation		
								Sequence components of *kabuli* genome	*Kabuli* gene accession IDs	NCBI-KOG	TFs	NCBI-nr database
CaPOPII_2192	Ca5	32605650	TAAAAAAAAAA/TAAAAAAAAA	TCAATCACAAATCAGGGTGC	ATCCAGATCCAATACGACGG	59.5	874	INTERGENIC				
CaPOPII_2193	Ca5	32610734	TAAAAAAAAA/TAAAAAAAAAA	ATCCAGCCGTAAAACAACG	GCTGGGGTTTGAAAAATCCT	60.0	832	INTERGENIC				
CaPOPII_2194	Ca5	32745764	TAAA/TAAAA	AGGCATCACATTGAGGGACT	CCTCGGTGACCCTGAATAAA	59.5	148	INTERGENIC				
CaPOPII_2195	Ca5	32879637	AGTAAACAATGTAAACAA/AGTAAACAA	TCCTGCTAAAGATGGTCATTCA	TTGTTGTAGGATGGATGGAGTG	59.7	778	INTERGENIC				
CaPOPII_2196	Ca5	32912336	AATATATATATATATATATATATATAT/AATATATATATATATATATATATATATATATATATATAT	ACCCCCTCACCTCCTTATTG	TTTTCTTGCGTCACTTGTTGA	60.2	499	INTERGENIC				
CaPOPII_2197	Ca5	33354598	TAAAAAAAAAA/TAAAAAAAAA	TGACAGTTACAAACCGCTGC	CAAATTTCTACCAATTCCCCA	59.9	363	INTERGENIC				
CaPOPII_2198	Ca5	33395960	AATTATTATTATTATTATT/AATTATTATTATTATT	TTTATCGGCTAAGGGACACG	GTGCTGCCTAGACAATCGGT	60.1	768	INTERGENIC				
CaPOPII_2199	Ca5	33444959	GATATA/GATATATA	TTATGGAGGCCACATTCACA	GAAATTAAGGCCTCTTGATCTTTTT	59.9	961	DRR	Ca_01912	O	MYB_related	SANT domain, DNA binding
CaPOPII_2200	Ca5	33456475	TTATGTTAAATATGTTAA/TTATGTTAAATATGTTAAATATGTTAA	GATGAAGTGCTGATCAGAGGC	ACCAGGAAACATTGCCAGAC	60.0	789	DRR	Ca_01911	O	MYB_related	SANT domain, DNA binding
CaPOPII_2201	Ca5	33457924	ATTTTTTT/ATTTTTTTT	CCGACACCCAAAATTTCAAC	GCCCTCAGATGAGAGTCAGG	60.2	680	DRR	Ca_01911	O	MYB_related	SANT domain, DNA binding
CaPOPII_2202	Ca5	33542100	GTTTATTTATTTATTTAT/GTTTATTTATTTATTTATTTATTTAT	AATTCGGTTTTTGGCGTGTA	GTTAAGGGACCACGAATGCT	60.4	569	INTERGENIC				
CaPOPII_2203	Ca5	33549168	TAAAAAAAAA/TAAAAAAAAAA	CAAAAACGGGTTTCACCAAC	GCTCTGCATCCAATTCCATT	60.2	447	INTERGENIC				
CaPOPII_2204	Ca5	33600042	CT/C	TAACCATTCCACAGCCACAA	CTTAGAGGTCGCTTCAACGG	60.0	680	DRR	Ca_01896			
CaPOPII_2205	Ca5	33606817	ATTTTACTTGTTTT/ATTTT	GACCTTGCCGGCTACTCTCT	ATATTTCTCATAGGGGCGGC	60.9	449	INTERGENIC				
CaPOPII_2206	Ca5	34191964	AAATAATAATAATAATAATAATAATAAT/AAATAATAATAATAATAATAATAATAATAATAAT	AGGCAAAATGTTTTTCACGG	TTTGTTTTGGCACATGCACT	60.0	561	DRR	Ca_01828			Domain of unknown function DUF2296
CaPOPII_2207	Ca5	34220162	GAAAAAAAAAAA/GAAAAAAAAAA	CCACTAACCTATTCGGAATCCA	GGCAATTGTCGGCACTTAAT	60.2	462	INTERGENIC				
CaPOPII_2208	Ca5	34263941	TTCAGCTATAGTGATATCAGCTATAGTGATAT/TTCAGCTATAGTGATATCAGCTATAGTGATATCAGCTATAGTGATAT	CAAGTGGATTCTTCCCCTCA	CCTTTTGTGGATTGCGTTTT	60.0	825	INTRON	Ca_01820			

INDEL marker IDs	Chromosomes /unanchored scaffolds	Physical positions (bp)	InDels (*Kabuli* reference genome-CDC Frontier/PI)	Forward primers (5'-3')	Reverse primers (5'-3')	Anneling temperature (0C)	Expected amplified product size (bp)	Structural annotation		Functional annotation		
								Sequence components of *kabuli* genome	*Kabuli* gene accession IDs	NCBI-KOG	TFs	NCBI-nr database
CaPOPII_2209	Ca5	34771204	AATATATATATATATATATATATATAT/AATATATATATATATATATATATATATATAT	TCTCATGTTTCCCAACCACA	CTGGACCACATTCTGGCTCT	59.9	487	DRR	Ca_01764		ERF	Pathogenesis-related transcriptional factor/ERF, DNA-binding
CaPOPII_2210	Ca5	34851417	TA/TAA	GACAGAGAGATGACGGGGAA	AAACCGAAAAGTTGAAAAACTGA	60.2	569	INTERGENIC				
CaPOPII_2211	Ca5	35070670	TATTAATTAATTAATTAATT/TATTAATTAATTAATTAATTAATT	TGTGACCTGCAGTGATATTTTG	ATAATGTTGCGAATGGAGGC	58.7	617	INTERGENIC				
CaPOPII_2212	Ca5	35149505	CTTTTTTTTTT/CTTTTTTTTTTT	CAATCACATGGCTTGGATGA	CAGCCACACCGATTACCTTT	60.5	400	INTERGENIC				
CaPOPII_2213	Ca5	35328160	ATTTTTTTTT/ATTTTTTTTTT	ACCACGTGACCCCTTAACTT	TGATGAAATTACGTTATATTGAGGATG	58.4	204	INTERGENIC				
CaPOPII_2214	Ca5	35485623	TAAAAAAAAA/TAAAAAAAAAA	CCCAACTTCTTCATTGCCTC	GGACCCAGCAGAGTTGGTAA	59.7	639	INTERGENIC				
CaPOPII_2215	Ca5	35595695	TTAATAATAATAATAATAATAATAATAATAATAATAATAATAAT/TTAATAATAATAATAATAATAATAATAATAATAATAATAAT	ACCATTCAAACGGCGAGTAG	ATGCAATCGTATCTCGTGGT	60.1	306	INTERGENIC				
CaPOPII_2216	Ca5	35689449	TC/T	CGTTGCAACAGAAGCAGAAG	GATCCAAAGCCCTCAATGAA	59.8	566	DRR	Ca_01660	M		Mechanosensitive ion channel MscS
CaPOPII_2217	Ca5	35751387	AATATATATATATATATATATATATATAT/AATATATATATATATATATATATATATATATAT	TCATTTTTCTTTCAATTTTGTCCTT	TTTTCACTTGGCATCCATCC	59.5	776	INTERGENIC				
CaPOPII_2218	Ca5	35872148	TAAAAAAAAAAA/TAAAAAAAAAA	ACCCTCACGTAAGCCTGATG	TCGCCTTCGTAAATAACGATT	60.1	357	INTERGENIC				
CaPOPII_2219	Ca5	35915901	ATTTTTTTTTT/ATTTTTTTTT	AGCATGTGTTCTTGTGGCAG	TCGGAAGTTTCACATTCATCC	59.9	589	INTERGENIC				
CaPOPII_2220	Ca5	36101390	TAAAAAAAAA/TAAAAAAAA	AGAGCAAAAATCCTTTCGTGA	TAGTGTCAATGGGCCCTCTC	59.0	711	INTERGENIC				
CaPOPII_2221	Ca5	36308359	TTTATT/TTTATTGGTATTATTATTATT	GTTTGTGGCGGATTTTGTTT	CCGATACAACGGAATTGTCC	59.8	423	DRR	Ca_01593	Z		Interferon-related developmental regulator, C-terminal
CaPOPII_2222	Ca5	36317817	CTTTTTTTTTTT/CTTTTTTTTTT	TCTCACCTCGGACACCAAAT	TGGAAGGTACAAGTGAGGGG	60.5	598	INTERGENIC				
CaPOPII_2223	Ca5	36583877	CATATATATATATATATAT/CATATATATATATATATATAT	TCACTTTTAGGATCCGTTTGA	CACAATGGACCATGCACTTC	57.4	809	INTERGENIC				
CaPOPII_2224	Ca5	36904833	GCTCTCTCTCTCTCTCTCTCTCTCTCTCTCTC/GCTCTCTCTCTCTCTCTCTCTCTCTCTCTCTCTC	TTCTGGAGAAAGAGGCGAAG	TGCGTAATTTGGTGCTGGTA	59.7	638	INTERGENIC				
CaPOPII_2225	Ca5	37086090	AGGGGGGGGGG/AGGGGGGGGG	CCGTGCAAAGGATGAAAAGT	TACTCGGCCCAAAATTAACG	60.1	614	INTRON	Ca_01507		G2-like	GRAM

INDEL marker IDs	Chromosomes /unanchored scaffolds	Physical positions (bp)	InDels (*Kabuli* reference genome-CDC Frontier/PI)	Forward primers (5'-3')	Reverse primers (5'-3')	Anneling temperature (0C)	Expected amplified product size (bp)	Structural annotation		Functional annotation		
								Sequence components of *kabuli* genome	*Kabuli* gene accession IDs	NCBI-KOG	TFs	NCBI-nr database
CaPOPII_2226	Ca5	37112216	CTTATTTATTTATTTATTTATTTATTTAT/CTTATTTATTTATTTATTTATTTATTTATTTATTTAT	TTCTTCCAGGGACATCCAAG	AGGAGGTGCCCATTTAGGAT	60.0	648	INTRON	Ca_01505	R		WD40 repeat
CaPOPII_2227	Ca5	37218586	TATACATACATACATACATACATA/TATACATACATACATACATA	CACTCGTATCATGCTTGTCG	CTTTTTCGCCACCATTTGTT	57.9	680	INTERGENIC				
CaPOPII_2228	Ca5	37235701	AATATATATATATATATATATATATA/AATATATATATATATATATATATA	GTTGGAGCCAAAAACTAGCG	ACATTCATTGGCCCTTTTGA	59.9	577	INTERGENIC				
CaPOPII_2229	Ca5	37633576	TAAAAAAAAAA/TAAAAAAAAAAA	TGCAATGTTAAAGATGGTTGG	GTGCGAATTTTGTGATTACTGA	58.5	264	INTERGENIC				
CaPOPII_2230	Ca5	37670957	GTTTTTTTTT/GTTTTTTTT	GCCTGCTCTTCTTTTTGGTG	TGAAAATATACATCTGTGGGGG	60.0	294	INTERGENIC				
CaPOPII_2231	Ca5	37905515	ATTTTTTTTTT/ATTTTTTTTT	TGATCGAAGACAATTTGTGTCA	CCGATCTCACATGCTTCCTT	59.2	670	INTERGENIC				
CaPOPII_2232	Ca5	38006809	G/GATCGTT	TGGAAGTGCATATGATGTTGG	CGAGGTGTTGCACATGACTT	59.4	585	INTERGENIC				
CaPOPII_2233	Ca5	38016624	TAAGAAA/TAA	ATCCTCGAGAAGGAAGGAGC	CCTTTTCCCTCCAATGTCAA	59.9	276	INTRON	Ca_01419	A		Pseudouridine synthase, RsuA and RluB/C/D/E/F
CaPOPII_2234	Ca5	38025852	AT/A	ATGCCAAAAATAAACGCCAG	TGAGGTTGAGAAGCCAAACC	60.0	647	INTERGENIC				
CaPOPII_2235	Ca5	38027475	T/TA	TGGAGTGATACACACTTTTGTGG	TGCTTACAAGGTATTCTTGCCA	59.9	446	INTERGENIC				
CaPOPII_2236	Ca5	38033573	GTTTTTTTTT/GTTTTTTTTTTT	AAAGCGGCATTATCTCGTTG	CGCGTCAAATAAAGCATTGA	60.2	520	INTERGENIC				
CaPOPII_2237	Ca5	38034465	AATTATTAT/AATTAT	TGCTATTGCGATCCCTCTCT	TTGTCGGTATAAATCGGACG	59.9	311	INTERGENIC				
CaPOPII_2238	Ca5	38034841	TTTA/TTTAATTA	TCGTCCGATTTATACCGACA	CGACGAGGTATATGCCAACA	59.0	856	INTERGENIC				
CaPOPII_2239	Ca5	38036769	TAA/TA	TGATAACTCGTCGGCTTGTG	TTTTTCCTTCCAAATAAGCCTC	59.9	633	INTERGENIC				
CaPOPII_2240	Ca5	38061782	ATTTT/ATT	CCCTCCGTGTTTATTTACCG	CACTCTAGCTTTGGGCTTGG	59.3	687	DRR	Ca_01416			
CaPOPII_2241	Ca5	38080854	A/AC	AATAGATCCTGATGGCCGTG	TCGATTCACCATGACAGCAT	59.9	813	INTRON	Ca_01411	D	WRKY	Protein kinase, catalytic domain
CaPOPII_2242	Ca5	38142286	GACACA/GACA	TTGTTCCCTTTTGGATGAGG	ACTTTGCTGGCAATGTGTGT	59.9	526	DRR	Ca_01403	R	NAC	Pentatricopeptide repeat

INDEL marker IDs	Chromosomes /unanchored scaffolds	Physical positions (bp)	InDels (*Kabuli* reference genome-CDC Frontier/PI)	Forward primers (5'-3')	Reverse primers (5'-3')	Anneling temperature (0C)	Expected amplified product size (bp)	Structural annotation		Functional annotation		
								Sequence components of *kabuli* genome	*Kabuli* gene accession IDs	NCBI-KOG	TFs	NCBI-nr database
CaPOPII_2243	Ca5	38155679	CTTA/CTTAATTA	TGTTGTTGTTGGATTGGCAT	AAAATGGAGCATTGGAAAAA	59.8	769	INTERGENIC				
CaPOPII_2244	Ca5	38255302	AAATAATAATAATAAT/AAATAATAATAAT	TGCCTCAACTCAACACTTGC	CAATTTACCAATCGCACGAA	60.0	762	INTERGENIC				
CaPOPII_2245	Ca5	38407176	AATATATATATATATATATATATATAT/AATATATATATATATATATATATATATATAT	TCTCAAAGTCTCAATGGTCCAA	TTGAAGTCAAATTTCGCCAG	59.7	658	INTERGENIC				
CaPOPII_2246	Ca5	38755924	TAAAA/TAAA	GTCTGCCACATCACCCTTTT	CGGATCAACTTTTCTTTGTGG	60.0	661	INTERGENIC				
CaPOPII_2247	Ca5	39537232	AATATATATATATATATATATATATATATATA/AATATATATATATATATATATATATATATATATA	TTGGCAACCAGTCAACAAAA	CACGTGAGAAAACCTCGACA	60.1	473	INTERGENIC				
CaPOPII_2248	Ca5	39557215	GATATATATATATATATATAT/GATATATATATATATATATATAT	GCAAAAAGGGGCACAATAAA	AATGTCAATCCTCCATTGCC	59.9	357	INTRON	Ca_07489	TBLD		Phosphatidylinositol 3-/4-kinase, catalytic
CaPOPII_2249	Ca5	39569272	ACTCTTNNNNNNNNNNNNCTCTT/ACTCTT	TAGACAAGCCCATTTCCAGG	TTCATGCAAACATTGGTGCT	60.1	450	INTRON	Ca_07489	TBLD		Phosphatidylinositol 3-/4-kinase, catalytic
CaPOPII_2250	Ca5	39793489	CAAAAAAAA/CAAAAAAA	AGGGCACTTTGTCTCCTCAA	ACCAAATCGATGGAATGACC	59.8	529	DRR	Ca_07506	S	bZIP	Armadillo
CaPOPII_2251	Ca5	40392907	GTATATATATATATATATATATATATATATAT/GTATATATATATATATATATATATATAT	GCATTATCACGAGCAACACG	TCAATGTGGTTCGCAAAAGA	60.3	192	INTERGENIC				
CaPOPII_2252	Ca5	40434180	A/ACATCTG	TTTCACCCAGTAGGAGGTCG	TGAACCGGAAGAACCTCAAC	60.1	500	INTERGENIC				
CaPOPII_2253	Ca5	40512625	GTATATATATATATATA/GTATATATATATATA	ATTTCCGTATCGTCAATGCC	TGCGGACAAAATTCAAAACA	59.8	626	INTERGENIC				
CaPOPII_2254	Ca5	40572884	ATTTTTTTT/ATTTTTTT	TTAGAGGAGCCACGTAAGCC	CCCTAGCCACTTTCTCTCCC	59.5	584	INTERGENIC				
CaPOPII_2255	Ca5	40747443	C/CT	TTGCAGAGAAAGAAAACTTGATATG	TGTGGAAGTTCCTAAAATACGC	59.0	237	INTERGENIC				
CaPOPII_2256	Ca5	40858234	GATATATATATATATATATATATATATATATATATATA/GATATATATATATATATATATATATATATATATATATATATATA	AGGCATGGTGTTGGAGTCAT	TCCGACAAGGAGAATAACCG	60.4	650	DRR	Ca_07607	K	M-type	Transcription factor, MADS-box
CaPOPII_2257	Ca5	41108441	GTTTTTTTTTTT/GTTTTTTTTTTTT	GGAGCGGAAAAGACTGAAAA	TTTTGTCTATGCGCACAAGG	59.4	586	INTERGENIC				
CaPOPII_2258	Ca5	41428807	TTATATATATATATATATATATATATATATATATATATATATAT/TTATATATATATATATATATATATATATATATATATATATATAT	TGAATTGAGTTTGGTTGATTTGA	AAAGGATGCAGTATGGGTTTTT	59.5	591	INTERGENIC				
CaPOPII_2259	Ca5	41678103	TCT/TCTTTCCCTTAAAGGGACT	CAGTGGGTCCTCTTCCACAT	CCCGAACTCCTGTTACTCCA	60.0	551	INTRON	Ca_19215	J	C3H	Argonaute/Dicer protein, PAZ

INDEL marker IDs	Chromosomes /unanchored scaffolds	Physical positions (bp)	InDels (*Kabuli* reference genome-CDC Frontier/PI)	Forward primers (5'-3')	Reverse primers (5'-3')	Anneling temperature (0C)	Expected amplified product size (bp)	Structural annotation		Functional annotation		
								Sequence components of *kabuli* genome	*Kabuli* gene accession IDs	NCBI-KOG	TFs	NCBI-nr database
CaPOPII_2260	Ca5	42231500	CC/CCGAGAACGTAC	TCAAAATCCACACCCCACTT	GCCATCTCCAATAGCACCAT	60.2	888	INTERGENIC				
CaPOPII_2261	Ca5	42425779	CATGTTTTATGTTTTA/CATGTTTTA	TCAATGGAATGGAGTCGTCA	CGACAATTTCATTATCCGTCC	60.0	749	INTERGENIC				
CaPOPII_2262	Ca5	42557966	TAAAAAA/TAAAAAAA	TGAAAGTGCATGTGACCGAT	CACAAACCAACGTGGAAGTG	60.1	613	INTERGENIC				
CaPOPII_2263	Ca5	42822040	AGCGNNNNNNNNNNGCG/AGCG	TCCATTGCAGTCAGCAACTC	GCTTGGTCCTAGAAGATGCG	60.0	433	INTRON	Ca_11327	R	MYB	Protein kinase, catalytic domain
CaPOPII_2264	Ca5	43298677	AATATATATATATATATATATATATATA/AATATATATATATATATATATATATATATATA	TCAAATTTGCAAAGACAGATTTTC	TTTTGGTGTCACTTAAGAGTGGAA	59.7	893	DRR	Ca_12727			
CaPOPII_2265	Ca5	43299130	TC/T	TTGGTTTACGAATTTTATGGTGAA	CTGCATAGGCAGGGTGGTAG	59.7	385	DRR	Ca_12727			
CaPOPII_2266	Ca5	43456554	TTCTTGGGCAGTCTTGGGCAG/TTCTTGGGCAGTCTTGGGCAGTCTTGGGCAG	TCTGGATGATAATTTCGGGC	TTGAATTCACCTTTGACCCC	59.9	520	INTRON	Ca_12708	O	MYB	Heat shock protein DnaJ, cysteine-rich domain
CaPOPII_2267	Ca5	43517939	AATATATATATATATATATATATA/AATATATATATATATATATATATATATA	TGAGATGTTCATTACTGCGTTG	AATTTTTGCGGCCAAGTATG	58.8	876	INTERGENIC				
CaPOPII_2268	Ca5	43796946	TTTATTATTATTATTATTATTATTATCATTATTATTATTATCATTATTATTATTATTATTATTATTATT/TTTATTATTATTATTATTATTATTATCATTATTATT	AGCCTGTACGCAAAAAGCAT	GGATAACGCGGAGGTATTGA	59.9	668	INTERGENIC				
CaPOPII_2269	Ca5	43847991	GT/GTGTGTGTGTCT	GGAAACTCGTTTTTGAGTGGA	TGTCAATGTGCCAAAGTATCG	59.2	679	INTERGENIC				
CaPOPII_2270	Ca5	43918951	CTTTT/CTTT	TTGCAAGGTAAAAAGCACGA	ACGATGCCCCATACAATGAT	59.5	783	INTERGENIC				
CaPOPII_2271	Ca5	44086162	CTTTTTTTTT/CTTTTTTTTTT	CGGGTCAACTTTGATAGACGA	GATGTGCCGTGAAAACAATG	60.1	594	INTERGENIC				
CaPOPII_2272	Ca5	44177714	GTATATATATATATATAT/GTATATATATATATATATATAT	GGTCACGAACATCCCAAAAT	CCATGGTGAGAGGGTCAAAT	59.7	377	INTERGENIC				
CaPOPII_2273	Ca5	44244203	AA/AATTTAGA	CAATGAACACATTTGCCACC	GGACGATCAAGCTCAACCAT	59.8	747	INTRON	Ca_03869	P		Natural resistance-associated macrophage protein
CaPOPII_2274	Ca5	44596809	ATT/ATTT	TTGGCACATGCATTTTTGTT	GACCAAATCTTGTTGTTGCAGA	60.0	582	INTERGENIC				
CaPOPII_2275	Ca5	44597112	CTATA/CTATATATA	GGGGTTAGTGCAAGCCAAAT	GTTCGCACGTGATGAACAAT	61.2	862	INTERGENIC				
CaPOPII_2276	Ca5	44914869	TTATATATATATATATATATATATATA/TTATATATATATATATATATATATATATATA	TTTCGAGCAATCTCATCCCT	CGGCAGCTTTTTACTACCCA	59.8	759	INTERGENIC				

INDEL marker IDs	Chromosomes /unanchored scaffolds	Physical positions (bp)	InDels (*Kabuli* reference genome-CDC Frontier/PI)	Forward primers (5'-3')	Reverse primers (5'-3')	Anneling temperature (0C)	Expected amplified product size (bp)	Structural annotation		Functional annotation		
								Sequence components of *kabuli* genome	*Kabuli* gene accession IDs	NCBI-KOG	TFs	NCBI-nr database
CaPOPII_2277	Ca5	45086826	TA/TAA	CAAACGCAAAATTAAACACACAA	AATACAGCTGGGTTTGTGGC	60.0	367	INTERGENIC				
CaPOPII_2278	Ca5	45094437	TG/T	AATCCATCCGAACCCTTTTC	TTGTCAATGTTGCATGTCTGAA	60.1	259	INTERGENIC				
CaPOPII_2279	Ca5	45372527	TTTATTATTATTATTATTATTATTATTATTATTATTATTATTATTATTATTATTATTA/TTTATTATTATTATTATTATTATTATTATTATTATTATTATTATTATTATTATTAT	CCCAGTGTCACCGTCTTTTT	TTCACACAAATGTCCACCAA	60.0	537	INTERGENIC				
CaPOPII_2280	Ca5	45557067	CATATATATATATATATATATATATATAT/CATATATATATATATATAT	CAATGAAAAAGTGTGGCCCT	ACGTTGATGGAACAATGCCT	60.0	873	INTERGENIC				
CaPOPII_2281	Ca5	45724530	AATTATTATTATTATTATTATTATTATTATTATTAT/AATTATTATTATTATTATTATTATTATTATTATTATTAT	GCGGTTGAATAAATGTGACAAA	TAGTTGGAGGTTGCTGACCC	59.9	616	INTERGENIC				
CaPOPII_2282	Ca5	45746975	ATT/ATTACACTCTTT	GGAGTTGCAAAAGAGCATGA	TGGTTAACACTGGAGAGGGG	59.0	136	DRR	Ca_04024	G		Glucosamine/galactosamine-6-phosphate isomerase
CaPOPII_2283	Ca5	46208612	AAT/AATAT	ATGCAGCACAAAATGCAAGT	TTCGAATCTTATTTTGACAATTTTT	59.4	502	INTERGENIC				
CaPOPII_2284	Ca5	46305506	GATATATATATATATATATATATATATATATATAT/GATATATATATATATATATATATATATATATATATATATATATATAT	TGCCCTCAATCTGAAAACAA	GAACAAGAGCGGTCTCACAA	59.2	876	DRR	Ca_04084			
CaPOPII_2285	Ca5	46393868	AG/AGTGATG	ACAGTTCAAAGTCCCTGGCA	TCAATCATTTAAAATTACTCTGAATCG	60.7	561	INTERGENIC				
CaPOPII_2286	Ca5	46661039	AATATATATATATATATATATAT/AATATATATATATATATATATATAT	TGGAATTCAAGGGACCAAAA	ACTTGCGAGATAATTTAGTAGGGA	60.3	863	INTERGENIC				
CaPOPII_2287	Ca5	46676212	ATTTTTTTTTT/ATTTTTTTTTTT	GCCACCACACCTCCAACTAT	TTGAGATGGGACTTTGGAGC	59.9	880	INTERGENIC				
CaPOPII_2288	Ca5	46946905	AATATATATATATATATAT/AATATATATATATATATATAT	CATCCGTGGTCCTTTGACTT	TGGTCATTGCATTTTCCTCA	60.0	482	DRR	Ca_04149	K	ERF	RNA recognition motif domain
CaPOPII_2289	Ca5	46985054	AATTATTATTATTATTATTATTATTATTATTATTATTATTATT/AATTATTATTATTATTATTATTATTATTATTATTATTATTATTATTATT	TCGCATTTTAAGGGAGGAAA	AGCAAGGAAGCATCATCCAA	59.7	212	INTERGENIC				
CaPOPII_2290	Ca5	47692025	ATTTTTTTTT/ATTTTTTTT	CACCAAAAGCAGGGTCTCTC	GCCTTAAAGCTTTGGGTGAA	59.8	346	DRR	Ca_04228			Protein of unknown function DUF3727
CaPOPII_2291	Ca5	47714017	GATATATATATATATATATATATATATA/GATATATATATATATATATATATATA	ATGCGTGTGAAATTGTGCAT	TCAGCTCAGTGGATGATTGG	60.0	402	INTRON	Ca_04232	G	C2H2	Glycoside hydrolase, family 35
CaPOPII_2292	Ca6	48925	ATTCTTCTTCTTCTTCTTCTTCTTCTT/ATTCTTCTTCTTCTTCTTCTTCTTCTTCTT	ATGTGTTTTCGTTCTTCGGG	TCCATGGGTTTATTGTGGCT	60.0	485	DRR	Ca_26378			
CaPOPII_2293	Ca6	112870	TG/T	AACTCCTTGGGACCGTGATT	ACCTTGCAACTGGAGCAAAT	60.7	242	INTERGENIC				

INDEL marker IDs	Chromosomes /unanchored scaffolds	Physical positions (bp)	InDels (*Kabuli* reference genome-CDC Frontier/PI)	Forward primers (5'-3')	Reverse primers (5'-3')	Anneling temperature (0C)	Expected amplified product size (bp)	Structural annotation		Functional annotation		
								Sequence components of *kabuli* genome	*Kabuli* gene accession IDs	NCBI-KOG	TFs	NCBI-nr database
CaPOPII_2294	Ca6	257739	CATATATATATATATATATATATATA/ CATATATATATATATATATATATATAT A	CGACACATGTGCTATTT AGCCT	ATGGGTTGCAGTTAGC TTGG	59.3	799	INTERGENIC				
CaPOPII_2295	Ca6	563674	AATATATATATATATATATATATATTT ATATATATATAT/AATATATATATATAT ATATATATTTATATATATATAT	GGATCAGATGGGTCGG TAGA	ATGCACGTTTGGTATG GTCA	59.9	441	DRR	Ca_13277	C		NADH:ubiquinone oxidoreductase, 75kDa subunit, conserved site
CaPOPII_2296	Ca6	850916	TCC/TCCC	AATCAAGTGCAAAAGT GGGG	ACCACACCCATATCCA GCAT	60.0	704	INTERGENIC				
CaPOPII_2297	Ca6	1257304	TTATATATATATATATATAT/TTATATA TATATATATATATAT	GGTGAGGATTGGTTCA CTCG	ATGAGTGGGGATCGAT CTTG	60.5	341	DRR	Ca_13331		NF-YB	Protein of unknown function DUF248, methyltransferase putative
CaPOPII_2298	Ca6	1343859	GAAAAA/GAAAAAA	CAACGAGTTTGATTTCT ATTCTAACG	TTGGGTAGCCAAGGTT TCAC	59.7	721	INTERGENIC				
CaPOPII_2299	Ca6	1350878	A/AGTCTGT	ACACATCAGCTTTTTGG CTG	AGCCCACTCACCTTCTT CCT	58.9	428	INTERGENIC				
CaPOPII_2300	Ca6	1354322	GAA/GAACCAA	AATTTGCATAGGCGGT GAAG	GGTGTTACAGCTTTCAT GAACAAA	60.1	420	INTERGENIC				
CaPOPII_2301	Ca6	1491943	CT/CTT	TGGTTTTCGTACCGGA CTCT	CAATGTCATCAAATAGC GGC	59.6	772	INTRON	Ca_27033	H		Folylpolyglutamate synthetase
CaPOPII_2302	Ca6	1492728	AT/ATT	TTGTTGTTTCAACGGTG CAT	ATGGTTGGGGGATAAA AAGG	60.0	651	INTRON	Ca_27033	H		Folylpolyglutamate synthetase
CaPOPII_2303	Ca6	1556869	TAAAAA/TAAAAAA	CAATTTGGTTGGGCGT AAAC	TTGCTTTATACTCATAT TTTGGCCT	60.2	528	INTERGENIC				
CaPOPII_2304	Ca6	1559285	GTTA/GTTATTA	CAGAGCAGTGTGACAG CAAGA	TCAAAAATTTCAAACAA AATCCA	60.4	456	INTERGENIC				
CaPOPII_2305	Ca6	1559581	ATTTTTTTTTTTT/ATTTTTTTTTT	TGAAGTTAAATTAAAGG ATCGCA	TATTTTATTCCCACCGC TGC	58.0	283	INTERGENIC				
CaPOPII_2306	Ca6	1560016	TTT/TTTAATT	AATATTACTGCAGCGG TGGG	GGACCACAAGAAATCC TCCA	60.0	719	INTERGENIC				
CaPOPII_2307	Ca6	1682722	GTGCATCTGTTT/G	GAGGCATTGTCTTTGG TGGT	GCAAATGTTTGGAATG GCTT	60.0	656	INTERGENIC				
CaPOPII_2308	Ca6	1683226	CAA/CA	AATAAGTCCATCCGGC TCCT	TTGTGGATGGCGTTTG ATAA	59.9	276	DRR	Ca_10299		HB-other	Rop nucleotide exchanger, PRONE
CaPOPII_2309	Ca6	1683327	AC/A	TTATCAAACGCCATCCA CAA	CACTTGCGTGTGGTAG TGCT	59.9	504	DRR	Ca_10299		HB-other	Rop nucleotide exchanger, PRONE
CaPOPII_2310	Ca6	1683470	C/CCAAAACAATCATAAC	TTATCAAACGCCATCCA CAA	CACTTGCGTGTGGTAG TGCT	59.9	504	DRR	Ca_10299		HB-other	Rop nucleotide exchanger, PRONE

INDEL marker IDs	Chromosomes /unanchored scaffolds	Physical positions (bp)	InDels (*Kabuli* reference genome-CDC Frontier/PI)	Forward primers (5'-3')	Reverse primers (5'-3')	Anneling temperature (0C)	Expected amplified product size (bp)	Structural annotation		Functional annotation		
								Sequence components of *kabuli* genome	*Kabuli* gene accession IDs	NCBI-KOG	TFs	NCBI-nr database
CaPOPII_2311	Ca6	1831168	AATATA/AATATATAAATAAAAGATATA	GGCAAAAGAACAAGGTCCAA	CCTGGATGAACCAAATACACAA	60.1	354	DRR	Ca_10310	G		Fructose-1,6-bisphosphatase class 1/Sedoheputulose-1,7-bisphosphatase
CaPOPII_2312	Ca6	1939700	AATATATATATATATAT/AATATATATATATATAT	TGATCAATTTACCAACGATTACAGA	ATTTTACATGCCCAAAAGCG	59.8	718	INTERGENIC				
CaPOPII_2313	Ca6	1976018	AAT/AATAGAT	GAACTAGATGCCGCAAAAGC	GTACGTGGTCCTTCCCTGAA	60.0	560	DRR	Ca_10322			
CaPOPII_2314	Ca6	1976736	TC/TCCC	TTATTCTCCATTGGGCATCC	TCCTGCGGGTAATGATAAGC	59.7	963	DRR	Ca_10322			
CaPOPII_2315	Ca6	1978360	A/ATCG	CGTGGAGCATTCCGAGTATT	GGAGTTAGGGCGTGTGATGT	60.1	575	INTRON	Ca_10322			
CaPOPII_2316	Ca6	2063027	GGAAAGAAAGAAAGAAAGAAAGAAAGAAA/GGAAAGAAAGAAAGAAAGAAAGAAAGAAA	AGGCAAAGAAAGGAGCAACA	GCATGACACGACAATGGAAG	60.0	807	INTRON	Ca_10326	A		Poly(A) polymerase, RNA-binding domain
CaPOPII_2317	Ca6	2128046	CTTTTTTTTT/CTTTTTTTT	AATTTGCTTTGATTTTGCCG	GTGCTCGTGCGACTTGTAAA	60.1	562	INTERGENIC				
CaPOPII_2318	Ca6	2384865	TTAATAATAATAATAATAATAATAATAATAATAATAA/TTAATAATAATAATAATAATAATAATAATAATAATAA	AGATTGCCCCACTTCAAAAA	TAACCTGCCAAAGAGGAGGA	59.5	866	INTERGENIC				
CaPOPII_2319	Ca6	2501071	TAACAACAACAACAACAACAA/TAACAACAACAACAACAA	CCCACCAACCCAAATTAAAA	TCACCATCGTGTGATGGACT	59.5	305	INTERGENIC				
CaPOPII_2320	Ca6	2518811	TA/TAA	AATCTGGCTCAAAATGCAGC	AATGCATTTGCCTGACTCAA	60.4	723	DRR	Ca_10384			Protein of unknown function DUF599
CaPOPII_2321	Ca6	2525217	TT/TTGCAT	AGTAACCGACCAGCCAATCA	CAAATTTTGGTCACATTCCG	60.5	741	INTERGENIC				
CaPOPII_2322	Ca6	2525428	TAAAAAAAAAA/TAAAAAAAAAAAA	CCTGAAATTTGATTCTGCGT	CAAATTTTGGTCACATTCCG	57.8	578	INTERGENIC				
CaPOPII_2323	Ca6	2528953	AATAT/AAT	TGGGTCAATGTTAAGAGGGC	CGTCAACGTCGTGCATTTAC	59.9	515	INTERGENIC				
CaPOPII_2324	Ca6	2639909	CTT/CT	AGCTGAAGCAATTTTCGCAT	GCAATGCAGCAGCAATAAAA	60.0	479	INTERGENIC				
CaPOPII_2325	Ca6	2674985	ATGTT/AT	GAAACTTCGTGCCTTTTTGC	ACGATAGTAGATGTGGCGGC	59.9	152	INTRON	Ca_10401	V		Protein of unknown function DUF1712, fungi
CaPOPII_2326	Ca6	3108108	AT/ATAATAATAATAATATT	CACCAGTGTCCAATCACCCT	CGTCTCATGTTCCTCCAGGT	60.8	508	INTERGENIC				
CaPOPII_2327	Ca6	3166147	TATCGGTTCGGCCATTTCCATCGGTTCGGCC/TATCGGTTCGGCC	TCAGATTCTATCGGTTCGGC	ATATGCTGCACCTTGGTTCC	60.2	202	INTERGENIC				

INDEL marker IDs	Chromosomes /unanchored scaffolds	Physical positions (bp)	InDels (*Kabuli* reference genome-CDC Frontier/PI)	Forward primers (5'-3')	Reverse primers (5'-3')	Anneling temperature (0C)	Expected amplified product size (bp)	Structural annotation		Functional annotation		
								Sequence components of *kabuli* genome	*Kabuli* gene accession IDs	NCBI-KOG	TFs	NCBI-nr database
CaPOPII_2328	Ca6	3176026	AGG/AGGG	GCAATGGAAGGTGAAAATGG	GCATAGGAGCGTGGAAATTG	60.3	522	INTERGENIC				
CaPOPII_2329	Ca6	3255826	AATATATATATATATAT/AATATATATATATATAT	TGGCTTGTTTGATTTAGGACA	ATTCGAATGATCAGTTGCCC	58.3	547	INTERGENIC				
CaPOPII_2330	Ca6	3331064	TAAAAAAA/TAAAAAA	CAAATTGAAAAGTGGGGCAT	TTAAAGTTGGGAGACCACCA	59.8	417	INTERGENIC				
CaPOPII_2331	Ca6	3340164	ATTTTTTTTT/ATTTTTTTTTT	AGGACAATGGGCAAAGACAC	TCATCACCTCAACCAATCCA	60.0	267	INTERGENIC				
CaPOPII_2332	Ca6	3351662	GATATAT/GATATATATAT	CGAAAATGGAAAACACCACC	TCGAGTTGCGTAACTGACATAG	60.2	687	INTERGENIC				
CaPOPII_2333	Ca6	3351745	CATATTATATATA/CATA	GGTTGATTTAGGCTTCACGG	TCGAGTTGCGTAACTGACATAG	59.6	580	INTERGENIC				
CaPOPII_2334	Ca6	3352912	AATTA/AA	AAAGTGGGTGGCAATTAACG	TTATCCAATTCCGTTGCACA	59.9	247	INTERGENIC				
CaPOPII_2335	Ca6	3355103	GCTATTGATCAATCTATTGAT/GCTATTGAT	TGAATAGATGTGCGCGAGAC	TCCACGGGAGTGAGAGAGAT	60.0	401	INTERGENIC				
CaPOPII_2336	Ca6	3355875	CTTT/CTTTT	AATTTGGCCCTATTGGCTTT	AGAAACGGAATGAGTCACGG	59.8	709	INTERGENIC				
CaPOPII_2337	Ca6	3377584	CTTTTT/CTT	ATGCGGTGATGGTGATATTG	TATGGCGAATATGGATGGGT	59.2	745	INTERGENIC				
CaPOPII_2338	Ca6	3378118	GAGAA/GA	ACCCATCCATATTCGCCATA	TTTGCCATCCTCTCTTTTTCA	60.0	653	INTERGENIC				
CaPOPII_2339	Ca6	3382789	CTTTTT/CTTT	ATCACCGACCACTCACCTTC	GTTCAATCAGTGCAGCGTGT	60.0	810	INTERGENIC				
CaPOPII_2340	Ca6	3382946	CAAAAA/CAAAA	TAACCACCAACATTCTGCCA	TGTTCCGTCCCACTCATACA	60.0	927	INTERGENIC				
CaPOPII_2341	Ca6	3387853	AGTACGTA/AGTA	GAGCCAACAGGATAAGAGCG	GAAAATTGAGTTCCATGACGTTT	60.0	477	INTERGENIC				
CaPOPII_2342	Ca6	3387937	A/AAT	GAGCCAACAGGATAAGAGCG	GAAAATTGAGTTCCATGACGTTT	60.0	477	INTERGENIC				
CaPOPII_2343	Ca6	3388065	GATATATATAT/GATATAT	GAGCCAACAGGATAAGAGCG	TTGTCTTTCACTTCTTTTTCCG	60.0	841	INTERGENIC				
CaPOPII_2344	Ca6	3393664	CAAAAAAA/CCAAAAAAAAA	CATTTTGGCCATCTAAAACGA	TGGAAAAATAAACGTTGCCC	60.0	603	INTERGENIC				

INDEL marker IDs	Chromosomes /unanchored scaffolds	Physical positions (bp)	InDels (*Kabuli* reference genome-CDC Frontier/PI)	Forward primers (5'-3')	Reverse primers (5'-3')	Anneling temperature (0C)	Expected amplified product size (bp)	Structural annotation		Functional annotation		
								Sequence components of *kabuli* genome	*Kabuli* gene accession IDs	NCBI-KOG	TFs	NCBI-nr database
CaPOPII_2345	Ca6	3394137	AT/ATT	CCATCCAGAGCACCTGAAAT	TCAATTGCCTTGCATGTTGT	60.1	371	INTERGENIC				
CaPOPII_2346	Ca6	3395635	GTT/GT	GGCAGGAACGATACCACAGT	TTGCCCGTACAAAAGAATCC	60.0	428	INTRON	Ca_05973	U	Trihelix	Ras GTPase
CaPOPII_2347	Ca6	3420698	TTATATATATATATATATATATATATAT/TTATATATATATATATATATATATATATATAT	TAGCCGACAACATTGGAACA	TGAACTCGCACATTTGAGGA	60.1	386	INTERGENIC				
CaPOPII_2348	Ca6	4498664	TAAAA/TAA	TCAGCCACATTGTTTCAAGC	AATAGTGTCAGCGGGTAGTTTT	59.8	373	DRR	Ca_05852		BES1	Glycoside hydrolase, family 14
CaPOPII_2349	Ca6	4635371	G/GAGAAAAATTCGGT	CCAAAGCCAACGGCTATAAT	ATGAATTGAGGGGGTAAGGG	59.1	302	INTERGENIC				
CaPOPII_2350	Ca6	4649762	AATAAGT/AATAAGTATAAGT	GGTCGAACCACTTGAGGTGT	AATCCACTCCCACTCCACTG	60.0	389	INTERGENIC				
CaPOPII_2351	Ca6	4699610	CG/CGG	TTCTCTTCAAAGCGTGAGCA	TTCCTTGTCCCATCTCCATC	59.9	304	INTERGENIC				
CaPOPII_2352	Ca6	4771733	TTATATATATATATATATATAT/TTATATATATATATATATAT	GGGCCACATGTATTTGTGTTT	GGAGGCAAGGCAATAAATCA	59.6	532	INTERGENIC				
CaPOPII_2353	Ca6	4964508	TAAAAAAAAAA/TAAAAAAAAA	GACAGAAGCGACATGGTTGA	AGTTGCTTAGTTGGCAACCC	59.8	767	INTERGENIC				
CaPOPII_2354	Ca6	5031791	ATTTTTTT/ATTTTTT	TGATGTCGCATGGGTATTGT	AAAAAGTTGAGGCCCAAAGG	59.8	459	INTERGENIC				
CaPOPII_2355	Ca6	5295925	TTTATTATTTATTA/TTTATTATTTATTATTATTTATTA	TTGACATTTCAAACCGGTCTAT	TGGAAAAGACTCCTGAATATGGA	58.5	359	INTERGENIC				
CaPOPII_2356	Ca6	5410706	ATTTTTTTT/ATTTTTTTTT	TTTGAAAAAGTGAGGGGTGG	CTTTGGTGGTGGTCAGAGGT	59.9	956	INTERGENIC				
CaPOPII_2357	Ca6	5645292	CTATATATATATATATATATATAT/CTATATATATATATATATATATATAT	GTAAACCCAAGAGAGGTGCG	TTCTAAATGGCGACTTTCCA	59.7	923	INTERGENIC				
CaPOPII_2358	Ca6	5765272	TTTTTTATTTTTATTTTTATTTTTATT/TTTTTTATTTTTATTTTTATT	CTCTTGGACATGTGGTGGTG	TCCCTTTCGTTTTGGTTGAC	60.0	541	INTERGENIC				
CaPOPII_2359	Ca6	5846506	TAAAAAAAAAAAAA/TAAAAAAAAAA	TGATTAGGCCTGAATTGTGGT	GCAAGTTGCCAAGAAAAAGC	59.4	461	INTERGENIC				
CaPOPII_2360	Ca6	6082681	TTATATATATATATATATATAT/TTATATATATATATATATATATATAT	CGCACTAGACTAAATGCGTGC	GCCCTTTGACGTCCAACTAA	61.0	442	INTERGENIC				
CaPOPII_2361	Ca6	6089953	ATTTTTTTTT/ATTTTTTTTTT	CGAATATCAAGCACGCACAT	GCAACGGCAAGGATTGTTAT	59.7	921	INTERGENIC				

INDEL marker IDs	Chromosomes /unanchored scaffolds	Physical positions (bp)	InDels (*Kabuli* reference genome-CDC Frontier/PI)	Forward primers (5'-3')	Reverse primers (5'-3')	Anneling temperature (0C)	Expected amplified product size (bp)	Structural annotation		Functional annotation		
								Sequence components of *kabuli* genome	*Kabuli* gene accession IDs	NCBI-KOG	TFs	NCBI-nr database
CaPOPII_2362	Ca6	6158112	GTATATATATATATAT/GTATATATATATATATAT	GATGAGTGGAAGGAGCCAAA	TGAAAGATAATCCAATTTTAAATGAGT	60.2	418	INTERGENIC				
CaPOPII_2363	Ca6	6163420	ATTTTTTTTT/ATTTTTTTTTT	CCGTCCCTTAGCAAAATTCA	AAGCAACTATGGCAAATGGG	60.1	443	DRR	Ca_05680	T	G2-like	Arf GTPase activating protein
CaPOPII_2364	Ca6	6411336	AAT/AATAT	ATCTCGTGGTGCAACAATCA	TTTGGGAAGGGTCAAGAAAA	60.1	628	INTERGENIC				
CaPOPII_2365	Ca6	7586339	GTGATGATGATGA/GTGATGATGATGATGA	GCACCTCTTGGACCATCAAT	ACAGTGTCAGTGCCTACCCC	59.9	454	CDS (large-effect mutations)	Ca_09626	C		Mitochondrial carrier protein
CaPOPII_2366	Ca6	7644876	ATGTGTGTGTGTGTGT/ATGTGTGTGTGTGT	GACTTCGTTCGTGCTTCCTC	GCCACCGTTGTGTTCCTTAT	60.0	495	INTERGENIC				
CaPOPII_2367	Ca6	7708884	AAGAG/AAG	CTCATCACACTGGGCAAATG	TGTTCATTTCCAATGCCAAA	60.1	983	INTERGENIC				
CaPOPII_2368	Ca6	7806679	TA/TACA	TCAAGGGATCCCCATAACAA	TTCACATTCCCCATTAAGCC	60.1	742	INTERGENIC				
CaPOPII_2369	Ca6	7972862	GTATATATATAT/GTATATATATATATAT	CTCATTGACTTGGGATCCGT	GAGAAAAGGTGGATCCTACATACC	59.9	921	INTERGENIC				
CaPOPII_2370	Ca6	7985102	TTT/TTTAATT	TCAACAGGATTTGCCAACTG	CACACCACCAAACGTTGAAG	59.7	473	INTRON	Ca_09577	G		Trehalose-phosphatase
CaPOPII_2371	Ca6	7997168	AAAAAATTAT/AAAAAATTATGAAAAATTAT	GCTTGCATTCAGTCAGACCA	GACACATGGTTGCACGATTC	60.0	625	INTERGENIC				
CaPOPII_2372	Ca6	8022121	TAATTAAAAATTAAAAAT/TAATTAAAAATTAAAAATTAAAAAT	TTTGTTTGCGATGGCTCATA	TGTTTTTCCGAGCGTACACA	60.2	818	INTERGENIC				
CaPOPII_2373	Ca6	8087365	ATAATTAA/ATAA	CGTTCAACTGAAATTTGCCA	ATCCACACTTCCAGCCTGAT	59.7	450	INTRON	Ca_09562	S	CAMTA	IQ motif, EF-hand binding site
CaPOPII_2374	Ca6	8410500	CAA/CA	AATCGATTGATTCAGCCCAG	TAATGCCCCGTTTTTCAAAG	60.0	795	INTERGENIC				
CaPOPII_2375	Ca6	8705473	AAGAGAGAGAGAG/AAGAGAGAGAG	GCTTGTGGGGATGTTAAACC	GAAGGGAATGAAATGCGAAA	59.3	492	INTERGENIC				
CaPOPII_2376	Ca6	8796967	A/AG	GCCAAACAATAGTTGAAACCAA	AACGTTACGCATGAACCTCC	59.0	588	INTRON	Ca_16754	E		Amino acid transporter, transmembrane
CaPOPII_2377	Ca6	8834153	ATTTTTTTTT/ATTTTTTTT	TTGCAGATTTTTGAGAAGTTCA	TTTGGTGGGTGATAGGCAAT	57.7	401	INTERGENIC				
CaPOPII_2378	Ca6	9014214	CA/CAA	TACTGGGGTGTCTGGTGTGA	AGCTGCACGCAGACTGTAAA	60.0	441	INTERGENIC				

INDEL marker IDs	Chromosomes /unanchored scaffolds	Physical positions (bp)	InDels (*Kabuli* reference genome-CDC Frontier/PI)	Forward primers (5'-3')	Reverse primers (5'-3')	Anneling temperature (0C)	Expected amplified product size (bp)	Structural annotation		Functional annotation		
								Sequence components of *kabuli* genome	*Kabuli* gene accession IDs	NCBI-KOG	TFs	NCBI-nr database
CaPOPII_2379	Ca6	9060428	TTATAAAACATATAAAA/TTATAAAA	AAAGTACAATGAGTCAAACAATTATCC	ATATGCTTCACTTGCCCCAC	58.2	555	DRR	Ca_16733	E		Amino acid transporter, transmembrane
CaPOPII_2380	Ca6	9203559	GAA/GTTATATTAA	TCGTGAAATAGTGGACGGTG	TGAACATCAAAATGCCCAAA	59.6	763	DRR	Ca_08676	OT		Peptidase, cysteine peptidase active site
CaPOPII_2381	Ca6	9873238	GTTCAAC/GTTCAACTTCAAC	AACTGTGGAACCAGACAGGG	AACATCGACCGGATTTAACG	60.0	544	INTRON	Ca_08602	F		Xanthine/uracil/vitamin C permease
CaPOPII_2382	Ca6	9998146	AATATATATATATATATA/AATATATATATATATATA	AGTGCATGTTCCATGACGTG	CTCACTTACCCTCGCTCCTG	60.6	116	INTERGENIC				
CaPOPII_2383	Ca6	10090816	ATTATTTATTTATTTATTTATTTATTTATTTATTTATTTATT/ATTATTTATTTATTTATTTATTTATTTATTTATTTATT	CCCGCAAAATTAAGTGGGTA	CCCATACCCGTTATCCACAC	59.8	560	DRR	Ca_08583	Z	Trihelix	Tubulin
CaPOPII_2384	Ca6	10127292	CAA/CA	TGGTTGCGACTTGTGTTGTT	CCGACTCAAATCAAAGGGAG	60.2	100	INTERGENIC				
CaPOPII_2385	Ca6	10141273	TAAAAAAAAAAA/TAAAAAAAAAA	GGAAATATCAAATGTTGTTACCGA	TCCAAAAACGACTTAGACCTCA	59.2	736	INTERGENIC				
CaPOPII_2386	Ca6	10501710	ACC/AC	TTGATTGCTTTTGTTGCTGC	GAACTGCAACTCCACCCAAT	60.0	461	INTRON	Ca_08546			Chalcone/stilbene synthase, N-terminal
CaPOPII_2387	Ca6	10503058	AATATATA/AATATA	AAACATGCTTTTGCATTTTTGTT	GACTTCAACCGGTAGCTGGA	59.9	407	INTERGENIC				
CaPOPII_2388	Ca6	10510710	AGG/AG	CGACTAGATGTCGCTGACCA	CACATTTTTGGTGTGCTTGG	60.0	385	INTRON	Ca_08544	S		Protein of unknown function DUF803
CaPOPII_2389	Ca6	10514411	CAAAAAAAAA/CAAAAAAAAAAA	TTCGCAATCTGTGTTTTTCG	CAAGAATTCGTATCGTCTAAATGC	59.8	642	INTERGENIC				
CaPOPII_2390	Ca6	10514563	TAAAAAAAAAA/TAAAAAAAAAAAAA	TCACCAATCCTATGCTGTTGTC	ACGAAGTGACACATGACCCA	60.0	924	INTERGENIC				
CaPOPII_2391	Ca6	10556820	ATTT/ATTTT	TATGACGGGCACACTTGAGA	CGTCATTTGCAAAAACGACTT	60.3	474	INTERGENIC				
CaPOPII_2392	Ca6	10572244	TAAA/TAA	CCGATGTAAGACTCTCCCCA	TGTTTCAACCTTGCATTGGA	60.1	790	INTERGENIC				
CaPOPII_2393	Ca6	10575764	ATTTTTTT/ATTTTTTTT	TTTGTGTTGTGTGTTGTCGC	CCCCATGATACCCTTTCCTT	59.2	294	INTERGENIC				
CaPOPII_2394	Ca6	10586822	CAAAAAAA/CAAAAAAAA	TTCCCAGTTCTTTCACCCAG	AACTGTAACACACGTGGCGA	60.1	573	INTERGENIC				
CaPOPII_2395	Ca6	10617092	C/CA	TTGTTTAATTGTTGGGGGAGA	AACGTAAGTTGGCACCATGC	59.3	222	INTERGENIC				

INDEL marker IDs	Chromosomes /unanchored scaffolds	Physical positions (bp)	InDels (*Kabuli* reference genome-CDC Frontier/PI)	Forward primers (5'-3')	Reverse primers (5'-3')	Anneling temperature (0C)	Expected amplified product size (bp)	Structural annotation		Functional annotation		
								Sequence components of *kabuli* genome	*Kabuli* gene accession IDs	NCBI-KOG	TFs	NCBI-nr database
CaPOPII_2396	Ca6	10620292	TAAAAAACA/TAAAAAACAAAAAAACA	CAGTTGTGGCGATTGATTTG	GGATCTGAAATCCCCTCTCC	60.1	836	INTERGENIC				
CaPOPII_2397	Ca6	10625658	AA/AATA	GCTCTCCAAGACTTCAACGG	TCCAACCTTCCCTCATCTTG	60.0	782	INTERGENIC				
CaPOPII_2398	Ca6	10642500	C/CCTATAGA	CGATTTGACAGCAAAAGCAA	GACAACCTTCGTTGCCCTAA	60.0	437	INTERGENIC				
CaPOPII_2399	Ca6	10649918	GTTTGTTTTGTTTTGTTTT/GTTTGTTTTGTTTTGTTTTGTTTT	GACGGTTATGGAGCCTTCAA	TTCGATATCTTGTGCCTCCC	60.1	363	DRR	Ca_08531	I		AMP-dependent synthetase/ligase
CaPOPII_2400	Ca6	10652240	TA/T	TTCGAATTTTCATGGTGGGT	GATAAGCGACGTCTTGGAGC	60.2	804	INTRON	Ca_08531	I		AMP-dependent synthetase/ligase
CaPOPII_2401	Ca6	10660543	G/GCC	TGATTGGACTGCCTCACAAG	GGCCTCAGATCAGTGGAAAA	59.8	617	INTRON	Ca_08531	I		AMP-dependent synthetase/ligase
CaPOPII_2402	Ca6	10700691	AG/A	ATAAAGAGAAGCGGAGGGGA	CAACGAAGAGCGTCGAGAAT	60.2	909	INTERGENIC				
CaPOPII_2403	Ca6	10708022	GCT/GCTGAATCT	CCAAGGGTTGGCTAGAACTG	TGGAAAATATGTGTGGGATCAG	59.7	689	DRR	Ca_08527	GC	HSF	UDP-glucuronosyl/UDP-glucosyltransferase
CaPOPII_2404	Ca6	10848271	TGGC/TGGCGGC	GGCCTCTTTCAACAAATGGA	ACAAATTTGGTGGTGGTGGT	60.1	311	CDS (large-effect mutations)	Ca_08513	K	MIKC	Transcription factor, MADS-box
CaPOPII_2405	Ca6	10968845	AATATATATATATATATATATATATAT/AATATATATATATATATATATAT	TGAAAACTTCATTTTCTTTATTGAGTC	GCACATGGTTGCATGAAAAA	58.5	637	INTERGENIC				
CaPOPII_2406	Ca6	11310241	GTTTTGCATA/G	CATGTCGAAGAAGCTCCACA	TCCTCCGGAACATAACTTGG	60.0	417	INTERGENIC				
CaPOPII_2407	Ca6	11316099	AC/A	GTTTGTTGTGGTTGCAGTGG	GCTGATTGGTTGCCTCTCTC	60.0	399	INTERGENIC				
CaPOPII_2408	Ca6	11546028	CAAA/CAAAA	GATCGATTTGGGCTACGAAA	ATCAGATTGCTGCAGGCTTT	60.0	651	INTERGENIC				
CaPOPII_2409	Ca6	11546493	TAAAAAAAAAAAAAAA/TAAAAAAAAAA	TCGGAAAGGTCATTCAAAGC	TTTGTGTCCAAAGCATTCTCC	60.2	472	INTERGENIC				
CaPOPII_2410	Ca6	11590997	CTTTT/CTTTTT	ATGTGCTCCCCACAAAAGTC	CCCTCCAGTAGCATCACCAT	60.0	538	INTERGENIC				
CaPOPII_2411	Ca6	11632067	GGTGTG/GGTG	CCAAAGTTAGCCCTTCCTCC	AGCACTGACCTTTTGAGCGT	60.1	445	DRR	Ca_25057	S		DNA methylase, N-6 adenine-specific, conserved site
CaPOPII_2412	Ca6	11685034	TGGG/TGG	ATCGCCAGCAACTTTAGCAT	GGGAACCAAAGTGTTGACCA	59.9	508	INTERGENIC				

INDEL marker IDs	Chromosomes /unanchored scaffolds	Physical positions (bp)	InDels (*Kabuli* reference genome-CDC Frontier/PI)	Forward primers (5'-3')	Reverse primers (5'-3')	Anneling temperature (0C)	Expected amplified product size (bp)	Structural annotation		Functional annotation		
								Sequence components of *kabuli* genome	*Kabuli* gene accession IDs	NCBI-KOG	TFs	NCBI-nr database
CaPOPII_2413	Ca6	11738985	TAAAAAAAA/TAAAAAAA	TTCAGCAGCTTCTTCGACAA	ATTACCGACATTGCAGAGGG	59.9	254	INTERGENIC				
CaPOPII_2414	Ca6	11746077	AATATATATATATATATATATA/AATATATATATATATATATATATATATA	TGCAAGTCGAAATTCACACG	TGGGTCAAGCCCAATAAAAC	60.8	538	INTERGENIC				
CaPOPII_2415	Ca6	11755125	GTCAAAGATTTTCTTCAA/GTCAA	CGATCATGTTCGTATTTTGTTCA	TGAAATTCCCTGAGCTGGTC	59.9	527	INTERGENIC				
CaPOPII_2416	Ca6	12233101	TG/TGG	CCTCAACGTGAGTTGCAGAA	TGCATGGCATTTGGATAAGA	60.0	344	CDS (FRAME SHIFT)	Ca_24847			
CaPOPII_2417	Ca6	12335628	TGAAG/TG	GGAAGGGTGTTGGTGTGTTT	TTGGTCACTAAGGGGGTGAG	59.7	968	DRR	Ca_24849			
CaPOPII_2418	Ca6	12534444	GTTTTTTTTTT/GTTTTTTTTT	GCTTTCTCTGCTGGAATTGG	TGGAGCCACGTGTTTGTATC	60.0	188	INTRON	Ca_05031	R		Sugar/inositol transporter
CaPOPII_2419	Ca6	12565656	TTTATTATTATTATTATTATTATTATTATTATTATTATTATT/TTTATTATTATTATTATTATTATTATTATTATTATTATTATTATT	ACATCCAATGAAATAGCCGC	AAAATAGCCTTTTCGCCACC	59.9	825	INTERGENIC				
CaPOPII_2420	Ca6	12787897	AATATATATATATATATATATATAT/AATATATATATATATATATAT	ATCAAACAAATGTTGCGAGC	CAAACCCCTATTTTTGTGATTGA	58.8	749	INTERGENIC				
CaPOPII_2421	Ca6	12861842	ATTTTTTT/ATTTTTT	TCAATCCCTTCATTCGGAAG	TCGATAGTGGGTTTCGTGTTC	60.0	612	INTERGENIC				
CaPOPII_2422	Ca6	12868319	TTGCG/T	GCATGTCAAGATAATGGGCA	CCGCATAACCAAACACTCAC	59.5	637	INTERGENIC				
CaPOPII_2423	Ca6	12870795	TAAAAAAAAA/TAAAAAAAAAAA	CATCCCAAGCAAAAACGAAT	CATTGAGAGGATCCAAAATCAA	59.9	533	INTERGENIC				
CaPOPII_2424	Ca6	12877302	CG/CGCTTTTTTTG	GTTCGTAAAGGACGGGATGA	CTTTTACAGCGCTTTCTGGG	59.9	235	INTERGENIC				
CaPOPII_2425	Ca6	12888429	T/TC	CCGTCAATTTATATCCTCCCA	AATCAGGGTGCCATTGTAGC	58.8	733	INTERGENIC				
CaPOPII_2426	Ca6	12898258	TAAAAAAAAAA/TAAAAAAAAAAA	ACTACCATGTTTGGTGGGGA	CACATTGAAAATCAACTTTACACCA	60.1	646	INTERGENIC				
CaPOPII_2427	Ca6	12905966	T/TGA	AGAGTTGTTCGCGAAAAGGA	TTACGCACCGGACATAATGA	60.0	203	INTRON	Ca_05064	T	bHLH	Protein kinase, catalytic domain
CaPOPII_2428	Ca6	12931395	CAAAAAAAAAA/CAAAAAAAAAAAA	CACGGTTTACATGGGCAATA	ATCTGGTATCAAATGCCCCA	59.3	493	INTERGENIC				
CaPOPII_2429	Ca6	12945433	G/GATTCAGAT	TGGTGGTGTAGGTCCACAGA	TTCACGGTGTTCAAATTCGT	60.0	415	INTERGENIC				

INDEL marker IDs	Chromosomes /unanchored scaffolds	Physical positions (bp)	InDels (*Kabuli* reference genome-CDC Frontier/PI)	Forward primers (5'-3')	Reverse primers (5'-3')	Anneling temperature (0C)	Expected amplified product size (bp)	Structural annotation		Functional annotation		
								Sequence components of *kabuli* genome	*Kabuli* gene accession IDs	NCBI-KOG	TFs	NCBI-nr database
CaPOPII_2430	Ca6	12957525	CAAA/CAAAA	CGGTTCACCTGTTTGCTTTT	TGAAAAGCTTGAGGAAGGGA	60.1	718	INTERGENIC				
CaPOPII_2431	Ca6	12964933	TCATTA/T	CTTGTTGCCAAGTAGGGTCG	CCAAGGGAGAGGTGCTTAAA	60.7	976	INTERGENIC				
CaPOPII_2432	Ca6	13128943	CTTTTTTTTTTT/CTTTTTTTTTT	CACCACGAGATCAAAACCCT	CCATTGGCAGTGGAACTCTT	60.0	482	INTERGENIC				
CaPOPII_2433	Ca6	13170216	AATATATA/AATATATATA	TCAACCAAGAATTGCTGCTAAA	AGGGAGTATTCACGGCACAC	59.9	502	INTERGENIC				
CaPOPII_2434	Ca6	13180103	TTTATTATTATTATTATTATTATTATTAT/TTTATTATTATTATTATTATTATTATTATTAT	TTTGAGTCAGTCAACGCAGG	TAAACCATTCAAACCCCCAA	60.0	830	INTERGENIC				
CaPOPII_2435	Ca6	13381796	TAAAAAAAAAAAA/TAAAAAAAAAAA	GGGTTCGTAGTTGTATGCGAA	CATGGATCAGAGTTGCCTTG	60.0	883	INTERGENIC				
CaPOPII_2436	Ca6	13475824	AAAGAAGAAGAAGAAGAAGAA/AAAGAAGAAGAAGAAGAA	TGTGGTCCCAAACAATCTCA	TTTTGGGTCAAGGAAAATGG	59.9	330	INTERGENIC				
CaPOPII_2437	Ca6	13838996	CCT/CCTTCT	TGTGTGATTGGTGTTGAGCA	GCAACAACGGGTGAAAATCT	59.7	563	INTERGENIC				
CaPOPII_2438	Ca6	14410304	TTTTATTTATTTATTTATTTATTTATTTATTTATTTATTTAT/TTTTATTTATTTATTTATTTATTTATTTAT	TTGTGCATGGTTGTGGATCT	TCTCAAGTTTGTAAGCGAGTCAA	60.0	193	INTERGENIC				
CaPOPII_2439	Ca6	14755534	AATATATATATATATATATATATATAT/AATATATATATATATATATATATATATATAT	TTTTAACCGTGGTTTGAAACTT	AGTCAGACGCTACTCGGCAT	57.8	212	INTERGENIC				
CaPOPII_2440	Ca6	14938058	TAAAAAAAAAA/TAAAAAAAAAAA	CCTCCTACATTGTGGGTGCT	ACATTTGGCCCATTTGGTTA	60.0	754	INTERGENIC				
CaPOPII_2441	Ca6	14959810	CTTTTTTTTTTT/CTTTTTTTTTT	GCGAAATGCTTCTCAAAACC	CGTTACAACGTTGCTGAATGA	59.8	745	INTERGENIC				
CaPOPII_2442	Ca6	15277411	AATTATTATTATTATTATTATTATTATTATTATTATTATTATT/AATTATTATTATTATTATTATTATTATTATTATTATTATTATT	CGTCTTTCTAATATTTTGCCGA	CATGAAAGTGGTCAGATGCG	58.5	700	INTERGENIC				
CaPOPII_2443	Ca6	15508917	TTATATATATATATATATATATATAT/TTATATATATATATATATATATATAT	TGTTTGATTGGGTTGGTTCA	TGGGTGCTTTATTAAGATGGG	59.8	849	DRR	Ca_05334			
CaPOPII_2444	Ca6	15555599	AATATATA/AATATA	TGTGGGCACATTAGTAGGCA	TCATCGTCGTTGCTTGAGTC	60.1	640	INTERGENIC				
CaPOPII_2445	Ca6	15559125	CTTT/CTT	TCACCAAACAAAAACTGGGG	CTACATTCCGGTTAGGGCAA	60.8	480	INTERGENIC				
CaPOPII_2446	Ca6	15597965	TAAA/TAAAA	CAGTTTCCACACGCACAGAT	TGGGAACAAGCATGGTGTAA	59.8	681	DRR	Ca_05341		bHLH	Terpene synthase-like

INDEL marker IDs	Chromosomes /unanchored scaffolds	Physical positions (bp)	InDels (*Kabuli* reference genome-CDC Frontier/PI)	Forward primers (5'-3')	Reverse primers (5'-3')	Anneling temperature (0C)	Expected amplified product size (bp)	Structural annotation		Functional annotation		
								Sequence components of *kabuli* genome	*Kabuli* gene accession IDs	NCBI-KOG	TFs	NCBI-nr database
CaPOPII_2447	Ca6	15727514	ATTTATTTTATTTTATTTTATTT/ATTTATTTTATTTTATTTTATTTTATTT	TTAGTTTGGGCTTGGGTTTT	TTGCTCCTACGTATCTCCCG	58.6	911	INTERGENIC				
CaPOPII_2448	Ca6	15730699	TAAA/TAA	ATTTGAACCTGGCAGCAAAA	GGTTTGAAAAAGGGGTGATG	60.6	175	INTERGENIC				
CaPOPII_2449	Ca6	15730843	T/TGA	ATTTGAACCTGGCAGCAAAA	GCTTGATGAATAAATCAATGGCT	60.6	525	INTERGENIC				
CaPOPII_2450	Ca6	15731057	TAAAAA/TAA	ATTTGAACCTGGCAGCAAAA	GGGACAAAAGGCTAGTTTGG	60.6	909	INTERGENIC				
CaPOPII_2451	Ca6	15946706	GATATATATATATATAT/GATATATATATATATATAT	TGTTGCAGTCATGCTTTTGA	TGAATTCTGCTATGCGCAAC	59.0	306	DRR	Ca_22027	C		Pyridine nucleotide-disulphide oxidoreductase, NAD-binding region
CaPOPII_2452	Ca6	15966204	GTTATTATTATTATTATTATTATTATTATTATTATTATTATTATTATTA/GTTATTATTATTATTATTATTATTATTATTATTATTA	CTGCCTGAAATTTGGTTGCT	AGATGCGACCTCCTACTTGC	60.2	229	INTERGENIC				
CaPOPII_2453	Ca6	15999975	A/AG	AAGTGTGGGCATTTCCTTTG	ATGGACCTCTGTATTCGCCA	60.0	137	INTERGENIC				
CaPOPII_2454	Ca6	16012701	TA/TAA	TAAGAGTTTGGTTCCGGTGG	TGTGCAGATAAATGGCCAAA	60.0	397	INTERGENIC				
CaPOPII_2455	Ca6	16093896	AAAAAATATATAAAAATATA/AAAAAATATA	TGCAAAAAGATGGCAATGAA	TTTCCTTAAATATCCATCCAGTCA	60.2	598	INTERGENIC				
CaPOPII_2456	Ca6	16547931	GA/GAGAGCTTA	TTCCTTCCTGCTCGTGCTAT	TTCTTTGGCTGAAATGGTCC	60.0	640	INTRON	Ca_06284	A		PSP, proline-rich
CaPOPII_2457	Ca6	17025431	ATTTTTTTTTTTT/ATTTTTTTTTTT	TTTTGCTCCACGTTTTCCTT	GGCTTATAGGCCCACGTTTT	59.7	562	INTERGENIC				
CaPOPII_2458	Ca6	17043003	CTTTTTTTTT/CTTTTTTTTTT	CGAGATTTAAAACAAAATTGGGA	TATCTTGGTGGGACTGGGAG	59.4	533	DRR	Ca_06325			Protein of unknown function DUF3223
CaPOPII_2459	Ca6	17044026	AAATAATAATAATAATAATAATAATAATAATAATAATAATAATAATAA/AAATAATAATAATAATAATAATAATAATAATAATAATAATAA	AATTGGGGCATGTGCTATTG	TGCTGCTGCAATTTGATTTC	60.7	874	DRR	Ca_06325			Protein of unknown function DUF3223
CaPOPII_2460	Ca6	17083127	AGA/AGAATTTTGA	TTAGCTGAATCACGAACCCC	TGATTCGCGAAAATAGTCCC	60.1	271	INTERGENIC				
CaPOPII_2461	Ca6	17085352	TTTTATTTATTT/TTTTATTTATTTATTT	CGCAAAAATGCAAATGACTG	TTCCCCATGCATCACTATCA	60.3	385	INTERGENIC				
CaPOPII_2462	Ca6	17092081	A/AG	TGCACCCCTAATTTTAAGCAA	GGATTTTGGGGAGCTTGAA	59.6	468	DRR	Ca_06327	C		Mitochondrial substrate/solute carrier
CaPOPII_2463	Ca6	17503155	GATATATATATATAT/GATATATATATATATAT	ATTCCCATTAAGTGCCCACA	TCCAAATGGCGACTTCCTAC	60.2	556	INTERGENIC				

INDEL marker IDs	Chromosomes /unanchored scaffolds	Physical positions (bp)	InDels (*Kabuli* reference genome-CDC Frontier/PI)	Forward primers (5'-3')	Reverse primers (5'-3')	Anneling temperature (0C)	Expected amplified product size (bp)	Structural annotation		Functional annotation		
								Sequence components of *kabuli* genome	*Kabuli* gene accession IDs	NCBI-KOG	TFs	NCBI-nr database
CaPOPII_2464	Ca6	17930276	ATTTTTTT/ATTTTTTTT	TCCTCCCTTAATTCATTCGTG	TCAGTTGGTGACCCATGAAC	59.0	391	INTERGENIC				
CaPOPII_2465	Ca6	18331022	G/GTTCCGGGTATC	TATATTGCTGCCACAATCGC	TTTCCTCCTTGGAATCATGG	59.7	402	INTRON	Ca_06449	QI	B3	Cytochrome P450
CaPOPII_2466	Ca6	18780292	TAAAAA/TAAAA	AAGGCACGTAACCAAATTGC	TGAATTAGGATGTGCTTCAAGG	60.0	771	INTERGENIC				
CaPOPII_2467	Ca6	19524549	GTTTTTTTTTTT/GTTTTTTTTTTTT	AAACTCGAAAAGCATGGTGG	GCCCAAAATTGCCTCTATGA	60.1	623	DRR	Ca_06559			
CaPOPII_2468	Ca6	20679328	CATATATATATATATAT/CATATATATATATATATAT	CAAATTTGTCACGGAAAGCA	CAATACTTGTGTTGTGGCGG	59.7	605	INTERGENIC				
CaPOPII_2469	Ca6	21021586	CTATTTATTTATTTATTTATTTATTT/CTATTTATTTATTTATTTATTT	CACCGTCGTGTTCATCTGTC	TTATTTCACCGTCGTGTCCA	60.2	458	INTERGENIC				
CaPOPII_2470	Ca6	21100809	ATTTTTTTTTTTT/ATTTTTTTTTTTTT	TGAGGGATTCCTCGAAGTTG	TGTGATTTGTTGTGGGAAGAA	60.2	490	INTERGENIC				
CaPOPII_2471	Ca6	21139884	ATTTTTTTT/ATTTTTTTTT	CAGAAGGATTGCTCGTGTGA	TGCATAGGCAAAAATGATTGA	60.0	528	INTERGENIC				
CaPOPII_2472	Ca6	21141563	GTTTTTT/GTTTTT	TCCGATAGGAAGAGCCAATG	ACGTTTATTTTTCGGACCCC	60.2	697	INTERGENIC				
CaPOPII_2473	Ca6	21401312	C/CT	AGCTCAGTCCTCACAACCGT	TTAGATCGGACAAGAAACCGA	59.9	344	INTERGENIC				
CaPOPII_2474	Ca6	21408015	ATTTTTTTT/ATTTTTTT	AGGCCTTTTATTTCGTTGCC	TTGAATCGCAAAACAACCAA	60.4	897	DRR	Ca_19860			Methyltransferase type 11
CaPOPII_2475	Ca6	21470474	CAAAAAAAAAA/CAAAAAAAAAAA	TTTCACATATCCTATCAAACACATTTT	TTCGTTGCTCCAAAGACTCC	59.2	367	INTERGENIC				
CaPOPII_2476	Ca6	21485595	GTTTTTTT/GTTTTTTTT	TGCGGGCTAGCTCACTTAAT	TCAAAACAAATAGGCAGGGG	60.0	115	INTERGENIC				
CaPOPII_2477	Ca6	21485767	ACCCC/ACCCCC	TGCGGGCTAGCTCACTTAAT	TTTGATTCTCTGGCCGAGTT	60.0	263	INTERGENIC				
CaPOPII_2478	Ca6	21739441	AATATATATATATATATATATATATATATATATAT/AATATATATATATATATATATATATATATATATATATATATATATAT	TGAAGAACCATGGTCATATTAGACA	AAACAAGGCTTCAACCCTGA	59.8	569	INTERGENIC				
CaPOPII_2479	Ca6	21777762	TCCTCT/TCCTCTTATAAGCCCCCTCT	GATTGTCAGCCCAAAAGAGC	TGAGGAACGACAGATCGAAA	59.8	753	DRR	Ca_11261		ERF	Pathogenesis-related transcriptional factor/ERF, DNA-binding
CaPOPII_2480	Ca6	21801686	GA/GAA	TCTTGAACCGATACCCTTTTT	GACGGATGAGCATCGGTTAT	57.7	311	INTERGENIC				

INDEL marker IDs	Chromosomes /unanchored scaffolds	Physical positions (bp)	InDels (*Kabuli* reference genome-CDC Frontier/PI)	Forward primers (5'-3')	Reverse primers (5'-3')	Anneling temperature (0C)	Expected amplified product size (bp)	Structural annotation		Functional annotation		
								Sequence components of *kabuli* genome	*Kabuli* gene accession IDs	NCBI-KOG	TFs	NCBI-nr database
CaPOPII_2481	Ca6	21891223	TTCTC/TTC	GGGAAGACCTTGGATTCACA	CAATACCGTCCTGCACATTG	59.9	517	INTRON	Ca_11250	S		Prefoldin subunit
CaPOPII_2482	Ca6	21919231	TTA/TTAATA	CTTGCATGGGTACGTTAGCA	ACTTAGCACGGGCTCGATAC	59.8	370	DRR	Ca_11246	O		
CaPOPII_2483	Ca6	22099449	ATTTTTTTTT/ATTTTTTTTTTTT	TGTGTTGCTTCTTGGCTTTG	ACGTGTGGGTTTCAGGTCTC	60.0	620	INTERGENIC				
CaPOPII_2484	Ca6	22124013	TTCTCTCTCTCTCTCTCTCTCT/TTCTCTCTCTCTCTCTCTCT	ATTCCAACGATCCAAGATGG	TGCTCCAATGATGTTAGAAACG	59.8	692	INTERGENIC				
CaPOPII_2485	Ca6	22128416	CTTTTTTTTTT/CTTTTTTTTT	CACACACACACAAAGAGGGG	ATAGGACCTGCTGCCTGAGA	60.0	557	INTERGENIC				
CaPOPII_2486	Ca6	23042823	CATATATATATATAT/CATATATATATAT	CAACGATCTGCACCACACAT	GGTGCAGCAAGTGAGCAATA	60.6	665	INTRON	Ca_11143		ERF	Glycoside hydrolase, family 17
CaPOPII_2487	Ca6	23042981	TAAA/TAA	CAACGATCTGCACCACACAT	GGTGCAGCAAGTGAGCAATA	60.6	665	INTRON	Ca_11143		ERF	Glycoside hydrolase, family 17
CaPOPII_2488	Ca6	23064745	CA/C	TGGGTTGATTTTCCCACATT	TCTCGAGCAAATCACAAGTTAAA	60.0	571	INTERGENIC				
CaPOPII_2489	Ca6	23078754	TGG/TGGG	TGGACAATCGCCCAAATTAT	TCTCGAGCAAATCACGAGTTA	60.2	716	INTERGENIC				
CaPOPII_2490	Ca6	23078938	T/TC	TGGACAATCGCCCAAATTAT	TCTCGAGCAAATCACGAGTTA	60.2	716	INTERGENIC				
CaPOPII_2491	Ca6	23090578	AC/A	GCCAAGCTTGTTTGTTGGTT	TGTGTGCACATGTTGTTCGT	60.2	490	INTERGENIC				
CaPOPII_2492	Ca6	23409253	CTTTTT/CTTTTTT	TGCCGTATAAAATGGGCTTC	CTTGCTCTTCCTTCGTTGCT	59.9	533	INTERGENIC				
CaPOPII_2493	Ca6	23642077	T/TTTCC	GATTACCATGGTTGGGATCG	CAATTTCGGTGCTTGTTCCT	60.0	137	INTRON	Ca_11091	R		
CaPOPII_2494	Ca6	24296638	CATATATATATATATATATATATA/CATATATATATATATATATATATA	GAGAAGGAGGGAAAAATGGC	ATGTTTTGGATTTTGGCTCG	60.0	421	INTERGENIC				
CaPOPII_2495	Ca6	24582806	CATATATATATATATATATATATATATAT/CATATATATATATATATATATATATATATATAT	TCGTTTTGTTTATTGGGGGA	GTTAAATGCGGGGTTTATCG	60.2	565	DRR	Ca_24169		bHLH	Protein kinase, catalytic domain
CaPOPII_2496	Ca6	24637049	TTTCTTCTTCTTCTTCTTCTTCTTCTT/TTTCTTCTTCTTCTTCTTCTTCTTCT	TGTTTTTGGTGAAACGTGGA	AGGATGAATGTGGTGTTGAAGA	60.0	376	INTERGENIC				
CaPOPII_2497	Ca6	24744388	TACACACACA/TACACA	TTTGGATTTTGACTCGGAGG	AATGGAGGAAGGAGGGAGAA	60.0	384	INTERGENIC				

INDEL marker IDs	Chromosomes /unanchored scaffolds	Physical positions (bp)	InDels (*Kabuli* reference genome-CDC Frontier/PI)	Forward primers (5'-3')	Reverse primers (5'-3')	Anneling temperature (0C)	Expected amplified product size (bp)	Structural annotation		Functional annotation		
								Sequence components of *kabuli* genome	*Kabuli* gene accession IDs	NCBI-KOG	TFs	NCBI-nr database
CaPOPII_2498	Ca6	25130968	A/AATAAAGCCT	CCAAAAAGGGTGGAGATTGA	CCTTTTACAGCGCTTTTCCA	59.9	275	DRR	Ca_26068			
CaPOPII_2499	Ca6	26277672	T/TG	TTAGGTGCGCTTGACTGATG	GTTTTGGCGTTGGTTTCACT	60.0	558	INTERGENIC				
CaPOPII_2500	Ca6	26354164	GT/GTT	TGTTGATTTGGGGAAAGAGG	GGTGCCAACATCCAACTTCT	59.9	650	DRR	Ca_16678		GRAS	Transcription factor GRAS
CaPOPII_2501	Ca6	26438751	ATTTTTTTTT/ATTTTTTTTTTT	TCAGACACTACCCAGCATCAA	CTTAGGCCGCCACAAAATA	59.3	960	INTERGENIC				
CaPOPII_2502	Ca6	26668470	T/TCTATATTTTTTTA	CGACGCAATGCATTAAGAGA	CCTTAATCGCCGAAAATCAT	60.0	490	DRR	Ca_16695			Thaumatin, pathogenesis-related
CaPOPII_2503	Ca6	26790864	GAAAAAAAA/GAAAAAAA	CCGAAAATTTGAAAATCCGA	CAAGTACAGGTGGAGTGGCA	59.9	728	INTERGENIC				
CaPOPII_2504	Ca6	26931819	TTATATATATATATATAT/TTATATATATATATATATATAT	GTACCACATTGCCGAGGTTT	GCTTGCCAAAATAAATGGCT	59.9	580	INTERGENIC				
CaPOPII_2505	Ca6	27165011	ATATTATTATTATTATTATTATTATTATT/ATATTATTATTATTATTATTATTATTATT	CATTGTTTGATCCATGTCGC	CCATCATTGACACTCGAAAGAA	59.9	675	INTERGENIC				
CaPOPII_2506	Ca6	27264510	ATTTTTTTTT/ATTTTTTTT	AACGTCCCATTAAGTTCGCA	TGACAAATTGCCTTTGCTTG	60.5	465	INTERGENIC				
CaPOPII_2507	Ca6	27289106	GATATATAT/GATATATATAT	CAATTCCCACAGGTACGCTT	AATTTCAAGGATTGGCGAAG	60.0	612	INTERGENIC				
CaPOPII_2508	Ca6	27315680	GAGTTTTCAATTTTAGTTTTCAATTTTA/GAGTTTTCAATTTTA	CATGCATCGGAAGTTGTGTT	TCACTGTCAGGCAGATTTCG	59.6	496	INTERGENIC				
CaPOPII_2509	Ca6	27348230	ATTTTTTTTTT/ATTTTTTTTTTT	TTGATCCCATATCCTCCCAA	GACACCCAAATCAAACCCAC	60.1	595	INTERGENIC				
CaPOPII_2510	Ca6	27447919	ATTTTTTTT/ATTTTTTTTT	CACCATGTGCCACTTCATTC	TGGGCGGCTATGTTAAAACT	60.0	359	INTERGENIC				
CaPOPII_2511	Ca6	27448301	TTATATATATATATATATATATATATATATATAT/TTATATATATATATATATATATATATATATATATATATAT	CACCATGTGCCACTTCATTC	CTAGTGCAAGGTCGCTCCTC	60.0	629	INTERGENIC				
CaPOPII_2512	Ca6	27691258	ATTTTTTTTTTT/ATTTTTTTTTTTT	CACTCGGTTGAGACGTGAGA	AGGTCGGCGTCATAAACATC	60.0	290	INTERGENIC				
CaPOPII_2513	Ca6	28190835	ATATTATTATTATTATT/ATATTATTATTATTATTATT	AGGTCTCATACCAGAGATGCG	AAGTTAAGGGACCGCAAATG	59.3	871	INTERGENIC				
CaPOPII_2514	Ca6	28193559	AGGG/AGG	AAGGACTAAGTCGGGAAGAAAAA	GATGGTTTTTGTCCATTGAATTT	59.7	131	INTERGENIC				

INDEL marker IDs	Chromosomes /unanchored scaffolds	Physical positions (bp)	InDels (*Kabuli* reference genome-CDC Frontier/PI)	Forward primers (5'-3')	Reverse primers (5'-3')	Anneling temperature (0C)	Expected amplified product size (bp)	Structural annotation		Functional annotation		
								Sequence components of *kabuli* genome	*Kabuli* gene accession IDs	NCBI-KOG	TFs	NCBI-nr database
CaPOPII_2515	Ca6	28194024	ATTTTTT/ATTTTTTT	GGACCACGAATGTAATTTAGCC	TGCGAGCATTAGAAGTTGTTG	59.7	737	INTERGENIC				
CaPOPII_2516	Ca6	28326755	AT/ATT	TTTGGGCTTGAAGATGAAAA	TTTAAAAGCAAGGGTGGTGG	58.3	336	DRR	Ca_25968	A		Ribonuclease T2
CaPOPII_2517	Ca6	28327723	CATATATATATATAT/CATATATATATAT	CAAGAGCATCGTGTCAAGGA	GTCCTGGCGTGACAAGAAAT	60.0	424	DRR	Ca_25968	A		Ribonuclease T2
CaPOPII_2518	Ca6	28332319	ATTTT/ATTTTT	TTTCAAAAAGTCCCAATCGC	GGTTTGGAGAAGAAGACGAAA	60.1	751	INTERGENIC				
CaPOPII_2519	Ca6	28332579	A/AG	GTCGGAGCTCTGCCATAAAA	GGTTTGGAGAAGAAGACGAAA	60.4	599	INTERGENIC				
CaPOPII_2520	Ca6	28332651	C/CT	GTCGGAGCTCTGCCATAAAA	GGTTTGGAGAAGAAGACGAAA	60.4	599	INTERGENIC				
CaPOPII_2521	Ca6	28332758	TAAA/TAAAA	TTTCGTCTTCTTCTCCAAACC	CCCAACATATTACCTGTTACCCA	58.4	502	INTERGENIC				
CaPOPII_2522	Ca6	28332973	A/AT	TTTCGTCTTCTTCTCCAAACC	CGTGCAAAGTCGAAGCGTAT	58.4	669	INTERGENIC				
CaPOPII_2523	Ca6	28354937	GTAT/GT	TTTTATGTGACCCGCTTTGC	AGAACGGCATCCAGAGAAGA	61.0	600	INTERGENIC				
CaPOPII_2524	Ca6	28443583	CTCTTTTGTTGTTA/CTCTTTTGTTGTTATATCTTTTGTTGTTA	AAAAACACCAAAAGCCCCTC	CATAAAATGCCATTGATCCCT	60.3	499	INTERGENIC				
CaPOPII_2525	Ca6	28513941	ANNNNNNNNNN/ANNNNN	TGAAGAAAATTTAAAGGGACGAA	GTGTTGCATCTCATTGGTGG	59.2	569	INTERGENIC				
CaPOPII_2526	Ca6	28520924	CAA/CAATTAAA	TTAGAAACAGGGACCCCTCC	TTGTCTGTGAACCGAAGCTG	60.3	577	INTERGENIC				
CaPOPII_2527	Ca6	28520971	TCTTGCCTTGCCTTG/TCTTGCCTTG	TTAGAAACAGGGACCCCTCC	TTGTCTGTGAACCGAAGCTG	60.3	577	INTERGENIC				
CaPOPII_2528	Ca6	28521027	A/ACATAG	TCTCATTCTTGCCTTGCCTT	TTGTCTGTGAACCGAAGCTG	60.0	172	INTERGENIC				
CaPOPII_2529	Ca6	28521076	AACTAT/A	TCTCATTCTTGCCTTGCCTT	TTGTCTGTGAACCGAAGCTG	60.0	172	INTERGENIC				
CaPOPII_2530	Ca6	28577470	GATATATATATATATATAT/GATATATATATATATATATAT	GGTGTTGGAGTCATGTCTTTGA	ATTGAACTAACCCGTGCGTC	60.0	235	DRR	Ca_17491	O	Trihelix	Peptidase, cysteine peptidase active site
CaPOPII_2531	Ca6	28878487	T/TCA	CTCCGTTTTCTGCACATCAA	TGATGTTGCCTCTGTTTTGC	59.8	396	INTERGENIC				

INDEL marker IDs	Chromosomes /unanchored scaffolds	Physical positions (bp)	InDels (*Kabuli* reference genome-CDC Frontier/PI)	Forward primers (5'-3')	Reverse primers (5'-3')	Anneling temperature (0C)	Expected amplified product size (bp)	Structural annotation		Functional annotation		
								Sequence components of *kabuli* genome	*Kabuli* gene accession IDs	NCBI-KOG	TFs	NCBI-nr database
CaPOPII_2532	Ca6	29085384	CAAAAAAAA/CAAAAAAA	CGCTAGCAACAGAATTGCAG	TTCGACCGATTGAAGAAAGC	59.8	300	DRR	Ca_17464	U	LSD	Snf7
CaPOPII_2533	Ca6	29231459	AAAGTCTCAAGTCTCAAGTCTCAA/AAAGTCTCAAGTCTCAAGTCTCAAGTCTCAA	ACTCCCACACACCTCAAAGG	TTTCAAGTCTAGTCGAAGTTCATCA	60.0	834	INTERGENIC				
CaPOPII_2534	Ca6	30456247	AATTAT/AAT	GCGTTTCGACACAATTTTCC	AAAATAACGGGCAAGCCTTT	60.5	882	INTERGENIC				
CaPOPII_2535	Ca6	30588229	AATCGC/A	GGACGTCTACAACATTGGGG	CCACATGAGTGTGTTGAGGG	60.2	948	DRR	Ca_16461		SAP	Aminotransferase-like, plant mobile domain
CaPOPII_2536	Ca6	30698837	GTTATTTATTTATTTATTTATTTATTTATTTATT/GTTATTTATTTATTTATTTATTTATTTATTTATTTATTTATT	CGTTTTCGGTTTCAGGGTAA	CCCATACCTATTACCCACGC	60.0	949	INTERGENIC				
CaPOPII_2537	Ca6	30724309	AATATATATATATATATATATAT/AATATATATATATATATATATATAT	CCGTTGGGAGTTTGGAAGTA	TGCGTATGCTTCAACGTCAT	60.0	587	INTERGENIC				
CaPOPII_2538	Ca6	31097180	ATTT/ATTTTT	TCCCAATTGAGTGACATCTTTC	CTTATAGCCGAATGGCCAAA	59.0	587	INTERGENIC				
CaPOPII_2539	Ca6	31097254	ATT/ATTTTT	CAACAATTTCCCAAACAAAACA	CTTATAGCCGAATGGCCAAA	59.8	492	INTERGENIC				
CaPOPII_2540	Ca6	31099504	TA/T	TGGGATGATGTTGGGAAAGT	TTGCAAAGAAAACCTCGCTT	60.2	129	INTERGENIC				
CaPOPII_2541	Ca6	31146150	TAAA/TAAAA	TGCACGAGATGATTTGTTTGA	TCAATGGTGGCTTGTGGTTA	60.3	374	INTERGENIC				
CaPOPII_2542	Ca6	31288919	AATATATATATATATATATATTTATATATATATATATTTATATATATATATATATATA/AATATATATATATATATATATTTATATATATATATATTTATATATATATATA	TGATTCTTGAAAGTTAGCAAACACA	ATCGACAAACCAACATGCAA	60.2	460	INTERGENIC				
CaPOPII_2543	Ca6	31331884	TTATATATATATATATATATATA/TTATATATATATATATATATA	TGACATTGTGTATTTCGTTGTTTT	GTTTGTTGAAATTGGGGGTG	58.5	874	DRR	Ca_15166			F-box domain, cyclin-like
CaPOPII_2544	Ca6	31471169	TTATATATATATATATATAT/TTATATATATATATATATATATAT	CAACGTCGTCGAAGACAAGA	CCTTCGGCAAACTTCTTCAG	60.0	731	INTERGENIC				
CaPOPII_2545	Ca6	31551380	A/ATTG	ATGGCATCGTTGTCACAAAA	CAACTAAACTGCGCCACCTT	60.0	699	INTERGENIC				
CaPOPII_2546	Ca6	31681100	ATTTTTTTTT/ATTTTTTTTTT	GAGCCCCGATCCATATACCT	GTCACACAATCACTGACCGC	60.1	635	INTERGENIC				
CaPOPII_2547	Ca6	31939689	G/GAT	CTGCGGTTCTTGCTGATGTA	AGGCAGCTTGATTTCTTGGA	60.0	382	INTERGENIC				
CaPOPII_2548	Ca6	32372827	CAA/CA	GAATACGCGGCTGATTCATT	CGCCCCGTTTTAACTCATTA	60.1	613	INTERGENIC				

INDEL marker IDs	Chromosomes /unanchored scaffolds	Physical positions (bp)	InDels (*Kabuli* reference genome-CDC Frontier/PI)	Forward primers (5'-3')	Reverse primers (5'-3')	Anneling temperature (0C)	Expected amplified product size (bp)	Structural annotation		Functional annotation		
								Sequence components of *kabuli* genome	*Kabuli* gene accession IDs	NCBI-KOG	TFs	NCBI-nr database
CaPOPII_2549	Ca6	32373522	AATA/AAAATATA	TAATGAGTTAAAACGGGGCG	TTGGTCCAACACGAAGAGA	60.0	382	INTERGENIC				
CaPOPII_2550	Ca6	32391786	AATATATATATATATA/AATATATATATATATATA	CGCTATGCCCCATTAGATGT	TTTGATTGACTCATTGCGTG	59.9	294	INTERGENIC				
CaPOPII_2551	Ca6	32519592	AG/A	CGCATGCCACTCACTCTAAA	CGCTGCCACTTTCTACATCA	60.0	611	INTERGENIC				
CaPOPII_2552	Ca6	32521477	GACATGATAC/GAC	TTGCCAACGTGTCAATCACT	TTTTCCCTACTATTTTGCAGCC	60.2	259	INTERGENIC				
CaPOPII_2553	Ca6	32522263	CA/CAA	TTGTTCATTTTTGGTCGTTCA	CCTTGGCGGATATAGGGAAT	59.0	347	INTERGENIC				
CaPOPII_2554	Ca6	32539826	TAAAAAAAAAA/TAAAAAAAAA	TATGAGCCTCTGCCAGCTTT	GTCCGCGTCCATTGATAAAC	60.1	562	INTERGENIC				
CaPOPII_2555	Ca6	32540257	TACATAA/TACATAAGAAACATAA	GTTTATCAATGGACGCGGAC	TTCCCCTAAAAACGGTTGTG	60.3	341	INTERGENIC				
CaPOPII_2556	Ca6	32540480	GTTTTTTTT/GTTTTTTT	CACAACCGTTTTTAGGGGAA	GAGGATTGCTATGGACAACCA	59.8	214	INTERGENIC				
CaPOPII_2557	Ca6	32540585	AATATATAT/AATATAT	CACAACCGTTTTTAGGGGAA	AAGGAGAACCAGTCAACCACA	59.8	278	INTERGENIC				
CaPOPII_2558	Ca6	32617100	AAA/AAATAA	GAACGGTCCATGCAAAAGTT	CCCCCGATCCTTATACGACT	60.0	554	INTERGENIC				
CaPOPII_2559	Ca6	32629991	TAATGCA/TA	CGCTCTCAATCGTGCAATAA	TAGTGATTGGAAAGGACGGC	60.0	719	INTERGENIC				
CaPOPII_2560	Ca6	32630376	AGGGG/AGGGGGG	GCCGTCCTTTCCAATCACTA	ATCGGGAAGAGGTTAGGGAA	60.1	123	INTERGENIC				
CaPOPII_2561	Ca6	33094621	ATTT/ATT	TCAAAATGTAGCGGGTTTCC	AAGCCTTCCTCAAAATCCAA	59.9	398	INTERGENIC				
CaPOPII_2562	Ca6	33094845	CAAAAA/CAAAAAA	TCAAAATGTAGCGGGTTTCC	AAGTTGGACAAAATTGGGGT	59.9	950	INTERGENIC				
CaPOPII_2563	Ca6	33113807	TTTATTATTATTATTATTATTATTATTATTATTATTATTATTATTATT/TTTATTATTATTATTATTATTATTATTATTATTATTATTATTATTATT	AGAGGCAAACAAGAACCGAA	TTGTGGCATTTTCCACTTCA	59.9	433	INTERGENIC				
CaPOPII_2564	Ca6	33229272	GAAAA/GAAA	TGTTGACGGTGTGTTTTTAAGG	AAATTTTAGACCGCGACACG	59.9	477	INTERGENIC				
CaPOPII_2565	Ca6	33229296	TG/TGG	CGGTGTGTTTTTAAGGATTGG	AAATTTTAGACCGCGACACG	59.4	471	INTERGENIC				

INDEL marker IDs	Chromosomes /unanchored scaffolds	Physical positions (bp)	InDels (*Kabuli* reference genome-CDC Frontier/PI)	Forward primers (5'-3')	Reverse primers (5'-3')	Anneling temperature (0C)	Expected amplified product size (bp)	Structural annotation		Functional annotation		
								Sequence components of *kabuli* genome	*Kabuli* gene accession IDs	NCBI-KOG	TFs	NCBI-nr database
CaPOPII_2566	Ca6	33229523	AAAAACGAAAA/AAAAA	CGGTGTGTTTTTAAGG ATTGG	GCGGATATCGTAGGGT GCTA	59.4	644	INTERGENIC				
CaPOPII_2567	Ca6	33229803	TAAAAAAAA/TAAAAAA	AAGCAAGCCAAAAACG AAAA	GCGGATATCGTAGGGT GCTA	59.9	382	INTERGENIC				
CaPOPII_2568	Ca6	33229847	T/TC	AAGCAAGCCAAAAACG AAAA	GCGGATATCGTAGGGT GCTA	59.9	382	INTERGENIC				
CaPOPII_2569	Ca6	33240261	A/AAAGAG	GTTTGGTATTGGGCTC CCTT	CACAATTCCTCCCTCTC CAA	60.2	314	INTERGENIC				
CaPOPII_2570	Ca6	33240380	A/AG	GTTTGGTATTGGGCTC CCTT	CGAAACTCACCCCTTG TTGT	60.2	750	INTERGENIC				
CaPOPII_2571	Ca6	33293827	TGGG/TGGGG	CCAAGGCTATGAGACC TATCTGA	TGAAAACCCTTTGAAG GTGC	59.8	510	INTERGENIC				
CaPOPII_2572	Ca6	33506970	AAAAGTTAA/AAA	CCATTTGATCTGCAACA TTGA	GAAAAGGGGTGATTTG GGAT	59.5	273	INTERGENIC				
CaPOPII_2573	Ca6	34164737	CCCACCAC/CCCAC	CCACTTTGTTGGGAAA AACC	AGAAGAACGGTTGCCA AAGA	59.3	504	INTERGENIC				
CaPOPII_2574	Ca6	34728984	TTATATATATATATATATATAT/TTATA TATATATATATATATATAT	TAGGCACATCAAGTCA ACCG	TCAGACCATCCGATAG AGGC	59.7	570	INTERGENIC				
CaPOPII_2575	Ca6	34816135	ATTTT/ATTTTT	ATGCAATGCGTATATG CCAA	GTGTTTTGGTGTGGAT CGTG	59.9	872	INTERGENIC				
CaPOPII_2576	Ca6	34827878	CATATATATATATATATATATATATAT ATATA/CATATATATATATATATATATA TATATATA	AAGTTTCGGGGTAGAG CGAT	TGTAGCATTCTCATGG CGAG	60.1	645	INTERGENIC				
CaPOPII_2577	Ca6	34859064	GATATATATATATATATATATATATA/ GATATATATATATATATATATATATAT A	GAAAGAGTTTTGAGCC ACGG	AAGTCCAATCTCGGTC AAACTT	59.9	625	INTERGENIC				
CaPOPII_2578	Ca6	35093227	GAGTGTTGACTTTTTCA/GAGTGTTG ACTTTTTCATAGTGTTGACTTTTTCA	AACCGCTATTGGGCTA GGTT	CTCTGATATGCGTGGC AAGA	60.0	646	INTERGENIC				
CaPOPII_2579	Ca6	35161798	TA/TAAATATAAAA	AGGAAGAGTGGAGTTG CGAA	CGGTTGAGAACCAAGG ATGT	60.0	649	INTERGENIC				
CaPOPII_2580	Ca6	35169658	GTATATATATATATATATATATATATA/ GTATATATATATATATATATATATATA TATATATATA	AGGAAGAGTGGAGTTG CGAA	TATTTTGGATTTTGGCT CGG	60.0	524	INTERGENIC				
CaPOPII_2581	Ca6	35399356	GTTTGCTTTATA/GAATTTGCTTTATA	TTGAAGCTCAGATGGC ACAC	CTCCCCCTTTTTGGCAT AAT	60.0	718	INTERGENIC				
CaPOPII_2582	Ca6	35616799	C/CCTT	GTTTCTGAGGCAAAAT CCCA	TGCATTTGTCTTCCCAT TGA	60.1	537	INTERGENIC				

INDEL marker IDs	Chromosomes /unanchored scaffolds	Physical positions (bp)	InDels (*Kabuli* reference genome-CDC Frontier/PI)	Forward primers (5'-3')	Reverse primers (5'-3')	Anneling temperature (0C)	Expected amplified product size (bp)	Structural annotation		Functional annotation		
								Sequence components of *kabuli* genome	*Kabuli* gene accession IDs	NCBI-KOG	TFs	NCBI-nr database
CaPOPII_2583	Ca6	35719325	AATAGAATACTTTATTAT/AATAGAATACTTTATTATAGAATACTTTATTAT	TGCATCGTTTTTCATCTTTCA	GCGGCTAATTTCAACCAAAT	59.3	630	INTRON	Ca_15773			
CaPOPII_2584	Ca6	35802672	TTTCT/TT	GAGCCTTAGAGTTGGGAGCA	ACCCAACTTGTCGAACCAAC	59.6	778	INTERGENIC				
CaPOPII_2585	Ca6	35810337	C/CT	TCATCGATTGCAACTTGCTT	CCCTAAAATTTTGTGGGCTT	59.4	672	INTERGENIC				
CaPOPII_2586	Ca6	35832130	TTCTCT/TTCT	TCGCCTTCTTCGAAATTAAGTC	AAAAGATTGGTTGGATTAAAAAGA	59.9	147	INTERGENIC				
CaPOPII_2587	Ca6	35922388	AT/ATT	TCTCCGTTTCATTCTCCACC	CCTGGGGGCTGTTTTACATA	60.1	450	INTERGENIC				
CaPOPII_2588	Ca6	36029920	CTTT/CTTTT	TTTGGAACCAAAAACTTCTTCAA	GGAGAAAAAGAATTAAAATCCTCTAAA	60.0	520	INTERGENIC				
CaPOPII_2589	Ca6	36722414	ACCC/ACC	CTTAGGTTTGCACTCAGCCC	TCGCGGATCTGCTCTTAAAT	59.9	685	INTERGENIC				
CaPOPII_2590	Ca6	36722630	TA/TAA	TTGCTCCTACGTATCCCCAG	TCGCGGATCTGCTCTTAAAT	60.1	295	INTERGENIC				
CaPOPII_2591	Ca6	36898169	AATATATATATAT/AATATATATATATAT	AAGAGAGGTCACTTTGGCGA	CAATTCCCACAGGTACGCTT	60.0	692	INTERGENIC				
CaPOPII_2592	Ca6	37741039	CAAA/CAA	AGGGTGAGTGATCCGTTGTC	CCACGAGTTCAGGGTTTCAT	60.0	558	INTERGENIC				
CaPOPII_2593	Ca6	37981858	TAAA/TAA	TGCAATGACCTTGAAATTCG	CTTTTAGCCTTCATCGCCAG	59.7	817	INTERGENIC				
CaPOPII_2594	Ca6	37982172	AATGAT/AAT	GGTACACTTTCCCATCAGCAA	CTTTTAGCCTTCATCGCCAG	60.0	468	INTERGENIC				
CaPOPII_2595	Ca6	39879844	GA/GAA	ATCAACCCGCCCTTTACTTT	CGGCGGCTCTTTATTTAGTG	59.8	497	INTERGENIC				
CaPOPII_2596	Ca6	41168319	ATATTATTATTATTATTATTATTATTATTATTATTATTATTATTATTATTATT/ATATTATTATTATTATTATTATTATTATTATTATTATTATTATT	TTGAATCGTGAGGGTGTCAG	TGATTTCAAAAGCAATGTCAAAA	59.7	600	INTERGENIC				
CaPOPII_2597	Ca6	41464281	CTTTGATTTGA/CTTTGA	AAGCAGAAGGTAGTGGCGAA	GTACGTGGTCCTTCCCTGAA	60.0	395	INTERGENIC				
CaPOPII_2598	Ca6	41574227	CATAATAATAATAATAAT/CATAATAATAATAATAAT	TGATTAATCGCAAATTGTCCA	TTTTTGGGGTCGTTTTATGC	59.0	471	INTERGENIC				
CaPOPII_2599	Ca6	41574282	TTTATTATTATTATTATT/TTTATTATTATTATTATT	TGATTAATCGCAAATTGTCCA	TTTTTGGGGTCGTTTTATGC	59.0	471	INTERGENIC				

INDEL marker IDs	Chromosomes /unanchored scaffolds	Physical positions (bp)	InDels (*Kabuli* reference genome-CDC Frontier/PI)	Forward primers (5'-3')	Reverse primers (5'-3')	Anneling temperature (0C)	Expected amplified product size (bp)	Structural annotation		Functional annotation		
								Sequence components of *kabuli* genome	*Kabuli* gene accession IDs	NCBI-KOG	TFs	NCBI-nr database
CaPOPII_2600	Ca6	41659483	CAAAA/CAAAAA	CCATCAGAAACTGGGATGGT	TGGCCCTTCATTTTTCAATC	59.8	495	INTERGENIC				
CaPOPII_2601	Ca6	41669872	ATT/ATTT	TGTAGAATCCACCAATGTCCA	ACCAATCATGTTTCGTGGAA	58.8	651	INTERGENIC				
CaPOPII_2602	Ca6	41957220	CTATATATATAT/CTATATATAT	TCCCCAACTTTTGAATTTGC	TGCTTCAGTCAATGGGACAG	59.9	877	DRR	Ca_21949	O		Armadillo
CaPOPII_2603	Ca6	42418850	ATATTATTATTATTATTATT/ATATTATTATTATTATT	CCGACGTCTCTCATTCTCAA	AACGACAACTTTCAACCCATTT	59.0	686	INTERGENIC				
CaPOPII_2604	Ca6	43043105	CA/C	TGATTTGGTCGCAACAGCTA	AAAAATTGTGCCAAAGTTATGTGA	60.4	556	INTERGENIC				
CaPOPII_2605	Ca6	43138332	GAAAA/GAAA	GTTCCCTTTGGAATGCTTGA	TCTTTGGGCCAGTGAAAATC	60.1	741	INTERGENIC				
CaPOPII_2606	Ca6	43909185	TTGAAG/TTGAAGCAAGTACAACTCATGAAG	TGATTGGGTTTGGGTTCATT	ACATAGATGCCGCCTTCATT	60.0	445	INTERGENIC				
CaPOPII_2607	Ca6	43909507	CTTTTTTTTTT/CTTTTT	GAAGGCGGCATCTATGTGTT	GGCTTTTGAATCACCGAAAA	60.1	447	INTERGENIC				
CaPOPII_2608	Ca6	44263063	TTATATATATATATATATATATATATAT/TTATATATATATATATATATATATAT	CCCTTCCTCCACCATCACTA	TTTAAAATTCGGCCGTGAAG	59.9	643	INTERGENIC				
CaPOPII_2609	Ca6	44301191	CTTTTT/CTTTTTT	TGCACATTTTACAGCGCTTC	TGAAATTATGGGTGGGCATT	60.0	254	INTERGENIC				
CaPOPII_2610	Ca6	45282323	TCCTTTTTAGAAAGTAC/TC	TGTTGGAGAGGCTCTGCTTT	CGTTTTTCTGCACTCTGCAA	60.1	214	INTERGENIC				
CaPOPII_2611	Ca6	45290500	AT/A	GGCACGCTATGGAAAGAGTC	TGAAATTCCATAGGAAGCGG	59.8	589	INTERGENIC				
CaPOPII_2612	Ca6	45329060	T/TTTAAAA	TTTTTGCTATTTTATTCTCGTGG	TCGATTATGCGTAGGGAAGG	57.6	762	INTERGENIC				
CaPOPII_2613	Ca6	45336183	TTTATTATTATTATTATTATTATTATTATTA/TTTATTATTATTATTATTATTATTATTATTA	TTGCCACAAACAGAACGTGT	CCAAAAACACCAACTGGCTT	60.2	688	INTRON	Ca_13834	T		Ubiquitin
CaPOPII_2614	Ca6	45895264	TTATATATATATATATATA/TTATATATATATATATA	TTGAAAGAACCAAACGAGGG	TTTGAGAAATCCCCATTGGA	60.1	470	INTERGENIC				
CaPOPII_2615	Ca6	46134674	TAAAAAAAAA/TAAAAAAAA	CCTGAAATTCCTAACGCCAA	GCAGCGAAAAATCACCAAGT	60.1	446	DRR	Ca_13879		MYB_related	Bromo adjacent homology (BAH) domain
CaPOPII_2616	Ca6	46399842	T/TCA	TAACCTTGCATGGGGAAAAG	TTCATGGTGTTTTCTTGGCA	59.9	619	INTERGENIC				

INDEL marker IDs	Chromosomes /unanchored scaffolds	Physical positions (bp)	InDels (*Kabuli* reference genome-CDC Frontier/PI)	Forward primers (5'-3')	Reverse primers (5'-3')	Anneling temperature (0C)	Expected amplified product size (bp)	Structural annotation		Functional annotation		
								Sequence components of *kabuli* genome	*Kabuli* gene accession IDs	NCBI-KOG	TFs	NCBI-nr database
CaPOPII_2617	Ca6	46772986	A/ATT	GATTATTCTGCCTCCCGTCA	AAACGCAATCCTTGAGTTCG	60.0	633	INTERGENIC				
CaPOPII_2618	Ca6	47001052	A/AG	GAAGCGGTACTTCCCATTGA	GGTTACAATGTGGGGACGAG	60.1	417	INTERGENIC				
CaPOPII_2619	Ca6	48038283	AATTATTATTATTATTATTATTATTATTATTATTATTATTATTATTATTATTATTATTATT/AATTATTATTATTATTATTATTATTATTATTATTATTATTATTATTATTATTATT	GTGGGTAATCCGTGCGTAAT	CCCAAATTTGTTTAAAGAAGGC	59.7	663	DRR	Ca_23440		bZIP	Protein of unknown function DUF247, plant
CaPOPII_2620	Ca6	48247002	TTTATTATTATTATTATTATTATTATTATTATTATTATTATTATTATTATTATTATTATCATTATTATTAT/TTTATTATTATTATTATTATTATTATTATTATT	GAATTTAGGGCAATGGCTCA	TTTTTGGCACCAGTAGGAGG	60.0	632	INTRON	Ca_24603	A	HD-ZIP	Queuine/other tRNA-ribosyltransferase
CaPOPII_2621	Ca6	48260819	GAAAATTAATAAAATA/GAAAATTAATAAAATAAAATTAATAAAATA	GTGGATGGTGGGGAATAGTG	CTGAGGTCCCAAGCTACGTC	60.1	504	INTERGENIC				
CaPOPII_2622	Ca6	48279466	G/GA	AACCAGATACTAACCCGCGA	TCTGGAAGTTTCTTGACCGTG	59.6	620	INTERGENIC				
CaPOPII_2623	Ca6	48279626	ATTTTTTT/ATTTTTT	AACCAGATACTAACCCGCGA	TCTGGAAGTTTCTTGACCGTG	59.6	620	INTERGENIC				
CaPOPII_2624	Ca6	48280097	GAAAAAAA/GAAAAAAAA	GACACGGTCAAGAAACTTCCA	TGCGTTGACTCTTCAATACACA	60.1	376	INTERGENIC				
CaPOPII_2625	Ca6	48280225	GTAT/GT	GACACGGTCAAGAAACTTCCA	TGTGGCACATTCCAAATTTAT	60.1	601	INTERGENIC				
CaPOPII_2626	Ca6	48280286	ATTTTTTTTTTTTTTT/ATTTTTTTTTTTT	GACACGGTCAAGAAACTTCCA	TGTGGCACATTCCAAATTTAT	60.1	601	INTERGENIC				
CaPOPII_2627	Ca6	48280545	ATT/ATTT	TGTGTATTGAAGAGTCAACGCAT	AAAAGTTGTGCCAAAGTTATGTGA	59.7	732	INTERGENIC				
CaPOPII_2628	Ca6	48280766	TCTA/TCTAAAACTA	TGTATTGATGAGTCGTTGAATGAT	GGAGACGTTAAAGTCCCACA	58.5	902	INTERGENIC				
CaPOPII_2629	Ca6	48281570	TA/TAATTAAA	TAGGGCAATTGTGCACATGA	CTCCAGGTAACGGTTGTGGT	61.1	335	INTERGENIC				
CaPOPII_2630	Ca6	48385345	G/GA	GGCTTTTGCAATGGTTGTTT	TGCACTCACTCGCCATTTAC	60.0	586	INTERGENIC				
CaPOPII_2631	Ca6	48792073	TAAATTACAAATTACAAAT/TAAATTACAAAT	TTTGCAGCCACAAAAGACTG	GAACTCTGCCTTCCAGCAAC	60.0	906	DRR	Ca_20702			
CaPOPII_2632	Ca6	48905387	GAAAAAA/GAAAAAAAA	CACGGTCCCTAGAGCCAATA	ATCGACTCCCGAACTCAAAA	60.1	666	INTERGENIC				
CaPOPII_2633	Ca6	49218883	TAAAAAA/TAAAAAAAA	CCCCTTCATATGCTTCCAGA	GTGCTTGCTTTTACTTCGCC	60.0	446	INTERGENIC				

INDEL marker IDs	Chromosomes /unanchored scaffolds	Physical positions (bp)	InDels (*Kabuli* reference genome-CDC Frontier/PI)	Forward primers (5'-3')	Reverse primers (5'-3')	Anneling temperature (0C)	Expected amplified product size (bp)	Structural annotation		Functional annotation		
								Sequence components of *kabuli* genome	*Kabuli* gene accession IDs	NCBI-KOG	TFs	NCBI-nr database
CaPOPII_2634	Ca6	49222205	T/TG	CCTGCGGAATTTCCTAATGA	AGGTAACACGGCAGGTAACG	60.0	197	INTERGENIC				
CaPOPII_2635	Ca6	49611302	AAG/AAGCTAG	CATTGAAGGCAACCAATGTG	CTGTAGGCCACACAAAATGC	60.0	951	INTERGENIC				
CaPOPII_2636	Ca6	49733827	TCC/TC	TTGCAATCTCATCAACTCGG	GTAAAATGAAGCGCTTTCGC	59.8	810	INTERGENIC				
CaPOPII_2637	Ca6	49749941	TATAATAATAATAATAATAATAATAAAAATAATAATAATAATAATAATAATAATAA/TATAATAATAATAATAATAATAATAATAATAAAAATAATAATAATAATAAT	GACGCACGGGTTACACACTA	ATGTGGATGGTGCAGTACGA	59.6	299	INTERGENIC				
CaPOPII_2638	Ca6	51785881	GTTTTTTTTTTT/GTTTTTTTTTT	AACATTGTTGCGGCCTAAAA	CAGAAGATCACTCGTGCCAA	60.5	382	INTERGENIC				
CaPOPII_2639	Ca6	52075058	GTTATTATTATT/GTTATTATTATTATT	GCGTGTCTTATTGGAGAGAGC	GGCTATCGCTTATGCAGGAC	59.1	782	DRR	Ca_17514		SAP	Aminotransferase-like, plant mobile domain
CaPOPII_2640	Ca6	52787964	CG/C	TTCTCACGAACGAACACGAG	GTGGCACCTCGGTAAACAGT	60.0	667	INTERGENIC				
CaPOPII_2641	Ca6	52848807	ACTCGCT/A	CTGCCAAATACTGCGACTCA	TCAATTTTGCTTGTAGGATGGA	60.0	381	INTERGENIC				
CaPOPII_2642	Ca6	52850074	ACTAATTTCTAATT/ACTAATTTCTAATTTCTAATT	TCTGATTCGTTAATGACAACCTTT	CGTGGGAGCAATTATGTCCT	59.1	218	INTERGENIC				
CaPOPII_2643	Ca6	52850184	TA/T	AGGACATAATTGCTCCCACG	TCCATCTCCAACAACATCAAA	60.0	305	INTERGENIC				
CaPOPII_2644	Ca6	52858291	AAAATAA/AAA	CACATGCTGCAAAATTGAATG	CCACAAAAATGGTGTTGCAG	60.1	190	INTERGENIC				
CaPOPII_2645	Ca6	52858643	GTATCTATCTATCTAT/GTATCTATCTATCTATCTAT	CTGCAACACCATTTTTGTGG	TTTGCTGAAAGCATGTTTGTTT	60.0	689	INTERGENIC				
CaPOPII_2646	Ca6	52859293	ATATATTATATT/ATATATT	AAGTGAATTCCACCTTCCCA	CGAGCCTCCAACTTCTCTTG	59.4	560	INTERGENIC				
CaPOPII_2647	Ca6	52862596	T/TTATAA	ACATGAAGGCTTGGGTTGAC	GAGGAGCATGATAGCAAGGC	60.0	193	INTERGENIC				
CaPOPII_2648	Ca6	52889876	ATT/ATTT	TAAAGGACCAAGTCGGGAAA	TGCTCATGGAGTTAGAAGCAA	59.5	471	INTERGENIC				
CaPOPII_2649	Ca6	52935026	CATATATATATATATATATATATATAT/CATATATATATATATATATATATATATAT	CTTTGTGTGCTTCCCATCAA	TGCACGAACTTGTGTATGAAAA	59.7	888	INTERGENIC				
CaPOPII_2650	Ca6	53305336	ATTTTTTTT/ATTTTTTTTT	CAAGATTTCGTGGTGCAAGA	TTGAGGACAAAAGCTGCTGA	59.8	660	INTRON	Ca_22925	G		Major intrinsic protein

INDEL marker IDs	Chromosomes /unanchored scaffolds	Physical positions (bp)	InDels (*Kabuli* reference genome-CDC Frontier/PI)	Forward primers (5'-3')	Reverse primers (5'-3')	Anneling temperature (0C)	Expected amplified product size (bp)	Structural annotation		Functional annotation		
								Sequence components of *kabuli* genome	*Kabuli* gene accession IDs	NCBI-KOG	TFs	NCBI-nr database
CaPOPII_2651	Ca6	53311252	AT/ATT	GATGGTGGCCATTGAAGAGT	CAACAGTGTATGCACCTGCC	59.9	721	INTERGENIC				
CaPOPII_2652	Ca6	53312302	AAAAATAAAATAAAATAAAATAAAA/AAAAATAAAATAAAATAAAATAAAATAAAA	ATAGCAATTGGTGCATCCCT	ATGACCAAGGCATTGAGGAC	59.4	761	INTERGENIC				
CaPOPII_2653	Ca6	53413835	ATT/ATTT	CGTCATTTGGGGGACTAAAA	GAGTTCGGGAGTAGGGAAGG	59.8	978	INTERGENIC				
CaPOPII_2654	Ca6	53799025	ATTT/ATTTT	GGCCCAATATTTGAAACCCT	CAACTTGCAAAGACCCATCA	60.0	704	INTERGENIC				
CaPOPII_2655	Ca6	54068748	CCATCAT/CCAT	GTAAGTGAAAGCGGGAGTGC	ACCACCAACTTGCTTGCTCT	59.9	527	INTERGENIC				
CaPOPII_2656	Ca6	54068881	TAATATAAAAA/TAATATAAAAATATAAAAA	GATCCCAACCATCATTGTCC	ACCACCAACTTGCTTGCTCT	60.0	258	INTERGENIC				
CaPOPII_2657	Ca6	54068968	GA/GATA	GATCCCAACCATCATTGTCC	ACCACCAACTTGCTTGCTCT	60.0	258	INTERGENIC				
CaPOPII_2658	Ca6	54069131	ATTTT/ATTTTTT	GATCCCAACCATCATTGTCC	AATTACCACGGCCTCTTGTG	60.0	824	INTERGENIC				
CaPOPII_2659	Ca6	54070088	ATT/ATTT	GCAATTGTCGTGATTTTATTTTTG	TTTTGGTGGGTTCCACTTTT	59.8	379	INTERGENIC				
CaPOPII_2660	Ca6	54070236	TAAAA/TAA	TTTCTTTCCTCATGCTTTAGAGG	TTTTGGTGGGTTCCACTTTT	59.1	123	INTERGENIC				
CaPOPII_2661	Ca6	54144071	ATTTT/ATTT	GCCGCCGAATCTTATATTACC	CGTGCATTAACATCGACAGG	59.8	580	INTERGENIC				
CaPOPII_2662	Ca6	54144443	ATAAACTAA/ATAAACTAAACTAA	TGTTTTGTCGGGTGGACATA	CGTGCATTAACATCGACAGG	59.8	450	INTERGENIC				
CaPOPII_2663	Ca6	54161011	TGAGAGA/TGAGA	TTGATTTGACAATTTTCATTGCTT	TCAGTTTTCCGGTTTCTTGC	59.9	703	INTERGENIC				
CaPOPII_2664	Ca6	54192734	ATTTTTTTT/ATTTTTTTTT	GGACGGCGAAAATAAAACAA	TTGAAAATGGCTAAGGCAAAA	59.9	642	INTERGENIC				
CaPOPII_2665	Ca6	54683653	TTAATAATAATAATAATAATAATAATAATAATAATAATAATAATAATAATAATAATAATAA/TTAATAATAATAATAATAATAATAATAATAATAATAATAATAATAA	TAGTCTCATGCTGACACGCC	TGCGAGAGAGAAGAAGGAGG	60.0	219	INTERGENIC				
CaPOPII_2666	Ca6	54754837	GCCAAATCGATTT/GCCAAATCGATTTCCAAATCGATTT	GTAAAGGCCACCAATCGAAA	TTGTCCGTGAGAGACTGCAC	59.9	939	INTERGENIC				
CaPOPII_2667	Ca6	55769150	C/CT	TCGATTATGCGTAGGGAAGG	TTTTTCCTTTGTAGGCCCAA	60.1	943	INTERGENIC				

INDEL marker IDs	Chromosomes /unanchored scaffolds	Physical positions (bp)	InDels (*Kabuli* reference genome-CDC Frontier/PI)	Forward primers (5'-3')	Reverse primers (5'-3')	Anneling temperature (0C)	Expected amplified product size (bp)	Structural annotation		Functional annotation		
								Sequence components of *kabuli* genome	*Kabuli* gene accession IDs	NCBI-KOG	TFs	NCBI-nr database
CaPOPII_2668	Ca6	55771133	GTC/GTCTTC	GGAAACGTGTGAACGG AAGT	GGTAACCAATCCGAAG ACGA	60.0	575	INTERGENIC				
CaPOPII_2669	Ca6	55771190	TAT/TATCGTCTTCGGAT	TTACCGATGCATTGATC GAC	GGTAACCAATCCGAAG ACGA	59.5	143	INTERGENIC				
CaPOPII_2670	Ca6	55771649	TAAAAAAAAA/TAAAAAAAAAAAAA	GGATTGATTATCCGAT GCGT	GAACGCGAGATATTGG GTGT	59.8	544	INTERGENIC				
CaPOPII_2671	Ca6	55987809	AATATATATATATATATATATATATAT ATATATATAT/AATATATATATATATAT ATATATATATATATATATATATAT	TTCAAGGTTCATTAACA TCAACG	AAAGTTGACTCATATCA AAACCCA	59.0	390	INTERGENIC				
CaPOPII_2672	Ca6	56087656	CTTT/CTTTT	TTGTGAGCATGAGAAG TTGTTG	TGAAAAATTATTGAAAA AGTTACTTCC	59.0	737	INTERGENIC				
CaPOPII_2673	Ca6	56139090	AGG/AAACGG	ACCCTCTCACCCCTCTT GAT	TCCTCATCATTCCCTCC TTG	59.9	387	INTERGENIC				
CaPOPII_2674	Ca6	56503979	AAACAA/AAA	GTTGGCGCAAACTCTT CTTC	TGCAACTTTCAGGTCC CTTT	60.0	583	INTERGENIC				
CaPOPII_2675	Ca6	56504001	ATTTTT/ATTTT	GTTGGCGCAAACTCTT CTTC	TGCAACTTTCAGGTCC CTTT	60.0	583	INTERGENIC				
CaPOPII_2676	Ca6	56504140	AAAAGAAA/AAAA	AAATGCAAAGTCGGAT GGTC	TGCGAAGAAGAATGAT CACG	59.9	896	INTERGENIC				
CaPOPII_2677	Ca6	56504753	ATTTTTTTTTTTTT/ATTTTTTTTTT	CGTGATCATTCTTCTTC GCA	TCGGTCACCAAGTTAC CACA	59.9	614	INTERGENIC				
CaPOPII_2678	Ca6	56505598	AC/A	TGGTAACTTGGTGACC GACA	AATTTTTGCAGAAGAAA GAAAAAG	60.0	485	INTERGENIC				
CaPOPII_2679	Ca6	56507452	TATCA/TA	CGTGATCATTCTTCTTC GCA	ATCCAAACATTGGCCAT TTC	59.9	685	INTERGENIC				
CaPOPII_2680	Ca6	56507723	GTTTTTTTTTTTTTT/GTTTTTTTTTTT TTTTTT	CGTGATCATTCTTCTTC GCA	CTCCCAATTTGCAGTG TCCT	59.9	966	INTERGENIC				
CaPOPII_2681	Ca6	56507798	CA/CAA	AAATTCAAGTCTCGACG CAAA	CTCCCAATTTGCAGTG TCCT	59.9	511	INTERGENIC				
CaPOPII_2682	Ca6	56688837	GTTTT/GTTTTT	TGTTGAGTCGTGGTTA TGGG	TGAACATGCATTCCGA AAAA	59.4	725	DRR	Ca_17406			
CaPOPII_2683	Ca6	56713561	ATT/ATATTT	TTCACGTTGGAACTTG CTTG	TTTTTGTGATTCAGGTG GCA	59.9	833	INTERGENIC				
CaPOPII_2684	Ca6	56727392	TAA/TAAA	AGGCAAAACATGTTCC CTCA	AATGTTAGTGCACCGT GTCG	60.5	706	INTERGENIC				

INDEL marker IDs	Chromosomes /unanchored scaffolds	Physical positions (bp)	InDels (*Kabuli* reference genome-CDC Frontier/PI)	Forward primers (5'-3')	Reverse primers (5'-3')	Anneling temperature (0C)	Expected amplified product size (bp)	Structural annotation		Functional annotation		
								Sequence components of *kabuli* genome	*Kabuli* gene accession IDs	NCBI-KOG	TFs	NCBI-nr database
CaPOPII_2685	Ca6	56767873	TAAAAAAAAAAAA/TAAAAAAAAAAAAA	TCATGGATTCAACCAGATGC	TTCATCTTTCAAACTTGAGACTCG	59.5	188	INTERGENIC				
CaPOPII_2686	Ca6	57039572	TAAAAAAAA/TAAAAAAAAA	GGTGTAGTGGATCGGTCTTGA	ATAGTGCCGAAGGAAGCAAA	60.0	334	INTERGENIC				
CaPOPII_2687	Ca6	57117976	AATATATATATATATATATATATATATATATATA/AATATATATATATATATATATATATATA	TTTCATGTGCGTGTGAGTGA	TCAACCAATTCAACAATTTGACA	59.9	519	INTERGENIC				
CaPOPII_2688	Ca6	58373671	GATATATATATATATATATATATA/GATATATATATATATATATATATATA	TCACGCAAAAGATTCATAAAAGA	AGTTTGTAAGCTGTGCGTCG	58.9	500	INTERGENIC				
CaPOPII_2689	Ca6	58539579	CATATATATATATATATATATATATATATATATAT/CATATATATATATATATATATATATATATATATATATAT	CGAGCCTAATAGTCGACCCA	AACGGGTCAACCAATCACAT	60.2	258	INTERGENIC				
CaPOPII_2690	Ca6	58594670	TAAAA/TAAAAA	GATGATGTGGCTGCTCAAGA	TTCATTTGTCCGTTGAGTGC	60.0	490	DRR	Ca_15401			
CaPOPII_2691	Ca6	58615933	GTAGTT/G	CAAATTGTCCGTGCAATCAC	GAAGAATGGTAGGCGGAACA	60.0	227	INTERGENIC				
CaPOPII_2692	Ca6	58758402	ATTTTTTTTTT/ATTTTTTTTTTT	TGGTCTGGCCTATTCTCACC	GATCCGTCAAGTCCCTCGTA	60.1	449	INTERGENIC				
CaPOPII_2693	Ca6	58785175	TTCTCTCTCTCTCTCT/TTCTCTCTCTCTCT	TCAAATATTGATGCGACAAAAA	TTGTCAAATGCTGTTTGCGT	58.2	522	DRR	Ca_15422	K	MYB	SANT domain, DNA binding
CaPOPII_2694	Ca6	58787039	TAATAAATAAATAAATAAATAAA/TAATAAATAAATAAATAAATAAATAAATAAA	GCAAAGCATCTAGGTGTCCC	GCCAATCGATGAGATCCAGT	59.7	233	DRR	Ca_15422	K	MYB	SANT domain, DNA binding
CaPOPII_2695	Ca6	58808278	T/TC	TGGTGCGTCCACTATTCAAA	ATTTTCCCACATGGACTGGT	60.1	622	INTERGENIC				
CaPOPII_2696	Ca6	58823708	GTAA/GTAAATAA	AATTTTAGATCACGTGGCGT	GCACTCATTAGCCGTTCCAT	57.2	830	DRR	Ca_15424	S		
CaPOPII_2697	Ca6	58827188	GAAAAA/GAAAAAA	GTGCAAACCAGTGGAATCCT	TTGGTTTGGGTTGGAACATT	60.0	600	INTRON	Ca_15424	S		
CaPOPII_2698	Ca6	58851448	AG/A	CTTGATTTGCGGATGGATTT	TTAAAGCGCTCCCATCAACT	59.9	896	INTERGENIC				
CaPOPII_2699	Ca6	58873196	GATATATATATATATATATATATATATA/GATATATATATATATATATATATATATA	TTGAGCGAGAATGTAGAGAGAATG	AGAGCAAGCTTAAGCCGACA	60.0	233	INTERGENIC				
CaPOPII_2700	Ca6	58874492	GTATATATATATATATATATATATATA/GTATATATATATATATATATA	TCTCAATGTGATTTTAATGTTCCC	GCCTCCTCATGCTGATTCAC	59.3	671	INTERGENIC				
CaPOPII_2701	Ca6	59066187	TAAAAAAAAA/TAAAAAAAAAA	TGTTTGTCACACTTTCAAATCCA	TGTGGGCTGATGTTGGTTTA	60.4	608	INTERGENIC				

INDEL marker IDs	Chromosomes /unanchored scaffolds	Physical positions (bp)	InDels (*Kabuli* reference genome-CDC Frontier/PI)	Forward primers (5'-3')	Reverse primers (5'-3')	Anneling temperature (0C)	Expected amplified product size (bp)	Structural annotation		Functional annotation		
								Sequence components of *kabuli* genome	*Kabuli* gene accession IDs	NCBI-KOG	TFs	NCBI-nr database
CaPOPII_2702	Ca6	59093194	ATTTTTTTTTTT/ATTTTTTTTTT	GGAGGCAACCCAACTCTATG	TCGCATAATTGGAACGTCAA	59.6	271	INTERGENIC				
CaPOPII_2703	Ca6	59273311	TA/T	TTTTTGGGCTGAAGCCATAC	GGTGGAGAATGAAACGGAGA	60.1	359	INTERGENIC				
CaPOPII_2704	Ca6	59349983	AATATATATATATATATATATATATATA/AATATATATATATATATATATATATATATATATA	TTTCCATTGCACTCACTCCA	CCTATCATGGCCTGACCTGT	60.2	777	INTERGENIC				
CaPOPII_2705	Ca6	59384361	AATATATATATATATA/AATATATATATATATATA	CGGCCTTATCCTAAAGACCC	AAAATAGGGAGACCAATTTCG	59.9	393	INTERGENIC				
CaPOPII_2706	Ca6	59434628	CAAAAA/CAAAAAA	ACAGCAGCCTTGCATTCTCT	TGTTGCCTCTGTTTTTGCAG	60.2	374	INTERGENIC				
CaPOPII_2707	Ca7	180427	C/CAGAGGCA	TCAACAGCAGCTTTGCATTC	TTTCGCAGCTGGTATGTGAG	60.1	280	INTERGENIC				
CaPOPII_2708	Ca7	585059	TAAA/TAA	GTTTAGGCCGCCACAAAATA	GAACTCTGCCTTCCAGCAAC	60.0	871	DRR	Ca_24416			Transposase, Ptta/En/Spm, plant
CaPOPII_2709	Ca7	586986	AA/AATTA	TTCCATTTCCTCCACAAAGC	CAACCGCATTGTCATCATTC	60.1	531	DRR	Ca_24415			
CaPOPII_2710	Ca7	760856	GTTGAAGATTG/GTTGAAGATTGAAGATTG	TAAACGCTTGTTGGCATTTG	AGTTCGCAGGCTCTGTTGTT	59.7	445	DRR	Ca_03403	R		Short-chain dehydrogenase/reductase SDR
CaPOPII_2711	Ca7	816979	AAATAAAAATTAATAAAAAT/AAATAAAAATTAATAAAAATTAATAAAAAT	CGACCAATGTTTTTCTTCTTCA	CAGATCATGGCACGTGAAAG	59.2	496	DRR	Ca_03396			
CaPOPII_2712	Ca7	1018745	C/CT	TTGCAAACATGATAGCTGCC	TTGTTGGCATTGTTGTGGTT	59.8	467	INTERGENIC				
CaPOPII_2713	Ca7	1018777	ATTGCAT/ATTGCATATTTGCAT	TTGTTCCCGTAAAAGATCGC	TTGTTGGCATTGTTGTGGTT	60.1	233	INTERGENIC				
CaPOPII_2714	Ca7	1245279	A/AATTTCAT	TGCACAAAACACACAACCCT	TCGCTCCTGTGTCAGTTTTG	60.0	468	INTERGENIC				
CaPOPII_2715	Ca7	1326824	T/TA	TCCAAAAATGCACTCAAATCA	ATGAGTGGGAGAACATTGCC	59.1	715	INTERGENIC				
CaPOPII_2716	Ca7	1339137	ATTTTTTTTT/ATTTTTTTTTT	CTTCACAACCAGAGCACCAA	TGCATTAAATTTTACGGAGAAACA	59.9	638	INTERGENIC				
CaPOPII_2717	Ca7	1423751	GTAATTAATTAA/GTAATTAATTAATTAA	CGAGCTTTAAGCCACCTTTG	GGATTCGACCCTTGTGCTTA	60.0	706	INTRON	Ca_03325			Plastocyanin-like
CaPOPII_2718	Ca7	1649022	ATTTTTT/ATTTT	CCTTAGTGATTTGGAGGGCA	CGAGAAGGTCGTGAGGTAGC	60.1	564	INTERGENIC				

INDEL marker IDs	Chromosomes /unanchored scaffolds	Physical positions (bp)	InDels (*Kabuli* reference genome-CDC Frontier/PI)	Forward primers (5'-3')	Reverse primers (5'-3')	Anneling temperature (0C)	Expected amplified product size (bp)	Structural annotation		Functional annotation		
								Sequence components of *kabuli* genome	*Kabuli* gene accession IDs	NCBI-KOG	TFs	NCBI-nr database
CaPOPII_2719	Ca7	1835089	CAA/CAAA	AACAAAAGTAGCGTGCGACA	ATTTGTCAACTCGGGGTTTG	59.5	632	INTERGENIC				
CaPOPII_2720	Ca7	1902358	GTTTTAATAATA/G	CAAGTTTATCTCTTCAATCGCGT	TCGCATATTGCTCTCCCTCT	59.8	318	INTERGENIC				
CaPOPII_2721	Ca7	2066755	CAAAAAAAAAAA/CAAAAAAAAAAAA	TCGTTGGAGAGTTGGTGATG	TGGGATGGTAAGCATTTGAA	59.7	563	INTERGENIC				
CaPOPII_2722	Ca7	2168224	CATA/CATATA	TTCTGAGATTGGCAAAAGGG	CGCGATCGTTCACTGATATG	60.2	820	INTRON	Ca_03253	P		Porin, eukaryotic type
CaPOPII_2723	Ca7	2169903	T/TA	CAGGGCCTTACTGCTACAGG	CAACGGAAAACATCGTTCAA	59.9	397	INTRON	Ca_03253	P		Porin, eukaryotic type
CaPOPII_2724	Ca7	2525335	CAATACAATTGATTAATA/CAATACAATTGATTAATACAATTGATTAATA	TGGTGGAAAAATAGCATTTGG	CCTCTCCCGAATGACTCAAG	59.8	868	INTERGENIC				
CaPOPII_2725	Ca7	2808679	GAAAAAAAAAA/GAAAAAAAAAAAA	ACTTCCCTGTGGAACGACAC	ATATGCACGGAACGAAAACC	60.0	431	INTERGENIC				
CaPOPII_2726	Ca7	2831340	TAAAAAAAAA/TAAAAAAAAAA	TGATAATCTCGCGGGATAGG	GAATTGCCGAAAAATGATTGA	60.0	465	DRR	Ca_03193	K	GATA	Zinc finger, GATA-type
CaPOPII_2727	Ca7	3076524	ATTTT/ATTT	GAAATGGATTGTGGAAGGGA	CCTCTTAGGTGTGCCTCTGC	59.7	742	INTERGENIC				
CaPOPII_2728	Ca7	3256595	CTCGTTCGT/CTCGT	AAAGAAAACGTCGCCTGAGA	CACGGACTTGGCTTGGTAAT	60.0	559	INTERGENIC				
CaPOPII_2729	Ca7	3377629	GTTTTTT/GTTTTTTTT	ATAACCGCAAGCAAAACCAA	GGGGATTTTGGAGTTGGAGT	60.5	649	INTRON	Ca_03127	T	C3H	NUDIX hydrolase domain
CaPOPII_2730	Ca7	3512361	AG/A	AATTCGCATGCATATTCAACA	GCAGAGATTTGATTCGAGGC	59.0	677	INTRON	Ca_03117	Z	MYB_related	Kinesin, motor domain
CaPOPII_2731	Ca7	3580383	ATTT/ATTTT	TGTCAACAAAAACCCAAGCA	TTTGACTGCTGGCTTGACTG	60.1	339	INTRON	Ca_03112	U	MYB	Sec34-like protein
CaPOPII_2732	Ca7	3608175	CA/CAA	TTTTCGTGGGATCAAATTCC	ATTCACGAACCTGCGATACC	59.7	495	INTERGENIC				
CaPOPII_2733	Ca7	3608219	GT/GTCCCAATTT	TTTTCGTGGGATCAAATTCC	ATTCACGAACCTGCGATACC	59.7	495	INTERGENIC				
CaPOPII_2734	Ca7	3612730	G/GTTTAA	CTATGGCATCGACATTGCAT	ATCGAGAGATCGCGAGAGAG	59.5	803	INTERGENIC				
CaPOPII_2735	Ca7	3612993	ATATAATT/AT	AACACTCCGTATGTCCCACC	ATCGAGAGATCGCGAGAGAG	59.7	148	INTERGENIC				

INDEL marker IDs	Chromosomes /unanchored scaffolds	Physical positions (bp)	InDels (*Kabuli* reference genome-CDC Frontier/PI)	Forward primers (5'-3')	Reverse primers (5'-3')	Anneling temperature (0C)	Expected amplified product size (bp)	Structural annotation		Functional annotation		
								Sequence components of *kabuli* genome	*Kabuli* gene accession IDs	NCBI-KOG	TFs	NCBI-nr database
CaPOPII_2736	Ca7	3613058	CAGAA/CAGAAGAA	AACACTCCGTATGTCCCACC	CAACCCAACACCCAACTACA	59.7	433	INTERGENIC				
CaPOPII_2737	Ca7	3649235	GAATCCAATATCTA/GAATCCAATATCTAAAATCCAATATCTA	GTCCGAGTCCTAACAGCAGC	TCATGGTGAGATTGGCAAAA	60.0	722	DRR	Ca_03103	U	LBD	SEC7-like
CaPOPII_2738	Ca7	3654337	GTGGGAATCTTTT/GTGGGAATCTTTTCATGGGAATCTTTT	AAAGCAAACTCAGCAACGGT	TATCAGAGCCTTCCATTGGG	59.9	427	INTRON	Ca_03103	U	LBD	SEC7-like
CaPOPII_2739	Ca7	3668391	GC/GCC	GCGGGTTTTTGACAATGTTT	TTACTTTTGACGGGAAACGG	59.8	618	INTERGENIC				
CaPOPII_2740	Ca7	3682108	TCGATTC/T	CCATTTGCAGGATTCAAGGT	GGTGCTTTGCTACCTAGCCTT	59.9	629	INTRON	Ca_03101			Tify
CaPOPII_2741	Ca7	3689798	TAGAGAG/TAGAG	ATGGTAGGGGGAAGAGGAGT	CTTTTCGTTTGGCCAATGTT	58.9	381	DRR	Ca_03100	S		Uncharacterised protein family UPF0136, Transmembrane
CaPOPII_2742	Ca7	3754420	GTCTCTCTCTCTCTCTCTCTCTCTCTCT/GTCTCTCTCTCTCTCTCTCTCTCTCTCTCTCTCTCT	AAATCGACGGCCACAGATAC	CACAATGTCCATGCTCCAAC	60.0	703	INTRON	Ca_03092	BK		SNF5/SMARCB1/INI1
CaPOPII_2743	Ca7	3976290	ATTTTTTTT/ATTTTTTT	AAAAAGTTGAGGTGGGTAAAAAT	TTGCAAGCTATTGTTCCACG	57.3	730	INTERGENIC				
CaPOPII_2744	Ca7	4118702	ATTTTTTTTT/ATTTTTTTT	TTGTTATTGCTTGGTGTGGTT	GCAAAAAGAGACCTGCCAAG	58.1	661	INTERGENIC				
CaPOPII_2745	Ca7	4339580	TTTTTTATTTTTATTTTTATT/TTTTTTATTTTTATTTTTATTTTTTATT	GCTAGTGCCTCCCTTGAGTG	TCACGGGTGCATTGAACTTA	60.0	614	INTERGENIC				
CaPOPII_2746	Ca7	4418125	A/ATC	CAAGTCAGCACCGACAAAGA	ATAAAAGGGTGACGTGGCAG	60.0	592	INTERGENIC				
CaPOPII_2747	Ca7	4562422	ATT/ATTT	TCTCCGATTTCCTCCTCAAA	ACATCTTGGATGATGTGGCA	59.7	283	INTERGENIC				
CaPOPII_2748	Ca7	4664736	ATTTT/ATTT	CAATTGTGTGTGAGTTTTTGATGA	ATGCACGATGGCACTTGTAG	59.9	604	INTERGENIC				
CaPOPII_2749	Ca7	4775529	TAAAAAAAAAA/TAAAAAAAAAAA	TGCACATTGCACACTACCAA	GTGCATTCGAAGAAAGAGCC	59.7	619	INTERGENIC				
CaPOPII_2750	Ca7	4867122	AATATATATATATATATATATATATATATTTATATATATATAT/AATATATATATATATATATATATATATATATATTTATATATATATAT	AGGTGGTGAGCGTTTTTGAA	TTGATGATGTGAAGGAATGACAG	60.7	670	INTERGENIC				
CaPOPII_2751	Ca7	5014340	CAA/CAAA	AATACCACCACCGACCACAT	AATGAGCTCCAACGGTCATC	60.0	787	INTERGENIC				
CaPOPII_2752	Ca7	5030934	ATTT/ATTTT	AACTGGTCAGATTGGTTGAGAAA	CATCCAATTCTTTCACTCCGA	60.0	585	INTERGENIC				

INDEL marker IDs	Chromosomes /unanchored scaffolds	Physical positions (bp)	InDels (*Kabuli* reference genome-CDC Frontier/PI)	Forward primers (5'-3')	Reverse primers (5'-3')	Anneling temperature (0C)	Expected amplified product size (bp)	Structural annotation		Functional annotation		
								Sequence components of *kabuli* genome	*Kabuli* gene accession IDs	NCBI-KOG	TFs	NCBI-nr database
CaPOPII_2753	Ca7	5034485	ATTTTTTTTT/ATTTTTTTTTT	CCCACTTGCAACGGTTTATT	TGAGGTAAGCTTTGCATCATGT	59.9	837	INTERGENIC				
CaPOPII_2754	Ca7	5387713	TATTTAAAATTTAAAAT/TATTTAAAAT	TGCATATAATTGGATCATTGGTG	ATGGAAATGGTGGTGATGGT	59.6	607	INTERGENIC				
CaPOPII_2755	Ca7	5388602	CTATATA/CTATA	CAACCTGTGACTCCCAACCT	GGAGTTTGGAATTCGTTTGG	60.0	398	INTRON	Ca_06831	R		Kelch repeat type 1
CaPOPII_2756	Ca7	5398164	CA/CAA	CATGGATTGCATTTGTTTCG	TTGAATCAGCTCAGCCAGTG	59.9	752	INTRON	Ca_06830	R		Kelch repeat type 1
CaPOPII_2757	Ca7	5405644	AATATAT/AATATATAT	GCGATGGAAGAAAAGTTGGA	AACGTGCAACAATATCGCAA	60.2	245	INTERGENIC				
CaPOPII_2758	Ca7	5407517	TCTAAC/TCTAACTAAC	AAATTAGGGAGTGGATTTTCACC	GTTTTGGGTTGAGACGTGGT	59.6	525	INTERGENIC				
CaPOPII_2759	Ca7	5414070	ACTCTCTCT/ACTCTCT	TGACAAATTCAAACTCCCGA	GGTGACCTGGGAAAATCCTT	59.1	290	INTERGENIC				
CaPOPII_2760	Ca7	5418013	ACTA/ACTAATGTATAATCTA	TGTGAACTCACGTTTCACCA	GGGACCTTCTACTTACCCGC	58.7	517	DRR	Ca_06827	T	WRKY	Protein kinase, catalytic domain
CaPOPII_2761	Ca7	5429372	ATT/ATTT	AAAGGCGAGATGGAAAGTTG	TTGGTGTGATTCGTGACCAT	59.3	741	INTERGENIC				
CaPOPII_2762	Ca7	5443791	TATAAATAAATAAATAA/TATAAATAAATAA	TATTTGCGAGCGTGTCTTCA	TTTTGATGCTTTTTCCACCA	60.5	516	INTERGENIC				
CaPOPII_2763	Ca7	5458536	ACTAG/ACTAGCTAG	ACAATTACGTGAAGGCCACA	TTCGGATTGCAACCTTTTTC	59.1	391	INTERGENIC				
CaPOPII_2764	Ca7	5480065	TAAAAAAAA/TAAAAAA	TGGAGTCGGTTACGTGAGTG	CCTACCCAAACCCTACCCAT	59.7	820	INTERGENIC				
CaPOPII_2765	Ca7	5481672	ATTTTTTT/ATTTTTTTT	GGTGTCCACTGAGTTTGGGT	TGCTTGTGATTTACCTTAATTTCC	59.9	812	INTERGENIC				
CaPOPII_2766	Ca7	5488147	TATTAATTAATT/TATTAATTAATTAATT	AACCTCCTTTTCCGTCACCT	CATTCCACAGCCTCCATTTT	60.0	781	INTRON	Ca_06817	CIQ		Phosphopantetheine attachment site
CaPOPII_2767	Ca7	5513596	TTATATATATAT/TTATATATAT	AGGCTGCTGTAGGAGATGGA	AATCAACCTTGGAATGGTGC	60.0	504	DRR	Ca_06816	BK	G2-like	SNF2-related
CaPOPII_2768	Ca7	5567501	TAAAAAAAAA/TAAAAAAAAAA	TCATGAAATGTCATTCGGTTG	TTTTTGGGCCTGAAAACAAG	59.4	200	DRR	Ca_06806			
CaPOPII_2769	Ca7	5572175	TAAAAAAAAAAAAAAA/TAAAAAAAAAAA	TGTCAAATGTGAGGAAGCCA	TAGGGACCCATATGCAAAAA	60.2	641	INTERGENIC				

INDEL marker IDs	Chromosomes /unanchored scaffolds	Physical positions (bp)	InDels (*Kabuli* reference genome-CDC Frontier/PI)	Forward primers (5'-3')	Reverse primers (5'-3')	Anneling temperature (0C)	Expected amplified product size (bp)	Structural annotation		Functional annotation		
								Sequence components of *kabuli* genome	*Kabuli* gene accession IDs	NCBI-KOG	TFs	NCBI-nr database
CaPOPII_2770	Ca7	5578208	AG/AGAATGAATG	ATGGGAAATTGTGAATGGGA	TGCCCGTTAGTCAATGTTTG	60.0	435	DRR	Ca_06805			
CaPOPII_2771	Ca7	5628630	GAAAAAAAAA/GAAAAAAAA	CGGGTTGGTATTTATTGGGA	ATTGAGTTGATCACTCGGGG	59.5	678	INTERGENIC				
CaPOPII_2772	Ca7	5633058	T/TAAG	GCAAGCCAAACCACAAAAAT	ACAGGCGCTTCCTTCTTGTA	60.0	386	DRR	Ca_06796	GC	HSF	UDP-glucuronosyl/UDP-glucosyltransferase
CaPOPII_2773	Ca7	5650363	AAATAATAATAATA/AAATAATAATAATAATA	TCGTTCCCTCTGAAATGAAA	TCGAGTGAACGTGGAAGATG	58.3	239	INTERGENIC				
CaPOPII_2774	Ca7	5663307	ATCCATTAGT/AT	CGGAGCCTATTGCTGCTATC	TATCCACGGACACGAGTTCA	60.0	524	INTERGENIC				
CaPOPII_2775	Ca7	5685708	T/TG	TTCGGTTTTAGCAGTTTGGG	GCGTCACACTGAGACTGGAA	60.1	788	DRR	Ca_06789	K		RNA polymerase I specific transcription initiation factor RRN3
CaPOPII_2776	Ca7	5694761	TAAAAAA/TAAAAAAA	GGAATTCTGTTTGCAAGGGA	AACCCATCACTTGCTGATCC	60.1	627	INTERGENIC				
CaPOPII_2777	Ca7	5697428	TAAAAAAAAAAA/TAAAAAAAAA	AATGTTGTAAGGTGGGCGAG	AAGTTAAGGGACCACGAGTGA	60.0	718	INTERGENIC				
CaPOPII_2778	Ca7	5713279	CAAAA/CAAA	GCCTGGTTGCTCCAATTAAA	GGGCTTCAGAAGTTGCTCAC	60.1	488	DRR	Ca_06787	Q		Copper amine oxidase
CaPOPII_2779	Ca7	5717190	CTTTTTTTTTTTTTT/CTTTTTTTTTTT	ACCAAAACTCTATCCCCTTAAAA	CCACTCTCAAAGTCAATGGC	57.4	620	INTERGENIC				
CaPOPII_2780	Ca7	5722804	A/AC	TGGGACAGTGAGTCAATCCA	TCCGATTTTTGCTCTCGACT	60.1	191	INTRON	Ca_06785	R	FAR1	Alpha/beta hydrolase fold-1
CaPOPII_2781	Ca7	5787059	AT/ATTGTTATT	TTTGACCTCAAGCAAATCCC	ATTTTGGATGAGATCGGGTG	60.1	527	INTERGENIC				
CaPOPII_2782	Ca7	5791147	AATTTTAAAGT/AATTTTAAAGTAATAATATTTTAAAGT	CGACATGAATAAATTGAGGATGA	TGATCTGGTTGGTGTTGTCC	59.0	988	DRR	Ca_06779	S		Cystathionine beta-synthase, core
CaPOPII_2783	Ca7	5799756	TGAGA/TGAGAGA	CCATCATACCCCTAGCGAAA	CAGCCTATGTTTGGAAACCC	59.9	730	DRR	Ca_06778	R		RNA recognition motif domain
CaPOPII_2784	Ca7	5841891	CTTT/CTTTT	TGCAACTTTGAAGGTGCTTG	CAACAAAGCCACAAAAGCAA	60.0	731	DRR	Ca_06775			
CaPOPII_2785	Ca7	5843643	AT/ATT	GGTGAGTTTTTCTCTCCCCC	TTCATCGTCTGAAGTCACGG	59.9	501	INTERGENIC				
CaPOPII_2786	Ca7	5867187	ATATTATTATTATTATTAT/ATATTATTATTATTATTATTAT	ACAACAGATGATGGCTGCTG	AATGTACGCACTCCACCACA	59.9	606	DRR	Ca_06771	P		Heavy metal transport/detoxification protein

INDEL marker IDs	Chromosomes /unanchored scaffolds	Physical positions (bp)	InDels (*Kabuli* reference genome-CDC Frontier/PI)	Forward primers (5'-3')	Reverse primers (5'-3')	Anneling temperature (0C)	Expected amplified product size (bp)	Structural annotation		Functional annotation		
								Sequence components of *kabuli* genome	*Kabuli* gene accession IDs	NCBI-KOG	TFs	NCBI-nr database
CaPOPII_2787	Ca7	5874353	GA/G	TTTGGTACAACGAGGTCCAC	TTGGCTTGTCACATCAGCTT	58.5	268	INTERGENIC				
CaPOPII_2788	Ca7	5884631	ATTTTTT/ATTTTTTTT	TCTTGTTTGCCTGTCTCGTG	ATGCGAAGGAGCAATTATGG	60.0	832	INTERGENIC				
CaPOPII_2789	Ca7	5884732	CAA/CA	ACATGGCTTTACTTGGTGGA	ATGCGAAGGAGCAATTATGG	58.1	425	INTERGENIC				
CaPOPII_2790	Ca7	5894442	ATTTTTTT/ATTTTTTTT	CAAGTCATGCACAACATTTGG	TGTCAACCTGATTGAACCGA	60.0	531	INTERGENIC				
CaPOPII_2791	Ca7	5897121	CT/CTTTGTT	CAAAGATTGCTTCAGGGACC	CCACTGTTTAAAATGTGCCG	59.7	451	INTERGENIC				
CaPOPII_2792	Ca7	5936337	GTTTT/GTT	AAGAGCAACTTAGTCCGCGA	AAGGTAGCGGCTCACAACAC	60.2	421	INTERGENIC				
CaPOPII_2793	Ca7	5947080	CCA/CCACA	AGGCTAACGTTGCTTTTCCA	GCTTCCTCTGAGTGCACCTT	59.9	572	INTRON	Ca_06762	L		DNA-dependent ATPase MCM
CaPOPII_2794	Ca7	6002838	A/AT	GAAAACCATCTTCAACCCGA	ACGGACGATACTTTGAACGC	59.9	551	INTERGENIC				
CaPOPII_2795	Ca7	6017174	TAA/TA	TGCACTTGTCAAAATGAGTTAAGG	CGTGTCTCGTAACTCAATCCA	60.5	734	INTERGENIC				
CaPOPII_2796	Ca7	6049845	T/TTTC	TCTTACTCCGAAATTCCCCC	TTATGCCCCAACCATCTCTC	60.3	560	INTERGENIC				
CaPOPII_2797	Ca7	6049898	GAAAAAA/GAAAAAAA	CCATGAGCTGCACATCAAAC	TGGATTTCCCTTTGAAACGA	60.3	453	INTERGENIC				
CaPOPII_2798	Ca7	6063507	ATT/ATTT	GCACGAGCTAAAACTATAGGCG	ATTGGTCATTTTCCCACGAA	60.4	660	DRR	Ca_06754	K	TALE	Homeobox
CaPOPII_2799	Ca7	6065331	TAAAAA/TAAAAAA	TTGTCCACCAAAGTTTCATTTC	ATCATGCTTTTTCCCGTCTG	59.0	492	DRR	Ca_06752	T	bZIP	Phox/Bem1p
CaPOPII_2800	Ca7	6080588	GAAAAAAAA/GAAAAAAAAA	CGGTGATGAGAGAAACAGCA	CGTGTGAATCATGTGCAAAA	60.0	930	INTERGENIC				
CaPOPII_2801	Ca7	6082788	AGTG/A	TGCAGTGCATATGACTCTGTGT	ATTTGACAGCAAAACGGTCC	59.4	477	INTERGENIC				
CaPOPII_2802	Ca7	6087806	CTTTTTTTTTTT/CTTTTTTTTT	ATGTTGGGGAGGTTAGGGAC	GGCCAACCAAACCTCAGTTA	60.1	337	INTERGENIC				
CaPOPII_2803	Ca7	6102449	TA/T	CGAATGAAACGTGTACTCGAAA	GGTCGTAAGATGGAGAAACGA	60.2	618	INTERGENIC				

INDEL marker IDs	Chromosomes /unanchored scaffolds	Physical positions (bp)	InDels (*Kabuli* reference genome-CDC Frontier/PI)	Forward primers (5'-3')	Reverse primers (5'-3')	Anneling temperature (0C)	Expected amplified product size (bp)	Structural annotation		Functional annotation		
								Sequence components of *kabuli* genome	*Kabuli* gene accession IDs	NCBI-KOG	TFs	NCBI-nr database
CaPOPII_2804	Ca7	6116383	GAAAAAAAAAAAA/GAAAAAAAAA	TGGAAAAACTTTTGATGCCA	CATGTGTGTCCAAGAGGGTG	59.1	352	INTRON	Ca_06745	T	MYB_related	Protein phosphatase 2C, manganese/magnesium aspartate binding site
CaPOPII_2805	Ca7	6121180	A/ACTTCC	TTAAATTTCACCGCACAACG	CGACGTTGTGGTTTCATGTC	59.6	771	INTERGENIC				
CaPOPII_2806	Ca7	6122315	TAGAGA/TAGA	TGGGTTCGAGCCTAATTTTG	CCGATTCATCCAAAGCCTTA	60.1	435	INTERGENIC				
CaPOPII_2807	Ca7	6126281	TAAAAA/TAAAA	TTTCTGCCTAGAAAAGTTCCAT	TGTAGCCACGTAAGGAAACCT	57.2	246	DRR	Ca_06744	O		Ubiquitin
CaPOPII_2808	Ca7	6126376	TAAAATAAAT/TAAAATAAATAAAAAATAAAT	CGGTCATTTTTCAACGTATTTT	TGTAGCCACGTAAGGAAACCT	58.1	151	DRR	Ca_06744	O		Ubiquitin
CaPOPII_2809	Ca7	6166062	TAAAAAAAA/TAAAAAAA	TCAACACATGAATTGCACAAAA	CTGTTTCCGTGTCTCCCATT	60.0	269	INTERGENIC				
CaPOPII_2810	Ca7	6614224	TAAAAAAAAAA/TAAAAAAAAAAA	AGCGCTGCAATAGGCTTTTA	TGAAGATGGTTTGCAATGGA	60.1	697	INTERGENIC				
CaPOPII_2811	Ca7	6710088	AAGAGAGAGAGAGAGAGAGAGAGAGAGA/AAGAGAGAGAGAGAGAGAGAGAGAGAGAGAGA	GCCATCCGATCAAAACAAGT	CAAAGGCAAAATTTCGTGGT	59.9	839	INTRON	Ca_06690	R		Zinc finger, UBP-type
CaPOPII_2812	Ca7	6746268	AATATATATATATATATA/AATATATATATATATATATA	TCAACAATGCTTCCTTTCCC	TTCGAAATGTGGGGATTAAAA	60.1	356	INTERGENIC				
CaPOPII_2813	Ca7	6799187	CAAAAAAAAA/CAAAAAAAAAA	AACGACTCTCGTCGTTCCAT	ATGTTGTCGCACTCTCATCG	59.7	647	INTERGENIC				
CaPOPII_2814	Ca7	6912264	TTATATATATATATATATATATAT/TTATATATATATATATATATATATAT	TGCACTTGCACATGTTGAATAA	AATCCCTGCAATTGTGGAAG	60.2	560	INTERGENIC				
CaPOPII_2815	Ca7	6986107	AATATATATATATATATATATAT/AATATATATATATATATATATATAT	CCCAAACTGCAACAAATCCT	TTTATTGTGAGCACGGGTGA	60.0	283	INTERGENIC				
CaPOPII_2816	Ca7	7512410	GATATATATATATATATATATA/GATATATATATATATATATATA	TCCGCCTTAGTGCCGTATTA	TTCCGTGCCATACCGTTTAT	60.6	431	INTERGENIC				
CaPOPII_2817	Ca7	7535397	GTTTTTTTTT/GTTTTTTTT	CGGTAAAAGGGTGCAAATGT	AAAAATTCCTTCCACCAAAGTC	59.9	840	INTERGENIC				
CaPOPII_2818	Ca7	7738038	TC/T	GATTCCATTGGGATTGGTTG	CACGTTTTAGAGATGTCCATTTTG	60.0	505	INTERGENIC				
CaPOPII_2819	Ca7	7738228	AAAAAA/AAAAAATAGAAAAA	GATTCCATTGGGATTGGTTG	CACGTTTTAGAGATGTCCATTTTG	60.0	505	INTERGENIC				
CaPOPII_2820	Ca7	7738595	GTATATATATATATATATATATATA/GTATATATATATATATATATATA	CAAAATGGACATCTCTAAAACGTG	TCTCTCACCCAATCACCAAA	59.9	583	INTERGENIC				

INDEL marker IDs	Chromosomes /unanchored scaffolds	Physical positions (bp)	InDels (*Kabuli* reference genome-CDC Frontier/PI)	Forward primers (5'-3')	Reverse primers (5'-3')	Anneling temperature (0C)	Expected amplified product size (bp)	Structural annotation		Functional annotation		
								Sequence components of *kabuli* genome	*Kabuli* gene accession IDs	NCBI-KOG	TFs	NCBI-nr database
CaPOPII_2821	Ca7	7751929	G/GT	AAGGTATCCCCTTCCCAAGA	TTGTTCGCCATTGATTTTGA	59.8	821	DRR	Ca_13244			
CaPOPII_2822	Ca7	7957533	GATATATATATATATATATAT/GATATATATATATATATATAT	AAGACTTTGCGAGGGTCTGA	ATCCTCACCTTTGATGCTCG	60.0	669	INTERGENIC				
CaPOPII_2823	Ca7	8148654	GATACATACATA/GATACATA	GCAAGTCATGCATGCTCCTA	TCTTGGGGGAATTGGTGTAA	60.0	483	INTERGENIC				
CaPOPII_2824	Ca7	8578840	AATATATATATATATATAT/AATATATATATATATAT	GAGTGGTGTTGGAGTCGATG	ATTTTTGCATGGGGTGTTGT	59.1	566	INTERGENIC				
CaPOPII_2825	Ca7	9180941	TA/TAGA	ATGATTTCCACGAGCCAAAC	TGCAACATTTCCTGATGAGC	59.9	322	INTERGENIC				
CaPOPII_2826	Ca7	9181858	CT/C	TATTACCCAACCAAGCCAGC	TGAACTTTGATTGCCGCTAA	60.0	554	INTERGENIC				
CaPOPII_2827	Ca7	9182870	CATATATATATATATATAT/CATATATATATATATAT	TGCAATTGATGCTTGGTGAT	AAAGGGCATATTTGCAATCG	60.1	857	INTERGENIC				
CaPOPII_2828	Ca7	9332729	T/TTCAAAATGA	ACCAATCCAAATGCCATCAT	TCATCTTTTGATTTGTGGCG	60.0	201	INTERGENIC				
CaPOPII_2829	Ca7	9404184	ATTT/ATT	TTTTCCTTGGGAACGAAGTG	AAACGAGTGCGTGAAACTGA	60.1	555	INTERGENIC				
CaPOPII_2830	Ca7	9578967	AAAAATTAAAA/AAAAATTAAAATTAAAA	ACTCCACAACACTTACGCCC	CGGAATACGGCGAATTAAGA	60.0	669	INTERGENIC				
CaPOPII_2831	Ca7	10192729	TTTTTTTCATATA/TTTTTTTCATATAATGTTTTTTCATATA	CCCATTCCGAATACATTGCT	GAGTTGGGTGCACGATTTTT	59.8	624	INTERGENIC				
CaPOPII_2832	Ca7	10240117	ATTTTTTT/ATTTTTTTT	CCTCAGGATGCGACCATCTA	CCAGCTTGAACAATGTTTCTT	61.2	687	INTERGENIC				
CaPOPII_2833	Ca7	10568493	TTATATATATATATATATATATATATAT/TTATATATATATATATATATATAT	ACGATGGAGGCCAAAGTATG	AAAATAAGGCATGGTGTTGGA	60.0	487	INTERGENIC				
CaPOPII_2834	Ca7	10667777	TA/T	AAACGTCATAGCAAATAGATGAAA	ACTCACTAGGCAACCGCAAT	57.2	713	INTERGENIC				
CaPOPII_2835	Ca7	10740094	TAAAAATTT/TAAAAATTTTAAAAAAATTT	TTTTTGCATTGCCACTTCTG	TTCTGATTTAAATTCCAACTTGTGAG	59.8	492	INTERGENIC				
CaPOPII_2836	Ca7	10821468	AAATAATAATAATAATAATAATAATAATAATAATAAT/AAATAATAATAATAATAATAATAATAATAATAATAATAAT	ACGTGACGTGGATGGGTTAT	TTTCGTCGTCTCAAATCGAA	60.1	816	INTERGENIC				
CaPOPII_2837	Ca7	10863846	TGGGGGGGG/TGGGGGGG	GGACAGGGAAGAGGAAAAGG	ACAACCTACAACAGTCCCCG	60.0	466	INTERGENIC				

INDEL marker IDs	Chromosomes /unanchored scaffolds	Physical positions (bp)	InDels (*Kabuli* reference genome-CDC Frontier/PI)	Forward primers (5'-3')	Reverse primers (5'-3')	Anneling temperature (0C)	Expected amplified product size (bp)	Structural annotation		Functional annotation		
								Sequence components of *kabuli* genome	*Kabuli* gene accession IDs	NCBI-KOG	TFs	NCBI-nr database
CaPOPII_2838	Ca7	11073789	CTTTTTTTTTTT/CTTTTTTTTTT	TTGCAGCATCAACTAGGCAC	CCTCTCCCAAATCGCTACAA	60.0	649	INTERGENIC				
CaPOPII_2839	Ca7	11083612	TAAAAAAAAAAA/TAAAAAAAAAAAA	ACTGGTTTGTCATCAACCTGA	TTTTTAGGCAGGTGGACATATT	58.0	702	INTERGENIC				
CaPOPII_2840	Ca7	11332314	AATATATATATATATATA/AATATATATATATATATATATA	AACCCAAGTTCCAAATGACG	ACCCAAGAGAGGTGCGACTA	59.8	569	INTERGENIC				
CaPOPII_2841	Ca7	11382209	ATTTTTTTTTT/ATTTTTTTTTTT	TGTGCTACTTGCCATGCTCT	CGACCGACTCACTTTTCCAT	59.6	826	INTERGENIC				
CaPOPII_2842	Ca7	11647837	GATTTTATTTT/GATTTTATTTTAATTTTATTTT	GCACCAAACAAGAAGGGAAA	GGAGATTCGTGCGCATATTT	60.1	166	INTERGENIC				
CaPOPII_2843	Ca7	12003043	TT/TTTAT	GATGGCAAAGACACACATGG	CGGTTGTGAGCATTGGACTA	60.0	884	INTERGENIC				
CaPOPII_2844	Ca7	12204671	ATTTTTTTTTTT/ATTTTTTTTTT	GCTTGGTTGGATAGCGAGAG	CACCTCAGCTACCGTTCACA	60.0	730	INTERGENIC				
CaPOPII_2845	Ca7	12365777	TTATATATATATATATATAT/TTATATATATATATATATATATAT	TTCGGGAACAGATTTTGGAG	TTCAAAAGAATCGGAGGAAGA	60.0	434	INTRON	Ca_09277	K	HSF	Heat shock factor (HSF)-type, DNA-binding
CaPOPII_2846	Ca7	12447118	TAAAAAAAA/TAAAAAAAAA	TTTGTCCCGAGTTCGAATGT	ATAAGGGGCAGCCTTAGGAA	60.5	301	INTERGENIC				
CaPOPII_2847	Ca7	12465968	TTATATATATATATATATATATATATATAT/TTATATATATATATATATATATATATATATATATAT	TTATGAACTCGGTTGCTTCG	CAGATGTGAGTCAGTCCGAGA	58.9	331	DRR	Ca_09270			
CaPOPII_2848	Ca7	12539752	TTTTATTTA/TTTTATTTTATTTTATTTA	TCCTACTGAAGTCGTGGCCT	TGCATTTTCACCAACATCGT	59.9	596	INTERGENIC				
CaPOPII_2849	Ca7	12691360	CAAAAATCATTTT/CAAAAATCATTTTATTAAAAAAATCATTTT	GTCAAGGGCCTATGGTCAGA	TTTGGTCATGGGATGTGCTA	60.1	909	INTERGENIC				
CaPOPII_2850	Ca7	13555513	C/CGTAAT	TTTCTAATCAGTGGCCTGGTC	TCAACGAGCATGTGGAAAAG	59.2	554	DRR	Ca_16040		bZIP	Basic-leucine zipper (bZIP) transcription factor
CaPOPII_2851	Ca7	13588964	AATATATATATATATAT/AATATATATATATATATAT	AAATCGATGAATGGGTGGAG	AGAAACGTGCGGTGTCTCTT	59.8	561	INTERGENIC				
CaPOPII_2852	Ca7	13891702	CTATATATATATATATAT/CTATATATATATATATATAT	TGCCTTTTACTCTTGGTGGG	GAGTGGGATTTTGGAGGTGA	60.1	596	INTERGENIC				
CaPOPII_2853	Ca7	14075293	CATATATATATATATAT/CATATATATATATAT	TTTGTTTAGCCACAACTTTTGA	CGTGCTCGGGATTTATTTGT	57.6	862	INTERGENIC				
CaPOPII_2854	Ca7	15467222	TTTATTATTATTATTATTATTATTATTATTATTATTATTATTAT/TTTATTATTATTATTATTATTATTATTATTATTATTATTATTATTATTAT	TGGGAAACGGTGCTAAACTT	TCCATTTACGGCAAATTCAA	59.6	716	INTERGENIC				

INDEL marker IDs	Chromosomes /unanchored scaffolds	Physical positions (bp)	InDels (*Kabuli* reference genome-CDC Frontier/PI)	Forward primers (5'-3')	Reverse primers (5'-3')	Anneling temperature (0C)	Expected amplified product size (bp)	Structural annotation		Functional annotation		
								Sequence components of *kabuli* genome	*Kabuli* gene accession IDs	NCBI-KOG	TFs	NCBI-nr database
CaPOPII_2855	Ca7	15562093	GAAAATAA/GAA	AATACACCACCTTCCACCCA	TTTTTAAGGGATCCGACGTG	60.1	161	INTERGENIC				
CaPOPII_2856	Ca7	15653714	CTTTTTT/CTTTTTTT	TTTCTTGCAAGGGTAATTTGC	GCTTGTGTGGCCGACTTAAT	59.2	641	INTERGENIC				
CaPOPII_2857	Ca7	16001405	AATTATTATTATTATTATTATTATTAT/AATTATTATTATTATTATTATTATTATTAT	AAAACACAACGTGCAAACGA	GGGTCACCAAGCACAGTTTT	60.2	679	INTERGENIC				
CaPOPII_2858	Ca7	16051093	TAA/TAAA	TGGTTCGTCGGATATGTGAA	TTGAGTTCACGTACGCGTTT	59.9	600	INTERGENIC				
CaPOPII_2859	Ca7	16126431	TAAAAAAAAAAA/TAAAAAAAAAAAA	TCACTATTTCATGCAACCGC	TTTGAAGATTTGCGTTACGA	59.7	494	INTERGENIC				
CaPOPII_2860	Ca7	16349610	CTTTTTTTTTTT/CTTTTTTTTTT	GCAGTGCTGAATTTTGGTGA	TTGGTTTCATGACTTGCATCT	59.8	740	INTERGENIC				
CaPOPII_2861	Ca7	16941588	TAAA/TAAAA	TGCCGTAGTATTCACCACCA	TGCCATGAAATTCTGCCATA	60.0	852	INTERGENIC				
CaPOPII_2862	Ca7	17133790	ATTTTTTT/ATTTTTT	GACACTACCCGGCATCAAGT	CCAACCCAAATAAATGAAAATCA	60.0	530	INTERGENIC				
CaPOPII_2863	Ca7	17172621	AATCATAT/AATCATATCATAT	GGCTAAACCTTGTCTTTGGC	AGACTCTGCTTGACCGGAAA	58.8	541	INTERGENIC				
CaPOPII_2864	Ca7	17374590	CTTTTTTTTTTTT/CTTTTTTTTT	GCATCTGTAAAACAAGACCCG	ACATCGATAAGAGGCAGCGT	59.6	525	INTERGENIC				
CaPOPII_2865	Ca7	17596965	GACTTAACTTAA/GACTTAA	CATCTATGCCACCTCCACCT	GATCCAGAAGAAGGCTGCAC	60.0	789	INTRON	Ca_15849			Protein of unknown function DUF1639
CaPOPII_2866	Ca7	17922655	TGGGGGG/TGGGGG	AAGTCGAGTGCTTGAGCCAT	TCTTTGCGTGCGTGCTATAC	60.0	606	DRR	Ca_15831		bHLH	Protein kinase, catalytic domain
CaPOPII_2867	Ca7	18007668	CTTATTATTATTATTATTAT/CTTATTATTATTATTAT	CTCGTGTTTCTCGGTGGTTT	AAGTCCATTGAAGTGTCGCC	60.1	264	INTERGENIC				
CaPOPII_2868	Ca7	18188416	CTTT/CTTTT	TGTCTTGAGCATGCCTTGTC	AAATGCAAACCCAAATGCTC	60.0	525	INTERGENIC				
CaPOPII_2869	Ca7	18333235	A/ATTAT	GCAAGTGTGTTTGTGGCACT	TTCAAGCACATAATGCAAGTCC	59.8	113	INTERGENIC				
CaPOPII_2870	Ca7	18439833	TAGAG/TAG	TATTTGGAGCAGCGGGTTAG	CCCACAACCACACACTCATC	60.2	813	INTERGENIC				
CaPOPII_2871	Ca7	18587623	AAAGGCGGAAGGCGG/AAAGGCGG	CGATGATGACTGATTGGGGT	TGTTTGTGTTTTGAGGCGTT	60.7	374	INTERGENIC				

INDEL marker IDs	Chromosomes /unanchored scaffolds	Physical positions (bp)	InDels (*Kabuli* reference genome-CDC Frontier/PI)	Forward primers (5'-3')	Reverse primers (5'-3')	Anneling temperature (0C)	Expected amplified product size (bp)	Structural annotation		Functional annotation		
								Sequence components of *kabuli* genome	*Kabuli* gene accession IDs	NCBI-KOG	TFs	NCBI-nr database
CaPOPII_2872	Ca7	18588675	TTATAT/TTATATAT	CCAAGACTCCTTGTAACACCC	GCACATATATACACGTAAAATCGCA	58.6	299	INTERGENIC				
CaPOPII_2873	Ca7	19095896	GTTTTTTTT/GTTTTTTT	GTTTTGCTTCCCACTTCCAA	CTCCTTGCAGATATAGCGCC	60.1	580	INTERGENIC				
CaPOPII_2874	Ca7	19534402	TTATATATATATATATATATATATATATATAT/TTATATATATATATATATATATATATAT	GCCCAAATTCTAAATGGCAA	CACCCTCGGTCTAAAGTCCA	59.9	448	INTRON	Ca_12348			Phosphoenolpyruvate carboxykinase, ATP-utilising
CaPOPII_2875	Ca7	19589014	AATTTATCTATTTATCTATTTAT/AATTTATCTATTTATCTATTTATCTATTTAT	GGTCGTCCAAATAGTACCCG	CCGATTTCCCAAAGGTTTTT	59.3	358	INTERGENIC				
CaPOPII_2876	Ca7	19589201	TTATATATATATATATATATATAT/TTATATATATATATATATATATAT	GGTCGTCCAAATAGTACCCG	AGGAAGCCCAAGTTCCAAAT	59.3	828	INTERGENIC				
CaPOPII_2877	Ca7	19718791	GTAATAATAATAATAATAATAATAATAATAATAATAATAATAA/GTAATAATAATAATAATAATAATAATAATAATAATAATAATAATAATAA	TTAGGGAGCTTACATCGCGT	TAGCTGTGAGTTGACGACGG	59.9	488	INTERGENIC				
CaPOPII_2878	Ca7	19857139	AAATAATAATAATAATAATAATAATAATAATAA/AAATAATAATAATAATAATAATAATAATAATAA	CCATATGTCATAGTGCGGTCA	AGAGGCAAACAAGAACCGAA	59.4	314	INTERGENIC				
CaPOPII_2879	Ca7	19860378	GT/G	CCTCAATATGTTGGGACATGC	TGATTCTCAGGGAGGGTGTC	60.2	464	INTERGENIC				
CaPOPII_2880	Ca7	20059991	TTGT/TTGTGT	TGATTCGCATCAAACCAATAA	TCATCATTTGAGTCATCTTGCC	59.0	487	INTERGENIC				
CaPOPII_2881	Ca7	20252580	GTAGAATA/GTAGAATAGAATA	ACCTGTCAGCTAAGAGCCCA	CACATGTGTGAACCTCCTGG	60.0	641	DRR	Ca_12383			Protein of unknown function DUF220
CaPOPII_2882	Ca7	20488727	TC/T	CAAACATGGTGTCCTTGTGC	ATTGCCCCATGTTTCAAGAG	60.0	731	CDS (FRAME SHIFT)	Ca_12399	T	M-type	Leucine-rich repeat
CaPOPII_2883	Ca7	20494272	CAA/CAAA	AGACGTGTCGATCGAGCTTT	AGCTGAATGCAAACCCTTGA	60.0	497	DRR	Ca_12400		AP2	Pathogenesis-related transcriptional factor/ERF, DNA-binding
CaPOPII_2884	Ca7	20505889	ATA/ATAGAAATTA	GGCATGGAAAATTCTGAAAAA	TTCATTTCTCGATTGAGGGC	59.0	803	INTERGENIC				
CaPOPII_2885	Ca7	20505916	GGA/G	GGCATGGAAAATTCTGAAAAA	TTCATTTCTCGATTGAGGGC	59.0	803	INTERGENIC				
CaPOPII_2886	Ca7	20510218	GTAAT/GTAATAAT	TTGAACAATGCAAGAGCCAC	CTGCTCATGTTTCCCGTTTT	59.8	933	INTERGENIC				
CaPOPII_2887	Ca7	20537234	CAAAAA/CAAAAAAA	TCGCACAGGATGAGAACTTG	TCAGCCTCTCCATCAAACCT	60.0	179	INTERGENIC				
CaPOPII_2888	Ca7	20566359	T/TA	GGTGACTGGTGGGATTGTTC	TGAAAGTTCTCTTTAATCTGTCACG	60.2	370	INTERGENIC				

INDEL marker IDs	Chromosomes /unanchored scaffolds	Physical positions (bp)	InDels (*Kabuli* reference genome-CDC Frontier/PI)	Forward primers (5'-3')	Reverse primers (5'-3')	Anneling temperature (0C)	Expected amplified product size (bp)	Structural annotation		Functional annotation		
								Sequence components of *kabuli* genome	*Kabuli* gene accession IDs	NCBI-KOG	TFs	NCBI-nr database
CaPOPII_2889	Ca7	20567011	TAAAAAAA/TAAAAAA	TGGAATTCATGTGTCTTTGGA	CACCTTCCTTGTGTTCCGAT	59.0	281	INTERGENIC				
CaPOPII_2890	Ca7	20574766	GTTT/GTTTT	GGGAGATCGGAATGCATAGA	ATGCGTGACGGTGCATAATA	60.0	276	INTERGENIC				
CaPOPII_2891	Ca7	20617780	C/CAAT	GGCGAGGGATGGAATTTTAG	GAATTGGGACTTCTGCCGTA	60.8	777	INTERGENIC				
CaPOPII_2892	Ca7	20623409	TAACAA/TAA	AGGTGTCGAAGGATCACCAC	CTCGAGGAGACGAGAAATGC	60.0	212	INTERGENIC				
CaPOPII_2893	Ca7	20626429	CTAATA/CTAATAATA	TCAGTTATCGTCGTTGGTCG	CGTGCAACATTTTTCTTCCA	59.7	623	INTERGENIC				
CaPOPII_2894	Ca7	21099731	T/TGCGCAA	GAGAAATCAAAATTGCCCCA	AGTGCCTCCTGTTCCTGAGA	59.9	500	INTERGENIC				
CaPOPII_2895	Ca7	21207750	GCATCCCCA/GCATCCCCATCCCCA	TTTTATCCACCAAAAATTCAAAAA	GAATGAGCAAGAAAGACGGC	58.9	505	INTERGENIC				
CaPOPII_2896	Ca7	21434053	ATTTTTTTT/ATTTTTTTTT	TTTTTGTGGGCTAAGTGGAG	GAAGCACAAAGAATAAAAGCAACA	57.9	939	INTERGENIC				
CaPOPII_2897	Ca7	21670841	A/AAGAT	TGACAAATCTTTGCAACCCA	ATTGCTCATTTCATGCCCTC	60.1	217	INTERGENIC				
CaPOPII_2898	Ca7	21685577	TT/TTAT	TTTATAAATTGGCCCCATCG	TAGGGGGACCAAGAAACTGG	59.6	501	INTERGENIC				
CaPOPII_2899	Ca7	21728387	CAAAA/CAAA	ATCTCTGCGCAATATCGCTT	AGCCGACTTTTCCTACCACA	60.0	674	INTERGENIC				
CaPOPII_2900	Ca7	21729097	TAAAAA/TAAAA	TGCTCAAAAGGGGAAGAAAA	GCCACGTGGTTGAAAGAAGT	59.8	240	INTERGENIC				
CaPOPII_2901	Ca7	21729303	T/TC	TTGCTCAAAAGGGGAAGAAA	TTGTTATTTTCTTTATGCCGTTG	59.8	426	INTERGENIC				
CaPOPII_2902	Ca7	21731192	ATTTTTTTTT/ATTTTTTTT	TGATGGATACCCTTTTGAACG	ACCGCTCCAGCTGAGAATAA	59.8	838	INTERGENIC				
CaPOPII_2903	Ca7	21743270	CAA/CA	TTGCCCCTCTAAATTCTTGG	TCCCTCGTATTTAGCCCTCA	59.2	686	DRR	Ca_14521			Transposase, Ptta/En/Spm, plant
CaPOPII_2904	Ca7	21743482	TGGCTAAATATGAGGGCTAAATA/TGGCTAAATA	TCGAAGCATAAGGTTGATCC	GGGATTGAGCGCCTATACAA	57.8	500	DRR	Ca_14521			Transposase, Ptta/En/Spm, plant
CaPOPII_2905	Ca7	21743627	ATTTTTTT/ATTTTTTTT	TGAGGGCTAAATACGAGGGA	GGGATTGAGCGCCTATACAA	59.7	461	DRR	Ca_14521			Transposase, Ptta/En/Spm, plant

INDEL marker IDs	Chromosomes /unanchored scaffolds	Physical positions (bp)	InDels (*Kabuli* reference genome-CDC Frontier/PI)	Forward primers (5'-3')	Reverse primers (5'-3')	Anneling temperature (0C)	Expected amplified product size (bp)	Structural annotation		Functional annotation		
								Sequence components of *kabuli* genome	*Kabuli* gene accession IDs	NCBI-KOG	TFs	NCBI-nr database
CaPOPII_2906	Ca7	21743947	AA/AAATA	CCTGGTGTGCATTGAAAAGA	AATTGCGGTTTTGGTTCAAG	59.7	410	DRR	Ca_14521			Transposase, Ptta/En/Spm, plant
CaPOPII_2907	Ca7	21745626	TTTTATATTTAT/TTTTAT	ATAGGGCCCATTTTACAGCG	CCAGTTGCCCTGTCCATACT	61.2	455	INTRON	Ca_14522	O	MYB	SANT domain, DNA binding
CaPOPII_2908	Ca7	22036269	GCTC/G	GCTCAGCCTTGAAATCGAAC	TTCATCATCATCTTCAAACAAAA	60.0	627	INTERGENIC				
CaPOPII_2909	Ca7	22038011	C/CT	CTCCAAATTTCCAGCCAAAA	ACACGGACGGGTCTTGTATC	60.0	822	INTERGENIC				
CaPOPII_2910	Ca7	22039243	ACC/AC	CGTTATTGCAACCACAACCA	CAATTCAACTCCGACGTGTG	60.4	622	INTERGENIC				
CaPOPII_2911	Ca7	22043317	T/TG	TGGTTCAAATGCTGTAATCTTCTC	ATTCGCTCAATACACGGGTC	59.7	884	INTERGENIC				
CaPOPII_2912	Ca7	22045478	ATTTTTTTT/ATTTTTTT	TGTGGTGTTCCCAAAAGACA	CAAAGCGAAGTGCAAAATGA	60.0	246	INTERGENIC				
CaPOPII_2913	Ca7	22046891	CATAATAATAATAATAATAATAATAATAATAATAATAATAATAAT/CATAATAATAATAATAATAATAATAATAATAATAATAATAATAATAATAATAATAAT	ATAATTTGCCTTTGCGGTTG	TTCTGTGAGCAAGGCTGATG	60.0	319	INTERGENIC				
CaPOPII_2914	Ca7	22046957	CTATATATATATATATATATATATAT/CTATATATATATATATATATATAT	ATAATTTGCCTTTGCGGTTG	TTCTGTGAGCAAGGCTGATG	60.0	319	INTERGENIC				
CaPOPII_2915	Ca7	22046996	TGCGCGC/TGC	ATAATTTGCCTTTGCGGTTG	ACCTGGTCGTAGATGCCACT	60.0	414	INTERGENIC				
CaPOPII_2916	Ca7	22091542	GAAAAAAAAAAA/GAAAAAAAAAA	CCCCAACCCTCCATTCTATT	AATTGCAAGATGAGGGCAAC	60.0	347	INTERGENIC				
CaPOPII_2917	Ca7	22126044	TAAAAAAAAA/TAAAAAAAAAA	TGAAGCAACAAAGCTGATGAA	TTGTTGCAACCACTTCCAAA	59.6	518	INTERGENIC				
CaPOPII_2918	Ca7	22327481	TC/T	GATTCATCGCAGCTCAAACA	GATCAAAACCGGGGAAAAAT	60.0	638	DRR	Ca_14548			
CaPOPII_2919	Ca7	22960245	AATATATATATATATATATATATATATATATATATATATAT/AATATATATATATATATATATATATATATAT	TTGGTATTGAACTCGGAGGC	CTTTCACGTTCGGTTACGGT	60.1	700	INTERGENIC				
CaPOPII_2920	Ca7	22969908	CTT/CTTT	AATGTGCTGATCTGTGAGCG	CCTTGGAGTATTCTCGGCAG	60.0	894	INTERGENIC				
CaPOPII_2921	Ca7	23075370	AAATAATAATAATAATAATAATAATAATA/AAATAATAATAATAATAATAATAATAATAATAATAATA	AACGGATGACCCTCTCCTCT	TTTTGGTTGGGATTTTTGGA	60.1	812	INTERGENIC				
CaPOPII_2922	Ca7	23667025	AAATAATAATAATAATAATA/AAATAATAATAATAATAATA	ACACTGATTTCCCAAAACGC	TTTCCTTCCCTTCTCATTGC	60.0	728	DRR	Ca_26877	G	FAR1	NAD-dependent epimerase/dehydratase

INDEL marker IDs	Chromosomes /unanchored scaffolds	Physical positions (bp)	InDels (*Kabuli* reference genome-CDC Frontier/PI)	Forward primers (5'-3')	Reverse primers (5'-3')	Anneling temperature (0C)	Expected amplified product size (bp)	Structural annotation		Functional annotation		
								Sequence components of *kabuli* genome	*Kabuli* gene accession IDs	NCBI-KOG	TFs	NCBI-nr database
CaPOPII_2923	Ca7	23780060	TTATATATATATATAT/TTATATATATATAT	CGCTCGTCTATGCGATTGTA	TAAAATCTCCGCTCGCAACT	60.0	946	INTERGENIC				
CaPOPII_2924	Ca7	23882859	AATATATATATATATATATA/AATATATATATATATATATATA	TATTTTGGATTTTGGCTCGG	ATGGGTTTCGCGTAGACAAC	59.9	400	INTERGENIC				
CaPOPII_2925	Ca7	23903739	TAAATAA/TAAATAAAAATAA	TGAGCATGGAATTTGGTTGT	TGAATTCATGGCTAAGAAGCCT	59.0	592	INTERGENIC				
CaPOPII_2926	Ca7	23907440	AATATATATAT/AATATATATATAT	TTGGAGAATTCAAAAATAAGGCA	CGTGACAAAGCACGAAAGAA	60.0	513	INTERGENIC				
CaPOPII_2927	Ca7	24027767	GATAATAATAATAATAATAATAATAA/GATAATAATAATAATAATAATAATAATAA	ACAATGGATGCTCACGACAC	GTTTCCCATCCTCCCTTTGT	59.6	633	INTERGENIC				
CaPOPII_2928	Ca7	24347972	TAAAA/TAAAAA	CTCTCATCTGAGGGCAAAGC	CGATTGAATTAAGCCCAGGA	60.1	356	INTERGENIC				
CaPOPII_2929	Ca7	24348355	TAA/TAAA	TCCTGGGCTTAATTCAATCG	CGTTCCTGAGGGAATTGTGT	60.0	378	INTERGENIC				
CaPOPII_2930	Ca7	24348515	CAA/CAAA	TCCTGGGCTTAATTCAATCG	CGTTCCTGAGGGAATTGTGT	60.0	378	INTERGENIC				
CaPOPII_2931	Ca7	24421160	AATATATATATATATATATATA/AATATATATATATATATA	AGGAAGCCCAAGTTCCAAAT	ACAGCCAACAACCACATCAA	59.9	469	INTERGENIC				
CaPOPII_2932	Ca7	24485265	CG/CAACTTTG	GTCCCACCCTAGGAGCTTTC	CGGACCAAAAATTAAAGCGA	60.1	986	INTERGENIC				
CaPOPII_2933	Ca7	24502290	T/TAAGCA	AATTTGGCAGAACAGGGATG	AGAAAAGCAGGGGAAGCAAT	59.9	575	INTERGENIC				
CaPOPII_2934	Ca7	24786712	AATATATATATATATATATATATAT/AATATATATATATATATATATATATAT	ACAGACCCGACCAAGTCAAC	TCAAACATTTAACGATCACCATT	60.0	447	DRR	Ca_19894	GC	HSF	UDP-glucuronosyl/UDP-glucosyltransferase
CaPOPII_2935	Ca7	25060530	GTTTTTTTT/GTTTTTTTTT	TTGTCGATTTGGCACTTCAG	TGAATGTGGATCCCCTCAAT	59.8	327	INTERGENIC				
CaPOPII_2936	Ca7	25342809	TAAAAAAAA/TAAAAAAAAA	CATTCAACAGCCACATGACC	CGGTACCGCATTCGAGTATT	60.0	642	INTERGENIC				
CaPOPII_2937	Ca7	25436582	GAAAAAAA/GAAAAAAAA	ATTTTTGAGAGCCAAGCCAA	GAACGTGTTCGGTAAGGGTG	59.8	786	INTERGENIC				
CaPOPII_2938	Ca7	25540307	CTTTT/CTTT	CCTCAGATGAGAGCCAGGAC	GAGCGCTATTACAGTTCGGC	59.9	472	INTERGENIC				
CaPOPII_2939	Ca7	25563600	AAGAGAGAGAGAGAGAGAGAGAGAGAGAGAGA/AAGAGAGAGAGAGAGAGAGAGAGAGAGAGAGA	CACAAACAAGAACGACGGTG	GAGGCTACGTGTTGTACGCA	60.2	603	CDS (FRAME SHIFT)	Ca_23609	KT		Zinc finger, PHD-type

**INDEL marker IDs**	**Chromosomes /unanchored scaffolds**	**Physical positions (bp)**	**InDels (*Kabuli* reference genome-CDC Frontier/PI)**	**Forward primers (5'-3')**	**Reverse primers (5'-3')**	**Anneling temperature (0C)**	**Expected amplified product size (bp)**	**Structural annotation**		**Functional annotation**		
								**Sequence components of *kabuli* genome**	***Kabuli* gene accession IDs**	**NCBI-KOG**	**TFs**	**NCBI-nr database**
CaPOPII_2940	Ca7	25626892	CGG/CGGG	TTGGCCAAGTCCCTGTTTAC	CCCGTGATAGGCAGTACGTT	60.0	420	INTERGENIC				
CaPOPII_2941	Ca7	26035041	TAAAAAAAA/TAAAAAAAAA	TAAAGAGATTCGCAGCGGTT	CATGTTTTCGAAGCTCACCA	60.0	407	INTERGENIC				
CaPOPII_2942	Ca7	26075420	TTATATATATAT/TTATATATATATAT	TATTTTGGATTTTGGCTCGG	TTTGGATCAAGGAGAAACGG	59.9	584	INTERGENIC				
CaPOPII_2943	Ca7	26160184	AG/A	GTGGCTACCTTCTTGGGTGA	TTTCATCGCCGAAACCTATC	60.1	141	INTERGENIC				
CaPOPII_2944	Ca7	26221720	AATATATATATATATATATAT/AATATATATATATAT	CTTGGGAATTGGAGAGCAAG	GGGTTTCTTTCCTTGAAGCC	59.8	671	INTERGENIC				
CaPOPII_2945	Ca7	26228410	GAAAAAAAAAA/GAAAAAAAAAAAA	ATGGTGCAAAATGGTGACAA	GAAAGTCGGTGCATGGAAGT	59.8	304	INTERGENIC				
CaPOPII_2946	Ca7	26235537	TTATATATATATA/TTATATATATATATATA	TGCGACCTCAGTTTCTCAAA	GGGGTCATAGCATCCCCTAT	59.6	157	INTERGENIC				
CaPOPII_2947	Ca7	26662956	CAA/CA	TCCAAGCTTCCTTCAATGGT	CTCAAAAGCGCTCCAATTTC	59.7	542	INTERGENIC				
CaPOPII_2948	Ca7	26694678	TCCCCC/TCCCCCCCCCC	CTCGGCCATCCGAGATATAA	GCTCACTAACTCTGCCTGGG	60.0	881	CDS (FRAME SHIFT)	Ca_16250			
CaPOPII_2949	Ca7	26701106	A/ACTAGGAATTACTCCC	CAATAGGGCGAGTCGAATGT	AAGTCCAGTAGCCGAAAGCA	60.1	838	DRR	Ca_16249			
CaPOPII_2950	Ca7	26701534	C/CG	AAGCTTCATCCTGGTCATGG	AAGTCCAGTAGCCGAAAGCA	60.1	381	DRR	Ca_16249			
CaPOPII_2951	Ca7	27031473	TTCTCTCTCTCTCTCTCTCTCTCTCTCTCTCTCTCTCTC/TTCTCTCTCTCTCTCTCTCTCTCTCTCTCTCTCTCTCTCTC	TCTTCCTTCCTTGCTCTCCA	GACTTGGCATGTCATGATGG	60.1	551	INTERGENIC				
CaPOPII_2952	Ca7	27341260	A/AG	CACGATCCACACCAAAACAC	TCTCCCTCCTTCTCCCATTT	59.9	382	INTERGENIC				
CaPOPII_2953	Ca7	27346895	TTATATATATATATATATATATAT/TTATATATATATATATATATATATATAT	TGCTTGATTGCTTGAGTTCC	CGCGCAGATTGTCAAATAGA	59.0	641	INTERGENIC				
CaPOPII_2954	Ca7	27521328	CAAA/CAA	GCCAAATTCCCTCTTTCTCC	CCTTTCCACACAACCACAGA	60.0	393	INTERGENIC				
CaPOPII_2955	Ca7	27761690	TTATATATATATATATATATATATAT/TTATATATATATATATATATATAT	AAATTCGCAGCACTTACGCT	GTTTGTGAGTAAGCTCGCCC	60.0	873	INTERGENIC				
CaPOPII_2956	Ca7	28089294	GAAAAA/GAAAAAA	CGTAAAGTCCACTTCCGTTTC	CAACAGATGTTGTGCGTGTG	58.8	500	INTERGENIC				

INDEL marker IDs	Chromosomes /unanchored scaffolds	Physical positions (bp)	InDels (*Kabuli* reference genome-CDC Frontier/PI)	Forward primers (5'-3')	Reverse primers (5'-3')	Anneling temperature (0C)	Expected amplified product size (bp)	Structural annotation		Functional annotation		
								Sequence components of *kabuli* genome	*Kabuli* gene accession IDs	NCBI-KOG	TFs	NCBI-nr database
CaPOPII_2957	Ca7	28129563	CAAAAAAAAAAAAA/CAAAAAAAAAAAAAA	TCTTCACCAAAACAAACCCA	GCATCGTAAAGTTGCACATCA	59.0	835	INTERGENIC				
CaPOPII_2958	Ca7	28190197	AGTTTATTTGG/AGTTTATTTGGTTTATTTGG	TTGGTTTATTTGGGTTGGGA	ACCAGGAAAAGAAGAAGGGC	60.0	519	INTERGENIC				
CaPOPII_2959	Ca7	28317021	ATTTTTTTTTTTT/ATTTTTTTTTTT	GATCCCATGTATGGTGAGGG	CTGTCTGTTGCAGCTGGTGT	60.0	481	INTERGENIC				
CaPOPII_2960	Ca7	28529637	ATTTTTTT/ATTTTTTTT	CAGACCAGACCGTGCTTTTT	ATGTGGCCTGTCATAGGCTT	60.3	498	INTERGENIC				
CaPOPII_2961	Ca7	28698838	TTATATATATATATATATATATAT/TTATATATATATATATATAT	TCCACACAAAAACCCAATCA	CCTGTGCAGAAGTTGCTTGA	59.8	691	INTRON	Ca_18957			Protein of unknown function DUF1639
CaPOPII_2962	Ca7	28702659	ATCCTCC/ATCC	TCCATTAACCCTCTTGAGGAAA	TCAGGATGAAGAAGGGGAGA	59.9	531	INTERGENIC				
CaPOPII_2963	Ca7	28707412	GAAATAACAATAAAAACAAATAACAATAAAA/GAAATAACAATAAAAACAAATAACAATAAAAACAAATAACAATAAAA	ATCCCACCATCAAAAACCAA	GATATGTTCCGAGTGCCGTT	60.0	491	INTERGENIC				
CaPOPII_2964	Ca7	29245826	AATATATATATATATATATATA/AATATATATATATATATATATATATATA	ATTTCCAGCATGCCAATTTC	GCAACATTGGGTGTTGTTTG	59.9	516	INTERGENIC				
CaPOPII_2965	Ca7	29311995	ATATTATTATTATTATTATTATTATTATTATTATTATTATTATT/ATATTATTATTATTATTATTATTATTATTATTATTATTATTATTATTATTATT	TCATCGAAAACTTCAAAAACTATGA	GATGCAGGTCATGAAACCAA	59.2	494	INTERGENIC				
CaPOPII_2966	Ca7	29416457	ACC/AC	AAAATCATTACTCACCATAAGTGGAA	TGCTCTCCTCCTTCAACAACT	59.3	436	INTERGENIC				
CaPOPII_2967	Ca7	29416537	ATATAAATTATAAATTATAAATTAT/ATATAAATTATAAATTATAAATTATAAATTAT	AAAATCATTACTCACCATAAGTGGAA	TGCTCTCCTCCTTCAACAACT	59.3	436	INTERGENIC				
CaPOPII_2968	Ca7	29416730	AAGAAGTTGTTGAAGGAG/AAG	ATTGTAGAAGGAAGAGGAGAAGCA	AAAAAGCTGATGTGTACCTGTTGA	59.9	100	INTERGENIC				
CaPOPII_2969	Ca7	29417874	ATTT/ATT	AGTTGTTGAAGGAGGAGAGCA	TTGTAGGCTGGACATCATGC	59.1	645	INTERGENIC				
CaPOPII_2970	Ca7	29418101	C/CT	CATGTACACGTCACATCGGA	TTGTAGGCTGGACATCATGC	59.0	329	INTERGENIC				
CaPOPII_2971	Ca7	29433568	T/TG	AGGCATGATTCAAGGTACGG	CAGGTGAAGGTCCATTTCGT	60.0	278	DRR	Ca_11776			
CaPOPII_2972	Ca7	29446201	ATTTTTTTTTT/ATTTTTTTTTTTT	CGTTGCGCATGTGTAATTTG	TCATTCCCAACATTATTTCGTG	61.1	639	DRR	Ca_11775	R		Alpha/beta hydrolase fold-1
CaPOPII_2973	Ca7	29610109	TCCC/TTCCCC	AACAGAAAGAAGGCCGTTTG	TGAGTTAAGCCCCGCTAGAA	59.4	134	DRR	Ca_11764			F-box domain, cyclin-like

INDEL marker IDs	Chromosomes /unanchored scaffolds	Physical positions (bp)	InDels (*Kabuli* reference genome-CDC Frontier/PI)	Forward primers (5'-3')	Reverse primers (5'-3')	Anneling temperature (0C)	Expected amplified product size (bp)	Structural annotation		Functional annotation		
								Sequence components of *kabuli* genome	*Kabuli* gene accession IDs	NCBI-KOG	TFs	NCBI-nr database
CaPOPII_2974	Ca7	29776063	TAAAAAAA/TAAAAAAAA	TGGTTACACTTCGTGGCAAA	CATGGGCACTACATCGTCAC	60.1	679	INTERGENIC				
CaPOPII_2975	Ca7	29987440	AAATAATAATAATAATAATAATAATA/AAATAATAATAATAATAATAATAATAATAATAATAATA	GAACATGGTTGATTGGATTGC	CTCCTCGTTGTGTTGGATCA	60.2	751	INTERGENIC				
CaPOPII_2976	Ca7	30053648	CAAAGT/C	GGACTGAAAAGAAAAATATGCGA	GTGCTCTCTTTTGCCGATTC	59.6	774	INTERGENIC				
CaPOPII_2977	Ca7	30059154	AGG/AGGG	GTGCAGTGCTGTTTTCTGGA	CAAATTGGCAACCTGGTTCT	60.0	481	INTRON	Ca_11722	P	NF-YB	ATPase, P-type, K/Mg/Cd/Cu/Zn/Na/Ca/ Na/H-transporter
CaPOPII_2978	Ca7	30078953	GAAAA/GAAA	ATGCCAAAACGTAGCAAAGC	CGCACCACTTTCCATCATAA	60.3	749	INTERGENIC				
CaPOPII_2979	Ca7	30102864	GA/G	TAAACTGGGGCAATCACACA	TGATTATGCAGCAACCAAAA	60.0	585	DRR	Ca_11717		bZIP	Protein of unknown function DUF760
CaPOPII_2980	Ca7	30130333	CTTT/CT	GGTACGGTACGGTGGCTAAA	TCTCCGATCCATAATAATTGACCTA	59.9	302	INTRON	Ca_11715	U		Citron-like
CaPOPII_2981	Ca7	30131573	ATTTTT/ATTT	TGTTTTCGGCTAACACATCG	TGCAATGTCCAACTCACTTGT	59.7	372	INTRON	Ca_11715	U		Citron-like
CaPOPII_2982	Ca7	30208302	CGA/C	TTGGACGATGTGGATTGAAA	CCATCTCAGCATGTGTTTGG	59.9	642	INTRON	Ca_11706			Mitochondrial inner membrane translocase complex, subunit Tim17/22
CaPOPII_2983	Ca7	30208770	ATTT/ATT	CCAAACACATGCTGAGATGG	CTTCCTTTGCACGACCGTAT	60.1	707	INTRON	Ca_11706			Mitochondrial inner membrane translocase complex, subunit Tim17/22
CaPOPII_2984	Ca7	30220020	CA/C	CGCTCTAGCGCAAACTAAGG	AACACCGACACCGAATTCAT	60.3	163	DRR	Ca_11705	GC	HSF	UDP-glucuronosyl/UDP-glucosyltransferase
CaPOPII_2985	Ca7	30221695	T/TGAA	CTTGTTGATGCAATGTTGGC	TTTGGCCACACAAAAACAAA	60.1	538	INTERGENIC				
CaPOPII_2986	Ca7	30415200	C/CG	CAATTCAAAAGAAACCCCAGA	GTTAAATCGCCTTTTCCACG	59.1	750	INTERGENIC				
CaPOPII_2987	Ca7	30737184	TTATATATATATATATATATATATATA/TTATATATATATATATATATATATA	TCCGCTTTTATTTGTGTGTCA	AATCTGAACAAGGGCCAATTA	59.2	717	INTERGENIC				
CaPOPII_2988	Ca7	30874290	TAA/TAAA	TTGTCTCAACAATCTCCCCC	ACCACCTCACTGCCCTACAC	59.9	734	DRR	Ca_23317			
CaPOPII_2989	Ca7	30967683	ATTTT/ATTTTT	CCCCATGATCTTCCCTTTTT	GCAACAAACCTATGGCAGGT	60.1	355	DRR	Ca_23312			
CaPOPII_2990	Ca7	30981750	TG/TGG	GGTAAGGGCTCCTTCCAAAC	GGGTCTGCACGATTTACAAAA	59.9	967	INTERGENIC				

INDEL marker IDs	Chromosomes /unanchored scaffolds	Physical positions (bp)	InDels (*Kabuli* reference genome-CDC Frontier/PI)	Forward primers (5'-3')	Reverse primers (5'-3')	Anneling temperature (0C)	Expected amplified product size (bp)	Structural annotation		Functional annotation		
								Sequence components of *kabuli* genome	*Kabuli* gene accession IDs	NCBI-KOG	TFs	NCBI-nr database
CaPOPII_2991	Ca7	31112471	TGG/TG	GCAAAGGCACTCATTCCATT	GCAAAAACACGAGAAGGAGG	60.1	493	INTERGENIC				
CaPOPII_2992	Ca7	31201587	T/TG	TGTCCGACATCGACACATTT	CTCACCTGATTCCATGAGCA	60.0	445	INTRON	Ca_10137		bHLH	Protein kinase, catalytic domain
CaPOPII_2993	Ca7	31210939	CTC/CTCAATC	CCTTTCTCAGCCCATATCCA	TCAACAATGCAAACGAAGGA	60.0	306	DRR	Ca_10136		ARF	AUX/IAA protein
CaPOPII_2994	Ca7	31217391	A/AT	GGGACAAAATTGAACTGGGA	CCCTGGTGAACAGGTGTCTT	59.8	938	INTRON	Ca_10135	G		Hexokinase
CaPOPII_2995	Ca7	31217521	GATACAT/GATACATCATACAT	GACCAGTGCAAAGGGATGTT	CCCTGGTGAACAGGTGTCTT	60.0	460	INTRON	Ca_10135	G		Hexokinase
CaPOPII_2996	Ca7	31232674	CTTTTTTTT/CTTTTTTTTT	GCAAAATAGCTCTTGCGGTC	CTCTTCTTCGTTGAAACGCC	60.0	565	INTERGENIC				
CaPOPII_2997	Ca7	31406570	CAATAATA/CAATA	GACCATCTTAAATGACCGCAA	ATGCGTCGGTGCAATAATAA	60.0	449	INTERGENIC				
CaPOPII_2998	Ca7	31745904	TCCCC/TCCC	TGCATGGAATGGTGAGTGAT	ATTGATGGGTTTACTTGGCG	59.9	219	INTERGENIC				
CaPOPII_2999	Ca7	31969575	TAAAAAAAAA/TAAAAAAAAAA	ACATTTAATTGCCCACTGGC	GATTGATCCTCGTCACCGTT	59.8	536	INTRON	Ca_10067		FAR1	
CaPOPII_3000	Ca7	32358180	TTGG/TTGGACAGGATGG	GTTTTGAGAGCAGCCTGGAG	GCGGGTCTTGGTTTTGATAA	60.1	246	INTERGENIC				
CaPOPII_3001	Ca7	32479028	AATATATATATATATATATATATAT/AATATATATATATATATATATATATAT	AGGGAGTTTAGTGGTGGGCT	CGGATGCATGAAAGTGAAAA	60.0	636	INTERGENIC				
CaPOPII_3002	Ca7	32601479	A/AGGAATGCT	CAGCAACAGCGCAAAGATTA	GGATGACAAAGAAAGGCCAA	60.2	543	INTRON	Ca_10025	TU		Calcium-binding EF-hand
CaPOPII_3003	Ca7	32613866	AT/ATT	AAAAGGTGGTTAGCTCTTTGACA	GGGGGCGAAAGATTTTATTT	59.4	377	INTERGENIC				
CaPOPII_3004	Ca7	32649545	T/TC	TTGCAGGATGCACAGAAAAG	AGGTGCATCCCCTGTAACTG	60.0	415	INTERGENIC				
CaPOPII_3005	Ca7	32650059	TAATAATGTAAAAAA/T	ATGGGACTGTGGGAGAGAGA	GAAAAAGGCTTCAAACATCAAA	59.6	398	INTERGENIC				
CaPOPII_3006	Ca7	32654894	AAATAATAATAATAATAAT/AAATAATAATAATAATAATAAT	AACGAAGTGAGACGTGAGCA	TGATGGCTATGGTGGAACAA	59.6	618	INTERGENIC				
CaPOPII_3007	Ca7	32655163	TTAATA/TTA	AACGAAGTGAGACGTGAGCA	TGATGGCTATGGTGGAACAA	59.6	618	INTERGENIC				

INDEL marker IDs	Chromosomes /unanchored scaffolds	Physical positions (bp)	InDels (*Kabuli* reference genome-CDC Frontier/PI)	Forward primers (5'-3')	Reverse primers (5'-3')	Anneling temperature (0C)	Expected amplified product size (bp)	Structural annotation		Functional annotation		
								Sequence components of *kabuli* genome	*Kabuli* gene accession IDs	NCBI-KOG	TFs	NCBI-nr database
CaPOPII_3008	Ca7	33175105	CTT/CT	TGAAGAAATCAATCATGCAAGT	AACTTGCGTTAGAGTCGGGA	57.4	598	INTERGENIC				
CaPOPII_3009	Ca7	33189130	TG/TGCAG	AGACATTTTGTTCAATGGATCTG	AGGAGAAATGCGAGGAAACA	58.1	462	INTERGENIC				
CaPOPII_3010	Ca7	33194117	TAAAAAAA/TAAAAAAAA	TTGACCTATATACAAAACCCAAAGA	CCCACGTGTCTTTTCAAGGT	58.2	796	INTERGENIC				
CaPOPII_3011	Ca7	33194624	ATT/ATTT	ACCTTGAAAAGACACGTGGG	GGATTTAATCGAAATACTGCAACA	60.0	532	INTERGENIC				
CaPOPII_3012	Ca7	33219414	GT/GTT	GGGGAAAGTTGTCGTTTTCA	AAGACCCAAAACAAAGAGCTAAAA	59.9	246	INTRON	Ca_16150			Nucleoside diphosphate kinase
CaPOPII_3013	Ca7	33219780	CA/CAA	GGGGAAAGTTGTCGTTTTCA	GAGAAAAATTTGCATGCCGT	59.9	848	INTRON	Ca_16150			Nucleoside diphosphate kinase
CaPOPII_3014	Ca7	33237542	CAT/CATGTTTAT	ACGTTGTCCTCTTCGCTGTC	TTGGGTGATAAGGCCAAAAC	60.5	433	INTERGENIC				
CaPOPII_3015	Ca7	33243964	GA/GAA	CAACGGTGCAGAGTGAAAAA	TGTTTCATAACTGAAGGGGGA	59.9	624	DRR	Ca_16151			
CaPOPII_3016	Ca7	33246780	ATGATGTTTG/ATGATGTTTGATGTTTG	GCATAATGTCTTGGGCAGGT	TTCATGACATTTTCCCCACA	60.0	471	INTERGENIC				
CaPOPII_3017	Ca7	33327221	TAAA/TAAAA	TGCACATAACTTGTGCGACC	TTCCATTTTCCCTTAGTGCG	60.7	658	INTERGENIC				
CaPOPII_3018	Ca7	33327595	T/TG	GCATTGATCCGCCTAAAGAA	TGGGCTGTCTATCCCAATTC	60.2	882	INTERGENIC				
CaPOPII_3019	Ca7	33336366	T/TA	AAGCCAGGCGATTTATGATG	ATCGGCAAATATCCGAGAAA	60.1	510	DRR	Ca_16158	R	G2-like	RNA recognition motif domain
CaPOPII_3020	Ca7	33366145	CTG/CTGTG	TGGGGAAAAGGTGTTTTCTG	TCGTGACTGCTCAAAAATGG	59.9	811	INTRON	Ca_16162	YU		
CaPOPII_3021	Ca7	33385317	AT/A	TTCATCGTTTTCCCGAACTC	CCTCTTTGTCTTCTTGTGGCA	60.1	723	INTERGENIC				
CaPOPII_3022	Ca7	33421552	AATATATATATATATATATA/AATATATATATATATATA	GTCTTGGGTGATGGCAAAGT	GATTCTGCCTACTCATGTTTCAA	60.0	479	INTRON	Ca_16165	U	Dof	Synaptobrevin
CaPOPII_3023	Ca7	33485739	ATATAT/ATATATCTTTTTATAT	TAGTTGCGAATGGATGGTCA	ATCGGAACCCACTTCTCCTT	60.1	246	INTRON	Ca_16169			Dormancyauxin associated
CaPOPII_3024	Ca7	33521411	ATT/ATTT	TGTTGAACATTGTAACAGCCG	GACGAAAAATGATTTAGACGTTCA	59.6	364	INTERGENIC				

INDEL marker IDs	Chromosomes /unanchored scaffolds	Physical positions (bp)	InDels (*Kabuli* reference genome-CDC Frontier/PI)	Forward primers (5'-3')	Reverse primers (5'-3')	Anneling temperature (0C)	Expected amplified product size (bp)	Structural annotation		Functional annotation		
								Sequence components of *kabuli* genome	*Kabuli* gene accession IDs	NCBI-KOG	TFs	NCBI-nr database
CaPOPII_3025	Ca7	33526924	A/ACGTAG	TGGCATTGGAAAAGAAAAGG	GATGCCACCACCTGAAAGAT	60.0	721	INTRON	Ca_16172	S	M-type	NADH:ubiquinone oxidoreductase intermediate-associated protein 30
CaPOPII_3026	Ca7	33535580	AT/A	TGCAATGAAAACACTGGGAG	AAAGTTCTCATTCAGCATTACGG	59.7	246	INTERGENIC				
CaPOPII_3027	Ca7	33539096	AA/AATA	GGTAAATATGTGCATGGCCC	GGATTAGCACCGTCCAATTT	60.0	722	INTERGENIC				
CaPOPII_3028	Ca7	33539636	GTTTT/GTTT	AAATTGGACGGTGCTAATCC	CACGAAAGGTTTCATCACAAA	58.9	243	INTERGENIC				
CaPOPII_3029	Ca7	33558467	A/AGCTT	TGAAGGACAACTCAGTGTCACC	TGCATTTTTCCTTCTTCCACTT	60.2	897	INTERGENIC				
CaPOPII_3030	Ca7	33562209	CTG/CTGTG	CATCTGGCAACACATCATCC	TGTGACAAAGATGAGCCAGG	59.9	599	INTRON	Ca_16175	QI	B3	Cytochrome P450
CaPOPII_3031	Ca7	33576122	GTTTTTTTTTT/GTTTTTTTTT	TAATTTAACGAACGGCCGAG	TTGGGTGACTCATGGTGAGA	60.1	410	DRR	Ca_16178	I		Synaptojanin, N-terminal
CaPOPII_3032	Ca7	33597364	CTAATTAATT/CTAATT	GATGTTAACGCACCTCCACC	CACACTTCGATCTCCGATCA	60.4	483	INTERGENIC				
CaPOPII_3033	Ca7	33639029	TAAAAAAAAA/TAAAAAAAAAA	TCAGAGCTTTGCAGTTATGTGA	GCACATGGTTTCGAATGTTG	58.7	555	DRR	Ca_16180		ERF	Pathogenesis-related transcriptional factor/ERF, DNA-binding
CaPOPII_3034	Ca7	33640029	TTA/T	CCAAATCGTCGGTGTGAATA	TTTTGGAGGAGATGTTTGGG	59.4	526	DRR	Ca_16180		ERF	Pathogenesis-related transcriptional factor/ERF, DNA-binding
CaPOPII_3035	Ca7	33640082	TAA/TAAA	CCAAATCGTCGGTGTGAATA	CCTTTGTTTGCTGGGATCAT	59.4	614	DRR	Ca_16180		ERF	Pathogenesis-related transcriptional factor/ERF, DNA-binding
CaPOPII_3036	Ca7	33642641	ATTTTTTTTTTT/ATTTTTTTTTT	CCAGGCAAAGCTAGGTGAAC	GGGTTCCTGCTGTCAATTTT	59.9	166	INTRON	Ca_16180		ERF	Pathogenesis-related transcriptional factor/ERF, DNA-binding
CaPOPII_3037	Ca7	33643277	CAAAAAAAAAAA/CAAAAAAAAAA	CCCAATAAAATGTGGGGAAA	ATCTTGTGGCCGAATTTGAG	59.5	312	INTRON	Ca_16180		ERF	Pathogenesis-related transcriptional factor/ERF, DNA-binding
CaPOPII_3038	Ca7	33648564	A/AT	TCCCAAATAAGCATTAGGATGAA	GGGGGATATATTGAATGAAGAAA	59.8	317	INTERGENIC				
CaPOPII_3039	Ca7	33660304	GAAAAA/GAAAAAA	TCGGCAGTACCAATTTCACA	TACAACGGTTTGATCGGTGA	60.1	610	INTERGENIC				
CaPOPII_3040	Ca7	33683250	CAA/CA	AAAAACGCAAGTCATCATCCT	GATTGAGCCGAAAGTGAAGC	58.7	427	INTERGENIC				
CaPOPII_3041	Ca7	33683634	C/CCTT	GCTTCACTTTCGGCTCAATC	CACAATCAAGGCCATCAAAA	60.0	214	INTERGENIC				

INDEL marker IDs	Chromosomes /unanchored scaffolds	Physical positions (bp)	InDels (*Kabuli* reference genome-CDC Frontier/PI)	Forward primers (5'-3')	Reverse primers (5'-3')	Anneling temperature (0C)	Expected amplified product size (bp)	Structural annotation		Functional annotation		
								Sequence components of *kabuli* genome	*Kabuli* gene accession IDs	NCBI-KOG	TFs	NCBI-nr database
CaPOPII_3042	Ca7	33732435	ATTTTTTTTTT/ATTTTTTTTTTT	GTTGATCGGGAAAAAGGGAT	GGAAGCGATAAGCACCAAAG	60.1	190	INTERGENIC				
CaPOPII_3043	Ca7	33746470	T/TAA	CAAAATTAAAATGGCACAGGG	TTTCCCAATCCACCACTCTC	59.4	332	DRR	Ca_16182			ARID/BRIGHT DNA-binding domain
CaPOPII_3044	Ca7	33768316	CTA/CTATA	GCTTCATTGGTGCATCCTTT	TCAGGCTAAAAGTGAAAACTCG	60.1	303	INTERGENIC				
CaPOPII_3045	Ca7	33772634	AAGA/AA	TGGATTTTGTTTGGATGCAG	TTCCCAACTTTTCAAAATATCCA	59.5	512	INTERGENIC				
CaPOPII_3046	Ca7	33802472	TTATATATATTCAT/TTATATATATTCATATATATATTCAT	TACTGACCTCTATCGCCGGT	CGTATGCGACTCATCCATTG	59.7	612	DRR	Ca_16188	P	GRAS	ATPase, P-type cation-transporter, N-terminal
CaPOPII_3047	Ca7	33803390	TACAC/TAC	TGAGGATGGAATGGGTTTTG	CTTTGGTTTCACATTGGGCT	60.7	555	DRR	Ca_16188	P	GRAS	ATPase, P-type cation-transporter, N-terminal
CaPOPII_3048	Ca7	33805672	TAAAATGAAAAATGAAAA/TAAAATGAAAAATGAAAAATGAAAA	TCACCGCAACTGACAAAGTC	GCGTTATTAGACGAATGACTTGAA	59.9	406	INTERGENIC				
CaPOPII_3049	Ca7	33815769	TAAA/TA	ATGGCTATTCACTGCCATGC	ATCATCCTTGGTCCCGAATA	61.0	434	DRR	Ca_16190	E		1-aminocyclopropane-1-carboxylate synthase
CaPOPII_3050	Ca7	33820036	GA/GATA	AGGGTATTGAGGGATGGGAC	ATAGAGCGAGCATGGCAGAT	60.0	452	INTRON	Ca_16190	E		1-aminocyclopropane-1-carboxylate synthase
CaPOPII_3051	Ca7	33846722	T/TA	GCTGTGCTCCAACCTCTTTC	CAGCGTGCACATGGATTAGT	60.0	544	INTRON	Ca_16192		NAC	Pectinesterase, catalytic
CaPOPII_3052	Ca7	33852197	CTATATATATAT/CTATATATATATAT	TGCATCAACTTTTTGGGTGA	TTGGCCATATTTGAACCACTC	60.1	303	INTERGENIC				
CaPOPII_3053	Ca7	33857243	GAAAAA/GAAAAAA	TTGGAAGCCAGAGCAAAAGT	GCCTCGTTTCATTTAATCTGC	60.0	691	INTERGENIC				
CaPOPII_3054	Ca7	33877621	TACAATG/TACAATGTGTACTACAACAATG	TTGTGCATGTATTGGGCTTG	CGGGGGTATTTCTGTGAAGA	60.5	544	INTRON	Ca_16194	O		Ubiquitin-conjugating enzyme, E2
CaPOPII_3055	Ca7	33894708	ATTTTTT/ATTTTT	TCACTATGACAACGGGGTGA	CCCGTGAAATGTCTTGGAGT	60.0	286	INTERGENIC				
CaPOPII_3056	Ca7	33895177	CTTTTT/CTTTT	ACTCCAAGACATTTCACGGG	GCATGCAATTCACGCATTAT	60.0	340	INTERGENIC				
CaPOPII_3057	Ca7	34080547	AT/A	AGGCAGGGGCAACTAAAGAT	TTTGTGGGGGTGCTTAGTTC	60.1	446	INTRON	Ca_16200			
CaPOPII_3058	Ca7	34116846	ATTTTTTT/ATTTTTTTT	GATTTGACGTCCTGTCTCGG	AGAATTGGCAGATTCGATGG	60.7	571	INTERGENIC				

INDEL marker IDs	Chromosomes /unanchored scaffolds	Physical positions (bp)	InDels (*Kabuli* reference genome-CDC Frontier/PI)	Forward primers (5'-3')	Reverse primers (5'-3')	Anneling temperature (0C)	Expected amplified product size (bp)	Structural annotation		Functional annotation		
								Sequence components of *kabuli* genome	*Kabuli* gene accession IDs	NCBI-KOG	TFs	NCBI-nr database
CaPOPII_3059	Ca7	34143500	CA/CAA	AATGAGATGCCGATTTTTGC	AATGTTACCTTGCAGGACCG	60.0	615	INTRON	Ca_21006	K	ERF	DNA-directed RNA polymerase, subunit 2, domain 6
CaPOPII_3060	Ca7	34144763	CTT/CTTT	GGCCATAGAGTGCAAGAAGC	CTCACCCAAAACCCTGAAAA	60.0	388	INTRON	Ca_21006	K	ERF	DNA-directed RNA polymerase, subunit 2, domain 6
CaPOPII_3061	Ca7	34152194	TAA/TAAAAGAA	AAGCTTGGAGCAGGACAGAA	CTGCAAGATCGCAGGTGTAA	60.1	775	INTRON	Ca_21006	K	ERF	DNA-directed RNA polymerase, subunit 2, domain 6
CaPOPII_3062	Ca7	34187425	GAA/GAAA	AGGTCTGGATTCGTGGTTTG	ACATCCGTGCAAGGAAAAAG	60.0	646	INTRON	Ca_21004	S		
CaPOPII_3063	Ca7	34190333	AAG/AAGAG	TCGAACTACTTGCGTGAGAA	TTGGTTCTTTTGCATCTTCG	57.2	559	DRR	Ca_21004	S		
CaPOPII_3064	Ca7	34195712	AT/ATTT	GAGGCTCCATATTGCGAAAA	AGGCTAACACCATTGCCAAC	60.2	189	DRR	Ca_21002		WRKY	Protein kinase, catalytic domain
CaPOPII_3065	Ca7	34195793	TAAAAAAAAAA/TAAAAAAAAA	GAGGCTCCATATTGCGAAAA	AGGCTAACACCATTGCCAAC	60.2	189	DRR	Ca_21003	O	FAR1	ATPase, AAA+ type, core
CaPOPII_3066	Ca7	34196242	GTTT/GTT	GTTGGCAATGGTGTTAGCCT	TTGAGGGTTGCTTTTGTTGTT	60.0	771	DRR	Ca_21002		WRKY	Protein kinase, catalytic domain
CaPOPII_3067	Ca7	34203381	A/AATATATAGGTAG	GTAAAGCTGGCAAGCACGAT	CCCAGAACAAAAGTTGGTGAA	60.4	725	INTERGENIC				
CaPOPII_3068	Ca7	34205414	ATTTT/ATTTTT	TTGAAATACACACACAAAATTGACA	TTGACCTTGAAATCAACTCACC	59.4	267	INTERGENIC				
CaPOPII_3069	Ca7	34216629	GTTTTTTTT/GTTTTTTT	TTTGTCCCTCCCAAGAAATG	ATCTCACCCCTGGTTGTCTG	59.9	427	DRR	Ca_20999	L		Metallo-dependent phosphatase
CaPOPII_3070	Ca7	34222973	A/AAAATAT	GCAAAACTGGGGAGAAACAA	TCGCTTACTGGCTTCCATCT	60.1	792	INTRON	Ca_20999	L		Metallo-dependent phosphatase
CaPOPII_3071	Ca7	34226505	AAATA/AA	AACACCAATCGCTCTTCCAC	GAGGGAACACCAACTCCAAA	60.1	889	INTERGENIC				
CaPOPII_3072	Ca7	34237238	GGTTGTTGTTGTTGTTGTTGTTGTTGT/GGTTGTTGTTGTTGT	TTCCCAATTGCAACAATCAA	GGTCTCATCTCATCCTCCCA	59.9	437	CDS (large-effect mutations)	Ca_20997		S1Fa-like	Protein kinase, catalytic domain
CaPOPII_3073	Ca7	34237601	TTG/TTGGTTTTGGTTATG	TGGGAGGATGAGATGAGACC	CAAGGAACCATCCATTTGCT	60.0	590	INTERGENIC				
CaPOPII_3074	Ca7	34278071	T/TC	TCTTCAAGTGGTCGCTTCCT	CCTTCGCCATTTAATTTTGG	60.0	915	INTERGENIC				
CaPOPII_3075	Ca7	34278231	ATTTTTT/ATTT	TGGACTGCTCGATTGTCAAA	CCTTCGCCATTTAATTTTGG	60.4	557	INTERGENIC				

INDEL marker IDs	Chromosomes /unanchored scaffolds	Physical positions (bp)	InDels (*Kabuli* reference genome-CDC Frontier/PI)	Forward primers (5'-3')	Reverse primers (5'-3')	Anneling temperature (0C)	Expected amplified product size (bp)	Structural annotation		Functional annotation		
								Sequence components of *kabuli* genome	*Kabuli* gene accession IDs	NCBI-KOG	TFs	NCBI-nr database
CaPOPII_3076	Ca7	34286325	A/AAATAT	ACGTCAGACATTGGATGTGC	TTGTTTTCAAGGAGTTGCGA	59.6	666	INTERGENIC				
CaPOPII_3077	Ca7	34290113	T/TAG	TTTTGAACAGAACCAAGGGC	GAAGAGAAAAGCCGGTGTTG	60.1	362	INTRON	Ca_20989	K	NAC	Endonuclease/exonuclease/phosphatase
CaPOPII_3078	Ca7	34309692	TA/T	TGTGAGTCGATGAATCAGCC	TCGAATTTCGTGTCCACTCA	59.8	417	INTERGENIC				
CaPOPII_3079	Ca7	34311495	TAAAA/TAAA	TCCAGCATGCAGCATAGTTT	TGCAGGACCAGATAGGGAAG	59.4	214	INTERGENIC				
CaPOPII_3080	Ca7	34312381	CTTT/CTTTTT	CTCCTTTCTCACACTCACTCCA	TTGGTGGACTTCCCAACTTC	59.5	515	INTERGENIC				
CaPOPII_3081	Ca7	34312433	GATAAATA/GATA	CTCCTTTCTCACACTCACTCCA	TTGGTGGACTTCCCAACTTC	59.5	515	INTERGENIC				
CaPOPII_3082	Ca7	34312678	ATTTTTTTTT/ATTTTTTTT	CTCCTTTCTCACACTCACTCCA	TTGGTGGACTTCCCAACTTC	59.5	515	INTERGENIC				
CaPOPII_3083	Ca7	34315074	GATATATA/GATATATACATATATATATA	TTCTGATAATTGGAATCACTTCTAACA	TTGCACCCGTAGTATTGTGG	59.5	561	INTERGENIC				
CaPOPII_3084	Ca7	34316435	ATAATTAAT/ATAATTAATTAAT	CATGGGTCTCACCCCTAGAA	AAGGTTGCCATTGTATTGGG	59.9	412	INTERGENIC				
CaPOPII_3085	Ca7	34317406	ATATA/ATATAGTATA	TGGAATTTGTATGCGAGACG	GCAGGGTTTTTGTTTTTGGA	59.7	896	INTERGENIC				
CaPOPII_3086	Ca7	34317535	AATATATATATATA/AATATATATATA	TTGTTTTTGGTGAAAAGTGTGTG	GCAGGGTTTTTGTTTTTGGA	60.0	429	INTERGENIC				
CaPOPII_3087	Ca7	34318595	CATTATTACAGATTT/CATTATTACAGATTTATTATTACAGATTT	TCAATGACTGTGACAAAATCCA	GCTAGCAGCCACCTTCTTTG	59.0	503	INTERGENIC				
CaPOPII_3088	Ca7	34320519	C/CTA	TTAACAAAACGGCTCCAACC	ACATGGCTTGGAGTTGGAAT	60.0	438	INTERGENIC				
CaPOPII_3089	Ca7	34321844	ATTTTTTTT/ATTTTTTTTT	TCGGTGCCTTCATATTTGACT	TTTTCTCATGGATCTTTTGGA	59.6	812	INTERGENIC				
CaPOPII_3090	Ca7	34328779	AT/ATTCAGT	AGGGAGGCACTTCGGAGTAT	GTGCAACCGATTGGTATCCT	60.1	793	INTERGENIC				
CaPOPII_3091	Ca7	34335084	TTATATA/TTATA	CCAATAACAATGGGGGTTCA	CCTCTTTGTTGGATGAGGGA	60.4	259	INTERGENIC				
CaPOPII_3092	Ca7	34336023	AA/AAAAAAGA	TATTTGACCCGTGAGACGTG	CAACCACAATTCAATTTATGCAA	59.6	539	INTERGENIC				

INDEL marker IDs	Chromosomes /unanchored scaffolds	Physical positions (bp)	InDels (*Kabuli* reference genome-CDC Frontier/PI)	Forward primers (5'-3')	Reverse primers (5'-3')	Anneling temperature (0C)	Expected amplified product size (bp)	Structural annotation		Functional annotation		
								Sequence components of *kabuli* genome	*Kabuli* gene accession IDs	NCBI-KOG	TFs	NCBI-nr database
CaPOPII_3093	Ca7	34342253	TTTTT/TTTTTATTTT	AATGATAGGTGGACGTTCAAA	GGGTAACCTCGCCACAACTA	57.1	725	INTERGENIC				
CaPOPII_3094	Ca7	34347667	A/AC	TCATTTAGCGGCTGGTTTCT	TGCCGCAATAAAATGAAACA	59.8	643	DRR	Ca_20986	C		Inorganic pyrophosphatase
CaPOPII_3095	Ca7	34350373	TAAA/TAA	AAAGAAGCGACGAATCTCCA	CATGCAGGTAAATTGTTGCC	60.0	599	INTRON	Ca_20986	C		Inorganic pyrophosphatase
CaPOPII_3096	Ca7	34375146	GTCCACTCCACTCCACTC/GTCCACTCCACTCCACTCCACTC	GAGGGGTTTGTGGAATGGTA	GTAATCACCAACCAATCGGG	59.6	271	INTERGENIC				
CaPOPII_3097	Ca7	34392098	ATTTTTTT/ATTTTTTTTT	GGCGAGCATGAAATACCAAT	TCTGCCAAAAACGAAGATCG	59.9	819	INTERGENIC				
CaPOPII_3098	Ca7	34394315	AG/A	TATTTGGCTATCTGCGGTCC	TCGGATGTAAATGCGATGAA	60.1	726	INTERGENIC				
CaPOPII_3099	Ca7	34396059	TATCTTATAAAA/TATCTTATAAAATCTTATAAAA	GTGCCAGAGGAGGTGGTAGA	TGACATTTGGGTCAGCATGT	60.3	944	INTRON	Ca_20981	QR	B3	Oxoglutarate/iron-dependent oxygenase
CaPOPII_3100	Ca7	34583170	CATATATATATATATATATATATATATATATAT/CATATATATATATATATATATATATATAT	TTCATCCACCAAGAAGCTCA	GAACTCGACCAGTGCAGACA	59.4	253	INTERGENIC				
CaPOPII_3101	Ca7	34663130	ATTTTTTTTTTT/ATTTTTTTTTTTT	AAATTTTGGTTGCGATTCCA	ACCCGGGTATAAGGTTCCAC	60.3	385	INTERGENIC				
CaPOPII_3102	Ca7	34735432	CAA/CAAA	GGTTTTGCCCCTATTTCCAT	TTGCAAGTTTAGATCGCTTGTC	60.0	435	INTERGENIC				
CaPOPII_3103	Ca7	34922873	AATTATTATTATTATTATTATTATTATTATTATTATTATTATTA/AATTATTATTATTATTATTATTATTATTATTATTATTATTATTATTA	GCCACCACCTTCTTGAACAT	TTGTCCAAGGTGCAATTCAA	60.0	604	INTERGENIC				
CaPOPII_3104	Ca7	35048654	TTATATAT/TTATATATAT	AGGTGGGCAACTTTGATGAC	GCATCTGGCTAGGAACTTGG	60.0	673	INTRON	Ca_17632	R		WD40 repeat
CaPOPII_3105	Ca7	35109014	ATTTTTTT/ATTTTTTTTT	CCTGGGATGGTGATGAGAGT	TCCCAACCAGACTGAACTGG	59.9	806	INTERGENIC				
CaPOPII_3106	Ca7	35131412	TAAAAAAA/TAAAAAAAA	TCGTGGTCGCATTGAACTTA	GAATCAAAATGGAGCGGAAA	60.3	376	INTERGENIC				
CaPOPII_3107	Ca7	35134144	TAAA/TAAAA	ACCATTCCATTTCTCCTTGC	CCTTCAATTTTGCCGTCAGT	59.0	494	DRR	Ca_17638		ERF	Pathogenesis-related transcriptional factor/ERF, DNA-binding
CaPOPII_3108	Ca7	35141972	TA/TAA	TTGTCCCACGAGGAGAAAAC	CGACAAACACAATCACTGGG	60.1	824	INTERGENIC				
CaPOPII_3109	Ca7	35156256	ATTTTTT/ATTTTTTT	TTCCTGTTTTGTAGCACCCC	CACCATTGTCCGATTGTCAG	60.0	757	INTERGENIC				

INDEL marker IDs	Chromosomes /unanchored scaffolds	Physical positions (bp)	InDels (*Kabuli* reference genome-CDC Frontier/PI)	Forward primers (5'-3')	Reverse primers (5'-3')	Anneling temperature (0C)	Expected amplified product size (bp)	Structural annotation		Functional annotation		
								Sequence components of *kabuli* genome	*Kabuli* gene accession IDs	NCBI-KOG	TFs	NCBI-nr database
CaPOPII_3110	Ca7	35166766	TAAAAAAAA/TAAAAAAA	GGCGCTATATGGTATTTTGAGTT	GGTTCTTCGCCTTCACAAAC	58.7	948	INTERGENIC				
CaPOPII_3111	Ca7	35177660	A/ATAAAAATAAAT	GCCAACCGACTCCATCTTTA	TTTAGCAGTTTTTCAATTTCCAA	60.1	690	INTERGENIC				
CaPOPII_3112	Ca7	35178628	TT/TTCT	TTTTCAAACTTTTCCCCTCG	TCGTTCATCAAATCACGAGAA	59.2	241	INTERGENIC				
CaPOPII_3113	Ca7	35230846	ATTTT/ATTT	AAGTAGCAAAGCCTCACCCA	CGGATAATTTCAAATGGATCACT	59.9	401	INTERGENIC				
CaPOPII_3114	Ca7	35232044	AA/AAATTA	TTTGACCCTCAGCCATTTTC	GAAAAATGGGAATGCAATGG	60.1	244	INTERGENIC				
CaPOPII_3115	Ca7	35256420	ATTTTTTTTTTTT/ATTTTTTTTTT	CATTGTGGACTTGTCGGAGA	CTCACACGGTGCTTCTCAAA	59.7	557	INTERGENIC				
CaPOPII_3116	Ca7	35302573	GTTTTT/GTTTTTT	TTTGAACAGCAATCCAACCA	AGTGAACAACCAACCCATTTT	60.1	299	INTERGENIC				
CaPOPII_3117	Ca7	35313643	TC/T	TTCGATAAAGCAAAAGCTCCA	AACTGAATATGGGCCAGTCG	60.0	805	INTERGENIC				
CaPOPII_3118	Ca7	35331728	A/AG	GTTGCTGATCTGAGGGAAGC	ATCTACATTGCAGGGGTCCA	60.0	727	INTRON	Ca_17647	BK	FAR1	PWWP
CaPOPII_3119	Ca7	35357002	CTTTTTTTTT/CTTTTTTTTTTT	TGGATGTCATGTTTCTTAATGGA	CACATACGGGGTTTTAGATTCG	59.3	657	INTERGENIC				
CaPOPII_3120	Ca7	35364737	AGATTAGGA/AGA	CGCATTCCTCTCGAAAAGTC	TTGGAGAAAGATCTCCGGTT	60.0	439	INTERGENIC				
CaPOPII_3121	Ca7	35369159	CAAAAAAAA/CAAAAAAAAA	CCAAAGGCATTTGAGGATTC	TTGCCATTCTTTGACACACA	59.5	308	DRR	Ca_17651			Conserved hypothetical protein CHP01589, plant
CaPOPII_3122	Ca7	35370294	TCTAC/TCTACTAC	ATAATTTGAGCCATGACGGG	TCTTGACAGGTTTGGTTGGA	59.8	128	DRR	Ca_17651			Conserved hypothetical protein CHP01589, plant
CaPOPII_3123	Ca7	35382567	G/GCTAA	CCGATCCAATTATATCGACAG	TTATCAAGGCTCCCACCATC	57.1	742	INTERGENIC				
CaPOPII_3124	Ca7	35605462	CATATATATATATATATATATA/CATATATATATATATATATATA	TCAACCATTCCGAGTGCATA	CGCCGTAAATGGCTTAAAAA	60.1	667	INTERGENIC				
CaPOPII_3125	Ca7	35786413	TTATTAATATTA/TTATTA	GGGAAACCCACCTTTTGATT	GGAGGGTGTGAATTGGAAGA	60.0	679	INTERGENIC				
CaPOPII_3126	Ca7	35787388	A/AG	CCTTCCAATGTAGCTGATGTTTT	TGGACGCAAATAGATGCAAA	59.6	540	INTERGENIC				

INDEL marker IDs	Chromosomes /unanchored scaffolds	Physical positions (bp)	InDels (*Kabuli* reference genome-CDC Frontier/PI)	Forward primers (5'-3')	Reverse primers (5'-3')	Anneling temperature (0C)	Expected amplified product size (bp)	Structural annotation		Functional annotation		
								Sequence components of *kabuli* genome	*Kabuli* gene accession IDs	NCBI-KOG	TFs	NCBI-nr database
CaPOPII_3127	Ca7	36117059	CTTT/CTTTTCTTTATTTT	TGGATTGGGCTTTCCAATAA	TGCCTTAACGTACCCAAACA	60.3	435	DRR	Ca_13721	G	Trihelix	Protein of unknown function DUF604
CaPOPII_3128	Ca7	36243491	TAAAAAAAA/TAAAAAAAAA	TGTATCGCGTTAATCTGCGT	TTTGGTTTCATGGATGTGGA	59.4	886	INTERGENIC				
CaPOPII_3129	Ca7	36439529	ATTTTTTT/ATTTTTTTT	CGCTTTTTCCGTCTTAGTGC	TCATTTCGATAATTTCACACAAA	60.0	628	INTERGENIC				
CaPOPII_3130	Ca7	36548120	TAAAA/TAAAAAA	ACAACCAAGCGTGTCGTGTA	TGTTTGTTGGAGAAATGCCA	60.2	213	DRR	Ca_13744	O		SKP1 component
CaPOPII_3131	Ca7	36717770	AATATATATATATATATA/AATATATATATATATATATA	TGCCTTTAACAATAATCGAGAATG	TGGTTTGGTTCCATCAGGTT	59.6	803	DRR	Ca_13757	S		Chromatin associated protein KTI12
CaPOPII_3132	Ca7	37595726	ATTTTTTTTTT/ATTTTTTTT	CAAAGTATCTACCGGGTTCCA	CAACTCGGTTCACACCTTCA	59.0	659	INTERGENIC				
CaPOPII_3133	Ca7	37622218	TCA/T	TACCCCACGGTAGCTCTTTG	TTCGATCATCACCCTCAACA	60.1	722	INTERGENIC				
CaPOPII_3134	Ca7	37956889	GAAAAAAAA/GAAAAAA	ATATTGTCGCGTTCGGTTTG	GGGACCGTAGGTGCAATTTA	60.9	468	INTERGENIC				
CaPOPII_3135	Ca7	38669994	TTTATTATTATTATTATTATTATTATTATT/TTTATTATTATTATTATTATTATTATTATT	CCTGCCGGTTTATTTTGTTC	GCGATCGAATGGCAATATCT	59.5	657	INTERGENIC				
CaPOPII_3136	Ca7	38743655	CTATTTATTTATTTATTTATTTATTTATTT/CTATTTATTTATTTATTTATTTATTTATTTATTTATTTATTT	CTTAGGTTTGCACTCAGCCC	CTCAATCGTGGGTGACCTTT	59.9	932	INTERGENIC				
CaPOPII_3137	Ca7	38757753	TAAAAAAAAA/TAAAAAAAA	GCCATCTTTATTCCAACCGA	TTTGATGAATTTGATGTTGTTGG	59.9	776	DRR	Ca_16426		SBP	Glutaredoxin
CaPOPII_3138	Ca7	38806740	GAAAAAAA/GAAAAAAAA	TGAGTGCCATTTTTCATCCA	CATGGGAGAAGATTTCAGGC	60.0	932	INTRON	Ca_16429	QR	B3	Oxoglutarate/iron-dependent oxygenase
CaPOPII_3139	Ca7	39029671	C/CTAAGTCTA	CAATGGCTTTGTTGAATTGG	TGCCGCTATGTACTTTTGGC	59.0	789	INTERGENIC				
CaPOPII_3140	Ca7	39518115	TAAACAAAAACAAAA/TAAACAAAAACAAAAACAAAA	GGTCCCTTGTAAGCCCAAAT	TTTACGAGCCCATTTGTTGA	60.2	450	INTERGENIC				
CaPOPII_3141	Ca7	39525063	ATTTTTTTTTT/ATTTTTTTTTTT	AACGACCAACTTGGGAAAAA	GGAGTGGGTCACCGAGAATA	59.4	520	INTERGENIC				
CaPOPII_3142	Ca7	39611392	AAATAATAATAATAATAATAATAATAATAATAA/AAATAATAATAATAATAATAATAATAATAATAATAATAA	CTGTGCGCTTGACTGATGTT	CTAAATTCGAAATTCGGCCA	60.1	930	INTERGENIC				
CaPOPII_3143	Ca7	40430153	AAGTAAGTA/AAGTAAGTAGTAAGTA	GTTGGTTCAAAAGCAACCGT	ACAAGGATACGTGCAGGGAC	60.0	493	CDS (FRAME SHIFT)	Ca_20258		Trihelix	Transposase, Ptta/En/Spm, plant

INDEL marker IDs	Chromosomes /unanchored scaffolds	Physical positions (bp)	InDels (*Kabuli* reference genome-CDC Frontier/PI)	Forward primers (5'-3')	Reverse primers (5'-3')	Anneling temperature (0C)	Expected amplified product size (bp)	Structural annotation		Functional annotation		
								Sequence components of *kabuli* genome	*Kabuli* gene accession IDs	NCBI-KOG	TFs	NCBI-nr database
CaPOPII_3144	Ca7	40965776	ATTTTTTTT/ATTTTTTT	TGGTATTGAAACACCC CAAA	AATATGAAGTTGCGGC TGCT	58.7	857	INTERGENIC				
CaPOPII_3145	Ca7	40985025	TAAAA/TAAAAA	GCAACTGCCTTAAAAAT GGAA	GGAACGTGCGTACATT GTTG	59.2	303	INTERGENIC				
CaPOPII_3146	Ca7	41008241	CAAAAAAAAAAAAA/CAAAAAAAAAAA	GCCAATCAGGACACAT CTCA	TCAGAACATTCCATCG GTGA	59.6	563	INTERGENIC				
CaPOPII_3147	Ca7	41008810	TTAA/TTAACTAA	TAGCGCCTACATCGGA GAGT	GTTCATTATGCGCAAG CATT	60.0	387	INTERGENIC				
CaPOPII_3148	Ca7	41009960	ATTTTTTTT/ATTTTTTTTTT	CATGACCTCGGTTTTCA ATAA	CCCTCACATTTTTAAGG CACA	57.6	814	INTERGENIC				
CaPOPII_3149	Ca7	41010142	CAA/CAAAA	TGCAATGAATGAAAGTA TGCTACC	GAAAAAGCGCTGTAAA TGGC	60.4	815	INTERGENIC				
CaPOPII_3150	Ca7	41251256	AG/A	CCAATAGTTTAGTGGG CCAAA	AAGGGTCCTCTTGAAG CGTT	59.0	213	INTERGENIC				
CaPOPII_3151	Ca7	41252327	TTAT/TT	TGCAATGCAATGTCCAT TTT	TTTTTCCTCATGGTGCA GTG	59.9	324	INTERGENIC				
CaPOPII_3152	Ca7	41484126	GTATATATATATATATATATATAT/GTAT ATATATATATATAT	AGATTCGCAGGCAGTT GAGT	CGAGAGAGTGTCACGC ATGT	60.0	701	INTERGENIC				
CaPOPII_3153	Ca7	41751259	TAAAAAAAAAAAAAA/TAAAAAAAAA AAAA	GAGATACCCACTGCCA CGAT	AGGTGACAAACCGCCA TAAC	60.0	542	INTERGENIC				
CaPOPII_3154	Ca7	42384922	C/CAAAATAA	TTTACAGGCAGCGGAA AAGT	GCTAAGGTGCATGCTT GGTT	59.9	340	INTERGENIC				
CaPOPII_3155	Ca7	42738535	TAAAAAAA/TAAAAAAAA	TCCCGCTACACTTTGA GGAC	TCAAAAGCAAGTGTTG CAGG	60.3	592	INTERGENIC				
CaPOPII_3156	Ca7	42757617	T/TGGTTTGAAGTTTGC	TTTGCTCTGAAAGCCCA AAT	AGTTCCTGCAACTCCG AAGA	59.8	527	INTRON	Ca_20201			Domain of unknown function DUF239, plant
CaPOPII_3157	Ca7	42832699	CATTATTATTATTATTATTATTATTAT TATTGTTATTATTATTATTATTATTAT TATTATTATTA/CATTATTATTATTATT ATTATTATTATTGTTATTATTATTATT	CCCAACATAGCAAGGT TGAAA	TTGTGGTGGGAAAAAG AAGG	60.0	816	INTERGENIC				
CaPOPII_3158	Ca7	42919520	AAT/AAAGAATCAT	TCAGCACTGCACCAAA ACAT	AAAAGGCGCATACGCT AAGA	60.3	621	INTERGENIC				
CaPOPII_3159	Ca7	43208355	CTTTTT/CTTTT	CACAGGCACAAAATTTT CTCAA	TGAATTGAGATCGTTG GCAC	60.1	568	INTERGENIC				
CaPOPII_3160	Ca7	43375115	CAAAA/CAAA	CCAATATCTCGTGTTCC CGT	CATTTCAAGTTGGGGC AGTT	59.8	335	INTERGENIC				

INDEL marker IDs	Chromosomes /unanchored scaffolds	Physical positions (bp)	InDels (*Kabuli* reference genome-CDC Frontier/PI)	Forward primers (5'-3')	Reverse primers (5'-3')	Anneling temperature (0C)	Expected amplified product size (bp)	Structural annotation		Functional annotation		
								Sequence components of *kabuli* genome	*Kabuli* gene accession IDs	NCBI-KOG	TFs	NCBI-nr database
CaPOPII_3161	Ca7	44001226	GAAA/GAA	GAACACCACACGCAAAGAAA	TTTGTCCAATGGCAGTTTCA	59.7	529	INTERGENIC				
CaPOPII_3162	Ca7	44001613	T/TG	TGAAACTGCCATTGGACAAA	CAATTTCTTTGCGTGTGGTG	60.1	476	INTERGENIC				
CaPOPII_3163	Ca7	44150096	T/TTTA	AGTCTTACCAGCGCCACACT	TCCCAACTACAACGAGGACC	59.9	567	DRR	Ca_21366	O		Prohibitin
CaPOPII_3164	Ca7	44150736	TAAAAA/TAAAAAAA	GGTCCTCGTTGTAGTTGGGA	CCAAGGCATCAGCTCACATA	60.0	467	DRR	Ca_21366	O		Prohibitin
CaPOPII_3165	Ca7	44395386	TAAAAAAA/TAAAAAAAA	AAATTTGGTTGAATCGCGTC	GCAGATAATGGGGGTTGCTA	59.9	779	INTERGENIC				
CaPOPII_3166	Ca7	44474070	AT/ATT	TAGCAACAAAAATGGGGGAG	TGGTAGCGTACCGTTTGACA	59.9	634	INTERGENIC				
CaPOPII_3167	Ca7	44885868	CA/CAGAAAAATA	TTTGGTTCAAACAAGGGGAG	ACGAAAATGTCAAAAACCGC	59.9	373	DRR	Ca_15709		FAR1	
CaPOPII_3168	Ca7	45169614	ATTTTTTTTT/ATTTTTTTT	TGTCCAATTGGCAATACAAAA	AAAGAAGCTCGCTTGTATTTGA	58.9	678	INTERGENIC				
CaPOPII_3169	Ca7	45170609	ATTTTTTTTT/ATTTTTTTTTT	AAAGGGTTATTGCCAGCTTTT	AGGCATAGTTAGACGCCCCT	59.2	778	INTERGENIC				
CaPOPII_3170	Ca7	45206871	GTATATATATATATA/GTATATATATATATATA	AGGAGGTTTGTGTTTGACGG	TTTGGATTTTGACTCGGAGG	60.0	617	INTERGENIC				
CaPOPII_3171	Ca7	45407833	TGG/TG	CCGTCCTTCCTTCTTTCTCA	GTGTGCATTGGACCAGATTG	59.4	445	INTERGENIC				
CaPOPII_3172	Ca7	45762039	ATT/ATTACTAGTTATTTGTT	TTTTAAGGCGTGTTTTTGGG	TCAACAAGTCACAGGCGATT	60.0	893	DRR	Ca_15751	J		Ribosomal protein L5
CaPOPII_3173	Ca7	46332309	GAACACAA/GAACACAATCAACACAA	AACGTACTGCCTATCACGGG	TTCGCAACTCCACTCTTCCT	60.0	649	INTERGENIC				
CaPOPII_3174	Ca7	46354851	CTATATATATATATATATATATATATATGTATATATATAT/CTATATATATATATATATATATATATATGTATATATATAT	TATCGTTGGACGAATGGACA	TTCATGAGCAACTTTCCACAA	59.9	486	INTERGENIC				
CaPOPII_3175	Ca7	46573334	TATAATAATAATAATAATAATAATAATAATAATAATAA/TATAATAATAATAATAATAATAATAATAATAATAATAATAATAATAA	GGCACGACATCATCATCATC	CTTTTAGGCCGGGTTACCAT	59.9	597	INTERGENIC				
CaPOPII_3176	Ca7	47155072	A/AAGAG	AGGAGGGGTGCGATTTTTAT	TTATGCGTGGTTGTTGCATT	59.8	166	INTERGENIC				
CaPOPII_3177	Ca7	47286553	GATAATAATAATAATAATAATAATAA/GATAATAATAATAATAATAATAATAATAATAA	CTGAAGGTGCAAACACGAGA	CAAGGACTAAATTGAAACCCAAA	60.0	661	INTERGENIC				

INDEL marker IDs	Chromosomes /unanchored scaffolds	Physical positions (bp)	InDels (*Kabuli* reference genome-CDC Frontier/PI)	Forward primers (5'-3')	Reverse primers (5'-3')	Anneling temperature (0C)	Expected amplified product size (bp)	Structural annotation		Functional annotation		
								Sequence components of *kabuli* genome	*Kabuli* gene accession IDs	NCBI-KOG	TFs	NCBI-nr database
CaPOPII_3178	Ca7	47367383	TAAAAATTGAAAAAAATTGAAA/TAAAAATTGAAA	CGTGTGAGAAAACCTCGACA	GTTCGAAATGCCGATTTCAC	59.9	435	INTRON	Ca_19497	L		DNA ligase, ATP-dependent, N-terminal
CaPOPII_3179	Ca7	47551388	C/CCA	TGGCATTGATGATGGCTTTA	CCAGGATAAAACAGCCCAGA	60.0	622	DRR	Ca_19500	O	MYB_related	Ubiquitin-conjugating enzyme, E2
CaPOPII_3180	Ca7	47551645	ATTCTAGAACGGTTTTCTA/ATTCTAGAACGGTTTTCTAGAACGGTTTTCTA	GCAATTTTGTGAAGGAGGGA	CCAGGATAAAACAGCCCAGA	60.1	497	DRR	Ca_19500	O	MYB_related	Ubiquitin-conjugating enzyme, E2
CaPOPII_3181	Ca7	48312562	TAAAA/TAAAAA	CGAAGCACAAGGAGATGACA	CAGTGATGCGGAAAAACTCA	60.0	196	INTERGENIC				
CaPOPII_3182	Ca7	48312616	AT/ATATCT	CGAAGCACAAGGAGATGACA	TTTCCGAGTGCTGTCAAATG	60.0	382	INTERGENIC				
CaPOPII_3183	Ca7	48392684	TAAAAAAAAA/TAAAAAAAAAA	TGATTCCCCAACATCCAACT	ATGATATTGGAGTTTGCGCC	60.2	165	INTERGENIC				
CaPOPII_3184	Ca7	48429143	AATATATATATATATATAT/AATATATATATATATATATAT	AACACCGGGTTCAGATGTGT	GGCCAAATACGTTGGAAATG	60.3	663	INTERGENIC				
CaPOPII_3185	Ca8	15508	G/GAAT	ACACGTGTGCAAAGTGCAA	TTTTTACGGGTACCTGGTCG	59.9	386	INTERGENIC				
CaPOPII_3186	Ca8	18339	CAAA/CAAAA	TGGCACACTGTCTTGTCCTC	GTGTGCCAATTGAGGAAGGT	59.9	268	INTERGENIC				
CaPOPII_3187	Ca8	24399	TGTTTAGGGTTTAGGGTTTAGG/TGTTTAGGGTTTAGG	CGGATTTAGGGTTTTGGGAT	GCGTCCGACAACCATTTACT	60.0	448	INTERGENIC				
CaPOPII_3188	Ca8	46677	TT/TTAT	CAATCGGACACCAATTCAAA	TCTGTCTTGTCCCACTCCAA	59.4	251	INTERGENIC				
CaPOPII_3189	Ca8	1184414	GAAAAAAAAAA/GAAAAAAAAA	TTCGTACCGTTCTCTCCTCAA	TTGTACACAACTCCCTTGCG	59.9	563	INTERGENIC				
CaPOPII_3190	Ca8	1185649	TAGAAGAAGAA/TAGAAGAA	GACTCGCCAACTCAACCATT	ACACATGCTCCATACCACCA	60.1	282	CDS (large-effect mutations)	Ca_15061			
CaPOPII_3191	Ca8	1355185	CTTTTTTTTT/CTTTTTTTTTT	TTTATTTCCTTCACGTGGGC	ATGTGTTGGGTGGGTTCACT	59.9	125	INTERGENIC				
CaPOPII_3192	Ca8	1452383	CAAAAAAAA/CAAAAAAAAA	TTGCAAGGGAAGGAAACAAC	CGGGCTACCTAGAATCCCTC	60.1	561	INTERGENIC				
CaPOPII_3193	Ca8	1524117	ATTTTTTTTTT/ATTTTTTTTT	TTCAATTGTATGGGCAAGGA	GTGAAGGAGCACGCATGTAA	59.0	276	INTERGENIC				
CaPOPII_3194	Ca8	1870873	CNNNNNNNNNN/CNNNNNNNNN	GCAAACCCATCCTTTTCTCA	TGATCTCTGTGAGTCCGTCG	60.1	463	INTERGENIC				

INDEL marker IDs	Chromosomes /unanchored scaffolds	Physical positions (bp)	InDels (*Kabuli* reference genome-CDC Frontier/PI)	Forward primers (5'-3')	Reverse primers (5'-3')	Anneling temperature (0C)	Expected amplified product size (bp)	Structural annotation		Functional annotation		
								Sequence components of *kabuli* genome	*Kabuli* gene accession IDs	NCBI-KOG	TFs	NCBI-nr database
CaPOPII_3195	Ca8	1881652	ATGTGTGTGTGTGTGTGTGTGT/ATGTGTGTGTGTGTGTGTGTGTGT	TGAAAAGTTTTGAATGAAAAGTGG	CCATCCAAATTACACATGCCT	59.6	549	DRR	Ca_02414	T	NAC	PAS
CaPOPII_3196	Ca8	2295637	TAAAAAAAAAA/TAAAAAAAAA	CCACCCTATCGAGAAGACCA	AACTGACAGCCATAAAAGCCA	60.1	864	INTERGENIC				
CaPOPII_3197	Ca8	2507807	TTATATATATATATATATATAT/TTATATATATATATATAT	CGGACTGGAATCAAAAGCTC	AGCTAAATGCGATTTGCAGG	59.8	852	INTERGENIC				
CaPOPII_3198	Ca8	2522963	ATTTTTTTTT/ATTTTTTTTTT	ACCAATGGGGAGGTTTTCAT	CTCAGTGACTTTCATTTGGCA	60.4	332	DRR	Ca_02333	L		DNA mismatch repair protein MutS, C-terminal domain
CaPOPII_3199	Ca8	2869406	TG/T	CAACTGGATAACCAGCTCCC	CGGCCAAATTGATCAGGTAT	59.6	565	INTRON	Ca_02297	U		Pleckstrin homology domain
CaPOPII_3200	Ca8	3261756	GAAAAAAAAAA/GAAAAAAAAAAA	TCCCTGCGAATTCTCATTCT	CCCCCACTTTGTCTCATCAT	59.8	561	INTERGENIC				
CaPOPII_3201	Ca8	3363189	GTATATATATATATATATATATAT/GTATATATATATATATATATATATATAT	CCAATTAATTTGTTAAAGACCATCTG	TAAAAGCAATTTTGGGTGGG	59.3	599	INTERGENIC				
CaPOPII_3202	Ca8	3804574	T/TC	AATGTTTGGGCACACATGAA	GATTTTCCTTCCCCACCAAT	59.8	337	DRR	Ca_02194	K		RNA polymerase, subunit H/Rpb5 C-terminal
CaPOPII_3203	Ca8	4071945	TG/T	GAGGGGTGGTAACTGACTCG	ATGCACAAATTCTCGCACAG	59.6	657	INTERGENIC				
CaPOPII_3204	Ca8	4114988	CTTTTTTTT/CTTTTTTT	GCAGCCCGAGTGATAAAGAA	TGGCTCAATCACACAAAGGA	60.4	261	INTERGENIC				
CaPOPII_3205	Ca8	4179867	AATATATATATATATATATATAT/AATATATATATATATATATATATAT	AGTAGGAGCGGTTGAGGGAT	ACCGGCAAAGATTGATGAAC	60.1	652	INTERGENIC				
CaPOPII_3206	Ca8	4264902	GTATATATATATATATATATATATATATAT/GTATATATATATATATATATATATATATATATAT	TAGCATTGGGGAACATGACA	TCCACCAATTCAATGGACAA	59.9	843	INTERGENIC				
CaPOPII_3207	Ca8	4384084	ATTTTTTTTTT/ATTTTTTTTT	TCCCTTTTCCATGAACATTTG	TTTGCGTTATGGATTTGATGA	59.8	554	INTERGENIC				
CaPOPII_3208	Ca8	4399889	AAATAATAATAATAATAATAATAATAATA/AAATAATAATAATAATAATAATAATA	CAACCTCCCAAAGGGTTTTT	AGGTGTGGTAAGATTGTTTTGAA	60.2	372	INTERGENIC				
CaPOPII_3209	Ca8	4399931	AAATAATAATAATAATAATAAT/AAATAATAATAATAATAATAATAAT	CAACCTCCCAAAGGGTTTTT	AGGTGTGGTAAGATTGTTTTGAA	60.2	372	INTERGENIC				
CaPOPII_3210	Ca8	4752741	TTATATATATATATATATATATATATATAT/TTATATATATATATATATATATATAT	TTTGGGCAGCATAAATAGGG	TGCTAAAATTTCCAGGGGTG	59.9	819	INTERGENIC				
CaPOPII_3211	Ca8	4859400	GAAAAAAAA/GAAAAAAAAAA	TTCATTGCACAAACGCTCTC	CCAACCACCCACTACCATTT	60.0	753	INTERGENIC				

INDEL marker IDs	Chromosomes /unanchored scaffolds	Physical positions (bp)	InDels (*Kabuli* reference genome-CDC Frontier/PI)	Forward primers (5'-3')	Reverse primers (5'-3')	Anneling temperature (0C)	Expected amplified product size (bp)	Structural annotation		Functional annotation		
								Sequence components of *kabuli* genome	*Kabuli* gene accession IDs	NCBI-KOG	TFs	NCBI-nr database
CaPOPII_3212	Ca8	4983898	AATATATATATAT/AATATATATAT	TATGTTCGTCTCTGCATGGC	TTGGAATGAGCAAATAGGCA	59.8	594	DRR	Ca_02055	Q	GeBP	Cytochrome P450
CaPOPII_3213	Ca8	5016373	TAAAAAA/TAAAAAAA	TTGTCCGTTATAACATTAAACACTTT	GAAAAGACAACCCCGCATTA	57.5	279	INTERGENIC				
CaPOPII_3214	Ca8	5363068	GATATATATATATATATATAT/GATATATATATATATATATATAT	TTTCAATGGCAGAGAAAGGG	TGTCACACCCTCAAGACACAA	60.2	823	INTERGENIC				
CaPOPII_3215	Ca8	5366435	GTTTTTTTTTTT/GTTTTTTTTTT	CTGATGAAGGGAAGGGAACA	CTTCATCATTTGGTGGGAGAA	60.0	652	INTERGENIC				
CaPOPII_3216	Ca8	5464595	TTTTTATTTTATTTTATTTTA/TTTTTATTTTATTTTATTTTATTTTA	ACTTCCCTTTCGAGACCGAT	CGAAGTACAATGGTTTGTAAGCA	60.1	870	INTERGENIC				
CaPOPII_3217	Ca8	5596170	GATATATATATATATATATATATATATAT/GATATATATATATATATATATATATATATATATATATAT	AGTCCTAACAATCTGGCACGA	CTATGGTGAATGCGCAAAAA	59.7	706	INTERGENIC				
CaPOPII_3218	Ca8	5601429	TAAAAAAAAA/TAAAAAAAAAA	ATGCGAAAATTGACAACGTG	CCGGTACCCTAAACTGCAAA	59.6	724	INTERGENIC				
CaPOPII_3219	Ca8	5719968	TTGTGTG/TTGTG	GTTCCATTCCAACCCCAAT	AAAGTCGTGTGCTCGTGTTG	59.4	579	INTERGENIC				
CaPOPII_3220	Ca8	5724554	TAATAAATAAATAAA/TAATAAATAAATAAATAAA	CTGCCGACAAGGACAATGTA	AACAAGAACATCCTGGTCGC	59.7	906	INTERGENIC				
CaPOPII_3221	Ca8	5733091	TAC/TACTTTTAAC	TGATTCTCAGGGAGGGTGTC	GAGGGGATGAGAACGACAAA	60.0	537	INTERGENIC				
CaPOPII_3222	Ca8	6124309	TAAAAAAAA/TAAAAAAAAA	AAAACCATGGCACCTCTTTG	GACGATCGCGGTTAAGAAGA	60.0	491	INTERGENIC				
CaPOPII_3223	Ca8	6136556	CTATTATTATTATTATTATTATT/CTATTATTATTATTATTATT	GAACGCAAAGGACATGGATT	CCAAAGCCATCAACATCAGA	59.9	715	INTERGENIC				
CaPOPII_3224	Ca8	6307113	ATAATACATGTAATACATGTAATA/ATAATACATGTAATA	GCAATTCAACGAAAATGCAA	CCACTAAGTCATTTGCAAGGC	59.7	942	INTERGENIC				
CaPOPII_3225	Ca8	6492029	A/ATCAACC	TCTGTTTTGCCCAACCTTTC	ATCTCAACGATCCAACGGTC	60.1	455	INTRON	Ca_10739	D		
CaPOPII_3226	Ca8	6492546	CAAAA/CAAA	CGTAAGCAACCCACCAATCT	TTGTCAACAACTCAGCGTAAAAA	60.0	142	INTERGENIC				
CaPOPII_3227	Ca8	6492603	ATTTTTTTTTTTTT/ATTTTTTTTTTT	CGTAAGCAACCCACCAATCT	TTGTCAACAACTCAGCGTAAAAA	60.0	142	INTERGENIC				
CaPOPII_3228	Ca8	6567143	AAGAGAGAGAGAGAGAGAGAGAGAGAGAGAGAGAGAGAGAG/AAGAGAGAGAGAGAGAGAGAGAGAGAGAGAGAG	GGAAAATTGAGGGAAGAGGG	GTGTGGGTCACTTCAGCAAA	59.9	227	INTERGENIC				

INDEL marker IDs	Chromosomes /unanchored scaffolds	Physical positions (bp)	InDels (*Kabuli* reference genome-CDC Frontier/PI)	Forward primers (5'-3')	Reverse primers (5'-3')	Anneling temperature (0C)	Expected amplified product size (bp)	Structural annotation		Functional annotation		
								Sequence components of *kabuli* genome	*Kabuli* gene accession IDs	NCBI-KOG	TFs	NCBI-nr database
CaPOPII_3229	Ca8	6976159	GTAATAATAATAATAATAATAATAATAATAAT/GTAATAATAATAATAATAATAATAATAATAATAATAATAAT	GCGAAAAAGACAACGAAAGC	TCATCGGATTGGTCCTCTTC	60.0	255	INTRON	Ca_10695	I		Diacylglycerol acyltransferase
CaPOPII_3230	Ca8	6991978	ATTTTTTTTTT/ATTTTTTTTTTT	TTCAACATCTTGGTTCTTTATGC	ATTTCCAGTGGGATGGTTGA	58.3	431	INTERGENIC				
CaPOPII_3231	Ca8	7203379	CATGTTA/CATGTTAATGTTA	TGCCTTCAATTGTGCTTTACC	TTTGGAGAAGCGACCTTGTT	60.1	423	INTERGENIC				
CaPOPII_3232	Ca8	7683519	TTGT/TT	AACCATTTGCTTTGGCACTC	CAAGTTTGATTTGCACATTTGTT	60.1	587	INTERGENIC				
CaPOPII_3233	Ca8	7777478	C/CATA	TCCAGTGGCACCATAACAAA	TGAATCCCCGTGACGTTTAT	60.0	588	INTRON	Ca_10607	Z	MYB_related	Kinesin, motor domain
CaPOPII_3234	Ca8	7777713	CACATAACATA/CACATA	TCCAGTGGCACCATAACAAA	CTCCCAAGAACCCTTCTTCC	60.0	744	INTRON	Ca_10607	Z	MYB_related	Kinesin, motor domain
CaPOPII_3235	Ca8	7896399	ATGTTGTT/ATGTTGTTGTT	TTTGATATGTTGCTTCCGCA	CGATGCAGCCTCTTAGGTGT	60.2	689	INTERGENIC				
CaPOPII_3236	Ca8	7924797	TA/T	CGTTTGTCCATCGTCTTTCA	TAATCTGCGCGTTAAGCAGA	59.7	518	INTERGENIC				
CaPOPII_3237	Ca8	7927288	CAAA/CAAAA	AGGACCGTGCAAATGTTTCT	TGGCCTACTTATGGTCTGGC	59.6	349	DRR	Ca_11431	J		Protein synthesis factor, GTP-binding
CaPOPII_3238	Ca8	7927539	GTATTGCTTTTAT/GTATTGCTTTTATTGCTTTTAT	GCCAGACCATAAGTAGGCCA	GCAACTGGATTGCTGGATTT	60.1	509	DRR	Ca_11430	J		Translation elongation factor EFTu/EF1A, C-terminal
CaPOPII_3239	Ca8	7927639	ATT/AT	GCCAGACCATAAGTAGGCCA	GCAACTGGATTGCTGGATTT	60.1	509	DRR	Ca_11430	J		Translation elongation factor EFTu/EF1A, C-terminal
CaPOPII_3240	Ca8	7938103	AT/ATTT	CACGGGGAAAAGTTGATTTG	GAAGTCGCCATTTGGAACTC	60.3	634	INTERGENIC				
CaPOPII_3241	Ca8	7938264	TTATATA/TTATATATA	CACGGGGAAAAGTTGATTTG	CACCAAAATACCCCCATTTG	60.3	697	INTERGENIC				
CaPOPII_3242	Ca8	7948182	TCA/TCACA	GCCGCAAAGAAGAGAAGAGA	TGCCCAAGTCACTAATGCTG	59.8	443	INTERGENIC				
CaPOPII_3243	Ca8	7950561	CAAAAAA/CAAAAA	GTCTTTGGCCTCCAATCAAA	TGGAGTTGGGCCAATAATTT	60.1	359	INTERGENIC				
CaPOPII_3244	Ca8	7950841	AAAT/AAATAAT	TGTGTTGACCCAACTCCAAA	AGCCACTTGGTTGTGTTTGA	60.0	450	INTERGENIC				
CaPOPII_3245	Ca8	7952607	GAAAAAA/GAAAAAAAA	CCTTCAAAATGGTGTTGCTCT	GTCATTTTGCCGATCACCTT	59.2	676	INTERGENIC				

INDEL marker IDs	Chromosomes /unanchored scaffolds	Physical positions (bp)	InDels (*Kabuli* reference genome-CDC Frontier/PI)	Forward primers (5'-3')	Reverse primers (5'-3')	Anneling temperature (0C)	Expected amplified product size (bp)	Structural annotation		Functional annotation		
								Sequence components of *kabuli* genome	*Kabuli* gene accession IDs	NCBI-KOG	TFs	NCBI-nr database
CaPOPII_3246	Ca8	7953093	T/TC	AAGGTGATCGGCAAAATGAC	CAGCCCTTCAGTGTCATGAGT	59.9	609	INTERGENIC				
CaPOPII_3247	Ca8	7953809	TACCTCAA/TACCTCAAACCTCAA	ACCTGCGACACGTGAACATA	GAAAACGAGGCGTAAACGAG	60.2	360	INTERGENIC				
CaPOPII_3248	Ca8	7954393	TAA/TA	CTCGTTTACGCCTCGTTTTC	CCGTAGCATGAGAGTGACCA	59.9	561	INTERGENIC				
CaPOPII_3249	Ca8	7954421	CATATATATATATATATATAT/CATATATATATATATATATATATAT	CTCGTTTACGCCTCGTTTTC	CCGTAGCATGAGAGTGACCA	59.9	561	INTERGENIC				
CaPOPII_3250	Ca8	7954496	CATATA/CATATATA	TTTCGTCGTATAAAGACGGAGAG	CCGTAGCATGAGAGTGACCA	59.8	545	INTERGENIC				
CaPOPII_3251	Ca8	7955526	TCTC/T	CATGCAAGAGAAGGGAAAGAA	GTGTGGGAGAGAAAGCATCC	59.4	296	INTERGENIC				
CaPOPII_3252	Ca8	7956165	GTTATATAATATTA/GTTATATAATATTATTATATAATATTA	TTGATTTGTCTAGAAGCAGCACA	CACTTCTTGGCTGAGTTGGA	60.1	476	DRR	Ca_11432		G2-like	Myb, DNA-binding
CaPOPII_3253	Ca8	8055205	CAATAAAGTAATA/CAATA	TGATAAACATGATTTTCCCTCATTT	TTGTTTGTTTGGAGAAGGATCA	60.0	403	INTERGENIC				
CaPOPII_3254	Ca8	8076052	TAAAA/TAAAAAA	TAATGCCATTTTGGTGTTGC	TTTTCAACAAAATGTGCGGA	59.4	219	INTERGENIC				
CaPOPII_3255	Ca8	8240879	TC/TCC	GTCGCCGCTACGAGATAAAC	TTTGTGCTGATGGTGATGGT	59.9	729	INTERGENIC				
CaPOPII_3256	Ca8	8312325	AAATAGGAATGTTTA/AAATAGGAATGTTTAATAGGAATGTTTA	CGGCGATCATTAAGGAGAAA	ATTTTGAACAAGCGGACCTG	60.2	561	INTERGENIC				
CaPOPII_3257	Ca8	8623323	ATTTTTTTT/ATTTTTTTTT	GGGGTGATTATGGAGCAAGA	GGTCACGTGTGTTCCAAGAA	59.9	963	INTERGENIC				
CaPOPII_3258	Ca8	8624257	ATTAAAAGC/ATCAACTTTAAAAGC	CTTTGATTGGGGTTTTGGAA	TGGTGACAATTATTTTTCCCGT	59.8	664	INTERGENIC				
CaPOPII_3259	Ca8	8624418	CAAAAATTTAGAAAAAT/CAAAAATTTAGAAAAATTTAGAAAAAT	TCCTTTCAATGGACTGGTGA	GTCCCGAGCATGACGTTTAT	59.1	888	INTERGENIC				
CaPOPII_3260	Ca8	8624597	TAAAAAAAAAA/TAAAAAAAAA	CAATTCTTGCATCCTAACCAAA	GTCCCGAGCATGACGTTTAT	59.1	384	INTERGENIC				
CaPOPII_3261	Ca8	8636114	ATTTTTT/ATTTTTTT	TTGAATTTGGTTTCAATTGGG	ACTCTCCTTTCCTCCCCTCA	59.7	514	INTERGENIC				
CaPOPII_3262	Ca8	8694777	GAA/GA	AATTGGCAGGGGAAGAAGAT	TAAGGCAAATGGTGCCTACC	59.9	715	INTRON	Ca_11501	V	NAC	NAD-dependent epimerase/dehydratase

INDEL marker IDs	Chromosomes /unanchored scaffolds	Physical positions (bp)	InDels (*Kabuli* reference genome-CDC Frontier/PI)	Forward primers (5'-3')	Reverse primers (5'-3')	Anneling temperature (0C)	Expected amplified product size (bp)	Structural annotation		Functional annotation		
								Sequence components of *kabuli* genome	*Kabuli* gene accession IDs	NCBI-KOG	TFs	NCBI-nr database
CaPOPII_3263	Ca8	8701927	AATATATATATATATATATATATATATATATATATAT/AATATATATATATATATATATATATATATATATATATATATATATATAT	GTGGAGTGCAAAGTTGGTGA	GCCTGAATATGCACACACAAA	59.7	795	INTERGENIC				
CaPOPII_3264	Ca8	8854983	TTAAAG/TTAAAGGAAAAATAAAG	GCCATATCCGGCTTACCTTT	CCCTTGACCAATTCCAGAGA	60.3	475	DRR	Ca_11515			
CaPOPII_3265	Ca8	8860098	GAAAA/GAAAAA	ATGCATCCACTCCCAGAAAC	TGTTTTGGTTGTGTCCCAGA	59.9	512	INTERGENIC				
CaPOPII_3266	Ca8	8868722	ATTTTTT/ATTTTT	TGGTGGTTCAAAGGTAAGGG	CAATTTTCAAAACATGTGCAA	59.8	317	INTERGENIC				
CaPOPII_3267	Ca8	8880565	ATTTTT/ATTTT	TTTCGACCCTTGAGCTTTGT	TTGCTCCCTTTGACTCTACCA	59.9	736	INTERGENIC				
CaPOPII_3268	Ca8	8894265	ATTTTTTTT/ATTTTTTT	GAGGCCCAAAATGAATAGCA	TTGTCGACGTCCTCGGTATT	60.0	485	DRR	Ca_11521	G		Major facilitator superfamily MFS-1
CaPOPII_3269	Ca8	8899206	AT/A	GTGGATGACAAAACCTCGTG	TTCTCATATGCCCCTCCAAC	59.0	633	INTRON	Ca_11521	G		Major facilitator superfamily MFS-1
CaPOPII_3270	Ca8	8907678	CAAA/CAAAA	TACCAAAGTTTTCCCCCTCC	GCAATGCATGAGAGGACAAG	60.2	122	INTERGENIC				
CaPOPII_3271	Ca8	8920440	TAAAAAAAA/TAAAAAAA	TGTTCATGTAAGCCATTGGG	TCCAAAACCCATTTCACCTC	59.4	540	INTRON	Ca_11522			Starch synthase, catalytic domain
CaPOPII_3272	Ca8	8954725	GAAAAAAAAA/GAAAAAAAA	ATCCATCGAGCAAGTCGTTC	GGCTTAGGGATAACTGCATCC	60.2	616	INTERGENIC				
CaPOPII_3273	Ca8	8956224	TATGCCATGCCATGCCAT/TATGCCATGCCAT	TGTGGGACCTTCCAAAACAT	CAAACAACGTCTATCTTCGGC	60.2	486	INTERGENIC				
CaPOPII_3274	Ca8	8956561	TG/TGATCG	GCCATGCCATCCTTGATAAT	ATGTCCAACATCTGCTTCCC	59.8	628	INTERGENIC				
CaPOPII_3275	Ca8	8956637	CATAAT/C	GCCATGCCATCCTTGATAAT	ATGTCCAACATCTGCTTCCC	59.8	628	INTERGENIC				
CaPOPII_3276	Ca8	8967849	TAAAAAAAA/TAAAAAAAAA	CTGCTGTTTGTGCCACTGTT	TCATCCGAATGGTATGCTCA	59.9	334	INTERGENIC				
CaPOPII_3277	Ca8	8968364	GTT/GTTT	TGTTGAGCATACCATTCGGA	TCGTGTCTCATGTTCTGCCT	60.1	780	INTERGENIC				
CaPOPII_3278	Ca8	8969339	GACACA/GACACACA	GCAACATTGGCAAAAAGTGA	CACATGGCCGTGGGTAAAT	59.7	534	INTERGENIC				
CaPOPII_3279	Ca8	8972356	TC/T	TCCCCATGACATCACACAAG	TGAGAAAATTTGGCAAAGGG	60.4	592	INTERGENIC				

INDEL marker IDs	Chromosomes /unanchored scaffolds	Physical positions (bp)	InDels (*Kabuli* reference genome-CDC Frontier/PI)	Forward primers (5'-3')	Reverse primers (5'-3')	Anneling temperature (0C)	Expected amplified product size (bp)	Structural annotation		Functional annotation		
								Sequence components of *kabuli* genome	*Kabuli* gene accession IDs	NCBI-KOG	TFs	NCBI-nr database
CaPOPII_3280	Ca8	8998003	TTGT/TTGTGT	TTTGATGACTCGTAAGCCCC	TGTAAGATGGAGGATCCAAAGTG	60.1	240	INTERGENIC				
CaPOPII_3281	Ca8	8998056	TG/T	TTTGATGACTCGTAAGCCCC	TGTAAGATGGAGGATCCAAAGTG	60.1	240	INTERGENIC				
CaPOPII_3282	Ca8	9023763	TTATATATATATATATATATA/TTATATATATATATATATATA	TAAGCGCATGTTCCATGTTG	TGACGATGTGCTAAGCATGA	60.7	359	INTERGENIC				
CaPOPII_3283	Ca8	9087628	ATATTATTATTATT/ATATTATTATTATTATT	TTGGACCCCATCTTACTTGG	TGTAGCATCCCATTTTCCACT	59.8	741	INTERGENIC				
CaPOPII_3284	Ca8	9098007	TTAT/TT	AACCGTTAAATCCGCAACTG	CCAAGGAAACAGCTCAAAGC	60.0	794	INTRON	Ca_11533		C2H2	Glycoside hydrolase, family 3, N-terminal
CaPOPII_3285	Ca8	9101623	CT/CTTT	CAAGGTTCTCCAGAAACCCA	ACACGTGTGCGCTCAAAATA	60.1	671	DRR	Ca_11533		C2H2	Glycoside hydrolase, family 3, N-terminal
CaPOPII_3286	Ca8	9201647	CA/CAA	GTCTCGATCGCAAGTCACAA	GATATATACGGCTGACGGCG	60.0	300	INTERGENIC				
CaPOPII_3287	Ca8	9201705	GATGTATTATGTAT/GATGTAT	GTCTCGATCGCAAGTCACAA	GATATATACGGCTGACGGCG	60.0	300	INTERGENIC				
CaPOPII_3288	Ca8	9208402	TAAAA/TAA	TTTTGGATTAGTTGGGCCAC	TGTCAACAATAGTGTTTGCGG	59.8	647	INTERGENIC				
CaPOPII_3289	Ca8	9218033	ATTTT/ATTTTT	CAAAATCAGGTGTTGGCTGA	CGGTGTCAAAGTCAGGGAAT	59.7	730	INTERGENIC				
CaPOPII_3290	Ca8	9364522	ATT/ATTT	TTGTCGGTGAGATTGTGTGAA	AAAGATTGTGATTTTGGCGG	60.1	802	INTERGENIC				
CaPOPII_3291	Ca8	9397792	ATTTTTT/ATTTTTTT	CCGCTCAAAATCATCGTACA	ACGGGCATCTAATGTTGAGG	59.7	244	INTERGENIC				
CaPOPII_3292	Ca8	9950946	CTT/CTTGTCGATT	ACCATGGCTTATTGGTCAGC	TGGCATAATGTCAGACGCAT	60.0	310	INTRON	Ca_18394			Protein of unknown function DUF1442
CaPOPII_3293	Ca8	9953316	TTGTGT/TTGTGTGT	AGCAGCAACAACCGTCTTTT	AAAAATCGATCGGGTGCATA	59.9	312	DRR	Ca_18394			Protein of unknown function DUF1442
CaPOPII_3294	Ca8	9960024	CATAATAATAATAAT/CATAATAATAATAATAATAATAAT	CCATTGGGATGTTTGCTTTT	GCTCATGTGGTGGTGTTTTG	59.8	280	INTRON	Ca_18395			Stress up-regulated Nod 19
CaPOPII_3295	Ca8	10301817	TTAATAATAATAATAATAA/TTAATAATAATAATAA	ACTGGGGAAGTTGGCTTTTT	ACGAGGTGTCAGATTTTGGC	60.0	689	DRR	Ca_18422	R	Trihelix	Pentatricopeptide repeat
CaPOPII_3296	Ca8	10366674	CTT/CT	CAAGAAGACGCCGATGAAAT	GTCGAACGTTCTCCGTTTGT	60.2	571	INTERGENIC				

INDEL marker IDs	Chromosomes /unanchored scaffolds	Physical positions (bp)	InDels (*Kabuli* reference genome-CDC Frontier/PI)	Forward primers (5'-3')	Reverse primers (5'-3')	Anneling temperature (0C)	Expected amplified product size (bp)	Structural annotation		Functional annotation		
								Sequence components of *kabuli* genome	*Kabuli* gene accession IDs	NCBI-KOG	TFs	NCBI-nr database
CaPOPII_3297	Ca8	10366831	GTTTT/GTTT	CCCTCAAACAAAAATGCACC	GTCGAACGTTCTCCGTTTGT	60.3	400	INTERGENIC				
CaPOPII_3298	Ca8	10375574	CG/CGG	TGGTCTCTCGATCAGATTTTTG	GATGATGTGGCACAGTGGAC	59.3	517	INTERGENIC				
CaPOPII_3299	Ca8	10381692	AGG/AGGG	CGTTTCCTCTGCCTCTTGAC	TTGTGAAAAACGAGCACGAC	60.0	677	INTRON	Ca_18429	R	NAC	Pentatricopeptide repeat
CaPOPII_3300	Ca8	10590161	ATTTTTT/ATTTTTTT	AACGGAGGAGGAACAAACAC	TTAGAAGGCCTTGCATACGA	59.0	212	INTERGENIC				
CaPOPII_3301	Ca8	10612849	CTTTT/CTTT	AGTGGGAGTTTCGTCCATTG	AGCACATGGAACCAAAGAGG	60.0	299	INTERGENIC				
CaPOPII_3302	Ca8	10686546	ATTTT/ATTT	TGCTATCGGTACCAATTTTGC	TCCGTTGTCTCATTCGATCA	60.0	628	INTERGENIC				
CaPOPII_3303	Ca8	10701972	ATGTT/AT	TCAACGGTTATACGGCAACA	TTTTATCTATGAAAAATGCCTCACA	60.0	373	INTERGENIC				
CaPOPII_3304	Ca8	10934374	AT/ATT	TCCGATTCATCCTCTTGGTC	AATGAAAAAGCGGGGAAACT	60.0	368	DRR	Ca_19747	R	NAC	Pentatricopeptide repeat
CaPOPII_3305	Ca8	11070760	AG/A	GCTTCTAGCTGGTGCTGCTT	AGGGAATCAATTGTAGGGGG	59.9	900	INTERGENIC				
CaPOPII_3306	Ca8	11113946	GA/GAATA	CCAGAAAACGAAAACCAAACA	TTTGATTTTGAGGCAGAGCA	60.0	480	INTERGENIC				
CaPOPII_3307	Ca8	11124508	CATAT/CAT	CAGTGGGAAAAGTCAACGGT	GGTTCCGACATCACAGGAGT	60.0	536	DRR	Ca_16840		WRKY	
CaPOPII_3308	Ca8	11145831	TCC/TC	TCTTTCTGGTTGCAAATTGTTTT	AAATTGAAGCAAACGGAACC	60.0	980	INTERGENIC				
CaPOPII_3309	Ca8	11146284	TGTGTCTTAGGT/TGT	TGTGATTGAGGCACTATCAAGC	CCGGGTGAAATGGTTATTTG	60.3	280	INTERGENIC				
CaPOPII_3310	Ca8	11146309	ATTTTTTTTTTT/ATTTTTTTTT	TGGTTTGCTGGAATTGTGTG	CCGGGTGAAATGGTTATTTG	60.6	167	INTERGENIC				
CaPOPII_3311	Ca8	11146772	TAAA/TAAAA	TAACCTGTGCGCTATTCGTG	TGCAAATAATTCCTCATCATTTTT	59.9	329	INTERGENIC				
CaPOPII_3312	Ca8	11168829	ATAG/ATAGTGAGCTAG	GAAACGTTGCAACTTGGTCA	GACCTTTGGTGGGGAAAAAT	59.7	227	INTERGENIC				
CaPOPII_3313	Ca8	11246794	TTATATATATATATATATATATATATATATAT/TTATATATATATATATATATATATATATATATATATATAT	TCAAATTCATTCTTTGCATCGT	GGCGAACCAAAAAGTTTGAA	59.6	766	INTERGENIC				

INDEL marker IDs	Chromosomes /unanchored scaffolds	Physical positions (bp)	InDels (*Kabuli* reference genome-CDC Frontier/PI)	Forward primers (5'-3')	Reverse primers (5'-3')	Anneling temperature (0C)	Expected amplified product size (bp)	Structural annotation		Functional annotation		
								Sequence components of *kabuli* genome	*Kabuli* gene accession IDs	NCBI-KOG	TFs	NCBI-nr database
CaPOPII_3314	Ca8	11280314	TAA/TA	TGTGTCATTTTCCTTGGAAGC	CATCATAACAAGTCGAATGTCCA	60.1	517	INTERGENIC				
CaPOPII_3315	Ca8	11359890	TC/T	CTCGAAAATTAGGCGAGCAG	CACGTGCATGACCCTAAATG	60.1	605	INTRON	Ca_16822	G	NAC	Glycoside hydrolase, family 1
CaPOPII_3316	Ca8	11536143	CATTATTATTATTATTATT/CATTATTATTATTATTATTATT	TCTTACACTGCAGCAATAAAAATCA	TGGTTGATGCCCAAGTACAA	60.2	576	DRR	Ca_16812	R	MYB	Integrase, catalytic core
CaPOPII_3317	Ca8	11546931	T/TCG	GCCATGTTCAGTCTTGGTCA	ACTCCACCATTGCTTGCTTT	59.7	247	INTRON	Ca_16811			Auxin efflux carrier
CaPOPII_3318	Ca8	11769671	C/CCCTTACCAA	TCATTGGTCCTACCCTCGTC	TTTGGCAATCGACATTGTGT	59.9	154	INTERGENIC				
CaPOPII_3319	Ca8	11955442	T/TG	GGCCGATTTACTGCTCACAT	GTTGCCACTTGGCGTAAAAT	60.1	430	INTERGENIC				
CaPOPII_3320	Ca8	11975713	CT/C	TATACTCGTGCACTACGCGG	GGATGGAGACAATGATGAGGA	59.9	135	INTERGENIC				
CaPOPII_3321	Ca8	12016131	ACCCC/ACCCCC	CAACTCTCGTCGCAACTCAA	ATGCAACTTGGGAATTGGAG	60.2	362	INTRON	Ca_23846	T		Apoptosis inhibitory 5
CaPOPII_3322	Ca8	12048375	ACC/ACCC	TTCCCCCTAAAACAGAACCA	GTTGTTGCTGCCCTTTGTTT	59.4	723	INTERGENIC				
CaPOPII_3323	Ca8	12875576	GGAT/GGATGGAGAT	TGTATGATCGATCTGGGCAA	TGCATGTGCCTTCTGAAATG	60.0	731	INTERGENIC				
CaPOPII_3324	Ca8	12979016	C/CG	ATATGTTGGGGGTGATGGAA	GGGGTCGGACATTTACTAGC	59.9	921	INTERGENIC				
CaPOPII_3325	Ca8	12979079	TTTA/TTTAATTA	CGTGCAGTTTAGGGAAGGAT	GGGGTCGGACATTTACTAGC	59.2	873	INTERGENIC				
CaPOPII_3326	Ca8	12983474	ATTTTT/ATTTT	TGCAGATGCACGAATCTCTT	TGCACTAATTTATCGGGACCA	59.6	496	INTERGENIC				
CaPOPII_3327	Ca8	12983910	TAA/TA	TTGGTCCCGATAAATTAGTGC	GCCCAAGTTAAGACAACCCA	59.0	442	INTERGENIC				
CaPOPII_3328	Ca8	12984097	CGTGTGT/CGTGT	AGTGTTAACCGGGATCAAACA	GCCCAAGTTAAGACAACCCA	59.3	301	INTERGENIC				
CaPOPII_3329	Ca8	12988606	AG/A	CTCGCTCAAGATTTCCAAGG	GTTGTGGGAGGATCGAAAGA	59.9	567	INTERGENIC				
CaPOPII_3330	Ca8	12990010	CTA/CTATA	TGGCTAATAAATTGTGCCCAT	TCATGTCCCCTCCTTGAATC	59.3	552	INTERGENIC				

INDEL marker IDs	Chromosomes /unanchored scaffolds	Physical positions (bp)	InDels (*Kabuli* reference genome-CDC Frontier/PI)	Forward primers (5'-3')	Reverse primers (5'-3')	Anneling temperature (0C)	Expected amplified product size (bp)	Structural annotation		Functional annotation		
								Sequence components of *kabuli* genome	*Kabuli* gene accession IDs	NCBI-KOG	TFs	NCBI-nr database
CaPOPII_3331	Ca8	12998724	ATTTTTTTT/ATTTTTTTTTT	TCACACCTCTTCACTTTCATGG	CAAAGTTGTTGGGCAAGGTT	60.1	138	INTERGENIC				
CaPOPII_3332	Ca8	13297997	TA/TAA	AAATTGATTGGATGGTGGGA	AAATGCAGAGGCACCACTCT	60.0	442	INTERGENIC				
CaPOPII_3333	Ca8	13298358	TTATATATATATATATATATAT/TTATATATATATATATATATATAT	AGGAGGGGTCCCTGTTTCTA	ACACGAAAATTGCTCAGCCT	59.9	548	INTERGENIC				
CaPOPII_3334	Ca8	13338881	ATTTTTTT/ATTTTTTTT	TGTGAGGTACCGAGTTCCAA	TTATAACGGGTGGAAACGGA	59.1	689	INTERGENIC				
CaPOPII_3335	Ca8	13757181	ACCCCCCCC/ACCCCCCC	AATTCAGCTCCTACGAGGCA	GCATTTTGGGCATTTTGATT	60.0	326	INTERGENIC				
CaPOPII_3336	Ca8	13844941	AATATATATATATATA/AATATATATATATATATA	GGCTCTTCCATCTCCTCAAA	TTTCTTCGAGAGGTCGCTTC	59.4	607	INTERGENIC				
CaPOPII_3337	Ca8	13993624	TTG/T	GCACAATTTGGCAAGAACAA	TTGGGATTGAGGTTGAGGAG	59.7	455	DRR	Ca_22739	K		CASP, C-terminal
CaPOPII_3338	Ca8	13993907	GTTTTT/GTTTT	CTCCTCAACCTCAATCCCAA	TCACCGGTCAATCAATTAAAAA	60.0	228	DRR	Ca_22739	K		CASP, C-terminal
CaPOPII_3339	Ca8	14035508	GA/GAATA	TGAAATATTTTAAGCATTCACTGATAA	CATGCCTTCTTGCTTCAACA	57.7	466	INTERGENIC				
CaPOPII_3340	Ca8	14064085	CATATATATATATATATA/CATATATATATATATA	TGTGTGTGTGTGTCCAGAGG	GGTATCCATGGGAGCTTTGA	59.1	804	INTERGENIC				
CaPOPII_3341	Ca8	14074282	CCGCG/CCG	AAACAAAATCCACCGCACTC	TAGGTTCCAAAGGTGCCAAC	60.0	606	CDS (FRAME SHIFT)	Ca_22744	U		Clathrin/coatomer adaptor, adaptin-like, N-terminal
CaPOPII_3342	Ca8	14077330	TAGAGAAAAGAAGAGAAA/TAGAGAAA	TTGATATGTCATCCCCGGTT	TTCTTTCCCACCCTTCTTCC	60.0	696	DRR	Ca_22744	U		Clathrin/coatomer adaptor, adaptin-like, N-terminal
CaPOPII_3343	Ca8	14077398	GA/GAAA	TTGATATGTCATCCCCGGTT	AGCTGCATCTTCCACGATTT	60.0	940	DRR	Ca_22744	U		Clathrin/coatomer adaptor, adaptin-like, N-terminal
CaPOPII_3344	Ca8	14111238	C/CA	TGAACGGTTGGTCACCATAA	CACCCCAACCATCAAATTCT	59.8	419	INTERGENIC				
CaPOPII_3345	Ca8	14111879	GTTAATT/GTT	GGATTTGATGATCGATTCGG	CAACCAGCAGTAGACGCAAA	60.2	796	INTERGENIC				
CaPOPII_3346	Ca8	14113684	TAAAAAA/TAAAAA	AACACGCCTCACACCACATA	CCAATTCACAAAATCGGTCC	60.0	447	INTERGENIC				
CaPOPII_3347	Ca8	14121334	ATAATATTAATATTAATATTAATA/ATAATATTAATATTAATATTAATATTAATA	AAGCTGAGGCCAAGACAAAA	TCGTCAGGAATCAAAGACCA	60.0	280	INTRON	Ca_13048		GRAS	Drug/metabolite transporter

INDEL marker IDs	Chromosomes /unanchored scaffolds	Physical positions (bp)	InDels (*Kabuli* reference genome-CDC Frontier/PI)	Forward primers (5'-3')	Reverse primers (5'-3')	Anneling temperature (0C)	Expected amplified product size (bp)	Structural annotation		Functional annotation		
								Sequence components of *kabuli* genome	*Kabuli* gene accession IDs	NCBI-KOG	TFs	NCBI-nr database
CaPOPII_3348	Ca8	14143645	ATTTTTTTT/ATTTTTTTTT	ATGCATGTGAAAGGGACACA	AAGGGCCCCACTATCACTTC	60.0	497	DRR	Ca_13045			Late embryogenesis abundant protein, group 2
CaPOPII_3349	Ca8	14157701	ATTTTTTTTTT/ATTTTTTTTTTT	CCTTGCATCAACATCAACCA	TGGTCGTCAGTAGTCAACGG	60.5	454	INTERGENIC				
CaPOPII_3350	Ca8	14182954	ATTTTTTTT/ATTTTTT	CGTAGAGGCATCAACACTCG	GAAGGTGCTTTGTTGTTTGGA	59.5	601	INTERGENIC				
CaPOPII_3351	Ca8	14218237	A/AT	TCTCCACAAATGCAAGGAAA	AAGCCTGGCCCAACTAATTT	59.2	465	INTERGENIC				
CaPOPII_3352	Ca8	14244514	CAAA/CAA	ATCTTAAGCATCCCCCTCGT	TCAATGGTGTCCATCCCTTT	59.9	670	INTERGENIC				
CaPOPII_3353	Ca8	14252510	C/CG	AATCTATATGCACCGCCGAC	CGCAACATTTGCATAACTGC	60.0	547	INTRON	Ca_13038			Kinesin, motor domain
CaPOPII_3354	Ca8	14257165	GTT/GTTCAGACCTTT	AGGCCGCAGATGAACTGTAT	TGGTTTGCTTTTAAGCGAGG	59.7	824	DRR	Ca_13038			Alpha/beta hydrolase fold-1
CaPOPII_3355	Ca8	14291937	CT/C	TTCAAAACGGTGTCTTATGCC	CCTTCCCACTTTACAACGTCA	60.0	388	INTERGENIC				
CaPOPII_3356	Ca8	14319609	A/AG	CCTTGATGCACCATGCTAGA	GTCATTTTCGGTGCAATGTG	59.8	257	INTERGENIC				
CaPOPII_3357	Ca8	14320978	TG/T	TGATGCTTTGGTTTCATGTCA	TCCCAAACAACTCAATTCCA	60.1	359	DRR	Ca_13032	Z	MYB_related	Protein of unknown function DUF789
CaPOPII_3358	Ca8	14334449	AC/A	CAATAGCCGTGTTGAAATGC	ACAACACCTTTGGTTCCAGC	59.2	468	INTRON	Ca_13031			
CaPOPII_3359	Ca8	14365858	A/AC	CCAGCCCAAACAGGAATTTA	CCTTTGTTCTTGCACCCATT	59.9	476	INTERGENIC				
CaPOPII_3360	Ca8	14568257	TAAAAAAAAA/TAAAAAAAAAAA	AACGCGCGGGTTATATTCTA	GGCAAAGGGTTTTTGCAATC	59.6	599	INTERGENIC				
CaPOPII_3361	Ca8	14574261	AGTG/AGTGTG	GTGGAATGGTGTGAAAGGGT	TAGCCAGAGAACAACCCTCC	59.7	520	DRR	Ca_13010			
CaPOPII_3362	Ca8	14576894	ATTTTTTT/ATTTTTT	TTTTGTTTGGTGGTTGATCATT	AGCAGCTCCAGAATTGACTTG	59.2	923	DRR	Ca_13009	Z	MYB_related	Kinesin, motor domain
CaPOPII_3363	Ca8	14583961	AT/ATATT	GTTGAAATACGCCGCTCAAT	TGGATCTGCGAACAAACTCA	60.1	778	INTRON	Ca_13009	Z	MYB_related	Kinesin, motor domain
CaPOPII_3364	Ca8	14636009	GATATATATATATATATATATATATAT/GATATATATATATATATATATAT	TTTGCAGGAAGCAAACACTG	GGATAATCCGGGATGCCTAT	60.0	587	INTRON	Ca_13004			Toprim domain

INDEL marker IDs	Chromosomes /unanchored scaffolds	Physical positions (bp)	InDels (*Kabuli* reference genome-CDC Frontier/PI)	Forward primers (5'-3')	Reverse primers (5'-3')	Anneling temperature (0C)	Expected amplified product size (bp)	Structural annotation		Functional annotation		
								Sequence components of *kabuli* genome	*Kabuli* gene accession IDs	NCBI-KOG	TFs	NCBI-nr database
CaPOPII_3365	Ca8	14647157	GTTT/GTTTT	TTGCTTGTCCATGACGTAGG	TCTGCAACGAAGATTAAACCAA	59.7	297	INTERGENIC				
CaPOPII_3366	Ca8	14661963	AT/A	TGTTGTTGTGAAATCGACGAA	TGTTAGGTGGCCAAAGGAAG	60.1	399	INTERGENIC				
CaPOPII_3367	Ca8	14665010	TAAAAAAA/TAAAAAAAA	AATCTCAAGCAAAGCCGAAA	AATATCAGGGGCATCAAGGA	60.0	636	INTERGENIC				
CaPOPII_3368	Ca8	14671445	TAAAAAAAAA/TAAAAAAAA	CCTTCCCGATTTGATCCTTT	TCGACAACTTACATTTCATCATCC	60.3	831	INTERGENIC				
CaPOPII_3369	Ca8	14705254	AATTTTATTTTAT/AATTTTAT	TAGAGGGACCAAAACTGCAA	CCAGGCGTAATTTTGGAAAC	58.4	366	INTRON	Ca_12998	R	SBP	Malate transporter, aliminium toerance
CaPOPII_3370	Ca8	14727145	TA/TAA	CATTGTGAAGTGTGAGTGGGA	TTGGTTAAGGTGGTTATGATTGG	59.6	690	INTRON	Ca_12995	O	NAC	Peptidase A1
CaPOPII_3371	Ca8	14750887	GTTTTTT/GTTTTTTT	ACTCAAAAACCGTGGACTCG	GGCACTTGGTTGGTCTTCAT	60.1	781	INTRON	Ca_12994			Uncharacterised protein family Cys-rich
CaPOPII_3372	Ca8	14751128	TC/T	TGATGCGTTGATAGTGTTGG	GGCACTTGGTTGGTCTTCAT	58.1	619	INTRON	Ca_12994			Uncharacterised protein family Cys-rich
CaPOPII_3373	Ca8	14751499	TGG/TG	ATGAAGACCAACCAAGTGCC	ACAAAGCTCGTTTGCTCACA	60.0	189	DRR	Ca_12994			Uncharacterised protein family Cys-rich
CaPOPII_3374	Ca8	14757174	TAAAAAAAA/TAAAAAAAAA	CATGGAATCCAAATCGATGA	ACCTCCCCTCCAAACAAATC	59.3	477	DRR	Ca_12993	O		Proteasome, subunit alpha/beta
CaPOPII_3375	Ca8	14759364	CA/CAA	TTTGGTTCCTTGTTTGGAGC	CAAATTCAGTTGGCTCAGCA	60.1	694	INTERGENIC				
CaPOPII_3376	Ca8	14776665	T/TTAA	CCAACTCTGGTGGTTGGTCT	GCTTGGGGGTTTGTAATACG	60.0	583	INTERGENIC				
CaPOPII_3377	Ca8	14776722	A/AT	CCAACTCTGGTGGTTGGTCT	GCTTGGGGGTTTGTAATACG	60.0	583	INTERGENIC				
CaPOPII_3378	Ca8	14821532	T/TC	GCATTGATTTCACCATTCCC	CATAGTCGGGTCGACCTTGT	60.1	457	INTRON	Ca_12987	D	bHLH	Mrp, conserved site
CaPOPII_3379	Ca8	14900024	CTAATAATAATA/CTAATAATAATAATAATA	AAATGACATAATCGCAATGAAAA	CGTGGACTTTTTGTTTCAAATG	58.6	561	INTERGENIC				
CaPOPII_3380	Ca8	14922109	GTTTTTTTT/GTTTTTTT	CTCCCAACTCCTCCACACAT	GAGTTTGTTGTCAGCGAGCA	60.0	569	INTERGENIC				
CaPOPII_3381	Ca8	15162330	TTAATAATAATAATAATAATAA/TTAATAATAATAATAATAA	TTCGTCGTGTAGGTCCAGTG	GGGAAACCCACCTCTTGATT	59.7	757	INTERGENIC				

INDEL marker IDs	Chromosomes /unanchored scaffolds	Physical positions (bp)	InDels (*Kabuli* reference genome-CDC Frontier/PI)	Forward primers (5'-3')	Reverse primers (5'-3')	Anneling temperature (0C)	Expected amplified product size (bp)	Structural annotation		Functional annotation		
								Sequence components of *kabuli* genome	*Kabuli* gene accession IDs	NCBI-KOG	TFs	NCBI-nr database
CaPOPII_3382	Ca8	15177027	GTTTTTTTTTT/GTTTTTTTTT	CCAAACGCATTATGGTCAACT	CATTTGTCTTGACACCTGTGTTT	59.9	714	INTERGENIC				
CaPOPII_3383	Ca8	15313993	ACC/ACCTCC	TGTTGTGGAGTTCTAGGGGC	TGGACCTTTTTGGTCCTTCT	60.1	180	INTERGENIC				
CaPOPII_3384	Ca8	15577896	CCA/CCATCA	TTTTCAGCAAACCCCAAAAG	TAGAGGCTTGTCATTCGGCT	60.1	665	CDS (large-effect mutations)	Ca_17886	T	EIL	Protein phosphatase 2C, manganese/magnesium aspartate binding site
CaPOPII_3385	Ca8	15580121	A/AATTTAATTT	TCCTCAATTTTGAGCACACG	TGGACACCACACTTCAAATCA	59.8	657	INTERGENIC				
CaPOPII_3386	Ca8	15685177	AGG/AG	GCCCAACAACAGAACCAAGT	GACTCCAAAACCCTGTGCAT	60.0	420	INTERGENIC				
CaPOPII_3387	Ca8	15685626	T/TA	ATGCACAGGGTTTTGGAGTC	ACTTGGTTCTGTTGTTGGGC	60.0	416	INTERGENIC				
CaPOPII_3388	Ca8	15685828	TAAAAAAAA/TAAAAAAAAAA	ATGCACAGGGTTTTGGAGTC	ACTTGGTTCTGTTGTTGGGC	60.0	416	INTERGENIC				
CaPOPII_3389	Ca8	15685888	A/AG	ATGCACAGGGTTTTGGAGTC	ACTTGGTTCTGTTGTTGGGC	60.0	416	INTERGENIC				
CaPOPII_3390	Ca8	15694311	GTATATATATATATATATATATATATATATATA/GTATATATATATATATATATATATATATATA	CCGTCTCTTTCACATGGGTT	AAAGGCTCGGGTCAGTCATA	60.0	438	INTRON	Ca_17876	A		RNA-dependent RNA polymerase, eukaryotic-type
CaPOPII_3391	Ca8	15788537	TAAAAAAAAAA/TAAAAAAAAAAA	TCGGTTCCTTTCAACTTTGG	ATCAATGCTTCCACCCTTTG	60.1	588	INTERGENIC				
CaPOPII_3392	Ca8	15788948	ATT/ATTT	GACATTCCAAAGGGTGGAAG	GGTTTCCAAAACCAGAATCG	59.4	422	INTERGENIC				
CaPOPII_3393	Ca8	15821014	CAAAAAAAAAA/CAAAAAAAAAAA	GTCGCGATCAAGAAAAACAA	TCAAGTGACAAAGAAAGGGAGTT	58.9	500	DRR	Ca_17863		NF-YB	Protein kinase, catalytic domain
CaPOPII_3394	Ca8	15876633	TAAAAAAAAAA/TAAAAAAAAAAA	AATGGAAATACAGCGCAACC	ACACGACGAAGGTTTGGTTC	60.0	487	INTRON	Ca_17857	T	CAMTA	HEAT
CaPOPII_3395	Ca8	15880009	CAAAAAA/CAAAAA	TACGTCGCACAGAAAGAACG	TGCCCACTTGATACAACAAAA	60.1	523	INTERGENIC				
CaPOPII_3396	Ca8	15881213	AC/ACC	TTATTAGGACGGGCAACAGC	ACCGTTGAGAATTGAGTGGC	60.1	903	INTERGENIC				
CaPOPII_3397	Ca8	15892786	AAAGA/AAAGAAGA	AGCAGTTGAACTTGCCTCGT	CGATGATTCATTCCCAAACC	60.1	513	INTERGENIC				
CaPOPII_3398	Ca8	15925648	TAAAATGAAAAAT/TAAAATGAAAAATGAAAAAT	GCTTGATTCTTTTGTGGGGA	AGCACTGCATTTTCTCACCC	60.1	650	INTERGENIC				

INDEL marker IDs	Chromosomes /unanchored scaffolds	Physical positions (bp)	InDels (*Kabuli* reference genome-CDC Frontier/PI)	Forward primers (5'-3')	Reverse primers (5'-3')	Anneling temperature (0C)	Expected amplified product size (bp)	Structural annotation		Functional annotation		
								Sequence components of *kabuli* genome	*Kabuli* gene accession IDs	NCBI-KOG	TFs	NCBI-nr database
CaPOPII_3399	Ca8	16132504	AATATATATATATATATATATATAT/AATATATATATATATATATATAT	CGTGGCGAGCATTATTTCAT	CTGGCTGGACTGACATAGCA	61.0	681	DRR	Ca_15525	J		Ribosomal protein L31e
CaPOPII_3400	Ca8	16315020	TAAAA/TAAA	AATGCAAAGCAACAAGGTTTC	TTGTATGCATGTGTGATGTGTG	59.3	504	INTERGENIC				
CaPOPII_3401	Ca8	16316035	CAAAA/CAAAAAA	GGCATCACCCCAAGACATAG	CCCTTGATTTTTCAATCACTCC	60.3	352	INTERGENIC				
CaPOPII_3402	Ca8	16467871	ATTTTTTTTTTTT/ATTTTTTTTTTTTT	TTTTTCCGACTTGGTCCTTT	AGTGCACATGAGTTTTGGTCA	58.7	439	INTERGENIC				
CaPOPII_3403	scaffold1003	41332	G/GAGCGGT	GGGATTTGAAGGTGCTGAAA	TAGTGGCCACAACCAACTCA	60.1	452	INTERGENIC				
CaPOPII_3404	scaffold1003	41354	TCA/T	GGGATTTGAAGGTGCTGAAA	TAGTGGCCACAACCAACTCA	60.1	452	INTERGENIC				
CaPOPII_3405	scaffold1006	109456	T/TC	TTGATTTCAGCCCAGGATTC	TTTACAAGCAGCGGGAAAGT	60.0	493	INTERGENIC				
CaPOPII_3406	scaffold1006	338954	GTGACTT/GT	GAAGCCCCAATTCAACAAAA	CCCAAAAACTCATGGCCTAA	59.9	312	INTRON	Ca_18027		BES1	Glycoside hydrolase, family 14B, plant
CaPOPII_3407	scaffold1006	553062	GTGCTGCTGCTGCTGCTGCT/GTGCTGCTGCTGCTGCT	CTTGCACCAGTATGGGAGGT	AGATTTTCACCCGTTGTTGC	60.0	376	CDS (large-effect mutations)	Ca_18040			
CaPOPII_3408	scaffold1006	621524	AATATATATATATATATATATATATA/AATATATATATATATATATATATATATA	TGGGCCACCACTATTTGACT	GTGATTTGCGCCATGATTAG	60.4	803	INTERGENIC				
CaPOPII_3409	scaffold1008	12678	TC/T	TCTTGGTTGTAACCCTTGGC	GGGCTGATGCCATAAACACT	60.0	727	INTERGENIC				
CaPOPII_3410	scaffold10180	748	AT/ATT	GGTGAGTTTGTGTCGTTGGA	AGGGAGGACGAAGATGACCT	59.6	448	INTERGENIC				
CaPOPII_3411	scaffold10227	6464	AATATATATATATATATATATATATATATATATA/AATATATATATATATATATATA	GGGAACGTGAAGAACTCAGC	TGCAACAACCACAACCACTT	59.9	518	INTRON	Ca_28086		bHLH	Helix-loop-helix DNA-binding domain
CaPOPII_3412	scaffold1036	205006	ATTGGAATTGGACTTGGAATTGGACTTGGAATTGGA/ATTGGAATTGGACTTGGAATTGGA	GCAAGCGACTATGGGTAAGG	TACATAGTTACGGAGCCGCC	59.7	273	INTERGENIC				
CaPOPII_3413	scaffold1038	720	TGG/TG	CGAAAATCGCCCAGAAAATA	TCCTGGTCCTCGATTTCATC	60.0	842	INTERGENIC				
CaPOPII_3414	scaffold1038	2468	AGGG/AGG	TCCAGATGAAATCGAGGACC	TTTTCGGACTAGACGATGGG	60.0	440	INTERGENIC				
CaPOPII_3415	scaffold1039	4419	GGTAATTT/G	TTGGTATTGAACTCGGAGGC	GTTGGTTCCCTAGCACCGTA	60.1	608	INTERGENIC				

INDEL marker IDs	Chromosomes /unanchored scaffolds	Physical positions (bp)	InDels (*Kabuli* reference genome-CDC Frontier/PI)	Forward primers (5'-3')	Reverse primers (5'-3')	Anneling temperature (0C)	Expected amplified product size (bp)	**Structural annotation**		**Functional annotation**		
								**Sequence components of *kabuli* genome**	***Kabuli* gene accession IDs**	**NCBI-KOG**	**TFs**	**NCBI-nr database**
CaPOPII_3416	scaffold1039	48817	ATTTTTTTTTTT/ATTTTTTTT	CGACATGCATGCCTTATTTG	CATGAGGAAACAATTGTGCAG	60.1	571	INTERGENIC				
CaPOPII_3417	scaffold1043	1901	GTATATATATATATATATATATATATATATATAT/GTATATATATATATATATATATATATATATAT	CGAAGCAAAAGAAAGAACCG	TGGATTACGGGTGAAATGGT	60.0	321	INTERGENIC				
CaPOPII_3418	scaffold1046	10422	TAAAAAAAA/TAAAAAAA	CATTGGATGCACAAGATTGG	TGCGTTTGTGTCATTGGATT	59.9	696	INTERGENIC				
CaPOPII_3419	scaffold1046	17706	ACC/AC	CATTGGATGCACAAGATTGG	AGGTCATTTTCGTCCTCCCT	59.9	871	INTERGENIC				
CaPOPII_3420	scaffold1046	20332	TAAAAAAAA/TAAAAAAA	GCGCAAACAAACCTATGTGA	TCAGGTCATTTTCGTCCTCC	59.7	788	INTERGENIC				
CaPOPII_3421	scaffold1046	31524	AGGGGGGGG/AGGGGGG	AGGGAGGACGAAAATGACCT	TTGTTGGCCAATTAGGGTTT	59.9	395	INTERGENIC				
CaPOPII_3422	scaffold1047	109934	TAAAAAAAAA/TAAAAAAAAAA	AAAGCAGTCCCACCATTTTG	CTAAGCGAAATCAATCCCCA	60.0	829	INTERGENIC				
CaPOPII_3423	scaffold1047	159189	CTTTTTTTTTT/CTTTTTTTTT	TTGGTGGGTGGTGGTAGATT	TTACCCCTTCCTTTTTCGTG	60.1	795	INTERGENIC				
CaPOPII_3424	scaffold1047	313122	GAAAAAAAAA/GAAAAAAAAAA	AAAATTCCACACGTCCGAAG	TCAAAAGATCGCAGTTGTGTG	60.0	354	INTRON	Ca_17199	I		Patatin/Phospholipase A2-related
CaPOPII_3425	scaffold1047	419626	C/CAAACATG	CCGATGAAAATTCACCCATC	CCATTGATAAAGCGTGTACGG	60.1	327	INTERGENIC				
CaPOPII_3426	scaffold1049	44931	CAA/CAAA	TCACACTTCGACAGATTGGC	TAAGGGCTCCTCCCAAACTT	59.8	461	INTERGENIC				
CaPOPII_3427	scaffold1049	75199	ACC/AC	ACGTTCCTGAGGGAATTGTG	GTAAAGGGTCACCAACCGAA	60.0	828	INTERGENIC				
CaPOPII_3428	scaffold1049	293745	AAAGAAG/AAAG	CGTTTTTCAAAGCGAGGGTA	GCCCTCAGATGAGAGTCAGG	60.2	439	INTERGENIC				
CaPOPII_3429	scaffold1050	149500	ACGC/AC	TTCGTTCTCCCATGACACAC	GGAGGTGCACCAACTCCTTA	59.5	676	INTRON	Ca_25917	I		Male sterility
CaPOPII_3430	scaffold1052	10999	TGGG/TGG	CCACATTGTAACCCTTGCCT	AATGATCGTCTTCGGATTGG	59.9	717	INTERGENIC				
CaPOPII_3431	scaffold1052	16426	T/TG	CCGTTGTTTCAACTTCCACC	GCAGCAGCCATTCATACAGA	60.4	323	INTERGENIC				
CaPOPII_3432	scaffold1052	233350	TAA/TA	TAACAAAACGCAACCGACAC	TGTCAAGAAAACACCATGAATTG	59.6	456	INTERGENIC				

INDEL marker IDs	Chromosomes /unanchored scaffolds	Physical positions (bp)	InDels (*Kabuli* reference genome-CDC Frontier/PI)	Forward primers (5'-3')	Reverse primers (5'-3')	Anneling temperature (0C)	Expected amplified product size (bp)	Structural annotation		Functional annotation		
								Sequence components of *kabuli* genome	*Kabuli* gene accession IDs	NCBI-KOG	TFs	NCBI-nr database
CaPOPII_3433	scaffold1055	339319	ATTTTT/ATTT	TTTAAGGTGTGGGACCAAGC	GGAAACAATAAACCGAGGCA	60.0	540	INTERGENIC				
CaPOPII_3434	scaffold1055	340051	GAAAAAAA/GAAAAAAAA	TGTTGTTCGGCATACGACAT	TGCACATACATAATGGACCACA	60.0	516	INTERGENIC				
CaPOPII_3435	scaffold1055	340491	TT/TATCT	TGCAATAAAGCTTTTGAAGTGG	ACATACTCCCTCACTCCCCC	59.4	413	INTERGENIC				
CaPOPII_3436	scaffold1055	340641	ATGGGGGAGTG/ATG	TGCAATAAAGCTTTTGAAGTGG	AAGTTGTGTCACCATTGCCA	59.4	813	INTERGENIC				
CaPOPII_3437	scaffold1056	142810	TAAAAAAAAAA/TAAAAAAAAAAA	TTGCTCCTACGTATCCCCAG	TGGCCACAAGAAGACAATGA	60.1	705	INTERGENIC				
CaPOPII_3438	scaffold1060	305887	CA/CAA	CGGTGACATAAATGACCGTG	CAAAATGGACAAAATTGGGG	59.8	392	INTERGENIC				
CaPOPII_3439	scaffold1061	21088	TGTTTAGGGTTTA/TGTTTAGGGTTTAGGGTTTA	GGTCCTCGATTTCATCTGGA	ATAACCCGAACCTCAAACCC	60.0	348	INTERGENIC				
CaPOPII_3440	scaffold1061	31501	ATTTTTTTT/ATTTTTTT	TCGTTATGCAGTCAAACCTCA	CGTGTTTGAAATATGGCACG	59.3	618	INTERGENIC				
CaPOPII_3441	scaffold1061	32882	T/TC	ACTAACCATCGTTTGGCCTG	GCAATTTTCGTACCCCTTGA	60.0	227	INTERGENIC				
CaPOPII_3442	scaffold1061	33010	ATT/ATTT	TGCCCGGAAAGTCTAGAAGA	TGTATTTTCCGACCACACGA	59.9	453	INTERGENIC				
CaPOPII_3443	scaffold1061	49764	TAAA/TAA	CCTTGTCACACAGTTGGCTG	TCTTGGTTGTAACCCTTGGC	60.3	150	INTERGENIC				
CaPOPII_3444	scaffold1061	76996	CGG/CG	TATTTGGACCAGAGGATGGC	CCCAAACAGTAGGACCAGGA	59.9	109	INTERGENIC				
CaPOPII_3445	scaffold1061	77938	GTTT/GTT	TCAACGAAATCGATTGGACA	TTTGGCAATGGGCTAAACTC	60.0	502	INTERGENIC				
CaPOPII_3446	scaffold1067	203101	ATTTTTTTT/AATTTTTTTTT	TGAAACTGGGAGGAGGAAGA	CAGTGCGATGGAGAAATCAA	59.8	418	DRR	Ca_24425			
CaPOPII_3447	scaffold1067	222485	C/CGACAGGGT	AAGCAACCCAACAAACAAGG	ACTCTTAGCGAATCCCAGCA	60.0	207	INTERGENIC				
CaPOPII_3448	scaffold1067	229129	CTTTT/CTTTTT	ACATGGTTCGGAAACGTAGG	TCCATGCAAGAAGAGCTCAA	59.9	341	INTERGENIC				
CaPOPII_3449	scaffold1067	238607	T/TCGAC	AGCATGCGGTATGTTAAGGG	CCAACCTCCGATAGCAAAAA	60.0	197	INTERGENIC				

INDEL marker IDs	Chromosomes /unanchored scaffolds	Physical positions (bp)	InDels (*Kabuli* reference genome-CDC Frontier/PI)	Forward primers (5'-3')	Reverse primers (5'-3')	Anneling temperature (0C)	Expected amplified product size (bp)	Structural annotation		Functional annotation		
								Sequence components of *kabuli* genome	*Kabuli* gene accession IDs	NCBI-KOG	TFs	NCBI-nr database
CaPOPII_3450	scaffold1067	238677	C/CT	AGCATGCGGTATGTTAAGGG	CCAACCTCCGATAGCAAAAA	60.0	197	INTERGENIC				
CaPOPII_3451	scaffold1067	243237	AGGGG/AGGGGG	TTATTGTTGTTGCTTGGGCA	TCTTGTGTTGGGTTAAGGGC	60.1	447	INTERGENIC				
CaPOPII_3452	scaffold1067	243320	TATCCAAAT/TATCCAAATCCAAAT	TTATTGTTGTTGCTTGGGCA	TCTTGTGTTGGGTTAAGGGC	60.1	447	INTERGENIC				
CaPOPII_3453	scaffold1067	243805	CAAGCCT/CAAGCCTAAAGCCT	GCCCTTAACCCAACACAAGA	AGGCGACCCTATACCGAAGT	60.0	643	INTERGENIC				
CaPOPII_3454	scaffold1067	263503	AA/AGCTAGTCAGCA	CAGTGAGTAGCAAGCAAGCG	CCCAGCCTAGTGACATCCAT	60.0	445	INTERGENIC				
CaPOPII_3455	scaffold1067	266043	CTTTTTTTT/CTTTTTTT	ATCCAGAATGGAGCAACTGG	CGGAGAAATCCGTTTTGCTA	60.1	575	INTERGENIC				
CaPOPII_3456	scaffold1081	86270	GAAAAAAA/GTAAAAAAAA	TTATTCGGGCCTGTCAAAAC	CATGCGTTCCTTGTGGTAGA	59.9	251	INTERGENIC				
CaPOPII_3457	scaffold1081	88052	TT/TTGT	TGAGCATGGCTAAGTTGCAC	AAGGGGAAGGGCATAATGTT	60.0	522	INTERGENIC				
CaPOPII_3458	scaffold1082	20939	CCGTTAACCCT/CCGTTAACCCTTAATCGTTAACCCT	TTGGACGAACTGCAATGAAT	GTTTAGGGTTTGGGGTTTGG	59.1	372	INTERGENIC				
CaPOPII_3459	scaffold1083	122050	C/CT	AAGCCTAGGGAGGACGAAAA	AACGAATCATGTGCATCCAA	60.2	772	INTERGENIC				
CaPOPII_3460	scaffold1087	13529	TTTTATTTATTTATTTATT/TTTTATTTATTTATT	TTCCGCATAACACTAAGTGGC	CCATGCTAATGGATGCTGTG	60.1	521	INTERGENIC				
CaPOPII_3461	scaffold1087	14399	CGAGAGA/CGAGA	TGGGTTGTACCGTTAATTGG	ACCCCAAATATTGCCATGAA	58.3	810	INTERGENIC				
CaPOPII_3462	scaffold1087	14921	TAAAAAA/TAAAAAAAAAAA	TTCATGGCAATATTTGGGGT	AACTTGATTTCTGGCCAACG	60.0	623	INTERGENIC				
CaPOPII_3463	scaffold1087	23029	CTTTTTTTTTT/CTTTTTTTT	TGCACCACGAGATCTTGAAA	CGTTGATACATAACAAAAATACAAACA	60.4	597	INTERGENIC				
CaPOPII_3464	scaffold1087	23220	T/TG	TGTGGGATATCAAGTTTTGGA	AAAAATGCACGGGACAAATA	57.9	704	INTERGENIC				
CaPOPII_3465	scaffold1089	12635	ACCCATATC/AC	CTCGTCGGACCATCATTTTT	ACTCTGATGCGCTTCCTGAT	59.9	534	INTERGENIC				
CaPOPII_3466	scaffold1092	83003	GTTTTTT/GTTTTT	TAACCAATCTTGTGCGTCCA	AATAGGCCTAGGGAGGACGA	60.1	280	INTERGENIC				

INDEL marker IDs	Chromosomes /unanchored scaffolds	Physical positions (bp)	InDels (*Kabuli* reference genome-CDC Frontier/PI)	Forward primers (5'-3')	Reverse primers (5'-3')	Anneling temperature (0C)	Expected amplified product size (bp)	Structural annotation		Functional annotation		
								Sequence components of *kabuli* genome	*Kabuli* gene accession IDs	NCBI-KOG	TFs	NCBI-nr database
CaPOPII_3467	scaffold1092	121415	AT/A	AGGTCATTTTCGTCCTCCCT	AATCCAATGGCACAAACACA	59.9	648	INTERGENIC				
CaPOPII_3468	scaffold1092	139231	GTTT/GTTTT	TCCTCGCCAGGCTTATTCTA	ATCCAACGACACGAAGGAAC	59.9	281	INTERGENIC				
CaPOPII_3469	scaffold1101	3795	TCC/TC	TTGGCTCATTGCTTGAGTTG	AGCCAAGGGAGGATGAAAAT	60.0	214	INTERGENIC				
CaPOPII_3470	scaffold1109	14028	ATTTTTTTTTTTTT/ATTTTTTTT	TTTGAAAACCTCACCAAGGG	TAAACTGTCAGGGGCGAAGA	59.9	416	INTERGENIC				
CaPOPII_3471	scaffold1109	14081	GTT/GTTAATT	TTTGAAAACCTCACCAAGGG	TATAAACTGTCAGGGGCGAA	59.9	418	INTERGENIC				
CaPOPII_3472	scaffold1116	42811	TC/TCC	GATGAAACCGAGGACCAAGA	GTGTTTTCGGACCAGACGAT	60.1	599	INTERGENIC				
CaPOPII_3473	scaffold1116	94918	TATAATAATAATA/TATAATAATAATAATA	TCTTCGTTATGCAGTCAAACCT	ACCCAACAAAAATGGACCAA	58.9	728	INTERGENIC				
CaPOPII_3474	scaffold1116	118274	AATAT/AAT	TGCAGTCAAACTTTAACAAGAAGAT	TTTGACAACTCAATTTTGTTCTATTT	58.6	608	INTERGENIC				
CaPOPII_3475	scaffold1118	29890	GCTTCTTCTTCTTCTTCTTCTTCTTCTTC/GCTTCTTCTTCTTCTTCTTCTTCTTCTTCTTC	AACGTTCTCCGTTTGTAGCG	TTTGGAGACCAATGGTGACA	60.3	752	INTERGENIC				
CaPOPII_3476	scaffold1118	34583	AATTATTATTATTATTATTATTATT/AATTATTATTATTATTATTATT	CGGGTTGAGTTGTTGTTCCT	TTGGTTTCAAGAAGGATGGC	60.0	721	INTERGENIC				
CaPOPII_3477	scaffold1120	11138	C/CA	TTCATCCAAAGAATCTGGGC	TGCAAGAGGAGTGCCTTTCT	60.0	486	URR	Ca_27900			
CaPOPII_3478	scaffold1120	44664	TCC/TC	AACCAATCTTGTGCATCCAA	AGGGACAAAGAAAATGGCCT	59.0	470	INTERGENIC				
CaPOPII_3479	scaffold1128	179315	CTTT/CTT	TTGGACAGTGGTTGCTCAAG	CCCATGGTCACTTCTCACCT	59.9	778	INTERGENIC				
CaPOPII_3480	scaffold1131	30056	AG/AGG	AGTCGGTACAACACCATCCC	TGTGGTGTTCTTGGTGGAGA	59.7	701	INTERGENIC				
CaPOPII_3481	scaffold1135	9704	GTTTGTGCT/GT	TTTCGGTTGGGTTTGCTTAC	TACCCGTGCTCAACATCAAG	60.0	704	INTERGENIC				
CaPOPII_3482	scaffold1135	14111	TTAAATAAATAAATAA/TTAAATAAATAAATAAATAA	TCTGGCACATGAACTTCAGC	CACTCGACAATTGGGAGGTT	60.0	532	INTRON	Ca_26843		SAP	Aminotransferase-like, plant mobile domain
CaPOPII_3483	scaffold1135	74988	TCTAAACCCTAAACCCTAA/TCTAAACCCTAAACCCTAAACCCTAA	ACCCAAACCCTTAACCTTCC	CGGTTCGGGTTTATGGTTTA	59.2	291	INTERGENIC				

**INDEL marker IDs**	**Chromosomes /unanchored scaffolds**	**Physical positions (bp)**	**InDels (*Kabuli* reference genome-CDC Frontier/PI)**	**Forward primers (5'-3')**	**Reverse primers (5'-3')**	**Anneling temperature (0C)**	**Expected amplified product size (bp)**	**Structural annotation**		**Functional annotation**		
								**Sequence components of *kabuli* genome**	***Kabuli* gene accession IDs**	**NCBI-KOG**	**TFs**	**NCBI-nr database**
CaPOPII_3484	scaffold1139	1393	CT/CTTT	TCATCCAGTGTGGGGTACAA	TTGTGTGTGTTGGAGATGAGC	59.8	313	INTERGENIC				
CaPOPII_3485	scaffold1139	1605	TAACAACAA/TAACAACAACAA	TCATCCAGTGTGGGGTACAA	TTTGAGAATTTGGGATGGAAA	59.8	785	INTERGENIC				
CaPOPII_3486	scaffold1142	4296	T/TGA	AGGTCATTTTCGTCCTCCCT	TTGCATGCACAAGATTGGTT	59.9	268	INTERGENIC				
CaPOPII_3487	scaffold1145	3948	ACTTCT/ACTTCTTCT	AATCCTCAAAGCCCTCACCT	TTTTGGAACCTTCGTCCTTT	60.1	750	INTERGENIC				
CaPOPII_3488	scaffold1145	15770	ATATTATTATTATTATTATTATTA/ATATTATTATTATTATTATTA	TGGCACAACATCATCATCATT	GCGCATTCTTCATCTTCTCC	59.8	496	INTERGENIC				
CaPOPII_3489	scaffold115	52073	GA/GACTA	CAATTAACACAAACGCCCCT	TTGGCCTCCCTAGGCTTATT	59.9	577	INTERGENIC				
CaPOPII_3490	scaffold1151	18791	CTTT/CTTTT	AGCCGTCATGACTTTTGTCC	ACGGACCCTTTGTTGAGACA	60.1	392	INTERGENIC				
CaPOPII_3491	scaffold1152	7028	CGGAGGA/CGGA	GAATTTGGCGTCAGAAATTGA	CTGTGCAAATGGTGGTTTTG	60.1	433	INTERGENIC				
CaPOPII_3492	scaffold1152	115530	T/TA	ACAAGATGGGACAAACAGCC	TCCTTTTCATCCTCCGTTTG	60.0	460	INTERGENIC				
CaPOPII_3493	scaffold1159	27402	TG/TGG	AGGGAGGACGAAAATGACCT	CCTTCCTTGGCTTGTTCTTG	59.9	345	INTERGENIC				
CaPOPII_3494	scaffold1174	23485	GTTTTTT/GTTTTTTT	TTGTGCCTCCAATAGGTGTG	AGGGAGGACGAAAATGACCT	59.6	464	INTERGENIC				
CaPOPII_3495	scaffold118	129054	CAAAAAAAAAAA/CAAAAAAAAAA	AGGGAGGTCGGCTAGATGAT	TGAGGACTGGACGTAGCCTT	60.1	350	INTERGENIC				
CaPOPII_3496	scaffold118	173557	TTATATATATATATATA/TTATATATATATATATA	TGTCATGCTAATATCCGCCA	TGGATGGATTTCTAGTTGCCTT	60.1	489	INTERGENIC				
CaPOPII_3497	scaffold118	188968	A/AC	AATAGGGGTGCAAACAGTCG	GGTGTTGCAAAGGGATCTGT	60.0	731	INTERGENIC				
CaPOPII_3498	scaffold1180	2599	ATT/ATTTT	GAGAGGTCATGGCACCCTTA	GAGCAACACATTGCATGTCC	60.1	166	INTERGENIC				
CaPOPII_3499	scaffold1180	2913	A/AC	GAGAGGTCATGGCACCCTTA	TCTTGCCCAACGTTTTACAT	60.1	513	INTERGENIC				
CaPOPII_3500	scaffold1180	3279	GAT/GATAT	ACACATGTAAAACGTTGGGC	GTACATGCAAATGACCGCAC	58.4	573	INTERGENIC				

INDEL marker IDs	Chromosomes /unanchored scaffolds	Physical positions (bp)	InDels (*Kabuli* reference genome-CDC Frontier/PI)	Forward primers (5'-3')	Reverse primers (5'-3')	Anneling temperature (0C)	Expected amplified product size (bp)	Structural annotation		Functional annotation		
								Sequence components of *kabuli* genome	*Kabuli* gene accession IDs	NCBI-KOG	TFs	NCBI-nr database
CaPOPII_3501	scaffold1180	3744	GA/G	GTGCACGAAAGTGTGGCTTA	ACTTTACCATTGCCTTCCCC	59.9	766	INTERGENIC				
CaPOPII_3502	scaffold1180	36749	CTATTTATTTATTTATTTATTTATTTATTT/CTATTTATTTATTTATTTATTTATTT	TACGGTTCGATCTCATGCAA	TGCCTCTTCCCCTGTTTCTA	60.2	419	DRR	Ca_25737		G2-like	Peptidase S8/S53, subtilisin/kexin/sedolisin
CaPOPII_3503	scaffold1180	135497	GTTTTTTT/GTTTTTTTTT	TTGTGTGCGTTTGACTGACA	AAGCGGAGAAGTCGGAGATT	59.9	789	INTERGENIC				
CaPOPII_3504	scaffold1180	206096	AATATATATATATATATA/AATATATATATATATATATA	CCATGTGGTTCTTTTGCATTT	AACTTCGCCATTTGTCATCC	59.9	548	INTERGENIC				
CaPOPII_3505	scaffold1180	208476	TA/T	TAAGAGGGGCTTGTTACCCC	CCAACCTAATATTCATGCCACA	60.3	216	INTERGENIC				
CaPOPII_3506	scaffold1180	226315	GTCCATTC/GTC	TGGGGTTTAGGGTGTCACAT	AACCGGCACGACTAACATTC	60.1	101	INTERGENIC				
CaPOPII_3507	scaffold1181	5941	TAACCCTAAACCCTAAACCCTAAACCCTAAACTCTATACCCTAAACCCTATACCCTAAACCCTAAACCCTAAA/TAACCCTAAACCCTAAACCCTAAACTCTAT	AAACCCTAAATCCCAAACCC	TTTAGGTCATTTTGGCCTTTTT	59.0	465	INTERGENIC				
CaPOPII_3508	scaffold1185	6345	CAAAAAA/CAAAAA	TGAATGCGAACCTAGTGTGG	TAAAGGACCAACTCGGGAAA	59.7	745	INTERGENIC				
CaPOPII_3509	scaffold1185	67322	GATATATATATAT/GATATATATAT	CAATTCCCACAGGTACGCTT	GACGCGAAAACAACATTTCA	60.0	683	INTERGENIC				
CaPOPII_3510	scaffold1185	67468	T/TAGGGTGA	CAATTCCCACAGGTACGCTT	GACGCGAAAACAACATTTCA	60.0	683	INTERGENIC				
CaPOPII_3511	scaffold119	35751	A/AG	ACTCATATGCAAAATGCCCC	TTATGCTGGAGATGGGAAGG	59.8	444	INTERGENIC				
CaPOPII_3512	scaffold1193	7288	TCAA/TCAATTTAAGCAA	AATGCTTTCCAGAAAACCGA	TGATCTGATGGCTGACTTTCA	59.7	367	INTERGENIC				
CaPOPII_3513	scaffold1193	241608	CAA/CA	AAAGAAAGGGAGTTGGACGG	CACACGAATACGATTCCACG	60.5	401	INTERGENIC				
CaPOPII_3514	scaffold1196	103885	GAAAAAA/GAAAAA	CAACTTGTTTCACCCGACCT	GGATTTGTGGACAAGGGAGA	60.0	478	INTERGENIC				
CaPOPII_3515	scaffold1196	117323	ATTTTTTTTTTT/ATTTTTTTTTT	TGGACTTTGCTTTCTGTTTGG	GTGCCCAACAAACACTTGAA	60.3	523	INTERGENIC				
CaPOPII_3516	scaffold1196	206626	ATT/ATTT	TTCTGCCAAGTTCCCATTTC	TAAAGGATTCCCACTGTGCC	60.1	699	URR	Ca_26186		Trihelix	Peptidase C48, SUMO/Sentrin/Ubl1
CaPOPII_3517	scaffold1197	5120	GT/GTT	TCGCAGCTCAATTGGTAGTG	GAGAGCTACCGTGCAACACA	60.0	486	INTERGENIC				

INDEL marker IDs	Chromosomes /unanchored scaffolds	Physical positions (bp)	InDels (*Kabuli* reference genome-CDC Frontier/PI)	Forward primers (5'-3')	Reverse primers (5'-3')	Anneling temperature (0C)	Expected amplified product size (bp)	Structural annotation		Functional annotation		
								Sequence components of *kabuli* genome	*Kabuli* gene accession IDs	NCBI-KOG	TFs	NCBI-nr database
CaPOPII_3518	scaffold1197	10258	AC/A	CGTTCTTGATCGGGATTGAT	GATATGGTTCCGCCAGAAAA	59.9	390	INTERGENIC				
CaPOPII_3519	scaffold1197	191244	TAT/TATGACTTGCGTCAT	GGCATCCCTATGCAAAACAT	TCCTTCATCTCCTTGGGTTG	59.8	532	INTERGENIC				
CaPOPII_3520	scaffold1197	244953	TAAAAAAA/TAAAAAAAA	CACTTTTTGTTTCATCGCCA	ACTGTGATCCGGTGATTTGA	59.7	574	INTERGENIC				
CaPOPII_3521	scaffold12	16589	TTATATATAT/TTATATATATAT	AGTTTTGCAATCACGTGCAG	GGTGCACAAAACTGCTCCTA	59.9	214	INTERGENIC				
CaPOPII_3522	scaffold12	16684	AAATTGTTTTCAATTGTTTTC/AAATTGTTTTC	AGTTTTGCAATCACGTGCAG	AAGCTGCATCTCAACCTCGT	59.9	611	INTERGENIC				
CaPOPII_3523	scaffold12	58042	TA/T	GTGAAAGCGCTGTAAAAGGC	TCTTGGAGGTGCCAACTTCT	60.0	459	INTERGENIC				
CaPOPII_3524	scaffold12	296676	ATT/ATTT	GGAACCAAGACTTTGGGTGA	CCAATACTTGCGTGGGAACT	59.9	715	INTERGENIC				
CaPOPII_3525	scaffold1202	25847	CTTTTTCTTTTTTTTTC/CTTTTTCTTTTTTTTTCTTTTTTTTTC	TTGTCCACAGTGGCATATTGA	TGGCAGATATGGGGGTGTAT	60.0	737	INTERGENIC				
CaPOPII_3526	scaffold1202	82238	AATATATATATATATA/AATATATATATATATATA	AACACATCCGGGTTCCATTA	TTTGATCCATTTGCCCTAGC	60.1	739	INTERGENIC				
CaPOPII_3527	scaffold1202	83479	GTATATATATATATATAT/GTATATATATATATAT	ACGGTGTAGGCAAGATGGAC	AGGATGCATGGGATGAAAAG	60.0	458	INTERGENIC				
CaPOPII_3528	scaffold1202	179415	TG/TGCCG	AATAGGTAGCACGGTGTGGC	TCACATGGAACTTTCCCCTC	60.0	431	INTRON	Ca_24666			
CaPOPII_3529	scaffold1202	179607	AG/AGG	AATAGGTAGCACGGTGTGGC	TCCCTTGCCTACATTGTTCC	60.0	814	INTRON	Ca_24666			
CaPOPII_3530	scaffold1215	12684	TAAAAAAAAA/TAAAAAAAAAAAA	TCTCTCCCGATTTGGTCATT	TTTGCTCCTCTTCTTCTGGC	59.5	588	INTERGENIC				
CaPOPII_3531	scaffold1215	13648	TAAAAAA/TAAAAA	TCGTGGGACTAAAACTGAGGA	CGCACATATGAATGATCCCA	59.7	576	INTERGENIC				
CaPOPII_3532	scaffold1215	41563	GATATATATATATATATATATATA/GATATATATATATATATATATA	CCCTTCATTTCCTTCGCATA	GAAGCAGGCTTCGATGGATA	60.0	415	INTRON	Ca_28164		G2-like	Peptidase S8/S53, subtilisin/kexin/sedolisin
CaPOPII_3533	scaffold1215	45663	ATT/ATTT	TGGCTTTTGTATTTCAGAGTTGG	AGTGGGACCATGATTTTTGAA	60.5	492	INTERGENIC				
CaPOPII_3534	scaffold1215	45939	ATTTT/ATTT	TTTCAAAAATCATGGTCCCAC	GGGGTGGTGTCTATCTTTGC	59.7	337	INTERGENIC				

INDEL marker IDs	Chromosomes /unanchored scaffolds	Physical positions (bp)	InDels (*Kabuli* reference genome-CDC Frontier/PI)	Forward primers (5'-3')	Reverse primers (5'-3')	Anneling temperature (0C)	Expected amplified product size (bp)	Structural annotation		Functional annotation		
								Sequence components of *kabuli* genome	*Kabuli* gene accession IDs	NCBI-KOG	TFs	NCBI-nr database
CaPOPII_3535	scaffold1219	40006	GTATATATATATATATAT/GTATATATATATATAT	TGGTTTAATGTTGGCAAATGG	ATCCAGTTGGTGAGGAAAAA	60.6	317	INTERGENIC				
CaPOPII_3536	scaffold1238	42907	AATATATATATATATATA/AATATATATATATATATA	TATTTTGGATTTTGGCTCGG	AGGAAGAGTGGAGTTGCGAA	59.9	502	INTERGENIC				
CaPOPII_3537	scaffold1239	4253	TC/TCC	TCGAGTGGTACGAAAATCCC	TCCCAGTCCTCGATTTCATC	59.9	539	INTERGENIC				
CaPOPII_3538	scaffold1240	105707	A/AG	ACATGGAACCCATCCAAAGA	GCCCTCAGATGAGAGTCAGG	60.2	268	INTERGENIC				
CaPOPII_3539	scaffold1244	3584	AGGGGGGG/AGGGGGG	CCTACGGAGGACGAAAATGA	TGCCTAATCGATTTGCTCAG	60.1	556	INTERGENIC				
CaPOPII_3540	scaffold1246	7947	TAAAA/TAAA	TGGATGCACAAGATGGGTTA	TCAGGTCATTTTCGTCCTCC	59.9	871	INTERGENIC				
CaPOPII_3541	scaffold1252	98018	CA/CAA	TTTGCCAATCGATTATCGTG	TGCACAAGTCAGTAGCACCC	59.5	561	INTERGENIC				
CaPOPII_3542	scaffold1256	617	ATTTTTTTTTTT/ATTTTTTTTTT	TCCAACACCCTCACAAATCA	TTGGACTACGGGTTAGTGGG	59.9	914	INTERGENIC				
CaPOPII_3543	scaffold127	20427	TAATAAA/TAATAAAATAAA	AAACCCAAAATCCTGAACCTT	CTGGACATTTTGGCGATTTT	58.9	350	INTERGENIC				
CaPOPII_3544	scaffold127	54036	TG/T	AAAAATGGGTGGTCAAATCAA	TGGCATTAAGGTTCTGTGTTCA	59.2	640	INTERGENIC				
CaPOPII_3545	scaffold127	111345	GAAAAAAAAAA/GAAAAAAAAA	TCATTGCTCCCCCGTAATAG	CGACACTCGCTTCACCAATA	59.9	504	DRR	Ca_26797			
CaPOPII_3546	scaffold127	144443	AGTGT/AGT	CCCGAAAAGTCCAGATGAAA	CAAGGATCAGAGGGGATTCA	60.0	581	INTERGENIC				
CaPOPII_3547	scaffold127	270423	AATTTTCATTTTCATTT/AATTTTCATTT	GCCCACATATCTTGTTGCCT	TTCAAGCACATACTGCACCAG	60.0	687	INTRON	Ca_26800			
CaPOPII_3548	scaffold1274	6685	T/TGTG	TCATTTTCGTCCTTCCTTGG	ACACACCTATTGGACGCACA	60.0	272	INTERGENIC				
CaPOPII_3549	scaffold1275	6195	CAA/CAAGAGATAA	TGTTGCCTCGTAATTCAACG	CAGGGACTAAAACTGTTTGCTG	59.7	556	INTERGENIC				
CaPOPII_3550	scaffold1276	1671	TT/TTATAT	AAGCATGGGACCAAAGTGAG	TTTCCCAATCGATTTGCTTC	60.1	942	INTERGENIC				
CaPOPII_3551	scaffold1276	52730	AAGGGTTTAGGGTTTAGGGTTT/AAGGGTTTAGGGTTTAGGGTTTAGGGTTT	CCTATTAGTGCAAAAGTGCCA	AGACCTAGGCCCTTAAACCA	58.0	491	INTERGENIC				

INDEL marker IDs	Chromosomes /unanchored scaffolds	Physical positions (bp)	InDels (*Kabuli* reference genome-CDC Frontier/PI)	Forward primers (5'-3')	Reverse primers (5'-3')	Anneling temperature (0C)	Expected amplified product size (bp)	Structural annotation		Functional annotation		
								Sequence components of *kabuli* genome	*Kabuli* gene accession IDs	NCBI-KOG	TFs	NCBI-nr database
CaPOPII_3552	scaffold1278	63103	GAAAAA/GAAAA	ACGAAGGGTGTAGGACGATG	ATTTGTTTGCCGATCGCTAC	60.0	611	INTERGENIC				
CaPOPII_3553	scaffold1278	63247	TAAAAAAAA/TAAAAAAA	ACGAAGGGTGTAGGACGATG	ATTTGTTTGCCGATCGCTAC	60.0	611	INTERGENIC				
CaPOPII_3554	scaffold1278	63301	ATT/ATTT	ACGAAGGGTGTAGGACGATG	ATTTGTTTGCCGATCGCTAC	60.0	611	INTERGENIC				
CaPOPII_3555	scaffold1278	65072	TTTATTATTATTATTATTATTATTATTATTATTATTATTA/TTTATTATTATTATTATTATTATTATTATTATTATTATTA	TGGGTAGCAGTAATAATGGCA	TGAGCAAGATGTGTTTTGTGG	58.2	341	INTERGENIC				
CaPOPII_3556	scaffold128	256571	ATT/ATTT	GCCAATCGATTACCCTCAAA	CACTTATGCAAAAAGGGGGA	59.9	451	INTERGENIC				
CaPOPII_3557	scaffold128	588046	TAA/TAAA	AGTACTGAGCCCCCTGGTTT	ATGATGTTGTAGCGATTGCG	60.0	308	INTERGENIC				
CaPOPII_3558	scaffold128	747253	G/GT	GGTGTGTTTGTGACGTTGGA	GGAGGACGAAAATGATCGAA	60.5	646	INTERGENIC				
CaPOPII_3559	scaffold128	853519	CCATACATA/CCATACATACATA	AACACATCCGGGATCCATTA	AATGACGACGACGATGATGA	60.0	551	INTRON	Ca_21452	D		Helicase, C-terminal
CaPOPII_3560	scaffold1281	1507	TAAAAAAAAAA/TAAAAAAAAAAA	CGATTTCCCACTCGAAATGT	TTTTGGTTTCTTTTGGGTTTTT	59.9	786	INTERGENIC				
CaPOPII_3561	scaffold1281	192341	TTATATATATATATATATATATATATATATA/TTATATATATATATATATATATATA	CGAAGAATCACTTTAAACCCCA	TCCTTGGTCCATGTATATAAGCC	60.3	916	INTRON	Ca_21513		bHLH	
CaPOPII_3562	scaffold1281	376337	GTTTTTTTTTT/GTTTTTTTT	TGTTTAGGCGCTAGTTTGGC	TCGATTATGCGTAGGGAAGG	60.4	665	INTERGENIC				
CaPOPII_3563	scaffold1281	469460	GAAAAAAAAAAAA/GAAAAAAAAAAA	TCTCTGGCAACCGTTCTTCT	ACCTCGTTGAGACACGCTTT	60.0	566	INTERGENIC				
CaPOPII_3564	scaffold1281	471713	AATATATATATATA/AATATATATATATATA	GAGCATTGTGACAAAATTGAGC	CGGGAATTCTTGATGAATCG	59.8	541	INTERGENIC				
CaPOPII_3565	scaffold1281	473339	GATATATATATATATATATATA/GATATATATATATATATATATATA	ACGCATGGATATTGCAGTCA	TCCGATTGTTGACCAGTTTTT	60.1	783	INTERGENIC				
CaPOPII_3566	scaffold1281	492209	TAAAAA/TAAAA	GGAATGGAAAACACATTCAACA	GAATGCTTGAGAAACTTTGGG	59.7	499	INTERGENIC				
CaPOPII_3567	scaffold1292	2932	CATATATA/CATATATATA	GCCTTAAGGTTCGACAGCAG	GGCTCGCTATGTATGGCAGT	60.0	613	INTERGENIC				
CaPOPII_3568	scaffold1307	6228	A/AG	ACACACCTATTGGACGCACA	AGGTCAATTTCGTCCTCCCT	60.0	377	INTERGENIC				

INDEL marker IDs	Chromosomes /unanchored scaffolds	Physical positions (bp)	InDels (*Kabuli* reference genome-CDC Frontier/PI)	Forward primers (5'-3')	Reverse primers (5'-3')	Anneling temperature (0C)	Expected amplified product size (bp)	Structural annotation		Functional annotation		
								Sequence components of *kabuli* genome	*Kabuli* gene accession IDs	NCBI-KOG	TFs	NCBI-nr database
CaPOPII_3569	scaffold1308	16327	ACCC/ACC	AGGGAGGACGAAAATGACCT	CCAATTGGGGTTTTTAACCA	59.9	488	INTERGENIC				
CaPOPII_3570	scaffold1308	79242	TC/T	AATCCAATGACGCAAACACA	GGTTTCGAGGAAATTGGACA	60.0	669	INTERGENIC				
CaPOPII_3571	scaffold1309	58470	CTTTT/CTTTTT	TGGGTAACGTAACCTACGGC	ATCCGGTAAAAATCTCCGCT	59.9	951	DRR	Ca_26763			
CaPOPII_3572	scaffold1312	65816	TCAATATTTGTAACAATATTT/TCAATATTT	CGCGGGGACTTAGAGATGTA	AAGCGAACATAATCCTTTACTTTTG	60.2	561	INTERGENIC				
CaPOPII_3573	scaffold1312	78648	TAAA/TAAAA	GCATTAAATGTGGAACAAGCA	TTACCTGTGTGGGACAACGA	58.7	526	INTERGENIC				
CaPOPII_3574	scaffold1312	78828	GAAAAAA/GAAAAAAA	GCATTAAATGTGGAACAAGCA	TTACCTGTGTGGGACAACGA	58.7	526	INTERGENIC				
CaPOPII_3575	scaffold1312	78884	TAAGAAGAAGAAGAAGAA/TAAGAAGAAGAAGAA	ATTTGACCTCGACACAACGG	GCCGCTTGATCGTTAATTGT	61.0	425	INTERGENIC				
CaPOPII_3576	scaffold1312	78931	CG/C	TCGACACAACGGACAAGAGT	GCCGCTTGATCGTTAATTGT	59.3	417	INTERGENIC				
CaPOPII_3577	scaffold1312	80554	ATTT/ATTTTTT	GCCAAGGTAAGGGTGAGACA	ATGAATTGTCCCAATCTCGG	60.1	322	INTERGENIC				
CaPOPII_3578	scaffold1312	89696	ATTTTTTTTTT/ATTTTTTTTT	TTCATTACGCCATTGACACTTT	ACAATGTGTGCCCAACAAAA	59.5	457	INTERGENIC				
CaPOPII_3579	scaffold1312	89732	ATTTTTTT/ATTTTTTTT	TTCATTACGCCATTGACACTTT	ACAATGTGTGCCCAACAAAA	59.5	457	INTERGENIC				
CaPOPII_3580	scaffold1313	43842	ATT/AT	AGGGGCTAGCAAAGGGTAAA	GACACCAGGAGGCATGTTTT	60.1	684	INTERGENIC				
CaPOPII_3581	scaffold1313	102973	GAAAAAAAAA/GAAAAAAAAAAA	GTGCTACAACGCATTTGGTG	CGGCTTGGTCTCATGATTTT	60.2	598	INTERGENIC				
CaPOPII_3582	scaffold1313	133844	TAAAAAAAAAA/TAAAAAAAA	TGAAGCAGTAAGCGGTTGTG	GCAAACTTGCTCCGTTTGAT	60.1	707	INTERGENIC				
CaPOPII_3583	scaffold1315	151607	ATTTT/ATTT	CAGTGGCTGCGATAAACAAA	TCTGGGCAACTACATTAAGCAA	59.9	765	INTERGENIC				
CaPOPII_3584	scaffold1315	182592	CTTTTTTTTTTTT/CTTTTTTTTTTTTTT	TGCTAGACTGATTTTCCCCG	TGCCTTGTTGATTCTTTGGTC	60.2	860	INTERGENIC				
CaPOPII_3585	scaffold1316	4181	TA/TAA	GTAAAGGGTCACCAACCGAA	TTGTCCGTGAGAGACTGCAC	59.8	813	INTERGENIC				

INDEL marker IDs	Chromosomes /unanchored scaffolds	Physical positions (bp)	InDels (*Kabuli* reference genome-CDC Frontier/PI)	Forward primers (5'-3')	Reverse primers (5'-3')	Anneling temperature (0C)	Expected amplified product size (bp)	Structural annotation		Functional annotation		
								Sequence components of *kabuli* genome	*Kabuli* gene accession IDs	NCBI-KOG	TFs	NCBI-nr database
CaPOPII_3586	scaffold1316	13036	AATTTATTTATTTATTTATTTATT/AATTTATTTATTTATTTATTTATTTATT	ACATGCACAGAGAATGCCAA	GTGCCAGTCGATTGCCTAAT	60.3	631	DRR	Ca_28021			
CaPOPII_3587	scaffold1317	22112	TATAATAATAATAATAA/TATAATAATAATAATAATAATAA	TTTGATTCATGCTTCTTTCTTTTC	TTTGATCGGGGAAGTTTGAG	59.0	382	INTERGENIC				
CaPOPII_3588	scaffold1317	44894	AT/A	GAGCAATGACAGCTCGTTGA	ATTGGATCGTTGGTTGTGGT	60.1	718	INTERGENIC				
CaPOPII_3589	scaffold1317	45149	GA/GAA	CGTTCTCACTCTCCTCCACC	TGTTGTGGTGTTCAAGCCAT	59.8	431	INTERGENIC				
CaPOPII_3590	scaffold1317	46249	AC/A	CATGGGTGAAGACTGTGGTG	ACCACTGTTGCATGCCTAAA	60.0	134	INTERGENIC				
CaPOPII_3591	scaffold1317	46362	AAATAAATAAAATTTTAATA/AAATA	CATGGGTGAAGACTGTGGTG	TTGGTTTGCTTCTTTTGAATTG	60.0	572	INTERGENIC				
CaPOPII_3592	scaffold1317	46601	TAAAAAAAAA/TAAAAAAAAAAA	CATGGGTGAAGACTGTGGTG	CAATTGCGAAAACATCTCCA	60.0	732	INTERGENIC				
CaPOPII_3593	scaffold1323	6638	ATTT/ATTTT	GGCCACTGACTTGCAGTGTA	TTGTGCATCCAATACGTGTG	59.9	717	INTERGENIC				
CaPOPII_3594	scaffold1324	7929	AATATATATAT/AATATATATATAT	AGCAGCTGGTCTCACCAAAT	TGACCGACAATGCAACAAAT	59.9	251	INTERGENIC				
CaPOPII_3595	scaffold1324	8519	ATTTT/ATTT	ATTTGTTGCATTGTCGGTCA	CTGCCTCACGATTTCACTCA	60.0	899	INTERGENIC				
CaPOPII_3596	scaffold1324	227334	TG/T	TTGGTGACATTTAGCATTTCTCA	GTCCTTTGTGTCGTGGGTCT	59.6	511	INTERGENIC				
CaPOPII_3597	scaffold1324	235091	CGTTGT/CGT	TCTTGGTTGTAACCCTTGGC	TTTCGACAAAAGGTTCTCCG	60.0	431	INTERGENIC				
CaPOPII_3598	scaffold1324	236638	GAAAAA/GAAAA	ATGCACCTTGAAAGGGTTTG	TTCGAGGAGGGTTTTGATTG	60.0	597	INTERGENIC				
CaPOPII_3599	scaffold1324	238280	CTTTTCCTTTTGTTTTTCCTTT/CTTTTCCTTT	ACGGTAGCTTTAACGAGCGA	ATGAGGGAACAACACCCTGA	60.0	900	INTERGENIC				
CaPOPII_3600	scaffold1324	238347	CATATATATATATATATATATATATATAT/CATATATATATATATATATATATATATATATAT	ACGGTAGCTTTAACGAGCGA	ACCCAACTATTGGAGATGCG	60.0	941	INTERGENIC				
CaPOPII_3601	scaffold1324	241946	AAAACAAAGAAACAAAGAAA/AAAACAAAGAAA	TGACTTTTGCAGCATCGTTC	GTTCATACCCTGTGGATGGG	60.0	809	INTERGENIC				
CaPOPII_3602	scaffold1324	252648	T/TTCTTTCAAC	CCTATAGGCCCACTAAGCCC	TTGCAAGCAAACTTCACGAT	59.9	587	INTERGENIC				

INDEL marker IDs	Chromosomes /unanchored scaffolds	Physical positions (bp)	InDels (*Kabuli* reference genome-CDC Frontier/PI)	Forward primers (5'-3')	Reverse primers (5'-3')	Anneling temperature (0C)	Expected amplified product size (bp)	Structural annotation		Functional annotation		
								Sequence components of *kabuli* genome	*Kabuli* gene accession IDs	NCBI-KOG	TFs	NCBI-nr database
CaPOPII_3603	scaffold1324	252816	CGTGAAGT/CGT	TGTTAACATGATCAGGCCCA	ACATAGCGCCACAACATGAA	59.9	789	INTERGENIC				
CaPOPII_3604	scaffold1324	253776	ATTTTTTTTTTTT/ATTTTTTTTTT	GAGTGCTCCTTGCACATGAA	ATGAAGACCAAGCCATCCAC	60.0	514	INTERGENIC				
CaPOPII_3605	scaffold1324	255649	CTTTTTTTTTTTT/CTTTTTTTTT	CCTTGGAGGCAGTGATTTGT	GAGCTACCCCAATGGTTGAA	60.1	613	INTERGENIC				
CaPOPII_3606	scaffold1324	255844	GAAAAAA/GAAAAAAA	CCTTGGAGGCAGTGATTTGT	GAGCTACCCCAATGGTTGAA	60.1	613	INTERGENIC				
CaPOPII_3607	scaffold1324	255890	ATTTTT/ATTTT	CCTTGGAGGCAGTGATTTGT	GAGCTACCCCAATGGTTGAA	60.1	613	INTERGENIC				
CaPOPII_3608	scaffold1324	256337	CAAAA/CAAA	TTCAACCATTGGGGTAGCTC	TTCACTTGAATATTTTGTGGCA	59.9	474	INTERGENIC				
CaPOPII_3609	scaffold1324	266092	CAA/CAAA	GAGGGGGTCCGAAAAATAAA	TCAATTTGAACTCAAACCCAT	60.1	396	INTERGENIC				
CaPOPII_3610	scaffold1324	266713	AATCTATTATCTATTATCTATTAT/AATCTATTATCTATTAT	TGTATGCATCAACAATGTCAAGA	TCAGAGGATGTGGCATAGAGG	59.1	160	INTERGENIC				
CaPOPII_3611	scaffold1324	266796	CA/CAA	TCCTCTATGCCACATCCTCTG	CACAAATAGTTGTACATTGGTTGC	60.2	238	INTERGENIC				
CaPOPII_3612	scaffold1324	267973	A/AT	CCAAGGCACACACAAATGAA	AGAGAATAACCCGCACGTTG	60.6	344	INTERGENIC				
CaPOPII_3613	scaffold1324	270833	GTT/GT	ACAAAATCGGCCACTGAATC	TCCCGTTCATCTCTCTCACC	59.9	500	INTERGENIC				
CaPOPII_3614	scaffold1324	272072	ATTTTTTTT/ATTTTTT	CAAATGTCGGGAGCTACCAT	TGACACCCTAAATCCCCAAA	60.0	522	INTERGENIC				
CaPOPII_3615	scaffold1324	272116	TGGGG/TGGG	CCCCGCTCTGATAGGTGATA	TGTTTGTCATGCGAGCATTT	60.1	767	INTERGENIC				
CaPOPII_3616	scaffold1324	272539	TAAAAAAAAA/TAAAAAAAA	TTTGGGGATTTAGGGTGTCA	AAATTCTTGGGGACTTTCGG	60.2	538	INTERGENIC				
CaPOPII_3617	scaffold1324	279339	ATTTT/ATTT	CATCATGAATTCGACCATGC	ATAGTTGCACCACTTTGGGC	59.9	722	DRR	Ca_19767		BES1	F-box domain, cyclin-like
CaPOPII_3618	scaffold1324	516063	ATTTTTTTTTTT/ATTTTTTTTTT	AAAACACGCTAGGCTCCAAT	TGGTCATGTGGTTTTTGCAT	58.9	642	INTERGENIC				
CaPOPII_3619	scaffold1324	520260	AATTATTATTATTATTATTATTATTATTATTATTATT/AATTATTATTATTATTATTATTATTATTATTATTATTATTATTATTATT	TTCTTTTCGCTATTTTATTTTCGTG	TCTTACGTATCTCCCGGTGC	60.0	734	INTERGENIC				

INDEL marker IDs	Chromosomes /unanchored scaffolds	Physical positions (bp)	InDels (*Kabuli* reference genome-CDC Frontier/PI)	Forward primers (5'-3')	Reverse primers (5'-3')	Anneling temperature (0C)	Expected amplified product size (bp)	Structural annotation		Functional annotation		
								Sequence components of *kabuli* genome	*Kabuli* gene accession IDs	NCBI-KOG	TFs	NCBI-nr database
CaPOPII_3620	scaffold1324	642408	ATTTTTT/ATTTTTTT	CCTTGCCCTTTTATTTCGGT	TCCTAGAACATGCATCACGC	60.3	496	INTERGENIC				
CaPOPII_3621	scaffold1324	700658	CATATATATATATATATATAT/CATATATATATATATATAT	ATCACACCAAGAATGGCACA	CACGTAAATAGTTATTTGATTTTGTGA	60.0	294	INTERGENIC				
CaPOPII_3622	scaffold1331	50640	GT/GTT	AATGGGCGCTGTTAAATGTC	TGGTGTCGTTGGCACTCTAC	60.0	411	INTERGENIC				
CaPOPII_3623	scaffold1331	79915	AATATATATATATATATATATATATA/AATATATATATATATATATATATATATA	TTTATGCCCAAATCTCAGCC	CCTCTGAAATTCCCTTTGGA	60.0	493	INTERGENIC				
CaPOPII_3624	scaffold1332	4491	TCCCC/TCCCCC	CCCTCCATATCTCCACGAAA	AGCACAAACCCAAATTCACA	59.9	512	INTERGENIC				
CaPOPII_3625	scaffold1332	8473	ATTTTTTT/ATTTTTTTT	CACAATTTCTTTTCTATGTGTGGA	AACCCGTCACACAGGAAGTC	58.2	894	INTERGENIC				
CaPOPII_3626	scaffold1335	45191	AAGCTAGCTA/AAGCTA	TGAAGAGTTGCACGAGATGG	ATGCCTCATTTCCCAACAAG	60.0	198	DRR	Ca_28106	S		
CaPOPII_3627	scaffold1335	46272	TC/T	TTCGTGCAACTGACGCTAAC	CCATTTGGATTTAAAAGAAGCC	60.1	345	DRR	Ca_28106	S		
CaPOPII_3628	scaffold1337	46228	AAAACCCTAAACCCTAAA/AAAACCCTAAACCCTAAACCCTAAA	AAACCCCAAACCTTAAACCC	TTGGGACATTTCGGTTTTTG	59.1	353	INTERGENIC				
CaPOPII_3629	scaffold134	365115	T/TG	TTTCCCCATGCAGAGTTAGG	AATCGCTAGCCACTTAGGCA	60.1	491	INTERGENIC				
CaPOPII_3630	scaffold134	424697	TTTATTATTATTATTATTATTATTATTATTA/TTTATTATTATTATTATTATTATTATTA	CACCTAAATTTTCTCCGGCA	TTACATCGAGCGTGAACGAG	60.1	623	INTERGENIC				
CaPOPII_3631	scaffold135	113512	A/AG	GCTTATTGGGAGAAGCGTTG	GCCCTCAGATGAGAGTCAGG	59.8	514	INTERGENIC				
CaPOPII_3632	scaffold1351	199024	TATAATAATAATAATAATAATAATAATAATAATAATGATAATAATAATAATAATAATAAT/TATAATAATAATAATAATAATAATAATAATAATAATGATAATAA	ACCTCCCACTGGGTTGTTTT	GCTAATAGGGGGTCGTTTCC	60.6	713	INTRON	Ca_21997	R		Carbonic anhydrase, alpha-class, catalytic domain
CaPOPII_3633	scaffold1359	74272	TAC/TCAC	CCAATTTGAATTGTCCGTCC	TTAAAGAGATTCGCTGCGGT	60.2	663	DRR	Ca_26683	KR	G2-like	Mitochodrial transcription termination factor-related
CaPOPII_3634	scaffold1359	74591	ATTTTTTTT/ATTTTTTTTT	TGGGTGGGAGTAATTTTTCG	TTAAAGAGATTCGCTGCGGT	59.8	297	DRR	Ca_26683	KR	G2-like	Mitochodrial transcription termination factor-related
CaPOPII_3635	scaffold1360	2778	GAAAAAAAAA/GAAAAAAAAAAA	GCGTTCACTTGTTGGGTTTT	TGTGGTGGAGGTCATGAGAA	60.0	549	INTERGENIC				
CaPOPII_3636	scaffold1365	74375	CATG/CATGATG	TATTGTGAAGAGGCGTGTGC	TGTTGGGATTTGGTTGTGAA	59.9	351	INTERGENIC				

INDEL marker IDs	Chromosomes /unanchored scaffolds	Physical positions (bp)	InDels (*Kabuli* reference genome-CDC Frontier/PI)	Forward primers (5'-3')	Reverse primers (5'-3')	Anneling temperature (0C)	Expected amplified product size (bp)	Structural annotation		Functional annotation		
								Sequence components of *kabuli* genome	*Kabuli* gene accession IDs	NCBI-KOG	TFs	NCBI-nr database
CaPOPII_3637	scaffold1369	80991	TAAAA/TAAAAA	CGTATTTCGGGGAAGAGTCA	GGCAGTAGTGGAATCTGGGA	60.1	425	INTERGENIC				
CaPOPII_3638	scaffold1369	145181	TTCTCTCTCTCTCTCTCTCTCTCT/TTCTCTCTCTCTCTCTCTCTCT	AAAGGGTGACGACGATTTCA	TGGCTTTAAGAAACATGGACAA	60.5	858	INTERGENIC				
CaPOPII_3639	scaffold1380	5644	ATTTTTTTT/ATTTTTTTTT	TTTTAGCGGATGTCGTAGGG	TCACAAGGGGGACTCTATGG	60.1	719	INTERGENIC				
CaPOPII_3640	scaffold1382	294316	CT/CTT	GCTCGTATTCATCCTCCCAA	ACACACCTATCGGATGCACA	60.0	523	INTERGENIC				
CaPOPII_3641	scaffold1386	13609	CAAAA/CAAAAA	TTTGTGGTAATTTATGGTTGCTACA	TTTCCGTTTCCTTTGTTTGC	59.7	424	INTERGENIC				
CaPOPII_3642	scaffold1390	38477	A/AC	TCGATGCACCAGATTGGTTA	AGTGTGTTTGTGGCATTGGA	60.1	787	INTERGENIC				
CaPOPII_3643	scaffold1393	2098	T/TG	GGTCACTTTCGTGCTCCCTA	TGGATGCACGAGATTGGTTA	60.3	873	INTERGENIC				
CaPOPII_3644	scaffold140	2406	CATATATATATATATATATATATATATATATAT/CATATATATATATATATATATATATATATATATAT	CTTGTAAAATCTCCGCTCGC	TTGGTATTGAACTCGGAGGC	60.0	665	INTERGENIC				
CaPOPII_3645	scaffold140	25774	AATTT/A	TCCTAGAACATGCATCACGC	CTCAAGTCGACAAGCCTTCC	59.8	666	INTERGENIC				
CaPOPII_3646	scaffold1408	24952	GTTTTTT/GTTTTT	TGTGCGTCCAATAGGTGTGT	GACACGAATAAGCCAAGGGA	60.0	478	INTERGENIC				
CaPOPII_3647	scaffold1408	70937	AGG/AGGG	TTTTCATCCTTCCCATGCTC	ACACACCTATCGGATGCACA	60.0	273	INTERGENIC				
CaPOPII_3648	scaffold1408	92293	GTTTTT/GTTTTTT	AGGTCATTTTCGTCCTCCCT	TAATTGGGCAAGAATGAGCC	59.9	640	INTERGENIC				
CaPOPII_3649	scaffold1414	38236	GTTATTATTAT/GTTATTAT	TGGTCCTAGATTTCATCGGG	CGTTATTAAGTACAACTACAAAACGAA	59.9	871	INTERGENIC				
CaPOPII_3650	scaffold1414	190587	GAA/GAAA	TCGGATCACGGTCTGATACA	GAGGCAAATGGGACAAGGTA	60.1	611	CDS (FRAME SHIFT)	Ca_25542	R		Zinc finger, CCHC-type
CaPOPII_3651	scaffold1417	39952	TAAAAAAAAAA/TAAAAAAAAAAA	AAAAGGATTCCATTCGCCTT	GGACACATTATTTCGTGGGG	59.9	577	INTERGENIC				
CaPOPII_3652	scaffold1417	123825	ACC/ACCC	CCCTCTGCAATGTCGGTAAT	ATTTGACAAACCTTTTGCGG	60.0	363	INTERGENIC				
CaPOPII_3653	scaffold1417	243147	A/AG	AGGTAAGGGCTCCTTCCAAA	ACGATCCACACCAAAACACA	60.1	351	INTERGENIC				

INDEL marker IDs	Chromosomes /unanchored scaffolds	Physical positions (bp)	InDels (*Kabuli* reference genome-CDC Frontier/PI)	Forward primers (5'-3')	Reverse primers (5'-3')	Anneling temperature (0C)	Expected amplified product size (bp)	Structural annotation		Functional annotation		
								Sequence components of *kabuli* genome	*Kabuli* gene accession IDs	NCBI-KOG	TFs	NCBI-nr database
CaPOPII_3654	scaffold1418	5209	TATAATAATAATAATAATAATAATAATAATAATAATAATAAT/TATAATAATAATAATAATAATAATAATAATAATAATAATAAT	CACAATTTCACGATTTTCACG	CGGCTAGGTTGACCCTTACA	59.1	819	INTERGENIC				
CaPOPII_3655	scaffold1418	17845	TAAAAAAAAAAA/TAAAAAAAAA	GCATTGTATCCATCCCCATC	TATTGGTGGCCATGTTCTCA	60.0	216	INTERGENIC				
CaPOPII_3656	scaffold1418	18174	AC/A	GCATTGTATCCATCCCCATC	ACCCACGTTTAAAGGTTCCC	60.0	797	INTERGENIC				
CaPOPII_3657	scaffold1418	21418	AG/A	GCCATAATTGCTTCTGCAGTTT	TCGCGGTGTGGTAAGTCATA	60.6	235	INTRON	Ca_25261	QR	B3	Oxoglutarate/iron-dependent oxygenase
CaPOPII_3658	scaffold1418	38513	CTTTTATTTTTTATTT/CTTTTATTTTTTATTTTTTATTT	CTTTCCCTAAAGCCCAATCC	TTGCTCCCTTTACTCATCTGC	59.9	811	INTRON	Ca_25262	S		Domain of unknown function DUF21
CaPOPII_3659	scaffold1419	10604	ATTTTTTTTTTTT/ATTTTTTTTTT	CCTCTTGCTGCAAACACAAA	AACTTTTTCCGCTGCTTGAA	60.0	294	INTERGENIC				
CaPOPII_3660	scaffold1419	21351	TAAAA/TAAAAA	AAAAACCTTTGGGAAATCGG	AATTCAAGTTCGAAATGCCG	60.2	207	INTERGENIC				
CaPOPII_3661	scaffold1419	30683	T/TG	TCCATCTCCAACAACATCCA	TAATTGCTCTTGCGGTCCTT	59.9	306	INTERGENIC				
CaPOPII_3662	scaffold1426	5828	TCC/TTCCC	AACGGGGTGTTACAATGAGC	CTGGTTTGACAATGCGTTTG	59.9	623	INTERGENIC				
CaPOPII_3663	scaffold1426	6734	TCATCAC/TCATCACCATCAC	TTGATACTATGCCACCACGC	GCATTTTGCTTGTGGACTCA	59.6	635	INTERGENIC				
CaPOPII_3664	scaffold1426	7221	TAAAAAA/TAAAAAAA	TGAGTCCACAAGCAAAATGC	GGTTCCTCTTGGTTTTGGGT	59.8	494	INTERGENIC				
CaPOPII_3665	scaffold143	67526	TA/TAA	GGATTGTTGGTTCATTTGGG	TTAGGAAATCGATTGGGGTG	60.0	495	INTERGENIC				
CaPOPII_3666	scaffold1430	1269	ATTTTT/ATTTT	TCATGTGCATTTCTACCACCA	TGATTCGAGTCAAACCACCA	60.0	315	INTERGENIC				
CaPOPII_3667	scaffold1432	80490	CTTTTTTTT/CTTTTTTT	GCCACTACCAACAACATTTTCA	ATTTGTGTTTGTGGGGGAAA	59.9	713	INTERGENIC				
CaPOPII_3668	scaffold1432	80541	AAATAATAATAATAATAATAATAAT/AAATAATAATAATAATAATAATAATAAT	GCCACTACCAACAACATTTTCA	ATTTGTGTTTGTGGGGGAAA	59.9	713	INTERGENIC				
CaPOPII_3669	scaffold1433	21283	ATTTTT/ATTTT	TCCAGATGAAATCGAGGACC	GAGATTTTCGTACCACCCGA	60.0	481	INTERGENIC				
CaPOPII_3670	scaffold1433	21769	AGCATTATTTTC/A	TCCAGATGAAATCGAGGACC	TCATATGTACGTTTCGGGCA	60.0	498	INTERGENIC				

INDEL marker IDs	Chromosomes /unanchored scaffolds	Physical positions (bp)	InDels (*Kabuli* reference genome-CDC Frontier/PI)	Forward primers (5'-3')	Reverse primers (5'-3')	Anneling temperature (0C)	Expected amplified product size (bp)	Structural annotation		Functional annotation		
								Sequence components of *kabuli* genome	*Kabuli* gene accession IDs	NCBI-KOG	TFs	NCBI-nr database
CaPOPII_3671	scaffold1433	21950	TAAAAAA/TAAAAA	GATGAAATGGAGGACCGAGA	TCATATGTACGTTTCGGGCA	60.0	332	INTERGENIC				
CaPOPII_3672	scaffold1433	33630	CAAAA/CAAAAA	AGGCGATTTTCGTACCACAC	CGTGTCATTTATGTCGGGTG	60.0	112	INTERGENIC				
CaPOPII_3673	scaffold1448	11427	CAAA/CAA	AATCCAATGACGCAAACACA	AGGTCATTTTCGTCCTCCCT	60.0	792	INTERGENIC				
CaPOPII_3674	scaffold1448	99427	GAATTTAATATAAATTAATTTAATATAAATT/GAATTTAATATAAATTAATTTAATATAAATTAATTTAATATAAATT	CCAGGAAATGACCCCCTATT	TTTATTTTAGCGCGGTGTCC	60.0	774	DRR	Ca_26688			
CaPOPII_3675	scaffold1449	3971	AATATATATATATATATATATATATATATAT/AATATATATATATATATATATATATATATAT	AGGAAGAGTGGAGTTGCGAA	CTGTAAACTGAAGCGCCCTC	60.0	456	INTERGENIC				
CaPOPII_3676	scaffold1450	10288	AATATATATATATATATATATATATATAT/AATATATATATATATATATATAT	CGTTGCACCAAGATGAAAAA	GAGACCGGCGTGAAAAATTA	59.7	463	INTRON	Ca_28008			Late embryogenesis abundant protein, group 1
CaPOPII_3677	scaffold1452	1227	GTATATATATATATATATATATAT/GTATATATATATATATATATATATATAT	CGAAATCTTTGGGTTTTGGA	ACCTAAATTGGGGAGCTCGT	59.9	618	INTERGENIC				
CaPOPII_3678	scaffold1452	29738	ATTT/ATTTT	GAAGCCTTTTCAGCAGGTTG	TAGTGTGCCACCACTCCAAA	60.0	648	URR	Ca_27551	R	ZF-HD	Pentatricopeptide repeat
CaPOPII_3679	scaffold1452	30210	TAAA/TAAAA	TGCCCAATTACCACAATCAA	ACCAACCCTTCATTTCCCTC	59.8	465	INTERGENIC				
CaPOPII_3680	scaffold1455	13166	TCCC/TCC	CGAAGGCTTTGTTGAGAAGG	CTTGCCTTGTGCAGATTTGA	60.0	220	INTERGENIC				
CaPOPII_3681	scaffold1457	19983	G/GA	TGGCTTTCAAATTCCACACA	GCGAGCAGAGATTTTTACCG	60.1	468	INTERGENIC				
CaPOPII_3682	scaffold1462	12076	G/GACCACCGAATGCC	CTGGGTCCTAGTTCGCTCAA	TCAGCTTTTTGGCTGATGTG	60.4	129	INTERGENIC				
CaPOPII_3683	scaffold1465	3149	AATATATATATATATATATAT/AATATATATATATATATATATATATATATATATAT	AAGGCTCAACCTTACGTGGT	TTTTTAGCAATGGCACAACATT	58.7	558	INTERGENIC				
CaPOPII_3684	scaffold1465	20135	TTA/TTAGATA	ATAGATCCCCTCAGCCCAAC	TTTCGGTAGTCCCGTTCATC	60.3	604	INTERGENIC				
CaPOPII_3685	scaffold1467	11911	AATATATATATATATATATATATATATAT/AATATATATATATATATATATATATATATAT	TCGCAGCTGTGATGCTAAGT	ATGGGAGGTCTTGTGCAGTT	59.8	359	DRR	Ca_26807	Q	GeBP	Cytochrome P450
CaPOPII_3686	scaffold1467	16133	GTC/GTCAATC	ACTTTCAAACGGTCAATCCG	ACATTATGTGAGGGCGCTTC	60.0	596	INTERGENIC				
CaPOPII_3687	scaffold1475	99881	AGG/AACTACTTTGG	GGGGTGTGAATAGCTACCGA	TATTGCAGGTCTTGCTGCAC	60.0	436	INTERGENIC				

INDEL marker IDs	Chromosomes /unanchored scaffolds	Physical positions (bp)	InDels (*Kabuli* reference genome-CDC Frontier/PI)	Forward primers (5'-3')	Reverse primers (5'-3')	Anneling temperature (0C)	Expected amplified product size (bp)	Structural annotation		Functional annotation		
								Sequence components of *kabuli* genome	*Kabuli* gene accession IDs	NCBI-KOG	TFs	NCBI-nr database
CaPOPII_3688	scaffold1483	6428	TAAAAAAAAAAAAA/TAAAAAAAA	TAACTTGCATGCCCATTGTC	CAAAAGACACACATATTTGGCA	59.5	573	INTERGENIC				
CaPOPII_3689	scaffold1483	6873	ATGT/ATGTGT	GCACCACGTGCGATAACA	TGTGTGAGCGTTATTGGTTTG	60.3	984	INTERGENIC				
CaPOPII_3690	scaffold1488	625	A/AG	ACTCAATGCCAAAAACTGGG	GCAGACCTTACACAATGCGA	60.0	776	INTERGENIC				
CaPOPII_3691	scaffold1490	1256	GAAAAA/GAAAAAA	CAAACACACCTGTTGGATGG	AGGTCATTTTCGTCCTCCCT	59.8	281	INTERGENIC				
CaPOPII_3692	scaffold1490	74253	TAAAA/TAAAAA	GGCATCACTCAGGTCCTGTT	CACTGGAATGATTGCCAAAA	60.1	304	INTERGENIC				
CaPOPII_3693	scaffold1493	65515	AC/A	AGGCAATTTGGGATTCCTCT	GGCTGCTTTGAGTTCCTTTTT	59.9	372	INTERGENIC				
CaPOPII_3694	scaffold1496	157204	CATATATATATATATATATATATATATATATATATATATAT/CATATATATATATATATATATATATATATATATATATATATATATAT	TCTTTTCCGTTTCCTTCCTTC	TGCGTTACGGTGAAATGGTA	59.7	165	INTERGENIC				
CaPOPII_3695	scaffold1496	236426	AATATATATATATATATA/AATATATATATATATA	TTCAACCTCGAATTTCAGCA	AGCGGTGGAGAGAACTCTGA	59.4	764	INTERGENIC				
CaPOPII_3696	scaffold1496	237245	CTTTTTTTTTT/CTTTTTTTTTTTT	ACCGCTAAATCGGTGTCAAC	CACCGGAAGAAATTGATTTGA	60.0	776	INTERGENIC				
CaPOPII_3697	scaffold1499	931	TAAAAA/TAAAA	TTCTTATTCACCTGGTTCCTTTT	TTGAAGGATACATTACGAGATTCA	58.3	734	INTERGENIC				
CaPOPII_3698	scaffold1499	65960	AGG/AGGG	TTTGCAGCCACAAAAGACAG	CCCTCACAAAGGAGGGATTT	60.0	151	INTERGENIC				
CaPOPII_3699	scaffold1499	112594	ATTTTTT/ATTTTT	AACGTAGCATTTCAATGGGG	ACCTCCTTTCTCGTGTTTGC	59.8	761	INTERGENIC				
CaPOPII_3700	scaffold15	98165	C/CTTTTTCACAAT	GGCAATCGATTCCAACAAGT	CCCAAGAAAGTAGCCACCAG	59.9	514	INTERGENIC				
CaPOPII_3701	scaffold15	131277	CAAAAAAA/CAAAAAA	CATTAGTTTCGTTTTGTAGCCG	TTTCTTTGACAGCTTAATTGTGTT	58.9	199	INTERGENIC				
CaPOPII_3702	scaffold15	143714	CATA/CATATA	TTCATCTGGACTTTTTGGGC	ATACCCCGATCCTCAAACCT	60.1	318	INTERGENIC				
CaPOPII_3703	scaffold15	144204	CAA/CAAA	AGGTTTGAGGATCGGGGTAT	CAAGATTTGGGCAATCTGCT	59.7	757	INTERGENIC				
CaPOPII_3704	scaffold1502	859	TAAAAAA/TAAAAA	AGCCTAGGGAGGACGAAATC	TCCCAATCTTGTGCATTCAA	59.7	476	INTERGENIC				

INDEL marker IDs	Chromosomes /unanchored scaffolds	Physical positions (bp)	InDels (*Kabuli* reference genome-CDC Frontier/PI)	Forward primers (5'-3')	Reverse primers (5'-3')	Anneling temperature (0C)	Expected amplified product size (bp)	Structural annotation		Functional annotation		
								Sequence components of *kabuli* genome	*Kabuli* gene accession IDs	NCBI-KOG	TFs	NCBI-nr database
CaPOPII_3705	scaffold1506	110236	CATATATATATATATATA/CATATATATATATATATATATA	GCTTTTTCGCAAACAGAAGG	GGGTTGCTGATGTGAGTGAA	60.0	269	INTERGENIC				
CaPOPII_3706	scaffold151	814	ATTTTTTTTT/ATTTTTTTTTT	GGAGACTCCCATACATTGGC	TGCTATCCCACTTTGAGCCT	59.4	332	INTERGENIC				
CaPOPII_3707	scaffold151	1278	TAAAAAA/TAAAAAAA	AGGCTCAAAGTGGGATAGCA	GAAGAGCTTCAAGGAAGGCA	59.8	534	INTERGENIC				
CaPOPII_3708	scaffold151	1987	GAAAA/GAAAAA	GGACATTTGTGAACAAGGCA	TTGGAGCTTATGTGTTCCTCAA	59.5	732	INTERGENIC				
CaPOPII_3709	scaffold151	11544	GAAAAAAAA/GAAAAAAA	TTACAGCGCTTGATTCCACA	AAGTGCTTCAAGGAAGGCAA	60.4	705	INTERGENIC				
CaPOPII_3710	scaffold151	35903	AAT/AATAT	ACACACGCATAACACACGAG	TTGGAGTGCATGTGACCATT	58.2	459	INTERGENIC				
CaPOPII_3711	scaffold151	42359	TCCC/TCC	CCTGCCACTAAGGCAACTTC	GGCTTTTCCCCACACAAATA	59.9	556	INTERGENIC				
CaPOPII_3712	scaffold151	68819	ATATTATTATTATTATTATTATTATTATTATTATTATTATT/ATATTATTATTATTATTATTATTATTATTATTATTATTATTATTATT	AAATGAATGGAAGACGCGAT	AAAATTCTCTTGAATCCCCATT	59.5	851	INTERGENIC				
CaPOPII_3713	scaffold151	221306	TCCCC/TCCCCC	TTCAAAGGAGATGGTTTGCC	ATGACAATTCCAATGCACCC	60.1	738	INTERGENIC				
CaPOPII_3714	scaffold1511	61385	TAAAAAAAA/TAAAAAAAAA	CGACACTACGCCAAAAATGA	TGTCCAAAAGCGCTGTAAAA	59.7	250	INTERGENIC				
CaPOPII_3715	scaffold1511	110106	TAA/TAAA	TTAATGCAGGGGTTTGTGCT	ATTGAAGCCCACTATGGCTG	60.5	396	INTERGENIC				
CaPOPII_3716	scaffold1515	15616	TAAA/TAA	TTATTTCGAAAGGTGGGTGG	CCATGTCCATGCTCCTTCTT	59.8	464	INTERGENIC				
CaPOPII_3717	scaffold1521	10780	CA/CATCGA	TCATGTGAACAATGCAACCA	CCACAGCTTGTCTGTCGGTA	59.5	296	DRR	Ca_26977		bHLH	Protein kinase, catalytic domain
CaPOPII_3718	scaffold1521	15206	T/TTTGAAATAAATTAT	GGGGTTAGGGCTCATACGAT	GTTGCAGCGTGTTGTTCTGT	60.2	821	INTERGENIC				
CaPOPII_3719	scaffold1521	15603	GAA/GAAA	TCGTGGTTCAATAATTGGCA	CAAAATAACGATTCGGGCTC	59.9	228	INTERGENIC				
CaPOPII_3720	scaffold1521	15896	TAAAAAAA/TAAAAAAAAA	TCGTGGTTCAATAATTGGCA	AAATTAGGCATACAGAAATGAAGGA	59.9	403	INTERGENIC				
CaPOPII_3721	scaffold1521	19418	CAAAA/CAAA	GAAGAATGGGAACAATGCGT	CAACGGGCAAAAATGAAACT	59.9	748	CDS (FRAME SHIFT)	Ca_26979		bHLH	Protein kinase, catalytic domain

INDEL marker IDs	Chromosomes /unanchored scaffolds	Physical positions (bp)	InDels (*Kabuli* reference genome-CDC Frontier/PI)	Forward primers (5'-3')	Reverse primers (5'-3')	Anneling temperature (0C)	Expected amplified product size (bp)	Structural annotation		Functional annotation		
								Sequence components of *kabuli* genome	*Kabuli* gene accession IDs	NCBI-KOG	TFs	NCBI-nr database
CaPOPII_3722	scaffold1521	21280	A/ACTTAAGGT	TTGAACCCGAAATTTTCCAC	TACGAGGGACGATGTTGTGA	59.8	264	DRR	Ca_26979		bHLH	Protein kinase, catalytic domain
CaPOPII_3723	scaffold153	81004	TT/TTGGTCAAT	ACTACCAATCGTCTGGTCCG	GGTCCTCGATTTCATCTGGA	60.0	399	INTERGENIC				
CaPOPII_3724	scaffold1530	10045	TAAAAA/TAAAA	TTCACAACACCACCATCGTT	CGTGGAATCGTATTCGTGTG	59.9	559	INTERGENIC				
CaPOPII_3725	scaffold1530	14211	GAATA/GA	TGATAGATGCAAAAAGGGGG	TTTAGCCATCAACTTTGGGG	59.9	606	INTERGENIC				
CaPOPII_3726	scaffold1530	16153	ACCCCC/ACCCCCC	TCTCACGGTTGTGAGGACTG	ACCGTTCTTGGTTTCTGACC	59.9	746	INTERGENIC				
CaPOPII_3727	scaffold1530	16923	GATATATATATATATATATA/GATATATATATATATATATATATATATA	CATTGTGAGTGGTGTGGACC	TAGCTTTCCACGATCTGGCT	59.8	299	INTERGENIC				
CaPOPII_3728	scaffold1530	17844	GATAATAATAATAATAA/GATAATAATAATAATAATAA	TGGAGAAGGGTTCCAAAGTG	CGTTTATCTAGGGCAACCGA	60.1	445	INTERGENIC				
CaPOPII_3729	scaffold1530	18676	AG/AGG	CAGTTGTTCACACCTTATGTTGC	CAAACAGATGAATGGCGATG	59.6	196	INTERGENIC				
CaPOPII_3730	scaffold1530	19506	GTTTTTTT/GTTTTTTTT	GATTCATGGAAGCAAGTTCGT	AGGGCCGATTTAACTTTGCT	59.2	527	INTERGENIC				
CaPOPII_3731	scaffold1530	20299	A/AG	AGCAAAGTTAAATCGGCCCT	CCCATATCCAAGAGTAAAACCAA	60.1	429	INTERGENIC				
CaPOPII_3732	scaffold1530	21107	TC/TCC	TCTAAAAGATTTTGAACCCCAAA	TCCAAGGGTGTTAGAAACGG	59.1	812	INTERGENIC				
CaPOPII_3733	scaffold1530	23128	AATATATATATATATATATA/AATATATATATATATATATATATATATATATATATA	TCTCCATTGCACACCCAATA	GGTGCTGAAATCGGAAAATC	59.9	189	INTERGENIC				
CaPOPII_3734	scaffold1530	24697	CTTTTTTTT/CTTTTTTTTTT	TGGAGAGTTCAGCACACGTC	CCTCCGGTACCATAAACCCT	60.0	249	INTERGENIC				
CaPOPII_3735	scaffold1530	25494	TAT/TATGTCAAAT	CTTTTTCCGTTTTCAGCCAC	TTTACCCCTCGGTAAAACCTC	59.7	607	INTERGENIC				
CaPOPII_3736	scaffold1530	25850	TTTTTTTATTTTTTATTT/TTTTTTTATTT	TCGATCTTGAGCGAATTTGA	CAATACCAATTTCACGAAAATTCA	59.5	482	INTERGENIC				
CaPOPII_3737	scaffold1530	26371	CTTTTTTTT/CTTTTTTTTT	CAAAATTTTCAAACAAAACCCA	AAAGCACCTTTACCAAAAGCA	58.9	454	INTERGENIC				
CaPOPII_3738	scaffold1530	26727	CT/CTT	TGCTTTTGGTAAAGGTGCTT	ATTCGCAAACCAAACTCGAT	57.6	742	INTERGENIC				

INDEL marker IDs	Chromosomes /unanchored scaffolds	Physical positions (bp)	InDels (*Kabuli* reference genome-CDC Frontier/PI)	Forward primers (5'-3')	Reverse primers (5'-3')	Anneling temperature (0C)	Expected amplified product size (bp)	Structural annotation		Functional annotation		
								Sequence components of *kabuli* genome	*Kabuli* gene accession IDs	NCBI-KOG	TFs	NCBI-nr database
CaPOPII_3739	scaffold1530	26900	AT/A	CCCATCATAACCTACAAATTCCA	ATTCGCAAACCAAACTCGAT	60.0	360	INTERGENIC				
CaPOPII_3740	scaffold1530	27041	AATAT/AATATAT	CGTTTTCGACATCAAGATGGTA	TCGCAAACCAAACTCGATAA	60.0	134	INTERGENIC				
CaPOPII_3741	scaffold1530	27186	CTTATTTAT/CTTAT	TCGAGTTTGGTTTGCGAATA	CGATTGCTCATAAAATCGCTC	59.3	538	INTERGENIC				
CaPOPII_3742	scaffold1530	27232	ATTTTTTT/ATTTTTTTT	TCGAGTTTGGTTTGCGAATA	CGATTGCTCATAAAATCGCTC	59.3	538	INTERGENIC				
CaPOPII_3743	scaffold1530	27815	ATTTTTTTT/ATTTTTTTTT	TTTGAGAGGGCCTTTGGTAA	TCGACAGGTTTGTTTGTTCG	59.7	405	INTERGENIC				
CaPOPII_3744	scaffold1530	27996	CCTATGGTG/C	TTTGAGAGGGCCTTTGGTAA	TCGACAGGTTTGTTTGTTCG	59.7	405	INTERGENIC				
CaPOPII_3745	scaffold1530	28367	CAATTAA/CAA	GGCCCTATGGTGTTATGGTG	TTCGAAGCCATTATGTTTGATG	60.1	817	INTERGENIC				
CaPOPII_3746	scaffold1531	24929	AT/ATTCT	TCAGGATTTTGGGTTTAGGG	TTTTGTAGTTGTACTCAATAACAAGGA	58.9	482	INTERGENIC				
CaPOPII_3747	scaffold1531	29443	AG/AGGG	TCCAGATGAAATCGAGGACC	GATTTTCGTTCCACCCTTCA	60.0	621	INTERGENIC				
CaPOPII_3748	scaffold1532	68652	CTTTTTTTT/CTTTTTTTTT	CATTGCACTGCATGCTCTTT	TCTTTGCTCACAACACAGCC	60.0	649	INTERGENIC				
CaPOPII_3749	scaffold1544	14620	CTTT/CTT	CCACTGGATGAATCTCGGTT	TAGAAGGAACGGAGGGGTTT	59.9	794	INTERGENIC				
CaPOPII_3750	scaffold1545	81448	GCC/GC	TTGCATGCGATTTATGCAAT	TCGTGATCTGCTGAGAGTGC	60.1	814	INTERGENIC				
CaPOPII_3751	scaffold1545	178426	CATATATATATATATATA/CATATATATATATATATATATA	ACGGCAAGACCCTAAAACCT	ACCAGCATGATGACCAGACA	60.0	763	INTERGENIC				
CaPOPII_3752	scaffold1548	887	TTT/TTTATACATT	CGTTTTCTTCTCCATTTGGG	CAGCCATAACCAAGCCATTT	59.5	600	CDS (FRAME SHIFT)	Ca_27706	QI		
CaPOPII_3753	scaffold1548	11049	GTTT/GTT	CAACTTTGTCCCCAATGCTT	CTGGTGCTAGTTTTGCCACA	60.0	454	INTERGENIC				
CaPOPII_3754	scaffold1550	16901	CA/C	GATGAAATCGAGGACCAGGA	TTTCATCTGGACTTTTCGGG	60.0	429	INTERGENIC				
CaPOPII_3755	scaffold1550	56017	TAAAAA/TAAA	AGGCGATTTTCGTACCACAC	TCCAGATGAAATCGAGGACC	60.0	322	INTERGENIC				

INDEL marker IDs	Chromosomes /unanchored scaffolds	Physical positions (bp)	InDels (*Kabuli* reference genome-CDC Frontier/PI)	Forward primers (5'-3')	Reverse primers (5'-3')	Anneling temperature (0C)	Expected amplified product size (bp)	Structural annotation		Functional annotation		
								Sequence components of *kabuli* genome	*Kabuli* gene accession IDs	NCBI-KOG	TFs	NCBI-nr database
CaPOPII_3756	scaffold1562	44885	GTTTTTTTT/GTTTTTTTTT	TTTTGCGCTTGACTGATGTT	TCCAACTCCCTTCCCTTTTT	59.5	566	INTERGENIC				
CaPOPII_3757	scaffold1566	2139	G/GGATCCAAT	CCGATTGGATTGTCAGGACT	CCAAGGAAGGACGAAAATGA	59.9	439	INTERGENIC				
CaPOPII_3758	scaffold1569	32526	ACC/ACCC	CAGGGCCTTAAACCATGAAA	GGTTTGGGGTTTGGGATTTA	59.9	601	INTERGENIC				
CaPOPII_3759	scaffold1569	32547	GCCC/GCC	CAGGGCCTTAAACCATGAAA	GGTTTGGGGTTTGGGATTTA	59.9	601	INTERGENIC				
CaPOPII_3760	scaffold1569	32980	TAA/TA	ATAAACCGAACCGCAAAACA	GGTTTTATGGTTTAACGGCCT	60.4	714	INTERGENIC				
CaPOPII_3761	scaffold157	139214	ATTTTTT/ATTTTTTT	ATGTTTTCATGACGCGAGTG	GCTCCATTTTTGTTTTTGGC	59.7	675	INTERGENIC				
CaPOPII_3762	scaffold157	255926	TA/T	TTTGGTCTTGCCTTTGATGA	TGCGGTTAAGGTTGGTTCTC	59.2	888	INTERGENIC				
CaPOPII_3763	scaffold157	289273	T/TATC	GCACATTCAGTGCCAAGAGA	ATTGAGGGCATTGTCACTCC	60.0	492	INTERGENIC				
CaPOPII_3764	scaffold157	352152	CTTTTTTT/CTTTTTTTTT	CGATGAGGAAGAATGACGGT	TGCGTTCCATTTCCATATCA	60.1	986	INTRON	Ca_21079		SBP	DEP domain
CaPOPII_3765	scaffold157	359689	CTATCTACATA/CTATCTACATATATCTACATA	AACAAATCAAACAGGCCAGG	TCTTGTTTTCCTGTATGTGTCCC	60.0	620	DRR	Ca_21079		SBP	DEP domain
CaPOPII_3766	scaffold1575	195052	CATATATATATATATA/CATATATATATATATATA	CTTGGTCTAACCCTCACCCA	CCTGCATGCTTGGTTACTGA	60.0	564	INTERGENIC				
CaPOPII_3767	scaffold1578	76801	ATCTCT/ATCTCTCT	CTGATAGTGACACCGGGTTG	CTTGTTTTCTCCATGGTCGC	59.0	664	INTERGENIC				
CaPOPII_3768	scaffold1579	7520	GTTTTT/GTTTT	GGTTCAGCTCATTGCCAAAT	TCCAGCTTCCTTGAAGCCTA	60.1	711	INTERGENIC				
CaPOPII_3769	scaffold1579	64472	A/AT	GAGCGAATTGAGAAACGAGG	TTTTCAAATAAAGTAACATTGACAACA	60.0	927	INTERGENIC				
CaPOPII_3770	scaffold158	131634	T/TAAC	GTGACTGGTGACTGGGGACT	TTATGCCTGCTGTTCTGCAC	60.0	227	INTRAGENIC				
CaPOPII_3771	scaffold1580	69955	ATTTTT/ATTTT	ATCACCAGCCACTTAGGCAA	TCCCTTATGACAATTCATGGTG	60.7	639	INTERGENIC				
CaPOPII_3772	scaffold1580	251123	CNNNNNNNNNN/C	TGTTCTCTTAGGGGGTGGTG	GGCCATCTTTTAAACCCCTT	60.0	569	CDS (FRAME SHIFT)	Ca_20799	B		DEK, C-terminal

INDEL marker IDs	Chromosomes /unanchored scaffolds	Physical positions (bp)	InDels (*Kabuli* reference genome-CDC Frontier/PI)	Forward primers (5'-3')	Reverse primers (5'-3')	Anneling temperature (0C)	Expected amplified product size (bp)	Structural annotation		Functional annotation		
								Sequence components of *kabuli* genome	*Kabuli* gene accession IDs	NCBI-KOG	TFs	NCBI-nr database
CaPOPII_3773	scaffold1580	472188	ATTT/ATTTT	TGATGCAAGAAGCCACATCT	CTTGAAAGCCATTGGAGAGC	59.4	677	INTERGENIC				
CaPOPII_3774	scaffold1580	472375	CATA/CATATA	TTTTGGTCAGGGGAAGGTTT	CTTGAAAGCCATTGGAGAGC	60.7	578	INTERGENIC				
CaPOPII_3775	scaffold1580	472426	CT/CTT	TTTTGGTCAGGGGAAGGTTT	CTTGAAAGCCATTGGAGAGC	60.7	578	INTERGENIC				
CaPOPII_3776	scaffold1585	62915	CATATATATATATATATATATATATAT/CATATATATATATATATATATATATATAT	AGGAAGAGTGGAGTTGCGAA	GTTTTGAATTTTGGCTCGGA	60.0	522	INTERGENIC				
CaPOPII_3777	scaffold1586	1838	AC/ACC	AGGGAGGACGAAAATGACCT	TGACCAATTTTGAGCATCCA	59.9	471	INTERGENIC				
CaPOPII_3778	scaffold1607	123790	TAAAAAAAAAA/TAAAAAAAAA	AGCTGACAATTTGTGTGGCTT	ATAAACGTCATGCACGGGAC	59.8	135	INTERGENIC				
CaPOPII_3779	scaffold1613	98434	A/AG	AAGAAAGTTGCTCCTTGCCA	AGGAGCACTTACCTCCGTTG	60.0	133	INTERGENIC				
CaPOPII_3780	scaffold1613	106669	C/CGATATCATAGCCAAG	ACAATTTACGCCACCGAAAG	AGAAGCGACCCCATAGGTTT	60.0	549	INTERGENIC				
CaPOPII_3781	scaffold1614	14076	TC/TCC	TTTTGGTCCCCTCTAGGCTT	AGGGAGGACGAAAATGACCT	60.1	690	INTERGENIC				
CaPOPII_3782	scaffold1614	131511	AATATATATATAT/AATATATATATATAT	CCGCGAGTAATTCCGATAAA	ATCAGCCTAAGGGGGAAGAA	60.1	875	INTERGENIC				
CaPOPII_3783	scaffold1614	164436	CTCTGAT/CT	AAAACCGCCAAAGAAATGTG	ATGACATGTGTGAGCCGTGT	60.0	161	INTERGENIC				
CaPOPII_3784	scaffold1615	725	ATT/ATTT	GATGAAATCGAGGACCAGGA	TTTCATCTGGACTTTTCGGG	60.0	334	INTERGENIC				
CaPOPII_3785	scaffold1630	6306	T/TA	GTACCACCCGACAAAAATGG	TCCAGATGAAATCGAGGACC	60.1	530	INTERGENIC				
CaPOPII_3786	scaffold1631	32889	TGGTTTAAAAA/T	GTCCACTTCCGTTTCGGTAA	GTCGCTTCAAAAGTTGGCTC	60.0	625	INTERGENIC				
CaPOPII_3787	scaffold1633	15743	AAATAATAATAATAATAATAATAATAATAATAAT/AAATAATAATAATAATAATAATAATAATAATAAT	AACGTTTTCGTCGAATGTCC	TCAATTTTGGATGCACCTGA	60.0	669	DRR	Ca_26202	UR		Pleckstrin homology domain
CaPOPII_3788	scaffold1635	74246	TCCC/TCCCC	GGTAAACGTGTGTTGCATGG	GGAGTACCATAGGCCAACGA	59.9	258	INTERGENIC				
CaPOPII_3789	scaffold1643	4999	CAAAAAAA/CAAAAAA	CTCCCAGCCTCTTGTACTGG	AGGCAAGCCATTGAAAGAAA	59.9	449	INTERGENIC				

INDEL marker IDs	Chromosomes /unanchored scaffolds	Physical positions (bp)	InDels (*Kabuli* reference genome-CDC Frontier/PI)	Forward primers (5'-3')	Reverse primers (5'-3')	Anneling temperature (0C)	Expected amplified product size (bp)	Structural annotation		Functional annotation		
								Sequence components of *kabuli* genome	*Kabuli* gene accession IDs	NCBI-KOG	TFs	NCBI-nr database
CaPOPII_3790	scaffold1643	156175	CTT/CTTT	ATTGTCCAACACCTCCAAGC	CTCCCCTTTCCTTCGTTTTC	60.0	386	INTERGENIC				
CaPOPII_3791	scaffold1643	163768	AATATATATATATATATATATATATATA/AATATATATATATATATATATATATATATATATATATA	ATTTCCAGCATGCCAATTTC	CTTCCAGAAACTAGCCGCAC	59.9	719	INTERGENIC				
CaPOPII_3792	scaffold1643	166873	CAAAAAAAA/CAAAAAAA	GCAACTAGGGGTGTTCAGGA	ATGCACACTTGTTGCACCTT	60.1	610	INTERGENIC				
CaPOPII_3793	scaffold1643	169454	CAAAAAAAAAAA/CAAAAAAAAA	GTCATTTTTGGCGTAGTGGG	TTTCCGGTCAACACTCATCA	60.4	133	INTERGENIC				
CaPOPII_3794	scaffold1643	171022	TTTA/TTTAATTA	TAACCCCATCAAATTTGGGA	TCTTGAAGGAGCGGGTAGAA	60.0	563	INTERGENIC				
CaPOPII_3795	scaffold1643	180974	ATGATGAATCTTGATGAATCTTGATGAAT/ATGATGAATCTTGATGAAT	CCCACGCTTCTTTAGCAGTC	TTTCACTTTCCCGCTTCTGT	60.0	859	INTERGENIC				
CaPOPII_3796	scaffold1643	192850	TATAAATA/TATA	GAGGGGGAGAAAGCTGAGAG	TGCTGACATGATTGTTGATGTG	60.5	535	INTERGENIC				
CaPOPII_3797	scaffold1643	210380	CTTT/CTTTT	AATACAGCGAAAAAGCTCGC	TCGAAAAGAAATCGAGACCG	59.6	710	INTERGENIC				
CaPOPII_3798	scaffold1643	210540	ATTTTTTTT/ATTTTTTTTTT	CACACAATCACAAATTTTTCTCAA	AAAGAAATCGAGACCGTTGC	59.1	419	INTERGENIC				
CaPOPII_3799	scaffold1643	211018	TGTA/T	TTCTCTCCACCGCTAACTCG	ATGGTGGGGGTATGAATGAA	60.5	611	INTERGENIC				
CaPOPII_3800	scaffold1643	211096	TTCT/TT	ACGGTCTCGATTTCTTTTCG	ATGGTGGGGGTATGAATGAA	59.3	585	INTERGENIC				
CaPOPII_3801	scaffold1643	213932	TAAAAAAAA/TATAAAAAAAAA	CGGATTTCGTCTCTTGAAGC	CATTGGTGCAGTTGTTCGTC	60.0	662	INTERGENIC				
CaPOPII_3802	scaffold1651	5134	GAAAAAAA/GAAAAAAAAAAA	ATGGCACAAGTCTCCCAACT	GGGGCCACTTAGGGTTGTAT	59.6	276	INTERGENIC				
CaPOPII_3803	scaffold1651	12361	GTTTTTTT/GTTTTTTTT	AAATTGTTGCTTGGGGACAA	TGACGTCATGACCTGCATTT	60.3	882	INTERGENIC				
CaPOPII_3804	scaffold1651	13370	TATTAATTAATTAATT/TATTAATTAATTAATTAATT	CCTTGAGTTCGACACTCGGT	TGGTGACCGTGTAATTGTTG	60.3	337	INTERGENIC				
CaPOPII_3805	scaffold1651	13586	CAAAAAAA/CAAAAAAAA	CCTTGAGTTCGACACTCGGT	CAGGATGTGAACCGTAAATCTTC	60.3	547	INTERGENIC				
CaPOPII_3806	scaffold1651	14250	ATATTTTAAGGTATTTTAAGGTAT/ATATTTTAAGGTAT	AAAAATGGAGAGATCGAAAGTG	TTGATGCTAGGGGGATTTTG	57.5	553	INTERGENIC				

INDEL marker IDs	Chromosomes /unanchored scaffolds	Physical positions (bp)	InDels (*Kabuli* reference genome-CDC Frontier/PI)	Forward primers (5'-3')	Reverse primers (5'-3')	Anneling temperature (0C)	Expected amplified product size (bp)	Structural annotation		Functional annotation		
								Sequence components of *kabuli* genome	*Kabuli* gene accession IDs	NCBI-KOG	TFs	NCBI-nr database
CaPOPII_3807	scaffold1651	16483	AGG/AG	GGGAAGGGATGTAAAAGGGA	TTTCGTCGTGTTTTCCTGTG	60.1	135	INTERGENIC				
CaPOPII_3808	scaffold1651	16500	CA/CAAA	GGGAAGGGATGTAAAAGGGA	ACGTCGTGATTTGTGCTTCA	60.1	231	INTERGENIC				
CaPOPII_3809	scaffold1651	16843	GACC/GACCACC	GGGAAGGGATGTAAAAGGGA	AAAATCTGGCCTGGACCTG	60.1	510	INTERGENIC				
CaPOPII_3810	scaffold1651	16897	TTA/T	TAAGGCATGGTATGGGGAAA	CTAAGCCTGAGTTTGGCCTG	60.1	921	INTERGENIC				
CaPOPII_3811	scaffold1651	17729	GATAT/GATATAT	TCTCTTGGGTGATTTTTCGG	ACACCCAAACTCTTGTTGGC	60.0	561	INTERGENIC				
CaPOPII_3812	scaffold1651	18006	A/AG	TGATTGGGGGCATACATTTT	ACACCCAAACTCTTGTTGGC	60.0	171	INTERGENIC				
CaPOPII_3813	scaffold1651	18574	CA/C	CTGGATTGGAATTGTCAGCA	TCACGCACACTCCATCTCTC	59.6	679	INTERGENIC				
CaPOPII_3814	scaffold1651	18823	AG/A	GAGAGATGGAGTGTGCGTGA	AACCCAGAACTCGAGCTGAA	60.0	461	INTERGENIC				
CaPOPII_3815	scaffold1659	19972	CAAAAAAAAAAAAA/CAAAAAAAAAAAAAAA	TTGGGGTTGCTAATGGGATA	TTTGGAGAACTTTCGGGTTG	60.1	289	INTERGENIC				
CaPOPII_3816	scaffold1659	96268	GTTT/GTTTT	TTGCTCACTACCACGCTACG	CACCAAAGCACATCCATTCA	60.1	382	INTERGENIC				
CaPOPII_3817	scaffold1659	269627	AATATATATATATATATATAT/AATATATATATATATATATATAT	CACCGTAGTGTGGTGGTGAG	CGGTGAATCCTTTACGGAGA	60.1	531	INTERGENIC				
CaPOPII_3818	scaffold166	24696	AATATATATATATATATATATATATATA/AATATATATATATATATATATATATATA	GCATTCTACCGTCAAAATGAAA	TTTTCTGTTGCAAACTTGTCTGA	59.1	696	INTERGENIC				
CaPOPII_3819	scaffold166	76470	AATATATATATATATATATATATATATATAT/AATATATATATATATATATATATATATATAT	ACACACGTGCTTCTTGGAAA	GCGATTGCTTTTACCATTTCA	59.3	582	INTERGENIC				
CaPOPII_3820	scaffold166	227311	TTATATATATATATATATATATATATATA/TTATATATATATATATATATATATATATATATA	CATCTCGTCGGTCATCATTG	TGATTCATGTAACACCCCAAA	60.1	551	INTERGENIC				
CaPOPII_3821	scaffold1661	91058	AATATATATATATATAT/AATATATATATATATAT	GTTGGATCCCTAGCACCGTA	AAAAATGAAAAGGGGAGCGT	60.0	905	INTERGENIC				
CaPOPII_3822	scaffold167	29049	CTTT/CTT	TTACCGGCTATTCATGCCTC	TCCCTTAATTTTCCTGCCCT	60.1	508	INTERGENIC				
CaPOPII_3823	scaffold167	66850	TAAAAAAA/TAAAAAAAAA	AAGAGAGGTCACTTTGGCGA	CAATTCCCACAGGTACGCTT	60.0	587	INTERGENIC				

**INDEL marker IDs**	**Chromosomes /unanchored scaffolds**	**Physical positions (bp)**	**InDels (*Kabuli* reference genome-CDC Frontier/PI)**	**Forward primers (5'-3')**	**Reverse primers (5'-3')**	**Anneling temperature (0C)**	**Expected amplified product size (bp)**	**Structural annotation**		**Functional annotation**		
								**Sequence components of *kabuli* genome**	***Kabuli* gene accession IDs**	**NCBI-KOG**	**TFs**	**NCBI-nr database**
CaPOPII_3824	scaffold167	219311	AC/ACTGC	AGGAAACACATGAAGGCACC	TCGGTATTTGCCATGAGACA	60.0	484	INTERGENIC				
CaPOPII_3825	scaffold167	288909	A/AGT	TCATCCTCCCCAATTTCAAC	CATTGTTCATGCATTTTGGC	59.7	705	INTRON	Ca_24156	C		Oxidoreductase, molybdopterin-binding domain
CaPOPII_3826	scaffold1679	2481	CGGGTTTAGGGTTTAGGGTTTAGGGTT/CGGGTTTAGGGTTTAGGGTT	TTACGGTTTTGCGTTTAGGG	AACCAAAATCCGACACCCTT	60.0	503	INTERGENIC				
CaPOPII_3827	scaffold1681	934	ATTTT/ATTTTT	GAGATTTTCGTACCACCCGA	TCCAGATGAAATCGAGGACC	59.9	797	INTERGENIC				
CaPOPII_3828	scaffold1682	8324	TTGTGT/TTGT	AGGTCGTTTTCGTCCTTCCT	CACCCAATGACACCAATGAA	60.1	693	INTERGENIC				
CaPOPII_3829	scaffold1684	6435	TA/TAA	TATCGCGGGTCTTGTTTTTC	ACCAAATCGATTGGCACTTC	60.1	330	INTERGENIC				
CaPOPII_3830	scaffold1684	69381	TAA/TA	CGGGTTGTACAAAAATCGCT	CAGCGATGGCTAGTGTTTGA	60.0	458	INTERGENIC				
CaPOPII_3831	scaffold1685	2214	ATATTATTATTATTATTATTATTATTATTATTATTATTATTATTATTATTAT/ATATTATTATTATTATTATTATTATTATTATTATTATTATTATTAT	TTATTTCACCGTCGTGTCCA	AGGTGTGTTTTGGGTTCTGC	60.0	526	INTERGENIC				
CaPOPII_3832	scaffold1685	110905	TTATAT/TTAT	AAAATTGGGTTGCCAAATAA	AAATGGACCGAAATCGAGTG	57.1	649	INTERGENIC				
CaPOPII_3833	scaffold1687	165308	GTTATTTATTTATTTATTTATTTA/GTTATTTATTTATTTATTTA	ACAAAATACCCGCATTGAGC	ATTTGAACTCACGACACCCC	60.0	484	INTERGENIC				
CaPOPII_3834	scaffold1696	4415	ATTTTTTTTTT/ATTTTTTTTT	ATAACCCGAACCTCAAACCC	TGTTTGAAATATGACGCCGA	60.1	645	INTERGENIC				
CaPOPII_3835	scaffold1696	5390	TGTTTCGGGTTTCGGGTTTCG/TGTTTCGGGTTTCG	TTGAAGTACGAAAATCGCCC	TTCGTTATGCGGTCAAACCT	60.1	813	INTERGENIC				
CaPOPII_3836	scaffold17	297467	AG/AGG	ACACCTTCAGCGGCATACTT	TGAGGCATGAACCTCAACTCT	59.8	852	INTERGENIC				
CaPOPII_3837	scaffold1701	101096	TT/TTCT	AGGTCATTTTCGTCCTCCCT	GCCCTCAGATGAGAGTCAGG	59.9	515	INTERGENIC				
CaPOPII_3838	scaffold1703	131019	TGAAGAAGAAGAAGAAGAAGAAG/TGAAGAAGAAGAAGAAGAAGAAGAAG	CCAAAACGATTTCCCTTCAA	TTTCCAAAAGTGACCGAACC	59.9	234	INTERGENIC				
CaPOPII_3839	scaffold1704	9435	GAAAAAAA/GAAAAAAAA	TTTTTGTGTGCTTGACTGATGTT	GAAATTTGGACGAAAACGGA	59.7	715	INTERGENIC				
CaPOPII_3840	scaffold1704	11771	AATATA/AATATATA	AACGATCAAGAGTCGCAAAA	ACCCTAAACCCTAAAGCCCA	58.5	347	INTERGENIC				

INDEL marker IDs	Chromosomes /unanchored scaffolds	Physical positions (bp)	InDels (*Kabuli* reference genome-CDC Frontier/PI)	Forward primers (5'-3')	Reverse primers (5'-3')	Anneling temperature (0C)	Expected amplified product size (bp)	Structural annotation		Functional annotation		
								Sequence components of *kabuli* genome	*Kabuli* gene accession IDs	NCBI-KOG	TFs	NCBI-nr database
CaPOPII_3841	scaffold1706	101986	GAAAAAAAAAA/GAAAAAAAAAAAA	TGTTAGGAGGATCTCACGGG	AACAAGATTCAACGGGCAAG	60.1	631	INTERGENIC				
CaPOPII_3842	scaffold1708	14932	GTTT/GT	GCCATTTGGGTGGTTGATAG	CTGCTTGAGGACAAGCAACA	60.2	901	INTERGENIC				
CaPOPII_3843	scaffold1708	68435	TCC/TCCC	GAAAAAGCGCTGTAAATGGC	CATTCGAGACGTGAGCAAAA	59.9	510	DRR	Ca_27642			RNA polymerase Rpb1, domain 5
CaPOPII_3844	scaffold1710	153888	TAAAAAAAAAA/TAAAAAAAAA	CAAAATCTTCATGTTGACCCC	GCACATGATATGCACTCCTAAA	59.3	401	INTERGENIC				
CaPOPII_3845	scaffold1722	124015	TACAC/TAATATAACAC	CGTCCTCGAAACTAAGAGGAAA	TGGAAGATGTGACGATCCTG	59.9	871	INTERGENIC				
CaPOPII_3846	scaffold1723	1064	ACTCTC/ACTC	CCTCATAGGTTCGACTGGGA	GCGCCCTCACATAATGTTTT	60.1	467	INTERGENIC				
CaPOPII_3847	scaffold1725	28680	TTT/TTTATT	GATTCAGTGGCCGATTTTGT	TTGGACGAAAAAGGAGGAAA	59.9	917	INTERGENIC				
CaPOPII_3848	scaffold1731	39857	AATATATATATATATATATAT/AATATATATATATATATAT	ATGTTTTGGATTTTGGCTCG	GCACCGTGAGTAAATTCGGT	59.9	704	INTERGENIC				
CaPOPII_3849	scaffold1731	49034	CATATATATATATATATATA/CATATATATATATATATA	ATGGGAAGCAACAACGATTC	CCGTACCGTTTCTGGGATAA	59.9	784	DRR	Ca_27041	IR		ABC transporter-like
CaPOPII_3850	scaffold1731	53952	AATATATATATATATAT/AATATATATATATATATAT	TTTGGATATTGGTTCGGAGG	AGGAGGTTTGTGTTTGACGG	59.8	514	INTERGENIC				
CaPOPII_3851	scaffold1731	65474	TTTATTATTATTATTATTAT/TTTATTATTATTATTATTAT	TAATCGGGGACTCAACATCC	CCCTTTGGATGCGAAATAAA	59.8	279	INTERGENIC				
CaPOPII_3852	scaffold1731	85329	CATATATATATATATATATA/CATATATATATATATATATA	TCTCCGCATAAGCTTTCGAT	GCTTCGACACTGTAGCACCTC	59.9	478	DRR	Ca_27043			
CaPOPII_3853	scaffold1731	86422	AATATATATATATATATATATAT/AATATATATATATATATATAT	TTTTGGGAATTCCTGCTCTG	GTGACACCCTAAACCCCAAA	60.2	299	DRR	Ca_27043			
CaPOPII_3854	scaffold174	8927	TAAAAAAAAAAAA/TAAAAAAAAAAAAAA	TTACCACTTCCAGACCCGAC	TTGGTTCCAACAATCTCGCT	60.0	609	INTERGENIC				
CaPOPII_3855	scaffold174	11363	CAAAAAAAAA/CAAAAAAAA	TTATGTCGTTCGTTAGGGGC	TGTGTGCAATAGGGATGGAA	60.0	744	INTERGENIC				
CaPOPII_3856	scaffold174	103191	ATTTTTTT/ATTTTTTTT	ACGCTTGTTAAAGGCAGAGG	GAAAATTTGCGAGAATCCCA	59.5	459	INTERGENIC				
CaPOPII_3857	scaffold174	143696	ATTTTTTTTTTTT/ATTTTTTTTTT	CCCCTTTTTGAAAGGCTTAAA	CAAACGGGTTAGCCAGAAAA	59.6	925	INTERGENIC				

INDEL marker IDs	Chromosomes /unanchored scaffolds	Physical positions (bp)	InDels (*Kabuli* reference genome-CDC Frontier/PI)	Forward primers (5'-3')	Reverse primers (5'-3')	Anneling temperature (0C)	Expected amplified product size (bp)	Structural annotation		Functional annotation		
								Sequence components of *kabuli* genome	*Kabuli* gene accession IDs	NCBI-KOG	TFs	NCBI-nr database
CaPOPII_3858	scaffold174	189673	GTTTTTTTTTTT/GTTTTTTTTTT	AACCCCATAAGCAGCATGAC	AAAGAAAAATGCGATCGGTG	60.0	615	INTRON	Ca_21713	R	C2H2	Zinc finger, C2H2-type
CaPOPII_3859	scaffold174	350080	TGCCTCGCCTCGCCTCG/TGCCTCGCCTCG	GTGTTGTTGATCCCTTGCCT	GCCATCATTCTAAAGCAGCC	60.0	576	INTERGENIC				
CaPOPII_3860	scaffold174	422115	TAAAAAAAAAAA/TAAAAAAAAAA	GGGCAAATCATATCCTCCAA	TGCATGCCACATTGTAAACC	59.7	638	INTERGENIC				
CaPOPII_3861	scaffold174	424440	CAACAAAAACAAAAACAAAAACAAAAACAAA/CAACAAAAACAAAAACAAAAACAAA	GGAGCTTTGATTCCCATGAA	GATGCATTTGGGAAAGATGG	60.0	560	INTERGENIC				
CaPOPII_3862	scaffold174	434745	CAA/CAAA	TTTTGCCATTCCAAGTTTCC	TTGATCTCGTGAAGTTTGCG	59.9	350	INTRON	Ca_21723	A	C3H	
CaPOPII_3863	scaffold174	450819	AT/A	AAGATCACCGTGCAAAAAGG	GCCTCCACGACCACTACAAT	60.1	433	INTRON	Ca_21723	A	C3H	
CaPOPII_3864	scaffold1740	2698	TAA/TA	CGATTTGGCCATACAGGAAT	CCCATCTACATACCCATCCG	59.8	498	INTERGENIC				
CaPOPII_3865	scaffold1756	5242	CGGTA/C	GGTGAGGTCTTTTGCCTCAA	TTGGATGAAGGCCATAAAGG	60.2	665	INTERGENIC				
CaPOPII_3866	scaffold1756	68155	CTTTTTTTTTT/CTTTTTTTTT	TCGGAATAATTAAGGGCAAGA	TTTGCAATGTGTCTATGCGA	58.7	791	INTERGENIC				
CaPOPII_3867	scaffold1757	1167	CTT/CTTATT	AAGCGTACCTGTGGGAATTG	TTAATGAGGGGCTGAATTGG	60.0	259	INTERGENIC				
CaPOPII_3868	scaffold1773	13836	TT/TTAATCGAGT	AATCCAATGACGCAAACACA	GCCCAATTCGGGTTTTTAAT	60.0	655	INTERGENIC				
CaPOPII_3869	scaffold1776	23561	CTTT/CTT	TCACAACTCAATTTTGCTTTATGTT	TTCGTTTGGTAGTTGTATTTAATAACG	59.2	519	INTERGENIC				
CaPOPII_3870	scaffold1777	69156	CAAAAAAAAAA/CAAAAAAAAA	TTGCCACTATTACGCATGGA	TGGAAAATGGGTGTGTAGCA	60.1	645	INTERGENIC				
CaPOPII_3871	scaffold1777	402796	CTTTTTTTTTT/CTTTTTTTTTTT	GGGTCCAAAGCCCATAAAGT	TATGGATGGTGAGCGTCAAA	60.2	594	INTERGENIC				
CaPOPII_3872	scaffold1777	521551	ATTTT/ATTT	CTTTGTTTTTCACCGACCGT	ACGCCTGACGGTTATTGTTC	60.0	497	INTERGENIC				
CaPOPII_3873	scaffold1778	1269	C/CG	ACTTCCGCGCTAGATTTCAA	TCCTGGTCCTCGATTTCATC	60.0	282	INTERGENIC				
CaPOPII_3874	scaffold1779	131414	CAAAAAAAAAA/CAAAAAAAAAAAA	TTGTTTCTCCCCAGTTTTGG	TTCCATATCACACGGAACCA	59.9	275	INTERGENIC				

INDEL marker IDs	Chromosomes /unanchored scaffolds	Physical positions (bp)	InDels (*Kabuli* reference genome-CDC Frontier/PI)	Forward primers (5'-3')	Reverse primers (5'-3')	Anneling temperature (0C)	Expected amplified product size (bp)	Structural annotation		Functional annotation		
								Sequence components of *kabuli* genome	*Kabuli* gene accession IDs	NCBI-KOG	TFs	NCBI-nr database
CaPOPII_3875	scaffold1779	131497	ATTTTTTTT/ATTTTTTT	TTGTTTCTCCCCAGTTTTGG	TTCCATATCACACGGAACCA	59.9	275	INTERGENIC				
CaPOPII_3876	scaffold178	605	AACACAC/AACAC	CAATAGGCCAAGGAAGGACA	ACATTGAGGCCATTTTCGTC	60.1	236	INTERGENIC				
CaPOPII_3877	scaffold1795	5623	A/AGCC	CAAATGTCGGGAGCTACCAT	TCGTTTAATTTCGGGATTGG	60.0	456	INTERGENIC				
CaPOPII_3878	scaffold1804	57835	CTGATT/C	CCTATTCCTTTTCCCCCAAA	GCAGCTGACCGCGTAATAAT	60.1	496	INTERGENIC				
CaPOPII_3879	scaffold1804	176118	AATATATATATATATATATATATAT/AATATATATATATATATATATATATAT	AGCCCTGTTTGATTCCCTTT	TGAGCGTTCTAGTATCCACCTC	59.9	765	INTERGENIC				
CaPOPII_3880	scaffold1806	3130	CAAAAAA/CAAAAAAAA	TGGACTTCTCGAGCGATTTT	TCCAGATGAAATCGAGGACC	60.0	656	INTERGENIC				
CaPOPII_3881	scaffold1806	6835	CAAAAAA/CAAAAA	TTTCATCTGGACTTTTCGGG	GATGAAATCGAGGACTGGGA	60.0	584	INTERGENIC				
CaPOPII_3882	scaffold1810	32448	GTATTATTATTATTATTATTATTATTATTATTATTATTATT/GTATTATTATTATTATTATTATTATTATTATTATTATTATTATT	TTCGCTTGAATTACAAATGTTTT	TCAATGATTGTTGACAAGGGA	58.0	469	INTERGENIC				
CaPOPII_3883	scaffold1810	98576	GGTTGT/GGTTGTTGT	TGACGATACGAAACGATGGA	AGCGATGCATGTCTCAAGTG	60.1	430	URR	Ca_26004			
CaPOPII_3884	scaffold1810	103255	GC/GCC	TTGCTAGTTGCTGATGTGGC	GGTGTCATCGGGAGTCTGTT	60.0	300	INTERGENIC				
CaPOPII_3885	scaffold1810	103852	GAAAA/GAAAAA	AACAGACTCCCGATGACACC	AACATTTATGACGATATTTATGAGTGA	60.0	438	INTERGENIC				
CaPOPII_3886	scaffold1815	23915	AAAAATTGAAAAT/AAAAATTGAAAATTGAAAAT	CATCTGAGCTTCTTTGGACG	GCGGGTGCATTTTCATTTAT	58.6	506	INTERGENIC				
CaPOPII_3887	scaffold1816	53607	ATATATAATTTATATAATTTATATAATTT/ATATATAATTTATATAATTT	TGGTGAGGAAATGAGCAACA	ACAAAATGAAGGTGCGGAAG	60.2	679	INTERGENIC				
CaPOPII_3888	scaffold1822	17416	GTT/GTTT	AAGCACCAATTACCGAGGTG	TTGTATTTGGCGTGTCCGTA	60.0	720	INTERGENIC				
CaPOPII_3889	scaffold1825	15825	CATATATATATATATAT/CATATATATATATATATAT	GTTGGTTCCCTAGCACCGTA	CGGGATGGTGGCATAGTATC	60.0	790	INTERGENIC				
CaPOPII_3890	scaffold1825	26286	CAAAGAAAAAAGAAAAA/CAAAGAAAAA	TGGCAAATCGATTGACAGAA	AGTTTCCTCCCCGAAACCTA	60.2	778	INTERGENIC				
CaPOPII_3891	scaffold1830	4465	CTATA/CTA	CCAAGGGAAGACGAAAATGA	TGCATCCAACAGGTGTGTTT	60.0	665	INTERGENIC				

INDEL marker IDs	Chromosomes /unanchored scaffolds	Physical positions (bp)	InDels (*Kabuli* reference genome-CDC Frontier/PI)	Forward primers (5'-3')	Reverse primers (5'-3')	Anneling temperature (0C)	Expected amplified product size (bp)	Structural annotation		Functional annotation		
								Sequence components of *kabuli* genome	*Kabuli* gene accession IDs	NCBI-KOG	TFs	NCBI-nr database
CaPOPII_3892	scaffold1835	8124	TCAACAACA/TCAACA	TCATTGCTCCTTTGAGTTGC	ATTTGCCACAGATACCGCTC	59.0	453	INTERGENIC				
CaPOPII_3893	scaffold1835	8173	CAA/CA	TCATTGCTCCTTTGAGTTGC	ATTTGCCACAGATACCGCTC	59.0	453	INTERGENIC				
CaPOPII_3894	scaffold1835	8463	TAAAAAAAA/TAAAAAAAAA	GAGCGGTATCTGTGGCAAAT	CGGTTTGTGGCTTTCCTAAA	60.1	486	INTERGENIC				
CaPOPII_3895	scaffold1835	10086	GCC/GCCC	TGGTTAACCTTGCACCACAA	GGATGCTTGAATTATGCCGT	60.0	672	INTERGENIC				
CaPOPII_3896	scaffold1844	2008	CTTTTTTTTTTT/CTTTTTTTTTTTTTT	AACATGGGACGAGCTGTTTC	TTCAAAGAGAGATAAATTCAAAGGTC	60.1	903	INTERGENIC				
CaPOPII_3897	scaffold1844	2095	AAA/AAAATAA	AACATGGGACGAGCTGTTTC	GGGATATTCAATAAGTGAAATTCAAA	60.1	923	INTERGENIC				
CaPOPII_3898	scaffold1844	2214	T/TC	TGCAATTATGATCATGCTTTATATG	GGGATATTCAATAAGTGAAATTCAAA	58.2	732	INTERGENIC				
CaPOPII_3899	scaffold1844	2526	CA/CAA	GACCTTTGAATTTATCTCTCTTTGAA	TTACAACATGTCAGGCCGAA	58.5	373	INTERGENIC				
CaPOPII_3900	scaffold1844	166750	TTTATTATTATTATTATTA/TTTATTATTATTATTA	GGCTAGACGAGTTCGACGAC	CTAGATGGTCGCATTCTGGG	60.0	418	DRR	Ca_24145			
CaPOPII_3901	scaffold1844	167253	CAAAAAA/CAAAA	CCCCAATTTGCGATCAATAA	TCAAAACTAAGAAACTCGCCAA	60.6	694	DRR	Ca_24145			
CaPOPII_3902	scaffold1845	79664	ANNNNNNNNNN/ANNN	CCTTGATATGCAATGCTCACA	ATCGAGCCTCTTGATATGGC	59.7	336	INTERGENIC				
CaPOPII_3903	scaffold1852	94526	ATTTTTT/ATTTTT	GGTCCAACATCGACGCTAAT	TCGGATGTTACACACCTCACA	60.0	515	INTERGENIC				
CaPOPII_3904	scaffold1852	94771	GGTGTGT/GGTGT	GGTCCAACATCGACGCTAAT	TTTCAGTCATTTGATTTCGGA	60.0	531	INTERGENIC				
CaPOPII_3905	scaffold1852	94935	ATTGTT/ATT	TACCATGGAGTGTGCTCGAA	TTTCAGAACACAAATCTGCATGT	60.3	765	INTERGENIC				
CaPOPII_3906	scaffold1852	102952	CAT/CATAT	GGTCAGGGCCTCTAAGGTCT	TTGACACTCATTGCTCCTCG	59.7	847	INTERGENIC				
CaPOPII_3907	scaffold1852	103507	ATTTTTTT/ATTTTTTTT	CGAGGAGCAATGAGTGTCAA	GTTAAATTCGGGGTCGAGGT	60.0	253	INTERGENIC				
CaPOPII_3908	scaffold186	252188	AATATATATATATATA/AATATATATATATATATA	TACTCCCAAATGGCGACTTC	CATTTCGAACTTGGGTTTGC	60.1	485	INTERGENIC				

INDEL marker IDs	Chromosomes /unanchored scaffolds	Physical positions (bp)	InDels (*Kabuli* reference genome-CDC Frontier/PI)	Forward primers (5'-3')	Reverse primers (5'-3')	Anneling temperature (0C)	Expected amplified product size (bp)	Structural annotation		Functional annotation		
								Sequence components of *kabuli* genome	*Kabuli* gene accession IDs	NCBI-KOG	TFs	NCBI-nr database
CaPOPII_3909	scaffold1864	2902	GT/GTAGTAAT	CGTGACAACCACCAACTCTTT	TCTGAAAGCCGTGAAAGACC	60.1	490	INTERGENIC				
CaPOPII_3910	scaffold1864	3660	TACCA/TA	ATGTTGTGAAGATTTGGGGG	TGCATTTGGGTCCCATAGA	59.6	369	INTERGENIC				
CaPOPII_3911	scaffold1864	4285	TCC/TC	ATGGGACCCAAATGCAATAA	ACGTAGCGTAGAAGTCCCCA	60.0	339	INTERGENIC				
CaPOPII_3912	scaffold1864	4508	ATCCTGTTTACT/AT	TTGGGGACTTCTACGCTACG	GCAACAGGGATGACTGACTG	60.3	713	INTERGENIC				
CaPOPII_3913	scaffold1864	4680	T/TC	TTGGGGACTTCTACGCTACG	TCGGTCAGAAGAAAACTGCC	60.3	883	INTERGENIC				
CaPOPII_3914	scaffold1864	5094	TAAA/TAAAA	CAGTCAGTCATCCCTGTTGC	TCGGTCAGAAGAAAACTGCC	59.3	190	INTERGENIC				
CaPOPII_3915	scaffold1864	5589	A/AT	TTCAGCGCTTAACGTGAGAA	TCGAGATGATGATGGATGGA	59.8	638	INTERGENIC				
CaPOPII_3916	scaffold1864	5884	CAAAAAAAA/CAAAAAAA	TTGCCCATTCTCCAAGTTTC	CGGATATAAGCATGGCATCA	60.1	659	INTERGENIC				
CaPOPII_3917	scaffold1864	5957	AATG/AATGATG	TTGCCCATTCTCCAAGTTTC	CGGATATAAGCATGGCATCA	60.1	659	INTERGENIC				
CaPOPII_3918	scaffold1864	5989	ATAGT/AT	TTGCCCATTCTCCAAGTTTC	CGGATATAAGCATGGCATCA	60.1	659	INTERGENIC				
CaPOPII_3919	scaffold1864	6249	TCC/TC	AGTGACATGTGCTGCCTTTG	AAATTGGGGTCTCAACGAAA	59.9	295	INTERGENIC				
CaPOPII_3920	scaffold1864	14836	TAAAAAAA/TAAAAAA	CGCTCTGTTAAGTCGGTTCA	CATCCACGGAGCAAAAACTT	59.1	251	INTERGENIC				
CaPOPII_3921	scaffold1864	14934	GTGTGATAAGATAT/GTGTGATAAGATATGTGATAAGATAT	AACTCGAACACGAAACCGAC	CATCCACGGAGCAAAAACTT	60.2	188	INTERGENIC				
CaPOPII_3922	scaffold1866	41886	CTTTTTTTTTT/CTTTTTTTTT	TGGAGTGGGGTACCTAGTGG	GGGACAGAAAGAAAGGGGAG	59.8	758	INTRON	Ca_26041	R		Uncharacterised protein family, ATP binding
CaPOPII_3923	scaffold1866	62792	AAATAAA/AAATAAATAGAATAAA	TTGCTCCTACGTATCTCCCG	TTAAGTTGGTTTGGGCTTGG	60.2	855	INTRON	Ca_26041	R		Uncharacterised protein family, ATP binding
CaPOPII_3924	scaffold1866	139647	TACACACACACACACACACACACACACA/TACACACACACACACACACACACACA	GCAAATGCTCCCCTAATTGA	CAATTTTCTGCACTCTCGCA	60.0	296	INTERGENIC				
CaPOPII_3925	scaffold1867	226694	ATTT/ATTTT	ATCGATTTTGTCCGTTTTGC	GGAGGGTTTTGGTTTGGAAT	59.9	473	INTERGENIC				

INDEL marker IDs	Chromosomes /unanchored scaffolds	Physical positions (bp)	InDels (*Kabuli* reference genome-CDC Frontier/PI)	Forward primers (5'-3')	Reverse primers (5'-3')	Anneling temperature (0C)	Expected amplified product size (bp)	Structural annotation		Functional annotation		
								Sequence components of *kabuli* genome	*Kabuli* gene accession IDs	NCBI-KOG	TFs	NCBI-nr database
CaPOPII_3926	scaffold1869	19531	GAAAA/GAAA	GACTTTTGGAGCGACTTTCG	AACAATGTTTCAGGTTCCGC	60.0	273	INTERGENIC				
CaPOPII_3927	scaffold1869	20055	CAA/CAAA	TTTCAGGCGATTTTCGTACC	ATTGTGTTTCGGGTTTCGAG	60.1	825	INTERGENIC				
CaPOPII_3928	scaffold1878	8070	CAAAAAAAA/CAAAAAAAAA	TTGGTTTGAAAAAGGGGTTG	TACGTTGCAATCGTCCAAAA	59.8	468	INTERGENIC				
CaPOPII_3929	scaffold1878	25991	TATAATAATAATAATAA/TATAATAATAATAATAATAA	CTAATGGGTTGATCCGCTGT	TTGACATGATTTGGTTTCGG	60.0	416	INTERGENIC				
CaPOPII_3930	scaffold1878	51803	T/TA	GATGCATATTTGCTTCTTTCCA	CCAAAATTAGCCTTGTCACCA	59.2	603	INTERGENIC				
CaPOPII_3931	scaffold1885	40287	GATATATATATATAT/GATATATATATATATATATATAT	CCATTGTGGTCGTTTAGGCT	CCCAAATGACATCAAAACCC	60.0	572	INTERGENIC				
CaPOPII_3932	scaffold1885	47510	A/AG	GTAGCTTCGGCTACACCGAC	ATTCCGTTGTGCACTTGATG	59.9	664	INTERGENIC				
CaPOPII_3933	scaffold1886	17993	TA/TAA	GAGAGAGGCATTGCACAACA	CGAAATTTCTTTAACGAGGGG	60.0	537	INTERGENIC				
CaPOPII_3934	scaffold1905	1617	CTG/CTCTGATG	ACTGATGTCAAGAGGGCCAG	AGAGGGTGGCAATGGTAATG	60.3	251	INTERGENIC				
CaPOPII_3935	scaffold1918	213558	CTTAATTA/CTTA	TGACATGCCAAAACTAGTCAAAA	TGCAAAAATAACACATGGACG	59.7	663	INTERGENIC				
CaPOPII_3936	scaffold1923	10722	ATTTTTTTTTT/ATTTTTTTTT	GGCCCCCAATATTTTGGTAA	ATTATTGGCCCCAACTCACA	60.7	383	INTERGENIC				
CaPOPII_3937	scaffold1928	15951	TG/T	ATCTACCCGGCAAACGTCTA	GCTGCAAAAGTCAACGTCAA	59.6	456	INTERGENIC				
CaPOPII_3938	scaffold193	79630	TAAAAAAA/TAAAAAA	AAAATAAATGCGCCGTATGC	AGATCATGTGCAAAAAGGGG	60.0	477	INTERGENIC				
CaPOPII_3939	scaffold193	79687	TAATAA/TAATAATAAAATAA	AAAATAAATGCGCCGTATGC	AGATCATGTGCAAAAAGGGG	60.0	477	INTERGENIC				
CaPOPII_3940	scaffold193	329576	TTATAAGTA/TTA	TCCATAAACCGTGTCCCAAT	TCAACCAAAATCCCCCAATA	60.1	342	INTERGENIC				
CaPOPII_3941	scaffold193	394517	ATAT/ATATTTAT	TTGGAAGGAGCCCTTACCTT	ACCCAATTACTGGAGATGCG	60.1	873	INTERGENIC				
CaPOPII_3942	scaffold1930	33808	TTATTAT/TT	CAACTGAATTTATTCCGTGCAA	CGTCATTAAGTACAACTATAAAACGAA	60.0	142	INTERGENIC				

INDEL marker IDs	Chromosomes /unanchored scaffolds	Physical positions (bp)	InDels (*Kabuli* reference genome-CDC Frontier/PI)	Forward primers (5'-3')	Reverse primers (5'-3')	Anneling temperature (0C)	Expected amplified product size (bp)	Structural annotation		Functional annotation		
								Sequence components of *kabuli* genome	*Kabuli* gene accession IDs	NCBI-KOG	TFs	NCBI-nr database
CaPOPII_3943	scaffold1943	2994	TAAAAAAAAA/TAAAAAAAA	TTTGTAGCCTGCTCGGATTT	TCCATCGAGACTCATTTATAAGCA	59.8	807	INTERGENIC				
CaPOPII_3944	scaffold1943	251763	TAAAAAAAAAAA/TAAAAAAAAAAAA	CCTTACCCCTCGCACTATGA	TGGGTCATGGTTCCTTCATT	60.1	623	INTERGENIC				
CaPOPII_3945	scaffold1943	282409	CATATA/CATATATATA	TTGGTTTATTTGGGTTGGGA	AGCGATGCATGTCTCAAGTG	60.0	912	DRR	Ca_21930		M-type	
CaPOPII_3946	scaffold1943	393620	ATC/ATCTTC	TGGTTCTCATGATACGTGTGG	GCGCAAACACAAACAACAAA	59.4	635	INTERGENIC				
CaPOPII_3947	scaffold1945	4916	GTTT/GTT	CGTCAAACACAAACCTCCCT	TGGTTTTCACGCCAACATTA	60.0	659	INTERGENIC				
CaPOPII_3948	scaffold1946	1226	TATATA/TATATAGATATA	ACATTAAAATTCGGGGGAGG	CACACTCTCACCCGGCTAAT	60.0	388	INTERGENIC				
CaPOPII_3949	scaffold1954	2359	ATTTT/ATTTTT	AAAGCCGACAAACTTCGCTA	CGAGGAGCAATGAGTGTCAA	60.0	752	INTERGENIC				
CaPOPII_3950	scaffold1956	63893	ATATTATTATTATTATTATTATTATT/ATATTATTATTATTATTATTATTATTATTATT	TTCGTTATGCAGTCAAATTTCTAA	AAAATGGACCAACGTCCGTA	58.1	719	INTERGENIC				
CaPOPII_3951	scaffold1959	47247	GC/GCC	TCCTCCTCAAGTTGGAGCAT	ATATTGCATTGCAGTGGTGG	59.8	458	DRR	Ca_26462	K	G2-like	Signal transduction response regulator, receiver domain
CaPOPII_3952	scaffold1959	83297	CAAAA/CAAAAA	TTCGGTGGCATAAATTGTGA	TTCAAAATGGTCGACATCCA	59.9	512	INTERGENIC				
CaPOPII_3953	scaffold1959	121649	TT/TCGT	GACACCAGGAGGCATGTTTT	GGAAGGCTTGTCAGCTTGAG	60.0	357	INTERGENIC				
CaPOPII_3954	scaffold1962	159146	AAATATAAGCAGAAAATTAA/AAATATAAGCAGAAAATTAATATAAGCAGAAAATTAA	CTTTTTACAGCGCTTGTGGG	AAGCCGTGTAATCTGAACCG	60.8	557	INTERGENIC				
CaPOPII_3955	scaffold1962	170714	GTTTTTTTTT/GTTTTTTTT	TCAAGGACCCAAAGCACATT	TACTCCCCCTTTGAGCTTGA	60.5	423	INTERGENIC				
CaPOPII_3956	scaffold1964	6786	GTTTTTT/GTTTTT	CCCTTGGAAGAAAAACCACA	TTGGGAAGGGAGATGTCAAG	59.9	577	INTERGENIC				
CaPOPII_3957	scaffold1964	8928	ATTTTT/ATTTT	CCTCCACTGCTGAGTTGTGA	AAGCCTAAGCTGCATTGGAA	60.0	651	INTERGENIC				
CaPOPII_3958	scaffold1964	11011	A/ACTC	ATGTTCCTCGCCAATCATTC	TTCCAAAAGAGGAATGGTGC	59.9	771	DRR	Ca_25209	G		Histidine phosphatase superfamily, clade-1
CaPOPII_3959	scaffold1964	11803	TAGAATTATGCAG/TAGAATTATGCAGAATTATGCAG	GCACCATTCCTCTTTTGGAA	GATGGACCACCTTCTCTCCA	60.1	553	CDS (FRAME SHIFT)	Ca_25209	G		Histidine phosphatase superfamily, clade-1

INDEL marker IDs	Chromosomes /unanchored scaffolds	Physical positions (bp)	InDels (*Kabuli* reference genome-CDC Frontier/PI)	Forward primers (5'-3')	Reverse primers (5'-3')	Anneling temperature (0C)	Expected amplified product size (bp)	Structural annotation		Functional annotation		
								Sequence components of *kabuli* genome	*Kabuli* gene accession IDs	NCBI-KOG	TFs	NCBI-nr database
CaPOPII_3960	scaffold1964	21448	CAAAATAAAAATAA/CAAAATAA	GGGTCAGTTCATCCCATGAC	TATGCATCTTTGTTGACGCC	60.2	292	INTERGENIC				
CaPOPII_3961	scaffold1964	172119	ATTGG/A	TTGTTTTGTTAGAAACGCCG	ACCCACATAGCGCAAGATTC	58.9	313	INTERGENIC				
CaPOPII_3962	scaffold198	435699	AATATATATATATATATATATATATATAT/AATATATATATATATATATATATATATATATATATAT	TTTTGAAATCAAATCAAATCATGT	AATTTATTGCGCATGCTCCT	57.6	511	INTERGENIC				
CaPOPII_3963	scaffold198	501505	CA/CAA	TTGGCCAAGTCCCTGTTTAC	CGTTCCTGAGGGAATTGTGT	60.0	498	INTERGENIC				
CaPOPII_3964	scaffold198	637738	CTTAATTATATTTAATTA/CTTAATTATATTTAATTATATTTAATTA	TGACCGCAACCAGTTCAATA	CAATTGAAACTCTCACATTCAAAA	60.1	722	INTERGENIC				
CaPOPII_3965	scaffold1981	116705	GATAATAATA/GATAATAATAATA	TTGTTGATCGCAACTTGAGG	AATTGAACCAATAAAACACGTTAAA	59.8	363	INTERGENIC				
CaPOPII_3966	scaffold1981	118399	TAAAAAAAAAAA/TAAAAAAAAAA	AGCGAAAAGCACCATTGAAC	ATCGGAATTTTTGTGCCTTG	60.3	332	INTERGENIC				
CaPOPII_3967	scaffold1981	149526	ATTTTTTTT/ATTTTTTTTT	CTCAAATTTTGTTTGGGGGA	TGTGAGTACGGATAAGGGGG	59.8	443	DRR	Ca_21257	C		
CaPOPII_3968	scaffold1981	233549	CTTTTTTTTTTT/CTTTTTTTTTTTT	CGTTTAGCCTCGTAGGTTCG	CAGCCATCTACTGCTCCTCC	59.9	756	INTERGENIC				
CaPOPII_3969	scaffold1981	239519	AATATATATATATATATATATATATATATA/AATATATATATATATATATATATATATATATATATATATATA	AAATTTGCGTTTAAATAACGGG	TGGATCCTCCCTTTTGTACG	59.3	779	INTERGENIC				
CaPOPII_3970	scaffold1981	317439	TAAA/TAAAA	TCTCATTTGCTCCCGACTCT	TTGTCTCAACAATCTCCCCC	60.0	501	DRR	Ca_21265			
CaPOPII_3971	scaffold1981	336889	ATTTT/ATTTTT	CTTGACGGTGGAGAGCTAGG	GCAAGGGTTTGAGATTTTGG	60.0	828	INTERGENIC				
CaPOPII_3972	scaffold1981	355366	A/AC	ACGATATTTTGAAAGGCCCC	CGCGGATCCAATATCTCACT	60.2	426	INTERGENIC				
CaPOPII_3973	scaffold1983	25976	CTTT/CTT	TCGCACTCCTGAGTCATGTT	TCAATTTCAACTGCAAACCAA	59.4	504	INTERGENIC				
CaPOPII_3974	scaffold1983	34254	G/GATAA	AGAAAGGCTCAAACGACCAA	CTCAAGTCGACAAGCCTTCC	59.9	301	INTERGENIC				
CaPOPII_3975	scaffold1988	7769	TACACACAATCAAA/TACACACACAATCAAA	ACCATACAAGTTCCCGCATC	GAAGGAGCAGTAAGCCATCG	59.8	725	INTERGENIC				
CaPOPII_3976	scaffold1996	1333	TG/TGG	CGGATGAATCATTGGGAAAT	AGTGAAACCAACGCCAAAAC	59.6	325	INTERGENIC				

INDEL marker IDs	Chromosomes /unanchored scaffolds	Physical positions (bp)	InDels (*Kabuli* reference genome-CDC Frontier/PI)	Forward primers (5'-3')	Reverse primers (5'-3')	Anneling temperature (0C)	Expected amplified product size (bp)	Structural annotation		Functional annotation		
								Sequence components of *kabuli* genome	*Kabuli* gene accession IDs	NCBI-KOG	TFs	NCBI-nr database
CaPOPII_3977	scaffold1996	5730	GAAAAAAAA/GAAAAAAAAAA	TGTTAGGAGGATCTCACGGG	ATTGTTGGAAAATTGGCTGC	60.1	823	INTERGENIC				
CaPOPII_3978	scaffold1999	29603	GACA/GACACA	AGGGAGGACGAAAATGACCT	AACCAATCTTGTGCATCCAA	59.9	670	INTERGENIC				
CaPOPII_3979	scaffold1999	29835	TAAAAAAA/TAAAAAA	AATCCAACGACAACGGAACT	CCGATGTTGTGCATCCAATA	59.5	541	INTERGENIC				
CaPOPII_3980	scaffold2	504035	CAAA/CAAAA	CGTTCTTTTTGGGTTCGTGT	ATCCCATTAGCCACATTCCA	60.0	265	INTERGENIC				
CaPOPII_3981	scaffold2000	1544	GAAAAAAA/GAAAAAAAA	GCCTAGGGATGACGAAAATG	TGTGTTTGCGTCATTGGATT	59.5	566	INTERGENIC				
CaPOPII_3982	scaffold2005	31637	ATTTTTTTTTT/ATTTTTTTTTTT	TGGTGAGTGAACCAAACGAG	CAGCCGTCTTCATTTTCATCT	59.7	302	INTERGENIC				
CaPOPII_3983	scaffold2010	3634	AT/ATT	AATCGATTGATTCAGCCCAG	TGATTTCCAACGGTCATCAA	60.0	869	DRR	Ca_27976			
CaPOPII_3984	scaffold2010	6749	T/TC	TTCCCTTTCGTTGTGTATCG	GGGCACCGATGTTACATAAA	58.6	857	URR	Ca_27976			
CaPOPII_3985	scaffold2010	7925	ATTTTTTTTT/ATTTTTTTTTT	AAAGTGCCTTCTTCTTGGCA	ATGTTTTCTTTTGGGTTGCG	60.0	564	URR	Ca_27976			
CaPOPII_3986	scaffold2018	4354	TGGAG/TG	CATGGTTTTGTTTGTCGTGC	TGTTTGCTTTAAGCGATGTCA	60.0	462	INTERGENIC				
CaPOPII_3987	scaffold2018	16998	CAATAATAATAATAATAATAATAATAATAATAATAATAATAATAATAATAATAAT/CAATAATAATAATAATAATAATAATAATAATAATAATAATAATAATAATAAT	AAGGCTCACTTGCAAAGACG	GGTATTGCTGCAGTTTTTGG	60.6	445	INTERGENIC				
CaPOPII_3988	scaffold202	17816	AAA/AAATAA	TCACAAGGGTAGTTTTGGGTTT	CTGCCAGCTCACCTCCTATC	59.8	685	INTERGENIC				
CaPOPII_3989	scaffold202	20536	CTA/C	TGCATCCAACTTTGGTGTGT	ACACCGTTGTTTATGCACGA	60.0	373	INTERGENIC				
CaPOPII_3990	scaffold202	22635	CG/C	GGATTACATTCGCTTCTCCG	GCAAACAATGCGAAACAGAA	59.7	804	INTERGENIC				
CaPOPII_3991	scaffold202	22768	AT/A	TTGCAATCATGAGGCAAGTC	GCAAACAATGCGAAACAGAA	59.8	235	INTERGENIC				
CaPOPII_3992	scaffold202	37860	TAAA/TAA	ATTGGAGGCTGGTTTTGTTG	ATCGTGGGGATGAATTTGAA	60.0	539	INTERGENIC				
CaPOPII_3993	scaffold202	37967	T/TC	ATTGGAGGCTGGTTTTGTTG	ATCGTGGGGATGAATTTGAA	60.0	539	INTERGENIC				

INDEL marker IDs	Chromosomes /unanchored scaffolds	Physical positions (bp)	InDels (*Kabuli* reference genome-CDC Frontier/PI)	Forward primers (5'-3')	Reverse primers (5'-3')	Anneling temperature (0C)	Expected amplified product size (bp)	Structural annotation		Functional annotation		
								Sequence components of *kabuli* genome	*Kabuli* gene accession IDs	NCBI-KOG	TFs	NCBI-nr database
CaPOPII_3994	scaffold202	39053	CTTTTTTTT/CTTTTTTT	GCACCGGAAAATACGTAGGA	ATTATCTTGTCGCGGCCTAA	60.0	359	INTERGENIC				
CaPOPII_3995	scaffold202	39185	TATG/TATGATG	GCACCGGAAAATACGTAGGA	ATTATCTTGTCGCGGCCTAA	60.0	359	INTERGENIC				
CaPOPII_3996	scaffold202	39293	CTTTT/CTTTTT	GCACCGGAAAATACGTAGGA	ATTATCTTGTCGCGGCCTAA	60.0	359	INTERGENIC				
CaPOPII_3997	scaffold202	61873	GTAAAATGAAATAAAATGA/GTAAAATGAAATAAAATGAAATAAAATGA	CAGCCCTTGAATTCGATCAT	GCTGTTTGGCTCAACCATTT	60.0	331	INTRON	Ca_27125			
CaPOPII_3998	scaffold202	103827	AGGG/AGG	TCAATAAGACGTGATAAGTTAATCCA	TTGGAGAAAGATATGATACGATAGGA	58.3	847	URR	Ca_27127	A		Like-Sm ribonucleoprotein (LSM) domain
CaPOPII_3999	scaffold202	103994	ATTTTTTTTTT/ATTTTTTTTTTTTT	AAATTGCTTATTTAGTAGTGTCAATCG	AACTCAGCAAAACCCAAAACA	58.5	409	URR	Ca_27127	A		Like-Sm ribonucleoprotein (LSM) domain
CaPOPII_4000	scaffold202	104373	TAAAAAAAAAAA/TAAAAAAAAA	TGTTTTGGGTTTTGCTGAGTT	TGGATGGATTGGATGAATGA	59.6	180	URR	Ca_27127	A		Like-Sm ribonucleoprotein (LSM) domain
CaPOPII_4001	scaffold202	104727	TTA/T	TCATTCATCCAATCCATCCA	TCGAGTGTCGATCTCAAGGA	59.7	489	INTERGENIC				
CaPOPII_4002	scaffold2023	2321	CAA/CAAA	AACACCAACTCCACCACACA	TGGTTTTGGTTGAGAGAGGG	59.9	544	INTERGENIC				
CaPOPII_4003	scaffold2024	1945	CAAA/CAAAA	GAATGTTAGGGTTTCCGGGT	GATGAAATCGAGGACTGGGA	60.1	338	INTERGENIC				
CaPOPII_4004	scaffold2027	955	GAAAAAAAA/GAAAAAAAAA	AGAAAGGCTCAAACGACCAA	ACAGAGGAAGCTGCCACCTA	59.9	672	INTERGENIC				
CaPOPII_4005	scaffold2030	4596	ATT/ATTTT	AGGGTTTAGGATTTCGCGTT	CATATCAAAAACCCAAATGACC	60.0	243	INTERGENIC				
CaPOPII_4006	scaffold2033	44747	GTTTTTTTT/GTTTTTTTTT	ATGAATACCCCTGCGAGTTG	GAACAAGCAATCGCAGACAA	60.0	549	INTERGENIC				
CaPOPII_4007	scaffold2033	183883	CGAAGAAGAAG/CGAAGAAGAAGAAG	CTTGGGGTGGCAAGTACAGT	TCGAATACATGAATTAACAAAAGGA	60.0	371	DRR	Ca_26143		bHLH	Lipase, GDSL
CaPOPII_4008	scaffold2040	159577	CA/CAGGTA	TGAAGAGGGGAATCCATCTG	TTAACAACTCCTGGTTCGGG	60.0	372	INTERGENIC				
CaPOPII_4009	scaffold205	41104	GT/GTCT	TAATGCGCTTCTGATTGTGC	AGGCCGTATTGCTTTTGAGA	60.0	634	INTERGENIC				
CaPOPII_4010	scaffold205	46882	GAGGA/GAGGAAGGA	GAGAGGATTTGTGTTCCCGA	GGAATGCTCAAAGGTTGCAT	60.1	235	INTERGENIC				

INDEL marker IDs	Chromosomes /unanchored scaffolds	Physical positions (bp)	InDels (*Kabuli* reference genome-CDC Frontier/PI)	Forward primers (5'-3')	Reverse primers (5'-3')	Anneling temperature (0C)	Expected amplified product size (bp)	Structural annotation		Functional annotation		
								Sequence components of *kabuli* genome	*Kabuli* gene accession IDs	NCBI-KOG	TFs	NCBI-nr database
CaPOPII_4011	scaffold206	162025	GTTTTT/GTTTT	CTTGGATTCATTGATCCGCT	GTTTGGTTTTCTTTGGGGGT	60.0	423	INTERGENIC				
CaPOPII_4012	scaffold206	175737	TTAAATAAA/TTAAA	GCCCTCAGATGAGAGTCAGG	TGACGCCATGGTGACATACT	59.9	558	INTERGENIC				
CaPOPII_4013	scaffold206	176077	T/TATAG	CTCTCGCTATGAAAAACGGG	TGGGTTCAATTGGCATTTTT	59.8	759	INTERGENIC				
CaPOPII_4014	scaffold206	190834	CATATATATATATATATAT/CATATATATATATATATATAT	ATCCCTAACGCCGTTTTTCT	ATATTGGCTCGGAGGCTTTT	60.0	480	INTERGENIC				
CaPOPII_4015	scaffold206	199524	T/TA	CCGAGCCAAAATCCAAAATA	TCTCAACGATAGCCATCACG	59.9	578	INTERGENIC				
CaPOPII_4016	scaffold207	6894	AC/A	GCAAACTCGACAGGAAAAGC	TTGGTGAAGATGTCAGCCAG	60.0	370	INTERGENIC				
CaPOPII_4017	scaffold208	15689	CTATATATA/CTATATATATA	TTTTGGCCATTTCAACTCAA	TCCAAATACTTAAATGCGAACAAA	59.1	452	INTERGENIC				
CaPOPII_4018	scaffold208	174233	GAAAA/GAAA	CCTTGGAGTATTCTCGGCAG	TGCTGATCCGTGAGAGATTG	59.8	894	INTERGENIC				
CaPOPII_4019	scaffold209	258029	TA/TAA	TCAGGCAAACAAGCAGACAC	ATCGAATGCCTCAATCTTGG	60.0	522	INTERGENIC				
CaPOPII_4020	scaffold209	378233	AATATATATATATATATAGATATATATATATAT/AATATATATATATATATATATAGATATATATATATATAT	AAATGGAGGAGAAGGAGGGA	ATTTTGGGTCGGAAGCTTTT	60.0	446	INTERGENIC				
CaPOPII_4021	scaffold2097	4977	GC/GCC	ACATTCCCATCCACAGGGTA	CATGCAGGAATTCAGCAAAA	60.1	480	INTERGENIC				
CaPOPII_4022	scaffold210	31686	CAA/CAAA	TTTTCTCGCAAAACATGCAG	TTCGCAACTCCACTCTTCCT	60.0	712	INTERGENIC				
CaPOPII_4023	scaffold210	330740	AATATATATATATATATATATATAT/AATATATATATATATATATATATATATATATATAT	ATGTTTTGGATTTTGGCTCG	AGGAAGAGTGGAGTTGCGAA	59.9	522	INTERGENIC				
CaPOPII_4024	scaffold2103	124055	AT/A	AACCCAATCACTGGAGATGC	AGGAAGAGTGGAGTTGCGAA	59.9	540	INTERGENIC				
CaPOPII_4025	scaffold2103	124113	AG/AGG	AACCCAATCACTGGAGATGC	AGGAAGAGTGGAGTTGCGAA	59.9	540	INTERGENIC				
CaPOPII_4026	scaffold2103	145039	GTATATATATATATATATAT/GTATATATATATATATATAT	GCCAGAACACCGACCATACT	CCAAGTGGTACAACTGGTGTCT	60.0	581	INTERGENIC				
CaPOPII_4027	scaffold212	463806	TAA/TA	AAACGCAATCCTTGAGTTCG	TGGGTCAAAGAAAAGATCGG	60.2	323	INTERGENIC				

INDEL marker IDs	Chromosomes /unanchored scaffolds	Physical positions (bp)	InDels (*Kabuli* reference genome-CDC Frontier/PI)	Forward primers (5'-3')	Reverse primers (5'-3')	Anneling temperature (0C)	Expected amplified product size (bp)	Structural annotation		Functional annotation		
								Sequence components of *kabuli* genome	*Kabuli* gene accession IDs	NCBI-KOG	TFs	NCBI-nr database
CaPOPII_4028	scaffold2126	3526	GATATATATATATA/GATATATATATATATA	CCATGAAAAGAAGAAGGGCA	GCCTTTTACTGCACTTTGGC	60.2	472	INTERGENIC				
CaPOPII_4029	scaffold2126	19827	AATATATATATATATATATATATATATATATA/AATATATATATATATATATATATATATATATATATATATA	GGAGAAGGAGGGGAAATCAG	TATTTTGGATTTTGGCTCGG	60.0	394	INTERGENIC				
CaPOPII_4030	scaffold2127	32525	TGATTGGTTTGTTTGAGATTGGTTTGTTTG/TGATTGGTTTGTTTG	TTTGCAAGAATTTTGCCTCA	TTACGCTACACCGTCACCAG	59.4	645	INTERGENIC				
CaPOPII_4031	scaffold2131	815	TCTATTGAAATAC/TCTATTGAAATACTATTGAAATAC	TGGTCGCAACAACTAGCAAC	CTGCAAATCGCAAAGTGAAA	59.9	975	INTERGENIC				
CaPOPII_4032	scaffold2134	5442	TTA/T	GAAAAAGGAACGAAGGGAGG	AACGGAACGAGCGAGTCTTA	60.0	566	INTERGENIC				
CaPOPII_4033	scaffold2141	17958	ATGTGT/ATGT	TCGATTGGCACGTTGATTTA	TCGTTGGTTTTTGGATCTGA	60.1	513	INTERGENIC				
CaPOPII_4034	scaffold2147	24244	GT/GTT	TGGTGGCACAAGATAGGTCA	CATTTTCGTCCTCCATAGGC	60.1	462	INTERGENIC				
CaPOPII_4035	scaffold2152	76335	ATTATTGTTATT/ATTATT	TTCATGGGAACACAAGCAAG	GAAGGAAAGGGTCAAGGCTC	59.7	258	INTERGENIC				
CaPOPII_4036	scaffold2152	76564	AATTATTATTATTATTA/AATTATTATTATTATTATTA	GAGCCTTGACCCTTTCCTTC	TGTCACCGCTTGCTTAAGAG	60.2	661	INTERGENIC				
CaPOPII_4037	scaffold2152	108576	CT/CTATATTTCATATCTT	TTTCAGCTTCATCGCATTCA	GAGATTGCCAAGAACAAGGC	60.5	448	INTERGENIC				
CaPOPII_4038	scaffold2152	340315	TAATCA/TAATCAAATCA	CTTGCCCCCTAATCATTTCA	AATGAATTCGGCGCTAGAGA	59.9	386	INTERGENIC				
CaPOPII_4039	scaffold2153	6068	TAAAAAAAAAAA/TAAAAAAAAAA	CCGCTGTCAATTCTCCATTT	TAACATGCGCTATTTGCAGG	60.1	583	INTERGENIC				
CaPOPII_4040	scaffold2158	64854	ATTTT/ATTTTT	CGTGGTAATCGCGTAGGAAT	CTCTCATCTGAGGGCAAAGC	60.0	515	INTERGENIC				
CaPOPII_4041	scaffold2158	71695	A/AATACCATTGT	TCGCGTAAGCAGACTCGTAA	GCTACCTCACGACCTTCTCG	59.8	212	INTERGENIC				
CaPOPII_4042	scaffold2161	111313	ATT/ATTT	AGAAATTGTTGATGGCGTCC	GGTTGCATCAGGGTCAATCT	59.9	240	INTERGENIC				
CaPOPII_4043	scaffold2163	71476	AC/A	AGGGAGGACGAAAATGACCT	TTGCGTTTGTGTCATTGGAT	59.9	547	INTERGENIC				
CaPOPII_4044	scaffold2166	41038	AG/AGG	GCTCGAAACTGGTGGCTACT	TCCACAACAACCTACGTCCA	59.5	245	INTERGENIC				

INDEL marker IDs	Chromosomes /unanchored scaffolds	Physical positions (bp)	InDels (*Kabuli* reference genome-CDC Frontier/PI)	Forward primers (5'-3')	Reverse primers (5'-3')	Anneling temperature (0C)	Expected amplified product size (bp)	Structural annotation		Functional annotation		
								Sequence components of *kabuli* genome	*Kabuli* gene accession IDs	NCBI-KOG	TFs	NCBI-nr database
CaPOPII_4045	scaffold2166	183523	AAGTCTCTTTAATTGA/AA	CAGAGGAAATCCAGGGACAA	GTTGTGGGATGAACTGACCC	60.0	666	INTERGENIC				
CaPOPII_4046	scaffold2166	202348	TAAAAAAAAAAA/TAAAAAAAAAA	GTGTCGCGACTGATTTGCTA	TCCTTTGTGTAACTCCTTGGC	60.0	769	INTERGENIC				
CaPOPII_4047	scaffold2170	5002	AACCTTACCTTACCTTACCT/AACCTTACCTTACCT	GCAAATCAAGGTTGCAGTCA	ACACTCTAATGGGGACAGCG	59.8	222	INTRON	Ca_26928	YU		
CaPOPII_4048	scaffold2170	5357	C/CTATTGGG	CGCTGTCCCCATTAGAGTGT	GTTTGCCAATTAGGGCACAT	60.1	387	INTRON	Ca_26928	YU		
CaPOPII_4049	scaffold2170	6851	AC/A	TTTGTGCTTCACTACCGCTG	TTGGCAAATTTTTCCCAGAG	60.1	547	INTRON	Ca_26928	YU		
CaPOPII_4050	scaffold2170	12258	CTTTTT/CTTTT	TGCTTTGTTCAATTGCTTGC	GGAGTCCCGGAAAATTTCAT	60.0	531	INTRON	Ca_26929	O		
CaPOPII_4051	scaffold2170	14347	AC/ACC	GCTCATTCTTGTCCCCAAAA	GCCTCTGCTAATTGGTCTCG	60.1	583	INTRON	Ca_26929	O		
CaPOPII_4052	scaffold2173	19619	TAAAAAA/TAAAAAAA	GGATGACACAAACGCACCTA	ATTTTTGTCCTCCCTTGGCT	59.6	385	INTERGENIC				
CaPOPII_4053	scaffold2173	19824	A/AG	CCAAGGGAGGACAAAAATGA	TTTTCGTCCTTCACAGGCTT	59.9	515	INTERGENIC				
CaPOPII_4054	scaffold2179	20727	CTTTT/CTTTTT	GTGAGGCCATCCTTGACATT	TGCTACCAGCCCAGTTCTCT	59.9	673	INTERGENIC				
CaPOPII_4055	scaffold2179	20793	TCCCCC/TCCCC	ATCAGCGAGGATGCAGAACT	CATCGCTCAGTTTGGACTCA	60.0	789	INTERGENIC				
CaPOPII_4056	scaffold2179	35934	TG/TGCG	GAATTTGAACCTCCAAGCCA	ATAGCGGAGAAGCCCATACA	60.1	404	INTERGENIC				
CaPOPII_4057	scaffold2179	35973	TC/TCC	GAATTTGAACCTCCAAGCCA	ATAGCGGAGAAGCCCATACA	60.1	404	INTERGENIC				
CaPOPII_4058	scaffold2179	36066	ATAG/A	GAATTTGAACCTCCAAGCCA	TGCATTCCACATACAAGGGA	60.1	582	INTERGENIC				
CaPOPII_4059	scaffold2183	19955	TATTAATTAATT/TATTAATT	TAGGGCGATTTTCGTACCAC	TTTTCGCATATTTATTTGACAACTC	60.0	468	INTERGENIC				
CaPOPII_4060	scaffold2183	65749	A/AG	CCCATCGATGATAATGGACC	CTAGTCATCGTCTGGTGCGA	60.0	380	INTERGENIC				
CaPOPII_4061	scaffold2183	67523	GT/G	AGTCGCTCGAAAAGTCCAAA	TCCTGGTCCTCGATTTCATC	60.0	528	INTERGENIC				

INDEL marker IDs	Chromosomes /unanchored scaffolds	Physical positions (bp)	InDels (*Kabuli* reference genome-CDC Frontier/PI)	Forward primers (5'-3')	Reverse primers (5'-3')	Anneling temperature (0C)	Expected amplified product size (bp)	Structural annotation		Functional annotation		
								Sequence components of *kabuli* genome	*Kabuli* gene accession IDs	NCBI-KOG	TFs	NCBI-nr database
CaPOPII_4062	scaffold2183	67873	ATTTTT/ATTTT	GATGAAATCGAGGACCAGGA	TTTCATCTGGACTTTTCGGG	60.0	333	INTERGENIC				
CaPOPII_4063	scaffold2192	8042	CATATATATATATATATATATATAT/CATATATATATATATATATATATATATAT	AGGAAGAGTGGAGTTGCGAA	ATGTTTTGGATTTTGGCTCG	60.0	524	INTERGENIC				
CaPOPII_4064	scaffold2192	93608	AAGAGAGAGAGAGAGAGAGAGAGA/AAGAGAGAGAGAGAGAGAGA	TCATCAAAAGCCACGAATGA	TTTGGTTGAGGGACTTGGAA	60.2	713	INTRON	Ca_26813	R		Major facilitator superfamily MFS-1
CaPOPII_4065	scaffold2192	106941	CTATTATTATTATTATTATTATTATTATT/CTATTATTATTATTATTATTATTATTATTATT	TGGTCTCCCTCTCCTTCTCA	TTCCATTGCGCAAAATCATA	59.9	483	INTERGENIC				
CaPOPII_4066	scaffold22	24507	AAT/A	ATGAGGCTCAAAATGCCATC	GCCAACTTTTCCAATGCCTA	60.0	727	INTERGENIC				
CaPOPII_4067	scaffold22	34524	GTTTTT/GTTTTTTT	GCAGCATCGCAGATCATAGA	CGAATTGGGTTTTCGTTCTC	60.1	181	INTERGENIC				
CaPOPII_4068	scaffold2205	49159	ATT/ATTT	TTTTTAACCAATCTTCGGCG	AGGGAGGACGAAAATGACCT	60.1	272	INTERGENIC				
CaPOPII_4069	scaffold2208	20005	CA/CATA	CAAACACACCTGTTGGATGC	CCCTAGGCTTATTCTTGCCC	60.0	362	INTERGENIC				
CaPOPII_4070	scaffold2210	15184	CG/CGGATTTG	TCCAGATGAAATCGAGGACC	AAATCCGACACGTAACTCGG	60.0	531	INTERGENIC				
CaPOPII_4071	scaffold2210	61082	AGTGTATTCGAATACA/AGTGTATTCGAATACACGTGTATTCGAATACA	TTTAGGGTTTAAGATTCGGGG	TCTTAAGATGCGTGGCCAAT	59.3	735	INTERGENIC				
CaPOPII_4072	scaffold223	95879	AGCGACTACT/A	TTGGTCTCCTTGTGTTGTGC	TGGAGACCCCTTGATGAAAC	59.7	502	INTERGENIC				
CaPOPII_4073	scaffold224	186566	GTTTT/GTTTTT	TGAAGTGTTCGAATTGCAGC	TTGCGTTGAAAGACTCGATG	60.0	462	DRR	Ca_26345			
CaPOPII_4074	scaffold2249	1002	CT/CTT	TATTCCCACCCACGATCATT	CAACCAAGCCATTTTCCATT	60.0	871	INTERGENIC				
CaPOPII_4075	scaffold227	34781	TAAAAAAAAAAA/TAAAAAAAAAA	TGAATAGCGAGTGTGTTGGG	TGCACATAGTAAAACTTGGCG	59.7	639	INTERGENIC				
CaPOPII_4076	scaffold227	135497	TCC/TCCC	CTTGGATGTGGTAGCCGTTT	GGTCTTGTGGTGGCTTTTGT	60.0	651	INTRON	Ca_25190			
CaPOPII_4077	scaffold227	140947	TTA/TTAAAAGTA	ACCCTTTTGTTCCACACGAG	TACGGATGTTGACGCAATGT	60.0	511	INTRON	Ca_25190			
CaPOPII_4078	scaffold227	195682	CAA/CAAA	AGACACAAACCCCGCAATAG	GGATGCCACCATTCAAGTTT	60.0	338	INTERGENIC				

INDEL marker IDs	Chromosomes /unanchored scaffolds	Physical positions (bp)	InDels (*Kabuli* reference genome-CDC Frontier/PI)	Forward primers (5'-3')	Reverse primers (5'-3')	Anneling temperature (0C)	Expected amplified product size (bp)	Structural annotation		Functional annotation		
								Sequence components of *kabuli* genome	*Kabuli* gene accession IDs	NCBI-KOG	TFs	NCBI-nr database
CaPOPII_4079	scaffold227	195989	ATTTTTTTTTTTTTT/ATTTTTTTTTT	AAACTTGAATGGTGGCATCC	AGTAATGAATGGGCGAGTGG	59.8	595	INTERGENIC				
CaPOPII_4080	scaffold227	196111	TAAAAAAA/TAAAAAAAAA	AAACTTGAATGGTGGCATCC	AGTAATGAATGGGCGAGTGG	59.8	595	INTERGENIC				
CaPOPII_4081	scaffold227	196280	TAAAA/TAAAAA	AAACTTGAATGGTGGCATCC	AGTAATGAATGGGCGAGTGG	59.8	595	INTERGENIC				
CaPOPII_4082	scaffold227	197474	TAAAAAAAA/TAAAAAAAAAA	CCTAATGGCGGAAATTGTGT	TAATCAGACCGTGTGGGTGA	59.8	626	INTERGENIC				
CaPOPII_4083	scaffold2275	15734	AATATATATATATATATATATATATAT/AATATATATATATATATATATAT	AGTGTATAACCCGTGCGTCG	TTGGAGTCATGCTTTTCATCA	61.0	229	INTERGENIC				
CaPOPII_4084	scaffold228	136549	A/AC	GATGAAATCGAGGACAGGGA	TGCGTCTTTCTCAAGTGCAT	60.0	445	INTERGENIC				
CaPOPII_4085	scaffold228	183161	ATTT/ATT	CACGCCTAAAAGAGTCGTGG	AAGTTTGACTGCAAAACGAAGA	60.8	783	INTERGENIC				
CaPOPII_4086	scaffold2289	12871	ATTT/ATTTT	TGTGTTTGTGCCATTGGATT	AGGGAGGATGAAAACGACCT	59.8	475	INTERGENIC				
CaPOPII_4087	scaffold2301	1549	GA/GAGAACCCAAA	TGCAACGTCGTTCTCTCTTG	CAGACTTTCCAGCGTCACAA	60.2	441	INTERGENIC				
CaPOPII_4088	scaffold2301	6372	AAAATG/AAAATGACCTAAATG	AGGGAGGACGAAAATGACCT	AACCAATCATGTGCATCCAA	59.9	557	INTERGENIC				
CaPOPII_4089	scaffold2301	22791	CTT/CT	CAAACACACCTGTTGGATGC	CCTCGCTTGGCTTATTCTTG	60.0	466	INTERGENIC				
CaPOPII_4090	scaffold2301	54382	ATG/ATGTG	CCAAAGGAGGACGAAAATGA	GCGTGTTTGTGACATTGGAG	60.0	349	INTERGENIC				
CaPOPII_4091	scaffold2301	54476	C/CA	TGGATGCACATGATTGGTTT	AGGTCATTTTCGTCCTCCCT	59.8	316	INTERGENIC				
CaPOPII_4092	scaffold2301	65310	A/AG	GCTTAATTTCGAAGAAGGCG	TGAAATTCAAATAAAGTCAACATATTC	59.1	224	INTERGENIC				
CaPOPII_4093	scaffold231	18150	AC/A	GTTTCGTACCCCTCGACAAA	TCCAGATGAAATCGAGGACC	60.0	475	INTERGENIC				
CaPOPII_4094	scaffold2325	58923	CA/C	TTTCCTTCCTCCCTAGGCTT	TCCTGAATGGATGCACAAGA	59.3	670	INTERGENIC				
CaPOPII_4095	scaffold2343	16033	ATTAGGGTTTAGGGTTTA/ATTAGGGTTTAGGGTTTAGGGTTTA	AGGCGAGTTTTTGTCATTGG	AGGGAGGACGAAAATGACCT	60.1	641	INTERGENIC				

INDEL marker IDs	Chromosomes /unanchored scaffolds	Physical positions (bp)	InDels (*Kabuli* reference genome-CDC Frontier/PI)	Forward primers (5'-3')	Reverse primers (5'-3')	Anneling temperature (0C)	Expected amplified product size (bp)	Structural annotation		Functional annotation		
								Sequence components of *kabuli* genome	*Kabuli* gene accession IDs	NCBI-KOG	TFs	NCBI-nr database
CaPOPII_4096	scaffold2345	11872	TG/T	TAGGTGGTACGAAAATCGCC	TTTCATCTGGACTTTTCGGG	60.0	364	INTERGENIC				
CaPOPII_4097	scaffold2346	28393	GA/GAA	TGGGCGATTTTTGTACTTCC	CCCGAAAAGTCCAGATGAAA	59.9	395	INTERGENIC				
CaPOPII_4098	scaffold2348	176388	TTTTATTTATTTATTT/TTTTATTTATTTATTTATTT	GGGAGGAAGTCTGTGCTGAG	CAACATGTTTGCAGTCCACC	60.0	564	DRR	Ca_25471			
CaPOPII_4099	scaffold235	146229	CTTTT/CTTT	CCTCAGATGAGAGCCAGGAC	TAACCAAAGGTTCCACAGCC	59.9	500	INTERGENIC				
CaPOPII_4100	scaffold2356	138194	TT/TTCT	CCTTGAACTGTTGTCTGGCA	CCAACAACATCAAATTCATCG	59.9	687	INTRON	Ca_24344	Q		Alcohol dehydrogenase superfamily, zinc-containing
CaPOPII_4101	scaffold236	25371	CATATATATATAT/CATATATATATATAT	GAGGTTGTCCAGCTCACACA	TTGGCACATTGATTTTGACC	59.9	772	INTERGENIC				
CaPOPII_4102	scaffold236	41897	TTATATATATATAT/TTATATATATAT	CCAGATTTCAGCCTTTCCAA	GATATGCGTCGACTGAGCAA	60.2	603	INTERGENIC				
CaPOPII_4103	scaffold2368	25942	CGTG/CGTGTG	TTGTTTGCATCCATGGGTAA	AAATCCACGTCATCCCAAAA	59.8	593	DRR	Ca_26718			
CaPOPII_4104	scaffold2368	53512	TGGG/TGGGG	AACCCTGGAGATGGGAAAGT	CTAGCCATTAGCTTCGACCG	59.8	607	DRR	Ca_26720	P		Zinc/iron permease
CaPOPII_4105	scaffold2368	54204	GTT/GTTT	CATCGGTCAAACACCATCTG	GCTTTGCAGAACATGCGTAA	60.0	490	DRR	Ca_26720	P		Zinc/iron permease
CaPOPII_4106	scaffold2368	55017	A/AG	TTGCATCATGTACGCCAAAT	ACTTTCAGTGGCTCGGAGAA	60.0	392	INTRON	Ca_26720	P		Zinc/iron permease
CaPOPII_4107	scaffold2368	59799	TC/TCTCC	CTCCAAATCCCAATCTCCAA	CTCACTGTCAAGGTGCCGTA	59.9	725	URR	Ca_26721		MYB	
CaPOPII_4108	scaffold2371	73153	TAAAAAAAAAA/TAAAAAAAA	TGTGTCGGAGAATCTTCCCT	ATCGACGATAAGTCATCGGG	59.7	950	INTERGENIC				
CaPOPII_4109	scaffold2371	73366	ATTTTTATTTT/ATTTTTATTTTTATTTTTTATTTT	CAAGAAAAAGTTTTGACTTCCGA	ATCCACGCCACATAAACCAT	59.8	919	INTERGENIC				
CaPOPII_4110	scaffold2371	73819	GTT/GT	ATGGTTTATGTGGCGTGGAT	TGAATGAAGCGAAAATGTGC	60.1	321	INTERGENIC				
CaPOPII_4111	scaffold2371	74028	AG/A	ATGGTTTATGTGGCGTGGAT	TGAATGAAGCGAAAATGTGC	60.1	321	INTERGENIC				
CaPOPII_4112	scaffold2371	150056	TGGG/TGG	GCATTCGGGGGTATGTTTTA	CATCTTCCTTCCACAATTTCTTT	59.7	970	INTERGENIC				

INDEL marker IDs	Chromosomes /unanchored scaffolds	Physical positions (bp)	InDels (*Kabuli* reference genome-CDC Frontier/PI)	Forward primers (5'-3')	Reverse primers (5'-3')	Anneling temperature (0C)	Expected amplified product size (bp)	Structural annotation		Functional annotation		
								Sequence components of *kabuli* genome	*Kabuli* gene accession IDs	NCBI-KOG	TFs	NCBI-nr database
CaPOPII_4113	scaffold2371	287452	CTTTTTTTTTT/CTTTTTTTTT	CGACTCGTGTCCTAAAACTCAA	TTTCATGCCATAAGTCATTGTG	59.4	884	INTERGENIC				
CaPOPII_4114	scaffold2373	39509	GAA/GA	CGAACCGCAAATCTACCAAC	ATGTGTTCTCCTCCACTGCC	60.5	170	INTERGENIC				
CaPOPII_4115	scaffold2373	64133	CAAGTCTAAGTCT/CAAGTCTAAGTCTAAGTCT	AAGCAGTATTTGACGGGGTG	ATGCATGGATTGATTGGGAT	60.0	802	INTRON	Ca_27075			Transposase, Ptta/En/Spm, plant
CaPOPII_4116	scaffold2385	5093	TCC/TC	GAGTGAATTGTAACAGCCCAAA	GGACTTGCGGTGAAGAAGAA	59.1	720	INTERGENIC				
CaPOPII_4117	scaffold2385	5335	TT/TTTCAAT	GAGTGAATTGTAACAGCCCAAA	TCATTTCGACGCTTTCACAC	59.1	968	INTERGENIC				
CaPOPII_4118	scaffold239	61563	A/AT	ATCCATTGTTGTGCATCCAA	GCGATGGAGGACGAAAGTTA	59.8	466	INTERGENIC				
CaPOPII_4119	scaffold239	64716	GTTTTTTTT/GTTTTTTTTTT	CGTCCTCCATCGCCTTAGTA	AGGGAGGACGAAAATGACCT	60.2	898	INTERGENIC				
CaPOPII_4120	scaffold239	109046	T/TGTCTGA	TGCCTTAACTGACAAAGGGG	CAATCGATTCCCTCAGCACT	60.1	560	INTERGENIC				
CaPOPII_4121	scaffold239	109266	CA/C	GGTTTCAACCCCAATGCTTA	TCGACTTGTCAATCGCTTTG	59.8	808	INTERGENIC				
CaPOPII_4122	scaffold2392	10736	TA/TATATAAAGA	TAGGCTCCACCACTTGGGTA	TTGGAAGGAGCCCTTACCTT	60.5	745	INTERGENIC				
CaPOPII_4123	scaffold2392	10820	TTTCTTCTTCTTCTTCT/TTTCTTCTTCTTCTTCTTCT	GGTAGGCCTTGGGCTAGTGT	TTGGAAGGAGCCCTTACCTT	60.5	729	INTERGENIC				
CaPOPII_4124	scaffold2392	259018	TTCTCTCTCTCTCTCTCT/TTCTCTCTCTCTCTCT	ATCCTCCACCACCATAACCA	GCAGATCGCACCCAAATAAT	60.1	362	INTERGENIC				
CaPOPII_4125	scaffold2392	326211	AATATTT/A	TCATTCCCCACCTACACAAG	GTGCATGTGGCTGAAAAACA	58.4	552	INTERGENIC				
CaPOPII_4126	scaffold240	38776	TTATATATATATATATATATAT/TTATATATATATATATATATATATAT	TATTTTGGATTTTGGCTCGG	TAGGAGAAACGGAGGGGTTT	59.9	575	INTERGENIC				
CaPOPII_4127	scaffold240	73279	AT/A	CAAGGTGTCGATGTTGGTTG	CTGGTGGCTACTTTCTTGGG	60.0	472	INTERGENIC				
CaPOPII_4128	scaffold240	148297	TAG/TAGAGGAG	TGTGAGGCATGTCAAAAAGG	CGGCTGTGTTTTTCTGTGTC	59.7	822	DRR	Ca_25660			Transposase, Ptta/En/Spm, plant
CaPOPII_4129	scaffold240	150038	GTATATATATATATATATATATAT/GTATATATATATATATATAT	GGAGGCTTTGAAATTAGGGC	CTCCAGCGCACAACATCTAA	60.0	783	INTERGENIC				

INDEL marker IDs	Chromosomes /unanchored scaffolds	Physical positions (bp)	InDels (*Kabuli* reference genome-CDC Frontier/PI)	Forward primers (5'-3')	Reverse primers (5'-3')	Anneling temperature (0C)	Expected amplified product size (bp)	Structural annotation		Functional annotation		
								Sequence components of *kabuli* genome	*Kabuli* gene accession IDs	NCBI-KOG	TFs	NCBI-nr database
CaPOPII_4130	scaffold240	151385	TAAAAAAA/TAAAAAAAA	AGATGCATGTGATGAAACCAA	TACTCCCATTTCCTCCCCTT	59.0	727	INTERGENIC				
CaPOPII_4131	scaffold240	156860	AAACAACA/AAACA	GACAATCGCAGGAAGTGGTT	GCATTGCACCAAAACACAAC	60.1	699	INTERGENIC				
CaPOPII_4132	scaffold2408	509	AATATATATATATATATATATA/AATATATATATATATATATA	TCGAAGTGAAACTCGTCCCT	ACATCACCAACACGGTCTGA	59.8	631	INTERGENIC				
CaPOPII_4133	scaffold2410	15231	TTATATATATATATATAT/TTATATATATATATAT	CTTGGGAACTGGAGAGCAAG	AAATCGGAGAAGGAGGGAAA	60.0	559	INTERGENIC				
CaPOPII_4134	scaffold242	307052	CTTTTTTTTTT/CTTTTTTTTTTTTT	TTGCGTACGAGCATATCCAT	ATGGGTGCTTTCTATGGTCG	59.2	177	INTERGENIC				
CaPOPII_4135	scaffold242	360673	CTTTTTTTTTTTTTT/CTTTTTTTTTTT	CCAGAAAATCATCGGAAAGC	CCCAAGGAAATGAAGCAAAC	59.6	761	INTERGENIC				
CaPOPII_4136	scaffold242	368643	CAAAAAAAAA/CAAAAAAAA	AATGCATTAATTGAGGGGGA	AGGCAAAAGCTTCGTTGAAA	59.2	384	INTERGENIC				
CaPOPII_4137	scaffold242	577314	GTTTTTTTTT/GTTTTTTTTTT	GCAAGTTAAATGCGATTGTGT	GAGTTTTTGAGATGTGAATCAAACA	57.8	744	INTERGENIC				
CaPOPII_4138	scaffold242	593752	ACCC/ACC	GGTTGTTTGAGCAAACTGCAT	TTTGATTTGTTTGATGAGCCA	60.2	634	INTERGENIC				
CaPOPII_4139	scaffold242	698186	GAAA/GAAAA	GGCATTTTAAGGTTGATCAATAA	GACATTGGAGGCGATTGTTT	57.3	363	INTERGENIC				
CaPOPII_4140	scaffold242	735562	A/AC	CGAACTTGAGAATTCACCGC	GTGGAAAAGGGGATTGGATT	60.8	371	INTERGENIC				
CaPOPII_4141	scaffold242	793808	TCCC/TCC	CCTTCTTTCTTAGGGTTCCACC	TAGACACTGAACGCCAATCG	60.3	320	INTERGENIC				
CaPOPII_4142	scaffold2433	2074	AATATATAT/AATATAT	ACGATACGTTGTCGGCTAGG	TTGTGTCAAGCGGGATCATA	60.2	432	URR	Ca_27860			
CaPOPII_4143	scaffold2434	76263	TGG/TG	TTCTTCGTTTTGTAGTTGTACTCAATG	GGCAAACAAGAACCGAAAAC	60.0	638	INTERGENIC				
CaPOPII_4144	scaffold2466	8204	CT/CTT	CGATTGTGTTTGTCACAGGG	TCATTTTCGTCCTCCAAAGG	60.0	769	INTERGENIC				
CaPOPII_4145	scaffold2489	2070	CATA/CA	TTGGATGCACATGATTGGTT	CGTCCCCTCTTGGCTTATTT	59.8	214	INTERGENIC				
CaPOPII_4146	scaffold249	144782	AAGG/AAGGTAAAAGAGG	TGGATGCTCAAGATTGGTGA	CATTTTCGTCCTCCATAGGC	60.2	510	INTERGENIC				

INDEL marker IDs	Chromosomes /unanchored scaffolds	Physical positions (bp)	InDels (*Kabuli* reference genome-CDC Frontier/PI)	Forward primers (5'-3')	Reverse primers (5'-3')	Anneling temperature (0C)	Expected amplified product size (bp)	Structural annotation		Functional annotation		
								Sequence components of *kabuli* genome	*Kabuli* gene accession IDs	NCBI-KOG	TFs	NCBI-nr database
CaPOPII_4147	scaffold249	145492	TGG/TG	AGCCTAGGGAGGACGAAAAT	TTTTCATTTGCGTCATTGGA	59.2	656	INTERGENIC				
CaPOPII_4148	scaffold2491	6330	TTGTGT/TTGT	TCGAGGACCGAGAGACACTT	GCATTTTTGGACCAGACGAT	60.0	863	INTERGENIC				
CaPOPII_4149	scaffold250	82632	AT/ATAATTTTCTT	CCGGATCACTTTTTCCTTGA	GTGATGATAGCCCTCCTCCA	60.0	235	INTERGENIC				
CaPOPII_4150	scaffold2501	7458	TG/T	AACCGATCTTGTCGATCCAA	AGGGAGGACGAAAATGACCT	60.5	373	INTERGENIC				
CaPOPII_4151	scaffold2505	1462	TCCC/TCCCC	ATTCCAAACCAAAACCCTCC	TTGCTCACTACCACGCTACG	60.0	884	INTERGENIC				
CaPOPII_4152	scaffold2505	1793	TG/TGGAAGG	CGTTTCCACACAACCACAAA	TTGCTCACTACCACGCTACG	60.4	112	INTERGENIC				
CaPOPII_4153	scaffold2511	7402	CT/C	TGTTGAAGAAGGTTTTGGGG	CCAATAACGATCAAGCAATCG	59.9	359	INTERGENIC				
CaPOPII_4154	scaffold2528	19502	GTTTTT/GTTTT	GTCCTCCGTTGGCTTATTCA	GGACGAAAATGACCAAAAGG	60.1	488	INTERGENIC				
CaPOPII_4155	scaffold2542	2207	TAGGAG/TAG	TCATTTGGACTTTTCGGGAG	TCAATTTGGGTTCAGGGTGT	60.0	816	INTERGENIC				
CaPOPII_4156	scaffold2557	49964	AA/AATTA	CTTGGGCAAACACCTCCTAA	TGCATTGGTATATGGAGGCT	60.1	907	INTERGENIC				
CaPOPII_4157	scaffold2557	61886	ATTTTTT/ATTTTTTT	GCTAGAAATTTCGCCACACC	TCGGCAAAATATTAGGGACA	59.7	671	INTERGENIC				
CaPOPII_4158	scaffold2557	79849	TTATATATATATATATATATATA/TTATATATATATATATATA	TTCATTAGGATTTTGTTGCGG	TCAACAAGACGACGAGATGC	60.0	586	INTERGENIC				
CaPOPII_4159	scaffold256	43420	ATTT/ATTTT	TCACCTATCACGGGTCAACA	TGGTTTTGTCGTGAAATTGG	60.0	621	INTERGENIC				
CaPOPII_4160	scaffold2560	60629	T/TA	GGAGAACATTATCCGTTTTCCA	TCAAATTTCATCCTCACCCA	60.2	536	INTERGENIC				
CaPOPII_4161	scaffold2560	61168	ACACGCACGCAC/ACACGCACGCACGCACGCAC	GGGTGAGGATGAAATTTGAAGA	ATTCGGTAGCACATTCGTCC	60.3	864	INTERGENIC				
CaPOPII_4162	scaffold2575	196132	C/CA	ACTTTTCACCACATCCCTCG	ATCCATTCCCCACATTGAAA	60.0	347	INTERGENIC				
CaPOPII_4163	scaffold2575	247902	G/GA	AGCCATAACTTGCTTGAGGC	CCATCCTCACGTGGTTTTCT	59.5	374	INTERGENIC				

INDEL marker IDs	Chromosomes /unanchored scaffolds	Physical positions (bp)	InDels (*Kabuli* reference genome-CDC Frontier/PI)	Forward primers (5'-3')	Reverse primers (5'-3')	Anneling temperature (0C)	Expected amplified product size (bp)	Structural annotation		Functional annotation		
								Sequence components of *kabuli* genome	*Kabuli* gene accession IDs	NCBI-KOG	TFs	NCBI-nr database
CaPOPII_4164	scaffold2575	279306	TAA/TA	TTTTTGTTGGATGTAGTCCCTG	CATGAACATTTAAGAGTTTGCATTT	59.0	583	INTERGENIC				
CaPOPII_4165	scaffold2575	280082	C/CATTGATA	TCCCAAGGTATCATTGTCTCTTT	TCGGAGTTTTGTGTGAGTGTG	59.0	316	INTERGENIC				
CaPOPII_4166	scaffold26	84614	CATATATATATATA/CATATATATATATATA	TACAGAAACGGAGGGGTTTG	GGAGGCTTTTAGGCCAAAGT	60.0	559	INTERGENIC				
CaPOPII_4167	scaffold26	506230	GTT/GTTT	ATTGGCGTGAAAACCGTTAG	TGACGGGTCTGCACAATTTA	60.0	538	INTERGENIC				
CaPOPII_4168	scaffold260	1842	TAAA/TAA	AATGCACTCCAGGACCAAAC	CCTTCTTCAGCCGTATCAGC	60.0	547	INTERGENIC				
CaPOPII_4169	scaffold2603	6448	ATCATAAGTGAT/A	AGCAGCTTCAAGCAAAGCTC	TGAACCAGTCCAATTCAACAGT	60.0	485	INTERGENIC				
CaPOPII_4170	scaffold2603	6501	CCA/CCACA	AGCAGCTTCAAGCAAAGCTC	TGAACCAGTCCAATTCAACAGT	60.0	485	INTERGENIC				
CaPOPII_4171	scaffold2603	67369	GTT/G	AATCCGCAACGGAGTTTATG	TGAACGAGCTGAGAGAAGCA	60.0	617	INTERGENIC				
CaPOPII_4172	scaffold2617	320997	TAAAA/TAAA	TGGGGTATTCCTTTACGTCTT	TGAAAGAGGGGAAAAGGAGC	57.6	296	DRR	Ca_21883		WRKY	DNA-binding WRKY
CaPOPII_4173	scaffold2617	321441	ATTTTTTTT/ATTTTTTTTTT	TGCTCCTTTTCCCCTCTTTC	TTATGTGCGCATGACCAATC	60.7	542	DRR	Ca_21883		WRKY	DNA-binding WRKY
CaPOPII_4174	scaffold2617	321825	TAAAAA/TAAAAAAAAAA	TTGGTCATGCGCACATAATA	GATGGCTGTGTCGGAGACTT	58.6	529	DRR	Ca_21883		WRKY	DNA-binding WRKY
CaPOPII_4175	scaffold2617	324497	TGTG/T	AAACAAGTTGGTGGTGGAGC	AAAGTTAATTCAAATGCACTGTCA	60.0	290	INTERGENIC				
CaPOPII_4176	scaffold2617	402483	GAA/GAAA	GTTGCAAGGGTGGAGTGATT	AACAATTGTGAAACGTCTCCG	60.0	640	INTERGENIC				
CaPOPII_4177	scaffold2617	402519	TG/T	GTTGCAAGGGTGGAGTGATT	AACAATTGTGAAACGTCTCCG	60.0	640	INTERGENIC				
CaPOPII_4178	scaffold2617	402584	AAATAATAATAATAATAATAATAATAATAATAATAATAATAATA/AAGTAATAATAATAATAATAATAATAATAATAATAATAATAATAATAATA	GTTGCAAGGGTGGAGTGATT	AACAATTGTGAAACGTCTCCG	60.0	640	INTERGENIC				
CaPOPII_4179	scaffold2617	402777	ATTTTTTT/ATTTTTTTTTT	GTTGCAAGGGTGGAGTGATT	AACAATTGTGAAACGTCTCCG	60.0	640	INTERGENIC				
CaPOPII_4180	scaffold2660	12451	TAAAA/TAAAAA	CCTAGGGACGACGAAAATGA	TGCATCCAACAGGTGTGTTT	60.1	462	INTERGENIC				

INDEL marker IDs	Chromosomes /unanchored scaffolds	Physical positions (bp)	InDels (*Kabuli* reference genome-CDC Frontier/PI)	Forward primers (5'-3')	Reverse primers (5'-3')	Anneling temperature (0C)	Expected amplified product size (bp)	Structural annotation		Functional annotation		
								Sequence components of *kabuli* genome	*Kabuli* gene accession IDs	NCBI-KOG	TFs	NCBI-nr database
CaPOPII_4181	scaffold2662	1313	T/TC	TGCACTTGCAACACATCTCA	TGGCTATTGATTATCCCATTTTG	60.0	866	INTERGENIC				
CaPOPII_4182	scaffold2662	26227	TT/TATTTCCT	AACACCATGAAACGCTCCTC	CGAAGCCGAGAAAATGACTC	60.1	589	INTERGENIC				
CaPOPII_4183	scaffold2662	31062	TAA/TA	AGAGGATGCGGATCAATGTC	GGACAACCTGGGAAAAGTGA	60.0	898	INTERGENIC				
CaPOPII_4184	scaffold2662	43828	CNNNNNNNNNN/CNNNNNNNN	TCACGGGTCAACACGATTTA	TGCCATGTGATTTGTTGGTT	60.0	103	INTERGENIC				
CaPOPII_4185	scaffold2669	16328	AATATTGATATTGAT/AATATTGAT	TTTTAGGCGATTCTACGTGC	TTGAGGGGTGGCAAGTTTAG	58.1	641	INTERGENIC				
CaPOPII_4186	scaffold2678	3506	AATATATATATATATATATATATATATATATATATATATATATAT/AATATATATATATATATATATATATATATATATAT	GTCTTCACCAACCACCGTCT	CCATGCGTTTCAATTCTCCT	60.0	458	INTERGENIC				
CaPOPII_4187	scaffold2692	1438	AAAAATATTATCAAAATATTAT/AAAAATATTAT	CCCTCAAGTCAAACATAGGCA	AAGGAAATCCGAGTCTGCAA	60.1	511	INTERGENIC				
CaPOPII_4188	scaffold2692	14565	ATTTTTTT/ATTTTTTTT	ACCTGTCAAAAGTTTTCAAGCA	TCCTTCACCAACCAAACAAA	58.9	420	INTERGENIC				
CaPOPII_4189	scaffold2692	14759	ATTTTTTT/ATTTTTT	ACCTGTCAAAAGTTTTCAAGCA	TTTCCTTCACCAACCAAACA	58.9	422	INTERGENIC				
CaPOPII_4190	scaffold271	57742	TGAGATGATTAG/TG	TGGGGTGTTTCATAGCATCA	TTCATCCCATGCATCAGAAA	59.9	444	INTERGENIC				
CaPOPII_4191	scaffold271	205973	GAA/GA	GCACCGTGAGTAAATTCGGT	TATTTTGGATTTTGGCTCGG	60.0	692	DRR	Ca_25334			
CaPOPII_4192	scaffold271	267926	GATATATATATATATATATATAT/GATATATATATATATATATAT	GGGGTGCTCAACTTGTTTGT	GGCCAAGGTAAGGGTGAGAT	60.0	223	INTERGENIC				
CaPOPII_4193	scaffold2726	4506	G/GA	CAAACACCCCTATTGGATGC	AGGTCATTTTCGTCCTCCCT	60.2	380	INTERGENIC				
CaPOPII_4194	scaffold2728	11268	ATTTTTTTTTT/ATTTTTTTTTTT	TGGCATCCGGAGTCTATTTT	TGATAAACATGATTTTCCGTCA	59.5	603	INTERGENIC				
CaPOPII_4195	scaffold2734	606	TGCAAG/TGCAAGCAAG	TTTCTTTTTCCCTCCAAGCA	GGGGGAGAGAACAAATCCAT	59.8	550	INTERGENIC				
CaPOPII_4196	scaffold2736	3536	GAAAAAA/GAAAAA	TTCAAGGAAAATGCCCAGTC	TGTTTGCGACAACACAACAA	60.1	385	INTERGENIC				
CaPOPII_4197	scaffold2737	9534	TAAA/TAA	TCTTCGTTTTACGGTTTTATTTCA	AACATATAACAAAATTGAGTTGTCAAA	59.2	419	INTERGENIC				

INDEL marker IDs	Chromosomes /unanchored scaffolds	Physical positions (bp)	InDels (*Kabuli* reference genome-CDC Frontier/PI)	Forward primers (5'-3')	Reverse primers (5'-3')	Anneling temperature (0C)	Expected amplified product size (bp)	Structural annotation		Functional annotation		
								Sequence components of *kabuli* genome	*Kabuli* gene accession IDs	NCBI-KOG	TFs	NCBI-nr database
CaPOPII_4198	scaffold2738	31131	TTTCTTTCTTCTTTCTTCTTTCTT/TTTCTTTCTTCTTTCTTCTTTCTTCTTTCTT	TCCTAGAACATGCATCACGC	AAACGCAATCCTTGAGTTCG	59.8	379	INTERGENIC				
CaPOPII_4199	scaffold2748	27802	TCA/TCACA	TCTCGTTTAGATCGTCCGCT	TGGAATTCTTCTCCAATGCC	60.0	551	INTRON	Ca_28188			
CaPOPII_4200	scaffold275	147158	AG/A	GGAGCAAACGGAAGGTATGA	ACATGAGTCTTTGTTGCCCC	60.1	331	INTERGENIC				
CaPOPII_4201	scaffold275	162362	TG/TGG	CGAAGCACAAGGAGATGACA	GGTTTGGGCAATCTGCTTTA	60.0	762	INTERGENIC				
CaPOPII_4202	scaffold275	355509	GAAAAAAA/GAAAAAA	GCTTAGGCAGCCACAAAATC	TTGCCCCTTGGATAAACTCA	59.9	510	INTERGENIC				
CaPOPII_4203	scaffold275	377072	AATATATATATATATATATATATATAT/AATATATATATATATATATATATATATATAT	TCAAGATACCCAGTGCACCA	AGCGACATGTGAACAACAGC	60.1	551	INTERGENIC				
CaPOPII_4204	scaffold275	377587	TAAAAA/TAAAA	GCTGTTGTTCACATGTCGCT	CAATTCCCACAGGTACGCTT	59.9	485	INTERGENIC				
CaPOPII_4205	scaffold2763	9760	C/CCTCACG	ATGCCAGTCGTTATTCCCAG	GAGATGCCATTGAAGCCATT	60.0	400	CDS (large-effect mutations)	Ca_24393	R		
CaPOPII_4206	scaffold2763	183619	TC/TCC	GCACTGAAAGCAACAACCAA	ACCAGGTTATGAATAGCCGC	59.9	422	INTERGENIC				
CaPOPII_4207	scaffold277	4447	TACTGA/TA	TAAAGGAGCATCCGTGGAAT	TGCATGTATCATAGACCGCC	59.5	472	INTERGENIC				
CaPOPII_4208	scaffold277	4861	AATATATAT/AATATAT	TGGAGAAACCTTGTGAACCA	GAAACGGAAAGGGGAACAAT	59.1	316	INTERGENIC				
CaPOPII_4209	scaffold277	205504	TTATATATATATATATATATAT/TGATATATATATATATATATATATATATATAT	GGTTTAGGTGTGGGAGTGGA	TGATACAAGGTTCAACCGCA	59.8	444	DRR	Ca_25752			
CaPOPII_4210	scaffold277	252454	CTCTT/CT	CCCATTGCATTGCCTTCTAT	TAAAGGACCATGTCGGGAAG	59.9	329	INTERGENIC				
CaPOPII_4211	scaffold2777	2240	ATT/ATTT	GACTTTTGCACTTATTGGACGA	CATTGCGGTTCAAAATCCAT	59.3	653	INTERGENIC				
CaPOPII_4212	scaffold2777	4052	GTTAAC/G	CATTGATTAAGAACGCAAATGG	AGTTGGTCCCAAAAGTGCAA	59.5	199	INTERGENIC				
CaPOPII_4213	scaffold2779	6967	GT/GTGTGTT	AGGTCATTTTCGTCCTCCCT	TCGAAATGTTCATCCAACGA	59.9	498	INTERGENIC				
CaPOPII_4214	scaffold2782	6224	AGG/AGGG	CTTTTCGGGCGATTTTCATA	GATGAAATCGAGGACTGGGA	60.0	483	INTERGENIC				

INDEL marker IDs	Chromosomes /unanchored scaffolds	Physical positions (bp)	InDels (*Kabuli* reference genome-CDC Frontier/PI)	Forward primers (5'-3')	Reverse primers (5'-3')	Anneling temperature (0C)	Expected amplified product size (bp)	Structural annotation		Functional annotation		
								Sequence components of *kabuli* genome	*Kabuli* gene accession IDs	NCBI-KOG	TFs	NCBI-nr database
CaPOPII_4215	scaffold280	52649	CTCAAT/CTCAATTGATTTATCAAT	TGGTCATGCAGAAACTCAGC	ACTCTCATCTGAGGGCGAAG	60.0	330	INTERGENIC				
CaPOPII_4216	scaffold280	107582	C/CCTCTTGGG	AGCTCCTGCTCAAGCAGTTC	AAAATGACCCCCTGATTTCC	59.9	653	INTERGENIC				
CaPOPII_4217	scaffold280	128741	CAA/CAAA	ATTCCAAACCAAAACCCTCC	TGATTTTGGGGGAAAATGAA	60.0	943	INTERGENIC				
CaPOPII_4218	scaffold280	302193	CAA/CAAA	TCACCTATCACGGGTCAACA	TCATCGTATTTCGCGTGTTC	60.0	593	INTERGENIC				
CaPOPII_4219	scaffold281	1263	CAA/CAATTAA	TGTTCGACACTCAGTCTTACGG	TGCATGCCCTTTGATTTACA	60.3	287	INTERGENIC				
CaPOPII_4220	scaffold281	1388	GAAAAAAAAAA/GAAAAAAAAA	TGTTCGACACTCAGTCTTACGG	TGCATGCCCTTTGATTTACA	60.3	287	INTERGENIC				
CaPOPII_4221	scaffold281	1467	GGCATGCATG/GGCATG	TGTTCGACACTCAGTCTTACGG	ATCGGAAAAGTGACATTGGG	60.3	515	INTERGENIC				
CaPOPII_4222	scaffold281	2224	CT/C	TGGGCAGATTTGTAACACGA	GCGAAACCCGATTCTATTGT	60.1	593	INTERGENIC				
CaPOPII_4223	scaffold281	3002	TAAAAA/TAAAAAA	ACAATAGAATCGGGTTTCGC	AGGACTATTTTTGGTCTCCCA	59.0	623	INTERGENIC				
CaPOPII_4224	scaffold281	3090	AATATATATATATAT/AATATATATAT	ACAATAGAATCGGGTTTCGC	AGGACTATTTTTGGTCTCCCA	59.0	623	INTERGENIC				
CaPOPII_4225	scaffold281	3414	GAAAAGAAAA/GAAAAGAAAAAGAAAA	TGGGAGACCAAAAATAGTCCT	CGTTGACCATTTTAAGCTTGG	57.7	534	INTERGENIC				
CaPOPII_4226	scaffold281	3735	A/AATT	TGGGAGACCAAAAATAGTCCT	CAAGAGGGGTTGCAAGTGTT	57.7	648	INTERGENIC				
CaPOPII_4227	scaffold281	4125	TGG/TG	AACACTTGCAACCCCTCTTG	ATGATGATTTTCTCCCCGTG	60.1	595	INTERGENIC				
CaPOPII_4228	scaffold281	4227	AG/AGGAATTATG	AACACTTGCAACCCCTCTTG	ATGATGATTTTCTCCCCGTG	60.1	595	INTERGENIC				
CaPOPII_4229	scaffold281	4869	TGCCGGCCG/TGCCG	ACACGGGGAGAAAATCATCA	GACGCGCTATAGAACTTCGC	60.3	711	INTERGENIC				
CaPOPII_4230	scaffold281	5058	TATAAT/TAT	CAATTGCAGTCAGGTTGTGC	GACGCGCTATAGAACTTCGC	60.3	297	INTERGENIC				
CaPOPII_4231	scaffold2812	16756	TAAAAAA/TAAAAAAA	CAATTGTTGTTGTTGACATTTGG	GGTTGATGCGTCAATGTTGT	60.2	871	INTERGENIC				

INDEL marker IDs	Chromosomes /unanchored scaffolds	Physical positions (bp)	InDels (*Kabuli* reference genome-CDC Frontier/PI)	Forward primers (5'-3')	Reverse primers (5'-3')	Anneling temperature (0C)	Expected amplified product size (bp)	Structural annotation		Functional annotation		
								Sequence components of *kabuli* genome	*Kabuli* gene accession IDs	NCBI-KOG	TFs	NCBI-nr database
CaPOPII_4232	scaffold2812	43005	TTATATATATATATATATATAT/TTATATATATATATATATATATAT	TCACACGAGAATCATGTCAAG	TTGCGAATTTGTTTGCAAGT	57.2	837	INTERGENIC				
CaPOPII_4233	scaffold2812	58880	TAAAAA/TAAAAAA	TTGCTCCCAAAACAAGCTCT	TACTTTGTGGCTGTTTCCCC	60.0	672	INTERGENIC				
CaPOPII_4234	scaffold2815	71273	TC/T	CGGTGCTAGTGGACCAATTT	GTTATTCATAGCCTCCGCCA	60.0	576	INTERGENIC				
CaPOPII_4235	scaffold284	3250	AGAATGAATGAATGAA/AGAATGAATGAATGAATGAA	TCACAATGGGCACAAGAAAA	TTTCCCATGCAACTTGAACA	60.1	146	INTERGENIC				
CaPOPII_4236	scaffold284	4461	CTCATCATCATCATCATCA/CTCCTCCTCCTCCTCATCATCATCATCATCATCA	TACGATGATGAACAGTCGGC	TTGGTTTCAAGACCTGCATCT	59.7	424	INTERGENIC				
CaPOPII_4237	scaffold284	21461	AT/A	AATGTTGTGGTTGGGTTGGT	ACAAGGCTAACTCAAGGCCA	60.0	517	INTERGENIC				
CaPOPII_4238	scaffold284	477291	AAATAATAATAATAATAATAATAATAATAATAATAATAATAATAATAATAA/AAATAATAATAATAATAATAATAATAATAATAATAATAATAATAATAATAA	TTTATGTGCCCTTGACCGAT	GTTTTGGCGTTGGTTTCACT	60.3	847	INTERGENIC				
CaPOPII_4239	scaffold284	702931	ATTTTTTTTT/ATTTTTTTT	CATTTTTGCCTCTATCTGCCA	TTTGCGATGCTTGATCGTTA	60.2	766	INTERGENIC				
CaPOPII_4240	scaffold2853	6530	CTTTTTTTT/CTTTTTTT	CCATTTCGGTTTTAACCAATGTA	GATTGATGCACTAATTCGACG	60.0	642	INTERGENIC				
CaPOPII_4241	scaffold287	22647	AA/AATTA	TTTTAACAGATGTTCCGCCC	GGAACAATGTAGGCAAGGGA	59.9	708	INTRON	Ca_25380			
CaPOPII_4242	scaffold2871	30418	AGT/AGTGT	GGTTTTGGATTTGGGCTACA	GGGTTATGGCTTTGATCATGT	59.8	521	INTERGENIC				
CaPOPII_4243	scaffold2887	7283	CTG/C	TGGTGCGTCTTAATGTGGAA	TTTGCCATGACCTTTTTGTTC	60.1	473	INTERGENIC				
CaPOPII_4244	scaffold2893	10683	AGG/AGGG	GCCAGATCTTTCGATTCAGC	CCTTCAATGGAACCGCATAC	59.9	255	INTERGENIC				
CaPOPII_4245	scaffold29	100652	C/CA	TCCCGATCTTCGATTTCATC	CCATCGTTCAGTCCGAAAAT	60.0	254	INTERGENIC				
CaPOPII_4246	scaffold290	206705	GATAT/GATCAATTAAATAT	TCCCTCTTTTCAAGTCAACTCA	TCGCTCTAGGCTAATCCAACT	59.0	318	INTRON	Ca_19830	F		Xanthine/uracil/vitamin C permease
CaPOPII_4247	scaffold290	293241	ATTATTTATTTATTTATTTATTTATT/ATTATTTATTTATTTATTTATTTATTTATT	TTGCTCTTAATTTTTCCTCGAA	GTGAGTCTGCGACCACTTGA	58.2	602	INTERGENIC				
CaPOPII_4248	scaffold290	400824	TAAAAAAAAAA/TAAAAAAAAAAAA	TTGTGTTGCATTGTGGATTCT	CACAGCCCCTCTAAACCAAC	59.0	650	INTERGENIC				

INDEL marker IDs	Chromosomes /unanchored scaffolds	Physical positions (bp)	InDels (*Kabuli* reference genome-CDC Frontier/PI)	Forward primers (5'-3')	Reverse primers (5'-3')	Anneling temperature (0C)	Expected amplified product size (bp)	Structural annotation		Functional annotation		
								Sequence components of *kabuli* genome	*Kabuli* gene accession IDs	NCBI-KOG	TFs	NCBI-nr database
CaPOPII_4249	scaffold290	452067	GAAA/GAA	GAAAAATTCACCACCGATTAGG	TACAATCAGGATGCGTCCAA	59.7	632	INTERGENIC				
CaPOPII_4250	scaffold290	820202	ATT/ATTT	ACCAAGCCTTTTGGACAATG	TCTTCGGTTGGAATTTGGTT	60.0	879	INTERGENIC				
CaPOPII_4251	scaffold290	892920	GAAAATAAAAAAATAAAAAAATA/GAAAATAAAAAAATA	ACCCGGGTATAAGGTTCCAC	TTCCCTACGCATAATCGGTC	59.9	467	INTERGENIC				
CaPOPII_4252	scaffold2900	7979	ATT/AT	TATGAAAATCGCCCGAAAAG	GGTCCTCGATTTCATCTGGA	60.0	355	INTERGENIC				
CaPOPII_4253	scaffold2922	7477	CGG/CG	TTTCATCTGGACTTTTCGGG	TAACTACGTTTCGGGTTCCG	60.0	447	INTERGENIC				
CaPOPII_4254	scaffold2922	8355	GAAAAA/GAAAAAA	GACTTTTCGGGCGATATTCA	ATATGCAACCGAAGACCAGG	60.0	647	INTERGENIC				
CaPOPII_4255	scaffold2924	8200	ATTTTT/ATTTTTT	TTGCTTCATGGAACTTGAGG	CGTCCTCGAAATTTGACGTG	58.8	295	INTERGENIC				
CaPOPII_4256	scaffold2928	22766	ATTTT/ATTTTT	AACCAATGATGTGCATCCAA	AGGGAGGACGAAAATGACCT	59.8	471	INTERGENIC				
CaPOPII_4257	scaffold2928	23701	TTGT/TT	AGACCTCGCTTGGCTGATTA	ACGGAGGAGGAAAATGACCT	60.0	210	INTERGENIC				
CaPOPII_4258	scaffold2931	1404	CTTTTTTT/CTTTTTTTT	GCCCTCAGATGAGAGTCAGG	GGAGGGTTTTGGTTTGGAAT	59.9	707	INTERGENIC				
CaPOPII_4259	scaffold2932	2304	GTTTT/GTTTTT	ATTGTCGTCCTCCCTAGGCT	ACACCTATTGGATGCCCAAG	60.1	670	INTERGENIC				
CaPOPII_4260	scaffold2940	1242	TC/T	TGTGCACCGAATAGGTGTGT	TCCTTCGGAGGATGAAAATG	60.0	266	INTERGENIC				
CaPOPII_4261	scaffold2950	1436	ATTTT/ATTTTT	GTCCTCTGAAACCTCCCACA	TTTTGGGTCGTGACACTTGA	60.1	337	INTERGENIC				
CaPOPII_4262	scaffold2950	6843	A/AG	CCAAATTACAACCGACCCAC	AAAGGCGCTCCTCTATCACA	60.1	650	INTERGENIC				
CaPOPII_4263	scaffold2950	6903	AAA/AAATAA	CCAAATTACAACCGACCCAC	AAAGGCGCTCCTCTATCACA	60.1	650	INTERGENIC				
CaPOPII_4264	scaffold2950	8204	T/TG	TGGTCAGAATGGGCTTTACC	TTCAGAAGGGCCAGAAGAAA	59.9	506	INTRON	Ca_27517	L	FAR1	BRCT
CaPOPII_4265	scaffold2950	10165	AGG/AGGG	AGTTGTTTGGATACACCGGC	AGTGCTGTGGCTGGACCTAT	59.9	662	INTRON	Ca_27517	L	FAR1	BRCT

INDEL marker IDs	Chromosomes /unanchored scaffolds	Physical positions (bp)	InDels (*Kabuli* reference genome-CDC Frontier/PI)	Forward primers (5'-3')	Reverse primers (5'-3')	Anneling temperature (0C)	Expected amplified product size (bp)	Structural annotation		Functional annotation		
								Sequence components of *kabuli* genome	*Kabuli* gene accession IDs	NCBI-KOG	TFs	NCBI-nr database
CaPOPII_4266	scaffold2950	10696	G/GT	TGATGATATCAGGCAACGGA	TGTCCCCACAGGTAGCTTTC	60.0	588	INTRON	Ca_27517	L	FAR1	BRCT
CaPOPII_4267	scaffold2955	9708	TTACTA/TCTACTACTA	ACTATGCAAAGGTGCAAGGC	CACCATTTTCCGTCCACTCT	60.3	374	INTERGENIC				
CaPOPII_4268	scaffold2955	10080	CT/CTT	AGAGTGGACGGAAAATGGTG	AAACTTGGTGCCCTGACTTG	60.0	621	INTERGENIC				
CaPOPII_4269	scaffold296	11136	GTTTT/GTTT	TTGAATCCTTTTCTGGGTGG	CTTGTGCTTCGGTTACAGCA	59.9	658	INTERGENIC				
CaPOPII_4270	scaffold296	11327	TCC/TCCC	TGGACGTAGTCCGAGTTGGT	CTTGTGCTTCGGTTACAGCA	60.6	517	INTERGENIC				
CaPOPII_4271	scaffold296	17248	AATATATATATATATATATATATATATATATATATAT/AATATATATATATATATATATATATATATATATATATATATATATATATAT	AGGAGGAGGGATCTGGAAAA	TTGCTCCTACGTATCTCCCG	60.0	965	INTERGENIC				
CaPOPII_4272	scaffold296	233039	AATATATATATATATATATATATAT/AATATATATATATATATATATAT	TTGAGTTGCGACGAGAGTACA	GAAACATGGAGGTTTGCGTT	59.6	488	INTERGENIC				
CaPOPII_4273	scaffold296	261628	TACACACACAC/TACACACACACAC	CATTGTGGGAGCGTAAGACA	TCGCAACTCTCATGTGTATGG	59.7	388	INTERGENIC				
CaPOPII_4274	scaffold296	261904	CAAAAA/CAAAA	CCATACACATGAGAGTTGCGA	TTGACTCAGCCTTAAATTTTTATTGA	59.7	534	DRR	Ca_20542			
CaPOPII_4275	scaffold296	262823	ATATTATTATTATTATTATTA/ATATTATTATTATTATTA	CACTTTTTAACCGGAGGAATG	GCAGTGGATAAGGCCGATAA	58.6	140	DRR	Ca_20542			
CaPOPII_4276	scaffold296	406805	GTTATTATTATTATTATTATTATTATTATTATTATTATTATTAATATTATTATTAT/GTTATTATTATTATTATTATTATTATTATTATTATTATTATTATTA	AGTTATTTACGACGCACGGG	GGCACGACATCATCATCATC	60.0	547	INTERGENIC				
CaPOPII_4277	scaffold298	8123	TGA/T	AAATGAAAAGTGGCGAGGAC	AGTTACCCATTTGTGGCAGC	59.2	740	INTERGENIC				
CaPOPII_4278	scaffold298	8968	ATT/ATTTT	GCTGCCACAAATGGGTAACT	CCAAAAACGGTGACGAAACT	60.0	771	INTERGENIC				
CaPOPII_4279	scaffold298	14439	ATTT/ATT	TTACACCAAATCCTTTGCCC	GAGCCCAACAGTTCCATGAT	59.8	792	INTERGENIC				
CaPOPII_4280	scaffold298	156072	GTTTTTT/GTTTTT	ACCCAATTACTGGAGATGCG	AGTCAGTGGCAAAAACAGCA	60.0	410	INTERGENIC				
CaPOPII_4281	scaffold298	208119	GTTTTTTTT/GTTTTTTT	AGTCGGAGGTTCCTAGCACA	ATTGTTGGGCTTGTCAGTCC	59.9	471	INTERGENIC				
CaPOPII_4282	scaffold298	208171	TTATATATATATA/TTATATATATATATATATA	AGTCGGAGGTTCCTAGCACA	ATTGTTGGGCTTGTCAGTCC	59.9	471	INTERGENIC				

INDEL marker IDs	Chromosomes /unanchored scaffolds	Physical positions (bp)	InDels (*Kabuli* reference genome-CDC Frontier/PI)	Forward primers (5'-3')	Reverse primers (5'-3')	Anneling temperature (0C)	Expected amplified product size (bp)	Structural annotation		Functional annotation		
								Sequence components of *kabuli* genome	*Kabuli* gene accession IDs	NCBI-KOG	TFs	NCBI-nr database
CaPOPII_4283	scaffold298	212123	CAAAAAAAAAAAAA/CAAAAAAAAAA	ATAGCCTCCCTTTTTGGCAT	TGCAGCTTCTGGTGTTCATC	59.9	371	INTRON	Ca_27492			
CaPOPII_4284	scaffold299	53102	CAAAAAAAAAA/CAAAAAAAAAAAA	TGAAGGGTGTCACAAACCAA	ATTCATGGTGCTTTCTTGGC	60.0	313	INTERGENIC				
CaPOPII_4285	scaffold299	158960	TA/TAA	TACGAACCGGTAGAACCGTC	CGTGGTAATCGCGTAGGAAT	60.0	574	INTRON	Ca_25996	AJ		RNA recognition motif domain
CaPOPII_4286	scaffold300	74648	TG/T	AAAACACGCCTATTGGATGC	AGGTCATTTTCGTCCTCCCT	60.0	382	INTERGENIC				
CaPOPII_4287	scaffold3015	68656	A/AACCGGCACAGCC	TTCGCAGACAAAACTCATGC	AGCATTATGGGCCATCTGAG	60.0	283	CDS (large-effect mutations)	Ca_27318			
CaPOPII_4288	scaffold3015	69170	AG/A	GGCCCATAATGCTCAAGTGT	TCATAGGCATGGGATTTGAA	60.0	472	DRR	Ca_27318			
CaPOPII_4289	scaffold3015	73264	TGGCATAG/TG	TTTGTGTTTGCGATGACGAT	TAAGAGCGGAGTTTCTCCCA	60.1	792	INTERGENIC				
CaPOPII_4290	scaffold3015	73454	TTTTGACTTTGACTTT/TTTTGACTTTGACTTTGACTTT	GTCTCGCGTTGAGTTAAGGG	TAAGAGCGGAGTTTCTCCCA	59.9	489	INTERGENIC				
CaPOPII_4291	scaffold3015	73940	TG/T	TGGGAGAAACTCCGCTCTTA	TGATGGCTCCAAGGTGTATG	59.9	538	INTERGENIC				
CaPOPII_4292	scaffold3015	74020	AAAATCATA/AA	TGGGAGAAACTCCGCTCTTA	TGATGGCTCCAAGGTGTATG	59.9	538	INTERGENIC				
CaPOPII_4293	scaffold3015	74169	TTT/TTTCTT	TTTTGAAATTTAGGGTGTCACG	TGATGGCTCCAAGGTGTATG	59.0	221	INTERGENIC				
CaPOPII_4294	scaffold3015	76583	CTTTTTTTT/CTTTTTTTTTT	GGCTAAATTACATCTGTGGTCCTT	TGAAAGAGAAAGGGCTTGGA	59.8	532	INTERGENIC				
CaPOPII_4295	scaffold3015	76843	CAAAA/CAA	TTTCCCGACTTGGTCCTTTA	TGAAAGAGAAAGGGCTTGGA	59.5	468	INTERGENIC				
CaPOPII_4296	scaffold3016	87899	TTATATATATATATATATATATATATATATA/TTATATATATATATATATATATATATATATATATATATATA	TCCTCACTCTTCGCACCTCT	CATTGGCTTTGTTGGTGATG	60.1	674	INTRON	Ca_26725	C	NAC	
CaPOPII_4297	scaffold3019	17640	GTTTT/GTTTTT	TGCATTATGGGTAGGTGCAA	GATGTCTACCAGTTGCGGGT	60.0	570	DRR	Ca_28069			XS domain
CaPOPII_4298	scaffold3019	20892	AT/AATGGTT	TGCATGAGTTGTTAAGGCGA	TATGCAAAACGGGACTTCCT	60.4	582	URR	Ca_28069			XS domain
CaPOPII_4299	scaffold3021	2998	ATTTTTTTT/ATTTTTTT	TGGTTTGTGCGATGAGAGAG	TCACGCAGAAAGCAAAACAC	60.0	649	INTERGENIC				

INDEL marker IDs	Chromosomes /unanchored scaffolds	Physical positions (bp)	InDels (*Kabuli* reference genome-CDC Frontier/PI)	Forward primers (5'-3')	Reverse primers (5'-3')	Anneling temperature (0C)	Expected amplified product size (bp)	**Structural annotation**		**Functional annotation**		
								**Sequence components of *kabuli* genome**	***Kabuli* gene accession IDs**	**NCBI-KOG**	**TFs**	**NCBI-nr database**
CaPOPII_4300	scaffold3029	14921	AGG/AGGG	GCGTTCAATTGGTGTG TTTG	GTCCTACCGGTTCGTA CCCT	60.0	721	INTERGENIC				
CaPOPII_4301	scaffold303	162542	AAGCGCTGT/AAGCGCTGTGGAAAC GAGCGCTGT	GCCATTTACAGCGCTTT TTC	TGAAATTATGGGTGGG CATT	59.9	357	DRR	Ca_26661		Trihelix	
CaPOPII_4302	scaffold3046	14446	AGGG/AGG	TTGGATGAACATTTCG GTCA	AAGCCTAGGGAGGACG AAAA	59.9	240	INTERGENIC				
CaPOPII_4303	scaffold306	128535	TA/T	ACCGAAAGGAGAACAC ATGG	TGCAAATGAAAGTTTAA AGACCA	60.0	240	INTERGENIC				
CaPOPII_4304	scaffold306	166393	TAAATCGATTG/TAAATCGATTGGGA AATCGATTG	TGCACAAGTCAGTAGC ACCC	GCCAATCGATTACCCT CAAA	59.9	138	INTERGENIC				
CaPOPII_4305	scaffold3082	10375	TAAAAAAAAAAAA/TAAAAAAAAAA	GCCCTCAGATGAGAGT CAGG	CCATCAGCATAACCCC ACAT	59.9	716	INTERGENIC				
CaPOPII_4306	scaffold3092	1124	TAAAAAAA/TAAAAAAAA	GAGGTATTTTACAGCG CTCTTTC	TTGGGTCTGAAGAATG GCTC	58.7	117	INTERGENIC				
CaPOPII_4307	scaffold3093	19732	GATATATATATATAT/GATATATATAT ATATAT	GAACCCTCACAATCACA CGA	GAGCGCTGTAAAACAC CTCC	59.5	863	INTERGENIC				
CaPOPII_4308	scaffold311	23991	TG/T	TTTTCGTCCTCCCTAGG CTT	GCCAATGACACAAACA CACC	60.2	455	INTERGENIC				
CaPOPII_4309	scaffold311	91768	CAAAAA/CAAAA	TGGGCAAGAAAAAGCC TATG	AGGTCATTTTCGTCCTC CCT	60.2	836	INTERGENIC				
CaPOPII_4310	scaffold311	131360	A/ACAT	GAAGCTCAGATGGCAC ACAA	CAATCTCCCCCTTGAG TTGA	60.0	716	DRR	Ca_26332			
CaPOPII_4311	scaffold3115	62578	ATTTTTTTTTTTT/ATTTTTTTTTTTTTT	CTTGGTGGTCCACTAT GTGC	GGAGGTCAAAATACTA ACTGCGA	59.0	594	INTERGENIC				
CaPOPII_4312	scaffold3115	62639	CTTTTTTT/CTTTTTTTT	CTTGGTGGTCCACTAT GTGC	TTCATAGGAGGCTGCC ATTC	59.0	632	INTERGENIC				
CaPOPII_4313	scaffold3115	62933	TTTATTATTATTATTATTATTATTATTA TTTTTATTATTATTATTATTATAATTAT TATTATTA/TTTATTATTATTATTATTA TTATTATTTTTATTATTATTATTATTAT	CTTGGTGGTCCACTAT GTGC	TTCATAGGAGGCTGCC ATTC	59.0	632	INTERGENIC				
CaPOPII_4314	scaffold3115	63986	AATATATATATATATATATATATATAT ATA/AATATATATATATATATATATATA TATATATA	TGAAGCATATGCCGTG AGAG	GCGTCAATGAACTTTA CATGC	60.0	559	INTERGENIC				
CaPOPII_4315	scaffold3127	2181	AC/A	GGAAGCACAAGATGGG GTAA	CGGTCATTTTTCTCCTC CCT	59.9	265	INTERGENIC				
CaPOPII_4316	scaffold3127	8863	A/AT	AATCCAATGGCACAAA CACA	GGACATTTTCGTCCTG CCTA	59.8	488	INTERGENIC				

**INDEL marker IDs**	**Chromosomes /unanchored scaffolds**	**Physical positions (bp)**	**InDels (*Kabuli* reference genome-CDC Frontier/PI)**	**Forward primers (5'-3')**	**Reverse primers (5'-3')**	**Anneling temperature (0C)**	**Expected amplified product size (bp)**	**Structural annotation**		**Functional annotation**		
								**Sequence components of *kabuli* genome**	***Kabuli* gene accession IDs**	**NCBI-KOG**	**TFs**	**NCBI-nr database**
CaPOPII_4317	scaffold3127	10029	TGAT/TGATCCACAAGATTGGAT	AGGGAGGACGAAAATGACCT	TGTGCGTCCAATAGGTGTGT	59.9	561	INTERGENIC				
CaPOPII_4318	scaffold3134	12154	ACATC/A	TAAACGAGGTAAACCGGTGC	GAAAGTCGCATGTGCAACAC	60.0	437	DRR	Ca_27104	O	C2H2	Peptidase A1
CaPOPII_4319	scaffold3134	13444	TA/TATTAAACAAA	CCAATCAGTTGGAGCCAGTT	CCAAGAGAAAGGCAGTCGAG	60.1	891	DRR	Ca_27104	O	C2H2	Peptidase A1
CaPOPII_4320	scaffold3134	15211	TAAA/TAAAAA	CCTGGTTGAGTCTGGATAGCA	GCATTGGCGTATCTTGTTCA	60.3	874	INTRON	Ca_27104	O	C2H2	Peptidase A1
CaPOPII_4321	scaffold3134	109334	AATATATATATATATATATATAT/AATATATATATATATAT	AGGAAGAGTGGAGTTGCGAA	TTAATGTGTTGCGGGTGAAA	60.0	342	INTERGENIC				
CaPOPII_4322	scaffold3135	4161	TCC/TC	TCCAGATGAAATCGAGGACC	CCCGAAAAGTCCAGATGAAA	60.0	882	INTERGENIC				
CaPOPII_4323	scaffold314	1870	GAAAAAAAAAA/GAAAAAAAAAAAAAA	GCCCTCAGATGAGAGTCAGG	AAACGCAATCCTTGAGTTCG	59.9	509	INTERGENIC				
CaPOPII_4324	scaffold314	404143	AT/ATAACAGT	TTTTGAAACTTGGGTGGGAG	CATTGCACTGCATGCTTTTT	59.9	651	DRR	Ca_21806	KR	G2-like	Mitochodrial transcription termination factor-related
CaPOPII_4325	scaffold3149	9644	TAG/TAGGAG	TTTCATCTGGACTTTTCGGG	ATCGTCGATGGGTACGAAAG	60.0	452	INTERGENIC				
CaPOPII_4326	scaffold3155	24057	GAAAAAAAA/GAAAAAAA	TGCATATTTTGAAACGAATGACA	TGCAACCATCGGTTAAAATG	60.4	505	INTRON	Ca_27114	R	NF-YB	
CaPOPII_4327	scaffold3160	9833	TGG/TG	AACCAGTCACTCAAGCGTCC	TGGCTGGTGATTTGTGTGAT	60.3	165	INTERGENIC				
CaPOPII_4328	scaffold3160	10499	AG/AGG	TGGCTTTTTAGTTAGAGCACCA	GCAAACAAAGGAAACGGAAA	59.1	629	INTERGENIC				
CaPOPII_4329	scaffold3168	514	CAGTAG/CAG	AGTGAAACCAACGCCAAAAC	ATTCCCCACCCAAATACCTC	60.0	761	INTERGENIC				
CaPOPII_4330	scaffold317	209733	T/TC	TGTTGTTGTGATTGTGCCCT	CAGGATGTCAGCAGCAAAAA	60.0	243	INTERGENIC				
CaPOPII_4331	scaffold3170	106196	CTATATATATATATATATAT/CTATATATATATATATATATAT	GGAATAAGGCGGGTGTATCA	TCACAATGAAACAAAGGGCA	59.8	529	INTERGENIC				
CaPOPII_4332	scaffold3171	2398	C/CTA	TGATGGAGGATTGAGGAAGG	GTTGACAAATGCAAAATGCG	60.0	431	INTERGENIC				
CaPOPII_4333	scaffold3171	2647	ATTTTTT/ATTTT	TGATGGAGGATTGAGGAAGG	GTTGACAAATGCAAAATGCG	60.0	431	INTERGENIC				

INDEL marker IDs	Chromosomes /unanchored scaffolds	Physical positions (bp)	InDels (*Kabuli* reference genome-CDC Frontier/PI)	Forward primers (5'-3')	Reverse primers (5'-3')	Anneling temperature (0C)	Expected amplified product size (bp)	Structural annotation		Functional annotation		
								Sequence components of *kabuli* genome	*Kabuli* gene accession IDs	NCBI-KOG	TFs	NCBI-nr database
CaPOPII_4334	scaffold321	36107	AC/A	AGGGAGGACGAAAATGACCT	CTTGGGCGTCCAATAGATGT	59.9	862	INTERGENIC				
CaPOPII_4335	scaffold3212	725	CTT/CTTTT	CGAGTCGTGTCTTGATAATGGA	CCCATTAGTGGTGATGCTCC	60.1	125	INTERGENIC				
CaPOPII_4336	scaffold3212	1519	GAAAAAAAA/GAAAAAAAAAA	AATGACATTTGTCCCCGTAGA	ATCGGTGGCATGTCCTTACT	59.3	741	INTERGENIC				
CaPOPII_4337	scaffold3212	1828	TAATCTAA/TA	GCAGAAATTAAAATGAGTGGTGTG	TGGAAGGTGGAGGTTCAGAG	59.9	715	INTERGENIC				
CaPOPII_4338	scaffold3212	2564	A/AC	TGAACCTCCACCTTCCATTC	CAAACAAATGAGTGGCGATG	59.9	439	INTERGENIC				
CaPOPII_4339	scaffold3212	3813	GTT/GTTT	GTGGCCATTGTTCCAAGAGT	GTATGCATGAATTACGGGGG	60.0	728	INTERGENIC				
CaPOPII_4340	scaffold3212	6334	ATTTTTTTT/ATTTTTTT	GAGCACATGATCTCGCAGAA	CACTGCTGAAGCGGAATACA	60.1	213	INTERGENIC				
CaPOPII_4341	scaffold3217	4699	TTA/TTACTATA	CGCTTTGCCCTTAGTTTGAG	ATTCCCCATCAATTTGGACA	60.0	298	INTERGENIC				
CaPOPII_4342	scaffold322	54117	AC/A	AGAAGATTAGCATGGCCCCT	TGCATTTTAACAATGTTGGCA	60.1	295	INTERGENIC				
CaPOPII_4343	scaffold3228	1858	CATA/CATATA	TCAGCTTTTTGGCTGATGTG	CTTGTGGATTGGGTCTTGTG	60.0	247	INTERGENIC				
CaPOPII_4344	scaffold3229	5378	TA/TATTGCA	AGCTGAAATTCCACAAGGCA	GATAATGGCCGCATGAAAAG	60.8	366	INTERGENIC				
CaPOPII_4345	scaffold3229	8934	GATATATATATATATATATATATATATAT/GATATATATATATATATATATATATATATATAT	TGAAGCACAATGTTGGTTTTG	TGTAAAGTGCAACACCCGTC	59.6	692	INTERGENIC				
CaPOPII_4346	scaffold3234	11746	CAAAA/CAAAAA	CATGCACCTTGAAAGGGTTT	TTGGCAAGTGTGGATGTTGT	60.0	561	INTERGENIC				
CaPOPII_4347	scaffold324	63390	TAAA/TAA	CGCGTGTCTTGTTTTTCTTG	TGCATAATCGATTGGCACAT	59.5	413	INTERGENIC				
CaPOPII_4348	scaffold324	350218	CAAAAA/CAAAA	GTACGAAAATTGCCCGAAAA	AGTTTCGAACCAAACGATGG	59.9	439	INTERGENIC				
CaPOPII_4349	scaffold3258	34164	TAAAAAAAAAA/TAAAAAAAAA	GGGTTTGCTTACCGAACTCA	TACGGAAAATTACCCACGGA	60.1	375	URR	Ca_27898			Transposase, Ptta/En/Spm, plant
CaPOPII_4350	scaffold3258	41416	AT/A	TTTTTGTGTTTGAATACACGGTT	TTTTTCCTCGAGTGCAGCTT	58.5	588	INTERGENIC				

INDEL marker IDs	Chromosomes /unanchored scaffolds	Physical positions (bp)	InDels (*Kabuli* reference genome-CDC Frontier/PI)	Forward primers (5'-3')	Reverse primers (5'-3')	Anneling temperature (0C)	Expected amplified product size (bp)	Structural annotation		Functional annotation		
								Sequence components of *kabuli* genome	*Kabuli* gene accession IDs	NCBI-KOG	TFs	NCBI-nr database
CaPOPII_4351	scaffold3258	46908	GAC/GACCGGCTACGTAC	ATGGCCCCATTATTGAGGTT	TGCACGAGTCTAATGAGGGA	60.4	696	INTERGENIC				
CaPOPII_4352	scaffold3258	48852	TTATGT/TT	ATGACACGGCTGAATGTCAA	ACGCACGTATGTTGTCCAAA	60.1	374	INTERGENIC				
CaPOPII_4353	scaffold3258	52201	TA/T	TTTTTGGGTCCCTATCAACTAA	TCCAACAATAATTTAAAACCTTACAA	57.7	500	INTERGENIC				
CaPOPII_4354	scaffold3258	66747	TACA/TA	GAAAGCGCTGTAAAAGGTGC	TGATTTGCTGGGAAATGACA	60.0	504	INTERGENIC				
CaPOPII_4355	scaffold3258	71440	CTATATATATATATATATATATATATATATATAT/CTATATATATATATATATATATATATATATATATATATAT	GCAAGCGATATGTGACCCTT	GATTCAACTGGCGATGGTTT	60.1	836	INTERGENIC				
CaPOPII_4356	scaffold3258	74031	ATTTTTTT/ATTTTTT	AACTTCCACACCTGTCTCCG	AATTCAAAAGAATGTCGGCG	60.2	726	INTERGENIC				
CaPOPII_4357	scaffold3258	74657	CTTTTTTTTTTT/CTTTTTTTTTT	AATGCAATCGTCAGGACACA	TATGGACCGGAAATGGATGT	60.1	630	INTERGENIC				
CaPOPII_4358	scaffold331	19367	ATT/AT	ACATTCCCATCCACAGGGTA	AAAAACATGCAGGGAAATCG	60.1	616	INTERGENIC				
CaPOPII_4359	scaffold331	155436	CATATATATATATATATAT/CATATATATATATATAT	TTGGAAGGAGCCCTTACCTT	TTGGTATTGAACTCGGAGGC	60.1	716	DRR	Ca_25647	FH		Permease, cytosine/purines, uracil, thiamine, allantoin
CaPOPII_4360	scaffold3317	3339	TGACGAC/TGAC	GCATCTGTGTAGCCATCCAA	TAGGTCATTTTCGTCCTCCG	59.7	738	INTERGENIC				
CaPOPII_4361	scaffold3321	25022	ATTTTTTTTT/ATTTTTTTT	TTTTCTGGTTGTAGGACGGG	CAACAACTTCTCATGCTCGC	60.0	711	DRR	Ca_25706	QR	B3	Oxoglutarate/iron-dependent oxygenase
CaPOPII_4362	scaffold3321	69689	TAA/TA	TCTTGCAGGAGCTTGGATTT	TGTGCCGACAATTAACCAAA	60.0	681	INTERGENIC				
CaPOPII_4363	scaffold333	8945	TCC/TC	GATGAAATCGAGGACCAGGA	GACTTTTCGAGCGATTTTCG	60.0	299	INTERGENIC				
CaPOPII_4364	scaffold333	21781	TGGG/TGG	GGTACGAAAATCGCCAGAAA	GACGTGGAACCCAAAAGAAA	60.1	585	INTERGENIC				
CaPOPII_4365	scaffold334	58371	CTATATATATATATA/CTATATATATATATATA	ACCTTGCTCCCTTACCCACT	TATTTTGGATTTTGGCTCGG	60.0	490	INTERGENIC				
CaPOPII_4366	scaffold335	88156	GTTATTATTATTATTATTATTATTATTATTATT/GTTATTATTATTATTATTATTATTATTATTATT	TTGTCTTCTTGTGGCCGACT	TTTTACAGGCGATTTGGAGC	60.8	785	INTERGENIC				
CaPOPII_4367	scaffold335	191468	ATT/AT	ACTCGAGTCGTTTCGCAAGT	GGAATAAAAGTCCAATGCCG	60.1	730	INTERGENIC				

INDEL marker IDs	Chromosomes /unanchored scaffolds	Physical positions (bp)	InDels (*Kabuli* reference genome-CDC Frontier/PI)	Forward primers (5'-3')	Reverse primers (5'-3')	Anneling temperature (0C)	Expected amplified product size (bp)	Structural annotation		Functional annotation		
								Sequence components of *kabuli* genome	*Kabuli* gene accession IDs	NCBI-KOG	TFs	NCBI-nr database
CaPOPII_4368	scaffold336	186479	AT/ATATATGT	CGCGTTAACAGAGGAAGAGG	GCGGTGAATCTTTTACGAGG	60.0	290	INTERGENIC				
CaPOPII_4369	scaffold336	617875	AATATATATATATATATA/AATATATATATATATATA	GGCAACGTGCATAAACAAAA	TGTGAGTGTGGGAAACGGTA	59.6	437	INTERGENIC				
CaPOPII_4370	scaffold336	633921	A/AC	GAGAAAGGCAAGACCAGTGC	TTGCAATTCAACTGATGGGA	60.0	365	INTERGENIC				
CaPOPII_4371	scaffold3364	43644	T/TG	AGCCAAGGGAGGATGAAAAT	CGTCCACCCTTTGATTATCC	59.9	214	INTERGENIC				
CaPOPII_4372	scaffold3369	18856	T/TA	TTCAACGTGACCTGCTCATC	ATGGGTCCAGGTCATCTCAG	59.8	671	INTERGENIC				
CaPOPII_4373	scaffold338	76520	GTATATATATATATATAT/GTATATATATATATATAT	CTTGGCCATAAAATCGGAAA	AGGATGCATGGGATGAAAAG	59.9	661	INTERGENIC				
CaPOPII_4374	scaffold3397	17764	AT/ATT	GATCAGATCTGGCAGCAACA	GAGGATGGAAATTGCTGCAT	60.0	366	DRR	Ca_26271			
CaPOPII_4375	scaffold3397	55203	ATA/ATACTATTA	TTACTCACTTGCAACACGCC	GGGAAGCAATGTTTTGATCC	59.9	859	INTERGENIC				
CaPOPII_4376	scaffold3397	88653	GTTATTATTATTATTATT/GTTATTATTATTATTATTATTATTATT	CGGCAAAATATCAGAAACACG	AGGATGCGACCATTTACTGC	60.5	477	INTERGENIC				
CaPOPII_4377	scaffold340	18699	CA/CAAA	GAATGCACACAGCAGAAGGA	CTAACTCTAATGGGCGGCAG	60.0	639	DRR	Ca_24954	DL		Protein of unknown function DUF688
CaPOPII_4378	scaffold340	60721	CTTTTTTTTTT/CTTTTTTTTTTT	TGCTACGCACTAGATGGCAC	TTGCAGCATTGTCTTTGTCC	60.0	803	INTERGENIC				
CaPOPII_4379	scaffold340	161360	ATTTTTTT/ATTTTTTTT	GCGGATATCGTAGGGTGCTA	TACATCCAGAAGGCTACCGC	60.1	539	INTERGENIC				
CaPOPII_4380	scaffold340	194683	GAAAAAAAAAA/GAAAAAAAAAAA	AGGTCGGAGGGGATAGTTTG	TTCAAGGTGGCATGATGAAA	60.3	628	INTERGENIC				
CaPOPII_4381	scaffold340	259875	CT/CTTTTTATTT	TAAAGGACCAAGTCGGGAAA	GAAAGAGCTTTTGATTGGACG	59.5	449	INTERGENIC				
CaPOPII_4382	scaffold340	316742	ATTTTTTT/ATTTTTTTT	CGGGAATTAACTGAAAGCCA	TGTTTCATGGATGCAAGTACG	60.1	610	INTERGENIC				
CaPOPII_4383	scaffold341	135303	GAAAA/GAAAAA	AATGGAGTTGCGACGAGAGT	TCTCCGTAAAGGATTCACCG	59.9	540	INTERGENIC				
CaPOPII_4384	scaffold342	310549	CATATATAT/CATAT	TTGTGACATTTCATCAAACACA	CAACCGACGTTGAAGAACAA	57.5	933	INTERGENIC				

INDEL marker IDs	Chromosomes /unanchored scaffolds	Physical positions (bp)	InDels (*Kabuli* reference genome-CDC Frontier/PI)	Forward primers (5'-3')	Reverse primers (5'-3')	Anneling temperature (0C)	Expected amplified product size (bp)	Structural annotation		Functional annotation		
								Sequence components of *kabuli* genome	*Kabuli* gene accession IDs	NCBI-KOG	TFs	NCBI-nr database
CaPOPII_4385	scaffold342	340574	TC/T	TGTTCCAGCATACCAAATGC	AGCAAAATCCGAGGAGGAAT	59.5	557	INTERGENIC				
CaPOPII_4386	scaffold342	370625	TTATCAT/TT	TGGGGTCCTTTCTCTTCTCC	TATGCCCATTGAGTTGGACA	60.6	387	INTERGENIC				
CaPOPII_4387	scaffold3422	131072	ATT/A	CCGAAATCATGTAATTTGACCA	TGTGTTGCACTTGACACGAA	59.7	668	INTERGENIC				
CaPOPII_4388	scaffold3432	23969	CAATAAT/CAAT	CCTGAATCCGAGACAAGACG	TCATGGGTTGTATGGAAGCA	60.8	487	INTERGENIC				
CaPOPII_4389	scaffold3432	35439	TTTATATT/TTTATATTATATT	TGATTGATTTTGCGGTTACG	AAAGAGATCGGTTCTACCCAAA	59.6	278	INTERGENIC				
CaPOPII_4390	scaffold3436	9281	GAGACTTAT/G	GGCCATGAATTCAACAACAA	TTGCCAAATACAAAAAGAAATGAA	59.4	570	INTERGENIC				
CaPOPII_4391	scaffold3436	9348	GTAATAATAATAATAATAATAATAATAATAATAATAATAATAATAATAATAATAATAATAATAATAATAGTAATAATAATAA/GTAATAATAATAATAATAATAATAA	TTTCACCATTTTGCATTTCG	CAGGTTCACGTTATTTTGTTTCA	59.5	635	INTERGENIC				
CaPOPII_4392	scaffold3436	9959	TAA/TTAATAATATACAAA	TGGAGTTCGGGCTAAGTACAG	CGTGCTTGCAGAAAAACAAA	59.4	309	INTERGENIC				
CaPOPII_4393	scaffold3436	13785	TGAGGA/TGA	GCGCTACATTTTACGGCTCT	TTCGTCCTCGAAATTCAACC	59.5	757	INTERGENIC				
CaPOPII_4394	scaffold3438	22632	TA/T	GTGCTGTTGAACCCCAAACT	TGAATACCCTCCCTTGAACG	60.0	711	INTERGENIC				
CaPOPII_4395	scaffold3453	1173	CA/CAAA	ATATGCCTCTACCGTGCAGC	TCGTGAAAGCAAACAAAGGA	60.3	507	INTERGENIC				
CaPOPII_4396	scaffold3472	6763	GTATATATATATATATATATATATATATATA/GTATATATATATATATATATATATATATATA	CAAGCATGATCCAGGGAGAT	CTCGGCGAGTTTGTGTGTAA	60.0	471	INTERGENIC				
CaPOPII_4397	scaffold3472	7148	TATTAAATAATT/TATTAAATAATTAAATAATT	CAGGCTAGCTAAATGCCTCG	ACCATGGTTGTTGCAAGTGA	60.1	720	INTERGENIC				
CaPOPII_4398	scaffold3472	9063	CAA/CAAA	GCTCCATGTGCTTCACACAC	GTGACACGTGACAATCCCAC	60.3	107	INTERGENIC				
CaPOPII_4399	scaffold3472	9396	TC/TCC	TTTTGACAGAGAAACCCGCT	CTGTGAGAGGCGAAGATGGT	59.9	223	INTERGENIC				
CaPOPII_4400	scaffold3472	51384	TAAAAAAAAA/TAAAAAAAAAA	GAAATCCATGTTAGAAATTTTGTTGA	TTGTGTTGGTTTTGCCTTCA	59.7	327	INTERGENIC				
CaPOPII_4401	scaffold3472	71251	CTACAGCGCTT/CTACAGCGCTTGTTTTACAGCGCTT	GAAAGCGCTTCCGTTTACAG	GACATTTAACAGCGCCCATT	60.0	159	INTERGENIC				

INDEL marker IDs	Chromosomes /unanchored scaffolds	Physical positions (bp)	InDels (*Kabuli* reference genome-CDC Frontier/PI)	Forward primers (5'-3')	Reverse primers (5'-3')	Anneling temperature (0C)	Expected amplified product size (bp)	Structural annotation		Functional annotation		
								Sequence components of *kabuli* genome	*Kabuli* gene accession IDs	NCBI-KOG	TFs	NCBI-nr database
CaPOPII_4402	scaffold3475	6139	AG/A	GGCCTGGAGACCCTTATAGC	ACCACTCGACGAAAATGGAC	60.1	616	INTERGENIC				
CaPOPII_4403	scaffold3475	31282	TG/TGG	GCTATTTTCGTACCACCCGA	GCCCAAAAAGTCCAGATGAA	60.0	326	INTERGENIC				
CaPOPII_4404	scaffold3489	1552	GTTT/GTT	ACTATCCATCGTCTGGTCCG	GAAAGGGTCCAACAGAACGA	60.0	452	INTERGENIC				
CaPOPII_4405	scaffold3489	2052	TAATAAAAAATAAAAAATAAAAAATAAAAAA/TAATAAAAAATAAAAAATAAAAAA	TCGTTCTGTTGGACCCTTTC	AGTGGAAAATATGCCGTTGC	60.1	521	INTERGENIC				
CaPOPII_4406	scaffold349	335267	ATT/ATTT	TTGGTCAATGTGGCTTCTCA	CTGAGCTTGTGACCAAATCG	60.2	531	INTERGENIC				
CaPOPII_4407	scaffold350	165550	ATGTGTG/ATGTGTGTG	CAATTCCATGGCTCCAAAGT	CAATGCCTCCCCATAACAAT	59.9	670	INTERGENIC				
CaPOPII_4408	scaffold3500	6141	C/CTGTA	GCGTTCGATTCCACATCTTT	TCATTATGCGTGTTCCGCTA	60.1	434	INTERGENIC				
CaPOPII_4409	scaffold3514	12548	CAAAAAAAAAA/CAAAAAAAAAAAA	CTCTTTAAGACGTTTCGCGG	TTGGTGTCCCACTGATTCAA	60.0	653	INTERGENIC				
CaPOPII_4410	scaffold3514	40711	GAAA/GAAAA	TGACCAAATCGGAATTGATTTT	CATTTCCGAATCTTCCCGTA	60.5	723	INTERGENIC				
CaPOPII_4411	scaffold352	133593	AAAGA/AA	TTTTGAGTTCGGGAGTCGAT	TTTCTTGAGTTTTGGGACACA	59.7	453	INTERGENIC				
CaPOPII_4412	scaffold352	177986	TAAAAAA/TAAAAAAA	AACGCATCTTTTTACCCACG	GGCCTGAATCTCATCCAAGA	60.0	568	INTERGENIC				
CaPOPII_4413	scaffold352	178010	ATTT/ATT	AACGCATCTTTTTACCCACG	GGCCTGAATCTCATCCAAGA	60.0	568	INTERGENIC				
CaPOPII_4414	scaffold352	178511	ATGAGTTGAGTTGAGTT/ATGAGTTGAGTT	TCGCAATGCAACATCTCTTT	GCTTGTGAGTGTGTGTGCCT	59.4	823	INTERGENIC				
CaPOPII_4415	scaffold3542	507	AGGGGGGG/AGGGGGG	GAAGAGTTACGCGGACAAGC	CCGAGCCAAAATCCAAAATA	60.0	566	INTERGENIC				
CaPOPII_4416	scaffold3542	25608	C/CGGCGCAT	CAGGTTAGGAAGCAAGCAGG	TACAGCGCTCATTTCCACAG	60.0	527	INTERGENIC				
CaPOPII_4417	scaffold3542	26037	TT/TCAGATTTAT	CTGTGGAAATGAGCGCTGTA	AGACCTTGGACACGTAACGG	60.0	464	INTERGENIC				
CaPOPII_4418	scaffold355	1384	GTTTTT/GTTTT	GGGGCGTTACATGTGGTATC	CAGATTCACGAAGGTCACACA	60.1	314	INTERGENIC				

INDEL marker IDs	Chromosomes /unanchored scaffolds	Physical positions (bp)	InDels (*Kabuli* reference genome-CDC Frontier/PI)	Forward primers (5'-3')	Reverse primers (5'-3')	Anneling temperature (0C)	Expected amplified product size (bp)	Structural annotation		Functional annotation		
								Sequence components of *kabuli* genome	*Kabuli* gene accession IDs	NCBI-KOG	TFs	NCBI-nr database
CaPOPII_4419	scaffold3562	1038	ATTT/ATT	AGGGAGGACGAAAATGACCT	CCAATTGGTGTGTTTGCATC	59.9	556	INTERGENIC				
CaPOPII_4420	scaffold3594	965	GAGCTAAAAAGCTAAAAAGCT/GAGCTAAAAAGCTAAAAAGCTAAAAAGCT	CGTTCAAAACGAGATCGTCA	TACAATGCCGAGCAAATCAA	59.8	575	INTERGENIC				
CaPOPII_4421	scaffold36	236312	ATTTTTTTTT/ATTTTTTTTTT	TAGGTCTCATAGCCCCGATG	GCCCTCAGATGAGAGTCAGG	60.1	640	INTERGENIC				
CaPOPII_4422	scaffold362	166434	TGA/T	CCCGGTATTGTTTGTGCTCT	CACATCAGCCACATTTGACC	60.0	774	DRR	Ca_24991	C		Aldehyde dehydrogenase NAD(P)-dependent
CaPOPII_4423	scaffold362	267543	A/AC	TATCTTGTTCCGACCCAACC	CTTGAATTCCATGCGTCCTT	59.8	698	INTERGENIC				
CaPOPII_4424	scaffold3637	13149	GATATATATATATATATA/GATATATATATATATATA	AATGTGAATCCCACCAGGAA	CAAGCGCTATGTGACCCTTT	60.2	628	INTERGENIC				
CaPOPII_4425	scaffold3659	2503	AT/ATT	GCCATTAATTATGTTAACGTTTGG	CAGAACATTTAACGTGAGCAAGA	58.8	665	INTERGENIC				
CaPOPII_4426	scaffold3663	4120	T/TG	TTTTATTGTTTTACTACATAACGACGA	AAGTTTGACTGCATAACAATGACA	57.7	631	INTERGENIC				
CaPOPII_4427	scaffold3663	4430	TAAA/TAAAA	TGTCATTGTTATGCAGTCAAACTT	TCTTCGTTTTATGGTTTTATTTCA	58.7	280	INTERGENIC				
CaPOPII_4428	scaffold3687	82563	G/GTAA	GAGTTTCCTCTACCACCCCC	GGGACTATGTTAGTGGCGGA	59.8	417	INTERGENIC				
CaPOPII_4429	scaffold3689	16220	TATTCA/TA	TCCCATCCCTATTTTGGACA	ACATGGCAAGCCAAATTTTC	60.1	350	INTERGENIC				
CaPOPII_4430	scaffold369	125737	ATTTTTTTTTTTT/ATTTTTTTTTTT	CGGAAGCTCGGATTACACAT	GGGTCGTGGGACTTTGATAG	60.1	771	INTERGENIC				
CaPOPII_4431	scaffold370	58169	TC/T	CCCTCGTCGTGTTTGAAAAT	TTTTGGGCACTTTTCGTACC	60.0	393	INTERGENIC				
CaPOPII_4432	scaffold370	75163	AC/A	GATTTTCGTACCCCTCGTCA	AGTCGCTCGAAAAGTCCAAA	59.9	533	INTERGENIC				
CaPOPII_4433	scaffold3705	4075	TC/TGGGCTAAAACCC	CAATAACCCTTCAATGGCTCA	GCTTTGCTTCCCTTTCCTCT	59.9	393	INTERGENIC				
CaPOPII_4434	scaffold373	25857	ACTTC/ACTTCTTC	AGCCACGAATTTAAGCTCCA	AGGGACAAAGGGCAAGAAAT	59.8	559	INTERGENIC				
CaPOPII_4435	scaffold374	13002	AC/ACC	CAAATTCAACGAGGTCACACA	ATTTGGAGCGTTACATTGGG	59.6	184	INTERGENIC				

INDEL marker IDs	Chromosomes /unanchored scaffolds	Physical positions (bp)	InDels (*Kabuli* reference genome-CDC Frontier/PI)	Forward primers (5'-3')	Reverse primers (5'-3')	Anneling temperature (0C)	Expected amplified product size (bp)	Structural annotation		Functional annotation		
								Sequence components of *kabuli* genome	*Kabuli* gene accession IDs	NCBI-KOG	TFs	NCBI-nr database
CaPOPII_4436	scaffold374	142006	TAAA/TAA	AATTCCTTCCCACACAACCA	AGCGGAGATTTTACCGGAAT	60.2	128	INTERGENIC				
CaPOPII_4437	scaffold374	154680	TAAAAAAAAAAAAAA/TAAAAAAAA	GATTTGATGTTGGGACCGTT	TCAAATGCTTCAACTTGTGCTT	59.7	477	INTERGENIC				
CaPOPII_4438	scaffold374	158468	ACACGATCAGCACGA/ACACGA	CCTATCACAGGTCAGCACGA	GGTGTGGATGGAATTTGGAG	59.9	531	INTERGENIC				
CaPOPII_4439	scaffold374	205813	CAT/CATCCTGAT	AGCTGTTTCTGCTAGGCTGG	TTTATTTACGCGAATGCCCT	59.8	941	DRR	Ca_26214	O		K Homology
CaPOPII_4440	scaffold3742	3802	TTAT/TTATAT	CGATAGACTCAAGTTGGACCTG	GTGTCTGCTTGTTTGCCTGA	58.9	632	INTERGENIC				
CaPOPII_4441	scaffold377	75306	C/CAT	TCACGGGTCGACACAATTTA	AACTGGACGTTGAGAATGGG	60.0	403	INTERGENIC				
CaPOPII_4442	scaffold377	263808	GTATATATATATATATATATATATAT/GTATATATATATATATATATAT	GTGTGTAGAAGCCTAGCCGC	TGCACTTTGTCCTTCAATCG	60.0	690	INTERGENIC				
CaPOPII_4443	scaffold3780	7994	C/CT	TCATTCGTGAAATTAGCCCC	CCCAATTCCCCCAACTTAAC	59.9	557	INTERGENIC				
CaPOPII_4444	scaffold3789	2620	AGG/AGGG	AATGGTTCTTGGACAGTGGC	GGCTGGTGGTTTTGATAGGA	60.0	548	INTERGENIC				
CaPOPII_4445	scaffold379	194523	AATATATATATATATATATA/AATATATATATATATATA	CCGAACTTTACTGGGCCTAA	ACCGTTTTGGTGTTTCTTGC	59.2	602	INTERGENIC				
CaPOPII_4446	scaffold3794	34380	GTAT/GTATTTCATTTATATTAT	TGTGAGAGGGACACTTGGCT	CAGTGTCGTGGTGGATTTTG	60.9	499	INTERGENIC				
CaPOPII_4447	scaffold3794	82476	CCAC/CC	CAAAGGGACCACACCTCTTT	TTGGCCCCTTTAGTGTATCG	59.0	554	INTERGENIC				
CaPOPII_4448	scaffold38	204585	GTTGTTTCT/GTTGTTTCTTTGTTTCT	CATCATTTCTTGCACATGCC	CAAACACAAATCAATCCCCC	60.1	528	INTERGENIC				
CaPOPII_4449	scaffold38	248171	CTTTTTTTTTTT/CTTTTTTTT	TAGCCTTGAGACCGAGCATT	CAAGGACTAAACTGAAGCCCA	60.0	372	DRR	Ca_23620			
CaPOPII_4450	scaffold38	413791	ATTTT/ATTTTT	CTTCGCTGCAAAATTTCCTC	TTGATGTCGGGTAGTGTCTGA	60.0	755	INTERGENIC				
CaPOPII_4451	scaffold38	474073	TAAAAAAAAAAAAA/TAAAAAAAAAAA	TGTGTCAGAAGGATCTTGCG	TGAGATTCGCCAGTTAACCC	60.0	652	INTERGENIC				
CaPOPII_4452	scaffold381	6157	CGTTTTAGGTTTTAGGTTTTAGGTTTTAGG/CGTTTTAGGTTTTAGGTTTTAGGTTTTAGGTTTTAGG	GTTGATGGTTTCGGGTTGAT	AACGCAAAACACTAAACCGC	59.7	304	INTERGENIC				

**INDEL marker IDs**	**Chromosomes /unanchored scaffolds**	**Physical positions (bp)**	**InDels (*Kabuli* reference genome-CDC Frontier/PI)**	**Forward primers (5'-3')**	**Reverse primers (5'-3')**	**Anneling temperature (0C)**	**Expected amplified product size (bp)**	**Structural annotation**		**Functional annotation**		
								**Sequence components of *kabuli* genome**	***Kabuli* gene accession IDs**	**NCBI-KOG**	**TFs**	**NCBI-nr database**
CaPOPII_4453	scaffold381	6830	AAG/A	TGCAAAAGGGTCACTTGATTT	CCCCAAACCCTAAACCCTAA	59.6	355	INTERGENIC				
CaPOPII_4454	scaffold3837	2371	ATTTTTTTTTTTTT/ATTTTTTTTTTT	CTTCTTGGTGGCATGGTTTT	TACCCTGTGGATGGGAATGT	60.0	591	INTERGENIC				
CaPOPII_4455	scaffold3837	5661	AT/ATT	GCCTCCGAGTCAATACCAAA	ATTGAGGGTAGGGGAGCAGT	60.1	415	INTERGENIC				
CaPOPII_4456	scaffold387	16023	CAT/C	CACACCCCTTGTCAACACTG	TTAAGCTGAGAGCCTTCCCA	60.0	439	INTERGENIC				
CaPOPII_4457	scaffold387	22975	AATC/AATCATC	CGCAATCTGAACAAATGACAA	TCCACTAACCACCTTGACCC	59.7	338	INTERGENIC				
CaPOPII_4458	scaffold387	23363	ATGT/ATGTGT	GGGTCAAGGTGGTTAGTGGA	TCCTCTGCCTTTCATGTGTG	59.8	496	INTERGENIC				
CaPOPII_4459	scaffold387	23485	ATTTTT/ATTTTTTT	GGGTCAAGGTGGTTAGTGGA	TCCTCTGCCTTTCATGTGTG	59.8	496	INTERGENIC				
CaPOPII_4460	scaffold387	23589	ATGTGTGTG/ATGTGTGTGTG	GGGTCAAGGTGGTTAGTGGA	TCCTCTGCCTTTCATGTGTG	59.8	496	INTERGENIC				
CaPOPII_4461	scaffold387	160969	TTA/TTACTA	AGCTCCAAATCCAGAGCAAA	GGGATATGATCTTCTACCCCA	60.0	835	INTERGENIC				
CaPOPII_4462	scaffold387	161432	ATAAAA/ATAAAAGTAAAA	TGGTACTCACATGAAAATCGAAA	GCGACTATTGCAATTTTGTGAA	59.5	281	INTERGENIC				
CaPOPII_4463	scaffold388	8749	C/CCTATA	GCCCCAGAATTCAAAGTTCA	GCAGGAATCCTCGTACCTCA	60.1	520	INTERGENIC				
CaPOPII_4464	scaffold39	40229	GAAAAAAA/GAAAAAAAAA	CGGCCATCTACGTTGTCTTT	GACATTGCATTTTGGACACG	60.1	924	INTERGENIC				
CaPOPII_4465	scaffold3900	4432	CG/CAG	TTCTGAATCAATGTAGTTCGTCG	AAACCCTAAATCCCAAACCC	59.3	294	INTERGENIC				
CaPOPII_4466	scaffold3900	4684	T/TC	GGGTTTGGGATTTAGGGTTT	CCTAGGAAAGACGAAAATGACC	59.0	250	INTERGENIC				
CaPOPII_4467	scaffold3918	4292	TAAAAAAAA/TAAAAAAAAAA	ATGCCTTCGTCCTCTCCTTT	ACATTGAAACTGCCCATTCC	60.2	612	INTERGENIC				
CaPOPII_4468	scaffold3918	4537	CTTAATT/CTT	ATGCCTTCGTCCTCTCCTTT	CGAGTGAAGGCATGGGTATT	60.2	914	INTERGENIC				
CaPOPII_4469	scaffold3923	3230	GA/GAA	TGCTGCAACTTCTACATCCG	AGAAAGCGTGAAAACTCCGA	60.0	695	INTERGENIC				

INDEL marker IDs	Chromosomes /unanchored scaffolds	Physical positions (bp)	InDels (*Kabuli* reference genome-CDC Frontier/PI)	Forward primers (5'-3')	Reverse primers (5'-3')	Anneling temperature (0C)	Expected amplified product size (bp)	Structural annotation		Functional annotation		
								Sequence components of *kabuli* genome	*Kabuli* gene accession IDs	NCBI-KOG	TFs	NCBI-nr database
CaPOPII_4470	scaffold3927	1574	ATTTTT/ATTTTTTT	GAATCCTGGGCTGAAATCAA	CGTGGTAATCGCGTAGGAAT	60.0	342	INTERGENIC				
CaPOPII_4471	scaffold3934	972	A/AAC	AAACGTTTTTGGTCCATCCA	ACGAGAGAGAAAGGCGTGAG	60.2	329	INTERGENIC				
CaPOPII_4472	scaffold3945	12983	G/GATCCTC	TTCCAACTCGATCCAGCTCT	ATACCGATTTGACTGGGCTG	60.0	531	INTRON	Ca_26970			
CaPOPII_4473	scaffold395	8212	TT/TTAAT	TTCCTGGTGGGATTCACATT	AGCGATGCATGTCTCAAGTG	60.2	440	INTERGENIC				
CaPOPII_4474	scaffold395	232446	AAAT/AAATTTCTTCAAT	TTCATTCCTAGCTTCGTCAACA	AAACTCCAAAACCGAAGAAAAA	59.9	789	INTERGENIC				
CaPOPII_4475	scaffold3950	6762	C/CAAGAAGTTTT	AGTGGCATAGGAGTGCCATC	ATCGGAGAAACGACATCTGG	60.1	510	INTERGENIC				
CaPOPII_4476	scaffold3950	7394	GTATATATATATATA/GTATATATATATATATA	CCAGATGTCGTTTCTCCGAT	TTGTATGACATCTCGCCACC	60.1	665	INTERGENIC				
CaPOPII_4477	scaffold3950	7697	C/CTT	TTTCTCTTTTGGTGGTTGCC	TCCACCAATATGCAAAAGCA	60.1	854	INTERGENIC				
CaPOPII_4478	scaffold3950	7767	TAAAAA/TAAAA	GGTGGCGAGATGTCATACAA	TCCACCAATATGCAAAAGCA	59.5	366	INTERGENIC				
CaPOPII_4479	scaffold3950	8477	TAAAAAAAAAA/TAAAAAAAA	TGCTTTTGCATATTGGTGGA	AAAACATGGCCTTCAACTGG	60.1	475	INTERGENIC				
CaPOPII_4480	scaffold3950	8667	AAATAATAATAATAA/AAATAATAATAA	CCAGTTGAAGGCCATGTTTT	TTTTATGGAAGCATGTGGCA	60.0	528	INTERGENIC				
CaPOPII_4481	scaffold3950	8791	TAAAAAAAAAA/TAAAAAAAA	CCAGTTGAAGGCCATGTTTT	TTTTATGGAAGCATGTGGCA	60.0	528	INTERGENIC				
CaPOPII_4482	scaffold3950	12638	T/TTCAGCA	CAAGAGCCTAAGGATGCTGC	AGCATGAAACGGTGGAGAAT	60.1	919	INTERGENIC				
CaPOPII_4483	scaffold3952	963	G/GT	GATGAAATCGAGGACCAGGA	TTTCATTTGGTCTTTTCGGC	60.0	470	INTERGENIC				
CaPOPII_4484	scaffold396	372299	CATATATATATATATATATATATATA/CATATATATATATATATATATATA	TGTATTTCTGCGCATTCTCG	TTGTTAGTTTGACGAGGGCA	60.0	357	INTERGENIC				
CaPOPII_4485	scaffold396	577559	GAA/GA	TCCCAACCCAAATAAACCAA	AAGCTCAACATGCAAAACCC	60.0	538	INTERGENIC				
CaPOPII_4486	scaffold396	577641	ACCC/ACCCC	TCCCAACCCAAATAAACCAA	AAGCTCAACATGCAAAACCC	60.0	538	INTERGENIC				

INDEL marker IDs	Chromosomes /unanchored scaffolds	Physical positions (bp)	InDels (*Kabuli* reference genome-CDC Frontier/PI)	Forward primers (5'-3')	Reverse primers (5'-3')	Anneling temperature (0C)	Expected amplified product size (bp)	Structural annotation		Functional annotation		
								Sequence components of *kabuli* genome	*Kabuli* gene accession IDs	NCBI-KOG	TFs	NCBI-nr database
CaPOPII_4487	scaffold397	23238	CATATATATATATAT/CATATATATATATATAT	TGGAGGCGGCTACTAGTGTT	ATTGAATTCGCCATAAACGG	59.9	411	INTERGENIC				
CaPOPII_4488	scaffold397	23446	A/AT	TGGAGGCGGCTACTAGTGTT	ATTGAATTCGCCATAAACGG	59.9	411	INTERGENIC				
CaPOPII_4489	scaffold398	33523	AATTA/AA	AGTGATCCAACTCCAACATCG	GGATTTCCCACATTCATAGCA	60.0	912	INTERGENIC				
CaPOPII_4490	scaffold398	33778	AATAT/AATATAT	TTTGATTTTGGATTGATTTATTCG	CATGGCCACTCAAAGCACTA	59.3	494	INTERGENIC				
CaPOPII_4491	scaffold398	33851	A/ATTTTT	TTTGATTTTGGATTGATTTATTCG	CATGGCCACTCAAAGCACTA	59.3	494	INTERGENIC				
CaPOPII_4492	scaffold398	45043	T/TGTA	CCCTACGACGTCCGTTAAAA	TCGCTCGTTGCACCATATTA	60.0	554	INTERGENIC				
CaPOPII_4493	scaffold398	75332	ATTTTTTTTTT/ATTTTTTTTT	AAACCCAAATCTCCGCTTTT	CAACCCCAATCTTTTGAGGA	59.9	445	INTERGENIC				
CaPOPII_4494	scaffold398	270743	TAAAAAAAAAA/TAAAAAAAAAAA	GTAGTGGTTGAAGTGGCGGT	CTACTCCATCGGGTCTCCAA	60.0	462	INTERGENIC				
CaPOPII_4495	scaffold398	275366	ATTTTT/ATTTTTT	GCACCACAGTCTTCCTCCTC	TCCGTGCCCATGATAAATAA	59.8	579	DRR	Ca_22289	B		
CaPOPII_4496	scaffold398	291477	CTTT/CTT	GGGCAAGAAAAGGGAGAATC	TTGAAAAACCAGCCTTTTGG	60.0	316	INTERGENIC				
CaPOPII_4497	scaffold398	301322	TTTTT/TTTTTATTTT	TCTAGATACGGGATGGGCAC	TCGCGAAGAAGGATAAAATG	59.9	523	DRR	Ca_22290	A		Helicase, C-terminal
CaPOPII_4498	scaffold398	309013	T/TC	TCACTTTGGAGGGTGCTTCT	TCGCAATAGGTTAGGATGGG	59.8	763	INTERGENIC				
CaPOPII_4499	scaffold398	345675	CATAAT/CAT	AGAATCACCACAGGCACACA	TGCCTCATTAGCTCAAGGGT	60.2	522	INTRON	Ca_22294	K	C3H	Endonuclease/exonuclease/phosphatase
CaPOPII_4500	scaffold398	347600	TAAAAAA/TAAAAA	TTTGGTCGGACATACTCGCT	CCAACTGCGCCTCATAATTT	60.7	685	INTERGENIC				
CaPOPII_4501	scaffold398	379143	AGAA/AGAAATTGAA	TCTGAGGAAGAGGAGGACGA	AGCGTGGCAGATTTTGAAAG	60.1	778	INTERGENIC				
CaPOPII_4502	scaffold398	459574	CAAAAAAAAAAA/CAAAAAAAAAA	TCGGGTTGTAATGGGGTTTA	TTGGTCGTTTGAGCCTTTCT	60.0	477	INTERGENIC				
CaPOPII_4503	scaffold398	466018	ACCCCC/ACCCC	TGCATGCAGACAAGAGTCAA	TTGGAAAAGCCCACCTACAC	59.1	468	INTERGENIC				

INDEL marker IDs	Chromosomes /unanchored scaffolds	Physical positions (bp)	InDels (*Kabuli* reference genome-CDC Frontier/PI)	Forward primers (5'-3')	Reverse primers (5'-3')	Anneling temperature (0C)	Expected amplified product size (bp)	Structural annotation		Functional annotation		
								Sequence components of *kabuli* genome	*Kabuli* gene accession IDs	NCBI-KOG	TFs	NCBI-nr database
CaPOPII_4504	scaffold398	474746	ATTTTTTTTT/ATTTTTTTTTTT	TGTGGGGCACAAGTTTGTTA	CCATCAAGTGGGAGTGTCCT	60.0	192	INTERGENIC				
CaPOPII_4505	scaffold40	56808	CATA/CATATA	ATTTGCACGGAAAGTCATCC	CCTCCACCTCCTCCTCCTAC	59.9	810	INTERGENIC				
CaPOPII_4506	scaffold40	496475	ATATTATTATTATTATTATTATTATTATTATTATT/ATATTATTATTATTATTATTATTATTATTATTATTATTATTATT	AGTATCCTGGGTATGCGACG	TTTGATAACAATTTTCGCTCCTC	60.0	692	DRR	Ca_18973	R		F-box domain, cyclin-like
CaPOPII_4507	scaffold40	846364	GC/GCC	CCTCAGCAGGATCAAACCAT	TTACACTGCTGGGGTTGTTG	60.1	642	INTRON	Ca_18992		MYB_related	Glycoside hydrolase, family 16
CaPOPII_4508	scaffold4024	4486	GTATATATATATATATATATATATAT/GTATATATATATATATATATATAT	TGAATGTTGCTTTGTTTTGCAT	GCCCTCAGATGAGAGTCAGG	60.5	852	INTERGENIC				
CaPOPII_4509	scaffold404	236122	GATAATAATAATAATAATAATAATAATAATAATAATAATAATA/GATAATAATAATAATAATAATAATAATAATAATAATAATAATAATA	ATTTGGTTTGACAATGGGGA	TTATTTCACCGTCGTGTCCA	60.0	349	INTERGENIC				
CaPOPII_4510	scaffold4041	994	CT/CTTT	TCATTCTAATTCCTGCCTGATG	GTCCAAACCCAGCACAATCT	59.2	494	INTERGENIC				
CaPOPII_4511	scaffold4041	1126	TTATATATATATATATATATAT/TTATATATATATATATATATATATATAT	GGGTTGGGATATGTTAGCAAAA	GTCCAAACCCAGCACAATCT	60.1	192	INTERGENIC				
CaPOPII_4512	scaffold4041	15570	AT/ATGTGTGTGT	AGATTGTGCTGGGTTTGGAC	AAGGAAATGAGGAAAATGGGA	60.0	803	INTERGENIC				
CaPOPII_4513	scaffold4041	15702	T/TG	AGGAATTGGATCGGGAGATT	AAGGAAATGAGGAAAATGGGA	59.7	626	INTERGENIC				
CaPOPII_4514	scaffold4041	15753	TAAAAAAAAA/TAAAAAAAAAAAAA	AGGAATTGGATCGGGAGATT	AAGGAAATGAGGAAAATGGGA	59.7	626	INTERGENIC				
CaPOPII_4515	scaffold4041	15867	AT/ATT	GGGTTGGGATATGTTAGCAAAA	GAATGCGAAAGCCACAATTT	60.1	851	INTERGENIC				
CaPOPII_4516	scaffold4041	15956	TAAAAAAAA/TAAAAAAA	TCCCATTTTCCTCATTTCCTT	GAATGCGAAAGCCACAATTT	59.8	493	INTERGENIC				
CaPOPII_4517	scaffold4041	16171	AAA/AAATTAA	GGCACGTCCCTATTTTTGAA	GAATGCGAAAGCCACAATTT	59.9	345	INTERGENIC				
CaPOPII_4518	scaffold4041	39114	A/AAG	GTGTGGTGGACGAACCTTTT	CGTGTCTCAAAACTCGAGGA	59.9	769	INTERGENIC				
CaPOPII_4519	scaffold4057	6605	T/TCTTTTA	CCTATGTGCTCTTCGTGCAA	AAAGCTTATATCCCAAGCTCAAA	60.0	667	DRR	Ca_27488			Uncharacterised protein family UPF0261
CaPOPII_4520	scaffold4064	805	TTATATATATATATATATATATATATATATATA/TTATATATATATATATATATATATATATATATATATA	ATGCTCTGCACTTGCAGCTA	TGACTTGACTCTTGTCCGCA	59.9	581	INTERGENIC				

INDEL marker IDs	Chromosomes /unanchored scaffolds	Physical positions (bp)	InDels (*Kabuli* reference genome-CDC Frontier/PI)	Forward primers (5'-3')	Reverse primers (5'-3')	Anneling temperature (0C)	Expected amplified product size (bp)	Structural annotation		Functional annotation		
								Sequence components of *kabuli* genome	*Kabuli* gene accession IDs	NCBI-KOG	TFs	NCBI-nr database
CaPOPII_4521	scaffold4064	1246	ATTTT/ATTTTT	TATGCATGCGGACAAGAGTC	GTGTTGCACTGCATGCTTTT	59.8	277	INTERGENIC				
CaPOPII_4522	scaffold4064	1366	TTAT/TT	AAAAGCATGCAGTGCAACAC	TCCCGTCTGAAAATTATGGC	59.9	367	INTERGENIC				
CaPOPII_4523	scaffold408	3569	TCCCC/TCCCCC	TAATACACGTCCGCTTTCCC	CGAACCTGCTGTCCAAAAAT	60.0	394	INTERGENIC				
CaPOPII_4524	scaffold408	35918	TTATATATATATATATATATATATATAT/TTATATATATATATATATATATATATATATATATAT	TTGTTAGTTTGACGAGGGCA	TTTTGTTGTGGTAGGGCACA	59.3	423	INTRON	Ca_25271		Trihelix	Peptidase C48, SUMO/Sentrin/Ubl1
CaPOPII_4525	scaffold408	130988	T/TG	GTGGTGATATTTGGGAAGCC	TCGTGGTTTTGGTGTTGGTA	59.2	662	INTERGENIC				
CaPOPII_4526	scaffold408	131029	A/AAG	GTGGTGATATTTGGGAAGCC	TCGTGGTTTTGGTGTTGGTA	59.2	662	INTERGENIC				
CaPOPII_4527	scaffold4088	5270	AT/A	ATCAACTTGCAAGGCAGCTT	TACCATTCAATTGGCGAACA	60.0	454	INTERGENIC				
CaPOPII_4528	scaffold409	2612	GTAAAATTT/G	AAGTCACAGTGGAACCCCAG	TCGATTGCAACCAAATTGAA	60.0	151	DRR	Ca_27582			
CaPOPII_4529	scaffold41	122306	ATT/ATTT	TCGCTACGGCTCTAGTGGTT	AAGTCCTGTAGCCGAAAGCA	60.0	294	INTERGENIC				
CaPOPII_4530	scaffold41	122382	C/CG	TCGCTACGGCTCTAGTGGTT	AAGTCCTGTAGCCGAAAGCA	60.0	294	INTERGENIC				
CaPOPII_4531	scaffold4107	9634	CT/CTAAATT	CCTAAAACCTAAAACCAAAAACC	TCGTCCAATAAGAACAAAAGTCAA	57.4	127	INTERGENIC				
CaPOPII_4532	scaffold4108	8749	CTT/CT	CAAAACCGGAAACGCTTAAC	TTTTGGGCTTCGAGTTTAGG	59.6	294	INTERGENIC				
CaPOPII_4533	scaffold411	298521	AT/AGATTGT	CTTGCATGGCAATTATCCAA	TTGGGCTGATGCAATAAACA	59.5	469	INTERGENIC				
CaPOPII_4534	scaffold414	21975	GAAAAA/GAAAA	TTCCTATTGCTCACCCCTTG	GAAAAACAAGACCCGCGATA	60.1	516	DRR	Ca_27849			
CaPOPII_4535	scaffold415	9749	T/TCTGA	TTCGAGCTCAAAGAGGTGCT	AGCGCTGTAAAATGTGCCTT	60.3	727	DRR	Ca_28252		Trihelix	
CaPOPII_4536	scaffold4155	7060	AATATATAT/AATATATATAT	CGGTTCAACGTAACCGACTT	TTGGGCAACAATCGATGTAA	60.0	402	INTERGENIC				
CaPOPII_4537	scaffold418	9574	TTATATATATATATATATATATATATATATA/TTATATATATATATATATATATATATATA	TGAATATGTGGCGGAAAACA	CATGTGTGAGGGTTGTGGAG	59.9	606	INTERGENIC				

INDEL marker IDs	Chromosomes /unanchored scaffolds	Physical positions (bp)	InDels (*Kabuli* reference genome-CDC Frontier/PI)	Forward primers (5'-3')	Reverse primers (5'-3')	Anneling temperature (0C)	Expected amplified product size (bp)	Structural annotation		Functional annotation		
								Sequence components of *kabuli* genome	*Kabuli* gene accession IDs	NCBI-KOG	TFs	NCBI-nr database
CaPOPII_4538	scaffold418	152839	AACACACACACACACACA/AAACACACACACACACACACA	CTGGGCAAGGGATCTGAATA	TGCATTTTACAGCGCTTTTG	60.0	596	DRR	Ca_24681			
CaPOPII_4539	scaffold419	148404	CTATATATATATATATATAT/CTATATATATATATATATATAT	GAGCAACGACCTATTCTGGC	GGTGAGGATCTGGTGTGGTT	59.8	750	INTERGENIC				
CaPOPII_4540	scaffold419	148627	CA/CAA	AACAAGCAAGCCATTCAAGC	GGTGAGGATCTGGTGTGGTT	60.4	657	INTERGENIC				
CaPOPII_4541	scaffold4192	3668	T/TC	ATCCAAGTTTGAGGCAATCG	CGATTTTCGCAGGTCTTGTT	60.1	484	INTERGENIC				
CaPOPII_4542	scaffold4197	16348	GAAA/GAA	AATGGCCACGAATCTGAAAG	GTGCGGAAGCCATGAGTAAT	60.1	483	INTERGENIC				
CaPOPII_4543	scaffold420	69655	CAA/CAAA	ATGGCGAAGAATTATGGTGC	CATGTACCGCCCATCTCTTT	59.9	265	INTERGENIC				
CaPOPII_4544	scaffold420	265273	GAAAAAAAA/GAAAAAAAAA	CGTGATCGTGTTTGGTTGTC	GGGAATGGCAGCAAATAGAA	60.0	587	INTERGENIC				
CaPOPII_4545	scaffold420	344571	CTTTTTTTTT/CTTTTTTTTTT	TCCTCTATGTTGCCGTTTCC	TCACTCTCCAAATGGAACCC	60.1	192	URR	Ca_22212	G	NAC	Glycoside hydrolase, family 1
CaPOPII_4546	scaffold4213	2577	GC/GCC	AGTGCCGGTGCATTTTATTC	CGCATTCCAAATTCAATTCC	60.0	780	INTERGENIC				
CaPOPII_4547	scaffold4221	569	AAAGAA/AAA	ATTGATTTTGAAATCGCCCA	CAGTTTCCTGCCAGCCTTAG	60.3	441	INTERGENIC				
CaPOPII_4548	scaffold4221	691	A/AG	CTAAGGCTGGCAGGAAACTG	CGCTTCTTTGGTCATGCAA	60.0	443	INTERGENIC				
CaPOPII_4549	scaffold4221	7762	TAAAAA/TAAAA	TGCCAGAACTGATTTTCTGCT	GAGCGCTGTAAAACACCTCC	60.0	540	INTERGENIC				
CaPOPII_4550	scaffold4221	54616	CAATAATAATAATAATAATAATAATAATAATAATAATAGTAATAATAATAATAATA/CAATAATAATAATAATAATAATAATAATAATAATAGTAATAATAATAATAATA	GAGCCCGAATCGTTATTTTG	TTGTGGTATACCCGACAAAGAA	59.5	695	INTERGENIC				
CaPOPII_4551	scaffold4243	1042	CGG/CG	GGTACGGAAATTGCCTGAAA	GGTCCTCGATTTCATCTGGA	59.9	204	INTERGENIC				
CaPOPII_4552	scaffold4243	14917	CT/CTT	TGCTTGATTGCTTGAGTTCC	GATGAAATCGAGGACTGGGA	59.0	537	INTERGENIC				
CaPOPII_4553	scaffold4243	17716	AGG/AGGG	AGGCGATTTTCGTACCACAC	AAACCGTGTTTCGGTTTCTG	60.0	589	INTERGENIC				
CaPOPII_4554	scaffold429	69799	TAA/TA	TGGATGCACGAGATTGGTTA	AGGTCATTTTCGTCCTCCCT	60.1	446	INTERGENIC				

INDEL marker IDs	Chromosomes /unanchored scaffolds	Physical positions (bp)	InDels (*Kabuli* reference genome-CDC Frontier/PI)	Forward primers (5'-3')	Reverse primers (5'-3')	Anneling temperature (0C)	Expected amplified product size (bp)	Structural annotation		Functional annotation		
								Sequence components of *kabuli* genome	*Kabuli* gene accession IDs	NCBI-KOG	TFs	NCBI-nr database
CaPOPII_4555	scaffold429	179314	CATATATATATATATATATATATATATATATATATATA/CATATATATATATATATATATATATATATATATA	AAGCTGCAACAATCAAAGCA	TAACCAATCTTGTGCACCCA	59.6	923	DRR	Ca_26240			
CaPOPII_4556	scaffold4319	1189	A/AGTCAAAT	CGATTTCCCTCACGACAAAT	TTTTGGACAGCTACAAGTGGTT	59.9	582	INTERGENIC				
CaPOPII_4557	scaffold4319	1245	TAAAAAA/TAAAA	CGATTTCCCTCACGACAAAT	TTTTGGACAGCTACAAGTGGTT	59.9	582	INTERGENIC				
CaPOPII_4558	scaffold433	70798	CGGG/CGG	GGGTACGTCGGTGAGTGTTT	GAACAAAAGAGAACGGCTGC	59.9	751	INTERGENIC				
CaPOPII_4559	scaffold433	86247	GTTT/GTT	GCCCTCAGATGAGAGTCAGG	AGAATTTACGAGCAGCGGAA	59.9	287	INTERGENIC				
CaPOPII_4560	scaffold433	146841	AATTATTATTATTATTATTATTATTATT/AATTATTATTATTATTATTATT	TGGTGACAATTTGATGGTTCT	CAGTGCGATGGAGAAATCAA	57.9	523	INTERGENIC				
CaPOPII_4561	scaffold433	157672	GATATATA/GATATA	GCATTTCAAGATGGGCAGTT	AATGATCGTCTTCGGATTGG	60.1	714	INTERGENIC				
CaPOPII_4562	scaffold433	165941	A/AG	AAAATTGAAACATCCGACGC	ACTTGTCGCTGGACGAATCT	59.9	512	DRR	Ca_26821			
CaPOPII_4563	scaffold433	169477	ATTATTTATTTATTTATTTA/ATTATTTATTTATTTATTTATTTA	CTAGTGGGTACCCGCAAAAA	GGTGGGCATATTACAACGCT	60.0	576	URR	Ca_26821			
CaPOPII_4564	scaffold4349	1831	GTTTT/GTTT	AGGTCATTTTCGTCCTCCCT	CAAACACACCTGTTGGATGC	59.9	182	INTERGENIC				
CaPOPII_4565	scaffold436	67143	A/AC	CAAACACACCTGTTGGATGC	AGGTCATTTTCGTCCTCCCT	60.0	478	INTERGENIC				
CaPOPII_4566	scaffold436	68438	A/AT	GGCCGGATGAAAATGAACTA	ATTTTCGTCCTCCAAAGGCT	59.9	513	INTERGENIC				
CaPOPII_4567	scaffold4366	1890	G/GC	GGACATGAATAGGGCAATGG	GGTCATTTTTGTCCACGCTT	60.2	532	INTERGENIC				
CaPOPII_4568	scaffold437	45718	CAATATTAAAAAATATTAAA/CAATATTAAAAAATATTAAAATATTAAAAAAATATTAAA	TCGAAGAGGACCATTCATCA	AGGTCGCACTCCAAATATCG	59.2	777	INTERGENIC				
CaPOPII_4569	scaffold4374	1159	CTAAACC/CTAAACCATAAACC	TAAACCCCAAATGACCGAAA	AGGGGTTAGGGGTTAGGGTT	60.2	444	INTERGENIC				
CaPOPII_4570	scaffold4377	6572	AA/AAGA	TCCAGATGAAATCGAGGACC	AGGCGATTTTCGTACCACAC	60.0	319	INTERGENIC				
CaPOPII_4571	scaffold4383	25907	TATAATAATAATAATAATAATAATAATAATAATAATAATAATAATAATAATAATAATAATAATAATAA/TATAATAATAATAATAATAATAATAATAATAATAATAAT	TGATCTCTAACGGACCACCC	TCATGTGCTTTTACTGCCCA	59.9	781	INTRON	Ca_28195			SWIB/MDM2 domain

INDEL marker IDs	Chromosomes /unanchored scaffolds	Physical positions (bp)	InDels (*Kabuli* reference genome-CDC Frontier/PI)	Forward primers (5'-3')	Reverse primers (5'-3')	Anneling temperature (0C)	Expected amplified product size (bp)	Structural annotation		Functional annotation		
								Sequence components of *kabuli* genome	*Kabuli* gene accession IDs	NCBI-KOG	TFs	NCBI-nr database
CaPOPII_4572	scaffold4387	5637	TTCT/TTCTCTCT	TCAAAAAGAGGCGAGCATTT	GGCGATTGTGGAAAGACAGT	60.0	191	INTRON	Ca_27838	U		Clathrin/coatomer adaptor, adaptin-like, N-terminal
CaPOPII_4573	scaffold44	287139	CA/CATGTTTTGAATA	TTAAAATTTGGCGGTTTTGG	AAAAATTTCGAAGCGGAAAG	59.8	166	INTERGENIC				
CaPOPII_4574	scaffold440	8696	CAAAAAAAA/CAAAAAAA	ATCAGCATCAAGTGCACAGC	TCCAGATTTGTGACGAGCAA	60.0	473	INTERGENIC				
CaPOPII_4575	scaffold442	20594	TGGGGGGGG/TGGGGGGG	TGGACGCACAAGATTGGTTA	AGGTCATTTTCGTCCTCCCT	60.1	370	INTERGENIC				
CaPOPII_4576	scaffold442	37151	TAAAAA/TAAAA	AGGGAGGACGAAAATGACCT	TGTGCGTCCAATAGGTGTGT	59.9	363	INTERGENIC				
CaPOPII_4577	scaffold446	67842	A/ATAT	GGACGGCCTAGTCTATTCCC	ACGACGTAGAACTATAAAACGGTAA	59.9	500	INTERGENIC				
CaPOPII_4578	scaffold446	163582	CAAATGAAAATTTAAA/CAAATGAAAATTTAAATGAAAATTTAAA	TTGCATCATGCTATTTCCCA	ACATAACTTTGTGGGCCAGC	60.0	685	INTERGENIC				
CaPOPII_4579	scaffold446	224556	AAATAATAATAATAATAATAATAATAATAATAATAATAATAATAATAATA/AAATAATAATAATAATAATAATAATAATAATAATAATAATAATAATAATA	TTCATCTGGTCTTTTTGGGC	CATTGAGTACAACTACAAAACGACG	60.1	771	INTERGENIC				
CaPOPII_4580	scaffold446	235656	TAA/TA	TCTGACAACTCAATTTTGCTCTATG	GTTGTACTTCATAACGAACACAATACT	59.8	521	INTERGENIC				
CaPOPII_4581	scaffold450	108759	AATATATATATATATATATATATAT/AATATATATATATATATATATATATATATAT	TTTTTCGAGTAGGTCGGGTG	AGCAAAAGGAAGCAACGAAA	60.1	620	INTERGENIC				
CaPOPII_4582	scaffold4508	33683	CTTTTTTT/CTTTTTT	TGAATGTCGTCAATGGGAGA	AAGAATCGAATGGGAAATCG	60.0	772	INTERGENIC				
CaPOPII_4583	scaffold4508	36727	TAA/TA	TTGTGTTTGTCACAGGCCAT	TCAGGAACAACAACTCAACCC	60.0	371	INTERGENIC				
CaPOPII_4584	scaffold451	288952	ACCCCCCCCCCC/ACCCCCCCCC	TTGGAAGGTCAGGTGCTTTT	CCAACAGAGCGATGTAACCA	59.7	516	INTERGENIC				
CaPOPII_4585	scaffold4511	22953	TAAAA/TAAAAA	CTCCCCCTGAGTTTTGGAAT	GACGACAATCACCCGAAGAT	60.3	753	DRR	Ca_28219		Trihelix	Peptidase C48, SUMO/Sentrin/Ubl1
CaPOPII_4586	scaffold4511	38356	GTTTTTTTTTT/GTTTTTTTTTTTT	AATGCCCACCCATAATTTCA	CTGGGCAAGGGATCTGAATA	60.0	642	INTRON	Ca_28219		Trihelix	Peptidase C48, SUMO/Sentrin/Ubl1
CaPOPII_4587	scaffold453	79397	AG/AATACCATCTAAGATTTG	TTGTTTGACCCATTGTACTTTGA	TTGCTAGTTGCTGATGTGGC	59.4	183	INTRON	Ca_21728		MYB_related	Toll-Interleukin receptor
CaPOPII_4588	scaffold453	80166	T/TCATG	AATTTAAACCGGCCAAAACC	TCACTCATAAATATCCTCATGGACA	60.1	578	INTRON	Ca_21728		MYB_related	Toll-Interleukin receptor

INDEL marker IDs	Chromosomes /unanchored scaffolds	Physical positions (bp)	InDels (*Kabuli* reference genome-CDC Frontier/PI)	Forward primers (5'-3')	Reverse primers (5'-3')	Anneling temperature (0C)	Expected amplified product size (bp)	Structural annotation		Functional annotation		
								Sequence components of *kabuli* genome	*Kabuli* gene accession IDs	NCBI-KOG	TFs	NCBI-nr database
CaPOPII_4589	scaffold453	147436	AAAATTCCCAAATTCCCAAATT/AAAATTCCCAAATT	AAGAGAGGTCACTTTGGCGA	AACCTCGAGACCACCATCAC	60.0	771	INTERGENIC				
CaPOPII_4590	scaffold453	497792	CAATAATAATAATAATA/CAATAATAATAATAATAATA	GCCGTGATTTGGAAAAAGAA	TGTCAGTTTTGAGCCCAATG	60.1	479	URR	Ca_21748			SAC3/GANP/Nin1/mts3/eIF-3 p25
CaPOPII_4591	scaffold453	498751	GATAT/GATATAT	GCCCCTCAAAGATAACCCAT	GACCATGATTTCTTACTAGCCGTT	60.2	838	URR	Ca_21748			SAC3/GANP/Nin1/mts3/eIF-3 p25
CaPOPII_4592	scaffold4551	6498	TAAAAAAA/TAAAAA	CTGGTGAGGTTGCTTGGAAT	CATGCCTTGGAGACCTTCAT	60.1	883	INTERGENIC				
CaPOPII_4593	scaffold4582	7612	A/AG	TCCAGATGAAATCGAGGACC	CGAAATTTGACACGGAACCT	60.0	258	INTERGENIC				
CaPOPII_4594	scaffold46	6079	CTTTT/CTTTTT	GGGCCGAGAGTCCCTTATAG	GGTCCTCGATTTCATCTGGA	60.1	304	INTERGENIC				
CaPOPII_4595	scaffold46	9094	ATTTT/ATTT	TTGAAATCGGACATGGAACA	GATGAAATCGAGGACCAGGA	59.9	852	INTERGENIC				
CaPOPII_4596	scaffold46	40359	ATTT/ATTTT	TGTGCCTGAGAAAGATGCAA	CAGAATCAATGTGCCAATCG	60.5	509	DRR	Ca_27600			
CaPOPII_4597	scaffold466	12138	AATATATA/AATATATATA	ATTCAGTTCCCTTGCAGCAT	TCTCCGCATAAGCTTTCGAT	59.7	661	INTERGENIC				
CaPOPII_4598	scaffold466	169717	A/ATGCAAAAGATTCTA	CTTTCGTGCCCTTGATTTGT	CGTGGCCTATCGATCCTTTA	60.1	740	INTRON	Ca_25699			
CaPOPII_4599	scaffold466	170442	CA/CACGGA	ACCCTTTTGTTCCACACGAG	AAGCTGACTTCGGTTGGCTA	60.0	270	INTRON	Ca_25699			
CaPOPII_4600	scaffold466	254290	TCTTCTCCACTT/TCTT	GGATTCCGCGTAAGATTTCA	ACTAGGTTTTGGGTTCCGCT	60.0	465	INTERGENIC				
CaPOPII_4601	scaffold466	286406	CATATATATATATATATATATATATATATATATATATAT/CATATATATATATATATATATATATATATATATAT	GGAAGAAGGAAGGAGTTGGG	TGCTGAAAATTGTGCCAGAC	60.0	445	INTERGENIC				
CaPOPII_4602	scaffold469	49590	AATATATATATATATATATATATATAT/AATATATATATATATATATATATATATATATATAT	CGGTGAATCCTTTACGGAGA	GAGGAGGGAGGGATTTTGAG	60.1	293	INTERGENIC				
CaPOPII_4603	scaffold469	58499	AATATATATATATAT/AATATATATATATATAT	CTTGGGAACTGGAGAGCAAG	GAGGAGGGAGGGATTTTGAG	60.0	530	INTERGENIC				
CaPOPII_4604	scaffold470	186075	ATGAACTGAACTGAACTGAA/ATGAACTGAACTGAA	ACCAGGAAAAGAAGAAGGGC	TTGGTTTATTTGGGTTGGGA	59.7	456	INTERGENIC				
CaPOPII_4605	scaffold470	241092	AATATATATATATATATATATATAT/AATATATATATATATATATATATATAT	AATCGGAAGGGTAAACTCGT	TTTCGTTTGCAAGGTTTTGA	57.7	660	INTERGENIC				

INDEL marker IDs	Chromosomes /unanchored scaffolds	Physical positions (bp)	InDels (*Kabuli* reference genome-CDC Frontier/PI)	Forward primers (5'-3')	Reverse primers (5'-3')	Anneling temperature (0C)	Expected amplified product size (bp)	Structural annotation		Functional annotation		
								Sequence components of *kabuli* genome	*Kabuli* gene accession IDs	NCBI-KOG	TFs	NCBI-nr database
CaPOPII_4606	scaffold470	336358	TTATGTAT/TTAT	GTCATCCTAGCCATCGTCGT	GTGTCCCAAAACTCAAGGGA	60.1	876	INTERGENIC				
CaPOPII_4607	scaffold470	365605	CTTTTTTTTTTTTT/CTTTTTTTTTTT	AGAAGCAAGCTAGCTCGTGG	ATTGGAGCACATTCAGGCTT	59.9	815	INTERGENIC				
CaPOPII_4608	scaffold470	370786	TAAAAAAA/TAAAAAA	CATGCCAGTTGCTACATGCT	GCTTGGACTCAGTGGCTAGG	59.9	949	INTERGENIC				
CaPOPII_4609	scaffold470	372719	GAAA/GAAAA	ATTTTATGCTGCGACATCCC	GCAAATCCCACACTTGGAGT	59.9	265	INTERGENIC				
CaPOPII_4610	scaffold470	375954	AATATATATATATATATATATATATATATATATATATAT/AATATATATATATATATATATATATATATATATATATATAT	AGGGAATCGAGGGAAAAATG	AGGTGGCAACCTTCTCATTG	60.3	463	INTERGENIC				
CaPOPII_4611	scaffold4711	5708	GTTTTTTTT/GTTTTTTT	AGGTCATTTTCGTCCTCCCT	TTGGATGCACAAGATTGGTT	59.9	271	INTERGENIC				
CaPOPII_4612	scaffold4711	6211	GTTTTTTT/GTTTTTTTT	TTTTCGTCCTCCCTAGGCTT	AGGAACTTATTGGATGCCCA	60.2	470	INTERGENIC				
CaPOPII_4613	scaffold4713	1946	TG/TGG	TCATCCAATGACACAAACGC	AGGTCATTTTCGTCCTCCCT	60.5	193	INTERGENIC				
CaPOPII_4614	scaffold4733	507	AT/ATT	ACAGTGAACGTGCACCCATA	CTGGTTATGCCGTTTGGAGT	60.0	697	INTERGENIC				
CaPOPII_4615	scaffold475	141188	A/AAAATGGGG	GAGGAGCGACCAGTTGTTTC	GAAACTAATGCGACCGGTGT	59.9	884	INTERGENIC				
CaPOPII_4616	scaffold475	174713	GTTTTTTT/GTTTTTTTT	AAGGTTGACTGCATAACGAAGA	CAACACCATTTGACAACACCA	58.9	730	INTERGENIC				
CaPOPII_4617	scaffold4769	4939	TAAAA/TAAAAA	ACAACTTCTCATGCTCGCAA	GGGAAAATCATGTTTATCACAATC	59.6	327	INTERGENIC				
CaPOPII_4618	scaffold477	11283	C/CA	TGTCATCTTGTTTGCCTTGG	TGATGAAACTCTGAACTCGTGG	59.7	114	INTERGENIC				
CaPOPII_4619	scaffold477	20714	TA/TGA	CCCCCTAATGCTCAATTTCA	TGCAACAAGAGGCAAAACAC	59.9	288	INTERGENIC				
CaPOPII_4620	scaffold477	28410	TAAAAA/TAAA	GTAGGAGGATCTCAAGGGGC	CCAAAATTAGGAAGCACCCA	60.0	567	INTERGENIC				
CaPOPII_4621	scaffold477	46653	GAAAAAA/GAAAAAAA	ATGCAACTGTCGTTTTAGCA	TTAGAGCGCCTTCTGATGGT	57.0	405	INTERGENIC				
CaPOPII_4622	scaffold4773	16885	TNNNNNNNNNN/TNNNNNNNNN	TGCGGCTATTCATAACATGG	GCATATCTGAGGCTGCCAAT	59.5	600	INTERGENIC				

INDEL marker IDs	Chromosomes /unanchored scaffolds	Physical positions (bp)	InDels (*Kabuli* reference genome-CDC Frontier/PI)	Forward primers (5'-3')	Reverse primers (5'-3')	Anneling temperature (0C)	Expected amplified product size (bp)	**Structural annotation**		**Functional annotation**		
								**Sequence components of *kabuli* genome**	***Kabuli* gene accession IDs**	**NCBI-KOG**	**TFs**	**NCBI-nr database**
CaPOPII_4623	scaffold4777	118728	CTTTTTTT/CTTTTTTTTT	AACGGTTTGGTTTGATCTGC	ACGTCGTCAAACAGATGCAC	60.0	231	INTRON	Ca_26711	R		WD40 repeat
CaPOPII_4624	scaffold4784	684	TG/TGATGGCTAG	TCCTGGTCCTCGATTTCATC	GGGCCGAGAGACCCTTATAG	60.0	339	INTERGENIC				
CaPOPII_4625	scaffold479	7594	TTA/TTAGGCATA	TGTGTTTGTGGCATTGGATT	AGGGAGGACGAAAATGACCT	59.8	329	INTERGENIC				
CaPOPII_4626	scaffold479	16760	ATTTTTTTTTTTTTT/ATTTTTTTTTTT	TGTTCATTGTAACACCCCGA	TTAAGCCAATGCCACAAACA	59.8	533	INTERGENIC				
CaPOPII_4627	scaffold479	125417	CTTTT/CTTTTT	ACAAACCAATCGATTTCCCA	CGGAGGACGAAAATGACCTA	60.2	180	INTERGENIC				
CaPOPII_4628	scaffold48	15048	AAATAATAATAATAATAATAATAATAATAATAATAATAATAATAATAATAATAATAATAATAATAA/AAATAATAATAATAATAATAATAATAATAATAATAATA	TCGGTTCATCATGTTGGAAAT	TCTTTTGATGTGGGTTAGTGAGAA	60.2	677	INTERGENIC				
CaPOPII_4629	scaffold480	43150	TCCC/TCCCC	AACGTACTGCCTATCACGGG	TGTTCATCGTATTTGGCGTG	60.0	836	INTERGENIC				
CaPOPII_4630	scaffold4804	1921	TAAG/TAAGCCAAG	TTGGATGCACAACATTGGTT	AGGTCATTTTCGTCCTCCCT	59.8	561	INTERGENIC				
CaPOPII_4631	scaffold4804	8867	CTT/CT	TGGATGCACCAGATTGGTTA	AGGTCATTTTCGTCCTCCCT	59.9	570	INTERGENIC				
CaPOPII_4632	scaffold4804	9853	CAAAAA/CAAAA	AAGCCTGAGGATGACGAAAA	TGCATCCAACAGGTGTGTTT	59.8	571	INTERGENIC				
CaPOPII_4633	scaffold4822	847	A/AGAATACT	CAGCGGATCCCCAGATATAC	AGGTCATTTTCGTCCTCCCT	59.4	545	INTERGENIC				
CaPOPII_4634	scaffold4829	6279	TCCC/TCCCC	GACCCGCATTTTCCTCATTA	GGAGAACGTTAAGGCAGCAG	59.9	507	INTERGENIC				
CaPOPII_4635	scaffold4829	8205	TAAAA/TAAA	CAATAGCATGCAGTTGGAGG	GGAGACTCCCATACATTGGC	59.3	500	INTERGENIC				
CaPOPII_4636	scaffold4833	740	TTTTTTGTTTTT/TTTTTT	TGGGAATGTAGTGGATGAACC	TCCCGAACACCTATGCTTTT	59.7	529	INTERGENIC				
CaPOPII_4637	scaffold4833	3774	T/TA	CCAAGCCAGTCAAACACAAA	GGGCTCGCTATGTACAGCA	59.7	839	INTERGENIC				
CaPOPII_4638	scaffold484	136302	TCCCC/TCCC	ACCGAGGTGCCACTTATCAC	ATAGTGGTGACTGGATGGGC	60.0	229	INTERGENIC				
CaPOPII_4639	scaffold484	319973	ATTTTTTTTTT/ATTTTTTTTTTT	TTGCACTCACGGAACGAATA	TCGCCATTTAAAATTGAGGTG	60.3	452	INTERGENIC				

INDEL marker IDs	Chromosomes /unanchored scaffolds	Physical positions (bp)	InDels (*Kabuli* reference genome-CDC Frontier/PI)	Forward primers (5'-3')	Reverse primers (5'-3')	Anneling temperature (0C)	Expected amplified product size (bp)	Structural annotation		Functional annotation		
								Sequence components of *kabuli* genome	*Kabuli* gene accession IDs	NCBI-KOG	TFs	NCBI-nr database
CaPOPII_4640	scaffold484	656687	AC/A	TATCACCTATCAGAGCGGGG	TGATTCCGGGGAAGTACAAC	60.1	472	INTERGENIC				
CaPOPII_4641	scaffold4851	3486	GTT/GT	CTATAAGGGTCTCTCGGCCC	CCGATGCTAAAAATGGCAGT	60.1	733	INTERGENIC				
CaPOPII_4642	scaffold4851	3617	TAAAAAAA/TAAAAAAAA	CGCGGAACCTGAAACACTAT	CCGATGCTAAAAATGGCAGT	60.1	616	INTERGENIC				
CaPOPII_4643	scaffold486	38993	CAAAAAAA/CAAAAAAAA	CTCCCCCTTTTGTCATTGAA	TGACCAGCTGAAGCACAATC	59.9	415	INTERGENIC				
CaPOPII_4644	scaffold487	3721	T/TG	AAGTGCATAAGTCCCAACGG	TGCCTAAGAACCAAAGTGGG	60.0	533	INTERGENIC				
CaPOPII_4645	scaffold487	87751	T/TC	ACATTGAGGTCATTTTCGCC	AGCCTAGGGAGGATGCAAAT	59.9	229	INTERGENIC				
CaPOPII_4646	scaffold4872	661	G/GTCTATCA	TGGGCACATGCTTCTCAGTA	TACTGGAACGGACCACACAA	60.4	498	INTERGENIC				
CaPOPII_4647	scaffold4902	8464	CG/C	CGTCTAGTCGAATGGATCTGC	AAAAATGGGTTTAACGTTATGCTT	59.9	321	INTERGENIC				
CaPOPII_4648	scaffold4902	39096	TAAAAAA/TAAAAA	AAGCCGCTGCTTACGAAATA	AGTTAGTGGCTGGGGGAGAT	60.0	494	DRR	Ca_26348	TU		ANTH
CaPOPII_4649	scaffold493	124059	ACTCTC/ACTC	TCTTTGGTTTTACGGCTGCT	TGAAATCGGGTCACTTGTCA	59.9	759	INTERGENIC				
CaPOPII_4650	scaffold495	111241	TTTTGAAAATAACCCTT/TTT	GGTTTGCCTTTGGTGTCTGT	GTGCTTTGCATGGAACAAGA	60.0	534	INTERGENIC				
CaPOPII_4651	scaffold496	121429	CT/C	TCCTGGTCCTCGATTTCATC	AATCGCCTGAAAAGACGAGA	60.0	379	INTERGENIC				
CaPOPII_4652	scaffold497	69995	GTTTTTTTTTTTTT/GTTTTTTTTTT	TGGTGCTTTTGGAAAAAGTG	ATTTGGTTTGGTTGCATTCG	58.8	446	INTERGENIC				
CaPOPII_4653	scaffold498	45313	CTTTTTTTTT/CTTTTTTTTTT	CAGGAAAAGGCGTTGTGTTT	CCTCAATTTTATCCGACCAA	60.1	348	INTERGENIC				
CaPOPII_4654	scaffold498	206145	GTTTTTTTT/GTTTTTTT	CACCGCCTCTGTTCCATATT	GAGATTTAGAACCATCAAATTGTCA	60.0	829	INTERGENIC				
CaPOPII_4655	scaffold498	214216	AA/AAAAGTCA	TTTCATGGTCATGTTTTGGC	GAAAGGCCAATGTCCAGAAA	59.4	781	INTERGENIC				
CaPOPII_4656	scaffold498	214228	CATAATAAAACTAATAATAAAACTAATAAT/CATAATAAAACTAATAATAAAACTAATAATAAAACTAATAAT	TTTCATGGTCATGTTTTGGC	GAAAGGCCAATGTCCAGAAA	59.4	781	INTERGENIC				

INDEL marker IDs	Chromosomes /unanchored scaffolds	Physical positions (bp)	InDels (*Kabuli* reference genome-CDC Frontier/PI)	Forward primers (5'-3')	Reverse primers (5'-3')	Anneling temperature (0C)	Expected amplified product size (bp)	Structural annotation		Functional annotation		
								Sequence components of *kabuli* genome	*Kabuli* gene accession IDs	NCBI-KOG	TFs	NCBI-nr database
CaPOPII_4657	scaffold498	214273	TACACACACACACAC/TACACACACACAC	TTTCATGGTCATGTTTTGGC	GAAAGGCCAATGTCCAGAAA	59.4	781	INTERGENIC				
CaPOPII_4658	scaffold498	214558	CTTTTT/CTTTTTT	CACACACACGTAAGAAGGCTG	TTGGACAGAATCAAGGCTCC	59.4	544	INTERGENIC				
CaPOPII_4659	scaffold4983	27196	AAT/AATAT	AAGGTGGTACGAAAATCCCC	GCACGTGGGAAAATGAGATT	60.1	893	INTERGENIC				
CaPOPII_4660	scaffold4983	27256	C/CT	AAGGTGGTACGAAAATCCCC	GCACGTGGGAAAATGAGATT	60.1	893	INTERGENIC				
CaPOPII_4661	scaffold4983	27276	ATTT/ATT	AAGGTGGTACGAAAATCCCC	GCACGTGGGAAAATGAGATT	60.1	893	INTERGENIC				
CaPOPII_4662	scaffold5	1535	CTT/CTTT	CGATTGTGTTTGTCACAGGG	GAAGCAAATCGATTGGGAAA	60.0	316	INTERGENIC				
CaPOPII_4663	scaffold50	165794	GATATATATATATATATATATATAT/GATATATATATATATATATATAT	GCGACTTGGAAACTGGAGAG	GCACCGTGAGTAAATTCGGT	60.0	846	INTERGENIC				
CaPOPII_4664	scaffold50	192238	A/AGTGATAGAAATT	CTGCCCATTAGCCCTTCATA	CTGAATGCAAGAGAGAGGGG	60.1	291	INTERGENIC				
CaPOPII_4665	scaffold50	196038	A/ACTG	CTGCCCATTAGCCCTTCATA	AGGAAGGAGTGATGCGAAGA	60.1	477	INTERGENIC				
CaPOPII_4666	scaffold50	299723	GC/GTAACGTACAACATC	GCGAAGCAAAGTCCATCATT	AAATTCGCTCGCTAGGTTGA	60.2	524	INTERGENIC				
CaPOPII_4667	scaffold50	333436	AATATATATATATATATAT/AATATATATATATATATATAT	TGGCGTGACAATAACCTCAA	AAATTGGTCCACTAGCACCG	60.1	878	INTERGENIC				
CaPOPII_4668	scaffold50	337379	CATATATATATATATATATATA/CATATATATATATATATATATATA	AACTTTTTCCGCTGCTTGAA	CGACTCTGTTGGGGATGTCT	60.0	383	INTERGENIC				
CaPOPII_4669	scaffold501	87430	CATATATATATATATAT/CATATATATATATATATAT	TGGGTGTGCCTATATACACGTAA	TTCTTTGCCACACACCATGT	59.3	519	INTERGENIC				
CaPOPII_4670	scaffold5030	906	AAATAATAAT/AAATAAT	TGGGGATACGTAGGAGCAAG	TGTTGGTCCATTTTTGACGA	60.1	432	INTERGENIC				
CaPOPII_4671	scaffold5030	11801	GCTT/GCTTGTACTT	AATGACATTTAACAGCGCCC	TGGCCGCACAGTATGTAGAG	60.0	459	INTERGENIC				
CaPOPII_4672	scaffold506	18975	TA/TAA	TCTCGATACCATCGTGGACA	TCCCATTTGATTGCTGAACA	60.1	489	INTERGENIC				
CaPOPII_4673	scaffold5094	2305	TAA/TA	TGGCACATGAATAGTTCCACA	GCCACCTCATTCAAATATTGC	60.0	669	INTERGENIC				

INDEL marker IDs	Chromosomes /unanchored scaffolds	Physical positions (bp)	InDels (*Kabuli* reference genome-CDC Frontier/PI)	Forward primers (5'-3')	Reverse primers (5'-3')	Anneling temperature (0C)	Expected amplified product size (bp)	Structural annotation		Functional annotation		
								Sequence components of *kabuli* genome	*Kabuli* gene accession IDs	NCBI-KOG	TFs	NCBI-nr database
CaPOPII_4674	scaffold510	13129	CATATATATATATATATATATATATA/CATATATATATATATATATATATATATATA	TGGGTGCTATTTGCAATCAA	CCTTCATGAACCGCAATACA	60.1	406	DRR	Ca_26546			Peptidase C48, SUMO/Sentrin/Ubl1
CaPOPII_4675	scaffold510	85609	TAACA/TA	TTGTCAAATAAAGTAACAACAAAACG	TTGCAACAATTGTACCCGAA	58.7	995	INTERGENIC				
CaPOPII_4676	scaffold511	193038	AATATATATATATATATATATA/AATATATATATATATATATATATATA	TTTCAGGTTATCGACTGGGG	AACAAGAAAGCGCAAGCAAT	59.9	729	INTERGENIC				
CaPOPII_4677	scaffold511	194615	AT/ATATT	AGGCCTGGGAAGTAGGAACA	TGCACAATAAACCTGCTTGC	61.0	557	INTERGENIC				
CaPOPII_4678	scaffold5115	16853	GAA/GAAA	AATGGCCACGAATCTGAAAG	GAAGTACGCCTAAGAGGGGG	60.1	538	INTERGENIC				
CaPOPII_4679	scaffold5120	13346	TTAATAATAATAATAATAATAATAA/TTAATAATAATAATAATAATAATAATAA	TTTGAAGCAAATTGCGAGTG	GGTGTGGTGTGGTGCAAGTA	60.0	382	INTERGENIC				
CaPOPII_4680	scaffold5120	14840	TTATATATATATATATATATATATAT/TTATATATATATATATATATATATATATAT	TATCGTGCCTGTTGAGAACG	CTAATTAGCGTCGGAGTCGG	59.9	527	INTERGENIC				
CaPOPII_4681	scaffold5120	15547	TAGAGTACGAGA/TAGA	CCGAAGTAATAGTGGGGCAA	GGACCAGGTTCTGCATCAAT	60.0	214	INTERGENIC				
CaPOPII_4682	scaffold515	28282	TA/T	CGCACGAAAAGTCTTGATGA	CGGTCGATTTTCGTACCACT	60.0	169	INTERGENIC				
CaPOPII_4683	scaffold515	43016	GAAAAA/GAAAA	CAAGAGTCTCCTGGTCCTCG	CTCCCGAAAAGTCCAAATGA	60.0	912	INTERGENIC				
CaPOPII_4684	scaffold515	61810	TCTC/T	CTCGAAACCCGAAACACAAT	GCGTCGTTTGAACCAAAAAT	60.0	560	INTERGENIC				
CaPOPII_4685	scaffold5158	6559	TAT/TATCATTATCAT	CCAAACAAATCTATTCCCCATC	CGGCTTATGTTGTTTGACCA	59.6	618	INTERGENIC				
CaPOPII_4686	scaffold5158	17065	TAAAAAAAA/TAAAAAAAAA	GGCCATCACGTTCATCTTCT	GATACTATGTCACCGCGCAA	60.1	832	INTERGENIC				
CaPOPII_4687	scaffold5186	8653	GA/G	GAGTGTTGGGGTCGTTGAAT	CCTAGGGAGGCTAAAATGGC	59.8	270	INTERGENIC				
CaPOPII_4688	scaffold5195	4588	TA/TCGCTATGTGCA	AATAACATGTGCAGGGAGCC	GGTTTGGAACGGAACCCTAT	60.0	766	INTERGENIC				
CaPOPII_4689	scaffold5211	3755	TAAAAAAAAA/TAAAAAAAA	GTTCTCGAAGCAGAAAACGG	CTTTGGTCAAGTCTGGCACA	60.0	702	INTERGENIC				
CaPOPII_4690	scaffold5234	584	ATTTTTTTTTT/ATTTTTT	ATGACGAAGCTGCTGGAGTT	AATCTCCCCCTGAGTTTTGG	60.0	664	INTERGENIC				

INDEL marker IDs	Chromosomes /unanchored scaffolds	Physical positions (bp)	InDels (*Kabuli* reference genome-CDC Frontier/PI)	Forward primers (5'-3')	Reverse primers (5'-3')	Anneling temperature (0C)	Expected amplified product size (bp)	Structural annotation		Functional annotation		
								Sequence components of *kabuli* genome	*Kabuli* gene accession IDs	NCBI-KOG	TFs	NCBI-nr database
CaPOPII_4691	scaffold5234	996	G/GTT	ATTCCAAAACTCAGGGGGAG	TTGGTTGATTCCTTGAGTTGC	60.3	246	INTERGENIC				
CaPOPII_4692	scaffold5235	1115	CTAT/CTATTATTAGTTATAT	CGGAAAATTTACACGCAGGT	AGCCTTTTTGGTTCCATGTG	60.0	640	INTERGENIC				
CaPOPII_4693	scaffold5251	1190	CTCTTCTT/CTCTT	TGGCCACCATTAGCATTACA	AGCGCAGTGGTGAAAAGAAT	60.0	383	INTERGENIC				
CaPOPII_4694	scaffold527	807	CAA/CAAA	TGCAGCCACATATTCAGCTT	GCGTTGAGTTTGAAACCAGG	59.4	507	INTERGENIC				
CaPOPII_4695	scaffold527	2067	CG/C	CCAGTCCATGTTTGTGCAAG	TTTGGCTCATTGTGCTTCAG	60.2	677	INTERGENIC				
CaPOPII_4696	scaffold527	4805	AT/ATTACTT	TCGTTGGTGCATTATTTCCA	TTGCAAGAAGGGGTTTTGAC	59.9	437	INTERGENIC				
CaPOPII_4697	scaffold527	81215	AC/ACACC	CAATGGGAGAGGAGTTCCAA	TGGTGCATAGAAGAAATTGGG	60.0	687	INTERGENIC				
CaPOPII_4698	scaffold528	183983	CAAAAAA/CAAAAA	TGAACTCAAAATTCGATTTGTCT	TGAAAAACATTTGCCTGAACA	57.9	651	INTERGENIC				
CaPOPII_4699	scaffold5291	5244	TAA/TAAA	CATGTGCGTCCATTAAGTGC	CCAAGGGAGAACGAAAATGA	60.1	165	INTERGENIC				
CaPOPII_4700	scaffold53	137170	CTT/CTTTT	GGACTGGTGGCTCAAAGGTA	TCGACCGGGAGTTTTGATTA	60.1	671	INTERGENIC				
CaPOPII_4701	scaffold530	10667	CG/C	ACCAAATTTGTCGAACCAGC	TTGAGTGTGAGTGTCCAGGG	60.0	342	INTRON	Ca_27188	J		Ribosomal protein L14b/L23e
CaPOPII_4702	scaffold531	11911	A/AT	ATTCCATTTGGCATGCTTTC	TTTGGGTACAAAATCAGAAAATTG	59.9	892	INTRON	Ca_22388			Domain of unknown function DUF231, plant
CaPOPII_4703	scaffold531	12542	CAAAAAAAAAAA/CAAAAAAA	AAACAATTTTCTGATTTTGTACCCA	GTTATGTCAACGAGGCCCAT	60.0	344	INTRON	Ca_22388			Domain of unknown function DUF231, plant
CaPOPII_4704	scaffold535	94648	GATATATATAT/GATATATATATAT	AATGGAGGAGAAGGGAGGAA	TGAAAAGGGGTAGGCAACAC	60.0	563	INTERGENIC				
CaPOPII_4705	scaffold537	258578	CTTTTTTATTT/CTTTTTTATTTTTTATTT	GATGTTTGGATTGGGGTTTG	AAGCACATATTTGTTGCCCC	60.0	288	URR	Ca_23292	R		Sugar/inositol transporter
CaPOPII_4706	scaffold5420	685	ACC/ACCC	AAGGGAGGATGGAAATGACC	ATTTTTGTCCTCCTTGGGCT	60.1	613	INTERGENIC				
CaPOPII_4707	scaffold543	28493	ATTTTTTTTTT/ATTTTTTTTTTT	GACAGCTCTCTCCAAATGCC	AAGGAATCCGCATGCTTAAA	60.0	507	DRR	Ca_24779	IR		ABC transporter-like

INDEL marker IDs	Chromosomes /unanchored scaffolds	Physical positions (bp)	InDels (*Kabuli* reference genome-CDC Frontier/PI)	Forward primers (5'-3')	Reverse primers (5'-3')	Anneling temperature (0C)	Expected amplified product size (bp)	Structural annotation		Functional annotation		
								Sequence components of *kabuli* genome	*Kabuli* gene accession IDs	NCBI-KOG	TFs	NCBI-nr database
CaPOPII_4708	scaffold543	69059	TGGGG/TGGGGG	TCGCTACTGAAGATGGGCTT	TTGATAACTTGAAAACGAAATGAAA	60.0	363	CDS (FRAME SHIFT)	Ca_24780			
CaPOPII_4709	scaffold543	69595	GATAATAATAATAATAATAATAATAATAAT/GATAATAATAATAATAATAATAATAAT	TTTCATTTCGTTTTCAAGTTATCAA	CCTCCTAATGCGTTGGTTTT	59.1	619	DRR	Ca_24780			
CaPOPII_4710	scaffold543	229568	ATTTTTT/ATTTT	TTTGCCCCCAACTCTACATC	ATGGGCACATGCTATCATCA	59.9	755	DRR	Ca_24786			
CaPOPII_4711	scaffold544	85733	GAAAAAAAAA/GAAAAAAAA	TTTATCTGGCTTCCTTCCCA	TTTAAGTTGGAACATGGTGGA	59.6	520	INTERGENIC				
CaPOPII_4712	scaffold544	146396	CTTTTTT/CTTT	TTCCTATTGCTCACCCCTTG	GAAAAACAAGACCCGCGATA	60.1	520	DRR	Ca_26540			
CaPOPII_4713	scaffold544	147706	CAAAAA/CAAAA	CCCCCTTTTTGCATAAGTGA	GAGGAGACATCTGCTGAGGG	59.9	582	DRR	Ca_26540			
CaPOPII_4714	scaffold544	192787	AG/A	TGGTTCAAAGCCCTTATTGG	AGGTCATTTTCGTCCTCCCT	59.9	554	INTERGENIC				
CaPOPII_4715	scaffold544	221561	GA/G	ACACACCTATGGGATGCACA	AGGTCATTTTCGTCCTCCCT	59.8	478	INTERGENIC				
CaPOPII_4716	scaffold544	269437	CTTTTTTTTTT/CTTTTTTTTT	ACGAGCGAGGACGAAGTAAA	GCTACGTGGGAAGACTCTGG	60.0	916	INTERGENIC				
CaPOPII_4717	scaffold544	343923	CAAAAAAAAAA/CAAAAAAAAAAA	GTGAAAATAAGGGGGAAGGG	TTTTCTTCCTCTTTCCTGCG	59.6	323	INTERGENIC				
CaPOPII_4718	scaffold545	193109	CAAAAA/CAAAAAA	AATCTCCCCCTGAGTTTTGG	ATCTTTGGACACCGTCGTTC	60.3	643	INTERGENIC				
CaPOPII_4719	scaffold547	96984	CAAAA/CAAAAA	CACGATGGCTAGTGCTTGAA	CGATCGGTATGAACATTGACC	60.0	276	INTERGENIC				
CaPOPII_4720	scaffold547	155914	ATCAAA/ATCAAAGTTCAAA	TCGATCTGTACGAACATTGACC	CGGAACCCGAAACACTTTTA	60.0	295	INTERGENIC				
CaPOPII_4721	scaffold547	156143	CAAA/CAAAA	TTCGGTCTATTTTTGTCGGG	CGGAACCCGAAACACTTTTA	59.9	219	INTERGENIC				
CaPOPII_4722	scaffold548	212010	G/GA	GGAACACTTCGGTGTGGACT	CATGTCCCGAAAAGAGCAAT	60.0	563	DRR	Ca_21489			
CaPOPII_4723	scaffold549	61007	GTATATATATATATATA/GTATATATATATATA	TGCCAATTCGAAGATCAAAA	CACCCCTTACCTCGACTGAA	59.2	570	INTERGENIC				
CaPOPII_4724	scaffold55	606	CA/CAA	GATGTGGCACACAAGGATGT	TGGTGGACTCAAATTGACGA	59.4	321	INTERGENIC				

INDEL marker IDs	Chromosomes /unanchored scaffolds	Physical positions (bp)	InDels (*Kabuli* reference genome-CDC Frontier/PI)	Forward primers (5'-3')	Reverse primers (5'-3')	Anneling temperature (0C)	Expected amplified product size (bp)	Structural annotation		Functional annotation		
								Sequence components of *kabuli* genome	*Kabuli* gene accession IDs	NCBI-KOG	TFs	NCBI-nr database
CaPOPII_4725	scaffold5504	1323	T/TTA	ACCAATCAAGTGCATCCCAT	TAAGCCTAAGGAGGACGCAA	60.2	175	INTERGENIC				
CaPOPII_4726	scaffold553	2669	CTT/CT	TTGGTTCACTCCCTGGATTC	AACATCAGTCAAGCGCACAA	59.9	484	INTERGENIC				
CaPOPII_4727	scaffold553	325514	CT/CTAT	ATCGGAAGTTGGACAGGTTG	TGGCAAATCAGCAGAGACAC	60.0	317	INTRON	Ca_19934	L		
CaPOPII_4728	scaffold553	527326	AGG/AG	CATTTCAAGTTGGGGCAGTT	TCGTCTTCGGGTTGGTTATC	60.0	837	INTERGENIC				
CaPOPII_4729	scaffold553	800299	ATTTTTTT/ATTTTTTTT	CAAAATCGGGGATAGGGTTT	CGGGGTCATTCTCTTCACAT	60.0	570	INTERGENIC				
CaPOPII_4730	scaffold553	927370	GAA/GAAA	TTGGCCAAGTCCCTGTTTAC	CGTGGTAATCGCGTAGGAAT	60.0	140	INTERGENIC				
CaPOPII_4731	scaffold5551	550	CTTTTTTT/CTTTTTTTT	GATACGTTGGAGCATCGACA	CACGAAACACCCTCTTCCAT	59.7	230	INTERGENIC				
CaPOPII_4732	scaffold559	93166	AAAATAAATAAATAAATAAATAAATAAATAAATAAATAAATAAATAAA/AAAATAAATAAATAAATAAATAAATAAATAAATAAATAAA	TTAGGTGCGCTTGACTGATG	TTTGGCATTGGTTTCACTCA	60.0	576	INTERGENIC				
CaPOPII_4733	scaffold5598	13569	ATTTTTTTTTTTTT/ATTTTTTTTTTT	CGGCATTGGATCATGGTTAG	TTTCGACTGCCTAGCGATTT	61.2	549	INTERGENIC				
CaPOPII_4734	scaffold560	134002	TTAAAAGAAT/TT	TGGCACGATATCATCATCATT	ATGAAGTTGACGTATGGGGG	58.8	918	INTERGENIC				
CaPOPII_4735	scaffold560	160364	TTATATATATATATATA/TTATATATATATATATATA	TCACCATGCACAATCAGTTTC	TCATGAAACGCACAACAACTT	59.6	744	INTERGENIC				
CaPOPII_4736	scaffold5600	533	TAAAAAAAAAAAA/TAAAAAAAAAA	TATAGGCCGGTCTGTCAAGC	TAACCTTCCGAGGATATGCG	60.2	470	INTERGENIC				
CaPOPII_4737	scaffold561	25431	CTCTTTCT/CTCT	TTGATTCATCAAACACCGGA	AAATTGGGTTCTGCCAATCA	59.9	399	DRR	Ca_26215		MYB_related	
CaPOPII_4738	scaffold561	66066	TTATATATATATATATAT/TTATATATATATATATAT	TGGCAACACCCCCTAAAATA	TTGACATCCCAAGTCAAACATC	60.2	713	INTERGENIC				
CaPOPII_4739	scaffold561	84146	AT/ATT	ATTCGACTAGTGGTGGTGCC	AGGCCGCCACAAAATATTAC	60.0	669	INTERGENIC				
CaPOPII_4740	scaffold562	299510	CTTTT/CTTT	GGCAACCTGTTTTCAGGCTA	TGTTAATGGTGTGCCAATCG	60.2	312	INTERGENIC				
CaPOPII_4741	scaffold564	5212	G/GAAC	TGGGATGCAAACAATTCTCA	TTGTCAATTTGGGAGTGCAA	60.0	560	INTERGENIC				

INDEL marker IDs	Chromosomes /unanchored scaffolds	Physical positions (bp)	InDels (*Kabuli* reference genome-CDC Frontier/PI)	Forward primers (5'-3')	Reverse primers (5'-3')	Anneling temperature (0C)	Expected amplified product size (bp)	Structural annotation		Functional annotation		
								Sequence components of *kabuli* genome	*Kabuli* gene accession IDs	NCBI-KOG	TFs	NCBI-nr database
CaPOPII_4742	scaffold564	8317	TAAAAAA/TAAAAAAA	CAACATCCAAATTTTGTTATAGAGC	CGACATGCATGCCTTATTTG	58.3	143	INTERGENIC				
CaPOPII_4743	scaffold564	12224	A/AC	GGTATTGGGCTCCCTTCCTA	CTCGTGAGGAGCAACAACAA	60.3	552	INTERGENIC				
CaPOPII_4744	scaffold564	14245	T/TTTAA	TGCCAATTGTTTTTATCTCCG	CCACCAAATTACCATTTAAGACA	60.0	403	INTERGENIC				
CaPOPII_4745	scaffold564	26112	A/AG	GTATCTGAGCCTCTGCCAGC	AATTGGTGTGTCTGCGTCAA	60.1	555	INTERGENIC				
CaPOPII_4746	scaffold571	11860	A/AACTGGACGC	CCAATTTGTTGTGTGCCAAG	TTTGGCGAGTTTGTGTGAAG	60.0	383	INTERGENIC				
CaPOPII_4747	scaffold571	18167	TTACATCTCATA/TTA	CCTCACGGTTTCAATTGCTT	CTTAATGCACAGCCTACCCC	60.1	670	INTRON	Ca_27520	T	NAC	Serine-threonine/tyrosine-protein kinase
CaPOPII_4748	scaffold571	79289	GTT/GT	AATAAGCCTAGGGAAGCCGA	CCTAGGAATATTCGCGTCCA	60.2	312	INTERGENIC				
CaPOPII_4749	scaffold5730	653	ATTT/ATTTT	ACAAAGGCAACCTTTTGTGG	CTGCCCACCGTTGTTATTCT	60.0	866	INTERGENIC				
CaPOPII_4750	scaffold575	65125	G/GT	TTGAAGTTAAGGGGTGCTGC	TCCATCCAACCTTGTGGATT	60.2	531	DRR	Ca_27003			
CaPOPII_4751	scaffold575	66804	CTTTTTTTT/CTTTTTTT	AACCGGATGTTGGGATGTAA	CCCATTCACTCCCACTCTGT	60.1	348	INTERGENIC				
CaPOPII_4752	scaffold575	67025	ATTTAGGGTA/ATTTAGGGTATGGTTTAGGGTA	ACAGAGTGGGAGTGAATGGG	CCTAAGCCTGACCCTACCCT	60.0	397	INTERGENIC				
CaPOPII_4753	scaffold575	67830	TGGTTTAGGGTTTAGGGTT/TGGTTTAGGGTT	GGGTTAGGGTAGGGTCAGGA	AGGCTGGAATCGGTAAGTGA	60.2	357	INTERGENIC				
CaPOPII_4754	scaffold575	89948	TAAAAAAA/TAAAAAAAAA	GGATTGGCCCCAAATAGAAT	CCCTTGTCATTGCTGCTGTA	60.0	722	INTERGENIC				
CaPOPII_4755	scaffold575	90216	G/GA	CCCAAATTGCAATAACCAGG	TACCGTTTCCCACACTCACA	60.2	637	INTERGENIC				
CaPOPII_4756	scaffold575	90829	ATTTTTTTT/ATTTTTTT	TGTGAGTGTGGGAAACGGTA	GGCAATGTGCATAAACAAAAA	60.0	426	INTERGENIC				
CaPOPII_4757	scaffold575	91034	AATATATATATATATATATATATAT/AATATATATATATATATATATAT	TGTGAGTGTGGGAAACGGTA	GGCAATGTGCATAAACAAAAA	60.0	426	INTERGENIC				
CaPOPII_4758	scaffold575	91358	TC/T	TTTTTGTTTATGCACATTGCC	TTCAAGACACCGTAAACACCA	58.6	690	INTERGENIC				

INDEL marker IDs	Chromosomes /unanchored scaffolds	Physical positions (bp)	InDels (*Kabuli* reference genome-CDC Frontier/PI)	Forward primers (5'-3')	Reverse primers (5'-3')	Anneling temperature (0C)	Expected amplified product size (bp)	Structural annotation		Functional annotation		
								Sequence components of *kabuli* genome	*Kabuli* gene accession IDs	NCBI-KOG	TFs	NCBI-nr database
CaPOPII_4759	scaffold575	92874	A/AT	GCCCTTGAAATTGACAAAGC	TTCGTGACCGTGCAATACAT	59.7	577	INTERGENIC				
CaPOPII_4760	scaffold575	98810	TC/T	TAAGCCAGGGCTTTCTTTCA	CCTCCACTCCAAACCTACCA	60.0	404	INTERGENIC				
CaPOPII_4761	scaffold575	99188	CT/CTGT	TGGTAGGTTTGGAGTGGAGG	GGCTAAGTCTGAAAGTGGCG	60.0	263	INTERGENIC				
CaPOPII_4762	scaffold575	99548	CGG/CGGG	CGCCACTTTCAGACTTAGCC	AGGAAGCAAGGGGGATTCTA	60.0	690	INTERGENIC				
CaPOPII_4763	scaffold575	103253	AGGGG/AGGGGG	AAACAACGAGTATGGGCGAC	AGCTTGCTTTGAAAGGGACA	60.0	113	INTERGENIC				
CaPOPII_4764	scaffold575	103640	ACCCCCC/ACCCC	AAACAACGAGTATGGGCGAC	CGCAATTAGCCTCTTTCGTC	60.0	707	INTERGENIC				
CaPOPII_4765	scaffold575	103819	AT/ATGCCTTATT	TCTTCTCGCTGACTTGGGAT	CGCAATTAGCCTCTTTCGTC	60.0	205	INTERGENIC				
CaPOPII_4766	scaffold575	110510	GT/GTGTACACT	CTTTCTGGTCAAGGCCTCAG	CTAGACCTGCGGAAGACAGG	60.0	446	INTERGENIC				
CaPOPII_4767	scaffold575	112885	GCCTGACCT/GCCT	CCTTACACTTTGCTCCTCGC	GGCTTGAGCCTAGCCTTTTT	60.0	419	INTERGENIC				
CaPOPII_4768	scaffold575	113273	ATT/AT	TTCGGCAACATTCCGTATTT	GCCTGACCTATGGCCTATCA	60.3	283	INTERGENIC				
CaPOPII_4769	scaffold575	113322	ATTTTAAATAGGCTT/ATTTTAAATAGGCTTTTAAATAGGCTT	TTCGGCAACATTCCGTATTT	TCCCAGTCTCTGGTCAATCC	60.3	652	INTERGENIC				
CaPOPII_4770	scaffold575	113457	CTGGC/CTGGCATGGC	TGATAGGCCATAGGTCAGGC	GCTAAGTGGTCTTTCCGCTG	60.1	440	INTERGENIC				
CaPOPII_4771	scaffold575	113710	GAAAAAA/GAAAAAAA	TGATAGGCCATAGGTCAGGC	GCTAAGTGGTCTTTCCGCTG	60.1	440	INTERGENIC				
CaPOPII_4772	scaffold5761	842	CTTTT/CTTTTT	GGTCCTCGATTTCATCTGGA	AACGCCTTAAAAGCCCAAAT	60.0	684	INTERGENIC				
CaPOPII_4773	scaffold5761	1101	C/CG	TCCTGGTCCTCGATTTCATC	AACGCCTTAAAAGCCCAAAT	60.0	362	INTERGENIC				
CaPOPII_4774	scaffold5777	16517	CTT/CTTT	TTGCAATTGGCACACAAAAT	TGAGTTGCTTCAAAGTGATGTG	60.0	742	INTERGENIC				
CaPOPII_4775	scaffold5777	16606	ACTT/ACTTTCTT	TTGCAATTGGCACACAAAAT	TGAGTTGCTTCAAAGTGATGTG	60.0	742	INTERGENIC				

INDEL marker IDs	Chromosomes /unanchored scaffolds	Physical positions (bp)	InDels (*Kabuli* reference genome-CDC Frontier/PI)	Forward primers (5'-3')	Reverse primers (5'-3')	Anneling temperature (0C)	Expected amplified product size (bp)	Structural annotation		Functional annotation		
								Sequence components of *kabuli* genome	*Kabuli* gene accession IDs	NCBI-KOG	TFs	NCBI-nr database
CaPOPII_4776	scaffold578	11617	CTTTTTTTT/CTTTTTT	TTCATTTGATCTTTTCGGGG	CCTCGAGAAAAATGGACCAA	59.9	513	INTERGENIC				
CaPOPII_4777	scaffold5780	2877	AC/A	GGAATCCGGTTTCTTTCCAT	GACCATTATTCACACGGTGG	60.1	765	INTERGENIC				
CaPOPII_4778	scaffold5780	5394	CAAAAAAA/CAAAAAAAAAA	CGTGTTCTCCCCGTGTATTT	ATGTGACACCCTAAACCCCA	59.9	488	INTERGENIC				
CaPOPII_4779	scaffold5780	5464	ATT/AT	CGTGTTCTCCCCGTGTATTT	ATGTGACACCCTAAACCCCA	59.9	488	INTERGENIC				
CaPOPII_4780	scaffold5780	8742	CTTTTT/CTTTT	AAGTTCCGCATTAGGCCTTT	GTTTGGGTCAAGGCTATGGA	60.1	444	INTERGENIC				
CaPOPII_4781	scaffold5780	9297	GAAAC/GAAACGTCAATAAAAAC	TCCATAGCCTTGACCCAAAC	CCCACTGCTTGCAATAATGA	59.9	404	INTERGENIC				
CaPOPII_4782	scaffold5801	854	GATT/GATTATT	AGACGCATTACACACCACCA	AAGGCGGCATACAAAACAAC	60.0	582	INTERGENIC				
CaPOPII_4783	scaffold5812	4416	TAAAA/TAAAAA	TGCAAACCTAAATGTGGCAA	CGAGCACCATATTGCATCAC	60.1	716	INTERGENIC				
CaPOPII_4784	scaffold583	89302	AATATATATATATATATATATATATATATAT/AATATATATATATATATATATATATAT	TCGAGTCCGTTGTGTTGGTA	TCTTTAGTAGCCCGCCAAGA	60.2	542	INTERGENIC				
CaPOPII_4785	scaffold583	106896	ATTT/ATTTT	CGAACGGAGATTTTATCGGA	TTTCCACGAAACATCCATCA	60.0	233	INTERGENIC				
CaPOPII_4786	scaffold584	29046	ATT/AT	CAATCCGAGACAGTCCCCTA	TTTGCGACGCTTGATCATTA	60.1	524	INTERGENIC				
CaPOPII_4787	scaffold5869	1633	TTTATTATTATTATTATTATTATTATTATTATT/TTTATTATTATTATTATTATTATTATTATT	GGGTAGGAAAAGCATCACGA	TGATTGATTTGTTGGACCGA	60.1	387	INTERGENIC				
CaPOPII_4788	scaffold5869	18300	GAAAAAAAAAAA/GAAAAAAAAA	TTCCAAATATGCTTGGGACAC	TGAACTCTTTTACACGGTTCTCA	59.8	601	INTERGENIC				
CaPOPII_4789	scaffold587	17163	AC/A	GGAAAATCCATGAGGCAAGA	AGGCCCCAAAGAAGAGTGAT	60.0	496	CDS (FRAME SHIFT)	Ca_27621			
CaPOPII_4790	scaffold587	18087	T/TCA	GTCCTTGCGCTTATGCTTGT	TGCTTGATTGCTTGAGATCC	60.4	347	DRR	Ca_27621			
CaPOPII_4791	scaffold587	35297	C/CA	TTCAAACGTTCTCTCACCCC	AAAAATGGGAATTGGAAGGG	60.1	530	INTERGENIC				
CaPOPII_4792	scaffold587	50853	TAAAAA/TAAAAAA	AGGGGAATTTTCGTACCACC	ATCGAAGACCAGGAGACCCT	60.1	638	INTERGENIC				

INDEL marker IDs	Chromosomes /unanchored scaffolds	Physical positions (bp)	InDels (*Kabuli* reference genome-CDC Frontier/PI)	Forward primers (5'-3')	Reverse primers (5'-3')	Anneling temperature (0C)	Expected amplified product size (bp)	Structural annotation		Functional annotation		
								Sequence components of *kabuli* genome	*Kabuli* gene accession IDs	NCBI-KOG	TFs	NCBI-nr database
CaPOPII_4793	scaffold587	51041	CAAAAAAAAAAA/CAAAAAAAA	AGGGGAATTTTCGTACCACC	ATCGAAGACCAGGAGACCCT	60.1	638	INTERGENIC				
CaPOPII_4794	scaffold587	53018	CAAAAA/CAAAA	TACACGAAACCCGAAACACA	CGAGGACCAGGAGACCATTA	60.0	749	INTERGENIC				
CaPOPII_4795	scaffold587	54888	AATATATATATATATATA/AATATATATATATATATA	CAAGACATGCGAAAGTCGAA	TCCAGCTTCCTCAAAGCCTA	60.0	484	INTERGENIC				
CaPOPII_4796	scaffold587	56115	GAA/GAAA	AACTGCCAAAGAGATGTGGG	TGCAGTTCCATCTCATGCTC	60.1	338	INTERGENIC				
CaPOPII_4797	scaffold590	3079	AT/ATT	AATAAAAGACCAAATCGGAAAAA	TGACAACCATTTATATTTGCCAC	58.2	393	INTERGENIC				
CaPOPII_4798	scaffold590	19258	TT/TTACCATAGT	TCCTAAGAAAGCTACCCCACC	TGGCAAAGCACAAGACACTC	59.6	398	INTERGENIC				
CaPOPII_4799	scaffold590	19474	AG/AGG	CTATCCATGCATGTGCAACAC	TGGCAAAGCACAAGACACTC	60.0	175	INTERGENIC				
CaPOPII_4800	scaffold590	46710	AATATATATATATATATAT/AATATATATATATAT	GAGAAGGCTGGGTGAAACAA	CGTAGCGTGGTAGTGAGCAA	60.2	874	INTERGENIC				
CaPOPII_4801	scaffold5904	22420	ATTTT/ATTTTT	TCAGTGTCAACGGTGTCGAT	CCCCCTTTTTCCTAAATTCG	60.2	636	INTERGENIC				
CaPOPII_4802	scaffold5916	851	GAAA/GAA	TCATTTTCGTCCTTCCTTGG	TGGGCCTAGAGAATGGTGTC	60.0	749	INTERGENIC				
CaPOPII_4803	scaffold5916	1178	CAAAA/CAAAAA	TGACACCATTCTCTAGGCCC	AGGGAGGACGAAAATGACCT	60.1	310	INTERGENIC				
CaPOPII_4804	scaffold593	6803	TAAAAAAAAAA/TAAAAAAAAAAA	CGTGTTTGGTGGGACTTTCT	AATGAGCCACTTTGGCAATC	60.0	925	INTERGENIC				
CaPOPII_4805	scaffold593	95845	AATATATATATATATATATA/AATATATATATATATATA	GATGATCCAATCGGTCTTGC	TTGTTGGAAGAAAAACAAAGTCA	60.4	392	INTERGENIC				
CaPOPII_4806	scaffold593	209468	TC/T	GCAAACCTTTGCAAAAAGAAA	GGAATAAAATATGCATCCGAGC	59.4	153	DRR	Ca_26412		C2H2	Glycoside hydrolase, family 28
CaPOPII_4807	scaffold593	209698	CT/CTTT	GCAAACCTTTGCAAAAAGAAA	GGAATGGAAATAATTGAAAAGTTTG	59.4	847	DRR	Ca_26412		C2H2	Glycoside hydrolase, family 28
CaPOPII_4808	scaffold593	210090	TT/TTATTTAAT	GAGCACAGTTTCAATTAACAGCA	GTCTCTGCTCAACCAGGAGG	59.5	862	DRR	Ca_26412		C2H2	Glycoside hydrolase, family 28
CaPOPII_4809	scaffold5954	716	GTT/GT	TGGATGCACAAGGTTGGTTA	TTCATTTTCGTCCTCCCTTG	60.0	767	INTERGENIC				

INDEL marker IDs	Chromosomes /unanchored scaffolds	Physical positions (bp)	InDels (*Kabuli* reference genome-CDC Frontier/PI)	Forward primers (5'-3')	Reverse primers (5'-3')	Anneling temperature (0C)	Expected amplified product size (bp)	**Structural annotation**		**Functional annotation**		
								**Sequence components of *kabuli* genome**	***Kabuli* gene accession IDs**	**NCBI-KOG**	**TFs**	**NCBI-nr database**
CaPOPII_4810	scaffold5954	1414	A/AG	TGGATGCACAAGGTTGGTTA	AGGTCATTTTCGTCCTCCCT	60.0	367	INTERGENIC				
CaPOPII_4811	scaffold598	94510	CTT/CTTT	CCCCAATGCTACACCAAAAG	TCTTCGCAAATCGACACAAG	60.4	193	INTERGENIC				
CaPOPII_4812	scaffold598	99722	ATTTTTTT/ATTTTTT	CATCGCCTGAATGGCTTAAT	TGACATGTACGGTGATGCCT	60.1	795	DRR	Ca_21751	R		Reverse transcriptase
CaPOPII_4813	scaffold598	420243	GATATATATATATAT/GATATATATATATATAT	GCCGAATAAAACCCTCACAA	GACATCTCATTCCCCCTTCA	59.9	683	INTERGENIC				
CaPOPII_4814	scaffold598	568507	CTCAAAATCAAAATCAAAATCAAAATCAAAATC/CTCAAAATCAAAATCAAAATCAAAATCAAAATCAAAATC	CAGCCACTACATTGGCCTTT	AGGATTCTCCAAGTTGGCCT	60.1	499	INTERGENIC				
CaPOPII_4815	scaffold599	96808	ATTTT/ATTTTT	CCCCAATTTTCAAACATCCTT	GGTCATTTTCGTCTTCCCAA	60.0	351	INTERGENIC				
CaPOPII_4816	scaffold599	307088	CTTATTATTATTATTATTATTATTATTATT/CTTATTATTATTATTATTATTATTATTATTATTATTATTATT	TCTTCCCATTCCTTGTCTGG	GGCGTAGCCCAAATATTCAA	60.0	120	INTERGENIC				
CaPOPII_4817	scaffold599	307530	AATATATATATATATATATATATATATATATAT/AATATATATATATATATATATATATATATATAT	TCTTCCCATTCCTTGTCTGG	TGTGTGCTTCCTATGATGTTCT	60.0	855	INTERGENIC				
CaPOPII_4818	scaffold599	307709	TAAAAAAAA/TAAAAAAAAAAA	TCCTCCAAACACCATCTAATTTT	CAACATTAAAAGAACGGCGA	58.9	949	INTERGENIC				
CaPOPII_4819	scaffold599	307947	AT/A	AGAACATCATAGGAAGCACACA	CAACATTAAAAGAACGGCGA	57.4	317	INTERGENIC				
CaPOPII_4820	scaffold599	312899	CTTTTTTTTTTT/CTTTTTTTTTTTTTT	CGTTGTCGTCGGTAGGATTT	TTCAAAAGTTATTCGGTGGAAA	60.0	521	INTERGENIC				
CaPOPII_4821	scaffold599	319689	CT/C	CACGCAAAAATTGACGATTG	TTTTTCTTCTCGATTTGGTATTTT	60.1	663	INTERGENIC				
CaPOPII_4822	scaffold599	322634	GTTTTTTTTTT/GTTTTTTTTTTTT	TTGATAAACCGCCATGACAA	TTTTGCTTTGCCGTATTTTT	59.9	563	INTERGENIC				
CaPOPII_4823	scaffold599	323301	TTATATATATATATATATATATATATA/TTATATATATATATATATATATATA	AAAAATACGGCAAAGCAAAA	TTGAGGTTGAGAGTCTTTTCCA	57.5	732	INTERGENIC				
CaPOPII_4824	scaffold599	329422	CAAAAAAAAA/CAAAAAAAA	CACGTAAAATCGCCTAAAATCT	CCGATAGTAAAACGACATCCTG	57.6	562	INTERGENIC				
CaPOPII_4825	scaffold599	335351	CTTTTTTTTTT/CTTTTTTTTTTTT	GCCAACCATGTAAATCGCAT	TCAATCATCAATTTTTGTGTGTTTC	60.7	327	INTERGENIC				
CaPOPII_4826	scaffold599	347393	CTTTTTTT/CTTTTTT	CCTCTTTTCTTCGCATTTCG	AACAACAGCCCCAATGTAGC	60.0	504	INTERGENIC				

INDEL marker IDs	Chromosomes /unanchored scaffolds	Physical positions (bp)	InDels (*Kabuli* reference genome-CDC Frontier/PI)	Forward primers (5'-3')	Reverse primers (5'-3')	Anneling temperature (0C)	Expected amplified product size (bp)	Structural annotation		Functional annotation		
								Sequence components of *kabuli* genome	*Kabuli* gene accession IDs	NCBI-KOG	TFs	NCBI-nr database
CaPOPII_4827	scaffold599	351148	ATAAAAAGC/ATAAAAAGCTAAAAAGC	CTGTTGGGCAAGGGATCTAA	CACGCTTGTTTTGCACATTT	60.1	641	DRR	Ca_25346			Peptidase C48, SUMO/Sentrin/Ubl1
CaPOPII_4828	scaffold599	352330	TAAAA/TAAA	AAAAATACGGCAAAGCAAAA	TCAATGGACATGAGTCTTCACA	57.5	887	DRR	Ca_25346			Peptidase C48, SUMO/Sentrin/Ubl1
CaPOPII_4829	scaffold599	364148	CTATATATATATATATATATATAT/CTATATATATATATATATAT	GCAAGCACCGTTGTGAGTAA	TCTTTAGTAGCCCGCCAAGA	59.9	784	INTERGENIC				
CaPOPII_4830	scaffold599	366068	ATTTTTTTTT/ATTTTTTTTTT	AGAGCGCAATCATTTCAGGT	CTTTTGGAAAGAAGCCCTCC	59.8	613	INTERGENIC				
CaPOPII_4831	scaffold599	371527	TAAAAAAA/TAAAAAA	CAGCGTTGTTGTTGTTGTTTTT	TTTCGAATGAACATGGATGA	60.1	531	INTERGENIC				
CaPOPII_4832	scaffold599	373801	TAAAAAAAAAAAA/TAAAAAAAAAAAAAAA	CCCCCTAAGTTCATTCCACA	GGCTTGTCCGTCGGTAAATA	59.8	567	DRR	Ca_25347			Transposase, Ptta/En/Spm, plant
CaPOPII_4833	scaffold599	392616	GAAAAAA/GAAAAA	AAAAAGATATGGCAAGGCAAAA	TGAGGTTGAGAGTCCTTTCCAT	60.0	689	INTERGENIC				
CaPOPII_4834	scaffold599	410067	GTTTT/GTTT	ATCGATTTCCCAATCGGTTA	TATACCCTATAGCCACGGCG	59.2	609	INTERGENIC				
CaPOPII_4835	scaffold5992	1551	G/GA	CGAGGTAAGGGGTGAGATGA	TCCCCGAATCACTAGGTTTG	60.1	544	INTERGENIC				
CaPOPII_4836	scaffold5993	18562	GTTTTTTT/GTTTTTT	TTGATGTACGAAAAGTTTTAGCAAG	GGGGGCACTTATTTATCACTT	59.0	267	INTERGENIC				
CaPOPII_4837	scaffold5993	18598	CTTATATTTTTATATTTTTATATTTT/CTTATATTTTTATATTTT	TTGATGTACGAAAAGTTTTAGCAAG	GGGGGCACTTATTTATCACTT	59.0	267	INTERGENIC				
CaPOPII_4838	scaffold601	49572	CTTTTTTT/CTTTTTT	GGGGACATTTCGAGAGTCAA	TCACAAGGGAGGCTCTATGG	60.1	519	INTERGENIC				
CaPOPII_4839	scaffold605	25215	TC/TCGGAGGGGGAC	CAGGCCCAGGTAATCTTTGA	CATGCCAGGAGCACACATAC	60.1	784	INTERGENIC				
CaPOPII_4840	scaffold6113	1438	GAAAA/GAAAAA	ACAAGCCTATCGTTTGGCAC	GATGCAACATTGGAGCATGA	60.1	589	INTERGENIC				
CaPOPII_4841	scaffold612	28576	GAAAAAA/GAAAAAAA	AAAAAGGGAGGGTCACATGAT	TCACCAATATCTTAACCACCAAAA	59.7	701	INTERGENIC				
CaPOPII_4842	scaffold6120	1380	ACTCTCTCT/ACTCTCT	CCAAAATGCTTCATTCTGCAA	CCACCAGACCGACTTTGATT	61.1	493	INTERGENIC				
CaPOPII_4843	scaffold6125	1553	TAAAGA/TA	ATGCGATCCTCTCGGTTCTA	GAGACCGAAGAGTATTGGGAGA	59.8	838	INTERGENIC				

**INDEL marker IDs**	**Chromosomes /unanchored scaffolds**	**Physical positions (bp)**	**InDels (*Kabuli* reference genome-CDC Frontier/PI)**	**Forward primers (5'-3')**	**Reverse primers (5'-3')**	**Anneling temperature (0C)**	**Expected amplified product size (bp)**	**Structural annotation**		**Functional annotation**		
								**Sequence components of *kabuli* genome**	***Kabuli* gene accession IDs**	**NCBI-KOG**	**TFs**	**NCBI-nr database**
CaPOPII_4844	scaffold6154	548	CCGCCTCA/CCGCCTCAGGCGCCTCA	AACCGCTCCAATGGTGTTAG	GAAGATGATGTGCGCTTGAA	60.0	221	INTERGENIC				
CaPOPII_4845	scaffold617	8886	A/AAC	CCAAAATGGACGAGAATAGACC	TGGTTTAGGGTTTAGGCCAAT	59.8	408	INTERGENIC				
CaPOPII_4846	scaffold6177	3708	AG/A	TTTTTGTCATCATCGGACCA	AAATCCGTTTGGATTTGCAG	59.9	613	INTERGENIC				
CaPOPII_4847	scaffold6179	967	TAAAAAAAA/TAAAAAAA	TGGGTGCACAAGATTGGTTA	AGGTCATTTTCGTCCTCCCT	60.0	468	INTERGENIC				
CaPOPII_4848	scaffold618	87840	AT/ATTATTTT	TGAGGGTGTTGGGGTATTTG	TATTGTACGTAGCGAGCCCC	60.6	599	INTERGENIC				
CaPOPII_4849	scaffold6185	3433	TAAAAA/TAAAA	GCAAGCCATTTGTCCATTTT	TCAGCTCACTCTCGCTTTGA	59.9	190	DRR	Ca_27924			Lipase, GDSL
CaPOPII_4850	scaffold6199	832	G/GTATTG	TCATCCAATGACCCAAACAA	TTACGTAATTTTCGTCGCCC	59.8	577	INTERGENIC				
CaPOPII_4851	scaffold62	150220	TAAAAAA/TAAAAAAA	AACATTTAAGAAAATTGAGTTGTCAAA	GTGTGTTGGGCTGTTTAGGG	58.7	405	INTERGENIC				
CaPOPII_4852	scaffold62	150949	CA/CAACTCTA	CAGCCCAACACACTAAACCC	CAACACAATTGAGCTGTCAAA	60.4	572	INTERGENIC				
CaPOPII_4853	scaffold62	338714	ATT/ATTT	CCATCGTCAAGTCCGAAAAT	CCAAAACTCGAAACCCAAAA	59.9	565	INTERGENIC				
CaPOPII_4854	scaffold622	10235	TAAAAAA/TAAAAAAA	ATAGGCCTAGGGAGGACGAA	TTTAGCCAACTTGTGCATCG	60.1	381	INTERGENIC				
CaPOPII_4855	scaffold622	30884	A/AC	TGGGTGCACAAGATTGGTTA	AGGGGTGTTTGAGTCATTGG	60.0	832	INTERGENIC				
CaPOPII_4856	scaffold622	48194	AC/A	TCCGATGACACAAACACACC	TTTGGTCATTTTCGTCCTCC	60.4	194	INTERGENIC				
CaPOPII_4857	scaffold622	61725	GACACACACACACAC/GACACACACACAC	CAAACCCACCTATTGGATGC	GGTCATTTTCGTCCTCGCTA	60.2	310	INTERGENIC				
CaPOPII_4858	scaffold622	79107	GC/GCC	AGGGAGGACGAAAATGACCT	TAACCAATCTTGTGCACCCA	59.9	271	INTERGENIC				
CaPOPII_4859	scaffold626	55533	CTATT/CT	TGAACTCCTTTGTCCTTTGGA	TCATTGCTCCCCCGTAATAG	59.7	320	DRR	Ca_25170			
CaPOPII_4860	scaffold626	172733	C/CAATTA	GTGAGCAAAATGCTGGGAAT	GTTGTTGAGTTGTTGGGGCT	60.1	393	INTERGENIC				

INDEL marker IDs	Chromosomes /unanchored scaffolds	Physical positions (bp)	InDels (*Kabuli* reference genome-CDC Frontier/PI)	Forward primers (5'-3')	Reverse primers (5'-3')	Anneling temperature (0C)	Expected amplified product size (bp)	Structural annotation		Functional annotation		
								Sequence components of *kabuli* genome	*Kabuli* gene accession IDs	NCBI-KOG	TFs	NCBI-nr database
CaPOPII_4861	scaffold626	219233	CAAA/CAAAA	GGATTTAGGGTTTTGGCCTT	GATGAAATCGAGGACTGGGA	59.3	654	INTERGENIC				
CaPOPII_4862	scaffold626	236880	AGGG/AGG	CCCATCCACAAAAATATGCC	GATGAAATCGAGGACCAGGA	60.0	302	INTERGENIC				
CaPOPII_4863	scaffold626	452023	A/AC	TAACTCGGAACCCGAAACAC	TCCAGATGAAATCGAGGACC	60.0	842	INTERGENIC				
CaPOPII_4864	scaffold626	512589	ATTTA/ATTTAGGGTTTA	CCCGACAAAAACGGACTAAA	ATAACCCGAACCTCAAACCC	60.0	359	INTERGENIC				
CaPOPII_4865	scaffold626	522371	CTTT/CTT	TTTGTCGAGGGGTACGAAAC	GGTCCTCGATTTCATCTGGA	60.0	691	INTERGENIC				
CaPOPII_4866	scaffold626	524724	ATTTTTTTTTTT/ATTTTTTTT	AAATCCCCCGAAAATACCAG	TCCAGATCAAATCGAGGACC	60.0	670	INTERGENIC				
CaPOPII_4867	scaffold626	527539	A/AC	CTCGGACGAAATCTAAAGCG	GATGAAATCGAGGACTGGGA	60.0	683	INTERGENIC				
CaPOPII_4868	scaffold63	59319	TAA/TAATAAA	CGAGGTAAGGGGTGAGATGA	AGGTTTGTCAGACCCTCCCT	60.1	481	INTERGENIC				
CaPOPII_4869	scaffold631	53707	GC/GCC	AAGCACACCTATTGGATGCC	TCATTTTCGTCCTCCCTTTG	60.0	477	INTERGENIC				
CaPOPII_4870	scaffold637	107635	ATT/ATTT	GCCTTGAGGAATCGGTATCA	GTGTGAATGCAACCTTCCCT	60.0	313	INTERGENIC				
CaPOPII_4871	scaffold6382	1419	CTCCCTATGCTT/CT	AGGTCATTTTCGTCCTCCCT	CAAACACACGTTTTGGATGC	59.9	181	INTERGENIC				
CaPOPII_4872	scaffold639	11325	T/TC	TGGTGCAATTTACTTTCTCGG	TTCGGTTCTTGTTTGCCTTT	60.1	783	INTERGENIC				
CaPOPII_4873	scaffold639	160230	GACCCTTAACCCTTAACCCTTAACC/GACCCTTAACCCTTAACCCTTAACCCTTAACC	TGGGTTTAGGGTGTGTTTTGA	TTTTGGGTTTCAGGTTTAAGG	60.2	392	INTERGENIC				
CaPOPII_4874	scaffold642	27247	TGATTGGTTTGTTTGAGATTGGTTTGTTTG/TGATTGGTTTGTTTGAGATTGGTTTGTTTGAGATTGGTTTGTTTG	GAGAAGCGAAAGCGAAGAGA	GGAAGGGTAGAACGTAGGGC	60.0	451	INTERGENIC				
CaPOPII_4875	scaffold642	60062	AATATATATATATATATATATATATATA/AATATATATATATATATATA	TATTTTGGGATCTTGGCTCG	CGTAGCGTGGTAGTGAGCAA	60.0	619	INTERGENIC				
CaPOPII_4876	scaffold642	285620	CTTTTTTT/CTTTTTTTT	TCATGCATACATGGGCCTTA	GGCGTTTAGTTTTTGGGTCA	59.9	670	INTERGENIC				
CaPOPII_4877	scaffold642	314115	GTTTTTTTT/GTTTTTTTTT	CTCCAGGTGAGATGGTGGAT	TCGAGCATGATTGGAACAAA	59.9	693	INTERGENIC				

INDEL marker IDs	Chromosomes /unanchored scaffolds	Physical positions (bp)	InDels (*Kabuli* reference genome-CDC Frontier/PI)	Forward primers (5'-3')	Reverse primers (5'-3')	Anneling temperature (0C)	Expected amplified product size (bp)	Structural annotation		Functional annotation		
								Sequence components of *kabuli* genome	*Kabuli* gene accession IDs	NCBI-KOG	TFs	NCBI-nr database
CaPOPII_4878	scaffold6429	4894	CCTA/C	GGGTGGACGAAAATGAGCTA	TGTGTCATTGGATTCGCATT	60.1	537	INTERGENIC				
CaPOPII_4879	scaffold643	71253	G/GC	CCACATTCTTAACGCCCAGT	CATTGCGAACGGAGATTTTT	60.0	446	INTERGENIC				
CaPOPII_4880	scaffold645	62567	CTTT/CTTTT	TACTCAGACGGCCAAAAAGC	CTTGCTTCGGTCTACAAGGG	60.4	609	INTERGENIC				
CaPOPII_4881	scaffold645	87205	AAATGCAATGCAATGCAATGCA/AAATGCAATGCAATGCAATGCAATGCAATGCA	ACTGCAATTTCACAAACGCA	GAGGACAAGGCCAATTTGAA	60.3	416	INTERGENIC				
CaPOPII_4882	scaffold645	224616	AAATAATAATAATAATAATAATAATAATAATAA/AAATAATAATAATAATAATAATAATAA	AGGGTTTGGAGGAGCGTAAT	CATCCTTCCCTCCCTCTTTC	60.0	143	INTERGENIC				
CaPOPII_4883	scaffold645	287773	TAAAAA/TAAAA	AGGGAGGACGAAAATGACCT	GCGTGTTTGTGTCATTGGAC	59.9	348	INTERGENIC				
CaPOPII_4884	scaffold647	200535	A/AT	CAAGCTGTCGGTTGAAGACA	AACAATCTCCCCCTTTTTGG	60.0	603	DRR	Ca_27495			
CaPOPII_4885	scaffold6480	530	ATTTT/ATTT	TAATCGATCATCCCAGACCC	TACAATTGGGCTCCTTCACC	59.7	900	INTERGENIC				
CaPOPII_4886	scaffold6480	672	CTTTTTTTTTTTT/CTTTTTTTT	AGAAACATTTGCACGGTCCT	TACAATTGGGCTCCTTCACC	59.6	612	INTERGENIC				
CaPOPII_4887	scaffold651	30345	CCAATCTTGTATATTCAAT/CCAATCTTGTATATTCAATCTTGTATATTCAAT	TTTTCGTCCTCCCTAGGCTT	TTGGATGCACAAGATTGGTT	60.2	162	INTERGENIC				
CaPOPII_4888	scaffold653	171379	CAAAAAAAA/CAAAAAAAAAAAA	GGCTATATCTTGCCCGCTTT	ATGTCACCCGTGGATCCTTA	60.6	368	INTERGENIC				
CaPOPII_4889	scaffold661	208026	TTATATATATATATATATATATATATAT/TTATATATATATATATATATATATATATATAT	GCGCCACAACTCCTACTCTT	TTTGTTTTGGCAGCTTGTTG	59.5	473	INTERGENIC				
CaPOPII_4890	scaffold661	474773	GTATATATATATATATATATATATAT/GTATATATATATATATATATATATAT	CGTAGCGTGGTAGTGAGCAA	GTTTTGGATTTTGGCTTGGA	60.1	668	INTERGENIC				
CaPOPII_4891	scaffold662	50819	GAAAAAA/GAAAAA	TGGGAAATCGATTGGGTAAG	TCATGTTAATCGGAGTGCCA	59.8	252	INTERGENIC				
CaPOPII_4892	scaffold665	16345	TA/T	GGTCCTCGATTTCATCTGGA	CCAGGTTCCGTGTCAGATTT	60.0	607	INTERGENIC				
CaPOPII_4893	scaffold6694	1671	TAAAAAAAA/TAAAAAAAAA	CCCACACTACGACCACGTTA	CGTAACTCGAATGAACGGAAA	59.5	671	INTERGENIC				
CaPOPII_4894	scaffold670	197970	AAAT/AAATAAT	TGGCCAATGATCCAAGAGAT	CCGACAAAAATGGACCAAAA	60.4	823	INTERGENIC				

INDEL marker IDs	Chromosomes /unanchored scaffolds	Physical positions (bp)	InDels (*Kabuli* reference genome-CDC Frontier/PI)	Forward primers (5'-3')	Reverse primers (5'-3')	Anneling temperature (0C)	Expected amplified product size (bp)	Structural annotation		Functional annotation		
								Sequence components of *kabuli* genome	*Kabuli* gene accession IDs	NCBI-KOG	TFs	NCBI-nr database
CaPOPII_4895	scaffold6719	505	GTTTGTTTT/GTTT	TGTTGGTTCCATTTTGTGGTT	GCTTTGCTGTTTCTTTTGCAT	60.1	532	INTERGENIC				
CaPOPII_4896	scaffold6720	536	TAAAAAAAAAA/TAAATAAAAAAAAAAA	CGATACTCACCCCTCGTTGT	ATGTGACACCCTAAACCCCA	60.0	338	INTERGENIC				
CaPOPII_4897	scaffold6720	5246	CA/CAA	TTTTCGTCCTCCCTAGGCTT	CAACTATTGGATGCGCAAGA	60.2	470	INTERGENIC				
CaPOPII_4898	scaffold674	150228	TAAAAAAAAAAAA/TAAAAAAAAA	CATGACCCCTTCATATTGGG	CTCGTTGTGGTTGTTGGTTG	60.0	294	INTERGENIC				
CaPOPII_4899	scaffold674	248531	GAT/GATAT	ACTTTTATGTGCGTTTGACTGA	TCCAACTCCCTTCCCTTTTT	57.5	534	INTERGENIC				
CaPOPII_4900	scaffold674	253414	CTTTTTT/CTTTTT	TGGCAAAATCAATACACCTCA	TTGGTTTCGGCTAGAAATGG	59.0	426	INTERGENIC				
CaPOPII_4901	scaffold674	401473	TAAAA/TAAA	TGCATTAGTCGATGTCCCAA	AATCGATTTCGCGTTTGTTC	60.1	134	INTERGENIC				
CaPOPII_4902	scaffold674	476011	GTATATATATATATATATATATATATAT/GTATATATATATATATATATATATATATATATATAT	TGAATTTGTGCTCTTGGTGG	AGTTTGGGAGGAAAGGGAAA	59.7	806	INTERGENIC				
CaPOPII_4903	scaffold674	529799	A/AC	CACCTAAGTTGGCGGGTTAC	CGCCTCGTGTGACCTAAGTT	59.5	290	INTERGENIC				
CaPOPII_4904	scaffold674	624095	TAA/TA	CCTCGGATCTGGATTCTTCA	GGTCAAGCTTAAAGCCACTCA	60.2	271	INTERGENIC				
CaPOPII_4905	scaffold674	630014	CATATATATATATATAT/CATATATATATATATATAT	GCGAGGATTGGTATCGTCAT	GCAAAACACATTCAAAATCCAA	59.9	263	DRR	Ca_20173	BD		
CaPOPII_4906	scaffold675	15473	TATAATAATAATAATAATAATAATAATAATAATAATAATAATAATAATAATAA/TATAATAATAATAATAATAATAATAATAATAATAATAATAATAATAAT	TGGCATGCTTGTGTACGAAT	TTGAGTTATCTCCCCAACCG	60.1	584	INTERGENIC				
CaPOPII_4907	scaffold675	81863	CTTTTT/CTTTTTT	TATCAGAGTTCTCCCCACCG	AAACATGACGAGGGCATTTA	60.1	895	INTERGENIC				
CaPOPII_4908	scaffold675	82372	T/TC	TAAATGCCCTCGTCATGTTT	CCCCTTTCCACGGCTATTAT	58.1	444	INTERGENIC				
CaPOPII_4909	scaffold675	89761	A/AT	AGATGCGCTTGCTTTTTCAT	GCTAAAATCACCGCAAGAGG	60.0	400	INTERGENIC				
CaPOPII_4910	scaffold676	14755	TC/TCTCC	TCCAGATGAAATCGAGGACC	CTTTTCGGGCAATTTTCGTA	60.0	450	INTERGENIC				
CaPOPII_4911	scaffold676	72988	TAA/TAAA	CTTTTCCGGCGATTTTTGTA	TCCTGATGAAATCGAGGACC	60.1	162	INTERGENIC				

INDEL marker IDs	Chromosomes /unanchored scaffolds	Physical positions (bp)	InDels (*Kabuli* reference genome-CDC Frontier/PI)	Forward primers (5'-3')	Reverse primers (5'-3')	Anneling temperature (0C)	Expected amplified product size (bp)	Structural annotation		Functional annotation		
								Sequence components of *kabuli* genome	*Kabuli* gene accession IDs	NCBI-KOG	TFs	NCBI-nr database
CaPOPII_4912	scaffold676	84534	GCAC/GCACAAAACAC	TTTTCGGATTAGACGATGGC	GATGAAATCGAGGACCAGGA	60.0	796	INTERGENIC				
CaPOPII_4913	scaffold6760	908	CTTCATTTT/CTT	TGTTTTCAAAAGGGGGTCAG	GGGCTGAGATGGACACATTT	59.9	621	INTERGENIC				
CaPOPII_4914	scaffold6760	1141	ATTTTTT/ATTTTT	AAATGTGTCCATCTCAGCCC	TCAAAAACTCAAGGGGCAAT	59.9	545	INTERGENIC				
CaPOPII_4915	scaffold678	39763	TAGTTATTAAGTTATT/TAGTTATT	AGGAGTTTGAGCATCGCATT	ATCCCACCCCTAAAACCAAG	59.8	470	INTERGENIC				
CaPOPII_4916	scaffold678	42464	TAAAAAA/TAAAAAAAA	GCGCACAATAGATGGGTTTA	TTTTCCAACGAGTCCACCTC	58.7	816	INTERGENIC				
CaPOPII_4917	scaffold678	81722	TAAAAAAA/TAAAAAA	AGGAAGCCCAAGTTCCAAAT	AGAAAGTGTTGACGGGTTGG	59.9	458	INTERGENIC				
CaPOPII_4918	scaffold6782	544	AC/A	ACTCACCCCACATCCAACAT	ATGGTACCGCAAAGAACGTC	60.1	337	INTERGENIC				
CaPOPII_4919	scaffold6782	758	ATTTTTTTT/ATTTTTTTTT	GACGTTCTTTGCGGTACCAT	TCAAACGCTCACACACACAA	60.0	292	INTERGENIC				
CaPOPII_4920	scaffold6782	1068	GA/GAGAAA	GACGTTCTTTGCGGTACCAT	CAAATGTCAACCACACATTTTG	60.0	428	INTERGENIC				
CaPOPII_4921	scaffold6782	1165	C/CA	GACGTTCTTTGCGGTACCAT	TTTAAATTGCTGCTAAGATTGGT	60.0	469	INTERGENIC				
CaPOPII_4922	scaffold6790	704	ACATCATC/ACATCATCATC	TAGTTATGCTGAGGGGACGG	CTACGAATTGGGGTCAAGGA	60.1	630	INTERGENIC				
CaPOPII_4923	scaffold6790	1414	C/CA	TCCTTGACCCCAATTCGTAG	TTTTGGGGTTTAGGGTGTCA	59.9	356	INTERGENIC				
CaPOPII_4924	scaffold6790	2258	ACC/ACCC	TTCAAATTGGCCTTGTCCTC	CATAGACCAACGAGGGGAGA	60.1	317	INTERGENIC				
CaPOPII_4925	scaffold682	88286	CAATAATAATAATAATAA/CAATAATAATAATAATAATAA	AGCCGAGTGAATATCAAAATGA	GTGAATGGAAGACGTCGGTT	58.7	555	INTERGENIC				
CaPOPII_4926	scaffold682	239210	ACC/AC	TCCAAAGCATCACGTATCCA	TGCGTGACAGGAACAAGAAG	60.1	667	DRR	Ca_23188	R	ERF	Integrase, catalytic core
CaPOPII_4927	scaffold682	239323	AGATGATG/AGATG	CAACTCCCCCTGCAAACTTA	TGCGTGACAGGAACAAGAAG	60.1	417	DRR	Ca_23188	R	ERF	Integrase, catalytic core
CaPOPII_4928	scaffold682	302365	A/AGAGAGAT	GCTGCAAAAGTCAACGTCAA	CACCCTTGAACTTTGGAGGA	60.0	177	INTERGENIC				

INDEL marker IDs	Chromosomes /unanchored scaffolds	Physical positions (bp)	InDels (*Kabuli* reference genome-CDC Frontier/PI)	Forward primers (5'-3')	Reverse primers (5'-3')	Anneling temperature (0C)	Expected amplified product size (bp)	Structural annotation		Functional annotation		
								Sequence components of *kabuli* genome	*Kabuli* gene accession IDs	NCBI-KOG	TFs	NCBI-nr database
CaPOPII_4929	scaffold682	506392	GATATATATATATATATATATATATAT ATATATAT/GATATATATATATATATAT ATATATATATATATATATATATATAT	AGGGAGGTTTGTGTTT GACG	TTTAATGTGTTGCGGG TGAA	60.0	408	INTERGENIC				
CaPOPII_4930	scaffold682	539867	CT/CTTT	TCGCCATTGTACTTGCT CTG	CCATGCCTCCAATCAA AAGT	60.0	886	INTERGENIC				
CaPOPII_4931	scaffold682	540419	TGAG/TGAGAG	ACTTTTGATTGGAGGC ATGG	TAATATGGGGAGAGGG GAGG	59.9	665	INTERGENIC				
CaPOPII_4932	scaffold682	540592	GTTTTTTTTTTT/GTTTTTTT	ACTTTTGATTGGAGGC ATGG	TAATATGGGGAGAGGG GAGG	59.9	665	INTERGENIC				
CaPOPII_4933	scaffold682	540642	T/TC	ACTTTTGATTGGAGGC ATGG	GAAGCAAAACCAGGGA ATGA	59.9	886	INTERGENIC				
CaPOPII_4934	scaffold682	540845	A/AT	AAGGACTGGTTCACCA GCAG	GAAGCAAAACCAGGGA ATGA	60.3	437	INTERGENIC				
CaPOPII_4935	scaffold682	540957	GAAA/GAAAA	CCTCCCCTCTCCCCATA TTA	GAAGCAAAACCAGGGA ATGA	60.1	241	INTERGENIC				
CaPOPII_4936	scaffold683	20681	TAA/TAAA	GAAGGATGAAAATGGG GGTT	TTGGAAAGGCTGAAAT CTGG	60.0	670	INTERGENIC				
CaPOPII_4937	scaffold684	40548	CATATATATATATATATATA/CATATA TATATATATATATATA	AGGAAGAGTGGAGTTG CGAA	TTAATTTAAGGCGTTTC GGG	60.0	375	INTERGENIC				
CaPOPII_4938	scaffold6846	522	T/TC	TGCATTGGTATATGGG GGTT	CGATCGACAGGAAGAG GAAG	59.9	538	INTERGENIC				
CaPOPII_4939	scaffold686	108971	GATATATATATATATATATATATA/GA TATATATATATATATATATATATA	GGGAGAAGGAGGAAG AATGG	CTTGGGAACTGGAGAG CAAG	60.0	564	INTERGENIC				
CaPOPII_4940	scaffold686	180622	GATATATATATATATATATATATGTAT ATATATATATATATATATATATATACA TATATATATATATA/GATATATATATAT ATATATATATATGTATATATATATATA	AATTTTACCGTGCAACT CGC	TCATCACAATGCAAATT CGAC	60.1	574	INTERGENIC				
CaPOPII_4941	scaffold686	183469	AT/ATT	TGGATTAAATCATTCCC CCA	ATCCCTGTTGATTTGCT TCG	60.0	617	INTERGENIC				
CaPOPII_4942	scaffold686	183725	TACAAAGAACTAGAAAACAAAGAACT AGAAAACAA/TACAAAGAACTAGAAA ACAA	TTACGAAGCAAATCAAC AGGG	GTGGCATAGTATCGTG GCCT	60.1	332	INTERGENIC				
CaPOPII_4943	scaffold6878	1840	TC/T	TGGACACGACGGTGAA ATAA	GTTTGAAATCTGACCC GGAA	60.0	704	URR	Ca_27881			
CaPOPII_4944	scaffold688	2385	GAATT/GATT	AGGTCATTTCCGTCCTT CCT	GACACGAACACACCTC ATGG	59.9	388	INTERGENIC				
CaPOPII_4945	scaffold6903	5314	A/AT	GCACACCACAACAAAAT GGA	GCATCCTCTGATTGTTT GGG	60.4	458	INTRON	Ca_28223			Domain of unknown function DUF239, plant

INDEL marker IDs	Chromosomes /unanchored scaffolds	Physical positions (bp)	InDels (*Kabuli* reference genome-CDC Frontier/PI)	Forward primers (5'-3')	Reverse primers (5'-3')	Anneling temperature (0C)	Expected amplified product size (bp)	Structural annotation		Functional annotation		
								Sequence components of *kabuli* genome	*Kabuli* gene accession IDs	NCBI-KOG	TFs	NCBI-nr database
CaPOPII_4946	scaffold696	25880	ATTTTTTT/ATTTT	TTGGAATTGGAATTGGAAGG	TTCAATTGAGTTGCGACGAC	59.7	559	INTERGENIC				
CaPOPII_4947	scaffold696	196380	CATATATATATATATATATATATAT/CATATATATATATATATATATATAT	AAATGGAGGAAGGAGGGAGA	TATTTTGGATTTTGGCTCGG	60.0	437	INTERGENIC				
CaPOPII_4948	scaffold7	215103	A/AC	TTGATCCTTGCAGTCACTCG	TGGCACTCCGATTAACATGA	60.0	805	INTERGENIC				
CaPOPII_4949	scaffold702	49940	ATATTATTA/ATATTA	GTTTTCGAATCAACGAGGGA	TTTGGCTCGAAGGCTTTTTA	60.1	452	INTERGENIC				
CaPOPII_4950	scaffold702	55997	CTACATACATACATACATACGTACATACATAC/CTACATACATACATACATACATACATACGTACATACATAC	GGGGTCCATCACATTTGTTC	CCATGTTAGGTTTGAACCAGG	60.0	114	INTERGENIC				
CaPOPII_4951	scaffold702	99680	ATTTTGCATAGTTTTTGCATAGT/ATTTTGCATAGT	CGCTCGATAACCCCAAAATA	ATTACCGACATTGCAGAGGG	59.9	577	INTERGENIC				
CaPOPII_4952	scaffold702	216004	GA/G	CTGTTTGGGCGTCGAATTAT	TATTTTCTCCCCTCGTTGGT	60.0	630	INTERGENIC				
CaPOPII_4953	scaffold705	30569	CAAAAAAAAAA/CAAAAAAAAA	GGTCCTCGATTTCATCTGGA	AGGGGTACGAAAATATCCCG	60.0	619	INTERGENIC				
CaPOPII_4954	scaffold705	34777	A/ATATTGTTATT	GGTTTGACTGCATAACCAAGA	GTACTTCATAACGAAGACAAATTAACA	57.7	608	INTERGENIC				
CaPOPII_4955	scaffold705	45291	CGGG/CGG	TTTCATCTGGTCTTTTCGGG	CGATCCATTTTTGTCGTGTG	60.0	215	INTERGENIC				
CaPOPII_4956	scaffold7081	2451	A/AC	TTAATGAGGGGCTGAATTGG	AAGCGTACCTGTGGGAATTG	59.9	259	INTERGENIC				
CaPOPII_4957	scaffold7081	2734	ATTTT/ATTTTT	CAATTCCCACAGGTACGCTT	TGGGGGTTAAAGGGATTAGG	60.0	465	INTERGENIC				
CaPOPII_4958	scaffold71	37235	ATTT/ATTTT	CCCTCATATTCAAAACAATCCC	GGCCATTCTCAGGAATTTCA	59.6	488	INTERGENIC				
CaPOPII_4959	scaffold7107	12825	AA/AAAGA	GGGGACTAAAATTGGGGAGA	TGCAGGCAATCAATGAAAGA	60.1	459	INTERGENIC				
CaPOPII_4960	scaffold7107	13331	TAAAAA/TAAAAAA	GACTCATTCAATGCACCCAA	TGCGATTGTTGTGGTTGTTT	59.5	627	INTERGENIC				
CaPOPII_4961	scaffold7107	13380	TTTTTGAAAATTTTGAA/TTTTTGAA	GACTCATTCAATGCACCCAA	TGCGATTGTTGTGGTTGTTT	59.5	627	INTERGENIC				
CaPOPII_4962	scaffold7107	14328	GTAT/GT	ATGCATTGTGAGGGGAAAAT	CGGGATGCGACCTTCTATTA	59.2	365	INTERGENIC				

INDEL marker IDs	Chromosomes /unanchored scaffolds	Physical positions (bp)	InDels (*Kabuli* reference genome-CDC Frontier/PI)	Forward primers (5'-3')	Reverse primers (5'-3')	Anneling temperature (0C)	Expected amplified product size (bp)	Structural annotation		Functional annotation		
								Sequence components of *kabuli* genome	*Kabuli* gene accession IDs	NCBI-KOG	TFs	NCBI-nr database
CaPOPII_4963	scaffold7107	14640	AA/AATTA	TAATAGAAGGTCGCATCCCG	TGTGTCAAACGAAATGATGTG	60.1	368	INTERGENIC				
CaPOPII_4964	scaffold7107	16409	CTATATATATATATATATA/CTATATATATATATATATATATATATATA	GGGGCTAGAACCTCCTACAA	CCATTTGCATGATTCCACAC	58.3	946	INTERGENIC				
CaPOPII_4965	scaffold7107	16449	TTTCAT/TTTCATCTTCAT	TCCAATGCTTACAATCCTCTCTC	CCATTTGCATGATTCCACAC	59.7	508	INTERGENIC				
CaPOPII_4966	scaffold711	204653	GATATATATATATATATA/GATATATATATATATA	AATGTGAATCCCACCAGGAA	TTTACAGCGCTTGTCCTCAA	60.2	796	INTERGENIC				
CaPOPII_4967	scaffold716	117351	CATATATATATATATATATATATATAT/CATATATATATATATATATATATATATATATAT	AGGAGGTTTGTGTTTGACGG	GCTGTTAGCCCAGTTTTTGG	60.0	612	INTERGENIC				
CaPOPII_4968	scaffold716	135403	AATTATTATTATTATTATTATTATTATTATTATTATTATTATTATTAT/AATTATTATTATTATTATTATTATTATTATTATTATTATTATTATTAT	TGATCATATAAAAACAAATGCTCACA	TGTGCAAGATTCCTGTTCCA	59.8	410	INTRON	Ca_24965		bHLH	Protein kinase, catalytic domain
CaPOPII_4969	scaffold716	189823	TAAA/TAAAA	AAGCTTGTGCAAAGGGAAAA	TGGTTTTGATGGGACCAAAT	59.9	113	INTERGENIC				
CaPOPII_4970	scaffold716	241616	CTTTTTTT/CTTTTTT	CCAACCACAACAAAATTCCC	CTTCCACCTCCTTTTTCCAA	60.1	564	INTERGENIC				
CaPOPII_4971	scaffold7187	901	A/ATAAC	TTTTAGGTTTGAGGTTCGGG	AATTATTTCATATTTATTTGGCAACTC	59.1	388	INTERGENIC				
CaPOPII_4972	scaffold719	20457	AAATAATAATAATAATAA/AAATAATAATAATAA	TGCTTATACCCATTGGTCCC	TGAGAAACTTTTCCGCTGCT	59.6	401	INTERGENIC				
CaPOPII_4973	scaffold719	123762	TAAAA/TAAA	AGGTCATTTTCGTCCTCCCT	TTCATCCAATGGCACAAAGA	59.9	695	INTERGENIC				
CaPOPII_4974	scaffold720	333511	CA/CATA	CGCTGTTAAAAATCGGGTGT	CTCATTTGATGCCTGCTTGA	60.0	709	INTERGENIC				
CaPOPII_4975	scaffold720	370256	GTT/GTTT	TTGCCTTAACGGAAATCCAC	GGTGGGTGTAGGGAGTGAGA	59.9	336	INTERGENIC				
CaPOPII_4976	scaffold720	370691	ATTGATTTTGATTTTGATTTT/ATTGATTTTGATTTT	TCTCACTCCCTACACCCACC	CAATTCACAATTGCACCGTC	60.0	625	INTERGENIC				
CaPOPII_4977	scaffold720	372071	T/TTA	ACCAACATTTCAGGCTCACC	TAGAGCGCCAAACCCTAATG	60.0	486	INTERGENIC				
CaPOPII_4978	scaffold7210	7073	GA/G	TTTTCGTCCTCCCTAGGCTT	TTGGATGCACATGATTGGTT	60.2	364	INTERGENIC				
CaPOPII_4979	scaffold724	21848	TATAAAAATAAAAATAAAAATAAAAATAAAAATAAAAATAAAA/TATAAAAATAAAAATAAAAATAAAAATAAAAATAAAAATAAAAATAAAAATAAAA	CAGAACAAACACGAGAGCCA	ATCAGAGCACATCATCGCAT	60.0	620	INTRON	Ca_25043	O		

INDEL marker IDs	Chromosomes /unanchored scaffolds	Physical positions (bp)	InDels (*Kabuli* reference genome-CDC Frontier/PI)	Forward primers (5'-3')	Reverse primers (5'-3')	Anneling temperature (0C)	Expected amplified product size (bp)	Structural annotation		Functional annotation		
								Sequence components of *kabuli* genome	*Kabuli* gene accession IDs	NCBI-KOG	TFs	NCBI-nr database
CaPOPII_4980	scaffold724	100088	ATTTTTTTT/ATTTTTTTTTT	ACAGGAGGGTGATCGAGAGA	TGCATACTTGTGTGGCATTTT	59.8	727	DRR	Ca_25051	Q	NAC	Short-chain dehydrogenase/reductase SDR
CaPOPII_4981	scaffold724	100193	TAAAAAAAA/TAAAAAAA	CTGTAAGCGGAGAAAATTGG	TTGCATACTTGTGTGGCATTT	57.5	319	DRR	Ca_25051	Q	NAC	Short-chain dehydrogenase/reductase SDR
CaPOPII_4982	scaffold724	100328	TAAAAAAA/TAAAAAA	CTGTAAGCGGAGAAAATTGG	AATGGTAGTCCCTCTGGCCT	57.5	690	DRR	Ca_25051	Q	NAC	Short-chain dehydrogenase/reductase SDR
CaPOPII_4983	scaffold724	100591	AATTTAACATAT/A	AAATGCCACACAAGTATGCAA	AATGGTAGTCCCTCTGGCCT	59.1	392	DRR	Ca_25051	Q	NAC	Short-chain dehydrogenase/reductase SDR
CaPOPII_4984	scaffold724	108519	ATATTATTATTATTATTATTAT/ATATTATTATTATTATTATTATTAT	GCGGTACTGGCATTTAGCAT	TTGAAAGGCCTGAGCCTAGA	60.1	792	INTERGENIC				
CaPOPII_4985	scaffold724	126741	CTTTTT/CTTTT	TCGCACTTCAATATGCTTCG	AAAGGCCATGCAAGAAGAGA	60.0	851	URR	Ca_25052	Q	NAC	Short-chain dehydrogenase/reductase SDR
CaPOPII_4986	scaffold728	32793	TC/T	ATGGAGACGAGTGAATTCGG	TGAAGAAGAGTTGGGAAACGA	60.1	502	INTERGENIC				
CaPOPII_4987	scaffold7280	1708	AC/ACC	GTTTCGTACCCCTCGACAAA	TCCAGATGAAATCGAGGACC	60.0	311	INTERGENIC				
CaPOPII_4988	scaffold7293	13170	AG/A	TGCCTCTGGATTGGTTGAAT	AATCTATCCACCCGGAAACC	60.5	471	INTERGENIC				
CaPOPII_4989	scaffold7293	15472	TT/TTAGAAAAGTGATAGT	TTCATGCCAGCAACTCTTTG	TGCTTTGCTATGCACCAGTC	60.0	626	INTERGENIC				
CaPOPII_4990	scaffold7293	20198	TC/T	TTGCTGCCTTCTCCTGTTTT	ATGATGCACTTTGGGCCTAC	60.0	661	INTERGENIC				
CaPOPII_4991	scaffold7299	551	AATATATATATATATATAAATATATATATA/AATATATATATATATATATATAAATATATATATA	CACCAAAACTTAATTTCCCCTTT	TGATTCCTATGCATCGTATTTTG	59.7	459	INTERGENIC				
CaPOPII_4992	scaffold731	94332	ATTTTTTTTTT/ATTTTTTTT	GAGCGCTGTAAAAGACCTCC	TGAAGATGGTTTGCAATGGA	59.1	188	DRR	Ca_23810		B3	
CaPOPII_4993	scaffold731	313013	AA/ATTGATAAAGAAGGA	TCAAAATTTTTCCGCTGCTT	AACTGTCTCAACTCTCGGGC	59.8	192	INTERGENIC				
CaPOPII_4994	scaffold731	315903	GATGA/GATGAATGA	AACTCGAAGTGCCATGCTTT	CAATACGCAATGGTGGTGAG	59.9	660	DRR	Ca_23814	J		Ribosomal protein S11
CaPOPII_4995	scaffold731	322781	T/TCAAATTCATTAAAA	ATCCCCGATTCCTCTCAAGT	AATTCCTACTGGATGCACGC	59.9	479	INTERGENIC				
CaPOPII_4996	scaffold731	323181	GATAAATCAATAT/GATAAATCAATATAAATCAATAT	GCAGGAATCCTCGTACCTCA	CTGCGAATTGCGTGAAAGTA	60.2	296	INTERGENIC				

INDEL marker IDs	Chromosomes /unanchored scaffolds	Physical positions (bp)	InDels (*Kabuli* reference genome-CDC Frontier/PI)	Forward primers (5'-3')	Reverse primers (5'-3')	Anneling temperature (0C)	Expected amplified product size (bp)	Structural annotation		Functional annotation		
								Sequence components of *kabuli* genome	*Kabuli* gene accession IDs	NCBI-KOG	TFs	NCBI-nr database
CaPOPII_4997	scaffold731	323712	T/TCTTCCGA	TACTTTCACGCAATTCGCAG	TTTATTTTCCGATTGCCTCG	60.0	584	INTERGENIC				
CaPOPII_4998	scaffold731	324476	CAA/CAAAA	TGTGATATGGTTGCGAAGGA	CAACCATCCCGTTTGGTATC	60.1	650	INTERGENIC				
CaPOPII_4999	scaffold731	324642	T/TA	TGTTTGGAAGACCGAAAACC	CAACCATCCCGTTTGGTATC	59.9	257	INTERGENIC				
CaPOPII_5000	scaffold731	335005	A/AGTGGA	CTATCAATAACCCAGCCGGA	TGGATCAGATCGAGTTGGAA	59.9	857	INTERGENIC				
CaPOPII_5001	scaffold731	339474	GT/GTTTCTCTT	TGTGCAAACGTCATTGGATT	CCTCAGATGAGAGGCAGGAC	60.0	544	INTERGENIC				
CaPOPII_5002	scaffold731	342154	CAAAAAAAA/CAAAAA	AAATGGAGGAGAAGGAGGGA	ACCCAATTACTGGAGATGCG	60.0	574	INTERGENIC				
CaPOPII_5003	scaffold731	354972	T/TATAATCATAC	ATAACCTTAGCGCATTCCCA	CCTCCCGTAAGATCCAACAA	59.6	308	INTERGENIC				
CaPOPII_5004	scaffold731	361613	TGG/TGGG	TGTGCTATTGCTCAAATCCG	AGCAGTCGTTTCCAGCTGTT	59.8	640	DRR	Ca_23818			Cytochrome f
CaPOPII_5005	scaffold731	366076	GAA/GAAA	TCTCGCGGATTTTCATAACC	CCATCCATTTCCGTATTGCT	60.0	100	INTERGENIC				
CaPOPII_5006	scaffold731	371953	T/TCACTTCCGC	CATCAGTTACTTCAGCGCCA	GTTTGCCTAGAAGCAGCCAC	60.0	641	INTRON	Ca_23820			
CaPOPII_5007	scaffold731	374028	AA/AAGGGAGATAGTGCA	TCCTTGGGGTGATCTCGTAG	ATGGACGGTAGTTGGAGTCG	60.1	222	INTRON	Ca_23820			
CaPOPII_5008	scaffold731	390482	C/CAA	TATCATAGGCCGTAGGCCAG	TTATTCGTCCAATTCCCTCG	60.1	482	INTRON	Ca_23820			
CaPOPII_5009	scaffold731	392660	TT/TTTTTGCTAATCTAAT	GAACGACGGAACCTGTGAAT	AGGAAGCCCAAGTTCCAAAT	60.0	665	INTRON	Ca_23820			
CaPOPII_5010	scaffold7330	2275	AG/A	TTGGTCGACAAGCAACTGAG	AAAATCCAACAATCTCCCCC	60.0	699	DRR	Ca_28207			
CaPOPII_5011	scaffold7355	18760	A/ATC	ATTTTGGCTAGGGGAGAGGA	GAGTGGGCTGATGCAAAAAT	60.0	439	INTERGENIC				
CaPOPII_5012	scaffold7364	2154	CAT/C	TCAATTTGGGTTTAGGGTGC	TGTAGTGGCATGTTTGGCAT	59.8	497	INTERGENIC				
CaPOPII_5013	scaffold7377	545	CT/CTT	TTTCTTCCCAACCTTCATCG	GCCGTTGGAACTTCATCATT	60.0	816	INTERGENIC				

INDEL marker IDs	Chromosomes /unanchored scaffolds	Physical positions (bp)	InDels (*Kabuli* reference genome-CDC Frontier/PI)	Forward primers (5'-3')	Reverse primers (5'-3')	Anneling temperature (0C)	Expected amplified product size (bp)	Structural annotation		Functional annotation		
								Sequence components of *kabuli* genome	*Kabuli* gene accession IDs	NCBI-KOG	TFs	NCBI-nr database
CaPOPII_5014	scaffold738	2137	T/TC	TGAATATCGCCCGAAAAGTC	TTTCATAGCAGACGAGGGCT	60.0	292	INTERGENIC				
CaPOPII_5015	scaffold738	5098	ATTTTTT/ATTTTT	TCGGGTGGTACTTAAATCGC	ATCTGACGTGGAAACCGAAC	60.0	274	INTERGENIC				
CaPOPII_5016	scaffold741	6784	AATATATATATATATATATATATATATATAT/AATATATATATATATATATATATATATATATAT	TTGGGATGGTGATTCTCACA	TTTACCACTTGGAGTTGGGC	59.9	215	DRR	Ca_28074		B3	
CaPOPII_5017	scaffold743	14244	AACACACA/AACACA	TGCTTGATTGCTTGAGTTCC	GTACCCTGATTGCCCTCTGA	59.0	321	INTERGENIC				
CaPOPII_5018	scaffold7448	21480	GAAA/GAAAA	AGGATCTCCCGATTCTCGAT	TTCCGGGTCAGATTTCAAAC	60.0	287	INTERGENIC				
CaPOPII_5019	scaffold7470	2428	TCCC/TCC	TGTGCATCCGATAGGTGTGT	GGGAGAATGAAAATGACCGA	60.0	760	INTERGENIC				
CaPOPII_5020	scaffold7477	2293	TAAA/TAA	TTGGTGGATTTGAGACCACA	ACTTCCCTTTGCACATTTGG	59.9	652	INTERGENIC				
CaPOPII_5021	scaffold7477	6564	TTTG/TTTGTTG	CCTGCAGAAAGCAAAACACA	ACCTTGGGAAGGAGGAGAAA	60.0	590	INTERGENIC				
CaPOPII_5022	scaffold7489	558	TAAAA/TA	AACCACAACAATGCCAACAA	TTTTGCTTTTTGGTCCAACC	59.9	539	INTERGENIC				
CaPOPII_5023	scaffold7489	828	GTATATATATATATATATA/GTATATATATATATATA	GGTTGGACCAAAAAGCAAAA	TCACCTTCTCATCACAACAGC	60.0	602	INTERGENIC				
CaPOPII_5024	scaffold749	1624	CTATATATATATATATATATATATATAT/CTATATATATATATATATATATAT	CTTGGGAACTGGAGAGCAAG	GAGGAGGGAGGGATTTTGAG	60.0	536	INTERGENIC				
CaPOPII_5025	scaffold750	31851	GAAAAAAAAA/GAAAAAA	TGAGTTTCTATGCAAGGGCA	TTCATGAAGACACCGGAACA	59.4	273	INTERGENIC				
CaPOPII_5026	scaffold750	38979	AT/A	CACTGATAACGCGACTAATTTGA	ACCGCCACAAATGATAAAGC	59.3	748	INTERGENIC				
CaPOPII_5027	scaffold750	59023	ATTGTTGTTGTT/ATTGTTGTTGTTGTT	CGTAGCATCAGCAACCTTCA	TCCCTCTTTTGTTTTTCCCA	60.0	989	INTERGENIC				
CaPOPII_5028	scaffold750	360259	GA/G	TGTTTGTCTTGCTGCTTTGC	AAAATCCAACAATCTCCCCC	60.2	626	DRR	Ca_25944			
CaPOPII_5029	scaffold752	269557	A/AC	AACGACACAATTTTACTCATCGT	TAAGGTTGTCTGGGGAGGTG	57.8	368	INTERGENIC				
CaPOPII_5030	scaffold752	505163	TTTTATTTTATTTATTTTAT/TTTTATTTTATTTATTTTATTTATTTTAT	AGCCATTGCAGTCTTCGTCT	TCGTGTCTCAAAACTCAAGGAA	60.0	854	INTERGENIC				

INDEL marker IDs	Chromosomes /unanchored scaffolds	Physical positions (bp)	InDels (*Kabuli* reference genome-CDC Frontier/PI)	Forward primers (5'-3')	Reverse primers (5'-3')	Anneling temperature (0C)	Expected amplified product size (bp)	Structural annotation		Functional annotation		
								Sequence components of *kabuli* genome	*Kabuli* gene accession IDs	NCBI-KOG	TFs	NCBI-nr database
CaPOPII_5031	scaffold7569	558	GAA/GA	ACTACCCCATTCTGCTGTGG	AGATGAAAAATGGCTTTGGC	60.0	489	INTERGENIC				
CaPOPII_5032	scaffold758	3569	CTATATATATATATATATATATATATA/CTATATATATATATATATATATATATATATA	CATGATCCTCCTACTCCCGA	CAAACAAGGTTGGTGTGCTG	60.0	348	INTERGENIC				
CaPOPII_5033	scaffold758	13400	AATATATATATATATATATATATA/AATATATATATATATATATATA	CTCGTCCAATTCCCCTTTTT	CGATTTTACGTGAATATACGAACAC	60.3	603	INTERGENIC				
CaPOPII_5034	scaffold7650	9735	T/TG	TATGGCATTGTGGTGCACTT	GGATTAAAATTGGAATTGTTCAAGA	60.0	482	URR	Ca_27809	O		SKP1 component
CaPOPII_5035	scaffold7650	9922	CA/CATA	CCGCAATTATATCTGCGACA	AGTTCAAAAATACGCGGGTG	59.7	857	URR	Ca_27809	O		SKP1 component
CaPOPII_5036	scaffold7657	660	CACAACAA/CACAA	AGGGAGGACGAAAATGACCT	GGTGTGTTTGTGTCGTTGGA	59.9	412	INTERGENIC				
CaPOPII_5037	scaffold766	243668	GTTA/GTTAATTA	CTCAACATGCATGCAAAACC	TCCCAACCCAAATAAACCAA	60.1	537	URR	Ca_24693			
CaPOPII_5038	scaffold768	560	T/TG	CCATGAGAGTCGAAATCGGT	TCCCACAGATCCACATTCAA	60.1	188	INTERGENIC				
CaPOPII_5039	scaffold769	1084	TTT/TAACTT	AAATGAACCAATTTAATGAGCTACC	CCCTCCCAAAACACCCTTAT	59.0	479	INTERGENIC				
CaPOPII_5040	scaffold769	2786	TGGCAGCAG/TG	ACGTACACGTCAGCGTTTTG	CCCAATTCTCAATCCCACTT	59.8	147	INTERGENIC				
CaPOPII_5041	scaffold769	5414	AATATATATATATATATATATATATATATATAT/AATATATATATATATATATATATAT	TCACTCGCGTTAATTTTGATG	GCGAGTGAATACATGTGCCA	58.8	518	INTERGENIC				
CaPOPII_5042	scaffold769	29353	AGC/AGCCCACTAATATGC	GCACTCTCATTCATCCCACA	CCACATGTCATTTAACCCTGC	59.6	370	INTERGENIC				
CaPOPII_5043	scaffold7697	589	G/GA	CTAGCCCTCGTCTGTTCCAG	AATCCGTATTTTCGCACCAG	60.0	521	INTERGENIC				
CaPOPII_5044	scaffold772	11892	TAAA/TA	GGCACACTCCCGTAAAATTC	TTTTTCGCACACCAATGAGA	59.4	539	INTERGENIC				
CaPOPII_5045	scaffold772	23600	G/GAGAGACATGTGTT	GGAAACAATGCCTCCAAGAA	GGAGGATGGTAACGACGAAA	60.1	191	CDS (FRAME SHIFT)	Ca_26413			
CaPOPII_5046	scaffold772	152481	GTT/GT	GGAGGATCTATGGTGCTGGA	GTGTTCTGCCGCTTTTTCTC	60.0	170	CDS (FRAME SHIFT)	Ca_26416			
CaPOPII_5047	scaffold772	211515	TCT/TCTTACAACTTCCCT	AATGGAGGAAGGAGGGAGAA	ACGTTTCCAAGTTCTGGTGG	60.0	452	INTERGENIC				

INDEL marker IDs	Chromosomes /unanchored scaffolds	Physical positions (bp)	InDels (*Kabuli* reference genome-CDC Frontier/PI)	Forward primers (5'-3')	Reverse primers (5'-3')	Anneling temperature (0C)	Expected amplified product size (bp)	Structural annotation		Functional annotation		
								Sequence components of *kabuli* genome	*Kabuli* gene accession IDs	NCBI-KOG	TFs	NCBI-nr database
CaPOPII_5048	scaffold772	213642	AGG/AGGG	ACTTGAGGGCGAAGAACAAA	TTGGATGACCTACGACTCCC	59.9	573	INTERGENIC				
CaPOPII_5049	scaffold775	110824	TAAA/TAA	GCCGAATCGATTTTGTCAGT	GGAGGGTTTTGGTTTGGAAT	60.1	521	INTERGENIC				
CaPOPII_5050	scaffold775	446063	TTATATATATATAT/TTATATATATATATAT	TTTTGCAAGATCTGCCACTG	TGGAAGGATCCGGAACTATG	60.0	867	INTERGENIC				
CaPOPII_5051	scaffold775	449733	CTTATCAATTGTGTTT/CTTATCAATTGTGTTTATCAATTGTGTTT	CTCTCTTCTGCGCATCAACT	TTGACCATGACACATGCCTT	58.3	459	DRR	Ca_23414		NAC	Pectinesterase, catalytic
CaPOPII_5052	scaffold776	224423	T/TTCCCTA	TGGGATACGTAGGAGCAAGG	TGTCCGTGAAAAATGAGTCG	60.1	310	INTERGENIC				
CaPOPII_5053	scaffold7765	977	ATTTTTTTTTT/ATTTTTTTTTTTTTTT	AGATGGCTGCAGCTCAAAGT	TTTCCAAGAGGTTTTGGGTC	60.2	464	INTERGENIC				
CaPOPII_5054	scaffold783	120194	CTATATATATATATATATATATATATAT/CTATATATATATATATATATATATATATATATAT	TTGGATTTTGGTTCAGAGGC	ACCGTAGTTTGGTGGTGAGC	60.1	662	INTERGENIC				
CaPOPII_5055	scaffold787	129918	AAATAATAATAATAATAATAATAATAATAATA/AAATAATAATAATAATAATAATAATAATA	AGAATCCACAACGCAACACA	AAGGCACGACATCATCATCA	60.2	461	INTERGENIC				
CaPOPII_5056	scaffold787	149556	ATTTTT/ATTTT	CGGGATGGTGGCATAATATC	AGCAGGGTTTCTCCCAAACT	60.0	343	INTERGENIC				
CaPOPII_5057	scaffold787	334329	ATTTTTTTTT/ATTTTTTTTTT	CTTCCTCTGTGCCACATCCT	TTCATTCGTGTCTGCTTTGG	60.3	598	INTERGENIC				
CaPOPII_5058	scaffold787	353218	A/AT	CCCCCTCTTAGGCGTACTTC	TACCTCCCGTCACCAAAAAC	60.1	529	INTERGENIC				
CaPOPII_5059	scaffold787	460684	CAT/CATAT	TATGGGAAGGAAATGTGGGA	TCACTCGCAACGAGAAGTTG	60.1	601	INTERGENIC				
CaPOPII_5060	scaffold787	462837	GGTGGCCATTCCCTAGTG/GGTGGCCATTCCCTAGTGGCCATTCCCTAGTG	ACCACCCTTTAACGTTTTGC	GTTACTCCCACGTGACACCC	59.0	282	INTERGENIC				
CaPOPII_5061	scaffold787	469394	CAGG/C	GGCGGTTGCTAAAGTAGTGC	ACGATCTTTCTCCACGATGC	59.9	331	INTERGENIC				
CaPOPII_5062	scaffold7890	680	TG/TGG	TTTCTCCCCTTTCTTTCCGT	GAAGGGACTGCGGCATAATA	60.0	162	INTERGENIC				
CaPOPII_5063	scaffold7891	3768	CAAA/CAAAA	TCTGGACTTTTCGAGCGATT	AGTGCATTTTAGTCGGGTGG	60.0	379	INTERGENIC				
CaPOPII_5064	scaffold7902	684	CTT/CTTT	GGGTAGCATGGCAAGTTGTT	CGAGTAAAGCGTTTTGACTAACG	60.0	363	INTERGENIC				

INDEL marker IDs	Chromosomes /unanchored scaffolds	Physical positions (bp)	InDels (*Kabuli* reference genome-CDC Frontier/PI)	Forward primers (5'-3')	Reverse primers (5'-3')	Anneling temperature (0C)	Expected amplified product size (bp)	Structural annotation		Functional annotation		
								Sequence components of *kabuli* genome	*Kabuli* gene accession IDs	NCBI-KOG	TFs	NCBI-nr database
CaPOPII_5065	scaffold7922	5215	GAAAAAAAA/GAAAAAAAAAA	GAAGGTTTCTGCATTTCGGA	TTATACCTCCCACCCCATCA	60.2	361	INTERGENIC				
CaPOPII_5066	scaffold7923	2123	AAGCTCA/AA	TCGGGGTCTTTAGTCGTCAT	GCGAGGAAGCAAAAGCTAGA	59.6	796	DRR	Ca_27506	O		
CaPOPII_5067	scaffold7923	22242	CAAAAAAAAAA/CAAAAAAAAA	TTTCCCGACTTGGTCCTTTA	TTTGCGTTACGAATTTGATGA	59.5	177	DRR	Ca_27507	QI	bHLH	Cytochrome P450
CaPOPII_5068	scaffold7923	25395	ATC/A	AAAATATGCCATTCGATCAAGA	AAATGGCTTGGTTATGGCTG	58.6	200	URR	Ca_27507	QI	bHLH	Cytochrome P450
CaPOPII_5069	scaffold793	207344	CATATATATATATATATATATATATATATAT/CATATATATATATATATATATATATATAT	TTAAGCCAAGACAACCCCTG	GTTGGTTCCCTAGCACCGTA	60.1	718	INTERGENIC				
CaPOPII_5070	scaffold794	138143	GA/G	CTGTCAAACCGGGTTCTGTT	TGCTTGCAGTGCTTCATCTT	60.0	378	INTERGENIC				
CaPOPII_5071	scaffold794	138803	AA/AATGCTTA	CAGAAATGGGTTGCCAAAAA	GCGGAAACATTAGCAAGACC	60.8	407	INTERGENIC				
CaPOPII_5072	scaffold796	26479	GTT/GTTT	TCATTTTCGTCCTCCTTTGG	GCCTATTGGATGCACAGGAT	60.0	470	INTERGENIC				
CaPOPII_5073	scaffold797	15287	CAAAAAA/CAAAAAAAA	GGTGATTTTTATGATGTGCTAGTTTT	CCCTTTCAATTCCCGTTTTT	59.0	689	INTERGENIC				
CaPOPII_5074	scaffold798	1261	A/AT	ACAGCGGAAGAGTTTCTCCA	GATTTGGCAACACAGGGACT	60.0	706	INTERGENIC				
CaPOPII_5075	scaffold798	16927	T/TC	ATGCAATGCGTATATGCCAA	TAAATCGTGTTGACCCGTGA	59.9	233	INTERGENIC				
CaPOPII_5076	scaffold80	43513	AGGG/AGG	CCGAGCCAAAATCCAAAATA	TTCTCGGGCTAAAATCATGG	59.9	352	INTERGENIC				
CaPOPII_5077	scaffold80	264906	ATTTTT/ATTTTTT	GTTGCTGGGGATTGTCATCT	CCATGTGGTGATGCTACCAG	59.9	671	INTERGENIC				
CaPOPII_5078	scaffold801	72301	CTTTTTTTTTTTT/CTTTTTTTTTTTTT	TGTTAGCGTAACAAATGGGG	TGTGTTTTATGGATTGCGGA	58.6	557	DRR	Ca_26176	OE	WRKY	Peptidase S10, serine carboxypeptidase
CaPOPII_5079	scaffold801	75364	GTTATTATTATTATTAT/GTTATTATTATTAT	TGCATTCCATTCCCCTTTTA	ACTAAAACCTATTCGGGGCG	60.3	397	INTERGENIC				
CaPOPII_5080	scaffold801	207271	CAAAAAAAA/CAAAAAAAAA	TCACAACTTGCTTATTTGCCA	TCACTTCATTCCTGCACTCCT	59.4	519	INTERGENIC				
CaPOPII_5081	scaffold801	260922	GTATATATATATATATAT/GTATATATATATATATATATATAT	TCGTCCGTAAAAGATACCGC	GGACCAAAATTTGAGCAACAA	60.1	658	INTERGENIC				

INDEL marker IDs	Chromosomes /unanchored scaffolds	Physical positions (bp)	InDels (*Kabuli* reference genome-CDC Frontier/PI)	Forward primers (5'-3')	Reverse primers (5'-3')	Anneling temperature (0C)	Expected amplified product size (bp)	Structural annotation		Functional annotation		
								Sequence components of *kabuli* genome	*Kabuli* gene accession IDs	NCBI-KOG	TFs	NCBI-nr database
CaPOPII_5082	scaffold801	260992	CATAT/CAT	TCGTCCGTAAAAGATACCGC	GGAGGACCAAAATTTGAGCA	60.1	661	INTERGENIC				
CaPOPII_5083	scaffold8054	1926	AT/A	TGTTAATCCAATGCCGATGA	GAATTTGGGATTTCAACCGA	59.9	447	INTERGENIC				
CaPOPII_5084	scaffold81	91419	A/AC	TGAGGAGAAACAAAATGGGG	ATGGAAGGGTCGTTGACATT	59.9	458	INTERGENIC				
CaPOPII_5085	scaffold811	56559	GATATATATATATATATATATATATAT/GATATATATATATATATATATATATATATAT	TTTAACGTGTCGCGTCTTTG	GAATAAAATGCACCGGCACT	59.9	748	INTERGENIC				
CaPOPII_5086	scaffold811	125062	AATATATATATATATATATATATATA/AATATATATATATATATATATATATATATATATA	TTGGTATTGAACTCGGAGGC	GGTTTTGCATGCATGTTGAG	60.1	628	INTERGENIC				
CaPOPII_5087	scaffold811	159571	TTATATATATATATATATATATATATATAT/TTATATATATATATATATATATATATAT	TTGCAACATGCTATTTCCCA	TGGGTGCTATTTGCAATCAA	60.1	613	INTRON	Ca_27462			
CaPOPII_5088	scaffold812	213139	TTTTTATTTTA/TTTTTATTTTATTTTA	GCGCACAAAGTCAAGAATGA	TAGGTTGGACGTCTTGTCCC	60.0	224	INTERGENIC				
CaPOPII_5089	scaffold812	215247	TGG/TG	AGAAGGAACAACGAGAACGC	TGTCCTTCTCATTGGGAACC	59.5	178	INTERGENIC				
CaPOPII_5090	scaffold812	245558	GAAAAAAAAA/GAAAAAAAAAA	CAAAGTCTTCACACACCGACA	TGTTGACTCCAGTCACCGAG	59.8	736	INTERGENIC				
CaPOPII_5091	scaffold812	471428	AT/ATT	TCTGATGCGTTTCTTGATGC	TTGGTGTGTCGAGTTGGGTA	60.0	580	INTERGENIC				
CaPOPII_5092	scaffold812	525957	AT/A	TCGACTTGTCAATCGCTTTG	CACTCCAGTGCTGAGGGAAT	60.0	215	INTERGENIC				
CaPOPII_5093	scaffold812	526031	TGG/TG	TCGACTTGTCAATCGCTTTG	CACTCCAGTGCTGAGGGAAT	60.0	215	INTERGENIC				
CaPOPII_5094	scaffold812	540816	TTATATATATATATATAT/TTATATATATATATATATATAT	GTAAAATGAAGCGCTTTCGC	TTGCAATCTCATCAACTCGG	60.0	916	INTERGENIC				
CaPOPII_5095	scaffold820	15078	A/AG	TCATCATGGGCTTACGACCT	GGCCAAACCCATTAAAACCT	60.5	343	INTERGENIC				
CaPOPII_5096	scaffold8200	526	T/TTC	CCAACTCATGCACCCCTAGT	TTCGATTTTGATGTGGGACA	60.0	388	INTERGENIC				
CaPOPII_5097	scaffold824	4509	T/TG	GAGGACGAAAATGACCCAAA	ATTTCCATCCTTCCTTGGCT	59.9	112	INTERGENIC				
CaPOPII_5098	scaffold824	6128	CA/C	CACCCCAGTTTTGAGGGTAA	CGATTGTGTTTGTCACAGGG	59.8	191	INTERGENIC				

INDEL marker IDs	Chromosomes /unanchored scaffolds	Physical positions (bp)	InDels (*Kabuli* reference genome-CDC Frontier/PI)	Forward primers (5'-3')	Reverse primers (5'-3')	Anneling temperature (0C)	Expected amplified product size (bp)	Structural annotation		Functional annotation		
								Sequence components of *kabuli* genome	*Kabuli* gene accession IDs	NCBI-KOG	TFs	NCBI-nr database
CaPOPII_5099	scaffold829	8235	CTTTT/CTTTTT	TGTTTGAAATATGACGCGGA	TTTCGGTCCATTTTAGTCGG	60.1	583	INTERGENIC				
CaPOPII_5100	scaffold829	10973	T/TA	TTTATTTGACAACTCGTTTTTGTT	GAGCGAATTGAGAAACGAGG	57.1	799	INTERGENIC				
CaPOPII_5101	scaffold8314	868	CAAAAAAAAAAAAA/CAAAAAAAAAAAA	TGAATGGTTGCATGGTTATCA	GTGGGAAGTGGTAGAGTGCG	59.8	415	INTERGENIC				
CaPOPII_5102	scaffold8314	1258	CTTTTTTTT/CTTTTTTT	AATTTAGCTCATTGCTGCCG	TACCTCCTTTGGAAGCATGG	60.4	353	INTERGENIC				
CaPOPII_5103	scaffold8315	18988	TAAAA/TTTTAAAAA	AAAAATTTATATTGCGTCAATTTGG	GTGCTGGCCATAGGAAATGT	59.6	565	INTERGENIC				
CaPOPII_5104	scaffold8315	20462	GTTT/GTT	AAAAGGATATTCAACTCTCCAAAAA	CGATTTTGCTACACAAAATTTCC	58.7	759	INTERGENIC				
CaPOPII_5105	scaffold8378	1988	C/CG	AGCAACCACTCGCTTCAAAT	CAATATTGCTAGGCGCAACA	59.9	458	INTERGENIC				
CaPOPII_5106	scaffold8412	961	TAAAAAAAA/TAAAAAAAAA	TTTTCACATTTCAAAAGCATGA	TCACATCTTCCTTATGTCCGC	58.3	342	INTERGENIC				
CaPOPII_5107	scaffold842	20647	TA/TAA	TGATTCCTGTAACACCCCAA	AACCAAACACACAAGGAAATATGA	58.8	332	INTERGENIC				
CaPOPII_5108	scaffold843	1852	GCC/G	TTTCGGTCCATTTTTGCTTC	TCCTGGTCCTCGATTTCATC	60.1	283	INTERGENIC				
CaPOPII_5109	scaffold8442	1501	CA/C	AACCAAACTGGTGCATCACA	AGCCTAGGGTGGACGAAAAT	60.0	676	INTERGENIC				
CaPOPII_5110	scaffold8445	5721	TAA/TA	CATCTACGCGTGTGTTCGTAA	TGCATAAAATCACCATGCCA	59.8	206	INTERGENIC				
CaPOPII_5111	scaffold845	2172	TT/TTCTTTAGT	CAACATTTTGGGTGCATTTG	TGGTTATTTTGCATGTAGCGA	59.8	657	INTERGENIC				
CaPOPII_5112	scaffold845	11900	TATATTTAAA/TATATTTAAATAATAATATTTAAA	GCTTCTTTGAGATACCGTGCC	TGTGAACACGACCTCTCCCT	61.1	535	INTERGENIC				
CaPOPII_5113	scaffold845	38901	CAAAATAAAATAAAAT/CAAAATAAAATAAAATAAAATAAAAT	GGTGCAAATGAGAAGCACAA	AAAAGCATGCTGTCAACCG	59.8	584	INTERGENIC				
CaPOPII_5114	scaffold845	41535	TAAAA/TAAAAA	GCATTTGCAACTTTGTGTGC	ACTTGGTGGGTCATGGAAAA	60.3	397	INTERGENIC				
CaPOPII_5115	scaffold845	47867	GAAAA/GAAAAA	AAGGGAAAAGGGTGTGTGTG	AATGCGACGTTTGATTTATCG	59.9	999	INTERGENIC				

INDEL marker IDs	Chromosomes /unanchored scaffolds	Physical positions (bp)	InDels (*Kabuli* reference genome-CDC Frontier/PI)	Forward primers (5'-3')	Reverse primers (5'-3')	Anneling temperature (0C)	Expected amplified product size (bp)	Structural annotation		Functional annotation		
								Sequence components of *kabuli* genome	*Kabuli* gene accession IDs	NCBI-KOG	TFs	NCBI-nr database
CaPOPII_5116	scaffold845	48050	ATT/A	TTGATAGGGATTGGAATAAACAA	TTTTGTGTTGGTGCGACATT	57.6	427	INTERGENIC				
CaPOPII_5117	scaffold845	64302	CT/CTGT	TGGATTCGTGTCCATCTGAA	ACAATTGCGCAAATTCCTCT	60.0	610	INTERGENIC				
CaPOPII_5118	scaffold845	69320	TAAAAAA/TAAAAA	TGAGTGTGAGTCCGAATGGA	GAACCCCAACCTGAAAACAA	60.2	493	DRR	Ca_22414	R		RNA recognition motif domain
CaPOPII_5119	scaffold845	116005	TAAAAAAAAAAAA/TAAAAAAAAAAA	GGGATCATGCTCATTGCTTT	TTTTGACCAAAAAGTTTGCTG	60.0	691	INTERGENIC				
CaPOPII_5120	scaffold845	132751	CAAATAAAT/CAAATAAATAAAT	AGTGTTGTTTGGGTTGGGAG	TCGATTTGACATTTGCGTGT	59.9	526	INTERGENIC				
CaPOPII_5121	scaffold845	170397	GATATAATATATAATATATA/GATATAATATATA	ATTTGGTGCGCATGATAAGA	TTTACAAGGGTATTTTTGTGATTTAC	59.1	469	INTERGENIC				
CaPOPII_5122	scaffold845	170625	ATTT/ATTTT	ATTTGGTGCGCATGATAAGA	AAAAATGAGGTGGGTTAGAAATTA	59.1	777	INTERGENIC				
CaPOPII_5123	scaffold845	175162	AA/AATTA	AAAGGGCTCCTGCAGGTAAT	TGATTAATTTTGCAACGGCA	60.1	435	INTERGENIC				
CaPOPII_5124	scaffold845	281210	TCCCC/TCCC	TGCAAAGATCAATCTTGGGTT	GTGGGTTACGTCCAGTCCTC	59.6	806	INTERGENIC				
CaPOPII_5125	scaffold845	331956	TG/TGG	TTGCTTGGAAAGGAAAATCG	GGGGCCAAATAATGTTGCTA	60.2	802	INTERGENIC				
CaPOPII_5126	scaffold8493	9496	A/AT	CGACCATGATTTAACGACCA	GTTTTCAAGAGGTGGCCTTG	59.4	360	INTERGENIC				
CaPOPII_5127	scaffold8493	18046	ATTTT/ATTT	TGCACAGTCAATAGGCTTCG	TGCGGGAGACATTGATGATA	60.0	547	INTERGENIC				
CaPOPII_5128	scaffold855	30893	ATT/ATTT	AGGCCGTGATGTTTTTGAAC	GCATCATATGTGTGGGCAAG	60.0	268	INTERGENIC				
CaPOPII_5129	scaffold855	31194	ACCC/ACC	CTCGTACGCTTTTGGTGTGA	GTAGGGGGTAGAAAACCCCA	59.9	556	INTERGENIC				
CaPOPII_5130	scaffold855	31470	TTAATATCC/T	AATTGGATACCCCTTTTGGC	GTAGGGGGTAGAAAACCCCA	60.0	384	INTERGENIC				
CaPOPII_5131	scaffold8565	6875	GTTTTTTTT/GTTTTTTTTTT	TGACCAAAATCCATCCCTTC	TTTACAGCGCTTGTTCCAAA	59.7	517	INTERGENIC				
CaPOPII_5132	scaffold859	19948	A/ATAATTATAT	TTTAACTAGACTGCCTTATCGTGTTT	AACTGGAACTCAACAGGTATGGA	59.0	589	INTERGENIC				

INDEL marker IDs	Chromosomes /unanchored scaffolds	Physical positions (bp)	InDels (*Kabuli* reference genome-CDC Frontier/PI)	Forward primers (5'-3')	Reverse primers (5'-3')	Anneling temperature (0C)	Expected amplified product size (bp)	Structural annotation		Functional annotation		
								Sequence components of *kabuli* genome	*Kabuli* gene accession IDs	NCBI-KOG	TFs	NCBI-nr database
CaPOPII_5133	scaffold859	27190	CTGT/CTATTGT	TGGAGGTTAAACTCAAGCCG	ACTTTGCATCCTCTGCATCC	60.2	579	INTERGENIC				
CaPOPII_5134	scaffold864	247808	TGGGG/TGGGGG	TTCTGCCCTCATGGATTTTC	CATGGCTCGAGTGAAACAGA	60.0	349	CDS (FRAME SHIFT)	Ca_24213			
CaPOPII_5135	scaffold8646	2745	TAAAAAA/TAAAAAAA	AGTCTACGACGAAGCGTTCC	TCCATAGGAGGTCGATGGTC	59.5	532	INTERGENIC				
CaPOPII_5136	scaffold866	36277	ACCC/ACC	GAACAACAAGTGGGATGCCT	TGTGAAAAATGAACACCCCA	60.0	360	INTERGENIC				
CaPOPII_5137	scaffold866	142556	TAAAA/TAAAAA	GCAGCGGTTCCAAAATAGAG	GGTGGCATAGTATCACGCCT	59.8	571	INTERGENIC				
CaPOPII_5138	scaffold866	142956	CTT/CT	GGATCCCCAATCGATAACCT	CTGTTGAGCCTTAGGGCAAG	60.0	450	INTERGENIC				
CaPOPII_5139	scaffold869	26934	CAAAAAA/CAAAAAAA	GCACTGCCCCAAGAATTTTA	GTGGAGATCACTCGGGTTGT	60.1	209	INTERGENIC				
CaPOPII_5140	scaffold87	521197	TAAAAAAAAAAA/TAAAAAAAAAAAA	TCTTGTGCATGAATCCCAAA	CATACGTCGTGGGACTGTTG	60.0	528	INTERGENIC				
CaPOPII_5141	scaffold870	32135	CTTTTTTTT/CTTTTTTTTTTTTT	CATGAGGCCACCAACTTTTT	TCGTGGTTTTCTTGGGAGAC	60.0	407	INTERGENIC				
CaPOPII_5142	scaffold870	39934	TTTATTATTATTATTATTATTATTATTATTATTATTATTATTATTAT/TTTATTATTATTATTATTATTATTATTATTATTATTATTATTAT	AGAGGCAAACAAGAACCGAA	GGTGAGCCACCTTCATTTGT	59.9	517	INTERGENIC				
CaPOPII_5143	scaffold870	82445	GT/GTT	TGGTGGTGCAATTCACATCT	TTTCACCGTCATGCTTTTTG	60.0	540	INTERGENIC				
CaPOPII_5144	scaffold873	18275	GATATATATATATATATATAT/GATATATATATATATATATAT	TGTTGGGTCCACAAAGAGAG	TGTTGTTCTCCTCTGACACAATG	58.7	402	INTERGENIC				
CaPOPII_5145	scaffold873	18311	GAAAAAAA/GAAAAAA	TGTTGGGTCCACAAAGAGAG	TGTTGTTCTCCTCTGACACAATG	58.7	402	INTERGENIC				
CaPOPII_5146	scaffold8734	801	TAA/TAAA	TTGGGCGAAAATGGATAAAG	CCCCGCTCTGATAGGTGATA	59.9	802	INTERGENIC				
CaPOPII_5147	scaffold8734	1079	TAAA/TAAAA	CTGTGACACCCTAAATCCCA	GATCTATGAGGACAGGGCCA	58.4	798	INTERGENIC				
CaPOPII_5148	scaffold876	5429	TTTATTATTATTATTATTATTATTATTATTATT/TTTATTATTATTATTATTATTATTATTATTATT	AGAGGCAAACAAGAACCGAA	TACGGCATAACAATTGCAGC	59.9	566	INTERGENIC				
CaPOPII_5149	scaffold876	369643	A/AC	AACGTACTGCCTATCACGGG	GGTAAGGGCTCCTTCCAAAC	60.0	836	INTERGENIC				

INDEL marker IDs	Chromosomes /unanchored scaffolds	Physical positions (bp)	InDels (*Kabuli* reference genome-CDC Frontier/PI)	Forward primers (5'-3')	Reverse primers (5'-3')	Anneling temperature (0C)	Expected amplified product size (bp)	Structural annotation		Functional annotation		
								Sequence components of *kabuli* genome	*Kabuli* gene accession IDs	NCBI-KOG	TFs	NCBI-nr database
CaPOPII_5150	scaffold876	622726	TAA/TAAA	TCAGGAACAACAATCCAATCC	TTACCCTCAAAACTGGGGTG	59.8	331	INTERGENIC				
CaPOPII_5151	scaffold877	10844	TAAAAA/TAAAA	GCAGCAGTTGCAGCAGTTTA	TGATTTAGCGTCGGTGTCAG	60.3	601	DRR	Ca_28083	F		Orotidine 5'-phosphate decarboxylase domain
CaPOPII_5152	scaffold878	43697	AC/A	AACTGCGATTTCCCAAACAC	ATTGGGAGCTACCATGCAAC	60.0	489	INTERGENIC				
CaPOPII_5153	scaffold8789	619	TGTTGTCTTTTTTAGTTGT/TGTTGT	AGGGTTTAGGGTTTCTGGTTT	GATTGATGCACTAATTCGACG	58.1	417	INTERGENIC				
CaPOPII_5154	scaffold88	86970	TTATATATATATATATATATATATATATATAT/TTATATATATATATATATATATATATATAT	AGGAAGAGTGGAGTTGCGAA	TATTTTGGATTTTGGCTCGG	60.0	373	INTERGENIC				
CaPOPII_5155	scaffold882	70530	CAAAAAA/CAAAAA	CACATCAACAGCAGCCTTGT	TGATGTTGCCTCTGTTTTGC	59.9	385	INTERGENIC				
CaPOPII_5156	scaffold882	446893	GTTT/GTTTT	TGGTTGGTTTTTGTGGTGAA	TCACGGGCAAACACAATTTA	59.8	644	INTERGENIC				
CaPOPII_5157	scaffold8825	968	TAAAAA/TAAAAAA	AATCGATTGGCATCAACACA	CCAGTCAAGCATTCCCATCT	59.9	475	INTERGENIC				
CaPOPII_5158	scaffold8836	981	GAAATAAAAA/GAAA	CAACACAACAAGAACGAGCAA	TCCAAGTAGGTCTGCCCAAT	59.9	288	INTERGENIC				
CaPOPII_5159	scaffold886	151276	AG/AGCTG	AGGGATGCTTGTAGTGGTTGA	TTTGACGTGCATCCAATGTT	59.6	162	DRR	Ca_26671			
CaPOPII_5160	scaffold89	212189	ATTTTTTTT/ATTTTTTT	CAACCCAGCAGCCAAATAAT	GCATAGGTGCACATGAAAGG	60.0	377	INTERGENIC				
CaPOPII_5161	scaffold892	26717	ATT/ATTT	TCAAGCTTCCTCTCCACACA	AATTAATATCCACGCAACGC	59.5	255	INTERGENIC				
CaPOPII_5162	scaffold892	29801	CAAAAAA/CAAAAA	GAAGCCATGAAAACCTTCCA	ATGCAAACATTTCCCACCTC	60.1	392	INTERGENIC				
CaPOPII_5163	scaffold892	33546	TTTCTT/TTTCTTCTT	TCCCAATTTGGTTTTGGTGT	TGGAATCTTCACCCTGACAA	60.1	713	DRR	Ca_27455	R		Sugar/inositol transporter
CaPOPII_5164	scaffold892	33776	TC/T	TCAAAAGACACCAAATGGAAAA	TGGAATCTTCACCCTGACAA	59.5	251	DRR	Ca_27455	R		Sugar/inositol transporter
CaPOPII_5165	scaffold892	34457	T/TTTTATTATG	CATGCCTAACGCACGAATTA	CCGACATGGTCTTCTTCCTT	59.7	426	DRR	Ca_27455	R		Sugar/inositol transporter
CaPOPII_5166	scaffold892	42751	TGGGG/TGGGGG	GACGATGAAAAATATGCGGG	TGTTGATTGGCGAACACAAT	60.3	387	DRR	Ca_27456			

INDEL marker IDs	Chromosomes /unanchored scaffolds	Physical positions (bp)	InDels (*Kabuli* reference genome-CDC Frontier/PI)	Forward primers (5'-3')	Reverse primers (5'-3')	Anneling temperature (0C)	Expected amplified product size (bp)	Structural annotation		Functional annotation		
								Sequence components of *kabuli* genome	*Kabuli* gene accession IDs	NCBI-KOG	TFs	NCBI-nr database
CaPOPII_5167	scaffold892	44352	TAAAAAAAA/TAAAAAAA	CTGAGTTGCGACGAGAGTTG	TTGGAAGGAGCCCTTACCTT	59.8	465	INTERGENIC				
CaPOPII_5168	scaffold895	93605	GTTATTATTATTATTATTATTATTATTATTATTATT/GTTATTATTATTATTATTATTATTATTATTATT	AGAGGCAAACAAGAACCGAA	GAAGCCGAGAAAACGACTTG	59.9	399	INTERGENIC				
CaPOPII_5169	scaffold896	47144	TAAA/TAAAA	AGGGAGGACGAAAATGACCT	TGTGCATCAAATAGGCGTGT	59.9	259	INTERGENIC				
CaPOPII_5170	scaffold9	34748	CACAACAA/CACAA	GGACAAACTACCCCTCCTTTTC	TCTTCCCCCATAAACCAACA	60.2	317	INTRON	Ca_27384		WOX	
CaPOPII_5171	scaffold9059	539	ATT/ATTT	TTAGGGCTCATACGATTCGG	GGAAAATTAGCCACGGATCA	60.1	443	INTERGENIC				
CaPOPII_5172	scaffold909	6745	TA/TAAA	TTTCATCTGAACCTTTCGGG	TAGGGTGGTACGAAAATCGC	60.0	365	INTERGENIC				
CaPOPII_5173	scaffold913	140774	CAT/CATAT	TTGAGTTATGGCTAATGAAGGTCA	AGCGAGCCGCTCAATTAGTA	60.0	269	INTERGENIC				
CaPOPII_5174	scaffold913	216659	CTATATATATATATA/CTATATATATATA	TCAGTTCTCACCGGATACGC	AAATTGCAGGGATGTGAACC	61.2	328	INTERGENIC				
CaPOPII_5175	scaffold913	282492	ATTTT/ATT	TTGGATTTCATACCCACACATC	AAAATCCGTTGGAAATTTTGG	59.6	466	INTERGENIC				
CaPOPII_5176	scaffold916	3252	AATATA/AATA	AAAGCGCTGTAAAATGGGTG	TATAGGGTCAAAACGCCCAC	60.1	726	DRR	Ca_21034	G	E2F/DP	Major facilitator superfamily MFS-1
CaPOPII_5177	scaffold916	3317	TTTAATT/TTT	ACATCTCATGCCATTGTGGA	GAGACAGTCAGAAGGCCACC	59.9	520	DRR	Ca_21034	G	E2F/DP	Major facilitator superfamily MFS-1
CaPOPII_5178	scaffold916	3519	AATATATATATATATATATAT/AATATATATATATATATAT	ACATCTCATGCCATTGTGGA	GAGACAGTCAGAAGGCCACC	59.9	520	DRR	Ca_21034	G	E2F/DP	Major facilitator superfamily MFS-1
CaPOPII_5179	scaffold916	4344	GAAAAA/GAAAAAAAA	TGTTGACGAATGTCCATGAAA	CCTCCAAAACCCAACTACCA	60.0	181	INTERGENIC				
CaPOPII_5180	scaffold916	4805	T/TACAATA	TGGTAGTTGGGTTTTGGAGG	TTCATGACATGCATCCATCC	59.8	907	INTERGENIC				
CaPOPII_5181	scaffold916	4868	GTTTTTTTT/GTTTTTTTTTT	TGGTAGTTGGGTTTTGGAGG	TTCATGACATGCATCCATCC	59.8	907	INTERGENIC				
CaPOPII_5182	scaffold916	5303	A/AT	TGTCTCTTTTACGCCGCAAT	CTAGGCATTGCAACAGGGAT	60.8	738	INTERGENIC				
CaPOPII_5183	scaffold916	7870	TTGAAAAATGAAAA/TTGAAAA	TGTCCTTCTGTTCAATCAAATACC	TGGTGGTGCATAGTTGGTGT	59.4	636	INTERGENIC				

INDEL marker IDs	Chromosomes /unanchored scaffolds	Physical positions (bp)	InDels (*Kabuli* reference genome-CDC Frontier/PI)	Forward primers (5'-3')	Reverse primers (5'-3')	Anneling temperature (0C)	Expected amplified product size (bp)	Structural annotation		Functional annotation		
								Sequence components of *kabuli* genome	*Kabuli* gene accession IDs	NCBI-KOG	TFs	NCBI-nr database
CaPOPII_5184	scaffold916	8185	TT/TTCGATTAAT	ACACCAACTATGCACCACCA	ATAGGCCCCAATTAACCCAC	59.9	376	INTERGENIC				
CaPOPII_5185	scaffold916	8308	ATT/AT	ACACCAACTATGCACCACCA	ATAGGCCCCAATTAACCCAC	59.9	376	INTERGENIC				
CaPOPII_5186	scaffold916	9278	CTTT/CTTTT	TCACATAGGGATTGCCAACA	TCCCACCACATTCAATCCTT	59.9	769	INTERGENIC				
CaPOPII_5187	scaffold916	9949	AAG/A	AGGATTGAATGTGGTGGGAA	GCAGAAAAATAAGGGCGATG	60.2	354	INTERGENIC				
CaPOPII_5188	scaffold916	134889	TTT/TTATT	AAATGTGGTCTCGGTTTTGC	TTGTTTTGTGAGTTTGAATGATGTT	60.0	770	INTERGENIC				
CaPOPII_5189	scaffold916	363659	TAAAATTAAAAATTAAAAATTAAAAA/TAAAATTAAAAATTAAAAATTAAAAATTAAAAA	CCACCCTCTACCATGCAACT	TGTTTTCCATGTGTGCCACT	60.0	569	INTERGENIC				
CaPOPII_5190	scaffold916	470919	TTTATTATTATTATTATTATTATTATTATT/TTTATTATTATTATTATTATTATTATTATT	TGATTAACACCATGCCGTGT	TTACAAGCAGGGAAAATGCC	59.8	564	INTERGENIC				
CaPOPII_5191	scaffold916	499308	ATAAAT/ATAAATTATCTAAAT	CCGTACGTAGTAAGCCCCAA	ACGAACCCATTGTTGCATTT	60.0	855	INTERGENIC				
CaPOPII_5192	scaffold918	49291	CAAA/CAAAA	TAGCGAGGACGAAAATGACC	CGTCCTCCCTGGGTTTATTC	60.2	820	INTERGENIC				
CaPOPII_5193	scaffold919	83832	AAAAAAAATAAAAAAA/AAAAAAAATAAAAAAATAAAAAAA	GTTTGAAGTTGTCCGCGTCT	GACACTTGCGCCCCTATAAA	60.3	479	INTERGENIC				
CaPOPII_5194	scaffold919	183431	ATGTTGTT/ATGTT	TGGCAAATACCGATTCACAA	TGCTTCCATCCTTTATTGGC	59.9	559	INTERGENIC				
CaPOPII_5195	scaffold919	724282	GCCC/GCC	CCAAAAAGTGCAGAAAGGGA	TGGGATGCAGTTGAAAATGA	60.2	491	INTERGENIC				
CaPOPII_5196	scaffold92	45246	ATATTATTATTATTATTATT/ATATTATTATTATTATTATTATTATTATT	TGCAATAAAACCATAATACGAAGA	TGTTTGACTGCATAACGAAGA	57.9	438	INTERGENIC				
CaPOPII_5197	scaffold9236	1447	ACCC/ACC	GGAGGACGAAAATGACCAAA	TCCCTTGGCTTAATCGTGTC	59.9	307	INTERGENIC				
CaPOPII_5198	scaffold924	202228	TTATATATATATATAT/TTATATATATATAT	AGGAAGAGTGGAGTTGCGAA	CAGGTTTGATGCAACCATTG	60.0	689	INTERGENIC				
CaPOPII_5199	scaffold93	141155	CAA/CAAA	TCGATTGGCTTAAAACCAGG	AGAATTTACGAGCAGCGGAA	60.1	617	INTERGENIC				
CaPOPII_5200	scaffold93	338023	TCCCC/TCCC	AAACTTGCAAGCCATTCCAG	TTTGAGAAATATGCTGGGGC	60.2	876	INTRON	Ca_23073	L	GRAS	

INDEL marker IDs	Chromosomes /unanchored scaffolds	Physical positions (bp)	InDels (*Kabuli* reference genome-CDC Frontier/PI)	Forward primers (5'-3')	Reverse primers (5'-3')	Anneling temperature (0C)	Expected amplified product size (bp)	Structural annotation		Functional annotation		
								Sequence components of *kabuli* genome	*Kabuli* gene accession IDs	NCBI-KOG	TFs	NCBI-nr database
CaPOPII_5201	scaffold93	573293	AATAT/AAT	AATTTAGCCCGATTTTGCCT	CCTAAACGGGCTAGAGGTCC	59.9	711	INTRON	Ca_23083			
CaPOPII_5202	scaffold93	662816	TC/T	AACGAGTGGAAGAAAAGAGCA	GCGCTGTAAAATGTGCAAAA	59.1	695	INTERGENIC				
CaPOPII_5203	scaffold9303	1186	CT/CTT	AAGACCACATCATTGTGTCACC	TGTTTGTTCGCGTCTTAATTTT	59.8	439	INTERGENIC				
CaPOPII_5204	scaffold931	100930	A/AC	TTTGAGCCCCCTTCAATATG	GATTCTCCTTGGGGATGGAT	59.9	616	INTERGENIC				
CaPOPII_5205	scaffold931	116282	GTTTTTTTTTTTTTTT/GTTTTTTTTTTTT	TCAGTTGCAATTTCCTTCCC	TAAAACCGATTTGGGAGTCG	60.1	519	INTERGENIC				
CaPOPII_5206	scaffold931	116416	ATTT/ATTTCCCTAGATGCTCTTT	CGACTCCCAAATCGGTTTTA	TTGGGCTGATGCAATAAACA	59.9	260	INTERGENIC				
CaPOPII_5207	scaffold931	116537	ATT/ATTT	CGACTCCCAAATCGGTTTTA	TTGGGCTGATGCAATAAACA	59.9	260	INTERGENIC				
CaPOPII_5208	scaffold931	117076	CAAA/CAA	TGCCACAACCAATCTTCATC	AGACAGTGGGTGTTCCAAGC	59.5	391	INTERGENIC				
CaPOPII_5209	scaffold931	118275	GAAAA/GAAAAA	TGCTCTGACTCATTCAACCG	CACCAACAATTGGCATCAAG	60.0	725	INTERGENIC				
CaPOPII_5210	scaffold931	180349	GATATATATATATATATA/GATATATATATATATA	TCGTCCCCATGTAATTGTCA	TGGATACCCAAAAACCATTCA	59.8	281	INTERGENIC				
CaPOPII_5211	scaffold931	180540	T/TG	TCGTCCCCATGTAATTGTCA	CACTCATTGCTTGTAAGAGTGCT	59.8	486	INTERGENIC				
CaPOPII_5212	scaffold931	181299	G/GTT	GTAGAGTGCCAACGACACCA	TGAACTATTGATTGGATCGTGC	59.8	665	INTERGENIC				
CaPOPII_5213	scaffold931	246666	TTATATATATATATATATATATAT/TTATATATATATATATATATAT	GGGTGTCACATCACCCTTTT	GCGGAAGACTTTATAGTGAAAGGA	59.7	191	INTERGENIC				
CaPOPII_5214	scaffold933	2760	GTCGCATATATGTGTCTCGCGTATATGTGTCTC/GTCGCGTATATGTGTCTC	CGTGGTGGTTTGATGTTGAG	GGCGCTAGCAACTCTATTGG	60.0	615	INTERGENIC				
CaPOPII_5215	scaffold933	4208	GTTTTTTTTT/GTTTTTTTTTTT	TCAAACCCAGTGGTCTCCTT	AGTCCGATGAAGGGTCACAG	59.5	654	INTERGENIC				
CaPOPII_5216	scaffold9371	631	TTTTT/TTTTTGTTTT	GCGGCTGAAAGAGAGAGAGA	AAAGATTTCCGGTACACACCA	60.0	687	INTERGENIC				
CaPOPII_5217	scaffold939	1093	ATTT/ATT	GTGACGTTGGGGGAGTTAAA	TCGAGCCTTATGGCAAGATT	59.8	317	INTERGENIC				

INDEL marker IDs	Chromosomes /unanchored scaffolds	Physical positions (bp)	InDels (*Kabuli* reference genome-CDC Frontier/PI)	Forward primers (5'-3')	Reverse primers (5'-3')	Anneling temperature (0C)	Expected amplified product size (bp)	Structural annotation		Functional annotation		
								Sequence components of *kabuli* genome	*Kabuli* gene accession IDs	NCBI-KOG	TFs	NCBI-nr database
CaPOPII_5218	scaffold9411	2892	TTAACT/TT	GGATATGTCAGAAAAGGAACGG	GAAGCATCTCCAACAGCTCA	59.8	393	INTERGENIC				
CaPOPII_5219	scaffold9411	3721	TAAAAA/TAAAA	TGAAGTGTTGCTCTTCGCAC	TTTTTCCCATTTCGGTCTATTT	60.2	536	INTERGENIC				
CaPOPII_5220	scaffold9411	3911	AATA/AATATATA	TGAAGTGTTGCTCTTCGCAC	GAAAGCCCATTTTACCCCAT	60.2	840	INTERGENIC				
CaPOPII_5221	scaffold943	267616	T/TTACC	CAAACACTCAAATGGGCAAA	GCTTTCCACAAACCGTGATT	59.6	151	INTERGENIC				
CaPOPII_5222	scaffold944	672	ATTTT/ATTT	TTGTAAGTTGAGTTGCGACGA	TGTGGTCGCAGGTTTTATGA	59.5	308	INTERGENIC				
CaPOPII_5223	scaffold944	2389	CAAAAAAA/CAAAAAA	GAACGGGAAATATTCACGTCAT	TCGGACCACACTCCTAATCC	60.1	495	INTERGENIC				
CaPOPII_5224	scaffold944	2557	TG/T	GGATTAGGAGTGTGGTCCGA	CAACTGGGTACCACTAAGCCA	59.9	450	INTERGENIC				
CaPOPII_5225	scaffold944	3122	GAAAAAAAAA/GAAAAAAAA	TGGCTTAGTGGTACCCAGTTG	TGGCGCTTATATTCAAAGCA	60.0	643	INTERGENIC				
CaPOPII_5226	scaffold944	3226	GTTTTTTT/GTTTTTTTT	TGGCTTAGTGGTACCCAGTTG	TGGCGCTTATATTCAAAGCA	60.0	643	INTERGENIC				
CaPOPII_5227	scaffold9444	3467	AG/A	TCCACAACGCAACCAAATTA	AGCAGGTCTGTCCAATCAGG	60.0	228	INTERGENIC				
CaPOPII_5228	scaffold949	3822	CAAAAAA/CAAAAAAA	AAGAGGGGCGTGAACTTTTT	GCTTCCATACCTGTTTTGGG	60.1	646	CDS (FRAME SHIFT)	Ca_25481			Photosystem antenna protein-like
CaPOPII_5229	scaffold950	3787	TG/T	AGGGAGGACGAAAATGACCT	TCCTCTCGAAGCCTATCCAA	59.9	707	INTERGENIC				
CaPOPII_5230	scaffold953	7421	A/AG	TTCCTCGGTTTAATTTTGGG	GCTGAAACCAGATGCAAACA	58.9	955	INTERGENIC				
CaPOPII_5231	scaffold953	79145	TGTN/T	CAGCACGATTTACCAAAGCA	GAATTTAATGCGTTGCGGAT	59.9	531	INTERGENIC				
CaPOPII_5232	scaffold953	123729	TA/TAAATTGAGGAACCAAA	GAGCCACCGACTTGAAGAAG	TTGTGTAGGTGGGCTTTTCC	60.0	542	INTERGENIC				
CaPOPII_5233	scaffold953	141896	ATTTTTTTTTTT/ATTTTTTTTT	TCGTTTTGCCATGTGTGTTT	GCGTGGGAAAAGGGAACTTA	60.0	594	INTERGENIC				
CaPOPII_5234	scaffold953	141960	GATATATATAT/GATATATATATATATAT	TCGTTTTGCCATGTGTGTTT	GCGTGGGAAAAGGGAACTTA	60.0	594	INTERGENIC				

INDEL marker IDs	Chromosomes /unanchored scaffolds	Physical positions (bp)	InDels (*Kabuli* reference genome-CDC Frontier/PI)	Forward primers (5'-3')	Reverse primers (5'-3')	Anneling temperature (0C)	Expected amplified product size (bp)	Structural annotation		Functional annotation		
								Sequence components of *kabuli* genome	*Kabuli* gene accession IDs	NCBI-KOG	TFs	NCBI-nr database
CaPOPII_5235	scaffold956	19709	G/GCTTCTT	TCCAGATGAAATCGAGGACC	GGATTAGCCTTTCGAGTCCC	60.0	381	INTERGENIC				
CaPOPII_5236	scaffold958	3567	AATATATATATATATATATATAT/AATATATATATATATATAT	GATGATGATGATGTCGTGCC	GAATGAAATCACAGACCGGC	59.9	328	INTERGENIC				
CaPOPII_5237	scaffold959	11831	TG/T	CAAGCGTTTCCATCTCCATT	GTGGTAATCGCGTTGAAGGT	60.1	817	INTERGENIC				
CaPOPII_5238	scaffold959	57749	GAAAAAAAAAA/GAAAAAAAA	AAGGCGTGCATATCCCATAC	TGTTCGTCAAAATGTCCCAA	59.8	553	INTRON	Ca_27234		Trihelix	Peptidase C48, SUMO/Sentrin/Ubl1
CaPOPII_5239	scaffold96	316614	A/AT	TCGACTATGAGTGATTACGTGGTT	CAATCAAGGTACAATGAGTCAAACA	60.0	426	INTERGENIC				
CaPOPII_5240	scaffold962	36020	AATATATATATATATTTATATATATAT/AATATATATATATATATATATATATTTATATATATAT	TGCACGTCTTGAGCAAAAAC	GTGCAGTCTCTCACGGACAA	60.0	859	INTRON	Ca_26498		Trihelix	Peptidase C48, SUMO/Sentrin/Ubl1
CaPOPII_5241	scaffold962	231201	AATATATATATATATATATATATAT/AATATATATATATATATATATATATATAT	ATGCGTTATGGGTGAAATGG	GTTGGTTCCCTAGCACCGTA	60.6	477	INTERGENIC				
CaPOPII_5242	scaffold963	6111	TAAA/TAA	GCGGGGAAGAACATTCATAA	GCAATTTTGTCAACGCACTG	59.9	400	INTERGENIC				
CaPOPII_5243	scaffold963	17319	AATATATATATATATATATATATATATATATATATATAT/AATATATATATATATATATATATATATAT	CCGGGGATAATGGCTTAAAT	GGGGTATGCATTTGTTGGAC	60.0	429	INTERGENIC				
CaPOPII_5244	scaffold972	18681	GTTTTT/GTTTTTT	CCTGAAGCCTGCACACATAA	ACCCGGGTTACGTCTAGTCC	59.9	695	INTERGENIC				
CaPOPII_5245	scaffold9740	598	TTATGATCTATGATCT/TTATGATCT	GGAGACACCTGTGGAGGGTA	TTTCCCAGTCGTTTGGTTTC	60.0	704	INTERGENIC				
CaPOPII_5246	scaffold9757	838	TAAAA/TAAAAA	TGTTAATCCAACGGCACAAA	TCCCCTGGCTTATTGTTGTC	60.0	279	INTERGENIC				
CaPOPII_5247	scaffold9784	548	A/AATTAG	CCGGTGCATCCAATTAGTTC	CACCCGTTGACACAAATGAA	60.3	632	INTERGENIC				
CaPOPII_5248	scaffold981	6691	ATAAACCCTAAACCCTAAACCCTAAACCCTAAACC/ATAAACCCTAAACCCTAAACCCTAAACC	CCCGAAACGATTAACCTGAA	TTTAGGGTGTCGGGATTCAA	59.9	567	INTERGENIC				
CaPOPII_5249	scaffold9813	1199	GAAA/GAAAA	AAAATCGCGCGAAAAGTCTA	TCCTGGTCCTCGATTTCATC	60.0	772	INTERGENIC				
CaPOPII_5250	scaffold982	766	GTTTTTT/GTTTTTTT	TCCTTGTCATTTTCCTGCAA	TATTGTGGCACTCCATCCAA	59.2	416	INTERGENIC				
CaPOPII_5251	scaffold982	874	ATT/ATTT	TTGGATGGAGTGCCACAATA	AACTTGGGGTTGAAGATGAAGA	59.9	164	INTERGENIC				

INDEL marker IDs	Chromosomes /unanchored scaffolds	Physical positions (bp)	InDels (*Kabuli* reference genome-CDC Frontier/PI)	Forward primers (5'-3')	Reverse primers (5'-3')	Anneling temperature (0C)	Expected amplified product size (bp)	Structural annotation		Functional annotation		
								Sequence components of *kabuli* genome	*Kabuli* gene accession IDs	NCBI-KOG	TFs	NCBI-nr database
CaPOPII_5252	scaffold982	1609	A/AAT	TAAAGGACCAAGTCGGGAAA	TTCACTTTTGAGAGTGTATCTTTGG	59.5	108	INTERGENIC				
CaPOPII_5253	scaffold982	5778	AT/A	GCAAAGCATTCAAATGCAGA	TTCATCTTCCTCCAATTCAAAAA	60.0	755	INTERGENIC				
CaPOPII_5254	scaffold983	88220	T/TG	CACCCACAAGGAGGAAGAAC	CATGGATCAAACAGGCTTCA	59.5	517	INTERGENIC				
CaPOPII_5255	scaffold983	88456	AGTG/AG	TTTATTCGCATGTTGTTGCC	CATGGATCAAACAGGCTTCA	59.6	274	INTERGENIC				
CaPOPII_5256	scaffold983	141925	T/TCATAAAGG	AGGTGTGACAACAAGATGCCT	CGTGACAATTACATGGGGACT	59.6	851	INTERGENIC				
CaPOPII_5257	scaffold983	255175	GTTTT/GTTT	TTACCGGATTTACTCACGGC	ACCCGTCAAATACGACGAAC	60.0	319	INTERGENIC				
CaPOPII_5258	scaffold983	287686	A/ATT	TTGCTCTGATTTTCATTGCG	AATTGTGCCAGTCGATTGCT	60.0	642	INTERGENIC				
CaPOPII_5259	scaffold99	81654	ATTTT/ATTT	CAGGGGCTGAGATATGCTGT	CTCTCATCTGAGGGCAAAGC	60.2	395	INTERGENIC				
CaPOPII_5260	scaffold992	40484	CA/CACCGAAA	TCAATGCCAAAACATGCAAT	TTCGGATGTCCCCTTAAACA	59.9	513	INTERGENIC				
CaPOPII_5261	scaffold992	42301	CTCT/CTTTCT	GGGAGCATGAAATTGAACAAA	TCTTTTGCGTCTGAATTTGG	59.9	708	INTERGENIC				
CaPOPII_5262	scaffold999	130386	TG/TGG	TGTAAACCTGCTTGCACACC	CTGCACAACAATCCCACAAC	59.8	160	INTERGENIC				
