# Supplementary material for: A High-Resolution InDel (Insertion–Deletion) Markers-Anchored Consensus Genetic Map Identifies Major QTLs Governing Pod Number and Seed Yield in Chickpea
Source: Front Plant Sci. 2016 Sep 16;7:1362. doi: 10.3389/fpls.2016.01362 (PMC5025440; doi:10.3389/fpls.2016.01362)
Supplement: Supplementary file 7 [file Table7.PDF]

Table S7. InDel markers (2049) polymorphic between found common between two mapping populations (Pusa 1103 x ILWC 46) and (Pusa 256 x ILWC 46) with reference to *kabuli* (CDC Frontier) genome

INDEL marker IDs	Chromosomes/ unanchored scaffolds	Physical positions (bp)	Forward primers (5'-3')	Reverse primers (5'-3')	Anneling temperature (0C)	Expected amplified product size (bp)	Structural annotation		Functional annotation	
							Sequence components of *kabuli* genome	*Kabuli* gene accession IDs	NCBI-KOG	TFs
CaPOPI_II_248	Ca2	35944964	TCCAACCATACCTGAAAGGC	CTCGTGCGAACTTGTGTGTT	59.93	313	CDS (large-effect mutations)	Ca_09769		
CaPOPI_II_592	Ca5	24097297	CAGAGCCAACACTACAGGCA	ATTTGAAGGGCCATGTGAAG	60.05	329	CDS (large-effect mutations)	Ca_17656	S	
CaPOPI_II_1490	scaffold3015	68656	TTCGCAGACAAAACTCATGC	AGCATTATGGGCCATCTGAG	60.00	283	CDS (large-effect mutations)	Ca_27318		
CaPOPI_II_470	Ca4	30538166	TTGTTCTTGCATTTCCATTTTG	CGCCATCTTCCTCTTCTTTG	59.98	380	CDS (large-effect mutations)	Ca_14204		NAC
CaPOPI_II_1445	scaffold2763	9760	ATGCCAGTCGTTATTCCCAG	GAGATGCCATTGAAGCCATT	59.96	400	CDS (large-effect mutations)	Ca_24393	R	
CaPOPI_II_275	Ca3	6838667	GGAACCCCACAATCATCATC	GCAGCCAATTTTGTTGCATA	60.00	547	CDS (large-effect mutations)	Ca_16550	R	bHLH
CaPOPI_II_998	Ca8	15577896	TTTTCAGCAAACCCCAAAAG	TAGAGGCTTGTCATTCGGCT	60.08	665	CDS (large-effect mutations)	Ca_17886	T	EIL
CaPOPI_II_1031	scaffold1067	203101	TGAAACTGGGAGGAGGAAGA	CAGTGCGATGGAGAAATCAA	59.77	418	DRR	Ca_24425		
CaPOPI_II_1065	scaffold1180	36749	TACGGTTCGATCTCATGCAA	TGCCTCTTCCCCTGTTTCTA	60.22	419	DRR	Ca_25737		G2-like
CaPOPI_II_1104	scaffold1309	58470	TGGGTAACGTAACCTACGGC	ATCCGGTAAAAATCTCCGCT	59.88	951	DRR	Ca_26763		
CaPOPI_II_1112	scaffold1316	13036	ACATGCACAGAGAATGCCAA	GTGCCAGTCGATTGCCTAAT	60.27	631	DRR	Ca_28021		
CaPOPI_II_1136	scaffold1335	46272	TTCGTGCAACTGACGCTAAC	CCATTTGGATTTAAAAGAAGCC	60.06	345	DRR	Ca_28106	S	
CaPOPI_II_1139	scaffold1359	74591	TGGGTGGGAGTAATTTTTCG	TTAAAGAGATTCGCTGCGGT	59.80	297	DRR	Ca_26683	KR	G2-like
CaPOPI_II_1166	scaffold1448	99427	CCAGGAAATGACCCCCTATT	TTTATTTTAGCGCGGTGTCC	60.01	774	DRR	Ca_26688		
CaPOPI_II_1174	scaffold1467	11911	TCGCAGCTGTGATGCTAAGT	ATGGGAGGTCTTGTGCAGTT	59.77	359	DRR	Ca_26807	Q	GeBP

INDEL marker IDs	Chromosomes/ unanchored scaffolds	Physical positions (bp)	Forward primers (5'-3')	Reverse primers (5'-3')	Anneling temperature (0C)	Expected amplified product size (bp)	Structural annotation		Functional annotation	
							Sequence components of *kabuli* genome	*Kabuli* gene accession IDs	NCBI-KOG	TFs
CaPOPI_II_1189	scaffold1521	10780	TCATGTGAACAATGCAACCA	CCACAGCTTGTCTGTCGGTA	59.52	296	DRR	Ca_26977		bHLH
CaPOPI_II_1260	scaffold1708	68435	GAAAAAGCGCTGTAAATGGC	CATTCGAGACGTGAGCAAAA	59.86	510	DRR	Ca_27642		
CaPOPI_II_1264	scaffold1731	49034	ATGGGAAGCAACAACGATTC	CCGTACCGTTTCTGGGATAA	59.94	784	DRR	Ca_27041	IR	
CaPOPI_II_1267	scaffold1731	85329	TCTCCGCATAAGCTTTCGAT	GCTTCGACACTGTAGCACCTC	59.95	478	DRR	Ca_27043		
CaPOPI_II_1268	scaffold1731	86422	TTTTGGGAATTCCTGCTCTG	GTGACACCCTAAACCCCAAA	60.18	299	DRR	Ca_27043		
CaPOPI_II_1288	scaffold1844	167253	CCCCAATTTGCGATCAATAA	TCAAAACTAAGAAACTCGCCAA	60.64	694	DRR	Ca_24145		
CaPOPI_II_129	Ca1	46531041	GCGAAAATAATGTTGATGCAGA	GTCTGCGGATAATTTCGCTT	60.10	139	DRR	Ca_21551	M	
CaPOPI_II_13	Ca1	2885377	GCCATTGTTAACTCCGCAAT	CATTTGGAGAGGGCATAGGA	59.97	360	DRR	Ca_00351	T	M-type
CaPOPI_II_1307	scaffold1959	47247	TCCTCCTCAAGTTGGAGCAT	ATATTGCATTGCAGTGGTGG	59.80	458	DRR	Ca_26462	K	G2-like
CaPOPI_II_1310	scaffold1964	11011	ATGTTCCTCGCCAATCATTC	TTCCAAAAGAGGAATGGTGC	59.90	771	DRR	Ca_25209	G	
CaPOPI_II_1316	scaffold1981	317439	TCTCATTTGCTCCCGACTCT	TTGTCTCAACAATCTCCCCC	59.95	501	DRR	Ca_21265		
CaPOPI_II_1324	scaffold2010	3634	AATCGATTGATTCAGCCCAG	TGATTTCCAACGGTCATCAA	60.04	869	DRR	Ca_27976		
CaPOPI_II_134	Ca2	753916	TTGCCCATTAACTTTACCCG	ACTTGTGCATTTGCATTAGTTC	59.83	555	DRR	Ca_17000		ARF
CaPOPI_II_1344	scaffold2033	183883	CTTGGGGTGGCAAGTACAGT	TCGAATACATGAATTAACAAAAGGA	60.03	371	DRR	Ca_26143		bHLH
CaPOPI_II_1391	scaffold2368	53512	AACCCTGGAGATGGGAAAGT	CTAGCCATTAGCTTCGACCG	59.80	607	DRR	Ca_26720	P	
CaPOPI_II_1392	scaffold2368	54204	CATCGGTCAAACACCATCTG	GCTTTGCAGAACATGCGTAA	59.96	490	DRR	Ca_26720	P	

INDEL marker IDs	Chromosomes/ unanchored scaffolds	Physical positions (bp)	Forward primers (5'-3')	Reverse primers (5'-3')	Anneling temperature (0C)	Expected amplified product size (bp)	Structural annotation		Functional annotation	
							Sequence components of *kabuli* genome	*Kabuli* gene accession IDs	NCBI-KOG	TFs
CaPOPI_II_142	Ca2	3901844	GTCGCTATCTCCCCAGTGAA	CATTCTGTGCCGCTATCTGA	60.22	248	DRR	Ca_10466	GT	
CaPOPI_II_1426	scaffold2617	320997	TGGGGTATTCCTTTACGTCTT	TGAAAGAGGGGAAAAGGAGC	57.62	296	DRR	Ca_21883		WRKY
CaPOPI_II_1427	scaffold2617	321825	TTGGTCATGCGCACATAATA	GATGGCTGTGTCGGAGACTT	58.58	529	DRR	Ca_21883		WRKY
CaPOPI_II_1438	scaffold271	205973	GCACCGTGAGTAAATTCGGT	TATTTTGGATTTTGGCTCGG	60.00	692	DRR	Ca_25334		
CaPOPI_II_144	Ca2	4812881	AACCAGTTCCGTTCCAAGTG	TCCCCCAAACAGAATTTGAG	60.01	731	DRR	Ca_21090		MYB
CaPOPI_II_1448	scaffold277	205504	GGTTTAGGTGTGGGAGTGGA	TGATACAAGGTTCAACCGCA	59.82	444	DRR	Ca_25752		
CaPOPI_II_1485	scaffold296	261904	CCATACACATGAGAGTTGCGA	TTGACTCAGCCTTAAATTTTTATTGA	59.73	534	DRR	Ca_20542		
CaPOPI_II_1486	scaffold296	262823	CACTTTTTAACCGGAGGAATG	GCAGTGGATAAGGCCGATAA	58.62	140	DRR	Ca_20542		
CaPOPI_II_1491	scaffold3015	69170	GGCCCATAATGCTCAAGTGT	TCATAGGCATGGGATTTGAA	59.96	472	DRR	Ca_27318		
CaPOPI_II_1498	scaffold303	162542	GCCATTTACAGCGCTTTTTC	TGAAATTATGGGTGGGCATT	59.86	357	DRR	Ca_26661		Trihelix
CaPOPI_II_1505	scaffold311	131360	GAAGCTCAGATGGCACACAA	CAATCTCCCCCTTGAGTTGA	59.99	716	DRR	Ca_26332		
CaPOPI_II_1511	scaffold3134	12154	TAAACGAGGTAAACCGGTGC	GAAAGTCGCATGTGCAACAC	60.00	437	DRR	Ca_27104	O	C2H2
CaPOPI_II_1573	scaffold362	166434	CCCGGTATTGTTTGTGCTCT	CACATCAGCCACATTTGACC	59.99	774	DRR	Ca_24991	C	
CaPOPI_II_1585	scaffold374	205813	AGCTGTTTCTGCTAGGCTGG	TTTATTTACGCGAATGCCCT	59.78	941	DRR	Ca_26214	O	
CaPOPI_II_1638	scaffold414	21975	TTCCTATTGCTCACCCCTTG	GAAAAACAAGACCCGCGATA	60.07	516	DRR	Ca_27849		
CaPOPI_II_1654	scaffold433	165941	AAAATTGAAACATCCGACGC	ACTTGTCGCTGGACGAATCT	59.95	512	DRR	Ca_26821		

INDEL marker IDs	Chromosomes/ unanchored scaffolds	Physical positions (bp)	Forward primers (5'-3')	Reverse primers (5'-3')	Anneling temperature (0C)	Expected amplified product size (bp)	Structural annotation		Functional annotation	
							Sequence components of *kabuli* genome	*Kabuli* gene accession IDs	NCBI-KOG	TFs
CaPOPI_II_1666	scaffold4511	22953	CTCCCCCTGAGTTTTGGAAT	GACGACAATCACCCGAAGAT	60.30	753	DRR	Ca_28219		Trihelix
CaPOPI_II_1739	scaffold543	229568	TTTGCCCCCAACTCTACATC	ATGGGCACATGCTATCATCA	59.93	755	DRR	Ca_24786		
CaPOPI_II_1740	scaffold544	147706	CCCCCTTTTTGCATAAGTGA	GAGGAGACATCTGCTGAGGG	59.93	582	DRR	Ca_26540		
CaPOPI_II_1745	scaffold548	212010	GGAACACTTCGGTGTGGACT	CATGTCCCGAAAAGAGCAAT	60.01	563	DRR	Ca_21489		
CaPOPI_II_1752	scaffold561	25431	TTGATTCATCAAACACCGGA	AAATTGGGTTCTGCCAATCA	59.90	399	DRR	Ca_26215		MYB_related
CaPOPI_II_1757	scaffold575	65125	TTGAAGTTAAGGGGTGCTGC	TCCATCCAACCTTGTGGATT	60.25	531	DRR	Ca_27003		
CaPOPI_II_1781	scaffold587	18087	GTCCTTGCGCTTATGCTTGT	TGCTTGATTGCTTGAGATCC	60.42	347	DRR	Ca_27621		
CaPOPI_II_1791	scaffold593	209468	GCAAACCTTTGCAAAAAGAAA	GGAATAAAATATGCATCCGAGC	59.39	153	DRR	Ca_26412		C2H2
CaPOPI_II_1792	scaffold593	209698	GCAAACCTTTGCAAAAAGAAA	GGAATGGAAATAATTGAAAAGTTTG	59.39	847	DRR	Ca_26412		C2H2
CaPOPI_II_1806	scaffold599	373801	CCCCCTAAGTTCATTCCACA	GGCTTGTCCGTCGGTAAATA	59.78	567	DRR	Ca_25347		
CaPOPI_II_1834	scaffold647	200535	CAAGCTGTCGGTTGAAGACA	AACAATCTCCCCCTTTTTGG	60.03	603	DRR	Ca_27495		
CaPOPI_II_1856	scaffold682	239323	CAACTCCCCCTGCAAACTTA	TGCGTGACAGGAACAAGAAG	60.10	417	DRR	Ca_23188	R	ERF
CaPOPI_II_1885	scaffold724	100088	ACAGGAGGGTGATCGAGAGA	TGCATACTTGTGTGGCATTTT	59.79	727	DRR	Ca_25051	Q	NAC
CaPOPI_II_1886	scaffold724	100193	CTGTAAGCGGAGAAAATTGG	TTGCATACTTGTGTGGCATTT	57.52	319	DRR	Ca_25051	Q	NAC
CaPOPI_II_1887	scaffold724	100328	CTGTAAGCGGAGAAAATTGG	AATGGTAGTCCCTCTGGCCT	57.52	690	DRR	Ca_25051	Q	NAC
CaPOPI_II_1888	scaffold724	100591	AAATGCCACACAAGTATGCAA	AATGGTAGTCCCTCTGGCCT	59.10	392	DRR	Ca_25051	Q	NAC

INDEL marker IDs	Chromosomes/ unanchored scaffolds	Physical positions (bp)	Forward primers (5'-3')	Reverse primers (5'-3')	Anneling temperature (0C)	Expected amplified product size (bp)	Structural annotation		Functional annotation	
							Sequence components of *kabuli* genome	*Kabuli* gene accession IDs	NCBI-KOG	TFs
CaPOPI_II_1895	scaffold731	94332	GAGCGCTGTAAAAGACCTCC	TGAAGATGGTTTGCAATGGA	59.08	188	DRR	Ca_23810		B3
CaPOPI_II_1896	scaffold731	315903	AACTCGAAGTGCCATGCTTT	CAATACGCAATGGTGGTGAG	59.88	660	DRR	Ca_23814	J	
CaPOPI_II_1900	scaffold731	361613	TGTGCTATTGCTCAAATCCG	AGCAGTCGTTTCCAGCTGTT	59.83	640	DRR	Ca_23818		
CaPOPI_II_1905	scaffold7330	2275	TTGGTCGACAAGCAACTGAG	AAAATCCAACAATCTCCCCC	60.03	699	DRR	Ca_28207		
CaPOPI_II_1918	scaffold750	360259	TGTTTGTCTTGCTGCTTTGC	AAAATCCAACAATCTCCCCC	60.18	626	DRR	Ca_25944		
CaPOPI_II_1934	scaffold775	449733	CTCTCTTCTGCGCATCAACT	TTGACCATGACACATGCCTT	58.34	459	DRR	Ca_23414		NAC
CaPOPI_II_1943	scaffold7923	2123	TCGGGGTCTTTAGTCGTCAT	GCGAGGAAGCAAAAGCTAGA	59.55	796	DRR	Ca_27506	O	
CaPOPI_II_1944	scaffold7923	22242	TTTCCCGACTTGGTCCTTTA	TTTGCGTTACGAATTTGATGA	59.54	177	DRR	Ca_27507	QI	bHLH
CaPOPI_II_1985	scaffold877	10844	GCAGCAGTTGCAGCAGTTTA	TGATTTAGCGTCGGTGTCAG	60.35	601	DRR	Ca_28083	F	
CaPOPI_II_1990	scaffold886	151276	AGGGATGCTTGTAGTGGTTGA	TTTGACGTGCATCCAATGTT	59.61	162	DRR	Ca_26671		
CaPOPI_II_1993	scaffold892	33546	TCCCAATTTGGTTTTGGTGT	TGGAATCTTCACCCTGACAA	60.07	713	DRR	Ca_27455	R	
CaPOPI_II_1994	scaffold892	33776	TCAAAAGACACCAAATGGAAAA	TGGAATCTTCACCCTGACAA	59.47	251	DRR	Ca_27455	R	
CaPOPI_II_1995	scaffold892	42751	GACGATGAAAAATATGCGGG	TGTTGATTGGCGAACACAAT	60.29	387	DRR	Ca_27456		
CaPOPI_II_20	Ca1	6205233	GGGTATTGGATAAAAATTTCAAAAA	TGAAAATTAAGGGACGGGTTT	58.74	604	DRR	Ca_07880	T	
CaPOPI_II_2002	scaffold916	3519	ACATCTCATGCCATTGTGGA	GAGACAGTCAGAAGGCCACC	59.93	520	DRR	Ca_21034	G	E2F/DP
CaPOPI_II_207	Ca2	30369374	TGTACTTCCTCCGATTCCAAA	TGAATGACGACGACGGTAAA	59.56	632	DRR	Ca_12515	Q	

INDEL marker IDs	Chromosomes/ unanchored scaffolds	Physical positions (bp)	Forward primers (5'-3')	Reverse primers (5'-3')	Anneling temperature (0C)	Expected amplified product size (bp)	Structural annotation		Functional annotation	
							Sequence components of *kabuli* genome	*Kabuli* gene accession IDs	NCBI-KOG	TFs
CaPOPI_II_210	Ca2	30449164	TGTTGCAAAGAGAAAGAACTTCA	TGACGGTGGCAATCGATATT	59.19	557	DRR	Ca_12507		
CaPOPI_II_220	Ca2	32173774	ATGTTTTGATTGGGGCTTTG	TAAATCAGCCCTTTTGGTGG	59.80	631	DRR	Ca_17806		WRKY
CaPOPI_II_273	Ca3	6772905	TCCCTTTGTCTTTTCGCACT	CACTGCCGTTCTTCACTTCA	59.85	383	DRR	Ca_16559	R	NAC
CaPOPI_II_274	Ca3	6774064	TTTGCTGGACTTTGAAGCCT	CCCGGTCCTAGAGAGAAAGA	59.99	640	DRR	Ca_16559	R	NAC
CaPOPI_II_283	Ca3	10398321	CGGATTGCCAAGTACAAGGT	CAAACCACAACCAGCACAAC	59.99	770	DRR	Ca_18819	J	
CaPOPI_II_296	Ca3	15072147	GAGTGGTGTTGGAGTCATGTTT	AGACATGTGTTTCCCTTCCG	58.97	424	DRR	Ca_17011		
CaPOPI_II_315	Ca3	19368565	GGTGATGCATGATGATTTGC	TGGGTTAGCTTTTGGGATTG	59.90	630	DRR	Ca_19336		
CaPOPI_II_318	Ca3	19383912	GTCAGGTCAGGCTGGAAAAA	TCGTCTTAATCCACTTGGTGC	60.23	436	DRR	Ca_19337		
CaPOPI_II_323	Ca3	23016776	GCACACCTATGTGGTCTTGC	CCATTTTGTCGAAGTTTGGTT	59.17	615	DRR	Ca_06208	QR	B3
CaPOPI_II_325	Ca3	25585812	TGTTTCGTTTTTGTTCCCTCT	TCCATTGCAATCGTACTTAATGA	58.73	735	DRR	Ca_05976		B3
CaPOPI_II_352	Ca3	38174447	TTCACATCCACACTCATCGG	CTGGCACTGGCATTCCTAAT	60.53	628	DRR	Ca_01161		NF-YA
CaPOPI_II_353	Ca3	38215897	TGTTGCCTGGAAATGAGTTG	TGCTGCATTGAAATCGAGTC	59.69	688	DRR	Ca_01165	R	
CaPOPI_II_358	Ca4	2035697	AGTCCAGTCTAAGCTGCCCA	TGGAATCATGCCATGCTAGA	60.01	436	DRR	Ca_07823	R	MYB
CaPOPI_II_359	Ca4	2035798	AGTCCAGTCTAAGCTGCCCA	TGGAATCATGCCATGCTAGA	60.01	436	DRR	Ca_07823	R	MYB
CaPOPI_II_361	Ca4	4196538	ATTGGCCGGAAATTGTACTG	TAACCCAAGGCCATATCCTG	59.82	841	DRR	Ca_03838	R	
CaPOPI_II_362	Ca4	4487412	TCAGACTCTCCCGAACGATT	CGTACGACGTCTTTCGTCAA	59.80	281	DRR	Ca_03808		

INDEL marker IDs	Chromosomes/ unanchored scaffolds	Physical positions (bp)	Forward primers (5'-3')	Reverse primers (5'-3')	Anneling temperature (0C)	Expected amplified product size (bp)	Structural annotation		Functional annotation	
							Sequence components of *kabuli* genome	*Kabuli* gene accession IDs	NCBI-KOG	TFs
CaPOPI_II_367	Ca4	8193266	GCATCTCTCCAAGATGTCTGC	TGGGAATTGGGAGCTATCAG	59.97	620	DRR	Ca_08309	R	
CaPOPI_II_382	Ca4	12571013	GGCTCACATCAGGAATGGAT	GGCTCTTGGTCATTGGATGT	59.89	968	DRR	Ca_04479	E	
CaPOPI_II_385	Ca4	13617448	GGGCCTTTTCTTCAGCTTTT	TGCATGTTATCCGCAATCTC	59.84	713	DRR	Ca_04579		
CaPOPI_II_389	Ca4	14405755	ATGTGCATGCCAAGTTGTGT	ACCTATAAGCCCTTTGCGCT	60.04	963	DRR	Ca_05663	E	MYB
CaPOPI_II_390	Ca4	14406133	TTGGTTCCACATTGTACCCC	CCATTGGTTGGGATATGGAG	60.47	892	DRR	Ca_05663	E	MYB
CaPOPI_II_414	Ca4	22134995	GCACCTATGGTGGCAAAAAC	TTTGATGCCCACATTGAAGA	60.37	506	DRR	Ca_20134	J	
CaPOPI_II_425	Ca4	25457334	CATTTTACTCATCGATCTTTGTGG	TGGACAAAATCATGTCTCTTTGA	59.90	336	DRR	Ca_16577		
CaPOPI_II_429	Ca4	25916811	ATGGTAACAGATATCGCCGC	ATTGTAGCATTGCCCAAAGG	59.95	627	DRR	Ca_16606	I	
CaPOPI_II_43	Ca1	24030518	CCAATTCAAAAGCTCCCAAA	CAGATCCTCCTTTCCCAACA	60.05	424	DRR	Ca_19291	OK	
CaPOPI_II_430	Ca4	25962811	TGGTTGGAAGCTCCAGAAGT	GAAAAGGTGAGGAATCGCTG	59.84	275	DRR	Ca_16607		B3
CaPOPI_II_449	Ca4	27267123	CCCTCAACTCAACTGAGATCC	TGCATTGGTTTGAATTTGGA	58.73	568	DRR	Ca_20461	R	
CaPOPI_II_450	Ca4	27283830	TATTGGTCGTCAAGCAGCAG	TTTCCCAATCGATTTGCTTC	60.01	681	DRR	Ca_20463		
CaPOPI_II_48	Ca1	28910299	GAAAATGATCGGTGGAGGAA	CTTGCGTGTTGCACGAGTAT	59.87	620	DRR	Ca_18486	R	
CaPOPI_II_488	Ca4	33308913	TCATTTTCCCCTCGTTTCTG	TTGGCCAAAACCTTTTGATG	60.04	183	DRR	Ca_24021	R	C2H2
CaPOPI_II_49	Ca1	28910492	GAGTCATTGGTGCCTTGGTT	CTTGCGTGTTGCACGAGTAT	59.97	385	DRR	Ca_18486	R	
CaPOPI_II_506	Ca4	39038460	CCGGTCACATTCAATAGCCT	ATTGCGTTGTTTCTTCCAGG	59.96	411	DRR	Ca_13078	A	

INDEL marker IDs	Chromosomes/ unanchored scaffolds	Physical positions (bp)	Forward primers (5'-3')	Reverse primers (5'-3')	Anneling temperature (0C)	Expected amplified product size (bp)	Structural annotation		Functional annotation	
							Sequence components of *kabuli* genome	*Kabuli* gene accession IDs	NCBI-KOG	TFs
CaPOPI_II_515	Ca4	44522624	GAAGTTGCTTGCTCCGATTC	CCCTAGCAAGTGCTAACCCA	59.96	675	DRR	Ca_09124		ERF
CaPOPI_II_521	Ca4	48314680	GTAATGCGTTTGACCTGCAA	ATAGGCGCATCTCAATCACC	59.74	649	DRR	Ca_10756		BBR-BPC
CaPOPI_II_527	Ca5	210200	TTAAACACCCGTGTTGTGGA	AAATGGCTTAGGGAGGGAGA	59.86	607	DRR	Ca_18176		C2H2
CaPOPI_II_528	Ca5	210510	TCTCCCTCCCTAAGCCATTT	GAATTATCCGCTGCTTGCAT	60.03	201	DRR	Ca_18176		C2H2
CaPOPI_II_532	Ca5	291688	AAAGAGCCAAAGAAGCACCA	AGCAAATGCGACTGTGATTG	59.99	722	DRR	Ca_18186		
CaPOPI_II_533	Ca5	291943	TTCGTGGCTTAATCCCACTC	GTCTGGTGCTCTTCCCAATC	60.07	517	DRR	Ca_18186		
CaPOPI_II_534	Ca5	292149	AAAGTGCCGAAGTTTGCAGT	TGCTTTCCCTTCAACAGTCA	59.92	485	DRR	Ca_18186		
CaPOPI_II_535	Ca5	360986	TGTGAGTTTAAGGGAGGGGA	GCATCCAGGATCAGAGGTGT	59.52	390	DRR	Ca_18192	P	
CaPOPI_II_569	Ca5	11983861	CTGCTGTCAACTCCACTCCA	TCAAAATCCCCCAATAACCA	60.02	713	DRR	Ca_17106		
CaPOPI_II_576	Ca5	16765808	CGATCGTGCTACGTCTTCAA	TGGTGGGAATGTTGTGATTG	60.01	677	DRR	Ca_17941	R	HSF
CaPOPI_II_58	Ca1	29846530	TGGATTGGGGATCATTCATT	TCAACATTTGGAAGCACAAGA	59.95	897	DRR	Ca_24388		
CaPOPI_II_588	Ca5	22919631	GATTTGGTGGAATCTCCGAA	GCCACCGACTACTTCTCCCT	59.87	336	DRR	Ca_21962		
CaPOPI_II_589	Ca5	22919722	GATTTGGTGGAATCTCCGAA	GCCACCGACTACTTCTCCCT	59.87	336	DRR	Ca_21962		
CaPOPI_II_59	Ca1	29846941	TGTTGCCAACTGAAAATCGT	ATACAGACACCGAATGGGGA	59.17	828	DRR	Ca_24388		
CaPOPI_II_594	Ca5	25803856	ATCCGATAAATCGCATTGTG	CCCACAAGGTCCGAGAATTA	58.46	203	DRR	Ca_09020		
CaPOPI_II_595	Ca5	26995171	GGAGCGGGAATATGACTGAA	GCGGCAGAAGAACCTATTTG	60.04	576	DRR	Ca_08931	R	MYB

INDEL marker IDs	Chromosomes/ unanchored scaffolds	Physical positions (bp)	Forward primers (5'-3')	Reverse primers (5'-3')	Anneling temperature (0C)	Expected amplified product size (bp)	Structural annotation		Functional annotation	
							Sequence components of *kabuli* genome	*Kabuli* gene accession IDs	NCBI-KOG	TFs
CaPOPI_II_603	Ca5	29938246	GCAAAAACAGCGACACTGAA	TTGTGTGGGAAAGGTGACAA	60.04	764	DRR	Ca_16674		
CaPOPI_II_610	Ca5	33444959	TTATGGAGGCCACATTCACA	GAAATTAAGGCCTCTTGATCTTTTT	59.92	961	DRR	Ca_01912	O	MYB_related
CaPOPI_II_611	Ca5	33456475	GATGAAGTGCTGATCAGAGGC	ACCAGGAAACATTGCCAGAC	59.97	789	DRR	Ca_01911	O	MYB_related
CaPOPI_II_612	Ca5	33457924	CCGACACCCAAAATTTCAAC	GCCCTCAGATGAGAGTCAGG	60.21	680	DRR	Ca_01911	O	MYB_related
CaPOPI_II_613	Ca5	33600042	TAACCATTCCACAGCCACAA	CTTAGAGGTCGCTTCAACGG	59.96	680	DRR	Ca_01896		
CaPOPI_II_615	Ca5	36308359	GTTTGTGGCGGATTTTGTTT	CCGATACAACGGAATTGTCC	59.85	423	DRR	Ca_01593	Z	
CaPOPI_II_641	Ca6	1683226	AATAAGTCCATCCGGCTCCT	TTGTGGATGGCGTTTGATAA	59.93	276	DRR	Ca_10299		HB-other
CaPOPI_II_642	Ca6	1683327	TTATCAAACGCCATCCACAA	CACTTGCGTGTGGTAGTGCT	59.93	504	DRR	Ca_10299		HB-other
CaPOPI_II_643	Ca6	1976018	GAACTAGATGCCGCAAAAGC	GTACGTGGTCCTTCCCTGAA	59.99	560	DRR	Ca_10322		
CaPOPI_II_644	Ca6	1976736	TTATTCTCCATTGGGCATCC	TCCTGCGGGTAATGATAAGC	59.72	963	DRR	Ca_10322		
CaPOPI_II_646	Ca6	2518811	AATCTGGCTCAAAATGCAGC	AATGCATTTGCCTGACTCAA	60.36	723	DRR	Ca_10384		
CaPOPI_II_665	Ca6	6163420	CCGTCCCTTAGCAAAATTCA	AAGCAACTATGGCAAATGGG	60.07	443	DRR	Ca_05680	T	G2-like
CaPOPI_II_668	Ca6	9203559	TCGTGAAATAGTGGACGGTG	TGAACATCAAAATGCCCAAA	59.57	763	DRR	Ca_08676	OT	
CaPOPI_II_671	Ca6	10649918	GACGGTTATGGAGCCTTCAA	TTCGATATCTTGTGCCTCCC	60.07	363	DRR	Ca_08531	I	
CaPOPI_II_672	Ca6	12335628	GGAAGGGTGTTGGTGTGTTT	TTGGTCACTAAGGGGGTGAG	59.72	968	DRR	Ca_24849		
CaPOPI_II_688	Ca6	21408015	AGGCCTTTTATTTCGTTGCC	TTGAATCGCAAAACAACCAA	60.44	897	DRR	Ca_19860		

INDEL marker IDs	Chromosomes/ unanchored scaffolds	Physical positions (bp)	Forward primers (5'-3')	Reverse primers (5'-3')	Anneling temperature (0C)	Expected amplified product size (bp)	Structural annotation		Functional annotation	
							Sequence components of *kabuli* genome	*Kabuli* gene accession IDs	NCBI-KOG	TFs
CaPOPI_II_701	Ca6	28326755	TTTGGGCTTGAAGATGAAAA	TTTAAAAGCAAGGGTGGTGG	58.33	336	DRR	Ca_25968	A	
CaPOPI_II_702	Ca6	28327723	CAAGAGCATCGTGTCAAGGA	GTCCTGGCGTGACAAGAAAT	59.98	424	DRR	Ca_25968	A	
CaPOPI_II_714	Ca6	30588229	GGACGTCTACAACATTGGGG	CCACATGAGTGTGTTGAGGG	60.23	948	DRR	Ca_16461		SAP
CaPOPI_II_751	Ca6	41957220	TCCCCAACTTTTGAATTTGC	TGCTTCAGTCAATGGGACAG	59.92	877	DRR	Ca_21949	O	
CaPOPI_II_767	Ca6	52075058	GCGTGTCTTATTGGAGAGAGC	GGCTATCGCTTATGCAGGAC	59.09	782	DRR	Ca_17514		SAP
CaPOPI_II_8	Ca1	2015582	CTCTAGAACCCCCAACTCCC	CTCCAATGTTGGGAGACTACG	59.93	499	DRR	Ca_00253	E	
CaPOPI_II_801	Ca7	586986	TTCCATTTCCTCCACAAAGC	CAACCGCATTGTCATCATTC	60.05	531	DRR	Ca_24415		
CaPOPI_II_802	Ca7	760856	TAAACGCTTGTTGGCATTTG	AGTTCGCAGGCTCTGTTGTT	59.74	445	DRR	Ca_03403	R	
CaPOPI_II_809	Ca7	3689798	ATGGTAGGGGGAAGAGGAGT	CTTTTCGTTTGGCCAATGTT	58.88	381	DRR	Ca_03100	S	
CaPOPI_II_815	Ca7	5578208	ATGGGAAATTGTGAATGGGA	TGCCCGTTAGTCAATGTTTG	59.99	435	DRR	Ca_06805		
CaPOPI_II_816	Ca7	5633058	GCAAGCCAAACCACAAAAAT	ACAGGCGCTTCCTTCTTGTA	59.98	386	DRR	Ca_06796	GC	HSF
CaPOPI_II_821	Ca7	6126281	TTTCTGCCTAGAAAAGTTCCAT	TGTAGCCACGTAAGGAAACCT	57.22	246	DRR	Ca_06744	O	
CaPOPI_II_836	Ca7	17922655	AAGTCGAGTGCTTGAGCCAT	TCTTTGCGTGCGTGCTATAC	60.02	606	DRR	Ca_15831		bHLH
CaPOPI_II_842	Ca7	20252580	ACCTGTCAGCTAAGAGCCCA	CACATGTGTGAACCTCCTGG	60.01	641	DRR	Ca_12383		
CaPOPI_II_851	Ca7	21743270	TTGCCCCTCTAAATTCTTGG	TCCCTCGTATTTAGCCCTCA	59.16	686	DRR	Ca_14521		
CaPOPI_II_852	Ca7	21743627	TGAGGGCTAAATACGAGGGA	GGGATTGAGCGCCTATACAA	59.66	461	DRR	Ca_14521		

INDEL marker IDs	Chromosomes/ unanchored scaffolds	Physical positions (bp)	Forward primers (5'-3')	Reverse primers (5'-3')	Anneling temperature (0C)	Expected amplified product size (bp)	Structural annotation		Functional annotation	
							Sequence components of *kabuli* genome	*Kabuli* gene accession IDs	NCBI-KOG	TFs
CaPOPI_II_853	Ca7	21743947	CCTGGTGTGCATTGAAAAGA	AATTGCGGTTTTGGTTCAAG	59.69	410	DRR	Ca_14521		
CaPOPI_II_862	Ca7	23667025	ACACTGATTTCCCAAAACGC	TTTCCTTCCCTTCTCATTGC	59.98	728	DRR	Ca_26877	G	FAR1
CaPOPI_II_876	Ca7	26701106	CAATAGGGCGAGTCGAATGT	AAGTCCAGTAGCCGAAAGCA	60.10	838	DRR	Ca_16249		
CaPOPI_II_877	Ca7	26701534	AAGCTTCATCCTGGTCATGG	AAGTCCAGTAGCCGAAAGCA	60.07	381	DRR	Ca_16249		
CaPOPI_II_88	Ca1	37412282	CGTGGATGATGCTTTTCTCA	TGCTTCTCATCTGCTTGGTG	59.80	580	DRR	Ca_21842	R	GRAS
CaPOPI_II_884	Ca7	29446201	CGTTGCGCATGTGTAATTTG	TCATTCCCAACATTATTTCGTG	61.09	639	DRR	Ca_11775	R	
CaPOPI_II_889	Ca7	30874290	TTGTCTCAACAATCTCCCCC	ACCACCTCACTGCCCTACAC	59.90	734	DRR	Ca_23317		
CaPOPI_II_890	Ca7	30967683	CCCCATGATCTTCCCTTTTT	GCAACAAACCTATGGCAGGT	60.12	355	DRR	Ca_23312		
CaPOPI_II_892	Ca7	31210939	CCTTTCTCAGCCCATATCCA	TCAACAATGCAAACGAAGGA	60.03	306	DRR	Ca_10136		ARF
CaPOPI_II_901	Ca7	33576122	TAATTTAACGAACGGCCGAG	TTGGGTGACTCATGGTGAGA	60.09	410	DRR	Ca_16178	I	
CaPOPI_II_903	Ca7	33640082	CCAAATCGTCGGTGTGAATA	CCTTTGTTTGCTGGGATCAT	59.40	614	DRR	Ca_16180		ERF
CaPOPI_II_904	Ca7	33815769	ATGGCTATTCACTGCCATGC	ATCATCCTTGGTCCCGAATA	61.03	434	DRR	Ca_16190	E	
CaPOPI_II_913	Ca7	36117059	TGGATTGGGCTTTCCAATAA	TGCCTTAACGTACCCAAACA	60.26	435	DRR	Ca_13721	G	Trihelix
CaPOPI_II_928	Ca7	44150096	AGTCTTACCAGCGCCACACT	TCCCAACTACAACGAGGACC	59.94	567	DRR	Ca_21366	O	
CaPOPI_II_940	Ca8	7927288	AGGACCGTGCAAATGTTTCT	TGGCCTACTTATGGTCTGGC	59.60	349	DRR	Ca_11431	J	
CaPOPI_II_941	Ca8	7927539	GCCAGACCATAAGTAGGCCA	GCAACTGGATTGCTGGATTT	60.10	509	DRR	Ca_11430	J	

INDEL marker IDs	Chromosomes/ unanchored scaffolds	Physical positions (bp)	Forward primers (5'-3')	Reverse primers (5'-3')	Anneling temperature (0C)	Expected amplified product size (bp)	Structural annotation		Functional annotation	
							Sequence components of *kabuli* genome	*Kabuli* gene accession IDs	NCBI-KOG	TFs
CaPOPI_II_942	Ca8	7927639	GCCAGACCATAAGTAGGCCA	GCAACTGGATTGCTGGATTT	60.10	509	DRR	Ca_11430	J	
CaPOPI_II_951	Ca8	7956165	TTGATTTGTCTAGAAGCAGCACA	CACTTCTTGGCTGAGTTGGA	60.07	476	DRR	Ca_11432		G2-like
CaPOPI_II_958	Ca8	9101623	CAAGGTTCTCCAGAAACCCA	ACACGTGTGCGCTCAAAATA	60.08	671	DRR	Ca_11533		C2H2
CaPOPI_II_961	Ca8	9953316	AGCAGCAACAACCGTCTTTT	AAAAATCGATCGGGTGCATA	59.92	312	DRR	Ca_18394		
CaPOPI_II_968	Ca8	10934374	TCCGATTCATCCTCTTGGTC	AATGAAAAAGCGGGGAAACT	60.01	368	DRR	Ca_19747	R	NAC
CaPOPI_II_975	Ca8	11536143	TCTTACACTGCAGCAATAAAAATCA	TGGTTGATGCCCAAGTACAA	60.19	576	DRR	Ca_16812	R	MYB
CaPOPI_II_987	Ca8	13993907	CTCCTCAACCTCAATCCCAA	TCACCGGTCAATCAATTAAAAA	60.04	228	DRR	Ca_22739	K	
CaPOPI_II_990	Ca8	14077330	TTGATATGTCATCCCCGGTT	TTCTTTCCCACCCTTCTTCC	60.01	696	DRR	Ca_22744	U	
CaPOPI_II_991	Ca8	14077398	TTGATATGTCATCCCCGGTT	AGCTGCATCTTCCACGATTT	60.01	940	DRR	Ca_22744	U	
CaPOPI_II_1148	scaffold1414	190587	TCGGATCACGGTCTGATACA	GAGGCAAATGGGACAAGGTA	60.07	611	CDS (FRAME SHIFT)	Ca_25542	R	
CaPOPI_II_1215	scaffold1580	251123	TGTTCTCTTAGGGGGTGGTG	GGCCATCTTTTAAACCCCTT	59.96	569	CDS (FRAME SHIFT)	Ca_20799	B	
CaPOPI_II_1311	scaffold1964	11803	GCACCATTCCTCTTTTGGAA	GATGGACCACCTTCTCTCCA	60.05	553	CDS (FRAME SHIFT)	Ca_25209	G	
CaPOPI_II_1738	scaffold543	69059	TCGCTACTGAAGATGGGCTT	TTGATAACTTGAAAACGAAATGAAA	59.98	363	CDS (FRAME SHIFT)	Ca_24780		
CaPOPI_II_183	Ca2	17551687	ATGTGGTTGATAAGGCCTCG	AGCAAATGCAATGCTCTGAA	59.96	398	CDS (FRAME SHIFT)	Ca_16000	OE	
CaPOPI_II_1975	scaffold864	247808	TTCTGCCCTCATGGATTTTC	CATGGCTCGAGTGAAACAGA	60.01	349	CDS (FRAME SHIFT)	Ca_24213		
CaPOPI_II_2037	scaffold949	3822	AAGAGGGGCGTGAACTTTTT	GCTTCCATACCTGTTTTGGG	60.11	646	CDS (FRAME SHIFT)	Ca_25481		

INDEL marker IDs	Chromosomes/ unanchored scaffolds	Physical positions (bp)	Forward primers (5'-3')	Reverse primers (5'-3')	Anneling temperature (0C)	Expected amplified product size (bp)	Structural annotation		Functional annotation	
							Sequence components of *kabuli* genome	*Kabuli* gene accession IDs	NCBI-KOG	TFs
CaPOPI_II_355	Ca4	2032207	TGCTGAGTTCGAGGAAAGGT	CACATCTCTTGTTCGCTCCA	59.99	517	CDS (FRAME SHIFT)	Ca_07822	D	
CaPOPI_II_435	Ca4	25975114	ACACATGTTGGCCAATGAGA	GAGTCCGATTTCCATGAGGA	59.97	621	CDS (FRAME SHIFT)	Ca_16608		
CaPOPI_II_591	Ca5	23672060	TGAGTAAGGGGGCTGAAATG	TGAGAGACGGTGGTTTACCC	60.07	822	CDS (FRAME SHIFT)	Ca_17683		
CaPOPI_II_9	Ca1	2073961	AGAGGCACGAGATTTCCAGA	AAATATCCCCGCAATAAGGG	59.95	336	CDS (FRAME SHIFT)	Ca_00263	T	M-type
CaPOPI_II_916	Ca7	40430153	GTTGGTTCAAAAGCAACCGT	ACAAGGATACGTGCAGGGAC	60.02	493	CDS (FRAME SHIFT)	Ca_20258		Trihelix
CaPOPI_II_989	Ca8	14074282	AAACAAAATCCACCGCACTC	TAGGTTCCAAAGGTGCCAAC	59.98	606	CDS (FRAME SHIFT)	Ca_22744	U	
CaPOPI_II_1	Ca1	18088	CCGCTTGTTTTTCTTTGCAG	TTGATTGCTTGTTGCTCCAG	60.90	543	INTERGENIC			
CaPOPI_II_10	Ca1	2663715	GTTGCCATGCCTATAACGGT	TGAAGACCATGGACACCAAC	59.85	547	INTERGENIC			
CaPOPI_II_100	Ca1	37927862	TGCAAAATCTGGTTGATTGAG	TCCCAGATCAAAATTGGGTT	58.76	745	INTERGENIC			
CaPOPI_II_1000	Ca8	15685177	GCCCAACAACAGAACCAAGT	GACTCCAAAACCCTGTGCAT	60.01	420	INTERGENIC			
CaPOPI_II_1001	Ca8	15685626	ATGCACAGGGTTTTGGAGTC	ACTTGGTTCTGTTGTTGGGC	59.97	416	INTERGENIC			
CaPOPI_II_1002	Ca8	15685828	ATGCACAGGGTTTTGGAGTC	ACTTGGTTCTGTTGTTGGGC	59.97	416	INTERGENIC			
CaPOPI_II_1003	Ca8	15685888	ATGCACAGGGTTTTGGAGTC	ACTTGGTTCTGTTGTTGGGC	59.97	416	INTERGENIC			
CaPOPI_II_1005	Ca8	15788537	TCGGTTCCTTTCAACTTTGG	ATCAATGCTTCCACCCTTTG	60.08	588	INTERGENIC			
CaPOPI_II_1006	Ca8	15892786	AGCAGTTGAACTTGCCTCGT	CGATGATTCATTCCCAAACC	60.06	513	INTERGENIC			
CaPOPI_II_1007	Ca8	16315020	AATGCAAAGCAACAAGGTTTC	TTGTATGCATGTGTGATGTGTG	59.26	504	INTERGENIC			

INDEL marker IDs	Chromosomes/ unanchored scaffolds	Physical positions (bp)	Forward primers (5'-3')	Reverse primers (5'-3')	Anneling temperature (0C)	Expected amplified product size (bp)	Structural annotation		Functional annotation	
							Sequence components of *kabuli* genome	*Kabuli* gene accession IDs	NCBI-KOG	TFs
CaPOPI_II_1008	Ca8	16316035	GGCATCACCCCAAGACATAG	CCCTTGATTTTTCAATCACTCC	60.34	352	INTERGENIC			
CaPOPI_II_1009	scaffold1003	41332	GGGATTTGAAGGTGCTGAAA	TAGTGGCCACAACCAACTCA	60.05	452	INTERGENIC			
CaPOPI_II_101	Ca1	37929646	TGTGACGGTTAATATGGTGGTC	TTGACTTGTTTTGCGGATTG	59.62	817	INTERGENIC			
CaPOPI_II_1010	scaffold1006	109456	TTGATTTCAGCCCAGGATTC	TTTACAAGCAGCGGGAAAGT	60.01	493	INTERGENIC			
CaPOPI_II_1011	scaffold1008	12678	TCTTGGTTGTAACCCTTGGC	GGGCTGATGCCATAAACACT	59.97	727	INTERGENIC			
CaPOPI_II_1012	scaffold10180	748	GGTGAGTTTGTGTCGTTGGA	AGGGAGGACGAAGATGACCT	59.57	448	INTERGENIC			
CaPOPI_II_1014	scaffold1038	720	CGAAAATCGCCCAGAAAATA	TCCTGGTCCTCGATTTCATC	60.03	842	INTERGENIC			
CaPOPI_II_1015	scaffold1039	48817	CGACATGCATGCCTTATTTG	CATGAGGAAACAATTGTGCAG	60.10	571	INTERGENIC			
CaPOPI_II_1016	scaffold1043	1901	CGAAGCAAAAGAAAGAACCG	TGGATTACGGGTGAAATGGT	59.99	321	INTERGENIC			
CaPOPI_II_1017	scaffold1046	17706	CATTGGATGCACAAGATTGG	AGGTCATTTTCGTCCTCCCT	59.92	871	INTERGENIC			
CaPOPI_II_1018	scaffold1046	20332	GCGCAAACAAACCTATGTGA	TCAGGTCATTTTCGTCCTCC	59.74	788	INTERGENIC			
CaPOPI_II_102	Ca1	37993665	GTGAGCCCTTTACCAATCCA	CAATTCCCACAGGTACGCTT	59.93	654	INTERGENIC			
CaPOPI_II_1020	scaffold1047	419626	CCGATGAAAATTCACCCATC	CCATTGATAAAGCGTGTACGG	60.13	327	INTERGENIC			
CaPOPI_II_1021	scaffold1049	75199	ACGTTCCTGAGGGAATTGTG	GTAAAGGGTCACCAACCGAA	59.97	828	INTERGENIC			
CaPOPI_II_1022	scaffold1052	16426	CCGTTGTTTCAACTTCCACC	GCAGCAGCCATTCATACAGA	60.39	323	INTERGENIC			
CaPOPI_II_1023	scaffold1055	339319	TTTAAGGTGTGGGACCAAGC	GGAAACAATAAACCGAGGCA	59.97	540	INTERGENIC			

INDEL marker IDs	Chromosomes/ unanchored scaffolds	Physical positions (bp)	Forward primers (5'-3')	Reverse primers (5'-3')	Anneling temperature (0C)	Expected amplified product size (bp)	Structural annotation		Functional annotation	
							Sequence components of *kabuli* genome	*Kabuli* gene accession IDs	NCBI-KOG	TFs
CaPOPI_II_1024	scaffold1055	340051	TGTTGTTCGGCATACGACAT	TGCACATACATAATGGACCACA	60.00	516	INTERGENIC			
CaPOPI_II_1025	scaffold1055	340491	TGCAATAAAGCTTTTGAAGTGG	ACATACTCCCTCACTCCCC	59.42	413	INTERGENIC			
CaPOPI_II_1026	scaffold1055	340641	TGCAATAAAGCTTTTGAAGTGG	AAGTTGTGTCACCATTGCCA	59.42	813	INTERGENIC			
CaPOPI_II_1027	scaffold1056	142810	TTGCTCCTACGTATCCCCAG	TGGCCACAAGAAGACAATGA	60.09	705	INTERGENIC			
CaPOPI_II_1028	scaffold1061	32882	ACTAACCATCGTTTGGCCTG	GCAATTTTCGTACCCCTTGA	59.99	227	INTERGENIC			
CaPOPI_II_1029	scaffold1061	49764	CCTTGTCACACAGTTGGCTG	TCTTGGTTGTAACCCTTGGC	60.35	150	INTERGENIC			
CaPOPI_II_103	Ca1	38070666	AACCATTGGGGGAGAATAGAA	TTGTTAGAACCATTGGGGGA	59.65	362	INTERGENIC			
CaPOPI_II_1030	scaffold1061	76996	TATTTGGACCAGAGGATGGC	CCCAAACAGTAGGACCAGGA	59.89	109	INTERGENIC			
CaPOPI_II_1032	scaffold1067	229129	ACATGGTTCGGAAACGTAGG	TCCATGCAAGAAGAGCTCAA	59.85	341	INTERGENIC			
CaPOPI_II_1033	scaffold1067	243320	TTATTGTTGTTGCTTGGGCA	TCTTGTGTTGGGTTAAGGGC	60.11	447	INTERGENIC			
CaPOPI_II_1034	scaffold1067	243805	GCCCTTAACCCAACACAAGA	AGGCGACCCTATACCGAAGT	59.97	643	INTERGENIC			
CaPOPI_II_1035	scaffold1067	263503	CAGTGAGTAGCAAGCAAGCG	CCCAGCCTAGTGACATCCAT	59.95	445	INTERGENIC			
CaPOPI_II_1036	scaffold1067	266043	ATCCAGAATGGAGCAACTGG	CGGAGAAATCCGTTTTGCTA	60.07	575	INTERGENIC			
CaPOPI_II_1037	scaffold1081	86270	TTATTCGGGCCTGTCAAAAC	CATGCGTTCCTTGTGGTAGA	59.94	251	INTERGENIC			
CaPOPI_II_1038	scaffold1081	88052	TGAGCATGGCTAAGTTGCAC	AAGGGGAAGGGCATAATGTT	60.02	522	INTERGENIC			
CaPOPI_II_1039	scaffold1083	122050	AAGCCTAGGGAGGACGAAAA	AACGAATCATGTGCATCCAA	60.20	772	INTERGENIC			

INDEL marker IDs	Chromosomes/ unanchored scaffolds	Physical positions (bp)	Forward primers (5'-3')	Reverse primers (5'-3')	Anneling temperature (0C)	Expected amplified product size (bp)	Structural annotation		Functional annotation	
							Sequence components of *kabuli* genome	*Kabuli* gene accession IDs	NCBI-KOG	TFs
CaPOPI_II_104	Ca1	38070895	CTCCCCCAATGGTTCTAACA	CACTGGAATCCGTTTCTTTTG	59.78	587	INTERGENIC			
CaPOPI_II_1040	scaffold1087	13529	TTCCGCATAACACTAAGTGGC	CCATGCTAATGGATGCTGTG	60.15	521	INTERGENIC			
CaPOPI_II_1041	scaffold1087	14399	TGGGTTGTACCGTTAATTGG	ACCCCAAATATTGCCATGAA	58.27	810	INTERGENIC			
CaPOPI_II_1042	scaffold1087	23029	TGCACCACGAGATCTTGAAA	CGTTGATACATAACAAAAATACAAACA	60.39	597	INTERGENIC			
CaPOPI_II_1043	scaffold1087	23220	TGTGGGATATCAAGTTTTGGA	AAAAATGCACGGGACAAATA	57.93	704	INTERGENIC			
CaPOPI_II_1044	scaffold1092	121415	AGGTCATTTTCGTCCTCCCT	AATCCAATGGCACAAACACA	59.94	648	INTERGENIC			
CaPOPI_II_1045	scaffold1101	3795	TTGGCTCATTGCTTGAGTTG	AGCCAAGGGAGGATGAAAAT	59.99	214	INTERGENIC			
CaPOPI_II_1046	scaffold1109	14028	TTTGAAAACCTCACCAAGGG	TAAACTGTCAGGGGCGAAGA	59.94	416	INTERGENIC			
CaPOPI_II_1047	scaffold1109	14081	TTTGAAAACCTCACCAAGGG	TATAAACTGTCAGGGGCGAA	59.94	418	INTERGENIC			
CaPOPI_II_1048	scaffold1116	42811	GATGAAACCGAGGACCAAGA	GTGTTTTCGGACCAGACGAT	60.05	599	INTERGENIC			
CaPOPI_II_1049	scaffold1116	94918	TCTTCGTTATGCAGTCAAACCT	ACCCAACAAAAATGGACCAA	58.91	728	INTERGENIC			
CaPOPI_II_105	Ca1	38071534	CTCCTCCTCATTCACCCAAC	ACACACCCACTCTCCTTTGC	59.51	189	INTERGENIC			
CaPOPI_II_1051	scaffold1135	9704	TTTCGGTTGGGTTTGCTTAC	TACCCGTGCTCAACATCAAG	59.98	704	INTERGENIC			
CaPOPI_II_1053	scaffold1139	1393	TCATCCAGTGTGGGGTACAA	TTGTGTGTGTTGGAGATGAGC	59.81	313	INTERGENIC			
CaPOPI_II_1054	scaffold1139	1605	TCATCCAGTGTGGGGTACAA	TTTGAGAATTTGGGATGGAAA	59.81	785	INTERGENIC			
CaPOPI_II_1055	scaffold1142	4296	AGGTCATTTTCGTCCTCCCT	TTGCATGCACAAGATTGGTT	59.94	268	INTERGENIC			

INDEL marker IDs	Chromosomes/ unanchored scaffolds	Physical positions (bp)	Forward primers (5'-3')	Reverse primers (5'-3')	Anneling temperature (0C)	Expected amplified product size (bp)	Structural annotation		Functional annotation	
							Sequence components of *kabuli* genome	*Kabuli* gene accession IDs	NCBI-KOG	TFs
CaPOPI_II_1056	scaffold1145	3948	AATCCTCAAAGCCCTCACCT	TTTTGGAACCTTCGTCCTTT	60.07	750	INTERGENIC			
CaPOPI_II_1057	scaffold1145	15770	TGGCACAACATCATCATCATT	GCGCATTCTTCATCTTCTCC	59.80	496	INTERGENIC			
CaPOPI_II_1058	scaffold115	52073	CAATTAACACAAACGCCCCT	TTGGCCTCCCTAGGCTTATT	59.86	577	INTERGENIC			
CaPOPI_II_1059	scaffold1151	18791	AGCCGTCATGACTTTTGTCC	ACGGACCCTTTGTTGAGACA	60.12	392	INTERGENIC			
CaPOPI_II_106	Ca1	38131605	CGTAACTCGTGTTGGGTGAA	GAAGCAAGAGGCAACTCCAC	59.61	379	INTERGENIC			
CaPOPI_II_1060	scaffold1159	27402	AGGGAGGACGAAAATGACCT	CCTTCCTTGGCTTGTTCTTG	59.94	345	INTERGENIC			
CaPOPI_II_1061	scaffold118	173557	TGTCATGCTAATATCCGCCA	TGGATGGATTTCTAGTTGCCTT	60.06	489	INTERGENIC			
CaPOPI_II_1062	scaffold1180	2599	GAGAGGTCATGGCACCCTTA	GAGCAACACATTGCATGTCC	60.07	166	INTERGENIC			
CaPOPI_II_1063	scaffold1180	2913	GAGAGGTCATGGCACCCTTA	TCTTGCCCAACGTTTTACAT	60.07	513	INTERGENIC			
CaPOPI_II_1064	scaffold1180	3744	GTGCACGAAAGTGTGGCTTA	ACTTTACCATTGCCTTCCCC	59.91	766	INTERGENIC			
CaPOPI_II_1066	scaffold1180	206096	CCATGTGGTTCTTTTGCATTT	AACTTCGCCATTTGTCATCC	59.85	548	INTERGENIC			
CaPOPI_II_1067	scaffold1180	208476	TAAGAGGGGCTTGTTACCCC	CCAACCTAATATTCATGCCACA	60.31	216	INTERGENIC			
CaPOPI_II_1068	scaffold1185	67322	CAATTCCCACAGGTACGCTT	GACGCGAAAACAACATTTCA	59.99	683	INTERGENIC			
CaPOPI_II_1069	scaffold1185	67468	CAATTCCCACAGGTACGCTT	GACGCGAAAACAACATTTCA	59.99	683	INTERGENIC			
CaPOPI_II_107	Ca1	39261977	GGTTTGCTGTCGAGCCTTAG	AAACACAAGTCCAATCAAAAGG	60.02	421	INTERGENIC			
CaPOPI_II_1070	scaffold119	35751	ACTCATATGCAAAATGCCCC	TTATGCTGGAGATGGGAAGG	59.79	444	INTERGENIC			

INDEL marker IDs	Chromosomes/ unanchored scaffolds	Physical positions (bp)	Forward primers (5'-3')	Reverse primers (5'-3')	Anneling temperature (0C)	Expected amplified product size (bp)	Structural annotation		Functional annotation	
							Sequence components of *kabuli* genome	*Kabuli* gene accession IDs	NCBI-KOG	TFs
CaPOPI_II_1071	scaffold1193	7288	AATGCTTTCCAGAAAACCGA	TGATCTGATGGCTGACTTTCA	59.69	367	INTERGENIC			
CaPOPI_II_1072	scaffold1197	5120	TCGCAGCTCAATTGGTAGTG	GAGAGCTACCGTGCAACACA	60.01	486	INTERGENIC			
CaPOPI_II_1073	scaffold1197	244953	CACTTTTTGTTTCATCGCCA	ACTGTGATCCGGTGATTTGA	59.71	574	INTERGENIC			
CaPOPI_II_1074	scaffold1202	25847	TTGTCCACAGTGGCATATTGA	TGGCAGATATGGGGGTGTAT	59.98	737	INTERGENIC			
CaPOPI_II_1075	scaffold1202	82238	AACACATCCGGGTTCCATTA	TTTGATCCATTTGCCCTAGC	60.05	739	INTERGENIC			
CaPOPI_II_1076	scaffold1202	83479	ACGGTGTAGGCAAGATGGAC	AGGATGCATGGGATGAAAAG	60.00	458	INTERGENIC			
CaPOPI_II_1078	scaffold1215	12684	TCTCTCCCGATTTGGTCATT	TTTGCTCCTCTTCTTCTGGC	59.48	588	INTERGENIC			
CaPOPI_II_1079	scaffold1215	13648	TCGTGGGACTAAAACTGAGGA	CGCACATATGAATGATCCCA	59.72	576	INTERGENIC			
CaPOPI_II_108	Ca1	40424994	TGCAAAACTTTGTTCATCGAG	GCATCATTCTCCTTCGAGACA	58.95	748	INTERGENIC			
CaPOPI_II_1081	scaffold1215	45663	TGGCTTTTGTATTTCAGAGTTGG	AGTGGGACCATGATTTTTGAA	60.51	492	INTERGENIC			
CaPOPI_II_1082	scaffold1215	45939	TTTCAAAAATCATGGTCCCAC	GGGGTGGTGTCTATCTTTGC	59.65	337	INTERGENIC			
CaPOPI_II_1083	scaffold1219	40006	TGGTTTAATGTTGGCAAATGG	ATCCAGTTGGTGAGGAAAAA	60.59	317	INTERGENIC			
CaPOPI_II_1084	scaffold1240	105707	ACATGGAACCCATCCAAAGA	GCCCTCAGATGAGAGTCAGG	60.17	268	INTERGENIC			
CaPOPI_II_1085	scaffold1252	98018	TTTGCCAATCGATTATCGTG	TGCACAAGTCAGTAGCACCC	59.52	561	INTERGENIC			
CaPOPI_II_1086	scaffold1256	617	TCCAACACCCTCACAAATCA	TTGGACTACGGGTTAGTGGG	59.94	914	INTERGENIC			
CaPOPI_II_1088	scaffold1274	6685	TCATTTTCGTCCTTCCTTGG	ACACACCTATTGGACGCACA	60.04	272	INTERGENIC			

INDEL marker IDs	Chromosomes/ unanchored scaffolds	Physical positions (bp)	Forward primers (5'-3')	Reverse primers (5'-3')	Anneling temperature (0C)	Expected amplified product size (bp)	Structural annotation		Functional annotation	
							Sequence components of *kabuli* genome	*Kabuli* gene accession IDs	NCBI-KOG	TFs
CaPOPI_II_1089	scaffold1275	6195	TGTTGCCTCGTAATTCAACG	CAGGGACTAAAACTGTTTGCTG	59.73	556	INTERGENIC			
CaPOPI_II_109	Ca1	40535882	GATGATGATGATGTCGTGCC	ATCACAGTTTGGCCAAGTCC	59.89	311	INTERGENIC			
CaPOPI_II_1090	scaffold1276	1671	AAGCATGGGACCAAAGTGAG	TTTCCCAATCGATTTGCTTC	60.11	942	INTERGENIC			
CaPOPI_II_1091	scaffold1278	63103	ACGAAGGGTGTAGGACGATG	ATTTGTTTGCCGATCGCTAC	59.99	611	INTERGENIC			
CaPOPI_II_1092	scaffold1278	63247	ACGAAGGGTGTAGGACGATG	ATTTGTTTGCCGATCGCTAC	59.99	611	INTERGENIC			
CaPOPI_II_1093	scaffold1278	63301	ACGAAGGGTGTAGGACGATG	ATTTGTTTGCCGATCGCTAC	59.99	611	INTERGENIC			
CaPOPI_II_1094	scaffold1278	65072	TGGGTAGCAGTAATAATGGCA	TGAGCAAGATGTGTTTTGTGG	58.20	341	INTERGENIC			
CaPOPI_II_1095	scaffold128	588046	AGTACTGAGCCCCCTGGTTT	ATGATGTTGTAGCGATTGCG	59.99	308	INTERGENIC			
CaPOPI_II_1096	scaffold128	747253	GGTGTGTTTGTGACGTTGGA	GGAGGACGAAAATGATCGAA	60.46	646	INTERGENIC			
CaPOPI_II_1097	scaffold1281	376337	TGTTTAGGCGCTAGTTTGGC	TCGATTATGCGTAGGGAAGG	60.40	665	INTERGENIC			
CaPOPI_II_1098	scaffold1281	469460	TCTCTGGCAACCGTTCTTCT	ACCTCGTTGAGACACGCTTT	59.99	566	INTERGENIC			
CaPOPI_II_1099	scaffold1281	471713	GAGCATTGTGACAAAATTGAGC	CGGGAATTCTTGATGAATCG	59.76	541	INTERGENIC			
CaPOPI_II_11	Ca1	2674516	TGTCGGAATATGGGAAGCTC	GCCGTTGTTTTCAGATCCAT	60.04	934	INTERGENIC			
CaPOPI_II_110	Ca1	40889769	GCATCTCTTGATGCCCTTTT	AACATACGCCATACCCAAGC	59.27	289	INTERGENIC			
CaPOPI_II_1100	scaffold1281	473339	ACGCATGGATATTGCAGTCA	TCCGATTGTTGACCAGTTTTT	60.10	783	INTERGENIC			
CaPOPI_II_1101	scaffold1281	492209	GGAATGGAAAACACATTCAACA	GAATGCTTGAGAAACTTTGGG	59.71	499	INTERGENIC			

INDEL marker IDs	Chromosomes/ unanchored scaffolds	Physical positions (bp)	Forward primers (5'-3')	Reverse primers (5'-3')	Anneling temperature (0C)	Expected amplified product size (bp)	Structural annotation		Functional annotation	
							Sequence components of *kabuli* genome	*Kabuli* gene accession IDs	NCBI-KOG	TFs
CaPOPI_II_1102	scaffold1307	6228	ACACACCTATTGGACGCACA	AGGTCAATTTCGTCCTCCCT	60.03	377	INTERGENIC			
CaPOPI_II_1103	scaffold1308	16327	AGGGAGGACGAAAATGACCT	CCAATTGGGGTTTTTAACCA	59.94	488	INTERGENIC			
CaPOPI_II_1105	scaffold1312	78648	GCATTAAATGTGGAACAAGCA	TTACCTGTGTGGGACAACGA	58.69	526	INTERGENIC			
CaPOPI_II_1106	scaffold1312	78828	GCATTAAATGTGGAACAAGCA	TTACCTGTGTGGGACAACGA	58.69	526	INTERGENIC			
CaPOPI_II_1107	scaffold1312	78884	ATTTGACCTCGACACAACGG	GCCGCTTGATCGTTAATTGT	60.95	425	INTERGENIC			
CaPOPI_II_1108	scaffold1312	78931	TCGACACAACGGACAAGAGT	GCCGCTTGATCGTTAATTGT	59.31	417	INTERGENIC			
CaPOPI_II_1109	scaffold1312	80554	GCCAAGGTAAGGGTGAGACA	ATGAATTGTCCCAATCTCGG	60.11	322	INTERGENIC			
CaPOPI_II_111	Ca1	41695402	TACTTCCATGCAAACCAGCA	CCAACTCTGAAATACAAAAGCCA	60.26	486	INTERGENIC			
CaPOPI_II_1110	scaffold1312	89732	TTCATTACGCCATTGACACTTT	ACAATGTGTGCCCAACAAAA	59.52	457	INTERGENIC			
CaPOPI_II_1111	scaffold1316	4181	GTAAAGGGTCACCAACCGAA	TTGTCCGTGAGAGACTGCAC	59.83	813	INTERGENIC			
CaPOPI_II_1113	scaffold1317	46249	CATGGGTGAAGACTGTGGTG	ACCACTGTTGCATGCCTAAA	60.00	134	INTERGENIC			
CaPOPI_II_1114	scaffold1323	6638	GGCCACTGACTTGCAGTGTA	TTGTGCATCCAATACGTGTG	59.91	717	INTERGENIC			
CaPOPI_II_1115	scaffold1324	8519	ATTTGTTGCATTGTCGGTCA	CTGCCTCACGATTTCACTCA	59.97	899	INTERGENIC			
CaPOPI_II_1116	scaffold1324	227334	TTGGTGACATTTAGCATTTCTCA	GTCCTTTGTGTCGTGGGTCT	59.64	511	INTERGENIC			
CaPOPI_II_1117	scaffold1324	235091	TCTTGGTTGTAACCCTTGGC	TTTCGACAAAAGGTTCTCCG	59.97	431	INTERGENIC			
CaPOPI_II_1118	scaffold1324	236638	ATGCACCTTGAAAGGGTTTG	TTCGAGGAGGGTTTTGATTG	59.97	597	INTERGENIC			

INDEL marker IDs	Chromosomes/ unanchored scaffolds	Physical positions (bp)	Forward primers (5'-3')	Reverse primers (5'-3')	Anneling temperature (0C)	Expected amplified product size (bp)	Structural annotation		Functional annotation	
							Sequence components of *kabuli* genome	*Kabuli* gene accession IDs	NCBI-KOG	TFs
CaPOPI_II_1119	scaffold1324	252648	CCTATAGGCCCACTAAGCCC	TTGCAAGCAAACTTCACGAT	59.95	587	INTERGENIC			
CaPOPI_II_112	Ca1	41704419	GGGGAATTTTTCATTTTGTGG	TGTTTTGGTTTCAAGGGACA	60.39	311	INTERGENIC			
CaPOPI_II_1120	scaffold1324	252816	TGTTAACATGATCAGGCCCA	ACATAGCGCCACAACATGAA	59.92	789	INTERGENIC			
CaPOPI_II_1121	scaffold1324	253776	GAGTGCTCCTTGCACATGAA	ATGAAGACCAAGCCATCCAC	59.99	514	INTERGENIC			
CaPOPI_II_1122	scaffold1324	255649	CCTTGGAGGCAGTGATTTGT	GAGCTACCCCAATGGTTGAA	60.11	613	INTERGENIC			
CaPOPI_II_1123	scaffold1324	255844	CCTTGGAGGCAGTGATTTGT	GAGCTACCCCAATGGTTGAA	60.11	613	INTERGENIC			
CaPOPI_II_1124	scaffold1324	255890	CCTTGGAGGCAGTGATTTGT	GAGCTACCCCAATGGTTGAA	60.11	613	INTERGENIC			
CaPOPI_II_1125	scaffold1324	256337	TTCAACCATTGGGGTAGCTC	TTCACTTGAATATTTTGTGGCA	59.93	474	INTERGENIC			
CaPOPI_II_1126	scaffold1324	266713	TGTATGCATCAACAATGTCAAGA	TCAGAGGATGTGGCATAGAGG	59.10	160	INTERGENIC			
CaPOPI_II_1127	scaffold1324	266796	TCCTCTATGCCACATCCTCTG	CACAAATAGTTGTACATTGGTTGC	60.23	238	INTERGENIC			
CaPOPI_II_1128	scaffold1324	267973	CCAAGGCACACACAAATGAA	AGAGAATAACCCGCACGTTG	60.55	344	INTERGENIC			
CaPOPI_II_1129	scaffold1324	270833	ACAAAATCGGCCACTGAATC	TCCCGTTCATCTCTCTCACC	59.94	500	INTERGENIC			
CaPOPI_II_113	Ca1	41704503	GGGGAATTTTTCATTTTGTGG	TGTTTTGGTTTCAAGGGACA	60.39	311	INTERGENIC			
CaPOPI_II_1130	scaffold1324	272116	CCCCGCTCTGATAGGTGATA	TGTTTGTCATGCGAGCATTT	60.05	767	INTERGENIC			
CaPOPI_II_1131	scaffold1324	516063	AAAACACGCTAGGCTCCAAT	TGGTCATGTGGTTTTTGCAT	58.86	642	INTERGENIC			
CaPOPI_II_1132	scaffold1324	520260	TTCTTTTCGCTATTTTATTTTCGTG	TCTTACGTATCTCCCGGTGC	60.04	734	INTERGENIC			

INDEL marker IDs	Chromosomes/ unanchored scaffolds	Physical positions (bp)	Forward primers (5'-3')	Reverse primers (5'-3')	Anneling temperature (0C)	Expected amplified product size (bp)	Structural annotation		Functional annotation	
							Sequence components of *kabuli* genome	*Kabuli* gene accession IDs	NCBI-KOG	TFs
CaPOPI_II_1133	scaffold1324	642408	CCTTGCCCTTTTATTTCGGT	TCCTAGAACATGCATCACGC	60.31	496	INTERGENIC			
CaPOPI_II_1134	scaffold1331	50640	AATGGGCGCTGTTAAATGTC	TGGTGTCGTTGGCACTCTAC	59.97	411	INTERGENIC			
CaPOPI_II_1135	scaffold1332	8473	CACAATTTCTTTTCTATGTGTGGA	AACCCGTCACACAGGAAGTC	58.24	894	INTERGENIC			
CaPOPI_II_1137	scaffold1337	46228	AAACCCCAAACCTTAAACCC	TTGGGACATTTCGGTTTTTG	59.09	353	INTERGENIC			
CaPOPI_II_1138	scaffold135	113512	GCTTATTGGGAGAAGCGTTG	GCCCTCAGATGAGAGTCAGG	59.85	514	INTERGENIC			
CaPOPI_II_114	Ca1	41718814	GGGTCGAATGTCGATTCTGT	TCATCTCTCAGCCCCACTCT	59.93	156	INTERGENIC			
CaPOPI_II_1140	scaffold1365	74375	TATTGTGAAGAGGCGTGTGC	TGTTGGGATTTGGTTGTGAA	59.87	351	INTERGENIC			
CaPOPI_II_1141	scaffold1380	5644	TTTTAGCGGATGTCGTAGGG	TCACAAGGGGGACTCTATGG	60.09	719	INTERGENIC			
CaPOPI_II_1142	scaffold1386	13609	TTTGTGGTAATTTATGGTTGCTACA	TTTCCGTTTCCTTTGTTTGC	59.74	424	INTERGENIC			
CaPOPI_II_1143	scaffold140	2406	CTTGTAAAATCTCCGCTCGC	TTGGTATTGAACTCGGAGGC	59.98	665	INTERGENIC			
CaPOPI_II_1144	scaffold140	25774	TCCTAGAACATGCATCACGC	CTCAAGTCGACAAGCCTTCC	59.83	666	INTERGENIC			
CaPOPI_II_1145	scaffold1408	24952	TGTGCGTCCAATAGGTGTGT	GACACGAATAAGCCAAGGGA	60.03	478	INTERGENIC			
CaPOPI_II_1146	scaffold1408	92293	AGGTCATTTTCGTCCTCCCT	TAATTGGGCAAGAATGAGCC	59.94	640	INTERGENIC			
CaPOPI_II_1147	scaffold1414	38236	TGGTCCTAGATTTCATCGGG	CGTTATTAAGTACAACTACAAAACGAA	59.89	871	INTERGENIC			
CaPOPI_II_1149	scaffold1418	5209	CACAATTTCACGATTTTCACG	CGGCTAGGTTGACCCTTACA	59.07	819	INTERGENIC			
CaPOPI_II_115	Ca1	44247311	TTCTCCAGACTCTAGCCCGA	AGCTTCTCAGTAGGGGGCTC	60.09	592	INTERGENIC			

INDEL marker IDs	Chromosomes/ unanchored scaffolds	Physical positions (bp)	Forward primers (5'-3')	Reverse primers (5'-3')	Anneling temperature (0C)	Expected amplified product size (bp)	Structural annotation		Functional annotation	
							Sequence components of *kabuli* genome	*Kabuli* gene accession IDs	NCBI-KOG	TFs
CaPOPI_II_1150	scaffold1418	18174	GCATTGTATCCATCCCCATC	ACCCACGTTTAAAGGTTCCC	59.98	797	INTERGENIC			
CaPOPI_II_1153	scaffold1419	10604	CCTCTTGCTGCAAACACAAA	AACTTTTTCCGCTGCTTGAA	60.03	294	INTERGENIC			
CaPOPI_II_1154	scaffold1419	30683	TCCATCTCCAACAACATCCA	TAATTGCTCTTGCGGTCCTT	59.89	306	INTERGENIC			
CaPOPI_II_1155	scaffold1426	5828	AACGGGGTGTTACAATGAGC	CTGGTTTGACAATGCGTTTG	59.86	623	INTERGENIC			
CaPOPI_II_1156	scaffold1426	6734	TTGATACTATGCCACCACGC	GCATTTTGCTTGTGGACTCA	59.57	635	INTERGENIC			
CaPOPI_II_1157	scaffold1426	7221	TGAGTCCACAAGCAAAATGC	GGTTCCTCTTGGTTTTGGGT	59.85	494	INTERGENIC			
CaPOPI_II_1158	scaffold143	67526	GGATTGTTGGTTCATTTGGG	TTAGGAAATCGATTGGGGTG	60.03	495	INTERGENIC			
CaPOPI_II_1159	scaffold1430	1269	TCATGTGCATTTCTACCACCA	TGATTCGAGTCAAACCACCA	59.98	315	INTERGENIC			
CaPOPI_II_116	Ca1	44650256	TCGTTTGCGTCATTGGATTA	AGGGAGGACGAAAATGACCT	60.07	444	INTERGENIC			
CaPOPI_II_1160	scaffold1432	80490	GCCACTACCAACAACATTTTCA	ATTTGTGTTTGTGGGGGAAA	59.90	713	INTERGENIC			
CaPOPI_II_1161	scaffold1432	80541	GCCACTACCAACAACATTTTCA	ATTTGTGTTTGTGGGGGAAA	59.90	713	INTERGENIC			
CaPOPI_II_1162	scaffold1433	21769	TCCAGATGAAATCGAGGACC	TCATATGTACGTTTCGGGCA	60.01	498	INTERGENIC			
CaPOPI_II_1163	scaffold1433	21950	GATGAAATGGAGGACCGAGA	TCATATGTACGTTTCGGGCA	60.01	332	INTERGENIC			
CaPOPI_II_1164	scaffold1433	33630	AGGCGATTTTCGTACCACAC	CGTGTCATTTATGTCGGGTG	60.00	112	INTERGENIC			
CaPOPI_II_1165	scaffold1448	11427	AATCCAATGACGCAAACACA	AGGTCATTTTCGTCCTCCCT	59.97	792	INTERGENIC			
CaPOPI_II_1167	scaffold1449	3971	AGGAAGAGTGGAGTTGCGAA	CTGTAAACTGAAGCGCCCTC	59.99	456	INTERGENIC			

INDEL marker IDs	Chromosomes/ unanchored scaffolds	Physical positions (bp)	Forward primers (5'-3')	Reverse primers (5'-3')	Anneling temperature (0C)	Expected amplified product size (bp)	Structural annotation		Functional annotation	
							Sequence components of *kabuli* genome	*Kabuli* gene accession IDs	NCBI-KOG	TFs
CaPOPI_II_1168	scaffold1452	1227	CGAAATCTTTGGGTTTTGGA	ACCTAAATTGGGGAGCTCGT	59.91	618	INTERGENIC			
CaPOPI_II_117	Ca1	44776081	TGGAATACCATATATTTTACGTTTTCA	TGACGGATCCTTTCTTGACA	59.13	795	INTERGENIC			
CaPOPI_II_1170	scaffold1452	30210	TGCCCAATTACCACAATCAA	ACCAACCCTTCATTTCCCTC	59.79	465	INTERGENIC			
CaPOPI_II_1171	scaffold1457	19983	TGGCTTTCAAATTCCACACA	GCGAGCAGAGATTTTTACCG	60.09	468	INTERGENIC			
CaPOPI_II_1172	scaffold1465	3149	AAGGCTCAACCTTACGTGGT	TTTTTAGCAATGGCACAACATT	58.72	558	INTERGENIC			
CaPOPI_II_1173	scaffold1465	20135	ATAGATCCCCTCAGCCCAAC	TTTCGGTAGTCCCGTTCATC	60.29	604	INTERGENIC			
CaPOPI_II_1175	scaffold1467	16133	ACTTTCAAACGGTCAATCCG	ACATTATGTGAGGGCGCTTC	59.97	596	INTERGENIC			
CaPOPI_II_1176	scaffold1483	6428	TAACTTGCATGCCCATTGTC	CAAAAGACACACATATTTGGCA	59.55	573	INTERGENIC			
CaPOPI_II_1177	scaffold1483	6873	GCACCACGTGCGATAACA	TGTGTGAGCGTTATTGGTTTG	60.28	984	INTERGENIC			
CaPOPI_II_1178	scaffold1490	1256	CAAACACACCTGTTGGATGG	AGGTCATTTTCGTCCTCCCT	59.85	281	INTERGENIC			
CaPOPI_II_1179	scaffold1493	65515	AGGCAATTTGGGATTCCTCT	GGCTGCTTTGAGTTCCTTTTT	59.90	372	INTERGENIC			
CaPOPI_II_118	Ca1	44847313	TGTTTTCTGCCTTTTTGCCT	AAAATCCAACAATCTCCCCC	59.86	274	INTERGENIC			
CaPOPI_II_1180	scaffold1496	236426	TTCAACCTCGAATTTCAGCA	AGCGGTGGAGAGAACTCTGA	59.40	764	INTERGENIC			
CaPOPI_II_1181	scaffold1499	931	TTCTTATTCACCTGGTTCCTTTT	TTGAAGGATACATTACGAGATTCA	58.26	734	INTERGENIC			
CaPOPI_II_1182	scaffold15	144204	AGGTTTGAGGATCGGGGTAT	CAAGATTTGGGCAATCTGCT	59.65	757	INTERGENIC			
CaPOPI_II_1183	scaffold151	814	GGAGACTCCCATACATTGGC	TGCTATCCCACTTTGAGCCT	59.37	332	INTERGENIC			

INDEL marker IDs	Chromosomes/ unanchored scaffolds	Physical positions (bp)	Forward primers (5'-3')	Reverse primers (5'-3')	Anneling temperature (0C)	Expected amplified product size (bp)	Structural annotation		Functional annotation	
							Sequence components of *kabuli* genome	*Kabuli* gene accession IDs	NCBI-KOG	TFs
CaPOPI_II_1184	scaffold151	1987	GGACATTTGTGAACAAGGCA	TTGGAGCTTATGTGTTCCTCAA	59.55	732	INTERGENIC			
CaPOPI_II_1185	scaffold151	35903	ACACACGCATAACACACGAG	TTGGAGTGCATGTGACCATT	58.20	459	INTERGENIC			
CaPOPI_II_1186	scaffold151	68819	AAATGAATGGAAGACGCGAT	AAAATTCTCTTGAATCCCCATT	59.53	851	INTERGENIC			
CaPOPI_II_1187	scaffold151	221306	TTCAAAGGAGATGGTTTGCC	ATGACAATTCCAATGCACCC	60.05	738	INTERGENIC			
CaPOPI_II_1188	scaffold1511	110106	TTAATGCAGGGGTTTGTGCT	ATTGAAGCCCACTATGGCTG	60.50	396	INTERGENIC			
CaPOPI_II_119	Ca1	45246034	CGATTTCGCTTTTAGCATCC	CAATTGCGATATTATGGGGG	59.82	253	INTERGENIC			
CaPOPI_II_1190	scaffold1521	15206	GGGGTTAGGGCTCATACGAT	GTTGCAGCGTGTTGTTCTGT	60.17	821	INTERGENIC			
CaPOPI_II_1191	scaffold1521	15603	TCGTGGTTCAATAATTGGCA	CAAAATAACGATTCGGGCTC	59.93	228	INTERGENIC			
CaPOPI_II_1192	scaffold1521	15896	TCGTGGTTCAATAATTGGCA	AAATTAGGCATACAGAAATGAAGGA	59.93	403	INTERGENIC			
CaPOPI_II_1193	scaffold1530	16153	TCTCACGGTTGTGAGGACTG	ACCGTTCTTGGTTTCTGACC	59.86	746	INTERGENIC			
CaPOPI_II_1194	scaffold1530	21107	TCTAAAAGATTTTGAACCCCAAA	TCCAAGGGTGTTAGAAACGG	59.06	812	INTERGENIC			
CaPOPI_II_1195	scaffold1530	23128	TCTCCATTGCACACCCAATA	GGTGCTGAAATCGGAAAATC	59.92	189	INTERGENIC			
CaPOPI_II_1196	scaffold1530	24697	TGGAGAGTTCAGCACACGTC	CCTCCGGTACCATAAACCCT	60.03	249	INTERGENIC			
CaPOPI_II_1197	scaffold1530	25494	CTTTTTCCGTTTTCAGCCAC	TTTACCCCTCGGTAAAACCTC	59.72	607	INTERGENIC			
CaPOPI_II_1198	scaffold1530	25850	TCGATCTTGAGCGAATTTGA	CAATACCAATTTCACGAAAATTCA	59.50	482	INTERGENIC			
CaPOPI_II_1199	scaffold1530	26727	TGCTTTTGGTAAAGGTGCTT	ATTCGCAAACCAAACTCGAT	57.57	742	INTERGENIC			

INDEL marker IDs	Chromosomes/ unanchored scaffolds	Physical positions (bp)	Forward primers (5'-3')	Reverse primers (5'-3')	Anneling temperature (0C)	Expected amplified product size (bp)	Structural annotation		Functional annotation	
							Sequence components of *kabuli* genome	*Kabuli* gene accession IDs	NCBI-KOG	TFs
CaPOPI_II_12	Ca1	2766077	AAAACAATTGATTGGGCTCC	CCAATCATACGCAGGTGAAA	58.88	561	INTERGENIC			
CaPOPI_II_120	Ca1	45609121	TCGCGTGAGAAAATAATCCC	CGAACCCTCGGTAAACAAAA	60.04	295	INTERGENIC			
CaPOPI_II_1200	scaffold1530	27186	TCGAGTTTGGTTTGCGAATA	CGATTGCTCATAAAATCGCTC	59.30	538	INTERGENIC			
CaPOPI_II_1201	scaffold1530	27815	TTTGAGAGGGCCTTTGGTAA	TCGACAGGTTTGTTTGTTCG	59.68	405	INTERGENIC			
CaPOPI_II_1202	scaffold1530	27996	TTTGAGAGGGCCTTTGGTAA	TCGACAGGTTTGTTTGTTCG	59.68	405	INTERGENIC			
CaPOPI_II_1203	scaffold1530	28367	GGCCCTATGGTGTTATGGTG	TTCGAAGCCATTATGTTTGATG	60.07	817	INTERGENIC			
CaPOPI_II_1204	scaffold1531	24929	TCAGGATTTTGGGTTTAGGG	TTTTGTAGTTGTACTCAATAACAAGGA	58.88	482	INTERGENIC			
CaPOPI_II_1205	scaffold1531	29443	TCCAGATGAAATCGAGGACC	GATTTTCGTTCCACCCTTCA	60.01	621	INTERGENIC			
CaPOPI_II_1206	scaffold1544	14620	CCACTGGATGAATCTCGGTT	TAGAAGGAACGGAGGGGTTT	59.93	794	INTERGENIC			
CaPOPI_II_1207	scaffold1550	56017	AGGCGATTTTCGTACCACAC	TCCAGATGAAATCGAGGACC	60.00	322	INTERGENIC			
CaPOPI_II_1208	scaffold1569	32526	CAGGGCCTTAAACCATGAAA	GGTTTGGGGTTTGGGATTTA	59.93	601	INTERGENIC			
CaPOPI_II_1209	scaffold1569	32980	ATAAACCGAACCGCAAAACA	GGTTTTATGGTTTAACGGCCT	60.36	714	INTERGENIC			
CaPOPI_II_121	Ca1	46088799	TAAGTCCGTAAGTGGTGCCC	GGGCTTTCCAAAGTGTTATCC	59.99	586	INTERGENIC			
CaPOPI_II_1210	scaffold157	289273	GCACATTCAGTGCCAAGAGA	ATTGAGGGCATTGTCACTCC	59.99	492	INTERGENIC			
CaPOPI_II_1211	scaffold1578	76801	CTGATAGTGACACCGGGTTG	CTTGTTTTCTCCATGGTCGC	59.01	664	INTERGENIC			
CaPOPI_II_1212	scaffold1579	64472	GAGCGAATTGAGAAACGAGG	TTTTCAAATAAAGTAACATTGACAACA	59.96	927	INTERGENIC			

INDEL marker IDs	Chromosomes/ unanchored scaffolds	Physical positions (bp)	Forward primers (5'-3')	Reverse primers (5'-3')	Anneling temperature (0C)	Expected amplified product size (bp)	Structural annotation		Functional annotation	
							Sequence components of *kabuli* genome	*Kabuli* gene accession IDs	NCBI-KOG	TFs
CaPOPI_II_1214	scaffold1580	69955	ATCACCAGCCACTTAGGCAA	TCCCTTATGACAATTCATGGTG	60.66	639	INTERGENIC			
CaPOPI_II_1216	scaffold1580	472375	TTTTGGTCAGGGGAAGGTTT	CTTGAAAGCCATTGGAGAGC	60.69	578	INTERGENIC			
CaPOPI_II_1217	scaffold1580	472426	TTTTGGTCAGGGGAAGGTTT	CTTGAAAGCCATTGGAGAGC	60.69	578	INTERGENIC			
CaPOPI_II_1218	scaffold1586	1838	AGGGAGGACGAAAATGACCT	TGACCAATTTTGAGCATCCA	59.94	471	INTERGENIC			
CaPOPI_II_1219	scaffold1613	98434	AAGAAAGTTGCTCCTTGCCA	AGGAGCACTTACCTCCGTTG	59.99	133	INTERGENIC			
CaPOPI_II_122	Ca1	46278771	ACCTTCAGTCAGGGGCCTAT	AAGTCTGGTCTGGCCTGAAA	59.96	538	INTERGENIC			
CaPOPI_II_1220	scaffold1613	106669	ACAATTTACGCCACCGAAAG	AGAAGCGACCCCATAGGTTT	60.00	549	INTERGENIC			
CaPOPI_II_1221	scaffold1614	131511	CCGCGAGTAATTCCGATAAA	ATCAGCCTAAGGGGGAAGAA	60.05	875	INTERGENIC			
CaPOPI_II_1222	scaffold1630	6306	GTACCACCCGACAAAAATGG	TCCAGATGAAATCGAGGACC	60.09	530	INTERGENIC			
CaPOPI_II_1223	scaffold1631	32889	GTCCACTTCCGTTTCGGTAA	GTCGCTTCAAAAGTTGGCTC	59.97	625	INTERGENIC			
CaPOPI_II_1224	scaffold1635	74246	GGTAAACGTGTGTTGCATGG	GGAGTACCATAGGCCAACGA	59.89	258	INTERGENIC			
CaPOPI_II_1225	scaffold1643	163768	ATTTCCAGCATGCCAATTTC	CTTCCAGAAACTAGCCGCAC	59.91	719	INTERGENIC			
CaPOPI_II_1226	scaffold1643	166873	GCAACTAGGGGTGTTCAGGA	ATGCACACTTGTTGCACCTT	60.11	610	INTERGENIC			
CaPOPI_II_1227	scaffold1643	169454	GTCATTTTTGGCGTAGTGGG	TTTCCGGTCAACACTCATCA	60.37	133	INTERGENIC			
CaPOPI_II_1228	scaffold1643	171022	TAACCCCATCAAATTTGGGA	TCTTGAAGGAGCGGGTAGAA	59.99	563	INTERGENIC			
CaPOPI_II_1229	scaffold1643	180974	CCCACGCTTCTTTAGCAGTC	TTTCACTTTCCCGCTTCTGT	60.02	859	INTERGENIC			

INDEL marker IDs	Chromosomes/ unanchored scaffolds	Physical positions (bp)	Forward primers (5'-3')	Reverse primers (5'-3')	Anneling temperature (0C)	Expected amplified product size (bp)	Structural annotation		Functional annotation	
							Sequence components of *kabuli* genome	*Kabuli* gene accession IDs	NCBI-KOG	TFs
CaPOPI_II_123	Ca1	46286614	CCAAGTTATGCTTGATCGCA	CGAGAACCATCCTGCATTTT	59.83	820	INTERGENIC			
CaPOPI_II_1230	scaffold1643	192850	GAGGGGGAGAAAGCTGAGAG	TGCTGACATGATTGTTGATGTG	60.47	535	INTERGENIC			
CaPOPI_II_1231	scaffold1643	210540	CACACAATCACAAATTTTTCTCAA	AAAGAAATCGAGACCGTTGC	59.08	419	INTERGENIC			
CaPOPI_II_1232	scaffold1643	211018	TTCTCTCCACCGCTAACTCG	ATGGTGGGGGTATGAATGAA	60.53	611	INTERGENIC			
CaPOPI_II_1233	scaffold1643	211096	ACGGTCTCGATTTCTTTTCG	ATGGTGGGGGTATGAATGAA	59.31	585	INTERGENIC			
CaPOPI_II_1234	scaffold1643	213932	CGGATTTCGTCTCTTGAAGC	CATTGGTGCAGTTGTTCGTC	59.96	662	INTERGENIC			
CaPOPI_II_1235	scaffold1651	5134	ATGGCACAAGTCTCCCAACT	GGGGCCACTTAGGGTTGTAT	59.58	276	INTERGENIC			
CaPOPI_II_1236	scaffold1651	12361	AAATTGTTGCTTGGGGACAA	TGACGTCATGACCTGCATTT	60.34	882	INTERGENIC			
CaPOPI_II_1237	scaffold1651	13370	CCTTGAGTTCGACACTCGGT	TGGTGACCGTGTAATTGTTG	60.30	337	INTERGENIC			
CaPOPI_II_1238	scaffold1651	13586	CCTTGAGTTCGACACTCGGT	CAGGATGTGAACCGTAAATCTTC	60.30	547	INTERGENIC			
CaPOPI_II_1239	scaffold1651	14250	AAAAATGGAGAGATCGAAAGTG	TTGATGCTAGGGGGATTTTG	57.49	553	INTERGENIC			
CaPOPI_II_124	Ca1	46286661	CCAAGTTATGCTTGATCGCA	CGAGAACCATCCTGCATTTT	59.83	820	INTERGENIC			
CaPOPI_II_1240	scaffold1651	16500	GGGAAGGGATGTAAAAGGGA	ACGTCGTGATTTGTGCTTCA	60.12	231	INTERGENIC			
CaPOPI_II_1241	scaffold1651	16843	GGGAAGGGATGTAAAAGGGA	AAAATCTGGCCTGGACCTG	60.12	510	INTERGENIC			
CaPOPI_II_1242	scaffold1651	17729	TCTCTTGGGTGATTTTTCGG	ACACCCAAACTCTTGTTGGC	60.04	561	INTERGENIC			
CaPOPI_II_1243	scaffold1651	18006	TGATTGGGGGCATACATTTT	ACACCCAAACTCTTGTTGGC	60.02	171	INTERGENIC			

INDEL marker IDs	Chromosomes/ unanchored scaffolds	Physical positions (bp)	Forward primers (5'-3')	Reverse primers (5'-3')	Anneling temperature (0C)	Expected amplified product size (bp)	Structural annotation		Functional annotation	
							Sequence components of *kabuli* genome	*Kabuli* gene accession IDs	NCBI-KOG	TFs
CaPOPI_II_1244	scaffold1651	18574	CTGGATTGGAATTGTCAGCA	TCACGCACACTCCATCTCTC	59.65	679	INTERGENIC			
CaPOPI_II_1245	scaffold1651	18823	GAGAGATGGAGTGTGCGTGA	AACCCAGAACTCGAGCTGAA	59.99	461	INTERGENIC			
CaPOPI_II_1246	scaffold1659	96268	TTGCTCACTACCACGCTACG	CACCAAAGCACATCCATTCA	60.07	382	INTERGENIC			
CaPOPI_II_1247	scaffold1659	269627	CACCGTAGTGTGGTGGTGAG	CGGTGAATCCTTTACGGAGA	60.06	531	INTERGENIC			
CaPOPI_II_1248	scaffold1661	91058	GTTGGATCCCTAGCACCGTA	AAAAATGAAAAGGGGAGCGT	59.96	905	INTERGENIC			
CaPOPI_II_1249	scaffold167	29049	TTACCGGCTATTCATGCCTC	TCCCTTAATTTTCCTGCCCT	60.06	508	INTERGENIC			
CaPOPI_II_125	Ca1	46352138	AGCGATGCATGTCTCAAGTG	TGCCCTTCTTCTTTTCCTAGTG	60.02	396	INTERGENIC			
CaPOPI_II_1250	scaffold1679	2481	TTACGGTTTTGCGTTTAGGG	AACCAAAATCCGACACCCTT	59.99	503	INTERGENIC			
CaPOPI_II_1251	scaffold1681	934	GAGATTTTCGTACCACCCGA	TCCAGATGAAATCGAGGACC	59.93	797	INTERGENIC			
CaPOPI_II_1252	scaffold1682	8324	AGGTCGTTTTCGTCCTTCCT	CACCCAATGACACCAATGAA	60.11	693	INTERGENIC			
CaPOPI_II_1253	scaffold1684	69381	CGGGTTGTACAAAAATCGCT	CAGCGATGGCTAGTGTTTGA	60.00	458	INTERGENIC			
CaPOPI_II_1254	scaffold1696	4415	ATAACCCGAACCTCAAACCC	TGTTTGAAATATGACGCCGA	60.05	645	INTERGENIC			
CaPOPI_II_1255	scaffold1696	5390	TTGAAGTACGAAAATCGCCC	TTCGTTATGCGGTCAAACCT	60.07	813	INTERGENIC			
CaPOPI_II_1256	scaffold1701	101096	AGGTCATTTTCGTCCTCCCT	GCCCTCAGATGAGAGTCAGG	59.94	515	INTERGENIC			
CaPOPI_II_1257	scaffold1703	131019	CCAAAACGATTTCCCTTCAA	TTTCCAAAAGTGACCGAACC	59.91	234	INTERGENIC			
CaPOPI_II_1258	scaffold1704	11771	AACGATCAAGAGTCGCAAAA	ACCCTAAACCCTAAAGCCCA	58.50	347	INTERGENIC			

INDEL marker IDs	Chromosomes/ unanchored scaffolds	Physical positions (bp)	Forward primers (5'-3')	Reverse primers (5'-3')	Anneling temperature (0C)	Expected amplified product size (bp)	Structural annotation		Functional annotation	
							Sequence components of *kabuli* genome	*Kabuli* gene accession IDs	NCBI-KOG	TFs
CaPOPI_II_1259	scaffold1708	14932	GCCATTTGGGTGGTTGATAG	CTGCTTGAGGACAAGCAACA	60.19	901	INTERGENIC			
CaPOPI_II_126	Ca1	46491737	GATGCATCTCACCAAGGGTT	CGGTGCAAACTCTACGTTCA	59.93	420	INTERGENIC			
CaPOPI_II_1261	scaffold1723	1064	CCTCATAGGTTCGACTGGGA	GCGCCCTCACATAATGTTTT	60.07	467	INTERGENIC			
CaPOPI_II_1262	scaffold1725	28680	GATTCAGTGGCCGATTTTGT	TTGGACGAAAAAGGAGGAAA	59.94	917	INTERGENIC			
CaPOPI_II_1263	scaffold1731	39857	ATGTTTTGGATTTTGGCTCG	GCACCGTGAGTAAATTCGGT	59.94	704	INTERGENIC			
CaPOPI_II_1265	scaffold1731	53952	TTTGGATATTGGTTCGGAGG	AGGAGGTTTGTGTTTGACGG	59.76	514	INTERGENIC			
CaPOPI_II_1266	scaffold1731	65474	TAATCGGGGACTCAACATCC	CCCTTTGGATGCGAAATAAA	59.75	279	INTERGENIC			
CaPOPI_II_1269	scaffold1740	2698	CGATTTGGCCATACAGGAAT	CCCATCTACATACCCATCCG	59.78	498	INTERGENIC			
CaPOPI_II_127	Ca1	46523548	GTGGACAAACCTGCATTCCT	ACCATGCACAGTTGCTGAGA	59.97	675	INTERGENIC			
CaPOPI_II_1270	scaffold1756	5242	GGTGAGGTCTTTTGCCTCAA	TTGGATGAAGGCCATAAAGG	60.23	665	INTERGENIC			
CaPOPI_II_1271	scaffold1757	1167	AAGCGTACCTGTGGGAATTG	TTAATGAGGGGCTGAATTGG	59.99	259	INTERGENIC			
CaPOPI_II_1272	scaffold1778	1269	ACTTCCGCGCTAGATTTCAA	TCCTGGTCCTCGATTTCATC	59.98	282	INTERGENIC			
CaPOPI_II_1273	scaffold1779	131414	TTGTTTCTCCCCAGTTTTGG	TTCCATATCACACGGAACCA	59.94	275	INTERGENIC			
CaPOPI_II_1274	scaffold1779	131497	TTGTTTCTCCCCAGTTTTGG	TTCCATATCACACGGAACCA	59.94	275	INTERGENIC			
CaPOPI_II_1275	scaffold178	605	CAATAGGCCAAGGAAGGACA	ACATTGAGGCCATTTTCGTC	60.07	236	INTERGENIC			
CaPOPI_II_1277	scaffold1810	103255	TTGCTAGTTGCTGATGTGGC	GGTGTCATCGGGAGTCTGTT	60.02	300	INTERGENIC			

INDEL marker IDs	Chromosomes/ unanchored scaffolds	Physical positions (bp)	Forward primers (5'-3')	Reverse primers (5'-3')	Anneling temperature (0C)	Expected amplified product size (bp)	Structural annotation		Functional annotation	
							Sequence components of *kabuli* genome	*Kabuli* gene accession IDs	NCBI-KOG	TFs
CaPOPI_II_1278	scaffold1810	103852	AACAGACTCCCGATGACACC	AACATTTATGACGATATTTATGAGTGA	59.97	438	INTERGENIC			
CaPOPI_II_1279	scaffold1816	53607	TGGTGAGGAAATGAGCAACA	ACAAAATGAAGGTGCGGAAG	60.24	679	INTERGENIC			
CaPOPI_II_128	Ca1	46528558	TTAAAATTCCCTCCTCGGCT	AACTCTGGGAACCCTCCTGT	60.03	398	INTERGENIC			
CaPOPI_II_1280	scaffold1822	17416	AAGCACCAATTACCGAGGTG	TTGTATTTGGCGTGTCCGTA	59.99	720	INTERGENIC			
CaPOPI_II_1281	scaffold1830	4465	CCAAGGGAAGACGAAAATGA	TGCATCCAACAGGTGTGTTT	60.04	665	INTERGENIC			
CaPOPI_II_1282	scaffold1835	8173	TCATTGCTCCTTTGAGTTGC	ATTTGCCACAGATACCGCTC	59.00	453	INTERGENIC			
CaPOPI_II_1283	scaffold1835	8463	GAGCGGTATCTGTGGCAAAT	CGGTTTGTGGCTTTCCTAAA	60.10	486	INTERGENIC			
CaPOPI_II_1284	scaffold1835	10086	TGGTTAACCTTGCACCACAA	GGATGCTTGAATTATGCCGT	60.00	672	INTERGENIC			
CaPOPI_II_1285	scaffold1844	2095	AACATGGGACGAGCTGTTTC	GGGATATTCAATAAGTGAAATTCAAA	60.12	923	INTERGENIC			
CaPOPI_II_1286	scaffold1844	2214	TGCAATTATGATCATGCTTTATATG	GGGATATTCAATAAGTGAAATTCAAA	58.20	732	INTERGENIC			
CaPOPI_II_1287	scaffold1844	2526	GACCTTTGAATTTATCTCTCTTTGAA	TTACAACATGTCAGGCCGAA	58.52	373	INTERGENIC			
CaPOPI_II_1289	scaffold1852	94526	GGTCCAACATCGACGCTAAT	TCGGATGTTACACACCTCACA	59.96	515	INTERGENIC			
CaPOPI_II_1290	scaffold1852	94771	GGTCCAACATCGACGCTAAT	TTTCAGTCATTTGATTTCGGA	59.96	531	INTERGENIC			
CaPOPI_II_1291	scaffold1852	94935	TACCATGGAGTGTGCTCGAA	TTTCAGAACACAAATCTGCATGT	60.26	765	INTERGENIC			
CaPOPI_II_1292	scaffold1864	5989	TTGCCCATTCTCCAAGTTTC	CGGATATAAGCATGGCATCA	60.05	659	INTERGENIC			
CaPOPI_II_1293	scaffold1864	14836	CGCTCTGTTAAGTCGGTTCA	CATCCACGGAGCAAAAACTT	59.07	251	INTERGENIC			

INDEL marker IDs	Chromosomes/ unanchored scaffolds	Physical positions (bp)	Forward primers (5'-3')	Reverse primers (5'-3')	Anneling temperature (0C)	Expected amplified product size (bp)	Structural annotation		Functional annotation	
							Sequence components of *kabuli* genome	*Kabuli* gene accession IDs	NCBI-KOG	TFs
CaPOPI_II_1295	scaffold1867	226694	ATCGATTTTGTCCGTTTTGC	GGAGGGTTTTGGTTTGGAAT	59.95	473	INTERGENIC			
CaPOPI_II_1296	scaffold1869	20055	TTTCAGGCGATTTTCGTACC	ATTGTGTTTCGGGTTTCGAG	60.07	825	INTERGENIC			
CaPOPI_II_1297	scaffold1885	47510	GTAGCTTCGGCTACACCGAC	ATTCCGTTGTGCACTTGATG	59.90	664	INTERGENIC			
CaPOPI_II_1298	scaffold1886	17993	GAGAGAGGCATTGCACAACA	CGAAATTTCTTTAACGAGGGG	59.99	537	INTERGENIC			
CaPOPI_II_1299	scaffold1905	1617	ACTGATGTCAAGAGGGCCAG	AGAGGGTGGCAATGGTAATG	60.26	251	INTERGENIC			
CaPOPI_II_130	Ca1	46539360	TCCCAAACAGCCTGATAACC	CTGATGACAAAAGGGGGAGA	59.93	744	INTERGENIC			
CaPOPI_II_1300	scaffold1923	10722	GGCCCCCAATATTTTGGTAA	ATTATTGGCCCCAACTCACA	60.72	383	INTERGENIC			
CaPOPI_II_1301	scaffold193	79630	AAAATAAATGCGCCGTATGC	AGATCATGTGCAAAAAGGGG	59.96	477	INTERGENIC			
CaPOPI_II_1302	scaffold193	79687	AAAATAAATGCGCCGTATGC	AGATCATGTGCAAAAAGGGG	59.96	477	INTERGENIC			
CaPOPI_II_1303	scaffold1943	2994	TTTGTAGCCTGCTCGGATTT	TCCATCGAGACTCATTTATAAGCA	59.85	807	INTERGENIC			
CaPOPI_II_1304	scaffold1943	393620	TGGTTCTCATGATACGTGTGG	GCGCAAACACAAACAACAAA	59.43	635	INTERGENIC			
CaPOPI_II_1305	scaffold1945	4916	CGTCAAACACAAACCTCCCT	TGGTTTTCACGCCAACATTA	60.01	659	INTERGENIC			
CaPOPI_II_1306	scaffold1956	63893	TTCGTTATGCAGTCAAATTTCTAA	AAAATGGACCAACGTCCGTA	58.10	719	INTERGENIC			
CaPOPI_II_1308	scaffold1962	159146	CTTTTTACAGCGCTTGTGGG	AAGCCGTGTAATCTGAACCG	60.79	557	INTERGENIC			
CaPOPI_II_1309	scaffold1962	170714	TCAAGGACCCAAAGCACATT	TACTCCCCCTTTGAGCTTGA	60.50	423	INTERGENIC			
CaPOPI_II_1312	scaffold1964	21448	GGGTCAGTTCATCCCATGAC	TATGCATCTTTGTTGACGCC	60.18	292	INTERGENIC			

INDEL marker IDs	Chromosomes/ unanchored scaffolds	Physical positions (bp)	Forward primers (5'-3')	Reverse primers (5'-3')	Anneling temperature (0C)	Expected amplified product size (bp)	Structural annotation		Functional annotation	
							Sequence components of *kabuli* genome	*Kabuli* gene accession IDs	NCBI-KOG	TFs
CaPOPI_II_1313	scaffold1964	172119	TTGTTTTGTTAGAAACGCCG	ACCCACATAGCGCAAGATTC	58.86	313	INTERGENIC			
CaPOPI_II_1314	scaffold198	501505	TTGGCCAAGTCCCTGTTTAC	CGTTCCTGAGGGAATTGTGT	59.97	498	INTERGENIC			
CaPOPI_II_1315	scaffold1981	239519	AAATTTGCGTTTAAATAACGGG	TGGATCCTCCCTTTTGTACG	59.34	779	INTERGENIC			
CaPOPI_II_1317	scaffold1981	355366	ACGATATTTTGAAAGGCCCC	CGCGGATCCAATATCTCACT	60.15	426	INTERGENIC			
CaPOPI_II_1318	scaffold1988	7769	ACCATACAAGTTCCCGCATC	GAAGGAGCAGTAAGCCATCG	59.82	725	INTERGENIC			
CaPOPI_II_1319	scaffold1996	1333	CGGATGAATCATTGGGAAAT	AGTGAAACCAACGCCAAAAC	59.58	325	INTERGENIC			
CaPOPI_II_132	Ca1	47630703	CGTGTCCGGCAAAGTAAAAT	GTGATGCACGCAGTAATTGG	60.00	517	INTERGENIC			
CaPOPI_II_1320	scaffold1996	5730	TGTTAGGAGGATCTCACGGG	ATTGTTGGAAAATTGGCTGC	60.07	823	INTERGENIC			
CaPOPI_II_1321	scaffold1999	29603	AGGGAGGACGAAAATGACCT	AACCAATCTTGTGCATCCAA	59.94	670	INTERGENIC			
CaPOPI_II_1322	scaffold1999	29835	AATCCAACGACAACGGAACT	CCGATGTTGTGCATCCAATA	59.45	541	INTERGENIC			
CaPOPI_II_1323	scaffold2000	1544	GCCTAGGGATGACGAAAATG	TGTGTTTGCGTCATTGGATT	59.53	566	INTERGENIC			
CaPOPI_II_1327	scaffold2018	4354	CATGGTTTTGTTTGTCGTGC	TGTTTGCTTTAAGCGATGTCA	60.01	462	INTERGENIC			
CaPOPI_II_1328	scaffold2018	16998	AAGGCTCACTTGCAAAGACG	GGTATTGCTGCAGTTTTTGG	60.57	445	INTERGENIC			
CaPOPI_II_1329	scaffold202	17816	TCACAAGGGTAGTTTTGGGTTT	CTGCCAGCTCACCTCCTATC	59.78	685	INTERGENIC			
CaPOPI_II_133	Ca2	42172	TGGCAGTCTGTTTCACGTTT	CAAGGGACCAGATTCTGCAT	59.34	553	INTERGENIC			
CaPOPI_II_1330	scaffold202	20536	TGCATCCAACTTTGGTGTGT	ACACCGTTGTTTATGCACGA	60.01	373	INTERGENIC			

INDEL marker IDs	Chromosomes/ unanchored scaffolds	Physical positions (bp)	Forward primers (5'-3')	Reverse primers (5'-3')	Anneling temperature (0C)	Expected amplified product size (bp)	Structural annotation		Functional annotation	
							Sequence components of *kabuli* genome	*Kabuli* gene accession IDs	NCBI-KOG	TFs
CaPOPI_II_1331	scaffold202	22635	GGATTACATTCGCTTCTCCG	GCAAACAATGCGAAACAGAA	59.67	804	INTERGENIC			
CaPOPI_II_1332	scaffold202	22768	TTGCAATCATGAGGCAAGTC	GCAAACAATGCGAAACAGAA	59.81	235	INTERGENIC			
CaPOPI_II_1333	scaffold202	37860	ATTGGAGGCTGGTTTTGTTG	ATCGTGGGGATGAATTTGAA	59.97	539	INTERGENIC			
CaPOPI_II_1334	scaffold202	37967	ATTGGAGGCTGGTTTTGTTG	ATCGTGGGGATGAATTTGAA	59.97	539	INTERGENIC			
CaPOPI_II_1335	scaffold202	39185	GCACCGGAAAATACGTAGGA	ATTATCTTGTCGCGGCCTAA	59.96	359	INTERGENIC			
CaPOPI_II_1336	scaffold202	39293	GCACCGGAAAATACGTAGGA	ATTATCTTGTCGCGGCCTAA	59.96	359	INTERGENIC			
CaPOPI_II_1340	scaffold202	104727	TCATTCATCCAATCCATCCA	TCGAGTGTCGATCTCAAGGA	59.66	489	INTERGENIC			
CaPOPI_II_1341	scaffold2024	1945	GAATGTTAGGGTTTCCGGGT	GATGAAATCGAGGACTGGGA	60.05	338	INTERGENIC			
CaPOPI_II_1342	scaffold2027	955	AGAAAGGCTCAAACGACCAA	ACAGAGGAAGCTGCCACCTA	59.85	672	INTERGENIC			
CaPOPI_II_1343	scaffold2030	4596	AGGGTTTAGGATTTCGCGTT	CATATCAAAAACCCAAATGACC	59.97	243	INTERGENIC			
CaPOPI_II_1345	scaffold2040	159577	TGAAGAGGGGAATCCATCTG	TTAACAACTCCTGGTTCGGG	60.00	372	INTERGENIC			
CaPOPI_II_1346	scaffold205	46882	GAGAGGATTTGTGTTCCCGA	GGAATGCTCAAAGGTTGCAT	60.05	235	INTERGENIC			
CaPOPI_II_1347	scaffold206	162025	CTTGGATTCATTGATCCGCT	GTTTGGTTTTCTTTGGGGGT	60.04	423	INTERGENIC			
CaPOPI_II_1348	scaffold206	176077	CTCTCGCTATGAAAAACGGG	TGGGTTCAATTGGCATTTTT	59.84	759	INTERGENIC			
CaPOPI_II_1349	scaffold206	190834	ATCCCTAACGCCGTTTTTCT	ATATTGGCTCGGAGGCTTTT	59.97	480	INTERGENIC			
CaPOPI_II_135	Ca2	1640062	AGATCATGTGCAAAAAGGGG	AGTAACCGTGAGGGAACGTG	59.93	509	INTERGENIC			

INDEL marker IDs	Chromosomes/ unanchored scaffolds	Physical positions (bp)	Forward primers (5'-3')	Reverse primers (5'-3')	Anneling temperature (0C)	Expected amplified product size (bp)	Structural annotation		Functional annotation	
							Sequence components of *kabuli* genome	*Kabuli* gene accession IDs	NCBI-KOG	TFs
CaPOPI_II_1350	scaffold207	6894	GCAAACTCGACAGGAAAAGC	TTGGTGAAGATGTCAGCCAG	60.00	370	INTERGENIC			
CaPOPI_II_1351	scaffold208	174233	CCTTGGAGTATTCTCGGCAG	TGCTGATCCGTGAGAGATTG	59.83	894	INTERGENIC			
CaPOPI_II_1352	scaffold210	31686	TTTTCTCGCAAAACATGCAG	TTCGCAACTCCACTCTTCCT	59.99	712	INTERGENIC			
CaPOPI_II_1353	scaffold2103	145039	GCCAGAACACCGACCATACT	CCAAGTGGTACAACTGGTGTCT	60.00	581	INTERGENIC			
CaPOPI_II_1354	scaffold2126	3526	CCATGAAAAGAAGAAGGGCA	GCCTTTTACTGCACTTTGGC	60.18	472	INTERGENIC			
CaPOPI_II_1355	scaffold2134	5442	GAAAAAGGAACGAAGGGAGG	AACGGAACGAGCGAGTCTTA	60.05	566	INTERGENIC			
CaPOPI_II_1356	scaffold2152	108576	TTTCAGCTTCATCGCATTCA	GAGATTGCCAAGAACAAGGC	60.49	448	INTERGENIC			
CaPOPI_II_1357	scaffold2166	41038	GCTCGAAACTGGTGGCTACT	TCCACAACAACCTACGTCCA	59.50	245	INTERGENIC			
CaPOPI_II_136	Ca2	2169056	AACCTCTCCAAGAGGATCGG	GGAGAGTGAAACGGATGGAA	60.59	648	INTERGENIC			
CaPOPI_II_1362	scaffold2173	19619	GGATGACACAAACGCACCTA	ATTTTTGTCCTCCCTTGGCT	59.57	385	INTERGENIC			
CaPOPI_II_1363	scaffold2173	19824	CCAAGGGAGGACAAAAATGA	TTTTCGTCCTTCACAGGCTT	59.90	515	INTERGENIC			
CaPOPI_II_1364	scaffold2179	20793	ATCAGCGAGGATGCAGAACT	CATCGCTCAGTTTGGACTCA	59.98	789	INTERGENIC			
CaPOPI_II_1365	scaffold2179	35934	GAATTTGAACCTCCAAGCCA	ATAGCGGAGAAGCCCATACA	60.05	404	INTERGENIC			
CaPOPI_II_1366	scaffold2179	35973	GAATTTGAACCTCCAAGCCA	ATAGCGGAGAAGCCCATACA	60.05	404	INTERGENIC			
CaPOPI_II_1367	scaffold2179	36066	GAATTTGAACCTCCAAGCCA	TGCATTCCACATACAAGGGA	60.05	582	INTERGENIC			
CaPOPI_II_1368	scaffold2183	19955	TAGGGCGATTTTCGTACCAC	TTTTCGCATATTTATTTGACAACTC	59.96	468	INTERGENIC			

INDEL marker IDs	Chromosomes/ unanchored scaffolds	Physical positions (bp)	Forward primers (5'-3')	Reverse primers (5'-3')	Anneling temperature (0C)	Expected amplified product size (bp)	Structural annotation		Functional annotation	
							Sequence components of *kabuli* genome	*Kabuli* gene accession IDs	NCBI-KOG	TFs
CaPOPI_II_1369	scaffold2183	67523	AGTCGCTCGAAAAGTCCAAA	TCCTGGTCCTCGATTTCATC	59.99	528	INTERGENIC			
CaPOPI_II_137	Ca2	2692293	AAAGGTGATGAAGCAGATTTTGA	TGGTGTCCTCTCTTCTCTAATCG	60.12	478	INTERGENIC			
CaPOPI_II_1370	scaffold22	24507	ATGAGGCTCAAAATGCCATC	GCCAACTTTTCCAATGCCTA	60.04	727	INTERGENIC			
CaPOPI_II_1371	scaffold22	34524	GCAGCATCGCAGATCATAGA	CGAATTGGGTTTTCGTTCTC	60.09	181	INTERGENIC			
CaPOPI_II_1372	scaffold2205	49159	TTTTTAACCAATCTTCGGCG	AGGGAGGACGAAAATGACCT	60.07	272	INTERGENIC			
CaPOPI_II_1373	scaffold2208	20005	CAAACACACCTGTTGGATGC	CCCTAGGCTTATTCTTGCCC	60.01	362	INTERGENIC			
CaPOPI_II_1374	scaffold2210	15184	TCCAGATGAAATCGAGGACC	AAATCCGACACGTAACTCGG	60.01	531	INTERGENIC			
CaPOPI_II_1375	scaffold223	95879	TTGGTCTCCTTGTGTTGTGC	TGGAGACCCCTTGATGAAAC	59.73	502	INTERGENIC			
CaPOPI_II_1376	scaffold2249	1002	TATTCCCACCCACGATCATT	CAACCAAGCCATTTTCCATT	60.01	871	INTERGENIC			
CaPOPI_II_1379	scaffold227	195682	AGACACAAACCCCGCAATAG	GGATGCCACCATTCAAGTTT	59.99	338	INTERGENIC			
CaPOPI_II_138	Ca2	2996172	TTCTAAGGACCAACGCAACA	GTATGATCGATTATGCGCGA	59.32	434	INTERGENIC			
CaPOPI_II_1380	scaffold227	196111	AAACTTGAATGGTGGCATCC	AGTAATGAATGGGCGAGTGG	59.80	595	INTERGENIC			
CaPOPI_II_1381	scaffold227	196280	AAACTTGAATGGTGGCATCC	AGTAATGAATGGGCGAGTGG	59.80	595	INTERGENIC			
CaPOPI_II_1382	scaffold227	197474	CCTAATGGCGGAAATTGTGT	TAATCAGACCGTGTGGGTGA	59.82	626	INTERGENIC			
CaPOPI_II_1383	scaffold2275	15734	AGTGTATAACCCGTGCGTCG	TTGGAGTCATGCTTTTCATCA	60.96	229	INTERGENIC			
CaPOPI_II_1384	scaffold228	136549	GATGAAATCGAGGACAGGGA	TGCGTCTTTCTCAAGTGCAT	60.01	445	INTERGENIC			

INDEL marker IDs	Chromosomes/ unanchored scaffolds	Physical positions (bp)	Forward primers (5'-3')	Reverse primers (5'-3')	Anneling temperature (0C)	Expected amplified product size (bp)	Structural annotation		Functional annotation	
							Sequence components of *kabuli* genome	*Kabuli* gene accession IDs	NCBI-KOG	TFs
CaPOPI_II_1385	scaffold2289	12871	TGTGTTTGTGCCATTGG ATT	AGGGAGGATGAAAACG ACCT	59.82	475	INTERGENIC			
CaPOPI_II_1386	scaffold2301	22791	CAAACACACCTGTTGGA TGC	CCTCGCTTGGCTTATTC TTG	60.01	466	INTERGENIC			
CaPOPI_II_1387	scaffold2301	54382	CCAAAGGAGGACGAAAA TGA	GCGTGTTTGTGACATTG GAG	60.04	349	INTERGENIC			
CaPOPI_II_1388	scaffold2301	54476	TGGATGCACATGATTGG TTT	AGGTCATTTTCGTCCTC CCT	59.78	316	INTERGENIC			
CaPOPI_II_1389	scaffold2346	28393	TGGGCGATTTTTGTACT TCC	CCCGAAAAGTCCAGATG AAA	59.94	395	INTERGENIC			
CaPOPI_II_1394	scaffold2371	73153	TGTGTCGGAGAATCTTC CCT	ATCGACGATAAGTCATC GGG	59.66	950	INTERGENIC			
CaPOPI_II_1395	scaffold2371	73366	CAAGAAAAAGTTTTGAC TTCCGA	ATCCACGCCACATAAAC CAT	59.80	919	INTERGENIC			
CaPOPI_II_1396	scaffold2371	73819	ATGGTTTATGTGGCGTG GAT	TGAATGAAGCGAAAATG TGC	60.08	321	INTERGENIC			
CaPOPI_II_1397	scaffold2371	150056	GCATTCGGGGGTATGTT TTA	CATCTTCCTTCCACAAT TTCTTT	59.66	970	INTERGENIC			
CaPOPI_II_1398	scaffold2371	287452	CGACTCGTGTCCTAAAA CTCAA	TTTCATGCCATAAGTCA TTGTG	59.42	884	INTERGENIC			
CaPOPI_II_1399	scaffold2385	5093	GAGTGAATTGTAACAGC CCAAA	GGACTTGCGGTGAAGA AGAA	59.13	720	INTERGENIC			
CaPOPI_II_14	Ca1	3020354	CAAGCAACATGCTGAAG GAA	ATGTCCTTAGTGTCACG GGG	59.99	808	INTERGENIC			
CaPOPI_II_140	Ca2	3031259	TCAATTGCGATGGATAG AGAAA	AATTGGTGTACCGATTG CGT	59.68	647	INTERGENIC			
CaPOPI_II_1400	scaffold2385	5335	GAGTGAATTGTAACAGC CCAAA	TCATTTCGACGCTTTCA CAC	59.13	968	INTERGENIC			
CaPOPI_II_1401	scaffold239	109266	GGTTTCAACCCCAATGC TTA	TCGACTTGTCAATCGCT TTG	59.80	808	INTERGENIC			
CaPOPI_II_1402	scaffold2392	10820	GGTAGGCCTTGGGCTA GTGT	TTGGAAGGAGCCCTTAC CTT	60.52	729	INTERGENIC			

INDEL marker IDs	Chromosomes/ unanchored scaffolds	Physical positions (bp)	Forward primers (5'-3')	Reverse primers (5'-3')	Anneling temperature (0C)	Expected amplified product size (bp)	Structural annotation		Functional annotation	
							Sequence components of *kabuli* genome	*Kabuli* gene accession IDs	NCBI-KOG	TFs
CaPOPI_II_1403	scaffold2392	326211	TCATTCCCCACCTACACAAG	GTGCATGTGGCTGAAAAACA	58.41	552	INTERGENIC			
CaPOPI_II_1404	scaffold240	38776	TATTTTGGATTTTGGCTCGG	TAGGAGAAACGGAGGGGTTT	59.90	575	INTERGENIC			
CaPOPI_II_1405	scaffold240	150038	GGAGGCTTTGAAATTAGGGC	CTCCAGCGCACAACATCTAA	60.04	783	INTERGENIC			
CaPOPI_II_1406	scaffold240	151385	AGATGCATGTGATGAAACCAA	TACTCCCATTTCCTCCCCTT	58.99	727	INTERGENIC			
CaPOPI_II_1407	scaffold240	156860	GACAATCGCAGGAAGTGGTT	GCATTGCACCAAAACACAAC	60.12	699	INTERGENIC			
CaPOPI_II_1408	scaffold242	593752	GGTTGTTTGAGCAAACTGCAT	TTTGATTTGTTTGATGAGCCA	60.17	634	INTERGENIC			
CaPOPI_II_1409	scaffold242	735562	CGAACTTGAGAATTCACCGC	GTGGAAAAGGGGATTGGATT	60.78	371	INTERGENIC			
CaPOPI_II_1410	scaffold2434	76263	TTCTTCGTTTTGTAGTTGTACTCAATG	GGCAAACAAGAACCGAAAAC	60.04	638	INTERGENIC			
CaPOPI_II_1411	scaffold2501	7458	AACCGATCTTGTCGATCCAA	AGGGAGGACGAAAATGACCT	60.46	373	INTERGENIC			
CaPOPI_II_1412	scaffold2505	1462	ATTCCAAACCAAAACCCTCC	TTGCTCACTACCACGCTACG	60.03	884	INTERGENIC			
CaPOPI_II_1413	scaffold2505	1793	CGTTTCCACACAACCACAAA	TTGCTCACTACCACGCTACG	60.44	112	INTERGENIC			
CaPOPI_II_1414	scaffold2511	7402	TGTTGAAGAAGGTTTTGGGG	CCAATAACGATCAAGCAATCG	59.94	359	INTERGENIC			
CaPOPI_II_1415	scaffold2528	19502	GTCCTCCGTTGGCTTATTCA	GGACGAAAATGACCAAAAGG	60.07	488	INTERGENIC			
CaPOPI_II_1416	scaffold2542	2207	TCATTTGGACTTTTCGGGAG	TCAATTTGGGTTCAGGGTGT	60.04	816	INTERGENIC			
CaPOPI_II_1417	scaffold2557	49964	CTTGGGCAAACACCTCCTAA	TGCATTGGTATATGGAGGCT	60.10	907	INTERGENIC			
CaPOPI_II_1418	scaffold2560	60629	GGAGAACATTATCCGTTTTCCA	TCAAATTTCATCCTCACCCA	60.18	536	INTERGENIC			

INDEL marker IDs	Chromosomes/ unanchored scaffolds	Physical positions (bp)	Forward primers (5'-3')	Reverse primers (5'-3')	Anneling temperature (0C)	Expected amplified product size (bp)	Structural annotation		Functional annotation	
							Sequence components of *kabuli* genome	*Kabuli* gene accession IDs	NCBI-KOG	TFs
CaPOPI_II_1419	scaffold2560	61168	GGGTGAGGATGAAATTTGAAGA	ATTCGGTAGCACATTCGTCC	60.30	864	INTERGENIC			
CaPOPI_II_1420	scaffold2575	196132	ACTTTTCACCACATCCCTCG	ATCCATTCCCCACATTGAAA	59.97	347	INTERGENIC			
CaPOPI_II_1421	scaffold2575	279306	TTTTTGTTGGATGTAGTCCCTG	CATGAACATTTAAGAGTTTGCATTT	58.99	583	INTERGENIC			
CaPOPI_II_1422	scaffold2575	280082	TCCCAAGGTATCATTGTCTCTTT	TCGGAGTTTTGTGTGAGTGTG	59.01	316	INTERGENIC			
CaPOPI_II_1423	scaffold26	506230	ATTGGCGTGAAAACCGTTAG	TGACGGGTCTGCACAATTTA	60.00	538	INTERGENIC			
CaPOPI_II_1424	scaffold2603	6501	AGCAGCTTCAAGCAAAGCTC	TGAACCAGTCCAATTCAACAGT	60.04	485	INTERGENIC			
CaPOPI_II_1425	scaffold2603	67369	AATCCGCAACGGAGTTTATG	TGAACGAGCTGAGAGAAGCA	59.96	617	INTERGENIC			
CaPOPI_II_1428	scaffold2617	324497	AAACAAGTTGGTGGTGGAGC	AAAGTTAATTCAAATGCACTGTCA	60.01	290	INTERGENIC			
CaPOPI_II_1429	scaffold2617	402483	GTTGCAAGGGTGGAGTGATT	AACAATTGTGAAACGTCTCCG	59.97	640	INTERGENIC			
CaPOPI_II_143	Ca2	4285697	CCAGTCCCGAAATTAAACGA	ACTTGCGATAATCGATTGGG	59.93	613	INTERGENIC			
CaPOPI_II_1430	scaffold2617	402519	GTTGCAAGGGTGGAGTGATT	AACAATTGTGAAACGTCTCCG	59.97	640	INTERGENIC			
CaPOPI_II_1431	scaffold2660	12451	CCTAGGGACGACGAAAATGA	TGCATCCAACAGGTGTGTTT	60.07	462	INTERGENIC			
CaPOPI_II_1432	scaffold2662	1313	TGCACTTGCAACACATCTCA	TGGCTATTGATTATCCCATTTTG	60.03	866	INTERGENIC			
CaPOPI_II_1433	scaffold2662	26227	AACACCATGAAACGCTCCTC	CGAAGCCGAGAAAATGACTC	60.12	589	INTERGENIC			
CaPOPI_II_1434	scaffold2662	31062	AGAGGATGCGGATCAATGTC	GGACAACCTGGGAAAAGTGA	60.04	898	INTERGENIC			
CaPOPI_II_1435	scaffold2669	16328	TTTTAGGCGATTCTACGTGC	TTGAGGGGTGGCAAGTTTAG	58.07	641	INTERGENIC			

INDEL marker IDs	Chromosomes/ unanchored scaffolds	Physical positions (bp)	Forward primers (5'-3')	Reverse primers (5'-3')	Anneling temperature (0C)	Expected amplified product size (bp)	Structural annotation		Functional annotation	
							Sequence components of *kabuli* genome	*Kabuli* gene accession IDs	NCBI-KOG	TFs
CaPOPI_II_1436	scaffold2692	14565	ACCTGTCAAAAGTTTTCAAGCA	TCCTTCACCAACCAAACAAA	58.93	420	INTERGENIC			
CaPOPI_II_1437	scaffold2692	14759	ACCTGTCAAAAGTTTTCAAGCA	TTTCCTTCACCAACCAAACA	58.93	422	INTERGENIC			
CaPOPI_II_1439	scaffold2726	4506	CAAACACCCCTATTGGATGC	AGGTCATTTTCGTCCTCCCT	60.19	380	INTERGENIC			
CaPOPI_II_1440	scaffold2734	606	TTTCTTTTTCCCTCCAAGCA	GGGGGAGAGAACAAATCCAT	59.80	550	INTERGENIC			
CaPOPI_II_1441	scaffold2736	3536	TTCAAGGAAAATGCCCAGTC	TGTTTGCGACAACACAACAA	60.05	385	INTERGENIC			
CaPOPI_II_1443	scaffold275	162362	CGAAGCACAAGGAGATGACA	GGTTTGGGCAATCTGCTTTA	59.98	762	INTERGENIC			
CaPOPI_II_1444	scaffold275	377072	TCAAGATACCCAGTGCACCA	AGCGACATGTGAACAACAGC	60.11	551	INTERGENIC			
CaPOPI_II_1446	scaffold277	4447	TAAAGGAGCATCCGTGGAAT	TGCATGTATCATAGACCGCC	59.53	472	INTERGENIC			
CaPOPI_II_1447	scaffold277	4861	TGGAGAAACCTTGTGAACCA	GAAACGGAAAGGGGAACAAT	59.11	316	INTERGENIC			
CaPOPI_II_1449	scaffold277	252454	CCCATTGCATTGCCTTCTAT	TAAAGGACCATGTCGGGAAG	59.92	329	INTERGENIC			
CaPOPI_II_145	Ca2	4892794	GCGGACATAAGGAAGATGTGA	AGGTCTGCGAAGATGGAAAA	60.09	606	INTERGENIC			
CaPOPI_II_1450	scaffold2777	4052	CATTGATTAAGAACGCAAATGG	AGTTGGTCCCAAAAGTGCAA	59.49	199	INTERGENIC			
CaPOPI_II_1451	scaffold2782	6224	CTTTTCGGGCGATTTTCATA	GATGAAATCGAGGACTGGGA	60.03	483	INTERGENIC			
CaPOPI_II_1452	scaffold280	52649	TGGTCATGCAGAAACTCAGC	ACTCTCATCTGAGGGCGAAG	59.99	330	INTERGENIC			
CaPOPI_II_1453	scaffold280	128741	ATTCCAAACCAAAACCCTCC	TGATTTTGGGGGAAAATGAA	60.03	943	INTERGENIC			
CaPOPI_II_1454	scaffold281	1263	TGTTCGACACTCAGTCTTACGG	TGCATGCCCTTTGATTTACA	60.35	287	INTERGENIC			

INDEL marker IDs	Chromosomes/ unanchored scaffolds	Physical positions (bp)	Forward primers (5'-3')	Reverse primers (5'-3')	Anneling temperature (0C)	Expected amplified product size (bp)	Structural annotation		Functional annotation	
							Sequence components of *kabuli* genome	*Kabuli* gene accession IDs	NCBI-KOG	TFs
CaPOPI_II_1455	scaffold281	1388	TGTTCGACACTCAGTCTTACGG	TGCATGCCCTTTGATTTACA	60.35	287	INTERGENIC			
CaPOPI_II_1456	scaffold281	1467	TGTTCGACACTCAGTCTTACGG	ATCGGAAAAGTGACATTGGG	60.35	515	INTERGENIC			
CaPOPI_II_1457	scaffold281	2224	TGGGCAGATTTGTAACACGA	GCGAAACCCGATTCTATTGT	60.11	593	INTERGENIC			
CaPOPI_II_1458	scaffold281	3002	ACAATAGAATCGGGTTTCGC	AGGACTATTTTTGGTCTCCCA	59.05	623	INTERGENIC			
CaPOPI_II_1459	scaffold281	3090	ACAATAGAATCGGGTTTCGC	AGGACTATTTTTGGTCTCCCA	59.05	623	INTERGENIC			
CaPOPI_II_146	Ca2	4894086	TTGAACTACAACCAGCGCAC	TTTTGTTCAATTTCTTGCTCCA	59.91	493	INTERGENIC			
CaPOPI_II_1460	scaffold281	3414	TGGGAGACCAAAAATAGTCCT	CGTTGACCATTTTAAGCTTGG	57.66	534	INTERGENIC			
CaPOPI_II_1461	scaffold281	3735	TGGGAGACCAAAAATAGTCCT	CAAGAGGGGTTGCAAGTGTT	57.66	648	INTERGENIC			
CaPOPI_II_1462	scaffold281	4125	AACACTTGCAACCCCTCTTG	ATGATGATTTTCTCCCCGTG	60.15	595	INTERGENIC			
CaPOPI_II_1463	scaffold281	4227	AACACTTGCAACCCCTCTTG	ATGATGATTTTCTCCCCGTG	60.15	595	INTERGENIC			
CaPOPI_II_1464	scaffold284	4461	TACGATGATGAACAGTCGGC	TTGGTTTCAAGACCTGCATCT	59.68	424	INTERGENIC			
CaPOPI_II_1465	scaffold2871	30418	GGTTTTGGATTTGGGCTACA	GGGTTATGGCTTTGATCATGT	59.80	521	INTERGENIC			
CaPOPI_II_1466	scaffold2893	10683	GCCAGATCTTTCGATTCAGC	CCTTCAATGGAACCGCATAC	59.92	255	INTERGENIC			
CaPOPI_II_1468	scaffold290	293241	TTGCTCTTAATTTTTCCTCGAA	GTGAGTCTGCGACCACTTGA	58.20	602	INTERGENIC			
CaPOPI_II_1469	scaffold290	400824	TTGTGTTGCATTGTGGATTCT	CACAGCCCCTCTAAACCAAC	59.04	650	INTERGENIC			
CaPOPI_II_147	Ca2	6505178	CGTAATAACGGCGAGGAAAA	TGAGCAGGGGGAAGAGTAGA	60.09	548	INTERGENIC			

INDEL marker IDs	Chromosomes/ unanchored scaffolds	Physical positions (bp)	Forward primers (5'-3')	Reverse primers (5'-3')	Anneling temperature (0C)	Expected amplified product size (bp)	Structural annotation		Functional annotation	
							Sequence components of *kabuli* genome	*Kabuli* gene accession IDs	NCBI-KOG	TFs
CaPOPI_II_1470	scaffold290	452067	GAAAAATTCACCACCGATTAGG	TACAATCAGGATGCGTCCAA	59.72	632	INTERGENIC			
CaPOPI_II_1471	scaffold2900	7979	TATGAAAATCGCCCGAAAAG	GGTCCTCGATTTCATCTGGA	60.03	355	INTERGENIC			
CaPOPI_II_1472	scaffold2928	22766	AACCAATGATGTGCATCCAA	AGGGAGGACGAAAATGACCT	59.78	471	INTERGENIC			
CaPOPI_II_1473	scaffold2928	23701	AGACCTCGCTTGGCTGATTA	ACGGAGGAGGAAAATGACCT	59.98	210	INTERGENIC			
CaPOPI_II_1474	scaffold2932	2304	ATTGTCGTCCTCCCTAGGCT	ACACCTATTGGATGCCCAAG	60.10	670	INTERGENIC			
CaPOPI_II_1475	scaffold2950	1436	GTCCTCTGAAACCTCCCACA	TTTTGGGTCGTGACACTTGA	60.09	337	INTERGENIC			
CaPOPI_II_1476	scaffold2950	6843	CCAAATTACAACCGACCCAC	AAAGGCGCTCCTCTATCACA	60.09	650	INTERGENIC			
CaPOPI_II_1477	scaffold2950	6903	CCAAATTACAACCGACCCAC	AAAGGCGCTCCTCTATCACA	60.09	650	INTERGENIC			
CaPOPI_II_148	Ca2	6640316	ATGGCCACGAATCTGAAGAG	ACGGTTGTTTTGGCATTGAT	60.22	890	INTERGENIC			
CaPOPI_II_1481	scaffold2955	9708	ACTATGCAAAGGTGCAAGGC	CACCATTTTCCGTCCACTCT	60.28	374	INTERGENIC			
CaPOPI_II_1482	scaffold2955	10080	AGAGTGGACGGAAAATGGTG	AAACTTGGTGCCCTGACTTG	59.97	621	INTERGENIC			
CaPOPI_II_1483	scaffold296	11327	TGGACGTAGTCCGAGTTGGT	CTTGTGCTTCGGTTACAGCA	60.57	517	INTERGENIC			
CaPOPI_II_1484	scaffold296	261628	CATTGTGGGAGCGTAAGACA	TCGCAACTCTCATGTGTATGG	59.72	388	INTERGENIC			
CaPOPI_II_1487	scaffold296	406805	AGTTATTTACGACGCACGGG	GGCACGACATCATCATCATC	60.02	547	INTERGENIC			
CaPOPI_II_1488	scaffold298	8123	AAATGAAAAGTGGCGAGGAC	AGTTACCCATTTGTGGCAGC	59.17	740	INTERGENIC			
CaPOPI_II_1489	scaffold298	208119	AGTCGGAGGTTCCTAGCACA	ATTGTTGGGCTTGTCAGTCC	59.87	471	INTERGENIC			

INDEL marker IDs	Chromosomes/ unanchored scaffolds	Physical positions (bp)	Forward primers (5'-3')	Reverse primers (5'-3')	Anneling temperature (0C)	Expected amplified product size (bp)	Structural annotation		Functional annotation	
							Sequence components of *kabuli* genome	*Kabuli* gene accession IDs	NCBI-KOG	TFs
CaPOPI_II_149	Ca2	6641737	GTATTCAGGGGCGAAGGAAT	AATGCAAACGACAAAGGAGG	60.29	500	INTERGENIC			
CaPOPI_II_1492	scaffold3015	73264	TTTGTGTTTGCGATGACGAT	TAAGAGCGGAGTTTCTCCCA	60.12	792	INTERGENIC			
CaPOPI_II_1493	scaffold3015	73454	GTCTCGCGTTGAGTTAAGGG	TAAGAGCGGAGTTTCTCCCA	59.88	489	INTERGENIC			
CaPOPI_II_1494	scaffold3015	73940	TGGGAGAAACTCCGCTCTTA	TGATGGCTCCAAGGTGTATG	59.95	538	INTERGENIC			
CaPOPI_II_1495	scaffold3015	74020	TGGGAGAAACTCCGCTCTTA	TGATGGCTCCAAGGTGTATG	59.95	538	INTERGENIC			
CaPOPI_II_1496	scaffold3015	74169	TTTTGAAATTTAGGGTGTCACG	TGATGGCTCCAAGGTGTATG	59.03	221	INTERGENIC			
CaPOPI_II_1499	scaffold3046	14446	TTGGATGAACATTTCGGTCA	AAGCCTAGGGAGGACGAAAA	59.90	240	INTERGENIC			
CaPOPI_II_15	Ca1	3783626	TTCACAATTACCACAAAGATCG	GCGACTATCCAGGAAATCCA	57.69	433	INTERGENIC			
CaPOPI_II_150	Ca2	6641916	GTTCTATGGGGACAGGGTCA	TATCAACTACCAGAGCGGGG	59.78	476	INTERGENIC			
CaPOPI_II_1500	scaffold306	128535	ACCGAAAGGAGAACACATGG	TGCAAATGAAAGTTTAAAGACCA	59.97	240	INTERGENIC			
CaPOPI_II_1501	scaffold306	166393	TGCACAAGTCAGTAGCACCC	GCCAATCGATTACCCTCAAA	59.91	138	INTERGENIC			
CaPOPI_II_1502	scaffold3092	1124	GAGGTATTTTACAGCGCTCTTTC	TTGGGTCTGAAGAATGGCTC	58.67	117	INTERGENIC			
CaPOPI_II_1503	scaffold3093	19732	GAACCCTCACAATCACACGA	GAGCGCTGTAAAACACCTCC	59.53	863	INTERGENIC			
CaPOPI_II_1504	scaffold311	91768	TGGGCAAGAAAAAGCCTATG	AGGTCATTTTCGTCCTCCCT	60.20	836	INTERGENIC			
CaPOPI_II_1506	scaffold3115	62578	CTTGGTGGTCCACTATGTGC	GGAGGTCAAAATACTAACTGCGA	59.01	594	INTERGENIC			
CaPOPI_II_1507	scaffold3115	62639	CTTGGTGGTCCACTATGTGC	TTCATAGGAGGCTGCCATTC	59.01	632	INTERGENIC			

INDEL marker IDs	Chromosomes/ unanchored scaffolds	Physical positions (bp)	Forward primers (5'-3')	Reverse primers (5'-3')	Anneling temperature (0C)	Expected amplified product size (bp)	Structural annotation		Functional annotation	
							Sequence components of *kabuli* genome	*Kabuli* gene accession IDs	NCBI-KOG	TFs
CaPOPI_II_1508	scaffold3115	62933	CTTGGTGGTCCACTATGTGC	TTCATAGGAGGCTGCCATTC	59.01	632	INTERGENIC			
CaPOPI_II_1509	scaffold3115	63986	TGAAGCATATGCCGTGAGAG	GCGTCAATGAACTTTACATGC	59.97	559	INTERGENIC			
CaPOPI_II_151	Ca2	6643843	ATGGAACAAGCAGCGAAGTT	GTTGATACGAGGGGGTTGAA	59.88	537	INTERGENIC			
CaPOPI_II_1510	scaffold3127	8863	AATCCAATGGCACAAACACA	GGACATTTTCGTCCTGCCTA	59.82	488	INTERGENIC			
CaPOPI_II_1513	scaffold3134	109334	AGGAAGAGTGGAGTTGCGAA	TTAATGTGTTGCGGGTGAAA	59.99	342	INTERGENIC			
CaPOPI_II_1514	scaffold3135	4161	TCCAGATGAAATCGAGGACC	CCCGAAAAGTCCAGATGAAA	60.01	882	INTERGENIC			
CaPOPI_II_1515	scaffold3149	9644	TTTCATCTGGACTTTTCGGG	ATCGTCGATGGGTACGAAAG	60.04	452	INTERGENIC			
CaPOPI_II_1517	scaffold3160	9833	AACCAGTCACTCAAGCGTCC	TGGCTGGTGATTTGTGTGAT	60.31	165	INTERGENIC			
CaPOPI_II_1518	scaffold3160	10499	TGGCTTTTTAGTTAGAGCACCA	GCAAACAAAGGAAACGGAAA	59.08	629	INTERGENIC			
CaPOPI_II_1519	scaffold3171	2398	TGATGGAGGATTGAGGAAGG	GTTGACAAATGCAAAATGCG	60.00	431	INTERGENIC			
CaPOPI_II_152	Ca2	6648530	TTATCGATCGTCGGTGTCAA	TTCCATATGCACCTCCCATT	60.07	612	INTERGENIC			
CaPOPI_II_1520	scaffold3171	2647	TGATGGAGGATTGAGGAAGG	GTTGACAAATGCAAAATGCG	60.00	431	INTERGENIC			
CaPOPI_II_1521	scaffold3212	725	CGAGTCGTGTCTTGATAATGGA	CCCATTAGTGGTGATGCTCC	60.13	125	INTERGENIC			
CaPOPI_II_1522	scaffold3212	1519	AATGACATTTGTCCCCGTAGA	ATCGGTGGCATGTCCTTACT	59.30	741	INTERGENIC			
CaPOPI_II_1523	scaffold3212	1828	GCAGAAATTAAAATGAGTGGTGTG	TGGAAGGTGGAGGTTCAGAG	59.94	715	INTERGENIC			
CaPOPI_II_1524	scaffold3212	2564	TGAACCTCCACCTTCCATTC	CAAACAAATGAGTGGCGATG	59.90	439	INTERGENIC			

INDEL marker IDs	Chromosomes/ unanchored scaffolds	Physical positions (bp)	Forward primers (5'-3')	Reverse primers (5'-3')	Anneling temperature (0C)	Expected amplified product size (bp)	Structural annotation		Functional annotation	
							Sequence components of *kabuli* genome	*Kabuli* gene accession IDs	NCBI-KOG	TFs
CaPOPI_II_1525	scaffold3212	6334	GAGCACATGATCTCGCAGAA	CACTGCTGAAGCGGAATACA	60.10	213	INTERGENIC			
CaPOPI_II_1526	scaffold3217	4699	CGCTTTGCCCTTAGTTTGAG	ATTCCCCATCAATTTGGACA	60.01	298	INTERGENIC			
CaPOPI_II_1527	scaffold3228	1858	TCAGCTTTTTGGCTGATGTG	CTTGTGGATTGGGTCTTGTG	59.99	247	INTERGENIC			
CaPOPI_II_1528	scaffold3229	5378	AGCTGAAATTCCACAAGGCA	GATAATGGCCGCATGAAAAG	60.78	366	INTERGENIC			
CaPOPI_II_1529	scaffold3229	8934	TGAAGCACAATGTTGGTTTTG	TGTAAAGTGCAACACCCGTC	59.62	692	INTERGENIC			
CaPOPI_II_153	Ca2	7171869	CGGCCATTAAATTTCTGACA	TTGCATTATGTTTCTGGCAAA	58.63	320	INTERGENIC			
CaPOPI_II_1531	scaffold3258	41416	TTTTTGTGTTTGAATACACGGTT	TTTTTCCTCGAGTGCAGCTT	58.52	588	INTERGENIC			
CaPOPI_II_1532	scaffold3258	46908	ATGGCCCCATTATTGAGGTT	TGCACGAGTCTAATGAGGGA	60.40	696	INTERGENIC			
CaPOPI_II_1533	scaffold3258	48852	ATGACACGGCTGAATGTCAA	ACGCACGTATGTTGTCCAAA	60.12	374	INTERGENIC			
CaPOPI_II_1534	scaffold3258	52201	TTTTTGGGTCCCTATCAACTAA	TCCAACAATAATTTAAAACCTTACAA	57.69	500	INTERGENIC			
CaPOPI_II_1535	scaffold3258	66747	GAAAGCGCTGTAAAAGGTGC	TGATTTGCTGGGAAATGACA	60.03	504	INTERGENIC			
CaPOPI_II_1536	scaffold3258	74031	AACTTCCACACCTGTCTCCG	AATTCAAAAGAATGTCGGCG	60.15	726	INTERGENIC			
CaPOPI_II_1537	scaffold3258	74657	AATGCAATCGTCAGGACACA	TATGGACCGGAAATGGATGT	60.12	630	INTERGENIC			
CaPOPI_II_1538	scaffold3317	3339	GCATCTGTGTAGCCATCCAA	TAGGTCATTTTCGTCCTCCG	59.68	738	INTERGENIC			
CaPOPI_II_1539	scaffold3321	69689	TCTTGCAGGAGCTTGGATTT	TGTGCCGACAATTAACCAAA	59.96	681	INTERGENIC			
CaPOPI_II_154	Ca2	7171912	CGGCCATTAAATTTCTGACA	TCATTTCATAACGAGTGACCCA	58.63	584	INTERGENIC			

INDEL marker IDs	Chromosomes/ unanchored scaffolds	Physical positions (bp)	Forward primers (5'-3')	Reverse primers (5'-3')	Anneling temperature (0C)	Expected amplified product size (bp)	Structural annotation		Functional annotation	
							Sequence components of *kabuli* genome	*Kabuli* gene accession IDs	NCBI-KOG	TFs
CaPOPI_II_1540	scaffold333	8945	GATGAAATCGAGGACCAGGA	GACTTTTCGAGCGATTTTCG	60.01	299	INTERGENIC			
CaPOPI_II_1541	scaffold335	88156	TTGTCTTCTTGTGGCCGACT	TTTTACAGGCGATTTGGAGC	60.83	785	INTERGENIC			
CaPOPI_II_1542	scaffold336	633921	GAGAAAGGCAAGACCAGTGC	TTGCAATTCAACTGATGGGA	60.00	365	INTERGENIC			
CaPOPI_II_1543	scaffold3364	43644	AGCCAAGGGAGGATGAAAAT	CGTCCACCCTTTGATTATCC	59.90	214	INTERGENIC			
CaPOPI_II_1544	scaffold3369	18856	TTCAACGTGACCTGCTCATC	ATGGGTCCAGGTCATCTCAG	59.84	671	INTERGENIC			
CaPOPI_II_1545	scaffold338	76520	CTTGGCCATAAAATCGGAAA	AGGATGCATGGGATGAAAAG	59.90	661	INTERGENIC			
CaPOPI_II_1546	scaffold340	161360	GCGGATATCGTAGGGTGCTA	TACATCCAGAAGGCTACCGC	60.08	539	INTERGENIC			
CaPOPI_II_1547	scaffold341	135303	AATGGAGTTGCGACGAGAGT	TCTCCGTAAAGGATTCACCG	59.87	540	INTERGENIC			
CaPOPI_II_1548	scaffold342	310549	TTGTGACATTTCATCAAACACA	CAACCGACGTTGAAGAACAA	57.51	933	INTERGENIC			
CaPOPI_II_1549	scaffold342	340574	TGTTCCAGCATACCAAATGC	AGCAAAATCCGAGGAGGAAT	59.55	557	INTERGENIC			
CaPOPI_II_155	Ca2	7172121	TTTGCCAGAAACATAATGCAA	TCATTTCATAACGAGTGACCCA	59.20	285	INTERGENIC			
CaPOPI_II_1550	scaffold342	370625	TGGGGTCCTTTCTCTTCTCC	TATGCCCATTGAGTTGGACA	60.57	387	INTERGENIC			
CaPOPI_II_1551	scaffold3432	23969	CCTGAATCCGAGACAAGACG	TCATGGGTTGTATGGAAGCA	60.79	487	INTERGENIC			
CaPOPI_II_1552	scaffold3436	9281	GGCCATGAATTCAACAACAA	TTGCCAAATACAAAAAGAAATGAA	59.38	570	INTERGENIC			
CaPOPI_II_1553	scaffold3436	9959	TGGAGTTCGGGCTAAGTACAG	CGTGCTTGCAGAAAAACAAA	59.39	309	INTERGENIC			
CaPOPI_II_1554	scaffold3453	1173	ATATGCCTCTACCGTGCAGC	TCGTGAAAGCAAACAAAGGA	60.27	507	INTERGENIC			

INDEL marker IDs	Chromosomes/ unanchored scaffolds	Physical positions (bp)	Forward primers (5'-3')	Reverse primers (5'-3')	Anneling temperature (0C)	Expected amplified product size (bp)	Structural annotation		Functional annotation	
							Sequence components of *kabuli* genome	*Kabuli* gene accession IDs	NCBI-KOG	TFs
CaPOPI_II_1555	scaffold3472	6763	CAAGCATGATCCAGGGAGAT	CTCGGCGAGTTTGTGTGTAA	60.03	471	INTERGENIC			
CaPOPI_II_1556	scaffold3472	7148	CAGGCTAGCTAAATGCCTCG	ACCATGGTTGTTGCAAGTGA	60.13	720	INTERGENIC			
CaPOPI_II_1557	scaffold3472	9063	GCTCCATGTGCTTCACACAC	GTGACACGTGACAATCCCAC	60.33	107	INTERGENIC			
CaPOPI_II_1558	scaffold3472	9396	TTTTGACAGAGAAACCCGCT	CTGTGAGAGGCGAAGATGGT	59.85	223	INTERGENIC			
CaPOPI_II_1559	scaffold3472	71251	GAAAGCGCTTCCGTTTACAG	GACATTTAACAGCGCCCATT	60.02	159	INTERGENIC			
CaPOPI_II_156	Ca2	7172172	TTTGCCAGAAACATAATGCAA	TTTTGGAAAAATGAAGGACCA	59.20	774	INTERGENIC			
CaPOPI_II_1560	scaffold3475	6139	GGCCTGGAGACCCTTATAGC	ACCACTCGACGAAAATGGAC	60.06	616	INTERGENIC			
CaPOPI_II_1561	scaffold3475	31282	GCTATTTTCGTACCACCCGA	GCCCAAAAAGTCCAGATGAA	59.96	326	INTERGENIC			
CaPOPI_II_1562	scaffold3489	2052	TCGTTCTGTTGGACCCTTTC	AGTGGAAAATATGCCGTTGC	60.09	521	INTERGENIC			
CaPOPI_II_1563	scaffold349	335267	TTGGTCAATGTGGCTTCTCA	CTGAGCTTGTGACCAAATCG	60.24	531	INTERGENIC			
CaPOPI_II_1564	scaffold350	165550	CAATTCCATGGCTCCAAAGT	CAATGCCTCCCCATAACAAT	59.93	670	INTERGENIC			
CaPOPI_II_1565	scaffold3500	6141	GCGTTCGATTCCACATCTTT	TCATTATGCGTGTTCCGCTA	60.08	434	INTERGENIC			
CaPOPI_II_1566	scaffold3514	40711	TGACCAAATCGGAATTGATTTT	CATTTCCGAATCTTCCCGTA	60.53	723	INTERGENIC			
CaPOPI_II_1567	scaffold352	178010	AACGCATCTTTTTACCCACG	GGCCTGAATCTCATCCAAGA	60.00	568	INTERGENIC			
CaPOPI_II_1568	scaffold352	178511	TCGCAATGCAACATCTCTTT	GCTTGTGAGTGTGTGTGCCT	59.43	823	INTERGENIC			
CaPOPI_II_1569	scaffold3542	507	GAAGAGTTACGCGGACAAGC	CCGAGCCAAAATCCAAAATA	60.02	566	INTERGENIC			

INDEL marker IDs	Chromosomes/ unanchored scaffolds	Physical positions (bp)	Forward primers (5'-3')	Reverse primers (5'-3')	Anneling temperature (0C)	Expected amplified product size (bp)	Structural annotation		Functional annotation	
							Sequence components of *kabuli* genome	*Kabuli* gene accession IDs	NCBI-KOG	TFs
CaPOPI_II_157	Ca2	7276294	TCCACCACAATCGTTAAGCA	AGCGGAGATTTTACCGGATT	60.11	676	INTERGENIC			
CaPOPI_II_1570	scaffold355	1384	GGGGCGTTACATGTGGTATC	CAGATTCACGAAGGTCACACA	60.08	314	INTERGENIC			
CaPOPI_II_1571	scaffold3562	1038	AGGGAGGACGAAAATGACCT	CCAATTGGTGTGTTTGCATC	59.94	556	INTERGENIC			
CaPOPI_II_1572	scaffold3594	965	CGTTCAAAACGAGATCGTCA	TACAATGCCGAGCAAATCAA	59.84	575	INTERGENIC			
CaPOPI_II_1574	scaffold362	267543	TATCTTGTTCCGACCCAACC	CTTGAATTCCATGCGTCCTT	59.79	698	INTERGENIC			
CaPOPI_II_1575	scaffold3659	2503	GCCATTAATTATGTTAACGTTTGG	CAGAACATTTAACGTGAGCAAGA	58.84	665	INTERGENIC			
CaPOPI_II_1576	scaffold3663	4120	TTTTATTGTTTTACTACATAACGACGA	AAGTTTGACTGCATAACAATGACA	57.67	631	INTERGENIC			
CaPOPI_II_1577	scaffold3663	4430	TGTCATTGTTATGCAGTCAAACTT	TCTTCGTTTTATGGTTTTATTTCA	58.75	280	INTERGENIC			
CaPOPI_II_1578	scaffold370	75163	GATTTTCGTACCCCTCGTCA	AGTCGCTCGAAAAGTCCAAA	59.93	533	INTERGENIC			
CaPOPI_II_1579	scaffold3705	4075	CAATAACCCTTCAATGGCTCA	GCTTTGCTTCCCTTTCCTCT	59.95	393	INTERGENIC			
CaPOPI_II_158	Ca2	7617731	CCAAGATCTCGTAGTGCAACA	TGGGTTTAGTCCGTTTTTCTTC	58.94	468	INTERGENIC			
CaPOPI_II_1580	scaffold373	25857	AGCCACGAATTTAAGCTCCA	AGGGACAAAGGGCAAGAAAT	59.85	559	INTERGENIC			
CaPOPI_II_1581	scaffold374	13002	CAAATTCAACGAGGTCACACA	ATTTGGAGCGTTACATTGGG	59.60	184	INTERGENIC			
CaPOPI_II_1582	scaffold374	142006	AATTCCTTCCCACACAACCA	AGCGGAGATTTTACCGGAAT	60.21	128	INTERGENIC			
CaPOPI_II_1583	scaffold374	154680	GATTTGATGTTGGGACCGTT	TCAAATGCTTCAACTTGTGCTT	59.65	477	INTERGENIC			
CaPOPI_II_1584	scaffold374	158468	CCTATCACAGGTCAGCACGA	GGTGTGGATGGAATTTGGAG	59.86	531	INTERGENIC			

INDEL marker IDs	Chromosomes/ unanchored scaffolds	Physical positions (bp)	Forward primers (5'-3')	Reverse primers (5'-3')	Anneling temperature (0C)	Expected amplified product size (bp)	Structural annotation		Functional annotation	
							Sequence components of *kabuli* genome	*Kabuli* gene accession IDs	NCBI-KOG	TFs
CaPOPI_II_1586	scaffold3742	3802	CGATAGACTCAAGTTGGACCTG	GTGTCTGCTTGTTTGCCTGA	58.86	632	INTERGENIC			
CaPOPI_II_1587	scaffold377	75306	TCACGGGTCGACACAATTTA	AACTGGACGTTGAGAATGGG	59.96	403	INTERGENIC			
CaPOPI_II_1588	scaffold379	194523	CCGAACTTTACTGGGCCTAA	ACCGTTTTGGTGTTTCTTGC	59.22	602	INTERGENIC			
CaPOPI_II_1589	scaffold3794	82476	CAAAGGGACCACACCTCTTT	TTGGCCCCTTTAGTGTATCG	59.04	554	INTERGENIC			
CaPOPI_II_159	Ca2	7657147	TTATGTGGGGGATTTGGATG	TTCCACGTCCTTTTCATTCC	60.38	705	INTERGENIC			
CaPOPI_II_1590	scaffold38	413791	CTTCGCTGCAAAATTTCCTC	TTGATGTCGGGTAGTGTCTGA	59.96	755	INTERGENIC			
CaPOPI_II_1591	scaffold38	474073	TGTGTCAGAAGGATCTTGCG	TGAGATTCGCCAGTTAACCC	59.98	652	INTERGENIC			
CaPOPI_II_1592	scaffold381	6830	TGCAAAAGGGTCACTTGATTT	CCCCAAACCCTAAACCCTAA	59.60	355	INTERGENIC			
CaPOPI_II_1593	scaffold3837	2371	CTTCTTGGTGGCATGGTTTT	TACCCTGTGGATGGGAATGT	59.97	591	INTERGENIC			
CaPOPI_II_1594	scaffold3837	5661	GCCTCCGAGTCAATACCAAA	ATTGAGGGTAGGGGAGCAGT	60.07	415	INTERGENIC			
CaPOPI_II_1595	scaffold387	16023	CACACCCCTTGTCAACACTG	TTAAGCTGAGAGCCTTCCCA	60.04	439	INTERGENIC			
CaPOPI_II_1596	scaffold387	22975	CGCAATCTGAACAAATGACAA	TCCACTAACCACCTTGACCC	59.72	338	INTERGENIC			
CaPOPI_II_1597	scaffold387	23485	GGGTCAAGGTGGTTAGTGGA	TCCTCTGCCTTTCATGTGTG	59.82	496	INTERGENIC			
CaPOPI_II_1598	scaffold387	23589	GGGTCAAGGTGGTTAGTGGA	TCCTCTGCCTTTCATGTGTG	59.82	496	INTERGENIC			
CaPOPI_II_1599	scaffold387	160969	AGCTCCAAATCCAGAGCAAA	GGGATATGATCTTCTACCCCA	59.96	835	INTERGENIC			
CaPOPI_II_16	Ca1	4742142	GACCGCGTAGATGTGGAACT	GGATGAGACCTCCTACTTGCC	60.14	677	INTERGENIC			

INDEL marker IDs	Chromosomes/ unanchored scaffolds	Physical positions (bp)	Forward primers (5'-3')	Reverse primers (5'-3')	Anneling temperature (0C)	Expected amplified product size (bp)	Structural annotation		Functional annotation	
							Sequence components of *kabuli* genome	*Kabuli* gene accession IDs	NCBI-KOG	TFs
CaPOPI_II_160	Ca2	7724864	ACCAGAGACACTGCAGCAAA	AACAAGATTCTCGAATGCTCC	59.62	464	INTERGENIC			
CaPOPI_II_1600	scaffold3918	4292	ATGCCTTCGTCCTCTCCTTT	ACATTGAAACTGCCCATTCC	60.21	612	INTERGENIC			
CaPOPI_II_1601	scaffold3918	4537	ATGCCTTCGTCCTCTCCTTT	CGAGTGAAGGCATGGGTATT	60.21	914	INTERGENIC			
CaPOPI_II_1602	scaffold3923	3230	TGCTGCAACTTCTACATCCG	AGAAAGCGTGAAAACTCCGA	60.01	695	INTERGENIC			
CaPOPI_II_1603	scaffold3927	1574	GAATCCTGGGCTGAAATCAA	CGTGGTAATCGCGTAGGAAT	60.01	342	INTERGENIC			
CaPOPI_II_1604	scaffold3950	7394	CCAGATGTCGTTTCTCCGAT	TTGTATGACATCTCGCCACC	60.07	665	INTERGENIC			
CaPOPI_II_1605	scaffold3950	7697	TTTCTCTTTTGGTGGTTGCC	TCCACCAATATGCAAAAGCA	60.09	854	INTERGENIC			
CaPOPI_II_1606	scaffold3950	7767	GGTGGCGAGATGTCATACAA	TCCACCAATATGCAAAAGCA	59.53	366	INTERGENIC			
CaPOPI_II_1607	scaffold3950	8477	TGCTTTTGCATATTGGTGGA	AAAACATGGCCTTCAACTGG	60.07	475	INTERGENIC			
CaPOPI_II_1608	scaffold3950	8667	CCAGTTGAAGGCCATGTTTT	TTTTATGGAAGCATGTGGCA	59.97	528	INTERGENIC			
CaPOPI_II_1609	scaffold3950	12638	CAAGAGCCTAAGGATGCTGC	AGCATGAAACGGTGGAGAAT	60.12	919	INTERGENIC			
CaPOPI_II_161	Ca2	7773617	GCTGAGCCCAAACAAGCTAA	CCACTGTGGAAAGGTAAATGC	60.52	845	INTERGENIC			
CaPOPI_II_1610	scaffold396	372299	TGTATTTCTGCGCATTCTCG	TTGTTAGTTTGACGAGGGCA	59.98	357	INTERGENIC			
CaPOPI_II_1611	scaffold396	577559	TCCCAACCCAAATAAACCAA	AAGCTCAACATGCAAAACCC	60.02	538	INTERGENIC			
CaPOPI_II_1612	scaffold396	577641	TCCCAACCCAAATAAACCAA	AAGCTCAACATGCAAAACCC	60.02	538	INTERGENIC			
CaPOPI_II_1613	scaffold397	23446	TGGAGGCGGCTACTAGTGTT	ATTGAATTCGCCATAAACGG	59.90	411	INTERGENIC			

INDEL marker IDs	Chromosomes/ unanchored scaffolds	Physical positions (bp)	Forward primers (5'-3')	Reverse primers (5'-3')	Anneling temperature (0C)	Expected amplified product size (bp)	Structural annotation		Functional annotation	
							Sequence components of *kabuli* genome	*Kabuli* gene accession IDs	NCBI-KOG	TFs
CaPOPI_II_1614	scaffold398	33778	TTTGATTTTGGATTGATTTATTCG	CATGGCCACTCAAAGCACTA	59.28	494	INTERGENIC			
CaPOPI_II_1615	scaffold398	33851	TTTGATTTTGGATTGATTTATTCG	CATGGCCACTCAAAGCACTA	59.28	494	INTERGENIC			
CaPOPI_II_1616	scaffold398	45043	CCCTACGACGTCCGTTAAAA	TCGCTCGTTGCACCATATTA	59.99	554	INTERGENIC			
CaPOPI_II_1617	scaffold398	270743	GTAGTGGTTGAAGTGGCGGT	CTACTCCATCGGGTCTCCAA	60.04	462	INTERGENIC			
CaPOPI_II_1618	scaffold398	379143	TCTGAGGAAGAGGAGGACGA	AGCGTGGCAGATTTTGAAAG	60.06	778	INTERGENIC			
CaPOPI_II_1619	scaffold398	459574	TCGGGTTGTAATGGGGTTTA	TTGGTCGTTTGAGCCTTTCT	60.05	477	INTERGENIC			
CaPOPI_II_162	Ca2	7780730	TTGTCTCCATCTCCAGCCTT	TGTTGTTTGAGTTGGGACACA	59.80	995	INTERGENIC			
CaPOPI_II_1620	scaffold398	466018	TGCATGCAGACAAGAGTCAA	TTGGAAAAGCCCACCTACAC	59.11	468	INTERGENIC			
CaPOPI_II_1621	scaffold40	56808	ATTTGCACGGAAAGTCATCC	CCTCCACCTCCTCCTCCTAC	59.94	810	INTERGENIC			
CaPOPI_II_1622	scaffold4041	994	TCATTCTAATTCCTGCCTGATG	GTCCAAACCCAGCACAATCT	59.18	494	INTERGENIC			
CaPOPI_II_1623	scaffold4041	15702	AGGAATTGGATCGGGAGATT	AAGGAAATGAGGAAAATGGGA	59.73	626	INTERGENIC			
CaPOPI_II_1624	scaffold4041	15753	AGGAATTGGATCGGGAGATT	AAGGAAATGAGGAAAATGGGA	59.73	626	INTERGENIC			
CaPOPI_II_1625	scaffold4041	15867	GGGTTGGGATATGTTAGCAAAA	GAATGCGAAAGCCACAATTT	60.08	851	INTERGENIC			
CaPOPI_II_1626	scaffold4041	15956	TCCCATTTTCCTCATTTCCTT	GAATGCGAAAGCCACAATTT	59.76	493	INTERGENIC			
CaPOPI_II_1627	scaffold4041	16171	GGCACGTCCCTATTTTTGAA	GAATGCGAAAGCCACAATTT	59.94	345	INTERGENIC			
CaPOPI_II_1628	scaffold4064	1366	AAAAGCATGCAGTGCAACAC	TCCCGTCTGAAAATTATGGC	59.92	367	INTERGENIC			

INDEL marker IDs	Chromosomes/ unanchored scaffolds	Physical positions (bp)	Forward primers (5'-3')	Reverse primers (5'-3')	Anneling temperature (0C)	Expected amplified product size (bp)	Structural annotation		Functional annotation	
							Sequence components of *kabuli* genome	*Kabuli* gene accession IDs	NCBI-KOG	TFs
CaPOPI_II_1629	scaffold408	3569	TAATACACGTCCGCTTTCCC	CGAACCTGCTGTCCAAAAAT	59.96	394	INTERGENIC			
CaPOPI_II_163	Ca2	7940136	TCTTGACCGTTAGATTGAACCA	CAAAATGGACAAAATTGGAGC	59.61	714	INTERGENIC			
CaPOPI_II_1630	scaffold408	130988	GTGGTGATATTTGGGAAGCC	TCGTGGTTTTGGTGTTGGTA	59.25	662	INTERGENIC			
CaPOPI_II_1631	scaffold408	131029	GTGGTGATATTTGGGAAGCC	TCGTGGTTTTGGTGTTGGTA	59.25	662	INTERGENIC			
CaPOPI_II_1632	scaffold4088	5270	ATCAACTTGCAAGGCAGCTT	TACCATTCAATTGGCGAACA	60.02	454	INTERGENIC			
CaPOPI_II_1633	scaffold41	122306	TCGCTACGGCTCTAGTGGTT	AAGTCCTGTAGCCGAAAGCA	60.04	294	INTERGENIC			
CaPOPI_II_1634	scaffold41	122382	TCGCTACGGCTCTAGTGGTT	AAGTCCTGTAGCCGAAAGCA	60.04	294	INTERGENIC			
CaPOPI_II_1635	scaffold4107	9634	CCTAAAACCTAAAACCAAAAACC	TCGTCCAATAAGAACAAAAGTCAA	57.42	127	INTERGENIC			
CaPOPI_II_1636	scaffold4108	8749	CAAAACCGGAAACGCTTAAC	TTTTGGGCTTCGAGTTTAGG	59.63	294	INTERGENIC			
CaPOPI_II_1637	scaffold411	298521	CTTGCATGGCAATTATCCAA	TTGGGCTGATGCAATAAACA	59.52	469	INTERGENIC			
CaPOPI_II_1639	scaffold4155	7060	CGGTTCAACGTAACCGACTT	TTGGGCAACAATCGATGTAA	60.03	402	INTERGENIC			
CaPOPI_II_164	Ca2	8420270	CCAAGTTTGAAATGCCGATT	GCTGCATGATCTTATGCTCG	59.94	451	INTERGENIC			
CaPOPI_II_1640	scaffold419	148627	AACAAGCAAGCCATTCAAGC	GGTGAGGATCTGGTGTGGTT	60.40	657	INTERGENIC			
CaPOPI_II_1641	scaffold4192	3668	ATCCAAGTTTGAGGCAATCG	CGATTTTCGCAGGTCTTGTT	60.07	484	INTERGENIC			
CaPOPI_II_1642	scaffold4197	16348	AATGGCCACGAATCTGAAAG	GTGCGGAAGCCATGAGTAAT	60.07	483	INTERGENIC			
CaPOPI_II_1643	scaffold420	69655	ATGGCGAAGAATTATGGTGC	CATGTACCGCCCATCTCTTT	59.93	265	INTERGENIC			

INDEL marker IDs	Chromosomes/ unanchored scaffolds	Physical positions (bp)	Forward primers (5'-3')	Reverse primers (5'-3')	Anneling temperature (0C)	Expected amplified product size (bp)	Structural annotation		Functional annotation	
							Sequence components of *kabuli* genome	*Kabuli* gene accession IDs	NCBI-KOG	TFs
CaPOPI_II_1644	scaffold420	265273	CGTGATCGTGTTTGGTTGTC	GGGAATGGCAGCAAATAGAA	60.01	587	INTERGENIC			
CaPOPI_II_1645	scaffold4221	569	ATTGATTTTGAAATCGCCCA	CAGTTTCCTGCCAGCCTTAG	60.27	441	INTERGENIC			
CaPOPI_II_1646	scaffold4221	691	CTAAGGCTGGCAGGAAACTG	CGCTTCTTTGGTCATGCAA	60.01	443	INTERGENIC			
CaPOPI_II_1647	scaffold4221	7762	TGCCAGAACTGATTTTCTGCT	GAGCGCTGTAAAACACCTCC	60.01	540	INTERGENIC			
CaPOPI_II_1648	scaffold4243	1042	GGTACGGAAATTGCCTGAAA	GGTCCTCGATTTCATCTGGA	59.94	204	INTERGENIC			
CaPOPI_II_1649	scaffold4243	14917	TGCTTGATTGCTTGAGTTCC	GATGAAATCGAGGACTGGGA	59.00	537	INTERGENIC			
CaPOPI_II_165	Ca2	8478800	CCCGTATGAATGAACGGAAC	CTACCCCCACATTTCAATCG	60.19	519	INTERGENIC			
CaPOPI_II_1650	scaffold4319	1245	CGATTTCCCTCACGACAAAT	TTTTGGACAGCTACAAGTGGTT	59.93	582	INTERGENIC			
CaPOPI_II_1651	scaffold433	86247	GCCCTCAGATGAGAGTCAGG	AGAATTTACGAGCAGCGGAA	59.95	287	INTERGENIC			
CaPOPI_II_1652	scaffold433	146841	TGGTGACAATTTGATGGTTCT	CAGTGCGATGGAGAAATCAA	57.91	523	INTERGENIC			
CaPOPI_II_1653	scaffold433	157672	GCATTTCAAGATGGGCAGTT	AATGATCGTCTTCGGATTGG	60.08	714	INTERGENIC			
CaPOPI_II_1655	scaffold4349	1831	AGGTCATTTTCGTCCTCCCT	CAAACACACCTGTTGGATGC	59.94	182	INTERGENIC			
CaPOPI_II_1656	scaffold436	67143	CAAACACACCTGTTGGATGC	AGGTCATTTTCGTCCTCCCT	60.01	478	INTERGENIC			
CaPOPI_II_1657	scaffold436	68438	GGCCGGATGAAAATGAACTA	ATTTTCGTCCTCCAAAGGCT	59.90	513	INTERGENIC			
CaPOPI_II_1658	scaffold4366	1890	GGACATGAATAGGGCAATGG	GGTCATTTTTGTCCACGCTT	60.16	532	INTERGENIC			
CaPOPI_II_1659	scaffold437	45718	TCGAAGAGGACCATTCATCA	AGGTCGCACTCCAAATATCG	59.17	777	INTERGENIC			

INDEL marker IDs	Chromosomes/ unanchored scaffolds	Physical positions (bp)	Forward primers (5'-3')	Reverse primers (5'-3')	Anneling temperature (0C)	Expected amplified product size (bp)	Structural annotation		Functional annotation	
							Sequence components of *kabuli* genome	*Kabuli* gene accession IDs	NCBI-KOG	TFs
CaPOPI_II_166	Ca2	8478910	CCCGTATGAATGAACGGAAC	CTACCCCCACATTTCAATCG	60.19	519	INTERGENIC			
CaPOPI_II_1660	scaffold4374	1159	TAAACCCCAAATGACCGAAA	AGGGGTTAGGGGTTAGGGTT	60.16	444	INTERGENIC			
CaPOPI_II_1661	scaffold4377	6572	TCCAGATGAAATCGAGGACC	AGGCGATTTTCGTACCACAC	60.01	319	INTERGENIC			
CaPOPI_II_1662	scaffold442	20594	TGGACGCACAAGATTGGTTA	AGGTCATTTTCGTCCTCCCT	60.11	370	INTERGENIC			
CaPOPI_II_1663	scaffold446	224556	TTCATCTGGTCTTTTTGGGC	CATTGAGTACAACTACAAAACGACG	60.05	771	INTERGENIC			
CaPOPI_II_1664	scaffold446	235656	TCTGACAACTCAATTTTGCTCTATG	GTTGTACTTCATAACGAACACAATACT	59.84	521	INTERGENIC			
CaPOPI_II_1665	scaffold4508	36727	TTGTGTTTGTCACAGGCCAT	TCAGGAACAACAACTCAACCC	60.01	371	INTERGENIC			
CaPOPI_II_167	Ca2	9494071	GAACTGCAACAACAACGACAA	GGTCTCGAAATCCCTGATGA	59.80	609	INTERGENIC			
CaPOPI_II_1671	scaffold4582	7612	TCCAGATGAAATCGAGGACC	CGAAATTTGACACGGAACCT	60.01	258	INTERGENIC			
CaPOPI_II_1672	scaffold46	6079	GGGCCGAGAGTCCCTTATAG	GGTCCTCGATTTCATCTGGA	60.05	304	INTERGENIC			
CaPOPI_II_1673	scaffold466	12138	ATTCAGTTCCCTTGCAGCAT	TCTCCGCATAAGCTTTCGAT	59.70	661	INTERGENIC			
CaPOPI_II_1676	scaffold466	254290	GGATTCCGCGTAAGATTTCA	ACTAGGTTTTGGGTTCCGCT	60.04	465	INTERGENIC			
CaPOPI_II_1677	scaffold470	336358	GTCATCCTAGCCATCGTCGT	GTGTCCCAAAACTCAAGGGA	60.10	876	INTERGENIC			
CaPOPI_II_1678	scaffold470	365605	AGAAGCAAGCTAGCTCGTGG	ATTGGAGCACATTCAGGCTT	59.92	815	INTERGENIC			
CaPOPI_II_1679	scaffold470	370786	CATGCCAGTTGCTACATGCT	GCTTGGACTCAGTGGCTAGG	59.90	949	INTERGENIC			
CaPOPI_II_168	Ca2	9500229	AGAGGTTGGGGGAGAAGAAA	GCAACAACAGCAGAGACAATG	60.05	213	INTERGENIC			

INDEL marker IDs	Chromosomes/ unanchored scaffolds	Physical positions (bp)	Forward primers (5'-3')	Reverse primers (5'-3')	Anneling temperature (0C)	Expected amplified product size (bp)	Structural annotation		Functional annotation	
							Sequence components of *kabuli* genome	*Kabuli* gene accession IDs	NCBI-KOG	TFs
CaPOPI_II_1680	scaffold470	372719	ATTTTATGCTGCGACATCCC	GCAAATCCCACACTTGGAGT	59.93	265	INTERGENIC			
CaPOPI_II_1681	scaffold470	375954	AGGGAATCGAGGGAAAAATG	AGGTGGCAACCTTCTCATTG	60.26	463	INTERGENIC			
CaPOPI_II_1682	scaffold4711	5708	AGGTCATTTTCGTCCTCCCT	TTGGATGCACAAGATTGGTT	59.94	271	INTERGENIC			
CaPOPI_II_1683	scaffold4711	6211	TTTTCGTCCTCCCTAGGCTT	AGGAACTTATTGGATGCCCA	60.20	470	INTERGENIC			
CaPOPI_II_1684	scaffold4713	1946	TCATCCAATGACACAAACGC	AGGTCATTTTCGTCCTCCCT	60.53	193	INTERGENIC			
CaPOPI_II_1685	scaffold4733	507	ACAGTGAACGTGCACCCATA	CTGGTTATGCCGTTTGGAGT	60.03	697	INTERGENIC			
CaPOPI_II_1686	scaffold475	141188	GAGGAGCGACCAGTTGTTTC	GAAACTAATGCGACCGGTGT	59.85	884	INTERGENIC			
CaPOPI_II_1687	scaffold475	174713	AAGGTTGACTGCATAACGAAGA	CAACACCATTTGACAACACCA	58.91	730	INTERGENIC			
CaPOPI_II_1688	scaffold477	11283	TGTCATCTTGTTTGCCTTGG	TGATGAAACTCTGAACTCGTGG	59.69	114	INTERGENIC			
CaPOPI_II_1689	scaffold477	20714	CCCCCTAATGCTCAATTTCA	TGCAACAAGAGGCAAAACAC	59.89	288	INTERGENIC			
CaPOPI_II_169	Ca2	10779124	CACTTTTCTTATGCACCGCA	GCAGCGAGTAGGAGAAATGC	59.87	663	INTERGENIC			
CaPOPI_II_1690	scaffold477	28410	GTAGGAGGATCTCAAGGGGC	CCAAAATTAGGAAGCACCCA	60.04	567	INTERGENIC			
CaPOPI_II_1691	scaffold477	46653	ATGCAACTGTCGTTTTAGCA	TTAGAGCGCCTTCTGATGGT	57.03	405	INTERGENIC			
CaPOPI_II_1692	scaffold4773	16885	TGCGGCTATTCATAACATGG	GCATATCTGAGGCTGCCAAT	59.54	600	INTERGENIC			
CaPOPI_II_1694	scaffold4784	684	TCCTGGTCCTCGATTTCATC	GGGCCGAGAGACCCTTATAG	60.01	339	INTERGENIC			
CaPOPI_II_1695	scaffold479	7594	TGTGTTTGTGGCATTGGATT	AGGGAGGACGAAAATGACCT	59.82	329	INTERGENIC			

INDEL marker IDs	Chromosomes/ unanchored scaffolds	Physical positions (bp)	Forward primers (5'-3')	Reverse primers (5'-3')	Anneling temperature (0C)	Expected amplified product size (bp)	Structural annotation		Functional annotation	
							Sequence components of *kabuli* genome	*Kabuli* gene accession IDs	NCBI-KOG	TFs
CaPOPI_II_1696	scaffold479	125417	ACAAACCAATCGATTTCCCA	CGGAGGACGAAAATGACCTA	60.17	180	INTERGENIC			
CaPOPI_II_1697	scaffold48	15048	TCGGTTCATCATGTTGGAAAT	TCTTTTGATGTGGGTTAGTGAGAA	60.19	677	INTERGENIC			
CaPOPI_II_1698	scaffold480	43150	AACGTACTGCCTATCACGGG	TGTTCATCGTATTTGGCGTG	60.02	836	INTERGENIC			
CaPOPI_II_1699	scaffold4804	1921	TTGGATGCACAACATTGGTT	AGGTCATTTTCGTCCTCCCT	59.82	561	INTERGENIC			
CaPOPI_II_170	Ca2	12048675	CAAACATGCTGATGTGGACC	CCATTGGTAAATGGCTAACAA	59.97	278	INTERGENIC			
CaPOPI_II_1700	scaffold4804	8867	TGGATGCACCAGATTGGTTA	AGGTCATTTTCGTCCTCCCT	59.92	570	INTERGENIC			
CaPOPI_II_1701	scaffold4822	847	CAGCGGATCCCCAGATATAC	AGGTCATTTTCGTCCTCCCT	59.37	545	INTERGENIC			
CaPOPI_II_1702	scaffold4829	6279	GACCCGCATTTTCCTCATTA	GGAGAACGTTAAGGCAGCAG	59.90	507	INTERGENIC			
CaPOPI_II_1703	scaffold4829	8205	CAATAGCATGCAGTTGGAGG	GGAGACTCCCATACATTGGC	59.30	500	INTERGENIC			
CaPOPI_II_1704	scaffold4833	740	TGGGAATGTAGTGGATGAACC	TCCCGAACACCTATGCTTTT	59.66	529	INTERGENIC			
CaPOPI_II_1705	scaffold4833	3774	CCAAGCCAGTCAAACACAAA	GGGCTCGCTATGTACAGCA	59.73	839	INTERGENIC			
CaPOPI_II_1706	scaffold484	136302	ACCGAGGTGCCACTTATCAC	ATAGTGGTGACTGGATGGGC	60.00	229	INTERGENIC			
CaPOPI_II_1707	scaffold484	656687	TATCACCTATCAGAGCGGGG	TGATTCCGGGGAAGTACAAC	60.05	472	INTERGENIC			
CaPOPI_II_1708	scaffold4851	3617	CGCGGAACCTGAAACACTAT	CCGATGCTAAAAATGGCAGT	60.13	616	INTERGENIC			
CaPOPI_II_1709	scaffold487	87751	ACATTGAGGTCATTTTCGCC	AGCCTAGGGAGGATGCAAAT	59.94	229	INTERGENIC			
CaPOPI_II_171	Ca2	12933106	AGGTTGAAAGAAGCCCCACT	ATCGGTTGGTTGGTGAAATG	60.11	834	INTERGENIC			

INDEL marker IDs	Chromosomes/ unanchored scaffolds	Physical positions (bp)	Forward primers (5'-3')	Reverse primers (5'-3')	Anneling temperature (0C)	Expected amplified product size (bp)	Structural annotation		Functional annotation	
							Sequence components of *kabuli* genome	*Kabuli* gene accession IDs	NCBI-KOG	TFs
CaPOPI_II_1710	scaffold4872	661	TGGGCACATGCTTCTCAGTA	TACTGGAACGGACCACACAA	60.41	498	INTERGENIC			
CaPOPI_II_1711	scaffold496	121429	TCCTGGTCCTCGATTTCATC	AATCGCCTGAAAAGACGAGA	60.01	379	INTERGENIC			
CaPOPI_II_1712	scaffold497	69995	TGGTGCTTTTGGAAAAAGTG	ATTTGGTTTGGTTGCATTCG	58.79	446	INTERGENIC			
CaPOPI_II_1713	scaffold498	206145	CACCGCCTCTGTTCCATATT	GAGATTTAGAACCATCAAATTGTCA	59.96	829	INTERGENIC			
CaPOPI_II_1714	scaffold498	214228	TTTCATGGTCATGTTTTGGC	GAAAGGCCAATGTCCAGAAA	59.38	781	INTERGENIC			
CaPOPI_II_1715	scaffold498	214273	TTTCATGGTCATGTTTTGGC	GAAAGGCCAATGTCCAGAAA	59.38	781	INTERGENIC			
CaPOPI_II_1716	scaffold498	214558	CACACACACGTAAGAAGGCTG	TTGGACAGAATCAAGGCTCC	59.42	544	INTERGENIC			
CaPOPI_II_1717	scaffold4983	27276	AAGGTGGTACGAAAATCCCC	GCACGTGGGAAAATGAGATT	60.05	893	INTERGENIC			
CaPOPI_II_1718	scaffold50	192238	CTGCCCATTAGCCCTTCATA	CTGAATGCAAGAGAGAGGGG	60.05	291	INTERGENIC			
CaPOPI_II_1719	scaffold501	87430	TGGGTGTGCCTATATACACGTAA	TTCTTTGCCACACACCATGT	59.33	519	INTERGENIC			
CaPOPI_II_172	Ca2	12933573	TCATTGACAAGGAAAGAACATGA	CGAATTGGTTTTGGGATTTG	59.60	413	INTERGENIC			
CaPOPI_II_1720	scaffold5030	906	TGGGGATACGTAGGAGCAAG	TGTTGGTCCATTTTTGACGA	60.09	432	INTERGENIC			
CaPOPI_II_1721	scaffold506	18975	TCTCGATACCATCGTGGACA	TCCCATTTGATTGCTGAACA	60.07	489	INTERGENIC			
CaPOPI_II_1722	scaffold5094	2305	TGGCACATGAATAGTTCCACA	GCCACCTCATTCAAATATTGC	59.98	669	INTERGENIC			
CaPOPI_II_1723	scaffold510	85609	TTGTCAAATAAAGTAACAACAAAACG	TTGCAACAATTGTACCCGAA	58.74	995	INTERGENIC			
CaPOPI_II_1724	scaffold511	193038	TTTCAGGTTATCGACTGGGG	AACAAGAAAGCGCAAGCAAT	59.93	729	INTERGENIC			

INDEL marker IDs	Chromosomes/ unanchored scaffolds	Physical positions (bp)	Forward primers (5'-3')	Reverse primers (5'-3')	Anneling temperature (0C)	Expected amplified product size (bp)	Structural annotation		Functional annotation	
							Sequence components of *kabuli* genome	*Kabuli* gene accession IDs	NCBI-KOG	TFs
CaPOPI_II_1725	scaffold5120	14840	TATCGTGCCTGTTGAGAACG	CTAATTAGCGTCGGAGTCGG	59.86	527	INTERGENIC			
CaPOPI_II_1726	scaffold5120	15547	CCGAAGTAATAGTGGGCAA	GGACCAGGTTCTGCATCAAT	59.95	214	INTERGENIC			
CaPOPI_II_1727	scaffold515	43016	CAAGAGTCTCCTGGTCCTCG	CTCCCGAAAAGTCCAAATGA	59.98	912	INTERGENIC			
CaPOPI_II_1728	scaffold5186	8653	GAGTGTTGGGGTCGTTGAAT	CCTAGGGAGGCTAAAATGGC	59.83	270	INTERGENIC			
CaPOPI_II_1729	scaffold5211	3755	GTTCTCGAAGCAGAAAACGG	CTTTGGTCAAGTCTGGCACA	59.99	702	INTERGENIC			
CaPOPI_II_173	Ca2	12934202	AGAAAGCAAGCAAATCCCAA	TTCCAACACCTCACGATTCA	59.82	488	INTERGENIC			
CaPOPI_II_1730	scaffold5251	1190	TGGCCACCATTAGCATTACA	AGCGCAGTGGTGAAAAGAAT	59.95	383	INTERGENIC			
CaPOPI_II_1731	scaffold527	4805	TCGTTGGTGCATTATTTCCA	TTGCAAGAAGGGGTTTTGAC	59.93	437	INTERGENIC			
CaPOPI_II_1732	scaffold528	183983	TGAACTCAAAATTCGATTTGTCT	TGAAAAACATTTGCCTGAACA	57.93	651	INTERGENIC			
CaPOPI_II_1733	scaffold53	137170	GGACTGGTGGCTCAAAGGTA	TCGACCGGGAGTTTTGATTA	60.11	671	INTERGENIC			
CaPOPI_II_1736	scaffold535	94648	AATGGAGGAGAAGGGAGGAA	TGAAAAGGGGTAGGCAACAC	60.01	563	INTERGENIC			
CaPOPI_II_1737	scaffold5420	685	AAGGGAGGATGGAAATGACC	ATTTTTGTCCTCCTTGGGCT	60.13	613	INTERGENIC			
CaPOPI_II_174	Ca2	12937651	GATTGCTTTCCTCTATGCCG	GTTTTGGCGTTGGTTTCACT	59.81	353	INTERGENIC			
CaPOPI_II_1741	scaffold545	193109	AATCTCCCCCTGAGTTTTGG	ATCTTTGGACACCGTCGTTC	60.30	643	INTERGENIC			
CaPOPI_II_1742	scaffold547	96984	CACGATGGCTAGTGCTTGAA	CGATCGGTATGAACATTGACC	60.01	276	INTERGENIC			
CaPOPI_II_1743	scaffold547	155914	TCGATCTGTACGAACATTGACC	CGGAACCCGAAACACTTTTA	60.00	295	INTERGENIC			

INDEL marker IDs	Chromosomes/ unanchored scaffolds	Physical positions (bp)	Forward primers (5'-3')	Reverse primers (5'-3')	Anneling temperature (0C)	Expected amplified product size (bp)	Structural annotation		Functional annotation	
							Sequence components of *kabuli* genome	*Kabuli* gene accession IDs	NCBI-KOG	TFs
CaPOPI_II_1744	scaffold547	156143	TTCGGTCTATTTTTGTC GGG	CGGAACCCGAAACACTT TTA	59.93	219	INTERGENIC			
CaPOPI_II_1746	scaffold549	61007	TGCCAATTCGAAGATCA AAA	CACCCCTTACCTCGACT GAA	59.23	570	INTERGENIC			
CaPOPI_II_1747	scaffold5504	1323	ACCAATCAAGTGCATCC CAT	TAAGCCTAAGGAGGAC GCAA	60.20	175	INTERGENIC			
CaPOPI_II_1748	scaffold553	2669	TTGGTTCACTCCCTGGA TTC	AACATCAGTCAAGCGCA CAA	59.90	484	INTERGENIC			
CaPOPI_II_175	Ca2	12939688	TTTTGGTGTTGAGGGTG TCA	TTAATCAAAGCGGCAAA AGAA	59.98	564	INTERGENIC			
CaPOPI_II_1750	scaffold5551	550	GATACGTTGGAGCATCG ACA	CACGAAACACCCTCTTC CAT	59.68	230	INTERGENIC			
CaPOPI_II_1751	scaffold5598	13569	CGGCATTGGATCATGGT TAG	TTTCGACTGCCTAGCGA TTT	61.25	549	INTERGENIC			
CaPOPI_II_1753	scaffold564	5212	TGGGATGCAAACAATTC TCA	TTGTCAATTTGGGAGTG CAA	60.05	560	INTERGENIC			
CaPOPI_II_1754	scaffold564	8317	CAACATCCAAATTTTGTT ATAGAGC	CGACATGCATGCCTTAT TTG	58.29	143	INTERGENIC			
CaPOPI_II_1755	scaffold564	26112	GTATCTGAGCCTCTGCC AGC	AATTGGTGTGTCTGCGT CAA	60.13	555	INTERGENIC			
CaPOPI_II_1758	scaffold575	66804	AACCGGATGTTGGGATG TAA	CCCATTCACTCCCACTC TGT	60.05	348	INTERGENIC			
CaPOPI_II_1759	scaffold575	67025	ACAGAGTGGGAGTGAAT GGG	CCTAAGCCTGACCCTAC CCT	59.96	397	INTERGENIC			
CaPOPI_II_176	Ca2	12941200	TGTGCTTCTTCCTGATT TTGG	GGCCAAAGACGGATAT GGTA	60.23	888	INTERGENIC			
CaPOPI_II_1760	scaffold575	67830	GGGTTAGGGTAGGGTC AGGA	AGGCTGGAATCGGTAA GTGA	60.18	357	INTERGENIC			
CaPOPI_II_1761	scaffold575	89948	GGATTGGCCCCAAATAG AAT	CCCTTGTCATTGCTGCT GTA	59.98	722	INTERGENIC			
CaPOPI_II_1762	scaffold575	90216	CCCAAATTGCAATAACC AGG	TACCGTTTCCCACACTC ACA	60.18	637	INTERGENIC			

INDEL marker IDs	Chromosomes/ unanchored scaffolds	Physical positions (bp)	Forward primers (5'-3')	Reverse primers (5'-3')	Anneling temperature (0C)	Expected amplified product size (bp)	Structural annotation		Functional annotation	
							Sequence components of *kabuli* genome	*Kabuli* gene accession IDs	NCBI-KOG	TFs
CaPOPI_II_1763	scaffold575	91034	TGTGAGTGTGGGAAACGGTA	GGCAATGTGCATAAACAAAAA	60.00	426	INTERGENIC			
CaPOPI_II_1764	scaffold575	98810	TAAGCCAGGGCTTTCTTTCA	CCTCCACTCCAAACCTACCA	59.95	404	INTERGENIC			
CaPOPI_II_1765	scaffold575	99188	TGGTAGGTTTGGAGTGGAGG	GGCTAAGTCTGAAAGTGGCG	59.96	263	INTERGENIC			
CaPOPI_II_1766	scaffold575	103253	AAACAACGAGTATGGGCGAC	AGCTTGCTTTGAAAGGGACA	60.00	113	INTERGENIC			
CaPOPI_II_1767	scaffold575	103640	AAACAACGAGTATGGGCGAC	CGCAATTAGCCTCTTTCGTC	60.00	707	INTERGENIC			
CaPOPI_II_1768	scaffold575	103819	TCTTCTCGCTGACTTGGGAT	CGCAATTAGCCTCTTTCGTC	59.95	205	INTERGENIC			
CaPOPI_II_1769	scaffold575	110510	CTTTCTGGTCAAGGCCTCAG	CTAGACCTGCGGAAGACAGG	59.98	446	INTERGENIC			
CaPOPI_II_177	Ca2	12941800	CATATCCGTCTTTGGCCATT	CCTTGAACAATTTGGGCAGT	59.78	643	INTERGENIC			
CaPOPI_II_1770	scaffold575	113273	TTCGGCAACATTCCGTATTT	GCCTGACCTATGGCCTATCA	60.32	283	INTERGENIC			
CaPOPI_II_1771	scaffold575	113710	TGATAGGCCATAGGTCAGGC	GCTAAGTGGTCTTTCCGCTG	60.06	440	INTERGENIC			
CaPOPI_II_1772	scaffold5761	842	GGTCCTCGATTTCATCTGGA	AACGCCTTAAAAGCCCAAAT	60.01	684	INTERGENIC			
CaPOPI_II_1773	scaffold5761	1101	TCCTGGTCCTCGATTTCATC	AACGCCTTAAAAGCCCAAAT	60.01	362	INTERGENIC			
CaPOPI_II_1774	scaffold5777	16517	TTGCAATTGGCACACAAAAT	TGAGTTGCTTCAAAGTGATGTG	59.98	742	INTERGENIC			
CaPOPI_II_1775	scaffold578	11617	TTCATTTGATCTTTTCGGGG	CCTCGAGAAAAATGGACCAA	59.87	513	INTERGENIC			
CaPOPI_II_1776	scaffold5780	2877	GGAATCCGGTTTCTTTCCAT	GACCATTATTCACACGGTGG	60.13	765	INTERGENIC			
CaPOPI_II_1777	scaffold5780	5394	CGTGTTCTCCCCGTGTATTT	ATGTGACACCCTAAACCCCA	59.85	488	INTERGENIC			

INDEL marker IDs	Chromosomes/ unanchored scaffolds	Physical positions (bp)	Forward primers (5'-3')	Reverse primers (5'-3')	Anneling temperature (0C)	Expected amplified product size (bp)	Structural annotation		Functional annotation	
							Sequence components of *kabuli* genome	*Kabuli* gene accession IDs	NCBI-KOG	TFs
CaPOPI_II_1778	scaffold5780	5464	CGTGTTCTCCCCGTGTATTT	ATGTGACACCCTAAACCCCA	59.85	488	INTERGENIC			
CaPOPI_II_1779	scaffold5780	8742	AAGTTCCGCATTAGGCCTTT	GTTTGGGTCAAGGCTATGGA	60.10	444	INTERGENIC			
CaPOPI_II_178	Ca2	12947970	TGCTTTTATGAGCATTACGGG	CGACTTCAAATCCCTTCGAG	60.10	777	INTERGENIC			
CaPOPI_II_1780	scaffold5780	9297	TCCATAGCCTTGACCCAAAC	CCCACTGCTTGCAATAATGA	59.93	404	INTERGENIC			
CaPOPI_II_1782	scaffold587	50853	AGGGGAATTTTCGTACCACC	ATCGAAGACCAGGAGACCCT	60.05	638	INTERGENIC			
CaPOPI_II_1783	scaffold587	51041	AGGGGAATTTTCGTACCACC	ATCGAAGACCAGGAGACCCT	60.05	638	INTERGENIC			
CaPOPI_II_1784	scaffold587	53018	TACACGAAACCCGAAACACA	CGAGGACCAGGAGACCATTA	60.00	749	INTERGENIC			
CaPOPI_II_1785	scaffold587	54888	CAAGACATGCGAAAGTCGAA	TCCAGCTTCCTCAAAGCCTA	59.99	484	INTERGENIC			
CaPOPI_II_1786	scaffold587	56115	AACTGCCAAAGAGATGTGGG	TGCAGTTCCATCTCATGCTC	60.11	338	INTERGENIC			
CaPOPI_II_1787	scaffold590	3079	AATAAAAGACCAAATCGGAAAAA	TGACAACCATTTATATTTGCCAC	58.20	393	INTERGENIC			
CaPOPI_II_1788	scaffold590	19258	TCCTAAGAAAGCTACCCCACC	TGGCAAAGCACAAGACACTC	59.60	398	INTERGENIC			
CaPOPI_II_1789	scaffold590	19474	CTATCCATGCATGTGCAACAC	TGGCAAAGCACAAGACACTC	60.01	175	INTERGENIC			
CaPOPI_II_179	Ca2	12952557	AAAGCATTTTATCCCGACCA	TTTTTCATCTCACTCTCTCCCA	59.41	787	INTERGENIC			
CaPOPI_II_1790	scaffold5916	1178	TGACACCATTCTCTAGGCCC	AGGGAGGACGAAAATGACCT	60.07	310	INTERGENIC			
CaPOPI_II_1793	scaffold5954	1414	TGGATGCACAAGGTTGGTTA	AGGTCATTTTCGTCCTCCCT	59.96	367	INTERGENIC			
CaPOPI_II_1794	scaffold598	568507	CAGCCACTACATTGGCCTTT	AGGATTCTCCAAGTTGGCCT	60.13	499	INTERGENIC			

INDEL marker IDs	Chromosomes/ unanchored scaffolds	Physical positions (bp)	Forward primers (5'-3')	Reverse primers (5'-3')	Anneling temperature (0C)	Expected amplified product size (bp)	Structural annotation		Functional annotation	
							Sequence components of *kabuli* genome	*Kabuli* gene accession IDs	NCBI-KOG	TFs
CaPOPI_II_1795	scaffold599	307088	TCTTCCCATTCCTTGTCTGG	GGCGTAGCCCAAATATTCAA	60.04	120	INTERGENIC			
CaPOPI_II_1796	scaffold599	307530	TCTTCCCATTCCTTGTCTGG	TGTGTGCTTCCTATGATGTTCT	60.04	855	INTERGENIC			
CaPOPI_II_1797	scaffold599	307947	AGAACATCATAGGAAGCACACA	CAACATTAAAAGAACGGCGA	57.36	317	INTERGENIC			
CaPOPI_II_1798	scaffold599	312899	CGTTGTCGTCGGTAGGATTT	TTCAAAAGTTATTCGGTGGAAA	59.99	521	INTERGENIC			
CaPOPI_II_1799	scaffold599	322634	TTGATAAACCGCCATGACAA	TTTTGCTTTGCCGTATTTTT	59.93	563	INTERGENIC			
CaPOPI_II_180	Ca2	12952944	TGGGAGAGAGTGAGATGAAAAA	ACGGATGTCTAATCCGTTGG	58.91	308	INTERGENIC			
CaPOPI_II_1800	scaffold599	323301	AAAAATACGGCAAAGCAAAA	TTGAGGTTGAGAGTCTTTTCCA	57.54	732	INTERGENIC			
CaPOPI_II_1801	scaffold599	329422	CACGTAAAATCGCCTAAAATCT	CCGATAGTAAAACGACATCCTG	57.57	562	INTERGENIC			
CaPOPI_II_1802	scaffold599	335351	GCCAACCATGTAAATCGCAT	TCAATCATCAATTTTTGTGTGTTTC	60.73	327	INTERGENIC			
CaPOPI_II_1803	scaffold599	347393	CCTCTTTTCTTCGCATTTCG	AACAACAGCCCCAATGTAGC	59.95	504	INTERGENIC			
CaPOPI_II_1804	scaffold599	366068	AGAGCGCAATCATTTCAGGT	CTTTTGGAAAGAAGCCCTCC	59.84	613	INTERGENIC			
CaPOPI_II_1805	scaffold599	371527	CAGCGTTGTTGTTGTTGTTTTT	TTTCGAATGAACATGGATGA	60.11	531	INTERGENIC			
CaPOPI_II_1807	scaffold599	410067	ATCGATTTCCCAATCGGTTA	TATACCCTATAGCCACGGCG	59.23	609	INTERGENIC			
CaPOPI_II_1808	scaffold5992	1551	CGAGGTAAGGGGTGAGATGA	TCCCCGAATCACTAGGTTTG	60.07	544	INTERGENIC			
CaPOPI_II_1809	scaffold5993	18562	TTGATGTACGAAAAGTTTTAGCAAG	GGGGGCACTTATTTATCACTT	59.01	267	INTERGENIC			
CaPOPI_II_181	Ca2	15589036	ATACGCCTCAACAAAATGGC	CTGCTTTGCTTGATTCCGTT	59.97	376	INTERGENIC			

INDEL marker IDs	Chromosomes/ unanchored scaffolds	Physical positions (bp)	Forward primers (5'-3')	Reverse primers (5'-3')	Anneling temperature (0C)	Expected amplified product size (bp)	Structural annotation		Functional annotation	
							Sequence components of *kabuli* genome	*Kabuli* gene accession IDs	NCBI-KOG	TFs
CaPOPI_II_1810	scaffold5993	18598	TTGATGTACGAAAAGTTTTAGCAAG	GGGGGCACTTATTTATCACTT	59.01	267	INTERGENIC			
CaPOPI_II_1811	scaffold601	49572	GGGGACATTTCGAGAGTCAA	TCACAAGGGAGGCTCTATGG	60.05	519	INTERGENIC			
CaPOPI_II_1812	scaffold6113	1438	ACAAGCCTATCGTTTGGCAC	GATGCAACATTGGAGCATGA	60.14	589	INTERGENIC			
CaPOPI_II_1813	scaffold612	28576	AAAAAGGGAGGGTCACATGAT	TCACCAATATCTTAACCACCAAAA	59.68	701	INTERGENIC			
CaPOPI_II_1814	scaffold6125	1553	ATGCGATCCTCTCGGTTCTA	GAGACCGAAGAGTATTGGGAGA	59.80	838	INTERGENIC			
CaPOPI_II_1815	scaffold6154	548	AACCGCTCCAATGGTGTTAG	GAAGATGATGTGCGCTTGAA	59.99	221	INTERGENIC			
CaPOPI_II_1816	scaffold6177	3708	TTTTTGTCATCATCGGACCA	AAATCCGTTTGGATTTGCAG	59.90	613	INTERGENIC			
CaPOPI_II_1817	scaffold6179	967	TGGGTGCACAAGATTGGTTA	AGGTCATTTTCGTCCTCCCT	59.96	468	INTERGENIC			
CaPOPI_II_1818	scaffold62	338714	CCATCGTCAAGTCCGAAAAT	CCAAAACTCGAAACCCAAAA	59.93	565	INTERGENIC			
CaPOPI_II_1819	scaffold622	10235	ATAGGCCTAGGGAGGACGAA	TTTAGCCAACTTGTGCATCG	60.05	381	INTERGENIC			
CaPOPI_II_182	Ca2	17061321	TTTGGCTCGGAAGCTTTTTA	AGGAAGAGTGGAGTTGCGAA	59.96	498	INTERGENIC			
CaPOPI_II_1820	scaffold622	30884	TGGGTGCACAAGATTGGTTA	AGGGGTGTTTGAGTCATTGG	59.96	832	INTERGENIC			
CaPOPI_II_1821	scaffold622	61725	CAAACCCACCTATTGGATGC	GGTCATTTTCGTCCTCGCTA	60.19	310	INTERGENIC			
CaPOPI_II_1822	scaffold626	172733	GTGAGCAAAATGCTGGGAAT	GTTGTTGAGTTGTTGGGGCT	60.08	393	INTERGENIC			
CaPOPI_II_1823	scaffold626	219233	GGATTTAGGGTTTTGGCCTT	GATGAAATCGAGGACTGGGA	59.31	654	INTERGENIC			
CaPOPI_II_1824	scaffold626	236880	CCCATCCACAAAAATATGCC	GATGAAATCGAGGACCAGGA	60.02	302	INTERGENIC			

INDEL marker IDs	Chromosomes/ unanchored scaffolds	Physical positions (bp)	Forward primers (5'-3')	Reverse primers (5'-3')	Anneling temperature (0C)	Expected amplified product size (bp)	Structural annotation		Functional annotation	
							Sequence components of *kabuli* genome	*Kabuli* gene accession IDs	NCBI-KOG	TFs
CaPOPI_II_1825	scaffold626	512589	CCCGACAAAAACGGACTAAA	ATAACCCGAACCTCAAACCC	59.97	359	INTERGENIC			
CaPOPI_II_1826	scaffold626	522371	TTTGTCGAGGGGTACGAAAC	GGTCCTCGATTTCATCTGGA	59.97	691	INTERGENIC			
CaPOPI_II_1827	scaffold626	524724	AAATCCCCCGAAAATACCAG	TCCAGATCAAATCGAGGACC	60.01	670	INTERGENIC			
CaPOPI_II_1828	scaffold63	59319	CGAGGTAAGGGGTGAGATGA	AGGTTTGTCAGACCCTCCCT	60.07	481	INTERGENIC			
CaPOPI_II_1829	scaffold637	107635	GCCTTGAGGAATCGGTATCA	GTGTGAATGCAACCTTCCCT	60.04	313	INTERGENIC			
CaPOPI_II_1830	scaffold6382	1419	AGGTCATTTTCGTCCTCCCT	CAAACACACGTTTTGGATGC	59.94	181	INTERGENIC			
CaPOPI_II_1831	scaffold639	11325	TGGTGCAATTTACTTTCTCGG	TTCGGTTCTTGTTTGCCTTT	60.12	783	INTERGENIC			
CaPOPI_II_1832	scaffold642	60062	TATTTTGGGATCTTGGCTCG	CGTAGCGTGGTAGTGAGCAA	60.03	619	INTERGENIC			
CaPOPI_II_1833	scaffold6429	4894	GGGTGGACGAAAATGAGCTA	TGTGTCATTGGATTCGCATT	60.07	537	INTERGENIC			
CaPOPI_II_1835	scaffold6480	530	TAATCGATCATCCCAGACCC	TACAATTGGGCTCCTTCACC	59.71	900	INTERGENIC			
CaPOPI_II_1836	scaffold6480	672	AGAAACATTTGCACGGTCCT	TACAATTGGGCTCCTTCACC	59.60	612	INTERGENIC			
CaPOPI_II_1837	scaffold661	474773	CGTAGCGTGGTAGTGAGCAA	GTTTTGGATTTTGGCTTGGA	60.07	668	INTERGENIC			
CaPOPI_II_1838	scaffold6694	1671	CCCACACTACGACCACGTTA	CGTAACTCGAATGAACGGAAA	59.49	671	INTERGENIC			
CaPOPI_II_1839	scaffold670	197970	TGGCCAATGATCCAAGAGAT	CCGACAAAAATGGACCAAAA	60.43	823	INTERGENIC			
CaPOPI_II_184	Ca2	18833380	TCGAAAAATCCCTTGAAAAAGA	GGACCTATCAAGTGAGCTTGTG	60.05	551	INTERGENIC			
CaPOPI_II_1840	scaffold6720	536	CGATACTCACCCCTCGTTGT	ATGTGACACCCTAAACCCCA	59.99	338	INTERGENIC			

INDEL marker IDs	Chromosomes/ unanchored scaffolds	Physical positions (bp)	Forward primers (5'-3')	Reverse primers (5'-3')	Anneling temperature (0C)	Expected amplified product size (bp)	Structural annotation		Functional annotation	
							Sequence components of *kabuli* genome	*Kabuli* gene accession IDs	NCBI-KOG	TFs
CaPOPI_II_1841	scaffold6720	5246	TTTTCGTCCTCCCTAGGCTT	CAACTATTGGATGCGCAAGA	60.20	470	INTERGENIC			
CaPOPI_II_1842	scaffold674	248531	ACTTTTATGTGCGTTTGACTGA	TCCAACTCCCTTCCCTTTTT	57.51	534	INTERGENIC			
CaPOPI_II_1843	scaffold674	401473	TGCATTAGTCGATGTCCCAA	AATCGATTTCGCGTTTGTTC	60.07	134	INTERGENIC			
CaPOPI_II_1844	scaffold675	81863	TATCAGAGTTCTCCCCACCG	AAACATGACGAGGGCATTTA	60.07	895	INTERGENIC			
CaPOPI_II_1845	scaffold675	82372	TAAATGCCCTCGTCATGTTT	CCCCTTTCCACGGCTATTAT	58.09	444	INTERGENIC			
CaPOPI_II_1846	scaffold676	84534	TTTTCGGATTAGACGATGGC	GATGAAATCGAGGACCAGGA	60.04	796	INTERGENIC			
CaPOPI_II_1847	scaffold6760	1141	AAATGTGTCCATCTCAGCCC	TCAAAAACTCAAGGGGCAAT	59.93	545	INTERGENIC			
CaPOPI_II_1848	scaffold678	39763	AGGAGTTTGAGCATCGCATT	ATCCCACCCCTAAAACCAAG	59.84	470	INTERGENIC			
CaPOPI_II_1849	scaffold678	42464	GCGCACAATAGATGGGTTTA	TTTTCCAACGAGTCCACCTC	58.65	816	INTERGENIC			
CaPOPI_II_185	Ca2	19219016	CCCGTGAGATCCTCCTAACA	TGAATCTACGGCTTTCTGGG	60.07	498	INTERGENIC			
CaPOPI_II_1850	scaffold6782	544	ACTCACCCCACATCCAACAT	ATGGTACCGCAAAGAACGTC	60.10	337	INTERGENIC			
CaPOPI_II_1851	scaffold6782	758	GACGTTCTTTGCGGTACCAT	TCAAACGCTCACACACACAA	60.00	292	INTERGENIC			
CaPOPI_II_1852	scaffold6782	1068	GACGTTCTTTGCGGTACCAT	CAAATGTCAACCACACATTTTG	60.00	428	INTERGENIC			
CaPOPI_II_1853	scaffold6782	1165	GACGTTCTTTGCGGTACCAT	TTTAAATTGCTGCTAAGATTGGT	60.00	469	INTERGENIC			
CaPOPI_II_1854	scaffold6790	1414	TCCTTGACCCCAATTCGTAG	TTTTGGGGTTTAGGGTGTCA	59.93	356	INTERGENIC			
CaPOPI_II_1855	scaffold6790	2258	TTCAAATTGGCCTTGTCCTC	CATAGACCAACGAGGGGAGA	60.05	317	INTERGENIC			

INDEL marker IDs	Chromosomes/ unanchored scaffolds	Physical positions (bp)	Forward primers (5'-3')	Reverse primers (5'-3')	Anneling temperature (0C)	Expected amplified product size (bp)	Structural annotation		Functional annotation	
							Sequence components of *kabuli* genome	*Kabuli* gene accession IDs	NCBI-KOG	TFs
CaPOPI_II_1857	scaffold682	302365	GCTGCAAAAGTCAACGTCAA	CACCCTTGAACTTTGGAGGA	60.04	177	INTERGENIC			
CaPOPI_II_1858	scaffold682	506392	AGGGAGGTTTGTGTTTGACG	TTTAATGTGTTGCGGGTGAA	60.01	408	INTERGENIC			
CaPOPI_II_1859	scaffold682	539867	TCGCCATTGTACTTGCTCTG	CCATGCCTCCAATCAAAAGT	60.01	886	INTERGENIC			
CaPOPI_II_186	Ca2	19791685	TAGGCCATTTTCGTCCTCAC	AATCCAATGACACAAACGCA	60.07	392	INTERGENIC			
CaPOPI_II_1860	scaffold682	540419	ACTTTTGATTGGAGGCATGG	TAATATGGGGAGAGGGGAGG	59.93	665	INTERGENIC			
CaPOPI_II_1861	scaffold682	540642	ACTTTTGATTGGAGGCATGG	GAAGCAAAACCAGGGAATGA	59.93	886	INTERGENIC			
CaPOPI_II_1862	scaffold682	540845	AAGGACTGGTTCACCAGCAG	GAAGCAAAACCAGGGAATGA	60.30	437	INTERGENIC			
CaPOPI_II_1863	scaffold683	20681	GAAGGATGAAAATGGGGGTT	TTGGAAAGGCTGAAATCTGG	60.00	670	INTERGENIC			
CaPOPI_II_1864	scaffold6846	522	TGCATTGGTATATGGGGGTT	CGATCGACAGGAAGAGGAAG	59.90	538	INTERGENIC			
CaPOPI_II_1865	scaffold686	108971	GGGAGAAGGAGGAAGAATGG	CTTGGGAACTGGAGAGCAAG	60.01	564	INTERGENIC			
CaPOPI_II_1866	scaffold686	183469	TGGATTAAATCATTCCCCCA	ATCCCTGTTGATTTGCTTCG	59.95	617	INTERGENIC			
CaPOPI_II_1867	scaffold688	2385	AGGTCATTTCCGTCCTTCCT	GACACGAACACACCTCATGG	59.94	388	INTERGENIC			
CaPOPI_II_1869	scaffold7	215103	TTGATCCTTGCAGTCACTCG	TGGCACTCCGATTAACATGA	59.98	805	INTERGENIC			
CaPOPI_II_187	Ca2	21314611	CAAACCCTTTCAAGGTGCAT	GCACTGCCCCAAGAATTTTA	59.97	443	INTERGENIC			
CaPOPI_II_1870	scaffold702	49940	GTTTTCGAATCAACGAGGGA	TTTGGCTCGAAGGCTTTTTA	60.05	452	INTERGENIC			
CaPOPI_II_1871	scaffold702	99680	CGCTCGATAACCCCAAAATA	ATTACCGACATTGCAGAGGG	59.92	577	INTERGENIC			

INDEL marker IDs	Chromosomes/ unanchored scaffolds	Physical positions (bp)	Forward primers (5'-3')	Reverse primers (5'-3')	Anneling temperature (0C)	Expected amplified product size (bp)	Structural annotation		Functional annotation	
							Sequence components of *kabuli* genome	*Kabuli* gene accession IDs	NCBI-KOG	TFs
CaPOPI_II_1872	scaffold702	216004	CTGTTTGGGCGTCGAATTAT	TATTTTCTCCCCTCGTTGGT	59.96	630	INTERGENIC			
CaPOPI_II_1873	scaffold7081	2451	TTAATGAGGGGCTGAATTGG	AAGCGTACCTGTGGGAATTG	59.89	259	INTERGENIC			
CaPOPI_II_1874	scaffold7081	2734	CAATTCCCACAGGTACGCTT	TGGGGGTTAAAGGGATTAGG	59.99	465	INTERGENIC			
CaPOPI_II_1875	scaffold7107	12825	GGGGACTAAAATTGGGGAGA	TGCAGGCAATCAATGAAAGA	60.12	459	INTERGENIC			
CaPOPI_II_1876	scaffold7107	14640	TAATAGAAGGTCGCATCCCG	TGTGTCAAACGAAATGATGTG	60.05	368	INTERGENIC			
CaPOPI_II_1877	scaffold7107	16409	GGGGCTAGAACCTCCTACAA	CCATTTGCATGATTCCACAC	58.27	946	INTERGENIC			
CaPOPI_II_1878	scaffold7107	16449	TCCAATGCTTACAATCCTCTCTC	CCATTTGCATGATTCCACAC	59.74	508	INTERGENIC			
CaPOPI_II_1879	scaffold711	204653	AATGTGAATCCCACCAGGAA	TTTACAGCGCTTGTCCTCAA	60.17	796	INTERGENIC			
CaPOPI_II_188	Ca2	23193466	CCACAAATGCTCATCGTGAC	TTTGCAGCCACAAAAGACAG	60.12	561	INTERGENIC			
CaPOPI_II_1881	scaffold716	189823	AAGCTTGTGCAAAGGGAAAA	TGGTTTTGATGGGACCAAAT	59.86	113	INTERGENIC			
CaPOPI_II_1882	scaffold719	123762	AGGTCATTTTCGTCCTCCCT	TTCATCCAATGGCACAAAGA	59.94	695	INTERGENIC			
CaPOPI_II_1883	scaffold720	372071	ACCAACATTTCAGGCTCACC	TAGAGCGCCAAACCCTAATG	59.97	486	INTERGENIC			
CaPOPI_II_1884	scaffold7210	7073	TTTTCGTCCTCCCTAGGCTT	TTGGATGCACATGATTGGTT	60.20	364	INTERGENIC			
CaPOPI_II_189	Ca2	23509535	CCCTCTTGAATTTTTGTTTTCC	CCCACACCCTCGACTAAAGA	59.01	542	INTERGENIC			
CaPOPI_II_1890	scaffold7280	1708	GTTTCGTACCCCTCGACAAA	TCCAGATGAAATCGAGGACC	59.97	311	INTERGENIC			
CaPOPI_II_1891	scaffold7293	13170	TGCCTCTGGATTGGTTGAAT	AATCTATCCACCCGGAAACC	60.46	471	INTERGENIC			

INDEL marker IDs	Chromosomes/ unanchored scaffolds	Physical positions (bp)	Forward primers (5'-3')	Reverse primers (5'-3')	Anneling temperature (0C)	Expected amplified product size (bp)	Structural annotation		Functional annotation	
							Sequence components of *kabuli* genome	*Kabuli* gene accession IDs	NCBI-KOG	TFs
CaPOPI_II_1892	scaffold7293	15472	TTCATGCCAGCAACTCTTTG	TGCTTTGCTATGCACCAGTC	59.99	626	INTERGENIC			
CaPOPI_II_1893	scaffold7293	20198	TTGCTGCCTTCTCCTGTTTT	ATGATGCACTTTGGGCCTAC	59.99	661	INTERGENIC			
CaPOPI_II_1894	scaffold7299	551	CACCAAAACTTAATTTCCCCTTT	TGATTCCTATGCATCGTATTTTG	59.67	459	INTERGENIC			
CaPOPI_II_1897	scaffold731	322781	ATCCCCGATTCCTCTCAAGT	AATTCCTACTGGATGCACGC	59.90	479	INTERGENIC			
CaPOPI_II_1898	scaffold731	342154	AAATGGAGGAGAAGGAGGGA	ACCCAATTACTGGAGATGCG	60.01	574	INTERGENIC			
CaPOPI_II_1899	scaffold731	354972	ATAACCTTAGCGCATTCCCA	CCTCCCGTAAGATCCAACAA	59.57	308	INTERGENIC			
CaPOPI_II_19	Ca1	6203281	CTCCTTGAGTTTTGAGACACGA	TCGATTATGCGTAGGGAAGG	59.52	453	INTERGENIC			
CaPOPI_II_190	Ca2	23932608	AAGGCATCCAATCAAAGCAC	CTCAAGATTTTAGCGCCGTC	60.08	460	INTERGENIC			
CaPOPI_II_1901	scaffold731	366076	TCTCGCGGATTTTCATAACC	CCATCCATTTCCGTATTGCT	60.04	100	INTERGENIC			
CaPOPI_II_1906	scaffold7364	2154	TCAATTTGGGTTTAGGGTGC	TGTAGTGGCATGTTTGGCAT	59.80	497	INTERGENIC			
CaPOPI_II_1907	scaffold7377	545	TTTCTTCCCAACCTTCATCG	GCCGTTGGAACTTCATCATT	60.04	816	INTERGENIC			
CaPOPI_II_1908	scaffold738	2137	TGAATATCGCCCGAAAAGTC	TTTCATAGCAGACGAGGGCT	60.04	292	INTERGENIC			
CaPOPI_II_1909	scaffold738	5098	TCGGGTGGTACTTAAATCGC	ATCTGACGTGGAAACCGAAC	59.96	274	INTERGENIC			
CaPOPI_II_191	Ca2	23989802	AATTCGTTGAGCCGGTCTAGT	AGCACGGGGAGTATGAGAAA	60.14	421	INTERGENIC			
CaPOPI_II_1910	scaffold743	14244	TGCTTGATTGCTTGAGTTCC	GTACCCTGATTGCCCTCTGA	59.00	321	INTERGENIC			
CaPOPI_II_1911	scaffold7448	21480	AGGATCTCCCGATTCTCGAT	TTCCGGGTCAGATTTCAAAC	60.00	287	INTERGENIC			

INDEL marker IDs	Chromosomes/ unanchored scaffolds	Physical positions (bp)	Forward primers (5'-3')	Reverse primers (5'-3')	Anneling temperature (0C)	Expected amplified product size (bp)	Structural annotation		Functional annotation	
							Sequence components of *kabuli* genome	*Kabuli* gene accession IDs	NCBI-KOG	TFs
CaPOPI_II_1912	scaffold7477	2293	TTGGTGGATTTGAGACCACA	ACTTCCCTTTGCACATTTGG	59.94	652	INTERGENIC			
CaPOPI_II_1913	scaffold7477	6564	CCTGCAGAAAGCAAAACACA	ACCTTGGGAAGGAGGAGAAA	60.03	590	INTERGENIC			
CaPOPI_II_1914	scaffold749	1624	CTTGGGAACTGGAGAGCAAG	GAGGAGGGAGGGATTTTGAG	59.98	536	INTERGENIC			
CaPOPI_II_1915	scaffold750	31851	TGAGTTTCTATGCAAGGGCA	TTCATGAAGACACCGGAACA	59.42	273	INTERGENIC			
CaPOPI_II_1916	scaffold750	38979	CACTGATAACGCGACTAATTTGA	ACCGCCACAAATGATAAAGC	59.33	748	INTERGENIC			
CaPOPI_II_1917	scaffold750	59023	CGTAGCATCAGCAACCTTCA	TCCCTCTTTTGTTTTTCCCA	60.01	989	INTERGENIC			
CaPOPI_II_1919	scaffold752	505163	AGCCATTGCAGTCTTCGTCT	TCGTGTCTCAAAACTCAAGGAA	60.02	854	INTERGENIC			
CaPOPI_II_192	Ca2	23990182	TCTCATACTCCCCGTGCTTT	ATCCCCTACACCAGATGCAC	59.69	565	INTERGENIC			
CaPOPI_II_1920	scaffold758	13400	CTCGTCCAATTCCCCTTTTT	CGATTTTACGTGAATATACGAACAC	60.29	603	INTERGENIC			
CaPOPI_II_1923	scaffold7657	660	AGGGAGGACGAAAATGACCT	GGTGTGTTTGTGTCGTTGGA	59.94	412	INTERGENIC			
CaPOPI_II_1924	scaffold768	560	CCATGAGAGTCGAAATCGGT	TCCCACAGATCCACATTCAA	60.07	188	INTERGENIC			
CaPOPI_II_1925	scaffold769	1084	AAATGAACCAATTTAATGAGCTACC	CCCTCCCAAAACACCCTTAT	58.96	479	INTERGENIC			
CaPOPI_II_1926	scaffold769	2786	ACGTACACGTCAGCGTTTTG	CCCAATTCTCAATCCCACTT	59.83	147	INTERGENIC			
CaPOPI_II_1927	scaffold769	5414	TCACTCGCGTTAATTTTGATG	GCGAGTGAATACATGTGCCA	58.83	518	INTERGENIC			
CaPOPI_II_1928	scaffold769	29353	GCACTCTCATTCATCCCACA	CCACATGTCATTTAACCCTGC	59.64	370	INTERGENIC			
CaPOPI_II_1929	scaffold7697	589	CTAGCCCTCGTCTGTTCCAG	AATCCGTATTTTCGCACCAG	60.01	521	INTERGENIC			

INDEL marker IDs	Chromosomes/ unanchored scaffolds	Physical positions (bp)	Forward primers (5'-3')	Reverse primers (5'-3')	Anneling temperature (0C)	Expected amplified product size (bp)	Structural annotation		Functional annotation	
							Sequence components of *kabuli* genome	*Kabuli* gene accession IDs	NCBI-KOG	TFs
CaPOPI_II_193	Ca2	24252139	GATCCTCAAATCGACCTCCA	TGTCACCATTGGTCTCCAAA	60.01	465	INTERGENIC			
CaPOPI_II_1930	scaffold772	11892	GGCACACTCCCGTAAAATTC	TTTTTCGCACACCAATGAGA	59.43	539	INTERGENIC			
CaPOPI_II_1931	scaffold772	213642	ACTTGAGGGCGAAGAACAAA	TTGGATGACCTACGACTCCC	59.85	573	INTERGENIC			
CaPOPI_II_1932	scaffold775	110824	GCCGAATCGATTTTGTCAGT	GGAGGGTTTTGGTTTGGAAT	60.08	521	INTERGENIC			
CaPOPI_II_1933	scaffold775	446063	TTTTGCAAGATCTGCCACTG	TGGAAGGATCCGGAACTATG	59.99	867	INTERGENIC			
CaPOPI_II_1935	scaffold776	224423	TGGGATACGTAGGAGCAAGG	TGTCCGTGAAAAATGAGTCG	60.09	310	INTERGENIC			
CaPOPI_II_1936	scaffold7765	977	AGATGGCTGCAGCTCAAAGT	TTTCCAAGAGGTTTTGGGTC	60.16	464	INTERGENIC			
CaPOPI_II_1937	scaffold787	149556	CGGGATGGTGGCATAATATC	AGCAGGGTTTCTCCCAAACT	60.00	343	INTERGENIC			
CaPOPI_II_1938	scaffold787	334329	CTTCCTCTGTGCCACATCCT	TTCATTCGTGTCTGCTTTGG	60.26	598	INTERGENIC			
CaPOPI_II_1939	scaffold787	460684	TATGGGAAGGAAATGTGGGA	TCACTCGCAACGAGAAGTTG	60.13	601	INTERGENIC			
CaPOPI_II_194	Ca2	24943252	AACCCCTCCAAAGAACGACT	AGGGCCCCACTAACTCCTTA	59.97	680	INTERGENIC			
CaPOPI_II_1940	scaffold7890	680	TTTCTCCCCTTTCTTTCCGT	GAAGGGACTGCGGCATAATA	60.05	162	INTERGENIC			
CaPOPI_II_1941	scaffold7891	3768	TCTGGACTTTTCGAGCGATT	AGTGCATTTTAGTCGGGTGG	59.96	379	INTERGENIC			
CaPOPI_II_1942	scaffold7902	684	GGGTAGCATGGCAAGTTGTT	CGAGTAAAGCGTTTTGACTAACG	60.00	363	INTERGENIC			
CaPOPI_II_1946	scaffold794	138143	CTGTCAAACCGGGTTCTGTT	TGCTTGCAGTGCTTCATCTT	60.01	378	INTERGENIC			
CaPOPI_II_1947	scaffold796	26479	TCATTTTCGTCCTCCTTTGG	GCCTATTGGATGCACAGGAT	60.04	470	INTERGENIC			

INDEL marker IDs	Chromosomes/ unanchored scaffolds	Physical positions (bp)	Forward primers (5'-3')	Reverse primers (5'-3')	Anneling temperature (0C)	Expected amplified product size (bp)	Structural annotation		Functional annotation	
							Sequence components of *kabuli* genome	*Kabuli* gene accession IDs	NCBI-KOG	TFs
CaPOPI_II_1948	scaffold797	15287	GGTGATTTTTATGATGTGCTAGTTTT	CCCTTTCAATTCCCGTTTTT	59.01	689	INTERGENIC			
CaPOPI_II_1949	scaffold798	16927	ATGCAATGCGTATATGCCAA	TAAATCGTGTTGACCCGTGA	59.95	233	INTERGENIC			
CaPOPI_II_195	Ca2	28465231	TCATCGCAGGTGTACCATGT	GGTATCAGAGCCCACCATGT	59.99	908	INTERGENIC			
CaPOPI_II_1950	scaffold801	260992	TCGTCCGTAAAAGATACCGC	GGAGGACCAAAATTTGAGCA	60.10	661	INTERGENIC			
CaPOPI_II_1951	scaffold81	91419	TGAGGAGAAACAAAATGGGG	ATGGAAGGGTCGTTGACATT	59.90	458	INTERGENIC			
CaPOPI_II_1952	scaffold811	56559	TTTAACGTGTCGCGTCTTTG	GAATAAAATGCACCGGCACT	59.91	748	INTERGENIC			
CaPOPI_II_1954	scaffold812	215247	AGAAGGAACAACGAGAACGC	TGTCCTTCTCATTGGGAACC	59.48	178	INTERGENIC			
CaPOPI_II_1955	scaffold812	245558	CAAAGTCTTCACACACCGACA	TGTTGACTCCAGTCACCGAG	59.78	736	INTERGENIC			
CaPOPI_II_1956	scaffold812	526031	TCGACTTGTCAATCGCTTTG	CACTCCAGTGCTGAGGGAAT	59.99	215	INTERGENIC			
CaPOPI_II_1957	scaffold820	15078	TCATCATGGGCTTACGACCT	GGCCAAACCCATTAAAACCT	60.48	343	INTERGENIC			
CaPOPI_II_1958	scaffold824	6128	CACCCCAGTTTTGAGGGTAA	CGATTGTGTTTGTCACAGGG	59.82	191	INTERGENIC			
CaPOPI_II_1959	scaffold829	10973	TTTATTTGACAACTCGTTTTTGTT	GAGCGAATTGAGAAACGAGG	57.13	799	INTERGENIC			
CaPOPI_II_196	Ca2	28465620	AGCTCCATTGCAAAAACACC	GCAATTCCCACAGGTATGCT	60.12	553	INTERGENIC			
CaPOPI_II_1960	scaffold8314	868	TGAATGGTTGCATGGTTATCA	GTGGGAAGTGGTAGAGTGCG	59.80	415	INTERGENIC			
CaPOPI_II_1961	scaffold8314	1258	AATTTAGCTCATTGCTGCCG	TACCTCCTTTGGAAGCATGG	60.36	353	INTERGENIC			
CaPOPI_II_1962	scaffold8315	18988	AAAAATTTATATTGCGTCAATTTGG	GTGCTGGCCATAGGAAATGT	59.60	565	INTERGENIC			

INDEL marker IDs	Chromosomes/ unanchored scaffolds	Physical positions (bp)	Forward primers (5'-3')	Reverse primers (5'-3')	Anneling temperature (0C)	Expected amplified product size (bp)	Structural annotation		Functional annotation	
							Sequence components of *kabuli* genome	*Kabuli* gene accession IDs	NCBI-KOG	TFs
CaPOPI_II_1963	scaffold8315	20462	AAAAGGATATTCAACTCTCCAAAAA	CGATTTTGCTACACAAAATTTCC	58.72	759	INTERGENIC			
CaPOPI_II_1964	scaffold8412	961	TTTTCACATTTCAAAAGCATGA	TCACATCTTCCTTATGTCCGC	58.33	342	INTERGENIC			
CaPOPI_II_1965	scaffold842	20647	TGATTCCTGTAACACCCCAA	AACCAAACACACAAGGAAATATGA	58.82	332	INTERGENIC			
CaPOPI_II_1966	scaffold8442	1501	AACCAAACTGGTGCATCACA	AGCCTAGGGTGGACGAAAAT	60.01	676	INTERGENIC			
CaPOPI_II_1967	scaffold845	2172	CAACATTTTGGGTGCATTTG	TGGTTATTTTGCATGTAGCGA	59.83	657	INTERGENIC			
CaPOPI_II_1968	scaffold845	132751	AGTGTTGTTTGGGTTGGGAG	TCGATTTGACATTTGCGTGT	59.86	526	INTERGENIC			
CaPOPI_II_1969	scaffold845	170625	ATTTGGTGCGCATGATAAGA	AAAAATGAGGTGGGTTAGAAATTA	59.14	777	INTERGENIC			
CaPOPI_II_197	Ca2	29220253	AGGCATGGTGTTGGAGTCAT	CTAGTTCGTAACCCGTGCGT	60.40	253	INTERGENIC			
CaPOPI_II_1970	scaffold845	331956	TTGCTTGGAAAGGAAAATCG	GGGGCCAAATAATGTTGCTA	60.18	802	INTERGENIC			
CaPOPI_II_1971	scaffold8493	9496	CGACCATGATTTAACGACCA	GTTTTCAAGAGGTGGCCTTG	59.40	360	INTERGENIC			
CaPOPI_II_1972	scaffold855	31194	CTCGTACGCTTTTGGTGTGA	GTAGGGGGTAGAAAACCCCA	59.90	556	INTERGENIC			
CaPOPI_II_1973	scaffold855	31470	AATTGGATACCCCTTTTGGC	GTAGGGGGTAGAAAACCCCA	60.02	384	INTERGENIC			
CaPOPI_II_1974	scaffold8565	6875	TGACCAAAATCCATCCCTTC	TTTACAGCGCTTGTTCCAAA	59.73	517	INTERGENIC			
CaPOPI_II_1976	scaffold8646	2745	AGTCTACGACGAAGCGTTCC	TCCATAGGAGGTCGATGGTC	59.50	532	INTERGENIC			
CaPOPI_II_1977	scaffold866	36277	GAACAACAAGTGGGATGCCT	TGTGAAAAATGAACACCCCA	59.97	360	INTERGENIC			
CaPOPI_II_1978	scaffold866	142956	GGATCCCCAATCGATAACCT	CTGTTGAGCCTTAGGGCAAG	59.98	450	INTERGENIC			

INDEL marker IDs	Chromosomes/ unanchored scaffolds	Physical positions (bp)	Forward primers (5'-3')	Reverse primers (5'-3')	Anneling temperature (0C)	Expected amplified product size (bp)	Structural annotation		Functional annotation	
							Sequence components of *kabuli* genome	*Kabuli* gene accession IDs	NCBI-KOG	TFs
CaPOPI_II_1979	scaffold870	39934	AGAGGCAAACAAGAACCGAA	GGTGAGCCACCTTCATTTGT	59.85	517	INTERGENIC			
CaPOPI_II_198	Ca2	29240150	CTTAGAGCATCTCCAACGGC	GAAGCATCGTTCCTTCCTGA	59.98	134	INTERGENIC			
CaPOPI_II_1980	scaffold870	82445	TGGTGGTGCAATTCACATCT	TTTCACCGTCATGCTTTTTG	59.97	540	INTERGENIC			
CaPOPI_II_1981	scaffold873	18311	TGTTGGGTCCACAAAGAGAG	TGTTGTTCTCCTCTGACACAATG	58.70	402	INTERGENIC			
CaPOPI_II_1982	scaffold8734	801	TTGGGCGAAAATGGATAAAG	CCCCGCTCTGATAGGTGATA	59.90	802	INTERGENIC			
CaPOPI_II_1983	scaffold8734	1079	CTGTGACACCCTAAATCCCA	GATCTATGAGGACAGGGCCA	58.41	798	INTERGENIC			
CaPOPI_II_1984	scaffold876	622726	TCAGGAACAACAATCCAATCC	TTACCCTCAAAACTGGGGTG	59.78	331	INTERGENIC			
CaPOPI_II_1986	scaffold882	70530	CACATCAACAGCAGCCTTGT	TGATGTTGCCTCTGTTTTGC	59.91	385	INTERGENIC			
CaPOPI_II_1987	scaffold882	446893	TGGTTGGTTTTTGTGGTGAA	TCACGGGCAAACACAATTTA	59.83	644	INTERGENIC			
CaPOPI_II_1988	scaffold8825	968	AATCGATTGGCATCAACACA	CCAGTCAAGCATTCCCATCT	59.93	475	INTERGENIC			
CaPOPI_II_1989	scaffold8836	981	CAACACAACAAGAACGAGCAA	TCCAAGTAGGTCTGCCCAAT	59.94	288	INTERGENIC			
CaPOPI_II_199	Ca2	29240382	CTTAGAGCATCTCCAACGGC	CCTTGCACTATCATTTTCGGA	59.98	803	INTERGENIC			
CaPOPI_II_1991	scaffold892	26717	TCAAGCTTCCTCTCCACACA	AATTAATATCCACGCAACGC	59.55	255	INTERGENIC			
CaPOPI_II_1992	scaffold892	29801	GAAGCCATGAAAACCTTCCA	ATGCAAACATTTCCCACCTC	60.05	392	INTERGENIC			
CaPOPI_II_1996	scaffold892	44352	CTGAGTTGCGACGAGAGTTG	TTGGAAGGAGCCCTTACCTT	59.77	465	INTERGENIC			
CaPOPI_II_1997	scaffold896	47144	AGGGAGGACGAAAATGACCT	TGTGCATCAAATAGGCGTGT	59.94	259	INTERGENIC			

INDEL marker IDs	Chromosomes/ unanchored scaffolds	Physical positions (bp)	Forward primers (5'-3')	Reverse primers (5'-3')	Anneling temperature (0C)	Expected amplified product size (bp)	Structural annotation		Functional annotation	
							Sequence components of *kabuli* genome	*Kabuli* gene accession IDs	NCBI-KOG	TFs
CaPOPI_II_1999	scaffold9059	539	TTAGGGCTCATACGATTCGG	GGAAAATTAGCCACGGATCA	60.05	443	INTERGENIC			
CaPOPI_II_2	Ca1	19168	CAGAACCGAGGAATTAAGCG	AGGCTTGAAGCGTGGTAATG	59.84	637	INTERGENIC			
CaPOPI_II_200	Ca2	29252004	TCAAAGGTGCACTCTTGCTATT	TGGCTTCCGGAAAGACTAAA	59.04	502	INTERGENIC			
CaPOPI_II_2000	scaffold913	140774	TTGAGTTATGGCTAATGAAGGTCA	AGCGAGCCGCTCAATTAGTA	60.02	269	INTERGENIC			
CaPOPI_II_2001	scaffold913	282492	TTGGATTTCATACCCACACATC	AAAATCCGTTGGAAATTTTGG	59.55	466	INTERGENIC			
CaPOPI_II_2003	scaffold916	4344	TGTTGACGAATGTCCATGAAA	CCTCCAAAACCCAACTACCA	59.96	181	INTERGENIC			
CaPOPI_II_2004	scaffold916	4805	TGGTAGTTGGGTTTTGGAGG	TTCATGACATGCATCCATCC	59.82	907	INTERGENIC			
CaPOPI_II_2005	scaffold916	4868	TGGTAGTTGGGTTTTGGAGG	TTCATGACATGCATCCATCC	59.82	907	INTERGENIC			
CaPOPI_II_2006	scaffold916	5303	TGTCTCTTTTACGCCGCAAT	CTAGGCATTGCAACAGGGAT	60.77	738	INTERGENIC			
CaPOPI_II_2007	scaffold916	8185	ACACCAACTATGCACCACCA	ATAGGCCCCAATTAACCCAC	59.88	376	INTERGENIC			
CaPOPI_II_2008	scaffold916	8308	ACACCAACTATGCACCACCA	ATAGGCCCCAATTAACCCAC	59.88	376	INTERGENIC			
CaPOPI_II_2009	scaffold916	9278	TCACATAGGGATTGCCAACA	TCCCACCACATTCAATCCTT	59.92	769	INTERGENIC			
CaPOPI_II_201	Ca2	29444244	AAATCCGACTGTCTAACCCG	TTTTCTCCTCCAATTTAGTCCAA	59.05	826	INTERGENIC			
CaPOPI_II_2010	scaffold916	9949	AGGATTGAATGTGGTGGGAA	GCAGAAAAATAAGGGCGATG	60.17	354	INTERGENIC			
CaPOPI_II_2011	scaffold916	134889	AAATGTGGTCTCGGTTTTGC	TTGTTTTGTGAGTTTGAATGATGTT	59.98	770	INTERGENIC			
CaPOPI_II_2012	scaffold916	363659	CCACCCTCTACCATGCAACT	TGTTTTCCATGTGTGCCACT	59.99	569	INTERGENIC			

INDEL marker IDs	Chromosomes/ unanchored scaffolds	Physical positions (bp)	Forward primers (5'-3')	Reverse primers (5'-3')	Anneling temperature (0C)	Expected amplified product size (bp)	Structural annotation		Functional annotation	
							Sequence components of *kabuli* genome	*Kabuli* gene accession IDs	NCBI-KOG	TFs
CaPOPI_II_2013	scaffold918	49291	TAGCGAGGACGAAAATGACC	CGTCCTCCCTGGGTTTATTC	60.21	820	INTERGENIC			
CaPOPI_II_2014	scaffold919	83832	GTTTGAAGTTGTCCGCGTCT	GACACTTGCGCCCCTATAAA	60.30	479	INTERGENIC			
CaPOPI_II_2015	scaffold92	45246	TGCAATAAAACCATAATACGAAGA	TGTTTGACTGCATAACGAAGA	57.88	438	INTERGENIC			
CaPOPI_II_2016	scaffold924	202228	AGGAAGAGTGGAGTTGCGAA	CAGGTTTGATGCAACCATTG	59.99	689	INTERGENIC			
CaPOPI_II_2017	scaffold93	141155	TCGATTGGCTTAAAACCAGG	AGAATTTACGAGCAGCGGAA	60.07	617	INTERGENIC			
CaPOPI_II_2019	scaffold9303	1186	AAGACCACATCATTGTGTCACC	TGTTTGTTCGCGTCTTAATTTT	59.75	439	INTERGENIC			
CaPOPI_II_202	Ca2	29445927	TGTCTAACCCGTCTAACCCG	CCCTACCTAGGGGTGGACAT	59.99	138	INTERGENIC			
CaPOPI_II_2020	scaffold931	100930	TTTGAGCCCCCTTCAATATG	GATTCTCCTTGGGGATGGAT	59.89	616	INTERGENIC			
CaPOPI_II_2021	scaffold931	116282	TCAGTTGCAATTTCCTTCCC	TAAAACCGATTTGGGAGTCG	60.05	519	INTERGENIC			
CaPOPI_II_2022	scaffold931	116416	CGACTCCCAAATCGGTTTTA	TTGGGCTGATGCAATAAACA	59.93	260	INTERGENIC			
CaPOPI_II_2023	scaffold931	116537	CGACTCCCAAATCGGTTTTA	TTGGGCTGATGCAATAAACA	59.93	260	INTERGENIC			
CaPOPI_II_2024	scaffold931	117076	TGCCACAACCAATCTTCATC	AGACAGTGGGTGTTCCAAGC	59.50	391	INTERGENIC			
CaPOPI_II_2025	scaffold931	118275	TGCTCTGACTCATTCAACCG	CACCAACAATTGGCATCAAG	59.98	725	INTERGENIC			
CaPOPI_II_2026	scaffold931	180349	TCGTCCCCATGTAATTGTCA	TGGATACCCAAAAACCATTCA	59.77	281	INTERGENIC			
CaPOPI_II_2027	scaffold931	181299	GTAGAGTGCCAACGACACCA	TGAACTATTGATTGGATCGTGC	59.75	665	INTERGENIC			
CaPOPI_II_2028	scaffold931	246666	GGGTGTCACATCACCCTTTT	GCGGAAGACTTTATAGTGAAAGGA	59.68	191	INTERGENIC			

INDEL marker IDs	Chromosomes/ unanchored scaffolds	Physical positions (bp)	Forward primers (5'-3')	Reverse primers (5'-3')	Anneling temperature (0C)	Expected amplified product size (bp)	Structural annotation		Functional annotation	
							Sequence components of *kabuli* genome	*Kabuli* gene accession IDs	NCBI-KOG	TFs
CaPOPI_II_2029	scaffold933	4208	TCAAACCCAGTGGTCTCCTT	AGTCCGATGAAGGGTCACAG	59.55	654	INTERGENIC			
CaPOPI_II_203	Ca2	29829940	CTTTTCGTCGGAGTTTGTCC	TCGTCCTCTGGTTTTCTCGT	59.71	476	INTERGENIC			
CaPOPI_II_2030	scaffold9371	631	GCGGCTGAAAGAGAGAGAGA	AAAGATTTCCGGTACACACCA	59.97	687	INTERGENIC			
CaPOPI_II_2031	scaffold939	1093	GTGACGTTGGGGGAGTTAAA	TCGAGCCTTATGGCAAGATT	59.83	317	INTERGENIC			
CaPOPI_II_2032	scaffold944	672	TTGTAAGTTGAGTTGCGACGA	TGTGGTCGCAGGTTTTATGA	59.52	308	INTERGENIC			
CaPOPI_II_2033	scaffold944	2389	GAACGGGAAATATTCACGTCAT	TCGGACCACACTCCTAATCC	60.08	495	INTERGENIC			
CaPOPI_II_2034	scaffold944	2557	GGATTAGGAGTGTGGTCCGA	CAACTGGGTACCACTAAGCCA	59.93	450	INTERGENIC			
CaPOPI_II_2035	scaffold944	3122	TGGCTTAGTGGTACCCAGTTG	TGGCGCTTATATTCAAAGCA	60.04	643	INTERGENIC			
CaPOPI_II_2036	scaffold944	3226	TGGCTTAGTGGTACCCAGTTG	TGGCGCTTATATTCAAAGCA	60.04	643	INTERGENIC			
CaPOPI_II_2038	scaffold956	19709	TCCAGATGAAATCGAGGACC	GGATTAGCCTTTCGAGTCCC	60.01	381	INTERGENIC			
CaPOPI_II_204	Ca2	30095996	CATTCCCGCTACATTTTGGT	GGAGAGAGGCCAATTTTTCA	59.82	226	INTERGENIC			
CaPOPI_II_2040	scaffold963	17319	CCGGGGATAATGGCTTAAAT	GGGGTATGCATTTGTTGGAC	60.00	429	INTERGENIC			
CaPOPI_II_2041	scaffold9757	838	TGTTAATCCAACGGCACAAA	TCCCCTGGCTTATTGTTGTC	59.97	279	INTERGENIC			
CaPOPI_II_2042	scaffold9813	1199	AAAATCGCGCGAAAAGTCTA	TCCTGGTCCTCGATTTCATC	59.99	772	INTERGENIC			
CaPOPI_II_2043	scaffold982	874	TTGGATGGAGTGCCACAATA	AACTTGGGGTTGAAGATGAAGA	59.92	164	INTERGENIC			
CaPOPI_II_2044	scaffold983	88220	CACCCACAAGGAGGAAGAAC	CATGGATCAAACAGGCTTCA	59.55	517	INTERGENIC			

INDEL marker IDs	Chromosomes/ unanchored scaffolds	Physical positions (bp)	Forward primers (5'-3')	Reverse primers (5'-3')	Anneling temperature (0C)	Expected amplified product size (bp)	Structural annotation		Functional annotation	
							Sequence components of *kabuli* genome	*Kabuli* gene accession IDs	NCBI-KOG	TFs
CaPOPI_II_2045	scaffold983	88456	TTTATTCGCATGTTGTTGCC	CATGGATCAAACAGGCTTCA	59.57	274	INTERGENIC			
CaPOPI_II_2046	scaffold983	255175	TTACCGGATTTACTCACGGC	ACCCGTCAAATACGACGAAC	59.96	319	INTERGENIC			
CaPOPI_II_2047	scaffold992	40484	TCAATGCCAAAACATGCAAT	TTCGGATGTCCCCTTAAACA	59.94	513	INTERGENIC			
CaPOPI_II_2048	scaffold992	42301	GGGAGCATGAAATTGAACAAA	TCTTTTGCGTCTGAATTTGG	59.93	708	INTERGENIC			
CaPOPI_II_2049	scaffold999	130386	TGTAAACCTGCTTGCACACC	CTGCACAACAATCCCACAAC	59.76	160	INTERGENIC			
CaPOPI_II_205	Ca2	30096276	CATTCCCGCTACATTTTGGT	GAAGCTGCTTGTGTGCCATA	59.82	876	INTERGENIC			
CaPOPI_II_206	Ca2	30341034	TGTCTTATCCTGAACTCAAACTCG	ATTTGGTCAACCACCCAAAA	59.82	537	INTERGENIC			
CaPOPI_II_208	Ca2	30395203	CCCAACAACAACAAAAGCCT	AAACCAGCATGACAAAAGCA	60.01	289	INTERGENIC			
CaPOPI_II_209	Ca2	30447611	CTTTTGTCTTCTCGGCCTTG	CGTGCTCATCTCCATTGTTG	59.99	891	INTERGENIC			
CaPOPI_II_21	Ca1	6267078	TTTCTTGCACTGGTTTGTGC	TGGGTCCCTCATTGTACTCC	59.89	560	INTERGENIC			
CaPOPI_II_211	Ca2	30553379	TTTTTATGAGGCGTGGAAGG	CCATGAACACGCCAAACATA	60.07	874	INTERGENIC			
CaPOPI_II_214	Ca2	30676782	GCTCCAGCAAAAATAGAAATGG	TCATCAATCAAACTACTTACGCAGA	60.10	504	INTERGENIC			
CaPOPI_II_215	Ca2	30890128	GATGATGTAAATGGGAGCCAA	CATTTGTTGAATGTTGCCCA	59.78	697	INTERGENIC			
CaPOPI_II_216	Ca2	31240228	CTGCCCCCACTATTTCTCAA	GAAACACTTGGCATGTTAAGGA	60.07	479	INTERGENIC			
CaPOPI_II_22	Ca1	7215414	AACCCCCAACTACAAGGTCC	CAATTTGACAAGCCCCCTAA	60.09	639	INTERGENIC			
CaPOPI_II_221	Ca2	32209131	GCAAAGATAGACACCACCCC	AACCATTCCCATGGAACAAA	59.41	577	INTERGENIC			

INDEL marker IDs	Chromosomes/ unanchored scaffolds	Physical positions (bp)	Forward primers (5'-3')	Reverse primers (5'-3')	Anneling temperature (0C)	Expected amplified product size (bp)	Structural annotation		Functional annotation	
							Sequence components of *kabuli* genome	*Kabuli* gene accession IDs	NCBI-KOG	TFs
CaPOPI_II_222	Ca2	32209334	TCCATGGGAATGGTTTTGAT	TTGAAGAGGAAGTTTTGGCA	59.99	342	INTERGENIC			
CaPOPI_II_223	Ca2	32209671	TCCATGGGAATGGTTTTGAT	TTTTAGTGTGCACCAATGTCAA	59.99	899	INTERGENIC			
CaPOPI_II_224	Ca2	32210099	TCAGCTTCAATTATCTCCAACAA	TCGGCGACAAAAATCACATA	58.89	644	INTERGENIC			
CaPOPI_II_225	Ca2	32213171	CAACATGACAAGGACTTTCCAA	TTACCGGTATCGCAAGGAAC	60.01	529	INTERGENIC			
CaPOPI_II_226	Ca2	32213424	AGGGATAGATAACGGCACGA	AAGGCCCTGCTAGAATGATG	59.55	504	INTERGENIC			
CaPOPI_II_227	Ca2	32379585	TGTGCGAGTTTATTGCGGTA	CATTTGGTTCAAAATGTCATGG	60.27	624	INTERGENIC			
CaPOPI_II_228	Ca2	32393117	TGCCCATCCTTATGCTCTTC	ATCACGTGCAACCACAAGTC	60.18	626	INTERGENIC			
CaPOPI_II_229	Ca2	32393187	TGCCCATCCTTATGCTCTTC	ATCACGTGCAACCACAAGTC	60.18	626	INTERGENIC			
CaPOPI_II_23	Ca1	9871519	CTTAGTCCACGTGCAAACGA	AAAGTGCGGGCCAATATCTA	59.90	600	INTERGENIC			
CaPOPI_II_230	Ca2	32393441	TCGCACTTTTTCGTGTAGGA	GGGCTTGTTTGACGCATTAT	59.46	588	INTERGENIC			
CaPOPI_II_231	Ca2	32393633	GACTTGTGGTTGCACGTGAT	GGGCTTGTTTGACGCATTAT	59.60	445	INTERGENIC			
CaPOPI_II_232	Ca2	33257195	ATCGCATTTTGCCTTTGTTT	GCTGTCGTTTGCAGCATAAA	59.59	801	INTERGENIC			
CaPOPI_II_233	Ca2	33405227	AAAGAGACATGACTTTGTCCATCA	TTTCGCTACCCATCATACCC	60.04	586	INTERGENIC			
CaPOPI_II_234	Ca2	33984310	TGGCTATGATCACAATTGGC	CCCTTTGCAAGAGACTCCAA	59.50	869	INTERGENIC			
CaPOPI_II_235	Ca2	34028835	AAAGGGGTTTTGGCTATGCT	CATGATCATAATTGCATGGAAAA	59.97	229	INTERGENIC			
CaPOPI_II_236	Ca2	34353805	AGTTGAAGGAGTGCATGAGGA	TAGGAGGCATATGTGAGGGG	59.86	633	INTERGENIC			

INDEL marker IDs	Chromosomes/ unanchored scaffolds	Physical positions (bp)	Forward primers (5'-3')	Reverse primers (5'-3')	Anneling temperature (0C)	Expected amplified product size (bp)	Structural annotation		Functional annotation	
							Sequence components of *kabuli* genome	*Kabuli* gene accession IDs	NCBI-KOG	TFs
CaPOPI_II_237	Ca2	34355206	TCTTTGATGCAATGCCAGAG	ATCATTCGAGGTGCCATCAT	59.95	975	INTERGENIC			
CaPOPI_II_238	Ca2	34355457	ATTCTTGGGCTGTGTTTTGG	GGGTGATGAGGACTGGTTGT	59.97	725	INTERGENIC			
CaPOPI_II_239	Ca2	34683764	GTTTTTCGCAACCACCTTTC	CGCTTTTTGCACTAACCTCC	59.59	682	INTERGENIC			
CaPOPI_II_24	Ca1	10057638	TGATGGGAGCAATGAATGAA	TCAAATTCCGACTTGAGGGA	60.01	542	INTERGENIC			
CaPOPI_II_240	Ca2	34684386	ACAATACTGCGGCCTGTTTC	GATTCCGATCGTTTTTCACG	60.14	830	INTERGENIC			
CaPOPI_II_241	Ca2	34684923	TCGTGAAAAACGATCGGAAT	TGCTTGATTTTAATGGATGCC	60.45	550	INTERGENIC			
CaPOPI_II_242	Ca2	34983521	CAAATGCATGCTCCCCTAAT	CTAAAAGCCCAAAGCTGCAC	59.92	450	INTERGENIC			
CaPOPI_II_243	Ca2	35258590	AAAAAGTTTTAACACGGAGGTTTTT	TGTTGTTGGATTGGACATGG	59.78	487	INTERGENIC			
CaPOPI_II_244	Ca2	35288109	TGCTTTATAGTGCTATTATGTGCCA	CGTCATTAAAAGGGAAACCAA	60.08	757	INTERGENIC			
CaPOPI_II_245	Ca2	35303488	TTCCGAATAAGGATTTCTTGG	ATGGTTTGGGGAAAAGAACC	58.19	137	INTERGENIC			
CaPOPI_II_246	Ca2	35304698	GCTTGGAAACAGCCTCAGAC	CAGGGTGGTACTGCAATGTG	60.00	536	INTERGENIC			
CaPOPI_II_247	Ca2	35329169	CAATTTTGGAAGACGATTTTGA	TGTCCCGAAAAAGTTCATCC	59.10	330	INTERGENIC			
CaPOPI_II_249	Ca2	36146623	AAACAGCAACAAAAATCCCAA	TTTGGAAATCAAGAGGTGGG	59.48	201	INTERGENIC			
CaPOPI_II_25	Ca1	12420239	AAATGCAATCTTGGTTTCCA	TTTTGGTAATGTGCCCATGATA	58.07	595	INTERGENIC			
CaPOPI_II_250	Ca3	454494	GGATTGGAGTGTTCAGCCAT	TTGTCATTAGTTTCAATCTCACCAA	59.93	460	INTERGENIC			
CaPOPI_II_251	Ca3	454633	GGATTGGAGTGTTCAGCCAT	TTGTCATTAGTTTCAATCTCACCAA	59.93	460	INTERGENIC			

INDEL marker IDs	Chromosomes/ unanchored scaffolds	Physical positions (bp)	Forward primers (5'-3')	Reverse primers (5'-3')	Anneling temperature (0C)	Expected amplified product size (bp)	Structural annotation		Functional annotation	
							Sequence components of *kabuli* genome	*Kabuli* gene accession IDs	NCBI-KOG	TFs
CaPOPI_II_252	Ca3	939073	ATTGGTTCTGCTCTGGGTTG	GGGACCACAGCACCTATGTT	60.11	296	INTERGENIC			
CaPOPI_II_253	Ca3	1533357	ACCCAAAAGCTAACTTGCCA	GCATCAACAAAGCAGCAGAA	59.75	533	INTERGENIC			
CaPOPI_II_254	Ca3	1534003	TTCTGCTGCTTTGTTGATGC	GTGGATGGGTGTTTTCCATC	60.14	774	INTERGENIC			
CaPOPI_II_255	Ca3	2855993	CTTGGTTGGAATTGCATGTG	CGTTTTGGAAGGTAGGGTGT	59.96	514	INTERGENIC			
CaPOPI_II_256	Ca3	3185857	CGCCTCTCTTTTTCTATGCG	TGTTGCTGTTGTTGCCTCTC	60.11	468	INTERGENIC			
CaPOPI_II_257	Ca3	3235341	TGGAGTGAGCTGTGGTTGAG	TGGTCACCACTTGGTGTTTG	60.02	840	INTERGENIC			
CaPOPI_II_258	Ca3	3235471	ATAGGCCAAGCAACCAAAGA	GACGATGGTGAAGATGACGA	59.71	663	INTERGENIC			
CaPOPI_II_259	Ca3	3818047	ATGCTCTGCACTTGCAGCTA	TTCATCGCCTCAAAGTGTTG	59.92	707	INTERGENIC			
CaPOPI_II_26	Ca1	12947634	AACTTTGTGGTCGTTTTGGC	TGCTTATTGTGGCTGCTTTG	60.02	647	INTERGENIC			
CaPOPI_II_260	Ca3	4073796	CTCAACATCCACCATGCAAT	TGCAAATTTCATCAAATGCATAA	59.37	746	INTERGENIC			
CaPOPI_II_261	Ca3	4689138	CAGCTTGCATTTGCTTTTGA	TTGCTCCTACGTATCTCCCG	60.13	888	INTERGENIC			
CaPOPI_II_262	Ca3	4965548	CGGGGAATGTGATTGATAGG	GTTGGAGAGATCGGTGGAAA	60.15	948	INTERGENIC			
CaPOPI_II_263	Ca3	4966098	TTTCCACCGATCTCTCCAAC	AAACCCGCTCCTTTCAAAAT	60.05	514	INTERGENIC			
CaPOPI_II_264	Ca3	4975195	CAAAGTTCAATACGCCAGCA	AGGTTTTGGCGTAACTCCCT	59.87	455	INTERGENIC			
CaPOPI_II_265	Ca3	5270478	ACGAAACGAAAGCGAGAAAA	CGACCTCGAAATAATCGGAA	60.00	521	INTERGENIC			
CaPOPI_II_266	Ca3	5286221	CACCCCTCCCAAAACATCT	TTGTGAGTGCATTTGATTTTGTT	59.76	876	INTERGENIC			

INDEL marker IDs	Chromosomes/ unanchored scaffolds	Physical positions (bp)	Forward primers (5'-3')	Reverse primers (5'-3')	Anneling temperature (0C)	Expected amplified product size (bp)	Structural annotation		Functional annotation	
							Sequence components of *kabuli* genome	*Kabuli* gene accession IDs	NCBI-KOG	TFs
CaPOPI_II_267	Ca3	5288032	AGAAGGTCGCATCTCCAAGA	TGGCACTTGTATTCACTCGC	59.95	456	INTERGENIC			
CaPOPI_II_268	Ca3	5347423	TTGCTCCTACGTATCTCCCG	TTGTGACAATTTGATGGTTCTAAA	60.23	563	INTERGENIC			
CaPOPI_II_269	Ca3	5502243	TGCGTTTTTCTGCATTTCTG	ATTCTGCGCGACTATCAACC	59.99	661	INTERGENIC			
CaPOPI_II_27	Ca1	12956423	TTGTCGGATACCTGCTCAGT	TTGCAAATTCGGACAAACAA	58.32	455	INTERGENIC			
CaPOPI_II_270	Ca3	5520403	CAAATGACGACGACGATGAC	GAATGATGACAATGCGGTTG	60.12	503	INTERGENIC			
CaPOPI_II_271	Ca3	5689325	TTGGTCAATGTGGCTTCTCA	TGAACTCTGAGCGTGTGACC	60.24	439	INTERGENIC			
CaPOPI_II_276	Ca3	7959750	TTGGCATAGGTCATGGAAGTT	TTTGCCAATGTTGCTAGCTG	59.44	848	INTERGENIC			
CaPOPI_II_277	Ca3	8245494	TGCGCCTCTTCTAAAATCGT	TATCGCAATCAGAGGAACCC	59.98	670	INTERGENIC			
CaPOPI_II_278	Ca3	8393314	GTTGGTTCCCTAGCACCGTA	TATTTTGGATTTTGGCTCGG	59.99	602	INTERGENIC			
CaPOPI_II_279	Ca3	8523296	CACATGACAAAATTGGTGGAA	CGTTAAGGCACATCTCACTCC	59.28	508	INTERGENIC			
CaPOPI_II_28	Ca1	13007507	CACGGGTCCAGATGGTTAGT	CCAAGCATCTTGGTGCATAA	59.84	322	INTERGENIC			
CaPOPI_II_280	Ca3	8942193	TGGGGAAAAACCCTTACCTT	CACATGATTTCAATTGGCTTTG	59.68	326	INTERGENIC			
CaPOPI_II_281	Ca3	9186815	TCCACAAACACGATAACATTGA	TGTAACAAGCATTTGTCGGG	58.94	693	INTERGENIC			
CaPOPI_II_282	Ca3	10159468	TGGGGCAAACAAAGCTATTC	CTAAGGGCGCTACAACAAGC	60.07	107	INTERGENIC			
CaPOPI_II_284	Ca3	10846962	CCAAATGACGACTTCCCACT	CAAGACGTCGTCAAAATCCC	59.97	683	INTERGENIC			
CaPOPI_II_285	Ca3	10923237	GCTGATTGAAATCGAGCACA	TTAAAACAAGCACCAAGGGC	59.96	347	INTERGENIC			

INDEL marker IDs	Chromosomes/ unanchored scaffolds	Physical positions (bp)	Forward primers (5'-3')	Reverse primers (5'-3')	Anneling temperature (0C)	Expected amplified product size (bp)	Structural annotation		Functional annotation	
							Sequence components of *kabuli* genome	*Kabuli* gene accession IDs	NCBI-KOG	TFs
CaPOPI_II_286	Ca3	12715466	TATTCCCGATTAATGCAGCC	GTCCCGAATGCAATGTCTCT	59.89	667	INTERGENIC			
CaPOPI_II_287	Ca3	12969818	TTACACCAATTCCCCCAAGA	CATGCAAGGTTGGGCTAGAT	60.16	400	INTERGENIC			
CaPOPI_II_288	Ca3	13097036	CCTTCTTTACGGGACCCAAT	TCATCAGTATCGTGGGTGGA	60.18	364	INTERGENIC			
CaPOPI_II_29	Ca1	14995847	CAGCGCTTTTCCCAAATAAG	CCAAAAAGGGTGGAGATTGA	59.85	359	INTERGENIC			
CaPOPI_II_290	Ca3	14843003	GAGAAAGAAGATATCGGATTCTCAA	AAAAATCAACGCAGTGGGAG	59.30	356	INTERGENIC			
CaPOPI_II_291	Ca3	14874833	AGGTCATTTTCGTCCTCCCT	CAAACACACCTGTTGGATGC	59.94	679	INTERGENIC			
CaPOPI_II_292	Ca3	14880871	TTTCGGTCATTTTCGTCCTC	ACACACCTATCGGATGCACA	60.05	616	INTERGENIC			
CaPOPI_II_293	Ca3	14902851	CGGTGGGGAGAACTCTGATA	TTCACATGACATCATCCCGT	60.07	474	INTERGENIC			
CaPOPI_II_294	Ca3	14919264	CGATTGTGTTTGTCACAGGG	GAAGCAAATCGATTGGGAAA	60.00	303	INTERGENIC			
CaPOPI_II_295	Ca3	15039270	CAACACGATCATGACAAGCA	GCATTTTGCATTTGTCAACG	59.25	677	INTERGENIC			
CaPOPI_II_297	Ca3	15660475	AAGTGAGCTAACGGGGGTTT	TGGACAGAGCAAGTGAGGTG	60.00	140	INTERGENIC			
CaPOPI_II_298	Ca3	16133157	CGTCTCTTGAAGCGATGAAA	CATTGTTGGTCACTAGCAAATCA	59.15	698	INTERGENIC			
CaPOPI_II_299	Ca3	16133384	CGTCTCTTGAAGCGATGAAA	ATAAGCCGTCAATCTGCCAC	59.15	807	INTERGENIC			
CaPOPI_II_3	Ca1	1397435	TGGAAACAGCAACGTGGTAG	CAAAGGGTTGGATTTTCCAC	59.76	555	INTERGENIC			
CaPOPI_II_30	Ca1	16629521	AACGTTTGAGGATTCCGTTG	GAAACTCCAGCTCCAAAACG	59.97	370	INTERGENIC			
CaPOPI_II_300	Ca3	16133499	TTTGCCCCATTATAAACAAAA	ATAAGCCGTCAATCTGCCAC	57.18	625	INTERGENIC			

INDEL marker IDs	Chromosomes/ unanchored scaffolds	Physical positions (bp)	Forward primers (5'-3')	Reverse primers (5'-3')	Anneling temperature (0C)	Expected amplified product size (bp)	Structural annotation		Functional annotation	
							Sequence components of *kabuli* genome	*Kabuli* gene accession IDs	NCBI-KOG	TFs
CaPOPI_II_301	Ca3	16137267	TGGCACATCTAAAAATAGGAGGA	AGAAAGTCGCATCCCAGAGA	59.98	544	INTERGENIC			
CaPOPI_II_302	Ca3	16166714	TCAACCATCTGGGTTGGAAT	CCCCTAGCAAAGGAAACAAA	60.17	674	INTERGENIC			
CaPOPI_II_303	Ca3	16171083	TACAGCGCTTCATTCCACAG	CGTGCATGAGCAAAAGAAAA	60.01	468	INTERGENIC			
CaPOPI_II_304	Ca3	16182804	CCGGCATCACCTATCAGAGT	TCCGACCTCCTTTGAGAAGA	60.10	583	INTERGENIC			
CaPOPI_II_305	Ca3	16190775	TCGCGGTTCTTGATGATGTA	AGCTCGACTTATCCTTCGCA	60.22	423	INTERGENIC			
CaPOPI_II_306	Ca3	16195131	TGATAAAAGACTAACGATGCAAACA	TAAACCATCAATCTGCCACG	60.07	655	INTERGENIC			
CaPOPI_II_307	Ca3	16195709	ACGTGGCAGATTGATGGTTT	GAACGTACGGAAGAGAAGCG	60.38	754	INTERGENIC			
CaPOPI_II_308	Ca3	16212378	ATGCACTTTGCCACTGTTGT	GAGGGGATGATGCTTCAAAA	59.22	879	INTERGENIC			
CaPOPI_II_309	Ca3	16544552	TGCTCGACATGTTTCTCCTT	CCACACAAACTGACAACTCCA	58.44	605	INTERGENIC			
CaPOPI_II_31	Ca1	18181716	CGAATACTCATGGTTTTTGATCTG	ACCCAACGTAGCACGTTACC	59.90	626	INTERGENIC			
CaPOPI_II_310	Ca3	17039245	TTCTAACTTTGCTGTCCCATTTT	GGGGTAAAACCAGGAGGAAG	59.22	750	INTERGENIC			
CaPOPI_II_311	Ca3	17491839	TGAATTCATGGCTAAAAAGCCT	GCAAAGATGATAATTCGGCAA	60.10	654	INTERGENIC			
CaPOPI_II_312	Ca3	18943181	TGGAGTGTTGATGTCGTTGTC	AAATCGAAGATCGCAACAGT	59.59	428	INTERGENIC			
CaPOPI_II_313	Ca3	19359038	CGTCCTTTGTTCATTCCCAC	TGAGGGCCTTTAGAATCGTG	60.35	670	INTERGENIC			
CaPOPI_II_314	Ca3	19359334	AGGGAAATCAACGACAGAGC	TGAGGGCCTTTAGAATCGTG	59.29	365	INTERGENIC			
CaPOPI_II_316	Ca3	19373696	GCTGAATGCGTGAAGAACAA	GGGTAGAAAACACGCGGAAT	60.00	339	INTERGENIC			

INDEL marker IDs	Chromosomes/ unanchored scaffolds	Physical positions (bp)	Forward primers (5'-3')	Reverse primers (5'-3')	Anneling temperature (0C)	Expected amplified product size (bp)	Structural annotation		Functional annotation	
							Sequence components of *kabuli* genome	*Kabuli* gene accession IDs	NCBI-KOG	TFs
CaPOPI_II_317	Ca3	19376077	TGACCTTGTGCATTGTTTTGA	GCAAACTCAACCTTGCCAAT	60.14	333	INTERGENIC			
CaPOPI_II_319	Ca3	21097843	GGCATTTTGATTTGATTCGC	CAAATTTTGTGTTTCAAAAACCATT	60.41	653	INTERGENIC			
CaPOPI_II_32	Ca1	18300800	AGCAAGAAAATTGAAGGCCA	GGAAGCTTTAAAGGGAGCAAA	59.82	161	INTERGENIC			
CaPOPI_II_320	Ca3	21311568	CACGTCACATCGCAATTTTT	CATGTCATTTCAACCCACGA	59.59	542	INTERGENIC			
CaPOPI_II_322	Ca3	21897869	GAGTTGAGTGTTGCCAGGGT	AGCTACCAAGCTGATGGTGAA	60.16	296	INTERGENIC			
CaPOPI_II_324	Ca3	24272959	GCATGTCCGTTACCTCCACT	GCGATTTATTATTCAGAGTTATTAGCG	60.00	646	INTERGENIC			
CaPOPI_II_326	Ca3	25888907	CAGGTCATGCCAGACTTTGA	TCTGACCCGACCTGGTCTAC	59.83	578	INTERGENIC			
CaPOPI_II_327	Ca3	25889145	GTAGACCAGGTCGGGTCAGA	TGATATGTCACATTGTCGCAC	60.11	403	INTERGENIC			
CaPOPI_II_33	Ca1	18364053	CCCGTAGCAAGACTTGAAGC	TTGGAGAAACTTTTCCGCTG	60.02	627	INTERGENIC			
CaPOPI_II_331	Ca3	26872317	CAATGTGCTCGATTTCTCCA	TTACATTCCTTGCCTCGTCA	59.80	389	INTERGENIC			
CaPOPI_II_332	Ca3	27854154	TCGATAATGAGTGATTAGGTGGG	ACCTCACCCTCGAATGTCAC	60.21	459	INTERGENIC			
CaPOPI_II_333	Ca3	27854804	GGATTGAGAGGGGAGAAAGG	TTTGAAAGCAAGAGCGTCAA	60.01	516	INTERGENIC			
CaPOPI_II_334	Ca3	27856755	CGAAGATGAAGAGGCTTGAAA	CAACAACTTCTGATGCTCGC	59.58	593	INTERGENIC			
CaPOPI_II_335	Ca3	28113252	TCCTTCTCCTCTTCCTTCCA	TGTTTGTGCAATTGAATTATAGTTATG	58.94	849	INTERGENIC			
CaPOPI_II_336	Ca3	28188438	ACCCATAACGCACTCGTTTC	TCCTCCATTTTTCGTTTTCG	60.00	434	INTERGENIC			
CaPOPI_II_337	Ca3	28797825	CATAGTCTGCGAAACCAGCA	GGTGGAATTGATCGGAGAGA	60.01	454	INTERGENIC			

INDEL marker IDs	Chromosomes/ unanchored scaffolds	Physical positions (bp)	Forward primers (5'-3')	Reverse primers (5'-3')	Anneling temperature (0C)	Expected amplified product size (bp)	Structural annotation		Functional annotation	
							Sequence components of *kabuli* genome	*Kabuli* gene accession IDs	NCBI-KOG	TFs
CaPOPI_II_34	Ca1	18868658	AATCGATTGATTCAGCCCAG	CGTTTTTCAAAGCGAGGGTA	60.04	707	INTERGENIC			
CaPOPI_II_341	Ca3	30930504	GCATGGACCGTTTAAAATTG	TTTAGGTGGAAAATTCATTAGAAAA	58.02	818	INTERGENIC			
CaPOPI_II_342	Ca3	31339236	CCAACCCATGTACCGACATT	AGAGACCGTTTCATGAGATTTG	60.50	525	INTERGENIC			
CaPOPI_II_343	Ca3	31519164	TACACGTGGCCTCAAACAAA	TCAAGCATGGTTCCAATCAG	60.15	364	INTERGENIC			
CaPOPI_II_344	Ca3	31560705	TGTTCACTGCCAAACTCTGC	GCAGTGCACAACTGATGGTT	60.03	585	INTERGENIC			
CaPOPI_II_345	Ca3	32563568	CGAAAGAAAAAGAAAACATCGC	GAAGGTGGCTCGGCATATAA	60.23	574	INTERGENIC			
CaPOPI_II_346	Ca3	33828272	AGAGGCAAACAAGAACCGAA	TGCATATAAGGAATTGACGCC	59.85	318	INTERGENIC			
CaPOPI_II_347	Ca3	33954528	GGCTTCAAAGCCAACATCAT	TCATACAAAAACCAAACGGG	60.08	622	INTERGENIC			
CaPOPI_II_348	Ca3	34474422	AAAAATCAATTTATGCCGTTGT	TTATGCCGGATTGACACTCTT	57.65	478	INTERGENIC			
CaPOPI_II_349	Ca3	36041204	TTCACACTCTCCAGTTCGGA	AAGTCGACGTTTGTGAAGAGTTC	59.39	699	INTERGENIC			
CaPOPI_II_35	Ca1	19498678	GCCCGAGTTAACAACAAACG	ATCGATTTCCTCAGCACTGG	60.53	374	INTERGENIC			
CaPOPI_II_350	Ca3	36420971	TGGCATCGTGATTTCTACCA	GGCCATGGTGATTGTAAAGG	60.07	746	INTERGENIC			
CaPOPI_II_351	Ca3	37017817	CAGAAGGCCAGGAGAAGAAA	TGTGCGTGAAGTTTCATGGT	59.55	707	INTERGENIC			
CaPOPI_II_360	Ca4	3017713	GACAAATTAGTGGGGCAACC	TTGTATTGTTGGTGAGGCCA	59.29	902	INTERGENIC			
CaPOPI_II_364	Ca4	6907181	TTTGAAATGGCGACTTCCTT	CCAAATGGCAACTTCCAACT	59.69	569	INTERGENIC			
CaPOPI_II_365	Ca4	7089091	CTTTGCATACCCCTCTCGAC	TTGGGAGGATTGGTTTCAAG	59.69	644	INTERGENIC			

INDEL marker IDs	Chromosomes/ unanchored scaffolds	Physical positions (bp)	Forward primers (5'-3')	Reverse primers (5'-3')	Anneling temperature (0C)	Expected amplified product size (bp)	Structural annotation		Functional annotation	
							Sequence components of *kabuli* genome	*Kabuli* gene accession IDs	NCBI-KOG	TFs
CaPOPI_II_366	Ca4	8169626	TATGGCACCTCCAGTTGAAA	TGTTAAATCCTCCAGACGGC	59.12	858	INTERGENIC			
CaPOPI_II_368	Ca4	8309234	GAATTTAAGAGGAAGGCGATGA	TTCACCCTAATTCGTAGGCG	59.70	450	INTERGENIC			
CaPOPI_II_369	Ca4	8315933	GGACCCGTGATGCTGTTTAT	TCTCGGTTGATGTCATTATGCT	59.82	586	INTERGENIC			
CaPOPI_II_37	Ca1	22131328	GTGTGGACATGCCTAAAACG	GTTGCAATTGAGGTGCTGAA	59.05	403	INTERGENIC			
CaPOPI_II_370	Ca4	8316304	GCATAATGACATCAACCGAGAG	AAAAGAAATGGTTGTTTCTCGTTC	59.60	235	INTERGENIC			
CaPOPI_II_375	Ca4	9066423	GCAACCCGTAGGTGTAGGAA	TGTGCGTTGGTAGTCAATGTG	59.99	546	INTERGENIC			
CaPOPI_II_376	Ca4	9371255	GGAGCATATGCCTCGTTGTT	TGGAAATTCAACTTTTTGGACA	60.10	549	INTERGENIC			
CaPOPI_II_377	Ca4	9630289	CCTCTTCCAAACAAATCTTCG	CCAGCTCATCAAAGCCATTT	58.82	550	INTERGENIC			
CaPOPI_II_378	Ca4	9632653	TGGATTGTCGTGATCTCAAA	CACACCCGTACTCAGCATTG	57.58	630	INTERGENIC			
CaPOPI_II_379	Ca4	10033685	CTAGTTCGTAACCCGTGCGT	AGGCATGGTGTTGGAGTCAT	60.19	248	INTERGENIC			
CaPOPI_II_38	Ca1	22131513	GTGTGGACATGCCTAAAACG	GTTGCAATTGAGGTGCTGAA	59.05	403	INTERGENIC			
CaPOPI_II_380	Ca4	10173137	CCCGTTATTTTTGACGAAACA	TGTGTTGAGTGGTGGCATTT	59.86	791	INTERGENIC			
CaPOPI_II_381	Ca4	10233239	TGTCGGTTGTTGCTTGAAGA	ATACAATCATTTTCCCGCCA	60.43	403	INTERGENIC			
CaPOPI_II_383	Ca4	13433311	CGAGCAAACACAATGAATGG	TTGGTTGGTTCATTGGTTGA	60.11	569	INTERGENIC			
CaPOPI_II_384	Ca4	13509527	CCTCTTCACGGTGCTCATTT	CCTCGTTTTAAGCCCAATCA	60.26	158	INTERGENIC			
CaPOPI_II_386	Ca4	13908536	CTCCTCCGATGTCTGGAAAA	CGCGGTTGCTTCAATTCTAT	60.19	232	INTERGENIC			

INDEL marker IDs	Chromosomes/ unanchored scaffolds	Physical positions (bp)	Forward primers (5'-3')	Reverse primers (5'-3')	Anneling temperature (0C)	Expected amplified product size (bp)	Structural annotation		Functional annotation	
							Sequence components of *kabuli* genome	*Kabuli* gene accession IDs	NCBI-KOG	TFs
CaPOPI_II_387	Ca4	13909254	CATCACGATTTGACGAGTTGA	AAAAACATGACTCCAAACACCA	59.71	636	INTERGENIC			
CaPOPI_II_388	Ca4	14397300	TAAATGCATGCTCCCCTGAT	AACACATCCGGGATCCATTA	60.44	273	INTERGENIC			
CaPOPI_II_39	Ca1	22886112	TGATTTGGGGTTTTGGGTAA	GGGTGAGTTCTTTGAGGGTG	60.02	531	INTERGENIC			
CaPOPI_II_391	Ca4	14677950	TGTTGGGCAAAATAAATTGAGA	CAAACCTTACCACCGTCTTG	59.47	424	INTERGENIC			
CaPOPI_II_392	Ca4	15048181	CAAAAGCTGTAACACCCCGT	AATCGATTGATTCAGCCCAG	60.03	528	INTERGENIC			
CaPOPI_II_393	Ca4	16943222	CTCAAGCTCAGTCCCAGTCC	TGCGTTTTGTTTGGTGTAGC	59.99	668	INTERGENIC			
CaPOPI_II_395	Ca4	17196085	CCTCAACCATGGCCTGTACT	AGTTGCATTGGAGACAAGGG	59.99	118	INTERGENIC			
CaPOPI_II_396	Ca4	17247653	TTTATGTTTGGCCAACTCCC	TTTTTAGGGACGCAATTCAAA	59.80	539	INTERGENIC			
CaPOPI_II_398	Ca4	17313521	GCTAGACAGGTTTGCCTTCG	AAGGCTCCTGCAAGCATTTA	60.02	966	INTERGENIC			
CaPOPI_II_4	Ca1	1841034	TCAAGGTGTTGATTCGGTAGG	TTTTGCAACCCGGTCTATTC	59.98	595	INTERGENIC			
CaPOPI_II_40	Ca1	23784964	GTCAACCAATTGACCCCAAC	TGCCAAGAAAACACCATGAA	60.07	663	INTERGENIC			
CaPOPI_II_401	Ca4	17475440	CGGGCAGTAGCATTAGATGG	GGATGAATGGGAGGTGGTTA	60.62	210	INTERGENIC			
CaPOPI_II_402	Ca4	17600797	AAGGGTGATGGACGAAAAAC	TTAAATGGTCGCATAGAGCAA	58.89	682	INTERGENIC			
CaPOPI_II_403	Ca4	17600811	AAGGGTGATGGACGAAAAAC	TCCAACAACATCAAATTCATCC	58.89	951	INTERGENIC			
CaPOPI_II_404	Ca4	17942733	CATCGTCAGCACATCACAAA	CTTAGGAGGTCTTGCCCCTC	59.25	447	INTERGENIC			
CaPOPI_II_406	Ca4	18196212	GTGGTCCAGATTCTCCCAAA	TGTCGCTTCAGCAAAAAGAA	59.90	549	INTERGENIC			

INDEL marker IDs	Chromosomes/ unanchored scaffolds	Physical positions (bp)	Forward primers (5'-3')	Reverse primers (5'-3')	Anneling temperature (0C)	Expected amplified product size (bp)	Structural annotation		Functional annotation	
							Sequence components of *kabuli* genome	*Kabuli* gene accession IDs	NCBI-KOG	TFs
CaPOPI_II_407	Ca4	19045820	GGGCTTGCTTGGTTTGAATA	TCAACTGCACGTGTTAAATGC	60.07	825	INTERGENIC			
CaPOPI_II_408	Ca4	19643688	GGTGCAGTTGTTTGATGCAC	ATGCGATTTACATGGTTGGC	60.17	255	INTERGENIC			
CaPOPI_II_409	Ca4	19678061	ATTTTGGCAACGGCTGATAC	CCACAATCCTCCGTTGTCTT	59.97	492	INTERGENIC			
CaPOPI_II_41	Ca1	23838777	AATGCCCACCCATAATTTCA	TGTACGAGAAGGTCGTGTGC	60.02	593	INTERGENIC			
CaPOPI_II_410	Ca4	20709240	ACTTTGCCCATCAGTTTTGC	TCCGTTGGTGAAAGTTTGAA	60.12	251	INTERGENIC			
CaPOPI_II_411	Ca4	20798832	TAGTGCTTTTGCATGCCTCA	TTTGTCCTGGGAAAATGCTC	60.55	316	INTERGENIC			
CaPOPI_II_412	Ca4	21529168	TGGGGGATTGTATTCCAAAA	TATGGAATGGACCTCCTGCT	59.99	499	INTERGENIC			
CaPOPI_II_413	Ca4	21804779	CCGCATCATAAACAGCCTTT	CTAAAGCTGGTGCTTTTGGC	60.10	159	INTERGENIC			
CaPOPI_II_415	Ca4	22441886	TCCAATGGTGGAAAATAAACG	TCAATCACAAATTTCAAGGAGG	59.69	848	INTERGENIC			
CaPOPI_II_416	Ca4	22546535	CATTTTGGTTTCAAAGCCGT	CCAGGGGATGAGATCATAGG	59.98	714	INTERGENIC			
CaPOPI_II_417	Ca4	22867454	GATCTTCATCCCCTGCAGAC	CACCACCACCACAAAACACT	59.62	292	INTERGENIC			
CaPOPI_II_418	Ca4	23002623	TTTATGGCGGCTGGTTTTAC	ACGATGCATTTGATTGATGTT	59.96	546	INTERGENIC			
CaPOPI_II_419	Ca4	24427573	CTGGAAAATCCTCCTCCTCC	TTCGTCTCAACAATCTCCCC	60.01	346	INTERGENIC			
CaPOPI_II_42	Ca1	23966814	AACTGTGGAGGAATCCATGC	GCTTCAAGTCTTGCTACGGG	59.93	490	INTERGENIC			
CaPOPI_II_420	Ca4	24478956	CGGTTCTACACAAGTTGCGA	TCGATTATGCGTAGGGAAGG	59.90	746	INTERGENIC			
CaPOPI_II_421	Ca4	24479035	CGGTTCTACACAAGTTGCGA	TCGATTATGCGTAGGGAAGG	59.90	746	INTERGENIC			

INDEL marker IDs	Chromosomes/ unanchored scaffolds	Physical positions (bp)	Forward primers (5'-3')	Reverse primers (5'-3')	Anneling temperature (0C)	Expected amplified product size (bp)	Structural annotation		Functional annotation	
							Sequence components of *kabuli* genome	*Kabuli* gene accession IDs	NCBI-KOG	TFs
CaPOPI_II_422	Ca4	24828297	TGGAATTTCAGACTGCCTTG	GGGCACGAAGAAGCACTATC	58.85	716	INTERGENIC			
CaPOPI_II_423	Ca4	25071336	TGAGACTGCTTGGCTACCCT	ATCTGAATCATCCCAAGCCA	60.01	488	INTERGENIC			
CaPOPI_II_424	Ca4	25352610	GGAAATGGTTCTTTGAAAGCC	AGCCTTACGCATGTATTGCC	59.94	719	INTERGENIC			
CaPOPI_II_426	Ca4	25596032	AGTGATGTGGGCAACAATGA	TGCATTTTACAGCGCTTTTG	59.97	515	INTERGENIC			
CaPOPI_II_427	Ca4	25822369	TGTTTGAACCCGAGACTTCAC	CGTGGCATTTGAAAAAGCAT	60.14	611	INTERGENIC			
CaPOPI_II_428	Ca4	25847698	CATGCTACCAAAGCGATTGA	ATCGATTTCGCGAGTCTTGT	59.83	370	INTERGENIC			
CaPOPI_II_431	Ca4	25967972	TTGTACCCTTGTGCAACCAA	TGGTTGGATACTTGCAATGG	60.00	180	INTERGENIC			
CaPOPI_II_432	Ca4	25968820	TTCTTCTGCCCAAAAATGCT	CCTCAAGCAGACCTTTGGAG	59.82	412	INTERGENIC			
CaPOPI_II_433	Ca4	25969511	TCCCTTGCCTCTAGCCAGTA	GCAGATGTTCTCAACAGCCA	59.97	350	INTERGENIC			
CaPOPI_II_434	Ca4	25970159	TGGTTGGGTCTTGGTTTGTT	CACCTGCTATGGATGCAATG	60.25	709	INTERGENIC			
CaPOPI_II_436	Ca4	25978445	TGCCTCAAAGGCTTCATCTT	AGCACTCATCCTCGGGTAGA	59.96	435	INTERGENIC			
CaPOPI_II_437	Ca4	25979689	GTCCTCGTCAGGAGGTTGAG	CCACTGTTGCTGGCAGATAA	59.84	707	INTERGENIC			
CaPOPI_II_438	Ca4	25984442	AGATCCTGTTGACACGGCTT	CGGTTGTTGTTGTGATTTGC	59.73	524	INTERGENIC			
CaPOPI_II_439	Ca4	25987622	TTCAACTGGGGTAAATGCCT	TGTTCTAGTCCAGATCAAAACGA	59.43	323	INTERGENIC			
CaPOPI_II_44	Ca1	26683611	CAATTGTGTAAAACGTGCGG	TTGCATTTGGTTTGACTCCA	60.03	538	INTERGENIC			
CaPOPI_II_440	Ca4	25988978	AAGCTGGATGTGGACTTTGC	AGTTCCAATTGGCCTCCTTT	60.26	404	INTERGENIC			

INDEL marker IDs	Chromosomes/ unanchored scaffolds	Physical positions (bp)	Forward primers (5'-3')	Reverse primers (5'-3')	Anneling temperature (0C)	Expected amplified product size (bp)	Structural annotation		Functional annotation	
							Sequence components of *kabuli* genome	*Kabuli* gene accession IDs	NCBI-KOG	TFs
CaPOPI_II_441	Ca4	25989281	AAGCTGGATGTGGACTTTGC	AGTTCCAATTGGCCTCCTTT	60.26	404	INTERGENIC			
CaPOPI_II_442	Ca4	25989322	GTACGCGATCTAGTCCCCAA	CCGGGGAGAAAGTTCTAGTG	60.10	168	INTERGENIC			
CaPOPI_II_443	Ca4	25994721	TTAAGATTGAGGGGTGTGGG	CACCACTTCATTCGGGACTT	59.78	516	INTERGENIC			
CaPOPI_II_444	Ca4	25995802	CCCTAAATCAAAGCAGCGTC	TGCACTGTTGTTGTTGCTGA	59.85	470	INTERGENIC			
CaPOPI_II_445	Ca4	26495554	AACGATTTATCCTGGTCCCC	CCCGAATACAAACCCAAGTC	60.02	844	INTERGENIC			
CaPOPI_II_446	Ca4	26847495	AGCTTCAAGTCGGATCGAAA	AAAAATCTTGTGGGAATAATTCAA	59.96	216	INTERGENIC			
CaPOPI_II_447	Ca4	26903304	ACTCGATTGGTCCATGCTTC	ATGTTTTGGACGCACACAGA	60.08	532	INTERGENIC			
CaPOPI_II_448	Ca4	27017251	CCTTTGCATCTCAGGGACAT	TGAGACCGGGTTTGAGTACC	60.07	453	INTERGENIC			
CaPOPI_II_45	Ca1	28550132	TGGAAGCACGCTTAAGGACT	TTTCCCAATCGATTTGCTTC	60.02	664	INTERGENIC			
CaPOPI_II_452	Ca4	27548804	TTCCCCATTCTTAACACCCA	TTCGCAACTCCACTCTTCCT	60.16	672	INTERGENIC			
CaPOPI_II_453	Ca4	27928820	AGCGATGCATGTCTCAAGTG	AGCGCTTTGTGACCTCCTTA	60.02	754	INTERGENIC			
CaPOPI_II_454	Ca4	28209971	ATTGATGGAGCGAATCGAAC	AATTCCTCTTTTGGTGGGCT	60.04	891	INTERGENIC			
CaPOPI_II_455	Ca4	28254483	GGGGAACTCTCCAAGTCACA	CGTGGAAACCAACCTTGTCT	60.09	703	INTERGENIC			
CaPOPI_II_456	Ca4	28346696	ACCACAGGTGAAAGAAACCG	TTTTCAACTCTATCCGTAGCCC	60.01	340	INTERGENIC			
CaPOPI_II_457	Ca4	28390462	GTCATCAAAAGCCGACGAAT	GGCCCACAATTTTGAAGAGA	60.08	786	INTERGENIC			
CaPOPI_II_458	Ca4	28714992	AGAGCCAACGCTCATAGGAA	TTAAAATTCCCTCCTCGGCT	59.98	531	INTERGENIC			

INDEL marker IDs	Chromosomes/ unanchored scaffolds	Physical positions (bp)	Forward primers (5'-3')	Reverse primers (5'-3')	Anneling temperature (0C)	Expected amplified product size (bp)	Structural annotation		Functional annotation	
							Sequence components of *kabuli* genome	*Kabuli* gene accession IDs	NCBI-KOG	TFs
CaPOPI_II_459	Ca4	28793395	CCAAAATTACACCAACGGTTC	TTGTTCGCATGCTCATTCTC	59.23	275	INTERGENIC			
CaPOPI_II_46	Ca1	28713213	TTCATTTCTCTGATTCCCCG	TCCGCATTATAACCTCTGCC	60.01	425	INTERGENIC			
CaPOPI_II_460	Ca4	29191237	CCAACTTGGTTCCAACAATCT	GGAGAAAAGTTAGGGGGTGC	58.95	119	INTERGENIC			
CaPOPI_II_461	Ca4	29418531	GGTGGTGTACGGTGGAAGTT	GTCAAGGCAGACCAGAAAGC	59.75	383	INTERGENIC			
CaPOPI_II_462	Ca4	29582409	AGCTGTATTGGGCCTCCTTT	GCCGAATAAAACCCTCACAA	60.10	212	INTERGENIC			
CaPOPI_II_463	Ca4	29933754	AGCCACACGATGTTGATTGA	TGATTAGGGAATCACCCGTT	60.12	845	INTERGENIC			
CaPOPI_II_464	Ca4	30086898	ACGCCGAAAAACACTTTCAC	TCCATTCGACTGTCCTTCAA	60.15	630	INTERGENIC			
CaPOPI_II_465	Ca4	30211443	ACATCAAACAAACAGCGCAC	CCGCTAAAATTGCCCAATAA	59.77	714	INTERGENIC			
CaPOPI_II_466	Ca4	30351459	TCTTTGAAACACATCACTTTCG	AAAATTTGATCGGTGGTGGA	57.93	314	INTERGENIC			
CaPOPI_II_467	Ca4	30404870	TTAACTGTTGGTGCGCAGTC	CGTCACATTTAACACGCCAT	59.91	798	INTERGENIC			
CaPOPI_II_468	Ca4	30535832	CCATTCAGAATTAGAAAATCACCA	CCAAATTTCCCAAGGGTTCT	59.40	582	INTERGENIC			
CaPOPI_II_47	Ca1	28906702	TATGGAGAGCAGCGACTCAA	TGTTGGGTTGCACAACATTC	59.70	184	INTERGENIC			
CaPOPI_II_471	Ca4	30928717	CAATGTCCATGTCGACCAAA	TCTGTCGCACCATCTCAGTC	60.37	949	INTERGENIC			
CaPOPI_II_472	Ca4	31172943	TCGCCTAAGGTAAGGGCTTT	TTACACCAATTCCCCCAAGA	60.22	421	INTERGENIC			
CaPOPI_II_473	Ca4	31192815	TTGAAGGAGAAGCCAGAGTCA	TGAGGCATGCAGAAACGTAG	60.12	238	INTERGENIC			
CaPOPI_II_475	Ca4	31445256	AGATCTATGCATCGGCAACC	AGGGTACTACAGCTGCCCCT	60.07	338	INTERGENIC			

INDEL marker IDs	Chromosomes/ unanchored scaffolds	Physical positions (bp)	Forward primers (5'-3')	Reverse primers (5'-3')	Anneling temperature (0C)	Expected amplified product size (bp)	Structural annotation		Functional annotation	
							Sequence components of *kabuli* genome	*Kabuli* gene accession IDs	NCBI-KOG	TFs
CaPOPI_II_476	Ca4	31611840	CGTTTTCAGCTGAGCAAATTC	TTGATCCATTGGGATTGTGA	60.01	392	INTERGENIC			
CaPOPI_II_477	Ca4	31611867	CGTTTTCAGCTGAGCAAATTC	TTGATCCATTGGGATTGTGA	60.01	392	INTERGENIC			
CaPOPI_II_479	Ca4	31806633	ACAAAAGCCTGGATAGCGAA	TCACATATACCCTTAAAATCGCA	59.85	410	INTERGENIC			
CaPOPI_II_480	Ca4	31859597	GTTGGAGCCCCCACTTTATT	TGCAAGCAGCATCAGAGAAC	60.19	793	INTERGENIC			
CaPOPI_II_481	Ca4	32179682	TTGCATACCTCATACCATCCC	TTGATGCATTACCTCCCACA	59.66	731	INTERGENIC			
CaPOPI_II_482	Ca4	32227293	TCTTCACAACCCCTTGAACC	TTCTTCCAAACGAGCCTCTC	59.94	868	INTERGENIC			
CaPOPI_II_483	Ca4	32264980	GCACTTACCCTTCAATTGGG	AGGAGAGGTCCATTTCTCAGG	59.43	262	INTERGENIC			
CaPOPI_II_484	Ca4	32335179	AAAGTCCGTTTGTGAATCCG	TGGGCATTCAAATCAAACAA	59.97	507	INTERGENIC			
CaPOPI_II_485	Ca4	32531473	TAAAATGGATGCATCGAGCC	ACAAATCAACCAGTGTCGCA	60.94	662	INTERGENIC			
CaPOPI_II_486	Ca4	32562355	AAATGGAGGAGAAGGAGGGA	CGGTGAATCCTTTACGGAGA	60.01	286	INTERGENIC			
CaPOPI_II_487	Ca4	32886390	AATCATCCCATGTTGCCATT	CGAGATTTCACACCAAGGGT	60.02	519	INTERGENIC			
CaPOPI_II_489	Ca4	33346959	TTTTCGGTTTGTTCAAAGGC	GGAAAACATTGGGAAACCCT	60.09	766	INTERGENIC			
CaPOPI_II_490	Ca4	33615467	GGTCCTTGTTGAGTCCAACG	CTCAAAAACCTGAGCATGGC	60.55	646	INTERGENIC			
CaPOPI_II_491	Ca4	33633372	ACGAGGAAACATATCGTCCG	ATGTCCACACATTGGGTGTC	59.96	726	INTERGENIC			
CaPOPI_II_492	Ca4	33714267	GAACACGTTGTTAGACCGGC	TGACAAAATGGGGAGAAAGG	60.56	713	INTERGENIC			
CaPOPI_II_493	Ca4	33714529	CGTTGTTAGACCGGCTCTTG	TGGGCTGACATAATCACCAA	60.82	940	INTERGENIC			

INDEL marker IDs	Chromosomes/ unanchored scaffolds	Physical positions (bp)	Forward primers (5'-3')	Reverse primers (5'-3')	Anneling temperature (0C)	Expected amplified product size (bp)	Structural annotation		Functional annotation	
							Sequence components of *kabuli* genome	*Kabuli* gene accession IDs	NCBI-KOG	TFs
CaPOPI_II_494	Ca4	34169705	TCAAAGTGGCTCACAAGTGC	TCGACTCAAGAAGGCAAGTG	60.03	781	INTERGENIC			
CaPOPI_II_495	Ca4	34388181	GTTGGTTCCCTAGCACCGTA	CTGTCAATTGAGTTGCGACG	59.99	484	INTERGENIC			
CaPOPI_II_496	Ca4	34566424	CCCTGAGACTCACGAAGAGG	AAAATCCAACAATCTCCCCC	59.98	612	INTERGENIC			
CaPOPI_II_497	Ca4	35432013	TGTTCAACATCCACGAACAA	TGCCATACTCATGTCGGAAA	58.51	669	INTERGENIC			
CaPOPI_II_498	Ca4	35432836	TTTCCGACATGAGTATGGCA	TTTGGAAAGTAACACTGCACG	60.07	785	INTERGENIC			
CaPOPI_II_499	Ca4	35457008	AATTGCAGAAAAATGCGGTC	TTGTACCAGATATTGGCCCG	60.08	776	INTERGENIC			
CaPOPI_II_5	Ca1	1841424	GAATAGACCGGGTTGCAAAA	CCTCTTTCCGGACAACACAT	59.94	302	INTERGENIC			
CaPOPI_II_50	Ca1	28941503	TGGGGTTTAGGGTGTCACAT	CTTTTGTAAAGGCAGCTCCG	60.09	207	INTERGENIC			
CaPOPI_II_500	Ca4	35822348	GATCAAATCGAGGACCGAAA	CGAACAAAACGATGGGAAGT	60.01	695	INTERGENIC			
CaPOPI_II_501	Ca4	36079126	ATTTTGATGCTCACGGTTCC	CGGAGATGATGGTTCAGGTT	59.94	595	INTERGENIC			
CaPOPI_II_502	Ca4	36336918	ACAACAAGGGTGACCTCCAC	TGCTTGTCTATTCTGCGTCAA	59.86	736	INTERGENIC			
CaPOPI_II_503	Ca4	36337139	TGCAATGTCAATATGAACCAAA	CCACAACCACTCATCACGAC	58.94	479	INTERGENIC			
CaPOPI_II_504	Ca4	36972358	ACAACCAACAAAATGCGACA	AGCCTTGAGCGACCACTTTA	60.01	484	INTERGENIC			
CaPOPI_II_505	Ca4	37443931	CCGCATTTTCTTCATTAGGG	ACGTTAAGGCATGAGTTGGG	59.54	364	INTERGENIC			
CaPOPI_II_507	Ca4	39165742	CGTATACCGCTGCTCATCAA	TGATGTGATTTGTGCCATGA	59.86	259	INTERGENIC			
CaPOPI_II_508	Ca4	39689187	ATTACCGACATTGCAGAGGG	CTCCCATTCTGCCCTTGATA	59.96	329	INTERGENIC			

INDEL marker IDs	Chromosomes/ unanchored scaffolds	Physical positions (bp)	Forward primers (5'-3')	Reverse primers (5'-3')	Anneling temperature (0C)	Expected amplified product size (bp)	Structural annotation		Functional annotation	
							Sequence components of *kabuli* genome	*Kabuli* gene accession IDs	NCBI-KOG	TFs
CaPOPI_II_509	Ca4	40649020	TGTGATTATCCGGTACCACAAC	TGTATTCAGGACCTCGCAGA	59.62	207	INTERGENIC			
CaPOPI_II_51	Ca1	28943560	ACTTGGGACAAATCACATGG	TGATCACCAGCTTTCCAATG	58.26	252	INTERGENIC			
CaPOPI_II_510	Ca4	40842793	CTGGATTGAGTTGAGGCCAT	TGGACCAAACCAAGCACATA	60.07	568	INTERGENIC			
CaPOPI_II_511	Ca4	41027713	TGAATCCCAAAATTTGAACCA	TTTGATCGCAAGGGTTTAGG	60.15	637	INTERGENIC			
CaPOPI_II_513	Ca4	42548370	CGTGCCTTTGTCGTCTCTTT	AGAGGCCCAATATCCTTGCT	60.43	528	INTERGENIC			
CaPOPI_II_514	Ca4	44428431	TGTTGCGGAAATGTTAGTTCA	TTCATCCATGCAATATTCAAAAA	59.22	699	INTERGENIC			
CaPOPI_II_516	Ca4	46001807	TTGCTCCTACGTATCCCCAG	CTCAATCGTGGGTGACCTTT	60.09	385	INTERGENIC			
CaPOPI_II_517	Ca4	46004838	TGATTTCAATGAGAGGATTTTGTT	GCTCGATTGATGTTAAGGGC	59.04	425	INTERGENIC			
CaPOPI_II_518	Ca4	46674801	GGATGACTCGAAACGGTGTT	TTACCCAAAACCCCAAATCA	59.97	667	INTERGENIC			
CaPOPI_II_519	Ca4	47238699	TTTTGATCGAATCTGACCCA	TCGGCCATTTTTCTTCAATC	59.05	659	INTERGENIC			
CaPOPI_II_52	Ca1	28944496	TTGGCACCAGACTTCATATTG	AAATGTACTTTGCGGCACAT	58.64	775	INTERGENIC			
CaPOPI_II_523	Ca4	48577228	AACACATCCGGGATCCATTA	TGATGACGAAGACGAAGACG	60.01	303	INTERGENIC			
CaPOPI_II_524	Ca5	132937	CCAAAGACACAGCAAAGCAA	CACTTCCACCAGTTGCAAAA	60.03	569	INTERGENIC			
CaPOPI_II_525	Ca5	133428	ACCTTTACATGGTGCCAAGC	GCGTTGTGAAAACAGGACAA	60.00	433	INTERGENIC			
CaPOPI_II_526	Ca5	158794	TGATGCGTCATGGACATAAA	TTTTAAAAATGTCCATTTACACCG	58.49	498	INTERGENIC			
CaPOPI_II_529	Ca5	218592	TAGCGTGGTGGTGTTGTGAT	CCTTTCAAGGTCAGCCACAT	60.03	739	INTERGENIC			

INDEL marker IDs	Chromosomes/ unanchored scaffolds	Physical positions (bp)	Forward primers (5'-3')	Reverse primers (5'-3')	Anneling temperature (0C)	Expected amplified product size (bp)	Structural annotation		Functional annotation	
							Sequence components of *kabuli* genome	*Kabuli* gene accession IDs	NCBI-KOG	TFs
CaPOPI_II_53	Ca1	29124803	AGTGGCATTGGGCAAATTAC	ACCAAATATACGCGTCTGCC	59.83	350	INTERGENIC			
CaPOPI_II_530	Ca5	236073	TTGAATTGACCGGAGGAATC	GGGATAGGGAGGGTTGTGTT	59.87	680	INTERGENIC			
CaPOPI_II_536	Ca5	927603	GGTCTTTTGAAAATAATCTTAACGC	AAGGGGGAAGTTAAGCCAGA	58.78	664	INTERGENIC			
CaPOPI_II_537	Ca5	2323461	GCGCCAGATTTCAAACACTA	ACCAGCAGACCAAAATGGAC	58.92	262	INTERGENIC			
CaPOPI_II_538	Ca5	2327252	TCGAAATGAAATCGAGGACC	ACAGACGAGGGCTAGGGATT	60.01	721	INTERGENIC			
CaPOPI_II_539	Ca5	3742109	ATTTACGAGCAGCGGAAAGA	TGGTCATGCAGAAACTCAGC	59.98	605	INTERGENIC			
CaPOPI_II_540	Ca5	4064742	TATGTTTGTTTTGCCGTCCA	AGGATGTACCATTCTTGCGG	59.97	375	INTERGENIC			
CaPOPI_II_541	Ca5	4179599	CGGTTTAGGGATTGGGTTTT	TCGATTGAGTCTTTTGCACTT	60.05	166	INTERGENIC			
CaPOPI_II_542	Ca5	5190851	AGCGCTATGTGACCCTTTTG	AATGTGAATCCCACCAGGAA	60.27	555	INTERGENIC			
CaPOPI_II_543	Ca5	5318962	ACGTTGACCAATTCAGCTCC	CACTAGGGACCCTTTGGTGA	60.12	720	INTERGENIC			
CaPOPI_II_544	Ca5	5836817	GTATGAGGAGGATCTTGCGG	TCAAAATGCACCATAAACCG	59.65	594	INTERGENIC			
CaPOPI_II_545	Ca5	5837067	AAAGAAAAACACTCGCGCTG	AGCGTTTTGGGTTTAGGGTT	60.56	825	INTERGENIC			
CaPOPI_II_546	Ca5	6200121	TTCGCAACTCCACTCTTCCT	GATCCATTTTCCCCCAAATC	59.99	342	INTERGENIC			
CaPOPI_II_547	Ca5	6369660	GAGGAAACACGGTGAATGGT	CGCTCACTAGTCGAACGAGA	59.83	486	INTERGENIC			
CaPOPI_II_548	Ca5	6532288	TGTCTTCAACTTCTGCGGTG	TTGCAACGTGTCATTGTCCT	60.03	831	INTERGENIC			
CaPOPI_II_549	Ca5	6772456	TGCACAACAAAAGCACAACA	TGTACCATCATAGTGGCGGA	59.92	512	INTERGENIC			

INDEL marker IDs	Chromosomes/ unanchored scaffolds	Physical positions (bp)	Forward primers (5'-3')	Reverse primers (5'-3')	Anneling temperature (0C)	Expected amplified product size (bp)	Structural annotation		Functional annotation	
							Sequence components of *kabuli* genome	*Kabuli* gene accession IDs	NCBI-KOG	TFs
CaPOPI_II_550	Ca5	6833700	GCATAAAATCACGATGCCAA	CGATTTTTCGTCTACATGTGC	59.53	211	INTERGENIC			
CaPOPI_II_551	Ca5	6833833	AAATCACGATGCCAAAGAAAA	AAGAAAGTTGTAAATGTGTTTTATGGA	59.58	616	INTERGENIC			
CaPOPI_II_552	Ca5	6833924	AAATCACGATGCCAAAGAAAA	AAGAAAGTTGTAAATGTGTTTTATGGA	59.58	616	INTERGENIC			
CaPOPI_II_553	Ca5	6834263	GCTTAATCCATAAAACACATTTACAA	TTCTCATGCGCTTGACTAATG	57.86	526	INTERGENIC			
CaPOPI_II_554	Ca5	6834706	TGCATAAAATCACCATGCCA	TTGTACGTTTCTTTTCTATGATTTGC	60.88	360	INTERGENIC			
CaPOPI_II_555	Ca5	6837967	TCGAGAACAACTCGTCTCGTG	AATTTGCAGTGTCGCAAAAA	61.57	254	INTERGENIC			
CaPOPI_II_556	Ca5	6872599	CGGCCATCACACAATGATAA	TCAGGATGTCACCACGAAGA	60.34	454	INTERGENIC			
CaPOPI_II_557	Ca5	6907615	GGTTGTGTTATCCCTCGACA	AGGGTGCCACATTTACTCCA	58.42	539	INTERGENIC			
CaPOPI_II_558	Ca5	6909501	AATGATGGAGGGAGTGAAGC	AACTGTCCATTTGTGAGGGC	59.09	405	INTERGENIC			
CaPOPI_II_559	Ca5	9441399	TGCGATGGAGAAATCAAAGC	CTAGTTTGGCTTGACGGGAA	61.26	865	INTERGENIC			
CaPOPI_II_56	Ca1	29791416	TCCGATAAAATCTCCGTTCG	AAAAACACGTTCCCCTCCTT	60.03	323	INTERGENIC			
CaPOPI_II_560	Ca5	10686981	GCTCCTTCTGCATCAACACA	TGCCCCACATTTTAGACCTC	59.99	509	INTERGENIC			
CaPOPI_II_561	Ca5	10689102	ATGGGCCACCTATTGAGACA	TGCCAAAGAAAAAGGTTTCG	60.34	623	INTERGENIC			
CaPOPI_II_562	Ca5	10689622	CGAAACCTTTTTCTTTGGCA	AACGGATTGTCCCTTCATTG	60.22	418	INTERGENIC			
CaPOPI_II_563	Ca5	10693789	CCAATTTGAATTGTCCGTCC	TTGCAAAAGCTGGGTCAAGT	60.17	406	INTERGENIC			
CaPOPI_II_564	Ca5	11168411	TTCAAACGGAACTGATGCTTT	TTGGTTGGGGGAGTTATTGA	59.74	954	INTERGENIC			

INDEL marker IDs	Chromosomes/ unanchored scaffolds	Physical positions (bp)	Forward primers (5'-3')	Reverse primers (5'-3')	Anneling temperature (0C)	Expected amplified product size (bp)	Structural annotation		Functional annotation	
							Sequence components of *kabuli* genome	*Kabuli* gene accession IDs	NCBI-KOG	TFs
CaPOPI_II_565	Ca5	11168904	TCAATAACTCCCCCAACCAA	TCACAATTGGGTTTGGATCA	60.16	227	INTERGENIC			
CaPOPI_II_566	Ca5	11169035	TCAATAACTCCCCCAACCAA	TGACGCCGTGAAAAACATTA	60.16	626	INTERGENIC			
CaPOPI_II_567	Ca5	11729570	CACGGTCCCTAGAGCCAATA	CGGGAGATACGTAGGAGCAA	60.09	672	INTERGENIC			
CaPOPI_II_568	Ca5	11879108	AAGATTTCCTCCCTCCTCCA	CTTTAACATGCGTTTCCGGT	60.01	327	INTERGENIC			
CaPOPI_II_57	Ca1	29842423	TCATCAAAGGCATGCAAATC	GACCAAAACACAACGCAATG	59.63	359	INTERGENIC			
CaPOPI_II_570	Ca5	13636401	ATCGACAAACCAACATGCAA	GGGAAACTCACTTGCGGTTA	59.97	735	INTERGENIC			
CaPOPI_II_571	Ca5	13636921	TAACCGCAAGTGAGTTTCCC	GCGGTGGCTGAGTTTCTTAC	60.11	387	INTERGENIC			
CaPOPI_II_572	Ca5	14022502	GGAAATGGATGTCTGCCAAT	TTGCAATCTCATCAACTCGG	59.76	318	INTERGENIC			
CaPOPI_II_573	Ca5	14394852	TCTTCCACCCAGTTTTCACC	TTGAGGGCTATGTTTTTCGG	59.94	337	INTERGENIC			
CaPOPI_II_574	Ca5	15756736	GCGCGATTTACCAAAAATGT	TTTGAGTGGTGGGTTGTGAA	59.97	431	INTERGENIC			
CaPOPI_II_575	Ca5	16319965	TTGTGTGCAGGATCCACATT	TTGACCATTGCACGAAACAT	59.97	502	INTERGENIC			
CaPOPI_II_577	Ca5	17494887	CTACGCCAAGAAACTGCTCC	GTACGCGTCCAGACATAGCA	60.02	245	INTERGENIC			
CaPOPI_II_578	Ca5	17876530	TTTCATCCATCACCGACAAA	CTCAACGAATGCCTGAAACA	59.90	573	INTERGENIC			
CaPOPI_II_579	Ca5	18049660	GCACGTTGTAAAGAGTGGACTG	AAAAGGCCCACAAGAGTTGA	59.84	694	INTERGENIC			
CaPOPI_II_581	Ca5	18944656	TCCTGTCATGTGCTTTGAGC	CTAACCTCCCAGGACCCAAT	59.99	131	INTERGENIC			
CaPOPI_II_582	Ca5	20547334	AGGAGATGGAGCGTTGTTTG	TTGGTTAGCGTGCATCTGAG	60.26	462	INTERGENIC			

INDEL marker IDs	Chromosomes/ unanchored scaffolds	Physical positions (bp)	Forward primers (5'-3')	Reverse primers (5'-3')	Anneling temperature (0C)	Expected amplified product size (bp)	Structural annotation		Functional annotation	
							Sequence components of *kabuli* genome	*Kabuli* gene accession IDs	NCBI-KOG	TFs
CaPOPI_II_583	Ca5	21085449	TTAGGCGACCACTGTATCCC	TGAGCCACTTTGATCAGCAC	59.96	758	INTERGENIC			
CaPOPI_II_584	Ca5	21623515	AGGTCATTTTCATCCACCCA	ACACACCTATGGGATGCACA	60.17	678	INTERGENIC			
CaPOPI_II_585	Ca5	21899576	TGACCTGACCTGGCCTATTC	CCCAAACAAAGCCTTATGGA	60.07	519	INTERGENIC			
CaPOPI_II_586	Ca5	21965060	ACGCTCAGAGTTCACTGGGT	GGCAAATCGATTGGACAAGT	59.91	365	INTERGENIC			
CaPOPI_II_587	Ca5	22918526	TTATCGGCCTTATCCACTGC	TGAAAATCCATGAACCGTGA	60.06	915	INTERGENIC			
CaPOPI_II_590	Ca5	23664574	CAAGAAGGAACGAGGTGCTC	TAGCCTAGAGCAGGGGTGAA	59.99	626	INTERGENIC			
CaPOPI_II_596	Ca5	26996806	TCGTTTTGGGTGAATTGTGA	TGCCTCCTCGTTCCTTTTTA	59.94	410	INTERGENIC			
CaPOPI_II_597	Ca5	27000752	TTGGCACTGAATAGTGCTCG	CGTCCCGAATAGACGAACAT	60.01	698	INTERGENIC			
CaPOPI_II_598	Ca5	27000834	TTGGCACTGAATAGTGCTCG	CTCAGCGGGTAGAGTATCGC	60.01	940	INTERGENIC			
CaPOPI_II_599	Ca5	27472549	GCATTATGCCGTCATCATTTT	TTCGCATTCAAAATTGTTGTT	59.82	491	INTERGENIC			
CaPOPI_II_6	Ca1	1876420	CGGGTCTTCAAAATGGAATG	CAAAGGAAACGTGCTTCCAG	60.30	861	INTERGENIC			
CaPOPI_II_600	Ca5	28413401	TTCTCCTTGAAGCTGTCGGT	ATTGCCAACATTTCCGGTAG	59.99	252	INTERGENIC			
CaPOPI_II_601	Ca5	28588926	CAACTGCTCCTGTTCTCCAA	CTAGGAGCATGTCGCTCTCC	59.01	412	INTERGENIC			
CaPOPI_II_602	Ca5	29933647	GACCTTTTCAAAGGATGCATTA	CGTGATGACAGACCAACACC	58.25	713	INTERGENIC			
CaPOPI_II_604	Ca5	30126893	GTGGCTTCCAAAGAGAAACG	TTCGGTACACAATTTTCCCC	59.85	612	INTERGENIC			
CaPOPI_II_605	Ca5	30128968	TGTTTCTCTCTCCGCTCACA	CCGCCGACTATACCTGTTTT	59.70	681	INTERGENIC			

INDEL marker IDs	Chromosomes/ unanchored scaffolds	Physical positions (bp)	Forward primers (5'-3')	Reverse primers (5'-3')	Anneling temperature (0C)	Expected amplified product size (bp)	Structural annotation		Functional annotation	
							Sequence components of *kabuli* genome	*Kabuli* gene accession IDs	NCBI-KOG	TFs
CaPOPI_II_606	Ca5	30131050	CAAATTTCGAGCAACAATGC	AACACAAATGTTAAATTGCGAAGA	59.31	571	INTERGENIC			
CaPOPI_II_607	Ca5	31621509	ATCCGAGTTTGCGTATGTCC	GGGACGTGGAGATTGTTGAT	59.96	635	INTERGENIC			
CaPOPI_II_608	Ca5	32745764	AGGCATCACATTGAGGGACT	CCTCGGTGACCCTGAATAAA	59.54	148	INTERGENIC			
CaPOPI_II_609	Ca5	32912336	ACCCCCTCACCTCCTTATTG	TTTTCTTGCGTCACTTGTTGA	60.18	499	INTERGENIC			
CaPOPI_II_61	Ca1	29853119	TCCACGAGATTTTCCTTTGG	TGTCATCATTCTCTGCTGGC	60.04	357	INTERGENIC			
CaPOPI_II_614	Ca5	35328160	ACCACGTGACCCCTTAACTT	TGATGAAATTACGTTATATTGAGGATG	58.43	204	INTERGENIC			
CaPOPI_II_616	Ca5	36583877	TCACTTTTAGGATCCGTTTGA	CACAATGGACCATGCACTTC	57.35	809	INTERGENIC			
CaPOPI_II_618	Ca5	38034841	TCGTCCGATTTATACCGACA	CGACGAGGTATATGCCAACA	58.99	856	INTERGENIC			
CaPOPI_II_619	Ca5	38755924	GTCTGCCACATCACCCTTTT	CGGATCAACTTTTCTTTGTGG	59.97	661	INTERGENIC			
CaPOPI_II_62	Ca1	29853730	TTGCATTCCTAGGTTCAGCC	TATCGCTCGACCTCTGCTTT	60.21	602	INTERGENIC			
CaPOPI_II_621	Ca5	40392907	GCATTATCACGAGCAACACG	TCAATGTGGTTCGCAAAAGA	60.29	192	INTERGENIC			
CaPOPI_II_623	Ca5	42231500	TCAAAATCCACACCCCACTT	GCCATCTCCAATAGCACCAT	60.21	888	INTERGENIC			
CaPOPI_II_624	Ca5	42425779	TCAATGGAATGGAGTCGTCA	CGACAATTTCATTATCCGTCC	60.05	749	INTERGENIC			
CaPOPI_II_625	Ca5	44596809	TTGGCACATGCATTTTTGTT	GACCAAATCTTGTTGTTGCAGA	59.98	582	INTERGENIC			
CaPOPI_II_626	Ca5	44597112	GGGGTTAGTGCAAGCCAAAT	GTTCGCACGTGATGAACAAT	61.23	862	INTERGENIC			
CaPOPI_II_627	Ca5	45086826	CAAACGCAAAATTAAACACACAA	AATACAGCTGGGTTTGTGGC	59.96	367	INTERGENIC			

INDEL marker IDs	Chromosomes/ unanchored scaffolds	Physical positions (bp)	Forward primers (5'-3')	Reverse primers (5'-3')	Anneling temperature (0C)	Expected amplified product size (bp)	Structural annotation		Functional annotation	
							Sequence components of *kabuli* genome	*Kabuli* gene accession IDs	NCBI-KOG	TFs
CaPOPI_II_628	Ca5	46208612	ATGCAGCACAAAATGCAAGT	TTCGAATCTTATTTTGACAATTTTT	59.35	502	INTERGENIC			
CaPOPI_II_629	Ca5	46661039	TGGAATTCAAGGGACCAAAA	ACTTGCGAGATAATTTAGTAGGGA	60.28	863	INTERGENIC			
CaPOPI_II_63	Ca1	29856647	GCATTCCCTGCTCAAGACTC	AGACAAGGGAGAGTCCCACA	59.96	812	INTERGENIC			
CaPOPI_II_630	Ca6	112870	AACTCCTTGGGACCGTGATT	ACCTTGCAACTGGAGCAAAT	60.75	242	INTERGENIC			
CaPOPI_II_631	Ca6	1343859	CAACGAGTTTGATTTCTATTCTAACG	TTGGGTAGCCAAGGTTTCAC	59.66	721	INTERGENIC			
CaPOPI_II_632	Ca6	1350878	ACACATCAGCTTTTTGGCTG	AGCCCACTCACCTTCTTCCT	58.93	428	INTERGENIC			
CaPOPI_II_633	Ca6	1354322	AATTTGCATAGGCGGTGAAG	GGTGTTACAGCTTTCATGAACAAA	60.10	420	INTERGENIC			
CaPOPI_II_636	Ca6	1556869	CAATTTGGTTGGGCGTAAAC	TTGCTTTATACTCATATTTTGGCCT	60.22	528	INTERGENIC			
CaPOPI_II_637	Ca6	1559285	CAGAGCAGTGTGACAGCAAGA	TCAAAAATTTCAAACAAAATCCA	60.39	456	INTERGENIC			
CaPOPI_II_638	Ca6	1559581	TGAAGTTAAATTAAAGGATCGCA	TATTTTATTCCCACCGCTGC	58.04	283	INTERGENIC			
CaPOPI_II_639	Ca6	1560016	AATATTACTGCAGCGGTGGG	GGACCACAAGAAATCCTCCA	59.98	719	INTERGENIC			
CaPOPI_II_64	Ca1	30475519	AGGATGCATGGGATGAAAAG	AAACTTCTACTCGGGCCCAT	59.89	766	INTERGENIC			
CaPOPI_II_640	Ca6	1682722	GAGGCATTGTCTTTGGTGGT	GCAAATGTTTGGAATGGCTT	59.97	656	INTERGENIC			
CaPOPI_II_647	Ca6	2528953	TGGGTCAATGTTAAGAGGGC	CGTCAACGTCGTGCATTTAC	59.93	515	INTERGENIC			
CaPOPI_II_649	Ca6	3176026	GCAATGGAAGGTGAAAATGG	GCATAGGAGCGTGGAAATTG	60.31	522	INTERGENIC			
CaPOPI_II_65	Ca1	30682856	GTTGGTTCCCTAGCACCGTA	TCCGCCTAGGAGATAGCAGA	59.99	672	INTERGENIC			

INDEL marker IDs	Chromosomes/ unanchored scaffolds	Physical positions (bp)	Forward primers (5'-3')	Reverse primers (5'-3')	Anneling temperature (0C)	Expected amplified product size (bp)	Structural annotation		Functional annotation	
							Sequence components of *kabuli* genome	*Kabuli* gene accession IDs	NCBI-KOG	TFs
CaPOPI_II_650	Ca6	3255826	TGGCTTGTTTGATTTAGGACA	ATTCGAATGATCAGTTGCCC	58.28	547	INTERGENIC			
CaPOPI_II_651	Ca6	3351662	CGAAAATGGAAAACACCACC	TCGAGTTGCGTAACTGACATAG	60.21	687	INTERGENIC			
CaPOPI_II_652	Ca6	3351745	GGTTGATTTAGGCTTCACGG	TCGAGTTGCGTAACTGACATAG	59.57	580	INTERGENIC			
CaPOPI_II_653	Ca6	3352912	AAAGTGGGTGGCAATTAACG	TTATCCAATTCCGTTGCACA	59.86	247	INTERGENIC			
CaPOPI_II_654	Ca6	3355103	TGAATAGATGTGCGCGAGAC	TCCACGGGAGTGAGAGAGAT	59.98	401	INTERGENIC			
CaPOPI_II_655	Ca6	3355875	AATTTGGCCCTATTGGCTTT	AGAAACGGAATGAGTCACGG	59.81	709	INTERGENIC			
CaPOPI_II_656	Ca6	3377584	ATGCGGTGATGGTGATATTG	TATGGCGAATATGGATGGGT	59.23	745	INTERGENIC			
CaPOPI_II_657	Ca6	3382789	ATCACCGACCACTCACCTTC	GTTCAATCAGTGCAGCGTGT	59.97	810	INTERGENIC			
CaPOPI_II_658	Ca6	3382946	TAACCACCAACATTCTGCCA	TGTTCCGTCCCACTCATACA	59.96	927	INTERGENIC			
CaPOPI_II_659	Ca6	3387853	GAGCCAACAGGATAAGAGCG	GAAAATTGAGTTCCATGACGTTT	59.98	477	INTERGENIC			
CaPOPI_II_66	Ca1	30770184	AGTGGCTTGGAGGAGTTGGT	CGTTCGGCTTTCTTCTTCTG	61.08	535	INTERGENIC			
CaPOPI_II_660	Ca6	3387937	GAGCCAACAGGATAAGAGCG	GAAAATTGAGTTCCATGACGTTT	59.98	477	INTERGENIC			
CaPOPI_II_661	Ca6	3388065	GAGCCAACAGGATAAGAGCG	TTGTCTTTCACTTCTTTTTCCG	59.98	841	INTERGENIC			
CaPOPI_II_662	Ca6	3420698	TAGCCGACAACATTGGAACA	TGAACTCGCACATTTGAGGA	60.11	386	INTERGENIC			
CaPOPI_II_663	Ca6	4649762	GGTCGAACCACTTGAGGTGT	AATCCACTCCCACTCCACTG	60.01	389	INTERGENIC			
CaPOPI_II_664	Ca6	5295925	TTGACATTTCAAACCGGTCTAT	TGGAAAAGACTCCTGAATATGGA	58.49	359	INTERGENIC			

INDEL marker IDs	Chromosomes/ unanchored scaffolds	Physical positions (bp)	Forward primers (5'-3')	Reverse primers (5'-3')	Anneling temperature (0C)	Expected amplified product size (bp)	Structural annotation		Functional annotation	
							Sequence components of *kabuli* genome	*Kabuli* gene accession IDs	NCBI-KOG	TFs
CaPOPI_II_666	Ca6	6411336	ATCTCGTGGTGCAACAATCA	TTTGGGAAGGGTCAAGAAAA	60.12	628	INTERGENIC			
CaPOPI_II_669	Ca6	10514411	TTCGCAATCTGTGTTTTTCG	CAAGAATTCGTATCGTCTAAATGC	59.85	642	INTERGENIC			
CaPOPI_II_67	Ca1	31163466	ACTTTTGCGGAGAAGCACAT	CGTTCGTCTTTTCCTCTTGC	59.88	360	INTERGENIC			
CaPOPI_II_670	Ca6	10642500	CGATTTGACAGCAAAAGCAA	GACAACCTTCGTTGCCCTAA	59.99	437	INTERGENIC			
CaPOPI_II_673	Ca6	12787897	ATCAAACAAATGTTGCGAGC	CAAACCCCTATTTTTGTGATTGA	58.78	749	INTERGENIC			
CaPOPI_II_674	Ca6	12877302	GTTCGTAAAGGACGGGATGA	CTTTTACAGCGCTTTCTGGG	59.93	235	INTERGENIC			
CaPOPI_II_675	Ca6	13838996	TGTGTGATTGGTGTTGAGCA	GCAACAACGGGTGAAAATCT	59.71	563	INTERGENIC			
CaPOPI_II_676	Ca6	15555599	TGTGGGCACATTAGTAGGCA	TCATCGTCGTTGCTTGAGTC	60.13	640	INTERGENIC			
CaPOPI_II_677	Ca6	15559125	TCACCAAACAAAAACTGGGG	CTACATTCCGGTTAGGGCAA	60.76	480	INTERGENIC			
CaPOPI_II_678	Ca6	15727514	TTAGTTTGGGCTTGGGTTTT	TTGCTCCTACGTATCTCCCG	58.60	911	INTERGENIC			
CaPOPI_II_679	Ca6	15730699	ATTTGAACCTGGCAGCAAAA	GGTTTGAAAAAGGGGTGATG	60.62	175	INTERGENIC			
CaPOPI_II_680	Ca6	15731057	ATTTGAACCTGGCAGCAAAA	GGGACAAAAGGCTAGTTTGG	60.62	909	INTERGENIC			
CaPOPI_II_681	Ca6	15999975	AAGTGTGGGCATTTCCTTTG	ATGGACCTCTGTATTCGCCA	59.97	137	INTERGENIC			
CaPOPI_II_682	Ca6	16012701	TAAGAGTTTGGTTCCGGTGG	TGTGCAGATAAATGGCCAAA	59.96	397	INTERGENIC			
CaPOPI_II_684	Ca6	21021586	CACCGTCGTGTTCATCTGTC	TTATTTCACCGTCGTGTCCA	60.16	458	INTERGENIC			
CaPOPI_II_685	Ca6	21139884	CAGAAGGATTGCTCGTGTGA	TGCATAGGCAAAAATGATTGA	59.98	528	INTERGENIC			

INDEL marker IDs	Chromosomes/ unanchored scaffolds	Physical positions (bp)	Forward primers (5'-3')	Reverse primers (5'-3')	Anneling temperature (0C)	Expected amplified product size (bp)	Structural annotation		Functional annotation	
							Sequence components of *kabuli* genome	*Kabuli* gene accession IDs	NCBI-KOG	TFs
CaPOPI_II_686	Ca6	21141563	TCCGATAGGAAGAGCCAATG	ACGTTTATTTTTCGGACCCC	60.17	697	INTERGENIC			
CaPOPI_II_687	Ca6	21401312	AGCTCAGTCCTCACAACCGT	TTAGATCGGACAAGAAACCGA	59.91	344	INTERGENIC			
CaPOPI_II_689	Ca6	21485595	TGCGGGCTAGCTCACTTAAT	TCAAAACAAATAGGCAGGGG	60.00	115	INTERGENIC			
CaPOPI_II_69	Ca1	31768486	ACACATTATATTAAATGCGCGA	TGCCAAACGATAGGTTGTTG	57.38	646	INTERGENIC			
CaPOPI_II_690	Ca6	22099449	TGTGTTGCTTCTTGGCTTTG	ACGTGTGGGTTTCAGGTCTC	60.03	620	INTERGENIC			
CaPOPI_II_693	Ca6	23064745	TGGGTTGATTTTCCCACATT	TCTCGAGCAAATCACAAGTTAAA	60.03	571	INTERGENIC			
CaPOPI_II_695	Ca6	24296638	GAGAAGGAGGGAAAAATGGC	ATGTTTTGGATTTTGGCTCG	60.02	421	INTERGENIC			
CaPOPI_II_696	Ca6	24637049	TGTTTTTGGTGAAACGTGGA	AGGATGAATGTGGTGTTGAAGA	59.98	376	INTERGENIC			
CaPOPI_II_697	Ca6	26277672	TTAGGTGCGCTTGACTGATG	GTTTTGGCGTTGGTTTCACT	60.01	558	INTERGENIC			
CaPOPI_II_698	Ca6	27289106	CAATTCCCACAGGTACGCTT	AATTTCAAGGATTGGCGAAG	59.99	612	INTERGENIC			
CaPOPI_II_699	Ca6	28190835	AGGTCTCATACCAGAGATGCG	AAGTTAAGGGACCGCAAATG	59.32	871	INTERGENIC			
CaPOPI_II_7	Ca1	1930262	GGCAACATTTTACAAAAGTTAAGG	AGTTTGATTAATGTCGGGCG	58.32	318	INTERGENIC			
CaPOPI_II_70	Ca1	32037334	TCCAGTCATGTGGCTTCTCA	CTGAGCTTGTGACCAAATCG	60.40	559	INTERGENIC			
CaPOPI_II_700	Ca6	28193559	AAGGACTAAGTCGGGAAGAAAAA	GATGGTTTTTGTCCATTGAATTT	59.67	131	INTERGENIC			
CaPOPI_II_703	Ca6	28332319	TTTCAAAAAGTCCCAATCGC	GGTTTGGAGAAGAAGACGAAA	60.05	751	INTERGENIC			
CaPOPI_II_704	Ca6	28332579	GTCGGAGCTCTGCCATAAAA	GGTTTGGAGAAGAAGACGAAA	60.35	599	INTERGENIC			

INDEL marker IDs	Chromosomes/ unanchored scaffolds	Physical positions (bp)	Forward primers (5'-3')	Reverse primers (5'-3')	Anneling temperature (0C)	Expected amplified product size (bp)	Structural annotation		Functional annotation	
							Sequence components of *kabuli* genome	*Kabuli* gene accession IDs	NCBI-KOG	TFs
CaPOPI_II_705	Ca6	28332651	GTCGGAGCTCTGCCATAAAA	GGTTTGGAGAAGAAGACGAAA	60.35	599	INTERGENIC			
CaPOPI_II_706	Ca6	28332758	TTTCGTCTTCTTCTCCAAACC	CCCAACATATTACCTGTTACCCA	58.42	502	INTERGENIC			
CaPOPI_II_707	Ca6	28332973	TTTCGTCTTCTTCTCCAAACC	CGTGCAAAGTCGAAGCGTAT	58.42	669	INTERGENIC			
CaPOPI_II_708	Ca6	28443583	AAAAACACCAAAAGCCCCTC	CATAAAATGCCATTGATCCCT	60.33	499	INTERGENIC			
CaPOPI_II_709	Ca6	28513941	TGAAGAAAATTTAAAGGGACGAA	GTGTTGCATCTCATTGGTGG	59.18	569	INTERGENIC			
CaPOPI_II_71	Ca1	32153452	GTTTTGGCGTTGGTTTCACT	TGGGTTTGACCCTATGTGTG	60.02	353	INTERGENIC			
CaPOPI_II_710	Ca6	28520924	TTAGAAACAGGGACCCCTCC	TTGTCTGTGAACCGAAGCTG	60.30	577	INTERGENIC			
CaPOPI_II_711	Ca6	28521027	TCTCATTCTTGCCTTGCCTT	TTGTCTGTGAACCGAAGCTG	59.96	172	INTERGENIC			
CaPOPI_II_712	Ca6	28521076	TCTCATTCTTGCCTTGCCTT	TTGTCTGTGAACCGAAGCTG	59.96	172	INTERGENIC			
CaPOPI_II_713	Ca6	29231459	ACTCCCACACACCTCAAAGG	TTTCAAGTCTAGTCGAAGTTCATCA	60.00	834	INTERGENIC			
CaPOPI_II_715	Ca6	31097180	TCCCAATTGAGTGACATCTTTC	CTTATAGCCGAATGGCCAAA	59.03	587	INTERGENIC			
CaPOPI_II_716	Ca6	31099504	TGGGATGATGTTGGGAAAGT	TTGCAAAGAAAACCTCGCTT	60.17	129	INTERGENIC			
CaPOPI_II_717	Ca6	32372827	GAATACGCGGCTGATTCATT	CGCCCCGTTTTAACTCATTA	60.07	613	INTERGENIC			
CaPOPI_II_718	Ca6	32391786	CGCTATGCCCCATTAGATGT	TTTGATTGACTCATTGCGTG	59.95	294	INTERGENIC			
CaPOPI_II_719	Ca6	32519592	CGCATGCCACTCACTCTAAA	CGCTGCCACTTTCTACATCA	60.01	611	INTERGENIC			
CaPOPI_II_720	Ca6	32521477	TTGCCAACGTGTCAATCACT	TTTTCCCTACTATTTTGCAGCC	60.16	259	INTERGENIC			

INDEL marker IDs	Chromosomes/ unanchored scaffolds	Physical positions (bp)	Forward primers (5'-3')	Reverse primers (5'-3')	Anneling temperature (0C)	Expected amplified product size (bp)	Structural annotation		Functional annotation	
							Sequence components of *kabuli* genome	*Kabuli* gene accession IDs	NCBI-KOG	TFs
CaPOPI_II_721	Ca6	32522263	TTGTTCATTTTTGGTCGTTCA	CCTTGGCGGATATAGGGAAT	59.05	347	INTERGENIC			
CaPOPI_II_722	Ca6	32540257	GTTTATCAATGGACGCGGAC	TTCCCCTAAAAACGGTTGTG	60.34	341	INTERGENIC			
CaPOPI_II_723	Ca6	32540480	CACAACCGTTTTTAGGGGAA	GAGGATTGCTATGGACAACCA	59.83	214	INTERGENIC			
CaPOPI_II_724	Ca6	32617100	GAACGGTCCATGCAAAAGTT	CCCCCGATCCTTATACGACT	59.98	554	INTERGENIC			
CaPOPI_II_725	Ca6	33094621	TCAAAATGTAGCGGGTTTCC	AAGCCTTCCTCAAAATCCAA	59.94	398	INTERGENIC			
CaPOPI_II_726	Ca6	33094845	TCAAAATGTAGCGGGTTTCC	AAGTTGGACAAAATTGGGGT	59.94	950	INTERGENIC			
CaPOPI_II_727	Ca6	33229272	TGTTGACGGTGTGTTTTTAAGG	AAATTTTAGACCGCGACACG	59.94	477	INTERGENIC			
CaPOPI_II_728	Ca6	33229296	CGGTGTGTTTTTAAGGATTGG	AAATTTTAGACCGCGACACG	59.37	471	INTERGENIC			
CaPOPI_II_729	Ca6	33229523	CGGTGTGTTTTTAAGGATTGG	GCGGATATCGTAGGGTGCTA	59.37	644	INTERGENIC			
CaPOPI_II_73	Ca1	33777775	CATGCTCAATACACGGATGG	AACGTTCTTGAGTGGCTTGG	59.95	870	INTERGENIC			
CaPOPI_II_730	Ca6	33229803	AAGCAAGCCAAAAACGAAAA	GCGGATATCGTAGGGTGCTA	59.87	382	INTERGENIC			
CaPOPI_II_731	Ca6	33240261	GTTTGGTATTGGGCTCCCTT	CACAATTCCTCCCTCTCCAA	60.19	314	INTERGENIC			
CaPOPI_II_732	Ca6	33240380	GTTTGGTATTGGGCTCCCTT	CGAAACTCACCCCTTGTTGT	60.19	750	INTERGENIC			
CaPOPI_II_733	Ca6	34728984	TAGGCACATCAAGTCAACCG	TCAGACCATCCGATAGAGGC	59.72	570	INTERGENIC			
CaPOPI_II_734	Ca6	34859064	GAAAGAGTTTTGAGCCACGG	AAGTCCAATCTCGGTCAAACTT	59.85	625	INTERGENIC			
CaPOPI_II_735	Ca6	35161798	AGGAAGAGTGGAGTTGCGAA	CGGTTGAGAACCAAGGATGT	59.99	649	INTERGENIC			

INDEL marker IDs	Chromosomes/ unanchored scaffolds	Physical positions (bp)	Forward primers (5'-3')	Reverse primers (5'-3')	Anneling temperature (0C)	Expected amplified product size (bp)	Structural annotation		Functional annotation	
							Sequence components of *kabuli* genome	*Kabuli* gene accession IDs	NCBI-KOG	TFs
CaPOPI_II_736	Ca6	35399356	TTGAAGCTCAGATGGCACAC	CTCCCCCTTTTTGGCATAAT	59.99	718	INTERGENIC			
CaPOPI_II_737	Ca6	35616799	GTTTCTGAGGCAAAATCCCA	TGCATTTGTCTTCCCATTGA	60.05	537	INTERGENIC			
CaPOPI_II_739	Ca6	35802672	GAGCCTTAGAGTTGGGAGCA	ACCCAACTTGTCGAACCAAC	59.57	778	INTERGENIC			
CaPOPI_II_74	Ca1	34386272	AATCTGGAGGGCGGTAAGTT	ATATCGGTCAAACGCACACA	59.96	413	INTERGENIC			
CaPOPI_II_740	Ca6	35810337	TCATCGATTGCAACTTGCTT	CCCTAAAATTTTGTGGGCTT	59.43	672	INTERGENIC			
CaPOPI_II_741	Ca6	36029920	TTTGGAACCAAAAACTTCTTCAA	GGAGAAAAAGAATTAAAATCCTCTAAA	60.00	520	INTERGENIC			
CaPOPI_II_742	Ca6	36722414	CTTAGGTTTGCACTCAGCCC	TCGCGGATCTGCTCTTAAAT	59.88	685	INTERGENIC			
CaPOPI_II_743	Ca6	36722630	TTGCTCCTACGTATCCCCAG	TCGCGGATCTGCTCTTAAAT	60.09	295	INTERGENIC			
CaPOPI_II_744	Ca6	36898169	AAGAGAGGTCACTTTGGCGA	CAATTCCCACAGGTACGCTT	59.99	692	INTERGENIC			
CaPOPI_II_745	Ca6	37981858	TGCAATGACCTTGAAATTCG	CTTTTAGCCTTCATCGCCAG	59.66	817	INTERGENIC			
CaPOPI_II_746	Ca6	37982172	GGTACACTTTCCCATCAGCAA	CTTTTAGCCTTCATCGCCAG	59.99	468	INTERGENIC			
CaPOPI_II_747	Ca6	41574227	TGATTAATCGCAAATTGTCCA	TTTTTGGGGTCGTTTTATGC	59.02	471	INTERGENIC			
CaPOPI_II_748	Ca6	41574282	TGATTAATCGCAAATTGTCCA	TTTTTGGGGTCGTTTTATGC	59.02	471	INTERGENIC			
CaPOPI_II_749	Ca6	41659483	CCATCAGAAACTGGGATGGT	TGGCCCTTCATTTTTCAATC	59.78	495	INTERGENIC			
CaPOPI_II_75	Ca1	34971020	AAATACCCCTTTTTCCCACG	TGAAAACGCTCAATGCCATA	60.05	689	INTERGENIC			
CaPOPI_II_750	Ca6	41669872	TGTAGAATCCACCAATGTCCA	ACCAATCATGTTTCGTGGAA	58.84	651	INTERGENIC			

INDEL marker IDs	Chromosomes/ unanchored scaffolds	Physical positions (bp)	Forward primers (5'-3')	Reverse primers (5'-3')	Anneling temperature (0C)	Expected amplified product size (bp)	Structural annotation		Functional annotation	
							Sequence components of *kabuli* genome	*Kabuli* gene accession IDs	NCBI-KOG	TFs
CaPOPI_II_752	Ca6	43043105	TGATTTGGTCGCAACAGCTA	AAAAATTGTGCCAAAGTTATGTGA	60.40	556	INTERGENIC			
CaPOPI_II_753	Ca6	43138332	GTTCCCTTTGGAATGCTTGA	TCTTTGGGCCAGTGAAAATC	60.05	741	INTERGENIC			
CaPOPI_II_754	Ca6	43909185	TGATTGGGTTTGGGTTCATT	ACATAGATGCCGCCTTCATT	60.03	445	INTERGENIC			
CaPOPI_II_755	Ca6	45329060	TTTTTGCTATTTTATTCTCGTGG	TCGATTATGCGTAGGGAAGG	57.64	762	INTERGENIC			
CaPOPI_II_756	Ca6	46772986	GATTATTCTGCCTCCCGTCA	AAACGCAATCCTTGAGTTCG	60.04	633	INTERGENIC			
CaPOPI_II_757	Ca6	47001052	GAAGCGGTACTTCCCATTGA	GGTTACAATGTGGGGACGAG	60.07	417	INTERGENIC			
CaPOPI_II_758	Ca6	48260819	GTGGATGGTGGGGAATAGTG	CTGAGGTCCCAAGCTACGTC	60.05	504	INTERGENIC			
CaPOPI_II_759	Ca6	48279626	AACCAGATACTAACCCGCGA	TCTGGAAGTTTCTTGACCGTG	59.59	620	INTERGENIC			
CaPOPI_II_76	Ca1	35261821	TGGATTGTCATGGTCTGGAA	AATGGTCTTGAATTTTGCCG	59.89	640	INTERGENIC			
CaPOPI_II_760	Ca6	48280097	GACACGGTCAAGAAACTTCCA	TGCGTTGACTCTTCAATACACA	60.14	376	INTERGENIC			
CaPOPI_II_761	Ca6	48280225	GACACGGTCAAGAAACTTCCA	TGTGGCACATTCCAAATTTAT	60.14	601	INTERGENIC			
CaPOPI_II_762	Ca6	48280286	GACACGGTCAAGAAACTTCCA	TGTGGCACATTCCAAATTTAT	60.14	601	INTERGENIC			
CaPOPI_II_763	Ca6	48280766	TGTATTGATGAGTCGTTGAATGAT	GGAGACGTTAAAGTCCCACA	58.52	902	INTERGENIC			
CaPOPI_II_764	Ca6	48281570	TAGGGCAATTGTGCACATGA	CTCCAGGTAACGGTTGTGGT	61.08	335	INTERGENIC			
CaPOPI_II_765	Ca6	49218883	CCCCTTCATATGCTTCCAGA	GTGCTTGCTTTTACTTCGCC	60.03	446	INTERGENIC			
CaPOPI_II_766	Ca6	49733827	TTGCAATCTCATCAACTCGG	GTAAAATGAAGCGCTTTCGC	59.80	810	INTERGENIC			

INDEL marker IDs	Chromosomes/ unanchored scaffolds	Physical positions (bp)	Forward primers (5'-3')	Reverse primers (5'-3')	Anneling temperature (0C)	Expected amplified product size (bp)	Structural annotation		Functional annotation	
							Sequence components of *kabuli* genome	*Kabuli* gene accession IDs	NCBI-KOG	TFs
CaPOPI_II_768	Ca6	52848807	CTGCCAAATACTGCGACTCA	TCAATTTTGCTTGTAGGATGGA	60.01	381	INTERGENIC			
CaPOPI_II_769	Ca6	52850074	TCTGATTCGTTAATGACAACCTTT	CGTGGGAGCAATTATGTCCT	59.10	218	INTERGENIC			
CaPOPI_II_770	Ca6	52850184	AGGACATAATTGCTCCCACG	TCCATCTCCAACAACATCAAA	59.96	305	INTERGENIC			
CaPOPI_II_771	Ca6	52862596	ACATGAAGGCTTGGGTTGAC	GAGGAGCATGATAGCAAGGC	59.97	193	INTERGENIC			
CaPOPI_II_772	Ca6	52889876	TAAAGGACCAAGTCGGGAAA	TGCTCATGGAGTTAGAAGCAA	59.54	471	INTERGENIC			
CaPOPI_II_774	Ca6	53311252	GATGGTGGCCATTGAAGAGT	CAACAGTGTATGCACCTGCC	59.93	721	INTERGENIC			
CaPOPI_II_775	Ca6	53312302	ATAGCAATTGGTGCATCCCT	ATGACCAAGGCATTGAGGAC	59.42	761	INTERGENIC			
CaPOPI_II_776	Ca6	53413835	CGTCATTTGGGGGACTAAAA	GAGTTCGGGAGTAGGGAAGG	59.80	978	INTERGENIC			
CaPOPI_II_777	Ca6	53799025	GGCCCAATATTTGAAACCCT	CAACTTGCAAAGACCCATCA	60.02	704	INTERGENIC			
CaPOPI_II_778	Ca6	54068748	GTAAGTGAAAGCGGGAGTGC	ACCACCAACTTGCTTGCTCT	59.88	527	INTERGENIC			
CaPOPI_II_779	Ca6	54068881	GATCCCAACCATCATTGTCC	ACCACCAACTTGCTTGCTCT	60.00	258	INTERGENIC			
CaPOPI_II_780	Ca6	54068968	GATCCCAACCATCATTGTCC	ACCACCAACTTGCTTGCTCT	60.00	258	INTERGENIC			
CaPOPI_II_781	Ca6	54069131	GATCCCAACCATCATTGTCC	AATTACCACGGCCTCTTGTG	60.00	824	INTERGENIC			
CaPOPI_II_782	Ca6	54070088	GCAATTGTCGTGATTTTATTTTTG	TTTTGGTGGGTTCCACTTTT	59.80	379	INTERGENIC			
CaPOPI_II_783	Ca6	54070236	TTTCTTTCCTCATGCTTTAGAGG	TTTTGGTGGGTTCCACTTTT	59.07	123	INTERGENIC			
CaPOPI_II_784	Ca6	54192734	GGACGGCGAAAATAAAACAA	TTGAAAATGGCTAAGGCAAAA	59.94	642	INTERGENIC			

INDEL marker IDs	Chromosomes/ unanchored scaffolds	Physical positions (bp)	Forward primers (5'-3')	Reverse primers (5'-3')	Anneling temperature (0C)	Expected amplified product size (bp)	Structural annotation		Functional annotation	
							Sequence components of *kabuli* genome	*Kabuli* gene accession IDs	NCBI-KOG	TFs
CaPOPI_II_785	Ca6	55769150	TCGATTATGCGTAGGGAAGG	TTTTTCCTTTGTAGGCCCAA	60.05	943	INTERGENIC			
CaPOPI_II_786	Ca6	55771133	GGAAACGTGTGAACGGAAGT	GGTAACCAATCCGAAGACGA	60.01	575	INTERGENIC			
CaPOPI_II_787	Ca6	55771190	TTACCGATGCATTGATCGAC	GGTAACCAATCCGAAGACGA	59.50	143	INTERGENIC			
CaPOPI_II_788	Ca6	56087656	TTGTGAGCATGAGAAGTTGTTG	TGAAAAATTATTGAAAAAGTTACTTCC	58.98	737	INTERGENIC			
CaPOPI_II_789	Ca6	56504001	GTTGGCGCAAACTCTTCTTC	TGCAACTTTCAGGTCCCTTT	60.00	583	INTERGENIC			
CaPOPI_II_79	Ca1	35283797	GGGTGTGTTTGATAGGAGGG	CTCCCCTTCCATCTCCTCTT	59.26	139	INTERGENIC			
CaPOPI_II_790	Ca6	56504140	AAATGCAAAGTCGGATGGTC	TGCGAAGAAGAATGATCACG	59.94	896	INTERGENIC			
CaPOPI_II_791	Ca6	56504753	CGTGATCATTCTTCTTCGCA	TCGGTCACCAAGTTACCACA	59.95	614	INTERGENIC			
CaPOPI_II_792	Ca6	56505598	TGGTAACTTGGTGACCGACA	AATTTTTGCAGAAGAAAGAAAAAG	60.00	485	INTERGENIC			
CaPOPI_II_793	Ca6	56507452	CGTGATCATTCTTCTTCGCA	ATCCAAACATTGGCCATTTC	59.95	685	INTERGENIC			
CaPOPI_II_794	Ca6	56507723	CGTGATCATTCTTCTTCGCA	CTCCCAATTTGCAGTGTCCT	59.95	966	INTERGENIC			
CaPOPI_II_795	Ca6	56507798	AAATTCAAGTCTCGACGCAAA	CTCCCAATTTGCAGTGTCCT	59.88	511	INTERGENIC			
CaPOPI_II_796	Ca6	56727392	AGGCAAAACATGTTCCCTCA	AATGTTAGTGCACCGTGTCG	60.50	706	INTERGENIC			
CaPOPI_II_797	Ca6	58373671	TCACGCAAAAGATTCATAAAAGA	AGTTTGTAAGCTGTGCGTCG	58.93	500	INTERGENIC			
CaPOPI_II_798	Ca6	59093194	GGAGGCAACCCAACTCTATG	TCGCATAATTGGAACGTCAA	59.55	271	INTERGENIC			
CaPOPI_II_799	Ca6	59273311	TTTTTGGGCTGAAGCCATAC	GGTGGAGAATGAAACGGAGA	60.07	359	INTERGENIC			

INDEL marker IDs	Chromosomes/ unanchored scaffolds	Physical positions (bp)	Forward primers (5'-3')	Reverse primers (5'-3')	Anneling temperature (0C)	Expected amplified product size (bp)	Structural annotation		Functional annotation	
							Sequence components of *kabuli* genome	*Kabuli* gene accession IDs	NCBI-KOG	TFs
CaPOPI_II_80	Ca1	35284017	GAGATGGAAGGGGAGGAAAG	GCACAAAAGAAAAATACAAGTCCA	60.01	273	INTERGENIC			
CaPOPI_II_800	Ca6	59349983	TTTCCATTGCACTCACTCCA	CCTATCATGGCCTGACCTGT	60.24	777	INTERGENIC			
CaPOPI_II_803	Ca7	1018745	TTGCAAACATGATAGCTGCC	TTGTTGGCATTGTTGTGGTT	59.84	467	INTERGENIC			
CaPOPI_II_804	Ca7	1018777	TTGTTCCCGTAAAAGATCGC	TTGTTGGCATTGTTGTGGTT	60.07	233	INTERGENIC			
CaPOPI_II_805	Ca7	1245279	TGCACAAAACACACAACCCT	TCGCTCCTGTGTCAGTTTTG	60.05	468	INTERGENIC			
CaPOPI_II_808	Ca7	2525335	TGGTGGAAAAATAGCATTTGG	CCTCTCCCGAATGACTCAAG	59.82	868	INTERGENIC			
CaPOPI_II_81	Ca1	35284253	GAGATGGAAGGGGAGGAAAG	TCTGATCAAATATGTGGCAGAAA	60.01	657	INTERGENIC			
CaPOPI_II_810	Ca7	4418125	CAAGTCAGCACCGACAAAGA	ATAAAAGGGTGACGTGGCAG	60.03	592	INTERGENIC			
CaPOPI_II_811	Ca7	4867122	AGGTGGTGAGCGTTTTTGAA	TTGATGATGTGAAGGAATGACAG	60.67	670	INTERGENIC			
CaPOPI_II_812	Ca7	5014340	AATACCACCACCGACCACAT	AATGAGCTCCAACGGTCATC	59.97	787	INTERGENIC			
CaPOPI_II_813	Ca7	5034485	CCCACTTGCAACGGTTTATT	TGAGGTAAGCTTTGCATCATGT	59.86	837	INTERGENIC			
CaPOPI_II_814	Ca7	5443791	TATTTGCGAGCGTGTCTTCA	TTTTGATGCTTTTTCCACCA	60.55	516	INTERGENIC			
CaPOPI_II_817	Ca7	5650363	TCGTTCCCTCTGAAATGAAA	TCGAGTGAACGTGGAAGATG	58.27	239	INTERGENIC			
CaPOPI_II_818	Ca7	5694761	GGAATTCTGTTTGCAAGGGA	AACCCATCACTTGCTGATCC	60.05	627	INTERGENIC			
CaPOPI_II_819	Ca7	5843643	GGTGAGTTTTTCTCTCCCCC	TTCATCGTCTGAAGTCACGG	59.91	501	INTERGENIC			
CaPOPI_II_82	Ca1	35977319	AACTCGTTGGAACTTCCCTCT	GCAAGCAGCCTTGTTGACTA	59.24	540	INTERGENIC			

INDEL marker IDs	Chromosomes/ unanchored scaffolds	Physical positions (bp)	Forward primers (5'-3')	Reverse primers (5'-3')	Anneling temperature (0C)	Expected amplified product size (bp)	Structural annotation		Functional annotation	
							Sequence components of *kabuli* genome	*Kabuli* gene accession IDs	NCBI-KOG	TFs
CaPOPI_II_820	Ca7	5884631	TCTTGTTTGCCTGTCTCGTG	ATGCGAAGGAGCAATTATGG	60.03	832	INTERGENIC			
CaPOPI_II_822	Ca7	6912264	TGCACTTGCACATGTTGAATAA	AATCCCTGCAATTGTGGAAG	60.18	560	INTERGENIC			
CaPOPI_II_823	Ca7	7738038	GATTCCATTGGGATTGGTTG	CACGTTTTAGAGATGTCCATTTTG	59.99	505	INTERGENIC			
CaPOPI_II_824	Ca7	7738595	CAAAATGGACATCTCTAAAACGTG	TCTCTCACCCAATCACCAAA	59.93	583	INTERGENIC			
CaPOPI_II_825	Ca7	9180941	ATGATTTCCACGAGCCAAAC	TGCAACATTTCCTGATGAGC	59.94	322	INTERGENIC			
CaPOPI_II_826	Ca7	9181858	TATTACCCAACCAAGCCAGC	TGAACTTTGATTGCCGCTAA	59.96	554	INTERGENIC			
CaPOPI_II_827	Ca7	10192729	CCCATTCCGAATACATTGCT	GAGTTGGGTGCACGATTTTT	59.78	624	INTERGENIC			
CaPOPI_II_828	Ca7	10568493	ACGATGGAGGCCAAAGTATG	AAAATAAGGCATGGTGTTGGA	59.96	487	INTERGENIC			
CaPOPI_II_829	Ca7	14075293	TTTGTTTAGCCACAACTTTTGA	CGTGCTCGGGATTTATTTGT	57.57	862	INTERGENIC			
CaPOPI_II_83	Ca1	36044248	CGATTTCCCTGCATGTTTTT	GCCTGTGATAGGCGGTACAT	59.94	540	INTERGENIC			
CaPOPI_II_830	Ca7	15467222	TGGGAAACGGTGCTAAACTT	TCCATTTACGGCAAATTCAA	59.61	716	INTERGENIC			
CaPOPI_II_831	Ca7	16001405	AAAACACAACGTGCAAACGA	GGGTCACCAAGCACAGTTTT	60.20	679	INTERGENIC			
CaPOPI_II_832	Ca7	16941588	TGCCGTAGTATTCACCACCA	TGCCATGAAATTCTGCCATA	59.99	852	INTERGENIC			
CaPOPI_II_833	Ca7	17133790	GACACTACCCGGCATCAAGT	CCAACCCAAATAAATGAAAATCA	60.00	530	INTERGENIC			
CaPOPI_II_834	Ca7	17172621	GGCTAAACCTTGTCTTTGGC	AGACTCTGCTTGACCGGAAA	58.84	541	INTERGENIC			
CaPOPI_II_835	Ca7	17374590	GCATCTGTAAAACAAGACCCG	ACATCGATAAGAGGCAGCGT	59.62	525	INTERGENIC			

INDEL marker IDs	Chromosomes/ unanchored scaffolds	Physical positions (bp)	Forward primers (5'-3')	Reverse primers (5'-3')	Anneling temperature (0C)	Expected amplified product size (bp)	Structural annotation		Functional annotation	
							Sequence components of *kabuli* genome	*Kabuli* gene accession IDs	NCBI-KOG	TFs
CaPOPI_II_837	Ca7	18587623	CGATGATGACTGATTGGGGT	TGTTTGTGTTTTGAGGCGTT	60.75	374	INTERGENIC			
CaPOPI_II_838	Ca7	18588675	CCAAGACTCCTTGTAACACCC	GCACATATATACACGTAAAATCGCA	58.56	299	INTERGENIC			
CaPOPI_II_839	Ca7	19589201	GGTCGTCCAAATAGTACCCG	AGGAAGCCCAAGTTCCAAAT	59.31	828	INTERGENIC			
CaPOPI_II_84	Ca1	37122520	TGCAATCATTGGTGCTTAGG	AAAAAGCGCCGTAAAATGAG	59.69	710	INTERGENIC			
CaPOPI_II_840	Ca7	19857139	CCATATGTCATAGTGCGGTCA	AGAGGCAAACAAGAACCGAA	59.44	314	INTERGENIC			
CaPOPI_II_841	Ca7	19860378	CCTCAATATGTTGGGACATGC	TGATTCTCAGGGAGGGTGTC	60.21	464	INTERGENIC			
CaPOPI_II_843	Ca7	20505889	GGCATGGAAAATTCTGAAAAA	TTCATTTCTCGATTGAGGGC	59.05	803	INTERGENIC			
CaPOPI_II_844	Ca7	20617780	GGCGAGGGATGGAATTTTAG	GAATTGGGACTTCTGCCGTA	60.76	777	INTERGENIC			
CaPOPI_II_845	Ca7	21099731	GAGAAATCAAAATTGCCCCA	AGTGCCTCCTGTTCCTGAGA	59.88	500	INTERGENIC			
CaPOPI_II_846	Ca7	21670841	TGACAAATCTTTGCAACCCA	ATTGCTCATTTCATGCCCTC	60.09	217	INTERGENIC			
CaPOPI_II_847	Ca7	21685577	TTTATAAATTGGCCCCATCG	TAGGGGGACCAAGAAACTGG	59.63	501	INTERGENIC			
CaPOPI_II_848	Ca7	21728387	ATCTCTGCGCAATATCGCTT	AGCCGACTTTTCCTACCACA	59.97	674	INTERGENIC			
CaPOPI_II_849	Ca7	21729097	TGCTCAAAAGGGGAAGAAAA	GCCACGTGGTTGAAAGAAGT	59.80	240	INTERGENIC			
CaPOPI_II_85	Ca1	37122699	GGAGGAGCGCAACTTAATCA	AAAAAGCGCCGTAAAATGAG	60.35	372	INTERGENIC			
CaPOPI_II_850	Ca7	21729303	TTGCTCAAAAGGGGAAGAAA	TTGTTATTTTCTTTATGCCGTTG	59.80	426	INTERGENIC			
CaPOPI_II_855	Ca7	22036269	GCTCAGCCTTGAAATCGAAC	TTCATCATCATCTTCAAACAAAA	59.96	627	INTERGENIC			

INDEL marker IDs	Chromosomes/ unanchored scaffolds	Physical positions (bp)	Forward primers (5'-3')	Reverse primers (5'-3')	Anneling temperature (0C)	Expected amplified product size (bp)	Structural annotation		Functional annotation	
							Sequence components of *kabuli* genome	*Kabuli* gene accession IDs	NCBI-KOG	TFs
CaPOPI_II_856	Ca7	22038011	CTCCAAATTTCCAGCCAAAA	ACACGGACGGGTCTTGTATC	60.05	822	INTERGENIC			
CaPOPI_II_857	Ca7	22043317	TGGTTCAAATGCTGTAATCTTCTC	ATTCGCTCAATACACGGGTC	59.67	884	INTERGENIC			
CaPOPI_II_858	Ca7	22045478	TGTGGTGTTCCCAAAAGACA	CAAAGCGAAGTGCAAAATGA	59.98	246	INTERGENIC			
CaPOPI_II_859	Ca7	22046891	ATAATTTGCCTTTGCGGTTG	TTCTGTGAGCAAGGCTGATG	59.96	319	INTERGENIC			
CaPOPI_II_86	Ca1	37122810	GGAGGAGCGCAACTTAATCA	AAAAAGCGCCGTAAAATGAG	60.35	372	INTERGENIC			
CaPOPI_II_860	Ca7	22046957	ATAATTTGCCTTTGCGGTTG	TTCTGTGAGCAAGGCTGATG	59.96	319	INTERGENIC			
CaPOPI_II_861	Ca7	22046996	ATAATTTGCCTTTGCGGTTG	ACCTGGTCGTAGATGCCACT	59.96	414	INTERGENIC			
CaPOPI_II_863	Ca7	23780060	CGCTCGTCTATGCGATTGTA	TAAAATCTCCGCTCGCAACT	60.00	946	INTERGENIC			
CaPOPI_II_864	Ca7	23907440	TTGGAGAATTCAAAAATAAGGCA	CGTGACAAAGCACGAAAGAA	59.96	513	INTERGENIC			
CaPOPI_II_865	Ca7	24347972	CTCTCATCTGAGGGCAAAGC	CGATTGAATTAAGCCCAGGA	60.10	356	INTERGENIC			
CaPOPI_II_866	Ca7	24348355	TCCTGGGCTTAATTCAATCG	CGTTCCTGAGGGAATTGTGT	60.03	378	INTERGENIC			
CaPOPI_II_867	Ca7	24421160	AGGAAGCCCAAGTTCCAAAT	ACAGCCAACAACCACATCAA	59.94	469	INTERGENIC			
CaPOPI_II_868	Ca7	24485265	GTCCCACCCTAGGAGCTTTC	CGGACCAAAAATTAAAGCGA	60.07	986	INTERGENIC			
CaPOPI_II_869	Ca7	25626892	TTGGCCAAGTCCCTGTTTAC	CCCGTGATAGGCAGTACGTT	59.97	420	INTERGENIC			
CaPOPI_II_87	Ca1	37128515	CAATTCCCACAGGTACGCTT	TTGGTCTCATCAATGATTGTAAAAA	59.99	568	INTERGENIC			
CaPOPI_II_870	Ca7	26035041	TAAAGAGATTCGCAGCGGTT	CATGTTTTCGAAGCTCACCA	59.98	407	INTERGENIC			

INDEL marker IDs	Chromosomes/ unanchored scaffolds	Physical positions (bp)	Forward primers (5'-3')	Reverse primers (5'-3')	Anneling temperature (0C)	Expected amplified product size (bp)	Structural annotation		Functional annotation	
							Sequence components of *kabuli* genome	*Kabuli* gene accession IDs	NCBI-KOG	TFs
CaPOPI_II_871	Ca7	26160184	GTGGCTACCTTCTTGGGTGA	TTTCATCGCCGAAACCTATC	60.11	141	INTERGENIC			
CaPOPI_II_872	Ca7	26221720	CTTGGGAATTGGAGAGCAAG	GGGTTTCTTTCCTTGAAGCC	59.81	671	INTERGENIC			
CaPOPI_II_873	Ca7	26228410	ATGGTGCAAAATGGTGACAA	GAAAGTCGGTGCATGGAAGT	59.82	304	INTERGENIC			
CaPOPI_II_874	Ca7	26235537	TGCGACCTCAGTTTCTCAAA	GGGGTCATAGCATCCCCTAT	59.57	157	INTERGENIC			
CaPOPI_II_875	Ca7	26662956	TCCAAGCTTCCTTCAATGGT	CTCAAAAGCGCTCCAATTTC	59.67	542	INTERGENIC			
CaPOPI_II_878	Ca7	27346895	TGCTTGATTGCTTGAGTTCC	CGCGCAGATTGTCAAATAGA	59.00	641	INTERGENIC			
CaPOPI_II_879	Ca7	27521328	GCCAAATTCCCTCTTTCTCC	CCTTTCCACACAACCACAGA	60.02	393	INTERGENIC			
CaPOPI_II_880	Ca7	28190197	TTGGTTTATTTGGGTTGGGA	ACCAGGAAAAGAAGAAGGGC	60.02	519	INTERGENIC			
CaPOPI_II_881	Ca7	28702659	TCCATTAACCCTCTTGAGGAAA	TCAGGATGAAGAAGGGGAGA	59.94	531	INTERGENIC			
CaPOPI_II_882	Ca7	28707412	ATCCCACCATCAAAAACCAA	GATATGTTCCGAGTGCCGTT	60.03	491	INTERGENIC			
CaPOPI_II_883	Ca7	29416537	AAAATCATTACTCACCATAAGTGGAA	TGCTCTCCTCCTTCAACAACT	59.29	436	INTERGENIC			
CaPOPI_II_885	Ca7	30053648	GGACTGAAAAGAAAAATATGCGA	GTGCTCTCTTTTGCCGATTC	59.64	774	INTERGENIC			
CaPOPI_II_888	Ca7	30221695	CTTGTTGATGCAATGTTGGC	TTTGGCCACACAAAAACAAA	60.12	538	INTERGENIC			
CaPOPI_II_89	Ca1	37420598	GGGAAAAGGAAACCCAAAAA	TGAGTGAGGGAGACAAAGGG	60.14	950	INTERGENIC			
CaPOPI_II_891	Ca7	31112471	GCAAAGGCACTCATTCCATT	GCAAAAACACGAGAAGGAGG	60.08	493	INTERGENIC			
CaPOPI_II_893	Ca7	31745904	TGCATGGAATGGTGAGTGAT	ATTGATGGGTTTACTTGGCG	59.93	219	INTERGENIC			

INDEL marker IDs	Chromosomes/ unanchored scaffolds	Physical positions (bp)	Forward primers (5'-3')	Reverse primers (5'-3')	Anneling temperature (0C)	Expected amplified product size (bp)	Structural annotation		Functional annotation	
							Sequence components of *kabuli* genome	*Kabuli* gene accession IDs	NCBI-KOG	TFs
CaPOPI_II_894	Ca7	32479028	AGGGAGTTTAGTGGTGGGCT	CGGATGCATGAAAGTGAAAA	59.99	636	INTERGENIC			
CaPOPI_II_896	Ca7	32649545	TTGCAGGATGCACAGAAAAG	AGGTGCATCCCCTGTAACTG	59.99	415	INTERGENIC			
CaPOPI_II_897	Ca7	32650059	ATGGGACTGTGGGAGAGAGA	GAAAAAGGCTTCAAACATCAAA	59.64	398	INTERGENIC			
CaPOPI_II_898	Ca7	32654894	AACGAAGTGAGACGTGAGCA	TGATGGCTATGGTGGAACAA	59.62	618	INTERGENIC			
CaPOPI_II_899	Ca7	32655163	AACGAAGTGAGACGTGAGCA	TGATGGCTATGGTGGAACAA	59.62	618	INTERGENIC			
CaPOPI_II_90	Ca1	37421082	ACTTGACGGGGACTCCACTA	ACATGTTCCTTGTGCTCGTG	59.57	637	INTERGENIC			
CaPOPI_II_900	Ca7	33539636	AAATTGGACGGTGCTAATCC	CACGAAAGGTTTCATCACAAA	58.91	243	INTERGENIC			
CaPOPI_II_902	Ca7	33597364	GATGTTAACGCACCTCCACC	CACACTTCGATCTCCGATCA	60.38	483	INTERGENIC			
CaPOPI_II_906	Ca7	34312433	CTCCTTTCTCACACTCACTCCA	TTGGTGGACTTCCCAACTTC	59.49	515	INTERGENIC			
CaPOPI_II_907	Ca7	34312678	CTCCTTTCTCACACTCACTCCA	TTGGTGGACTTCCCAACTTC	59.49	515	INTERGENIC			
CaPOPI_II_908	Ca7	34318595	TCAATGACTGTGACAAAATCCA	GCTAGCAGCCACCTTCTTTG	59.02	503	INTERGENIC			
CaPOPI_II_909	Ca7	34342253	AATGATAGGTGGACGTTCAAA	GGGTAACCTCGCCACAACTA	57.08	725	INTERGENIC			
CaPOPI_II_91	Ca1	37424329	AAAAAGTATTGAACAAACACGCT	TGAAATCCAAATTAAATTGCTGAA	57.18	549	INTERGENIC			
CaPOPI_II_910	Ca7	35177660	GCCAACCGACTCCATCTTTA	TTTAGCAGTTTTTCAATTTCCAA	60.07	690	INTERGENIC			
CaPOPI_II_911	Ca7	35313643	TTCGATAAAGCAAAAGCTCCA	AACTGAATATGGGCCAGTCG	59.97	805	INTERGENIC			
CaPOPI_II_912	Ca7	35364737	CGCATTCCTCTCGAAAAGTC	TTGGAGAAAGATCTCCGGTT	59.96	439	INTERGENIC			

INDEL marker IDs	Chromosomes/ unanchored scaffolds	Physical positions (bp)	Forward primers (5'-3')	Reverse primers (5'-3')	Anneling temperature (0C)	Expected amplified product size (bp)	Structural annotation		Functional annotation	
							Sequence components of *kabuli* genome	*Kabuli* gene accession IDs	NCBI-KOG	TFs
CaPOPI_II_914	Ca7	38743655	CTTAGGTTTGCACTCAGCCC	CTCAATCGTGGGTGACCTTT	59.88	932	INTERGENIC			
CaPOPI_II_915	Ca7	39525063	AACGACCAACTTGGGAAAAA	GGAGTGGGTCACCGAGAATA	59.45	520	INTERGENIC			
CaPOPI_II_917	Ca7	40985025	GCAACTGCCTTAAAAATGGAA	GGAACGTGCGTACATTGTTG	59.24	303	INTERGENIC			
CaPOPI_II_918	Ca7	41008241	GCCAATCAGGACACATCTCA	TCAGAACATTCCATCGGTGA	59.64	563	INTERGENIC			
CaPOPI_II_919	Ca7	41009960	CATGACCTCGGTTTTCAATAA	CCCTCACATTTTTAAGGCACA	57.62	814	INTERGENIC			
CaPOPI_II_92	Ca1	37424983	GCAATTTAATTTGGATTTCATGG	TCACATCCATCCACGGTACA	59.60	761	INTERGENIC			
CaPOPI_II_920	Ca7	41010142	TGCAATGAATGAAAGTATGCTACC	GAAAAAGCGCTGTAAATGGC	60.38	815	INTERGENIC			
CaPOPI_II_921	Ca7	41251256	CCAATAGTTTAGTGGGCCAAA	AAGGGTCCTCTTGAAGCGTT	59.00	213	INTERGENIC			
CaPOPI_II_922	Ca7	41252327	TGCAATGCAATGTCCATTTT	TTTTTCCTCATGGTGCAGTG	59.94	324	INTERGENIC			
CaPOPI_II_923	Ca7	41484126	AGATTCGCAGGCAGTTGAGT	CGAGAGAGTGTCACGCATGT	60.02	701	INTERGENIC			
CaPOPI_II_924	Ca7	42738535	TCCCGCTACACTTTGAGGAC	TCAAAAGCAAGTGTTGCAGG	60.26	592	INTERGENIC			
CaPOPI_II_926	Ca7	43375115	CCAATATCTCGTGTTCCCGT	CATTTCAAGTTGGGGCAGTT	59.81	335	INTERGENIC			
CaPOPI_II_927	Ca7	44001613	TGAAACTGCCATTGGACAAA	CAATTTCTTTGCGTGTGGTG	60.09	476	INTERGENIC			
CaPOPI_II_929	Ca7	44474070	TAGCAACAAAAATGGGGGAG	TGGTAGCGTACCGTTTGACA	59.93	634	INTERGENIC			
CaPOPI_II_93	Ca1	37425153	GCAATTTAATTTGGATTTCATGG	TCACATCCATCCACGGTACA	59.60	761	INTERGENIC			
CaPOPI_II_930	Ca7	45206871	AGGAGGTTTGTGTTTGACGG	TTTGGATTTTGACTCGGAGG	60.01	617	INTERGENIC			

INDEL marker IDs	Chromosomes/ unanchored scaffolds	Physical positions (bp)	Forward primers (5'-3')	Reverse primers (5'-3')	Anneling temperature (0C)	Expected amplified product size (bp)	Structural annotation		Functional annotation	
							Sequence components of *kabuli* genome	*Kabuli* gene accession IDs	NCBI-KOG	TFs
CaPOPI_II_931	Ca7	46332309	AACGTACTGCCTATCACGGG	TTCGCAACTCCACTCTTCCT	60.02	649	INTERGENIC			
CaPOPI_II_932	Ca7	48312616	CGAAGCACAAGGAGATGACA	TTTCCGAGTGCTGTCAAATG	59.98	382	INTERGENIC			
CaPOPI_II_933	Ca7	48392684	TGATTCCCCAACATCCAACT	ATGATATTGGAGTTTGCGCC	60.17	165	INTERGENIC			
CaPOPI_II_934	Ca8	1184414	TTCGTACCGTTCTCTCCTCAA	TTGTACACAACTCCCTTGCG	59.86	563	INTERGENIC			
CaPOPI_II_935	Ca8	5016373	TTGTCCGTTATAACATTAAACACTTT	GAAAAGACAACCCCGCATTA	57.47	279	INTERGENIC			
CaPOPI_II_937	Ca8	6492546	CGTAAGCAACCCACCAATCT	TTGTCAACAACTCAGCGTAAAAA	59.99	142	INTERGENIC			
CaPOPI_II_938	Ca8	6492603	CGTAAGCAACCCACCAATCT	TTGTCAACAACTCAGCGTAAAAA	59.99	142	INTERGENIC			
CaPOPI_II_94	Ca1	37425620	TGTACCGTGGATGGATGTGA	TATGCATCCTCACGGTTTTG	60.82	675	INTERGENIC			
CaPOPI_II_943	Ca8	7948182	GCCGCAAAGAAGAGAAGAGA	TGCCCAAGTCACTAATGCTG	59.84	443	INTERGENIC			
CaPOPI_II_944	Ca8	7950561	GTCTTTGGCCTCCAATCAAA	TGGAGTTGGGCCAATAATTT	60.05	359	INTERGENIC			
CaPOPI_II_945	Ca8	7952607	CCTTCAAAATGGTGTTGCTCT	GTCATTTTGCCGATCACCTT	59.23	676	INTERGENIC			
CaPOPI_II_946	Ca8	7953093	AAGGTGATCGGCAAAATGAC	CAGCCCTTCAGTGTCATGAGT	59.94	609	INTERGENIC			
CaPOPI_II_947	Ca8	7953809	ACCTGCGACACGTGAACATA	GAAAACGAGGCGTAAACGAG	60.18	360	INTERGENIC			
CaPOPI_II_948	Ca8	7954393	CTCGTTTACGCCTCGTTTTC	CCGTAGCATGAGAGTGACCA	59.88	561	INTERGENIC			
CaPOPI_II_949	Ca8	7954496	TTTCGTCGTATAAAGACGGAGAG	CCGTAGCATGAGAGTGACCA	59.80	545	INTERGENIC			
CaPOPI_II_95	Ca1	37433380	AATAAATTAGTGCAGCAAACCAA	GGCAAAGAGAGGAAAGTGGA	57.98	756	INTERGENIC			

INDEL marker IDs	Chromosomes/ unanchored scaffolds	Physical positions (bp)	Forward primers (5'-3')	Reverse primers (5'-3')	Anneling temperature (0C)	Expected amplified product size (bp)	Structural annotation		Functional annotation	
							Sequence components of *kabuli* genome	*Kabuli* gene accession IDs	NCBI-KOG	TFs
CaPOPI_II_950	Ca8	7955526	CATGCAAGAGAAGGGAAAGAA	GTGTGGGAGAGAAAGCATCC	59.44	296	INTERGENIC			
CaPOPI_II_952	Ca8	8240879	GTCGCCGCTACGAGATAAAC	TTTGTGCTGATGGTGATGGT	59.87	729	INTERGENIC			
CaPOPI_II_953	Ca8	8312325	CGGCGATCATTAAGGAGAAA	ATTTTGAACAAGCGGACCTG	60.17	561	INTERGENIC			
CaPOPI_II_954	Ca8	8623323	GGGGTGATTATGGAGCAAGA	GGTCACGTGTGTTCCAAGAA	59.89	963	INTERGENIC			
CaPOPI_II_955	Ca8	8624257	CTTTGATTGGGGTTTTGGAA	TGGTGACAATTATTTTTCCCGT	59.77	664	INTERGENIC			
CaPOPI_II_956	Ca8	8624418	TCCTTTCAATGGACTGGTGA	GTCCCGAGCATGACGTTTAT	59.06	888	INTERGENIC			
CaPOPI_II_957	Ca8	9087628	TTGGACCCCATCTTACTTGG	TGTAGCATCCCATTTTCCACT	59.78	741	INTERGENIC			
CaPOPI_II_959	Ca8	9201647	GTCTCGATCGCAAGTCACAA	GATATATACGGCTGACGGCG	59.99	300	INTERGENIC			
CaPOPI_II_96	Ca1	37489959	ATTGTCCAGTTTCCTCACCG	CGATTGGTAAATCGATTGGG	59.97	594	INTERGENIC			
CaPOPI_II_960	Ca8	9364522	TTGTCGGTGAGATTGTGTGAA	AAAGATTGTGATTTTGGCGG	60.14	802	INTERGENIC			
CaPOPI_II_963	Ca8	10366674	CAAGAAGACGCCGATGAAAT	GTCGAACGTTCTCCGTTTGT	60.21	571	INTERGENIC			
CaPOPI_II_964	Ca8	10590161	AACGGAGGAGGAACAAACAC	TTAGAAGGCCTTGCATACGA	59.04	212	INTERGENIC			
CaPOPI_II_965	Ca8	10612849	AGTGGGAGTTTCGTCCATTG	AGCACATGGAACCAAAGAGG	59.97	299	INTERGENIC			
CaPOPI_II_966	Ca8	10686546	TGCTATCGGTACCAATTTTGC	TCCGTTGTCTCATTCGATCA	59.98	628	INTERGENIC			
CaPOPI_II_967	Ca8	10701972	TCAACGGTTATACGGCAACA	TTTTATCTATGAAAAATGCCTCACA	59.99	373	INTERGENIC			
CaPOPI_II_969	Ca8	11070760	GCTTCTAGCTGGTGCTGCTT	AGGGAATCAATTGTAGGGGG	59.93	900	INTERGENIC			

INDEL marker IDs	Chromosomes/ unanchored scaffolds	Physical positions (bp)	Forward primers (5'-3')	Reverse primers (5'-3')	Anneling temperature (0C)	Expected amplified product size (bp)	Structural annotation		Functional annotation	
							Sequence components of *kabuli* genome	*Kabuli* gene accession IDs	NCBI-KOG	TFs
CaPOPI_II_97	Ca1	37490066	ATTGTCCAGTTTCCTCACCG	TGAACTCTGAGCGTGTGACC	59.97	775	INTERGENIC			
CaPOPI_II_970	Ca8	11113946	CCAGAAAACGAAAACCAAACA	TTTGATTTTGAGGCAGAGCA	60.00	480	INTERGENIC			
CaPOPI_II_971	Ca8	11145831	TCTTTCTGGTTGCAAATTGTTTT	AAATTGAAGCAAACGGAACC	60.03	980	INTERGENIC			
CaPOPI_II_972	Ca8	11146284	TGTGATTGAGGCACTATCAAGC	CCGGGTGAAATGGTTATTTG	60.28	280	INTERGENIC			
CaPOPI_II_973	Ca8	11146309	TGGTTTGCTGGAATTGTGTG	CCGGGTGAAATGGTTATTTG	60.55	167	INTERGENIC			
CaPOPI_II_974	Ca8	11146772	TAACCTGTGCGCTATTCGTG	TGCAAATAATTCCTCATCATTTTT	59.90	329	INTERGENIC			
CaPOPI_II_976	Ca8	11955442	GGCCGATTTACTGCTCACAT	GTTGCCACTTGGCGTAAAAT	60.10	430	INTERGENIC			
CaPOPI_II_977	Ca8	11975713	TATACTCGTGCACTACGCGG	GGATGGAGACAATGATGAGGA	59.92	135	INTERGENIC			
CaPOPI_II_979	Ca8	12048375	TTCCCCCTAAAACAGAACCA	GTTGTTGCTGCCCTTTGTTT	59.40	723	INTERGENIC			
CaPOPI_II_98	Ca1	37917967	TCCACAAGTTATTCGACGAAAC	TTCCATTGATCTACGTTAATGTTTG	59.13	558	INTERGENIC			
CaPOPI_II_980	Ca8	12979016	ATATGTTGGGGGTGATGGAA	GGGGTCGGACATTTACTAGC	59.87	921	INTERGENIC			
CaPOPI_II_981	Ca8	12979079	CGTGCAGTTTAGGGAAGGAT	GGGGTCGGACATTTACTAGC	59.20	873	INTERGENIC			
CaPOPI_II_982	Ca8	12983474	TGCAGATGCACGAATCTCTT	TGCACTAATTTATCGGGACCA	59.55	496	INTERGENIC			
CaPOPI_II_983	Ca8	12983910	TTGGTCCCGATAAATTAGTGC	GCCCAAGTTAAGACAACCCA	58.96	442	INTERGENIC			
CaPOPI_II_984	Ca8	12984097	AGTGTTAACCGGGATCAAACA	GCCCAAGTTAAGACAACCCA	59.35	301	INTERGENIC			
CaPOPI_II_985	Ca8	12990010	TGGCTAATAAATTGTGCCCAT	TCATGTCCCCTCCTTGAATC	59.34	552	INTERGENIC			

INDEL marker IDs	Chromosomes/ unanchored scaffolds	Physical positions (bp)	Forward primers (5'-3')	Reverse primers (5'-3')	Anneling temperature (0C)	Expected amplified product size (bp)	Structural annotation		Functional annotation	
							Sequence components of *kabuli* genome	*Kabuli* gene accession IDs	NCBI-KOG	TFs
CaPOPI_II_986	Ca8	13297997	AAATTGATTGGATGGTGGGA	AAATGCAGAGGCACCACTCT	59.99	442	INTERGENIC			
CaPOPI_II_988	Ca8	14064085	TGTGTGTGTGTGTCCAGAGG	GGTATCCATGGGAGCTTTGA	59.11	804	INTERGENIC			
CaPOPI_II_99	Ca1	37927331	ATTTTGATCGCGGAAGTTTG	TCAATCAACCAGATTTTGCATT	60.07	553	INTERGENIC			
CaPOPI_II_992	Ca8	14113684	AACACGCCTCACACCACATA	CCAATTCACAAAATCGGTCC	60.03	447	INTERGENIC			
CaPOPI_II_993	Ca8	14218237	TCTCCACAAATGCAAGGAAA	AAGCCTGGCCCAACTAATTT	59.25	465	INTERGENIC			
CaPOPI_II_994	Ca8	14647157	TTGCTTGTCCATGACGTAGG	TCTGCAACGAAGATTAAACCAA	59.72	297	INTERGENIC			
CaPOPI_II_995	Ca8	14671445	CCTTCCCGATTTGATCCTTT	TCGACAACTTACATTTCATCATCC	60.26	831	INTERGENIC			
CaPOPI_II_997	Ca8	15162330	TTCGTCGTGTAGGTCCAGTG	GGGAAACCCACCTCTTGATT	59.75	757	INTERGENIC			
CaPOPI_II_999	Ca8	15580121	TCCTCAATTTTGAGCACACG	TGGACACCACACTTCAAATCA	59.84	657	INTERGENIC			
CaPOPI_II_1213	scaffold158	131634	GTGACTGGTGACTGGGGACT	TTATGCCTGCTGTTCTGCAC	60.01	227	INTRAGENIC			
CaPOPI_II_580	Ca5	18683897	ATGGCCGTTCTTAGTTGGTG	GTACAAAGGGCAGGGACGTA	59.99	395	INTRAGENIC			
CaPOPI_II_1004	Ca8	15694311	CCGTCTCTTTCACATGGGTT	AAAGGCTCGGGTCAGTCATA	59.97	438	INTRON	Ca_17876	A	
CaPOPI_II_1013	scaffold10227	6464	GGGAACGTGAAGAACTCAGC	TGCAACAACCACAACCACTT	59.85	518	INTRON	Ca_28086		bHLH
CaPOPI_II_1019	scaffold1047	313122	AAAATTCCACACGTCCGAAG	TCAAAAGATCGCAGTTGTGTG	59.97	354	INTRON	Ca_17199	I	
CaPOPI_II_1052	scaffold1135	14111	TCTGGCACATGAACTTCAGC	CACTCGACAATTGGGAGGTT	59.99	532	INTRON	Ca_26843		SAP
CaPOPI_II_1077	scaffold1202	179607	AATAGGTAGCACGGTGTGGC	TCCCTTGCCTACATTGTTCC	60.02	814	INTRON	Ca_24666		

INDEL marker IDs	Chromosomes/ unanchored scaffolds	Physical positions (bp)	Forward primers (5'-3')	Reverse primers (5'-3')	Anneling temperature (0C)	Expected amplified product size (bp)	Structural annotation		Functional annotation	
							Sequence components of *kabuli* genome	*Kabuli* gene accession IDs	NCBI-KOG	TFs
CaPOPI_II_1080	scaffold1215	41563	CCCTTCATTTCCTTCGCATA	GAAGCAGGCTTCGATGGATA	60.03	415	INTRON	Ca_28164		G2-like
CaPOPI_II_1087	scaffold127	270423	GCCCACATATCTTGTTGCCT	TTCAAGCACATACTGCACCAG	59.96	687	INTRON	Ca_26800		
CaPOPI_II_1151	scaffold1418	21418	GCCATAATTGCTTCTGCAGTTT	TCGCGGTGTGGTAAGTCATA	60.62	235	INTRON	Ca_25261	QR	B3
CaPOPI_II_1152	scaffold1418	38513	CTTTCCCTAAAGCCCAATCC	TTGCTCCCTTTACTCATCTGC	59.90	811	INTRON	Ca_25262	S	
CaPOPI_II_1294	scaffold1866	62792	TTGCTCCTACGTATCTCCCG	TTAAGTTGGTTTGGGCTTGG	60.23	855	INTRON	Ca_26041	R	
CaPOPI_II_131	Ca1	46831943	GTGGTTGGTTTGCAAGAGGT	AACTGGGTGAGAGCCTGAGA	60.01	725	INTRON	Ca_25116	U	
CaPOPI_II_1337	scaffold202	61873	CAGCCCTTGAATTCGATCAT	GCTGTTTGGCTCAACCATTT	60.04	331	INTRON	Ca_27125		
CaPOPI_II_1358	scaffold2170	5002	GCAAATCAAGGTTGCAGTCA	ACACTCTAATGGGGACAGCG	59.85	222	INTRON	Ca_26928	YU	
CaPOPI_II_1359	scaffold2170	5357	CGCTGTCCCCATTAGAGTGT	GTTTGCCAATTAGGGCACAT	60.13	387	INTRON	Ca_26928	YU	
CaPOPI_II_1360	scaffold2170	12258	TGCTTTGTTCAATTGCTTGC	GGAGTCCCGGAAAATTTCAT	60.00	531	INTRON	Ca_26929	O	
CaPOPI_II_1361	scaffold2170	14347	GCTCATTCTTGTCCCCAAAA	GCCTCTGCTAATTGGTCTCG	60.05	583	INTRON	Ca_26929	O	
CaPOPI_II_1377	scaffold227	135497	CTTGGATGTGGTAGCCGTTT	GGTCTTGTGGTGGCTTTTGT	59.99	651	INTRON	Ca_25190		
CaPOPI_II_1378	scaffold227	140947	ACCCTTTTGTTCCACACGAG	TACGGATGTTGACGCAATGT	60.01	511	INTRON	Ca_25190		
CaPOPI_II_139	Ca2	3009059	ATTCGCTTGCCTCTTTGGTA	CCCGAAACTTTCTGTGCATT	59.85	658	INTRON	Ca_10544	G	
CaPOPI_II_1390	scaffold2356	138194	CCTTGAACTGTTGTCTGGCA	CCAACAACATCAAATTCATCG	59.87	687	INTRON	Ca_24344	Q	
CaPOPI_II_141	Ca2	3042033	TGCCAAATCATGCGTTTTTA	CTCTGATGCGAAGGTTGACA	60.07	100	INTRON	Ca_10543		

INDEL marker IDs	Chromosomes/ unanchored scaffolds	Physical positions (bp)	Forward primers (5'-3')	Reverse primers (5'-3')	Anneling temperature (0C)	Expected amplified product size (bp)	Structural annotation		Functional annotation	
							Sequence components of *kabuli* genome	*Kabuli* gene accession IDs	NCBI-KOG	TFs
CaPOPI_II_1442	scaffold2748	27802	TCTCGTTTAGATCGTCCGCT	TGGAATTCTTCTCCAATGCC	59.98	551	INTRON	Ca_28188		
CaPOPI_II_1467	scaffold290	206705	TCCCTCTTTTCAAGTCAACTCA	TCGCTCTAGGCTAATCCAACT	58.96	318	INTRON	Ca_19830	F	
CaPOPI_II_1478	scaffold2950	8204	TGGTCAGAATGGGCTTTACC	TTCAGAAGGGCCAGAAGAAA	59.93	506	INTRON	Ca_27517	L	FAR1
CaPOPI_II_1479	scaffold2950	10165	AGTTGTTTGGATACACCGGC	AGTGCTGTGGCTGGACCTAT	59.86	662	INTRON	Ca_27517	L	FAR1
CaPOPI_II_1480	scaffold2950	10696	TGATGATATCAGGCAACGGA	TGTCCCCACAGGTAGCTTTC	60.03	588	INTRON	Ca_27517	L	FAR1
CaPOPI_II_1512	scaffold3134	15211	CCTGGTTGAGTCTGGATAGCA	GCATTGGCGTATCTTGTTCA	60.26	874	INTRON	Ca_27104	O	C2H2
CaPOPI_II_1516	scaffold3155	24057	TGCATATTTTGAAACGAATGACA	TGCAACCATCGGTTAAAATG	60.36	505	INTRON	Ca_27114	R	NF-YB
CaPOPI_II_1667	scaffold4511	38356	AATGCCCACCCATAATTTCA	CTGGGCAAGGGATCTGAATA	60.02	642	INTRON	Ca_28219		Trihelix
CaPOPI_II_1668	scaffold453	80166	AATTTAAACCGGCCAAAACC	TCACTCATAAATATCCTCATGGACA	60.06	578	INTRON	Ca_21728		MYB_related
CaPOPI_II_1674	scaffold466	169717	CTTTCGTGCCCTTGATTTGT	CGTGGCCTATCGATCCTTTA	60.11	740	INTRON	Ca_25699		
CaPOPI_II_1675	scaffold466	170442	ACCCTTTTGTTCCACACGAG	AAGCTGACTTCGGTTGGCTA	60.01	270	INTRON	Ca_25699		
CaPOPI_II_1693	scaffold4777	118728	AACGGTTTGGTTTGATCTGC	ACGTCGTCAAACAGATGCAC	59.98	231	INTRON	Ca_26711	R	
CaPOPI_II_17	Ca1	5803481	GCTCATGAGTTCCCTACCCA	GTCAGGGGCTCGTGTATGAT	60.07	560	INTRON	Ca_00671	O	FAR1
CaPOPI_II_1734	scaffold530	10667	ACCAAATTTGTCGAACCAGC	TTGAGTGTGAGTGTCCAGGG	59.98	342	INTRON	Ca_27188	J	
CaPOPI_II_1735	scaffold531	11911	ATTCCATTTGGCATGCTTTC	TTTGGGTACAAAATCAGAAAATTG	59.91	892	INTRON	Ca_22388		
CaPOPI_II_1749	scaffold553	325514	ATCGGAAGTTGGACAGGTTG	TGGCAAATCAGCAGAGACAC	59.97	317	INTRON	Ca_19934	L	

INDEL marker IDs	Chromosomes/ unanchored scaffolds	Physical positions (bp)	Forward primers (5'-3')	Reverse primers (5'-3')	Anneling temperature (0C)	Expected amplified product size (bp)	Structural annotation		Functional annotation	
							Sequence components of *kabuli* genome	*Kabuli* gene accession IDs	NCBI-KOG	TFs
CaPOPI_II_1756	scaffold571	18167	CCTCACGGTTTCAATTGCTT	CTTAATGCACAGCCTACCCC	60.11	670	INTRON	Ca_27520	T	NAC
CaPOPI_II_18	Ca1	5804081	AATAACACGGGCACAAGAGG	AGGTCCACCAGGTTTGTCAG	59.99	457	INTRON	Ca_00671	O	FAR1
CaPOPI_II_1868	scaffold6903	5314	GCACACCACAACAAAATGGA	GCATCCTCTGATTGTTTGGG	60.41	458	INTRON	Ca_28223		
CaPOPI_II_1880	scaffold716	135403	TGATCATATAAAAACAAATGCTCACA	TGTGCAAGATTCCTGTTCCA	59.82	410	INTRON	Ca_24965		bHLH
CaPOPI_II_1902	scaffold731	374028	TCCTTGGGGTGATCTCGTAG	ATGGACGGTAGTTGGAGTCG	60.07	222	INTRON	Ca_23820		
CaPOPI_II_1903	scaffold731	390482	TATCATAGGCCGTAGGCCAG	TTATTCGTCCAATTCCCTCG	60.07	482	INTRON	Ca_23820		
CaPOPI_II_1904	scaffold731	392660	GAACGACGGAACCTGTGAAT	AGGAAGCCCAAGTTCCAAAT	59.97	665	INTRON	Ca_23820		
CaPOPI_II_1953	scaffold811	159571	TTGCAACATGCTATTTCCCA	TGGGTGCTATTTGCAATCAA	60.07	613	INTRON	Ca_27462		
CaPOPI_II_1998	scaffold9	34748	GGACAAACTACCCCTCCTTTTC	TCTTCCCCCATAAACCAACA	60.22	317	INTRON	Ca_27384		WOX
CaPOPI_II_2018	scaffold93	338023	AAACTTGCAAGCCATTCCAG	TTTGAGAAATATGCTGGGGC	60.25	876	INTRON	Ca_23073	L	GRAS
CaPOPI_II_2039	scaffold959	57749	AAGGCGTGCATATCCCATAC	TGTTCGTCAAAATGTCCCAA	59.81	553	INTRON	Ca_27234		Trihelix
CaPOPI_II_212	Ca2	30622722	CTTGGTCAGAATCACGGAGC	CACGTTGACCCTTTTTGTCA	60.80	380	INTRON	Ca_12490	R	
CaPOPI_II_213	Ca2	30665443	TCATGGTGGACCGTAATCTG	CTACTGCCTTGGATTCCTTTT	59.37	403	INTRON	Ca_12489	QI	bHLH
CaPOPI_II_217	Ca2	31882952	TTTCTGGTGGGATCAGCTCT	GACATCCTGCGCCAGTTATT	59.80	591	INTRON	Ca_17830	D	LBD
CaPOPI_II_218	Ca2	31883010	TTTCTGGTGGGATCAGCTCT	GACATCCTGCGCCAGTTATT	59.80	591	INTRON	Ca_17830	D	LBD
CaPOPI_II_219	Ca2	32127033	TGCAAGTTGTGATGGGTTGT	TGTCTGTTCTGCACGGAGTC	60.01	650	INTRON	Ca_17812	O	

INDEL marker IDs	Chromosomes/ unanchored scaffolds	Physical positions (bp)	Forward primers (5'-3')	Reverse primers (5'-3')	Anneling temperature (0C)	Expected amplified product size (bp)	Structural annotation		Functional annotation	
							Sequence components of *kabuli* genome	*Kabuli* gene accession IDs	NCBI-KOG	TFs
CaPOPI_II_272	Ca3	6386934	TGTACGAGAAGGTCGTG TGC	GCCCAACAGCAAGCTAT TTC	59.91	574	INTRON	Ca_24834		Trihelix
CaPOPI_II_289	Ca3	14653349	AGGAGCTTCTGCCATTG AAA	TAAAAGGAGCAAAGGCC AGA	59.96	408	INTRON	Ca_22505	O	
CaPOPI_II_321	Ca3	21635589	AAGCATGCCAAACCAAA AAC	TGAACATCAAAGATCTG CGG	59.98	521	INTRON	Ca_09516		
CaPOPI_II_328	Ca3	26071877	TGCTCCAATCACGTTGA CAT	AGACGCTGCAACATCAA AGC	60.12	397	INTRON	Ca_08292		MYB_relate d
CaPOPI_II_329	Ca3	26114282	TTTATCCTGGGGACTTC GTG	ATACACCGATGGAGGG CTTT	59.93	838	INTRON	Ca_08292		MYB_relate d
CaPOPI_II_330	Ca3	26115029	TGGCATGGTACTCAACC AGA	TTCACATGAATGATTGT TGGGT	60.11	545	INTRON	Ca_08292		MYB_relate d
CaPOPI_II_338	Ca3	28994933	CAAATTAAGCAGAGCCC GTC	TTTTGGACAATGTCAAC CCA	59.85	165	INTRON	Ca_07207	KTDL	
CaPOPI_II_339	Ca3	28995118	CAAATTAAGCAGAGCCC GTC	CGGAATTTGAACAAAAC GCT	59.85	769	INTRON	Ca_07207	KTDL	
CaPOPI_II_340	Ca3	28999636	TCTGAATTTCTGTACCG CCC	GGGATAGGGCATGTGT GAGT	60.07	536	INTRON	Ca_07207	KTDL	
CaPOPI_II_354	Ca4	1677374	TCCAAACTAGTCCGACG TGA	AGCTGCTGGAGGAGTT GGTA	59.29	450	INTRON	Ca_07795		
CaPOPI_II_356	Ca4	2032547	TGCTGAGTTCGAGGAAA GGT	CACATCTCTTGTTCGCT CCA	59.99	517	INTRON	Ca_07822	D	
CaPOPI_II_357	Ca4	2035282	CGCGCCAGAAGTATGT GATA	TGGGCAGCTTAGACTG GACT	59.86	275	INTRON	Ca_07822	D	
CaPOPI_II_36	Ca1	21746056	CTATTGCCCCCTCCTCC TAC	TACCGATACCTCCAAGT GCC	59.92	585	INTRON	Ca_22116	S	
CaPOPI_II_363	Ca4	4891737	TGCCTTGTATCGTTCCA TCA	TTCCTGCTTTTTCTTTCC GA	60.07	540	INTRON	Ca_03769	Z	NAC
CaPOPI_II_371	Ca4	8381452	CAATCCGTTAAGGACCT GGA	GATGATTCCCTTGCTGG AAA	59.93	388	INTRON	Ca_08327	KL	
CaPOPI_II_372	Ca4	8441953	AGCAGGAGAGCTGAGA TTGG	TACTGTGTATCGGGGCA ACA	59.70	731	INTRON	Ca_08335	R	

INDEL marker IDs	Chromosomes/ unanchored scaffolds	Physical positions (bp)	Forward primers (5'-3')	Reverse primers (5'-3')	Anneling temperature (0C)	Expected amplified product size (bp)	Structural annotation		Functional annotation	
							Sequence components of *kabuli* genome	*Kabuli* gene accession IDs	NCBI-KOG	TFs
CaPOPI_II_373	Ca4	8497257	AGCGTGAGAGGAACCTGTGT	GACTGGCAACAGCAATTTGA	59.91	564	INTRON	Ca_08341		CPP
CaPOPI_II_374	Ca4	8698891	AAGCTGGCCACTGTAAGCAT	TCACAATCTCGACAACCCAA	59.90	246	INTRON	Ca_08365	G	
CaPOPI_II_394	Ca4	17081756	TACCGGAAGATTCGGAAATG	CTGCAGGTAAGCATCCCAAT	59.89	694	INTRON	Ca_05415		
CaPOPI_II_397	Ca4	17286686	TGCATTTCATGCCTTTGCTA	TGATTAGCCCGTATTTTGGG	60.36	648	INTRON	Ca_05393	R	
CaPOPI_II_399	Ca4	17318086	CTCAAAAGTGGGATAGGGCA	ACAGCGTGAAACAAGATCCC	60.07	429	INTRON	Ca_05390	M	
CaPOPI_II_400	Ca4	17431572	TCTGTCATTCCTGCCATTTTT	TCGCAATGTAGCTCCCTCTT	59.56	858	INTRON	Ca_05384	K	
CaPOPI_II_405	Ca4	18121565	GCTGCACCATGAGATCTTGA	ATGGTGCTGGAAGTTGCTTT	59.95	440	INTRON	Ca_18652		
CaPOPI_II_451	Ca4	27376069	ATCCTCTCCATTCCTTGCCT	TTGCTTTCACAGTCCAATGC	60.04	304	INTRON	Ca_21323		
CaPOPI_II_469	Ca4	30537523	TTTGATTGCCATTGTATCCG	CAAAATGGAAATGCAAGAACAA	59.38	803	INTRON	Ca_14204		NAC
CaPOPI_II_474	Ca4	31303190	GAGGTCCCGTCCAACCTAAC	TCAGCACCGAGAGTGTCAAC	60.75	488	INTRON	Ca_14231		bHLH
CaPOPI_II_478	Ca4	31649942	CGTGCCCTGCAGGTATTTAT	ATTGACCGGTGGAAGACATT	59.98	454	INTRON	Ca_15442	U	EIL
CaPOPI_II_512	Ca4	42120439	CACAAGCATTGGAGTCCCTT	AACTCGTGGGTTGGATTTTG	60.11	495	INTRON	Ca_10910		C2H2
CaPOPI_II_520	Ca4	47982672	CCAAACTCCAATCAAATGGG	CAAATGAGAATTTTCTGGGCA	60.16	483	INTRON	Ca_10790	U	ARF
CaPOPI_II_522	Ca4	48576925	ATCTCACCCCAAGACAACCA	TCATTCCAGAAGGAGATGGG	60.36	211	INTRON	Ca_23007	P	
CaPOPI_II_531	Ca5	291054	TGCTTCATATGCTTGACCACA	TGGTGCTTCTTTGGCTCTTT	60.27	476	INTRON	Ca_18185	R	B3
CaPOPI_II_54	Ca1	29636835	TCACAAATCTCAACCCTCCC	ATCAGTTGGAGCCTGGTTGT	59.90	497	INTRON	Ca_24382	AR	

INDEL marker IDs	Chromosomes/ unanchored scaffolds	Physical positions (bp)	Forward primers (5'-3')	Reverse primers (5'-3')	Anneling temperature (0C)	Expected amplified product size (bp)	Structural annotation		Functional annotation	
							Sequence components of *kabuli* genome	*Kabuli* gene accession IDs	NCBI-KOG	TFs
CaPOPI_II_55	Ca1	29637072	ACAACCAGGCTCCAACTGAT	ATGCTGCCAGCCTACAAAAT	59.58	503	INTRON	Ca_24382	AR	
CaPOPI_II_593	Ca5	25600693	CGTGCACTTTCACGACAAAT	TTGCTGTACTTGGTTGCTCG	59.76	801	INTRON	Ca_09030	E	
CaPOPI_II_60	Ca1	29848026	TCTTTTCATCAGGCCCAATC	GGAAAGTGAGGGAACCAATG	60.01	228	INTRON	Ca_24388		
CaPOPI_II_617	Ca5	38016624	ATCCTCGAGAAGGAAGGAGC	CCTTTTCCCTCCAATGTCAA	59.92	276	INTRON	Ca_01419	A	
CaPOPI_II_620	Ca5	39557215	GCAAAAAGGGGCACAATAAA	AATGTCAATCCTCCATTGCC	59.94	357	INTRON	Ca_07489	TBLD	
CaPOPI_II_622	Ca5	41678103	CAGTGGGTCCTCTTCCACAT	CCCGAACTCCTGTTACTCCA	59.96	551	INTRON	Ca_19215	J	C3H
CaPOPI_II_634	Ca6	1491943	TGGTTTTCGTACCGGACTCT	CAATGTCATCAAATAGCGGC	59.59	772	INTRON	Ca_27033	H	
CaPOPI_II_635	Ca6	1492728	TTGTTGTTTCAACGGTGCAT	ATGGTTGGGGGATAAAAAGG	60.01	651	INTRON	Ca_27033	H	
CaPOPI_II_645	Ca6	1978360	CGTGGAGCATTCCGAGTATT	GGAGTTAGGGCGTGTGATGT	60.10	575	INTRON	Ca_10322		
CaPOPI_II_648	Ca6	2674985	GAAACTTCGTGCCTTTTTGC	ACGATAGTAGATGTGGCGGC	59.87	152	INTRON	Ca_10401	V	
CaPOPI_II_667	Ca6	8796967	GCCAAACAATAGTTGAAACCAA	AACGTTACGCATGAACCTCC	59.03	588	INTRON	Ca_16754	E	
CaPOPI_II_68	Ca1	31592114	TCAGCTGCAGTTCCAACATC	GCACAAGTCCTTGAGGAAGC	59.99	375	INTRON	Ca_21868	F	
CaPOPI_II_683	Ca6	16547931	TTCCTTCCTGCTCGTGCTAT	TTCTTTGGCTGAAATGGTCC	59.98	640	INTRON	Ca_06284	A	
CaPOPI_II_691	Ca6	23042823	CAACGATCTGCACCACACAT	GGTGCAGCAAGTGAGCAATA	60.60	665	INTRON	Ca_11143		ERF
CaPOPI_II_692	Ca6	23042981	CAACGATCTGCACCACACAT	GGTGCAGCAAGTGAGCAATA	60.60	665	INTRON	Ca_11143		ERF
CaPOPI_II_694	Ca6	23642077	GATTACCATGGTTGGGATCG	CAATTTCGGTGCTTGTTCCT	60.01	137	INTRON	Ca_11091	R	

INDEL marker IDs	Chromosomes/ unanchored scaffolds	Physical positions (bp)	Forward primers (5'-3')	Reverse primers (5'-3')	Anneling temperature (0C)	Expected amplified product size (bp)	Structural annotation		Functional annotation	
							Sequence components of *kabuli* genome	*Kabuli* gene accession IDs	NCBI-KOG	TFs
CaPOPI_II_72	Ca1	33336930	CAATGGCAACAAGGTCAATG	TCACATCTGTTGGTAACGGG	59.96	856	INTRON	Ca_19441		MIKC
CaPOPI_II_738	Ca6	35719325	TGCATCGTTTTTCATCTTTCA	GCGGCTAATTTCAACCAAAT	59.29	630	INTRON	Ca_15773		
CaPOPI_II_77	Ca1	35281570	ATGACACGTCATTCGTTTCG	GGTTCGAGACATCCATAATTCA	59.57	733	INTRON	Ca_26099	QI	bHLH
CaPOPI_II_773	Ca6	53305336	CAAGATTTCGTGGTGCAAGA	TTGAGGACAAAAGCTGCTGA	59.84	660	INTRON	Ca_22925	G	
CaPOPI_II_78	Ca1	35281940	AAACGTGATCTATACAGTTTCCAAAA	TTCACATCTGGAAACAACGC	59.44	529	INTRON	Ca_26099	QI	bHLH
CaPOPI_II_806	Ca7	2168224	TTCTGAGATTGGCAAAAGGG	CGCGATCGTTCACTGATATG	60.18	820	INTRON	Ca_03253	P	
CaPOPI_II_807	Ca7	2169903	CAGGGCCTTACTGCTACAGG	CAACGGAAAACATCGTTCAA	59.89	397	INTRON	Ca_03253	P	
CaPOPI_II_854	Ca7	21745626	ATAGGGCCCATTTTACAGCG	CCAGTTGCCCTGTCCATACT	61.17	455	INTRON	Ca_14522	O	MYB
CaPOPI_II_886	Ca7	30208302	TTGGACGATGTGGATTGAAA	CCATCTCAGCATGTGTTTGG	59.90	642	INTRON	Ca_11706		
CaPOPI_II_887	Ca7	30208770	CCAAACACATGCTGAGATGG	CTTCCTTTGCACGACCGTAT	60.11	707	INTRON	Ca_11706		
CaPOPI_II_895	Ca7	32601479	CAGCAACAGCGCAAAGATTA	GGATGACAAAGAAAGGCCAA	60.16	543	INTRON	Ca_10025	TU	
CaPOPI_II_905	Ca7	34187425	AGGTCTGGATTCGTGGTTTG	ACATCCGTGCAAGGAAAAAG	59.97	646	INTRON	Ca_21004	S	
CaPOPI_II_925	Ca7	42757617	TTTGCTCTGAAAGCCCAAAT	AGTTCCTGCAACTCCGAAGA	59.82	527	INTRON	Ca_20201		
CaPOPI_II_936	Ca8	6492029	TCTGTTTTGCCCAACCTTTC	ATCTCAACGATCCAACGGTC	60.09	455	INTRON	Ca_10739	D	
CaPOPI_II_939	Ca8	7777713	TCCAGTGGCACCATAACAAA	CTCCCAAGAACCCTTCTTCC	59.96	744	INTRON	Ca_10607	Z	MYB_related
CaPOPI_II_962	Ca8	9960024	CCATTGGGATGTTTGCTTTT	GCTCATGTGGTGGTGTTTTG	59.80	280	INTRON	Ca_18395		

INDEL marker IDs	Chromosomes/ unanchored scaffolds	Physical positions (bp)	Forward primers (5'-3')	Reverse primers (5'-3')	Anneling temperature (0C)	Expected amplified product size (bp)	Structural annotation		Functional annotation	
							Sequence components of *kabuli* genome	*Kabuli* gene accession IDs	NCBI-KOG	TFs
CaPOPI_II_978	Ca8	12016131	CAACTCTCGTCGCAACTCAA	ATGCAACTTGGGAATTGGAG	60.18	362	INTRON	Ca_23846	T	
CaPOPI_II_996	Ca8	14751128	TGATGCGTTGATAGTGTTGG	GGCACTTGGTTGGTCTTCAT	58.13	619	INTRON	Ca_12994		
CaPOPI_II_1050	scaffold1120	11138	TTCATCCAAAGAATCTGGGC	TGCAAGAGGAGTGCCTTTCT	60.01	486	URR	Ca_27900		
CaPOPI_II_1169	scaffold1452	29738	GAAGCCTTTTCAGCAGGTTG	TAGTGTGCCACCACTCCAAA	59.99	648	URR	Ca_27551	R	ZF-HD
CaPOPI_II_1276	scaffold1810	98576	TGACGATACGAAACGATGGA	AGCGATGCATGTCTCAAGTG	60.07	430	URR	Ca_26004		
CaPOPI_II_1325	scaffold2010	6749	TTCCCTTTCGTTGTGTATCG	GGGCACCGATGTTACATAAA	58.62	857	URR	Ca_27976		
CaPOPI_II_1326	scaffold2010	7925	AAAGTGCCTTCTTCTTGGCA	ATGTTTTCTTTTGGGTTGCG	59.99	564	URR	Ca_27976		
CaPOPI_II_1338	scaffold202	103994	AAATTGCTTATTTAGTAGTGTCAATCG	AACTCAGCAAAACCCAAAACA	58.49	409	URR	Ca_27127	A	
CaPOPI_II_1339	scaffold202	104373	TGTTTTGGGTTTTGCTGAGTT	TGGATGGATTGGATGAATGA	59.64	180	URR	Ca_27127	A	
CaPOPI_II_1393	scaffold2368	59799	CTCCAAATCCCAATCTCCAA	CTCACTGTCAAGGTGCCGTA	59.86	725	URR	Ca_26721		MYB
CaPOPI_II_1497	scaffold3019	20892	TGCATGAGTTGTTAAGGCGA	TATGCAAAACGGGACTTCCT	60.40	582	URR	Ca_28069		
CaPOPI_II_1530	scaffold3258	34164	GGGTTTGCTTACCGAACTCA	TACGGAAAATTACCCACGGA	60.11	375	URR	Ca_27898		
CaPOPI_II_1669	scaffold453	497792	GCCGTGATTTGGAAAAAGAA	TGTCAGTTTTGAGCCCAATG	60.05	479	URR	Ca_21748		
CaPOPI_II_1670	scaffold453	498751	GCCCCTCAAAGATAACCCAT	GACCATGATTTCTTACTAGCCGTT	60.15	838	URR	Ca_21748		
CaPOPI_II_1889	scaffold724	126741	TCGCACTTCAATATGCTTCG	AAAGGCCATGCAAGAAGAGA	59.98	851	URR	Ca_25052	Q	NAC
CaPOPI_II_1921	scaffold7650	9735	TATGGCATTGTGGTGCACTT	GGATTAAAATTGGAATTGTTCAAGA	60.00	482	URR	Ca_27809	O	

INDEL marker IDs	Chromosomes/ unanchored scaffolds	Physical positions (bp)	Forward primers (5'-3')	Reverse primers (5'-3')	Anneling temperature (0C)	Expected amplified product size (bp)	Structural annotation		Functional annotation	
							Sequence components of *kabuli* genome	*Kabuli* gene accession IDs	NCBI-KOG	TFs
CaPOPI_II_1922	scaffold7650	9922	CCGCAATTATATCTGCGACA	AGTTCAAAAATACGCGGGTG	59.69	857	URR	Ca_27809	O	
CaPOPI_II_1945	scaffold7923	25395	AAAATATGCCATTCGATCAAGA	AAATGGCTTGGTTATGGCTG	58.59	200	URR	Ca_27507	QI	bHLH
