# Supplementary material for: A High-Resolution InDel (Insertion–Deletion) Markers-Anchored Consensus Genetic Map Identifies Major QTLs Governing Pod Number and Seed Yield in Chickpea
Source: Front Plant Sci. 2016 Sep 16;7:1362. doi: 10.3389/fpls.2016.01362 (PMC5025440; doi:10.3389/fpls.2016.01362)
Supplement: Supplementary file 8 [file Table8.PDF]

Table S8. Structural and functional annotation of three major pod number and seed yield robust QTL regions mapped on an InDel markers-based consensus iner-specific genetic linkage map of chickpea

Major pod number and seed yield QTLs delineated	INDEL marker IDs	Chromosomes/ unanchored scaffolds	Physical positions (bp)	Forward primers (5'-3')	Reverse primers (5'-3')	Anneling temperature (0C)	Expected amplified product size (bp)	Structural annotation		Functional annotation		Putative Function
								Sequence components of kabuli	*Kabuli* gene accession IDs	NCBI-KOG	TFs	
CaqcPN2.1	CaPOPI_II_203	Ca2	29829940	CTTTTCGTCGGAGTTTGTCC	TCGTCCTCTGGTTTTCTCGT	59.71	476	INTERGENIC	NA	NA	NA	**NA**
	CaPOPI_II_204	Ca2	30095996	CATTCCCGCTACATTTTGGT	GGAGAGAGGCCAATTTTTCA	59.82	226	INTERGENIC	NA	NA	NA	NA
	CaPOPI_II_205	Ca2	30096276	CATTCCCGCTACATTTTGGT	GAAGCTGCTTGTGTGCCATA	59.82	876	INTERGENIC	NA	NA	NA	NA
	CaPOPI_II_206	Ca2	30341034	TGTCTTATCCTGAACTCAAACTCG	ATTTGGTCAACCACCCAAAA	59.82	537	INTERGENIC	NA	NA	NA	NA
	CaPOPI_II_207	Ca2	30369374	TGTACTTCCTCCGATTCCAAA	TGAATGACGACGACGGTAAA	59.56	632	DRR	Ca_12515	Q	NA	Multicopper oxidase
	CaPOPI_II_208	Ca2	30395203	CCCAACAACAACAAAAGCCT	AAACCAGCATGACAAAAGCA	60.01	289	INTERGENIC	NA	NA	NA	NA
	CaPOPI_II_209	Ca2	30447611	CTTTTGTCTTCTCGGCCTTG	CGTGCTCATCTCCATTGTTG	59.99	891	INTERGENIC	NA	NA	NA	NA
	CaPOPI_II_210	Ca2	30449164	TGTTGCAAAGAGAAAGAACTTCA	TGACGGTGGCAATCGATATT	59.19	557	DRR	Ca_12507	NA	NA	NA
	CaPOPI_II_211	Ca2	30553379	TTTTTATGAGGCGTGGAAGG	CCATGAACACGCCAAACATA	60.07	874	INTERGENIC	NA	NA	NA	NA
	CaPOPI_II_212	Ca2	30622722	CTTGGTCAGAATCACGGAGC	CACGTTGACCCTTTTTGTCA	60.80	380	INTRON	Ca_12490	R	NA	Yippee-like protein
	CaPOPI_II_213	Ca2	30665443	TCATGGTGGACCGTAATCTG	CTACTGCCTTGGATTCCTTTT	59.37	403	INTRON	Ca_12489	QI	bHLH	Cytochrome P450
	CaPOPI_II_214	Ca2	30676782	GCTCCAGCAAAAATAGAAATGG	TCATCAATCAAACTACTTACGCAGA	60.10	504	INTERGENIC	NA	NA	NA	NA
	CaPOPI_II_215	Ca2	30890128	GATGATGTAAATGGGAGCCAA	CATTTGTTGAATGTTGCCCA	59.78	697	INTERGENIC	NA	NA	NA	NA
	CaPOPI_II_216	Ca2	31240228	CTGCCCCCACTATTTCTCAA	GAAACACTTGGCATGTTAAGGA	60.07	479	INTERGENIC	NA	NA	NA	NA

Major pod number and seed yield QTLs delineated	INDEL marker IDs	Chromosomes/ unanchored scaffolds	Physical positions (bp)	Forward primers (5'-3')	Reverse primers (5'-3')	Anneling temperature (0C)	Expected amplified product size (bp)	Structural annotation		Functional annotation		Putative Function
								Sequence components of *kabuli*	*Kabuli* gene accession IDs	NCBI-KOG	TFs	
	CaPOPI_II_217	Ca2	31882952	TTTCTGGTGGGATCAGCTCT	GACATCCTGCGCCAGTTATT	59.80	591	INTRON	Ca_17830	D	LBD	RZZ complex, subunit Zw10
	CaPOPI_II_218	Ca2	31883010	TTTCTGGTGGGATCAGCTCT	GACATCCTGCGCCAGTTATT	59.80	591	INTRON	Ca_17830	D	LBD	RZZ complex, subunit Zw10
	CaPOPI_II_219	Ca2	32127033	TGCAAGTTGTGATGGGTTGT	TGTCTGTTCTGCACGGAGTC	60.01	650	INTRON	Ca_17812	O		Ubiquitin
	CaPOPI_II_220	Ca2	32173774	ATGTTTTGATTGGGGCTTTG	TAAATCAGCCCTTTTGGTGG	59.80	631	DRR	Ca_17806		WRKY	Glycoside hydrolase, family 9
	CaPOPI_II_221	Ca2	32209131	GCAAAGATAGACACCACCCC	AACCATTCCCATGGAACAAA	59.41	577	INTERGENIC	NA	NA	NA	NA
	CaPOPI_II_222	Ca2	32209334	TCCATGGGAATGGTTTTGAT	TTGAAGAGGAAGTTTTGGCA	59.99	342	INTERGENIC	NA	NA	NA	NA
	CaPOPI_II_223	Ca2	32209671	TCCATGGGAATGGTTTTGAT	TTTTAGTGTGCACCAATGTCAA	59.99	899	INTERGENIC	NA	NA	NA	NA
	CaPOPI_II_224	Ca2	32210099	TCAGCTTCAATTATCTCCAACAA	TCGGCGACAAAAATCACATA	58.89	644	INTERGENIC	NA	NA	NA	NA
	CaPOPI_II_225	Ca2	32213171	CAACATGACAAGGACTTTCCAA	TTACCGGTATCGCAAGGAAC	60.01	529	INTERGENIC	NA	NA	NA	NA
	CaPOPI_II_226	Ca2	32213424	AGGGATAGATAACGGCACGA	AAGGCCCTGCTAGAATGATG	59.55	504	INTERGENIC	NA	NA	NA	NA
	CaPOPI_II_227	Ca2	32379585	TGTGCGAGTTTATTGCGGTA	CATTTGGTTCAAAATGTCATGG	60.27	624	INTERGENIC	NA	NA	NA	NA
	CaPOPI_II_228	Ca2	32393117	TGCCCATCCTTATGCTCTTC	ATCACGTGCAACCACAAGTC	60.18	626	INTERGENIC	NA	NA	NA	NA
	CaPOPI_II_229	Ca2	32393187	TGCCCATCCTTATGCTCTTC	ATCACGTGCAACCACAAGTC	60.18	626	INTERGENIC	NA	NA	NA	NA
	CaPOPI_II_230	Ca2	32393441	TCGCACTTTTTCGTGTAGGA	GGGCTTGTTTGACGCATTAT	59.46	588	INTERGENIC	NA	NA	NA	NA
	CaPOPI_II_231	Ca2	32393633	GACTTGTGGTTGCACGTGAT	GGGCTTGTTTGACGCATTAT	59.60	445	INTERGENIC	NA	NA	NA	NA

Major pod number and seed yield QTLs delineated	INDEL marker IDs	Chromosomes/ unanchored scaffolds	Physical positions (bp)	Forward primers (5'-3')	Reverse primers (5'-3')	Anneling temperature (0C)	Expected amplified product size (bp)	Structural annotation		Functional annotation		Putative Function
								Sequence components of *kabuli*	*Kabuli* gene accession IDs	NCBI-KOG	TFs	
CaqcPN4.1	CaPOPI_II_385	Ca4	13617448	GGGCCTTTTCTTCAGCTTTT	TGCATGTTATCCGCAATCTC	59.84	713	DRR	Ca_04579	NA	NA	Protein of unknown function DUF1677
	CaPOPI_II_386	Ca4	13908536	CTCCTCCGATGTCTGGAAAA	CGCGGTTGCTTCAATTCTAT	60.19	232	INTERGENIC	NA	NA	NA	NA
	CaPOPI_II_387	Ca4	13909254	CATCACGATTTGACGAGTTGA	AAAAACATGACTCCAAACACCA	59.71	636	INTERGENIC	NA	NA	NA	NA
	CaPOPI_II_388	Ca4	14397300	TAAATGCATGCTCCCCTGAT	AACACATCCGGGATCCATTA	60.44	273	INTERGENIC	NA	NA	NA	NA
CaqcPN4.2	CaPOPI_II_480	Ca4	31859597	GTTGGAGCCCCCACTTTATT	TGCAAGCAGCATCAGAGAAC	60.19	793	INTERGENIC	NA	NA	NA	NA
	CaPOPI_II_481	Ca4	32179682	TTGCATACCTCATACCATCCC	TTGATGCATTACCTCCCACA	59.66	731	INTERGENIC	NA	NA	NA	NA
	CaPOPI_II_482	Ca4	32227293	TCTTCACAACCCCTTGAACC	TTCTTCCAAACGAGCCTCTC	59.94	868	INTERGENIC	NA	NA	NA	NA
	CaPOPI_II_483	Ca4	32264980	GCACTTACCCTTCAATTGGG	AGGAGAGGTCCATTTCTCAGG	59.43	262	INTERGENIC	NA	NA	NA	NA
	CaPOPI_II_484	Ca4	32335179	AAAGTCCGTTTGTGAATCCG	TGGGCATTCAAATCAAACAA	59.97	507	INTERGENIC	NA	NA	NA	NA
	CaPOPI_II_485	Ca4	32531473	TAAAATGGATGCATCGAGCC	ACAAATCAACCAGTGTCGCA	60.94	662	INTERGENIC	NA	NA	NA	NA
	CaPOPI_II_486	Ca4	32562355	AAATGGAGGAGAAGGAGGGA	CGGTGAATCCTTTACGGAGA	60.01	286	INTERGENIC	NA	NA	NA	NA
	CaPOPI_II_487	Ca4	32886390	AATCATCCCATGTTGCCATT	CGAGATTTCACACCAAGGGT	60.02	519	INTERGENIC	NA	NA	NA	NA
	CaPOPI_II_488	Ca4	33308913	TCATTTTCCCCTCGTTTCTG	TTGGCCAAAACCTTTTGATG	60.04	183	DRR	Ca_24021	R	C2H2	Zinc finger, C2H2-type
	CaPOPI_II_489	Ca4	33346959	TTTTCGGTTTGTTCAAAGGC	GGAAAACATTGGGAAACCCT	60.09	766	INTERGENIC	NA	NA	NA	NA
	CaPOPI_II_490	Ca4	33615467	GGTCCTTGTTGAGTCCAACG	CTCAAAAACCTGAGCATGGC	60.55	646	INTERGENIC	NA	NA	NA	NA

Major pod number and seed yield QTLs delineated	INDEL marker IDs	Chromosomes/ unanchored scaffolds	Physical positions (bp)	Forward primers (5'-3')	Reverse primers (5'-3')	Anneling temperature (0C)	Expected amplified product size (bp)	Structural annotation		Functional annotation		Putative Function
								Sequence components of *kabuli*	*Kabuli* gene accession IDs	NCBI-KOG	TFs	
	CaPOPI_II_491	Ca4	33633372	ACGAGGAAACATATCGTCCG	ATGTCCACACATTGGGTGTC	59.96	726	INTERGENIC	NA	NA	NA	NA
	CaPOPI_II_492	Ca4	33714267	GAACACGTTGTTAGACCGGC	TGACAAAATGGGGAGAAAGG	60.56	713	INTERGENIC	NA	NA	NA	NA
CaqcSYP2.1	CaPOPI_II_203	Ca2	29829940	CTTTTCGTCGGAGTTTGTCC	TCGTCCTCTGGTTTTCTCGT	59.71	476	INTERGENIC				
	CaPOPI_II_204	Ca2	30095996	CATTCCCGCTACATTTTGGT	GGAGAGAGGCCAATTTTTCA	59.82	226	INTERGENIC				
	CaPOPI_II_205	Ca2	30096276	CATTCCCGCTACATTTTGGT	GAAGCTGCTTGTGTGCCATA	59.82	876	INTERGENIC				
	CaPOPI_II_206	Ca2	30341034	TGTCTTATCCTGAACTCAAACTCG	ATTTGGTCAACCACCCAAAA	59.82	537	INTERGENIC				
	CaPOPI_II_207	Ca2	30369374	TGTACTTCCTCCGATTCCAAA	TGAATGACGACGACGGTAAA	59.56	632	DRR	Ca_12515	Q		Multicopper oxidase
	CaPOPI_II_208	Ca2	30395203	CCCAACAACAACAAAAGCCT	AAACCAGCATGACAAAAGCA	60.01	289	INTERGENIC				
	CaPOPI_II_209	Ca2	30447611	CTTTTGTCTTCTCGGCCTTG	CGTGCTCATCTCCATTGTTG	59.99	891	INTERGENIC				
	CaPOPI_II_210	Ca2	30449164	TGTTGCAAAGAGAAAGAACTTCA	TGACGGTGGCAATCGATATT	59.19	557	DRR	Ca_12507			
	CaPOPI_II_211	Ca2	30553379	TTTTTATGAGGCGTGGAAGG	CCATGAACACGCCAAACATA	60.07	874	INTERGENIC				
	CaPOPI_II_212	Ca2	30622722	CTTGGTCAGAATCACGGAGC	CACGTTGACCCTTTTTGTCA	60.80	380	INTRON	Ca_12490	R		Yippee-like protein
	CaPOPI_II_213	Ca2	30665443	TCATGGTGGACCGTAATCTG	CTACTGCCTTGGATTCCTTTT	59.37	403	INTRON	Ca_12489	QI	bHLH	Helix-loop-helix DNA-binding domain
	CaPOPI_II_214	Ca2	30676782	GCTCCAGCAAAAATAGAAATGG	TCATCAATCAAACTACTTACGCAGA	60.10	504	INTERGENIC				
	CaPOPI_II_215	Ca2	30890128	GATGATGTAAATGGGAGCCAA	CATTTGTTGAATGTTGCCCA	59.78	697	INTERGENIC				

Major pod number and seed yield QTLs delineated	INDEL marker IDs	Chromosomes/ unanchored scaffolds	Physical positions (bp)	Forward primers (5'-3')	Reverse primers (5'-3')	Anneling temperature (0C)	Expected amplified product size (bp)	Structural annotation		Functional annotation		Putative Function
								Sequence components of *kabuli*	*Kabuli* gene accession IDs	NCBI-KOG	TFs	
	CaPOPI_II_216	Ca2	31240228	CTGCCCCCACTATTTCTCAA	GAAACACTTGGCATGTTAAGGA	60.07	479	INTERGENIC				
	CaPOPI_II_217	Ca2	31882952	TTTCTGGTGGGATCAGCTCT	GACATCCTGCGCCAGTTATT	59.80	591	INTRON	Ca_17830	D	LBD	RZZ complex, subunit Zw10
	CaPOPI_II_218	Ca2	31883010	TTTCTGGTGGGATCAGCTCT	GACATCCTGCGCCAGTTATT	59.80	591	INTRON	Ca_17830	D	LBD	RZZ complex, subunit Zw10
	CaPOPI_II_219	Ca2	32127033	TGCAAGTTGTGATGGGTTGT	TGTCTGTTCTGCACGGAGTC	60.01	650	INTRON	Ca_17812	O		Ubiquitin
	CaPOPI_II_220	Ca2	32173774	ATGTTTTGATTGGGGCTTTG	TAAATCAGCCCTTTTGGTGG	59.80	631	DRR	Ca_17806		WRKY	Glycoside hydrolase, family 9
	CaPOPI_II_221	Ca2	32209131	GCAAAGATAGACACCACCCC	AACCATTCCCATGGAACAAA	59.41	577	INTERGENIC				
	CaPOPI_II_222	Ca2	32209334	TCCATGGGAATGGTTTTGAT	TTGAAGAGGAAGTTTTGGCA	59.99	342	INTERGENIC				
	CaPOPI_II_223	Ca2	32209671	TCCATGGGAATGGTTTTGAT	TTTTAGTGTGCACCAATGTCAA	59.99	899	INTERGENIC				
	CaPOPI_II_224	Ca2	32210099	TCAGCTTCAATTATCTCCAACAA	TCGGCGACAAAAATCACATA	58.89	644	INTERGENIC				
	CaPOPI_II_225	Ca2	32213171	CAACATGACAAGGACTTTCCAA	TTACCGGTATCGCAAGGAAC	60.01	529	INTERGENIC				
	CaPOPI_II_226	Ca2	32213424	AGGGATAGATAACGGCACGA	AAGGCCCTGCTAGAATGATG	59.55	504	INTERGENIC				
	CaPOPI_II_227	Ca2	32379585	TGTGCGAGTTTATTGCGGTA	CATTTGGTTCAAAATGTCATGG	60.27	624	INTERGENIC				
CaqcSYP4.1	CaPOPI_II_468	Ca4	30535832	CCATTCAGAATTAGAAAATCACCA	CCAAATTTCCCAAGGGTTCT	59.40	582	INTERGENIC				
	CaPOPI_II_469	Ca4	30537523	TTTGATTGCCATTGTATCCG	CAAAATGGAAATGCAAGAACAA	59.38	803	INTRON	Ca_14204		NAC	No apical meristem (NAM) protein
	CaPOPI_II_470	Ca4	30538166	TTGTTCTTGCATTTCCATTTTG	CGCCATCTTCCTCTTCTTTG	59.98	380	CDS (large-effect mutations)	Ca_14204		NAC	No apical meristem (NAM) protein

Major pod number and seed yield QTLs delineated	INDEL marker IDs	Chromosomes/ unanchored scaffolds	Physical positions (bp)	Forward primers (5'-3')	Reverse primers (5'-3')	Anneling temperature (0C)	Expected amplified product size (bp)	Structural annotation		Functional annotation		Putative Function
								Sequence components of *kabuli*	*Kabuli* gene accession IDs	NCBI-KOG	TFs	
	CaPOPI_II_471	Ca4	30928717	CAATGTCCATGTCGACCAAA	TCTGTCGCACCATCTCAGTC	60.37	949	INTERGENIC				
	CaPOPI_II_472	Ca4	31172943	TCGCCTAAGGTAAGGGCTTT	TTACACCAATTCCCCCAAGA	60.22	421	INTERGENIC				
	CaPOPI_II_473	Ca4	31192815	TTGAAGGAGAAGCCAGAGTCA	TGAGGCATGCAGAAACGTAG	60.12	238	INTERGENIC				
	CaPOPI_II_474	Ca4	31303190	GAGGTCCCGTCCAACCTAAC	TCAGCACCGAGAGTGTCAAC	60.75	488	INTRON	Ca_14231		bHLH	Helix-loop-helix DNA-binding domain
	CaPOPI_II_475	Ca4	31445256	AGATCTATGCATCGGCAACC	AGGGTACTACAGCTGCCCCT	60.07	338	INTERGENIC				
	CaPOPI_II_476	Ca4	31611840	CGTTTTCAGCTGAGCAAATTC	TTGATCCATTGGGATTGTGA	60.01	392	INTERGENIC				
	CaPOPI_II_477	Ca4	31611867	CGTTTTCAGCTGAGCAAATTC	TTGATCCATTGGGATTGTGA	60.01	392	INTERGENIC				
	CaPOPI_II_478	Ca4	31649942	CGTGCCCTGCAGGTATTTAT	ATTGACCGGTGGAAGACATT	59.98	454	INTRON	Ca_15442	U	EIL	SPX, N-terminal
	CaPOPI_II_479	Ca4	31806633	ACAAAAGCCTGGATAGCGAA	TCACATATACCCTTAAAATCGCA	59.85	410	INTERGENIC				
	CaPOPI_II_480	Ca4	31859597	GTTGGAGCCCCCACTTTATT	TGCAAGCAGCATCAGAGAAC	60.19	793	INTERGENIC				
	CaPOPI_II_481	Ca4	32179682	TTGCATACCTCATACCATCCC	TTGATGCATTACCTCCCACA	59.66	731	INTERGENIC				
	CaPOPI_II_482	Ca4	32227293	TCTTCACAACCCCTTGAACC	TTCTTCCAAACGAGCCTCTC	59.94	868	INTERGENIC				
CaqcSYP6.1	CaPOPI_II_674	Ca6	12877302	GTTCGTAAAGGACGGGATGA	CTTTTACAGCGCTTTCTGGG	59.93	235	INTERGENIC				
	CaPOPI_II_675	Ca6	13838996	TGTGTGATTGGTGTTGAGCA	GCAACAACGGGTGAAAATCT	59.71	563	INTERGENIC				
	CaPOPI_II_676	Ca6	15555599	TGTGGGCACATTAGTAGGCA	TCATCGTCGTTGCTTGAGTC	60.13	640	INTERGENIC				

Major pod number and seed yield QTLs delineated	INDEL marker IDs	Chromosomes/ unanchored scaffolds	Physical positions (bp)	Forward primers (5'-3')	Reverse primers (5'-3')	Anneling temperature (0C)	Expected amplified product size (bp)	Structural annotation		Functional annotation		Putative Function
								Sequence components of *kabuli*	*Kabuli* gene accession IDs	NCBI-KOG	TFs	
	CaPOPI_II_677	Ca6	15559125	TCACCAAACAAAAACTGGGG	CTACATTCCGGTTAGGGCAA	60.76	480	INTERGENIC				
	CaPOPI_II_678	Ca6	15727514	TTAGTTTGGGCTTGGGTTTT	TTGCTCCTACGTATCTCCCG	58.60	911	INTERGENIC				
	CaPOPI_II_679	Ca6	15730699	ATTTGAACCTGGCAGCAAAA	GGTTTGAAAAAGGGGTGATG	60.62	175	INTERGENIC				
	CaPOPI_II_680	Ca6	15731057	ATTTGAACCTGGCAGCAAAA	GGGACAAAAGGCTAGTTTGG	60.62	909	INTERGENIC				
	CaPOPI_II_681	Ca6	15999975	AAGTGTGGGCATTTCCTTTG	ATGGACCTCTGTATTCGCCA	59.97	137	INTERGENIC				
	CaPOPI_II_682	Ca6	16012701	TAAGAGTTTGGTTCCGGTGG	TGTGCAGATAAATGGCCAAA	59.96	397	INTERGENIC				
	CaPOPI_II_683	Ca6	16547931	TTCCTTCCTGCTCGTGCTAT	TTCTTTGGCTGAAATGGTCC	59.98	640	INTRON	Ca_06284	A		PSP, proline-rich
