# Supplementary material for: A High-Resolution InDel (Insertion–Deletion) Markers-Anchored Consensus Genetic Map Identifies Major QTLs Governing Pod Number and Seed Yield in Chickpea
Source: Front Plant Sci. 2016 Sep 16;7:1362. doi: 10.3389/fpls.2016.01362 (PMC5025440; doi:10.3389/fpls.2016.01362)
Supplement: Supplementary file 1 [file Table1.PDF]

Table S1. InDel markers (25477) differentiating high pod number-containing parental accession (Pusa 1103) and homozygous bulk (HPNB) of a mapping population (Pusa 1103 x ILWC 46) from reference *kabuli* accession (CDC Frontier)

INDEL marker IDs	Chromosomes/ unanchored scaffolds	Physical positions (bp)	InDels (*Kabuli* reference genome-CDC Frontier/PI)	Forward primers (5'-3')	Reverse primers (5'-3')	Anneling temperature (0C)	Expected amplified product size (bp)	Structural annotation		Functional annotation	
								Sequence components of *kabuli* genome	*Kabuli* gene accession IDs	NCBI-KOG	TFs
C11060010_P1_682	C11060010	682	AATTATT/AATTATTATT	TCCGATATGCACCAATGAAA	AGGCGTCGGTTTTTCTTTCT	59.9	413	INTERGENIC	--	--	--
C11109468_P1_1121	C11109468	1121	TTATTTGT/TTCTCAAAAAAATATTTGT	TGTTTTGCAGGAACGAAATG	TGTGGTGGTGACAAATCAAGA	59.7	517	INTERGENIC	--	--	--
C11119398_P1_1161	C11119398	1161	TTATATATATA/TTATATATA	CATGCATGCTGAGGGATATG	TGTTTGTTTCGTCAATCACTTT	60.1	268	INTERGENIC	--	--	--
C11119398_P1_1428	C11119398	1428	GACACACA/GACACA	CATGCATGCTGAGGGATATG	CGGTACCCACACTTAATTTAACAA	60.1	822	INTERGENIC	--	--	--
C11154862_P1_690	C11154862	690	GAA/GA	TGGCAATCAGAAGCAGTGAG	TGACAGAACGAAGCAAATCG	60.1	561	INTERGENIC	--	--	--
C11154966_P1_887	C11154966	887	GAAAAAAAAAAA/GAAAAAAAAAA	TTCAAGAGTCTCCTAGGGCG	TTGCTTGTGTCGTTAGCTGC	59.6	669	DRR	Ca_28249	E	MYB
C11155258_P1_2950	C11155258	2950	TAAGAAGAAGAAGAAGAAGAA/TAAGAAGAAGAAGAA	TTGCAATGCACTTTATTGGC	AGGGATGTTAAGGTGGTCCC	59.7	921	DRR	Ca_28113	--	--
C11155258_P1_5339	C11155258	5339	TTAAT/TT	CCCACATTTCATGATTTCCC	GCTTCAATGAAATCGCAATACA	60.0	456	INTERGENIC	--	--	--
C11159426_P1_3587	C11159426	3587	TAA/TA	CACATTGAAGGTGCATCGAG	TTGTGACGCTAACGACTTGG	60.3	462	DRR	Ca_27806	--	--
C11164954_P1_502	C11164954	502	CATATATATATATATATATATATATATAT/CATATATATATATATATATATATATAT	TTTTTCTTAACACACTTTGGTGAAA	TTTTAATTTATGTAATTTGGTCAACGA	59.2	476	INTERGENIC	--	--	--
C11168930_P1_5756	C11168930	5756	TCCC/TCC	TGAGCAAAAGCAAACAGTGG	ATTAAGGGGCGCATTTTCTT	60.0	434	INTERGENIC	--	--	--

INDEL marker IDs	Chromosomes/ unanchored scaffolds	Physical positions (bp)	InDels (*Kabuli* reference genome-CDC Frontier/PI)	Forward primers (5'-3')	Reverse primers (5'-3')	Anneling temperature (0C)	Expected amplified product size (bp)	Structural annotation		Functional annotation	
								Sequence components of *kabuli* genome	*Kabuli* gene accession IDs	NCBI-KOG	TFs
C11168930_P1_7154	C11168930	7154	TG/T	CGGCAACGTCCAAGATAGTT	TCAGCGTCTTCAACATTTGC	60.1	640	INTERGENIC	--	--	--
C11169592_P1_3333	C11169592	3333	CTTTT/CTTTTT	TCTTTTTCCGATGTTTTCACG	GGGTGCCATCAACACATACA	60.1	125	INTERGENIC	--	--	--
C11172910_P1_8105	C11172910	8105	AATATATATATATAT/AATATATATATATATAT	TTCCATCACCAACAAGACCA	GTATTTGCAGGTCCCGTGTC	59.9	287	INTRON	Ca_27557	P	--
C11177592_P1_10688	C11177592	10688	ATTTTT/ATTTT	TAATTCCCAAGCCTGCCATA	GGGTGGGAGATGAAGGAGTT	60.4	749	DRR	Ca_27746	O	bHLH
C11178328_P1_11882	C11178328	11882	GAC/G	AAATCGGAGGAATACCCTGG	GAGGAGGCCTAAAAGATGGG	60.1	678	DRR	Ca_28266	T	M-type
C11178328_P1_12753	C11178328	12753	CAT/CATTAT	CCATCTTTTAGGCCTCCTCC	GAAGGCAACCATTCTAGGCA	60.0	925	DRR	Ca_28266	T	M-type
C11178532_P1_10916	C11178532	10916	TTATATATATATATATATATAT/TTATATATATATATATATAT	CTTCTATAGGCCCAAAGCCC	AACAAAGGCTTGCAAAATCG	60.1	637	INTERGENIC	--	--	--
C11181782_P1_15288	C11181782	15288	ATTTTTTTT/ATTTTTTT	AACCACGTATCAAAGCCCAA	TTCAAAAGTGTTTGAGCCGT	60.4	650	INTERGENIC		--	--
Ca1_P1_10040103	Ca1	10040103	ATTT/ATT	ATGCATCGAAAACCAACACA	TGCAATCCGGGCTTACTATC	60.0	378	INTRON	Ca_02784	LK	--
Ca1_P1_10057638	Ca1	10057638	CATATATATATATATATAT/CATATATATATATAT	TGATGGGAGCAATGAATGAA	TCAAATTCCGACTTGAGGGA	60.0	542	INTERGENIC	--	--	--
Ca1_P1_10060949	Ca1	10060949	T/TATATATATATATATA	AATCCAGAAATGCATCAGGG	ATTGGTTTAACCCCTCGTGA	59.9	225	INTERGENIC	--	--	--
Ca1_P1_10064752	Ca1	10064752	TTATATATATATATATATA/TTATATATATATATATATATATATATATA	ATGCAAATTCTCCCCCTCTT	TCATTTGATTTTAATTGGACGA	59.9	750	INTERGENIC	--	--	--

INDEL marker IDs	Chromosomes/ unanchored scaffolds	Physical positions (bp)	InDels (*Kabuli* reference genome-CDC Frontier/PI)	Forward primers (5'-3')	Reverse primers (5'-3')	Anneling temperature (0C)	Expected amplified product size (bp)	Structural annotation		Functional annotation	
								Sequence components of *kabuli* genome	*Kabuli* gene accession IDs	NCBI-KOG	TFs
Ca1_P1_10070211	Ca1	10070211	GAATAATAATAATAATAATAATAATAA/GAATAATAATAATAATAATAATAATAATAA	TGCTGATGCGTCTGTCCTAC	TTATATGGGAAACCGGAGGG	60.0	410	DRR	Ca_02782	--	--
Ca1_P1_10104142	Ca1	10104142	TTATATATATATATATATATATAT/TTATATATATATATATATATATATAT	TTCCAATGAGGACATGTGTGA	GGAGCTTTTTGTTGATAACGG	60.0	383	INTERGENIC	--	--	--
Ca1_P1_10168572	Ca1	10168572	GTTTTTTTTTT/GTTTTTTTTT	AGACAGGCATGGAGAAATGG	TTTAAGGCGTGGATGTCGTT	60.1	632	INTERGENIC	--	--	--
Ca1_P1_1029183	Ca1	1029183	TTTAT/TTTATTAT	TCAAAGAATAGGACGGAAGCA	CCACCTTGATTCGGTTGAGT	59.8	783	INTERGENIC	--	--	--
Ca1_P1_10323177	Ca1	10323177	G/GAGCT	TTGAGTGCTTCACCATTTGC	GTTGTATTGGCTCTCCTCGC	59.8	463	INTRON	Ca_02746	B	C2H2
Ca1_P1_10360863	Ca1	10360863	TTAATAATAATAATAATAATAATAAT/TTAATAATAATAATAATAATAAT	CATTGGTATGGCTAAGGCGT	TGGCTTGAATTTGGTGATGA	60.0	667	INTERGENIC	--	--	--
Ca1_P1_10397505	Ca1	10397505	ATCTCTCTCTCTCTCTCTCTCTCTCTCTCTCTCTCT/ATCTCTCTCTCTCTCTCTCTCTCTCTCTCTCTCT	TGCTGCTGATGTTCAAAACC	TCCGAATTTGTTTCGTTTCC	59.8	168	INTERGENIC	--	--	--
Ca1_P1_10414677	Ca1	10414677	CAGGAATNNNNNNNNNNAGGAAT/CAGGAAT	TTCTAAACGAGGCTTTTCAGTTG	GCATGTTGCTGATGATGGTC	59.9	489	DRR	Ca_02735	R	ERF
Ca1_P1_10458891	Ca1	10458891	TACACACA/TACACA	CCAATTTCAGTAGGCCCAAA	AGCATTGAAACCAAACCAGC	59.9	583	DRR	Ca_02729	--	HSF
Ca1_P1_10471260	Ca1	10471260	TTATATATATATATATATATA/TTATATATATATATATATATATA	TTCTCCATTCGCACTCACAC	TCAAATTTGGAGGCTGGAAC	59.8	312	INTERGENIC	--	--	--
Ca1_P1_10513925	Ca1	10513925	CTTATTTATTTATTTATTTATTTATTTATTTATTTATTTATT/CTTATTTATTTATTTATTTATTTATTTATTTATT	CCAGATTTCCTTTTCCCCTC	GGGTAGGCCACAGACTGTCTTA	59.9	634	INTERGENIC	--	--	--
Ca1_P1_10767761	Ca1	10767761	GTAACTATTTAACTATTTAA/GTAACTATTTAA	TTTACAAATATTCTCTCGGCTCAA	CAATGGCGTAATGGTGTGAG	59.3	509	DRR	Ca_02684	--	--

INDEL marker IDs	Chromosomes/ unanchored scaffolds	Physical positions (bp)	InDels (*Kabuli* reference genome-CDC Frontier/PI)	Forward primers (5'-3')	Reverse primers (5'-3')	Anneling temperature (0C)	Expected amplified product size (bp)	Structural annotation		Functional annotation	
								Sequence components of *kabuli* genome	*Kabuli* gene accession IDs	NCBI-KOG	TFs
Ca1_P1_10800904	Ca1	10800904	GAAAAAAAA/GAAAAAAAAA	AAACAAAGGACCGTGATTGC	CGCGCCAAACTACCATATTT	60.0	598	INTERGENIC	--	--	--
Ca1_P1_1084300	Ca1	1084300	A/AC	TCCCAAATGACTTAATCCGC	ATCGTCGGAGAAATTTGACG	59.9	608	INTRON	Ca_00134	DO	LBD
Ca1_P1_10843362	Ca1	10843362	CATATATATATATATA/CATATATATATATATA	CCGATATCTTCCTGCGATGT	GGAGATGTTGCAGTTGACCA	60.1	760	INTRON	Ca_02674	G	NAC
Ca1_P1_1084582	Ca1	1084582	TTT/TTTAACCCAATGGGTT	TTCCAACAGGACAGACCTCC	CTGCGAGTGTTTTCTGGTCA	60.1	719	INTRON	Ca_00134	DO	LBD
Ca1_P1_10846457	Ca1	10846457	CAAAAAA/CAAAAAAA	TTCCCACCTAAACGATCGAC	CAATGAACCTGAGTTTTGTCCC	59.9	865	INTERGENIC	--	--	--
Ca1_P1_1084940	Ca1	1084940	CTTT/CTTTT	CGTCAAATTTCTCCGACGAT	CTGCGAGTGTTTTCTGGTCA	60.1	290	INTRON	Ca_00134	DO	LBD
Ca1_P1_1085307	Ca1	1085307	AAT/AATAT	TGACCAGAAAACACTCGCAG	TCAGTGCTTGTGAAGATGGC	60.0	710	INTRON	Ca_00134	DO	LBD
Ca1_P1_1088158	Ca1	1088158	TCCAC/TCCACCAC	TCGAAAGCTGAATTCGTGTG	GCCCATACTTGAAACCACTAGC	60.0	482	DRR	Ca_00135	--	C2H2
Ca1_P1_1088478	Ca1	1088478	A/AC	GGCATCACATTTGTGTCGAA	TCACCCCACTATCCCGTTTA	60.5	717	DRR	Ca_00135	--	C2H2
Ca1_P1_10934337	Ca1	10934337	ATTTTTTTTTTT/ATTTTTTTTTT	CACTTGCATTTGATTGGTGG	CAAAACACCCATTTTCACCC	60.0	436	INTERGENIC	--	--	--
Ca1_P1_10944660	Ca1	10944660	TAAAAAAAAAA/TAAAAAAAAAAA	GCGACCTCGTGAATGAAAAA	GGCATAATTGTTCCCACAAA	61.1	534	INTERGENIC	--	--	--
Ca1_P1_1094498	Ca1	1094498	AC/A	TTGAGGAATGGTGCAAGATG	TGTACTTGCTATTCATTCTCGTTGA	59.6	254	INTERGENIC	--	--	--

INDEL marker IDs	Chromosomes/ unanchored scaffolds	Physical positions (bp)	InDels (*Kabuli* reference genome-CDC Frontier/PI)	Forward primers (5'-3')	Reverse primers (5'-3')	Anneling temperature (0C)	Expected amplified product size (bp)	Structural annotation		Functional annotation	
								Sequence components of *kabuli* genome	*Kabuli* gene accession IDs	NCBI-KOG	TFs
Ca1_P1_1094637	Ca1	1094637	TAAAAAAA/TAAAAAAAA	TTGAGGAATGGTGCAAGATG	TTTGCTAATAAAAGTAAATGAGTTGGA	59.6	394	INTERGENIC	--	--	--
Ca1_P1_10950044	Ca1	10950044	AATATATATATATATATA/AATATATATATATATATATATATATATATATATA	TGACGGGTTGTGATTTTCAA	AAAAAGTTTTGCGTACAAGTCAA	59.9	388	INTERGENIC	--	--	--
Ca1_P1_1095503	Ca1	1095503	GTTTT/GTTT	TGTAGGAGAGAGGAACTGAGGAA	TTGTTGTGATGGATTGGGTG	59.5	324	INTERGENIC	--	--	--
Ca1_P1_1095824	Ca1	1095824	TTAAAATAAAATAAAATAAAAT/TTAAAATAAAATAAAAT	CACCCAATCCATCACAACAA	GCTTCTCCTTGCCATCAAAG	60.2	540	INTERGENIC	--	--	--
Ca1_P1_1097365	Ca1	1097365	AAA/AAACAA	GGGACAAACTTGGAGTTGGA	TGCATGCCAATTAAATAAATCA	59.9	691	INTERGENIC	--	--	--
Ca1_P1_1102337	Ca1	1102337	CTT/CTTT	CTTAAGCCATGCCACAAATG	TCTCTTTCATCAATCAGATGCC	59.2	432	INTERGENIC	--	--	--
Ca1_P1_11056418	Ca1	11056418	TAAAAAAAAAAAAA/TAAAAAAAAAAAA	GGATCACAGGGAAGTTGCAC	CCCCCTTGTTTGCACTCTTA	60.5	288	INTERGENIC	--	--	--
Ca1_P1_11102279	Ca1	11102279	ATTTTTTTTTT/ATTTTTTTTT	TGCCGCGAATTTAGATCTTT	TCGGTGTTGGTGTCGTTATC	59.8	255	DRR	Ca_02649	C	--
Ca1_P1_11145101	Ca1	11145101	TTATATATATATATATATATATATAT/TTATATATATATATATATATAT	TTTGAGTCCAAATTGAAGGCT	CGGATCAAAATTCGCACAAT	58.8	837	INTERGENIC	--	--	--
Ca1_P1_11148737	Ca1	11148737	CAATAATAATAAT/CAATAATAAT	CGTGTAATTGGTAATGTGGCA	GGCTAACTTGAAGTCGTGGC	59.4	693	INTERGENIC	--	--	--
Ca1_P1_11152932	Ca1	11152932	T/TCAATAATAA	CTCAATCTCCCAATCCCAAA	TGAATCTGGTCCAAACATGAA	59.9	236	INTERGENIC	--	--	--
Ca1_P1_11170203	Ca1	11170203	CATA/CATATATA	ATGAGGGTATCCGAGCCATA	CCACAAGAGTTTTGCCATTG	59.4	378	DRR	Ca_02641	--	--

INDEL marker IDs	Chromosomes/ unanchored scaffolds	Physical positions (bp)	InDels (*Kabuli* reference genome-CDC Frontier/PI)	Forward primers (5'-3')	Reverse primers (5'-3')	Anneling temperature (0C)	Expected amplified product size (bp)	Structural annotation		Functional annotation	
								Sequence components of *kabuli* genome	*Kabuli* gene accession IDs	NCBI-KOG	TFs
Ca1_P1_11190216	Ca1	11190216	ATTACT/ATTACTTACT	TATGGCTAGCTGGTGAAGCA	TAGAATCCCACGCAGCTCTT	59.6	304	INTRON	Ca_02639	--	--
Ca1_P1_11195317	Ca1	11195317	TTCTCTCTCTCTCTCTCTCTCTCTC/TTCTCTCTCTCTCTCTCTCTC	AGGTGGTAAAGACACGTGGG	TGAACTCGTCACGAACCTTG	59.9	338	DRR	Ca_02638	--	--
Ca1_P1_11324975	Ca1	11324975	GAAGA/GAAGAAAGA	CTGCATCGGGGATACTTCAC	TTTTGACCGAGCCAGATACC	60.5	317	INTRON	Ca_02622	--	--
Ca1_P1_11331703	Ca1	11331703	TTTTA/TTTTAGTTTA	CCTCTGCTCACACCCTTTTC	CACCATTTATCTGTTTTGCGTT	59.8	860	INTERGENIC	--	--	--
Ca1_P1_11354233	Ca1	11354233	CTTTTTTTTTTT/CTTTTTTTTTT	TTGATTGATCCGGTTTTTCC	CGAGTTGCATACAAAAGATTGG	59.7	852	INTERGENIC	--	--	--
Ca1_P1_11425487	Ca1	11425487	TTATATATATATATA/TTATATATATATATATA	TTGTCTCACGTGAATCTGGG	GGAGTTTGGAGAGAAGGAGTGA	59.7	745	INTERGENIC	--	--	--
Ca1_P1_1145463	Ca1	1145463	AATATATATATATATATATATATATATATA/AATATATATATATATATATATATATATA	TTCACAAACAACACCACACAA	GTGTCTCCTCCTACCACCCA	58.5	485	INTERGENIC	--	--	--
Ca1_P1_11456505	Ca1	11456505	TTATATATATATATATATATA/TTATATATATATATATATATA	AACCGAGTGCCCTTTAACAA	TTGTTTTGCATTTTGGGACA	59.6	273	INTERGENIC	--	--	--
Ca1_P1_11457087	Ca1	11457087	ATTTTTTTTTTT/ATTTTTTTTTT	TGTCCCAAAATGCAAAACAA	GGAGATGACACATCCTATAACTTTTT	59.9	667	INTERGENIC	--	--	--
Ca1_P1_11563390	Ca1	11563390	ATT/AT	AATGAACATGCGGACCAAAT	TTATACTGTCTCCCGCCACC	60.2	303	INTERGENIC	--	--	--
Ca1_P1_1160199	Ca1	1160199	GATATATATATATATATATATATATA/GATATATATATATATATATATATATATATA	ATATTCGGGGAATCACCTCC	AGCCGACCTTCCCACTTAAT	60.0	578	DRR	Ca_00145	--	--
Ca1_P1_11766477	Ca1	11766477	TTATATATATATATATATATA/TTATATATATATATATATATA	TCGTCGACCTTTCTTAGAATTT	TCGATTTTTACAGGGTTCGG	57.2	858	INTRON	Ca_02576	--	YABBY

INDEL marker IDs	Chromosomes/ unanchored scaffolds	Physical positions (bp)	InDels (*Kabuli* reference genome-CDC Frontier/PI)	Forward primers (5'-3')	Reverse primers (5'-3')	Anneling temperature (0C)	Expected amplified product size (bp)	Structural annotation		Functional annotation	
								Sequence components of *kabuli* genome	*Kabuli* gene accession IDs	NCBI-KOG	TFs
Ca1_P1_11788825	Ca1	11788825	AATATATATATATATA/AATATATATATATATATA	TGGGAATAGGTCAGACCAGG	CCAAGTTGTTTGAGAGAATTTGTTT	59.9	696	INTERGENIC	--	--	--
Ca1_P1_11810350	Ca1	11810350	CTTTTTTTT/CTTTTTTTTT	CGGTTATAAGGCAACATCAGC	GAATGGCGAAAAATCCTCAA	59.6	696	INTERGENIC	--	--	--
Ca1_P1_11814946	Ca1	11814946	CATATATATATATATATATATATATA/CATATATATATATATATATATATATATATATATATA	CTTGTTGGCCAAAACGAAAT	TTCGAAAAACAAACAACGACA	60.0	647	INTERGENIC	--	--	--
Ca1_P1_11830305	Ca1	11830305	TTATATATATATATATATATATATATATA/TTATATATATATATATATATATATATATA	CCTTGCCTTCTTCTCCCTCT	GCTCATCGCTATCAAGCACA	60.0	543	INTERGENIC	--	--	--
Ca1_P1_11834197	Ca1	11834197	AA/AATAATA	TGCATTGCGGAGAGCTAATA	TTGAGTTTGTTAGCCGGTGA	59.6	674	INTERGENIC	--	--	--
Ca1_P1_1186147	Ca1	1186147	CTTTTT/CTTTTTTT	TATCGCAGAATTTTCGTCCC	AAGAGTTGCACCAATGGACC	60.0	320	INTRON	Ca_00147	EH	bHLH
Ca1_P1_11868314	Ca1	11868314	TTATATATATATATATAT/TTATATATATATATATATATATAT	GCAAGTATGCAACAACCTTCC	TTGCATATATTGTCACACCGC	59.6	346	INTERGENIC	--	--	--
Ca1_P1_11948916	Ca1	11948916	AATATATATATATATATATATATATATA/AATATATATATATATATATATATATATATATATA	AGGCAAACATGGCAAAATTC	TCTTTGCCTATGCCTATGAGC	59.9	746	INTERGENIC	--	--	--
Ca1_P1_11977187	Ca1	11977187	CAAAAAA/CAAAAAAA	GCAATTGGATGACATTGCATA	ACAGACAGGGCACAGGATCT	59.4	479	INTERGENIC	--	--	--
Ca1_P1_11994844	Ca1	11994844	CATATATATATATATAT/CATATATATATATAT	TGACTGGTGAGCGTTCTTTG	GGCAATGAATGAAACGGTCT	60.0	602	INTERGENIC	--	--	--
Ca1_P1_12090006	Ca1	12090006	TTGGAGAATGGAGAATGGAGAATGGAGAATGGAG/TTGGAGAATGGAGAATGGAGAATGGAG	GTAGTGTCGATGCTGCTGGA	AAGCTTCAATAAATGGTCCGT	60.0	277	INTERGENIC	--	--	--
Ca1_P1_12092743	Ca1	12092743	AATATATATATATATATATAT/AATATATATATATATATATATATATATAT	CGGCAATTTTGAGTTGATTTG	CATCGGATGGTCCAGAAAGT	60.5	825	INTERGENIC	--	--	--

INDEL marker IDs	Chromosomes/ unanchored scaffolds	Physical positions (bp)	InDels (*Kabuli* reference genome-CDC Frontier/PI)	Forward primers (5'-3')	Reverse primers (5'-3')	Anneling temperature (0C)	Expected amplified product size (bp)	Structural annotation		Functional annotation	
								Sequence components of *kabuli* genome	*Kabuli* gene accession IDs	NCBI-KOG	TFs
Ca1_P1_1210052	Ca1	1210052	TC/T	AAATACCCTTTTTCCGTGGC	CCTTGTTTCTCTGCTTTGCC	60.2	486	INTERGENIC	--	--	--
Ca1_P1_12102708	Ca1	12102708	AT/A	AATGTGCTTGACACTCGTGG	CACAACCCAAGTCAAACCAA	59.8	245	INTRON	Ca_02545	--	--
Ca1_P1_12135797	Ca1	12135797	GATATATATATATATATATATATATATATATATA/GATATATATATATATATATATATATATATATATATATATATA	GGTGTTGGAGTCATGCCTTT	GTCGCACGGAAACAACGTA	60.0	234	INTERGENIC	--	--	--
Ca1_P1_12165802	Ca1	12165802	AATATATATATATATAT/AATATATATATATATATATATATATATAT	AGGTGGACGTTACAAAAACG	AACATTCGCAAACAACATCG	57.7	613	INTERGENIC	--	--	--
Ca1_P1_12195477	Ca1	12195477	CTTTTTTTTT/CTTTTTTTTTT	TTGTTTGTTTCCTTGTGGCA	AGAAAAATGCGATGGTTCTTT	60.1	250	INTERGENIC	--	--	--
Ca1_P1_12208944	Ca1	12208944	ATTTTTTTTTTT/ATTTTTTTTTT	GGGTACCCCAAAATTTATTTGA	GACTAACGAGTGCTGCTAAGAAAA	59.0	807	DRR	Ca_02535	--	--
Ca1_P1_1225540	Ca1	1225540	ATAATAATTAATAATTAATAAT/ATAATAATTAATAATTAATAATTAATAAT	ATGCCGATAACACCCTCATC	CCCGGTCAAAACGTTACTGT	59.8	688	INTRON	Ca_00151	O	--
Ca1_P1_1230729	Ca1	1230729	AATATATATATATATATATATATATATATATATAT/AATATATATATATATATATATATATATATATATAT	ATCACTCACCGAAAGCGTCT	TTTCCCATTCTTTGCCTCAC	59.9	867	INTRON	Ca_00152	--	--
Ca1_P1_12340166	Ca1	12340166	TCCGTCTCTCTTCACCG/TCCG	TCGCATCACATTCTATCCCA	GCGCTCATGTGTTGAAAAGA	60.0	738	DRR	Ca_02515	K	ERF
Ca1_P1_1236378	Ca1	1236378	AATTATTATTATTATTATTATTATTATTATTATTATTATCATTATTATTATTATTATTATTATT/AATTATTATTATTATTATTATTATTATTATTATCATTATTATTATTATTATTATTATT	AACCATAATCTCCTTCACTCCTTG	ACCCCCAAGAACAAAGTGAA	59.9	828	INTERGENIC	--	--	--
Ca1_P1_12375128	Ca1	12375128	ATAGTGTATT/ATAGTGTATTTTTAGTGTATT	CAACGTAACATTTTCGGGCT	TTGTCTCGATTGAGAGGTGG	60.0	695	INTERGENIC	--	--	--
Ca1_P1_1237664	Ca1	1237664	GTC/G	CATCATCAATGAACGAGCCA	CGTAACTTGTGTTTTGCATGAGA	60.6	626	INTERGENIC	--	--	--

INDEL marker IDs	Chromosomes/ unanchored scaffolds	Physical positions (bp)	InDels (*Kabuli* reference genome-CDC Frontier/PI)	Forward primers (5'-3')	Reverse primers (5'-3')	Anneling temperature (0C)	Expected amplified product size (bp)	Structural annotation		Functional annotation	
								Sequence components of *kabuli* genome	*Kabuli* gene accession IDs	NCBI-KOG	TFs
Ca1_P1_12376905	Ca1	12376905	GGTTGTT/GGTT	GAAGGTTTGCTACGCAGGAG	TCCACATTGCTTCTCCAAGA	60.0	143	INTERGENIC	--	--	--
Ca1_P1_12420239	Ca1	12420239	CAAAAAAAAA/CAAAAAAAAAA	AAATGCAATCTTGGTTTCCA	TTTTGGTAATGTGCCCATGATA	58.1	595	INTERGENIC	--	--	--
Ca1_P1_12445428	Ca1	12445428	TAAA/TAAAA	TAGGTCACAACATCCCACCA	ATGGCCCAAAATATGGGTTT	59.8	142	INTERGENIC	--	--	--
Ca1_P1_12550318	Ca1	12550318	ATTTTTTTTTTT/ATTTTTTTTTT	CGTCTTTATTTTATGCGAGCG	TAATGCCGATGGTGTCAAAA	59.9	252	INTERGENIC	--	--	--
Ca1_P1_12580190	Ca1	12580190	TAAAAAAAAAA/TAAAAAAAAAAA	TAATTCACTCACCATCGCCA	TTCATGTTTGGTGTGGCACT	60.1	476	INTERGENIC	--	--	--
Ca1_P1_12644727	Ca1	12644727	TGTA/TGTACGTA	TTGATTTTCAATTGGGAGCC	TGCCAGGATGTAACCCTTTC	59.9	807	INTERGENIC	--	--	--
Ca1_P1_12644922	Ca1	12644922	AC/A	ACCCAACCGCAAAAACAATA	TGCCAGGATGTAACCCTTTC	60.2	440	INTERGENIC	--	--	--
Ca1_P1_12645139	Ca1	12645139	T/TTCAAAG	GAAAGGGTTACATCCTGGCA	TCAACATTGAAGAGGGTGTCC	59.9	149	INTERGENIC	--	--	--
Ca1_P1_12690899	Ca1	12690899	TTAAATAAATAAATAAATAA/TTAAATAAATAAATAAATAAATAA	TGCTTCATACCAACGTTCCA	CAGAGTTTTAAACGTATGACGGTG	60.1	516	INTERGENIC	--	--	--
Ca1_P1_12722291	Ca1	12722291	TC/T	TGCAAAACAACACCTCTGGA	TGGTTTTTAGTGGATTCGGC	60.3	519	INTERGENIC	--	--	--
Ca1_P1_12727627	Ca1	12727627	TTATATATATATATATATATATAT/TTATATATATATATATATAT	CCTTACGGACAACCAAGCAT	GCAAAAGAATTTGCAACAAAGA	60.0	419	INTERGENIC	--	--	--
Ca1_P1_12767960	Ca1	12767960	ATTTTTTTTT/ATTTTTTTTTT	TGGTTCGGTGATTTTAGAGAAGA	GACTGCACACAGTAAGCCCA	60.1	744	INTERGENIC	--	--	--

INDEL marker IDs	Chromosomes/ unanchored scaffolds	Physical positions (bp)	InDels (*Kabuli* reference genome-CDC Frontier/PI)	Forward primers (5'-3')	Reverse primers (5'-3')	Anneling temperature (0C)	Expected amplified product size (bp)	Structural annotation		Functional annotation	
								Sequence components of *kabuli* genome	*Kabuli* gene accession IDs	NCBI-KOG	TFs
Ca1_P1_12774149	Ca1	12774149	TTAAT/TTAATCGTTATAAT	GAAGCAGCCTCAACCTCAAC	TCCGCCACCTCTAAATATGAA	60.0	782	INTERGENIC	--	--	--
Ca1_P1_12776177	Ca1	12776177	T/TC	ATCTCCCCGTCAGATATCCC	GACAATAACCACCCAGTGCC	60.1	225	INTERGENIC	--	--	--
Ca1_P1_1280971	Ca1	1280971	TTATATATATATATATATATATA/TTATATATATATATATATATATATA	GGGGAAAACCTTGCTCCTAC	AGGGGGAGCAAAACAAAAAT	59.9	379	INTRON	Ca_00158	--	--
Ca1_P1_12816686	Ca1	12816686	CAAAAAAAAAA/CAAAAAAAAAAA	CCTTCATTGGACTTGATGGG	TAAAATAGGACGGGTGCAGG	60.3	818	DRR	Ca_02467	U	WOX
Ca1_P1_12876413	Ca1	12876413	AAGGGTAGG/AAGG	AAGCACGGCCTTCAAACTTA	CCAAAACATGCTGCTAACCC	59.9	574	DRR	Ca_02459	R	C2H2
Ca1_P1_12879368	Ca1	12879368	T/TG	CATAAATTGCATGTCGTGGC	GATCTCTTCGTTGCGTCACA	60.0	375	DRR	Ca_02458	A	--
Ca1_P1_12927388	Ca1	12927388	GAAAAAAA/GAAAAAA	ACCTACGACATCGGGTTTTG	TGGAAGAAACCAGAGTTGGG	59.9	919	INTERGENIC	--	--	--
Ca1_P1_12947634	Ca1	12947634	ATAAATTTAAA/ATAAATTTAAAATTTAAATTTAAA	AACTTTGTGGTCGTTTTGGC	TGCTTATTGTGGCTGCTTTG	60.0	647	INTERGENIC	--	--	--
Ca1_P1_12950830	Ca1	12950830	TTT/TTTATT	GCATTGAGTGCATTTGGTTG	ACGCCATGCATACTGACAGA	60.1	130	INTERGENIC	--	--	--
Ca1_P1_12956423	Ca1	12956423	CAAAAAAA/CAAAAA	TTGTCGGATACCTGCTCAGT	TTGCAAATTCGGACAAACAA	58.3	455	INTERGENIC	--	--	--
Ca1_P1_1297317	Ca1	1297317	ATTTTTTTTTT/ATTTTTTTTT	TGCATGAACAAAGGGACAAG	TAACATCCCGCGTAACATGA	59.7	625	INTERGENIC	--	--	--
Ca1_P1_12988934	Ca1	12988934	C/CGGG	ATCGGCATCCTTCCTTCTTT	GACGCTAAGCCCCTTTCTCT	60.0	354	INTERGENIC	--	--	--

INDEL marker IDs	Chromosomes/ unanchored scaffolds	Physical positions (bp)	InDels (*Kabuli* reference genome-CDC Frontier/PI)	Forward primers (5'-3')	Reverse primers (5'-3')	Anneling temperature (0C)	Expected amplified product size (bp)	Structural annotation		Functional annotation	
								Sequence components of *kabuli* genome	*Kabuli* gene accession IDs	NCBI-KOG	TFs
Ca1_P1_13005887	Ca1	13005887	TAAAAAAAAAAAAA/TAAAAAAAAAAA	TCGTCATCTAGCACCAGCAC	TCCCTCGTAAAGACCCATCT	60.0	347	INTERGENIC	--	--	--
Ca1_P1_13007507	Ca1	13007507	TTAT/TTATATAT	CACGGGTCCAGATGGTTAGT	CCAAGCATCTTGGTGCATAA	59.8	322	INTERGENIC	--	--	--
Ca1_P1_13012418	Ca1	13012418	TCC/TC	TTGATGAGGATCTTTCTATGGGA	TGCGATAATATTCATCCCGTG	59.9	306	INTERGENIC	--	--	--
Ca1_P1_13052834	Ca1	13052834	ACCCCCCC/ACCCCCC	CCAAAAGCCAAAACCAAAAA	GGAGTCATTGTGAGGGAGGA	60.0	513	INTERGENIC	--	--	--
Ca1_P1_13117590	Ca1	13117590	TAATAAAAATAAAAATAAAA/TAATAAAAATAAAA	TGTTGTTTACGGAATTTGGTTTT	TTTCGTTGTATATTAAAGCACGG	59.7	547	INTERGENIC	--	--	--
Ca1_P1_13134589	Ca1	13134589	TAAAAAAA/TAAAAAAAA	CGATGGATGACTTCCCGTAT	TTTACGTTGACGCACGAGAC	59.8	587	INTERGENIC	--	--	--
Ca1_P1_1319197	Ca1	1319197	AATATATATATATATATATATATATATATATATATATATATA/AATATATATATATATATATATATATATATATATATATA	AACCATGGCATTTTCAAATTA	GAGCACCAAAAAGTTGCACA	57.1	567	INTERGENIC	--	--	--
Ca1_P1_13196182	Ca1	13196182	GCTCTCTCTCTCTCTCTCTCTCTCTC/GCTCTCTCTCTCTCTCTCTCTCTCTC	GGAAATGCGGTTCAATCACT	ATGTGGCGAAGATCCAAACT	59.9	199	INTERGENIC	--	--	--
Ca1_P1_13302943	Ca1	13302943	TTTA/TTACTTTCATCTTA	AAGATGGATTCAATGCAGGG	TTTTGTCTGAACCTTTGATTTTGA	59.9	373	INTERGENIC	--	--	--
Ca1_P1_13303635	Ca1	13303635	AA/AATTA	TTCAAAATCAAAGGTTCAGACAAA	CTACGGGGTTGTGGTATTGG	60.0	429	INTERGENIC	--	--	--
Ca1_P1_13342022	Ca1	13342022	TTATATATATATATATATATATATATAT/TTATATATATATATATATATATAT	GTCCCATTGTGGAAACCAGT	TCGGATCGGATCAAATTTCT	59.7	633	INTRON	Ca_14096	--	--
Ca1_P1_13342900	Ca1	13342900	GATATATATATATATATATATATATATATATATA/GATATATATATATATATATATATATATATATA	GGTACAACATGTTTAGACGCTTG	CAATCCCGCGGTTATTCTTA	58.8	278	INTRON	Ca_14096	--	--

INDEL marker IDs	Chromosomes/ unanchored scaffolds	Physical positions (bp)	InDels (*Kabuli* reference genome-CDC Frontier/PI)	Forward primers (5'-3')	Reverse primers (5'-3')	Anneling temperature (0C)	Expected amplified product size (bp)	Structural annotation		Functional annotation	
								Sequence components of *kabuli* genome	*Kabuli* gene accession IDs	NCBI-KOG	TFs
Ca1_P1_13552505	Ca1	13552505	AT/A	GAAGAAGGGTTCTTGGGAGG	GCGTGGTAGACGTGGAAACT	60.0	569	DRR	Ca_14122	F	MYB_related
Ca1_P1_13570743	Ca1	13570743	AT/ATCTCCTCCT	CCGGAAGAACATTCAAAGGA	TTCAAACCCAAACCTCTTCG	60.0	460	INTERGENIC	--	--	--
Ca1_P1_13671430	Ca1	13671430	T/TC	CGAGGAAGAGGTGGATTCAA	TGGTTTATGATAGGTTCAATTTGG	60.2	348	INTERGENIC	--	--	--
Ca1_P1_13671513	Ca1	13671513	AACA/AA	CGAGGAAGAGGTGGATTCAA	TGGTTTATGATAGGTTCAATTTGG	60.2	348	INTERGENIC	--	--	--
Ca1_P1_13671962	Ca1	13671962	ATTT/ATT	CCAAATTGAACCTATCATAAACCA	AAAAGACCAAATCGGGAAAAA	59.2	748	INTERGENIC	--	--	--
Ca1_P1_13672025	Ca1	13672025	TA/T	CCAAATTGAACCTATCATAAACCA	AAAAGACCAAATCGGGAAAAA	59.2	748	INTERGENIC	--	--	--
Ca1_P1_13672495	Ca1	13672495	CAAAAAAAAA/CAAAAAAAA	TTTTTCCCGATTTGGTCTTTT	TTTCCCGATTTGCTCCTTTA	59.8	448	INTERGENIC	--	--	--
Ca1_P1_13672951	Ca1	13672951	TAAAAAAAAA/TAAAAAAAAAA	TAAAGGAGCAAATCGGGAAA	AAGCCCCGTGAATAATTGTG	59.7	506	INTERGENIC	--	--	--
Ca1_P1_13673338	Ca1	13673338	TTCCCCA/TTCCCCAATCCCCA	CACAATTATTCACGGGGCTT	TGTTCAGCCCTAGTCCAACC	59.8	187	INTERGENIC	--	--	--
Ca1_P1_13674092	Ca1	13674092	ATTTT/ATTT	AACTGGTTCGATTCGGTTTTT	TGAAAATTTCGCATTCACCA	59.9	382	INTERGENIC	--	--	--
Ca1_P1_13676235	Ca1	13676235	T/TC	CCGCTACAAGGCGATAGAAG	GGGTGTCATCATAGGTGGCT	60.0	856	DRR	Ca_14132	--	--
Ca1_P1_13676261	Ca1	13676261	ACT/ACTCT	CCGCTACAAGGCGATAGAAG	GGGTGTCATCATAGGTGGCT	60.0	856	DRR	Ca_14132	--	--

INDEL marker IDs	Chromosomes/ unanchored scaffolds	Physical positions (bp)	InDels (*Kabuli* reference genome-CDC Frontier/PI)	Forward primers (5'-3')	Reverse primers (5'-3')	Anneling temperature (0C)	Expected amplified product size (bp)	Structural annotation		Functional annotation	
								Sequence components of *kabuli* genome	*Kabuli* gene accession IDs	NCBI-KOG	TFs
Ca1_P1_13677531	Ca1	13677531	TAAAAAA/TAAAAAAA	ATCTGTCATTTGTGGCTCCC	TTGGTTGAGAGGGTATTCCG	59.9	657	INTERGENIC	--	--	--
Ca1_P1_13677982	Ca1	13677982	AAAC/A	CGGAATACCCTCTCAACCAA	GATTTTCCGAAAGGGAACAA	59.9	314	DRR	Ca_14133	--	ERF
Ca1_P1_13678266	Ca1	13678266	TGGGGG/TGGGG	CGGAATACCCTCTCAACCAA	TCCAAAGTATTTCTGCATTGAA	59.9	969	DRR	Ca_14133	--	ERF
Ca1_P1_13678320	Ca1	13678320	CATA/CATAAACGAGCCTATA	TTGTTCCCTTTCGGAAAATC	TCCAAAGTATTTCTGCATTGAA	59.0	675	DRR	Ca_14133	--	ERF
Ca1_P1_13679562	Ca1	13679562	TAAAA/TAAA	TAACCCCTATCCATGCCAAA	GCAAATTCCGAGTCCATGAT	60.1	524	DRR	Ca_14133	--	ERF
Ca1_P1_13680810	Ca1	13680810	ATTTTT/ATTTT	TGGTTGAACTGTGAACAGGC	CCAATTTCCCCTACCCAAAT	59.7	559	INTERGENIC	--	--	--
Ca1_P1_13682432	Ca1	13682432	CAA/CA	TTTCTTCCTCGGCCTCTCTT	TTCGGCGTTACACTAGGGAC	60.5	812	INTERGENIC	--	--	--
Ca1_P1_13683741	Ca1	13683741	CTTTTTTTTTTT/CTTTTTTTTTT	TCTAGGGCGTGCATGTGTTA	GACTTTGCACCCATTTCGAT	60.3	245	DRR	Ca_14134	--	MYB_related
Ca1_P1_13685328	Ca1	13685328	CTATA/CTA	CACCCATTGCTTGGTCTTTT	TGGTGAACCTTACTCGGTCC	60.0	650	INTRON	Ca_14134	--	MYB_related
Ca1_P1_13685418	Ca1	13685418	CATATATAT/CATAT	CACCCATTGCTTGGTCTTTT	TGGTGAACCTTACTCGGTCC	60.0	650	INTRON	Ca_14134	--	MYB_related
Ca1_P1_13685880	Ca1	13685880	TTAGATA/TTA	GGACCGAGTAAGGTTCACCA	TTTTGACACCACCCGTACAA	60.0	205	INTRON	Ca_14134	--	MYB_related
Ca1_P1_13686356	Ca1	13686356	T/TCTC	TTGTACGGGTGGTGTCAAAA	GAGGGTGACTCTGCTGGAAC	59.9	655	INTRON	Ca_14134	--	MYB_related

INDEL marker IDs	Chromosomes/ unanchored scaffolds	Physical positions (bp)	InDels (*Kabuli* reference genome-CDC Frontier/PI)	Forward primers (5'-3')	Reverse primers (5'-3')	Anneling temperature (0C)	Expected amplified product size (bp)	Structural annotation		Functional annotation	
								Sequence components of *kabuli* genome	*Kabuli* gene accession IDs	NCBI-KOG	TFs
Ca1_P1_13688361	Ca1	13688361	C/CG	TCACACGTTGTCATTTGGAA	GGCTACATTGTGGTGGAGGT	58.5	760	INTERGENIC	--	--	--
Ca1_P1_13688857	Ca1	13688857	CCCGCACACC/CCC	ACCTCCACCACAATGTAGCC	GGGTAGCGGACTCAGGTGTA	59.9	312	INTERGENIC	--	--	--
Ca1_P1_13690477	Ca1	13690477	CAA/CAATCGGTTTTATAA	CCGGTTATTTAACCATAACCAA	TGCAAGGAACAAATGGCATA	57.9	200	INTERGENIC	--	--	--
Ca1_P1_13692764	Ca1	13692764	CTTTT/CTTTTT	CGCCAGTGGTTATGCTCTTA	AAGGAACCACACTTGAACCG	58.9	875	INTERGENIC	--	--	--
Ca1_P1_13694461	Ca1	13694461	GTA/GTAATA	CCACTTCGCCACGTTTTTAT	CGGTTTATCGTCAAAGCGAT	60.0	379	INTERGENIC	--	--	--
Ca1_P1_13694751	Ca1	13694751	TA/TAA	CCACTTCGCCACGTTTTTAT	TTTCAATTTCAACCCCAACA	60.0	882	INTERGENIC	--	--	--
Ca1_P1_13696144	Ca1	13696144	TAAAAA/TAAAA	TTTGCGTTACGAATTTGATGA	AGAAGTTGAACTCACGAATCACA	59.2	757	INTERGENIC	--	--	--
Ca1_P1_13697223	Ca1	13697223	CAA/C	GCAAGCATTTTTGGCAAACT	GGGCATTATTCTTAAACCATACAA	60.3	546	DRR	Ca_14135	--	bHLH
Ca1_P1_13697453	Ca1	13697453	TAAAAAAA/TAAAAAAAA	TCTGAAAACAATTTCCCCTTG	CTAGACCATGGCCGTTCAAT	59.1	865	DRR	Ca_14135	--	bHLH
Ca1_P1_13698221	Ca1	13698221	TG/T	TTGAACGGCCATGGTCTAGT	ATGGAATTATGGTGGCGTGT	60.5	611	DRR	Ca_14135	--	bHLH
Ca1_P1_13699506	Ca1	13699506	GAAAAAAAAAA/GAAAAAAAAA	GGCAAGACCATTCAGCTCTC	CGGTTATGGGATGATTTAGGAA	60.0	921	INTRON	Ca_14135	--	bHLH
Ca1_P1_13701840	Ca1	13701840	AGAATGTATAT/A	TGCAAAACCAAGTCTGTCCA	ACCTTGCCCCTGAAGTAGGT	60.3	632	INTRON	Ca_14135	--	bHLH

INDEL marker IDs	Chromosomes/ unanchored scaffolds	Physical positions (bp)	InDels (*Kabuli* reference genome-CDC Frontier/PI)	Forward primers (5'-3')	Reverse primers (5'-3')	Anneling temperature (0C)	Expected amplified product size (bp)	Structural annotation		Functional annotation	
								Sequence components of *kabuli* genome	*Kabuli* gene accession IDs	NCBI-KOG	TFs
Ca1_P1_13704708	Ca1	13704708	A/AT	CAAACCGACACAAATTTTCAA	GGATATGCAATTGGGATGCT	58.6	725	INTRON	Ca_14135	--	bHLH
Ca1_P1_13754063	Ca1	13754063	CATATATATATATATATATATAT/CATATATATATATATATATATATAT	CCATATCAATTTGGAAAATTTGTG	AAATGATAACGCTTTTGCCG	59.5	387	INTERGENIC	--	--	--
Ca1_P1_13767969	Ca1	13767969	TA/TACA	TGTTCCTTTCACGTGCTTTG	CTCAACAAACGAGCCCTCTC	59.9	655	INTRON	Ca_14138	BK	bHLH
Ca1_P1_13814671	Ca1	13814671	TT/TTCCT	TGCTGATTTCAGGAATTCGAT	TGTGCAGCCAATAAACCAAA	59.7	184	INTRON	Ca_14146	G	Trihelix
Ca1_P1_13822052	Ca1	13822052	GAA/GA	AAGCCGTGTCTCTCAAGCAT	CGAAGCAACGAAAACAAACA	60.0	503	INTERGENIC	--	--	--
Ca1_P1_13841782	Ca1	13841782	GTTTTTTTTTT/GTTTTTTTTTTT	CATCTCCTATTTGCGGCCTA	TGAATCAATGCAACTTTTTATGC	60.2	465	INTERGENIC	--	--	--
Ca1_P1_13887259	Ca1	13887259	ATTTTTTTTTTTT/ATTTTTTTTTTTTTT	GTAAGGCCCAAGCTATCGGT	CCCAAAAACACCTGTATCGG	60.5	516	INTERGENIC	--	--	--
Ca1_P1_13939746	Ca1	13939746	TA/TACTTTATTAAAAAA	TCAGGTGTCTCATCCACTGC	TGGATTTTCAATGCCAGACA	59.8	755	DRR	Ca_07137	--	--
Ca1_P1_13950738	Ca1	13950738	TAAAAAAAAAA/TAAAAAAAAAAA	CACAAAACCATTATCCCCGT	TTGCGACAAATTAAGACCCC	59.5	593	INTERGENIC	--	--	--
Ca1_P1_1397435	Ca1	1397435	CA/C	TGGAAACAGCAACGTGGTAG	CAAAGGGTTGGATTTTCCAC	59.8	555	INTERGENIC	--	--	--
Ca1_P1_14094110	Ca1	14094110	TA/TAA	GAATTTCTTTTTGGGAGGAGAA	CCAACTACATTGTTTGTGCCA	58.7	442	INTERGENIC	--	--	--
Ca1_P1_14146591	Ca1	14146591	TTATATATATATATATATATATATATATATATATATATAT/TTATATATATATATATATATATATATATATATATAT	GGGGAGAGTTCTGAGGAACA	TCAAATTCCCTCCCATTTCA	59.2	711	INTERGENIC	--	--	--

INDEL marker IDs	Chromosomes/ unanchored scaffolds	Physical positions (bp)	InDels (*Kabuli* reference genome-CDC Frontier/PI)	Forward primers (5'-3')	Reverse primers (5'-3')	Anneling temperature (0C)	Expected amplified product size (bp)	Structural annotation		Functional annotation	
								Sequence components of *kabuli* genome	*Kabuli* gene accession IDs	NCBI-KOG	TFs
Ca1_P1_14180657	Ca1	14180657	ATCTCTCTCTCTCTCTCTCTCTCTCTCTCTCTCTCTCT/ATCTCTCTCTCTCTCTCTCTCTCTCTCTCTCTCTCTCT	TTGCGGCTCTGTGTACAGTC	TCCCCTGCCATTTGATAAAC	60.1	531	INTERGENIC	--	--	--
Ca1_P1_1427442	Ca1	1427442	TTATATATATATATATATATA/TTATATATATATATATATA	TTTGCGTAATTGTTTGTGTCAA	TCCACCCTCACCTCACTACC	59.2	554	DRR	Ca_00173	O	--
Ca1_P1_14318628	Ca1	14318628	GGATATA/G	ATCATCATATCTGGGGCCAA	CCTTCAACATTTGGGCTTGT	60.1	331	INTERGENIC	--	--	--
Ca1_P1_14385063	Ca1	14385063	CAA/CAAA	AATCGTCTCCGTCACACACA	GCGAATTGCAGACTGAAACA	60.2	662	DRR	Ca_07096	--	SAP
Ca1_P1_14400629	Ca1	14400629	TCAAAGCAA/TCAA	TGATGCGGCATTGAAGTTAG	TGGGAAACACGGATACAAGA	59.8	290	INTERGENIC	--	--	--
Ca1_P1_14441702	Ca1	14441702	CNNNNNNNNNN/CNNNNNNN	TTTCCATTTTCCTCTGCCAC	TTGGTGTTGGAATTCGTTGA	60.1	408	CDS (large-effect mutations)	Ca_07092	--	--
Ca1_P1_14541002	Ca1	14541002	GATATATATATAT/GATATATATATATAT	CTAGTTCGTAAGCCGTGCGT	AGGCATGGTGTTGGAGTCAT	60.5	259	INTERGENIC	--	--	--
Ca1_P1_14682464	Ca1	14682464	CTTTT/CTTT	TCCATTGTTGAGCGAAAACA	TGGATGAAGGATCAACCACA	60.2	659	INTERGENIC	--	--	--
Ca1_P1_14777698	Ca1	14777698	TAAAAAAAA/TAAAAAAA	CAATGACCTTGGTCCTCCTG	TTAACTGCAGCCATGCATCT	60.5	799	DRR	Ca_07052	R	--
Ca1_P1_14800179	Ca1	14800179	AATATATATATATATATATA/AATATATATATATATATATATA	TTGGATGTTGATAAATTGAAGCA	GAGGAATTGTTATTGGTTGTTGG	59.5	284	DRR	Ca_07050	R	MYB
Ca1_P1_14893455	Ca1	14893455	GTTTTTTTT/GTTTTTTT	AGGGACAAGGTTGGGTTTTT	TTAAAACTTTGCACGGAGGG	59.7	291	INTERGENIC	--	--	--
Ca1_P1_14940500	Ca1	14940500	GTTT/GTTTT	GAGGGTATGCCCCAAGTTTT	TTGACCGATGGACAAACAAA	60.2	493	INTERGENIC	--	--	--

INDEL marker IDs	Chromosomes/ unanchored scaffolds	Physical positions (bp)	InDels (*Kabuli* reference genome-CDC Frontier/PI)	Forward primers (5'-3')	Reverse primers (5'-3')	Anneling temperature (0C)	Expected amplified product size (bp)	Structural annotation		Functional annotation	
								Sequence components of *kabuli* genome	*Kabuli* gene accession IDs	NCBI-KOG	TFs
Ca1_P1_14995847	Ca1	14995847	ACATGCATGCA/ACATGCA	CAGCGCTTTTCCCAAATAAG	CCAAAAAGGGTGGAGATTGA	59.8	359	INTERGENIC	--	--	--
Ca1_P1_15037301	Ca1	15037301	CATATATATATATATA/CATATATATATATATATA	GTCACACGAGCATGGAAAGT	TAACCTGGCCAGTCACCATT	58.7	578	INTERGENIC	--	--	--
Ca1_P1_15051879	Ca1	15051879	GATATATATATATATATATATATATAT/GATATATATATATATATATATATATATATAT	GCGACCACAAGCAGGTTAAG	CGACGAGAGTAATTGTGTCCA	60.8	803	INTERGENIC	--	--	--
Ca1_P1_15188200	Ca1	15188200	TTATATATATATATATAT/TTATATATATATATAT	TCAGCCAAACCACAATTTGA	TCCTAGTGCGAGGTCGTTTT	60.1	661	INTERGENIC	--	--	--
Ca1_P1_15326926	Ca1	15326926	CATATATATATAT/CATATATATAT	GTTAAGCATCCCAAGGTGGA	AGCCAAGAGTTTGTGGAGTTG	59.9	626	INTERGENIC	--	--	--
Ca1_P1_15380342	Ca1	15380342	ATTCTTCTTCTTCTTCTTCTT/ATTCTTCTTCTTCTTCTTCTTCTTCTT	GGCTAAGCCATAAGTTCCCA	TGAACAGGATTTGGACGACA	59.2	238	INTERGENIC	--	--	--
Ca1_P1_1540452	Ca1	1540452	CTTTTTTT/CTTTTTTTTTTTT	AGTCTAAAGCCCCCGATTGT	ATTTGGCGGGTCTTGTGTAG	60.0	476	INTERGENIC	--	--	--
Ca1_P1_15437403	Ca1	15437403	CTATTATTATTATTATTATTATTATTATTATTATTATTATTATTATTATTATTA/CTATTATTATTATTATTATTATTATTATTATTATTATTATTATTATTATTATTATTATTATTA	GAGGACCAAAACGGCAATTA	GAGTTTGATAGAGTGGGGCG	59.9	323	DRR	Ca_06994	GMW	NAC
Ca1_P1_1559946	Ca1	1559946	GTATTATTATTATTATTATTATTATTATTATTATTATTATTATTATTATTATT/GTATTATTATTATTATTATTATTATTATTATTATTATTATTATTATTATTATTATTATTATT	AAAGCACACCCCATGCTAAC	ATATTGCAGAAGCACCACCC	60.0	551	INTRON	Ca_00189	--	--
Ca1_P1_15641150	Ca1	15641150	GA/GGACAACCCATATA	AGGGAAAGACAAAAGCAGCA	GGAGTCAAGCAAAGGCACTC	60.0	528	INTERGENIC	--	--	--
Ca1_P1_15667505	Ca1	15667505	AC/A	TCGGGCAAAAATTCTGACAT	CAATTCCCACAGGTACGCTT	60.4	447	INTERGENIC	--	--	--
Ca1_P1_15811388	Ca1	15811388	TAAAAAAA/TAAAAAAAA	GCGAGATTTTCGACTTCTGC	TGCGGCAAACGTAATTGTAA	60.1	590	INTERGENIC	--	--	--

INDEL marker IDs	Chromosomes/ unanchored scaffolds	Physical positions (bp)	InDels (*Kabuli* reference genome-CDC Frontier/PI)	Forward primers (5'-3')	Reverse primers (5'-3')	Anneling temperature (0C)	Expected amplified product size (bp)	Structural annotation		Functional annotation	
								Sequence components of *kabuli* genome	*Kabuli* gene accession IDs	NCBI-KOG	TFs
Ca1_P1_15821141	Ca1	15821141	GTTATT/GTTATTCATTATT	GGGGGAATTGGTGAAAAGTT	ATTAAACCAGCCACACAGGC	60.0	530	INTERGENIC	--	--	--
Ca1_P1_15835234	Ca1	15835234	TTTATTATTATTATTATTATTATTATTATTATT/TTTATTATTATTATTATTATTATTATTATT	TCGTTTGCCGTTAACTAGGG	TTGGCCTAACCTGAAAGCAT	60.1	768	INTERGENIC	--	--	--
Ca1_P1_15849492	Ca1	15849492	CAAAAAAAAA/CAAAAAAAAAA	CCCCATTTTGTTTCTCTCCA	CCACTTTCCGAAAACAACGA	59.9	335	INTERGENIC	--	--	--
Ca1_P1_15849587	Ca1	15849587	AATATATATATATATATATATATATATATATATATA/AATATATATATATATATATATATATATATATATA	CCCCATTTTGTTTCTCTCCA	ATTCTTGGAGGAGCGGGTAT	59.9	872	INTERGENIC	--	--	--
Ca1_P1_15852981	Ca1	15852981	CAA/CA	TGGAGGAAAAATGTACAACATCC	CGCTCGCGGTCATCTAGTA	60.1	400	INTERGENIC	--	--	--
Ca1_P1_15865372	Ca1	15865372	TTATATATATATATATATATA/TTATATATATATATATATATATA	AAAATGGGTTGGTGTCAAGC	ACAATAATGAAGGGGGAGCC	59.8	654	INTRON	Ca_06964	QR	B3
Ca1_P1_15890229	Ca1	15890229	ATTTTT/ATTTTTT	AATTCAAATGCGCTGCTTCT	TTGTCAATTACTTGCATCTCCC	60.0	707	DRR	Ca_06963	--	--
Ca1_P1_15947037	Ca1	15947037	TTCTCTCTCTCTCTCTCTCTCTCTCTCT/TTCTCTCTCTCTCTCTCTCTCTCTCTCTCTCT	AAGAATGGCGTGAGCTTCTC	TGCATTTGCACTGAAACGA	59.6	210	INTERGENIC	--	--	--
Ca1_P1_15952461	Ca1	15952461	GAAAAAAAAA/GAAAAAAAAAA	CCTTGGATTTGGCTACCTCA	CATTGGCCTCTCCATCAACT	60.1	422	INTERGENIC	--	--	--
Ca1_P1_16002199	Ca1	16002199	CTATATATATATATATATATATATATATAT/CTATATATATATATATATATATATATATATAT	TGGGAAAATTGGTGCCTAAA	TGCTTTTGCCACACAAAAAG	60.3	612	INTERGENIC	--	--	--
Ca1_P1_1606579	Ca1	1606579	CTATTATTATTATTATTAT/CTATTATTATTATTAT	TTCATCTGTCTCGCTCGATG	TGCAATTTTGACTCTTTCAAAC	60.1	557	DRR	Ca_00196	S	--
Ca1_P1_16078538	Ca1	16078538	CTTTTTTTTT/CTTTTTTTT	TGCATGTTGTTGGTAGAGTCAA	GCACAAGGTATGCCAAATGA	59.2	632	INTERGENIC	--	--	--

INDEL marker IDs	Chromosomes/ unanchored scaffolds	Physical positions (bp)	InDels (*Kabuli* reference genome-CDC Frontier/PI)	Forward primers (5'-3')	Reverse primers (5'-3')	Anneling temperature (0C)	Expected amplified product size (bp)	Structural annotation		Functional annotation	
								Sequence components of *kabuli* genome	*Kabuli* gene accession IDs	NCBI-KOG	TFs
Ca1_P1_16087465	Ca1	16087465	AGG/AGGG	GACGAAAGCAGAACAGAACAGA	AATCGAGCATTGCATTTTCA	59.7	836	DRR	Ca_06942	--	bHLH
Ca1_P1_16092158	Ca1	16092158	CTATATATATATATATAT/CTATATATATATATATAT	AGCTACGGTGACTCGGTTTG	CAACATGTCCATGCAGCATAC	60.3	710	INTERGENIC	--	--	--
Ca1_P1_16096244	Ca1	16096244	TG/TGG	ATGTAAGATTGGAAGCGGCA	CAATTGTTCCCCACTTGCTT	60.6	298	INTERGENIC	--	--	--
Ca1_P1_16096283	Ca1	16096283	TATTTAATTT/TATTT	ATGTAAGATTGGAAGCGGCA	CAATTGTTCCCCACTTGCTT	60.6	298	INTERGENIC	--	--	--
Ca1_P1_16106451	Ca1	16106451	ACCC/ACCCC	AGCCTTTGGCTGTCACTCAT	CCATAATACAAACTGTAGCCGC	59.9	341	INTERGENIC	--	--	--
Ca1_P1_16106759	Ca1	16106759	ATTTTTTT/ATTTTTT	TCAGCATTTCACGCACCTAC	AAGGGGATCAAATTGAAGTAAAAA	59.9	723	INTERGENIC	--	--	--
Ca1_P1_16108380	Ca1	16108380	AT/A	CGAGAGCGATTCACATTTTC	ACGTGGGTTGGCAGATAGAG	58.5	861	INTERGENIC	--	--	--
Ca1_P1_16110091	Ca1	16110091	GATATATAT/GATATATATATATAT	AAATGTTATACGTTCGGGAAAGA	AAAGGACGGGTGTAGTCTTACG	59.0	375	INTRON	Ca_06940	R	--
Ca1_P1_16116299	Ca1	16116299	AT/ATT	AATGCAACGGTTGGTTTGAT	GAATTCTTGTTATAAATTTGGCGA	60.2	444	DRR	Ca_06940	R	--
Ca1_P1_16116426	Ca1	16116426	GA/GAA	AATGCAACGGTTGGTTTGAT	GCACAAATGAAAAGCAGAACA	60.2	826	DRR	Ca_06940	R	--
Ca1_P1_16118156	Ca1	16118156	AT/A	ATGCCATTTTGAAGACTGCC	TGGGGGCAGTTACCCTTATT	60.1	489	INTERGENIC	--	--	--
Ca1_P1_16145317	Ca1	16145317	TAAA/TAAAA	TGGACATAATTAAATGGCGG	TTGTATTGTGCAACTCAGGGA	58.4	341	INTERGENIC	--	--	--

INDEL marker IDs	Chromosomes/ unanchored scaffolds	Physical positions (bp)	InDels (*Kabuli* reference genome-CDC Frontier/PI)	Forward primers (5'-3')	Reverse primers (5'-3')	Anneling temperature (0C)	Expected amplified product size (bp)	Structural annotation		Functional annotation	
								Sequence components of *kabuli* genome	*Kabuli* gene accession IDs	NCBI-KOG	TFs
Ca1_P1_16155793	Ca1	16155793	ATT/AT	GGCAACTCCATTGAATTCGT	AATCCCCACCCTAAATGACC	59.9	333	INTERGENIC	--	--	--
Ca1_P1_16155879	Ca1	16155879	TAAAAAAAA/TAAAAAAAAAA	GGCAACTCCATTGAATTCGT	AATCCCCACCCTAAATGACC	59.9	333	INTERGENIC	--	--	--
Ca1_P1_16158386	Ca1	16158386	TAAAA/TAAAAAA	TAGAGCCGTCAAAATGGACC	CACTAAATGGGTTGCGGACT	60.1	314	INTERGENIC	--	--	--
Ca1_P1_16158489	Ca1	16158489	TAAAAA/TAAAAAAAA	TAGAGCCGTCAAAATGGACC	CACTAAATGGGTTGCGGACT	60.1	314	INTERGENIC	--	--	--
Ca1_P1_16159274	Ca1	16159274	AAAAA/AAAAACAAAA	ATGGTTTTAAGCATTCCCCA	TTAAGTTGCGAGGTTGCTCA	59.3	457	INTERGENIC	--	--	--
Ca1_P1_16160051	Ca1	16160051	ATTTTTTTTT/ATTTTTTTTTT	AATGTCCTTTGCAATTTGGG	TACGTGCGCAAAGACAAATC	59.8	363	INTERGENIC	--	--	--
Ca1_P1_16160389	Ca1	16160389	TT/TTCT	CATGTGGATTTGTCTTTGCG	GCACAAGATCCTCAAAACTGG	60.1	238	INTERGENIC	--	--	--
Ca1_P1_16162054	Ca1	16162054	CTT/CTTT	TCCTCATGGCTTTTCATTCC	TCAATCTTCATAGGCTGAACGA	60.0	796	INTRON	Ca_06938	R	--
Ca1_P1_16162316	Ca1	16162316	TAAAAAAAAA/TAAAAAAAA	CGTTGGCTCTTTGATTGGTT	CCGCAAATAGTTTTCTTGGC	60.1	833	INTRON	Ca_06938	R	--
Ca1_P1_16164443	Ca1	16164443	AT/ATTAATAATGAGCACTT	ACATTCCCGAAGGGGTTATC	TCCCAAATATCCCCAAAACA	60.0	444	CDS (large-effect mutations)	Ca_06938	R	--
Ca1_P1_16166882	Ca1	16166882	AC/A	CCAGAGGTGTCCACTGATTACA	TTTTTAAAACGTGCCATGCT	60.0	495	DRR	Ca_06938	R	--
Ca1_P1_16167140	Ca1	16167140	TAAAA/TAAAAA	TTTTTAGCATGGCACGTTTT	CGGGTTGGATTAGATTTCAAG	58.4	378	INTERGENIC	--	--	--

INDEL marker IDs	Chromosomes/ unanchored scaffolds	Physical positions (bp)	InDels (*Kabuli* reference genome-CDC Frontier/PI)	Forward primers (5'-3')	Reverse primers (5'-3')	Anneling temperature (0C)	Expected amplified product size (bp)	Structural annotation		Functional annotation	
								Sequence components of *kabuli* genome	*Kabuli* gene accession IDs	NCBI-KOG	TFs
Ca1_P1_1617458	Ca1	1617458	AC/ACC	TGAAATCAAGTTTGGAAGAGTCA	TAAAAAGGAAATTGCGTCCG	58.9	350	INTERGENIC	--	--	--
Ca1_P1_16175034	Ca1	16175034	A/AAT	CCACGCGGTATTGTTTTTCT	TATGGAACGCGCGACATAAT	60.0	751	INTERGENIC	--	--	--
Ca1_P1_16182458	Ca1	16182458	TAAAAAAA/TAAAAAA	ATGTTGGATTCGCGTTGTTT	ACAAACGGTTGTTGTGGGAT	60.4	292	INTERGENIC	--	--	--
Ca1_P1_16184284	Ca1	16184284	A/AT	AAACCATTTCCAACCAAACG	TGGTCACAAGGAATGGATCA	59.7	862	DRR	Ca_06937	--	--
Ca1_P1_16187503	Ca1	16187503	AGTTTATGACG/AG	GTGTCACCATTGGCAATACG	TTCCTTCGACCTCACAATCC	59.8	382	CDS (large-effect mutations)	Ca_06937	--	--
Ca1_P1_16195304	Ca1	16195304	CAA/CAAA	AAAAAGATGGAGCCATAAGCC	CAGGTAAAATTGGGGTGGTG	59.6	562	INTERGENIC	--	--	--
Ca1_P1_16197519	Ca1	16197519	TA/TAA	TTGGTAAGTGGAAGAAGATGAGA	TTGGAGGCAACCAACAAAAT	58.0	789	INTERGENIC	--	--	--
Ca1_P1_16197580	Ca1	16197580	AAATAA/AAA	TTGGTAAGTGGAAGAAGATGAGA	TTGGAGGCAACCAACAAAAT	58.0	789	INTERGENIC	--	--	--
Ca1_P1_16199745	Ca1	16199745	AATATATATATATA/AATATATATATATATATATA	TCAAGTCCCGTCTTACCTCG	AGATAGGGACATGGCCTCCT	60.2	310	INTERGENIC	--	--	--
Ca1_P1_16199818	Ca1	16199818	G/GA	TCAAGTCCCGTCTTACCTCG	AGATAGGGACATGGCCTCCT	60.2	310	INTERGENIC	--	--	--
Ca1_P1_16201985	Ca1	16201985	CCTA/CCTACTA	CCAACCGTGACCAAAAGACT	AGTGTTTTTGTGAATCCGGC	60.0	877	INTERGENIC	--	--	--
Ca1_P1_16203089	Ca1	16203089	TC/T	CTTGTGTGGGGAAGGTCACT	TGTGTGACCCCTGCTGAATA	60.0	685	INTERGENIC	--	--	--

INDEL marker IDs	Chromosomes/ unanchored scaffolds	Physical positions (bp)	InDels (*Kabuli* reference genome-CDC Frontier/PI)	Forward primers (5'-3')	Reverse primers (5'-3')	Anneling temperature (0C)	Expected amplified product size (bp)	Structural annotation		Functional annotation	
								Sequence components of *kabuli* genome	*Kabuli* gene accession IDs	NCBI-KOG	TFs
Ca1_P1_16206365	Ca1	16206365	AA/AATTA	CTCGCAAGTTGTAGGTTCAATC	GGGGCTTACGAGTACAGACTCA	58.9	128	INTERGENIC	--	--	--
Ca1_P1_16206512	Ca1	16206512	GATA/GA	GAGTCTGTACTCGTAAGCCCCT	CGTCATCAACCACATTGATTT	58.9	603	INTERGENIC	--	--	--
Ca1_P1_16210891	Ca1	16210891	TA/T	CCAAGGTGGGAAAACACTTG	GCCTCTTCCAGCTGTTTGTT	60.4	280	INTERGENIC	--	--	--
Ca1_P1_16211868	Ca1	16211868	CT/CTATAT	TGGTGGGACACATCAAGAAA	GGCCAAAATCGCACTTACTC	59.9	465	INTRON	Ca_06936	--	bHLH
Ca1_P1_16212109	Ca1	16212109	CAA/CAATCTGTTAATCCAAA	TGGATTGGAATCAACCACCT	TTGACCTTGACATAACCCCA	60.2	539	INTRON	Ca_06936	--	bHLH
Ca1_P1_16214320	Ca1	16214320	ATAGTAGTA/ATAGTA	TGCCCTTTTACCAGGTTTTG	CCCCCACTCTCTCTTTTTCC	60.0	538	INTRON	Ca_06936	--	bHLH
Ca1_P1_16215379	Ca1	16215379	TTTGAAGGAATCATTGAA/TTTGAA	GCGTGGAATTAGGAAGGACA	TGAGAGTTTGTGGTCCCCTC	60.1	634	DRR	Ca_06936	--	bHLH
Ca1_P1_16254186	Ca1	16254186	TAAAAAAAAAA/TAAAAAAAAA	AAAAGGGCCATTTAGTTGGG	TTTTGCCGGCTAATTGAAAC	60.2	321	INTERGENIC	--	--	--
Ca1_P1_16259144	Ca1	16259144	GATATATATATATATATATATA/GATATATATATATATATATA	AAGTGTTTTGGGGAGATGAGAA	GCGTGAACTCCTAAAGCCTG	60.0	386	INTERGENIC	--	--	--
Ca1_P1_16259872	Ca1	16259872	AATATATATATATATATATATATATATATATATATAT/AATATATATATATATATATATATATATATATATATAT	GGCCAAACAGCTTTCCAATA	TTCCCTCCTAATTTTTCCGC	60.1	760	INTERGENIC	--	--	--
Ca1_P1_16398704	Ca1	16398704	TAAAAAAA/TAAAAAA	ATTCGCCAGAGATTGTGCTT	TCATATCCTTATTTACATTCTGACAAA	59.8	684	DRR	Ca_06924	--	--
Ca1_P1_16438862	Ca1	16438862	TAAAA/TAAA	TTTGTGGGAAACTTTGGAAT	TGCTTCAATTTTGAGGGGAC	57.0	996	INTERGENIC	--	--	--

INDEL marker IDs	Chromosomes/ unanchored scaffolds	Physical positions (bp)	InDels (*Kabuli* reference genome-CDC Frontier/PI)	Forward primers (5'-3')	Reverse primers (5'-3')	Anneling temperature (0C)	Expected amplified product size (bp)	Structural annotation		Functional annotation	
								Sequence components of *kabuli* genome	*Kabuli* gene accession IDs	NCBI-KOG	TFs
Ca1_P1_16475601	Ca1	16475601	TATAATAATAATAATAATAATAATAATAATAATAATAATAATAATAATAATAAAAATAATAATAAAAATAATAATAAT/TATAATAATAATAATAATAATAATAATAATAATAATAATAATAATAATAATAAAAATA[illegible]	TGGAAAAACCAAAAACCCAA	TTCTCCGGTATTGGAGGATG	60.2	753	INTRON	Ca_06916	Z	WRKY
Ca1_P1_16509431	Ca1	16509431	CA/C	AAATACGGTGCTTTTGGTGC	TCCGACGTGTAACTTCCCAT	60.0	714	INTRON	Ca_06913	--	--
Ca1_P1_16583164	Ca1	16583164	AATATATATATATATATATATATA/AATATATATATATATATATA	TTTGGCACTTTCTCATTCCC	GCCATGTATGGTCCAAGGTC	60.1	793	INTERGENIC	--	--	--
Ca1_P1_16629521	Ca1	16629521	TTTAATTAATTAATTAATT/TTTAATTAATTAATTAATTAATTAATT	AACGTTTGAGGATTCCGTTG	GAAACTCCAGCTCCAAAACG	60.0	370	INTERGENIC	--	--	--
Ca1_P1_16707141	Ca1	16707141	TTATATATATATATATATATATATATAT/TTATATATATATATATATATATATATAT	CTTTACAACCGCCACAAACC	CGAACAAAGTGCTTGTGGAA	60.4	755	INTERGENIC	--	--	--
Ca1_P1_16739207	Ca1	16739207	TTAATAATAATAATAATAATAATAATAATAATA/TTAATAATAATAATAATAATAATAATAATA	CCACTCATCGTGTAAGCTTTTC	TTCACAATTTGGGAGGGAAG	58.9	630	INTERGENIC	--	--	--
Ca1_P1_16782782	Ca1	16782782	TAAAAAAAAAA/TAAAAAAAAA	TTTTGAATCCAAGGACACCC	GCATCCAACCTAATTGCACA	59.8	627	INTERGENIC	--	--	--
Ca1_P1_16866662	Ca1	16866662	AATATATATATAT/AATATATATATATATAT	CAATTTGGGTCGTCCATCTC	TTAAGACCAACCCGAACCTG	60.3	614	DRR	Ca_06887	--	--
Ca1_P1_16901920	Ca1	16901920	T/TA	AATTTCCCCCTTTATGCGAC	GCCAGTGTTTGGAAGTTGGT	60.2	422	CDS (FRAME SHIFT)	Ca_06882	R	GRAS
Ca1_P1_16943629	Ca1	16943629	TTTTAAATTTA/TTTTAAATTTAAATTTA	TTTTCTTAAGACGGGCATGA	TGAAGGGTATACGGCACCTC	58.4	776	DRR	Ca_06876	O	--
Ca1_P1_16976485	Ca1	16976485	AATATATATATAT/AATATATATATATATAT	AAAATAGAGTCCATAAAACAACAAAAT	TGAGAAGTTGCATTGGCTTG	57.2	602	INTERGENIC	--	--	--
Ca1_P1_17001643	Ca1	17001643	TTAATAATAATAATAATAATAATAATAATAATAATAATAATAA/TTAATAATAATAATAATAATAATAATAATAATAATAATAATAATAA	TCAATTTTGTCATCCAGCAAA	GGCGTGGCACAATAAGTGTA	59.1	732	INTERGENIC	--	--	--

INDEL marker IDs	Chromosomes/ unanchored scaffolds	Physical positions (bp)	InDels (*Kabuli* reference genome-CDC Frontier/PI)	Forward primers (5'-3')	Reverse primers (5'-3')	Anneling temperature (0C)	Expected amplified product size (bp)	Structural annotation		Functional annotation	
								Sequence components of *kabuli* genome	*Kabuli* gene accession IDs	NCBI-KOG	TFs
Ca1_P1_17038860	Ca1	17038860	GATATATATATATATATATATATA/GATATATATATATATATATATATA	TCCTTCGTCTCCATTCATCC	CGATTTGATTTAGGCATGTTTG	60.0	833	INTERGENIC	--	--	--
Ca1_P1_1707710	Ca1	1707710	TAAAAAAAAAAAA/TAAAAAAAAAAA	TGAATTGAAGGGTTGGAGGA	TAATCCATTGCACCCCCTTA	60.4	479	INTERGENIC	--	--	--
Ca1_P1_17120792	Ca1	17120792	TAAAAAAA/TAAAAAAAA	TGTCATGGTAGTATATTTTCGCAGA	ACATTCAGGGGTGCAGGTAG	59.9	596	INTERGENIC	--	--	--
Ca1_P1_17121112	Ca1	17121112	CTT/CTTCTTTTT	CCTGCACCCCTGAATGTAGT	CGATCACAAATTTTGACTTATGTTG	60.0	557	INTERGENIC	--	--	--
Ca1_P1_17163326	Ca1	17163326	AATATATATATATATATATATATAT/AATATATATATATATATATATAT	ATGGCATGGTGTGACACTTC	ATCGAGGCATTGTAGGCTCA	59.4	402	INTERGENIC	--	--	--
Ca1_P1_17185713	Ca1	17185713	TTTGATATTTGATTAT/TTTGATATTTGATTATTGATATTTGATTAT	GCGTTAAAGTTGGAAACCCA	TGACGTGACATCGAGAGTCTG	60.0	594	INTERGENIC	--	--	--
Ca1_P1_17215249	Ca1	17215249	ATT/AT	GGTTGGGTTGAGGAGGAAAT	TGTGAACGAACACCTTCGAC	60.2	447	INTERGENIC	--	--	--
Ca1_P1_1723303	Ca1	1723303	TTGTAT/TT	ATGTCATCTGTGACCAAGCG	TGGATGAGTGAAAATTGATTGG	59.7	420	DRR	Ca_00216	R	--
Ca1_P1_1729960	Ca1	1729960	AAATAATAATAATAATAATAATAATAATAATAATAATAATAATAATAATAATAATAA/AAATAATAATAATAATAATAATAATAATAATAATAATAATAATAATAATAATAATAATAATAATAATAA	GGAACGACCGTTGACTGATT	TTAACTCAGAGGAGCGCAGC	60.0	618	DRR	Ca_00217	--	TALE
Ca1_P1_1732737	Ca1	1732737	TT/TTGT	GGAAGCTGGCCCTAAAAGTT	TGGAGAAGGTGGATTTGGTC	59.7	257	INTRON	Ca_00217	--	TALE
Ca1_P1_1736394	Ca1	1736394	C/CCTAT	GGCACTACTCCAAGATGGAAA	TTTTCCAGCACTCATTGACG	59.2	738	INTERGENIC	--	--	--
Ca1_P1_1737545	Ca1	1737545	CTATATAT/CTATAT	TGAGGTGAAGTTTTGGGTCC	TGCGTCAACTTGATAAAATATCG	59.9	786	INTERGENIC	--	--	--

INDEL marker IDs	Chromosomes/ unanchored scaffolds	Physical positions (bp)	InDels (*Kabuli* reference genome-CDC Frontier/PI)	Forward primers (5'-3')	Reverse primers (5'-3')	Anneling temperature (0C)	Expected amplified product size (bp)	Structural annotation		Functional annotation	
								Sequence components of *kabuli* genome	*Kabuli* gene accession IDs	NCBI-KOG	TFs
Ca1_P1_1744310	Ca1	1744310	ATGATGGATTGATGG/ATGATGG	TTGGCCTTCAATCAACCTTC	ATGGTATTGTGCCACAGGGT	60.1	397	DRR	Ca_00218	--	TALE
Ca1_P1_1745242	Ca1	1745242	AG/A	ATTGTGATTGCATTGCATGG	GAGTAGGTGCCTTCCCACAA	60.4	548	INTRON	Ca_00219	--	TALE
Ca1_P1_17478284	Ca1	17478284	TTATATATATATATATAT/TTATATATATATATATAT	CTTTCATTCCCATGTGACCC	TGTTTGGGAGGAAACAAGGT	60.2	573	INTRON	Ca_14787	--	--
Ca1_P1_17532277	Ca1	17532277	CTTTTTT/CTTTTTTT	AAAATTGGCAAATGTTTGTGTG	TCAATATTGGGGCAAAGCAT	59.8	700	INTERGENIC	--	--	--
Ca1_P1_17595576	Ca1	17595576	AATATATATATATATATATATATATATATAT/AATATATATATATATATATATATATATATATATATAT	CGTGGCCATTTTATTGTCTCT	TCACAAAAGTCATTGGTGGAA	59.1	581	INTERGENIC	--	--	--
Ca1_P1_17655526	Ca1	17655526	CTTATTTTTTTTATTTTTTTTAT/CTTATTTTTTTTAT	GTAGCATGCATCGCTTTTCA	TTGAGGGTCGAGTGAGCTTT	60.0	301	INTERGENIC	--	--	--
Ca1_P1_17666536	Ca1	17666536	TTTATTCATTATTAATTATTCATTATTAA/TTTATTCATTATTAA	GATGCCACCGTTGAGGTAAT	TGCACAAAAAGGGAGATGAA	59.8	661	INTERGENIC	--	--	--
Ca1_P1_17761773	Ca1	17761773	ATTTTTTTT/ATTTTTTT	ATAGGTTGTCGGGCACTCAC	GTCAGACAAGCAGCTGCAAA	60.0	563	INTRON	Ca_14761	IOT	--
Ca1_P1_1779893	Ca1	1779893	A/AAGT	GGTGAATGTTTGCTTCCTCAA	TGCATCGTCACTTTATACGAAA	60.1	222	INTERGENIC	--	--	--
Ca1_P1_1780788	Ca1	1780788	TTT/TTTAAAAAAAAAGATATT	AACCGAAATGCGACCTTTTA	CTGCGTCCCATAACCAAGTT	59.6	424	INTERGENIC	--	--	--
Ca1_P1_17816780	Ca1	17816780	TCC/TC	TGGCCATCAAGTGTCTTCTC	TTTCCAGTCCTTCTGATGCC	58.8	568	INTERGENIC	--	--	--
Ca1_P1_1783341	Ca1	1783341	T/TTA	CTGAACCTTTTTGCTCTGCC	TGACCTTCACACTGTTTGCC	60.0	940	INTRON	Ca_00224	S	--

**INDEL marker IDs**	**Chromosomes/ unanchored scaffolds**	**Physical positions (bp)**	**InDels (*Kabuli* reference genome-CDC Frontier/PI)**	**Forward primers (5'-3')**	**Reverse primers (5'-3')**	**Anneling temperature (0C)**	**Expected amplified product size (bp)**	**Structural annotation**		**Functional annotation**	
								**Sequence components of *kabuli* genome**	***Kabuli* gene accession IDs**	**NCBI-KOG**	**TFs**
Ca1_P1_1784592	Ca1	1784592	GTTT/GTTTT	TTGCAACAAGGCGTGTAAAG	CCGCAAGTAGAACTTTTGGC	59.9	892	DRR	Ca_00224	S	--
Ca1_P1_1785166	Ca1	1785166	AG/A	TCTACTTGCGGCAACTTGTG	CATCATTCCAATCAAGCACG	60.1	319	DRR	Ca_00224	S	--
Ca1_P1_17920630	Ca1	17920630	CATATATATATATATATATATATATA/CATATATATATATATATATATATA	CCAAAAATCAAAAGGGTGGA	TTTCTTCTTTCTGCTCCCCA	59.8	574	INTERGENIC	--	--	--
Ca1_P1_179359	Ca1	179359	AATATATATATATATATATATATATATATATATAT/AATATATATATATATATATATATATATATATATATATATATAT	CCTCAAACAATCGCAACCAT	ATTTTGGGGAAGTTGGATTC	60.9	726	INTERGENIC	--	--	--
Ca1_P1_1794406	Ca1	1794406	GTTT/GTT	TCTTGAACGAGAAAACCGCT	ACGGTTTCTTGCTTTTTCCC	60.0	365	INTERGENIC	--	--	--
Ca1_P1_17969327	Ca1	17969327	NNA/A,C,G,T	AAATAAGTGCTGGTGTGGGG	AGGTGCTTTCGTTGAGGAGT	59.9	492	INTERGENIC	--	--	--
Ca1_P1_1797134	Ca1	1797134	ACACCAC/ACAC	CCCTTTCATCAACCTCGTGT	CTGGAACACTCCATGGTCCT	60.0	501	INTRON	Ca_00227	P	--
Ca1_P1_1800007	Ca1	1800007	AGTT/A	TGTTGCACAAGGTCCAGAAG	TTTTGGCCAAGAGATGGATG	59.9	601	DRR	Ca_00227	P	--
Ca1_P1_18007777	Ca1	18007777	TTTCTTCTTCTTCTTCTTCTTCTT/TTTCTTCTTCTTCTTCTTCTT	CTTCCACGTGCTTCACTCAA	GAACGAATGTGATGGTCACG	60.0	524	CDS (large-effect mutations)	Ca_14742	--	--
Ca1_P1_18022154	Ca1	18022154	AATTATTATTATTATTATTATTATTATTATTA/AATTATTATTATTATTATTATTATTATTATTATTA	GGGGACTAAACGGGAAACAT	TTCGACTGAAACTCACTCCCT	60.1	438	INTERGENIC	--	--	--
Ca1_P1_1805050	Ca1	1805050	TAAA/TAAAA	TTCAGTTGACCTTTCTGCATTT	GGTTTGGGATTTTTGCATCA	58.9	727	DRR	Ca_00228	F	MYB_related
Ca1_P1_18078016	Ca1	18078016	TTCTCTCTCTCTCTCTCTCTCTCTCTCTCTCTCTCT/TTCTCTCTCTCTCTCTCTCTCTCTCTCTCTCTCT	CCCTTTTCTACCACCATCCA	CAACAATGTCTTTCCTTGCG	59.8	791	INTERGENIC	--	--	--

INDEL marker IDs	Chromosomes/ unanchored scaffolds	Physical positions (bp)	InDels (*Kabuli* reference genome-CDC Frontier/PI)	Forward primers (5'-3')	Reverse primers (5'-3')	Anneling temperature (0C)	Expected amplified product size (bp)	Structural annotation		Functional annotation	
								Sequence components of *kabuli* genome	*Kabuli* gene accession IDs	NCBI-KOG	TFs
Ca1_P1_18088	Ca1	18088	TA/TAA	CCGCTTGTTTTTCTTTGCAG	TTGATTGCTTGTTGCTCCAG	60.9	543	INTERGENIC	--	--	--
Ca1_P1_1811628	Ca1	1811628	AATGT/AATGTTATGT	TGTGAACGTACTTTGAGGCG	TGGCACTTACCTCTGGCTTT	59.9	469	INTERGENIC	--	--	--
Ca1_P1_1817297	Ca1	1817297	ATTTTT/ATTTT	TTCCAATTTCAACACATGCC	TGGAAAAATGGCCAGAGAAG	59.4	504	INTERGENIC	--	--	--
Ca1_P1_1817917	Ca1	1817917	G/GGAA	CTTCTCTGGCCATTTTTCCA	ATTGATTCGGCATAGCCAAG	60.2	752	INTERGENIC	--	--	--
Ca1_P1_18181716	Ca1	18181716	AATATATATATATATATATATATATA/AATATATATATATATATATATATATATATA	CGAATACTCATGGTTTTTGATCTG	ACCCAACGTAGCACGTTACC	59.9	626	INTERGENIC	--	--	--
Ca1_P1_18191422	Ca1	18191422	CAAA/CAAAA	CCAATGCTCAACTGCCCTAT	ATGAAGTGCTGGTACTGGGG	60.1	486	INTERGENIC	--	--	--
Ca1_P1_1820515	Ca1	1820515	AT/ATT	TGTTGACATTAAGGAAGACCGTT	GCTGTTACTACATGCTCTTCCAA	59.9	823	INTERGENIC	--	--	--
Ca1_P1_18298335	Ca1	18298335	TCC/TC	GGTAATGCCTCGTACAAAACAA	CATTCCTTATGCCTCCTACACC	59.1	751	INTERGENIC	--	--	--
Ca1_P1_18299880	Ca1	18299880	TTCCTATCCTATCC/TTCCTATCC	TGGATCACTCACCTCTCTTCC	TGGAAGTGGTGGTTTTGTTG	59.2	185	INTERGENIC	--	--	--
Ca1_P1_18300800	Ca1	18300800	ATTT/ATT	AGCAAGAAAATTGAAGGCCA	GGAAGCTTTAAAGGGAGCAAA	59.8	161	INTERGENIC	--	--	--
Ca1_P1_18301437	Ca1	18301437	AATT/A	TGATAAGCTAACAGGCCAAAAA	ACATGGGCTAGCAAGAAACC	58.9	417	INTERGENIC	--	--	--
Ca1_P1_18317638	Ca1	18317638	T/TG	TCATTTTCAACCCTTAGCAACA	TGGTAAGGTATACGCGGGAG	59.6	690	INTERGENIC	--	--	--

INDEL marker IDs	Chromosomes/ unanchored scaffolds	Physical positions (bp)	InDels (*Kabuli* reference genome-CDC Frontier/PI)	Forward primers (5'-3')	Reverse primers (5'-3')	Anneling temperature (0C)	Expected amplified product size (bp)	Structural annotation		Functional annotation	
								Sequence components of *kabuli* genome	*Kabuli* gene accession IDs	NCBI-KOG	TFs
Ca1_P1_18317709	Ca1	18317709	TAAAAAAA/TAAAAAA	TCATTTTCAACCCTTAG CAACA	TGGTAAGGTATACGCG GGAG	59.6	690	INTERGENIC	--	--	--
Ca1_P1_18325523	Ca1	18325523	AGG/AG	CGGATCATTACACACT TGCG	GCCAAAGCCATGTTTT CAAT	60.1	324	INTERGENIC	--	--	--
Ca1_P1_18325896	Ca1	18325896	TC/T	ATTGAAAACATGGCTT TGGC	TTTTGGAGGAACTACG GCAC	59.9	648	INTERGENIC	--	--	--
Ca1_P1_18328170	Ca1	18328170	GTTTTTT/GTTTTTTT	TTAAAACCACAAAATG GGGC	GAGGCAAAGCTTCCAA ATCA	59.7	475	INTERGENIC	--	--	--
Ca1_P1_1835247	Ca1	1835247	ATGAC/A	TCATAAGCCAGAACCC AAGC	GGTGGTGGAGATTCC AAGAA	60.2	448	INTERGENIC	--	--	--
Ca1_P1_18364053	Ca1	18364053	GAAA/GAAAA	CCCGTAGCAAGACTTG AAGC	TTGGAGAAACTTTTCC GCTG	60.0	627	INTERGENIC	--	--	--
Ca1_P1_1836845	Ca1	1836845	TATTTTAATT/TATTTTAATTTTAATT	CATTTGCATACCGTTT CACG	AGCACCACTTCTTCAT TCAAAA	60.0	704	INTERGENIC	--	--	--
Ca1_P1_1841034	Ca1	1841034	TTTCTTCT/TTTCT	TCAAGGTGTTGATTCG GTAGG	TTTTGCAACCCGGTCT ATTC	60.0	595	INTERGENIC	--	--	--
Ca1_P1_1841061	Ca1	1841061	TAAAA/TAAAAAAAA	TCAAGGTGTTGATTCG GTAGG	TTTTGCAACCCGGTCT ATTC	60.0	595	INTERGENIC	--	--	--
Ca1_P1_1841424	Ca1	1841424	A/AT	GAATAGACCGGGTTGC AAAA	CCTCTTTCCGGACAAC ACAT	59.9	302	INTERGENIC	--	--	--
Ca1_P1_1841547	Ca1	1841547	G/GATTATTCGTT	ATGTGTTGTCCGGAAA GAGG	TGAATCCGGAATAGAA CCGA	60.0	147	INTERGENIC	--	--	--
Ca1_P1_1843109	Ca1	1843109	C/CG	AAGCCATAATACGCCC ACAG	TGACGAGTCACCATCT TTGC	60.0	600	INTERGENIC	--	--	--

INDEL marker IDs	Chromosomes/ unanchored scaffolds	Physical positions (bp)	InDels (*Kabuli* reference genome-CDC Frontier/PI)	Forward primers (5'-3')	Reverse primers (5'-3')	Anneling temperature (0C)	Expected amplified product size (bp)	Structural annotation		Functional annotation	
								**Sequence components of *kabuli* genome**	***Kabuli* gene accession IDs**	**NCBI-KOG**	**TFs**
Ca1_P1_1843387	Ca1	1843387	ATATT/AT	CAACCAAAAATGCGACTTGA	GCCAAGCTAAGCTGACGAGT	59.7	526	INTERGENIC	--	--	--
Ca1_P1_18441371	Ca1	18441371	CACAAGAGAC/CAC	CCCTCTGTATGCCAGTCGAT	TGGCATTGATGATGGCTTTA	60.1	771	INTERGENIC	--	--	--
Ca1_P1_18441535	Ca1	18441535	CCGGACGG/CCGG	TTGACTGAGCAAGGCACAAG	TGGCATTGATGATGGCTTTA	60.2	382	INTERGENIC	--	--	--
Ca1_P1_18472041	Ca1	18472041	CAAAAAAAAAA/CAAAAAAAAAAA	CCTACATTCGCACGAGTTCA	TTTTTCTCCAGTTTTGGTCCT	59.9	404	INTERGENIC	--	--	--
Ca1_P1_1849075	Ca1	1849075	AATTATTATTATTATTATTATTAT/AATTATTATTATTATTATTAT	GAATTCAACGGTGTGGGACT	TGTTTGAGCCAAATGTGGAA	59.8	271	CDS (large-effect mutations)	Ca_00232	K	MYB
Ca1_P1_18495696	Ca1	18495696	A/AT	CGGCATTATTTTGGTCTCGT	TTTGATGTTCACGGCTTCAA	60.0	679	INTERGENIC	--	--	--
Ca1_P1_1850030	Ca1	1850030	CAATTAATTA/CAATTA	GTTGTCCGTCCTTCCTGGTA	GGACAACCGAAGAGGATCAA	60.0	563	INTRON	Ca_00232	K	MYB
Ca1_P1_1852604	Ca1	1852604	TA/TAA	ACACGGCGTTCATTTTCTTC	CAAACCATGGGAGAGAGAGC	60.1	843	INTERGENIC	--	--	--
Ca1_P1_18552012	Ca1	18552012	ATTTTTTTT/ATTTTTTTTT	TCTGATGCAGTGTTTGGCTC	GCTTTCTTTGTCTGCCTTGG	60.0	331	INTERGENIC	--	--	--
Ca1_P1_18609095	Ca1	18609095	ATATTATTATTAT/ATATTATTATTATTAT	CCCTCTACGAATTCCAAAAGAA	CGATTTGCTTTCAATTTTGG	59.6	540	INTERGENIC	--	--	--
Ca1_P1_186383	Ca1	186383	T/TC	TTCTGCATCACCATCTGAGG	TGATGAAGATCCCCCAAGAG	59.8	783	INTRON	Ca_00014	--	--
Ca1_P1_18688865	Ca1	18688865	TTGT/TT	TAGCAGCTGGAGGAACATGA	GCACCCAGTGTAAGACCCAT	59.5	301	INTERGENIC	--	--	--

INDEL marker IDs	Chromosomes/ unanchored scaffolds	Physical positions (bp)	InDels (*Kabuli* reference genome-CDC Frontier/PI)	Forward primers (5'-3')	Reverse primers (5'-3')	Anneling temperature (0C)	Expected amplified product size (bp)	Structural annotation		Functional annotation	
								Sequence components of *kabuli* genome	*Kabuli* gene accession IDs	NCBI-KOG	TFs
Ca1_P1_18703001	Ca1	18703001	CAAAA/CAAA	AATGGTGGCTCTACCG AGTG	TGGTTTTTGTTGACTG CTGC	60.1	536	DRR	Ca_17385	--	WRKY
Ca1_P1_1874080	Ca1	1874080	AC/ACC	AACATCGTGCCCTCAT ATCC	CCCGAGATCAAAACAG AAGC	59.8	316	INTERGENIC	--	--	--
Ca1_P1_18756557	Ca1	18756557	AATATATATATATATATATATATATA TATATATATATATATA/AATATATAT ATATATATATATATATATATATATAT ATATATATATA	TGACTTGATATTTTGCC TCCA	CACCCAAAGTTTAATT GGTTCA	58.2	671	INTERGENIC	--	--	--
Ca1_P1_1875806	Ca1	1875806	T/TATA	TCCAAGGTGGGTCAAA AGAC	ACGTTGTTTTTAATTC GCCG	59.9	574	INTERGENIC	--	--	--
Ca1_P1_1876420	Ca1	1876420	AT/ATACGTAGTTCGGTTGAGT	CGGGTCTTCAAAATGG AATG	CAAAGGAAACGTGCTT CCAG	60.3	861	INTERGENIC	--	--	--
Ca1_P1_1882571	Ca1	1882571	TC/T	ATATCGTTTTTCACACC CGC	GGTGCAAATGCAAGA GTCAA	59.8	282	INTERGENIC	--	--	--
Ca1_P1_1882757	Ca1	1882757	CTA/CTATA	TTGACTCTTGCATTTG CACC	ATGCAGGGAATGTTGG GTAA	59.8	140	DRR	Ca_00237	A	--
Ca1_P1_1882903	Ca1	1882903	GT/GTCAGCTTCTAT	TTGACTCTTGCATTTG CACC	ACATATGCGATTGGGA CACA	59.8	581	DRR	Ca_00237	A	--
Ca1_P1_1883092	Ca1	1883092	C/CT	GCACGTGGCCCACTTA TACT	ACATATGCGATTGGGA CACA	60.0	244	DRR	Ca_00237	A	--
Ca1_P1_1883148	Ca1	1883148	G/GA	GCACGTGGCCCACTTA TACT	ACATATGCGATTGGGA CACA	60.0	244	DRR	Ca_00237	A	--
Ca1_P1_18868658	Ca1	18868658	CGG/CGGGAATGG	AATCGATTGATTCAGC CCAG	CGTTTTTCAAAGCGAG GGTA	60.0	707	INTERGENIC	--	--	--
Ca1_P1_18880691	Ca1	18880691	ATATTAT/ATATTATTAT	TCATTTCTTGCGTCTC CCTT	CAAAAACCACATTCGC ACAC	59.8	195	INTERGENIC	--	--	--

**INDEL marker IDs**	**Chromosomes/ unanchored scaffolds**	**Physical positions (bp)**	**InDels (*Kabuli* reference genome-CDC Frontier/PI)**	**Forward primers (5'-3')**	**Reverse primers (5'-3')**	**Anneling temperature (0C)**	**Expected amplified product size (bp)**	**Structural annotation**		**Functional annotation**	
								**Sequence components of *kabuli* genome**	***Kabuli* gene accession IDs**	**NCBI-KOG**	**TFs**
Ca1_P1_1888179	Ca1	1888179	TAAAAAAA/TAAAAAAAA	CCTCACCCCATTTTCCTTTT	ACCATGAACCATTGCCAAGT	60.2	455	INTERGENIC	--	--	--
Ca1_P1_1888709	Ca1	1888709	TAAAA/TAAA	CCAATGTGCCACTTGTTGAC	CATTTGGCTTGTTTGGCATA	60.0	627	INTERGENIC	--	--	--
Ca1_P1_1888957	Ca1	1888957	GGA/G	CCTTACCCCACCCAACACTA	GGGTCAAAGTTCAATTGGGA	59.7	622	INTERGENIC	--	--	--
Ca1_P1_18899804	Ca1	18899804	CTATATATATATATATATATAT/CTATATATATATATATATATATAT	GCTTCAATGTGCACGATACG	TTCTTTGTTGGTAGGGCACC	60.3	237	INTERGENIC	--	--	--
Ca1_P1_18904416	Ca1	18904416	ATTGTTGT/ATTGTTGTTGT	TTTCAGAGCACAATTGCCAG	AACAAGCCGAGAGCTTACCA	60.0	767	INTRON	Ca_17369	--	--
Ca1_P1_18928985	Ca1	18928985	CTTATTTATTTATTTATTTATTTAT/CTTATTTATTTATTTATTTATTTATTTAT	TTTACCCTATCCCACACCCC	TCCAATGATTCCTGTTTTTGC	60.8	613	INTERGENIC	--	--	--
Ca1_P1_18957444	Ca1	18957444	C/CA	AACACCCCTATGTGGGATGA	ATAACCCAACACCCAATCCA	60.1	425	INTERGENIC	--	--	--
Ca1_P1_1903048	Ca1	1903048	GTT/GT	ATTGTGACCCAAGCAAGACC	ATGTTTGCTGATTCAAGGGG	60.0	479	INTRON	Ca_00238	R	--
Ca1_P1_19040329	Ca1	19040329	TTATATATATATATATATATATATATAT A/TTATATATATATATATATATATATATATATATATA	TTGTGCGAATGAACTCTTAATAATG	CGATTGTGTCTTCCCACATCT	59.6	715	INTERGENIC	--	--	--
Ca1_P1_19084336	Ca1	19084336	TGCAGG/TG	ACAACAAACCGTGCAATCAA	ACCCTGGATGCTTGTATGAA	60.0	631	CDS (FRAME SHIFT)	Ca_17356	--	NF-X1
Ca1_P1_1909356	Ca1	1909356	T/TTTA	TGACAAATGCACAGTTGGTTT	TGGAAGGAGGAATGTTTTGC	59.1	695	INTRON	Ca_00239	--	--
Ca1_P1_19093958	Ca1	19093958	GTTATTATTATTATTATTATTATTAT/GTTATTATTATTATTATTATTATTATTAT	ATGCTAGGTCGTTCGTCAGG	AGTATGGCATGGCCTGAAAG	60.3	510	INTERGENIC	--	--	--

INDEL marker IDs	Chromosomes/ unanchored scaffolds	Physical positions (bp)	InDels (*Kabuli* reference genome-CDC Frontier/PI)	Forward primers (5'-3')	Reverse primers (5'-3')	Anneling temperature (0C)	Expected amplified product size (bp)	Structural annotation		Functional annotation	
								Sequence components of *kabuli* genome	*Kabuli* gene accession IDs	NCBI-KOG	TFs
Ca1_P1_1909441	Ca1	1909441	T/TTAAAAA	TGACAAATGCACAGTTGGTTT	TGGAAGGAGGAATGTTTTGC	59.1	695	INTRON	Ca_00239	--	--
Ca1_P1_1910096	Ca1	1910096	TTATATATATATATATATATATA/TTATATATATATATATATA	GCAAAACATTCCTCCTTCCA	AGGTCGCTGTTCTCACCACT	60.1	511	INTRON	Ca_00239	--	--
Ca1_P1_1913927	Ca1	1913927	AATATATATATATATATA/AATATATATATATATA	TCTTTCACGTGGTTGCATCT	TGACGTGACAAAATATTAAATGAGG	59.3	795	INTERGENIC	--	--	--
Ca1_P1_19150973	Ca1	19150973	CTT/CT	AACTAGCCCGTCTTTGGGTT	GAAAACAAAAGCTTTACGGTCA	60.0	488	INTERGENIC	--	--	--
Ca1_P1_1915229	Ca1	1915229	CTCAT/CTCATCTGTTTCAT	TGGATACACAAAAACCGAACA	CACCTTTTCCATCCATCGAC	58.9	709	INTERGENIC	--	--	--
Ca1_P1_19168	Ca1	19168	GCTCTCT/GCTCT	CAGAACCGAGGAATTAAGCG	AGGCTTGAAGCGTGGTAATG	59.8	637	INTERGENIC	--	--	--
Ca1_P1_1917237	Ca1	1917237	GAAAAA/GAAAA	TGGTTGATGAAAGAGAGATGGA	TGTAACAAGCAGATGGAATCTTG	59.7	247	INTERGENIC	--	--	--
Ca1_P1_1926263	Ca1	1926263	TAA/TAAA	AGCCTATGTTTGGTTCCACG	ATGCTCGAATCTGTTTTGGG	60.0	797	INTRON	Ca_00240	--	C3H
Ca1_P1_1930262	Ca1	1930262	ATTTTTTTT/ATTTTTTTTTT	GGCAACATTTTACAAAAGTTAAGG	AGTTTGATTAATGTCGGGCG	58.3	318	INTERGENIC	--	--	--
Ca1_P1_19308287	Ca1	19308287	GA/GAA	TGTTTGACTCATTGTACCTCGATT	CAACAACTTCTCATGCTCGC	59.9	244	INTERGENIC	--	--	--
Ca1_P1_19394808	Ca1	19394808	TAGAGAGAGAGAGAGAGAGAGAGAGAGA/TAGAGAGAGAGAGAGAGAGAGAGAGAGAGA	CAAATCACTGGGAACTGGTG	AAAAGGGGTCAATTTGAGGG	59.0	428	INTERGENIC	--	--	--
Ca1_P1_19422873	Ca1	19422873	CTTATTTATTT/CTTATTT	GCCCATAACCTAGCCCATTT	TCGTGTCCCAAAACTCAATG	60.2	713	INTERGENIC	--	--	--

INDEL marker IDs	Chromosomes/ unanchored scaffolds	Physical positions (bp)	InDels (*Kabuli* reference genome-CDC Frontier/PI)	Forward primers (5'-3')	Reverse primers (5'-3')	Anneling temperature (0C)	Expected amplified product size (bp)	Structural annotation		Functional annotation	
								Sequence components of *kabuli* genome	*Kabuli* gene accession IDs	NCBI-KOG	TFs
Ca1_P1_19423044	Ca1	19423044	TAAAAAAA/TAAAAAA	TCTTTGAGTTGTTAGGATACAAGTGG	TCGATTATGCGTAGGGAAGG	60.0	728	INTERGENIC	--	--	--
Ca1_P1_19423865	Ca1	19423865	ATTATTTATTTATTTATTTATTTA/ATTATTTATTTATTTATTTATTTATTTATTTA	ACCGTTTTTAACGGATGTCG	TGTTCAAACCCGGGAGTAAG	59.9	525	INTERGENIC	--	--	--
Ca1_P1_19431535	Ca1	19431535	TCA/T	TCTGATGCAGTGTTTGGCTC	GTGATAAAGGCGTCAACGGT	60.0	688	INTERGENIC	--	--	--
Ca1_P1_19431572	Ca1	19431572	TAAAAAAA/TAAAAAAAA	TCTGATGCAGTGTTTGGCTC	GTGATAAAGGCGTCAACGGT	60.0	688	INTERGENIC	--	--	--
Ca1_P1_19493855	Ca1	19493855	AC/A	TAGCAGACTGCCACGATCAG	TGTGACATTGGGTGTCGAAT	60.2	446	INTERGENIC	--	--	--
Ca1_P1_19498678	Ca1	19498678	TTTTGATCCTTTGAT/TTTTGAT	GCCCGAGTTAACAACAAACG	ATCGATTTCCTCAGCACTGG	60.5	374	INTERGENIC	--	--	--
Ca1_P1_1950794	Ca1	1950794	CAAAA/CAAAAA	TCGATCGCTAGTGTGACTGAA	TTGAAGAATGTGTGGGTGGA	59.6	625	INTERGENIC	--	--	--
Ca1_P1_19508791	Ca1	19508791	TT/TTATAT	CAGCATAGAGGCAAACGACA	TCATGTGGTCGCTTTCTCAG	60.0	817	INTERGENIC	--	--	--
Ca1_P1_1951397	Ca1	1951397	CATT/CATTATT	TATCCTCCACTCTACGCGCT	GCTAGCATGAGTGGCCAAAT	60.0	172	INTERGENIC	--	--	--
Ca1_P1_19536862	Ca1	19536862	TCCCC/TCCCCC	TAGCACCTTCCTCCCAGAAA	CTACCAACAGAAAGGCCCAA	59.8	352	INTERGENIC	--	--	--
Ca1_P1_19559477	Ca1	19559477	GTATATATATATATATATATATATAT/GTATATATATATATATATATAT	AAGTTGAGGCCACAATGTCC	TAACCATGTCAATTCCCCCT	60.0	274	INTERGENIC	--	--	--
Ca1_P1_19632190	Ca1	19632190	AATATATATATATATATAT/AATATATATATATATAT	CAATTTGCCACATTTTGACG	CAATGAACATGTTGAACCCG	60.0	679	INTERGENIC	--	--	--

INDEL marker IDs	Chromosomes/ unanchored scaffolds	Physical positions (bp)	InDels (*Kabuli* reference genome-CDC Frontier/PI)	Forward primers (5'-3')	Reverse primers (5'-3')	Anneling temperature (0C)	Expected amplified product size (bp)	Structural annotation		Functional annotation	
								Sequence components of *kabuli* genome	*Kabuli* gene accession IDs	NCBI-KOG	TFs
Ca1_P1_1963997	Ca1	1963997	ATATTTATTT/ATATTTATTTATTT	AGGCAAACAACTTTTGCACC	TTACAATGTGCATAGCGGGA	60.2	317	DRR	Ca_00244	--	B3
Ca1_P1_1966252	Ca1	1966252	TTCTCT/TTCTCTCT	GGCAAGAGAGCATCAAAAGG	CAAGTGAAGTTTTGATTCAAGGG	60.0	703	DRR	Ca_00245	U	bZIP
Ca1_P1_1968368	Ca1	1968368	CAAAGAAAGAAAGAAAGA/CAAAGAAAGAAAGA	TCCAATCCATTCCACCATTT	TGCATTCCATTTAGCAGCAG	60.0	775	INTERGENIC	--	--	--
Ca1_P1_19687346	Ca1	19687346	TTTTTATTTT/TTTTTATTTTATTTT	TCACCACTGATATTTATTGAAGAACC	TCGATGTTGTTTGCGAATGT	59.7	393	INTERGENIC	--	--	--
Ca1_P1_1969241	Ca1	1969241	AAATTAA/AAA	AGAAGAGAACGGTTGCGTGT	GAAAACTACCACGGCCAAAA	59.9	604	INTERGENIC	--	--	--
Ca1_P1_1970241	Ca1	1970241	CAA/CAAAA	CGGATTTGGGCAGTGATATT	TTCGAGGTGGTTCATTTCTT	59.8	870	INTERGENIC	--	--	--
Ca1_P1_1974312	Ca1	1974312	A/AG	TTCAAATGACCAACCAGCAA	CAAGAGGAAGCAACACAGCA	60.1	450	INTRON	Ca_00246	A	--
Ca1_P1_1975964	Ca1	1975964	TAA/TAAA	CGTGCAACTTCTTTGACTGG	CAGATTCAATTGCCTGCTCA	59.5	743	INTRON	Ca_00246	A	--
Ca1_P1_1979258	Ca1	1979258	C/CCTGA	TACGCCTTTTCAGGTTGGAC	CCGTGCTAGTAGCCCACATT	60.1	621	INTERGENIC	--	--	--
Ca1_P1_1979311	Ca1	1979311	AATATATATATATA/AATATATATATA	TACGCCTTTTCAGGTTGGAC	CCGTGCTAGTAGCCCACATT	60.1	621	INTERGENIC	--	--	--
Ca1_P1_1985681	Ca1	1985681	TATG/T	TGAAAAGACCCTACTCACCCA	TGGGGCCTACAATTTATCCA	59.6	209	INTERGENIC	--	--	--
Ca1_P1_1988980	Ca1	1988980	TC/TCCC	ACCTGCCAAAAGCAGAAGAA	CACTGAGTTTTCGGGGAGAA	60.0	776	INTRON	Ca_00248	T	--

INDEL marker IDs	Chromosomes/ unanchored scaffolds	Physical positions (bp)	InDels (*Kabuli* reference genome-CDC Frontier/PI)	Forward primers (5'-3')	Reverse primers (5'-3')	Anneling temperature (0C)	Expected amplified product size (bp)	Structural annotation		Functional annotation	
								Sequence components of *kabuli* genome	*Kabuli* gene accession IDs	NCBI-KOG	TFs
Ca1_P1_19903627	Ca1	19903627	TA/TAATTGGCAGCA	TGGGCTGAAAAGCAACTTCT	ACCAGTGCTGTTCCCAAATC	60.0	233	INTRON	Ca_08847	K	--
Ca1_P1_1990497	Ca1	1990497	TAA/TA	TGGGTCAACGTTGGTGTCTA	CCACTCAAAGGCTCACACAA	60.0	769	INTERGENIC	--	--	--
Ca1_P1_19909063	Ca1	19909063	TTATATATATATATATATATATATATATATAT/TTATATATATATATATATATATATAT	CCATAATTTTTGGATCCTCCT	TTTGCTTATGAGAAAAAGGAGATGT	57.1	235	INTERGENIC	--	--	--
Ca1_P1_1991447	Ca1	1991447	GATATATATATATATATATATATATATAT/GATATATATATATATATATATATAT	CCAACCCTTAGGGGACAAAT	CATGTGCCAGAAAGCTCAAA	60.0	505	DRR	Ca_00249	CR	HB-other
Ca1_P1_1992977	Ca1	1992977	TAC/TACAC	AGCCTGCACCTGCACTTAAT	ATTATCGGCATTCAAGGTGG	59.9	185	INTRON	Ca_00249	CR	HB-other
Ca1_P1_19939016	Ca1	19939016	AATATATATATATAT/AATATATATATATATAT	CATCAGTTTGCTTCCCATCA	TGCAACTCAGGGATTAAAATG	59.6	654	INTERGENIC	--	--	--
Ca1_P1_1995874	Ca1	1995874	G/GT	CGAGTATTTGCCACGGTTTT	TTCACTTGATTTTGGGAGGG	60.0	595	INTRON	Ca_00250	CR	HB-other
Ca1_P1_19962858	Ca1	19962858	CTTTTTTTTTT/CTTTTTTTTTTT	TGGGATCGCCACATGTATATT	TTTTCTGTGAAATCATTGTCAACTC	60.1	486	INTERGENIC	--	--	--
Ca1_P1_1999397	Ca1	1999397	AGTGT/AGT	GACGAATATCGGTCGAATACAA	GTCCAAACATCGGAAAAGGA	59.0	706	INTERGENIC	--	--	--
Ca1_P1_2001214	Ca1	2001214	GAAAAA/GAAAAAA	CATCATACAAAGCACCGGG	GTTCCGTGGTTTTCTTCCAA	59.9	410	DRR	Ca_00251	J	--
Ca1_P1_2001586	Ca1	2001586	TAAG/TAAGAAG	AGCACTGGGCAGAAAGAAAA	TATCCCTGGCTGATGTAGCA	60.0	538	DRR	Ca_00251	J	--
Ca1_P1_2006509	Ca1	2006509	ATT/AT	ATGAAACGAATTGCTCAGGG	TGTGGTTTGGGTGTGAGAGA	60.1	924	DRR	Ca_00252	--	TCP

INDEL marker IDs	Chromosomes/ unanchored scaffolds	Physical positions (bp)	InDels (*Kabuli* reference genome-CDC Frontier/PI)	Forward primers (5'-3')	Reverse primers (5'-3')	Anneling temperature (0C)	Expected amplified product size (bp)	Structural annotation		Functional annotation	
								Sequence components of *kabuli* genome	*Kabuli* gene accession IDs	NCBI-KOG	TFs
Ca1_P1_2007237	Ca1	2007237	GGTG/GG	TCTCTCACACCCAAACCACA	TGTGAATTTTTGCCACTCAAA	60.1	741	DRR	Ca_00252	--	TCP
Ca1_P1_2008759	Ca1	2008759	GCCCC/GCC	ATCTTGTCACCGCGAGTCTT	TGGTATGTATGAAGAAATCCGAA	59.9	181	INTERGENIC	--	--	--
Ca1_P1_20097334	Ca1	20097334	CNNNNNNNNNNNNNN/CNNN	TTTCGTTTGCGATAGTGATCC	TCATTTCAAGATCCTGGTTCC	60.1	250	INTERGENIC	--	--	--
Ca1_P1_2011618	Ca1	2011618	AGG/AG	ACCATCAGGGCAAAGATCAC	GCCCCTCAACCAATTACAAA	59.9	969	INTRON	Ca_00253	E	--
Ca1_P1_2015582	Ca1	2015582	TTGT/TT	CTCTAGAACCCCCAACTCCC	CTCCAATGTTGGGAGACTACG	59.9	499	DRR	Ca_00253	E	--
Ca1_P1_2023898	Ca1	2023898	TAA/TAAA	TAATGAGGGCTTGAATTCGC	TCCGTTGTTGAGGATGATGA	60.2	386	DRR	Ca_00255	--	--
Ca1_P1_2028913	Ca1	2028913	CT/CTT	ACCTGTTCCCGTCTTCTCAA	CCCCTTCTCTGTAAGTCCCC	59.7	597	INTRON	Ca_00256	U	M-type
Ca1_P1_203179	Ca1	203179	CAATAATAATAATAATAATAATAATAATAACAATAATAATAA/CAATAATAATAATAATAATAATAATAACAATAATAATAA	CAGCGAAGAAAATGAGGAGG	GCTTTATCCTGCCAAAACCA	59.9	719	CDS (large-effect mutations)	Ca_00016	A	C3H
Ca1_P1_2049868	Ca1	2049868	ATT/ATTT	TGATGTTTCGTTTTCGTAAGGA	ATTGGCGTCAACCTTGTTTC	59.6	619	INTERGENIC	--	--	--
Ca1_P1_2057712	Ca1	2057712	TAAAAAAA/TAAAAAA	CGCCACTTTCTTTGAATCGT	ACCTCTGCGGTAACTTTTCG	60.2	560	DRR	Ca_00261	--	--
Ca1_P1_2057962	Ca1	2057962	GAAAAGTTA/GA	TGTGCATGAAAGCAAAATTGA	GGCCGTGAAAGATATTTGGA	60.2	712	DRR	Ca_00261	--	--
Ca1_P1_2067917	Ca1	2067917	ACGCCG/ACGCCGCCG	GTGCAGCTTCCCTCTTCATC	TTTGCATCATGAGCCACATT	60.0	344	INTRON	Ca_00262	U	M-type

**INDEL marker IDs**	**Chromosomes/ unanchored scaffolds**	**Physical positions (bp)**	**InDels (*Kabuli* reference genome-CDC Frontier/PI)**	**Forward primers (5'-3')**	**Reverse primers (5'-3')**	**Anneling temperature (0C)**	**Expected amplified product size (bp)**	**Structural annotation**		**Functional annotation**	
								**Sequence components of *kabuli* genome**	***Kabuli* gene accession IDs**	**NCBI-KOG**	**TFs**
Ca1_P1_2073961	Ca1	2073961	CTTTT/CTTT	AGAGGCACGAGATTTCCAGA	AAATATCCCCGCAATAAGGG	60.0	336	CDS (FRAME SHIFT)	Ca_00263	T	M-type
Ca1_P1_2085633	Ca1	2085633	CTTT/CTT	TGGGTTTGTAAAATGAAAAGTGA	TTGAGATTTTCCAAGCTCAAAG	58.6	737	INTERGENIC	--	--	--
Ca1_P1_2088365	Ca1	2088365	AAAACA/AA	ACAGCAACCAAGAGTTGTGC	CAGAAAAAGCTATTCAGGCCA	58.9	529	INTERGENIC	--	--	--
Ca1_P1_2088423	Ca1	2088423	ATT/AT	ACAGCAACCAAGAGTTGTGC	GCAGGTGGCATCAATTTACC	58.9	548	INTERGENIC	--	--	--
Ca1_P1_2088597	Ca1	2088597	TTTTT/TTTTTTTAAAAACTTTT	ACAGCAACCAAGAGTTGTGC	GCATCCGGCCAAAATAAATA	58.9	654	INTERGENIC	--	--	--
Ca1_P1_2088953	Ca1	2088953	ATATTTATTTATTT/ATATTTATTTATTTATTTATTT	GGTAAATTGATGCCACCTGC	TTTTGCTGCCACCATGATTA	60.3	469	INTERGENIC	--	--	--
Ca1_P1_2108793	Ca1	2108793	GTT/GTTTT	GTTTTATCTGCCGAACCTGC	CAATGGCAGTGATCTGCTCT	59.7	678	INTRON	Ca_00267	D	NAC
Ca1_P1_2116628	Ca1	2116628	A/AC	TTGCGTTCATTTACGTTTGA	TCAATTCACTTCATGCCCAA	57.8	636	INTERGENIC	--	--	--
Ca1_P1_2118876	Ca1	2118876	TATCTTTT/TATCTTTTGGAATCTTTT	CCGGCCATCAGAGTATCATT	AAGCGGTCTTTAAGTGCCAA	59.9	273	INTERGENIC	--	--	--
Ca1_P1_2131502	Ca1	2131502	TAAAAAAA/TAAAAAAAAAA	GGCCAACCTTGCTCAAAATA	ACATGTTACGGTGTAGCGCA	60.1	451	INTERGENIC	--	--	--
Ca1_P1_2135161	Ca1	2135161	AGTGTG/AGTGTGTG	AGAATTTACTCCACGTCCCG	TGAACCTCGTGCATGTAATTCT	59.1	437	INTERGENIC	--	--	--
Ca1_P1_2136478	Ca1	2136478	CAA/CAAA	CTGCTCAGAACGCTGCATAA	TCATCAAATTCGTAACGCAAA	60.3	480	INTERGENIC	--	--	--

INDEL marker IDs	Chromosomes/ unanchored scaffolds	Physical positions (bp)	InDels (*Kabuli* reference genome-CDC Frontier/PI)	Forward primers (5'-3')	Reverse primers (5'-3')	Anneling temperature (0C)	Expected amplified product size (bp)	Structural annotation		Functional annotation	
								Sequence components of *kabuli* genome	*Kabuli* gene accession IDs	NCBI-KOG	TFs
Ca1_P1_2139237	Ca1	2139237	ATTTTTTT/ATTTTTTTT	CCAACGTGGTTGGTTTCTTT	ATCGTTGAGGTCCGTTTCAC	59.9	623	INTERGENIC	--	--	--
Ca1_P1_21655501	Ca1	21655501	AAACAA/AAA	CTCCCCCTTTTTGGCATAAT	TTGAAGCTCAGATGGCACAC	60.1	721	INTERGENIC	--	--	--
Ca1_P1_21683923	Ca1	21683923	AATATATATATATATATATATATATATATATATATATA/AATATATATATATATATATATATATATATATATATATA	GGCGCTAGATACTACATCGCTT	GCAAATGGAAAGGTTTCACG	59.9	738	DRR	Ca_22118	--	--
Ca1_P1_2169501	Ca1	2169501	TT/TATTAT	TCGCTGAAAAAGAAAGGTGA	ATGTAGGTGATGGGATGGGA	58.6	509	DRR	Ca_00273	Z	--
Ca1_P1_21746056	Ca1	21746056	AC/ACACATATCAGCTTTC	CTATTGCCCCCTCCTCCTAC	TACCGATACCTCCAAGTGCC	59.9	585	INTRON	Ca_22116	S	--
Ca1_P1_21762018	Ca1	21762018	AATATATATATATATATATATATATATATATA/AATATATATATATATATATATATATATATATA	ATTCTTGTCGGACGGTGATT	TTTGTGTTGAACGGCAAAAG	59.4	639	INTERGENIC	--	--	--
Ca1_P1_21772275	Ca1	21772275	CATATATATATATATAT/CATATATATATATATATAT	CTTTTGTCTCCCCATCCTCA	AAAACTGAGACGAAAAAGTTGTCC	60.0	342	DRR	Ca_22115	--	MYB
Ca1_P1_21850480	Ca1	21850480	TTAATAATAATAATAATA/TTAATAATAATAATAATAATA	CATTAGTGGCGTTGGTTGTG	TGTGACATGGGAGGCTCATA	60.0	456	INTERGENIC	--	--	--
Ca1_P1_21915840	Ca1	21915840	C/CG	GTGCTCCGGTCTATGTTGGT	TGAATGCTTCTTTGTGCTGG	60.0	166	INTERGENIC	--	--	--
Ca1_P1_21938049	Ca1	21938049	CACTGAGTCCAGA/CACTGAGTCCAGACTGAGTCCAGA	TGTCAAAGGCCAAGATTCCT	CCTCACATCAAGAGCAGCAA	59.7	526	INTRON	Ca_22106	--	MIKC
Ca1_P1_21987016	Ca1	21987016	TTATATATATATATATATAT/TTATATATATATATATATATATAT	AGAAACGGAGGAGGTAGTTTTT	AAAGAATTTTGCATAAAAAGGACAA	57.6	636	INTRON	Ca_22102	--	--
Ca1_P1_22029011	Ca1	22029011	TTATATATATATATATATATATATATATA/TTATATATATATATATATATA	CGAATCTTCACCCTCTTTGG	GCCGGATCCAAAATCAAAAT	59.7	839	INTERGENIC	--	--	--

INDEL marker IDs	Chromosomes/ unanchored scaffolds	Physical positions (bp)	InDels (*Kabuli* reference genome-CDC Frontier/PI)	Forward primers (5'-3')	Reverse primers (5'-3')	Anneling temperature (0C)	Expected amplified product size (bp)	Structural annotation		Functional annotation	
								Sequence components of *kabuli* genome	*Kabuli* gene accession IDs	NCBI-KOG	TFs
Ca1_P1_22091873	Ca1	22091873	AATATATATATATATATATATA/AATATATATATATATATA	TTTCCAAATGGGCATTTCTC	CATTTCGAACTTGGGCTTGT	59.9	331	INTERGENIC	--	--	--
Ca1_P1_22131328	Ca1	22131328	AATATATAT/AATATATATAT	GTGTGGACATGCCTAAAACG	GTTGCAATTGAGGTGCTGAA	59.1	403	INTERGENIC	--	--	--
Ca1_P1_22131513	Ca1	22131513	GAAAAA/GAAAA	GTGTGGACATGCCTAAAACG	GTTGCAATTGAGGTGCTGAA	59.1	403	INTERGENIC	--	--	--
Ca1_P1_22132039	Ca1	22132039	G/GC	GGATGGTAGTTCAGCACCTCA	GAGCATTAGTGCTTGGCACA	60.1	774	INTERGENIC	--	--	--
Ca1_P1_22183065	Ca1	22183065	CATCGCAG/C	TCCCACTGGAAAGAAGATCG	CCATTAGATATGGGCCACCA	60.2	192	INTERGENIC	--	--	--
Ca1_P1_22189988	Ca1	22189988	AATATATATATATATATAT/AATATATATATATATAT	GTATTGGGTCGGTCTTGGAA	AAGGAATTTCATCAATCGTCG	59.8	547	INTERGENIC	--	--	--
Ca1_P1_22194021	Ca1	22194021	TTATATATATATATATATATATATATATATATATATATATATATATATA/TTATATATATATATATATATATATATATATATATATATATATA	CCCCCTTCATCTTCGACAA	TTATGAAAAAGAAATCAAATTGACA	61.0	587	INTERGENIC	--	--	--
Ca1_P1_22194556	Ca1	22194556	TTATATATATATATAT/TTATATATATATATATATAT	ATTGTCAATTTGATTTCTTTTTCAT	CATTTGTGACCATTTGCACC	57.4	747	INTERGENIC	--	--	--
Ca1_P1_22195947	Ca1	22195947	CATATATATATATATATATATATATATAT/CATATATATATATATATATATATATAT	TTTCATTGGCACTTGGTAGG	TCGTCGCGTCACTATGAATC	58.6	838	INTERGENIC	--	--	--
Ca1_P1_2223979	Ca1	2223979	TATAATAATAATAATAATAATAATAATAATAATAA/TATAATAATAATAATAATAATAATAATAATAATAA	AATAGGTCAGGCCAGGCTTT	TATGGATGGGAATAGGCTCG	60.1	659	INTERGENIC	--	--	--
Ca1_P1_22298046	Ca1	22298046	CTAA/CTAATAA	TGAAATTTAGATGATTGCCGC	GATTTGCATATGGGTGGTCC	60.1	821	DRR	Ca_20631	--	SAP
Ca1_P1_22335552	Ca1	22335552	AATATATATATATATATATATATATATATATATA/AATATATATATATATATATATATATATATATATATA	TTGAATTGCGAATTTGAATCTG	TGATGCACCAAAAGACTATTTCA	60.1	796	INTERGENIC	--	--	--

INDEL marker IDs	Chromosomes/ unanchored scaffolds	Physical positions (bp)	InDels (*Kabuli* reference genome-CDC Frontier/PI)	Forward primers (5'-3')	Reverse primers (5'-3')	Anneling temperature (0C)	Expected amplified product size (bp)	Structural annotation		Functional annotation	
								Sequence components of *kabuli* genome	*Kabuli* gene accession IDs	NCBI-KOG	TFs
Ca1_P1_22384776	Ca1	22384776	G/GAACAAGTGTGT	CTTCAGATCCTTGAGCTGCC	TGAATCTTTGTGTCCCACCC	60.1	539	INTERGENIC	--	--	--
Ca1_P1_2239989	Ca1	2239989	AATATATATATATATATATATAT/AATATATATATATATATAT	ATGGTGGCAACACAACTTGA	TGGTTTTGATTTGAGCTTTCC	60.0	775	DRR	Ca_00280	I	--
Ca1_P1_22415512	Ca1	22415512	AATATATATATATA/AATATATATATATATA	TTTTTGATGTACAAGTTAGCACCA	CCACAGTTGCAACAAAGAATG	58.8	519	INTERGENIC	--	--	--
Ca1_P1_22457190	Ca1	22457190	CAAAAAAAAAA/CAAAAAAAAAAA	TGATTTGGATTGATCGAATGTG	AAACATGGCCTCATCTGGAG	60.7	331	INTERGENIC	--	--	--
Ca1_P1_226454	Ca1	226454	AATATATATATATATATA/AATATATATATATATATATATA	TGGCATGTGAAATATGAACGA	TGTCCACGTCACCCTAATCA	59.9	560	INTERGENIC	--	--	--
Ca1_P1_22663919	Ca1	22663919	A/AAACAACACAATGAACCTC	CAAATCAGCAACAGCAGGAA	CGATCCCACCTCTAACCAAC	60.0	666	INTRON	Ca_20616	--	bHLH
Ca1_P1_22666073	Ca1	22666073	AATTTTTTATTTTTTATTT/AATTTTTTATTTTTTATTTTTTATTT	TGAATTTTGGCCTAGTCCCC	AGGCGCCAAATTAGAATGAG	61.2	417	INTRON	Ca_20616	--	bHLH
Ca1_P1_22684322	Ca1	22684322	AAATAATAATAATAATAATAATAATAATAATAATAATAATAATAATAATAATAATAATAAT/AAATAATAATAATAATAATAATAATAATAATAATAATAATAATAATAATAATAATAATAATAATAATAATAAT	GGATTCGATAAACGTAATCGGT	AGGAACGGTGTTCTTTCCCT	59.3	381	INTRON	Ca_20616	--	bHLH
Ca1_P1_22737650	Ca1	22737650	TAAAAA/TAAAA	TTTGCGCCAAAACCATTTAT	AAGGGGATGCTCATTTTGTG	60.3	570	INTERGENIC	--	--	--
Ca1_P1_22745256	Ca1	22745256	AATATATATATATATATATATATATATA/AAAATATATATATATATATATATATATATATATA	CTCCAGAAATTTTGCCTCCA	GTTAGGCCGAGGCTCTCTTT	60.2	480	INTRON	Ca_20613	BD	bHLH
Ca1_P1_22757866	Ca1	22757866	CATATATATATATATATAT/CATATATATATATATATATAT	TGTAAATATTAATGATTCGTTTGATGC	ACCTGGTAATGTCGGGAAAA	59.6	959	INTERGENIC	--	--	--
Ca1_P1_22778168	Ca1	22778168	ATTTT/ATTTTT	TCCAACATCCACCAATTCAA	TCAAGGCAACTCATCAACCA	59.8	590	INTERGENIC	--	--	--

INDEL marker IDs	Chromosomes/ unanchored scaffolds	Physical positions (bp)	InDels (*Kabuli* reference genome-CDC Frontier/PI)	Forward primers (5'-3')	Reverse primers (5'-3')	Anneling temperature (0C)	Expected amplified product size (bp)	Structural annotation		Functional annotation	
								Sequence components of *kabuli* genome	*Kabuli* gene accession IDs	NCBI-KOG	TFs
Ca1_P1_22808194	Ca1	22808194	TTCTCTCTCTCTCTCTCTCTCTCTCT/TTCTCTCTCTCTCTCTCTCTCTCTCTCT	AGGCAGGGAGAAGGAAGAAG	TGTGGAAATTTGTGGGTGAA	60.0	179	INTERGENIC	--	--	--
Ca1_P1_22886112	Ca1	22886112	GAA/GAAA	TGATTTGGGGTTTTGGGTAA	GGGTGAGTTCTTTGAGGGTG	60.0	531	INTERGENIC	--	--	--
Ca1_P1_22896758	Ca1	22896758	CTTTTTTTTTTTTTTT/CTTTTTTTTTTT	GCACCGTTTACAACGCTTTT	GATGAAACCGAGGACCAGAA	60.2	400	INTERGENIC	--	--	--
Ca1_P1_22957875	Ca1	22957875	CT/C	GTACATTTTGTGGCCGCTTT	GGCAGAGAGAAGGTCGAAAA	60.0	147	INTERGENIC	--	--	--
Ca1_P1_22963814	Ca1	22963814	TATA/T	ATATGGAGCTTTTGGCCCTT	AAGCCAAGACTACCGCTGAA	59.9	802	INTRON	Ca_22347	S	--
Ca1_P1_2299530	Ca1	2299530	AATATATATATATATATATATATATATATA/AATATATATATATATATATATATATATATATATA	CAGCAACTGCGATGAGATGT	TCACGGCGAGTCAATAAAAA	60.0	835	DRR	Ca_00288	R	--
Ca1_P1_23012426	Ca1	23012426	TTATATATATATATATATATATATATAT/TTATATATATATATATATATAT	ACCATTAAGTCGACCTTCACA	ATATCGAGGCTGGTCCAACA	57.2	729	INTERGENIC	--	--	--
Ca1_P1_23033164	Ca1	23033164	TA/TAATAATAA	AGGCAAACTGAGACACAACG	CTCTTTGTCCCGTGTTCGAT	58.9	784	INTERGENIC	--	--	--
Ca1_P1_23107865	Ca1	23107865	TTTATTTACTTATTTA/TTTATTTACTTATTTACTTATTTA	TGATGAAGAGTCATCAATATGCC	TCCGCGAGAGAGTTATAGCC	59.1	733	INTERGENIC	--	--	--
Ca1_P1_23116854	Ca1	23116854	AATATATATATATATATATAT/AATATATATATATATATATATAT	CATCGATCCAAGTTCAAGGG	ATCATCCGCAAAACTGGTTC	60.5	497	INTERGENIC	--	--	--
Ca1_P1_23140612	Ca1	23140612	G/GTAGGTCCGTAATA	TGCCATGTGATCTGTTGGTT	TGCATGATTCCAGAATTCCA	60.0	362	INTERGENIC	--	--	--
Ca1_P1_23192284	Ca1	23192284	TTAATAATAATAATAATAATAATAATAATAATAATAATAATAATAATAATAATAATAA/TTAATAATAATAATAATAATAATAATAATAATAATAATAATAATAATAATAATAATAATAATAATAA	TTCGAAACTTTTGGCCACTT	TTGGATTTTGGCCACATTTT	59.7	527	INTERGENIC	--	--	--

**INDEL marker IDs**	**Chromosomes/ unanchored scaffolds**	**Physical positions (bp)**	**InDels (*Kabuli* reference genome-CDC Frontier/PI)**	**Forward primers (5'-3')**	**Reverse primers (5'-3')**	**Anneling temperature (0C)**	**Expected amplified product size (bp)**	**Structural annotation**		**Functional annotation**	
								**Sequence components of *kabuli* genome**	***Kabuli* gene accession IDs**	**NCBI-KOG**	**TFs**
Ca1_P1_23215487	Ca1	23215487	AT/ATTATT	ACGTGGAAAAGAATGCAACC	CTTAAGATCCGCCTTGTGGA	60.0	440	INTERGENIC	--	--	--
Ca1_P1_2322027	Ca1	2322027	CTTAATATTTATAAAAT/CTTAATATTTATAAAATATTAATATTTATAAAAT	TCTTAAACAAACGGGTTCATCA	AAAAATCACAAATCATGTTCGG	59.5	205	INTRON	Ca_00291	V	NAC
Ca1_P1_23222434	Ca1	23222434	AGTTATGTTATGTTATGTTATGTTAT/AGTTATGTTATGTTATGTTAT	CACTCGACAGTTGGGAGGTT	TCGAGCAACAAAGCATCAAG	60.2	423	INTERGENIC	--	--	--
Ca1_P1_23297134	Ca1	23297134	AACACACACACACACACACACA/AACACACACACACACACACACA	TTTTTCAATGGGAGTGTTTTTG	TGCCATAGAGAAGACGGAATG	59.0	280	INTERGENIC	--	--	--
Ca1_P1_23305993	Ca1	23305993	AATATATATATATATATATATATAT/AATATATATATATATAT	TCTGAAGCAGAACCCAACCT	CACGAGCCTTTCTTCTACCG	59.8	797	INTERGENIC	--	--	--
Ca1_P1_23355067	Ca1	23355067	TTATATATATATATATATATATATATATATA/TTATATATATATATATATATATATATATATATATATATATA	GGAGTCTGAAAGTCCAAGCG	CGTTGAAGTTTGCCGGTAGT	60.0	732	INTERGENIC	--	--	--
Ca1_P1_23386171	Ca1	23386171	GTTATTATTATTATTATTATTATTATTATTATTATTATTAT/GTTATTATTATTATTATTATTATTATTATTATTATTATTAT	ACAAGAATGGCAAATGGAGC	TTTGCCCCACTAACTCTTGG	60.1	537	INTERGENIC	--	--	--
Ca1_P1_23421371	Ca1	23421371	CTTT/CTT	TGATTCCATGAGCAAACAGC	ATTTGAGTGAAACCAACGCC	59.8	221	INTERGENIC	--	--	--
Ca1_P1_23426642	Ca1	23426642	TAAA/TAAAA	AAGTTAAGGAACCACGAGTGC	AGAGAAGTGAATGGTGCATACAA	58.4	790	DRR	Ca_19317	--	--
Ca1_P1_23450310	Ca1	23450310	CTTTT/CTTTTT	GCTTGAGCTCCTTGTTGACC	ATCACCACATGGGATGACCT	60.0	728	INTERGENIC	--	--	--
Ca1_P1_23496241	Ca1	23496241	CT/CTTT	AGGGTGGATCAGTTCGATTG	TTATATTAATTGGGTGCGCGT	59.9	376	DRR	Ca_19314	--	--
Ca1_P1_23516838	Ca1	23516838	CAAAAAA/CAAAAA	GGAATCCAAACCAAAACCCT	TTGGTGTGGTTTTTGTGGAA	60.0	634	INTERGENIC	--	--	--

INDEL marker IDs	Chromosomes/ unanchored scaffolds	Physical positions (bp)	InDels (*Kabuli* reference genome-CDC Frontier/PI)	Forward primers (5'-3')	Reverse primers (5'-3')	Anneling temperature (0C)	Expected amplified product size (bp)	Structural annotation		Functional annotation	
								Sequence components of *kabuli* genome	*Kabuli* gene accession IDs	NCBI-KOG	TFs
Ca1_P1_23566751	Ca1	23566751	GATATATATATATATATATATAT/GATATATATATATATATATATATATAT	TTTTGACCTTGTTCAAATGGC	CGAGGGATATGCAAATGACA	60.0	511	INTERGENIC	--	--	--
Ca1_P1_23571644	Ca1	23571644	AA/AAGTCTTCCGATCTTTTGA	TGTGTTGGCTTAAAACATTTGAT	GGGCAATGTTGGTGGTACTC	58.6	810	INTERGENIC	--	--	--
Ca1_P1_23590385	Ca1	23590385	ATCTCTCTCTCTCTCTCTCTCTCTCTCTCTCTCTCT/ATCTCTCTCTCTCTCTCTCTCTCTCTCTCTCT	GCCTTTTAATGATTTGCGGA	TGCGTTTTGGTAATTGTGGA	60.0	373	INTERGENIC	--	--	--
Ca1_P1_236262	Ca1	236262	TAAAAAAAAA/TAAAAAAAA	TGAAAGAACAATATTCCACTAAACCA	GCAGCAATTGATGGTGTGAT	60.2	129	INTERGENIC	--	--	--
Ca1_P1_23643612	Ca1	23643612	TAGAG/TAG	TCGGAATGGTCTGACATCAA	CAATAGGTGGAGGTGCGATT	60.0	830	INTERGENIC	--	--	--
Ca1_P1_23651952	Ca1	23651952	TTATATATATATATATATATATATAT/TTATATATATATATATATATATAT	GCGTAGTGGTAGCCTTTTGG	AGTTGGATGTTGGGGAATCA	59.8	582	INTERGENIC	--	--	--
Ca1_P1_2368297	Ca1	2368297	GTAATAATAATAATAATAATAATAATAATAATAATAATAATAATAATAACAATAATAATAATAAT/GTAATAATAATAATAATAATAATAATAATAATAATAATAATAATAATAATAACAATAATAATAATAAT	AAAAATAACCCAGAAGCATCG	TTCAATTTCGGACATTCGTT	58.3	729	DRR	Ca_00301	DR	--
Ca1_P1_23721175	Ca1	23721175	GAA/GA	GGTTTTGCATGCATGTTGAG	TTTGAATCCCATGATGCGTA	60.1	682	INTERGENIC	--	--	--
Ca1_P1_23784964	Ca1	23784964	ATTTTTTTT/ATTTTTT	GTCAACCAATTGACCCCAAC	TGCCAAGAAAACACCATGAA	60.1	663	INTERGENIC	--	--	--
Ca1_P1_23824031	Ca1	23824031	AAG/AAGCCTAG	CCAAGGAAGGACGAAAATGA	TTTTCGGTGATTCAAAAGCC	60.0	390	INTERGENIC	--	--	--
Ca1_P1_23838777	Ca1	23838777	ATGTGTGTGTGT/ATGTGTGT	AATGCCCACCCATAATTTCA	TGTACGAGAAGGTCGTGTGC	60.0	593	INTERGENIC	--	--	--
Ca1_P1_23881803	Ca1	23881803	CA/CAA	CCATTTTCATTCGTCGCTTT	TACCGCGACCGACTCTACTT	60.1	747	INTERGENIC	--	--	--

INDEL marker IDs	Chromosomes/ unanchored scaffolds	Physical positions (bp)	InDels (*Kabuli* reference genome-CDC Frontier/PI)	Forward primers (5'-3')	Reverse primers (5'-3')	Anneling temperature (0C)	Expected amplified product size (bp)	Structural annotation		Functional annotation	
								Sequence components of *kabuli* genome	*Kabuli* gene accession IDs	NCBI-KOG	TFs
Ca1_P1_23966814	Ca1	23966814	GA/GAA	AACTGTGGAGGAATCCATGC	GCTTCAAGTCTTGCTACGGG	59.9	490	INTERGENIC	--	--	--
Ca1_P1_24030518	Ca1	24030518	CG/CGTGTTG	CCAATTCAAAAGCTCCCAAA	CAGATCCTCCTTTCCCAACA	60.0	424	DRR	Ca_19291	OK	--
Ca1_P1_24098971	Ca1	24098971	GATATATATATATATATATATATATATATA/GATATATATATATATATATATATATATATA	AGGCATGGTGTTGGAGTCAT	TGCATTTTTCCACGTTCAAG	60.4	526	INTERGENIC	--	--	--
Ca1_P1_24154735	Ca1	24154735	ATTTTT/ATTTTTT	GGGTGCCATCATAAGCAAGT	CAGATACTGCAGCCTCCACA	60.0	516	INTERGENIC	--	--	--
Ca1_P1_24180187	Ca1	24180187	TAAAAAAAA/TAAAAAAA	TTGCATCGATCATTGTTGAC	GAGTTGTTGTTCTTCCAACCAA	58.0	642	INTERGENIC	--	--	--
Ca1_P1_24199765	Ca1	24199765	CTATATATATATATATATA/CTATATATATATATA	TTGGCTCATTCTTTCGTTGA	CCCTTGTAATTTCAACCCCA	59.4	401	INTERGENIC	--	--	--
Ca1_P1_24242300	Ca1	24242300	TAAAAAAAAAAA/TAAAAAAAAAA	ACCAACTCATCCCTTTCCCT	ATGATTGCAGCATTTCACGA	59.8	638	INTERGENIC	--	--	--
Ca1_P1_24252089	Ca1	24252089	G/GC	TTTGCGCATCACGCTATAAG	CGCTCGATAACCCCAAAATA	60.0	469	INTERGENIC	--	--	--
Ca1_P1_24301670	Ca1	24301670	AATATATATATATATATATATATA/AATATATATATATATATATATATA	TCAAGCATGTCAAATTCCCA	GGGGTGCTACAGTGTCGAAT	60.0	633	INTERGENIC	--	--	--
Ca1_P1_24516831	Ca1	24516831	TTATATATATATATATAT/TTATATATATATATATATATATAT	TTCCGAGTTGTTTGAGAGGA	GGCCTATTATGGCCTGACCT	58.4	563	INTERGENIC	--	--	--
Ca1_P1_24611579	Ca1	24611579	AATATATATATATATA/AATATATATATATATA	TGCCCACTTGATACAACAAAA	CATCGCCTTTGACGAATACA	59.1	330	INTERGENIC	--	--	--
Ca1_P1_24636488	Ca1	24636488	ATTTT/ATTTATTTTT	GATGGTGTGAGTGGAGCTGA	AAACACGTTGGCATTTGTGA	59.8	906	DRR	Ca_18338	--	--

INDEL marker IDs	Chromosomes/ unanchored scaffolds	Physical positions (bp)	InDels (*Kabuli* reference genome-CDC Frontier/PI)	Forward primers (5'-3')	Reverse primers (5'-3')	Anneling temperature (0C)	Expected amplified product size (bp)	Structural annotation		Functional annotation	
								Sequence components of *kabuli* genome	*Kabuli* gene accession IDs	NCBI-KOG	TFs
Ca1_P1_24648509	Ca1	24648509	TTATATATATATATATATATATATA/TTATATATATATATATATATATA	CACTTGAATTTGTTTCTCTCGC	TGCTTTTTCTGGATTGGTCC	59.0	464	INTERGENIC	--	--	--
Ca1_P1_24676884	Ca1	24676884	A/ATATATATATATAT	AGTTCTCACCGGATACGCTC	GCAGTTGCCGCATATCTTCT	59.3	481	INTERGENIC	--	--	--
Ca1_P1_24719200	Ca1	24719200	AATATATATATATATATATATATATATATATATA/AATATATATATATATATATATATATATATATATA	TCTTTGTTTCAACGGTCAATG	ATTATTTCCTGTTCGGGGGA	58.7	661	DRR	Ca_18334	--	--
Ca1_P1_24723587	Ca1	24723587	ATTATTTATTTA/ATTATTTA	CGGAGGCTATGAGAAGGAGA	TTGTGCTGCCAACTAAATGC	59.5	890	INTRON	Ca_18333	--	Dof
Ca1_P1_24771219	Ca1	24771219	TCT/TCTCTTTCCCTCTTCCT	TTGCAAGCACGATTTGAAAC	TGGGTCCAAAGTGGATGAAT	59.9	528	INTERGENIC	--	--	--
Ca1_P1_24782596	Ca1	24782596	CTATATATATATATATATAT/CTATATATATATATAT	GCTCAACAATGCATCACACC	GAATTTTCGGGGAACACAAA	60.1	678	INTERGENIC	--	--	--
Ca1_P1_24797994	Ca1	24797994	GTTTT/GTTT	TGATTTTCGAAGACTTGGGG	TGCATCGTTCTCCTCTTCCT	60.0	894	DRR	Ca_18329	T	--
Ca1_P1_24801961	Ca1	24801961	CTTTTTTTTT/CTTTTTTTTTT	TTTCAATGATGTTGGGTTGC	CAGCATATCCCCAACAAAAGA	59.4	998	INTERGENIC	--	--	--
Ca1_P1_24935055	Ca1	24935055	TTATATATATATATATATATAT/TTATATATATATATATATAT	TGTGTGTTTTCTACAATGTGCTAGTT	TTGGGTTTGACCCATGAAAT	59.3	730	INTERGENIC	--	--	--
Ca1_P1_25002005	Ca1	25002005	GT/GTTACCTTACACT	AACACGGGTTACGTCCAGTC	ATCTGGACCATTTCCAACGA	59.9	496	INTERGENIC	--	--	--
Ca1_P1_25058286	Ca1	25058286	AATATATATATATATATATATATATAT/AATATATATATATATATATATATATATATATATAT	CATCCTGCAACACATGCTCT	TCATGCAAATTCAAAAGAAATGA	59.9	794	DRR	Ca_18316	--	--
Ca1_P1_25064793	Ca1	25064793	AATATATATATATATATATATATATATA/AATATATATATATATATATATATATATATA	GTGAGGGTGCTTGTCTCACA	AATGGGCGCTGTTAAATGTC	59.9	613	INTERGENIC	--	--	--

**INDEL marker IDs**	**Chromosomes/ unanchored scaffolds**	**Physical positions (bp)**	**InDels (*Kabuli* reference genome-CDC Frontier/PI)**	**Forward primers (5'-3')**	**Reverse primers (5'-3')**	**Anneling temperature (0C)**	**Expected amplified product size (bp)**	**Structural annotation**		**Functional annotation**	
								**Sequence components of *kabuli* genome**	***Kabuli* gene accession IDs**	**NCBI-KOG**	**TFs**
Ca1_P1_25121975	Ca1	25121975	TCAGGATGTGCAAGTGCACAGGATGTGCAAGTGCA/TCAGGATGTGCAAGTGCA	CCCCATTTGCTTGTAGGAGA	TTTGTATTGCACGCCTTTCA	60.1	608	CDS (FRAME SHIFT)	Ca_18311	--	--
Ca1_P1_2518243	Ca1	2518243	CATATATATATATATATATA/CATATATATATATATATATATA	AACGGGATAAATGTTTGTGCTT	GCGCTTTTACGGGACAACTA	59.8	794	INTERGENIC	--	--	--
Ca1_P1_25185186	Ca1	25185186	TTATATATATATATATATATATAT/TTATATATATATATATATATATATATAT	CCGCCTAGAAGACTCCAAGA	AAAAAGTCTTGTATATAGAGCCGAA	59.6	869	INTERGENIC	--	--	--
Ca1_P1_2519911	Ca1	2519911	AATATATATATATATATATATAT/AATATATATATATATATAT	TTTTGACAGCAGTCATTTGTGA	TGGTTTTGGGATTTTGTGGT	59.4	683	INTERGENIC	--	--	--
Ca1_P1_25261945	Ca1	25261945	CT/CATTGGT	GTGGTGGTCAACAGTGAACG	CCTTACCGTGAAGCCAATGT	60.0	463	INTERGENIC	--	--	--
Ca1_P1_25296361	Ca1	25296361	AAAAAAAA/AAAAAAAAAAA	GGCGATTCTATGTCCATTGC	TCAACAAAATCACAGGCCAC	60.4	697	INTERGENIC	--	--	--
Ca1_P1_25358096	Ca1	25358096	AACCA/AA	TCTGGAGCCCATAGGAGTTG	CCAACGGGATGGTGGTATAG	60.2	716	INTERGENIC	--	--	--
Ca1_P1_25382145	Ca1	25382145	AC/A	GAATCACTAGGCTTGCCAGG	GGGGTGAGAGAACGTTTGAA	59.8	714	INTERGENIC	--	--	--
Ca1_P1_25392070	Ca1	25392070	TC/TTGACTTCATTAAC	AGCCCCCTCTAAACCAACAT	ACCTTTTGATTGGGCTTGTG	59.8	588	INTERGENIC	--	--	--
Ca1_P1_25437512	Ca1	25437512	AATATATATATATATATATATATATATATATATATATATATATATA/AATATATATATATATATATATATATATATATATATATATATATATATATATA	GACTTTGATGCAAGTCACCG	CTCCCATCAGTTTAATCGGC	59.3	875	INTERGENIC	--	--	--
Ca1_P1_25458751	Ca1	25458751	AATATATATATATATATATATATATATATATATA/AATATATATATATATATATATATATATATATATATATATA	GACGTCGCTTCCGAAGTTAG	TCACTCAGGTTGCCAAAACTT	60.0	538	INTERGENIC	--	--	--
Ca1_P1_25477131	Ca1	25477131	AATTATT/AATTATTAAAATTATATTATT	CCCATGCATGCTTCATAGAG	TTCTCGAAGATCCGACAGGT	59.3	782	INTERGENIC	--	--	--

INDEL marker IDs	Chromosomes/ unanchored scaffolds	Physical positions (bp)	InDels (*Kabuli* reference genome-CDC Frontier/PI)	Forward primers (5'-3')	Reverse primers (5'-3')	Anneling temperature (0C)	Expected amplified product size (bp)	Structural annotation		Functional annotation	
								Sequence components of *kabuli* genome	*Kabuli* gene accession IDs	NCBI-KOG	TFs
Ca1_P1_25507843	Ca1	25507843	CAATAATAATAATAATAATAATAATAATAATAATAATA/CAATAATAATAATAATAATAATAATAATAATAATA	TCTCTGGCAACCGTTCTTCT	GAATGAAGTCCAAGGGTCCA	60.0	341	INTERGENIC	--	--	--
Ca1_P1_25526955	Ca1	25526955	A/ACG	TTCGTAATGGACGGGATGAT	GGGTTTTGCATGTTGAGCTT	60.2	510	INTERGENIC	--	--	--
Ca1_P1_25625962	Ca1	25625962	GTATATATATATATATA/GTATATATATATATATA	TTGGAAGGAGCCCTTACCTT	GGTGCTCTTTTGTTCAAGGG	60.1	835	INTERGENIC	--	--	--
Ca1_P1_25688981	Ca1	25688981	TAAAAAAA/TAAAAAA	AATGTCCCCAACAGAGTTGC	TCGTATCGAACTTGCCCATT	60.0	542	INTERGENIC	--	--	--
Ca1_P1_25796612	Ca1	25796612	GAAAAAAAAAAA/GAAAAAAAAA	GTGCCCCCAAACTACTTTCA	AAGTCCCGGTAAGGTTGAGC	60.0	638	INTERGENIC	--	--	--
Ca1_P1_25846966	Ca1	25846966	CAGTAATA/C	GCGGATAATCAATTTCCCAA	GGTCAGATTAGTGGGCGTGT	59.7	578	INTERGENIC	--	--	--
Ca1_P1_26023746	Ca1	26023746	ATTTTTT/ATTTTTTT	AGGGGCCTTACTCCAAGTCA	TTGGAATGATCTAGTGAAGATTGAA	61.0	547	INTERGENIC	--	--	--
Ca1_P1_26075548	Ca1	26075548	T/TAAA	CATGCTTACCCTCTGGCCTA	TGCTGCTTTGGTTCAAAATG	60.2	664	INTERGENIC	--	--	--
Ca1_P1_26133146	Ca1	26133146	CTT/CTTGGCTTT	GCCGAACCCTTTTCTCCTAC	CCACAACAAAACGAAAGATCAA	60.1	241	INTERGENIC	--	--	--
Ca1_P1_26142349	Ca1	26142349	TTAC/T	CTTCGCACAACCATTGACAC	TTCCTGCTGTTGCTGATTTG	60.2	206	CDS (large-effect mutations)	Ca_18596	R	ZF-HD
Ca1_P1_26148944	Ca1	26148944	CG/CGTATTTG	AATGGCCTTCTTTCCCAATC	GAATCTCGGTAACCGCTCAA	60.3	423	DRR	Ca_18597	--	NF-YB
Ca1_P1_26165648	Ca1	26165648	AC/A	CACTAAGAGTGAGGACTGCACG	CCGGACTCGCTTTACAAAAA	60.1	706	INTERGENIC	--	--	--

INDEL marker IDs	Chromosomes/ unanchored scaffolds	Physical positions (bp)	InDels (*Kabuli* reference genome-CDC Frontier/PI)	Forward primers (5'-3')	Reverse primers (5'-3')	Anneling temperature (0C)	Expected amplified product size (bp)	Structural annotation		Functional annotation	
								Sequence components of *kabuli* genome	*Kabuli* gene accession IDs	NCBI-KOG	TFs
Ca1_P1_26192996	Ca1	26192996	CAAAAAAAA/CAAAAAAA	ACTAGTGGCGACGATGTCCT	TGTTAGGAGGATCTCACGGG	59.8	466	INTERGENIC	--	--	--
Ca1_P1_26208589	Ca1	26208589	CATATATATATATATATATATATATATATATA/CATATATATATATATATATATATA	CAGCCACCCCAAACTTTTTA	TCACCACCAGAGACAAACCA	60.0	419	INTERGENIC	--	--	--
Ca1_P1_26263650	Ca1	26263650	ATATTATTATTATTATTATTATTATTA/ATATTATTATTATTATTATTATTA	TCACCCAACAAGATCGTGAA	CCAAGCCCAATATCCAACAT	60.1	780	INTERGENIC	--	--	--
Ca1_P1_26298447	Ca1	26298447	TC/TCCC	ATCCATTCCCCACATTGAAA	GGGTTTTGAGGAGGTTGTCA	60.0	306	INTERGENIC	--	--	--
Ca1_P1_26309126	Ca1	26309126	C/CGGTCCTAAA	AAACACCTAGTGTGGGCTGG	AGGACCATGGAAGCCTCTTT	60.0	302	CDS (large-effect mutations)	Ca_18601	--	--
Ca1_P1_26358637	Ca1	26358637	TTTTAGT/TTTTAGTTTAGT	TGTGTTCATCCTTTCCACCA	GGGTTCAACTTCAAAAGCCA	59.9	754	INTRON	Ca_18604	U	LSD
Ca1_P1_26358840	Ca1	26358840	AATTATTATTATTATTATTATTATTATTATTATTATTATTATTATTATT/AATTATTATTATTATTATTATTATTATTATTATTATTATTATTATTATT	GCCTAGATCTTCCCCAAACC	AAAGAGATCCCAGCCCATCT	59.9	654	INTRON	Ca_18604	U	LSD
Ca1_P1_26400101	Ca1	26400101	TA/T	AAGAATCGCGCATTTGACTT	GATTGAGGACCGAAAGGACA	59.9	123	INTERGENIC	--	--	--
Ca1_P1_26408419	Ca1	26408419	AC/A	TCTTCCACCCAGTTTTGACC	TGTGGCCTGTGATTTTGGTA	59.9	543	INTERGENIC	--	--	--
Ca1_P1_26410717	Ca1	26410717	TTATATATATATATATATATAT/TTATATATATATATATATATAT	GCTTGTGTTTAAAGCGCTCC	CAGGTGTTCAGGATGTGACG	60.0	673	INTERGENIC	--	--	--
Ca1_P1_26413202	Ca1	26413202	C/CCTTTAGG	AAGCGTACCTGTGGGAATTG	TTAATGAGGGGCTGAATTGG	60.0	252	INTERGENIC	--	--	--
Ca1_P1_2655633	Ca1	2655633	AGG/AG	TCCGTGGAAATGAACCTAGC	CATGGACTCAAAAACAGCCA	60.1	812	INTERGENIC	--	--	--

INDEL marker IDs	Chromosomes/ unanchored scaffolds	Physical positions (bp)	InDels (*Kabuli* reference genome-CDC Frontier/PI)	Forward primers (5'-3')	Reverse primers (5'-3')	Anneling temperature (0C)	Expected amplified product size (bp)	Structural annotation		Functional annotation	
								Sequence components of *kabuli* genome	*Kabuli* gene accession IDs	NCBI-KOG	TFs
Ca1_P1_2656577	Ca1	2656577	TAGCAG/TAG	TCGTAGAATGGGGATG CAAT	CAACTGCTTCTGAAAC CGGA	60.3	141	INTERGENIC	--	--	--
Ca1_P1_26589205	Ca1	26589205	ATTTTTTTTT/ATTTTTTTT	TTCCTAACATTCTCCG CTGG	TGATGTTGCATCAACC ATTTT	60.2	168	INTERGENIC	--	--	--
Ca1_P1_26592081	Ca1	26592081	C/CTGACAGTGA	ACACACAAATTTTGAC CGCA	GCAATTTTCTCCAATT GCGT	60.0	801	INTERGENIC	--	--	--
Ca1_P1_2659614	Ca1	2659614	TAAA/TAA	CAATCAGGATCGGCCA ATTA	CGTGTTTAGCGGTGA GTTGA	60.8	642	INTERGENIC	--	--	--
Ca1_P1_26605020	Ca1	26605020	CT/C	AAAGGCATCCTACAAT TCACA	TGGAGTTTGGTGGTGA AAAA	57.2	587	INTERGENIC	--	--	--
Ca1_P1_2660557	Ca1	2660557	TTCTCTCTCT/TTCTCTCTCTCTCTCT CT	CGACCACTGATTTGAC CCTT	AACAACATGGACAACG ACGA	60.0	388	INTERGENIC	--	--	--
Ca1_P1_2663715	Ca1	2663715	TGAAAAGAAAA/TGAAAA	GTTGCCATGCCTATAA CGGT	TGAAGACCATGGACAC CAAC	59.9	547	INTERGENIC	--	--	--
Ca1_P1_2664568	Ca1	2664568	GATGATGTGAATGATGTGAA/GATGAT GTGAA	CCAAAAATTGATGAATT GACGTT	TGAATCATGAAGCGGA AAAA	60.1	324	INTERGENIC	--	--	--
Ca1_P1_26681887	Ca1	26681887	AAAAAAACAAAAAACAAAAAACAAAA/ AAAAAAACAAAAAACAAAA	CCAGACACAACTCAAC CCCT	CTTTTGGCTCCCCCTA AGTC	60.0	476	INTERGENIC	--	--	--
Ca1_P1_26683611	Ca1	26683611	TG/T	CAATTGTGTAAAACGT GCGG	TTGCATTTGGTTTGAC TCCA	60.0	538	INTERGENIC	--	--	--
Ca1_P1_2670194	Ca1	2670194	CAA/CAAAA	CTGAATTTAAAGCCCG TTGG	TGTGTTGATAGTGGGC GAAA	59.6	779	DRR	Ca_00327	--	ERF
Ca1_P1_26726431	Ca1	26726431	AAATAATAATAATAATAATAATAATAAT AA/AAATAATAATAATAATAATAATAAT AATAATAA	GCCTTTGATATGAGGA GCCA	TGGCTTGTGATGCAAC ATTT	60.2	870	INTERGENIC	--	--	--

INDEL marker IDs	Chromosomes/ unanchored scaffolds	Physical positions (bp)	InDels (*Kabuli* reference genome-CDC Frontier/PI)	Forward primers (5'-3')	Reverse primers (5'-3')	Anneling temperature (0C)	Expected amplified product size (bp)	Structural annotation		Functional annotation	
								Sequence components of *kabuli* genome	*Kabuli* gene accession IDs	NCBI-KOG	TFs
Ca1_P1_2673583	Ca1	2673583	ACT/A	TTTTGGAACCTTTGCTCACA	GAGCTTCCCATATTCCGACA	59.3	908	INTERGENIC	--	--	--
Ca1_P1_2674516	Ca1	2674516	CTAGATTGGAT/CTAGATTGGATTAGATTGGAT	TGTCGGAATATGGGAAGCTC	GCCGTTGTTTTCAGATCCAT	60.0	934	INTERGENIC	--	--	--
Ca1_P1_2676035	Ca1	2676035	TC/T	CATCCGGTTGGTTTTGGTAG	GACGACGTGAAAGTATTTGGC	60.2	570	INTERGENIC	--	--	--
Ca1_P1_26774086	Ca1	26774086	GTTTTTTTTTT/GTTTTTTTTTTT	TGCTTATAATGTTTCTTTTTCAAAGC	GCACATGGATCACTTTCTCCT	59.4	509	INTERGENIC	--	--	--
Ca1_P1_26774824	Ca1	26774824	TCTTTCCNNNNNNNNNNCTTTCC/TCTTTCC	TTGCACCCTTCTTAGCACCT	ACAATGAGTCGTCGCACAAA	59.9	212	INTERGENIC	--	--	--
Ca1_P1_2679246	Ca1	2679246	CTTTTTTTT/CTTTTTTTTTT	CATCCTTATCCCTCATTCCTTG	ATGGTGGCCGGTAGACCTAT	59.8	723	INTERGENIC	--	--	--
Ca1_P1_26801684	Ca1	26801684	TGGGGGGG/TGGGGG	AATTTCGAAAGAGGGGTGAA	AGCAGGCATGCCATTTCTAT	58.6	500	DRR	Ca_20286	--	--
Ca1_P1_26805963	Ca1	26805963	AA/AATA	TTTTAAACGGGTTAGGTGCG	AAAGCCATGTGCCAAGTTTC	60.0	337	INTERGENIC	--	--	--
Ca1_P1_2683771	Ca1	2683771	CTATA/CTA	TTGAATTAAACTCTGAAAATAACGC	AAGATTGGACGGTTGTGAGG	58.1	580	INTERGENIC	--	--	--
Ca1_P1_26866750	Ca1	26866750	ATTTTTTTTTT/ATTTTTTTTTTT	TCTCAGTGGAAAACGAACCC	TCTCCACCATCTTCCTCTTCA	60.1	679	INTERGENIC	--	--	--
Ca1_P1_2686910	Ca1	2686910	AGTATTTGGGT/AGT	AATGTGATGCACGTTTTGGA	CAAATTTCACGCAACAATCG	60.0	444	DRR	Ca_00328	--	MIKC
Ca1_P1_26876468	Ca1	26876468	C/CG	TTGTGCATCCGTCTATCCAC	TCGGTGGAAATTGTAATGAGAG	59.5	937	INTERGENIC	--	--	--

INDEL marker IDs	Chromosomes/ unanchored scaffolds	Physical positions (bp)	InDels (*Kabuli* reference genome-CDC Frontier/PI)	Forward primers (5'-3')	Reverse primers (5'-3')	Anneling temperature (0C)	Expected amplified product size (bp)	Structural annotation		Functional annotation	
								Sequence components of *kabuli* genome	*Kabuli* gene accession IDs	NCBI-KOG	TFs
Ca1_P1_26879148	Ca1	26879148	G/GCC	AAAACCCAACAGATCGGAGTT	CGGACCCTATGATCTGGAAA	59.9	206	INTERGENIC	--	--	--
Ca1_P1_26894935	Ca1	26894935	GAAAAAAAAA/GAAAAAAAA	CGAAGAAGAAAATGCGACAA	AAAAACAAAATCACAGGTAACCAAA	59.0	512	INTERGENIC	--	--	--
Ca1_P1_2691545	Ca1	2691545	CCATGCATG/CCATG	TTAGCTCAGCTTGCACAGGA	GCATTGTACATTCTCGTGCG	59.9	541	DRR	Ca_00330	--	--
Ca1_P1_2692223	Ca1	2692223	TAAAAA/TAAAAAA	ACAATGCCATTTTCAAGGCT	CGTGAAGACTAAAGGTCAGAAAAA	59.6	532	DRR	Ca_00330	--	--
Ca1_P1_26945131	Ca1	26945131	ATATTATTATTATTATTATTATTATTATTATTATTAT/ATATTATTATTATTATTATTATTATTATTATTAT	AAACAAATAAACCATTTCACCAAA	TAACAACGGCTGGGTAAAGG	58.8	384	INTERGENIC	--	--	--
Ca1_P1_26958642	Ca1	26958642	TT/TTTGAT	TGCTCCATTTGATTGCTGAC	CCGACCAATAGACTTTTGTTGA	59.8	663	INTERGENIC	--	--	--
Ca1_P1_26958726	Ca1	26958726	GTTT/GTTTT	TGAGCGTGAAAAATTGTGGT	CCGACCAATAGACTTTTGTTGA	59.2	204	INTERGENIC	--	--	--
Ca1_P1_26958895	Ca1	26958895	CAAAAAAAAA/CAAAAAAAA	TGAGCGTGAAAAATTGTGGT	TTAGAGCGTTCAAATTATAGTTACACA	59.2	709	INTERGENIC	--	--	--
Ca1_P1_26987414	Ca1	26987414	ATTTTTTTTTTTTTT/ATTTTTTTTTTTTT	ATATGGCCGTATTTCCGTCA	CTCGGTCATCGAAGGAGAAA	60.2	510	INTERGENIC	--	--	--
Ca1_P1_26989616	Ca1	26989616	AAATAATAATAATAATAATAATAATAATAATAATAATAATAA/AAATAATAATAATAATAATAATAATAATAATAATAA	GAGGTCGTTGACCGGATATG	CGTGTGATTGGTAATGTGGC	60.3	639	INTERGENIC	--	--	--
Ca1_P1_26990827	Ca1	26990827	TGGCGGC/TGGC	AAATCCTTTGGGAGGTCACAT	TGAGCCTAATAGGAGGTCGC	59.7	109	INTERGENIC	--	--	--
Ca1_P1_2701362	Ca1	2701362	CTTTTTTTTT/CTTTTTTTTTTT	CCGGCAAAAATTTGGTTAAA	TGGTCTTGCAATGTCTCTGG	59.8	727	DRR	Ca_00331	--	--

INDEL marker IDs	Chromosomes/ unanchored scaffolds	Physical positions (bp)	InDels (*Kabuli* reference genome-CDC Frontier/PI)	Forward primers (5'-3')	Reverse primers (5'-3')	Anneling temperature (0C)	Expected amplified product size (bp)	Structural annotation		Functional annotation	
								Sequence components of *kabuli* genome	*Kabuli* gene accession IDs	NCBI-KOG	TFs
Ca1_P1_27019589	Ca1	27019589	TC/TCC	TTGGCCAACGTTAGTT AGGG	TGCAGTTCCATGTCCA TTGT	60.0	726	INTRON	Ca_20299	C	--
Ca1_P1_2702474	Ca1	2702474	CTT/CTTATT	CTGGCAGCATTGTTGT TGTT	ACTTCCTTCCACCACC ACTG	59.8	228	CDS (large-effect mutations)	Ca_00331	--	--
Ca1_P1_2704415	Ca1	2704415	CATA/CATATA	TAATCACACCGCTCAT CGAA	ATTACTCGCCATGCCT GTTC	60.2	222	INTERGENIC	--	--	--
Ca1_P1_2708925	Ca1	2708925	GTTT/GTTTTT	AAAGCAACAACCAGGC ACTC	CGGTAGTCATAGATGG CGGT	60.3	754	INTRON	Ca_00333	--	--
Ca1_P1_27104652	Ca1	27104652	CTTCTTCAAT/CTTCTTCAATATTCTTC AAT	AATTTTTCTTCCCACAC CCC	CGAAACCCAGATTTGG AAAA	60.0	335	INTERGENIC	--	--	--
Ca1_P1_27107121	Ca1	27107121	AAAC/A	TTTTCCACTTCGGAGT CCAC	TAGCACATTCGGTTGC AGAG	60.1	764	INTERGENIC	--	--	--
Ca1_P1_27107315	Ca1	27107315	TAA/TAAA	TTTTCCACTTCGGAGT CCAC	TAGCACATTCGGTTGC AGAG	60.1	764	DRR	Ca_20308	--	--
Ca1_P1_2719697	Ca1	2719697	AGTAAG/A	ATAGACATCCGGGACA GTCG	TGATACCCATCCATCC CTGT	60.0	613	INTRON	Ca_00334	G	--
Ca1_P1_27219176	Ca1	27219176	CTATTATTATTATTATTATTATTATTAT TATTATTATTATTATTGTTATTATTATT ATTATTATTATTATTATTATTATTA/CTA TTATTATTATTATTATTATTATTATTAT TATTATTATTATTATTGTTATTATTATT [illegible]	TGAGGGAGATATTTGG TGGG	TACGGCGCTGTTCTTT TCTT	59.7	277	INTERGENIC	--	--	--
Ca1_P1_2727935	Ca1	2727935	AT/A	CAAACGCAAATTGAGC GATA	CATTTTGAAAGATTCA CATTCATTT	59.8	717	INTERGENIC	--	--	--
Ca1_P1_27282873	Ca1	27282873	GTATA/GTATATA	AAAATCCAACAATCTCC CCC	CCCTGAGACTCACGAA GAGG	60.0	561	INTERGENIC	--	--	--
Ca1_P1_27349054	Ca1	27349054	C/CGCG	ACCTATTACGGGTCAG CACG	CCACGAGATGATTCCA TGTG	60.0	203	INTERGENIC	--	--	--

INDEL marker IDs	Chromosomes/ unanchored scaffolds	Physical positions (bp)	InDels (*Kabuli* reference genome-CDC Frontier/PI)	Forward primers (5'-3')	Reverse primers (5'-3')	Anneling temperature (0C)	Expected amplified product size (bp)	Structural annotation		Functional annotation	
								Sequence components of *kabuli* genome	*Kabuli* gene accession IDs	NCBI-KOG	TFs
Ca1_P1_27475132	Ca1	27475132	ATTTTTTTTTTTT/ATTTTTTTTTTTTT	TGGAATCAAAAAGACGTAAGCC	GAGGAAACATCATTCAATCGC	60.5	632	INTERGENIC	--	--	--
Ca1_P1_27595250	Ca1	27595250	AT/ATT	CGAGGTAATGACATCCCTTCA	TGGACGTAGGTTGTTGTGGA	59.9	566	INTERGENIC	--	--	--
Ca1_P1_2765307	Ca1	2765307	C/CAATGTG	CAGCCTCTGATGTTCAATGG	CTATGTGCATATCGGCCCTT	59.2	423	INTERGENIC	--	--	--
Ca1_P1_2766077	Ca1	2766077	CTTATGTT/CTTATGTTATGTT	AAAACAATTGATTGGGCTCC	CCAATCATACGCAGGTGAAA	58.9	561	INTERGENIC	--	--	--
Ca1_P1_2766176	Ca1	2766176	A/AG	AAAACAATTGATTGGGCTCC	CCAATCATACGCAGGTGAAA	58.9	561	INTERGENIC	--	--	--
Ca1_P1_27665473	Ca1	27665473	C/CCCTAA	GCGTTTGAATGATTGTTGGA	TTGAGAGACTTGTGCCCTAGC	59.5	819	INTRON	Ca_25073	--	--
Ca1_P1_27708434	Ca1	27708434	CAAAAAAAAAA/CAAAAAAAAA	TGGCATGAAATGGGTCTACA	TTCCATGCCCAGAAAATAGC	59.9	589	INTERGENIC	--	--	--
Ca1_P1_27771248	Ca1	27771248	TAAAA/TAAA	CAACATCAATTGAAAATGCGA	GGCGGGTTTGTTAGTCTTGA	59.6	285	INTERGENIC	--	--	--
Ca1_P1_2779559	Ca1	2779559	AATATATATATATATAT/AATATATATATAT	AAGGGTCCTCTCAATCGTCA	GTGAGGTTGGTCAACTCCGT	59.7	715	INTERGENIC	--	--	--
Ca1_P1_27802503	Ca1	27802503	ATTTTTTT/ATTTTTTTT	TCACCAGCTCCTTGTTAGCC	CCATGGTTCTGCATTCCTTT	60.4	587	INTERGENIC	--	--	--
Ca1_P1_27902256	Ca1	27902256	AATATATATATATATATATATATATATATATATATATAT/AATATATATATATATATATATATATATATATATATATATATATAT	TGCAACTCAGCATAAGACGG	CATTTGACGCTCTTGCAAAATA	60.0	340	INTERGENIC	--	--	--
Ca1_P1_2791561	Ca1	2791561	ATTT/AT	ATCGAAAGCAACCATTTTGG	TCATTCAAATGGTGACTCGTG	59.9	419	INTERGENIC	--	--	--

**INDEL marker IDs**	**Chromosomes/ unanchored scaffolds**	**Physical positions (bp)**	**InDels (*Kabuli* reference genome-CDC Frontier/PI)**	**Forward primers (5'-3')**	**Reverse primers (5'-3')**	**Anneling temperature (0C)**	**Expected amplified product size (bp)**	**Structural annotation**		**Functional annotation**	
								**Sequence components of *kabuli* genome**	***Kabuli* gene accession IDs**	**NCBI-KOG**	**TFs**
Ca1_P1_2796283	Ca1	2796283	T/TG	GGTGGACAGAGGCATCAACT	AAAAAGCGAGTTTCAGGGTG	60.1	530	INTERGENIC	--	--	--
Ca1_P1_2800448	Ca1	2800448	ATTTTTTTTT/ATTTTTTT	GAGAGCAAGAGCGAGCAAGT	ACCACATGAGGGGCATTTTA	60.0	439	INTERGENIC	--	--	--
Ca1_P1_2800724	Ca1	2800724	TTTT/TTTTTTTATTT	GAGAGCAAGAGCGAGCAAGT	TCCATTTTCTCCTTCAAGCG	60.0	754	INTRON	Ca_00344	--	NAC
Ca1_P1_2801241	Ca1	2801241	ATTATTATTTTTT/ATT	CGCTTGAAGGAGAAAATGGA	TTACACGGCATGACAATCAAA	60.3	352	INTRON	Ca_00344	--	NAC
Ca1_P1_2810482	Ca1	2810482	CAT/CATAT	TGCATAACCAACTGAATGCC	GCGTCGTAAAAGACGTGCTA	59.5	813	INTERGENIC	--	--	--
Ca1_P1_28170186	Ca1	28170186	CTTTTTTTTTT/CTTTTTTTTTTT	TCCAACGCCTTCTTTTCAAT	GTGCGATCCGTTGAAGTTCT	59.7	811	INTERGENIC	--	--	--
Ca1_P1_28225122	Ca1	28225122	TTCTCTCTCTCTCTCTCTCTCTCTCTCT/TTCTCTCTCTCTCTCTCTCTCTCTCT	ACTCAACGCATGAAGAGGCT	GCAAAGCTTTCCGCTAACTG	60.0	596	INTERGENIC	--	--	--
Ca1_P1_2828614	Ca1	2828614	T/TCAAA	TTGCAATTTTGGGGAAAAAG	TGACGCATGTAAAACGTGGT	59.9	369	INTERGENIC	--	--	--
Ca1_P1_2829969	Ca1	2829969	T/TTA	ATCCGGGTCCAAAGTTTCTT	AAAAGCATGCAAAAGCATCA	59.8	774	INTERGENIC	--	--	--
Ca1_P1_2831879	Ca1	2831879	TAAAAA/TAAAAAA	TTTGAGTCTTCGAGCCACAG	CTGGTCCTTTCCTTTCCCTC	59.2	342	DRR	Ca_00347	--	ARF
Ca1_P1_28322869	Ca1	28322869	GAAAAAAAAAA/GAAAAAAAAA	GAGAACAACAACTTGACCCAAA	GTTACCAGCCCATCAATGCT	59.1	376	INTERGENIC	--	--	--
Ca1_P1_2837552	Ca1	2837552	TTCTCTCTCTCTCTCTCTCTCTCT/TTCTCTCTCTCTCTCTCT	CAGCACAAGCATGCCATAGT	TGCATCTGTTCCTGCTTCAC	59.9	671	INTERGENIC	--	--	--

INDEL marker IDs	Chromosomes/ unanchored scaffolds	Physical positions (bp)	InDels (*Kabuli* reference genome-CDC Frontier/PI)	Forward primers (5'-3')	Reverse primers (5'-3')	Anneling temperature (0C)	Expected amplified product size (bp)	Structural annotation		Functional annotation	
								Sequence components of *kabuli* genome	*Kabuli* gene accession IDs	NCBI-KOG	TFs
Ca1_P1_2840591	Ca1	2840591	ATTTTATTTTTATTTTTAT/ATTTTATTTTTATTTTTATTTTTAT	GCACCCCTATTACCCCTCAT	GGCAGTAGCAAGCAATTGAA	60.0	757	INTERGENIC	--	--	--
Ca1_P1_2843204	Ca1	2843204	AAA/AAACAA	AACAAGCTGTTGCAAGCTCA	TTTCCGTTTGATTCTCCGTC	59.8	431	INTERGENIC	--	--	--
Ca1_P1_28449282	Ca1	28449282	ACGAATATTTTCCGAATATTTTCCGAATATTT/ACGAATATTTTCCGAATATTT	TGGATTGGTTGAAGGAGGTC	CTCTGTCACTGGCGTCACAT	59.9	695	DRR	Ca_18508	--	--
Ca1_P1_28458887	Ca1	28458887	AAATAATAATAATAATAATAATAATA/AAATAATAATAATAATAATAATAATAATAATA	CGTCGTTTGTTTTGACACATC	CGTTGTTTGTTTCGACACATCT	59.1	612	INTERGENIC	--	--	--
Ca1_P1_2846167	Ca1	2846167	AAT/A	GTTTGAACACGGGTGCTTTT	ATTTTAGACGCTTGCCCTCA	60.0	165	INTERGENIC	--	--	--
Ca1_P1_28501524	Ca1	28501524	CGGG/CGG	GATTTGCATTACGAATTTGATGA	GCTGCTACTTTGCAAATCCAT	59.0	479	INTERGENIC	--	--	--
Ca1_P1_2851127	Ca1	2851127	TAT/TATAAT	TGTCGTTTTCAATGGACTGG	AGAGAGTATGGGGCAGTCAAA	59.5	226	INTERGENIC	--	--	--
Ca1_P1_28533128	Ca1	28533128	CATAATAAT/CATAATAATAAT	CGGAGACAACACGAGACTGA	GTCACCAGAATTGGCTCGTT	60.0	391	INTERGENIC	--	--	--
Ca1_P1_28550132	Ca1	28550132	ATTTTTTTTTT/ATTTTTT	TGGAAGCACGCTTAAGGACT	TTTCCCAATCGATTTGCTTC	60.0	664	INTERGENIC	--	--	--
Ca1_P1_28550236	Ca1	28550236	T/TG	CTGACACAGGGGGAGAAAAA	TTTCCCAATCGATTTGCTTC	60.1	159	INTERGENIC	--	--	--
Ca1_P1_28573613	Ca1	28573613	AATATATATATATATATATATATATATATATATATAT/AATATATATATATATATATATATATATATATATATATATATATATATATAT	AGGTTTGGAGAACCGAAGGT	ACCATGGTTGTCCTGAAAGG	60.0	270	INTERGENIC	--	--	--
Ca1_P1_2861840	Ca1	2861840	CAAA/CAAAA	CGCCTTGAAAACCCTCAGTA	CTAATCCCTTGCCCTCAACA	60.2	666	INTRON	Ca_00348	R	--

INDEL marker IDs	Chromosomes/ unanchored scaffolds	Physical positions (bp)	InDels (*Kabuli* reference genome-CDC Frontier/PI)	Forward primers (5'-3')	Reverse primers (5'-3')	Anneling temperature (0C)	Expected amplified product size (bp)	Structural annotation		Functional annotation	
								Sequence components of *kabuli* genome	*Kabuli* gene accession IDs	NCBI-KOG	TFs
Ca1_P1_2862989	Ca1	2862989	CAA/CA	TAATGGCCAAATTCTCTCCG	GCTTTCACCAATTCCACGTT	60.0	691	INTRON	Ca_00348	R	--
Ca1_P1_2867067	Ca1	2867067	GAAA/GAAATAAAA	AGGAGCCTTTGGTTTTGGTT	TCCCTCTTCCCCTTTCTCAT	60.0	555	DRR	Ca_00348	R	--
Ca1_P1_28670946	Ca1	28670946	CATATATATATATATATATATATATAT/CATATATATATATATATATATATATATATATAT	GGAAGCATCCACGCCTATTA	TAACCACTGCCACCTCAACA	60.1	453	INTERGENIC	--	--	--
Ca1_P1_2867118	Ca1	2867118	CA/CAA	AGGAGCCTTTGGTTTTGGTT	TCCCTCTTCCCCTTTCTCAT	60.0	555	DRR	Ca_00348	R	--
Ca1_P1_2868551	Ca1	2868551	TTTGTT/TTTGTTGTT	CACCTCACTGTTCACCCCTT	CATCCACTGTCGACAACACC	60.0	697	INTRON	Ca_00349	--	--
Ca1_P1_2868921	Ca1	2868921	CAT/CATCGCGGAT	GTGTTGTCGACAGTGGATGG	TCTAGCTCGGTGCACCCTAT	60.0	199	INTRON	Ca_00349	--	--
Ca1_P1_28713213	Ca1	28713213	TTA/TTAGAAAGAGTGTA	TTCATTTCTCTGATTCCCCG	TCCGCATTATAACCTCTGCC	60.0	425	INTERGENIC	--	--	--
Ca1_P1_28716048	Ca1	28716048	CATATATATATATATATAT/CATATATATATATAT	CGCAAAGATTTGGGTTTGAT	TGGACCCTGAATTACGAAGG	59.9	776	INTERGENIC	--	--	--
Ca1_P1_28728261	Ca1	28728261	ATT/AT	GCCGGTGTTTGAAATTAACC	TAAGGCAGCAAACTTCCGTC	59.3	496	INTERGENIC	--	--	--
Ca1_P1_2878797	Ca1	2878797	TGTTGAGTT/TGTTGAGTTGAGTT	TCAATGCGGAAATGTGTTGT	TTTGTGTGGTCGCAACTCTC	60.0	413	DRR	Ca_00350	GC	SBP
Ca1_P1_28790237	Ca1	28790237	AAATGGAGATAATGGAGAT/AAATGGAGAT	GAACGCAGCAGCAACAATAA	CATGTCTTAACCGTCAAACGAA	60.0	364	DRR	Ca_18497	OU	--
Ca1_P1_2885377	Ca1	2885377	ATTTTTTT/ATTTTTTTT	GCCATTGTTAACTCCGCAAT	CATTTGGAGAGGGCATAGGA	60.0	360	DRR	Ca_00351	T	M-type

INDEL marker IDs	Chromosomes/ unanchored scaffolds	Physical positions (bp)	InDels (*Kabuli* reference genome-CDC Frontier/PI)	Forward primers (5'-3')	Reverse primers (5'-3')	Anneling temperature (0C)	Expected amplified product size (bp)	Structural annotation		Functional annotation	
								Sequence components of *kabuli* genome	*Kabuli* gene accession IDs	NCBI-KOG	TFs
Ca1_P1_28871254	Ca1	28871254	AAAATAAATAAA/AAAATAAATAAATAAA	ACGCCTCAAGCTATTCCTGA	AAGTTCGGGCGTAACTAGCA	60.0	495	INTERGENIC	--	--	--
Ca1_P1_28906702	Ca1	28906702	TAAAAAAA/TAAAAAA	TATGGAGAGCAGCGACTCAA	TGTTGGGTTGCACAACATTC	59.7	184	INTERGENIC	--	--	--
Ca1_P1_28910299	Ca1	28910299	CATT/CATTAATT	GAAAATGATCGGTGGAGGAA	CTTGCGTGTTGCACGAGTAT	59.9	620	DRR	Ca_18486	R	--
Ca1_P1_28910424	Ca1	28910424	TA/T	GAGTCATTGGTGCCTTGGTT	CTTGCGTGTTGCACGAGTAT	60.0	385	DRR	Ca_18486	R	--
Ca1_P1_28910492	Ca1	28910492	GATTAA/GATTAATTAA	GAGTCATTGGTGCCTTGGTT	CTTGCGTGTTGCACGAGTAT	60.0	385	DRR	Ca_18486	R	--
Ca1_P1_28911682	Ca1	28911682	A/AAAAAAAAT	CGTGAATGCTTTATTCACAACC	CCACATCCTAGTGAAAAGCCA	59.5	633	INTERGENIC	--	--	--
Ca1_P1_28929310	Ca1	28929310	G/GAGAT	AAATTCGAACTGGGCATTTG	CTTGGGCTATACCTTTTCTATTGT	59.9	107	INTERGENIC	--	--	--
Ca1_P1_28930954	Ca1	28930954	A/ATT	TGAAAAGTTTCTTTGTCCCTTTG	TGGTGTTGCCTTCAAATTCA	59.7	349	INTERGENIC	--	--	--
Ca1_P1_28935215	Ca1	28935215	ATTTTTTTT/ATTTTTTTTT	TTTTGTTGGCAGTTTGTTTG	CAGTCAACATTGATGTGACGTTAT	57.3	490	INTERGENIC	--	--	--
Ca1_P1_28936099	Ca1	28936099	ATTTTTTTT/ATTTTTTTTTTT	GAAATTTGACCAGAGCTGCC	TCGGTCACCGTTTTTAGTTT	59.8	465	INTERGENIC	--	--	--
Ca1_P1_28936233	Ca1	28936233	AT/ATGTAATT	GAAATTTGACCAGAGCTGCC	GGACCAATTACTCTGGTGGAA	59.8	650	INTERGENIC	--	--	--
Ca1_P1_28939471	Ca1	28939471	AAAATAAAACAAA/AAAA	CCATGTGTGAACCCTAATTGTT	GTTTTGGCGTTGGTTTCACT	58.7	226	INTERGENIC	--	--	--

INDEL marker IDs	Chromosomes/ unanchored scaffolds	Physical positions (bp)	InDels (*Kabuli* reference genome-CDC Frontier/PI)	Forward primers (5'-3')	Reverse primers (5'-3')	Anneling temperature (0C)	Expected amplified product size (bp)	Structural annotation		Functional annotation	
								Sequence components of *kabuli* genome	*Kabuli* gene accession IDs	NCBI-KOG	TFs
Ca1_P1_28941503	Ca1	28941503	CAA/CAAAA	TGGGGTTTAGGGTGTCACAT	CTTTTGTAAAGGCAGCTCCG	60.1	207	INTERGENIC	--	--	--
Ca1_P1_28941872	Ca1	28941872	GAAAAAAA/GAAAAAAAAAA	TGGGGTTTAGGGTGTCACAT	TCAATTTGGATTTCTTGGTGG	60.1	847	INTERGENIC	--	--	--
Ca1_P1_28943560	Ca1	28943560	GG/GGTG	ACTTGGGACAAATCACATGG	TGATCACCAGCTTTCCAATG	58.3	252	INTERGENIC	--	--	--
Ca1_P1_28944045	Ca1	28944045	TAAAAAA/TAAAAA	TTGCCTTTAATGGACCAAGG	CAATATGAAGTCTGGTGCCAA	59.9	345	INTERGENIC	--	--	--
Ca1_P1_28944496	Ca1	28944496	TTATATATAT/TTATATAT	TTGGCACCAGACTTCATATTG	AAATGTACTTTGCGGCACAT	58.6	775	INTERGENIC	--	--	--
Ca1_P1_28944803	Ca1	28944803	AACTA/AA	TTTCAATTGCATTAATCCGTTC	CAATAAAAATTGCGGTCCGA	59.0	963	INTERGENIC	--	--	--
Ca1_P1_28945159	Ca1	28945159	GTTT/GTTTT	TGCCGCAAAGTACATTTATCA	CAATAAAAATTGCGGTCCGA	59.2	369	INTERGENIC	--	--	--
Ca1_P1_2898163	Ca1	2898163	CAAAAAAAAAAA/CAAAAAAAAA	TGGTGTTTACCACTCCGTCA	TTAAATCACAGTGGCGGTCA	60.0	368	INTRON	Ca_00354	--	bHLH
Ca1_P1_2901135	Ca1	2901135	TTCTGAGAAAGTTATCT/TTCTGAGAAAGTTATCTGAGAAAGTTATCT	AGCTTTCCACGAAAATCAGC	GCACATTTACAGTTGGGGCT	59.5	565	INTRON	Ca_00354	--	bHLH
Ca1_P1_29077918	Ca1	29077918	TATATATATATAGATATATA/TATATATA	GACGAAGTCCTTATTGCATCG	TTTATTGAATTTGGTTGGGTG	59.7	817	INTERGENIC	--	--	--
Ca1_P1_29102832	Ca1	29102832	TTATATATATATATATATATATATA/TTATATATATATATATATATATATATATATATA	ATGTCAATTGAGTTGCGACG	CAAGCACCGTAGTGTGGAGA	59.7	340	INTERGENIC	--	--	--
Ca1_P1_2910422	Ca1	2910422	TAAGAAGAAGAAGAA/TAAGAAGAAGAA	TCCAACACCAGAATCACCAA	CCATTCCCCAAAACAAAACA	59.9	382	INTERGENIC	--	--	--

INDEL marker IDs	Chromosomes/ unanchored scaffolds	Physical positions (bp)	InDels (*Kabuli* reference genome-CDC Frontier/PI)	Forward primers (5'-3')	Reverse primers (5'-3')	Anneling temperature (0C)	Expected amplified product size (bp)	Structural annotation		Functional annotation	
								Sequence components of *kabuli* genome	*Kabuli* gene accession IDs	NCBI-KOG	TFs
Ca1_P1_29124803	Ca1	29124803	ATTT/ATTTT	AGTGGCATTGGGCAAATTAC	ACCAAATATACGCGTCTGCC	59.8	350	INTERGENIC	--	--	--
Ca1_P1_2917579	Ca1	2917579	TGT/TGTTGTGGT	TGGTTGAGCATGAACCATGT	AAGCTTCGCCATCTTCAAAA	60.0	450	CDS (large-effect mutations)	Ca_00357	--	WOX
Ca1_P1_2919258	Ca1	2919258	CAGAGAGAGAGAGAGAGAGAGAGAGAGAGAGAGAGAGAG/CAGAGAGAGAGAGAGAGAG	GAAGCAAAGGGAACATGGAA	CTTTGCCGAAAATAACCGAA	60.1	443	INTERGENIC	--	--	--
Ca1_P1_2921755	Ca1	2921755	A/ATATG	CAACTGGGCCAAATGCTTAT	TGATGATCACACGAGGGAGA	60.0	570	INTERGENIC	--	--	--
Ca1_P1_29219534	Ca1	29219534	AATATATATATATATATATATATATATATATATA/AATATATATATATATATATATATATATATA	AATAATGCTCCAAGCCTCCA	TCGCCATGTTGTTGGAGTTA	59.7	277	DRR	Ca_22567	D	WRKY
Ca1_P1_2922769	Ca1	2922769	CAAAA/CAAAAAA	TTTGTGGATCGTATTGCGAA	AAATGCATTCCTTCATTCTTTCA	60.1	604	INTERGENIC	--	--	--
Ca1_P1_2923053	Ca1	2923053	TATGAA/TATGAATGAATGAA	CGCGAAATCTTATTTTCATGG	TGATGCGATTGTAGTCCCAA	59.6	606	INTERGENIC	--	--	--
Ca1_P1_2923274	Ca1	2923274	A/ATATTAAAATAAT	CGCGAAATCTTATTTTCATGG	TGATGCGATTGTAGTCCCAA	59.6	606	INTERGENIC	--	--	--
Ca1_P1_2924550	Ca1	2924550	CTT/CTTT	GTTCAGCAAATCGGGTCAAT	TGAGTTAATGCGGCCTAACC	59.9	595	INTERGENIC	--	--	--
Ca1_P1_29246694	Ca1	29246694	CATATATATATATATATAT/CATATATATATATAT	TTTTGTCGCACATTGTTGGT	GAGGTGCACAAATAGTACGTCG	60.0	797	INTERGENIC	--	--	--
Ca1_P1_2925914	Ca1	2925914	ATTTT/ATT	GTTGGCCGTTGTTTTGTTCT	CCCTTCATAGATGATGGTGGA	60.0	372	INTERGENIC	--	--	--
Ca1_P1_2933961	Ca1	2933961	AAGAGA/AAGA	CTACCAACCGGTGAATGCTT	CCCCCACTTTCATTCAGTAAA	60.0	641	INTRON	Ca_00358	E	--

INDEL marker IDs	Chromosomes/ unanchored scaffolds	Physical positions (bp)	InDels (*Kabuli* reference genome-CDC Frontier/PI)	Forward primers (5'-3')	Reverse primers (5'-3')	Anneling temperature (0C)	Expected amplified product size (bp)	Structural annotation		Functional annotation	
								Sequence components of *kabuli* genome	*Kabuli* gene accession IDs	NCBI-KOG	TFs
Ca1_P1_2937747	Ca1	2937747	AT/ATT	CGGATGGAACAAGTGGAAGT	AACCACTCATCTTTCAAACCAAA	60.0	646	INTERGENIC	--	--	--
Ca1_P1_2938157	Ca1	2938157	AAATAA/AAATAATAA	TTGGTTTGAAAGATGAGTGGTTT	TTCGATTTTGTCTATCCGCC	59.9	575	INTERGENIC	--	--	--
Ca1_P1_29402165	Ca1	29402165	TG/TGTAG	GGCCGTAAACCAGACTCAAA	GGGGTAAGTTTCCTTGACCA	60.1	627	INTERGENIC	--	--	--
Ca1_P1_29412426	Ca1	29412426	T/TATATA	ATCTACCGATTGAATTGCGG	CAGCGTGACAATGAGCAACT	59.9	429	INTERGENIC	--	--	--
Ca1_P1_2946069	Ca1	2946069	TA/TAA	ATTCCATCGCCATAGCATTC	GGGACCAACAAATTCAAACAA	59.9	553	INTERGENIC	--	--	--
Ca1_P1_29472015	Ca1	29472015	TTTATTATTATTATTATTATTATTATTATTATTATTATTATT/TTTATTATTATTATTATTATTATTATTATTATTATTATTATTATTATT	TGGCCTGACCTACTTATGGC	AGCTTGAGTCTGGCCTACGA	60.1	589	INTERGENIC	--	--	--
Ca1_P1_2948444	Ca1	2948444	TTTCCAATTCCAATTCCAA/TTTCCAATTCCAATTCCAATTCCAA	CTGAAGAAGCTGCTTTGGCT	TTTCCCTTTGATTTGTTGCAC	59.9	376	CDS (large-effect mutations)	Ca_00359	--	ERF
Ca1_P1_29524255	Ca1	29524255	ATTTTTTTTTTT/ATTTTTTTTTTTTT	GGCTTCTTTCCAAAATGCAG	CACCATATTCCCCCAAAACA	59.8	669	INTERGENIC	--	--	--
Ca1_P1_2952486	Ca1	2952486	CAATAAATAAATAAATA/CAATAAATAAATAAATAAATA	ACCTCAAATCCGCAAAGATG	GTGCAAGAGTTTGGCTTGGT	60.1	571	DRR	Ca_00360	--	ERF
Ca1_P1_29613286	Ca1	29613286	TCACA/TCA	AGGTGCAAAAAGGAATGTGG	CGAACTCAAGAACCAGCCTT	60.0	147	INTERGENIC	--	--	--
Ca1_P1_29613542	Ca1	29613542	GATTATTATTATTATTATTATTATTATTATTATTATTATTATTATTATTATTATTATTATTATTATTATTAT/GATTATTATTATTATTATTATTATTATTATTATTATTATTATTATTATTATTATTATTATTATTATTATTATTATTATTAT	AGGTGCAAAAAGGAATGTGG	ACACAATTGGTGCAAGGACA	60.0	681	INTERGENIC	--	--	--
Ca1_P1_2961377	Ca1	2961377	TTAATA/TTA	TGACAAACATGTGTCATTTATTTTG	TGGAACACATAATTTTCACCGA	58.9	692	INTERGENIC	--	--	--

**INDEL marker IDs**	**Chromosomes/ unanchored scaffolds**	**Physical positions (bp)**	**InDels (*Kabuli* reference genome-CDC Frontier/PI)**	**Forward primers (5'-3')**	**Reverse primers (5'-3')**	**Anneling temperature (0C)**	**Expected amplified product size (bp)**	**Structural annotation**		**Functional annotation**	
								**Sequence components of *kabuli* genome**	***Kabuli* gene accession IDs**	**NCBI-KOG**	**TFs**
Ca1_P1_29636835	Ca1	29636835	AA/AAGA	TCACAAATCTCAACCC TCCC	ATCAGTTGGAGCCTGG TTGT	59.9	497	INTRON	Ca_24382	AR	--
Ca1_P1_29637072	Ca1	29637072	GAAAAAAAAA/GAAAAAAAAAAA	ACAACCAGGCTCCAAC TGAT	ATGCTGCCAGCCTACA AAAT	59.6	503	INTRON	Ca_24382	AR	--
Ca1_P1_2965539	Ca1	2965539	CAAAA/CAAA	ACATGCTGTGATTTGG GTCA	AAAGCGAATTTACAGA CGTGG	60.0	873	INTERGENIC	--	--	--
Ca1_P1_29667241	Ca1	29667241	AACACACACACACACACA/AACACACA CACACACACACA	CCGAGCCAAAATCCAA AATA	TTGGGTGCACATTTTA CAGC	59.9	436	INTERGENIC	--	--	--
Ca1_P1_29709028	Ca1	29709028	ACC/AC	AAAGATTTGTTGAGGC GTGG	GACGAGAATGGAGAT GGGAA	60.1	580	INTERGENIC	--	--	--
Ca1_P1_2973113	Ca1	2973113	TATA/TATAATA	TTTGATGTGGCGGCAT AGTA	TGTTTGCCAATCACAG CTTC	60.1	281	INTRON	Ca_00361	--	--
Ca1_P1_29738380	Ca1	29738380	GTATATATATATATATATATAT/GTATA TATATATATATATATAT	TATTTTGGATTTTGGCT CGG	AAACCGGTAAAATCTC CGCT	59.9	699	INTERGENIC	--	--	--
Ca1_P1_2975302	Ca1	2975302	ATT/AT	TCCTGCAACCAAATTT CACA	AGCCAAGTGGAATGA GTTGG	60.1	662	INTRON	Ca_00361	--	--
Ca1_P1_29791416	Ca1	29791416	GAAAA/GAAAAA	TCCGATAAAATCTCCG TTCG	AAAAACACGTTCCCCT CCTT	60.0	323	INTERGENIC	--	--	--
Ca1_P1_2979509	Ca1	2979509	A/ATTATTT	GGAATTCCAAAACTGA TCCAA	TGCCAACAAATCCTGA ACAA	58.9	653	INTRON	Ca_00361	--	--
Ca1_P1_2981588	Ca1	2981588	GC/G	TGTTCGGCCATTAGAC ATCA	AATCGCCCATTTCTCC TCTT	60.1	345	DRR	Ca_00361	--	--
Ca1_P1_29817198	Ca1	29817198	GTTTTTT/GTTTTTTTTT	TGTTAGGAGGATCTCA CGGG	TTGTACAGGTGTCTGC CGAG	60.1	522	INTERGENIC	--	--	--

INDEL marker IDs	Chromosomes/ unanchored scaffolds	Physical positions (bp)	InDels (*Kabuli* reference genome-CDC Frontier/PI)	Forward primers (5'-3')	Reverse primers (5'-3')	Anneling temperature (0C)	Expected amplified product size (bp)	Structural annotation		Functional annotation	
								Sequence components of *kabuli* genome	*Kabuli* gene accession IDs	NCBI-KOG	TFs
Ca1_P1_29818737	Ca1	29818737	ACTA/ACTAAATTACCGAGGTGCTA	CTCGGAATTCCAAACCAAAA	ATCGATGGCTCAATTGGTTC	59.9	359	INTERGENIC	--	--	--
Ca1_P1_29825526	Ca1	29825526	ATTTTT/ATTT	CAAGACTTAAGGCTCCCCCT	CCCATTTGCCTAACCAAGAA	59.7	500	INTERGENIC	--	--	--
Ca1_P1_29842423	Ca1	29842423	AC/A	TCATCAAAGGCATGCAAATC	GACCAAAACACAACGCAATG	59.6	359	INTERGENIC	--	--	--
Ca1_P1_29843805	Ca1	29843805	G/GA	ATGGATCAAGAGAAGGGGCT	CCTATGCGAAGTGGTGGTTT	60.0	535	INTERGENIC	--	--	--
Ca1_P1_29844164	Ca1	29844164	TTTTATTT/TTTT	ATGGATCAAGAGAAGGGGCT	CCTATGCGAAGTGGTGGTTT	60.0	535	INTERGENIC	--	--	--
Ca1_P1_29844704	Ca1	29844704	A/ACCTT	AAAAACGCTCCCCTCATTTT	TGAGAGATGGTCGTGGAGTG	59.9	733	INTERGENIC	--	--	--
Ca1_P1_29846530	Ca1	29846530	CTTTT/CTTTTT	TGGATTGGGGATCATTCATT	TCAACATTTGGAAGCACAAGA	60.0	897	DRR	Ca_24388	--	--
Ca1_P1_29846735	Ca1	29846735	A/AATATATATATAT	AGAACGGTTTTCTGTCCGTG	TCAACATTTGGAAGCACAAGA	60.1	689	DRR	Ca_24388	--	--
Ca1_P1_29846941	Ca1	29846941	TAAAAAAAAAA/TAAAAAAAA	TGTTGCCAACTGAAAATCGT	ATACAGACACCGAATGGGGA	59.2	828	DRR	Ca_24388	--	--
Ca1_P1_29848026	Ca1	29848026	ATGT/AT	TCTTTTCATCAGGCCCAATC	GGAAAGTGAGGGAACCAATG	60.0	228	INTRON	Ca_24388	--	--
Ca1_P1_29853119	Ca1	29853119	TAA/TA	TCCACGAGATTTTCCTTTGG	TGTCATCATTCTCTGCTGGC	60.0	357	INTERGENIC	--	--	--
Ca1_P1_29853730	Ca1	29853730	GTGGAAGTCT/GTGGAAGTCTGGAAGTCT	TTGCATTCCTAGGTTCAGCC	TATCGCTCGACCTCTGCTTT	60.2	602	INTERGENIC	--	--	--

INDEL marker IDs	Chromosomes/ unanchored scaffolds	Physical positions (bp)	InDels (*Kabuli* reference genome-CDC Frontier/PI)	Forward primers (5'-3')	Reverse primers (5'-3')	Anneling temperature (0C)	Expected amplified product size (bp)	Structural annotation		Functional annotation	
								Sequence components of *kabuli* genome	*Kabuli* gene accession IDs	NCBI-KOG	TFs
Ca1_P1_29856647	Ca1	29856647	CTTT/CTTTT	GCATTCCCTGCTCAAGACTC	AGACAAGGGAGAGTCCCACA	60.0	812	INTERGENIC	--	--	--
Ca1_P1_2993163	Ca1	2993163	ATACAATACATATTT/AT	AGCTGCTTCTTGCCATGTTT	GGAAGCAACAGAGAACAGCC	60.0	534	INTRON	Ca_00363	PET	NAC
Ca1_P1_2993633	Ca1	2993633	TACACA/TACACACA	GCTGTTCTCTGTTGCTTCCC	TGACCATTCCCTACCCCATA	60.0	278	INTERGENIC	--	--	--
Ca1_P1_2994411	Ca1	2994411	TAAATAAACAAAAATA/TAAATA	CCTCCTCGGATCTATCTTGG	CGCCATTGTTCTCTCCTCTC	58.7	388	INTERGENIC	--	--	--
Ca1_P1_29975624	Ca1	29975624	GTTTTTT/GTTTTTTT	ACCAAACCTGTGCATCCAAT	GGGAGGACGAAAATGACTGA	60.2	468	INTERGENIC	--	--	--
Ca1_P1_30036117	Ca1	30036117	TCC/TC	TGGACCCACATGATTGGTTA	TTTCCGTCCTCCCTAGGTTT	59.6	661	INTERGENIC	--	--	--
Ca1_P1_3003710	Ca1	3003710	AATATATATATATATATA/AATATATATATATATATATATATA	GAAAGGCATGCAATATGGGT	AATCTGCCGACGCAAACTAT	59.8	440	DRR	Ca_00364	A	bHLH
Ca1_P1_30044156	Ca1	30044156	AACACACAC/AACACAC	CCAAGGAAGGACGAAAATGA	TCGCCCTCCTTAGGCTTATT	60.0	815	INTERGENIC	--	--	--
Ca1_P1_30058506	Ca1	30058506	T/TC	GAACGGGAGGCTCATACAAG	TGCTGAGGAGTTCTTGGACA	59.7	475	INTERGENIC	--	--	--
Ca1_P1_30067411	Ca1	30067411	TAAAAAAAAAA/TAAAAAAAAAAAA	GGATCGAAGTTTGGGACAGA	AAGCGTGGTGTGGGAAAATA	60.1	710	INTERGENIC	--	--	--
Ca1_P1_3013948	Ca1	3013948	GTTTTT/GTTTT	TGCGGCTATTACATTGGTGA	AACCGACAAGGCAAGAAAGA	60.1	182	INTERGENIC	--	--	--
Ca1_P1_30167193	Ca1	30167193	ATTT/ATT	ATGTGGGCCAATATCCTTCA	TGCATTTCATCTCAATCCCA	60.2	260	INTERGENIC	--	--	--

INDEL marker IDs	Chromosomes/ unanchored scaffolds	Physical positions (bp)	InDels (*Kabuli* reference genome-CDC Frontier/PI)	Forward primers (5'-3')	Reverse primers (5'-3')	Anneling temperature (0C)	Expected amplified product size (bp)	Structural annotation		Functional annotation	
								Sequence components of *kabuli* genome	*Kabuli* gene accession IDs	NCBI-KOG	TFs
Ca1_P1_3017274	Ca1	3017274	TTATTA/TTATTACTATTA	TGCTAACAAATTTATTCGACCTTTT	GGCCACATTGTGCTTCATATT	59.5	179	INTERGENIC	--	--	--
Ca1_P1_30183116	Ca1	30183116	T/TG	TTCAACATGACCGAACCAAA	CCCCTCCTCAAGTTGGTACA	59.9	203	INTRON	Ca_22469	R	--
Ca1_P1_3020354	Ca1	3020354	AGG/AGGG	CAAGCAACATGCTGAAGGAA	ATGTCCTTAGTGTCACGGGG	60.0	808	INTERGENIC	--	--	--
Ca1_P1_3020722	Ca1	3020722	GGCTT/G	CCCCGTGACACTAAGGACAT	GAAGGTCGCATCTCCATGAT	59.8	513	INTERGENIC	--	--	--
Ca1_P1_30213031	Ca1	30213031	TGAGAGAGAGAGAGAGAGA/TGAGAGAGAGAGAGAGAGAGA	TTGTTAATTGAGTTGCGACGA	GCCCTCAGATGAGAGTCAGG	59.4	636	INTERGENIC	--	--	--
Ca1_P1_3024882	Ca1	3024882	GTTTTTT/GTTTTTTT	ATGGTCCCGTAGCTCAGTTG	TGAAAACCATGACCGACAAA	60.1	761	INTERGENIC	--	--	--
Ca1_P1_30287511	Ca1	30287511	GTTTT/GTTT	CCATCAAACATCGTCATGGA	ACTCAGATTGGTCTGTGGGG	60.3	305	INTERGENIC	--	--	--
Ca1_P1_3033831	Ca1	3033831	ATATCTATCTATC/ATATCTATC	GTGTTTGGCTTTGCAACTGA	AGGAGAAGCTGACGGAACAA	59.9	675	DRR	Ca_00368	L	--
Ca1_P1_30352443	Ca1	30352443	CACA/CACAACA	TCATCTTGGGAGGTCTCGAT	TTGTGGTCTAGGCGTCAAAA	59.6	673	INTERGENIC	--	--	--
Ca1_P1_3037452	Ca1	3037452	AT/ATTTAAAGTATTTGTT	ACACCAGCCGTTGAATAAGG	ATAACGGTGGAGCCTGTTTG	60.0	916	INTRON	Ca_00370	--	ARF
Ca1_P1_3039653	Ca1	3039653	GTTTTT/GTTTT	CGGAATTTAGCTTTGGTTCTTG	GGCGTTTTGTTGATTCTGTG	60.1	902	INTERGENIC	--	--	--
Ca1_P1_30398618	Ca1	30398618	GTTTTTTTTTT/GTTTTTTTTTTTT	TTAGGCCCAGTACAGTTCGG	GGGGTGCTCAACTTGTTTGT	60.1	901	INTERGENIC	--	--	--

INDEL marker IDs	Chromosomes/ unanchored scaffolds	Physical positions (bp)	InDels (*Kabuli* reference genome-CDC Frontier/PI)	Forward primers (5'-3')	Reverse primers (5'-3')	Anneling temperature (0C)	Expected amplified product size (bp)	Structural annotation		Functional annotation	
								Sequence components of *kabuli* genome	*Kabuli* gene accession IDs	NCBI-KOG	TFs
Ca1_P1_3040639	Ca1	3040639	ATTT/ATTTT	TGACAAATCAGATGGGGGAT	CAATTTGCAGCCACTCAATG	60.1	773	INTERGENIC	--	--	--
Ca1_P1_30406481	Ca1	30406481	ATGTGTGTGTGTG/ATGTGTGTGTGTGTG	GTTGCGGGATTGAGAGATGT	TGGTTGGAAGGAAGCAGACT	60.1	459	INTERGENIC	--	--	--
Ca1_P1_3042228	Ca1	3042228	ATTT/ATTTT	GAGCCAAGATCGATGGGAT	TTTGTATGAACACAACCCCAAA	60.0	502	INTERGENIC	--	--	--
Ca1_P1_30432201	Ca1	30432201	CTTTTTTT/CTTTTTT	CCACAATATCAAAGGGCCAC	AGAGAAAACAAAAGGCGCAA	60.2	471	INTERGENIC	--	--	--
Ca1_P1_3043596	Ca1	3043596	GTATATATATATATAT/GTATATATATATATATAT	GGACCGAAATGTTGCCTAAA	GACTTGGTCAATTTGCCGAT	59.9	531	INTERGENIC	--	--	--
Ca1_P1_30440874	Ca1	30440874	AAATAATAATAATAATAATAATAATAATAATAATAATAATAATAATAATAATAATAATAATAAT/AAATAATAATAATAATAATAATAATAATAATAATAATAATAATAATAATAATAATAATAATAATAAT	GAAATGCGGATGTGAGGTTT	ATGCTTTTGCCAATGAGGTC	59.9	612	INTERGENIC	--	--	--
Ca1_P1_30452929	Ca1	30452929	TT/TTAT	GAAGAGTTACGCGGACAAGC	AGGAAGAGTGGAGTTGCGAA	60.0	768	INTERGENIC	--	--	--
Ca1_P1_3045718	Ca1	3045718	TCAC/TCACTACAC	CGTAATCACATACATAGGGCTCA	CATTGCACGGTCAATTCTTG	59.0	479	DRR	Ca_00371	I	--
Ca1_P1_3045928	Ca1	3045928	TGC/TGCGGC	ACCGTGCAATGCAGATGTAA	TGATGCAGCCTAAGTGGATG	60.1	398	DRR	Ca_00371	I	--
Ca1_P1_30475519	Ca1	30475519	ATTT/ATTTT	AGGATGCATGGGATGAAAAG	AAACTTCTACTCGGGCCCAT	59.9	766	INTERGENIC	--	--	--
Ca1_P1_30483540	Ca1	30483540	ATTTAGAAGTCT/A	TGACCCACTAAGGCACATGA	CGGTATGGAATTTTTGGTGG	60.1	430	INTRON	Ca_22457	--	--
Ca1_P1_3049357	Ca1	3049357	ATT/AT	ATCCTTAAAGAGAGGGGCCA	AAACCCAGGAAAGGGTCACT	60.0	888	INTRON	Ca_00371	I	--

INDEL marker IDs	Chromosomes/ unanchored scaffolds	Physical positions (bp)	InDels (*Kabuli* reference genome-CDC Frontier/PI)	Forward primers (5'-3')	Reverse primers (5'-3')	Anneling temperature (0C)	Expected amplified product size (bp)	Structural annotation		Functional annotation	
								Sequence components of *kabuli* genome	*Kabuli* gene accession IDs	NCBI-KOG	TFs
Ca1_P1_30508503	Ca1	30508503	ATTTTTTTT/ATTTTTTTTTT	TGAGAAAAAGAAAGCTGTCAGATG	AAGATTCAGTCAAGGGGGCT	60.1	329	INTERGENIC	--	--	--
Ca1_P1_30530345	Ca1	30530345	ATTTT/ATTT	GCAACTACGGTGAAGGCAAT	CCCTTGTGAAACTCGGCATA	60.1	830	INTERGENIC	--	--	--
Ca1_P1_30603090	Ca1	30603090	TTATATATATATATATATATATATATAT/TTATATATATATATATATATAT	AAACCAACAAAGGTTGCGTC	TTGGAAGGAGCCCTTACCTT	60.0	813	INTERGENIC	--	--	--
Ca1_P1_30603494	Ca1	30603494	TAA/TAAA	GGTAAGGGCTCCTTCCAAAC	GCAGCTTGAGTGCAACATTT	59.9	323	INTERGENIC	--	--	--
Ca1_P1_30603511	Ca1	30603511	GTTTTT/GTTTT	GGTAAGGGCTCCTTCCAAAC	GCAGCTTGAGTGCAACATTT	59.9	323	INTERGENIC	--	--	--
Ca1_P1_30627831	Ca1	30627831	AATATATATATATATATAT/AATATATATATATATATATATATATAT	CTTGGGAACTGGAGAGCAAG	CCAACGTCACTTCCAAGGTT	60.0	797	INTERGENIC	--	--	--
Ca1_P1_30667611	Ca1	30667611	GAACGAAAAAATAAACGAAAAAA/GAACGAAAAAA	CATCGGGATCAAACAACACA	GGCGCCCCTAAAGTCTAAGT	60.4	410	INTERGENIC	--	--	--
Ca1_P1_30682856	Ca1	30682856	CATATATATATATATATATAT/CATATATATATATATATAT	GTTGGTTCCCTAGCACCGTA	TCCGCCTAGGAGATAGCAGA	60.0	672	INTERGENIC	--	--	--
Ca1_P1_3068309	Ca1	3068309	TAAAAAAAAA/TAAAAAAAA	TCATTGGGTTGGGTTTTCAT	TTATCAGTTGCAGCCTGTGC	60.0	626	INTRON	Ca_00374	EH	bHLH
Ca1_P1_30684955	Ca1	30684955	GTATATATATAT/GTATATATAT	GGCCATAAATCGAAGGCATA	ATTCGGTTTGGGTTCACGTA	59.9	184	INTERGENIC	--	--	--
Ca1_P1_3072719	Ca1	3072719	A/ATAAG	GCGAATGGTTGAAACTGAAA	CGTTGGAACTCCCAATGACT	58.7	563	INTERGENIC	--	--	--
Ca1_P1_3073093	Ca1	3073093	CTTTTT/CTTTTTT	CCTCCTTACGGCGAAACATA	CGTTGGAACTCCCAATGACT	60.1	234	INTRON	Ca_00375	--	bHLH

INDEL marker IDs	Chromosomes/ unanchored scaffolds	Physical positions (bp)	InDels (*Kabuli* reference genome-CDC Frontier/PI)	Forward primers (5'-3')	Reverse primers (5'-3')	Anneling temperature (0C)	Expected amplified product size (bp)	Structural annotation		Functional annotation	
								Sequence components of *kabuli* genome	*Kabuli* gene accession IDs	NCBI-KOG	TFs
Ca1_P1_30740126	Ca1	30740126	CTGT/CT	TCGGAGCACAACCTACACAA	ACATTTGCCACCGTTAGAGG	60.3	901	DRR	Ca_22454	OE	WRKY
Ca1_P1_30745547	Ca1	30745547	C/CT	TTTTTGGGTTTCCTTTTCCC	TGCAATATGTATCGTGGATGG	60.1	817	INTERGENIC	--	--	--
Ca1_P1_3075271	Ca1	3075271	TAA/TAAA	GAGGTGTATGAATGTTCACACGTA	TCATGGCAGTCTGCAAAAAG	59.0	542	INTRON	Ca_00375	--	bHLH
Ca1_P1_30755262	Ca1	30755262	CAAAAAAAAAAA/CAAAAAAAAAA	AAGAGAGGTCACTTTGGCGA	TGACTGAGACAGCTCGATGG	60.0	489	INTERGENIC	--	--	--
Ca1_P1_30766528	Ca1	30766528	GA/GAA	CTGCATTTCGTCCCAAGAAT	TAATTCCAGCTCCCGATTTG	60.1	318	DRR	Ca_22451	R	ZF-HD
Ca1_P1_30766663	Ca1	30766663	CA/CATGGA	CTGCATTTCGTCCCAAGAAT	GTTCACATTAATCGGTGCCC	60.1	702	DRR	Ca_22451	R	ZF-HD
Ca1_P1_30767234	Ca1	30767234	ATTTTTTTTT/ATTTTTTTTTT	GGGCACCGATTAATGTGAAC	GAAGGTGATGTTTGGCGATT	60.2	549	DRR	Ca_22451	R	ZF-HD
Ca1_P1_30770184	Ca1	30770184	TAAA/TAAAA	AGTGGCTTGGAGGAGTTGGT	CGTTCGGCTTTCTTCTTCTG	61.1	535	INTERGENIC	--	--	--
Ca1_P1_30790839	Ca1	30790839	TGTAAC/T	TTTTTGATGGTTCAACGAGAA	GGTTGAATAATTGAAAAGAGGTGG	58.3	250	INTERGENIC	--	--	--
Ca1_P1_30828576	Ca1	30828576	C/CTATATATATA	CCTCACTGAGCCAAAAGCTC	CACACAAACACACACACACACA	60.1	543	INTERGENIC	--	--	--
Ca1_P1_3083516	Ca1	3083516	AT/ATT	TCATTTGACTGAGGCTGCAT	GGACGAGGAGTTGGATTTGA	59.4	884	INTRON	Ca_00376	--	bHLH
Ca1_P1_30844081	Ca1	30844081	AATATATATATATATATA/AATATATATATATATATA	TGAAAAGGGATGGAATGGTT	AAGCATTATGAAGATGACGCC	59.2	387	INTERGENIC	--	--	--

INDEL marker IDs	Chromosomes/ unanchored scaffolds	Physical positions (bp)	InDels (*Kabuli* reference genome-CDC Frontier/PI)	Forward primers (5'-3')	Reverse primers (5'-3')	Anneling temperature (0C)	Expected amplified product size (bp)	Structural annotation		Functional annotation	
								Sequence components of *kabuli* genome	*Kabuli* gene accession IDs	NCBI-KOG	TFs
Ca1_P1_30846710	Ca1	30846710	TAAAAAAA/TAAAAAAAA	AAAATCCGATTATGAG GCCC	TAGGATGGGTCGTAG GGTTG	60.1	604	INTERGENIC	--	--	--
Ca1_P1_3086710	Ca1	3086710	GGAGAG/GGAG	AAATCCTCCTCTCAAC CCGT	TTTGAGCGATAGAATT GCCC	59.9	710	DRR	Ca_00377	J	--
Ca1_P1_30874605	Ca1	30874605	A/AT	TGTGATGCAGTTGCTC CATT	CAAGCGTCATGCCAAA CTTA	60.3	624	INTERGENIC	--	--	--
Ca1_P1_3088406	Ca1	3088406	AAACAACA/AAACA	GCCAATGGCTCCTTCA AAT	TTCCCGATAAGACGTT TTGC	60.0	439	DRR	Ca_00376	--	bHLH
Ca1_P1_3089369	Ca1	3089369	AT/ATT	AAAATCGGTCTTGGGC TCTT	CCGTGTTAGTCGTCTA TTTTTACCA	60.1	713	DRR	Ca_00378	I	--
Ca1_P1_30918162	Ca1	30918162	TTATATA/TTATA	GTCGACTCTCAATCCA TCCC	AAATCATCGGCTTCCA TGTC	59.5	613	INTERGENIC	--	--	--
Ca1_P1_30918807	Ca1	30918807	AGGGG/AGGG	GACATGGAAGCCGATG ATTT	CATTGGGATGGAAACC AAGT	59.9	744	INTERGENIC	--	--	--
Ca1_P1_30938421	Ca1	30938421	TCCC/TCC	CCCTCTGCAATGTCGG TAAT	GGTGAAAACTGGGTG GAAGA	60.0	526	INTERGENIC	--	--	--
Ca1_P1_30945218	Ca1	30945218	TCC/TCCC	ATATTGGCTCGGAGGC TTTT	CCGCGAGTAAATCCGA TAAA	60.1	661	INTERGENIC	--	--	--
Ca1_P1_3094552	Ca1	3094552	TA/T	GGTGCTCTTAGAGGCA AAGG	TTGGAACAATGACGGT GTTG	59.1	633	INTERGENIC	--	--	--
Ca1_P1_30968989	Ca1	30968989	A/ATACAT	ATGCATGACCATTGAA CCAA	GCCAATTAACCCGGAT AACA	59.8	733	INTERGENIC	--	--	--
Ca1_P1_30969047	Ca1	30969047	ATTTTTTGGT/AT	ATGCATGACCATTGAA CCAA	GCCAATTAACCCGGAT AACA	59.8	733	INTERGENIC	--	--	--

INDEL marker IDs	Chromosomes/ unanchored scaffolds	Physical positions (bp)	InDels (*Kabuli* reference genome-CDC Frontier/PI)	Forward primers (5'-3')	Reverse primers (5'-3')	Anneling temperature (0C)	Expected amplified product size (bp)	Structural annotation		Functional annotation	
								Sequence components of *kabuli* genome	*Kabuli* gene accession IDs	NCBI-KOG	TFs
Ca1_P1_3096944	Ca1	3096944	ATTTTT/ATTT	ATTTGGGCTACCATGCAAAA	AACCGAGTTGGCATTACTGG	60.3	463	INTERGENIC	--	--	--
Ca1_P1_30969550	Ca1	30969550	ATA/ATACAATTTGTTA	GTTATCCGGGTTAATTGGCA	AAAACGCTCGCTTTGTTAGC	59.7	631	INTERGENIC	--	--	--
Ca1_P1_30983343	Ca1	30983343	GTTTTTTTT/GTTTTTTT	GGGAGACAAAACTTGCAATGA	AACCACGCCTCTTTGTGTTC	60.1	857	INTERGENIC	--	--	--
Ca1_P1_3099270	Ca1	3099270	CATA/CATATATA	ACCGAATTTCCTTTCGCTTT	TGTGATTGTGGATGTTCGGT	60.1	624	INTERGENIC	--	--	--
Ca1_P1_30993040	Ca1	30993040	AAAAATTAAAATTAAAATTAA/AAAAATTAAAATTAAAATTAAAATTAA	TCCTTCCGATCGAGTAGTGG	AAACATGAAATGGACAATACAAAAA	60.2	398	INTERGENIC	--	--	--
Ca1_P1_30997388	Ca1	30997388	T/TGG	CCACGAAGTCCCCAAGATAA	CTTGGTGTCCGGTCAAAAGT	59.9	381	INTERGENIC	--	--	--
Ca1_P1_31014916	Ca1	31014916	ATTT/ATTTTTATTTCATTTTTTTTT	GAGCATGAAGCAACCTCTCC	TGATGAATCTGTGTGTCCATGA	60.0	198	INTERGENIC	--	--	--
Ca1_P1_31022339	Ca1	31022339	GTTTT/GTTT	GTTTCAACCGCATCCAATTT	TCCCAATCGAATCTACGAGG	59.8	442	INTERGENIC	--	--	--
Ca1_P1_3103121	Ca1	3103121	AT/ATT	GGGTGCAATGCTGGATTATT	GTTAAGTGAGGTCCGTCCCA	59.8	462	DRR	Ca_00380	--	--
Ca1_P1_3107570	Ca1	3107570	ATGTGTGTG/ATGTGTGTGTG	TCCTTGCACGCTTTTAATCA	AAACGTGATCGAACGAATCC	59.4	440	INTERGENIC	--	--	--
Ca1_P1_3107729	Ca1	3107729	ATT/ATTT	GTGCGTGCGTGTGAACTATT	TGGCAGTGAAAATAAAATCGC	59.8	616	INTERGENIC	--	--	--
Ca1_P1_31090559	Ca1	31090559	CAA/CA	CGCTCGCTCATTGAATAACA	CTCCACGACCATCCTGAAAT	60.0	417	INTRON	Ca_25826	S	--

INDEL marker IDs	Chromosomes/ unanchored scaffolds	Physical positions (bp)	InDels (*Kabuli* reference genome-CDC Frontier/PI)	Forward primers (5'-3')	Reverse primers (5'-3')	Anneling temperature (0C)	Expected amplified product size (bp)	Structural annotation		Functional annotation	
								Sequence components of *kabuli* genome	*Kabuli* gene accession IDs	NCBI-KOG	TFs
Ca1_P1_31093582	Ca1	31093582	GTCAATTCTTCAATTCT/GTCAATTCT	TGCGTGAAAAACTCAATCCT	ATTAAGTTCTACCGCGCCAC	58.4	897	INTERGENIC	--	--	--
Ca1_P1_3109985	Ca1	3109985	ATTTTTTTTTT/ATTTTTTTT	TGTCTACAATGGTGTTTGGCA	AAGAAAAAGTGCTAGTCCATGGTT	60.0	696	INTERGENIC	--	--	--
Ca1_P1_31102577	Ca1	31102577	ATTTTTTTT/ATTTTTTTTT	ACTGGGAGGTGTTTGAGTGG	CATGCACAACCTCATGGTTC	60.0	782	DRR	Ca_25825	--	--
Ca1_P1_3111391	Ca1	3111391	AGG/AGGG	GTTGGCTTTGCCGAATGTAT	CGTTGCATTTTCAAGTCTCG	60.0	254	INTERGENIC	--	--	--
Ca1_P1_31119469	Ca1	31119469	CAAGT/CAAGTAAGT	AATCCGTTGACTATTGCGAA	CACTACAACCGCCACTTCAA	58.2	108	INTERGENIC	--	--	--
Ca1_P1_3112331	Ca1	3112331	AC/A	AATCTTCCACGGAACAATCAA	GGGAGATGGAAATTGGGAAT	59.4	877	INTERGENIC	--	--	--
Ca1_P1_3112449	Ca1	3112449	AT/ATGT	CCAAGATCATAGGTGCAAAACA	GGGAGATGGAAATTGGGAAT	60.0	469	INTERGENIC	--	--	--
Ca1_P1_3113743	Ca1	3113743	GATATATATATAT/GATATATATAT	TGATTACAATCCTTTAAGCACG	GGAATCGAACTCGAGACTGC	57.1	564	DRR	Ca_00381	--	--
Ca1_P1_3113861	Ca1	3113861	TA/TAA	TGATTACAATCCTTTAAGCACG	GGAATCGAACTCGAGACTGC	57.1	564	DRR	Ca_00381	--	--
Ca1_P1_31141128	Ca1	31141128	C/CA	ATTATTGAGGTTGGCCCCTC	TCCACTTTTTGTGCTTTAGGC	60.2	376	INTERGENIC	--	--	--
Ca1_P1_31148066	Ca1	31148066	A/AC	CCAACCCCCTTCCTTGTAAT	TTGGGTACATCATGGGGAGT	60.0	468	INTERGENIC	--	--	--
Ca1_P1_31162887	Ca1	31162887	T/TG	CCGGGGATTGCCATATAACT	CTAATAGGCTGCATCGGAGG	60.9	342	INTERGENIC	--	--	--

INDEL marker IDs	Chromosomes/ unanchored scaffolds	Physical positions (bp)	InDels (*Kabuli* reference genome-CDC Frontier/PI)	Forward primers (5'-3')	Reverse primers (5'-3')	Anneling temperature (0C)	Expected amplified product size (bp)	Structural annotation		Functional annotation	
								Sequence components of *kabuli* genome	*Kabuli* gene accession IDs	NCBI-KOG	TFs
Ca1_P1_31163466	Ca1	31163466	TGG/TAGAGG	ACTTTTGCGGAGAAGCACAT	CGTTCGTCTTTTCCTCTTGC	59.9	360	INTERGENIC	--	--	--
Ca1_P1_3119826	Ca1	3119826	CAA/CAAA	TGTCTACAATGGTGTTTGGCA	GCGGACCATTAAATTGTTCAAG	60.0	451	DRR	Ca_00382	--	--
Ca1_P1_31205485	Ca1	31205485	AGTGTGTGTGTGTGTGTGTGTGTGTG/AGTGTGTGTGTGTGTGTGTGTGTGTGTGTG	AGACTCAAACTGAAGGCCCA	GACTCATCAGAGGCATGCAA	59.8	504	INTERGENIC	--	--	--
Ca1_P1_31216866	Ca1	31216866	AG/A	GCCCAAAGAGCAAACACATT	GCGATTTTTCTTAGGGACCA	60.1	785	INTERGENIC	--	--	--
Ca1_P1_31235357	Ca1	31235357	TAATGGAATGGAATG/TAATGGAATGGAATGGAATG	GACAACAGGTGGACACATCG	AACACAAAGGGTTGAGTCGG	60.0	562	INTERGENIC	--	--	--
Ca1_P1_31238518	Ca1	31238518	ATT/AT	CATCGTAAGCATGTGGTTGG	TAATGGCCCCATAGCAGAAG	60.0	861	DRR	Ca_21848	R	FAR1
Ca1_P1_3125340	Ca1	3125340	GATAAATAAATAAATAAATAAATAAA/GATAAATAAATAAATAAATAAATAAATAAATAAA	ACCACTGAATCATGCACCAA	CAGTCTGCAGTGACCTTCCA	60.0	103	INTRON	Ca_00382	--	--
Ca1_P1_31259507	Ca1	31259507	ATT/AT	ATCTTAAAACCCTGCGAGGC	TCCGACTTGGTCCTTTATTCC	60.6	501	INTERGENIC	--	--	--
Ca1_P1_31306839	Ca1	31306839	AAATAATAATAATAATAATAATAATAATAATAATAATAATAATAATAATAATAATA/AAATAATAATAATAATAATAATAATAATAATAATAATAATAATAATAATAATAATAATAATA	CTAACATGCGTGTTGCGTG	CGGCGAGTAATTCTAAACGG	59.9	541	INTERGENIC	--	--	--
Ca1_P1_31310549	Ca1	31310549	GCC/GCCC	GTTTGGTTTTCTTTGGGGGT	CTCCAGTCAAGCATTCCCAT	60.1	187	INTERGENIC	--	--	--
Ca1_P1_3131301	Ca1	3131301	TATA/TATACTCATA	GGCCAGACTCAGACCTTTCA	ACCCATTTTTCTGACAACCG	60.4	506	INTERGENIC	--	--	--
Ca1_P1_31324079	Ca1	31324079	AAA/AAATAA	TATTCCGTTGTCGAGTTCCC	AGGGATAACCTGGTGCTGTG	59.9	604	INTERGENIC	--	--	--

INDEL marker IDs	Chromosomes/ unanchored scaffolds	Physical positions (bp)	InDels (*Kabuli* reference genome-CDC Frontier/PI)	Forward primers (5'-3')	Reverse primers (5'-3')	Anneling temperature (0C)	Expected amplified product size (bp)	Structural annotation		Functional annotation	
								Sequence components of *kabuli* genome	*Kabuli* gene accession IDs	NCBI-KOG	TFs
Ca1_P1_31341315	Ca1	31341315	CTT/CTTT	TGTCCATTTGAAACTTCGGTT	AGAGCAAAATCGCTCCAAAA	59.5	772	INTERGENIC	--	--	--
Ca1_P1_3134264	Ca1	3134264	CGGGAAGTGCTG/CG	CCTTCGCTTCCTCTCCTTTT	TTCCATTTGCAACATCAACC	60.0	268	INTRON	Ca_00383	--	--
Ca1_P1_31362029	Ca1	31362029	TTATAAAGTGAGTAAAATATAAAGTGAG/TTATAAAGTGAG	TTCCAATGACTCTCACCTTCC	CGCACGACATTGCTAGAGAA	59.1	375	INTERGENIC	--	--	--
Ca1_P1_31386654	Ca1	31386654	TG/TGAAG	TTTCAACCGCTCAACATCAA	TGTCGCGCTACCTTTCTTCT	60.2	445	INTERGENIC	--	--	--
Ca1_P1_31396918	Ca1	31396918	GAAAATTAATTAAAAATTAAT/GAAAATTAAT	GGGACAATTATGGCAATGCT	ACAATTATTTTGTGGGGGCA	59.8	569	INTERGENIC	--	--	--
Ca1_P1_31401462	Ca1	31401462	CTATATTATATA/CTATA	ATTCGTAGTAGGCCATGCCA	TTTGCGGCCTTCAAAATATC	60.5	501	INTERGENIC	--	--	--
Ca1_P1_31428294	Ca1	31428294	ATATTTTTATTTTTATTTTTATTTTTAT/ATATTTTTATTTTTATTTTTAT	TCAACAGGTTGATGGGTGAA	TGAGATGGAGGTGATGGTGA	59.9	370	INTERGENIC	--	--	--
Ca1_P1_31442895	Ca1	31442895	T/TA	AATTTCCGAGCACGCATAAC	TCCACGAGTATGATTTGTGGTT	60.1	299	INTERGENIC	--	--	--
Ca1_P1_31451888	Ca1	31451888	AATATATATATATATATATATATATATATATATATATATAT/AATATATATATATATATATATATATATATATATATATATATATATAT	GTGGACCACCGTCAAAAATC	CTTGTTGAGGACCCTTTTCG	60.2	799	INTERGENIC	--	--	--
Ca1_P1_3147144	Ca1	3147144	AATATATATATATATATATATATA/AATATATATATATATA	TCCCAATGCTGCATGTTTTA	TGGGTTGAGTTGTGAGTTCG	60.1	650	INTRON	Ca_00385	--	--
Ca1_P1_31477691	Ca1	31477691	CAAAA/CAAAAA	AAGGGAAACGGGTACCAAAC	ACCCGTAAACCCCAAATCTC	60.1	629	INTERGENIC	--	--	--
Ca1_P1_31477842	Ca1	31477842	CTTTTTTTTTTTT/CTTTTTTTTTT	AAGGGAAACGGGTACCAAAC	ACCCGTAAACCCCAAATCTC	60.1	629	INTERGENIC	--	--	--

INDEL marker IDs	Chromosomes/ unanchored scaffolds	Physical positions (bp)	InDels (*Kabuli* reference genome-CDC Frontier/PI)	Forward primers (5'-3')	Reverse primers (5'-3')	Anneling temperature (0C)	Expected amplified product size (bp)	Structural annotation		Functional annotation	
								Sequence components of *kabuli* genome	*Kabuli* gene accession IDs	NCBI-KOG	TFs
Ca1_P1_31498666	Ca1	31498666	TAAAAAA/TAAAAAAA	TTGGAGCTTCAGCATTTGTG	CCTCTTTTCTTCGCATTTCG	60.0	747	DRR	Ca_21863	--	M-type
Ca1_P1_31505143	Ca1	31505143	GTATATATATATATATATATA/GTATATATATATATATATA	TCCCATTCATATTTATTGCGG	CATTTGACTATGCCACTAAATGC	59.6	831	INTERGENIC	--	--	--
Ca1_P1_31506311	Ca1	31506311	A/AC	CACAACTTGGTGTAGCCGAA	GATCAATGGCCATCATTCCT	59.8	249	INTERGENIC	--	--	--
Ca1_P1_3150964	Ca1	3150964	GCT/GCTCCT	AAGGTGAATGCAATTTCCTCAT	TTTGTCGGGAGAATTTGGAC	59.8	420	DRR	Ca_00386	S	--
Ca1_P1_3156764	Ca1	3156764	TAA/TAAA	TGGCTGTGATATTGTCGCAT	CCTTCATGTCCACATACAATTTC	60.1	696	INTERGENIC	--	--	--
Ca1_P1_3157078	Ca1	3157078	T/TG	TTGTTTGAAGTTGTGTGGTTTTT	CAGAGTCGATCCGTAGTTCG	58.6	701	INTERGENIC	--	--	--
Ca1_P1_31581161	Ca1	31581161	AAATAATAATAATAATAAT/AAATAATAATAATAATAATAATAATAATAAT	CAACCCTTTTCGATGCATTT	GGTTTTGGCATTGTTTAGGC	59.9	467	INTERGENIC	--	--	--
Ca1_P1_31587638	Ca1	31587638	TTATATATATATATATATATAT/TTATATATATATATATATATAT	CGTCGCTACGGTTTAGCATT	GGCAAACATTTTTATGCATGG	60.3	520	INTERGENIC	--	--	--
Ca1_P1_31592114	Ca1	31592114	CT/CTTT	TCAGCTGCAGTTCCAACATC	GCACAAGTCCTTGAGGAAGC	60.0	375	INTRON	Ca_21868	F	--
Ca1_P1_31592347	Ca1	31592347	GAA/GAAAA	CCTGTGTAGCCAGCTTCCTC	TAGCCTCATTCACTCTCGCA	60.0	618	INTRON	Ca_21868	F	--
Ca1_P1_31600651	Ca1	31600651	AC/A	GGATGGAAGAGAAATTGGGA	AGGATCATGTTTGTGGTTGC	58.9	899	INTERGENIC	--	--	--
Ca1_P1_31651609	Ca1	31651609	CTTTTTTTT/CTTTTTTTTT	ATGTGAAGTTGTCCATGCCA	GCACACGTGGTTCCTTAACTT	60.0	894	INTERGENIC	--	--	--

INDEL marker IDs	Chromosomes/ unanchored scaffolds	Physical positions (bp)	InDels (*Kabuli* reference genome-CDC Frontier/PI)	Forward primers (5'-3')	Reverse primers (5'-3')	Anneling temperature (0C)	Expected amplified product size (bp)	Structural annotation		Functional annotation	
								**Sequence components of *kabuli* genome**	***Kabuli* gene accession IDs**	**NCBI-KOG**	**TFs**
Ca1_P1_31716302	Ca1	31716302	A/AC	TCGGTATTTGCCATGAGACA	AAACAACCTTCATGCCTTGG	60.1	482	INTERGENIC	--	--	--
Ca1_P1_31741031	Ca1	31741031	GTATATATATATATATATATATA/GTATATATATATATATATATATATATA	CTTGGGCTTTACCCATGAAA	CGATCGGCCATCATATCTCT	59.9	781	INTERGENIC	--	--	--
Ca1_P1_31768486	Ca1	31768486	TTATA/TTTTTTATATA	ACACATTATATTAAATGCGCGA	TGCCAAACGATAGGTTGTTG	57.4	646	INTERGENIC	--	--	--
Ca1_P1_31783488	Ca1	31783488	AATATATATATATATATATATAT/AATATATATATATATATATAT	TGGATTGGGCTGGAATTAAA	TATAACCGACGAGGGACGAG	60.3	466	INTERGENIC	--	--	--
Ca1_P1_31803627	Ca1	31803627	TAAA/TAAAA	CTTACGGTGAAGGAGAACGG	ATCATTCTTTTATGCGTTTGACT	59.7	793	INTERGENIC	--	--	--
Ca1_P1_3194081	Ca1	3194081	ACGAATGGTCAG/A	GCAAGGCTTGATTCCAAAAA	GGTTGCACCTTCCATGAACT	60.2	799	DRR	Ca_00390	O	E2F/DP
Ca1_P1_31995451	Ca1	31995451	T/TCAC	TGCTTTGAAGACCATGTGGA	AAAAGGATTCATGCGTGTCC	60.2	354	INTERGENIC	--	--	--
Ca1_P1_31995500	Ca1	31995500	CTTT/CTTTT	TGCTTTGAAGACCATGTGGA	AAAAGGATTCATGCGTGTCC	60.2	354	INTERGENIC	--	--	--
Ca1_P1_32019371	Ca1	32019371	AATATATATATATATATATATATATATATATATATA/AATATATATATATATATATATATATATATATATATATATATATATATATATA	GGTGCACGTGTAGCACAAAC	TTGCCACAGTTAACCACCAA	60.2	422	INTERGENIC	--	--	--
Ca1_P1_32030892	Ca1	32030892	AATATATATATATATATATATATATATATATAT/AATATATATATATATATATATATATATATATATATATAT	CATGGATGGGTTTTGTACCC	TTGGAAGGAGCCCTTACCTT	59.9	771	INTERGENIC	--	--	--
Ca1_P1_32037255	Ca1	32037255	CA/CAA	GGGAAAACTTTTGTGTGGGA	TTGGCAAATCGATTAGACTGG	59.8	802	INTERGENIC	--	--	--
Ca1_P1_32037334	Ca1	32037334	CTTTTT/CTTTT	TCCAGTCATGTGGCTTCTCA	CTGAGCTTGTGACCAAATCG	60.4	559	INTERGENIC	--	--	--

INDEL marker IDs	Chromosomes/ unanchored scaffolds	Physical positions (bp)	InDels (*Kabuli* reference genome-CDC Frontier/PI)	Forward primers (5'-3')	Reverse primers (5'-3')	Anneling temperature (0C)	Expected amplified product size (bp)	Structural annotation		Functional annotation	
								Sequence components of *kabuli* genome	*Kabuli* gene accession IDs	NCBI-KOG	TFs
Ca1_P1_32037356	Ca1	32037356	CTTTTTT/CTTTTT	TCCAGTCATGTGGCTTCTCA	CTGAGCTTGTGACCAAATCG	60.4	559	INTERGENIC	--	--	--
Ca1_P1_32068343	Ca1	32068343	TAA/TA	AAAAAGTCGATCATGGCACC	CCACACAGAAGCGTCTTGAA	59.9	291	INTERGENIC	--	--	--
Ca1_P1_32080106	Ca1	32080106	T/TC	TTCCCCATGTCGATCTAAGC	AACCCTGAACAGATTGTCGG	60.0	612	INTRON	Ca_26405	--	--
Ca1_P1_32080183	Ca1	32080183	C/CGGCGATTACT	TTCCCCATGTCGATCTAAGC	AACCCTGAACAGATTGTCGG	60.0	612	INTRON	Ca_26405	--	--
Ca1_P1_32080315	Ca1	32080315	G/GA	TTCATGTAGGCGAGTTGCAG	ACCCCAGTAGTCCTAGCCGT	60.0	541	CDS (FRAME SHIFT)	Ca_26405	--	--
Ca1_P1_32140157	Ca1	32140157	AT/ATTATT	GGCCAAAACCCTAATTGACA	TGGCAAAGGGGAAGTCAGTA	59.8	505	INTERGENIC	--	--	--
Ca1_P1_32147050	Ca1	32147050	T/TA	CGGCCTCCAAATCTTTAACA	TTGTCATGAAAGTCTCGCCT	60.1	670	INTERGENIC	--	--	--
Ca1_P1_32151710	Ca1	32151710	TAAAAAAAAAAA/TAAAAAAAAAAAAAAAA	TGAAGGTGTGAGCAGATTCG	TCGGGTGGAGAATTCCTATG	60.0	593	INTERGENIC	--	--	--
Ca1_P1_32153054	Ca1	32153054	ACTCT/ACTCTCT	TATTTCGACGCCTTCACACA	AGTGAAACCAACGCCAAAAC	60.3	241	INTERGENIC	--	--	--
Ca1_P1_32153452	Ca1	32153452	TTTATTTTAT/TTTATTTTATATTATTTTAT	GTTTTGGCGTTGGTTTCACT	TGGGTTTGACCCTATGTGTG	60.0	353	INTERGENIC	--	--	--
Ca1_P1_32153737	Ca1	32153737	TTATATATATATAT/TTATATATATATATAT	CACACATAGGGTCAAACCCA	ATACATGGATTGGTTGGGGA	59.3	585	INTERGENIC	--	--	--
Ca1_P1_32155666	Ca1	32155666	AATATATATATATATATATATATATATATATATATATAT/AATATATATATATATATATATATATATATATATAT	TTTTAGTTTAACGGGAGAATCCA	TGTCAAAGAAGGTGGCCTTT	59.1	471	INTERGENIC	--	--	--

INDEL marker IDs	Chromosomes/ unanchored scaffolds	Physical positions (bp)	InDels (*Kabuli* reference genome-CDC Frontier/PI)	Forward primers (5'-3')	Reverse primers (5'-3')	Anneling temperature (0C)	Expected amplified product size (bp)	Structural annotation		Functional annotation	
								Sequence components of *kabuli* genome	*Kabuli* gene accession IDs	NCBI-KOG	TFs
Ca1_P1_32155872	Ca1	32155872	TTATATATATATATATATATATATA/TTATATATATATATATATATATATATATATATATA	TTACCCAAGATGGAGGAAAAA	TGTTGTCCCTTGTGCTGGTA	58.5	554	INTERGENIC	--	--	--
Ca1_P1_32163457	Ca1	32163457	CAAA/CAAAA	TTTCCCCCAAATCAACACTC	AGGTAAGGGCTCCTTCCAAA	59.8	100	INTERGENIC	--	--	--
Ca1_P1_32174815	Ca1	32174815	TA/T	GGACTTTCAACCTTGTGGGA	ACCCAACACATCAACCCATT	59.9	580	INTERGENIC	--	--	--
Ca1_P1_32187346	Ca1	32187346	TTAAAAATAAAAAT/TTAAAAATAAAAATAAAAAT	GGCAACCAAGAGGTATGGAA	GCCCAAGTAGTTGCAAGAGC	59.9	641	INTERGENIC	--	--	--
Ca1_P1_32189060	Ca1	32189060	ATTTT/ATTTTT	ATTAACGCCATTGCCTTGTC	GCTTTTTCCTCGCTTCCTTT	60.0	632	DRR	Ca_26126	QI	bHLH
Ca1_P1_32193944	Ca1	32193944	ACAC/ACACACACCCACGCAC	TGGGCATAATCCTTCACACA	AAGGATGACAGGGTTGTTGC	59.9	376	INTERGENIC	--	--	--
Ca1_P1_32194223	Ca1	32194223	G/GT	TGGGCATAATCCTTCACACA	AAGGATGACAGGGTTGTTGC	59.9	376	INTERGENIC	--	--	--
Ca1_P1_32222272	Ca1	32222272	ATTTT/ATTTTT	ACAACTTCTCATGCTCGCAA	GGTGGCATACCTCAACGAGT	59.6	620	INTRON	Ca_26127	--	Trihelix
Ca1_P1_32239314	Ca1	32239314	GTTT/GTT	GCGTTCATGGAACACACAAC	TCACCTGAGGCACATTTCAA	60.0	654	DRR	Ca_26127	--	Trihelix
Ca1_P1_32243358	Ca1	32243358	ATT/ATTTTTT	TCATGGCCATTAGAGTTTTTCA	ACATTTGCAAAGTCTTGGGG	59.6	895	INTERGENIC	--	--	--
Ca1_P1_32244195	Ca1	32244195	A/AG	TGAATAGAGGGAAACGGGTG	CAGTGACCTTGTGGAGAGCA	59.9	640	INTERGENIC	--	--	--
Ca1_P1_32245598	Ca1	32245598	GTTTTTTT/GTTTTTTTT	ATTTTGATGGTCAAGCCTGG	TCCCTGCTTCAGTTCGACTT	59.9	581	INTERGENIC	--	--	--

INDEL marker IDs	Chromosomes/ unanchored scaffolds	Physical positions (bp)	InDels (*Kabuli* reference genome-CDC Frontier/PI)	Forward primers (5'-3')	Reverse primers (5'-3')	Anneling temperature (0C)	Expected amplified product size (bp)	Structural annotation		Functional annotation	
								Sequence components of *kabuli* genome	*Kabuli* gene accession IDs	NCBI-KOG	TFs
Ca1_P1_32245801	Ca1	32245801	ACCCC/ACCCCC	ATTTTGATGGTCAAGCCTGG	TCCCTGCTTCAGTTCGACTT	59.9	581	INTERGENIC	--	--	--
Ca1_P1_32257387	Ca1	32257387	TAAATCTTGATAAAATCTT/TAAATCTT	CATGCCTAGCTTGGTCAACA	TTTGATCAAGATTTATCGCAAGA	59.9	554	INTERGENIC	--	--	--
Ca1_P1_32257635	Ca1	32257635	AGATAAATCTTGATAAATCTT/AGATAAATCTT	GCTTTGATATGGGCAGGAAC	CAACAAGACGAGCGATGTGT	59.5	863	INTERGENIC	--	--	--
Ca1_P1_32261065	Ca1	32261065	CTTTTTTTTT/CTTTTTTTTTT	CCAAATGTTTGACAAATTGCAT	GTTAGAGTTTTGGCCCGACA	59.7	243	INTERGENIC	--	--	--
Ca1_P1_32265621	Ca1	32265621	AT/A	GGTGTGAATGGACCAAAACC	TTTGCCCCTCCTCATCATTA	60.1	731	INTERGENIC	--	--	--
Ca1_P1_32267410	Ca1	32267410	T/TTTAA	TTCGTGTTAAAATAGTGTTAGCCA	AAAAATCACATTCCTTGACATAGC	58.0	635	INTERGENIC	--	--	--
Ca1_P1_32267954	Ca1	32267954	T/TG	AAGCTATGTCAAGGAATGTGATTT	CCGTGTTTTTGAAAATTGACAC	58.3	378	INTERGENIC	--	--	--
Ca1_P1_32268687	Ca1	32268687	TAA/TA	AAATGGGCGGGATAACAAAT	TTTTTAAAAGGAAGGGCCAC	60.4	505	INTERGENIC	--	--	--
Ca1_P1_32269031	Ca1	32269031	TTGATGA/TTGA	GCATATAATGGTGCCCCTTG	CGCATTGAACACTATGATCG	60.2	978	INTERGENIC	--	--	--
Ca1_P1_32281261	Ca1	32281261	CAAA/CAAAA	CACCATGTTATGCAGGGTTG	ACCAAAATTAGGGAGTAAAATTCA	59.8	505	INTRON	Ca_26129	--	--
Ca1_P1_32430396	Ca1	32430396	TG/T	TCGTTTGAGCCTTTCGAACT	GCAATCACAATGCAGCAAAC	60.0	473	INTERGENIC	--	--	--
Ca1_P1_32454365	Ca1	32454365	A/AG	AGGAAACACATGAAGGCACC	TCTTCGTTGTCACTCCATGC	60.0	572	INTERGENIC	--	--	--

INDEL marker IDs	Chromosomes/ unanchored scaffolds	Physical positions (bp)	InDels (*Kabuli* reference genome-CDC Frontier/PI)	Forward primers (5'-3')	Reverse primers (5'-3')	Anneling temperature (0C)	Expected amplified product size (bp)	Structural annotation		Functional annotation	
								Sequence components of *kabuli* genome	*Kabuli* gene accession IDs	NCBI-KOG	TFs
Ca1_P1_32485193	Ca1	32485193	CAAAAAAAAAA/CAAAAAAAAAAA	ACAATGTAGCAAAGACGGGG	CTTGTGCGATGCACAGATAA	60.0	692	DRR	Ca_26796	Z	--
Ca1_P1_32494116	Ca1	32494116	TTATATATATATAT/TTATATATATAT	TGCAAAGGAAAAGACCACAA	GGATAAAAACCCCAAAAGCA	59.3	477	INTERGENIC	--	--	--
Ca1_P1_32536205	Ca1	32536205	TAATATATAAATATATAAATATATAAATATAT/TAATATATAAATATATAAATATAT	GATTAGATCGAACGGCTGGA	ACCCAGGCTACAAAACGATG	60.2	551	INTERGENIC	--	--	--
Ca1_P1_32541628	Ca1	32541628	GATTTTATATTTATTTTATATTTATTTTA/GATTTTATATTTATTTTA	TGCGACTTGGAGTTAGCACTT	AGGTGTTGTTGGTTTCCTCG	60.1	897	INTERGENIC	--	--	--
Ca1_P1_32566008	Ca1	32566008	TTATATATATATATATATATATATATA/TTATATATATATATATATATATATA	TGAGCGGTTACCAAATGTGA	CCCACAAGGCTAACACCAAT	60.1	606	INTERGENIC	--	--	--
Ca1_P1_32743551	Ca1	32743551	AA/AATAATAATAATAAATA	GTCTGGCTTGACCTGAAAGC	TTCATGAAGACCCCAAAACC	60.0	604	INTERGENIC	--	--	--
Ca1_P1_32749816	Ca1	32749816	CA/C	CTGAATTTCAAAATCCCCGA	GGTGCATCTCACCAAGGTTT	59.9	440	INTERGENIC	--	--	--
Ca1_P1_32847990	Ca1	32847990	CTTTTT/CTTTT	GCAATCACAATGCAGCAAAC	TTGGGGTGATGATTCTCAAA	60.3	299	INTERGENIC	--	--	--
Ca1_P1_3285133	Ca1	3285133	TTATATATATATATATATATATATA/TTATATATATATATATATATA	AAATGGGACTTGCATAGCCA	CTGTTTTCCTCGTTGCACCT	60.5	379	INTRON	Ca_00398	--	LBD
Ca1_P1_32875368	Ca1	32875368	ATTTTTTTTTT/ATTTTTTTTT	TACGGGTCTTACACAACGCA	TAGCGGTGGGAAGAACTCTG	60.2	343	INTERGENIC	--	--	--
Ca1_P1_32911601	Ca1	32911601	AAATAATAATAATAA/AAATAATAATAATAATAA	CAAAATGGAGCAAGAAACAGG	CAAGTTCAACGAGCGCCTAT	59.7	892	INTERGENIC	--	--	--
Ca1_P1_32911818	Ca1	32911818	GATAATAATAATAATAATAATAATAATAATAATAATAA/GATAATAATAATAATAATAATAATAATAATAA	TGTGGTTGACTAAATGTGCAA	ATGAGTCGGCTTACGCTGTT	57.7	452	INTERGENIC	--	--	--

INDEL marker IDs	Chromosomes/ unanchored scaffolds	Physical positions (bp)	InDels (*Kabuli* reference genome-CDC Frontier/PI)	Forward primers (5'-3')	Reverse primers (5'-3')	Anneling temperature (0C)	Expected amplified product size (bp)	Structural annotation		Functional annotation	
								Sequence components of *kabuli* genome	*Kabuli* gene accession IDs	NCBI-KOG	TFs
Ca1_P1_32917662	Ca1	32917662	ATTTTTTTT/ATTTTTTTTT	TCTCCAAAATAGGAGGGACA	TTTGTTGTTGCAGGAATACGA	57.2	566	INTERGENIC	--	--	--
Ca1_P1_32922285	Ca1	32922285	CTT/CTTTT	CCCTTTGCTTTTTGGAGTGA	ATGCTCCCATGGAAAGATTG	60.2	386	INTERGENIC	--	--	--
Ca1_P1_32960646	Ca1	32960646	CA/CACATAAAAATGTA	CCGAGCCAAAATCCAAAATA	ACTTCATTGTCCTCCCATGC	59.9	215	INTERGENIC	--	--	--
Ca1_P1_33034774	Ca1	33034774	AATATATATATATATA/AATATATATATA	ACCCCAACTTGTAAAGGCAA	TCACGTTGATTGAGGGATGA	59.5	618	INTERGENIC	--	--	--
Ca1_P1_33055263	Ca1	33055263	CTTTTTTTTT/CTTTTTTTTTT	CAACAATGTTCAAGTTCAATGC	TTTTCATGCACGGATTATGC	58.2	342	INTERGENIC	--	--	--
Ca1_P1_33102523	Ca1	33102523	TTT/TTTATT	AAGGCTAAATCACATTCGTGG	TCACTTCCGTCCATGTCAAA	59.1	835	INTERGENIC	--	--	--
Ca1_P1_33104165	Ca1	33104165	AACCGC/A	TGCACATTACTCCCAAACCA	AGAGACGGAAGAGTGGCAAA	60.0	199	DRR	Ca_19439	--	--
Ca1_P1_3310529	Ca1	3310529	AATATATATATATATA/AATATATATATATATATA	TCCACATTAGTGGCCAAACA	GTAATCCACCCACCGTCAAG	60.0	690	DRR	Ca_00401	T	YABBY
Ca1_P1_33105635	Ca1	33105635	TC/T	TGCCAAGAACGATAAAAGCA	TTCTGCATTTTCCTTGAGCA	59.4	101	DRR	Ca_19438	--	--
Ca1_P1_33117706	Ca1	33117706	TAAAAAAAA/TAAAAAAAAA	TATAAAGGACCCTGCCCAAA	TGCCCTTTTCACCGATCTAC	59.4	706	INTRON	Ca_19439	--	--
Ca1_P1_33122230	Ca1	33122230	TGATGTGTACATGAAGATGTG/TGATGTG	CAATTTGAGTTGCAATTGACG	TTTTCAGAAATTGTTAAGGGAAAAA	59.2	141	INTRON	Ca_19439	--	--
Ca1_P1_33134129	Ca1	33134129	CTATA/CTATATATA	CGTTGACTATTTCAATCGTTGG	CACCTGTAGTCCAACACTCGTT	59.5	265	INTERGENIC	--	--	--

INDEL marker IDs	Chromosomes/ unanchored scaffolds	Physical positions (bp)	InDels (*Kabuli* reference genome-CDC Frontier/PI)	Forward primers (5'-3')	Reverse primers (5'-3')	Anneling temperature (0C)	Expected amplified product size (bp)	Structural annotation		Functional annotation	
								Sequence components of *kabuli* genome	*Kabuli* gene accession IDs	NCBI-KOG	TFs
Ca1_P1_33167519	Ca1	33167519	CTTTTTT/CTTTTTTT	GCGGCATTATTTGAACGATT	TGGCACCATTTTCTTTCACA	59.9	654	INTERGENIC	--	--	--
Ca1_P1_33187249	Ca1	33187249	GTAT/GTATAT	TAAAGGTTCCCTCGCATCTG	CACCACAGAAGAACTTGGCA	60.2	530	INTRON	Ca_19440	A	--
Ca1_P1_33228816	Ca1	33228816	AATATATATATATATATATATATATATATAT/AATATATATATATATATATATATATATATATATATATATAT	TACCTTTGTGCTTCCCCATT	ACCAAACACCCCCAATATGA	59.4	842	INTERGENIC	--	--	--
Ca1_P1_33237244	Ca1	33237244	GATGTATGTATGTATGTATGTATGT/GATGTATGTATGTATGTATGT	TGCTGCGAGAAATAACATCG	ACGTTGCTTTCAGAGGGAAA	60.0	550	INTERGENIC	--	--	--
Ca1_P1_33244510	Ca1	33244510	ATTTTTT/ATTTTTTTT	AATTCAGACGCGAAAGAGGA	CAAGTCTCCCAACTTCCCAA	60.0	221	INTERGENIC	--	--	--
Ca1_P1_33247147	Ca1	33247147	TA/T	GTGTTGGACCGGTTCTTGAT	GTCTGATGACAAAAGGGGGA	59.8	643	INTERGENIC	--	--	--
Ca1_P1_33252560	Ca1	33252560	TAAAAAGAA/TAAAAAGAACCCAAAAAAGAA	TCTCCTTGTTGGTGCACTTG	ACATGCTGTTTTTGCTGCTG	59.9	603	INTERGENIC	--	--	--
Ca1_P1_33269540	Ca1	33269540	AGGTTTTTGTGGGTTTTTG/AGGTTTTTGTGGGTTTTTGTGGGTTTTTG	CTCCACGTCAAAACCACCTT	CTGTCTGCACTCCACTCCAA	60.0	629	INTERGENIC	--	--	--
Ca1_P1_33289762	Ca1	33289762	TAAAAAA/TAAAAAAA	GCGAACTCGAGGATGGTTAT	GAGGTTGATCCTTCTGCTCG	59.2	585	INTERGENIC	--	--	--
Ca1_P1_33300693	Ca1	33300693	TCAACAACAACAACAACAA/TCAACAACAACAA	CTCAACCATCAGAGCACCAA	TTATACGGCGGAGGAATGAG	59.8	382	INTERGENIC	--	--	--
Ca1_P1_33301285	Ca1	33301285	CATATATATATATATATAT/CATATATATATATATATATATATATATATATAT	ATGGGAGGAAGAAACCGAAG	TTGGAATTTCAGTGCCATCA	60.4	838	INTERGENIC	--	--	--
Ca1_P1_33328448	Ca1	33328448	GTTTTTTTTT/GTTTTTTTTTTT	CGTTTCATTGTCTATCCATCTCA	TGACTTTGATGAAAGCCTTGT	59.1	130	INTRON	Ca_19441	--	MIKC

INDEL marker IDs	Chromosomes/ unanchored scaffolds	Physical positions (bp)	InDels (*Kabuli* reference genome-CDC Frontier/PI)	Forward primers (5'-3')	Reverse primers (5'-3')	Anneling temperature (0C)	Expected amplified product size (bp)	Structural annotation		Functional annotation	
								Sequence components of *kabuli* genome	*Kabuli* gene accession IDs	NCBI-KOG	TFs
Ca1_P1_33335208	Ca1	33335208	AAGAGAGAGA/AAGAGAGAGAGA	TCCAAATCGACTTTCCAACC	CAACCATTGTTGCTCCCTTT	59.9	777	INTRON	Ca_19441	--	MIKC
Ca1_P1_33336930	Ca1	33336930	GAAAAA/GAAAA	CAATGGCAACAAGGTCAATG	TCACATCTGTTGGTAACGGG	60.0	856	INTRON	Ca_19441	--	MIKC
Ca1_P1_33353185	Ca1	33353185	ATTT/ATT	GGGAGATTGTTGGAACCAAGT	CCGGGCTTCAATTTAGTCCT	60.2	601	INTRON	Ca_19441	--	MIKC
Ca1_P1_33354017	Ca1	33354017	ATTCA/ATTCAACCCCCTTTTCA	GGAGGGTCAGGTGCTTTGTA	CAGCCTCCATCCTTCTTCTG	60.1	516	INTRON	Ca_19441	--	MIKC
Ca1_P1_33356994	Ca1	33356994	TCC/TC	CGAGTGGTTTTGTGTGTCGT	ATCATTATCCTCGTGTGGGC	59.6	345	INTRON	Ca_19441	--	MIKC
Ca1_P1_33379812	Ca1	33379812	GTTTTTTTT/GTTTTTTT	AGAATTGGTGGTTGAGGACG	TTGGGAAAGAAATCGAAAACA	60.0	588	INTERGENIC	--	--	--
Ca1_P1_33401507	Ca1	33401507	AAGAGAGAG/AAGAGAGAGAG	AGCTCTTTGATGACCGTTGG	GGAGGATGGCAGACCTATGA	60.3	303	INTERGENIC	--	--	--
Ca1_P1_33452743	Ca1	33452743	CCTTCTTCTTCTTCTTCTTCTTCTTCTTCTTCTTCTTCTT/CCTTCTTCTTCTTCTTCTTCTTCTTCTTCTTCTT	TTGGATCCAGAGGCAGAGTT	TGAAGAAGAGGAGCCCAAGA	59.8	477	INTERGENIC	--	--	--
Ca1_P1_33527629	Ca1	33527629	AGG/AG	TCCTTCCAATTGCTTGCTTT	GAGGATGTTGGAACCAAGTAGG	59.8	521	INTERGENIC	--	--	--
Ca1_P1_33546385	Ca1	33546385	TAAAAAAA/TAAAAAAAA	TGTGTCTGAAGCGCAAATTC	CTCATCGCAAGCATTCGTTA	60.0	445	INTERGENIC	--	--	--
Ca1_P1_33560144	Ca1	33560144	TAAAAAA/TAAAAA	CTCAAATTCTGTTTGGGGGA	GAAGTCGGGGTGTTACTCCA	59.9	515	INTERGENIC	--	--	--
Ca1_P1_33567505	Ca1	33567505	CATATATATATATATATATAT/CATATATATATATAT	GCATTCCCTCATTGTAACTCG	GGGTTTGAGTTTGATGACAGG	59.6	711	INTERGENIC	--	--	--

INDEL marker IDs	Chromosomes/ unanchored scaffolds	Physical positions (bp)	InDels (*Kabuli* reference genome-CDC Frontier/PI)	Forward primers (5'-3')	Reverse primers (5'-3')	Anneling temperature (0C)	Expected amplified product size (bp)	Structural annotation		Functional annotation	
								Sequence components of *kabuli* genome	*Kabuli* gene accession IDs	NCBI-KOG	TFs
Ca1_P1_33568041	Ca1	33568041	ATT/ATTT	CCTGTCATCAAACTCAAACCC	TCGCAATGTGGTGTTAAGGA	59.4	367	INTERGENIC	--	--	--
Ca1_P1_33579938	Ca1	33579938	GTT/GT	TTCATTATTTACCCGCCCAA	TTCCATAGAGTTGGAACCCG	60.1	530	INTERGENIC	--	--	--
Ca1_P1_33597493	Ca1	33597493	ATT/ATTT	CGGCTTTGGTCCCTTCTTA	CTGGATCATGAAAGCCCTGT	60.2	498	INTERGENIC	--	--	--
Ca1_P1_33603009	Ca1	33603009	C/CCCTACAAGAAG	CAAATCTTCTTCAAATCTTGACCAT	AGGCACATGTTGGCTTCTTC	59.9	285	INTERGENIC	--	--	--
Ca1_P1_33630707	Ca1	33630707	ATCGTCTTC/ATCGTCTTCAACATAGTCGTCTTC	CGAGATACCGGGACTACCTC	AGTGTGAGACATGGGGGAAG	58.6	475	INTERGENIC	--	--	--
Ca1_P1_33648367	Ca1	33648367	AATTTTTTATTTTTTATTTTTT/AATTTTTTATTTTTTATTTTTTATTTTTT	GCCTAAAGGGGCTTTTGTTC	GCTGAGGAAAAACCTGTTGC	60.1	617	DRR	Ca_19452	--	--
Ca1_P1_33658726	Ca1	33658726	ATTT/ATT	ATGTGAATCCCACCAGGAAA	TTGCACAGCCAAGTTCGTAG	60.2	778	INTERGENIC	--	--	--
Ca1_P1_33665321	Ca1	33665321	ATTTTTTT/ATTTTTT	AGTGCAAACTGCAAATCACG	CTGTACGGGTTGGATCGAGT	59.9	420	INTERGENIC	--	--	--
Ca1_P1_33728733	Ca1	33728733	TA/TAA	ATTGGGAGAAATTGGTGACG	TGTTCATCCAACCGAGTTCA	59.8	815	INTERGENIC	--	--	--
Ca1_P1_33736245	Ca1	33736245	AATATATATATA/AATATATATATATA	TTGAATTTCGGTTATGAAACCA	TTTTTGTTGTCTCACTCGATCA	59.3	587	INTERGENIC	--	--	--
Ca1_P1_33736521	Ca1	33736521	TC/T	TTGAATTTCGGTTATGAAACCA	TGGACGTAGGTTGTTGTGGA	59.3	908	INTERGENIC	--	--	--
Ca1_P1_33746473	Ca1	33746473	GTATATATATATATATAT/GTATATATATAT	CCAATCGATTTCCTCCAAAA	TTTCATCAAAAACCCCCTTG	59.9	779	INTERGENIC	--	--	--

INDEL marker IDs	Chromosomes/ unanchored scaffolds	Physical positions (bp)	InDels (*Kabuli* reference genome-CDC Frontier/PI)	Forward primers (5'-3')	Reverse primers (5'-3')	Anneling temperature (0C)	Expected amplified product size (bp)	Structural annotation		Functional annotation	
								Sequence components of *kabuli* genome	*Kabuli* gene accession IDs	NCBI-KOG	TFs
Ca1_P1_33765021	Ca1	33765021	AAATAAAATTAATAAATAATAAAAT/AAATAAAAT	GCGACCGAATATGGAACAGT	TAGGCTAGGTCGTCCGTTTG	60.0	301	INTERGENIC	--	--	--
Ca1_P1_33777775	Ca1	33777775	ATTT/ATT	CATGCTCAATACACGGATGG	AACGTTCTTGAGTGGCTTGG	59.9	870	INTERGENIC	--	--	--
Ca1_P1_33795058	Ca1	33795058	ATTTTTTTTTT/ATTTTTTTT	TTGGGGTTTGGCAGTTTTAG	CTTGTTCGGGTACCTCGTGT	60.0	502	INTERGENIC	--	--	--
Ca1_P1_33863283	Ca1	33863283	GAAAAAAAAAAAA/GAAAAAAAAAAAAA	GCATGATGAGGTCAAACGAA	GCTATTTTTCCCAACACCCA	59.7	426	INTERGENIC	--	--	--
Ca1_P1_33923831	Ca1	33923831	GTTTT/GTTT	AAGGATGGATTGAATGCTCG	GATCTTCATGCATCGCTCAA	60.0	546	INTERGENIC	--	--	--
Ca1_P1_33926178	Ca1	33926178	AATATATATATATATATATATATATATATAT/AATATATATATATATATATATATATATATATAT	AGGTGGAAGAGAGCTCGACA	TGGACGAGAATGTGATCCAA	60.1	322	INTERGENIC	--	--	--
Ca1_P1_3396002	Ca1	3396002	AATATATATATATATATATATATATATATATATATATATATAT/AATATATATATATATATATATATATATATATATATATATATATATATAT	TTTCCGCGTTGTTGTAGTTG	TTTTGGGAGTGTATACGGTTGA	59.8	876	INTERGENIC	--	--	--
Ca1_P1_34006382	Ca1	34006382	AGGG/AGG	CAATTAACGATCAAGCGTCG	TTGACCCTTTCTCGCTCTGT	59.3	497	DRR	Ca_27634	--	--
Ca1_P1_34057382	Ca1	34057382	C/CA	GGGGATTAAGTGCTTGGACA	AAAGAAAGGCACTAAGGCCC	59.9	156	INTERGENIC	--	--	--
Ca1_P1_34128656	Ca1	34128656	GT/GTT	TTTTCGGTTGTGTAATGTTTGA	AAACCCTTGAGGATCGGAAT	58.1	586	INTERGENIC	--	--	--
Ca1_P1_34130270	Ca1	34130270	ATT/ATTT	TGATTCAAATCCTCAAGCTTTTT	GACACGGTCTCCAATGGTTT	59.3	667	INTERGENIC	--	--	--
Ca1_P1_34140644	Ca1	34140644	ACT/ACTCT	TGGAAAAGACGGTTTTGGTC	TGGTTGTTGTTTGATTACCCA	59.9	381	INTERGENIC	--	--	--

INDEL marker IDs	Chromosomes/ unanchored scaffolds	Physical positions (bp)	InDels (*Kabuli* reference genome-CDC Frontier/PI)	Forward primers (5'-3')	Reverse primers (5'-3')	Anneling temperature (0C)	Expected amplified product size (bp)	Structural annotation		Functional annotation	
								Sequence components of *kabuli* genome	*Kabuli* gene accession IDs	NCBI-KOG	TFs
Ca1_P1_3414496	Ca1	3414496	GATATATATA/GATATATATATA	AGCTAGCATGCAGTCGACAC	TATGTTTTTGGAAGAGGCGG	59.2	654	INTERGENIC	--	--	--
Ca1_P1_34145329	Ca1	34145329	TTATATATATATATA/TTATATATATATATATA	TTTTTCAACCTCGAATTTTACCA	TTTGATGCTAAGGCTTAATGTG	59.9	169	INTERGENIC	--	--	--
Ca1_P1_34147315	Ca1	34147315	C/CTG	TCAAATCGATTGGGACATCA	TACTCGCCAAACTCTCACGA	59.9	900	INTERGENIC	--	--	--
Ca1_P1_34159249	Ca1	34159249	CATGAATATCCTCTGA/CA	CCATGGAAATTCCGTAGCAT	TCGCTTCAAACTCGTATCCC	59.8	451	INTERGENIC	--	--	--
Ca1_P1_34160007	Ca1	34160007	C/CAT	TGGAGACACTTGCCCTCAAT	TTTTATGTCAGGGCGAAACC	60.7	501	INTERGENIC	--	--	--
Ca1_P1_34160353	Ca1	34160353	ATT/AT	GGTTTCGCCCTGACATAAAA	ACTCTGGACACTTGGGTTGC	59.9	773	DRR	Ca_25473	O	--
Ca1_P1_34167121	Ca1	34167121	GT/GTT	CCCCCTTGTTTTATCCCAAT	CTGTTCGTTAGTGACTGGCCT	59.9	481	INTERGENIC	--	--	--
Ca1_P1_34177476	Ca1	34177476	TAAAAAAAAA/TAAAAAAAA	AAGAACATGGATGGGGATGA	TTCATAAACGCAACGTGCTC	60.1	412	INTERGENIC	--	--	--
Ca1_P1_34178856	Ca1	34178856	AC/A	AGAACGTTTTGCCTTGCACT	ATGTCACAAGTTGGGAAGGG	59.9	719	INTERGENIC	--	--	--
Ca1_P1_34194633	Ca1	34194633	TAAA/TAAAA	CCGCGGTATGCTTAGTTTGT	TCAAACCCCCATTTTTGTGT	60.2	578	INTERGENIC	--	--	--
Ca1_P1_34194737	Ca1	34194737	GTATAT/GTAT	CCGCGGTATGCTTAGTTTGT	TCAAACCCCCATTTTTGTGT	60.2	578	INTERGENIC	--	--	--
Ca1_P1_34225442	Ca1	34225442	ATTATTTATTTATTTATTTATTT/ATTATTTATTTATTT	ATCGGGTTCCTTTGCTTTTT	CGCAACTTATAACTAAAAATTGGTCA	59.9	475	INTERGENIC	--	--	--

INDEL marker IDs	Chromosomes/ unanchored scaffolds	Physical positions (bp)	InDels (*Kabuli* reference genome-CDC Frontier/PI)	Forward primers (5'-3')	Reverse primers (5'-3')	Anneling temperature (0C)	Expected amplified product size (bp)	Structural annotation		Functional annotation	
								Sequence components of *kabuli* genome	*Kabuli* gene accession IDs	NCBI-KOG	TFs
Ca1_P1_34227071	Ca1	34227071	CAAAAAAAAAAAA/CAAAAAAAAAA	TTTTTCGATTTGGTCCTTTATTTT	TCCGTCTCAGTGGTGCTCTA	59.3	759	INTERGENIC	--	--	--
Ca1_P1_34279803	Ca1	34279803	TAATAAGCA/T	AGGTTGGTCTGGCTCAAAAA	AAGTTGGGTCGTCGTCAGAG	59.7	418	INTERGENIC	--	--	--
Ca1_P1_34281146	Ca1	34281146	AAATAA/AAATAAAAATAATAA	TTCGTACCCTCATCCCTCAC	AGAGTAGCAGCCCTATGCCA	59.9	566	INTERGENIC	--	--	--
Ca1_P1_34286273	Ca1	34286273	ATATTTTTTATTTTTTATTTTTT/ATATTTTTTATTTTTT	TATGCATGCGGACAAGAGTC	TTGGAAAAGCCCACCTACAC	59.8	448	INTERGENIC	--	--	--
Ca1_P1_34301461	Ca1	34301461	GTATATATATATAT/GTATATATATATATATAT	TCGGTTGGAGAAGATGAAAGA	CAATATTGTGTTTGGGCCGT	59.8	368	INTERGENIC	--	--	--
Ca1_P1_34302342	Ca1	34302342	GACACTCACACTCACAC/GACACTCACACTCACACTCACACTCACAC	CTTTTCCATTTTGTCCCCAA	ATGTGGGAGTTTGTGCTTCA	59.8	354	INTERGENIC	--	--	--
Ca1_P1_34310442	Ca1	34310442	TCACAC/TCAC	TTTTGCTTCCCACACCTCTC	ACACTTTTGCTTCCCACACC	60.2	985	INTERGENIC	--	--	--
Ca1_P1_34331823	Ca1	34331823	CTCA/CTCAAGTCA	TGATGAATTTGATGTTGTTGGA	AAAACCCCTGCTTCCAATTT	58.9	407	INTERGENIC	--	--	--
Ca1_P1_34332252	Ca1	34332252	GA/G	AATTGGAAGCAGGGGTTTTT	GGATTGTACCCTTGGGGTTT	59.8	849	INTERGENIC	--	--	--
Ca1_P1_34345300	Ca1	34345300	CGGG/CGGGG	TGATCAAACCTCCTATATTTATGCC	TGAAATAATTGGTCGGAGCC	59.7	566	INTERGENIC	--	--	--
Ca1_P1_34345340	Ca1	34345340	ATTT/ATTTT	CTACACGAATTCCCGTTCACT	TGAAATAATTGGTCGGAGCC	59.1	456	INTERGENIC	--	--	--
Ca1_P1_34359052	Ca1	34359052	CAAAAAAACTAAAAAAACTA/CAAAAAAACTA	CGCCATTCCAAATGAAAACT	TTGTGGCGCTTTGATAGTTG	59.9	701	INTERGENIC	--	--	--

INDEL marker IDs	Chromosomes/ unanchored scaffolds	Physical positions (bp)	InDels (*Kabuli* reference genome-CDC Frontier/PI)	Forward primers (5'-3')	Reverse primers (5'-3')	Anneling temperature (0C)	Expected amplified product size (bp)	Structural annotation		Functional annotation	
								Sequence components of *kabuli* genome	*Kabuli* gene accession IDs	NCBI-KOG	TFs
Ca1_P1_34363515	Ca1	34363515	GCC/GCCC	GCGTTGTAAAAGACGCACTG	TGGAAATGATGAGCTTTTGG	59.5	463	INTERGENIC	--	--	--
Ca1_P1_34371294	Ca1	34371294	AAAGAAG/AAAGAAGAAG	AAACGGGTGCAAGAAGATTG	TCTTTACGACCTTTGTGGGC	60.1	233	INTERGENIC	--	--	--
Ca1_P1_34373289	Ca1	34373289	CTTTTTTTTTTTTTTT/CTTTTTTTTTTTTT	TAGGCGGGAAGATAATGTCG	TCTGATGACAAAAGGGGGAG	60.1	780	INTERGENIC	--	--	--
Ca1_P1_34383914	Ca1	34383914	CTTTTTTTTTTTT/CTTTTTTTTT	GGGGCACATGATAAAATGTCT	GATTTGCGTCACGAATTTGA	58.8	645	INTERGENIC	--	--	--
Ca1_P1_34384331	Ca1	34384331	T/TTA	TTTCATCAAATTCGTGACGC	AACCACCAAAATACCTTTCAAGA	59.7	460	INTERGENIC	--	--	--
Ca1_P1_34386272	Ca1	34386272	GTTT/GTTTT	AATCTGGAGGGCGGTAAGTT	ATATCGGTCAAACGCACACA	60.0	413	INTERGENIC	--	--	--
Ca1_P1_34390427	Ca1	34390427	TTATATATATATATATATATATATATATATAT/TTATATATATATATATATATATATATATATATATATATAT	GCGACTATCCAGGAAATCCA	AAAGAGACATGACTTTGTCCATCA	60.0	564	INTERGENIC	--	--	--
Ca1_P1_34390964	Ca1	34390964	TAA/T	TGATCGAGTGAGAGAACAAAAA	TGATCTTTGTGGTAACTGCGA	57.6	382	INTERGENIC	--	--	--
Ca1_P1_34398918	Ca1	34398918	AAGCAG/AAGCAGCAG	GATATCGCACGTGAGTTGGA	TGTTTGGTCCACACGCTTTA	59.7	487	INTERGENIC	--	--	--
Ca1_P1_34434177	Ca1	34434177	AC/A	TGGCCAATGCTAATGTGGTA	TGAAGCGCTGTAAAATGTGC	60.0	386	INTERGENIC	--	--	--
Ca1_P1_34505064	Ca1	34505064	TTCTCTCTCTCTCTCTCTCTCTCTCTCTCTCTCTCTCTC/TTCTCTCTCTCTCTCTCTCTCTCTCTCTCTCTCTCTCTCTC	ACAAGTCCATGTGGCAATGA	CGGGTGGTGAAATAAAATGG	60.0	124	INTERGENIC	--	--	--
Ca1_P1_34647055	Ca1	34647055	CAAAAAA/CAAAAAAA	TGGATGAATGCTTGGATGAA	TCAACCAACTTCTTGCTCCC	60.0	866	INTERGENIC	--	--	--

INDEL marker IDs	Chromosomes/ unanchored scaffolds	Physical positions (bp)	InDels (*Kabuli* reference genome-CDC Frontier/PI)	Forward primers (5'-3')	Reverse primers (5'-3')	Anneling temperature (0C)	Expected amplified product size (bp)	Structural annotation		Functional annotation	
								Sequence components of *kabuli* genome	*Kabuli* gene accession IDs	NCBI-KOG	TFs
Ca1_P1_34647251	Ca1	34647251	CAT/CATACTGTTAAT	TTGCCACAATTATTCCAAGG	TAGTCCAACAATGGCCAACA	58.5	527	INTERGENIC	--	--	--
Ca1_P1_34647577	Ca1	34647577	CCA/CCATTAACA	GGGAGCAAGAAGTTGGTTGA	TAGTCCAACAATGGCCAACA	60.2	132	INTERGENIC	--	--	--
Ca1_P1_34666828	Ca1	34666828	TAAAAAAAAA/TAAAAAAAA	TGTTTCAGGATGTCGACCAA	GTTACCGCAGAGGGACGTAA	60.1	640	INTERGENIC	--	--	--
Ca1_P1_34667053	Ca1	34667053	ATTTTTTTTTT/ATTTTTTTTT	AGACTGCTCGGTCCAAAGAA	GTTACCGCAGAGGGACGTAA	60.0	264	INTERGENIC	--	--	--
Ca1_P1_34682417	Ca1	34682417	AATATATATATATATATATATATATATATATAT/AATATATATATATATATATATATATATATATATATATATATATATAT	TATTTTGGATTTTGGCTCGG	CCAACACCGTTTTTCTTCGT	59.9	500	INTERGENIC	--	--	--
Ca1_P1_34694319	Ca1	34694319	AGCAAGTG/AGCAAGTGTATTGATCGTTGCAAGTG	GTCGGCATTTTCGCAATTAT	CGCAATCTTTGAGTTCGACA	59.9	507	INTERGENIC	--	--	--
Ca1_P1_34694405	Ca1	34694405	TAAAAAAAAAAA/TAAAAAAAAAA	TGACAATTTCTGGCAACAGC	CCAAGGTTGGATTCGAAGTG	59.8	247	INTERGENIC	--	--	--
Ca1_P1_34754150	Ca1	34754150	CATAT/CAT	TATCCAGCCAAACATGTCCA	TCAAATGCATGTCCACAGGT	59.9	699	DRR	Ca_21418	--	C3H
Ca1_P1_34808789	Ca1	34808789	TAAAAAAA/TAAAAAA	CCTTCTTGGCCGTATCACAT	TGCCTTTGAGCACATAGTGG	60.0	698	INTERGENIC	--	--	--
Ca1_P1_34812970	Ca1	34812970	ATTTTTTTTTTT/ATTTTTTTTTT	CACAACAATGTGGGTTGAGG	ACCAAGAACAAAATGGTGCC	59.8	428	INTERGENIC	--	--	--
Ca1_P1_34845186	Ca1	34845186	CTGTATATTGCTTAGAAGTGTA/CTGTA	TTGGGTGGAAGGTTAGTCCA	TTGTTCAAACGGTTATGCCA	60.3	391	INTERGENIC	--	--	--
Ca1_P1_34854240	Ca1	34854240	TTATATATATATATATATATATATATATATATATATATATAT/TTATATATATATATATATATATATATATATATATATATAT	AGTTTGGCTAGATGGGCCTT	CCATGCACGTTTTGGACTTA	60.1	886	INTERGENIC	--	--	--

INDEL marker IDs	Chromosomes/ unanchored scaffolds	Physical positions (bp)	InDels (*Kabuli* reference genome-CDC Frontier/PI)	Forward primers (5'-3')	Reverse primers (5'-3')	Anneling temperature (0C)	Expected amplified product size (bp)	Structural annotation		Functional annotation	
								Sequence components of *kabuli* genome	*Kabuli* gene accession IDs	NCBI-KOG	TFs
Ca1_P1_34886508	Ca1	34886508	AAAATAATAAAT/AAAATAATAAATAATAAAT	AGTGAACTTGAACCAACGCA	CACCACCTTCACCTCCAGTT	59.3	573	INTERGENIC	--	--	--
Ca1_P1_34907456	Ca1	34907456	CTTTTTTTTTTTTTTTT/CTTTTTTTTTTTTTTTTTT	TAGCGTTGTGTCGGAAAGTG	TATAAGGTTGGCTTGGTGGC	59.9	372	INTRON	Ca_21409	--	--
Ca1_P1_34935340	Ca1	34935340	GAAAAAAA/GAAAAAAAA	CCGTATGAATCATGTATGGGA	TGCGTCCGTACAAATCTAGG	58.2	515	INTERGENIC	--	--	--
Ca1_P1_34971020	Ca1	34971020	CAAAAAAA/CAAAAA	AAATACCCCTTTTTCCCACG	TGAAAACGCTCAATGCCATA	60.0	689	INTERGENIC	--	--	--
Ca1_P1_34978712	Ca1	34978712	GT/GTT	ATGGCATGGTGGATTTTGTT	CTAGAAAGTTGCAGCCCAGG	60.1	525	INTRON	Ca_21407	--	--
Ca1_P1_34983014	Ca1	34983014	TGAAGAAG/TGAAG	ACGCAAAGCTTCCTTTTTGA	TTCAACGCATCCCTTAAACC	60.0	617	INTERGENIC	--	--	--
Ca1_P1_34989112	Ca1	34989112	CAT/CATAT	CAGGATAGTCGACGTGCAAA	TCTTGGCATAGCAAGAAGCA	59.9	406	INTERGENIC	--	--	--
Ca1_P1_34989444	Ca1	34989444	AATA/AATATA	CAGGATAGTCGACGTGCAAA	CCCATGAGTCAAGCTTCTCA	59.9	804	INTERGENIC	--	--	--
Ca1_P1_35054161	Ca1	35054161	ATCACATTCACTT/A	ATGGACCATTCACGTTTAGAA	AACATCCCTAAAGGATTGCTCA	57.1	721	INTERGENIC	--	--	--
Ca1_P1_35059019	Ca1	35059019	TC/T	TTTCGAACAAATGGTGGGTT	AGGCTTTTTCTTTGTGGTCAG	60.2	413	INTERGENIC	--	--	--
Ca1_P1_35059495	Ca1	35059495	TAAAAAAAAA/TAAAAAAAAAAAAA	CTGACCACAAAGAAAAAGCCT	ATGGGTTTCCAACCACAAAA	58.5	480	INTERGENIC	--	--	--
Ca1_P1_35061932	Ca1	35061932	TAAA/TAA	GGCCTTGAGAAGCATTTGTT	TTCTAAGGCCTCTCACCATGA	59.3	660	INTERGENIC	--	--	--

INDEL marker IDs	Chromosomes/ unanchored scaffolds	Physical positions (bp)	InDels (*Kabuli* reference genome-CDC Frontier/PI)	Forward primers (5'-3')	Reverse primers (5'-3')	Anneling temperature (0C)	Expected amplified product size (bp)	Structural annotation		Functional annotation	
								Sequence components of *kabuli* genome	*Kabuli* gene accession IDs	NCBI-KOG	TFs
Ca1_P1_35076462	Ca1	35076462	ATTTTTTTTTTTTTT/ATTTTTTTTTTT	CATGCTATGCGCTGTGTCTC	ATTGATTCCTCATGCAAGGC	60.6	760	INTERGENIC	--	--	--
Ca1_P1_35076840	Ca1	35076840	AA/AATATTTGA	CCAAAACTTTGGCCCTTTTA	ATTGATTCCTCATGCAAGGC	59.1	239	INTERGENIC	--	--	--
Ca1_P1_35093952	Ca1	35093952	CGT/CGTATGT	GCAACATACTAACTGTGTCCCC	TTCCCTGTCAATGAGTGCAT	58.5	462	INTERGENIC	--	--	--
Ca1_P1_35096364	Ca1	35096364	AT/A	TTGATGTGGTCGGAGATTGA	ACAATTTTCAGCAACCCGAG	60.0	588	INTERGENIC	--	--	--
Ca1_P1_35112845	Ca1	35112845	ATTATTTATTTATTT/ATTATTTATTTATTTATTT	TCCTGCATCAACACATAACACA	TGAGCTTATACCGTTTTTCTGAG	60.0	495	INTRON	Ca_21404	K	HD-ZIP
Ca1_P1_35117113	Ca1	35117113	TAAAAAA/TAAAAAAA	CCAAAAAGAACAGTTTTTAAACCA	CGCCGATGCTATCTAATGCT	58.7	680	INTERGENIC	--	--	--
Ca1_P1_35117250	Ca1	35117250	ATATATTATATTA/ATATATTATATTATATTA	GGAAGGAATATCATTTTCACCG	GAATGCGACTTGCATCTGAA	59.7	565	INTERGENIC	--	--	--
Ca1_P1_35130212	Ca1	35130212	GAAAAAAAA/GAAAAAAA	AAAAATCCCCCATAGGCATC	GGAAGTGTTCCAGAAACCCA	60.0	722	INTERGENIC	--	--	--
Ca1_P1_35132021	Ca1	35132021	GTTTTT/GTTTTTT	GAGCTTCGATGGCAGTTTTC	CCACCTAGAAAGGCTGCTTG	60.0	642	INTERGENIC	--	--	--
Ca1_P1_35147370	Ca1	35147370	CTTT/CTT	TCTATCGTGGTTCATTGCCA	TCTGAATGTCAATCCTCCGA	60.1	631	INTERGENIC	--	--	--
Ca1_P1_35150306	Ca1	35150306	CTATAAAAGG/C	TGCAACATTCCTCCAAGGAT	CATGGACACGATCGTCACTT	60.5	302	INTERGENIC	--	--	--
Ca1_P1_35150770	Ca1	35150770	TC/TCC	GTGACGATCGTGTCCATGTC	CAAGCCTCTCCAAGAAATCG	60.0	715	INTERGENIC	--	--	--

INDEL marker IDs	Chromosomes/ unanchored scaffolds	Physical positions (bp)	InDels (*Kabuli* reference genome-CDC Frontier/PI)	Forward primers (5'-3')	Reverse primers (5'-3')	Anneling temperature (0C)	Expected amplified product size (bp)	Structural annotation		Functional annotation	
								Sequence components of *kabuli* genome	*Kabuli* gene accession IDs	NCBI-KOG	TFs
Ca1_P1_35150983	Ca1	35150983	TAGAGAG/TAGAG	AATCTGCGCAAATCAATCAA	CAAGCCTCTCCAAGAAATCG	59.3	467	INTERGENIC	--	--	--
Ca1_P1_35151847	Ca1	35151847	TTATG/TTATGTATG	TCGCTATGGGATTGTTAGGC	GTTTGTTTTGGCGTTGGAAT	60.1	361	INTERGENIC	--	--	--
Ca1_P1_35153435	Ca1	35153435	GTTTTTTTTTTTTT/GTTTTTT	TTTGGTTTCCGATTGCTAGG	GGTTGTATGGCTCCCAAGAA	60.1	409	INTERGENIC	--	--	--
Ca1_P1_35165919	Ca1	35165919	GTTTTTT/GTTTTTTTTTTTT	TCATCGCAGGTGTACCATGT	CACCTAAGGGAATCGGTCAA	60.0	377	INTERGENIC	--	--	--
Ca1_P1_35169496	Ca1	35169496	T/TC	GAAATCGAGAAGAGGCGTTG	TGTCTCGAAAAAGCGAGATG	60.0	636	INTERGENIC	--	--	--
Ca1_P1_35169802	Ca1	35169802	AC/A	CATCTCGCTTTTTCGAGACAT	TGTCACGGTCAACCCAATAA	59.5	377	INTERGENIC	--	--	--
Ca1_P1_35178139	Ca1	35178139	TAA/TA	ACAAGGTTTGCCTTAGCCCT	TTCCACGTGCATTTTTGTTC	60.1	777	INTERGENIC	--	--	--
Ca1_P1_35178440	Ca1	35178440	GA/GATA	AAAATGAAAACACAAAGGAAGCA	GAATGGAAGGGAATAACTGCC	60.0	816	INTERGENIC	--	--	--
Ca1_P1_35178731	Ca1	35178731	AG/A	CAAAAATGCACGTGGAAGAG	GAATGGAAGGGAATAACTGCC	59.3	378	INTERGENIC	--	--	--
Ca1_P1_35180764	Ca1	35180764	CAAA/CAA	ATGCCCCAATGAATTGTGAT	GTGCCTTCAACAATTTGGGT	60.0	755	INTERGENIC	--	--	--
Ca1_P1_35181144	Ca1	35181144	AACACACAC/AACACACACAC	TACATCGAGTACCTTCGCCC	GTGCCTTCAACAATTTGGGT	60.1	188	INTERGENIC	--	--	--
Ca1_P1_35182197	Ca1	35182197	TGAAGTTATACG/TGAAGTTATACGGGAAGTTATACG	GGGAAGATGACGAGGAATGA	GATTGGAGACGCCACCTAGA	60.0	489	INTERGENIC	--	--	--

INDEL marker IDs	Chromosomes/ unanchored scaffolds	Physical positions (bp)	InDels (*Kabuli* reference genome-CDC Frontier/PI)	Forward primers (5'-3')	Reverse primers (5'-3')	Anneling temperature (0C)	Expected amplified product size (bp)	Structural annotation		Functional annotation	
								Sequence components of *kabuli* genome	*Kabuli* gene accession IDs	NCBI-KOG	TFs
Ca1_P1_35182430	Ca1	35182430	TTT/TTTGTTTTTGTT	GGGAAGATGACGAGG AATGA	TGTACGGGTCATGTG GCTTA	60.0	731	INTERGENIC	--	--	--
Ca1_P1_35186204	Ca1	35186204	ATTTTTTTTT/ATTTTTTT	CCAAGAAACCCTAGCA CCAA	AGCCTCGAAAATGAAC TCCA	60.1	287	INTERGENIC	--	--	--
Ca1_P1_35234438	Ca1	35234438	T/TG	GAGGCATATGCAATTC ACGA	CAACCATTGTTGCTCC CTTT	59.7	481	INTERGENIC	--	--	--
Ca1_P1_35235861	Ca1	35235861	CATTATTATTATT/CATTATTATTATTA TT	GGGATGCGACCTTGTA CTGT	TCCTCCTCTTTTGGCA CATT	60.0	463	INTERGENIC	--	--	--
Ca1_P1_35236185	Ca1	35236185	ATTTTT/ATTTT	ATGTGCCAAAAGAGGA GGAA	GGGATGCGACCTTGTA CTGT	59.7	704	INTERGENIC	--	--	--
Ca1_P1_35236411	Ca1	35236411	ATTATT/ATTATTGTTATT	ATGTGCCAAAAGAGGA GGAA	GGGATGCGACCTTGTA CTGT	59.7	704	INTERGENIC	--	--	--
Ca1_P1_35236504	Ca1	35236504	ATTTTTTT/ATTTTTTTT	CAACTTCCTCCTCTTTT GGC	GGGATGCGACCTTGTA CTGT	58.9	522	INTERGENIC	--	--	--
Ca1_P1_35238941	Ca1	35238941	CTTTTT/CTTTTTT	AAAATGCATTGCCTCT GGTC	TTTATTTACCCAAACG AAAACAA	60.1	824	INTERGENIC	--	--	--
Ca1_P1_35240205	Ca1	35240205	ATAT/ATATTAT	GTAGCATTTTTGGCCT CCCT	GTCGAGAAGAAGGCTT GCAT	60.5	782	DRR	Ca_26097	QI	bHLH
Ca1_P1_35244173	Ca1	35244173	TAAAA/TAAA	ATTTGGATCGGTGTGG TTTT	TCGGAAATGTTGCTGA GATG	59.2	553	INTERGENIC	--	--	--
Ca1_P1_35245008	Ca1	35245008	CAT/C	TGCATTTGTTTTGGCA TGTT	TAAAGGACCAAGTCGG GAAA	60.0	667	INTERGENIC	--	--	--
Ca1_P1_35257433	Ca1	35257433	CTTTTTT/CTTTTTTT	CCCAATTCCAAAACAT GACC	GCGTACTCCCTGTTGA ATTTG	60.0	467	INTERGENIC	--	--	--

INDEL marker IDs	Chromosomes/ unanchored scaffolds	Physical positions (bp)	InDels (*Kabuli* reference genome-CDC Frontier/PI)	Forward primers (5'-3')	Reverse primers (5'-3')	Anneling temperature (0C)	Expected amplified product size (bp)	Structural annotation		Functional annotation	
								Sequence components of *kabuli* genome	*Kabuli* gene accession IDs	NCBI-KOG	TFs
Ca1_P1_35261821	Ca1	35261821	ATT/AT	TGGATTGTCATGGTCTGGAA	AATGGTCTTGAATTTTGCCG	59.9	640	INTERGENIC	--	--	--
Ca1_P1_35266220	Ca1	35266220	ATAT/ATATTAT	CGCGGTAGAGTTATTGGAAAA	TGGGGATACGTAGGAGCAAG	59.3	682	INTERGENIC	--	--	--
Ca1_P1_35281490	Ca1	35281490	ATTTTTTT/ATTTTTT	CTAAGGCACCCTTCTCCACA	TTTTTGATGTCTCTCTGTTCAAAT	60.2	543	INTRON	Ca_26099	QI	bHLH
Ca1_P1_35281570	Ca1	35281570	TC/T	ATGACACGTCATTCGTTTCG	GGTTCGAGACATCCATAATTCA	59.6	733	INTRON	Ca_26099	QI	bHLH
Ca1_P1_35281940	Ca1	35281940	TA/TAA	AAACGTGATCTATACAGTTTCCAAAA	TTCACATCTGGAAACAACGC	59.4	529	INTRON	Ca_26099	QI	bHLH
Ca1_P1_35283249	Ca1	35283249	TT/TTTAAATGT	ATGCATTTGTTTTGGCATGT	TGAAGTGTTCTGCAAAATTAGTGA	58.9	612	INTERGENIC	--	--	--
Ca1_P1_35283797	Ca1	35283797	TGGGGG/TGGGG	GGGTGTGTTTGATAGGAGGG	CTCCCCTTCCATCTCCTCTT	59.3	139	INTERGENIC	--	--	--
Ca1_P1_35284017	Ca1	35284017	ATTT/ATT	GAGATGGAAGGGGAGGAAAG	GCACAAAAGAAAAATACAAGTCCA	60.0	273	INTERGENIC	--	--	--
Ca1_P1_35284253	Ca1	35284253	TT/TTGT	GAGATGGAAGGGGAGGAAAG	TCTGATCAAATATGTGGCAGAAA	60.0	657	INTERGENIC	--	--	--
Ca1_P1_35284860	Ca1	35284860	CAAAAA/CAA	TGAAGGGAGTGCTATTTGGT	TCACCTTTTGACTTCCTCATGTT	57.3	108	INTERGENIC	--	--	--
Ca1_P1_35294425	Ca1	35294425	TA/TAGA	CCATAGGATTGATTGCGCTT	TTCGCATCAGCCATATTTTG	60.1	835	INTERGENIC	--	--	--
Ca1_P1_35301437	Ca1	35301437	ATTTTTTTTTTT/ATTTTTTTTT	GCTTTCATGGTGTGCTCTGA	TTAGGCCCAGTGAAGTTTGG	60.0	560	INTERGENIC	--	--	--

INDEL marker IDs	Chromosomes/ unanchored scaffolds	Physical positions (bp)	InDels (*Kabuli* reference genome-CDC Frontier/PI)	Forward primers (5'-3')	Reverse primers (5'-3')	Anneling temperature (0C)	Expected amplified product size (bp)	Structural annotation		Functional annotation	
								Sequence components of *kabuli* genome	*Kabuli* gene accession IDs	NCBI-KOG	TFs
Ca1_P1_35304454	Ca1	35304454	CAA/CAAA	TTGACTTGAGTGTGCTTCCG	GGTCATGTTTTGGAATTGGG	60.0	213	INTERGENIC	--	--	--
Ca1_P1_35310269	Ca1	35310269	AT/ATATTTATGATT	GTTGATGGTCCATGGGGTAG	CATTGCAATTCCTGCACAAC	60.1	432	INTERGENIC	--	--	--
Ca1_P1_35312742	Ca1	35312742	TC/T	CTCTTGGCTGATCCCAAGTC	AAAATCCACCTTTGTGGCTG	59.8	841	INTERGENIC	--	--	--
Ca1_P1_35315582	Ca1	35315582	AT/ATGT	CTATCCATTTGTGACGGCCT	TTTTGTGGCCGACTAAAACC	60.0	417	INTERGENIC	--	--	--
Ca1_P1_35317313	Ca1	35317313	CTTTTTTT/CTTTTTTTT	CCTGATCCTCATCGTCCATT	GCCAGGTTCACTCGATTCAT	59.9	464	DRR	Ca_26100	QI	bHLH
Ca1_P1_35321165	Ca1	35321165	GTTT/GTTTT	CATCATTCGTTTCAAATTTGTTG	ACGAAGGTTTGAGACATCCA	59.4	586	INTRON	Ca_26101	QI	bHLH
Ca1_P1_35332961	Ca1	35332961	ATTTTTTTTTTT/ATTTTTTTTTT	CACTCCGCTCCCAACTACAT	AACTCCCCCTCAATATGTGC	60.1	595	INTERGENIC	--	--	--
Ca1_P1_35335691	Ca1	35335691	ATTTT/ATTT	TTCTAGGCATGTTTCCCCTG	GAGGCTGTGCATTTTGGAAT	60.1	615	INTERGENIC	--	--	--
Ca1_P1_35339506	Ca1	35339506	TC/TCACGC	TGCACCCAATCTTTCAAAAAC	AACTTGTGGCTGGGTTTGAC	60.0	701	INTERGENIC	--	--	--
Ca1_P1_35344651	Ca1	35344651	T/TC	TGCAACTGACCCTGAATCTG	ATTTGCTTGCCTTTGCATCT	59.8	418	INTERGENIC	--	--	--
Ca1_P1_35349237	Ca1	35349237	ATTT/ATT	CCACTTAGGTGCTTTGTGGG	CAAGGCTTCTTGAACTTGGG	60.5	219	INTERGENIC	--	--	--
Ca1_P1_35353052	Ca1	35353052	G/GTTGA	TCAGCAGCAAATAGGCTTCA	AGCCCATTCAGATTTTGTGG	59.7	352	INTERGENIC	--	--	--

INDEL marker IDs	Chromosomes/ unanchored scaffolds	Physical positions (bp)	InDels (*Kabuli* reference genome-CDC Frontier/PI)	Forward primers (5'-3')	Reverse primers (5'-3')	Anneling temperature (0C)	Expected amplified product size (bp)	Structural annotation		Functional annotation	
								Sequence components of *kabuli* genome	*Kabuli* gene accession IDs	NCBI-KOG	TFs
Ca1_P1_35370041	Ca1	35370041	T/TG	TTGTTCAGCAGCCCTTTCTT	ATGACTATGGCAGCCACTCC	60.0	269	INTERGENIC	--	--	--
Ca1_P1_35372027	Ca1	35372027	CA/CAA	TGTGAGGATGGTGAGTTGTGA	AAGCGTAAAAGAGGGGGTGT	60.1	309	INTERGENIC	--	--	--
Ca1_P1_35403368	Ca1	35403368	C/CT	CACGTCCACCTCTTTGTTCA	TGCAATTTAACGTTTATTCACACA	59.7	892	INTERGENIC	--	--	--
Ca1_P1_35404558	Ca1	35404558	TAAAAAAA/TAAAAAAAA	TGTTTTTCATTCAAAACTGCCA	TGAATCAAACAATTCAATGTGAG	60.5	158	INTERGENIC	--	--	--
Ca1_P1_35405146	Ca1	35405146	ATCCTG/A	TCCTCAAGCTTTTTATTCACTTTTT	CACAAGTGGTAGTTTTGAAGGAAA	59.0	752	INTERGENIC	--	--	--
Ca1_P1_35429375	Ca1	35429375	T/TG	GTGGAGATCACTCGGGTTGT	GCACTCTCTTGGAAAGGCAT	60.0	528	INTERGENIC	--	--	--
Ca1_P1_35438100	Ca1	35438100	AC/A	GCTTAAAGGGCGAGTAAGCA	GATCATTGGCAATCGTTCCT	59.6	565	INTERGENIC	--	--	--
Ca1_P1_35442355	Ca1	35442355	GGTGTGTGTGTGTGTGTGTGTG/GGTGTGTGTGTGTGTGTGTGTGTGTGTGTGTG	AGGCCGAGAAACCCTAGAAG	CATTTTGGATTCAACTCGGG	59.8	309	INTERGENIC	--	--	--
Ca1_P1_35453813	Ca1	35453813	AGTG/AG	GGGGTGAAAAATCAAAACGA	CTACGCAGTTGTATTCGGCT	59.8	458	INTERGENIC	--	--	--
Ca1_P1_3550137	Ca1	3550137	CA/CATA	TTTTTCACCCACATCGATCA	CCTCCAACTGTTTTTGCCAT	59.9	607	DRR	Ca_00432	K	ERF
Ca1_P1_35506098	Ca1	35506098	CTTTTTTTT/CTTTTTTT	CAGATTGCAACTCCCTCCAT	CACCGAATGGGTCAGTCTTT	60.1	228	INTERGENIC	--	--	--
Ca1_P1_35540202	Ca1	35540202	AT/ATT	TTGTTTATGCCCCTTTGAGC	ACAAACAAGTTGAGCACCCC	60.1	146	INTERGENIC	--	--	--

INDEL marker IDs	Chromosomes/ unanchored scaffolds	Physical positions (bp)	InDels (*Kabuli* reference genome-CDC Frontier/PI)	Forward primers (5'-3')	Reverse primers (5'-3')	Anneling temperature (0C)	Expected amplified product size (bp)	Structural annotation		Functional annotation	
								Sequence components of *kabuli* genome	*Kabuli* gene accession IDs	NCBI-KOG	TFs
Ca1_P1_35540299	Ca1	35540299	AA/AATA	GGGGTGCTCAACTTGTTTGT	GGCCAAGGTAAGGGTGAGAT	60.0	254	INTERGENIC	--	--	--
Ca1_P1_35547757	Ca1	35547757	TAAAAAAAAAAAAAA/TAAAAAAAAAAAA	GCCTCTTCCAATTCCAAACA	TCACCACGAACTTTTGTCTCA	60.1	570	INTERGENIC	--	--	--
Ca1_P1_35648390	Ca1	35648390	CATGTCATGTATTCTCAGTATGTCATGTATTCTCAGTATGTCATGTA/CATGTCATGTATTCTCAGTATGTCATGTA	CATTTGGCACCGGTAAGACT	ATTTTGGCCCAACCATAACA	60.0	753	INTERGENIC	--	--	--
Ca1_P1_35679606	Ca1	35679606	CAAAAAAAAAAA/CAAAAAAAAAA	GTGCACAACCTTGTGGTGTC	TAGCCAACATGCCATACGTC	60.1	604	INTERGENIC	--	--	--
Ca1_P1_35695109	Ca1	35695109	A/AAATCTT	CTTGTTGTTTTGATTGGCCC	GATCGGTTGCTTTTGGAAAC	60.3	528	INTERGENIC	--	--	--
Ca1_P1_35698580	Ca1	35698580	TC/TCGGTGCTAC	CAAACTCGTTTTCTCTCGCC	AACTTTGGGCCTTATCCACC	60.0	459	INTERGENIC	--	--	--
Ca1_P1_35712938	Ca1	35712938	A/AT	GGGTGTGACAGTCGAGACAA	ATTCATCGCCATCATCATCA	59.7	281	DRR	Ca_24110	K	--
Ca1_P1_35738288	Ca1	35738288	AGTTTAGCTTATCTAT/AGTTTAGCTTATCTATGTTTAGCTTATCTAT	GAGATTGTGCTCTGATGCCA	ACCCATATTGGGTGAACCAG	60.0	292	INTERGENIC	--	--	--
Ca1_P1_35899211	Ca1	35899211	TTTTAGACAC/TTTTAGACACATTTAGACAC	CACCTCCCCTGAGTTCGTTA	GCAAGCTCCAAATGGATTACA	60.1	404	INTERGENIC	--	--	--
Ca1_P1_35908875	Ca1	35908875	TAAAAAAAAAAAA/TAAAAAAAAAA	TTTAATGGGGCCTAAGGGAG	AATTTAGATGGGGGATTGGC	60.3	745	INTERGENIC	--	--	--
Ca1_P1_35910761	Ca1	35910761	ATTTTTTTTTTTTTT/ATTTTTTTTTTTT	TCTGTCTTGTCCCACTCGAA	CAAGCAACTGTGCGAAAAAC	59.4	358	INTERGENIC	--	--	--
Ca1_P1_35913252	Ca1	35913252	TA/TAAA	CATCATTGTTTGGGCTTGTG	TTTATTGAGGATGGCGAAGG	60.0	798	INTERGENIC	--	--	--

INDEL marker IDs	Chromosomes/ unanchored scaffolds	Physical positions (bp)	InDels (*Kabuli* reference genome-CDC Frontier/PI)	Forward primers (5'-3')	Reverse primers (5'-3')	Anneling temperature (0C)	Expected amplified product size (bp)	Structural annotation		Functional annotation	
								Sequence components of *kabuli* genome	*Kabuli* gene accession IDs	NCBI-KOG	TFs
Ca1_P1_35918154	Ca1	35918154	TGGGGG/TGGGG	GGTCCCCCTATTTTAGCTGG	AGTGAAGGGAAATCGGTGTG	59.8	576	INTERGENIC	--	--	--
Ca1_P1_35973449	Ca1	35973449	TTATATATATATATATATATATATATATATATATATAT/TTATATATATATATATATATATATATATATATAT	GCCCACCCAACAAATACATC	TTGGCACCAGAGAAAAACAA	60.1	733	INTERGENIC	--	--	--
Ca1_P1_35977319	Ca1	35977319	TA/TAA	AACTCGTTGGAACTTCCCTCT	GCAAGCAGCCTTGTTGACTA	59.2	540	INTERGENIC	--	--	--
Ca1_P1_36035161	Ca1	36035161	AAGAGGGAGAGGGAGAGGGAGAGG/AAGAGGGAGAGGGAGAGGGAGAGGGAGAGG	TTCGTGATCCGTGAATTTGA	AACGAAACAATGCCGAACAT	60.0	518	INTERGENIC	--	--	--
Ca1_P1_36044248	Ca1	36044248	CGG/CG	CGATTTCCCTGCATGTTTTT	GCCTGTGATAGGCGGTACAT	59.9	540	INTERGENIC	--	--	--
Ca1_P1_36063199	Ca1	36063199	TCC/TTTTTCCCC	CGTTCGTGGTCCCTTAACTT	TTGCGTTACGGATTTGATGA	59.1	227	INTERGENIC	--	--	--
Ca1_P1_36124030	Ca1	36124030	GTTTTT/GTTTT	TTTTCAGCTTTAAGTTCCGCA	TTTAGGAAGACTGGCTCGGA	60.0	683	INTERGENIC	--	--	--
Ca1_P1_36158144	Ca1	36158144	AATATATATATATATATAT/AATATATATATATATATATATATATATATAT	CCGGCACATAGTAAAATTTGG	GAACGTTCGTCATGATTTTGAA	59.4	321	INTERGENIC	--	--	--
Ca1_P1_36166710	Ca1	36166710	CATTATTATTATTATTATTATTATTATTA/CATTATTATTATTATTATTATTATTA	TCGTTGGCCTATGGTACTCC	CCAACGATTCATACAGGGCT	60.0	574	INTERGENIC	--	--	--
Ca1_P1_36203203	Ca1	36203203	TGG/TG	TGGTACCATCCCCACAAACT	AGAATGAAAGGCGATGGATG	60.1	373	INTERGENIC	--	--	--
Ca1_P1_36243785	Ca1	36243785	TT/TTCTTCTTTCCCTAGACT	ATTTGCCACATCGAAGAACC	GCAGAAATCAAGAATGGGGA	59.9	477	INTERGENIC	--	--	--
Ca1_P1_36249439	Ca1	36249439	TTTATTATTATTATTATTATTATTATTATTATTATTATTATT/TTTATTATTATTATTATTATTATTATTATTATTATTATT	TCCCCCTTTGTCTTCTTGTG	TCCATCTAATTGCTACCGGC	60.1	930	INTERGENIC	--	--	--

INDEL marker IDs	Chromosomes/ unanchored scaffolds	Physical positions (bp)	InDels (*Kabuli* reference genome-CDC Frontier/PI)	Forward primers (5'-3')	Reverse primers (5'-3')	Anneling temperature (0C)	Expected amplified product size (bp)	Structural annotation		Functional annotation	
								Sequence components of *kabuli* genome	*Kabuli* gene accession IDs	NCBI-KOG	TFs
Ca1_P1_36283336	Ca1	36283336	ATT/AT	GAGCATAGTGAGTGCGCTTG	TGATGGCTGAAATGGAATGA	59.8	531	INTERGENIC	--	--	--
Ca1_P1_36283621	Ca1	36283621	GAAAAAAAAA/GAAAAAAA	GAGCATAGTGAGTGCGCTTG	TGATGGCTGAAATGGAATGA	59.8	531	INTERGENIC	--	--	--
Ca1_P1_36355714	Ca1	36355714	A/ATTTTTGGAAG	GTGGCACCTCGGTAAACAGT	TTAGCGAAGTCCCTGCAACT	60.0	520	INTERGENIC	--	--	--
Ca1_P1_36416188	Ca1	36416188	ATTTTATTTTTTTATTTTTTTATTTTTTTATTTTT/ATTTTATTTTTTTATTTTTTTATTTTT	GCATGCGAACAAGAGTCAAA	CTCATTGGAAAAGCCCACAT	60.0	477	INTERGENIC	--	--	--
Ca1_P1_36427911	Ca1	36427911	ATTTTTTT/ATTTTTT	CATTGTTAATCTGCCGGCTT	AAACTCCTTAATGCCATGCG	60.1	843	INTERGENIC	--	--	--
Ca1_P1_36431556	Ca1	36431556	T/TTATAA	CCATTCCACTCCAACCTAGC	GTGTCTACACGGAAAGGGGA	59.6	849	DRR	Ca_21688	--	--
Ca1_P1_36624175	Ca1	36624175	TT/TTATTATTTGT	TCAAAAATGGGCTGCTTACC	CCACCCCTAATTGCGAGATA	60.1	525	INTERGENIC	--	--	--
Ca1_P1_36726460	Ca1	36726460	GAAA/GAAAA	ACAGGAACGACCGAGAGAAA	ATAGTTTTTGGGCGGGCTAC	59.8	502	INTERGENIC	--	--	--
Ca1_P1_36727739	Ca1	36727739	TCC/TC	AGGCTGCATTTTAGTCCACG	ACGACCACTGCAAACACAAA	60.3	218	INTERGENIC	--	--	--
Ca1_P1_36728375	Ca1	36728375	GTTTTT/GTTTTTT	TGGGAGGAGTTGAGAAGGAA	TGGATCCAAATAAAAGGGGA	59.8	111	INTERGENIC	--	--	--
Ca1_P1_36869128	Ca1	36869128	CA/CAAA	TCCACAACAACCTACGTCCA	CCCAGAAATCGATTAGGCAA	60.0	555	INTERGENIC	--	--	--
Ca1_P1_36876798	Ca1	36876798	AGGG/AGGGG	GTTTCCAGCCATCCAACACT	AATTGGTTTCATGACCTGCAC	60.0	503	INTERGENIC	--	--	--

INDEL marker IDs	Chromosomes/ unanchored scaffolds	Physical positions (bp)	InDels (*Kabuli* reference genome-CDC Frontier/PI)	Forward primers (5'-3')	Reverse primers (5'-3')	Anneling temperature (0C)	Expected amplified product size (bp)	Structural annotation		Functional annotation	
								Sequence components of *kabuli* genome	*Kabuli* gene accession IDs	NCBI-KOG	TFs
Ca1_P1_36897436	Ca1	36897436	TTATATATATATATATATATATATA/TTATATATATATATATATATATATA	CACTAGCATCGTAGCGTGGA	TGTGGAAATTTGTGGGTGAA	60.0	415	INTERGENIC	--	--	--
Ca1_P1_36993209	Ca1	36993209	GATCAT/GAT	TGGCCTTGTAAAAACTGCAAG	TTCGCATGTTCTTGATTTGC	60.3	645	INTRON	Ca_21824	J	Trihelix
Ca1_P1_37031419	Ca1	37031419	TNNNNNNNNNN/TNNNNNNNNN	GGGTGAATGGATTTGTCCAC	TTGAAATGTCATGGATGGGA	60.0	537	INTERGENIC	--	--	--
Ca1_P1_37034378	Ca1	37034378	AC/A	ATTTTTGTCAATCCCTCCCC	GCAGCAGTAGGTCTCGGAAG	60.0	521	INTERGENIC	--	--	--
Ca1_P1_37075088	Ca1	37075088	TTAAA/T	GCCCTCAGATGAGAGTCAGG	ATTCTGCAGCACGGATCTCT	59.9	830	INTERGENIC	--	--	--
Ca1_P1_37078511	Ca1	37078511	TTAT/TTATAT	CCATTCCAAAAAGGAGTTGG	TGAAAATGTTGGAGTGATGTGA	59.4	938	INTERGENIC	--	--	--
Ca1_P1_37078904	Ca1	37078904	TTAAATAAATAAATA/TTAAATAAATAAATAAATAAATA	TGTGACACCCTAAACCCTAAGAA	TTGATTGCTTGCGTGTTCTC	59.9	499	INTERGENIC	--	--	--
Ca1_P1_37092021	Ca1	37092021	CTTTTTTTTTT/CTTTTTTT	TATCAGAGTTCTCCCCACCG	TTGAGGTGTGGTTTGTGGTG	60.1	759	INTERGENIC	--	--	--
Ca1_P1_37122051	Ca1	37122051	CAAAAAAAA/CAAAAAAA	CGTATCTCGTATCACCGACG	AGTTGCGCTCCTCCTAATCA	59.2	873	INTERGENIC	--	--	--
Ca1_P1_37122104	Ca1	37122104	CAAAAAAAAAA/CAAAAAAAAAAAAA	CGTATCTCGTATCACCGACG	AGTTGCGCTCCTCCTAATCA	59.2	873	INTERGENIC	--	--	--
Ca1_P1_37122520	Ca1	37122520	AGG/AG	TGCAATCATTGGTGCTTAGG	AAAAAGCGCCGTAAAATGAG	59.7	710	INTERGENIC	--	--	--
Ca1_P1_37122699	Ca1	37122699	GTTT/GTT	GGAGGAGCGCAACTTAATCA	AAAAAGCGCCGTAAAATGAG	60.4	372	INTERGENIC	--	--	--

INDEL marker IDs	Chromosomes/ unanchored scaffolds	Physical positions (bp)	InDels (*Kabuli* reference genome-CDC Frontier/PI)	Forward primers (5'-3')	Reverse primers (5'-3')	Anneling temperature (0C)	Expected amplified product size (bp)	Structural annotation		Functional annotation	
								Sequence components of *kabuli* genome	*Kabuli* gene accession IDs	NCBI-KOG	TFs
Ca1_P1_37122810	Ca1	37122810	TACAAC/TACAACAAC	GGAGGAGCGCAACTTAATCA	AAAAAGCGCCGTAAAATGAG	60.4	372	INTERGENIC	--	--	--
Ca1_P1_37125192	Ca1	37125192	GATGGAGATGGTTTGCAATGGAGATG/GATGGAGATG	AGCGGTTTAGGGTTTAGGGA	CCAAATGTGCATACGAACCA	60.0	113	INTERGENIC	--	--	--
Ca1_P1_37128515	Ca1	37128515	ATTTTTTT/ATTTT	CAATTCCCACAGGTACGCTT	TTGGTCTCATCAATGATTGTAAAAA	60.0	568	INTERGENIC	--	--	--
Ca1_P1_37129039	Ca1	37129039	CTTTTTTT/CTTTTTT	CAATCATTGATGAGACCAAAAA	AATGGCACATAGTTAATTGAGCA	57.6	780	INTERGENIC	--	--	--
Ca1_P1_3717542	Ca1	3717542	ATT/AT	AGTGATGGTGTTGGGTGGAT	CAATAATGTCCTCTCTATGCACCTT	60.1	597	INTERGENIC	--	--	--
Ca1_P1_37197766	Ca1	37197766	GTTT/GTT	TGAGAGGAAAGAAGACAACCATC	TTTTCTTTTTAAAGCGGCGA	59.7	605	DRR	Ca_21831	L	C3H
Ca1_P1_37243078	Ca1	37243078	AAATTAT/AAATTATAGAATTAT	TCATCCACATCAGCCAAAAA	TTTGTGCATTTTAATAATTGGAAG	60.0	490	INTERGENIC	--	--	--
Ca1_P1_37389641	Ca1	37389641	AT/ATTATT	GAGAGAGAGCCTGTTGGTCG	AGGCCAAAAATCACCATTTC	60.1	757	INTERGENIC	--	--	--
Ca1_P1_37391477	Ca1	37391477	TTATATATATATATATATATATAT/TTATATATATATATATATAT	CTGTCAATTGAGATGCGACG	GCGATTACGGTACCACGAGT	60.4	600	INTERGENIC	--	--	--
Ca1_P1_37391521	Ca1	37391521	AATATATATATATATATATATATATATATATATATATATATATATAT/AATATATATATATATATATATATATATATATATATATATATATATAT	CTGTCAATTGAGATGCGACG	GCGATTACGGTACCACGAGT	60.4	600	INTERGENIC	--	--	--
Ca1_P1_37410363	Ca1	37410363	GAAAAAA/GAAAAAAA	TGAATGAGGAGCACGTCTTG	CTGTTGTTGCCAACGGTTTT	60.0	445	INTERGENIC	--	--	--
Ca1_P1_37412282	Ca1	37412282	CCATG/CCATGCATG	CGTGGATGATGCTTTTCTCA	TGCTTCTCATCTGCTTGGTG	59.8	580	DRR	Ca_21842	R	GRAS

INDEL marker IDs	Chromosomes/ unanchored scaffolds	Physical positions (bp)	InDels (*Kabuli* reference genome-CDC Frontier/PI)	Forward primers (5'-3')	Reverse primers (5'-3')	Anneling temperature (0C)	Expected amplified product size (bp)	Structural annotation		Functional annotation	
								Sequence components of *kabuli* genome	*Kabuli* gene accession IDs	NCBI-KOG	TFs
Ca1_P1_37420494	Ca1	37420494	ATTTTTTTTT/ATTTTTTT	GGGAAAAGGAAACCCAAAAA	TAGTGGAGTCCCCGTCAAGT	60.1	630	INTERGENIC	--	--	--
Ca1_P1_37420598	Ca1	37420598	ATTTT/ATTT	GGGAAAAGGAAACCCAAAAA	TGAGTGAGGGAGACAAAGGG	60.1	950	INTERGENIC	--	--	--
Ca1_P1_37420725	Ca1	37420725	TCC/TCCC	ACTTCACGGGGACTCCACTA	TGAGTGAGGGAGACAAAGGG	59.6	403	INTERGENIC	--	--	--
Ca1_P1_37421082	Ca1	37421082	CCTCACTCACTCACTCACTCAC/CCTCACTCACTCACTCAC	ACTTGACGGGGACTCCACTA	ACATGTTCCTTGTGCTCGTG	59.6	637	INTERGENIC	--	--	--
Ca1_P1_37424329	Ca1	37424329	CATATATATATATATATATATAT/CATATATATATATATATAT	AAAAAGTATTGAACAAACACGCT	TGAAATCCAAATTAAATTGCTGAA	57.2	549	INTERGENIC	--	--	--
Ca1_P1_37424741	Ca1	37424741	ATTTTT/ATTTTTT	GCAATTTAATTTGGATTTCATGG	TGCAAATCTAGAAATTTTGATGACA	59.6	179	INTERGENIC	--	--	--
Ca1_P1_37424983	Ca1	37424983	AC/A	GCAATTTAATTTGGATTTCATGG	TCACATCCATCCACGGTACA	59.6	761	INTERGENIC	--	--	--
Ca1_P1_37425153	Ca1	37425153	G/GAT	GCAATTTAATTTGGATTTCATGG	TCACATCCATCCACGGTACA	59.6	761	INTERGENIC	--	--	--
Ca1_P1_37425620	Ca1	37425620	ACG/A	TGTACCGTGGATGGATGTGA	TATGCATCCTCACGGTTTTG	60.8	675	INTERGENIC	--	--	--
Ca1_P1_37426537	Ca1	37426537	ATT/AT	CTTCAAAACCGTGAGGATGC	TCTGAGCTTCTGATGCTTGC	60.6	916	INTERGENIC	--	--	--
Ca1_P1_37433380	Ca1	37433380	CAA/CAACGTAAA	AATAAATTAGTGCAGCAAACCAA	GGCAAAGAGAGGAAAGTGGA	58.0	756	INTERGENIC	--	--	--
Ca1_P1_37433540	Ca1	37433540	TATAATAGATAATAGATAATA/TATAATAGATAATA	TGAAAAACATCTCTCTAATAGGCG	ACCACTGCACCAAGGGTAAG	59.0	532	INTERGENIC	--	--	--

INDEL marker IDs	Chromosomes/ unanchored scaffolds	Physical positions (bp)	InDels (*Kabuli* reference genome-CDC Frontier/PI)	Forward primers (5'-3')	Reverse primers (5'-3')	Anneling temperature (0C)	Expected amplified product size (bp)	Structural annotation		Functional annotation	
								Sequence components of *kabuli* genome	*Kabuli* gene accession IDs	NCBI-KOG	TFs
Ca1_P1_37489932	Ca1	37489932	CAAAAA/CAAAA	ATTGTCCAGTTTCCTC ACCG	CGATTGGTAAATCGAT TGGG	60.0	594	INTERGENIC	--	--	--
Ca1_P1_37489959	Ca1	37489959	AT/ATT	ATTGTCCAGTTTCCTC ACCG	CGATTGGTAAATCGAT TGGG	60.0	594	INTERGENIC	--	--	--
Ca1_P1_37490066	Ca1	37490066	AT/AATGTATT	ATTGTCCAGTTTCCTC ACCG	TGAACTCTGAGCGTGT GACC	60.0	775	INTERGENIC	--	--	--
Ca1_P1_37516914	Ca1	37516914	TAAAAAA/TAAAAA	AGGGAGGACGAAAATG ACCT	ACCAATCTCGTGCATC CAAT	59.9	567	INTERGENIC	--	--	--
Ca1_P1_37558126	Ca1	37558126	TAAAAAAAAAAA/TAAAAAAAAAAAA	GCAAACACGTGAAAAG GGAT	GCTTTCATTGGTTGTT GCCT	60.0	139	INTERGENIC	--	--	--
Ca1_P1_37685004	Ca1	37685004	AC/A	GCTAAATAGCACCCGC AGTC	TGCATTCAAACAAATC CAACA	59.9	718	INTERGENIC	--	--	--
Ca1_P1_37686545	Ca1	37686545	TCCCC/TCCCCC	GAACTCCCTTCATGAC CCAA	TTTGTTGGTGGTGGAG AACA	59.9	527	INTERGENIC	--	--	--
Ca1_P1_37692598	Ca1	37692598	TT/TTCT	ACCATGGCACAAGGTA GAGG	GTGACGCAGAGAATGT TCCA	60.0	708	DRR	Ca_23533	--	G2-like
Ca1_P1_37694938	Ca1	37694938	TAAAAAAAA/TAAAAAAAAA	GCTTGCCTTCCAGCTT GTTA	TTTCTTGCCAACTTGC AGTG	60.5	235	INTERGENIC	--	--	--
Ca1_P1_37696033	Ca1	37696033	T/TC	GGACTCGAGCAAGTTT GAGG	TTTTGGTATCTTTCTC GGGG	60.0	640	INTERGENIC	--	--	--
Ca1_P1_37697426	Ca1	37697426	GTTTTTT/GTTTTTTTT	TATTGGGTAAGTCCAC GGCT	TGGGGTGTACTGGAAA ACTTG	59.5	644	INTERGENIC	--	--	--
Ca1_P1_37697662	Ca1	37697662	A/AT	CAAAGGAAAGCCCTTG AGGT	GCATGCCCCTCCAAAG TAT	60.6	871	INTERGENIC	--	--	--

INDEL marker IDs	Chromosomes/ unanchored scaffolds	Physical positions (bp)	InDels (*Kabuli* reference genome-CDC Frontier/PI)	Forward primers (5'-3')	Reverse primers (5'-3')	Anneling temperature (0C)	Expected amplified product size (bp)	Structural annotation		Functional annotation	
								Sequence components of *kabuli* genome	*Kabuli* gene accession IDs	NCBI-KOG	TFs
Ca1_P1_37701521	Ca1	37701521	TAAAAAA/TAAAAA	TCCGAAAAACTCGCAATAGG	TCACGTGATGCGACCATAAT	60.2	807	INTERGENIC	--	--	--
Ca1_P1_37710483	Ca1	37710483	AAAT/A	GCGCTCTTTTGCTTGTTGAT	GGATACAGTGGTCGCCAAAT	60.5	740	INTERGENIC	--	--	--
Ca1_P1_37712787	Ca1	37712787	A/ACATGTG	GGTGTTGGAGGTGTAATGGG	CATGTCCCGTATTCGCTTTT	60.1	639	INTERGENIC	--	--	--
Ca1_P1_37714757	Ca1	37714757	ATTT/ATTTT	TCGCTCACATATAAGGTTGCAT	TTCGCCCATATTTCCTTTTG	59.6	640	INTERGENIC	--	--	--
Ca1_P1_37718461	Ca1	37718461	CT/CTCCACTT	TTTTTGAAAGTCCCGGTTCA	TCCATATATTTGAGGGGGCA	60.5	454	INTERGENIC	--	--	--
Ca1_P1_37718748	Ca1	37718748	CCTACTA/CCTACTACTA	AAATTTTTGCCCCCTCAAAT	CCACGCATGAAATGATTCAA	59.7	510	INTERGENIC	--	--	--
Ca1_P1_37722513	Ca1	37722513	A/ATTTGGT	ATTCGATTCTCGCCTAAGCA	GAGTTCAGCACAAATGGAAGAA	59.9	595	INTRON	Ca_23534	--	--
Ca1_P1_37724930	Ca1	37724930	CCACACACAC/CCACACAC	ACCCTGTTGCTGCCAAATAC	GCTCCAATTCCCTCAGATCA	60.0	664	INTRON	Ca_23534	--	--
Ca1_P1_37724987	Ca1	37724987	A/ATT	ACCCTGTTGCTGCCAAATAC	GCTCCAATTCCCTCAGATCA	60.0	664	INTRON	Ca_23534	--	--
Ca1_P1_37729113	Ca1	37729113	CA/CAA	TCAATCCATCTTAGCGGAGG	CCTTTGTGAGAGCTTTTGGC	60.2	464	INTERGENIC	--	--	--
Ca1_P1_37733160	Ca1	37733160	TTTGTT/TTT	CTCACCTGCAACAAATCGAA	ACTAGCGCTTTTTCAACGGA	59.8	661	INTERGENIC	--	--	--
Ca1_P1_37737691	Ca1	37737691	AAAATAAATAAATAAATAAATAAA/AAAATAAATAAATAAATAAA	AGGGACCACTATGACCGTTT	TCCTGCAATCACCCTTTTTC	58.4	481	INTERGENIC	--	--	--

INDEL marker IDs	Chromosomes/ unanchored scaffolds	Physical positions (bp)	InDels (*Kabuli* reference genome-CDC Frontier/PI)	Forward primers (5'-3')	Reverse primers (5'-3')	Anneling temperature (0C)	Expected amplified product size (bp)	Structural annotation		Functional annotation	
								Sequence components of *kabuli* genome	*Kabuli* gene accession IDs	NCBI-KOG	TFs
Ca1_P1_37740010	Ca1	37740010	TAAAAAAA/TAAAAAAAA	GGGTGAGTTGGTTTGCTCAT	AGAACGTGTGGGAGTGCATA	60.0	545	INTERGENIC	--	--	--
Ca1_P1_37741814	Ca1	37741814	AATATATATATATAT/AATATATATAT	CGAATTGGGAGAATCCTTGA	CTGGATCTTGTGGCTTTGGT	60.0	566	INTERGENIC	--	--	--
Ca1_P1_37744829	Ca1	37744829	AGGG/AGG	CTTCCGAGAAACAAGAACGG	AGTCTGGGACGAATGACACC	59.8	657	DRR	Ca_23535	--	--
Ca1_P1_37747193	Ca1	37747193	GTTTTT/GTTTTTT	TCCATTAAAAATTATGTTGAAAGTCG	TTCTAATCGCCATCAAACCC	59.7	443	INTERGENIC	--	--	--
Ca1_P1_37747367	Ca1	37747367	GTTTTTTTTT/GTTTTTTTTTTTT	ATGGGTTTGATGGCGATTAG	TGCCACGTCTTAGTTTAATTAGCA	59.8	497	INTERGENIC	--	--	--
Ca1_P1_37747508	Ca1	37747508	A/AC	AATTGAATGGGTTTGATGGC	GCTCATTGCTTCACACCGTA	59.6	780	INTERGENIC	--	--	--
Ca1_P1_37748225	Ca1	37748225	C/CATAT	TACGGTGTGAAGCAATGAGC	TTACGCCTGATGATTAGCCA	59.9	380	INTERGENIC	--	--	--
Ca1_P1_37758416	Ca1	37758416	AATATATATAT/AATATATAT	AATGCAAATTTTTCCCACCA	AGCGAATGATTTCAACCACC	60.2	791	INTERGENIC	--	--	--
Ca1_P1_37758491	Ca1	37758491	TA/TAA	TGACGAGGTTCGTGATGATG	AGCGAATGATTTCAACCACC	60.7	489	INTERGENIC	--	--	--
Ca1_P1_37759669	Ca1	37759669	A/AG	TCCATCTTGATCCTTTGCCT	TGAGCAAATGATGTTGGAGC	59.6	787	INTERGENIC	--	--	--
Ca1_P1_37769132	Ca1	37769132	AA/AATAGGAAAAAATA	TGATGCAGCCTAAATTGCAG	TTAGTTTCGTTGGAGTCGGG	60.0	569	INTERGENIC	--	--	--
Ca1_P1_37771437	Ca1	37771437	CTTTTT/CTTTT	CTGTGGCCCGACAAAGTATT	TCGTATATGCAGCAAGTGGC	60.0	627	INTERGENIC	--	--	--

INDEL marker IDs	Chromosomes/ unanchored scaffolds	Physical positions (bp)	InDels (*Kabuli* reference genome-CDC Frontier/PI)	Forward primers (5'-3')	Reverse primers (5'-3')	Anneling temperature (0C)	Expected amplified product size (bp)	Structural annotation		Functional annotation	
								Sequence components of *kabuli* genome	*Kabuli* gene accession IDs	NCBI-KOG	TFs
Ca1_P1_37784588	Ca1	37784588	AATTATTATTATTATTATTATTATTATTATTATT/AATTATTATTATTATTATTATTATTATTATT	TTGATTTCTCCATCGCACTG	CCATAGGAGTCGCCAACTGT	59.8	336	INTERGENIC	--	--	--
Ca1_P1_37794018	Ca1	37794018	GAAA/GAA	TGCACACATTTTAGGCGAAG	CCTGCTGAATGCAAATCAAA	59.9	651	DRR	Ca_23537	--	NAC
Ca1_P1_37794896	Ca1	37794896	CAAAAAA/CAAAAAAA	TTTGATTTGCATTCAGCAGG	GAAGCAAATGGATATGGGGA	59.8	513	DRR	Ca_23537	--	NAC
Ca1_P1_37796757	Ca1	37796757	ATAATTAAT/ATAAT	CGTGAGGAAAATGGGGAGTA	AAATTGGTGGGGTCATAGCA	59.9	543	INTRON	Ca_23537	--	NAC
Ca1_P1_37798793	Ca1	37798793	TATTA/TATTATTAATATCTGATTA	TGTGGAACTTGATTTTGGTGA	TATCGCCTCATGGAATCACA	59.0	583	INTRON	Ca_23537	--	NAC
Ca1_P1_37799193	Ca1	37799193	TAAAA/TAAAAA	TGTGATTCCATGAGGCGATA	TTGATTGCAACAGTGGCCTA	60.0	785	INTRON	Ca_23537	--	NAC
Ca1_P1_37799228	Ca1	37799228	CACTAATTTA/CA	TGTGATTCCATGAGGCGATA	TTGATTGCAACAGTGGCCTA	60.0	785	INTRON	Ca_23537	--	NAC
Ca1_P1_37806398	Ca1	37806398	AC/A	GGGGAATTTGATGTGCAAAC	TGCAATTGCCCAAATCTACA	60.2	631	INTERGENIC	--	--	--
Ca1_P1_37809250	Ca1	37809250	ACTTTTCCTTTTCCT/ACTTTTCCT	TGCCTTTGGGGATTTTATCA	TTTTGCATTAAAAATGGATGAA	60.3	579	INTERGENIC	--	--	--
Ca1_P1_37810398	Ca1	37810398	TA/TAA	AAGCTTGATGGGCCATAGTG	CGGCAAAAGACTCAAGAACC	60.1	810	INTERGENIC	--	--	--
Ca1_P1_3782006	Ca1	3782006	CAAAA/CAAA	GGTTTCAAAATCGAAATCCAA	ACCGTGGTAATTGACATGAAA	58.9	397	INTERGENIC	--	--	--
Ca1_P1_37829328	Ca1	37829328	AC/ACC	GTCGCTCTTGAGATCGTTCC	GGGAACCGCAACAAATAGAA	60.0	780	INTERGENIC	--	--	--

INDEL marker IDs	Chromosomes/ unanchored scaffolds	Physical positions (bp)	InDels (*Kabuli* reference genome-CDC Frontier/PI)	Forward primers (5'-3')	Reverse primers (5'-3')	Anneling temperature (0C)	Expected amplified product size (bp)	Structural annotation		Functional annotation	
								Sequence components of *kabuli* genome	*Kabuli* gene accession IDs	NCBI-KOG	TFs
Ca1_P1_3783477	Ca1	3783477	AAATAATAA/AAATAA	AATGATGATAATTCATTTGCCA	GCGACTATCCAGGAAATCCA	57.0	522	INTERGENIC	--	--	--
Ca1_P1_3783626	Ca1	3783626	TATTTGCATTTG/TATTTG	TTCACAATTACCACAAAGATCG	GCGACTATCCAGGAAATCCA	57.7	433	INTERGENIC	--	--	--
Ca1_P1_37836904	Ca1	37836904	CAGTTAGTTAGTTAGTTAGTTAGTTA/ CAGTTAGTTAGTTAGTTAGTTAGTTAGTTAGTTA	ATGGATGTAGCGATTACGCC	CCAATACTCCACCTGCGATT	60.0	442	INTERGENIC	--	--	--
Ca1_P1_37846054	Ca1	37846054	T/TG	GGAAGAGGAGGAGGTTTTGG	TGCATTTTGCAAACCAATGT	60.0	648	INTRON	Ca_23538	T	E2F/DP
Ca1_P1_37849378	Ca1	37849378	G/GA	CCTAAGGGCCGTAGTAACCTG	AGGATCCACCAAGCAAATCA	60.0	154	INTRON	Ca_23538	T	E2F/DP
Ca1_P1_3784964	Ca1	3784964	TTAAATAAATAAATAAATAAATAAATAAATAAATAAA/TTAAATAAATAAATAAATAAATAAATAAATAAATAAATAAATAAATAAATAAA	CTCCTTCTATGGGGCATCAA	ATAAGCCCCTTCCAAAGCAT	60.0	272	INTERGENIC	--	--	--
Ca1_P1_37851381	Ca1	37851381	GGTTAATA/GGTTAATAGTTAATA	GGGGGTGACAAATTTAGGTG	ACCTGGCCTCTTCAACCTTT	59.1	630	INTRON	Ca_23538	T	E2F/DP
Ca1_P1_37851702	Ca1	37851702	AAATAA/AAA	AAAGGTTGAAGAGGCCAGGT	AACATGCTATGGGCAACCTC	60.1	568	INTRON	Ca_23538	T	E2F/DP
Ca1_P1_37852400	Ca1	37852400	T/TAAGA	TGTGGTATGTGCGGTTCCTA	GCTCCGGTGTATTTGGTTCT	60.0	687	INTERGENIC	--	--	--
Ca1_P1_37852569	Ca1	37852569	TAAAAAAA/TAAAAAA	TGTGGTATGTGCGGTTCCTA	TTTGATGGGTTCATGTTCCA	60.0	814	INTERGENIC	--	--	--
Ca1_P1_37852817	Ca1	37852817	AAGCA/AAGCAGCA	CACGTAGCAAAAATAATTGGCA	TCATCCTGCCGGTTAGTACC	60.0	765	INTERGENIC	--	--	--
Ca1_P1_37867707	Ca1	37867707	TCAAACA/TCAAACAAACA	ATTGCAGCCTTACACCCAAG	AACTTGAACCATCCAGCACC	60.1	410	INTRON	Ca_23539	KT	--

INDEL marker IDs	Chromosomes/ unanchored scaffolds	Physical positions (bp)	InDels (*Kabuli* reference genome-CDC Frontier/PI)	Forward primers (5'-3')	Reverse primers (5'-3')	Anneling temperature (0C)	Expected amplified product size (bp)	Structural annotation		Functional annotation	
								Sequence components of *kabuli* genome	*Kabuli* gene accession IDs	NCBI-KOG	TFs
Ca1_P1_37869799	Ca1	37869799	AC/ACC	TTCCTGATTCCAGATTTGCC	AAAGATGGGGTCAGTGCATC	60.0	371	INTRON	Ca_23539	KT	--
Ca1_P1_37872834	Ca1	37872834	GT/GTT	TTAAGTCTTGGCTCTGCGGT	CAAAGCGTGCACTGGATAGA	60.0	419	INTRON	Ca_23539	KT	--
Ca1_P1_37881963	Ca1	37881963	T/TC	GAATGGGCATGTCATTTGTG	ACTAGAGGCCCTCACAAGCA	59.8	657	INTRON	Ca_23539	KT	--
Ca1_P1_37891631	Ca1	37891631	TAACAA/TAA	GTATGACCGGCTTGAGTGGT	GCAGTGAGTGATTTGCTGGA	60.0	772	DRR	Ca_23539	KT	--
Ca1_P1_37909579	Ca1	37909579	TAAAAAAAAA/TAAAAAAAAAAA	CCGAATGTCGAACTCAAAGA	AGCGATGAGTGAAACAAGATGA	58.8	590	INTERGENIC	--	--	--
Ca1_P1_37911541	Ca1	37911541	CAA/CAAA	TGATTTTGCCTGTTCCACAA	TGTGTTAATGCAGGTTGCCT	60.1	589	INTERGENIC	--	--	--
Ca1_P1_37913622	Ca1	37913622	AATATATATATATA/AATATATATATATATA	ACGTTCGCCACCTATCTCAT	CCTTATGCGGTAATCCCTGA	59.6	563	INTERGENIC	--	--	--
Ca1_P1_37913733	Ca1	37913733	TAAA/TAA	ACGTTCGCCACCTATCTCAT	CCTTATGCGGTAATCCCTGA	59.6	563	INTERGENIC	--	--	--
Ca1_P1_37913861	Ca1	37913861	GTATATATATATAT/GTATATATATATATAT	GGAGTGAATTTGTGTTGCTCC	TGAGGGTAACAGGGAAGGTG	59.6	611	INTERGENIC	--	--	--
Ca1_P1_37917967	Ca1	37917967	ATATGACAGTTA/ATA	TCCACAAGTTATTCGACGAAAC	TTCCATTGATCTACGTTAATGTTTG	59.1	558	INTERGENIC	--	--	--
Ca1_P1_37919008	Ca1	37919008	TCCCCC/TCCCC	TTTGCGAAAGTCACATCCAG	ATCTCCTTCAAGAGGTCGCA	59.8	756	INTERGENIC	--	--	--
Ca1_P1_37926020	Ca1	37926020	TCCC/TCC	GATCTATGAGGACAGGGCCA	AAGGTTCAAGACGTCACATTCA	60.0	374	INTERGENIC	--	--	--

**INDEL marker IDs**	**Chromosomes/ unanchored scaffolds**	**Physical positions (bp)**	**InDels (*Kabuli* reference genome-CDC Frontier/PI)**	**Forward primers (5'-3')**	**Reverse primers (5'-3')**	**Anneling temperature (0C)**	**Expected amplified product size (bp)**	**Structural annotation**		**Functional annotation**	
								**Sequence components of *kabuli* genome**	***Kabuli* gene accession IDs**	**NCBI-KOG**	**TFs**
Ca1_P1_37927331	Ca1	37927331	TAAA/TAAATTGTATCAAA	ATTTTGATCGCGGAAGTTTG	TCAATCAACCAGATTTTGCATT	60.1	553	INTERGENIC	--	--	--
Ca1_P1_37927862	Ca1	37927862	G/GTA	TGCAAAATCTGGTTGATTGAG	TCCCAGATCAAAATTGGGTT	58.8	745	INTERGENIC	--	--	--
Ca1_P1_37928451	Ca1	37928451	TTTTGATG/T	CACCAAATCATACCACCACG	GAATTGACATTGTTGCACGG	59.7	394	INTERGENIC	--	--	--
Ca1_P1_37928853	Ca1	37928853	CT/CTGAAT	CCGTGCAACAATGTCAATTC	GACCACCATATTAACCGTCACA	60.0	571	INTERGENIC	--	--	--
Ca1_P1_37929646	Ca1	37929646	TAAAAAAAA/TAAAAAAAAAA	TGTGACGGTTAATATGGTGGTC	TTGACTTGTTTTGCGGATTG	59.6	817	INTERGENIC	--	--	--
Ca1_P1_37980254	Ca1	37980254	GTAATAATAATAATAATAATAATAATAATAATAATAATAATAATAATAATA/GTAATAATAATAATAATAATAATAATAATAATAATAATAATAATAATAATA	CCATTTTGTCCATGTTTCCC	AATTGGGGTGTCCATTTTGA	60.0	348	INTERGENIC	--	--	--
Ca1_P1_37980508	Ca1	37980508	AAATAATAATAATAATAATAATAATAA/AAATAATAATAATAATAATAATAA	CCATTTTGTCCATGTTTCCC	AATCGGACGCTAAATCAACG	60.0	903	INTERGENIC	--	--	--
Ca1_P1_37993665	Ca1	37993665	CAAAAAA/CAAAAA	GTGAGCCCTTTACCAATCCA	CAATTCCCACAGGTACGCTT	59.9	654	INTERGENIC	--	--	--
Ca1_P1_38024874	Ca1	38024874	TATGAATGAAT/TATGAATGAATGAAT	GCAAGCTCAGTTCCAGTTCC	TGCATCAATTATGGCTCCAA	60.0	655	INTRON	Ca_27443	S	--
Ca1_P1_38030335	Ca1	38030335	TTAAAATAAAATAAAATAAAA/TTAAAATAAAATAAAATAAAATAAAA	AGCTCGAAACATGGTATCCAA	CTAGCCATCCTCAGCGAGAG	59.6	662	INTERGENIC	--	--	--
Ca1_P1_38043395	Ca1	38043395	GATATATATATATATATATATATATATATATATATATATATAT/GATATATATATATATATATATATATATATATATATATATATATAT	TCTGAATTTGGCAACATCATT	CTTTCGCGATTCAGTCAAAA	58.1	846	INTERGENIC	--	--	--
Ca1_P1_38060326	Ca1	38060326	CGG/CG	GGTTTGTCCGTTCTTCTCCA	CTTGTTGCGACGATGAAAAA	60.1	310	INTERGENIC	--	--	--

INDEL marker IDs	Chromosomes/ unanchored scaffolds	Physical positions (bp)	InDels (*Kabuli* reference genome-CDC Frontier/PI)	Forward primers (5'-3')	Reverse primers (5'-3')	Anneling temperature (0C)	Expected amplified product size (bp)	Structural annotation		Functional annotation	
								Sequence components of *kabuli* genome	*Kabuli* gene accession IDs	NCBI-KOG	TFs
Ca1_P1_38067157	Ca1	38067157	ATT/AT	TTCGTAAATCATTCGCCCAT	ACAAACAGGGTCCTTTCCAA	60.3	655	INTERGENIC	--	--	--
Ca1_P1_38070666	Ca1	38070666	T/TA	AACCATTGGGGGAGAATAGAA	TTGTTAGAACCATTGGGGGA	59.7	362	INTERGENIC	--	--	--
Ca1_P1_38070895	Ca1	38070895	ATGTGTGT/ATGTGT	CTCCCCCAATGGTTCTAACA	CACTGGAATCCGTTTCTTTTG	59.8	587	INTERGENIC	--	--	--
Ca1_P1_38071534	Ca1	38071534	TACACACAC/TACACAC	CTCCTCCTCATTCACCCAAC	ACACACCCACTCTCCTTTGC	59.5	189	INTERGENIC	--	--	--
Ca1_P1_38088606	Ca1	38088606	TAGAG/TAG	GCAAATCGGGGAAAATCTCT	GGCAGTGGAGCCATTACATT	60.4	820	INTERGENIC	--	--	--
Ca1_P1_38090984	Ca1	38090984	CAT/CATATATAT	GGAATAAAGGACTAAATCGGGAA	TGATTTGATGCCGTGAGTGT	59.7	545	INTERGENIC	--	--	--
Ca1_P1_38091862	Ca1	38091862	TAAAAAAAA/TAAAAAAAAA	TATGGCAATTTTGGCATCCT	AAACCCTTTGAATAGACCCCA	60.3	521	INTERGENIC	--	--	--
Ca1_P1_38106060	Ca1	38106060	TATA/T	AGGAGAAAATTGCAACACCC	TGCATATACACGTAAATTCGCC	59.0	291	INTERGENIC	--	--	--
Ca1_P1_38106752	Ca1	38106752	CAA/CAAA	TTTTGTTGGTGTTAAAATGCC	TTGCGGTTGCTCTTCTTCTT	57.6	540	INTERGENIC	--	--	--
Ca1_P1_38109587	Ca1	38109587	ATTT/ATTTT	ATTATGAAATTCCCTTGGCG	TCTCTCACCCCCTACCTCAA	58.9	597	INTERGENIC	--	--	--
Ca1_P1_38110776	Ca1	38110776	AT/ATTTT	GGGAGCATCCATAACATTGC	GCCATTTCAAACACATCACG	60.3	188	INTERGENIC	--	--	--
Ca1_P1_38115001	Ca1	38115001	ATT/ATTT	TCACCTCATTCGTTGTTTGTG	TCAAAAGTCCACCAAGGTCC	59.6	601	INTERGENIC	--	--	--

INDEL marker IDs	Chromosomes/ unanchored scaffolds	Physical positions (bp)	InDels (*Kabuli* reference genome-CDC Frontier/PI)	Forward primers (5'-3')	Reverse primers (5'-3')	Anneling temperature (0C)	Expected amplified product size (bp)	Structural annotation		Functional annotation	
								Sequence components of *kabuli* genome	*Kabuli* gene accession IDs	NCBI-KOG	TFs
Ca1_P1_38127589	Ca1	38127589	ATTTTTTTT/ATTTTTTTTTT	AACGACTACCCATGCTCCAC	CCTGTCAGAACTCGCATTGA	60.0	544	INTERGENIC	--	--	--
Ca1_P1_38130303	Ca1	38130303	C/CG	AAAGGGGGAGAACCATCTGT	TGCATTTGCTTGACTTGGAG	59.8	680	INTERGENIC	--	--	--
Ca1_P1_38131605	Ca1	38131605	C/CT	CGTAACTCGTGTTGGGTGAA	GAAGCAAGAGGCAACTCCAC	59.6	379	INTERGENIC	--	--	--
Ca1_P1_38132160	Ca1	38132160	GTC/GTCTC	AGGCGAATTTTAAACCGAGA	TTCCATGGCACGTGTAAAAA	58.8	150	INTERGENIC	--	--	--
Ca1_P1_38137457	Ca1	38137457	TG/T	CACTCTTTTGCTTCCACCTCA	TTCAAAAATGATGGACAAAGTCA	60.4	478	INTERGENIC	--	--	--
Ca1_P1_38141452	Ca1	38141452	ATTT/ATTTT	AGCAAAGAAACCTGTCAGAAAA	TTTGAAGATTTGCATTACGGA	58.2	485	INTERGENIC	--	--	--
Ca1_P1_38142065	Ca1	38142065	A/AGT	GCTCTAACAGCGTAGCAAAAA	TATGAGGTGGCAGCAAGAGA	57.6	321	INTERGENIC	--	--	--
Ca1_P1_38146747	Ca1	38146747	T/TG	TTTTTGGATTGCCAACATCA	ACGTTGCGCGAATTTATTTT	59.9	764	INTERGENIC	--	--	--
Ca1_P1_38147070	Ca1	38147070	ATTTTTTTTT/ATTTTTTTTTT	GACAACCCATTATCCAAACGA	ACGTTGCGCGAATTTATTTT	59.7	481	INTERGENIC	--	--	--
Ca1_P1_38147657	Ca1	38147657	TC/TCC	TGTTGACACATCACATCCTTAACA	TTTCATGTCTCATTTCCCCA	60.3	858	INTERGENIC	--	--	--
Ca1_P1_38148667	Ca1	38148667	GAAA/GAAAA	GCAACGTGCGGGTTATATTT	GAAAAAGCGCTGTAAATGGC	59.9	228	INTERGENIC	--	--	--
Ca1_P1_38163218	Ca1	38163218	G/GC	TTTTTCCCGACTTGGTCATT	ACATATACGCGAAAATCGGG	59.4	812	INTERGENIC	--	--	--

INDEL marker IDs	Chromosomes/ unanchored scaffolds	Physical positions (bp)	InDels (*Kabuli* reference genome-CDC Frontier/PI)	Forward primers (5'-3')	Reverse primers (5'-3')	Anneling temperature (0C)	Expected amplified product size (bp)	Structural annotation		Functional annotation	
								Sequence components of *kabuli* genome	*Kabuli* gene accession IDs	NCBI-KOG	TFs
Ca1_P1_38170055	Ca1	38170055	C/CT	GAAAAAGGGAGCTTCATCCC	TTGGAGCTCAAAGAGGAGGA	60.0	935	INTERGENIC	--	--	--
Ca1_P1_38185784	Ca1	38185784	ACC/ACCC	TCCATGCTCCATGCATTAAA	GGAAGTGTGATTCAGTTGGTGA	60.0	686	INTERGENIC	--	--	--
Ca1_P1_38187356	Ca1	38187356	AT/ATT	ATCACTCCACCGACTTCCAC	GAGTAGGGCTGTGCATGGTT	60.0	602	INTERGENIC	--	--	--
Ca1_P1_38190589	Ca1	38190589	ATTTTT/ATTTT	GTGGAGTACCCGCAGAAAAA	GAACCCCGAATGAAATGTTG	60.1	567	INTERGENIC	--	--	--
Ca1_P1_38193113	Ca1	38193113	CAAAAAAAAAA/CAAAAAAAAAAAA	CGAACAAACTCACCGGATCT	CCAATGGAGTCCCAAAAAGA	60.1	724	INTERGENIC	--	--	--
Ca1_P1_38193154	Ca1	38193154	TAAAAAAAAAAA/TAAAAAAAAA	CGAACAAACTCACCGGATCT	CCAATGGAGTCCCAAAAAGA	60.1	724	INTERGENIC	--	--	--
Ca1_P1_38194482	Ca1	38194482	GTAATTAATTAATTAATTAATTA/GTAATTAATTAATTAATTA	GCACCTAGTGGAGCACATGA	CTGCGAGAATTAGGGCTGTC	59.9	285	INTRON	Ca_13974	L	C2H2
Ca1_P1_38197697	Ca1	38197697	TAAAAAAA/TAAAAAA	TTAGCGACCAATATACCGGC	AACCAAATGTTTCGGGATCA	60.0	289	DRR	Ca_13974	L	C2H2
Ca1_P1_38217037	Ca1	38217037	TAAAAAAA/TAAAAAAAA	CCGTGGAACAGGTGAAAACT	TTTTGACGTGTTGGGTTTGA	60.0	181	INTERGENIC	--	--	--
Ca1_P1_38225545	Ca1	38225545	ATTTTTTT/ATTTTTTTT	CACCTCCCCTTTCAGTTTCA	GAATTGCTTTTGGGTTCGTG	60.1	951	INTERGENIC	--	--	--
Ca1_P1_38225631	Ca1	38225631	GAACCGTATAAGGTAA/GAA	TGGTTTGGATAAGCATTTCAC	GAATTGCTTTTGGGTTCGTG	57.6	595	INTERGENIC	--	--	--
Ca1_P1_38229860	Ca1	38229860	TC/T	TGGCAATCACCATCTCACAT	ATTGCCTCAAGGGTCCTTCT	59.9	716	INTERGENIC	--	--	--

INDEL marker IDs	Chromosomes/ unanchored scaffolds	Physical positions (bp)	InDels (*Kabuli* reference genome-CDC Frontier/PI)	Forward primers (5'-3')	Reverse primers (5'-3')	Anneling temperature (0C)	Expected amplified product size (bp)	Structural annotation		Functional annotation	
								**Sequence components of *kabuli* genome**	***Kabuli* gene accession IDs**	**NCBI-KOG**	**TFs**
Ca1_P1_38241608	Ca1	38241608	TGTCACTA/T	GTTGGGAGACTTGTGCCCTA	ACCGAGTGTCGAACTCAAGG	60.1	763	INTERGENIC	--	--	--
Ca1_P1_38244609	Ca1	38244609	GAAAAAAAAT/GAAAAAAAATTTGAAAAAAAAAT	GCGGAAATATTTTTGCAGGA	AACCAATTGCATCACCCTTC	60.0	523	INTERGENIC	--	--	--
Ca1_P1_38247533	Ca1	38247533	CAAAAAAAAAAAA/CAAAAAAAAAA	AAGGGCCAAGTGAAGAGGAT	TTGCATACGTCTTGATCCCA	60.1	802	INTERGENIC	--	--	--
Ca1_P1_38262001	Ca1	38262001	GTTTTTTTTTTT/GTTTTTTTTTTTTTT	TTAAATGTAAGGGTGGGCACT	TAACCCACGTGTTACGCAGA	58.5	677	INTERGENIC	--	--	--
Ca1_P1_38267264	Ca1	38267264	CTTTTTTT/CTTTTTTTT	GATTAGTCCGCCAAAAACCA	TGTAAACTTGCAACCACGCT	59.9	369	INTERGENIC	--	--	--
Ca1_P1_38273940	Ca1	38273940	CAAAAAAAAAAA/CAAAAAAAAAA	TATTTGGGGACCAAAACTGC	CATGGTTTGAATGCGAGTTG	59.8	637	INTERGENIC	--	--	--
Ca1_P1_38284193	Ca1	38284193	TC/T	GCCAACACATGGTGAAAGTG	AACAAAGTTTGCATTTGGGC	60.0	939	INTERGENIC	--	--	--
Ca1_P1_38287252	Ca1	38287252	TACA/TACACA	ATTTGGAGGTCACGTCCTTG	CTAAGGCACAACGGGGATAA	60.0	529	INTERGENIC	--	--	--
Ca1_P1_38303301	Ca1	38303301	TTATATA/TTATA	GGTCGTGCTGCAAGGATTAT	GGAATTGAGTGGGTGATTGTT	60.1	404	INTERGENIC	--	--	--
Ca1_P1_38303361	Ca1	38303361	TTTATT/TTT	GGTCGTGCTGCAAGGATTAT	GGAATTGAGTGGGTGATTGTT	60.1	404	INTERGENIC	--	--	--
Ca1_P1_38303577	Ca1	38303577	TT/TTGT	GGTCGTGCTGCAAGGATTAT	GTGTGGTACTCATGCGATGC	60.1	873	INTERGENIC	--	--	--
Ca1_P1_38303616	Ca1	38303616	ATTTTTTTTTT/ATTTTTTTTTTTTT	GGTCGTGCTGCAAGGATTAT	GTGTGGTACTCATGCGATGC	60.1	873	INTERGENIC	--	--	--

INDEL marker IDs	Chromosomes/ unanchored scaffolds	Physical positions (bp)	InDels (*Kabuli* reference genome-CDC Frontier/PI)	Forward primers (5'-3')	Reverse primers (5'-3')	Anneling temperature (0C)	Expected amplified product size (bp)	Structural annotation		Functional annotation	
								Sequence components of *kabuli* genome	*Kabuli* gene accession IDs	NCBI-KOG	TFs
Ca1_P1_38319158	Ca1	38319158	CTTTT/CTTT	TGTGGCGCTTTCATTAAAAA	GAAGATGATGATCAATTTTTGGG	59.3	395	INTERGENIC	--	--	--
Ca1_P1_38319972	Ca1	38319972	TAAAAAAAAA/TAAAAAAAAAAA	CATTTCCATCAAGAACAACATCA	TTCACCGTTCATACATTTGTCA	59.9	676	INTERGENIC	--	--	--
Ca1_P1_38335041	Ca1	38335041	GC/G	TCCACACCAATGGATGCTAA	TCGCGAACTACGTGACAATC	59.9	459	INTERGENIC	--	--	--
Ca1_P1_38340566	Ca1	38340566	AT/A	ATTTTAATGAATGCCGACGC	TTCAAATCCTCGGCCATAAG	59.9	913	INTERGENIC	--	--	--
Ca1_P1_38345058	Ca1	38345058	ATTTTTTTTTTT/ATTTTTTTTT	TTTGAGAAACATTGTGCATGG	TGGGGTGTTGGATTGTTTCT	59.6	866	DRR	Ca_13966	--	--
Ca1_P1_38347901	Ca1	38347901	AAT/A	TCAGCCGTTGATTAAGTGATT	TGCATCAACCTGGTGATTTT	57.4	274	INTERGENIC	--	--	--
Ca1_P1_38429887	Ca1	38429887	CAAAAAAAAAAAA/CAAAAAAAAAAAAAA	TTGTTGGGATTTTTCTTTTTGTT	GAAGCGGTTGTGTGCAGTTA	59.0	911	INTERGENIC	--	--	--
Ca1_P1_38450592	Ca1	38450592	ATGTGTGTGTGTGTGTG/ATGTGTGTGTGTGTG	TGCTTGACAATCTCCTGCAC	TTATCTTCGATCCCACCAGG	60.0	454	INTERGENIC	--	--	--
Ca1_P1_38476810	Ca1	38476810	TTAT/TTATAT	TTGCAAGTACGTTCTCCAAAA	GTGTTACACGCCCTTCATCC	58.5	280	INTERGENIC	--	--	--
Ca1_P1_38479463	Ca1	38479463	AATATATATATATATATATATATATATATA/AAAAAAATATATATATATATATATATATATATATATA	AGCGTGAATGTGACGTCTTG	TTAAAGAGATTCGCAGCGGT	59.9	100	INTERGENIC	--	--	--
Ca1_P1_38487463	Ca1	38487463	ATTTTTTTTTT/ATTTTTTTTT	TGCTACCTGCAACCAGTGAG	GTCAACTTTAAGGGGGCGTC	60.0	872	DRR	Ca_13962	--	--
Ca1_P1_3849371	Ca1	3849371	AATATATATATATATATATATATA/AATATATATATATATATATATATATA	CATGGCATCCATAGAAGGAGA	GGGCTTGAGCGTATAAACAAA	60.0	669	INTERGENIC	--	--	--

INDEL marker IDs	Chromosomes/ unanchored scaffolds	Physical positions (bp)	InDels (*Kabuli* reference genome-CDC Frontier/PI)	Forward primers (5'-3')	Reverse primers (5'-3')	Anneling temperature (0C)	Expected amplified product size (bp)	Structural annotation		Functional annotation	
								Sequence components of *kabuli* genome	*Kabuli* gene accession IDs	NCBI-KOG	TFs
Ca1_P1_38495552	Ca1	38495552	AATTATTATTATTATTATTATTATTATTATTATTATTATTGTTATTATTATTATTATTA/AATTATTATTATTATTATTATTATTATTATTATTATTATTATTGTTATTATTATTATTA	AACACCTTCCTGATCCTCCC	TGGCGAACTTTTAAGAATGGA	60.3	659	INTERGENIC	--	--	--
Ca1_P1_38503999	Ca1	38503999	A/AAT	TCACAATTCTCAAATGCGGA	AGTTCAAGTTCGAAATGCCG	60.2	350	INTERGENIC	--	--	--
Ca1_P1_38527402	Ca1	38527402	ATTTTTTTTTTT/ATTTTTTTTT	GCATACCACTGCTTGGGTTT	TTAAGCTGAGAGCCTTCCCA	60.0	551	INTERGENIC	--	--	--
Ca1_P1_38528019	Ca1	38528019	ATTTTTTTTTT/ATTTTTTTTTTTT	TGGGGATCCTCACCTACAAG	ATTTGACTCGTGTGCGACTG	59.9	796	INTERGENIC	--	--	--
Ca1_P1_38537861	Ca1	38537861	TAAATCGATTATGAAATCGATT/TAAATCGATTATGAAATCGATTATGAAATCGATT	AAACAAAGCTGACCCAATCG	CCTGTGGCCAGATAGGAAAA	60.1	357	INTERGENIC	--	--	--
Ca1_P1_38559279	Ca1	38559279	CGTT/CGTTGTT	CCTAACCTGTGTCCTTCCCA	GCATCGCTGGGTAATTTGTT	60.0	826	DRR	Ca_13957	--	--
Ca1_P1_38560129	Ca1	38560129	CT/CTT	CTCCTCTCCTTTTGGTTCCC	CAAAAGACTTCCCGATTCCA	60.0	729	DRR	Ca_13957	--	--
Ca1_P1_38562322	Ca1	38562322	AT/ATATTTT	GTCCATGGTTTGGGTGTTTC	AAGTTAAAGGACCACATGAGCAA	60.1	458	INTERGENIC	--	--	--
Ca1_P1_38562406	Ca1	38562406	TG/TGG	GTCCATGGTTTGGGTGTTTC	AAGTTAAAGGACCACATGAGCAA	60.1	458	INTERGENIC	--	--	--
Ca1_P1_38565927	Ca1	38565927	TAAAAA/TAAAA	TGGACGATGAATTTGAAGCA	TTTCCTACCAATTTTTGCCG	60.2	494	INTERGENIC	--	--	--
Ca1_P1_38567121	Ca1	38567121	A/ATT	TATGTCCTCCCACAAGCTCC	CCCTTCTCCATTTTCCATGA	60.1	773	INTERGENIC	--	--	--
Ca1_P1_38567687	Ca1	38567687	T/TTA	AGGCGAACTTACAAGGACGA	TGTTCGAGTTCAATCTAAACGG	59.9	409	INTERGENIC	--	--	--

INDEL marker IDs	Chromosomes/ unanchored scaffolds	Physical positions (bp)	InDels (*Kabuli* reference genome-CDC Frontier/PI)	Forward primers (5'-3')	Reverse primers (5'-3')	Anneling temperature (0C)	Expected amplified product size (bp)	Structural annotation		Functional annotation	
								Sequence components of *kabuli* genome	*Kabuli* gene accession IDs	NCBI-KOG	TFs
Ca1_P1_38567886	Ca1	38567886	A/ACAC	AGGCGAACTTACAAGGACGA	GCAACGGTCATTTCAAGGTT	59.9	612	INTERGENIC	--	--	--
Ca1_P1_38576779	Ca1	38576779	TAT/TATTTTGGATGACAT	ATGGTGCGAATGGTAAAAGC	TGAAAGGGGACAGAAAATGG	60.0	447	INTERGENIC	--	--	--
Ca1_P1_38577185	Ca1	38577185	GTTTTT/GTTTT	CCATTTTCTGTCCCCTTTCA	GCATTGGCTAAGCATGGATT	59.9	760	INTERGENIC	--	--	--
Ca1_P1_38577834	Ca1	38577834	T/TA	CCAATCCATGCTTAGCCAAT	GGTGGCGCATAAGATTGTTT	59.9	315	INTERGENIC	--	--	--
Ca1_P1_38584480	Ca1	38584480	AAAATC/AAAATCGAAATC	GAAAATCAACCAATCCCGAA	CTCAAACGATCAAACGAGCA	59.7	888	INTERGENIC	--	--	--
Ca1_P1_38597585	Ca1	38597585	GAT/GATAT	ACCGTATCCCAATGCTTGTC	AACAACAAGGGAGGCAAATG	59.8	345	DRR	Ca_13956	--	--
Ca1_P1_38597715	Ca1	38597715	ATAATTTTAATTTTAATTT/ATAATTTTAATTT	CATTTGCCTCCCTTGTTGTT	TGTAGTTGCTGCAAACGAGG	60.0	551	DRR	Ca_13956	--	--
Ca1_P1_38597934	Ca1	38597934	CAAAAA/CAAAAAA	CATTTGCCTCCCTTGTTGTT	TGTAGTTGCTGCAAACGAGG	60.0	551	DRR	Ca_13956	--	--
Ca1_P1_38608326	Ca1	38608326	CAAA/CA	TATGCGTCCATCTTCAATCG	GGGGTGTTACAGTTGGAGGA	59.6	459	INTERGENIC	--	--	--
Ca1_P1_38623512	Ca1	38623512	ATTTTTT/ATTTT	AGCAACTGCTTCAACCCACT	CGTCACGGCTTCCATTTATT	59.9	858	INTERGENIC	--	--	--
Ca1_P1_38636029	Ca1	38636029	TAAAAAAAAA/TAAAAAAAAAAAAA	GCCACAAAATTGGTCCAAAA	CGTGAGGCTGTTGGGATATT	60.7	530	INTERGENIC	--	--	--
Ca1_P1_38641343	Ca1	38641343	GTTTTTTT/GTTTTTTTT	TTCTACCCTACGTTCACCGC	GGAAGTTGCCTTAGTGGCAG	60.1	342	INTERGENIC	--	--	--

INDEL marker IDs	Chromosomes/ unanchored scaffolds	Physical positions (bp)	InDels (*Kabuli* reference genome-CDC Frontier/PI)	Forward primers (5'-3')	Reverse primers (5'-3')	Anneling temperature (0C)	Expected amplified product size (bp)	Structural annotation		Functional annotation	
								Sequence components of *kabuli* genome	*Kabuli* gene accession IDs	NCBI-KOG	TFs
Ca1_P1_38657360	Ca1	38657360	TAAAAAAA/TAAAAAA	CCATCAGTGATAACTTGTCCCA	CACAGGACAAGTGCAATCTCA	59.9	451	INTERGENIC	--	--	--
Ca1_P1_38670857	Ca1	38670857	TG/TGG	TGTCTTGAGCATGCCTTGTC	TGCTATGCATGAGCTTGGAC	60.0	583	INTERGENIC	--	--	--
Ca1_P1_38684636	Ca1	38684636	GTTTTTTTTTTTT/GTTTTTTTTT	TTGGGTGCACAGTTTACAGC	CAAAACGCAACGCAATGTAA	59.8	772	INTERGENIC	--	--	--
Ca1_P1_38710610	Ca1	38710610	AAATAATAATA/AAATAATAATAATA	TGGAAAACTGTGTTTGGAAGAA	TTCCAACTCCCTTCCCTTTT	59.6	183	INTERGENIC	--	--	--
Ca1_P1_3872384	Ca1	3872384	TTATATATATATATATATATATATA/TTATATATATATATATATATATATA	GGGCAGAGCAAGAAAACAAG	CTGACCGTTTTTCGATTTCC	60.0	393	INTERGENIC	--	--	--
Ca1_P1_38746856	Ca1	38746856	CA/CAAA	CATCAATGCTTTACACGGGTT	TGTTCGAACGGTTAAAATGGA	59.9	124	INTERGENIC	--	--	--
Ca1_P1_38747434	Ca1	38747434	AAT/A	AGGGTAATTTCTCCACAACGA	AGGTCTGTCCAATCAGGTCG	58.6	481	INTERGENIC	--	--	--
Ca1_P1_38757375	Ca1	38757375	GA/GAA	TGTTGGATGTCCAAGTGGTG	TGGAATTCCCTCCTTCCTCT	60.4	622	INTERGENIC	--	--	--
Ca1_P1_38764037	Ca1	38764037	GTTTTTTTT/GTTTTTTTTT	TGATCAAATTCCCCCACACT	TGGAAGCATGTGGTAGTGCT	60.2	302	INTERGENIC	--	--	--
Ca1_P1_38766832	Ca1	38766832	ACT/ACTCT	GAGCTTCTTCAAGTCGGTGG	GTTTGGAATACCCTGAGCCA	60.0	222	INTERGENIC	--	--	--
Ca1_P1_38773402	Ca1	38773402	AT/A	GTTTTGGCGTTGGTTTCACT	TGGGTGCTTTCTATAATGTGC	60.0	977	INTERGENIC	--	--	--
Ca1_P1_38773629	Ca1	38773629	TAAAAAA/TAAAAAAA	TCCCTCCAATTTTTCCAATTT	TGGGTGCTTTCTATAATGTGC	59.6	569	INTERGENIC	--	--	--

INDEL marker IDs	Chromosomes/ unanchored scaffolds	Physical positions (bp)	InDels (*Kabuli* reference genome-CDC Frontier/PI)	Forward primers (5'-3')	Reverse primers (5'-3')	Anneling temperature (0C)	Expected amplified product size (bp)	Structural annotation		Functional annotation	
								Sequence components of *kabuli* genome	*Kabuli* gene accession IDs	NCBI-KOG	TFs
Ca1_P1_38773798	Ca1	38773798	ATTTTT/ATTTTTT	TCCCTCCAATTTTTCCAATTT	ATTCCAACAATGCCCCTTTT	59.6	874	INTERGENIC	--	--	--
Ca1_P1_38775530	Ca1	38775530	ACC/AC	AGGGTCAAATCAATTGCCTG	GATCTTTGCATATCCGAGGC	59.9	556	INTERGENIC	--	--	--
Ca1_P1_38779608	Ca1	38779608	CAAAAAAAA/CAAAAAAA	CCATGAGAGCTCTGTGCTTG	TATGCATGCGGACAAGAGTC	59.7	523	INTERGENIC	--	--	--
Ca1_P1_38790509	Ca1	38790509	AC/A	AGAGCTCTGAAGTCAAGCGG	GAAATCCTTGTTGCCTTGGA	59.9	163	DRR	Ca_13953	--	--
Ca1_P1_38798126	Ca1	38798126	CAAAA/CAAAAA	AGGACTGGACGTAGCCTTCA	GTGTCCCAGAGCTCGTTAGC	59.9	626	INTERGENIC	--	--	--
Ca1_P1_38844259	Ca1	38844259	TAAAAAAAAA/TAAAAAAAAAAA	TGAAAGTCACATTCCAGCTCA	CAAACCTCGTGCAAATGCTA	59.4	673	INTERGENIC	--	--	--
Ca1_P1_38844389	Ca1	38844389	CG/C	TGAAAGTCACATTCCAGCTCA	CAAACCTCGTGCAAATGCTA	59.4	673	INTERGENIC	--	--	--
Ca1_P1_38852972	Ca1	38852972	AATTAGGAT/AAT	TGGGTTTGTGTTGAGCCATA	TTTGAGTTCCTAAGGTCCCG	60.0	440	INTERGENIC	--	--	--
Ca1_P1_38853204	Ca1	38853204	CTT/CTTAATT	TGGGTTTGTGTTGAGCCATA	TTTGAGTTCCTAAGGTCCCG	60.0	440	INTERGENIC	--	--	--
Ca1_P1_38854619	Ca1	38854619	ATT/ATTT	TGTCCCCATTAAGATCCGAC	GATTCCTGTGCCCCATCTAA	59.8	703	INTERGENIC	--	--	--
Ca1_P1_38855131	Ca1	38855131	TGGGGG/TGGGGGG	TTAGATGGGGCACAGGAATC	TTCCCTTGTCATTGGTCCTC	59.9	526	INTERGENIC	--	--	--
Ca1_P1_38872590	Ca1	38872590	TTA/TTATA	TGTTGTTTCAATTGATTTTTGC	TTGTCTCTAAGGTGTTAGTTTTTGTGA	57.7	209	INTERGENIC	--	--	--

INDEL marker IDs	Chromosomes/ unanchored scaffolds	Physical positions (bp)	InDels (*Kabuli* reference genome-CDC Frontier/PI)	Forward primers (5'-3')	Reverse primers (5'-3')	Anneling temperature (0C)	Expected amplified product size (bp)	Structural annotation		Functional annotation	
								Sequence components of *kabuli* genome	*Kabuli* gene accession IDs	NCBI-KOG	TFs
Ca1_P1_38874049	Ca1	38874049	TAAAAAAA/TAAAAAAAAA	GTTTGAGTGCGACACCTCCT	TGCAACGGGTTGAAATCTAA	60.3	370	INTERGENIC	--	--	--
Ca1_P1_38882967	Ca1	38882967	GTTAGGA/GTTAGGACTTAGGA	ATTAGGAACCCTGGCATGTG	GGCAGCATTTGGTGAACTTT	59.8	464	DRR	Ca_13949	G	--
Ca1_P1_38886459	Ca1	38886459	AAAATAAA/AAAA	GCATGTTGTATGCTTTCTTCCA	ATTTATCCATCCATGTCGCC	60.1	863	INTERGENIC	--	--	--
Ca1_P1_38896842	Ca1	38896842	TAA/TAAA	AGTACCAAAGAGGCAATGCG	CCAAGAACTGCACGATCTCA	60.3	221	INTERGENIC	--	--	--
Ca1_P1_38899645	Ca1	38899645	CTTTTTT/CTTTTT	GGTAATGGCAAGAAACGAGC	GAAAAAGCGCTGTAAATGGC	59.7	881	INTERGENIC	--	--	--
Ca1_P1_38911281	Ca1	38911281	TAAA/TAAAA	AATGGGCGCTGTTAAATGTC	ACAAGCCTGAGCCTGACCTA	60.0	720	INTERGENIC	--	--	--
Ca1_P1_38918913	Ca1	38918913	GTATATATATATATATAT/GTATATATATATATATATATATAT	GTGTTGGCAGAACAAGCTGA	GGTAACCAATCCGAAGACGA	60.0	691	INTERGENIC	--	--	--
Ca1_P1_38923411	Ca1	38923411	TAT/TATCTTGAT	GCACATTCGAACCTTTCCAT	TTAGGGCACATGCATTTCAA	59.9	642	INTERGENIC	--	--	--
Ca1_P1_38931061	Ca1	38931061	CAAAAAAAAAA/CAAAAAAAAAAAA	GCATAAATCGTGCTCGACAA	CTCCATCCTCCCAATCTTCA	59.8	430	INTERGENIC	--	--	--
Ca1_P1_38931341	Ca1	38931341	TTGGGAGGATGG/TTGGGAGGATGGGAGGATGG	GCATAAATCGTGCTCGACAA	CAAATGAAGGGCAATAGCGT	59.8	516	INTERGENIC	--	--	--
Ca1_P1_38931461	Ca1	38931461	CTTTTTTTT/CTTTTTTTTTT	TGAAGATTGGGAGGATGGAG	TTCATGGATTGGTGAGGTGA	60.0	423	INTERGENIC	--	--	--
Ca1_P1_38933493	Ca1	38933493	ACCC/ACCCC	TGTCGCAACCTAACATTTCG	ATACGCATAATCGACTCCCG	59.7	167	INTERGENIC	--	--	--

INDEL marker IDs	Chromosomes/ unanchored scaffolds	Physical positions (bp)	InDels (*Kabuli* reference genome-CDC Frontier/PI)	Forward primers (5'-3')	Reverse primers (5'-3')	Anneling temperature (0C)	Expected amplified product size (bp)	Structural annotation		Functional annotation	
								Sequence components of *kabuli* genome	*Kabuli* gene accession IDs	NCBI-KOG	TFs
Ca1_P1_38943612	Ca1	38943612	ATTTAAA/ATTTAAATGTTCTCTTTAAA	GCTCTTGTGAGTTGGTGTTGC	TGGCCTCTTCGGTACTGATT	60.9	571	INTERGENIC	--	--	--
Ca1_P1_38951140	Ca1	38951140	CAA/CAAAA	AACTTTCCCAAGGCTCCAGT	GTTGTTGCTTCTGCTCCTCC	60.1	680	INTERGENIC	--	--	--
Ca1_P1_38952882	Ca1	38952882	TAAAAAAAAAAAAAAA/TAAAAAAAAAAA	TGATATCCACCTCCTCCTCC	TGAATCGCGAAAGTAGCCTT	58.9	564	INTERGENIC	--	--	--
Ca1_P1_38955335	Ca1	38955335	CA/C	TTTTTGGCTCATAGTCCCCA	GCCTCCAACACTAAAAAGCC	60.4	341	INTERGENIC	--	--	--
Ca1_P1_38955475	Ca1	38955475	AT/A	TTTTTGGCTCATAGTCCCCA	CTAGTTGGGCTTGAAGCAGC	60.4	656	INTERGENIC	--	--	--
Ca1_P1_38955594	Ca1	38955594	ATTTTTTTTT/ATTTTTTTTTTT	TTTTTGGCTCATAGTCCCCA	CTAGTTGGGCTTGAAGCAGC	60.4	656	INTERGENIC	--	--	--
Ca1_P1_39013076	Ca1	39013076	GAATAAATAAATA/GAATAAATAAATAAATA	TTGCTCCTACGTATCTCCCG	GGGCTTGGGTTTCAACAATA	60.2	893	INTERGENIC	--	--	--
Ca1_P1_39019011	Ca1	39019011	GTTTTTT/GTTTTTTT	CAACGTGTGTATCCAGGCAC	ATCAATGGAGGGAGGAGGAG	60.0	685	INTRON	Ca_13946	--	--
Ca1_P1_39019566	Ca1	39019566	ATA/ATAAATTA	CTCCTCCTCCCTCCATTGAT	ACGAACACCCTTCGTCATTC	60.4	684	INTRON	Ca_13946	--	--
Ca1_P1_39027902	Ca1	39027902	CA/C	CACCAATCTCATGGTGTTCG	GCATTTTCAGGAAAAGAAAAAGG	60.0	618	INTRON	Ca_13946	--	--
Ca1_P1_39028142	Ca1	39028142	AATATATATATAT/AATATATATAT	TCATGGAAGGAGCAATCACA	TAAGGTTTAGGATGCCCCAA	60.2	764	INTRON	Ca_13946	--	--
Ca1_P1_39028216	Ca1	39028216	TAAAAAAAAAA/TAAAAAAAAAAAA	TCATGGAAGGAGCAATCACA	TAAGGTTTAGGATGCCCCAA	60.2	764	INTRON	Ca_13946	--	--

INDEL marker IDs	Chromosomes/ unanchored scaffolds	Physical positions (bp)	InDels (*Kabuli* reference genome-CDC Frontier/PI)	Forward primers (5'-3')	Reverse primers (5'-3')	Anneling temperature (0C)	Expected amplified product size (bp)	Structural annotation		Functional annotation	
								Sequence components of *kabuli* genome	*Kabuli* gene accession IDs	NCBI-KOG	TFs
Ca1_P1_39038586	Ca1	39038586	GTATATATATATATATATATATATATATATATATATATA/GTATATATATATATATATATATATATATATATATATATATATATATATATA	CGATTTTGAATTTGGGTCTCA	TTAAATAGGCTTCCAGGCCA	59.9	708	INTRON	Ca_13946	--	--
Ca1_P1_39041778	Ca1	39041778	T/TG	GCACTTTTTGGACAAGCGAT	GCAATCTCCTCAACTCGGTC	60.3	879	INTRON	Ca_13946	--	--
Ca1_P1_39056293	Ca1	39056293	T/TCG	CCTTTTACCGCGCTTATTTG	AAACGCTGTAGAATCGAGCG	59.7	256	INTRON	Ca_13946	--	--
Ca1_P1_39058204	Ca1	39058204	AA/AATA	GAGGATCTTGCCACCCATTA	TGGTGCTTACAACCATGGAA	59.9	694	INTRON	Ca_13946	--	--
Ca1_P1_39059526	Ca1	39059526	CATATATATATATATATATATATATATATATATATATATATATA/CATATATATATATATATATATATATATATATATATATATATATATATATA	TGTGATTTCGCAAACGTAAGA	GGTACTTTTTCGGCAAGAATCA	59.4	565	INTRON	Ca_13946	--	--
Ca1_P1_39061533	Ca1	39061533	CTTTT/CTTT	CTAGAGCACCACCACTCCGT	TGAATCCAAATCTTCCTCAACC	60.3	615	INTRON	Ca_13946	--	--
Ca1_P1_39061987	Ca1	39061987	TTTGTT/TTT	AAAAGGTTGAGGAAGATTTGGA	CGTGTGACCACAAGAAGTGG	59.1	672	INTRON	Ca_13946	--	--
Ca1_P1_39062678	Ca1	39062678	CTTT/CTT	TTCTACTCCTCCTCCCTCCA	ATGCATATGGGACACGAACA	58.8	674	INTRON	Ca_13946	--	--
Ca1_P1_39073326	Ca1	39073326	GTT/GTTT	GAAAACATGCCCATGGATCT	GCATTAAGGTGCGATGAATG	59.8	578	DRR	Ca_13946	--	--
Ca1_P1_39073809	Ca1	39073809	A/AC	CATTCATCGCACCTTAATGC	TTGAAGAAGCATCCCTCTGA	59.1	309	DRR	Ca_13946	--	--
Ca1_P1_39074119	Ca1	39074119	ATTTTT/ATTTTTT	CATTCATCGCACCTTAATGC	TGGTTGAGAACCATTGACAAA	59.1	556	DRR	Ca_13946	--	--
Ca1_P1_39075214	Ca1	39075214	T/TA	TGCTCCTCTTTCCTGGATTG	TTCTGCACACGGGACACTTA	60.3	489	INTERGENIC	--	--	--

INDEL marker IDs	Chromosomes/ unanchored scaffolds	Physical positions (bp)	InDels (*Kabuli* reference genome-CDC Frontier/PI)	Forward primers (5'-3')	Reverse primers (5'-3')	Anneling temperature (0C)	Expected amplified product size (bp)	Structural annotation		Functional annotation	
								**Sequence components of *kabuli* genome**	***Kabuli* gene accession IDs**	**NCBI-KOG**	**TFs**
Ca1_P1_39075429	Ca1	39075429	TA/T	TGAATGTGTTCTTTGAGCCG	CCTTGCTTCAACTTCTTCAGG	59.8	541	INTERGENIC	--	--	--
Ca1_P1_39076657	Ca1	39076657	TGT/TGTTTTTATTAAAAATAGAGT	ATCATCACTTCTTTCGCGGT	TGTCGCATTTTTCCTTTCGT	59.7	427	INTERGENIC	--	--	--
Ca1_P1_39077224	Ca1	39077224	CTTTTTTTT/CTTTTTT	ACGAAAGGAAAAATGCGACA	TTTTGGTTGTTTTTGCATAAGG	60.6	634	INTERGENIC	--	--	--
Ca1_P1_39077300	Ca1	39077300	AA/AACA	CCATCTCTTGGATTCTTGACG	AGGTTTTGGTTGTTTTTGCAT	59.7	532	INTERGENIC	--	--	--
Ca1_P1_39082963	Ca1	39082963	CTA/CTATA	GCAAATGTGGCATTTTTGAA	CAAATGGCGAGGTTCGTAAT	59.5	501	INTERGENIC	--	--	--
Ca1_P1_39110990	Ca1	39110990	ATT/ATTT	TTTTATGGGTCTGTGTAATGCG	ACACGGGGCACTTATTTTGA	59.9	273	INTERGENIC	--	--	--
Ca1_P1_39135722	Ca1	39135722	ACAAAATAAATTTTTTCAAAATAAATTT/ACAAAATAAATTTTTTCAAAATAAATTTTTTCAAAATAAATTT	ACGCGTTCTTTCGACAAGTT	GCATATTGTGCATTGTGGGA	59.9	611	INTERGENIC	--	--	--
Ca1_P1_39144961	Ca1	39144961	CACGTAC/CAC	CATCCTTTGCAGGATTGGTT	TTGTGCTTTGGAAAATGCAG	59.9	494	INTRON	Ca_13942	--	ARF
Ca1_P1_39146284	Ca1	39146284	AATA/AA	GCGAGCATGAGAAGTTGTTG	AAAATAGATTCCGATGCCACA	59.6	101	DRR	Ca_13942	--	ARF
Ca1_P1_39146441	Ca1	39146441	AATATATAT/AATATAT	TGTTGTGGCATCGGAATCTA	TTTCCCTGATCATTTTAATTTTCC	60.1	646	DRR	Ca_13942	--	ARF
Ca1_P1_39147028	Ca1	39147028	AT/ATT	AAAATGATCAGGGAAAATCATGT	ACAACTTCTCATGCTCGCAA	58.7	339	DRR	Ca_13942	--	ARF
Ca1_P1_39166873	Ca1	39166873	GTTTTATTTTATTTTAT/GTTTTATTTTAT	ATAATTGCGTTAGCGATGCC	GGTGTCATCGACCACACATC	60.1	428	INTRON	Ca_13941	--	Trihelix

INDEL marker IDs	Chromosomes/ unanchored scaffolds	Physical positions (bp)	InDels (*Kabuli* reference genome-CDC Frontier/PI)	Forward primers (5'-3')	Reverse primers (5'-3')	Anneling temperature (0C)	Expected amplified product size (bp)	Structural annotation		Functional annotation	
								Sequence components of *kabuli* genome	*Kabuli* gene accession IDs	NCBI-KOG	TFs
Ca1_P1_39167382	Ca1	39167382	TCAACC/T	GTGTGGTCGATGACACCAAG	TCCCACCATTCAAAATTTCAT	60.0	580	INTRON	Ca_13941	--	Trihelix
Ca1_P1_39251292	Ca1	39251292	CAAA/CAAAA	GATCACCTTCACATCCCACC	TTGTCGGCAGGGGTAGTAAC	60.2	651	INTERGENIC	--	--	--
Ca1_P1_39254298	Ca1	39254298	AAATAATAAT/AAATAAT	TTCAATTTTTCCGCCCTATG	ATGGCTACACCAATGCATCC	59.9	589	INTERGENIC	--	--	--
Ca1_P1_39261977	Ca1	39261977	A/AT	GGTTTGCTGTCGAGCCTTAG	AAACACAAGTCCAATCAAAAGG	60.0	421	INTERGENIC	--	--	--
Ca1_P1_39265880	Ca1	39265880	AT/A	GCATGTTCCTTGGCCATTAT	GGCTAAGTTTTCTGCCTTGC	59.8	552	INTERGENIC	--	--	--
Ca1_P1_3929921	Ca1	3929921	AT/ATTATTATTATTATTATTTT	TTGTCTTTTGTGGTAGCCCC	GGATCATGTCCGAGCATTTT	60.0	412	DRR	Ca_00475	LK	Nin-like
Ca1_P1_39303293	Ca1	39303293	CTT/CT	TTCACCGATTTCTTTCCCAC	CACTTGAAAATGTGGATGGTTG	59.9	621	INTERGENIC	--	--	--
Ca1_P1_39384808	Ca1	39384808	T/TA	GGGCCAAGAAACAACAAAAA	TTCCACCAATCAATTAGGCA	60.0	105	INTERGENIC	--	--	--
Ca1_P1_39386660	Ca1	39386660	TAA/TAACAACTAAA	AACGGGGACATTAACCAAAA	GGACGCGTCTTTACTTTCCA	59.2	285	INTERGENIC	--	--	--
Ca1_P1_39390988	Ca1	39390988	TA/TAA	GCCAGCTCACGTCTAATGAA	GCCTAATTTTGCCACCGATA	59.0	566	INTERGENIC	--	--	--
Ca1_P1_39391672	Ca1	39391672	GTTTTTTT/GTTTTTTTT	AGCTACCCCATGGTAAGCCT	TCTTCTTAATCTTTTGGGTCCTTC	60.0	192	INTERGENIC	--	--	--
Ca1_P1_39392297	Ca1	39392297	CAAAA/CAAAAA	GAAGGACCCAAAAGATTAAGAAGA	CAATCAGCGCTGGAGATACA	59.2	916	INTERGENIC	--	--	--

INDEL marker IDs	Chromosomes/ unanchored scaffolds	Physical positions (bp)	InDels (*Kabuli* reference genome-CDC Frontier/PI)	Forward primers (5'-3')	Reverse primers (5'-3')	Anneling temperature (0C)	Expected amplified product size (bp)	Structural annotation		Functional annotation	
								Sequence components of *kabuli* genome	*Kabuli* gene accession IDs	NCBI-KOG	TFs
Ca1_P1_39399195	Ca1	39399195	CAT/CATAT	CATTCGCCCAAGAATCATCT	GGATCTTTTGGCAGGTTGAA	60.0	278	INTERGENIC	--	--	--
Ca1_P1_39406657	Ca1	39406657	CA/CAAA	ACCATGAAACCTTCGACCTG	AAAAAGGTGACGTGGCAGAC	60.0	634	INTERGENIC	--	--	--
Ca1_P1_39423396	Ca1	39423396	TAA/TA	GGAAAGTTGAGTTGGAAGATGTTT	CCCCTCTCTCCCAAATTCTC	59.9	632	INTERGENIC	--	--	--
Ca1_P1_39430506	Ca1	39430506	ATT/ATTTT	TGCATCTCCCTCAAGCTTTT	TGGAAAATTTTGGGGTTCAA	60.0	976	INTERGENIC	--	--	--
Ca1_P1_39432122	Ca1	39432122	CTTTTTTTT/CTTTTTTTTTTTTT	CAATTGCACAAATCATTGTTGA	CAGGTGCATTATTCGGTGTG	59.5	870	INTERGENIC	--	--	--
Ca1_P1_39432866	Ca1	39432866	TG/TGTAGCAGAAGCAG	CACACCGAATAATGCACCTG	GAGAAAATGGCAGGGTGGTA	60.0	556	INTERGENIC	--	--	--
Ca1_P1_39433392	Ca1	39433392	CTTTTTTT/CTTTTTT	TATCCATACCACCCTGCCAT	CTCAGGATCCTCAACCCAAA	60.0	378	INTRON	Ca_13931	Z	NAC
Ca1_P1_39433677	Ca1	39433677	A/ATATCATAG	AGGATCCTGAGATTGCATGG	CATGAACATCGGACACAACC	60.0	360	INTRON	Ca_13931	Z	NAC
Ca1_P1_39436808	Ca1	39436808	T/TG	TGGCAGGATTGTGGATAAGA	ACCAGGTTTCTGCATGTGTG	59.1	796	INTRON	Ca_13931	Z	NAC
Ca1_P1_39437989	Ca1	39437989	CAAA/CAA	AGCCGAAACTTTCTCGAACA	CCGTTGTGGCAGTGTGTATC	60.0	646	INTRON	Ca_13931	Z	NAC
Ca1_P1_39441175	Ca1	39441175	T/TG	TTGAAGAAGCTAAGGGGCAA	CTGCTGTTTGCATGGATTGT	60.0	445	INTRON	Ca_13931	Z	NAC
Ca1_P1_39444170	Ca1	39444170	TAAAA/TAA	AATGATGGAAAAGCCGAATG	GGAGGGACCTGAAGTCACAA	59.9	838	INTRON	Ca_13931	Z	NAC

INDEL marker IDs	Chromosomes/ unanchored scaffolds	Physical positions (bp)	InDels (*Kabuli* reference genome-CDC Frontier/PI)	Forward primers (5'-3')	Reverse primers (5'-3')	Anneling temperature (0C)	Expected amplified product size (bp)	Structural annotation		Functional annotation	
								Sequence components of *kabuli* genome	*Kabuli* gene accession IDs	NCBI-KOG	TFs
Ca1_P1_39447104	Ca1	39447104	TTAATG/TTAATGTTGATCTAATG	CAACTTTATTGGGGCGAAAA	CCCTCGATTAATTCTCTTGAGGT	59.9	253	DRR	Ca_13931	Z	NAC
Ca1_P1_39451528	Ca1	39451528	T/TA	GTTCGTACCCTTCTCACCCA	ACAGTGAGCACAACAGTGGC	60.0	718	INTRON	Ca_13930	--	--
Ca1_P1_39457632	Ca1	39457632	AAGTGA/AA	GGAAATCCAAGTTCAGCCAA	CGGCAACTTGTGGGTAGTCT	60.1	407	DRR	Ca_13930	--	--
Ca1_P1_39462665	Ca1	39462665	TGGG/TGG	AGCGGCGCTTTATACCTTTT	GGGCCGGGAATTAATTAGAA	60.2	642	INTERGENIC	--	--	--
Ca1_P1_39463854	Ca1	39463854	AAATTTAAT/AAAT	ATATTGAGAGGTTGCGTGGG	ACCATCTCGATGCAACACAA	60.0	556	INTERGENIC	--	--	--
Ca1_P1_39463879	Ca1	39463879	AG/AGG	ATATTGAGAGGTTGCGTGGG	ACCATCTCGATGCAACACAA	60.0	556	INTERGENIC	--	--	--
Ca1_P1_39463964	Ca1	39463964	TAAA/TAAAAA	ATATTGAGAGGTTGCGTGGG	ACCATCTCGATGCAACACAA	60.0	556	INTERGENIC	--	--	--
Ca1_P1_39464239	Ca1	39464239	T/TA	TTGTGTTGCATCGAGATGGT	CAAAGCCGGAAAAGTTTCAA	60.1	560	INTERGENIC	--	--	--
Ca1_P1_39464317	Ca1	39464317	ATAAGAGACTAAGAG/ATAAGAG	TTGTGTTGCATCGAGATGGT	CAAAGCCGGAAAAGTTTCAA	60.1	560	INTERGENIC	--	--	--
Ca1_P1_39464986	Ca1	39464986	ATT/ATTCACTT	ATTTGCTATGCTGGGGAGTG	TCTAAACGGCATCCAAGGAC	60.1	502	INTERGENIC	--	--	--
Ca1_P1_39466068	Ca1	39466068	TTATATATATAT/TTATATATATATAT	CCGCTGGATTTTCATGTTTT	TGAACCTTTAAGAGGACCCCA	59.9	370	INTERGENIC	--	--	--
Ca1_P1_39472871	Ca1	39472871	CAAAAAAAAAA/CAAAAAAAA	CATCAAACTCTAGACGGGGG	TTTTAGGCGAATATGGAGCAA	59.5	587	INTERGENIC	--	--	--

INDEL marker IDs	Chromosomes/ unanchored scaffolds	Physical positions (bp)	InDels (*Kabuli* reference genome-CDC Frontier/PI)	Forward primers (5'-3')	Reverse primers (5'-3')	Anneling temperature (0C)	Expected amplified product size (bp)	Structural annotation		Functional annotation	
								Sequence components of *kabuli* genome	*Kabuli* gene accession IDs	NCBI-KOG	TFs
Ca1_P1_39490521	Ca1	39490521	CACGAATACGAAT/CACGAAT	ACAGCAGGTTCCCAACATTC	AAAGTGGATGGAGTGATGCC	60.0	571	INTERGENIC	--	--	--
Ca1_P1_39492770	Ca1	39492770	A/AC	ACCGAGTGTCGAACTCAAGG	GCAGTGCTGAATTTTGGTGA	60.3	902	INTERGENIC	--	--	--
Ca1_P1_39508433	Ca1	39508433	GAA/GA	ACCTTTAGACTTGGGGCACA	TTTGGTTAATCCATTAGCTCTCA	59.6	566	INTERGENIC	--	--	--
Ca1_P1_39508687	Ca1	39508687	TAAAAAAAAAA/TAAAAAAAAA	AACGTGTCGTCTAAGGGAAAAA	TTGGTTGTCAGAGGTGTCCA	60.0	725	INTERGENIC	--	--	--
Ca1_P1_39541335	Ca1	39541335	AATATATATATATATATATATA/AATATATATATATATATATATA	TTCCTTTTCCTCCTGACCCT	CAAAAGATTCGGGCATTTGT	60.0	627	INTERGENIC	--	--	--
Ca1_P1_39559516	Ca1	39559516	GACACA/GACACACA	CCGGTAACGCAGGAAAAATA	TCCATTTCCCTTTTACCACG	60.0	611	INTERGENIC	--	--	--
Ca1_P1_39568953	Ca1	39568953	TATAATAATAATAAT/TATAAT	TGCCTAACTTTCCTCCCTCA	ATTGGAAAAAGGGTTCCTTG	59.8	479	INTERGENIC	--	--	--
Ca1_P1_39594942	Ca1	39594942	TAAAAAAA/TAAAAAA	TGAACCCAAAGGTCCATCAT	TTGGTGTTGATGGGATTTCA	60.2	660	INTERGENIC	--	--	--
Ca1_P1_39597878	Ca1	39597878	TAAAAAAAAA/TAAAAAAA	GGAAAGCTCGTGGTGTCATT	TTGGATTTCTACCACACTTGCTT	60.1	257	INTERGENIC	--	--	--
Ca1_P1_39598601	Ca1	39598601	TC/TCCC	TTTCGTTATTTAAGCTACAACGG	GCGAAGATGGTGCCAATAAT	57.7	930	INTERGENIC	--	--	--
Ca1_P1_39605532	Ca1	39605532	ATTTTTTTTT/ATTTTTTT	AACACATGGGTTGAGTAAGTGA	CACCGAAAACCGAGATCATT	57.1	300	INTERGENIC	--	--	--
Ca1_P1_39605985	Ca1	39605985	CTTTT/CTTTTT	TTGGCCAAATTTTCACAACA	TTGGTACTATGCACGGCTTG	59.9	439	INTERGENIC	--	--	--

INDEL marker IDs	Chromosomes/ unanchored scaffolds	Physical positions (bp)	InDels (*Kabuli* reference genome-CDC Frontier/PI)	Forward primers (5'-3')	Reverse primers (5'-3')	Anneling temperature (0C)	Expected amplified product size (bp)	Structural annotation		Functional annotation	
								Sequence components of *kabuli* genome	*Kabuli* gene accession IDs	NCBI-KOG	TFs
Ca1_P1_39606006	Ca1	39606006	CTT/CT	TTGGCCAAATTTTCACAACA	TTGGTACTATGCACGGCTTG	59.9	439	INTERGENIC	--	--	--
Ca1_P1_39609113	Ca1	39609113	AATAT/AAT	ATGGCCTTTCATTTTCAACG	TTGTCTCAAGCGTTCAATTTTT	59.9	725	INTERGENIC	--	--	--
Ca1_P1_39611784	Ca1	39611784	CTTTTT/CTTTTTT	GGTGAAGCCACATCTTCCAT	TGAGGGATTTGAAATTGGGA	59.9	216	INTERGENIC	--	--	--
Ca1_P1_39615435	Ca1	39615435	GAAAAAAAA/GAAAAAAAAA	GGAGTGGACCGTTAGGACAA	GCTGGAACCCAGTCAACAAT	60.0	435	INTRON	Ca_13924	F	YABBY
Ca1_P1_39619759	Ca1	39619759	TAA/TAAA	CATATTCACGTTGTGTGCCC	GGCCAGTCAGCCCCTATTAT	59.8	405	DRR	Ca_13924	F	YABBY
Ca1_P1_39652228	Ca1	39652228	ACCC/ACCCC	GGAGTGACAATGCCCTCAAT	CGGGAGATACGTAGGAGCAA	59.9	774	INTERGENIC	--	--	--
Ca1_P1_39654601	Ca1	39654601	CT/CTGTT	CAGGGTTTGACAGTGAACAAAG	CCAAATTGCCTCAAACACCT	59.7	696	INTERGENIC	--	--	--
Ca1_P1_39657790	Ca1	39657790	GTTTTTTT/GTTTTTT	ATTACCTCTCGCGTGCAATC	GACAGAAAAGGGGGAAGAGG	60.2	390	INTERGENIC	--	--	--
Ca1_P1_39675431	Ca1	39675431	CATATATATATATATAT/CATATATATATATATATATATATATAT	CACCAAAGACAGGACCACCT	CCTAGGGTTTTTGGTTGGGT	60.0	784	DRR	Ca_13920	--	--
Ca1_P1_39680160	Ca1	39680160	ATTTTTTTTTT/ATTTTTTTTTTTT	CTCAGCAATCGGTCATGAAA	GCATGACTTAACTGCAAGCG	59.8	762	INTERGENIC	--	--	--
Ca1_P1_39680939	Ca1	39680939	GTT/GT	ATTCCGATGAGTGTACCGCT	GCTAGCTTCTCAATGGGTGC	59.6	716	INTERGENIC	--	--	--
Ca1_P1_39682708	Ca1	39682708	GCT/G	TCAATTGGCATTTTGCACAT	AAATTCAGCACTGCACCAAA	59.9	802	INTERGENIC	--	--	--

INDEL marker IDs	Chromosomes/ unanchored scaffolds	Physical positions (bp)	InDels (*Kabuli* reference genome-CDC Frontier/PI)	Forward primers (5'-3')	Reverse primers (5'-3')	Anneling temperature (0C)	Expected amplified product size (bp)	Structural annotation		Functional annotation	
								Sequence components of *kabuli* genome	*Kabuli* gene accession IDs	NCBI-KOG	TFs
Ca1_P1_39696784	Ca1	39696784	GATAATAATAATAATAAT/GATAATAATAATAATAATAAT	TTTTCTTAAAGATCAAACCACCC	TGTGGAAAACTGGCAACAAA	58.6	482	INTERGENIC	--	--	--
Ca1_P1_39718152	Ca1	39718152	GAAGAAAAAAAG/GAAGAAAAAAAAGAAAAAAAG	AGGATCAATTTTATGAGCCGA	TGCTGCTTCCATCCTTTTTC	58.7	748	INTERGENIC	--	--	--
Ca1_P1_39726359	Ca1	39726359	CATGAA/CA	TGTGGAAAGGTTTACAGAACCC	TAAAAGCCCAAACAAATGGG	60.3	537	INTRON	Ca_13917	C	--
Ca1_P1_39730486	Ca1	39730486	TA/T	TGGAAAACTGCGAAAGTGTG	CGCGCGGACCATAATCTAGT	59.9	242	INTERGENIC	--	--	--
Ca1_P1_39763220	Ca1	39763220	ATTTTTTTTTTTT/ATTTTTTTTT	GCATTGGATCCTTGTTGGTT	AGAGGCCAACCTCTGTCTCA	59.8	695	INTERGENIC	--	--	--
Ca1_P1_39766448	Ca1	39766448	AT/ATCTT	AAAGCCACTATCAGGGTGGA	TCCAAATCCATTTCCAATCC	59.6	701	INTERGENIC	--	--	--
Ca1_P1_39778752	Ca1	39778752	ATTTT/ATTTTT	TCGAATACACCTCTGCAACG	TGATCAAGTTGCAAGCTCAAA	59.9	622	INTERGENIC	--	--	--
Ca1_P1_39779833	Ca1	39779833	TAAA/TAAAA	GCCTATGGCCTGACCTAACA	TTCATTGCAACAACCTTCCA	60.1	850	INTERGENIC	--	--	--
Ca1_P1_39780204	Ca1	39780204	ATTTTTTTTTT/ATTTTTTTT	CATTGGTATTGGTATGTGGGG	GGGTGGGGATAAGTCAGGTT	59.8	286	INTERGENIC	--	--	--
Ca1_P1_39782012	Ca1	39782012	TA/T	TTTTCCTAGACCATCTGCGG	CGATCAAATATTTTTCACCGAA	60.2	678	INTERGENIC	--	--	--
Ca1_P1_39783200	Ca1	39783200	ATT/ATTGACCTT	TGGACGGATGGTTATTGTTG	GTCGTTGTCAGGTTGTGTGG	59.2	453	INTERGENIC	--	--	--
Ca1_P1_39793280	Ca1	39793280	CAAAAAA/CAAAAA	CCTGTCGCATCACTTCTCAA	TGGCTTGTTTTCAAATTCACC	60.0	535	INTERGENIC	--	--	--

INDEL marker IDs	Chromosomes/ unanchored scaffolds	Physical positions (bp)	InDels (*Kabuli* reference genome-CDC Frontier/PI)	Forward primers (5'-3')	Reverse primers (5'-3')	Anneling temperature (0C)	Expected amplified product size (bp)	Structural annotation		Functional annotation	
								Sequence components of *kabuli* genome	*Kabuli* gene accession IDs	NCBI-KOG	TFs
Ca1_P1_39816730	Ca1	39816730	ATTTTTTTT/ATTTTTTT	TTGTGTGAGTTCATGGTGGAA	CCAGTAATACTTTCGGTGGCA	60.0	920	INTERGENIC	--	--	--
Ca1_P1_39845179	Ca1	39845179	TATATAAAAATATAAAA/TATATAAAAATATAAAAATATAAAA	CACCTTTGGAGGACGATGTT	TTTCGATTTTCCGTTCCAAG	60.0	900	INTERGENIC	--	--	--
Ca1_P1_39847720	Ca1	39847720	CATATATAT/CATATATATAT	GCTGAGCACACCTACATTGG	TCCGCAACTGATTTATGCAA	59.3	556	INTERGENIC	--	--	--
Ca1_P1_39871055	Ca1	39871055	TAAA/TAAAA	CATTGGCCACCTTTAGGAAA	TGCATTGCTTCAGTCATGGT	59.9	329	INTERGENIC	--	--	--
Ca1_P1_39871464	Ca1	39871464	AATATATATAT/AATATATATATAT	AATTTTCAACCCCATTGTCG	GAGTTGAGGTATGACCATTGGA	59.7	982	INTERGENIC	--	--	--
Ca1_P1_39898174	Ca1	39898174	GAAAAA/GAAAA	GGAATCCCATCAAGCACAGT	GCGAAGTTGAGACACGTGAA	59.9	633	INTERGENIC	--	--	--
Ca1_P1_39904718	Ca1	39904718	TAA/TAACAA	AAAACAAAATCAGCCCTCCA	GTTAATGAAACAAGGCCGGA	59.5	367	INTERGENIC	--	--	--
Ca1_P1_39905247	Ca1	39905247	TTATATATATAT/TTATATATAT	TCCGGCCTTGTTTCATTAAC	CCGTTCATCCCACGTTATTT	59.9	751	INTERGENIC	--	--	--
Ca1_P1_39949927	Ca1	39949927	AACACA/AACA	TTCATTCTCGTGTTTGCACC	GGAAAGCCGTCACAAATGTT	59.7	421	INTERGENIC	--	--	--
Ca1_P1_39954761	Ca1	39954761	GAAAAAAAAAAA/GAAAAAAAAA	CTCCCCTCCATGAGGATACA	CCTTTTCCTCTTCATCCATTTG	59.9	649	INTERGENIC	--	--	--
Ca1_P1_39976327	Ca1	39976327	CA/CATA	TTTAAGAGAAGCGTTTGAGCG	CTTTGACTACGTTTCCCCGA	59.8	211	INTERGENIC	--	--	--
Ca1_P1_39990400	Ca1	39990400	TGAGAGAGAGAGAGAGAGAGAGAGAGAGAGAGAGA/TGAGAGAGAGAGAGAGAGAGAGAGA	TCAAACTCTCGACCACATGC	CCTTCTTCTCCATTGATCCG	59.8	575	INTERGENIC	--	--	--

INDEL marker IDs	Chromosomes/ unanchored scaffolds	Physical positions (bp)	InDels (*Kabuli* reference genome-CDC Frontier/PI)	Forward primers (5'-3')	Reverse primers (5'-3')	Anneling temperature (0C)	Expected amplified product size (bp)	Structural annotation		Functional annotation	
								Sequence components of *kabuli* genome	*Kabuli* gene accession IDs	NCBI-KOG	TFs
Ca1_P1_39990569	Ca1	39990569	TAA/TA	CGGATCAATGGAGAAG AAGG	ATACATCTCGCGCTCA CCTC	59.6	252	INTERGENIC	--	--	--
Ca1_P1_40004361	Ca1	40004361	GTTTTTTT/GTTTTTT	TGGTTGGGGTGTTCTA GGAG	AGGCCAAATCAATGGT CAAG	60.0	755	INTERGENIC	--	--	--
Ca1_P1_40010867	Ca1	40010867	CTTTTTTTTTTTT/CTTTTTTTTTT	CCAACAATCTCCCCAT TTGT	TGTCAACAACAGAGGA TGGG	59.6	591	INTERGENIC	--	--	--
Ca1_P1_40011794	Ca1	40011794	TC/T	TCCTCTTTTGGGTTTTT GGA	CACAGGGCTACAGTCA GCAA	59.5	673	INTERGENIC	--	--	--
Ca1_P1_40015677	Ca1	40015677	CTTTT/CTTTTT	CAACTTGGTCAAAGAT TTTCTTCA	CCAATAGTGGTTGCCT TCGT	59.7	573	INTERGENIC	--	--	--
Ca1_P1_40023860	Ca1	40023860	C/CA	AATCAAGGTCGGTCCA ACAC	TGATACCCACGAAACA ACAGA	59.8	537	INTERGENIC	--	--	--
Ca1_P1_40033572	Ca1	40033572	TCC/TC	TCCAAGAATATGCCGG ACTC	TTTTATCACAACGCTG CCAA	60.0	575	DRR	Ca_13904	OE	WRKY
Ca1_P1_40047203	Ca1	40047203	T/TTTG	AAACGAGCTATGTTCC CCTTT	GTCGTCTTGGTGCGG TATTT	59.1	682	INTERGENIC	--	--	--
Ca1_P1_40049510	Ca1	40049510	TTAAGTA/TTA	GCAAATTTGGATAGGG ACCA	ATGGCTTCTTGCGTTG ACTT	59.8	563	INTERGENIC	--	--	--
Ca1_P1_40052680	Ca1	40052680	TAAAAA/TAAAA	TTGCACCTACAAACCG TTGA	TACAAGCTTTGAATCG CGTG	60.1	610	INTERGENIC	--	--	--
Ca1_P1_40086233	Ca1	40086233	GAAAA/GAAA	ACAACTCCTCACACGG GAAA	TTGACTCAATCCACGA GCAG	60.5	407	INTERGENIC	--	--	--
Ca1_P1_40092028	Ca1	40092028	TAAGAAGA/TAAGA	TGTTTGTCGCAGTCTT TTTGA	CAACGCACACCACATA CACA	59.5	388	INTERGENIC	--	--	--

INDEL marker IDs	Chromosomes/ unanchored scaffolds	Physical positions (bp)	InDels (*Kabuli* reference genome-CDC Frontier/PI)	Forward primers (5'-3')	Reverse primers (5'-3')	Anneling temperature (0C)	Expected amplified product size (bp)	Structural annotation		Functional annotation	
								Sequence components of *kabuli* genome	*Kabuli* gene accession IDs	NCBI-KOG	TFs
Ca1_P1_40092197	Ca1	40092197	CAAAAA/CAAAAAAA	GGCAGCAAAACTAATCAGCA	CAACGCACACCACATACACA	59.1	221	INTERGENIC	--	--	--
Ca1_P1_4009762	Ca1	4009762	AT/ATT	CGCACACACTTTCTACGCAT	TTGATCTGGTCAAATAAGCCA	59.9	841	INTERGENIC	--	--	--
Ca1_P1_40100944	Ca1	40100944	A/AT	TCTCGCAGTGCAAGTCTTTG	TTTTGCATTCTGGCTCTCTG	60.3	303	INTERGENIC	--	--	--
Ca1_P1_40115020	Ca1	40115020	ATT/ATTT	TCCAACTCAAATTCAGGATAAGAA	GGCTAGATCCGACCAAAACA	59.2	778	INTERGENIC	--	--	--
Ca1_P1_40125641	Ca1	40125641	GTTTTTTTTTTT/GTTTTTTTTTTTTT	TTTTTGTGGATACCCAATGAG	TCAAACTTTTCCAACTCGTCG	57.5	896	INTERGENIC	--	--	--
Ca1_P1_40126750	Ca1	40126750	CATA/CA	AGGCGCCACAATCTAGAAGA	TTCTCCATCACAGCGTTCAG	60.0	544	INTERGENIC	--	--	--
Ca1_P1_40135781	Ca1	40135781	ATTTTT/ATTTT	GTTTTGGTCGTTCCCTTGAA	TCGTTAGAGGTTTATCTCCAGAGG	59.9	881	INTERGENIC	--	--	--
Ca1_P1_40138981	Ca1	40138981	TCC/TC	AAAGGGCTTGTAATCATGCG	GAGCTCACGAATGCACAAAA	60.1	125	DRR	Ca_13900	J	--
Ca1_P1_40166165	Ca1	40166165	TTCTCTCTCTCTCTCTCT/TTCTCTCTCTCTCTCTCTCT	CTCCAGGAGGATTGGTTCAA	TTTAGTGCGAGCCATTTGTG	60.0	660	INTERGENIC	--	--	--
Ca1_P1_40178554	Ca1	40178554	TAGAGAGA/TAGAGAGAGA	TTTGTGTACGGGAAAATTTGAA	ACCCCAAAACAAAAATGGTG	59.4	669	INTERGENIC	--	--	--
Ca1_P1_40195336	Ca1	40195336	TTGG/T	TGTTTTCTTGTGATTCGGTCA	TTCTGCATTTCTTTGCATCG	59.2	471	INTERGENIC	--	--	--
Ca1_P1_40196351	Ca1	40196351	CAAA/CAA	ACATGCCAGGTGACAAAACA	TGTTAACCGATTCGTCCAGA	60.0	912	INTERGENIC	--	--	--

INDEL marker IDs	Chromosomes/ unanchored scaffolds	Physical positions (bp)	InDels (*Kabuli* reference genome-CDC Frontier/PI)	Forward primers (5'-3')	Reverse primers (5'-3')	Anneling temperature (0C)	Expected amplified product size (bp)	Structural annotation		Functional annotation	
								Sequence components of *kabuli* genome	*Kabuli* gene accession IDs	NCBI-KOG	TFs
Ca1_P1_40216460	Ca1	40216460	GTT/GTTCTT	GTCTCTTCTGATTGGGGCAA	TGGGTGGAAAGCAATAGAGG	60.2	738	INTERGENIC	--	--	--
Ca1_P1_40219453	Ca1	40219453	TT/TTAAT	CCAATGGAGAAGGAAGTGGA	TTTTGCACACATAGCGGGTA	60.0	782	INTERGENIC	--	--	--
Ca1_P1_40224121	Ca1	40224121	ATTTTTTTT/ATTTTTTTTT	CATTTGCATCTGATTGGTGG	TCTTTGCCTCATAGGAGCGT	59.9	664	INTERGENIC	--	--	--
Ca1_P1_40225071	Ca1	40225071	ATTATTT/ATTATTTATTTATTT	GTTGGAACTGTCCATTGGCT	AAGTCCGTTTCAGGGTTGTG	60.0	355	INTERGENIC	--	--	--
Ca1_P1_40231594	Ca1	40231594	ATTTTTTTTTT/ATTTTTTTT	GCGGTTATTCACTTTTATGGG	CTCAAATCCAAACACCTTGG	58.5	743	INTERGENIC	--	--	--
Ca1_P1_40232307	Ca1	40232307	CAAAAA/CAAA	TCCAAGGTGTTTGGATTTGAG	CAGAAATTTGGTTGAGGGGA	60.0	717	INTERGENIC	--	--	--
Ca1_P1_40234096	Ca1	40234096	ATTTTTTTTTTTTTT/ATTTTTTTTTTTT	GAACGTGGAGGTGAGGAAAA	AACAAAATTCAACAACCAAAGAA	60.1	575	DRR	Ca_13896	--	--
Ca1_P1_40246846	Ca1	40246846	AGG/AG	TTTTCGAATCTTGGGAACCA	TCGCATGATGATCACTTTGAA	60.4	594	INTERGENIC	--	--	--
Ca1_P1_40247281	Ca1	40247281	TCTTTTCNNNNNNNNN/T	ATGCAACATGCACCATTACG	ATTTACACGAGGTTGCGGTC	60.4	422	INTERGENIC	--	--	--
Ca1_P1_40251561	Ca1	40251561	CAA/CAACAAA	GTTGCGCTCACTACGTGAAA	TGCATTTTACAGCGCTTTTG	60.1	340	INTERGENIC	--	--	--
Ca1_P1_40260077	Ca1	40260077	TC/TCC	TTTGGTTTTATGCATTGCGA	ACATCAATGGCCAACAATCC	60.1	525	INTERGENIC	--	--	--
Ca1_P1_4026071	Ca1	4026071	CAAAAAAAAAAAAA/CAAAAAAAAAAAAAA	GCATCATGTTTGGTTATGCG	CAAGGTCTCGAGCAATAGCC	60.0	692	INTERGENIC	--	--	--

INDEL marker IDs	Chromosomes/ unanchored scaffolds	Physical positions (bp)	InDels (*Kabuli* reference genome-CDC Frontier/PI)	Forward primers (5'-3')	Reverse primers (5'-3')	Anneling temperature (0C)	Expected amplified product size (bp)	Structural annotation		Functional annotation	
								Sequence components of *kabuli* genome	*Kabuli* gene accession IDs	NCBI-KOG	TFs
Ca1_P1_40266793	Ca1	40266793	AACT/AT	GCACCGGAAGATACGTAGGA	TCGATTATGCGTAGGGAAGG	60.1	243	INTERGENIC	--	--	--
Ca1_P1_40278889	Ca1	40278889	TAAAAAAT/TAAAAAATAAAAAAAAT	TTTGATCGGCCTCAAAAGAC	GGAGTGTTTACCGATGCGTT	60.2	601	INTERGENIC	--	--	--
Ca1_P1_40297702	Ca1	40297702	TAAAAAAAAA/TAAAAAAAAAAAA	TCCTCAGGTTTAGTTTCTTCAAA	ATTGCCACGTTTTGGACTTC	57.3	396	INTERGENIC	--	--	--
Ca1_P1_40298582	Ca1	40298582	TAAAAAAAAAAA/TAAAAAAAAA	TTTTAAAGATGCTGAAACAAACAAA	AGCCGTGTTGATTTTCGCTA	59.3	251	INTERGENIC	--	--	--
Ca1_P1_40303709	Ca1	40303709	TGG/TG	TGACCAATGCCTTGAGAACA	CAAGGAGAGGCGAGCTTAAA	60.2	602	INTERGENIC	--	--	--
Ca1_P1_40304519	Ca1	40304519	T/TCCAACGAAG	TGCAATTTTATCGAACACCG	CCGACACCTCTTAATGGGAA	59.6	527	INTERGENIC	--	--	--
Ca1_P1_40312634	Ca1	40312634	A/AC	TGAGGGTCCTTTGAAATTGG	AACCGCGTAAACTCAATTCG	59.9	560	INTERGENIC	--	--	--
Ca1_P1_40312693	Ca1	40312693	GAAAAAAAAA/GAAAAAAAAAAA	TGAGGGTCCTTTGAAATTGG	AACCGCGTAAACTCAATTCG	59.9	560	INTERGENIC	--	--	--
Ca1_P1_40329164	Ca1	40329164	AT/A	GCCGATTGTCTCAGGAAGAG	TACGCAAATCCCACACAAAA	60.0	419	INTERGENIC	--	--	--
Ca1_P1_40337281	Ca1	40337281	CACACATA/CA	AGCAACATTGGTTCCCTGAC	GGCAATCTTCCCGAGGTAAT	60.0	553	INTRON	Ca_13893	S	--
Ca1_P1_40338655	Ca1	40338655	CGG/CGGGG	CATGCCAACAAACTACCCCT	CGACACAACCACTTCCATTG	59.9	657	INTRON	Ca_13893	S	--
Ca1_P1_40357264	Ca1	40357264	ACTAACAACCTA/ACTA	ACGCAAGAGCAGACACACTC	CAGCTGAGGGGAGCATAAAG	59.2	663	INTRON	Ca_13893	S	--

INDEL marker IDs	Chromosomes/ unanchored scaffolds	Physical positions (bp)	InDels (*Kabuli* reference genome-CDC Frontier/PI)	Forward primers (5'-3')	Reverse primers (5'-3')	Anneling temperature (0C)	Expected amplified product size (bp)	Structural annotation		Functional annotation	
								Sequence components of *kabuli* genome	*Kabuli* gene accession IDs	NCBI-KOG	TFs
Ca1_P1_40363234	Ca1	40363234	TTAT/TTATAT	CATGTTTAAACGCCGATCCT	TTTGGTGGCATTTGTTGCTA	60.0	405	INTRON	Ca_13893	S	--
Ca1_P1_40366814	Ca1	40366814	ATTT/ATT	CAAAAGGGGAAAGCAAACAA	ATAGCATGGCGGATTTTCTG	60.1	461	INTRON	Ca_13893	S	--
Ca1_P1_40374065	Ca1	40374065	ATTTTTT/ATTTTTTT	TACCAAAACTCAGCATTGCG	CAGCTTGTCCGTCGGTAAAT	59.9	865	DRR	Ca_13892	--	Trihelix
Ca1_P1_40380273	Ca1	40380273	CTATA/CTATATATA	CCTGGTCCCACTTTTACGAA	GAAAAAGCGCTGTAAATGGC	60.0	487	INTRON	Ca_13892	--	Trihelix
Ca1_P1_40391433	Ca1	40391433	ACCCCC/ACCCCCC	TCCTGAAGGGGATTCATCAG	GTTAGGCGATCGCGAATACT	60.0	610	INTERGENIC	--	--	--
Ca1_P1_40392920	Ca1	40392920	AT/A	AATTGCTTCTTGCTTGCCTT	TTGACCAACTATCACCAAGCC	59.1	594	INTERGENIC	--	--	--
Ca1_P1_40398387	Ca1	40398387	CT/C	TGAAAAATACTTTTGGGCCG	AAAACGCCATGGATTGTCAT	59.9	858	INTERGENIC	--	--	--
Ca1_P1_40408351	Ca1	40408351	CAACGTATCAAA/CAA	TCACAAACAATTGCGAAAACA	CCACCTCCATGCCTCTAAAA	60.1	657	INTERGENIC	--	--	--
Ca1_P1_40408801	Ca1	40408801	ATT/ATTGAGCCAACTT	TTTTAGAGGCATGGAGGTGG	TTTTGCAAATGTTGGGTTCA	60.1	343	INTERGENIC	--	--	--
Ca1_P1_40421450	Ca1	40421450	ATTTTTT/ATTTTTTT	GGTGGTCTTGAGCGAGTAGC	CGGTCTTTGGCTTGGAAATA	60.0	583	DRR	Ca_13890	A	--
Ca1_P1_40423276	Ca1	40423276	GTTTTTTTTTTT/GTTTTTTTTT	TCGGTTTTGCTTCTTTTGAA	CCACCTCCATGCCTCTAAAA	58.5	528	INTERGENIC	--	--	--
Ca1_P1_40423335	Ca1	40423335	TA/TAA	TCGGTTTTGCTTCTTTTGAA	CCACCTCCATGCCTCTAAAA	58.5	528	INTERGENIC	--	--	--

INDEL marker IDs	Chromosomes/ unanchored scaffolds	Physical positions (bp)	InDels (*Kabuli* reference genome-CDC Frontier/PI)	Forward primers (5'-3')	Reverse primers (5'-3')	Anneling temperature (0C)	Expected amplified product size (bp)	Structural annotation		Functional annotation	
								Sequence components of *kabuli* genome	*Kabuli* gene accession IDs	NCBI-KOG	TFs
Ca1_P1_40423480	Ca1	40423480	ATTTT/A	TCGGTTTTGCTTCTTTTGAA	CCACCTCCATGCCTCTAAAA	58.5	528	INTERGENIC	--	--	--
Ca1_P1_40423667	Ca1	40423667	CAGAAAGTAAAGAAAGT/CAGAAAGT	CCAAACCATTGTTGCATTGT	TCCAAGAGGTCAAACGTCAA	59.3	600	INTERGENIC	--	--	--
Ca1_P1_40423792	Ca1	40423792	CAAAAAAA/CAAAAA	TTTTAGAGGCATGGAGGTGG	TCCAAGAGGTCAAACGTCAA	60.1	432	INTERGENIC	--	--	--
Ca1_P1_40424994	Ca1	40424994	C/CCAAAATATTATAA	TGCAAAACTTTGTTCATCGAG	GCATCATTCTCCTTCGAGACA	59.0	748	INTERGENIC	--	--	--
Ca1_P1_40425232	Ca1	40425232	AAA/AAATAA	CATTTTTAAGCTTTTGTTGCCA	GCATCATTCTCCTTCGAGACA	59.3	513	INTERGENIC	--	--	--
Ca1_P1_40425317	Ca1	40425317	GTCTC/GTCTCTC	CATTTTTAAGCTTTTGTTGCCA	TTTCATTAAGGTGAATCGTTCAGA	59.3	608	INTERGENIC	--	--	--
Ca1_P1_40426155	Ca1	40426155	ATTTTTTTT/ATTTTTTTTTT	CTTTACTGCGTTGGACCACA	ATAAATTGGATTTGCACCGC	59.8	544	INTERGENIC	--	--	--
Ca1_P1_40431867	Ca1	40431867	G/GGTCATAATCGCT	CTTGGGAGATGGCAAGAGAG	GACCTAGGGCAGCACAAGAC	59.9	540	INTERGENIC	--	--	--
Ca1_P1_40433642	Ca1	40433642	T/TTTTC	CGTGAGACCAACTAAACTGCC	CGGGGCGGTTTTCTATTTAT	59.8	766	INTERGENIC	--	--	--
Ca1_P1_40434120	Ca1	40434120	GAAAA/GAAAAA	TCCTTTCACATCCCACTTTCT	GCCCTTAGAGCCCTCTCACT	58.6	487	INTERGENIC	--	--	--
Ca1_P1_40450419	Ca1	40450419	ATATTATTATTATTATTATTATTATTATTATTATTATTATTATTATTATTAT/ATATTATTATTATTATTATTATTATTATTATTATTATTATTAT	TCTATGGTCAAACCAACCCC	AGAGCTTCCAACCCCACTTT	59.6	427	INTERGENIC	--	--	--
Ca1_P1_40534315	Ca1	40534315	GAAA/GAAAA	CAATTTTGACCATGGAACCC	TGAATTGACCTCGCAGATTG	60.0	730	INTERGENIC	--	--	--

INDEL marker IDs	Chromosomes/ unanchored scaffolds	Physical positions (bp)	InDels (*Kabuli* reference genome-CDC Frontier/PI)	Forward primers (5'-3')	Reverse primers (5'-3')	Anneling temperature (0C)	Expected amplified product size (bp)	Structural annotation		Functional annotation	
								Sequence components of *kabuli* genome	*Kabuli* gene accession IDs	NCBI-KOG	TFs
Ca1_P1_40535465	Ca1	40535465	TTAATAATAATAATAATAATA/TTAATAATAATAATAATAATAATA	CATGCGTTCCTTGTGGTAGA	ATCACAGTTTGGCCAAGTCC	59.7	599	INTERGENIC	--	--	--
Ca1_P1_40535882	Ca1	40535882	AATATATATATATATATATATAT/AATATATATATATATATATATATATAT	GATGATGATGATGTCGTGCC	ATCACAGTTTGGCCAAGTCC	59.9	311	INTERGENIC	--	--	--
Ca1_P1_4053855	Ca1	4053855	GTATATAT/GTATATATAT	CAAGCACGGTACCCCTGTAT	AGCCGCTGACTATGAAGCAT	59.9	260	INTERGENIC	--	--	--
Ca1_P1_40795125	Ca1	40795125	AATATATATATATATATATATATAT/AATATATATATATATATATAT	TGAAATCAATTGAGGACATTCG	ACGTAGCGAGCCTCGAATTA	59.9	547	INTERGENIC	--	--	--
Ca1_P1_40861492	Ca1	40861492	GTTTTTTTT/GTTTTTTTTTTTTT	ATTTCTAAACCCGCAAACCC	TCCACCCACGATACTGATGA	60.2	941	INTERGENIC	--	--	--
Ca1_P1_40872577	Ca1	40872577	GTTTTTTTTTTTT/GTTTTTTTTTTT	CGGATCTTTCAGTTGTCTTGG	ATGGGTGTTTGTGTATCGCA	59.7	381	DRR	Ca_18460	--	--
Ca1_P1_40889769	Ca1	40889769	TTATATA/TTA	GCATCTCTTGATGCCCTTTT	AACATACGCCATACCCAAGC	59.3	289	INTERGENIC	--	--	--
Ca1_P1_40890013	Ca1	40890013	ATT/ATTT	GCTTGGGTATGGCGTATGTT	TACGAACACTTGCAAATCCG	59.9	243	INTERGENIC	--	--	--
Ca1_P1_40890559	Ca1	40890559	ATTTTT/ATTTTTT	CGGATTTGCAAGTGTTCGTA	TGGGGTTTAGGATATCACATCA	59.7	707	INTERGENIC	--	--	--
Ca1_P1_40899161	Ca1	40899161	GTTTTTTTTTTTTT/GTTTTTTTTT	ATTTGGTTCCATTTGATCCG	TTATGTTAGGGCAGGGTTTGA	59.6	360	INTERGENIC	--	--	--
Ca1_P1_40966090	Ca1	40966090	A/AG	CTTTGGTTTCAAAGTTGCCC	CGTGTTTATACGCACACCCA	59.6	571	INTERGENIC	--	--	--
Ca1_P1_4105789	Ca1	4105789	A/AGCACT	AAAAGTTTGCTGAGGAGCCA	AAAGGGCAAGGATGTGTTTG	60.0	163	INTRON	Ca_00493	D	TCP

INDEL marker IDs	Chromosomes/ unanchored scaffolds	Physical positions (bp)	InDels (*Kabuli* reference genome-CDC Frontier/PI)	Forward primers (5'-3')	Reverse primers (5'-3')	Anneling temperature (0C)	Expected amplified product size (bp)	Structural annotation		Functional annotation	
								Sequence components of *kabuli* genome	*Kabuli* gene accession IDs	NCBI-KOG	TFs
Ca1_P1_41058981	Ca1	41058981	CTTTTTTTTTTT/CTTTTTTTTTT	GCATGGGAAATGGATTCTCT	TGGCTCATACACAGTTTTCTGG	58.9	679	INTERGENIC	--	--	--
Ca1_P1_41198339	Ca1	41198339	ATCTCTCTCTCTCTCTCTCTCT/ATCTCTCTCTCTCTCTCTCTCT	TCGTACGAGCTTCTTCTCTTCTC	ACCCCCAGTTTCCAATTTTC	59.4	436	INTERGENIC	--	--	--
Ca1_P1_41225712	Ca1	41225712	ACCC/ACC	GTGCAATTAGCAATGGGCTT	TTCCTTTGTGGCGGTTTTAC	60.1	658	INTERGENIC	--	--	--
Ca1_P1_41248476	Ca1	41248476	GAAAAAAA/GAAAA	CCAAAAAGAAGGACGACCAA	AAACAATTGGATAGCGGCAC	60.1	547	INTERGENIC	--	--	--
Ca1_P1_41304267	Ca1	41304267	CAAAA/CAA	ATAGGCCATGTTTGGCACTC	CTAATGGCTGCTGGGATCAT	60.0	472	INTRON	Ca_23105	O	B3
Ca1_P1_4131582	Ca1	4131582	AAAATAAATAAATAAATAAATAAATAAA/AAAATAAATAAATAAATAAATAAATAAATAAATAAATAAATAAA	TCCGAATAAATCGCATACCC	CCAGAGAGACAGATGCACCA	59.8	631	INTERGENIC	--	--	--
Ca1_P1_41493960	Ca1	41493960	TAAAAAAAA/TAAAAAAAAA	TCGGCATAAAAATGAAAACAA	GAAGATGCCTCTGGCCAATA	58.2	388	INTERGENIC	--	--	--
Ca1_P1_41503912	Ca1	41503912	TCC/TC	TCGATTATGCGTAGGGAAGG	CTTGGTGGTTTTGAAACTCAAT	60.1	540	INTERGENIC	--	--	--
Ca1_P1_41535010	Ca1	41535010	TCCCC/TCCC	GGAACTCCCGGATTAAATTG	GCGACCATCTGTTGCAGTTA	58.4	635	INTERGENIC	--	--	--
Ca1_P1_41549433	Ca1	41549433	CAGAGAGAGAGAGAGAGAGAGAGAGA/CAGAGAGAGAGAGAGAGAGAGA	TCAGCAACAACAACCGTAGC	AACACAAAGAAGGGCCAAGA	59.9	460	INTERGENIC	--	--	--
Ca1_P1_4157854	Ca1	4157854	TA/T	GTGGACACGCGTATGATTTG	TAACAGGCGATGCAATTGAG	60.0	307	INTERGENIC	--	--	--
Ca1_P1_4158639	Ca1	4158639	TAAAAAAAA/TAAAAAAA	GGTCCAAAACTCCAAATCCA	GAAAATGAACGTGAAGATGCTG	59.8	579	INTERGENIC	--	--	--

INDEL marker IDs	Chromosomes/ unanchored scaffolds	Physical positions (bp)	InDels (*Kabuli* reference genome-CDC Frontier/PI)	Forward primers (5'-3')	Reverse primers (5'-3')	Anneling temperature (0C)	Expected amplified product size (bp)	Structural annotation		Functional annotation	
								Sequence components of *kabuli* genome	*Kabuli* gene accession IDs	NCBI-KOG	TFs
Ca1_P1_41659303	Ca1	41659303	GATAATAATAATAATAATAATAATAATAATAATA/GATAATAATAATAATAATAATAATAATAATA	ATGCATAAGCAGCAGACACG	CAGAAGATTTTCTTCCTGCCA	60.0	773	INTERGENIC	--	--	--
Ca1_P1_41695402	Ca1	41695402	CTA/CTATA	TACTTCCATGCAAACCAGCA	CCAACTCTGAAATACAAAAGCCA	60.3	486	INTERGENIC	--	--	--
Ca1_P1_41696972	Ca1	41696972	TAAAAAAAAA/TAAAAAAAAAA	ATGCCACATGAGCAACTCTG	TCGTTCTCCTTCGTCGTCTT	59.9	524	INTERGENIC	--	--	--
Ca1_P1_41704419	Ca1	41704419	CATAATAAT/CATAAT	GGGGAATTTTTCATTTTGTGG	TGTTTTGGTTTCAAGGGACA	60.4	311	INTERGENIC	--	--	--
Ca1_P1_41704503	Ca1	41704503	CTTTTTTT/CTTTTTT	GGGGAATTTTTCATTTTGTGG	TGTTTTGGTTTCAAGGGACA	60.4	311	INTERGENIC	--	--	--
Ca1_P1_41705653	Ca1	41705653	ATTTTTTTT/ATTTTTTT	CATCACCGTAACATGACTTACCA	TCCCAATAATTTTTCCTTTTCG	59.8	723	INTERGENIC	--	--	--
Ca1_P1_41709578	Ca1	41709578	AGGGGG/AGGGG	TTGTGGAAGCAAGAGGTGAA	TCCCCTCATCATATTTTGCC	59.4	136	INTERGENIC	--	--	--
Ca1_P1_4171296	Ca1	4171296	TTTTA/TCATTTTTTAGCTTATTTA	CAACAAAACGCGCTAATGAC	CGAGATTTTGAGGCATGTTG	59.4	522	INTERGENIC	--	--	--
Ca1_P1_41718814	Ca1	41718814	T/TG	GGGTCGAATGTCGATTCTGT	TCATCTCTCAGCCCCACTCT	59.9	156	INTERGENIC	--	--	--
Ca1_P1_41722278	Ca1	41722278	TAA/TA	GGGAAAATCATGTTTATCACAATC	ACAACTTCTCATGTCCGCAA	58.7	756	INTERGENIC	--	--	--
Ca1_P1_41727767	Ca1	41727767	ACCCCC/ACCCC	TAACGTGGAAAACCCCAAAA	TGCTGACTAGAAGTGGGGCT	60.2	586	INTERGENIC	--	--	--
Ca1_P1_41817073	Ca1	41817073	AC/ACTC	AATCAGCTTATTGGGGCAAC	GGTACGAGGAAAGCATGCAA	59.0	212	INTERGENIC	--	--	--

INDEL marker IDs	Chromosomes/ unanchored scaffolds	Physical positions (bp)	InDels (*Kabuli* reference genome-CDC Frontier/PI)	Forward primers (5'-3')	Reverse primers (5'-3')	Anneling temperature (0C)	Expected amplified product size (bp)	Structural annotation		Functional annotation	
								Sequence components of *kabuli* genome	*Kabuli* gene accession IDs	NCBI-KOG	TFs
Ca1_P1_41875055	Ca1	41875055	TA/TAA	AGGGGCCAAAAACAAAGAGT	AAAACAAAATGCCAAATTGC	60.0	618	INTERGENIC	--	--	--
Ca1_P1_41935770	Ca1	41935770	AGGG/AGGGG	TCGATCAGATAAAGCAACCG	CAATGCTTCATACGTCCTGC	58.9	410	DRR	Ca_22531	--	--
Ca1_P1_41951410	Ca1	41951410	AATATATATATATATATATATATA/AATATATATATATATATATATA	GGTGCTTCTTTGGGATGTGT	TTGCAATCAAACTCAACAACG	60.0	934	INTERGENIC	--	--	--
Ca1_P1_4196063	Ca1	4196063	TCTACT/TCT	GCTGCAAAATGCAAAACTGA	TGGAACGAGGAATTGGAGTC	60.0	694	INTERGENIC	--	--	--
Ca1_P1_42079737	Ca1	42079737	AATATATATATATATATATATATA/AATATATATATATATATATATATATA	GTTGCAACGAACCAACCTTT	GCTACTTTGCAAGTGGATGAAG	60.0	730	INTERGENIC	--	--	--
Ca1_P1_4211915	Ca1	4211915	TTATATATATATATATATA/TTATATATATATATATATATATA	ATCTCTCACGCGACGAAGTT	CCAAATCCGTTCCGCTAGTA	60.0	276	INTERGENIC	--	--	--
Ca1_P1_42206965	Ca1	42206965	T/TCATGC	AGACTCCGATACCACACCAA	GGGAAAATCATGTTTATCACAATC	58.0	363	INTERGENIC	--	--	--
Ca1_P1_4233360	Ca1	4233360	TTTAAAATTAAAATTAAAATT/TTTAAAATTAAAATT	TAATGGGCTTCGTGGGTTAG	GTGCAGCACATTCGAGAGAA	60.0	683	DRR	Ca_00504	--	--
Ca1_P1_42334747	Ca1	42334747	AATATATATATATATAT/AATATATATATATATAT	ACACCTAATTCACCCCCTCC	TGTCACATCGCTTGCACATA	60.1	409	DRR	Ca_22670	--	--
Ca1_P1_42384198	Ca1	42384198	AATTATTATTATTATTATTATTATTATTATTATT/AATTATTATTATTATTATTATTATTATTATTATT	CAAGATCGATGTCGGTTTGA	ACTAGATGGTCGCATCCTGG	59.6	410	INTERGENIC	--	--	--
Ca1_P1_4238785	Ca1	4238785	ATTT/ATT	TGAAGATGGCTTTGCAGTTG	TGCCGATAAATGACCAAACA	60.0	681	INTERGENIC	--	--	--
Ca1_P1_4241058	Ca1	4241058	TC/T	TTTCCAACGCCTTTTCAAAC	ATTGGGACCCACTGAGATGT	60.1	957	INTERGENIC	--	--	--

INDEL marker IDs	Chromosomes/ unanchored scaffolds	Physical positions (bp)	InDels (*Kabuli* reference genome-CDC Frontier/PI)	Forward primers (5'-3')	Reverse primers (5'-3')	Anneling temperature (0C)	Expected amplified product size (bp)	Structural annotation		Functional annotation	
								Sequence components of *kabuli* genome	*Kabuli* gene accession IDs	NCBI-KOG	TFs
Ca1_P1_4242614	Ca1	4242614	CTATTATTATTATTATTATTATTATTATTATTATTATTATTATTAT/CTATTATTATTATTATTATTATTATTATTATTATTATTATTAT	TCTGGTCCAGCTTGTAACCC	ACACTGGGAAAGGAATGGAA	60.1	489	DRR	Ca_00505	--	--
Ca1_P1_42432936	Ca1	42432936	AATATATATATATATATATATAT/AATATATATATATATAT	CAAATTCCTCTTGCTCCACC	TGAAAGCTGATGAAAATGGTC	59.7	695	INTERGENIC	--	--	--
Ca1_P1_4249114	Ca1	4249114	T/TC	GATAACCATTCATGAGCCGC	TCATGTTTCAGCAGCTTTGG	60.4	870	INTRON	Ca_00506	R	bZIP
Ca1_P1_42493814	Ca1	42493814	TTTA/TTTAACATTA	AGCTGTTGGGGTTTTCCTTT	TCCTCCACTACTCTCGAGCAC	60.0	473	INTERGENIC	--	--	--
Ca1_P1_42572608	Ca1	42572608	AAGATTA/AA	TTCCTCGTATTTCCACCGTC	ACGATGATGACGATGACGAA	59.9	351	INTERGENIC	--	--	--
Ca1_P1_4261803	Ca1	4261803	GAAAAA/GAAAA	TTTGCGGTCATTTAAGATGATTT	TGGTTCCATGCACTTTGAAT	59.9	414	INTERGENIC	--	--	--
Ca1_P1_42642962	Ca1	42642962	TA/TAA	TGTGCATGCAATAAGTGGGT	AAAAACCCGGAATGGGTAAG	60.0	588	INTERGENIC	--	--	--
Ca1_P1_42767298	Ca1	42767298	AAAAAAATTTCAAAAAA/AAAAAAATTTCAAAAAATTTCAAAAAA	ACATGTTGGGGGAGTCAAAT	TACACAAAAGCCCCCAACTC	59.1	513	INTERGENIC	--	--	--
Ca1_P1_42810991	Ca1	42810991	AATATATATATATATATATATATA/AATATATATATATATATATATA	GTCCACATCATTCAAACCCC	AATGGGTCTTTCACGAATCAA	60.0	837	INTERGENIC	--	--	--
Ca1_P1_42822054	Ca1	42822054	AATATATATATATATATATATATAT/AATATATATATATATATATATATATATATAT	AAAGGCATGACCAACTCCAT	CACCTGATTCCTCGTTGGTT	59.4	671	DRR	Ca_13599	--	C2H2
Ca1_P1_4282317	Ca1	4282317	AATATATATATATATATATATATATA/AATATATATATATATATATATATATATA	TGCACCACAAAGTAGTTGCAG	GTATGGAAAGCGGCATTTGT	60.0	899	DRR	Ca_00509	J	--
Ca1_P1_42848958	Ca1	42848958	T/TACTGA	GGTTGAATGATTTTGGACGC	AAAAATCCCACCATTGGACA	60.3	215	INTERGENIC	--	--	--

**INDEL marker IDs**	**Chromosomes/ unanchored scaffolds**	**Physical positions (bp)**	**InDels (*Kabuli* reference genome-CDC Frontier/PI)**	**Forward primers (5'-3')**	**Reverse primers (5'-3')**	**Anneling temperature (0C)**	**Expected amplified product size (bp)**	**Structural annotation**		**Functional annotation**	
								**Sequence components of *kabuli* genome**	***Kabuli* gene accession IDs**	**NCBI-KOG**	**TFs**
Ca1_P1_4286158	Ca1	4286158	ATT/ATTCTT	GGTGCTGCTATTTGCACCTT	GCAATTTCAGGTATTGTTCATGC	60.3	980	INTERGENIC	--	--	--
Ca1_P1_43108269	Ca1	43108269	TTCTCTCTCTCTCTCTCTCTCTCTCTCTCTCTCTCTCTCTCTCTCTCTCT/TTCTCTCTCTCTCTCTCTCTCTCTCTCTCTCTCTCTCTCTCT	CTAGCAGCATGTCGTTGAGG	AAATCAACGCTCCAAAAACG	59.6	666	INTERGENIC	--	--	--
Ca1_P1_43119967	Ca1	43119967	AATATATATATATATATATATA/AATATATATATATATATA	TTTCCGGGAATAAATCCCTC	GAAGGAGAGTTCGCGTTGAC	60.1	361	INTERGENIC	--	--	--
Ca1_P1_4316126	Ca1	4316126	CTCTCTAT/CT	TCATGTCAAATGGGTCAAGC	ATGGGGCCAAATATGGAGTT	59.5	931	INTERGENIC	--	--	--
Ca1_P1_43188274	Ca1	43188274	AATATATATATATATAT/AATATATATATATAT	CACCTTTTAACAAATTGACCATTG	AGCAAGAACAGCCGCATAGT	59.7	916	INTERGENIC	--	--	--
Ca1_P1_43236855	Ca1	43236855	GAAAAAAAAAAA/GAAAAAAAAAAAAA	GGCTCAAATGGGCTTAAACA	TTGGAAAAGCCCACCTACAC	60.1	538	INTERGENIC	--	--	--
Ca1_P1_43259961	Ca1	43259961	CTATATATATATATATATATAT/CTATATATATATATATATAT	TCCCCCATCCTTTTAGTTCC	TCTTGTGATCGAATCCCTCC	60.1	768	INTERGENIC	--	--	--
Ca1_P1_43262365	Ca1	43262365	GAA/GA	CGTATCTCCTAGTGCGATGGA	AAAAGGGGTTGATTTCCTTCA	60.2	792	INTERGENIC	--	--	--
Ca1_P1_4327787	Ca1	4327787	TAGAAG/T	TTGTATAGACCAGCAGGCCC	TTTTCATACCCGTTCCAATGA	60.1	371	DRR	Ca_00516	KR	--
Ca1_P1_43319690	Ca1	43319690	A/AC	AACCACCTTGACCTGCAAAC	GGGAGCATGAGTGTTTTGGT	60.0	512	INTERGENIC	--	--	--
Ca1_P1_43335241	Ca1	43335241	AATATATATATATATATATATATATATATATATATAT/AATATATATATATATATATATATATATATAT	CGTGTATATATGCGCACCCA	CACAAACGGAAAAGTCGGAT	60.4	788	INTERGENIC	--	--	--
Ca1_P1_43438412	Ca1	43438412	CGTCGTGTCGTGT/CGTCGTGT	CATGAACGACGTTTGATTGG	TGGCCACCAAATTAATGACA	60.0	575	INTERGENIC	--	--	--

INDEL marker IDs	Chromosomes/ unanchored scaffolds	Physical positions (bp)	InDels (*Kabuli* reference genome-CDC Frontier/PI)	Forward primers (5'-3')	Reverse primers (5'-3')	Anneling temperature (0C)	Expected amplified product size (bp)	Structural annotation		Functional annotation	
								Sequence components of *kabuli* genome	*Kabuli* gene accession IDs	NCBI-KOG	TFs
Ca1_P1_43439444	Ca1	43439444	T/TATTTTA	CAAAATTGATTTTCGTTTTATTTCT	TCAATGATTGTTGACAAGGGA	57.2	293	INTERGENIC	--	--	--
Ca1_P1_43454769	Ca1	43454769	TGGG/TGG	TGAAGCCATTCTCCATTTCC	GCAGGGAAGTTGCACTTGA	60.0	295	CDS (FRAME SHIFT)	Ca_13574	QR	B3
Ca1_P1_43469250	Ca1	43469250	A/ATATATTATT	GAGGGTTTGCAGCTCTTGTC	AACGCATCGGTAAACACTCC	60.0	547	INTERGENIC	--	--	--
Ca1_P1_43534972	Ca1	43534972	CTTTTTTTTTT/CTTTTTTTTTTTT	GTTGTTTGCGTTTTCCCCTA	TGTGTTAAATCCACCGCAAA	60.0	460	INTERGENIC	--	--	--
Ca1_P1_43550360	Ca1	43550360	ATTT/ATT	TGCAGAGTAATTCGGTTTGTTTT	GGCATTAAGCCTCAAATCCA	60.1	458	INTERGENIC	--	--	--
Ca1_P1_43595794	Ca1	43595794	AAAGAAGAAGAAGAAGAAGAAGAAGAAGAA/AAAGAAGAAGAAGAAGAAGAAGAAGAAGAAGAA	GGAAGCCTGGAATGACACAT	TTCAATTCGGATTTTGACCC	59.9	555	INTERGENIC	--	--	--
Ca1_P1_43638401	Ca1	43638401	A/ATATGTC	CGCTTCATGTGTGTTGCTTT	ACCCCTCCTAAGTGGGTTGA	59.9	857	INTERGENIC	--	--	--
Ca1_P1_43649275	Ca1	43649275	GAAAAAAAAAAA/GAAAAAAAAAA	GCATGTGAAGTTCTATGGCTGA	AAAATTTTGTTTGGGGGACC	60.3	825	INTERGENIC	--	--	--
Ca1_P1_43690044	Ca1	43690044	AATATATATATATATATATATATAT/AATATATATATATATATATATAT	AGACATATGTTCGCTGGCCT	TGAGTTTGTTTGAGAGGATTTGTT	59.7	781	INTERGENIC	--	--	--
Ca1_P1_43700268	Ca1	43700268	CTT/CT	ATAGCTCGCCTCAACGTCAT	TAGCAGCAGCAGTTCACCAC	59.9	427	DRR	Ca_13557	R	--
Ca1_P1_43862504	Ca1	43862504	GTATAATA/GTA	CCTGGACAATTAAAACTCGCA	CGCAATTTTTGAGTTCGACA	60.1	500	INTERGENIC	--	--	--
Ca1_P1_43867975	Ca1	43867975	AATATATATATATATATATATATATATA/AATATATATATATATATATATATATATATATATATA	TTGCTGGCATAAGTGAATCG	CCGTGAATTTTCATGGAATAAA	59.8	711	INTERGENIC	--	--	--

INDEL marker IDs	Chromosomes/ unanchored scaffolds	Physical positions (bp)	InDels (*Kabuli* reference genome-CDC Frontier/PI)	Forward primers (5'-3')	Reverse primers (5'-3')	Anneling temperature (0C)	Expected amplified product size (bp)	**Structural annotation**		**Functional annotation**	
								**Sequence components of *kabuli* genome**	***Kabuli* gene accession IDs**	**NCBI-KOG**	**TFs**
Ca1_P1_43954938	Ca1	43954938	AATATATATATATATATATATATATATATATATATAT/AATATATATATATATATATATATATATATATAT	CAGGTGGCTGCACTCAAGTA	CCTTTCCAAGGTGCGTGTAT	60.0	326	INTRON	Ca_13537	--	NAC
Ca1_P1_44050778	Ca1	44050778	TAAAA/TAAAAA	AAGCAGGCAAACCAGAGAAA	CGCATCGGTAAACACTCTGA	60.0	467	INTERGENIC	--	--	--
Ca1_P1_44129266	Ca1	44129266	TTGTGT/TAATGTGT	GGTAAGGGCTCCTTCCAAAC	TCACGGGTCGACACAATTTA	59.9	460	INTERGENIC	--	--	--
Ca1_P1_44172001	Ca1	44172001	GTTTTT/GTTTTTT	ACCTATGCCCACTGATGAGG	GCAATTGCCAATGACGACTA	60.0	750	INTERGENIC	--	--	--
Ca1_P1_44187356	Ca1	44187356	CTCTTCTTC/CTCTTC	CCGACTTTTCCAATGCCTAA	TAAAGCAGATTGCCCAAACC	60.1	336	DRR	Ca_23493	--	--
Ca1_P1_44247311	Ca1	44247311	GTTTTTTTTTT/GTTTTTTTTT	TTCTCCAGACTCTAGCCCGA	AGCTTCTCAGTAGGGGGCTC	60.1	592	INTERGENIC	--	--	--
Ca1_P1_44351850	Ca1	44351850	TTATATATATATATATA/TTATATATATATATATATA	TGAAATTCGGAAGAACCAGG	CGCATTCCCTTCACCTCTAA	60.0	111	INTERGENIC	--	--	--
Ca1_P1_44365208	Ca1	44365208	A/ACGCC	AGTCAACCCAAGTGCAAAGG	TTGGTTTCATGACCTGCATC	60.1	642	INTERGENIC	--	--	--
Ca1_P1_44368738	Ca1	44368738	GATAATAATAATAATAATAATAATAATAATAATAATAATAATAATAATAA/GATAATAATAATAATAATAATAATAATAATAATAATAATAA	TTGTAACTCCCCGAATTGTTG	AGAGGCAAACAAGAACCGAA	59.8	534	INTERGENIC	--	--	--
Ca1_P1_44372843	Ca1	44372843	CNNNNNNNNNNNN/CNNNNNN	ACTGGCCGTGAAGGTAATTG	TGAACATGCACAAGCAACAA	60.0	764	INTERGENIC	--	--	--
Ca1_P1_44417905	Ca1	44417905	GAAAAAAAAAA/GAAAAAAAAAAA	TATCGGCCCCACAATTAAAA	TTAGATCTCGCACCAATCCC	60.1	179	INTERGENIC	--	--	--
Ca1_P1_4444544	Ca1	4444544	AATATATATATATATATATATA/AATATATATATATATATATA	AATCGATTGGCTGAAATCCA	GACATTTAACAGCGCCCATT	60.4	663	INTERGENIC	--	--	--

INDEL marker IDs	Chromosomes/ unanchored scaffolds	Physical positions (bp)	InDels (*Kabuli* reference genome-CDC Frontier/PI)	Forward primers (5'-3')	Reverse primers (5'-3')	Anneling temperature (0C)	Expected amplified product size (bp)	Structural annotation		Functional annotation	
								Sequence components of *kabuli* genome	*Kabuli* gene accession IDs	NCBI-KOG	TFs
Ca1_P1_44467119	Ca1	44467119	GTT/GTTT	ATGCAGCTCAAAAACAGCCT	GGTTCACCCAAAGATGGCTA	60.0	537	INTERGENIC	--	--	--
Ca1_P1_44650256	Ca1	44650256	T/TC	TCGTTTGCGTCATTGGATTA	AGGGAGGACGAAAATGACCT	60.1	444	INTERGENIC	--	--	--
Ca1_P1_44734189	Ca1	44734189	TC/T	TTTCACCATTGGTCTCCACA	GTGGCGAATCATCACAAATG	59.9	429	INTERGENIC	--	--	--
Ca1_P1_44745225	Ca1	44745225	A/AC	TCCGGATATAGTCCTGCCAC	TCCTTGTGAACGACTTGGTG	59.9	632	INTERGENIC	--	--	--
Ca1_P1_44776081	Ca1	44776081	AATATATATATATATATATATATATATATATATATATATATA/AATATATATATATATATATATATATATATATATATATATATATATATA	TGGAATACCATATATTTTACGTTTTCA	TGACGGATCCTTTCTTGACA	59.1	795	INTERGENIC	--	--	--
Ca1_P1_44847079	Ca1	44847079	ATTTTTTTTTTT/ATATTTTTTTTTTTTTT	GACATGCTCAGTAATTGCCC	AAAATCCAACAATCTCCCCC	58.2	373	INTERGENIC	--	--	--
Ca1_P1_44847252	Ca1	44847252	ATCTGATGTTCTGATG/ATCTGATGTTCTGATGTTCTGATG	TGTTTTCTGCCTTTTTGCCT	AAAATCCAACAATCTCCCCC	59.9	274	INTERGENIC	--	--	--
Ca1_P1_44847313	Ca1	44847313	TATAATAATAATAATAATAATA/TATAATAATAATAATAATA	TGTTTTCTGCCTTTTTGCCT	AAAATCCAACAATCTCCCCC	59.9	274	INTERGENIC	--	--	--
Ca1_P1_4485491	Ca1	4485491	CATATATATATATATATA/CATATATATATATATA	TTTGATGTTGTTGGAGATGAAA	TGCCATGCATCAGTTGTTTT	58.1	792	INTERGENIC	--	--	--
Ca1_P1_44921329	Ca1	44921329	A/ATGATGCTAATCTT	CCTCAAACGATTTTCTGGGA	CTTTCCCAATGCGTGAAGAT	60.0	478	INTERGENIC	--	--	--
Ca1_P1_45003074	Ca1	45003074	AA/AATTCCGAATCA	TCTTCCACATTGATTTTGTTGC	TGCTTATTCCCCAGATACCG	60.0	988	DRR	Ca_25714	--	--
Ca1_P1_45029559	Ca1	45029559	TTATATATATATATATATATATATATATAT/TTATATATATATATATATATATATATATAT	TTCTGTAGGACAAGGGGTGG	TTCGCTAAACCAAAAACGCT	60.0	180	DRR	Ca_25715	I	--

INDEL marker IDs	Chromosomes/ unanchored scaffolds	Physical positions (bp)	InDels (*Kabuli* reference genome-CDC Frontier/PI)	Forward primers (5'-3')	Reverse primers (5'-3')	Anneling temperature (0C)	Expected amplified product size (bp)	Structural annotation		Functional annotation	
								Sequence components of *kabuli* genome	*Kabuli* gene accession IDs	NCBI-KOG	TFs
Ca1_P1_45033030	Ca1	45033030	ATTTTTTTT/ATTTTTTTTTT	TTTCCCCATGCAGAGT TAGG	TAAACCAGCCACTAAG GCGT	60.1	894	INTERGENIC	--	--	--
Ca1_P1_45058076	Ca1	45058076	TAAAAAA/TAAAAAAA	AAACTAGATTATGGCC TGCG	TGCTCGCAATTAAAAC ATGC	57.0	426	INTERGENIC	--	--	--
Ca1_P1_45104438	Ca1	45104438	TTAATAATAATAATAATAATAATAATAA TAATAATAATAATAATAATAATAATAAT AATAATAATAATAA/TTAATAATAATAA TAATAATAATAATAATAATAATAATAAT AATAATAATAATAATAATAATAATAATA ATAA	TTTCAGGCGATTTTCG TACC	AAGTGGGGAACGTTG ACTTG	60.1	744	INTERGENIC	--	--	--
Ca1_P1_45124635	Ca1	45124635	GTATATATATATATATATATATATA/GT ATATATATATATATATATATATATATAT A	TCCGAGACACAAGACC AACA	TTACGAACACAATTGT TGCCA	60.3	836	INTERGENIC	--	--	--
Ca1_P1_45238172	Ca1	45238172	T/TGACA	ATTCTTTTTGGAGCGC AGAA	CCAATTAGGTTTACGG CGAA	60.0	401	INTERGENIC	--	--	--
Ca1_P1_45241605	Ca1	45241605	A/ACCC	GTTTTGGGAGTATCCC GGTT	ATACGAAAACGGAAAC CGAA	60.1	522	INTERGENIC	--	--	--
Ca1_P1_45243785	Ca1	45243785	TA/TAGACA	TGTGCCAAGGTTTCAG AACA	CTTTTATTGCAAGACC CCGA	60.3	605	DRR	Ca_26020	--	--
Ca1_P1_45244574	Ca1	45244574	TC/TCATAAAGCTCAC	TCGGGGTCTTGCAATA AAAG	GACAATGGGAATTCGC CTAA	60.1	532	CDS (FRAME SHIFT)	Ca_26020	--	--
Ca1_P1_45246034	Ca1	45246034	C/CGTCTTGTT	CGATTTCGCTTTTAGC ATCC	CAATTGCGATATTATG GGGG	59.8	253	INTERGENIC	--	--	--
Ca1_P1_45251376	Ca1	45251376	C/CCTATGA	CTAGCTCTGGGTTCGA GTGG	ATCCGACTAGTTCCGG GTTC	60.0	247	INTERGENIC	--	--	--
Ca1_P1_45255790	Ca1	45255790	CATA/CA	ATTTACAAATCTGCCAC CGC	CGTCAAAGACTTCACT GCCA	60.0	726	INTERGENIC	--	--	--
Ca1_P1_45332822	Ca1	45332822	TG/TGCTGATTG	GCTCAAAGCACATGAC AGGA	CAATGCGTTTATGCCA CAAC	60.0	248	INTERGENIC	--	--	--

INDEL marker IDs	Chromosomes/ unanchored scaffolds	Physical positions (bp)	InDels (*Kabuli* reference genome-CDC Frontier/PI)	Forward primers (5'-3')	Reverse primers (5'-3')	Anneling temperature (0C)	Expected amplified product size (bp)	Structural annotation		Functional annotation	
								Sequence components of *kabuli* genome	*Kabuli* gene accession IDs	NCBI-KOG	TFs
Ca1_P1_45339273	Ca1	45339273	TA/TAA	TGATTGGGAAATCGATTGGT	TTGATCCTTGCAGTCACTCG	60.1	566	DRR	Ca_24431	--	--
Ca1_P1_45385323	Ca1	45385323	AGTGG/AG	TCCAACAAATGAAGAGCCAA	TCACGAATGGTCCGTGAGTA	59.2	411	DRR	Ca_24432	--	--
Ca1_P1_45395070	Ca1	45395070	AT/A	CACGAACGACAGAAGAACCA	CCTCAGATGAGAGCCAGGAC	59.9	481	INTERGENIC	--	--	--
Ca1_P1_45398138	Ca1	45398138	CATATATATATATATATATATATATATATAT/CATATATATATATATATATATAT	GTTGGTTCCCTAGCACCGTA	TTAATGCGTTGTGGGTGAAA	60.0	524	INTERGENIC	--	--	--
Ca1_P1_45497100	Ca1	45497100	CTTTTTTTTTTT/CTTTTTTTTTTTTT	AGTATAAGTGGGGCCGTGTG	TGGATTGTGCCAACTTTTCA	59.9	237	INTERGENIC	--	--	--
Ca1_P1_45507115	Ca1	45507115	CATATATATATATATATATATATATATATATATATATATATATATAT/CATATATATATATATATATATATATATATATATATATATATATATATAT	CCATGGGTGTGACTAGTTTGAA	AATGTGATTTGGGATGTGCT	59.9	695	INTERGENIC	--	--	--
Ca1_P1_4551372	Ca1	4551372	GTGAATTT/GT	AACCACGGACTAATTGTCTCA	AGTGTTCAACGTGGCTGTTG	57.2	187	INTERGENIC	--	--	--
Ca1_P1_45553320	Ca1	45553320	GGAA/GGAACACGAA	TTCCTGGTGGGATTCACATT	ACCAAAGAACAAGTGGGTCG	60.2	440	INTERGENIC	--	--	--
Ca1_P1_45609121	Ca1	45609121	C/CT	TCGCGTGAGAAAATAATCCC	CGAACCCTCGGTAAACAAAA	60.0	295	INTERGENIC	--	--	--
Ca1_P1_45635524	Ca1	45635524	AATATATATATATATATATATATATATATAT/AATATATATATATATATATATATATATATATATAT	ATGTTTTGGATTTTGGCTCG	GGAGAAGGAGGGAGAAATGG	59.9	433	INTERGENIC	--	--	--
Ca1_P1_45663376	Ca1	45663376	TAATATATATA/TAATATATATAAATATATATA	CTGTTTGCCCAGTTTTTGGT	GTTGGTTCCCTAGCACCGTA	60.0	697	DRR	Ca_24443	--	--
Ca1_P1_45685135	Ca1	45685135	CTTTTTTTTTTTT/CTTTTTTTTTTT	CCGTGCAACGGAGTGTTAT	AAAATTGTTTATGTGGCATTGCT	59.6	536	INTERGENIC	--	--	--

INDEL marker IDs	Chromosomes/ unanchored scaffolds	Physical positions (bp)	InDels (*Kabuli* reference genome-CDC Frontier/PI)	Forward primers (5'-3')	Reverse primers (5'-3')	Anneling temperature (0C)	Expected amplified product size (bp)	Structural annotation		Functional annotation	
								Sequence components of *kabuli* genome	*Kabuli* gene accession IDs	NCBI-KOG	TFs
Ca1_P1_45749239	Ca1	45749239	GAAAAAAAAAA/GAAAAAAAAA	TTGTGCGCTTGACTGATGTT	GTTTTGGCGTTGGTTTCACT	60.5	857	INTERGENIC	--	--	--
Ca1_P1_45869301	Ca1	45869301	GTATATATATATATATATATATAT/GTATATATATATATATATAT	TCATGGTCATTCAAACCCAA	CAAATGACTATGTGTTGCCGA	59.8	575	INTERGENIC	--	--	--
Ca1_P1_45914806	Ca1	45914806	ATT/AT	AGTGCTGTAAAATGCCCACC	CTGGGCAAGGGATCTGAATA	60.0	548	DRR	Ca_21533	--	Trihelix
Ca1_P1_45941271	Ca1	45941271	AATATATATATATA/AATATATATATATATA	GAAAGGAGATCACGAGCAGC	GTTGAATGGAACAGGCGAGT	60.1	723	INTERGENIC	--	--	--
Ca1_P1_45941295	Ca1	45941295	GATATATATATATATATATATATAT/GATATATATATATATATATATATATAT	GAAAGGAGATCACGAGCAGC	CAAGTCGTGGGGAGCATTAT	60.1	846	INTERGENIC	--	--	--
Ca1_P1_45951051	Ca1	45951051	GTTTTTTTT/GTTTTTTT	GCACCGGATTTTTAATCCAA	TCTTCGTGGAAGTTGCCTTT	59.8	714	INTERGENIC	--	--	--
Ca1_P1_45958085	Ca1	45958085	TAAA/TAA	ATCACCGAAAATGTAACGCC	CAGTGGTCGATTTTGTGTGG	59.8	687	INTERGENIC	--	--	--
Ca1_P1_46003289	Ca1	46003289	ATTTTTTTTTT/ATTTTTTTTTTT	ATGGCAACATGGGATGATTT	CTGGTTTGGCAAAACGATCT	60.0	653	INTERGENIC	--	--	--
Ca1_P1_46023584	Ca1	46023584	CACTAGTTACTAGTTACTAGTTACTAGTTACT/CACTAGTTACTAGTTACTAGTTACT	TTTTCCACCAACAAACCTCA	AGTTGTGGAATCTGCCTGCT	59.0	330	INTERGENIC	--	--	--
Ca1_P1_46088799	Ca1	46088799	CTATATATATATATATATATATATATATAT/CTATATATATATATATATATATATATATATAT	TAAGTCCGTAAGTGGTGCCC	GGGCTTTCCAAAGTGTTATCC	60.0	586	INTERGENIC	--	--	--
Ca1_P1_4612179	Ca1	4612179	CAA/CAAA	GCCATTTGGAATGACGACTT	CGTTTTTGCAACTTCCCAAC	59.9	327	INTERGENIC	--	--	--
Ca1_P1_46122011	Ca1	46122011	CATATATATATATATATATATATATATATATATATATA/CATATATATATATATATATATATATATATATATATATATATATA	CCTATTGGGAAATGAAGCGA	CCGTACCGTTTCTGGGATTA	60.0	637	DRR	Ca_21537	--	--

INDEL marker IDs	Chromosomes/ unanchored scaffolds	Physical positions (bp)	InDels (*Kabuli* reference genome-CDC Frontier/PI)	Forward primers (5'-3')	Reverse primers (5'-3')	Anneling temperature (0C)	Expected amplified product size (bp)	Structural annotation		Functional annotation	
								Sequence components of *kabuli* genome	*Kabuli* gene accession IDs	NCBI-KOG	TFs
Ca1_P1_4613210	Ca1	4613210	TTATATATATATATATATATATAT/TTATATATATATATATATATATATAT	CCGTGTTATGTGGTGCATGT	CTCTGTCAAATGCATGAAATGTT	60.3	230	INTERGENIC	--	--	--
Ca1_P1_46177937	Ca1	46177937	GAAAAAAA/GAAAAAAAAAAA	TTGACACTCATTGCTCCTTCA	GCATGTTTGAGAGGGGAGAA	59.4	309	INTERGENIC	--	--	--
Ca1_P1_46178255	Ca1	46178255	AATTTATAATTTAT/AAT	TCAAACATGCTCTGAATGCC	AACCAATACATAACATCACAGTGC	59.8	570	INTERGENIC	--	--	--
Ca1_P1_46186559	Ca1	46186559	AGTGATTGGTTTGTTTGTGATTGGTTTGTTTG/AGTGATTGGTTTGTTTGTGATTGGTTTGTTTGTGATTGGTTTGTTTG	AAAGCACACGATTTTCACCC	TTCGGCATCTAGCACTTCCT	60.0	775	INTERGENIC	--	--	--
Ca1_P1_46201366	Ca1	46201366	TA/TACA	GGGGTACCTCTAGTGGGGAA	GCCAAACACAAACAACGAGA	60.2	424	INTERGENIC	--	--	--
Ca1_P1_46207586	Ca1	46207586	ATATTATTATTATTATTATTATTATTATTATTATTATTATTATT/ATATTATTATTATTATTATTATTATTATTATTATT	CCACATAACAAAATTCGTGGG	CACATTTACTGCCGTGTTGAA	60.1	545	INTERGENIC	--	--	--
Ca1_P1_46259102	Ca1	46259102	CACACCCCAGACACCCCAG/CACACCCCAG	GACAAAGCTGCAACCACAGA	ATCCCTTAGCGTGGGAGAAT	60.0	405	INTERGENIC	--	--	--
Ca1_P1_46259340	Ca1	46259340	AATATATATATATATATATATATATATATATATATATA/AATATATATATATATATATATATATATATATATATA	TCAGACAAAGCTGCAACCAC	GTTACCGGTTCCTTGCTTCA	60.0	845	INTERGENIC	--	--	--
Ca1_P1_46268212	Ca1	46268212	TA/T	AAACGCAATCCTTGAGTTCG	CATGACTTGGATTGGTCGTG	60.2	226	INTERGENIC	--	--	--
Ca1_P1_46278771	Ca1	46278771	ATGGCCTGGCCTGGCCTGGCCTG/ATGGCCTGGCCTG	ACCTTCAGTCAGGGGCCTAT	AAGTCTGGTCTGGCCTGAAA	60.0	538	INTERGENIC	--	--	--
Ca1_P1_46283206	Ca1	46283206	C/CTAATTATAA	AGCTGCCATGCCTAATGAAT	CAATGGGCTTCTTACCTGGA	59.7	425	INTERGENIC	--	--	--
Ca1_P1_46284274	Ca1	46284274	C/CTACA	CAGATGGCACACCTCTCTCA	TCTATCTCACAATGCGGCAG	60.0	544	INTERGENIC	--	--	--

INDEL marker IDs	Chromosomes/ unanchored scaffolds	Physical positions (bp)	InDels (*Kabuli* reference genome-CDC Frontier/PI)	Forward primers (5'-3')	Reverse primers (5'-3')	Anneling temperature (0C)	Expected amplified product size (bp)	Structural annotation		Functional annotation	
								Sequence components of *kabuli* genome	*Kabuli* gene accession IDs	NCBI-KOG	TFs
Ca1_P1_46286614	Ca1	46286614	CTTTT/CTTT	CCAAGTTATGCTTGATCGCA	CGAGAACCATCCTGCATTTT	59.8	820	INTERGENIC	--	--	--
Ca1_P1_46286661	Ca1	46286661	GTAGGGATA/GTA	CCAAGTTATGCTTGATCGCA	CGAGAACCATCCTGCATTTT	59.8	820	INTERGENIC	--	--	--
Ca1_P1_46309103	Ca1	46309103	CAATAATAATAATAATAATAATAATAATAATAATAATAATAATAATAATAATAATAATA/CAATAATAATAATAATAATAATAATAATAATAATAATAATAATAATAATAATAATAATAATAATAATAATA	CAGGAATTACAATCCGTTCCA	ATCGATTCCGGTGGTCTATG	59.8	778	INTERGENIC	--	--	--
Ca1_P1_46318101	Ca1	46318101	TATT/TATTATTATTATTATCATT	AGGCCAAGAGACTTGGTTGA	ATTATGCCGGTGGTGTGAAT	59.8	335	INTRON	Ca_21543	--	--
Ca1_P1_46352138	Ca1	46352138	CACAAC/CACAACAAC	AGCGATGCATGTCTCAAGTG	TGCCCTTCTTCTTTTCCTAGTG	60.0	396	INTERGENIC	--	--	--
Ca1_P1_46382197	Ca1	46382197	CA/CATATATATA	TCTTGAGCTGGGAAACTCGT	CCCAACCATGCTAGTGTGC	60.0	671	INTERGENIC	--	--	--
Ca1_P1_46409324	Ca1	46409324	ATTTTTTTTT/ATTTTTTTT	CCCATGAGCAACCAAAAACT	TGATCATCCAGGCATGTGTT	60.0	289	INTERGENIC	--	--	--
Ca1_P1_4648276	Ca1	4648276	TTTATTATTATTATTATT/TTTATTATTATTATTATTATT	CTTTCACAACGGAGTGTCCC	TTGTACCAGTTTCGCTCCTTT	60.5	529	INTERGENIC	--	--	--
Ca1_P1_46491737	Ca1	46491737	CAAG/CAAGAAG	GATGCATCTCACCAAGGGTT	CGGTGCAAACTCTACGTTCA	59.9	420	INTERGENIC	--	--	--
Ca1_P1_46523548	Ca1	46523548	CAAAAAAAAAAAAAAAA/CAAAAAAAAAAAAA	GTGGACAAACCTGCATTCCT	ACCATGCACAGTTGCTGAGA	60.0	675	INTERGENIC	--	--	--
Ca1_P1_46524349	Ca1	46524349	TAAAAAAAAA/TAAAAAAAAAAA	TGAAGCATGCACAAATCCTC	TTGATGTAGCAACGGCTGAG	59.8	843	INTERGENIC	--	--	--
Ca1_P1_46525859	Ca1	46525859	GAAAAAA/GAAAAAAA	CCCACCTGGATACAAGGCTA	AGTAGAGGGAGTTCGGGTGC	59.9	711	INTERGENIC	--	--	--

INDEL marker IDs	Chromosomes/ unanchored scaffolds	Physical positions (bp)	InDels (*Kabuli* reference genome-CDC Frontier/PI)	Forward primers (5'-3')	Reverse primers (5'-3')	Anneling temperature (0C)	Expected amplified product size (bp)	Structural annotation		Functional annotation	
								Sequence components of *kabuli* genome	*Kabuli* gene accession IDs	NCBI-KOG	TFs
Ca1_P1_46526481	Ca1	46526481	CAAAAAAA/CAAAAAAAA	TTAAAATTCCGAGGCGAAAA	ACATGGTTGTGGTGGTGATG	59.7	698	INTERGENIC	--	--	--
Ca1_P1_46526676	Ca1	46526676	TAAAA/TAAA	TTAAAATTCCGAGGCGAAAA	ACATGGTTGTGGTGGTGATG	59.7	698	INTERGENIC	--	--	--
Ca1_P1_46528558	Ca1	46528558	CT/CTT	TTAAAATTCCCTCCTCGGCT	AACTCTGGGAACCCTCCTGT	60.0	398	INTERGENIC	--	--	--
Ca1_P1_46528735	Ca1	46528735	GTGACAGCCTGACAG/GTGACAG	TTAAAATTCCCTCCTCGGCT	GGGGAAAACATCCCGTTATT	60.0	865	INTERGENIC	--	--	--
Ca1_P1_46530950	Ca1	46530950	CAAATTGAAAATTGAAAATT/CAAATTGAAAATT	ATTTGAGAATGTTCGGGCAA	GTCTGCGGATAATTTCGCTT	60.4	665	DRR	Ca_21551	M	--
Ca1_P1_46531041	Ca1	46531041	T/TG	GCGAAAATAATGTTGATGCAGA	GTCTGCGGATAATTTCGCTT	60.1	139	DRR	Ca_21551	M	--
Ca1_P1_46539360	Ca1	46539360	CAAA/CAAAA	TCCCAAACAGCCTGATAACC	CTGATGACAAAAGGGGGAGA	59.9	744	INTERGENIC	--	--	--
Ca1_P1_46598729	Ca1	46598729	GATATATATATAT/GATATATATATATAT	TGATTTGTTAGCTTGTGGCAA	TGGAAGCATGATCCTCAACA	59.4	477	INTERGENIC	--	--	--
Ca1_P1_46633354	Ca1	46633354	GTATATATATATATATATATAT/GTATATATATATATATATAT	CGACATGGCTCTGATACCAA	TCAGACCGCCTTTACCAATC	59.7	615	INTERGENIC	--	--	--
Ca1_P1_46662012	Ca1	46662012	AAT/AATAT	GCATTTACGTCGATATTTATGAGTG	TCAGCCAAAAAGCTGATGTG	59.1	617	INTERGENIC	--	--	--
Ca1_P1_46728277	Ca1	46728277	ATTT/ATT	TTCTTTAACCTGGCCAAACG	CCGTCTTCGAATCACCGTAT	60.1	681	DRR	Ca_25109	--	--
Ca1_P1_46782070	Ca1	46782070	AAT/AATTAT	CTCCCTTTCTAATCCCCGAC	AATGTCTCACGACATCAGCG	59.9	829	INTERGENIC	--	--	--

INDEL marker IDs	Chromosomes/ unanchored scaffolds	Physical positions (bp)	InDels (*Kabuli* reference genome-CDC Frontier/PI)	Forward primers (5'-3')	Reverse primers (5'-3')	Anneling temperature (0C)	Expected amplified product size (bp)	Structural annotation		Functional annotation	
								Sequence components of *kabuli* genome	*Kabuli* gene accession IDs	NCBI-KOG	TFs
Ca1_P1_46831943	Ca1	46831943	CTTTT/CTTT	GTGGTTGGTTTGCAAGAGGT	AACTGGGTGAGAGCCTGAGA	60.0	725	INTRON	Ca_25116	U	--
Ca1_P1_46834640	Ca1	46834640	AATATATATATATATATATATATATATATAT/AATATATATATATATATATATATATATAT	TGAATTACAGCTATAAAACGAAGATAA	GATCAGGATGAACCGGAAGA	57.1	435	DRR	Ca_25116	U	--
Ca1_P1_46856347	Ca1	46856347	GCT/GCTGAACT	ATGCAATCCGATAAGCAACC	ACGATTTTAGAAGAGGCGCA	59.9	980	INTERGENIC	--	--	--
Ca1_P1_46867290	Ca1	46867290	CTT/C	GCCCTCAGATGAGAGTCAGG	CGTTTTTCAAAGCGAGGGTA	59.9	439	DRR	Ca_25118	--	--
Ca1_P1_46867363	Ca1	46867363	CTTTT/CTTT	GCCCTCAGATGAGAGTCAGG	CAAACCTTTCATGCGGGTAT	59.9	836	DRR	Ca_25118	--	--
Ca1_P1_46900592	Ca1	46900592	ATTT/ATTTT	GATGCCTTGACCATCCTTGT	TGGGTACACGCATATTAACCAA	59.9	555	INTERGENIC	--	--	--
Ca1_P1_4690205	Ca1	4690205	CATATATATATATATATAT/CATATATATATATATAT	GGCAAGCAATTCCAGTAGGA	ACTTTGACATCCACCCCAAC	60.2	524	INTRON	Ca_00553	F	--
Ca1_P1_46916401	Ca1	46916401	GATATATATATATATATATATA/GATATATATATATATATATATATATA	CAGCAAGCACCAACCACTTA	TTGCCAGTTATTTTTGCAAGG	59.9	363	DRR	Ca_12949	--	--
Ca1_P1_46947905	Ca1	46947905	ATTATTTATTTATTTATTTATTTATTTATTTATTTATTTATTTATTTATTTATT/ATTATTTATTTATTTATTTATTTATTTATTTATTTATTTATTTATTTATT	CAAGCGACACTACAAGCCAA	CCCTACTCATACCCGTTACCAA	60.1	984	INTERGENIC	--	--	--
Ca1_P1_47009174	Ca1	47009174	TTCTTCTTCTTCTTCTTCTTCTTCTTCTTCTTCTTCTTCTTCTTCTTCTTCTTC/TTCTTCTTCTTCTTCTTCTTCTTCTTCTTCTTCTTCTTCTTCTTCTTCTTCTTCTTCTTC	TTACCCCATGCTGCCTAATC	TGCGATCATGAAAATTGGAA	59.9	585	INTERGENIC	--	--	--
Ca1_P1_47016631	Ca1	47016631	ACTCTCTCTCTCTCT/ACTCTCTCTCTCTCTCT	ATTGCCTCTCAGTCGCGTTA	AAAATTCGACATCGTGAGGG	60.9	531	INTERGENIC	--	--	--
Ca1_P1_47063529	Ca1	47063529	TTATATATATATATATATATATAT/TTATATATATAT	GAATGTTGTCGTATGCCACG	CGCAAATTCAAAAGAATTCAAA	60.0	711	INTERGENIC	--	--	--

INDEL marker IDs	Chromosomes/ unanchored scaffolds	Physical positions (bp)	InDels (*Kabuli* reference genome-CDC Frontier/PI)	Forward primers (5'-3')	Reverse primers (5'-3')	Anneling temperature (0C)	Expected amplified product size (bp)	Structural annotation		Functional annotation	
								Sequence components of *kabuli* genome	*Kabuli* gene accession IDs	NCBI-KOG	TFs
Ca1_P1_47116478	Ca1	47116478	AATATATATATATATATATATATAT/AATATATATATATATATATATAT	ACACGCTTCCCTGTTGACAT	TAGGCCTGAACTGGGATGAC	60.6	772	INTRON	Ca_12935	--	ERF
Ca1_P1_47124845	Ca1	47124845	A/ACACTCT	GAGACAGAGGATTGCCGAAG	CGGGTTGAAAATTAATCGGA	60.0	459	DRR	Ca_12934	--	TALE
Ca1_P1_4714640	Ca1	4714640	AAG/A	AACGGTGATTATTTGGCGAC	TCACTTGTTGCTTTCGAGGA	59.8	795	DRR	Ca_00555	T	MYB_related
Ca1_P1_4720443	Ca1	4720443	AT/A	TCAGAGTTCATTGCAAACGG	GGGATTCCCATTTTCCATCT	59.8	691	INTRON	Ca_00555	T	MYB_related
Ca1_P1_47255442	Ca1	47255442	TTATATATATATATATATATATATATAT A/TTATATATATATATATATATATATATA	AACTCGTTCAACCCATTTGC	GGCAGCAAAAGAACAAGGAG	60.0	512	INTERGENIC	--	--	--
Ca1_P1_47266775	Ca1	47266775	CATACTTCGTAATAAAA/CA	GAATGGACCTATTCGTTGTGC	CAATTCGAAACGCAATCCTT	59.4	409	INTERGENIC	--	--	--
Ca1_P1_4730002	Ca1	4730002	TAAAAAAAAAA/TAAAAAAAAA	TCGAGATCAAATCACCGGAT	CCCCAACACTTTAAGTCGGA	60.4	429	INTERGENIC	--	--	--
Ca1_P1_47314380	Ca1	47314380	AATTATTATTATTATTATTATTATTATT ATTATTATTATTATTAT/AATTATTATTA TTATTATTATTATTATTATTATTATTAT TATTATTAT	CCGTAGGAATTGCATGTTTG	GATCAGACCTAAGCAACGGG	59.0	669	INTERGENIC	--	--	--
Ca1_P1_47418486	Ca1	47418486	TTATATATATATATATATAT/TTATATATATATATATATATAT	CACTGATTGACAAGCACCTCA	AAGCCCACGTTTCAAATGAC	59.9	288	DRR	Ca_12915	--	WRKY
Ca1_P1_4742142	Ca1	4742142	TAAA/TAA	GACCGCGTAGATGTGGAACT	GGATGAGACCTCCTACTTGCC	60.1	677	INTERGENIC	--	--	--
Ca1_P1_47455552	Ca1	47455552	TTAATAATAATAATAATAA/TTAATAATAATAATAA	TTGGGGTCAGTCAAACACAA	CGGTGAAATAGGTATGCCAAA	60.0	600	INTERGENIC	--	--	--
Ca1_P1_47455633	Ca1	47455633	GAAAAAAAA/GAAAAAAAAA	TTGGGGTCAGTCAAACACAA	CGGTGAAATAGGTATGCCAAA	60.0	600	INTERGENIC	--	--	--

INDEL marker IDs	Chromosomes/ unanchored scaffolds	Physical positions (bp)	InDels (*Kabuli* reference genome-CDC Frontier/PI)	Forward primers (5'-3')	Reverse primers (5'-3')	Anneling temperature (0C)	Expected amplified product size (bp)	Structural annotation		Functional annotation	
								Sequence components of *kabuli* genome	*Kabuli* gene accession IDs	NCBI-KOG	TFs
Ca1_P1_47464200	Ca1	47464200	TTATATATATATATATATATATAT/TTATATATATATATATATATATATAT	AGTTTCCGCCGATAAGGAAT	GAGAGTCATCGGCTGCTACC	59.9	416	INTERGENIC	--	--	--
Ca1_P1_47478012	Ca1	47478012	AATTATTATTATTATTATTATTATTATTATT/AATTATTATTATTATTATTATTATTATTATTATT	CCGGTAAATCTGTAGCTCACG	TGACTATTGCAATCAAAACCTCTT	59.8	283	INTERGENIC	--	--	--
Ca1_P1_47498704	Ca1	47498704	ATTTTTTTTTT/ATTTTTTTTTTT	TGTTGGGGTGACATGTGATT	CACAAAACGACAACATGCAA	59.7	427	INTERGENIC	--	--	--
Ca1_P1_47533952	Ca1	47533952	TG/T	TTCACCAAAACCCCGTAGAG	TTTCCCGACCATGTCCTTTA	60.0	363	INTERGENIC	--	--	--
Ca1_P1_47580698	Ca1	47580698	ATTTTTTTTTTT/ATTTTTTTTTT	TTTGAATTAGCCAAGGGTCG	TCGATTCGAAATGGATGTCA	60.1	615	INTERGENIC	--	--	--
Ca1_P1_47630703	Ca1	47630703	TAAAAAGAAAAAGAAAAAGAAA/TAAAAAGAAAAAGAAAAAGAAAAAGAAA	CGTGTCCGGCAAAGTAAAAT	GTGATGCACGCAGTAATTGG	60.0	517	INTERGENIC	--	--	--
Ca1_P1_47630977	Ca1	47630977	AC/ACC	GGCCTTCCATCGTGAATAAA	TTTCGAATTCCAGAACTGCC	59.9	916	INTERGENIC	--	--	--
Ca1_P1_47673639	Ca1	47673639	CTTTTTTTTTT/CTTTTTTTTTTT	GGTTTATGAATTGAACCGCA	TTCATGGTGCAAAAATCGAA	58.5	549	INTRON	Ca_12903	--	--
Ca1_P1_47676057	Ca1	47676057	ATTTTTTTTTTT/ATTTTTTTTTTTT	AATCCCTCCCTGCCTAAAAA	ATGTGGTGGTCCAATCAACA	59.9	619	INTRON	Ca_12903	--	--
Ca1_P1_47680022	Ca1	47680022	C/CATATATATA	TCCACTACTTGGCTTTTATTGG	TCGACTCACCAAAGCTACAAA	58.3	849	INTERGENIC	--	--	--
Ca1_P1_47680035	Ca1	47680035	CATATATATATATATATATATATAT/CATATATATATATATATATATAT	TCCACTACTTGGCTTTTATTGG	TCGACTCACCAAAGCTACAAA	58.3	849	INTERGENIC	--	--	--
Ca1_P1_47731732	Ca1	47731732	AATATATATATATATATATATATATAT/AATATATATATATATAT	GAGAGTCAAGAAAGGTCGCC	TCATTTGTGGTTGTGGGATG	59.0	689	INTERGENIC	--	--	--

**INDEL marker IDs**	**Chromosomes/ unanchored scaffolds**	**Physical positions (bp)**	**InDels (*Kabuli* reference genome-CDC Frontier/PI)**	**Forward primers (5'-3')**	**Reverse primers (5'-3')**	**Anneling temperature (0C)**	**Expected amplified product size (bp)**	**Structural annotation**		**Functional annotation**	
								**Sequence components of *kabuli* genome**	***Kabuli* gene accession IDs**	**NCBI-KOG**	**TFs**
Ca1_P1_47843354	Ca1	47843354	TAAAAAAAAA/TAAAAAAAA	AGTTCGTGTTGCATGTTCAAA	CAAGGAAGAACCCTATCGCA	59.2	677	INTERGENIC	--	--	--
Ca1_P1_47875198	Ca1	47875198	AT/ATT	GAAAAACAAGACCCGCGATA	TTCCTATTGCTCACCCCTTG	60.1	466	INTERGENIC	--	--	--
Ca1_P1_47905221	Ca1	47905221	AATATATATATATATATATATATATATATATATA/AATATATATATATATATATATATATATATATA	TGACACTCCCTTGACTGCAT	CGAGAAATCAAACAAACACCAA	59.3	570	INTERGENIC	--	--	--
Ca1_P1_47916750	Ca1	47916750	ATT/ATTTCAGGTTT	GTTGGTGATGCCACTGAATG	GAGAGAAAGGCAAGTGTGGG	60.0	979	DRR	Ca_12885	O	M-type
Ca1_P1_47921129	Ca1	47921129	AAATAATAATAATAATAATAATAATAATAATAATAAT/AAATAATAATAATAATAATAATAATAATAATAATAATAAT	TGCCAAAACGAATGAAGTTG	TTTCCACATAGAACATTCTAAATACAG	59.7	545	INTERGENIC	--	--	--
Ca1_P1_47945221	Ca1	47945221	TCAAGCCA/TCA	AGGACAGTGCCAAAAGATGG	GTCATGTATGCATTGGGCAG	60.1	226	DRR	Ca_12882	R	--
Ca1_P1_48004686	Ca1	48004686	ATTTTTTTT/ATTTTTTT	GGGTTCCTAAAAATTCCCCA	TCAACCCACAACAGTGGAAA	60.0	614	INTERGENIC	--	--	--
Ca1_P1_48064288	Ca1	48064288	TTATATATATATATATATATATATATA/TTATATATATATATATATATA	TGGGTGGATAGATGAGAGGC	TCTGAATAGGTTTGGTCGGC	60.0	404	INTERGENIC	--	--	--
Ca1_P1_48091761	Ca1	48091761	TAAAAAAAA/TAAAAAAA	TCTGGAGATGGGACCTCTTG	CCAAATCGGAGGAAGTTGTT	60.2	700	INTERGENIC	--	--	--
Ca1_P1_48114146	Ca1	48114146	TTATATATATATATATATATATATAT/TTATATATATATATATATATATAT	TGATTGTTGTTGGGCCACTA	TTTGTTCTCCATTTTGCTTTTG	60.0	807	INTERGENIC	--	--	--
Ca1_P1_48172069	Ca1	48172069	AAATAATAATAATAATAATAATAATAATAATAATAATAATA/AAATAATAATAATAATAATAATAATAATAATAATAATAATAATAATAATA	TAAATGGCAGGTAAAGGCGA	AGACTTTGCTGCCACCTCAT	60.6	315	DRR	Ca_12862	R	WRKY
Ca1_P1_48200764	Ca1	48200764	AT/A	TTTATTTGGTCAACAAAGTAGTATCAA	TCATTTTCTCAAGTCGGGCT	57.6	322	INTERGENIC	--	--	--

INDEL marker IDs	Chromosomes/ unanchored scaffolds	Physical positions (bp)	InDels (*Kabuli* reference genome-CDC Frontier/PI)	Forward primers (5'-3')	Reverse primers (5'-3')	Anneling temperature (0C)	Expected amplified product size (bp)	Structural annotation		Functional annotation	
								Sequence components of *kabuli* genome	*Kabuli* gene accession IDs	NCBI-KOG	TFs
Ca1_P1_48236817	Ca1	48236817	TAA/TA	TGGGTGTTGCCCATATATCA	TTTGGATCCATTTCATTTGATT	59.6	626	INTERGENIC	--	--	--
Ca1_P1_48245778	Ca1	48245778	TAAAAAAAAAAAAAA/TAAAAAAAAAAAAA	CGGCCCTATTCATGTTTGTT	TGTGGAGGAAAGGAGGTGTC	59.8	484	DRR	Ca_12858	--	--
Ca1_P1_48277687	Ca1	48277687	TTTATTATTATTATTATTATTATTATTATTATTATTATTATTATTATTATTATTATTATTATTATTATT/TTTATTATTATTATTATTATTATTATTATTATTATTATTATTATTATTATTATTATTATTATTATT	TCCTTCTTTGGAAGAGGCAA	ACCGTGCCCTACTACCATGA	59.9	662	INTERGENIC	--	--	--
Ca1_P1_48318880	Ca1	48318880	AATATATATATATATATATATATATAT/AATATATATATATATATATATATAT	CGCCTTTATTGTGAGATTCCA	GGTGGTCTAACCCGATTCATT	60.1	480	INTERGENIC	--	--	--
Ca1_P1_48331419	Ca1	48331419	AATTATTATTATTATTATTATTATTATTATTATT/AATTATTATTATTATTATTATTATTATTATT	CATTGCGTTGTCACAAAATCA	AAGTAACTGGCACCACCAGG	60.6	395	INTERGENIC	--	--	--
Ca1_P1_4837643	Ca1	4837643	GAATAATAATAATAATAATAATAATAATAATAATAATAATAATAATAATAATAA/GAATAATAATAATAATAATAATAATAATAATAATAATAATAATAATAATAATAATAATAA	ACCAAAAACTTGACTTGCCC	TATTTTCCTGCCACGCCTAC	59.1	208	INTERGENIC	--	--	--
Ca1_P1_4933923	Ca1	4933923	CATGATGATG/CATGATGATGATG	TGCCTAGGATGAGAGGTGCT	GGACATCCTCCTCCAACTGA	60.0	252	CDS (large-effect mutations)	Ca_00581	--	--
Ca1_P1_4934416	Ca1	4934416	ATTATTTGTTTAATGTTATTT/ATTATTTGTTTAATGTTATTTGTTTAATGTTATTT	AGCCTCAGATGGAGGAGGAT	AGGCCGAAGAAAGAGGTAGC	60.2	653	DRR	Ca_00581	--	--
Ca1_P1_4957879	Ca1	4957879	CAAAAAAAAAAAA/CAAAAAAAAAAA	TCATGACAATGCAAATTCGAC	TTATTCGTGTGTGCCTTGGA	59.5	459	DRR	Ca_00586	T	WRKY
Ca1_P1_5106336	Ca1	5106336	CT/CTCTAT	CGAGTGATTTGCTTCATTGG	TGTTACGCATTGTTTGGGAA	59.3	651	INTERGENIC	--	--	--
Ca1_P1_5252022	Ca1	5252022	AACAC/AAC	GGGAGGGGTCTAAGGTTCAG	TCGACCATCATTGCCAAATA	59.9	924	DRR	Ca_00613	--	--
Ca1_P1_5275130	Ca1	5275130	AATATATATATATATATA/AATATATATATATATATATA	CCATTCTCACCCGCAATAGT	CCAGCCTCAAATCATCCCTA	60.0	445	INTERGENIC	--	--	--

INDEL marker IDs	Chromosomes/ unanchored scaffolds	Physical positions (bp)	InDels (*Kabuli* reference genome-CDC Frontier/PI)	Forward primers (5'-3')	Reverse primers (5'-3')	Anneling temperature (0C)	Expected amplified product size (bp)	Structural annotation		Functional annotation	
								Sequence components of *kabuli* genome	*Kabuli* gene accession IDs	NCBI-KOG	TFs
Ca1_P1_5344971	Ca1	5344971	CATAGT/C	CATGGGGATTGGAATACTGG	ACATAAATGGCTCTCGGTGG	60.0	632	DRR	Ca_00621	J	--
Ca1_P1_5350735	Ca1	5350735	AATATAT/AATAT	CGTTCTACCACAATACCTGCC	GGCAAGCTATGCAAGTCACA	59.5	632	INTERGENIC	--	--	--
Ca1_P1_5351038	Ca1	5351038	CATATATA/CATATATATATA	TGTGGCAGACAGCCAACTAA	TTTGGCAATCGAAAATTGTG	60.4	607	INTERGENIC	--	--	--
Ca1_P1_5375317	Ca1	5375317	A/AAAAGTTT	TATGATGTGCTTGTGGAGCC	GCCCTAATAAGCCAACCCAT	59.7	515	INTERGENIC	--	--	--
Ca1_P1_5376097	Ca1	5376097	ATGGTGGTG/ATGGTG	AAAAGATTAGTGGCTGCCGA	TTTGTGGTGGTCAAAGTCCA	59.8	219	INTERGENIC	--	--	--
Ca1_P1_5392823	Ca1	5392823	TCA/T	TATTTGGCATTGGTGGTTGA	GAGGCCATGTCAAGGAAGTT	59.8	829	INTERGENIC	--	--	--
Ca1_P1_5393298	Ca1	5393298	ATTTTTTTT/ATTTTTTTTTT	AACTTCCTTGACATGGCCTC	TGATGTTGTTGGAGATGGAAA	59.1	505	INTERGENIC	--	--	--
Ca1_P1_5396397	Ca1	5396397	CATATATATATATATATATATAT/CATATATATATATATATATAT	GCTTTAGGCCCAAACAAAAA	AGTTGGTGGCCATTGCTAAG	59.2	570	INTERGENIC	--	--	--
Ca1_P1_5403862	Ca1	5403862	C/CTTTATTATT	ACGGTTGACTAATTGTGCCA	TCGCATCCCTACGATACGAT	59.1	629	DRR	Ca_00627	--	--
Ca1_P1_5419424	Ca1	5419424	C/CA	TTTGTGGTGGACTCCAATGA	AAAGCAATTGCCCCCTAAAT	59.9	581	INTERGENIC	--	--	--
Ca1_P1_5422335	Ca1	5422335	TA/T	CGGTGAGGGTTACTTTAGAAAAGA	TGGTTTTTACTCATCGCACA	60.1	782	INTERGENIC	--	--	--
Ca1_P1_5423307	Ca1	5423307	CTTTT/CTTTTT	CGGGGTAGTTTTTGTATTCCC	GGTGACGCCTTTTAATTTGC	59.6	640	INTERGENIC	--	--	--

INDEL marker IDs	Chromosomes/ unanchored scaffolds	Physical positions (bp)	InDels (*Kabuli* reference genome-CDC Frontier/PI)	Forward primers (5'-3')	Reverse primers (5'-3')	Anneling temperature (0C)	Expected amplified product size (bp)	Structural annotation		Functional annotation	
								Sequence components of *kabuli* genome	*Kabuli* gene accession IDs	NCBI-KOG	TFs
Ca1_P1_5425033	Ca1	5425033	TTTTATTT/TTTTATTTATTTATTT	TCACGGTGAAAGTTCTGTGC	AAATCCCAATAAGCAAGGCA	59.9	427	INTERGENIC	--	--	--
Ca1_P1_5425222	Ca1	5425222	CTTATT/CTTATTTATT	TCACGGTGAAAGTTCTGTGC	TCCCGAACCTGTGAGGTTAT	59.9	864	INTERGENIC	--	--	--
Ca1_P1_5425431	Ca1	5425431	TAA/TAAGAA	TGCCTTGCTTATTGGGATTT	TCCCGAACCTGTGAGGTTAT	59.5	457	INTERGENIC	--	--	--
Ca1_P1_5426267	Ca1	5426267	TTATATATATATATATATATATAT/TTATATATATATATATATATATATAT	ACCAACCGACCCACTTGATA	TCAAACACACGAGATGGTCC	60.2	317	INTERGENIC	--	--	--
Ca1_P1_5428904	Ca1	5428904	TCACA/TCA	TTAATGGGTAGGTGGGTGGA	TTTTCAGCGGTGTCACTGAG	60.0	839	INTERGENIC	--	--	--
Ca1_P1_5437282	Ca1	5437282	CATATATATATATATATATATA/CATATATATATATATATATA	TGCAATGTTAATTAAGCGGG	GAACTGCATTGTGATGGGTG	58.7	456	INTERGENIC	--	--	--
Ca1_P1_5454188	Ca1	5454188	GCAGGGAAT/G	GAGAATTCCAGCACAGCCTC	ACACGTCCTTTTCCGAACAC	60.0	415	INTERGENIC	--	--	--
Ca1_P1_5454590	Ca1	5454590	TA/TATAA	GTGTTCGGAAAAGGACGTGT	AATGGTCCCAGTTCAACCAC	60.0	655	INTERGENIC	--	--	--
Ca1_P1_546650	Ca1	546650	TATCTTCATTTTGA/T	TGAAATTTTAAACAAACAAACCCA	TTGTAAACGTTTGCCGACAC	59.7	420	INTERGENIC	--	--	--
Ca1_P1_5471011	Ca1	5471011	ATTGTT/ATT	GTTGAGGTGGGTTGTGGACT	CACAGGATGTCGGGAAAGAT	59.9	566	INTRON	Ca_00632	O	--
Ca1_P1_5481892	Ca1	5481892	AACAAGTGGA/AA	ACATGGTATCATTCACGGCA	GTGTTTCGATCCGGATTTGT	59.8	711	DRR	Ca_00633	--	S1Fa-like
Ca1_P1_5484796	Ca1	5484796	TAA/TAAA	CGCGCGTTGTGTTTATAAGT	TTACCAAGATTGCCCCTCTG	58.9	444	INTERGENIC	--	--	--

INDEL marker IDs	Chromosomes/ unanchored scaffolds	Physical positions (bp)	InDels (*Kabuli* reference genome-CDC Frontier/PI)	Forward primers (5'-3')	Reverse primers (5'-3')	Anneling temperature (0C)	Expected amplified product size (bp)	Structural annotation		Functional annotation	
								Sequence components of *kabuli* genome	*Kabuli* gene accession IDs	NCBI-KOG	TFs
Ca1_P1_5491696	Ca1	5491696	GTATCTG/GTATCTGGCTATCTG	GTTTCGGTAACTGCCTGCAT	CTGCAATTTCAGCCTCACAA	60.1	375	INTRON	Ca_00634	--	--
Ca1_P1_5493909	Ca1	5493909	CAAAAA/CAAAA	TGGTTCAGGCTGTGAACAAG	GACCAACCACCACCTAGCAT	59.9	585	INTRON	Ca_00634	--	--
Ca1_P1_5495609	Ca1	5495609	C/CAA	TACTCAATCACGCCTTGCTG	TTTCCATTTGTGGGTCCAAT	60.0	184	DRR	Ca_00634	--	--
Ca1_P1_5500920	Ca1	5500920	CTAT/CTATAAACGTCATAT	GGAAGGGAATCGATGAGAAA	TATTCATTGGTTGGGGGAAA	59.1	456	INTERGENIC	--	--	--
Ca1_P1_5529052	Ca1	5529052	AT/ATGT	AGCCCGTGAGCATACAGTTC	ATCCATCCCACAACTCAAGC	60.3	599	INTERGENIC	--	--	--
Ca1_P1_5531229	Ca1	5531229	CAGATAAGATAA/CAGATAA	TGCTTCTCTTGGCTTTTTCC	CCAAACTTCCCACATTGGAG	59.6	265	INTERGENIC	--	--	--
Ca1_P1_5611256	Ca1	5611256	ATTTTTTTTT/ATTTTTTTT	TCTCAGAACCGACACTGTTCA	TGAATTCAGGTGAGCGTGAG	59.5	886	INTRON	Ca_00645	--	G2-like
Ca1_P1_5616025	Ca1	5616025	CTATATATATATATATATATA/CTATATATATATATATATATATA	TTCTCGAGAGAGACAATCCGA	TTCCTTTTAATGTCTGCGGG	60.1	495	DRR	Ca_00646	--	--
Ca1_P1_5672055	Ca1	5672055	TTATATATATATATATATATATAT/TTATATATATATATATATATATATATAT	AAAAGCTTCAAACACCCCCT	GAATGGTGACTTCACGAAGGA	60.0	560	INTERGENIC	--	--	--
Ca1_P1_5679064	Ca1	5679064	GTATTATTATTATTATTATTATTATTATTATTATTATTATTATTATTATTATTATTATTATTATT/GTATTATTATTATTATTATTATTATTATTATTATTATTATTATTATTATTATTATTATTATT	GAATAAGAGGTGCGGCAAAA	ATTTGAGCAATTGGGAGCAA	60.2	656	INTERGENIC	--	--	--
Ca1_P1_5689290	Ca1	5689290	AATATATATATATATATATATATATA/AATATATATATATATATATATATATATATATATA	TAGAGGAGGGCGACTTTCAA	TCTCAGAGCACATATATTTCAAGGA	59.9	671	INTERGENIC	--	--	--
Ca1_P1_5710967	Ca1	5710967	GATAT/GAT	TCCTCCAATGCTTTTGGAAC	GTTGCAAAGAAATCCGCAAT	60.1	471	CDS (FRAME SHIFT)	Ca_00663	K	--

INDEL marker IDs	Chromosomes/ unanchored scaffolds	Physical positions (bp)	InDels (*Kabuli* reference genome-CDC Frontier/PI)	Forward primers (5'-3')	Reverse primers (5'-3')	Anneling temperature (0C)	Expected amplified product size (bp)	Structural annotation		Functional annotation	
								Sequence components of *kabuli* genome	*Kabuli* gene accession IDs	NCBI-KOG	TFs
Ca1_P1_5741642	Ca1	5741642	CACACATA/CA	ATCCCTCAATTTTCCCAACC	ATCTAACGTGGTCGTCCTGC	60.0	927	INTERGENIC	--	--	--
Ca1_P1_5803481	Ca1	5803481	TTAAGATAAGAT/TTAAGAT	GCTCATGAGTTCCCTACCCA	GTCAGGGGCTCGTGTATGAT	60.1	560	INTRON	Ca_00671	O	FAR1
Ca1_P1_5804081	Ca1	5804081	AT/ATT	AATAACACGGGCACAAGAGG	AGGTCCACCAGGTTTGTCAG	60.0	457	INTRON	Ca_00671	O	FAR1
Ca1_P1_5809942	Ca1	5809942	TAAAAAAAAAA/TAAAAAAAAAAA	TGTGGAGATGGGAGCATACA	CACTTCCGGCCTTTTATTGA	60.1	463	INTERGENIC	--	--	--
Ca1_P1_5843197	Ca1	5843197	GAAAAA/GAAAAAA	GTGTCGGGAGGTGTTGGTAT	TTGATCCTCGTGTCAATCCA	59.7	126	INTERGENIC	--	--	--
Ca1_P1_5875747	Ca1	5875747	TTAATTTAAATT/TTAATTTAAATTAAATAATTTAAATT	TGATGGATGTCCTGGAAACA	CATCCATGCAAAAGCAGAGA	59.9	504	INTERGENIC	--	--	--
Ca1_P1_5904030	Ca1	5904030	TAAAAAAAAAAAA/TAAAAAAAAAAAAA	ACACCTTGCCTTTAGGGTCA	CGGCACCTCTAAACCAACAT	59.6	423	INTERGENIC	--	--	--
Ca1_P1_5951191	Ca1	5951191	ATTTTTTTTTT/ATTTTTTTTTTT	AGTTTGAAGGAATTGTTGACCA	ACCCCAACCCACACTTATGA	58.6	699	INTERGENIC	--	--	--
Ca1_P1_5955465	Ca1	5955465	AATATATATATATATATATATATATA/AATATATATATATATATATATATATATA	TGCAGTGGAAGTGTTGTTGA	GAACGAGGTCGGCAAAAGTA	58.8	686	INTERGENIC	--	--	--
Ca1_P1_6015644	Ca1	6015644	CTATATATATATATATATATATATATATATATATATATATATAT/CTATATATATATATATATATATATATATATATATATATATAT	AAATTTGAAGTCGCTGTTTGC	TGACCTCAAGTTTGCAGCAC	59.4	562	DRR	Ca_00689	I	--
Ca1_P1_6105440	Ca1	6105440	GTATTATTATTATTATTATTATTATTATTATTATTATTATTATTATTATTATTATTATT/GTATTATTATTATTATTATTATTATTATTATTATTATTATTATTATTATTATTATTATTATTATTATTATTATT	ATGTGGCAAAAGAGGAGGAA	GGGATGCGACCTTGTACTGT	59.7	396	INTERGENIC	--	--	--
Ca1_P1_6128515	Ca1	6128515	ATTTTCTTTTTTCTTTTTT/ATTTTCTTTTTT	ATGGAAATGGATTGGACCAC	TCCGACCAAGATACATGACG	59.5	567	DRR	Ca_00697	P	WRKY

INDEL marker IDs	Chromosomes/ unanchored scaffolds	Physical positions (bp)	InDels (*Kabuli* reference genome-CDC Frontier/PI)	Forward primers (5'-3')	Reverse primers (5'-3')	Anneling temperature (0C)	Expected amplified product size (bp)	Structural annotation		Functional annotation	
								Sequence components of *kabuli* genome	*Kabuli* gene accession IDs	NCBI-KOG	TFs
Ca1_P1_6132213	Ca1	6132213	TAA/TAAA	TTTTGCATATGCTCCATCCA	ATAGACATGGCCGGTCTTTG	60.0	519	DRR	Ca_00698	--	--
Ca1_P1_6136087	Ca1	6136087	AATTATCTTAAGAGATA/AATTATCTTAAGAGATATTATCTTAAGAGATA	TCGAAATTCCGTTGTTGATG	TGACATTTTACCGCCATTCA	59.5	907	INTERGENIC	--	--	--
Ca1_P1_6137600	Ca1	6137600	T/TC	TCTCATAGAAGGCGACGGTT	CATTTCCGGAGCCAGTAAAA	59.8	412	INTERGENIC	--	--	--
Ca1_P1_6157606	Ca1	6157606	A/AT	TGGAGTGTCATTGAGGACCA	GGAACGTTACATGACCGGAA	60.1	789	INTERGENIC	--	--	--
Ca1_P1_6158982	Ca1	6158982	TA/TAA	GAGATTAAATTGTCGCGGTTT	GGGTTGATACGGGATTGAAA	58.3	606	DRR	Ca_00701	T	M-type
Ca1_P1_6163758	Ca1	6163758	G/GTTTCTTAAATACAAA	TCTTTCATGTAGGATGCGATAAA	ATGGAACCTTCCACTCATGG	58.8	767	INTERGENIC	--	--	--
Ca1_P1_6166074	Ca1	6166074	TA/T	AACGTGACACATTCGCACTC	CTTTGGTTCCAACGTATAGTTTTT	59.8	862	INTERGENIC	--	--	--
Ca1_P1_6166331	Ca1	6166331	ATGTGT/ATGT	AAATCACAAGCATGAACTCTTCTTT	CCATATTCTTCTGATGCTTACCA	59.7	827	INTERGENIC	--	--	--
Ca1_P1_6174612	Ca1	6174612	TTAT/TTATAT	CCTCTCATCCACATCCATCC	GATATCCATCGGCTTACGACA	60.3	538	INTERGENIC	--	--	--
Ca1_P1_6176982	Ca1	6176982	A/AAT	GGTGGTAATAGTTCAGCCCG	AATCTTTGCCAATGGTTTGT	59.5	564	INTERGENIC	--	--	--
Ca1_P1_6178179	Ca1	6178179	ATTTTAATTTTTAATT/ATTTTAATT	AATTTTGGCATGCGACTACC	TTTCCTTCCCAATATCTGCC	60.0	514	INTERGENIC	--	--	--
Ca1_P1_6202713	Ca1	6202713	CTTTTT/CTTTTTT	CGCTTCCTTCTTCACACACA	AAAAGGCAATACATCCAATCAA	60.0	640	INTERGENIC	--	--	--

INDEL marker IDs	Chromosomes/ unanchored scaffolds	Physical positions (bp)	InDels (*Kabuli* reference genome-CDC Frontier/PI)	Forward primers (5'-3')	Reverse primers (5'-3')	Anneling temperature (0C)	Expected amplified product size (bp)	Structural annotation		Functional annotation	
								Sequence components of *kabuli* genome	*Kabuli* gene accession IDs	NCBI-KOG	TFs
Ca1_P1_6203281	Ca1	6203281	GTTTTTTTTT/GTTTTTTTTTT	CTCCTTGAGTTTTGAG ACACGA	TCGATTATGCGTAGGG AAGG	59.5	453	INTERGENIC	--	--	--
Ca1_P1_6204822	Ca1	6204822	AAGA/AA	TGGTTTGGTTGCATCA CTTT	TCGAAATTTGTGTTCC AAAAGA	59.0	543	DRR	Ca_07880	T	--
Ca1_P1_6205233	Ca1	6205233	CAAAAAAAAAAA/CAAAAAAAAAAAA	GGGTATTGGATAAAAA TTTCAAAAA	TGAAAATTAAGGGACG GGTTT	58.7	604	DRR	Ca_07880	T	--
Ca1_P1_6206568	Ca1	6206568	CTTTTTT/CTTTTTTTT	TTATGCTTTCAACCAGT GCG	TGTCGGATAAGTGTGA TATATTCG	59.9	747	INTERGENIC	--	--	--
Ca1_P1_6212978	Ca1	6212978	AATATATATATAT/AATATATATATATA T	GTGCGCAGTACAATTC TCCA	CAACCGCTCCCAAATA GAAA	59.9	573	INTERGENIC	--	--	--
Ca1_P1_6214084	Ca1	6214084	TAAACTAATTTTTTAATTGAAACTA/TA AACTAATTTTTTAATTGAAACTAATTTT TTAATTGAAACTA	TGTCACCGTTATACGA GTTTTAGA	TTGGCGTAGTGAAACG AACA	58.0	618	INTERGENIC	--	--	--
Ca1_P1_6219100	Ca1	6219100	TTACGGTAT/TT	TGGGAAAACTTGTTCC ATGAG	TTGATCTCCACATCAC CAAGA	60.0	587	INTERGENIC	--	--	--
Ca1_P1_6226167	Ca1	6226167	CT/CTT	CCTATTTCGCCTTGCA TTGT	CAACCATGTGTCAAAC CAGG	60.1	385	DRR	Ca_07881	S	bZIP
Ca1_P1_6262149	Ca1	6262149	TATGTATAATGTATAATGT/TATGTATA ATGT	CATCACAACCATTCTC CCCT	CGTTGCGTTTGAATTC TGTG	59.8	576	DRR	Ca_07886	--	--
Ca1_P1_6267078	Ca1	6267078	GAAA/GA	TTTCTTGCACTGGTTT GTGC	TGGGTCCCTCATTGTA CTCC	59.9	560	INTERGENIC	--	--	--
Ca1_P1_6268048	Ca1	6268048	GAACA/GAACACTAATAACA	ATTAGCCGTGGCAGTG TAGG	TAACATGTGCTTTCGG TTGC	60.2	446	INTERGENIC	--	--	--
Ca1_P1_6269904	Ca1	6269904	CAGAAC/CAGAACAGAATAGAAC	AACCACTATTATGGCG GTGC	CAGGAATCTCTGCCAC ACAA	59.9	415	INTERGENIC	--	--	--

INDEL marker IDs	Chromosomes/ unanchored scaffolds	Physical positions (bp)	InDels (*Kabuli* reference genome-CDC Frontier/PI)	Forward primers (5'-3')	Reverse primers (5'-3')	Anneling temperature (0C)	Expected amplified product size (bp)	Structural annotation		Functional annotation	
								Sequence components of *kabuli* genome	*Kabuli* gene accession IDs	NCBI-KOG	TFs
Ca1_P1_6277414	Ca1	6277414	ATT/ATTT	TACTCCCATCCATGCAATCA	CCAATATGGAGTCCACGTCA	59.9	491	DRR	Ca_07888	--	--
Ca1_P1_6279076	Ca1	6279076	CAGAAGAAGAAGA/CA	TCAATCGCGAAATGTGGATA	ATCACCGGCGTTTCTGTTAC	60.0	783	DRR	Ca_07888	--	--
Ca1_P1_6284031	Ca1	6284031	CATATAT/CATAT	CCTCACTCCTCCCAACAAAA	CCTTTCGAGATGCTGGACTC	60.1	484	INTERGENIC	--	--	--
Ca1_P1_6286237	Ca1	6286237	CAAAAAAAAAAAAAAAA/CAAAAAAAAAAAA	ATGCAGGCAAGAACAAATCC	TTAGGATTCATAATCGCGGC	60.1	713	DRR	Ca_07889	--	--
Ca1_P1_6293780	Ca1	6293780	TAAA/TAAAA	ACACACTGATCGATGATCCC	TATCTTTTTGGTGGGCCAAG	58.3	716	INTERGENIC	--	--	--
Ca1_P1_6294144	Ca1	6294144	TAAAAAA/TAAAAAAA	CTTGGCCCACCAAAAAGATA	GGGGATGAATGAAAAACACG	59.9	351	INTERGENIC	--	--	--
Ca1_P1_6298941	Ca1	6298941	GTT/GTATTT	CTCGTCGTCGTCGTCAATTA	TTCAATGACATGATTGGAGACTTT	59.9	110	DRR	Ca_07890	--	--
Ca1_P1_6311246	Ca1	6311246	GTTATTATT/GTTATT	CGGAGAACCAATCACTCACA	TGCAGTCAACAGTCACAAAAA	59.7	457	INTERGENIC	--	--	--
Ca1_P1_6311738	Ca1	6311738	ATAGT/AT	TTTTTGTGACTGTTGACTGCAT	TTTTCCCCTCATCGTTTGTC	58.3	275	INTERGENIC	--	--	--
Ca1_P1_6313903	Ca1	6313903	TAAA/TAAAA	GGAGTCTTGTGTCATGTGCAA	TTCACGTGCTTTTTAATTTTGTG	59.7	529	INTERGENIC	--	--	--
Ca1_P1_6314409	Ca1	6314409	ATAAATAAAT/A	CACAAAATTAAAAAGCACGTGAA	GCGACATACGAAACGAGGAT	59.2	592	INTERGENIC	--	--	--
Ca1_P1_6316633	Ca1	6316633	CATA/C	CTGTTGGAGCTGAGGTGACA	CTAATGGCGGTTACGCAAAT	60.0	296	INTRON	Ca_07892	B	MYB_related

INDEL marker IDs	Chromosomes/ unanchored scaffolds	Physical positions (bp)	InDels (*Kabuli* reference genome-CDC Frontier/PI)	Forward primers (5'-3')	Reverse primers (5'-3')	Anneling temperature (0C)	Expected amplified product size (bp)	Structural annotation		Functional annotation	
								Sequence components of *kabuli* genome	*Kabuli* gene accession IDs	NCBI-KOG	TFs
Ca1_P1_6317430	Ca1	6317430	TTAAAG/TTAAAGCCATAAATAATAAAG	GCTGAGCATGTTGGAACAAA	AAGATCCCCCATGGAAACTC	59.8	606	INTRON	Ca_07892	B	MYB_related
Ca1_P1_6321573	Ca1	6321573	TTATATATATATATATATATATATATAT/TTATATATATATATATATATATATATATATATAT	CAGGTAATTGCAGAATGGGG	TTTTCATGGCACATTTGGTG	60.3	697	DRR	Ca_07892	B	MYB_related
Ca1_P1_633425	Ca1	633425	AATATATATATATATATATATATATATATATATATATATATATAT/AATATATATATATATATATATATATATATATAT	GGACCACGAATGTAATTTAGCC	TCGCATCAACAATAATGCCT	59.7	652	INTERGENIC	--	--	--
Ca1_P1_6337777	Ca1	6337777	CATACAATACAA/CATACAA	CGGACACAAGCCTCTCTCTT	TTTTATTTTGACCCAATCAACG	59.6	674	INTERGENIC	--	--	--
Ca1_P1_6337857	Ca1	6337857	TAAAAA/TAAAAAA	CGGACACAAGCCTCTCTCTT	TTTTATTTTGACCCAATCAACG	59.6	674	INTERGENIC	--	--	--
Ca1_P1_6338411	Ca1	6338411	AAATTAA/AAA	CAACGTTGATTGGGTCAAAA	CCCTTCATACACGGACCATC	59.4	662	INTERGENIC	--	--	--
Ca1_P1_6345643	Ca1	6345643	AATGATGATGATGATGATGATG/AATGATGATGATGATGATGATGATGATGATG	CACCTTGTTCACACACACCC	TTTGTTGCCAATGTTGTTTCA	59.9	450	CDS (large-effect mutations)	Ca_07895	--	--
Ca1_P1_6352421	Ca1	6352421	CTATATATATAT/CTATATATAT	TCAACAATCGAATCAAAATCAAA	ACGGATTGTAGAACCGTTGG	59.5	872	DRR	Ca_07897	--	--
Ca1_P1_6362852	Ca1	6362852	TC/T	AGTTTGGAGCGCATGTTTCT	TTGTGTGTCGTAGTCCCCAA	59.9	358	INTERGENIC	--	--	--
Ca1_P1_6363129	Ca1	6363129	CAATAATAATAATAAT/CAATAATAATAATAAT	TTGGGGACTACGACACACAA	GATATTGAGGTGGCGGCTAA	60.0	558	INTERGENIC	--	--	--
Ca1_P1_6374117	Ca1	6374117	CAA/CA	TGCCATTCAAAAATAAGGCA	TCCATCTTGGACCAATCTCA	59.1	792	INTERGENIC	--	--	--
Ca1_P1_6374330	Ca1	6374330	AAAGAAGAAGAAGAA/AAAGAAGAAGAA	CAAAATGATGGACACCCTTG	TCCATCTTGGACCAATCTCA	58.8	291	INTERGENIC	--	--	--

INDEL marker IDs	Chromosomes/ unanchored scaffolds	Physical positions (bp)	InDels (*Kabuli* reference genome-CDC Frontier/PI)	Forward primers (5'-3')	Reverse primers (5'-3')	Anneling temperature (0C)	Expected amplified product size (bp)	Structural annotation		Functional annotation	
								Sequence components of *kabuli* genome	*Kabuli* gene accession IDs	NCBI-KOG	TFs
Ca1_P1_637710	Ca1	637710	ATTTTTTTTTT/ATTTTTTTTT	GCCCAATACCTTTGCCATAA	TGAGCAGATTTAACCCCAAAA	59.8	672	DRR	Ca_00074	--	--
Ca1_P1_6403212	Ca1	6403212	AA/AATAATA	TCACGCACGTTAAACTTCCA	TGCACCGACCTACACACATT	60.3	501	INTERGENIC	--	--	--
Ca1_P1_6408616	Ca1	6408616	CTATACA/CTATACAATTATACA	GGTCATGCACTACCACATGC	GGTCTCACTTTGCCTTCTGG	60.0	438	INTERGENIC	--	--	--
Ca1_P1_6426099	Ca1	6426099	AAAA/AAAATAAA	TCAAATTTACGTGCGGTTTG	AGCGGATGGTGAGAAAGAAA	59.6	395	INTERGENIC	--	--	--
Ca1_P1_6429504	Ca1	6429504	AATTA/AA	ATGGGTTTAACTCGTGGTGC	TTGTAGGGGTTGGAAAATCA	59.9	816	INTERGENIC	--	--	--
Ca1_P1_6444712	Ca1	6444712	A/AG	GTGAAGGTCTTTCGACGCTC	TGTTCCTGGCTAAATGCTGA	60.0	531	DRR	Ca_07908	K	Trihelix
Ca1_P1_6453816	Ca1	6453816	AT/ATT	GGTCTTGCTGACCTTGAAGC	CACGTTAAAATCTCTAACGATGAA	60.0	644	DRR	Ca_07909	--	Trihelix
Ca1_P1_6454462	Ca1	6454462	T/TGTA	TTTCGTGTGCAACGTAGGAT	TGCGTTGTGGTAATTGGAAG	59.2	521	DRR	Ca_07909	--	Trihelix
Ca1_P1_6461935	Ca1	6461935	AAAA/AAAATAAA	AAACAAAGTATCAACGCGGC	TGGGAAATTTTTGTGTCGTG	60.1	612	INTERGENIC	--	--	--
Ca1_P1_6464068	Ca1	6464068	GAA/GA	AGCCTGAAATGTCTACCCACA	TAGGGGTATTCGTGGTGTGC	59.6	708	INTERGENIC	--	--	--
Ca1_P1_6467793	Ca1	6467793	GGAG/GGAGAG	AAAGGGCAAGATTTGTCCAC	AAGGGATAATGTGTTCCCCC	59.0	197	DRR	Ca_07910	S	--
Ca1_P1_6476072	Ca1	6476072	GAAT/GAATTAAT	CAATTCAAACCCCCTTCCTT	AAATGATGCTCAAGCGACAA	60.2	623	INTERGENIC	--	--	--

INDEL marker IDs	Chromosomes/ unanchored scaffolds	Physical positions (bp)	InDels (*Kabuli* reference genome-CDC Frontier/PI)	Forward primers (5'-3')	Reverse primers (5'-3')	Anneling temperature (0C)	Expected amplified product size (bp)	Structural annotation		Functional annotation	
								Sequence components of *kabuli* genome	*Kabuli* gene accession IDs	NCBI-KOG	TFs
Ca1_P1_6525194	Ca1	6525194	TCACACA/TCACACACA	ATGGACCTGCAATCACACAA	AAGGCCTACCAATCCCAATC	60.0	611	INTRON	Ca_07917	I	Trihelix
Ca1_P1_6532843	Ca1	6532843	CAAA/CAAAA	CAGCACCGATATTGATAAGCA	TTTTTGGCTCCTACCACCAC	58.8	396	INTERGENIC	--	--	--
Ca1_P1_6535430	Ca1	6535430	GTTATAGTTTTTATAGTT/GTTATAGTT	GCGATGATCGATGACACTCTT	CAAAGTCGAGTATCACCGCC	60.2	236	INTERGENIC	--	--	--
Ca1_P1_6535635	Ca1	6535635	TA/TATACA	CACTCAATGGTGGCACACTC	CAAAATTGTAAAATGATTGGTGCT	60.2	480	INTERGENIC	--	--	--
Ca1_P1_6541418	Ca1	6541418	TAAA/TAA	CTGCAGGGAAAATTTTGGAA	ATGGAGTTCTGTCCTGGTGG	60.0	622	INTRON	Ca_07919	R	bZIP
Ca1_P1_6543503	Ca1	6543503	AAAGAAG/AAAGAAGAAG	ACGTGATTCTGCCAAACTCC	TATGGTAATCCCTCACCCCA	60.1	579	INTERGENIC	--	--	--
Ca1_P1_6546764	Ca1	6546764	TCCCC/TCCC	TGGGACATGGCATCTCATAA	ATTCCAAACCACCGCTATTG	59.9	265	INTERGENIC	--	--	--
Ca1_P1_6553971	Ca1	6553971	GAAAAA/GAAAAAA	TTGTTTTATCCGCATCTCCC	CGGGGATGGTATACTGGATG	59.9	371	INTRON	Ca_07920	P	MYB_related
Ca1_P1_6565458	Ca1	6565458	CCTGTCTCAACTGT/CCTGT	GACTGCTGCTGGACAAATGA	CATAGCATGCTCAGCCAAAA	60.0	654	INTRON	Ca_07921	H	--
Ca1_P1_6657702	Ca1	6657702	TTATATATATATATATATAT/TTATATATATATATATATATATAT	CACCCCAATTCCAATCAAAC	ATGATTTGTTTGCCTGGCTC	60.0	691	INTERGENIC	--	--	--
Ca1_P1_6847788	Ca1	6847788	TATT/TATTATTATAATT	GCGTATGGGTTGATTTTGCT	GGAATGTGTCACGACAGTGG	60.0	738	INTRON	Ca_07951	U	--
Ca1_P1_6883190	Ca1	6883190	CTTTTTTTTT/CTTTTTTTTTTT	CGAAAATGATGAATATGCGAA	CACTCCAATGCAATAGTCCAAA	58.6	688	INTERGENIC	--	--	--

INDEL marker IDs	Chromosomes/ unanchored scaffolds	Physical positions (bp)	InDels (*Kabuli* reference genome-CDC Frontier/PI)	Forward primers (5'-3')	Reverse primers (5'-3')	Anneling temperature (0C)	Expected amplified product size (bp)	Structural annotation		Functional annotation	
								Sequence components of *kabuli* genome	*Kabuli* gene accession IDs	NCBI-KOG	TFs
Ca1_P1_6902783	Ca1	6902783	ATATTATTATTATTATTATTATTATTATTATTATTATTA/ATATTATTATTATTATTATTATTATTATTATTATTA	ATGAGTGGAAGAATACGCCG	GGACAGAACCCTGCATAGGA	60.1	669	INTRON	Ca_07956	--	bHLH
Ca1_P1_6965841	Ca1	6965841	CTTTTTT/CTTTTTTT	GTTTTCGTTCTCTCCTCCCC	TGTCATCAGGGAAACCACAA	60.1	759	INTERGENIC	--	--	--
Ca1_P1_6967420	Ca1	6967420	TAAAAA/TAAAA	CAGCGACCACATTTGCTTTA	CCTATCCTTGTGATTCCCGA	59.9	353	INTERGENIC	--	--	--
Ca1_P1_6995429	Ca1	6995429	CTATATATATATATATATA/CTATATATATATATATA	TTTTTACTCACTCACGTGTTTTCA	ATTTCATCCCTCACACCCAA	58.9	366	INTERGENIC	--	--	--
Ca1_P1_6999458	Ca1	6999458	TTATATATATATATATATATATATAT/TTATATATATATATATATATATATATAT	AACAACGGCTTTCATTGACTTT	TTTTTCCTGAAACAGGCAGT	60.0	795	INTERGENIC	--	--	--
Ca1_P1_7215414	Ca1	7215414	AA/AACA	AACCCCCAACTACAAGGTCC	CAATTTGACAAGCCCCCTAA	60.1	639	INTERGENIC	--	--	--
Ca1_P1_7274145	Ca1	7274145	AATATATATATATATATATATATATATA/AATATATATATATATATATATATATA	TGAATGAACTTTTATCCTGCCA	CAGCGTGAACGACTTCAAAA	59.6	539	INTERGENIC	--	--	--
Ca1_P1_7422088	Ca1	7422088	GGAAGAAGAAGAAGAAGAAGAAGAAGAAGAA/GGAAGAAGAAGAAGAAGAAGAAGAAGAAGAAGAAGAA	AGAGCCACCACTTTCCTTCA	TCTCTCTCGAGATTCGTTTGTG	59.8	117	INTERGENIC	--	--	--
Ca1_P1_7423449	Ca1	7423449	CTTTTTTTTTTTT/CTTTTTTTTTTT	CCATTTGGTTGCTTGGTTTT	TTTTGCTTATATGTTTGGTTCTTGA	59.8	118	INTERGENIC	--	--	--
Ca1_P1_7573998	Ca1	7573998	TAGAGAGAGAGAGAGAGAGAGAGAGAGA/TAGAGAGAGAGAGAGAGAGAGAGAGAGAGA	AAATGGGCAATGGATTGAAG	GAATTGGACCCAGAGAACCA	59.8	260	INTRON	Ca_08012	--	--
Ca1_P1_7630412	Ca1	7630412	G/GACAAAA	TCGTTGCGAAGATAGTGTGG	AGCATGAGTTGCACAAGTGG	59.9	768	CDS (large-effect mutations)	Ca_08016	--	HD-ZIP
Ca1_P1_7634571	Ca1	7634571	GT/GTT	AAAGTGAATTGCTCAATTTCGTC	AATTGACACGTGAAGATTTGTTT	59.7	777	INTERGENIC	--	--	--

INDEL marker IDs	Chromosomes/ unanchored scaffolds	Physical positions (bp)	InDels (*Kabuli* reference genome-CDC Frontier/PI)	Forward primers (5'-3')	Reverse primers (5'-3')	Anneling temperature (0C)	Expected amplified product size (bp)	Structural annotation		Functional annotation	
								Sequence components of *kabuli* genome	*Kabuli* gene accession IDs	NCBI-KOG	TFs
Ca1_P1_7634633	Ca1	7634633	ATTTTTTTT/ATTTTTTTTTT	TCCAGCACAAATACCTTTTTACC	TGAATTCGCGTTGTTTGTGT	59.4	666	INTERGENIC	--	--	--
Ca1_P1_7635339	Ca1	7635339	ATGTCATCCCT/AT	GCACGCGTTTTCAATGTAGA	AAATGTCATCCCTCAGCGAC	59.9	207	INTERGENIC	--	--	--
Ca1_P1_7639400	Ca1	7639400	AAAATAAATAAATAAA/AAAATAAATAAATAAATAAA	TCCGGAAAATTTGCACCTAC	GGTCTGTTGCCCAAACAACT	59.9	287	INTERGENIC	--	--	--
Ca1_P1_7642434	Ca1	7642434	TA/TAA	TGCCACACAAAAATCTTGGA	TAACCTACGCTTGTGGGGTC	60.1	219	INTERGENIC	--	--	--
Ca1_P1_7652722	Ca1	7652722	TAATTAATCAATCAATTAAT/TAATTAAT	CAAGCTTCAAAGTCCGGAAG	CAGATCTTCCGTCCCGATAA	60.0	500	INTRON	Ca_08019	Z	--
Ca1_P1_7653498	Ca1	7653498	TAAAAAAAAAAAA/TAAAAAAAAAA	AGTGTTGAGGGCATTTTTGG	TGAGTCCAAAAGCATAAATGAGA	60.0	573	INTERGENIC	--	--	--
Ca1_P1_7654198	Ca1	7654198	AG/A	CAAAGTCATTTGTGGTGCACTT	CGGTAGACAAATCACGTGGG	60.1	280	INTERGENIC	--	--	--
Ca1_P1_7655351	Ca1	7655351	TA/T	ATCCGTGGTGAAATCTCGTC	TGGTGTTGCATCCTTTTCAA	59.9	123	INTERGENIC	--	--	--
Ca1_P1_7655921	Ca1	7655921	ATTAAATTTAAATTT/ATTAAATTT	AAACGCTTTTAACATCTGCCA	ATTCCAATCTGGCAATCAGC	59.8	929	INTERGENIC	--	--	--
Ca1_P1_7660688	Ca1	7660688	GAAAAAAAA/GAAAAAAAAAA	TTTCTGATTGGTGGACTTGTTC	GGTTGGTGGGTATCCTTTGA	59.1	205	INTERGENIC	--	--	--
Ca1_P1_7661306	Ca1	7661306	CTT/CTTT	AGTGTGAGCTTCTTTTCCGC	CACGAAATGAATAAACAGACATTTG	59.6	266	INTERGENIC	--	--	--
Ca1_P1_7661630	Ca1	7661630	GAAA/GAAAA	AGTGTGAGCTTCTTTTCCGC	TCCCACACAAGAATCCATCA	59.6	777	INTERGENIC	--	--	--

INDEL marker IDs	Chromosomes/ unanchored scaffolds	Physical positions (bp)	InDels (*Kabuli* reference genome-CDC Frontier/PI)	Forward primers (5'-3')	Reverse primers (5'-3')	Anneling temperature (0C)	Expected amplified product size (bp)	Structural annotation		Functional annotation	
								Sequence components of *kabuli* genome	*Kabuli* gene accession IDs	NCBI-KOG	TFs
Ca1_P1_7665875	Ca1	7665875	TA/T	GAATGGAATCAATTGG GTGG	AGGAAGAGACCCAACA AGCA	60.0	256	DRR	Ca_08020	--	--
Ca1_P1_7669378	Ca1	7669378	AATAT/AATATAT	CGTTCTTTGCGTGTTG CTTA	ACTGTTGTGCAGTGAG TGGG	60.1	828	INTERGENIC	--	--	--
Ca1_P1_7671768	Ca1	7671768	ACCCC/ACCCCC	GGCCTTGAACGTTTAA CCAA	TGTCGTCATAAGGTGG TGGA	60.0	699	INTERGENIC	--	--	--
Ca1_P1_7675946	Ca1	7675946	GAAAAAAA/GAAAAAA	TGAGACCCAAGTTAAA TCCGA	AATCCAAATAGTAGCC GCAA	59.6	632	INTERGENIC	--	--	--
Ca1_P1_7677005	Ca1	7677005	TCA/T	TTGCGAATGTCGACCT TAAA	TGAATTTTGTTCGCAT TTCATT	59.3	870	INTERGENIC	--	--	--
Ca1_P1_7677905	Ca1	7677905	CTATTATTATTATTATTATTATT/CTATT ATTATTATTATTATTATTATT	ATCGGAATCAAACCAC TTCG	TGAGCCGGAATATTGA GATTG	59.9	567	INTERGENIC	--	--	--
Ca1_P1_7678036	Ca1	7678036	TAAAAAAAA/TAAAAAAA	ATCGGAATCAAACCAC TTCG	ACTTTGTCATCTGTGT GCGG	59.9	852	INTERGENIC	--	--	--
Ca1_P1_7687290	Ca1	7687290	ATT/AT	AATCGAACGAAGGCTG ACCT	AGGATGGACTTCATGC TTCC	61.2	126	INTERGENIC	--	--	--
Ca1_P1_7688188	Ca1	7688188	C/CTA	AAGCCAAAATAATAAG GCTCAAGA	CAATAAATGCCCTCAT CCGT	59.7	630	INTERGENIC	--	--	--
Ca1_P1_7688347	Ca1	7688347	T/TCAAAA	AAGCCAAAATAATAAG GCTCAAGA	AGCACAACAGGGATTT ACGG	59.7	895	INTERGENIC	--	--	--
Ca1_P1_7695907	Ca1	7695907	G/GC	CCAACACCAAACTCGT TCCT	TGAAGAGATTGGCCTG CTTT	60.0	722	DRR	Ca_08022	--	GRAS
Ca1_P1_7699454	Ca1	7699454	CCA/C	GACAGAGACCAATCAT GCGA	GCAATGGGATAAAAAG GTGG	59.8	117	INTERGENIC	--	--	--

INDEL marker IDs	Chromosomes/ unanchored scaffolds	Physical positions (bp)	InDels (*Kabuli* reference genome-CDC Frontier/PI)	Forward primers (5'-3')	Reverse primers (5'-3')	Anneling temperature (0C)	Expected amplified product size (bp)	Structural annotation		Functional annotation	
								Sequence components of *kabuli* genome	*Kabuli* gene accession IDs	NCBI-KOG	TFs
Ca1_P1_7706813	Ca1	7706813	AATAT/AATATAT	TTTGGTGTCGGTTTGACAAT	AAATTTCCAGACACATGCCC	58.9	685	INTERGENIC	--	--	--
Ca1_P1_7712070	Ca1	7712070	GCTACTAC/GCTAC	CGCATCAGCCAACTACTCAA	TGGAAATAATAATGGACCGCA	60.0	530	DRR	Ca_08023	K	bHLH
Ca1_P1_7712937	Ca1	7712937	CAAA/CAAAA	CACGACCTAAAACCGAATGC	CCCCTTCATGTTGTTCGTTT	60.5	428	INTERGENIC	--	--	--
Ca1_P1_7713145	Ca1	7713145	AC/A	AAACGAACAACATGAAGGGG	AGACGACGATGATTGTCACAT	59.8	233	INTERGENIC	--	--	--
Ca1_P1_7713182	Ca1	7713182	TAA/TAAA	AAACGAACAACATGAAGGGG	AGACGACGATGATTGTCACAT	59.8	233	INTERGENIC	--	--	--
Ca1_P1_7728817	Ca1	7728817	ATTTTTTTT/ATTTTTTTTT	CATCTGATCCAAAATTGAGCC	AAAACCCATTTTGCAAATCAA	59.5	390	INTERGENIC	--	--	--
Ca1_P1_7745366	Ca1	7745366	A/ATAT	TGGAGAAACTCTTCCGCTGT	ACTTTGGGGAGAGGCTTTGT	60.0	175	INTERGENIC	--	--	--
Ca1_P1_7748427	Ca1	7748427	CTTTT/CTTTTT	TAGTGGGTTCACTTGGGGAG	TTCTTTTTGTGCATTAAGTTGGAA	60.0	460	DRR	Ca_08026	--	--
Ca1_P1_7748872	Ca1	7748872	CTATATATATATATATATATATATATATATATATATATATATAT/CTATATATATATATATATATATATATATATATATATATATATATATATATAT	TTCCAACTTAATGCACAAAAAGAA	TCTCCATCACTCTCCTTTACGA	60.0	234	INTERGENIC	--	--	--
Ca1_P1_7755830	Ca1	7755830	TTATATATATATATATATATATATATATATAT/TTATATATATATATATATATATATATATATATATAT	GGAGCCAGAAAAATGGTGTG	TGTGTTGCAATTCGAAGGAA	60.5	604	INTERGENIC	--	--	--
Ca1_P1_7763628	Ca1	7763628	ATGAAA/ATGAAAATCTGTGAAA	CAGCTCCTCCCAACATCATT	AGCTGCAGTGTTGTATGCCA	60.1	364	INTERGENIC	--	--	--
Ca1_P1_7775058	Ca1	7775058	C/CCTTAAT	AGCCCAACCCTTGACCTAAT	GCACGGAAACAACATGTGAC	59.8	838	INTERGENIC	--	--	--

INDEL marker IDs	Chromosomes/ unanchored scaffolds	Physical positions (bp)	InDels (*Kabuli* reference genome-CDC Frontier/PI)	Forward primers (5'-3')	Reverse primers (5'-3')	Anneling temperature (0C)	Expected amplified product size (bp)	Structural annotation		Functional annotation	
								Sequence components of *kabuli* genome	*Kabuli* gene accession IDs	NCBI-KOG	TFs
Ca1_P1_7778630	Ca1	7778630	CTTT/C	CCACACACATCAACCACCAC	GGGCGATTGACTTGAGACAT	60.8	444	INTERGENIC	--	--	--
Ca1_P1_7781067	Ca1	7781067	CATTATTATTATTATTATTATTATTATTATTATTATTATTAT/CATTATTATTATTATTATTATTATTATTATTATTATTATTATTAT	CATGTGTGATTGGTGGGGTA	TCTGTGTGCAACGAAGGAAG	60.1	465	DRR	Ca_08030	--	GRAS
Ca1_P1_7781791	Ca1	7781791	CAA/C	TGCAATCTCCTTCACACTCG	AAGTTGCTTTGTTTGAACGC	60.0	324	DRR	Ca_08030	--	GRAS
Ca1_P1_7784640	Ca1	7784640	T/TG	ATTGGGCATGTTTCGTCTGT	TGGGTAGACCTCTTTGACCG	60.4	411	INTERGENIC	--	--	--
Ca1_P1_7784813	Ca1	7784813	CA/C	ATTGGGCATGTTTCGTCTGT	TGGGTAGACCTCTTTGACCG	60.4	411	INTERGENIC	--	--	--
Ca1_P1_7789823	Ca1	7789823	ATTTTTT/ATTTTTTT	CAAAACGCGGGTTAACATCT	TGAAGGGAGGAGATTTGGTG	60.0	779	INTERGENIC	--	--	--
Ca1_P1_7790046	Ca1	7790046	AATATATATATA/AATATATATATATA	ATACCAAAAACTTGTGCGGG	TGAAGGGAGGAGATTTGGTG	59.9	561	INTERGENIC	--	--	--
Ca1_P1_7790700	Ca1	7790700	AAAA/AAAACTGCAAA	TCCATAATGTCAAGTTTTGAGCC	ACATCCACAGCACTTGCATC	60.4	949	INTERGENIC	--	--	--
Ca1_P1_7793042	Ca1	7793042	TAAAA/TAAA	TGATGAGACCGAGCACTTTG	TGGAAAACTCAAGTGCCACA	60.0	353	INTERGENIC	--	--	--
Ca1_P1_7794125	Ca1	7794125	TA/T	TGATTTGGGGGTGATGATCT	TCATTGACCAAATTTCCGGT	60.1	572	INTERGENIC	--	--	--
Ca1_P1_7810637	Ca1	7810637	G/GAA	AACGATTGGTTGAGACCCAG	ACCTTGTGTCTATCTGGCCG	60.0	505	DRR	Ca_08031	--	FAR1
Ca1_P1_7811994	Ca1	7811994	C/CTAGAA	TGCACTGCTTTGAGTGGTTC	CGGTAAGTGTTTGTGCATGG	60.0	291	INTRON	Ca_08032	--	--

INDEL marker IDs	Chromosomes/ unanchored scaffolds	Physical positions (bp)	InDels (*Kabuli* reference genome-CDC Frontier/PI)	Forward primers (5'-3')	Reverse primers (5'-3')	Anneling temperature (0C)	Expected amplified product size (bp)	Structural annotation		Functional annotation	
								Sequence components of *kabuli* genome	*Kabuli* gene accession IDs	NCBI-KOG	TFs
Ca1_P1_7813845	Ca1	7813845	ACT/A	AACCAGAAGGCACACAGCTT	AAGCAGCTAGACTGCAAGCC	59.9	602	INTRON	Ca_08032	--	--
Ca1_P1_7841172	Ca1	7841172	A/AC	TTTTGGCGTTGTTGATGTGT	GTACCACACGGGAGCTTCAT	60.0	839	INTERGENIC	--	--	--
Ca1_P1_7841241	Ca1	7841241	TAAAAAAA/TAAAAAAAAAA	CACGACCGTCCGATTAAGAT	GTTGTACCACACGGGAGCTT	60.0	473	INTERGENIC	--	--	--
Ca1_P1_7841312	Ca1	7841312	TT/TTGT	CACGACCGTCCGATTAAGAT	GTTGTACCACACGGGAGCTT	60.0	473	INTERGENIC	--	--	--
Ca1_P1_7845858	Ca1	7845858	GTTT/GTTTT	AACGAATTTTCAGCAAAGCA	GAGGCAGGAAAAGATGTTGC	58.6	569	DRR	Ca_08034	--	GRAS
Ca1_P1_7865713	Ca1	7865713	C/CT	TGCACTCACTCCAACAAACA	CAAAGCATACCAACCACAGC	58.8	799	INTERGENIC	--	--	--
Ca1_P1_7883822	Ca1	7883822	TAAAAAAAAA/TAAAAAAAAAAAAAAA	AGGATGCGACCTACATCTGG	AAAAACGCAGGCATTCTTTG	60.1	440	DRR	Ca_08037	I	bHLH
Ca1_P1_7887908	Ca1	7887908	TCC/TC	TGTGAATAGGCTAGGCCGTC	GGCCTGAGTCTGACCTACGA	60.2	604	INTERGENIC	--	--	--
Ca1_P1_7889640	Ca1	7889640	TT/TTCT	TCACCTTCAAAATTAACGGGAC	GATGGAAAAGGACGGAGACA	60.2	462	INTERGENIC	--	--	--
Ca1_P1_7895366	Ca1	7895366	GAAA/GAA	TTGCCACAAGCTAAAAGGCT	TGGTCAGTTTGGCCTATTCC	60.0	514	DRR	Ca_08038	--	--
Ca1_P1_7904061	Ca1	7904061	TCC/TC	ATCAAATGTGGTCAGAGCCC	AATAAATTGCGGCCACTACG	59.9	411	DRR	Ca_08039	R	--
Ca1_P1_7915897	Ca1	7915897	GAAAA/GAAAATAAAA	CTAACCTCTGGAGCACCGAC	GCACAATACAAGGCTGAGCA	59.9	459	INTERGENIC	--	--	--

INDEL marker IDs	Chromosomes/ unanchored scaffolds	Physical positions (bp)	InDels (*Kabuli* reference genome-CDC Frontier/PI)	Forward primers (5'-3')	Reverse primers (5'-3')	Anneling temperature (0C)	Expected amplified product size (bp)	Structural annotation		Functional annotation	
								Sequence components of *kabuli* genome	*Kabuli* gene accession IDs	NCBI-KOG	TFs
Ca1_P1_7926977	Ca1	7926977	TG/T	GCATTGGCATTCATTG TGAG	CCCTTACCTTGAGGCA AACA	60.1	758	DRR	Ca_08042	--	HB-other
Ca1_P1_7929093	Ca1	7929093	GA/G	AATGCCTAAAAGCAAT GCAAA	AACACGATGAGATGGA AGGG	59.7	527	INTERGENIC	--	--	--
Ca1_P1_7933454	Ca1	7933454	A/ATG	CGCAACTAATTTCCTT CAACAA	CCTTCACAAACTTGAA GGACC	59.3	591	INTERGENIC	--	--	--
Ca1_P1_7942966	Ca1	7942966	TAAA/TAAAA	GCACTTTCCATCCTTTT GGA	GAAAAGGCCTCCAACA CAAA	60.1	378	INTRON	Ca_08044	QI	B3
Ca1_P1_7943283	Ca1	7943283	CA/CAA	TGTTGGAGGCCTTTTC CTTA	TTATCACCTGGCCAAA AAGC	59.7	521	INTRON	Ca_08044	QI	B3
Ca1_P1_7946141	Ca1	7946141	GTT/GTTT	GGCATGCATGACTTTG TGAA	TTTTGTGTTAGATTCAT GTCTCCAG	60.7	471	INTERGENIC	--	--	--
Ca1_P1_7947587	Ca1	7947587	AC/ACC	TCTTCCTCCAAATTCCA ATCA	TTTTTATTCTCGTGGG AGTGAG	59.5	578	INTERGENIC	--	--	--
Ca1_P1_7947965	Ca1	7947965	TAAAAAAAAA/TAAAAAAA	TCATGGTATTACCCGT ACACTTACA	TTTTTATTCTCGTGGG AGTGAG	59.6	540	DRR	Ca_08045	QI	B3
Ca1_P1_7948441	Ca1	7948441	AATATATATATATATATA/AATATATAT ATA	CTCACTCCCACGAGAA TAAAAA	TGGAAGAGGAGAGAT GCAAAA	58.4	661	DRR	Ca_08045	QI	B3
Ca1_P1_7948690	Ca1	7948690	ATTTTTTT/ATTTTTTTT	TTTTGCATCTCTCCTCT TCCA	CCAACAGTTCCGACAT CTCA	59.9	453	DRR	Ca_08045	QI	B3
Ca1_P1_7954789	Ca1	7954789	TTAT/TT	CCCCTCGGAACTTTCA TTTT	TCGTAATTGCTTTCTG GCCT	60.3	558	INTRON	Ca_08045	QI	B3
Ca1_P1_7954891	Ca1	7954891	TAAAAAAAA/TAAAAAAA	CCCCTCGGAACTTTCA TTTT	TCGTAATTGCTTTCTG GCCT	60.3	558	INTRON	Ca_08045	QI	B3

INDEL marker IDs	Chromosomes/ unanchored scaffolds	Physical positions (bp)	InDels (*Kabuli* reference genome-CDC Frontier/PI)	Forward primers (5'-3')	Reverse primers (5'-3')	Anneling temperature (0C)	Expected amplified product size (bp)	Structural annotation		Functional annotation	
								Sequence components of *kabuli* genome	*Kabuli* gene accession IDs	NCBI-KOG	TFs
Ca1_P1_7955292	Ca1	7955292	TAAA/TAA	AGGCCAGAAAGCAATTACGA	TGAAGCCAGATCCACAAGAA	59.8	655	INTRON	Ca_08045	QI	B3
Ca1_P1_7963074	Ca1	7963074	TAAAAAAAAAAAA/TAAAAAAAAAA	TGAACGAAACAGGATGACCA	CCTAACTCGTGAGGGTTCCA	60.1	907	INTERGENIC	--	--	--
Ca1_P1_7964977	Ca1	7964977	ATATTCTTT/AT	TGGTTGGTCCTATCGAGGAG	ATGGAGACCAAGTGCCAAAT	60.1	279	INTERGENIC	--	--	--
Ca1_P1_7968628	Ca1	7968628	GAAA/GAAAA	TGTGGGGGTGAAAAAGCTAA	ATAGGACGGTGAAATCGTGC	60.5	768	INTERGENIC	--	--	--
Ca1_P1_7974056	Ca1	7974056	TTT/TTTATTTTATT	CATTGTCTCATCTGTCCCGA	TCATGAGAGCATGCAAAGGT	59.6	205	INTRON	Ca_08047	U	MYB
Ca1_P1_7986734	Ca1	7986734	ATAT/ATATGTTGGTGTTAATTAT	ACACGGAAATTTGGACTTGC	ACTTTTACTGCCATGGGTGG	60.0	175	INTERGENIC	--	--	--
Ca1_P1_7987932	Ca1	7987932	TAAAAAAAAAAAAAAA/TAAAAAAAAAAA	CCAATTGATTTCACCAATCTTTT	TTCTGGACTTGTTTGGATTGA	59.3	618	INTERGENIC	--	--	--
Ca1_P1_7991091	Ca1	7991091	T/TTGAATGGTGAAAA	TAGATGCAACTGATGCGACC	TGGTTTGTATTCCTGCACCA	59.8	624	INTRON	Ca_08049	--	WRKY
Ca1_P1_7992031	Ca1	7992031	GTAA/GTAATTAA	TGATTCTCTCCCATGCCATT	TTCTTCAGCTGGTGTTGCAC	60.4	450	INTRON	Ca_08049	--	WRKY
Ca1_P1_7992471	Ca1	7992471	TGTAGTA/TGTAGTAGTA	GTGCAACACCAGCTGAAGAA	CAACATCATGATCAATGGCA	60.0	310	CDS (large-effect mutations)	Ca_08049	--	WRKY
Ca1_P1_7994189	Ca1	7994189	AATATTTTTCCATA/AATA	AACAAAAGAGGCGGCAAGTA	GAAGGTGGCACGTTAAAACA	59.9	115	INTERGENIC	--	--	--
Ca1_P1_7995324	Ca1	7995324	ATAGTAGT/ATAGTAGTAGT	CGGATTGTTTTCGATATGGG	AAGGGATTTTTATTCACAAAACAA	60.1	527	INTERGENIC	--	--	--

INDEL marker IDs	Chromosomes/ unanchored scaffolds	Physical positions (bp)	InDels (*Kabuli* reference genome-CDC Frontier/PI)	Forward primers (5'-3')	Reverse primers (5'-3')	Anneling temperature (0C)	Expected amplified product size (bp)	Structural annotation		Functional annotation	
								Sequence components of *kabuli* genome	*Kabuli* gene accession IDs	NCBI-KOG	TFs
Ca1_P1_7996926	Ca1	7996926	CAA/CAAGTTAATAAA	TCCCCGGTCACACAATAATC	TTCTCAAGCACCAAGCACAC	60.6	185	INTERGENIC	--	--	--
Ca1_P1_7997866	Ca1	7997866	AATATAT/AATAT	ATGTTGTGGGGACAAAGAGG	CTCATGATTTTGGCGAGGAT	59.8	315	INTERGENIC	--	--	--
Ca1_P1_7999912	Ca1	7999912	CTATATATATATAT/CTATATATATATATAT	AAAATTCTGTTTGGGGGACC	AAACGAGTGTGAAGGGTTGG	60.0	842	INTERGENIC	--	--	--
Ca1_P1_8000612	Ca1	8000612	T/TG	CCAACCCTTCACACTCGTTT	TGACACCAAACTCAGTCCTTG	60.0	489	INTERGENIC	--	--	--
Ca1_P1_8000857	Ca1	8000857	ATGT/AT	CGCCATCTTCACGTCACTTA	TCCAACCCAAATTTCAAACA	59.9	699	INTERGENIC	--	--	--
Ca1_P1_8001697	Ca1	8001697	AATATATATATA/AATATATATATATATA	TAAAGGTATCCGTACCCGCA	AGATGGCATGCATGATCAAA	60.3	275	INTERGENIC	--	--	--
Ca1_P1_8001965	Ca1	8001965	ATT/AT	AGTAGGGGGCCACTTCAAAT	TGGTCCATAGCCTGCATAAA	59.8	286	INTERGENIC	--	--	--
Ca1_P1_8002384	Ca1	8002384	TA/T	TTTAAATCGGGTAGGGTGGG	TGAGCCAATTCCTCTTTTGTC	60.9	424	INTERGENIC	--	--	--
Ca1_P1_8002969	Ca1	8002969	ACTT/ACTTCTT	CGCCACAAATTTGACTCTGA	GGGAATGCGATGTGAAGAAT	59.8	310	INTERGENIC	--	--	--
Ca1_P1_8006358	Ca1	8006358	GTT/GT	TCTTCCCTCCTTCCTTGGAT	TGCTCTCCAACCCAACTCTT	60.0	193	INTERGENIC	--	--	--
Ca1_P1_8006710	Ca1	8006710	TG/TGG	TCTTCCCTCCTTCCTTGGAT	TAACACAAGTAGTCGCCCCC	60.0	748	INTERGENIC	--	--	--
Ca1_P1_8015388	Ca1	8015388	CAAAAAAAAA/CAAAAAAA	CAGCAATTTGACAAGCGAAA	TTGCCCCCACTTATCAGTTC	60.0	459	INTRON	Ca_08050	R	MYB

INDEL marker IDs	Chromosomes/ unanchored scaffolds	Physical positions (bp)	InDels (*Kabuli* reference genome-CDC Frontier/PI)	Forward primers (5'-3')	Reverse primers (5'-3')	Anneling temperature (0C)	Expected amplified product size (bp)	Structural annotation		Functional annotation	
								Sequence components of *kabuli* genome	*Kabuli* gene accession IDs	NCBI-KOG	TFs
Ca1_P1_8016686	Ca1	8016686	CAAAAAAAAAA/CAAAAAAAAAAAAA	ACGAATTTTCTTAAGCGCGA	TTTTTGTTTGCGATGGAACA	60.0	207	INTERGENIC	--	--	--
Ca1_P1_8017454	Ca1	8017454	ATT/ATTTT	GGCGAAACTCGTGAGGATAC	TCAAACCATCGAACCAAACA	59.7	754	INTERGENIC	--	--	--
Ca1_P1_8022656	Ca1	8022656	AG/A	TACGGGCCTGAATTTCTTTG	CCTCCATAATTGGGGGTCTT	60.1	703	INTERGENIC	--	--	--
Ca1_P1_8031692	Ca1	8031692	TTA/TTATA	ATCTTCTTTTTGTGGGGCCT	CTTGGCAGATCTCGAGTATGAA	59.9	397	INTERGENIC	--	--	--
Ca1_P1_8031727	Ca1	8031727	CG/C	ATCTTCTTTTTGTGGGGCCT	CTTGGCAGATCTCGAGTATGAA	59.9	397	INTERGENIC	--	--	--
Ca1_P1_8035534	Ca1	8035534	T/TTA	CAAATGCAAATGCAATGACC	AGGCATCTTTTCAGGTTCCC	59.9	782	INTRON	Ca_08051	M	FAR1
Ca1_P1_8043772	Ca1	8043772	AAA/AAACAA	GACTCATGATTTGCTCTTGGC	GAAAATCTTGTTTGCTTCCCA	59.8	781	DRR	Ca_08052	U	--
Ca1_P1_8046858	Ca1	8046858	CTT/CTTT	AGGACCAACTCCATGACAGG	GGGTTTGGAAATGGTGTTTG	60.0	579	INTRON	Ca_08052	U	--
Ca1_P1_8048852	Ca1	8048852	CGAAG/C	CTGAAGAGGTGTAGGAGCGG	GGCCCAAGTCCAATTGATAA	60.0	372	INTERGENIC	--	--	--
Ca1_P1_8049957	Ca1	8049957	ATAACCATTAACCAT/ATAACCAT	AAAATGTTGCTCTTGGAAACAAA	TGGTTGTGTTGAGTTTGGGA	60.0	526	INTERGENIC	--	--	--
Ca1_P1_8061451	Ca1	8061451	TAAAA/TAA	CAAATGCACGGGTCCTAAAC	CGTTTCAACTTTCAATGCCA	60.4	622	INTERGENIC	--	--	--
Ca1_P1_8061730	Ca1	8061730	TGT/TGTTTGCAGT	AAAATGCAAGAACGGGTCAA	CTTGTGTTGTTGGGCGAATA	60.5	100	INTERGENIC	--	--	--

INDEL marker IDs	Chromosomes/ unanchored scaffolds	Physical positions (bp)	InDels (*Kabuli* reference genome-CDC Frontier/PI)	Forward primers (5'-3')	Reverse primers (5'-3')	Anneling temperature (0C)	Expected amplified product size (bp)	Structural annotation		Functional annotation	
								Sequence components of *kabuli* genome	*Kabuli* gene accession IDs	NCBI-KOG	TFs
Ca1_P1_8062208	Ca1	8062208	TCCTCCCTC/TCCTC	TTATTCGCCCAACAACACAA	TCATTGGTAGTACGTGAACCGA	60.0	758	INTERGENIC	--	--	--
Ca1_P1_8063349	Ca1	8063349	CTGGTTGGT/CTGGT	GCAGTATGGCAAGAGCAACA	TCATCACATTAAGGCCAGCA	60.0	486	INTRON	Ca_08053	O	MYB_related
Ca1_P1_8065415	Ca1	8065415	TAAAA/TAAAAA	ATTGCCACCAACATTCCATT	TCCACATTCTCGTGATGCTT	60.1	641	DRR	Ca_08053	O	MYB_related
Ca1_P1_8074446	Ca1	8074446	A/ACT	AGGGCTAAAGCAAGGGTTGT	GCTTCTCCGAATGGATTGAA	60.1	440	DRR	Ca_08054	O	--
Ca1_P1_8074695	Ca1	8074695	GAAAAA/GAAAAAA	AGGGCTAAAGCAAGGGTTGT	TGCAGCTGAAGTAGCGAAAA	60.1	652	DRR	Ca_08054	O	--
Ca1_P1_8092031	Ca1	8092031	CTTTT/CTTT	TCATCTTCGCCAGAGTTTCA	AAGGCATTGGGAAATGGTAA	59.5	480	INTERGENIC	--	--	--
Ca1_P1_8093889	Ca1	8093889	AAACAACAA/AAACAA	CAGGACCACATTTTAGGCAAA	TGGCTTTGTGGTACATTGGA	60.0	764	INTERGENIC	--	--	--
Ca1_P1_8095747	Ca1	8095747	AATATATATA/AATATATA	GGTTTTGAAGGCTTGTGTCG	GGAACAGCGATGATTGTCATT	60.7	680	INTERGENIC	--	--	--
Ca1_P1_8097574	Ca1	8097574	AAAAAATAAAAATAAAA/AAAAAATAAAA	TGGTGAGAAAGAAAATGCGA	TCAAGCATCCATCAAAACCA	59.4	738	INTERGENIC	--	--	--
Ca1_P1_8102400	Ca1	8102400	CAA/CA	CCCACCATCCATCTTCCTTA	CCTGCTGTTACACCCCTCAT	59.7	403	INTRON	Ca_08056	C	--
Ca1_P1_8114379	Ca1	8114379	AATATATATATATATATAT/AATATATATATATATATATAT	GGGAAAAGTGATTCTGGCTG	CCTGGGTTTCCCAAGTTTTC	59.7	657	DRR	Ca_08057	A	--
Ca1_P1_8118293	Ca1	8118293	TTCC/TTCCTCC	TTTTGACACATTAACTCAACCACA	GCCTTATGTTGCTTCGGGTA	59.5	656	INTERGENIC	--	--	--

INDEL marker IDs	Chromosomes/ unanchored scaffolds	Physical positions (bp)	InDels (*Kabuli* reference genome-CDC Frontier/PI)	Forward primers (5'-3')	Reverse primers (5'-3')	Anneling temperature (0C)	Expected amplified product size (bp)	Structural annotation		Functional annotation	
								Sequence components of *kabuli* genome	*Kabuli* gene accession IDs	NCBI-KOG	TFs
Ca1_P1_8119637	Ca1	8119637	ATTTT/ATT	TCAGTGGTGGCAGTTGTGAT	AAACTCAGGGGCTCCTTCAT	60.2	314	INTRON	Ca_08058	R	--
Ca1_P1_8124056	Ca1	8124056	AATATATATATATA/AATATATATATATATATATA	TGTTGAGAAGCATGCGAAAG	TCGAATCGAACACAATCCAA	60.1	918	DRR	Ca_08058	R	--
Ca1_P1_8124850	Ca1	8124850	TATTT/TATTTAAATTT	GTTCGATTCGATTGGCGTAT	GCGAACAACTTGAGTAGGGC	59.9	447	DRR	Ca_08058	R	--
Ca1_P1_8125888	Ca1	8125888	G/GACATATTAAAA	TGTTGGGTTACGAGGGAGTC	TTGCGGGTTTTTAACCTGAG	60.0	526	INTERGENIC	--	--	--
Ca1_P1_8127338	Ca1	8127338	CTT/CTTT	CCCTCCTTCCAGAAAACCTT	AATCCTGCAGTGAAACCACC	59.5	708	INTERGENIC	--	--	--
Ca1_P1_8130349	Ca1	8130349	ATTTT/ATTT	TTACTCACCAGGAACCGTCC	GTTGCCGGGTAAGTTTTCAA	60.0	557	INTRON	Ca_08059	P	--
Ca1_P1_8130436	Ca1	8130436	ATTTTT/ATTTT	TTACTCACCAGGAACCGTCC	GTTGCCGGGTAAGTTTTCAA	60.0	557	INTRON	Ca_08059	P	--
Ca1_P1_8132050	Ca1	8132050	AT/ATTT	TCTGCCAATGCTCAAATCAG	TGGCAATGTGGAGTCTATGC	59.9	181	DRR	Ca_08060	--	--
Ca1_P1_8132296	Ca1	8132296	TTACTACTA/TTACTACTACTA	TCTGCCAATGCTCAAATCAG	TCCACACAACTGCAAAGTCTAAA	59.9	768	DRR	Ca_08060	--	--
Ca1_P1_8301326	Ca1	8301326	TTATATATATATATATATATATAT/TTATATATATATATATATAT	TTTCACCACCCTCCTCAAAA	TTGCTCTGGGAATTGGAATC	60.5	620	INTERGENIC	--	--	--
Ca1_P1_8304983	Ca1	8304983	ATTTTTT/ATTTTTTT	CAAAGGCAGGTGAGTTCAAA	GCTTGTGGAGTTGTGGTCCT	58.9	652	DRR	Ca_08070	QR	B3
Ca1_P1_830680	Ca1	830680	AAATAATAATAATAATAATAATAATAATAATAATAATAAT/AAATAATAATAATAATAATAATAATAATAATAATAATAATAATAATAAT	AAGGAGTGTGCTTATTTGGCA	TCCGGCTCTATATATCACAAGACA	59.8	492	DRR	Ca_00101	--	--

INDEL marker IDs	Chromosomes/ unanchored scaffolds	Physical positions (bp)	InDels (*Kabuli* reference genome-CDC Frontier/PI)	Forward primers (5'-3')	Reverse primers (5'-3')	Anneling temperature (0C)	Expected amplified product size (bp)	Structural annotation		Functional annotation	
								**Sequence components of *kabuli* genome**	***Kabuli* gene accession IDs**	**NCBI-KOG**	**TFs**
Ca1_P1_831015	Ca1	831015	T/TCTCTTATAAATGA	GCCGGATTAAATATAAATCCACA	TGACCTAATAAGCCTGTTTGTTT	59.2	595	DRR	Ca_00101	--	--
Ca1_P1_8319139	Ca1	8319139	TGGGCGCG/TGGGCGCGCTCGGGCGCG	CCCAAGTTCCCAAGAGTCAA	CAAAGACACCGGAACCAAAT	60.1	292	INTERGENIC	--	--	--
Ca1_P1_8327619	Ca1	8327619	AATATATATATATATATATATATATATATATATATATATA/AATATATATATATATATATATATATATATATATATATATA	AATAGCTCCCGCAAGTTTGA	CATGACATCTTTCAATCCAATCA	59.8	425	INTERGENIC	--	--	--
Ca1_P1_8338184	Ca1	8338184	AATATATATAT/AATATATATATAT	CGAAATAGGCACTGGTTTCA	TCTCCATTCACACCATGGAA	58.8	594	INTERGENIC	--	--	--
Ca1_P1_8391335	Ca1	8391335	T/TG	TGGAAGCTGAAATTTGGACC	TTAAAAGTTCATCCGCACAAA	60.1	364	DRR	Ca_08075	--	--
Ca1_P1_839232	Ca1	839232	AGTGT/AGTGTGT	AAGGATTGTGAAATCGCCAC	TAGATGCCCGTCATTGTTGA	59.9	680	INTERGENIC	--	--	--
Ca1_P1_8403563	Ca1	8403563	TTAATAATAATAATAATAATAATAATAATAATAATAATAATAATAATAATAATA/TTAATAATAATAATAATAATAATAATAATAATAATAATAATAATA	CCCCTAAAAATTAGTCGAACTCC	GAGTCTTTGTCCCCAGCAAG	59.4	474	INTRON	Ca_08076	--	--
Ca1_P1_8415420	Ca1	8415420	AG/A	ATGGGTGAAACGAGCAACTC	TTTTCCTTTTGCATCATCTTTG	60.1	910	INTERGENIC	--	--	--
Ca1_P1_8428429	Ca1	8428429	AAT/A	TGCACGTAGAATCGCCTAAA	TGTTAACACGCCCATTTTTAT	59.5	263	INTERGENIC	--	--	--
Ca1_P1_8430466	Ca1	8430466	ATTT/ATT	AGGTCATGGGCTGGATGTAG	ACCCTTTCACTGCGCTCTTA	60.0	612	INTERGENIC	--	--	--
Ca1_P1_8430733	Ca1	8430733	C/CCTAGGCCTTT	GGGCTATTAAGAGCGCAGTG	TTGGCATAAGGATCAAAGGG	60.0	117	INTERGENIC	--	--	--
Ca1_P1_8433346	Ca1	8433346	AAAAAAAAAAATTAAAAAAA/AAAAAAAA	GGATCGCGGAGACTATTTTG	AGATCCAAGAGCGGATGAGA	59.7	290	DRR	Ca_08078	--	--

INDEL marker IDs	Chromosomes/ unanchored scaffolds	Physical positions (bp)	InDels (*Kabuli* reference genome-CDC Frontier/PI)	Forward primers (5'-3')	Reverse primers (5'-3')	Anneling temperature (0C)	Expected amplified product size (bp)	Structural annotation		Functional annotation	
								Sequence components of *kabuli* genome	*Kabuli* gene accession IDs	NCBI-KOG	TFs
Ca1_P1_8558148	Ca1	8558148	TCCC/TCC	CCTTCTCATTGCGAACCCTA	GTTCGACACTGTAGCACCCA	60.2	256	INTERGENIC	--	--	--
Ca1_P1_8661436	Ca1	8661436	CAAAAAAAAAA/CAAAAAAAAAAAA	ATGCTTTCACAAAAGCCCAC	GTGCCTTCACATCTTTGGGT	60.1	529	INTERGENIC	--	--	--
Ca1_P1_8669425	Ca1	8669425	TA/T	AGCCAGGCCATGTAACAAAC	GATGGAGAGGGTCATCCAGA	60.0	234	INTRON	Ca_02944	P	GRAS
Ca1_P1_8830262	Ca1	8830262	TTATATATATATATATATATATATATA/TTATATATATATATATATATATATA	GCTACCTTAGCCTCTCCATGC	GGAATGCCATAGAAACACCAA	60.4	811	INTERGENIC	--	--	--
Ca1_P1_8833857	Ca1	8833857	TAAAAAAAA/TAAAAAAA	TTTTATGTGAAATCTCTGAAGTGACA	AGTCCTCGTGCTCATTGCTT	59.3	593	INTERGENIC	--	--	--
Ca1_P1_8909387	Ca1	8909387	T/TCC	AGGCAAAAGGTTTTTGGGTT	GTGAGGCGTGAAGTGGTTTT	59.9	806	DRR	Ca_02914	--	--
Ca1_P1_8927735	Ca1	8927735	AATATATATATATATATATATATATAT/AATATATATATATATATATATATATATAT	TTGCTTCGCCAAGTATTGTG	AGGATGGGTGAAGAAACGTG	59.9	653	INTRON	Ca_02911	--	bHLH
Ca1_P1_89785	Ca1	89785	ATTTTTTTTTTTTT/ATTTTTTTTTTTTTT	CACGAGTTGACATTTCACGG	TCCATCAAGATCATTTCCAGC	60.2	510	INTERGENIC	--	--	--
Ca1_P1_9130103	Ca1	9130103	TTATATATATATATATATATAT/TTATATATATATATATATAT	GGGTCAAAATTCGTTGACCAT	TACGGGTCAAGACAGGTTCG	61.0	550	INTERGENIC	--	--	--
Ca1_P1_9189087	Ca1	9189087	TTCTCTCTCTCTCTCTCTCTCTCTCTCTCTCTCTCTCTCT/TTCTCTCTCTCTCTCTCTCTCTCTCTCTCTCTCTCTCTCTCTCTCT	TCACTCACTCAACAAAGCGG	CCCACTTCACTGTAGCTCCC	60.0	268	INTERGENIC	--	--	--
Ca1_P1_9225279	Ca1	9225279	GTTTTTTT/GTTTTTT	AGGATTCCACAGATCAGCGT	TCAATTCACAAAGTTCGAGGG	59.7	646	INTERGENIC	--	--	--
Ca1_P1_9233850	Ca1	9233850	GTTTTTTTTTTTTT/GTTTTTTTTTT	TGAGTTCTTTTGAATTTGCGTG	GTTGCGTCTAGCAACTTTCTG	60.3	637	INTERGENIC	--	--	--

INDEL marker IDs	Chromosomes/ unanchored scaffolds	Physical positions (bp)	InDels (*Kabuli* reference genome-CDC Frontier/PI)	Forward primers (5'-3')	Reverse primers (5'-3')	Anneling temperature (0C)	Expected amplified product size (bp)	Structural annotation		Functional annotation	
								Sequence components of *kabuli* genome	*Kabuli* gene accession IDs	NCBI-KOG	TFs
Ca1_P1_9374824	Ca1	9374824	CAGAG/CAG	TTTTGTTCGTGCTGAATTGC	TGCATTCATGTGCTGGAAGT	59.9	780	INTRON	Ca_02861	--	C3H
Ca1_P1_9394926	Ca1	9394926	CG/C	CAAGTCCCCACTTGGAAAAA	CGGGCTCTAAATATCACCTACG	59.9	441	INTERGENIC	--	--	--
Ca1_P1_9396	Ca1	9396	CAAAAA/CAAAA	TTGGCAAATCGATTGAAACA	TTGATCCTTGCAGTCACTCG	60.1	906	DRR	Ca_00001	--	--
Ca1_P1_9729415	Ca1	9729415	TGG/TG	TGGGAAACTGGAGAATGGAG	GATTGCCCATGCAAAAATCT	60.0	407	DRR	Ca_02824	Z	MYB_related
Ca1_P1_9871519	Ca1	9871519	ATT/A	CTTAGTCCACGTGCAAACGA	AAAGTGCGGGCCAATATCTA	59.9	600	INTERGENIC	--	--	--
Ca1_P1_9930936	Ca1	9930936	TAATAC/TAATACAACAATAC	TTTGGAATGCCATGCAGTTA	ATGGATGCTGTGGTGAATGA	60.1	424	DRR	Ca_02792	O	WRKY
Ca1_P1_9968650	Ca1	9968650	TTATATATATATATATATATATATATATATAT/TTATATATATATATATATATATATAT	GCAGCTCGTAAAGCCAAAAG	GGTGGGCATCTCGTAGTGTT	60.2	582	INTERGENIC	--	--	--
Ca2_P1_10013835	Ca2	10013835	ATTTTTTTTT/ATTTTTTTT	TACTCACCCAAATCCGCTTC	TAGACCCTCATCCTCATCCG	60.1	393	INTERGENIC	--	--	--
Ca2_P1_10030734	Ca2	10030734	TGCGTCAAG/TG	TTGTCACGTGTCAGCCAAAT	GGCCATTAACTTCTCAACGG	60.2	273	INTERGENIC	--	--	--
Ca2_P1_10032701	Ca2	10032701	AATATATATATATA/AATATATATATATA	TTCTTCAGAGAAAAGAGTGGTTTTT	CCGTGTTTACGCTCTGTTGA	59.1	392	INTERGENIC	--	--	--
Ca2_P1_1012870	Ca2	1012870	T/TG	TTGCGGTGGTTAAGAAAAGG	ACGGCAGCAGAAGAAAATGT	60.1	834	INTERGENIC	--	--	--
Ca2_P1_10151537	Ca2	10151537	CATATATATATATATATATATATATA/CATATATATATATATATATATATATA	CAATTTGATAACACCATGAGGG	TTGCAGTTTGATGCGAAGAG	59.2	337	INTERGENIC	--	--	--

INDEL marker IDs	Chromosomes/ unanchored scaffolds	Physical positions (bp)	InDels (*Kabuli* reference genome-CDC Frontier/PI)	Forward primers (5'-3')	Reverse primers (5'-3')	Anneling temperature (0C)	Expected amplified product size (bp)	Structural annotation		Functional annotation	
								Sequence components of *kabuli* genome	*Kabuli* gene accession IDs	NCBI-KOG	TFs
Ca2_P1_10187103	Ca2	10187103	AATATATATATATATATATATATATA/AATATATATATATATATATATATATATATA	AAGGCGAGAACAAGCCATTA	CCTGAATATGCGGCGTTATC	59.8	314	INTERGENIC	--	--	--
Ca2_P1_10206326	Ca2	10206326	CATATATATATATAT/CATATATATATATATAT	CTGACAATTTGCCACGTTTG	GGTATGCCATCATGTGTTGC	60.1	348	INTERGENIC	--	--	--
Ca2_P1_1023421	Ca2	1023421	TTATATATATATATATATA/TTATATATATATATATATATA	TCCACGTCCTAAAGCCATTC	ACAAGACAGACCCTTTTGACA	60.1	639	INTERGENIC	--	--	--
Ca2_P1_10240635	Ca2	10240635	AACA/AA	CCCAAAAATAAATAACAAAATTCAA	GGTTTGTGGTTGGCACTTCT	57.7	727	DRR	Ca_17599	--	--
Ca2_P1_10272016	Ca2	10272016	TC/T	GTGGTTATGGCGCAATTCTT	GATGCAATGCAACTACGGTG	60.0	596	INTERGENIC	--	--	--
Ca2_P1_10281029	Ca2	10281029	CTATTTATTTATTTATTTATTTATTTATTT/CTATTTATTTATTTATTTATTTATTTATTTATTT	GGCGATATTCACGGACAGTT	TTCGAATCCAACATCACACAA	60.0	313	INTRON	Ca_17595	T	FAR1
Ca2_P1_1035507	Ca2	1035507	GTTACTT/GTT	TTTGCGAGTGCAATTCATGT	TCCAACATTTCACCAAACCA	60.3	514	INTERGENIC	--	--	--
Ca2_P1_10381921	Ca2	10381921	GAAAAAAAAAAAA/GAAAAAAAAAAA	AGTTGGTTTGATGGGAGGTG	TGAAGGGACATTACAAGGGC	59.8	713	INTERGENIC	--	--	--
Ca2_P1_10498057	Ca2	10498057	CTTTTTTTTTTT/CTTTTTTTTTTTT	TGACCAAATCGGAAAGAAAAA	AGGGAATGAGAGCAATGACG	59.6	454	INTERGENIC	--	--	--
Ca2_P1_10500780	Ca2	10500780	TTATATATATATATAT/TTATATATATATATATAT	TTCCTCCTCATTTGGTTTGTC	GTTCGGCAATTTGGTTCAGT	59.0	570	INTERGENIC	--	--	--
Ca2_P1_10535352	Ca2	10535352	GNNNNNNNNNNNN/GNNNNNNN	CCTTTCTTTCAATCCCCGAT	TTAAAACGCAACACGACTGC	60.3	527	INTERGENIC	--	--	--
Ca2_P1_10586644	Ca2	10586644	C/CGG	GAATCGAATCGGTGAGGAAA	AACACACCATGGGATCGACT	60.0	714	DRR	Ca_17575	O	--

INDEL marker IDs	Chromosomes/ unanchored scaffolds	Physical positions (bp)	InDels (*Kabuli* reference genome-CDC Frontier/PI)	Forward primers (5'-3')	Reverse primers (5'-3')	Anneling temperature (0C)	Expected amplified product size (bp)	Structural annotation		Functional annotation	
								Sequence components of *kabuli* genome	*Kabuli* gene accession IDs	NCBI-KOG	TFs
Ca2_P1_10591835	Ca2	10591835	TAAAAAAAAAAA/TAAAAAAAAAA	CCCCTCATTGGGCTTAATTT	TCAGGTTCGCTGTTGAAAAA	60.1	481	INTERGENIC	--	--	--
Ca2_P1_10663075	Ca2	10663075	TTATATATATATATATATATATATAT/TTATATATATATATATATATATATAT	CGACTTCACTGGGGACATTT	CCAGAAGGCTACCACTACGC	60.0	374	INTERGENIC	--	--	--
Ca2_P1_10720836	Ca2	10720836	ATTTTGTTTTTGTTTTTGTTTTTGTTTTT/ATTTTGTTTTTGTTTTTGTTTTTGTTTTTGTTTTT	GTTTGTCCCCAAGCAACAAT	TTGGTCGTTTGAGCCTTTCT	59.8	796	INTERGENIC	--	--	--
Ca2_P1_1074389	Ca2	1074389	CATTTATTTTATTTATTT/CATTTATTTTATTTATTTTATTTATTT	CCTCCTCGGTCTCTTCTCCT	AAATTGAGTTTCAAAACCACCAA	59.9	397	INTERGENIC	--	--	--
Ca2_P1_1074420	Ca2	1074420	ATT/ATTTTTT	CCTCCTCGGTCTCTTCTCCT	AAATTGAGTTTCAAAACCACCAA	59.9	397	INTERGENIC	--	--	--
Ca2_P1_10773522	Ca2	10773522	CTATATATATATATATATA/CTATATATATATATATATATA	ACATGTTGTTGCAAATCGGA	TGTCGATTGGATGGAGATGA	60.0	233	INTERGENIC	--	--	--
Ca2_P1_10779124	Ca2	10779124	TTA/TTACATA	CACTTTTCTTATGCACCGCA	GCAGCGAGTAGGAGAAATGC	59.9	663	INTERGENIC	--	--	--
Ca2_P1_1078289	Ca2	1078289	TAAAAAAA/TAAAAAAAA	TGTAAAATTCACCCGAAGCC	ACGATGGAGTGGACGAAAAC	59.9	453	INTERGENIC	--	--	--
Ca2_P1_1083231	Ca2	1083231	GAAAAAAAAAAA/GAAAAAAAAAAAA	ACGCGAAAAGCAGCATAGTT	TCTTCTCTGGCAACCGTTCT	60.0	449	DRR	Ca_20670	--	--
Ca2_P1_10853311	Ca2	10853311	ATTTTTTTTTT/ATTTTTTTTT	CAACTCAACCCCTAAACCGA	TGAGAAATTTTTCCGCTGCT	60.0	261	INTERGENIC	--	--	--
Ca2_P1_1087914	Ca2	1087914	GAAA/GAAAA	TGCGGCATACAAACAAACAT	AATGCAGCAGTGTCGAACTTT	60.0	361	INTERGENIC	--	--	--
Ca2_P1_10913431	Ca2	10913431	GAAAAAAAAAAA/GAAAAAAAAAA	TGCATAATCGATTGGCACAT	TCACTTACCGATTCCAGCCT	59.9	838	DRR	Ca_17557	--	--

INDEL marker IDs	Chromosomes/ unanchored scaffolds	Physical positions (bp)	InDels (*Kabuli* reference genome-CDC Frontier/PI)	Forward primers (5'-3')	Reverse primers (5'-3')	Anneling temperature (0C)	Expected amplified product size (bp)	Structural annotation		Functional annotation	
								Sequence components of *kabuli* genome	*Kabuli* gene accession IDs	NCBI-KOG	TFs
Ca2_P1_10928556	Ca2	10928556	ATTTTTTTT/ATTTTTTTTT	CATCCGTGGTCCCTTAACTT	GCTAAATTACATTCGTGGTCCC	58.9	370	INTERGENIC	--	--	--
Ca2_P1_10948001	Ca2	10948001	ATTA/ATTACACTGACGTTA	TGATTCTTGATCATGTTGTTTTCA	CCATCCTCACTCCAAAGACAA	59.5	702	INTERGENIC	--	--	--
Ca2_P1_1107319	Ca2	1107319	ATAATA/ATAATAATATTAATA	AAAAGTTTTGTGCACCTCCA	ATGCCATGGTTGTGGAACTT	58.3	459	INTERGENIC	--	--	--
Ca2_P1_11144082	Ca2	11144082	CTTTTT/CTTTT	TGCGAGCTTTCCTTTTTCAT	AAGGAGCTCTGGGACACTGA	60.0	214	INTERGENIC	--	--	--
Ca2_P1_11144239	Ca2	11144239	T/TACTC	TCAGTGTCCCAGAGCTCCTT	TCAATTGGCATCAGAGCAAG	60.0	402	INTERGENIC	--	--	--
Ca2_P1_11146648	Ca2	11146648	GAAAAAAAAAA/GAAAAAAAAAAA	TGCGGATGTGTTGATTGATT	CTACCGCTAGAGCGTCCACT	59.9	513	INTERGENIC	--	--	--
Ca2_P1_11170816	Ca2	11170816	AG/A	GTCAATGGTGGCTGCTCTTT	AACGGTATCACGCTCTCCAC	60.3	182	CDS (FRAME SHIFT)	Ca_17548	--	C2H2
Ca2_P1_11226197	Ca2	11226197	GATATATATATATATATATATA/GATATATATATATATATATATATA	CGCGATCAAAACAAAACGTA	CCAACATGGCATGAGTTGAC	59.7	757	INTERGENIC	--	--	--
Ca2_P1_11240445	Ca2	11240445	ATTTTTT/ATTTTTTT	TCGACCGAATCAAACAATGA	ACGTTTCCTCACGTTTCCAC	60.0	622	INTERGENIC	--	--	--
Ca2_P1_11281126	Ca2	11281126	GAAAAAAAAAAA/GAAAAAAAAAAAA	GATCACGCTTGCAATCCTTT	GGCCAAAGTTGTGTTCCACT	60.2	673	INTERGENIC	--	--	--
Ca2_P1_1136843	Ca2	1136843	GTTTTTTTTTTT/GTTTTTTTTTTTT	GGCCGAGATCAACAAATGTA	TGAGAAGCAGAAGCAGCGTA	58.6	504	INTERGENIC	--	--	--
Ca2_P1_11497600	Ca2	11497600	AG/A	TGATGAATGGTTGTGGCACT	ACGGGGTGTTACATTGTGGT	60.0	480	INTERGENIC	--	--	--

INDEL marker IDs	Chromosomes/ unanchored scaffolds	Physical positions (bp)	InDels (*Kabuli* reference genome-CDC Frontier/PI)	Forward primers (5'-3')	Reverse primers (5'-3')	Anneling temperature (0C)	Expected amplified product size (bp)	Structural annotation		Functional annotation	
								Sequence components of *kabuli* genome	*Kabuli* gene accession IDs	NCBI-KOG	TFs
Ca2_P1_11530366	Ca2	11530366	CTTGCATCTTTGCATCTT/CTTGCATCTT	TTTTGGTTCCATTTGCATCA	CAACCAATATCGAGTTGCCA	59.9	171	INTERGENIC	--	--	--
Ca2_P1_11625278	Ca2	11625278	AATATATATATATATATATATATATATA/AATATATATATATATATATATATATATATATATA	GTTTAGCGCTCCTCCATCAT	CTGCCCGTAAAAATCTTCCA	59.3	839	INTERGENIC	--	--	--
Ca2_P1_11648268	Ca2	11648268	AATATATATATATATATATATA/AATATATATATATATATATATATATA	TGAGTGCAAGCCTAGTGTGG	TGCACGGAAAACTGACTCTG	60.0	521	INTERGENIC	--	--	--
Ca2_P1_11671380	Ca2	11671380	G/GT	CCACTAGGTACCCCACTCCA	ATAGCCGGTGGTGCTGTATC	59.8	579	INTERGENIC	--	--	--
Ca2_P1_11711787	Ca2	11711787	ATATTATTATTATTATTATTATTATTATTATTATTA/ATATTATTATTATTATTATTATTATTATTATTATTATTA	TTCAAAATTGCTGGTTTGGTC	TGAAAGAAGCGCGGTAAAAC	60.0	596	INTERGENIC	--	--	--
Ca2_P1_11715547	Ca2	11715547	TC/T	AGTTCAATATGCAGCAGGGC	TCCACTCCTACCAATCAATGC	60.2	824	INTERGENIC	--	--	--
Ca2_P1_11722953	Ca2	11722953	AATATATATATATATA/AATATATATATATATATA	TTTGAAATGGCGACTTTCTT	AATTGGCATTTCGAACTTGG	57.5	534	INTERGENIC	--	--	--
Ca2_P1_11728038	Ca2	11728038	CAT/CATAT	TTTTAGAATTGATTGGCGGC	TCGGAGGATGTGGAATTTCT	60.0	671	INTERGENIC	--	--	--
Ca2_P1_11829316	Ca2	11829316	TTATATATATATATATATATATATA/TTATATATATATATATATATATA	TTCCCCTCAACAATTTTCAA	CTCAAGGGCCTTCACCTTTT	58.0	549	INTERGENIC	--	--	--
Ca2_P1_11869889	Ca2	11869889	TTAACTATAACTAT/TTAACTAT	TGCAAGGAAGCAGATCGAAT	AGCAAACATCCAAACAAGCC	60.9	537	INTERGENIC	--	--	--
Ca2_P1_11940630	Ca2	11940630	TTATATATATATATATAT/TTATATATATATATATATAT	GTAAAATGAAGCGCTTTCGC	ATTGGCAGACATCCATTTCC	60.0	634	INTERGENIC	--	--	--
Ca2_P1_11986064	Ca2	11986064	GTTT/GTT	TGGAGTGCAACCACTGACAT	TCCCACGATACCTTCTCCTG	60.2	184	INTRON	Ca_18763	--	--

INDEL marker IDs	Chromosomes/ unanchored scaffolds	Physical positions (bp)	InDels (*Kabuli* reference genome-CDC Frontier/PI)	Forward primers (5'-3')	Reverse primers (5'-3')	Anneling temperature (0C)	Expected amplified product size (bp)	Structural annotation		Functional annotation	
								Sequence components of *kabuli* genome	*Kabuli* gene accession IDs	NCBI-KOG	TFs
Ca2_P1_12040012	Ca2	12040012	CAA/CAAA	TGTCAATCCATTTATAATTTCTTTCAA	GTGGCATTCGGAGTCTGTTT	59.3	436	INTERGENIC	--	--	--
Ca2_P1_12040246	Ca2	12040246	TTATATATATATATATATAT/TTATATATATATATAT	TGTCAATCCATTTATAATTTCTTTCAA	CAGCTTTTTGGTTGATGTGG	59.3	490	INTERGENIC	--	--	--
Ca2_P1_12040519	Ca2	12040519	ATT/AT	CCACATCAACCAAAAAGCTG	GTGAGGTGGCATTCGAGAGT	59.2	665	INTERGENIC	--	--	--
Ca2_P1_12041702	Ca2	12041702	GTTTTTTT/GTTTTTT	TCATCCTTTCTCCCCTCTCA	GAGGAGAGGTCCACATCAGC	59.7	768	INTERGENIC	--	--	--
Ca2_P1_12048675	Ca2	12048675	AATAATTTATAATTTATA/AATAATTTATA	CAAACATGCTGATGTGGACC	CCATTGGTAAATGGCTAACAA	60.0	278	INTERGENIC	--	--	--
Ca2_P1_12167727	Ca2	12167727	GTATATATATATATATATATA/GTATATATATATATATATATATATATA	AAGGTCGTAAGCTTGTCGATG	TAGCACCGTAGCGGAGAGAT	59.4	715	INTERGENIC	--	--	--
Ca2_P1_1221818	Ca2	1221818	ATTTTTTTTTT/ATTTTTTTTTTT	TAGGTGCCCAACATAGCACA	ACAAAATGGTTCTCGAAATGA	60.1	978	INTERGENIC	--	--	--
Ca2_P1_12307967	Ca2	12307967	GAAA/GAAAA	TGTCTTTGGTCCCTGCAAAT	TCAAACCCCCATTTTTGTGT	60.5	755	INTERGENIC	--	--	--
Ca2_P1_12326965	Ca2	12326965	CAA/CAAA	GAATTCCACACGGAGTCCAC	TTGATTCTCAGCCAAAAAGGA	60.4	867	INTERGENIC	--	--	--
Ca2_P1_1233900	Ca2	1233900	C/CG	CGTTTTTGGAAGCCATTTGT	CTGCCATAAAGGCTGCAGAT	60.0	830	INTERGENIC	--	--	--
Ca2_P1_12360564	Ca2	12360564	TAAAAAAAAAAAA/TAAAAAAAAAAA	TCGCACTCACACAACAAAATC	CCTCTTCACTTTTTGGAAGGG	59.8	155	INTERGENIC	--	--	--
Ca2_P1_12435719	Ca2	12435719	TA/TAA	AAACGCAATCCTTGAGTTCG	TCCCTCAATCATGCGACATA	60.2	365	INTERGENIC	--	--	--

INDEL marker IDs	Chromosomes/ unanchored scaffolds	Physical positions (bp)	InDels (*Kabuli* reference genome-CDC Frontier/PI)	Forward primers (5'-3')	Reverse primers (5'-3')	Anneling temperature (0C)	Expected amplified product size (bp)	Structural annotation		Functional annotation	
								Sequence components of *kabuli* genome	*Kabuli* gene accession IDs	NCBI-KOG	TFs
Ca2_P1_12475607	Ca2	12475607	TTGTATTTGATGTAT/TTGTAT	TCAAAGAGAAATGACTTTGTCCA	TCACTTTGCTTTGTGTGTCTCA	58.9	852	INTERGENIC	--	--	--
Ca2_P1_12502809	Ca2	12502809	AAATAATAATAATAATAATAATAAT/AAATAATAATAATAATAATAAT	TGTCCTACTTTTTGGGTCCG	TCCAATAGGAAAGGTCGCTG	60.0	361	DRR	Ca_18077	--	G2-like
Ca2_P1_12553140	Ca2	12553140	ATTTTTTTTTT/ATTTTTTTTTTT	CCCCCTCCAACCTTTCATAC	ATCCAAATTCAATGCATCCC	60.5	811	INTERGENIC	--	--	--
Ca2_P1_12653249	Ca2	12653249	GTTTTTTTTTT/GTTTTTTTTTTT	TCTTCGATGGAACAACCTCC	GTGGAGATTGTGCTCCGTTT	60.1	842	INTERGENIC	--	--	--
Ca2_P1_12707442	Ca2	12707442	GAATTTCAATTTCAAT/GAATTTCAATTTCAATTTCAAT	AGCAGCTGACCGAGCATATT	GGATGGGTGATTGAAAATCG	60.0	902	INTRON	Ca_18086	R	--
Ca2_P1_12758393	Ca2	12758393	TAAAAAAAAAAAAAAA/TAAAAAAAAAAAAAA	GCAAAACAATTGGAGAACATGA	CTCGTCGCTGAACAAAATCA	60.0	503	INTERGENIC	--	--	--
Ca2_P1_12933106	Ca2	12933106	A/AT	AGGTTGAAAGAAGCCCCACT	ATCGGTTGGTTGGTGAAATG	60.1	834	INTERGENIC	--	--	--
Ca2_P1_12933573	Ca2	12933573	CACCAACCAACC/CACCAACC	TCATTGACAAGGAAAGAACATGA	CGAATTGGTTTTGGGATTTG	59.6	413	INTERGENIC	--	--	--
Ca2_P1_12934202	Ca2	12934202	AAA/AAACAA	AGAAAGCAAGCAAATCCCAA	TTCCAACACCTCACGATTCA	59.8	488	INTERGENIC	--	--	--
Ca2_P1_12934953	Ca2	12934953	TTCTATCTATCTATCTATCTATCTATC/TTCTATCTATCTATCTATCTATCTATCTATC	TGCCTCAAAAATCTGGTGTG	CTGCTCCAAACTGATCACGA	59.7	516	INTERGENIC	--	--	--
Ca2_P1_12937651	Ca2	12937651	ATTTT/ATTTTT	GATTGCTTTCCTCTATGCCG	GTTTTGGCGTTGGTTTCACT	59.8	353	INTERGENIC	--	--	--
Ca2_P1_12939688	Ca2	12939688	AATATATA/AATATA	TTTTGGTGTTGAGGGTGTCA	TTAATCAAAGCGGCAAAAGAA	60.0	564	INTERGENIC	--	--	--

INDEL marker IDs	Chromosomes/ unanchored scaffolds	Physical positions (bp)	InDels (*Kabuli* reference genome-CDC Frontier/PI)	Forward primers (5'-3')	Reverse primers (5'-3')	Anneling temperature (0C)	Expected amplified product size (bp)	Structural annotation		Functional annotation	
								Sequence components of *kabuli* genome	*Kabuli* gene accession IDs	NCBI-KOG	TFs
Ca2_P1_12941200	Ca2	12941200	CAAAA/CAAA	TGTGCTTCTTCCTGATTTTGG	GGCCAAAGACGGATATGGTA	60.2	888	INTERGENIC	--	--	--
Ca2_P1_12941800	Ca2	12941800	T/TA	CATATCCGTCTTTGGCCATT	CCTTGAACAATTTGGGCAGT	59.8	643	INTERGENIC	--	--	--
Ca2_P1_12947738	Ca2	12947738	TTATATAT/TTATAT	TGCTTTTATGAGCATTACGGG	GTTGGAGCATCAACCTGTCC	60.1	618	INTERGENIC	--	--	--
Ca2_P1_12947970	Ca2	12947970	G/GT	TGCTTTTATGAGCATTACGGG	CGACTTCAAATCCCTTCGAG	60.1	777	INTERGENIC	--	--	--
Ca2_P1_12952557	Ca2	12952557	ACATATCATATCA/ACATATCA	AAAGCATTTTATCCCGACCA	TTTTTCATCTCACTCTCTCCCA	59.4	787	INTERGENIC	--	--	--
Ca2_P1_12952944	Ca2	12952944	TGTCATGATTAT/TGTCATGATTATAGTCATGATTAT	TGGGAGAGAGTGAGATGAAAAA	ACGGATGTCTAATCCGTTGG	58.9	308	INTERGENIC	--	--	--
Ca2_P1_12963805	Ca2	12963805	ATTTTTTTTTTT/ATTTTTTTTTT	TGATTGTGCTATCTTACCTTTGGA	GATCCCATCAAAACCACCAG	60.0	867	INTERGENIC	--	--	--
Ca2_P1_13079939	Ca2	13079939	ATTTTTTT/ATTTTTTTT	GCAGCAAAATGAAAATTGGG	CGCAACAAAATGGAAGATCA	60.4	541	INTERGENIC	--	--	--
Ca2_P1_13220102	Ca2	13220102	CAAAAAAAAAAAA/CAAAAAAAAAAAAA	GACCCTTTGTTGGACAGCAT	TCAGAACACGTGGCTTTACG	60.0	436	INTRON	Ca_11681	R	--
Ca2_P1_13247575	Ca2	13247575	ATTTTTTTT/ATTTTTTT	TGAAAAGCTAAATGCATGGAA	TCATGACCATGGACCCACTA	58.4	937	INTERGENIC	--	--	--
Ca2_P1_13258978	Ca2	13258978	TAAA/TAA	TGTGAATGCGTTGATTGCTT	GCACGCCAACTGTTTGTTAG	60.3	951	INTERGENIC	--	--	--
Ca2_P1_13284989	Ca2	13284989	TGG/TGGG	GTCGGCTGTCGGTTGTTATT	GGTCTGAGGTGGTTTAACGC	60.0	803	INTERGENIC	--	--	--

INDEL marker IDs	Chromosomes/ unanchored scaffolds	Physical positions (bp)	InDels (*Kabuli* reference genome-CDC Frontier/PI)	Forward primers (5'-3')	Reverse primers (5'-3')	Anneling temperature (0C)	Expected amplified product size (bp)	Structural annotation		Functional annotation	
								Sequence components of *kabuli* genome	*Kabuli* gene accession IDs	NCBI-KOG	TFs
Ca2_P1_1336064	Ca2	1336064	CTATATATATATATATATATATATATATATATAT/CTATATATATATATATATATATATATATATAT	TGTGGGTGTGGCTAGTTTGA	ATGTGCTTAAGGATGCAGGG	60.2	716	INTERGENIC	--	--	--
Ca2_P1_13514762	Ca2	13514762	TAAAAAAAAAAA/TAAAAAAAAAAAA	AGGGGGTATTATTGGATTAGGA	TTTGGAAAATAAAATCAAGGGG	57.8	963	INTERGENIC	--	--	--
Ca2_P1_13569948	Ca2	13569948	ATTTTTTTTTTTTT/ATTTTTTTTTTTT	TGACGCATGTACAAAATCGTG	CCCCCAAATCTTGGTGTTTT	60.6	661	INTERGENIC	--	--	--
Ca2_P1_13619074	Ca2	13619074	AATATATATATATATATATAT/AATATATATATATATATATATATAT	TGGGATGAAGAATGCCCTAA	TTGACTCGTTGTTGCTCTGG	60.4	678	INTERGENIC	--	--	--
Ca2_P1_13662185	Ca2	13662185	CATATATATATATATATATATA/CATATATATATATATATATATATA	GGGGGACCAATTCCATAAAT	AAGTGAATCAATTGTGTGGTCTTT	59.7	724	DRR	Ca_11645	R	C2H2
Ca2_P1_13701671	Ca2	13701671	AATATATATATATATATATATAT/AATATATATATATATATATAT	CTATCCCCTACCCTCCAACC	GCTGATCAAGACAAATGGCA	59.6	284	DRR	Ca_11644	--	WRKY
Ca2_P1_13721929	Ca2	13721929	AATATATATATATATATA/AATATATATATATATATATA	CATTTCATGCAATTGTTGTCA	AAAAACCAGTCGTGTGGAGG	58.0	528	INTERGENIC	--	--	--
Ca2_P1_13728931	Ca2	13728931	TTTATTATTATTATTATTATTATTATTATTATTATTATTATTATT/TTTATTATTATTATTATTATTATTATTATTATTATTATTATTATT	AGAGGCAAACAAGAACCGAA	GAGGACCAAAGACCTCCACA	59.9	417	INTERGENIC	--	--	--
Ca2_P1_13794561	Ca2	13794561	CTTTTTTT/CTTTTTT	GATTCGGTGGAGAATCCAAA	CACCGGTGGTAACGAGAACT	59.9	834	INTRON	Ca_11639	--	SAP
Ca2_P1_13797357	Ca2	13797357	GAAAAAA/GAAAAA	TTCGCAACTCCACTCTTCCT	CGCAAGAAATTCCGGTAAAA	60.0	204	INTRON	Ca_11639	--	SAP
Ca2_P1_13852126	Ca2	13852126	GAAAAAAAAAA/GAAAAAAAAA	CACAGTATGGGTTTTTGATCCAT	TTTCTCCTCTTTGGCACGTT	60.0	512	INTERGENIC	--	--	--
Ca2_P1_13923559	Ca2	13923559	ATGCAGCTTTCTAAA/ATGCAGCTTTCTAAATTCTGCAGCTTTCTAAA	TTTGACCAAAACCTTGGAGG	CAGGGGCGTACAAACCTTTA	59.9	436	INTERGENIC	--	--	--

INDEL marker IDs	Chromosomes/ unanchored scaffolds	Physical positions (bp)	InDels (*Kabuli* reference genome-CDC Frontier/PI)	Forward primers (5'-3')	Reverse primers (5'-3')	Anneling temperature (0C)	Expected amplified product size (bp)	Structural annotation		Functional annotation	
								Sequence components of *kabuli* genome	*Kabuli* gene accession IDs	NCBI-KOG	TFs
Ca2_P1_13956607	Ca2	13956607	ATTTTTTTTTT/ATTATTTTTTTTTTTT	TGAAATTTGTGATTTAGTTGCTTAAAA	AAACAAGTTGGTGGTGGAGC	59.6	686	INTERGENIC	--	--	--
Ca2_P1_13957739	Ca2	13957739	TGG/TG	ATTATCCCCACCCCCACTAC	TTTCAAAAACGGTGGTGTGA	59.8	755	INTERGENIC	--	--	--
Ca2_P1_13957855	Ca2	13957855	TACA/TACACA	GCTCCACCACCAACTTGTTT	CAGCTACGCACAATGGCTAA	60.0	196	INTERGENIC	--	--	--
Ca2_P1_13959549	Ca2	13959549	ATTTTTTTTTT/ATTTTTTTTTTT	TGAAATTTGTGATTTAGTTGCTTAAAA	CATAGATTCCCCACCCAATG	59.6	752	INTERGENIC	--	--	--
Ca2_P1_13983591	Ca2	13983591	ATTTTTTTTTT/ATTTTTTTTTTT	ACACGAGGCGGAGAAATAAA	TGCAATTGTGGTCCATTTGT	59.7	893	INTERGENIC	--	--	--
Ca2_P1_14086143	Ca2	14086143	C/CT	GCTTCTGATGGTGCACTTGA	TGGCCTAAAGCAAGCTCCTA	60.0	371	INTERGENIC	--	--	--
Ca2_P1_14192522	Ca2	14192522	GAAAAAAAAAA/GAAAAAAAAAAA	TACAAATTGGATGCGGTTGA	CCCTCTGCAATGTCGGTAAT	59.9	755	INTERGENIC	--	--	--
Ca2_P1_15189592	Ca2	15189592	TTATATATATATATATATATATAT/TTATATATATATATATATATATATATAT	GGAAGGAGGGAAACCTCTTG	AAGCTCATCATGTGGTGCAA	60.0	721	INTERGENIC	--	--	--
Ca2_P1_15349212	Ca2	15349212	TAAAAA/TAAAA	GACAACTTTATGGCCCCTGA	ACATCATTGGGCAGGAGTTC	59.9	731	INTERGENIC	--	--	--
Ca2_P1_15454134	Ca2	15454134	AG/AGTGACTTG	CGATAATCATGAAATTGATGGG	GCTCACGAGGCAGTAATGAA	59.1	765	INTERGENIC	--	--	--
Ca2_P1_15479768	Ca2	15479768	ATTTTTTTTTTT/ATTTTTTTTTTTT	GGGGTTCTTCAAAATTGGGT	CAAACTTGGGGATGAAAACG	60.0	461	INTERGENIC	--	--	--
Ca2_P1_15557649	Ca2	15557649	AATATATATATATATATATATATATATA/AATATATATATATATATATATATATATATA	GAAAGCAATTGGGCTACTGG	CCAAAAACGTCTTTTCAATGG	59.7	251	INTERGENIC	--	--	--

INDEL marker IDs	Chromosomes/ unanchored scaffolds	Physical positions (bp)	InDels (*Kabuli* reference genome-CDC Frontier/PI)	Forward primers (5'-3')	Reverse primers (5'-3')	Anneling temperature (0C)	Expected amplified product size (bp)	Structural annotation		Functional annotation	
								Sequence components of *kabuli* genome	*Kabuli* gene accession IDs	NCBI-KOG	TFs
Ca2_P1_1558409	Ca2	1558409	TCGGAAGNNNNNNNNNNCGGAAG/TCGGAAG	TCGAAATTTTAGGCCACGAC	TGAGGTTGTTGCTTTGACCA	60.1	631	INTERGENIC	--	--	--
Ca2_P1_15589036	Ca2	15589036	ATTT/ATTTT	ATACGCCTCAACAAAATGGC	CTGCTTTGCTTGATTCCGTT	60.0	376	INTERGENIC	--	--	--
Ca2_P1_15691178	Ca2	15691178	TTATATATATATATATATATATATAT/TTATATATATATATATATATATATATAT	GACTGTCCAAATTTGTGGGG	TTTTTGGCATCAAGTTGTCTTTT	60.2	581	DRR	Ca_18540	--	--
Ca2_P1_15812866	Ca2	15812866	TAAAAAAAAA/TAAAAAAAAAA	CATTGGCGGAGCTTTACAAT	CCATGGCCACTTCAAGATTT	60.1	298	INTERGENIC	--	--	--
Ca2_P1_15904424	Ca2	15904424	TTAAAATTAAAATAAAATTAAAAT/TTAAAATTAAAATAAAATTAAAATAAAATTAAAAT	TTAGGCCGCCACAAAGTATC	GAGGGTAAAGGCATCCACAA	60.1	524	INTERGENIC	--	--	--
Ca2_P1_15918350	Ca2	15918350	CATATATATATATATATAT/CATATATATATATATATATAT	CTTGGCCAGAAAATCGGTAA	AGGATGCATGGGATGAAAAG	60.1	691	INTERGENIC	--	--	--
Ca2_P1_16003270	Ca2	16003270	AAT/A	GGTACACTTCCGGCAAGAAA	AAGAGAGGTCACTTTGGCGA	60.1	482	INTERGENIC	--	--	--
Ca2_P1_16003324	Ca2	16003324	TTTT/TTTTATTT	GGTACACTTCCGGCAAGAAA	AAGAGAGGTCACTTTGGCGA	60.1	482	INTERGENIC	--	--	--
Ca2_P1_16093747	Ca2	16093747	CATATATATATATATATATATATATATATATA/CATATATATATATATATATATATATATA	CGTGAGCAACTATTCGTTGAA	GGGAAGGGAGAAAGAGGAGA	59.0	429	INTERGENIC	--	--	--
Ca2_P1_16312234	Ca2	16312234	GAAAAAAAAAA/GAAAAAAAAAAA	TTTTTCCGACTTGGTCCTTT	TGTCCCAAAATATGAACATCCA	58.7	494	INTERGENIC	--	--	--
Ca2_P1_16398494	Ca2	16398494	ATTTTTTTTTT/ATTTTTTTTT	CAATTTGCATCTCCAAAGCA	TGATCTTGATCGGGATGTGA	59.8	381	INTERGENIC	--	--	--
Ca2_P1_1640062	Ca2	1640062	ATTTTTTTTT/ATTTTTT	AGATCATGTGCAAAAAGGGG	AGTAACCGTGAGGGAACGTG	59.9	509	INTERGENIC	--	--	--

INDEL marker IDs	Chromosomes/ unanchored scaffolds	Physical positions (bp)	InDels (*Kabuli* reference genome-CDC Frontier/PI)	Forward primers (5'-3')	Reverse primers (5'-3')	Anneling temperature (0C)	Expected amplified product size (bp)	Structural annotation		Functional annotation	
								Sequence components of *kabuli* genome	*Kabuli* gene accession IDs	NCBI-KOG	TFs
Ca2_P1_16424392	Ca2	16424392	AC/ACGACGCGAAAATTCCC	GCACTCTAATGCAATGCGAA	TAGCGGTGGGGAGAACTATG	60.0	699	INTERGENIC	--	--	--
Ca2_P1_16514436	Ca2	16514436	AC/A	TGTGCTGGAAAGGCACATAG	GTCTCCCCCACTACAGTCCA	59.9	812	INTERGENIC	--	--	--
Ca2_P1_16698930	Ca2	16698930	TTCA/TTCATTCCACATCA	AGCGAAATTTCCAACGTGTC	AGGTTGAATGTGGCAAAACA	60.1	321	INTERGENIC	--	--	--
Ca2_P1_16961941	Ca2	16961941	AATATATATATATATATATATATATATATATATATATATA/AATATATATATATATATATATATATATATATATATATA	AAATGGTTCATTCATCGGTGT	TGAATAATTCTTGACCATCCATGT	59.2	499	INTERGENIC	--	--	--
Ca2_P1_17046294	Ca2	17046294	AATAAATT/AAAATTTATAAATT	CTATTCCATCCACGAATGCC	TCCATGGAGGATATTTATGTCG	60.3	523	DRR	Ca_24768	--	G2-like
Ca2_P1_17049910	Ca2	17049910	TAA/TA	AGAGAAGCTCGTTGTGGCAT	TCGTCGAGCATGTGACTTTC	60.0	479	DRR	Ca_24767	--	--
Ca2_P1_17050299	Ca2	17050299	TC/TCC	ACCCCTTGATGAGACAAACG	ATGCTGCCGAGTTTCTGAAT	60.0	556	DRR	Ca_24767	--	--
Ca2_P1_17061321	Ca2	17061321	TAATATATA/TAATATATAAATATATA	TTTGGCTCGGAAGCTTTTTA	AGGAAGAGTGGAGTTGCGAA	60.0	498	INTERGENIC	--	--	--
Ca2_P1_17061679	Ca2	17061679	T/TCCA	TTCGCAACTCCACTCTTCCT	CGGTAAAAATCTCTGCTCGC	60.0	190	INTERGENIC	--	--	--
Ca2_P1_1728113	Ca2	1728113	AATTATTATTATTATTATTATTATTATTATTATTATT/AATTATTATTATTATTATTATTATTATTATTATT	GAAAAGACACACATATTTGGCA	GTTTGTTGTGGTTGCAGTGG	57.7	491	INTERGENIC	--	--	--
Ca2_P1_17318657	Ca2	17318657	TT/TTGAT	GATGGCTCTCATGAGGGAAG	TCACATGTCATTTGGGGAGA	59.8	169	INTERGENIC	--	--	--
Ca2_P1_17368825	Ca2	17368825	AA/AATTTATTTTTAGGA	TGACATATCCGCTATGAACGA	ATCCATCAAACGCTTTCACC	59.2	592	INTERGENIC	--	--	--

INDEL marker IDs	Chromosomes/ unanchored scaffolds	Physical positions (bp)	InDels (*Kabuli* reference genome-CDC Frontier/PI)	Forward primers (5'-3')	Reverse primers (5'-3')	Anneling temperature (0C)	Expected amplified product size (bp)	Structural annotation		Functional annotation	
								**Sequence components of *kabuli* genome**	***Kabuli* gene accession IDs**	**NCBI-KOG**	**TFs**
Ca2_P1_17388033	Ca2	17388033	GTT/GTTT	TGGACACGACGGTGAAATAA	GTCGGCCACAAGAAGACAAT	60.0	578	INTERGENIC	--	--	--
Ca2_P1_17551687	Ca2	17551687	G/GC	ATGTGGTTGATAAGGCCTCG	AGCAAATGCAATGCTCTGAA	60.0	398	CDS (FRAME SHIFT)	Ca_16000	OE	--
Ca2_P1_17619991	Ca2	17619991	ATTTTTTTTTTTTT/ATTTTTTTTTTT	CATCAAGATTTCCGATGGCT	CATGTGGTTCAGCTCGATTT	60.0	608	INTERGENIC	--	--	--
Ca2_P1_17850204	Ca2	17850204	GAAAAAAAA/GAAAAAAAAA	TCTCCTTTGGCCCAACTATG	TTGTTTGACATTCCAAGGGT	60.1	745	DRR	Ca_15990	--	B3
Ca2_P1_17893254	Ca2	17893254	ATTTTTT/ATTTTTTTT	TCGCTTCCTATGGAATTTCG	CATCATTTCCAACAATCCCC	60.2	298	INTERGENIC	--	--	--
Ca2_P1_17893359	Ca2	17893359	GAAAAAAAA/GAAAAAAA	GGGGATTGTTGGAAATGATG	CAATGGATCACCAGAACGTG	60.0	585	INTERGENIC	--	--	--
Ca2_P1_17994837	Ca2	17994837	AATATATATATATATATATATATATAT/ AATATATATATATATATATATATATATAT	TGTGGCCCTATGTACTGCTG	TTGGAAGGAGCCCTTACCTT	59.7	799	INTERGENIC	--	--	--
Ca2_P1_18086083	Ca2	18086083	ATATTATTATTATTATTATTATTATTATTATTATTATTATTATT/ATATTATTATTATTATTATTATTATTATTATTATT	AAAAAGCGGATGGAGTGATG	GGGAGTTGGGTTCTTCCTTC	60.1	688	INTRON	Ca_15983	--	--
Ca2_P1_18160173	Ca2	18160173	TATAATAATAATAATAATAATAATAATAATAAT/TATAATAATAATAATAATAATAATAATAATAAT	AGGGACACCTCATTTGTTCG	GAAGCGACCACCTGAAGAAC	60.0	510	INTERGENIC	--	--	--
Ca2_P1_18211722	Ca2	18211722	CCCCAAGAATCCCAAGAATC/CCCCAAGAATC	CAACCTACACGCCGATTTTT	TGGGTCCATGTTCCAATTTT	60.0	321	INTERGENIC	--	--	--
Ca2_P1_18223174	Ca2	18223174	TTAATAATAATAATAATAATAATAATAATAATAATAA/TTAATAATAATAATAATAATAATAATAATAA	CTCAGGATGCGACTTGTTGA	AAGTGATTTGTGGGTGATTGG	60.0	558	INTERGENIC	--	--	--
Ca2_P1_18225059	Ca2	18225059	GTTTTTTTTT/GTTTTTTTTTT	TTGTTTTGATGGATGATTTGGA	AAGCAAACATTTGGAATGGC	60.2	460	INTERGENIC	--	--	--

INDEL marker IDs	Chromosomes/ unanchored scaffolds	Physical positions (bp)	InDels (*Kabuli* reference genome-CDC Frontier/PI)	Forward primers (5'-3')	Reverse primers (5'-3')	Anneling temperature (0C)	Expected amplified product size (bp)	Structural annotation		Functional annotation	
								Sequence components of *kabuli* genome	*Kabuli* gene accession IDs	NCBI-KOG	TFs
Ca2_P1_18225968	Ca2	18225968	AATT/AATTAATTTCATT	CGGGAAATCGTTATTAGCCA	AACAACGCAGAAGAGAAGCC	59.9	127	INTERGENIC	--	--	--
Ca2_P1_18241747	Ca2	18241747	C/CTA	GACGTTTCCATCGTCAAGGT	TTGTTGCGATGGAGTCAGAG	60.0	551	INTERGENIC	--	--	--
Ca2_P1_1835747	Ca2	1835747	TTATATATATATATATATATATATATAT/TTATATATATATATATATATATAT	CACAATGCACCATGTGACAA	TGGAACTGAAGCCATATCCC	60.0	831	INTRON	Ca_12601	--	--
Ca2_P1_18391802	Ca2	18391802	AATATATATATATATATATA/AATATATATATATATATA	CAAAGCGGGTAGCAAGGATA	GCCGCTCCTCTTCAAGTAAA	60.2	524	INTERGENIC	--	--	--
Ca2_P1_18474690	Ca2	18474690	GATATTTAATA/GATA	TTCGGAGCAAACTTCCACTT	CCAAGGCCAACTCAAATTGT	59.9	692	INTERGENIC	--	--	--
Ca2_P1_18496873	Ca2	18496873	AATATATATATATATATATATATATATATATATATATATAT/AATATATATATATATATATATATATATATATATATATAT	AAAAATCCCACGTTTGTCCA	TAAGGTTGTGCGAGAAGTGC	60.2	914	INTERGENIC	--	--	--
Ca2_P1_18574917	Ca2	18574917	ATTTTT/ATTTT	CAATTCCCACAGGTACGCTT	CAAACCCTAATTTTTCAAACACG	60.0	349	INTERGENIC	--	--	--
Ca2_P1_18584340	Ca2	18584340	GAAAAAAAAAA/GAAAAAAAAAAA	ATGTACCTCCGCCTCATTTG	TCTCTGGCAACCGTTCTTCT	60.0	257	INTERGENIC	--	--	--
Ca2_P1_18610007	Ca2	18610007	TAA/TAACAA	TTGATCCCTTATTTCCTGCG	TTATTTCACCGTCGTGTCCA	60.0	492	INTERGENIC	--	--	--
Ca2_P1_18611453	Ca2	18611453	GT/G	TGCCCCTTGGATAGACTCAT	CATGACACCCTTACCGAACA	59.5	508	INTERGENIC	--	--	--
Ca2_P1_18719612	Ca2	18719612	ATATG/A	ATGGAAAATTGTCGTTTCGG	TTGTGTGTGTTTCCCACGTT	59.8	591	INTERGENIC	--	--	--
Ca2_P1_18742446	Ca2	18742446	GATATATATATATATATATATATATATATATATATA/GATATATATATATATATATATATATATATATA	GATACAAATTGGCATTGGGG	TCAATTGAAATTGTGTGGAGAAA	60.0	679	INTERGENIC	--	--	--

INDEL marker IDs	Chromosomes/ unanchored scaffolds	Physical positions (bp)	InDels (*Kabuli* reference genome-CDC Frontier/PI)	Forward primers (5'-3')	Reverse primers (5'-3')	Anneling temperature (0C)	Expected amplified product size (bp)	Structural annotation		Functional annotation	
								Sequence components of *kabuli* genome	*Kabuli* gene accession IDs	NCBI-KOG	TFs
Ca2_P1_18764616	Ca2	18764616	GAATAATAATAATAATAATAATA/GAATAATAATAATAATAATA	ATTTGCCTCCCTCAACCTTT	TGTCTTCATGGGTGCATTGT	59.9	841	INTERGENIC	--	--	--
Ca2_P1_18768813	Ca2	18768813	AG/AGG	TGGAACATCTCAATCACTCCA	AGCACTAGAAGCATCGGGAA	59.1	391	INTERGENIC	--	--	--
Ca2_P1_1879905	Ca2	1879905	CAAAAAAA/CAAAAAAAA	AACATCTCTAGCGTGTGCCA	CACACCCACTCCCTTGTCTT	59.5	711	DRR	Ca_12598	YU	--
Ca2_P1_18833380	Ca2	18833380	TAAAAAAAAAAA/TAAAAAAAAAAAA	TCGAAAAATCCCTTGAAAAAGA	GGACCTATCAAGTGAGCTTGTG	60.0	551	INTERGENIC	--	--	--
Ca2_P1_19008979	Ca2	19008979	TAAAAAA/TAAAAAAAA	GGCTATATCTCCCATGCCAA	GCAAGAAACGAGGAAAGAAGG	59.9	663	INTRON	Ca_15954	--	bHLH
Ca2_P1_19015976	Ca2	19015976	T/TA	AAACACTTATGGGCGAGGTG	ATTGAATTGGTGGTCGAAGC	60.0	256	INTRON	Ca_15954	--	bHLH
Ca2_P1_19101541	Ca2	19101541	ATTT/ATT	TTGGTCATAAGGGGAACCAG	GCCTAAGAGTGGGGGTGAAT	59.8	153	INTERGENIC	--	--	--
Ca2_P1_19122153	Ca2	19122153	TCC/TCCC	ACGGAGAGGTTTGTGTTTGG	ATTTTGGCTCGGAGGTTTTT	60.0	562	INTERGENIC	--	--	--
Ca2_P1_19130519	Ca2	19130519	TATAATAATAATAATAATAATAATAATAATAATAATAATAATAATAATA/TATAATAATAATAATAATAATAATAATAATAATAATAATAATAATAATA	GGTACCGCGAGTAACCGATA	TGTCTATTGAGTTGCGACGAG	60.0	575	INTERGENIC	--	--	--
Ca2_P1_19138559	Ca2	19138559	C/CGACGCGACA	CAATTCCAAAACATCCACCC	CCACGAGATGATTCCATGTG	60.0	517	INTERGENIC	--	--	--
Ca2_P1_1919646	Ca2	1919646	TC/TCTCC	TTGAAGCGATGTGGTTGAAG	CCAAAACAACTATGCCCGTT	59.8	339	INTERGENIC	--	--	--
Ca2_P1_19207562	Ca2	19207562	TAAAAAAA/TAAAAAAAA	ACAAACCAAAACCCAACCAA	TTTCACCGACCCAAACAAAT	60.1	663	INTERGENIC	--	--	--

INDEL marker IDs	Chromosomes/ unanchored scaffolds	Physical positions (bp)	InDels (*Kabuli* reference genome-CDC Frontier/PI)	Forward primers (5'-3')	Reverse primers (5'-3')	Anneling temperature (0C)	Expected amplified product size (bp)	Structural annotation		Functional annotation	
								Sequence components of *kabuli* genome	*Kabuli* gene accession IDs	NCBI-KOG	TFs
Ca2_P1_19219016	Ca2	19219016	ATTTT/ATTT	CCCGTGAGATCCTCCTAACA	TGAATCTACGGCTTTCTGGG	60.1	498	INTERGENIC	--	--	--
Ca2_P1_1935888	Ca2	1935888	CAAAAAAAAAAAA/CAAAAAAAAAAA	CGAATTTTAAGACCACCCAAA	ACATGCAATTGTGTGCCTTC	59.0	398	INTERGENIC	--	--	--
Ca2_P1_19384298	Ca2	19384298	GGAGAGAGAGAGAGAGAGAGAGAGAGAGAGAGAG/GGAGAGAGAGAGAGAGAGAGAGAGAGAGAG	TGGTTTGGTCAATTTGAGGC	GAAGCCCAAGTTCCAAATGA	60.9	679	INTERGENIC	--	--	--
Ca2_P1_19465818	Ca2	19465818	TAAAA/TAAAAA	GCCACAAAACACAGAAAGCA	CAGTGTCACCGTCATCGAAC	59.9	469	INTERGENIC	--	--	--
Ca2_P1_19563753	Ca2	19563753	TAAAAAAAAAA/TAAAAAAAAAAA	GCTCATGCAGTCCCAAAACT	GCATAATTGCTCATGCGGTA	60.3	381	INTERGENIC	--	--	--
Ca2_P1_19573107	Ca2	19573107	TTATATATATATATATATATATATAT/TTATATATATATATATATATATATAT	TTGTCCAAATGGTACAGCCA	AAGGAAGGCCTACTCCCAAA	60.0	647	INTERGENIC	--	--	--
Ca2_P1_19620908	Ca2	19620908	CAAAAAAA/CAAAAA	TGGGGTCCATGTACCACTTT	CCCCTTTCTGATTTGAAGGA	60.1	334	INTERGENIC	--	--	--
Ca2_P1_19652227	Ca2	19652227	AAA/AAAATAA	AGATGTTTGTTTTGCCGTCC	TGCTGTTGAGTTGTTGGGAG	60.0	544	INTRON	Ca_24537	--	SAP
Ca2_P1_19705772	Ca2	19705772	GAAAA/GAAAAA	TATTTTGGATTTTGGCTCGG	AGGAAGAGTGGAGTTGCGAA	59.9	475	INTERGENIC	--	--	--
Ca2_P1_19772797	Ca2	19772797	CT/CTT	GTTTAGGCACGTAGGGACCA	CGTTTAATTTCGGGACTGGA	60.0	417	INTERGENIC	--	--	--
Ca2_P1_19791685	Ca2	19791685	C/CG	TAGGCCATTTTCGTCCTCAC	AATCCAATGACACAAACGCA	60.1	392	INTERGENIC	--	--	--
Ca2_P1_19860997	Ca2	19860997	GTTTTT/GTTTT	TTGTGCATCCGGTAAGTTCA	AAGCCTAGGGAGGACGAAAA	60.1	269	INTERGENIC	--	--	--

INDEL marker IDs	Chromosomes/ unanchored scaffolds	Physical positions (bp)	InDels (*Kabuli* reference genome-CDC Frontier/PI)	Forward primers (5'-3')	Reverse primers (5'-3')	Anneling temperature (0C)	Expected amplified product size (bp)	Structural annotation		Functional annotation	
								Sequence components of *kabuli* genome	*Kabuli* gene accession IDs	NCBI-KOG	TFs
Ca2_P1_19893185	Ca2	19893185	TAAAA/TAAA	ACACCATTTTCGATTCCGAG	CCATGTACCATTCGTTGCTG	59.9	474	INTERGENIC	--	--	--
Ca2_P1_1990862	Ca2	1990862	GATTATTATTATTATTATTATTATTATTATTATTATTATTATTATT/GATTATTATTATTATTATTATTATTATTATTATTATTATTATTATTATTATT	GAGGATTAAGCCAATGCCAA	ACTTGTGTTTGGTCCCATGA	60.0	635	INTRON	Ca_12580	GE	HB-other
Ca2_P1_20175480	Ca2	20175480	GATATATATATATATATATA/GATATATATATATATATATATA	TTTCAGAGTGTCTGTGGTTAGCA	GATCTGAGGTGTCATGGGCT	60.0	669	INTERGENIC	--	--	--
Ca2_P1_20196959	Ca2	20196959	TGGTG/TGGTGGGTG	TTCTAAGTTTGGGGTTGCTCA	TCCTAGAACATGCATCACGC	59.7	385	INTERGENIC	--	--	--
Ca2_P1_20249774	Ca2	20249774	AAT/AATGAT	GCAACAACAACAACAACAACAA	TGTGATTGGTAATGTGGCAAA	59.6	334	INTERGENIC	--	--	--
Ca2_P1_2026250	Ca2	2026250	GTTTTTTTTTTT/GTTTTTTTTTT	ACGGTGGTTGGTGACTGTTA	TTTTCTGCATAGGCAAAGCC	58.9	506	INTERGENIC	--	--	--
Ca2_P1_20280019	Ca2	20280019	TAAAAAAAAAAA/TAAAAAAAAAA	GCAAGGTTTGACAGTGAGCA	CATCGACAACCAATCCACAG	60.0	554	INTERGENIC	--	--	--
Ca2_P1_20353586	Ca2	20353586	T/TG	TCTCCCTCCTTCCTCCATTT	CATTAAGAGGTTCCCCCTCC	60.0	844	INTERGENIC	--	--	--
Ca2_P1_20407750	Ca2	20407750	TTCTCTCTCTCTCTCTCTCTCTCTCTCTCT/TTCTCTCTCTCTCTCTCTCTCTCTCTCT	CTAACGGCGACTTCTTGGAG	TGGAAGTTGCACTTTCCTTTG	60.0	640	INTERGENIC	--	--	--
Ca2_P1_20423156	Ca2	20423156	ATGT/AT	CATTTTGTTCAAAGTGGGCA	AGAAAGGCTCAAACGACCAA	59.6	702	INTERGENIC	--	--	--
Ca2_P1_20439742	Ca2	20439742	TTT/TTTATATATTCTT	CAGCGTTTTGGCATTTACAA	TGCTGTCTTCTCCCGAGTTT	59.7	761	INTRON	Ca_25923	LT	--
Ca2_P1_20451589	Ca2	20451589	CAAAA/CA	ACCAAATTGACTCGGTGCTC	TGTGGGATGACCCTTTTTGT	60.1	460	INTERGENIC	--	--	--

INDEL marker IDs	Chromosomes/ unanchored scaffolds	Physical positions (bp)	InDels (*Kabuli* reference genome-CDC Frontier/PI)	Forward primers (5'-3')	Reverse primers (5'-3')	Anneling temperature (0C)	Expected amplified product size (bp)	Structural annotation		Functional annotation	
								Sequence components of *kabuli* genome	*Kabuli* gene accession IDs	NCBI-KOG	TFs
Ca2_P1_20561228	Ca2	20561228	TNNNNNNNNNNNNN/TNNNNNNNNNNNN	AAGCCATCCAATACGCAAAC	TGTGGAGGGTTGTTTCAAGA	60.0	922	INTERGENIC	--	--	--
Ca2_P1_20647114	Ca2	20647114	AATATATATATATATATATAT/AATATATATATATATATATAT	ATCACTCGCACAACAAACCA	TTGGAAGGAGCCCTTACCTT	60.2	920	INTERGENIC	--	--	--
Ca2_P1_20673165	Ca2	20673165	GT/G	TCGACTTGTCAATCGCTTTG	TACATGCCATAGTGGGGGAT	60.0	667	INTERGENIC	--	--	--
Ca2_P1_20715795	Ca2	20715795	T/TCATCA	GACATTGCATTTTGGACACG	AAATGGATTTCAGCCAATCG	60.0	539	INTERGENIC	--	--	--
Ca2_P1_2072389	Ca2	2072389	ATTTTTTTTTTT/ATTTTTTTTTTTT	TTCTGGACTTTTCACATAGCGA	CACACGATTGTGAGGACTGG	59.9	366	INTERGENIC	--	--	--
Ca2_P1_20907810	Ca2	20907810	AATATATATATATATATATATATATATATATATATATAT/AATATATATATATATATATATATATATAT	GTCGATGAGTCTGTGCGTGT	CATAAAACGTTGTCCGATCA	59.9	363	INTERGENIC	--	--	--
Ca2_P1_20919626	Ca2	20919626	ATTTTTTTTTTT/ATTTTTTTTTT	ATTCATGGATGCGGGTATGT	TTACATCAATGAGCCAATCG	60.0	552	INTERGENIC	--	--	--
Ca2_P1_20970910	Ca2	20970910	ATTCCCT/A	AACAAAGGAACAAAATGGCG	AGGGACAAAGGGCAAGAAAT	60.0	853	INTERGENIC	--	--	--
Ca2_P1_21000306	Ca2	21000306	TTCCAATC/TTC	GCCATGTCAAAGTATCGCCT	GCCCCGTATTTACCAATGTG	60.1	431	INTERGENIC	--	--	--
Ca2_P1_21028365	Ca2	21028365	TTTATTATTATTATTATTATTATTATTATTATTATTATTATTATTATTATTA/TTTATTATTATTATTATTATTATTATTATTATTATTATTATTATTATTATTATTA	GGGGAACTGGATAAGGTGGT	CCGTAATGCTGTTTCCCTGT	60.1	441	INTERGENIC	--	--	--
Ca2_P1_21084107	Ca2	21084107	AT/ATT	ACATAATATCTGGCCGGCTG	CTGGCTTTGAAGAGCTTGGT	59.9	469	INTERGENIC	--	--	--
Ca2_P1_21088001	Ca2	21088001	TA/TAAAAGCATCA	AGAACGATGACACGTTGGGT	CTTCTGTCCAAAAAGCAGCC	60.4	812	INTERGENIC	--	--	--

INDEL marker IDs	Chromosomes/ unanchored scaffolds	Physical positions (bp)	InDels (*Kabuli* reference genome-CDC Frontier/PI)	Forward primers (5'-3')	Reverse primers (5'-3')	Anneling temperature (0C)	Expected amplified product size (bp)	Structural annotation		Functional annotation	
								Sequence components of *kabuli* genome	*Kabuli* gene accession IDs	NCBI-KOG	TFs
Ca2_P1_21208615	Ca2	21208615	T/TCTCAA	TTTGATGAGACCTATG CCCC	ATGAAAATGCCTTGCT TGCT	59.9	329	INTERGENIC	--	--	--
Ca2_P1_21308629	Ca2	21308629	CAAA/CAA	TTTCGAAGCAACGAGG AAGT	TTGGTATTGAACTCGG AGGC	60.0	474	INTERGENIC	--	--	--
Ca2_P1_21312312	Ca2	21312312	GTTTTTT/GTTTTTTTT	TATCAGAGTTCTCCCC ACCG	AGAAACCACAACGCAA CACA	60.1	356	INTERGENIC	--	--	--
Ca2_P1_21314611	Ca2	21314611	AG/AGG	CAAACCCTTTCAAGGT GCAT	GCACTGCCCCAAGAAT TTTA	60.0	443	INTERGENIC	--	--	--
Ca2_P1_21439321	Ca2	21439321	TATAATAATAATAATAATAATAATAATA ATAATAATAATAATAATAATAATAATAA TAA/TATAATAATAATAATAATAATAAT AATAATAATAATAATAATAATAATAATA ATAA	TGGAAATCACTCAAAT GCAAG	AAGATAGCAACCACAT TCCCA	58.8	751	INTERGENIC	--	--	--
Ca2_P1_21440030	Ca2	21440030	TTATATATATATATATATATAT/TTATAT ATATATATATATAT	TGGGAATGTGGTTGCT ATCTT	CAAGAGAATCTCCATC GTCTTTT	59.4	389	INTERGENIC	--	--	--
Ca2_P1_21538005	Ca2	21538005	T/TG	TTCGCAACTCCACTCT TCCT	TCAAGTATGGACACGC CAAA	60.0	468	INTERGENIC	--	--	--
Ca2_P1_21553655	Ca2	21553655	TTTATTATTATTATTATTATTATTATTA TTACTATTATTTTTATTATTATTATTAT TATTATTATTATTATTATTATTATT/TTT ATTATTATTATTATTATTATTATTATTA TTATTATTACTATTATTTTTATTATTAT [illegible]	TGTGCTCTCAATTGTG CTCC	AATTACCCGCATGTTG AAGC	60.0	792	INTERGENIC	--	--	--
Ca2_P1_21596317	Ca2	21596317	TTTATTATTATTATTATTATTATTATTA TTATT/TTTATTATTATTATTATTATTAT TATTATTATTATT	AATCCCTCCCTTCGTC ACTT	TCCCTTTCTACAAGCG TTCA	59.9	210	INTERGENIC	--	--	--
Ca2_P1_21672716	Ca2	21672716	ATT/AT	TGATGCCGTGCCATAA ATAG	TAACCTTGCCTTTGGG TGAG	59.5	595	INTERGENIC	--	--	--
Ca2_P1_2169056	Ca2	2169056	C/CG	AACCTCTCCAAGAGGA TCGG	GGAGAGTGAAACGGA TGGAA	60.6	648	INTERGENIC	--	--	--
Ca2_P1_2169072	Ca2	2169072	TTCTCTCTC/TTCTCTCTCTC	AACCTCTCCAAGAGGA TCGG	GGAGAGTGAAACGGA TGGAA	60.6	648	INTERGENIC	--	--	--

INDEL marker IDs	Chromosomes/ unanchored scaffolds	Physical positions (bp)	InDels (*Kabuli* reference genome-CDC Frontier/PI)	Forward primers (5'-3')	Reverse primers (5'-3')	Anneling temperature (0C)	Expected amplified product size (bp)	Structural annotation		Functional annotation	
								Sequence components of *kabuli* genome	*Kabuli* gene accession IDs	NCBI-KOG	TFs
Ca2_P1_21714880	Ca2	21714880	TAAAAAAAA/TAAAAAAAAA	GCAGAAAAGAGGTGTTTGCC	ATTCGTCCGATGTGAAGTCC	59.9	600	INTERGENIC	--	--	--
Ca2_P1_21717951	Ca2	21717951	CTATATATATATATATAT/CTATATATATATATATAT	ACAGTTGCCAAAAAGGCTGT	TGATCATTAGCTCCACTGTCAAA	59.8	312	INTERGENIC	--	--	--
Ca2_P1_21724533	Ca2	21724533	TTATATATATATATATATATATATATATATATATATAT/TTATATATATATATATATATATATATATATATATAT	TTGTTCCCCGCTCTCTTCTA	GGGTTGCATTGAGTAATTGGA	59.9	331	INTERGENIC	--	--	--
Ca2_P1_2174282	Ca2	2174282	ATTTTTTTTTTTTT/ATTTTTTTTTTT	TGACATCCATCTTATGTGGGTC	GGTCTACTCATGTTGAAAGACGC	59.7	674	INTERGENIC	--	--	--
Ca2_P1_21783996	Ca2	21783996	ATTTTTT/ATTTTTTTT	ATCTCGTGGGTGGATCTGAC	GCTATGGGACCTATCAGGCA	59.9	381	DRR	Ca_23057	--	--
Ca2_P1_21804815	Ca2	21804815	CAT/CATAAAT	CGCCATAGACCATTGGAAAC	TCCATTGATCACACAACGGT	60.3	860	INTERGENIC	--	--	--
Ca2_P1_21943275	Ca2	21943275	AGCGCG/AGCG	GCAGATTTGGAAGGTGTGGT	ACAATCAGCACGCAAGAATG	60.0	461	INTERGENIC	--	--	--
Ca2_P1_22057378	Ca2	22057378	T/TC	GGACAATTGGTGGAGGAGAA	TGGGTGAAAGTTACCTTGGG	59.9	250	INTERGENIC	--	--	--
Ca2_P1_22100604	Ca2	22100604	CTTAGTTAGTTAGTTAGTTAGTTAGTTAGTTA/CTTAGTTAGTTAGTTAGTTAGTTAGTTAGTTA	GGTTTGTGACTGAAAAACGGA	TGACCCTCACACTTCACTCG	60.0	350	INTERGENIC	--	--	--
Ca2_P1_22103224	Ca2	22103224	GAA/G	AAGAGAAGGCACTGCCACAT	TGTAGGAATCCCTTGGCAAC	59.9	804	INTERGENIC	--	--	--
Ca2_P1_22219214	Ca2	22219214	GTGGTGGTCG/GTGGTGGTCGTTGGTGGTCG	CAAAGCGGTCGAAGTAGAGG	GTTGTTCGGGGTTGTGAGTT	60.0	391	INTERGENIC	--	--	--
Ca2_P1_22286038	Ca2	22286038	GAAGCGC/GAAGCGCACCCAAGCGC	GGGAGGCAACCCAATTTTAT	GCTTAGATCATCCGCTCCTG	60.0	368	INTERGENIC	--	--	--

INDEL marker IDs	Chromosomes/ unanchored scaffolds	Physical positions (bp)	InDels (*Kabuli* reference genome-CDC Frontier/PI)	Forward primers (5'-3')	Reverse primers (5'-3')	Anneling temperature (0C)	Expected amplified product size (bp)	Structural annotation		Functional annotation	
								Sequence components of *kabuli* genome	*Kabuli* gene accession IDs	NCBI-KOG	TFs
Ca2_P1_2235889	Ca2	2235889	TTATTA/TTATTACTATTA	TCTTGCACAACTCATTTCGG	AAAAAGCAAAGAAATTGTCAGAA	59.8	330	INTERGENIC	--	--	--
Ca2_P1_22455964	Ca2	22455964	GTATATATATA/GTATATATATATA	AGCGCTGTAAAATGTCCACC	CTGGGCAAGGGATCTGAATA	60.1	470	DRR	Ca_14999	--	Trihelix
Ca2_P1_224634	Ca2	224634	TCATTTATAGCATTTATAG/TCATTTATAG	GTTGGGTCACCGTTTCACTT	GCTCAACATTGGTTGCCTTT	59.9	657	INTRON	Ca_16974	--	--
Ca2_P1_22468520	Ca2	22468520	CAAAAAAAAAA/CAAAAAAAAAAA	TGGCCATGTGCATTGTTTAT	AGGCATCAACGCCTAAATTG	59.8	674	INTERGENIC	--	--	--
Ca2_P1_22579550	Ca2	22579550	CT/CTTT	CTAAGCCCTCACAAAGGCAG	AGTTGAAGCTTCTGGGACGA	60.0	292	DRR	Ca_14994	--	--
Ca2_P1_22650634	Ca2	22650634	TG/T	GAGAGGAGGGTTCCCAGAGT	GTGCATCCTCCATCTTTGGT	59.7	550	DRR	Ca_14991	--	--
Ca2_P1_2278774	Ca2	2278774	T/TG	GGGCTTGTTTGATTGTGTTTG	GGAAGAGAGGAAGGCTGGAT	60.4	812	INTERGENIC	--	--	--
Ca2_P1_228183	Ca2	228183	GTTTTTTTTTT/GTTTTTTTTT	TTCGAAACGTTGGATGAATG	GCAACCATCTGACCAAGTGA	59.5	418	INTERGENIC	--	--	--
Ca2_P1_22846943	Ca2	22846943	ATTTTTTTTTT/ATTTTTTTTTTT	GGTTCCAAACTCTCGGTTGA	GCACGTGGCTAGGGTTATGT	60.1	569	DRR	Ca_14984	--	--
Ca2_P1_22890617	Ca2	22890617	TTATATATATATATATATATATATAT/TTATATATATATATATATATATATATAT	GCCACCCTTGTGAGAGTTTC	CACGAGTCCGATAAGGGTGT	59.7	554	INTERGENIC	--	--	--
Ca2_P1_22939976	Ca2	22939976	TATTTAAATTTAA/TATTTAAATTTAAATTTAA	GCACCCACAAAGTAGGCATT	CTCGGCCTCAATACTTGCAT	60.0	893	INTERGENIC	--	--	--
Ca2_P1_2301289	Ca2	2301289	CATATATATATA/CATATATATATATA	TGCAAAATCAAGCGAATGAG	TTTTTGCAGGTCTACCCAGG	60.0	418	INTERGENIC	--	--	--

INDEL marker IDs	Chromosomes/ unanchored scaffolds	Physical positions (bp)	InDels (*Kabuli* reference genome-CDC Frontier/PI)	Forward primers (5'-3')	Reverse primers (5'-3')	Anneling temperature (0C)	Expected amplified product size (bp)	Structural annotation		Functional annotation	
								Sequence components of *kabuli* genome	*Kabuli* gene accession IDs	NCBI-KOG	TFs
Ca2_P1_23025137	Ca2	23025137	TTCTCTCTCTCTCTCTCTCTCTCTCTCTCTCTCTCTCT/TTCTCTCTCTCTCTCTCTCTCTCTCTCTCTCTCT	CGACTTTTGAGGTAGCCAGC	TTACTTGTCTCGCTGTTGCG	60.0	668	INTERGENIC	--	--	--
Ca2_P1_23042658	Ca2	23042658	A/AATT	CTCAAGTCGACAAGCCTTCC	TATCCCATGAGCAACCACAA	60.0	480	INTERGENIC	--	--	--
Ca2_P1_23058191	Ca2	23058191	GTTTTTTTTTTT/GTTTTTTTTTTTT	GAAACTGTGTCACGAGGCAA	AGAGCCGTCCAGAAAAATCA	59.9	121	INTERGENIC	--	--	--
Ca2_P1_23167563	Ca2	23167563	TACCTCCGACCT/TACCT	AGCGAGTCATTCAGTGGACC	TCTATATATAAGACCCCGTTGATTAAA	60.3	572	INTERGENIC	--	--	--
Ca2_P1_23193466	Ca2	23193466	TATTTAATA/TTTTAATA	CCACAAATGCTCATCGTGAC	TTTGCAGCCACAAAAGACAG	60.1	561	INTERGENIC	--	--	--
Ca2_P1_23208938	Ca2	23208938	ATTTTTTTTTTTTTTT/ATTTTTTTTTTTTTTTTT	TTGTTTCTCCCCAGTTTTGG	TTGTTTCTCCCCAGTTTTGG	59.9	228	INTERGENIC	--	--	--
Ca2_P1_23361316	Ca2	23361316	GTTATTATTATTATTATTATTATTATTATTATTATT/GTTATTATTATTATTATTATTATTATTATTATTATT	AGAAGAAGGGTTGGCTGACA	AGCCCACCCAACAACTACTG	59.8	620	INTERGENIC	--	--	--
Ca2_P1_23408235	Ca2	23408235	AATATATATATATATATATATATAT/AATATATATATATATATATATATATAT	GGTAGGAATTGGGTGTGGTG	AGGAAGAGTGGAGTTGCGAA	60.1	710	INTERGENIC	--	--	--
Ca2_P1_23472039	Ca2	23472039	CTTTATTTATTTATTTATTTATTTATTTATTTATTTAT/CTTTATTTATTTATTTATTTATTTATTTATTTATTTATTTAT	TTTTGCTAACCACACCCCTC	TGACCACGTGTTTTTGGGTA	60.0	434	INTRON	Ca_14958	--	--
Ca2_P1_23509535	Ca2	23509535	AAAAACTTAAAACTTAAAACTTA/AAAAACTTAAAACTTA	CCCTCTTGAATTTTTGTTTTCC	CCCACACCCTCGACTAAAGA	59.0	542	INTERGENIC	--	--	--
Ca2_P1_23601524	Ca2	23601524	AATATATATATATATATATATATATATATATAT/AATATATATATATATATATATATATATATATAT	GTTCTTTTGGGGGTTGGTTT	CCGCTAACACTCTGACCCAT	60.1	511	INTERGENIC	--	--	--
Ca2_P1_23643863	Ca2	23643863	ATCTCTCTCTCTCTCTCTCTCT/ATCTCTCTCTCTCTCTCTCTCTCTCT	TTCAGCCAAGTAGTGGAGGG	GCTTCCAACAAAAGCAAAGC	60.2	523	INTERGENIC	--	--	--

INDEL marker IDs	Chromosomes/ unanchored scaffolds	Physical positions (bp)	InDels (*Kabuli* reference genome-CDC Frontier/PI)	Forward primers (5'-3')	Reverse primers (5'-3')	Anneling temperature (0C)	Expected amplified product size (bp)	Structural annotation		Functional annotation	
								Sequence components of *kabuli* genome	*Kabuli* gene accession IDs	NCBI-KOG	TFs
Ca2_P1_23718665	Ca2	23718665	TA/T	GATTCTCGGACTTTTGGGGT	TTCAACCATAGGAATTTAAGGCA	60.3	265	INTERGENIC	--	--	--
Ca2_P1_2373367	Ca2	2373367	AATATATATATATATATATATATATA/AATATATATATATATATATATATATATATA	AAAATTGTCAGGTTCACCGC	GGTGGTGATTTGGTGCTCTT	60.0	896	DRR	Ca_12541	R	DBB
Ca2_P1_23792320	Ca2	23792320	GGAGTAAGAGAGTAA/GGAGTAAGAGAGTAAGAGAGTAA	TCAGAATCCCTGATCCCAAG	GCAACCAGGAGGCAAGTCTA	60.0	490	INTERGENIC	--	--	--
Ca2_P1_23819744	Ca2	23819744	ATTT/ATT	TTGTTGTAGGGCACATTGGA	TGTTCGTTTGACGAGAGCAC	60.0	391	DRR	Ca_14945	--	--
Ca2_P1_23820380	Ca2	23820380	TTATATATATATATAT/TTATATATATATATATAT	GTGCTCTCGTCAAACGAACA	AAGCGCTGTAAATGGTGCAT	60.0	716	DRR	Ca_14945	--	--
Ca2_P1_23838318	Ca2	23838318	AG/A	CAACAATCTCTCCCGCTCTC	TGGCGACTAGGAGGTTTTGA	60.0	665	INTERGENIC	--	--	--
Ca2_P1_23851284	Ca2	23851284	GATATATATATATATATATATATATATA/GATATATATATATATATATATATATATATATA	TGCCTTCATCATGTGGTTGT	ATGGCAATTTCTCAGGATGG	60.0	501	INTERGENIC	--	--	--
Ca2_P1_23874695	Ca2	23874695	TTATATATATATATAT/TTATATATATATATATAT	TTTTTAAGGATAAATTACGAAACCAA	CCCGTATTTGCTGCAACTTT	58.6	431	DRR	Ca_14942	--	--
Ca2_P1_23932608	Ca2	23932608	TAAATTAATGAATAAAAATTAAT/TAAATTAAT	AAGGCATCCAATCAAAGCAC	CTCAAGATTTTAGCGCCGTC	60.1	460	INTERGENIC	--	--	--
Ca2_P1_23989802	Ca2	23989802	AAAAACAAAACAAAA/AAAAACAAAACAAAACAAAA	AATTCGTTGAGCCGGTCTAGT	AGCACGGGGAGTATGAGAAA	60.1	421	INTERGENIC	--	--	--
Ca2_P1_23990182	Ca2	23990182	AAAAACAAAACAAAA/AAAAACAAAACAAAACAAAA	TCTCATACTCCCCGTGCTTT	ATCCCCTACACCAGATGCAC	59.7	565	INTERGENIC	--	--	--
Ca2_P1_24019689	Ca2	24019689	TAAAAAAAAAAAAAA/TAAAAAAAAAAAAA	TGCCTTGAACAACCACTGTC	CCTTTCAGAGAATCGTGGGA	59.7	584	INTERGENIC	--	--	--

INDEL marker IDs	Chromosomes/ unanchored scaffolds	Physical positions (bp)	InDels (*Kabuli* reference genome-CDC Frontier/PI)	Forward primers (5'-3')	Reverse primers (5'-3')	Anneling temperature (0C)	Expected amplified product size (bp)	Structural annotation		Functional annotation	
								Sequence components of *kabuli* genome	*Kabuli* gene accession IDs	NCBI-KOG	TFs
Ca2_P1_24102058	Ca2	24102058	TCC/TC	ACCAAAATCACTGCTCACCC	GATTGAATCAAACACGCCCT	60.0	467	INTRON	Ca_21670	S	--
Ca2_P1_24102909	Ca2	24102909	GT/GTT	TGGGGGATCCAAGTTAAAAA	GAAAACTCTCCGGCATTCAA	59.2	428	CDS (FRAME SHIFT)	Ca_21670	S	--
Ca2_P1_24124699	Ca2	24124699	ATTTTTTTTTTTTTTT/ATTTTTTTTTTTTT	ATATATCGGGGTTCATGGCA	CCATGCTTCACCCTTGGTAT	60.0	603	INTERGENIC	--	--	--
Ca2_P1_24151703	Ca2	24151703	T/TATGGAA	ATTTGATGTTTTGCTTCGGG	GGTGCAGCAAGATTGGATTT	59.9	360	INTERGENIC	--	--	--
Ca2_P1_24241560	Ca2	24241560	ATCT/AT	TTAATGTGTTGCGGGTGAAA	GTTGGTTCCCTAGCACCGTA	60.0	447	INTERGENIC	--	--	--
Ca2_P1_24252139	Ca2	24252139	TAA/TAAGAAA	GATCCTCAAATCGACCTCCA	TGTCACCATTGGTCTCCAAA	60.0	465	INTERGENIC	--	--	--
Ca2_P1_24259838	Ca2	24259838	TAA/TAAA	TCTTCTCCCTCCCTAGCCTC	GCGAATTGCAGACTGAAACA	59.9	825	DRR	Ca_21665	--	--
Ca2_P1_24259982	Ca2	24259982	CAA/CAAA	CATCATCAAAAAGGGGGAGA	GCGAATTGCAGACTGAAACA	59.9	535	DRR	Ca_21665	--	--
Ca2_P1_24349766	Ca2	24349766	AATTTAATT/AATTTAATTATATTTAATT	TCTTCACGCCAAGAAGAAAAA	AAAAACGATTGCGAATGGAG	60.0	614	INTERGENIC	--	--	--
Ca2_P1_24563119	Ca2	24563119	TCCCC/TCCC	CCATCTACTGCCCTTGGAAA	GATCCAGGCAAGGTCAATGT	60.1	260	INTERGENIC	--	--	--
Ca2_P1_24653257	Ca2	24653257	GATATATATATATATATATATATATATATATATATAT/GATATATATATATATATATATATATATATATATATATATATATATAT	TGTAGGGTTTGAACGTGGTG	TGGCTACAATCTGTTTATCCCTC	59.5	564	INTERGENIC	--	--	--
Ca2_P1_24798855	Ca2	24798855	AA/AACAAATAATAATAATA	TTCTCAAACTCGTTGCACCA	CACTTTTCTGCAAAGGCACA	60.4	481	INTERGENIC	--	--	--

INDEL marker IDs	Chromosomes/ unanchored scaffolds	Physical positions (bp)	InDels (*Kabuli* reference genome-CDC Frontier/PI)	Forward primers (5'-3')	Reverse primers (5'-3')	Anneling temperature (0C)	Expected amplified product size (bp)	Structural annotation		Functional annotation	
								Sequence components of *kabuli* genome	*Kabuli* gene accession IDs	NCBI-KOG	TFs
Ca2_P1_24940560	Ca2	24940560	CAAA/CAAAA	GTGAACAATGACCAAGCCAA	CCTAACTCTGCATGGGGAAA	59.5	186	INTERGENIC	--	--	--
Ca2_P1_24943252	Ca2	24943252	CTTT/CTT	AACCCCTCCAAAGAACGACT	AGGGCCCCACTAACTCCTTA	60.0	680	INTERGENIC	--	--	--
Ca2_P1_24948166	Ca2	24948166	CTTTTT/CTTTT	GGTGAGACTTTCGAGGGCTA	GGCATTGAACGTTTTCACAA	59.4	295	INTERGENIC	--	--	--
Ca2_P1_24948276	Ca2	24948276	GAAAA/GAAA	AAACGTCCAAGGTAAGGGCT	GGCATTGAACGTTTTCACAA	60.0	255	INTERGENIC	--	--	--
Ca2_P1_25025231	Ca2	25025231	TAA/TA	CAGCCACCCACCTACAAAGT	ATAGCACCAACAAACCCAGC	60.0	829	INTRON	Ca_14236	--	CO-like
Ca2_P1_25120783	Ca2	25120783	ATTTTTTTTTTTT/ATTTTTTTTTTTTT	CGATTCAGATTGAACTCGAACA	GGTGGGAGAGCAATAATGGA	60.2	445	INTERGENIC	--	--	--
Ca2_P1_25171520	Ca2	25171520	TCAACAA/TCAA	GGAAATGAGCTGAATGGGAA	CCAGAGGGGATGTTGAGAAA	60.0	551	INTRON	Ca_14250	T	M-type
Ca2_P1_25241576	Ca2	25241576	GATATATATATATATA/GATATATATATATATATATA	TATTTTGGATTTTGGCTCGG	AAATGGAGGAAGGAGGGAGA	59.9	433	INTERGENIC	--	--	--
Ca2_P1_25352886	Ca2	25352886	ATTTTTTTTTT/ATTTTTTTTTTT	AAAGAGACACATGACATAACTCCTC	ATGGGGGATGGACCTAGAAC	57.9	685	DRR	Ca_14268	--	--
Ca2_P1_25411964	Ca2	25411964	ATTT/A	CAAAATCATTACTCACCATAAGTGG	TCACATCAGCCAAAAAGCTG	58.9	405	INTERGENIC	--	--	--
Ca2_P1_25427657	Ca2	25427657	TTATATATATATATATATATATATATATATAT/TTATATATATATATATATATATATATATATAT	TGTTGTTGAAGATGAAATATTGGC	TTTGGCGTAGTGAAGTTGACA	60.2	723	INTERGENIC	--	--	--
Ca2_P1_2546989	Ca2	2546989	AT/ATCATAGTTT	CCCGTCGATCAAAAGAAAAA	TTGAATTGCTTTCCAGTGTCC	60.0	705	INTRON	Ca_10592	R	--

INDEL marker IDs	Chromosomes/ unanchored scaffolds	Physical positions (bp)	InDels (*Kabuli* reference genome-CDC Frontier/PI)	Forward primers (5'-3')	Reverse primers (5'-3')	Anneling temperature (0C)	Expected amplified product size (bp)	Structural annotation		Functional annotation	
								Sequence components of *kabuli* genome	*Kabuli* gene accession IDs	NCBI-KOG	TFs
Ca2_P1_25529091	Ca2	25529091	TA/TAGA	CCATTGAATTTACCGACGAAA	TTTACCGGGATTCTGCAAAG	59.8	663	INTERGENIC	--	--	--
Ca2_P1_25529860	Ca2	25529860	CTTT/CTTTTTT	AGCAGCCCAAGCAAGACTTA	TCATGCCGATTGATAGAGGTT	60.2	553	INTERGENIC	--	--	--
Ca2_P1_25535050	Ca2	25535050	CTTTTTTTTT/CTTCTTTTTTTTTTTTTTT	CCAGATTTGAACTGGGGAAA	AATCAAGTGGGTTCGAGTGG	59.9	551	DRR	Ca_14283	R	MYB
Ca2_P1_25615480	Ca2	25615480	A/ATCCACTCTTTCCTCCAC	TTCTCCACCAACCCAAGAAC	GCAAGTTGCTATGTGAGCCA	59.9	489	INTERGENIC	--	--	--
Ca2_P1_25619256	Ca2	25619256	CTTTTTT/CTTTTTTT	TTGAGAGACCAAAGTGTCCAAA	AAGTGACTATTTCCTCGGTCCTT	59.8	829	INTERGENIC	--	--	--
Ca2_P1_25661456	Ca2	25661456	AATATATATATATATATATATATATA/AATATATATATATATATATATATATATATATA	CCCATAGGGATTGCTAACGA	TGCCCCAGTTATCCAAAAGT	59.9	652	INTERGENIC	--	--	--
Ca2_P1_25713315	Ca2	25713315	CA/CATGAGATA	TCAGCCTTGCTTGTCATTTG	TGCTTGCAATGTCTTCTCTCA	60.0	528	INTERGENIC	--	--	--
Ca2_P1_25767704	Ca2	25767704	GAAGTTAAGTT/GAAGTT	ATGAATTTTGGCATCCAACC	CGTGGACAAAGATCGTGATG	59.6	654	INTRON	Ca_14294	G	NAC
Ca2_P1_25791084	Ca2	25791084	ATTTTTTTTTTTTTTT/ATTTTTTTTTTTTT	GCAAGTTGAGCCTCATGGAT	GCCTCGGTTGAAAATTGAAA	60.2	452	INTERGENIC	--	--	--
Ca2_P1_25795167	Ca2	25795167	GAATTTTTAAATTTTTAAA/GAATTTTTAAATTTTTAAATTTTTAAA	AGCCACGGCTCTAAAGATCA	TTGGTTTCATTGTTTTGCATTT	60.0	674	INTERGENIC	--	--	--
Ca2_P1_25809875	Ca2	25809875	CAATAATAATAATAATAATAATAATAATAATAATAATAATAATAATAATAATAATAATAATAATAA/CAATAATAATAATAATAATAATAATAATAATAATAATAATAATAATAATAATAATAATAATAATAATAATAATAATAATAA	TGACAAAAATATCCGTAAGAAGCA	AGCTCAAAAGACAAATGCCG	60.0	535	INTERGENIC	--	--	--
Ca2_P1_25847633	Ca2	25847633	ATATCTAT/ATAT	TTTTAACAGAAGTTGGGATGACA	CAGAACAAAGTTCAAATCTTCTCG	58.7	762	INTERGENIC	--	--	--

INDEL marker IDs	Chromosomes/ unanchored scaffolds	Physical positions (bp)	InDels (*Kabuli* reference genome-CDC Frontier/PI)	Forward primers (5'-3')	Reverse primers (5'-3')	Anneling temperature (0C)	Expected amplified product size (bp)	Structural annotation		Functional annotation	
								Sequence components of *kabuli* genome	*Kabuli* gene accession IDs	NCBI-KOG	TFs
Ca2_P1_26106556	Ca2	26106556	CAAAAAA/CAAAAAAA	TCTAGCGAAAAGACCGAAGC	TCCATTTTGGATCAACCCAT	59.7	699	INTERGENIC	--	--	--
Ca2_P1_26112745	Ca2	26112745	CATATATATATATATATATATATATATATATATATATA/CATATATATATATATATATATATATATATATATATA	TCGTTGCCACTCAATAAAAACTT	TCAAATTCCGAAACCTTTGC	60.1	195	INTERGENIC	--	--	--
Ca2_P1_2612391	Ca2	2612391	CTCTTTCTTTCTTTCTTTCTTTCTTTCTTTCTTTCTTTCTT/CTCTTTCTTTCTTTCTTTCTTTCTTTCTTTCTTTCTTTCTTTCTT	CCATCTTCCTTCTCAACATGC	ATGGAGGTGCGTAATTGACA	59.7	449	INTERGENIC	--	--	--
Ca2_P1_2624920	Ca2	2624920	CGGCTGGCT/CGGCT	GGCCAAGTTGCTGAAGGATA	CGCTAAGAGGCCGTTATCAA	60.2	610	INTRON	Ca_10586	--	--
Ca2_P1_26500379	Ca2	26500379	CAATAATAATAATAATAATAATAATAATAATAATAATAATAATAATAATAATAATAATAATAATAATA/CAATAATAATAATAATAATAATAATAATAATAATAATAATAATAATAATAATAATAATAATAATA	ATTGATTTCAACGGCTCCAC	TGAACTTGGCCAATATGTGTG	59.9	516	INTERGENIC	--	--	--
Ca2_P1_26504489	Ca2	26504489	CAA/CAAAA	CCGACGTCCAGTCAGAATTT	CTCAATTGGACACCGTTCCT	60.1	772	INTERGENIC	--	--	--
Ca2_P1_26509024	Ca2	26509024	AT/ATT	GCTTTTTGAAGGTTTCGACG	GGGTTAAGCCCATATGGAAGA	59.9	547	DRR	Ca_17250	C	--
Ca2_P1_26556425	Ca2	26556425	TTATATATATATATATATATATATATATATA/TTATATATATATATATATATATATATATA	TCAGGTGCAGATCTTTTCACA	TATTTGGAGAGTTGGGAGCG	59.4	781	INTERGENIC	--	--	--
Ca2_P1_26570798	Ca2	26570798	GAAAAAAAAAAA/GAAAAAAAAAA	GGTGGGCATGAGATGAGAGT	TTTACGGGGAAGAGAAGTGC	60.1	782	INTERGENIC	--	--	--
Ca2_P1_26691947	Ca2	26691947	AATATATATATATATATATATATA/AATATATATATATATATATATATATA	AGTTTTACAGCGCTTTTCGG	TTATTGGCGTGCATGTTTTT	59.5	643	INTERGENIC	--	--	--
Ca2_P1_26715780	Ca2	26715780	ATTTTTT/ATTTTTTT	AAGGTGAGGTGTGGTTGAGG	TTGGGAAAGGCGATTTGTAG	60.0	518	INTERGENIC	--	--	--
Ca2_P1_26716017	Ca2	26716017	GC/GCC	AAGGTGAGGTGTGGTTGAGG	TTGGGAAAGGCGATTTGTAG	60.0	518	INTERGENIC	--	--	--

INDEL marker IDs	Chromosomes/ unanchored scaffolds	Physical positions (bp)	InDels (*Kabuli* reference genome-CDC Frontier/PI)	Forward primers (5'-3')	Reverse primers (5'-3')	Anneling temperature (0C)	Expected amplified product size (bp)	Structural annotation		Functional annotation	
								Sequence components of *kabuli* genome	*Kabuli* gene accession IDs	NCBI-KOG	TFs
Ca2_P1_26839360	Ca2	26839360	TAAAAAAAAAA/TAAAAAAAAA	TTGCCTTTTGAATACCCGAG	TGAGTATGGACACGGAGACG	60.1	606	INTERGENIC	--	--	--
Ca2_P1_26914055	Ca2	26914055	ATTTTTTTTTT/ATTTTTTTTTTTT	AGGTTTGAACCGTCACCAAC	TTTGCATGTTCAATCACTCCA	59.9	255	INTERGENIC	--	--	--
Ca2_P1_2692293	Ca2	2692293	ATTTTTTTT/ATTTTTTT	AAAGGTGATGAAGCAGATTTTGA	TGGTGTCCTCTCTTCTCTAATCG	60.1	478	INTERGENIC	--	--	--
Ca2_P1_27153936	Ca2	27153936	TTATATATATATATATATATATATAT/TTATATATATATATATATATAT	TCCAAACTAGGTGGCAACAA	TTGCCCACTCATTTCCTTTC	59.2	478	INTERGENIC	--	--	--
Ca2_P1_27347942	Ca2	27347942	TT/TTATTAT	TTTTGGAATGTGCTGCAAAG	TGAAAAAGGGAAACCAAATGA	59.8	603	INTERGENIC	--	--	--
Ca2_P1_27366969	Ca2	27366969	CAAAAAAAAAAAA/CAAAAAAAAAAA	TTACCGTTGAATTTGCCACA	ATGGGTGTTCATAGCGGAAA	60.0	756	DRR	Ca_15658	DT	--
Ca2_P1_27398740	Ca2	27398740	TTTTAAATTTAAATTTAAATTT/TTTTAAATTTAAATTTAAATTTAAATTT	TTCATGGTTGGATTGACTTTTG	AACCTCCTCCTGCTTGTGAA	59.8	697	INTERGENIC	--	--	--
Ca2_P1_27399194	Ca2	27399194	CTTCAATAACTAATAATTCAA/CTTCAA	TCACAAGCAGGAGGAGGTTT	ACCGTCCATCTGAAATGAGG	59.8	188	INTERGENIC	--	--	--
Ca2_P1_27401550	Ca2	27401550	TAAAAAAAAAAAA/TAAAAAAAAAAAAA	GCCGTTGGGTTTGAGTTAGA	CCAAATCACCTCCAGTCACC	60.1	678	INTERGENIC	--	--	--
Ca2_P1_27408634	Ca2	27408634	CATATATATATATATATATATATAT/CATATATATATATATATATAT	ATTGCAATTTCATGGGTGTT	TGTTGCATGTTTAGACGACAA	57.8	519	INTERGENIC	--	--	--
Ca2_P1_27420609	Ca2	27420609	CATATATATATATATATATATATATATATAT/CATATATATATATATATATATATATATATATATATATATATAT	TTTAGATCCCTTGCACGACC	CTTCGACACTGTAGCACCCA	60.1	204	DRR	Ca_15661	--	--
Ca2_P1_27436271	Ca2	27436271	CAAA/CAA	TCCTACGTATCTTTTGATGCGA	CGTGCCCAATCTCTCAATCT	59.7	382	INTERGENIC	--	--	--

INDEL marker IDs	Chromosomes/ unanchored scaffolds	Physical positions (bp)	InDels (*Kabuli* reference genome-CDC Frontier/PI)	Forward primers (5'-3')	Reverse primers (5'-3')	Anneling temperature (0C)	Expected amplified product size (bp)	Structural annotation		Functional annotation	
								Sequence components of *kabuli* genome	*Kabuli* gene accession IDs	NCBI-KOG	TFs
Ca2_P1_27482007	Ca2	27482007	AATTTGTATGGCATAATT/AATTTGTATGGCATAATTCATTTGTATGGCATAATT	TTGATGCTGGTTGCTGACTC	CGGTTCAAACTCTTCCCGTA	60.0	583	INTERGENIC	--	--	--
Ca2_P1_2749945	Ca2	2749945	CTTT/CTT	ATATGGATTCATGCCCCAAA	TTGCATTTTTCAGAGGGAGG	60.0	580	INTRON	Ca_10569	GMO	E2F/DP
Ca2_P1_27538165	Ca2	27538165	CTGATGCATAATGATGCATAAT/CTGATGCATAAT	CATTCCCCCTACCTCCATTT	TTGAGGATGATTGAGAGGGG	60.0	432	INTERGENIC	--	--	--
Ca2_P1_27549994	Ca2	27549994	AATATATATATATATATATAT/AATATATATATATATAT	GCACAGCACCTTTCTTCCTC	TCAGCAAATCATGGTGGAAA	60.0	754	INTERGENIC	--	--	--
Ca2_P1_27560889	Ca2	27560889	TTCCCTCCTATCCCTC/TTCCCTC	GGTGCATTTGGGAACCTTTA	CTGCAGCATCCCTACACTCA	59.8	767	INTERGENIC	--	--	--
Ca2_P1_27614728	Ca2	27614728	AAATAATAATAATAATAATAATAATAATAATAATAATAATAATGCTAATAATAATAATAATAATTGTAATAATAATAATAATAATAACAATAATAATAATAATAA/AAATAATAATAATAATAATAATAATAATAA[illegible]	CGCGCGAGCCATAATCTAGT	CGACGCCAATCTGCTTATTT	62.6	625	INTERGENIC	--	--	--
Ca2_P1_27618744	Ca2	27618744	GAAAAAAAAA/GAAAAAAAAAA	CAACTGTGGGCAATGTCATC	ACAAATGTCGTTGGATGGGT	60.0	822	INTERGENIC	--	--	--
Ca2_P1_27647945	Ca2	27647945	T/TC	ATGATTTGGAGCCATTCCAG	CAAGTCAGTGGCCAAAATCC	59.9	244	INTERGENIC	--	--	--
Ca2_P1_27668293	Ca2	27668293	TC/T	AGGTTGGGTTTAGCGACTCA	TTCCCGATCTAGTCCTTTATTCC	59.7	365	INTERGENIC	--	--	--
Ca2_P1_27692325	Ca2	27692325	TG/T	AGACAAAACGTTCAAGTTGTCG	TCTTTAATTGCATGCGCAAA	59.3	762	DRR	Ca_15671	--	ERF
Ca2_P1_27694535	Ca2	27694535	TGAGAGAGAGAGAGAGAGAGAGAGAGA/TGAGAGAGAGAGAGAGAGAGAGAGAGA	TTGATTTGCTTGATGGGTCA	TCACACTCCATTTTCCCTCC	60.0	733	INTERGENIC	--	--	--
Ca2_P1_27715428	Ca2	27715428	AT/ATATTT	AAGTCGCATCACCGATATTGT	CAAGGCGATTTGAATTGGTT	59.5	372	INTERGENIC	--	--	--

INDEL marker IDs	Chromosomes/ unanchored scaffolds	Physical positions (bp)	InDels (*Kabuli* reference genome-CDC Frontier/PI)	Forward primers (5'-3')	Reverse primers (5'-3')	Anneling temperature (0C)	Expected amplified product size (bp)	Structural annotation		Functional annotation	
								Sequence components of *kabuli* genome	*Kabuli* gene accession IDs	NCBI-KOG	TFs
Ca2_P1_27742692	Ca2	27742692	C/CTTGA	CTCCGTTTTCTGCACATCAA	TATGAATGAGCAGCAGTGGC	59.8	331	DRR	Ca_15675	--	--
Ca2_P1_27776478	Ca2	27776478	GTTTTTTTTTT/GTTTTTTTTTTT	CAATGAACTTGGTCAACCCC	GCCCATGTCTGCATAGTAACAA	60.2	407	INTERGENIC	--	--	--
Ca2_P1_27804750	Ca2	27804750	TCC/TC	CAGGAAACTCTGCCTTTTCG	TAGCAGCTGTATGGTGCAGG	60.0	137	INTERGENIC	--	--	--
Ca2_P1_2798745	Ca2	2798745	TCAATAACAATAACAATAACAATAACA/TCAATAACAATAACAATAACA	CAGCACGTGAAGAACCAAAA	TTTGCTCCGCTCTCTCTCTC	59.9	444	INTRON	Ca_10562	B	WRKY
Ca2_P1_28064133	Ca2	28064133	GTATATATATATATATATAT/GTATATATATATATATATAT	TCAAAATGCCACAATTCCAA	ACATATACCATTGGGGCGAT	59.9	612	INTERGENIC	--	--	--
Ca2_P1_28153616	Ca2	28153616	CTT/CT	ACGCCTTAGAGGGTTGGTTT	TTGAGTTTCCAACTTTGGGG	60.0	323	DRR	Ca_14386	--	--
Ca2_P1_28157957	Ca2	28157957	TTATATATATATATATATATATAT/TTATATATATATATATATATATATATATAT	GCATGGACATTCAAAAATGAGA	CCATGTCTAAGTGGCAGGGT	59.9	583	DRR	Ca_14385	O	MYB_related
Ca2_P1_28202546	Ca2	28202546	TATAATAATAATAATAATAATAATAATAATAATAATAATAAT/TATAATAATAATAATAATAATAATAATAATAATAAT	TGAACCACATCCCTTATGCTT	CCATGAATGAGAGTGCAAAGG	59.4	632	INTERGENIC	--	--	--
Ca2_P1_28235085	Ca2	28235085	GTTTTTTTTTT/GTTTTTTTTTTT	TCCAAATAGCGACTTCCCAC	TCCAAATGTTACAGCCAGCTC	60.1	567	INTERGENIC	--	--	--
Ca2_P1_28235280	Ca2	28235280	AATATATATATATATATATA/AATATATATATATATATATATA	TCCAAATAGCGACTTCCCAC	ACAATCCTCAAATGCGGAGA	60.1	701	INTERGENIC	--	--	--
Ca2_P1_28235432	Ca2	28235432	AATATATATATATATATATATA/AATATATATATATATATATATATA	TCCAAATAGCGACTTCCCAC	GCTGCATGTTCTTATGCTCG	60.1	849	INTERGENIC	--	--	--
Ca2_P1_28296041	Ca2	28296041	AGTATG/A	GATGCGATCTTGCTTGTTGA	TCGACATGGCATGGTGAATA	60.0	399	INTERGENIC	--	--	--

INDEL marker IDs	Chromosomes/ unanchored scaffolds	Physical positions (bp)	InDels (*Kabuli* reference genome-CDC Frontier/PI)	Forward primers (5'-3')	Reverse primers (5'-3')	Anneling temperature (0C)	Expected amplified product size (bp)	Structural annotation		Functional annotation	
								Sequence components of *kabuli* genome	*Kabuli* gene accession IDs	NCBI-KOG	TFs
Ca2_P1_28304737	Ca2	28304737	TAAAAAAAAAAAAA/TAAAAAAAAA	CATGATTAACGTGGACGCAA	TCGTCTCGAAATTTGATGTTTG	60.5	675	INTERGENIC	--	--	--
Ca2_P1_28412021	Ca2	28412021	ATATTATTATTATTATTATTATTATTATTATTATTATTATTATTATTATTATTATTATTA/ATATTATTATTATTATTATTATTATTATTATTATTATTATTATTATTATTATTATTATTA	TGAAATGCACTAAGTGGCTCC	CATGATCAAAACATCACCGC	60.3	786	INTERGENIC	--	--	--
Ca2_P1_28422143	Ca2	28422143	TAAAAAAA/TAAAAAA	TGAGGCTGGTCCACAATACA	TCCAAAATATGCACACTCACA	60.1	413	INTERGENIC	--	--	--
Ca2_P1_28465109	Ca2	28465109	AA/AATA	TCATCGCAGGTGTACCATGT	AACATTGCACCGGTTTGATT	60.0	414	INTERGENIC	--	--	--
Ca2_P1_28465231	Ca2	28465231	CAAAAA/CAAAA	TCATCGCAGGTGTACCATGT	GGTATCAGAGCCCACCATGT	60.0	908	INTERGENIC	--	--	--
Ca2_P1_28465336	Ca2	28465336	AAT/A	AAGAGAGGTCACTTTGGCGA	GCAATTCCCACAGGTATGCT	60.0	886	INTERGENIC	--	--	--
Ca2_P1_28465620	Ca2	28465620	A/AT	AGCTCCATTGCAAAAACACC	GCAATTCCCACAGGTATGCT	60.1	553	INTERGENIC	--	--	--
Ca2_P1_28482218	Ca2	28482218	CATA/CATATA	TCCATAGAATTAATGGTCCCC	TTTGCTTTATCAAGCCACCC	57.7	198	INTERGENIC	--	--	--
Ca2_P1_28556774	Ca2	28556774	GAAAAAAAA/GAAAAAAA	TCCTAGCAAATTCCACACGA	GACAATGAAATTGGGGTTGG	59.3	450	INTERGENIC	--	--	--
Ca2_P1_285679	Ca2	285679	AAATAATAATAATAATAATAATAATAATAATAATAATAATACTAATAATAATAATAATAATAA/AAATAATAATAATAATAATAATAATAATAATAATAATAATACTAATAATAATAATAATAATAA	AAACGACTCACACCCGAAAC	GCCATTGATTCATTTCTTCG	60.0	614	INTERGENIC	--	--	--
Ca2_P1_28569036	Ca2	28569036	TGG/TGGG	TCTCCAAATGATTGGAAGGC	GACTGGACGTAACCCACGTT	60.0	913	INTERGENIC	--	--	--
Ca2_P1_28569281	Ca2	28569281	T/TC	CATCTGTAATGATGCGCCAC	GACTGGACGTAACCCACGTT	60.1	503	INTERGENIC	--	--	--

INDEL marker IDs	Chromosomes/ unanchored scaffolds	Physical positions (bp)	InDels (*Kabuli* reference genome-CDC Frontier/PI)	Forward primers (5'-3')	Reverse primers (5'-3')	Anneling temperature (0C)	Expected amplified product size (bp)	Structural annotation		Functional annotation	
								Sequence components of *kabuli* genome	*Kabuli* gene accession IDs	NCBI-KOG	TFs
Ca2_P1_28582452	Ca2	28582452	TATT/TATTGAATT	TCGTCGCAAACTTTCATCAG	AAAGGACCAAATCGGAAAAA	60.0	761	INTERGENIC	--	--	--
Ca2_P1_28582611	Ca2	28582611	TTATTGTATTGTATTGTATT/TTATTGTATTGTATT	ACCTTCATCAGTTCGTTGCC	AAAGGACCAAATCGGAAAAA	60.1	499	INTERGENIC	--	--	--
Ca2_P1_28617666	Ca2	28617666	TTTTATTTATTTATTTATT/TTTTATTTATTTATTTATTTATTTATT	ATCGACTCCCGAACTCAAAA	CCACGGTTCTTAGAGCCATTA	59.7	621	INTERGENIC	--	--	--
Ca2_P1_28709767	Ca2	28709767	AG/A	TCATCAAATTCGTAACGCAAA	TGAACACTCTTGTGGATTCAATG	59.2	715	INTERGENIC	--	--	--
Ca2_P1_28723744	Ca2	28723744	AAAAACACACACACA/AACACACA	AAAAAGCGCTGTTCATTTCAA	AAAAAGCCTCCACCAAAGGT	59.9	511	DRR	Ca_14359	R	--
Ca2_P1_28812761	Ca2	28812761	TATTAACTTGAATTGACGA/TATTAACTTGAATTGACGATTAACTTGAATTGACGA	AAGGCGATGATCAAATCCAA	CCAGCATTGTACGAGCTTCA	60.4	316	INTERGENIC	--	--	--
Ca2_P1_2881711	Ca2	2881711	CTTTTGTTACTCTTT/CTTTTGTTACTCTTTTTGTTACTCTTT	TTCATTTCATCAATTCGATCCTT	GGCAATGCCACCGTAAAATA	59.8	776	INTERGENIC	--	--	--
Ca2_P1_28873803	Ca2	28873803	GTTTT/GTTTTT	GGCAGGCAACAAGACAAAAT	TCCGTGCATGCCATAATCTA	60.1	656	INTERGENIC	--	--	--
Ca2_P1_28874011	Ca2	28874011	CTTT/CTT	TGATGGAATGCAATGAAATGA	TCCGTGCATGCCATAATCTA	59.9	386	INTERGENIC	--	--	--
Ca2_P1_2888796	Ca2	2888796	AAATAATAATAATAATAATAATAA/AAATAATAATAATAATAATAATAATAA	CTTGCCAATACGTGTGTTCC	TGCCTCAATTGTTCAGGAGA	59.1	442	INTERGENIC	--	--	--
Ca2_P1_28905309	Ca2	28905309	TAAAAAAAAAAA/TAAAAAAAAAA	AGGTGTGGATATTTCGGTCG	TTGTGCACCTTCATTTTACCA	59.8	970	INTERGENIC	--	--	--
Ca2_P1_29029807	Ca2	29029807	AAATAATAATAATAATAATAATAATAATAATAATAATAATAATAATAA/AAATAATAATAATAATAATAATAATAATAATAATAATAATAA	AGTTGGCTTTTGTACCCCCT	AATGTGCCACAAACAAAACG	59.9	732	INTERGENIC	--	--	--

INDEL marker IDs	Chromosomes/ unanchored scaffolds	Physical positions (bp)	InDels (*Kabuli* reference genome-CDC Frontier/PI)	Forward primers (5'-3')	Reverse primers (5'-3')	Anneling temperature (0C)	Expected amplified product size (bp)	Structural annotation		Functional annotation	
								Sequence components of *kabuli* genome	*Kabuli* gene accession IDs	NCBI-KOG	TFs
Ca2_P1_29051306	Ca2	29051306	CTTTTTTTT/CTTTTTTTTT	TTGTTGTGAGCAACCACCAT	TCGTGAAACCTTGCACTTCA	60.0	837	INTERGENIC	--	--	--
Ca2_P1_29065437	Ca2	29065437	GAAAAAAAAAAAA/GAAAAAAAAAAA	ACAAAATGCATGGTGCAATC	CACCAGCAATCCTAATTTCAA	59.4	452	INTERGENIC	--	--	--
Ca2_P1_2907015	Ca2	2907015	AATATATATATATATATATATATA/AATATATATATATATATATATATATA	TTTCTCTGTGTTTATTGTGCGTT	TTTTTGACTGACGCCTCAAAT	58.9	577	INTERGENIC	--	--	--
Ca2_P1_29072040	Ca2	29072040	ATCTGCAGACTTT/ATCTGCAGACTTTCTGCAGACTTT	TTGCTGGCAGAACAATCAAG	TGTGGGAAGCACCATGTTTA	60.0	546	DRR	Ca_14332	--	--
Ca2_P1_29170521	Ca2	29170521	TTTATTATTATTATTATTATTATTATTATTATTATTATTATTATTATTATTATTATTATTATTATTA/TTTATTATTATTATTATTATTATTATTATTATTATTATTATTATTATTATTATTATTATTATTATTATTATTA	CCTCTGATTAGGGGTGGACA	GCTGGATTGAGAGTGTGGGT	59.9	446	INTERGENIC	--	--	--
Ca2_P1_29175467	Ca2	29175467	CTATATATATATATATATATATATA/CTATATATATATATATATATATATATA	TAGCGGGTTATGGTGGACAT	CTCCTGATAACGCCATTGGT	60.2	749	INTERGENIC	--	--	--
Ca2_P1_29212961	Ca2	29212961	TTATATATATATATATATATATATATATATATAT/TTATATATATATATATATATATATATATATATATATATATAT	TAGTTTGTAACCCGTGCGTC	CAAAAATAAGACATGGTGTTGGA	58.7	273	INTERGENIC	--	--	--
Ca2_P1_29215176	Ca2	29215176	T/TTTGATATTCTCTC	ACGGTTGAGAATTGAGTGGC	CGCACACCTCTAACAACCAA	60.1	508	INTERGENIC	--	--	--
Ca2_P1_29215461	Ca2	29215461	TTAT/TTATAT	TACTTTAGCAACCACCCGCT	ATGACTCCAACACCATGCCT	59.8	873	INTERGENIC	--	--	--
Ca2_P1_29220253	Ca2	29220253	GATATATATATATATATATATATA/GATATATATATATATATATATATATATATATA	AGGCATGGTGTTGGAGTCAT	CTAGTTCGTAACCCGTGCGT	60.4	253	INTERGENIC	--	--	--
Ca2_P1_29220546	Ca2	29220546	GATAATAATAATAATAATAATAATAATAAT/GATAATAATAATAATAATAATAATAATAAT	CACGTGTCAAACTTGCCAAT	GTAACCGTTGGAGCACCACT	59.6	534	INTERGENIC	--	--	--
Ca2_P1_29238503	Ca2	29238503	A/AGTT	TGTGGAACACCAACGAATGT	ACCTCCCGCTAATGTCAATG	59.9	746	DRR	Ca_14317	--	--

INDEL marker IDs	Chromosomes/ unanchored scaffolds	Physical positions (bp)	InDels (*Kabuli* reference genome-CDC Frontier/PI)	Forward primers (5'-3')	Reverse primers (5'-3')	Anneling temperature (0C)	Expected amplified product size (bp)	Structural annotation		Functional annotation	
								Sequence components of *kabuli* genome	*Kabuli* gene accession IDs	NCBI-KOG	TFs
Ca2_P1_29238544	Ca2	29238544	AAATAATAATAATAATAATAATAATAATAATAATAATAATAATAATAATAA/AAATAATAATAATAATAATAATAATAATAATAATAATAA	TGTGGAACACCAACGAATGT	ACCTCCCGCTAATGTCAATG	59.9	746	DRR	Ca_14317	--	--
Ca2_P1_29239999	Ca2	29239999	AAT/A	GGCTGCTGATCCTGTTCTTC	CTGACGGACGGACTCATTTT	60.0	482	INTERGENIC	--	--	--
Ca2_P1_29240150	Ca2	29240150	TC/TCACC	CTTAGAGCATCTCCAACGGC	GAAGCATCGTTCCTTCCTGA	60.0	134	INTERGENIC	--	--	--
Ca2_P1_29240273	Ca2	29240273	ATTTTTTTT/ATTTTTTT	CTTAGAGCATCTCCAACGGC	CAACGGTGAACTCAATATGGG	60.0	671	INTERGENIC	--	--	--
Ca2_P1_29240382	Ca2	29240382	TCCC/TCC	CTTAGAGCATCTCCAACGGC	CCTTGCACTATCATTTTCGGA	60.0	803	INTERGENIC	--	--	--
Ca2_P1_29252004	Ca2	29252004	GTT/GT	TCAAAGGTGCACTCTTGCTATT	TGGCTTCCGGAAAGACTAAA	59.0	502	INTERGENIC	--	--	--
Ca2_P1_29253047	Ca2	29253047	TTTATTATTATTATTATTATTATTATTATTATTATTATTATTATTATTATTATTATTATT/TTTATTATTATTATTATTATTATTATTATTATTATTATTATTATTATTATTATTATTATT	GACACCCAAATATAGTGTCAACGA	CCCGGGTATAGGGTCTCACT	60.2	583	INTERGENIC	--	--	--
Ca2_P1_29314373	Ca2	29314373	GATC/GATCATC	CCAAAGCAAATCACAGAGCA	AGGTGACATTCATGGGCAGT	60.0	433	CDS (large-effect mutations)	Ca_25184	TR	FAR1
Ca2_P1_29339791	Ca2	29339791	CAAA/CAAAA	CCACAGCCCTACCTGAACAT	CCCAAGGTATGCAGTGGATT	60.0	502	DRR	Ca_25183	TZ	--
Ca2_P1_29348083	Ca2	29348083	TTATATATATATATATATATATAT/TTATATATATATATATATATATAT	TTTTCCAACACCAGAGTCCC	GATGCCTCATCGTGGAGATT	59.9	629	INTERGENIC	--	--	--
Ca2_P1_2941315	Ca2	2941315	GGG/GGGCCGG	CAAACCCATTGGTCCCTTTA	AAGTTTGAATCTTGAGGGTGG	59.7	695	INTERGENIC	--	--	--
Ca2_P1_29431087	Ca2	29431087	CATAATAATAATAATAATAATAATAATA/CATAATAATAATAATAATAATAATAATAATA	CATGCGTTCCTTGTGGTAGA	CCGATATTTATGGCACGACA	59.7	321	INTERGENIC	--	--	--

INDEL marker IDs	Chromosomes/ unanchored scaffolds	Physical positions (bp)	InDels (*Kabuli* reference genome-CDC Frontier/PI)	Forward primers (5'-3')	Reverse primers (5'-3')	Anneling temperature (0C)	Expected amplified product size (bp)	Structural annotation		Functional annotation	
								Sequence components of *kabuli* genome	*Kabuli* gene accession IDs	NCBI-KOG	TFs
Ca2_P1_29444244	Ca2	29444244	ATTTT/ATTTTT	AAATCCGACTGTCTAACCCG	TTTTCTCCTCCAATTTAGTCCAA	59.1	826	INTERGENIC	--	--	--
Ca2_P1_29445825	Ca2	29445825	TGTCTAACCCGTCTAACCCG/TGTCTAACCCG	TCACCCACACTCTCAATCCA	CCCTACCTAGGGGTGGACAT	60.1	419	INTERGENIC	--	--	--
Ca2_P1_29445927	Ca2	29445927	AACCCGAACCC/AACCCGAACCCACCCGAACCC	TGTCTAACCCGTCTAACCCG	CCCTACCTAGGGGTGGACAT	60.0	138	INTERGENIC	--	--	--
Ca2_P1_29520346	Ca2	29520346	TTAATAATAATAATAATAATAATAATAATAATAATAATAATAA/TTAATAATAATAATAATAATAATAATAATAATAA	ACACGTTTTGTTTGTGGCAA	GTTGGCTTTTGTACCCCTCA	60.1	599	DRR	Ca_16133	H	--
Ca2_P1_29534597	Ca2	29534597	ATTTTTTTT/ATTTTTTT	AGAGTCGCTCCCAAGCATTA	GGAGTGGTTGACGTTGGATT	60.0	472	INTERGENIC	--	--	--
Ca2_P1_29551226	Ca2	29551226	CCAC/CC	CCATGAACAAGTCAAAATCCG	AGCAAGACAACCAACAACCC	60.4	743	INTERGENIC	--	--	--
Ca2_P1_29563734	Ca2	29563734	C/CA	TTTAGAAATCTGAAAATGCAATCAA	CGGGACAACTCTTTCAACAAA	59.2	715	INTRON	Ca_16130	--	--
Ca2_P1_29564147	Ca2	29564147	AATATATATATATAT/AATATATATATAT	CTCCATGATGTCAACCATGC	AATTTCCGGGAGGAATGAGT	59.9	890	INTRON	Ca_16130	--	--
Ca2_P1_29608982	Ca2	29608982	TAAAAAAA/TAAAAAA	TTTGCCACAAACAAAACGTG	TGAAATTTCCCTACGATGCC	60.6	504	INTERGENIC	--	--	--
Ca2_P1_29621106	Ca2	29621106	ATTTTTTTTTTT/ATTTTTTTTTTTT	CGATGGTAAGCGCAATTAAA	CAAAAACAGAAGCACAGGCA	58.8	490	INTERGENIC	--	--	--
Ca2_P1_29659079	Ca2	29659079	CAAAAAAAAAAA/CAAAAAAAAAA	CGACGATCTCATTTAGCAATCA	TTTGCATTACGGATTTGATGA	60.2	384	INTERGENIC	--	--	--
Ca2_P1_29679294	Ca2	29679294	T/TTAAC	TGCATGAACTCACCACCATT	AGGAGTGTGTCGTTTTTGGA	60.0	885	INTERGENIC	--	--	--

INDEL marker IDs	Chromosomes/ unanchored scaffolds	Physical positions (bp)	InDels (*Kabuli* reference genome-CDC Frontier/PI)	Forward primers (5'-3')	Reverse primers (5'-3')	Anneling temperature (0C)	Expected amplified product size (bp)	Structural annotation		Functional annotation	
								Sequence components of *kabuli* genome	*Kabuli* gene accession IDs	NCBI-KOG	TFs
Ca2_P1_2969263	Ca2	2969263	C/CAAATTAAT	TTCCAAAATCCCTCACATCC	CGGAACATCGTGTTCTCCTT	59.7	408	DRR	Ca_10546	--	--
Ca2_P1_2975084	Ca2	2975084	GTATTATT/GTATT	ATCTACGAAGACGTGGTGGG	TGAGGATTAATTGGCTGCTCT	60.0	852	INTERGENIC	--	--	--
Ca2_P1_2976254	Ca2	2976254	TTA/T	TTCATCCTTCTTTTGGTCGG	GGGGCTGATTAATCCACCTTA	60.0	528	INTERGENIC	--	--	--
Ca2_P1_2977761	Ca2	2977761	A/ACTT	CAACTTGCCAAACAGCTTCA	TGGTGCAAGGATAGTACGACA	60.0	620	INTERGENIC	--	--	--
Ca2_P1_2978014	Ca2	2978014	CTAT/CTATAT	TTGCTGTAATTGTTGCACATGA	CCTCCCCAACTTGTAATTTCTG	60.2	542	INTERGENIC	--	--	--
Ca2_P1_29789566	Ca2	29789566	AATTATTATTATTATTATTATTATTATTATTATTATTATTAT/AATTATTATTATTATTATTATTATTATTATTATTAT	TCCCATTTGGGGTATTTTGA	CTCCGCTACCTAAATCGACC	60.0	743	INTERGENIC	--	--	--
Ca2_P1_29802522	Ca2	29802522	GATATATATATATATATATATATATATA/GATATATATATATATATATATATATATATA	TTCTCTGGGAAATCGACCAC	TCTTGCAATTTTCCAAGTTCG	60.1	728	INTERGENIC	--	--	--
Ca2_P1_29819377	Ca2	29819377	GTTTTTTTTTTTTTT/GTTTTTTTTTTTTT	TAATGGGGACCAATTTCTCG	CTAAAACCTCGCGATAACGG	59.8	157	INTERGENIC	--	--	--
Ca2_P1_29829880	Ca2	29829880	T/TA	CTTTTCGTCGGAGTTTGTCC	TCGTCCTCTGGTTTTCTCGT	59.7	476	INTERGENIC	--	--	--
Ca2_P1_29829940	Ca2	29829940	TT/TTTCGAAAAAAT	CTTTTCGTCGGAGTTTGTCC	TCGTCCTCTGGTTTTCTCGT	59.7	476	INTERGENIC	--	--	--
Ca2_P1_29835133	Ca2	29835133	GC/G	AAGGCAACAACAAACAAGCC	TGTGACCGTTGGAAAATTCA	60.2	402	INTERGENIC	--	--	--
Ca2_P1_2984306	Ca2	2984306	GTTTTTTT/GTTTTTT	GCTCACAACAGCAAAAACCA	TCTCTTTCTTAGACCGGAGCA	59.9	443	INTERGENIC	--	--	--

INDEL marker IDs	Chromosomes/ unanchored scaffolds	Physical positions (bp)	InDels (*Kabuli* reference genome-CDC Frontier/PI)	Forward primers (5'-3')	Reverse primers (5'-3')	Anneling temperature (0C)	Expected amplified product size (bp)	Structural annotation		Functional annotation	
								Sequence components of *kabuli* genome	*Kabuli* gene accession IDs	NCBI-KOG	TFs
Ca2_P1_2984359	Ca2	2984359	CTTTTTTTTTTTT/CTTTTTTTTTT	GCTCACAACAGCAAAAACCA	TCTCTTTCTTAGACCGGAGCA	59.9	443	INTERGENIC	--	--	--
Ca2_P1_2984403	Ca2	2984403	GAT/GATATAT	GCTCACAACAGCAAAAACCA	TCTCTTTCTTAGACCGGAGCA	59.9	443	INTERGENIC	--	--	--
Ca2_P1_2984623	Ca2	2984623	CATTA/CA	TGCCAAACCAATGTCTTCTT	GAGTCTCATGTCTCAACGAGGA	58.2	663	INTERGENIC	--	--	--
Ca2_P1_2985774	Ca2	2985774	ATGT/AT	GTGAAGCTGTATGGCCCAAT	AGATCTGGGTCGGGAAAAGT	60.0	540	INTERGENIC	--	--	--
Ca2_P1_2986282	Ca2	2986282	TTATATAT/TTATAT	TCAATCAACTTTTCCCGACC	GGACCAACTTTTGAACACATGA	59.9	712	INTERGENIC	--	--	--
Ca2_P1_2987500	Ca2	2987500	TAAAAAAAAA/TAAAAAAAAAAA	GCCAAATGATTCGGTTGCTA	GCCGACCATGGAGTAATTGT	61.0	700	INTERGENIC	--	--	--
Ca2_P1_2996094	Ca2	2996094	T/TA	TTCTAAGGACCAACGCAACA	GTATGATCGATTATGCGCGA	59.3	434	INTERGENIC	--	--	--
Ca2_P1_2996172	Ca2	2996172	ATTT/ATT	TTCTAAGGACCAACGCAACA	GTATGATCGATTATGCGCGA	59.3	434	INTERGENIC	--	--	--
Ca2_P1_29962779	Ca2	29962779	G/GC	AGCCAACGGACCACTAACAC	TTCGCCATTCTTCTTCGTCT	60.0	237	INTERGENIC	--	--	--
Ca2_P1_29962869	Ca2	29962869	TAACAACAA/TAACAA	TGGAAGCCATGATTAGGAGG	TCTCCCATTCGTTGTTGATG	60.0	182	INTERGENIC	--	--	--
Ca2_P1_29964324	Ca2	29964324	ATTTTTTT/ATTTTTTTT	TGACAAAACCAACACACAAAAA	TGTCTTTCATGAGCATCCAGA	59.0	178	INTERGENIC	--	--	--
Ca2_P1_29976673	Ca2	29976673	ATTTTTTTT/ATTTTTTTTT	AAAAGCGCGCTCATTAGAGT	AAAGCTTTGAAAGTCAAATCCTAC	59.3	118	INTERGENIC	--	--	--

INDEL marker IDs	Chromosomes/ unanchored scaffolds	Physical positions (bp)	InDels (*Kabuli* reference genome-CDC Frontier/PI)	Forward primers (5'-3')	Reverse primers (5'-3')	Anneling temperature (0C)	Expected amplified product size (bp)	Structural annotation		Functional annotation	
								Sequence components of *kabuli* genome	*Kabuli* gene accession IDs	NCBI-KOG	TFs
Ca2_P1_3000587	Ca2	3000587	ATTTTTTT/ATTTTTT	CCTGTCATTCTAATTAACGTGTTTTG	TGTGAGACCATTTAACCCATCA	60.2	999	INTERGENIC	--	--	--
Ca2_P1_3000644	Ca2	3000644	ATTTTTT/ATTTT	TGATTCAATTGTGAAGTTTTAATGTG	GGAGAAACATCACGTTGACC	59.4	949	INTERGENIC	--	--	--
Ca2_P1_3000948	Ca2	3000948	GT/GTT	GACTCTTAGACTTTGTGAAGCTTTGA	GAAGTTGCCGAAAATTGGAA	59.7	471	INTERGENIC	--	--	--
Ca2_P1_3001687	Ca2	3001687	CAAA/CAAAAA	TTCCAATTTTCGGCAACTTC	GCTGATGAAGGAGAGGAACG	60.1	915	INTERGENIC	--	--	--
Ca2_P1_3003816	Ca2	3003816	GTTTTTT/GTTTTTTT	ACGGTTTTAGGCCCAATTCT	GCTTATTCGCCCATGTGTTT	59.8	457	INTERGENIC	--	--	--
Ca2_P1_3005316	Ca2	3005316	TAAAAAAAAA/TAAAAAAAA	ATTGATGGGCCAACAGATTT	AAAGCGGTTTTTGTCACACC	59.2	692	INTERGENIC	--	--	--
Ca2_P1_3006782	Ca2	3006782	CAAAAAAA/CAAAAAAAA	TGAAGGGGAAGAGAATGGTG	GTGGCGATGTGTTTCTCTGA	60.0	665	DRR	Ca_10544	G	--
Ca2_P1_3009059	Ca2	3009059	ACCCCC/ACCCC	ATTCGCTTGCCTCTTTGGTA	CCCGAAACTTTCTGTGCATT	59.8	658	INTRON	Ca_10544	G	--
Ca2_P1_30095996	Ca2	30095996	TAAAAAAA/TAAAAAAAAA	CATTCCCGCTACATTTTGGT	GGAGAGAGGCCAATTTTTCA	59.8	226	INTERGENIC	--	--	--
Ca2_P1_30096276	Ca2	30096276	TA/TAAA	CATTCCCGCTACATTTTGGT	GAAGCTGCTTGTGTGCCATA	59.8	876	INTERGENIC	--	--	--
Ca2_P1_30096869	Ca2	30096869	CATATATATATATATATATATATATATATATATATAT/CATATATATATATATATATATATATAT	CATTGCAGGTGCTATGGTTG	TTCTCTTATGAAGCGGAGGA	60.1	759	INTERGENIC	--	--	--
Ca2_P1_30117060	Ca2	30117060	CTTTTTTTTTT/CTTTTTTTTTTT	AAGTGACATTGGTGGTGGGT	AGGGAAAGCGGAAAACAGAT	60.1	246	INTERGENIC	--	--	--

INDEL marker IDs	Chromosomes/ unanchored scaffolds	Physical positions (bp)	InDels (*Kabuli* reference genome-CDC Frontier/PI)	Forward primers (5'-3')	Reverse primers (5'-3')	Anneling temperature (0C)	Expected amplified product size (bp)	Structural annotation		Functional annotation	
								Sequence components of *kabuli* genome	*Kabuli* gene accession IDs	NCBI-KOG	TFs
Ca2_P1_3011716	Ca2	3011716	G/GA	TGATTTGGGGTGTTCAGAAA	TCCGCACACCAACATTAAAA	58.9	657	INTERGENIC	--	--	--
Ca2_P1_3011986	Ca2	3011986	GTTTTTTTTTTT/GTTTTTTTTTT	TGTGTGTGGAAAAATTGGTGA	TCCGCACACCAACATTAAAA	59.8	577	INTERGENIC	--	--	--
Ca2_P1_30125989	Ca2	30125989	TAAAAAA/TAAAAAAA	TTCCACTTATGGTGAGTAATGATTTT	TTGTGAGCATGAGAAGTTGTTG	59.3	641	INTERGENIC	--	--	--
Ca2_P1_30146454	Ca2	30146454	CAAAAAA/CAAAAAAAA	GCCCAAAACCAAATTTAGCA	AAGACCCAGAAACTTCCCGT	59.9	452	INTERGENIC	--	--	--
Ca2_P1_30165012	Ca2	30165012	T/TGA	TTGTTGTGACATCGAACGTCT	CAACAATTTCTCATGCTCGC	59.2	560	INTERGENIC	--	--	--
Ca2_P1_3016524	Ca2	3016524	GAAAAAAAA/GAAAAAAAAA	TGGGAGAGATAAAACGCTGC	TCTTTCACAACCTCAAAAGTGC	60.4	353	INTERGENIC	--	--	--
Ca2_P1_30171972	Ca2	30171972	AAT/AATAATTATTAT	TGAAAGGTTAGAGAAAAATGACATC	TTGGGTAGCCAAGGTTTCAC	58.0	293	INTERGENIC	--	--	--
Ca2_P1_30174020	Ca2	30174020	CTT/CTTT	TTATCCCCGCATATTGGACT	AGTCCTTGGGACCGAAAAGT	59.3	227	INTERGENIC	--	--	--
Ca2_P1_30187523	Ca2	30187523	GATATATATATATATATATATATATA/GATATATATATATATATATATA	GGACCATAGGTCAGGGGTTT	GAGGTCTGGTAACTCAAATAGGAA	60.1	870	INTERGENIC	--	--	--
Ca2_P1_30198484	Ca2	30198484	GAAAAAAAAAA/GAAAAAAAAAAA	GCTCGCTTATTCAAATACCCA	CATTGGCAACGACTTTTTCA	59.2	267	INTERGENIC	--	--	--
Ca2_P1_3020857	Ca2	3020857	AT/ATT	GCATAATCTATATCGGCTTCTTCA	ATTTCGGAAACGTTTTGCAG	58.9	628	INTERGENIC	--	--	--
Ca2_P1_3021004	Ca2	3021004	TAAAAAAAAA/TAAAAAAAAAA	ATCAACAACGCATACCAACA	ATTTCGGAAACGTTTTGCAG	57.5	380	INTERGENIC	--	--	--

INDEL marker IDs	Chromosomes/ unanchored scaffolds	Physical positions (bp)	InDels (*Kabuli* reference genome-CDC Frontier/PI)	Forward primers (5'-3')	Reverse primers (5'-3')	Anneling temperature (0C)	Expected amplified product size (bp)	Structural annotation		Functional annotation	
								Sequence components of *kabuli* genome	*Kabuli* gene accession IDs	NCBI-KOG	TFs
Ca2_P1_3021370	Ca2	3021370	CACTA/CA	TGCAAAACGTTTCCGAAATA	TCAAAAATTGATTTCCACCCA	59.2	241	INTERGENIC	--	--	--
Ca2_P1_3022120	Ca2	3022120	TCC/TC	CATAAGTCTGGCCTACGGCT	AGGACCAGGACCACTTGTGT	59.4	756	INTERGENIC	--	--	--
Ca2_P1_3022370	Ca2	3022370	T/TAGTCTGTAAGA	TCCTGAAACAAATGCACCAA	TCTGTCCTATGGCCTGACCT	60.1	379	INTERGENIC	--	--	--
Ca2_P1_3022599	Ca2	3022599	GTGGTCCTGGTCCT/GTGGTCCT	TCCTGAAACAAATGCACCAA	TGAGAAACTTTCGAGTCGGG	60.1	614	INTERGENIC	--	--	--
Ca2_P1_3022835	Ca2	3022835	TTAA/TTAATAA	CCCGACTCGAAAGTTTCTCA	AAAATTGACACGGCTTCGTC	60.4	544	INTERGENIC	--	--	--
Ca2_P1_3023037	Ca2	3023037	TAAAAAAA/TAAAAAAAA	CCCGACTCGAAAGTTTCTCA	AAAATTGACACGGCTTCGTC	60.4	544	INTERGENIC	--	--	--
Ca2_P1_3023630	Ca2	3023630	ATTTTTTT/ATTTTTT	GACGAAGCCGTGTCAATTTT	TCGACTCCAACAACAACGTAA	60.1	508	INTERGENIC	--	--	--
Ca2_P1_30243585	Ca2	30243585	CTT/CT	TTCCTACTCCCACATTTTAACGA	GCCTGTATCCAGCCATTTGT	59.9	467	INTERGENIC	--	--	--
Ca2_P1_3024878	Ca2	3024878	ATTTTTTTT/ATTTTTTTTTT	CTGGTTTTCAAACGCGCTAC	CGGGGTCGGTATTATTTTGA	60.8	799	INTERGENIC	--	--	--
Ca2_P1_3029449	Ca2	3029449	TTTAATAATTATT/TTTAATAATTATTAATAATTATT	ACCGCTCTATTAAGGGCGAG	TCCATCGCAATTGAATAATGA	60.7	765	INTERGENIC	--	--	--
Ca2_P1_3029534	Ca2	3029534	ATAAT/A	ACCGCTCTATTAAGGGCGAG	TCCATCGCAATTGAATAATGA	60.7	765	INTERGENIC	--	--	--
Ca2_P1_3030942	Ca2	3030942	CAAGA/C	TTTTCATTTTCTCCCTTCCCT	TCCATCGCAATTGAATAATGA	59.1	592	INTERGENIC	--	--	--

INDEL marker IDs	Chromosomes/ unanchored scaffolds	Physical positions (bp)	InDels (*Kabuli* reference genome-CDC Frontier/PI)	Forward primers (5'-3')	Reverse primers (5'-3')	Anneling temperature (0C)	Expected amplified product size (bp)	Structural annotation		Functional annotation	
								Sequence components of *kabuli* genome	*Kabuli* gene accession IDs	NCBI-KOG	TFs
Ca2_P1_3031259	Ca2	3031259	ATTTTT/ATTTTTT	TCAATTGCGATGGATAGAGAAA	AATTGGTGTACCGATTGCGT	59.7	647	INTERGENIC	--	--	--
Ca2_P1_3031554	Ca2	3031554	C/CCAAAATATCT	CATCTTGTCCATCAAACGGA	GGTGTACCGATTGCGTATCC	59.5	279	INTERGENIC	--	--	--
Ca2_P1_30328574	Ca2	30328574	ATTTTTTTTTTTTT/ATTTTTTTTTTTT	TTTGAAGAAATGCGCAAGTG	TCATCCTCTCCATTCACGTTT	60.0	466	DRR	Ca_12520	--	MIKC
Ca2_P1_3033081	Ca2	3033081	TA/TAA	AACGCAACAAGCTCCAAAAG	CGTTAGGCCTTATCCTAGCC	60.4	902	INTERGENIC	--	--	--
Ca2_P1_30341034	Ca2	30341034	TAA/TAAA	TGTCTTATCCTGAACTCAAACTCG	ATTTGGTCAACCACCCAAAA	59.8	537	INTERGENIC	--	--	--
Ca2_P1_30342173	Ca2	30342173	CA/CATA	TGAATCATTGACAAAAGACTCAATC	CTAGCCCAAGATCCAAACCA	59.5	572	INTERGENIC	--	--	--
Ca2_P1_30342240	Ca2	30342240	ACTCT/ACT	AAAAATGAAATTGAGGCATTTTA	CTAGCCCAAGATCCAAACCA	57.0	104	INTERGENIC	--	--	--
Ca2_P1_30342844	Ca2	30342844	ATTTTT/ATTT	TGCCTAATTTTGACTTCCCC	TCTCTAATCTGCTACGCATCCA	59.0	439	INTERGENIC	--	--	--
Ca2_P1_30342955	Ca2	30342955	TAAA/TAAAA	TGCCTAATTTTGACTTCCCC	TCTCTAATCTGCTACGCATCCA	59.0	439	INTERGENIC	--	--	--
Ca2_P1_30343063	Ca2	30343063	ATTTTTTTTTTT/ATTTTTTTTTT	TGCCTAATTTTGACTTCCCC	TGCCTAACACATTCAACTCCTTT	59.0	898	INTERGENIC	--	--	--
Ca2_P1_30343145	Ca2	30343145	TAAA/TAA	TGGATGCGTAGCAGATTAGAGA	CGTCACTTGGTCACTTGGTG	60.0	581	INTERGENIC	--	--	--
Ca2_P1_30343226	Ca2	30343226	C/CT	TGGATGCGTAGCAGATTAGAGA	CGTCACTTGGTCACTTGGTG	60.0	581	INTERGENIC	--	--	--

INDEL marker IDs	Chromosomes/ unanchored scaffolds	Physical positions (bp)	InDels (*Kabuli* reference genome-CDC Frontier/PI)	Forward primers (5'-3')	Reverse primers (5'-3')	Anneling temperature (0C)	Expected amplified product size (bp)	Structural annotation		Functional annotation	
								Sequence components of *kabuli* genome	*Kabuli* gene accession IDs	NCBI-KOG	TFs
Ca2_P1_30343408	Ca2	30343408	TTAATAAA/T	TGGATGCGTAGCAGATTAGAGA	CGTCACTTGGTCACTTGGTG	60.0	581	INTERGENIC	--	--	--
Ca2_P1_30343585	Ca2	30343585	CACCAAGTGACCAAGTGAC/CACCAAGTGAC	CAATCAATAATTTTGACGTCGG	CATTTCACGGGGCTCTTAAA	59.4	658	INTERGENIC	--	--	--
Ca2_P1_30344009	Ca2	30344009	A/AC	TTTAAGAGCCCCGTGAAATG	AACGATGGAACTTTGTTGCAC	60.1	311	INTERGENIC	--	--	--
Ca2_P1_30344081	Ca2	30344081	CAAAAAAAAA/CAAAAAAAAAA	TTTAAGAGCCCCGTGAAATG	AACGATGGAACTTTGTTGCAC	60.1	311	INTERGENIC	--	--	--
Ca2_P1_30345200	Ca2	30345200	TG/T	TAAGTTGGGCGAAATCATCC	TGGGGTGGGTGTTTAGAAAA	59.9	524	INTERGENIC	--	--	--
Ca2_P1_30352364	Ca2	30352364	ATTTTTTTT/ATTTTTT	TAAAAAGTGGTTCGGTTCGG	TTATTTTTCCCTTTTGGCCC	60.0	826	INTERGENIC	--	--	--
Ca2_P1_30352714	Ca2	30352714	GAAAA/GAAAAA	GGGCCAAAAGGGAAAAATAA	CCTGTCACTCTCCACTTCACC	60.1	369	INTERGENIC	--	--	--
Ca2_P1_30356368	Ca2	30356368	AATATATA/AATATATATA	CCACATGGCTGCTTATGATG	AGCGCATGGTAATGTTGAGA	60.1	459	DRR	Ca_12517	--	ERF
Ca2_P1_30356995	Ca2	30356995	GAAA/GAA	TCTCAACATTACCATGCGCT	GCCTTGATGTGGTGTTGATG	59.3	736	INTERGENIC	--	--	--
Ca2_P1_30361193	Ca2	30361193	GAAAAAAAAAA/GAAAAAAAAA	GAAACGGAGACGGGTGTAGA	TCTGCCACTATTAGCGGATAAG	60.1	543	INTERGENIC	--	--	--
Ca2_P1_30361475	Ca2	30361475	TATC/TATCGATC	TCCGCTAATAGTGGCAGATAAA	ACCAATTTGTGAAAGCTGGC	58.9	617	INTERGENIC	--	--	--
Ca2_P1_30365282	Ca2	30365282	GATATATATATATAT/GATATATATATAT	AATTCCCAGGGCCAACTATC	AAGCTGTTTGATTCCATGCC	60.2	539	INTERGENIC	--	--	--

INDEL marker IDs	Chromosomes/ unanchored scaffolds	Physical positions (bp)	InDels (*Kabuli* reference genome-CDC Frontier/PI)	Forward primers (5'-3')	Reverse primers (5'-3')	Anneling temperature (0C)	Expected amplified product size (bp)	Structural annotation		Functional annotation	
								Sequence components of *kabuli* genome	*Kabuli* gene accession IDs	NCBI-KOG	TFs
Ca2_P1_30367772	Ca2	30367772	TA/TATTTGTTAAA	CTTCCACCACCAAATGACCT	TACCTGGCGCGTTTTATTTT	59.8	300	DRR	Ca_12515	Q	--
Ca2_P1_30367815	Ca2	30367815	CA/CAA	CTTCCACCACCAAATGACCT	ATCAATTGAGCGCCTGTAGC	59.8	371	DRR	Ca_12515	Q	--
Ca2_P1_30369374	Ca2	30369374	TAAAA/TAAAAA	TGTACTTCCTCCGATTCCAAA	TGAATGACGACGACGGTAAA	59.6	632	DRR	Ca_12515	Q	--
Ca2_P1_30373739	Ca2	30373739	TAAAAAAAAAA/TAAAAAAAAA	CGGGAATGAAAAGTGGAAGA	CCAGGGGCAAAACAAAGTAA	60.0	518	DRR	Ca_12514	S	--
Ca2_P1_30375028	Ca2	30375028	AC/A	AATTGAATCAACCCCCTTCC	AAGGAGGGACTAGGTTTGGG	60.0	308	DRR	Ca_12514	S	--
Ca2_P1_30375161	Ca2	30375161	ACCCCC/ACCCC	AATTGAATCAACCCCCTTCC	GGTTTGAAGCTAATGTCGTGC	60.0	737	DRR	Ca_12514	S	--
Ca2_P1_30375213	Ca2	30375213	TAAAAAAA/TAAAAAA	AATTGAATCAACCCCCTTCC	GGTTTGAAGCTAATGTCGTGC	60.0	737	DRR	Ca_12514	S	--
Ca2_P1_30375265	Ca2	30375265	CT/C	AACCCCCTTCCTTTTGTGTC	TTGATTTTTGGGGTGGCTAA	60.2	963	DRR	Ca_12514	S	--
Ca2_P1_30380354	Ca2	30380354	GTTTTTTTTTT/GTTTTTTTTTTT	ACCTTTGGGGTGTTTTAGGC	TTTTCAGACAGCTCAATCGAA	60.2	273	INTERGENIC	--	--	--
Ca2_P1_3038248	Ca2	3038248	GAAAAAAA/GAAAAAAAA	ACTTGCACTTTGGAGTGCCT	CCCTTGTGCCTTATGGTTTG	59.9	784	INTERGENIC	--	--	--
Ca2_P1_30383360	Ca2	30383360	AC/A	TCATAAGGTGGGTCCAAACC	TGATGGCTTGCCATATTCTT	59.6	805	INTERGENIC	--	--	--
Ca2_P1_30385121	Ca2	30385121	A/AAT	TGATTTGGCCCAACACCTAT	GCAAATACAAAGGACCGTCAA	60.2	381	INTERGENIC	--	--	--

INDEL marker IDs	Chromosomes/ unanchored scaffolds	Physical positions (bp)	InDels (*Kabuli* reference genome-CDC Frontier/PI)	Forward primers (5'-3')	Reverse primers (5'-3')	Anneling temperature (0C)	Expected amplified product size (bp)	Structural annotation		Functional annotation	
								Sequence components of *kabuli* genome	*Kabuli* gene accession IDs	NCBI-KOG	TFs
Ca2_P1_30386487	Ca2	30386487	AGTGGG/AGTGGGTGTGGG	CAAATACAGGCACATGGTGG	AAAGCTGCAAAAAGTTGGGA	59.8	392	DRR	Ca_12512	R	--
Ca2_P1_30389809	Ca2	30389809	CG/C	CGACAACTGAGCTTTCCTCC	CCACCAAACACTTTTCTGCC	60.0	586	INTRON	Ca_12512	R	--
Ca2_P1_30391104	Ca2	30391104	AT/ATT	TTGCATTCTCAAGGCTCAGA	TTTTGGGAGTGCTTGGTTTC	59.7	461	INTRON	Ca_12512	R	--
Ca2_P1_3039238	Ca2	3039238	ATTTTTTT/ATTTTTTTT	CAGATAAAGAGAGCGTGGGC	TGAGTGGACAATCCAACCATT	60.0	218	INTERGENIC	--	--	--
Ca2_P1_3039311	Ca2	3039311	CTTTT/CTTT	AGATAAAGAGAGCGTGGGCA	GGAATAAAGGACGGGGAAAA	60.0	595	INTERGENIC	--	--	--
Ca2_P1_30393573	Ca2	30393573	CT/CTT	TCCCAAACAGGAATGAAACC	AGTGCAAGCATCATGGAGAA	59.8	714	INTERGENIC	--	--	--
Ca2_P1_3039362	Ca2	3039362	CCACTCACTCA/CCACTCA	AGATAAAGAGAGCGTGGGCA	GGAATAAAGGACGGGGAAAA	60.0	595	INTERGENIC	--	--	--
Ca2_P1_30393638	Ca2	30393638	TAAAAAAAAAA/TAAAAAAAAAAA	TCCCAAACAGGAATGAAACC	AGTGCAAGCATCATGGAGAA	59.8	714	INTERGENIC	--	--	--
Ca2_P1_30393886	Ca2	30393886	TC/T	ATTCTCCGATTTTTCCCCAT	TGTTTTCAGTGCAAGCATCA	59.6	475	INTERGENIC	--	--	--
Ca2_P1_3039423	Ca2	3039423	AT/A	AGATAAAGAGAGCGTGGGCA	GGAATAAAGGACGGGGAAAA	60.0	595	INTERGENIC	--	--	--
Ca2_P1_30394906	Ca2	30394906	CAAAA/CAAAAA	GAGAGAGGGGCAAGGTCAC	AGGCTTTTGTTGTTGTTGGG	59.8	392	INTERGENIC	--	--	--
Ca2_P1_30394922	Ca2	30394922	AA/AATATA	GAGAGAGGGGCAAGGTCAC	AGGCTTTTGTTGTTGTTGGG	59.8	392	INTERGENIC	--	--	--

**INDEL marker IDs**	**Chromosomes/ unanchored scaffolds**	**Physical positions (bp)**	**InDels (*Kabuli* reference genome-CDC Frontier/PI)**	**Forward primers (5'-3')**	**Reverse primers (5'-3')**	**Anneling temperature (0C)**	**Expected amplified product size (bp)**	**Structural annotation**		**Functional annotation**	
								**Sequence components of *kabuli* genome**	***Kabuli* gene accession IDs**	**NCBI-KOG**	**TFs**
Ca2_P1_30395203	Ca2	30395203	CATATATATA/CATATATATATA	CCCAACAACAACAAAAGCCT	AAACCAGCATGACAAAAGCA	60.0	289	INTERGENIC	--	--	--
Ca2_P1_30395542	Ca2	30395542	CAAAAA/CAAAAAA	CCCAACAACAACAAAAGCCT	CCAAATGAAACAATCGAGGAA	60.0	780	INTERGENIC	--	--	--
Ca2_P1_30399984	Ca2	30399984	CATATATATATATATATAT/CATATATATATATATAT	AGGTGGGCCCATTACTTGAT	AGTGGAGGACCACTTCCTCA	60.6	836	INTERGENIC	--	--	--
Ca2_P1_30401833	Ca2	30401833	ATTT/ATT	GGTCGAGTTTGCAATGATGA	TGACCACAAGTTGCCAAAAA	59.7	761	INTERGENIC	--	--	--
Ca2_P1_30403193	Ca2	30403193	ATTGAGTT/ATTGAGTTGAGTT	AAAGTTGCACCAAGAGGTGG	TCTCTGGCCTTCGTTAGTCAA	60.1	989	DRR	Ca_12511	--	--
Ca2_P1_30415385	Ca2	30415385	AAAACAT/A	CAAAACAGGGGGCTGTAAAA	AATCTTCAACGGGTTGTTGC	60.0	506	INTRON	Ca_12510	--	--
Ca2_P1_30419465	Ca2	30419465	ATAATTT/ATAATTTATTATTCGTAATTT	AGTGGTTGCAATCATCACCA	CGGTGGAGAACAGAATTTCAA	60.0	654	DRR	Ca_12509	C	M-type
Ca2_P1_3042033	Ca2	3042033	TTTTGTTTGCTT/TTT	TGCCAAATCATGCGTTTTTA	CTCTGATGCGAAGGTTGACA	60.1	100	INTRON	Ca_10543	--	--
Ca2_P1_30420544	Ca2	30420544	CTTTTTTTTTTTTTTT/CTTTTTTTTTTT	AGGCTTTGGCTTTGTAAGCA	AACTCGGGACCTCACAGTTG	60.0	417	INTRON	Ca_12509	C	M-type
Ca2_P1_30432943	Ca2	30432943	GTTTTTTTTTT/GTTTTTTTT	ATGAATCAAACGAACACCGA	TTACAGTTAAGGGCCAACCG	59.0	778	INTERGENIC	--	--	--
Ca2_P1_30433128	Ca2	30433128	TAAAAAAAAA/TAAAAAAAA	GAAATTGCATTTATCTCCCCC	TTACAGTTAAGGGCCAACCG	59.6	737	INTERGENIC	--	--	--
Ca2_P1_30433778	Ca2	30433778	TA/TAA	CGGTTGGCCCTTAACTGTAA	TCCTCAAACCCAAAATGGTC	60.0	674	INTERGENIC	--	--	--

INDEL marker IDs	Chromosomes/ unanchored scaffolds	Physical positions (bp)	InDels (*Kabuli* reference genome-CDC Frontier/PI)	Forward primers (5'-3')	Reverse primers (5'-3')	Anneling temperature (0C)	Expected amplified product size (bp)	Structural annotation		Functional annotation	
								Sequence components of *kabuli* genome	*Kabuli* gene accession IDs	NCBI-KOG	TFs
Ca2_P1_30439365	Ca2	30439365	GAAAAAAAAAA/GAAAAAAAAA	CCAAGTTCAACACTCATATGCC	ATGGAGAATTCAGCCCAACA	59.5	406	INTERGENIC	--	--	--
Ca2_P1_30447611	Ca2	30447611	A/AT	CTTTTGTCTTCTCGGCCTTG	CGTGCTCATCTCCATTGTTG	60.0	891	INTERGENIC	--	--	--
Ca2_P1_30447653	Ca2	30447653	AC/A	CTTTTGTCTTCTCGGCCTTG	ATCTCCTCAGCATTGTCGCT	60.0	943	INTERGENIC	--	--	--
Ca2_P1_30447804	Ca2	30447804	TTG/TTGTG	AACAAAAGGGGTTGGAAAGC	ATCTCCTCAGCATTGTCGCT	60.3	809	INTERGENIC	--	--	--
Ca2_P1_30448097	Ca2	30448097	GGTGGC/GGTGGCAGTGGC	CAACAATGGAGATGAGCACG	TTTTCCTCTTCCCACCCTCT	60.3	111	CDS (large-effect mutations)	Ca_12507	--	--
Ca2_P1_30448973	Ca2	30448973	GAAAAAA/GAAAAAAA	GCTTTTGATTTTCTAAAGGATGG	TCTTCCTTGTTGGTTCTTTGG	58.4	759	DRR	Ca_12507	--	--
Ca2_P1_30449164	Ca2	30449164	TAAAAAAA/TAAAAAAAA	TGTTGCAAAGAGAAAGAACTTCA	TGACGGTGGCAATCGATATT	59.2	557	DRR	Ca_12507	--	--
Ca2_P1_30450495	Ca2	30450495	TAAAAAAAAAA/TAAAAAAAA	AGAATTTGGATTTGTTGCGG	ACTCTCCTTCACCGTTGTGG	59.9	413	INTERGENIC	--	--	--
Ca2_P1_30456719	Ca2	30456719	TAAAAAAAA/TAAAAAAA	TTCCTGATTCCGATCTCACC	CATACCTTGGGGATGCAAAG	60.0	601	DRR	Ca_12504	--	WOX
Ca2_P1_30457303	Ca2	30457303	A/AT	TGCATCCCCAAGGTATGACT	CCACCATTAGATTTGCGGTT	60.3	649	DRR	Ca_12504	--	WOX
Ca2_P1_30461111	Ca2	30461111	TC/T	ACGTTTTGGTCTTGGAGCAT	TTTTTGTCTCACATTGATTGTCTTT	59.6	918	INTERGENIC	--	--	--
Ca2_P1_30461396	Ca2	30461396	CTTTTTTT/CTTTTTTTTT	AAAAAGAAGTGGTTGTCCGC	AAAACGATGCTTATCCGTGAA	59.2	929	INTERGENIC	--	--	--

INDEL marker IDs	Chromosomes/ unanchored scaffolds	Physical positions (bp)	InDels (*Kabuli* reference genome-CDC Frontier/PI)	Forward primers (5'-3')	Reverse primers (5'-3')	Anneling temperature (0C)	Expected amplified product size (bp)	Structural annotation		Functional annotation	
								Sequence components of *kabuli* genome	*Kabuli* gene accession IDs	NCBI-KOG	TFs
Ca2_P1_30464150	Ca2	30464150	CTT/CTTT	CCACCTCCCAAACAAACACT	GGCAAGTTGCTTTTACCAAAAC	59.9	602	INTERGENIC	--	--	--
Ca2_P1_30465917	Ca2	30465917	GAAAAAAAAA/GAAAAAAAAAA	TCCATGATTGAGTGACTAGGGA	GCAGTCAACCAGCGACAATA	59.6	322	DRR	Ca_12503	J	--
Ca2_P1_30467933	Ca2	30467933	AT/A	GCAAGAGCCAAAAGCAAGTC	TGGAGAGGGCACCATAAAAG	60.1	387	INTRON	Ca_12503	J	--
Ca2_P1_3047152	Ca2	3047152	CTTT/CTTTT	TGAAATTTAACGGGCCTTCA	GTGGAAAAACTGGACCCTCA	60.4	643	INTRON	Ca_10543	--	--
Ca2_P1_30473938	Ca2	30473938	TTTTTTA/T	AGATGAGGAGCAAAATCAGCA	TGTGTTCCTGGAGCATTTGA	60.0	624	INTERGENIC	--	--	--
Ca2_P1_3047767	Ca2	3047767	TGG/TGGG	TGAGGGTCCAGTTTTTCCAC	AACCAAGTTGGCCCAAAAA	59.9	266	DRR	Ca_10543	--	--
Ca2_P1_30478526	Ca2	30478526	CTTTTTTTT/CTTTTTTT	GTGCCTTGAAGAAGTGAGGG	CATCATAGCCCCGAGAAAAA	59.8	390	DRR	Ca_12502	G	--
Ca2_P1_30478867	Ca2	30478867	AATATATATAT/AATATATATATATAT	TTTTTCTCGGGGCTATGATG	GTAGGGCATGTGTGTGTTGC	60.0	640	DRR	Ca_12502	G	--
Ca2_P1_3048024	Ca2	3048024	CTTTTTT/CTTTTTTTT	TGAGGGTCCAGTTTTTCCAC	TGTCGAATTAAGTGGATAGAGCA	59.9	905	DRR	Ca_10543	--	--
Ca2_P1_30480817	Ca2	30480817	TAAA/TAAAA	CTGCGTCCCATTTCAAGAAT	ATGTGTCTTTGGGAGCCAAC	60.1	880	INTRON	Ca_12502	G	--
Ca2_P1_30481523	Ca2	30481523	TTTAAAAAAT/TTTAAAAAATATGTTAAAAAAT	GTTGGCTCCCAAAGACACAT	TCGATTTTCATTAAAGTGTTGGA	60.0	631	INTERGENIC	--	--	--
Ca2_P1_30481892	Ca2	30481892	AACATA/AA	TCAATGCATGGAAAATAATCTCT	TGGTCCTCATCCATCAAACA	57.8	401	INTERGENIC	--	--	--

INDEL marker IDs	Chromosomes/ unanchored scaffolds	Physical positions (bp)	InDels (*Kabuli* reference genome-CDC Frontier/PI)	Forward primers (5'-3')	Reverse primers (5'-3')	Anneling temperature (0C)	Expected amplified product size (bp)	Structural annotation		Functional annotation	
								Sequence components of *kabuli* genome	*Kabuli* gene accession IDs	NCBI-KOG	TFs
Ca2_P1_30484744	Ca2	30484744	GG/GGACG	ACATGAGACCGTCAATGCAA	CGAAAAATCAAAATAACATCGC	60.1	386	INTERGENIC	--	--	--
Ca2_P1_30484802	Ca2	30484802	A/ATT	ACATGAGACCGTCAATGCAA	CGAAAAATCAAAATAACATCGC	60.1	386	INTERGENIC	--	--	--
Ca2_P1_30489361	Ca2	30489361	TTAT/TT	TGCAAAGTTTGATGGAGTGC	TAGAACCCCAATTTTGTCCG	59.8	363	INTERGENIC	--	--	--
Ca2_P1_30490084	Ca2	30490084	ATTTTTTTTTTTTTTT/ATTTTTTTTTTT	CCCCCTACTACTTGACGATCC	TTCCCAAATGCAATTTTTCC	59.8	393	INTERGENIC	--	--	--
Ca2_P1_30490221	Ca2	30490221	T/TA	GGAAAAATTGCATTTGGGAA	GTTGGCTTACACATTGGGCT	59.8	578	INTERGENIC	--	--	--
Ca2_P1_30490465	Ca2	30490465	ATTTTTTT/ATTTTTTTT	GGAAAAATTGCATTTGGGAA	GTTGGCTTACACATTGGGCT	59.8	578	INTERGENIC	--	--	--
Ca2_P1_30490798	Ca2	30490798	AA/AACA	AGCCCAATGTGTAAGCCAAC	AAGCTTTTGCAAATGGGAGA	60.0	410	INTERGENIC	--	--	--
Ca2_P1_30503807	Ca2	30503807	TAA/TAAA	GTTCTCACTATGCCCCCAAA	AGTAATTAGCCCGAATCGCA	59.9	404	INTERGENIC	--	--	--
Ca2_P1_30507919	Ca2	30507919	CT/CTT	TCATCTTTGATGCATTTCGC	TGCATTTGCGACAAGAAAAG	59.8	503	INTRON	Ca_12501	GMW	--
Ca2_P1_30510130	Ca2	30510130	ATTTTTTTTTTTTTTT/ATTTTTTTTTTTTT	AGACCCCACGGTTAACATGA	AGCACCTTACAAGGGGGAGT	60.2	700	DRR	Ca_12500	--	--
Ca2_P1_30513922	Ca2	30513922	T/TG	TTGAATGGTTTGTGATCCGA	TCCATTGGAAGCATTCACTTC	59.9	483	INTERGENIC	--	--	--
Ca2_P1_30514265	Ca2	30514265	AAGA/AA	CACCAAATCACCCACAATCA	ATGGGCTGGTATATCATGGG	60.2	972	INTERGENIC	--	--	--

INDEL marker IDs	Chromosomes/ unanchored scaffolds	Physical positions (bp)	InDels (*Kabuli* reference genome-CDC Frontier/PI)	Forward primers (5'-3')	Reverse primers (5'-3')	Anneling temperature (0C)	Expected amplified product size (bp)	Structural annotation		Functional annotation	
								Sequence components of *kabuli* genome	*Kabuli* gene accession IDs	NCBI-KOG	TFs
Ca2_P1_30514604	Ca2	30514604	ATTTTTTT/ATTTTT	TGGTTTGTGAACCACTGTGAA	AAACTTTCAAATGCATGGGC	60.0	336	INTERGENIC	--	--	--
Ca2_P1_30516125	Ca2	30516125	GATATA/GATA	GAGATGAAAACGGATGGGTG	CATGACAATCATTTGCAGCC	60.3	831	INTERGENIC	--	--	--
Ca2_P1_3051651	Ca2	3051651	GTTTTTTTTTTTT/GTTTTTTTTTTTTTT	TCCGAGAAAATCAAAATCGG	TACAGATTCCAAACACGGCA	60.0	533	INTERGENIC	--	--	--
Ca2_P1_30516532	Ca2	30516532	GT/G	CACAAGGTTTGTGCACTGAAA	CATGACAATCATTTGCAGCC	59.8	440	INTERGENIC	--	--	--
Ca2_P1_3051893	Ca2	3051893	CATATAGATGTGATATAGATGTGAT/CATATAGATGTGAT	TCCGAGAAAATCAAAATCGG	TACAGATTCCAAACACGGCA	60.0	533	INTERGENIC	--	--	--
Ca2_P1_30522321	Ca2	30522321	ATTTTTTTT/ATTTTTT	GAACAGTGGCAATTCATCCA	CGATGTGTGCATGCTCTCTAA	59.5	865	INTERGENIC	--	--	--
Ca2_P1_30523186	Ca2	30523186	CT/CTTT	TCATCGTTTGCAAGGTTTTG	TAATGCACTTCATTGCTGGC	59.7	249	INTERGENIC	--	--	--
Ca2_P1_30525393	Ca2	30525393	TTA/TTATA	CAGCCACAGCATAGCTTTCA	GACGTGTGTTGCTTTAAGGG	60.2	516	INTERGENIC	--	--	--
Ca2_P1_30526387	Ca2	30526387	TATCTCAT/TAT	CAATTTCGTGCGTGAATCAG	CCGACCTAATCCTTCACGAG	60.3	994	INTERGENIC	--	--	--
Ca2_P1_30526988	Ca2	30526988	GAAAA/GAAA	CTCGTGAAGGATTAGGTCGG	TTGCAACGCATCTCAATCTC	59.7	353	INTERGENIC	--	--	--
Ca2_P1_30527508	Ca2	30527508	TAAAAAAAAAAAA/TAAAAAAAAAA	TTGAGATGCGTTGCAATAGG	CGTGCCACTTTCTTCCTTTC	59.8	681	INTERGENIC	--	--	--
Ca2_P1_30527572	Ca2	30527572	A/AT	TTGAGATGCGTTGCAATAGG	CGTGCCACTTTCTTCCTTTC	59.8	681	INTERGENIC	--	--	--

INDEL marker IDs	Chromosomes/ unanchored scaffolds	Physical positions (bp)	InDels (*Kabuli* reference genome-CDC Frontier/PI)	Forward primers (5'-3')	Reverse primers (5'-3')	Anneling temperature (0C)	Expected amplified product size (bp)	Structural annotation		Functional annotation	
								Sequence components of *kabuli* genome	*Kabuli* gene accession IDs	NCBI-KOG	TFs
Ca2_P1_30550518	Ca2	30550518	GAAAAAAA/GAAAAAAAA	CGCTAGCTATTTCTCTGGCGT	TGCATTCGATCAAGCTCAAC	61.0	189	INTERGENIC	--	--	--
Ca2_P1_30553119	Ca2	30553119	T/TTAGAC	TTTTTATGAGGCGTGGAAGG	AAAACTAACACACCCCCGTG	60.1	223	INTERGENIC	--	--	--
Ca2_P1_30553227	Ca2	30553227	A/AG	TTTTTATGAGGCGTGGAAGG	CAACTTGAGGGAGGGTATTGA	60.1	688	INTERGENIC	--	--	--
Ca2_P1_30553379	Ca2	30553379	GAAAA/GAAAAA	TTTTTATGAGGCGTGGAAGG	CCATGAACACGCCAAACATA	60.1	874	INTERGENIC	--	--	--
Ca2_P1_30555764	Ca2	30555764	AAG/AAGACACGTGTAG	AAAACATAACCTTTTGCCCG	TCACTCCAAGCTTCCCAAAT	59.0	257	INTERGENIC	--	--	--
Ca2_P1_30558124	Ca2	30558124	TAAA/TAAAA	TGTTTAAGGGTGATGCGACA	AGGCTTCATAGATGGGGTGA	60.1	948	INTERGENIC	--	--	--
Ca2_P1_30558600	Ca2	30558600	CTAT/CTATATAT	GATGCATACAAAAGTCACCCC	GGATTGATGAAGTGGTTGGG	59.3	254	INTERGENIC	--	--	--
Ca2_P1_30559202	Ca2	30559202	AT/ATT	CCCAACCACTTCATCAATCC	AAAAATTATGGGCTTTGGGC	60.2	750	INTERGENIC	--	--	--
Ca2_P1_30563316	Ca2	30563316	GAAAAAAA/GAAAAAAAA	ATTCATTGGGTGAAATGGGA	AAAGCATGACACCACAACCA	60.0	573	INTERGENIC	--	--	--
Ca2_P1_30563758	Ca2	30563758	ATGTGT/ATGTGTGT	TGGTTGTGGTGTCATGCTTT	ATGCCAACCAAAACCTGAAG	60.0	427	INTERGENIC	--	--	--
Ca2_P1_30568142	Ca2	30568142	AT/ATGT	CTTGTTTGTCGCAGTCGCTA	GGTCATACCAAGGCCAAGAA	60.2	453	INTRON	Ca_12495	E	--
Ca2_P1_30569240	Ca2	30569240	AT/ATT	AGTTTTGCAGCTTTTGCCTC	CCGTCCAATTTTCTCTTGGA	59.6	609	INTRON	Ca_12495	E	--

INDEL marker IDs	Chromosomes/ unanchored scaffolds	Physical positions (bp)	InDels (*Kabuli* reference genome-CDC Frontier/PI)	Forward primers (5'-3')	Reverse primers (5'-3')	Anneling temperature (0C)	Expected amplified product size (bp)	Structural annotation		Functional annotation	
								Sequence components of *kabuli* genome	*Kabuli* gene accession IDs	NCBI-KOG	TFs
Ca2_P1_30569354	Ca2	30569354	TCACA/TCA	AGTTTTGCAGCTTTTG CCTC	CCGTCCAATTTTCTCT TGGA	59.6	609	INTRON	Ca_12495	E	--
Ca2_P1_30572769	Ca2	30572769	ATATTATTATTATTATTATTATTATTAT TA/ATATTATTATTATTATTATTATTATT ATTATTATTA	TTTGGCAATTGGCATG TAGA	GATTCCCTACGGCTGA TGAA	60.1	601	DRR	Ca_12495	E	--
Ca2_P1_30573212	Ca2	30573212	GA/GAA	TTCATCAGCCGTAGGG AATC	CGGACAAGTTCCAAGA TTCAA	60.0	477	DRR	Ca_12495	E	--
Ca2_P1_30573602	Ca2	30573602	CGGG/CGGGG	TGGCTCATGACCCCTT TATC	GGCCCCTTTTCATTTT CATT	59.9	709	DRR	Ca_12495	E	--
Ca2_P1_30573670	Ca2	30573670	TAAAAAAAAAA/TAAAAAAAAA	TGCATGCAAAAGAATT GTCC	GGCCCCTTTTCATTTT CATT	59.7	534	DRR	Ca_12495	E	--
Ca2_P1_30574200	Ca2	30574200	ATTTTTT/ATTTTTTT	AAATGAAAAGGGGCCA AAGT	AATTTCCCGTTGCAAT GTCT	59.8	859	INTERGENIC	--	--	--
Ca2_P1_30574328	Ca2	30574328	TAAAAA/TAAAAAAA	CCATTGAGGAGTGGAG AACTTT	CTTGTGTGTTCCCAAT GCAA	59.6	783	INTERGENIC	--	--	--
Ca2_P1_30575054	Ca2	30575054	AT/A	TGCAGCAACAATTATC CAGC	GACACACACCTCGTTT GCAT	59.8	858	INTERGENIC	--	--	--
Ca2_P1_30576047	Ca2	30576047	ACCCCCC/ACCCCC	GAATGGATGCTGCTTT GGAT	CAATAAAGGGCCATCC TTGA	60.0	214	INTRON	Ca_12494	--	--
Ca2_P1_30577110	Ca2	30577110	CTTT/CTT	GTTCTCGTGGCTCAGG ACTC	CCATTGGCTTTTGCTC ATTT	60.0	388	DRR	Ca_12493	--	--
Ca2_P1_30590146	Ca2	30590146	A/AG	TGTTCGAACTCGGAAC ATGA	AACCAAAACGACTACG ACGG	60.2	705	DRR	Ca_12492	--	--
Ca2_P1_30592392	Ca2	30592392	AATTATTATTATTATTAT/AATTATTATT ATTATTATTATTATTATTATTATTAT	TGAGTTGGACTCGTGA ATCG	CCGAAAATGTAGGGAC AACG	59.8	274	INTERGENIC	--	--	--

INDEL marker IDs	Chromosomes/ unanchored scaffolds	Physical positions (bp)	InDels (*Kabuli* reference genome-CDC Frontier/PI)	Forward primers (5'-3')	Reverse primers (5'-3')	Anneling temperature (0C)	Expected amplified product size (bp)	Structural annotation		Functional annotation	
								Sequence components of *kabuli* genome	*Kabuli* gene accession IDs	NCBI-KOG	TFs
Ca2_P1_3062029	Ca2	3062029	C/CAGTATAA	TTTGTGCCTTTGTGAAGCTG	CCCGACCCTTCTGTTTATCA	60.0	222	INTRON	Ca_10542	Z	--
Ca2_P1_30621875	Ca2	30621875	TT/TTCT	TAAGCTTTCCCGTGTCTGCT	TTGGGGTCAAATGGTTGATT	60.0	813	INTRON	Ca_12490	R	--
Ca2_P1_30622722	Ca2	30622722	A/ACCGTAATCC	CTTGGTCAGAATCACGGAGC	CACGTTGACCCTTTTTGTCA	60.8	380	INTRON	Ca_12490	R	--
Ca2_P1_30622746	Ca2	30622746	AC/A	CTTGGTCAGAATCACGGAGC	CACGTTGACCCTTTTTGTCA	60.8	380	INTRON	Ca_12490	R	--
Ca2_P1_30622806	Ca2	30622806	AATCA/AATCATCA	CTTGGTCAGAATCACGGAGC	CACGTTGACCCTTTTTGTCA	60.8	380	INTRON	Ca_12490	R	--
Ca2_P1_30623473	Ca2	30623473	TAAA/TAAAAA	TGACAAAAAGGGTCAACGTG	CCGCAATCAATTTAGCGAAT	59.6	686	INTERGENIC	--	--	--
Ca2_P1_30623537	Ca2	30623537	TAAAAAAA/TAAAAAAAAA	AGTAGAGCCCGACAAGATGC	CCGCAATCAATTTAGCGAAT	59.5	483	INTERGENIC	--	--	--
Ca2_P1_30623593	Ca2	30623593	TTATGTTTTAT/TTATGTTTTATATGTTTTAT	AGTAGAGCCCGACAAGATGC	CCGCAATCAATTTAGCGAAT	59.5	483	INTERGENIC	--	--	--
Ca2_P1_30623846	Ca2	30623846	TAAAA/TAAAAAAA	ATTCGCTAAATTGATTGCGG	TGTTGCAAGCAAACTCAAGC	60.1	606	INTERGENIC	--	--	--
Ca2_P1_30624016	Ca2	30624016	A/AAT	ATTCGCTAAATTGATTGCGG	TGTTGCAAGCAAACTCAAGC	60.1	606	INTERGENIC	--	--	--
Ca2_P1_30624454	Ca2	30624454	C/CT	TCATGAATGCAACGTGGTTT	TTTTGCTGTTTGTTTTACGTCAT	60.0	646	INTERGENIC	--	--	--
Ca2_P1_30630371	Ca2	30630371	ATTTA/ATTTATTTTA	TGCGTGTATCATTTTCAACTCA	GGAGATCCAATAGATGGCGA	59.2	841	INTERGENIC	--	--	--

INDEL marker IDs	Chromosomes/ unanchored scaffolds	Physical positions (bp)	InDels (*Kabuli* reference genome-CDC Frontier/PI)	Forward primers (5'-3')	Reverse primers (5'-3')	Anneling temperature (0C)	Expected amplified product size (bp)	Structural annotation		Functional annotation	
								Sequence components of *kabuli* genome	*Kabuli* gene accession IDs	NCBI-KOG	TFs
Ca2_P1_30630925	Ca2	30630925	CT/CTGAT	TGTTGATCCGACAAGGGTTT	ATTCTCCCCGTCCCAAATTA	60.4	410	INTERGENIC	--	--	--
Ca2_P1_30631182	Ca2	30631182	AG/A	TGTTGATCCGACAAGGGTTT	AACCTCGTGAGCGTTCTTGT	60.4	719	INTERGENIC	--	--	--
Ca2_P1_30637842	Ca2	30637842	TGG/TG	GGAAAGGGAGCCTGAAGAGA	GGGTCTTTTTGCAAATTTGATT	60.8	455	INTERGENIC	--	--	--
Ca2_P1_30641002	Ca2	30641002	TTTCTT/TTT	CAAATTGAAGGTGTGGCCTT	TGATTCATTGAGGCTCACCA	60.0	656	INTERGENIC	--	--	--
Ca2_P1_30642500	Ca2	30642500	GAAAAAAAAA/GAAAAAAAAAAA	TGCAGTTGCCGACAATTTTAT	TCCTCGGTAACCAAACAAGC	60.5	128	INTERGENIC	--	--	--
Ca2_P1_30645883	Ca2	30645883	TAAAAAAAAAA/TAAAAAAAAAAA	TGCTGGGTTGATGGATGTAA	TGCATGCATGGATCCCTAA	59.9	816	INTERGENIC	--	--	--
Ca2_P1_30647034	Ca2	30647034	CAT/CATAT	TTGACGTACCCAACCATTGA	CCCTCACTTAAGCCCACAAA	59.8	443	INTERGENIC	--	--	--
Ca2_P1_30653450	Ca2	30653450	TATATAGAAAATTGATATAGAAAA/TATATAGAAAA	TGAACTTTCCTTGGTCGAATG	GGGTGGTGAAGGTTTCAAGA	60.1	450	INTERGENIC	--	--	--
Ca2_P1_30665443	Ca2	30665443	ATTTTTTTTTT/ATTTTTTTT	TCATGGTGGACCGTAATCTG	CTACTGCCTTGGATTCCTTTT	59.4	403	INTRON	Ca_12489	QI	bHLH
Ca2_P1_30665911	Ca2	30665911	ATTTTTTTTTTT/ATTTTTTTTTTTT	AAAAGGAATCCAAGGCAGTAG	TCCATTACCATCATACGCCA	57.5	788	INTRON	Ca_12489	QI	bHLH
Ca2_P1_30665994	Ca2	30665994	CTTTTTTTT/CTTTTTTTTT	AAAAGGAATCCAAGGCAGTAG	ACGAACGCAGATCCATTACC	57.5	799	INTRON	Ca_12489	QI	bHLH
Ca2_P1_30667046	Ca2	30667046	GTGATTATGA/GTGA	TGAATTTGTGGTGTGTGCAA	GCGGTGGAGAAAACAAGAAA	59.6	721	INTRON	Ca_12489	QI	bHLH

INDEL marker IDs	Chromosomes/ unanchored scaffolds	Physical positions (bp)	InDels (*Kabuli* reference genome-CDC Frontier/PI)	Forward primers (5'-3')	Reverse primers (5'-3')	Anneling temperature (0C)	Expected amplified product size (bp)	Structural annotation		Functional annotation	
								Sequence components of *kabuli* genome	*Kabuli* gene accession IDs	NCBI-KOG	TFs
Ca2_P1_30667297	Ca2	30667297	TATTAAT/TAT	TTTCTTGTTTTCTCCACCGC	TGAAGCAGACTGCAGAATCG	60.2	461	INTRON	Ca_12489	QI	bHLH
Ca2_P1_30667379	Ca2	30667379	T/TCAC	TTTCTTGTTTTCTCCACCGC	TGAAGCAGACTGCAGAATCG	60.2	461	INTRON	Ca_12489	QI	bHLH
Ca2_P1_30669761	Ca2	30669761	ATTTTTTTTTTT/ATTTTTTTTTT	AGGCCCACTTCACAAATGTC	AGGGCTCGATACTTCCGAAT	60.0	879	INTRON	Ca_12489	QI	bHLH
Ca2_P1_30669952	Ca2	30669952	AATATAT/AATATATAT	AGACCCAATCATGCAAATCC	AAAACGGTGAAGAAACACGG	59.8	776	INTRON	Ca_12489	QI	bHLH
Ca2_P1_30672058	Ca2	30672058	ATTTT/ATT	GGGCTGATCCAACACGTTAT	CCATGGGAAACGTTGGTAAC	59.8	396	DRR	Ca_12489	QI	bHLH
Ca2_P1_30672151	Ca2	30672151	TGGGG/TGGG	GGGCTGATCCAACACGTTAT	CCATGGGAAACGTTGGTAAC	59.8	396	DRR	Ca_12489	QI	bHLH
Ca2_P1_30675995	Ca2	30675995	A/AC	TTAAGTGGGTTGCGCTTTTT	AAGAACTTGGACGTTGGTGG	59.8	536	INTERGENIC	--	--	--
Ca2_P1_30676386	Ca2	30676386	GAAAAAAAAAA/GAAAAAAAAAAA	ATCCACCAACGTCCAAGTTC	CCTTGGTATGACCCTAATTCCTT	59.8	532	INTERGENIC	--	--	--
Ca2_P1_30676782	Ca2	30676782	ACTAGC/ACTAGCTAGC	GCTCCAGCAAAAATAGAAATGG	TCATCAATCAAACTACTTACGCAGA	60.1	504	INTERGENIC	--	--	--
Ca2_P1_30677085	Ca2	30677085	TAAAAAAAA/TTATTAAAAAAAAA	TCTGCGTAAGTAGTTTGATTGATGA	TTGCGCCTTTTCAGTATTTTC	60.2	668	INTERGENIC	--	--	--
Ca2_P1_30678831	Ca2	30678831	TT/TTTTAT	CAAACCCAAAACCTTTTCCA	CTCACGCATCTCCAAACTCA	59.8	369	INTERGENIC	--	--	--
Ca2_P1_30679543	Ca2	30679543	TAAAAA/TAAAA	ATATCCAACGCGTCAACCTC	TGTTAAACGCAATCGATCACA	60.0	282	INTERGENIC	--	--	--

INDEL marker IDs	Chromosomes/ unanchored scaffolds	Physical positions (bp)	InDels (*Kabuli* reference genome-CDC Frontier/PI)	Forward primers (5'-3')	Reverse primers (5'-3')	Anneling temperature (0C)	Expected amplified product size (bp)	Structural annotation		Functional annotation	
								Sequence components of *kabuli* genome	*Kabuli* gene accession IDs	NCBI-KOG	TFs
Ca2_P1_30680495	Ca2	30680495	TAAAAAAAAAA/TAAAAAAAAAAAA	TCATGCTACAATTTGAAGGGAA	TCCAAATCCATAGTCAGCCC	59.6	500	INTERGENIC	--	--	--
Ca2_P1_30680775	Ca2	30680775	AATATATAT/AATATAT	GGGCTGACTATGGATTTGGA	TATTTGGCTGACGTGGAACA	59.9	225	INTERGENIC	--	--	--
Ca2_P1_30689429	Ca2	30689429	CAAGAAA/CAAGAAAAAGAAA	CGTACCTTGAGTGGGAAAGG	TTGCTTCGCAACATGAGAAC	59.6	381	INTERGENIC	--	--	--
Ca2_P1_30689624	Ca2	30689624	CAATTGTAATT/CAATTGTAATTGTAATT	CCGTACCTTGAGTGGGAAAG	ACGTTCCCCTAAACCCAAAC	59.6	677	INTERGENIC	--	--	--
Ca2_P1_30689650	Ca2	30689650	TGCAAA/TGCAAAGCAAA	CCGTACCTTGAGTGGGAAAG	ACGTTCCCCTAAACCCAAAC	59.6	677	INTERGENIC	--	--	--
Ca2_P1_30690091	Ca2	30690091	CT/CTTT	GCGGTGTTTGTTTGGGTAGT	GGAGCACACAGATGCGAATA	59.9	335	INTERGENIC	--	--	--
Ca2_P1_30690274	Ca2	30690274	A/AT	TTAGGGGAACGTGAAATTGG	CGGTCATACTGCACACACAA	59.8	444	INTERGENIC	--	--	--
Ca2_P1_30690413	Ca2	30690413	AT/ATAAATT	TATTCGCATCTGTGTGCTCC	CGTGGTTGAGTAGATGTGCC	59.8	670	INTERGENIC	--	--	--
Ca2_P1_30690532	Ca2	30690532	CTATTTTATT/CTATT	TATTCGCATCTGTGTGCTCC	CGTGGTTGAGTAGATGTGCC	59.8	670	INTERGENIC	--	--	--
Ca2_P1_30690585	Ca2	30690585	AAATAATAATAATAATAA/AAATAATAATAATAATAATAATAATAA	TATTCGCATCTGTGTGCTCC	CGTGGTTGAGTAGATGTGCC	59.8	670	INTERGENIC	--	--	--
Ca2_P1_30691906	Ca2	30691906	TAAAAAAAAAAA/TAAAAAAAAA	ACAGCTGCAAACACCACATC	ATCAAGGTCGGGGAATAACC	59.8	305	INTERGENIC	--	--	--
Ca2_P1_30695617	Ca2	30695617	CTTTTTTTTTT/CTTTTTTTTTTT	CCCATGCATATCACGTTTTG	ACGCAGGGCCATGATACTAC	59.8	361	DRR	Ca_12487	KLB	YABBY

INDEL marker IDs	Chromosomes/ unanchored scaffolds	Physical positions (bp)	InDels (*Kabuli* reference genome-CDC Frontier/PI)	Forward primers (5'-3')	Reverse primers (5'-3')	Anneling temperature (0C)	Expected amplified product size (bp)	Structural annotation		Functional annotation	
								Sequence components of *kabuli* genome	*Kabuli* gene accession IDs	NCBI-KOG	TFs
Ca2_P1_30695733	Ca2	30695733	T/TGAAATTAA	CCCATGCATATCACGT TTTG	ACGCAGGGCCATGAT ACTAC	59.8	361	DRR	Ca_12487	KLB	YABBY
Ca2_P1_30701503	Ca2	30701503	T/TAATC	TTATCTGCCCTGGATT CTGC	TTGGAAGAGGCCAAAT ATGC	60.2	753	INTRON	Ca_12487	KLB	YABBY
Ca2_P1_30702625	Ca2	30702625	GAAAAA/GAAAAAA	CTTCCGCCATCAATGA CAC	AATCTCTTCAATCCCC CGTT	60.1	566	INTRON	Ca_12487	KLB	YABBY
Ca2_P1_30702934	Ca2	30702934	ATTTTTTTTTT/ATTTTTTTTT	AACGGGGGATTGAAGA GATT	GCAAATTCTGCGGGTG TTAT	59.8	508	INTERGENIC	--	--	--
Ca2_P1_30702980	Ca2	30702980	TATTTAATTTAATTT/TATTTAATTT	AACGGGGGATTGAAGA GATT	GCAAATTCTGCGGGTG TTAT	59.8	508	INTERGENIC	--	--	--
Ca2_P1_30703181	Ca2	30703181	TAAAAAAA/TAAAAAA	AACGGGGGATTGAAGA GATT	CGCATACATACGGTGA TTGC	59.8	882	INTERGENIC	--	--	--
Ca2_P1_30703635	Ca2	30703635	TAAAAAAAAAA/TAAAAAAAA	ACCCGCAGAATTTGCT AAGA	GGAGTTTTCAAGAGGT GCCA	59.8	498	INTERGENIC	--	--	--
Ca2_P1_30703947	Ca2	30703947	ATTTTTTTTTT/ATTTTTTTTT	CCGTATGTATGCGTGA CACC	TCGAATTGGATTTCAC ATATTTTC	59.9	423	INTERGENIC	--	--	--
Ca2_P1_30704006	Ca2	30704006	TAAAA/TAAA	CCGTATGTATGCGTGA CACC	GGAAGGTGAATGATCT TAAAATAACG	59.9	902	INTERGENIC	--	--	--
Ca2_P1_30704638	Ca2	30704638	TA/TAA	AAATTCAGTGCAACGC ACAT	CCCACTTTGGATAACA ATTCC	59.2	445	INTERGENIC	--	--	--
Ca2_P1_30704814	Ca2	30704814	CAA/CAAA	AAATTCAGTGCAACGC ACAT	CCCACTTTGGATAACA ATTCC	59.2	445	INTERGENIC	--	--	--
Ca2_P1_30713265	Ca2	30713265	A/AG	TTCTGCTGGTCTGGCT TTTT	TGAAAGGGAATTGGGT GAAG	60.0	233	INTRON	Ca_12485	T	RAV

INDEL marker IDs	Chromosomes/ unanchored scaffolds	Physical positions (bp)	InDels (*Kabuli* reference genome-CDC Frontier/PI)	Forward primers (5'-3')	Reverse primers (5'-3')	Anneling temperature (0C)	Expected amplified product size (bp)	Structural annotation		Functional annotation	
								Sequence components of *kabuli* genome	*Kabuli* gene accession IDs	NCBI-KOG	TFs
Ca2_P1_30719499	Ca2	30719499	AT/A	CACGGTTGTGGGGACTAAAC	AAGAACCATCCAGCGGAAC	60.3	988	INTERGENIC	--	--	--
Ca2_P1_30719828	Ca2	30719828	AA/AATA	CAACGGACAAGAAAATCTCACA	TCACAAATTAACGGCCACAA	60.1	804	INTERGENIC	--	--	--
Ca2_P1_30720315	Ca2	30720315	TATTA/T	TTGTGGCCGTTAATTTGTGA	GTTGATGTTATCAAATAAACAATTTCA	60.0	344	INTERGENIC	--	--	--
Ca2_P1_30720521	Ca2	30720521	CAAAAAAAA/CAAAAAAA	TTGTGGCCGTTAATTTGTGA	CGAGTTTCTTTTTCCCGTCA	60.0	787	INTERGENIC	--	--	--
Ca2_P1_30720601	Ca2	30720601	TAAAAA/TAAAAAA	TTGTGGCCGTTAATTTGTGA	TTGTTGTGGATGCGGTTAAA	60.0	917	INTERGENIC	--	--	--
Ca2_P1_30732861	Ca2	30732861	A/AG	TCACAGAAGGGGTTTTGGTC	AAAATTGTGAACGGAGACCG	59.9	394	INTRON	Ca_12484	T	RAV
Ca2_P1_30737308	Ca2	30737308	TAAAAAAAAA/TAAAAAAAAAAA	TTTTGCCATGGTCTTGGATT	TCAAGAAAATTGCCAACCCT	60.3	618	DRR	Ca_12484	T	RAV
Ca2_P1_30737463	Ca2	30737463	TAAAAAA/TAAAAAAA	TTTTGCCATGGTCTTGGATT	TGGGCCCTCATGATATTGAT	60.3	720	DRR	Ca_12484	T	RAV
Ca2_P1_30739062	Ca2	30739062	AC/ACC	TTGTGTGTGGGCTCAACTTC	TGGCCTACCTCTTGTGATGA	59.7	569	DRR	Ca_12483	T	FAR1
Ca2_P1_30742309	Ca2	30742309	CAGAGAGAG/CAGAGAGAGAGAGAG	CGTTGCGGTAACAAACAATG	TTGGAGAAAAGGCAGGCTTA	60.0	349	INTERGENIC	--	--	--
Ca2_P1_30742679	Ca2	30742679	TAAAAAA/TAAAAAAAA	TGTTTGCTTCATTGGCAGAG	AAATTTGATGTAGAATATAACCCAAAA	60.0	674	INTERGENIC	--	--	--
Ca2_P1_30773455	Ca2	30773455	GAAAAAAAAA/GAAAAAAAAAA	TGACTCCATCCACTTATCCCA	CCCATGAGTTTATGTTGGGG	60.3	158	INTERGENIC	--	--	--

INDEL marker IDs	Chromosomes/ unanchored scaffolds	Physical positions (bp)	InDels (*Kabuli* reference genome-CDC Frontier/PI)	Forward primers (5'-3')	Reverse primers (5'-3')	Anneling temperature (0C)	Expected amplified product size (bp)	Structural annotation		Functional annotation	
								Sequence components of *kabuli* genome	*Kabuli* gene accession IDs	NCBI-KOG	TFs
Ca2_P1_30775811	Ca2	30775811	ATTTTTT/ATTTTTTT	TGGAACTAGTAGAATTGAAAGGAAAAA	TTGCCTTAGTGGCTTTGAGTC	60.0	349	INTERGENIC	--	--	--
Ca2_P1_30783115	Ca2	30783115	CTTTTTTTT/CTTTTTTT	AGGCATGAAGAATGTGGAGC	GCTGGTGGTACCATGTCCTT	60.2	712	INTERGENIC	--	--	--
Ca2_P1_30788585	Ca2	30788585	CTTTTTTTTTT/CTTTTTTTTTTTT	TCTATCCTCACCACCTCAAGC	TGCAAATTGGATTTCACAAGA	59.3	432	INTERGENIC	--	--	--
Ca2_P1_30788694	Ca2	30788694	TTTTATTTATTTATT/TTTTATTTATTTATTTATT	TCTATCCTCACCACCTCAAGC	TGCATGTCAAGTTGGATTCAT	59.3	774	INTERGENIC	--	--	--
Ca2_P1_30790808	Ca2	30790808	GAAAAAAAA/GAAAAAAAAA	CACTCAGGGGATGAATGTACG	CGAAGTTTGGACATGAGCTG	60.4	480	INTERGENIC	--	--	--
Ca2_P1_30792756	Ca2	30792756	ATTTTTTTT/ATTTTTT	ACGTGCCAATTATGATGCTG	GAATGCTGATGTGTGCATAAGA	59.6	396	INTERGENIC	--	--	--
Ca2_P1_30794341	Ca2	30794341	ATTTTT/ATTTTTT	GAGTTGAATGCAGCTGACCA	CCAAAAACAAATTTTTCGGGA	60.0	678	INTERGENIC	--	--	--
Ca2_P1_30794367	Ca2	30794367	ATTTTTTT/ATTTTTTTT	GAGTTGAATGCAGCTGACCA	TGCATTTCACTGGAGACGTT	60.0	741	INTERGENIC	--	--	--
Ca2_P1_30794400	Ca2	30794400	CTTTTTTT/CTTTTTT	GAGTTGAATGCAGCTGACCA	TGCATTTCACTGGAGACGTT	60.0	741	INTERGENIC	--	--	--
Ca2_P1_30794493	Ca2	30794493	A/AT	GAGTTGAATGCAGCTGACCA	TGCATTTCACTGGAGACGTT	60.0	741	INTERGENIC	--	--	--
Ca2_P1_30794649	Ca2	30794649	TAAAAAAAAA/TAAAAAAAA	TGATTCTCGACTGAAAACATGAG	TTGCATTTCACTGGAGACGTT	59.4	641	INTERGENIC	--	--	--
Ca2_P1_30794993	Ca2	30794993	TAATAAATA/TAATA	ACGTCTCCAGTGAAATGCAA	AGCCTTGATGTTGTTTGCG	59.3	506	INTERGENIC	--	--	--

INDEL marker IDs	Chromosomes/ unanchored scaffolds	Physical positions (bp)	InDels (*Kabuli* reference genome-CDC Frontier/PI)	Forward primers (5'-3')	Reverse primers (5'-3')	Anneling temperature (0C)	Expected amplified product size (bp)	Structural annotation		Functional annotation	
								Sequence components of *kabuli* genome	*Kabuli* gene accession IDs	NCBI-KOG	TFs
Ca2_P1_30795414	Ca2	30795414	TAAAA/TAAA	GCAAACAACATCAAGGCTCA	TGAAAATAGTTTTTGGTCCCTG	59.8	558	INTERGENIC	--	--	--
Ca2_P1_30796973	Ca2	30796973	CAAA/CAAAA	CGTGTCCCTGATATTTTGCC	GGGACAGTTTTGTTTGTGGC	60.3	108	INTERGENIC	--	--	--
Ca2_P1_30799097	Ca2	30799097	ATTTTTT/ATTTTTTT	ACGTTTTGCCAAACAACCTC	TGCTCCTGGGTAAATCGAAC	60.0	359	DRR	Ca_12478	R	MYB
Ca2_P1_30799752	Ca2	30799752	T/TGAC	CCATGAAGTTGAAGGCAAGA	TCTCTGGTGGGAAGGTTCAG	58.8	556	DRR	Ca_12478	R	MYB
Ca2_P1_30799865	Ca2	30799865	CAAAAAAAAAA/CAAAAAAAA	TGCTCAATCCTTCCAATTCA	AATCAACCCCAAAAGAAGGC	59.2	333	DRR	Ca_12478	R	MYB
Ca2_P1_30800347	Ca2	30800347	TTGTGT/TTGTGTGT	TGCTGATCTGTAGGTGCTGC	AAAAACGGCAAAATGTCCTG	60.2	606	INTRON	Ca_12478	R	MYB
Ca2_P1_30807589	Ca2	30807589	CTTTTTTTTTT/CTTTTTTTTTTT	TTCCCTCTTACCTTTGTTCCC	AATTTGCAATTTACGCACCC	59.4	406	INTERGENIC	--	--	--
Ca2_P1_30842603	Ca2	30842603	CCATCAT/CCAT	ACGTTAAGGGGGCAATTACA	AACAATTCCATGGTCACTCTCA	59.3	269	INTERGENIC	--	--	--
Ca2_P1_30842775	Ca2	30842775	AT/ATGATAGGAGCT	ACGTTAAGGGGGCAATTACA	CGTGACTTCAGGGGCAATAG	59.3	728	INTERGENIC	--	--	--
Ca2_P1_30844156	Ca2	30844156	TTTAA/TTTAATTAA	TCCACACTCTTGTAGGTTGCAT	CAGAATCAGCACGCAAACAT	59.7	854	INTERGENIC	--	--	--
Ca2_P1_30846096	Ca2	30846096	GAAAA/GAAAAA	CCTTTGCGAACTTGAAGACC	TCCTCCCTCTTGCAGGTTTA	59.9	815	INTERGENIC	--	--	--
Ca2_P1_30846540	Ca2	30846540	AATATATATATATATATATA/AATATATATATATA	CGGATTGCAAACATGGTAGA	TAAGGCTTGCAAAGATTGGG	59.5	529	INTERGENIC	--	--	--

INDEL marker IDs	Chromosomes/ unanchored scaffolds	Physical positions (bp)	InDels (*Kabuli* reference genome-CDC Frontier/PI)	Forward primers (5'-3')	Reverse primers (5'-3')	Anneling temperature (0C)	Expected amplified product size (bp)	Structural annotation		Functional annotation	
								Sequence components of *kabuli* genome	*Kabuli* gene accession IDs	NCBI-KOG	TFs
Ca2_P1_30848508	Ca2	30848508	TTGATAAT/TTGATAATAATGATAAT	TTGAAGGACCAAAATCATGAAC	CATACAACGCAAGCTAGGCA	58.9	533	INTERGENIC	--	--	--
Ca2_P1_30849743	Ca2	30849743	ATT/ATTT	TCAACTTAGATATGCCGTCCG	TGGGTTGGTTTGAATCATCA	60.1	410	INTERGENIC	--	--	--
Ca2_P1_30850146	Ca2	30850146	TAAAAAAAA/TAAAAAAA	TGATGATTCAAACCAACCCA	ATTTAGGTGGCAAAAGACGC	59.8	345	INTERGENIC	--	--	--
Ca2_P1_30860320	Ca2	30860320	TAA/TAAA	TGAACAAAAAGGTTGGGTGA	TGCTCAACTTTGACCCATTG	59.0	487	INTERGENIC	--	--	--
Ca2_P1_30860618	Ca2	30860618	ATTTT/ATTTTTT	TGGGTCAAAGTTGAGCATGA	CACCTTTGGAAAGAGCTGGA	60.2	557	INTERGENIC	--	--	--
Ca2_P1_30860644	Ca2	30860644	CAAA/CA	TGGGTCAAAGTTGAGCATGA	CACCTTTGGAAAGAGCTGGA	60.2	557	INTERGENIC	--	--	--
Ca2_P1_30860700	Ca2	30860700	AC/A	TGGGTCAAAGTTGAGCATGA	CACCTTTGGAAAGAGCTGGA	60.2	557	INTERGENIC	--	--	--
Ca2_P1_30861287	Ca2	30861287	AA/AATA	CCAAAGGTGGGTGAAGAAGA	CAGGCCCAATTCACAAAGTT	60.1	554	INTERGENIC	--	--	--
Ca2_P1_30861798	Ca2	30861798	A/AAT	AACTTTGTGAATTGGGCCTG	TTTGCATGCCGATAATTTGA	60.0	365	INTERGENIC	--	--	--
Ca2_P1_30861870	Ca2	30861870	A/AT	AACTTTGTGAATTGGGCCTG	TTTGCATGCCGATAATTTGA	60.0	365	INTERGENIC	--	--	--
Ca2_P1_30862282	Ca2	30862282	T/TCA	TTATCGGCATGCAAAACAAA	GTTGGGTGACAGACAGCACA	60.1	794	INTERGENIC	--	--	--
Ca2_P1_30864086	Ca2	30864086	AATATATATATA/AATATATATATATATA	TGGTTGTCAACATGAGTTCCA	CAAACTTTTGAATTTTGGTGAATG	60.0	278	INTERGENIC	--	--	--

INDEL marker IDs	Chromosomes/ unanchored scaffolds	Physical positions (bp)	InDels (*Kabuli* reference genome-CDC Frontier/PI)	Forward primers (5'-3')	Reverse primers (5'-3')	Anneling temperature (0C)	Expected amplified product size (bp)	Structural annotation		Functional annotation	
								Sequence components of *kabuli* genome	*Kabuli* gene accession IDs	NCBI-KOG	TFs
Ca2_P1_30864595	Ca2	30864595	ATTTTTTTTT/ATTTTTTTTTTT	TCAAAGAGCGGATAAT AGGTTTT	CTTTCGAAATCCCTCA GCTT	58.4	769	INTERGENIC	--	--	--
Ca2_P1_30864873	Ca2	30864873	TAAAAAA/TAAAAAAA	GATTTTAGTTATTCACG ACGTCCA	CTTTCGAAATCCCTCA GCTT	59.5	375	INTERGENIC	--	--	--
Ca2_P1_30872234	Ca2	30872234	CAT/CATAT	CAAAAGCACAACAAGC CAGA	AATTTAAAGAAAGGCC GGGA	60.0	268	INTRON	Ca_12473	U	--
Ca2_P1_3088291	Ca2	3088291	GTTTT/GTTT	CCGCGCTACTCAAAAA TCAT	GTGCCCTTGAATGGAT GTCT	60.2	440	INTERGENIC	--	--	--
Ca2_P1_30883523	Ca2	30883523	TGCG/TG	CACATCAACAGGGCAA AATG	GTGTGTGCAATCTCTG GCAT	60.0	682	INTRON	Ca_12473	U	--
Ca2_P1_30883879	Ca2	30883879	CACACAGACACAGACAC/CACACAGA CAC	GCCTGTTTCTCAAATC CCAA	GTGTGTGCAATCTCTG GCAT	60.1	514	INTRON	Ca_12473	U	--
Ca2_P1_30885844	Ca2	30885844	TAAAAAAAAA/TAAAAAAAA	TTGGTTTTGTTCCAGG CTTC	GTTGCATCCTTCACCA ATGA	60.1	703	INTERGENIC	--	--	--
Ca2_P1_30887613	Ca2	30887613	AT/A	TCAAGGGAGGATTACA TGGG	AAAGGGAAAGTTGCAT CACG	59.7	826	INTERGENIC	--	--	--
Ca2_P1_30887902	Ca2	30887902	T/TC	GCATCGCACGGATTCT AATA	GGGCTAGTGGCAGTC TTCAG	58.7	796	INTERGENIC	--	--	--
Ca2_P1_30890128	Ca2	30890128	AAAATAAATAAATAAATAAA/AAAATAA ATAAATAAA	GATGATGTAAATGGGA GCCAA	CATTTGTTGAATGTTG CCCA	59.8	697	INTERGENIC	--	--	--
Ca2_P1_30890411	Ca2	30890411	AAATGAATGAATGAA/AAATGAATGAA TGAATGAA	GATGATGGATGGGCAA CATT	GCGGTGAGACTCGAA AAGAG	60.6	330	INTERGENIC	--	--	--
Ca2_P1_30890896	Ca2	30890896	ATTTTTT/ATTTTTTT	CTCTTTTCGAGTCTCA CCGC	ATGCCATGACGTGAAC AAAA	60.1	566	INTERGENIC	--	--	--

INDEL marker IDs	Chromosomes/ unanchored scaffolds	Physical positions (bp)	InDels (*Kabuli* reference genome-CDC Frontier/PI)	Forward primers (5'-3')	Reverse primers (5'-3')	Anneling temperature (0C)	Expected amplified product size (bp)	Structural annotation		Functional annotation	
								Sequence components of *kabuli* genome	*Kabuli* gene accession IDs	NCBI-KOG	TFs
Ca2_P1_30892153	Ca2	30892153	A/ATGTAC	AGGATGGGTTTCTGTGTTGC	CTAAGGGGCAAAAGCGTACA	60.0	617	INTRON	Ca_12472	--	--
Ca2_P1_30897629	Ca2	30897629	TAAAAAAAA/TAAAAAAA	CCAACAGATCAAGGGGAATG	ATCGAGATCTCACCGTGGAA	60.3	548	INTERGENIC	--	--	--
Ca2_P1_30897792	Ca2	30897792	AAT/AATATAT	CCAACAGATCAAGGGGAATG	ATTGGGGTTTGGTGAAATGA	60.3	708	INTERGENIC	--	--	--
Ca2_P1_30900645	Ca2	30900645	T/TA	GATGAAGGGTCCACGGAATA	TTCAGAACCGGAGTGGTTTC	59.8	668	INTRON	Ca_12471	--	--
Ca2_P1_30901717	Ca2	30901717	TG/T	TCATTGGTGTTTACCGGGAT	TAGCCTTTTGCAATATGGGG	60.1	886	DRR	Ca_12470	DO	--
Ca2_P1_30902299	Ca2	30902299	CTT/CT	CCCCATATTGCAAAAGGCTA	CAAATGGGTTTGCAAGTGTG	59.9	385	DRR	Ca_12470	DO	--
Ca2_P1_30902626	Ca2	30902626	T/TC	CCCCATATTGCAAAAGGCTA	TGGGGAATCGCAAGATAAAC	59.9	839	DRR	Ca_12471	--	--
Ca2_P1_30905223	Ca2	30905223	ATTTTTTTTT/ATTTTTTT	AGGAAAGTGCTCAATCAGGC	GCCAGAGAGTGATGACCGAT	59.4	599	INTRON	Ca_12470	DO	--
Ca2_P1_30907113	Ca2	30907113	TAAA/TAA	ACTGGCACCCAATAGGATCA	AGTTCCATTGCTGCTTTGCT	60.3	812	INTRON	Ca_12470	DO	--
Ca2_P1_3090922	Ca2	3090922	TA/T	GAGACTTTTGGGCATGGAAA	TGGTCAGTCAAATTCTCCGA	60.1	368	INTRON	Ca_10541	T	HD-ZIP
Ca2_P1_30915268	Ca2	30915268	A/ACAATCACTAAG	GAAGTGAAGTGGTTCTTTGCG	CAAGACCTTGTGGCTCATCA	59.9	783	INTRON	Ca_12470	DO	--
Ca2_P1_30920554	Ca2	30920554	ATTTTTT/ATTTTTTT	TCCACAACTCAAACCCATGA	AGTTTCCTTGTGGGTCGATG	59.9	344	INTRON	Ca_12470	DO	--

INDEL marker IDs	Chromosomes/ unanchored scaffolds	Physical positions (bp)	InDels (*Kabuli* reference genome-CDC Frontier/PI)	Forward primers (5'-3')	Reverse primers (5'-3')	Anneling temperature (0C)	Expected amplified product size (bp)	Structural annotation		Functional annotation	
								Sequence components of *kabuli* genome	*Kabuli* gene accession IDs	NCBI-KOG	TFs
Ca2_P1_30931555	Ca2	30931555	GCATCATC/GCATC	CAAAAACGTCCCCAAAAGAA	CGTAGGACGAAGGAAGATGC	59.9	857	INTRON	Ca_12470	DO	--
Ca2_P1_30931955	Ca2	30931955	ACC/AC	GCATCTTCCTTCGTCCTACG	AAGAAGTTGGATGCCAAGGA	59.8	116	INTRON	Ca_12470	DO	--
Ca2_P1_30933751	Ca2	30933751	TC/T	TCGAAACGTGTGGTGATGAT	ACAATTCCACGCAATGTGAG	60.0	895	INTERGENIC	--	--	--
Ca2_P1_30937234	Ca2	30937234	CTTTTTT/CTTTTTTTT	TTGAATTGTGTGCGGTTGAT	CGTTGATGTGTCACACGTTTC	60.0	467	INTERGENIC	--	--	--
Ca2_P1_30938413	Ca2	30938413	CTTTTTTT/CTTTTTTTT	TGCAAAATAGAATGGAGGTGA	TTTTTCATTTGGTCCATCCC	58.2	319	INTERGENIC	--	--	--
Ca2_P1_30938733	Ca2	30938733	AC/A	GGGATGGACCAAATGAAAAA	AAGGCCCTAGTAGCAGGCAT	59.6	570	INTERGENIC	--	--	--
Ca2_P1_30940538	Ca2	30940538	ATGTGTGTGT/ATGTGTGTGTGT	CATAAGTGGGTATCCAACCGA	CATTTGCAAGCAAAATTGACA	59.7	620	INTERGENIC	--	--	--
Ca2_P1_30943416	Ca2	30943416	TAAAAA/TAAA	CGAACTCACCTGATGCTCAA	GCCAATATGTTCATTGACGG	60.0	607	INTERGENIC	--	--	--
Ca2_P1_30945167	Ca2	30945167	GT/GTCT	GACCATGGAATCGGACAAGT	CTTCTTGCATCCCCTTGTGT	59.8	684	INTRON	Ca_12469	U	bHLH
Ca2_P1_30949563	Ca2	30949563	GTTATTATTATTATTATTATTATTATTATTATTATTATTATTATTATTATTATTATTATTATT/GTTATTATTATTATTATTATTATTATTATTATTATTATTATTATTATTATTATTATTATTATTATTATTATT	GGCGACTGCGTAATAGCTTC	CCACAACCTGCAATGACAAC	60.0	149	INTRON	Ca_12469	U	bHLH
Ca2_P1_3095070	Ca2	3095070	ATT/ATTT	AGGGTCAGCCTGTAAGAGCA	CCAAAACTATGCTTGAGCCC	60.0	899	DRR	Ca_10541	T	HD-ZIP
Ca2_P1_30957679	Ca2	30957679	ATTTTTT/ATTTTTTTT	TCGGTTGTAGCCGAGCTAGT	TTTTGCTTGGGTCGAGAGAT	60.0	791	DRR	Ca_12469	U	bHLH

INDEL marker IDs	Chromosomes/ unanchored scaffolds	Physical positions (bp)	InDels (*Kabuli* reference genome-CDC Frontier/PI)	Forward primers (5'-3')	Reverse primers (5'-3')	Anneling temperature (0C)	Expected amplified product size (bp)	Structural annotation		Functional annotation	
								Sequence components of *kabuli* genome	*Kabuli* gene accession IDs	NCBI-KOG	TFs
Ca2_P1_30957722	Ca2	30957722	CCA/CCACA	TCGGTTGTAGCCGAGCTAGT	TTTTGCTTGGGTCGAGAGAT	60.0	791	DRR	Ca_12469	U	bHLH
Ca2_P1_30963140	Ca2	30963140	GT/G	TAAGCCCATTGAACTGCTCC	CACAATTGCCTACACCATCG	60.2	780	INTERGENIC	--	--	--
Ca2_P1_30964401	Ca2	30964401	T/TC	TGCACAAGTATTTTCCACCAA	TCGCCTTACAACATAAGCGTT	59.1	918	INTERGENIC	--	--	--
Ca2_P1_3096490	Ca2	3096490	ATTTT/ATTTTT	TGGTTTTCCTTAAACATGCAA	CTTTATCTTCATTTCGGCGG	58.2	134	DRR	Ca_10541	T	HD-ZIP
Ca2_P1_30965491	Ca2	30965491	GAAAA/GAAAAAAAA	GCGAAACCCTAATCACCAAA	CCGTTCCGACAATTTGAAGT	59.9	474	INTERGENIC	--	--	--
Ca2_P1_30979063	Ca2	30979063	GATCA/GA	CTCGCCAACTTACCGATTGT	TCATTATTGTGAAATAGGGCCA	60.1	554	DRR	Ca_12466	O	TCP
Ca2_P1_3098006	Ca2	3098006	GTTTTTTTTTTTT/GTTTTTTTTTT	TCGCTGACTATTATGCGGCT	CACGTTGTTTCATTGCATCC	60.9	435	INTERGENIC	--	--	--
Ca2_P1_30980417	Ca2	30980417	ATT/ATTT	AAAGGGGTTGGTTCCTTTTG	GGAAACGGAATGTTGCATCT	60.2	469	DRR	Ca_12466	O	TCP
Ca2_P1_30983172	Ca2	30983172	GAAAA/GAAA	GTAGCATCGCCTTGGGATTA	TCGACATTCCCATTTTATTGC	60.1	267	INTERGENIC	--	--	--
Ca2_P1_30983322	Ca2	30983322	GTTTTT/GTTTT	GTAGCATCGCCTTGGGATTA	TGTCCACCCATAAGTAATCATTTG	60.1	744	INTERGENIC	--	--	--
Ca2_P1_3098512	Ca2	3098512	TTAAAAATT/TTAAAAATTATATTTGATCTAAAAATT	GGATGCAATGAAACAACGTG	ACCCTTTCGTGAAGCATTTG	60.0	514	INTERGENIC	--	--	--
Ca2_P1_30987559	Ca2	30987559	ATTTTTT/ATTTTTTT	TCAAATCATGCTCTAAGCGG	GTTGATAAACACCGGCCATT	59.0	848	INTERGENIC	--	--	--

INDEL marker IDs	Chromosomes/ unanchored scaffolds	Physical positions (bp)	InDels (*Kabuli* reference genome-CDC Frontier/PI)	Forward primers (5'-3')	Reverse primers (5'-3')	Anneling temperature (0C)	Expected amplified product size (bp)	Structural annotation		Functional annotation	
								Sequence components of *kabuli* genome	*Kabuli* gene accession IDs	NCBI-KOG	TFs
Ca2_P1_3098929	Ca2	3098929	TTTTCTT/TTT	CAAATGCTTCACGAAAGGGT	AGCCACAAAACCACAACACA	60.1	361	INTERGENIC	--	--	--
Ca2_P1_3099476	Ca2	3099476	ATTT/ATTTT	GTTGTGGTTTTGTGGCTGTG	CCAATGCTTGACCCATCTTT	60.0	649	INTERGENIC	--	--	--
Ca2_P1_3099613	Ca2	3099613	ATATTATTA/ATATTA	GTTGTGGTTTTGTGGCTGTG	CCAATGCTTGACCCATCTTT	60.0	649	INTERGENIC	--	--	--
Ca2_P1_30997163	Ca2	30997163	GT/G	TGAAAACGGTTCGACAAACA	ATGTGGCCATTGACAAGTGA	60.1	655	DRR	Ca_12465	--	ERF
Ca2_P1_30997225	Ca2	30997225	A/AT	TCACCCTCGTATCCACAACA	ATGTGGCCATTGACAAGTGA	60.0	245	DRR	Ca_12465	--	ERF
Ca2_P1_30999439	Ca2	30999439	T/TC	CCCAAACCTTTTTGGAGTTG	TAGCTTGAGCGTGGTTTCCT	59.4	625	INTERGENIC	--	--	--
Ca2_P1_31004156	Ca2	31004156	TAAA/TAAAA	AACGGTCCTCTTAGGACATCA	ACATGGGAATCCAAAGACCA	58.7	562	DRR	Ca_12464	--	LBD
Ca2_P1_3101145	Ca2	3101145	GTTGGAA/GTTGGAACTTTGGAA	TTGTCATGGTGGTTGCATCT	TCTTCGACAGCCTTCGCTAT	60.0	657	INTRON	Ca_10540	C	--
Ca2_P1_31013539	Ca2	31013539	GAAAAAAAAAAAA/GAAAAAAAAAAA	TGGTCACGGAGACAAATCAA	TTGTGCCTTCCCTCTACCAC	60.1	706	INTERGENIC	--	--	--
Ca2_P1_31013894	Ca2	31013894	TAAAAAAAAA/TAAAAAAAAAAAA	GTGGTAGAGGGAAGGCACAA	TCATGGATTTGTGGTGTGCT	60.1	615	INTERGENIC	--	--	--
Ca2_P1_31017880	Ca2	31017880	A/ATTG	TCCTTGATGCTAAGGTTGCC	GACCCATCTCCAATAGCCCT	60.2	584	DRR	Ca_12462	R	G2-like
Ca2_P1_3102410	Ca2	3102410	AATATAT/AATAT	ACACGTGTCGATGTCGGATA	TGGCCTTTTGAAAGTGCTTC	60.0	621	DRR	Ca_10540	C	--

INDEL marker IDs	Chromosomes/ unanchored scaffolds	Physical positions (bp)	InDels (*Kabuli* reference genome-CDC Frontier/PI)	Forward primers (5'-3')	Reverse primers (5'-3')	Anneling temperature (0C)	Expected amplified product size (bp)	Structural annotation		Functional annotation	
								Sequence components of *kabuli* genome	*Kabuli* gene accession IDs	NCBI-KOG	TFs
Ca2_P1_3102450	Ca2	3102450	AAAATA/AAAATAAATA	ACACGTGTCGATGTCGGATA	TGGCCTTTTGAAAGTGCTTC	60.0	621	DRR	Ca_10540	C	--
Ca2_P1_31031179	Ca2	31031179	CTCTTCTTCTTC/CTCTTCTTCTTCTTC	TGTTCCATTTGGGGCATATT	TTCATTTTCAGGTGTGCCAA	60.0	403	CDS (large-effect mutations)	Ca_12461	--	LBD
Ca2_P1_31033011	Ca2	31033011	CTT/CTTT	TGGGAGAGAAACCAACAAGG	GGCACCAGAGCTAGTGAACC	60.1	409	INTRON	Ca_12461	--	LBD
Ca2_P1_31033700	Ca2	31033700	A/AT	GGTTCACTAGCTCTGGTGCC	AAGTGGTGACCCTTTTGGTG	59.9	655	INTRON	Ca_12461	--	LBD
Ca2_P1_31035260	Ca2	31035260	TAAGAAA/TAA	TCGACTTTAACAAACGCAAA	AAAGTGAAGTCACGGGGATG	57.1	478	INTERGENIC	--	--	--
Ca2_P1_31036015	Ca2	31036015	TAAA/TAAAAAAA	GGCTTTGTCTCACTTCAAACC	TATCACATGCCATGCAACAA	58.8	516	INTERGENIC	--	--	--
Ca2_P1_31038690	Ca2	31038690	ATTTTT/ATTTT	CCTTGGGCTTTTTGATTGAA	CACTGGGATCGTGGCATAAT	60.0	938	INTERGENIC	--	--	--
Ca2_P1_31040473	Ca2	31040473	ATTTTTTTTT/ATTTTTTTTTT	CCGGACTTACGGTGAAGAAA	GTTAAGGAACCGCCGATGTA	60.1	567	INTERGENIC	--	--	--
Ca2_P1_31041115	Ca2	31041115	TTATTTGAAT/TTATTTGAATATTTGAAT	TACATCGGCGGTTCCTTAAC	CAAAATTTTCAAACAAAACCCA	60.0	379	INTERGENIC	--	--	--
Ca2_P1_31041300	Ca2	31041300	GGAA/GGAAGAAGAA	TACATCGGCGGTTCCTTAAC	GGCTAAATTACATCTGTGGTCCTT	60.0	553	INTERGENIC	--	--	--
Ca2_P1_3104148	Ca2	3104148	TAAAAAAA/TAAAAAAAA	GGAAACGCCGAGACATACAT	GCACAACCTTTTTGGCGTAT	60.0	360	DRR	Ca_10539	P	WOX
Ca2_P1_31041722	Ca2	31041722	TA/T	AAAGGACCAAATCAGGAAAAA	TACCGCCACTAGAGGCTACG	57.7	848	INTERGENIC	--	--	--

INDEL marker IDs	Chromosomes/ unanchored scaffolds	Physical positions (bp)	InDels (*Kabuli* reference genome-CDC Frontier/PI)	Forward primers (5'-3')	Reverse primers (5'-3')	Anneling temperature (0C)	Expected amplified product size (bp)	Structural annotation		Functional annotation	
								Sequence components of *kabuli* genome	*Kabuli* gene accession IDs	NCBI-KOG	TFs
Ca2_P1_31042129	Ca2	31042129	CGG/CGGG	AACCCTTGTGGTAATGATTCG	CATGGAATATCAAGTGGGGG	58.8	346	INTERGENIC	--	--	--
Ca2_P1_31047371	Ca2	31047371	TAAAAAA/TAAAAAAA	TTTCCTTGTCTTTTTGGAGCA	GCGGTGATGATTCCAAAGTT	59.8	369	INTERGENIC	--	--	--
Ca2_P1_31051512	Ca2	31051512	GTATATATATATATAT/GTATATATATATATATATATAT	CCAAACGGCCACTAATGTCT	CTGCATTTTCCATCCCAAGT	60.0	614	INTRON	Ca_12459	D	--
Ca2_P1_31060815	Ca2	31060815	GATAA/GATAATAA	TTTGACCGAGTGGAGCTTTT	CCACTCCAACAGCCATACCT	59.9	426	INTERGENIC	--	--	--
Ca2_P1_31068357	Ca2	31068357	GGCAATG/GG	TTCAAAGGTGTTTGTGCTTCA	TCACCATAGCAGTTTCCATCC	59.4	877	DRR	Ca_12458	M	--
Ca2_P1_31073018	Ca2	31073018	AAG/AAGAG	TAAACACCGTACACGACGGA	AATGGGAGGGCTGGACTAAT	60.0	603	INTRON	Ca_12457	--	NF-YB
Ca2_P1_31075019	Ca2	31075019	CTTT/CT	GCTGGAAGGTTGTGGCTAAG	TGTCCTTGTTGAACTCGTCG	59.9	585	INTRON	Ca_12457	--	NF-YB
Ca2_P1_31077102	Ca2	31077102	AAAA/AAAATTTAAA	TCCTTTTCAAATCACCCAAGA	AGAGGAAAGCTTGCAGACCA	59.5	747	DRR	Ca_12456	--	--
Ca2_P1_31077356	Ca2	31077356	A/AT	TCCTATGAGAAAGTTTTTGGCA	AGAGGAAAGCTTGCAGACCA	58.9	481	DRR	Ca_12456	--	--
Ca2_P1_31077411	Ca2	31077411	TAAAAA/TAAAAAA	CAATCCAACACACCAAAGAGAA	AGAGGAAAGCTTGCAGACCA	60.0	115	DRR	Ca_12456	--	--
Ca2_P1_31081453	Ca2	31081453	CAGAG/CAGAGAG	ATGAGGTGTGTTCCCCAAAC	TCCTTGGTGCATCTCATCCT	59.7	258	INTERGENIC	--	--	--
Ca2_P1_31085057	Ca2	31085057	T/TTAAA	CACCAACATAAACAATGTCGAA	ACCACAAATGGAGAAGGTGC	58.5	942	DRR	Ca_12455	--	--

INDEL marker IDs	Chromosomes/ unanchored scaffolds	Physical positions (bp)	InDels (*Kabuli* reference genome-CDC Frontier/PI)	Forward primers (5'-3')	Reverse primers (5'-3')	Anneling temperature (0C)	Expected amplified product size (bp)	Structural annotation		Functional annotation	
								Sequence components of *kabuli* genome	*Kabuli* gene accession IDs	NCBI-KOG	TFs
Ca2_P1_31088258	Ca2	31088258	GT/GTAT	ACAGGCGAGAGGATTGAAGA	TAGCATAGCGGGATTTGACC	60.0	518	INTRON	Ca_12454	J	--
Ca2_P1_31103366	Ca2	31103366	CGTGTG/CGTG	CCACACCTAAATCCTCGCAT	TCATGTCTTGTTGTGGGCAT	60.0	414	INTRON	Ca_12452	F	--
Ca2_P1_31105983	Ca2	31105983	TAAAAAA/TAAAAAAA	TGTGGATTATCACCACTCCAA	TTTTTCCCGCTTAAAATGTAGC	58.8	557	INTERGENIC	--	--	--
Ca2_P1_31108104	Ca2	31108104	C/CT	CGAAATAGAACTTTGCGGGT	TGGATTGACACTTGGTTGGA	59.2	516	INTERGENIC	--	--	--
Ca2_P1_31108593	Ca2	31108593	ATTTTT/ATTTTTT	TCCAACCAAGTGTCAATCCA	TGCTAGATTGGCCAGCATAA	59.9	792	INTERGENIC	--	--	--
Ca2_P1_31109532	Ca2	31109532	ATTTTTTTTT/ATTTTTTTT	ACATAAGAAGCTGTGCGCCT	TTGGTTTCATGACCTGCATC	60.0	559	DRR	Ca_12451	F	--
Ca2_P1_31110207	Ca2	31110207	TC/T	CCAAGGTTGGATTCGAAGTG	AGGCTTTCAGGTCAGACCAA	60.5	396	DRR	Ca_12451	F	--
Ca2_P1_31113980	Ca2	31113980	CT/CTATTTTATACTATT	ATGCCCACAACAAGACATGA	TGTTTTGGCCACCAGGTAAT	60.0	825	INTRON	Ca_12451	F	--
Ca2_P1_31115854	Ca2	31115854	CAACTCTGTAACTCTGT/CAACTCTGT	TAATGGAAATGCGACCATGA	CACCTTCCAATAGGTGAGTGC	59.9	429	INTERGENIC	--	--	--
Ca2_P1_31116827	Ca2	31116827	T/TA	GTCACCCCTCGGTCAAAGTA	GCATGACATGAAAAACCCAA	60.0	541	INTERGENIC	--	--	--
Ca2_P1_31118846	Ca2	31118846	ATATTATTATTATTATTAT/ATATTATTATTATTAT	AACGCCAAGTCAAGACGAGT	AAGTATGTTGCATTGCGATTTTT	59.9	352	INTERGENIC	--	--	--
Ca2_P1_31122289	Ca2	31122289	ATTTTTTTT/ATTTTTTTTT	TGTGTTGAATTTACTAAAACACCCTT	TGCCTCACATGATTCAAAGC	59.4	918	INTERGENIC	--	--	--

INDEL marker IDs	Chromosomes/ unanchored scaffolds	Physical positions (bp)	InDels (*Kabuli* reference genome-CDC Frontier/PI)	Forward primers (5'-3')	Reverse primers (5'-3')	Anneling temperature (0C)	Expected amplified product size (bp)	Structural annotation		Functional annotation	
								Sequence components of *kabuli* genome	*Kabuli* gene accession IDs	NCBI-KOG	TFs
Ca2_P1_31122580	Ca2	31122580	GTATATATA/GTATATA	AAAATTAAAACACCGGAGGTAGT	TGCCTCACATGATTCAAAGC	57.4	217	INTERGENIC	--	--	--
Ca2_P1_31125141	Ca2	31125141	TTCT/TT	TCTCTTCCGTCTGCCATTTT	CCACATTGGGTATTTTTGGG	59.8	581	INTERGENIC	--	--	--
Ca2_P1_31130324	Ca2	31130324	TAA/TAAAAAA	TGCACATATTTTGAGCATTGAA	TGATGTTGAGGAAACACGGA	59.2	520	INTERGENIC	--	--	--
Ca2_P1_31131673	Ca2	31131673	GAATAATAA/GAATAATAATAA	TTTGGACAGCAAGAAGATGG	CATGCAAGAAATGCAAGGAA	58.8	473	INTERGENIC	--	--	--
Ca2_P1_31133031	Ca2	31133031	AC/A	GGTGATCAAACACATCGTCG	TTAGGTGGAGAGGGTGAGGA	60.0	807	INTERGENIC	--	--	--
Ca2_P1_31133111	Ca2	31133111	CAA/CAAA	GGTGATCAAACACATCGTCG	TTAGGTGGAGAGGGTGAGGA	60.0	807	INTERGENIC	--	--	--
Ca2_P1_3113326	Ca2	3113326	AACTTATACTT/AACTTATACTTATACTT	ATTTGTTCTGGCCCACCTTT	AGCTTGATGCTGCAATTCCT	60.7	535	INTRON	Ca_10538	--	--
Ca2_P1_3113498	Ca2	3113498	GTT/GT	TACGACGGCAATTGTAGCTG	ACCTGATCAATGTGTTGGCA	59.9	538	INTRON	Ca_10538	--	--
Ca2_P1_31138425	Ca2	31138425	AA/AATA	TGATGAGACGTTGGTCAAACA	TTTCCTCCTTGTTCACCTCC	60.1	464	INTRON	Ca_12449	--	--
Ca2_P1_31141727	Ca2	31141727	CTTTTTTTT/CTTTTTT	GGGGTTTGCAGAAAGATGAA	TCCAAATAAAATGATGACGCA	60.1	747	INTERGENIC	--	--	--
Ca2_P1_31146139	Ca2	31146139	TTACTATTAATA/TTA	GCGGTGAGAGTTGAGAGGAG	ATTGAAAGCAACTTGCCGAT	60.1	579	INTERGENIC	--	--	--
Ca2_P1_31146373	Ca2	31146373	CTTTTT/CTTTTTT	CTAGAAAGTCCGGCGTATCG	TTGGCCTGAAGAATGACCTC	59.9	557	INTERGENIC	--	--	--

INDEL marker IDs	Chromosomes/ unanchored scaffolds	Physical positions (bp)	InDels (*Kabuli* reference genome-CDC Frontier/PI)	Forward primers (5'-3')	Reverse primers (5'-3')	Anneling temperature (0C)	Expected amplified product size (bp)	Structural annotation		Functional annotation	
								Sequence components of *kabuli* genome	*Kabuli* gene accession IDs	NCBI-KOG	TFs
Ca2_P1_31146520	Ca2	31146520	CA/CACTAATTTTAGA	CTAGAAAGTCCGGCGTATCG	TTGGCCTGAAGAATGACCTC	59.9	557	INTERGENIC	--	--	--
Ca2_P1_31146694	Ca2	31146694	CTTTT/CTTTTT	CTAGAAAGTCCGGCGTATCG	CATCCCATTTGGTTCACTCC	59.9	866	INTERGENIC	--	--	--
Ca2_P1_31146777	Ca2	31146777	CTC/CTCGTC	ATCGGCAAGTTGCTTTCAAT	CATCCCATTTGGTTCACTCC	59.7	850	INTERGENIC	--	--	--
Ca2_P1_31151939	Ca2	31151939	A/AT	AGCCAAAGTGAGAAAGGCAA	AAGGATCCAAGCCCACTTCT	60.0	740	INTERGENIC	--	--	--
Ca2_P1_31153085	Ca2	31153085	C/CT	AGCCAATTGTCTGTGGAACA	GCCAGACCATAGGTTCCTGA	59.1	238	DRR	Ca_12447	L	ERF
Ca2_P1_31153445	Ca2	31153445	GAAAAAAAAAAAAAAAAA/GAAAAAAAAAAAA	AGGAACCTATGGTCTGGCCT	AAGTCTGGCCTACGGCCTAT	60.0	421	DRR	Ca_12447	L	ERF
Ca2_P1_31157909	Ca2	31157909	CG/CGG	CGACCTCCCGAAAGTTGATA	CTGGAATCGACATATGCGTG	60.1	581	INTRON	Ca_12447	L	ERF
Ca2_P1_31158685	Ca2	31158685	GAAAA/GAAA	GGGAACCTGGATTACCACCT	TCCATTCAATGCTCACCGTA	60.1	179	INTRON	Ca_12447	L	ERF
Ca2_P1_31160151	Ca2	31160151	AATATATATATATATATATATATAT/AATATATATATATATATATATATATATAT	AGCCTTGAAGTTCGTGCAGT	TGCGTGTTTTCCTCTCAAACT	60.1	445	INTERGENIC	--	--	--
Ca2_P1_31160632	Ca2	31160632	ATTTTTTTTTTT/ATTTTTT	AGTTTGAGAGGAAAACACGCA	TTTTGAACGTCACTTTATGATCG	59.9	426	INTERGENIC	--	--	--
Ca2_P1_31164745	Ca2	31164745	TTTTTG/TTTTTGTATTTTG	TGTTATGCACATCCCCTCAA	TTGGGCCTATCTGAAAATGG	59.9	842	DRR	Ca_12446	--	ERF
Ca2_P1_31165324	Ca2	31165324	ATT/AT	CCATTTTCAGATAGGCCCAA	TGTTGTTGATTCGAGTTGGC	59.9	349	DRR	Ca_12446	--	ERF

INDEL marker IDs	Chromosomes/ unanchored scaffolds	Physical positions (bp)	InDels (*Kabuli* reference genome-CDC Frontier/PI)	Forward primers (5'-3')	Reverse primers (5'-3')	Anneling temperature (0C)	Expected amplified product size (bp)	Structural annotation		Functional annotation	
								Sequence components of *kabuli* genome	*Kabuli* gene accession IDs	NCBI-KOG	TFs
Ca2_P1_31165502	Ca2	31165502	CTTT/CTT	CCATTTTCAGATAGGCCCAA	CTCTGTGGCCACTTTTGTCA	59.9	712	DRR	Ca_12446	--	ERF
Ca2_P1_31168865	Ca2	31168865	CCA/C	AGCGTCCTTCTGGATGCTAA	TGTCGGGAAAGTGAGAGAAGA	60.0	134	INTERGENIC	--	--	--
Ca2_P1_31169306	Ca2	31169306	TAA/TAACAA	TCTTCTCTCACTTTCCCGACA	TGCAAATATGTTCCGTCAAAA	60.0	489	INTERGENIC	--	--	--
Ca2_P1_31170304	Ca2	31170304	TC/T	TCAACGAAGTCCTTTCTTCCA	TAAGTTTCCCTTGCCCAATC	59.8	312	INTERGENIC	--	--	--
Ca2_P1_31170446	Ca2	31170446	CG/C	TCAACGAAGTCCTTTCTTCCA	TAAGTTTCCCTTGCCCAATC	59.8	312	INTERGENIC	--	--	--
Ca2_P1_31170922	Ca2	31170922	ATTTTTTT/ATTTTTTTTT	GATTGGGCAAGGGAAACTTA	TCGGATATGCGAATGCAATA	59.0	617	INTERGENIC	--	--	--
Ca2_P1_31172482	Ca2	31172482	TATTTAAT/TAT	CCTTGAATAAAAATATGGGGCA	TCAATAACTTTTAACAGTCCGACAA	60.0	526	INTERGENIC	--	--	--
Ca2_P1_31172734	Ca2	31172734	CA/C	TGGGGCAAGATTTATAGGAA	TAGTGCGCGACATTTTATGC	57.2	751	INTERGENIC	--	--	--
Ca2_P1_31172925	Ca2	31172925	AATATATATATA/AATATATATATATA	TTGTCGGACTGTTAAAAGTTATTGA	TAGTGCGCGACATTTTATGC	59.2	265	INTERGENIC	--	--	--
Ca2_P1_31176924	Ca2	31176924	CATATAT/CATAT	TCCAATTTGATATCGGTCTCTTG	CATGGGGATAAAATTTGTCAATG	60.3	508	INTERGENIC	--	--	--
Ca2_P1_31177108	Ca2	31177108	ATT/ATTT	TCCAATTTGATATCGGTCTCTTG	AAACATAAAACAACGTGAGACAAA	60.3	970	INTERGENIC	--	--	--
Ca2_P1_31177967	Ca2	31177967	CTTTTTTTT/CTTTTTTTTTT	TCTGTGACAAGTAGCTGTCTCGTAT	TGAGACGCATAAGGGTTATTCA	59.6	322	INTERGENIC	--	--	--

INDEL marker IDs	Chromosomes/ unanchored scaffolds	Physical positions (bp)	InDels (*Kabuli* reference genome-CDC Frontier/PI)	Forward primers (5'-3')	Reverse primers (5'-3')	Anneling temperature (0C)	Expected amplified product size (bp)	Structural annotation		Functional annotation	
								Sequence components of *kabuli* genome	*Kabuli* gene accession IDs	NCBI-KOG	TFs
Ca2_P1_31178385	Ca2	31178385	CA/C	TGAATAACCCTTATGCGTCTCA	TCCTCCCTTTTTGTTACATTTTTC	59.6	581	INTERGENIC	--	--	--
Ca2_P1_31178815	Ca2	31178815	CTTTTTTTTT/CTTTTTTT	TGTAACAAAAAGGGAGGAAAAA	ACGCAGATGCCAAAGTTGTA	57.9	691	INTERGENIC	--	--	--
Ca2_P1_31179014	Ca2	31179014	TAAG/TAAGAAG	TTGAAGCTCAAATTTCAACAGAA	TTAACACGCAGATGCCAAAG	59.0	423	INTERGENIC	--	--	--
Ca2_P1_31179559	Ca2	31179559	GAAAAAAAAA/GAAAAAAAA	TTTGGCATCTGCGTGTTAAA	CAAAGATCAACATGGGTTGG	60.3	474	INTERGENIC	--	--	--
Ca2_P1_31180202	Ca2	31180202	AAT/A	CCAACCCATGTTGATCTTTG	TCCACTTAGCAATTGGCCTT	58.8	797	INTERGENIC	--	--	--
Ca2_P1_31180458	Ca2	31180458	TATAGATA/TATA	TTGATCTGTCTTACGCCTTTTTC	TCCACTTAGCAATTGGCCTT	59.8	572	INTERGENIC	--	--	--
Ca2_P1_31180507	Ca2	31180507	GATATATATA/GATATA	CACACTTGTTGTAATTTTCACGG	TCCACTTAGCAATTGGCCTT	59.5	429	INTERGENIC	--	--	--
Ca2_P1_31180716	Ca2	31180716	TAA/TAAA	GGCCAATTGCTAAGTGGATG	GCTTTGATTCTTGCAATGTCC	60.5	364	INTERGENIC	--	--	--
Ca2_P1_31188890	Ca2	31188890	AG/A	CAATAGGGTTTGCATCCCAC	TTCATGGTTGAGAGCCACTG	60.2	532	INTRON	Ca_12445	Q	--
Ca2_P1_31189040	Ca2	31189040	GATCA/GATCATCA	CAATAGGGTTTGCATCCCAC	TTCATGGTTGAGAGCCACTG	60.2	532	INTRON	Ca_12445	Q	--
Ca2_P1_3118907	Ca2	3118907	TAAAAA/TAAAAAAA	TGCACCAGAATGCCTTGTTA	TGGCCTTCACATTTCAAACA	60.3	752	INTRON	Ca_10537	--	AP2
Ca2_P1_31189398	Ca2	31189398	ACC/ACCC	CAGCCCATCCTGTTCTGATT	ATGGACAATTTCCAGGTCCA	60.1	403	INTRON	Ca_12445	Q	--

INDEL marker IDs	Chromosomes/ unanchored scaffolds	Physical positions (bp)	InDels (*Kabuli* reference genome-CDC Frontier/PI)	Forward primers (5'-3')	Reverse primers (5'-3')	Anneling temperature (0C)	Expected amplified product size (bp)	Structural annotation		Functional annotation	
								Sequence components of *kabuli* genome	*Kabuli* gene accession IDs	NCBI-KOG	TFs
Ca2_P1_31189472	Ca2	31189472	T/TTA	CAGCCCATCCTGTTCTGATT	ATGGACAATTTCCAGGTCCA	60.1	403	INTRON	Ca_12445	Q	--
Ca2_P1_3118956	Ca2	3118956	ATATTA/ATATTATTA	ATTTCCTTTGCAGCATCGTT	GGCCCAATTTATGTGGTGAC	59.7	804	INTRON	Ca_10537	--	AP2
Ca2_P1_31191262	Ca2	31191262	CC/CCATGTATTCAAC	AAAAAGTGTCCGCAATTTGG	TTGTGGGTGAGAGCACTACG	60.0	577	INTERGENIC	--	--	--
Ca2_P1_31191785	Ca2	31191785	GAA/GA	CGTAGTGCTCTCACCCACAA	AAACCACAAGAGGATACCACAGA	59.9	614	INTERGENIC	--	--	--
Ca2_P1_31193349	Ca2	31193349	TCTTAC/TC	CGCGTTTGAGAATTTTGATG	TGGATCAAGCCTTCCATTTT	59.3	136	INTERGENIC	--	--	--
Ca2_P1_31194189	Ca2	31194189	A/ATACACTCAT	GTGGGTGCGAACTTAGGAAA	TTTTCAATGATCCTAGCCCG	60.1	334	INTERGENIC	--	--	--
Ca2_P1_31194356	Ca2	31194356	CAAA/CAAAA	GTGGGTGCGAACTTAGGAAA	TTTTCAATGATCCTAGCCCG	60.1	334	INTERGENIC	--	--	--
Ca2_P1_31196009	Ca2	31196009	GC/GCTC	ACGAGAATCATGAAACGGAA	CCGTAGCTCATTTACTCGGG	58.1	313	INTERGENIC	--	--	--
Ca2_P1_31196347	Ca2	31196347	TA/TAA	ATTGAATTTGCACACCACCA	TTTTCGGCAAAGCTATGGTT	59.8	685	INTERGENIC	--	--	--
Ca2_P1_31197321	Ca2	31197321	C/CTTG	CACCTCTCCGCTTACCCATA	AAATTGCACTCGCGTCTTTT	60.1	555	INTERGENIC	--	--	--
Ca2_P1_31197911	Ca2	31197911	TTATA/TTA	AAAAGACGCGAGTGCAATTT	TGCCAATTTTCGAACTGGAT	59.9	841	INTERGENIC	--	--	--
Ca2_P1_31203854	Ca2	31203854	CTTTTT/CTTTT	ATCCAAAGCTTCCCCAAAAT	TTTTATGTTCCTTGCTCCCG	59.8	418	DRR	Ca_12443	--	ERF

INDEL marker IDs	Chromosomes/ unanchored scaffolds	Physical positions (bp)	InDels (*Kabuli* reference genome-CDC Frontier/PI)	Forward primers (5'-3')	Reverse primers (5'-3')	Anneling temperature (0C)	Expected amplified product size (bp)	Structural annotation		Functional annotation	
								Sequence components of *kabuli* genome	*Kabuli* gene accession IDs	NCBI-KOG	TFs
Ca2_P1_31211587	Ca2	31211587	GAA/GA	AGCCCCTATTTCAATCACCC	GGAACAAATTTGATGAGTGTGC	60.2	714	INTERGENIC	--	--	--
Ca2_P1_31211740	Ca2	31211740	TAAAAA/TAAAA	AATGTGACAACGTAGCCCCT	CGGGAGAGAAGTGAAAAAGAGA	59.5	949	INTERGENIC	--	--	--
Ca2_P1_3122073	Ca2	3122073	TCCC/TCCCC	GCACACAATCCAAATACCCTTT	TTGAAGTTGGTGCATGGAAA	60.1	238	DRR	Ca_10536	--	--
Ca2_P1_31232642	Ca2	31232642	GTTAATTAATTA/GTTAATTA	TGTCATCAAGATGCGTCAAAG	CCATTGTGATGTTGGTGGAA	59.9	830	INTERGENIC	--	--	--
Ca2_P1_31232923	Ca2	31232923	GTT/GTTT	ACCCGTGATGGAAAGAATGA	CCATTGTGATGTTGGTGGAA	60.3	323	INTERGENIC	--	--	--
Ca2_P1_31233960	Ca2	31233960	AG/A	GGGTGGAAATCATGGTGAAC	TTGGTTGGAAGGAATGGAAG	60.0	436	INTRON	Ca_12440	--	--
Ca2_P1_31235021	Ca2	31235021	TAAAAAAAAAAAAAA/TAAAAAAAAAAA	CACGAGATCTTGAGGCATGA	TTCTGAGGATTTTCCAACGG	59.9	607	INTRON	Ca_12440	--	--
Ca2_P1_31240228	Ca2	31240228	TCAA/T	CTGCCCCCACTATTTCTCAA	GAAACACTTGGCATGTTAAGGA	60.1	479	INTERGENIC	--	--	--
Ca2_P1_3124197	Ca2	3124197	T/TTTG	TGCAGCCATTGGATTAGGAT	TTTTGCTTCCACATTTTCCC	60.4	680	INTRON	Ca_10536	--	--
Ca2_P1_31244339	Ca2	31244339	AGG/AG	CAAGACTCAGGGTGGGTTGT	AAATCCATTGGGAAATGCAG	60.0	547	INTRON	Ca_12439	--	C3H
Ca2_P1_31245574	Ca2	31245574	TTCTCTCTCTCTCTCT/TTCTCTCTCTCTCTCTCTCTCT	AACCCCATCATCATCATCGT	GAAACGAACCAACCTGGAGA	60.0	262	INTRON	Ca_12439	--	C3H
Ca2_P1_31247048	Ca2	31247048	CGGCA/CGGCATATTGGCA	CGTTGGCTATGGAGACCCTA	TCGTGTTCAAATGCCGATTA	60.1	251	DRR	Ca_12439	--	C3H

INDEL marker IDs	Chromosomes/ unanchored scaffolds	Physical positions (bp)	InDels (*Kabuli* reference genome-CDC Frontier/PI)	Forward primers (5'-3')	Reverse primers (5'-3')	Anneling temperature (0C)	Expected amplified product size (bp)	Structural annotation		Functional annotation	
								Sequence components of *kabuli* genome	*Kabuli* gene accession IDs	NCBI-KOG	TFs
Ca2_P1_3126083	Ca2	3126083	CTATATATAT/CTATATATATAT	AACGATTCCATGACTAATTTGAA	TTGAACGGTTTAAATGTGGCT	57.7	518	INTERGENIC	--	--	--
Ca2_P1_3126941	Ca2	3126941	TAT/TATAAT	ATGTGCATGCAAAATTTCAA	ATTTAAGGGACACGTGGCAA	57.6	571	INTERGENIC	--	--	--
Ca2_P1_3127696	Ca2	3127696	ATTTTTTT/ATTTTTTTTT	TAGTTAATGAATCGGGGGCA	TTTAAGGAACTTTTGCGCTTC	60.3	488	INTERGENIC	--	--	--
Ca2_P1_31280508	Ca2	31280508	GC/GCC	CCACTGTCGCACACAAAATC	TGCTTCCTTTTCCATCATCC	60.2	834	INTERGENIC	--	--	--
Ca2_P1_31286944	Ca2	31286944	A/ATAATG	TGGTATATTTGCACGGACCA	AAATGCGAGTCTAAGAGGATGC	59.8	330	INTERGENIC	--	--	--
Ca2_P1_3128864	Ca2	3128864	CATATATATATA/CATATATATA	TGTGCTTTGGAATGGAGACA	CATCACATTCGGGTACACACA	60.2	727	INTERGENIC	--	--	--
Ca2_P1_31294019	Ca2	31294019	AG/AGAGG	AGACCGTCGGTTTCAATGAC	AAAAATCTTTAACAAGCGGGT	60.0	494	INTERGENIC	--	--	--
Ca2_P1_31294501	Ca2	31294501	TAAAAAAAAAAA/TAAAAAAAAA	TGAGAAGCTCTTCCCAAAATTA	TGATCACAATCATTGGAGCCT	58.1	262	INTERGENIC	--	--	--
Ca2_P1_31299022	Ca2	31299022	ATTTTTTT/ATTTTTTTT	TGAGCAGAGGTGTCAACGAT	TTATGCATTTCCATTGCAGC	59.4	227	INTERGENIC	--	--	--
Ca2_P1_31299649	Ca2	31299649	TAAAAAAAAAA/TAAAAAAAA	GCTGCAATGGAAATGCATAA	GCGAATTTCACAATCCCACT	59.7	972	INTERGENIC	--	--	--
Ca2_P1_31300005	Ca2	31300005	A/AG	CATTCCAATGTGCAACCTCT	GCGAATTTCACAATCCCACT	58.6	535	INTERGENIC	--	--	--
Ca2_P1_31300563	Ca2	31300563	TAA/TAAA	GGGATTGTGAAATTCGCCTA	CGTCTCCTCCTTCATCTTCG	59.9	510	DRR	Ca_12434	P	--

INDEL marker IDs	Chromosomes/ unanchored scaffolds	Physical positions (bp)	InDels (*Kabuli* reference genome-CDC Frontier/PI)	Forward primers (5'-3')	Reverse primers (5'-3')	Anneling temperature (0C)	Expected amplified product size (bp)	Structural annotation		Functional annotation	
								Sequence components of *kabuli* genome	*Kabuli* gene accession IDs	NCBI-KOG	TFs
Ca2_P1_31300865	Ca2	31300865	CTTTTTT/CTTTTT	CGAAGATGAAGGAGGAGACG	CAACCAAGCCAGTCAACAAA	59.9	294	DRR	Ca_12435	--	--
Ca2_P1_31302964	Ca2	31302964	ATGT/AT	TTCTTCAGTGGTGACGTTGC	CTTGCACTGCCCTTTTCTTC	59.9	524	INTRON	Ca_12434	P	--
Ca2_P1_31312554	Ca2	31312554	A/AGG	AAGAAATATTTTGCTGTGTGTATTTCA	CAACCCAACGTACCACAATG	59.5	132	INTERGENIC	--	--	--
Ca2_P1_31312751	Ca2	31312751	TAGAAAA/TA	CATTGTGGTACGTTGGGTTG	GGGAGGAATGAAAGAAAATTGA	59.7	614	INTERGENIC	--	--	--
Ca2_P1_31313203	Ca2	31313203	TA/TAA	CATGCATCAATTTGACTTTCG	GCGAGAGAAGACATCAAGGG	59.2	558	INTERGENIC	--	--	--
Ca2_P1_31317559	Ca2	31317559	GTT/GTTT	ATGGCAAACTTATGGCCAAG	GAGTGGGTTGGTTGTTTGGT	60.0	831	DRR	Ca_12431	U	--
Ca2_P1_31322875	Ca2	31322875	CAAAAAAAAAAAAA/CAAAAAAAAAAA	TGTTCACCACCAAACTGGAA	TTTGGTTCAACTTGCTACAACA	60.0	291	INTERGENIC	--	--	--
Ca2_P1_31323113	Ca2	31323113	GATATATATATATATATATA/GATATATATATATATATATATA	TGTTCACCACCAAACTGGAA	TTCATTTTAAAGGCATCGTGA	60.0	901	INTERGENIC	--	--	--
Ca2_P1_31325223	Ca2	31325223	TAAAAAAAA/TAAAAAAAAA	CTGCCTTGGTTAATATCGGC	CCACCAGTGGATACCAAACC	59.6	815	INTRON	Ca_12430	E	--
Ca2_P1_31336462	Ca2	31336462	GTTTTTTTTT/GTTTTTTTTTTT	TGGTCCAAACAAAGGATGAAG	AAAAATGTCCGGTAATGTTTTTAG	60.0	493	INTERGENIC	--	--	--
Ca2_P1_31341838	Ca2	31341838	CAATACA/C	TTTCTACGGTGAGGAGTGGG	TGTTTGGACTGCAAATTAAAGAAA	60.1	455	INTERGENIC	--	--	--
Ca2_P1_31347826	Ca2	31347826	ATATCTAATCTAT/ATAT	GGAGCCAACAACAAAGAGGA	ACCTCGCTTAGGAAAGCACA	60.2	820	DRR	Ca_12428	E	--

INDEL marker IDs	Chromosomes/ unanchored scaffolds	Physical positions (bp)	InDels (*Kabuli* reference genome-CDC Frontier/PI)	Forward primers (5'-3')	Reverse primers (5'-3')	Anneling temperature (0C)	Expected amplified product size (bp)	Structural annotation		Functional annotation	
								Sequence components of *kabuli* genome	*Kabuli* gene accession IDs	NCBI-KOG	TFs
Ca2_P1_31350663	Ca2	31350663	A/AGGGAT	CAGGGTCCTCTCCGATCATA	TCTGGGGTGACAGAGGAATC	60.0	440	INTRON	Ca_12427	R	--
Ca2_P1_31352669	Ca2	31352669	CTTTT/CTTT	CCCTTGCTGTGGATGGTAGT	TCCAAAATCTGCAAGAGGCT	60.0	569	INTERGENIC	--	--	--
Ca2_P1_31355084	Ca2	31355084	AT/ATT	GCATGGAAAAATGAAGGCAT	GAATGCATTGCTCATTTGGA	59.9	674	INTERGENIC	--	--	--
Ca2_P1_31363732	Ca2	31363732	AACGTAC/AAC	CATCATCGACATCGTGGAAC	AGAGAGGGATTCGTGAGGGT	59.9	422	INTERGENIC	--	--	--
Ca2_P1_31363878	Ca2	31363878	TTATATATAT/TTATATATATAT	CATCATCGACATCGTGGAAC	TGTGTAATTCATTGTGAGACGGA	59.9	461	INTERGENIC	--	--	--
Ca2_P1_31365474	Ca2	31365474	CT/C	TTTACGGCAGAATTTGACCA	TGGCTTTTTCCCTACTTCCA	59.2	361	INTERGENIC	--	--	--
Ca2_P1_31365682	Ca2	31365682	CTTTTTTT/CTTTTTTTTTT	TGGAAGTAGGGAAAAAGCCA	AGCATGAGATGATTGAGGCA	59.7	343	INTERGENIC	--	--	--
Ca2_P1_31367430	Ca2	31367430	TA/TAGGCAA	ATTGTAATGCGGTTCCGAAG	CTCCACACGTGCCATCATAC	60.0	485	INTERGENIC	--	--	--
Ca2_P1_31367840	Ca2	31367840	CTATA/CTA	GTATGATGGCACGTGTGGAG	CCTTCCCTTTCCCTTGAAAA	60.0	399	INTERGENIC	--	--	--
Ca2_P1_31368120	Ca2	31368120	AATATATAT/AATATATATAT	TCTTTCGATGCCATTTTTCC	GGCATGCATCTGAGCAAATA	60.0	801	INTERGENIC	--	--	--
Ca2_P1_31368502	Ca2	31368502	TAAAA/TAAAAAA	AATTAATCGAGTGGCGTGGT	CCGTTCCATTATCGGAGAGA	59.5	339	INTERGENIC	--	--	--
Ca2_P1_31370208	Ca2	31370208	ATTT/AT	TGGTTGATGGCAGGTAAACA	TACCTTGGCTGCTTTTTGCT	60.0	452	INTRON	Ca_12425	--	bHLH

INDEL marker IDs	Chromosomes/ unanchored scaffolds	Physical positions (bp)	InDels (*Kabuli* reference genome-CDC Frontier/PI)	Forward primers (5'-3')	Reverse primers (5'-3')	Anneling temperature (0C)	Expected amplified product size (bp)	Structural annotation		Functional annotation	
								Sequence components of *kabuli* genome	*Kabuli* gene accession IDs	NCBI-KOG	TFs
Ca2_P1_31370241	Ca2	31370241	ATT/ATTT	TGGTTGATGGCAGGTAAACA	TACCTTGGCTGCTTTTTGCT	60.0	452	INTRON	Ca_12425	--	bHLH
Ca2_P1_3137278	Ca2	3137278	CCTTAAAAGAGAT/C	CGTCTTTCTTGCATTTTGCAT	ATGGCTATGTTTCCGTTTGC	60.3	276	DRR	Ca_10535	A	C2H2
Ca2_P1_31373746	Ca2	31373746	CA/CACAA	AGAGATCGCAACATACATGGG	TTGCCACCAAATGACAAGAA	60.0	700	DRR	Ca_12425	--	bHLH
Ca2_P1_31375000	Ca2	31375000	CAAA/CAAAA	TTGCCTTTTCATATGTGTCTGG	TGGTGGTTACACTCTGGTGC	60.0	890	DRR	Ca_12424	--	--
Ca2_P1_31376623	Ca2	31376623	CTTTTTTTTTTTT/CTTTTTTTT	TGTGGGTGAAGTCGTCAAAG	TCCAGATGGAAGAGTGGGAG	59.7	438	DRR	Ca_12424	--	--
Ca2_P1_31380888	Ca2	31380888	TT/TTCTGT	AACACCCGAGTCTTGGTCAC	AGAGGCTGCCTGAAATGAAA	60.0	242	INTRON	Ca_12423	--	--
Ca2_P1_31385073	Ca2	31385073	GAAA/GAAAA	TGTTCTTTGGGGTCTTGGAC	CACTTCAACTTGTCGATCCG	59.9	314	INTERGENIC	--	--	--
Ca2_P1_31385803	Ca2	31385803	ATTT/A	TGCCGATACCGTTAGTCTTTG	AAGGTGATTTGTGTCTCCCG	60.1	774	INTERGENIC	--	--	--
Ca2_P1_31385863	Ca2	31385863	T/TG	TGGTTTTATCATGGTGCAAAA	AAGGTGATTTGTGTCTCCCG	58.9	694	INTERGENIC	--	--	--
Ca2_P1_31386815	Ca2	31386815	TAAA/TAA	CAGTCGAATCCGCCATATCT	TTGGGAATCGCATTTTTCTC	60.1	587	INTRON	Ca_12422	--	MYB_related
Ca2_P1_31386870	Ca2	31386870	GCTACATACTACATA/GCTACATA	CAGTCGAATCCGCCATATCT	TTGGGAATCGCATTTTTCTC	60.1	587	INTRON	Ca_12422	--	MYB_related
Ca2_P1_31388110	Ca2	31388110	TAAAA/TAAA	TCAGGGGACAATAACTTGGC	TCCATTTACCCACTAGACCCA	59.9	919	DRR	Ca_12422	--	MYB_related

INDEL marker IDs	Chromosomes/ unanchored scaffolds	Physical positions (bp)	InDels (*Kabuli* reference genome-CDC Frontier/PI)	Forward primers (5'-3')	Reverse primers (5'-3')	Anneling temperature (0C)	Expected amplified product size (bp)	Structural annotation		Functional annotation	
								Sequence components of *kabuli* genome	*Kabuli* gene accession IDs	NCBI-KOG	TFs
Ca2_P1_31388227	Ca2	31388227	TAAAA/TAAAAA	CATCAGTCCCCTCACTGGAT	TCCATTTACCCACTAGACCCA	59.9	707	DRR	Ca_12422	--	MYB_related
Ca2_P1_31388368	Ca2	31388368	TTT/TTTTCTT	CATCAGTCCCCTCACTGGAT	CGAAACAGTTCCCGAAAGAA	59.9	866	DRR	Ca_12422	--	MYB_related
Ca2_P1_31406637	Ca2	31406637	TAAAAAAAAAA/TAAAAAAAA	AAGTCCAAACAAAACCTAGGACA	CTTTATACGGCTGGGTTCCA	59.1	488	INTRON	Ca_12421	--	ERF
Ca2_P1_31407170	Ca2	31407170	GAAAAAAA/GAAAAAAAA	TGGAACCCAGCCGTATAAAG	TCTTGCTCAAGGGTCATTCA	60.0	223	INTRON	Ca_12421	--	ERF
Ca2_P1_31412396	Ca2	31412396	TAAA/TAAAA	TGAAAATCGAGCGCTAACAA	GTGCACAAGAATGCCCTTTT	59.6	463	INTRON	Ca_12420	--	LBD
Ca2_P1_31412866	Ca2	31412866	ATTATTTTTTA/ATTA	GCTGCACAAGGTGTTGTTGT	CATGCCTGAATGTCCAGAGA	59.8	333	INTERGENIC	--	--	--
Ca2_P1_31412999	Ca2	31412999	GGAGAGAGAGAGAGAGAG/GGAGAGAGAGAGAG	GCTGCACAAGGTGTTGTTGT	CATGCCTGAATGTCCAGAGA	59.8	333	INTERGENIC	--	--	--
Ca2_P1_31415262	Ca2	31415262	GATAT/GATATATAT	GGAAGTTTGACTTTGACCCG	GCTATCAATACACGCGCTGA	59.6	482	INTERGENIC	--	--	--
Ca2_P1_31415795	Ca2	31415795	AG/A	TCAGCGCGTGTATTGATAGC	TCATCATGAACCTGCATCTG	60.0	741	INTERGENIC	--	--	--
Ca2_P1_31419525	Ca2	31419525	T/TGG	GGCTTTCCATTTTGGGTTTT	ATTCCAATGTGGAAGGACAA	60.2	258	INTERGENIC	--	--	--
Ca2_P1_31421303	Ca2	31421303	GGACGAGGACGATGACGAGGACGA/GGACGAGGACGA	TGTGGATTGCATTTCCCTTT	CCCTTACACCTCCACCTTCA	60.3	582	INTERGENIC	--	--	--
Ca2_P1_31426034	Ca2	31426034	GCTTTCTACTTTCTACTTTCTACTTTC/GCTTTCTACTTTCTACTTTC	CTTTTCCTTCCACCAACCAA	AAACGGGTAACCGCTTCTTT	59.9	384	INTERGENIC	--	--	--

**INDEL marker IDs**	**Chromosomes/ unanchored scaffolds**	**Physical positions (bp)**	**InDels (*Kabuli* reference genome-CDC Frontier/PI)**	**Forward primers (5'-3')**	**Reverse primers (5'-3')**	**Anneling temperature (0C)**	**Expected amplified product size (bp)**	**Structural annotation**		**Functional annotation**	
								**Sequence components of *kabuli* genome**	***Kabuli* gene accession IDs**	**NCBI-KOG**	**TFs**
Ca2_P1_31430059	Ca2	31430059	TTTTATTTATTTATTTATTT/TTTTATTTATTT	ACTGAACCGAACCACCTTTG	AAAAAGACGGTAAGGCGACA	60.0	542	DRR	Ca_12419	--	--
Ca2_P1_31439958	Ca2	31439958	GAAAAAAA/GAAAAAAAA	CAAATGTCAAGGTGAAAATCCA	TGCTCCTTATGCCAACTCCT	59.8	492	INTERGENIC	--	--	--
Ca2_P1_3144381	Ca2	3144381	TAGGTAGTTAGTTATTGAGGTAGTTAGTT/TAGGTAGTTAGTT	TTATGGTGGTGCCTGCAATA	GACTTTGATCATGTCAAGGGAA	60.0	590	INTRON	Ca_10533	--	AP2
Ca2_P1_31450111	Ca2	31450111	TCC/TC	TTCCCAATCGATCCTACGAG	ATTGTGGAGAATCTGCAGGG	60.0	313	INTERGENIC	--	--	--
Ca2_P1_31450760	Ca2	31450760	AC/ACC	TACAAATTGGATGCGGTTGA	GTTTTGACTAGCACTCCCGC	59.9	221	INTERGENIC	--	--	--
Ca2_P1_3145519	Ca2	3145519	AGAAG/A	TCGAACTTCGCTTTGTTGTG	TCTTGAATTCCCCGTAATCG	60.0	385	CDS (FRAME SHIFT)	Ca_10533	--	AP2
Ca2_P1_31456735	Ca2	31456735	ATTTTT/ATTTTTT	AGCGCGCAATATGTCTACCT	CCACTCTAGCTCGGTCCTTG	59.9	300	DRR	Ca_12418	--	Trihelix
Ca2_P1_31459870	Ca2	31459870	ATTTT/ATTT	TGTTGGGGGTTTGAGGATAA	CCTATGCCTACGACATGCCT	60.2	519	INTERGENIC	--	--	--
Ca2_P1_3146989	Ca2	3146989	T/TG	CTGTTTAGAAATTCCGGCCA	AATGTATTCATGTTCGCGGC	60.1	543	DRR	Ca_10532	R	C3H
Ca2_P1_31474139	Ca2	31474139	ATTTTTTTTT/ATTTTTTTT	TGTGACAACTCCTAGCGGTG	CGTATGGTGGTCCCCTTCTA	59.9	715	INTERGENIC	--	--	--
Ca2_P1_31534378	Ca2	31534378	CG/CGG	AGGAAAGCAACGTGAACAGC	CCAAGACAGGCAAAGAAAGC	60.4	519	INTERGENIC	--	--	--
Ca2_P1_31534854	Ca2	31534854	TT/TTCT	GCTTTCTTTGCCTGTCTTGG	ACTTTGTCCACTGCCAAACC	60.0	331	INTERGENIC	--	--	--

INDEL marker IDs	Chromosomes/ unanchored scaffolds	Physical positions (bp)	InDels (*Kabuli* reference genome-CDC Frontier/PI)	Forward primers (5'-3')	Reverse primers (5'-3')	Anneling temperature (0C)	Expected amplified product size (bp)	Structural annotation		Functional annotation	
								Sequence components of *kabuli* genome	*Kabuli* gene accession IDs	NCBI-KOG	TFs
Ca2_P1_31540387	Ca2	31540387	AATATATATATATATATATATATATATATATATATAT/AATATATATATATATATATATATATATATAT	CATGTCCATAGTCCCCCAAA	ATCGTGCGATTGGATCTTTC	60.6	403	INTERGENIC	--	--	--
Ca2_P1_31540844	Ca2	31540844	TAAAATAATTCAAA/TAAAATAATTCAAAAAATAATTCAAA	ACGATCCAGCAATCTTTCGT	CAATTCCCTGATTAATTTGTTTGA	59.7	582	INTERGENIC	--	--	--
Ca2_P1_3154111	Ca2	3154111	A/AT	TTCGTCCTGTCATCCAAAAT	TTTGACGGTGAAAAAGTGATATG	58.0	975	INTERGENIC	--	--	--
Ca2_P1_3154882	Ca2	3154882	TAACA/TAACAACTGCTTTAAAAACA	TGGACTCAACATTCCAACCA	CCGATGCCTGTTTTAAGGAA	59.9	496	INTERGENIC	--	--	--
Ca2_P1_31550288	Ca2	31550288	CTTTTT/CTTTTTT	ACAAATATCCCATCCCACGA	TGCACGTAAAATTGCCTAAAA	60.0	721	INTERGENIC	--	--	--
Ca2_P1_31551913	Ca2	31551913	TTGTG/TTG	TTGAAGTTGAAGTCGGACCC	CTTGGCACTCAATATCGCAC	60.1	521	INTERGENIC	--	--	--
Ca2_P1_31562756	Ca2	31562756	ACC/ACCC	CACTTAGGAATTCCCCACCA	AGCTTGAAACCCTTGACCCT	59.8	476	INTERGENIC	--	--	--
Ca2_P1_3156648	Ca2	3156648	GTCT/GTCTCT	GGTCAGAACAAAGGGTTGGA	GTTTGTTCCCTTCTAGGCCC	59.9	488	INTERGENIC	--	--	--
Ca2_P1_31577288	Ca2	31577288	CTAATAATAATAATAATAATAATAATAATAATAATAATAATAATAATAATA/CTAATAATAATAATAATAATAATAATAATAATAATAATAATA	TTGCATTTGCCTCACTTGAA	TGGTGAGAAACGTTGGATGA	60.4	805	INTERGENIC	--	--	--
Ca2_P1_31581118	Ca2	31581118	GTTTTTTTTTTTTTT/GTTTTTTTTTT	GAATTCATACCGAGTCCAACAA	CCATTATGCACATGCAACCT	59.0	604	DRR	Ca_17856	E	--
Ca2_P1_31581278	Ca2	31581278	ATTTTT/ATTTTTT	GAATTCATACCGAGTCCAACAA	CCATTATGCACATGCAACCT	59.0	604	DRR	Ca_17856	E	--
Ca2_P1_3158875	Ca2	3158875	GTATTATTATTATTATTAT/GTATTATTATTATTATTATTAT	CGCAAAAGTTGCATGAAGAA	TCTAAGAAATTTGCCCCAATG	60.0	295	INTERGENIC	--	--	--

INDEL marker IDs	Chromosomes/ unanchored scaffolds	Physical positions (bp)	InDels (*Kabuli* reference genome-CDC Frontier/PI)	Forward primers (5'-3')	Reverse primers (5'-3')	Anneling temperature (0C)	Expected amplified product size (bp)	Structural annotation		Functional annotation	
								Sequence components of *kabuli* genome	*Kabuli* gene accession IDs	NCBI-KOG	TFs
Ca2_P1_31591314	Ca2	31591314	CTATATATATATATAT/CTATATATATATATATATATATAT	AATTTTTAGGGATGTGGCCC	TTGGTCTGACTTGGAAGCCT	60.0	825	DRR	Ca_17855	U	bHLH
Ca2_P1_3159298	Ca2	3159298	ATTTTTTTT/ATTTTTTTTTT	CGCAAAAGTTGCATGAAGAA	TGCAGGACGGTCTAACAATG	60.0	600	INTERGENIC	--	--	--
Ca2_P1_31595446	Ca2	31595446	ATTTTTT/ATTTTTTT	GCCGAATGAACTACGTTTCTC	CCGTTTGATGCATATTTGATG	58.9	677	INTERGENIC	--	--	--
Ca2_P1_31595806	Ca2	31595806	TAGATACAATAAGATA/TAGATA	GAGATGTGATGGTTGTATGTGGA	CCGTTTGATGCATATTTGATG	59.7	252	INTERGENIC	--	--	--
Ca2_P1_31596346	Ca2	31596346	ATTTTTT/ATTTTT	GCATCAAATATGCATCAAACG	TGACGCTCCAACAGTCTTTC	59.0	567	INTERGENIC	--	--	--
Ca2_P1_31601656	Ca2	31601656	ATT/AT	TTGCAATGGTTGCATGATTT	CTTTGTTCAACTGCTTGCCA	59.9	553	INTRON	Ca_17854	--	SAP
Ca2_P1_3162145	Ca2	3162145	CAAAAAA/CAAAAA	CTCTTGCTTCTCTCTCCCCC	GGCCTTCTGTGAGAAGCATT	60.5	788	INTRON	Ca_10531	S	--
Ca2_P1_31622880	Ca2	31622880	CAAT/C	TCCTTCTAAAAGGTTGGTGTTGA	CTATTGTGTTGGGATTGGGG	60.0	677	DRR	Ca_17853	--	--
Ca2_P1_31624259	Ca2	31624259	AATATAT/AATATATATAT	AAAGGGCACCACTTTTGTTG	TTGAGTTAACTGAGGAACTATAACTGC	60.0	823	INTERGENIC	--	--	--
Ca2_P1_31624499	Ca2	31624499	ATCTTT/A	AATTAAAAAGTACAATCACATGTCAAA	TGTAATAGTTTCACCGAACAACA	57.5	589	INTERGENIC	--	--	--
Ca2_P1_31634150	Ca2	31634150	GAAAAAAAA/GAAAAAAAAA	GGGTGAAGGTGAAGAAGTGC	TTGGGCTTGATAAAAGTGGG	59.7	524	DRR	Ca_17852	--	--
Ca2_P1_31636405	Ca2	31636405	GTGATGA/GTGA	TCATGAGATGGTCGAGTTGC	TCCTCGTAATCAGGCAAACA	59.8	203	INTERGENIC	--	--	--

INDEL marker IDs	Chromosomes/ unanchored scaffolds	Physical positions (bp)	InDels (*Kabuli* reference genome-CDC Frontier/PI)	Forward primers (5'-3')	Reverse primers (5'-3')	Anneling temperature (0C)	Expected amplified product size (bp)	Structural annotation		Functional annotation	
								Sequence components of *kabuli* genome	*Kabuli* gene accession IDs	NCBI-KOG	TFs
Ca2_P1_3165078	Ca2	3165078	A/AT	CCACATTGTGTGTGTG TTGG	TGCAACTATGCTTTCA CACCA	59.3	728	DRR	Ca_10531	S	--
Ca2_P1_31657254	Ca2	31657254	CTTTTTTTT/CTTTTTTTTT	GAACGTGGAAAACTTC CATCA	GTTGGGAGGGTTCAC TTCTG	60.0	570	INTERGENIC	--	--	--
Ca2_P1_3166543	Ca2	3166543	T/TA	CATGTTTTCAGCCCAA CAAA	TTCCGCACATCAAACA CAAT	59.6	849	INTERGENIC	--	--	--
Ca2_P1_31670124	Ca2	31670124	ATAA/ATAATTAA	CGACATAATTCAGATG CACGA	GAGACTACAATGGTGG CGGT	59.7	824	DRR	Ca_17848	--	--
Ca2_P1_31670858	Ca2	31670858	CGTCGCTGTCGCTGTCGCTGTCGCT G/CGTCGCTGTCGCTG	ACCGCCACCATTGTAG TCTC	CCGAGCCATCAAAAGA TGAT	60.0	434	CDS (large-effect mutations)	Ca_17848	--	--
Ca2_P1_31678680	Ca2	31678680	TC/T	GGAGCGCTTTAAACAC AAGC	TGGAAACCAAGGTCCA AGTC	60.0	517	INTERGENIC	--	--	--
Ca2_P1_31680004	Ca2	31680004	GATATATATATATATATATATATATA TATAT/GATATATATATATATATATATA TATATAT	ATCCACGTGGTCTTCA AAGC	GCGTGGGAAAATGAG ACTTT	60.1	601	INTERGENIC	--	--	--
Ca2_P1_31682953	Ca2	31682953	ATTTTTTT/ATTTTTT	ACATGGCATGACCCAA AAAT	TCTAGCAGGTTTGTTG TGCG	60.1	333	INTERGENIC	--	--	--
Ca2_P1_31685454	Ca2	31685454	TAAAAAAA/TAAAAAA	AGCTTTCGATCCAACA AATGA	TCTCTTTTCGAGCCAG CATT	59.7	422	INTERGENIC	--	--	--
Ca2_P1_31688320	Ca2	31688320	TC/T	CCAACCTTTCATTGTTA CCGA	GATGCAAAGAGGCCAA CCTA	59.8	508	INTERGENIC	--	--	--
Ca2_P1_31688716	Ca2	31688716	TAAAAAAAA/TAAAAAAAAAA	TCCAACCTTTCATTGTT ACCG	CCGAGCCAAAATCCAA AATA	59.8	843	INTERGENIC	--	--	--
Ca2_P1_31692825	Ca2	31692825	AACTTCATACTTCATA/AACTTCATA	AAAACGGGGTGTCACA CATT	TTGTTTGCGAATGTTT TTGC	60.1	631	INTERGENIC	--	--	--

INDEL marker IDs	Chromosomes/ unanchored scaffolds	Physical positions (bp)	InDels (*Kabuli* reference genome-CDC Frontier/PI)	Forward primers (5'-3')	Reverse primers (5'-3')	Anneling temperature (0C)	Expected amplified product size (bp)	Structural annotation		Functional annotation	
								Sequence components of *kabuli* genome	*Kabuli* gene accession IDs	NCBI-KOG	TFs
Ca2_P1_31693043	Ca2	31693043	TAA/TA	CCAATTAACGGAATTGGTCC	TCGATGTTGTTTGCGAATGT	59.1	539	INTERGENIC	--	--	--
Ca2_P1_31693913	Ca2	31693913	CTT/CTTT	GGGAGTTGGCTAGTACACCTTG	AATGGGCTGGTTCATTTCAA	60.1	312	INTERGENIC	--	--	--
Ca2_P1_31694754	Ca2	31694754	A/AAT	GCTTTTCATAAGAAGCGTTGTAA	AATTGAGCTTGTTGATGTGGA	57.9	305	INTERGENIC	--	--	--
Ca2_P1_3169610	Ca2	3169610	TAAA/TAA	TTGTCGGTTTAAACTTGTGTGC	TGACATCGATATGTATGGACTCG	60.1	536	INTERGENIC	--	--	--
Ca2_P1_3169671	Ca2	3169671	ATTTTTTT/ATTTTTT	TTGTCGGTTTAAACTTGTGTGC	TTTTCCAAGGGATTTCAACTCT	60.1	892	INTERGENIC	--	--	--
Ca2_P1_31697056	Ca2	31697056	TTTT/TTTTTATTT	GTGTGCAGGTAAGGCTGACA	ATTTGGCATTGGGTTGGATA	59.9	759	INTERGENIC	--	--	--
Ca2_P1_3169869	Ca2	3169869	ATTTTTTTTT/ATTTTTTT	TCGATGTCATTGTAGTCACCAA	TCAAAGCCAGTTTCAAATGC	59.1	553	INTERGENIC	--	--	--
Ca2_P1_3170030	Ca2	3170030	ATTTTTTTTTT/ATTTTTTT	TCGATGTCATTGTAGTCACCAA	TCAAAGCCAGTTTCAAATGC	59.1	553	INTERGENIC	--	--	--
Ca2_P1_3170210	Ca2	3170210	TTAT/TTATAT	GAGTTGAAATCCCTTGGAAAA	AGGCACAATAGCTCATGCAGT	58.1	555	INTERGENIC	--	--	--
Ca2_P1_3171321	Ca2	3171321	TTCT/TT	ATGGCGAAGAAGGTATGCAC	CAGTTGAATCGGTCAGTCCA	60.1	381	INTERGENIC	--	--	--
Ca2_P1_31713656	Ca2	31713656	TAAG/TAAGAAG	TCGGTTTCTCCTTCACCATC	TTGTCAAACACGCAATCCAT	60.1	595	INTERGENIC	--	--	--
Ca2_P1_31714242	Ca2	31714242	ATTTTTTTTTTTTT/ATTTTTTTTTTT	CAATGGATTGCGTGTTTGAC	TTCGGTGAACCCATCTTACC	60.0	427	INTERGENIC	--	--	--

INDEL marker IDs	Chromosomes/ unanchored scaffolds	Physical positions (bp)	InDels (*Kabuli* reference genome-CDC Frontier/PI)	Forward primers (5'-3')	Reverse primers (5'-3')	Anneling temperature (0C)	Expected amplified product size (bp)	Structural annotation		Functional annotation	
								Sequence components of *kabuli* genome	*Kabuli* gene accession IDs	NCBI-KOG	TFs
Ca2_P1_31716115	Ca2	31716115	CAT/CATTATAT	AAGGAATTGCAGGCAGCTTA	GGAACGTAGAGGGAAGAGGG	60.0	451	INTERGENIC	--	--	--
Ca2_P1_31718772	Ca2	31718772	TTTATTAT/TTTATTATTAT	ATCACCGGTTCTCACCAAAG	GGGTGCCATCATAAGCAAGT	60.0	236	INTERGENIC	--	--	--
Ca2_P1_31725700	Ca2	31725700	TGG/TGGG	TTTGAATTTATTTATTGGCTTTGTT	GGTAATTACGGTCACGGTGG	57.5	883	INTERGENIC	--	--	--
Ca2_P1_31729891	Ca2	31729891	TAAAAA/TAAAAAA	ATGTTCCAATTGCCTTGCTT	TTTCATGCCTCATTTGTCCA	59.6	580	INTERGENIC	--	--	--
Ca2_P1_31732328	Ca2	31732328	TCCCC/TCCC	AAATTCGCAATTCGGTCAAG	TGACCCATGTGTGAACCCTA	60.1	415	INTERGENIC	--	--	--
Ca2_P1_3174472	Ca2	3174472	ATTTT/ATTT	TTTTGACTGAGGGTATTTAAAGGT	AAGCGAATGTGTGACGACAG	57.5	623	INTERGENIC	--	--	--
Ca2_P1_31744890	Ca2	31744890	ATTTTTTT/ATTTTT	TGTGTTCCTCAACCCTGTCA	ATTTGGCATTGGGTTGGTTA	60.1	580	DRR	Ca_17844	--	--
Ca2_P1_31745963	Ca2	31745963	AATATATATATATATATATAT/AATATATATATATATATAT	TCCATAGAGCCATGGAAAGG	ATAATGTCAGCATGTATGCAAAA	60.0	448	INTERGENIC	--	--	--
Ca2_P1_31746177	Ca2	31746177	AATAT/AATATAT	CGAGTTGTAAAATTTGGCTTCC	GGGGATGTGTATTAAAGGGGA	60.0	914	INTERGENIC	--	--	--
Ca2_P1_31746486	Ca2	31746486	TTAATAATAATAATAATAATAATAATAATAATAATAATAATAATAATAA/TTAATAATAATAATAATAATAATAATAATAATAATAATAATAATAATAATAA	CGACGTTTAATTAATTCATCCCA	CTCACCCAAGTCTTTGCTCC	60.1	714	INTERGENIC	--	--	--
Ca2_P1_31753054	Ca2	31753054	GA/GAAAA	TGAACGCATGTGGACACTTT	CACATTTGGAGCAACGACAC	60.2	328	INTERGENIC	--	--	--
Ca2_P1_31757753	Ca2	31757753	TCCC/TCC	TCCGTTATCGTGGTTTCCTC	TTCGCAATTGGCCATAGAAT	59.9	424	INTERGENIC	--	--	--

INDEL marker IDs	Chromosomes/ unanchored scaffolds	Physical positions (bp)	InDels (*Kabuli* reference genome-CDC Frontier/PI)	Forward primers (5'-3')	Reverse primers (5'-3')	Anneling temperature (0C)	Expected amplified product size (bp)	Structural annotation		Functional annotation	
								Sequence components of *kabuli* genome	*Kabuli* gene accession IDs	NCBI-KOG	TFs
Ca2_P1_31758644	Ca2	31758644	TAAAAAA/TAAAAAAA	CGACGTTTAATTAATTCATCCCA	CGTGTATTTCCAGCGTCTGA	60.1	463	INTERGENIC	--	--	--
Ca2_P1_31781318	Ca2	31781318	TGGGGG/TGGGG	TGGTGTTGGTTAGCATCCTTC	ATGACGGCAAACACTCAACA	60.0	134	INTERGENIC	--	--	--
Ca2_P1_31788666	Ca2	31788666	AT/A	ACATCGCATGGTTTAGAGGC	GCATGTCTTGTTGCCAAATG	60.1	606	INTERGENIC	--	--	--
Ca2_P1_31793722	Ca2	31793722	GATA/GATATATA	TCAGTTGAATTAAGGCGTAAGGA	TCCATGCACACGTACACACA	60.1	702	INTRON	Ca_17840	--	--
Ca2_P1_31794099	Ca2	31794099	TAA/TAAA	TGTGTACGTGTGCATGGAAG	ATCCTTGGCCTTTTGGCTAT	59.2	247	INTRON	Ca_17840	--	--
Ca2_P1_31794610	Ca2	31794610	GAAAAA/GAAAAAAA	ATAGCCAAAAGGCCAAGGAT	CTGGTCTGTCACTTTCAGCG	59.9	804	INTERGENIC	--	--	--
Ca2_P1_31795559	Ca2	31795559	CATTAATT/CATT	CACCGTCAAACAAAAACCAA	CAGCCGTGTAACTCGTCAAA	59.5	885	INTERGENIC	--	--	--
Ca2_P1_31799253	Ca2	31799253	TAAAAAA/TAAAAA	AAATGTCAACGTGGCGTACA	GCACGAGGATTGCTATGTGA	60.0	832	INTERGENIC	--	--	--
Ca2_P1_31814032	Ca2	31814032	TAAAAAAAA/TAAAAAAA	TGGTCCTGGCTCAAGTCTTT	CTCCTTGTTGTTGCCTCCAT	59.8	573	INTRON	Ca_17839	--	--
Ca2_P1_31814308	Ca2	31814308	CAAAAA/CAAAA	ATGGAGGCAACAACAAGGAG	TCTTAGCTGCAACAATGAGGA	60.1	612	INTRON	Ca_17839	--	--
Ca2_P1_31814386	Ca2	31814386	CAAAAAAA/CAAAAAA	GGAGGCAACAACAAGGAGAG	CCATCTTTCAAGCCAACTCC	59.8	685	INTRON	Ca_17839	--	--
Ca2_P1_31814597	Ca2	31814597	CTTTTTTT/CTTTTTTTTT	GGAGGCAACAACAAGGAGAG	GACGAGTTGCCATCCATCTT	59.8	698	INTRON	Ca_17839	--	--

INDEL marker IDs	Chromosomes/ unanchored scaffolds	Physical positions (bp)	InDels (*Kabuli* reference genome-CDC Frontier/PI)	Forward primers (5'-3')	Reverse primers (5'-3')	Anneling temperature (0C)	Expected amplified product size (bp)	Structural annotation		Functional annotation	
								Sequence components of *kabuli* genome	*Kabuli* gene accession IDs	NCBI-KOG	TFs
Ca2_P1_31814639	Ca2	31814639	TTATA/TTATATA	GGAGGCAACAACAAGGAGAG	GACGAGTTGCCATCCATCTT	59.8	698	INTRON	Ca_17839	--	--
Ca2_P1_31814698	Ca2	31814698	A/ATGATG	GGAGGCAACAACAAGGAGAG	GACGAGTTGCCATCCATCTT	59.8	698	INTRON	Ca_17839	--	--
Ca2_P1_31816754	Ca2	31816754	T/TTA	TGAATCCCAAAGCTCCTTCA	TGTTTAGCGAATAATTGGGATG	60.7	574	DRR	Ca_17839	--	--
Ca2_P1_31818875	Ca2	31818875	A/AG	TTTGTGATTGGTTTGCATTG	TTCCATGCCTTCAACCTTTC	58.0	511	INTERGENIC	--	--	--
Ca2_P1_31824019	Ca2	31824019	A/ACT	TGTCGACGTGTCAGTGTCAA	GGTTTGCGTCTTCCACTCAT	59.9	599	CDS (FRAME SHIFT)	Ca_17837	J	--
Ca2_P1_31824240	Ca2	31824240	TATAA/TATAATAA	ATGAGTGGAAGACGCAAACC	GTGATGACTGCTCTGTGGGA	60.1	466	INTRON	Ca_17837	J	--
Ca2_P1_31828278	Ca2	31828278	ATTGTT/ATTGTTGTT	AAAAATCGCCATTGTTTTGG	AAAAATAGGGAATCCGGTGC	59.8	510	INTRON	Ca_17836	--	MYB_related
Ca2_P1_31828392	Ca2	31828392	CTTTTTTT/CTTTTTT	AAAAATCGCCATTGTTTTGG	AAAAATAGGGAATCCGGTGC	59.8	510	INTRON	Ca_17836	--	MYB_related
Ca2_P1_31828649	Ca2	31828649	ATT/ATTT	CACAATGGATGACCCTTTCC	ACTTGGCCCAATTTCTTGTG	60.2	455	INTRON	Ca_17836	--	MYB_related
Ca2_P1_31830758	Ca2	31830758	CTTTTT/CTTTT	CACATGCATGCACTTCCTCT	TTGCTGACAGTTTGGACTGC	59.9	544	INTRON	Ca_17836	--	MYB_related
Ca2_P1_31833407	Ca2	31833407	GTTT/GTT	TATCACGTGCCAAACCAAGA	TTGAGAAGCTCAGAGCGTGA	60.1	626	INTERGENIC	--	--	--
Ca2_P1_31835632	Ca2	31835632	GTTTTTT/GTTTTTTT	GCCATTAGGCGTTTCCATTA	TATCGCATATGGCATTGTGG	59.9	542	INTRON	Ca_17835	V	bHLH

INDEL marker IDs	Chromosomes/ unanchored scaffolds	Physical positions (bp)	InDels (*Kabuli* reference genome-CDC Frontier/PI)	Forward primers (5'-3')	Reverse primers (5'-3')	Anneling temperature (0C)	Expected amplified product size (bp)	Structural annotation		Functional annotation	
								Sequence components of *kabuli* genome	*Kabuli* gene accession IDs	NCBI-KOG	TFs
Ca2_P1_31838256	Ca2	31838256	GTGCGGTG/GTGCGGTGGAAATATGCGGTG	ACTTGACGATGGTTTTTGGC	CTAACCGCGAAACCAAACAT	60.0	393	DRR	Ca_17834	--	--
Ca2_P1_31839662	Ca2	31839662	GCC/GC	TGATCGAATATTTTTAACCGCA	TGCTTTTGTTTTCCAAGTTGC	59.5	840	DRR	Ca_17835	V	bHLH
Ca2_P1_3184069	Ca2	3184069	GTATA/GTA	TCTGGAGGTTAGAGACAGCCA	ACTTGCCAAAGATGCTCCAG	60.0	432	INTRON	Ca_10528	T	HD-ZIP
Ca2_P1_31841304	Ca2	31841304	GATATATATATATATATATAT/GATATATATATATATATATATAT	GGAGAACTCAAGTTCATACCGTG	AGGTCAGTAGTTTGGCGTGT	60.0	534	INTERGENIC	--	--	--
Ca2_P1_31842513	Ca2	31842513	TAAAAAAAAA/TAAAAAAAAAA	AATCGCAAAGGCCAAATAGA	TTTTTCGGGGTTTGAACAAC	59.7	828	INTERGENIC	--	--	--
Ca2_P1_3184404	Ca2	3184404	AAAA/AAAATAAA	TGCATATCCTTGTGTTCCCA	CTTCCATGGGCTGTGAGAAT	59.9	486	INTRON	Ca_10528	T	HD-ZIP
Ca2_P1_31844278	Ca2	31844278	T/TA	TAGGTGGGGTGCATTTTTCT	TGATCATCCATGTATTATTGTGTGG	59.4	714	INTERGENIC	--	--	--
Ca2_P1_3184768	Ca2	3184768	CAAAAAAA/CAAAAAAAA	CTTTTGCAGCACCAAGCATA	TCTAGGTGCCCTCCAACATC	60.0	314	INTRON	Ca_10528	T	HD-ZIP
Ca2_P1_31849610	Ca2	31849610	TAA/TAAA	GTGGCATCTCCGAGTCGTAT	GGTTGGATGCGACCAACTAT	60.1	179	DRR	Ca_17833	--	--
Ca2_P1_31850651	Ca2	31850651	TATGCATG/TATG	TCCATTTCTTTTGAGAAGACGA	GTAGTTTGGGGGCCAATTTT	58.9	489	INTERGENIC	--	--	--
Ca2_P1_3185239	Ca2	3185239	AAGA/AA	GATGTTGGAGGGCACCTAGA	GTGAAGGAGGGTTTGGAACA	60.1	661	INTRON	Ca_10528	T	HD-ZIP
Ca2_P1_31853745	Ca2	31853745	ATTT/ATTATATTTT	CAACCGTAGCCCACCTAAAA	AATGCGCAAAGAAAGTTTGG	60.0	610	INTERGENIC	--	--	--

INDEL marker IDs	Chromosomes/ unanchored scaffolds	Physical positions (bp)	InDels (*Kabuli* reference genome-CDC Frontier/PI)	Forward primers (5'-3')	Reverse primers (5'-3')	Anneling temperature (0C)	Expected amplified product size (bp)	Structural annotation		Functional annotation	
								Sequence components of *kabuli* genome	*Kabuli* gene accession IDs	NCBI-KOG	TFs
Ca2_P1_31853792	Ca2	31853792	AA/AAGA	CAACCGTAGCCCACCTAAAA	AATGCGCAAAGAAAGTTTGG	60.0	610	INTERGENIC	--	--	--
Ca2_P1_31856792	Ca2	31856792	CTT/CTTT	CCCACAATTGAAAGCCTTGT	GTCTAGGCCAGCCCTACCTC	60.0	576	INTERGENIC	--	--	--
Ca2_P1_31860805	Ca2	31860805	AC/ACC	TCTCATTCACAAATTGGCCTC	TGTGCGACATACGACATACG	60.1	241	INTERGENIC	--	--	--
Ca2_P1_31861522	Ca2	31861522	ATTTT/ATTT	TTGCAAAATGCTTGAGAGAAAA	ATGGCCTATGCCAATGAAAC	60.0	459	INTERGENIC	--	--	--
Ca2_P1_3186542	Ca2	3186542	GATA/GATAATA	TTTGGGGTGTTTCAAAGTCA	TTCACTTGACACCTCTCTCCC	59.0	397	INTRON	Ca_10528	T	HD-ZIP
Ca2_P1_3187450	Ca2	3187450	AC/A	AAAGGGACCAGCCATTTCTT	TCCATGTGGGGCTAATTCAT	59.9	276	INTERGENIC	--	--	--
Ca2_P1_3187597	Ca2	3187597	TAAA/TAAAA	AAAGGGACCAGCCATTTCTT	TCCATGTGGGGCTAATTCAT	59.9	276	INTERGENIC	--	--	--
Ca2_P1_3187729	Ca2	3187729	CTTTTTTTT/CTTTTTTTTTTTTTTT	AAAGGGACCAGCCATTTCTT	GCTCAGAAAATGCATCCCTC	59.9	594	INTERGENIC	--	--	--
Ca2_P1_31882952	Ca2	31882952	CTTTATTTATTTAT/CTTTATTTATTTATTTAT	TTTCTGGTGGGATCAGCTCT	GACATCCTGCGCCAGTTATT	59.8	591	INTRON	Ca_17830	D	LBD
Ca2_P1_31883010	Ca2	31883010	AAGAG/AAG	TTTCTGGTGGGATCAGCTCT	GACATCCTGCGCCAGTTATT	59.8	591	INTRON	Ca_17830	D	LBD
Ca2_P1_3188322	Ca2	3188322	TG/T	GAGGGATGCATTTTCTGAGC	TTTTGGCCATTTTACTGCTG	59.8	727	INTERGENIC	--	--	--
Ca2_P1_31892832	Ca2	31892832	TC/T	CATTTGGTCGGTCAAAAGAAA	TTCTACCCAAGAAAACATGCG	60.0	473	INTERGENIC	--	--	--

INDEL marker IDs	Chromosomes/ unanchored scaffolds	Physical positions (bp)	InDels (*Kabuli* reference genome-CDC Frontier/PI)	Forward primers (5'-3')	Reverse primers (5'-3')	Anneling temperature (0C)	Expected amplified product size (bp)	Structural annotation		Functional annotation	
								Sequence components of *kabuli* genome	*Kabuli* gene accession IDs	NCBI-KOG	TFs
Ca2_P1_31896971	Ca2	31896971	TAGAAGAAGAA/TAGAAGAA	TGCAACCAATGTTCCCTACA	CTTAGAAGAGTGCATCCGCC	60.0	326	INTRON	Ca_17828	I	C3H
Ca2_P1_31898002	Ca2	31898002	TAAAAA/TAAAAAA	TAAATTGGCCGACCAAAAAG	CCAAGGGGTAGACATGGAAA	59.9	838	INTRON	Ca_17828	I	C3H
Ca2_P1_31905190	Ca2	31905190	ATTTT/ATTTTT	AATAAGGTGGTTTGGGGGAG	GGTATTCGTTGCGTCATGTG	60.0	450	INTRON	Ca_17827	--	C3H
Ca2_P1_31905735	Ca2	31905735	CTACTATACT/CTACTATACTATACTATACT	TTCCAACGGTGAAAACAACA	CGATATAGGGTTGGTTTGCG	60.0	207	DRR	Ca_17826	O	--
Ca2_P1_31911927	Ca2	31911927	TGGGGGG/TGGGGG	GGCTCTTGAGTTCGCTTGTC	GGCTCAGTCTCTGGTTGGAG	60.1	627	INTRON	Ca_17826	O	--
Ca2_P1_31912393	Ca2	31912393	TTATCTA/TTATCTATCTA	TGAGAAATCTCCATCCCCAG	CGCATCCTTGACAACTCTGA	60.0	650	INTRON	Ca_17826	O	--
Ca2_P1_3191565	Ca2	3191565	T/TATTTAAATA	TGTGCATCGATGAAATGAGG	TAGGGGCTCTTGCCTCTACA	60.6	585	INTERGENIC	--	--	--
Ca2_P1_31920016	Ca2	31920016	AATTATAATCAATTAT/AATTATAATCAATTATTATAATCAATTAT	TTCAGAAGAAGGAAACAGCACA	GATCCACAAAATTGGTTTTTCA	60.0	877	INTERGENIC	--	--	--
Ca2_P1_31921391	Ca2	31921391	TAAAAAA/TAAAAAAA	CATCGTTCAAACTTTAATTGCG	TCTGTATCGCATGGTTGCTT	59.7	827	INTERGENIC	--	--	--
Ca2_P1_31921422	Ca2	31921422	GTTTTTT/GTTTTTTT	GGAAAGTCAAACTCTTAAATGGGA	TCTGTATCGCATGGTTGCTT	59.9	533	INTERGENIC	--	--	--
Ca2_P1_31921460	Ca2	31921460	TA/TAA	GGAAAGTCAAACTCTTAAATGGGA	TCTGTATCGCATGGTTGCTT	59.9	533	INTERGENIC	--	--	--
Ca2_P1_31924339	Ca2	31924339	TTTATT/TTT	TGGATTTTGTTGTTTGGTGC	ACATCCAAACCGCAAGGTAA	59.4	438	INTERGENIC	--	--	--

INDEL marker IDs	Chromosomes/ unanchored scaffolds	Physical positions (bp)	InDels (*Kabuli* reference genome-CDC Frontier/PI)	Forward primers (5'-3')	Reverse primers (5'-3')	Anneling temperature (0C)	Expected amplified product size (bp)	Structural annotation		Functional annotation	
								Sequence components of *kabuli* genome	*Kabuli* gene accession IDs	NCBI-KOG	TFs
Ca2_P1_31925279	Ca2	31925279	GTTTTTTTTT/GTTTTTTTT	GACCAGCCTCCCTAACCTTC	TGTTGCCGATAGACCATGAA	60.1	538	INTERGENIC	--	--	--
Ca2_P1_31925325	Ca2	31925325	CT/CTTT	GACCAGCCTCCCTAACCTTC	TGTTGCCGATAGACCATGAA	60.1	538	INTERGENIC	--	--	--
Ca2_P1_31925644	Ca2	31925644	TG/T	TTCATGGTCTATCGGCAACA	ACTTGGATTGCATCTCCCAC	60.1	503	INTERGENIC	--	--	--
Ca2_P1_31929627	Ca2	31929627	AATA/AA	ACACAGCAACACATCATCACA	CAGGTCACTTCCCCAAAGAG	58.6	580	INTERGENIC	--	--	--
Ca2_P1_31929726	Ca2	31929726	ACC/ACCC	ACGGTTTGCAGCATTGTTTT	AAAAATCCTTTGGGAGGTCG	60.5	469	INTERGENIC	--	--	--
Ca2_P1_31929759	Ca2	31929759	CAAAAAAAAAA/CAAAAAAAAAAA	ACGGTTTGCAGCATTGTTTT	AAAAATCCTTTGGGAGGTCG	60.5	469	INTERGENIC	--	--	--
Ca2_P1_31929843	Ca2	31929843	ACC/AC	ACGGTTTGCAGCATTGTTTT	AAAAATCCTTTGGGAGGTCG	60.5	469	INTERGENIC	--	--	--
Ca2_P1_31931439	Ca2	31931439	TGAGAGAGA/TGAGAGAGAGA	TCGTAACGCAAATTTTCCAA	TTTTTCACTTCTTTCATCTCTCG	59.2	611	INTERGENIC	--	--	--
Ca2_P1_31933069	Ca2	31933069	ATTTTTTTTTT/ATTTTTTTT	ACGGAGCCAAACAAGAAAAA	ACGAATTGGCATGGCTAAAG	59.7	706	INTERGENIC	--	--	--
Ca2_P1_31933554	Ca2	31933554	CAAAA/CAAAAA	CATGAATTGCACGACCACTC	CCACTGTGGACCAATTGTAAA	60.1	249	INTERGENIC	--	--	--
Ca2_P1_31934723	Ca2	31934723	TAAAA/TAA	GCAACCGCTTAAGGAATTTG	TGGTGAGTGTAAGCCACTTTTT	59.7	491	INTERGENIC	--	--	--
Ca2_P1_31934777	Ca2	31934777	T/TG	GCAACCGCTTAAGGAATTTG	TGGTGAGTGTAAGCCACTTTTT	59.7	491	INTERGENIC	--	--	--

INDEL marker IDs	Chromosomes/ unanchored scaffolds	Physical positions (bp)	InDels (*Kabuli* reference genome-CDC Frontier/PI)	Forward primers (5'-3')	Reverse primers (5'-3')	Anneling temperature (0C)	Expected amplified product size (bp)	Structural annotation		Functional annotation	
								Sequence components of *kabuli* genome	*Kabuli* gene accession IDs	NCBI-KOG	TFs
Ca2_P1_31939044	Ca2	31939044	ATTTTTTTT/ATTTTTTT	ATCACATTGCAAAGCAACGA	TGTATTCACGGTTTGGGTTTT	60.3	586	DRR	Ca_17823	R	C3H
Ca2_P1_31943137	Ca2	31943137	CAAAAAAAA/CAAAAAAAAA	GGCAGTTACTCCCGTAACCA	CATTGATGATGATGGCTTGC	60.0	802	INTRON	Ca_17823	R	C3H
Ca2_P1_31945989	Ca2	31945989	ATTTTTTTTT/ATTTTTTT	CACATGGAAGGCTAAAGGAA	CCACAAGTGTGGATGACAGG	57.8	838	INTERGENIC	--	--	--
Ca2_P1_31946131	Ca2	31946131	TA/TAA	TGAACGTCATGTCCGGATT	CCACAAGTGTGGATGACAGG	59.9	351	INTERGENIC	--	--	--
Ca2_P1_31950824	Ca2	31950824	C/CGAA	CCAATTAATAAGTTAGCGGGTGA	TGTGTGAGGAGGACAACAAAA	59.4	870	INTERGENIC	--	--	--
Ca2_P1_3195583	Ca2	3195583	CTTTTTTTTTTTTTTTT/CTTTTTTTTTTTT	CGGGACATGTAAGGATTGTTG	TTCTCCGATTTGGTCCGTTA	60.2	773	INTERGENIC	--	--	--
Ca2_P1_31977188	Ca2	31977188	TTAGTAG/TTAG	CTCCTTTTCCCTGTCTTCCC	GTCCCATGTTAAATGCTCCG	60.0	826	INTRON	Ca_17820	--	--
Ca2_P1_31981432	Ca2	31981432	ATTTT/ATTT	CCCTCCGGACTCAATAAACA	GTGTGCGGCTGGCTATGTAT	59.9	408	INTERGENIC	--	--	--
Ca2_P1_31981661	Ca2	31981661	TA/TAAA	CCCTCCGGACTCAATAAACA	GTGCAGATCGCTAGTGTCCA	59.9	663	INTERGENIC	--	--	--
Ca2_P1_31983903	Ca2	31983903	CTA/CTATA	GACACTTGTTGCGTATGGGA	AGCCTTGCAGTGATGTGTGA	59.6	858	INTERGENIC	--	--	--
Ca2_P1_3199849	Ca2	3199849	TT/TTATTATTAGT	GAGCATGTGTTGGTGAATGG	TCACCGGAGGTTAGATCTCG	60.0	309	INTERGENIC	--	--	--
Ca2_P1_3200120	Ca2	3200120	CATATATATATA/CATATATATATATA	GAGCATGTGTTGGTGAATGG	CCTCTGTTTTGGTTGATACTCCTT	60.0	925	DRR	Ca_10527	K	HD-ZIP

INDEL marker IDs	Chromosomes/ unanchored scaffolds	Physical positions (bp)	InDels (*Kabuli* reference genome-CDC Frontier/PI)	Forward primers (5'-3')	Reverse primers (5'-3')	Anneling temperature (0C)	Expected amplified product size (bp)	Structural annotation		Functional annotation	
								Sequence components of *kabuli* genome	*Kabuli* gene accession IDs	NCBI-KOG	TFs
Ca2_P1_3200285	Ca2	3200285	TCA/T	CGAGATCTAACCTCCGGTGA	TTGATGCATGCTGCTACAAAT	60.2	811	DRR	Ca_10527	K	HD-ZIP
Ca2_P1_3200442	Ca2	3200442	CAT/CATAT	GGAGTTGAATTAGCCTCTTTATCC	TTGATGCATGCTGCTACAAAT	58.8	503	DRR	Ca_10527	K	HD-ZIP
Ca2_P1_3200504	Ca2	3200504	AA/AAAAAGATA	CATTTCCCGATTTTAGATTATGC	AATTGAAGGAAACATTTGCCA	58.9	511	DRR	Ca_10527	K	HD-ZIP
Ca2_P1_3200565	Ca2	3200565	AT/ATT	AAGGAGTATCAACCAAAACAGAGG	AATTGAAGGAAACATTTGCCA	59.9	423	DRR	Ca_10527	K	HD-ZIP
Ca2_P1_3200632	Ca2	3200632	CAATT/CAATTAATT	AAGGAGTATCAACCAAAACAGAGG	AATTGAAGGAAACATTTGCCA	59.9	423	DRR	Ca_10527	K	HD-ZIP
Ca2_P1_3201024	Ca2	3201024	AATATA/AATATATATA	TTTGGCAAATGTTTCCTTCA	GAGTGGGGGTTGGAGGTAGT	59.1	405	DRR	Ca_10527	K	HD-ZIP
Ca2_P1_32013591	Ca2	32013591	TCCC/TCC	TCTCCCTCCAAACACCAAAC	ATGATTGAACGAAGGCCAAG	59.9	480	INTERGENIC	--	--	--
Ca2_P1_32013861	Ca2	32013861	CATGATGATGATGATGATGATG/CATGATGATGATGATG	TCCCCCTTTCCCTTTCTCTA	ATGATTGAACGAAGGCCAAG	60.0	419	CDS (large-effect mutations)	Ca_17819	V	GeBP
Ca2_P1_3203039	Ca2	3203039	AT/A	TGGATTTCTGACACTGCCAC	ATGCTCTTCCCTGTCTCCAA	59.7	348	INTRON	Ca_10527	K	HD-ZIP
Ca2_P1_32032083	Ca2	32032083	ATATTTTTATTTTT/ATATTTTTATTTTTATTTTT	TGACAACGCATACGTGGATT	AAATAACACGAAATTCTAGCCCA	60.0	312	INTERGENIC	--	--	--
Ca2_P1_3204807	Ca2	3204807	CAAAGATAAAGATAAAGATAAAGATA/CAAAGATAAAGATAAAGATA	TGGAAAGGAAGGGAAGGAGT	CACCATGAGAGTGAGAGCCA	60.0	281	INTERGENIC	--	--	--
Ca2_P1_3205162	Ca2	3205162	TTTT/TTTTCTTT	TGGCTCTCACTCTCATGGTG	TGCACATTTTGGTCGGTCTA	60.0	487	INTERGENIC	--	--	--

INDEL marker IDs	Chromosomes/ unanchored scaffolds	Physical positions (bp)	InDels (*Kabuli* reference genome-CDC Frontier/PI)	Forward primers (5'-3')	Reverse primers (5'-3')	Anneling temperature (0C)	Expected amplified product size (bp)	Structural annotation		Functional annotation	
								Sequence components of *kabuli* genome	*Kabuli* gene accession IDs	NCBI-KOG	TFs
Ca2_P1_3205505	Ca2	3205505	TTTAATTAAT/TTTAAT	TTTAGACCGACCAAAATGTGC	GGCGGTTATGTCTCCACAAT	60.0	364	INTERGENIC	--	--	--
Ca2_P1_3205849	Ca2	3205849	TC/T	ATTGTGGAGACATAACCGCC	ACATCGAGACGTACGGGAAA	59.8	284	INTERGENIC	--	--	--
Ca2_P1_32079086	Ca2	32079086	AATATATATATATATATATATATATAT/ AATATATATATATATATATATATATATATATATAT	CCGGCTCTCATCTCAAAAAG	CAAGGGTGAATCACTCACATC	59.9	411	INTERGENIC	--	--	--
Ca2_P1_32079368	Ca2	32079368	CAA/CAACAATAATAATAATAA	AGATGTGAGTGATTCACCCTTG	AACCATGCTGGCTTCTTTTC	59.1	494	INTERGENIC	--	--	--
Ca2_P1_32083677	Ca2	32083677	TAAAAAAAAAA/TAAAAAAAAA	CCCATTATTTCATGGGGCTT	GGGCCTGTAGACCCACTTTT	60.9	331	INTERGENIC	--	--	--
Ca2_P1_32084077	Ca2	32084077	TTTAATTAATTA/TTTAATTA	GTGGGTCTACAGGCCCATTA	TGAATTCAATGACCCCAACA	59.8	309	INTERGENIC	--	--	--
Ca2_P1_32084348	Ca2	32084348	ATTT/ATT	GTGGGTCTACAGGCCCATTA	AAGCAAAGTGGGATCCTGAA	59.8	556	INTERGENIC	--	--	--
Ca2_P1_32088009	Ca2	32088009	TTAATAATAATAATAATAATAATAATA/ TTAATAATAATAATAATAATAATAATAATAATA	TCAGCAAGCAAGCATTATGG	ATGGGGAACGTTGACAGAAG	60.0	605	INTERGENIC	--	--	--
Ca2_P1_32102006	Ca2	32102006	A/AC	TTGCGTTTTATGGAGGAACTTT	GCGCACTAGGCTATGGTCTC	60.0	458	INTERGENIC	--	--	--
Ca2_P1_32120308	Ca2	32120308	CTT/C	TGTCCACCACCAGAATCAAA	CAATGGTGTGCACGATGAAT	59.9	843	INTRON	Ca_17813	--	ERF
Ca2_P1_32127033	Ca2	32127033	TGC/TGCGC	TGCAAGTTGTGATGGGTTGT	TGTCTGTTCTGCACGGAGTC	60.0	650	INTRON	Ca_17812	O	--
Ca2_P1_32146442	Ca2	32146442	AT/ATT	ATTCCCACGCCACTTAGATG	CACTGTCACAGAGATAAGATGCAA	60.0	807	INTRON	Ca_17808	--	G2-like

INDEL marker IDs	Chromosomes/ unanchored scaffolds	Physical positions (bp)	InDels (*Kabuli* reference genome-CDC Frontier/PI)	Forward primers (5'-3')	Reverse primers (5'-3')	Anneling temperature (0C)	Expected amplified product size (bp)	Structural annotation		Functional annotation	
								Sequence components of *kabuli* genome	*Kabuli* gene accession IDs	NCBI-KOG	TFs
Ca2_P1_32152511	Ca2	32152511	TTAATAATAATAATAATAA/TTAATAATAATAATAA	ACAAAAACAATCGGTAGCGG	GTGTTATGGGGTTTTTCCCC	60.0	736	INTERGENIC	--	--	--
Ca2_P1_32161753	Ca2	32161753	TCCCC/TCCC	AGGCCATTAAGCATGACACC	TTCTTGGAACCCTGCATACC	60.0	867	INTERGENIC	--	--	--
Ca2_P1_32163428	Ca2	32163428	CATA/CATATATATATA	TCAAAGAGACATGATTTTGTCCA	GGTCAATCAGCTGTGAGCAA	59.6	415	INTERGENIC	--	--	--
Ca2_P1_32167519	Ca2	32167519	TGG/TG	AGAAATGGGAGCTTGTTCCA	GAGGATGGGTGAAAAAGCAA	59.7	588	INTERGENIC	--	--	--
Ca2_P1_32170366	Ca2	32170366	TTCAAATCAA/TTCAA	GGGGATCATGTGAAATTTGG	GTCCTCTGCTCTTTGCCAAC	60.0	337	INTRON	Ca_17806	--	WRKY
Ca2_P1_32173774	Ca2	32173774	TC/T	ATGTTTTGATTGGGGCTTTG	TAAATCAGCCCTTTTGGTGG	59.8	631	DRR	Ca_17806	--	WRKY
Ca2_P1_32209131	Ca2	32209131	T/TG	GCAAAGATAGACACCACCCC	AACCATTCCCATGGAACAAA	59.4	577	INTERGENIC	--	--	--
Ca2_P1_32209334	Ca2	32209334	CTATATATA/CTATA	TCCATGGGAATGGTTTTGAT	TTGAAGAGGAAGTTTTGGCA	60.0	342	INTERGENIC	--	--	--
Ca2_P1_32209671	Ca2	32209671	TC/TCC	TCCATGGGAATGGTTTTGAT	TTTTAGTGTGCACCAATGTCAA	60.0	899	INTERGENIC	--	--	--
Ca2_P1_32210099	Ca2	32210099	CA/CAAATA	TCAGCTTCAATTATCTCCAACAA	TCGGCGACAAAAATCACATA	58.9	644	INTERGENIC	--	--	--
Ca2_P1_32210241	Ca2	32210241	TA/T	TGACATTGGTGCACACTAAAAA	CCACGTCATCTTCCTTTGGT	59.1	505	INTERGENIC	--	--	--
Ca2_P1_32211817	Ca2	32211817	ATATTATTATTATTATTATTATTATTATTATTATTATTATTAT/ATATTATTATTATTATTATTATTATTATTATTATTATTAT	ATGGAATTCTGAATGGTGGG	CATGTGGATCGAAATGATTTTT	59.6	365	INTERGENIC	--	--	--

INDEL marker IDs	Chromosomes/ unanchored scaffolds	Physical positions (bp)	InDels (*Kabuli* reference genome-CDC Frontier/PI)	Forward primers (5'-3')	Reverse primers (5'-3')	Anneling temperature (0C)	Expected amplified product size (bp)	Structural annotation		Functional annotation	
								Sequence components of *kabuli* genome	*Kabuli* gene accession IDs	NCBI-KOG	TFs
Ca2_P1_32211970	Ca2	32211970	A/AG	ATGGAATTCTGAATGGTGGG	AAAACCACAAAATGGGGTCA	59.6	763	INTERGENIC	--	--	--
Ca2_P1_32213171	Ca2	32213171	GTGAT/GTGATGAT	CAACATGACAAGGACTTTCCAA	TTACCGGTATCGCAAGGAAC	60.0	529	INTERGENIC	--	--	--
Ca2_P1_32213265	Ca2	32213265	AT/ATGGT	GAAGAGGCCAATTAAGCTTTCA	CTCGTGCCGTTATCTATCCC	59.9	299	INTERGENIC	--	--	--
Ca2_P1_32213424	Ca2	32213424	CAAA/CAAAA	AGGGATAGATAACGGCACGA	AAGGCCCTGCTAGAATGATG	59.6	504	INTERGENIC	--	--	--
Ca2_P1_32214370	Ca2	32214370	GTTTTTTTTTTT/GTTTTTTTTTTTTT	GGGTGGGATAGGGGACTAGA	GCAAATGTTTCACATGCTCG	60.1	656	INTERGENIC	--	--	--
Ca2_P1_32233566	Ca2	32233566	TAAAAAAAAA/TAAAAAAAAAA	AAAGATGGCAAGGCAAGATG	ACGAATACGGATGCGATGAT	60.2	953	INTERGENIC	--	--	--
Ca2_P1_32240521	Ca2	32240521	TGC/TGCGC	GGCGTCTTAAGCAAGGTGTC	CTGAGGGTGACGTCTCACAA	59.9	525	DRR	Ca_10293	--	--
Ca2_P1_32243132	Ca2	32243132	TCAA/TCAACAA	TCTGAGGGGATTCGTTATGC	TCCATAACTCTTGCCAACCC	60.0	524	CDS (large-effect mutations)	Ca_10292	--	bHLH
Ca2_P1_32250351	Ca2	32250351	GTCT/GT	AAGGCGCGCAGAATTTAATA	TTGCATCCCAACAAAGACAA	59.8	581	DRR	Ca_10291	E	C3H
Ca2_P1_32259695	Ca2	32259695	TCAC/TCACAC	AAATGCAACTCCTTCCTCCA	TCAGCTTAAGGCTCTTGCTGT	59.7	833	INTRON	Ca_10289	K	TALE
Ca2_P1_32263139	Ca2	32263139	A/AAAAATGG	GAAAACAAAATGTCACCGGC	ATTGCCAACAACACACCAAA	60.4	282	INTERGENIC	--	--	--
Ca2_P1_3226421	Ca2	3226421	TATGATGATGATGATGATGA/TATGATGATGATGATGATGATGATGATGA	GCCAAAGGTTTTTGTTGGAA	GGGCACCTTATTAGCCCATT	60.0	282	CDS (large-effect mutations)	Ca_10526	O	--

INDEL marker IDs	Chromosomes/ unanchored scaffolds	Physical positions (bp)	InDels (*Kabuli* reference genome-CDC Frontier/PI)	Forward primers (5'-3')	Reverse primers (5'-3')	Anneling temperature (0C)	Expected amplified product size (bp)	Structural annotation		Functional annotation	
								Sequence components of *kabuli* genome	*Kabuli* gene accession IDs	NCBI-KOG	TFs
Ca2_P1_32264515	Ca2	32264515	TAAA/TAAAA	CACGAATCATTGACCATTTGA	TCCCGTTTTTCAAACACACA	59.4	772	INTERGENIC	--	--	--
Ca2_P1_32282292	Ca2	32282292	CATTAGTGATT/CATTAGTGATTAGTGATT	ACCCTCCAGGGTGCTAAGAT	GCCAACATTTTCTGGTTGCT	60.0	726	INTRON	Ca_10287	S	--
Ca2_P1_32293632	Ca2	32293632	AAGAAATTCAGAAATTCA/AAGAAATTCAGAAATTCAGAAATTCA	AAACCCTACGAAGTTTGGCA	TTGTCACCCGTCCAAATACA	59.6	237	INTERGENIC	--	--	--
Ca2_P1_32332565	Ca2	32332565	AAAATAAATAAATAAATA/AAAATAAATAAATAAATAAATA	CGCCAAAGTCATTGATTGAA	CATCTTGTCAGCCATAGCCA	59.7	338	INTERGENIC	--	--	--
Ca2_P1_32340350	Ca2	32340350	TGG/TG	TCCATTCAATGCTTTCAAATTC	TTGTGGTCATTTAAGGTGGTTG	59.1	530	INTERGENIC	--	--	--
Ca2_P1_32340944	Ca2	32340944	GTTTTTTTT/GTTTTTTT	CAACCACCTTAAATGACCACAA	AAAAACAATGTCATGCAGAGG	59.8	682	INTERGENIC	--	--	--
Ca2_P1_32341023	Ca2	32341023	GAAT/GAATGTTAAAAT	CAACCACCTTAAATGACCACAA	AAAAACAATGTCATGCAGAGG	59.8	682	INTERGENIC	--	--	--
Ca2_P1_32346783	Ca2	32346783	CAAAAAACA/CAAAAAACATAAAAAACA	TCCACACCAGAAGCATTCAA	GTTCTGGAGGGAACAGCTTG	60.2	628	INTERGENIC	--	--	--
Ca2_P1_32348784	Ca2	32348784	TTTATT/TTT	TATCTCGCTCGGTGGAGTCT	ATGCAATCCTTTTGCTGCTT	60.0	216	INTRON	Ca_10283	--	--
Ca2_P1_32349770	Ca2	32349770	TTTTTTTT/TTTTTTTTTTATTTTTTT	AAGTTGGAGGCTCAAAAGCA	AAAATTGCAAATGCACACCA	60.0	661	INTRON	Ca_10283	--	--
Ca2_P1_32353326	Ca2	32353326	G/GA	GAACGACAACAATTTCGCCT	CAATGGATTTCTTTGGCCTT	60.1	513	DRR	Ca_10283	--	--
Ca2_P1_32353407	Ca2	32353407	TGAAC/T	GAACGACAACAATTTCGCCT	CAATGGATTTCTTTGGCCTT	60.1	513	DRR	Ca_10283	--	--

INDEL marker IDs	Chromosomes/ unanchored scaffolds	Physical positions (bp)	InDels (*Kabuli* reference genome-CDC Frontier/PI)	Forward primers (5'-3')	Reverse primers (5'-3')	Anneling temperature (0C)	Expected amplified product size (bp)	Structural annotation		Functional annotation	
								Sequence components of *kabuli* genome	*Kabuli* gene accession IDs	NCBI-KOG	TFs
Ca2_P1_32355990	Ca2	32355990	ATTTTTTT/ATTTTT	ACCAAAAGCTCCATGACCAC	TGCCATTCACAGCAACTAGC	60.0	180	INTERGENIC	--	--	--
Ca2_P1_32357617	Ca2	32357617	CTTT/CTTTT	TCCCAACTCTTCGAAATCGT	TTGGATGATAGACATTGAAATTTTG	59.7	367	INTERGENIC	--	--	--
Ca2_P1_32358261	Ca2	32358261	CAAAAAAA/CAAAAAA	TCAAAATTTCAATGTCTATCATCCA	GCGAGGATGAGAAGTTGTTGT	59.7	658	INTERGENIC	--	--	--
Ca2_P1_32359679	Ca2	32359679	GACACA/GACACACA	TTGATGAGTTCGGTCATGGA	AGCCGCTTAATTGATCGTTG	60.0	195	INTERGENIC	--	--	--
Ca2_P1_32359951	Ca2	32359951	TAAAAAAAA/TAAAAAAAAAAA	TTGATGAGTTCGGTCATGGA	TGGATTCAAATCCCAAGTCC	60.0	410	INTERGENIC	--	--	--
Ca2_P1_32361822	Ca2	32361822	ATTT/ATT	ACGTTGTATTCGCAAGAGGG	TCACCTTTTCATCAATGCTCA	60.1	263	INTERGENIC	--	--	--
Ca2_P1_32362034	Ca2	32362034	ATTTT/ATTTTT	ACGTTGTATTCGCAAGAGGG	TTGGATGTTGTCATTGTTCAGA	60.1	737	INTERGENIC	--	--	--
Ca2_P1_32362657	Ca2	32362657	TAAAAAAA/TAAAAAAAA	GCAAAAGCACAACCAAATCA	AATGACCACAATTTGCATTGA	59.7	260	INTERGENIC	--	--	--
Ca2_P1_32363795	Ca2	32363795	TA/T	TGTGTCAACGAAGAAGGTTGA	TGCACCAATAATGCAATGAAG	59.3	652	INTERGENIC	--	--	--
Ca2_P1_32365887	Ca2	32365887	ATTTTTT/ATTTTTTT	TCGTGACTTTGTCCATCATTTC	TCACGACTTTTTGAATTTTGGTT	60.0	380	INTERGENIC	--	--	--
Ca2_P1_32369826	Ca2	32369826	CT/CTCGT	TGGTTTCAAGGTCTTCCACA	GAAAAGTTAAAATCCAAAATAACATTC	59.1	794	INTERGENIC	--	--	--
Ca2_P1_32379585	Ca2	32379585	AATTAATTTT/AATTAATTTTGATTAATTTT	TGTGCGAGTTTATTGCGGTA	CATTTGGTTCAAAATGTCATGG	60.3	624	INTERGENIC	--	--	--

INDEL marker IDs	Chromosomes/ unanchored scaffolds	Physical positions (bp)	InDels (*Kabuli* reference genome-CDC Frontier/PI)	Forward primers (5'-3')	Reverse primers (5'-3')	Anneling temperature (0C)	Expected amplified product size (bp)	Structural annotation		Functional annotation	
								Sequence components of *kabuli* genome	*Kabuli* gene accession IDs	NCBI-KOG	TFs
Ca2_P1_32380306	Ca2	32380306	GTTTGAATTTAATTA/GTTTGAATTTAATTATTTGAATTTAATTA	AACTAACTTTTCGCAGCCCA	GGCAACTTGGCCAACACTAT	59.9	587	INTERGENIC	--	--	--
Ca2_P1_32388015	Ca2	32388015	TATAAT/TATAATAAT	CTTGGAGGCCACCATACCTA	TGACGAATGCTCTCATTTGC	59.9	663	INTRON	Ca_10280	--	--
Ca2_P1_32393042	Ca2	32393042	ATTTTTTTT/ATTTTTTTTT	TGCCCATCCTTATGCTCTTC	ATCACGTGCAACCACAAGTC	60.2	626	INTERGENIC	--	--	--
Ca2_P1_32393117	Ca2	32393117	T/TC	TGCCCATCCTTATGCTCTTC	ATCACGTGCAACCACAAGTC	60.2	626	INTERGENIC	--	--	--
Ca2_P1_32393187	Ca2	32393187	AATAT/A	TGCCCATCCTTATGCTCTTC	ATCACGTGCAACCACAAGTC	60.2	626	INTERGENIC	--	--	--
Ca2_P1_32393441	Ca2	32393441	TGG/TGTGGG	TCGCACTTTTTCGTGTAGGA	GGGCTTGTTTGACGCATTAT	59.5	588	INTERGENIC	--	--	--
Ca2_P1_32393633	Ca2	32393633	TAACATAATAA/TA	GACTTGTGGTTGCACGTGAT	GGGCTTGTTTGACGCATTAT	59.6	445	INTERGENIC	--	--	--
Ca2_P1_32394714	Ca2	32394714	TAAAAAAAAAAA/TAAAAAAAAAAAAA	GAGGGTAATATGGCCAAACG	TGCATTGTGAAAACCTGCAT	59.3	614	INTERGENIC	--	--	--
Ca2_P1_32395005	Ca2	32395005	ATTTTTT/ATTTTTTTT	AATGCAGGTTTTCACAATGC	GTGGGGTTTGTGGTCCATAG	58.6	418	INTERGENIC	--	--	--
Ca2_P1_32396308	Ca2	32396308	TTTTTT/TTTTTTATTTTT	CCCCAACCCATATTCTTCCT	TTTTTGGTGGCAAAATCCAT	60.0	740	INTERGENIC	--	--	--
Ca2_P1_32398798	Ca2	32398798	CTTTT/CTTTTT	TGCGAGAGATCAAGATCCAA	TGCCCCAAATTATTGTTGAA	59.5	779	INTERGENIC	--	--	--
Ca2_P1_32399194	Ca2	32399194	T/TATTAATGTCTAA	TCTTGCACGGGTGAGAACTA	CCTATTTGAAGAGTGCCCCA	59.4	587	INTERGENIC	--	--	--

INDEL marker IDs	Chromosomes/ unanchored scaffolds	Physical positions (bp)	InDels (*Kabuli* reference genome-CDC Frontier/PI)	Forward primers (5'-3')	Reverse primers (5'-3')	Anneling temperature (0C)	Expected amplified product size (bp)	Structural annotation		Functional annotation	
								Sequence components of *kabuli* genome	*Kabuli* gene accession IDs	NCBI-KOG	TFs
Ca2_P1_32399302	Ca2	32399302	ATTTTTTTTT/ATTTTTTTTTT	AATTTGGGGCACTCTTCAAA	GCCTCTTCAAAAGTCGGTCA	59.5	219	INTERGENIC	--	--	--
Ca2_P1_32400013	Ca2	32400013	CACTGAGATAGAGAC/CAC	TTCCACTCCTCCACCAAATC	GACGCCCAAAAGGAGACATA	59.9	278	INTERGENIC	--	--	--
Ca2_P1_32402263	Ca2	32402263	A/AG	GGCCGCGATCATTAATTCTA	TTCGGTTCTTGTTTGCCTCT	60.0	789	INTERGENIC	--	--	--
Ca2_P1_32403254	Ca2	32403254	GGAT/GGATCGAT	ATCACATGGTGAGCAATGGA	ATGTAAAGATCGTGGTCGGC	59.9	355	INTERGENIC	--	--	--
Ca2_P1_32406448	Ca2	32406448	TTTG/T	GGTGGAAAAGGGAGGAGAAG	GTCAGCTTGGAAAGGGTCTG	60.0	832	INTERGENIC	--	--	--
Ca2_P1_32409946	Ca2	32409946	ATTTTTT/ATTTT	TGGAGAGTTGAGAAGCCAGG	TACTCGAGGCCTCTCGCTAC	60.5	850	INTRON	Ca_10279	--	bHLH
Ca2_P1_32415535	Ca2	32415535	T/TCC	GGGATTGTGTTCATTCCAGG	ATCGATATACCAACGGCTGC	60.2	398	INTRON	Ca_10278	--	bHLH
Ca2_P1_32416839	Ca2	32416839	CAG/CAGTCATATTTAAG	GGACAAACATAGGTGCGGTT	TTCAAGGCCTCTTGCTGAAT	59.9	566	INTRON	Ca_10278	--	bHLH
Ca2_P1_32417284	Ca2	32417284	ATTTTTTTT/ATTTTTTTTT	AAATTTGCGTGGAAAGGTTG	CTCTGGTGGCATGTATCCAA	60.0	207	INTRON	Ca_10278	--	bHLH
Ca2_P1_32417636	Ca2	32417636	TTT/TTTTGTT	AAATTTGCGTGGAAAGGTTG	GCCTAGGATCCACGAGTTTG	60.0	788	INTRON	Ca_10278	--	bHLH
Ca2_P1_32418128	Ca2	32418128	A/AT	GTCTTCGCAAACTCGTGGAT	CTCTCCCAAGTTGAGCCATC	60.3	622	INTRON	Ca_10278	--	bHLH
Ca2_P1_32422496	Ca2	32422496	TT/TTGAT	AGCGAAAATTGTAGTTGCGG	GCATCACAACACTCCACACC	60.3	649	DRR	Ca_10277	--	--

INDEL marker IDs	Chromosomes/ unanchored scaffolds	Physical positions (bp)	InDels (*Kabuli* reference genome-CDC Frontier/PI)	Forward primers (5'-3')	Reverse primers (5'-3')	Anneling temperature (0C)	Expected amplified product size (bp)	Structural annotation		Functional annotation	
								Sequence components of *kabuli* genome	*Kabuli* gene accession IDs	NCBI-KOG	TFs
Ca2_P1_32423881	Ca2	32423881	CAAAA/CAAAAA	TGAGGCATGATAATTGAGCG	TGTTGTCATCAATCACGCAG	59.8	536	DRR	Ca_10277	--	--
Ca2_P1_32427550	Ca2	32427550	TATG/TATGATGCTGATG	CCACATGAGTGTGTTGAGGG	TCTCATCTTCAGGACCACCC	60.0	232	DRR	Ca_10276	--	SAP
Ca2_P1_3245422	Ca2	3245422	GATATATATATATATATATATATA/GATATATATATATATATATA	TTTTCAACGGAACCACAACA	CATGTCAGGCAGTTACCCCT	60.0	541	INTERGENIC	--	--	--
Ca2_P1_32476919	Ca2	32476919	ATGT/AT	CATGGATGGTGCTTCAAATG	CAACTGCAATGTTCCATATTCG	59.9	885	DRR	Ca_10273	R	C3H
Ca2_P1_32477611	Ca2	32477611	TAAAAAA/TAAAAAAA	TTCGAATATGGAACATTGCAG	GGCTAGGGGAGAATTCCTTG	58.6	473	DRR	Ca_10273	R	C3H
Ca2_P1_32478862	Ca2	32478862	TAAAAAAAAA/TAAAAAAAA	AAATTGGTAATCGGACGCTG	TGTCCACATGGGAAGTTTGA	60.0	471	INTERGENIC	--	--	--
Ca2_P1_32479419	Ca2	32479419	TAAAAAAAA/TAAAAAA	AATTACAATGCGAAGCTGGC	TCCTATACCACATTTAAAGCAAAA	60.2	803	INTERGENIC	--	--	--
Ca2_P1_32479799	Ca2	32479799	CTATATA/CTATA	CCATATTTATACTCATAGCCCACA	TGCTAACATCACTAAGACATAAGACAA	57.3	749	INTERGENIC	--	--	--
Ca2_P1_32480635	Ca2	32480635	GAAAAAAAA/GAAAAAAA	TCCACATGCTTCCAATCAAA	ACCACTCCAACCAGTCGTTT	60.0	584	INTERGENIC	--	--	--
Ca2_P1_32480670	Ca2	32480670	T/TA	TCCACATGCTTCCAATCAAA	ACCACTCCAACCAGTCGTTT	60.0	584	INTERGENIC	--	--	--
Ca2_P1_32480992	Ca2	32480992	GTTT/GTT	CATGCTTCCAATCAAACACG	CAACGCCTCAAGTAGGGGTA	60.1	740	INTERGENIC	--	--	--
Ca2_P1_32481790	Ca2	32481790	ATATAT/A	GGGTGGAAATGCAGAAGAAA	AAGCTTTCGGTGATTGCTGT	60.1	535	INTERGENIC	--	--	--

INDEL marker IDs	Chromosomes/ unanchored scaffolds	Physical positions (bp)	InDels (*Kabuli* reference genome-CDC Frontier/PI)	Forward primers (5'-3')	Reverse primers (5'-3')	Anneling temperature (0C)	Expected amplified product size (bp)	Structural annotation		Functional annotation	
								Sequence components of *kabuli* genome	*Kabuli* gene accession IDs	NCBI-KOG	TFs
Ca2_P1_32540405	Ca2	32540405	CATAATAATAATAATAAT/CATAATAATAAT	CGACATAACTTCCCACCCCT	AGCGCTGTTGTATTCTCCGT	61.1	482	INTERGENIC	--	--	--
Ca2_P1_32615052	Ca2	32615052	AATATATATATATATATATATATATA/AATATATATATATATATATATATA	GCCGCCTCAATCTAGTGTTT	TCTCCAAGAATTTTCGCCAC	59.3	602	INTERGENIC	--	--	--
Ca2_P1_32637444	Ca2	32637444	CTTATTTTATTTTATTT/CTTATTTTATTT	TGGACAAAAATCCTATTTGGAGA	GTAGGGGATTCGGGTGAAAT	59.8	395	INTERGENIC	--	--	--
Ca2_P1_32641659	Ca2	32641659	TGGG/TGG	GAAGGAAAGCGCAGAAGAGA	TCGACATCAGTTGGTGCTTC	59.8	849	DRR	Ca_10258	--	--
Ca2_P1_32642955	Ca2	32642955	ATTTTTTTTTT/ATTTTTTTTT	CAAAGTTGAGGAAAGGCCAG	GGAATCACCCATCCACATTC	59.8	869	DRR	Ca_10258	--	--
Ca2_P1_32644144	Ca2	32644144	ATTTTTTTT/ATTTTTTTTT	TAGGGCTGGCCCAAATAAGT	GAAGCCTGATAGCATTGTTTCTC	60.8	577	DRR	Ca_10257	O	--
Ca2_P1_32644321	Ca2	32644321	ATTTTTT/ATTTTT	AGGGCTGGCCCAAATAAGTA	CGCAGCAACGACAAATAAAA	60.8	932	DRR	Ca_10257	O	--
Ca2_P1_32648770	Ca2	32648770	GC/G	TGCCAAAACACAAAGTCACC	ATGGGTCAAGATTCGTCCAC	59.6	728	INTERGENIC	--	--	--
Ca2_P1_32666924	Ca2	32666924	ACCAATGATCTCATC/AC	CCCCATCACTCTTGGATCAT	AAGTACCCCACAGCTCCCTT	59.7	548	INTERGENIC	--	--	--
Ca2_P1_32713700	Ca2	32713700	TTTTTTTTT/TTTTTTTTTTT	AACGCATCGGTAAACACTCC	GAGGTACTACAGCTTGCCCC	60.0	234	INTERGENIC	--	--	--
Ca2_P1_32713895	Ca2	32713895	CAAAAAAA/CAAAACAAAAAAAA	AACGCATCGGTAAACACTCC	TGTTTTGCAGCAAGATGTCC	60.0	695	INTERGENIC	--	--	--
Ca2_P1_32745343	Ca2	32745343	TTCTCTCTCTCTCTCTCTCT/TTCTCTCTCTCTCTCTCT	AGCAGGCCAGCAACAGTATT	TTAGAAATGGTGACGGAGCC	59.9	522	INTRON	Ca_10250	O	ERF

INDEL marker IDs	Chromosomes/ unanchored scaffolds	Physical positions (bp)	InDels (*Kabuli* reference genome-CDC Frontier/PI)	Forward primers (5'-3')	Reverse primers (5'-3')	Anneling temperature (0C)	Expected amplified product size (bp)	Structural annotation		Functional annotation	
								Sequence components of *kabuli* genome	*Kabuli* gene accession IDs	NCBI-KOG	TFs
Ca2_P1_32757563	Ca2	32757563	TTATATATATATATATATATATATATAT/TTATATATATATATATATATATATATATATAT	CATGAAGCACACTCCGAAAA	TTTTGGTTGGATATGTGGGTT	59.8	594	INTERGENIC	--	--	--
Ca2_P1_32812104	Ca2	32812104	TTTATTATTATTATTATTATTAT/TTTATTATTATTATTATTATTATTATTAT	CAAAAGTCCAAGGCCAGAAA	CATGTCCATAAATGGTTGCG	60.2	375	INTERGENIC	--	--	--
Ca2_P1_32817821	Ca2	32817821	GAGAGAG/GAGAGAGATAGAGAG	CGGGAATGGAGTTAGTGGTG	GGCAAATCTGATCCCTCAAA	60.4	593	INTERGENIC	--	--	--
Ca2_P1_3282888	Ca2	3282888	TAA/TAAA	TGTAGCTTTTGGCATGTGGA	TGGGTGAAGGTATTTAATGTCAA	60.3	593	INTERGENIC	--	--	--
Ca2_P1_32843861	Ca2	32843861	TATAATAATAATAATAATAATAATAATAATAATAATAA/TATAATAATAATAATAATAATAATAATAATAATAATAA	AAAAATGCATTCGGTAATTGG	GGGGTCTGTTAAAACGTGGA	58.9	769	DRR	Ca_10238	--	--
Ca2_P1_32938114	Ca2	32938114	AATATATATATATATATATATAT/AATATATATATATATATATATATAT	ATTTGATGTGGGTGTGCGTA	ATTAAAGGGCAACCCCAAAT	59.8	303	INTERGENIC	--	--	--
Ca2_P1_32985262	Ca2	32985262	GTTTT/GTTTTT	GCGCACGTAAGCAACTAACA	GAAATGGTTCCCATGAAGGA	60.1	758	INTRON	Ca_10226	R	--
Ca2_P1_33036944	Ca2	33036944	GAAAAAAAAA/GAAAAAAAAAA	AATTGCAAGCAAGGAACTGC	CGAACCCTAGATATGCACCAA	60.4	333	INTERGENIC	--	--	--
Ca2_P1_33055348	Ca2	33055348	AATATATATATATATATATATATATATATATATATA/AATATATATATATATATATATATATATATATATATATATATA	TTTCAATACAAAGCCAAAAATCAA	ATCTGTTTGAGAGGTTCGGC	59.9	579	INTERGENIC	--	--	--
Ca2_P1_33058448	Ca2	33058448	CATATTAGTATATTAGTA/CATATTAGTA	CCTTACCATGACCCCAAATG	TGCCTGGGTTTCTTTAATGG	60.0	630	DRR	Ca_10218	R	B3
Ca2_P1_33107336	Ca2	33107336	TTATATATATATATATATATATATAT/TTATATATATATATATATATATATATATAT	TTTCAGACAAAGGTGCGTTG	TGGGTGTATTTCGAAGGCTC	59.9	684	INTERGENIC	--	--	--
Ca2_P1_33123353	Ca2	33123353	AATATATATATATATATATATATATATATATATATAT/AATATATATATATATATATATATATATATATATATATATATATAT	GCAACCGTTGTCCAGATTTT	TGGGTTTCCTTCGTAACAGG	60.0	931	INTRON	Ca_10209	--	S1Fa-like

INDEL marker IDs	Chromosomes/ unanchored scaffolds	Physical positions (bp)	InDels (*Kabuli* reference genome-CDC Frontier/PI)	Forward primers (5'-3')	Reverse primers (5'-3')	Anneling temperature (0C)	Expected amplified product size (bp)	Structural annotation		Functional annotation	
								Sequence components of *kabuli* genome	*Kabuli* gene accession IDs	NCBI-KOG	TFs
Ca2_P1_33152090	Ca2	33152090	TAAAAAAAA/TAAAAAAAAAA	AAAGACCAAGTCGGCAAAAA	TCGTTGAGGAAAAACAAACAAA	59.7	638	INTERGENIC	--	--	--
Ca2_P1_3321638	Ca2	3321638	CTTTTTTTTT/CCTTTTTTTTTTTTT	AGGTACTTGTCAGGTCCGCA	ACCACAACGGACAAAAGAGG	60.7	689	INTRON	Ca_10520	O	--
Ca2_P1_33233654	Ca2	33233654	TTATTATCAAAAATATT/TTATT	CGAACATTGTTTTGCCCACT	GTTGAATTCCGGGTTCGATT	60.9	388	INTERGENIC	--	--	--
Ca2_P1_33257195	Ca2	33257195	ATTTTTTTTT/ATTTTTTTTTT	ATCGCATTTTGCCTTTGTTT	GCTGTCGTTTGCAGCATAAA	59.6	801	INTERGENIC	--	--	--
Ca2_P1_33289844	Ca2	33289844	T/TGAACTTTCTCTAC	CGGTCGATTAGGCAAGATGT	TGCCGCAATTCAAATAACAA	60.1	417	DRR	Ca_10198	A	--
Ca2_P1_3329816	Ca2	3329816	AATATATATATATATATATATATATATATA/AATATATATATATATATATATA	CTTGGCCGTGACCACTCTAT	TTGCTCCAAATTTGTGTAATGC	60.1	565	INTERGENIC	--	--	--
Ca2_P1_33306373	Ca2	33306373	TTATATATATATATATATA/TTATATATATATATA	GAATCCGTTTCCGAGTTTGA	TCAATGGAAATCAAAGTTGAAGG	60.1	644	INTERGENIC	--	--	--
Ca2_P1_33345639	Ca2	33345639	GTT/GT	CTCCATTTTGGGCAGGTTTA	GGATGATCTGTTTTTCGCGT	59.9	573	INTRON	Ca_10191	T	FAR1
Ca2_P1_33346809	Ca2	33346809	C/CA	ATGCACATGAACCGATTGAA	AAACAAGGTTGAATCACGCC	59.9	718	INTRON	Ca_10191	T	FAR1
Ca2_P1_33398441	Ca2	33398441	TACACAC/TACAC	AGAGCCTACAGCAAGCCAAA	AAATGCAGGGTCACAACACA	60.2	205	DRR	Ca_10186	T	M-type
Ca2_P1_33405227	Ca2	33405227	AATATATATATA/AATATATATA	AAAGAGACATGACTTTGTCCATCA	TTTCGCTACCCATCATACCC	60.0	586	INTERGENIC	--	--	--
Ca2_P1_33523362	Ca2	33523362	GTTTTTTTTTT/GTTTTTTTTT	TCAGCTGGAGTGGATGAGTG	TGCAAGCATTGGTTGAAATG	60.0	447	DRR	Ca_10181	--	--

INDEL marker IDs	Chromosomes/ unanchored scaffolds	Physical positions (bp)	InDels (*Kabuli* reference genome-CDC Frontier/PI)	Forward primers (5'-3')	Reverse primers (5'-3')	Anneling temperature (0C)	Expected amplified product size (bp)	Structural annotation		Functional annotation	
								Sequence components of *kabuli* genome	*Kabuli* gene accession IDs	NCBI-KOG	TFs
Ca2_P1_33579121	Ca2	33579121	GAA/GAAA	ATCCAAATTCATTCGCCTTG	TAACTTTTGGCTGGCATGTG	59.9	608	INTERGENIC	--	--	--
Ca2_P1_33614018	Ca2	33614018	ATTTTTTTTTT/ATTTTTTTTTTT	TGCACTGACACACAAAAGCA	AATCAGATTGCATGGAAGGG	60.1	698	INTERGENIC	--	--	--
Ca2_P1_33615244	Ca2	33615244	ATATTTATTTATTTATTTATTTATTTATTTATTTATT/ATATTTATTTATTTATTTATTTATTTATTTATTTATT	CCATTTTGTTTTAATTGGCTGC	TTGAGATCACATACCCCGCT	60.7	552	INTERGENIC	--	--	--
Ca2_P1_33639211	Ca2	33639211	GAA/GAAA	TGGCAAACATGTTGTCCTAAA	AGAGAGCAAACTATTCCGTCG	59.1	230	INTERGENIC	--	--	--
Ca2_P1_33639777	Ca2	33639777	TAAAAAAAA/TAAAAAAA	CGACGGAATAGTTTGCTCTCT	CGGAAAACCGTAGCTTAATACG	58.6	892	INTERGENIC	--	--	--
Ca2_P1_33666707	Ca2	33666707	GTTTTTTTTTTT/GTTTTTTTTTT	GCACAATGGCCAGGAACTAT	AATTCCTCGTGGAGGATCTG	60.0	617	INTERGENIC	--	--	--
Ca2_P1_33715359	Ca2	33715359	ANNNNNNNNNN/ANNNNNNNN	CAAAAGGCGTGCATGAATTA	CTTGCCTTATTTTTCCTGCG	59.7	271	DRR	Ca_10165	K	G2-like
Ca2_P1_33809112	Ca2	33809112	GATATACATATACATATACATATACATATACATAT/GATATACATATACATATACATATACATAT	TTAATCAAATGCACCGACCA	GCGATCGTGAGTGAGTTCAA	59.9	462	DRR	Ca_10155	--	--
Ca2_P1_33922831	Ca2	33922831	GAAAAAAAAAA/GAAAAAAAAAAA	CACACGTGCCTCTCCCTATT	TCCTTCAAATCCAGAAGTTTCA	60.1	173	INTERGENIC	--	--	--
Ca2_P1_33939646	Ca2	33939646	CTTTTTTTTTTT/CTTTTTTTTTTTT	GAGTTTGACGATGGGCCTAA	CCCCTAAAATCTCAATTGGAAG	60.1	552	INTERGENIC	--	--	--
Ca2_P1_33947237	Ca2	33947237	TTATATATATATATATATATATATA/TTATATATATATATATATATATATATA	TCCTTCGCCAATATTTTGTTTT	TTTGAGTCGGAGTTGTCGGT	59.9	426	INTERGENIC	--	--	--
Ca2_P1_33955298	Ca2	33955298	AAAATAAATAAATAAATAAATAAATAAA/AAAATAAATAAATAAATAAATAAA	AATTTCGACTATTCTCTAATGATGG	TCGTTTTGGTGTTGGTTTCA	57.1	520	INTERGENIC	--	--	--

INDEL marker IDs	Chromosomes/ unanchored scaffolds	Physical positions (bp)	InDels (*Kabuli* reference genome-CDC Frontier/PI)	Forward primers (5'-3')	Reverse primers (5'-3')	Anneling temperature (0C)	Expected amplified product size (bp)	Structural annotation		Functional annotation	
								Sequence components of *kabuli* genome	*Kabuli* gene accession IDs	NCBI-KOG	TFs
Ca2_P1_33959397	Ca2	33959397	ATATTTATTTATTT/ATATTTATTT	GGCTAATTTTCGTTATATGGATGC	ACTAGTGATGGCCGAAATGG	60.1	764	INTERGENIC	--	--	--
Ca2_P1_33984310	Ca2	33984310	CTATATATATATATATATATATATA/CCATATATATATATATATATATATATATA	TGGCTATGATCACAATTGGC	CCCTTTGCAAGAGACTCCAA	59.5	869	INTERGENIC	--	--	--
Ca2_P1_33986085	Ca2	33986085	TAC/TTTTTCCCAAAATTGAC	CGAGACAAGTTTTCCAAGCA	ACTCCAAGATCATCATCGGC	59.0	818	INTERGENIC	--	--	--
Ca2_P1_3400163	Ca2	3400163	TTATATATATATATATAT/TTATATATATATATATATAT	TCATCCACCATGAAAGCAAA	TGTCTCGTCTCAATCACTTTCAG	60.0	490	INTERGENIC	--	--	--
Ca2_P1_34028835	Ca2	34028835	AC/A	AAAGGGGTTTTGGCTATGCT	CATGATCATAATTGCATGGAAAA	60.0	229	INTERGENIC	--	--	--
Ca2_P1_3406322	Ca2	3406322	TAAAAAAAAAA/TAAAAAAAAA	CCAACAGAATTCACACCCAA	TTTTCTTCTTCTCCCCGTCA	59.4	578	INTERGENIC	--	--	--
Ca2_P1_34089719	Ca2	34089719	CAAAAAAAA/CAAAAAAAAA	CATGAAATGGTGAGGCAAGTT	TGGGATGTAGTTGGTCTCCTTT	60.0	643	INTERGENIC	--	--	--
Ca2_P1_3415074	Ca2	3415074	TTATTATTATTATTATTATTATTATTATTATTATTATTATTATTATTATTATTATTATTATTATTATTATTATTATTATTATTATTATTATTATTATTATTATTATTATTATTATTATTATTATTATTATTATTAT/TTATTATTATTATTATTATTATTATTATTATTATTATTATTATTATTATTATTAT[illegible]	TGCTCAATTCTGGCAGATCA	AGTTTCATCACGGCAAAACC	60.5	447	INTERGENIC	--	--	--
Ca2_P1_34213186	Ca2	34213186	T/TAAG	GACACCATTATCGAAGGCGT	AGTATCGGACACCAAAAGCG	60.0	249	INTERGENIC	--	--	--
Ca2_P1_34353539	Ca2	34353539	CATATATATATATATATATATATATATAT/CATATATATATATATATATATATATAT	CATGAGCTTGAGTCGCAAAA	TAGGAGGCATATGTGAGGGG	60.1	760	INTERGENIC	--	--	--
Ca2_P1_34353805	Ca2	34353805	GTTTTTTTT/GTTTTTTTTT	AGTTGAAGGAGTGCATGAGGA	TAGGAGGCATATGTGAGGGG	59.9	633	INTERGENIC	--	--	--
Ca2_P1_34355206	Ca2	34355206	CTTGGGCT/CTTGGGCTTTGTTGGGCT	TCTTTGATGCAATGCCAGAG	ATCATTCGAGGTGCCATCAT	59.9	975	INTERGENIC	--	--	--

INDEL marker IDs	Chromosomes/ unanchored scaffolds	Physical positions (bp)	InDels (*Kabuli* reference genome-CDC Frontier/PI)	Forward primers (5'-3')	Reverse primers (5'-3')	Anneling temperature (0C)	Expected amplified product size (bp)	Structural annotation		Functional annotation	
								Sequence components of *kabuli* genome	*Kabuli* gene accession IDs	NCBI-KOG	TFs
Ca2_P1_34355457	Ca2	34355457	TAAAAAA/TAAAAAAA	ATTCTTGGGCTGTGTTTGG	GGGTGATGAGGACTGGTTGT	60.0	725	INTERGENIC	--	--	--
Ca2_P1_34392003	Ca2	34392003	CTATATATATATATATATATATAT/CTATATATATATATATATATATAT	TTTGGTGGATGTGAATCAAAA	CACGCACATACCATTCAAACA	58.9	576	INTERGENIC	--	--	--
Ca2_P1_3448246	Ca2	3448246	CAAAAAAAAA/CAAAAAAAAAA	TTGTGAATCTAAACCAAAACAAACA	AAAATCGCACAATTTTGTGTTAAA	59.8	308	INTERGENIC	--	--	--
Ca2_P1_34515091	Ca2	34515091	TTTATTATTATTATTATTATTATTATTATTATTATTATTATTATTATTATTATTATTA/TTTATTATTATTATTATTATTATTATTATTATTATTATTATTATTATTATTATTATTATTATTATTATTATTA	TGCCAATGTAATGATTCAGGA	AGCGTAAGTTTCGAGGACCA	59.0	766	INTERGENIC	--	--	--
Ca2_P1_34562775	Ca2	34562775	TCTTTA/TCTTTACTTTA	ATAGCGTGCAACGATCCTCT	CATAGCGCCCATAAAAGGAA	59.9	515	INTERGENIC	--	--	--
Ca2_P1_34576817	Ca2	34576817	TTATATATATATATATATATATA/TTATATATATATATATATA	CCTCTGCTGCATGAATTCTC	TGAGTGAAGATTGAATGATGACG	58.5	242	INTERGENIC	--	--	--
Ca2_P1_34654101	Ca2	34654101	GTATATATATATATATATATATATAT/GTATATATATATATATATATATATAT	AGGACGAGACAAAATGAGCC	TGGAAACCAAGGTCCAAGTC	59.3	219	INTERGENIC	--	--	--
Ca2_P1_34683764	Ca2	34683764	TC/TCC	GTTTTTCGCAACCACCTTTC	CGCTTTTTGCACTAACCTCC	59.6	682	INTERGENIC	--	--	--
Ca2_P1_34684386	Ca2	34684386	CAACGTTAAC/CAAC	ACAATACTGCGGCCTGTTTC	GATTCCGATCGTTTTTCACG	60.1	830	INTERGENIC	--	--	--
Ca2_P1_34684591	Ca2	34684591	TTATATATATATATATATATATATATA/TTATATATATATATATATATATATATA	TGGAGTTTTGTGTGATGGACA	GATTCCGATCGTTTTTCACG	60.0	758	INTERGENIC	--	--	--
Ca2_P1_34684688	Ca2	34684688	CATCTT/C	CGTACGTAGCAAATGCAACAA	ATTCCGATCGTTTTTCACGA	59.8	229	INTERGENIC	--	--	--
Ca2_P1_34684923	Ca2	34684923	ATTTTTTT/ATTTTTTTT	TCGTGAAAAACGATCGGAAT	TGCTTGATTTTAATGGATGCC	60.4	550	INTERGENIC	--	--	--

INDEL marker IDs	Chromosomes/ unanchored scaffolds	Physical positions (bp)	InDels (*Kabuli* reference genome-CDC Frontier/PI)	Forward primers (5'-3')	Reverse primers (5'-3')	Anneling temperature (0C)	Expected amplified product size (bp)	Structural annotation		Functional annotation	
								Sequence components of *kabuli* genome	*Kabuli* gene accession IDs	NCBI-KOG	TFs
Ca2_P1_34684966	Ca2	34684966	TAAAAA/TAAAA	CGTGAAAAACGATCGGAATC	TTGCCCCTTAAATGACATGC	60.4	593	INTERGENIC	--	--	--
Ca2_P1_34685252	Ca2	34685252	CATAAATAAATAAATAAA/CATAAATAAA	TCGTGAAAAACGATCGGAAT	TGGAGAGAAACAAAATGGGG	60.4	816	INTERGENIC	--	--	--
Ca2_P1_34685695	Ca2	34685695	ATTTTTT/ATTTTTTT	CCCCATTTTGTTTCTCTCCA	TTGTTTCTCCCCAGTTTTGG	59.9	234	INTERGENIC	--	--	--
Ca2_P1_34685921	Ca2	34685921	TAAAAAAA/TAAAAA	CCAAAACTGGGGAGAAACAA	TACGATAGGGGGATCTCACG	59.9	503	INTERGENIC	--	--	--
Ca2_P1_34854539	Ca2	34854539	ANNNNNNNNNN/ANNNNNNNNN	GTTCTTTTGCCCGTCATCAT	TCAAATTGAATGCGGGTTTT	59.9	387	INTERGENIC	--	--	--
Ca2_P1_34864878	Ca2	34864878	GTATA/GTA	CTTCAGTCTGACGCTCTCCC	TTGCTGCCAAAATAAACAACA	60.1	651	DRR	Ca_16882	--	FAR1
Ca2_P1_34983521	Ca2	34983521	AATATATATATATATATATATATATATATATATATATA/AATATATATATATATATATATATATATATATATATATATATA	CAAATGCATGCTCCCCTAAT	CTAAAAGCCCAAAGCTGCAC	59.9	450	INTERGENIC	--	--	--
Ca2_P1_35043159	Ca2	35043159	GATATATATATATATAT/GATATATATATATATATAT	CGATGGATCTGGAGGTTGTT	TGTTTGAAAGAGCGTGAGGA	59.9	927	INTRON	Ca_16869	A	--
Ca2_P1_35125170	Ca2	35125170	ATCTCTCTCTCTCTCTCTCTCTCTCTCTC/ATCTCTCTCTCTCTCTCTCTCTCTCTCTCTCTC	GATGGCTTTGAGGCAGAGAG	TTGAAGAAGCAAGCAGTGGA	60.1	141	INTERGENIC	--	--	--
Ca2_P1_35218069	Ca2	35218069	TTAATAATAATAATAATAATAATAATAATAA/TTAATAATAATAATAATAATAATAATAATAATAA	GTTTGGACACGGTTTGGTCT	GAATGCGTCCGTTTTGTTTT	59.9	548	INTERGENIC	--	--	--
Ca2_P1_35258590	Ca2	35258590	A/ATT	AAAAAGTTTTAACACGGAGGTTTTT	TGTTGTTGGATTGGACATGG	59.8	487	INTERGENIC	--	--	--
Ca2_P1_35259201	Ca2	35259201	ATTTTTTT/ATTTTTTTTTT	CCATGTCCAATCCAACAACA	GTTGCGAAAAAGTTCATCCG	60.2	744	INTERGENIC	--	--	--

INDEL marker IDs	Chromosomes/ unanchored scaffolds	Physical positions (bp)	InDels (*Kabuli* reference genome-CDC Frontier/PI)	Forward primers (5'-3')	Reverse primers (5'-3')	Anneling temperature (0C)	Expected amplified product size (bp)	Structural annotation		Functional annotation	
								Sequence components of *kabuli* genome	*Kabuli* gene accession IDs	NCBI-KOG	TFs
Ca2_P1_35273988	Ca2	35273988	CTAGATATTT/CTAGATATTTAGATATT T	GATTAAGCGATGGAGG ACCA	CCTTTCCTTTTCCCTC CAAA	60.0	555	INTERGENIC	--	--	--
Ca2_P1_35288109	Ca2	35288109	ATTTTTT/ATTTTTTT	TGCTTTATAGTGCTATT ATGTGCCA	CGTCATTAAAAGGGAA ACCAA	60.1	757	INTERGENIC	--	--	--
Ca2_P1_35288455	Ca2	35288455	TT/TTAT	TTCGATGGTATGCAGT AATTGG	TTCCCATGAAAGAGAG GTGG	59.9	433	INTERGENIC	--	--	--
Ca2_P1_3528916	Ca2	3528916	AATATATATATATATATATA/AATATAT ATATATATATATATA	CAGCATCGGGATCCAG TTAT	TTTGTAACGAAACCAC TCATCC	59.9	284	INTRON	Ca_10506	--	--
Ca2_P1_35289402	Ca2	35289402	ACCCCC/ACCCC	ACCCACCTCTTTTTCTT GGG	TGGTCCTCGAAATTTG TAATGA	60.3	609	INTERGENIC	--	--	--
Ca2_P1_35291896	Ca2	35291896	GTTTT/GTTTTT	AAAAAGTTCCGAAGCG TATTC	TTCATTTAAATTGCACA AACCAA	57.6	579	INTERGENIC	--	--	--
Ca2_P1_35297777	Ca2	35297777	C/CTGAACTGTAT	ACCAGGAAAAGAAGAA GGGC	TTTTGGGTTGGGATGA CATT	59.7	535	INTERGENIC	--	--	--
Ca2_P1_35298057	Ca2	35298057	AT/AATAGCGCTT	AATGTCATCCCAACCC AAAA	TCGCAATAAAAACATA TAAAACCA	60.0	190	INTERGENIC	--	--	--
Ca2_P1_35298866	Ca2	35298866	AA/AACA	TGGGGGCTTAGAGCTT TGTA	CGATGTTGTTGATCTG ATATCGT	59.8	527	INTERGENIC	--	--	--
Ca2_P1_35303488	Ca2	35303488	ATTTTTT/ATTTTTTT	TTCCGAATAAGGATTT CTTGG	ATGGTTTGGGGAAAAG AACC	58.2	137	INTERGENIC	--	--	--
Ca2_P1_35304698	Ca2	35304698	C/CG	GCTTGGAAACAGCCTC AGAC	CAGGGTGGTACTGCA ATGTG	60.0	536	INTERGENIC	--	--	--
Ca2_P1_35329169	Ca2	35329169	AATATATATATATATATATATATAT/ AATATATATATATATATATATAT	CAATTTTGGAAGACGA TTTTGA	TGTCCCGAAAAAGTTC ATCC	59.1	330	INTERGENIC	--	--	--

INDEL marker IDs	Chromosomes/ unanchored scaffolds	Physical positions (bp)	InDels (*Kabuli* reference genome-CDC Frontier/PI)	Forward primers (5'-3')	Reverse primers (5'-3')	Anneling temperature (0C)	Expected amplified product size (bp)	Structural annotation		Functional annotation	
								Sequence components of *kabuli* genome	*Kabuli* gene accession IDs	NCBI-KOG	TFs
Ca2_P1_35388862	Ca2	35388862	GTATTATTATTATTATTATTATTATTATTATTATTATTATTATTATTATTATTATTA/GTATTATTATTATTATTATTATTATTATTATTATTATTATTATTATTA	TTCTCACATTGAGCCAGCAG	TGACTTGGTATACGCCCACA	60.1	458	INTERGENIC	--	--	--
Ca2_P1_35510651	Ca2	35510651	CATATATATATATATATATATATATATAT/CATATATATATATATATATATATATATATATATAT	TTATCTGGCAAAACCGTTGA	TGTCGGCATTGCAAATTAAA	59.2	535	DRR	Ca_09714	--	--
Ca2_P1_35559233	Ca2	35559233	CAA/CAAA	CCAAAATATCCACCGTGTTT	TTCCTGGACGAAAATGAACC	57.3	582	DRR	Ca_09721	K	NF-YB
Ca2_P1_35628453	Ca2	35628453	TTAATAATAATAATAATAATAATAATAATAATAATAATAATAATAATAATAATAATAATAATAATAA/TTAATAATAATAATAATAATAATAATAATAATAATAATAATAATAATAATAATAATAATAATAA	TTGGGGTAGGAGGGATTTTT	CGTGAAATGAACTGAGTAACACC	59.6	844	INTERGENIC	--	--	--
Ca2_P1_3563291	Ca2	3563291	CTTTTTTTTT/CTTTTTTTT	GAACGTTCCATTTTTGCGAT	TTCGGTGGTAGTTCTAGGCTTT	59.9	584	INTERGENIC	--	--	--
Ca2_P1_35656426	Ca2	35656426	AATATATATATATATATATATATATA/AATATATATATATATATATATA	TGGTGTTGGAGTCATATTTTTCA	AACTAGTGGTAACCCGTGCG	59.4	231	INTERGENIC	--	--	--
Ca2_P1_35657396	Ca2	35657396	AAGAGAGAGA/AAGAGAGAGAGA	GCCGTGATTTGGTGGTAAAT	CCTCCAACAAAAAGGAACCA	59.7	295	INTERGENIC	--	--	--
Ca2_P1_3568270	Ca2	3568270	TC/T	TGATAGGCCGTAGGTCAGGT	AAACAACCGTCTTTGTTTGC	59.6	591	INTERGENIC	--	--	--
Ca2_P1_35822980	Ca2	35822980	AATATATATATATATATATA/AATATATATATATATATATATA	TTAATCGCAGCGTGCAATAG	GAAAATTAGATTGCCGGAAGAA	60.0	261	INTERGENIC	--	--	--
Ca2_P1_35856415	Ca2	35856415	TGAGAGAGAGAGAGAGAGAGAGA/TGAGAGAGAGAGAGAGAGAGAGAGAGA	GTGCTGGTGGACATGTTGAG	ATTGGGTTGGTCTCCATTTC	60.2	578	INTERGENIC	--	--	--
Ca2_P1_35856739	Ca2	35856739	AATATATATATATATAT/AATATATATATATATATAT	TCCTGGAAATGGAGACCAAC	GATGGAGGAAGGCAATCAAA	59.9	400	INTERGENIC	--	--	--
Ca2_P1_35857180	Ca2	35857180	GTATATATATATATATATATAT/GTATATATATATATATATAT	TTTGATTGCCTTCCTCCATC	TTCGGGATTAAAACATTCGG	60.0	509	DRR	Ca_09762	R	--

INDEL marker IDs	Chromosomes/ unanchored scaffolds	Physical positions (bp)	InDels (*Kabuli* reference genome-CDC Frontier/PI)	Forward primers (5'-3')	Reverse primers (5'-3')	Anneling temperature (0C)	Expected amplified product size (bp)	Structural annotation		Functional annotation	
								Sequence components of *kabuli* genome	*Kabuli* gene accession IDs	NCBI-KOG	TFs
Ca2_P1_35859193	Ca2	35859193	GAAAAAAAA/GAAAAAAAAAA	AACAATGCCAACATTACGCA	TACTGGAGGCTGTTGGAAGG	60.0	720	INTRON	Ca_09762	R	--
Ca2_P1_35864202	Ca2	35864202	GGA/G	GCAAATTCTGCCACGCTATT	AATCCTCCACCGTCTTTCCT	60.2	675	INTRON	Ca_09762	R	--
Ca2_P1_35866711	Ca2	35866711	CATATAT/CATAT	AAGGGCATGCATGAAAGTCT	TCGAGATGTCCCCTTAATGC	59.7	578	INTERGENIC	--	--	--
Ca2_P1_35871808	Ca2	35871808	AACTC/A	GATGAAATCCAATCCATGCC	TTTCCATTCCATGGGAAAAA	60.1	871	INTERGENIC	--	--	--
Ca2_P1_35872708	Ca2	35872708	T/TC	CCCTGACCTTGAAATATGCC	TTTGTTTGCAAATCATTCACG	59.4	645	INTERGENIC	--	--	--
Ca2_P1_35899592	Ca2	35899592	TAA/TAAA	GGTGGAACATCTCCAAGGAA	CTCAGACTACAACCTCGCCC	59.9	768	DRR	Ca_09764	T	--
Ca2_P1_35900083	Ca2	35900083	ATTAATTTAA/ATTAA	GGCGAGGTTGTAGTCTGAGG	TCCTCTCTTCCACTGCGATT	59.9	407	DRR	Ca_09764	T	--
Ca2_P1_35915881	Ca2	35915881	ATGATGTTGATGTTGATGTTGA/ATGATGTTGATGTTGA	CTTTCAGGTGCAGGAAGAGG	CCAGCACCACCTCCAAGTAT	60.0	425	INTRON	Ca_09767	M	--
Ca2_P1_35924859	Ca2	35924859	C/CGTAT	TTTGGACATGGCTGATGTTG	GCTTGCAGGTCACTGAACAA	60.5	537	INTERGENIC	--	--	--
Ca2_P1_35925409	Ca2	35925409	CCTGT/CCTGTGGCTGT	TTGTTCAGTGACCTGCAAGC	TGGTGGGTTATCCCAATAAA	60.0	750	INTERGENIC	--	--	--
Ca2_P1_35925583	Ca2	35925583	TAAAAA/TAAAAAA	TTGTTCAGTGACCTGCAAGC	TCACGTGTTGGTGGGTTATC	60.0	758	INTERGENIC	--	--	--
Ca2_P1_35925644	Ca2	35925644	TG/T	TTGTTCAGTGACCTGCAAGC	TCACGTGTTGGTGGGTTATC	60.0	758	INTERGENIC	--	--	--

INDEL marker IDs	Chromosomes/ unanchored scaffolds	Physical positions (bp)	InDels (*Kabuli* reference genome-CDC Frontier/PI)	Forward primers (5'-3')	Reverse primers (5'-3')	Anneling temperature (0C)	Expected amplified product size (bp)	Structural annotation		Functional annotation	
								Sequence components of *kabuli* genome	*Kabuli* gene accession IDs	NCBI-KOG	TFs
Ca2_P1_35926372	Ca2	35926372	T/TA	ATTGGGATAACCCACCAACA	ATGCGCAAAGAGCCTTGTAT	59.9	691	INTERGENIC	--	--	--
Ca2_P1_35939640	Ca2	35939640	CTTTT/CTTT	TCTCCGGTTTCTAAGGCAAA	GGGCTCCACAAAAATCTCAA	59.8	413	INTERGENIC	--	--	--
Ca2_P1_3593968	Ca2	3593968	AATTATTATTCTTATTATTATT/AATTATTATT	GTTCCAACCCATACCAAACG	GGAGATGCGACTTGGTGAAT	60.1	621	INTERGENIC	--	--	--
Ca2_P1_35939936	Ca2	35939936	TG/T	TTGAGATTTTTGTGGAGCCC	CAATTTTAATCTTTGGCGGC	60.1	461	INTERGENIC	--	--	--
Ca2_P1_35942518	Ca2	35942518	ATT/ATTT	GAAAATCTTGGATGGCGAAA	TTTTTGCCGTTATGCGTGTA	60.0	264	DRR	Ca_09769	--	--
Ca2_P1_35942715	Ca2	35942715	GCCCC/GCCC	GAAAATCTTGGATGGCGAAA	AAATGCGCAGTTGTGTCTTG	60.0	870	DRR	Ca_09769	--	--
Ca2_P1_35944964	Ca2	35944964	CAAAA/CA	TCCAACCATACCTGAAAGGC	CTCGTGCGAACTTGTGTGTT	59.9	313	CDS (large-effect mutations)	Ca_09769	--	--
Ca2_P1_35945318	Ca2	35945318	CCAA/C	AACATCATACTTTTGCGGGC	GCCTGAAATATGCCGAGGTA	60.0	417	INTRON	Ca_09769	--	--
Ca2_P1_35945786	Ca2	35945786	CTATA/CTATATATA	TACCTCGGCATATTTCAGGC	ATGCAGGTTTTGGATTTTGC	60.1	776	INTRON	Ca_09769	--	--
Ca2_P1_35955086	Ca2	35955086	CTATATATATATATATA/CTATATATATATATA	GGATGGAAGGGATATACGAGG	TACCCCACACTGCAAATTCA	59.6	407	INTERGENIC	--	--	--
Ca2_P1_35958458	Ca2	35958458	GAAAAA/GAAAAAA	CATGCATAGAGACATGCCCA	GTGGGGTGGGGTTTTAAGTT	60.7	785	INTERGENIC	--	--	--
Ca2_P1_35960202	Ca2	35960202	CAAAAAA/CAAAAAAA	AGAGGACTTGTGTTGCATCC	TCCATTATAAGAGAATGTCCCCA	57.7	707	DRR	Ca_09770	--	--

INDEL marker IDs	Chromosomes/ unanchored scaffolds	Physical positions (bp)	InDels (*Kabuli* reference genome-CDC Frontier/PI)	Forward primers (5'-3')	Reverse primers (5'-3')	Anneling temperature (0C)	Expected amplified product size (bp)	Structural annotation		Functional annotation	
								Sequence components of *kabuli* genome	*Kabuli* gene accession IDs	NCBI-KOG	TFs
Ca2_P1_35960498	Ca2	35960498	TAA/TA	TGTGGCAAGTTTTAAA CAAATGA	TGAGTGGAGTGTGAAT GAAAATG	59.6	663	DRR	Ca_09770	--	--
Ca2_P1_35960819	Ca2	35960819	CTAT/CTATAT	AAACTTCCTCTCTTCCA CAAAGAA	TGAAAGCTTCAGTTGC AAAAA	59.8	537	DRR	Ca_09770	--	--
Ca2_P1_35961921	Ca2	35961921	TAAAA/TAAA	AGACGCTCATCACTCA ATTTCA	TTCGACAATATGGCCA ATGA	59.9	251	INTERGENIC	--	--	--
Ca2_P1_35962118	Ca2	35962118	ATAATT/ATAATTAATT	GGCCATATTGTCGAAA AGGA	TCGTCGATCTCATGAC TCAATC	59.9	550	INTERGENIC	--	--	--
Ca2_P1_35965637	Ca2	35965637	CTT/CT	TGGAAGAATGCTTTCA AGGG	ACATTCCATGGGCTGC TTAG	60.2	636	DRR	Ca_09771	J	NAC
Ca2_P1_35967436	Ca2	35967436	AATTTATA/AA	TTGAGGGGAGGTAAGC CTTT	TGATAGGAGGAAGCG TGACC	60.1	583	INTRON	Ca_09771	J	NAC
Ca2_P1_35970659	Ca2	35970659	GAA/GAAA	GATGGTCTCCATGCGT ACCT	ATCCAGTCATATCCCC TCCC	60.0	447	INTERGENIC	--	--	--
Ca2_P1_35972465	Ca2	35972465	GGCTTG/GGCTTGCTTG	ATGGCGCTTACGTCAA AGAC	AGCTCTTGTTGCAGCC AAAT	60.3	477	INTERGENIC	--	--	--
Ca2_P1_35982827	Ca2	35982827	CT/CTTT	ACAGAGACTCCCGAGC TGAG	TATCCTCCAAAGTGCA TCCC	59.7	349	INTRON	Ca_09772	T	LBD
Ca2_P1_35989118	Ca2	35989118	TGG/TG	TGTTGTGCAAACATGT CCCT	CCTTCTTTTCAAGCCA ATGC	60.0	547	INTRON	Ca_09773	T	LBD
Ca2_P1_35996189	Ca2	35996189	AATATATATATATATAT/AATATATATA TATATATATATATATAT	GCAAAAATTGAAGCAA ACCC	AAAATCAACGACGATG AATAAGAA	59.6	602	INTERGENIC	--	--	--
Ca2_P1_35996261	Ca2	35996261	TTCTCT/TTCT	CCATTCGTCAACGGCA TAAT	AAAATCAACGACGATG AATAAGAA	60.7	330	INTERGENIC	--	--	--

INDEL marker IDs	Chromosomes/ unanchored scaffolds	Physical positions (bp)	InDels (*Kabuli* reference genome-CDC Frontier/PI)	Forward primers (5'-3')	Reverse primers (5'-3')	Anneling temperature (0C)	Expected amplified product size (bp)	Structural annotation		Functional annotation	
								Sequence components of *kabuli* genome	*Kabuli* gene accession IDs	NCBI-KOG	TFs
Ca2_P1_36001001	Ca2	36001001	AAA/AAATAAATAA	CGCGTTGGTACTTTCATGTG	TCCCAAGTTAGGGGTAAGGC	60.2	386	INTERGENIC	--	--	--
Ca2_P1_36001500	Ca2	36001500	T/TC	GCCTTACCCCTAACTTGGGA	AAAAAGTCGTCGGGTCAATG	60.3	333	INTERGENIC	--	--	--
Ca2_P1_36004750	Ca2	36004750	TAAAAAAAAAA/TAAAAAAAA	TCTTTCCCGTTGATAGGTATCTT	CACACACAAATCAAATGAGACAA	58.2	473	INTERGENIC	--	--	--
Ca2_P1_36005038	Ca2	36005038	TAAA/TAA	TCTTTCCCGTTGATAGGTATCTTA	TGATTTCCAAAAATCCATCATCT	58.3	567	INTERGENIC	--	--	--
Ca2_P1_36005345	Ca2	36005345	TTTTAAAATTTAAAAT/TTTTAAAATTTAAAATTTAAAAT	TTGTCTCATTTGATTTGTGTGTG	TTAGTTGACATAGCGGTTGAAA	58.6	641	INTERGENIC	--	--	--
Ca2_P1_36005478	Ca2	36005478	AT/ATATT	TTGTCTCATTTGATTTGTGTGTG	TTAGTTGACATAGCGGTTGAAA	58.6	641	INTERGENIC	--	--	--
Ca2_P1_36006597	Ca2	36006597	GGGATGGATG/GGGATGGATGGATG	TCATAATCCGATCAAACCGA	ATCCAACTCCACGTGACACA	58.9	473	INTERGENIC	--	--	--
Ca2_P1_36114502	Ca2	36114502	CATATATATATAT/CATATATATATATAT	GCCACAACTATGCATTTTTCA	TGTGAGAGAAAGACAGCCGA	58.7	772	INTERGENIC	--	--	--
Ca2_P1_36146623	Ca2	36146623	GAAAAAA/GAAAAA	AAACAGCAACAAAAATCCCAA	TTTGGAAATCAAGAGGTGGG	59.5	201	INTERGENIC	--	--	--
Ca2_P1_36146917	Ca2	36146917	TCCCCC/TCCCCCC	AGGGAAACCCACCTCTTGAT	AAATCCAAAGTGCTTGCGTT	59.8	623	INTERGENIC	--	--	--
Ca2_P1_36146995	Ca2	36146995	ACAACCCCA/ACA	AGGGAAACCCACCTCTTGAT	AAATCCAAAGTGCTTGCGTT	59.8	623	INTERGENIC	--	--	--
Ca2_P1_36147608	Ca2	36147608	T/TCATTTGTA	AACGCAAGCACTTTGGATTT	GGTCTATGGACTTTCGGTGAA	59.8	429	INTERGENIC	--	--	--

INDEL marker IDs	Chromosomes/ unanchored scaffolds	Physical positions (bp)	InDels (*Kabuli* reference genome-CDC Frontier/PI)	Forward primers (5'-3')	Reverse primers (5'-3')	Anneling temperature (0C)	Expected amplified product size (bp)	Structural annotation		Functional annotation	
								Sequence components of *kabuli* genome	*Kabuli* gene accession IDs	NCBI-KOG	TFs
Ca2_P1_36177227	Ca2	36177227	TGGGTTTAGGGTTTAGGGTTTAGGGTTTAGG/TGGGTTTAGGGTTTAGGGTTTAGGGTTTAGGGTTTAGGGTTTAGG	TTGATGTTTGCATGATGGGT	TCTTGCATGGCCTTCAACTA	59.8	427	INTERGENIC	--	--	--
Ca2_P1_36224008	Ca2	36224008	CTTTTTTTTTT/CTTTTTTTTTTT	TTTTCACCACGACACACAAA	TGCACGTTTATAAGGAATCGG	58.6	354	INTERGENIC	--	--	--
Ca2_P1_36242074	Ca2	36242074	AAAGAAGAAGAAGAAGAAGAAGA/AAAGAAGAAGAAGAAGAAGAAGAAGA	CCCTCAAATCAAAGCCAAAA	AAGTGTGCGTGTAGCTCGTG	60.0	398	INTERGENIC	--	--	--
Ca2_P1_36297095	Ca2	36297095	TTATATATATATATATATATATATATATATATATATAT/TTATATATATATATATATATATATATATATATATATATATATAT	TGTTGACTCCTTGAGTAACTTTGTG	TGTTGCTTTTTGTTATTGTTTTTCA	59.8	534	DRR	Ca_09815	--	MYB_related
Ca2_P1_3630204	Ca2	3630204	CATATATATATATATATATATATATAT/CATATATATATATATATATATATAT	TGAAACAAGCATATGCGTCAA	TTTGCCTTCAATAATTGCACA	60.3	158	INTRON	Ca_10502	P	ERF
Ca2_P1_36304630	Ca2	36304630	AATATATATATATATATATATATATATATATATAT/AATATATATATATATATATATATATATATATATATAT	TGAACCGCATGAATCTTGTC	TCACAAGCTTTAGCATGACACA	59.7	542	INTERGENIC	--	--	--
Ca2_P1_36372982	Ca2	36372982	GATTATTATTATTATTAATATTATTATTA/GATTATTATTATTATTATTATTAATATTATTATTA	TGAGCTGAAATTGTTCGTCG	CCAATCCTCGTTGTCAAGGT	60.0	274	CDS (large-effect mutations)	Ca_09826	--	HD-ZIP
Ca2_P1_36390282	Ca2	36390282	GAGAAG/GAG	TACGGGAACGTTAAAGACCG	GGTTTTGCAGCTTGCTTTTC	60.0	599	CDS (large-effect mutations)	Ca_09828	--	MIKC
Ca2_P1_36631768	Ca2	36631768	TTATATATATATATAT/TTATATATATATATATAT	TCTCCTACCTCTGGCCAACA	CTCCAACATATGCATGGCAC	60.8	100	INTERGENIC	--	--	--
Ca2_P1_366324	Ca2	366324	ATTTTTTTTTTT/ATTTTTTTTTTTT	CAAGGGGCATTACCTTACCA	CTCCTCTCTTGCTTTTGGCA	59.8	727	INTERGENIC	--	--	--
Ca2_P1_3704285	Ca2	3704285	CAAAAAAA/CAAAAAAAAA	TTTTACATTCCCCACGCAAT	GGGCGACTTTGTGGTACTTC	60.2	512	DRR	Ca_10491	D	WRKY
Ca2_P1_371546	Ca2	371546	TTATATATATATATATATATA/TTATATATATATATATATA	TGCACTCAGCTGCTTCACAT	CTTTTGTTCATTGTCCGCAA	60.8	552	INTERGENIC	--	--	--

INDEL marker IDs	Chromosomes/ unanchored scaffolds	Physical positions (bp)	InDels (*Kabuli* reference genome-CDC Frontier/PI)	Forward primers (5'-3')	Reverse primers (5'-3')	Anneling temperature (0C)	Expected amplified product size (bp)	Structural annotation		Functional annotation	
								Sequence components of *kabuli* genome	*Kabuli* gene accession IDs	NCBI-KOG	TFs
Ca2_P1_3723233	Ca2	3723233	TATTATCAATTATCAATTA/TATTATCAATTA	ATTTCGTTGCGTTTTGGACT	TGGTCAATTTTTGTTTGCCA	59.6	234	INTERGENIC	--	--	--
Ca2_P1_3723573	Ca2	3723573	G/GCA	TGGCAAACAAAAATTGACCA	TTCCTCAAATTGCAACACCA	59.9	752	INTERGENIC	--	--	--
Ca2_P1_3723695	Ca2	3723695	TAAAAAA/TAAAAAAA	TGGCAAACAAAAATTGACCA	TTCCTCAAATTGCAACACCA	59.9	752	INTERGENIC	--	--	--
Ca2_P1_3724652	Ca2	3724652	TA/TAAGGCAA	ATTGGGATTGTGATGGAGGA	TGCCTGTAAAGCAAAAGTTTTAGAC	60.1	693	INTERGENIC	--	--	--
Ca2_P1_3732620	Ca2	3732620	ATT/AT	CTGTGCCATGTCCATAATGC	CCTGCCAAAGGAAACTTCTG	60.0	581	INTRON	Ca_10490	G	C3H
Ca2_P1_3733191	Ca2	3733191	TTAGAGACTAA/T	ACCTGCAACATGTGCGATAA	TGCATGAGATTGGAAATGGA	60.1	349	DRR	Ca_10489	G	C3H
Ca2_P1_3740423	Ca2	3740423	CATAT/CAT	CGGTGATGGTGAGAAAACCT	AACTTACGCCGCTTCTTCAA	60.0	792	INTRON	Ca_10488	--	WRKY
Ca2_P1_3740721	Ca2	3740721	CAAAAATAAAAA/CAAAAATAAAAATAAAAA	TCACTCGTTGCAACAGCTTC	TCTCATCATCCCTCTCCACC	60.2	267	DRR	Ca_10487	--	--
Ca2_P1_3741294	Ca2	3741294	CT/CTTT	GGTGGAGAGGGATGATGAGA	GCAGAGCACCAAACACAAGA	60.0	605	DRR	Ca_10487	--	--
Ca2_P1_3742318	Ca2	3742318	TAAAAAA/TAAAA	TCATTCCTCCATTGGTCCAT	AACGGCTAAATCCAGTGGTG	60.1	433	DRR	Ca_10487	--	--
Ca2_P1_3743015	Ca2	3743015	A/AATACAAATGGTG	CACCACTGGATTTAGCCGTT	TCCTTTTCGCTGATGAAACA	60.0	722	INTRON	Ca_10487	--	--
Ca2_P1_3743740	Ca2	3743740	TAAAAAAAAAA/TAAAAAAAAAAAAAAA	TGATGAATGGCATACTTGGC	CAGCAAGTCCCACGAAAGAT	59.5	326	INTRON	Ca_10487	--	--

INDEL marker IDs	Chromosomes/ unanchored scaffolds	Physical positions (bp)	InDels (*Kabuli* reference genome-CDC Frontier/PI)	Forward primers (5'-3')	Reverse primers (5'-3')	Anneling temperature (0C)	Expected amplified product size (bp)	Structural annotation		Functional annotation	
								Sequence components of *kabuli* genome	*Kabuli* gene accession IDs	NCBI-KOG	TFs
Ca2_P1_3743829	Ca2	3743829	CTTTTTT/CTTTTT	TTGGCCAATACTCCAACCAT	CAGCAAGTCCCACGAAAGAT	60.2	311	INTRON	Ca_10487	--	--
Ca2_P1_3745143	Ca2	3745143	TAAAA/TAAA	GAAGCACTGGACCTCCCATA	TGACTTGGAATAGATTGGACCAT	60.1	630	INTERGENIC	--	--	--
Ca2_P1_3746503	Ca2	3746503	TTA/TTAAACATA	TCCACATTGCACCAACACTT	TTGCTCCTCTGGTCCTCACT	60.0	925	INTERGENIC	--	--	--
Ca2_P1_3746722	Ca2	3746722	ATTTTTT/ATTTTTTT	CCGGAAAAGTGAATGTTGGT	TTGCTCCTCTGGTCCTCACT	59.8	252	INTERGENIC	--	--	--
Ca2_P1_3751543	Ca2	3751543	TTCT/TT	GGTGTTCCTCCCAAACTTGA	AGGCACAGCACTGGAAGAAT	59.9	798	INTERGENIC	--	--	--
Ca2_P1_3754940	Ca2	3754940	GA/GATA	TCCTTCAACTGTGGAACCAA	GCTTCTTGTGTGGGCTTAGG	59.1	751	DRR	Ca_10486	DL	--
Ca2_P1_3761698	Ca2	3761698	TAA/TAAA	ACCCTCCATTGCTGTTTTTG	CTCGGAACATGCATAGAATGA	60.0	262	INTERGENIC	--	--	--
Ca2_P1_3766617	Ca2	3766617	A/AT	GCCGCACGAAGTGAATTAG	ATACCAGGACCACCCTACCC	59.4	762	INTERGENIC	--	--	--
Ca2_P1_3766913	Ca2	3766913	ATTTTTT/ATTTTTTT	AGAACCATGTGCTTCATCCC	CAACACAATTGAATGGCAGG	59.9	478	INTERGENIC	--	--	--
Ca2_P1_3767986	Ca2	3767986	TAT/TATGAT	TGAATGCTAGTTCCACCGTT	AAAATTGGAATAAAACGCGG	58.2	488	INTERGENIC	--	--	--
Ca2_P1_3768123	Ca2	3768123	GAT/GATAT	CCGCGTTTTATTCCAATTTT	TCGACAGTTTTGTTCCTTCAG	59.0	126	INTERGENIC	--	--	--
Ca2_P1_3769682	Ca2	3769682	CA/CAAA	CCCTTAGGGGTAGCTCCATC	AGCTGAGGTCCTGTTGGCTA	59.9	537	INTERGENIC	--	--	--

INDEL marker IDs	Chromosomes/ unanchored scaffolds	Physical positions (bp)	InDels (*Kabuli* reference genome-CDC Frontier/PI)	Forward primers (5'-3')	Reverse primers (5'-3')	Anneling temperature (0C)	Expected amplified product size (bp)	Structural annotation		Functional annotation	
								Sequence components of *kabuli* genome	*Kabuli* gene accession IDs	NCBI-KOG	TFs
Ca2_P1_3771310	Ca2	3771310	TTATATATATATATATA/TTATATATATATA	GCTCGACGGAATAGCATTTT	TGGCCAAGCATGATCTAACA	59.3	612	DRR	Ca_10483	E	--
Ca2_P1_3771400	Ca2	3771400	ATTTTTTT/ATTTTTTTT	GCTCGACGGAATAGCATTTT	TGGCCAAGCATGATCTAACA	59.3	612	DRR	Ca_10483	E	--
Ca2_P1_3771545	Ca2	3771545	AT/A	GCTCGACGGAATAGCATTTT	TGGCCAAGCATGATCTAACA	59.3	612	DRR	Ca_10483	E	--
Ca2_P1_3777372	Ca2	3777372	AATATA/AATATATA	AATTTTTGGGGGAAAAATGC	CTCTGATAGACCCTCCCACCT	60.0	646	INTERGENIC	--	--	--
Ca2_P1_3782950	Ca2	3782950	TAAAAAAA/TAAAAAAAA	TGCCATGGGAGCTCTATGAT	CGCACTTGGAGTTGATGTGT	60.6	554	INTERGENIC	--	--	--
Ca2_P1_3787678	Ca2	3787678	TC/T	AATGGGCTATGTAACGACGC	CGCAGTTATTTGTGTTCGATG	60.0	483	DRR	Ca_10481	O	--
Ca2_P1_3787811	Ca2	3787811	AATATA/AATA	CTCCTGGCATTGTTTTTGGT	CCCTTTAACAATCCACCTCTTTC	60.0	834	DRR	Ca_10481	O	--
Ca2_P1_3799399	Ca2	3799399	A/AGTC	ATTTCCCACACCTCATTTGG	TAGCTCCGACACAACGAGAA	59.6	275	INTERGENIC	--	--	--
Ca2_P1_3800163	Ca2	3800163	TA/TAA	TTCCTCATTTTTCTCTTGGTTTG	CCACACTGCGCTTCAGATAA	59.6	377	INTERGENIC	--	--	--
Ca2_P1_3804034	Ca2	3804034	TA/TAA	TGGAAGAAAATGTGGCTGTG	TCACTTGTGTTGAGGTCCAAG	59.7	529	DRR	Ca_10480	--	--
Ca2_P1_3804111	Ca2	3804111	A/AC	AACCATGGCCTTGCAATTTA	CATTCAAGAAGCATAATCCACG	60.3	390	DRR	Ca_10480	--	--
Ca2_P1_3805996	Ca2	3805996	TAAAAA/TAAAAAA	GAACAAGAGCAGGCGTTAGG	CCACCCCTTGAATCAAAAGA	60.0	567	INTRON	Ca_10479	--	--

INDEL marker IDs	Chromosomes/ unanchored scaffolds	Physical positions (bp)	InDels (*Kabuli* reference genome-CDC Frontier/PI)	Forward primers (5'-3')	Reverse primers (5'-3')	Anneling temperature (0C)	Expected amplified product size (bp)	Structural annotation		Functional annotation	
								Sequence components of *kabuli* genome	*Kabuli* gene accession IDs	NCBI-KOG	TFs
Ca2_P1_3810502	Ca2	3810502	GTTT/GTT	TGAGATGAGGGAATCCAAGG	TCCTCTATTGTTTGGTGCCTG	60.0	431	DRR	Ca_10479	--	--
Ca2_P1_3810880	Ca2	3810880	ATT/A	CCCCACTTGATGATTTTCCA	CGCGTTTTCTTAGTTTCCAA	60.7	733	INTERGENIC	--	--	--
Ca2_P1_3812277	Ca2	3812277	TATTAATTAATTAATTA/TATTAATTAATTA	TGTTCTGCAAAATCGCAAAG	TGGTGACATGACTAATTTTGGA	60.0	414	INTERGENIC	--	--	--
Ca2_P1_3813617	Ca2	3813617	CAA/CAAA	TTGAAGCATACCTTGAGTGGTG	TTAATCTGGTTTTGGTGGGC	60.2	570	INTERGENIC	--	--	--
Ca2_P1_3813973	Ca2	3813973	TAAA/TAAAA	TCCAAAATTCTATGGCCCAC	GGTCACACCCTCTTAACCCA	59.8	720	INTERGENIC	--	--	--
Ca2_P1_3901844	Ca2	3901844	AC/ACTTCACGAGC	GTCGCTATCTCCCCAGTGAA	CATTCTGTGCCGCTATCTGA	60.2	248	DRR	Ca_10466	GT	--
Ca2_P1_3937629	Ca2	3937629	GATAAATAAATAAATAAATAAATAAATAAATAA/GATAAATAAATAAATAAATAAATAAATAAATAAATAAATAA	AAGTGTTTTGGTGCTGTCCC	GACTTCTATTGCCCCCACAA	60.0	154	INTERGENIC	--	--	--
Ca2_P1_3949908	Ca2	3949908	TAAAAAAAAAAAA/TAAAAAAAAAAAAA	TCAAAGAATAGAGGTATTGTCTCCG	CTAAAGCCTGCCCATTTCTG	60.0	795	INTERGENIC	--	--	--
Ca2_P1_3955516	Ca2	3955516	TATAATAATAATAATAATAATAA/TATAATAATAATAATAATAATAATAATAA	TTTCCGATTTCGAGGTGTTC	TGAATAGTGGAAAACACAACAATG	60.1	385	INTERGENIC	--	--	--
Ca2_P1_4010671	Ca2	4010671	AAAG/AAAGAAG	CCATCAATAGCTGCATGGTG	TTTGGGTAAGACTTCCACCG	60.1	400	INTERGENIC	--	--	--
Ca2_P1_4013903	Ca2	4013903	ATTTTTTT/ATTTTTTTT	CGGTCACAAGCATCTCATGT	TGATGCTTAAAAACTATGGGACAA	59.7	669	INTERGENIC	--	--	--
Ca2_P1_4014195	Ca2	4014195	AATATATATATATATATATAT/AATATATATATATATATATATATAT	TGGAAATTATGAGTCCCTTGA	TGAATACCAACGACACACCG	57.1	642	INTERGENIC	--	--	--

INDEL marker IDs	Chromosomes/ unanchored scaffolds	Physical positions (bp)	InDels (*Kabuli* reference genome-CDC Frontier/PI)	Forward primers (5'-3')	Reverse primers (5'-3')	Anneling temperature (0C)	Expected amplified product size (bp)	Structural annotation		Functional annotation	
								Sequence components of *kabuli* genome	*Kabuli* gene accession IDs	NCBI-KOG	TFs
Ca2_P1_4023004	Ca2	4023004	TTATATATATATATATATATATATATATAT/TTATATATATATATATATATATATATATATATATATAT	GCATCCAAGTGGACAACTGA	GTCTCATTTTCACACCCGCT	59.7	547	INTERGENIC	--	--	--
Ca2_P1_4028180	Ca2	4028180	TAATTATTAAATTATTAAA/TAATTATTAAA	GGAGCAACCGTTCTTCTTGA	AAATCCAACCACACCACCAT	60.4	431	INTERGENIC	--	--	--
Ca2_P1_404702	Ca2	404702	TTTTTAATATTTTAATATTTTAAT/TTTTTAATATTTTAATATTTTAATATTTTAAT	ATGGATCAGCTCAACCCAAT	AACCTAAACGCAAGGTCTCG	59.4	225	INTERGENIC	--	--	--
Ca2_P1_405077	Ca2	405077	CTTTTTTTTTTT/CTTTTTTTTTTTT	GACCAGGTGGCTCTCACTGT	GGTTTTGTCTCCTTACCCCC	60.3	528	INTERGENIC	--	--	--
Ca2_P1_4057856	Ca2	4057856	AATATATATATATATATA/AATATATATATATATA	TGCAAAATGGAGATTTGTGG	TGATGGGAGCATGTGACACT	59.5	712	INTERGENIC	--	--	--
Ca2_P1_4060085	Ca2	4060085	AATATATATATATATATATATATATATATA/AATATATATATATATATATATATATATA	GACCTTGCATGGAGTGTTGA	GGCATAGCCATGAAGAACTTG	59.7	690	INTERGENIC	--	--	--
Ca2_P1_408895	Ca2	408895	TTATATATATATATATATATA/TTATATATATATATATATA	AATGGTGGCAATTGTTGTGA	AACATGCCATGCATCAAAAA	59.8	586	INTERGENIC	--	--	--
Ca2_P1_4098274	Ca2	4098274	AGAGTGGAAAATGAGTGGA/AGAGTGGA	CCATGGGTTTATTGTGGCTT	TTGAAAGTCGATCCACCACA	59.7	404	INTERGENIC	--	--	--
Ca2_P1_4116151	Ca2	4116151	GTTTTTTTTTT/GTTTTTTTTTTT	TTTTTATGACGCAGATGCCC	TGGATCCTTACATGGTCGGT	61.0	665	INTERGENIC	--	--	--
Ca2_P1_4123258	Ca2	4123258	AATATATATATATATA/AATATATATATATA	GCCCAATCATTTTCAAGGAA	CGTTCAACTCCCACAGGATT	59.9	667	INTERGENIC	--	--	--
Ca2_P1_416248	Ca2	416248	TTATATATATATATATATATATATATATAT/TTATATATATATATATATATATAT	ATAAATGAAGGGGTGGGAGG	GTTTCCCATAAACAGCACCC	60.0	882	INTERGENIC	--	--	--
Ca2_P1_4184153	Ca2	4184153	TTATATATATATATATATATATA/TTATATATATATATATATATATATATA	ACGTTGATCTCTTCGCCATC	TCTGAGGGAATTTTTCCACG	60.2	661	INTERGENIC	--	--	--

INDEL marker IDs	Chromosomes/ unanchored scaffolds	Physical positions (bp)	InDels (*Kabuli* reference genome-CDC Frontier/PI)	Forward primers (5'-3')	Reverse primers (5'-3')	Anneling temperature (0C)	Expected amplified product size (bp)	Structural annotation		Functional annotation	
								Sequence components of *kabuli* genome	*Kabuli* gene accession IDs	NCBI-KOG	TFs
Ca2_P1_4192077	Ca2	4192077	ATTTT/ATTTTT	GAGGAAGGGGGAGAAGAGAA	TCTGGTCCAGCTTGTAACCC	59.7	645	INTERGENIC	--	--	--
Ca2_P1_4204531	Ca2	4204531	GTATATATATATATATATATATATATA/GTATATATATATATATATATATA	GGGTTTGAACCGTCCCTACT	CTTTCTCCATCTCCTTCCCC	60.2	633	INTRON	Ca_21114	--	--
Ca2_P1_42172	Ca2	42172	TTATATATATATATATATATATATA/TTATATATATATATATA	TGGCAGTCTGTTTCACGTTT	CAAGGGACCAGATTCTGCAT	59.3	553	INTERGENIC	--	--	--
Ca2_P1_422540	Ca2	422540	G/GTCTTCCT	AGAGTTGGAGGGTGCAGCTA	CTTCGAATCCAACCTTGGTG	60.0	829	INTERGENIC	--	--	--
Ca2_P1_4254708	Ca2	4254708	CAATAATAATAATAATAATAATAATAATAATAATAACAACAATAACAATAATAATAATAATAATAATAATAATAATAATAATAATAATAATAATAATAATAATAATAATAATAATA/CAATAATAATAATAATAATAATAAT[illegible]	AGCATCTCGAGACGAACTCA	TGAAGGCCCAAAAACAATTC	58.7	562	DRR	Ca_21112	--	--
Ca2_P1_4266265	Ca2	4266265	A/ATTAATCCGAAAG	TGGACGTTTAACGGTTGACA	CCCAGTAGAAAAGAAGGGGC	60.0	705	INTERGENIC	--	--	--
Ca2_P1_4267670	Ca2	4267670	TTATATATATATATATATATAT/TTATATATATATATATATATAT	GTGTGCGAATGTGGTTTTTG	GGACGGACAACAGCTATTTATG	60.0	505	INTERGENIC	--	--	--
Ca2_P1_4285697	Ca2	4285697	TAAAA/TAAA	CCAGTCCCGAAATTAAACGA	ACTTGCGATAATCGATTGGG	59.9	613	INTERGENIC	--	--	--
Ca2_P1_4364666	Ca2	4364666	TC/T	GGACGAACGATCCAAAGAAA	TCCATTCGAAGTGTCAGTGC	60.1	806	INTRON	Ca_21108	GC	HSF
Ca2_P1_4451851	Ca2	4451851	GTGCAATG/GTG	TCGTCCACTTGTGGTCCTATC	ATTGCAACTCGGAATTGTCC	60.0	169	INTERGENIC	--	--	--
Ca2_P1_4527479	Ca2	4527479	GAAGACAAAAAAAACATAAGACAAAAAAAACAT/GAAGACAAAAAAAACATAAGACAAAAAAAACATAAGACAAAAAAAACAT	GCAATTTCACCTCTTCTGCC	TGACGAAGCCCACAATAACA	59.8	523	INTERGENIC	--	--	--
Ca2_P1_453297	Ca2	453297	TTATATATATATATATATAT/TTATATATATATATATATATAT	TTCAAACCAAATGATCCAATGA	ATAAATGTTGGGTGGCCAAA	60.2	685	DRR	Ca_16985	L	--

INDEL marker IDs	Chromosomes/ unanchored scaffolds	Physical positions (bp)	InDels (*Kabuli* reference genome-CDC Frontier/PI)	Forward primers (5'-3')	Reverse primers (5'-3')	Anneling temperature (0C)	Expected amplified product size (bp)	Structural annotation		Functional annotation	
								Sequence components of *kabuli* genome	*Kabuli* gene accession IDs	NCBI-KOG	TFs
Ca2_P1_4539194	Ca2	4539194	AATATATATATATATATATATAT/AATATATATATATATATATATAT	TTTGCCTTCCACTTTTGTCC	ACCAACCTGCATACCCTGTC	60.1	890	INTERGENIC	--	--	--
Ca2_P1_4595333	Ca2	4595333	CAATAATAATAATAATAATAATAATAATAATAATAATAATA/CAATAATAATAATAATAATAATAATAATAATAATAATAATA	GTGGACCAAGCCCATTAAAA	TCACTCCTCAAGTGGAACCC	59.8	124	INTERGENIC	--	--	--
Ca2_P1_4625527	Ca2	4625527	TT/TTATTATTAT	TTTCAAATTTTGTTTTGCACCTT	CGTTTGGGTCTTCAGTTGGT	59.9	416	INTERGENIC	--	--	--
Ca2_P1_4655023	Ca2	4655023	AATATATATATATATATATATATATA/AATATATATATATATATATATATA	AATATGCGGAATTTGGTCCT	TTGTGTTTCCGTAGCACGAG	58.4	891	INTERGENIC	--	--	--
Ca2_P1_469349	Ca2	469349	TA/TAA	CCCAAGACAACCTTGCAGAT	ATGAAGTGCAAACCATGCTG	60.1	674	DRR	Ca_16986	--	--
Ca2_P1_4700407	Ca2	4700407	ATTATTTATTTATTTATTTATTTATT/ATTATTTATTTATTTATTTATT	TCTCACGGTTGTGAGGACTG	CCTACCTCTGGAGTGTCCGT	59.9	926	INTERGENIC	--	--	--
Ca2_P1_4749260	Ca2	4749260	TTTTATTTATTTATTTATTTATTTATTTATTTATTT/TTTTATTTATTTATTTATTTATTTATTTATTTATTTATTTATTTATTTATTT	TCCTAGAACATGCATCACGC	TATCCCTTCGGAACTTGCAC	59.8	633	INTERGENIC	--	--	--
Ca2_P1_4812881	Ca2	4812881	ATTTTTTT/ATTTTTTTTT	AACCAGTTCCGTTCCAAGTG	TCCCCCAAACAGAATTTGAG	60.0	731	DRR	Ca_21090	--	MYB
Ca2_P1_4876489	Ca2	4876489	CCATTTCACGCCTAAAATCATTTCACGCCT/CCATTTCACGCCTAAAATCATTTCACGCCTAAAATCATTTCACGCCT	CCAACAAGGATGAAATGTGC	AAGCCGTGAAATGGTTTTTG	59.0	419	INTERGENIC	--	--	--
Ca2_P1_4891335	Ca2	4891335	AAATTGAAG/A	AACGGTGTGAAAGGGAAATG	TGAATTGAATTTGGAAGGGG	59.8	515	INTERGENIC	--	--	--
Ca2_P1_4892794	Ca2	4892794	CATATATATATATATATATATATATATATATAT/CATATATATATATATATATATATATATATATATATATATATATAT	GCGGACATAAGGAAGATGTGA	AGGTCTGCGAAGATGGAAAA	60.1	606	INTERGENIC	--	--	--
Ca2_P1_4894086	Ca2	4894086	TA/TAGACAACTCAA	TTGAACTACAACCAGCGCAC	TTTTGTTCAATTTCTTGCTCCA	59.9	493	INTERGENIC	--	--	--

INDEL marker IDs	Chromosomes/ unanchored scaffolds	Physical positions (bp)	InDels (*Kabuli* reference genome-CDC Frontier/PI)	Forward primers (5'-3')	Reverse primers (5'-3')	Anneling temperature (0C)	Expected amplified product size (bp)	Structural annotation		Functional annotation	
								**Sequence components of *kabuli* genome**	***Kabuli* gene accession IDs**	**NCBI-KOG**	**TFs**
Ca2_P1_4894836	Ca2	4894836	ATGATT/AT	AAAGGGAAGAAATTTCGGGA	AACCGGACGATCATTTGAAG	59.9	498	INTERGENIC	--	--	--
Ca2_P1_4895677	Ca2	4895677	AA/AATATGA	CCAGGATAAAACAGCCCAGA	TGGATGCTTTCCCAACTTTC	60.1	467	INTERGENIC	--	--	--
Ca2_P1_4915954	Ca2	4915954	AAATAATAATAATAATAATAATAATAATAATAATAATAATAATAATAATAA/AAATCATAATAATAATAATAATAATAATAATAATAATAATAATAATAATAATAATAATAA	TTATCGGACATTTTCCCGAG	CTAGGTCGCCCTTTCAGGTT	59.9	504	INTERGENIC	--	--	--
Ca2_P1_4921648	Ca2	4921648	AAAATAAATAAATAAATAAATA/AAAATAAATAAATAAATAAATAAATA	TCCTGGATCACTGCACCATA	CATTATTGTTGTTGCGTCCG	60.1	696	INTERGENIC	--	--	--
Ca2_P1_4966099	Ca2	4966099	A/ATGTTTTGCGCGGC	GGATGACATGGCTTTCAACA	GATGTTTCTTCTTAGCGGCG	59.5	455	INTERGENIC	--	--	--
Ca2_P1_5026096	Ca2	5026096	TAAATAAAATAAAATAA/TAAATAAAATAA	TTGAGACAAAATTAAGAAACATAAGCA	TCGTGCTCCGTAAACGGTAT	59.7	532	INTERGENIC	--	--	--
Ca2_P1_5096452	Ca2	5096452	AATATATATATATATATATATATAT/AATATATATATATATATATATATATAT	GTTTTGTGGTGCAGTTTGGA	TTTGAAAGAGTCCGAATTCAAATA	59.6	570	INTERGENIC	--	--	--
Ca2_P1_5112695	Ca2	5112695	CGAATGAAT/CGAAT	TTGCTGTTTTCCTGTTGCTG	GGACAAAGCCCAGATTTTCA	60.0	381	INTERGENIC	--	--	--
Ca2_P1_5135372	Ca2	5135372	AATATATATATATATATATATATATATATATATATATATATATAT/AATATATATATATATATATATATATATATATATATATATATAT	TGATTTATTTCCACGCCTACG	TTGTTTCTCCCCAGTTTTGG	60.0	952	INTERGENIC	--	--	--
Ca2_P1_5154866	Ca2	5154866	AAAATTG/A	TGTGTCGTGGTTTCTGTCTTG	TGAATTCATGGCTAAGAAGCCT	59.8	516	INTERGENIC	--	--	--
Ca2_P1_5163788	Ca2	5163788	CAAAAAAAAA/CAAAAAAAAAA	TGCGAGAAAGTGAATTCCAG	GGAATGATGATGGGGTTTTG	59.0	658	DRR	Ca_14696	--	--
Ca2_P1_5286155	Ca2	5286155	AATATATATATATATATATATATATATATATA/AATATATATATATATATATATATATATATATATATA	TTCATGCCTCCCATTTCTTC	CGCTCTGTCAACATGTCAGC	60.0	752	INTRON	Ca_14679	T	--

INDEL marker IDs	Chromosomes/ unanchored scaffolds	Physical positions (bp)	InDels (*Kabuli* reference genome-CDC Frontier/PI)	Forward primers (5'-3')	Reverse primers (5'-3')	Anneling temperature (0C)	Expected amplified product size (bp)	Structural annotation		Functional annotation	
								Sequence components of *kabuli* genome	*Kabuli* gene accession IDs	NCBI-KOG	TFs
Ca2_P1_5410265	Ca2	5410265	TGA/TGAAGA	CGAAACCCAGATTTGGAAAG	CAATTTTGTTTCCCACACCC	59.5	212	INTERGENIC	--	--	--
Ca2_P1_55390	Ca2	55390	GACACACACACACACACA/GACACACACACACACACACA	GCACCGGGAGATACGTAAGA	TCACGGCTTCACAAAACAAG	60.1	798	INTERGENIC	--	--	--
Ca2_P1_5566169	Ca2	5566169	GAAAAAAAAAAA/GAAAAAAAAAAAA	TGGGAGTATCCTTCACTGCC	TTCGGATTTTCCTTCCCTTT	60.1	836	INTERGENIC	--	--	--
Ca2_P1_5620818	Ca2	5620818	ATTTTTT/ATTTTTTT	TTCGATTTGACATAAAAGACACAAA	TGTTTTAATTGTAAGCATGAGAAGTTG	59.9	526	INTERGENIC	--	--	--
Ca2_P1_5626731	Ca2	5626731	ATTTTTTTTTTT/ATTTTTTTTTT	GAACCCAATATAGCCGCAAA	TTTGCTGCAACAACTCCAAG	59.9	782	INTRON	Ca_14650	I	C3H
Ca2_P1_5656182	Ca2	5656182	AAA/AAATAA	CGCAAAACATTTCCAAACCT	CAATTCCCACAGGTACGCTT	60.0	464	INTERGENIC	--	--	--
Ca2_P1_5674299	Ca2	5674299	TCTTTACTTTACTT/TCTTTACTTTACTTTACTT	ATTAGTCCACTGTGCTGGGG	GCTTCCAAATTGCTTGTTCC	60.0	815	INTRON	Ca_14646	O	FAR1
Ca2_P1_5753062	Ca2	5753062	ATCTCTCTCT/ATCTCTCT	TCATCTCGCAAGTCTACCCA	AAAATTGGAATGTCACACACCTT	59.4	539	INTERGENIC	--	--	--
Ca2_P1_5818662	Ca2	5818662	TTCAT/TT	CGGTTGGAAACGAAGAATGT	CTGGGTAGGCACGTTTGATT	60.0	524	INTRON	Ca_19687	--	--
Ca2_P1_5860585	Ca2	5860585	TTATATATATATATATATATATATATATATATAT/TTATATATATATATATATATATATATATATATATATATATAT	TGGGTAGTTTTGGATACCCC	CCCAAAAAGGAAAAAGAAACAA	58.6	833	INTERGENIC	--	--	--
Ca2_P1_5969703	Ca2	5969703	CTATATATATATATATATATATAT/CTATATATATATATATATATAT	TGTCGTCCGTCAATGGTCTA	AAAGTCGTCATTCCAAACGG	60.1	446	INTERGENIC	--	--	--
Ca2_P1_5999646	Ca2	5999646	ATTTTTTTTTTT/ATTTTTTTTTT	GGAGTGACATGCCACATTTT	AAAAATAAAATAGGGGCCAGAA	58.4	672	INTERGENIC	--	--	--

INDEL marker IDs	Chromosomes/ unanchored scaffolds	Physical positions (bp)	InDels (*Kabuli* reference genome-CDC Frontier/PI)	Forward primers (5'-3')	Reverse primers (5'-3')	Anneling temperature (0C)	Expected amplified product size (bp)	**Structural annotation**		**Functional annotation**	
								**Sequence components of *kabuli* genome**	***Kabuli* gene accession IDs**	**NCBI-KOG**	**TFs**
Ca2_P1_6026121	Ca2	6026121	AATATATATATATAT/AATATATATATAT	TACAAACATGGGCCAAGTGA	TGTGTGTTTGTCTGTTCGCA	60.0	325	INTERGENIC	--	--	--
Ca2_P1_6179167	Ca2	6179167	AATATATATATATATATATAT/AATATATATATATATATATATATATAT	TACACCTGGACAGAGAGGGC	GGAAATGTTGGCGAGTTGTT	60.3	928	INTERGENIC	--	--	--
Ca2_P1_6306996	Ca2	6306996	GTT/GT	CTGTTCAGCTTTTTCGTCCC	CTCCCCACCCTTAACTTGGT	59.9	922	INTERGENIC	--	--	--
Ca2_P1_6317566	Ca2	6317566	GTATATATATATATATATATATATATATATAT/GTATATATATATATATATATATATATAT	CACCAAATGATGACGACGAC	ATTCACGTCGTTCCTGTTCC	60.0	324	INTERGENIC	--	--	--
Ca2_P1_6357510	Ca2	6357510	ATTTTTTTTT/ATTTTTTTT	CGTAAATGTCCTCCAATTCCA	GCAAACGTTTTTCAAATTTTCTG	59.8	529	INTERGENIC	--	--	--
Ca2_P1_6436294	Ca2	6436294	TTTATTATTATTATTATTATTATTATTATTATTAT/TTTATTATTATTATTATTATTATTATTATTATTAT	CCCCATCTCCCATTCCTTAT	TTTATGGGCATGAAACCACTT	60.0	863	INTERGENIC	--	--	--
Ca2_P1_6489870	Ca2	6489870	AAATAATAATAATAATAATAATAAT/AAATAATAATAATAATAAT	ATGGTCCAAGGAAATTGTGC	GTCTGAATGGGAAACTTGGC	59.8	369	INTERGENIC	--	--	--
Ca2_P1_6505178	Ca2	6505178	TA/TAA	CGTAATAACGGCGAGGAAAA	TGAGCAGGGGGAAGAGTAGA	60.1	548	INTERGENIC	--	--	--
Ca2_P1_6513260	Ca2	6513260	AAATAATAATAATAATAATAATAATAATAATAAT/AAATAATAATAATAATAATAATAATAATAAT	ATGGTCCAAGGAAATTGTGC	AGTCGTCGAGGCCAAATCTA	59.8	265	INTERGENIC	--	--	--
Ca2_P1_6522206	Ca2	6522206	AT/ATT	GGAAGTGAAGGTCACGGTGT	TGAGCACGAATAATTGCAGC	60.0	593	DRR	Ca_20947	--	--
Ca2_P1_6638407	Ca2	6638407	GAATAACTGCATGTCTAATAACT/GAATAACT	GGGACCGCAAGTGCTATTTA	TAGGGCAAAAATTCAGGTGG	60.1	344	INTERGENIC	--	--	--
Ca2_P1_6640316	Ca2	6640316	TAAAAAA/TAAAAAAA	ATGGCCACGAATCTGAAGAG	ACGGTTGTTTTGGCATTGAT	60.2	890	INTERGENIC	--	--	--

INDEL marker IDs	Chromosomes/ unanchored scaffolds	Physical positions (bp)	InDels (*Kabuli* reference genome-CDC Frontier/PI)	Forward primers (5'-3')	Reverse primers (5'-3')	Anneling temperature (0C)	Expected amplified product size (bp)	Structural annotation		Functional annotation	
								Sequence components of *kabuli* genome	*Kabuli* gene accession IDs	NCBI-KOG	TFs
Ca2_P1_6640452	Ca2	6640452	GAAAA/GAAAAA	AAAAAGGTCCTAGGGTGGGA	ACGGTTGTTTTGGCATTGAT	59.8	473	INTERGENIC	--	--	--
Ca2_P1_6641737	Ca2	6641737	CATATAT/CATATATATAT	GTATTCAGGGGCGAAGGAAT	AATGCAAACGACAAAGGAGG	60.3	500	INTERGENIC	--	--	--
Ca2_P1_6641916	Ca2	6641916	TG/T	GTTCTATGGGGACAGGGTCA	TATCAACTACCAGAGCGGGG	59.8	476	INTERGENIC	--	--	--
Ca2_P1_6642515	Ca2	6642515	TTATATATATATATATA/TTATATATATATATATATA	CCCCGCTCTGGTAGTTGATA	ACCCACTTTGAGAAACGTGC	60.1	479	INTERGENIC	--	--	--
Ca2_P1_6643765	Ca2	6643765	AT/ATT	ATGGAACAAGCAGCGAAGTT	GTTGATACGAGGGGGTTGAA	59.9	537	INTERGENIC	--	--	--
Ca2_P1_6643843	Ca2	6643843	CTTTTTTTTTTT/CTTTTTTTT	ATGGAACAAGCAGCGAAGTT	GTTGATACGAGGGGGTTGAA	59.9	537	INTERGENIC	--	--	--
Ca2_P1_6647662	Ca2	6647662	GTTTT/GTTT	GGCTACTGCTGTACGTAGCGA	AGTGAAACCAACGCCAAAAC	60.6	356	INTERGENIC	--	--	--
Ca2_P1_6648530	Ca2	6648530	TAAAAAA/TAAAAAAAA	TTATCGATCGTCGGTGTCAA	TTCCATATGCACCTCCCATT	60.1	612	INTERGENIC	--	--	--
Ca2_P1_6648980	Ca2	6648980	ATTTTTTTTTT/ATTTTTTTTTTT	AGCCTAATGGGAGGTGCATA	TATGAGGGGGTTGAATTGGA	59.6	563	INTERGENIC	--	--	--
Ca2_P1_6656104	Ca2	6656104	CG/CGGG	GAGGACAAGGCCAATTTGAA	CCACCCACTTCGAGAAACAT	60.1	955	INTERGENIC	--	--	--
Ca2_P1_6659290	Ca2	6659290	GTTT/GTTTT	TGTTTGACAAATGTTAAGTGCG	GGTGTGACTGGCCGTATTTT	58.8	621	INTERGENIC	--	--	--
Ca2_P1_6680911	Ca2	6680911	AAATAATAATAAT/AAATAATAATAATAAT	GAACGAAAATGTGTTTTATGGC	GGCGTTGCCTACACTCAAAT	58.5	381	INTERGENIC	--	--	--

INDEL marker IDs	Chromosomes/ unanchored scaffolds	Physical positions (bp)	InDels (*Kabuli* reference genome-CDC Frontier/PI)	Forward primers (5'-3')	Reverse primers (5'-3')	Anneling temperature (0C)	Expected amplified product size (bp)	Structural annotation		Functional annotation	
								Sequence components of *kabuli* genome	*Kabuli* gene accession IDs	NCBI-KOG	TFs
Ca2_P1_6714211	Ca2	6714211	AT/ATATATATATATATATTT	CTCCATTGTTGGCATCCTTT	ACCGCGAAGATGTGGTTTAC	59.9	860	INTERGENIC	--	--	--
Ca2_P1_6753713	Ca2	6753713	TTATATATATATATATATATATATATAT/TTATATATATATATATATATATATATATATATATATAT	TGATGCCATGATATGAATGCT	GGCCAAGAGAAGTTCGAGTG	59.0	415	INTERGENIC	--	--	--
Ca2_P1_6769002	Ca2	6769002	ACCCC/ACCC	TGACCCGTCGTGACTACAAA	AGCGGTGATGCTTTCTCAAT	60.2	265	INTERGENIC	--	--	--
Ca2_P1_6772415	Ca2	6772415	TAAAAAAAAAAAAA/TAAAAAAAAAAAAAA	GGCTTTTCACTACAAGCCCA	TCGGCAATTATTTGTTTCCA	60.2	153	INTERGENIC	--	--	--
Ca2_P1_6815005	Ca2	6815005	ATTATTTATTTATTTATTTATTTA/ATTATTTATTTATTTATTTA	GAAATTTTAGGCGCAACAGC	TCCAATCCCATTGTCCACTT	59.9	703	INTERGENIC	--	--	--
Ca2_P1_6831436	Ca2	6831436	AC/A	CCTGTGACATGTTGCCTTTG	AAATGGGCTGAGTTTGAACA	60.2	360	INTERGENIC	--	--	--
Ca2_P1_6867609	Ca2	6867609	CAAAAAAAAAAA/CAAAAAAAAAA	TAACCACATTGCAAGCCAAA	CAATCAAAATGCAACAGACACA	60.1	451	INTERGENIC	--	--	--
Ca2_P1_699070	Ca2	699070	ATTATTATT/ATTATTATTCTTATTATT	CCGATTGTGTCTTGACTTTTGA	CTAATCCGCCCGACAAATAA	60.1	412	INTERGENIC	--	--	--
Ca2_P1_700339	Ca2	700339	ATTTTTTTTT/ATTTTTTTTTT	AAACTAGGTCAGAAACCGATAGAA	CCAACACAAACCTAAACCGA	57.7	896	INTERGENIC	--	--	--
Ca2_P1_7109849	Ca2	7109849	AATATATATATATATAT/AATATATATATATATATAT	CATCTTCGCAGGATGCATTA	TTACCACTAAGGCGCGAACT	59.8	273	INTERGENIC	--	--	--
Ca2_P1_7144237	Ca2	7144237	A/AG	GGACCGGAATCATAATGCAA	TTTTTCCGTAAAAGTCCCCC	60.7	743	INTERGENIC	--	--	--
Ca2_P1_7171869	Ca2	7171869	TTAT/TT	CGGCCATTAAATTTCTGACA	TTGCATTATGTTTCTGGCAAA	58.6	320	INTERGENIC	--	--	--

INDEL marker IDs	Chromosomes/ unanchored scaffolds	Physical positions (bp)	InDels (*Kabuli* reference genome-CDC Frontier/PI)	Forward primers (5'-3')	Reverse primers (5'-3')	Anneling temperature (0C)	Expected amplified product size (bp)	Structural annotation		Functional annotation	
								Sequence components of *kabuli* genome	*Kabuli* gene accession IDs	NCBI-KOG	TFs
Ca2_P1_7171912	Ca2	7171912	TATTTAATTTAATTTAATT/TATTTAATT TAATT	CGGCCATTAAATTTCT GACA	TCATTTCATAACGAGT GACCCA	58.6	584	INTERGENIC	--	--	--
Ca2_P1_7172121	Ca2	7172121	CTATATATATATAT/CTATATATATAT	TTTGCCAGAAACATAAT GCAA	TCATTTCATAACGAGT GACCCA	59.2	285	INTERGENIC	--	--	--
Ca2_P1_7172172	Ca2	7172172	ATTTTTTTTT/ATTTTTTTTTT	TTTGCCAGAAACATAAT GCAA	TTTTGGAAAAATGAAG GACCA	59.2	774	INTERGENIC	--	--	--
Ca2_P1_7173102	Ca2	7173102	GAAAAAA/GAAAAA	CTCAATCCAAACACAC GCTT	AATGACCACCTCTTGT TGCC	58.8	787	INTERGENIC	--	--	--
Ca2_P1_7276294	Ca2	7276294	CAAAA/CAAAAA	TCCACCACAATCGTTA AGCA	AGCGGAGATTTTACCG GATT	60.1	676	INTERGENIC	--	--	--
Ca2_P1_7302005	Ca2	7302005	TTATATATATATATATATATA/TTATATA TATATATATATATATA	TGTAGCAAAACGTGCA GACC	GGATATTTTGCTCTCT CGCAA	59.9	159	INTERGENIC	--	--	--
Ca2_P1_7380468	Ca2	7380468	AATATATATATATATATATATA/AATAT ATATATATATATATATATA	ATGGTGTTGCTTGGAA GGAC	ATGTGGAAGCAAATTG GGAA	60.0	305	INTERGENIC	--	--	--
Ca2_P1_7464860	Ca2	7464860	GACTTAGT/G	GCTGATGCATGAGGAA ATACC	AAAAGCCACGTCATCC AAAG	59.5	448	INTERGENIC	--	--	--
Ca2_P1_7531659	Ca2	7531659	ATTTTTTT/ATTTTTTTT	TCCATAACCGGATCCA AATC	CGTATGGAACAACTCC CGAT	59.6	730	INTERGENIC	--	--	--
Ca2_P1_7535934	Ca2	7535934	TAAAAAAAAAA/TAAAAAAAAAAA	AACCGGACATTTTGTT GGAG	TGCACAAATTGGTGGA AAAA	59.8	626	INTERGENIC	--	--	--
Ca2_P1_753916	Ca2	753916	CTTTTTTTTTT/CTTTTTTTTT	TTGCCCATTAACTTTAC CCG	ACTTGTGCATTTGCAT TAGTTC	59.8	555	DRR	Ca_17000	--	ARF
Ca2_P1_7540916	Ca2	7540916	TATAGTAATAAATAGTAATAAAT/TATA GTAATAAAT	AATATTCGAACCTCCC ACCC	TTAATCGTCCAACTCC CACC	60.0	890	INTERGENIC	--	--	--

INDEL marker IDs	Chromosomes/ unanchored scaffolds	Physical positions (bp)	InDels (*Kabuli* reference genome-CDC Frontier/PI)	Forward primers (5'-3')	Reverse primers (5'-3')	Anneling temperature (0C)	Expected amplified product size (bp)	Structural annotation		Functional annotation	
								Sequence components of *kabuli* genome	*Kabuli* gene accession IDs	NCBI-KOG	TFs
Ca2_P1_7555681	Ca2	7555681	GTTTTTTTTTTT/GTTTTTTTTTT	AATTTTGCGTTCATCGGAGA	AATCACACACGACACCGAGA	60.6	178	INTERGENIC	--	--	--
Ca2_P1_7572211	Ca2	7572211	TTATATATATATATATATATATATATATATATATATATAT/TTATATATATATATATATATATATATATATATATAT	ATCTATCATGCAAAGGCCCA	TCGAACCTAAGAATGCCCTT	60.4	347	DRR	Ca_18115	D	LBD
Ca2_P1_7592333	Ca2	7592333	AATATATATATATATATATATATATATA/AATATATATATATATATATATATATATATATATATA	CCAATTGTTCCTTTGCGTCT	CCAAAGAAAGACAATCCAAATG	60.1	778	INTERGENIC	--	--	--
Ca2_P1_7610276	Ca2	7610276	T/TTGTACTTGTG	CGCTTCGTGATTGTAAGCAA	TTCTCTCTCCTCGCATTTCC	60.0	386	INTERGENIC	--	--	--
Ca2_P1_7617731	Ca2	7617731	CTAAAATAAAATAAAA/CTAAAATAAAATAAAATAAAATAAAA	CCAAGATCTCGTAGTGCAACA	TGGGTTTAGTCCGTTTTTCTTC	58.9	468	INTERGENIC	--	--	--
Ca2_P1_7620237	Ca2	7620237	ATT/ATTGTTGTTGTTGTTGTT	CCTTGCGTGAGTTTGACGTA	TGGTGAATTTATGCCGATGA	59.9	463	INTERGENIC	--	--	--
Ca2_P1_7654401	Ca2	7654401	ATTAAACTTAAA/ATTAAACTTAAACTTAAA	CATGGGATGCAGTTTTAGCA	GGTATGTCAGGAAGGAACGG	59.7	590	INTERGENIC	--	--	--
Ca2_P1_7655014	Ca2	7655014	AA/AAATA	CCGTTCCTTCCTGACATACC	GCAAAATTGGCTTCTTACGG	59.4	516	INTERGENIC	--	--	--
Ca2_P1_7655513	Ca2	7655513	GTTTTTTTTTTT/GTTTTTTTTT	CCCACAATAGCACCACTCTTG	TCAAGTTGAAGCATCATGGTC	60.6	596	INTERGENIC	--	--	--
Ca2_P1_7657147	Ca2	7657147	CTTTTT/CTTTTTT	TTATGTGGGGGATTTGGATG	TTCCACGTCCTTTTCATTCC	60.4	705	INTERGENIC	--	--	--
Ca2_P1_7658403	Ca2	7658403	CAAAA/CAA	TTGACTCTGCCACGTCACAT	AAAACCACTTGTCCCTCCCT	60.3	434	DRR	Ca_18120	--	--
Ca2_P1_7658826	Ca2	7658826	ATTTT/ATTTTT	TTCGTCATGCACCAAATCAT	CAGCTTGTCCGTCGGTAAAT	59.9	360	DRR	Ca_18120	--	--

INDEL marker IDs	Chromosomes/ unanchored scaffolds	Physical positions (bp)	InDels (*Kabuli* reference genome-CDC Frontier/PI)	Forward primers (5'-3')	Reverse primers (5'-3')	Anneling temperature (0C)	Expected amplified product size (bp)	Structural annotation		Functional annotation	
								Sequence components of *kabuli* genome	*Kabuli* gene accession IDs	NCBI-KOG	TFs
Ca2_P1_7667610	Ca2	7667610	TAAAAAAAAA/TAAAAAAAA	CCTTACCTAAAGGGCGGTTC	CTCTGGCCGGTGTAATTTGT	60.0	512	INTERGENIC	--	--	--
Ca2_P1_7668820	Ca2	7668820	GTTATTATTATTATTATTAT/GTTATTATTATTAT	CCAACACAAGCACCAACATC	CACTGATGAAGAGGCGAACA	60.0	725	INTERGENIC	--	--	--
Ca2_P1_7671677	Ca2	7671677	ATGATT/A	TACACCCACCATGGGAATCT	ACCCCCATAACCCTCCATTA	60.1	227	INTERGENIC	--	--	--
Ca2_P1_7672516	Ca2	7672516	CGG/CG	TGCAAAAGTTTTTGTTGGCA	CCATTGCAATTCTTGAAGTCAG	60.3	437	INTERGENIC	--	--	--
Ca2_P1_7675590	Ca2	7675590	TAAAAAAA/TAAAAAAAA	ACTCCGGATTTGGAGAAAGG	AAAATGAGTTTACATTCACGGTACA	60.4	882	INTERGENIC	--	--	--
Ca2_P1_7676116	Ca2	7676116	TTTTTTATTTTTATTTTTAT/TTTTTTATTTTTATTTTTATTTTTTAT	TGTACCGTGAATGTAAACTCATTT	TGGCATTTGCACCTTCATTA	57.7	263	INTERGENIC	--	--	--
Ca2_P1_7676173	Ca2	7676173	TAAAAAAAAAA/TAAAAAAAAA	TGTACCGTGAATGTAAACTCATTT	TGGCATTTGCACCTTCATTA	57.7	263	INTERGENIC	--	--	--
Ca2_P1_7679286	Ca2	7679286	AA/AAGACCAATTTCA	TCACGATGGAAATCGAATGA	ATAAATCCTCGGGTTGTCCC	60.0	628	INTRON	Ca_18121	R	ERF
Ca2_P1_7680326	Ca2	7680326	AG/A	TCCGCTGCTTCTGGAGTTAT	AGCAAAGAAAGCCAATGGAA	60.0	732	INTRON	Ca_18121	R	ERF
Ca2_P1_7680341	Ca2	7680341	ATTTTTTTT/ATTTTTTT	TCCGCTGCTTCTGGAGTTAT	AGCAAAGAAAGCCAATGGAA	60.0	732	INTRON	Ca_18121	R	ERF
Ca2_P1_7681881	Ca2	7681881	TAAAAA/TAAAA	GAAAAAGAGCCACGGTGGTA	GCGAGAAAAGATTTGTTGAGAA	60.1	383	INTRON	Ca_18121	R	ERF
Ca2_P1_7682063	Ca2	7682063	CTTTTTTT/CTTTTTT	GAAAAAGAGCCACGGTGGTA	TTTCCAGCTCCAAGCTCATT	60.1	643	INTRON	Ca_18121	R	ERF

INDEL marker IDs	Chromosomes/ unanchored scaffolds	Physical positions (bp)	InDels (*Kabuli* reference genome-CDC Frontier/PI)	Forward primers (5'-3')	Reverse primers (5'-3')	Anneling temperature (0C)	Expected amplified product size (bp)	Structural annotation		Functional annotation	
								Sequence components of *kabuli* genome	*Kabuli* gene accession IDs	NCBI-KOG	TFs
Ca2_P1_7684165	Ca2	7684165	CAAAAAAAAAAAA/CAAAAAAAAAAA	TGTGAAGAGCACTGGGAAGA	GGTGCGCCTTTTACAATGTT	59.5	755	DRR	Ca_18121	R	ERF
Ca2_P1_7684658	Ca2	7684658	CAA/CAAA	GCGCTTTTGGGTGTACATTT	GGGAAATAGCATGTTGTGGG	60.0	553	DRR	Ca_18122	--	--
Ca2_P1_7684720	Ca2	7684720	ATTTTTTTT/ATTTTTTTTT	GCGCTTTTGGGTGTACATTT	GGGAAATAGCATGTTGTGGG	60.0	553	DRR	Ca_18122	--	--
Ca2_P1_7695766	Ca2	7695766	TG/T	GGCGGGACAATTATTTTGAA	CCATATAGGAAGTACGAGTACGTGA	59.8	401	INTERGENIC	--	--	--
Ca2_P1_7697412	Ca2	7697412	CAAAAAAAAAA/CAAAAAAAAAAA	GGTTAAACGTCATGTTTCGAATTT	TCACTGCAACAACCACAACA	60.5	590	INTERGENIC	--	--	--
Ca2_P1_7699107	Ca2	7699107	TAAAAAAA/TAAAAAAAA	CCAAAAATTGTGGAGATTTGG	GCTGATGATGCCTCCATTTT	59.3	846	INTERGENIC	--	--	--
Ca2_P1_7699126	Ca2	7699126	TAAAAAAAA/TAAAAAAA	CCAAAAATTGTGGAGATTTGG	GCTGATGATGCCTCCATTTT	59.3	846	INTERGENIC	--	--	--
Ca2_P1_7703622	Ca2	7703622	GATAA/GA	ATTCGTTGTGACTGGGAAGG	CTTGCCCGACTTTGGAAGTA	60.0	546	DRR	Ca_18123	R	ERF
Ca2_P1_7703919	Ca2	7703919	AAATAAAATAATAAAATA/AAATAAAATA	TCTCTTTTATTGGGGCATGG	AGATGTAAGGGGTGTGGAAAA	59.9	910	DRR	Ca_18123	R	ERF
Ca2_P1_7705481	Ca2	7705481	AATTGTGA/AA	ATTCCTGCGGTACAACCATC	GGTGTTTGTGGAAATGGGTC	59.8	897	INTERGENIC	--	--	--
Ca2_P1_7706169	Ca2	7706169	CTTTTTTTTTTT/CTTTTTTTTTT	GACCCATTTCCACAAACACC	GCAGGAAAAGCAAAATAGGC	60.1	288	INTERGENIC	--	--	--
Ca2_P1_7706488	Ca2	7706488	AAAATAATTAAATAATTAAAT/AAAATAATTAAATAATTAAATAATTAAAT	GCCTATTTTGCTTTTCCTGC	TCATCCACTTTCTTGCCTGT	59.0	689	INTERGENIC	--	--	--

INDEL marker IDs	Chromosomes/ unanchored scaffolds	Physical positions (bp)	InDels (*Kabuli* reference genome-CDC Frontier/PI)	Forward primers (5'-3')	Reverse primers (5'-3')	Anneling temperature (0C)	Expected amplified product size (bp)	Structural annotation		Functional annotation	
								Sequence components of *kabuli* genome	*Kabuli* gene accession IDs	NCBI-KOG	TFs
Ca2_P1_7707861	Ca2	7707861	AT/ATT	TTTAAAATCGGTTATGCGGG	CTTTTAGGAGGTCCCAAGGC	59.8	469	INTERGENIC	--	--	--
Ca2_P1_7707926	Ca2	7707926	T/TA	GCCTTGGGACCTCCTAAAAG	GTTCTTGAGAAGCTCCGCC	60.1	454	INTERGENIC	--	--	--
Ca2_P1_7708059	Ca2	7708059	ATTTTTTTT/ATTTTTTT	GCCTTGGGACCTCCTAAAAG	AGAGGGAGAGAGGAAGGCTG	60.1	638	INTERGENIC	--	--	--
Ca2_P1_7709881	Ca2	7709881	TAAAAAAAA/TAAAAAAA	TGACACATGCAAAACACCCT	AGAAGCACGAAGATATGCACTT	60.0	619	INTERGENIC	--	--	--
Ca2_P1_7714111	Ca2	7714111	CT/CTT	CTCACTCGATCGATGGTTCA	TGACAATGCTGGCTGTTAGC	59.8	588	INTERGENIC	--	--	--
Ca2_P1_7716515	Ca2	7716515	TA/TACCTGA	ATGGGCTTTAAATGTGGCAG	ACCCCGTTTGGCTTCTATTT	60.0	105	DRR	Ca_18124	--	--
Ca2_P1_7718496	Ca2	7718496	C/CT	CCTGCCACCACTCTTAGCTT	TGGGATGCTACCTCCTATGG	59.5	511	INTERGENIC	--	--	--
Ca2_P1_7718955	Ca2	7718955	TGAAA/T	GAGCTTCATTGAAATCGGGA	TCATATAGGAGGTCGCACCC	60.2	821	INTERGENIC	--	--	--
Ca2_P1_7720508	Ca2	7720508	TAAA/TAA	TAATCCGCCTAAATGGATGC	CTCCCATAGGATGAAGGGGT	59.9	530	INTERGENIC	--	--	--
Ca2_P1_7720908	Ca2	7720908	AAT/A	TTCACCCCTTCATCCTATGG	TCCAAATGTTTGTGATTGTGC	59.7	490	INTERGENIC	--	--	--
Ca2_P1_7721082	Ca2	7721082	TAAAAAAAA/TAAAAAAAAA	TTCACCCCTTCATCCTATGG	CATACCATTTGGTGGGTTTG	59.7	671	INTERGENIC	--	--	--
Ca2_P1_7722723	Ca2	7722723	ATTTTTTTT/ATTTTTTT	AAAGCAGTGTCCTTCGGAGA	TCCTACCCTTTTGGCTTAAAAA	60.0	408	INTERGENIC	--	--	--

INDEL marker IDs	Chromosomes/ unanchored scaffolds	Physical positions (bp)	InDels (*Kabuli* reference genome-CDC Frontier/PI)	Forward primers (5'-3')	Reverse primers (5'-3')	Anneling temperature (0C)	Expected amplified product size (bp)	Structural annotation		Functional annotation	
								Sequence components of *kabuli* genome	*Kabuli* gene accession IDs	NCBI-KOG	TFs
Ca2_P1_7723559	Ca2	7723559	TAAAAAAAAA/TAAAAAAAA	CGTGTCTTATAAAAATTGCGGTT	TTTGCAAGCCAAGCACATAC	59.5	558	INTERGENIC	--	--	--
Ca2_P1_7724864	Ca2	7724864	TA/TAAAGAGATTGAGA	ACCAGAGACACTGCAGCAAA	AACAAGATTCTCGAATGCTCC	59.6	464	INTERGENIC	--	--	--
Ca2_P1_7725038	Ca2	7725038	ATTTT/ATTT	ACCAGAGACACTGCAGCAAA	AGCCATTTGAGACTGTGCAA	59.6	789	INTERGENIC	--	--	--
Ca2_P1_7727178	Ca2	7727178	TAAAAAAAA/TAAAAAAAAA	ATTGCACTGTCTCCTCGCTT	CAATGCACCCCTTACTGCTT	60.0	527	INTERGENIC	--	--	--
Ca2_P1_7727585	Ca2	7727585	GAAAAAAA/GAAAAAAAA	CTTGCAAAAGCAGTAAGGGG	CCTGACAATCCCTGAATGAAG	59.9	831	INTERGENIC	--	--	--
Ca2_P1_7728939	Ca2	7728939	ATTTTTT/ATTTTT	TCTCATGCTGCAACAGGTTC	AATGCACCCCTTCTTCCTTT	60.0	385	INTERGENIC	--	--	--
Ca2_P1_7731377	Ca2	7731377	GAAAAA/GAA	GTGGTTGTGATGTTGAACGC	CTCCCGCTAACTCCTCTTCC	60.0	697	INTERGENIC	--	--	--
Ca2_P1_7737212	Ca2	7737212	ATTATATAATTTATATAATTT/ATTATATAATTT	CTTCCAGTTTTGGTCTCCCA	GTTTGCAAAAGGGATGAGGA	60.1	542	INTERGENIC	--	--	--
Ca2_P1_7737860	Ca2	7737860	TTCT/TT	TTGCCCTTTTACCCATTGTC	AAAAATCTCATTCCACCACTCA	59.8	319	INTERGENIC	--	--	--
Ca2_P1_7738853	Ca2	7738853	TTGT/TT	GGTTCTACGGCAACGTCAAT	GAGACAAAAAGTGAACACATTGC	60.0	183	INTERGENIC	--	--	--
Ca2_P1_7739214	Ca2	7739214	AACTTACTTA/AACTTA	GGTTCTACGGCAACGTCAAT	TTACGCGTGTCATTTTCTCG	60.0	790	INTERGENIC	--	--	--
Ca2_P1_7743405	Ca2	7743405	CG/C	ATCCATCATGTTCATGCCCT	ATCTCCACCCGTTCTCTTGA	60.2	579	INTERGENIC	--	--	--

INDEL marker IDs	Chromosomes/ unanchored scaffolds	Physical positions (bp)	InDels (*Kabuli* reference genome-CDC Frontier/PI)	Forward primers (5'-3')	Reverse primers (5'-3')	Anneling temperature (0C)	Expected amplified product size (bp)	Structural annotation		Functional annotation	
								Sequence components of *kabuli* genome	*Kabuli* gene accession IDs	NCBI-KOG	TFs
Ca2_P1_7743611	Ca2	7743611	G/GTT	TCTTTGAACCCAAGACACGA	TTTGGTTTGGATTGATTGGA	59.3	393	INTERGENIC	--	--	--
Ca2_P1_7743661	Ca2	7743661	AATC/AATCGATC	TCAAGAGAACGGGTGGAGAT	GATTCAAAATGTGAGCGCAA	59.7	530	INTERGENIC	--	--	--
Ca2_P1_7745257	Ca2	7745257	CAAAA/CAAAAAA	TGGCATGACATGAATGAAGAA	GAGCAATTTGTGTGGACCCT	60.1	760	INTERGENIC	--	--	--
Ca2_P1_7747929	Ca2	7747929	TAAAAAAAA/TAAAAAAA	TGGTGAAATGCTGCAGAAAA	CTCAACTTGTCGATGCCTCA	60.4	147	INTERGENIC	--	--	--
Ca2_P1_7749962	Ca2	7749962	TT/TTAT	GGAGCTGCAACCATCTTCTC	TGGATTTGGGCTGAATTAGG	60.0	321	INTERGENIC	--	--	--
Ca2_P1_7751325	Ca2	7751325	GTTTTTTTTTTT/GTTTTTTTTTTTTTTTT	TGTCCACTTGAGCTTGCAAAT	ACCTCAAATGCTTCCACACC	60.8	698	INTRON	Ca_18125	--	MYB_related
Ca2_P1_7753009	Ca2	7753009	A/AC	AAGTGGCGAAGCTTGTTGAT	AGCTTGCCATTGCAGCTACT	59.9	797	INTRON	Ca_18125	--	MYB_related
Ca2_P1_7753358	Ca2	7753358	C/CAAT	CGGAGGTTTGGTGTAAACTGA	AGCTTGCCATTGCAGCTACT	60.0	447	INTRON	Ca_18125	--	MYB_related
Ca2_P1_7756359	Ca2	7756359	CAAA/CAAAA	AACCAGCATGTGTTGTTGGA	TGTGGTGGGGGAATTAAAAA	60.0	399	INTERGENIC	--	--	--
Ca2_P1_7771490	Ca2	7771490	ATTTTTTTTT/ATTTTTTTTTTT	ACCATTTATTATTTTCGTGTTAATGA	GGTACCATGGATGATATTTAGCC	57.3	188	INTERGENIC	--	--	--
Ca2_P1_7772671	Ca2	7772671	AAAAAAATAAAAA/AAAAAA	CAATTGGTGGCCGTTATTTT	TGCCCTAAGGGGAATAAGCTA	59.7	750	INTERGENIC	--	--	--
Ca2_P1_7772892	Ca2	7772892	ATTTT/ATTT	CAATTGGTGGCCGTTATTTT	TTGGGGGAAGAAAATTGAGA	59.7	958	INTERGENIC	--	--	--

INDEL marker IDs	Chromosomes/ unanchored scaffolds	Physical positions (bp)	InDels (*Kabuli* reference genome-CDC Frontier/PI)	Forward primers (5'-3')	Reverse primers (5'-3')	Anneling temperature (0C)	Expected amplified product size (bp)	Structural annotation		Functional annotation	
								Sequence components of *kabuli* genome	*Kabuli* gene accession IDs	NCBI-KOG	TFs
Ca2_P1_7773243	Ca2	7773243	T/TC	GCTGAGCCCAAACAAGCTAA	TTGGGGGAAGAAAATTGAGA	60.5	198	INTERGENIC	--	--	--
Ca2_P1_7773617	Ca2	7773617	TTAAATAAAAT/TTAAATAAAATAAATAAAAT	GCTGAGCCCAAACAAGCTAA	CCACTGTGGAAAGGTAAATGC	60.5	845	INTERGENIC	--	--	--
Ca2_P1_7778320	Ca2	7778320	ATT/ATTT	AGACCTCTTACTGTGCGCGT	TGCACTTTTGGTCCCTCAAT	60.1	321	INTERGENIC	--	--	--
Ca2_P1_7780730	Ca2	7780730	ATTTTTTTTTTT/ATTTTTTTTTT	TTGTCTCCATCTCCAGCCTT	TGTTGTTTGAGTTGGGACACA	59.8	995	INTERGENIC	--	--	--
Ca2_P1_7782164	Ca2	7782164	AG/A	TCATTTCAATCCCCCATCAT	TTTGATGCTATGCGAAGGAG	60.0	388	INTERGENIC	--	--	--
Ca2_P1_7786133	Ca2	7786133	AC/A	GCTCCTTTGGTGTATCGCTC	CATTCCATTCCATGTTGCAC	59.8	369	INTERGENIC	--	--	--
Ca2_P1_7788989	Ca2	7788989	TC/T	GCTCTTTTACGTTTGCCAGC	TGTTTTAGATTGTCTTCTATTTTACGG	60.0	566	INTERGENIC	--	--	--
Ca2_P1_7789494	Ca2	7789494	TAAAAAA/TAAAAAAA	ACATGAACATGTTAAATTTTCTGAAC	ACCAATTGGCCAACTCTCAC	57.7	580	INTERGENIC	--	--	--
Ca2_P1_7790064	Ca2	7790064	CA/CATGTTA	AGAGTTGGCCAATTGGTTTG	GAATGTCAAGCATTGTGAAGGA	60.0	467	INTERGENIC	--	--	--
Ca2_P1_7792884	Ca2	7792884	TTC/T	ATGAGCAACTTCGGGTTGAC	CTAGTTCCTCGTTGGAGGGG	60.1	478	INTERGENIC	--	--	--
Ca2_P1_7793040	Ca2	7793040	A/AT	CAGTGCCTTGAACCACTTGA	GGGCCCATTATATAAGTGATAAACC	59.9	465	INTERGENIC	--	--	--
Ca2_P1_7793102	Ca2	7793102	TAAAAAAAA/TAAAAA	CAGTGCCTTGAACCACTTGA	AAAACTTTACGTTAGAATGGATTGC	59.9	944	INTERGENIC	--	--	--

INDEL marker IDs	Chromosomes/ unanchored scaffolds	Physical positions (bp)	InDels (*Kabuli* reference genome-CDC Frontier/PI)	Forward primers (5'-3')	Reverse primers (5'-3')	Anneling temperature (0C)	Expected amplified product size (bp)	Structural annotation		Functional annotation	
								Sequence components of *kabuli* genome	*Kabuli* gene accession IDs	NCBI-KOG	TFs
Ca2_P1_7793328	Ca2	7793328	GAAAAA/GAAAA	ATATCCCCTCCAACGAGGAA	TTTATTGCGGTTTCCCTTCA	60.7	877	INTERGENIC	--	--	--
Ca2_P1_7795001	Ca2	7795001	ATA/ATAAATTTTAAAAATTA	TATATGCATGGCTTGCCAAA	TTGTTCTGCGGGTTTCTTTT	60.1	440	INTERGENIC	--	--	--
Ca2_P1_7796926	Ca2	7796926	T/TC	GGGGACTCCAAGACTTCACA	GCCCGTACTCAGTTCTTGCT	60.1	699	INTERGENIC	--	--	--
Ca2_P1_7798797	Ca2	7798797	TTTTCTTT/TTTT	GGAACAACGGATTCTGTCCA	TGGTCTCGGTCACTATTTAAGTTTT	60.9	603	INTERGENIC	--	--	--
Ca2_P1_7799040	Ca2	7799040	CTAGT/CT	CGAACCAACGATTATCACTAACA	TGTTGTGTTTGTGTAGCCACC	59.1	848	INTERGENIC	--	--	--
Ca2_P1_7800945	Ca2	7800945	TT/TGTTATTAT	GGGTAAAAAGGGGATTTCCA	CCCTTTTCATTCTTCACAGGA	60.0	611	DRR	Ca_18127	--	ERF
Ca2_P1_7806065	Ca2	7806065	TAAAAAAAA/TAAAAAAAAA	TGCTCTTCATGTGAGATGGTG	TCACCGGAGTTTAATGCAAA	59.8	805	INTERGENIC	--	--	--
Ca2_P1_7806316	Ca2	7806316	CTATTTATTTATTTATTT/CTATTTATTTATTT	TCACAGGCACTTTATTTTAGTGG	TCACCGGAGTTTAATGCAAA	58.4	206	INTERGENIC	--	--	--
Ca2_P1_7811963	Ca2	7811963	CAAAAAAA/CAAAAAAAA	ACTAACTCGGTGGGGAACAA	TCCCTTGTTCTTGCTGTGAA	59.4	330	INTERGENIC	--	--	--
Ca2_P1_7815016	Ca2	7815016	TAATAACAAATAACAAA/TAATAACAAATAACAAATAACAAA	TTTTCACCAACACCGTCAAA	AAATAATCGTGGCCTTGCAC	60.0	901	INTERGENIC	--	--	--
Ca2_P1_7816834	Ca2	7816834	TAAAAAAAA/TAAAAAAA	TCAATTGAAAACCACAAAGTTGA	TCAACGGGAAAGATTTTCTATTTC	59.5	657	INTERGENIC	--	--	--
Ca2_P1_7817769	Ca2	7817769	TTATGATATATATAT/TTAT	TGGACATAGAAAAACCAAATGGA	ATGTGGAAGTTGAACCCCAA	60.6	598	DRR	Ca_18128	QR	B3

INDEL marker IDs	Chromosomes/ unanchored scaffolds	Physical positions (bp)	InDels (*Kabuli* reference genome-CDC Frontier/PI)	Forward primers (5'-3')	Reverse primers (5'-3')	Anneling temperature (0C)	Expected amplified product size (bp)	Structural annotation		Functional annotation	
								Sequence components of *kabuli* genome	*Kabuli* gene accession IDs	NCBI-KOG	TFs
Ca2_P1_7822688	Ca2	7822688	AC/A	CGAAACATCAAGCTCCACAA	AGGATTCTTCCTGAGGCGTT	59.8	445	INTRON	Ca_18129	R	CPP
Ca2_P1_7823811	Ca2	7823811	AT/A	GAAAGTTTTGCCACCAATTCA	TGCTTTTGAAAACGACACCA	60.0	309	INTERGENIC	--	--	--
Ca2_P1_7824116	Ca2	7824116	T/TG	TGTCTCGTGGATCTGGTTTG	TGACTTTGTGTCGGAATTTG	59.7	258	INTERGENIC	--	--	--
Ca2_P1_7836957	Ca2	7836957	T/TC	TGGGCCCTAAACTTTCTCAA	CGGATGAACGTGGCTTTATT	59.7	156	INTERGENIC	--	--	--
Ca2_P1_7855224	Ca2	7855224	TGCGGTGGTGTGAATAGCGGTGGTGTGAATAGCGGTG/TGCGGTGGTGTGAATAGCGGTG	AGCATGTGGATTGTTGTGGA	TTACTACTCCCACCTCCCCC	60.0	192	CDS (large-effect mutations)	Ca_18131	U	--
Ca2_P1_7881274	Ca2	7881274	GTAT/GTATCGTTAT	GCCACTCCAACACCAATTTC	TGGAGACCACATGGAGATGA	60.4	423	INTERGENIC	--	--	--
Ca2_P1_7881305	Ca2	7881305	TTAT/TT	GCCACTCCAACACCAATTTC	TGGAGACCACATGGAGATGA	60.4	423	DRR	Ca_18136	--	--
Ca2_P1_7881740	Ca2	7881740	ATATTCC/ATATTCCTATTCC	TCATCTCCATGTGGTCTCCA	TGCAAATGACCACCCACTTA	60.0	642	DRR	Ca_18136	--	--
Ca2_P1_7887346	Ca2	7887346	AATAT/AATATAT	GTGCAGAGGAGCAAAAGGAC	TGGAATTTGAGACATGTCAACC	60.0	889	DRR	Ca_18137	E	--
Ca2_P1_7887518	Ca2	7887518	ATTTTTTT/ATTTTTTTT	AAGCAAAATACATCTCGAAGTTCT	TCCCAAGAAAAGCTTCAACC	57.8	550	DRR	Ca_18137	E	--
Ca2_P1_7887647	Ca2	7887647	CAACATA/C	AAGCAAAATACATCTCGAAGTTCT	TCCCAAGAAAAGCTTCAACC	57.8	550	DRR	Ca_18137	E	--
Ca2_P1_7893168	Ca2	7893168	CTATA/CTA	AAACAAATTCGCGGTGCTAC	CGAACGGTGTCATGATTTGA	60.1	525	INTERGENIC	--	--	--

INDEL marker IDs	Chromosomes/ unanchored scaffolds	Physical positions (bp)	InDels (*Kabuli* reference genome-CDC Frontier/PI)	Forward primers (5'-3')	Reverse primers (5'-3')	Anneling temperature (0C)	Expected amplified product size (bp)	Structural annotation		Functional annotation	
								Sequence components of *kabuli* genome	*Kabuli* gene accession IDs	NCBI-KOG	TFs
Ca2_P1_7894741	Ca2	7894741	TA/TAA	GTCATGGTTGTAGCATCCCC	GATGGGGATGGAAGATGAAA	60.2	639	INTERGENIC	--	--	--
Ca2_P1_7894801	Ca2	7894801	TTTGCAATTGCA/TTTGCA	GTCATGGTTGTAGCATCCCC	GATGGGGATGGAAGATGAAA	60.2	639	INTERGENIC	--	--	--
Ca2_P1_7899504	Ca2	7899504	TTTCGAAGGTTTCAAATGTTCGAAGGTTT/TTTCGAAGGTTTCAAATGTTCGAAGGTTTCAAATGTTCGAAGGTTT	AAGGCCAAACTTCCAAACCT	TGGATTGTGGGTTTGACTCC	60.0	604	INTERGENIC	--	--	--
Ca2_P1_7916006	Ca2	7916006	A/AC	CTCATTTGCATCCGATTGGT	TATCGAAAGTTTCCGCGTTT	60.9	525	INTERGENIC	--	--	--
Ca2_P1_7917956	Ca2	7917956	ATT/ATTGAATTT	GTCGGAGATCCCACAAAAGA	TGGAACCAAATCTTCGTTGA	60.1	884	INTERGENIC	--	--	--
Ca2_P1_7919145	Ca2	7919145	CAA/CAAA	CGTGACCGTACCAACAATGA	ATTGTGAGACGGATGAAGCA	60.4	460	INTRON	Ca_18141	--	bHLH
Ca2_P1_7922625	Ca2	7922625	TAA/TA	GAAGCATCACTAGGTGGGGA	CAACCAAAACCATTTCAAAACT	60.1	369	DRR	Ca_18141	--	bHLH
Ca2_P1_7923182	Ca2	7923182	TGAGAG/TGAG	AAAAGAAATCTCCCTATGATGCC	CGTAATTGCTCAATGGTGGA	59.8	103	DRR	Ca_18141	--	bHLH
Ca2_P1_7924174	Ca2	7924174	AT/A	ATGCACCCACCAATGAAAAT	TGTTCGAGTTTTGCCGCTAT	60.1	401	DRR	Ca_18141	--	bHLH
Ca2_P1_7925944	Ca2	7925944	TATGAATGAAT/TATGAATGAATGAAT	TCATGCTCCACACGATGTTT	CTTATCCCTTCACTTCGCCA	60.1	628	INTRON	Ca_18142	U	--
Ca2_P1_7927251	Ca2	7927251	C/CT	GTATTCGTTCCCCACGCTTA	CAAATGACCCATCAGGAGGT	60.0	563	INTRON	Ca_18142	U	--
Ca2_P1_7932313	Ca2	7932313	ATTTTTTTT/ATTTTTTT	ATTTTCTTTGACGGACGGAA	GGAGAAATTTGCCAAAGGAA	59.5	596	INTERGENIC	--	--	--

INDEL marker IDs	Chromosomes/ unanchored scaffolds	Physical positions (bp)	InDels (*Kabuli* reference genome-CDC Frontier/PI)	Forward primers (5'-3')	Reverse primers (5'-3')	Anneling temperature (0C)	Expected amplified product size (bp)	Structural annotation		Functional annotation	
								Sequence components of *kabuli* genome	*Kabuli* gene accession IDs	NCBI-KOG	TFs
Ca2_P1_7933550	Ca2	7933550	TAAAAAAAA/TAAAAAAAAA	TTCCTTAAGCGTGCTTGTCA	CCAAAATAACCCACAATCGG	59.6	539	INTERGENIC	--	--	--
Ca2_P1_7933926	Ca2	7933926	ATT/AT	TTCCTTAAGCGTGCTTGTCA	CCAAAATAACCCACAATCGG	59.6	539	INTERGENIC	--	--	--
Ca2_P1_7934318	Ca2	7934318	T/TG	CCGATTGTGGGTTATTTTGG	TCAGCTTTTGGCCTTCAAAT	60.0	401	INTERGENIC	--	--	--
Ca2_P1_7935850	Ca2	7935850	TAATTA/TAATTAAATTA	GTTGGTAGATTTGGGAGGCA	TCGAACTTGCATTGCCATAA	59.9	511	DRR	Ca_18144	--	--
Ca2_P1_7938788	Ca2	7938788	AA/AACA	TGATCAAATAATTGAAACAAGCG	GCCGTATTGGGAGAAGATGA	59.2	589	INTRON	Ca_18144	--	--
Ca2_P1_7940136	Ca2	7940136	ATTTTTTT/ATTTTTTTT	TCTTGACCGTTAGATTGAACCA	CAAAATGGACAAAATTGGAGC	59.6	714	INTERGENIC	--	--	--
Ca2_P1_7948944	Ca2	7948944	TTA/T	TTCCTTAGTGCCCATGGTTT	CGTGTCTTGCAACAAATGCT	59.4	916	INTERGENIC	--	--	--
Ca2_P1_8001528	Ca2	8001528	ATTTTTTTTT/ATTTTTTTT	TGTTATTGCTTTGCAAGCGT	AGAGTGAGAAGCCACTCCGA	59.5	635	INTERGENIC	--	--	--
Ca2_P1_8166930	Ca2	8166930	AAATAATAATAATAATAATAATAATAAT/AAATAATAATAATAATAATAATAATAATAATAAT	CCTAGGGATGCGACCATCTA	TCCTCCTCTTTTGGCACATT	60.1	385	INTERGENIC	--	--	--
Ca2_P1_8180532	Ca2	8180532	CATAATAATAATAATAATAATAATAATAATAATAATAATAATAATA/CATAATAATAATAATAATAATAATAATAATAATAATAATAATAATAATAATAATA	GCCATGACTCTGAACCACCT	CATTTTTCATTTGCCAACCC	60.1	805	INTERGENIC	--	--	--
Ca2_P1_8299859	Ca2	8299859	TTATATATATATATATAT/TTATATATATATATATATAT	CCTCCCTCCCTCTCTTCATC	TAAGCAGACGTCCCACACAC	60.1	757	INTERGENIC	--	--	--
Ca2_P1_8332435	Ca2	8332435	GTTATTATTATTATTATTATTATTATTATTATTATTATT/GTTATTATTATTATTATTATTATTATTATTATTATTATTATT	CTACGGAGGATGGAACTGGA	TTTGGTGATCTGGGGAGAAG	60.1	734	INTRON	Ca_21468	TU	--

INDEL marker IDs	Chromosomes/ unanchored scaffolds	Physical positions (bp)	InDels (*Kabuli* reference genome-CDC Frontier/PI)	Forward primers (5'-3')	Reverse primers (5'-3')	Anneling temperature (0C)	Expected amplified product size (bp)	Structural annotation		Functional annotation	
								Sequence components of *kabuli* genome	*Kabuli* gene accession IDs	NCBI-KOG	TFs
Ca2_P1_833993	Ca2	833993	TAATAAAATAAAATAAAATAAAATA/TAATAAAATAAAATA	TTGCATCATGGTTATGCGAC	CATAGCCTCGTACATGCAACA	60.5	490	INTERGENIC	--	--	--
Ca2_P1_8343398	Ca2	8343398	AATATATATATATAT/AATATATATATATATAT	CCAGTGTTTGACCTCCAAGC	TTTGCAACAACGCGAAATAG	60.7	743	INTERGENIC	--	--	--
Ca2_P1_8344698	Ca2	8344698	AC/A	AGTGAAGGAGAAATGGGGCT	TCAGACCTTGTCTTGACCCC	60.1	874	INTRON	Ca_21467	U	C2H2
Ca2_P1_8374636	Ca2	8374636	AATATATATATATATATATATAT/AATATATATATATATATATATAT	AAAGGGACAATGAAGGCAAG	TTGAAAATGTGATGTTTCTTCCA	59.2	636	INTERGENIC	--	--	--
Ca2_P1_8401872	Ca2	8401872	CTTTT/CTTTTT	AAGATCCCCTTTCCATGTCA	GAACTTCCACCAGACCATCC	59.3	470	DRR	Ca_21459	--	WRKY
Ca2_P1_8420270	Ca2	8420270	AACA/AACACA	CCAAGTTTGAAATGCCGATT	GCTGCATGATCTTATGCTCG	59.9	451	INTERGENIC	--	--	--
Ca2_P1_8425795	Ca2	8425795	TTATATATATATATATATATATATATATATAT/TTATATATATATATATATATATATATATATATATATAT	CGTGTGAGAAAACCTCGACA	AATCCAAGTTCCAAATGGGC	59.9	472	INTERGENIC	--	--	--
Ca2_P1_8462463	Ca2	8462463	AATATATA/AATATA	CCTACGTACGGGAATGGAGA	CTATTAGCGTTGGCTCTCGG	59.9	666	INTERGENIC	--	--	--
Ca2_P1_8478800	Ca2	8478800	AATTT/ATT	CCCGTATGAATGAACGGAAC	CTACCCCCACATTTCAATCG	60.2	519	INTERGENIC	--	--	--
Ca2_P1_8478910	Ca2	8478910	ACT/A	CCCGTATGAATGAACGGAAC	CTACCCCCACATTTCAATCG	60.2	519	INTERGENIC	--	--	--
Ca2_P1_8480032	Ca2	8480032	TTAT/TT	AAGAAAGCACTCAAAGCCCA	TGCGCATTGTATTTTTCAGG	60.0	716	INTERGENIC	--	--	--
Ca2_P1_8597078	Ca2	8597078	CCT/C	TCACGTTTTTGAATTTGCGA	GACCCATCCATGTGGAGTTT	60.2	514	DRR	Ca_22316	A	--

INDEL marker IDs	Chromosomes/ unanchored scaffolds	Physical positions (bp)	InDels (*Kabuli* reference genome-CDC Frontier/PI)	Forward primers (5'-3')	Reverse primers (5'-3')	Anneling temperature (0C)	Expected amplified product size (bp)	Structural annotation		Functional annotation	
								Sequence components of *kabuli* genome	*Kabuli* gene accession IDs	NCBI-KOG	TFs
Ca2_P1_8708914	Ca2	8708914	ATTTTTTTT/ATTTTTTTTT	GCCCACACCGAAGAAATAAA	TTCATTTTGCTCCATCACTTTC	59.9	714	INTERGENIC	--	--	--
Ca2_P1_8777880	Ca2	8777880	ATTTTTTTTT/ATTTTTTTTTT	CATGTCCTCAGGTGGTTGTG	TTTGTCGTGGGATGAACTGA	60.0	807	INTERGENIC	--	--	--
Ca2_P1_8801338	Ca2	8801338	TC/T	CCCTTGTCCTTCCCACCTAT	AATGAAATGTGGTGGGGGTA	60.2	573	INTERGENIC	--	--	--
Ca2_P1_8820736	Ca2	8820736	ATTTTTTTTTTT/ATTTTTTTTTT	TTACCATGATGTCGGTGGTG	GTGAGCAAATGTTTCGGGAT	60.2	690	INTERGENIC	--	--	--
Ca2_P1_904938	Ca2	904938	GGTGT/GGTGTGT	TGAGCAGGTTGTTGCAATTC	CAGGGTTCTGTGGCATTTCT	59.8	575	INTERGENIC	--	--	--
Ca2_P1_9259474	Ca2	9259474	GAAAAAAAAAA/GAAAAAAAAA	TCTCTTTGGTCGAGCTTTGC	AAGGCTCCGTTGCTGTTAAA	60.7	637	INTERGENIC	--	--	--
Ca2_P1_9276851	Ca2	9276851	TAAAAAAAAAAA/TAAAAAAAAAA	GCTTCGATTTTCTTTGCGTC	TGACAGGTTGATTGCACGAT	60.0	381	DRR	Ca_20035	--	--
Ca2_P1_9277232	Ca2	9277232	AGG/AG	ATCGTGCAATCAACCTGTCA	GTTTCATGCAAATTGGGTCC	60.1	439	DRR	Ca_20035	--	--
Ca2_P1_9277595	Ca2	9277595	CATATA/CATA	CCCAATTTGCATGAAACAAG	CGGGCCTATTGATACTTGGA	59.0	728	DRR	Ca_20035	--	--
Ca2_P1_9287043	Ca2	9287043	ATTTTTTTTTT/ATTTTTTTTT	CTCTTTCTCCTCCCGAAACA	AAGGGTTTTGTTGAAAGGGG	59.4	706	INTERGENIC	--	--	--
Ca2_P1_9305222	Ca2	9305222	GTTTTTTTT/GTTTTTTTTT	GTTTGCTGCATTGTGATTGC	CCCCAAGCAACAATCAATTT	60.3	320	INTERGENIC	--	--	--
Ca2_P1_9379100	Ca2	9379100	ATTTTTTTTTTT/ATTTTTTTTTT	GTGCAACCACTTGAAGCAAA	GCCCTCAGATGAGAGACAGG	59.9	620	DRR	Ca_20030	F	--

INDEL marker IDs	Chromosomes/ unanchored scaffolds	Physical positions (bp)	InDels (*Kabuli* reference genome-CDC Frontier/PI)	Forward primers (5'-3')	Reverse primers (5'-3')	Anneling temperature (0C)	Expected amplified product size (bp)	Structural annotation		Functional annotation	
								Sequence components of *kabuli* genome	*Kabuli* gene accession IDs	NCBI-KOG	TFs
Ca2_P1_9408358	Ca2	9408358	CTT/CTTTCTATTATT	CATCAAAAACAGCAGC GAAA	TCGTGTCTTCTTTCCA TCCA	60.0	934	INTERGENIC	--	--	--
Ca2_P1_9427423	Ca2	9427423	TAAAAAAAAA/TAAAAAAAAAA	AATATTGCGACAACGC ATGT	TGAAATGTTTAATTGTA GGTTCGG	59.1	598	INTERGENIC	--	--	--
Ca2_P1_9485330	Ca2	9485330	TTTTTATTTTATTTTATTTT/TTTTTATT TTATTTT	TTATGCGCCACATCAC ATTT	CGCCGAAAAGGTTACA CAAT	60.0	604	INTRON	Ca_20024	T	M-type
Ca2_P1_9494071	Ca2	9494071	T/TA	GAACTGCAACAACAAC GACAA	GGTCTCGAAATCCCTG ATGA	59.8	609	INTERGENIC	--	--	--
Ca2_P1_9498553	Ca2	9498553	AT/A	CAGGGATTTGGAGACC GTTA	TCGGTTGATCAAACCT TCAA	59.9	423	INTERGENIC	--	--	--
Ca2_P1_9500229	Ca2	9500229	CGTATGCTAGACCTGG/CGTATGCTA GACCTGGGTATGCTAGACCTGG	AGAGGTTGGGGGAGA AGAAA	GCAACAACAGCAGAGA CAATG	60.0	213	INTERGENIC	--	--	--
Ca2_P1_961092	Ca2	961092	TAAAAAAAAAAA/TAAAAAAAAAAAA	TGCATGCATGTAGCCA TTCT	TTGGATACTGGTGGGA GGTC	60.3	525	INTERGENIC	--	--	--
Ca2_P1_9641060	Ca2	9641060	TTAATAGT/TTAATAGTTATGATTAAAT AATAGT	ATGGGGATGTAATGCG TGAT	ATGGATTGGCTTTTCG TGAC	60.0	827	INTERGENIC	--	--	--
Ca2_P1_9650270	Ca2	9650270	CATATATATATATATATA/CATATATAT ATATATA	GAAGGGGTATGAACGA AGCA	TGAGTAGGGATGGAG GACCA	60.1	590	INTERGENIC	--	--	--
Ca2_P1_9689510	Ca2	9689510	TTATATATATATATATATATATATA/TTA TATATATATATATATATATATA	GTGAGTTGAGCATCGC AAAA	GATTTGCGGTAGATAT TTGCATC	60.0	928	INTERGENIC	--	--	--
Ca2_P1_976095	Ca2	976095	ATTTTTTTT/ATTTTTTTTT	TTTGATGAGCAAACGA AAAA	TGATGATTGGAAGGGA TGAA	57.0	575	INTERGENIC	--	--	--
Ca2_P1_9877069	Ca2	9877069	TTATATATATATATATATATATATAT ATATA/TTATATATATATATATATATATA TATATATATATATA	CCGACTTTCCTTCATTA ACCA	GAAGCAATTTGGGATG CAAT	59.1	724	INTERGENIC	--	--	--

INDEL marker IDs	Chromosomes/ unanchored scaffolds	Physical positions (bp)	InDels (*Kabuli* reference genome-CDC Frontier/PI)	Forward primers (5'-3')	Reverse primers (5'-3')	Anneling temperature (0C)	Expected amplified product size (bp)	Structural annotation		Functional annotation	
								Sequence components of *kabuli* genome	*Kabuli* gene accession IDs	NCBI-KOG	TFs
Ca2_P1_9884034	Ca2	9884034	AATATATATAT/AATATATAT	TAAGTTGTTGTGGCATCGGA	CCGACCTAATCACTCATAAATATCG	60.1	322	INTERGENIC	--	--	--
Ca2_P1_9923359	Ca2	9923359	GTTTTTTT/GTTTTTTTT	CAAGTTGCGAATGCAAAACA	TTTGCAAGAAACGTGTGACAT	60.8	648	INTERGENIC	--	--	--
Ca2_P1_9938789	Ca2	9938789	ATTTTTTTTTTT/ATTTTTTTTTT	TTGTGGCAGAACCATTTTCA	ATATTTTTGGACGGTCGCAG	60.1	148	INTERGENIC	--	--	--
Ca3_P1_10030728	Ca3	10030728	AATATATATATATATAT/AATATATATATATATATAT	TCGTTTGAGCAAAAGATGTCC	TGATTGTATGTGGCCTATGGT	60.2	838	INTERGENIC	--	--	--
Ca3_P1_10068347	Ca3	10068347	CATATATATATATATATAT/CATATATATATATATATATAT	CAATTGAAAGCTCAATGCCA	TGTGTCTTCTGGTCAATGCC	59.8	539	INTERGENIC	--	--	--
Ca3_P1_10159468	Ca3	10159468	T/TA	TGGGGCAAACAAAGCTATTC	CTAAGGGCGCTACAACAAGC	60.1	107	INTERGENIC	--	--	--
Ca3_P1_10170287	Ca3	10170287	GTTT/GTTTT	CTATTAGCGTTGGCTCTCGG	ATGTGTTGCTGTGGTTGGAA	60.0	127	INTERGENIC	--	--	--
Ca3_P1_10188650	Ca3	10188650	ATTT/ATT	AGCGAAAAAGCGCTGTAAAA	GGAATCGAGCGCTGTAAAAG	60.2	236	DRR	Ca_18812	--	Trihelix
Ca3_P1_10194898	Ca3	10194898	ATTTTTTTTTTT/ATTTTTTTTTT	TGGAGAAGAGAGGAGCCAAA	CCGTTTTCTGTTTTCACAATCA	60.1	759	INTERGENIC	--	--	--
Ca3_P1_10230251	Ca3	10230251	TTAATAATAATAATAATAATAATAAT/TTAATAATAATAATAATAATAATAATAAT	AGGAGCAAATTCAGTGAGAACA	TCTCCTTCGCGTTCCTCTAA	59.0	806	INTERGENIC	--	--	--
Ca3_P1_10312456	Ca3	10312456	TAAAA/TAAAAA	TGACACGCAATATAGAACCG	CATCTTGGTGTGTGAAAGCG	57.3	598	INTERGENIC	--	--	--
Ca3_P1_10350276	Ca3	10350276	CAAGAAG/CAAGAAGAAG	ATTGGCAAATCGGTTCAGTC	GCCAAACCCTTACGAGATCA	59.9	459	DRR	Ca_18818	--	--

INDEL marker IDs	Chromosomes/ unanchored scaffolds	Physical positions (bp)	InDels (*Kabuli* reference genome-CDC Frontier/PI)	Forward primers (5'-3')	Reverse primers (5'-3')	Anneling temperature (0C)	Expected amplified product size (bp)	Structural annotation		Functional annotation	
								Sequence components of *kabuli* genome	*Kabuli* gene accession IDs	NCBI-KOG	TFs
Ca3_P1_1038019	Ca3	1038019	AAT/AATAATAATCAT	GGAGCAAAATGTGTTTTATGGA	GGAAACGGTCTTCCCTTTTT	59.0	173	INTERGENIC	--	--	--
Ca3_P1_10398321	Ca3	10398321	G/GATTGATCTTTCCC	CGGATTGCCAAGTACAAGGT	CAAACCACAACCAGCACAAC	60.0	770	DRR	Ca_18819	J	--
Ca3_P1_10449599	Ca3	10449599	ATTTTTTTTTT/ATTTTTTTTTTT	ACCAACATCCACCACCTCAT	GATAAAAGCCGCCGTATGAA	60.1	430	INTERGENIC	--	--	--
Ca3_P1_10684192	Ca3	10684192	CTATTATTATTATTATTATTATTATTATTATTATTA/CTATTATTATTATTATTATTATTATTATTATTA	TTTCATCCAATGCTTCAGGA	TTGTGGTTGCATCCCTAACA	59.2	822	DRR	Ca_18828	O	--
Ca3_P1_10774426	Ca3	10774426	TTATATATATATATATATATATATATATATATAT/TTATATATATATATATATATATATATATATATATATATAT	ATCAGCTTTGGCTCACCACT	CCGTGGCAGGGTACTATTCT	59.9	845	DRR	Ca_18834	O	--
Ca3_P1_10846962	Ca3	10846962	AATATATATATATATATATATATATATATA/AATATATATATATATATATATATATATATATATATATA	CCAAATGACGACTTCCCACT	CAAGACGTCGTCAAAATCCC	60.0	683	INTERGENIC	--	--	--
Ca3_P1_10860554	Ca3	10860554	TG/T	GCACACTGGTGTTGGATTTG	TCAATGGTAACTTCCCCAGC	60.0	851	INTRON	Ca_19358	G	--
Ca3_P1_10911530	Ca3	10911530	TTT/TTTATT	CCTGACATGGCCTGACCTAT	AAGCCTGGCCTACGGTCTAT	60.0	528	INTERGENIC	--	--	--
Ca3_P1_10923237	Ca3	10923237	T/TC	GCTGATTGAAATCGAGCACA	TTAAAACAAGCACCAAGGGC	60.0	347	INTERGENIC	--	--	--
Ca3_P1_10970095	Ca3	10970095	TAAAAAAAAA/TAAAAAAAA	CTCGTGTCTCAAAACTCAAGGA	ACACAAACAAACCTCTCGGC	59.5	756	INTERGENIC	--	--	--
Ca3_P1_11044475	Ca3	11044475	CAAAAAAAAAA/CAAAAAAAAA	GCATCCATCCAAACGACTTT	CATTACATCTTATTTGAGGGAGAGC	59.9	286	INTERGENIC	--	--	--
Ca3_P1_11095476	Ca3	11095476	TAAAAAAAAAAAA/TAAAAAAAAAAAAA	GCCCTCAGATGAGAGTCAGG	CATCGCTGCCACATTTATTG	59.9	789	INTERGENIC	--	--	--

INDEL marker IDs	Chromosomes/ unanchored scaffolds	Physical positions (bp)	InDels (*Kabuli* reference genome-CDC Frontier/PI)	Forward primers (5'-3')	Reverse primers (5'-3')	Anneling temperature (0C)	Expected amplified product size (bp)	Structural annotation		Functional annotation	
								Sequence components of *kabuli* genome	*Kabuli* gene accession IDs	NCBI-KOG	TFs
Ca3_P1_11182328	Ca3	11182328	GAAAAAAAAAA/GAAAAAAAAAAA	AGGTGCCCTTCTTTTTGGTT	CCATCGTCGACCTTGAGAAT	60.0	527	INTERGENIC	--	--	--
Ca3_P1_11273271	Ca3	11273271	ATTTTTTTTT/ATTTTTTTT	GCGCGCGGATAAAATAAATA	TCATGACGCAGCACTTTTTC	60.0	738	INTERGENIC	--	--	--
Ca3_P1_11307658	Ca3	11307658	CATATATATATATATATATATATAT/CATATATATATATATATATATAT	AATAAAAGGGGGCATTTTGG	GCAATCTTCCTCCTCCCTTT	60.0	713	INTERGENIC	--	--	--
Ca3_P1_11442472	Ca3	11442472	AAAATAAATAAATAAATAAATAAATAAA/AAAATAAATAAATAAATAAATAAATAAATAAATAAA	AATGTTTGGATGATTCCCCA	ATACGCATAATCGACTCCCG	60.0	543	INTERGENIC	--	--	--
Ca3_P1_11462251	Ca3	11462251	ATTTTTTTTT/ATTTTTTTTTT	ATGATTCCCCAACATCCAAA	CACGAGTCCGATAAGGGTGT	60.0	149	INTERGENIC	--	--	--
Ca3_P1_1149110	Ca3	1149110	TAAAAAAAAAAAA/TAAAAAAAAAAA	TGTGCAATCGTTTTAACCCA	TGAGGGGTAATTTGAACCGA	60.0	272	INTERGENIC	--	--	--
Ca3_P1_1149320	Ca3	1149320	ATTTTTTTTT/ATTTTTTTTTT	TGTGCAATCGTTTTAACCCA	CCCTTACCACAAAATCAACTCA	60.0	719	INTERGENIC	--	--	--
Ca3_P1_11497481	Ca3	11497481	ATTATTTATTTATTTATTTATTTATTTATGTATTTATGTATTTATGTATTTATTTATTCATTTATTTATTTATT/ATTATTTATTTATTTATTTATTTATTTATGTATTTATGTATTTATGTATTTATTTATTCATT[illegible]	GGGTTTGCACTTGAAGGTGT	CCTCCGAGCCAAAATACAAA	60.0	617	INTERGENIC	--	--	--
Ca3_P1_11498015	Ca3	11498015	AATATATATATAT/AATATATATAT	TTTGTATTTTGGCTCGGAGG	AGGAAGAGTGGAGTTGCGAA	60.1	438	INTERGENIC	--	--	--
Ca3_P1_11531936	Ca3	11531936	TAAAA/TAAAAA	CGACTGCATCGGAAAGGTAT	GGTGCGATGGAGAAATCAAA	60.1	890	INTERGENIC	--	--	--
Ca3_P1_11558186	Ca3	11558186	CTCTGTTTGTT/CT	TGAGACCTATGCCATTTTTGG	TGAAAGCGCTACTTATGCCT	59.9	510	INTERGENIC	--	--	--
Ca3_P1_11587128	Ca3	11587128	CAAAA/CAAAAA	TTTGGGCTTACTTTCCATGC	GGTGTCAATTTCTTTGCGGT	60.1	543	DRR	Ca_19379	--	--

INDEL marker IDs	Chromosomes/ unanchored scaffolds	Physical positions (bp)	InDels (*Kabuli* reference genome-CDC Frontier/PI)	Forward primers (5'-3')	Reverse primers (5'-3')	Anneling temperature (0C)	Expected amplified product size (bp)	Structural annotation		Functional annotation	
								Sequence components of *kabuli* genome	*Kabuli* gene accession IDs	NCBI-KOG	TFs
Ca3_P1_11590201	Ca3	11590201	CAAG/C	TTGCTCCTACGTATCTCCCG	CCTTTTGTCATTTTGGGCTC	60.2	800	DRR	Ca_19380	--	--
Ca3_P1_11615635	Ca3	11615635	TTATATATATATATATATATATATATAT/TTATATATATATATATATATATAT	TTGAAAGAAAATCTTGCCGC	GTAACACCACACGCCTCCTT	60.3	764	INTERGENIC	--	--	--
Ca3_P1_11649200	Ca3	11649200	TAAAAAA/TAAAAA	ATTCATCGCTGTCTTCCACC	GGTCCCTTAGCTTAATTTCCG	60.1	656	INTERGENIC	--	--	--
Ca3_P1_11765228	Ca3	11765228	CTTT/CTTTT	GATCAGATCTGGCAGCAACA	TGGCATACATCTAGGGGGAG	60.0	574	DRR	Ca_19390	--	--
Ca3_P1_11765428	Ca3	11765428	ATTT/ATTTT	GATCAGATCTGGCAGCAACA	TGGCATACATCTAGGGGGAG	60.0	574	DRR	Ca_19390	--	--
Ca3_P1_11773772	Ca3	11773772	CAAA/CAAAA	GGTTCACCCAAAGATGGCTA	TTGTCCACACCACCTCAAAA	59.9	558	INTERGENIC	--	--	--
Ca3_P1_11877050	Ca3	11877050	TAAAAAAAAA/TAAAAAAAA	TTCAAACCTTTCAATTTTGCTC	GCGATTTTGGTGAAATCCAT	58.4	594	INTERGENIC	--	--	--
Ca3_P1_11903699	Ca3	11903699	TA/TAA	CAACCGGTTCATTCAAAACA	AGGAATTTGCAATCCTCAATG	59.4	221	DRR	Ca_22253	GE	--
Ca3_P1_11919605	Ca3	11919605	GAA/GA	CGGAAATTGAAATCGGAAAA	ATCGCGAAAGTAGTCTCCCA	59.9	633	INTERGENIC	--	--	--
Ca3_P1_11919676	Ca3	11919676	CNNNNNNNNNNNNNNNNNNNNN/CNNNNNNNNN	CGGAAATTGAAATCGGAAAA	ATCGCGAAAGTAGTCTCCCA	59.9	633	INTERGENIC	--	--	--
Ca3_P1_11991484	Ca3	11991484	GAAAAAAAAAA/GAAAAAAAAAAA	TTTCCCGACTTGGTCCTTTA	TCATCAAATTCGTAACGCAAA	59.5	503	INTERGENIC	--	--	--
Ca3_P1_11991649	Ca3	11991649	CTTTTTTTTTT/CTTTTTTTTT	TTTCCCGACTTGGTCCTTTA	TCATCAAATTCGTAACGCAAA	59.5	503	INTERGENIC	--	--	--

INDEL marker IDs	Chromosomes/ unanchored scaffolds	Physical positions (bp)	InDels (*Kabuli* reference genome-CDC Frontier/PI)	Forward primers (5'-3')	Reverse primers (5'-3')	Anneling temperature (0C)	Expected amplified product size (bp)	Structural annotation		Functional annotation	
								Sequence components of *kabuli* genome	*Kabuli* gene accession IDs	NCBI-KOG	TFs
Ca3_P1_11996299	Ca3	11996299	GAAAAAAAAAA/GAAAAAAAAAAA	TGACAATTTCGTCTCCGTACC	TGCCATTGAAGAGTTCTGGA	60.0	351	INTERGENIC	--	--	--
Ca3_P1_12014385	Ca3	12014385	TATATATATATATATA/TATATATATATATATATA	AAACATTTAAGGGGTGTGGC	TGGGAAGAGGATTGAAATGG	58.8	411	INTERGENIC	--	--	--
Ca3_P1_1206045	Ca3	1206045	C/CTTT	GGCCGCCACAAAATATTAAC	TTTGAATTGGGGAGAGATGG	59.3	683	INTERGENIC	--	--	--
Ca3_P1_12083114	Ca3	12083114	TTATATATATATATATATA/TTATATATATATATATATA	GCTCACACCCCTGTGGTATT	GGCCAAGGTTTCAAAATCAA	59.9	604	INTERGENIC	--	--	--
Ca3_P1_12140676	Ca3	12140676	A/AT	TTCCTATTGCTCACCCCTTG	GAAAAACAAGACCCGCGATA	60.1	525	INTERGENIC	--	--	--
Ca3_P1_12288956	Ca3	12288956	TAAAAAAAAAAA/TAAAAAAAAAA	TAGCAGGGTGCATTGGTGTA	GTCGCACAAGGCTACAGTCA	60.1	605	INTERGENIC	--	--	--
Ca3_P1_12600811	Ca3	12600811	GAAAAAAAAAA/GAAAAAAAAAAA	AGAGAGAAGAGCCCTAGCCC	TGTGACACTTCCACCGAAGA	59.2	419	INTERGENIC	--	--	--
Ca3_P1_12661298	Ca3	12661298	CGTGTG/CGTGTGTG	TGTTGGCGTGAAAACCATTA	TGCTGAGTTTCTGTGTTGCC	60.0	844	INTERGENIC	--	--	--
Ca3_P1_12697436	Ca3	12697436	GTTTTTATTTTTATTTTTATTTTTATTTTTATTTT/GTTTTTATTTTTATTTTTATTTTTATTTTTATTTTTATTTT	AGCTTGCCAACTTCTGGGTA	TGCAAAACGTGTCATCCATT	59.9	670	INTERGENIC	--	--	--
Ca3_P1_12715466	Ca3	12715466	T/TACACATTAAAG	TATTCCCGATTAATGCAGCC	GTCCCGAATGCAATGTCTCT	59.9	667	INTERGENIC	--	--	--
Ca3_P1_12734144	Ca3	12734144	CAAG/CAAGAAAG	GATGGGATCCGAACAAGAAA	TCCCATCATGCTCATTTCAA	59.9	740	INTERGENIC	--	--	--
Ca3_P1_12747032	Ca3	12747032	TGGG/TGG	CCTACGGACGGGAATGTAGA	CGTTTTTCTAAGCGAGGGTG	59.9	398	INTERGENIC	--	--	--

INDEL marker IDs	Chromosomes/ unanchored scaffolds	Physical positions (bp)	InDels (*Kabuli* reference genome-CDC Frontier/PI)	Forward primers (5'-3')	Reverse primers (5'-3')	Anneling temperature (0C)	Expected amplified product size (bp)	Structural annotation		Functional annotation	
								Sequence components of *kabuli* genome	*Kabuli* gene accession IDs	NCBI-KOG	TFs
Ca3_P1_12747377	Ca3	12747377	ATAAGT/ATAAGTAAGT	CACCCTCGCTTAGAAAAACG	AGTCTCCGGATCTTGCTGAA	59.9	548	INTERGENIC	--	--	--
Ca3_P1_12819743	Ca3	12819743	GAAAAAAAAA/GAAAAAAAAAA	TTGGAAGAAGATTGGACAAAGA	CTCCGCATCAATTTATCCCT	58.8	509	INTERGENIC	--	--	--
Ca3_P1_1288242	Ca3	1288242	GTTTTTTTTTTT/GTTTTTTTTTT	TGGTGAGATCAACGATACGC	TGGTAACAAATCTCCGAAGGA	59.7	371	INTRON	Ca_19421	R	--
Ca3_P1_12911294	Ca3	12911294	C/CCT	TATCGAGTGTTTCCGGTTCC	CAGGTTCACGTGAGCAAGAA	59.9	337	INTERGENIC	--	--	--
Ca3_P1_12951849	Ca3	12951849	ATTTTTTTTTT/ATTTTTTTTTTT	GCCACCTCTATCTTTGTCGC	GCTGCTTTGTCTTTCAAATCG	59.8	608	INTERGENIC	--	--	--
Ca3_P1_12969818	Ca3	12969818	TAAAAAAA/TAAAAAAAA	TTACACCAATTCCCCCAAGA	CATGCAAGGTTGGGCTAGAT	60.2	400	INTERGENIC	--	--	--
Ca3_P1_13000551	Ca3	13000551	G/GAA	TCATCTTTCTCACCAAGGGG	TATGGGGTGGTGGAAGATGT	60.0	544	INTERGENIC	--	--	--
Ca3_P1_13072537	Ca3	13072537	C/CTGTGATAG	TCAGATCGTTCCCTGTTTCC	TGAAGCATATTCTGAGGGGG	60.1	376	INTERGENIC	--	--	--
Ca3_P1_13097015	Ca3	13097015	ATTTTTTT/ATTTTTT	CCTTCTTTACGGGACCCAAT	TCATCAGTATCGTGGGTGGA	60.2	364	INTERGENIC	--	--	--
Ca3_P1_13097036	Ca3	13097036	A/AT	CCTTCTTTACGGGACCCAAT	TCATCAGTATCGTGGGTGGA	60.2	364	INTERGENIC	--	--	--
Ca3_P1_13105438	Ca3	13105438	CAC/CACTCTTTCACCCCTTAC	ATCCAACATCCCGATAACGA	AAAGGCTATTTGCTCGTGGA	60.2	288	INTERGENIC	--	--	--
Ca3_P1_13185454	Ca3	13185454	AT/ATTT	GCACACGACCTTCTCGTACA	AATCTGGATCGGGGAAAATC	59.9	302	CDS (FRAME SHIFT)	Ca_22687	--	Trihelix

INDEL marker IDs	Chromosomes/ unanchored scaffolds	Physical positions (bp)	InDels (*Kabuli* reference genome-CDC Frontier/PI)	Forward primers (5'-3')	Reverse primers (5'-3')	Anneling temperature (0C)	Expected amplified product size (bp)	Structural annotation		Functional annotation	
								Sequence components of *kabuli* genome	*Kabuli* gene accession IDs	NCBI-KOG	TFs
Ca3_P1_13201289	Ca3	13201289	GTTTTTTTTTT/GTTTTTTTTTTT	AATGCCCACCCATAATTTCA	CTGGGCAAGGGATCTGAATA	60.0	600	DRR	Ca_22688	--	Trihelix
Ca3_P1_1320883	Ca3	1320883	CAAAAAAAAA/CAAAAAAAA	CATGAATTGATTGGATTGCG	AAAAATGAAAGGTTGGCACG	59.9	750	INTERGENIC	--	--	--
Ca3_P1_13236644	Ca3	13236644	AATATATATATATATATATATATAT/AATATATATATATATATATATATAT	AAGACCTGTGGGATCATTGC	TTTTGTGATAGGTTCCTGATGC	59.9	928	INTERGENIC	--	--	--
Ca3_P1_13313491	Ca3	13313491	CATTATTATTATTATTATTATTATTATTATTATTAATATTATTATTAT/CATTATTATTATTATTATTATTATTATTATTAATATTATTATTAT	AACCCACCTCTTGATTTCCA	CGACAATTTTGTTGGTGACG	59.4	637	INTERGENIC	--	--	--
Ca3_P1_13450095	Ca3	13450095	GAAAAAAAAA/GAAAAAAAAAA	TCGTGTCCCAAAATTCAAGG	CGGTGAAGCTGAGGATCATT	60.9	823	INTERGENIC	--	--	--
Ca3_P1_13507407	Ca3	13507407	ATTTTTTTTT/ATTTTTTTT	CCAACAATGTCCCTATCGCT	TGCTCAAAATCCCAATTTCC	60.0	194	INTERGENIC	--	--	--
Ca3_P1_135372	Ca3	135372	CA/C	GTTGTAATGGGGCTTAGGCA	TTGGTCGTTTGAGCCTTTCT	60.0	478	INTERGENIC	--	--	--
Ca3_P1_13647076	Ca3	13647076	AATATATATATATATATATAT/AATATATATATATATATATAT	TTGCACCTTATTTGATGCGT	TCTCTCTCTCATGCCACCCT	59.2	183	INTERGENIC	--	--	--
Ca3_P1_13647375	Ca3	13647375	AAGAGAGAGAGAGAGAGAGAGAGAGA/AAGAGAGAGAGAGAGAGAGAGAGAGA	AGGGTGGCATGAGAGAGAGA	TCATAATGGTGTGTTTGATTTGC	59.9	518	INTERGENIC	--	--	--
Ca3_P1_13698505	Ca3	13698505	CAAA/CA	ATGGTCCCCAATATCGAACA	AAAAGGGAGAGATTGTTGGGA	60.0	742	INTERGENIC	--	--	--
Ca3_P1_1371048	Ca3	1371048	CAAAAAAAAAAAA/CAAAAAAAAAAA	CGGATCTCAGTTCGTGGGTA	CAGAGAGAGCGAGAGGGAGA	61.1	567	INTERGENIC	--	--	--
Ca3_P1_13796912	Ca3	13796912	CAAAAAAAAAAA/CAAAAAAAAAAAA	TATCATGCATTTGCGGTTGT	CCTGAGAGAGAAAGGGGCTT	60.0	650	INTERGENIC	--	--	--

INDEL marker IDs	Chromosomes/ unanchored scaffolds	Physical positions (bp)	InDels (*Kabuli* reference genome-CDC Frontier/PI)	Forward primers (5'-3')	Reverse primers (5'-3')	Anneling temperature (0C)	Expected amplified product size (bp)	Structural annotation		Functional annotation	
								Sequence components of *kabuli* genome	*Kabuli* gene accession IDs	NCBI-KOG	TFs
Ca3_P1_13801244	Ca3	13801244	A/ATAT	TGGTTGTTGGATTATCAAATGC	CTTGCGAAATGGCTGAAGTT	59.7	416	INTERGENIC	--	--	--
Ca3_P1_13817165	Ca3	13817165	AATATATATATATATATATATATATATATATATATATATATAT/AATATATATATATATATATATATATATATATATATAT	TCGGACAAATCCAACCTCTC	TTGTTATCTGATTCGTTCTCAAGC	60.1	745	INTERGENIC	--	--	--
Ca3_P1_13827207	Ca3	13827207	TTATATATATATATATATAT/TTATATATATATATATATATAT	CTACTCCCGACACTGAAGCC	TTTTCCAGGAAGGAATCGTG	59.9	527	DRR	Ca_25325	--	--
Ca3_P1_13836046	Ca3	13836046	TAAAAAAAAA/TAAAAAAAAAA	TGAGCTGATGGTGAAGTGATTT	TGGTTGAGCTTCAGACATGC	59.7	530	INTERGENIC	--	--	--
Ca3_P1_13935710	Ca3	13935710	GAT/GATAAT	CAGTACCCCTTTGGAGCTTG	ATTTTCGGGCAATTTCACTG	59.7	127	INTERGENIC	--	--	--
Ca3_P1_13972287	Ca3	13972287	ATTT/ATTTTTT	TTTCATCATTTATTTATTCATGCACTT	GCGAGGGAATTATAGCCACA	59.3	664	INTERGENIC	--	--	--
Ca3_P1_1403982	Ca3	1403982	GAA/GAAGAAA	TCCCCCGTAATAGGTGCATA	TCTTTGTTTCTGCGAACCCT	60.2	521	INTERGENIC	--	--	--
Ca3_P1_14085019	Ca3	14085019	GAAAAAAAAAAA/GAAAAAAAAAAAA	GGGCCCCACTCTATACACAA	CGTTGGCTTCAAGCAGTGTA	59.8	783	INTERGENIC	--	--	--
Ca3_P1_14147273	Ca3	14147273	GAAAAAAAAAA/GAAAAAAA	TCAAGTCGACAAGCCTTCCT	TTTCTGAGAATCACCACCCC	60.0	521	INTERGENIC	--	--	--
Ca3_P1_14226734	Ca3	14226734	CAAATAAATAAATAAATAAATAAATAAATAAATAAATAAATAAATAAATAAATAA/CAAATAAATAAATAAATAAATAAATAAATAAATAAATAAATAAATAAATAAATAAATAA	TTTTGGGGTTTAGGGTGTCA	CGACCTCTCTTGCAGATCAA	60.2	865	INTERGENIC	--	--	--
Ca3_P1_14420408	Ca3	14420408	AATATATATATATATATATATATATATATATATATATATA/AATATATATATATATATATATATATATATATATATATATATATATATA	GCGGGGAGGTGTGAGTATTA	ACGACGCACCTTCTTGTTTT	60.0	397	INTERGENIC	--	--	--
Ca3_P1_14429203	Ca3	14429203	ATTTTTTTTTTT/ATTTTTTTTTT	TGCGATGTTATCCAAAACCA	TGCAAATATCAATTGGGGGT	59.9	610	INTERGENIC	--	--	--

INDEL marker IDs	Chromosomes/ unanchored scaffolds	Physical positions (bp)	InDels (*Kabuli* reference genome-CDC Frontier/PI)	Forward primers (5'-3')	Reverse primers (5'-3')	Anneling temperature (0C)	Expected amplified product size (bp)	Structural annotation		Functional annotation	
								Sequence components of *kabuli* genome	*Kabuli* gene accession IDs	NCBI-KOG	TFs
Ca3_P1_14441213	Ca3	14441213	AAT/AATGAT	CGGCATACAAATAACATGCG	TGTTTGTTTGTGGTGCCATT	60.0	626	INTERGENIC	--	--	--
Ca3_P1_1446842	Ca3	1446842	GC/GCAC	TTGAAACATTTGAAGTATCCGC	TGCATTTGAGGAATTTCACG	59.1	488	INTERGENIC	--	--	--
Ca3_P1_14493863	Ca3	14493863	GTATATATATATATATATATATATAT/GTATATATATATATATATATATATATATATATATAT	ATCGGAAAATCCCTACCAGC	TTGGTGGAACAAAACGACAA	60.3	762	INTERGENIC	--	--	--
Ca3_P1_14520599	Ca3	14520599	GTA/GTATA	GACGAAATGTGCTTTTGGGT	CCACCAGTTTTGTGATGCAA	60.0	222	DRR	Ca_22510	--	--
Ca3_P1_14561964	Ca3	14561964	TCCCCC/TCCCC	GCTGAATGGGTCCTTGATGT	GGTAACAACGGGAAGCAAAA	59.9	729	INTERGENIC	--	--	--
Ca3_P1_14597299	Ca3	14597299	TAAAAAAAA/TAAAAAAA	GGCATAATTGTTCATGCGGT	TGCAACACATTTTCAGCCTT	60.7	736	INTERGENIC	--	--	--
Ca3_P1_14615218	Ca3	14615218	ATTTTTTTTTT/ATTTTTTTTT	CCCATCCAAAACCTCATTTG	AGGCATGGTTGTCTTTCCAG	60.2	557	INTERGENIC	--	--	--
Ca3_P1_14653349	Ca3	14653349	A/AG	AGGAGCTTCTGCCATTGAAA	TAAAAGGAGCAAAGGCCAGA	60.0	408	INTRON	Ca_22505	O	--
Ca3_P1_14720724	Ca3	14720724	CTTTT/CTTT	TCGCCGTTTCTTCTTCACTT	GCTCACAATTCAAACCCCAT	60.0	510	INTERGENIC	--	--	--
Ca3_P1_14755374	Ca3	14755374	ATTTTTTTTTT/ATTTTTTTTTTT	TTTGGAAATGGCTAAGGGTG	ATTTGCATTCCCATGTTGGT	59.9	535	INTERGENIC	--	--	--
Ca3_P1_14764340	Ca3	14764340	TTAATAATAATAATAATAATAATAATAATAAT/TTAATAATAATAATAATAATAATAATAATAATAAT	AACGCGTCCCTAACATTTTG	GGGGATGCGACCATCTATTA	60.0	852	INTRON	Ca_22501	G	C2H2
Ca3_P1_14775765	Ca3	14775765	TTATATATATATATATATAT/TTATATATATATATATAT	GTCGCTTGAGATGTGCAAAA	GGAGGTTTGTGTTTGACGGT	60.0	834	INTERGENIC	--	--	--

INDEL marker IDs	Chromosomes/ unanchored scaffolds	Physical positions (bp)	InDels (*Kabuli* reference genome-CDC Frontier/PI)	Forward primers (5'-3')	Reverse primers (5'-3')	Anneling temperature (0C)	Expected amplified product size (bp)	Structural annotation		Functional annotation	
								Sequence components of *kabuli* genome	*Kabuli* gene accession IDs	NCBI-KOG	TFs
Ca3_P1_14801451	Ca3	14801451	GATATATATATATATATATATAT/GATATATATATATATATATATAT	ATGAGCCACAAGCTCCAAGT	TGCCATTTGGTGAACGATTA	59.9	357	INTERGENIC	--	--	--
Ca3_P1_14843003	Ca3	14843003	AATATATATATATATATATATATATATA/AATATATATATATATATATATATATATATA	GAGAAAGAAGATATCGGATTCTCAA	AAAAATCAACGCAGTGGGAG	59.3	356	INTERGENIC	--	--	--
Ca3_P1_14874833	Ca3	14874833	GCC/GCCC	AGGTCATTTTCGTCCTCCCT	CAAACACACCTGTTGGATGC	59.9	679	INTERGENIC	--	--	--
Ca3_P1_14880871	Ca3	14880871	TG/TGTCG	TTTCGGTCATTTTCGTCCTC	ACACACCTATCGGATGCACA	60.1	616	INTERGENIC	--	--	--
Ca3_P1_14902851	Ca3	14902851	GAA/GAAA	CGGTGGGGAGAACTCTGATA	TTCACATGACATCATCCCGT	60.1	474	INTERGENIC	--	--	--
Ca3_P1_14906453	Ca3	14906453	TGGGGG/TGGGGGG	TTGGTCTTGGGAAGTCAAGC	AACTCTGTTGATTGGCGACC	60.2	449	INTERGENIC	--	--	--
Ca3_P1_14919264	Ca3	14919264	GTTT/GTTTT	CGATTGTGTTTGTCACAGGG	GAAGCAAATCGATTGGGAAA	60.0	303	INTERGENIC	--	--	--
Ca3_P1_1499066	Ca3	1499066	G/GTAAGGA	CCTGAAACAGGTTGGCATTT	ATCTGGAGGTCATTTGTGGC	60.0	180	DRR	Ca_19414	--	--
Ca3_P1_15039160	Ca3	15039160	GTTTTT/GTTTT	CAACACGATCATGACAAGCA	GCATTTTGCATTTGTCAACG	59.2	677	INTERGENIC	--	--	--
Ca3_P1_15039270	Ca3	15039270	TC/T	CAACACGATCATGACAAGCA	GCATTTTGCATTTGTCAACG	59.2	677	INTERGENIC	--	--	--
Ca3_P1_15047295	Ca3	15047295	TA/T	TACTTTGTGCCATGCTCTGC	ACACAAGCCAACACATCCAA	60.0	117	INTERGENIC	--	--	--
Ca3_P1_15072147	Ca3	15072147	AATATATATATATATATATATATATA/AATATATATATATATATATATA	GAGTGGTGTTGGAGTCATGTTT	AGACATGTGTTTCCCTTCCG	59.0	424	DRR	Ca_17011	--	--

INDEL marker IDs	Chromosomes/ unanchored scaffolds	Physical positions (bp)	InDels (*Kabuli* reference genome-CDC Frontier/PI)	Forward primers (5'-3')	Reverse primers (5'-3')	Anneling temperature (0C)	Expected amplified product size (bp)	Structural annotation		Functional annotation	
								Sequence components of *kabuli* genome	*Kabuli* gene accession IDs	NCBI-KOG	TFs
Ca3_P1_15193812	Ca3	15193812	TTATATATATATATATATATATATATATAT/TTATATATATATATATATATATATATATATATATAT	TTGTAGGTGGTAGGGTTCGG	TGCCCATCAATTGTGGTTAAT	59.8	609	INTERGENIC	--	--	--
Ca3_P1_1521871	Ca3	1521871	GTTTTTTTTTTTTT/GTTTTTTTTTTTT	TGACCGAGCAAACTTCACTG	GGACACGTCTCCCAATGTCT	60.0	452	INTERGENIC	--	--	--
Ca3_P1_15232531	Ca3	15232531	TAAAAA/TAAAA	CTTTTACAGCGCTTTTTGGG	TTCGTAATGGACGGGATGAT	59.9	245	INTERGENIC	--	--	--
Ca3_P1_15318264	Ca3	15318264	TAAAAAAA/TAAAAAA	CGAAGGAGCTTTTCTTGTGG	CGCATTTTGCATTTGTCAAC	60.0	651	INTERGENIC	--	--	--
Ca3_P1_15320734	Ca3	15320734	GGAGAGAGAGAGAGAGAGAGAG/GGAGAGAGAGAGAGAGAGAG	TGTGCCTTCCTTACTGTGATG	GCATTGTGCATTTCATTTGC	58.8	675	INTERGENIC	--	--	--
Ca3_P1_1533357	Ca3	1533357	TCAAAA/TCAAAACTCCAAAA	ACCCAAAAGCTAACTTGCCA	GCATCAACAAAGCAGCAGAA	59.7	533	INTERGENIC	--	--	--
Ca3_P1_1534003	Ca3	1534003	AAACTGCAACTAACT/AAACTGCAACTAACTGCAACTAACT	TTCTGCTGCTTTGTTGATGC	GTGGATGGGTGTTTTCCATC	60.1	774	INTERGENIC	--	--	--
Ca3_P1_15342204	Ca3	15342204	T/TA	ATGATAATGCGGCGGTTAAG	CATGCTCCCATTTTTGGATT	60.0	637	INTERGENIC	--	--	--
Ca3_P1_15388201	Ca3	15388201	CATATATATAT/CATATATAT	AAACACATGCGCTTTCATTG	AATCGATTGGCTGAAATCCA	59.7	479	INTERGENIC	--	--	--
Ca3_P1_15396881	Ca3	15396881	TATAA/TATATATATAAATAA	TCAATGCGGTCTCTGAAGTG	CACATGCTAATCGACACCGT	60.0	412	DRR	Ca_17029	--	Trihelix
Ca3_P1_15463471	Ca3	15463471	CATATATATATATATATATATA/CATATATATATATATATATA	TTTGTGGATTTGCATTGATGA	TGAATTCACCGTAAACGGAA	59.9	783	INTERGENIC	--	--	--
Ca3_P1_15463577	Ca3	15463577	TTTTATTTATTTATTTATTTATTTATTTATTTATTTATTT/TTTTATTTATTTATTTATTTATTTATTTATTTATTT	GGGACTAGACTTTTGTTGGTGG	TGAATTCACCGTAAACGGAA	59.9	449	INTERGENIC	--	--	--

INDEL marker IDs	Chromosomes/ unanchored scaffolds	Physical positions (bp)	InDels (*Kabuli* reference genome-CDC Frontier/PI)	Forward primers (5'-3')	Reverse primers (5'-3')	Anneling temperature (0C)	Expected amplified product size (bp)	Structural annotation		Functional annotation	
								Sequence components of *kabuli* genome	*Kabuli* gene accession IDs	NCBI-KOG	TFs
Ca3_P1_15503247	Ca3	15503247	C/CTAAAATATTAGGGAA	ATTGGGAAGTGGGGAAACTC	CGGGGTACATTGATAGCGTT	60.2	800	INTERGENIC	--	--	--
Ca3_P1_15519811	Ca3	15519811	TAT/TATGCAAT	CCAATCCCATGACCAATTTC	TAACGCACCGTGCCTAATTT	60.0	502	INTERGENIC	--	--	--
Ca3_P1_15524418	Ca3	15524418	GTTTTTT/GTTTTTTT	GACGTGTGTGTGGAAAGAGC	CGGCCAAAGTAATGGATTGT	59.3	720	INTERGENIC	--	--	--
Ca3_P1_15579523	Ca3	15579523	ATTATAT/A	TCATATCACCAACATCAAAATTCA	TGATCCAACACTCGTCCAAA	59.2	572	INTERGENIC	--	--	--
Ca3_P1_15658487	Ca3	15658487	TTCCA/TTCCACTCCA	AAAGCATTCCCACTACGGTG	TTGTCACAGCCTAAAACCCC	60.0	343	DRR	Ca_17043	--	--
Ca3_P1_15659729	Ca3	15659729	CA/CCTTCCAAGA	AATTAAAGGGTCCTCGCGTT	GGTCTCAACAAAGAGCTCCG	60.0	748	DRR	Ca_17043	--	--
Ca3_P1_15660475	Ca3	15660475	TA/TAA	AAGTGAGCTAACGGGGGTTT	TGGACAGAGCAAGTGAGGTG	60.0	140	INTERGENIC	--	--	--
Ca3_P1_15694473	Ca3	15694473	TAAAAAAAAA/TAAAAAAAAAA	TTATCACCAACGAATGTGCC	GCATCCAACTTGCTCACTCA	59.4	340	INTERGENIC	--	--	--
Ca3_P1_1573297	Ca3	1573297	TAAAAAAAAAAA/TAAAAAAAAAA	ATAAGACCAAAGAAGCGGCA	GCATGCAATTGGTTTGACAC	59.8	607	INTERGENIC	--	--	--
Ca3_P1_15844609	Ca3	15844609	C/CTAGG	ATGGGTAGTAAGCCCCGTCT	TCTTTCCAAAGGATGGATGG	59.8	831	INTERGENIC	--	--	--
Ca3_P1_15868847	Ca3	15868847	TATTATTATTATTATTATTATTATTATTATTATTAT/TATTATTATTATAATTATTATTATTATTATTATTATTATTATTATTAT	AATCGTGCGTTTTTGCTTTT	AAATTTCGATGATCCAACGG	59.8	833	INTERGENIC	--	--	--
Ca3_P1_16094863	Ca3	16094863	CTTTTTT/CTTTTTTT	TCTCCGCATAAGCTTTCGAT	TGATTGCAAATAGCACCCAA	59.9	248	DRR	Ca_17065	--	--

INDEL marker IDs	Chromosomes/ unanchored scaffolds	Physical positions (bp)	InDels (*Kabuli* reference genome-CDC Frontier/PI)	Forward primers (5'-3')	Reverse primers (5'-3')	Anneling temperature (0C)	Expected amplified product size (bp)	Structural annotation		Functional annotation	
								Sequence components of *kabuli* genome	*Kabuli* gene accession IDs	NCBI-KOG	TFs
Ca3_P1_16107009	Ca3	16107009	TAAAA/TAAAAA	CCAAAATCTTGATGTGGCAA	CAGATGGAAATCCTCCTCCA	59.5	654	INTERGENIC	--	--	--
Ca3_P1_16133157	Ca3	16133157	GTTTTATTTTATTTTATTT/GTTTTATTTTATTTTATTTTATTT	CGTCTCTTGAAGCGATGAAA	CATTGTTGGTCACTAGCAAATCA	59.1	698	INTERGENIC	--	--	--
Ca3_P1_16133384	Ca3	16133384	CAATAATAATAATAATAATAATAATAATAATAATAATA/CAATAATAATAATAATAATAATAATAATAATAATAATAATA	CGTCTCTTGAAGCGATGAAA	ATAAGCCGTCAATCTGCCAC	59.1	807	INTERGENIC	--	--	--
Ca3_P1_16133499	Ca3	16133499	TA/TACACAAAGAA	TTTGCCCCATTATAAACAAAA	ATAAGCCGTCAATCTGCCAC	57.2	625	INTERGENIC	--	--	--
Ca3_P1_16137267	Ca3	16137267	CATATATATATATATATA/CATATATATATATATATATATA	TGGCACATCTAAAAATAGGAGGA	AGAAAGTCGCATCCCAGAGA	60.0	544	INTERGENIC	--	--	--
Ca3_P1_16138352	Ca3	16138352	TTATATATATATATATATATATATATA/TTATATATATATATATATATATATATATATATATATA	GCCACAATCTGTCCCCAATA	TAGTGAAGGCATTGGCAGAA	60.7	855	INTERGENIC	--	--	--
Ca3_P1_16151347	Ca3	16151347	AT/ATT	GGGCATGAAGGTAGTTTGGA	TAAACCATCAATCTGCCACG	59.9	842	INTERGENIC	--	--	--
Ca3_P1_16154986	Ca3	16154986	ATTTTTTT/ATTTTTTTT	TTGAAGTGGCAAAATCTTCG	AAGGGACAACTATTTCTTGGCA	58.9	585	INTERGENIC	--	--	--
Ca3_P1_16160970	Ca3	16160970	ATTTT/ATTT	TTATTTTGGGACGGATGGAG	TCTCCCGATTTGCTCCTTTA	59.8	263	INTERGENIC	--	--	--
Ca3_P1_16166714	Ca3	16166714	TTTTG/TTTTGCTTTG	TCAACCATCTGGGTTGGAAT	CCCCTAGCAAAGGAAACAAA	60.2	674	INTERGENIC	--	--	--
Ca3_P1_16168531	Ca3	16168531	AG/A	TGGAGGAGGATTTCCATCTG	TGCTTTCTTGCCCTGTTTTT	60.0	547	INTERGENIC	--	--	--
Ca3_P1_16171083	Ca3	16171083	T/TG	TACAGCGCTTCATTCCACAG	CGTGCATGAGCAAAAGAAAA	60.0	468	INTERGENIC	--	--	--

INDEL marker IDs	Chromosomes/ unanchored scaffolds	Physical positions (bp)	InDels (*Kabuli* reference genome-CDC Frontier/PI)	Forward primers (5'-3')	Reverse primers (5'-3')	Anneling temperature (0C)	Expected amplified product size (bp)	Structural annotation		Functional annotation	
								Sequence components of *kabuli* genome	*Kabuli* gene accession IDs	NCBI-KOG	TFs
Ca3_P1_16182804	Ca3	16182804	ATTTTTTTT/ATTTTTTTTTT	CCGGCATCACCTATCAGAGT	TCCGACCTCCTTTGAGAAGA	60.1	583	INTERGENIC	--	--	--
Ca3_P1_16182832	Ca3	16182832	AAATAATAATAATAATAATAATAATAATAATAATAATAATAATAATAATAATAATAATAATAATAA/AAATAATAATAATAATAATAATAATAATAATAATAATAATAATAATAATAA	CCGGCATCACCTATCAGAGT	TCCGACCTCCTTTGAGAAGA	60.1	583	INTERGENIC	--	--	--
Ca3_P1_16190775	Ca3	16190775	CA/C	TCGCGGTTCTTGATGATGTA	AGCTCGACTTATCCTTCGCA	60.2	423	INTERGENIC	--	--	--
Ca3_P1_16195131	Ca3	16195131	AT/ATT	TGATAAAAGACTAACGATGCAAACA	TAAACCATCAATCTGCCACG	60.1	655	INTERGENIC	--	--	--
Ca3_P1_16195368	Ca3	16195368	CATATAT/CATATATAT	ACGTGGCAGATTGATGGTTT	TTTGATGTTGGCGTAGAGGA	60.4	350	INTERGENIC	--	--	--
Ca3_P1_16195709	Ca3	16195709	AC/A	ACGTGGCAGATTGATGGTTT	GAACGTACGGAAGAGAAGCG	60.4	754	INTERGENIC	--	--	--
Ca3_P1_16195934	Ca3	16195934	AAATTAAT/AAAT	TCCTCTACGCCAACATCAAA	GAACGTACGGAAGAGAAGCG	59.3	424	INTERGENIC	--	--	--
Ca3_P1_16197261	Ca3	16197261	TCTTAA/T	CTGCATCGAATTAATGCAACA	AAGGGGGATCATGGAAAACT	59.7	316	DRR	Ca_18251	--	--
Ca3_P1_16212096	Ca3	16212096	TAA/TAAA	AATGCACTTTGCCACTGTTG	CCAAGATTTTGCCAGATCAA	59.8	550	INTERGENIC	--	--	--
Ca3_P1_16212378	Ca3	16212378	A/AACATTC	ATGCACTTTGCCACTGTTGT	GAGGGGATGATGCTTCAAAA	59.2	879	INTERGENIC	--	--	--
Ca3_P1_16230171	Ca3	16230171	ATTTTTTTTTTT/ATTTTTTTTTT	TAGAGTTCTCCCCACCGCTA	TCTAAGACACGGAGGAGGGA	59.8	568	INTERGENIC	--	--	--
Ca3_P1_16236332	Ca3	16236332	CACATAATACATA/CACATA	TATCACCTATCAGAGCGGGG	CCACGCAACACACGTTTATC	60.1	533	INTERGENIC	--	--	--

INDEL marker IDs	Chromosomes/ unanchored scaffolds	Physical positions (bp)	InDels (*Kabuli* reference genome-CDC Frontier/PI)	Forward primers (5'-3')	Reverse primers (5'-3')	Anneling temperature (0C)
Ca3_P1_16267741	Ca3	16267741	AATATATATATATAT/AATATATATATAT	CAAATCCCTTCATGCAGACC	TGTGTACAATGGCAAAGGGA	60.5
Ca3_P1_16328087	Ca3	16328087	TTATATATATATATATATATATATATATATATAT/TTATATATATATATATATATATATATATATATATATATATATATAT	GTGAATCATGCATTTGGTGC	GATGAACGCGATGAACCTTT	59.9
Ca3_P1_16331942	Ca3	16331942	TAA/TA	AAACGCAATCCTTGAGTTCG	TCAAGATTTGCCCCTCAATC	60.2
Ca3_P1_16347875	Ca3	16347875	CAAAAAA/CAAAAAAA	GCGTTTTTATGGTTGCTGGT	TATGGAAGGTGAGACAGGGG	60.0
Ca3_P1_16423935	Ca3	16423935	AATATATATATATATATATATAT/AATATATATATATATATATATATAT	TCCAGAAAATAAATCTTCTTCACG	CAAGGATGGGTTGATCGTCT	58.9
Ca3_P1_16441391	Ca3	16441391	A/AAGACATG	CGCTCGATAACCCCAAAATA	ATGTCTCGTCATTCGGGAAG	59.9
Ca3_P1_16448740	Ca3	16448740	TATT/TATTATTATTATTATTATCATT	GAGGACATTGACGGGAGAGA	CTTCTTTGTGCGTCCACTGA	60.2
Ca3_P1_16471674	Ca3	16471674	CA/CAA	GTGAGGACTGGACGTAACCC	TCCTTGCGGAGTTGTTCTTT	59.4
Ca3_P1_16495502	Ca3	16495502	AATATATATATATATATATATATATATATATATATATATATATATAT/AATATATATATATATATATATATATATATATATATATATATATAT	CATCAATGCCACCTCTGAAA	TGCACAACAACAATCATGTCA	59.6
Ca3_P1_16518599	Ca3	16518599	A/AACC	CTTTGGAGCAAGCTGGGTAG	TTTTGCGATGTTTGATCGTT	60.0
Ca3_P1_16544552	Ca3	16544552	GTTATTATTATTATTATTATTATTATTATTATTATTATTA/GTTATTATTATTATTATTATTATTATTATTATTATTA	TGCTCGACATGTTTCTCCTT	CCACACAAACTGACAACTCCA	58.4
Ca3_P1_16645497	Ca3	16645497	AATATATATATATATATATATATATATATATATATATATATATATA/AATATATATATATATATATATATATATATATATATATA	CCACTATCAGGCCCATGAAT	TGGTTGGATGGAGAAACCTC	59.8

INDEL marker IDs	Expected amplified product size (bp)	Structural annotation		Functional annotation	
		Sequence components of *kabuli* genome	*Kabuli* gene accession IDs	NCBI-KOG	TFs
Ca3_P1_16267741	624	INTERGENIC	--	--	--
Ca3_P1_16328087	607	INTERGENIC	--	--	--
Ca3_P1_16331942	373	INTERGENIC	--	--	--
Ca3_P1_16347875	690	INTERGENIC	--	--	--
Ca3_P1_16423935	702	INTERGENIC	--	--	--
Ca3_P1_16441391	441	INTERGENIC	--	--	--
Ca3_P1_16448740	568	INTERGENIC	--	--	--
Ca3_P1_16471674	505	INTERGENIC	--	--	--
Ca3_P1_16495502	806	INTERGENIC	--	--	--
Ca3_P1_16518599	544	INTERGENIC	--	--	--
Ca3_P1_16544552	605	INTERGENIC	--	--	--
Ca3_P1_16645497	590	INTERGENIC	--	--	--

INDEL marker IDs	Chromosomes/ unanchored scaffolds	Physical positions (bp)	InDels (*Kabuli* reference genome-CDC Frontier/PI)	Forward primers (5'-3')	Reverse primers (5'-3')	Anneling temperature (0C)	Expected amplified product size (bp)	Structural annotation		Functional annotation	
								Sequence components of *kabuli* genome	*Kabuli* gene accession IDs	NCBI-KOG	TFs
Ca3_P1_16688486	Ca3	16688486	GGAGAGAGAGAGAGAGAGAGAGA/GGAGAGAGAGAGAGAGAGAGAGA	TTGCAACAAAGGGGATCAAT	TTTCCATCGACACAATTCCA	60.3	142	INTERGENIC	--	--	--
Ca3_P1_16730062	Ca3	16730062	TTTATTATTATTATTATTATTATTATTATTATTATTATTACTATTATTATTATTATTA/TTTATTATTATTATTATTATTATTATTATTATTATTATTATTATTACTATTATTATTATTATTA	TTGCAACATTTGTAGGCATTTC	TAGGTTTTTGCTCCTTCCGA	60.0	706	INTRON	Ca_18280	F	MYB_related
Ca3_P1_16763989	Ca3	16763989	ATTTTTTTTTT/ATTTTTTTTTTT	GGCCATGTAAGCCTTTGGTA	ACTGGCATCAACAGCATCAA	60.0	571	INTRON	Ca_18282	O	--
Ca3_P1_16778238	Ca3	16778238	GAAAAAAA/GAAAAAA	TCCTATGGTGGCTCATCTCC	TTTTGCCTCAACAATCTCCC	60.0	359	INTERGENIC	--	--	--
Ca3_P1_16820332	Ca3	16820332	AATATATATATATATATA/AATATATATATATATATA	AGAGGACATCGGTTAGCTGC	AAGAAAGAAGGGGTCCCAAA	59.5	633	INTERGENIC	--	--	--
Ca3_P1_16875543	Ca3	16875543	GTATATATATATATATATATATATA/GTATATATATATATATATATATA	TTGATGCTAATGCATGGTGTC	CCATCGCTTACCCATTGTTT	59.6	204	INTERGENIC	--	--	--
Ca3_P1_16879725	Ca3	16879725	T/TCCCACTGGTCA	AAGCGTACCTGTGGGAATTG	GCGGATCCACTCACTAAAGG	60.0	818	DRR	Ca_18286	R	MYB
Ca3_P1_16885390	Ca3	16885390	CTATATATATATATATATATA/CTATATATATATATATATA	CATTTTCATCAAAGGTCGCA	CAAATTTGCCACTGTCTTGC	59.7	244	INTERGENIC	--	--	--
Ca3_P1_16942182	Ca3	16942182	AAATAATAATAATAATAATAATAATAATAATAATAA/AAATAATAATAATAATAATAATAATAATAATAATAA	TCCTCCGTTTTGCCAAATTA	CTCCATCACTTTACGCGGTC	60.4	278	DRR	Ca_18292	--	ERF
Ca3_P1_16954725	Ca3	16954725	CATATATATATATATATATATATATA/CATATATATATATATATATATATATATA	TAAATGACCAAGCCGGAAAA	CGTTTGATCGATTGCAGTTC	60.4	573	INTERGENIC	--	--	--
Ca3_P1_17033658	Ca3	17033658	ATT/AT	ATGCGATCATGTGGATGAGA	TGGCTAATGCAGTTCACACA	60.0	648	INTERGENIC	--	--	--
Ca3_P1_17033772	Ca3	17033772	AAATAATAATAATAATAATAATAATAATAATAATAATAATAAT/AAATAATAATAATAATAATAATAATAATAATAATAATAATAATAAT	ATGCGATCATGTGGATGAGA	TACCCCTTGTTGGTCCTGAG	60.0	840	INTERGENIC	--	--	--

INDEL marker IDs	Chromosomes/ unanchored scaffolds	Physical positions (bp)	InDels (*Kabuli* reference genome-CDC Frontier/PI)	Forward primers (5'-3')	Reverse primers (5'-3')	Anneling temperature (0C)	Expected amplified product size (bp)	Structural annotation		Functional annotation	
								Sequence components of *kabuli* genome	*Kabuli* gene accession IDs	NCBI-KOG	TFs
Ca3_P1_17039245	Ca3	17039245	CAAAAAA/CAAAAAAAA	TTCTAACTTTGCTGTCCCATTTT	GGGGTAAAACCAGGAGGAAG	59.2	750	INTERGENIC	--	--	--
Ca3_P1_17039468	Ca3	17039468	C/CA	TGTTTTGAGGACAAATGTTTTGA	GGGGTAAAACCAGGAGGAAG	59.5	301	INTERGENIC	--	--	--
Ca3_P1_17044346	Ca3	17044346	TAAAAAAAAAAAA/TAAAAAAAAAAA	AGCTGGGAGAGTTCAAAGCA	TCACACAATCTGAGTTCCCG	60.1	555	INTERGENIC	--	--	--
Ca3_P1_17122676	Ca3	17122676	ATTTTTTTTTT/ATTTTTTTTTTT	AATGCACCACTTTATGCACG	CCAGTTTTCATTCAGGCACA	59.6	358	INTERGENIC	--	--	--
Ca3_P1_17130457	Ca3	17130457	A/ATC	AGTGCCACAACCATTCATCA	TGGAGTCCAAGGGAGAAATG	60.0	426	INTERGENIC	--	--	--
Ca3_P1_17138095	Ca3	17138095	CAAA/CAAAA	GACAGCCCCTAGCCCTTTAC	GAAATTGGTGGAGCCACTGT	60.1	672	INTERGENIC	--	--	--
Ca3_P1_17187813	Ca3	17187813	CT/CTTT	AAACAAATAAGCCTGGCGTG	GGAGAGACCAAGCTCTCGAA	60.1	462	INTRON	Ca_24618	S	B3
Ca3_P1_17201946	Ca3	17201946	TTATATATATATATATATATATATATATATATATAT/TTATATATATATATATATATATATATATAT	AAAATCAAACATGGTGGGGA	AAGGCATGATGTTGGAGTCA	60.0	398	DRR	Ca_24619	S	--
Ca3_P1_17217773	Ca3	17217773	TCC/TC	ACAATCGGACACCGTTTCAT	TCATGTGGCAACACTGTTCA	60.2	253	INTERGENIC	--	--	--
Ca3_P1_17244270	Ca3	17244270	AATATATATATATATATATATATA/AATATATATATATATATATATA	AAGAGCGGTGTTGGAGTCAT	ATCTAGCCGCCTCCATTTCT	59.7	624	INTERGENIC	--	--	--
Ca3_P1_17260587	Ca3	17260587	ATTTTT/AT	GCATGTTTCAGAGGGGAGAA	AATGGAATTGGAAGGGGAAC	60.2	472	INTRON	Ca_23345	T	WRKY
Ca3_P1_1732184	Ca3	1732184	ATTTTTTTTTTT/ATTTTTTTTTTTT	CAACCCCAAATTGTTGAAGG	AAGCTCATTGTTGACGAAGC	60.2	322	INTERGENIC	--	--	--

INDEL marker IDs	Chromosomes/ unanchored scaffolds	Physical positions (bp)	InDels (*Kabuli* reference genome-CDC Frontier/PI)	Forward primers (5'-3')	Reverse primers (5'-3')	Anneling temperature (0C)	Expected amplified product size (bp)	Structural annotation		Functional annotation	
								Sequence components of *kabuli* genome	*Kabuli* gene accession IDs	NCBI-KOG	TFs
Ca3_P1_17356427	Ca3	17356427	CAAAAAAAAAA/CAAAAAAAAAAA	CACAGTCATTTAAATTAGCAAGCC	ACGCCATGTGTTTCATTCAA	59.3	428	INTERGENIC	--	--	--
Ca3_P1_17413597	Ca3	17413597	TTTAATTAATTA/TTTAATTA	AAGTTGGTTCGTCCACCAAG	GAAATCGGAGAGAGGAAATCG	60.0	830	INTERGENIC	--	--	--
Ca3_P1_17491839	Ca3	17491839	AATATATATATATATA/AATATATATATATA	TGAATTCATGGCTAAAAAGCCT	GCAAAGATGATAATTCGGCAA	60.1	654	INTERGENIC	--	--	--
Ca3_P1_1749212	Ca3	1749212	AATAGATATATA/AATATATA	AGGGATTGAACGAGGGAACT	GCATGATGTATCCATCGCAA	59.9	599	INTERGENIC	--	--	--
Ca3_P1_17516292	Ca3	17516292	TA/TATATAAAAA	GGTGTTGGAGTCATGTCTTTGA	CACGATCCATCTCTGCTTCA	60.0	287	INTERGENIC	--	--	--
Ca3_P1_17522127	Ca3	17522127	ATTTTT/ATTTT	TTCATTGGCACTTGGTTTGA	GCTTGGGGACAAGCAATAGA	60.1	330	INTERGENIC	--	--	--
Ca3_P1_17579559	Ca3	17579559	TAAAA/TAAATAAAAA	TGCATTTTCACCAACATCGT	AATCACCGAGCTGAAATCAAA	60.0	912	INTERGENIC	--	--	--
Ca3_P1_17692049	Ca3	17692049	GATATATATATATATA/GATATATATATATATATA	AAATCTCTCACCCTCTCATCG	GGGATTACTTTCGCGATTCA	58.3	407	INTERGENIC	--	--	--
Ca3_P1_17712986	Ca3	17712986	T/TA	TGCAACAACTTTAGATTCGGA	ATGGAGATAATCGACGTGCC	58.4	505	INTERGENIC	--	--	--
Ca3_P1_17714411	Ca3	17714411	G/GGAAATC	CATGCTCTCATGGTTGGTTG	TGCACATGCTTTTCCGTTAG	60.1	287	INTERGENIC	--	--	--
Ca3_P1_17739152	Ca3	17739152	GATATATATATATATATATATAT/GATATATATATATATATATATAT	TGTATGAATTTCGAATGGCG	ACTTGCCATTTTGCCAACTC	59.5	166	INTERGENIC	--	--	--
Ca3_P1_17739793	Ca3	17739793	TTGGATATATAT/TTAT	GCGTGGGGCACTACTATGAT	GTGGAAGAATGTCGGCTGAT	60.0	350	INTERGENIC	--	--	--

INDEL marker IDs	Chromosomes/ unanchored scaffolds	Physical positions (bp)	InDels (*Kabuli* reference genome-CDC Frontier/PI)	Forward primers (5'-3')	Reverse primers (5'-3')	Anneling temperature (0C)	Expected amplified product size (bp)	Structural annotation		Functional annotation	
								Sequence components of *kabuli* genome	*Kabuli* gene accession IDs	NCBI-KOG	TFs
Ca3_P1_17760054	Ca3	17760054	CTTTTTTTTTTTTT/CTTTTTTTTTTTT	GGCTAAATTACATTTGTGGTCCC	TTTGTGTTACGAAATTGATGAAA	60.8	247	INTERGENIC	--	--	--
Ca3_P1_17819480	Ca3	17819480	AATATATATATATATATATATATATATATATA/AATATATATATATATATATATATATATA	AACGATCTGATTGTAATGTGTTTCTT	GTCGTGAACGGTGCTACATC	59.4	884	INTERGENIC	--	--	--
Ca3_P1_17940619	Ca3	17940619	ATTTTTTTTTT/ATTTTTTTTTTT	GGCTAAATTACATTTGTGGTCCC	TTTTTCCGACTTGGTCCTTT	60.8	317	INTERGENIC	--	--	--
Ca3_P1_17940721	Ca3	17940721	GAAAAAAAAAAA/GAAAAAAAAAA	GGCTAAATTACATTTGTGGTCCC	GGCTAAATTATATTCGTGGTCCC	60.8	382	INTERGENIC	--	--	--
Ca3_P1_18015978	Ca3	18015978	TTATATATATATATATATATATATATATAT/TTATATATATATATATATATATATATAT	TCAGCAATAGAAGCCAAATGC	CGAACTTTGCCGTACGTCTT	60.4	470	INTERGENIC	--	--	--
Ca3_P1_18019115	Ca3	18019115	TAAAAAA/TAGAAAAAA	AACGCTGTAAAAAGCTTGCG	GCCACAGAAATACGAACGGT	60.6	724	INTERGENIC	--	--	--
Ca3_P1_18027762	Ca3	18027762	TTATATATATATATATATATATAT/TTATATATATATATATATATATAT	TTGGTTTATGCACCATGCAC	CTTTTCCCAGTTGGATTGGA	60.4	299	INTERGENIC	--	--	--
Ca3_P1_18089824	Ca3	18089824	AATATATATATATATATATATATATATATATA/AATATATATATATATATATATATATATA	TGCGTGATTATCTGTTTAACGAA	CCGACAGCGGATGATTTTAT	59.7	791	DRR	Ca_21136	--	MYB_related
Ca3_P1_18128285	Ca3	18128285	CT/CTT	ACACAAGCCAACACATCCAA	GGAGAGATTTTGGGGCAGTT	60.0	456	INTERGENIC	--	--	--
Ca3_P1_18241779	Ca3	18241779	CTATATATATATATA/CTATATATATATATATA	CTCTACCACGCGATTACGGT	GGAAGTGATGGGTGAAATGG	60.2	390	INTERGENIC	--	--	--
Ca3_P1_18268210	Ca3	18268210	GAAAAAAAAAA/GAAAAAAAAAAA	AACGAAGAAGTTGAGGTGGC	CCGAATTGCTCTTCATTGGT	59.3	940	DRR	Ca_19613	O	--
Ca3_P1_18345308	Ca3	18345308	TAAAAAAA/TAAAAAAAA	GGTTTCCCTCCTAAGTTAAGGGT	GCGGTGCAATCAGTAAACCT	60.1	271	DRR	Ca_19619	O	--

INDEL marker IDs	Chromosomes/ unanchored scaffolds	Physical positions (bp)	InDels (*Kabuli* reference genome-CDC Frontier/PI)	Forward primers (5'-3')	Reverse primers (5'-3')	Anneling temperature (0C)	Expected amplified product size (bp)	Structural annotation		Functional annotation	
								Sequence components of *kabuli* genome	*Kabuli* gene accession IDs	NCBI-KOG	TFs
Ca3_P1_18360628	Ca3	18360628	GAAAAAAAAAAA/GAAAAAAAAAA	GGCCTATGGCCTGACCTACT	GCCAAACCATAGACCCTTGA	60.5	679	INTERGENIC	--	--	--
Ca3_P1_18527193	Ca3	18527193	ATACTTACTTA/ATACTTA	TGAAAGGGATTGGTGGTCTC	ATAGGGGAGACGGGTGTTTT	59.9	392	INTERGENIC	--	--	--
Ca3_P1_18568373	Ca3	18568373	ATTTTTTTTT/ATTTTTTTT	AGGAATGGACTCCCTCCAAT	TGGTGGAATCTCAACCACAA	59.8	146	INTERGENIC	--	--	--
Ca3_P1_18647131	Ca3	18647131	ATTTTTTTTT/ATTTTTTTTTT	AATTGATTTAAGCCCAAAAATAGA	AGAAGTGGCAAGATGGATGG	57.5	576	DRR	Ca_19638	Q	--
Ca3_P1_18864031	Ca3	18864031	T/TC	AGCTTCTGAATCGCCAAGAG	GGACTCCCAACCCTTTTAGG	59.7	634	INTERGENIC	--	--	--
Ca3_P1_18943181	Ca3	18943181	AAT/A	TGGAGTGTTGATGTCGTTGTC	AAATCGAAGATCGCAACAGT	59.6	428	INTERGENIC	--	--	--
Ca3_P1_1901381	Ca3	1901381	GAAAAAAAAAA/GAAAAAAAAAAA	GGCCGAATTTTGTTGTATGG	AGAAAACCAAATTGCAACGG	60.2	637	INTERGENIC	--	--	--
Ca3_P1_19056367	Ca3	19056367	ATATTATTATTATTATTATTATTATTATTATTATTATTGTTATTATTATTATTATTATTATTAT/ATATTATTATTATTATTATTATTATTATTATTATTATTATTGTTATTATTATTATTATTATTAT	CAACACAACGATAATGGGCA	GACATTGGTTGGGGTTTGAC	60.4	717	INTERGENIC	--	--	--
Ca3_P1_19062819	Ca3	19062819	AATATATATATATATATATATATATATATATATATATATATA/AATATATATATATATATATATATATATATATATATATATA	CATGCTAAATAACATGAAGAATGC	GCAGGTTTGACAGTGAGCAA	57.9	637	INTERGENIC	--	--	--
Ca3_P1_19062976	Ca3	19062976	TAA/TAAA	TTGTTTCATCAATTTACAATGCG	GCAGGTTTGACAGTGAGCAA	59.9	185	INTERGENIC	--	--	--
Ca3_P1_19096971	Ca3	19096971	CTTTATTTATTTATTTATTTATTTATTTATTTATTTATTTATTT/CTTTATTTATTTATTTATTTATTTATTTATTTATTTATTTATTT	AGCTGTCAAACTCAGCCCAT	TCGTGTCCCAAAACTCAAGA	59.9	652	INTERGENIC	--	--	--
Ca3_P1_19124579	Ca3	19124579	TAAAAAAAAAAA/TAAAAAAAAAA	CACTTGTTGTTGGGTGGTGA	GGGCAAACAAGCAAGACAAT	60.5	302	DRR	Ca_19329	--	MYB_related

INDEL marker IDs	Chromosomes/ unanchored scaffolds	Physical positions (bp)	InDels (*Kabuli* reference genome-CDC Frontier/PI)	Forward primers (5'-3')	Reverse primers (5'-3')	Anneling temperature (0C)	Expected amplified product size (bp)	Structural annotation		Functional annotation	
								Sequence components of *kabuli* genome	*Kabuli* gene accession IDs	NCBI-KOG	TFs
Ca3_P1_19142417	Ca3	19142417	TTCCTGNNNNNNNNNNNTCCTG/TTCCTG	GACCCAACCAATGTACACCC	TTCACGAATAAGCCACCACA	60.0	698	INTERGENIC	--	--	--
Ca3_P1_19148213	Ca3	19148213	CATATATATATATATA/CATATATATATATATATA	GCACAAGACTTTACGTCCATCTC	TATTTTGTGTCGTGCGCATT	60.2	691	INTERGENIC	--	--	--
Ca3_P1_19163962	Ca3	19163962	GGTGTGTGTGTGT/GGTGTGTGTGT	AGTTAACCGACGAAACCCCT	TTAGCAATCAAACCAACGCA	59.9	321	INTERGENIC	--	--	--
Ca3_P1_19170970	Ca3	19170970	GAAAAAAAAAA/GAAAAAAAAAAA	TGCAGTTCTGAATTGGTTTGA	TTTTGGAAATTTGTTTTTCTCG	59.3	391	INTERGENIC	--	--	--
Ca3_P1_19196479	Ca3	19196479	CTATATAT/CTATATATATAT	GGTTCATTGCCATGTTGTGA	AAATGTGAATCCCACCAGGA	60.4	633	INTERGENIC	--	--	--
Ca3_P1_19250997	Ca3	19250997	AATATATATATATATATATAT/AATATATATATATATATATATAT	AAATCGATCGTGGAATCTGC	CCAACTTCTTGATGTCCCGT	60.0	423	INTERGENIC	--	--	--
Ca3_P1_19269186	Ca3	19269186	CAAAAAAAA/CAAAAAAAAA	CAATTGCAATGTTGTTTGCC	CCGTCGTTTTGCAGTATTTG	60.0	592	INTERGENIC	--	--	--
Ca3_P1_19299532	Ca3	19299532	ATTTTTTTTTT/ATTTTTTTTT	CGCATTACAACTAAAATGGACG	TGTGCTTCCACCTTTCATCA	59.6	936	INTERGENIC	--	--	--
Ca3_P1_19320997	Ca3	19320997	ATTTTTTTTTTTTT/ATTTTTTTTTTTT	TGAATTTGGTTTTGCCATCA	GGAATCGGATCAAGGAGACA	59.9	771	INTERGENIC	--	--	--
Ca3_P1_19358466	Ca3	19358466	ATTTTTTTT/ATTTTTTTTT	AAGATTTGGTCCGTTATGCG	GTGGGAATGAACAAAGGACG	60.0	517	INTERGENIC	--	--	--
Ca3_P1_19358625	Ca3	19358625	GTTTTTTTTTTTTT/GTTTTTTTT	AAGATTTGGTCCGTTATGCG	GTGGGAATGAACAAAGGACG	60.0	517	INTERGENIC	--	--	--
Ca3_P1_19359038	Ca3	19359038	TAAAA/TAAA	CGTCCTTTGTTCATTCCCAC	TGAGGGCCTTTAGAATCGTG	60.4	670	INTERGENIC	--	--	--

INDEL marker IDs	Chromosomes/ unanchored scaffolds	Physical positions (bp)	InDels (*Kabuli* reference genome-CDC Frontier/PI)	Forward primers (5'-3')	Reverse primers (5'-3')	Anneling temperature (0C)	Expected amplified product size (bp)	Structural annotation		Functional annotation	
								Sequence components of *kabuli* genome	*Kabuli* gene accession IDs	NCBI-KOG	TFs
Ca3_P1_19359334	Ca3	19359334	CCAA/CA	AGGGAAATCAACGACAGAGC	TGAGGGCCTTTAGAATCGTG	59.3	365	INTERGENIC	--	--	--
Ca3_P1_19361284	Ca3	19361284	TAAAAAAAAAA/TAAAAAAAAA	GATTGCAACTTGCCTTGGTT	ACAATGCATCCCAACCATCT	60.1	539	INTERGENIC	--	--	--
Ca3_P1_19367869	Ca3	19367869	TGCTAGCT/TGCT	ATTGATTCAAGGATGAGCGG	GCTGCAACATTTTCAAAGCA	60.0	665	DRR	Ca_19336	--	--
Ca3_P1_19368426	Ca3	19368426	GTTTTTTTT/GTTTTTTTTT	GGCACTCAAAGTGTTGGGTT	TGGGTTAGCTTTTGGGATTG	60.0	920	DRR	Ca_19336	--	--
Ca3_P1_19368565	Ca3	19368565	GTTTTT/GTTTTTT	GGTGATGCATGATGATTTGC	TGGGTTAGCTTTTGGGATTG	59.9	630	DRR	Ca_19336	--	--
Ca3_P1_19369038	Ca3	19369038	CCAACAAC/CCAAC	CAATCCCAAAAGCTAACCCA	AAGAATCTGCGCCAACATCT	59.9	173	INTERGENIC	--	--	--
Ca3_P1_19371062	Ca3	19371062	TAAAAAAAA/TAAAAAAAAA	TTCATTGGATTGTTTGTGGG	GAATTCTGCGTTTTTCCTGC	59.2	845	INTERGENIC	--	--	--
Ca3_P1_19373696	Ca3	19373696	TAA/TA	GCTGAATGCGTGAAGAACAA	GGGTAGAAAACACGCGGAAT	60.0	339	INTERGENIC	--	--	--
Ca3_P1_19375145	Ca3	19375145	AGAGAAAGGAGAAA/AGAGAAAGGAGAAAGGAGAAA	TCTCCGTTCCATTTACACGA	AACTCCAAATCCCTGCTCAA	59.1	365	INTERGENIC	--	--	--
Ca3_P1_19376077	Ca3	19376077	A/ATTT	TGACCTTGTGCATTGTTTTGA	GCAAACTCAACCTTGCCAAT	60.1	333	INTERGENIC	--	--	--
Ca3_P1_19376858	Ca3	19376858	GAAAAAAAAA/GAAAAAAA	TGGGGAGGCATCTTAGTTTG	AATGTCAATCGAATGGGAGG	60.1	128	INTERGENIC	--	--	--
Ca3_P1_19376958	Ca3	19376958	T/TTAAA	TGGGGAGGCATCTTAGTTTG	GCGTGGGAGAATGAAACCT	60.1	616	INTERGENIC	--	--	--

INDEL marker IDs	Chromosomes/ unanchored scaffolds	Physical positions (bp)	InDels (*Kabuli* reference genome-CDC Frontier/PI)	Forward primers (5'-3')	Reverse primers (5'-3')	Anneling temperature (0C)	Expected amplified product size (bp)	Structural annotation		Functional annotation	
								Sequence components of *kabuli* genome	*Kabuli* gene accession IDs	NCBI-KOG	TFs
Ca3_P1_19381557	Ca3	19381557	GTTATTATTAT/GTTATTATTATTAT	GGAGAAAAGCATCGAAAAGC	ACCTAACCCTCCAAGCCAGT	59.0	338	INTERGENIC	--	--	--
Ca3_P1_19383239	Ca3	19383239	AATATATATATATATATATATATATATATAT/AATATATATATATATATATATATATAT	TTTGTGCGTTTGTTTCAGGA	ACCACATGATGCTCTCCACA	60.3	179	INTERGENIC	--	--	--
Ca3_P1_19383912	Ca3	19383912	CAAA/CAA	GTCAGGTCAGGCTGGAAAAA	TCGTCTTAATCCACTTGGTGC	60.2	436	DRR	Ca_19337	--	--
Ca3_P1_19388471	Ca3	19388471	AATGATGATGATGAT/AATGATGATGATGATGAT	GACAACTTGGTTGACGTGGA	TGTCAGGAGGAAGGGAAGTG	59.6	723	DRR	Ca_19338	--	--
Ca3_P1_19402891	Ca3	19402891	AGA/AGATGA	ACCCAATCCAAGTTTGTCCA	GAAGGCGACCTCAAAGACTG	60.2	632	INTERGENIC	--	--	--
Ca3_P1_19496219	Ca3	19496219	CTTTTTTTTT/CTTTTTTTTTT	GCTCAACTCCCTCCATCATT	TGGTCCCTTAACTTATTTTCCG	59.1	742	INTERGENIC	--	--	--
Ca3_P1_19560633	Ca3	19560633	TAAAAAAAAA/TAAAAAAAAAA	TCAGTGCGATGGAGAAATCA	TGACATTTTAAACCTTGAGTTGC	60.4	453	INTERGENIC	--	--	--
Ca3_P1_19639547	Ca3	19639547	CT/CTTATAT	TGCAATCAAGAGAGGACACAA	CGAGAAATAGCTCACGAGGC	59.4	521	INTERGENIC	--	--	--
Ca3_P1_19643393	Ca3	19643393	AG/A	TTGGTGGCCACTTTGAATTT	TTTTTCGAGTTGGTCCTTCA	60.3	783	INTERGENIC	--	--	--
Ca3_P1_19883302	Ca3	19883302	AATGATGATGATGATGATGATGATGATGATGATGAT/AATGATGATGATGATGATGATGATGATGAT	CATTCAGCCCCAAAACCTAA	AAACCCTCCAACAAAGCTCA	59.9	512	DRR	Ca_09392	YU	--
Ca3_P1_19969942	Ca3	19969942	GATCAACGCATC/GATCAACGCATCAACGCATC	CATAAGGATCGGGGAACAAA	AGGATCATCCATCGTCTTCG	59.8	541	INTERGENIC	--	--	--
Ca3_P1_19989742	Ca3	19989742	CAAAAAAAAAA/CAAAAAAAAA	TGGGCAATGCATGAAAAGTA	CCCATGCATGCAATACAAAG	60.1	582	INTERGENIC	--	--	--

INDEL marker IDs	Chromosomes/ unanchored scaffolds	Physical positions (bp)	InDels (*Kabuli* reference genome-CDC Frontier/PI)	Forward primers (5'-3')	Reverse primers (5'-3')	Anneling temperature (0C)	Expected amplified product size (bp)	Structural annotation		Functional annotation	
								Sequence components of *kabuli* genome	*Kabuli* gene accession IDs	NCBI-KOG	TFs
Ca3_P1_20000721	Ca3	20000721	AGG/AGGG	TGCTTTCCTTGGTGGATTTC	TCCAATTTCACATTCTGCCA	60.1	835	INTRON	Ca_09402	--	--
Ca3_P1_20005707	Ca3	20005707	AATATATATATATATATA/AATATATATATATATA	CCACAACAGAACGATGTTGG	TTCCCAAAACTATTTATGCATGG	60.0	312	INTERGENIC	--	--	--
Ca3_P1_20011560	Ca3	20011560	GTTGACACTT/GTTGACACTTGACACTT	GCAATTGCAAGAGCGTGATA	TTTGGCATGCAATCAAACAT	60.0	277	DRR	Ca_09404	--	--
Ca3_P1_20012287	Ca3	20012287	CAT/CATAT	GATGGATCTCGAATGCTGGT	GAACGATGGTGCAAAGGAAT	60.0	424	DRR	Ca_09404	--	--
Ca3_P1_20052639	Ca3	20052639	AATTATTATTATTATTATTATTATTATTATTATTATTATTATTAT/AATTATTATTATTATTATTATTATTATTAT	ATCGTGTGCAAAGGAAGGAT	TGTGCTCAAATAAATAACGTGTCTC	59.6	856	INTERGENIC	--	--	--
Ca3_P1_20074437	Ca3	20074437	CTTTTTTTTTT/CTTTTTTTTT	CAAATACATGTGCCACACAAA	TTTAACCAAATTGTGCAAGGAA	58.0	525	INTERGENIC	--	--	--
Ca3_P1_20118748	Ca3	20118748	TCCCNNNNNNNNNNCCC/TCCC	AAGGGACGAATAACAGCCAT	GCTGGTACATGGGAGCAAAT	58.5	487	INTERGENIC	--	--	--
Ca3_P1_20258210	Ca3	20258210	GATATATATATATATATATATAT/GATATATATATATATATATATATATATAT	AGCAGAGTGTTGAAGCAGAGC	AACATCCAAAAGATGAGCGG	60.0	637	INTERGENIC	--	--	--
Ca3_P1_20295616	Ca3	20295616	CTNNNNNNNNNNNNNNNT/CT	TTTTCGAGTGATTGGGAAGC	GGGGCTGTCACTCAATTGTT	60.2	604	INTRON	Ca_09429	R	--
Ca3_P1_20315844	Ca3	20315844	ATTTTTT/ATTTTTTT	CGCAAACACACTTGAACTGC	AAAGCATGTCACCAAATAAGCA	60.5	184	INTERGENIC	--	--	--
Ca3_P1_20322285	Ca3	20322285	CTTTTTTT/CTTTTTTTT	AGAGCTCTCGGTGGTTTTCA	AAGTTTTCTTTTGCTGCGGA	60.0	532	INTERGENIC	--	--	--
Ca3_P1_20435798	Ca3	20435798	ATATTATTATTATTATTATTATTATTATTATTATTATTATTATTATTATTATTATTA/ATATTATTATTATTATTATTATTATTATTATTATTATTATTATTATTATTATTATTATTATTATTATTA	TAAAATAAATTGGCGGCGAC	GCCACAAAGTCATGTGATGC	59.9	512	INTERGENIC	--	--	--

INDEL marker IDs	Chromosomes/ unanchored scaffolds	Physical positions (bp)	InDels (*Kabuli* reference genome-CDC Frontier/PI)	Forward primers (5'-3')	Reverse primers (5'-3')	Anneling temperature (0C)	Expected amplified product size (bp)	Structural annotation		Functional annotation	
								Sequence components of *kabuli* genome	*Kabuli* gene accession IDs	NCBI-KOG	TFs
Ca3_P1_20445164	Ca3	20445164	TTATATATATATATATATATATATAT/TTATATATATATATATATATATAT	AGTGCCACATGTAACACCCC	GTTTTGGCGTTGGTTTCACT	60.7	815	INTERGENIC	--	--	--
Ca3_P1_20468595	Ca3	20468595	ATTTTTTTTTTTT/ATTTTTTTTTTT	ATTCTACCCCCACCATTTTT	TGCATTTCTGCCCTGTGTTA	57.3	500	INTERGENIC	--	--	--
Ca3_P1_20526351	Ca3	20526351	TTATATATATATATATATATATATATATAT/TTATATATATATATATATATATAT	TGCATGTTCTTACGCTCGTC	AATTCACAAGACGCCATTCC	60.0	504	INTERGENIC	--	--	--
Ca3_P1_20604662	Ca3	20604662	AATATATATATATATATATATAT/AATATATATATATATATAT	TTCATGAAGACCGACTCAACA	TGGTTTATGAAGGTAATACGGAAAA	59.3	863	INTERGENIC	--	--	--
Ca3_P1_20617847	Ca3	20617847	AATATATATATATATATATATATA/AATATATATATATATATATATATATA	AATCGATTGGCTGAAATCCA	TCGAGTTTGAGCATGTGAGG	60.4	809	INTERGENIC	--	--	--
Ca3_P1_2065051	Ca3	2065051	AATTATTATTATTATTATTATTATTATTATTA/AATTATTATTATTATTATTATTATTATTA	CGACCATGTAGTGAGGCAAA	AATAATTGCCCTACACCAACAA	59.7	685	INTERGENIC	--	--	--
Ca3_P1_20696013	Ca3	20696013	TTATATATATATATATAT/TTATATATATATATAT	CACTCTTTTCTCACCCCCAC	TTTTTGCCCATTCAAACACA	59.5	687	INTERGENIC	--	--	--
Ca3_P1_20709467	Ca3	20709467	CATATATATATATATATATATATATATA/CATATATATATATATATATATATATA	ATGGGCTTGGTAGGGACTTT	CAATCCAAGCATGCAATCC	59.8	638	INTERGENIC	--	--	--
Ca3_P1_20750487	Ca3	20750487	CTTTATTTATTTTATTTATTTTATTTATTTTATTTATTTTATTTATTTTATTT/CTTTATTTATTTTATTTATTTTATTTATTTTATTTATTTTATTTATTTTATTTATTTTATTT	TGTTATGTTGGGTTTGGGCT	TGCAGATGGGTTGAGTGTGT	60.2	875	INTERGENIC	--	--	--
Ca3_P1_20868628	Ca3	20868628	GTTTTTTTTTT/GTTTTTTTTT	TTTTACCGTTGCTGCATGTC	GCAATAGAAACCGGAAGTGTG	59.7	964	INTRON	Ca_09461	--	--
Ca3_P1_20947306	Ca3	20947306	TTATATATATATATATATATA/TTATATATATATATATATATA	GCATAAATGATGCAAAAGGTGA	ACGACCTGGTTGATAGGTGC	60.0	577	INTERGENIC	--	--	--
Ca3_P1_20969920	Ca3	20969920	AATATATATATATATATATATAT/AATATATATATATATATATATATAT	TCATTGCTTCAATAAATTTCATCAT	TAGAAGGCATCCCTTTGGAA	59.0	813	INTRON	Ca_09468	--	M-type

INDEL marker IDs	Chromosomes/ unanchored scaffolds	Physical positions (bp)	InDels (*Kabuli* reference genome-CDC Frontier/PI)	Forward primers (5'-3')	Reverse primers (5'-3')	Anneling temperature (0C)	Expected amplified product size (bp)	Structural annotation		Functional annotation	
								Sequence components of *kabuli* genome	*Kabuli* gene accession IDs	NCBI-KOG	TFs
Ca3_P1_20970314	Ca3	20970314	ATTTTTTTTTTT/ATTTTTTTTTTTT	TCTTTCCCGATCAAATTGAGA	CCCAAAATCAACCAAAGCTC	59.6	553	INTRON	Ca_09468	--	M-type
Ca3_P1_20988305	Ca3	20988305	TAAAAAAA/TAAAAAA	CATTTGCGGTCCCTTAATTT	TGTGGAAGTCCTTCAGCTTG	58.9	569	INTERGENIC	--	--	--
Ca3_P1_20988635	Ca3	20988635	AATATATATATATATATATATATATATATATATATATAT/AATATATATATATATATATATATATATATATATATATATATAT	CAAGCTGAAGGACTTCCACA	ATTATGTGGCGAGTGACGGT	59.0	477	INTERGENIC	--	--	--
Ca3_P1_21034670	Ca3	21034670	AATATATATATATATATATATATATATAT/AATATATATATATATATATATATATATATATATAT	TCCATCAAACAAAGTTGATGTAAAA	AAGACATTGGACGGAACCAG	59.8	387	DRR	Ca_09471	IG	--
Ca3_P1_21097843	Ca3	21097843	ATTTTTTTTT/ATTTTTTTTTT	GGCATTTTGATTTGATTCGC	CAAATTTTGTGTTTCAAAAACCATT	60.4	653	INTERGENIC	--	--	--
Ca3_P1_21114306	Ca3	21114306	CTAGTA/CTAGTAGTA	TCATGCGATCATCCATTCTAA	ATTTGCAACGATGTGAACCA	59.1	594	INTERGENIC	--	--	--
Ca3_P1_21186305	Ca3	21186305	A/ACTACG	CTTTGGGATTGTTTTGTGGG	TTGGCGAAGAAAAGCAAACT	60.2	709	INTRON	Ca_09480	T	NAC
Ca3_P1_21207756	Ca3	21207756	TAAAAAAAAAA/TAAAAAAAAA	TAACCATTGAACCGGCTTTT	GAGACTTCCTCCCCATCTCC	59.5	501	INTERGENIC	--	--	--
Ca3_P1_21208119	Ca3	21208119	CTATATATATATATATATATATATAT/CTATATATATATATATATATATATAT	GGAGATGGGGAGGAAGTCTC	TCTTGAAGCTAACGGATTTCG	60.0	300	INTERGENIC	--	--	--
Ca3_P1_21243884	Ca3	21243884	CTTTTTTTTT/CTTTTTTTTTT	GGAAGGAACGGTGCTTCATA	CTCCAACGGTGAACTCAACA	60.1	522	INTERGENIC	--	--	--
Ca3_P1_21276082	Ca3	21276082	AT/ATTATTTT	TTGATCTTTTGTTCGGTTGC	TCTTGGACATCATCAAAGCG	58.7	530	DRR	Ca_09490	--	S1Fa-like
Ca3_P1_21311568	Ca3	21311568	GAAAAAAAAA/GAAAAAAAAAA	CACGTCACATCGCAATTTTT	CATGTCATTTCAACCCACGA	59.6	542	INTERGENIC	--	--	--

INDEL marker IDs	Chromosomes/ unanchored scaffolds	Physical positions (bp)	InDels (*Kabuli* reference genome-CDC Frontier/PI)	Forward primers (5'-3')	Reverse primers (5'-3')	Anneling temperature (0C)	Expected amplified product size (bp)	Structural annotation		Functional annotation	
								Sequence components of *kabuli* genome	*Kabuli* gene accession IDs	NCBI-KOG	TFs
Ca3_P1_21319728	Ca3	21319728	AACTTACTTA/AACTTA	TGCTGTTCTAGTGTTCTAATTGCTC	GCCTGCCAGACGTTTTACAT	59.2	562	INTERGENIC	--	--	--
Ca3_P1_21328627	Ca3	21328627	CTTTTTTTTTTTT/CTTTTTTTTTTT	TGTCGGGACTTCACATGCTA	CTTGCCTGTGTCGAAGATGA	60.3	499	INTERGENIC	--	--	--
Ca3_P1_21355992	Ca3	21355992	TAAAAAAA/TAAAAAA	GTTGGTCCTTTTGGACCTCA	ACAATTTTCAGCAACCCGAG	59.9	545	INTERGENIC	--	--	--
Ca3_P1_213670	Ca3	213670	AATATATATATATATATATAT/AATATATATATATATATATAT	TTCCTTTTCCAATTTGCAGG	TCACGTAGAATGCTATCACTTTGA	60.0	544	DRR	Ca_22935	M	MYB_related
Ca3_P1_21424576	Ca3	21424576	CTTTTTT/CTTTTT	ACGTAGTTGGGTCTTGGCAC	CAAATTCTGTCCCGTCTGGT	60.0	385	INTERGENIC	--	--	--
Ca3_P1_21481670	Ca3	21481670	CTATTATTATTATTATTATTATTATTATTATTATTATTATTATTATTATTAT/CTATTATTATTATTATTATTATTATTATTATTATTATTATTATTATTATTATTAT	GCGTAGTAAATCCTTCTTCCACA	TGAGACAAAGAATAACACCCGA	59.7	568	INTERGENIC	--	--	--
Ca3_P1_21491510	Ca3	21491510	ATTTTTTTTTTT/ATTTTTTTTTTTT	CAAAGAACGGCATTTGGAAT	TGATGGACTTGCCTCTACTTGA	59.9	860	INTERGENIC	--	--	--
Ca3_P1_21545808	Ca3	21545808	AATATATATATATATATATATATATATATA/AATATATATATATATATATATATA	AAATATTTTTAAGCAACCCCAAAA	TGGCACCTCAACCAATGTTA	59.3	542	INTERGENIC	--	--	--
Ca3_P1_21546918	Ca3	21546918	GTATATATATATATATA/GTATATATATATATATA	GTCCAAAATCATCCCCCTTT	TGGCACTGTCATGGTTTGTT	60.0	403	INTERGENIC	--	--	--
Ca3_P1_21599415	Ca3	21599415	CAAAAAA/CAAAAAAA	GCAGCAGTTGTTTGCAGAAG	TCTCTTGCATGCCTAACACG	59.8	941	INTERGENIC	--	--	--
Ca3_P1_21635589	Ca3	21635589	GCATCATCA/GCATCA	AAGCATGCCAAACCAAAAAC	TGAACATCAAAGATCTGCGG	60.0	521	INTRON	Ca_09516	--	--
Ca3_P1_21757473	Ca3	21757473	CTTTTTTTTTTTT/CTTTTTTTTTTT	AAAATCTCAATTTTCGAGCCAA	AGGGCTAGGCAGCCATAAAT	60.1	393	INTERGENIC	--	--	--

INDEL marker IDs	Chromosomes/ unanchored scaffolds	Physical positions (bp)	InDels (*Kabuli* reference genome-CDC Frontier/PI)	Forward primers (5'-3')	Reverse primers (5'-3')	Anneling temperature (0C)	Expected amplified product size (bp)	Structural annotation		Functional annotation	
								Sequence components of *kabuli* genome	*Kabuli* gene accession IDs	NCBI-KOG	TFs
Ca3_P1_2176981	Ca3	2176981	GATTATTATTATTATTATTATTATTATTATTATTATTATTATTATGATTATTATTATTA/GATTATTATTATTATTATTATTATTATTATTATTATTATTATTATGATTATTATTATTA	ACGGATTGTGTTTTGCTTCT	CCTCTTCTTCGGTGATCTTTTG	57.3	906	INTERGENIC	--	--	--
Ca3_P1_21782272	Ca3	21782272	AATATATATATATATATATATATATATATATATAT/AATATATATATATATATATATATATATAT	TTGGTGAAAAATATTTGAGAGGC	CTTGACATCGATCCCACAAA	59.5	597	INTERGENIC	--	--	--
Ca3_P1_21870840	Ca3	21870840	TTTATTATTATTATTATTATTATTATTATTATTATTATTATTATTAT/TTTATTATTATTATTATTATTATTATTATTATTATTATTATTATTATTATTATTAT	ATGTTAGACCGTCCGTCAGG	GGTCTAAATCTGGCTTACGGC	60.0	655	INTRON	Ca_20433	I	--
Ca3_P1_21884477	Ca3	21884477	CATATATATATATATATAT/CATATATATATATATAT	CCGCGAGTAATTCCGATAAA	AGGGGCAACTCCTTTCTCAT	60.1	948	INTERGENIC	--	--	--
Ca3_P1_21897869	Ca3	21897869	ATATTATTATTATTATTATTATTATTA/ATATTATTATTATTATTATTATTA	GAGTTGAGTGTTGCCAGGGT	AGCTACCAAGCTGATGGTGAA	60.2	296	INTERGENIC	--	--	--
Ca3_P1_21910992	Ca3	21910992	AAATAATAATAATAATAATAATAATAATAATAA/AAATAATAATAATAATAATAATAATAATAA	AATGAGGCGATTTGTCGTTC	CTCACAATTGCAAGGAGCAA	60.1	934	INTERGENIC	--	--	--
Ca3_P1_2194698	Ca3	2194698	TG/TGG	ATTTTAGGGGGTCATCGTCC	ATGGAGACAGAACCAATGCC	60.0	351	INTERGENIC	--	--	--
Ca3_P1_2194841	Ca3	2194841	TT/TTAT	GTTGTTGCTTCTGCTCCTCC	TACAAGATGTTTGGTGGCGA	60.0	930	INTERGENIC	--	--	--
Ca3_P1_21959890	Ca3	21959890	GTTTTTTTTTTTT/GTTTTTTTTTTT	CATGATTCAATTCTTTTCGTGA	AATTGTTGGCAAAGACGACC	57.8	267	INTERGENIC	--	--	--
Ca3_P1_21989057	Ca3	21989057	CTATATATATATATATATATATATA/CTATATATATATATATATATATATATATATATA	GGGACAACTGATGTAATTTAGCC	ACAAAGATTCGCGGATTGAC	58.9	515	INTERGENIC	--	--	--
Ca3_P1_21989858	Ca3	21989858	TTATATATATATATATATATATATATATATATAT/TTATATATATATATATATATATATATAT	TGAAGCAAACCAAATAAATCCA	GACCGGTGGTAGACCTTTGA	59.5	592	INTERGENIC	--	--	--
Ca3_P1_22042563	Ca3	22042563	CTTTTTT/CTTTTTTT	AGGAAAGTGCCATGTGAAAAA	TCGTAGGTCAAGTTCAGACCG	59.6	764	INTERGENIC	--	--	--

INDEL marker IDs	Chromosomes/ unanchored scaffolds	Physical positions (bp)	InDels (*Kabuli* reference genome-CDC Frontier/PI)	Forward primers (5'-3')	Reverse primers (5'-3')	Anneling temperature (0C)	Expected amplified product size (bp)	Structural annotation		Functional annotation	
								Sequence components of *kabuli* genome	*Kabuli* gene accession IDs	NCBI-KOG	TFs
Ca3_P1_22057836	Ca3	22057836	GTTTTTTTTTTT/GTTTTTTTTTT	TGCATTCAATCAAGGGATCA	AAACAGGGGTAGCAAAATCG	60.0	631	INTERGENIC	--	--	--
Ca3_P1_22125691	Ca3	22125691	GAAAA/GAAAAA	TAACCCCTTGATCCTTGCAG	AGGGTTCAGCTAAGTGCCAA	60.1	647	DRR	Ca_20414	--	--
Ca3_P1_22249668	Ca3	22249668	GTTTTTTTTTTTTT/GTTTTTTTTTTTTTT	GTAAATAGTGCCACCGGCAT	TGAATTTTCTCCAAGGGCAC	59.9	561	DRR	Ca_20405	--	--
Ca3_P1_2225131	Ca3	2225131	GAAAAAAAAA/GAAAAAAAAAA	CACACAATACATGTAGAGTCGCC	GGGAGAATTGTAACACCCCA	59.6	296	INTERGENIC	--	--	--
Ca3_P1_22259485	Ca3	22259485	CTTTTTTTTTT/CTTTTTTTTTTT	AGAACCGCAAGGGAGGTATT	AGATGACTTTGCAACCACCA	60.0	493	INTERGENIC	--	--	--
Ca3_P1_22294368	Ca3	22294368	GACAACAACAACAA/GACAACAACAACAACAA	TTCACCATAACTTCCGTCCA	TCCCTAGCAGCCTCTTTTGA	59.0	722	DRR	Ca_06280	K	MIKC
Ca3_P1_22377503	Ca3	22377503	TGTT/TGTTAGTT	TTCATTAACGCTTATTCCCTCA	TGCAACTCTAACAGCGACAA	58.8	694	INTERGENIC	--	--	--
Ca3_P1_22378443	Ca3	22378443	AAATAATAATAATAATAATAATAATAATAATAATAATAA/AAATAATAATAATAATAATAATAATAATAATAATAA	TTCGAGATAGCCTTGCGAAT	TTGTTTGATGAGGCATGACA	59.9	721	INTERGENIC	--	--	--
Ca3_P1_22473112	Ca3	22473112	TTATATATATATATAT/TTATATATATATAT	TTACAAACAAGCTGGCAACG	CACTCGACACAATCTTAACCCA	59.9	820	INTERGENIC	--	--	--
Ca3_P1_22487747	Ca3	22487747	TACAGTGA/TA	GTAATGCCCCGTGAGTAGGA	CCCAATTGATTCCAAAAGAAA	60.0	778	INTERGENIC	--	--	--
Ca3_P1_22548422	Ca3	22548422	CATAAATAAATAAATAAATAAAT/CATAAATAAATAAATAAAT	CATGGACAAACCGAGTGATG	GCTGTGGCGTATCTGTTGAA	60.0	527	INTERGENIC	--	--	--
Ca3_P1_22553987	Ca3	22553987	CGACTTATT/CGACTTATTCAGACTTATT	CCACATGAGTGTGTTGAGGG	GGCTATCGCTTATGCAGGAC	60.0	588	DRR	Ca_06259	--	SAP

INDEL marker IDs	Chromosomes/ unanchored scaffolds	Physical positions (bp)	InDels (*Kabuli* reference genome-CDC Frontier/PI)	Forward primers (5'-3')	Reverse primers (5'-3')	Anneling temperature (0C)	Expected amplified product size (bp)	Structural annotation		Functional annotation	
								Sequence components of *kabuli* genome	*Kabuli* gene accession IDs	NCBI-KOG	TFs
Ca3_P1_22661182	Ca3	22661182	AATTATTATTATTATTATTATTATTATT ATTATTATTATTATTAT/AATTATTATTA TTATTATTATTATTATTATTATTATTAT TAT	AGAGAAGGTGAGGGG GTGTT	AGCCTGATAGGTGGT GGAAA	60.0	763	INTRON	Ca_06245	R	--
Ca3_P1_22720292	Ca3	22720292	TAAAAAACAAAAAAACAA/TAAAAAACA A	AGATCCAGCAGGGAAA TCCT	TGACAAAATTCGCGAG AAAA	60.0	661	DRR	Ca_06239	--	--
Ca3_P1_2273008	Ca3	2273008	CAAAAAAAAAAA/CAAAAAAAAAAAA	TAAAGGATCGGACCCA CTTG	GGCGATCCAAGAGTTC AATC	59.9	822	INTERGENIC	--	--	--
Ca3_P1_22744769	Ca3	22744769	AATATATATATATATATATATATAT/AA TATATATATATATATAT	TATAAGGCCTAACAGG CCGA	ACAATTAACCAAGAAC GGCG	59.7	776	INTERGENIC	--	--	--
Ca3_P1_22798410	Ca3	22798410	ATA/ATATTA	CAATAGAAAGGCGACG GAAG	GAGCTTCGGAATATCC CCTC	59.8	335	INTERGENIC	--	--	--
Ca3_P1_22819995	Ca3	22819995	TTTATTATTATTATTATTATTATTATTA TTATTATT/TTTATTATTATTATTATTAT TATTATTATT	GCATGCACAAGTCTCT GGAA	TTCTCCCCAGTTGCTT ATCG	60.0	642	DRR	Ca_06229	TD	--
Ca3_P1_2285204	Ca3	2285204	TCCCC/TCCCCC	TTGCAACATTCAGGTC TTCG	TCCCTCCTAGAGGCAA ACAA	59.8	761	INTERGENIC	--	--	--
Ca3_P1_22934054	Ca3	22934054	G/GCCGATACAATTTGT	CCACATTATGCCTTCG GTTT	GGTTTTCGCGATATTT TCCA	59.8	210	INTERGENIC	--	--	--
Ca3_P1_22936637	Ca3	22936637	TTAATAATAATAATAATAATAATAATAA TAATAAAAATAAAAATAATAATAATAAT AATAATATTAATATTAATAATAATAATA ATAATAATAATAATAATAATAATAATAA TAATAATAATAATAATAATAATAATAAT [illegible]	ACCACAAATTTCGAGG ACGA	TGAAAGATGTGGGTAG CAATAA	60.5	778	INTERGENIC	--	--	--
Ca3_P1_22949188	Ca3	22949188	TAAAAAAAAAAA/TAAAAAAAAAA	ATTGAGTTCGACGCTC GGTA	TCCAACCACTTGAGTC ATGC	60.8	416	INTERGENIC	--	--	--
Ca3_P1_22951325	Ca3	22951325	GCC/GCCC	CAAACTGTGCAATTAC GGATT	AGTGGGAGACGAGGT GTCTG	57.7	795	INTERGENIC	--	--	--
Ca3_P1_22976984	Ca3	22976984	AGGTTGGTTGGTTGGTTGG/AGGTTG GTTGGTTGGTTGGTTGG	TCCAATCGATGGGTCT AAGC	ATCCATCTAAAGGCAG GGGT	60.0	203	INTRON	Ca_06211	I	BES1

INDEL marker IDs	Chromosomes/ unanchored scaffolds	Physical positions (bp)	InDels (*Kabuli* reference genome-CDC Frontier/PI)	Forward primers (5'-3')	Reverse primers (5'-3')	Anneling temperature (0C)	Expected amplified product size (bp)	Structural annotation		Functional annotation	
								Sequence components of *kabuli* genome	*Kabuli* gene accession IDs	NCBI-KOG	TFs
Ca3_P1_23016776	Ca3	23016776	TCCTACCT/TCCT	GCACACCTATGTGGTCTTGC	CCATTTTGTCGAAGTTTGGTT	59.2	615	DRR	Ca_06208	QR	B3
Ca3_P1_23019440	Ca3	23019440	T/TCACTAC	TGACCCATCCAAACATTTCA	AAACGTGCAAGGTCCAACTC	59.8	153	INTERGENIC	--	--	--
Ca3_P1_23063614	Ca3	23063614	TTATATATATATATATATATATATATA/TTATATATATATATATATATATATATATA	GTAACTTGCACCGACCCAAT	ATGACGGTGTTTGGAGACAA	59.9	599	INTERGENIC	--	--	--
Ca3_P1_23102761	Ca3	23102761	CTATATATAT/CTATATATATAT	CACCTTGTCACATTCACAGACA	CCTTGAAGGTTAACAATTTCGT	59.6	543	INTERGENIC	--	--	--
Ca3_P1_23112762	Ca3	23112762	TT/TTTCCTTGATACTTCGT	TGATGCCAATTTGGTTCTGA	TTCGAATCCGTCACAAATGA	60.0	628	INTERGENIC	--	--	--
Ca3_P1_23201611	Ca3	23201611	TAGGTCGATCAG/TAG	AAAAAGGAGAGTTCTCCGGC	ATCGAGCGAACACATTGTCA	59.8	677	INTERGENIC	--	--	--
Ca3_P1_23213458	Ca3	23213458	TAAAAAAAAAA/TAAAAAAAAA	TGTTCTCGATCGATGTATTTTATCA	TGACGGTGTAAAACTGTCGG	59.9	150	DRR	Ca_06196	R	--
Ca3_P1_2327052	Ca3	2327052	CAAAAAAAAAAAAAA/CAAAAAAAAAAAAA	GATACGGAACCATCGCACTT	TTGGTTTCGTTCTCCATTCA	60.0	343	DRR	Ca_19393	K	MYB
Ca3_P1_23284742	Ca3	23284742	TTATATATATATATATATATATA/TTATATATATATATATATATATATA	GTAGCACATGCTTCAGTGGC	TCTCATGGAAGGAGCAAACA	59.5	445	INTERGENIC	--	--	--
Ca3_P1_23308159	Ca3	23308159	CTTTTTTTTTT/CTTTTTTTTTTT	TTGCCTTCATGCTTTCTCAA	CATGTTCCGATATGCCACTG	59.5	461	INTERGENIC	--	--	--
Ca3_P1_23325506	Ca3	23325506	A/AAAT	ATCAACACAACATCCACCCA	TTTGCAGGGTCTTAATGGACTT	59.7	809	DRR	Ca_06187	R	ERF
Ca3_P1_23329861	Ca3	23329861	A/AGGGTTG	GGGCTTGCAATAGACCAAAG	GCTGCAGGAATCTTGGTGTT	59.7	678	INTRON	Ca_06187	R	ERF

INDEL marker IDs	Chromosomes/ unanchored scaffolds	Physical positions (bp)	InDels (*Kabuli* reference genome-CDC Frontier/PI)	Forward primers (5'-3')	Reverse primers (5'-3')	Anneling temperature (0C)	Expected amplified product size (bp)	Structural annotation		Functional annotation	
								Sequence components of *kabuli* genome	*Kabuli* gene accession IDs	NCBI-KOG	TFs
Ca3_P1_23424382	Ca3	23424382	TATTAAAAAAT/TATTAAAAAATTAAAAAAT	CATTGTCTGGACTACATTGTGTGA	GAAGGGATCAATTTCTACCCA	60.0	674	INTERGENIC	--	--	--
Ca3_P1_23434692	Ca3	23434692	ATTTTTTTTTTT/ATTTTTTTTTTTT	GAGGGGACTCAGGGGACTTA	TAATTATTGGTCCCAGCCCA	60.4	381	INTERGENIC	--	--	--
Ca3_P1_23475694	Ca3	23475694	AAGAGAGAGAGAGAGAGAGAGAGAGAGAGAGAGAGAGAGAGAGAGAGAGAGA/AAGAGAGAGAGAGAGAGAGAGAGAGAGAGAGAGAGAGAGAGAGAGAGAGAGAGA	CCATAAACGGAGACCGAAGA	CAGCACTTCTTGCTGGCTTA	60.1	495	INTERGENIC	--	--	--
Ca3_P1_23530917	Ca3	23530917	AATATATATATATATATATATATATAT/AATATATATATATATATATATATATATAT	ACCCATTAGCGTAGGAGTGC	AACGTAAATGCACTTGAACCG	59.2	786	INTERGENIC	--	--	--
Ca3_P1_23541848	Ca3	23541848	TTATATATATATATATATATATATATATAT/TTATATATATATATATATATATATATATATATATATATATATATAT	ATAAGTTGGCGTTGCCTCTG	CGTCATATGGCGAAATAGATTG	60.3	518	INTERGENIC	--	--	--
Ca3_P1_23551096	Ca3	23551096	AACAATTTACAATTTACGATTTACAATTT/AACAATTTACAATTTACAATTTACGATTTACAATTT	TCCCATTTTGATTCCCTTCA	TCCCTAAATCGCTGAATCTGA	60.2	363	INTERGENIC	--	--	--
Ca3_P1_23595652	Ca3	23595652	GAAAAAAAAAA/GAAAAAAAAA	CCACCTACACAAAAACACGG	CGTGGCTTTATTCGTTCCAC	58.9	646	DRR	Ca_06168	T	--
Ca3_P1_23602746	Ca3	23602746	AA/AAAATA	AAGTTGCAAATTGTGCAGGA	CCTCCCTCCAAATCAAGTCA	59.3	474	INTERGENIC	--	--	--
Ca3_P1_23619757	Ca3	23619757	AAATAATAATAATAATAATAATAATAATAATAA/AAATAATAATAATAATAATAA	TCTCCATCAAATCCAGAGCC	ACTGCCTTGTGTTGTTGTGC	60.2	902	INTERGENIC	--	--	--
Ca3_P1_23623793	Ca3	23623793	CA/C	AGCTGCTGGTGGTAGCTGAT	GATGAAGTCAGCGAAAAGGTT	60.0	683	DRR	Ca_06167	--	WRKY
Ca3_P1_23651722	Ca3	23651722	AAT/AATAATTTAT	GGTACGTTTGGTCTTGCAGC	TGAGCGCTTATGATCCATTTT	60.7	529	DRR	Ca_06162	--	NAC
Ca3_P1_23656005	Ca3	23656005	CTTTTTTTTTT/CTTTTTTTTTTT	CGAAGAGCAATGATATTTGAACA	CATTGTGGGGAAGAAGGAAA	59.3	285	INTERGENIC	--	--	--

INDEL marker IDs	Chromosomes/ unanchored scaffolds	Physical positions (bp)	InDels (*Kabuli* reference genome-CDC Frontier/PI)	Forward primers (5'-3')	Reverse primers (5'-3')	Anneling temperature (0C)	Expected amplified product size (bp)	Structural annotation		Functional annotation	
								Sequence components of *kabuli* genome	*Kabuli* gene accession IDs	NCBI-KOG	TFs
Ca3_P1_23691313	Ca3	23691313	CTTTTTTTT/CTTTTTTTTT	TGTCAGCAACCCTTGTCTTG	TTGACGTCACCAATTCAACC	59.9	590	INTERGENIC	--	--	--
Ca3_P1_23759284	Ca3	23759284	GA/GAA	CCATCTCAAACGTGCTCTCA	TTTTGGGTGATGTGGGAAAT	60.0	181	DRR	Ca_06153	GC	--
Ca3_P1_23830553	Ca3	23830553	ACTCTCTCTCTCTCTCTCTCTCTCTCTC/ACTCTCTCTCTCTCTCTCTCTCTCTCTCTCTCTC	TCACGAAATTGGTCCCCTTA	GGGACCAAAACTGGAGAGAA	60.3	878	INTERGENIC	--	--	--
Ca3_P1_23881356	Ca3	23881356	TTATATATATATATATAT/TTATATATATATATATATATATAT	ACAGCCAACAACCACATCAA	CGTAAGTGTGACCGACGCTA	60.0	864	INTERGENIC	--	--	--
Ca3_P1_23881409	Ca3	23881409	ATTTTTTTT/ATTTTTTTTT	TGGTACTGGAGGCCACCTAC	CGTAAGTGTGACCGACGCTA	60.0	522	INTERGENIC	--	--	--
Ca3_P1_23905533	Ca3	23905533	CTTTTTTTTTTT/CTTTTTTTTTT	CCTTGAGGAATGGAATCGAA	GATGGGCTAGACCGATACACA	60.0	738	INTERGENIC	--	--	--
Ca3_P1_23972065	Ca3	23972065	CAAAAAAAAAAAA/CAAAAAAAAAAA	TTTCTATGAAATTAAAGCAAGCAA	GAAATGGGAATAAAATGATGGA	57.5	298	INTERGENIC	--	--	--
Ca3_P1_24011215	Ca3	24011215	ATT/ATTT	TCATGAAGATTTGGAAGAGAAAGA	TGTTGCTACGCTGAAACTGG	59.4	272	INTERGENIC	--	--	--
Ca3_P1_24015049	Ca3	24015049	CTTTTTTTTT/CTTTTTTTTTT	CTGACCTGACATGGAAGCCT	TATGGTCTGGTTTGGCCTGT	60.3	600	INTERGENIC	--	--	--
Ca3_P1_24033300	Ca3	24033300	TTAATAATAATAATAATAATAATAATA/TTAATAATAATAATAATAATAATA	TCCAAGAAAAGTGTTGGAAATG	CCTTCCTACGCCTCAAATCA	59.1	376	INTERGENIC	--	--	--
Ca3_P1_24071503	Ca3	24071503	CATATATATATATATATATATATATATATATATA/CATATATATATATATATATATATATATATATATATATA	CAGTTATCACTTGATACGCCCA	ACGAAATGAATAGCAACCCG	60.0	395	INTERGENIC	--	--	--
Ca3_P1_24117383	Ca3	24117383	TTACATACATACATAC/TTACATACATAC	GAAGAGGTGCTTGGTGGAGA	TACACCAGAATTTGCTCCCC	60.4	342	DRR	Ca_06107	O	--

INDEL marker IDs	Chromosomes/ unanchored scaffolds	Physical positions (bp)	InDels (*Kabuli* reference genome-CDC Frontier/PI)	Forward primers (5'-3')	Reverse primers (5'-3')	Anneling temperature (0C)	Expected amplified product size (bp)	Structural annotation		Functional annotation	
								Sequence components of *kabuli* genome	*Kabuli* gene accession IDs	NCBI-KOG	TFs
Ca3_P1_2414966	Ca3	2414966	A/AC	TTAATGAGGGGCTGAATTGG	AAGCGTACCTGTGGGAATTG	59.9	259	INTERGENIC	--	--	--
Ca3_P1_24156923	Ca3	24156923	TTATATATATATATATATATATATAT/TTATATATATATATATATATATATATAT	CAATAGCCCAATTTTGAAAGAA	TGCTTGGGTGAGTGAGAGTG	58.3	397	INTERGENIC	--	--	--
Ca3_P1_24187708	Ca3	24187708	A/ACAG	ATTGCATGGAACGACAATGA	CCATGAGTGTTTCGTGGATG	59.9	607	DRR	Ca_06101	J	--
Ca3_P1_24193117	Ca3	24193117	ATTT/ATT	TAAGGTTCAGGTTATGGCCG	CGCCACTATGTCGGCTATTT	60.0	695	INTRON	Ca_06100	J	--
Ca3_P1_24232615	Ca3	24232615	CAAAAAAAAAA/CAAAAAAAAA	AGCAATTTTTCGGCTAAGCA	TCTACGGCACGAATGATGAA	60.0	706	INTERGENIC	--	--	--
Ca3_P1_24272959	Ca3	24272959	GAATAATAA/GAATAATAATAA	GCATGTCCGTTACCTCCACT	GCGATTTATTATTCAGAGTTATTAGCG	60.0	646	INTERGENIC	--	--	--
Ca3_P1_24305831	Ca3	24305831	ATTTTTTTTTT/ATTTTTTTTTTT	TTCGCATATCGAGTTTTCCC	AGGTGGAGGAAGGACCTGAT	60.0	771	INTRON	Ca_06090	--	--
Ca3_P1_24359168	Ca3	24359168	GAAAAAAAAAAAAA/GAAAAAAAAAAA	GGCTAAATTACATCGCGGTC	TCTCCAAATTGCGATCAACA	59.6	617	INTERGENIC	--	--	--
Ca3_P1_24399333	Ca3	24399333	TAAAAAAAAAAAA/TAAAAAAAAAAA	TCTTAGCAACAATGGGAGGG	TGCAACATCATGTAATCGGA	60.1	750	DRR	Ca_06077	R	--
Ca3_P1_24410514	Ca3	24410514	GAAAAAAAAAAA/GAAAAAAAAAAAA	CAAAAGCACTGGGAGGAGAG	TCCCTGCCTTAACCTTGACA	60.0	519	INTERGENIC	--	--	--
Ca3_P1_24414589	Ca3	24414589	AATATATATATATATATATATATA/AATATATATATATATATATA	TGGAGGCAAATACACTCCCT	TGCATCAAAAATGAACTCGAA	59.6	588	INTERGENIC	--	--	--
Ca3_P1_24511988	Ca3	24511988	TTATATATATATATATATATAT/TTATATATATATATATATAT	CACGGACATGGTTGGTGTAA	AGCTGCCCTTCCAATTACCT	60.3	849	INTERGENIC	--	--	--

INDEL marker IDs	Chromosomes/ unanchored scaffolds	Physical positions (bp)	InDels (*Kabuli* reference genome-CDC Frontier/PI)	Forward primers (5'-3')	Reverse primers (5'-3')	Anneling temperature (0C)	Expected amplified product size (bp)	Structural annotation		Functional annotation	
								Sequence components of *kabuli* genome	*Kabuli* gene accession IDs	NCBI-KOG	TFs
Ca3_P1_24576379	Ca3	24576379	ATTCT/AT	AGGTGTTGAGCAAAATTGGG	TTTTAAAAACAGGATCAAAAGGG	60.0	463	DRR	Ca_06059	--	ERF
Ca3_P1_24584302	Ca3	24584302	TAAAAAA/TAAAAAAA	TGAATTTCGAATTTAGTCACGG	TAGGTCAGACCATATGCCCC	59.1	892	DRR	Ca_06058	--	ERF
Ca3_P1_24594655	Ca3	24594655	AATATATATATATATATAT/AATATATATATATATATAT	TCAAAAATCACTATGGTGTCGC	TGGACGTGTTGAGGTTGGTA	60.0	560	INTERGENIC	--	--	--
Ca3_P1_24618388	Ca3	24618388	TC/T	AAATCTTCACAAGCCACCGA	TCCTCTTAGGTCAGTCATCGTG	60.6	280	DRR	Ca_06055	--	ERF
Ca3_P1_24619541	Ca3	24619541	CTCTATAT/CT	GCCTATGGTCTGACCTGCTT	TTTTGAGAAATGGACACTCAACT	59.3	574	DRR	Ca_06055	--	ERF
Ca3_P1_24701158	Ca3	24701158	ATTTTTTTTTT/ATTTTTTTTT	TTTGGCACATCTATTCAACTGC	CGCATTCTTGGGATGCTTAT	60.1	136	INTERGENIC	--	--	--
Ca3_P1_24791040	Ca3	24791040	GAAAAAA/GAAAAA	GGGACCACGAATGTAATTTAGC	TGCGAGCATAAGAAGTTGTTG	59.7	740	INTERGENIC	--	--	--
Ca3_P1_24870328	Ca3	24870328	C/CGT	TGTGTCACAAAATGGCGTCT	TGCAAACATGTTGGGATAGC	60.2	315	INTERGENIC	--	--	--
Ca3_P1_24984768	Ca3	24984768	CTTTTTTTTT/CTTTTTTTT	TATATGGGCTGCTCTCCCTG	TTTTGCTTTTGAAGCCAACC	60.2	663	INTRON	Ca_06031	--	SAP
Ca3_P1_25030440	Ca3	25030440	ATTTTTTTTTTT/ATTTTTTTTTT	TTGATTCCACGTCAATGTCA	TACACACACCTTGCTCGCTC	58.5	514	INTERGENIC	--	--	--
Ca3_P1_251021	Ca3	251021	CTTTTTTTTTT/CTTTTTTTTT	TTATAACGTGTTGGACCCGC	TGAAGATGATAGTGGCGTCG	60.8	845	INTERGENIC	--	--	--
Ca3_P1_25341015	Ca3	25341015	GAAAAAAAAAAA/GAAAAAAAAAA	ATCAACCCACACAAACCACA	GACATCGCAGCTTTTTCACA	59.7	372	INTERGENIC	--	--	--

INDEL marker IDs	Chromosomes/ unanchored scaffolds	Physical positions (bp)	InDels (*Kabuli* reference genome-CDC Frontier/PI)	Forward primers (5'-3')	Reverse primers (5'-3')	Anneling temperature (0C)	Expected amplified product size (bp)	Structural annotation		Functional annotation	
								Sequence components of *kabuli* genome	*Kabuli* gene accession IDs	NCBI-KOG	TFs
Ca3_P1_25344299	Ca3	25344299	ATTT/ATT	ACTTCAGCTCTAGGCAACCG	TGCAAATGGTGTGGTCTGTT	59.6	304	INTRON	Ca_05995	--	LSD
Ca3_P1_25345900	Ca3	25345900	AATATATATATATATATATATATATATATATA/AATATATATATATATATATATATA	AGGTTCATGTGAATTGGGTTG	GCGAGATTGAAGTTGGAAGG	59.7	343	INTERGENIC	--	--	--
Ca3_P1_25444677	Ca3	25444677	ATTTTTTTTTT/ATTTTTTTTTTT	TCCAACACCTTCATTTCCAA	CAATTGCGGGTTGTTGTTC	58.9	658	INTERGENIC	--	--	--
Ca3_P1_25483288	Ca3	25483288	AATATATATATATATATATATATATATATATATATAT/AATATATATATATATATATATATATATATATAT	AAAAATCGAGAGCCCATAAAA	CCCATTCTTCTGGTTGTCGT	57.9	564	DRR	Ca_05987	R	--
Ca3_P1_25521658	Ca3	25521658	ACTCTCTCTCTCTCTCTCTCTCTCTC/ACTCTCTCTCTCTCTCTCTCTCTCTC	TACGCAACGCACGAATTAAC	ATCAGCATCCGAATTTCCAG	59.8	819	INTERGENIC	--	--	--
Ca3_P1_25585812	Ca3	25585812	TAAAAAAAA/TAAAAAAA	TGTTTCGTTTTTGTTCCCTCT	TCCATTGCAATCGTACTTAATGA	58.7	735	DRR	Ca_05976	--	B3
Ca3_P1_25758872	Ca3	25758872	TAAAAAAAAA/TAAAAAAAA	GGATCCGGTGCTACTCCATA	ATTTGAGGTTGTGATTATGCAA	59.9	713	INTERGENIC	--	--	--
Ca3_P1_25776431	Ca3	25776431	CATATATATATATATATATAT/CATATATATATATATATATAT	GGGGAAATTCCTACTTGACCA	TTGCAGCAGAATTGGATCTG	60.2	754	INTERGENIC	--	--	--
Ca3_P1_25866798	Ca3	25866798	AATATATATATATATATATATATATATATATATATA/AATATATATATATATATATATATATATATATATATATATATATATA	AGCCAATTAGGCAACCACAA	TTCCCCATTCAGAGTTAGGC	60.5	871	INTERGENIC	--	--	--
Ca3_P1_25885756	Ca3	25885756	TTCTTTTACCAT/TTCTTTTACCATCTTTTACCAT	TTGAGGGAACAAAGCGAGTT	CAATTCAGTTTGAGGCACGA	59.9	411	INTERGENIC	--	--	--
Ca3_P1_25888060	Ca3	25888060	AT/ATT	TGTGGAAGACATCCTTTACTGC	ACCTGACCTATTTCCACCCC	59.2	551	INTERGENIC	--	--	--
Ca3_P1_25888907	Ca3	25888907	GTTTTTTT/GTTTTTTTT	CAGGTCATGCCAGACTTTGA	TCTGACCCGACCTGGTCTAC	59.8	578	INTERGENIC	--	--	--

INDEL marker IDs	Chromosomes/ unanchored scaffolds	Physical positions (bp)	InDels (*Kabuli* reference genome-CDC Frontier/PI)	Forward primers (5'-3')	Reverse primers (5'-3')	Anneling temperature (0C)	Expected amplified product size (bp)	Structural annotation		Functional annotation	
								Sequence components of *kabuli* genome	*Kabuli* gene accession IDs	NCBI-KOG	TFs
Ca3_P1_25889145	Ca3	25889145	ATTTTTTTT/ATTTTTTTTT	GTAGACCAGGTCGGGTCAGA	TGATATGTCACATTGTCGCAC	60.1	403	INTERGENIC	--	--	--
Ca3_P1_25891751	Ca3	25891751	TAAA/TAAAA	CGGTTCCTAATTGAAAAGGC	CAACGCTTTGTTCTCTCAACA	58.7	341	INTERGENIC	--	--	--
Ca3_P1_25916961	Ca3	25916961	AATATATATATATATATATATATA/AATATATATATATATATATATATATA	CAAAAATCCGCACTCACCTT	TCGTCTTAATTTATGCGGGG	60.1	427	INTERGENIC	--	--	--
Ca3_P1_26020458	Ca3	26020458	CATATATATATATATATAT/CATATATATATATATATATATAT	CCCACTAAAGGCCCATATCA	TTCAAATTCACGCCCCTAAC	59.8	789	INTERGENIC	--	--	--
Ca3_P1_26071275	Ca3	26071275	ATTTTT/ATTTTTTT	TGTGTTTGTTTGGTTTCATCTTG	TGTCAACGTGATTGGAGCAT	59.9	563	INTRON	Ca_08292	--	MYB_related
Ca3_P1_26071877	Ca3	26071877	TAAA/TAAAA	TGCTCCAATCACGTTGACAT	AGACGCTGCAACATCAAAGC	60.1	397	INTRON	Ca_08292	--	MYB_related
Ca3_P1_26102027	Ca3	26102027	AATATATATATATATATA/AATATATATATATATATATATA	AATTCAAGTTCGAAATGCCG	AGCCCAAGTTTCAAATGACG	60.1	578	INTRON	Ca_08292	--	MYB_related
Ca3_P1_26102453	Ca3	26102453	TAAAAAAAAAAAA/TAAAAAAAAAAAAAA	AATTCAAGTTCGAAATGCCG	AGCCCAAGTTTCAAATGACG	60.1	418	INTRON	Ca_08292	--	MYB_related
Ca3_P1_26103278	Ca3	26103278	ATTTTT/ATTTTTTT	TGTGTTTGTTTGGTTTCATCTTG	CAATGTCCACGTGATTGGAG	59.9	519	INTRON	Ca_08292	--	MYB_related
Ca3_P1_26114065	Ca3	26114065	AG/A	TTTATCCTGGGGACTTCGTG	AGGAAAATACACGGGCAAGA	59.9	364	INTRON	Ca_08292	--	MYB_related
Ca3_P1_26114282	Ca3	26114282	ATTATAGTTTTTATT/AT	TTTATCCTGGGGACTTCGTG	ATACACCGATGGAGGGCTTT	59.9	838	INTRON	Ca_08292	--	MYB_related
Ca3_P1_26115029	Ca3	26115029	AACG/A	TGGCATGGTACTCAACCAGA	TTCACATGAATGATTGTTGGGT	60.1	545	INTRON	Ca_08292	--	MYB_related

INDEL marker IDs	Chromosomes/ unanchored scaffolds	Physical positions (bp)	InDels (*Kabuli* reference genome-CDC Frontier/PI)	Forward primers (5'-3')	Reverse primers (5'-3')	Anneling temperature (0C)	Expected amplified product size (bp)	Structural annotation		Functional annotation	
								Sequence components of *kabuli* genome	*Kabuli* gene accession IDs	NCBI-KOG	TFs
Ca3_P1_26155781	Ca3	26155781	AATATATATATATATATATATATATATATAT/AATATATATATATATATATATATATATATATATAT	TTTTATGCATCCCAACCACA	CCTTCTATACAAATATTCAACGGTTTG	59.8	389	INTERGENIC	--	--	--
Ca3_P1_26179982	Ca3	26179982	TA/TATATCTTAAAA	TCCAAATGCATATTCTGGCA	TAATGGTGGAAGTGGGTGGT	60.0	701	INTERGENIC	--	--	--
Ca3_P1_26245714	Ca3	26245714	C/CA	CGCCTGTACCGGTTGATACT	ACCCATTCCCAGTGTTTTCA	60.0	678	INTRON	Ca_08279	QI	B3
Ca3_P1_26349764	Ca3	26349764	GTTAACA/G	TCTGCATTGTCCAAAACCAA	TTATCCATGATTTGGGAGGG	60.1	737	INTERGENIC	--	--	--
Ca3_P1_263872	Ca3	263872	TAAAAAA/TAAA	GGGGTGGTTAAGGATAAGGC	TGGTGCGTTTTATTCCACAA	59.7	605	INTERGENIC	--	--	--
Ca3_P1_264555	Ca3	264555	TGG/TGGG	TTGTGGAATAAAACGCACCA	GTCTCAAAAGCGTTGCCTTC	60.0	574	INTERGENIC	--	--	--
Ca3_P1_26664368	Ca3	26664368	AATATATATATATATAT/AATATATATATATATATAT	TCACACCTTATGAGTTCGTTTGA	ATTGGAATCAAAGGCATGGA	59.7	893	INTERGENIC	--	--	--
Ca3_P1_26777304	Ca3	26777304	GTATATATATATATATAT/GTATATATATATATATAT	CACTCATCACAACGGGACAC	CAATGAATCGATGAAATGAATTG	60.0	754	DRR	Ca_08228	P	--
Ca3_P1_26866686	Ca3	26866686	AATATATATATATATATATATATATATA/AATATATATATATATATATATATA	CTTAAGGAACCTCGCGTTTG	TTTTTGGTTGGACGGAGAAG	59.9	295	INTERGENIC	--	--	--
Ca3_P1_26872317	Ca3	26872317	CAGAGAGAGAGAGAGAGAGAGAGAG/CAGAGAGAGAGAGAGAGAGAGAGAGAG	CAATGTGCTCGATTTCTCCA	TTACATTCCTTGCCTCGTCA	59.8	389	INTERGENIC	--	--	--
Ca3_P1_2691552	Ca3	2691552	TTATATATATATATATATATATATATATATATAT/TTATATATATATATATATATATATATATATATATATAT	TGCGAAATCGCAATTGTTAC	TTGTTAGGATTTTGGGGTGG	59.7	838	INTERGENIC	--	--	--
Ca3_P1_2692030	Ca3	2692030	ATTTTTTTT/ATTTTTTTTT	TTTATTGCCACCCCAAAATC	GCTTCATTCATATACCGGTTGTT	59.6	303	INTERGENIC	--	--	--

INDEL marker IDs	Chromosomes/ unanchored scaffolds	Physical positions (bp)	InDels (*Kabuli* reference genome-CDC Frontier/PI)	Forward primers (5'-3')	Reverse primers (5'-3')	Anneling temperature (0C)	Expected amplified product size (bp)	Structural annotation		Functional annotation	
								Sequence components of *kabuli* genome	*Kabuli* gene accession IDs	NCBI-KOG	TFs
Ca3_P1_26922565	Ca3	26922565	TAAAAAAAAAA/TAAAAAAAAAAA	GGCCATTTTTCCCCCTAATA	ATTGAGGGATCTGGAGGCTT	60.0	494	INTERGENIC	--	--	--
Ca3_P1_26961684	Ca3	26961684	ATTTTTTTTTTTT/ATTTTTTTTTTT	CACGGTTGTGATGACTCGAC	GTCGCGTCAACGTTTGTTTA	60.2	538	INTERGENIC	--	--	--
Ca3_P1_26998517	Ca3	26998517	AATATATATATATATATATATATATATATAT/AATATATATATATATATATATATATATATATATATATATATAT	TGGGATGTTTCAAATTTCTGC	AAAATGGCATTGGTGTGGTT	59.9	576	INTERGENIC	--	--	--
Ca3_P1_27080809	Ca3	27080809	AATATATATATATATATATATATATATATATATATATATATATATA/AATATATATATATATATATATATATATATATATATATATATATATATATATATATATATA	ACCATTTTGCTCTGGGTACG	TTTCGGATAATCTCATCGGC	60.0	765	INTERGENIC	--	--	--
Ca3_P1_27111407	Ca3	27111407	AATATATATATATATATATATATATATAT/AATATATATATATATATATATATATATATATAT	GTACGCCGTTTGGAAAGGTA	TGATTTGCGTGCAACTTCTA	60.0	304	INTERGENIC	--	--	--
Ca3_P1_27157195	Ca3	27157195	TAAAAAAAAAAAA/TAAAAAAAAAAA	AAGAGTTTCAAATGGCGATGA	CTATTCGTGGGAGTGCCAAT	59.7	337	INTERGENIC	--	--	--
Ca3_P1_27188795	Ca3	27188795	AATATATATATATATATATAT/AATATATATATATATATATATAT	CCAAATGACGACTTCCCACT	TTACTACATTTCTGGCCCCG	60.0	865	INTERGENIC	--	--	--
Ca3_P1_27273898	Ca3	27273898	GCTCTCTCTCTCTCTCTCTCT/GCTCTCTCTCTCTCTCTCTCTCTCT	TCCTTCATCCACCAGACTCC	TGCCACATCCAACACTTTGT	60.0	500	INTERGENIC	--	--	--
Ca3_P1_27277791	Ca3	27277791	CATATATATATATATATATATATA/CATATATATATATATATATATATATATATATATA	GCGAAGCGCCATACTAACTC	AAGCCCAAACTCCCTTTTTC	60.0	858	INTERGENIC	--	--	--
Ca3_P1_27299542	Ca3	27299542	ATTTTTTTTTTT/ATTTTTTTTTT	TGCTTTTTCAAAGGGAATGG	TGTCGAGTTCGGTCATTTTG	60.0	531	INTERGENIC	--	--	--
Ca3_P1_27300846	Ca3	27300846	CTTTTTTTTTTTT/CTTTTTTTTTTT	TGATCCCTTTCCTCATTTCAA	AAATTTAGTTTATGCGCTTTGTCA	59.5	359	INTERGENIC	--	--	--
Ca3_P1_27305744	Ca3	27305744	TTATATATATATATATATATATATAT/TTATATATATATATATATATATAT	TTGCCAAAAATTAATATGTCACG	ACTCCTTGGGAACGCAATTA	59.0	543	INTERGENIC	--	--	--

INDEL marker IDs	Chromosomes/ unanchored scaffolds	Physical positions (bp)	InDels (*Kabuli* reference genome-CDC Frontier/PI)	Forward primers (5'-3')	Reverse primers (5'-3')	Anneling temperature (0C)	Expected amplified product size (bp)	Structural annotation		Functional annotation	
								Sequence components of *kabuli* genome	*Kabuli* gene accession IDs	NCBI-KOG	TFs
Ca3_P1_27382854	Ca3	27382854	GTTTTTTTTTTTT/GTTTTTTTTTTT	GCGGTAAAAGAAGGCAAGTG	TGTGGGTACCTTAAACCCTAAAAA	59.9	591	INTERGENIC	--	--	--
Ca3_P1_27428592	Ca3	27428592	TA/TAACA	GAGACATATGAAAGGTGCCACA	ATAAGCTCAGACGCCTCGTT	60.0	562	INTERGENIC	--	--	--
Ca3_P1_27490383	Ca3	27490383	CATATAT/CATAT	TTCCCTTGCACTCAAGAACA	GGGCAAATCATTGTTTCGTT	59.4	390	DRR	Ca_08157	Q	B3
Ca3_P1_27551834	Ca3	27551834	TTATATATATATATATATA/TTATATATATATATATATATA	TCTTTGGATGGGTTCCATGT	TTGTTGACACCGAATTAGCG	60.2	672	INTERGENIC	--	--	--
Ca3_P1_27572338	Ca3	27572338	TAAAAAAAAAAA/TAAAAAAAAAA	ACATGCCTACTCGTGTCGTG	GGTGAGTCTTTAGTTGTTGTTGTTG	59.8	704	INTERGENIC	--	--	--
Ca3_P1_27582382	Ca3	27582382	AT/ATTACT	GGACTGACGATGGACTTGGT	CAAATGGATCAGGATGTCCC	60.0	553	INTRON	Ca_08151	--	--
Ca3_P1_27601862	Ca3	27601862	ATT/AT	CGTCCGAAAAAGAAATGGAA	CAACCTTTTTCATCGCCACT	60.0	570	INTERGENIC	--	--	--
Ca3_P1_27602646	Ca3	27602646	TAAAAAAAAAAA/TAAAAAAAAAA	AGTGGCGATGAAAAAGGTTG	TGGAGTGAGTGCAATGGAAA	60.1	791	DRR	Ca_08148	DO	Trihelix
Ca3_P1_27629559	Ca3	27629559	TAAAAAAAAAAA/TAAAAAAAAAAAA	CAAAATGCATTATTTTGGAGAGAA	AGCACCAAGAATCCAACACC	59.5	556	INTERGENIC	--	--	--
Ca3_P1_27648871	Ca3	27648871	AATATATATATATATATATATAT/AATATATATATATATATATATATAT	AATCCCATGGCACTGAGAAC	AGCGTGATCACATTCACAGC	59.9	670	INTERGENIC	--	--	--
Ca3_P1_27761788	Ca3	27761788	GAAAAAAAAA/GAAAAAAAAAA	ACGCAAGCTCTACTTCGCTC	ACAAGTTCTGCCCACAATCC	59.9	606	DRR	Ca_08131	--	--
Ca3_P1_27792784	Ca3	27792784	TGAGAGAGAGAGAGAGAGAGAGAGAG/TGAGAGAGAGAGAGAGAGAGAGAG	GCACAGGGCTGAATACCAAT	TCATCATCGTCGAAGAATCG	60.0	357	INTRON	Ca_08126	R	--

INDEL marker IDs	Chromosomes/ unanchored scaffolds	Physical positions (bp)	InDels (*Kabuli* reference genome-CDC Frontier/PI)	Forward primers (5'-3')	Reverse primers (5'-3')	Anneling temperature (0C)	Expected amplified product size (bp)	Structural annotation		Functional annotation	
								Sequence components of *kabuli* genome	*Kabuli* gene accession IDs	NCBI-KOG	TFs
Ca3_P1_27824179	Ca3	27824179	CAAAAAAAAAAAAAA/CAAAAAAAAAAAAA	ACAACTTTGACACGCTGCAC	GTTTTGGTTCTGCCGGTTTA	60.0	695	DRR	Ca_08124	--	--
Ca3_P1_27854154	Ca3	27854154	TGG/TG	TCGATAATGAGTGATTAGGTGGG	ACCTCACCCTCGAATGTCAC	60.2	459	INTERGENIC	--	--	--
Ca3_P1_27854804	Ca3	27854804	T/TA	GGATTGAGAGGGGAGAAAGG	TTTGAAAGCAAGAGCGTCAA	60.0	516	INTERGENIC	--	--	--
Ca3_P1_27854997	Ca3	27854997	ATTTTTTTTTTT/ATTTTTTTTT	GGATTGAGAGGGGAGAAAGG	TTTGAAAGCAAGAGCGTCAA	60.0	516	INTERGENIC	--	--	--
Ca3_P1_27856755	Ca3	27856755	TAAAAAAAAA/TAAAAAAAA	CGAAGATGAAGAGGCTTGAAA	CAACAACTTCTGATGCTCGC	59.6	593	INTERGENIC	--	--	--
Ca3_P1_27863472	Ca3	27863472	AT/A	TGCTTGGTTCTTTCATTCCC	TGGGGACCCATACTTGTTGT	60.1	681	INTERGENIC	--	--	--
Ca3_P1_27866552	Ca3	27866552	TTAATAATAATAATAATAATAATAATAATAATAATAATAATAATAGTAATAATAATAATA/TTAATAATAATAATAATAATAATAATAATAATAATAGTAATAATAATAATA	TCAGAATTTTGGTGAGTTGGC	GGGATTCGCTGGTTATCTCA	60.1	552	INTERGENIC	--	--	--
Ca3_P1_27871493	Ca3	27871493	GAATAATAATAATAATAATAATAATAATAATAATAATAATAATAATAATAATAATAA/GAATAATAATAATAATAATAATAATAATAATAATAATAATAATAATAATAATAATAATAATAATAA	CACCTTTCCTTTCACATTCACA	ATTTCTTGACTCTGCCTGCC	60.0	561	INTERGENIC	--	--	--
Ca3_P1_2788384	Ca3	2788384	CATATATATATATATATATATAT/CATATATATATATATATATATATATATATAT	AGTCAGTGGCAAAAACAGCA	TGCGTTACGGTGAAATGGTA	59.5	451	INTERGENIC	--	--	--
Ca3_P1_27949908	Ca3	27949908	AAATAATAATAATAATAATAATAATA/AAATAATAATAATAATAATAATAATAATAATA	ATAGATGGTCGCATTCCGAG	CCAAAAGAGGAGCAAGTTGT	60.1	925	INTERGENIC	--	--	--
Ca3_P1_27959892	Ca3	27959892	CTTATTTATTTATTTATTTATTTATTTATTT/CTTATTTATTTATTTATTTATTTATTT	TGAATTGTTGGATCAGCAAAA	GCGAACAAAATCACAACATCC	59.1	779	INTERGENIC	--	--	--
Ca3_P1_27975195	Ca3	27975195	TAAAAAAAAA/TAAAAAAAA	CTCCTTCTCCTCCTCCGAGT	TTGGCAAAATTACATCCAAAA	59.9	745	INTERGENIC	--	--	--

INDEL marker IDs	Chromosomes/ unanchored scaffolds	Physical positions (bp)	InDels (*Kabuli* reference genome-CDC Frontier/PI)	Forward primers (5'-3')	Reverse primers (5'-3')	Anneling temperature (0C)	Expected amplified product size (bp)	Structural annotation		Functional annotation	
								Sequence components of *kabuli* genome	*Kabuli* gene accession IDs	NCBI-KOG	TFs
Ca3_P1_27993552	Ca3	27993552	ATTTTTTTTT/ATTTTTTTTTT	TTCAATGGATGCAAAAAGGTT	CGAATTGGGCCAAATAAATG	59.4	561	INTERGENIC	--	--	--
Ca3_P1_28052364	Ca3	28052364	TCA/T	ACTTCCGTTCACACGTTTCC	CGATGCAGGTGAAGATCAAA	60.0	481	INTERGENIC	--	--	--
Ca3_P1_28113252	Ca3	28113252	ATTTTTTTT/ATTTTTTT	TCCTTCTCCTCTTCCTTCCA	TGTTTGTGCAATTGAATTATAGTTATG	58.9	849	INTERGENIC	--	--	--
Ca3_P1_28138567	Ca3	28138567	TG/TGG	TGGTGATGGCAATAATCGAA	ACAATGTTACACGATGGGCA	59.9	561	INTERGENIC	--	--	--
Ca3_P1_28188438	Ca3	28188438	CAAAA/CAAAAA	ACCCATAACGCACTCGTTTC	TCCTCCATTTTTCGTTTTCG	60.0	434	INTERGENIC	--	--	--
Ca3_P1_28191077	Ca3	28191077	CTTTTTTTTT/CTTTTTTTTTT	TAAGATGCGTGGGTTTAGGG	GCTTGTGTTTAAAGCGCTCC	60.0	659	INTERGENIC	--	--	--
Ca3_P1_28211047	Ca3	28211047	ATTT/ATTTT	AGCAATGCATTTTTGAAGGG	TGCGAAACTCTTTGAAACCC	60.1	304	INTERGENIC	--	--	--
Ca3_P1_2825319	Ca3	2825319	T/TCCAG	GGGGTTTCAATTCCAGAACA	AAAGACGGGAAAACCGAACT	59.8	140	DRR	Ca_22150	--	B3
Ca3_P1_28293830	Ca3	28293830	TAAAAAAAAAA/TAAAAAAAAA	GATTACTGCTAGGGCCCAAAT	ATCCCACTTGCAACACTTCC	59.5	147	INTERGENIC	--	--	--
Ca3_P1_28294648	Ca3	28294648	AATATATATATATATATATAT/AATATATATATATATATATATAT	TACCAGTACCAGGAGGCAGG	TGGTGGGTAATCCGATGAAT	60.1	670	INTERGENIC	--	--	--
Ca3_P1_28340520	Ca3	28340520	CTATATATATATATATATATATA/CTATATATATATATATATATA	CAGAGGTGATAGGGGCGATA	ACCAACGTCAATCATCGTCA	60.1	586	INTERGENIC	--	--	--
Ca3_P1_28354473	Ca3	28354473	CTATATATATATATATATATATATATATAT/CTATATATATATATATATATATATATATATATAT	ACCACATGACGATCTCCACA	GGTCGTTAGGGATGAGATGG	60.0	445	INTERGENIC	--	--	--

INDEL marker IDs	Chromosomes/ unanchored scaffolds	Physical positions (bp)	InDels (*Kabuli* reference genome-CDC Frontier/PI)	Forward primers (5'-3')	Reverse primers (5'-3')	Anneling temperature (0C)	Expected amplified product size (bp)	Structural annotation		Functional annotation	
								Sequence components of *kabuli* genome	*Kabuli* gene accession IDs	NCBI-KOG	TFs
Ca3_P1_28357344	Ca3	28357344	TTTATTATTATTATTATTATTATTATTATTATTATTAT/TTTATTATTATTATTATTATTATTATTATTAT	CGTGTCTCCAATATTTTGCCT	TCCTCATTGAAATTTCCCCA	59.1	440	INTERGENIC	--	--	--
Ca3_P1_28428889	Ca3	28428889	TTATATATATATATATATATA/TTATATATATATATATATATATA	AGGCAACTCGATTCAAGCAT	AAACAAACAAGCCCACCAAC	59.8	923	INTERGENIC	--	--	--
Ca3_P1_28479426	Ca3	28479426	CTTTTTTTTTTTT/CTTTTTTTTTTT	GCATTTGGTTGTCATGTGGA	CATTGCAAATGGCGACTTCT	60.4	331	DRR	Ca_07159	K	bHLH
Ca3_P1_28509480	Ca3	28509480	CATAATAATAATAATAATAAT/CATAATAATAATAATAAT	TTGACATGTGGGCATAGGAA	TTTTGGAACAACAAGTCAACAA	59.9	716	INTERGENIC	--	--	--
Ca3_P1_28514570	Ca3	28514570	ATATTATTATTATTATTATTATTATTATTATT/ATATTATTATTATTATTATTATTATTATT	CAAAGAAAATGGTGGGGATG	AAATTGGACTATGCCACCGA	60.2	841	INTERGENIC	--	--	--
Ca3_P1_28535226	Ca3	28535226	CATATATATATATATATATATATATATA/CATATATATATATATATATATATA	AAGCGCCAATCTAGTTTCATTT	AAAATGAGTCGTTGGATTCAAAA	59.3	411	INTERGENIC	--	--	--
Ca3_P1_2855780	Ca3	2855780	G/GA	TGGGAAACAAAATGATGTCAA	CACATGCAATTCCAACCAAG	58.9	591	INTERGENIC	--	--	--
Ca3_P1_2855993	Ca3	2855993	TAAA/TAA	CTTGGTTGGAATTGCATGTG	CGTTTTGGAAGGTAGGGTGT	60.0	514	INTERGENIC	--	--	--
Ca3_P1_2859847	Ca3	2859847	AACTA/AA	CGCAAGGACATTGAAAGGAT	ATTGGGCTCTGTGGTTCTTG	60.1	652	DRR	Ca_22151	R	NAC
Ca3_P1_28672365	Ca3	28672365	ATATTATTATTATTATTATTATTATTATTA/ATATTATTATTATTATTATTATTATTATTA	CGAGAGAGGGGACAAAACTG	ATCGTCAAAATGATGGGACC	59.8	255	INTRON	Ca_07175	E	C3H
Ca3_P1_28684295	Ca3	28684295	TTTTATTT/TTTTATTTTATTTATTT	GCCCCTACTTGTAGCACTGG	CAAACAATAGATGGCACCGA	59.8	253	INTERGENIC	--	--	--
Ca3_P1_2879647	Ca3	2879647	CAAACATCTG/CAAACATCTGACCACATTAAACATCTG	GGTGCAGCTGAATTTGAACA	GGTGGTGCTGTTGTGTATCG	59.8	361	DRR	Ca_22154	T	--

INDEL marker IDs	Chromosomes/ unanchored scaffolds	Physical positions (bp)	InDels (*Kabuli* reference genome-CDC Frontier/PI)	Forward primers (5'-3')	Reverse primers (5'-3')	Anneling temperature (0C)	Expected amplified product size (bp)	Structural annotation		Functional annotation	
								Sequence components of *kabuli* genome	*Kabuli* gene accession IDs	NCBI-KOG	TFs
Ca3_P1_28797139	Ca3	28797139	CAA/CAATTCGATAA	CAAGGAGCTGTTTTGCTTCA	GTAAAATGGCACGCGGTAGT	59.2	609	INTERGENIC	--	--	--
Ca3_P1_28797825	Ca3	28797825	A/AG	CATAGTCTGCGAAACCAGCA	GGTGGAATTGATCGGAGAGA	60.0	454	INTERGENIC	--	--	--
Ca3_P1_28799476	Ca3	28799476	G/GTC	TCTTGGACCTCACTGGGTTT	TGTCCTTGATCTTGTTTCCG	59.5	836	INTERGENIC	--	--	--
Ca3_P1_28799571	Ca3	28799571	AG/A	TCTTGGACCTCACTGGGTTT	TGTCCTTGATCTTGTTTCCG	59.5	836	INTERGENIC	--	--	--
Ca3_P1_2884020	Ca3	2884020	AA/AATAATAATAATTA	TCGAGAAAATTGAACGGACA	AACAAAATAAAGCGGACAGTCA	59.2	565	INTERGENIC	--	--	--
Ca3_P1_28859122	Ca3	28859122	CAAAAAAAAA/CAAAAAAAAAA	TTTCCCGACTTGGTCCTTTA	ATGACATTTCGCCAAACACA	59.5	432	INTERGENIC	--	--	--
Ca3_P1_2886351	Ca3	2886351	AATATATATATATATATATAT/AATATATATATATATATAT	TGTTTTTGTAAAGTAAAAGCCCC	CATGTGTTTGACGATGTGAATTT	58.7	548	INTERGENIC	--	--	--
Ca3_P1_28966806	Ca3	28966806	TTATATATATATATATATATATAT/TTATATATATATATATATATATATATAT	TCGATATTTAGAATTTACGAACGAGA	ATTCAAGAAAGTTCGCCGTG	59.6	850	INTERGENIC	--	--	--
Ca3_P1_28984656	Ca3	28984656	GTCTCTCT/GTCTCT	GAGAACGCAAGCTAACGGAC	GGATCAGAGAAAACGTGGGA	60.0	326	INTERGENIC	--	--	--
Ca3_P1_28994933	Ca3	28994933	AC/A	CAAATTAAGCAGAGCCCGTC	TTTTGGACAATGTCAACCCA	59.8	165	INTRON	Ca_07207	KTDL	--
Ca3_P1_28995118	Ca3	28995118	CAA/CAAA	CAAATTAAGCAGAGCCCGTC	CGGAATTTGAACAAAACGCT	59.8	769	INTRON	Ca_07207	KTDL	--
Ca3_P1_28996658	Ca3	28996658	ATT/ATTT	AGGGCTTTGGTCGTGTATTG	AACAATTGCGGGTGTAGAGG	60.0	861	INTRON	Ca_07207	KTDL	--

**INDEL marker IDs**	**Chromosomes/ unanchored scaffolds**	**Physical positions (bp)**	**InDels (*Kabuli* reference genome-CDC Frontier/PI)**	**Forward primers (5'-3')**	**Reverse primers (5'-3')**	**Anneling temperature (0C)**	**Expected amplified product size (bp)**	**Structural annotation**		**Functional annotation**	
								**Sequence components of *kabuli* genome**	***Kabuli* gene accession IDs**	**NCBI-KOG**	**TFs**
Ca3_P1_28999636	Ca3	28999636	CT/CTT	TCTGAATTTCTGTACCGCCC	GGGATAGGGCATGTGTGAGT	60.1	536	INTRON	Ca_07207	KTDL	--
Ca3_P1_2901635	Ca3	2901635	TG/T	GCGGTGTTGTAACGGAGTTT	TCACGCTCCTTTCACCTTCT	60.0	473	INTERGENIC	--	--	--
Ca3_P1_29053872	Ca3	29053872	TTATATATATATATATATATA/TTATATATATATATATATATA	TCCTCCGTCTTTTCATTTGG	GGTTGGGGGAGTGTTAGCTT	60.0	456	INTERGENIC	--	--	--
Ca3_P1_29087546	Ca3	29087546	AATATATATATATATATATATATATATATATATATATATA/AATATATATATATATATATATATATATATATATATATATA	GCTGGAAGCTTCTAATGGCA	AAACCGCCTTTTCAGAACAT	60.5	471	INTERGENIC	--	--	--
Ca3_P1_29091788	Ca3	29091788	TAAAAAA/TAAAAAAA	TTTTGGAAAAGCGCTGTAAAA	CTTCGACACTGTAGCACCCA	59.9	852	INTERGENIC	--	--	--
Ca3_P1_29123888	Ca3	29123888	AATATATATATATATATATATATATAT/AATATATATATATATATATATATATAT	AGGCCAACACAGAATCTTGG	GTGCTGCTTTGTGTCTGCAT	60.1	548	DRR	Ca_07216	R	--
Ca3_P1_29147395	Ca3	29147395	TTTATTATTATTATTATTATTATTATTATTATTATTATTATTATTATTATTATTATTATTA/TTTATTATTATTATTATTATTATTATTATTATTATTATTATTATTATTATTATTATTATTATTATTATTATTA	TGTTGAGGGAAAATAAAAGGGA	TGTGGATCCAAACCTTGTTTA	59.8	704	DRR	Ca_07218	L	bHLH
Ca3_P1_29162412	Ca3	29162412	GACAACAAC/GACAACAACGAAATTACAACAAC	TCAAATTCACGACTCACGGA	GGTTGGTTTGGTTCAAGCAT	60.2	668	INTERGENIC	--	--	--
Ca3_P1_29224433	Ca3	29224433	ATTTTTTTTTT/ATTTTTTTTTTT	TGCAATTTGTTGGAGCGTTA	TTGCTCCAAATTTCAAAGGC	60.3	394	INTERGENIC	--	--	--
Ca3_P1_29228366	Ca3	29228366	TTATATATATATATATATATAT/TTATATATATATATATAT	TCGGAGGCAGAGACAAGTTT	TTAATTGGGGGAAATCCACA	60.0	364	INTERGENIC	--	--	--
Ca3_P1_2930472	Ca3	2930472	ATCTC/ATCTCTC	AGGCGATGGAGGATAAAACC	CGGAAACGAGTGAAACCAAT	60.3	502	INTRON	Ca_22158	D	--
Ca3_P1_293228	Ca3	293228	AGG/A	GCTGAAAACTACCCCGATCA	AACGAGCACTTATGTGCCAA	60.1	803	INTERGENIC	--	--	--

INDEL marker IDs	Chromosomes/ unanchored scaffolds	Physical positions (bp)	InDels (*Kabuli* reference genome-CDC Frontier/PI)	Forward primers (5'-3')	Reverse primers (5'-3')	Anneling temperature (0C)	Expected amplified product size (bp)	Structural annotation		Functional annotation	
								Sequence components of *kabuli* genome	*Kabuli* gene accession IDs	NCBI-KOG	TFs
Ca3_P1_29334875	Ca3	29334875	TTATATATATATATATATATAT/TTATATATATATATATATATATATATAT	GGGTGGCTCATGAAGCTTAG	TCTCAACCGTTTTGGTGACA	59.8	576	INTERGENIC	--	--	--
Ca3_P1_29446926	Ca3	29446926	GCA/GCACA	CAGCAGGGGCATACGTTAAT	GATTCCCCTCCCTGATTCTC	60.0	306	INTERGENIC	--	--	--
Ca3_P1_29506460	Ca3	29506460	CGTT/C	TGCCCCTGTCATCAATGTTA	TCAATCCCCATCAATCATCA	59.9	598	DRR	Ca_07256	T	FAR1
Ca3_P1_29511325	Ca3	29511325	AAAGAAGAAGAAGAAGAAGAAGAAGAA/AAAGAAGAAGAAGAAGAAGAAGAAGAAGAAGAAGAAGAAGAA	TCCACTGAAAACAACCCTCC	GTGAACTAGGTCGAGGCGAG	59.9	798	INTRON	Ca_07256	T	FAR1
Ca3_P1_29563741	Ca3	29563741	TATTCAAATAAATT/TATTCAAATAAATTCAAATAAATT	GCACATGCGGTCCTTTAACT	TTCCCCTCTCTCCCTCTCTC	60.1	648	INTERGENIC	--	--	--
Ca3_P1_29572508	Ca3	29572508	TTTA/TTTAGCCTAAAAAGTATTA	GGACCAATTCGGAAAAAGAA	TTGGCACCCCAAATTTTAAG	59.0	359	DRR	Ca_07262	R	bHLH
Ca3_P1_2962592	Ca3	2962592	TTTATTATTATTATTATTATTATTATTATT/TTTATTATTATTATTATTATTATTATT	AGAGGCAAACAAGAACCGAA	AACTCGGTGCCAACAGTCTT	59.9	596	INTERGENIC	--	--	--
Ca3_P1_29657495	Ca3	29657495	AAT/A	CGTTTGTCGAATATCTGCTCA	TCTCTCCCTCGAATGTACGG	58.9	385	INTERGENIC	--	--	--
Ca3_P1_29658789	Ca3	29658789	ATTTTTTT/ATTTTTTTT	TGGCCTACTTATGGCCTGAC	AAGTCTGGCCTACGGCCTAT	60.1	619	INTERGENIC	--	--	--
Ca3_P1_29686720	Ca3	29686720	TAAAAAAAAA/TAAAAAAAAAA	TTAGCAGAGCTGCATTGGTG	TGACAATGGTTAAAAATGAGGAGA	60.2	792	INTERGENIC	--	--	--
Ca3_P1_29693307	Ca3	29693307	GTTTTTTTTTTT/GTTTTTTTTTT	TGGAATCGCTACCGAATATG	TGCTCAAGAATCAAGATGCG	58.6	572	INTERGENIC	--	--	--
Ca3_P1_29740808	Ca3	29740808	A/ATATATAT	TTTGGCCCGTTGTTATTCTC	GAAGCACCAGTCCTTACCACA	59.9	906	INTERGENIC	--	--	--

INDEL marker IDs	Chromosomes/ unanchored scaffolds	Physical positions (bp)	InDels (*Kabuli* reference genome-CDC Frontier/PI)	Forward primers (5'-3')	Reverse primers (5'-3')	Anneling temperature (0C)	Expected amplified product size (bp)	Structural annotation		Functional annotation	
								Sequence components of *kabuli* genome	*Kabuli* gene accession IDs	NCBI-KOG	TFs
Ca3_P1_29748543	Ca3	29748543	CATTATTATTATTATTATTATTATTATTATTATTATTATTATTATTATTATTATTATTATTATTATTATTATTATTAT/CATTATTATTATTATTATTATTATTATTATTATTATTATTATTATTATTATTATTATTATTATTATTAT	TGCACCAAGTCACTAACAAAAA	GGTGGCCCTCAACTTTCTTT	58.4	404	INTERGENIC	--	--	--
Ca3_P1_29749714	Ca3	29749714	TTATATATATATATATATATATATATATAT/TTATATATATATATATATATATATATATAT	TCAAATTCATGTGGTTTGAGTGA	ACCCATCGAGGAACAAACAG	60.4	946	INTERGENIC	--	--	--
Ca3_P1_29752847	Ca3	29752847	TAAAAAAAAAA/TAAAAAAAAAAA	AATCGAGATGCCTTAGTGCG	CCTATTCCCACCCCCTTTTA	60.4	868	INTERGENIC	--	--	--
Ca3_P1_29771272	Ca3	29771272	CTTTTTTTTTTT/CTTTTTTTTTT	GGATTGCATTATTGCCAAGTT	GCGAGACATTTTGGGTGAAT	59.0	264	INTERGENIC	--	--	--
Ca3_P1_29772076	Ca3	29772076	ATTTTTTTTTTTT/ATTTTTTTTTTTTTT	CCAGCTCCACAACCATCTTT	GCAAATGTTCTGGTCGCATA	60.1	391	INTERGENIC	--	--	--
Ca3_P1_29779379	Ca3	29779379	TTATATATATATATATATAT/TTATATATATATATATATATAT	GAGGAAAGCTTGTTGCTTGG	CCGAATATAAATCATTTGTGGAAA	60.0	323	DRR	Ca_07285	--	--
Ca3_P1_29809142	Ca3	29809142	ATAT/ATATAATTAT	GCTCATGCCTCCTTCAAAAA	AAGCAGCCATAATGACAGGG	60.3	856	INTERGENIC	--	--	--
Ca3_P1_29834248	Ca3	29834248	CAAAA/CAA	TCCAATAATTTTGGCCCATC	CCAAATTGTAAAATCGGTCG	59.6	335	DRR	Ca_07291	--	NF-YB
Ca3_P1_29845748	Ca3	29845748	CATAAAATAAAATAAAATAAAATAAAA/CATAAAATAAAATAAAATAAAA	CCAAAAGTGAAACCCAAGGA	CTCTGTCCCCGGAACTTACA	59.9	345	INTRON	Ca_07292	--	bZIP
Ca3_P1_29927483	Ca3	29927483	ATTTTTTTTT/ATTTTTTTTTT	CGGTGATCGAATTGATGTTG	GGCTGGATCAAAATTGGTGT	59.9	373	INTERGENIC	--	--	--
Ca3_P1_29949453	Ca3	29949453	TAAAAAAA/TAAAAAA	TGTGGAGGTTATTTTTGGGC	AAGCCGGCAGTGTAGTGTCT	59.8	692	INTERGENIC	--	--	--
Ca3_P1_29970428	Ca3	29970428	CATATAATATAATATAATAT/CATATAATATAATAT	GCAAATAACATCGAGGTTCAGG	AACGACTCAGATGAAATGCAA	60.8	454	INTERGENIC	--	--	--

INDEL marker IDs	Chromosomes/ unanchored scaffolds	Physical positions (bp)	InDels (*Kabuli* reference genome-CDC Frontier/PI)	Forward primers (5'-3')	Reverse primers (5'-3')	Anneling temperature (0C)	Expected amplified product size (bp)	Structural annotation		Functional annotation	
								Sequence components of *kabuli* genome	*Kabuli* gene accession IDs	NCBI-KOG	TFs
Ca3_P1_30032330	Ca3	30032330	CTTTTTTTTTTT/CTTTTTTTTTTTT	CGTGACAATAAAAACGGGCT	TTTGCGTTACGAATTTGATGA	60.0	574	INTERGENIC	--	--	--
Ca3_P1_30054625	Ca3	30054625	GAAAAAAAAAA/GAAAAAAAAAAA	AGCTGCTCAGATTTGGAATCA	GAGGCTTGCATGGACTTAGG	60.0	498	INTERGENIC	--	--	--
Ca3_P1_30061370	Ca3	30061370	CTTT/CT	CCAAAAATGACATTAATCTTGGC	ATCACAATTTGGTGCAGTTTT	59.7	194	INTERGENIC	--	--	--
Ca3_P1_30071057	Ca3	30071057	TAAAAAAAAAA/TAAAAAAAAA	TAAAAGCCAATGGAACTGGG	ATCGCTCTCCTCCTCAAACA	59.9	247	INTERGENIC	--	--	--
Ca3_P1_30100381	Ca3	30100381	ATATTATTATTATTATTATTATT/ATATTATTATTATTATTATT	TTGCACATTTACTCAACCGC	ATTCCAGAAATGCGACCTTG	59.7	319	INTERGENIC	--	--	--
Ca3_P1_30152540	Ca3	30152540	TTTATTATTATTATTATTATTATTATTATTAT/TTTATTATTATTATTATTATTATTATTATTATTATTAT	CCCTATTGGTGAGTGCTCCA	CAACACTCAAACATGCCAAAA	61.1	877	INTERGENIC	--	--	--
Ca3_P1_30159874	Ca3	30159874	ATTTTTTTT/ATTTTTTT	TGGATCAAACTTAACTCCCTCC	TTGCAATCAATGCCCTCATA	59.5	490	INTERGENIC	--	--	--
Ca3_P1_30177912	Ca3	30177912	CTATATATATATAT/CTATATATATATATAT	GAAATTGCAAAATTTAATGCAACA	TTGTTGATCTTTGTGGGCAA	60.2	868	INTERGENIC	--	--	--
Ca3_P1_3022233	Ca3	3022233	A/ATTTTCC	AGTTTTGGTAATTGTTGGTTTTATTT	CTCTAAGCCCCACAGAGTCG	57.8	328	INTERGENIC	--	--	--
Ca3_P1_30331116	Ca3	30331116	ATTTTTTTTT/ATTTTTTTT	CGTGTTCAAAGCCACCACTA	TGAAGGTTGGGTTGGTGAAT	59.8	847	INTERGENIC	--	--	--
Ca3_P1_30339041	Ca3	30339041	AAA/AAATAA	CCTCCTCCAACCATGAAATG	CCTGTGTGGTCAGAAAGGGT	60.3	496	INTERGENIC	--	--	--
Ca3_P1_30404235	Ca3	30404235	AAATAATAATAATAATAATAATAATAA/AAATAATAATAATAATAATAATAATAATAA	CAAGTGTCCTTAGCATTTCCC	CGGAGTAGCAAGCCAGAATC	58.7	469	DRR	Ca_07361	R	--

**INDEL marker IDs**	**Chromosomes/ unanchored scaffolds**	**Physical positions (bp)**	**InDels (*Kabuli* reference genome-CDC Frontier/PI)**	**Forward primers (5'-3')**	**Reverse primers (5'-3')**	**Anneling temperature (0C)**	**Expected amplified product size (bp)**	**Structural annotation**		**Functional annotation**	
								**Sequence components of *kabuli* genome**	***Kabuli* gene accession IDs**	**NCBI-KOG**	**TFs**
Ca3_P1_30415056	Ca3	30415056	AG/AGAAAGAGTG	TGGATACAAATGTCAACAAACTATCA	CGCATATTGTTTTATGCACCA	59.7	505	INTERGENIC	--	--	--
Ca3_P1_30415596	Ca3	30415596	TG/TGCGGG	TGGTGCATAAAACAATATGCG	CCCAAACTAGGCGACTATCA	59.5	704	INTERGENIC	--	--	--
Ca3_P1_30421988	Ca3	30421988	CTTTTTT/CTTTTTTT	TTCAATGCCTTCCAATCCTC	CAAGCAGGGAATCCTTTGAC	60.0	255	INTERGENIC	--	--	--
Ca3_P1_30450479	Ca3	30450479	ATCTTCTTCTTCTTCTTCTTCT/ATCTTCTTCTTCTTCTTCT	TCATGGGCCACAGCATATAA	GTCGTGCCAGCACAAGAGTA	59.9	366	INTERGENIC	--	--	--
Ca3_P1_3050180	Ca3	3050180	TGG/TG	GCTCATGATGATCCACCAAA	AAAGGGAGAAGCTTAGGCCA	59.5	958	INTERGENIC	--	--	--
Ca3_P1_3051012	Ca3	3051012	A/ATATATATATC	TGGCCTAAGCTTCTCCCTTT	TGTGCTCCAATTACTTCTTTAGCA	60.3	598	INTERGENIC	--	--	--
Ca3_P1_3054800	Ca3	3054800	TAAAAAAAAAA/TAAAAAAAAA	TCTACGTATCTCTCGGTGCG	TGTCTTCTTCGTTGTTGCCA	59.1	639	INTERGENIC	--	--	--
Ca3_P1_30586754	Ca3	30586754	CTATTATTATTATTATTATTATTATTATTATTATTATTATTATTATTATTAT/CTATTATTATTATTATTATTATTATTATTATTATTATTATTATTATTATTAT	CGTTCTCTCCTCCCTTCCTC	TTTTCTTTGATTCCGAACCG	60.3	530	INTERGENIC	--	--	--
Ca3_P1_30602335	Ca3	30602335	TTGA/TA	GCCCTCAGATGAGAGTCAGG	AAATTAAACCCTCATCCCCG	59.9	842	INTERGENIC	--	--	--
Ca3_P1_3063406	Ca3	3063406	AG/AGAGGATGG	GGAAGGGGTTTGATTTGGTT	GAACCACCATTCCCAAAATG	60.0	705	INTERGENIC	--	--	--
Ca3_P1_3066200	Ca3	3066200	AAATAATAATAATAATAATAATAATAATAATAATAATAATAATAATAATAATAAT/AATAATAATAATAATAATAATAATAATAATAATAATAATAATAATAATAATAATAAT	CACGGTCCCTAGAGCCAATA	TAAATTTTGGTGGCGATTCC	60.1	596	INTERGENIC	--	--	--
Ca3_P1_30708614	Ca3	30708614	ATTTTTTTTT/ATTTTTTTTTT	CGTCAGTCAACATGTATGTGC	GCACACAAAAATTATGCAAGGA	57.6	655	INTERGENIC	--	--	--

INDEL marker IDs	Chromosomes/ unanchored scaffolds	Physical positions (bp)	InDels (*Kabuli* reference genome-CDC Frontier/PI)	Forward primers (5'-3')	Reverse primers (5'-3')	Anneling temperature (0C)	Expected amplified product size (bp)	Structural annotation		Functional annotation	
								Sequence components of *kabuli* genome	*Kabuli* gene accession IDs	NCBI-KOG	TFs
Ca3_P1_30760598	Ca3	30760598	ATC/A	GCACCAAGTTCTTCTTTGCC	GGGAATTGGAGCACTTGAAA	59.9	258	INTERGENIC	--	--	--
Ca3_P1_30773948	Ca3	30773948	ACTTCTTC/ACTTC	AAGGTTGACTTGGTGGTTGC	ATGATGGCGATCAAAAGGAG	60.0	560	INTRON	Ca_07399	E	--
Ca3_P1_3080503	Ca3	3080503	AATATATATATATATATATATATATATAT/AATATATATATATATATATATATATATATATAT	CAGGTAAGCAAAAGTAAGTGCG	TCTCTAGGTTGAGGGGCAAA	59.1	916	INTERGENIC	--	--	--
Ca3_P1_30818445	Ca3	30818445	ATTTTTTTTTT/ATTTTTTTTTTT	GCAGCCGTTCTCTTTCATTC	CTTGCCTGTGTCGAAGATGA	60.0	258	INTERGENIC	--	--	--
Ca3_P1_30841154	Ca3	30841154	AATATATATATATATATAT/AATATATATATATATATATATATATATAT	CAACGGTGAGAAGCAACTGA	TCATGCAAATTCAAAAGAAATGA	60.0	825	DRR	Ca_12181	--	--
Ca3_P1_30899185	Ca3	30899185	G/GACA	CTTTAGTGGGGAGCCATGAA	GAAATTCGCCATTTTCTGGA	60.1	520	CDS (large-effect mutations)	Ca_12186	O	--
Ca3_P1_30930504	Ca3	30930504	GTTTTTATTTTTATTTTTA/GTTTTTATTTTTA	GCATGGACCGTTTAAAATTG	TTTAGGTGGAAAATTCATTAGAAAA	58.0	818	INTERGENIC	--	--	--
Ca3_P1_30936561	Ca3	30936561	ATTTTTTTT/ATTTTTTTTT	TGAGGCAGAACTTCTCGTTG	TGTTACGGGGTATTTAGCGG	59.2	427	INTERGENIC	--	--	--
Ca3_P1_30942170	Ca3	30942170	ATTTTTTTTT/ATTTTTTTTTT	ACACTCAAGAGGTGGCATCC	TGTCTTGCGACTATTTCCAATTT	60.1	570	INTERGENIC	--	--	--
Ca3_P1_3094451	Ca3	3094451	CTTTTTTTTTTTT/CTTTTTTTTTTT	TTTGGAATCTTGTGAGGAGAA	TCAACAAGCCCCCATGTAAT	57.4	167	INTERGENIC	--	--	--
Ca3_P1_30964476	Ca3	30964476	TTATATATATATATATATA/TTATATATATATATATATATA	TGGACAATTTTCTTTGTCTTTTGA	GCAAGAACACATTTACGCGA	60.0	800	INTERGENIC	--	--	--
Ca3_P1_30965764	Ca3	30965764	AATCTCACATCTCACAT/AATCTCACATCTCACATCTCACAT	CGCAAATTCGCAGTGTTCTA	TCACCCGCTCCTTAAAATTG	60.0	371	INTERGENIC	--	--	--

INDEL marker IDs	Chromosomes/ unanchored scaffolds	Physical positions (bp)	InDels (*Kabuli* reference genome-CDC Frontier/PI)	Forward primers (5'-3')	Reverse primers (5'-3')	Anneling temperature (0C)	Expected amplified product size (bp)	Structural annotation		Functional annotation	
								Sequence components of *kabuli* genome	*Kabuli* gene accession IDs	NCBI-KOG	TFs
Ca3_P1_31083415	Ca3	31083415	CAATAATTATAATAATTAT/CAATAATTATAATAATTATAATAATTAT	CTTAGATTCAAAGGACGGCG	AGTTAGTATCACCGTGGCGG	59.8	655	INTERGENIC	--	--	--
Ca3_P1_31101603	Ca3	31101603	ATTTTTTTTTTT/ATTTTTTTTTTTT	ATGGTCCCAATGAAATCCAA	TGTGTTGTCTCTGGCCTTTG	60.0	843	INTRON	Ca_12207	--	--
Ca3_P1_31126519	Ca3	31126519	ATTTTTTTTT/ATTTTTTTT	TATCGCTTTAAGGTCGCGTT	AAAATTCATCACCCCATTCG	59.9	406	DRR	Ca_12210	R	HD-ZIP
Ca3_P1_31138102	Ca3	31138102	GAAAAAAAAAA/GAAAAAAAAA	TTTTTGCAACGGTACATTTTT	GAATATGGGCTTCTTTGGCA	57.3	745	INTERGENIC	--	--	--
Ca3_P1_31201179	Ca3	31201179	TTAATAATAATAATAATAATAATAATAATAATA/TTAATAATAATAATAATAATAATAATAATAATAATAATAATA	AAGCTATTTTTAGTGGGAGAACTTTT	TTGGTGACACTCTAGCAAAGGA	58.6	371	INTERGENIC	--	--	--
Ca3_P1_31262764	Ca3	31262764	ATTTTTTTT/ATTTTTTT	AAGCACCGTACGCTTGAACT	AAGGGTCAACATTTTGCGTC	59.9	855	INTERGENIC	--	--	--
Ca3_P1_31266098	Ca3	31266098	TC/T	TGCGTGCGTCTTTGATTAAC	TAGCCCAAGTCAACCGAATC	59.9	446	INTERGENIC	--	--	--
Ca3_P1_31272282	Ca3	31272282	TA/T	CTGCAATCATTCTCAGGCAA	TTACGCGGTTTTCTGGATTT	59.9	785	INTRON	Ca_12223	Z	bHLH
Ca3_P1_31278038	Ca3	31278038	C/CGACTG	AAAAACAGCGTGGAAAGTTATGA	GTAAGGATATCCGGAGGGGA	60.1	494	INTERGENIC	--	--	--
Ca3_P1_31290021	Ca3	31290021	T/TTGA	GGTTCAAACGTGTTCCCTTG	ACGCTCTCAAATCGACCATC	60.4	929	DRR	Ca_12225	P	ERF
Ca3_P1_31290969	Ca3	31290969	CAAAAAAAAAA/CAAAAAAAAA	GATGGTCGATTTGAGAGCGT	TTCATAGGGTCACGGCAAAT	60.2	812	DRR	Ca_12225	P	ERF
Ca3_P1_31291433	Ca3	31291433	GAAAAAAAAA/GAAAAAAAA	ATTTGCCGTGACCCTATGAA	AAATGTTAAGCTAATTAAAGTGTTTGG	60.3	625	INTERGENIC	--	--	--

**INDEL marker IDs**	**Chromosomes/ unanchored scaffolds**	**Physical positions (bp)**	**InDels (*Kabuli* reference genome-CDC Frontier/PI)**	**Forward primers (5'-3')**	**Reverse primers (5'-3')**	**Anneling temperature (0C)**	**Expected amplified product size (bp)**	**Structural annotation**		**Functional annotation**	
								**Sequence components of *kabuli* genome**	***Kabuli* gene accession IDs**	**NCBI-KOG**	**TFs**
Ca3_P1_31292004	Ca3	31292004	TTCT/TT	CCAAACACTTTAATTAGCTTAACATTT	AATTGGCTTGATGTCCCAAC	57.8	419	INTERGENIC	--	--	--
Ca3_P1_31293455	Ca3	31293455	TG/TGG	TGGGATGTTTGAGAAAAGCA	ATCCCATCCCACCAACAATA	59.2	522	INTERGENIC	--	--	--
Ca3_P1_31293569	Ca3	31293569	AT/ATT	TGGGATGTTTGAGAAAAGCA	CCCACATCGTTTATCCCATC	59.2	534	INTERGENIC	--	--	--
Ca3_P1_31295638	Ca3	31295638	AT/A	GGACCATGGACTCCAGAAGA	TGTTTCCCAACATTTGATGC	60.0	361	INTRON	Ca_12226	K	MYB
Ca3_P1_31295880	Ca3	31295880	AT/ATT	GGACCATGGACTCCAGAAGA	TCGTCCTCCATCAGTTCGAT	60.0	688	INTRON	Ca_12226	K	MYB
Ca3_P1_31295937	Ca3	31295937	AAAAAA/AAAAAACAAAAA	GGACCATGGACTCCAGAAGA	TCGTCCTCCATCAGTTCGAT	60.0	688	INTRON	Ca_12226	K	MYB
Ca3_P1_31296714	Ca3	31296714	ATTTTTTT/ATTTTTT	TCGGTTTTAAGGGAAGCAAA	ACACACGCGTCAAACTCAAA	59.7	365	INTRON	Ca_12226	K	MYB
Ca3_P1_31296813	Ca3	31296813	CGTGTGT/CGTGT	TCGGTTTTAAGGGAAGCAAA	TGCTGCAATAGCTGACCATC	59.7	806	INTRON	Ca_12226	K	MYB
Ca3_P1_31298126	Ca3	31298126	GTTATTATTATTAT/GTTATTATTATTATTAT	GGACTTTTGGCACGATGTTT	TTTGCAACTTTTGACGGACA	60.0	716	DRR	Ca_12226	K	MYB
Ca3_P1_31298291	Ca3	31298291	CT/CTTTT	TGAGAGGTCGATTTTGGAGG	TTTGCAACTTTTGACGGACA	60.2	506	DRR	Ca_12226	K	MYB
Ca3_P1_31299543	Ca3	31299543	AGG/AG	CATGGCAACAGCTTCAGGTA	GGGGATTAATTTCCTTCCCA	59.9	717	DRR	Ca_12226	K	MYB
Ca3_P1_31303733	Ca3	31303733	TTTTATTTATTTATTT/TTTTATTTATTTATTTATTT	TCCCTCAATTGCATCAAACA	ACAACAGATGGCTGCACAAG	60.0	482	DRR	Ca_12228	--	--

INDEL marker IDs	Chromosomes/ unanchored scaffolds	Physical positions (bp)	InDels (*Kabuli* reference genome-CDC Frontier/PI)	Forward primers (5'-3')	Reverse primers (5'-3')	Anneling temperature (0C)	Expected amplified product size (bp)	Structural annotation		Functional annotation	
								Sequence components of *kabuli* genome	*Kabuli* gene accession IDs	NCBI-KOG	TFs
Ca3_P1_31307955	Ca3	31307955	CTT/CTTT	TCGTTTCCAAATCCAACACA	AATGCCCTGTTAAATTGGTCC	59.9	893	INTRON	Ca_12229	--	Dof
Ca3_P1_31311879	Ca3	31311879	TTATA/TTATATA	TTTCACGTTCTTCAAACCCA	TTATTCATTCGCTCGTGCAG	59.1	352	DRR	Ca_12230	--	Dof
Ca3_P1_31317909	Ca3	31317909	TA/T	CCATCAAACACACTGTTGCC	GCCACAGCAGACAAGATGAA	60.0	607	INTRON	Ca_12231	--	Dof
Ca3_P1_31319681	Ca3	31319681	GAAAA/GAAAAA	CGTGGCTCCTCAAAAGTCTC	ATGCACAAGGCTTCCATACC	60.0	599	DRR	Ca_12232	U	bHLH
Ca3_P1_31323955	Ca3	31323955	CTTTT/CTTT	AATCCATGTAACCGATCCCA	GTGGTCACACTAAGGGGGAA	60.0	510	INTRON	Ca_12232	U	bHLH
Ca3_P1_31326387	Ca3	31326387	GTTTT/GTTT	CGGGTACAGCCGGTAAGTAA	GGGTGCCTTTAAAGTGTGGA	60.0	639	INTERGENIC	--	--	--
Ca3_P1_31327857	Ca3	31327857	TAAAAAAAA/TAAAAAAA	ATTATGGTCGCGAATGATCC	AATTGCGGTTAGTTGGATGC	59.8	528	INTERGENIC	--	--	--
Ca3_P1_31327991	Ca3	31327991	AAT/AATATAT	ATGGAGGCATCCAACTAACC	CATGGCAAATTTAGGATGTTGA	58.9	455	INTERGENIC	--	--	--
Ca3_P1_31328317	Ca3	31328317	ATTTTTT/ATTTTTTT	TCCAACTAACCGCAATTCAA	TCATGGCAAATTTAGGATGTTG	59.2	447	INTERGENIC	--	--	--
Ca3_P1_31333366	Ca3	31333366	ATTTT/ATTT	TTCTCCGTGAAGGAGCTGTT	GACAACCAATGTTTCTGCCC	60.0	703	INTRON	Ca_12233	S	bZIP
Ca3_P1_31333717	Ca3	31333717	CAAAAA/CAAA	AAATTTTTGTCGGTTTGCAT	TCATGATTTTCCCTTCCCTG	57.2	560	INTRON	Ca_12233	S	bZIP
Ca3_P1_31334400	Ca3	31334400	CTTTTTTTT/CTTTTTTT	AAACCATTGGTGTTTAGGCG	CAAATTATGGGGGTCTCGTG	59.9	738	DRR	Ca_12233	S	bZIP

INDEL marker IDs	Chromosomes/ unanchored scaffolds	Physical positions (bp)	InDels (*Kabuli* reference genome-CDC Frontier/PI)	Forward primers (5'-3')	Reverse primers (5'-3')	Anneling temperature (0C)	Expected amplified product size (bp)	Structural annotation		Functional annotation	
								Sequence components of *kabuli* genome	*Kabuli* gene accession IDs	NCBI-KOG	TFs
Ca3_P1_31336604	Ca3	31336604	T/TC	AGCAGGCCTGGACCTTTACT	GGAATGCCAACGAATGAAAA	60.3	555	INTERGENIC	--	--	--
Ca3_P1_31338630	Ca3	31338630	ATTT/ATT	TATCGTTCGTTGCTCTCGTG	GGCCGAAAGTTATTACGTGG	60.0	870	INTERGENIC	--	--	--
Ca3_P1_31339236	Ca3	31339236	T/TC	CCAACCCATGTACCGACATT	AGAGACCGTTTCATGAGATTTG	60.5	525	INTERGENIC	--	--	--
Ca3_P1_31342385	Ca3	31342385	CATTATTATTATT/CATTATTATTATTATTATT	AAAGAAAGCGTCCACCATTG	GGAAGTGAAAACTTGCCACAA	60.1	646	DRR	Ca_12234	--	--
Ca3_P1_31342738	Ca3	31342738	CAAAAAAA/CAAAA	CGCAGTGGACTGGAAAAGTT	TGCATTGCACACAAGAATGA	60.3	640	DRR	Ca_12234	--	--
Ca3_P1_31343075	Ca3	31343075	CAATAAAAATAAAAATAAAAAT/CAATAAAAATAAAAAT	TGGCAAGTTTTCACTTCCATT	GATCACGGGAGGATCGACTA	59.6	792	DRR	Ca_12234	--	--
Ca3_P1_31343767	Ca3	31343767	G/GT	AATAGTCGATCCTCCCGTGAT	CCATGCTCAAGTCTAGGTTCG	59.8	510	DRR	Ca_12234	--	--
Ca3_P1_31344725	Ca3	31344725	CATGATTTAGCTCTATGATT/CATGATT	TTGTCACAAGCAAAACCCAA	TTCTTCAGCTGGCTTTCCAT	60.1	633	INTERGENIC	--	--	--
Ca3_P1_31344900	Ca3	31344900	GTAATAA/GTAA	TGCTTGCCATTGATCGTCTA	TTCTTCAGCTGGCTTTCCAT	60.4	280	INTERGENIC	--	--	--
Ca3_P1_31347476	Ca3	31347476	CAAAAAAAAAA/CAAAAAAAA	TCTTACGAAATTGCACACGC	CGCAATTGATGTTGTTGACC	59.9	173	INTERGENIC	--	--	--
Ca3_P1_31349079	Ca3	31349079	CAT/CATGAT	TAAAGGCATTTGCGTGTTGA	TTTTGGTGAAAGCCAATAACG	60.3	512	INTERGENIC	--	--	--
Ca3_P1_31353167	Ca3	31353167	TAAAA/TAAAAAA	GGCACTATTTTGGAAATTTTAAGG	GAATGACATATTGTGGCAGCA	59.4	517	INTERGENIC	--	--	--

INDEL marker IDs	Chromosomes/ unanchored scaffolds	Physical positions (bp)	InDels (*Kabuli* reference genome-CDC Frontier/PI)	Forward primers (5'-3')	Reverse primers (5'-3')	Anneling temperature (0C)	Expected amplified product size (bp)	Structural annotation		Functional annotation	
								Sequence components of *kabuli* genome	*Kabuli* gene accession IDs	NCBI-KOG	TFs
Ca3_P1_31353219	Ca3	31353219	CAAAAA/CAAAAAA	TCCCTAAAAGTTTCTTCATCCAA	GAATGACATATTGTGGCAGCA	59.2	225	INTERGENIC	--	--	--
Ca3_P1_31364505	Ca3	31364505	CTTTTTTTT/CTTTTTTTTT	ACCAATGTCTCGCAAGCTCT	GGTAAACATTTGCATCGTTGG	60.0	458	INTERGENIC	--	--	--
Ca3_P1_31366021	Ca3	31366021	A/ATT	CCCTCTTGTGCCTGTCTTTC	CAACGGGAATCTTCCGTCTA	59.8	469	INTERGENIC	--	--	--
Ca3_P1_31371532	Ca3	31371532	TATCAT/TATCATCAT	GGATCAGCCCAATTGGTAAA	TAGACCATAAGCCCCTGACG	59.8	322	INTERGENIC	--	--	--
Ca3_P1_31375853	Ca3	31375853	TAATAAATAAATAA/TAATAAATAAATAAATAA	TGGTTGTCAGAGCAGGTTCA	CATATGTGAACCATGGCAGC	60.4	334	INTERGENIC	--	--	--
Ca3_P1_31376681	Ca3	31376681	GTAT/GTATTTTTCATTAATAT	AGGGGCATGCTTGTGTAAAT	GACCAACTTTATGCGTGCAA	59.5	581	INTERGENIC	--	--	--
Ca3_P1_31379455	Ca3	31379455	TTT/TTTATT	AGTGTTAGATTGGGTTGGCG	CAATTCATAAAAATCAAAGACGATG	60.0	770	INTERGENIC	--	--	--
Ca3_P1_31381469	Ca3	31381469	TC/T	TAAACCTTGGCTGGGTCAAC	TTGTTTATTTTGTTCGTCGTGTT	60.0	403	INTERGENIC	--	--	--
Ca3_P1_31381485	Ca3	31381485	AATAT/AAT	TAAACCTTGGCTGGGTCAAC	TTGTTTATTTTGTTCGTCGTGTT	60.0	403	INTERGENIC	--	--	--
Ca3_P1_31381715	Ca3	31381715	GATATA/GATA	TAAACCTTGGCTGGGTCAAC	TTGTTTATTTTGTTCGTCGTGTT	60.0	403	INTERGENIC	--	--	--
Ca3_P1_31381813	Ca3	31381813	AT/A	TAAACCTTGGCTGGGTCAAC	TTGCTACCATTGTAAAGCTTGTTT	60.0	611	INTERGENIC	--	--	--
Ca3_P1_31383053	Ca3	31383053	CTTTTT/CTTTT	CCCTAAATCAACGGGAGTCA	AGCCGTCACTCTTTCTCCAA	59.9	285	INTERGENIC	--	--	--

INDEL marker IDs	Chromosomes/ unanchored scaffolds	Physical positions (bp)	InDels (*Kabuli* reference genome-CDC Frontier/PI)	Forward primers (5'-3')	Reverse primers (5'-3')	Anneling temperature (0C)	Expected amplified product size (bp)	Structural annotation		Functional annotation	
								Sequence components of *kabuli* genome	*Kabuli* gene accession IDs	NCBI-KOG	TFs
Ca3_P1_31383299	Ca3	31383299	GATAAAAA/GATAAAAATAAAAA	TTGGAGAAAGAGTGACGGCT	GAGGTGTTTGTTTCGCGTCT	60.0	671	INTERGENIC	--	--	--
Ca3_P1_31383550	Ca3	31383550	TAAAAAAAA/TAAAAAAA	TTGGAGAAAGAGTGACGGCT	AATGCTGACTTGGCTCGAAT	60.0	783	INTERGENIC	--	--	--
Ca3_P1_31384078	Ca3	31384078	CAAAAA/CAAAAAA	TTCGACTCGGGTCCATAAAC	TGTCTACACCGTCAACAGCC	59.9	564	INTERGENIC	--	--	--
Ca3_P1_31385280	Ca3	31385280	ATTTTTTTTTTT/ATTTTTTTTT	TTTCGATTGATTTTGCATTTTG	TTGACATGACATGCATTAACGA	59.9	381	INTERGENIC	--	--	--
Ca3_P1_31387066	Ca3	31387066	ATTTTTTTT/ATTTTTTT	CCACACACAAAACACTGGCT	AGAGACGGTAGCGTCGAGAG	59.6	511	INTERGENIC	--	--	--
Ca3_P1_31391982	Ca3	31391982	ATTTT/ATTT	CGTGCCCACATTATCAACAA	TCTTGCTCATGGTGCTTCTG	60.4	793	INTERGENIC	--	--	--
Ca3_P1_31392563	Ca3	31392563	TAC/T	CAGAAGCACCATGAGCAAGA	TGTCGGCAGTTAAGTTGGTG	60.1	439	INTERGENIC	--	--	--
Ca3_P1_31393335	Ca3	31393335	CTATA/CTA	CAACCACGTTGGTGTCAAAA	GAAAGGCGAGAGGACTTCAA	60.4	521	INTERGENIC	--	--	--
Ca3_P1_31401163	Ca3	31401163	TAAAAAA/TAAAAA	ATTTGCTTCTGGACCCTTCA	ATAGACACGGGTGCATTGTG	59.7	235	INTERGENIC	--	--	--
Ca3_P1_31402077	Ca3	31402077	TAAAA/TAAAAA	TGTACACGTCGGTGAACAACT	TGTGGCGACAACTAACTTTGA	59.1	634	INTERGENIC	--	--	--
Ca3_P1_31413249	Ca3	31413249	AA/AATA	TGTTGCACGTTGGACAAGTT	GACTGCTACCAACCCAGCAT	60.2	374	DRR	Ca_12238	A	--
Ca3_P1_31416689	Ca3	31416689	C/CT	TGTTGGTTTGGGTCTCTTGA	CGTCGTCTGTTGAGGTGCTA	59.1	352	INTERGENIC	--	--	--

INDEL marker IDs	Chromosomes/ unanchored scaffolds	Physical positions (bp)	InDels (*Kabuli* reference genome-CDC Frontier/PI)	Forward primers (5'-3')	Reverse primers (5'-3')	Anneling temperature (0C)	Expected amplified product size (bp)	Structural annotation		Functional annotation	
								Sequence components of *kabuli* genome	*Kabuli* gene accession IDs	NCBI-KOG	TFs
Ca3_P1_31426177	Ca3	31426177	C/CCCAACAAGTGT	TGAGTCCTCATCGGTCACAA	TCATCAATGACTAAAATTTGTTTTGTA	60.2	573	DRR	Ca_12239	R	YABBY
Ca3_P1_31434070	Ca3	31434070	ATTTTTTTT/ATTTTTTT	GTTGGCTGTGTACATTTGCG	ACATAAAATGCGTCTGCGTG	60.2	607	DRR	Ca_12240	T	E2F/DP
Ca3_P1_31441706	Ca3	31441706	AT/ATGAAATT	AACAATTTTTGGAACCCCTAA	TTTGCAAGTGCAACTTCTGC	57.5	669	DRR	Ca_12242	--	--
Ca3_P1_31443800	Ca3	31443800	ATCAAGT/AT	GGACCATCCCATCCAATATG	TTGATTTGCTACATTCGTCCC	59.8	720	INTERGENIC	--	--	--
Ca3_P1_31444145	Ca3	31444145	G/GC	GGCCTTTAAGGAGACCCTTC	CCGTGTGATGCTTGGAGTTA	59.2	744	INTERGENIC	--	--	--
Ca3_P1_31444395	Ca3	31444395	AC/A	TGGTCGAAATTTCAGGTGTG	CCGTGTGATGCTTGGAGTTA	59.5	121	DRR	Ca_12243	G	--
Ca3_P1_31444569	Ca3	31444569	TTAATA/TTAATATAATA	TAACTCCAAGCATCACACGG	AACTCAACTAATCCGGCCAA	59.7	443	DRR	Ca_12243	G	--
Ca3_P1_31446074	Ca3	31446074	CTTTTT/CTTTT	CATCCCAATTTGCATTGGTT	TGGAGGCTATTGGTACAGGG	60.6	531	DRR	Ca_12243	G	--
Ca3_P1_31448365	Ca3	31448365	TTTATT/TTTATTATT	GACCTTACATGCTCCAACGC	TGGCCACACAAAAAGTACCA	60.7	440	INTERGENIC	--	--	--
Ca3_P1_31449194	Ca3	31449194	TGGGGG/TGGGGGG	GAGTTTTCTACCACGCACCC	TTGGCCATATGAACCGAAAT	59.6	803	INTERGENIC	--	--	--
Ca3_P1_31450308	Ca3	31450308	CAAA/CAAAA	CCAAACACTTAACAACTGTCAGC	TCAATCACGTCCTTGCCATA	58.9	571	INTERGENIC	--	--	--
Ca3_P1_31450328	Ca3	31450328	AATAT/AATATAT	CCAAACACTTAACAACTGTCAGC	TCAATCACGTCCTTGCCATA	58.9	571	INTERGENIC	--	--	--

INDEL marker IDs	Chromosomes/ unanchored scaffolds	Physical positions (bp)	InDels (*Kabuli* reference genome-CDC Frontier/PI)	Forward primers (5'-3')	Reverse primers (5'-3')	Anneling temperature (0C)	Expected amplified product size (bp)	Structural annotation		Functional annotation	
								Sequence components of *kabuli* genome	*Kabuli* gene accession IDs	NCBI-KOG	TFs
Ca3_P1_31451366	Ca3	31451366	CTTTT/CTTT	TGGATTTTACAAACGCCCTC	TCTGGCCTGACCTGAGCTAT	59.9	947	INTERGENIC	--	--	--
Ca3_P1_31452285	Ca3	31452285	AGCCTG/A	GGTGGGAATAGCTCAGGTCA	TTGAATGGTGATGCAATGCT	60.1	877	INTERGENIC	--	--	--
Ca3_P1_31453640	Ca3	31453640	AAATTCGAAATCGAAT/AAAT	GACGCGGGTTTATGTTTGTT	GGATCAATGGCAAGAGCATT	59.9	539	INTERGENIC	--	--	--
Ca3_P1_31458789	Ca3	31458789	C/CACTAA	ATTGCGTGGTGCAAATGTAA	CAGCATGAAGTGCAAAGGAA	60.0	525	DRR	Ca_12244	P	--
Ca3_P1_31466998	Ca3	31466998	CTTTTTTTT/CTTTTTTTTT	GAAACCACGAGCAAGAAAGC	GCTCTCGCAAGAACAAAACC	60.0	759	INTERGENIC	--	--	--
Ca3_P1_31471223	Ca3	31471223	TAAAAA/TAAAAAA	CTCTTCTCCCCCTTCTGCTT	CGATTCAGCTTTTGGGTTTT	60.0	505	INTERGENIC	--	--	--
Ca3_P1_31471455	Ca3	31471455	CTTATTTATTTATTT/CTTATTTATTTATTTATTT	AAAACCCAAAAGCTGAATCG	CCCAAAGTTGGCATGAAGTT	59.2	624	INTERGENIC	--	--	--
Ca3_P1_31472024	Ca3	31472024	TAAAAAAAAAAAAAAAAA/TAAAAAAAAAAAA	TGCCAACTTTGGGAGGAATA	AACTCTGGTTGACGTTGTTGG	60.4	250	INTERGENIC	--	--	--
Ca3_P1_31472070	Ca3	31472070	GAAAAAATTCAGTA/GA	TGCCAACTTTGGGAGGAATA	AACTCTGGTTGACGTTGTTGG	60.4	250	INTERGENIC	--	--	--
Ca3_P1_31473018	Ca3	31473018	ATTTTTT/ATTTTTTT	GCATATAGCGACGGGTAGGA	TGACTGCAATCAATACAAATCTCA	60.1	182	INTERGENIC	--	--	--
Ca3_P1_31475626	Ca3	31475626	TAT/TATTCATATAGAT	ATCTTGTCGTGCCGTCATTT	TCACCAAACAAATCTGAACCA	60.5	540	INTERGENIC	--	--	--
Ca3_P1_31475946	Ca3	31475946	CT/CTGGTATTT	CGTGCCGTCATTTTCAAGTT	TCACCAAACAAATCTGAACCA	61.1	533	INTERGENIC	--	--	--

INDEL marker IDs	Chromosomes/ unanchored scaffolds	Physical positions (bp)	InDels (*Kabuli* reference genome-CDC Frontier/PI)	Forward primers (5'-3')	Reverse primers (5'-3')	Anneling temperature (0C)	Expected amplified product size (bp)	Structural annotation		Functional annotation	
								Sequence components of *kabuli* genome	*Kabuli* gene accession IDs	NCBI-KOG	TFs
Ca3_P1_31476091	Ca3	31476091	TAAT/TAATCATTAGTTTTCAAAT	TGGTTCAGATTTGTTTGGTGA	CAAACGTGAGTGAAACAACCA	59.0	568	INTERGENIC	--	--	--
Ca3_P1_31476304	Ca3	31476304	ATTTTT/ATTTT	TGGTTCAGATTTGTTTGGTGA	CAAACGTGAGTGAAACAACCA	59.0	568	INTERGENIC	--	--	--
Ca3_P1_31478334	Ca3	31478334	TCC/TCCC	TGGTGTGTGTTAGCCATAAAGG	GGGGCCTCTTCTTTTGTTTC	59.9	614	INTRON	Ca_12246	--	--
Ca3_P1_31480271	Ca3	31480271	ATTAAT/A	AAGTTGGGAAGCAAAAGGGT	AATGATTGGATCTCATGGGC	60.0	669	DRR	Ca_12246	--	--
Ca3_P1_31480294	Ca3	31480294	GATAATAATAATAATAATAACAATAATAATAAT/GATAATAATAATAATAATAATAACAATAATAATAAT	AAGTTGGGAAGCAAAAGGGT	AATGATTGGATCTCATGGGC	60.0	669	DRR	Ca_12246	--	--
Ca3_P1_31495232	Ca3	31495232	CTTT/CTT	CCAAAATGGTGAGAGACTTCG	TTCGGTGCTTTGTCTCTTCC	59.7	809	INTERGENIC	--	--	--
Ca3_P1_31498007	Ca3	31498007	GT/G	GGACACCACTCCCTCTGGTA	AAACCATGCGTCAGAAAACC	60.0	455	INTRON	Ca_12248	T	--
Ca3_P1_31498407	Ca3	31498407	ATTT/ATTTT	CTAGCTCGGGTTTTCTGACG	TTGGCCACAGTTTAACCACA	60.0	531	DRR	Ca_12248	T	--
Ca3_P1_31498522	Ca3	31498522	GC/G	CTAGCTCGGGTTTTCTGACG	TTGGCCACAGTTTAACCACA	60.0	531	DRR	Ca_12248	T	--
Ca3_P1_31498938	Ca3	31498938	ATTTTTTTT/ATTTTTTTTT	GGCCAAGTTTTTGTATTGGC	GTCCTTCCTGCCTCGAAAAT	59.5	348	DRR	Ca_12249	I	--
Ca3_P1_31499028	Ca3	31499028	ATTTTTTTTT/ATTTTTTTTTT	GGCCAAGTTTTTGTATTGGC	GTCCTTCCTGCCTCGAAAAT	59.5	348	DRR	Ca_12249	I	--
Ca3_P1_31502467	Ca3	31502467	TTAATA/TTA	GCAATACTCCCCTACCCTCC	TCCATCCTGATGCAAAATCA	59.8	441	INTRON	Ca_12249	I	--

INDEL marker IDs	Chromosomes/ unanchored scaffolds	Physical positions (bp)	InDels (*Kabuli* reference genome-CDC Frontier/PI)	Forward primers (5'-3')	Reverse primers (5'-3')	Anneling temperature (0C)	Expected amplified product size (bp)	Structural annotation		Functional annotation	
								Sequence components of *kabuli* genome	*Kabuli* gene accession IDs	NCBI-KOG	TFs
Ca3_P1_31513337	Ca3	31513337	CTT/CT	TCACCATGATTATGCC GAAA	GCTAGGTCGCTGTGA CATGA	59.9	687	DRR	Ca_12250	E	TALE
Ca3_P1_31514573	Ca3	31514573	CTTA/C	GATGTTGGTTCAGCAT CGTG	TTACTTGGGGGTTATG GGGT	60.1	540	DRR	Ca_12250	E	TALE
Ca3_P1_31519164	Ca3	31519164	C/CT	TACACGTGGCCTCAAA CAAA	TCAAGCATGGTTCCAA TCAG	60.1	364	INTERGENIC	--	--	--
Ca3_P1_31519218	Ca3	31519218	AA/AACAGGA	TACACGTGGCCTCAAA CAAA	AAGCCTGCAATGGATG AAAG	60.1	800	INTERGENIC	--	--	--
Ca3_P1_31520896	Ca3	31520896	ATTTTTTTTTT/ATTTTTTTTTTTT	GGCAACAACACTTGCA GCTA	GCAGCAGTCAGCACTT GTTC	60.1	428	INTRON	Ca_12251	--	--
Ca3_P1_31530376	Ca3	31530376	AAT/AATGCAT	CCAATCGTAGGAAGGC CATA	TGGGAAATAGATCGGT CTCG	59.9	730	INTRON	Ca_12252	E	TALE
Ca3_P1_31530560	Ca3	31530560	TTGGGT/TTGGGTGGGT	AACTCCTTCTGCACAT TGGG	TGGGAAATAGATCGGT CTCG	60.1	449	INTRON	Ca_12252	E	TALE
Ca3_P1_31531107	Ca3	31531107	T/TC	CGAGACCGATCTATTT CCCA	GCCGTTATCGGACCTT TGTA	60.0	893	INTRON	Ca_12252	E	TALE
Ca3_P1_31531338	Ca3	31531338	CATGTAATGTAAT/CATGTAAT	CCGTACCAATCATCCC AATC	GCCGTTATCGGACCTT TGTA	60.0	427	INTRON	Ca_12252	E	TALE
Ca3_P1_31540731	Ca3	31540731	CTATATATATATATATATATA/CTAT ATATATATATATATATATA	CTGTTCCATTGTCCAA CACG	CACATGGGTGACATCT GAGG	60.0	566	INTRON	Ca_12253	LK	--
Ca3_P1_31542450	Ca3	31542450	GC/GCC	CATTGGTCACACGTGG ATTC	CGCCCTCACCCAATAG TTTA	59.8	495	DRR	Ca_12253	LK	--
Ca3_P1_31560705	Ca3	31560705	A/AAG	TGTTCACTGCCAAACT CTGC	GCAGTGCACAACTGAT GGTT	60.0	585	INTERGENIC	--	--	--

INDEL marker IDs	Chromosomes/ unanchored scaffolds	Physical positions (bp)	InDels (*Kabuli* reference genome-CDC Frontier/PI)	Forward primers (5'-3')	Reverse primers (5'-3')	Anneling temperature (0C)	Expected amplified product size (bp)	Structural annotation		Functional annotation	
								Sequence components of *kabuli* genome	*Kabuli* gene accession IDs	NCBI-KOG	TFs
Ca3_P1_31560796	Ca3	31560796	GAAAAAAAA/GAAAAAAA	TGTTCACTGCCAAACTCTGC	GCAGTGCACAACTGATGGTT	60.0	585	INTERGENIC	--	--	--
Ca3_P1_31563003	Ca3	31563003	CATA/CATATA	GAATGGTGTTCCTTTGTCCC	AATTTCACCCAAAGACACGG	59.2	274	INTERGENIC	--	--	--
Ca3_P1_31563843	Ca3	31563843	CTTTTTTTTT/CTTTTTTTTTT	TGCTTCCAACTCCTTATCCAA	GATGATGGCTTACTTCGGGA	59.7	357	INTERGENIC	--	--	--
Ca3_P1_31564129	Ca3	31564129	ATTACACATAAAT/A	AAGTTTGACGCTATCCCGAA	AATGAAGCATTTTGGGGACTT	59.7	542	INTERGENIC	--	--	--
Ca3_P1_31565430	Ca3	31565430	GACGCCA/GA	GATTAACATGGTTGGGGTGG	TGCAAGCATGTGGTTCATTT	59.9	258	DRR	Ca_12255	--	G2-like
Ca3_P1_31565687	Ca3	31565687	TA/TAA	GATTAACATGGTTGGGGTGG	CTATTTTCCCCATGCCCTTT	59.9	695	DRR	Ca_12255	--	G2-like
Ca3_P1_31566099	Ca3	31566099	CG/C	AAAGGGCATGGGGAAAATAG	TTTTCATGCATTTGAGACGC	60.1	338	DRR	Ca_12255	--	G2-like
Ca3_P1_31566507	Ca3	31566507	CTTTTT/CTTTT	GCGTCTCAAATGCATGAAAA	CAGGATACCTCCAAAAGCCA	59.8	790	DRR	Ca_12255	--	G2-like
Ca3_P1_31567532	Ca3	31567532	TC/TCTCTGC	TGGAGGTACCTGCCTTGTTC	ATCCCCTTCGCTAGTACGGT	60.1	754	INTRON	Ca_12255	--	G2-like
Ca3_P1_31574859	Ca3	31574859	CTTTTTTTTTT/CTTTTTTTTT	TATCTACGTCGCTGCGATTG	TGTGGGGGTCACATGTATTT	60.0	689	INTERGENIC	--	--	--
Ca3_P1_31575384	Ca3	31575384	CTTTTTTTTTT/CTTTTTTTTT	CACAAATACATGTGACCCCC	AGGAAAGATGACGGTGTGATG	58.5	457	INTERGENIC	--	--	--
Ca3_P1_31576727	Ca3	31576727	AATATATATATATATATATATATATA/AATATATATATATATATA	GGGGTTGTGTTTGGATGAAG	AACCTCCATGGACATAGTTGAAA	60.2	946	INTERGENIC	--	--	--

INDEL marker IDs	Chromosomes/ unanchored scaffolds	Physical positions (bp)	InDels (*Kabuli* reference genome-CDC Frontier/PI)	Forward primers (5'-3')	Reverse primers (5'-3')	Anneling temperature (0C)	Expected amplified product size (bp)	Structural annotation		Functional annotation	
								Sequence components of *kabuli* genome	*Kabuli* gene accession IDs	NCBI-KOG	TFs
Ca3_P1_31578353	Ca3	31578353	CTTTTTTTT/CTTTTTTTTTT	TGTGGGGGTCACATGTATTT	CCCAAATCTTCATGTCCTGC	58.5	681	INTERGENIC	--	--	--
Ca3_P1_31578868	Ca3	31578868	TAAAAA/TAAAAAA	GCAGGACATGAAGATTTGGG	GCCCGTTAGGATGTGGAGTA	60.5	273	DRR	Ca_12256	--	--
Ca3_P1_31583466	Ca3	31583466	ACCCCC/ACCC	GAAACCAAAATCCAGGCAAA	TTGCAAGTGCTGCTAATGCT	59.9	512	DRR	Ca_12257	J	WRKY
Ca3_P1_31594774	Ca3	31594774	GATTT/GATTTTTTTATTT	TTGTGAGAAAAGAGGGGTGG	TTTTTGCACCAATAAATAACATCAA	60.1	567	INTERGENIC	--	--	--
Ca3_P1_31595214	Ca3	31595214	CATTTATAAACAT/C	TTGATGTTATTTATTGGTGCAAAA	AGTAACGACAACCTCCGTGG	58.5	623	INTERGENIC	--	--	--
Ca3_P1_31601843	Ca3	31601843	ATTT/ATT	TACTGGGCCTTGGATTTCTG	TTGAAGTTGTGCTCGTGCTT	60.1	583	INTERGENIC	--	--	--
Ca3_P1_31602428	Ca3	31602428	GAGTGCTAGTGCTA/GAGTGCTAGTGCTAGTGCTA	GCACGAGCACAACTTCAAAA	AAAGAACCATCATCGCAGGT	60.0	618	INTERGENIC	--	--	--
Ca3_P1_31602911	Ca3	31602911	ATGTT/ATGTTGTT	ACCGAATATTCCATGCTCCA	CCAAGTGATGTGTCATGGCT	60.3	565	INTERGENIC	--	--	--
Ca3_P1_31607270	Ca3	31607270	ATTTTTTTT/ATTTTTTT	TACCCTAACGGTGCTCCATC	AAGATCATTCCCCAAAACCC	60.0	567	INTRON	Ca_12260	--	--
Ca3_P1_31609545	Ca3	31609545	T/TTA	GACAAAGCCAGAACCTTTGC	ACCCAAGCAGGCATACAAAC	59.9	538	DRR	Ca_12260	--	--
Ca3_P1_31610075	Ca3	31610075	GAGAAGAAGAAGAAGAAGAA/GAGAAGAAGAAGAAGAA	GTTTGTATGCCTGCTTGGGT	CCCATCAAAATCCTTTCCTTC	60.0	557	INTERGENIC	--	--	--
Ca3_P1_31611126	Ca3	31611126	GAAAAGTAAAAGTAAAA/GAAAAGTAAAA	TTTGGATTAGCCATAACATCCA	CTTCCACCTTCGTGGAAAGA	59.3	617	INTERGENIC	--	--	--

INDEL marker IDs	Chromosomes/ unanchored scaffolds	Physical positions (bp)	InDels (*Kabuli* reference genome-CDC Frontier/PI)	Forward primers (5'-3')	Reverse primers (5'-3')	Anneling temperature (0C)	Expected amplified product size (bp)	Structural annotation		Functional annotation	
								Sequence components of *kabuli* genome	*Kabuli* gene accession IDs	NCBI-KOG	TFs
Ca3_P1_31624803	Ca3	31624803	AC/ACC	TCTGCCATTTTATTTTCGGG	AATAATGCACCAGTGCCACA	59.9	608	INTRON	Ca_12261	A	--
Ca3_P1_31629115	Ca3	31629115	ATTTTTT/ATTTTT	GTTTGTTTCGCAGGTTGGTT	ATTTTGGAGGGATTTGGAGG	60.0	498	INTRON	Ca_12262	T	--
Ca3_P1_31631967	Ca3	31631967	AATAT/AAT	ATTTCAGCAGTCAACCCCAG	TCGAATTCCACAAATGCGTA	60.1	738	INTRON	Ca_12262	T	--
Ca3_P1_31633229	Ca3	31633229	ATTT/ATT	TGGACCCAAGAACAGTGTCA	GCAATTGCAGAAATGCAAGA	60.1	496	INTRON	Ca_12262	T	--
Ca3_P1_31633512	Ca3	31633512	GTT/GTTT	AAATGCCTGGTTTTTGTTGC	CCTCCAGTTTCTTGGCATGT	60.0	288	INTRON	Ca_12262	T	--
Ca3_P1_31690391	Ca3	31690391	CAAAAAAAAAAA/CAAAAAAAAAAAA	TGGTCGCAACTGTCAAATGT	CAAGAGACGTCGAATTCCGT	60.2	756	INTERGENIC	--	--	--
Ca3_P1_31705223	Ca3	31705223	TAAAAAAAAAA/TAAAAAAAAA	TCTGTCGAACATGTGGCTGT	CATGCAAATGGTCCACGTTA	60.3	496	INTERGENIC	--	--	--
Ca3_P1_31754642	Ca3	31754642	TTATATATATATATATAT/TTATATATATATATATATAT	TCTGAATTTCGCTGATGCTG	GGCCAATCTTCTTTAGCTGC	60.1	854	INTERGENIC	--	--	--
Ca3_P1_31755384	Ca3	31755384	CTATATATATATATATATATATAT/CTATATATATATATATATATATATAT	GCAGCTAAAGAAGATTGGCCT	TTTGCGATATGAACCAAATCA	60.0	467	INTERGENIC	--	--	--
Ca3_P1_31778793	Ca3	31778793	ATTTTTTTTTTTTT/ATTTTTTTTTTT	TGCAAAATTCACATAGGCCA	ATTTGGGGATTTAGGGTTCG	60.1	541	INTERGENIC	--	--	--
Ca3_P1_31810634	Ca3	31810634	AAC/AACCTTTGAC	TGTGTGATTGCACAGGAACA	TTAGCGTGGGCAAATAGTCA	59.7	253	INTERGENIC	--	--	--
Ca3_P1_31852962	Ca3	31852962	TTTATTATTATTATTATTATTATTATTATTATTATTATTATTATTATTATTATTAT T/TTTATTATTATTATTATTATTATTATTATTATTATTATTATTATTATTATTATTATTATT	TTTAGGGAAATGGGTCATGG	TCAATCTGTCTCGTTTTGATTGA	59.6	576	DRR	Ca_12289	G	STAT

INDEL marker IDs	Chromosomes/ unanchored scaffolds	Physical positions (bp)	InDels (*Kabuli* reference genome-CDC Frontier/PI)	Forward primers (5'-3')	Reverse primers (5'-3')	Anneling temperature (0C)	Expected amplified product size (bp)	Structural annotation		Functional annotation	
								Sequence components of *kabuli* genome	*Kabuli* gene accession IDs	NCBI-KOG	TFs
Ca3_P1_3185857	Ca3	3185857	TAAAAAAAAAA/TAAAAAAAA	CGCCTCTCTTTTTCTATGCG	TGTTGCTGTTGTTGCCTCTC	60.1	468	INTERGENIC	--	--	--
Ca3_P1_31895120	Ca3	31895120	CTAATATCTTAA/CTAATATCTTAATATCTTAA	GCCCATCAAACTCTGTGGAT	ATCGGGTGTCAATTGTCGAT	59.9	771	INTERGENIC	--	--	--
Ca3_P1_31908295	Ca3	31908295	TAAAAAAAAAAAA/TAAAAAAAAAA	GAGGTGAAAATGGTGTAGATTAGAG	TCGTTCAACACCAAATGGAA	57.5	863	INTERGENIC	--	--	--
Ca3_P1_31912229	Ca3	31912229	GAAAA/GAAA	CTCGGATACTGCCGATTGAT	TGCCTGTTCTCTGTGATTGC	60.1	958	INTRON	Ca_12292	--	--
Ca3_P1_31982546	Ca3	31982546	AATATATATATATATATATATAT/AATATATATATATATATATAT	TGATTCCTTTGCTGGCTTCT	TGATGACAAACAAACGCACA	60.0	592	DRR	Ca_12299	O	FAR1
Ca3_P1_3235341	Ca3	3235341	AGCTTCAGTCGCT/AGCT	TGGAGTGAGCTGTGGTTGAG	TGGTCACCACTTGGTGTTTG	60.0	840	INTERGENIC	--	--	--
Ca3_P1_3235471	Ca3	3235471	TAAA/TAA	ATAGGCCAAGCAACCAAAGA	GACGATGGTGAAGATGACGA	59.7	663	INTERGENIC	--	--	--
Ca3_P1_3235549	Ca3	3235549	GCACACA/GCACACACA	ATAGGCCAAGCAACCAAAGA	GACGATGGTGAAGATGACGA	59.7	663	INTERGENIC	--	--	--
Ca3_P1_32399895	Ca3	32399895	CATATATATATATATATATATATATATATATATATAT/CATATATATATATATATATATATATATATATATATATATAT	TTTCTTTCCGAAAATGGAGA	TGTGAATCCCGCTTTAACAA	57.4	523	INTERGENIC	--	--	--
Ca3_P1_32486522	Ca3	32486522	AAATAATAATAATAATAATAATAATAATAATAATAAT/AAATAATAATAATAATAATAATAATAATAATAAT	AACCACTTTTAAATCGATGCG	TGCAGTAGTTTGCTGCCATC	59.1	498	DRR	Ca_11974	--	--
Ca3_P1_32502813	Ca3	32502813	GAAAAAAAAAAAAAA/GAAAAAAAAAAAAA	TTGCGTTACGGATTTGATGA	CCGCATAAGCATTTACTGGTG	60.1	457	INTERGENIC	--	--	--
Ca3_P1_32506989	Ca3	32506989	GTTTTTTTTTTTT/GTTTTTTTTTTT	ATTGCGAGAATTGTGGGATG	TTTGAAAAATGCACATAGCCC	60.9	495	INTERGENIC	--	--	--

INDEL marker IDs	Chromosomes/ unanchored scaffolds	Physical positions (bp)	InDels (*Kabuli* reference genome-CDC Frontier/PI)	Forward primers (5'-3')	Reverse primers (5'-3')	Anneling temperature (0C)	Expected amplified product size (bp)	Structural annotation		Functional annotation	
								Sequence components of *kabuli* genome	*Kabuli* gene accession IDs	NCBI-KOG	TFs
Ca3_P1_32563432	Ca3	32563432	TTATATATATATATATATATATAT/TTATATATATATATATATAT	AAAATACGCGATGGCAATTC	GGGATTGGTGGAAAGGAGAT	59.9	854	INTERGENIC	--	--	--
Ca3_P1_32563568	Ca3	32563568	TTATATATATATATATAT/TTATATATATATATATATATAT	CGAAAGAAAAAGAAAACATCGC	GAAGGTGGCTCGGCATATAA	60.2	574	INTERGENIC	--	--	--
Ca3_P1_32604694	Ca3	32604694	CTTCTTGATTTATTTATTTCTTGAT/CTTCTTGATTTATTTATTTCTTGATTTATTTATTTCTTGAT	TCGGCACATCACAATCATTT	TTGGCTTGCTTCACACATTC	59.9	677	DRR	Ca_11988	NI	--
Ca3_P1_32623510	Ca3	32623510	TTATATATATATATATA/TTATATATATATATATA	CTTCTGAATACCGGGCTCAC	AAATTTGATGGTGGGTGGTG	59.7	719	INTERGENIC	--	--	--
Ca3_P1_32629755	Ca3	32629755	TAAAAAAAA/TAAAAAAA	TTTTTGCGGTCCTTTGAATTA	GCGGTATTCACTCCAAACTGA	59.6	819	INTERGENIC	--	--	--
Ca3_P1_32659972	Ca3	32659972	AATATATATATATATATATATATAT/AATATATATATATATATATAT	TTCTCCTTGAAGCTGTCGGT	AAATTGGGCTTACTGGGCTT	60.0	604	INTERGENIC	--	--	--
Ca3_P1_3272993	Ca3	3272993	TTATATATATATATATATATATATATATA/TTATATATATATATATATATATATATA	TTGTAGTATCCCGACAATGTAAAA	TGATTCAACAATGATCCCCA	57.7	687	INTERGENIC	--	--	--
Ca3_P1_32756341	Ca3	32756341	TAAAAAAA/TAAAAAA	CCCACGAAGTCATCCAACTT	CCCCCTTACCCTTATAGGCA	60.0	495	INTERGENIC	--	--	--
Ca3_P1_32780242	Ca3	32780242	AAGAGAGAGAGA/AAGAGAGAGA	TTTTGTTTCCAACATTTTCCC	AATTGAGGGACCAAAACTGC	58.8	561	INTERGENIC	--	--	--
Ca3_P1_32865267	Ca3	32865267	ACC/ACCCC	TGTCAACTGCATTTTCTCGC	CTTTGTCCAAAGCAAGGAGC	60.0	441	INTRON	Ca_12023	A	--
Ca3_P1_32938450	Ca3	32938450	ACCA/ACCACCA	TGGGCATGATGATAGTTCCA	CCACAACGGTGACTATGACG	59.9	379	INTERGENIC	--	--	--
Ca3_P1_32980149	Ca3	32980149	GTATATATATATATATATATATA/GTATATATATATATATATATA	CATGGAGAGTTCGTAGGCGT	GGCGAATGATTGGAAGAAAA	60.3	550	INTRON	Ca_12037	O	HB-other

INDEL marker IDs	Chromosomes/ unanchored scaffolds	Physical positions (bp)	InDels (*Kabuli* reference genome-CDC Frontier/PI)	Forward primers (5'-3')	Reverse primers (5'-3')	Anneling temperature (0C)	Expected amplified product size (bp)	Structural annotation		Functional annotation	
								Sequence components of *kabuli* genome	*Kabuli* gene accession IDs	NCBI-KOG	TFs
Ca3_P1_33152812	Ca3	33152812	CTTTTTTTTT/CTTTTTTTTTT	TCCAAATATAAAATTGGGGATCA	GGCAAGGACAGCTTTGTTTC	59.4	253	DRR	Ca_12051	TO	--
Ca3_P1_3317901	Ca3	3317901	AATATATATATATATATATATA/AATATATATATATATATATA	TGTTTTGAATTGCACCGTTTA	GGGGATCAGTTGACTTCAAGA	59.1	773	INTERGENIC	--	--	--
Ca3_P1_33196113	Ca3	33196113	TAAAAAAA/TAAAAAA	CAGGGGCACAAATACATCAA	TTGCAATTCCTTGTGAACCA	59.4	334	INTERGENIC	--	--	--
Ca3_P1_33204885	Ca3	33204885	TATATAATAATA/TATA	GCACATGCCAAACTCGTAAA	AAATGCTGCGTTCCATCTTT	59.7	295	INTERGENIC	--	--	--
Ca3_P1_33243720	Ca3	33243720	CTT/CT	GATGTGGCTGCAACAGCTTA	TGACAGACTCGTCGTTCAGG	60.0	462	CDS (FRAME SHIFT)	Ca_12059	--	--
Ca3_P1_33271403	Ca3	33271403	CAAAAAAAAAA/CAAAAAAAAA	ATGACTCCAACACCATGCCT	TTTGCCACTTGATACAGCAAA	60.4	317	INTERGENIC	--	--	--
Ca3_P1_33346296	Ca3	33346296	GATATATATATATATATATATATA/GATATATATATATATATATATATATA	ATTTTGCCACATGATACGCA	GCTTCACACGGAAACAACAA	60.0	531	DRR	Ca_23675	--	--
Ca3_P1_33387153	Ca3	33387153	AATATATATATATATATATATATATATAT/AATATATATATATATATATATATATATATATATATATATAT	ATACCTACGGATTTGTGCCG	TTTGTTGTTGAAGATGGCAA	59.8	553	INTERGENIC	--	--	--
Ca3_P1_33399144	Ca3	33399144	GTTTTTTTTT/GTTTTTTTTTT	TCCGTAGATGGAGGTTGGAC	CCCAAACCAGTAAGCATCGT	59.9	772	INTERGENIC	--	--	--
Ca3_P1_33407661	Ca3	33407661	GTTTTTTTTTTT/GTTTTTTTTTTTT	TCCTAGAACATGCATCACGC	TAACCCTTTGCTAGCCCCTT	59.8	458	DRR	Ca_23673	--	B3
Ca3_P1_33424355	Ca3	33424355	TTAATAATAATAATAATAATAATAATAATAATAATAATAATAATAA/TTAATAATAATAATAATAATAATAATAATAATAATAATAATAATAATAATAATAA	TGACAGTTTAAGTCCTTCCCTCA	CATGATGGCAAGTTTGTTGG	60.2	684	DRR	Ca_23672	R	C2H2
Ca3_P1_33531472	Ca3	33531472	CAAA/CAAAA	TTGCCTTTGTATCACTGCCA	CTTGCCACTTGGTTGGAAAT	60.3	228	INTRON	Ca_23669	S	--

INDEL marker IDs	Chromosomes/ unanchored scaffolds	Physical positions (bp)	InDels (*Kabuli* reference genome-CDC Frontier/PI)	Forward primers (5'-3')	Reverse primers (5'-3')	Anneling temperature (0C)	Expected amplified product size (bp)	Structural annotation		Functional annotation	
								Sequence components of *kabuli* genome	*Kabuli* gene accession IDs	NCBI-KOG	TFs
Ca3_P1_33531533	Ca3	33531533	TAAAAAAAA/TAAAAAAA	TTGCCTTTGTATCACTGCCA	CTTGCCACTTGGTTGGAAAT	60.3	228	INTRON	Ca_23669	S	--
Ca3_P1_33531818	Ca3	33531818	C/CA	ATTTCCAACCAAGTGGCAAG	TGTTGCGTAAATTGCTTTGC	60.0	554	INTRON	Ca_23669	S	--
Ca3_P1_33545135	Ca3	33545135	C/CTATCAGTAAACATT	ACGTCATCAGTCGAAGTCCC	AGTTTGCGTAGGTTGTTGGG	60.1	329	INTERGENIC	--	--	--
Ca3_P1_33576979	Ca3	33576979	AATATATATATATATATATATATATATATATA/AATATATATATATATATATATATATATATATATATATATA	GCTTAGGCAGCCACAAAATC	TCTTGTTGCCTTGTCACTCG	59.9	909	INTERGENIC	--	--	--
Ca3_P1_33590873	Ca3	33590873	ATTTTTTTTTTT/ATTTTTTTTTT	GTTCCCCGATCCTTATGTGA	GAACGGTCCATGCAAAAGTT	59.8	558	INTRON	Ca_23666	GC	--
Ca3_P1_33630464	Ca3	33630464	ATT/ATTGTT	ATAGTGACATCCAGGCCCAG	TCATCGCAGGTGTACCATGT	60.0	651	INTERGENIC	--	--	--
Ca3_P1_33732050	Ca3	33732050	TTATATATATATATATATATAAATATATATATATATAT/TTATATATATATATATATAAATATATATATATATAT	CGCACGGAAACAACAATTT	TTTTCATCAATAACATTGAGCG	59.6	220	INTERGENIC	--	--	--
Ca3_P1_33748722	Ca3	33748722	CTTTTTTTTTTT/CTTTTTTTTTTTT	AGGTGCCAAAATTGATGAGG	CAAGTTGCGATGAACACCTG	59.9	399	INTERGENIC	--	--	--
Ca3_P1_33748791	Ca3	33748791	TAGTTTCAG/TAGTTTCAGTTTCAG	AGGTGCCAAAATTGATGAGG	CCTAGAAGGTCGCATCTCCA	59.9	417	INTERGENIC	--	--	--
Ca3_P1_33792073	Ca3	33792073	CTT/CT	CTTTCTCGCTTGCATCAACA	CAGTGCTTCCAATCCAACCT	60.1	619	INTRON	Ca_20877	--	--
Ca3_P1_33828272	Ca3	33828272	TTTATTATTATTATTATTATTATTATTATTATTATTATTATTATTATTATTATT/TTTATTATTATTATTATTATTATTATTATTATTATTATTATTATTATTATTATTATTA TT	AGAGGCAAACAAGAACCGAA	TGCATATAAGGAATTGACGCC	59.9	318	INTERGENIC	--	--	--
Ca3_P1_33831754	Ca3	33831754	ATTTTTTTTT/ATTTTTTTTTT	TGGGTTTATGATTACCCGTGA	AAAACATTTTCGAAACTTTCAGC	60.1	607	INTRON	Ca_20879	R	--

INDEL marker IDs	Chromosomes/ unanchored scaffolds	Physical positions (bp)	InDels (*Kabuli* reference genome-CDC Frontier/PI)	Forward primers (5'-3')	Reverse primers (5'-3')	Anneling temperature (0C)	Expected amplified product size (bp)	Structural annotation		Functional annotation	
								Sequence components of *kabuli* genome	*Kabuli* gene accession IDs	NCBI-KOG	TFs
Ca3_P1_33859343	Ca3	33859343	TT/TTATTATTAT	CAATTGAATTCATTCTTCATACAGG	TTGGAAAATGTTGCAAGTTCA	59.0	978	INTERGENIC	--	--	--
Ca3_P1_33884499	Ca3	33884499	TAAAAAAA/TAAAAAA	CAATTTTCGCAAGTCACACAA	CGAACCTTAATGGTCGGAAA	59.8	704	INTERGENIC	--	--	--
Ca3_P1_3392479	Ca3	3392479	TTG/TTGAAAGAGTG	AATGCATTTGGGTTTTCCAA	GGCAATCCTCGTTCACACTT	60.2	542	INTERGENIC	--	--	--
Ca3_P1_33954528	Ca3	33954528	ATATTATTATTATTATTATTATTATTATTATTATTATTATTATTATTATTATTATTATTATT/ATATTATTATTATTATTATTATTATTATTATTATTATTATTATTATTATTATTATTATTATTATTATTATTATTATT	GGCTTCAAAGCCAACATCAT	TCATACAAAAACCAAACGGG	60.1	622	INTERGENIC	--	--	--
Ca3_P1_33955943	Ca3	33955943	TAAAAAAAAAA/TAAAAAAAAAAA	CTGGATGTCATCGAAACCCT	TCGCAAAAATTGATGCACAC	59.9	725	INTERGENIC	--	--	--
Ca3_P1_33983043	Ca3	33983043	TA/TAGACACTGA	GGTGTTTTACGAATGGGGTG	GGGACTCATTTATGCCGATG	60.1	353	INTERGENIC	--	--	--
Ca3_P1_34032860	Ca3	34032860	CCAAAGC/CC	TAAATGTTGCGAACCTGCTG	CGTCGCGGTTATCACTTACA	59.9	678	INTERGENIC	--	--	--
Ca3_P1_3407409	Ca3	3407409	TAAAAAAAAAAAA/TAAAAAAAAAAA	TGGTTAGTAGCGTAACCTTTTTAAG	TGGACCACATGTGTGAATCTC	57.5	509	INTERGENIC	--	--	--
Ca3_P1_34089272	Ca3	34089272	GTCTTT/GTCTTTCTTT	TCGGTATTTGCCATGAGACA	AAACACATGAAGGCACCACA	60.1	724	INTERGENIC	--	--	--
Ca3_P1_3411224	Ca3	3411224	CATATATATATATATATATAT/CATATATATATATATATATATATATATATAT	TGAATTTTTAAATGATTTTCTGCAA	ATAGTCGGCAAAACCCTCCT	59.1	785	INTERGENIC	--	--	--
Ca3_P1_34128563	Ca3	34128563	TTATATATATATATA/TTATATATATATA	TCAAGTTTGGGAGGAACACC	CGGGTCAGGACTACATTTGC	59.9	590	INTERGENIC	--	--	--
Ca3_P1_34161169	Ca3	34161169	GAAAA/GAAAAA	GAAGCAAATCGATTGGGAAA	CTGGGGTTCCACTGTGACTT	60.0	239	INTERGENIC	--	--	--

INDEL marker IDs	Chromosomes/ unanchored scaffolds	Physical positions (bp)	InDels (*Kabuli* reference genome-CDC Frontier/PI)	Forward primers (5'-3')	Reverse primers (5'-3')	Anneling temperature (0C)	Expected amplified product size (bp)	Structural annotation		Functional annotation	
								Sequence components of *kabuli* genome	*Kabuli* gene accession IDs	NCBI-KOG	TFs
Ca3_P1_34192695	Ca3	34192695	TAAAAA/TAAAA	TCCAAATCCATTTTGTTGTCA	TGCGAGTATGCGAAGTTGTT	58.9	787	INTERGENIC	--	--	--
Ca3_P1_34219011	Ca3	34219011	TTCAATATCAATATCAATATCAATATCAATATCAATAT/TTCAATATCAATATCAATATCAATATCAATAT	CAATGAACATTGCCAACACA	CATCATCCTCCAATGATCCA	59.0	478	INTERGENIC	--	--	--
Ca3_P1_342264	Ca3	342264	GTTTTTTTTTTT/GTTTTTTTTTT	CTTCAGGCAAACATGGGAAT	TTCCAATAAATGCCGAAACA	59.9	702	INTERGENIC	--	--	--
Ca3_P1_3424341	Ca3	3424341	CAAAAAAAAA/CAAAAAAAAAA	TTGCTCATGCGGTCTATCAA	CGTTGACAAAGGGATCGAGT	60.4	600	INTERGENIC	--	--	--
Ca3_P1_34297920	Ca3	34297920	GAATAAATAAATAAATAAATAAATAAATAAA/GAATAAATAAATAAATAAATAAATAAATAAATAAATAAATAAA	CCTCATGGGGAGCAAAATAA	TTCAGTTCACTGCGCATTTC	59.9	607	INTERGENIC	--	--	--
Ca3_P1_34365238	Ca3	34365238	T/TC	TGACCCAAAATGTGACTTGC	ATGCCAAGAATCCAACTCGT	59.5	865	INTERGENIC	--	--	--
Ca3_P1_34405777	Ca3	34405777	T/TA	CCAATCAACTCATCATTGCG	CGATGATGTGGAGGATGATG	60.1	686	DRR	Ca_00717	J	--
Ca3_P1_34425187	Ca3	34425187	AATATATATATATATATATATATATATATAT/AATATATATATATATATATATATATATATATATATATATAT	GGACGATTGTAAAACAAATTTGAAC	AAGTCGGCGAATTTGATTATTT	60.0	474	INTRON	Ca_00720	R	--
Ca3_P1_34430332	Ca3	34430332	AATATATATATATATATATATATATATATAT/AATATATATATATATATATATATATATATAT	GTGATGATCAAATTCGGGCT	TTGTGGGGTTCACTTGTTCA	59.9	481	INTERGENIC	--	--	--
Ca3_P1_34456382	Ca3	34456382	AGG/AG	TCCCCTGTTTGGTTGTTGAT	GTAGTGTTGGTTTGTGGGGG	60.2	418	INTERGENIC	--	--	--
Ca3_P1_34474422	Ca3	34474422	TAAAAAAAA/TAAAAAAA	AAAAATCAATTTATGCCGTTGT	TTATGCCGGATTGACACTCTT	57.7	478	INTERGENIC	--	--	--
Ca3_P1_34520648	Ca3	34520648	GAA/GAAA	AAAGACGAAAGGGAACACCA	GAAATTGGAAACAAAGTCCGA	59.6	411	INTERGENIC	--	--	--

INDEL marker IDs	Chromosomes/ unanchored scaffolds	Physical positions (bp)	InDels (*Kabuli* reference genome-CDC Frontier/PI)	Forward primers (5'-3')	Reverse primers (5'-3')	Anneling temperature (0C)	Expected amplified product size (bp)	Structural annotation		Functional annotation	
								Sequence components of *kabuli* genome	*Kabuli* gene accession IDs	NCBI-KOG	TFs
Ca3_P1_34521509	Ca3	34521509	CTTTTTT/CTTTTTTT	ACCCATCTTGCGTTTTTCAG	TTAAATGGCGGCAAATTGAT	60.1	310	INTERGENIC	--	--	--
Ca3_P1_34540759	Ca3	34540759	GAAAAAA/GAAAAAAA	GCAAGCAATTGAACAAAGCA	TGCTATCACTTCCGTCCACA	60.0	521	INTRON	Ca_00726	S	--
Ca3_P1_34556153	Ca3	34556153	CTTTTTTTTT/CTTTTTTTT	CGTTGGCTGGATTTTCATTT	GACTCCGCATCATTGTTCCT	59.9	573	INTRON	Ca_00726	S	--
Ca3_P1_34557168	Ca3	34557168	TGAGAGAGAGAGAGAGAGAGAGAGAGAGAGAG/TGAGAGAGAGAGAGAGAG	TGTTCAATTATTTTGGGATGGA	TCCTCCAATTTTCTTTCCCC	59.2	582	DRR	Ca_00726	S	--
Ca3_P1_34557690	Ca3	34557690	GTTCCATTCCATT/GTTCCATTCCATTCCATT	AGCTCCATTCCATCGCATAC	TTCCCAACTTTGTTTCTAGGGA	60.1	553	DRR	Ca_00726	S	--
Ca3_P1_34558499	Ca3	34558499	GTTTTTTT/GTTTTTTTT	CTTCCCACGGATGTTTTTGT	GGATCTTGCCAATCCAGAAA	59.8	677	INTERGENIC	--	--	--
Ca3_P1_34559360	Ca3	34559360	CAAAAAAAAAAAAAAAA/CAAAAAAAAA	TGCTCGATTTTTCGGTGAAT	ATCATGCATGCCAAGAAACA	60.6	813	INTERGENIC	--	--	--
Ca3_P1_34561246	Ca3	34561246	ATTTTTT/ATTTTTTTTT	TGGTGGGGAATAGTGTCCTC	CCGTCACTAAATTGTGTGGCTA	59.8	588	INTERGENIC	--	--	--
Ca3_P1_34561739	Ca3	34561739	CAA/CAAA	GCCACACAATTTAGTGACGG	CATGGATTGGGGTAGAATCG	59.1	571	INTERGENIC	--	--	--
Ca3_P1_34565708	Ca3	34565708	CATTGATTGA/CATTGATTGATTGA	TGCTTTAGTTTGGCTTTGGC	TCAAACACGTCTGTATATCAAGAAAA	60.4	350	DRR	Ca_00727	GC	HSF
Ca3_P1_34565771	Ca3	34565771	CTAAT/CTAATTGAGTAAT	TGCTTTAGTTTGGCTTTGGC	TCAAACACGTCTGTATATCAAGAAAA	60.4	350	DRR	Ca_00727	GC	HSF
Ca3_P1_34565949	Ca3	34565949	AA/AAGA	TGCTTTAGTTTGGCTTTGGC	TGATAGGATTTGTTGGAAATCG	60.4	876	DRR	Ca_00727	GC	HSF

INDEL marker IDs	Chromosomes/ unanchored scaffolds	Physical positions (bp)	InDels (*Kabuli* reference genome-CDC Frontier/PI)	Forward primers (5'-3')	Reverse primers (5'-3')	Anneling temperature (0C)	Expected amplified product size (bp)	Structural annotation		Functional annotation	
								Sequence components of *kabuli* genome	*Kabuli* gene accession IDs	NCBI-KOG	TFs
Ca3_P1_34566898	Ca3	34566898	ATTTTT/ATTTTTT	TTATAGCCAAGCGTGTCATGT	CCGGTGAGTGACAGAATTGA	58.3	373	INTERGENIC	--	--	--
Ca3_P1_34568370	Ca3	34568370	AATAT/AAT	GTTTGTGCCCAGTCCAAAGT	ACAAACACAACGCCAAACAA	60.0	549	INTERGENIC	--	--	--
Ca3_P1_34568477	Ca3	34568477	CCTCCGA/CCTCCGACTCCGA	GTTTGTGCCCAGTCCAAAGT	ACAAACACAACGCCAAACAA	60.0	549	INTERGENIC	--	--	--
Ca3_P1_34572752	Ca3	34572752	TAAAA/TAA	ATCGTTTTTCTCCACGGTGT	AAAAGGCGAACGAACAAAAA	59.5	412	DRR	Ca_00728	GC	HSF
Ca3_P1_34582473	Ca3	34582473	TG/T	GGGGTGTTGAAGAATCGAGA	GGGAAATAGCGGTTTGTTCA	60.1	614	INTERGENIC	--	--	--
Ca3_P1_34588571	Ca3	34588571	A/ATAT	GTCGGAGTGATGGTTGGAGT	CTGCTGAGGGATGGAATGAT	60.0	988	DRR	Ca_00731	--	NAC
Ca3_P1_34590067	Ca3	34590067	TAAAAAAAAAAAAAAA/TAAAAAAAAAA	AACTCTCAATGCCACTGCCT	GCTACTCGTTTGGCGTCTTT	59.9	616	DRR	Ca_00730	GC	HSF
Ca3_P1_34590662	Ca3	34590662	CAA/CAAA	AAAGACGCCAAACGAGTAGC	GAAGACAAAATGGGCTTCCA	59.5	805	INTRON	Ca_00731	--	NAC
Ca3_P1_34592017	Ca3	34592017	ATTTTTTTTTTTTTT/ATTTTTTTTT	GAGGTCGGAAGATGGATACG	TGTTGGAATAGCGGTGGATT	59.5	243	INTERGENIC	--	--	--
Ca3_P1_34604942	Ca3	34604942	AA/AAGA	GGTAAATGGCAAAGCCGTAA	CTCTGCCCAACCAAAGACAT	60.0	906	INTERGENIC	--	--	--
Ca3_P1_34605240	Ca3	34605240	ATTTTTTTTTTTT/ATTTTTTTTT	CCGTTTACGTGGGTTTCTTG	CTCTGCCCAACCAAAGACAT	60.4	269	INTERGENIC	--	--	--
Ca3_P1_34605719	Ca3	34605719	CTTTTTAATTTTTTAATTTTTTAAT/CTTTTTAATTTTTTAAT	ATGTCTTTGGTTGGGCAGAG	GGGTTTGGAGGAGAGGAGAA	60.1	404	INTERGENIC	--	--	--

INDEL marker IDs	Chromosomes/ unanchored scaffolds	Physical positions (bp)	InDels (*Kabuli* reference genome-CDC Frontier/PI)	Forward primers (5'-3')	Reverse primers (5'-3')	Anneling temperature (0C)	Expected amplified product size (bp)	Structural annotation		Functional annotation	
								Sequence components of *kabuli* genome	*Kabuli* gene accession IDs	NCBI-KOG	TFs
Ca3_P1_34605818	Ca3	34605818	TACA/TA	ATGTCTTTGGTTGGGCAGAG	TTTGTTTAAGGGAAGGGGAGA	60.1	555	INTERGENIC	--	--	--
Ca3_P1_34608040	Ca3	34608040	GTCTCT/GTCT	TGGAGAAACAAAATACGGGA	TTGGGTAGACATTTTCCCTTG	58.1	575	INTERGENIC	--	--	--
Ca3_P1_34608178	Ca3	34608178	ATTG/A	TGGAGAAACAAAATACGGGA	TTGGGTAGACATTTTCCCTTG	58.1	575	INTERGENIC	--	--	--
Ca3_P1_34619372	Ca3	34619372	CAAA/CAA	CGGCTCAGTGATCCTCTTTC	CCAAGGGAAAAGATAACCAACA	60.0	334	INTERGENIC	--	--	--
Ca3_P1_34619391	Ca3	34619391	CTTTTTTT/CTTTTTT	CGGCTCAGTGATCCTCTTTC	CCAAGGGAAAAGATAACCAACA	60.0	334	INTERGENIC	--	--	--
Ca3_P1_34619904	Ca3	34619904	GTATATATATATA/GTATATATA	TGTTGGTTATCTTTTCCCTTGG	TGTGCGGATAAAAAGCTTCC	60.2	782	INTERGENIC	--	--	--
Ca3_P1_34620121	Ca3	34620121	ATTTT/ATTT	TTTTTCTTCCAATTTTCTGGAG	TGTGCGGATAAAAAGCTTCC	57.6	432	INTERGENIC	--	--	--
Ca3_P1_34620159	Ca3	34620159	TA/TAAA	TTTTTCTTCCAATTTTCTGGAG	TGTGCGGATAAAAAGCTTCC	57.6	432	INTERGENIC	--	--	--
Ca3_P1_34621297	Ca3	34621297	A/AC	CATCACCACCACCATTTCAG	AGGAAAAATGGCATCCAATG	59.8	910	INTERGENIC	--	--	--
Ca3_P1_34621352	Ca3	34621352	TAAAAAAA/TAAAAAAAA	GTATTGGCAAACCGTCGTAA	AGGAAAAATGGCATCCAATG	58.6	446	INTERGENIC	--	--	--
Ca3_P1_34623535	Ca3	34623535	CAT/CATAT	CCAAGCAAGTGGCATTATGA	ACGATTAGATTTGACCGAAAAA	59.7	457	INTERGENIC	--	--	--
Ca3_P1_34623936	Ca3	34623936	ATTTTT/ATTTT	CAGATAATTTGGGCAAGAGAA	GGAGCCAAGCTCCAGTTATT	57.0	716	INTERGENIC	--	--	--

INDEL marker IDs	Chromosomes/ unanchored scaffolds	Physical positions (bp)	InDels (*Kabuli* reference genome-CDC Frontier/PI)	Forward primers (5'-3')	Reverse primers (5'-3')	Anneling temperature (0C)	Expected amplified product size (bp)	Structural annotation		Functional annotation	
								Sequence components of *kabuli* genome	*Kabuli* gene accession IDs	NCBI-KOG	TFs
Ca3_P1_34625966	Ca3	34625966	TC/T	CCCCCTTTAAACTTGTGAGC	TGTTTGAAGAATTTGGTTTGC	58.7	693	DRR	Ca_00734	FH	--
Ca3_P1_34626093	Ca3	34626093	TTA/TTAAAAGTA	GGGCTAAGATCAACACCCTC	TGTTTGAAGAATTTGGTTTGC	58.6	233	DRR	Ca_00734	FH	--
Ca3_P1_34626343	Ca3	34626343	ATTTTTTT/ATTTTTTTT	GGGCTAAGATCAACACCCTC	CTTGTGCAATATCCGAAACG	58.6	771	DRR	Ca_00734	FH	--
Ca3_P1_34626639	Ca3	34626639	C/CAAATGATT	TTTGCAAACCAAATTCTTCAAA	GATGGGTCCTATTGGTGGAA	59.6	938	DRR	Ca_00734	FH	--
Ca3_P1_34630411	Ca3	34630411	TTAAAAATATATAAAAAT/TTAAAAAT	AATTCTCGATCCCCATTTCC	TCTTGATGTTTGATATGACGCC	60.1	644	INTERGENIC	--	--	--
Ca3_P1_34630494	Ca3	34630494	GAAAAAAA/GAAAAAAAA	AATTCTCGATCCCCATTTCC	TCTTGATGTTTGATATGACGCC	60.1	644	INTERGENIC	--	--	--
Ca3_P1_34633037	Ca3	34633037	T/TC	AATACGTCGACCCCACTCAA	TCAGGACCGGATTTGACTTC	60.4	524	DRR	Ca_00735	--	--
Ca3_P1_34633648	Ca3	34633648	AATAT/AAT	AAGTCAAATCCGGTCCTGAA	GCTTGCAGCAACAACAAAAA	59.5	223	DRR	Ca_00735	--	--
Ca3_P1_34634307	Ca3	34634307	TAAA/TAA	CACGCTGGAAAGCAATCTCT	AAGGGGTTTTGTCCAAGGTC	60.5	854	DRR	Ca_00735	--	--
Ca3_P1_34634374	Ca3	34634374	TAAAAA/TAAAAAA	AAAAAGTGTGGCCAATCCAT	GAAGGGGTTTTGTCCAAGGT	59.3	428	DRR	Ca_00735	--	--
Ca3_P1_34634718	Ca3	34634718	AAATAATAATAATAATAATAATAATAAT/AAATAATAATAATAATAATAATAATAATAATAAT	CCAAATCATGGACCTTGGAC	CAAGCTTTGGTTTGGATGGT	60.2	391	DRR	Ca_00735	--	--
Ca3_P1_34637083	Ca3	34637083	ATTTTTTT/AATTTTTTTT	GAGATTGGCGCTTACGCTAC	CCATTAAAAACATACTTTAGCAAGCA	60.0	669	INTERGENIC	--	--	--

INDEL marker IDs	Chromosomes/ unanchored scaffolds	Physical positions (bp)	InDels (*Kabuli* reference genome-CDC Frontier/PI)	Forward primers (5'-3')	Reverse primers (5'-3')	Anneling temperature (0C)	Expected amplified product size (bp)	Structural annotation		Functional annotation	
								Sequence components of *kabuli* genome	*Kabuli* gene accession IDs	NCBI-KOG	TFs
Ca3_P1_34637980	Ca3	34637980	T/TA	TTTGCACTTTTGATTTCTTTTGA	TCGACCTACGTTATCCGCTT	58.9	408	INTERGENIC	--	--	--
Ca3_P1_34638267	Ca3	34638267	AAATAA/AAATAATAA	AGCGGATAACGTAGGTCGAA	ATAACTATGCGGATGGCCTG	59.7	475	INTERGENIC	--	--	--
Ca3_P1_34640884	Ca3	34640884	TA/T	TTGACCGTGTGCTTTTTGAG	GATATTAGGGGCAACTCGCA	59.9	285	INTRON	Ca_00736	S	--
Ca3_P1_34641463	Ca3	34641463	ACT/ACTCT	TGCGAGTTGCCCCTAATATC	GCATCAGCAACAAAAGCAAA	60.1	879	INTRON	Ca_00736	S	--
Ca3_P1_34641614	Ca3	34641614	C/CTT	GACACGTGGAACTGGAACCT	GCATCAGCAACAAAAGCAAA	60.0	415	INTRON	Ca_00736	S	--
Ca3_P1_34643759	Ca3	34643759	TCCCCCCC/TCCCCCC	TCCTCTTCATCACTCACCCC	TCCTTGAAAGCCACAATTCC	60.0	135	DRR	Ca_00737	--	--
Ca3_P1_34646219	Ca3	34646219	ATTTTTTTTTTT/ATTTTTTTTTT	TTTTGTTTGCGGTGCATAGA	TGGGCCAAGACTCCTTTTTA	60.3	602	INTERGENIC	--	--	--
Ca3_P1_34648295	Ca3	34648295	AT/ATT	CTTCTATGCATGTTTCGGCA	GGAATGGAGAGAAAATGGTTTG	59.8	317	DRR	Ca_00738	J	--
Ca3_P1_34648505	Ca3	34648505	CATAT/CAT	CTTCTATGCATGTTTCGGCA	CCCATTAAATCATCTTTTCCAA	59.8	927	DRR	Ca_00738	J	--
Ca3_P1_34648548	Ca3	34648548	AATAT/AAT	CTTCTATGCATGTTTCGGCA	TCTTCCACGGCCAATAGTTC	59.8	979	DRR	Ca_00738	J	--
Ca3_P1_34648802	Ca3	34648802	T/TCA	CAAACCATTTTCTCTCCATTCC	TCTTCCACGGCCAATAGTTC	59.8	684	DRR	Ca_00738	J	--
Ca3_P1_34650529	Ca3	34650529	TG/T	TGGATTGGTGTTTTCCCATT	TCCCAAACAATCATCCCAAT	60.0	609	INTRON	Ca_00739	O	C2H2

INDEL marker IDs	Chromosomes/ unanchored scaffolds	Physical positions (bp)	InDels (*Kabuli* reference genome-CDC Frontier/PI)	Forward primers (5'-3')	Reverse primers (5'-3')	Anneling temperature (0C)	Expected amplified product size (bp)	Structural annotation		Functional annotation	
								Sequence components of *kabuli* genome	*Kabuli* gene accession IDs	NCBI-KOG	TFs
Ca3_P1_34658227	Ca3	34658227	GTTATTAT/GTTAT	TTTGGCAGTGTGTTTTCATTG	GGGTGGTGTGTGTGAGAGTG	59.6	741	INTERGENIC	--	--	--
Ca3_P1_34658557	Ca3	34658557	CCTCTCTCTCTCTCTCTCTCT/CCTCTCTCTCTCTCTCTCTCTCT	TATGTAATGGCGCGTGAGAA	CGTAAAGAGAAGCCGGAGTG	60.2	332	INTERGENIC	--	--	--
Ca3_P1_34660669	Ca3	34660669	GAA/GAAA	TCTTGCACCAGCAATTTGAG	TAAACCCAATGGATAGCCGA	60.0	824	INTRON	Ca_00740	AJ	NAC
Ca3_P1_34661031	Ca3	34661031	GAAA/GAAAA	TGTGTGCTTCTCCACTCCTG	TAAACCCAATGGATAGCCGA	60.0	554	INTRON	Ca_00740	AJ	NAC
Ca3_P1_34665269	Ca3	34665269	TGGGGG/TGGGG	CAAGAAGGTGGGAGGATTCA	TTGGTACAAGCCCAGACAAA	60.0	593	INTERGENIC	--	--	--
Ca3_P1_34671849	Ca3	34671849	CGGAAT/C	AGTCCGCTCTGCAGATGAAT	CATTGCTGCCATCTTTCTCA	60.0	925	INTRON	Ca_00741	T	--
Ca3_P1_34673107	Ca3	34673107	AAAGAA/AAA	GGTACGGTCCTCCAAGAACA	GAAATTTTCACGGCCATTTG	60.0	585	DRR	Ca_00741	T	--
Ca3_P1_34674040	Ca3	34674040	AG/AGG	ATGTTTCACGTGATTGGGGT	TTGTGGGTTTGAGTTCCTCC	60.1	684	DRR	Ca_00741	T	--
Ca3_P1_34674867	Ca3	34674867	TAAAAAA/TAAAAAAA	GGAGGAACTCAAACCCACAA	GTTTCGACTGGTGCAGATCA	59.9	659	INTERGENIC	--	--	--
Ca3_P1_34681160	Ca3	34681160	ATAAAATTAAAAT/ATAAAATTAAAATTAAAAT	AGGGGCCATGCTAATCTTCT	TTTGAAGGGAGAAATCCATCA	60.1	552	DRR	Ca_00743	V	GRAS
Ca3_P1_34681675	Ca3	34681675	GTTTTTTT/GTTTTTT	TGATGGATTTCTCCCTTCAAA	TTGGTTGTGGACCTGTGGTA	59.5	475	DRR	Ca_00743	V	GRAS
Ca3_P1_34682350	Ca3	34682350	GAACAA/GAA	TGCAACTCTTTTCGTCACCA	TTGCCCGAGTCTTCTCAGTT	60.4	539	DRR	Ca_00743	V	GRAS

INDEL marker IDs	Chromosomes/ unanchored scaffolds	Physical positions (bp)	InDels (*Kabuli* reference genome-CDC Frontier/PI)	Forward primers (5'-3')	Reverse primers (5'-3')	Anneling temperature (0C)	Expected amplified product size (bp)	Structural annotation		Functional annotation	
								Sequence components of *kabuli* genome	*Kabuli* gene accession IDs	NCBI-KOG	TFs
Ca3_P1_34682477	Ca3	34682477	CTAGCTCTG/C	GGGTGGTTGGTCCCTTATTT	TTGCCCGAGTCTTCTCAGTT	59.9	303	DRR	Ca_00743	V	GRAS
Ca3_P1_34685671	Ca3	34685671	CAAA/CAAAA	TTATGTCTCGTTTGGTGGACA	TTAGTGACATGCGTGGCTTC	59.0	586	INTERGENIC	--	--	--
Ca3_P1_34685742	Ca3	34685742	TAAAAAAA/TAAAAAA	TTATGTCTCGTTTGGTGGACA	TTAGTGACATGCGTGGCTTC	59.0	586	INTERGENIC	--	--	--
Ca3_P1_34703599	Ca3	34703599	ATTT/ATTTTTT	GCATCCATGGAAAATCAGGA	ATTTTGTGGCTCAAACCGAG	60.8	541	INTERGENIC	--	--	--
Ca3_P1_34715451	Ca3	34715451	AT/ATT	GTTCTCGTAAACTCCGCTGC	ATAATCCCACGCTTTTGTGC	60.0	819	INTERGENIC	--	--	--
Ca3_P1_34717035	Ca3	34717035	CAAAATTAAAATT/CAAAATTAAAATTAAAATT	AAAACATGTAGATCCATTTGAGTGA	AACTTTTTGGCTTTCCGGTT	59.0	640	INTERGENIC	--	--	--
Ca3_P1_34721625	Ca3	34721625	ACTATCATTCTATCA/ACTATCA	CAATCCGTTTAGAACTCCCG	TGTTGCACGTAGCCTAAGAAA	59.6	625	DRR	Ca_00746	GMW	ERF
Ca3_P1_34724989	Ca3	34724989	CTGTCATCTCATTGTCATCTCAT/CTGTCATCTCAT	AAAAGAAAATTGAAATTCACCTTGA	TGTGTCCCACAACTTACTTACTAACA	59.5	537	DRR	Ca_00747	--	--
Ca3_P1_34726048	Ca3	34726048	TAAAAAAAAAAAA/TAAAAAAAAAA	TATTGATGATGTTGCACGGG	TTCACGTGAGAGCAGGACAC	60.3	391	INTERGENIC	--	--	--
Ca3_P1_34730322	Ca3	34730322	AAA/AAAATTTGAA	ATTTTGGGCCCTCTTCTCAT	AGCCAGCCAAAACAGACACT	59.9	647	INTRON	Ca_00748	--	--
Ca3_P1_34731798	Ca3	34731798	TAAAAAAAA/TAAAAAAAAAA	TTTTCTGCCAAAATCATCCC	AAGAAAGAGTACCGTCGCCA	59.9	671	DRR	Ca_00748	--	--
Ca3_P1_34731939	Ca3	34731939	TAAAAAAAAAAAAAA/TAAAAAAAAAAAAA	TTTTCTGCCAAAATCATCCC	AAGAAAGAGTACCGTCGCCA	59.9	671	DRR	Ca_00748	--	--

INDEL marker IDs	Chromosomes/ unanchored scaffolds	Physical positions (bp)	InDels (*Kabuli* reference genome-CDC Frontier/PI)	Forward primers (5'-3')	Reverse primers (5'-3')	Anneling temperature (0C)	Expected amplified product size (bp)	Structural annotation		Functional annotation	
								Sequence components of *kabuli* genome	*Kabuli* gene accession IDs	NCBI-KOG	TFs
Ca3_P1_34732162	Ca3	34732162	TAAA/TAAAAA	TGGCGACGGTACTCTTTCTT	CACACTTTTAGGCCACCCAT	59.9	389	DRR	Ca_00748	--	--
Ca3_P1_34732224	Ca3	34732224	TCCC/TCCCC	TGGCGACGGTACTCTTTCTT	CACACTTTTAGGCCACCCAT	59.9	389	DRR	Ca_00748	--	--
Ca3_P1_34737485	Ca3	34737485	CTTTTTTT/CTTTTTT	TGCTCTTCGGTTCGTTTCTT	ACCCAAGGATACAATGCCAA	60.0	650	DRR	Ca_00749	J	--
Ca3_P1_34737907	Ca3	34737907	T/TTA	TTGGCATTGTATCCTTGGGT	GGGGGTATTTGTGCTTTTCA	60.2	301	DRR	Ca_00749	J	--
Ca3_P1_34739285	Ca3	34739285	TT/TTGGT	AGGTAGAGCCAGTGAGGTGG	TCTTTGGATGTGGTGGAACA	59.3	841	DRR	Ca_00749	J	--
Ca3_P1_34739482	Ca3	34739482	ATTTT/ATTT	AGGCAATTCAAAGTTAAAGGTG	TCTTTGGATGTGGTGGAACA	57.5	508	INTERGENIC	--	--	--
Ca3_P1_34745696	Ca3	34745696	ATTTT/ATTT	GGTGCAACCCATTTAAAAACA	GGGTCTTTAAACCATTTTCTGG	59.7	592	INTERGENIC	--	--	--
Ca3_P1_34745831	Ca3	34745831	A/AAGG	TGAATCTTCCAGAAAATGGTTT	AAAAACAAACGTGTAGACTAAAAAGC	57.7	349	INTERGENIC	--	--	--
Ca3_P1_34746354	Ca3	34746354	GAT/GATCAT	TGGTGTATAACTAAGAGACACGTCAA	GGGGAGGAGGAAGAAGCTC	59.2	810	INTERGENIC	--	--	--
Ca3_P1_34749435	Ca3	34749435	TC/T	TGGGGGAGAGACGTCATAAT	TGTCCTCGACATTTCTCTCCA	59.4	834	INTERGENIC	--	--	--
Ca3_P1_34749745	Ca3	34749745	A/ACCTTAT	CGTCAATATTTGTTGCAGGC	TATGGGTGGTTCCATTCCAC	59.2	332	INTERGENIC	--	--	--
Ca3_P1_34752760	Ca3	34752760	GTT/GTTGCGACTTGCTACTT	GACCCCTGTCTCTCTGCTTG	GGTTGTTGTGGTTTTGGACC	60.0	256	INTERGENIC	--	--	--

INDEL marker IDs	Chromosomes/ unanchored scaffolds	Physical positions (bp)	InDels (*Kabuli* reference genome-CDC Frontier/PI)	Forward primers (5'-3')	Reverse primers (5'-3')	Anneling temperature (0C)	Expected amplified product size (bp)	Structural annotation		Functional annotation	
								Sequence components of *kabuli* genome	*Kabuli* gene accession IDs	NCBI-KOG	TFs
Ca3_P1_34753362	Ca3	34753362	T/TA	GGTCCAAAACCACAACAACC	CTAAAATGTCCCCCGTCTCA	60.1	691	INTERGENIC	--	--	--
Ca3_P1_34753443	Ca3	34753443	TAA/TAACAA	TCCAAAACCACAACAACCAA	CTAAAATGTCCCCCGTCTCA	59.8	689	INTERGENIC	--	--	--
Ca3_P1_34755355	Ca3	34755355	ATTT/ATTTTT	TTTTTCCATTTTACCGGTCG	TTTTCAAGTGGCATCAACCA	59.8	587	DRR	Ca_00751	--	HD-ZIP
Ca3_P1_34755544	Ca3	34755544	T/TA	TTTTTCCATTTTACCGGTCG	TTTTCAAGTGGCATCAACCA	59.8	587	DRR	Ca_00751	--	HD-ZIP
Ca3_P1_34759868	Ca3	34759868	GTGATGATGATGATGATGATGATGATGATGATGATG/GTGATGATGATGATGATGATGATGATGATG	CTGCAAAACCCTAACACCGT	GAACGCAGTAACGTTGACGA	60.0	644	INTERGENIC	--	--	--
Ca3_P1_34762413	Ca3	34762413	T/TA	GGTGCTCAAAATGGTCGAGT	GCTCCTGGTTTTGTCTTTGC	60.1	360	INTERGENIC	--	--	--
Ca3_P1_34765170	Ca3	34765170	CTA/CTAAGTA	CCCTCCACATTTTACCCTCA	GTGGCTTGTTGGTTTTGGAT	59.8	619	INTERGENIC	--	--	--
Ca3_P1_34765275	Ca3	34765275	CAA/CA	CCCTCCACATTTTACCCTCA	GTGGCTTGTTGGTTTTGGAT	59.8	619	INTERGENIC	--	--	--
Ca3_P1_34765478	Ca3	34765478	AAACAACAACAACAACAACAACAA/AAACAACAACAACAACAACAACAACAA	CCCTCCACATTTTACCCTCA	CACCTGCTTCATCACCAATG	59.8	784	INTERGENIC	--	--	--
Ca3_P1_34785245	Ca3	34785245	ATTT/AT	TAAGCTGGCATGATTGTGCT	TCATGCCACCATCTATCCAA	59.4	560	INTERGENIC	--	--	--
Ca3_P1_34786759	Ca3	34786759	CTTTTAACTTTTTTTT/CTTTTAACTTTTTTTTTAACTTTTTTTT	CCAAGCGATAAAACAAAAGCA	GAACTTTCCGAAGTTGAGCG	60.2	355	INTERGENIC	--	--	--
Ca3_P1_34788996	Ca3	34788996	CTT/CTTT	CCATCTGGAATGCTTACGGT	CATCAGTGTCCCAACATTGC	60.0	309	INTERGENIC	--	--	--

INDEL marker IDs	Chromosomes/ unanchored scaffolds	Physical positions (bp)	InDels (*Kabuli* reference genome-CDC Frontier/PI)	Forward primers (5'-3')	Reverse primers (5'-3')	Anneling temperature (0C)	Expected amplified product size (bp)	Structural annotation		Functional annotation	
								Sequence components of *kabuli* genome	*Kabuli* gene accession IDs	NCBI-KOG	TFs
Ca3_P1_34789552	Ca3	34789552	AATATATATATATATATATAT/AATATATATATAT	GCAATGTTGGGACACTGATG	TGTATGTTGACGTGCTTTTGC	60.0	790	INTERGENIC	--	--	--
Ca3_P1_34790577	Ca3	34790577	CATATTAATATTAAT/CATATTAATATTAATATTAAT	CCTCACAGCTGCCCCTATTA	AAAACACACAGGGCATGATAA	60.2	692	INTERGENIC	--	--	--
Ca3_P1_34791543	Ca3	34791543	TC/T	AGCAAGAGAATGACACCCCA	CTTATCTCCCCAGTTGCCAG	60.7	494	INTERGENIC	--	--	--
Ca3_P1_34794610	Ca3	34794610	TAA/TATTATATAACTCATTATAAA	CACACAAAATGAATGAAGCGA	CATTTTGACAAGCCCGTGTA	59.7	575	DRR	Ca_00755	Q	B3
Ca3_P1_34794843	Ca3	34794843	T/TCC	TACACGGGCTTGTCAAAATG	AAACAGATGAACTGCCACGA	59.6	617	DRR	Ca_00755	Q	B3
Ca3_P1_34796244	Ca3	34796244	TA/TATGCCA	TGTTTTTGAACCGTGACTGAA	TCCGTTTCCAACCTGAACTC	59.2	639	INTERGENIC	--	--	--
Ca3_P1_34797632	Ca3	34797632	GTT/GTTT	GAGCGGCCTATCAAGTCATT	TCAAATCATGCAATGAGAATTTTT	59.3	887	INTERGENIC	--	--	--
Ca3_P1_34797791	Ca3	34797791	GAAAA/GAAAAA	TTGTCAAACATTGCAACAAGAA	CACACTGAAGGTCTCCGGTT	59.3	902	INTERGENIC	--	--	--
Ca3_P1_34797856	Ca3	34797856	GTTTTTTTT/GTTTTTT	TTGTCAAACATTGCAACAAGAA	CACACTGAAGGTCTCCGGTT	59.3	902	INTERGENIC	--	--	--
Ca3_P1_34798242	Ca3	34798242	TAAAA/TAAAAA	TCATTAATAGATCCGCATTTGAA	CACACTGAAGGTCTCCGGTT	58.6	235	DRR	Ca_00756	--	--
Ca3_P1_34798838	Ca3	34798838	CA/C	AACTAGGGTCCGGGTGTTTT	CACAGGACCTTTGAACCCAT	59.7	565	DRR	Ca_00756	--	--
Ca3_P1_34798929	Ca3	34798929	CTTTTTTTTTTT/CTTTTTTT	AACTAGGGTCCGGGTGTTTT	AAGTGTGGCAAGCTCTTGGT	59.7	803	DRR	Ca_00756	--	--

INDEL marker IDs	Chromosomes/ unanchored scaffolds	Physical positions (bp)	InDels (*Kabuli* reference genome-CDC Frontier/PI)	Forward primers (5'-3')	Reverse primers (5'-3')	Anneling temperature (0C)	Expected amplified product size (bp)	Structural annotation		Functional annotation	
								Sequence components of *kabuli* genome	*Kabuli* gene accession IDs	NCBI-KOG	TFs
Ca3_P1_34799954	Ca3	34799954	ACC/AC	ATGCAGACACTCCAATGCAA	GTTTGGCTCCTCTTGCACAT	60.3	518	DRR	Ca_00756	--	--
Ca3_P1_34803571	Ca3	34803571	AC/ACC	TGCGAATAATTTTGTTTTCATCA	CCTCGACCTAAATCAAGGGG	59.5	660	INTERGENIC	--	--	--
Ca3_P1_34805836	Ca3	34805836	AAAACTTC/A	AAAGCCTTTGTGAGTTCCTCAG	AAAAATACAAATTTTCGGTGGTT	59.9	698	DRR	Ca_00757	--	--
Ca3_P1_34806419	Ca3	34806419	ATTTT/ATTTTT	TTTGAAATTTTAACCACCGAAAA	GAAACCAAATTGCAAAGGGA	59.8	627	DRR	Ca_00757	--	--
Ca3_P1_34807994	Ca3	34807994	CATATATATATATATATATATATATATATATAT/CATATATATATATATATATATATATATATAT	AAATTGGTGTTTGCTGGCTT	CTCCTGCTGTAACTGTGCCA	59.6	785	INTERGENIC	--	--	--
Ca3_P1_34808849	Ca3	34808849	GAAT/GAATCTGTAAAT	TGGGAGTTAGGGCAATTGAA	GGGTTGACAATCGGTCAAAT	60.4	567	INTERGENIC	--	--	--
Ca3_P1_34809563	Ca3	34809563	CTTTTTT/CTTTTTTT	AGCTGGCCCTTACGAAGTTT	TTTGCTGCATCATCATTTCC	60.3	453	INTERGENIC	--	--	--
Ca3_P1_34816300	Ca3	34816300	TAAAACATTAA/TAAAACATTAAAAACATTAA	TCCACTGACCCAACAATCAA	ACCAAAAATGGAACGCTACG	59.9	536	INTRON	Ca_00758	--	--
Ca3_P1_34818875	Ca3	34818875	AAATAATAATAAT/AAATAATAATAATAATAAT	TTGTTGTCGCCATCATTGTT	ACTCCCGCAGTCCCTATTCT	60.0	455	INTRON	Ca_00758	--	--
Ca3_P1_34821669	Ca3	34821669	C/CGTTGAGTGA	TGTCCTTTGATGTGCAGGAC	CCAGCAACTCACGTGACAAT	59.7	521	DRR	Ca_00758	--	--
Ca3_P1_34825531	Ca3	34825531	GTTATTTTT/GTTATTTTTTTAATTTTATTTTT	TTGGATGGAGGATTTTGGAG	CTCAATTCCCCTTGGTTGAA	59.9	247	DRR	Ca_00759	R	B3
Ca3_P1_34835388	Ca3	34835388	A/ACG	TGTTACGGTGTTAAATTTTGCG	CGCAGGTTTGTTGATGCTAA	59.9	148	INTERGENIC	--	--	--

INDEL marker IDs	Chromosomes/ unanchored scaffolds	Physical positions (bp)	InDels (*Kabuli* reference genome-CDC Frontier/PI)	Forward primers (5'-3')	Reverse primers (5'-3')	Anneling temperature (0C)	Expected amplified product size (bp)	Structural annotation		Functional annotation	
								Sequence components of *kabuli* genome	*Kabuli* gene accession IDs	NCBI-KOG	TFs
Ca3_P1_34836958	Ca3	34836958	TTTTTTTAAATTTTTTA/TTTTTTTA	GTGGTAATCCAACCGTCGAG	GAACCGATCCAACATGCTTT	60.4	443	INTRON	Ca_00761	G	--
Ca3_P1_34840506	Ca3	34840506	GTTTTTTTTTT/GTTTTTTTT	GTTGGTGATGAAGGTGGCTT	ATCCGGTCCCCATAACTTTC	60.0	457	INTRON	Ca_00761	G	--
Ca3_P1_34845314	Ca3	34845314	GCAAA/G	ATGGGTACACACGAAAAGCA	ACACCCCCGTACTTCAAACA	59.1	563	INTERGENIC	--	--	--
Ca3_P1_34847492	Ca3	34847492	ATCTCTCTCTCTCTCTCTCTCTC/ATCTCTCTCTCTCTCTC	GAATCTCTTGACCCCGACAA	ATTCATTAGGGCATTGGCAG	60.1	365	INTRON	Ca_00762	A	C3H
Ca3_P1_34847796	Ca3	34847796	TTG/TTGTG	CAATTTGGCAGATCACATGG	ACCAGTAAAGGCAACATCGG	59.9	680	INTRON	Ca_00762	A	C3H
Ca3_P1_34847842	Ca3	34847842	AATATATATATATATAT/AATATATATATATATATATATATATAT	CAATTTGGCAGATCACATGG	ACCAGTAAAGGCAACATCGG	59.9	680	INTRON	Ca_00762	A	C3H
Ca3_P1_34848161	Ca3	34848161	TTAT/TT	CCGATGTTGCCTTTACTGGT	ATCGGCCGACCTAGAAGAAT	60.0	391	INTRON	Ca_00762	A	C3H
Ca3_P1_34849447	Ca3	34849447	CAAAA/CAAAAA	AGCATGGGAAACAGTTTTGG	TTTGAAAGCAACGTCAATGC	60.0	330	DRR	Ca_00762	A	C3H
Ca3_P1_34850005	Ca3	34850005	GC/G	AAACGGATTGGAATGGATCA	TGTTTGAGGATGATGGTGGA	60.1	502	DRR	Ca_00763	--	C2H2
Ca3_P1_34851179	Ca3	34851179	AC/A	TGTGAATCTGTGATGTGCTAAGG	CAAATGTGCTGTTGGGTTTG	60.2	447	DRR	Ca_00763	--	C2H2
Ca3_P1_34851298	Ca3	34851298	GA/GAA	TTGATCAAACCCAACAGCAC	GTGCCAAGTGAAGGGTTTGT	59.5	400	DRR	Ca_00763	--	C2H2
Ca3_P1_34851854	Ca3	34851854	TATAA/TATAATAA	ACAAACCCTTCACTTGGCAC	ACCGCAAACTCTATGTTGGG	60.0	648	INTRON	Ca_00763	--	C2H2

INDEL marker IDs	Chromosomes/ unanchored scaffolds	Physical positions (bp)	InDels (*Kabuli* reference genome-CDC Frontier/PI)	Forward primers (5'-3')	Reverse primers (5'-3')	Anneling temperature (0C)	Expected amplified product size (bp)	Structural annotation		Functional annotation	
								Sequence components of *kabuli* genome	*Kabuli* gene accession IDs	NCBI-KOG	TFs
Ca3_P1_34852048	Ca3	34852048	CTACTTTA/CTACTTTACTTTA	ACAAACCCTTCACTTGGCAC	ACCGCAAACTCTATGTTGGG	60.0	648	INTRON	Ca_00763	--	C2H2
Ca3_P1_34852406	Ca3	34852406	T/TC	CCCAACATAGAGTTTGCGGT	TGATGATGAGGAAACACCGA	60.0	499	INTRON	Ca_00763	--	C2H2
Ca3_P1_34856790	Ca3	34856790	TACA/TACACA	ATGGCGGTGAAACGGTATTA	AATCGAACTTTGCCCTCTTTT	60.2	738	INTERGENIC	--	--	--
Ca3_P1_34857034	Ca3	34857034	AG/AGG	ACGGCCAATGTTGTTCTGAT	AATCGAACTTTGCCCTCTTTT	60.4	428	INTERGENIC	--	--	--
Ca3_P1_34866347	Ca3	34866347	CTATT/CTATTATT	TACCATGATGATGAAGCGGA	TCAACCGAATCAACAACCAA	60.0	308	DRR	Ca_00764	A	C2H2
Ca3_P1_34867020	Ca3	34867020	GAAAA/GAAA	CCTTTAATTTTGCCGACGAC	AAGAGGTTGGAATGTGACCG	59.6	684	DRR	Ca_00764	A	C2H2
Ca3_P1_34869536	Ca3	34869536	AAT/A	TGGATTTTAATTTTGTTGGTTTGA	TTTTTCTTTTGCTAGGGCCA	59.7	651	INTERGENIC	--	--	--
Ca3_P1_34872921	Ca3	34872921	ATTTTTT/ATTTTTTT	CACAAATGCGATTAAGGGCT	CATGTTGAATGGAAGCACACA	60.1	685	INTERGENIC	--	--	--
Ca3_P1_34873766	Ca3	34873766	CAAAAAAA/CAAAAAA	AACACATTTTTGCAAGTGCG	GGCTTCTGAAAAACACAGCC	59.8	728	INTERGENIC	--	--	--
Ca3_P1_34877669	Ca3	34877669	TA/T	TTGATGTGACCCTCCTCTCC	TTGTTACGGTTGTGAAGCAAA	60.0	720	DRR	Ca_00765	--	--
Ca3_P1_34878239	Ca3	34878239	CTACTTACTT/CTACTT	TTTGCTTCACAACCGTAACAA	GACCCATCAAGCCCATTTTA	59.3	635	DRR	Ca_00765	--	--
Ca3_P1_34879554	Ca3	34879554	ATT/AT	TGTATCAAGGGGAGGGAGTG	ACGCTCTTTTTGTGCTGGAT	59.9	925	INTERGENIC	--	--	--

INDEL marker IDs	Chromosomes/ unanchored scaffolds	Physical positions (bp)	InDels (*Kabuli* reference genome-CDC Frontier/PI)	Forward primers (5'-3')	Reverse primers (5'-3')	Anneling temperature (0C)	Expected amplified product size (bp)	Structural annotation		Functional annotation	
								Sequence components of *kabuli* genome	*Kabuli* gene accession IDs	NCBI-KOG	TFs
Ca3_P1_34881596	Ca3	34881596	TTAT/TTATATAT	CACCCTCATCCTTGCATCTT	CTTTGGCAATCGCTTGAAGT	60.1	485	INTERGENIC	--	--	--
Ca3_P1_34886229	Ca3	34886229	CTTTTTT/CTTTTT	ACCATGGACACAAGTGCAAA	ACGTTCTAAATTGAGCCCCC	60.0	488	INTERGENIC	--	--	--
Ca3_P1_34886716	Ca3	34886716	ACC/AC	GGGGCTCAATTTAGAACGTG	GGAGGGTTTTGTGTGGAAGA	59.6	651	INTERGENIC	--	--	--
Ca3_P1_34887394	Ca3	34887394	GT/GTT	TCTTCCACACAAAACCCTCC	ACGTGCATCACAAAACCAAA	59.9	571	INTRON	Ca_00767	G	ERF
Ca3_P1_34892570	Ca3	34892570	TA/TAA	TGAGGGTTGTGGTTTCAATTT	GCTTGAATAAGGTCCACCCA	59.3	223	INTERGENIC	--	--	--
Ca3_P1_34893456	Ca3	34893456	AAAA/AAAATAAAAAAATAAA	CGAACATTGTGTCTGAGTGAAA	TGAAAAATGTGGTTGACCGA	58.8	229	INTERGENIC	--	--	--
Ca3_P1_34896331	Ca3	34896331	ATTTTT/ATTT	ATGAAGAGAACGAGGAGGCA	AACCCCACCTCCTAAGCTGT	60.0	546	INTERGENIC	--	--	--
Ca3_P1_34897346	Ca3	34897346	TAAAAAAA/TAAAAAAAA	AGAACAAATTTTGGCGCAAC	AGTTTTGTCGGTCCATGAGC	60.1	537	INTERGENIC	--	--	--
Ca3_P1_34900915	Ca3	34900915	T/TG	CCATGCACAAGCATTGAATC	TTTCTTTTGTTGGTGGTCATTT	60.1	762	INTERGENIC	--	--	--
Ca3_P1_34901511	Ca3	34901511	TAGAGACAGAG/TAGAG	AAATGACCACCAACAAAAGAAA	GCCGTGTATGAATTTGTGTCA	58.5	714	INTERGENIC	--	--	--
Ca3_P1_34901560	Ca3	34901560	AGGGG/AGGG	TGTTACTGCTTCTTGACGTTTCA	GCCGTGTATGAATTTGTGTCA	60.0	393	INTERGENIC	--	--	--
Ca3_P1_34904847	Ca3	34904847	TAA/TA	TGTGCTGTCGTCACCACTAA	GGCCAGGAACCATGATAAGA	58.9	569	INTERGENIC	--	--	--

INDEL marker IDs	Chromosomes/ unanchored scaffolds	Physical positions (bp)	InDels (*Kabuli* reference genome-CDC Frontier/PI)	Forward primers (5'-3')	Reverse primers (5'-3')	Anneling temperature (0C)	Expected amplified product size (bp)	Structural annotation		Functional annotation	
								Sequence components of *kabuli* genome	*Kabuli* gene accession IDs	NCBI-KOG	TFs
Ca3_P1_34906102	Ca3	34906102	CTTTTTTT/CTTTTTTTTT	TGGATGGTGATTATCC GACTC	TCCAAGTCTTTCAATA CAAAACACA	59.8	605	INTERGENIC	--	--	--
Ca3_P1_34910960	Ca3	34910960	GTTTTTT/GTTTTTTT	TCAAGGTCAAGTGTGG CAAA	CTTTGGTCGACGATGA GGAT	60.3	819	INTERGENIC	--	--	--
Ca3_P1_34911794	Ca3	34911794	ATATTA/ATA	TGGGTGTCTGAAATTC GTGA	TCCAGAGGTTTGGATC ATCA	60.1	481	CDS (large-effect mutations)	Ca_00768	--	ERF
Ca3_P1_34913670	Ca3	34913670	TATAGAATAGAA/TA	ACCCGTTTCTTTGTTG TTGG	CTATCCAAGAAGCATC CCCA	59.9	427	DRR	Ca_00768	--	ERF
Ca3_P1_34913784	Ca3	34913784	GTATATATATATA/GTATATATATATAT ATA	ACCCGTTTCTTTGTTG TTGG	CTATCCAAGAAGCATC CCCA	59.9	427	DRR	Ca_00768	--	ERF
Ca3_P1_34913867	Ca3	34913867	AA/AACACTTGA	TGGGGATGCTTCTTGG ATAG	AAAATTTCGAGGCCCA TACC	60.0	154	DRR	Ca_00768	--	ERF
Ca3_P1_34914599	Ca3	34914599	T/TCGGG	TGGGAAGATGGAGGAA GAAA	GCCGGTTTCACACTTT TGAT	59.6	387	INTERGENIC	--	--	--
Ca3_P1_34916105	Ca3	34916105	GTTTT/GTTTTTT	TCGACCATCGTCATAC TCCA	TCTCCCCTTCATATGA CCCTT	60.1	671	INTERGENIC	--	--	--
Ca3_P1_34917623	Ca3	34917623	CTTATGTTATGT/CTTATGT	GGTGGTAAGGTCCTGT GGAA	TGACCAGCATGGTTGA AAGA	59.8	292	INTERGENIC	--	--	--
Ca3_P1_34922472	Ca3	34922472	TTATATA/TTATA	CAAAAGCGACATTTCA GCAA	AAAGCCCTTTCCGTTT TGTT	60.0	240	INTRON	Ca_00769	--	--
Ca3_P1_34925163	Ca3	34925163	AAT/AATAT	TTTGAATGTGTGAGAC ACCGA	AAGGGGCGAATTGAAA GATT	60.1	795	INTERGENIC	--	--	--
Ca3_P1_34930539	Ca3	34930539	TGAA/T	TTTTCGTTTGCGATGA TTGA	CACGAAGCCTATGCTT TCAGA	60.2	367	INTERGENIC	--	--	--

INDEL marker IDs	Chromosomes/ unanchored scaffolds	Physical positions (bp)	InDels (*Kabuli* reference genome-CDC Frontier/PI)	Forward primers (5'-3')	Reverse primers (5'-3')	Anneling temperature (0C)	Expected amplified product size (bp)	Structural annotation		Functional annotation	
								Sequence components of *kabuli* genome	*Kabuli* gene accession IDs	NCBI-KOG	TFs
Ca3_P1_34930591	Ca3	34930591	GTATATATAT/GTATATATATAT	TTTTCGTTTGCGATGATTGA	CACGAAGCCTATGCTTTCAGA	60.2	367	INTERGENIC	--	--	--
Ca3_P1_34930930	Ca3	34930930	AA/AATTA	CCCAACGTTGTTTTAACGGT	TGTCCTTAGTGGTGGAAGGG	59.8	839	INTERGENIC	--	--	--
Ca3_P1_34932929	Ca3	34932929	TAAAAAAA/TAAAAAAAA	ACTTGCTTTGCACCTCGTTT	TTGAAAGATGACTCTTCACCGA	59.9	631	INTERGENIC	--	--	--
Ca3_P1_34933291	Ca3	34933291	GATATATAT/GATATATATAT	TCGGTGAAGAGTCATCTTTCAA	GGGCCTTCACGTGTCTAAAA	59.9	687	INTERGENIC	--	--	--
Ca3_P1_34942560	Ca3	34942560	GCAACAACAA/GCAACAA	TCAAGGTGATAAATTGCCGA	TGTGCACACACAATCACAAAA	59.1	480	INTERGENIC	--	--	--
Ca3_P1_34943170	Ca3	34943170	AAATA/AA	TGCATTTTTGTGATTGTGTGTG	GCTGGCTCTTTGGGTATGAG	60.5	454	INTERGENIC	--	--	--
Ca3_P1_34943202	Ca3	34943202	ATTT/ATTTT	TGCATTTTTGTGATTGTGTGTG	GCTGGCTCTTTGGGTATGAG	60.5	454	INTERGENIC	--	--	--
Ca3_P1_34943765	Ca3	34943765	ATTTTTTTTT/ATTTTTTTT	GTGTTTGCTTGCTTTGACCA	CGGAAACTACTCCATCGTGAA	59.9	384	DRR	Ca_00771	--	S1Fa-like
Ca3_P1_34945416	Ca3	34945416	GAAAA/GAAAAA	ATTGGTCTTTGGCACACACA	GAAGAAGCTGGAGGAGCTGA	60.0	419	DRR	Ca_00771	--	S1Fa-like
Ca3_P1_34946326	Ca3	34946326	C/CT	GCAGGTATGACCAAGTGGCT	GCGCTGGTTTATCAAGGAAA	60.1	611	INTRON	Ca_00771	--	S1Fa-like
Ca3_P1_34948778	Ca3	34948778	TG/T	TGCATCATAAAATGTTTCATCAATTA	ATTACGGGGAGAGGGAGAGA	59.7	674	INTERGENIC	--	--	--
Ca3_P1_34949397	Ca3	34949397	ATTT/ATTTT	TTATCTCTCCCTCTCCCCGT	CACACATTAAAATTGGAGGTTCA	60.0	587	INTERGENIC	--	--	--

INDEL marker IDs	Chromosomes/ unanchored scaffolds	Physical positions (bp)	InDels (*Kabuli* reference genome-CDC Frontier/PI)	Forward primers (5'-3')	Reverse primers (5'-3')	Anneling temperature (0C)	Expected amplified product size (bp)	Structural annotation		Functional annotation	
								Sequence components of *kabuli* genome	*Kabuli* gene accession IDs	NCBI-KOG	TFs
Ca3_P1_34953897	Ca3	34953897	TAAAAAA/TAAAAAAA	CCCGTGATTCCTCTACAACA	GAACTCGCTTGAGGACGAAG	58.6	395	INTERGENIC	--	--	--
Ca3_P1_34954280	Ca3	34954280	TC/T	CTTCGTCCTCAAGCGAGTTC	TGCATGCAGGAGAAGTATTTATT	60.1	681	INTERGENIC	--	--	--
Ca3_P1_34958868	Ca3	34958868	ACCCC/ACCC	GCAACACCATGATAACGTGC	GATGAGATGTTCGCCCTGTT	60.0	220	INTERGENIC	--	--	--
Ca3_P1_34962522	Ca3	34962522	CATATA/CATA	GACAAATTGTTGCTTGCGAA	AGATTTGTCCTGTGTACGACGA	59.9	259	INTERGENIC	--	--	--
Ca3_P1_34964095	Ca3	34964095	GCC/GCCCC	GGTGACCATGAAGCTCGTTT	GCAATCAACAAACAAGTGTGC	60.1	488	INTERGENIC	--	--	--
Ca3_P1_34964151	Ca3	34964151	ATATTTATTTATTTATTT/ATATTTATTTATTT	GGTGACCATGAAGCTCGTTT	GCAATCAACAAACAAGTGTGC	60.1	488	INTERGENIC	--	--	--
Ca3_P1_34967166	Ca3	34967166	TAATAAATAAA/TAATAAA	TTATTTCATGGAGGATGACGC	CATCAAGTCCAAAACCACCA	59.9	630	INTERGENIC	--	--	--
Ca3_P1_34967536	Ca3	34967536	TAAAAAAA/TAAAAAA	TGGTGGTTTTGGACTTGATG	TTTCTTCTTCTGCCTTCCACA	59.4	626	INTERGENIC	--	--	--
Ca3_P1_34967661	Ca3	34967661	GTATTTTTATTTGATATATTTT/GTATTTT	TGGTGGTTTTGGACTTGATG	ATCTGTAATTGGCAGGCAGG	59.4	773	INTERGENIC	--	--	--
Ca3_P1_34970200	Ca3	34970200	CTTTTTTTT/CTTTTTTT	AACACCAACAGATTCCCCAG	CAAGCTAAAATGGCTTCCCA	59.8	234	INTRON	Ca_00772	--	GRF
Ca3_P1_34972470	Ca3	34972470	TA/T	ATGAAGCTTTGGATTGTGGG	TCCACAGCGTACCAACAAAA	59.9	491	INTRON	Ca_00772	--	GRF
Ca3_P1_34973875	Ca3	34973875	GTTTTTTTTTT/GTTTTTTTTTTTTTTTT	AATGGGTGGACCACTAGCAG	TCAAGGCTTGTTTGAGAGTTCA	60.0	772	DRR	Ca_00773	G	--

INDEL marker IDs	Chromosomes/ unanchored scaffolds	Physical positions (bp)	InDels (*Kabuli* reference genome-CDC Frontier/PI)	Forward primers (5'-3')	Reverse primers (5'-3')	Anneling temperature (0C)	Expected amplified product size (bp)	Structural annotation		Functional annotation	
								Sequence components of *kabuli* genome	*Kabuli* gene accession IDs	NCBI-KOG	TFs
Ca3_P1_34974019	Ca3	34974019	AAATTAATTA/AAATTA	TGTTGACTTGTTGTTG GGGA	TCAAGGCTTGTTTGAG AGTTCA	60.0	527	DRR	Ca_00773	G	--
Ca3_P1_34976076	Ca3	34976076	TC/T	AACGGAAAAACCATGA CAGC	CAATTCAGCAAAGCGC AATA	60.0	717	INTRON	Ca_00773	G	--
Ca3_P1_34976442	Ca3	34976442	TT/TTTAGTCCT	TATTGCGCTTTGCTGA ATTG	TGCTCATCATTGGATT TGGA	60.0	554	INTRON	Ca_00773	G	--
Ca3_P1_34976806	Ca3	34976806	G/GC	TATTGCGCTTTGCTGA ATTG	TGCTCATCATTGGATT TGGA	60.0	554	INTRON	Ca_00773	G	--
Ca3_P1_34982689	Ca3	34982689	T/TG	ATCCAATGATAACCCC GTGA	GTGGGACTTAGGCAC CCATA	60.0	479	INTRON	Ca_00773	G	--
Ca3_P1_34988591	Ca3	34988591	A/AT	AGAATGGCATGGATCG GTAG	TGTGAAACATTTGGCA GCTC	59.9	433	INTRON	Ca_00773	G	--
Ca3_P1_35002476	Ca3	35002476	TA/T	GCATTGGGAGAAAGCC AATA	TTCCATGATGGGGTTT CAAT	60.0	339	INTERGENIC	--	--	--
Ca3_P1_35011286	Ca3	35011286	TT/TTCT	AGGCCTGATGTTGGTT CATC	GGAAAGACAACCCTCA TGGA	59.9	664	INTRON	Ca_00775	Q	Nin-like
Ca3_P1_35014215	Ca3	35014215	AATT/AATTATT	GGAACAGCAAGCTCAT CTCC	GGTGGACCAGCAAAG ACACT	60.0	345	INTERGENIC	--	--	--
Ca3_P1_35014454	Ca3	35014454	T/TTA	GGAACAGCAAGCTCAT CTCC	AACACCCAACCCATTC TCCT	60.0	474	INTERGENIC	--	--	--
Ca3_P1_35015622	Ca3	35015622	GTCA/GTCATGTCATCA	AATTGCTTCACGGTCT TGCT	AACTTCTCTTATGCGC GTGT	59.9	272	INTERGENIC	--	--	--
Ca3_P1_35017729	Ca3	35017729	AAA/AAATATTTAA	TCAATGCCATGTTAAG GCAA	GCACTCCCATATGTAT TGGTAGAA	60.1	890	INTERGENIC	--	--	--

INDEL marker IDs	Chromosomes/ unanchored scaffolds	Physical positions (bp)	InDels (*Kabuli* reference genome-CDC Frontier/PI)	Forward primers (5'-3')	Reverse primers (5'-3')	Anneling temperature (0C)	Expected amplified product size (bp)	Structural annotation		Functional annotation	
								Sequence components of *kabuli* genome	*Kabuli* gene accession IDs	NCBI-KOG	TFs
Ca3_P1_35017784	Ca3	35017784	TAAGAA/TAAGAAGAA	TGACCGAAAAGTGGTCAAAA	GCCTAGAAAGTAGGGGTGTTACAA	59.1	760	INTERGENIC	--	--	--
Ca3_P1_35019374	Ca3	35019374	A/AG	CCCCTGAAAATCGTATCACAA	GCTGAGTTTAATGACCCGGA	59.8	765	DRR	Ca_00776	Q	E2F/DP
Ca3_P1_35021922	Ca3	35021922	TAGA/TA	TGCAACAAGCATCATTCCAT	GTTCTTTTTCTTGGCGCAAC	60.1	594	INTRON	Ca_00776	Q	E2F/DP
Ca3_P1_35023048	Ca3	35023048	TAAA/TAAAGAAA	CAAGCTTATGTGAGTGGCGA	CTTGTGGGACTTCCAGGTGT	60.0	411	INTRON	Ca_00776	Q	E2F/DP
Ca3_P1_35030161	Ca3	35030161	GC/GCTACTAAAGTTC	GATGTCCAACTCCGACGACT	GAAACCGGATTTTCCCACTT	60.1	370	INTERGENIC	--	--	--
Ca3_P1_35031349	Ca3	35031349	CAA/C	ACTCATCTGTTCCCACAGCC	ATTCATGATGGTCCCTCAGC	60.1	584	INTERGENIC	--	--	--
Ca3_P1_35032146	Ca3	35032146	A/AAACATCTT	GGAATCTCAATGCTGAGGGA	ACTGTCGATTCTCACCGGAT	60.2	563	INTERGENIC	--	--	--
Ca3_P1_3503254	Ca3	3503254	TC/TCCATACTTGCTAC	GTTTCTCTGAGCCCAGTTCG	CTTTGCCTCTTCCTGCAAAC	60.0	419	INTERGENIC	--	--	--
Ca3_P1_35033028	Ca3	35033028	GATATA/GATA	TTCAAACTAGTCACACCAACGG	TTTCATGTTGTTCCCCCAAT	60.1	477	DRR	Ca_00777	R	--
Ca3_P1_35033877	Ca3	35033877	GTTTTTTTTTTT/GTTTTTTTTTTTT	TGCTTCGGCTGTATTTTTCA	CACACACACACACACGAAGG	59.4	410	DRR	Ca_00777	R	--
Ca3_P1_35034968	Ca3	35034968	CATA/CA	GTCTACGACTCCCTGGTGGA	CAAGTGGAGCGTTTGTTTGA	60.1	564	INTRON	Ca_00777	R	--
Ca3_P1_35035644	Ca3	35035644	TA/TAA	GCTGAACTCCGGATTACCAA	TCATGCTGCTTCAAAGGTTG	60.1	701	INTRON	Ca_00777	R	--

INDEL marker IDs	Chromosomes/ unanchored scaffolds	Physical positions (bp)	InDels (*Kabuli* reference genome-CDC Frontier/PI)	Forward primers (5'-3')	Reverse primers (5'-3')	Anneling temperature (0C)	Expected amplified product size (bp)	Structural annotation		Functional annotation	
								Sequence components of *kabuli* genome	*Kabuli* gene accession IDs	NCBI-KOG	TFs
Ca3_P1_35036449	Ca3	35036449	AC/ATC	CACAATGGTGCCAAAAACAG	ACGGATGCCTATTGTTCAGG	60.0	566	INTRON	Ca_00777	R	--
Ca3_P1_35040031	Ca3	35040031	GA/GAA	CATTCCAACCACACTTCCCT	GAACAACCACCATCTTTGGG	59.8	521	INTRON	Ca_00777	R	--
Ca3_P1_35040555	Ca3	35040555	ATT/AAAAAAAAGTT	AGCAAGGCCATCTTTCTTCA	TCAAGCTCTGCATATGGGTG	60.0	259	INTRON	Ca_00777	R	--
Ca3_P1_35044914	Ca3	35044914	ACTTCATCTTCATCTTCATCT/ACTTCATCTTCATCT	ATGGTGTTAAATCGGAAGCG	TCAAAAGCGAGAAATGAAATGA	60.0	194	INTRON	Ca_00777	R	--
Ca3_P1_35048156	Ca3	35048156	T/TTG	TCTCAACGGACATCGTGTGT	CGGGATGCGACCATCTATTA	60.2	504	INTERGENIC	--	--	--
Ca3_P1_35048266	Ca3	35048266	TATA/T	TCTCAACGGACATCGTGTGT	CGGGATGCGACCATCTATTA	60.2	504	INTERGENIC	--	--	--
Ca3_P1_35048468	Ca3	35048468	GTTTATTATTTATTATTT/GTTTATTATTT	AGCTGATCGCAACTTAGGGA	TTATGGGCAAAATAGCAGGG	60.0	536	INTERGENIC	--	--	--
Ca3_P1_35049322	Ca3	35049322	ATAGTA/ATA	TCCCTGCTATTTTGCCCATA	GGGAAGCGTAGTGACATCGT	60.4	687	INTERGENIC	--	--	--
Ca3_P1_35049614	Ca3	35049614	CAAAAAAAA/CAAAAAAA	ACGATGTCACTACGCTTCCC	CAGCAATTTCCATCATCACG	60.1	334	INTERGENIC	--	--	--
Ca3_P1_35051711	Ca3	35051711	TTTT/TTTTATTT	TCCAATTTATAGGTCTTTTCGTTG	GACATCACCAAATCATCCCC	58.7	815	INTERGENIC	--	--	--
Ca3_P1_35051804	Ca3	35051804	CTT/CT	TCCAATTTATAGGTCTTTTCGTTG	GACATCACCAAATCATCCCC	58.7	815	INTERGENIC	--	--	--
Ca3_P1_35051853	Ca3	35051853	CTTTT/CTTTTT	TTTTTGACTGTTTTTCGATTGAG	GACATCACCAAATCATCCCC	58.5	316	INTERGENIC	--	--	--

INDEL marker IDs	Chromosomes/ unanchored scaffolds	Physical positions (bp)	InDels (*Kabuli* reference genome-CDC Frontier/PI)	Forward primers (5'-3')	Reverse primers (5'-3')	Anneling temperature (0C)	Expected amplified product size (bp)	Structural annotation		Functional annotation	
								Sequence components of *kabuli* genome	*Kabuli* gene accession IDs	NCBI-KOG	TFs
Ca3_P1_35051973	Ca3	35051973	AT/ATT	TTTTTGACTGTTTTTCGATTGAG	GACATCACCAAATCATCCCC	58.5	316	INTERGENIC	--	--	--
Ca3_P1_35053371	Ca3	35053371	GTTTTTTTTTTT/GTTTTTTTTT	TCGCACATATGATTCGTCATT	ACGGGACAAATGTTTTGACA	59.0	381	INTRON	Ca_00778	Q	E2F/DP
Ca3_P1_35061749	Ca3	35061749	A/AACTGT	TCAATACCCCAGCACCAAAT	TTCTGCCTTCCATTCAAACC	60.2	710	CDS (FRAME SHIFT)	Ca_00778	Q	E2F/DP
Ca3_P1_35074656	Ca3	35074656	TAAATAAAATAAAATA/TAAATAAAATAAAATAAAATA	GTGCCAGAGAATGAACCCAT	CAACCTTTTGTTTTTGCGGT	59.9	719	INTERGENIC	--	--	--
Ca3_P1_35077901	Ca3	35077901	ATTTTT/ATTTTTTT	GTCCATCACGACTTGGACCT	TGCAGTCCAGCATTGGTAAC	60.0	281	INTERGENIC	--	--	--
Ca3_P1_35079401	Ca3	35079401	CT/CTTCCTGT	CGTCTTCCTTCTGCTATGCC	ATGGGTGTGATTGGTTTGGT	60.0	330	CDS (large-effect mutations)	Ca_00780	--	bZIP
Ca3_P1_35084445	Ca3	35084445	TAA/TAAA	GTGGTCATGAGGGCAGATTT	CTGCAGGCAAAAACATTGAA	59.9	551	INTRON	Ca_00781	--	G2-like
Ca3_P1_35086678	Ca3	35086678	ATTTTTT/ATTTTT	TCTGCTTCATGTGGCAAAAG	CATGTTGGAGACTGCGATTG	60.0	487	INTRON	Ca_00781	--	G2-like
Ca3_P1_35086999	Ca3	35086999	TGTTGCAGTTGCAGTTG/TGTTGCAGTTGCAGTTGCAGTTG	TGTTGTGGTGGTGTTGTTCC	TCACTCATTTCGCGTTCTTG	60.3	458	CDS (large-effect mutations)	Ca_00781	--	G2-like
Ca3_P1_35090910	Ca3	35090910	ATTTTTTTT/ATTTTTTTTT	TTTTCGGGGTACAAATTGATG	AGCTTTGGTCGCAGACACTT	59.7	493	INTERGENIC	--	--	--
Ca3_P1_35091039	Ca3	35091039	GATAAAAATAA/GATAA	TTTTCGGGGTACAAATTGATG	AGCTTTGGTCGCAGACACTT	59.7	493	INTERGENIC	--	--	--
Ca3_P1_35091334	Ca3	35091334	GAAAAAA/GAAAAAAA	CAAAAGTGTCTGCGACCAAA	CGGCAGTTTGGTTCAGTTTT	59.9	380	INTERGENIC	--	--	--

INDEL marker IDs	Chromosomes/ unanchored scaffolds	Physical positions (bp)	InDels (*Kabuli* reference genome-CDC Frontier/PI)	Forward primers (5'-3')	Reverse primers (5'-3')	Anneling temperature (0C)	Expected amplified product size (bp)	Structural annotation		Functional annotation	
								Sequence components of *kabuli* genome	*Kabuli* gene accession IDs	NCBI-KOG	TFs
Ca3_P1_35093634	Ca3	35093634	AAAATAAATAAATAAATAAAT/AAAATAAATAAAT	TTTCATTTCAAATATATTCATGGCA	TATTGCGCGTCAAAATACCA	59.6	153	INTERGENIC	--	--	--
Ca3_P1_35093805	Ca3	35093805	TTA/T	ATCCAAATAGATGCATGCCC	TGGTCCATAATTTTGTTGTTTTT	59.8	245	INTERGENIC	--	--	--
Ca3_P1_35100602	Ca3	35100602	TAAAAAAA/TAAAAAAAA	TTATAAAGTTTGGGGCACCG	CAACAGCGGTACAATCATCG	59.8	701	INTERGENIC	--	--	--
Ca3_P1_35100722	Ca3	35100722	TAAAAAA/TAAAAAAA	AAAAAGAGCATAAAAACGAAAGC	CAACAGCGGTACAATCATCG	58.3	554	INTERGENIC	--	--	--
Ca3_P1_35101262	Ca3	35101262	TTAAAAAGTAAAA/TTAAAAAGTAAAAAGTAAAA	CGATGATTGTACCGCTGTTG	CGAAGGAAGTGTTATCCGAA	60.1	885	INTERGENIC	--	--	--
Ca3_P1_35101590	Ca3	35101590	CTTCAATTC/CTTC	TGCAATTATAAGACCATCGCA	CGAAGGAAGTGTTATCCGAA	59.2	591	INTERGENIC	--	--	--
Ca3_P1_35103816	Ca3	35103816	ATT/ATTTT	TATGTGCATCCAACATGTGC	GTACACACGGGCGATGTATG	58.9	693	INTERGENIC	--	--	--
Ca3_P1_35106644	Ca3	35106644	AATATATATATATA/AATATATATATA	TGTCAAATGGGAAAGGGAAG	TTTGCTGCAATACATTTTCAGG	59.9	608	INTRON	Ca_00783	S	--
Ca3_P1_35117225	Ca3	35117225	T/TA	GGTGCACCATACAATAACAACAA	CATGGCATCGTAACAACGAC	59.7	580	DRR	Ca_00785	G	C3H
Ca3_P1_35117240	Ca3	35117240	CTTT/CTT	GGTGCACCATACAATAACAACAA	CATGGCATCGTAACAACGAC	59.7	580	DRR	Ca_00785	G	C3H
Ca3_P1_35119008	Ca3	35119008	G/GCACTTTTA	AGTTGGACCCACTTTGTTGG	AAAATGGATGGGTCTGTTGC	59.9	545	INTRON	Ca_00785	G	C3H
Ca3_P1_35119260	Ca3	35119260	CAAA/CAAAA	AGTTGGGCAGCACAATTAGG	TTCACAACAGATTGGAAAGGG	60.1	565	INTRON	Ca_00785	G	C3H

INDEL marker IDs	Chromosomes/ unanchored scaffolds	Physical positions (bp)	InDels (*Kabuli* reference genome-CDC Frontier/PI)	Forward primers (5'-3')	Reverse primers (5'-3')	Anneling temperature (0C)	Expected amplified product size (bp)	Structural annotation		Functional annotation	
								**Sequence components of *kabuli* genome**	***Kabuli* gene accession IDs**	**NCBI-KOG**	**TFs**
Ca3_P1_35121317	Ca3	35121317	GC/G	TTAGGTGAATTTTGGGCAGG	TATCGTGATTGATCGTGCGT	59.9	451	INTERGENIC	--	--	--
Ca3_P1_35131690	Ca3	35131690	CG/C	GCGTGGAAATTCGTGTAATG	AGTGGGAACAACTTTTGAGCA	59.0	494	INTERGENIC	--	--	--
Ca3_P1_35134115	Ca3	35134115	CAAAAAAA/CAAAAAA	TGTTGAAAAACCTGCGTCTG	ACTAGGCTGTCTATCGGGGC	59.9	659	INTERGENIC	--	--	--
Ca3_P1_35135744	Ca3	35135744	CTTAAAAATTT/CTTAAAAATTTACTATTAAAAATTT	TTTCATGGTTGACATTCAATTTTT	CGTGTGCGTGCTAGTGGTAT	59.6	896	INTERGENIC	--	--	--
Ca3_P1_35136413	Ca3	35136413	CTAT/CTATATAT	ATACCACTAGCACGCACACG	TTTATGCATATCACATCAACAAAAA	59.8	660	INTERGENIC	--	--	--
Ca3_P1_35138044	Ca3	35138044	TA/TAA	TTTTCAAAATTGGCCGGT	ACCAAGAGAATCTGGCCCTT	58.9	265	INTERGENIC	--	--	--
Ca3_P1_35138675	Ca3	35138675	TAA/TAAA	AGGGCCAGATTCTCTTGGTT	GTCAGATGAATTCCCGCATC	60.1	563	INTRON	Ca_00787	G	C3H
Ca3_P1_35143342	Ca3	35143342	ATTT/ATTTT	CACATGCACTTTGGAGTTGG	TCTTCGTCTCCCTCCATTTC	60.2	428	DRR	Ca_00788	G	C3H
Ca3_P1_35147082	Ca3	35147082	T/TAAAGA	GTCGGAGCCAAAAATGAAAA	TGTGTTTCTTTCCAGCAGAGAC	60.1	317	INTERGENIC	--	--	--
Ca3_P1_35155087	Ca3	35155087	CAA/CA	ACACGTCCTTACCCCTACCC	CCTTTTGAAAGGAATTGGAAGA	60.1	173	INTERGENIC	--	--	--
Ca3_P1_35166785	Ca3	35166785	TAGAAAG/TAG	TGGCATGTGGTTTGTCATTT	AATGTGACCAACTTCCTCGG	59.8	859	INTERGENIC	--	--	--
Ca3_P1_35168074	Ca3	35168074	ATGT/AT	TGTCATTTGCACCAACAGGT	CAATTCAAAACACCTCACAATCA	60.0	536	INTERGENIC	--	--	--

INDEL marker IDs	Chromosomes/ unanchored scaffolds	Physical positions (bp)	InDels (*Kabuli* reference genome-CDC Frontier/PI)	Forward primers (5'-3')	Reverse primers (5'-3')	Anneling temperature (0C)	Expected amplified product size (bp)	Structural annotation		Functional annotation	
								Sequence components of *kabuli* genome	*Kabuli* gene accession IDs	NCBI-KOG	TFs
Ca3_P1_35173626	Ca3	35173626	GATATATAT/GATATATATAT	CGACATTAATTTGGACGTGA	GGGTTTGCCTTCATTTTTCA	57.1	738	DRR	Ca_00790	R	--
Ca3_P1_35176138	Ca3	35176138	GTTAATT/GTT	GAAAGAACACATGGAATGCG	ATTCAATTCTGCCATCTCGC	59.1	383	INTERGENIC	--	--	--
Ca3_P1_35176384	Ca3	35176384	ATGTT/AT	TGGCAGAATTGAATGTTTCG	GCTTTTTGTGAGCTGGGAAG	59.7	560	INTERGENIC	--	--	--
Ca3_P1_35176570	Ca3	35176570	TAAA/TAA	TGGCAGAATTGAATGTTTCG	GCTTTTTGTGAGCTGGGAAG	59.7	560	INTERGENIC	--	--	--
Ca3_P1_35178095	Ca3	35178095	CA/CATCAGA	TTCCAATTTTGAAATTTTCGC	GGTTGGGTTCTTCGAAATCA	59.1	318	INTERGENIC	--	--	--
Ca3_P1_35180182	Ca3	35180182	TAAAA/TAAA	ATTTTCCCAAATTGAACCCC	GGATCAGCCGATGATTATTTTT	59.9	607	INTERGENIC	--	--	--
Ca3_P1_35180444	Ca3	35180444	A/AAT	GGCCAACCTTTAAACAATCG	GGATCAGCCGATGATTATTTTT	59.5	477	INTERGENIC	--	--	--
Ca3_P1_35181524	Ca3	35181524	ATTT/ATT	ATGTCGGTCATTTACCCCAC	CTTTGCCTGCACCCATATTT	59.5	485	INTERGENIC	--	--	--
Ca3_P1_35186643	Ca3	35186643	TTATATATA/TTATATATATA	GCATGAGTGCATCAAGAACC	TGCCTTTTGTTTTAGTGCCC	59.3	802	DRR	Ca_00792	--	--
Ca3_P1_35187164	Ca3	35187164	GTCT/GTCTCT	GGGCACTAAAACAAAAGGCA	TCAAATCCTGTGGTTGTGGA	60.1	588	DRR	Ca_00791	--	bZIP
Ca3_P1_35190026	Ca3	35190026	TATTTCA/TATTTCAATTTCA	CGATCAAGCCAACCCATTAT	AGGAGATAGAGAGGCTGGGG	59.8	408	INTERGENIC	--	--	--
Ca3_P1_35191149	Ca3	35191149	T/TC	TTTTAGAGGGTTTTGGCGG	AACAAAAATCTTAATGTGACGCAA	60.1	579	INTERGENIC	--	--	--

INDEL marker IDs	Chromosomes/ unanchored scaffolds	Physical positions (bp)	InDels (*Kabuli* reference genome-CDC Frontier/PI)	Forward primers (5'-3')	Reverse primers (5'-3')	Anneling temperature (0C)	Expected amplified product size (bp)	Structural annotation		Functional annotation	
								Sequence components of *kabuli* genome	*Kabuli* gene accession IDs	NCBI-KOG	TFs
Ca3_P1_35191444	Ca3	35191444	CTT/CTTT	TTTCAAAATTCAACTCACAAACAGA	ACCATTTCAGGCCATCTTGA	60.1	813	INTERGENIC	--	--	--
Ca3_P1_35191903	Ca3	35191903	AT/AAAACTTTTT	ATCAAGATGGCCTGAAATGG	CCTCTTGTGGGAGGCCTTAT	59.9	149	INTERGENIC	--	--	--
Ca3_P1_35192442	Ca3	35192442	CATATATATATAT/CATATATAT	ATAAGGCCTCCCACAAGAGG	AACCTTTTAGATCAGTCCGAACC	60.5	663	INTERGENIC	--	--	--
Ca3_P1_3519617	Ca3	3519617	AGTTTAGGGTTTAGGGTTTA/AGTTTAGGGTTTAGGGTTTAGGGTTTA	GGGTTTAGGGTTTCGGGATA	ATCACGTCCATGTGGGTAGA	60.0	936	INTERGENIC	--	--	--
Ca3_P1_35198592	Ca3	35198592	ACAAAATTGAGTACCCAAAATTG/ACAAAATTG	ACCTACCTCTCGTCGGGTTT	ACCATCGGTGAAAGTGGAAG	60.0	668	INTRON	Ca_00793	--	GATA
Ca3_P1_35200631	Ca3	35200631	A/ATT	TCAAATTGTTCGCACATCAAA	AAAAGGCCCCTTCTTCAGAG	60.1	749	DRR	Ca_00793	--	GATA
Ca3_P1_35204680	Ca3	35204680	AT/ATCT	TGGCCAAATCACAAACCTATT	AATACCAATTTGGCGAGTGC	59.3	560	INTERGENIC	--	--	--
Ca3_P1_35205215	Ca3	35205215	A/ATC	GCACTCGCCAAATTGGTATT	TTTGAGTCCAATGGGTGTGA	60.0	316	DRR	Ca_00795	R	B3
Ca3_P1_3520633	Ca3	3520633	TA/TAAA	TGTCGAATCATACAGGCCAA	CGAACTGGGCTCAGAGAAAC	60.1	569	INTRON	Ca_21350	J	--
Ca3_P1_35213093	Ca3	35213093	CTTTT/CTTT	GCCATCACATTCATGACAAAA	TGATATTGCAAGAAACCCCA	59.4	758	INTERGENIC	--	--	--
Ca3_P1_35213687	Ca3	35213687	TAAAAAAAA/TAAAAAAAAAA	TGGGGTTTCTTGCAATATCA	TCACCCATGAACAATATTTTAGGA	59.0	556	INTERGENIC	--	--	--
Ca3_P1_35215168	Ca3	35215168	TTTTTCCTTTCATTTTCCTTT/TTTTTCCTTTCATTTTCCTTTCATTTTCCTTT	GCGAAGAAAAAGAGGTCGTG	GTAATCGACAGCCTGCAACA	60.0	316	INTRON	Ca_00796	C	--

INDEL marker IDs	Chromosomes/ unanchored scaffolds	Physical positions (bp)	InDels (*Kabuli* reference genome-CDC Frontier/PI)	Forward primers (5'-3')	Reverse primers (5'-3')	Anneling temperature (0C)	Expected amplified product size (bp)	Structural annotation		Functional annotation	
								Sequence components of *kabuli* genome	*Kabuli* gene accession IDs	NCBI-KOG	TFs
Ca3_P1_35218092	Ca3	35218092	GTTTTTTTTT/GTTTTTTTT	AAGCCTGCGCAAATAGTGTT	GGCAACGTATTGCTTCCATT	59.9	557	INTERGENIC	--	--	--
Ca3_P1_35227118	Ca3	35227118	AGG/AGGG	TGATCGTTGCCCATGTGTAT	TTGTGCATTAGGGGAAAACC	59.8	490	INTRON	Ca_00797	I	--
Ca3_P1_35227262	Ca3	35227262	TAAA/TAA	TGATCGTTGCCCATGTGTAT	TTTGTCGAGCAAAACAGCAG	59.8	762	INTRON	Ca_00797	I	--
Ca3_P1_35230228	Ca3	35230228	CAA/CAAA	ACTCGCCTAAATTCTGCACG	GAAACTCCACGCTGAAAAGC	60.4	502	INTERGENIC	--	--	--
Ca3_P1_35232760	Ca3	35232760	TCC/TC	AATTGATGGAGGTTTGCCTG	TGTTGACGAGTTGACGTGGT	59.9	557	DRR	Ca_00798	CU	--
Ca3_P1_35235106	Ca3	35235106	CAAA/CAAAA	TTCTTGATTGCAGCGTTGAG	AACAACTTTGGCGTGTGGAT	60.1	432	DRR	Ca_00799	M	--
Ca3_P1_35241980	Ca3	35241980	CTT/CTTAGTT	GGGACGCTCATACAATGGTT	CGCACAGTGAAGTGCAAAGT	59.8	331	INTRON	Ca_00801	--	--
Ca3_P1_35247466	Ca3	35247466	CTTTT/CTT	CAACGATCGCTTGAATGAGA	ACAACCGCCCAAAGATAGTG	59.9	239	INTERGENIC	--	--	--
Ca3_P1_35247528	Ca3	35247528	TTATATA/TTATA	CAACGATCGCTTGAATGAGA	ACAACCGCCCAAAGATAGTG	59.9	239	INTERGENIC	--	--	--
Ca3_P1_35247904	Ca3	35247904	CATATATAT/CATATAT	CACTATCTTTGGGCGGTTGT	TTCTCTGACCGTGAAAGTGG	60.0	382	INTERGENIC	--	--	--
Ca3_P1_35248340	Ca3	35248340	AATTATATT/AATT	ACCACTTTCACGGTCAGAGA	TACAATTTGCACTCCACCCA	58.3	870	INTERGENIC	--	--	--
Ca3_P1_35248471	Ca3	35248471	TTAT/TTATAT	AACAACATTTTTGTGTTGTTTCG	TACAATTTGCACTCCACCCA	59.0	847	INTERGENIC	--	--	--

INDEL marker IDs	Chromosomes/ unanchored scaffolds	Physical positions (bp)	InDels (*Kabuli* reference genome-CDC Frontier/PI)	Forward primers (5'-3')	Reverse primers (5'-3')	Anneling temperature (0C)	Expected amplified product size (bp)	Structural annotation		Functional annotation	
								Sequence components of *kabuli* genome	*Kabuli* gene accession IDs	NCBI-KOG	TFs
Ca3_P1_35251230	Ca3	35251230	GTTTTT/GTTTTTT	TTCGTCCCTCACCATACACA	GCCTCTCCACTTTACGATGC	60.0	303	INTERGENIC	--	--	--
Ca3_P1_35256982	Ca3	35256982	AT/ATT	CAAACATTTGACAACGGGTTTA	CGCACATGTAGATTGCGAAG	59.8	956	INTERGENIC	--	--	--
Ca3_P1_35257517	Ca3	35257517	TCCC/TCC	ATTGAGTTAACGCGTTGCAG	ACTCCTCATTCCCATACCCC	59.0	513	INTERGENIC	--	--	--
Ca3_P1_35260612	Ca3	35260612	ATTTT/ATTT	TGGATTTTGGGATGGTTTGT	GGGCAATCACATAGTGCCTT	60.0	509	DRR	Ca_00803	O	--
Ca3_P1_35260978	Ca3	35260978	TACT/TACTGTAACTGCACT	ATGGGAACCAATTTGTTGGA	GCCTTGTGTTTGGGCTATGT	60.0	537	DRR	Ca_00803	O	--
Ca3_P1_35261808	Ca3	35261808	CGTCATAATGTCATAATGT/CGTCATAATGT	ACATAGCCCAAACACAAGGC	GAACAGTTTCACCCCCAAAA	60.0	584	DRR	Ca_00803	O	--
Ca3_P1_35264280	Ca3	35264280	ACTC/ACTCTC	ATGTCAACGCACCTCCATTT	TTTCATATCAAGAAACTCCACAACA	60.4	478	INTERGENIC	--	--	--
Ca3_P1_35265195	Ca3	35265195	TC/TCC	TTGGAGTCAGGAAATATCCCA	GCAACGAGGATTGACCGTAT	59.4	820	INTERGENIC	--	--	--
Ca3_P1_35268599	Ca3	35268599	TTATATATATATATATATAT/TTATATATATATATATAT	CCTAGTTGGCCTGAAACCAC	AGATGAATCGGAAATCCACG	59.6	521	DRR	Ca_00804	--	--
Ca3_P1_35279420	Ca3	35279420	GAATAATA/GAATA	TCGGGATACTGGTGAAGGAG	GGCTATGGTATGGTTGGTGG	60.1	570	INTRON	Ca_00806	A	bZIP
Ca3_P1_35284220	Ca3	35284220	ACT/A	TCACGTTCAACGATTGGCTA	CCTCGCTGATGCTTGTTGTA	60.3	151	INTERGENIC	--	--	--
Ca3_P1_35289189	Ca3	35289189	CAAA/CAA	AAACGAAGCGGCAGTTAAGA	TTGCTGACTCGACATCAAGG	60.0	927	DRR	Ca_00808	U	--

INDEL marker IDs	Chromosomes/ unanchored scaffolds	Physical positions (bp)	InDels (*Kabuli* reference genome-CDC Frontier/PI)	Forward primers (5'-3')	Reverse primers (5'-3')	Anneling temperature (0C)	Expected amplified product size (bp)	Structural annotation		Functional annotation	
								Sequence components of *kabuli* genome	*Kabuli* gene accession IDs	NCBI-KOG	TFs
Ca3_P1_35291358	Ca3	35291358	GAAAAA/GAAAA	ATGCATGCAGATCAGACGAC	TGCACAAATGGGTTCTGTGT	59.8	283	INTRON	Ca_00808	U	--
Ca3_P1_35299387	Ca3	35299387	TAAA/TAAAA	CCTCTGGCATCGAAAGAAAG	ATTGAAGGCCCTCTACAGCA	59.9	639	INTRON	Ca_00809	T	--
Ca3_P1_35300047	Ca3	35300047	CAAAAAAA/CAAAAAA	AAAAGGAATGTTTGGCCTGA	GGCTGGCCTTCTGATGTTAG	59.5	773	INTRON	Ca_00809	T	--
Ca3_P1_35302305	Ca3	35302305	ATTTTTTTTT/ATTTTTTTTTTT	GAACTCCATGGGCACTTGTT	TTGGATTTGGGACATGGATT	60.0	708	INTERGENIC	--	--	--
Ca3_P1_35303259	Ca3	35303259	AAAG/A	ACTAACCATTCGCTGCCATC	CACCACCACCTCATTGTTTG	60.1	821	INTERGENIC	--	--	--
Ca3_P1_35303338	Ca3	35303338	TAAAAAAAAAA/TAAAAAAAA	AGTAAGGAATTGGGGAAGCC	CACCACCACCTCATTGTTTG	59.4	683	INTERGENIC	--	--	--
Ca3_P1_35303453	Ca3	35303453	A/AATTTTGAGATG	AGGAATTGGGGAAGCCAATA	CACCACCACCTCATTGTTTG	60.6	679	INTERGENIC	--	--	--
Ca3_P1_35303767	Ca3	35303767	GAAAAAA/GAAAAA	CAAACAATGAGGTGGTGGTG	CCCTCCCTCCAATTTGATTT	59.8	627	INTERGENIC	--	--	--
Ca3_P1_35304886	Ca3	35304886	G/GAAAT	GGCGTTCTGCCTTATTTTCA	TGCATGCTGCTTTTCTCAAG	60.2	816	INTERGENIC	--	--	--
Ca3_P1_35305215	Ca3	35305215	TTAT/TTATCATCATAT	GATGCGTACATGTGGTGGTT	TGAGTAGTTTCTTTGCGGGG	59.3	426	DRR	Ca_00810	A	--
Ca3_P1_35306575	Ca3	35306575	TAAAAAA/TAAAAA	GTTTGATTCGTACCGCCAGT	CCCTGCTGGGTGTAAAATGT	60.0	769	DRR	Ca_00810	A	--
Ca3_P1_35307396	Ca3	35307396	TAAA/TAA	CAATCATGCGATTTGCATTC	ATTTGGTTTTGCGATGAAGC	60.0	636	INTRON	Ca_00810	A	--

INDEL marker IDs	Chromosomes/ unanchored scaffolds	Physical positions (bp)	InDels (*Kabuli* reference genome-CDC Frontier/PI)	Forward primers (5'-3')	Reverse primers (5'-3')	Anneling temperature (0C)	Expected amplified product size (bp)	Structural annotation		Functional annotation	
								Sequence components of *kabuli* genome	*Kabuli* gene accession IDs	NCBI-KOG	TFs
Ca3_P1_35307898	Ca3	35307898	TAA/TAAA	GCTTCATCGCAAAACCAAAT	TATAATCGCAAAAATGCCGC	60.1	212	INTRON	Ca_00810	A	--
Ca3_P1_35307977	Ca3	35307977	AT/ATT	GCTTCATCGCAAAACCAAAT	TATAATCGCAAAAATGCCGC	60.1	212	INTRON	Ca_00810	A	--
Ca3_P1_35311817	Ca3	35311817	CTTTTT/CTTTTTT	TTCCGATCGAGTTGCAATAA	CTTTGTCGCTCCTCTCCAAC	59.3	518	INTERGENIC	--	--	--
Ca3_P1_35312037	Ca3	35312037	ATATCCT/AT	AGCATGCACAAAATAGCCAA	CTTTGTCGCTCCTCTCCAAC	59.3	328	INTERGENIC	--	--	--
Ca3_P1_35312512	Ca3	35312512	AACACACACA/AACACACA	GTTGGAGAGGAGCGACAAAG	TGCAAACACTCTCACGTTCA	60.0	637	INTERGENIC	--	--	--
Ca3_P1_35315660	Ca3	35315660	TAAAAAAA/TAAAAAA	GATGTTGTGTCATCCTTCGC	CAACCACGTGATCAAAGGTG	59.1	681	DRR	Ca_00811	--	--
Ca3_P1_35317314	Ca3	35317314	TATATAATATAATAT/TATATAATAT	TGACGTTGTTGGAGATGAAAA	TGTCGGTCCACTCTCTACCC	59.2	590	INTRON	Ca_00811	--	--
Ca3_P1_35319485	Ca3	35319485	TGG/T	GATGACGTGTTGGAGTGGTG	TGCACATGGTGGTTGATTTT	60.0	518	INTERGENIC	--	--	--
Ca3_P1_35319796	Ca3	35319796	TAAA/TAA	GCCACCAACTGTTCGAGTTT	TTTTTAGTGTACACATGTCGGC	60.2	533	INTERGENIC	--	--	--
Ca3_P1_35327371	Ca3	35327371	ATA/ATATTA	CATCGACAGGGTTTTCACCT	CTTAACAGCCCTGGGAATCA	60.0	594	INTRON	Ca_00812	Z	MYB_related
Ca3_P1_35329937	Ca3	35329937	TA/TAA	ATAATATGCGCCTGCTTGCT	TCCAAATCGGCTCGTAAAAC	59.9	473	INTRON	Ca_00812	Z	MYB_related
Ca3_P1_35334965	Ca3	35334965	GTTTTT/GTTTTTT	GAATTGGATCAACGGTTCGT	ATTAGCATAACCGCCATTCC	59.8	448	INTRON	Ca_00813	G	--

INDEL marker IDs	Chromosomes/ unanchored scaffolds	Physical positions (bp)	InDels (*Kabuli* reference genome-CDC Frontier/PI)	Forward primers (5'-3')	Reverse primers (5'-3')	Anneling temperature (0C)	Expected amplified product size (bp)	Structural annotation		Functional annotation	
								Sequence components of *kabuli* genome	*Kabuli* gene accession IDs	NCBI-KOG	TFs
Ca3_P1_35335583	Ca3	35335583	GAAA/GAA	TGGTGTCAAGAGGGACAGTTT	TGAATGTGAGTCGGGTTCAA	59.6	916	INTRON	Ca_00813	G	--
Ca3_P1_35336928	Ca3	35336928	GT/GTT	TCTAAAACTCTCCGCCCTTG	ACCGAACCTTTTGGGTTGTA	59.4	607	INTRON	Ca_00813	G	--
Ca3_P1_35339135	Ca3	35339135	GTTTTT/GTTTTTT	TTTTGGGCTTTTGACTTTGG	GCGGTTGAACTTGGGATTTA	60.1	539	INTRON	Ca_00813	G	--
Ca3_P1_35341956	Ca3	35341956	ACTTTCTT/ACTT	TGAGTCATTGATGAGGCAAAA	AAGGTTGTGTTTGAAACCGC	59.3	660	DRR	Ca_00813	G	--
Ca3_P1_35347719	Ca3	35347719	ATTTTT/ATTTTTT	GTCCTTATTGCGGACCACAT	CGTCAACAAAAGAAAGAAAATGC	59.8	493	DRR	Ca_00814	QR	B3
Ca3_P1_35349190	Ca3	35349190	A/AAAATAG	TTTGGAGATCGAAGCAAAGG	TTGCTTCCATTGGGAAGGT	60.3	280	DRR	Ca_00815	J	YABBY
Ca3_P1_35351387	Ca3	35351387	TC/TCCAAAC	CCCAGTTAGGGTTTTGACGA	CCGGGTAAAAGTTGGGTTTT	60.0	331	DRR	Ca_00816	R	--
Ca3_P1_35358226	Ca3	35358226	TA/T	CGCATCTCATTATCGAAGCA	CCTGCATATCATGTCTTGCG	59.9	323	INTERGENIC	--	--	--
Ca3_P1_35361414	Ca3	35361414	AAATAATAATAATAATAA/AAATAATAATAATAA	GGAAGAAGAAGCTACGCCAA	AGAACCAAACCAACCACTCG	59.6	696	INTERGENIC	--	--	--
Ca3_P1_35361616	Ca3	35361616	TTATC/TTATCCCCGCCCTACATATC	AAATCGAGTGGTTGGTTTGG	TTGTTTAAACCGTGATGAGTCAA	59.8	439	INTERGENIC	--	--	--
Ca3_P1_35362095	Ca3	35362095	ATATTATT/ATATTATTATT	CGAGTGGTTGGTTTGGTTCT	TGCTTAATTTTCAACAAAAGTTGC	60.0	789	INTERGENIC	--	--	--
Ca3_P1_35362688	Ca3	35362688	AATATTTTTCTATTT/A	AAGCAACTTTTGTTGAAAATTAAGC	CATCAACATTCATCTTTATGTCATTTT	59.4	791	INTERGENIC	--	--	--

INDEL marker IDs	Chromosomes/ unanchored scaffolds	Physical positions (bp)	InDels (*Kabuli* reference genome-CDC Frontier/PI)	Forward primers (5'-3')	Reverse primers (5'-3')	Anneling temperature (0C)	Expected amplified product size (bp)	Structural annotation		Functional annotation	
								Sequence components of *kabuli* genome	*Kabuli* gene accession IDs	NCBI-KOG	TFs
Ca3_P1_35362840	Ca3	35362840	ATTTTTT/ATTTT	AAGCAACTTTTGTTGAAAATTAAGC	CATCAACATTCATCTTTATGTCATTTT	59.4	791	INTERGENIC	--	--	--
Ca3_P1_35365941	Ca3	35365941	ATTTTTTTTT/ATTTTTTTTTTT	AACAACGGGGTGTCACCTAC	TTGGTGGGTATCTTGTGAAGC	59.7	681	DRR	Ca_00817	--	--
Ca3_P1_35367888	Ca3	35367888	ATAT/ATATTAAAATATTAT	CGGAGTGGTCGATAGTGGTT	TTGGATTGGTGGTGAGTTGA	60.0	572	INTERGENIC	--	--	--
Ca3_P1_35368592	Ca3	35368592	TA/T	TCAACTCACCACCAATCCAA	GCCGATTCTGAATATGTGTAAGG	59.9	617	INTERGENIC	--	--	--
Ca3_P1_35369772	Ca3	35369772	ATTTTTTTT/ATTTTTTT	TTTATGGTCCGCTTCCTCTT	TCATTTGTTTGACAATGTTTTGC	58.8	569	INTERGENIC	--	--	--
Ca3_P1_35370329	Ca3	35370329	TAAAA/TAAA	TTGCAAAACATTGTCAAACAAA	TGTGCTTGCCTAAATGTCCA	59.1	856	INTERGENIC	--	--	--
Ca3_P1_35370358	Ca3	35370358	ATATTATTATTATTATTATTA/ATATTATTATTATTATTA	TTGCAAAACATTGTCAAACAAA	TGTGCTTGCCTAAATGTCCA	59.1	856	INTERGENIC	--	--	--
Ca3_P1_35370619	Ca3	35370619	GAAAAAAAAA/GAAAAAAAA	AAAATGTTTTACAGTCCCGTTG	CCAAATCTTTGGCACTCGAT	58.1	388	INTERGENIC	--	--	--
Ca3_P1_35370943	Ca3	35370943	A/AATTATGTT	TGGAAATGCATCAATTGGAA	TCATTGTTGGACGTTGGTTT	59.9	170	INTERGENIC	--	--	--
Ca3_P1_35376752	Ca3	35376752	ATTT/ATTTT	TCACTCCTCAAGTGGAACCC	CCGGAACCAAACCCTTATTT	60.1	165	DRR	Ca_00818	--	NAC
Ca3_P1_35383780	Ca3	35383780	GT/GGTGGGAATT	ACTCACCTGATAGCATGGGC	TTCAGAGAACCATCCCCTTG	60.1	504	INTRON	Ca_00819	O	--
Ca3_P1_35390580	Ca3	35390580	CG/C	TAGATCGGACGGTCGAAATC	CACGCACCTAGAACTTGCAG	60.0	704	INTRON	Ca_00819	O	--

INDEL marker IDs	Chromosomes/ unanchored scaffolds	Physical positions (bp)	InDels (*Kabuli* reference genome-CDC Frontier/PI)	Forward primers (5'-3')	Reverse primers (5'-3')	Anneling temperature (0C)	Expected amplified product size (bp)	Structural annotation		Functional annotation	
								Sequence components of *kabuli* genome	*Kabuli* gene accession IDs	NCBI-KOG	TFs
Ca3_P1_35390727	Ca3	35390727	CAAA/CAA	CAGCTCCAGTGTGCTTCAAC	CACGCACCTAGAACTTGCAG	59.6	423	INTRON	Ca_00819	O	--
Ca3_P1_35393263	Ca3	35393263	CTTTTT/CTTTTTT	AAGGAGATGCATGCCCTATG	CACCATCCCATATTTGGACC	60.1	715	INTRON	Ca_00820	--	--
Ca3_P1_35395647	Ca3	35395647	TAAAAA/TAAAAAA	CATGCCCATGTCTAACAACG	AGTTTGGAGGTGGTGCACTT	60.0	540	DRR	Ca_00820	--	--
Ca3_P1_35395855	Ca3	35395855	TAAAA/TAAAAA	GCTCGCCAGGTTAATGAAGA	TGAAGACATGCAAATGTTTACCA	60.4	931	DRR	Ca_00820	--	--
Ca3_P1_35395949	Ca3	35395949	AG/A	AAGTGCACCACCTCCAAACT	TGAAGACATGCAAATGTTTACCA	59.6	575	DRR	Ca_00820	--	--
Ca3_P1_35396014	Ca3	35396014	CAAAAA/CAAAA	AAGTGCACCACCTCCAAACT	ATCTGTGTCGACCGAAAAGG	59.6	721	DRR	Ca_00820	--	--
Ca3_P1_35397271	Ca3	35397271	TGTC/T	GATTCTCTTCACCGCTGCTC	ACAAACCGTTATTTTCCGCA	60.1	663	INTRON	Ca_00821	KA	--
Ca3_P1_35403982	Ca3	35403982	ATCT/AT	TTCGCCTGCTCTCTTTTTGT	TTGGTCAAATCATTCAATCAGA	60.1	730	INTERGENIC	--	--	--
Ca3_P1_35409303	Ca3	35409303	TTAT/TT	TTCATTTTCACTGGCAGCAG	CCAGATGCGCACAGACTAAA	60.0	426	INTERGENIC	--	--	--
Ca3_P1_35418774	Ca3	35418774	GAAAAAA/GAAAAA	TCGCCCTGTCTGAGCTTTAT	CAGGGGTAACGAAAAGCAAA	60.0	355	INTRON	Ca_00824	S	--
Ca3_P1_35423352	Ca3	35423352	ATCTTCTT/ATCTT	TTGGGTAACATGCCAAAACA	CAGAACCGTTGGCATTTCTT	59.8	818	DRR	Ca_00824	S	--
Ca3_P1_35423411	Ca3	35423411	A/AAAT	AAAAGATAGGGGCCACAACA	CAGAACCGTTGGCATTTCTT	59.4	318	DRR	Ca_00824	S	--

INDEL marker IDs	Chromosomes/ unanchored scaffolds	Physical positions (bp)	InDels (*Kabuli* reference genome-CDC Frontier/PI)	Forward primers (5'-3')	Reverse primers (5'-3')	Anneling temperature (0C)	Expected amplified product size (bp)	Structural annotation		Functional annotation	
								Sequence components of *kabuli* genome	*Kabuli* gene accession IDs	NCBI-KOG	TFs
Ca3_P1_35432127	Ca3	35432127	ACTCTCT/ACTCT	TTCCCTTTTAGGCAGGGTTT	AATATTGCAATCGGGGATGA	59.9	366	INTERGENIC	--	--	--
Ca3_P1_35436004	Ca3	35436004	ATTTTTTTTT/ATTTTTTTT	GAAATAACGTTCCGCGTGTT	CATCATTGCCAAGTGCAAGT	60.0	454	INTERGENIC	--	--	--
Ca3_P1_35438296	Ca3	35438296	TG/TGG	GGTGTGTGCCCATTCCTAGT	GGAAAGGGTGTCTCAACGAA	59.9	243	INTERGENIC	--	--	--
Ca3_P1_35438547	Ca3	35438547	TTATATATATATAT/TTATATATATAT	TTCGTTGAGACACCCTTTCC	TGTGTGTGTCATGTTTATGATTTTG	60.1	545	INTERGENIC	--	--	--
Ca3_P1_35440193	Ca3	35440193	T/TCA	AACTGTGCAGCATATCCATAAA	CAAGGGCGGGTCTAGTATGT	57.4	410	INTERGENIC	--	--	--
Ca3_P1_35440856	Ca3	35440856	ATTTTTTTTTTTTTTTTT/ATTTTTTTTTTTTTT	TGTCAGGAGACCAGGAGAGG	CCGGGGTCTTTGATGACTAC	60.4	540	INTERGENIC	--	--	--
Ca3_P1_35444296	Ca3	35444296	A/AT	ATGAGGCCCAATAGAAACCC	CAGTCATCAAGGCGAATCAA	60.2	629	INTERGENIC	--	--	--
Ca3_P1_35451740	Ca3	35451740	ATTTT/ATTT	ACGGACATTAGGCGTGTCTC	GAAACGCAAGCATGACTGAA	60.1	454	INTERGENIC	--	--	--
Ca3_P1_35452484	Ca3	35452484	T/TGA	TCACACCACATGCTAGTTACCC	GCCTGACCTATTTCCACCCT	59.9	482	INTERGENIC	--	--	--
Ca3_P1_35455506	Ca3	35455506	AAAATA/AAAATAAATAAATA	TACAATTGGCGGCTTTTACC	AAACCCTCGATTCACACGTC	60.0	667	INTERGENIC	--	--	--
Ca3_P1_35457861	Ca3	35457861	TTATTCTATATAAATATTCTATATAAAT/TTATTCTATATAAAT	CGTGGAAGGAAGAGTTTTGG	TTTTGGTCTCAAGCCAGATTTT	59.7	543	INTERGENIC	--	--	--
Ca3_P1_35458127	Ca3	35458127	AACA/AA	CGTGGAAGGAAGAGTTTTGG	GAAACTCGTAAAAAGCCGCA	59.7	802	INTERGENIC	--	--	--

**INDEL marker IDs**	**Chromosomes/ unanchored scaffolds**	**Physical positions (bp)**	**InDels (*Kabuli* reference genome-CDC Frontier/PI)**	**Forward primers (5'-3')**	**Reverse primers (5'-3')**	**Anneling temperature (0C)**	**Expected amplified product size (bp)**	**Structural annotation**		**Functional annotation**	
								**Sequence components of *kabuli* genome**	***Kabuli* gene accession IDs**	**NCBI-KOG**	**TFs**
Ca3_P1_35459352	Ca3	35459352	CACC/CACCAAAACC	ACTCGCATACGATTTCACCC	GATCGGACAAATGAGGAGGA	60.0	528	INTERGENIC	--	--	--
Ca3_P1_35463051	Ca3	35463051	GTTTTTTTTTTTTTTT/GTTTTTTTT	ATTAGCGGTGAGTTTGGTGG	GAATCATGCTCCAACCCTGT	60.0	526	DRR	Ca_00828	--	--
Ca3_P1_35468557	Ca3	35468557	TG/T	CGAACCATGCTTTGGAGATT	GCAGATGGTCCTTTTTCAGC	60.1	472	INTERGENIC	--	--	--
Ca3_P1_35477744	Ca3	35477744	T/TC	GAACTCAATCCTAAATCAGCAACA	TATGCTAGGTGGCCTCTCGT	59.7	803	INTERGENIC	--	--	--
Ca3_P1_35479461	Ca3	35479461	ATTTTTTT/ATTTTTT	ATTTGCTCATTGATGGAGGC	ATAGGGTGGGAAGGGATGAG	60.0	473	DRR	Ca_00830	R	bHLH
Ca3_P1_35479811	Ca3	35479811	CTATGCAGTAT/CTATGCAGTATGCAGTAT	TTTGGTCCTCGCCACTTATC	AAATCCAACCGTTGATCCAG	60.1	608	DRR	Ca_00830	R	bHLH
Ca3_P1_35485860	Ca3	35485860	GAAAAAAA/GAAAAAA	AAGGGGTGCAACTAGCAAAA	AGACATGGACACTGATGCCA	59.7	435	INTRON	Ca_00831	R	--
Ca3_P1_35488328	Ca3	35488328	GAAA/GAA	CTCGCTGGAATTTGCTAACC	AGGACGTTGCTGCAAGATTT	59.8	502	INTRON	Ca_00832	S	--
Ca3_P1_35490841	Ca3	35490841	TAA/TA	CAAATCACGGTGCCCTACTT	ATGCATGTTGCACTGGTGAT	60.0	772	INTRON	Ca_00832	S	--
Ca3_P1_35502556	Ca3	35502556	ATTTTTT/ATTTTTTT	GCCTTGAAATAACCCAAGCA	CACTCCCAAAGGCAAATTGT	60.1	447	INTERGENIC	--	--	--
Ca3_P1_35502607	Ca3	35502607	TAAAAAAAAA/TAAAAAAAAAA	GCCTTGAAATAACCCAAGCA	CACTCCCAAAGGCAAATTGT	60.1	447	INTERGENIC	--	--	--
Ca3_P1_35504104	Ca3	35504104	AAT/AATAT	TTTTGAACCTTTTTGCACCC	ATATTTCGTTCAACGCTCGC	60.0	461	INTERGENIC	--	--	--

INDEL marker IDs	Chromosomes/ unanchored scaffolds	Physical positions (bp)	InDels (*Kabuli* reference genome-CDC Frontier/PI)	Forward primers (5'-3')	Reverse primers (5'-3')	Anneling temperature (0C)	Expected amplified product size (bp)	Structural annotation		Functional annotation	
								Sequence components of *kabuli* genome	*Kabuli* gene accession IDs	NCBI-KOG	TFs
Ca3_P1_35504410	Ca3	35504410	AT/ATTT	TTTTGAACCTTTTTGCACCC	ATATTTCGTTCAACGCTCGC	60.0	461	INTERGENIC	--	--	--
Ca3_P1_35505847	Ca3	35505847	TAAAA/TAAA	CCGTCCTCCAATATAACCCA	TGTTTGGAGGGAAAACAAGG	59.6	334	INTERGENIC	--	--	--
Ca3_P1_35524754	Ca3	35524754	AGTGTGTGT/AGTGTGT	CCATCGGAAAATCGGAAGTA	GCCCCATCCTCTCTTTATGTT	59.9	871	INTERGENIC	--	--	--
Ca3_P1_35524864	Ca3	35524864	GTCAAATGACTCAAAT/GTCAAAT	CCATCGGAAAATCGGAAGTA	TAGACTTGAGAGGCTTCGCC	59.9	894	INTERGENIC	--	--	--
Ca3_P1_35528890	Ca3	35528890	ATTAT/ATTATTTTTAT	AACGTCGACCAGCCTACACT	ATCCACACAAAACTCGGCAT	59.8	422	INTERGENIC	--	--	--
Ca3_P1_35531180	Ca3	35531180	GTA/GTATACAACTA	TCAACGGCATTGCATAATTT	TCGTTTTTGTCACAGTAAAACCT	59.0	510	DRR	Ca_00835	--	--
Ca3_P1_35532345	Ca3	35532345	TCTGCACC/TCTGCACCTGCACC	TTCGAGATGTCAGATTCCCC	CCAGTTTGCTTCTCTGGACC	60.0	348	DRR	Ca_00835	--	--
Ca3_P1_35532624	Ca3	35532624	TG/T	CACATAATGTTGGCGTGGTC	ACCGTATGTGGACTCTCGCT	59.8	797	INTERGENIC	--	--	--
Ca3_P1_35533699	Ca3	35533699	TAAAAAAAAAAAAA/TAAAAAAAAA	GCCATTCAAATTCGAAAAACA	CACGACGAATCGAACCTAAAA	59.9	431	INTERGENIC	--	--	--
Ca3_P1_35543329	Ca3	35543329	AATATATATAT/AATATATATATATATATAT	ACCCTCTCATGACCCAACAG	GTGCTGCACATTTTGGTGAC	60.0	552	DRR	Ca_00836	--	E2F/DP
Ca3_P1_35554678	Ca3	35554678	ACAA/ACAACTCAA	TTTGGACGGAAAAGTTCGAG	GAAGAAAATGTTGGGGCAGA	60.2	416	INTERGENIC	--	--	--
Ca3_P1_35557750	Ca3	35557750	CTT/CT	AGGGCAAACATCAATGTGGT	CCGCTTTGTGTGAAGTGCTA	60.2	813	INTERGENIC	--	--	--

INDEL marker IDs	Chromosomes/ unanchored scaffolds	Physical positions (bp)	InDels (*Kabuli* reference genome-CDC Frontier/PI)	Forward primers (5'-3')	Reverse primers (5'-3')	Anneling temperature (0C)	Expected amplified product size (bp)	Structural annotation		Functional annotation	
								Sequence components of *kabuli* genome	*Kabuli* gene accession IDs	NCBI-KOG	TFs
Ca3_P1_35558006	Ca3	35558006	TAA/TA	ATCCCGCGTAATTCCTCTCT	ATCCCCTAACATTTCCGACC	60.1	403	INTERGENIC	--	--	--
Ca3_P1_35558074	Ca3	35558074	AT/ATT	ATCCCGCGTAATTCCTCTCT	ATCCCCTAACATTTCCGACC	60.1	403	INTERGENIC	--	--	--
Ca3_P1_35562721	Ca3	35562721	A/AT	TGATCTATTTGGTGGATATGAGG	TGCAATAGGTAAAGCCACCT	58.0	795	INTERGENIC	--	--	--
Ca3_P1_35568276	Ca3	35568276	TAACA/TAACAACA	AGTTGGATGGACCTTTCGTG	ACACTGGTTGCTGAGTGCAG	60.0	914	DRR	Ca_00841	--	--
Ca3_P1_35568618	Ca3	35568618	AACGTACGT/AACGT	TGTGGCATGCAAATGCTTAT	TCCTGTGTTGTTGTGTCCGT	60.1	572	DRR	Ca_00841	--	--
Ca3_P1_35571015	Ca3	35571015	TAA/TAATTTACTTTTAGCAA	ATTTGCCCTCGTTTGTGAAC	ATCTAGTACAGGGCGTCCCC	60.0	585	INTERGENIC	--	--	--
Ca3_P1_35572051	Ca3	35572051	AAAAAGAAA/AAAA	TCCCTTCTCTCGTCATCCTC	AAAATCTTCGAAACATGAGTTGT	59.3	599	INTERGENIC	--	--	--
Ca3_P1_35574602	Ca3	35574602	ACT/AT	CAAAATGCGTCCACACTCAG	TGGTGTTTCCACAACACTCAAT	60.3	988	INTERGENIC	--	--	--
Ca3_P1_35574829	Ca3	35574829	AG/A	TTCAAGATTGAAAATGCTTTGTG	AATCGCATCGGCTAAACAAT	59.3	412	INTERGENIC	--	--	--
Ca3_P1_35580737	Ca3	35580737	CTTAATTAA/CTTAA	CGGTGTTGCAAAGAACTTGA	TCTTCTCCACCCTCCATAAAA	59.9	423	INTERGENIC	--	--	--
Ca3_P1_35584605	Ca3	35584605	TAATACAA/TAATACAAAATACAA	CATCCCTTTCATTTTCCCCT	TTTTTATGGGTTATTGGCCG	60.1	230	INTERGENIC	--	--	--
Ca3_P1_35591231	Ca3	35591231	TAAAA/T	CACACCTTTGAAGATGGGTCT	TGTCTCCGGATAGATGACATTG	59.1	287	DRR	Ca_00843	--	bZIP

INDEL marker IDs	Chromosomes/ unanchored scaffolds	Physical positions (bp)	InDels (*Kabuli* reference genome-CDC Frontier/PI)	Forward primers (5'-3')	Reverse primers (5'-3')	Anneling temperature (0C)	Expected amplified product size (bp)	Structural annotation		Functional annotation	
								Sequence components of *kabuli* genome	*Kabuli* gene accession IDs	NCBI-KOG	TFs
Ca3_P1_35591631	Ca3	35591631	T/TG	TCCGGAGACAAAATTTTCAA	ATTAAATGGGCCCTCGAGAC	58.2	544	DRR	Ca_00843	--	bZIP
Ca3_P1_35595513	Ca3	35595513	TTATA/TTATATA	GCTTTTTCCAAATTCGTGGA	ACATGACAATGGCTTGGTGA	60.1	670	DRR	Ca_00844	--	bZIP
Ca3_P1_35595652	Ca3	35595652	TTATATATATATATA/TTATATATATATA	GCTTTTTCCAAATTCGTGGA	ACATGACAATGGCTTGGTGA	60.1	670	DRR	Ca_00844	--	bZIP
Ca3_P1_35596803	Ca3	35596803	ATTTTTTTT/ATTTTTTTTT	GTCAACGCAAGCGAAAACTA	CGAAAAATCATCCGGAGAAA	59.1	524	DRR	Ca_00844	--	bZIP
Ca3_P1_35599210	Ca3	35599210	ATTTTTTT/ATTTTTTTTTT	TGATTGGGTGCGAAAATACC	AGGCCAATTTGGTTTGAAAG	60.7	872	DRR	Ca_00845	--	--
Ca3_P1_35599539	Ca3	35599539	TGTA/TGTACGTA	TCGCTAGATACAACCTCATTGG	GCCCAAAGCAAGCAACTAAA	59.2	330	DRR	Ca_00845	--	--
Ca3_P1_35600191	Ca3	35600191	CT/CTT	TTTAGTTGCTTGCTTTGGGC	CCAAGTGCGATGTTGAGTTTT	60.4	919	DRR	Ca_00845	--	--
Ca3_P1_35600454	Ca3	35600454	TAA/TAAA	AATTTCACAGTCCCGTCACA	CAATGCGACTGGGTTTACCT	59.0	334	DRR	Ca_00845	--	--
Ca3_P1_35602083	Ca3	35602083	CATGTTTTAT/CAT	GGGTGCGTTAGTGCATTTTT	GCCATGCTTTAACGAAGAGG	60.0	940	INTERGENIC	--	--	--
Ca3_P1_35607478	Ca3	35607478	AACACA/AACA	ACCGACATGCTTTAAGGTGG	CCTAAATCATCCAGGCTTGC	60.0	657	INTERGENIC	--	--	--
Ca3_P1_35614019	Ca3	35614019	ATTTTTTTTT/ATTTTTTTTTT	TGATCAAGATTTGAATTTTTGTTTTC	ATCCCCGTGTGTTGTTGTTT	59.8	256	INTERGENIC	--	--	--
Ca3_P1_35615422	Ca3	35615422	CTTT/CTTTT	GCTACCTGGATTGTTGGGAA	TGAATTTGTAGGGATGCATGA	59.9	965	DRR	Ca_00847	--	--

INDEL marker IDs	Chromosomes/ unanchored scaffolds	Physical positions (bp)	InDels (*Kabuli* reference genome-CDC Frontier/PI)	Forward primers (5'-3')	Reverse primers (5'-3')	Anneling temperature (0C)	Expected amplified product size (bp)	Structural annotation		Functional annotation	
								Sequence components of *kabuli* genome	*Kabuli* gene accession IDs	NCBI-KOG	TFs
Ca3_P1_35616363	Ca3	35616363	TAA/TAAA	CCGTAGTGGCTACAAGGGAA	CGCATCAAATTTTTGGGTTT	60.1	430	DRR	Ca_00847	--	--
Ca3_P1_35618022	Ca3	35618022	GTTTTTTTT/GTTTTTTT	TGCGTACCTTAATCAGGAACAA	TTTACGCGGGCGTATTTTAC	59.6	882	INTERGENIC	--	--	--
Ca3_P1_35618119	Ca3	35618119	T/TC	TGTGAAACGAACAAAGCTTAAA	TTTACGCGGGCGTATTTTAC	57.7	555	INTERGENIC	--	--	--
Ca3_P1_35620254	Ca3	35620254	CAA/CAATGAAAGAAA	GCTAGCGACATAAACGCACA	CGTTTCTTCCTCTGTCGCTC	60.0	191	DRR	Ca_00848	--	--
Ca3_P1_35621583	Ca3	35621583	AAAACATCATAGTAAACATCATAG/AAAACATCATAG	ATCAATTACGCACCTCCCAG	ATACTGGGGGTAATCCCGTC	60.0	872	DRR	Ca_00848	--	--
Ca3_P1_35621870	Ca3	35621870	AGGGGG/AGGGG	TGTCGATTAAGAAACATGCGA	GGATGTGAAAACCTCGTCGT	59.3	821	DRR	Ca_00848	--	--
Ca3_P1_35626631	Ca3	35626631	ATTTTTT/ATTTTTTT	TTGAATCTGAATCACCGCTTT	CCAAAAGGAGTATGGATCAGTAA	59.7	459	DRR	Ca_00850	--	--
Ca3_P1_35627331	Ca3	35627331	TTTA/T	CTGAAAACTCCGACACAAACA	TGCCTAATGGGTGGAAGCTA	58.8	238	DRR	Ca_00850	--	--
Ca3_P1_35627589	Ca3	35627589	AGTTACATAC/A	TGAAATGCTAGCTTCCACCC	GGGTTGTGGGAGAAGTTGAA	60.2	595	DRR	Ca_00850	--	--
Ca3_P1_35632758	Ca3	35632758	CAAAAAAAAAAA/CAAAAAAAA	ACCGGAACACGCTTTACAAC	CTTTTCCACCTCGATTTCCA	60.0	370	DRR	Ca_00851	--	--
Ca3_P1_35634064	Ca3	35634064	TAAAAAAA/TAAAAAA	TCACGAGGAAGTATGGGGAG	GCAATGAATGGTGCATTTTT	60.1	864	INTERGENIC	--	--	--
Ca3_P1_35635369	Ca3	35635369	GTTTTTTTTTT/GTTTTTTTT	TTGTAACCAAAATGTAGCCCAA	AACACCAGCGCCAAAATTAC	59.4	526	INTERGENIC	--	--	--

INDEL marker IDs	Chromosomes/ unanchored scaffolds	Physical positions (bp)	InDels (*Kabuli* reference genome-CDC Frontier/PI)	Forward primers (5'-3')	Reverse primers (5'-3')	Anneling temperature (0C)	Expected amplified product size (bp)	Structural annotation		Functional annotation	
								Sequence components of *kabuli* genome	*Kabuli* gene accession IDs	NCBI-KOG	TFs
Ca3_P1_35635776	Ca3	35635776	CTTTT/CTTT	GTAATTTTGGCGCTGGTGTT	GCGAATGCATAGAGCCTACC	60.0	635	INTERGENIC	--	--	--
Ca3_P1_35643302	Ca3	35643302	ATCT/A	TGGATGCCATACAACCAAAA	TAATCAGCCGACGAAACACA	59.8	491	INTRON	Ca_00852	S	--
Ca3_P1_35643731	Ca3	35643731	AAAAGC/A	TGTGTTTCGTCGGCTGATTA	TGGTTCGCTGTTTGTCACTC	60.3	747	INTRON	Ca_00852	S	--
Ca3_P1_35644626	Ca3	35644626	TTCTC/TTCTCTCTC	CTCCCAAGAGTGAGAGCACC	CAGTTGGAGGTACCACCGTT	60.0	269	INTRON	Ca_00852	S	--
Ca3_P1_35652779	Ca3	35652779	GTT/GTTTT	AAGTGTTTGGTGGTGGTGGT	CCACTGTCCAGTCTCTGCAA	60.2	499	INTRON	Ca_00853	--	--
Ca3_P1_35653670	Ca3	35653670	ATTTGCAGTTTGCAG/ATTTGCAG	TCCCCAGTAGCACTGGATTT	ATCAATTAAGGGTACCCGGC	59.6	338	INTRON	Ca_00853	--	--
Ca3_P1_35654304	Ca3	35654304	GAAAA/GAAA	GCCGGGTACCCTTAATTGAT	TGCATCCACCACACCTTTTA	60.0	702	DRR	Ca_00853	--	--
Ca3_P1_35654347	Ca3	35654347	GAT/GATAT	GCCGGGTACCCTTAATTGAT	TGCATCCACCACACCTTTTA	60.0	702	DRR	Ca_00853	--	--
Ca3_P1_35655389	Ca3	35655389	AT/ATT	GGTCGTTCTTCAGGAATGGA	TGAATCATCCAAGTGCCAAA	60.1	717	DRR	Ca_00853	--	--
Ca3_P1_35661405	Ca3	35661405	TGTGGTGGTGGTGGTGGT/TGTGGTGGTGGTGGTGGTGGTGGT	TTTAGCGACGGTAATGGACC	CAACCCAACTCTCACACCCT	60.0	513	INTERGENIC	--	--	--
Ca3_P1_35663002	Ca3	35663002	ATTTATTTTTAT/ATTTATTTTTATTTTTAT	CGGTCCTATGCCTTCAAAAA	TGAGGTAATGTGAAGAGTGCG	60.1	564	INTERGENIC	--	--	--
Ca3_P1_35665190	Ca3	35665190	TTATA/TTA	CTAGTGGTGGAGGGGTTCAA	GAATTTGACGGGATTCATGTG	60.0	286	INTERGENIC	--	--	--

**INDEL marker IDs**	**Chromosomes/ unanchored scaffolds**	**Physical positions (bp)**	**InDels (*Kabuli* reference genome-CDC Frontier/PI)**	**Forward primers (5'-3')**	**Reverse primers (5'-3')**	**Anneling temperature (0C)**	**Expected amplified product size (bp)**	**Structural annotation**		**Functional annotation**	
								**Sequence components of *kabuli* genome**	***Kabuli* gene accession IDs**	**NCBI-KOG**	**TFs**
Ca3_P1_35665406	Ca3	35665406	TAGAG/TAGAGAG	CTAGTGGTGGAGGGG TTCAA	CAATTTTTCACACGGA CAACA	60.0	505	INTERGENIC	--	--	--
Ca3_P1_35668235	Ca3	35668235	ATTTT/ATT	CTTCCAGCACATGGAA GGAT	GGTTTCATACAGAGGC CCAA	60.1	472	INTERGENIC	--	--	--
Ca3_P1_35668453	Ca3	35668453	ATTAGAA/ATTAGAATTTAGAA	TTGGGCCTCTGTATGA AACC	CATGCTCGTCAACGTG ACTT	59.9	258	INTERGENIC	--	--	--
Ca3_P1_35668840	Ca3	35668840	TG/T	TTCAAGTCACGTTGAC GAGC	GGAGAACTTGCATCTG CCAT	60.0	576	INTERGENIC	--	--	--
Ca3_P1_35669679	Ca3	35669679	T/TTATTG	TTTATGAGAGTGCTCG TGCG	TTTTCGGGCACATACT CCTC	60.2	410	INTERGENIC	--	--	--
Ca3_P1_35675509	Ca3	35675509	TAAAAA/TAAAA	GGTTCCATGGCTTTGT GAAT	CCACCAAGCAATTCGT ACCT	59.8	348	INTRON	Ca_00855	--	--
Ca3_P1_35678085	Ca3	35678085	TTCT/TT	TTGAGCACGACAGAAT CACC	ACAATGGCCCAACTCT GTTC	59.8	441	DRR	Ca_00855	--	--
Ca3_P1_35679181	Ca3	35679181	ATTTTTTTT/ATTTTTTT	TTCATTCTTTGGCTTG GGAC	CGTCATGGTCGTGGC TTTAT	60.1	959	DRR	Ca_00856	R	SBP
Ca3_P1_35681374	Ca3	35681374	A/ACAAT	GAGGCCGTTCTTGAAT TTTG	AGGCAATTCGCCATAA AGAT	59.7	525	INTRON	Ca_00856	R	SBP
Ca3_P1_35685429	Ca3	35685429	AA/AACA	GAAATAGGCAGAGACA GGTTCAA	TGTGAAGGAGAGATC GATTTTG	59.8	570	INTERGENIC	--	--	--
Ca3_P1_35687870	Ca3	35687870	TAAAAAAAA/TAAAAAAA	AAGTTTTCGGACAAGT CAAACA	TTCCACCATGCCACTT GATA	58.8	412	DRR	Ca_00857	J	--
Ca3_P1_35692535	Ca3	35692535	TAAAA/TAAA	GTGCAAATCTCCAATT CGGT	CACTCTATTTGGGCCG TGTT	59.9	615	DRR	Ca_00858	R	ZF-HD

INDEL marker IDs	Chromosomes/ unanchored scaffolds	Physical positions (bp)	InDels (*Kabuli* reference genome-CDC Frontier/PI)	Forward primers (5'-3')	Reverse primers (5'-3')	Anneling temperature (0C)	Expected amplified product size (bp)	Structural annotation		Functional annotation	
								Sequence components of *kabuli* genome	*Kabuli* gene accession IDs	NCBI-KOG	TFs
Ca3_P1_3569666	Ca3	3569666	TAAAAAAAAAAAAA/TAAAAAAAAAAAA	AACCTGAACGATGAGGGATG	GTTTTGGTGTTTCCCTTGGA	59.9	618	INTERGENIC	--	--	--
Ca3_P1_35697688	Ca3	35697688	TAAAAAAA/TAAAAAAAAA	TTTCATATCCACCCGTCAAA	TGAATTTGCCTAATTTGGTCC	58.8	628	INTERGENIC	--	--	--
Ca3_P1_35698718	Ca3	35698718	CA/CAA	CAGGCCACATTAATGCCATA	CCGATGGTTGAGAGATTCGT	59.4	364	INTERGENIC	--	--	--
Ca3_P1_35698781	Ca3	35698781	T/TG	ACGAATCTCTCAACCATCGG	TTGTTTGTCCTACCAGGAAGC	60.1	277	INTERGENIC	--	--	--
Ca3_P1_35699071	Ca3	35699071	CTGTAATGTAAT/CTGTAAT	ACGAATCTCTCAACCATCGG	TGAGGCTCTCTTTGTGACTTTG	60.1	711	INTERGENIC	--	--	--
Ca3_P1_35699306	Ca3	35699306	TAAAAA/TAAAAAA	GCTTCCTGGTAGGACAAACAA	TGAGGCTCTCTTTGTGACTTTG	59.2	455	INTERGENIC	--	--	--
Ca3_P1_35703116	Ca3	35703116	ATCTGTCTGT/ATCTGT	CGGATATGCGGACAAAAACT	TTAGCTTTGGGGTTTGATGG	60.0	600	INTERGENIC	--	--	--
Ca3_P1_35703336	Ca3	35703336	CAAAAAA/CAAAAAAA	CGGATATGCGGACAAAAACT	TTAGCTTTGGGGTTTGATGG	60.0	600	INTERGENIC	--	--	--
Ca3_P1_35703988	Ca3	35703988	CA/CATAATAA	CCATCAAACCCCAAAGCTAA	CACTGTTTCTTTCACAACTCTCG	59.9	942	INTERGENIC	--	--	--
Ca3_P1_35705840	Ca3	35705840	GAT/GATAT	TCAAAACATTCCACGACCAA	GTGGAAGAAATAGTGGCCGA	59.9	619	INTERGENIC	--	--	--
Ca3_P1_35706702	Ca3	35706702	AAAATTAAATT/AAAATT	TCGGCCACTATTTCTTCCAC	CGTTGTGCCTAAATTGGGAT	60.1	985	INTERGENIC	--	--	--
Ca3_P1_35707421	Ca3	35707421	GTTGTTAC/GTTGTTACTTGTTAC	GCCAAAAGTTGGAGCACAAT	CTCATCTTCCCCTTCCAACA	60.1	360	INTERGENIC	--	--	--

INDEL marker IDs	Chromosomes/ unanchored scaffolds	Physical positions (bp)	InDels (*Kabuli* reference genome-CDC Frontier/PI)	Forward primers (5'-3')	Reverse primers (5'-3')	Anneling temperature (0C)	Expected amplified product size (bp)	Structural annotation		Functional annotation	
								Sequence components of *kabuli* genome	*Kabuli* gene accession IDs	NCBI-KOG	TFs
Ca3_P1_35711521	Ca3	35711521	AG/A	AAACCACCGTCCCTAAATCC	TGCGGTTTGATTCCATTTTT	60.1	388	INTERGENIC	--	--	--
Ca3_P1_35715888	Ca3	35715888	TA/T	GGGACCACTTATTTAGGGGC	GTGGTTGGATGCCTGAGTTT	59.7	447	INTERGENIC	--	--	--
Ca3_P1_35715912	Ca3	35715912	GTATATATATATATATA/GTATATATATATATATATA	GGGACCACTTATTTAGGGGC	GTGGTTGGATGCCTGAGTTT	59.7	447	INTERGENIC	--	--	--
Ca3_P1_35721797	Ca3	35721797	GTCCCAT/GTCCCATCCCAT	TGGCTAATTGGGGATGAAGA	CTGGCATGACCCGAAGTATT	60.4	624	DRR	Ca_00860	--	--
Ca3_P1_35724316	Ca3	35724316	TAAA/TAAAA	GACCTTCCTGAGTGCGTAGC	AGATCCATTCGCTTTTCCCT	60.0	300	INTRON	Ca_00860	--	--
Ca3_P1_35725827	Ca3	35725827	TAAAAAA/TAAAAAAA	AAATCATAAGAACAAGGAGGATCG	GCACCCCTCCTTTAGAGTCC	59.9	549	INTERGENIC	--	--	--
Ca3_P1_35725900	Ca3	35725900	CTAAA/CTAAAAGTAAA	TGTGTGTGTCTACCAAAATACTCG	TTTCCACACTCTAATCGGGG	59.1	743	INTERGENIC	--	--	--
Ca3_P1_35726016	Ca3	35726016	ACT/ACTCT	GGACTCTAAAGGAGGGGTGC	TTTCCACACTCTAATCGGGG	60.1	326	INTERGENIC	--	--	--
Ca3_P1_35726533	Ca3	35726533	TTATATA/TTATATATATA	CCCCGATTAGAGTGTGGAAA	GCACTTTCACATGGTGTGGT	59.9	410	INTERGENIC	--	--	--
Ca3_P1_35729511	Ca3	35729511	ATTTTTTT/ATTTTTTTT	ATTGCACGGATGACACATTG	TTCTTTCAATTTTGCACGCT	60.4	526	INTERGENIC	--	--	--
Ca3_P1_35729686	Ca3	35729686	TAT/TATGCATGAT	CAATGACAACGATTTATCCCG	TGTTCCACCGTAAAAATCAACA	60.2	934	INTERGENIC	--	--	--
Ca3_P1_35730551	Ca3	35730551	CATT/CATTAATT	TTAAAAGGCCATGGGCATAA	TGGTGGTGCTGGACAATTAG	60.3	125	INTERGENIC	--	--	--

INDEL marker IDs	Chromosomes/ unanchored scaffolds	Physical positions (bp)	InDels (*Kabuli* reference genome-CDC Frontier/PI)	Forward primers (5'-3')	Reverse primers (5'-3')	Anneling temperature (0C)	Expected amplified product size (bp)	Structural annotation		Functional annotation	
								Sequence components of *kabuli* genome	*Kabuli* gene accession IDs	NCBI-KOG	TFs
Ca3_P1_35732982	Ca3	35732982	TC/T	CGGCATTCGGGTAAAGTTAG	AAATGAAAGGAAAGGGGAGA	59.6	643	INTERGENIC	--	--	--
Ca3_P1_35733605	Ca3	35733605	CTTT/CTT	TCTCCCCTTTCCTTTCATTT	AAAAACGTTAATTTGAGACAGCAA	57.7	360	INTERGENIC	--	--	--
Ca3_P1_35734692	Ca3	35734692	TATACATAC/TATAC	CTGCGTGCGTCATTGTATTC	TGTTCATCCCAAATTGAAAATG	60.3	860	INTERGENIC	--	--	--
Ca3_P1_35735323	Ca3	35735323	G/GA	AGCAGCCGAGATGCATAACT	TTCTTAACATGGTATCGGAACAA	60.0	503	INTERGENIC	--	--	--
Ca3_P1_35737476	Ca3	35737476	TA/T	TTTCAAAACACTCAAATATCCCA	TTGTTTTGGCACACTTCAAAA	58.5	593	INTERGENIC	--	--	--
Ca3_P1_35740020	Ca3	35740020	T/TA	CATGCTAGGGTTTGGAGGAA	TGAAAGGGGAAATTCACGAC	60.1	826	DRR	Ca_00861	--	CPP
Ca3_P1_35740342	Ca3	35740342	GTTTTTT/GTTT	GCCAATTCATTTCTAAGGTGG	TGAAAGGGGAAATTCACGAC	58.6	246	DRR	Ca_00861	--	CPP
Ca3_P1_35740803	Ca3	35740803	GATT/GATTAATT	GTCGTGAATTTCCCCTTTCA	TGCCGCATTGATTCATTTTA	59.9	547	DRR	Ca_00861	--	CPP
Ca3_P1_35743048	Ca3	35743048	GTTTT/GTTTTTT	GCAACTTGGGTTAGGTCCAA	TCGTCACATACAAAGCAATCA	60.0	541	INTERGENIC	--	--	--
Ca3_P1_35744042	Ca3	35744042	GAAAAAA/GAAAAAAAA	AATTTGGTCTTTGTCACAGTCA	GATGCTTTGATATGCCCTCG	57.2	701	INTERGENIC	--	--	--
Ca3_P1_35744681	Ca3	35744681	AACA/AACACA	TGTGGATGCCACACGATTTA	TGGGAGAAATGCATTGAAAA	60.9	462	INTERGENIC	--	--	--
Ca3_P1_35745191	Ca3	35745191	ATTTTTTTT/ATTTTTTTTTT	TTTCAATGCATTTCTCCCAA	AAACCATTTCTTTCTCGGAAAAC	59.1	527	INTERGENIC	--	--	--

INDEL marker IDs	Chromosomes/ unanchored scaffolds	Physical positions (bp)	InDels (*Kabuli* reference genome-CDC Frontier/PI)	Forward primers (5'-3')	Reverse primers (5'-3')	Anneling temperature (0C)	Expected amplified product size (bp)	Structural annotation		Functional annotation	
								Sequence components of *kabuli* genome	*Kabuli* gene accession IDs	NCBI-KOG	TFs
Ca3_P1_35745632	Ca3	35745632	TTAAGTAAG/TTAAG	TGTTTTCCGAGAAAGAAATGG	GGGTTTGTTATGCGACGAGT	59.2	401	INTERGENIC	--	--	--
Ca3_P1_35745687	Ca3	35745687	GTTTTTTTTT/GTTTTTTT	TGTTTTCCGAGAAAGAAATGG	GGGTTTGTTATGCGACGAGT	59.2	401	INTERGENIC	--	--	--
Ca3_P1_35745972	Ca3	35745972	ATTTTTTTT/ATTTTTTT	CCAACTCGTCGCATAACAAA	GGGTTTGAAGATGCCGTATG	59.7	108	INTERGENIC	--	--	--
Ca3_P1_35746055	Ca3	35746055	A/ACGT	ACTCGTCGCATAACAAACCC	TTCAGATCGAGCCCTAACAAA	60.0	236	INTERGENIC	--	--	--
Ca3_P1_35759102	Ca3	35759102	CAAAA/CAA	GGAACTTTTGGCATGCACTT	TTCACAGCTTCTGCAAATGG	60.1	410	INTRON	Ca_00862	D	--
Ca3_P1_35763405	Ca3	35763405	TCCAACCAACCAACC/TCCAACCAACC	TGTGTCTGCTTTGCACATGA	ACCAAAGTTTTGCACAAGGG	60.0	533	INTRON	Ca_00862	D	--
Ca3_P1_35767362	Ca3	35767362	AAAAA/AAAAAATAAAA	GGGTGCTTCCTCAATCTTCA	GACTTTGGAAGTCGGCAAAA	60.2	798	INTERGENIC	--	--	--
Ca3_P1_35767772	Ca3	35767772	CAAA/CAA	TTTTGTACTCACTTTTGCCGAC	CATGGTTTTCACCGAATGTG	59.3	491	INTERGENIC	--	--	--
Ca3_P1_35768780	Ca3	35768780	T/TC	CCTTATCCCCGAAATTTGAA	GCGGATAGCAAATCGTTCAT	58.9	271	INTERGENIC	--	--	--
Ca3_P1_35768860	Ca3	35768860	TAAAAAA/TAAAAAAA	CCTTATCCCCGAAATTTGAA	GCGGATAGCAAATCGTTCAT	58.9	271	INTERGENIC	--	--	--
Ca3_P1_35770921	Ca3	35770921	GTAAT/GT	GCATGGTGCAGTGAAAGAGA	AATTGAGGTCCCGTCAGTTG	60.0	448	DRR	Ca_00863	U	bZIP
Ca3_P1_35771124	Ca3	35771124	GTTAATT/GTT	ATGGCTGTTTTGGTTGAAGC	CCCAACTCACCCAAACTTCT	60.1	871	DRR	Ca_00863	U	bZIP

INDEL marker IDs	Chromosomes/ unanchored scaffolds	Physical positions (bp)	InDels (*Kabuli* reference genome-CDC Frontier/PI)	Forward primers (5'-3')	Reverse primers (5'-3')	Anneling temperature (0C)	Expected amplified product size (bp)	Structural annotation		Functional annotation	
								Sequence components of *kabuli* genome	*Kabuli* gene accession IDs	NCBI-KOG	TFs
Ca3_P1_35771869	Ca3	35771869	TAAA/TAAAA	AGAAGTTTGGGTGAGTTGGG	AAATACTCCCTCCCGTGTTG	59.0	763	DRR	Ca_00863	U	bZIP
Ca3_P1_35772573	Ca3	35772573	CAA/CAAA	CAACACGGGAGGGAGTATTT	ATTTTGACCTCGAGCCACAT	58.9	347	DRR	Ca_00863	U	bZIP
Ca3_P1_35772721	Ca3	35772721	ATCT/AT	ATGTGGCTCGAGGTCAAAAT	AAAATGTTACGTTGGCATATCA	59.6	238	DRR	Ca_00863	U	bZIP
Ca3_P1_35776584	Ca3	35776584	TTATATAT/TTATATATAT	GGTGGTTTTGCACTTTTGCT	TCACATACTCGCCAGACTGC	60.2	712	DRR	Ca_00865	R	G2-like
Ca3_P1_35776934	Ca3	35776934	CA/CAA	GCAGTCTGGCGAGTATGTGA	TGATGGCAACATTTTTCGAG	60.0	407	DRR	Ca_00864	L	--
Ca3_P1_35778885	Ca3	35778885	TC/TCC	CCTGTGCAACCTGGATTTTT	GTATGGCAGGCCAACTTTGT	60.0	238	INTRON	Ca_00865	R	G2-like
Ca3_P1_35779416	Ca3	35779416	CTTTTTTTTTTTTT/CTTTTTTTTTTT	GCAAATCCTCTGCTCCTGTC	TTCCATTTCACTCACCACCA	60.0	548	DRR	Ca_00866	K	NF-YA
Ca3_P1_35781578	Ca3	35781578	CAAAACAAAA/CAAA	CACTGCAATTGTTGACCACC	CCATGGAATCATTAGGCGTC	60.0	517	INTRON	Ca_00866	K	NF-YA
Ca3_P1_35783476	Ca3	35783476	ACTATTC/AC	TGGAAAGTCAACTCAATGCG	CTTACATCGAGCCGACACAA	59.8	745	INTRON	Ca_00866	K	NF-YA
Ca3_P1_35783609	Ca3	35783609	ATGT/ATGTGT	TGGAAAGTCAACTCAATGCG	CTTACATCGAGCCGACACAA	59.8	745	INTRON	Ca_00866	K	NF-YA
Ca3_P1_35784715	Ca3	35784715	TAAAAAA/TAAAAA	GGTGCTGCTCCTGAATTAGC	GGCTCCAATCTAATGGGGTT	60.0	798	INTERGENIC	--	--	--
Ca3_P1_35786958	Ca3	35786958	TTA/TTACCTA	AGCATTAGGGTGAATCGTGC	GTATGCATTGGGATGTGTGC	60.1	625	INTERGENIC	--	--	--

INDEL marker IDs	Chromosomes/ unanchored scaffolds	Physical positions (bp)	InDels (*Kabuli* reference genome-CDC Frontier/PI)	Forward primers (5'-3')	Reverse primers (5'-3')	Anneling temperature (0C)	Expected amplified product size (bp)	Structural annotation		Functional annotation	
								Sequence components of *kabuli* genome	*Kabuli* gene accession IDs	NCBI-KOG	TFs
Ca3_P1_35787562	Ca3	35787562	TTAGAATAATAGTAGTAATAGAATA/TTAGAATA	ATGGAGAAGCATGTCCCTTG	TCAATGGCCTAAGGTGAAGG	60.1	305	INTERGENIC	--	--	--
Ca3_P1_35788163	Ca3	35788163	GATAAATATTTTATAAA/GATAAATATTTTATAAATATTTTATAAA	CCTTCACCTTAGGCCATTGA	CACCTTGATTTTACCACACTCG	60.1	645	INTERGENIC	--	--	--
Ca3_P1_35789954	Ca3	35789954	TTAAATTATAAAT/TTAAATTATAAATTATAAAT	TCATTCCTAAAATGTGCCCC	TTGATGGGGAGATTCTCTTGA	59.8	269	INTERGENIC	--	--	--
Ca3_P1_35790927	Ca3	35790927	CTAATCAAATT/CTAATCAAATTAATCAAATT	AGCCTATGGGTATAGAGGTATCG	CGAATCGACCGATGAAACTT	57.8	377	INTERGENIC	--	--	--
Ca3_P1_35794433	Ca3	35794433	ATATATAGTA/ATA	TTTGCCGTATAAAGAACGGG	CCCACGCTCTCATCTTCTTC	60.0	563	INTERGENIC	--	--	--
Ca3_P1_35796430	Ca3	35796430	CTTTT/CTTT	GCAATCTTGAGCTGGAGACC	GGCACAAGTCAAATGGTCAA	60.0	909	INTERGENIC	--	--	--
Ca3_P1_35796459	Ca3	35796459	ATTTTTT/ATTTTTTT	ACAAGTTGGTGGGTTGGTTC	GGCACAAGTCAAATGGTCAA	59.7	541	INTERGENIC	--	--	--
Ca3_P1_35799677	Ca3	35799677	ATTTTT/ATTTT	TAACTGCATTTGATTGCGGA	TGCTTTTGCAAGTGTTTTTGA	60.2	610	INTERGENIC	--	--	--
Ca3_P1_35800062	Ca3	35800062	AAA/AAATAA	TCAAAAACACTTGCAAAAGCA	TCGTCAAATAACACGACAAGC	59.5	350	INTERGENIC	--	--	--
Ca3_P1_35801457	Ca3	35801457	CATTAATG/C	CCGAGTTGAATTGCATCAAA	TGGTGCCTCAATCATGGTAA	59.7	399	INTERGENIC	--	--	--
Ca3_P1_35801766	Ca3	35801766	TAA/TA	TTACCATGATTGAGGCACCA	CCTTGTTTGGTTTTGGTTGG	59.9	395	INTERGENIC	--	--	--
Ca3_P1_35802868	Ca3	35802868	TATGCATG/TATG	TTGGTTGTGCTAACATGGGA	CACACAGCTACCGACCAAAA	60.0	822	DRR	Ca_00867	O	--

INDEL marker IDs	Chromosomes/ unanchored scaffolds	Physical positions (bp)	InDels (*Kabuli* reference genome-CDC Frontier/PI)	Forward primers (5'-3')	Reverse primers (5'-3')	Anneling temperature (0C)	Expected amplified product size (bp)	Structural annotation		Functional annotation	
								Sequence components of *kabuli* genome	*Kabuli* gene accession IDs	NCBI-KOG	TFs
Ca3_P1_35803883	Ca3	35803883	TC/T	TGGAATGTTAGTGGTGCCAA	TGGTTTCCAATTGAAATAAAATACG	60.0	658	DRR	Ca_00867	O	--
Ca3_P1_35809791	Ca3	35809791	CTTTTT/CTTTTTT	CCGGAAGGAAAATTCCAAAT	TGCGGTTTGTTTTGTGTGTT	60.1	297	INTRON	Ca_00868	--	--
Ca3_P1_35811115	Ca3	35811115	TTTTTATTTTATTTTATTTTATTTTATTTTA/TTTTTATTTTATTTTATTTTATTTTA	TTTTGGGTTTCTCACTTGCC	TGGTAGTAAATCACTCGCAGTTG	60.1	455	INTERGENIC	--	--	--
Ca3_P1_35811467	Ca3	35811467	CTATAT/CTAT	TGAACAAATAAAATTCAACTGCG	TCTGAATTTCTCCCGTGGAT	59.2	450	INTERGENIC	--	--	--
Ca3_P1_35815533	Ca3	35815533	AAAATTTTAAGATGCGA/AA	TATTGGGGGTAGCTCAGTGC	AAAGATCGACAACAATGTCACG	60.1	731	INTERGENIC	--	--	--
Ca3_P1_35818398	Ca3	35818398	TCCGTCCTCACCGTCCTCACCGTC/TCCGTCCTCACCGTC	CAAGTCCCTTGCTCGTTCTC	CTGAAAAACCCACCTCCAAA	60.0	586	CDS (large-effect mutations)	Ca_00869	O	GeBP
Ca3_P1_35823679	Ca3	35823679	CAAAA/CAAAAAA	CAAAATTTCATTTATGCCCCA	GCCAAAATACCTCATGAAATCC	59.7	300	INTRON	Ca_00869	O	GeBP
Ca3_P1_35828873	Ca3	35828873	CTTT/CTTTT	TCAATTTCGTTCATCATCGG	TTCACATGCAATGCAAAACA	59.5	571	INTRON	Ca_00870	--	--
Ca3_P1_35833491	Ca3	35833491	TTA/T	TGTGAGAAAGCATCAAAACCT	CAAACTACGGTCGGTGTCCT	57.5	238	DRR	Ca_00871	--	--
Ca3_P1_35839944	Ca3	35839944	ATT/ATTTT	TGTAGCGGACAAAATTCAACC	TTGCAATTAGAGTTGACTTGAGG	60.0	214	DRR	Ca_00872	--	--
Ca3_P1_35848294	Ca3	35848294	GC/GCC	TAATCGCAACCTCCCATCTC	GAATGTTCAAGAAGGCTCGC	60.0	645	DRR	Ca_00873	S	--
Ca3_P1_35852072	Ca3	35852072	ATCGTTTGACGTTC/ATC	CCGGCCTGTCAGACCTAATA	TGGCCAACAAGACAATTTCA	60.1	639	INTERGENIC	--	--	--

INDEL marker IDs	Chromosomes/ unanchored scaffolds	Physical positions (bp)	InDels (*Kabuli* reference genome-CDC Frontier/PI)	Forward primers (5'-3')	Reverse primers (5'-3')	Anneling temperature (0C)	Expected amplified product size (bp)	Structural annotation		Functional annotation	
								Sequence components of *kabuli* genome	*Kabuli* gene accession IDs	NCBI-KOG	TFs
Ca3_P1_35852219	Ca3	35852219	AAGGAATTGACTAG/AAG	CCGGCCTGTCAGACCTAATA	TGGCCAACAAGACAATTTCA	60.1	639	INTERGENIC	--	--	--
Ca3_P1_35856021	Ca3	35856021	ATTTTTTTTTTTT/ATTTTTTTTTTTTTTT	CTTTGACCGAAGCATTAGGC	ACCCCAAAAAGGAGGTGTTC	59.8	435	INTERGENIC	--	--	--
Ca3_P1_35859927	Ca3	35859927	TGGG/TGGGG	GAAATCAATTGAAAGAAAAGCCA	TGTCAACAATGTTATCTTCACAAAAA	59.6	808	INTERGENIC	--	--	--
Ca3_P1_35859979	Ca3	35859979	ATCTTCTT/ATCTT	AAACGTCATAATTGCTTATGGG	TGGCTTTTGTTTGAAGATTTTG	58.1	205	INTERGENIC	--	--	--
Ca3_P1_35861277	Ca3	35861277	ATTTTTTTTT/ATTTTTTTT	TGCAAACTTGGAGAAAAGGG	ATGGAGACAAATGGCTGTCA	60.2	417	INTERGENIC	--	--	--
Ca3_P1_35861839	Ca3	35861839	CA/CAA	TCGATTCGAAGAAATGCAAG	TGGAAAATTTTTATGTTGCCG	59.0	500	INTERGENIC	--	--	--
Ca3_P1_35861894	Ca3	35861894	TAAAAAA/TAAAAAAA	ATACAGATGAGGCAGCCGTT	TGGAAAATTTTTATGTTGCCG	59.7	373	INTERGENIC	--	--	--
Ca3_P1_35862188	Ca3	35862188	AAAAAT/AAAAATCTCATGTAAAAT	ATACAGATGAGGCAGCCGTT	CGAGTTTGCGCAATAAAACA	59.7	790	INTERGENIC	--	--	--
Ca3_P1_35862589	Ca3	35862589	ACCC/ACC	TTCAACTGTTCTACGGCAACA	TGGGGTTTCCAAAAGTTCAG	59.4	654	INTERGENIC	--	--	--
Ca3_P1_35867669	Ca3	35867669	TGAAG/TG	TGAAGGCATTGAAGGTTTCC	CCATTGAGCTAGGCCTTACG	60.1	659	INTRON	Ca_00876	--	--
Ca3_P1_35873093	Ca3	35873093	TC/T	TTCAATGGGAATGGGAATGT	GTCTCCGACACCGACACTTT	60.0	663	INTERGENIC	--	--	--
Ca3_P1_35879113	Ca3	35879113	AATATATATATAT/AATATATATATATAT	TATCCCCGCGAAACATAAAA	TGGATCATCATGCAAAAATTG	60.3	258	INTERGENIC	--	--	--

INDEL marker IDs	Chromosomes/ unanchored scaffolds	Physical positions (bp)	InDels (*Kabuli* reference genome-CDC Frontier/PI)	Forward primers (5'-3')	Reverse primers (5'-3')	Anneling temperature (0C)	Expected amplified product size (bp)	Structural annotation		Functional annotation	
								Sequence components of *kabuli* genome	*Kabuli* gene accession IDs	NCBI-KOG	TFs
Ca3_P1_35879421	Ca3	35879421	CTTT/CTTTT	TATCCCCGCGAAACATAAAA	GGTACAAAACCAAAGGGGGT	60.3	734	INTERGENIC	--	--	--
Ca3_P1_35884614	Ca3	35884614	TA/TACTCTATTAAA	CCCATCTGAAAAGGGAGACA	TTGATTAACATTACACTACACTGCACA	60.0	715	INTERGENIC	--	--	--
Ca3_P1_35885490	Ca3	35885490	T/TTTTAAAA	TCAATTGATTGACGAAAGACTATTTT	TTTTTAATGTGAAGCGCACAA	59.5	891	INTERGENIC	--	--	--
Ca3_P1_35887062	Ca3	35887062	CT/CTAATAATT	GCAACATGAAAGCACGTTTG	CGTTGGAACTTGGAACCATT	60.3	507	INTERGENIC	--	--	--
Ca3_P1_35889000	Ca3	35889000	TAAAAT/TAAAATTAAAAAAGATGAAAAT	TGTGTTTTGAATATGTGGTGACA	TGGGGTCCTCTGTGTGTGTA	58.9	548	DRR	Ca_00880	--	--
Ca3_P1_35894033	Ca3	35894033	AACCTTTTACC/AACC	CACACAAATTGGTTGGTGCT	TCAAAAGTTTGACTCCTAAACGG	59.5	508	INTERGENIC	--	--	--
Ca3_P1_35894525	Ca3	35894525	GTATATATATATATA/GTATATATATATATATATATATA	TGAAACCTTTTACCAGACTCCAA	GTTAATCAAAGGGCCGACAA	60.0	952	INTERGENIC	--	--	--
Ca3_P1_35894994	Ca3	35894994	AT/ATT	TTGTCGGCCCTTTGATTAAC	AATTGGAGGACACTATGCCG	59.9	521	INTERGENIC	--	--	--
Ca3_P1_35895341	Ca3	35895341	CAAAAAA/CAAAAAAAA	TTTCCTTAGGACGCGAAGAA	CGGAAGTTCCAATGGAGAAA	60.0	553	INTERGENIC	--	--	--
Ca3_P1_35897729	Ca3	35897729	GTATCTA/GTATCTATCTA	AGCGAAAATGTGGCTGAAGT	TGCTGTGGAACAAGAACGAG	59.9	821	DRR	Ca_00881	DO	WRKY
Ca3_P1_35900260	Ca3	35900260	ATT/ATTTT	GTGACGTTGTATTGCGATGG	GGGGCTCTTATTTAGGGGTG	60.0	221	DRR	Ca_00882	Q	Nin-like
Ca3_P1_35902087	Ca3	35902087	ATAT/ATATATAT	GGTTGAATTTTCATCCACCG	TTTGGTTGTGTTTGATTTGGA	60.2	650	INTRON	Ca_00882	Q	Nin-like

INDEL marker IDs	Chromosomes/ unanchored scaffolds	Physical positions (bp)	InDels (*Kabuli* reference genome-CDC Frontier/PI)	Forward primers (5'-3')	Reverse primers (5'-3')	Anneling temperature (0C)	Expected amplified product size (bp)	Structural annotation		Functional annotation	
								Sequence components of *kabuli* genome	*Kabuli* gene accession IDs	NCBI-KOG	TFs
Ca3_P1_35902145	Ca3	35902145	ATTTT/ATTT	GGTTGAATTTTCATCCACCG	TTTGGTTGTGTTTGATTTGGA	60.2	650	INTRON	Ca_00882	Q	Nin-like
Ca3_P1_35907437	Ca3	35907437	AAT/AATTTAT	CAACCATCGAATGTTGTTCG	TGTTTGTGTGGGGTACATGG	60.0	582	INTRON	Ca_00882	Q	Nin-like
Ca3_P1_35908543	Ca3	35908543	ATTT/ATTTTT	CCCTTTCCGTTGTAGAACCA	TCGGAGAAATGACAAAAAGAAAA	60.0	925	INTRON	Ca_00882	Q	Nin-like
Ca3_P1_35908613	Ca3	35908613	CATATATAT/CATAT	AGAGGTGGTTGGTTGGTTTG	TCGGAGAAATGACAAAAAGAAAA	59.9	858	INTRON	Ca_00882	Q	Nin-like
Ca3_P1_35909062	Ca3	35909062	TAAA/TAA	TTTTCTTTTTGTCATTTCTCCGA	TGGCAATACGGTAACCACAA	60.1	551	INTRON	Ca_00882	Q	Nin-like
Ca3_P1_35909810	Ca3	35909810	ATTTTTT/ATTTTT	TTGTGGTTACCGTATTGCCA	CCAAACCATTCAGTGATGATG	59.8	699	INTRON	Ca_00882	Q	Nin-like
Ca3_P1_35910142	Ca3	35910142	A/ATG	TTTTTCGGTCTCGCATTTTT	TCTTTAAAATCGCCACCAGG	59.7	705	INTRON	Ca_00882	Q	Nin-like
Ca3_P1_35911527	Ca3	35911527	TAA/TAATGGAA	GTGAATGGTTGGATTTGGCT	TCGAATTATTTTTGAGTTGGTTTG	59.8	784	INTERGENIC	--	--	--
Ca3_P1_35911655	Ca3	35911655	AG/A	GCATGCGACTCATTTACCAA	TCGAATTATTTTTGAGTTGGTTTG	59.7	599	INTERGENIC	--	--	--
Ca3_P1_35915051	Ca3	35915051	TTATATAT/TTATAT	TTTCATCCAAGATTACCGGC	TGACAAAACAATCTGCACACAA	59.9	720	INTERGENIC	--	--	--
Ca3_P1_35915426	Ca3	35915426	ATCTCT/ATCT	GTCCTCGTCCTTCCTATCCC	CAAAGTCTGGCCTATTCCCA	59.9	648	INTERGENIC	--	--	--
Ca3_P1_35917238	Ca3	35917238	AC/A	ATCTTAGCCCACTCAATCCG	GTGTCGTAAGCCTCCTCGAA	59.2	522	INTERGENIC	--	--	--

INDEL marker IDs	Chromosomes/ unanchored scaffolds	Physical positions (bp)	InDels (*Kabuli* reference genome-CDC Frontier/PI)	Forward primers (5'-3')	Reverse primers (5'-3')	Anneling temperature (0C)	Expected amplified product size (bp)	Structural annotation		Functional annotation	
								Sequence components of *kabuli* genome	*Kabuli* gene accession IDs	NCBI-KOG	TFs
Ca3_P1_35918318	Ca3	35918318	GCCCCCC/GCCCCCCC	TTTTGCTGAGATGAAC CGTG	AATCCTCCTCCTTCCT TCCA	59.8	623	INTERGENIC	--	--	--
Ca3_P1_35918699	Ca3	35918699	GTTTTTTTTT/GTTTTTTTTTT	TGGAAGGAAGGAGGA GGATT	CCAAACTGATCACCAT CCCT	60.0	620	INTERGENIC	--	--	--
Ca3_P1_35919265	Ca3	35919265	CC/CCGATTTTC	GGATCCGACCCAGTTG TTTA	GAAACTCCATGAGAGC TCGG	59.8	502	INTERGENIC	--	--	--
Ca3_P1_35928726	Ca3	35928726	AATATATATATAT/AATATATATAT	GGCATGGCTACTCAGC TCTT	TTCAGTTGCACCTCAA CGTC	59.6	801	DRR	Ca_00886	R	NAC
Ca3_P1_35928795	Ca3	35928795	ATTTTTTT/ATTTTTT	GATGGGCATCATTAGT GTTTGA	TTCAGTTGCACCTCAA CGTC	59.8	336	DRR	Ca_00885	--	--
Ca3_P1_35935420	Ca3	35935420	GTTT/GTT	TGGAACCTGGGTTGAG GTAG	GAAAATTGACCCAAAA GCCA	60.0	633	DRR	Ca_00888	--	--
Ca3_P1_35938883	Ca3	35938883	GTCTCTCTCTCTCT/GTCTCTCTCTCT	TTGTTTGGTTTGGAGG GAAG	TATGGGGGAAATGATG GTGT	59.9	317	INTERGENIC	--	--	--
Ca3_P1_35939043	Ca3	35939043	TTACC/T	TTGTTTGGTTTGGAGG GAAG	TATGGGGGAAATGATG GTGT	59.9	317	INTERGENIC	--	--	--
Ca3_P1_35939562	Ca3	35939562	T/TA	ACACCATCATTTCCCC CATA	TTTCCAGGAGATCACG AAGG	59.9	930	INTERGENIC	--	--	--
Ca3_P1_35943363	Ca3	35943363	ATTTTTTTTT/ATTTTTTTTTTTT	CTCAATTCGGTTGGTC GTTT	ACACAATATGGGAGTT TTTGCT	60.0	589	INTERGENIC	--	--	--
Ca3_P1_35944137	Ca3	35944137	ATTTTTTT/ATTTTTT	TGTAGTCAAACGCACA CGAA	GAAAATGCCATGGTTT CCAC	58.9	365	INTERGENIC	--	--	--
Ca3_P1_35946096	Ca3	35946096	T/TC	AAAAGCAAAAGTCGGG TGTG	ACCCTTGAGCTTCGAA AACA	60.1	406	INTRON	Ca_00889	J	--

INDEL marker IDs	Chromosomes/ unanchored scaffolds	Physical positions (bp)	InDels (*Kabuli* reference genome-CDC Frontier/PI)	Forward primers (5'-3')	Reverse primers (5'-3')	Anneling temperature (0C)	Expected amplified product size (bp)	Structural annotation		Functional annotation	
								Sequence components of *kabuli* genome	*Kabuli* gene accession IDs	NCBI-KOG	TFs
Ca3_P1_35947883	Ca3	35947883	CTTT/CT	TCCTGTCTAGCCGTAGTCCTG	TTGAAATTTTGACCGCATCA	59.5	434	DRR	Ca_00889	J	--
Ca3_P1_35948027	Ca3	35948027	A/AC	TCGATGCACATGTGATGTTG	CAGGTTTCGGAGCTAAGGTG	60.1	296	DRR	Ca_00889	J	--
Ca3_P1_35952084	Ca3	35952084	TAAAAAAAAA/TAAAAAAAAAAA	TTATTCCGTGAAAGAACCGC	CGTACGTGCGCCTAAATGAT	60.1	620	INTERGENIC	--	--	--
Ca3_P1_35955953	Ca3	35955953	ATAAACTATTTTAA/ATAA	CGGTTGATTGTGAATTTGTGA	AGCTTGATCCCATTTTGGTG	59.4	700	DRR	Ca_00890	V	--
Ca3_P1_35959985	Ca3	35959985	CAAAAAAAAAAAAAAA/CAAAAAAAAAAAA	TCTGAACAAATGATCGGGTG	ACATGCAATTTCCATCCACA	59.5	164	DRR	Ca_00891	V	GRAS
Ca3_P1_35960562	Ca3	35960562	AGTTTGATT/AGTTTGATTGTTTGATT	TCCGACTTGGTAAAACAGCA	TTTGGACGAAATAAATTTGAAAAA	59.3	398	DRR	Ca_00891	V	GRAS
Ca3_P1_35960868	Ca3	35960868	TAA/TAAA	GGATGCTACATTTCTTGGTGG	TGAAGGAGGGAGAAACATGG	59.4	835	DRR	Ca_00891	V	GRAS
Ca3_P1_35963451	Ca3	35963451	AAA/AAATAA	AGCTGTCTCAAGCACCGAAT	GACATCACAGTTCTGTGTCTAGCTT	60.0	272	INTERGENIC	--	--	--
Ca3_P1_35965206	Ca3	35965206	ATTTTTT/ATTTTT	TGATTGGTGCTTTTGTTGGA	TGCTATTGGCACCACCACTA	60.1	106	INTERGENIC	--	--	--
Ca3_P1_35967790	Ca3	35967790	GAAAATAGAAA/GAAAATAGAAAACAAAATAGAAA	TTGATTATGAGCACCAAGCG	TGTCTGGATTTTTGTTTTTGAGA	59.8	335	DRR	Ca_00892	C	--
Ca3_P1_35973469	Ca3	35973469	AC/A	TGCCAGTGATCATCGAAAAA	AGGGGCAAAGGTAACAAAGC	60.2	374	INTRON	Ca_00892	C	--
Ca3_P1_35973692	Ca3	35973692	CG/C	TGCCAGTGATCATCGAAAAA	TTCTGTGAAGGATGCAGCAG	60.2	778	INTRON	Ca_00892	C	--

INDEL marker IDs	Chromosomes/ unanchored scaffolds	Physical positions (bp)	InDels (*Kabuli* reference genome-CDC Frontier/PI)	Forward primers (5'-3')	Reverse primers (5'-3')	Anneling temperature (0C)	Expected amplified product size (bp)	Structural annotation		Functional annotation	
								Sequence components of *kabuli* genome	*Kabuli* gene accession IDs	NCBI-KOG	TFs
Ca3_P1_35974404	Ca3	35974404	GAA/GGAAA	TAGCAACATCTGCTGCATCC	ATCACACGGGAAATCTGAGC	60.0	721	INTRON	Ca_00892	C	--
Ca3_P1_35976313	Ca3	35976313	TTTTA/T	TAATGAACCAGAGCAGCGTG	AGGGTCACACTTTATCCCCC	60.0	514	INTRON	Ca_00892	C	--
Ca3_P1_35981391	Ca3	35981391	CAAAA/CAAAAA	CTCTCCCCTCCAAGTCTTCC	TTGGGTCCATTTGGTTTGAT	60.2	166	DRR	Ca_00893	--	--
Ca3_P1_35982476	Ca3	35982476	TGG/TG	TCGAAAAGAACTTTATCCGCA	TGAATTGCTGTAGCCTGTGG	59.8	408	DRR	Ca_00893	--	--
Ca3_P1_35982520	Ca3	35982520	AG/A	TCGAAAAGAACTTTATCCGCA	TGAATTGCTGTAGCCTGTGG	59.8	408	DRR	Ca_00893	--	--
Ca3_P1_35986133	Ca3	35986133	TACTATATT/TACTATATTTTAATAACTATATT	CCTTCATCAGCTCGGAGAAA	TTGGAAGCAAAGAATCCACC	60.5	379	INTRON	Ca_00893	--	--
Ca3_P1_35987287	Ca3	35987287	TAAATAAAATAAAATAAAATAAAATAAAA/TAAATAAAATAAAATAAAA	ACACAACACATACCTGGGCA	CGCGTTCACTTTCCTTCTTC	59.9	467	INTERGENIC	--	--	--
Ca3_P1_35990406	Ca3	35990406	ACTG/A	TGAATTTACACCCTGCCCTC	GCTCGAGATGAAGGGACTTG	59.9	331	INTERGENIC	--	--	--
Ca3_P1_35992570	Ca3	35992570	TAAAAA/TAAAAAA	TATTTCCGCTTTGCAACCTC	TTCAGGGGACTTGTGCTTCT	60.2	204	DRR	Ca_00894	--	--
Ca3_P1_35993956	Ca3	35993956	TAAAAAAAAAA/TAAAAAAAAAAAAA	TGGGTTCGAATCAATATCTTACAA	AAATCTTGATGGAATGAAACATCA	59.7	341	INTERGENIC	--	--	--
Ca3_P1_35994489	Ca3	35994489	CT/CTAAATATT	TGAATGATGTTTCATTCCATCA	GAATTCGGAACAAAACGTTCA	58.8	311	INTERGENIC	--	--	--
Ca3_P1_35994788	Ca3	35994788	TCAC/T	AAATTTGGTGCTCCATGGTC	GTGCTCCATTTTCTTCACGG	59.8	464	INTERGENIC	--	--	--

INDEL marker IDs	Chromosomes/ unanchored scaffolds	Physical positions (bp)	InDels (*Kabuli* reference genome-CDC Frontier/PI)	Forward primers (5'-3')	Reverse primers (5'-3')	Anneling temperature (0C)	Expected amplified product size (bp)	Structural annotation		Functional annotation	
								Sequence components of *kabuli* genome	*Kabuli* gene accession IDs	NCBI-KOG	TFs
Ca3_P1_36000767	Ca3	36000767	TTTATATTATATTATATTAT/TTTATATTATATTAT	TGTGTCCAAAAACTTATTTACCGA	TGGTGATGGCTTTGTGGTTA	59.8	514	INTERGENIC	--	--	--
Ca3_P1_36005419	Ca3	36005419	AATGGACTATGGATGGAC/AATGGAC	GTTACTGGAATTTGGGGCCT	CATCAACTTCTTTGGCAGCA	60.2	459	INTRON	Ca_00896	OPR	--
Ca3_P1_36005517	Ca3	36005517	TAAAA/TAAAAA	GTTACTGGAATTTGGGGCCT	CATCAACTTCTTTGGCAGCA	60.2	459	INTRON	Ca_00896	OPR	--
Ca3_P1_36006199	Ca3	36006199	TCATGGCATGGC/TCATGGC	GGTTGCTGAGCCAACTCTTC	ATGTTGAATTTCTGCCAGCC	60.0	549	INTRON	Ca_00896	OPR	--
Ca3_P1_36011212	Ca3	36011212	ATT/ATTT	CCAGTTACCGCCAACTGTTT	AGCTATGCTAGCGAAAACGC	60.0	559	DRR	Ca_00898	O	CAMTA
Ca3_P1_36016442	Ca3	36016442	TAAAA/TAAA	ATGCTCAACCTCCACGTTTC	CAACCCATCGAATTTGGTTC	60.1	444	DRR	Ca_00900	O	CAMTA
Ca3_P1_36019541	Ca3	36019541	AT/ATGT	ACCTTTGGATTCATGGTTGG	CCGATAGCCATTTGGACACT	59.6	614	INTERGENIC	--	--	--
Ca3_P1_36019725	Ca3	36019725	GAAA/GAA	ACCTTTGGATTCATGGTTGG	CCGATAGCCATTTGGACACT	59.6	614	INTERGENIC	--	--	--
Ca3_P1_36020115	Ca3	36020115	ATTTTT/ATTTT	AGTGTCCAAATGGCTATCGG	TCACTAACGCAACCTTCACCT	60.0	487	INTERGENIC	--	--	--
Ca3_P1_36020456	Ca3	36020456	CAAA/CAAAA	ACCACAACCGTCCAATGTTT	TTTCAAGATGCTTGACGAAGAA	60.1	736	INTERGENIC	--	--	--
Ca3_P1_36020478	Ca3	36020478	ATT/ATTT	AGGTGAAGGTTGCGTTAGTGA	TCAAATTGTGTGGGAGAAACA	59.8	536	INTERGENIC	--	--	--
Ca3_P1_36021252	Ca3	36021252	GAT/G	GTTTGCGGATTTGGTTGAGT	TATTTTAGGACCGCCTGACG	60.0	442	DRR	Ca_00901	O	CAMTA

INDEL marker IDs	Chromosomes/ unanchored scaffolds	Physical positions (bp)	InDels (*Kabuli* reference genome-CDC Frontier/PI)	Forward primers (5'-3')	Reverse primers (5'-3')	Anneling temperature (0C)	Expected amplified product size (bp)	Structural annotation		Functional annotation	
								Sequence components of *kabuli* genome	*Kabuli* gene accession IDs	NCBI-KOG	TFs
Ca3_P1_36022210	Ca3	36022210	AAATAATA/AAATAATAATA	TTGAAACTGAAACTTCTCCCAA	TGGAGATGGAAATGGAGAGG	58.9	833	DRR	Ca_00901	O	CAMTA
Ca3_P1_36023752	Ca3	36023752	GCAATATTACACAAT/GCAAT	TGCACGATTGAAATGTGTGA	CGATCGAGGGTTGGTATGTT	59.7	611	INTRON	Ca_00901	O	CAMTA
Ca3_P1_36026486	Ca3	36026486	CATATATATATAT/CATATATATATATATATAT	ATGCACTTAGATTCGCCGTT	ACGAAATTGAAACCTGCACC	59.7	249	INTERGENIC	--	--	--
Ca3_P1_36031164	Ca3	36031164	TG/T	TCATCAAATCCGTAACGCAA	CCGACCTGCAAATATCATGTAA	60.1	417	INTERGENIC	--	--	--
Ca3_P1_36032382	Ca3	36032382	ATTTTTTTTT/ATTTTTT	GCTTTGCTCCCACATCTCTC	AACGCTGAACCAAAATGACC	60.0	217	DRR	Ca_00902	S	bZIP
Ca3_P1_36032624	Ca3	36032624	CAAA/CAAAA	GGTCATTTTGGTTCAGCGTT	CCATGCATGAATCCTTGAAA	60.0	614	DRR	Ca_00902	S	bZIP
Ca3_P1_36032758	Ca3	36032758	ATTTTTT/ATTTTTTT	GGTCATTTTGGTTCAGCGTT	TCGATTGGATAGTGCCATCA	60.0	705	DRR	Ca_00902	S	bZIP
Ca3_P1_36033507	Ca3	36033507	TAGAGA/TAGAGAGA	CTTCTCACCCCCTCAAAACA	AGGCTTGATGACACGTGATG	60.1	696	DRR	Ca_00902	S	bZIP
Ca3_P1_36040732	Ca3	36040732	ATTTT/ATTT	CCACCACTGCAGAAGTGAGA	TCCGAACTGGAGAGTGTGAA	60.0	461	INTRON	Ca_00902	S	bZIP
Ca3_P1_36041204	Ca3	36041204	ATT/AT	TTCACACTCTCCAGTTCGGA	AAGTCGACGTTTGTGAAGAGTTC	59.4	699	INTERGENIC	--	--	--
Ca3_P1_36043188	Ca3	36043188	AC/ATC	TGGAATGAATTAAACCATCACG	GCCACAAAATCATGCTGAAA	59.7	245	INTERGENIC	--	--	--
Ca3_P1_36044328	Ca3	36044328	CAT/CATAT	TGTCAAATATCAAGGGGGATG	TGATGCTTTAAGCCGTCACA	59.6	541	INTERGENIC	--	--	--

INDEL marker IDs	Chromosomes/ unanchored scaffolds	Physical positions (bp)	InDels (*Kabuli* reference genome-CDC Frontier/PI)	Forward primers (5'-3')	Reverse primers (5'-3')	Anneling temperature (0C)	Expected amplified product size (bp)	Structural annotation		Functional annotation	
								Sequence components of *kabuli* genome	*Kabuli* gene accession IDs	NCBI-KOG	TFs
Ca3_P1_36045650	Ca3	36045650	ATT/ATTT	TGTGATTGAAAAGATG TGTCAAAA	GGGGTACGGAGTGGA GGTAT	59.5	622	INTERGENIC	--	--	--
Ca3_P1_36049035	Ca3	36049035	CAAAA/CAAA	ACTTATCCTCTGCTTC GGCA	CAGTTCGGTTTGGTTT TCGT	60.0	473	INTRON	Ca_00903	A	--
Ca3_P1_36051861	Ca3	36051861	TAA/TAAAAA	CCGCTATCTGAACCGA ACAT	TGTATGGGTCGTGCAT TGAT	60.1	793	DRR	Ca_00903	A	--
Ca3_P1_36052998	Ca3	36052998	A/AC	GTGTTCACGCTATCGG GTTT	TGGTGGATTGTGGATT TCCT	60.0	372	INTERGENIC	--	--	--
Ca3_P1_36053206	Ca3	36053206	TAAAAAA/TAAAAAAA	GTGTTCACGCTATCGG GTTT	TCTGGCAAACAACCCT TTTT	60.0	499	INTERGENIC	--	--	--
Ca3_P1_36053289	Ca3	36053289	ATGTATCTT/AT	CAAATGGATGCGCTCA CTTA	TCGATTTTACTCATCT CGCAAA	59.8	642	INTERGENIC	--	--	--
Ca3_P1_36053346	Ca3	36053346	CG/C	CAAATGGATGCGCTCA CTTA	AACGGGATTAGGGATT TTGG	59.8	887	INTERGENIC	--	--	--
Ca3_P1_36053714	Ca3	36053714	C/CTCGGT	AAAAAGGGTTGTTTGC CAGA	AACGGGATTAGGGATT TTGG	59.6	581	INTERGENIC	--	--	--
Ca3_P1_36073350	Ca3	36073350	TAATAAATAAATA/TAATAAATAAATAA ATA	CCCAAACTATGTCGCC CTAA	GGTGGATTCTTTGCCT TTGA	60.0	488	INTRON	Ca_00905	R	HSF
Ca3_P1_36076083	Ca3	36076083	ATAAAA/ATAAAAATTAAAA	TGTCAAAATTTGGATT GCCA	CCTTGTTGAAACAAAT GCAAGA	59.9	539	INTERGENIC	--	--	--
Ca3_P1_36076512	Ca3	36076512	AGAATT/A	GCATTTGTTTCAACAA GGTAAGG	TTTGGAGGACAAAAAC TGGG	59.9	395	INTERGENIC	--	--	--
Ca3_P1_36086627	Ca3	36086627	AC/ACC	ATTCCAACGGAAAACA TCCA	CTCCCAAACAAAGCCT ACGA	60.2	605	INTRON	Ca_00907	I	--

INDEL marker IDs	Chromosomes/ unanchored scaffolds	Physical positions (bp)	InDels (*Kabuli* reference genome-CDC Frontier/PI)	Forward primers (5'-3')	Reverse primers (5'-3')	Anneling temperature (0C)	Expected amplified product size (bp)	Structural annotation		Functional annotation	
								Sequence components of *kabuli* genome	*Kabuli* gene accession IDs	NCBI-KOG	TFs
Ca3_P1_36086749	Ca3	36086749	CACAAC/CACAACAAC	ATTCCAACGGAAAACATCCA	CTCCCAAACAAAGCCTACGA	60.2	605	INTRON	Ca_00907	I	--
Ca3_P1_36088731	Ca3	36088731	TATTAATTAATTA/TATTAATTAATTAATTA	CAGCCGTGGGTAATCTGTCT	TCCAAAACAAAACAAAGCCC	60.1	757	INTERGENIC	--	--	--
Ca3_P1_36089072	Ca3	36089072	GAAAAAAAAAAA/GAAAAAAAAAA	CACCTACTAAAAAGCGTGCTGA	TCCAAAACAAAACAAAGCCC	59.6	146	INTERGENIC	--	--	--
Ca3_P1_36089763	Ca3	36089763	TGAG/TG	TACGTTTAAGTGTTGGCCCC	AAACTGGTTTGAGTGACGGC	59.9	786	INTERGENIC	--	--	--
Ca3_P1_36089824	Ca3	36089824	AAT/AATTCCATCTAT	GGGATCAACAAGTAAAAGCTCC	AAACTGGTTTGAGTGACGGC	59.1	709	INTERGENIC	--	--	--
Ca3_P1_36090153	Ca3	36090153	AGTGTGTGTG/AGTGTGTG	ATGAATCCATTTGGCGCTAC	GAAGCGTCAAGTATCCTCCG	59.9	541	INTERGENIC	--	--	--
Ca3_P1_36090792	Ca3	36090792	ATTTTT/ATTTTTT	CCCCGTAACGGAGGATACTT	TTCAAATAGATCGTGCACAAAAA	60.2	618	INTERGENIC	--	--	--
Ca3_P1_36092177	Ca3	36092177	CAAAAAAAAA/CAAAAAAAAAA	ACATTCGTGACACGGTGAAA	GGTCTAGCCTATTCCACCCC	60.0	756	INTERGENIC	--	--	--
Ca3_P1_36096471	Ca3	36096471	ATTTT/ATTT	TGAAAGACCCAAGAGAAGAGG	TCAATTTTTGTGTTCCTCCCTT	58.5	462	INTERGENIC	--	--	--
Ca3_P1_36098742	Ca3	36098742	TC/T	TATGCTGATTCGGCCATTTT	CTCGGTTGTTGTTGGAGGTT	60.4	379	DRR	Ca_00908	K	--
Ca3_P1_36099581	Ca3	36099581	TAAA/TAAAAAA	CATTTCGGAGGTAGGTGGAA	TGCCTTACTGATTTGCCTCC	59.9	421	DRR	Ca_00908	K	--
Ca3_P1_36101033	Ca3	36101033	CCAAC/CCAACAAC	TATTCATGCAGCAGCGAAAC	TATTGCGTCCTCACAGCAAG	60.0	527	INTRON	Ca_00908	K	--

INDEL marker IDs	Chromosomes/ unanchored scaffolds	Physical positions (bp)	InDels (*Kabuli* reference genome-CDC Frontier/PI)	Forward primers (5'-3')	Reverse primers (5'-3')	Anneling temperature (0C)	Expected amplified product size (bp)	Structural annotation		Functional annotation	
								Sequence components of *kabuli* genome	*Kabuli* gene accession IDs	NCBI-KOG	TFs
Ca3_P1_36102139	Ca3	36102139	TCC/T	GGTGCATGATGGCCTC TATT	TTACGATGAATTTTTC GCCC	59.9	905	INTRON	Ca_00908	K	--
Ca3_P1_36102329	Ca3	36102329	TAAAAA/TAAAA	CCTGCAGGCAAGCAAA TAAT	GGTATTGTGGTGGGAT GGTC	60.2	486	INTRON	Ca_00908	K	--
Ca3_P1_36102523	Ca3	36102523	GAAAAA/GAAAA	TACCTGTGAACCCACC ATCA	GTATTGTGGTGGGATG GTCC	59.8	319	INTRON	Ca_00908	K	--
Ca3_P1_36107876	Ca3	36107876	ATTTT/ATTT	CAATCCCTTTAACTTC GCCA	GGTGGGTGACAATGAT TTCC	60.1	658	INTERGENIC	--	--	--
Ca3_P1_36109027	Ca3	36109027	CTTTTT/CTTTTTT	TCCATGCCATCAAAAT CAAA	TTTCGTGAAGGCATAC TCCA	59.9	316	DRR	Ca_00909	J	--
Ca3_P1_36109313	Ca3	36109313	TTATC/TTATCCTTCTATGTATC	TGGAGTATGCCTTCAC GAAA	TGCCGTGGTTAAGGG AATAG	59.3	652	DRR	Ca_00909	J	--
Ca3_P1_36109369	Ca3	36109369	CTTG/CTTGGTTG	TGGAGTATGCCTTCAC GAAA	TGCCGTGGTTAAGGG AATAG	59.3	652	DRR	Ca_00909	J	--
Ca3_P1_36111673	Ca3	36111673	CTT/CTTT	CTAGGGTTTTTCTTTC GCCC	GATGCATTGCAATTTG TTCA	60.1	462	INTERGENIC	--	--	--
Ca3_P1_36113689	Ca3	36113689	G/GA	TGTCCGTTGAAAGGAG GACT	GCCACTTGCTTCTGCA CATA	59.7	214	DRR	Ca_00910	--	--
Ca3_P1_36114251	Ca3	36114251	CTACATCTTTTTTA/CTACATCTTTTTT ACATCTTTTTTA	TATGTGCAGAAGCAAG TGGC	TTCCACCCAAATCAAC ACAA	60.0	678	INTRON	Ca_00910	--	--
Ca3_P1_36114572	Ca3	36114572	GAAAAAAAA/GAAAAAAAAAA	CATTTTCGACTCCTCC CAAA	CCATTTGGCACGTGTT ACAG	60.0	842	INTRON	Ca_00910	--	--
Ca3_P1_36119763	Ca3	36119763	ACTT/ACTTCTT	GCATATTCATCACCCC CATC	TCCTTCTTGCAGGTGC TTTT	60.0	376	CDS (large-effect mutations)	Ca_00911	D	--

INDEL marker IDs	Chromosomes/ unanchored scaffolds	Physical positions (bp)	InDels (*Kabuli* reference genome-CDC Frontier/PI)	Forward primers (5'-3')	Reverse primers (5'-3')	Anneling temperature (0C)	Expected amplified product size (bp)	Structural annotation		Functional annotation	
								Sequence components of *kabuli* genome	*Kabuli* gene accession IDs	NCBI-KOG	TFs
Ca3_P1_36120455	Ca3	36120455	TGGG/TGG	AAAGCACCTGCAAGAAGGAA	GGCTTGGTCTCTTCCAATCA	60.0	479	INTRON	Ca_00911	D	--
Ca3_P1_36120818	Ca3	36120818	TA/TAA	TGATTGGAAGAGACCAAGCC	CAATGGTGCAACAAACTTGG	60.2	680	INTRON	Ca_00911	D	--
Ca3_P1_36123248	Ca3	36123248	ACAG/ACAGGAGTAGCAG	TTGTTGTAGGGGAGACTGGG	TACTAGCCGATTTTGTGGGG	60.0	549	INTERGENIC	--	--	--
Ca3_P1_36126613	Ca3	36126613	A/AT	GACCCATCAATGAAGCGACT	GTAGGCATGTGGAGCCAAGT	60.1	612	INTERGENIC	--	--	--
Ca3_P1_36128710	Ca3	36128710	ATT/ATTT	ACTAACGGAACGACGAATGG	AAAGTCGAGGTGCTTTTTGC	60.0	657	INTERGENIC	--	--	--
Ca3_P1_36129655	Ca3	36129655	GATATA/GATA	TTGCTCGTTTGATTGGTGTG	TTTTTGTGATTTTACGTAAGGGTA	60.7	589	DRR	Ca_00912	YU	--
Ca3_P1_36129790	Ca3	36129790	T/TC	TTGCTCGTTTGATTGGTGTG	TTTTTGTGATTTTACGTAAGGGTA	60.7	589	DRR	Ca_00912	YU	--
Ca3_P1_36132835	Ca3	36132835	A/ATTTCCC	CCTGCATCTTCTTCGTCACA	GAAAGGCTAAACTCCCAGCC	60.0	574	INTRON	Ca_00912	YU	--
Ca3_P1_36133101	Ca3	36133101	TAACAC/TAACACAACAC	TAATTCCAAACGCAACCACA	CGCGGATCATCAGGTATTTT	60.0	945	INTRON	Ca_00912	YU	--
Ca3_P1_36135327	Ca3	36135327	A/AAT	ATTTGTTTGATTCGGCTTGG	GCATCAATATGGTCGCGTTT	59.9	638	DRR	Ca_00913	--	M-type
Ca3_P1_36135574	Ca3	36135574	ATT/AT	CAAATTAATTGGTTTGGATTTGA	TTTAGCACCGTTCTGGTGTG	58.0	873	DRR	Ca_00913	--	M-type
Ca3_P1_36136545	Ca3	36136545	CA/CATACATACATA	GCTTTGACACGTTTGCTTCA	ATTGTGGGAAGTGCTTACGG	60.0	368	INTRON	Ca_00913	--	M-type

INDEL marker IDs	Chromosomes/ unanchored scaffolds	Physical positions (bp)	InDels (*Kabuli* reference genome-CDC Frontier/PI)	Forward primers (5'-3')	Reverse primers (5'-3')	Anneling temperature (0C)	Expected amplified product size (bp)	Structural annotation		Functional annotation	
								Sequence components of *kabuli* genome	*Kabuli* gene accession IDs	NCBI-KOG	TFs
Ca3_P1_36136795	Ca3	36136795	AAATTAATTAATT/AAATTAATTAATTA ATT	TACCCGTAAGCACTTC CCAC	ATGGATCGGTTTTGTT GGAG	60.0	306	INTRON	Ca_00913	--	M-type
Ca3_P1_36137157	Ca3	36137157	CACA/CACACTAGTAACATAACA	TACCCGTAAGCACTTC CCAC	AGAGCTACCAGCCAAG TTGC	60.0	697	INTRON	Ca_00913	--	M-type
Ca3_P1_36138195	Ca3	36138195	TAAAAAAAA/TAAAAAAAAA	AGCTAATGAGCGGAAG CAAA	AATCTCCAAATTGGTG GTGG	60.1	558	INTERGENIC	--	--	--
Ca3_P1_36138844	Ca3	36138844	AG/A	TGGCTTGAGTGTGATG ATGAA	GGGTGTTGGCAATATT GGAT	60.3	806	INTERGENIC	--	--	--
Ca3_P1_36139492	Ca3	36139492	A/ATT	ATGCAAATTACCCAAG ACGC	TTATACCGGCTTCGTT GACC	60.0	580	INTERGENIC	--	--	--
Ca3_P1_36139755	Ca3	36139755	TTAT/TTATATAT	ATGCAAATTACCCAAG ACGC	TTATACCGGCTTCGTT GACC	60.0	580	INTERGENIC	--	--	--
Ca3_P1_36140163	Ca3	36140163	TAAAAAAAA/TAAAAAAAAAAA	GGTCAACGAAGCCGGT ATAA	CGAAAAATAACACCAA TGCAAA	60.0	551	INTERGENIC	--	--	--
Ca3_P1_36144562	Ca3	36144562	CAAAA/CAAA	GGTTTTTGCCCCAAAT ATGA	GTTTGCAAAGTGATCA TGCG	59.6	590	INTRON	Ca_00914	T	--
Ca3_P1_36148820	Ca3	36148820	TAAAA/TAAA	TTGCTTATGTCTCACG GCTG	CACCTTTGGAAGCAAA CAGAG	60.0	901	INTERGENIC	--	--	--
Ca3_P1_36149000	Ca3	36149000	GAAAAAAAAAA/GAAAAAAAA	TCCAAGGGGTTAAGAT TCATTG	CACCTTTGGAAGCAAA CAGAG	60.2	679	INTERGENIC	--	--	--
Ca3_P1_36154063	Ca3	36154063	TAAA/TAAAA	TTATTTTTCGGGCTTTT GTCA	CTTCCATTTGCAGGGT TTGT	59.6	839	DRR	Ca_00916	A	GeBP
Ca3_P1_36158143	Ca3	36158143	CTCTCTTTCTCTTTCTCTTTCTCT/CTC TCTTTCTCTTTCTCTTTCTCTTTCTCT	TTCCGTCATGTGTTTC GGTA	ATTAAAGCGCGTGGCT AAGA	60.0	631	INTERGENIC	--	--	--

INDEL marker IDs	Chromosomes/ unanchored scaffolds	Physical positions (bp)	InDels (*Kabuli* reference genome-CDC Frontier/PI)	Forward primers (5'-3')	Reverse primers (5'-3')	Anneling temperature (0C)	Expected amplified product size (bp)	Structural annotation		Functional annotation	
								Sequence components of *kabuli* genome	*Kabuli* gene accession IDs	NCBI-KOG	TFs
Ca3_P1_36159082	Ca3	36159082	TTATATA/TTA	TTCTTTTGCTTGGATCAAATGT	ATCACCGCAAACTGACGAA	58.7	193	INTERGENIC	--	--	--
Ca3_P1_36165450	Ca3	36165450	ATTTT/ATTT	GAAATGCATCGGTTCTTGGT	GTGGAGGAGCTAGTGTTGGG	59.9	634	INTRON	Ca_00917	A	--
Ca3_P1_36165658	Ca3	36165658	TC/TCTTTAAC	GAAATGCATCGGTTCTTGGT	GTGCTGCACTGTTTTCAGGA	59.9	661	INTRON	Ca_00917	A	--
Ca3_P1_36168208	Ca3	36168208	CAA/CAAA	ATCAATTCTGCTTCCCGATG	GAACTTTGTCTTTCGCAGGC	60.0	559	DRR	Ca_00918	KTDL	--
Ca3_P1_36168799	Ca3	36168799	CTCATAAGTCA/CTCA	GCCTGCGAAAGACAAAGTTC	ATTTGCAGGAGCTGCTGTTT	60.0	610	INTRON	Ca_00918	KTDL	--
Ca3_P1_36169333	Ca3	36169333	TAGAGGCA/TAGAGGCAGAGGCA	AAACAGCAGCTCCTGCAAAT	TACTGCGTCAAGCTCAATGG	60.0	723	INTRON	Ca_00918	KTDL	--
Ca3_P1_36178103	Ca3	36178103	GAAAA/GAAAAA	CCAAAGTGAAGATCTCACCGA	GCATTCTCCGGTCTTATCCA	60.2	499	DRR	Ca_00921	Q	B3
Ca3_P1_36178527	Ca3	36178527	GTTAAAAAT/GTTAAAAATTTAAAAAT	TGGATAAGACCGGAGAATGC	GCAGCAAGATTGCAGAATAGAA	60.0	147	DRR	Ca_00921	Q	B3
Ca3_P1_36178743	Ca3	36178743	TAA/TAAA	TGGATAAGACCGGAGAATGC	TCCTTATAAAATTATCATGCACTCAA	60.0	690	DRR	Ca_00921	Q	B3
Ca3_P1_36178800	Ca3	36178800	TATTTTAATTTTAATTT/TATTTTAATTTTAATTTTAATTT	TGGATAAGACCGGAGAATGC	TCTCGAAACTAGAAAAGAACAAAAA	60.0	812	DRR	Ca_00921	Q	B3
Ca3_P1_36179789	Ca3	36179789	CATTTTATTTTATTTTATTTT/CATTTTATTTTATTTTATTTTATTTT	TGACTTTTGGTGCCACTTTTT	GTTACCCCATGCATTGGAAG	59.6	713	DRR	Ca_00921	Q	B3
Ca3_P1_36179937	Ca3	36179937	CTTTAATTT/CTTT	TGACTTTTGGTGCCACTTTTT	GTTACCCCATGCATTGGAAG	59.6	713	DRR	Ca_00921	Q	B3

INDEL marker IDs	Chromosomes/ unanchored scaffolds	Physical positions (bp)	InDels (*Kabuli* reference genome-CDC Frontier/PI)	Forward primers (5'-3')	Reverse primers (5'-3')	Anneling temperature (0C)	Expected amplified product size (bp)	Structural annotation		Functional annotation	
								Sequence components of *kabuli* genome	*Kabuli* gene accession IDs	NCBI-KOG	TFs
Ca3_P1_36180636	Ca3	36180636	ATTTTTT/ATTTTT	TTCCAATGCATGGGGTAACT	TGATATGCAGGTGTTTCCGA	60.2	865	INTRON	Ca_00921	Q	B3
Ca3_P1_36180666	Ca3	36180666	CATA/CATATATA	ACTCGGCTTGGGAATACCAT	AGATCGACGCATAAGTGGCT	60.7	736	INTRON	Ca_00921	Q	B3
Ca3_P1_36183657	Ca3	36183657	TAAAAAAAA/TAAAAAAAAA	TGATTGATGATTCAACTTCACAAA	AAAACCTAACCCGTGCTTGA	59.5	808	INTERGENIC	--	--	--
Ca3_P1_36185975	Ca3	36185975	ATTTTTTTT/ATTTTTTT	CGGAATGTAGTGTTGACCCC	CAGTGACAGTGAGACGTGGG	60.2	535	INTERGENIC	--	--	--
Ca3_P1_36186523	Ca3	36186523	CAAA/CAAAA	CCACGTCTCACTGTCACTGC	CACCCTACCAAACAAGGCAT	60.5	500	INTERGENIC	--	--	--
Ca3_P1_36190637	Ca3	36190637	ATTTTTTTT/ATTTTTTTTT	TGGTTTGCTAAACCGTCCTC	ACCCAACAGCACTTGATTCC	60.1	870	INTRON	Ca_00922	Q	B3
Ca3_P1_36190883	Ca3	36190883	ATTTTTT/ATTTTTTTT	TGTTGTCTTTAGGGATCATATATTGG	ACCCAACAGCACTTGATTCC	59.6	650	INTRON	Ca_00922	Q	B3
Ca3_P1_36192321	Ca3	36192321	A/AT	ACGTTGAATGAGGACAAGGG	TGGACCAATTTTCGTATTCACTC	60.0	581	INTRON	Ca_00922	Q	B3
Ca3_P1_36192497	Ca3	36192497	GAAAAAAAAAA/GAAAAAAAAAAAAA	TGACATCTCCTCCAACCACA	TGGACCAATTTTCGTATTCACTC	60.1	332	INTRON	Ca_00922	Q	B3
Ca3_P1_36192941	Ca3	36192941	TAAAAA/TAAAA	CGAAAATTGGTCCATAAAATTG	ATGCTGGGGTTTTGTTGAAG	58.4	746	INTRON	Ca_00922	Q	B3
Ca3_P1_36194243	Ca3	36194243	TAT/TATATGTAAAAAAAT	TGCCATAAAGATGCGAAACA	TGAAATATGGCTTCCCTTCG	60.2	249	DRR	Ca_00923	--	bHLH
Ca3_P1_36195467	Ca3	36195467	TTTTATTTATTTATTTATTTATTTATTTATTTATTT/TTTTATTTATTTATTTATTTATTT	TATTGCCGACGTGAGTCTGA	TTCCCTTTCTTTTCGTCTGC	60.4	283	DRR	Ca_00923	--	bHLH

INDEL marker IDs	Chromosomes/ unanchored scaffolds	Physical positions (bp)	InDels (*Kabuli* reference genome-CDC Frontier/PI)	Forward primers (5'-3')	Reverse primers (5'-3')	Anneling temperature (0C)	Expected amplified product size (bp)	Structural annotation		Functional annotation	
								Sequence components of *kabuli* genome	*Kabuli* gene accession IDs	NCBI-KOG	TFs
Ca3_P1_36197056	Ca3	36197056	TTGTGT/TTGT	GTGGCGCAAAAAGAAAAGAG	TGCAGTAACTTCAACGGGTCT	60.0	756	INTRON	Ca_00923	--	bHLH
Ca3_P1_36198452	Ca3	36198452	AAATAA/AAA	TTCTTGCAATCAAAGCATCG	AACAGAGGCGGTGTCACTCT	60.0	469	DRR	Ca_00924	--	--
Ca3_P1_36199218	Ca3	36199218	TTTCTTCTT/TTTCTTCTTCTT	CACTCAAGGGACTAGGAGCG	GGTTTATGGGTCTTGGGGAT	60.0	482	DRR	Ca_00924	--	--
Ca3_P1_36200491	Ca3	36200491	GAACAACAACA/GAACAACA	TGCATTTCATTCATTCCCCT	ACTTTGTGCAATCCTTTGGC	60.3	489	INTRON	Ca_00924	--	--
Ca3_P1_36201927	Ca3	36201927	TGTGCTAG/TGTGCTAGTGCTAG	ATGCCACTTCAACAAAAGGG	ATCGAAGATCGTGAACCCTG	60.0	563	INTERGENIC	--	--	--
Ca3_P1_36203025	Ca3	36203025	CACTTAC/CAC	ATCGAAGCCGGTTAGGTTTT	TACATCTTAATTGGGCCGGA	60.0	168	INTERGENIC	--	--	--
Ca3_P1_36203687	Ca3	36203687	GGTG/GGTGTG	CACCGTGAAGATTGTGGATG	TGTGAGGGATTTGTGCATGT	60.0	292	INTERGENIC	--	--	--
Ca3_P1_36204918	Ca3	36204918	ATTT/ATTTATTAAGTTT	GTCGTTGGAGTCTTTGCGAT	TTCACCAAATGTTGACCGAA	60.3	452	DRR	Ca_00925	--	--
Ca3_P1_36207784	Ca3	36207784	GA/GAA	TGGGTTCATTAGCCACATCA	GTTCAAACTTCGTTGCCTCC	59.9	237	INTRON	Ca_00925	--	--
Ca3_P1_36208259	Ca3	36208259	CTT/CTTACATATAATTTTT	GAGGCAACGAAGTTTGAACC	GGTTTCGTCGTCGTTTGAGT	59.7	675	INTRON	Ca_00925	--	--
Ca3_P1_36209486	Ca3	36209486	AATATAT/AATATATATAT	GCGCCAAAGCAGAAGATACT	TTGGGCTGATTTTTACTGGC	59.6	630	INTERGENIC	--	--	--
Ca3_P1_36210315	Ca3	36210315	ATTTTT/ATTTTTT	CATTCAAATAAATGGGTATTGACG	TCATTGACCGTATGGATAGCA	59.5	542	INTERGENIC	--	--	--

INDEL marker IDs	Chromosomes/ unanchored scaffolds	Physical positions (bp)	InDels (*Kabuli* reference genome-CDC Frontier/PI)	Forward primers (5'-3')	Reverse primers (5'-3')	Anneling temperature (0C)	Expected amplified product size (bp)	Structural annotation		Functional annotation	
								Sequence components of *kabuli* genome	*Kabuli* gene accession IDs	NCBI-KOG	TFs
Ca3_P1_36213542	Ca3	36213542	GTGATGATGAT/GTGATGAT	AAAATTATTGTGGCCGAGCA	CCGCACCAAGTATGAAGAGC	60.5	414	DRR	Ca_00926	--	LSD
Ca3_P1_36213829	Ca3	36213829	ATG/ATGAAAAAGTG	AAAATTATTGTGGCCGAGCA	ACAGGGGATGCAGACTTTTT	60.5	845	DRR	Ca_00926	--	LSD
Ca3_P1_36215864	Ca3	36215864	CTTTT/CTT	TGATGACATTCAAAATTGCG	CCTGTTTGTTTGGCTTGGAT	58.1	582	DRR	Ca_00927	--	CO-like
Ca3_P1_36218063	Ca3	36218063	CAAAAAAAA/CAAAAAAAAAA	TTGATGTCTCCCACAAGCTG	TAATCAGATGATCGCGCAAG	59.8	480	INTRON	Ca_00927	--	CO-like
Ca3_P1_36218738	Ca3	36218738	TGAGAGAGAGAGAGAGAGA/TGAGAGAGAGAGAGAGA	ACGCCTCGATCAAATCATCT	CACTCTCTTCGCCACATGAA	59.7	708	INTRON	Ca_00927	--	CO-like
Ca3_P1_36219342	Ca3	36219342	GTTT/GTT	CATGTGGCGAAGAGAGTGAA	GTATTTCCTTTGGGCGTGAA	60.0	531	INTERGENIC	--	--	--
Ca3_P1_36219993	Ca3	36219993	TCA/T	TTCACGCCCAAAGGAAATAC	ACCGGTGATGACTCCAGTTC	59.9	757	DRR	Ca_00928	--	--
Ca3_P1_36220254	Ca3	36220254	T/TG	TTGGCTCTCTTATTGGTGGG	ACCGGTGATGACTCCAGTTC	60.1	265	DRR	Ca_00928	--	--
Ca3_P1_36220304	Ca3	36220304	GAAAAAAAAAAAAAA/GAAAAAAAAAAA	TTGGCTCTCTTATTGGTGGG	AATTCGGCGCTTTAGGATTT	60.1	446	DRR	Ca_00928	--	--
Ca3_P1_36221155	Ca3	36221155	CAAAA/CAAA	TTTTCACAATGTGCCAGCTC	AAATCCATCCCCGTATCTCC	59.8	405	DRR	Ca_00928	--	--
Ca3_P1_36222093	Ca3	36222093	TAAAAA/TAAAAAA	CTCGGCATGGTTTATGGTCT	CAATCGAATTTCCTTAGCCG	60.0	695	INTRON	Ca_00928	--	--
Ca3_P1_36223404	Ca3	36223404	TTGT/TT	ACTCTGGAATCAGCAAGCGT	ACAACAACTATGCCTTGCCC	60.0	386	INTRON	Ca_00928	--	--

INDEL marker IDs	Chromosomes/ unanchored scaffolds	Physical positions (bp)	InDels (*Kabuli* reference genome-CDC Frontier/PI)	Forward primers (5'-3')	Reverse primers (5'-3')	Anneling temperature (0C)	Expected amplified product size (bp)	Structural annotation		Functional annotation	
								Sequence components of *kabuli* genome	*Kabuli* gene accession IDs	NCBI-KOG	TFs
Ca3_P1_36223694	Ca3	36223694	GTGTCCAAAA/GTGTCCAAAAAAACTGTCCAAAA	GGGCAAGGCATAGTTGTTGT	GGTGAGCAAGGTGAAACCAT	60.0	694	INTRON	Ca_00928	--	--
Ca3_P1_36224870	Ca3	36224870	AAGAGAGAGAGAGAGAGAGAGAG/AAGAGAGAGAGAGAGAGAGAGAGAGAGAG	ACCTGTGTGACTCTGAGGGG	CCCAGTAACCGCTCTCTCTG	60.2	721	INTERGENIC	--	--	--
Ca3_P1_36225511	Ca3	36225511	TAAA/TAAAA	CAGAGAGAGCGGTTACTGGG	TTTGCTTTATTTCGATGGGC	60.0	733	INTERGENIC	--	--	--
Ca3_P1_36226234	Ca3	36226234	AATAGAT/AATAGATAGAT	TGGAGCCCATCGAAATAAAG	CCACATGGTGAAGAGCAGAA	60.0	729	INTERGENIC	--	--	--
Ca3_P1_36226833	Ca3	36226833	CGAGGAGG/CGAGG	CACGAAATCCGAAATGGAAC	TTGTTTGCTCTGATTTTGGAGA	60.3	537	INTERGENIC	--	--	--
Ca3_P1_36230990	Ca3	36230990	T/TA	TCCAGTCCTTTCCTTTTGGA	TGAGTTAAAGGACCACATGCC	59.6	353	DRR	Ca_00929	J	--
Ca3_P1_36233110	Ca3	36233110	AAATAAGA/AA	GAGGGCTGCAGTTTAATTCG	AGCTGCCTCATAATCCACCA	59.8	824	DRR	Ca_00930	O	--
Ca3_P1_36233556	Ca3	36233556	ATTTTTTTT/ATTTTTTTTTT	ATTGCTCAAGTTTTGCTGGG	AATCACACCTTTAAATCGGCA	60.2	690	DRR	Ca_00930	O	--
Ca3_P1_36234544	Ca3	36234544	GA/GAA	ACCACACCCTTTTCAGATGC	TCTGCTGGTTTGGGAGTTCT	60.0	157	INTERGENIC	--	--	--
Ca3_P1_36237362	Ca3	36237362	ATTT/ATTTT	TCGCAAGATGAGTCTTGGTG	AATGGGCTTTGAAGGGTCTT	60.0	596	DRR	Ca_00931	O	--
Ca3_P1_36238162	Ca3	36238162	TTCA/T	TGACCCTAATGCCATGAACA	AATCGTGGAGTTCGGTCAAT	59.9	522	INTERGENIC	--	--	--
Ca3_P1_36239497	Ca3	36239497	ATT/AT	TGAAATGCGTTTCCACTTTT	TTGGGCTTGTGGTCTCTAGG	58.3	657	INTERGENIC	--	--	--

INDEL marker IDs	Chromosomes/ unanchored scaffolds	Physical positions (bp)	InDels (*Kabuli* reference genome-CDC Frontier/PI)	Forward primers (5'-3')	Reverse primers (5'-3')	Anneling temperature (0C)	Expected amplified product size (bp)	Structural annotation		Functional annotation	
								Sequence components of *kabuli* genome	*Kabuli* gene accession IDs	NCBI-KOG	TFs
Ca3_P1_36241503	Ca3	36241503	AAT/AATGAT	TAATGAAGGGGACTGCAACC	TTCGACTCCGAGATATAACATTCA	59.9	789	INTERGENIC	--	--	--
Ca3_P1_36241653	Ca3	36241653	CC/CCAC	TTCGCAGAAAAACTATGCCC	TCCGTATTCGAATTTGAGACAA	60.2	808	INTERGENIC	--	--	--
Ca3_P1_36241823	Ca3	36241823	AG/A	TTTCGGTTTGCATGTTGAGT	AGGTGCGGTGTTTTGTTGTT	59.2	904	INTERGENIC	--	--	--
Ca3_P1_36243079	Ca3	36243079	T/TGAG	CAATGGAGGAAACCCAAGAA	AAGTGGTGGTGCTGTTTTCC	59.9	318	CDS (large-effect mutations)	Ca_00932	TO	--
Ca3_P1_36246276	Ca3	36246276	GTTTTTTTT/GTTTTTTTTT	TCGGCTCGTCTAGTTCCAGT	TGTCAAAACGCTCAAAAATGA	60.0	321	DRR	Ca_00932	TO	--
Ca3_P1_36246846	Ca3	36246846	ATGTTATT/AT	TCATTTTTGAGCGTTTTGACA	TCGGTTTTCAGCATACCACA	59.3	915	DRR	Ca_00932	TO	--
Ca3_P1_36249669	Ca3	36249669	C/CAAGATGT	TTCCATCTATTTGGTTCGCC	ACGACCCTTAATTGGCTCCT	59.9	984	DRR	Ca_00933	O	--
Ca3_P1_36250776	Ca3	36250776	AAATAATAATAATAATAATAA/AAATAATAATAATAATAA	GCAAAGCCAAACCGCTATAA	AACGCTAACGTTGGTTCCAC	60.2	935	DRR	Ca_00933	O	--
Ca3_P1_36251365	Ca3	36251365	TAA/TA	GGTAAATTCGGTGGAACCAA	TCTGCCTGTTGTTTTTGACG	59.7	336	INTERGENIC	--	--	--
Ca3_P1_36251816	Ca3	36251816	TTATAT/TTAT	CGTCAAAAACAACAGGCAGA	CAGAAGCTTGCAGCTACCCT	59.9	718	INTERGENIC	--	--	--
Ca3_P1_36253466	Ca3	36253466	TAAA/TAAAA	AGAATGCCACTTGGACAACC	TGACCGGAAGAAGAAGAAGAA	60.0	383	DRR	Ca_00934	O	--
Ca3_P1_36262526	Ca3	36262526	CTTTTT/CTTTT	GGCAGAGCTATCCCTCAAGA	AAAGTGCAGCAACCACACAC	59.5	591	INTRON	Ca_00936	--	MYB_related

INDEL marker IDs	Chromosomes/ unanchored scaffolds	Physical positions (bp)	InDels (*Kabuli* reference genome-CDC Frontier/PI)	Forward primers (5'-3')	Reverse primers (5'-3')	Anneling temperature (0C)	Expected amplified product size (bp)	Structural annotation		Functional annotation	
								Sequence components of *kabuli* genome	*Kabuli* gene accession IDs	NCBI-KOG	TFs
Ca3_P1_36264091	Ca3	36264091	CA/CAA	ATGAACAGAGACGGGTCGAT	ATGATCCATGTTGCAGGTGA	59.5	423	INTERGENIC	--	--	--
Ca3_P1_36266350	Ca3	36266350	C/CT	TTTGTTGATGATATTTATCCCGTG	GACACGGATATGAGAGCGGT	60.0	599	INTERGENIC	--	--	--
Ca3_P1_36269264	Ca3	36269264	TTCTCTCTC/TTCTCTC	TTCCCCATTTTTCAAAACCA	TCCAGAGGATCACTGGGAAG	60.1	219	INTRON	Ca_00937	J	Dof
Ca3_P1_36270011	Ca3	36270011	TACACACA/TACACA	CTGGAATATGGGCTGGTGAT	CAGAGCATGAGATGGCAGAA	59.8	330	INTRON	Ca_00937	J	Dof
Ca3_P1_36274277	Ca3	36274277	T/TA	TGGGTTTTCATTGGTTTGGT	AAGCCGACTTCAAGAAGGAC	60.1	419	DRR	Ca_00937	J	Dof
Ca3_P1_36274634	Ca3	36274634	AAT/AATAT	TGGAAGTTCTTGATGTGCAA	TCATGCGAATTTTTAAAGCA	57.8	420	DRR	Ca_00937	J	Dof
Ca3_P1_36274695	Ca3	36274695	ATAAAATATA/ATAAAATATATAATTAAAATATA	TGAGTTGCTTTAAAAATTCGCA	GGAGACAAATATAATTCACCACCA	59.9	541	DRR	Ca_00937	J	Dof
Ca3_P1_36275195	Ca3	36275195	AAT/AATAT	TTTTGGTGGTGAATTATATTTGTCTC	TGCATGACAAGTGGCTCTTC	59.6	392	DRR	Ca_00937	J	Dof
Ca3_P1_36276278	Ca3	36276278	T/TG	AGAAGCTTTCGCTGAAGCAC	TCCTCTCGATCCACCTATGG	59.9	610	INTRON	Ca_00938	R	--
Ca3_P1_36277496	Ca3	36277496	A/AG	GGCTTGGGAAGATAAGAGGG	CAGATCCAAAACCACCGAAT	60.0	602	INTRON	Ca_00938	R	--
Ca3_P1_36279187	Ca3	36279187	TG/TGG	AACAGGTAAGCCGTCCATTG	CGTTGACCAGAAAAATGGCT	60.0	268	INTRON	Ca_00938	R	--
Ca3_P1_36283272	Ca3	36283272	CAAA/CAAAA	GCAGCCTGACCCAGACTTAG	GTACCTGTCACACGACCGC	60.0	554	DRR	Ca_00938	R	--

INDEL marker IDs	Chromosomes/ unanchored scaffolds	Physical positions (bp)	InDels (*Kabuli* reference genome-CDC Frontier/PI)	Forward primers (5'-3')	Reverse primers (5'-3')	Anneling temperature (0C)	Expected amplified product size (bp)	Structural annotation		Functional annotation	
								Sequence components of *kabuli* genome	*Kabuli* gene accession IDs	NCBI-KOG	TFs
Ca3_P1_36283526	Ca3	36283526	ATTTTTTTTT/ATTTTTTTTTTTT	GCGGTCGTGTGACAGGTACT	TCTTATGCCGTTTTCCTTCG	61.2	654	DRR	Ca_00938	R	--
Ca3_P1_36284548	Ca3	36284548	ATATTATTATTAATATTATTATTATTATTATTATTATTATTATTAATATTATTATTATTATTAT/ATATTATTATTATTAATATTATTATTATTATTATTATTATTATTAATATTATTATTATTATTAT	ACCCCTCCTGTCGTGTATTG	AGCCACATTCACAAACCACA	59.8	449	INTERGENIC	--	--	--
Ca3_P1_36286597	Ca3	36286597	ATTTTTTT/ATTTTTTTT	TCATTTTGGCAACTTACCACC	TGCCTGAATTTATTGGGAATG	59.9	754	INTERGENIC	--	--	--
Ca3_P1_36286726	Ca3	36286726	TA/TATAAAATTGTCTGTTAA	TCATTTTGGCAACTTACCACC	CGGGCATTAGAGATCCACAT	59.9	935	INTERGENIC	--	--	--
Ca3_P1_36287550	Ca3	36287550	AAAGACAAGACAA/AAAGACAA	ATGTGGATCTCTAATGCCCG	CGTGGTTGAGTTTCGAACAG	59.9	413	INTERGENIC	--	--	--
Ca3_P1_36289119	Ca3	36289119	TTTCAC/TTTCACATTAATTCAC	TCCTTGTCCGTGTTAGCCTT	TCTTGGTCGTGTCCATGAAA	59.7	307	INTERGENIC	--	--	--
Ca3_P1_3628980	Ca3	3628980	TAAAAAA/TAAAAAAA	CCACCATTAGTTAACGGAGTCA	TTGTCTAAAGAAATTGAAATCATGTTG	59.0	267	INTERGENIC	--	--	--
Ca3_P1_36290753	Ca3	36290753	CA/C	AAAATCTCAAAATTCGCCTTTG	TGAGTGGGGTAAATAAACTCTGAA	59.6	416	INTERGENIC	--	--	--
Ca3_P1_36291066	Ca3	36291066	ATTTTTTT/ATTTTTTTT	TCCCTATCTCATTTTACCGTGA	GGTTTGGTTCCTTCGGTTTT	58.6	745	INTERGENIC	--	--	--
Ca3_P1_36295270	Ca3	36295270	TTGTTTTCATGTTT/TTGTTT	TGTAGAGAACGGAAATGGGG	TAAGGTGGCGAGGATTGAAC	59.9	593	INTRON	Ca_00939	--	--
Ca3_P1_36307490	Ca3	36307490	AT/ATATT	GTGTTCCGCGGTTAACAACT	TGGCTAATAAGCCTACGCAAA	60.0	409	INTERGENIC	--	--	--
Ca3_P1_36308085	Ca3	36308085	ATTTTTTTTT/ATTTTTTTTTTTT	CGTAAATAGCAGTGGTTGTTCG	TCTGCTGTATCAAGTGGCAAA	59.7	573	INTERGENIC	--	--	--

INDEL marker IDs	Chromosomes/ unanchored scaffolds	Physical positions (bp)	InDels (*Kabuli* reference genome-CDC Frontier/PI)	Forward primers (5'-3')	Reverse primers (5'-3')	Anneling temperature (0C)	Expected amplified product size (bp)	Structural annotation		Functional annotation	
								Sequence components of *kabuli* genome	*Kabuli* gene accession IDs	NCBI-KOG	TFs
Ca3_P1_36308195	Ca3	36308195	T/TC	TTTGCCACTTGATACAGCAGA	TTGAACCTAAACGATGACACCTT	59.5	309	INTERGENIC	--	--	--
Ca3_P1_36312854	Ca3	36312854	AAA/AAATTAAGTAA	CTATGCACGAGTTTGGGGTT	CAATCAAGAAGCGCATTGAA	60.0	586	DRR	Ca_00941	D	TCP
Ca3_P1_36315506	Ca3	36315506	ATTT/ATT	CACATTCTTCCTCCAACCGT	CCTGATGCTTTTTGGCATTT	60.0	753	INTRON	Ca_00941	D	TCP
Ca3_P1_36319172	Ca3	36319172	A/AT	AAAGGAATTGCAAGGACCAA	CGCGGTGATTTTATTGCTCT	59.5	616	INTRON	Ca_00941	D	TCP
Ca3_P1_36320561	Ca3	36320561	CTTTTTTTT/CTTTTTTTTTTT	GTCTCAAGCAATCCTCAGCC	CAACGAGAGAGTGGATGGGT	60.0	442	INTRON	Ca_00941	D	TCP
Ca3_P1_36321434	Ca3	36321434	TAAAAAAAA/TAAAAAAAAA	GCTGGCCTTGCATAAGAAAA	AGCTAGCTGATGGCTGGAAA	60.3	582	INTRON	Ca_00941	D	TCP
Ca3_P1_36322784	Ca3	36322784	CAAAAA/CAAAA	CAGCTTTCCTAGCATTTGCC	ATAATGCTGCAGCGACAATG	60.0	890	INTRON	Ca_00941	D	TCP
Ca3_P1_36324257	Ca3	36324257	ATTTTTTT/ATTTTTT	TGGTTGGTCTTTGCTTGTGT	GGAATGTTTCATGGACACCC	59.2	770	INTERGENIC	--	--	--
Ca3_P1_3632559	Ca3	3632559	ATTTTTTTTTTTTTT/ATTTTTTTTTTTTT	TTTGAGTCTAACCCGAACCAA	GTGGGGTAGGCAGAGACAAA	59.6	765	INTERGENIC	--	--	--
Ca3_P1_36326321	Ca3	36326321	ACT/ACTCT	TTGCAAAATGGTCCATCAAA	GCAGTGTGATGCAAAAATGG	59.9	680	INTERGENIC	--	--	--
Ca3_P1_36327814	Ca3	36327814	TAA/TAAA	CAAGAATTGGGGGAAAACAA	ATACATCTCGCCACAATGGG	59.8	296	INTERGENIC	--	--	--
Ca3_P1_36328306	Ca3	36328306	CTT/CT	TTATATCACCCATTGTGGCG	CGAGAGAGACTTAGGCCGTG	59.3	681	INTERGENIC	--	--	--

INDEL marker IDs	Chromosomes/ unanchored scaffolds	Physical positions (bp)	InDels (*Kabuli* reference genome-CDC Frontier/PI)	Forward primers (5'-3')	Reverse primers (5'-3')	Anneling temperature (0C)	Expected amplified product size (bp)	Structural annotation		Functional annotation	
								Sequence components of *kabuli* genome	*Kabuli* gene accession IDs	NCBI-KOG	TFs
Ca3_P1_36328528	Ca3	36328528	AATGTTATGTTA/AATGTTA	TTATATCACCCATTGTGGCG	CGTTCCAGACCTCCATTGTT	59.3	856	INTERGENIC	--	--	--
Ca3_P1_36328591	Ca3	36328591	GAAA/GAA	CACGATGAAATCACTATGAAAAGC	CGTTCCAGACCTCCATTGTT	60.0	440	INTERGENIC	--	--	--
Ca3_P1_36331408	Ca3	36331408	TGG/TG	TGTAGGTTTTTGACCGGAGG	TCACCAAGACCAAACAGGCT	60.0	494	INTRON	Ca_00942	--	HB-other
Ca3_P1_36333048	Ca3	36333048	TGGGG/TGGGGG	CAGCACAGAATGTTGATGGG	TTGGCCATTGCAAAAGTACA	60.1	192	INTRON	Ca_00942	--	HB-other
Ca3_P1_36334629	Ca3	36334629	TAAAAAAAAA/TAAAAAAAA	AACGGCGAATACTAACACGG	GCCCAAACGAAACCTATCAA	60.0	372	INTRON	Ca_00942	--	HB-other
Ca3_P1_36334754	Ca3	36334754	ATTTTTTCCATATTTTTT/ATTT	AACGGCGAATACTAACACGG	GCGGAATACCACGAGAAAAA	60.0	697	INTRON	Ca_00942	--	HB-other
Ca3_P1_36336902	Ca3	36336902	TAAA/TAA	CCCATCTCCAACTCCTTCAA	GGTGCTAAGTTTTGCCGTGT	60.0	818	INTRON	Ca_00942	--	HB-other
Ca3_P1_36338300	Ca3	36338300	ATTTTT/ATTTT	GAATCATGGAGTCCGGAGAA	ACGCAAACTCTGATCGTGTG	60.0	703	DRR	Ca_00942	--	HB-other
Ca3_P1_36350064	Ca3	36350064	TAT/TATAAT	GGTTTCCCCAATATTCCGTT	GATGGCAAGCTTTTGTCCTT	59.9	492	INTERGENIC	--	--	--
Ca3_P1_36350826	Ca3	36350826	TAAAAAA/TAAAAAAA	AAGGACAAAAGCTTGCCATC	TTGTCCCCAATGTTGGTCTT	59.3	663	INTERGENIC	--	--	--
Ca3_P1_36351161	Ca3	36351161	TAAAAA/TAAAA	AAGACCAACATTGGGGACAA	CGATTCAAGTGTCGCATGTC	60.2	473	INTERGENIC	--	--	--
Ca3_P1_36351703	Ca3	36351703	GATAAAATAATATAAAATAA/GATAAAATAA	GACATGCGACACTTGAATCG	GCCTGCAATGAACCGTAAGT	60.3	420	INTERGENIC	--	--	--

INDEL marker IDs	Chromosomes/ unanchored scaffolds	Physical positions (bp)	InDels (*Kabuli* reference genome-CDC Frontier/PI)	Forward primers (5'-3')	Reverse primers (5'-3')	Anneling temperature (0C)	Expected amplified product size (bp)	Structural annotation		Functional annotation	
								Sequence components of *kabuli* genome	*Kabuli* gene accession IDs	NCBI-KOG	TFs
Ca3_P1_36352882	Ca3	36352882	AT/ATT	TCACCTCGAGCAAACAAGTG	AAGCACCATTTGTAGTTTTCATGT	60.0	380	INTERGENIC	--	--	--
Ca3_P1_36356666	Ca3	36356666	CAAAAAAAAAAAA/CAAAAAAAAAAA	ATTTAAAAGTCACGCGGAGC	TCCAGGTATGCGGTAGTTCC	59.4	373	DRR	Ca_00944	--	--
Ca3_P1_36361587	Ca3	36361587	CAA/CA	ACTCATCTCCAACACGAGGG	CTGTCAACAAATCGCAGTCG	60.1	465	INTERGENIC	--	--	--
Ca3_P1_36363081	Ca3	36363081	CG/C	TGTGTAGCGATGTCACACTTG	TTCCGTCCATATGCATTGATT	58.3	426	INTERGENIC	--	--	--
Ca3_P1_36370241	Ca3	36370241	GAAAAAAA/GAAAAAAAAAA	TGGAGAAAATGCGAGATTCC	AAAACGGATCCCCTACCAAC	60.2	541	INTERGENIC	--	--	--
Ca3_P1_36371742	Ca3	36371742	ATTTTTTTT/ATTTTTTT	GGGTTTTCTAGTCCCCCAAG	TGTTTTTCATTATGTGCCCG	59.8	483	INTERGENIC	--	--	--
Ca3_P1_36381407	Ca3	36381407	ATTTTTT/ATTTTTTT	TTTTGAGAGCGCATTTTGTG	CCATGCTTCCCACGTCTAAT	60.0	352	INTERGENIC	--	--	--
Ca3_P1_36381967	Ca3	36381967	AACACAC/AACAC	ATTAGACGTGGGAAGCATGG	CATTGACGTTACAACCGACG	60.0	798	INTERGENIC	--	--	--
Ca3_P1_36384084	Ca3	36384084	TTAAA/T	TGGCACTGTTTTGGTCTTTG	TGGACCCAAAATTTCCCATA	59.7	605	INTERGENIC	--	--	--
Ca3_P1_36392229	Ca3	36392229	A/ACTT	TTTTGGACTGAGGGAGGTTG	TGCCAATTGGAATCACAAGA	60.1	698	INTERGENIC	--	--	--
Ca3_P1_36392375	Ca3	36392375	ATTTTTT/ATTTTTTT	AACGTGATTCTCAGAACGGC	TCCCCAAACAAGTTTTCAGC	60.3	736	INTERGENIC	--	--	--
Ca3_P1_36396966	Ca3	36396966	AAAATT/AAAATTTCAAATT	CTCACATCTGATCGAAGGCA	ACTGTAACCTTGTGCTCCCG	59.9	332	INTRON	Ca_00950	P	--

INDEL marker IDs	Chromosomes/ unanchored scaffolds	Physical positions (bp)	InDels (*Kabuli* reference genome-CDC Frontier/PI)	Forward primers (5'-3')	Reverse primers (5'-3')	Anneling temperature (0C)	Expected amplified product size (bp)	Structural annotation		Functional annotation	
								Sequence components of *kabuli* genome	*Kabuli* gene accession IDs	NCBI-KOG	TFs
Ca3_P1_36397163	Ca3	36397163	TTATATATAT/TTATATATATATAT	CTCACATCTGATCGAAGGCA	TGCAGCAGCAGAACCATTAC	59.9	708	INTRON	Ca_00950	P	--
Ca3_P1_36399143	Ca3	36399143	ATTTTTT/ATTTTTTT	ATAATTGGGCCACACATCGT	TGCAATGTGGAAATCAGAGAA	60.1	208	DRR	Ca_00950	P	--
Ca3_P1_36399326	Ca3	36399326	ATAATATGATATTAATATGATA/ATAATATGATA	ATAATTGGGCCACACATCGT	AACAAGCAAGCGCAACACTA	60.1	712	DRR	Ca_00950	P	--
Ca3_P1_36399524	Ca3	36399524	T/TG	ATAATTGGGCCACACATCGT	AACAAGCAAGCGCAACACTA	60.1	712	DRR	Ca_00950	P	--
Ca3_P1_36402333	Ca3	36402333	TA/T	GGCCTCACACTCCATCAAAT	GCTCAATGTTGCGTTTAGCA	59.9	316	INTRON	Ca_00951	H	--
Ca3_P1_36403180	Ca3	36403180	TA/T	AACCGGAAGGATCTGTGTTG	TTTTCACATTTCAACCGCAC	60.0	953	DRR	Ca_00951	H	--
Ca3_P1_36404085	Ca3	36404085	GTT/GT	CGAATTGAGGGGTTGTCACT	TGAATCTTATTGCGTGAGCG	60.0	803	DRR	Ca_00951	H	--
Ca3_P1_36406783	Ca3	36406783	AT/ATT	CAGTGACTATGGCTTTGCGA	ATTCTGCTGGCGATACAACC	60.0	357	INTRON	Ca_00952	--	--
Ca3_P1_36410249	Ca3	36410249	GAAAAAAAAAA/GAAAAAAAAAAAA	CGGAAATGTCATGTGTGGTC	ATGTTTGTCCACTTGCTCCC	59.8	458	DRR	Ca_00953	--	HSF
Ca3_P1_36410296	Ca3	36410296	GTTTTTTT/GTTTTTTTTT	ATGGTTGTTCATCGTCCCAT	ATGTTTGTCCACTTGCTCCC	60.1	126	DRR	Ca_00953	--	HSF
Ca3_P1_36410627	Ca3	36410627	TG/T	GGGAGCAAGTGGACAAACAT	CTTTGTTCCCCTCGTGTTGT	60.0	431	DRR	Ca_00954	G	--
Ca3_P1_36412760	Ca3	36412760	TAAAA/TAAA	TGCTTTGCTTTGGGTCTTCT	TCTCACGCGAATTCCCTATT	60.0	307	INTRON	Ca_00954	G	--

INDEL marker IDs	Chromosomes/ unanchored scaffolds	Physical positions (bp)	InDels (*Kabuli* reference genome-CDC Frontier/PI)	Forward primers (5'-3')	Reverse primers (5'-3')	Anneling temperature (0C)	Expected amplified product size (bp)	Structural annotation		Functional annotation	
								Sequence components of *kabuli* genome	*Kabuli* gene accession IDs	NCBI-KOG	TFs
Ca3_P1_36415214	Ca3	36415214	AAAGAAG/AAAG	GAGTCAACCTACCGGACCAA	TGGGAATGACCTTTGCTTTC	60.0	466	INTRON	Ca_00954	G	--
Ca3_P1_36418728	Ca3	36418728	T/TTTG	TCCTCCTTACCACATCGGTC	CGCATTTGTCCCCTGTAAGT	59.9	790	INTRON	Ca_00954	G	--
Ca3_P1_36418781	Ca3	36418781	GAAAA/GAAA	TCCTCCTTACCACATCGGTC	ACGTAGAAACAATGGCCGTC	59.9	929	INTRON	Ca_00954	G	--
Ca3_P1_36419771	Ca3	36419771	ATTTTTTT/ATTTTTT	GTCGTCTTGGGCTCTTTCAC	TTGCCGTTGAGAAAATGAGA	59.9	477	INTERGENIC	--	--	--
Ca3_P1_36420133	Ca3	36420133	TTGA/TA	TGTTTTTCGCTCATCCATTG	CCCCTTTTTCAGCCACACTA	59.7	464	INTERGENIC	--	--	--
Ca3_P1_36420292	Ca3	36420292	GGAGC/G	TGTTTTTCGCTCATCCATTG	TGGTAGAAATCACGATGCCA	59.7	710	INTERGENIC	--	--	--
Ca3_P1_36420495	Ca3	36420495	GTGTGAT/GTGTGATGTGAT	TAGTGTGGCTGAAAAAGGGG	TGGTAGAAATCACGATGCCA	60.1	266	INTERGENIC	--	--	--
Ca3_P1_36420971	Ca3	36420971	AATATATATATATAT/AATATATATAT	TGGCATCGTGATTTCTACCA	GGCCATGGTGATTGTAAAGG	60.1	746	INTERGENIC	--	--	--
Ca3_P1_36427493	Ca3	36427493	TAAG/TAAGATCAAG	GAAACCAGAGAAGCGTTTGC	TGAGTTGTGATAAAGGGCCA	60.0	690	INTERGENIC	--	--	--
Ca3_P1_36430053	Ca3	36430053	ACC/ACCGCATCC	AGGGATGCACCAGCTGTAAC	AGAGAAGAAACCTGGTGCGA	60.1	593	INTRON	Ca_00957	H	--
Ca3_P1_36435265	Ca3	36435265	AA/AAAAACACA	ATTCCCGATCCATCCTTTTT	AAGGTGGAGGAGGGAAAAGA	59.6	811	DRR	Ca_00957	H	--
Ca3_P1_36436313	Ca3	36436313	CAAAAAAAA/CAAAAAAAAA	TCTTTTCCCTCCTCCACCTT	ATCGCAATTAACCAAATGCC	60.0	654	INTRON	Ca_00958	A	--

INDEL marker IDs	Chromosomes/ unanchored scaffolds	Physical positions (bp)	InDels (*Kabuli* reference genome-CDC Frontier/PI)	Forward primers (5'-3')	Reverse primers (5'-3')	Anneling temperature (0C)	Expected amplified product size (bp)	Structural annotation		Functional annotation	
								Sequence components of *kabuli* genome	*Kabuli* gene accession IDs	NCBI-KOG	TFs
Ca3_P1_36437168	Ca3	36437168	C/CAT	AATTTGGCAACGTAGGATGG	CGGCTCTTGGTTTCTTCTTG	59.8	514	INTRON	Ca_00958	A	--
Ca3_P1_36438761	Ca3	36438761	CGTAGAAA/CGTAGAAAAGACGGTAGAAA	TTGGGTAGTTGGAATGGAGG	ATGTCTCCTCCTCGCATGAT	59.8	425	INTRON	Ca_00958	A	--
Ca3_P1_36438982	Ca3	36438982	T/TTG	AATTTGGCTTTGGTGGTTTG	GTAGCAAACTGGCCCCATTA	59.8	686	INTRON	Ca_00958	A	--
Ca3_P1_36440379	Ca3	36440379	AT/A	TCTTTTCAACCCCAACTCAAA	CATGACCAACTTACCTTTCGG	59.6	783	DRR	Ca_00959	R	--
Ca3_P1_36443050	Ca3	36443050	ATG/A	TTGCAATTGAAGTTCCCACA	GGCAAGCTCATTTCTGCTTC	60.1	427	INTRON	Ca_00959	R	--
Ca3_P1_36443105	Ca3	36443105	GCATCAT/GCAT	TTGCAATTGAAGTTCCCACA	GGCAAGCTCATTTCTGCTTC	60.1	427	INTRON	Ca_00959	R	--
Ca3_P1_36446253	Ca3	36446253	CAG/CAGAG	ATCCCAATCACCTCACTTGC	CCCAGATTTCTATGCGGAAA	59.9	533	DRR	Ca_00960	O	--
Ca3_P1_36448609	Ca3	36448609	CAAAA/CAAA	GAGCCAGACATCAACTGCAA	CAATGTAGGAGTGCGAAGCA	60.0	710	INTRON	Ca_00960	O	--
Ca3_P1_36448626	Ca3	36448626	TAACAACAA/TAACAACAACAA	GAGCCAGACATCAACTGCAA	CAATGTAGGAGTGCGAAGCA	60.0	710	INTRON	Ca_00960	O	--
Ca3_P1_36451224	Ca3	36451224	ATTT/ATTTT	CCTCTTCTTAGACGCCGATG	GTCGATGGTTTCAAGCACCT	60.0	828	INTRON	Ca_00961	--	--
Ca3_P1_36454087	Ca3	36454087	CGAT/CGATCATATTGGAT	TGATTCGGTTTGTGCTGTGT	CGTGACAGCCCATAGTTGAA	60.2	931	INTERGENIC	--	--	--
Ca3_P1_36455748	Ca3	36455748	AC/AGTTTTACTCTTAATACATC	TCAAATTACAACAAGGGCGA	CGTAAGGGAGCAAAGTGGTC	59.2	792	INTERGENIC	--	--	--

INDEL marker IDs	Chromosomes/ unanchored scaffolds	Physical positions (bp)	InDels (*Kabuli* reference genome-CDC Frontier/PI)	Forward primers (5'-3')	Reverse primers (5'-3')	Anneling temperature (0C)	Expected amplified product size (bp)	Structural annotation		Functional annotation	
								Sequence components of *kabuli* genome	*Kabuli* gene accession IDs	NCBI-KOG	TFs
Ca3_P1_36456540	Ca3	36456540	TATAATAAT/TATAAT	GACCACTTTGCTCCCTTACG	AAGTCTCAAACAAACCACCGA	59.7	585	INTERGENIC	--	--	--
Ca3_P1_36457575	Ca3	36457575	TGG/TG	TGGTACGCTTAATTGAGAGCTG	TGTGCTGTACATGTGGCTGA	59.6	314	INTERGENIC	--	--	--
Ca3_P1_36458050	Ca3	36458050	AG/A	TGGGTCCCAAAAGTGGTATC	CAATTTGGCCACTTGAATGA	59.6	772	INTERGENIC	--	--	--
Ca3_P1_36458326	Ca3	36458326	ATTTTTT/ATTTTTTT	TTTGCAAAGTGGCGGTAGTT	TGTTTTGCAAGGTGAATTGG	60.7	525	INTERGENIC	--	--	--
Ca3_P1_36464808	Ca3	36464808	TGTTGACGTTGA/TGTTGA	ACAATCGTTCACCGTCTTCC	AAGAAGACCCACGTGATTGG	60.0	659	CDS (large-effect mutations)	Ca_00963	--	--
Ca3_P1_36465253	Ca3	36465253	CAA/CAAGAAGAAGAAGAA	TCGAGTTCCTGCTGTTGATG	CACGCGCTTCGTTTATTTTT	60.0	553	INTERGENIC	--	--	--
Ca3_P1_36466048	Ca3	36466048	ATTTTTTTTT/ATTTTTTT	ATCATTGTGAGCAGGAGGAA	AATTGAACCAACGATGTCCAC	58.2	362	INTERGENIC	--	--	--
Ca3_P1_36468198	Ca3	36468198	GA/G	TTCATAGATTTTGTCAAAGTTGCAT	TTGTCAATGTCGCTGTCCTC	59.1	584	INTERGENIC	--	--	--
Ca3_P1_36473807	Ca3	36473807	ACAAC/A	ACAAATGCATAGCCAGGAGG	GGCGATATCCGTGAAAGAGA	60.1	532	INTRON	Ca_00964	A	B3
Ca3_P1_36476148	Ca3	36476148	A/AATACAC	TGGCAAATCGATTGACAAAA	ACATCCAGTCCTGCAAATCC	60.1	844	INTERGENIC	--	--	--
Ca3_P1_36476326	Ca3	36476326	GA/GAA	GCGAGAAAAGCCAGCTAGAA	ACATCCAGTCCTGCAAATCC	59.9	239	INTERGENIC	--	--	--
Ca3_P1_36481031	Ca3	36481031	TAATAAAGAAAT/TAAT	AAATAGAAGGGGGTGTTGGG	AAAAATGGGAGTGACGATGC	60.0	607	INTERGENIC	--	--	--

INDEL marker IDs	Chromosomes/ unanchored scaffolds	Physical positions (bp)	InDels (*Kabuli* reference genome-CDC Frontier/PI)	Forward primers (5'-3')	Reverse primers (5'-3')	Anneling temperature (0C)	Expected amplified product size (bp)	Structural annotation		Functional annotation	
								Sequence components of *kabuli* genome	*Kabuli* gene accession IDs	NCBI-KOG	TFs
Ca3_P1_36482503	Ca3	36482503	AAAAAAA/AAAAAAAAAATTAAAAAA	AATGAAAGGGGGAATTGGAC	GATGTGTGTCATCAACCCCA	60.0	764	INTERGENIC	--	--	--
Ca3_P1_36485126	Ca3	36485126	TC/TCC	CCAAACACTTTTCTACCGGC	GGGTGGCGAATGAGAACTTA	59.6	233	INTERGENIC	--	--	--
Ca3_P1_36485511	Ca3	36485511	GAA/GA	GCCGTGATGGTTTCATTTTT	TCGGGTCGGAAGTTGAATAA	59.8	547	INTERGENIC	--	--	--
Ca3_P1_36485658	Ca3	36485658	ATTCTAA/ATTCTAATTTCTAA	GCCGTGATGGTTTCATTTTT	TCGGGTCGGAAGTTGAATAA	59.8	547	INTERGENIC	--	--	--
Ca3_P1_36485880	Ca3	36485880	TTAGTATTTTAT/TT	TTATTCAACTTCCGACCCGA	ACCCCACAAAATCACGAAAT	60.4	461	INTERGENIC	--	--	--
Ca3_P1_36489573	Ca3	36489573	CGG/C	ATTGTGGGTTGGAATTTGGA	TTGTGGATTGAAAGGAAGCC	60.0	519	DRR	Ca_00966	I	bHLH
Ca3_P1_36490446	Ca3	36490446	T/TCAC	CAGGGTCACCTGCTCAAAAT	TCCAAGATCAGTTGACTGCG	60.1	333	DRR	Ca_00965	--	--
Ca3_P1_36492586	Ca3	36492586	AA/AATTA	GCACCATGAAAAAGAGGGAG	TATCATGGCTTTTGGCATCA	59.7	552	INTRON	Ca_00966	I	bHLH
Ca3_P1_36499924	Ca3	36499924	TAAAAAAAAA/TAAAAAA	CAGGAATTAATCCTTAGCGGTG	ACATGCTGTGAACTCGTTGG	60.0	785	DRR	Ca_00967	S	--
Ca3_P1_36508488	Ca3	36508488	ATTTTTT/ATTTTT	TAGTTGGAAATCGCTGACCC	TTTGTTATCAACCGTCATTTGAA	60.1	474	INTERGENIC	--	--	--
Ca3_P1_36508559	Ca3	36508559	AAATAATAATAATAATAA/AAATAATAATAATAATAATAA	TAGTTGGAAATCGCTGACCC	TTTGTTATCAACCGTCATTTGAA	60.1	474	INTERGENIC	--	--	--
Ca3_P1_36512162	Ca3	36512162	AAAATAAA/AAAA	TTAAGCATGCTCCCCAAAAC	GTCGAAGGCATAGTGTGCAA	60.1	348	DRR	Ca_00968	A	--

INDEL marker IDs	Chromosomes/ unanchored scaffolds	Physical positions (bp)	InDels (*Kabuli* reference genome-CDC Frontier/PI)	Forward primers (5'-3')	Reverse primers (5'-3')	Anneling temperature (0C)	Expected amplified product size (bp)	Structural annotation		Functional annotation	
								Sequence components of *kabuli* genome	*Kabuli* gene accession IDs	NCBI-KOG	TFs
Ca3_P1_36517092	Ca3	36517092	CAATAATT/CAATAATTTTTTTAAATAAAATAATT	AGGTCATCTTTCATTTGGCA	CGATGTGCGTGAGACTTTGT	58.1	473	INTERGENIC	--	--	--
Ca3_P1_36519114	Ca3	36519114	A/AC	TGATCAAGGAGTATCACGGG	AGTAGAACAAAGCAACGGCG	58.5	852	INTERGENIC	--	--	--
Ca3_P1_36519638	Ca3	36519638	TAAA/TATATTCAAAATTTTGAAA	CACCCTAATTAAAGTCGCCG	GTGGTCTAAGCGCCATGTTT	59.6	466	INTERGENIC	--	--	--
Ca3_P1_36523573	Ca3	36523573	TAAAAAA/TAAAAAAA	CCCTCTCTTTCCCATTCACA	AAACACTTCGTTAAATCCTTTTCAA	60.0	337	INTERGENIC	--	--	--
Ca3_P1_36523661	Ca3	36523661	T/TTAAA	ATGGACAAGGTTCGTGGAAG	CCACTTGGCGTGAAATCATA	60.0	875	INTERGENIC	--	--	--
Ca3_P1_36524052	Ca3	36524052	AG/A	TTTTTGAAAAGGATTTAACGAAGTG	TTAAGATGTGAGTTGGGGGC	59.9	585	INTERGENIC	--	--	--
Ca3_P1_36525432	Ca3	36525432	TAAAAAAAA/TAAAAAAA	TCCAAGGACTAAAACGTCCAA	TCCAAAGAGACTCATCCCAAA	59.6	519	DRR	Ca_00969	--	--
Ca3_P1_36525538	Ca3	36525538	AAT/AAAATCTTTAT	TCCAAGGACTAAAACGTCCAA	TCCAAAGAGACTCATCCCAAA	59.6	519	DRR	Ca_00969	--	--
Ca3_P1_36526855	Ca3	36526855	TA/T	AAGTCGCCAACGCAATAAAC	CTCTTTGTGCAAACAGGCAA	60.1	827	DRR	Ca_00970	--	--
Ca3_P1_36527172	Ca3	36527172	ATTTTTT/ATTTTTTT	TTTGGTGTACCCAACCCACT	AAATTTTGCCGTTTGGTGAG	60.1	654	DRR	Ca_00970	--	--
Ca3_P1_36528973	Ca3	36528973	GTTTTTTTTT/GTTTTTTTTTT	GTGGCACCTTGTGGAAGAAT	CCCATGAATGTTGGATAGGG	60.0	875	DRR	Ca_00971	U	--
Ca3_P1_36529799	Ca3	36529799	AAAGA/AA	CCCTATCCAACATTCATGGG	ACCAAACTGGTGGAACTTGC	60.0	722	DRR	Ca_00971	U	--

INDEL marker IDs	Chromosomes/ unanchored scaffolds	Physical positions (bp)	InDels (*Kabuli* reference genome-CDC Frontier/PI)	Forward primers (5'-3')	Reverse primers (5'-3')	Anneling temperature (0C)	Expected amplified product size (bp)	Structural annotation		Functional annotation	
								Sequence components of *kabuli* genome	*Kabuli* gene accession IDs	NCBI-KOG	TFs
Ca3_P1_36530182	Ca3	36530182	T/TC	GATTTAGCAGGAGTGCCGAG	TCGGATCGACTCTTTGGAAC	60.0	686	DRR	Ca_00971	U	--
Ca3_P1_36533590	Ca3	36533590	ATTT/ATTTT	TTGTTATACCCCATACTTTCTTGTCA	TGCGAACGATGGATGAGTAA	60.1	442	INTERGENIC	--	--	--
Ca3_P1_36548101	Ca3	36548101	CATCAA/CA	TCAATTTGGAGGACCAAAGC	TTGGATTGAACTGTCCCACA	60.1	737	DRR	Ca_00973	I	B3
Ca3_P1_36548331	Ca3	36548331	ATTTTT/ATTTT	TCAATTTGGAGGACCAAAGC	CCGGTGAAATTTATTGTGGG	60.1	992	DRR	Ca_00973	I	B3
Ca3_P1_36549137	Ca3	36549137	C/CA	TTTGATCTGTTCTCCGGTCC	TATTATCGCGCTCTCGACCT	60.1	350	INTRON	Ca_00973	I	B3
Ca3_P1_36570861	Ca3	36570861	A/AT	TACCGAATCCTTTGTCCTGC	GAGTTTGCGCGTTTAAGAGG	60.1	772	DRR	Ca_00977	--	NAC
Ca3_P1_36575996	Ca3	36575996	GTAT/GTATAT	CCATACTCAATGCAAAATCGTT	AGAAAAACATGCGTTGTCCC	59.0	590	INTERGENIC	--	--	--
Ca3_P1_36582696	Ca3	36582696	T/TC	ACCCTCCGTCTGTTTCACAC	AAACATCAAAACCAACACACTCA	60.0	328	INTERGENIC	--	--	--
Ca3_P1_36583128	Ca3	36583128	AAATTTTAA/AAA	TCTGTTTCACACCCATCGAA	GAGCCAACAAGGGTGATGTT	60.1	794	INTERGENIC	--	--	--
Ca3_P1_36595951	Ca3	36595951	TA/TAAA	AAAGTTTTTGGCAAGTCGGA	AGAGCAAGGAAACCGAATCA	59.7	596	INTERGENIC	--	--	--
Ca3_P1_36596139	Ca3	36596139	GAAAAAA/GAAAAAAA	AAAGTTTTTGGCAAGTCGGA	AGAGCAAGGAAACCGAATCA	59.7	596	INTERGENIC	--	--	--
Ca3_P1_36597966	Ca3	36597966	TAAAAAAAA/TAAAAAAA	TAACCAACAACCGCATGAAA	TCAATTTCTTAAAAGAGGTGGCA	60.0	242	INTERGENIC	--	--	--

INDEL marker IDs	Chromosomes/ unanchored scaffolds	Physical positions (bp)	InDels (*Kabuli* reference genome-CDC Frontier/PI)	Forward primers (5'-3')	Reverse primers (5'-3')	Anneling temperature (0C)	Expected amplified product size (bp)	Structural annotation		Functional annotation	
								Sequence components of *kabuli* genome	*Kabuli* gene accession IDs	NCBI-KOG	TFs
Ca3_P1_36599433	Ca3	36599433	ATGT/ATGTTGT	CAAAGACCACTGCGTGAAAA	GGACGCACCACACAACTATG	59.9	311	INTERGENIC	--	--	--
Ca3_P1_3660457	Ca3	3660457	AAATAATAATAATAATAATAATAATAATAATAATAATAATAATA/AAATAATAATAATAATAATAATAATAATAATAATAATAATAATAATAATAATA	TGAGACCGAAATTGATTTTCCT	ATGGTTGTGGGTCGGAATAG	59.9	675	INTERGENIC	--	--	--
Ca3_P1_36608541	Ca3	36608541	T/TA	TGGCTCAAAGATAGGATGGG	CCCTTCCTCCATCAATCTCA	60.0	315	DRR	Ca_00981	--	--
Ca3_P1_36609637	Ca3	36609637	CATATAT/CATAT	ATTGAAATGCGGGTTCTCAG	TTGATCACAACGAGGAGCTG	60.1	519	DRR	Ca_00981	--	--
Ca3_P1_36614124	Ca3	36614124	TGTA/T	TTGCATTTCAAAAGTTTCGC	TGTGGCTCAACGAGAGATTT	58.9	277	INTERGENIC	--	--	--
Ca3_P1_36621274	Ca3	36621274	T/TA	TTTGAAGGTAACTTGCCGCT	AACACGTGGACTCCATCACA	59.9	581	INTRON	Ca_00983	T	--
Ca3_P1_36622989	Ca3	36622989	ACC/AC	GCAACAGCTTTAGAGTGGGC	TCGTATTGTCAGGGGTGTGA	60.0	518	INTERGENIC	--	--	--
Ca3_P1_36624385	Ca3	36624385	CAAAAAAAAAAA/CAAAAAAAA	TGATTTCCACTGCACCACAT	TGCAACACAAACTCCTTTGC	60.0	517	INTERGENIC	--	--	--
Ca3_P1_36624455	Ca3	36624455	ATTTTTTT/ATTTTTTTT	TGATTTCCACTGCACCACAT	TGCAACACAAACTCCTTTGC	60.0	517	INTERGENIC	--	--	--
Ca3_P1_36624904	Ca3	36624904	GTTT/GTTTCTTTTTT	TGCAAAGGAGTTTGTGTTGC	AACATAAAAGAACAGAGCGGAGA	59.9	211	INTERGENIC	--	--	--
Ca3_P1_36625021	Ca3	36625021	ATTTT/ATTTTT	TGCAAAGGAGTTTGTGTTGC	TCAACGAATTGGTGAAAGAAAA	59.9	733	INTERGENIC	--	--	--
Ca3_P1_36625899	Ca3	36625899	TTATTTATTTA/T	ATTTCACTGACTGTCGCCCT	CAACAGGAAGGGGATTAGAAAA	59.7	356	INTERGENIC	--	--	--

INDEL marker IDs	Chromosomes/ unanchored scaffolds	Physical positions (bp)	InDels (*Kabuli* reference genome-CDC Frontier/PI)	Forward primers (5'-3')	Reverse primers (5'-3')	Anneling temperature (0C)	Expected amplified product size (bp)	Structural annotation		Functional annotation	
								Sequence components of *kabuli* genome	*Kabuli* gene accession IDs	NCBI-KOG	TFs
Ca3_P1_36625958	Ca3	36625958	ATTTTTTT/ATTTTTT	ATTTCACTGACTGTCGCCCT	CAACAGGAAGGGGATTAGAAAA	59.7	356	INTERGENIC	--	--	--
Ca3_P1_36626461	Ca3	36626461	TAAAAAAA/TAAAAAAAAA	TTTTCTAATCCCCTTCCTGTTG	GAAGCACTCATCATGGATCAAA	59.5	820	INTERGENIC	--	--	--
Ca3_P1_36626552	Ca3	36626552	ATTT/AT	TTTTCTAATCCCCTTCCTGTTG	GCACTCACCATGCATCAAAA	59.5	901	INTERGENIC	--	--	--
Ca3_P1_36628231	Ca3	36628231	TCGTCGAAC/TC	TGAACAAAATGCACTCCAACA	TGCACGAATCACCGAGATAG	60.1	344	DRR	Ca_00984	C	GATA
Ca3_P1_36629946	Ca3	36629946	GTATT/GTATTATT	TCCTCGGTAAAAGCCAGCTA	GTTGCTATTGCCTCATCGGT	60.0	518	INTRON	Ca_00984	C	GATA
Ca3_P1_36634950	Ca3	36634950	TAAAAAAAAA/TAAAAAAA	TCTCGCTGGCTCCTTATTGT	CATGCCACGTCATTTAAAACAT	60.0	470	INTERGENIC	--	--	--
Ca3_P1_36636712	Ca3	36636712	CTTTTTT/CTTTTT	CGGATAACACTTGTTTGTGAGTTT	GTCTTTTCTTGATCCCGACG	59.5	776	INTERGENIC	--	--	--
Ca3_P1_36638153	Ca3	36638153	TG/T	TCGTCAACTCGCAACTCATC	AAAAAGCCAAACAATTCTAGAAACA	60.0	487	INTERGENIC	--	--	--
Ca3_P1_36638232	Ca3	36638232	AATTTT/A	TCGTCAACTCGCAACTCATC	AAAAAGCCAAACAATTCTAGAAACA	60.0	487	INTERGENIC	--	--	--
Ca3_P1_36639180	Ca3	36639180	TTATATATATATATATATATATATAT/TTATATATATATATATATATAT	CAACGCGTGAAAGAAACAAA	TCGGATGGGAAAACTACTCAA	59.9	626	INTERGENIC	--	--	--
Ca3_P1_36640741	Ca3	36640741	TAAAA/TAAA	TTTACCGGAAGTAACACCGC	AGCATTTGTGAAACAGAGGGA	60.0	439	INTERGENIC	--	--	--
Ca3_P1_36641621	Ca3	36641621	CAAAA/CAAA	TGATCTTTGAAATACGAGACAAATG	CATAAAAAGGAGGGCGACAC	59.6	557	INTERGENIC	--	--	--

INDEL marker IDs	Chromosomes/ unanchored scaffolds	Physical positions (bp)	InDels (*Kabuli* reference genome-CDC Frontier/PI)	Forward primers (5'-3')	Reverse primers (5'-3')	Anneling temperature (0C)	Expected amplified product size (bp)	Structural annotation		Functional annotation	
								Sequence components of *kabuli* genome	*Kabuli* gene accession IDs	NCBI-KOG	TFs
Ca3_P1_36647741	Ca3	36647741	CAAAA/CAAA	TTGCCATCCATTCTGGTACA	TGGTGTTGGAGGAATGTGAA	59.9	882	DRR	Ca_00985	U	--
Ca3_P1_36652224	Ca3	36652224	AATAT/A	TTCTGGTGCAAAACCACAAG	TGAGCTGGCTTTCTTTGTGA	59.7	935	DRR	Ca_00986	--	bZIP
Ca3_P1_36652485	Ca3	36652485	CAAAA/CAAA	TACGGATTGCCATTCTCGAT	CCACAACACGTCACGCTAGT	60.4	760	DRR	Ca_00986	--	bZIP
Ca3_P1_36652542	Ca3	36652542	ATTTTTTT/ATTTTTTTT	TACGGATTGCCATTCTCGAT	CCACAACACGTCACGCTAGT	60.4	760	DRR	Ca_00986	--	bZIP
Ca3_P1_36652791	Ca3	36652791	TGGG/TG	TCACAAAGAAAGCCAGCTCA	TCCAGAGCTTTTCATGGCTT	59.7	451	DRR	Ca_00986	--	bZIP
Ca3_P1_36652888	Ca3	36652888	CAATTAATTAATTAATTAATT/CAATTAATTAATTAATT	ACTAGCGTGACGTGTTGTGG	TCCAGAGCTTTTCATGGCTT	59.8	296	DRR	Ca_00986	--	bZIP
Ca3_P1_36653994	Ca3	36653994	CAAAAAAA/CAAAAAA	ACTGACAACTGGCATTTGGA	TGAATCCCATAATGAAGCCA	59.1	393	INTERGENIC	--	--	--
Ca3_P1_36654116	Ca3	36654116	TAGACTAA/T	CTGGCATTTGGAATGTTGAG	GATGGCTCCAAAAATAGCCA	59.1	867	INTERGENIC	--	--	--
Ca3_P1_36654185	Ca3	36654185	ACT/ACTCT	TGGCTTCATTATGGGATTCA	GGCTCCAAAAATAGCCAACTT	58.9	499	INTERGENIC	--	--	--
Ca3_P1_36654407	Ca3	36654407	CTT/CTTT	TGGCTTCATTATGGGATTCA	GGCTCCAAAAATAGCCAACTT	58.9	499	INTERGENIC	--	--	--
Ca3_P1_36656553	Ca3	36656553	CG/C	GAAATGCACGTTGCAATCAC	CGAATGAGCAACTTTTGTCG	60.1	278	INTERGENIC	--	--	--
Ca3_P1_36670189	Ca3	36670189	CATA/CA	CAGAGGCCCACAAAATTCAT	GTTGGTTATGCGCAGACTGA	59.9	604	INTERGENIC	--	--	--

INDEL marker IDs	Chromosomes/ unanchored scaffolds	Physical positions (bp)	InDels (*Kabuli* reference genome-CDC Frontier/PI)	Forward primers (5'-3')	Reverse primers (5'-3')	Anneling temperature (0C)	Expected amplified product size (bp)	Structural annotation		Functional annotation	
								Sequence components of *kabuli* genome	*Kabuli* gene accession IDs	NCBI-KOG	TFs
Ca3_P1_36675129	Ca3	36675129	GC/G	TTCAAGCTTCCTTTGCGTTT	GCATGAACGCTAACAAATGG	60.0	417	INTERGENIC	--	--	--
Ca3_P1_36682594	Ca3	36682594	GTTTTTTTTT/GTTTTTTTTTT	TTCATCGATGTTCGGACAAA	CCAACTCGGTTCATTTACTCG	60.0	536	INTERGENIC	--	--	--
Ca3_P1_36688981	Ca3	36688981	TTCTTTATCTTTATCTTTATC/TTCTTTATCTTTATCTTTATCTTTATC	AGGGGAGACAACCATAGGCT	GTGATGTGATTGGCTGGTTG	60.0	862	DRR	Ca_00990	O	C3H
Ca3_P1_36696855	Ca3	36696855	TA/TAA	CGGTACATGGACCTCACTCC	CAAGCAAATATGGTCACTACCAG	60.4	395	INTERGENIC	--	--	--
Ca3_P1_36697642	Ca3	36697642	ATT/AT	CGACGCATATTTCAAATGACTT	TCTTTTGGCAACAGGTTGGT	59.1	634	INTERGENIC	--	--	--
Ca3_P1_36697708	Ca3	36697708	AC/A	CGACGCATATTTCAAATGACTT	AGTTGCATGCTTCTTTTGGC	59.1	645	INTERGENIC	--	--	--
Ca3_P1_36699717	Ca3	36699717	TAAAAAA/TAAAAA	TGCCGTATAATCATGCAACC	TGTGTGTTTGTGAATCTTAGCG	59.4	759	INTERGENIC	--	--	--
Ca3_P1_36700036	Ca3	36700036	TAG/TAGTAGGGGGTTAAG	CACAGGGAATAGAGATAAAGGCA	CTCCAACATCAAACGCAAGA	59.6	484	INTERGENIC	--	--	--
Ca3_P1_36702720	Ca3	36702720	CATTACTTTT/CATTACTTTTATGGAATTACTTTT	GTCGTGGATTCAAATTCCGT	AGGGCCGACTCTACCAGATT	59.8	662	INTERGENIC	--	--	--
Ca3_P1_36707043	Ca3	36707043	CT/C	CTCCTCCCAGTGGTAACAAA	CAGTGAAAAATTGAATCCCCA	57.6	595	INTERGENIC	--	--	--
Ca3_P1_36707318	Ca3	36707318	TA/TAAA	CAAAAACAGTTTGGGGATTCA	CATTTGACCTTACGTCCATCC	59.8	502	INTERGENIC	--	--	--
Ca3_P1_36707636	Ca3	36707636	TTTTATTTATT/TTTTATTTATTTATTTATTT	CAAAAACAGTTTGGGGATTCA	GATGATGGAAGGGGATGAGA	59.8	868	INTERGENIC	--	--	--

INDEL marker IDs	Chromosomes/ unanchored scaffolds	Physical positions (bp)	InDels (*Kabuli* reference genome-CDC Frontier/PI)	Forward primers (5'-3')	Reverse primers (5'-3')	Anneling temperature (0C)	Expected amplified product size (bp)	Structural annotation		Functional annotation	
								Sequence components of *kabuli* genome	*Kabuli* gene accession IDs	NCBI-KOG	TFs
Ca3_P1_36713668	Ca3	36713668	TTT/TTTCTT	TGAGAAAGGCATGCAAGATG	AACGCATCCAGTGATCAACA	59.9	568	INTRON	Ca_00995	O	MYB
Ca3_P1_36716273	Ca3	36716273	CAAAAAAA/CAAAAAAAAAA	TTTGTCCTGCCATAGCCATT	CGGTCACCCCTCAAATAAAA	60.5	734	DRR	Ca_00995	O	MYB
Ca3_P1_36716479	Ca3	36716479	GTTGAGATTGAGATTGAGA/GTTGAGATTGAGA	CCACCATTGAAGAGAGTGGG	CGGTCACCCCTCAAATAAAA	60.5	512	DRR	Ca_00995	O	MYB
Ca3_P1_36716843	Ca3	36716843	GC/G	TTTTATTTGAGGGGTGACCG	GATGGATTGGTCGTGAGGTT	59.8	554	DRR	Ca_00995	O	MYB
Ca3_P1_36724118	Ca3	36724118	AG/A	AATAGGGGAGAGAAAGCCCA	TGACTGCAATCATGTGGTGA	60.0	787	DRR	Ca_00996	--	--
Ca3_P1_36724181	Ca3	36724181	TAA/TAAA	AATAGGGGAGAGAAAGCCCA	TGACTGCAATCATGTGGTGA	60.0	787	DRR	Ca_00996	--	--
Ca3_P1_36726574	Ca3	36726574	TCC/TC	CATTTTTCATCCCAACCACC	TGTCACACGAACATCAAATCAA	60.0	598	INTERGENIC	--	--	--
Ca3_P1_36727040	Ca3	36727040	ATTTTTT/ATTTTTTT	TGATTTGATGTTCGTGTGACAA	GCATCTCACGAGGGCTACAT	60.0	513	INTERGENIC	--	--	--
Ca3_P1_36728882	Ca3	36728882	CTA/C	TGACGTGTAATGGTGTGATGA	AAAAAGAATGCAACATGGGC	58.4	533	INTERGENIC	--	--	--
Ca3_P1_36729235	Ca3	36729235	TAAAAAAAAA/TAAAAAAAAAAA	TTGGGCCAAATTAACCTCTC	AAAAAGAATGCAACATGGGC	59.0	454	INTERGENIC	--	--	--
Ca3_P1_36729751	Ca3	36729751	GAACTCCCTTAA/GAA	ACTTGCAAGCAAGGAAAGGA	ACAGTATTGCCCTCGTTTGG	60.0	622	INTERGENIC	--	--	--
Ca3_P1_36731868	Ca3	36731868	TATAATAATAATAATAATAATAAT/TATAATAATAATAATAATAATAAT	CAGCTCCAGTCCTTTTCCTG	CAAACAAAATGCGAAAGGGT	60.0	333	DRR	Ca_00997	--	--

INDEL marker IDs	Chromosomes/ unanchored scaffolds	Physical positions (bp)	InDels (*Kabuli* reference genome-CDC Frontier/PI)	Forward primers (5'-3')	Reverse primers (5'-3')	Anneling temperature (0C)	Expected amplified product size (bp)	Structural annotation		Functional annotation	
								Sequence components of *kabuli* genome	*Kabuli* gene accession IDs	NCBI-KOG	TFs
Ca3_P1_36735649	Ca3	36735649	T/TA	TACGTGGCCTATTAGGTGGC	TCCATGGCATATAAGTATTTGGTT	60.0	388	INTERGENIC	--	--	--
Ca3_P1_36735770	Ca3	36735770	AATAAATAAATATAAATAAATATAAATA/AATAAATAAATATAAATA	TACGTGGCCTATTAGGTGGC	GCATATGGTGATTGCTGACTCT	60.0	648	INTERGENIC	--	--	--
Ca3_P1_36735874	Ca3	36735874	TAAAAAAA/TAAAAAA	TACGTGGCCTATTAGGTGGC	CGGCGGAGTCAGGTAATAAA	60.0	834	INTERGENIC	--	--	--
Ca3_P1_36735945	Ca3	36735945	AT/ATT	TACGTGGCCTATTAGGTGGC	ATGGGTAACCGGGTGTATCA	60.0	874	INTERGENIC	--	--	--
Ca3_P1_36736757	Ca3	36736757	ATTTTTTTT/ATTTTTTT	ATACACCCGGTTACCCATCA	CCACTTTTCTTCGCCTCTTG	59.9	486	INTERGENIC	--	--	--
Ca3_P1_36743589	Ca3	36743589	AT/A	AAAAGGGACCATGCATTTTG	CACTTTGCCTCCTTCTCGAC	59.8	516	INTERGENIC	--	--	--
Ca3_P1_36744974	Ca3	36744974	ATTT/ATTTGTTTT	TGTGGACAGGTGCTTAGCTG	GCAATCAAATCAAAATACGGG	60.0	629	INTERGENIC	--	--	--
Ca3_P1_36746091	Ca3	36746091	CTCTTGA/CTCTTGAATCTTGA	AACGGGGCGAAGATCTTTAT	TGAGTTTTGTACACCGCTGC	59.9	199	INTERGENIC	--	--	--
Ca3_P1_36746609	Ca3	36746609	AT/ATCT	GCAGCGGTGTACAAAACTCA	ATCCAGAAGGTCAACGTCCA	59.9	854	DRR	Ca_00998	--	C2H2
Ca3_P1_36749120	Ca3	36749120	GTATTATTATTATTA/GTATTATTATTATTATTA	GTCACAGGTAGCTTGCCTCC	TGTCTTTAACAGTTTCCAAAACCA	59.9	516	INTRON	Ca_00998	--	C2H2
Ca3_P1_36749610	Ca3	36749610	TAAAAA/TAAAA	TGGTTTTGGAAACTGTTAAAGACA	AATGATCCTCGAATTGCTGG	59.9	251	INTRON	Ca_00998	--	C2H2
Ca3_P1_36750142	Ca3	36750142	CTTTTTTTT/CTTTTTT	GCCCACAAGATACCAGCAAT	AAACACTGGTCCCACTTTGC	60.0	815	INTRON	Ca_00998	--	C2H2

INDEL marker IDs	Chromosomes/ unanchored scaffolds	Physical positions (bp)	InDels (*Kabuli* reference genome-CDC Frontier/PI)	Forward primers (5'-3')	Reverse primers (5'-3')	Anneling temperature (0C)	Expected amplified product size (bp)	Structural annotation		Functional annotation	
								Sequence components of *kabuli* genome	*Kabuli* gene accession IDs	NCBI-KOG	TFs
Ca3_P1_36750458	Ca3	36750458	CT/CTT	ACTGTTGCATCTGCTTGACG	AAACACTGGTCCCACTTTGC	60.1	614	INTRON	Ca_00998	--	C2H2
Ca3_P1_36751667	Ca3	36751667	GTTTTTTTTTT/GTTTTTTTTTTT	TAACATCCATGGCCTCACAA	TGAAGTCACTAAAGCAACCGC	59.9	376	INTRON	Ca_00998	--	C2H2
Ca3_P1_36751775	Ca3	36751775	C/CCTATTAACAAGGAAA	TAACATCCATGGCCTCACAA	CGGTTTGTCTTTGGATAATGG	59.9	835	INTRON	Ca_00998	--	C2H2
Ca3_P1_36751932	Ca3	36751932	AT/A	TTTTATTCAACCGGACCGAC	CGTAGGCCAAGTTGATTCGT	59.8	801	INTRON	Ca_00998	--	C2H2
Ca3_P1_36752520	Ca3	36752520	AAGTAGTAGTAGTAGTA/AAGTAGTAGTAGTAGTAGTA	ACGAATCAACTTGGCCTACG	AGGGCAATGTACAATGGAGG	60.1	602	INTRON	Ca_00998	--	C2H2
Ca3_P1_36756795	Ca3	36756795	TAAAAAA/TAAAAAAA	AAGTGCTTGTTGCTGGAGTG	TCGTCGCATCTACAAACAAAA	59.1	529	INTERGENIC	--	--	--
Ca3_P1_36759627	Ca3	36759627	ATTTT/ATTTTT	GATGTTGTCGCAGATGAGGA	TGCCTTCAAGCAAAAACTGA	59.8	644	INTERGENIC	--	--	--
Ca3_P1_36761749	Ca3	36761749	TATAAAAACCTAATAAAA/TATAAAA	TGGGCTGTGAAATACAACGA	CCGAATTTAAGCATTTGGGA	60.1	816	INTERGENIC	--	--	--
Ca3_P1_36768446	Ca3	36768446	GTTTTTT/GTTTTTTT	TGCACGGAGGCTTTTATTTT	TCAAGGGTCACTTGCTTTTT	59.7	568	INTERGENIC	--	--	--
Ca3_P1_36768741	Ca3	36768741	AAA/AAAGTAATAAAGGGAA	CCACATGCAAACAAAATTCA	CTTTTCTCCGTGTGTTGCAC	58.0	877	INTERGENIC	--	--	--
Ca3_P1_36770528	Ca3	36770528	AT/ATATGAGCAGAAGT	GGCTGCCACAATTTTGATTT	TCATCTGAACAGCACTGCAA	59.9	344	INTERGENIC	--	--	--
Ca3_P1_36770611	Ca3	36770611	TTAT/TTATAT	GGCTGCCACAATTTTGATTT	TCATCTGAACAGCACTGCAA	59.9	344	INTERGENIC	--	--	--

INDEL marker IDs	Chromosomes/ unanchored scaffolds	Physical positions (bp)	InDels (*Kabuli* reference genome-CDC Frontier/PI)	Forward primers (5'-3')	Reverse primers (5'-3')	Anneling temperature (0C)	Expected amplified product size (bp)	Structural annotation		Functional annotation	
								Sequence components of *kabuli* genome	*Kabuli* gene accession IDs	NCBI-KOG	TFs
Ca3_P1_36771840	Ca3	36771840	TC/T	ACTTAGTCATCGGCCCACAC	TGGTCCGTTAAATGGTGTCA	60.0	571	INTERGENIC	--	--	--
Ca3_P1_36772843	Ca3	36772843	CATATATATATATATA/CATATATATATA	GTTAGTGCCGACCACTTGCT	TTTCAATTTTGTCTGCCGTG	60.3	301	INTERGENIC	--	--	--
Ca3_P1_36773118	Ca3	36773118	TTAT/TTATCTAT	ATTTGCTTTCCTTCACACGG	CAAGATTATCCGATAGACAATATCCA	60.1	393	INTERGENIC	--	--	--
Ca3_P1_36877810	Ca3	36877810	TTATATATATATATATATATATATATATATATAT/TTATATATATATATATATATATATATAT	CGTGCTTTCTACCCGTTCAT	TAGTTTCCTTGCGCTCAGGT	60.1	874	INTERGENIC	--	--	--
Ca3_P1_36932559	Ca3	36932559	GAAAAAAAAAAAAA/GAAAAAAAAAAAA	GTTACGATTTTCATTGGCGA	CTTTGGTATGTTGGGCCTGT	58.6	917	DRR	Ca_01018	--	WRKY
Ca3_P1_36986782	Ca3	36986782	AACT/A	TTTCAAGGTGTGTTCACCGA	TCTTACACCTCTTCGGGTGC	60.1	502	DRR	Ca_01027	U	C2H2
Ca3_P1_37000758	Ca3	37000758	TTAACTAACTAACTAACTAAC/TTAACTAACTAACTAAC	TGTGTGCTAACCAAATGCAAA	TGAATTGGATGCATCATCGT	60.2	664	INTRON	Ca_01028	--	--
Ca3_P1_37017817	Ca3	37017817	AATATATATATATATATATATATATATATATATA/AATATATATATATATATATATATATATATATATATATATATATATATATATA	CAGAAGGCCAGGAGAAGAAA	TGTGCGTGAAGTTTCATGGT	59.5	707	INTERGENIC	--	--	--
Ca3_P1_37030222	Ca3	37030222	AATATATATATATATATATATATATA/AATATATATATATATATATATATATATATA	GAAAAGCCCATTTTGTCTCG	AAAGGTTTGAAAATGGATGGTC	59.7	593	DRR	Ca_01031	G	C3H
Ca3_P1_3704345	Ca3	3704345	TAAAAAA/TAAAAA	TGGCAAGGAAATGACTCACA	GCAGATTGTGGGTTTGATTG	60.2	944	DRR	Ca_21343	A	--
Ca3_P1_37047269	Ca3	37047269	ATTTTTTTTT/ATTTTTTTT	CAGTTTTCAGAGTTGCATTTTCA	ATGAATGAAGGGGCTGAATG	59.4	474	INTERGENIC	--	--	--
Ca3_P1_370773	Ca3	370773	CTT/CT	GCGTTTTCCAGAACTGCTTC	TCAAGGCTAACTCCCAAGGA	60.0	796	INTERGENIC	--	--	--

INDEL marker IDs	Chromosomes/ unanchored scaffolds	Physical positions (bp)	InDels (*Kabuli* reference genome-CDC Frontier/PI)	Forward primers (5'-3')	Reverse primers (5'-3')	Anneling temperature (0C)	Expected amplified product size (bp)	Structural annotation		Functional annotation	
								Sequence components of *kabuli* genome	*Kabuli* gene accession IDs	NCBI-KOG	TFs
Ca3_P1_37078968	Ca3	37078968	CT/C	GGAAAGTAGCTGCTGTCGCT	AATGGAGTGCCAGACAAACC	59.8	556	INTRON	Ca_01034	R	--
Ca3_P1_3712841	Ca3	3712841	CATATATATATATATATATATAT/CATATATATATATATATATAT	TGACACACCCACCCACTAAA	GACTGAGATGATGATTCACTTTGC	59.8	520	INTERGENIC	--	--	--
Ca3_P1_37150587	Ca3	37150587	GAAAAAAAAAAA/GAAAAAAAAAA	TGCTTGCGTGAAAATTGAAA	TCCCATGTTCCGTATATCCAA	60.4	554	INTERGENIC	--	--	--
Ca3_P1_37150967	Ca3	37150967	ATTTTTTTT/ATTTTTTTTT	TTTTGCTTGCGTGAAAATTG	CCAAATGATAATCCATGGGC	59.9	604	INTERGENIC	--	--	--
Ca3_P1_37191129	Ca3	37191129	TAAAAAAAAAA/TAAAAAAAAA	TGGTTTCATTCATCTTTTGCAG	GCCCAAATCAACCCATTTTA	60.1	445	INTERGENIC	--	--	--
Ca3_P1_37208667	Ca3	37208667	TAAAAAA/TAAAAAAA	CTCAACCCTTACGGTGCAAT	TCAATTGGTCACGTAATTAACAAAA	60.0	301	INTERGENIC	--	--	--
Ca3_P1_3731004	Ca3	3731004	A/ACAGGTT	CAGCTCCCAGTTGCAATGTA	GCCCTCTATGGGTTGAAACA	59.9	656	INTERGENIC	--	--	--
Ca3_P1_37356302	Ca3	37356302	TGG/TG	TGGTTGATCACCCGAATACA	AACAAACCCCCACCACATTA	59.8	544	INTRON	Ca_01069	--	--
Ca3_P1_37360401	Ca3	37360401	AATATATATATATATATATATAT/AATATATATATATATATATATATATATATATATATAT	TTGGCCCATACTTAGGTTGC	CAATGGTTTGGAACCTGCTT	60.0	809	DRR	Ca_01069	--	--
Ca3_P1_37377986	Ca3	37377986	TCTCTCTCT/TCTCTCTCTCACTCTCTCT	ACCTCAGATTCCAAACGTGC	GCTGCATTGTGGATAAGGGT	60.1	425	INTERGENIC	--	--	--
Ca3_P1_37384200	Ca3	37384200	GAAAAAAAAAA/GAAAAAAAAA	GGTTGACCGACAAAATCGAG	TCTTGTAGCCAATCCAACCA	60.5	826	INTERGENIC	--	--	--
Ca3_P1_37428682	Ca3	37428682	T/TGGA	TTGAGGATCAGCTACACCCC	TGGAACAATTTTGGTGGTGA	60.1	546	INTERGENIC	--	--	--

INDEL marker IDs	Chromosomes/ unanchored scaffolds	Physical positions (bp)	InDels (*Kabuli* reference genome-CDC Frontier/PI)	Forward primers (5'-3')	Reverse primers (5'-3')	Anneling temperature (0C)	Expected amplified product size (bp)	Structural annotation		Functional annotation	
								Sequence components of *kabuli* genome	*Kabuli* gene accession IDs	NCBI-KOG	TFs
Ca3_P1_37475199	Ca3	37475199	TAAAAAAAAAA/TAAAAAAAAA	TGGCATTGGTTGAATCAAGA	ATACTTTAAGCTGCCGCCAA	60.0	519	DRR	Ca_01082	D	--
Ca3_P1_37484993	Ca3	37484993	TATAATAATAATAATAATAATAATATTAATAATAATA/TATAATAATAATAATAATAATATTAATAATAATA	CCCGCGTAAATATCAATGCT	GGTGGTGGTTTGGACGTAAC	60.0	482	INTERGENIC	--	--	--
Ca3_P1_37494243	Ca3	37494243	GATATATATATATATATATA/GATATATATATATATATA	GCAATTGGAATCAAATGTTGG	TTTCTACGCACCCTCAAAGC	60.2	441	INTERGENIC	--	--	--
Ca3_P1_37495861	Ca3	37495861	AATATATATATATATATATATATATATATA/AATATATATATATATATATATATATATATATATATATATATATATA	GGTGTGTGTTGTTTGGCATT	TGTTGCGAGATAGATCTTCACCT	59.3	464	INTERGENIC	--	--	--
Ca3_P1_37552722	Ca3	37552722	ATATTATTATTATTATTATTATTATTATTGTTATTATCATTATTATTATTATTATTATT/ATATTATTATTATTATTATTATTATTGTTATTATCATTATTATTATTATTATTATT	ATTGTTGCTGGGCTTTATGC	TGAACTTGGCGTGAGAGTTG	60.1	727	DRR	Ca_01090	--	ERF
Ca3_P1_37594905	Ca3	37594905	TTATATATATATATATATATATA/TTATATATATATATATATATATATATA	GTCCGCCCTAAAAAGGTAGC	TCAAAACACCACATCAAAACG	60.1	188	INTERGENIC	--	--	--
Ca3_P1_37700532	Ca3	37700532	TAAAAAAAAAAAAA/TAAAAAAAAAAAA	CCCTCCAATTTCAAGGGTTT	GCTTGTTGCTTGTTCCTTCC	60.2	714	INTERGENIC	--	--	--
Ca3_P1_37719525	Ca3	37719525	GCCC/GCC	TTGGGTCAAGAATTCATCTCG	CCCAAAATGTGGCTTGAAAT	60.1	581	INTERGENIC	--	--	--
Ca3_P1_3777234	Ca3	3777234	AATAATAATAATAATA/AATAATAATAATAATAATA	TCTCTGGCAACCGTTCTTCT	GAGCTGTGTCTTGGCAATCA	60.0	594	DRR	Ca_21336	--	--
Ca3_P1_37828643	Ca3	37828643	CCAGAC/CC	GGGAATAGGCTAGACCGTCC	GCTTGGCCTACGACCTATCA	59.9	512	INTERGENIC	--	--	--
Ca3_P1_378382	Ca3	378382	CTTTTTTTT/CTTTTTTTTT	ATTCCAACCAAAAACCCTCC	TTAAATAGGCTTCCAGGCCA	60.0	220	INTERGENIC	--	--	--
Ca3_P1_37902152	Ca3	37902152	GT/GTGCCT	GCTTTCAAATGCAATTGGTTC	TTGTTGAGTGTTGTGGTTGGA	59.6	744	INTERGENIC	--	--	--

INDEL marker IDs	Chromosomes/ unanchored scaffolds	Physical positions (bp)	InDels (*Kabuli* reference genome-CDC Frontier/PI)	Forward primers (5'-3')	Reverse primers (5'-3')	Anneling temperature (0C)	Expected amplified product size (bp)	Structural annotation		Functional annotation	
								Sequence components of *kabuli* genome	*Kabuli* gene accession IDs	NCBI-KOG	TFs
Ca3_P1_37955104	Ca3	37955104	TAAAAAAAAAA/TAAAAAAAAAAA	AACTGCTTCCAGAACAATGG	GCTTCAAATGACATCGCTGA	57.8	543	INTERGENIC	--	--	--
Ca3_P1_37977625	Ca3	37977625	AAATAATAATAATAATAATAATAATAATAAT/AAATAATAATAATAATAATAATAATAAT	TCCTGGTCTAGAGTGCGACC	AGAGGCAAGCAGAAACCAAA	60.4	435	INTERGENIC	--	--	--
Ca3_P1_37989883	Ca3	37989883	GTATATATATATATATATATA/GTATATATATATATATATATATATATATA	TGTCACGGGTGGACAAATTA	TAGGCGATTGTAGGGCTTTG	59.8	749	INTERGENIC	--	--	--
Ca3_P1_38001736	Ca3	38001736	CTTTTTTTTTTT/CTTTTTTTTTT	TTTACCAAGCACAGCTCCAA	GGATGATGATTAATTTTATGGGG	59.5	480	INTERGENIC	--	--	--
Ca3_P1_38007052	Ca3	38007052	ATATATATGTATAT/ATATAT	CCATGATCCAACTTGCAAAC	CTTCTCCAACAAGAGCGACC	59.0	693	INTERGENIC	--	--	--
Ca3_P1_38007387	Ca3	38007387	TTCTCTCTCTCTCTCTCTCTCTCTCT/TTCTCTCTCTCTCTCTCTCTCTCTCTCTCT	AAACAATACGCAAACCGGAG	CTTCTCCAACAAGAGCGACC	60.0	245	INTERGENIC	--	--	--
Ca3_P1_38073514	Ca3	38073514	CAAAAAAAAAAAAA/CAAAAAAAAAAAAAA	CTTCTGAATGGGTTTGGTGG	ATGGACTCAATTGGGAGTGG	60.3	439	DRR	Ca_01151	DL	--
Ca3_P1_38129457	Ca3	38129457	CATATATATATATATA/CATATATATATATATATA	CAAAAGCCGGACCAAAATTA	GGGCATGGGCAATAATCTAA	59.9	398	INTERGENIC	--	--	--
Ca3_P1_38139076	Ca3	38139076	A/AC	TTGCATTAAACTTGAACGTTTTT	TCAGGCCAACCCAGTACATT	58.0	416	INTERGENIC	--	--	--
Ca3_P1_38141259	Ca3	38141259	TTA/T	GGAAAGTCATGATGTCCTTTTT	GTCATTTGTAAATGGCGGCT	57.2	436	INTERGENIC	--	--	--
Ca3_P1_38141514	Ca3	38141514	ATTTTTT/ATTTTTTT	CCGCCATTTACAAATGACACT	TCGTCAAAATTATTTAGCGCAT	59.9	536	INTERGENIC	--	--	--
Ca3_P1_38141558	Ca3	38141558	CTT/CTTT	CCGCCATTTACAAATGACACT	TGGTTATGGAAAATTGCACG	59.9	602	INTERGENIC	--	--	--

INDEL marker IDs	Chromosomes/ unanchored scaffolds	Physical positions (bp)	InDels (*Kabuli* reference genome-CDC Frontier/PI)	Forward primers (5'-3')	Reverse primers (5'-3')	Anneling temperature (0C)	Expected amplified product size (bp)	Structural annotation		Functional annotation	
								Sequence components of *kabuli* genome	*Kabuli* gene accession IDs	NCBI-KOG	TFs
Ca3_P1_38145525	Ca3	38145525	AT/ATT	CTGATCTGGAGGTCTCTCGC	AAATTGGCACAGAAGGGTTG	60.1	545	INTRON	Ca_01158	C	--
Ca3_P1_38148366	Ca3	38148366	AA/AAAGAGTATA	TGCTAATTCTCCGTATGGGC	CAAACAGCAAAAGCTGTCCA	60.1	572	INTRON	Ca_01158	C	--
Ca3_P1_38149944	Ca3	38149944	GACTTTACTTTACT/GACTTTACT	AGTTATTACATGCGGCAGGG	TTAACCATGTGTCAAGGTCG	60.0	287	DRR	Ca_01158	C	--
Ca3_P1_38149993	Ca3	38149993	ATTA/ATTAACTTA	AGTTATTACATGCGGCAGGG	GCCAAACAGTGTTGTAATTCG	60.0	752	DRR	Ca_01158	C	--
Ca3_P1_38154708	Ca3	38154708	CGGAGGA/CGGAGGAGGA	AGAGCGGAAAAGTTATCGCA	TTCATGTTGTCCCTTTTGACA	60.0	477	DRR	Ca_01160	L	MYB_related
Ca3_P1_38162716	Ca3	38162716	GTTTT/GTT	GCAAGTTCGGATGTTCGATT	AGGCGGAAAATTCCTTCAAT	60.1	748	INTRON	Ca_01160	L	MYB_related
Ca3_P1_38164329	Ca3	38164329	TG/TGAGAGAGAG	TATTCCCCAATTCCTAGCCC	CAAGATGCTGGCACAGAAAA	60.1	740	INTRON	Ca_01160	L	MYB_related
Ca3_P1_38170265	Ca3	38170265	TAA/TA	CCCAGATCTGCCTAATGGAA	GATGGGTTTGGAACATCCAG	60.0	594	INTRON	Ca_01160	L	MYB_related
Ca3_P1_38170383	Ca3	38170383	CAGTAGTA/CAGTA	ATAGAATCTGGCATGGGGTG	TTCAGCCACTGCATTTGTTC	59.8	789	INTRON	Ca_01160	L	MYB_related
Ca3_P1_38171480	Ca3	38171480	TTTC/TTTCTAGTAAGAAGTATTC	ATCTACAAGCTTCCTCGCCA	GCTGGTTCAACGAGACAACA	60.0	645	INTRON	Ca_01160	L	MYB_related
Ca3_P1_38172415	Ca3	38172415	ACC/AC	TTTCAAACGTAAATGCGGAA	CCAGAAAACTTGCTTGCACA	59.2	743	INTRON	Ca_01160	L	MYB_related
Ca3_P1_38174447	Ca3	38174447	A/AT	TTCACATCCACACTCATCGG	CTGGCACTGGCATTCCTAAT	60.5	628	DRR	Ca_01161	--	NF-YA

INDEL marker IDs	Chromosomes/ unanchored scaffolds	Physical positions (bp)	InDels (*Kabuli* reference genome-CDC Frontier/PI)	Forward primers (5'-3')	Reverse primers (5'-3')	Anneling temperature (0C)	Expected amplified product size (bp)	Structural annotation		Functional annotation	
								Sequence components of *kabuli* genome	*Kabuli* gene accession IDs	NCBI-KOG	TFs
Ca3_P1_3818047	Ca3	3818047	GT/GGAT	ATGCTCTGCACTTGCAGCTA	TTCATCGCCTCAAAGTGTTG	59.9	707	INTERGENIC	--	--	--
Ca3_P1_38182684	Ca3	38182684	ATTT/ATTTATTAAAATATTTTTT	TCGTGTGTGCACCCTGTTAT	GGTTTTGCCAACATCTGCTT	60.0	647	INTERGENIC	--	--	--
Ca3_P1_38182761	Ca3	38182761	TTGATATGATAT/TTGATAT	AAACAACGTCATGAAGAAAATTGA	GGTTTTGCCAACATCTGCTT	59.9	606	INTERGENIC	--	--	--
Ca3_P1_38182839	Ca3	38182839	AGTGTGTGTGTGTGTGT/AGTGTGTGTGTGT	AAAGCGTCTTTCATTAATAGTGTGTG	GGTTTTGCCAACATCTGCTT	60.0	319	INTERGENIC	--	--	--
Ca3_P1_38183210	Ca3	38183210	TTAAATTAATAAATTAATAAA/TTAAATTAATAAA	AATCAAGCAAGCCGTTGACT	TAAAAGGTTTGTGCGTGTCG	59.9	356	INTERGENIC	--	--	--
Ca3_P1_38184945	Ca3	38184945	T/TG	AAGAAGAGAGGTTGGAGCCC	CTGGCTTGTGAACCCAAAAT	59.8	143	DRR	Ca_01162	--	--
Ca3_P1_38185117	Ca3	38185117	TTAT/TT	ATTTTGGGTTCACAAGCCAG	CAATTGGTGTTAGGCCACCT	60.0	497	DRR	Ca_01162	--	--
Ca3_P1_38187108	Ca3	38187108	C/CT	GTAAAGGGTTCCTCCTTCGC	GAAGACTCCAGCTTGCCATC	60.1	400	INTRON	Ca_01162	--	--
Ca3_P1_38198339	Ca3	38198339	CAATAATAAT/CAATAAT	TCGCACAATTCAACCTCAAG	CTTATGTGCCGCTCAACGTA	59.8	412	INTERGENIC	--	--	--
Ca3_P1_38201045	Ca3	38201045	CTTTTTTTT/CTTTTTTT	CCCCCTTTTCCCTGTGTTAT	TTGTGCTGCCAAACTGAAAG	60.0	952	INTRON	Ca_01163	E	--
Ca3_P1_38204385	Ca3	38204385	TTCTC/TTC	GCCCGGATACCATCCTACTT	TGCCCAGGTTACATATGGGT	60.2	396	INTRON	Ca_01163	E	--
Ca3_P1_38204961	Ca3	38204961	GAAAAAAAAA/GAAAAAAAA	TTTTGCTGTGTGTTGCATGA	GACAAGCAGCTACTGGAGGG	59.9	904	INTRON	Ca_01163	E	--

INDEL marker IDs	Chromosomes/ unanchored scaffolds	Physical positions (bp)	InDels (*Kabuli* reference genome-CDC Frontier/PI)	Forward primers (5'-3')	Reverse primers (5'-3')	Anneling temperature (0C)	Expected amplified product size (bp)	Structural annotation		Functional annotation	
								Sequence components of *kabuli* genome	*Kabuli* gene accession IDs	NCBI-KOG	TFs
Ca3_P1_38205894	Ca3	38205894	TAAAAAAAAA/TAAAAAAAA	ACAACACTCGGCCTATCACC	CTCCCCCTTCTCCAGGATAG	60.0	768	DRR	Ca_01163	E	--
Ca3_P1_38206155	Ca3	38206155	TGGGGGG/TGGGGGGG	GCATGCTGCCCATACCTATT	CTCCCCCTTCTCCAGGATAG	60.0	691	DRR	Ca_01163	E	--
Ca3_P1_38206248	Ca3	38206248	GCCC/GCCCC	GGCATTCAAATCCAATTGCT	CTCCCCCTTCTCCAGGATAG	59.9	179	DRR	Ca_01163	E	--
Ca3_P1_38206711	Ca3	38206711	GAAAAAAAAAAA/GAAAAAAAAAAAAA	CTATCCTGGAGAAGGGGGAG	GCTATTGCATTGGAGTTGCTC	60.0	706	DRR	Ca_01163	E	--
Ca3_P1_38206767	Ca3	38206767	AAGAG/AAG	CTATCCTGGAGAAGGGGGAG	GCTATTGCATTGGAGTTGCTC	60.0	706	DRR	Ca_01163	E	--
Ca3_P1_38212764	Ca3	38212764	CTTGTTT/CTT	ATGCAGACTTTGCTGCTCAA	TCAGAGGGATCGGACATAGG	59.7	267	INTRON	Ca_01164	T	WRKY
Ca3_P1_38215897	Ca3	38215897	CTT/CTTCTCTTCATT	TGTTGCCTGGAAATGAGTTG	TGCTGCATTGAAATCGAGTC	59.7	688	DRR	Ca_01165	R	--
Ca3_P1_38219148	Ca3	38219148	A/AC	CTACCGGCTACTTTCTTGCG	TATGCATGGTGAAAAAGCGA	60.0	463	DRR	Ca_01166	Z	B3
Ca3_P1_38221107	Ca3	38221107	TCTCCTC/TCTC	CCACTGGTGGATTCAGAGGT	GAGAACTCATGTGCCGGAAT	60.0	566	CDS (large-effect mutations)	Ca_01166	Z	B3
Ca3_P1_38225159	Ca3	38225159	TAGAGAGAGAGAG/TAGAGAGAGAGAGAG	TTAGAGAACGGAAACCTGGC	ACAGGTTGGTTTCTTGTCCG	59.3	363	INTRON	Ca_01166	Z	B3
Ca3_P1_38225828	Ca3	38225828	TAA/TA	CCACCATCCTTGTGCTTTTT	CCCCTCTCCCACGTTTAGTT	60.0	444	DRR	Ca_01167	R	--
Ca3_P1_38226041	Ca3	38226041	GTTTTTTTT/GTTTTTTTTT	AACTAAACGTGGGAGAGGGG	TTTGAGAGGTTGGATGAGCC	60.4	565	DRR	Ca_01167	R	--

INDEL marker IDs	Chromosomes/ unanchored scaffolds	Physical positions (bp)	InDels (*Kabuli* reference genome-CDC Frontier/PI)	Forward primers (5'-3')	Reverse primers (5'-3')	Anneling temperature (0C)	Expected amplified product size (bp)	Structural annotation		Functional annotation	
								Sequence components of *kabuli* genome	*Kabuli* gene accession IDs	NCBI-KOG	TFs
Ca3_P1_38228425	Ca3	38228425	A/AGTGTGAATAC	ATGATCCACTGAGATTCCGC	CCAGAGTGTGACGCTTTTGA	60.0	472	DRR	Ca_01168	R	bHLH
Ca3_P1_38240356	Ca3	38240356	TCCCC/TCCC	GTGGTGAGTTCAGTTCCGGT	ACCGTTAGAGGAGAGGAGCC	60.0	475	INTERGENIC	--	--	--
Ca3_P1_38241149	Ca3	38241149	A/AAT	GGCTCCTCTCCTCTAACGGT	GACAAGTGCAACAATTTTCATCA	59.8	527	INTERGENIC	--	--	--
Ca3_P1_38241297	Ca3	38241297	TTTATT/TTT	TTGATGAAAATTGTTGCACTTG	TTGCTAACACAAAAGTATTTGAATGTC	58.7	189	INTERGENIC	--	--	--
Ca3_P1_38305310	Ca3	38305310	CATATATATATATATATA/CATATATATATATATATATATA	ACCCACTCGTCCAAAAATCA	CCAAAAATCGTCTCCTTGGT	60.4	190	INTERGENIC	--	--	--
Ca3_P1_38373609	Ca3	38373609	TAATAAATA/TAATAAATAAATA	GCATTTGACAAGGTATCGCA	TCAGTTGCTGCAATCGAAAC	59.7	626	INTRON	Ca_01188	S	--
Ca3_P1_38406107	Ca3	38406107	AATATATATATATATATATATAT/AATATATATATATATATATATATATATAT	CTCTCAAAGTGCTGAAGAGACC	GGGACTGCATACCAATAGCTG	58.3	775	INTERGENIC	--	--	--
Ca3_P1_3841127	Ca3	3841127	A/AAT	CATGAAATAATGGGGGTTGC	TGGGGTCCATGTACCACTTT	60.0	410	INTERGENIC	--	--	--
Ca3_P1_3847338	Ca3	3847338	CATATATATATAT/CATATATATATATATAT	AGGAAGAGTGGAGTTGCGAA	ATTTTGGTTCGGAGGCTTTT	60.0	496	INTERGENIC	--	--	--
Ca3_P1_38618211	Ca3	38618211	TAAAAAAAAA/TAAAAAAAAAA	ATGAGGAACGAAACCGACAG	TTTTCAGCACATTCTTTTTGG	60.1	632	INTERGENIC	--	--	--
Ca3_P1_38690322	Ca3	38690322	AATATATATATATATATATATA/AATATATATATATATATATATATATATATA	GAAGCAGGGAGGAGGAAAAC	TGTCAATGAGGATTATGCCAA	60.2	910	DRR	Ca_01224	K	Trihelix
Ca3_P1_38710158	Ca3	38710158	TAAAAAAAAAAA/TAAAAAAAAAA	AAAGAGTGAAGCACGAGGGA	TTTCAACTGACCCGCATGTA	60.0	666	INTERGENIC	--	--	--

INDEL marker IDs	Chromosomes/ unanchored scaffolds	Physical positions (bp)	InDels (*Kabuli* reference genome-CDC Frontier/PI)	Forward primers (5'-3')	Reverse primers (5'-3')	Anneling temperature (0C)	Expected amplified product size (bp)	Structural annotation		Functional annotation	
								Sequence components of *kabuli* genome	*Kabuli* gene accession IDs	NCBI-KOG	TFs
Ca3_P1_3874106	Ca3	3874106	ATTTTTT/ATTTTTTT	TATGGGGATCGGAAATCAAA	GATGTGCGAAAGCAAGACAA	60.1	687	INTERGENIC	--	--	--
Ca3_P1_3876327	Ca3	3876327	AAATAATAATAATAATAATAATAGTAATAATAATAATAATAATAATAAT/AAATAATAATAATAATAATAATAATAATAGTAATAATAATAATAATAATAATAAT	TCAAACCCGGGTGTAAGGT	CAAAATTTTAGGCCGCGATA	60.2	371	INTERGENIC	--	--	--
Ca3_P1_38764047	Ca3	38764047	TAAAAATAAAA/TAAAAATAAAAAATAAAA	TGGGCTCCGAGAGTCTTATG	CGTGGATTAATCGCTACCGT	60.4	253	DRR	Ca_01234	--	bZIP
Ca3_P1_38788717	Ca3	38788717	GTATATATATATATATATAT/GTATATATATATATATAT	GGCTAAACAAAGGGAACGGT	TTTATTGCCAACAGCAACCA	60.4	581	DRR	Ca_01237	R	--
Ca3_P1_38801200	Ca3	38801200	CTTTTTTTTTT/CTTTTTTTTT	TCTTGGGAAAAAGTTACGAATCTC	CGTCAATGAAGAAGGTGCAA	60.0	905	INTERGENIC	--	--	--
Ca3_P1_3881329	Ca3	3881329	AAAAATATATAAAATATATAAAATA/AAAAATATATAAAATATATAAAATATATAAAATA	AGGTAGGCAAGTCTGAGGCA	CAAACGCATGACCAAGACAG	60.0	773	INTERGENIC	--	--	--
Ca3_P1_38870959	Ca3	38870959	AAATAATAATAATAATAATAATAATAATAATAATAATAATAATAATAA/AAATAATAATAATAATAATAATAATAATAATAATAATAATAATAA	TTTGCATATGACATTGAAAGCA	TGAAACAGGCAATCTTTCCC	59.2	292	INTERGENIC	--	--	--
Ca3_P1_38900910	Ca3	38900910	AATTTAT/AATTTATTTAT	ACCAGGTACCGCTACTGCAA	ATGCCGCAGACTGATGTTTT	60.7	592	INTERGENIC	--	--	--
Ca3_P1_38901208	Ca3	38901208	TAA/TAAA	CTTGCACATGCAACCAGGTA	ATTGTTCATGTTGTGTGGCG	60.7	625	INTERGENIC	--	--	--
Ca3_P1_3898691	Ca3	3898691	CAAAAAAAAAAAA/CAAAAAAAAAAAAA	TTGGTTACATTTCGAAAACGG	CCTGGAAAATCAAAAGCCAA	59.9	839	INTRON	Ca_21332	E	--
Ca3_P1_38998926	Ca3	38998926	AATAAATGT/A	GTATTGGGGCGTCACTTTTC	CACAAACTAAAACTGTGTGCAAAA	59.4	400	INTERGENIC	--	--	--
Ca3_P1_39010533	Ca3	39010533	AG/AGG	CGGGCTTTCCTGTTACACAT	GCAGAGAAAGGAACAGAGCG	60.0	309	INTERGENIC	--	--	--

INDEL marker IDs	Chromosomes/ unanchored scaffolds	Physical positions (bp)	InDels (*Kabuli* reference genome-CDC Frontier/PI)	Forward primers (5'-3')	Reverse primers (5'-3')	Anneling temperature (0C)	Expected amplified product size (bp)	Structural annotation		Functional annotation	
								Sequence components of *kabuli* genome	*Kabuli* gene accession IDs	NCBI-KOG	TFs
Ca3_P1_39079459	Ca3	39079459	ACCCCCC/ACCCCC	TAACCATCTTTGGGCGAATC	TTGAGAAGTGGCATATGAAACAA	59.9	566	DRR	Ca_01271	--	--
Ca3_P1_3921303	Ca3	3921303	AATATATATATATATATATATATATATATATATAT/AATATATATATATATATATATATATATATATATATATATATATATAT	ACTCCACCGCTATATGCACC	TTTTGTGTGGCATTTGGATG	60.0	534	DRR	Ca_21330	O	WRKY
Ca3_P1_39355383	Ca3	39355383	CATATATATATATATATATATATATAT/CATATATATATATATATATATATAT	TCGTAGGACCTCAAGTTGTATGAG	TTGGCCAAATCTTCGATCTC	59.7	338	INTERGENIC	--	--	--
Ca3_P1_39413210	Ca3	39413210	CATATATATATATATATATATATATATATATAT/CATATATATATATATATATATATATATAT	GGCATTCAATGGATATTGGG	ACGTTATCCGATCAAAACCG	60.0	357	INTERGENIC	--	--	--
Ca3_P1_39419619	Ca3	39419619	TAATATGAATATGAATAT/TAATATGAATAT	CCTAACTCAGCATGGGGAAA	GCGTGACAATAGTAAGATAATTGTTCC	60.1	506	INTERGENIC	--	--	--
Ca3_P1_39465898	Ca3	39465898	GTTATTATTATTATTATTATTATTATTATTATTATTATTATTATTAT/GTTATTATTATTATTATTATTATTATTATTATTATTATTATTAT	GAAGTTCGCTTCCTCCAAAA	TTGGCAGTACCAACGAATGA	59.4	358	DRR	Ca_01313	--	--
Ca3_P1_39571556	Ca3	39571556	GAAAAAAAAA/GAAAAAAAA	AATTTGTTTCGGCAATTCCA	GCAAAAGTGGCTGATGACAA	60.3	553	INTERGENIC	--	--	--
Ca3_P1_39611074	Ca3	39611074	T/TC	ACCGGACACGATATCAGGAG	TGCTTCAACAAAGGCAAACA	60.0	474	INTRON	Ca_01338	--	--
Ca3_P1_39611240	Ca3	39611240	C/CCT	ACCGGACACGATATCAGGAG	TGGTCCAATGCACAATCAAT	60.0	923	INTRON	Ca_01338	--	--
Ca3_P1_39611324	Ca3	39611324	TA/T	TGTTTGCCTTTGTTGAAGCA	CCCAATTTCGAAGCTCTCTG	60.4	198	INTRON	Ca_01338	--	--
Ca3_P1_3962696	Ca3	3962696	AAAAATATTT/AAAAATATTTAATATAAAATATTT	AGTTCCATGGGCTTTTCCTT	AGACTGTGGCACACAATGGA	59.9	552	INTERGENIC	--	--	--
Ca3_P1_39663553	Ca3	39663553	TAAAAAAAAA/TAAAAAAAA	GTGGCCACGTATCGAGGTAG	TCCAAAAGAAAATCATGGGG	60.5	516	INTERGENIC	--	--	--

INDEL marker IDs	Chromosomes/ unanchored scaffolds	Physical positions (bp)	InDels (*Kabuli* reference genome-CDC Frontier/PI)	Forward primers (5'-3')	Reverse primers (5'-3')	Anneling temperature (0C)	Expected amplified product size (bp)	Structural annotation		Functional annotation	
								Sequence components of *kabuli* genome	*Kabuli* gene accession IDs	NCBI-KOG	TFs
Ca3_P1_39713077	Ca3	39713077	GAAA/GAAAA	CACAAAGTTTCATCAATCCAAGA	TCGACTCCTGTTCAACCAAG	59.1	442	DRR	Ca_01353	J	--
Ca3_P1_39763068	Ca3	39763068	CAGAGAGAGAGAGAGAGAGA/CAGAGAGAGAGAGAGAGA	GGTCGCACATTGTCATCATC	AAAGGTTTCGACCACGTCAC	59.9	522	INTRON	Ca_01363	R	--
Ca3_P1_39783688	Ca3	39783688	CTCTT/C	ATCACGCTTTCAGCCACTCT	CAGAGGGCAAAGAATCCAAG	60.0	386	INTRON	Ca_01365	O	MYB_related
Ca3_P1_39806623	Ca3	39806623	A/ATATATAT	ACAAAGTGTGTGATGCCCAA	TTTGGAAATTCAAAGCCAGG	60.0	615	INTERGENIC	--	--	--
Ca3_P1_39866286	Ca3	39866286	GCATTAAGTTTAAAGAACT/G	TTGAGTCGGTTGAGCTGTTG	CCCATCGAACCAAAGACTGT	60.0	562	INTRON	Ca_01372	--	WRKY
Ca3_P1_39866735	Ca3	39866735	C/CT	ACAGTCTTTGGTTCGATGGG	CTGCCCCAAAAGACACAAAT	60.0	288	INTRON	Ca_01372	--	WRKY
Ca3_P1_39866810	Ca3	39866810	ATGG/ATGGTGG	ACAGTCTTTGGTTCGATGGG	CTGCCCCAAAAGACACAAAT	60.0	288	INTRON	Ca_01372	--	WRKY
Ca3_P1_39873548	Ca3	39873548	TAAAAAAAA/TAAAAAAAAA	TTTTCAACAACTCTTCTCATTACAAA	AAACGTTGTCTTGATTTTAATTTGC	58.5	573	INTERGENIC	--	--	--
Ca3_P1_39892163	Ca3	39892163	A/ACGTAGGG	ATTATCGAATGCCAAGTCGG	GGTCATTGGCATTGCTTCTT	59.9	461	INTRON	Ca_01375	YUJ	--
Ca3_P1_39966266	Ca3	39966266	TAAAAAAAAAA/TAAAAAAAAAAA	CAAAGTCTTCAAACAAAACCCA	TCGCTGCAAAATCATCACTC	59.2	730	INTERGENIC	--	--	--
Ca3_P1_39988476	Ca3	39988476	GTTTT/GTTT	AGAACAGAGCTGGCCTCAAA	ATTGCAAGAAAACCAGCACC	60.1	148	INTERGENIC	--	--	--
Ca3_P1_4006367	Ca3	4006367	A/AATGGTGTACT	TGTTCCAAGAGGGCATTACA	GACGTGATGCTCACTCCAGA	59.1	590	INTERGENIC	--	--	--

INDEL marker IDs	Chromosomes/ unanchored scaffolds	Physical positions (bp)	InDels (*Kabuli* reference genome-CDC Frontier/PI)	Forward primers (5'-3')	Reverse primers (5'-3')	Anneling temperature (0C)	Expected amplified product size (bp)	Structural annotation		Functional annotation	
								Sequence components of *kabuli* genome	*Kabuli* gene accession IDs	NCBI-KOG	TFs
Ca3_P1_4031026	Ca3	4031026	ATAT/ATATGTAT	GTTGGTTCCCTAGCACCGTA	ACCCAATTACTGGAGATGCG	60.0	706	INTERGENIC	--	--	--
Ca3_P1_4042829	Ca3	4042829	GATATATATATATATATATATATAT/GATATATATATATATATATATATATAT	ACACGAAGAGCAGGATCGTT	AAACAAAAATAAAAACTCGGAGACA	59.9	743	DRR	Ca_21325	QR	--
Ca3_P1_4058711	Ca3	4058711	A/ATT	ACTCTCGCCTTCATCTTCCA	GAGCGCTTTGTGAGGAAAAC	60.0	527	INTERGENIC	--	--	--
Ca3_P1_406033	Ca3	406033	ATT/ATTT	AGCCAATTTTGGAAGTGACG	CAATTCCCATGACCTCATCC	60.1	595	INTERGENIC	--	--	--
Ca3_P1_4073796	Ca3	4073796	CT/CTT	CTCAACATCCACCATGCAAT	TGCAAATTTCATCAAATGCATAA	59.4	746	INTERGENIC	--	--	--
Ca3_P1_4076583	Ca3	4076583	ATATTATTATTATTATTATTATTATTATTATTATTATTATTATTATTATT/ATATTATTATTATTATTATTATTATTATTATTATTATTATTATTATTATT	ATAGTCCTCACCCACGCATC	AAGCGTACCTGTGGGAATTG	60.0	607	INTERGENIC	--	--	--
Ca3_P1_4132252	Ca3	4132252	GATATATATATATATATATAT/GATATATATATATATATATATATAT	CGTGTGAGAAAACCTCGACA	CCGATTTCCCAAAGGTTTTT	59.9	242	INTERGENIC	--	--	--
Ca3_P1_414861	Ca3	414861	T/TG	CTATGCAACCACAGCTTCCA	GCAGATTCCGGTTCAAAAGA	59.9	438	INTRON	Ca_22928	O	--
Ca3_P1_415189	Ca3	415189	AATCAATGTTA/AA	TCTTTTGAACCGGAATCTGC	AAGAATGTATTGGGGCTTGG	60.2	229	INTRON	Ca_22928	O	--
Ca3_P1_4194452	Ca3	4194452	GTTTTTTT/GTTTTTT	TGGCCTCTTTAGACAATGGC	TCGGTGGTTTGGAATCATTT	60.2	159	INTERGENIC	--	--	--
Ca3_P1_4200504	Ca3	4200504	GAAAAAAAAA/GAAAAAAAAAA	CTTTCCGACTCCCAAACTCA	TCGAATTCAGAATCTCACAGTTG	60.2	546	DRR	Ca_22381	--	SAP
Ca3_P1_4227642	Ca3	4227642	TTATATATATATATATATATATAT/TTATATATATATATATATAT	TTTGATTTCAACGCTACGTGAT	TGACCCAAAAGTGACGACTG	59.7	632	INTERGENIC	--	--	--

INDEL marker IDs	Chromosomes/ unanchored scaffolds	Physical positions (bp)	InDels (*Kabuli* reference genome-CDC Frontier/PI)	Forward primers (5'-3')	Reverse primers (5'-3')	Anneling temperature (0C)	Expected amplified product size (bp)	Structural annotation		Functional annotation	
								Sequence components of *kabuli* genome	*Kabuli* gene accession IDs	NCBI-KOG	TFs
Ca3_P1_4372036	Ca3	4372036	ATTTTTTTTTT/ATTTTTTTTTTT	CTTGAGAGCAACAATTGGCA	CCTCCAGGCTTTGAAAATCA	60.0	857	INTERGENIC	--	--	--
Ca3_P1_4470002	Ca3	4470002	ATTTTTTTTTTTT/ATTTTTTTTTTTTTT	CATTATTAAAAGTCCATCGACACAA	TTTGAAATTTTGGTTTTGGTCC	59.4	302	INTERGENIC	--	--	--
Ca3_P1_451560	Ca3	451560	ATAACGTGATCTTA/ATA	AGCAGAAAGGGAGAATGGGT	ACGGTGAAGCTCCGAAAATA	60.1	734	INTERGENIC	--	--	--
Ca3_P1_454494	Ca3	454494	AGGG/AGG	GGATTGGAGTGTTCAGCCAT	TTGTCATTAGTTTCAATCTCACCAA	59.9	460	INTERGENIC	--	--	--
Ca3_P1_454633	Ca3	454633	GATATATATATATAT/GATATATATATAT	GGATTGGAGTGTTCAGCCAT	TTGTCATTAGTTTCAATCTCACCAA	59.9	460	INTERGENIC	--	--	--
Ca3_P1_457655	Ca3	457655	G/GTT	TTATGCTTATGCAGCTTGCG	TCCAAAATTAAAGGAAACCGC	60.1	527	INTERGENIC	--	--	--
Ca3_P1_4622794	Ca3	4622794	ATATTATTATTATTATTATTATTATTATTATTATTATTATTATTA/ATATTATTATTATTATTATTATTATTATTATTATTATTATTATTATTATTA	TTGTCAAGTTAGGGCACATTTTT	TCTTGAAGTGTGTGGCCATT	59.9	627	INTERGENIC	--	--	--
Ca3_P1_4626820	Ca3	4626820	TTGTG/TTG	GGAACGCTCTTTCTTCTCCC	GCGATCAGAGTTCCCATGTT	60.3	573	INTRON	Ca_22084	R	--
Ca3_P1_4688067	Ca3	4688067	GAA/GA	TCTGATGCAGTGTTTGGCTC	GCTTTCTTTGTCTGCCTTGG	60.0	332	INTERGENIC	--	--	--
Ca3_P1_4688180	Ca3	4688180	GAAAA/G	CCAAGGCAGACAAAGAAAGC	AGTAAAAGGCAGCAAGGCAA	60.0	442	INTERGENIC	--	--	--
Ca3_P1_4688550	Ca3	4688550	TA/TAA	CCAAGGCAGACAAAGAAAGC	ATGCCTATTCATCCCTGCAC	60.0	579	INTERGENIC	--	--	--
Ca3_P1_4689138	Ca3	4689138	TAAAAAA/TAAAAAAA	CAGCTTGCATTTGCTTTTGA	TTGCTCCTACGTATCTCCCG	60.1	888	INTERGENIC	--	--	--

INDEL marker IDs	Chromosomes/ unanchored scaffolds	Physical positions (bp)	InDels (*Kabuli* reference genome-CDC Frontier/PI)	Forward primers (5'-3')	Reverse primers (5'-3')	Anneling temperature (0C)	Expected amplified product size (bp)	Structural annotation		Functional annotation	
								Sequence components of *kabuli* genome	*Kabuli* gene accession IDs	NCBI-KOG	TFs
Ca3_P1_4694918	Ca3	4694918	GGAACTTTGAA/GGAACTTTGAACTTTGAA	ATGTGCAGGTCAAGTTGTCG	TCCGCTGCCATTTCTCTACT	59.8	484	INTRON	Ca_22085	--	--
Ca3_P1_474304	Ca3	474304	GTTTTTT/GTTTTTTT	ACGTTGCGAGCAGAGATTTT	TTGGCTTCTGAATTTGGTCC	60.0	655	INTERGENIC	--	--	--
Ca3_P1_4806714	Ca3	4806714	AACA/AA	AGGCCATGATCTCAAATGGA	TCGGCCAACCTACACCTTAC	60.4	704	INTRON	Ca_22089	D	WRKY
Ca3_P1_4817660	Ca3	4817660	ATGTGTGTGTGTGTGTGT/ATGTGTGTGTGTGTGTGT	ATTACGCAATCGCAATCCA	CGTTTTGTAGTTGGTCGCAT	60.1	237	INTERGENIC	--	--	--
Ca3_P1_4850058	Ca3	4850058	TT/TTGATAT	CCTTTTGCACAAGCTTCACA	TTTTCTGGGAAGCGAAAGAA	60.0	216	INTERGENIC	--	--	--
Ca3_P1_4897380	Ca3	4897380	AA/AAGA	AGCCATAACTTGCTTGAGGG	ACCTCGAATGAAAATCGTCG	59.3	723	INTERGENIC	--	--	--
Ca3_P1_4929398	Ca3	4929398	ATTTTTTTTT/ATTTTTTTTTT	GGCTTGTCACATCAGCTCAA	TCAGAACCATGTCAACGAGTTC	60.0	681	INTERGENIC	--	--	--
Ca3_P1_4938478	Ca3	4938478	ATTT/ATTTT	TCAACTCAACCCGAAACACA	TGTTTGAATGTGTGGCATTTG	60.1	813	INTERGENIC	--	--	--
Ca3_P1_4965548	Ca3	4965548	ATTTT/ATTTTT	CGGGGAATGTGATTGATAGG	GTTGGAGAGATCGGTGGAAA	60.1	948	INTERGENIC	--	--	--
Ca3_P1_4965627	Ca3	4965627	T/TGAG	TGCAAATCTGGAGCAGAGAA	GTTGGAGAGATCGGTGGAAA	59.7	510	INTERGENIC	--	--	--
Ca3_P1_4966098	Ca3	4966098	CTTTTTTTT/CTTTTTTT	TTTCCACCGATCTCTCCAAC	AAACCCGCTCCTTTCAAAAT	60.1	514	INTERGENIC	--	--	--
Ca3_P1_4975195	Ca3	4975195	AATATATATATATATA/AATATATATATA	CAAAGTTCAATACGCCAGCA	AGGTTTTGGCGTAACTCCCT	59.9	455	INTERGENIC	--	--	--

INDEL marker IDs	Chromosomes/ unanchored scaffolds	Physical positions (bp)	InDels (*Kabuli* reference genome-CDC Frontier/PI)	Forward primers (5'-3')	Reverse primers (5'-3')	Anneling temperature (0C)	Expected amplified product size (bp)	Structural annotation		Functional annotation	
								Sequence components of *kabuli* genome	*Kabuli* gene accession IDs	NCBI-KOG	TFs
Ca3_P1_5013857	Ca3	5013857	ATTTTTTTTTTT/ATTTTTTTTTT	GTGCGGGTATAATTTGGTGG	TGGGTGAAGTTTTCCGGTAG	60.1	854	INTERGENIC	--	--	--
Ca3_P1_5070771	Ca3	5070771	ATTTTTTTTTTTTTT/ATTTTTTTTTTTTT	AACCTTGATGCTCACATGAAA	TTACACTTGTTAGCCGGATGC	58.2	696	DRR	Ca_23878	--	--
Ca3_P1_5182639	Ca3	5182639	ATTTTTTTTT/ATTTTTTTTTT	TCTCTTTCTAAAACTAGGCAACCA	TTGAGGTGGCCAAAGCTACT	58.7	492	INTERGENIC	--	--	--
Ca3_P1_5239462	Ca3	5239462	AATATATATATATATATATATATATAT/AATATATATATATATATATATATATATAT	TGAGATCAGTTTTTGTGGAGGA	CAGTCACCACCCTTGCTTTT	59.7	482	INTERGENIC	--	--	--
Ca3_P1_5270072	Ca3	5270072	GAAA/GAAAA	TGGCAAATCGATTGAAAGAA	TTCTAGCTGGCTTTTCTCGC	59.2	519	INTERGENIC	--	--	--
Ca3_P1_5270123	Ca3	5270123	GAA/GA	ACGAAACGAAAGCGAGAAAA	CGACCTCGAAATAATCGGAA	60.0	521	INTERGENIC	--	--	--
Ca3_P1_5270478	Ca3	5270478	TATCAATC/TATC	ACGAAACGAAAGCGAGAAAA	CGACCTCGAAATAATCGGAA	60.0	521	INTERGENIC	--	--	--
Ca3_P1_5286221	Ca3	5286221	ATTT/ATT	CACCCCTCCCAAAACATCT	TTGTGAGTGCATTTGATTTTGTT	59.8	876	INTERGENIC	--	--	--
Ca3_P1_5288032	Ca3	5288032	AATATATATATATATATATATATAT/AATATATATATATATATATAT	AGAAGGTCGCATCTCCAAGA	TGGCACTTGTATTCACTCGC	60.0	456	INTERGENIC	--	--	--
Ca3_P1_5347423	Ca3	5347423	CAATAATAATAATAATAATAATAATAATAATAATAA/CAATAATAATAATAATAATAATAATAATAA	TTGCTCCTACGTATCTCCCG	TTGTGACAATTTGATGGTTCTAAA	60.2	563	INTERGENIC	--	--	--
Ca3_P1_5486984	Ca3	5486984	ATTTTTTTTTTT/ATTTTTTTTTT	AGGCCTGATCTACGGCCTAT	CAACAGGGGCCTATGGTATG	60.1	637	INTERGENIC	--	--	--
Ca3_P1_5502243	Ca3	5502243	TG/T	TGCGTTTTTCTGCATTTCTG	ATTCTGCGCGACTATCAACC	60.0	661	INTERGENIC	--	--	--

INDEL marker IDs	Chromosomes/ unanchored scaffolds	Physical positions (bp)	InDels (*Kabuli* reference genome-CDC Frontier/PI)	Forward primers (5'-3')	Reverse primers (5'-3')	Anneling temperature (0C)	Expected amplified product size (bp)	Structural annotation		Functional annotation	
								Sequence components of *kabuli* genome	*Kabuli* gene accession IDs	NCBI-KOG	TFs
Ca3_P1_5507746	Ca3	5507746	AATATATATATATATATATATATATATATATATAT/AATATATATATATATATATATATATATATATATATATATATATATATAT	TATTTTGGATTTTGGGCTCG	GGAGGGATAAGGTTTCTCGC	59.9	427	INTERGENIC	--	--	--
Ca3_P1_5520403	Ca3	5520403	GTATATATATATATATATATATATATA/GTATATATATATATATATATATATA	CAAATGACGACGACGATGAC	GAATGATGACAATGCGGTTG	60.1	503	INTERGENIC	--	--	--
Ca3_P1_5619451	Ca3	5619451	TAAAAAAAAAAAA/TAAAAAAAAAAA	TTCCGGGAAGTGATTTATCG	TGAAAAATTCCCAGGATGGA	59.9	966	INTERGENIC	--	--	--
Ca3_P1_5677125	Ca3	5677125	TTATTATTATTATTATTATTATTATTATTATTATTATTATTATTATTATTATTAT/TTATTATTATTATTATTATTATTATTATTATTATTATTATTATTATTATTATTATTAT	GCAACAAAATTCACACACCCT	GAAAGTCGATTTGGCGAGTT	59.9	819	DRR	Ca_20649	K	MIKC
Ca3_P1_5689325	Ca3	5689325	ATTT/ATT	TTGGTCAATGTGGCTTCTCA	TGAACTCTGAGCGTGTGACC	60.2	439	INTERGENIC	--	--	--
Ca3_P1_5689863	Ca3	5689863	GTTT/GTTTT	GGTCACACGCTCAGAGTTCA	GAAGCAAATCGATTGGGAAA	60.0	543	INTERGENIC	--	--	--
Ca3_P1_5692378	Ca3	5692378	T/TGACAATC	TGTGCAATTAAGAGAGGACACAA	CGAGAAATAGCTCACGAGGC	59.8	268	DRR	Ca_20648	--	--
Ca3_P1_571479	Ca3	571479	GTT/GTTGATT	GTGAGTGGTGAGGGCCTAAT	TTGCATACCTCGTATCATCCC	59.0	407	INTERGENIC	--	--	--
Ca3_P1_5763639	Ca3	5763639	GTTTTTTTTTT/GTTTTTTTTTTT	CCGAAGAGGCTTTGAAAACA	CCTCACCGCATGAGATTTTT	60.4	864	INTERGENIC	--	--	--
Ca3_P1_5805263	Ca3	5805263	GT/GTT	CAATTGGTCGAAAGACGGAT	TTCGAATGAACATAATTGAACTTT	59.9	164	INTERGENIC	--	--	--
Ca3_P1_5805325	Ca3	5805325	TAAAAAA/TAAAAAAA	CAATTGGTCGAAAGACGGAT	TTCATCTTCGTCAACAATGAAAA	59.9	554	INTERGENIC	--	--	--
Ca3_P1_5994220	Ca3	5994220	TGAGTAAAG/TGAGTAAAGAGTAAAG	AAAGCAGTCTGCAAGGGAAA	GTGCAACCATCATGCAAAAG	60.0	382	INTRON	Ca_27241	--	--

INDEL marker IDs	Chromosomes/ unanchored scaffolds	Physical positions (bp)	InDels (*Kabuli* reference genome-CDC Frontier/PI)	Forward primers (5'-3')	Reverse primers (5'-3')	Anneling temperature (0C)	Expected amplified product size (bp)	Structural annotation		Functional annotation	
								Sequence components of *kabuli* genome	*Kabuli* gene accession IDs	NCBI-KOG	TFs
Ca3_P1_6062601	Ca3	6062601	AATATATATATATATATAT/AATATATATATATATATATAT	AATCATCTCGCCCTGAATTG	CCACTAGCCTTGGGTTTCAA	60.0	621	INTERGENIC	--	--	--
Ca3_P1_611609	Ca3	611609	CATATATATATATATATATATATATATATATATATATATATATA/CATATATATATATATATATATATATATATATATATATATATA	CACAAACCCTAACCCGAAAG	GCCCTCATTCCATGAAACAT	59.5	650	INTERGENIC	--	--	--
Ca3_P1_613347	Ca3	613347	ATTTTTTTTTTT/ATTTTTTTTTT	TGGAGCTTTGTTTTGGACCT	AGTAAGTGTCGTCGTTGGGC	59.7	409	INTERGENIC	--	--	--
Ca3_P1_6200763	Ca3	6200763	AATATATATATATATATATATATAT/AATATATATATATATATATATATATATAT	TTGGTACATGCTTCACACGA	TGTGCCACGTGAAAATGAAT	58.7	569	INTERGENIC	--	--	--
Ca3_P1_6243942	Ca3	6243942	CTTATT/CTT	TGATGGACTTCGTGCTCTTG	CCTCAGGATAAAACAGCCCA	60.0	298	INTERGENIC	--	--	--
Ca3_P1_6249650	Ca3	6249650	ATTCTTCTTCTTCTTCTTCTTCTT/ATTCTTCTTCTTCTTCTTCTT	TGATGGACTTCGTGCTCTTG	CCCTCAGTATGCCAGTCGAT	60.0	225	INTERGENIC	--	--	--
Ca3_P1_6289426	Ca3	6289426	CATATATATATAT/CATATATATATATATAT	CGGTTCAGAACTGCATTGAA	CCTTACAAGGCCCACCAATA	59.8	457	INTERGENIC	--	--	--
Ca3_P1_62942	Ca3	62942	AATATATATATATATATATATATA/AATATATATATATATATATATATATATATA	TTCTCTCGAGGATAGTCCGA	AGTTAAACGAGGGGTTTGCC	57.6	936	INTERGENIC	--	--	--
Ca3_P1_6301895	Ca3	6301895	TCCCCC/TCCCC	CATTGGAAGCTTTAGGCTCG	GGGTTTTGAGGAGGTTGTCA	60.0	372	INTERGENIC	--	--	--
Ca3_P1_6314718	Ca3	6314718	GTTATTATTATTATTATTATTATTATTATTATTATTATTATTATTATTATTAT/GTTATTATTATTATTATTATTATTATTATTATTATTATTATTATTATTATTATTAT	AAAGGTTGACTTTTCCGGCT	ACTCGGTTCACACTTGAGGG	60.1	676	INTERGENIC	--	--	--
Ca3_P1_6386934	Ca3	6386934	TGG/TGGG	TGTACGAGAAGGTCGTGTGC	GCCCAACAGCAAGCTATTTC	59.9	574	INTRON	Ca_24834	--	Trihelix
Ca3_P1_6432046	Ca3	6432046	TAAAAAAAAAAA/TAAAAAAAAAA	TCCTGATGGCAATGCAAATA	AATAAGGTTGTCGGTTTGCG	60.0	172	INTERGENIC	--	--	--

INDEL marker IDs	Chromosomes/ unanchored scaffolds	Physical positions (bp)	InDels (*Kabuli* reference genome-CDC Frontier/PI)	Forward primers (5'-3')	Reverse primers (5'-3')	Anneling temperature (0C)	Expected amplified product size (bp)	Structural annotation		Functional annotation	
								Sequence components of *kabuli* genome	*Kabuli* gene accession IDs	NCBI-KOG	TFs
Ca3_P1_6448123	Ca3	6448123	AATATATATATATATATATATATATATATATATATATATA/AATATATATATATATATATATATATATATATATATATA	TTTTAATGAAGGATTGCCAATGT	TGAACTCGACAAAAGAACAACAA	59.7	421	INTERGENIC	--	--	--
Ca3_P1_6482071	Ca3	6482071	ATTTTTTTTTT/ATTTTTTTTTTT	CTAGCCTCCTTCCCTTGCTT	CGAACTTTACACCGGACCAC	60.0	693	INTERGENIC	--	--	--
Ca3_P1_6581424	Ca3	6581424	AATATATATATATATATATAT/AATATATATATATATATATATATATAT	AGCAAAAGGAAGCAACGAAA	CAATTGAGTTGCGACGAGAG	60.0	251	INTERGENIC	--	--	--
Ca3_P1_6587330	Ca3	6587330	T/TA	TTGAAGGGTTGCATGAATGA	GGAGGTCGAAAATGACCAAA	60.0	741	INTERGENIC	--	--	--
Ca3_P1_6599941	Ca3	6599941	ATTTTTTTTT/ATTTTTTTTTT	TCCATTTTGTGCGTTTGTGT	ACGGAGGACCAAAAAGACCT	60.0	657	INTERGENIC	--	--	--
Ca3_P1_666105	Ca3	666105	T/TG	CAGTCCCTCAACCTGACCAC	GTCTTCCCAATGGTGCATTT	60.6	235	INTERGENIC	--	--	--
Ca3_P1_6663794	Ca3	6663794	TAAAAAAAAAAAA/TAAAAAAAAAA	AAGGCTCAACATGCAAAACC	AATGCCCACCCATAATTTCA	60.1	324	INTERGENIC	--	--	--
Ca3_P1_6664320	Ca3	6664320	TAAAAAA/TAAAAAAA	TGAAATTATGGGTGGGCATT	GCTGAAAAAGTGCCGAAGAA	60.0	569	INTERGENIC	--	--	--
Ca3_P1_6666596	Ca3	6666596	ATT/AT	ATGCAAAATTGACCACCACA	CCAGACCTTCCTTATTGCCA	59.8	168	INTERGENIC	--	--	--
Ca3_P1_6672422	Ca3	6672422	TTTTATTTATTTATTTATTTATTTATTTAT/TTTTATTTATTTATTTATTTATTTATTTATTTATTTAT	GCGCCGCAATAGAATAGAAG	CAACAAAGGGAGGCAAATGT	60.0	672	INTRON	Ca_25772	A	--
Ca3_P1_6694845	Ca3	6694845	ATTTTTTTTT/ATTTTTTTTTT	TTTGTCACGTCAGACAAGGC	AATCGGTGTAGAGGCAATGG	59.9	660	INTERGENIC	--	--	--
Ca3_P1_6721297	Ca3	6721297	GAAAAAAAA/GAAAAAAA	ATATGGGTGATGAGGCATCC	GAGTTGTCCCTTCCTCACCA	59.6	737	INTERGENIC	--	--	--

INDEL marker IDs	Chromosomes/ unanchored scaffolds	Physical positions (bp)	InDels (*Kabuli* reference genome-CDC Frontier/PI)	Forward primers (5'-3')	Reverse primers (5'-3')	Anneling temperature (0C)	Expected amplified product size (bp)	Structural annotation		Functional annotation	
								Sequence components of *kabuli* genome	*Kabuli* gene accession IDs	NCBI-KOG	TFs
Ca3_P1_6728905	Ca3	6728905	TTGAAT/TT	CCGCGAGTAATTCCGATAAA	ATGCTTGGTGGGTGAGAAAG	60.1	243	INTERGENIC	--	--	--
Ca3_P1_6730727	Ca3	6730727	CTTTTTTTTTTTT/CTTTTTTTTTTTTTT	TTTAGGATTTTCCAACCACCA	TGGGGAGATAGGGTTGATTTT	59.3	655	INTERGENIC	--	--	--
Ca3_P1_6772905	Ca3	6772905	GTTT/GTT	TCCCTTTGTCTTTTCGCACT	CACTGCCGTTCTTCACTTCA	59.9	383	DRR	Ca_16559	R	NAC
Ca3_P1_6774064	Ca3	6774064	T/TTAA	TTTGCTGGACTTTGAAGCCT	CCCGGTCCTAGAGAGAAAGA	60.0	640	DRR	Ca_16559	R	NAC
Ca3_P1_6780454	Ca3	6780454	AAAA/AAAACAAA	GGATGCTTCGTTTGGTGTTT	TTTGAACCAAGATGCAGCAG	60.0	832	INTRON	Ca_16557	R	NAC
Ca3_P1_6788233	Ca3	6788233	AATTTATTAT/AAT	TTTTTCACTATTGTGTGAAATCAGT	TTTCCTACCATATTCCCGCT	57.0	826	DRR	Ca_16556	R	NAC
Ca3_P1_6821158	Ca3	6821158	CTTTT/CTTTTT	GATCATCCCTCACCACAACC	TTGCCACGTTGTTACTCGAT	60.2	409	INTERGENIC	--	--	--
Ca3_P1_6821985	Ca3	6821985	TTATATATATATATATATATATATATATA/TTATATATATATATATATATATATATATATATATATA	GCGTGATCCCAAGCTACAAT	CATCTTTTAAGGTGCGGGTG	60.1	139	INTERGENIC	--	--	--
Ca3_P1_6829727	Ca3	6829727	AATATATATATATATATATATATATATATATA/AATATATATATATATATATATATATATATATATA	AAATAAGCCGGTTGCATGAG	ACAGCGTCACGGAAGATAAA	60.1	445	INTERGENIC	--	--	--
Ca3_P1_6838667	Ca3	6838667	AGCA/AGCAGGATCCAATGCAATGGCA	GGAACCCCACAATCATCATC	GCAGCCAATTTTGTTGCATA	60.0	547	CDS (large-effect mutations)	Ca_16550	R	bHLH
Ca3_P1_6867569	Ca3	6867569	TC/T	TCAAACCATTTTTACCAGGCA	TTGTTATTCGACAAAGTTTCGTG	60.3	781	INTERGENIC	--	--	--
Ca3_P1_6959714	Ca3	6959714	TCCCCC/TCCCC	GCACTTTTGGTCCCCCTATT	GATTCTAGTCTCTTGCGCCG	60.2	420	DRR	Ca_16543	--	G2-like

INDEL marker IDs	Chromosomes/ unanchored scaffolds	Physical positions (bp)	InDels (*Kabuli* reference genome-CDC Frontier/PI)	Forward primers (5'-3')	Reverse primers (5'-3')	Anneling temperature (0C)	Expected amplified product size (bp)	Structural annotation		Functional annotation	
								Sequence components of *kabuli* genome	*Kabuli* gene accession IDs	NCBI-KOG	TFs
Ca3_P1_7076805	Ca3	7076805	CG/CCCGAATGTG	TCTTCGATTCCATTCCAACC	TTGGGGGAGACGATATTCTG	59.9	701	INTERGENIC	--	--	--
Ca3_P1_7202917	Ca3	7202917	ATG/A	TCATTGCGCTTTGTCAACAT	AACTGGTTTCTCCCCTCGTT	60.3	650	INTERGENIC	--	--	--
Ca3_P1_7258511	Ca3	7258511	CAAAAAAAAAAA/CAAAAAAAAAA	TGGAGATGACTTGCTTGCAG	TTTTTGTCGGTCAATCACCA	60.1	515	INTERGENIC	--	--	--
Ca3_P1_726429	Ca3	726429	ATTTTTTT/ATTTTTTTT	TCCTGTATCTTACCAAACAAGGC	GTTTGTTGTCGGCTTACGGT	59.6	715	INTERGENIC	--	--	--
Ca3_P1_7283088	Ca3	7283088	TAAAAAAAAAAAA/TAAAAAAAAAAA	TCACGGCATCTCAAGTAGGA	ATTTGGAAATCCAACCCACA	59.4	544	INTERGENIC	--	--	--
Ca3_P1_7367436	Ca3	7367436	CGAAGAAGA/CGAAGA	TGATTGTTTGTCTGGTGGGA	CACTCTCTTCCTTGGCCTTG	59.9	331	INTERGENIC	--	--	--
Ca3_P1_7368184	Ca3	7368184	TTAATAAATAATAA/TTAATAA	ATTGGGGCAATAACCAACAA	TGGTGCGATTGTGGAAAATA	60.1	854	INTERGENIC	--	--	--
Ca3_P1_746590	Ca3	746590	ATTTTTTTTT/ATTTTTTTTTT	TGCAATGCAATGTCCATTTT	CCACCGACTTGAAGAAGCTC	59.9	387	INTERGENIC	--	--	--
Ca3_P1_7467677	Ca3	7467677	AACATTAGA/AACATTAGACATTAGA	CCACACCCCTCACACTTTCT	AGCAAAGGGGATGAAAAGGT	60.0	461	INTERGENIC	--	--	--
Ca3_P1_7550712	Ca3	7550712	T/TAGAATGATATAG	TTGATTTGGTCGCAATTTTT	TGCACTAGTGTTGTACTCCGTTT	58.1	411	INTERGENIC	--	--	--
Ca3_P1_7580880	Ca3	7580880	TAAAAAAAAA/TAAAAAAAAAA	GTGGTTCCGGAAAAGATGAA	CCCCATCCATTTCCCTATCT	59.9	373	INTERGENIC	--	--	--
Ca3_P1_7623505	Ca3	7623505	TAAAAAAA/TAAAAAAAA	AATCTGGTTTTCTTGCACGG	ATGTGATTGGATTGTGCGAG	60.1	767	DRR	Ca_16514	--	bZIP

INDEL marker IDs	Chromosomes/ unanchored scaffolds	Physical positions (bp)	InDels (*Kabuli* reference genome-CDC Frontier/PI)	Forward primers (5'-3')	Reverse primers (5'-3')	Anneling temperature (0C)	Expected amplified product size (bp)	Structural annotation		Functional annotation	
								Sequence components of *kabuli* genome	*Kabuli* gene accession IDs	NCBI-KOG	TFs
Ca3_P1_7636444	Ca3	7636444	TATTATTATTAT/TATTATTATTATGATTATTATTAT	TGGCCTTGTGTGTATTGCAT	CTGAATTGCGTGTGTCTGGT	60.0	566	INTERGENIC	--	--	--
Ca3_P1_7768323	Ca3	7768323	AG/AGG	ATTTCAAGGCGCTCTTTGTG	TGTTGGGTTGGTTGAAGTGA	60.4	539	INTERGENIC	--	--	--
Ca3_P1_7773838	Ca3	7773838	AATATATATATATATAT/AATATATATATATAT	CATGCGAGTTGTAACACCCT	CGTGCATACTTAATATCAGTCAAGTG	58.7	365	INTERGENIC	--	--	--
Ca3_P1_7792015	Ca3	7792015	TAT/TATTATTCTTTCCCTAAT	TAGGTAAAGGTGGTTTGCCG	TGAGGGATAGATGCAGGTCA	60.0	611	INTERGENIC	--	--	--
Ca3_P1_781722	Ca3	781722	CAA/CAAAA	AAAACGCGCCACTAATGACT	AGCAAGAGTGCAAAGTCGGT	59.8	546	DRR	Ca_22643	V	B3
Ca3_P1_7880432	Ca3	7880432	TCTAG/T	AGGAAGAGTGGAGTTGCGAA	TATTTTGGATTTTGGCTCGG	60.0	503	INTERGENIC	--	--	--
Ca3_P1_7899910	Ca3	7899910	ACC/AC	GTACTTGGCACCCGAGTCAT	TCTCATCTCTGTGCTTCATGC	60.0	643	INTERGENIC	--	--	--
Ca3_P1_7922530	Ca3	7922530	TTATATATATATATATAT/TTATATATATATATATAT	AGGAAGAGTGGAGTTGCGAA	TATTTTGGATTTTGGCTCGG	60.0	504	INTERGENIC	--	--	--
Ca3_P1_794982	Ca3	794982	GTTTTTTTTTTT/GTTTTTTTTTT	TAGAGGGAGGAGGTGCAAGA	GGTGATATAGACGGGGCAGA	59.9	686	INTERGENIC	--	--	--
Ca3_P1_7959750	Ca3	7959750	TTAATTTGATTATGTTAAGTAATTTGATTATGTTAA/TTAATTTGATTATGTTAAGTAATTTGATTATGTTAAGTAATTTGATTATGTTAA	TTGGCATAGGTCATGGAAGTT	TTTGCCAATGTTGCTAGCTG	59.4	848	INTERGENIC	--	--	--
Ca3_P1_8059631	Ca3	8059631	GTTT/GTT	TGTGCGACCATCTACTGCTC	GCAATCTCCTCAACTCGGTC	60.0	813	INTERGENIC	--	--	--
Ca3_P1_8069482	Ca3	8069482	AATAT/AAT	ATGCTGCAAGGATCTGAAT	AAGCGCTGTAAAAGGTGCAT	59.7	425	DRR	Ca_24370	--	--

INDEL marker IDs	Chromosomes/ unanchored scaffolds	Physical positions (bp)	InDels (*Kabuli* reference genome-CDC Frontier/PI)	Forward primers (5'-3')	Reverse primers (5'-3')	Anneling temperature (0C)	Expected amplified product size (bp)	Structural annotation		Functional annotation	
								Sequence components of *kabuli* genome	*Kabuli* gene accession IDs	NCBI-KOG	TFs
Ca3_P1_8088284	Ca3	8088284	C/CACA	TCTTCCACCCAGTTTTGACC	ACATAACACGTGGCCTGTGA	59.9	477	INTERGENIC	--	--	--
Ca3_P1_8188815	Ca3	8188815	ACTCTCTCTCTCTCTCTCTCTCTCTCTCTCTCTCTCTCTCTCTCTCTCTCTC/ACTCTCTCTCTCTCTCTCTCTCTCTCTCTCTCTCTCTCTCTC	TGGCTTGGAGAGTGATTTACC	GCGGCTAAAGGTAGATATGGG	59.2	465	INTERGENIC	--	--	--
Ca3_P1_8245494	Ca3	8245494	TCT/TCTTCTGCT	TGCGCCTCTTCTAAAATCGT	TATCGCAATCAGAGGAACCC	60.0	670	INTERGENIC	--	--	--
Ca3_P1_8290057	Ca3	8290057	T/TC	AACGATGAAGAATGGGTTCG	CGTGGATTAGCTTCGAGGAA	59.9	426	INTRON	Ca_24378	--	--
Ca3_P1_8381552	Ca3	8381552	TAA/TAAA	AGCAGCGGAAAAGTTTCTCA	ATGGGAATGCTTGACTGGAG	60.1	266	INTERGENIC	--	--	--
Ca3_P1_8393314	Ca3	8393314	GTATATATATATATATATAT/GTATATATATATATATATATAT	GTTGGTTCCCTAGCACCGTA	TATTTTGGATTTTGGCTCGG	60.0	602	INTERGENIC	--	--	--
Ca3_P1_8437169	Ca3	8437169	ATTATTTATTTA/ATTATTTA	TGCCAAGAAAACACCATGAA	GTTTAGAACGTGACCCCGAA	60.1	662	INTERGENIC	--	--	--
Ca3_P1_8443852	Ca3	8443852	AATATATATATATATATATATATATAT/AATATATATATATATATATATATATATATAT	ACCGTAGCATGGAAAGTTGG	ATGCGACTTGGGAATTTGAG	60.0	699	INTERGENIC	--	--	--
Ca3_P1_8523296	Ca3	8523296	ATTTTTTTTTT/ATTTTTTTT	CACATGACAAAATTGGTGGAA	CGTTAAGGCACATCTCACTCC	59.3	508	INTERGENIC	--	--	--
Ca3_P1_8535141	Ca3	8535141	ATTTTTTTTTTT/ATTTTTTTTTT	GGACAAAGCCTAATGGTGGA	AATGGATTTGGCACAAAAGG	59.9	589	INTERGENIC	--	--	--
Ca3_P1_8536795	Ca3	8536795	CTA/CTATATGATA	TTAACACACCCCTCATGCAA	CGGTCACCGAAAGACAAGTT	60.0	731	INTERGENIC	--	--	--
Ca3_P1_8754025	Ca3	8754025	GTATATATATATATATATATATATATAT/GTATATATATATATATATATATATATATAT	AAAATCCTTGGCCTTTCGAT	GATGAGGTATGTGCGTGTGG	59.9	863	INTRON	Ca_23720	--	B3

INDEL marker IDs	Chromosomes/ unanchored scaffolds	Physical positions (bp)	InDels (*Kabuli* reference genome-CDC Frontier/PI)	Forward primers (5'-3')	Reverse primers (5'-3')	Anneling temperature (0C)	Expected amplified product size (bp)	Structural annotation		Functional annotation	
								Sequence components of *kabuli* genome	*Kabuli* gene accession IDs	NCBI-KOG	TFs
Ca3_P1_8756604	Ca3	8756604	CT/CTGTTTTAAAAT	TGGGTTGGAACACTTCATCA	ATTCAAGCATTCGATGGAGG	59.9	249	INTERGENIC	--	--	--
Ca3_P1_8814472	Ca3	8814472	CATATATATATATATATATATATTTATATATATATAT/CATATATATATATATATATATATATATTTATATATATATAT	GGATGGCTTGTGGATAGGTG	GGATTGAAGATGAAGGTTGTTGA	60.3	602	INTERGENIC	--	--	--
Ca3_P1_8826227	Ca3	8826227	CAAAAAAAAAAA/CAAAAAAAAAA	CTTGCGGTTGGAATGAAGTT	TATACACGGAACGAAAGCCC	60.1	589	INTERGENIC	--	--	--
Ca3_P1_888801	Ca3	888801	ATT/ATTT	AAATGTGCCCAGTTTTGGAG	CCTTGATTGATTGGCTGGTT	60.0	273	INTERGENIC	--	--	--
Ca3_P1_8900151	Ca3	8900151	TAAAAAA/TAAAAAAA	GTGAAAATCCACCCTCGACT	TCAAACCTGCCATTTTCTCA	59.0	156	DRR	Ca_25279	R	--
Ca3_P1_8942193	Ca3	8942193	AT/ATT	TGGGGAAAAACCCTTACCTT	CACATGATTTCAATTGGCTTTG	59.7	326	INTERGENIC	--	--	--
Ca3_P1_8950675	Ca3	8950675	TTGTGTGTGTGTGTGTGTATGTGTGTGTGT/TTGTGTGTGTGTGTGTGTGTATGTGTGTGT	GAATCAGGCCAAAAGGTTGA	GTGAGAGCGCTAGTTGACCC	60.1	422	INTERGENIC	--	--	--
Ca3_P1_914099	Ca3	914099	CGAAATCGATTT/CGAAATCGATTTGGAAATCGATTT	TGGCAAAATTGAAAACACGA	GTGAAAGTGGGTTGCGAAAT	60.1	615	INTERGENIC	--	--	--
Ca3_P1_9153929	Ca3	9153929	ATTTTTTT/ATTTTTTTT	TCTGATGCCAATTGAAGGTG	GCTTGTACGGTCAAGGTGGT	59.6	616	INTERGENIC	--	--	--
Ca3_P1_9186815	Ca3	9186815	CTATATATATATATATATATATATA/CTATATATATATATATATATATATATA	TCCACAAACACGATAACATTGA	TGTAACAAGCATTTGTCGGG	58.9	693	INTERGENIC	--	--	--
Ca3_P1_9189477	Ca3	9189477	AATATATATATATATATATATATATATATATAT/AATATATATATATATATATATATATATATATATATAT	AAAGGTTTGGTTGGAGATACG	TTGGCTGCAAAATATCCCTC	58.1	856	INTERGENIC	--	--	--
Ca3_P1_9226047	Ca3	9226047	CAAAAAAAAAA/CAAAAAAAAAAAA	CCGGAGAACAAAACAACCAT	GAGGAAAAGCAAAGGGTGTG	59.8	246	INTERGENIC	--	--	--

INDEL marker IDs	Chromosomes/ unanchored scaffolds	Physical positions (bp)	InDels (*Kabuli* reference genome-CDC Frontier/PI)	Forward primers (5'-3')	Reverse primers (5'-3')	Anneling temperature (0C)	Expected amplified product size (bp)	Structural annotation		Functional annotation	
								Sequence components of *kabuli* genome	*Kabuli* gene accession IDs	NCBI-KOG	TFs
Ca3_P1_9367428	Ca3	9367428	TG/TGAG	TTTATGTGGTGAATTGGCGA	TGATGTGCCTTCAATTTCCA	59.9	289	INTERGENIC	--	--	--
Ca3_P1_9374112	Ca3	9374112	CTATATATATATATATATATATATATATATATATAT/CTATATATATATATATATATATATATATATATATATATATATAT	GCGAAATGACCGATTTTTCA	AATGGGCGCTGTTAAATGTC	61.0	411	INTERGENIC	--	--	--
Ca3_P1_9389984	Ca3	9389984	GATATATATATATATATATATA/GATATATATATATATATATATATA	TACCCTGGTGCTCTTTTGCT	GGACCCTTTTGAGTTGTCCA	59.9	853	INTERGENIC	--	--	--
Ca3_P1_939073	Ca3	939073	GTT/GTTTT	ATTGGTTCTGCTCTGGGTTG	GGGACCACAGCACCTATGTT	60.1	296	INTERGENIC	--	--	--
Ca3_P1_9396146	Ca3	9396146	CATATATATATATAT/CATATATATATATATAT	TCAAAGCAAATCATGTCCCA	TTGTAGGGGGAGAGATCGTG	60.0	757	DRR	Ca_20968	--	ERF
Ca3_P1_9402454	Ca3	9402454	CATAATAATAATAATAATAATAATAATAATAAT/CATAATAATAATAATAATAATAATAATAAT	TCAGGGGCATATAGCCTGAC	AAAGTCTGCCCTGACATGGA	60.1	581	INTERGENIC	--	--	--
Ca3_P1_9414969	Ca3	9414969	CATAT/CAT	TGTCTTGAGCATGCCTTGTC	CGTTCTCCATGCTGTTCTCA	60.0	372	INTERGENIC	--	--	--
Ca3_P1_9416399	Ca3	9416399	AG/AGG	CCCAAGAGAACGCAATTCAT	TCTCACGGTTGTGAGGACTG	60.1	811	INTERGENIC	--	--	--
Ca3_P1_9427257	Ca3	9427257	TTTA/T	CATGCGTTCCTTGTGGTAGA	GGCCTAAGATCATAGCAATTCAA	59.7	165	INTERGENIC	--	--	--
Ca3_P1_943806	Ca3	943806	ATCT/ATCTCT	GTGAATTCATCCCATGTCCC	AGCCCTGGTAAGCCTTTTGT	60.0	515	INTERGENIC	--	--	--
Ca3_P1_9471126	Ca3	9471126	AATATATATATATATATATATATATATATAT/AATATATATATATATATATATATATATATATAT	CAGGTGGTGGGGACTGTTAC	GGATGCAACAAAAGGTGGAT	60.3	752	DRR	Ca_20972	K	HSF
Ca3_P1_9487765	Ca3	9487765	AAAAATATATA/AATA	GAAAGCCCAAGTTCCAAATG	CGGTTAAGTGGCCATACTCG	59.5	233	INTERGENIC	--	--	--

INDEL marker IDs	Chromosomes/ unanchored scaffolds	Physical positions (bp)	InDels (*Kabuli* reference genome-CDC Frontier/PI)	Forward primers (5'-3')	Reverse primers (5'-3')	Anneling temperature (0C)	Expected amplified product size (bp)	Structural annotation		Functional annotation	
								Sequence components of *kabuli* genome	*Kabuli* gene accession IDs	NCBI-KOG	TFs
Ca3_P1_9491740	Ca3	9491740	TTATATATATATATATATATAT/TTATATATATATATATATAT	ACTAGAGGCCACCTTCGACA	TTAAGCCTCCCAGAACCAAA	59.9	696	INTERGENIC	--	--	--
Ca3_P1_9577853	Ca3	9577853	C/CTTGACG	TTGCGTTCAAAGACTCGATG	TGAATTGCAGAATCCCATGA	60.0	575	INTRON	Ca_20977	--	--
Ca3_P1_9577976	Ca3	9577976	AGG/AGGG	TTGCGTTCAAAGACTCGATG	TGAATTGCAGAATCCCATGA	60.0	575	INTRON	Ca_20977	--	--
Ca3_P1_9578317	Ca3	9578317	CAAAA/CAAAAA	TTGCGTTCAAAGACTCGATG	CTACGTCCCTGCCCTTTGTA	60.0	543	INTRON	Ca_20977	--	--
Ca3_P1_9735716	Ca3	9735716	CTTTTTTTTTTT/CTTTTTTTTTT	GGAATATTGATCCACTCGCC	GAAAAAGCGCTGTAAATGGC	59.3	410	INTERGENIC	--	--	--
Ca3_P1_9767209	Ca3	9767209	GTTTT/GTTT	TCAAGTATGGACACGCCAAA	TTCGCAACTCCACTCTTCCT	60.1	487	INTERGENIC	--	--	--
Ca3_P1_9940470	Ca3	9940470	AAATAAATAATAATA/AAATA	ACCGGTATTGTAACACCCCA	CCTTATGCGGTAATCCCTGA	60.0	475	INTERGENIC	--	--	--
Ca3_P1_9940560	Ca3	9940560	A/AT	ACCGGTATTGTAACACCCCA	CCTTATGCGGTAATCCCTGA	60.0	475	INTERGENIC	--	--	--
Ca4_P1_10033685	Ca4	10033685	TTATATATATATATATATAT/TTATATATATATATATATAT	CTAGTTCGTAACCCGTGCGT	AGGCATGGTGTTGGAGTCAT	60.2	248	INTERGENIC	--	--	--
Ca4_P1_10035522	Ca4	10035522	GT/GTT	AAGGGAGACGCAAGAAATGA	CATCACGATCTTTGTCCACG	59.8	666	INTERGENIC	--	--	--
Ca4_P1_10057169	Ca4	10057169	AAAATTCAAATTCAAATTCAAATTCAAA/AAAATTCAAATTCAAATTCAAA	AACGTGCATCGACACTTTGA	AACGGAAGAATACGCCCTTTA	60.3	542	INTERGENIC	--	--	--
Ca4_P1_10108054	Ca4	10108054	T/TAA	AACCGAATGAAATGAAACGG	AAGCGCACAGGAAAACCTTA	59.8	551	INTERGENIC	--	--	--

INDEL marker IDs	Chromosomes/ unanchored scaffolds	Physical positions (bp)	InDels (*Kabuli* reference genome-CDC Frontier/PI)	Forward primers (5'-3')	Reverse primers (5'-3')	Anneling temperature (0C)	Expected amplified product size (bp)	Structural annotation		Functional annotation	
								Sequence components of *kabuli* genome	*Kabuli* gene accession IDs	NCBI-KOG	TFs
Ca4_P1_10139275	Ca4	10139275	CATATATATATATATATATATATATATATATATATA/CATATATATATATATATATATATATATATATATATATATATA	GGTATGGCGACTCCTTGGTA	CAAAATCGCACCATTCAACA	60.0	622	INTERGENIC	--	--	--
Ca4_P1_10147217	Ca4	10147217	GTTTTTTTTT/GTTTTTTTT	TGAATGCGCGTATTGTGAAT	AAAAATGACAAACGAAACATAAAA	60.1	728	INTERGENIC	--	--	--
Ca4_P1_10173137	Ca4	10173137	ATTTTTTTT/ATTTTTTTTT	CCCGTTATTTTTGACGAAACA	TGTGTTGAGTGGTGGCATTT	59.9	791	INTERGENIC	--	--	--
Ca4_P1_10223331	Ca4	10223331	AATATATATATATATATATATATATATATAT/AATATATATATATATATATATATATAT	ACGTGAAGTCCAAATCTGCC	TTCGACTGTCCACGTATATCTCA	60.1	586	INTERGENIC	--	--	--
Ca4_P1_10233239	Ca4	10233239	GACAATTATTTGGTACAATTAT/GACAATTATTTGGTACAATTATTTGGTACAATTAT	TGTCGGTTGTTGCTTGAAGA	ATACAATCATTTTCCCGCCA	60.4	403	INTERGENIC	--	--	--
Ca4_P1_10261671	Ca4	10261671	ATTTTTT/ATTTTT	TGGATTTTGGACTTGGTCTTG	AACAACACGTGATTTGAATTGC	60.0	654	INTERGENIC	--	--	--
Ca4_P1_10270991	Ca4	10270991	ATATTATTATTATTATTATTATTATT/ATATTATTATTATTATTATTATTATT	GTCACTGCCGGAGTACCAAT	CCCTCCTGCTGTTGTGAAAT	60.0	223	INTRON	Ca_08494	I	NF-YB
Ca4_P1_10330675	Ca4	10330675	GTTTTTTTTT/GTTTTTTTTTT	TGATTTGTGATGCACATTTGG	TTGCATGAAAAATTGTGAATGAT	60.4	747	INTERGENIC	--	--	--
Ca4_P1_10336098	Ca4	10336098	TTAATAATAATAATAATAATAATAATAATAATAATAATAATAATAATAA/TTAATAATAATAATAATAATAATAATAATAATAATAATAATAATAATAATAATAATAATAA	ACCAATCCCACAAGTGCTTC	CGTCTTCACATCAACAACAACA	60.0	832	INTERGENIC	--	--	--
Ca4_P1_10350088	Ca4	10350088	ATTTTTTTT/ATTTTTTT	ATATTGTCCCGCAAACGAAC	TGGGTTGAAGACGAACATTG	59.8	559	INTERGENIC	--	--	--
Ca4_P1_10439513	Ca4	10439513	GAAAAAAAAAA/GAAAAAAAAA	CCTCTCTGCCTGAAGTGACC	GCAAGGATGCATTTGAAACA	60.0	516	INTRON	Ca_04281	Z	--
Ca4_P1_10460957	Ca4	10460957	CATATATATATATATATATATATAT/CATATATATATATATATATATATATATATAT	TTAATTCCAATGGCGGGTAG	TCACATTCAGCTTTTGGTCG	59.8	428	INTERGENIC	--	--	--

INDEL marker IDs	Chromosomes/ unanchored scaffolds	Physical positions (bp)	InDels (*Kabuli* reference genome-CDC Frontier/PI)	Forward primers (5'-3')	Reverse primers (5'-3')	Anneling temperature (0C)	Expected amplified product size (bp)	Structural annotation		Functional annotation	
								Sequence components of *kabuli* genome	*Kabuli* gene accession IDs	NCBI-KOG	TFs
Ca4_P1_10612683	Ca4	10612683	TAAAAAAAAAA/TAAAAAAAAA	AATCACGCAAACAAGTGTCG	AGTCCAGCCGGCAGATATAA	59.8	959	INTERGENIC	--	--	--
Ca4_P1_10624424	Ca4	10624424	TTAATAATAATAATAATAATA/TTAATAATAATAATAATA	GGATGTCCCAAAGGGTTTTT	GGATGATTCTGGAGCGAGAC	60.0	303	INTERGENIC	--	--	--
Ca4_P1_10629265	Ca4	10629265	ATTTTTTTTT/ATTTTTTTTTT	GAGGGCCACCCCATATTACT	TGATGAGTTTTCCCTCCAAAA	60.0	654	INTERGENIC	--	--	--
Ca4_P1_10655355	Ca4	10655355	TTATATATATATATATATA/TTATATATATATATATATATATA	TCGAAGCAGCAAAACAAATG	AAGGTCGCATCTCTCTCTCG	60.0	566	INTERGENIC	--	--	--
Ca4_P1_10657750	Ca4	10657750	T/TCTAACACTCTTTCC	ATACAAAAACATTTGGGCCG	TTCACATGAAGTGGCGAGAC	59.7	417	DRR	Ca_04301	--	--
Ca4_P1_10738318	Ca4	10738318	CA/CACATATA	CCGTCATAAAAGGTTCCGAC	TTTGTTTTCAATGCCCCAAT	59.4	733	INTERGENIC	--	--	--
Ca4_P1_10875155	Ca4	10875155	CTATATATATATATATATATATA/CTATATATATATATATATATA	CACGAAGACAAATCTGCATGT	TCGGTTTTGAAACCAAAATG	58.8	651	INTERGENIC	--	--	--
Ca4_P1_10885811	Ca4	10885811	TCAATCCAGCAATC/TCAATC	TCACACACACGAAGGGTGTT	ATGCATTTTCCACGCACATA	60.0	420	INTERGENIC	--	--	--
Ca4_P1_10939792	Ca4	10939792	G/GAGTGTTGGTC	GCAGCAAAAAGGGATAACCA	AAGAAACCTCGTGTGCCATC	60.1	231	DRR	Ca_04328	GMW	NAC
Ca4_P1_11046179	Ca4	11046179	TAAAATAAAATAAAA/TAAAATAAAATAAAATAAAATAAAA	CTCTCCGTTCCGATCTTCAG	TTTTTACGTACCACATCCATTATGA	59.9	454	INTERGENIC	--	--	--
Ca4_P1_11198337	Ca4	11198337	A/ATCTT	CTTTCAAACGACCCTTCCAA	ATTTCGTCAAGCATTCCCTG	60.1	570	INTERGENIC	--	--	--
Ca4_P1_11209323	Ca4	11209323	TAAAAAAA/TAAAAAA	TTAGTGTTGAATTGACGGCG	CAATGCCTAGAATCACGGGT	59.7	960	INTERGENIC	--	--	--

INDEL marker IDs	Chromosomes/ unanchored scaffolds	Physical positions (bp)	InDels (*Kabuli* reference genome-CDC Frontier/PI)	Forward primers (5'-3')	Reverse primers (5'-3')	Anneling temperature (0C)	Expected amplified product size (bp)	Structural annotation		Functional annotation	
								Sequence components of *kabuli* genome	*Kabuli* gene accession IDs	NCBI-KOG	TFs
Ca4_P1_11216142	Ca4	11216142	CGAGA/CGA	GCATAACCAATGCTCTTGGAA	ATTAAGACACTGGCGCGAAG	60.1	523	DRR	Ca_04353	U	M-type
Ca4_P1_11217933	Ca4	11217933	AAAATAAA/AAAA	CGCCTCGTGGACATCTCTAT	CCTCTTCAACACCGCAAGAT	60.2	232	INTERGENIC	--	--	--
Ca4_P1_11220000	Ca4	11220000	AAATAATAATAA/AAATAATAATAATAA	ATTTCTTCGTGTACCGCACC	CTGATAGACCGACACACACACA	60.0	465	INTERGENIC	--	--	--
Ca4_P1_11227743	Ca4	11227743	ATCTCTCT/ATCTCTCTCTCTCT	CGGGAGGTCCACTATTTTCA	ATTGCACCAAACATCGTTGA	59.9	211	INTRON	Ca_04355	G	--
Ca4_P1_11228298	Ca4	11228298	CTT/CTTT	TTTTGTTCTCATGTCACCGC	CCCCCTCTTGAATAAAATGAAA	59.7	893	INTRON	Ca_04355	G	--
Ca4_P1_11234449	Ca4	11234449	GCC/GC	TCATTGCAGCCTTTGTTTTG	GCAAATCATTTTTGCTGGGT	59.8	693	INTRON	Ca_04356	M	--
Ca4_P1_11238142	Ca4	11238142	ATATTATTATTATTATTATT/ATATTATTATTATTATT	CCCTTTGTCCTACAGTGAACG	TTCTTCCGGAACCATTATAAAAA	59.6	327	DRR	Ca_04357	O	NAC
Ca4_P1_11243897	Ca4	11243897	GC/GCC	CAATGGCTGCTTCAAAGGAT	TCAACTTGCTGCTTCTGCAT	60.2	361	INTERGENIC	--	--	--
Ca4_P1_11245216	Ca4	11245216	GCTTGTATCTCTTGCTCTTGTATCTC/ GCTTGTATCTCTTGCTCTTGTATCTCTTGCTCTTGTATCTC	TGGCTTGATTCTCCCATTTC	AAGCTCTGCCAAGACAGGAA	60.0	876	INTERGENIC	--	--	--
Ca4_P1_11251984	Ca4	11251984	CTA/CTATA	TCTGATTGTGTTCCTTGGCA	GGCCGAGATTTTCAATTTCA	60.2	293	DRR	Ca_04358	R	--
Ca4_P1_11263983	Ca4	11263983	CAAAAAAAAA/CAAAAAAAAAAAA	CGCATGTGTGCTTGAATCTC	TCCACCAATTTTGCATTGAA	60.4	786	INTERGENIC	--	--	--
Ca4_P1_11271331	Ca4	11271331	GTATTTT/GTATTTTATTTATTTT	AGATGGCATTGATGGTGGAT	CCTGGCAAACAGAGCAAAAT	60.2	678	INTERGENIC	--	--	--

INDEL marker IDs	Chromosomes/ unanchored scaffolds	Physical positions (bp)	InDels (*Kabuli* reference genome-CDC Frontier/PI)	Forward primers (5'-3')	Reverse primers (5'-3')	Anneling temperature (0C)	Expected amplified product size (bp)	Structural annotation		Functional annotation	
								Sequence components of *kabuli* genome	*Kabuli* gene accession IDs	NCBI-KOG	TFs
Ca4_P1_11281622	Ca4	11281622	TG/T	TTTAAGGCATGTGAGTTCAGC	TGCAAAATCAATACATGGTTCG	58.0	641	DRR	Ca_04359	--	--
Ca4_P1_11285447	Ca4	11285447	C/CAA	TCCACATGCACCTGTGAACT	AGTAGTGCCTCAAACCACGG	60.2	382	DRR	Ca_04361	GE	GATA
Ca4_P1_11301484	Ca4	11301484	GTTTTT/GTTTTTT	CCATTGCGCACATTAAGAAA	TGCTTCAATGCAACAACACA	59.7	561	INTERGENIC	--	--	--
Ca4_P1_11305082	Ca4	11305082	T/TG	CCTTGTAATCCCAGAGCCAA	TTCATCGTCTGAACCATCCA	60.1	497	INTRON	Ca_04362	--	--
Ca4_P1_11305685	Ca4	11305685	CTGTGAGAT/CTGTGAGATGTGAGAT	CTGCGAACACAATCCATCAC	TACAAGAAATCAATCGCCCC	60.1	600	DRR	Ca_04362	--	--
Ca4_P1_11317243	Ca4	11317243	ATCTCTCTCTCTCTCTC/ATCTCTCTCTCTCTC	CCTTCCACTGCCAAAACATT	CAATTGGTCGGTTTCTGGTT	60.0	430	DRR	Ca_04364	D	WRKY
Ca4_P1_11321691	Ca4	11321691	ATT/ATTT	AACATGTGGCTAACAACCCTT	CAGAGAATCCGACTCCGAAA	58.5	316	INTERGENIC	--	--	--
Ca4_P1_11322366	Ca4	11322366	ATTT/ATTTTT	TTGTGAGGAATGCAAACTGC	ACAATCATCATGCCATCGAA	59.8	501	INTERGENIC	--	--	--
Ca4_P1_11322531	Ca4	11322531	TA/TAA	TTGTGAGGAATGCAAACTGC	ACAATCATCATGCCATCGAA	59.8	501	INTERGENIC	--	--	--
Ca4_P1_11324806	Ca4	11324806	CTTT/CTTTT	ATATCCCATCAGATCACGCC	TTGTGGAAGATGTGGTCGAA	59.7	625	DRR	Ca_04365	--	M-type
Ca4_P1_11327468	Ca4	11327468	GAAAAAA/GAAAAAAAA	GCGCATCATTTTGTCTCTCA	CTCGCACCATCCCAAATAAT	60.0	339	INTERGENIC	--	--	--
Ca4_P1_11331229	Ca4	11331229	CTT/CT	ATGATGGCGGCGAGTATAAC	TCTCTTCTCGCCCCAGTTTA	60.0	838	DRR	Ca_04367	U	FAR1

INDEL marker IDs	Chromosomes/ unanchored scaffolds	Physical positions (bp)	InDels (*Kabuli* reference genome-CDC Frontier/PI)	Forward primers (5'-3')	Reverse primers (5'-3')	Anneling temperature (0C)	Expected amplified product size (bp)	Structural annotation		Functional annotation	
								Sequence components of *kabuli* genome	*Kabuli* gene accession IDs	NCBI-KOG	TFs
Ca4_P1_11331252	Ca4	11331252	TAAA/TAAAA	AGAGGGAAAAAGGGG TTGAA	TCTCTTCTCGCCCCAG TTTA	59.9	742	DRR	Ca_04367	U	FAR1
Ca4_P1_11337470	Ca4	11337470	TG/TGTGAG	TTTCCCTCAAACGAAT CCTG	CGTCCGTGTTATTCCT CGAT	60.0	509	INTERGENIC	--	--	--
Ca4_P1_11339513	Ca4	11339513	ACAATTTTAGCTTCAATTTTA/ACAATT TTA	AAGGAAGCAAACCCAC TCAC	TGCTGACATTTGTCAA TCAAAA	59.2	525	INTERGENIC	--	--	--
Ca4_P1_11340703	Ca4	11340703	CG/C	TCATCACCGTCCAATT TCAA	CTCCTTGCCCAATTTG TTGT	59.9	333	INTERGENIC	--	--	--
Ca4_P1_11344769	Ca4	11344769	TTGTTACT/TT	TCCTGGTGCTCCTCTG TCTT	TAGTCCACCCACAAAC CACA	60.0	602	INTRON	Ca_04368	R	--
Ca4_P1_11352038	Ca4	11352038	GAGAGAGAGA/GAGAGAGAGAAAGAG AGAGA	TGCCCATGTTTACCAC AAAA	CCCTCTCTCTCTCTCT CTCGG	59.8	898	INTERGENIC	--	--	--
Ca4_P1_11354394	Ca4	11354394	CAACAATAACAATAACAATAACAATAA CA/CAACAATAACAATAACAATAACA	GATGTTCCTTTGCCCC ATAA	AGAGACAAATTCAGGG TGGG	59.8	720	INTERGENIC	--	--	--
Ca4_P1_11356453	Ca4	11356453	T/TCAAA	GCACTATTGCTGACTT GGCA	TTTTGGGTAATCAACG AGGC	60.0	304	INTERGENIC	--	--	--
Ca4_P1_11356736	Ca4	11356736	AG/AGTG	GCACTATTGCTGACTT GGCA	TTGGTAGAAAAATGTC ATCCAAAA	60.0	386	INTERGENIC	--	--	--
Ca4_P1_11356854	Ca4	11356854	A/AT	GCACTATTGCTGACTT GGCA	AAATCTCCTTCAATCAT GTTTATTTTT	60.0	712	INTERGENIC	--	--	--
Ca4_P1_11356900	Ca4	11356900	TTATATATATATATATATA/TTATATATA TATATATATATA	ATCCAAACTTTGCCTC GTTG	AAATCTCCTTCAATCAT GTTTATTTTT	60.1	439	INTERGENIC	--	--	--
Ca4_P1_11357023	Ca4	11357023	C/CA	GCCTCGTTGATTACCC AAAA	GGCATGGACGTAGGC TAGAG	59.9	809	INTERGENIC	--	--	--

INDEL marker IDs	Chromosomes/ unanchored scaffolds	Physical positions (bp)	InDels (*Kabuli* reference genome-CDC Frontier/PI)	Forward primers (5'-3')	Reverse primers (5'-3')	Anneling temperature (0C)	Expected amplified product size (bp)	Structural annotation		Functional annotation	
								Sequence components of *kabuli* genome	*Kabuli* gene accession IDs	NCBI-KOG	TFs
Ca4_P1_11360175	Ca4	11360175	CTGATTTTG/CTGATTTTGATTTTG	AAATGAATCCCCCAAT GTCA	TTATCAATCGAAAAAG AAACGC	60.0	212	INTERGENIC	--	--	--
Ca4_P1_11361302	Ca4	11361302	AATTA/AATTATTA	CAAATAGCGTTTGTGA TCCG	CCCATATTGAGCTCCA TCCA	59.2	417	DRR	Ca_04370	--	ERF
Ca4_P1_11371424	Ca4	11371424	T/TTA	TTTCTCTCATCCCCTC CCTT	TTCAGTCCACATGTTT GATGG	60.0	484	INTERGENIC	--	--	--
Ca4_P1_11375704	Ca4	11375704	TG/T	CACAATTAAACTGGGG TGGG	CATCTCAAACAACACA AGCCA	60.1	616	INTERGENIC	--	--	--
Ca4_P1_11380730	Ca4	11380730	GTA/GTATA	TAGTCAGTGGCCCTTC TGCT	GGATTTCGAAGCCATG AAAC	60.0	660	DRR	Ca_04371	--	--
Ca4_P1_11384567	Ca4	11384567	CTTTT/CTTTTT	AAGTATTGCATATTTGT GGGCA	TGGATTTGAATCCGGT TTTG	59.4	486	DRR	Ca_04372	A	E2F/DP
Ca4_P1_11391824	Ca4	11391824	CAAAA/CAAAAAA	ATGCAATGAGATAAAC CGGC	CTGAAATTTCGACATG CCCT	59.9	743	INTERGENIC	--	--	--
Ca4_P1_11392166	Ca4	11392166	AG/AGCATGCG	CTCAGAGCTCCAAAAT CGCT	TTTGTTGGTGGCATGT TTGT	59.7	347	INTERGENIC	--	--	--
Ca4_P1_11393203	Ca4	11393203	GCAT/GCATCAT	ACAAACATGCCACCAA CAAA	TCCATTGCTCACATTT GGAA	59.9	585	INTERGENIC	--	--	--
Ca4_P1_11398641	Ca4	11398641	CAA/CA	CCTTTTCCTCTGTGAC GAGC	CCTTTCAACCATCAAT TCCG	60.0	338	INTERGENIC	--	--	--
Ca4_P1_11416574	Ca4	11416574	ATATGT/AT	TTTTGCGGATAAGACT TTTTGTT	GCCATTGTTAACGGTG GTTT	59.2	547	INTERGENIC	--	--	--
Ca4_P1_11417977	Ca4	11417977	TGAA/TGAAGAA	CAAAGCTCGACAAATG TAAGAGAA	CCACATCCCAATTCAA GGAC	60.0	783	INTERGENIC	--	--	--

INDEL marker IDs	Chromosomes/ unanchored scaffolds	Physical positions (bp)	InDels (*Kabuli* reference genome-CDC Frontier/PI)	Forward primers (5'-3')	Reverse primers (5'-3')	Anneling temperature (0C)	Expected amplified product size (bp)	Structural annotation		Functional annotation	
								Sequence components of *kabuli* genome	*Kabuli* gene accession IDs	NCBI-KOG	TFs
Ca4_P1_11424081	Ca4	11424081	GTTT/GTTTT	CGGTACCCCTTAGCTCACAA	GAGATGGAGTTGGCAGTGGT	60.1	896	INTRON	Ca_04376	GC	bHLH
Ca4_P1_11435815	Ca4	11435815	AAC/A	CGGAGGTTTCAGTTCAGCTC	GTGAATTGGTCGAGCGGTAT	60.0	906	DRR	Ca_04378	S	--
Ca4_P1_11441070	Ca4	11441070	TAAAAAA/TAAAAAAA	GCTGGCCACAATCCATATTC	ACCACCGAGGTTTGAGACAC	60.3	243	DRR	Ca_04379	O	MYB_related
Ca4_P1_11443015	Ca4	11443015	AATT/A	GGCTTGGTGTGGGTTTACAT	AGGCCCCTTAGTTGTCTCGT	59.7	614	INTERGENIC	--	--	--
Ca4_P1_11447148	Ca4	11447148	TAA/TA	ACGTGGTTCCATTTTAGCCA	GCATTGCTATCATTACTGTGCG	60.4	687	INTERGENIC	--	--	--
Ca4_P1_11447220	Ca4	11447220	ATTTT/ATTT	ACGTGGTTCCATTTTAGCCA	GCATTGCTATCATTACTGTGCG	60.4	687	INTERGENIC	--	--	--
Ca4_P1_11447874	Ca4	11447874	CAGAG/CAG	TCGCACAGTAATGATAGCAATG	TGGATAATATGGGCGGTGAT	58.9	461	INTERGENIC	--	--	--
Ca4_P1_1144979	Ca4	1144979	CATATATATATATATATATATATATATA/CATATATATATATATATATATATATATA	AACGCATGCAATCTATTCCC	ATTGTTGGCGTCTTCCAAAC	59.9	813	INTERGENIC	--	--	--
Ca4_P1_11451772	Ca4	11451772	TGTAA/T	TGACGGATACTCCAACACCA	CAAACCCACCAGATCCAACT	60.0	845	INTRON	Ca_04381	--	--
Ca4_P1_11452051	Ca4	11452051	AG/AGG	TGGTGGTTTTGCTTTGGTAA	GCAACACGAAGCTGAAAACA	59.1	913	INTRON	Ca_04381	--	--
Ca4_P1_11459465	Ca4	11459465	TATCTAATCTAATCTA/TATCTAATCTAATCTAATCTA	GTTGGGAACTTGCACACCTT	TGCCCTTGATTTTGGATCTC	60.0	299	INTERGENIC	--	--	--
Ca4_P1_11468207	Ca4	11468207	CCTA/CCTAGCTA	GGTGGAATGTCATGATGTGG	TTGACGAGGAACTTTGAATGAA	59.6	962	INTERGENIC	--	--	--

INDEL marker IDs	Chromosomes/ unanchored scaffolds	Physical positions (bp)	InDels (*Kabuli* reference genome-CDC Frontier/PI)	Forward primers (5'-3')	Reverse primers (5'-3')	Anneling temperature (0C)	Expected amplified product size (bp)	Structural annotation		Functional annotation	
								Sequence components of *kabuli* genome	*Kabuli* gene accession IDs	NCBI-KOG	TFs
Ca4_P1_11471153	Ca4	11471153	CAAA/CAAAA	TTCGATTGTTCAAACTCATTCAA	TTGGCCATCGTTTGAATTTT	59.6	787	INTERGENIC	--	--	--
Ca4_P1_11472411	Ca4	11472411	CATATATATATATATATATATATATATATATATATAT/CATATATATATATATATATATATATATATATAT	TTTCTTGTCTCCTCGGTTGG	AATTATTTGATGTCGGAACGAA	60.2	474	INTERGENIC	--	--	--
Ca4_P1_11479120	Ca4	11479120	AT/ATTTT	AATTGAAGGTTATTTTTGGAATCA	AGTGTCATGCGCACACAAAT	58.1	372	INTERGENIC	--	--	--
Ca4_P1_11482459	Ca4	11482459	TA/TAA	TGGGGAGACAAGGGAGTATG	TGGGATTAAAGCCCTTGACA	59.9	252	INTERGENIC	--	--	--
Ca4_P1_11492800	Ca4	11492800	AAAACAA/AAAACAATAAACAAACAA	CTCACACACCCAAAGAAGCA	AACATCATGGTCAGCACCAA	59.9	543	INTERGENIC	--	--	--
Ca4_P1_11493423	Ca4	11493423	ATTTTTT/ATTTTTTT	CAAAGAAGCTGACCCTGGAG	CCCATAACTGGATGGGAGAA	60.0	679	INTERGENIC	--	--	--
Ca4_P1_11494266	Ca4	11494266	GAA/GAAAAA	TAACGACGCAAGCATTTCTG	GGTGCGAGAGAGAAAACCTG	60.0	598	INTERGENIC	--	--	--
Ca4_P1_11494600	Ca4	11494600	ATAAATTTAAATTTAAATTTA/ATAAATTTAAATTTA	CAGGTTTTCTCTCTCGCACC	CAACACAACCCGTGAACATC	60.0	398	INTERGENIC	--	--	--
Ca4_P1_11504328	Ca4	11504328	TTATATATATATATATATAT/TTATATATATATATATATATAT	AACCTTTTAAAAATTGACATTTCG	AAGGAAGGCCTACTCCCAAA	57.9	359	INTERGENIC	--	--	--
Ca4_P1_11505377	Ca4	11505377	GAT/GATAT	CAAGTGCCTTCGGCATTAGT	GATACACCGTCATCATTGCG	60.3	618	INTERGENIC	--	--	--
Ca4_P1_11506583	Ca4	11506583	CTTA/CTTAATTA	CTCGACATCATGGTTCATGG	CGGATCTATGCATGGGATAA	59.9	507	INTERGENIC	--	--	--
Ca4_P1_11507043	Ca4	11507043	ATTT/ATTTT	TTATCCCATGCATAGATCCG	TTATGGCAAGAATAGTTTTATCATTTT	58.4	796	INTERGENIC	--	--	--

INDEL marker IDs	Chromosomes/ unanchored scaffolds	Physical positions (bp)	InDels (*Kabuli* reference genome-CDC Frontier/PI)	Forward primers (5'-3')	Reverse primers (5'-3')	Anneling temperature (0C)	Expected amplified product size (bp)	Structural annotation		Functional annotation	
								Sequence components of *kabuli* genome	*Kabuli* gene accession IDs	NCBI-KOG	TFs
Ca4_P1_11511300	Ca4	11511300	TTATATATATATATATATATATATATATATATATATAT/TTATATATATATATATATATATATATATATATATATATATATATAT	TGGAGACGGATTTAAGGGTG	ACGGAGGGATTTTGTCTCCT	59.9	436	INTERGENIC	--	--	--
Ca4_P1_11514990	Ca4	11514990	T/TGATTCC	TCAGTCGCTGATTGATTTCG	AAGCCACATTTTGGGTCAAG	59.9	582	INTERGENIC	--	--	--
Ca4_P1_11539961	Ca4	11539961	TAAAAA/TAAAAAA	GGATCATGAACCGTTGTCAAT	TTATGTCGGTCACCCCAAAT	59.7	787	INTERGENIC	--	--	--
Ca4_P1_11541528	Ca4	11541528	TAAAAGAAAAAAAAGAA/TAAAAGAA	CACATGGATCTTCATCCAAAGA	AATGGTCAGCGTCGGTTATC	59.9	565	INTERGENIC	--	--	--
Ca4_P1_11547482	Ca4	11547482	ATTTTTTTTT/ATTTTT	TCCCCAAGCCTACAAACAAC	TGTTCATCGCAGATCCTTTCT	60.0	552	INTERGENIC	--	--	--
Ca4_P1_11550574	Ca4	11550574	AT/ATTTAATTT	CTCATTATGCACACCGTTGG	CCCCAAAATGTTATGCCCTA	60.0	809	INTERGENIC	--	--	--
Ca4_P1_11551467	Ca4	11551467	TTTTTTTTAATTTTTTTA/TTTTTTTTA	ATCCAAAAGCCCCCAATAAC	TCACTGTTGCATCTCCAAGC	60.0	844	INTERGENIC	--	--	--
Ca4_P1_11552635	Ca4	11552635	AG/A	ATGGGGTGCCATACTCCATA	TGCGTTTCGATGATAACCAA	60.0	563	INTRON	Ca_04385	K	Trihelix
Ca4_P1_11554701	Ca4	11554701	CTTTTTTTTTTTTTTT/CTTTTTTTTTTT	ATGGTGCAACCTGAACAACA	GTTTTGCTTTGCTGGTCCAT	60.0	823	DRR	Ca_04385	K	Trihelix
Ca4_P1_11565393	Ca4	11565393	TAAA/TA	CAACGTGCAACAAAGGTTTT	TTGTGTATGAATATGCAACCG	58.7	670	INTERGENIC	--	--	--
Ca4_P1_11567821	Ca4	11567821	CA/CACGAATA	TTTCTTTTGCCCTCATTTCAA	TTCCACCTGATCATCATTTCA	59.7	463	INTERGENIC	--	--	--
Ca4_P1_11568915	Ca4	11568915	ATTTTTTTTTTT/ATTTTTTTTT	GGGATAAGTCGCACATGCTT	TCATGATGAATCGGTCCAAA	60.1	685	INTERGENIC	--	--	--

INDEL marker IDs	Chromosomes/ unanchored scaffolds	Physical positions (bp)	InDels (*Kabuli* reference genome-CDC Frontier/PI)	Forward primers (5'-3')	Reverse primers (5'-3')	Anneling temperature (0C)	Expected amplified product size (bp)	Structural annotation		Functional annotation	
								Sequence components of *kabuli* genome	*Kabuli* gene accession IDs	NCBI-KOG	TFs
Ca4_P1_11570032	Ca4	11570032	CAATTAAT/CAAT	TGGGTCCGACAAAGGAATAG	TGTGGCAAATGCAACAAATAA	59.9	848	INTERGENIC	--	--	--
Ca4_P1_11594941	Ca4	11594941	TAA/TAAAA	TCAAGACGGACACATTTGCT	GGGATTAAAAATGCCGATCA	59.3	512	INTERGENIC	--	--	--
Ca4_P1_11663675	Ca4	11663675	AATATATATATATATATATATATATATA/AATATATATATATATATATATATATATATATATA	TCACTTGTGCTTGGATTTGC	TGTTTCCTATCAACAATTTTGGTCT	59.8	845	INTERGENIC	--	--	--
Ca4_P1_1169667	Ca4	1169667	TTTATTATTATTATTATTATTATTATTATTATTATTATTATTATTAT/TTTATTATTATTATTTTATTATTATTATTATTATTATTATTATTATTATTATTATTATTAT	TTTTAGCCCCGTTTTGTTCA	TAAGGTGTCCGACATCAGATT	60.5	701	DRR	Ca_07748	OU	--
Ca4_P1_11753197	Ca4	11753197	AAATAATAATAATAATAATAATAATAATAATAATAATAATAATAATAATAATAATAATA/AAATAATAATAATAATAATAATAATAATAATAATAATAATAATAATAATAATAATAATA	CATGCCGGAAGAAGGAATAA	TGTTCTCTCCTCCCCTTCCT	60.0	492	INTERGENIC	--	--	--
Ca4_P1_11769762	Ca4	11769762	TA/T	TCCATAGTGGAGATGCGACA	GGAACATGCGAATCACAAAA	60.2	666	INTERGENIC	--	--	--
Ca4_P1_11809196	Ca4	11809196	TAATTAAATTAAATT/TAATTAAATTAAATTAAATT	TGATCGAGTGAGAAAACAAACAA	GGTTTCAAAATCGAAATCCAA	59.8	297	INTERGENIC	--	--	--
Ca4_P1_11818956	Ca4	11818956	CAAA/CAAAA	CCAAAACATGAAAGCAACAAAA	TAACATTGATGGCAGAGGCA	60.0	170	INTERGENIC	--	--	--
Ca4_P1_1185780	Ca4	1185780	AATATATATATATATATATATATATATAT/AATATATATATATATATATATATATAT	AATCATGGGCCTGTAACTGC	CTGGTTGCAATCCTCCATTT	60.0	439	INTRON	Ca_07749	D	--
Ca4_P1_11866901	Ca4	11866901	TATAATAATAATAATAA/TATAATAATAA	GAATAGACCAGGCCAGACCA	CTATTAGGCCTGACAAGGCG	60.1	493	INTERGENIC	--	--	--
Ca4_P1_11927566	Ca4	11927566	A/AAAAAACT	AACTCAACCCATTCAATGCC	TGTCTATATGCCAAGATGATGGA	59.8	427	DRR	Ca_04414	O	Trihelix
Ca4_P1_11988786	Ca4	11988786	TAAAATTAAATAAAAAA/TAAAATTAAATAAAAAATTAAATAAAAAA	TCTTTAACAAGTGTCGTGCCA	GGCAACTGGGAAGCAAATAA	59.4	654	INTERGENIC	--	--	--

INDEL marker IDs	Chromosomes/ unanchored scaffolds	Physical positions (bp)	InDels (*Kabuli* reference genome-CDC Frontier/PI)	Forward primers (5'-3')	Reverse primers (5'-3')	Anneling temperature (0C)	Expected amplified product size (bp)	Structural annotation		Functional annotation	
								Sequence components of *kabuli* genome	*Kabuli* gene accession IDs	NCBI-KOG	TFs
Ca4_P1_12015084	Ca4	12015084	CATATATATATATATATATATATAT/CATATATATATATATATATATATATATAT	ACCATAAAACAGCCTCCACG	GTGTTGCGATTTTGGTGTTG	60.0	499	INTERGENIC	--	--	--
Ca4_P1_12027795	Ca4	12027795	AAAAAAGAAATAAAAAGAAATAAA/AAAAAAGAAATAAA	GATGTCGTCATGGCTGTTTG	TCATTGCGTTCTTTAGTGCG	60.1	510	INTERGENIC	--	--	--
Ca4_P1_12063127	Ca4	12063127	GTTTTTTT/GTTTTTTTT	TTTTGTGTGCGTTTGACTGA	CAGCACATAGTAAAACTTGGCG	58.9	483	INTERGENIC	--	--	--
Ca4_P1_12078532	Ca4	12078532	GTTTTTTTTTT/GTTTTTTTTTTT	CAAAACGGTTCTGTTCCAAAA	GAGTTCAACCGATTCTTCCG	60.0	838	DRR	Ca_04432	--	C2H2
Ca4_P1_12098577	Ca4	12098577	GAAA/GAA	TGGATTTGAGTGCTTCCACA	TGCGCATGAAAAGAAAATGA	60.2	761	INTERGENIC	--	--	--
Ca4_P1_12313260	Ca4	12313260	G/GA	ATATTTTCGATGTGCAGGGC	TGAAGGAGGCTTCTAGGACG	59.9	545	DRR	Ca_04455	J	--
Ca4_P1_12334767	Ca4	12334767	C/CTATT	GTCCTCCAACTGACCCAAAA	AGGTTAGGTCGTCCGTCAAG	59.9	377	INTERGENIC	--	--	--
Ca4_P1_12351718	Ca4	12351718	TAAAAAAAAA/TAAAAAAAA	CTGCAACAAGTGGCATATGAT	AACGAGATCGCTTCTCCAAA	58.7	328	INTRON	Ca_04459	M	--
Ca4_P1_1237884	Ca4	1237884	AATATATATATATATATATATATATA/AATATATATATATATATATA	GTGGTCTGGAACATCGTCCT	TTGCAGTTGCAATATCGTTG	60.0	863	INTERGENIC	--	--	--
Ca4_P1_12469703	Ca4	12469703	ATTTTTTTTTT/ATTTTTTTTT	CATTTTCTTGTTTTGGGATTTCA	GATGCTGCGTTTGCCTAAAT	60.2	470	INTRON	Ca_04469	Z	bHLH
Ca4_P1_12472732	Ca4	12472732	GAAAAAAAAAA/GAAAAAAAAAAAA	GGGGAGTTTGGTTCCTTATTTT	GATCAACGGCGAGAGAAAAG	59.6	465	DRR	Ca_04469	Z	bHLH
Ca4_P1_12479687	Ca4	12479687	GA/GAA	GATGCCCCACAACCACTACT	CGAGACCAAGAAACGGAAAA	59.9	164	INTERGENIC	--	--	--

INDEL marker IDs	Chromosomes/ unanchored scaffolds	Physical positions (bp)	InDels (*Kabuli* reference genome-CDC Frontier/PI)	Forward primers (5'-3')	Reverse primers (5'-3')	Anneling temperature (0C)	Expected amplified product size (bp)	Structural annotation		Functional annotation	
								Sequence components of *kabuli* genome	*Kabuli* gene accession IDs	NCBI-KOG	TFs
Ca4_P1_12489843	Ca4	12489843	AAG/A	CCATAAGCAGAAAATTGCGG	GCTTAGCTGGGATTCCTTCC	60.6	351	DRR	Ca_04472	R	NAC
Ca4_P1_12490666	Ca4	12490666	TGTAGTAGTAGTAGTAGTAGTAGTAGTA/TGTAGTAGTAGTAGTAGTAGTAGTAGTAGTAGTAGTAGTAGTAGTA	GGAATATGGCAGCAGACGAT	GCCTGCATATGGCTAAGAGC	60.1	734	DRR	Ca_04472	R	NAC
Ca4_P1_12493651	Ca4	12493651	ATTTTTT/ATTTTTTTT	ATTCCTCGTCCGTTTTCAGA	TTTTTCTTCCTGATTTGGTCC	59.7	293	DRR	Ca_04473	--	--
Ca4_P1_12493732	Ca4	12493732	AAGATA/AA	ATTCCTCGTCCGTTTTCAGA	GAAAGTGACATTGACCTTCTCCT	59.7	699	DRR	Ca_04473	--	--
Ca4_P1_12493934	Ca4	12493934	TAAAAAAAA/TAAAAAAAAA	ATTCCTCGTCCGTTTTCAGA	GAAAGTGACATTGACCTTCTCCT	59.7	699	DRR	Ca_04473	--	--
Ca4_P1_12498624	Ca4	12498624	TAAAAAA/TAAAAAAAA	TAAATTGGCCTCAAATTGCC	CCATGCACACGGATCAAATA	59.9	389	INTRON	Ca_04473	--	--
Ca4_P1_12504646	Ca4	12504646	TAAAAAAATAAA/TAAAAAAATAAAAAAATAAA	TGGCACCAAAGATACCATCA	TGCAACCGCAATATTTACCA	59.9	328	INTRON	Ca_04474	--	MIKC
Ca4_P1_12504806	Ca4	12504806	CAAA/CAAAAA	TGGCACCAAAGATACCATCA	TGCAACCGCAATATTTACCA	59.9	328	INTRON	Ca_04474	--	MIKC
Ca4_P1_12505498	Ca4	12505498	ATATATATATA/ATATATATATGTATATATATA	GCAAGGACAAGGGATGTAGG	TCAACCGCAATTGAGATTTTC	59.6	677	INTERGENIC	--	--	--
Ca4_P1_12505639	Ca4	12505639	GTCTTAAA/GTCTTAAATATCTTAAA	GCAAGGACAAGGGATGTAGG	TCAACCGCAATTGAGATTTTC	59.6	677	INTERGENIC	--	--	--
Ca4_P1_12506017	Ca4	12506017	ATTTTTTT/ATTTTTTTT	TCAATTGCGGTTGAATTATCA	AAGCTGCTCTCAACACCCAT	59.0	485	INTERGENIC	--	--	--
Ca4_P1_12507863	Ca4	12507863	AAAAGAAA/AAAA	ATTCAGCACATGCCACTGTC	CCTTCAATGTGGTGGTAGGG	59.7	827	INTERGENIC	--	--	--

INDEL marker IDs	Chromosomes/ unanchored scaffolds	Physical positions (bp)	InDels (*Kabuli* reference genome-CDC Frontier/PI)	Forward primers (5'-3')	Reverse primers (5'-3')	Anneling temperature (0C)	Expected amplified product size (bp)	Structural annotation		Functional annotation	
								Sequence components of *kabuli* genome	*Kabuli* gene accession IDs	NCBI-KOG	TFs
Ca4_P1_12508938	Ca4	12508938	AACTA/AACTACTA	TGGACGAGCAAAAAGAGACA	GGCAAACAATAGCCACCATT	59.6	810	INTERGENIC	--	--	--
Ca4_P1_12509526	Ca4	12509526	CTTTTTT/CTTTTTTT	AATGGTGGCTATTGTTTGCC	CGGATTTTAAGACCGATCCA	59.8	726	INTERGENIC	--	--	--
Ca4_P1_12518595	Ca4	12518595	ATTTTTTTT/ATTTTTTTTT	GGAGCCTTACACATGTGGCT	TCATTTGTGAGATTGTCACGTT	60.1	364	INTERGENIC	--	--	--
Ca4_P1_12520618	Ca4	12520618	AATATATATATATAT/AATATATATATATATAT	TCAAAGCTGCATTTTGTTGC	TTCGTATGCTATGCACGCTC	60.0	441	INTERGENIC	--	--	--
Ca4_P1_12525819	Ca4	12525819	GTT/GTTT	GCTGCGTTCAAGAATCACAA	AGACAAGGCCGGAACTTTTT	60.0	768	INTRON	Ca_04475	C	bZIP
Ca4_P1_12526163	Ca4	12526163	T/TTA	GCGTTTTCAAACACACGCTA	GAGAAGCTGTGCCAATAGCC	59.9	530	INTRON	Ca_04475	C	bZIP
Ca4_P1_12526734	Ca4	12526734	CTAATTAATTAAT/CTAATTAATTAATTAAT	GGCTATTGGCACAGCTTCTC	AAAGCCACCACCTGTTTGTC	60.0	557	DRR	Ca_04475	C	bZIP
Ca4_P1_12528293	Ca4	12528293	AT/ATT	TTCCTTCGACCAACAAAACC	CATGGGACCCTAGCGTTAAA	59.9	573	DRR	Ca_04475	C	bZIP
Ca4_P1_12528908	Ca4	12528908	GTATA/GTA	TAACGCTAGGGTCCCATGTC	TCGTTTTGATCATTTATCGTTTTT	60.0	720	INTERGENIC	--	--	--
Ca4_P1_12529378	Ca4	12529378	CTTTTTTT/CTTTTTTTT	AAAACGATAAATGATCAAAACGAA	TAGGCATGACAAATTGACCG	59.0	565	INTERGENIC	--	--	--
Ca4_P1_12529895	Ca4	12529895	TAA/TAAA	AACGTGTTCAAACTCGGAGC	ACAACACCTTATTCTGCGCC	60.3	630	INTERGENIC	--	--	--
Ca4_P1_12529954	Ca4	12529954	CTTTT/CTTTTT	AACGTGTTCAAACTCGGAGC	AGCTTAGCTGGGTGGGTTTT	60.3	707	INTERGENIC	--	--	--

INDEL marker IDs	Chromosomes/ unanchored scaffolds	Physical positions (bp)	InDels (*Kabuli* reference genome-CDC Frontier/PI)	Forward primers (5'-3')	Reverse primers (5'-3')	Anneling temperature (0C)	Expected amplified product size (bp)	Structural annotation		Functional annotation	
								Sequence components of *kabuli* genome	*Kabuli* gene accession IDs	NCBI-KOG	TFs
Ca4_P1_12529978	Ca4	12529978	T/TC	AACGTGTTCAAACTCGGAGC	AGCTTAGCTGGGTGGGTTTT	60.3	707	INTERGENIC	--	--	--
Ca4_P1_12531006	Ca4	12531006	AATATATATATA/AATATATATATATATATATATA	CAACCTCAACCTGACCCAGT	ATCACCCAATTGGAAGCAAA	60.0	255	INTRON	Ca_04476	--	--
Ca4_P1_12532996	Ca4	12532996	C/CA	ATGAATTTGATGAGCAGGGC	GTTCCAGTTGCCCTGTGTTT	60.0	349	DRR	Ca_04477	K	MYB
Ca4_P1_12535193	Ca4	12535193	GAAAAAAAA/GAAAAAA	CGAAGCCTGCAACTTTTACC	CCAAACATGCAGGTTCCTCT	59.9	231	DRR	Ca_04476	--	--
Ca4_P1_12542560	Ca4	12542560	TAAAAAAA/TAAAAAAAA	TGCCAAATTGTGTTTGTCGT	TATGACGGGAATGCATTTGA	60.0	631	INTERGENIC	--	--	--
Ca4_P1_12544410	Ca4	12544410	TAATAAATAAATAAATAAATAAA/TAATAAATAAATAAATAAA	ATCAATCTTCAGACGGCCAC	ATTTGACGGTGCATTCCTTC	60.1	658	INTERGENIC	--	--	--
Ca4_P1_12545942	Ca4	12545942	GTTTTTTTTTTT/GTTTTTTTTT	GTCATTTCCTTCCCCTGACA	CATTTTTGACAACCGATTGTTTT	59.9	444	INTERGENIC	--	--	--
Ca4_P1_12546019	Ca4	12546019	TAAAAAAAAA/TAAAAAAAA	GTCATTTCCTTCCCCTGACA	TTGTTGAATTGCAGCTTGAGA	59.9	883	INTERGENIC	--	--	--
Ca4_P1_12548954	Ca4	12548954	TAAAA/TAAAAA	AAGCATGCGAATGTATTACTGC	CATGAGGGCTTAAACAAGGAA	59.3	487	INTERGENIC	--	--	--
Ca4_P1_12554922	Ca4	12554922	GTTTTTTTTTT/GTTTTTTTTTTTTT	TCGGCTTACCCTAAGAAGCA	GTGATTGCGCTGCAGTTTTA	60.0	701	DRR	Ca_04478	A	EIL
Ca4_P1_12555007	Ca4	12555007	TC/TCAAAC	TCGGCTTACCCTAAGAAGCA	GTGATTGCGCTGCAGTTTTA	60.0	701	DRR	Ca_04478	A	EIL
Ca4_P1_12559779	Ca4	12559779	ACCC/ACC	TCAGGACACAACCAAATCCA	AGCCCAAGATGGAGAAGGAT	59.9	428	INTRON	Ca_04478	A	EIL

INDEL marker IDs	Chromosomes/ unanchored scaffolds	Physical positions (bp)	InDels (*Kabuli* reference genome-CDC Frontier/PI)	Forward primers (5'-3')	Reverse primers (5'-3')	Anneling temperature (0C)	Expected amplified product size (bp)	Structural annotation		Functional annotation	
								Sequence components of *kabuli* genome	*Kabuli* gene accession IDs	NCBI-KOG	TFs
Ca4_P1_12559873	Ca4	12559873	TATAATAATAATAATA/TATAATAATAATAATAATAATA	TCAGGACACAACCAAATCCA	GGGACGGACAGACAGTGATT	59.9	661	INTRON	Ca_04478	A	EIL
Ca4_P1_12565536	Ca4	12565536	ATTTTTTTTTT/ATTTTTTTT	CATGAATCTCAATTGGTGCG	GGCCAAGTTGATTCACAACA	60.1	534	INTERGENIC	--	--	--
Ca4_P1_12565888	Ca4	12565888	AC/A	GTTGTGAATCAACTTGGCCC	TAGGTCATGACGGACGGTCT	60.4	206	INTERGENIC	--	--	--
Ca4_P1_12566497	Ca4	12566497	CTTTTTTT/CTTTTTTTTT	CCGTCCGTCATGACCTATAA	CATCTCAAACAAACTCGGACA	58.4	718	INTERGENIC	--	--	--
Ca4_P1_12566654	Ca4	12566654	AG/A	TCAGTCTTTCCATATTCTCTCCG	AAAACCCAGGAGGTCAGCTT	59.7	619	INTERGENIC	--	--	--
Ca4_P1_12567928	Ca4	12567928	AATATATATAT/AATATATAT	ATTCGGCAATTCTACCCCTT	GAGGTAGAGCTCGACGATGG	59.8	446	INTERGENIC	--	--	--
Ca4_P1_12569665	Ca4	12569665	CTTTTT/CTTTT	ATCTCCTTTCCGGAGCAAAT	GGGGCAGTTGTAGCCTTGTA	60.0	174	DRR	Ca_04479	E	--
Ca4_P1_12571013	Ca4	12571013	GTATATA/GTATA	GGCTCACATCAGGAATGGAT	GGCTCTTGGTCATTGGATGT	59.9	968	DRR	Ca_04479	E	--
Ca4_P1_12571941	Ca4	12571941	AA/AATTTA	ACATCCAATGACCAAGAGCC	TTGTTTTGCATGCTCTTCCA	59.9	820	INTERGENIC	--	--	--
Ca4_P1_12572622	Ca4	12572622	TAAAAAAAAA/TAAAAAAAAAA	TGGAAGAGCATGCAAAACAA	TGAGATCTTGAGGCATGGTG	60.4	644	INTERGENIC	--	--	--
Ca4_P1_12578358	Ca4	12578358	TAT/TATACCAGCAT	AGGCTTCTTAGGCATTGGGT	ATTCTTGTAATCCCGCGTTG	60.1	346	INTRON	Ca_04480	L	--
Ca4_P1_12581069	Ca4	12581069	TAA/TA	TTTTAGTTGCCGCCTTTTTG	CTTCAGGAGGGTGATACCGA	60.2	468	INTRON	Ca_04480	L	--

INDEL marker IDs	Chromosomes/ unanchored scaffolds	Physical positions (bp)	InDels (*Kabuli* reference genome-CDC Frontier/PI)	Forward primers (5'-3')	Reverse primers (5'-3')	Anneling temperature (0C)	Expected amplified product size (bp)	Structural annotation		Functional annotation	
								Sequence components of *kabuli* genome	*Kabuli* gene accession IDs	NCBI-KOG	TFs
Ca4_P1_12582939	Ca4	12582939	TTAATATAA/TTAA	CAAGCTTGTACCTGGGGTGT	CCGAAAAAGCATCACTGGTT	60.0	503	INTRON	Ca_04480	L	--
Ca4_P1_12587309	Ca4	12587309	TT/TTGT	GCAAACAAGGTACGGGTCAT	ACAAAAGTGTTGGCGGTTTC	59.9	102	INTERGENIC	--	--	--
Ca4_P1_12588722	Ca4	12588722	ATTTTTTTTT/ATTTTTTTT	GGTTCATCAACGCTTCCACT	CACGCATCAGATCTCGAAAG	60.1	361	INTERGENIC	--	--	--
Ca4_P1_12588936	Ca4	12588936	CTTTTTTT/CTTTTTTTTTT	CTTCTCGTGGGTTCTGAAGG	AGCATTACCAAAATGCCACA	59.8	861	INTERGENIC	--	--	--
Ca4_P1_12589173	Ca4	12589173	ATTTTTTTTTT/ATTTTTTTTT	CTTTCGAGATCTGATGCGTG	ACAACGTCTTCCATTACGCC	59.5	753	INTERGENIC	--	--	--
Ca4_P1_12589473	Ca4	12589473	G/GA	TGTGGCATTTTGGTAATGCT	ACAACGTCTTCCATTACGCC	59.0	200	INTERGENIC	--	--	--
Ca4_P1_12591540	Ca4	12591540	CAAA/CAA	GTCCAAGCGTGTTTGGTTTT	ATTCGTAATGACAAAGCGGC	60.0	495	DRR	Ca_04481	R	--
Ca4_P1_12592291	Ca4	12592291	ATA/ATACAAATACTTGATGTA	TGACTGCCGCTTTGTCATTA	TTTCGATTCAAACCCAAACC	60.4	588	DRR	Ca_04481	R	--
Ca4_P1_12593132	Ca4	12593132	AT/ATGTTGT	TCACTGTAAGTCCATGCCTCC	GCCCTTGTCAAAACAAGTTGA	60.1	454	INTERGENIC	--	--	--
Ca4_P1_12593182	Ca4	12593182	TAAAA/TAAA	TCACTGTAAGTCCATGCCTCC	GCCCTTGTCAAAACAAGTTGA	60.1	454	INTERGENIC	--	--	--
Ca4_P1_12593930	Ca4	12593930	AATATATAT/AATATATATAT	TCAACTTGTTTTGACAAGGGC	TCTCCCATAGCTGCACACTG	60.1	733	INTERGENIC	--	--	--
Ca4_P1_12594218	Ca4	12594218	TCTCT/TCTCTCTCCCTCT	TGAAAAGGCTAAAAACCAAACC	TCTCCCATAGCTGCACACTG	59.5	270	INTERGENIC	--	--	--

**INDEL marker IDs**	**Chromosomes/ unanchored scaffolds**	**Physical positions (bp)**	**InDels (*Kabuli* reference genome-CDC Frontier/PI)**	**Forward primers (5'-3')**	**Reverse primers (5'-3')**	**Anneling temperature (0C)**	**Expected amplified product size (bp)**	**Structural annotation**		**Functional annotation**	
								**Sequence components of *kabuli* genome**	***Kabuli* gene accession IDs**	**NCBI-KOG**	**TFs**
Ca4_P1_12594574	Ca4	12594574	TTTTTT/TTTTTTTCTTTTCTTTTT	CAGTGTGCAGCTATGGGAGA	TCAACATGGAGGAGCAACAA	60.0	463	INTRON	Ca_04482	R	--
Ca4_P1_12595116	Ca4	12595116	TAA/TA	AATGGTTGCAGCCCAATAAG	GGTCCACCAGGTCCATAAAA	60.0	576	INTRON	Ca_04482	R	--
Ca4_P1_12596654	Ca4	12596654	AAGA/AA	TTTCCTAAGCCAGGCAAAAA	TCATGCATTTATCCCCACAA	59.8	488	DRR	Ca_04482	R	--
Ca4_P1_12597000	Ca4	12597000	AT/ATCTAATGGT	CACATGGTTCCAGGTTCCTT	CTTATGGCGTGGCCTTAAAC	59.8	820	DRR	Ca_04482	R	--
Ca4_P1_12604165	Ca4	12604165	CTTTTTTTTTTTTT/CTTTTTTTTTTT	CGATTTCACTTCCCCAACTT	AGCAGCAGAAAGCATGGACT	59.0	209	INTERGENIC	--	--	--
Ca4_P1_12604453	Ca4	12604453	ATTTT/ATT	AGTCCATGCTTTCTGCTGCT	ATTTCACAAATGGGGCTGAG	60.2	669	INTERGENIC	--	--	--
Ca4_P1_12605843	Ca4	12605843	ATTTTTTT/ATTTTTT	TGAGAAGTTTCATGGATTCATTTT	AATGGAACAACGGAGGTGAG	59.0	154	INTERGENIC	--	--	--
Ca4_P1_12609866	Ca4	12609866	TA/TAA	ACAGGTAGTTGGCGATGCTT	GTGGTTCCCACTGCCTAAAA	59.8	339	DRR	Ca_04483	O	TCP
Ca4_P1_12641907	Ca4	12641907	CAAAAAAAAA/CAAAAAAAAAA	TGGTGGAAAATGGTGGAGTT	TTTTTCGCCTGGTGGTACTT	60.2	883	INTERGENIC	--	--	--
Ca4_P1_12644877	Ca4	12644877	CATATATATATATATATAT/CATATATATATATATATATATAT	TCACTTGTTAGTATGACCACAACA	TTTGAGGCCATTAGTTTGGG	57.3	877	INTERGENIC	--	--	--
Ca4_P1_12660952	Ca4	12660952	ATTTTTTTTTTTTT/ATTTTTTTTTTTT	CCCATCCTGGGTCAATACAT	TGATGAAGTTGTGTGCATTTGA	59.5	713	DRR	Ca_04489	--	STAT
Ca4_P1_12665673	Ca4	12665673	ATCT/ATCTCT	TTACCCCAATCCCCTACACA	TTTGTATGAACGGAAATCTTTTG	60.0	524	INTERGENIC	--	--	--

INDEL marker IDs	Chromosomes/ unanchored scaffolds	Physical positions (bp)	InDels (*Kabuli* reference genome-CDC Frontier/PI)	Forward primers (5'-3')	Reverse primers (5'-3')	Anneling temperature (0C)	Expected amplified product size (bp)	Structural annotation		Functional annotation	
								Sequence components of *kabuli* genome	*Kabuli* gene accession IDs	NCBI-KOG	TFs
Ca4_P1_12665730	Ca4	12665730	CAAAAAAAAAAAA/CAAAAAAAAAAA	TTACCCCAATCCCCTACACA	GATGAATTGATTTCTCTGTGCAA	60.0	642	INTERGENIC	--	--	--
Ca4_P1_12667466	Ca4	12667466	GTTTTTTTTTTT/GTTTTTTTTTTTTT	TCTCTTTCCTAGCTTGCATCG	TGGTTGCTAGAAAATTGAACGA	59.7	633	INTERGENIC	--	--	--
Ca4_P1_12672011	Ca4	12672011	GTTTTTTTTTTTT/GTTTTTTTTTTT	AGATTGGGCCACATTCATTT	AAATCAAAATGTTGGCAGAAAAA	59.2	205	INTERGENIC	--	--	--
Ca4_P1_12676299	Ca4	12676299	CATATGTGATATGTGATA/CATATGTGATATGTGATATGTGATA	GATCTCGGGTTAGATGGTCG	TCCACTCTCGCTCTCAATCC	59.5	740	INTERGENIC	--	--	--
Ca4_P1_12697543	Ca4	12697543	ATTTTTTTT/ATTTTTTTTT	AACCCAAGAAAGGTGCAACA	ATCGGTTAGAGCCAACAACG	60.5	882	INTERGENIC	--	--	--
Ca4_P1_12699163	Ca4	12699163	TAAAAAAAAAA/TAAAAAAAAA	TTGTAACCGCTTAAGCACATTT	GCTTCACATTTAAAGCAATTTTCA	58.9	595	INTERGENIC	--	--	--
Ca4_P1_12715012	Ca4	12715012	TA/TATAGAAAGA	AGAAGCGATGCAGACAACTTT	AGGGGCGAATATCGCTAACT	59.1	373	INTERGENIC	--	--	--
Ca4_P1_12724267	Ca4	12724267	GAAAAAAAAAAA/GAAAAAAAAAAAAA	CCAAGTGCAATGACGAGCTA	CAAGGATATGGATCCAATGTGA	60.0	610	INTERGENIC	--	--	--
Ca4_P1_12741131	Ca4	12741131	GTTATTATTATTATTATTATTATTATTATTATTATT/GTTATTATTATTATTATTATTATTATTATTATTATTATTATTATT	AACAACGGTTCAACCTCACG	ATACGCACATCGCCACATTA	61.0	681	INTERGENIC	--	--	--
Ca4_P1_12760776	Ca4	12760776	TAAAAAAAAAAA/TAAAAAAAAAA	TTCTAACAACATGGGAGAGAAAAA	AAGGCCCAAAGTCATAACCC	59.2	215	INTERGENIC	--	--	--
Ca4_P1_12796042	Ca4	12796042	TAAAAAA/TAAAAA	ATTTACGGCGCTTTTTCAGA	GCATGCTGCAAGGGATAGTT	59.9	514	INTERGENIC	--	--	--
Ca4_P1_12832103	Ca4	12832103	GTT/GT	GCGTTTCCAATGGATGAAGT	ATGCCGAAAAAGACTGGTCA	59.9	203	DRR	Ca_04501	T	ARR-B

INDEL marker IDs	Chromosomes/ unanchored scaffolds	Physical positions (bp)	InDels (*Kabuli* reference genome-CDC Frontier/PI)	Forward primers (5'-3')	Reverse primers (5'-3')	Anneling temperature (0C)	Expected amplified product size (bp)	Structural annotation		Functional annotation	
								Sequence components of *kabuli* genome	*Kabuli* gene accession IDs	NCBI-KOG	TFs
Ca4_P1_12853488	Ca4	12853488	TGCACAGCACAGCACAGCACA/TGCACAGCACAGCACAGCACAGCACA	CCCCTTGAAGGACACAAGTT	TTTGAAGGAAAGAGGCATGG	59.0	693	INTERGENIC	--	--	--
Ca4_P1_12866217	Ca4	12866217	GGG/GGGAGCTTTATTTTTTGG	TAGACTCCACTAAGCCCCCA	CCTTCCAACGCAACATTTCT	59.7	294	DRR	Ca_04506	T	FAR1
Ca4_P1_12885868	Ca4	12885868	ATTTTTTTTTT/ATTTTTTTTT	AGGTCATTGGTGTGCAAGGT	TGAAGCTGACTGCACTTTGG	60.4	666	DRR	Ca_04508	R	--
Ca4_P1_1291755	Ca4	1291755	CTTGTTGTT/CTTGTT	ATAGCCTCACGCGGTTCATA	CCTCACACTTAAATTTCGGCA	60.6	310	INTERGENIC	--	--	--
Ca4_P1_12929423	Ca4	12929423	AATATATATATATATATATATAT/AATATATATATATATATATATATATATATAT	ATATCGCGCATCCCAAATAC	TCAACTCATGTGTATCAATCGAA	59.8	506	INTERGENIC	--	--	--
Ca4_P1_13017972	Ca4	13017972	AATATATATATATATATAT/AATATATATATATATATAT	CCAATATTGCTTTGGGACTG	TTTGTGGAAGGGGGTTATCA	58.1	466	DRR	Ca_04523	V	GRAS
Ca4_P1_13028368	Ca4	13028368	CTTGTTG/CTTGTTGTTG	TGCACGCTCCACATTATTTC	CTTTTCTGGGGCAAATTGAA	59.7	367	INTRON	Ca_04525	T	bHLH
Ca4_P1_13068436	Ca4	13068436	AATATATATATATATATATATATAT/AATATATATATATATATATATATATATATATATATAT	GCTCAATTTTTCTTAGGGCG	TGTCGCAACAATTTGTCTCTT	59.0	432	INTERGENIC	--	--	--
Ca4_P1_1309061	Ca4	1309061	GAAAAAA/GAAAAAAA	AGCTACAAAATGGGCAATGG	CCACGGTACCGAAAAATGAT	60.0	237	INTERGENIC	--	--	--
Ca4_P1_1310245	Ca4	1310245	ATTTTTTTTTTT/ATTTTTTTTTT	TGAAATTGCTTGATTTTGCTTT	CGACCGATTTTATCCGTCTC	58.9	825	INTERGENIC	--	--	--
Ca4_P1_13133292	Ca4	13133292	ATTTT/ATATATTTTTT	CAGCCGCCTCCAACTATTAC	CCATCCATGCAATTGAACAC	59.7	408	INTERGENIC	--	--	--
Ca4_P1_13210889	Ca4	13210889	G/GCCATCTGATTCT	TTGTCTTTTTGGGCTGCTCT	GGTCACACACAAATGCCAAG	60.0	388	INTERGENIC	--	--	--

INDEL marker IDs	Chromosomes/ unanchored scaffolds	Physical positions (bp)	InDels (*Kabuli* reference genome-CDC Frontier/PI)	Forward primers (5'-3')	Reverse primers (5'-3')	Anneling temperature (0C)	Expected amplified product size (bp)	Structural annotation		Functional annotation	
								Sequence components of *kabuli* genome	*Kabuli* gene accession IDs	NCBI-KOG	TFs
Ca4_P1_13268721	Ca4	13268721	AAGAGAGAGAGAGAGAGAGAGAGAGAGAGAGAGA/AAGAGAGAGAGAGAGAGAGAGAGAGAGAGAGAGAGA	GGGGGATGATAATGAAGCAC	CCCAATCCCCAACTAACCTT	59.2	623	INTRON	Ca_04549	TU	--
Ca4_P1_13300050	Ca4	13300050	CATATATATATATATATATATATA/CATATATATATATATATATATATATA	TTGTACCCATTTGCAGACCA	CGTGGGTATCCACCAAAATC	60.0	611	INTERGENIC	--	--	--
Ca4_P1_13320887	Ca4	13320887	AATATATATATATATATATATA/AATATATATATATATATATATATATA	TATGCGCCTCAGCTTATCCT	CCGAGGAAACAATTGGATGT	60.0	600	INTERGENIC	--	--	--
Ca4_P1_13325790	Ca4	13325790	ATTTTTT/ATTT	TTGATCATCCTATTTCAAAAGTTCA	ATTTTCGACTCCACACACGG	59.1	640	INTERGENIC	--	--	--
Ca4_P1_13388133	Ca4	13388133	GTATTATTATTATTATTATTATTATTATTATTATTATTATTATTATTATTATTATTATT/GTATTATTATTATTATTATTATTATTATTATTATTATTATTATTATTATTATTATTATTATT	CGGTTGAGAGACCAAAAGGA	TGCACAGTAGAGATGATATAGCGA	60.2	503	INTERGENIC	--	--	--
Ca4_P1_13433311	Ca4	13433311	TA/T	CGAGCAAACACAATGAATGG	TTGGTTGGTTCATTGGTTGA	60.1	569	INTERGENIC	--	--	--
Ca4_P1_13446467	Ca4	13446467	CAAAAAAATAAAAAAAAATAAAAA/CAAAAAAATAAAAA	TGAGATGCTGCATTACCTGC	ATCCAATGTTCTGGACGGTG	60.0	484	INTERGENIC	--	--	--
Ca4_P1_13509527	Ca4	13509527	ATTTT/ATTTTATTAAAAAATTTATTTTT	CCTCTTCACGGTGCTCATTT	CCTCGTTTTAAGCCCAATCA	60.3	158	INTERGENIC	--	--	--
Ca4_P1_13578890	Ca4	13578890	CATATATATAT/CATATATATATAT	TTTTCCCGAAATTTCTGCTG	GCTGCCCCACTAAACCACTA	60.2	687	INTERGENIC	--	--	--
Ca4_P1_13579808	Ca4	13579808	CAA/CAAA	TTCCCAATTTTTCTTTCAATTTT	GCACTCACGACTCACGAGAA	58.2	579	INTERGENIC	--	--	--
Ca4_P1_13582638	Ca4	13582638	A/AT	GTATCGCAGCACCATGATTG	GAAAGCGGTTTGTGATTGGT	60.1	803	INTERGENIC	--	--	--
Ca4_P1_13604124	Ca4	13604124	GA/G	TTCAGACCCCAGTTTCATCA	GACATGTATCGTGTGCGGTC	59.1	631	INTERGENIC	--	--	--

INDEL marker IDs	Chromosomes/ unanchored scaffolds	Physical positions (bp)	InDels (*Kabuli* reference genome-CDC Frontier/PI)	Forward primers (5'-3')	Reverse primers (5'-3')	Anneling temperature (0C)	Expected amplified product size (bp)	Structural annotation		Functional annotation	
								Sequence components of *kabuli* genome	*Kabuli* gene accession IDs	NCBI-KOG	TFs
Ca4_P1_13604501	Ca4	13604501	TGG/TGGG	GCACCGTTGCAATCTTTGAT	GACATGCCCCTGTTTCACTT	61.0	763	INTERGENIC	--	--	--
Ca4_P1_13612180	Ca4	13612180	CTAT/CTATAT	TGTTTCGACATTCCTCGTTG	CCAAATTCACGAGAACCAAAA	59.7	782	INTERGENIC	--	--	--
Ca4_P1_13612409	Ca4	13612409	AACAATCAACACAATCA/AACAATCA	GCACCCTAACACTTCTTGCAT	CACCAAATTCACGAGAACCA	59.3	329	INTERGENIC	--	--	--
Ca4_P1_13614309	Ca4	13614309	C/CTCACCTG	CATTTGATTCGAGTGGCACA	GTGGTCGGTTTAAGTGCGTT	60.7	373	INTERGENIC	--	--	--
Ca4_P1_13614386	Ca4	13614386	TAAAAAAA/TAAAAAAAA	CATTTGATTCGAGTGGCACA	GTGGTCGGTTTAAGTGCGTT	60.7	373	INTERGENIC	--	--	--
Ca4_P1_13617448	Ca4	13617448	TG/TGG	GGGCCTTTTCTTCAGCTTTT	TGCATGTTATCCGCAATCTC	59.8	713	DRR	Ca_04579	--	--
Ca4_P1_13624309	Ca4	13624309	CTTT/CTT	CTGGAAAAGAAGAGGGGGAC	CAGGTTGGTCGAATCATGTG	60.0	748	DRR	Ca_04580	--	--
Ca4_P1_13626293	Ca4	13626293	TTAATA/TTA	TTTCAACTCGAGCATTGTGC	AGCACCTTAACTTCCACCCC	60.0	374	INTERGENIC	--	--	--
Ca4_P1_13629046	Ca4	13629046	AAAAATAAAAT/AAAAATAAAATAAAAT	AGCGTTTGTGGATGGAAGAC	CGAAGGTTCAAAAAGACACGA	60.1	399	INTERGENIC	--	--	--
Ca4_P1_13630632	Ca4	13630632	AA/AAGAGTCTA	TATGCAGGCCCAAACTCTCT	TTTTCACAATCAATGCCCAA	59.8	370	INTERGENIC	--	--	--
Ca4_P1_13631062	Ca4	13631062	GCC/GC	TTGGGCATTGATTGTGAAAA	TCGCAACTCGTATAGGACCA	59.9	271	INTERGENIC	--	--	--
Ca4_P1_13632354	Ca4	13632354	TC/TCC	ACACTGTTTTAACGGGGCTG	TGCGGTCCTTTAATTTTATTTCA	60.0	814	INTERGENIC	--	--	--

INDEL marker IDs	Chromosomes/ unanchored scaffolds	Physical positions (bp)	InDels (*Kabuli* reference genome-CDC Frontier/PI)	Forward primers (5'-3')	Reverse primers (5'-3')	Anneling temperature (0C)	Expected amplified product size (bp)	Structural annotation		Functional annotation	
								Sequence components of *kabuli* genome	*Kabuli* gene accession IDs	NCBI-KOG	TFs
Ca4_P1_13636270	Ca4	13636270	TAAA/TAA	CTTGGCAATTGGACACCTTT	ATTGACCCATTTCATTCCCA	60.0	570	DRR	Ca_04581	--	--
Ca4_P1_13641454	Ca4	13641454	TTATCTATC/TTATC	TTGATCAACCTCAGCCTCAA	GGCACAGTTGGAGGAATGAT	59.4	687	INTRON	Ca_04582	--	--
Ca4_P1_13642522	Ca4	13642522	GCACAGACCACAG/GCACAG	CTTAAGGCGCAGAAAAGCAT	AATCGCGGGTGTTAAAGATG	59.6	571	INTRON	Ca_04582	--	--
Ca4_P1_13643715	Ca4	13643715	GTT/GTTT	ATAGTTGTTGAAGGGGCACG	GGGTGAAGAATGTATGCCAAA	60.0	798	INTERGENIC	--	--	--
Ca4_P1_13654399	Ca4	13654399	CAA/CA	AATCAACGACAAGGGCAAAG	TGGTTCAATGGAATGGTTGA	60.1	462	DRR	Ca_04584	--	--
Ca4_P1_13655282	Ca4	13655282	TTATA/TTATAGGATATA	AATTCACAAGGTCCAGTGGC	GGGCATGATTTTTCCCCTAT	60.0	790	INTERGENIC	--	--	--
Ca4_P1_13655663	Ca4	13655663	CCAAATCAAAT/CCAAAT	GGCACGATTTGACATTTGTG	GGCAACTCAATGCAAAAACA	60.0	576	INTERGENIC	--	--	--
Ca4_P1_13657824	Ca4	13657824	ATATT/AT	TGCATGCATGAATAGTGTTTGA	TCTCCCAATGTGATTGCTCA	60.1	395	INTERGENIC	--	--	--
Ca4_P1_13663032	Ca4	13663032	TAAAAA/TAAAA	AGTACCTTCGTCGTGTTCGC	GGACTTTTCAAAACGGCTCA	60.3	231	DRR	Ca_04585	R	bHLH
Ca4_P1_13666161	Ca4	13666161	ACTG/ACTGCTG	CCCATTTCACCTCTTTGGAA	TTCCCGCAACAAATAGATCC	59.9	840	CDS (large-effect mutations)	Ca_04586	Q	B3
Ca4_P1_13669796	Ca4	13669796	ATT/AT	AACGTTATTTAAACGAGGATTTCA	CCCTTTGTGGGTTCCCTTAT	58.3	555	INTERGENIC	--	--	--
Ca4_P1_13670198	Ca4	13670198	CAAAA/CAAA	ATAAGGGAACCCACAAAGGG	CATGAGATCGTATGTCGCGT	60.0	643	INTERGENIC	--	--	--

INDEL marker IDs	Chromosomes/ unanchored scaffolds	Physical positions (bp)	InDels (*Kabuli* reference genome-CDC Frontier/PI)	Forward primers (5'-3')	Reverse primers (5'-3')	Anneling temperature (0C)	Expected amplified product size (bp)	Structural annotation		Functional annotation	
								Sequence components of *kabuli* genome	*Kabuli* gene accession IDs	NCBI-KOG	TFs
Ca4_P1_13674474	Ca4	13674474	CTT/CT	GGTCTCGTGTCTGTCAGCAA	ATGTTGAAGGGAATTCGTCG	60.0	520	DRR	Ca_04587	Q	B3
Ca4_P1_13675043	Ca4	13675043	CAAA/CAAAAA	CGACGAATTCCCTTCAACAT	GTGGGGGATTGTGAGAGAAA	59.9	459	DRR	Ca_04587	Q	B3
Ca4_P1_13689699	Ca4	13689699	CTAAAAATACAATAAAAATACAATAA/CTAAAAATACAATAA	GTCGTGTTCCCGCTTAAGAT	GCAAATGGATAAAAGTCACATCA	59.2	684	INTERGENIC	--	--	--
Ca4_P1_13691259	Ca4	13691259	GTT/GT	CCATTTATTTCCGGCATTTC	AGCCGACCAAACACTATTGG	59.3	271	INTERGENIC	--	--	--
Ca4_P1_13692964	Ca4	13692964	TGTATCAGTAT/TGTAT	TGTATCAAGTTCGTCCACCG	TTGATTTACGCCTGTTTCCC	59.6	247	DRR	Ca_04590	--	--
Ca4_P1_13694721	Ca4	13694721	TA/TAA	AATGCCTTCCAGGTTGTGAC	GGTTAAGTTTGCTGGGGACA	60.0	566	DRR	Ca_04590	--	--
Ca4_P1_13700242	Ca4	13700242	GCC/GC	ATTTGTTTCCCAAACCCTCC	CCTGTTGGTGCCCTTTAGAA	60.0	863	INTRON	Ca_04591	--	--
Ca4_P1_13703757	Ca4	13703757	GAA/GAATAA	CTCCTTCCTTCCCCTCAAAC	CCTGGTGGTGAGTTTCCTGT	60.0	576	INTRON	Ca_04591	--	--
Ca4_P1_13705518	Ca4	13705518	GAAAA/GAAAAA	TCTTCGAAAATTCAAATGAACAA	TCCGTGTTCCAAATCCTTTT	58.8	607	DRR	Ca_04592	R	--
Ca4_P1_13710634	Ca4	13710634	CAAAAAAAA/CAAAAAAA	GCTCCAAATGTTGGACCCTA	GGGTTAAATGCTTGCGTGTT	59.9	855	DRR	Ca_04593	R	--
Ca4_P1_1371815	Ca4	1371815	TATAATAATAATAATAATAA/TATAATAATAATAATAATAA	ATGAAGTTCCCCATGAGTGC	AGGCAAAAGGGAAAGACCAC	59.9	463	INTERGENIC	--	--	--
Ca4_P1_13730284	Ca4	13730284	TTATATATATATATATATATATATAT/TTATATATATATATATATATATATAT	AACACATAACCACTTCGCCC	TGCAGCGAAATTATTCGAGA	59.9	458	INTERGENIC	--	--	--

INDEL marker IDs	Chromosomes/ unanchored scaffolds	Physical positions (bp)	InDels (*Kabuli* reference genome-CDC Frontier/PI)	Forward primers (5'-3')	Reverse primers (5'-3')	Anneling temperature (0C)	Expected amplified product size (bp)	Structural annotation		Functional annotation	
								Sequence components of *kabuli* genome	*Kabuli* gene accession IDs	NCBI-KOG	TFs
Ca4_P1_13731764	Ca4	13731764	GAAAATAA/GAAAATAAAAAAATAA	CGTTACATGACGCACCAATC	GTCCAAGTAATCTGGCGCAT	60.0	659	INTERGENIC	--	--	--
Ca4_P1_13743876	Ca4	13743876	GAA/GAAGCATATGCAA	CTGGTTCATGCCCCTACAAT	TCAAAGCACTAGCCACATGC	59.8	740	DRR	Ca_04598	R	MYB_related
Ca4_P1_13746289	Ca4	13746289	AT/A	TATTGGTCAAGCCCTTACGC	TGGAAAAGAAAACCAGTGCC	60.1	618	DRR	Ca_04599	R	MIKC
Ca4_P1_13750785	Ca4	13750785	ATTTTTTT/ATTTTTTTTT	GAGCATGTTTGGATTTACTTTGC	TTTTTATGTAAAGGACGAGGATGA	60.0	721	INTERGENIC	--	--	--
Ca4_P1_13757687	Ca4	13757687	ACTTGC/AC	TAACCTGTTGCCATGGTTCA	AAGATGGGGAGAGGGAAAGA	60.0	649	INTRON	Ca_04600	--	NF-YB
Ca4_P1_13758154	Ca4	13758154	TCCC/TCC	TTCTTTCCCTCTCCCCATCT	TTTTGGAAATGTTGGTGGCT	60.0	683	INTRON	Ca_04600	--	NF-YB
Ca4_P1_13770434	Ca4	13770434	AGTAC/AGTACGTAC	AAGCACCCATCCTGAAATTG	TTTCACCGCGATAAAAATCA	59.9	586	INTERGENIC	--	--	--
Ca4_P1_13770493	Ca4	13770493	TAAAAAA/TAAAAAAA	AAGCACCCATCCTGAAATTG	TTTCACCGCGATAAAAATCA	59.9	586	INTERGENIC	--	--	--
Ca4_P1_13770687	Ca4	13770687	TAA/TA	AAGCACCCATCCTGAAATTG	TTTCACCGCGATAAAAATCA	59.9	586	INTERGENIC	--	--	--
Ca4_P1_13776143	Ca4	13776143	A/AG	AACTGTGCGGCCTAAATAGC	GTATGGAGCCCAATTTGGAA	59.4	201	INTERGENIC	--	--	--
Ca4_P1_13776936	Ca4	13776936	GTATATATATATA/GTATATATATA	TGTGTCTGCCCATGAGTGTT	ATATGCGGTTTCATCCTTGC	60.2	301	INTERGENIC	--	--	--
Ca4_P1_13777793	Ca4	13777793	CAGA/CAGAGA	ATTCCCTTTAACCCCCAATG	GAAAGGCTGCAGTGATGGAT	59.9	741	INTERGENIC	--	--	--

INDEL marker IDs	Chromosomes/ unanchored scaffolds	Physical positions (bp)	InDels (*Kabuli* reference genome-CDC Frontier/PI)	Forward primers (5'-3')	Reverse primers (5'-3')	Anneling temperature (0C)	Expected amplified product size (bp)	Structural annotation		Functional annotation	
								Sequence components of *kabuli* genome	*Kabuli* gene accession IDs	NCBI-KOG	TFs
Ca4_P1_13779676	Ca4	13779676	CTTTTTTTTTT/CTTTTTTTTTTTTTTTT	CATTTTTCTCGAAATGGGGA	ACCAGCCACATAAGCCAAAC	59.9	685	INTRON	Ca_04602	I	--
Ca4_P1_13792182	Ca4	13792182	T/TA	GGCAACATAGATCCTCTCACC	GCTCACTCCGTCAGGTTAGG	58.6	719	INTRON	Ca_04604	Q	--
Ca4_P1_13792402	Ca4	13792402	ATTAAAATTTTAAAATTTT/ATTAAAATTTT	GGCAACATAGATCCTCTCACC	GCTCACTCCGTCAGGTTAGG	58.6	719	INTRON	Ca_04604	Q	--
Ca4_P1_13795734	Ca4	13795734	TTGAT/TT	GCCGGAAATACACCGACTAA	AAAGGGTAGGAACAACCGCT	60.0	599	INTERGENIC	--	--	--
Ca4_P1_13798734	Ca4	13798734	GAAAAAAA/GAAAAA	AATTTCGGAAACCTCGTGTG	TGCTAATTCACCCCTTAACCC	60.0	522	DRR	Ca_04605	R	--
Ca4_P1_13805156	Ca4	13805156	TTATA/TTA	TGGGGAAGAAGACAAGCCTA	AAGAACTTTTCATTCGACGGTT	59.8	545	INTRON	Ca_04606	--	NF-YA
Ca4_P1_13805476	Ca4	13805476	ATTTTTTTT/ATTTTTTT	TGGGGAAGAAGACAAGCCTA	AAGAACTTTTCATTCGACGGTT	59.8	545	INTRON	Ca_04606	--	NF-YA
Ca4_P1_13807289	Ca4	13807289	TAAAAAA/TAAAAA	ACAAGAAAAGCACAGGCACC	TGATCAAAGCCGTCATGTCT	60.3	576	INTERGENIC	--	--	--
Ca4_P1_13807671	Ca4	13807671	CAAAAAAA/CAAAAAA	GCCACAACTGGGTAGGTTTT	GGTTTCAACTCGAGTCGTCG	59.0	885	INTERGENIC	--	--	--
Ca4_P1_13807898	Ca4	13807898	ACTTCTTCTT/ACTTCTT	TGACGGCTTTGATCATTGTT	GGTTTCAACTCGAGTCGTCG	59.1	416	INTERGENIC	--	--	--
Ca4_P1_13808750	Ca4	13808750	A/AGTGATTCAGACTC	ATTCCACGTTCACTTCTCCG	CGCCACTCTTCTACGGTTCT	60.1	376	INTERGENIC	--	--	--
Ca4_P1_13809545	Ca4	13809545	ATT/ATTT	AAAAGGGGATGAGTTTTTCCT	TCGTAATTAATCCACATGAATCTGA	58.1	923	INTERGENIC	--	--	--

INDEL marker IDs	Chromosomes/ unanchored scaffolds	Physical positions (bp)	InDels (*Kabuli* reference genome-CDC Frontier/PI)	Forward primers (5'-3')	Reverse primers (5'-3')	Anneling temperature (0C)	Expected amplified product size (bp)	Structural annotation		Functional annotation	
								Sequence components of *kabuli* genome	*Kabuli* gene accession IDs	NCBI-KOG	TFs
Ca4_P1_13810357	Ca4	13810357	CTAAGG/C	ATAAAGATCGACTGCGTGGG	GCGTCAGGCAACACCTAATC	60.1	611	INTERGENIC	--	--	--
Ca4_P1_13826117	Ca4	13826117	ATTTTT/ATTTTTT	AGGTGGTTGTTGCACTTTGA	CAAATTTGTTCCGCTTCACA	59.2	474	INTERGENIC	--	--	--
Ca4_P1_13826465	Ca4	13826465	TAAAAAAA/TAAAAAA	TGGAACTATGTGAAGCGGAA	TTGCGGAGATCATACATTGC	59.3	643	INTERGENIC	--	--	--
Ca4_P1_13826902	Ca4	13826902	CAAAA/CAAA	GCAATGTATGATCTCCGCAA	GTTGAGGAAAACGCAGGAAG	59.7	275	INTERGENIC	--	--	--
Ca4_P1_13828032	Ca4	13828032	GTTATTATTATTATTATTATT/GTTATTATTATTATTATTATTATTATTATTATT	GTAATTGCCGGGGTCCTTAT	CAATAGTGTTTGCACGTGGG	60.0	430	INTERGENIC	--	--	--
Ca4_P1_13829467	Ca4	13829467	AT/ATT	TGAGAATTTGCATGCCTCAC	AGCGAAGTGCTAGCAACACA	59.8	671	INTERGENIC	--	--	--
Ca4_P1_13833376	Ca4	13833376	TA/T	TTTGCCTCAATTTGTCATGC	AAGCAGGCATTGTTTCGATT	59.7	307	INTERGENIC	--	--	--
Ca4_P1_13834744	Ca4	13834744	CAAAAAAAAAAAAAAAA/CAAAAAAAAAAAA	GCATTTCGCTATGTAATGCAA	TCCCAATTAGATGCCGTCTC	58.9	570	INTERGENIC	--	--	--
Ca4_P1_13836956	Ca4	13836956	GAAA/GAA	CGTGGGATGCTGACAGTAGA	AGGCTGCAAGAGCAGTAAGC	59.9	464	DRR	Ca_04608	--	--
Ca4_P1_13850248	Ca4	13850248	TAA/TA	GAAAAATAAAAGACCATTTCCCCT	TCCACTTTTGTTGGGAAAAA	60.0	548	INTERGENIC	--	--	--
Ca4_P1_13851521	Ca4	13851521	CAATA/CA	GGAAACAACTCATGACATTTTGA	TGGGTTGCATATTCAATGCTC	59.0	640	INTERGENIC	--	--	--
Ca4_P1_13862277	Ca4	13862277	AAT/AATATAT	AAAATCGGCATTGTCAGAGG	GGACAGCTAAATAAATTGCAAGC	60.1	714	DRR	Ca_04609	R	--

INDEL marker IDs	Chromosomes/ unanchored scaffolds	Physical positions (bp)	InDels (*Kabuli* reference genome-CDC Frontier/PI)	Forward primers (5'-3')	Reverse primers (5'-3')	Anneling temperature (0C)	Expected amplified product size (bp)	Structural annotation		Functional annotation	
								Sequence components of *kabuli* genome	*Kabuli* gene accession IDs	NCBI-KOG	TFs
Ca4_P1_13865484	Ca4	13865484	T/TTTC	TCAGATGTTTATTTGATCCAGCA	CTTTGAGGATCCGTTTGTCA	59.6	590	INTRON	Ca_04610	R	--
Ca4_P1_13866963	Ca4	13866963	G/GAT	GCAGTGCCAGTTTTCCTTTC	AAAGTTCGAAGACTTCCCCC	59.9	827	INTRON	Ca_04610	R	--
Ca4_P1_13885715	Ca4	13885715	GAAAAAAAAAAA/GAAAAAAAAAA	CTCTTCTGCAAAAGGGATGC	TCCGGTGTTGGGTTGTATTT	60.0	599	INTRON	Ca_04612	I	G2-like
Ca4_P1_13896280	Ca4	13896280	CGAAGAAGAAGAAGAA/CGAAGAAGAAGAA	TACACTGGCCCATCATCTTG	GCCTCGTTGGAATGATTTGT	59.5	482	INTERGENIC	--	--	--
Ca4_P1_13908536	Ca4	13908536	TC/T	CTCCTCCGATGTCTGGAAAA	CGCGGTTGCTTCAATTCTAT	60.2	232	INTERGENIC	--	--	--
Ca4_P1_13909254	Ca4	13909254	CA/CAA	CATCACGATTTGACGAGTTGA	AAAAACATGACTCCAAACACCA	59.7	636	INTERGENIC	--	--	--
Ca4_P1_13909691	Ca4	13909691	AATATATATATATATATATATATA/AATATATATATATATATATA	TGGTGTTTGGAGTCATGTTTTT	CGCTGCACGGAAACTATCTT	59.4	209	INTERGENIC	--	--	--
Ca4_P1_13909852	Ca4	13909852	GT/G	CAGCGCACAGGTTATCCACT	GGCTCATCTGCCAAACAAAT	61.3	503	INTERGENIC	--	--	--
Ca4_P1_13912134	Ca4	13912134	GTCT/GT	TTGTTCTGTTGGTGTTGCGT	ACAACCAAACTCCATGGCTC	60.2	448	INTERGENIC	--	--	--
Ca4_P1_13979065	Ca4	13979065	AT/ATTATT	TTTCACGCTCACGAAGTCAC	GGGAATTTACTCAAAAAGAGGC	60.0	741	DRR	Ca_04623	S	--
Ca4_P1_13988720	Ca4	13988720	TAATAAATAAATAAATAAATAAATA/TAATAAATAAATAAATAAATAAATAAATA	TCATCAAATTCGTAACGCAAA	GATCCAAATCACGTAAACACCA	59.2	605	INTERGENIC	--	--	--
Ca4_P1_14000969	Ca4	14000969	AATATATATATATATATATAT/AATATATATATATATATATATAT	CCTTGTCGGATTAACAGGGA	TAAAAACAGGATCCCATGCC	59.9	542	INTERGENIC	--	--	--

INDEL marker IDs	Chromosomes/ unanchored scaffolds	Physical positions (bp)	InDels (*Kabuli* reference genome-CDC Frontier/PI)	Forward primers (5'-3')	Reverse primers (5'-3')	Anneling temperature (0C)	Expected amplified product size (bp)	Structural annotation		Functional annotation	
								Sequence components of *kabuli* genome	*Kabuli* gene accession IDs	NCBI-KOG	TFs
Ca4_P1_14011652	Ca4	14011652	TAA/TAACAA	GCACCGACTTGCTGATGTAA	GCCTCATGCTCCAATTTTGT	59.9	755	INTERGENIC	--	--	--
Ca4_P1_1407458	Ca4	1407458	CATTTTTCATGAT/CATTTTTCATGATTTTTCATGAT	TTCAACGTAAAACCGGGAAC	ACCGGATTTTTGGTCCCTAT	59.8	557	INTERGENIC	--	--	--
Ca4_P1_14182954	Ca4	14182954	AATATATATATATATATATATATATATATAT/AATATATATATATATATATATATATATATATATATATATATAT	CACCGCATACACCCCTAGAT	TAAGATCCTTGCGCCAAACT	59.8	453	INTRON	Ca_04651	--	TALE
Ca4_P1_14196867	Ca4	14196867	CTATATATATATATATAT/CTATATATATATATAT	CACTCGTGTTTCCCAAGGTT	TTGCAAGTGCATCCAAATTC	60.0	584	INTERGENIC	--	--	--
Ca4_P1_14238993	Ca4	14238993	TTATATATATATA/TTATATATATA	TGCTTTACACAAGCAAGCCA	CGACAGAGACGAGGGAAGTC	60.6	170	DRR	Ca_04659	K	M-type
Ca4_P1_14396557	Ca4	14396557	TTAGTAGT/TTAGT	AGTCAGACGCTACTCGGCAT	GTGTGTGTGTGTGTGTGTGTGT	60.0	590	INTERGENIC	--	--	--
Ca4_P1_14397068	Ca4	14397068	GATACAT/GATACATACATACATATACAT	ACACACACACACACACACACAC	AACACATCCGGGATCCATTA	58.8	676	INTERGENIC	--	--	--
Ca4_P1_14397300	Ca4	14397300	GATATATATATATATATATATGTATATATATATATA/GATATATATATATATATATATATATATATGTATATATATATATA	TAAATGCATGCTCCCCTGAT	AACACATCCGGGATCCATTA	60.4	273	INTERGENIC	--	--	--
Ca4_P1_14405625	Ca4	14405625	TGTTGTT/TGTTGTTGTT	CGGTGGTTGCTCTCTCTTTC	GGGGTACAATGTGGAACCAA	60.0	440	DRR	Ca_05663	E	MYB
Ca4_P1_14405755	Ca4	14405755	ATCTCTCTCT/ATCTCTCT	ATGTGCATGCCAAGTTGTGT	ACCTATAAGCCCTTTGCGCT	60.0	963	DRR	Ca_05663	E	MYB
Ca4_P1_14406037	Ca4	14406037	GTCTTT/GT	TTGGTTCCACATTGTACCCC	TTGTTGCTGGAGTTGGATCA	60.5	639	DRR	Ca_05663	E	MYB
Ca4_P1_14406133	Ca4	14406133	CAAA/CAAAA	TTGGTTCCACATTGTACCCC	CCATTGGTTGGGATATGGAG	60.5	892	DRR	Ca_05663	E	MYB

INDEL marker IDs	Chromosomes/ unanchored scaffolds	Physical positions (bp)	InDels (*Kabuli* reference genome-CDC Frontier/PI)	Forward primers (5'-3')	Reverse primers (5'-3')	Anneling temperature (0C)	Expected amplified product size (bp)	Structural annotation		Functional annotation	
								Sequence components of *kabuli* genome	*Kabuli* gene accession IDs	NCBI-KOG	TFs
Ca4_P1_14416755	Ca4	14416755	TTATATATATATA/TTATATATATATATA	TCATTTCAAACCCTTAAACTCCA	TTGCGGTCAATTGTATGAAGAC	59.9	392	INTERGENIC	--	--	--
Ca4_P1_14487983	Ca4	14487983	AATATATATATATATATATATATA/AATATATATATATATATATA	CATGTGCATATGCGTTTCAA	TTTCCCTTGTTCTTCGGATG	59.1	762	INTERGENIC	--	--	--
Ca4_P1_14618637	Ca4	14618637	TTATATATATATATA/TTATATATATATATATA	TGGGGGACTATTTTCGTGAT	TTTGGTTTTAGTGTCATGCGA	59.2	665	INTERGENIC	--	--	--
Ca4_P1_14624427	Ca4	14624427	ATTTTTTTTTTT/ATTTTTTTTTT	AAACATGCAAAACACGCAAA	ATCGCGTGCCTGTTTAATTT	60.2	768	INTERGENIC	--	--	--
Ca4_P1_1463289	Ca4	1463289	TAAAAAAAAAAA/TAAAAAAAAAA	CCCACTTTTACGGAAGACGA	CCCCAACCACTTCCTCACTA	60.1	511	INTERGENIC	--	--	--
Ca4_P1_14652419	Ca4	14652419	TTTATTATTATTATTATTATTATTATTATTATTATTATTATTAT/TTTATTATTATTATTATTATTATTATTATTATTATTATTATTATTATTATTAT	ATCCAAGCCGTAAGGGTCAT	AAGTCCGACATATCACTCGTCA	60.7	733	INTERGENIC	--	--	--
Ca4_P1_14665183	Ca4	14665183	TTATATATATATATATAT/TTATATATATATATAT	CGTGCCCTTTTATTCTTAAGGT	TGTTTATGCGTGTGGCATTT	58.8	735	INTERGENIC	--	--	--
Ca4_P1_14677950	Ca4	14677950	CAAAAAAA/CAAAAAAAA	TGTTGGGCAAAATAAATTGAGA	CAAACCTTACCACCGTCTTG	59.5	424	INTERGENIC	--	--	--
Ca4_P1_14771240	Ca4	14771240	ATATATATAT/ATATATATATATTTATATATAT	TCAATGCGGTCTCTGAAGTG	TATTCAGATCCCTTGCCCAG	60.0	576	INTRON	Ca_05627	--	Trihelix
Ca4_P1_14800141	Ca4	14800141	TAAAAAAAAAAAA/TAAAAAAAAAA	TTGTACACGTAGTAGCGCCG	AAGATGGAATTCAAGGGGCT	60.0	475	INTERGENIC	--	--	--
Ca4_P1_1482356	Ca4	1482356	TAAAAAAAAAAA/TAAAAAAAAAAAAA	TGGGTCTGCATACCAATGAG	CAAGTAGGGTGGTCTGGAGG	59.5	474	INTERGENIC	--	--	--
Ca4_P1_14967549	Ca4	14967549	GTAATAATAATAATAATAATAATAATAATAA/GTAATAATAATAATAATAATAATAATAATAATAATAA	CAAAGTGCTTTTGTACCCCC	CCGTTTGTTTTGGGACATCT	59.5	899	INTERGENIC	--	--	--

INDEL marker IDs	Chromosomes/ unanchored scaffolds	Physical positions (bp)	InDels (*Kabuli* reference genome-CDC Frontier/PI)	Forward primers (5'-3')	Reverse primers (5'-3')	Anneling temperature (0C)	Expected amplified product size (bp)	Structural annotation		Functional annotation	
								Sequence components of *kabuli* genome	*Kabuli* gene accession IDs	NCBI-KOG	TFs
Ca4_P1_14995963	Ca4	14995963	TTATATATATATATATATATATATATAT/TTATATATATATATATATATATATATATATAT	TCAACACATACCCGCAAAAA	TTTAAGTTCAAATGCACCCAAT	60.0	167	INTERGENIC	--	--	--
Ca4_P1_15012755	Ca4	15012755	AATATATATATATATA/AATATATATATATATATATA	TACTCCCAAATGGCGACTTC	GACATTTCGAACTTGAATTTCCT	60.1	322	INTERGENIC	--	--	--
Ca4_P1_15020189	Ca4	15020189	CATATATATATATATATATATATATATATA/CATATATATATATATATATATATATATA	TTGGCCACTTGTGTTTGTGT	TGTCAAAATTGAAAAGCAAAATG	60.0	487	INTERGENIC	--	--	--
Ca4_P1_15020792	Ca4	15020792	GTTTTTTTT/GTTTTTTTTT	TTCCTCCATAAATTTCCAACG	AGCCACCGGTCTCAGTAAAT	58.9	288	INTERGENIC	--	--	--
Ca4_P1_15045995	Ca4	15045995	CTATATATATATATATATATATATATATATATATATA/CTATATATATATATATATATATATATATATATATATATATATA	GAAAATTATATTCTTTCCCAAATGC	GGAGGAGAGAGACGAAGGCT	58.5	402	INTERGENIC	--	--	--
Ca4_P1_15048181	Ca4	15048181	TA/TAA	CAAAAGCTGTAACACCCCGT	AATCGATTGATTCAGCCCAG	60.0	528	INTERGENIC	--	--	--
Ca4_P1_15120844	Ca4	15120844	CATATATATA/CATATATATATATA	ATTCCGGTAAAATCTCCGCT	TATTTTGGATTTTGGCTCGG	59.9	688	INTERGENIC	--	--	--
Ca4_P1_15146205	Ca4	15146205	TTTATTATTATTATTATTATTATTATTATTATTATTATTATTATTATTATTATTATTATTATT/TTTATTATTATTATTATTATTATTATTATTATTATTATTATTATTATTATTATTATTATTATTATTATT	GGATTCTCCTTGTTTCTGCG	TTTTCCGCAAAAATTCAAAAA	59.8	690	DRR	Ca_05586	Z	--
Ca4_P1_15168496	Ca4	15168496	CAAAAAAAAAAAA/CAAAAAAAAAAAAA	TCATTACTTCCCAGTCCCAAA	TCCTCATGGAACACATGAAA	59.4	608	INTERGENIC	--	--	--
Ca4_P1_15216075	Ca4	15216075	TAAAAAAAAAA/TAAAAAAAAAAA	TGAACCAAAAATCATCGCAA	AGGCTCTTGTTAAGCGACCA	60.1	402	INTERGENIC	--	--	--
Ca4_P1_15221737	Ca4	15221737	ATTTTTT/ATTTTTTT	AGGGTGCGATCAGGTAAATG	AACAAGTGGTGCGATTGTGA	60.0	430	INTERGENIC	--	--	--
Ca4_P1_15251377	Ca4	15251377	AATA/AATATATA	TCCTGAAACAAACGCATTCA	AAATAGGTTAGGCCGTCCGT	60.2	685	INTERGENIC	--	--	--

INDEL marker IDs	Chromosomes/ unanchored scaffolds	Physical positions (bp)	InDels (*Kabuli* reference genome-CDC Frontier/PI)	Forward primers (5'-3')	Reverse primers (5'-3')	Anneling temperature (0C)	Expected amplified product size (bp)	Structural annotation		Functional annotation	
								Sequence components of *kabuli* genome	*Kabuli* gene accession IDs	NCBI-KOG	TFs
Ca4_P1_15252487	Ca4	15252487	GTA/GTACATATA	TTTTTGAGTGAAACCAGTTTGG	CCAACCATGTTGGTGTGCTA	59.2	533	INTERGENIC	--	--	--
Ca4_P1_15272177	Ca4	15272177	AG/A	GTTTCGGCTAATGGGTTGAA	TTGCTTGTAATTTTGGGTTTTG	59.9	878	DRR	Ca_05572	A	--
Ca4_P1_15288869	Ca4	15288869	TAAAAAA/TAAAAAAA	TACGCTACCAGCTATCGCCT	AGAACCACCGTAACGACCTG	60.0	556	CDS (FRAME SHIFT)	Ca_05570	R	--
Ca4_P1_15392307	Ca4	15392307	TA/TACACTAA	GGTCTCACCAACACTGTGGA	CGGTTTTGGCGTCTTTATGT	59.6	428	DRR	Ca_05557	R	B3
Ca4_P1_15406107	Ca4	15406107	CCATA/C	TAAGCACACGGAGATTGCAT	GTGGTACCGCAGATTCTGGT	59.3	884	INTERGENIC	--	--	--
Ca4_P1_1543745	Ca4	1543745	CATATATATATATATATATATATATA/CATATATATATATATATATATATA	CCTTTCCATCCCTGTGAAAA	CCACACTATAGCATTGGTGCAT	59.9	377	INTRON	Ca_07783	S	--
Ca4_P1_15473254	Ca4	15473254	TTATATATATATATATAT/TTATATATATATATAT	TTGGACCGAAGAAATTGTCA	CTCCGTTAATCCTCATGCGT	59.1	702	INTERGENIC	--	--	--
Ca4_P1_15481101	Ca4	15481101	AATA/AA	TAGTAGGGCTCTTGCTCCCA	CCAATTGTACCGGGATCTTC	60.0	933	INTERGENIC	--	--	--
Ca4_P1_15507374	Ca4	15507374	CTTTTTTTTTT/CTTTTTTTTT	CACATCCACCAATGAGATGG	TCAACAACATCAAATAAAGAATGTCA	59.8	373	INTERGENIC	--	--	--
Ca4_P1_15578288	Ca4	15578288	TTATTCTAAGGT/TT	TTTTCATCCTCCTCCACCTG	TGGACGTAAATATAGGGCGG	60.0	689	DRR	Ca_05544	K	ARR-B
Ca4_P1_15590624	Ca4	15590624	GTATTATTATTATTATTATTATTATTAT/GTATTATTATTATTATTATTATTATT	CGACCTTGCATTAGCAGTGA	ATATGTCAGCCGGCCTAGC	60.0	397	INTRON	Ca_05543	E	--
Ca4_P1_15609692	Ca4	15609692	TAAAAAAAAAAAAAA/TAAAAAAAAAAAAA	TTGTCTCAACAATCTCCCCC	CTCCGTCATCTGATGGGTCT	59.9	357	DRR	Ca_05541	--	--

INDEL marker IDs	Chromosomes/ unanchored scaffolds	Physical positions (bp)	InDels (*Kabuli* reference genome-CDC Frontier/PI)	Forward primers (5'-3')	Reverse primers (5'-3')	Anneling temperature (0C)	Expected amplified product size (bp)	Structural annotation		Functional annotation	
								Sequence components of *kabuli* genome	*Kabuli* gene accession IDs	NCBI-KOG	TFs
Ca4_P1_15643039	Ca4	15643039	T/TTACTGTAGGGA	TGCACCGACAAACTGAAGTC	ACGCGTGAGTTTGCTCACTA	59.9	592	INTERGENIC	--	--	--
Ca4_P1_15669145	Ca4	15669145	AT/ATATATATATGTGTGT	TTCCATTCAACATTGACATCCT	GCATTATACAGCGAGGGCTT	59.3	513	DRR	Ca_05537	I	--
Ca4_P1_15734407	Ca4	15734407	TAGATGAAGAT/TAGAT	CAACTCCAAAATTGCCGTTTA	GGTTTGGGAATTGTGGTTCTT	60.0	208	INTERGENIC	--	--	--
Ca4_P1_15745437	Ca4	15745437	ATTTTTT/ATTTTTTT	GTGAACCAAATGACGTGTGC	AAAACAAACAAGCCCATTATTTTC	60.0	773	DRR	Ca_05530	--	--
Ca4_P1_15780356	Ca4	15780356	CATATATATATATATATATA/CATATATATATATATATATATA	CAACTTTAAAAAGGACCAAAATGAA	TTGTGCCACGACCAATTAAA	59.8	537	INTERGENIC	--	--	--
Ca4_P1_157836	Ca4	157836	GAAAAAAAAAAA/GAAAAAAAAAA	GTAAGCCGCCTCTAATGGGT	GGTTTTGATCGCGGAATAAG	60.5	483	INTERGENIC	--	--	--
Ca4_P1_15881990	Ca4	15881990	TAAAAAAAAAA/TAAAAAAAA	CAAAACGTACTTTTCCATGCC	AAAAACAACTCCCAATGCAA	59.5	465	DRR	Ca_05519	--	--
Ca4_P1_15888264	Ca4	15888264	ATT/AT	TTCCTCCTTTTAGTTATTGGAAAA	TCCAGTTTGACATTGGAACAA	57.6	542	INTERGENIC	--	--	--
Ca4_P1_15888971	Ca4	15888971	T/TG	TGCATCAGATTTTTGAAGGTTG	AAAATTGGTGGGCGTTGTTA	60.1	669	INTERGENIC	--	--	--
Ca4_P1_15891944	Ca4	15891944	CTTT/CTTTT	CGACGTCAACACAAATTGC	AGATGCGCGGTGATTAGAGT	58.7	802	INTERGENIC	--	--	--
Ca4_P1_15892004	Ca4	15892004	AC/ACCGACTC	CGACGTCAACACAAATTGC	AGATGCGCGGTGATTAGAGT	58.7	802	INTERGENIC	--	--	--
Ca4_P1_15897548	Ca4	15897548	GGAAGAAAGA/GGAAGAAAGATGAAGAAAGA	AAAATCAGCCCGATTCAATG	TGCCATTTGATATGAACCACTT	59.9	577	INTERGENIC	--	--	--

INDEL marker IDs	Chromosomes/ unanchored scaffolds	Physical positions (bp)	InDels (*Kabuli* reference genome-CDC Frontier/PI)	Forward primers (5'-3')	Reverse primers (5'-3')	Anneling temperature (0C)	Expected amplified product size (bp)	Structural annotation		Functional annotation	
								Sequence components of *kabuli* genome	*Kabuli* gene accession IDs	NCBI-KOG	TFs
Ca4_P1_15898293	Ca4	15898293	T/TA	CCTTCAAAATTTGTGAGTCCG	ATGGGAATTGGTGAATGGAA	59.6	941	INTERGENIC	--	--	--
Ca4_P1_15908669	Ca4	15908669	ATTTTT/ATTTTTT	TTCTTTGACATGCACCTTCG	CAAGAGACAAATTAGCAATCTTCG	59.8	476	INTERGENIC	--	--	--
Ca4_P1_15908734	Ca4	15908734	TGAAAAGAAAA/TGAAAAGAAAAGAAAA	TTCTTTGACATGCACCTTCG	CATCGCATGAGAATTGGTCTT	59.8	652	INTERGENIC	--	--	--
Ca4_P1_15920083	Ca4	15920083	AGGTG/AGGTGGTG	TGATTTCATGCCTAATGCCA	AATGAAAATGGGGTCCAATG	60.0	495	DRR	Ca_05514	--	SBP
Ca4_P1_1592013	Ca4	1592013	GATATATATATATATATA/GATATATATATATATATATATA	CCCTAATGGGTGAGCAGTTC	GTTGGTCAATCCACTCGGTC	59.6	706	INTERGENIC	--	--	--
Ca4_P1_15925083	Ca4	15925083	TTAAAA/TTAAAATAAAA	TCGAGATTATGAGGCATCCA	ATGTTGCAAACAGCCAGACA	59.2	545	DRR	Ca_05513	R	--
Ca4_P1_15926036	Ca4	15926036	AAAGA/AAAGAAGA	GTTTCCCTTACGCCAGATGA	GCATTGCTGTATTGGTGCAT	60.1	532	DRR	Ca_05513	R	--
Ca4_P1_15926090	Ca4	15926090	ACT/ACTCT	AGCAGCACCCACCAAATATC	TGCTGGTGTTCTAGTTGGCA	60.0	674	DRR	Ca_05513	R	--
Ca4_P1_15930046	Ca4	15930046	GAAAAAAAAAAA/GAAAAAAAAAA	TCTCTCGAGCTGGATGGAAT	AGGTGATCATTGTGTGGGGT	59.9	702	INTRON	Ca_05513	R	--
Ca4_P1_15936591	Ca4	15936591	AC/A	AATTTTTCCCCACAACCCAT	CACCAAAAATCTCCCTCCAA	60.3	596	INTERGENIC	--	--	--
Ca4_P1_15937152	Ca4	15937152	AT/ATT	TTGGAGGGAGATTTTTGGTG	TTGAAAATGGGGAAATGAGG	59.9	573	INTERGENIC	--	--	--
Ca4_P1_15939075	Ca4	15939075	AT/ATT	GTGCACCGGTAACGAAGTTT	TTCGTTGGAGGTTTGGATTC	60.0	452	INTERGENIC	--	--	--

INDEL marker IDs	Chromosomes/ unanchored scaffolds	Physical positions (bp)	InDels (*Kabuli* reference genome-CDC Frontier/PI)	Forward primers (5'-3')	Reverse primers (5'-3')	Anneling temperature (0C)	Expected amplified product size (bp)	Structural annotation		Functional annotation	
								Sequence components of *kabuli* genome	*Kabuli* gene accession IDs	NCBI-KOG	TFs
Ca4_P1_15939183	Ca4	15939183	ATATTGGTAACTTTTATTG/ATATTG	GTGCACCGGTAACGAAGTTT	GTGGTTTCCTGGGAGGGTAT	60.0	711	INTERGENIC	--	--	--
Ca4_P1_15939264	Ca4	15939264	AAAAATAAAATAAAA/AAAAATAAAATAAAATAAAA	GTGCACCGGTAACGAAGTTT	GTGGTTTCCTGGGAGGGTAT	60.0	711	INTERGENIC	--	--	--
Ca4_P1_159440	Ca4	159440	TAGAGAGAGAGAAAGAGAGAGAGA/TAGAGAGAGAGAGAAAGAGAGAGAGA	CCCAAGTCATTATGGTGTTTGA	CAATATGGCGAAGAAAATATTGAA	59.7	718	INTERGENIC	--	--	--
Ca4_P1_15945430	Ca4	15945430	AAAA/AAAAATCAAA	AGTCCAGCCTCTGCACAAAT	GGAGCAAGATGGAACAATCAA	59.9	212	DRR	Ca_05512	O	C2H2
Ca4_P1_15945824	Ca4	15945824	CAAAAAA/CAAAA	AGTCCAGCCTCTGCACAAAT	CCCCAACACTAAAAACCCCT	59.9	742	DRR	Ca_05512	O	C2H2
Ca4_P1_15948936	Ca4	15948936	TTATA/TTA	AATCTGCCACAAAACCCAAG	TTCAGTCCAATTGAAAGTCGG	60.0	795	INTERGENIC	--	--	--
Ca4_P1_15950510	Ca4	15950510	GTTATTTTTATT/GTTATTTTTATTATTATTTTTATT	AAAAGGGATTGTCTTCCCAGA	CAAAGGTAGCCCTGCTTCAG	59.9	601	INTERGENIC	--	--	--
Ca4_P1_15950827	Ca4	15950827	ATTTT/ATTTTT	AGCGACGTGAGACTGGATCT	CAAAGGTAGCCCTGCTTCAG	60.0	303	INTERGENIC	--	--	--
Ca4_P1_15957579	Ca4	15957579	A/AT	TGTACCCAGATTCAAACATCCA	ATGCGATCATGTGAACCAAA	60.2	445	DRR	Ca_05511	--	C2H2
Ca4_P1_15962571	Ca4	15962571	ACTT/ACTTCTCTT	CAGGAAATTGTGCATCATGG	TGGTGCATGACTGAATGGTT	59.9	723	INTERGENIC	--	--	--
Ca4_P1_15963289	Ca4	15963289	TA/T	AACCATTCAGTCATGCACCA	CAGATGCACGCTAGGAAACA	60.0	828	INTERGENIC	--	--	--
Ca4_P1_15963503	Ca4	15963503	ATTTT/ATTTTGTTTTT	CCATGATCAGGCTTTTTGGT	CAGATGCACGCTAGGAAACA	59.9	747	INTERGENIC	--	--	--

**INDEL marker IDs**	**Chromosomes/ unanchored scaffolds**	**Physical positions (bp)**	**InDels (*Kabuli* reference genome-CDC Frontier/PI)**	**Forward primers (5'-3')**	**Reverse primers (5'-3')**	**Anneling temperature (0C)**	**Expected amplified product size (bp)**	**Structural annotation**		**Functional annotation**	
								**Sequence components of *kabuli* genome**	***Kabuli* gene accession IDs**	**NCBI-KOG**	**TFs**
Ca4_P1_15967752	Ca4	15967752	AATA/AA	GGTCCTGCACCATCAGAAGT	CACAGGCTTGTAACGTGTTAGTG	60.1	220	DRR	Ca_05510	J	NF-YA
Ca4_P1_15968625	Ca4	15968625	ATTTTTTTTT/ATTTTTTTT	CCTCCTTCGTCAACCGAATA	TGCTCACACTCCCAGGACTT	60.1	559	DRR	Ca_05510	J	NF-YA
Ca4_P1_15969051	Ca4	15969051	CAA/CA	TAAGGCGTTAACTTGCACCC	TGGTTGTGGCGATTACAAGA	60.1	494	DRR	Ca_05510	J	NF-YA
Ca4_P1_15981742	Ca4	15981742	GGCACAA/GGCACAAGCACAA	AGGTGAGCCAAAGCAACAAT	CTAGCATTCCCATGCTCCAT	59.7	205	INTERGENIC	--	--	--
Ca4_P1_15983115	Ca4	15983115	AATT/AATTATT	TGATGTTGATGATATGACGTGG	ATTGTTCTGCAAATGAGGGG	59.3	729	INTERGENIC	--	--	--
Ca4_P1_15986801	Ca4	15986801	ATTTTTTTTTT/ATTTTTTTTT	AGATTTTGATAAAGATTTGTTTTGAAG	TCGCACATTTATCACAAGGA	57.7	285	INTERGENIC	--	--	--
Ca4_P1_15991574	Ca4	15991574	ATTTTTT/ATTTTTTT	ATTTGAAACTAACGGCCACG	GGAATTATATTAAAAACAGCCAAAAA	60.0	459	INTERGENIC	--	--	--
Ca4_P1_15993940	Ca4	15993940	CT/CTAT	TTCTGCTGATGCGTACAAGG	AAGCTAAATTTTCGCCACGA	60.0	507	DRR	Ca_05509	--	--
Ca4_P1_16000633	Ca4	16000633	TACA/TA	CGATGTGATTTTTGTAGTCAAGC	TATTTTCCGCACACCAATGA	58.8	392	INTERGENIC	--	--	--
Ca4_P1_16027847	Ca4	16027847	A/AG	AATTTTGGCTTTTTGCGTTG	ATCATATCCATCAAACGGGC	60.1	536	INTERGENIC	--	--	--
Ca4_P1_16028817	Ca4	16028817	CGTGTGT/CGTGTGTGT	TGATTTTACACAAGGGTAATTTTG	CGCCGCATACTGTAATGGTA	57.3	951	INTERGENIC	--	--	--
Ca4_P1_16033478	Ca4	16033478	ATAACTTAA/ATAA	TAACCCTTTTCGGGTTTTCC	TGGTCCAAGGCTTGTGATTT	60.2	496	INTERGENIC	--	--	--

INDEL marker IDs	Chromosomes/ unanchored scaffolds	Physical positions (bp)	InDels (*Kabuli* reference genome-CDC Frontier/PI)	Forward primers (5'-3')	Reverse primers (5'-3')	Anneling temperature (0C)	Expected amplified product size (bp)	Structural annotation		Functional annotation	
								Sequence components of *kabuli* genome	*Kabuli* gene accession IDs	NCBI-KOG	TFs
Ca4_P1_16044624	Ca4	16044624	GTTTTTTTT/GTTTTTTTATTTTTGTTTTTTTTTT	TTTTACGTTGACGGCCTTTT	AGGTTGGTCCGATTCCTTTT	59.6	335	INTERGENIC	--	--	--
Ca4_P1_16048595	Ca4	16048595	TG/T	GACCATTTTTGGTGATTGGG	AGGTACCACCAACCAAGTGC	60.0	258	INTRON	Ca_05504	J	bHLH
Ca4_P1_16051344	Ca4	16051344	CTG/CTGTG	GAGGCATGACTTCAACAGCA	TGGAAAAGGCACCTGTCAGT	60.0	600	INTRON	Ca_05504	J	bHLH
Ca4_P1_16055399	Ca4	16055399	AACTTAGTTG/AACTTAGTTGTAGCACTTAGTTG	ATGTTGTGAATGCACATGGA	CCAGTGGCGGAGAAAAATAA	58.3	345	DRR	Ca_05504	J	bHLH
Ca4_P1_16056793	Ca4	16056793	TAAAAAA/TAAAAAAA	CTGCAATGCAACAACCAATC	TCAAAAAGAATCCCGACCTG	60.1	657	INTERGENIC	--	--	--
Ca4_P1_16057030	Ca4	16057030	GTTAATTTTTT/GTTAATTTTTTTTAATTTTTT	CAGGTCGGGATTCTTTTTGA	TTGCATTGAGTCACACGTCA	60.0	344	INTERGENIC	--	--	--
Ca4_P1_16058853	Ca4	16058853	AA/AAATA	TTTGCCATAATTCCACAATGA	CGTCACTGACAGCGTAGCAC	58.9	802	INTERGENIC	--	--	--
Ca4_P1_16060305	Ca4	16060305	TAAAAAAAAAAAA/TAAAAAAAAAA	TTGGATGATACTTCTTCCGTCTC	AGAACACCCTGCAGGTTCAC	59.6	559	INTERGENIC	--	--	--
Ca4_P1_16061988	Ca4	16061988	ATT/AT	TAGCCTATACCGGTGGTTGC	TTTTGTTGGAATTGAAAGAGACA	60.0	507	DRR	Ca_05503	K	MYB
Ca4_P1_16064766	Ca4	16064766	TTAAAT/TTAAATAGGAATATAAAT	CAAAAATTGACATGGGTACGG	AGGTGAGGTGAGGTGATTGA	60.1	736	INTERGENIC	--	--	--
Ca4_P1_16065621	Ca4	16065621	AT/A	TTAGTCCAATGGAAGTCGCC	ATGCAAAACCCTGCTTCATC	60.1	706	INTERGENIC	--	--	--
Ca4_P1_16065708	Ca4	16065708	ACATCAT/ACAT	TTAGTCCAATGGAAGTCGCC	ATGCAAAACCCTGCTTCATC	60.1	706	INTERGENIC	--	--	--

INDEL marker IDs	Chromosomes/ unanchored scaffolds	Physical positions (bp)	InDels (*Kabuli* reference genome-CDC Frontier/PI)	Forward primers (5'-3')	Reverse primers (5'-3')	Anneling temperature (0C)	Expected amplified product size (bp)	Structural annotation		Functional annotation	
								Sequence components of *kabuli* genome	*Kabuli* gene accession IDs	NCBI-KOG	TFs
Ca4_P1_16066680	Ca4	16066680	CAAAA/CAAA	TCTCGATTGAAGTGCATTGG	CATTAGAGTTCACTTGGCGG	59.8	514	INTERGENIC	--	--	--
Ca4_P1_16066890	Ca4	16066890	ATTTTT/ATTTT	AGAACCGATTTTGGTGATGC	GGGACCAGTGATTTAATTTAGCC	59.9	705	INTERGENIC	--	--	--
Ca4_P1_16072318	Ca4	16072318	CTACA/CTACATACA	TGCGTGATCGTCTTCAGAAC	GGCTTCGGAGTAGAATCCCT	60.0	813	INTERGENIC	--	--	--
Ca4_P1_16072416	Ca4	16072416	ACT/ACTAAGCCT	GTCACAGCTCTGATGCCAAA	GTTTGCCGGACAGTTGAGTT	60.0	641	INTERGENIC	--	--	--
Ca4_P1_16072673	Ca4	16072673	AA/AACA	GTCACAGCTCTGATGCCAAA	GCGATACGGAGTGGACATCT	60.0	787	INTERGENIC	--	--	--
Ca4_P1_16073050	Ca4	16073050	TTG/TTGTTTAGCTG	TGCAAAAGGGATTTGTGTCA	TTGAAGGGAACGTTTGATGA	60.1	736	INTERGENIC	--	--	--
Ca4_P1_16074542	Ca4	16074542	T/TTTG	GGCTGCCTAACATGGGTTTA	GACAGGCAACCCTGTGAAAT	60.0	473	INTRON	Ca_05502	T	--
Ca4_P1_16074616	Ca4	16074616	TA/TAA	GGCTGCCTAACATGGGTTTA	GACAGGCAACCCTGTGAAAT	60.0	473	DRR	Ca_05501	--	TCP
Ca4_P1_16079766	Ca4	16079766	TAAAAAAAAAA/TAAAAAAAAA	TGGCTACTTAAAGGGCCAAA	GAAAGGGATCACGTGAGGAA	59.7	916	INTERGENIC	--	--	--
Ca4_P1_16080327	Ca4	16080327	CTTTTTTTTTTTTT/CTTTTTTTTTTT	TTCCTCACGTGATCCCTTTC	ACAATTGCTCCAACGGTCAT	60.1	567	INTERGENIC	--	--	--
Ca4_P1_16085050	Ca4	16085050	TAAAAAAA/TAAAAAAAA	ATCGGAGTTCTATTGCACCG	GGGGTGTGCTCCAAGAGATA	60.1	452	INTERGENIC	--	--	--
Ca4_P1_16088405	Ca4	16088405	ATTTATTTT/ATTTATTTTTCTTTATTTT	AGCGTTGATTAACTGCGTGA	TCCGAAAAGCAGCAATTCTC	59.5	860	INTERGENIC	--	--	--

INDEL marker IDs	Chromosomes/ unanchored scaffolds	Physical positions (bp)	InDels (*Kabuli* reference genome-CDC Frontier/PI)	Forward primers (5'-3')	Reverse primers (5'-3')	Anneling temperature (0C)	Expected amplified product size (bp)	Structural annotation		Functional annotation	
								Sequence components of *kabuli* genome	*Kabuli* gene accession IDs	NCBI-KOG	TFs
Ca4_P1_16088431	Ca4	16088431	GAT/GATCAT	AGCGTTGATTAACTGCGTGA	CCCTCACTCACGATCTTTCC	59.5	906	INTERGENIC	--	--	--
Ca4_P1_16095440	Ca4	16095440	TA/TAA	AGCCCAAGAAAGAAAACGCT	AAGGCAAAAGTCGTCCAATC	60.4	726	INTERGENIC	--	--	--
Ca4_P1_16095909	Ca4	16095909	TTAAT/TT	TACAGAATCGCTCCCTCCAC	TACATGGTTCGGTTGGGTTT	60.2	665	INTERGENIC	--	--	--
Ca4_P1_16095927	Ca4	16095927	GATAATATAATATAATATA/GATAATATAATATAATATAATATA	TACAGAATCGCTCCCTCCAC	TACATGGTTCGGTTGGGTTT	60.2	665	INTERGENIC	--	--	--
Ca4_P1_16096225	Ca4	16096225	TA/TAA	AAACCCAACCGAACCATGTA	TACGTGCAAACAAATTGGGA	60.1	383	INTERGENIC	--	--	--
Ca4_P1_16098449	Ca4	16098449	C/CT	CAATTCCAAAATATGACAACAACC	TCATACATGAGGGGAAAGCA	59.5	528	DRR	Ca_05500	--	--
Ca4_P1_16099826	Ca4	16099826	GAAC/G	GGAAAACAAAGTTCCCAACC	TTTTGTTTGATGGCTTGCAG	58.4	748	INTERGENIC	--	--	--
Ca4_P1_16103329	Ca4	16103329	AT/ATCT	CAATCCATTTATTTGTTTCCCA	CGGATGTGAGGCTTGGTATT	58.7	786	DRR	Ca_05499	--	--
Ca4_P1_16120236	Ca4	16120236	AATATATATATATATATATA/AATATATATATATATATA	TCGAAAGCATTCTTCTCCAAA	TGGTTTGACCTATTTATGGTTGG	59.9	550	INTERGENIC	--	--	--
Ca4_P1_16120686	Ca4	16120686	AATATATATATATAT/AATATATATATAT	CTCCCTTTTTGGGAGTTTTT	GCATTTCAAATACGCGATCA	57.3	531	INTERGENIC	--	--	--
Ca4_P1_16121131	Ca4	16121131	GATATATATATATATATATATATA/GATATATATATATATATATATATATA	CGCATCCCAGTAATGTAGCC	TTGCAGTCTCTGAATGGCTG	60.5	369	INTERGENIC	--	--	--
Ca4_P1_16131048	Ca4	16131048	TATAAATAAATAAATAAATAAATAAATAAATAAATAAA/TATAAATAAATAAATAAATAAATAAATAAATAAATAAATAAATAAA	CGGTCCTTAGACGAGATGGT	TCGGTGACAAATATTCATCTCATT	59.2	705	INTERGENIC	--	--	--

INDEL marker IDs	Chromosomes/ unanchored scaffolds	Physical positions (bp)	InDels (*Kabuli* reference genome-CDC Frontier/PI)	Forward primers (5'-3')	Reverse primers (5'-3')	Anneling temperature (0C)	Expected amplified product size (bp)	Structural annotation		Functional annotation	
								Sequence components of *kabuli* genome	*Kabuli* gene accession IDs	NCBI-KOG	TFs
Ca4_P1_16138106	Ca4	16138106	TTTATTATTATTATTATTATTATTATTATTATTATTTT/TTTATTATTATTATTATTATTATTATTATTATTATTATT	CAATGACATTACTTGGGCCTT	GGGAGAAGAAAAGAATGGGAA	59.0	391	INTERGENIC	--	--	--
Ca4_P1_16146645	Ca4	16146645	CTATTATTATTATTATTATTAT/CTATTATTATTATTATTATTATTATTATTATTATTAT	AAAGAAGCAGAGGAAGCGTG	TGGCGGTGTGTTTGACTAAA	59.8	667	DRR	Ca_05497	I	bHLH
Ca4_P1_16168307	Ca4	16168307	ATTTTTTTTT/ATTTTTTTT	TCCGTAAGTAAAAACAACTATCCA	TGCAGCCCCATATTCTTACC	57.1	652	INTERGENIC	--	--	--
Ca4_P1_16186698	Ca4	16186698	T/TC	TTGTTTACAAGAGATGTTTTGTCATTT	AAACCACGACTCCAAGCAAT	59.5	372	DRR	Ca_05493	Z	--
Ca4_P1_16245511	Ca4	16245511	NNT/A,C,G,T	GTTGAAGATTGCGTGGGAAT	AAGCAGTACGTCATCGGTCC	59.9	277	INTERGENIC	--	--	--
Ca4_P1_16329274	Ca4	16329274	A/AAGCATAC	TTGGACGATAGTACCGTATTCAA	CGAAGCAAATAGCAAGACTGC	58.6	815	INTERGENIC	--	--	--
Ca4_P1_16337268	Ca4	16337268	ATTTTTTTTTTTT/ATTTTTTTTTTT	AACGTTTAGCATGGAGGTGG	TGACCGGTGATTCTTTTCCT	60.0	374	INTERGENIC	--	--	--
Ca4_P1_1637025	Ca4	1637025	TA/T	GCATTATGGCAGACAACGAA	GGGTGGTTAAACAATCCTTTG	59.7	391	INTERGENIC	--	--	--
Ca4_P1_16430711	Ca4	16430711	ATTTTTTTTTT/ATTTTTTTTTTTT	GGAAGGTCGTCGATTGAAAA	TTTTTCCGACTTGGTCATTT	60.1	389	DRR	Ca_05484	A	--
Ca4_P1_16468176	Ca4	16468176	TTTATTATTATTATTATTATTATTATT/TTTATTATTATTATTATTATTATTATTATT	ATTGGAATCCGATGCTGAAC	AAACACTGACAGGGACAGGG	59.9	416	INTRON	Ca_05478	A	MYB
Ca4_P1_16568360	Ca4	16568360	CTTTTTT/CTTTTT	GAGCATTGTAGACCCTTTCCA	TCACTTTCGTTTCTTTCCTATGC	59.2	165	INTERGENIC	--	--	--
Ca4_P1_16605966	Ca4	16605966	TTATA/TTA	GGAATCAAATTCGAAGCCAA	CCTCTGACTCCGACGCTAAC	60.0	364	INTERGENIC	--	--	--

INDEL marker IDs	Chromosomes/ unanchored scaffolds	Physical positions (bp)	InDels (*Kabuli* reference genome-CDC Frontier/PI)	Forward primers (5'-3')	Reverse primers (5'-3')	Anneling temperature (0C)	Expected amplified product size (bp)	Structural annotation		Functional annotation	
								Sequence components of *kabuli* genome	*Kabuli* gene accession IDs	NCBI-KOG	TFs
Ca4_P1_1665800	Ca4	1665800	CATATATATATATATATATA/CATATATATATATATATATATATA	CATTGAAAGCGCGTTGATAA	GGTGAATTGATTTATGCTGGAA	59.8	394	INTERGENIC	--	--	--
Ca4_P1_16670902	Ca4	16670902	T/TG	ATACAGCGGCGAGCTACAGT	AAACCTTGCCCATGATGGTA	60.1	936	INTRON	Ca_05457	--	--
Ca4_P1_16693431	Ca4	16693431	ATTTTTTTTTT/ATTTTTTTTTTT	TCGCGAGGATCACATAAATCT	TCGATTTGGATTCTTTTCGG	59.7	797	DRR	Ca_05455	E	--
Ca4_P1_16711599	Ca4	16711599	TTATATATATATATATATATATATATATAT/TTATATATATATATATATATATATATATATATATATATAT	GCCCTCAATACGGTACCTCAC	TATCGTTGCTTGCCAACTCA	60.8	726	INTERGENIC	--	--	--
Ca4_P1_1676869	Ca4	1676869	ATA/ATACGACATTTA	AACAATGCTATAACGCCGCT	TCACGTCGGACTAGTTTGGA	59.8	771	INTRON	Ca_07795	--	--
Ca4_P1_1677374	Ca4	1677374	TAAAAAAA/TAAAAA	TCCAAACTAGTCCGACGTGA	AGCTGCTGGAGGAGTTGGTA	59.3	450	INTRON	Ca_07795	--	--
Ca4_P1_1677611	Ca4	1677611	GA/GAAGCCACTA	TCCAAACTAGTCCGACGTGA	AGCTGCTGGAGGAGTTGGTA	59.3	450	CDS (FRAME SHIFT)	Ca_07795	--	--
Ca4_P1_16785536	Ca4	16785536	CATATATATATATATATATATATATATA/CATATATATATATATATATA	AATATGGGCCCACTAAACCC	TTTTGGCCTAACTGGCCTAC	59.9	611	INTERGENIC	--	--	--
Ca4_P1_16787893	Ca4	16787893	CTTTTTTTTT/CTTTTTTT	CCCTTTAAAATGAGAGAATTGAAG	ATCGTCACGTGCAAACAATC	57.7	628	INTERGENIC	--	--	--
Ca4_P1_16826129	Ca4	16826129	A/AAATT	TTTTATCCACCAAAAATTCAAAAA	TGATTTTTCAGGAGGCCACT	58.9	521	DRR	Ca_05439	--	--
Ca4_P1_16826292	Ca4	16826292	CAAAAAAA/CAAAAAA	GCGACCATCTGTTGCAGTTA	TGATTTTTCAGGAGGCCACT	59.9	459	DRR	Ca_05439	--	--
Ca4_P1_16827901	Ca4	16827901	AATATTGATTATA/AATATTGATTATATTGATTATA	ACGCCAACATTTGTGAGTGA	TCGGATTCACTTAGTTGCACA	60.2	853	INTERGENIC	--	--	--

INDEL marker IDs	Chromosomes/ unanchored scaffolds	Physical positions (bp)	InDels (*Kabuli* reference genome-CDC Frontier/PI)	Forward primers (5'-3')	Reverse primers (5'-3')	Anneling temperature (0C)	Expected amplified product size (bp)	Structural annotation		Functional annotation	
								Sequence components of *kabuli* genome	*Kabuli* gene accession IDs	NCBI-KOG	TFs
Ca4_P1_16846815	Ca4	16846815	CACCTTCTTTT/CACCTTCTTTTACCTTCTTTT	ACATGTGCCTTGGGACTAGC	GCGAAAAGTCGAAGATCCTG	60.1	571	CDS (FRAME SHIFT)	Ca_05437	--	--
Ca4_P1_16846897	Ca4	16846897	C/CTT	ACATGTGCCTTGGGACTAGC	GCGAAAAGTCGAAGATCCTG	60.1	571	INTRON	Ca_05437	--	--
Ca4_P1_16870773	Ca4	16870773	ATTTTTTTTTT/ATTTTTTTTT	CGTTTGAAGAAATCACGCAA	CCTGACCTACCTGCCATGTT	59.8	916	INTRON	Ca_05436	--	--
Ca4_P1_16925907	Ca4	16925907	TAAAAAAAAAAAA/TAAAAAAAAAAA	TGCAAAACGTCGCTGTCTAC	TGGTCACTGGTCACTCTTGG	60.1	566	DRR	Ca_05430	R	C2H2
Ca4_P1_16933042	Ca4	16933042	TAAAAAAAAAA/TAAAAAAAAA	CCAAATAAACTACTTTTGTGAGACGA	CACACTCTGCCTCCACTGAA	60.0	388	INTERGENIC	--	--	--
Ca4_P1_16943222	Ca4	16943222	TATGAATGAAT/TATGAATGAATGAAT	CTCAAGCTCAGTCCCAGTCC	TGCGTTTTGTTTGGTGTAGC	60.0	668	INTERGENIC	--	--	--
Ca4_P1_16960625	Ca4	16960625	GTT/GTTT	GCGAATTGCAGACTGAAACA	CTGGAAAATCCTCCTCCTCC	60.0	925	DRR	Ca_05428	--	--
Ca4_P1_16984107	Ca4	16984107	ATTTTTTTT/ATTTTTTT	TAACAAGGGTGCCATGTGTG	ATTGATTTGGCAATGGTGGT	60.4	822	INTRON	Ca_05426	U	--
Ca4_P1_16988445	Ca4	16988445	ATTT/ATT	GTGTAGGTCCGTGAGCCAAT	ATTTCTGGTTGCCACGAATC	60.0	665	INTRON	Ca_05426	U	--
Ca4_P1_16990316	Ca4	16990316	ATTTTTTTTTT/ATTTTTTTTTTT	TTGCCCAAGATTTTCCATTT	TGAGGGCTTTGGGATGTAAC	59.4	501	INTRON	Ca_05426	U	--
Ca4_P1_16999887	Ca4	16999887	AGAG/AGAGGAG	GACCAAACCATCAAACCACC	TTCAGGAAAATTGCTACGGG	60.1	408	INTRON	Ca_05425	Z	--
Ca4_P1_17011803	Ca4	17011803	CTT/CTTT	TTTGCCCAAAGAAAAGCACT	TAAAGACCGTGGGACACCTT	59.9	321	INTRON	Ca_05422	--	G2-like

INDEL marker IDs	Chromosomes/ unanchored scaffolds	Physical positions (bp)	InDels (*Kabuli* reference genome-CDC Frontier/PI)	Forward primers (5'-3')	Reverse primers (5'-3')	Anneling temperature (0C)	Expected amplified product size (bp)	Structural annotation		Functional annotation	
								Sequence components of *kabuli* genome	*Kabuli* gene accession IDs	NCBI-KOG	TFs
Ca4_P1_17012786	Ca4	17012786	TAAAAAAAA/TAAAAAAAAA	ATATAGAATGCACGGGCAGC	CATTTCCACAACATACCCCC	60.1	554	INTRON	Ca_05422	--	G2-like
Ca4_P1_17017274	Ca4	17017274	T/TC	TCCTTCTCCAACAACGTCAA	ATCGGCACAAGTCAACTTCC	59.3	720	INTERGENIC	--	--	--
Ca4_P1_17017613	Ca4	17017613	ATTTTT/ATTTTTT	GGAAGTTGACTTGTGCCGAT	TTGTTCTGAAAGAAACGGGG	60.1	527	INTERGENIC	--	--	--
Ca4_P1_17017739	Ca4	17017739	AATAT/AATATAT	GGAAGTTGACTTGTGCCGAT	TTGTTCTGAAAGAAACGGGG	60.1	527	INTERGENIC	--	--	--
Ca4_P1_17027285	Ca4	17027285	CAAA/CAA	AAAGAACATCCCATGCAAGC	GCTGCATGTGGTAGCAGAAA	60.1	452	DRR	Ca_05421	C	--
Ca4_P1_17029753	Ca4	17029753	ATTTTTTTTT/ATTTTTTTT	ACCATTTCCTCCTGTGTTGC	CAAAAATTTGAGGGTTCAAACA	60.0	728	INTERGENIC	--	--	--
Ca4_P1_17030352	Ca4	17030352	TAA/TTGGAAATCAA	AAGATTTTGTGTTTGAACCCTCA	CTTCATCGTTGAATTTGGGG	59.9	596	DRR	Ca_05420	R	bHLH
Ca4_P1_17031185	Ca4	17031185	ATT/ATTT	TCTTTCCTTCGGAATTGGTG	CTAACAGTGACGGGGGAAGA	60.0	464	DRR	Ca_05420	R	bHLH
Ca4_P1_17039202	Ca4	17039202	AC/A	TTGGCGAGTCTTTCCTTGTT	TGTCTGTCCATCCCATTTCA	59.9	674	INTRON	Ca_05419	C	bHLH
Ca4_P1_17040189	Ca4	17040189	T/TC	TGGGGTCCATCCATTAGTGT	GCAGGAGGCACAGCTTTTAG	60.1	332	INTRON	Ca_05419	C	bHLH
Ca4_P1_17043933	Ca4	17043933	CAAAAAAAAAA/CAAAAAAAAAAAA	GTGGTGGCGTCAAGTAGGAT	GGCATTCGCGTAAATTGTTT	60.0	391	INTERGENIC	--	--	--
Ca4_P1_17047766	Ca4	17047766	ATTTTTTTTTTT/ATTTTTTTTTT	GAACCCAGTGCACAACTCCT	AAGCGACCAGTTGAAGGAAA	60.2	662	DRR	Ca_05418	DA	--

INDEL marker IDs	Chromosomes/ unanchored scaffolds	Physical positions (bp)	InDels (*Kabuli* reference genome-CDC Frontier/PI)	Forward primers (5'-3')	Reverse primers (5'-3')	Anneling temperature (0C)	Expected amplified product size (bp)	Structural annotation		Functional annotation	
								Sequence components of *kabuli* genome	*Kabuli* gene accession IDs	NCBI-KOG	TFs
Ca4_P1_17047788	Ca4	17047788	ATTTTTTTTT/ATTTTTTTTTT	GAACCCAGTGCACAAC TCCT	AAGCGACCAGTTGAAG GAAA	60.2	662	DRR	Ca_05418	DA	--
Ca4_P1_17049502	Ca4	17049502	CGT/CGTGT	TTTGTTATCCGTCAATG GAAGA	GCAGGAGAAGAACATT TCGC	59.5	601	DRR	Ca_05418	DA	--
Ca4_P1_17049556	Ca4	17049556	CAAAAAAAAA/CAAAAAAAA	CATTGGAGCACAATCC AAAA	GCAGGAGAAGAACATT TCGC	59.5	381	DRR	Ca_05418	DA	--
Ca4_P1_17051753	Ca4	17051753	A/AGT	TGAACGAAGGGGAAAA ACTG	CTTCCTCCTTGGCTTC TCCT	60.1	718	INTRON	Ca_05418	DA	--
Ca4_P1_17052864	Ca4	17052864	C/CAA	GGAGCCTCCATCCTCT TTTT	ATGCATGATGACAGAC CCAA	59.7	282	INTRON	Ca_05418	DA	--
Ca4_P1_17062902	Ca4	17062902	A/AG	CCTTTACCCCCTCCTA GGCT	TCTCAATAACAAATCAA TCCGTTT	60.8	706	INTERGENIC	--	--	--
Ca4_P1_17063264	Ca4	17063264	ATTTTT/ATTTT	AAACGGATTGATTTGT TATTGAGA	TCACCGTATAAAGCTT TTCTTTTG	59.0	595	INTERGENIC	--	--	--
Ca4_P1_17063499	Ca4	17063499	GTTTTTTTTTATAATTTTTTTTT/GTTTTT TTT	AAACGGATTGATTTGT TATTGAGA	TCACCGTATAAAGCTT TTCTTTTG	59.0	595	INTERGENIC	--	--	--
Ca4_P1_17063810	Ca4	17063810	TAAAAAAAA/TAAAAAAAAAA	CAAAAGAAAAGCTTTAT ACGGTGA	TTCGGTTTATGTAGCA GCCC	59.0	558	INTERGENIC	--	--	--
Ca4_P1_17066139	Ca4	17066139	TAAAAAAAA/TAAAAAAAAA	AATGCTAAACACACGA GGCA	CCTCACCTTCTTCCAT CACC	59.3	322	INTERGENIC	--	--	--
Ca4_P1_17066351	Ca4	17066351	GGAG/GGAGAG	AATGCTAAACACACGA GGCA	CGATTTGAAGTCGAAC ACACA	59.3	556	INTERGENIC	--	--	--
Ca4_P1_17067066	Ca4	17067066	TAAAAAAA/TAAAAAAAA	CGACTTCAAATCGAAC CAAA	CTCATTTTCGGATTTT CGGA	58.7	653	INTERGENIC	--	--	--

INDEL marker IDs	Chromosomes/ unanchored scaffolds	Physical positions (bp)	InDels (*Kabuli* reference genome-CDC Frontier/PI)	Forward primers (5'-3')	Reverse primers (5'-3')	Anneling temperature (0C)	Expected amplified product size (bp)	Structural annotation		Functional annotation	
								Sequence components of *kabuli* genome	*Kabuli* gene accession IDs	NCBI-KOG	TFs
Ca4_P1_17067149	Ca4	17067149	AATAT/AAT	TTCATCCTCTTTTTGTGGTTG	TCCTCATTTTCGGATTTTCG	57.7	190	INTERGENIC	--	--	--
Ca4_P1_17067171	Ca4	17067171	CATATAT/CATAT	TTCATCCTCTTTTTGTGGTTG	TCCTCATTTTCGGATTTTCG	57.7	190	INTERGENIC	--	--	--
Ca4_P1_17071722	Ca4	17071722	ATTTTTTTTT/ATTTTTTTTTT	GGGCTGCTACATAAACCGAA	TCCGACATGCCATTTTGTTA	60.1	189	INTERGENIC	--	--	--
Ca4_P1_17074454	Ca4	17074454	CTCTTCTTCTTCTTCTTCTTC/CTCTTCTTCTTCTTCTTC	TTGTTGTGGTGGGAGTGAAA	AAGGACGGTGTGGCTCATAG	60.0	332	CDS (large-effect mutations)	Ca_05416	--	bHLH
Ca4_P1_17081659	Ca4	17081659	TG/TGG	TACCGGAAGATTCGGAAATG	CTGCAGGTAAGCATCCCAAT	59.9	694	INTERGENIC	--	--	--
Ca4_P1_17081756	Ca4	17081756	TTATA/TTATATA	TACCGGAAGATTCGGAAATG	CTGCAGGTAAGCATCCCAAT	59.9	694	INTRON	Ca_05415	--	--
Ca4_P1_17081772	Ca4	17081772	TAATA/T	TACCGGAAGATTCGGAAATG	CTGCAGGTAAGCATCCCAAT	59.9	694	INTRON	Ca_05415	--	--
Ca4_P1_17082004	Ca4	17082004	TGAT/TGATCGAT	TTTTGACAATGGGCACTCAA	CTGCAGGTAAGCATCCCAAT	60.1	295	DRR	Ca_05415	--	--
Ca4_P1_17082915	Ca4	17082915	CTTTTT/CTTTTTT	TCAGCTCACTCTCGCTTTGA	TGATGGAAGAATAAGGGGTCC	60.0	503	DRR	Ca_05415	--	--
Ca4_P1_17090015	Ca4	17090015	ATATTTATTTATTTATTTATTT/ATATTTATTTATTTATTT	CCAACATTAAAACGGCCCTA	GCATTTTCACAATGTGGTGC	59.8	698	INTERGENIC	--	--	--
Ca4_P1_17094592	Ca4	17094592	TA/T	TGCTTCAAAGAAAAATCGGA	TGGGATTACCTGACCCCATA	58.5	190	INTERGENIC	--	--	--
Ca4_P1_17095065	Ca4	17095065	TAAAAAAAA/TAAAAAAA	GGAGATGATTGCCCAAAGAA	CTCAATGATGACCGTTGTGG	60.0	764	INTERGENIC	--	--	--

INDEL marker IDs	Chromosomes/ unanchored scaffolds	Physical positions (bp)	InDels (*Kabuli* reference genome-CDC Frontier/PI)	Forward primers (5'-3')	Reverse primers (5'-3')	Anneling temperature (0C)	Expected amplified product size (bp)	Structural annotation		Functional annotation	
								Sequence components of *kabuli* genome	*Kabuli* gene accession IDs	NCBI-KOG	TFs
Ca4_P1_17095718	Ca4	17095718	TAAAAAAA/TAAAAAA	CGGTCATCATTGAGGGCTAT	TCCATATGTGCCCATTGCTA	59.9	694	INTERGENIC	--	--	--
Ca4_P1_17096036	Ca4	17096036	CTTTCAA/CTTTCAAGTTTCAA	CTGCAATTGTCAAGCTCCAA	TCCATATGTGCCCATTGCTA	60.0	276	INTERGENIC	--	--	--
Ca4_P1_17096313	Ca4	17096313	AACACACACACACA/AACACACACACA	CTGCAATTGTCAAGCTCCAA	AGTGACACAACCCCTCCATC	60.0	750	INTERGENIC	--	--	--
Ca4_P1_17096800	Ca4	17096800	T/TA	GATGGAGGGGTTGTGTCACT	TCAAAGGCAAAAACTCGACA	59.8	566	INTERGENIC	--	--	--
Ca4_P1_17097131	Ca4	17097131	AATATATATATATATATATATAT/AATATATATATATATATATATAT	GATGGAGGGGTTGTGTCACT	AATGTCGGACAAAAAGCCAC	59.8	883	INTERGENIC	--	--	--
Ca4_P1_17101140	Ca4	17101140	TAACAAC/TAACAACAAC	TGGTTTGATTTATTGTGGTGC	CATTGGTTTTAACAAGTGTTGTTGT	58.4	472	DRR	Ca_05414	--	--
Ca4_P1_17101199	Ca4	17101199	A/AC	TGGTTTGATTTATTGTGGTGC	GTTCTTTGAAATTGCACGAATA	58.4	812	DRR	Ca_05414	--	--
Ca4_P1_17101268	Ca4	17101268	TAAAAAA/TAAAAAAA	TGGTTTGATTTATTGTGGTGC	TCGAGAGTTGGGTCATGGTC	58.4	881	DRR	Ca_05414	--	--
Ca4_P1_17101334	Ca4	17101334	TACAACAACA/TACAACA	TGGTTTGATTTATTGTGGTGC	GTCGAGAGTTGGGTCATGGT	58.4	882	DRR	Ca_05414	--	--
Ca4_P1_17101437	Ca4	17101437	TAAAAAAAAA/TAAAAAAAA	TTGAAAAACGTCAAAAGTACGTG	GTCGAGAGTTGGGTCATGGT	59.3	787	DRR	Ca_05414	--	--
Ca4_P1_17101583	Ca4	17101583	AG/A	TGATGTTAACTGACAAATGCTGTTT	GTCGAGAGTTGGGTCATGGT	60.0	322	DRR	Ca_05414	--	--
Ca4_P1_17101658	Ca4	17101658	ATTTT/ATTTTT	TGATGTTAACTGACAAATGCTGTTT	GTCGAGAGTTGGGTCATGGT	60.0	322	DRR	Ca_05414	--	--

INDEL marker IDs	Chromosomes/ unanchored scaffolds	Physical positions (bp)	InDels (*Kabuli* reference genome-CDC Frontier/PI)	Forward primers (5'-3')	Reverse primers (5'-3')	Anneling temperature (0C)	Expected amplified product size (bp)	Structural annotation		Functional annotation	
								Sequence components of *kabuli* genome	*Kabuli* gene accession IDs	NCBI-KOG	TFs
Ca4_P1_17115270	Ca4	17115270	ATTTTT/ATTTT	GGGACCGTTCGTTTCAAATA	AACATGATTCGAATTCAATTTCTC	59.8	221	DRR	Ca_05412	CR	Nin-like
Ca4_P1_17129875	Ca4	17129875	CAT/CATTAT	CCCATCCTCTAAAGCACAGC	GCTAGGGATGAAGGAAGCCT	59.8	530	INTRON	Ca_05411	CR	Nin-like
Ca4_P1_17131392	Ca4	17131392	TC/T	CATGCCTTCTAACACAGCGA	AAATTGCCGAGTCATCGAAC	60.0	300	INTRON	Ca_05411	CR	Nin-like
Ca4_P1_17131868	Ca4	17131868	GTTTTTTTTT/GTTTTTTTT	AATTTGTTCGATGACTCGGC	GCGCTATGGCAAAAGAAAAA	60.1	662	INTRON	Ca_05411	CR	Nin-like
Ca4_P1_17137650	Ca4	17137650	C/CCTCACCA	AGTGTGGTGGCACATCGTAA	TTGTGTTGAAAAGGTTGGCA	60.0	544	INTERGENIC	--	--	--
Ca4_P1_17137872	Ca4	17137872	ATTTTTT/ATTTTTTT	AGTGTGGTGGCACATCGTAA	TTGTGTTGAAAAGGTTGGCA	60.0	544	INTERGENIC	--	--	--
Ca4_P1_17141313	Ca4	17141313	T/TC	TTTCAATCGTATCAAGTTTTTGC	AAACTGAAAGGCCTAATTCCAA	58.4	682	DRR	Ca_05410	CR	Nin-like
Ca4_P1_17141850	Ca4	17141850	TAA/TAACAA	TTGGAATTAGGCCTTTCAGTTT	ATAATTTGGCAGGGTTCGTG	59.2	646	DRR	Ca_05410	CR	Nin-like
Ca4_P1_17143680	Ca4	17143680	TAAAA/TAAAAA	TTCGACCAAGCATGATTTTG	GGTTAGGATTCAAGGAGGCA	59.7	736	INTRON	Ca_05410	CR	Nin-like
Ca4_P1_17147517	Ca4	17147517	G/GACGCTCATCA	ATTTCCCAACAAGATTCCCC	GGCGTTCCAAGTGAATGTTT	60.0	627	DRR	Ca_05409	K	--
Ca4_P1_17157766	Ca4	17157766	TTAAT/TTAATAAT	AGAACCTTGCTATGCGTCGT	CATGGTGTGGACAAGATTGG	59.9	501	DRR	Ca_05407	--	--
Ca4_P1_17159337	Ca4	17159337	G/GC	GCCACGCTCTAGGTTGAGAT	CCTCAGGCCTGCAACTGTAT	59.5	222	DRR	Ca_05407	--	--

INDEL marker IDs	Chromosomes/ unanchored scaffolds	Physical positions (bp)	InDels (*Kabuli* reference genome-CDC Frontier/PI)	Forward primers (5'-3')	Reverse primers (5'-3')	Anneling temperature (0C)	Expected amplified product size (bp)	Structural annotation		Functional annotation	
								Sequence components of *kabuli* genome	*Kabuli* gene accession IDs	NCBI-KOG	TFs
Ca4_P1_17166977	Ca4	17166977	A/AC	CACACACAAGTCGAAGAGCAA	GGAAATGGTGGAGAGTGGAA	60.1	117	INTERGENIC	--	--	--
Ca4_P1_17167273	Ca4	17167273	T/TC	TTCCACTCTCCACCATTTCC	CACCAACAAGGATCGGATTT	59.9	429	INTERGENIC	--	--	--
Ca4_P1_17167756	Ca4	17167756	TAA/TA	AAATCCGATCCTTGTTGGTG	CACACACTCTCAAGGGTAGAGG	59.8	583	INTERGENIC	--	--	--
Ca4_P1_17170709	Ca4	17170709	GTTTTTT/GTTTTTTT	TGATTGGCACAAATGTCAGC	ACACGTTACTGCCCTCCATC	60.7	635	INTERGENIC	--	--	--
Ca4_P1_17171401	Ca4	17171401	AGTGTGT/AGTGTGTGT	GATGGAGGGCAGTAACGTGT	GTGCTCTCAAAGTCGCACAA	60.0	465	INTERGENIC	--	--	--
Ca4_P1_17171598	Ca4	17171598	TAA/TAAA	GATGGAGGGCAGTAACGTGT	GTGCTCTCAAAGTCGCACAA	60.0	465	INTERGENIC	--	--	--
Ca4_P1_17188428	Ca4	17188428	CTTTTTTTT/CTTTTTTTTTT	TATCGATGAGGGGTGTTGGT	TGAATCCGTCGAGGTATGTTC	60.2	543	INTERGENIC	--	--	--
Ca4_P1_17188486	Ca4	17188486	A/AT	TATCGATGAGGGGTGTTGGT	TGAATCCGTCGAGGTATGTTC	60.2	543	INTERGENIC	--	--	--
Ca4_P1_17193639	Ca4	17193639	ATTTT/ATTT	AAGCAGCAAGGATCACCAAT	CCCCATTAGTATTGCCGCTA	59.7	532	DRR	Ca_05405	T	--
Ca4_P1_17195362	Ca4	17195362	TAA/TAAA	TGTTGAACAAAATTCAAAATTCAAA	AAAAACTATTCATCGAAATATCACCC	59.8	194	INTERGENIC	--	--	--
Ca4_P1_17196085	Ca4	17196085	TTCTCTCTCTCTCTCTCTCTCTCTCTCT/TTCTCTCTCTCTCTCTCTCTCTCTCTCT	CCTCAACCATGGCCTGTACT	AGTTGCATTGGAGACAAGGG	60.0	118	INTERGENIC	--	--	--
Ca4_P1_17203357	Ca4	17203357	TAAGA/TAAGAAGA	TGTGCTCCATGTCTCTTTGC	CTCTTGGCTGGTAGCTTTGG	60.0	611	INTRON	Ca_05403	--	--

INDEL marker IDs	Chromosomes/ unanchored scaffolds	Physical positions (bp)	InDels (*Kabuli* reference genome-CDC Frontier/PI)	Forward primers (5'-3')	Reverse primers (5'-3')	Anneling temperature (0C)	Expected amplified product size (bp)	Structural annotation		Functional annotation	
								Sequence components of *kabuli* genome	*Kabuli* gene accession IDs	NCBI-KOG	TFs
Ca4_P1_17206399	Ca4	17206399	ATTTTTT/ATTTTT	AACCGTTTGATGCGAAAAAT	ATCTATTGCCATCTGCCAGG	59.5	925	INTERGENIC	--	--	--
Ca4_P1_17206661	Ca4	17206661	T/TA	AATCAATCAACCAAGAACGGA	ATCTATTGCCATCTGCCAGG	59.4	598	INTERGENIC	--	--	--
Ca4_P1_17206719	Ca4	17206719	ATTTTTTTTT/ATTTTTTTTTT	AATCAATCAACCAAGAACGGA	ATCTATTGCCATCTGCCAGG	59.4	598	INTERGENIC	--	--	--
Ca4_P1_17206854	Ca4	17206854	GAAAAAAAAAAAAAAA/GAAAAAAAAAA	CCTGGCAGATGGCAATAGAT	GAAGGGGAACGTGTACGAGA	60.1	529	INTERGENIC	--	--	--
Ca4_P1_17206969	Ca4	17206969	TAAAAAAAA/TAAAAAAA	CCTGGCAGATGGCAATAGAT	GAAAATGGATCCGACGAAGA	60.1	593	INTERGENIC	--	--	--
Ca4_P1_17207193	Ca4	17207193	TA/T	CCTGGCAGATGGCAATAGAT	GAAAATGGATCCGACGAAGA	60.1	593	INTERGENIC	--	--	--
Ca4_P1_17208586	Ca4	17208586	TACAC/TACACAC	TGTAAGATGGGTGTGGCTCA	GGTCCCTAATGCAGTTGACC	60.1	487	INTRON	Ca_05402	C	--
Ca4_P1_17209722	Ca4	17209722	CT/CTT	CCATTCAGAAAAGGGCAAGA	AAGCCTCGGGTAAAATTGGT	60.2	442	INTRON	Ca_05402	C	--
Ca4_P1_17210535	Ca4	17210535	CTT/CT	TTCAGGGAGTTCCATCAACC	TAGCCTTCTGAATTCCCCCT	59.9	448	INTRON	Ca_05402	C	--
Ca4_P1_17210802	Ca4	17210802	ATG/ATGGTG	AGGGGGAATTCAGAAGGCTA	CCTCTTCTCCACCCTGCATA	60.0	581	INTRON	Ca_05402	C	--
Ca4_P1_17214540	Ca4	17214540	A/ATATAG	GCTTTTTAGGCTGGGGAATC	TTGGTCAATGCGGAAGTGTA	60.0	627	DRR	Ca_05402	C	--
Ca4_P1_17219514	Ca4	17219514	CTTATTTATTTATTTAT/CTTATTTATTTATTTATTTATTTAT	GGAATGAGAGAGAGCGTTGG	TTATGCGATGCCAATTCAAA	60.0	563	INTRON	Ca_05401	O	--

INDEL marker IDs	Chromosomes/ unanchored scaffolds	Physical positions (bp)	InDels (*Kabuli* reference genome-CDC Frontier/PI)	Forward primers (5'-3')	Reverse primers (5'-3')	Anneling temperature (0C)	Expected amplified product size (bp)	Structural annotation		Functional annotation	
								Sequence components of *kabuli* genome	*Kabuli* gene accession IDs	NCBI-KOG	TFs
Ca4_P1_17226014	Ca4	17226014	GTTTTTTTTT/GTTTTTTTT	GCTTGGCAGCTTTAGAATGG	ACGCTCTGCTAACCCTGGTA	60.0	322	INTRON	Ca_05400	YU	--
Ca4_P1_17235137	Ca4	17235137	CAAAAAAAAAAAA/CAAAAAAAAA	CATTTCATGCAACCTTGGTG	TAGTCTTCATTGTGCGCAGG	60.0	369	INTRON	Ca_05400	YU	--
Ca4_P1_17238317	Ca4	17238317	CTTTATTT/CTTTATTTATTT	AAAAATGGGGTGGATGAACA	TTCCAATGTTGCAGAAGCAG	60.0	439	DRR	Ca_05400	YU	--
Ca4_P1_17242170	Ca4	17242170	GTCAT/GT	CTACATGAAGGGGCTCAACC	TCCAAGGACCGAGCTCATTA	59.6	515	INTERGENIC	--	--	--
Ca4_P1_17242500	Ca4	17242500	T/TAA	CTTGATGGCTTCAACTTGGG	AACTAATTTTTGGTTCCACTATTTTT	60.6	766	INTERGENIC	--	--	--
Ca4_P1_17243538	Ca4	17243538	AT/A	TTCACACATGCATTGTTTACCA	TGCAAAACTGCTCTACCCAA	59.9	422	INTERGENIC	--	--	--
Ca4_P1_17244811	Ca4	17244811	CGTC/CGTCCATCAATTAAGTC	AACAACAAAGCCAGGCCATA	CCCCGATTAAAAACATAACCA	60.5	138	INTERGENIC	--	--	--
Ca4_P1_17247653	Ca4	17247653	GAAAAAAAA/GAAAAAAAAAA	TTTATGTTTGGCCAACTCCC	TTTTTAGGGACGCAATTCAAA	59.8	539	INTERGENIC	--	--	--
Ca4_P1_17247848	Ca4	17247848	TAAAAAAAAA/TAAAAAAAAAA	TTTATGTTTGGCCAACTCCC	TTTTTAGGGACGCAATTCAAA	59.8	539	INTERGENIC	--	--	--
Ca4_P1_17247905	Ca4	17247905	GT/G	CAAGACATTGGGGACAAACA	TCTGGCCAAAAATTAGCTTCA	59.4	851	INTERGENIC	--	--	--
Ca4_P1_17248318	Ca4	17248318	G/GA	TTTGAATTGCGTCCCTAAAAA	TTTTGGCATGAATTTGGGAT	59.6	653	INTERGENIC	--	--	--
Ca4_P1_17250847	Ca4	17250847	ATTTT/ATTT	GCCGAAAAACCAAAGTGTGT	CGTTGGATTCCTTTCCTCTC	60.0	514	INTERGENIC	--	--	--

INDEL marker IDs	Chromosomes/ unanchored scaffolds	Physical positions (bp)	InDels (*Kabuli* reference genome-CDC Frontier/PI)	Forward primers (5'-3')	Reverse primers (5'-3')	Anneling temperature (0C)	Expected amplified product size (bp)	Structural annotation		Functional annotation	
								Sequence components of *kabuli* genome	*Kabuli* gene accession IDs	NCBI-KOG	TFs
Ca4_P1_17251218	Ca4	17251218	TTTT/TTTTACATCTCGATTT	GAGAGGAAAGGAATCCAACG	CAAATTGTCGAACCATGTCG	58.7	653	INTERGENIC	--	--	--
Ca4_P1_17251694	Ca4	17251694	T/TAATC	GCTCGATAAAGCAGTCGTCC	GCCAAATCGAACCAACTTTT	60.0	577	INTERGENIC	--	--	--
Ca4_P1_17252021	Ca4	17252021	ATTT/ATT	GCTCGATAAAGCAGTCGTCC	CAACGTAACACGCCTCTTGA	60.0	661	INTERGENIC	--	--	--
Ca4_P1_17253546	Ca4	17253546	CAAAAAAAAAA/CAAAAAAAAAAA	CCGTTTTCAACAGAAAACCAA	TTTTTCGACATAAAAAGTTTCCA	60.0	673	INTERGENIC	--	--	--
Ca4_P1_17253834	Ca4	17253834	AAATAA/AAA	TGCAAATATCATTTCATGTTCTTG	TGATGAAGTGAGTCGCTTGG	59.0	843	INTERGENIC	--	--	--
Ca4_P1_17253939	Ca4	17253939	T/TA	TCAACATCTAGTTAGAGGTGCAAAA	TGATGAAGTGAGTCGCTTGG	59.4	780	INTERGENIC	--	--	--
Ca4_P1_17254027	Ca4	17254027	TAATT/TAATTCAATT	TGGAAACTTTTTATGTCGAAAAAT	TGATGAAGTGAGTCGCTTGG	58.3	470	INTERGENIC	--	--	--
Ca4_P1_17254750	Ca4	17254750	GTAATAA/GTAA	CAAATATTGTGCCAGCCAAA	TCTTAATCTTGACCAGCCCG	59.6	898	INTERGENIC	--	--	--
Ca4_P1_17255615	Ca4	17255615	AT/A	CGGGCTGGTCAAGATTAAGA	TCGGCAGGAAATACCAAAAG	60.2	678	INTERGENIC	--	--	--
Ca4_P1_17257056	Ca4	17257056	ATTTT/ATTT	CCAACAAGCATTACATTGCG	ACGTTGACATTCCAGCATGA	60.1	423	INTERGENIC	--	--	--
Ca4_P1_17257974	Ca4	17257974	AATA/AATATA	ACATGGCAGGTTGGATTCAT	TCATGGGTACCCCACCTTAG	60.2	605	INTERGENIC	--	--	--
Ca4_P1_17259358	Ca4	17259358	TAAA/TAAAA	ATGGGTGCATTTGTTTCAGA	GCCTGAGTTTGGCATACGAT	59.0	338	INTERGENIC	--	--	--

INDEL marker IDs	Chromosomes/ unanchored scaffolds	Physical positions (bp)	InDels (*Kabuli* reference genome-CDC Frontier/PI)	Forward primers (5'-3')	Reverse primers (5'-3')	Anneling temperature (0C)	Expected amplified product size (bp)	Structural annotation		Functional annotation	
								Sequence components of *kabuli* genome	*Kabuli* gene accession IDs	NCBI-KOG	TFs
Ca4_P1_17260936	Ca4	17260936	CTT/CT	AGCAACCGAATAACGAAACG	GCAGGGACGCTTATGAAGAA	60.1	453	INTRON	Ca_05398	R	--
Ca4_P1_17264905	Ca4	17264905	TACA/TA	ACTGCCTTCAATGGGAGATG	AATACAGCCATCAAGCCACC	60.1	250	INTRON	Ca_05398	R	--
Ca4_P1_17266850	Ca4	17266850	GCATTGAGTG/GCATTGAGTGGTAAAACATTGAGTG	CATTGCATCCAAACAAGTCG	TGAACGGTGCATCTTCTGAC	60.1	519	DRR	Ca_05397	S	--
Ca4_P1_17267236	Ca4	17267236	GT/GTT	GCTTAATGTGCCTTGGGCTA	ATAGGAGGAGGAGCACGGAT	60.2	274	DRR	Ca_05397	S	--
Ca4_P1_17267471	Ca4	17267471	T/TC	ATCCGTGCTCCTCCTCCTAT	CCCGGAAAAATGAAGAACAA	60.1	507	DRR	Ca_05397	S	--
Ca4_P1_17271006	Ca4	17271006	CAAAAAAAAAAA/CAAAAAA	GCATCCTGACCCCACAAAT	CGTCTACCTTCTTCCAAGCG	60.7	734	INTRON	Ca_05397	S	--
Ca4_P1_17271174	Ca4	17271174	GATAATAATAATAATAAT/GATAATAATAATAAT	GAGAGGCGTCGATGAGAAAG	CGTCTACCTTCTTCCAAGCG	60.1	191	INTRON	Ca_05397	S	--
Ca4_P1_17284874	Ca4	17284874	GATA/GA	TTGAATATGAGCCTCCAAGAGA	CTTGTTGAGGCTTTGGGTGT	58.9	794	DRR	Ca_05393	R	--
Ca4_P1_17285160	Ca4	17285160	ACT/A	CATTTCCTTTGAGTCATGAGAGG	CTTGTTGAGGCTTTGGGTGT	60.1	310	DRR	Ca_05393	R	--
Ca4_P1_17286530	Ca4	17286530	TA/T	GGCCCCTGTTATCCCTAAAG	CTAGGGACCATCGCAAGTTC	59.8	551	INTRON	Ca_05393	R	--
Ca4_P1_17286686	Ca4	17286686	CATTGGATTGGATTG/CATTGGATTG	TGCATTTCATGCCTTTGCTA	TGATTAGCCCGTATTTTGGG	60.4	648	INTRON	Ca_05393	R	--
Ca4_P1_17287338	Ca4	17287338	AC/A	CCCAAAATACGGGCTAATCA	TCTTCACTTGGCTGGAAAGC	59.8	508	INTRON	Ca_05393	R	--

INDEL marker IDs	Chromosomes/ unanchored scaffolds	Physical positions (bp)	InDels (*Kabuli* reference genome-CDC Frontier/PI)	Forward primers (5'-3')	Reverse primers (5'-3')	Anneling temperature (0C)	Expected amplified product size (bp)	Structural annotation		Functional annotation	
								Sequence components of *kabuli* genome	*Kabuli* gene accession IDs	NCBI-KOG	TFs
Ca4_P1_17289085	Ca4	17289085	GAAA/GAAAAA	TGGGGTTAAACTTGAAGAATGC	GGAGCATGGAGTTTGTTGGT	60.3	629	INTERGENIC	--	--	--
Ca4_P1_17290871	Ca4	17290871	GTTTAATT/GTTTAATTTTAATT	CCAAGTATGGCAGGTGGAAT	TCATGAGCTAGAATGGTTGTAAAAA	59.8	615	INTERGENIC	--	--	--
Ca4_P1_17291290	Ca4	17291290	TAAAAAA/TAAAAAAA	AAAATTATCGCGTGACGCTT	TAGATCACCCACACCAACGA	59.7	590	INTERGENIC	--	--	--
Ca4_P1_17293581	Ca4	17293581	GTAT/GTATAT	TCACATCCGTTGAACGAAGA	TTTGTTTTATTAGTTTTGGTGTGG	60.2	652	DRR	Ca_05392	R	--
Ca4_P1_17297028	Ca4	17297028	TA/TAA	TTTGCCTCAGCTGTGTTGAC	TCATCAGCTGGACAACTTCG	60.0	607	INTRON	Ca_05392	R	--
Ca4_P1_17313521	Ca4	17313521	TAAA/TAA	GCTAGACAGGTTTGCCTTCG	AAGGCTCCTGCAAGCATTTA	60.0	966	INTERGENIC	--	--	--
Ca4_P1_17314228	Ca4	17314228	CAAAAAAA/CAAAAAA	TAAATGCTTGCAGGAGCCTT	CCTTTTGTTCTCAATCGCGT	60.0	366	INTERGENIC	--	--	--
Ca4_P1_17314621	Ca4	17314621	ATTTTTT/ATTTTT	ACGCGATTGAGAACAAAAGG	CTTCCCCACTCACAGTCCAT	60.2	604	DRR	Ca_05390	M	--
Ca4_P1_17314777	Ca4	17314777	TAAAAAA/TAAA	ACGCGATTGAGAACAAAAGG	CTTCCCCACTCACAGTCCAT	60.2	604	DRR	Ca_05390	M	--
Ca4_P1_17314848	Ca4	17314848	CAA/CAAACCGAACTAGAA	TTGTAAAAACTGCATCAATCGAA	CTTCCCCACTCACAGTCCAT	59.7	488	DRR	Ca_05390	M	--
Ca4_P1_17315192	Ca4	17315192	TAAA/TAAAA	ATGGACTGTGAGTGGGGAAG	TTGGTCCAACAGCTTGATGA	60.0	716	DRR	Ca_05390	M	--
Ca4_P1_17318086	Ca4	17318086	A/AG	CTCAAAAGTGGGATAGGGCA	ACAGCGTGAAACAAGATCCC	60.1	429	INTRON	Ca_05390	M	--

INDEL marker IDs	Chromosomes/ unanchored scaffolds	Physical positions (bp)	InDels (*Kabuli* reference genome-CDC Frontier/PI)	Forward primers (5'-3')	Reverse primers (5'-3')	Anneling temperature (0C)	Expected amplified product size (bp)	Structural annotation		Functional annotation	
								Sequence components of *kabuli* genome	*Kabuli* gene accession IDs	NCBI-KOG	TFs
Ca4_P1_17321001	Ca4	17321001	AATGGTATG/ATGGTATGGTATG	CATCGGCAAAGACTGAGTGA	CGATGGTGCAGATAGCAGAA	60.0	582	INTERGENIC	--	--	--
Ca4_P1_17324120	Ca4	17324120	TTTATTATTAT/TTTATTATTATTATTATTATTAT	TGAAACCCACAACCACAAGA	CCCGACTTCTTTCTCAGCAC	60.0	567	INTERGENIC	--	--	--
Ca4_P1_17324609	Ca4	17324609	TGTC/TGTCTCGTTTTCCGTC	GTGCTGAGAAAGAAGTCGGG	TGTCGACTTGTCCTTTCGTG	60.0	339	INTERGENIC	--	--	--
Ca4_P1_17325230	Ca4	17325230	C/CATAT	TCCTCTAACACCACGAAAGGA	TCACCAATAAATATAAAAACATGGGA	59.7	889	INTERGENIC	--	--	--
Ca4_P1_17325708	Ca4	17325708	CCAATT/C	TCCCATGTTTTTATATTTATTGGTGA	GAATCCGTTGTTGTGTGCAA	59.8	124	INTERGENIC	--	--	--
Ca4_P1_17327182	Ca4	17327182	CAAAAAAAAA/CAAAAAAAA	ATTGGTGCATTTGAAGTGGG	CGAGATCTTATCGTTGTGTTCG	60.8	352	INTERGENIC	--	--	--
Ca4_P1_17329245	Ca4	17329245	TCACA/TCA	GGCATGATAAAGAGGGAGGA	TAACCTCTCACGTGCAATCG	59.1	386	INTERGENIC	--	--	--
Ca4_P1_17332259	Ca4	17332259	GAAAAAAAAAAAA/GAAAAAAAAAA	CGGCAAAATTACATCTGTGG	GCGCATTGTCTAGCATTGAA	59.0	704	INTERGENIC	--	--	--
Ca4_P1_17339892	Ca4	17339892	ATTT/ATT	TTTTGGGGAACACAACCTTT	GACATTTCTCATAGGGGCGA	59.3	297	INTERGENIC	--	--	--
Ca4_P1_17343658	Ca4	17343658	GTT/GT	GCATGAGACCAAGCTAAGCC	GACCATGCTTGTGTTGTTGG	60.0	408	DRR	Ca_05389	A	--
Ca4_P1_17353052	Ca4	17353052	ATTTTTTTTTT/ATTTTTTTTT	TGCTTTAAGGCCAAATTCAAA	CGATCCATTATTTCAGTGGGA	59.7	625	INTERGENIC	--	--	--
Ca4_P1_17353713	Ca4	17353713	A/AGAATA	TCCCACTGAAATAATGGATCG	TTGGTGTCCACAGAAGAACAA	59.8	770	INTERGENIC	--	--	--

INDEL marker IDs	Chromosomes/ unanchored scaffolds	Physical positions (bp)	InDels (*Kabuli* reference genome-CDC Frontier/PI)	Forward primers (5'-3')	Reverse primers (5'-3')	Anneling temperature (0C)	Expected amplified product size (bp)	Structural annotation		Functional annotation	
								Sequence components of *kabuli* genome	*Kabuli* gene accession IDs	NCBI-KOG	TFs
Ca4_P1_17354782	Ca4	17354782	TAAAAAAAA/TAAAAAAA	TATTGATTCGGTCAATTATGACA	TGGATTAGGCCTTGAAGCAT	57.1	728	INTERGENIC	--	--	--
Ca4_P1_17360464	Ca4	17360464	TTATATATATATATATAT/TTATATATATATATATATATATATATAT	GTGTGCGATTGAGTTGAACG	CTTGCTGGGCTTTTCAAGAC	60.3	579	INTRON	Ca_05388	T	NAC
Ca4_P1_17360539	Ca4	17360539	TAA/TAAA	GTGTGCGATTGAGTTGAACG	CTTGCTGGGCTTTTCAAGAC	60.3	579	INTRON	Ca_05388	T	NAC
Ca4_P1_17364120	Ca4	17364120	CAA/CAAA	AAATTTTCAGATCCACCCCC	CTTTGTGCAGGTTCATCCCT	60.0	550	INTRON	Ca_05388	T	NAC
Ca4_P1_17365284	Ca4	17365284	TA/TAA	CCCCGGCTTACACATTCTTA	TATTGGTGTAACCGAACGCA	60.0	557	INTRON	Ca_05388	T	NAC
Ca4_P1_17366666	Ca4	17366666	ACTCATAATG/A	GTGAGCCCAAAGTTGATCGT	TGGCACTCTCCTTTAGCTGC	60.1	852	INTERGENIC	--	--	--
Ca4_P1_17366794	Ca4	17366794	CTTTATCTTT/CTT	AAGCACGATGTAATGCGATG	TGGCACTCTCCTTTAGCTGC	59.7	652	INTERGENIC	--	--	--
Ca4_P1_17367149	Ca4	17367149	AATAT/AAT	GCAGCTAAAGGAGAGTGCCA	AGACAGTTCGCTCGCAAATC	60.7	506	INTERGENIC	--	--	--
Ca4_P1_17367667	Ca4	17367667	AC/ACC	TAAATGTGATTTGCGAGCGA	ATGCATGGAGGTGATGATGA	60.4	284	INTERGENIC	--	--	--
Ca4_P1_17368867	Ca4	17368867	CTATA/CTA	GCTTTTCAGCCGATTTTGTC	GGGACATCATTCTCGCTCAC	59.8	380	INTERGENIC	--	--	--
Ca4_P1_17369103	Ca4	17369103	GATATAT/GATAT	GTGAGCGAGAATGATGTCCC	TGACTTAGCATCTTCCCCGA	60.6	243	INTERGENIC	--	--	--
Ca4_P1_17376716	Ca4	17376716	ATTTTTTTTT/ATTTTTTTTTTT	CCCCCATCTTCAAATGTGTT	AGATGTAGCAACGCAAACCA	59.6	698	DRR	Ca_05387	R	--

INDEL marker IDs	Chromosomes/ unanchored scaffolds	Physical positions (bp)	InDels (*Kabuli* reference genome-CDC Frontier/PI)	Forward primers (5'-3')	Reverse primers (5'-3')	Anneling temperature (0C)	Expected amplified product size (bp)	Structural annotation		Functional annotation	
								Sequence components of *kabuli* genome	*Kabuli* gene accession IDs	NCBI-KOG	TFs
Ca4_P1_17388357	Ca4	17388357	TAAAAAAAAAAAAA/TAAAAAAAAA	AAATCGCCATTTGGAACTTG	CACGATCATGTGTTCCACAA	59.9	308	INTERGENIC	--	--	--
Ca4_P1_17395103	Ca4	17395103	TAAAAAA/TAAAAAAA	GCGTGGGTAGCAACTTTCAT	TCGATGGTCATTTTTGTTGC	60.1	378	INTRON	Ca_05386	K	--
Ca4_P1_17396241	Ca4	17396241	GTTT/GTT	CCAAGCATGGTTTAGTGGCT	TTTCTCTGGCCACCATTTTC	60.1	371	INTRON	Ca_05386	K	--
Ca4_P1_17420097	Ca4	17420097	TA/TAA	TCGTTCAACCATTGCATCAT	TCCGAATGAGTGACCAAAATC	59.9	612	INTERGENIC	--	--	--
Ca4_P1_17420687	Ca4	17420687	GAAAAAA/GAAAAAAAAA	GATTTTGGTCACTCATTCGGA	AACTTCCAACAACTCACGGG	59.9	837	INTERGENIC	--	--	--
Ca4_P1_17421137	Ca4	17421137	GT/GTT	CCCGTGAGTTGTTGGAAGTT	ATGCCCTCATTGGAACTTGA	60.0	351	INTERGENIC	--	--	--
Ca4_P1_17421248	Ca4	17421248	G/GTTTAA	CCCGTGAGTTGTTGGAAGTT	TCGATAATTTGAAACCGCCT	60.0	569	INTERGENIC	--	--	--
Ca4_P1_17423254	Ca4	17423254	ATAAGATT/ATAAGATTAAGATT	GTGTGGGTTGGCTTTCATCT	GAAACGCTATGATGCCGTAAA	60.0	492	INTERGENIC	--	--	--
Ca4_P1_17423293	Ca4	17423293	TTATATA/TTA	GTGTGGGTTGGCTTTCATCT	GAAACGCTATGATGCCGTAAA	60.0	492	INTERGENIC	--	--	--
Ca4_P1_17423564	Ca4	17423564	TC/T	TATTTTGCATTGTTTGCGGA	TCCATCCCTCATCACTCACTC	60.1	719	INTERGENIC	--	--	--
Ca4_P1_17425855	Ca4	17425855	C/CT	AGAGCATTGGAGTCGTGACC	GAATTGAATTTCGATGTCGC	60.3	527	INTERGENIC	--	--	--
Ca4_P1_17430506	Ca4	17430506	GTTTTTTT/GTTTTTTTT	TGTTGCTTTTGCATCCTCTG	CAAGGTTAAGAGCGCCTTTG	60.0	485	INTRON	Ca_05384	K	--

INDEL marker IDs	Chromosomes/ unanchored scaffolds	Physical positions (bp)	InDels (*Kabuli* reference genome-CDC Frontier/PI)	Forward primers (5'-3')	Reverse primers (5'-3')	Anneling temperature (0C)	Expected amplified product size (bp)	Structural annotation		Functional annotation	
								Sequence components of *kabuli* genome	*Kabuli* gene accession IDs	NCBI-KOG	TFs
Ca4_P1_17431572	Ca4	17431572	GC/G	TCTGTCATTCCTGCCATTTTT	TCGCAATGTAGCTCCCTCTT	59.6	858	INTRON	Ca_05384	K	--
Ca4_P1_17431622	Ca4	17431622	TG/TGG	TGTCCCTCATTATAAGCACCC	TCGCAATGTAGCTCCCTCTT	58.9	606	INTRON	Ca_05384	K	--
Ca4_P1_17432501	Ca4	17432501	AC/A	ATTCTTCGGATGGTGCTTTG	CTCATTTCTGTTCCCCTCCA	60.1	531	INTRON	Ca_05384	K	--
Ca4_P1_17436350	Ca4	17436350	CTTTTT/CTTT	AAGATCCTGCCCCTCAAGAT	TGCTCCAACTGAGTGGTGAC	60.0	459	INTRON	Ca_05384	K	--
Ca4_P1_17439269	Ca4	17439269	T/TCTA	TCCTCACCAAGTCCGAAATC	GGGTGTGGGGATAGTTGATG	60.1	534	INTRON	Ca_05384	K	--
Ca4_P1_17443455	Ca4	17443455	TAA/TA	GTTGGTGTTGGCTTTTGCTT	ACATAGAGCTGTGCGTGGTG	60.2	246	DRR	Ca_05384	K	--
Ca4_P1_17448021	Ca4	17448021	TATTTTCATTTTC/TATTTTCATTTTCATTTTC	GAGTTGAGGAAACAATTATGTCAAA	TTCTTTTTCTGCGATTTCTCG	58.7	654	INTERGENIC	--	--	--
Ca4_P1_17448099	Ca4	17448099	TAAAAAAAA/TAAAAAA	TCGTTGATTATTGGTATTGTCTTTTC	CGACGGTAGCCCTACAACTC	59.7	460	INTERGENIC	--	--	--
Ca4_P1_17461972	Ca4	17461972	TAAAAAAAAAAA/TAAAAAAAAAA	TCCAATTTGAATTTGATGGG	TCATGTACATCAAACAAATCCTAAAAA	58.3	301	INTERGENIC	--	--	--
Ca4_P1_17462763	Ca4	17462763	CAAAA/CAAA	TGATGGCATAGTGACATAATAAACA	TTCAGACCGTATCAAATTCCAA	58.5	890	INTERGENIC	--	--	--
Ca4_P1_17470871	Ca4	17470871	GTGGCTA/GTGGCTAATGGCTA	GTCCCACCAAAGAAAGCAAA	CACTTGAAGAGGCTCCATCC	60.1	343	DRR	Ca_05380	AB	--
Ca4_P1_17470896	Ca4	17470896	TTATTACTATTA/TTATTA	GTCCCACCAAAGAAAGCAAA	CACTTGAAGAGGCTCCATCC	60.1	343	DRR	Ca_05380	AB	--

**INDEL marker IDs**	**Chromosomes/ unanchored scaffolds**	**Physical positions (bp)**	**InDels (*Kabuli* reference genome-CDC Frontier/PI)**	**Forward primers (5'-3')**	**Reverse primers (5'-3')**	**Anneling temperature (0C)**	**Expected amplified product size (bp)**	**Structural annotation**		**Functional annotation**	
								**Sequence components of *kabuli* genome**	***Kabuli* gene accession IDs**	**NCBI-KOG**	**TFs**
Ca4_P1_17471120	Ca4	17471120	C/CCTTAA	CACAGTGGCTGAGAGTGGAA	CGGATACACCCATTGGCTAC	60.0	502	DRR	Ca_05380	AB	--
Ca4_P1_17475440	Ca4	17475440	GT/GTTGTTATT	CGGGCAGTAGCATTAGATGG	GGATGAATGGGAGGTGGTTA	60.6	210	INTERGENIC	--	--	--
Ca4_P1_17484481	Ca4	17484481	ATTTTTTTTT/ATTTTTTTTTT	TCGCCTGATGCTTGTACTTG	AGGAGGCTTTTCCACAGGTT	60.0	361	INTRON	Ca_05379	O	--
Ca4_P1_17487582	Ca4	17487582	TAATA/TAATAAATA	TGGGAAATTGCAAGACAACA	TCTTTCCACGGATCCTGTTC	60.1	291	INTRON	Ca_05379	O	--
Ca4_P1_17491763	Ca4	17491763	TAAAA/TAAAAA	TTTTCCCGTAGATTCCATGC	GCTGGAGAAAGAGTTGGTGC	59.9	705	INTRON	Ca_05378	--	--
Ca4_P1_17494891	Ca4	17494891	GAAAAAAAAAAAA/GAAAAAAAAAA	AGATCTCCGAACGATGATGG	AAGCAAACCCCACTTCACAC	60.0	245	DRR	Ca_05377	R	B3
Ca4_P1_17495331	Ca4	17495331	TATACTCA/TA	GTGTGAAGTGGGGTTTGCTT	CGAAATTCATGCATTCAACG	60.0	476	DRR	Ca_05377	R	B3
Ca4_P1_17495949	Ca4	17495949	C/CTT	TCATTGCTGAAGAGCACGAT	TGCTCAACAACAGGAAGTGG	59.6	728	DRR	Ca_05377	R	B3
Ca4_P1_17518444	Ca4	17518444	TAAAAAAAAAA/TAAAAAAAA	CAATTTGGGGGTGTTGGTAG	TGGTGTTTGGATGTTACCTTTTC	60.1	440	INTERGENIC	--	--	--
Ca4_P1_17524638	Ca4	17524638	GTATTATTATTATTA/GTATTATTATTATTATTA	GGAGATGGAGATGGCTGTGT	GGGTATCAAGCCGAATCAAA	60.1	862	DRR	Ca_05374	R	NAC
Ca4_P1_17532742	Ca4	17532742	TAAAA/TAAAAA	CCCATCTTGTTTGGTTCACA	CTGAAAGGGCATAAAGGCAA	59.4	954	INTERGENIC	--	--	--
Ca4_P1_17533392	Ca4	17533392	CTT/CTTT	CTTTTTGCTGGTGCCATTTT	GGCATTGTGCTGAGTGATTTT	60.1	679	INTERGENIC	--	--	--

INDEL marker IDs	Chromosomes/ unanchored scaffolds	Physical positions (bp)	InDels (*Kabuli* reference genome-CDC Frontier/PI)	Forward primers (5'-3')	Reverse primers (5'-3')	Anneling temperature (0C)	Expected amplified product size (bp)	Structural annotation		Functional annotation	
								Sequence components of *kabuli* genome	*Kabuli* gene accession IDs	NCBI-KOG	TFs
Ca4_P1_17533529	Ca4	17533529	GT/G	CTTTTTGCTGGTGCCATTTT	TGCAAGAAGAAGGAAGCCAT	60.1	713	INTERGENIC	--	--	--
Ca4_P1_17534928	Ca4	17534928	TAAAAAAAAA/TAAAAAAAA	TTTTTGTGTGTGTGCGGTTT	TGTGTCAAAGATGGTATTTCAATG	60.1	410	INTERGENIC	--	--	--
Ca4_P1_17535175	Ca4	17535175	ACT/ACTCT	TTTTTGTGTGTGTGCGGTTT	GGCTGGAAGTGAGCTAGTGG	60.1	811	INTERGENIC	--	--	--
Ca4_P1_17535397	Ca4	17535397	TG/T	CATTGAAATACCATCTTTGACACA	GGCTGGAAGTGAGCTAGTGG	58.9	425	INTERGENIC	--	--	--
Ca4_P1_17545627	Ca4	17545627	TAAAAAAAA/TAAAAAAA	AGTTTAGGGGCAAGCACTGA	TGCATCGGAGATCACATCTT	59.9	363	INTERGENIC	--	--	--
Ca4_P1_17552174	Ca4	17552174	GTT/GTTT	AAGTGAGGCCACTGGAAAGA	ATGTGTTGGAACTTTTCCGC	59.8	204	INTERGENIC	--	--	--
Ca4_P1_17600797	Ca4	17600797	TC/T	AAGGGTGATGGACGAAAAAC	TTAAATGGTCGCATAGAGCAA	58.9	682	INTERGENIC	--	--	--
Ca4_P1_17600811	Ca4	17600811	TAAAAAA/TAAAAAAA	AAGGGTGATGGACGAAAAAC	TCCAACAACATCAAATTCATCC	58.9	951	INTERGENIC	--	--	--
Ca4_P1_17601510	Ca4	17601510	TC/T	GGATGAATTTGATGTTGTTGGA	CCGTGTCTATCCCCATTTTG	59.7	515	INTERGENIC	--	--	--
Ca4_P1_17601888	Ca4	17601888	TAAA/TAAAA	CAAAATGGGGATAGACACGG	TTTCAAATGAACGTTTGCTACA	60.2	440	INTERGENIC	--	--	--
Ca4_P1_17602358	Ca4	17602358	GT/GTT	TTTGAATGTGTATTGTAGCAAACG	CCTATTCTCGAGAGCAACCG	59.2	322	INTERGENIC	--	--	--
Ca4_P1_17606665	Ca4	17606665	ATTTTT/ATTTTTT	GCATTGTGAACAAATTATCGTAGC	GCGGAAGTAGCGATCTCAAG	59.9	767	DRR	Ca_05371	S	--

INDEL marker IDs	Chromosomes/ unanchored scaffolds	Physical positions (bp)	InDels (*Kabuli* reference genome-CDC Frontier/PI)	Forward primers (5'-3')	Reverse primers (5'-3')	Anneling temperature (0C)	Expected amplified product size (bp)	Structural annotation		Functional annotation	
								Sequence components of *kabuli* genome	*Kabuli* gene accession IDs	NCBI-KOG	TFs
Ca4_P1_17609228	Ca4	17609228	CTA/CTATA	TACACAAGAACGGGGTGACA	GCAGGGCTATTCCAAAAACA	60.0	890	DRR	Ca_05370	O	--
Ca4_P1_17610544	Ca4	17610544	TC/T	TGGTGTGATTTCATCATGGC	TGAACCGAACTGGTTTTTCA	60.3	379	DRR	Ca_05369	E	--
Ca4_P1_17613177	Ca4	17613177	CAAAAAAAAAAA/CAAAAAAAAAAAA	CATTGGCGCAACAATATGAC	TGGCATATCAGGTGAGTGGA	60.0	354	INTRON	Ca_05369	E	--
Ca4_P1_17617805	Ca4	17617805	GAAAAAAAA/GAAAAAAA	CATCTAGTGTTGGGGAATGTGA	ACGTTTCGCCCTACATCATT	59.9	838	INTERGENIC	--	--	--
Ca4_P1_17618031	Ca4	17618031	TAAAAAAAA/TAAAAAAAAA	GAAATGTAGGGCGAAACGTC	TCGAGTTCAGTTTTGGTGTGTC	59.6	828	INTERGENIC	--	--	--
Ca4_P1_17618568	Ca4	17618568	TAAAAAAAAAA/TAAAAAAAAAAA	TAATGATGTAGGGCGAAACG	GGTTGAAGTTGAAGATCGGC	58.7	693	INTERGENIC	--	--	--
Ca4_P1_17618987	Ca4	17618987	TATCATCAT/TATCAT	GCCGATCTTCAACTTCAACC	TCGAGCTAGAAGCACCAACA	59.7	470	INTERGENIC	--	--	--
Ca4_P1_17619173	Ca4	17619173	TAAAAAAAA/TAAAAAAA	GCCGATCTTCAACTTCAACC	TCGAGCTAGAAGCACCAACA	59.7	470	INTERGENIC	--	--	--
Ca4_P1_17622678	Ca4	17622678	ATTTTTT/ATTTTT	CCTGAAAGGCATGGCTGTAT	TGCTTCCTTCCTTGAAGCAT	60.1	289	INTRON	Ca_05368	F	--
Ca4_P1_17624755	Ca4	17624755	ATTTTTTTTTT/ATTTTTTTTTTT	TGAATTAGCTGCATGCTTGG	TTATTTGGCGGTTTATTGCC	60.0	312	DRR	Ca_05368	F	--
Ca4_P1_17626407	Ca4	17626407	AGGT/AGGTGGT	TGTCCCTTTCTCGTGCTCTT	GTCCAACTCCGGTTTAATGC	60.0	285	DRR	Ca_05368	F	--
Ca4_P1_17626510	Ca4	17626510	ATTT/AT	TGTCCCTTTCTCGTGCTCTT	CGGAAGAGTGTGAATCTCCA	60.0	809	DRR	Ca_05368	F	--

INDEL marker IDs	Chromosomes/ unanchored scaffolds	Physical positions (bp)	InDels (*Kabuli* reference genome-CDC Frontier/PI)	Forward primers (5'-3')	Reverse primers (5'-3')	Anneling temperature (0C)	Expected amplified product size (bp)	Structural annotation		Functional annotation	
								Sequence components of *kabuli* genome	*Kabuli* gene accession IDs	NCBI-KOG	TFs
Ca4_P1_17629803	Ca4	17629803	ATTTTTTTT/ATTTTTTTTTTT	CTTTTCCGCGAACACATTTT	GTACATACATGGACGCGACG	60.1	587	INTERGENIC	--	--	--
Ca4_P1_17631582	Ca4	17631582	GTT/GTTAGAATT	TGCACTTTTCATCGCTTTTG	ACATGGGATGGGATCTGTGT	60.0	347	INTRON	Ca_05367	Z	WRKY
Ca4_P1_17636026	Ca4	17636026	C/CCAAGATATA	GGCCTCATCTCTAGCCAATG	CCAAGGAAGTTTCACCAGGA	59.8	297	INTRON	Ca_05367	Z	WRKY
Ca4_P1_17637226	Ca4	17637226	CTATT/C	ACACAAGTTAGGTGGGGCAG	CTGCATCTGTAGTGAGGCCA	60.0	346	INTRON	Ca_05367	Z	WRKY
Ca4_P1_17637458	Ca4	17637458	GAC/G	ACACAAGTTAGGTGGGGCAG	GCATGGCTTGATGTGAGAGA	60.0	475	INTRON	Ca_05367	Z	WRKY
Ca4_P1_17637631	Ca4	17637631	CTCTTAGGT/CT	CCAAGCCTTCCATTCAACAT	CGCAAACTTTTGGTGAGACA	59.9	724	INTRON	Ca_05367	Z	WRKY
Ca4_P1_17638859	Ca4	17638859	TC/T	GTTGCTTCAGGGGTTACCAA	GACAACTGCAGCATCAATGG	60.0	301	INTRON	Ca_05367	Z	WRKY
Ca4_P1_17640054	Ca4	17640054	TTTATTATTATTATTATTATT/TTTATTATTATTATTATT	TATGAGTGCTGGTTTGAGCG	GAACCCTTTCCATCGAGTCA	60.0	779	DRR	Ca_05367	Z	WRKY
Ca4_P1_17640423	Ca4	17640423	CTTTTTTT/CTTTTTT	CAAGAAGAAGTGGTGGGGAA	GAACCCTTTCCATCGAGTCA	60.1	524	DRR	Ca_05367	Z	WRKY
Ca4_P1_17640878	Ca4	17640878	T/TC	GACTCGATGGAAAGGGTTCA	AGCCAACAAAACCACCAAAG	60.1	634	DRR	Ca_05367	Z	WRKY
Ca4_P1_1764308	Ca4	1764308	AT/ATGT	AGAGGCATTGCTCATCAACC	TCGAAAAGATGCAAGACCAA	60.2	754	INTERGENIC	--	--	--
Ca4_P1_17649020	Ca4	17649020	TTTTAAA/TTTTAAATTTAAA	CCAAAGAGCTGAAATGCACA	GGAAGCCGAAAAACTAAGGC	60.0	399	DRR	Ca_05365	H	--

INDEL marker IDs	Chromosomes/ unanchored scaffolds	Physical positions (bp)	InDels (*Kabuli* reference genome-CDC Frontier/PI)	Forward primers (5'-3')	Reverse primers (5'-3')	Anneling temperature (0C)	Expected amplified product size (bp)	Structural annotation		Functional annotation	
								Sequence components of *kabuli* genome	*Kabuli* gene accession IDs	NCBI-KOG	TFs
Ca4_P1_17652245	Ca4	17652245	ATTTTTTTTTTT/ATTTTTTTTTT	CAGGTATGGGGTTGTT TTGG	GAGAAATGCTGCGGA GAAAC	60.1	774	INTRON	Ca_05364	S	--
Ca4_P1_1765263	Ca4	1765263	CTTTTA/C	GGTCATGCGTGGGCTA CTAT	TTTTCTGAAATTTTGGA TGAATG	60.0	497	INTERGENIC	--	--	--
Ca4_P1_17654597	Ca4	17654597	TCACACAC/TCACACACAC	TCCAATTGACACCACT GCTC	TTTTCAACATTCTAAAA ATGGGC	59.7	542	DRR	Ca_05364	S	--
Ca4_P1_17654627	Ca4	17654627	AT/ATAAAACTTT	TCCAATTGACACCACT GCTC	TTTTCAACATTCTAAAA ATGGGC	59.7	542	DRR	Ca_05364	S	--
Ca4_P1_17656569	Ca4	17656569	GATA/GATATA	AATTTGGGGCGTTACA AACA	GTGCAAGATCCGCTAC CAGT	60.2	427	INTERGENIC	--	--	--
Ca4_P1_17656991	Ca4	17656991	ATTTTTTTTT/ATTTTTTTT	ACTGGTAGCGGATCTT GCAC	GAACCAACAAACTCTA ATGCCC	60.3	276	INTERGENIC	--	--	--
Ca4_P1_17658038	Ca4	17658038	A/ACTATCCACCACAATAG	TCTTGTTGCTCCCCTT CATC	GGCCCAACGATGAGT GATAA	60.2	475	INTERGENIC	--	--	--
Ca4_P1_17658409	Ca4	17658409	GT/GTT	TCTTGTTGCTCCCCTT CATC	ACACACGTGCATTCGG TAAA	60.2	702	INTERGENIC	--	--	--
Ca4_P1_17661910	Ca4	17661910	ATTTTTT/ATTTTTTT	AACATGTGAAATGGGA AGGC	TTAACGGTTCGTTTTC CAGG	59.8	982	DRR	Ca_05363	R	WRKY
Ca4_P1_17662474	Ca4	17662474	ATTATTGTTATT/ATTATT	TTATCCTGGAAAACGA ACCG	GCGATTAAGCGAAGAA CCTG	59.9	477	DRR	Ca_05363	R	WRKY
Ca4_P1_17663581	Ca4	17663581	CGTGGTGGTGGTGGTGGTGG/CGTG GTGGTGGTGGTGGTGGTGG	TGCCGTGTGGACATGA GTAT	CCAACTAGGCAAAAGC TTCG	60.0	338	CDS (large-effect mutations)	Ca_05362	O	--
Ca4_P1_17665452	Ca4	17665452	A/AC	AAGGTTCACAGCGTTT ACCG	GTGGAGAATGGCCAC TTGAT	60.2	782	DRR	Ca_05362	O	--

INDEL marker IDs	Chromosomes/ unanchored scaffolds	Physical positions (bp)	InDels (*Kabuli* reference genome-CDC Frontier/PI)	Forward primers (5'-3')	Reverse primers (5'-3')	Anneling temperature (0C)	Expected amplified product size (bp)	Structural annotation		Functional annotation	
								Sequence components of *kabuli* genome	*Kabuli* gene accession IDs	NCBI-KOG	TFs
Ca4_P1_17666197	Ca4	17666197	CAAAAAAAAAAA/CAAAAAAAAAAAA	CATTCTAAGCACCATTGCCA	TCTTACCATATTGCCCCCAC	59.7	912	DRR	Ca_05361	--	--
Ca4_P1_17666821	Ca4	17666821	A/ATT	TTCACATGCAAGCCATGTTT	GCATTCACACATAACCCCGT	60.1	215	DRR	Ca_05361	--	--
Ca4_P1_17666834	Ca4	17666834	C/CT	TTCACATGCAAGCCATGTTT	GCATTCACACATAACCCCGT	60.1	215	DRR	Ca_05361	--	--
Ca4_P1_17667851	Ca4	17667851	T/TC	GACATGTCAATCGAACGTGG	ACCTGCAGTGAGATTGGGAC	60.0	540	INTRON	Ca_05361	--	--
Ca4_P1_17670952	Ca4	17670952	T/TG	TGTTGACTCTTGGCTGTTGC	CGACATCATGGATTGTTTGG	60.0	659	INTERGENIC	--	--	--
Ca4_P1_17673781	Ca4	17673781	GAAAAAAA/GAAAAAA	TGCCATGTCACCAAGGTTTA	CCATTCAAGTTTCTTCCCCA	60.0	878	DRR	Ca_05360	--	--
Ca4_P1_17674751	Ca4	17674751	A/AC	TGCTCCCAATCTCAATTTCC	GCCTGATTCTTCAGGCAAGT	60.0	474	INTRON	Ca_05360	--	--
Ca4_P1_17678728	Ca4	17678728	ATTTTTTTTTTT/ATTTTTTTTTTTTTTT	CACCATGAAACCTTGCTCCT	GTTGCAGAGATGAATGGGGT	60.1	499	INTERGENIC	--	--	--
Ca4_P1_17690587	Ca4	17690587	TGGG/TGGGG	CGTTCTCAGAGGGAAATGGA	TCCCAAAGTGAAATGCATCA	60.2	611	INTRON	Ca_05356	TU	--
Ca4_P1_17697196	Ca4	17697196	TAAAAAAAAA/TAAAAAAAAAA	GGAAATTTGGAAAGTTGGCA	ATAAGGTTCCCCCTGCAATC	59.9	564	INTERGENIC	--	--	--
Ca4_P1_17705462	Ca4	17705462	GAAAAA/GAAAAAA	GTGCTTTCCCAAGCAATCAT	TCTGAGATGGGTTTGCCTTT	60.1	885	DRR	Ca_05355	R	SBP
Ca4_P1_17707066	Ca4	17707066	ATTTTTT/ATTTTTTT	TTGCATGCTTGGATTCAAAA	CCATCGAAAAGGAATGGCTA	60.2	413	DRR	Ca_05354	O	--

INDEL marker IDs	Chromosomes/ unanchored scaffolds	Physical positions (bp)	InDels (*Kabuli* reference genome-CDC Frontier/PI)	Forward primers (5'-3')	Reverse primers (5'-3')	Anneling temperature (0C)	Expected amplified product size (bp)	Structural annotation		Functional annotation	
								Sequence components of *kabuli* genome	*Kabuli* gene accession IDs	NCBI-KOG	TFs
Ca4_P1_1771183	Ca4	1771183	TATATATA/TATATATAACATATATA	GATCAATATCTCCATGCGGG	GTTGGGCACCGTCATAAAGT	60.3	423	INTERGENIC	--	--	--
Ca4_P1_17713411	Ca4	17713411	TAA/TA	TCCGTCGTTTATTCTTTGGC	TGAATCGTTTTGGAAAATTCG	60.1	673	INTERGENIC	--	--	--
Ca4_P1_17715218	Ca4	17715218	GAAAAAAAAAA/GAAAAAAAA	CTGTTCCTCACATCACGCAT	GGAGTCACCCTCCAAATTGA	59.7	143	INTERGENIC	--	--	--
Ca4_P1_17715291	Ca4	17715291	CTT/CT	TCAATTTGGAGGGTGACTCC	TACCAGCTTTGGTTTCTGCC	59.9	250	INTERGENIC	--	--	--
Ca4_P1_17715354	Ca4	17715354	AATAA/AATAACAATATTCCATAA	TCAATTTGGAGGGTGACTCC	TACCAGCTTTGGTTTCTGCC	59.9	250	INTERGENIC	--	--	--
Ca4_P1_17715629	Ca4	17715629	AT/ATATATT	TCAATTTGGAGGGTGACTCC	GCGCTGATGCAATACATACAC	59.9	532	INTERGENIC	--	--	--
Ca4_P1_17717044	Ca4	17717044	TGG/TG	TCTGTCAAAGTCGGTGCAAG	ATAATGGCGCCACAGAAAAG	60.0	821	INTERGENIC	--	--	--
Ca4_P1_17717288	Ca4	17717288	T/TG	GCAAACATGTTAACCAGCGA	ATAATGGCGCCACAGAAAAG	59.7	577	INTERGENIC	--	--	--
Ca4_P1_17720367	Ca4	17720367	CTGATCTTGATC/CTGATCTTGATCTTGATC	ACCCTCAGCATGGTCAAAAG	TAGTGGTGGCTCCCCTAATG	60.1	480	DRR	Ca_05352	KL	bHLH
Ca4_P1_17721817	Ca4	17721817	AGCCAACTTG/AG	CACAATTTGGCAGGGATTTT	TAATTTGCGGGTATGCTTCC	59.8	874	DRR	Ca_05352	KL	bHLH
Ca4_P1_17735002	Ca4	17735002	T/TA	TTTTTAAAGGGCCCGATTG	TGGACGGTCAAAAATAGAAGAAA	59.9	345	INTERGENIC	--	--	--
Ca4_P1_17735379	Ca4	17735379	ATATT/AT	TTTTTAAAGGGCCCGATTG	TGATTGTCACGTCATCAAAATTC	59.9	554	INTERGENIC	--	--	--

INDEL marker IDs	Chromosomes/ unanchored scaffolds	Physical positions (bp)	InDels (*Kabuli* reference genome-CDC Frontier/PI)	Forward primers (5'-3')	Reverse primers (5'-3')	Anneling temperature (0C)	Expected amplified product size (bp)	Structural annotation		Functional annotation	
								Sequence components of *kabuli* genome	*Kabuli* gene accession IDs	NCBI-KOG	TFs
Ca4_P1_17735574	Ca4	17735574	T/TA	TCACGATCGAAAAACCTATTTG	AAACATGACAAAAGTTGATGTTGAA	59.1	916	INTERGENIC	--	--	--
Ca4_P1_17735792	Ca4	17735792	TC/TCC	AAGAATTTTGATGACGTGACAATC	TGGGAGTGCATAATGTTTGA	59.4	787	INTERGENIC	--	--	--
Ca4_P1_17738735	Ca4	17738735	A/AT	GGAGTCATGGAATTTGGTCG	TTGCTGTATCAAGTGGCAAAA	60.3	810	INTERGENIC	--	--	--
Ca4_P1_17740594	Ca4	17740594	GTTTTTTTT/GTTTTTTT	ATATGGCAAGGAACAACGGA	TACCTTGGAATTGGCTGGTC	60.3	664	INTERGENIC	--	--	--
Ca4_P1_17742132	Ca4	17742132	CTTTTT/CTTTTTT	CAGCTTCACCGGAGAAAAGA	ATCACGGAATTGATTTTGGG	60.5	569	INTERGENIC	--	--	--
Ca4_P1_17742452	Ca4	17742452	A/ATAAT	CCCAAAATCAATTCCGTGAT	GTTGACGATCCATCTCAGCA	59.6	391	INTERGENIC	--	--	--
Ca4_P1_17743058	Ca4	17743058	ATGATTGAT/ATGATTGATTGAT	ATGTTACAGGGCTTTGGTGC	TGATGTGGTTGTGGTTGTGA	60.0	353	INTERGENIC	--	--	--
Ca4_P1_17748105	Ca4	17748105	GTTT/GTTTT	CCCATTTACGAAGAATCCGA	CGTACCTTTCGTTGCCATTT	59.9	287	INTERGENIC	--	--	--
Ca4_P1_17752864	Ca4	17752864	CTACTTACTT/CTACTT	ACGTTGGAGCGCACTAGATT	TCTATTGCATTGTCCGTCCA	59.9	723	INTERGENIC	--	--	--
Ca4_P1_1775612	Ca4	1775612	TT/TTTCAATTTTTGCT	GAGAAATGGAGAGGGAAGGG	TCGTCTGACACCACCAACAT	60.0	718	DRR	Ca_07800	K	HSF
Ca4_P1_17757375	Ca4	17757375	T/TC	TAAACCAACCCCACACCATT	ACAATGCCTTTTTGCCACTC	59.9	252	DRR	Ca_05349	O	--
Ca4_P1_17758892	Ca4	17758892	GTTTTTTTT/GTTTTTTT	TTTGATGCAAACAAGTTCCTG	CGATGAAGGAGTTCTCAGCC	58.8	499	DRR	Ca_05349	O	--

INDEL marker IDs	Chromosomes/ unanchored scaffolds	Physical positions (bp)	InDels (*Kabuli* reference genome-CDC Frontier/PI)	Forward primers (5'-3')	Reverse primers (5'-3')	Anneling temperature (0C)	Expected amplified product size (bp)	Structural annotation		Functional annotation	
								Sequence components of *kabuli* genome	*Kabuli* gene accession IDs	NCBI-KOG	TFs
Ca4_P1_17765512	Ca4	17765512	GAA/GA	TAAACCGTGGTAAAGGCTGG	TCCGATCCCGATGTACTCTC	60.0	602	INTRON	Ca_05349	O	--
Ca4_P1_17766305	Ca4	17766305	TAAAAAAA/TAAAAAAAA	ATGCGACCAATGTTTAAGCC	ACAAGTGGAGCACAATGCAA	60.0	366	DRR	Ca_05348	--	--
Ca4_P1_17766593	Ca4	17766593	TAAAA/TAAA	ATTGCATTGTGCTCCACTTG	AGGGGTTGGTATAGTAGAAGGAA	59.7	512	DRR	Ca_05348	--	--
Ca4_P1_17766645	Ca4	17766645	CTTTTTT/CTTTTTTT	ATTGCATTGTGCTCCACTTG	GGCTCTGTTTGATACAGCCA	59.7	744	DRR	Ca_05348	--	--
Ca4_P1_17766999	Ca4	17766999	TAA/TAAA	TTCCTTCTACTATACCAACCCCT	AAAAGCCACACAGGATGGAC	57.4	421	DRR	Ca_05348	--	--
Ca4_P1_17768735	Ca4	17768735	ATTT/ATT	ATGATATGCACGGCCTTCTC	TCATCTTTTGGAATGGGCTC	60.1	278	INTRON	Ca_05348	--	--
Ca4_P1_17769133	Ca4	17769133	TAAAAAA/TAAAAAAA	GAGCCCATTCCAAAAGATGA	ACATCCGTCCCATCAAAGAC	60.0	708	INTRON	Ca_05348	--	--
Ca4_P1_17769321	Ca4	17769321	GAACAAACAAACAAACAAACAA/GAACAAACAAACAAACAA	GAGCCCATTCCAAAAGATGA	TTCAGCCTCGACCAGTTTTT	60.0	814	INTRON	Ca_05348	--	--
Ca4_P1_17772365	Ca4	17772365	AACTT/AACTTACTT	TCTCATCCCGATTCCAAAAG	CGTAAGTTTCCCGTTCCAAA	60.0	387	INTRON	Ca_05347	R	--
Ca4_P1_17774752	Ca4	17774752	ATTTTTTTTT/ATTTTTTT	ACATTTGTTTTCACTCCGGC	TGCAGCCACGTGTTTATTTC	60.0	736	DRR	Ca_05347	R	--
Ca4_P1_17775093	Ca4	17775093	A/AC	AAGAGTTGCCAAAGTGGTGC	ACGCGGAGAAATTTGCTATT	60.3	519	DRR	Ca_05347	R	--
Ca4_P1_17775890	Ca4	17775890	TAAA/TA	TGCATTCAGCAAAAAGTACCA	GGCACTGCCGTCAAAAGTAT	59.4	525	DRR	Ca_05347	R	--

INDEL marker IDs	Chromosomes/ unanchored scaffolds	Physical positions (bp)	InDels (*Kabuli* reference genome-CDC Frontier/PI)	Forward primers (5'-3')	Reverse primers (5'-3')	Anneling temperature (0C)	Expected amplified product size (bp)	Structural annotation		Functional annotation	
								Sequence components of *kabuli* genome	*Kabuli* gene accession IDs	NCBI-KOG	TFs
Ca4_P1_17777571	Ca4	17777571	TTTATTATTATTATTATTATTATTATT/TTTATTATTATTATTATTATTATTATTATT	TTTGCATTCTTCCTTAGGGG	TGCAGATAAATGGCCAAACA	59.2	863	INTERGENIC	--	--	--
Ca4_P1_17777647	Ca4	17777647	CATCTGAATCTGAA/CATCTGAA	CCTTAGGGGAGATTTGCATTC	TGCAGATAAATGGCCAAACA	59.9	852	INTERGENIC	--	--	--
Ca4_P1_17778073	Ca4	17778073	GTTGATTTG/GTTGATTTGATTTG	AAGATTGGTTCTGGTGGTGG	ATTCACCAGGTTCCCAATCA	59.8	660	INTERGENIC	--	--	--
Ca4_P1_17778807	Ca4	17778807	TG/T	TGATTGGGAACCTGGTGAAT	CATGCCAAAATCAACAGCAC	60.2	671	INTERGENIC	--	--	--
Ca4_P1_17786377	Ca4	17786377	T/TG	CGCGCCAACTTTACTTCTTC	TTTGGTAACGGTCTTCTGCC	60.0	176	INTERGENIC	--	--	--
Ca4_P1_1780181	Ca4	1780181	GAAAAA/GAAAA	AATTCAAGAAGACTGGGGGAA	TTTTTGCCGGTTGAACTGAG	59.9	444	INTERGENIC	--	--	--
Ca4_P1_1780772	Ca4	1780772	TTAATTAT/TTAATTATAATTAT	CAGTTCAACCGGCAAAAAGT	TGTGGTGGAAAGGAGAAACC	60.1	801	INTERGENIC	--	--	--
Ca4_P1_17819452	Ca4	17819452	T/TTGAGAAGA	ACGAAGAAAACGGCAGAAGA	TGAGGGAGTGCACGATACAA	60.0	294	INTERGENIC	--	--	--
Ca4_P1_17821019	Ca4	17821019	AAGA/AA	GGAACTGTCGTATTGTTCCCA	AGATGTGGGCATGTGTTTCA	59.8	805	INTERGENIC	--	--	--
Ca4_P1_17822319	Ca4	17822319	TAA/TA	CCAAGTTGGAACGCTAAAGG	AAAAATACCAGCGTGGTTGG	59.7	464	INTERGENIC	--	--	--
Ca4_P1_1782351	Ca4	1782351	TAA/TA	TGGTTTGGATTTGGAGCTGT	TAGATCCGGAATGAAGACCG	60.5	361	INTRON	Ca_07801	T	--
Ca4_P1_17824069	Ca4	17824069	ATTTTTTTTTTT/ATTTTTTTT	TTGACCAATTTCACTTCGTG	TTGAGGGACCAAAACTGGAG	57.2	630	INTERGENIC	--	--	--

INDEL marker IDs	Chromosomes/ unanchored scaffolds	Physical positions (bp)	InDels (*Kabuli* reference genome-CDC Frontier/PI)	Forward primers (5'-3')	Reverse primers (5'-3')	Anneling temperature (0C)	Expected amplified product size (bp)	Structural annotation		Functional annotation	
								Sequence components of *kabuli* genome	*Kabuli* gene accession IDs	NCBI-KOG	TFs
Ca4_P1_17825598	Ca4	17825598	ACC/ACCC	AATCCATCAAACTCGGTTGC	GGGCCCCGTAAGTATTCAGT	59.9	679	INTERGENIC	--	--	--
Ca4_P1_17825940	Ca4	17825940	CGGGG/CGGG	AAAAGGCCAACCAAATAGCA	CCTCAATTCTCGATTTCCCA	59.6	555	INTERGENIC	--	--	--
Ca4_P1_17825981	Ca4	17825981	ATTTTTTTT/ATTTTTTTTT	GGGGCCCCATAATCATATTT	CCTCAATTCTCGATTTCCCA	59.7	164	INTERGENIC	--	--	--
Ca4_P1_17826443	Ca4	17826443	ATTTTT/ATTTTTT	TGGGAAATCGAGAATTGAGG	AAACATCCCACGACAAAAGC	60.0	552	INTERGENIC	--	--	--
Ca4_P1_17827610	Ca4	17827610	A/AT	TCAACTTCGGTGCTTGACAG	TTGGGGTACACGAACAATGA	60.0	141	INTERGENIC	--	--	--
Ca4_P1_17830486	Ca4	17830486	ATTTTTTTTT/ATTTTTTTTTT	TGGCTTGCATTCTCATTCAG	TTTGGATGCATAGGCCATTT	59.9	296	INTERGENIC	--	--	--
Ca4_P1_17831112	Ca4	17831112	TAA/TA	CACTCCAAGCCTCTTATGGG	GGAAAGATGGACACACGAAAA	59.7	605	INTERGENIC	--	--	--
Ca4_P1_17842849	Ca4	17842849	CTTTTTTTT/CTTTTTTT	GGCAAATCATAAAGCAAGCC	TTGTGCCTATGGGAATCCTT	59.7	858	INTERGENIC	--	--	--
Ca4_P1_17845214	Ca4	17845214	TAAAAAAAA/TAAAAAAAAA	TACCATTCACCCGTTCACCT	CTGGGGTCATTTTGGAGAAA	60.2	535	DRR	Ca_18621	J	--
Ca4_P1_17846018	Ca4	17846018	AAATA/AA	TTCACCAAATTCATGTCAAAGC	ACAACATGGGTCGGTCTCTC	60.0	737	DRR	Ca_18621	J	--
Ca4_P1_17847500	Ca4	17847500	CTCCTTTTCATC/CTCCTTTTCATCCTTTTCATC	TGAACGATGGTGGAAAGACA	GCAGAGCTCTTTGGCAGAGT	60.1	391	INTRON	Ca_18621	J	--
Ca4_P1_17849398	Ca4	17849398	AGGG/AGGGG	AGTGTAAGTGCCCAAGGTGG	CCGTTCGTTAAGTGCGAAAT	60.0	156	INTRON	Ca_18621	J	--

INDEL marker IDs	Chromosomes/ unanchored scaffolds	Physical positions (bp)	InDels (*Kabuli* reference genome-CDC Frontier/PI)	Forward primers (5'-3')	Reverse primers (5'-3')	Anneling temperature (0C)	Expected amplified product size (bp)	Structural annotation		Functional annotation	
								Sequence components of *kabuli* genome	*Kabuli* gene accession IDs	NCBI-KOG	TFs
Ca4_P1_1785807	Ca4	1785807	A/AC	AGCTTCCGCTCTGATCATGT	ACAATCGCGTACAACTTCCC	60.0	423	INTRON	Ca_07801	T	--
Ca4_P1_17862487	Ca4	17862487	CTTT/CTT	TTTTCTTCGGATGGAAATGC	ACAAAAATGGCAATGCACAA	60.0	571	INTRON	Ca_18623	--	WOX
Ca4_P1_17862773	Ca4	17862773	AGT/AGTGT	TTTTCTTCGGATGGAAATGC	ACAAAAATGGCAATGCACAA	60.0	571	INTRON	Ca_18623	--	WOX
Ca4_P1_1786291	Ca4	1786291	GTTAGTTAGTT/GTTAGTTAGTTTGTTATTAGTTAGTT	AAATCGTGTCAACCGCTACC	ATCGTCGTCTCTCACCATCC	60.0	870	INTRON	Ca_07801	T	--
Ca4_P1_17863620	Ca4	17863620	CTTATTTGGAATTATTTGGAATTATT/CTTATTTGGAATTATT	TTTGATTGTGGCTTTTGCAG	ACGAGCTAAAGGATGGGTTG	59.8	540	DRR	Ca_18623	--	WOX
Ca4_P1_17864334	Ca4	17864334	ATTTTTTT/ATTTTTTTT	GAGGCATGATTGGCAGATTT	TCGTCATTCCAAATAGCGAC	60.0	536	DRR	Ca_18623	--	WOX
Ca4_P1_17864537	Ca4	17864537	GTTTTTT/GTTTTTTT	GAGGCATGATTGGCAGATTT	CGGCGACTTTCCATATGATT	60.0	872	DRR	Ca_18623	--	WOX
Ca4_P1_17865546	Ca4	17865546	TAAAAAA/TAAAAAAA	GAGGTGTTTTATTGCCTCGG	TGTTAATCCCAATTCAATATCCC	59.6	133	INTERGENIC	--	--	--
Ca4_P1_17866250	Ca4	17866250	TAAAAAAAAAA/TAAAAAAAAAAAAA	GACTTTTGTTTTTATGGAGATTGC	AGGTAATCGTAATCGGCACG	58.3	448	INTERGENIC	--	--	--
Ca4_P1_17871181	Ca4	17871181	TAAAAAAAAAA/TAAAAAAAAA	GGGGTATTTGGACGAATGTG	CGAAAGCGATTGTTTACGGT	60.1	651	DRR	Ca_18624	TU	WOX
Ca4_P1_17872084	Ca4	17872084	TGA/TGAATGCAGA	TTGTGTTGAGCGAAAACGTC	AGCCTATGACCCACATTTCG	59.9	738	INTERGENIC	--	--	--
Ca4_P1_1789480	Ca4	1789480	ATT/ATTT	TCAAATCAGGATGAGGGAGG	CTGGTTGGCCTTGAGTGAAT	60.0	270	INTRON	Ca_07801	T	--

INDEL marker IDs	Chromosomes/ unanchored scaffolds	Physical positions (bp)	InDels (*Kabuli* reference genome-CDC Frontier/PI)	Forward primers (5'-3')	Reverse primers (5'-3')	Anneling temperature (0C)	Expected amplified product size (bp)	Structural annotation		Functional annotation	
								Sequence components of *kabuli* genome	*Kabuli* gene accession IDs	NCBI-KOG	TFs
Ca4_P1_1791824	Ca4	1791824	GA/G	CGATATTGTCAAATGGCGTG	TTTTGGATTCCTCCCTCCTT	60.0	644	INTRON	Ca_07801	T	--
Ca4_P1_1791916	Ca4	1791916	AGTTAGTTTGTTA/AGTTAGTTTGTTAGTTTGTTA	CGATATTGTCAAATGGCGTG	CAATGATCACACCAAGGTCG	60.0	741	INTRON	Ca_07801	T	--
Ca4_P1_1794143	Ca4	1794143	TAA/TAAA	TTGGTAGCAAAGGACCCAAC	CAGCTTCTTTCCACTGAGGG	60.0	385	INTRON	Ca_07801	T	--
Ca4_P1_17942733	Ca4	17942733	CTATATATATATATAT/CTATATATATATATATAT	CATCGTCAGCACATCACAAA	CTTAGGAGGTCTTGCCCCTC	59.2	447	INTERGENIC	--	--	--
Ca4_P1_1795184	Ca4	1795184	AATAT/AATATAT	GGTTTGAAAGATCCACCCAA	CAACCCATCATCCCAGTTTC	59.8	317	INTRON	Ca_07801	T	--
Ca4_P1_17954288	Ca4	17954288	CAAAAAAAAAAA/CAAAAAAAAAAAA	GGCCAAATGAATGGAAGAGA	TCGTAAGTCACGCACCGTTA	60.0	823	INTERGENIC	--	--	--
Ca4_P1_18033913	Ca4	18033913	CT/CTT	CTGAGTGACCTCCCTTCCAA	TCATTCCAGAAGGAGATGGG	60.2	433	INTRON	Ca_18645	--	--
Ca4_P1_18060757	Ca4	18060757	CTTTTTTTTTTT/CTTTTTTTTTTTT	GAAGCGACCACCTGAAGAAC	AACGGGTCAAAACAAATGATG	59.9	482	INTERGENIC	--	--	--
Ca4_P1_18101227	Ca4	18101227	GTTTTTTTTT/GTTTTTTTTTT	TTCTCACCGACAGTTACCCC	TGGTTTCAGGCTGGGAGTAG	60.0	874	INTERGENIC	--	--	--
Ca4_P1_18110946	Ca4	18110946	ATTTTTTTTTT/ATTTTTTTTT	TTCTTTGGAGACTGAGTTGGTTC	GACTCGATGAAAATGATGGTGA	59.8	377	INTERGENIC	--	--	--
Ca4_P1_18121565	Ca4	18121565	TAAGAAG/TAAG	GCTGCACCATGAGATCTTGA	ATGGTGCTGGAAGTTGCTTT	60.0	440	INTRON	Ca_18652	--	--
Ca4_P1_18178929	Ca4	18178929	GATATATATATATATATATATATATATATAT/GATATATATATATATATATATAT	TGTATGCAGGCTGTACCCAA	TTCATTATACGTGGCCGTGA	60.1	415	INTERGENIC	--	--	--

INDEL marker IDs	Chromosomes/ unanchored scaffolds	Physical positions (bp)	InDels (*Kabuli* reference genome-CDC Frontier/PI)	Forward primers (5'-3')	Reverse primers (5'-3')	Anneling temperature (0C)	Expected amplified product size (bp)	Structural annotation		Functional annotation	
								Sequence components of *kabuli* genome	*Kabuli* gene accession IDs	NCBI-KOG	TFs
Ca4_P1_1818459	Ca4	1818459	CACAGAGAGAGAGAGAGAGAGAGAGAGAGA/CAGAGAGAGAGAGA	CCCTAAACCCAGAAAGCAAA	GGTGTCCAACACACCAACAG	59.2	590	INTERGENIC	--	--	--
Ca4_P1_1818718	Ca4	1818718	AATATATATATA/AATATATATA	TTGCACTGTTTGGTGAAAGG	CAACCGATCCAACTTCAACA	59.7	218	INTERGENIC	--	--	--
Ca4_P1_18196212	Ca4	18196212	ATTT/ATTTAATCTTTTT	GTGGTCCAGATTCTCCCAAA	TGTCGCTTCAGCAAAAAGAA	59.9	549	INTERGENIC	--	--	--
Ca4_P1_1819824	Ca4	1819824	T/TA	TCTGGACCACTCCTACAGTCC	ATTGCATTTGGAACCCTGAC	59.2	513	INTERGENIC	--	--	--
Ca4_P1_1824490	Ca4	1824490	ATTTTTTTT/ATTTTTTTTT	TTGAATTTTCATTGGTCCAAAA	CGAGCCAAAACCGTACAAAT	59.3	788	INTERGENIC	--	--	--
Ca4_P1_18247265	Ca4	18247265	A/ATCACTTT	TGGCATGTTGCTTTTGTTGT	CACGCAGAGGATCATGCTTA	60.2	280	INTRON	Ca_18659	S	--
Ca4_P1_1825530	Ca4	1825530	TAAAAAAAAAAA/TAAAAAAAAA	TCACACCTTGCATCCTTCAA	TGTTGCAATGTTGGTTCTTCA	60.2	926	INTERGENIC	--	--	--
Ca4_P1_1828978	Ca4	1828978	AGGGGG/AGGGG	TTGAATGTGAATTGCAGCGT	CATCGTGAGCAAACCAAATG	60.3	914	INTRON	Ca_07803	T	--
Ca4_P1_18295337	Ca4	18295337	A/ATT	TTCCTATTGCTCACCCCTTG	GAAAAACAAGACCCGCGATA	60.1	539	DRR	Ca_18662	--	--
Ca4_P1_1831456	Ca4	1831456	TTA/TTATA	TCCAATAAGGTTGCCTAAGGA	GAGATTAAGCCGGAAGACCC	58.7	256	DRR	Ca_07804	--	--
Ca4_P1_1831550	Ca4	1831550	AGATG/AG	GGGTCTTCCGGCTTAATCTC	TCAACCTTTCCTTCCCCTTT	60.0	357	DRR	Ca_07804	--	--
Ca4_P1_1836494	Ca4	1836494	TTT/TTTATT	GTGACATGATGCGTTCAAGG	GTTTCAACCGTTTTGCCATT	60.1	572	INTERGENIC	--	--	--

INDEL marker IDs	Chromosomes/ unanchored scaffolds	Physical positions (bp)	InDels (*Kabuli* reference genome-CDC Frontier/PI)	Forward primers (5'-3')	Reverse primers (5'-3')	Anneling temperature (0C)	Expected amplified product size (bp)	Structural annotation		Functional annotation	
								Sequence components of *kabuli* genome	*Kabuli* gene accession IDs	NCBI-KOG	TFs
Ca4_P1_18388650	Ca4	18388650	ATATTA/ATA	AGTTATCGATCACGGATGCC	CGTGTATATTCGCGCACTCA	59.9	514	INTERGENIC	--	--	--
Ca4_P1_18407838	Ca4	18407838	AATATATATATATATATATATATATATA/AATATATATATATATATATATATATATA	TAATAGGAACCCACACCCCA	ATGCTCCGGTGAACAATCTC	60.0	360	INTERGENIC	--	--	--
Ca4_P1_1840930	Ca4	1840930	CTTTTTTT/CTTTTTT	AGCCAGCAGATGGAAGAGAA	CAAGCGACCTCCAACTTTGT	60.1	150	DRR	Ca_07805	--	--
Ca4_P1_1841092	Ca4	1841092	TTC/T	AGCCAGCAGATGGAAGAGAA	GCCAAAGCATTTGACATTGA	60.1	638	DRR	Ca_07805	--	--
Ca4_P1_1843195	Ca4	1843195	GAAAAAAAAA/GAAAAAAAA	AGGTAAGAGCGGAGGTTGGT	TTCTGAGATGCCCACACTTG	60.1	850	DRR	Ca_07806	R	WRKY
Ca4_P1_18446227	Ca4	18446227	GTTTTTT/GTTTTTTT	TCTCACGGTTGTGAGGACTG	GGTGGAGGTGAAGGTAAACAA	59.9	304	INTERGENIC	--	--	--
Ca4_P1_1847483	Ca4	1847483	CACTT/CACTTTACTT	TTCACAATAGCCGATGACGA	TCAAGAAGATCCGACTTGCC	60.2	796	INTRON	Ca_07806	R	WRKY
Ca4_P1_1848187	Ca4	1848187	TATAATAATAA/TATAATAATAATAA	AGAAGCAAAAGAGCCCTTCC	ATAGGAATATAGGCGCGGGT	60.0	323	INTERGENIC	--	--	--
Ca4_P1_1849410	Ca4	1849410	TAAAAAAA/TAAAAAA	CCTCTTTGTTCTCACGCGAT	ATCAACTGAAACGACCTGGC	60.4	879	INTERGENIC	--	--	--
Ca4_P1_1849451	Ca4	1849451	CTTTTTTTT/CTTTTTTTTT	GGATTTTCCATGAGAAAGGCTA	ATCAACTGAAACGACCTGGC	59.6	689	INTERGENIC	--	--	--
Ca4_P1_1849594	Ca4	1849594	CT/CTAAT	GGATTTTCCATGAGAAAGGCTA	ATCAACTGAAACGACCTGGC	59.6	689	INTERGENIC	--	--	--
Ca4_P1_18515805	Ca4	18515805	TATAAATAAATAAATAAATAAATAAAT/TATAAATAAATAAATAAATAAATAAATAAAT	TTTTTGAGATTGGTACGGGA	TCAGCGTTTGTTTGTTGACTT	58.1	738	INTERGENIC	--	--	--

INDEL marker IDs	Chromosomes/ unanchored scaffolds	Physical positions (bp)	InDels (*Kabuli* reference genome-CDC Frontier/PI)	Forward primers (5'-3')	Reverse primers (5'-3')	Anneling temperature (0C)	Expected amplified product size (bp)	Structural annotation		Functional annotation	
								Sequence components of *kabuli* genome	*Kabuli* gene accession IDs	NCBI-KOG	TFs
Ca4_P1_18518709	Ca4	18518709	ATTTTTTTTTTTTTTT/ATTTTTTTTTTTTT	ATGTGACACGTTAGCCACCA	TGCATTTCGGAACTTTCCAT	60.0	569	INTERGENIC	--	--	--
Ca4_P1_18530309	Ca4	18530309	TTAATAATAATAATAATAAT/TTAATAATAATAATAATAATAATAAT	TGCGTTGATAATCCAGAGGA	AGGTAACAAGGGCAATGACG	59.2	403	INTERGENIC	--	--	--
Ca4_P1_1855105	Ca4	1855105	TTTAT/TTTATTAT	CACGACCCTTTCGATTGTTT	AAACCAGCTGCCAATTCTGT	60.0	430	INTERGENIC	--	--	--
Ca4_P1_1856395	Ca4	1856395	CACATACTA/CA	CAAACTTTTGACTTCCTTTCTCAA	TCCATATTCGGTAGCCGTTC	59.0	435	INTERGENIC	--	--	--
Ca4_P1_18620373	Ca4	18620373	TAAAAAAAAA/TAAAAAAAAAA	GCATATGCATTGGGTAGAAAAA	ATCGGTCACTCCACGCTCTA	59.0	837	INTERGENIC	--	--	--
Ca4_P1_1863645	Ca4	1863645	TGA/T	CCCAATATTTGAATGGGCAC	CCCAACATGGAACCCATTAG	60.0	336	INTRON	Ca_07807	P	MIKC
Ca4_P1_18649901	Ca4	18649901	AATATATATATATATATATATATATATATATAT/AATATATATATATATATATATATATATATAT	CTCTTTGGTTCTACGGCTGC	TATGGCTTACGAGGGACTGG	60.0	911	INTERGENIC	--	--	--
Ca4_P1_18674772	Ca4	18674772	ATATATAT/ATATATATCTATATAT	CATGAGGGTTAGGTTTCGCT	ACTTGCATCCACGTACTCCC	59.2	649	INTERGENIC	--	--	--
Ca4_P1_186963	Ca4	186963	AATATATATATATATATATATATATATAT/AATATATATATATATATATATATATATATAT	CACGTCAAGTTTGCAATCAG	TTTTCAAGGTGGTAGAGCCG	57.9	156	INTERGENIC	--	--	--
Ca4_P1_18705110	Ca4	18705110	AAT/AATTAT	TTGTGCATTCGACAACATCC	ATGCACAATCAAAAACAGCG	60.5	553	INTERGENIC	--	--	--
Ca4_P1_18746243	Ca4	18746243	T/TG	CTGATGTATGGGGTCCTGCT	TCCTTTCTGCAATCCCATTC	60.0	305	INTRON	Ca_13979	M	MYB_related
Ca4_P1_18781017	Ca4	18781017	TAAAAAAAAAAA/TAAAAAAAAAA	GAGAAACAACGCAGGGAAAC	ATTATTGCCGCCAATTCAAA	59.7	350	INTERGENIC	--	--	--

INDEL marker IDs	Chromosomes/ unanchored scaffolds	Physical positions (bp)	InDels (*Kabuli* reference genome-CDC Frontier/PI)	Forward primers (5'-3')	Reverse primers (5'-3')	Anneling temperature (0C)	Expected amplified product size (bp)	Structural annotation		Functional annotation	
								Sequence components of *kabuli* genome	*Kabuli* gene accession IDs	NCBI-KOG	TFs
Ca4_P1_18821207	Ca4	18821207	CATTATTATTATTATTATTATTATTATT/CATTATTATTATTATTATTATTATTATTATT	AAAGTCGCATTCTGGGGATA	ATGTGCCAACTTCGACAATG	59.5	722	INTERGENIC	--	--	--
Ca4_P1_18853639	Ca4	18853639	CTT/CTTT	TTTGTGTCAAGGCTGCAAAG	CAACACCTCCTGTAGGGGAA	60.0	681	INTRON	Ca_13984	J	Nin-like
Ca4_P1_18860078	Ca4	18860078	TTATATATATATATATATATATATAT/TTATATATATATATATATATATATATATATATAT	GAGAAACTCCGCTCTTGTGG	GAACTCCCCGTCCAACACTA	60.0	700	INTERGENIC	--	--	--
Ca4_P1_18917609	Ca4	18917609	T/TCCC	ATGGGTTTAGAGCGCTGTTC	TGGCAAAGTTGGTATGACGA	59.3	502	DRR	Ca_13990	G	--
Ca4_P1_18918605	Ca4	18918605	GTATATATATATATATATATATATATATATAT/GTATATATATATATATATATATATATATATATAT	TGCTTAAAAATTCGGCAACA	TTCCAATTCTTCATCCCAGC	59.3	791	DRR	Ca_13990	G	--
Ca4_P1_18919761	Ca4	18919761	GTTATTATTATTATTATTATTATTATTATTATTATTATTATTATTATTATTATT/GTTATTATTATTATTATTATTATTATTATTATTATTATTATTATT	CTCCGACTCATTGAATTGACA	GCGTGCACCGATTTTTATTT	58.7	545	INTERGENIC	--	--	--
Ca4_P1_18943390	Ca4	18943390	TATAATAATAATAATAATA/TATAATAATAATAATAATA	CGACCATCACATACTGTGCC	TTGAAATATTGGGCTCAGGC	60.0	700	DRR	Ca_13992	--	--
Ca4_P1_18993059	Ca4	18993059	GTTTTTTT/GTTTTTT	CGTTAAGAATGCGTTGCAGA	TCGCAACTTTTGTTGGTTTTT	60.0	302	INTERGENIC	--	--	--
Ca4_P1_19045820	Ca4	19045820	A/AT	GGGCTTGCTTGGTTTGAATA	TCAACTGCACGTGTTAAATGC	60.1	825	INTERGENIC	--	--	--
Ca4_P1_19046060	Ca4	19046060	CAAAAAAA/CAAAAAAAA	TGAAAATGAGATTAGAACCACCAA	TCTTCACCTGCATCGAAATG	59.9	677	INTERGENIC	--	--	--
Ca4_P1_1907150	Ca4	1907150	TATAAAAAATAAAAA/TATAAAAAATAAAAAATAAAAA	AAAGAGTTTAAAGGCGGGGA	GCGGTCATATTCCGAATGAA	60.1	437	INTERGENIC	--	--	--
Ca4_P1_19081618	Ca4	19081618	CAATAATAATAATAATAATAATAA/CAATAATAATAATAATAATAATAATAA	CGACACTTCAACACAAAATTCA	TGTTGAAAACAACATGTTCCC	58.7	893	INTERGENIC	--	--	--

INDEL marker IDs	Chromosomes/ unanchored scaffolds	Physical positions (bp)	InDels (*Kabuli* reference genome-CDC Frontier/PI)	Forward primers (5'-3')	Reverse primers (5'-3')	Anneling temperature (0C)	Expected amplified product size (bp)	Structural annotation		Functional annotation	
								Sequence components of *kabuli* genome	*Kabuli* gene accession IDs	NCBI-KOG	TFs
Ca4_P1_19090156	Ca4	19090156	CTCATTCATTCATTCATTCATTCATTCA/CTCATTCATTCATTCATTCATTCA	GTTGCAGCAGATGCCTATGA	ATTGCAGGGAAGGTGTTTTG	60.0	561	INTRON	Ca_14010	R	C2H2
Ca4_P1_19119005	Ca4	19119005	CAAAAAAAAAAAA/CAAAAAAAAAAA	GCACGTGGGAAAATGAGACT	TAACGCCCCAAATTTTTGAT	60.1	442	INTERGENIC	--	--	--
Ca4_P1_1913477	Ca4	1913477	T/TATTATTATTATG	AAGCTTTTTGCCGTCAATTC	CAAGCGTCATTTCACATCTCA	59.3	708	INTERGENIC	--	--	--
Ca4_P1_19138283	Ca4	19138283	TAAAAA/TATATAAAAAAA	AACCCATGTTAATCGTTAAAGC	CCTAATTTGGGAGGTACGCA	57.3	673	INTERGENIC	--	--	--
Ca4_P1_19159916	Ca4	19159916	GTG/GTGTGAGAACTATGATTG	ACGTGTTTGAGAGTGCGTTG	CCTTTCCAGTATCAGCGAGC	59.9	297	CDS (large-effect mutations)	Ca_14014	L	--
Ca4_P1_19186411	Ca4	19186411	CATAAATTTATTAAATAAATTTATTA/CATAAATTTATTA	GCAATTCCACTTCCATTCGT	GTCAAATACGTTCGTTCGCC	59.9	645	DRR	Ca_14018	S	--
Ca4_P1_19198535	Ca4	19198535	CTTTTTTTTTTT/CTTTTTTTTTTTTT	AGAGGCGGGTGAGAAATTGT	CGTGCTATTCGGCAATCATA	61.0	707	INTRON	Ca_14019	O	--
Ca4_P1_19228202	Ca4	19228202	TATG/TATGATCGTGATG	GCAATCTGGAAAGATGGGAA	AAATTCGCCCTGTGTTTTTG	60.0	288	CDS (large-effect mutations)	Ca_14021	T	WRKY
Ca4_P1_1928030	Ca4	1928030	AATATATATATATATATATATATATATATA/AATATATATATATATATATATATATATATATA	CAATTCCTTCATCGATCCACT	ACAACCCATTCACCCTGGTA	59.0	475	INTERGENIC	--	--	--
Ca4_P1_19294536	Ca4	19294536	TTATATATATATATATATATATATATAT/TTATATATATATATATATATATATATATATATAT	GCATACTCTGTCCTTTCGTCC	GGCTGCCATCACTTCATCTC	58.8	586	INTERGENIC	--	--	--
Ca4_P1_19331435	Ca4	19331435	GCTTCTTCTTCTTCTTCTTCTTCTTCTTCTTCTTCTTCTTCTTCTTCTTC/GCTTCTTCTTCTTCTTCTTCTTCTTCTTCTTCTTCTTCTTC	CCAATCTCACACGAGAAGCA	ACGAGCGTAATCCAAACACC	60.0	680	CDS (large-effect mutations)	Ca_14030	R	MYB_related
Ca4_P1_19342121	Ca4	19342121	TAAAAAAAA/TAAAAAAAAA	TTCCGAAAATTCACATGCAC	GAATTTGGATTATTCGATTCTCTGA	59.5	388	INTERGENIC	--	--	--

INDEL marker IDs	Chromosomes/ unanchored scaffolds	Physical positions (bp)	InDels (*Kabuli* reference genome-CDC Frontier/PI)	Forward primers (5'-3')	Reverse primers (5'-3')	Anneling temperature (0C)	Expected amplified product size (bp)	Structural annotation		Functional annotation	
								Sequence components of *kabuli* genome	*Kabuli* gene accession IDs	NCBI-KOG	TFs
Ca4_P1_19369951	Ca4	19369951	CAAAAA/CAAAAAA	CCCATGTTTAAGGGTGGTTG	AATTTTTCACGACCTTCTTCAA	60.1	561	INTERGENIC	--	--	--
Ca4_P1_19397426	Ca4	19397426	GAAAA/GAAA	GCCAACATATGCTTGATGGA	TTGAACTTTCTCCGTTTGGG	59.5	667	INTERGENIC	--	--	--
Ca4_P1_19406743	Ca4	19406743	AAAACCAAATACACACTACAA/AAAACCAAATACACACTACAAACCAAATACACACTACAA	ACCATCCGTCACAAAAACAA	CAACTCCACCAAACCACCTT	58.9	576	INTERGENIC	--	--	--
Ca4_P1_1943645	Ca4	1943645	CATATATATATATATATATATATAT/CATATATATATATATATATATAT	GCACATGGTGTGAAACTGCT	TTGGGGAGATGGAAGAAAAA	59.8	662	INTERGENIC	--	--	--
Ca4_P1_19445900	Ca4	19445900	GA/GAA	ACAGATTCGTGGAGGTGGAG	ATGCCAAGCGTTTCACTTTT	60.1	429	INTERGENIC	--	--	--
Ca4_P1_19468672	Ca4	19468672	TAAAAAAAAAAA/TAAAAAAAAAA	CATGAAATTGAAATAAACGCA	TTGCGAAACCAAAGAAAAGG	57.8	469	INTERGENIC	--	--	--
Ca4_P1_19565032	Ca4	19565032	GTTTTT/GTTTT	GGTTCTTTTACTCCGTGGCA	GGCCAAAAGTCAATTCGCTA	60.1	454	INTERGENIC	--	--	--
Ca4_P1_19642653	Ca4	19642653	TAA/TA	TCAAACTTTTGGTGCCATTG	GGTAGACCTCCCACCTGGAT	59.6	389	INTERGENIC	--	--	--
Ca4_P1_19643688	Ca4	19643688	CTTTTT/CTTTTTT	GGTGCAGTTGTTTGATGCAC	ATGCGATTTACATGGTTGGC	60.2	255	INTERGENIC	--	--	--
Ca4_P1_19678061	Ca4	19678061	CAA/CGGCTGGTACAAAA	ATTTTGGCAACGGCTGATAC	CCACAATCCTCCGTTGTCTT	60.0	492	INTERGENIC	--	--	--
Ca4_P1_19680920	Ca4	19680920	AATATATATATATATATATAT/AATATATATATATATATATATAT	TTTGTTCACTTTTATGATGTGGTG	TTATATAGCCCCAGCCGTTG	59.0	610	DRR	Ca_14054	S	--
Ca4_P1_19708013	Ca4	19708013	TAAAAAAAAAAA/TAAAAAAAAAA	CAATTGCCAGAAGTTGTTGG	GATTGCTGGATTTGGAAGGA	59.2	842	DRR	Ca_14058	J	NF-YB

INDEL marker IDs	Chromosomes/ unanchored scaffolds	Physical positions (bp)	InDels (*Kabuli* reference genome-CDC Frontier/PI)	Forward primers (5'-3')	Reverse primers (5'-3')	Anneling temperature (0C)	Expected amplified product size (bp)	Structural annotation		Functional annotation	
								**Sequence components of *kabuli* genome**	***Kabuli* gene accession IDs**	**NCBI-KOG**	**TFs**
Ca4_P1_19780699	Ca4	19780699	CTTGTAATTTGTAATTTGT/CTTGTAATTTGTAATTTGTAATTTGT	AGCAAACCACACAACCACAA	TTTGTGACCACCACTTCCAA	60.0	484	INTERGENIC	--	--	--
Ca4_P1_19808086	Ca4	19808086	CAAAAAAAAAAAAAAAAAA/CAAAAAAAAAAAAA	CTAGGGGATTGCACTCCAAA	TTGGAATTGGTGGAACAACA	60.1	561	INTERGENIC	--	--	--
Ca4_P1_19825308	Ca4	19825308	T/TTATTATTATTAA	AATTTTGGGAAAAACCCCAG	TCACGACCAAAATTGTCAAAA	60.0	749	INTERGENIC	--	--	--
Ca4_P1_19883616	Ca4	19883616	ATTTTTTT/ATTTTTTTT	CATGTGCAATTTCTTGTGGG	TTTTTGTGTGCAGAGATGGG	60.0	572	INTERGENIC	--	--	--
Ca4_P1_20051846	Ca4	20051846	GTAT/GTATGTGATTAGTGACTAT	CACGAAGCGTATTGACTCCA	AAATTTGAAGGCGCTTTTTG	59.9	432	INTERGENIC	--	--	--
Ca4_P1_20097108	Ca4	20097108	GTTTT/GTTT	CAAGGTAAGGGCTTTTTCCC	AATCGCTAGCCACTTAGGCA	59.9	591	INTERGENIC	--	--	--
Ca4_P1_2013087	Ca4	2013087	CTATATATATATATATATATATATATATATATATATAT/CTATATATATATATATATATATATATATATATATATATATATATATAT	GCGCAGGACTAGAAATCTGC	TCATAGAAGCGGGACCAATC	60.1	441	DRR	Ca_07820	--	B3
Ca4_P1_20155743	Ca4	20155743	TTTATTATTATTATTATTATTATTATTATTATTATTATT/TTTATTATTATTATTATTATTATTATTATTATTATTATTATTATT	TCGGTTACGGTTGTAAATTCG	CATCAGCTTTGCGATCATGT	59.9	660	INTERGENIC	--	--	--
Ca4_P1_20212880	Ca4	20212880	AATCAT/AAT	TGAAAGAAATTATAAATGGATTGACAT	TCAGCCAAAAAGCTGATGTG	58.5	481	INTERGENIC	--	--	--
Ca4_P1_2022623	Ca4	2022623	AAATAATAATAATAATAATAATAATAATAATAATAA/AAATAATAATAATAATAATAATAATAATAATAA	AACGTGTTTGAGTGCGAAAA	TCGAGATGCGATTATGTGGA	59.4	819	INTERGENIC	--	--	--
Ca4_P1_2030157	Ca4	2030157	GAAAAAAA/GAAAAAAAA	TGAAAGTGCCAGAAAACCCT	CGAATGTTTATGGTTGGGGT	59.7	354	INTERGENIC	--	--	--
Ca4_P1_2032207	Ca4	2032207	TC/T	TGCTGAGTTCGAGGAAAGGT	CACATCTCTTGTTCGCTCCA	60.0	517	CDS (FRAME SHIFT)	Ca_07822	D	--

INDEL marker IDs	Chromosomes/ unanchored scaffolds	Physical positions (bp)	InDels (*Kabuli* reference genome-CDC Frontier/PI)	Forward primers (5'-3')	Reverse primers (5'-3')	Anneling temperature (0C)	Expected amplified product size (bp)	Structural annotation		Functional annotation	
								Sequence components of *kabuli* genome	*Kabuli* gene accession IDs	NCBI-KOG	TFs
Ca4_P1_2032547	Ca4	2032547	CTGTGTGTGT/CTGTGTGT	TGCTGAGTTCGAGGAAAGGT	CACATCTCTTGTTCGCTCCA	60.0	517	INTRON	Ca_07822	D	--
Ca4_P1_2034424	Ca4	2034424	CTTTT/CTTTTT	ACCAGATACCGCACCAAAAG	TTCTGCATACTTGCTCCACG	60.0	352	INTRON	Ca_07822	D	--
Ca4_P1_2034667	Ca4	2034667	GA/GATA	ACCAGATACCGCACCAAAAG	AGTTGGCAAGTTTGTCAGGC	60.0	570	INTRON	Ca_07822	D	--
Ca4_P1_2035282	Ca4	2035282	GTTTTTT/GTTTTTTT	CGCGCCAGAAGTATGTGATA	TGGGCAGCTTAGACTGGACT	59.9	275	INTRON	Ca_07822	D	--
Ca4_P1_2035697	Ca4	2035697	ATTT/AT	AGTCCAGTCTAAGCTGCCCA	TGGAATCATGCCATGCTAGA	60.0	436	DRR	Ca_07823	R	MYB
Ca4_P1_2035798	Ca4	2035798	A/AGTGT	AGTCCAGTCTAAGCTGCCCA	TGGAATCATGCCATGCTAGA	60.0	436	DRR	Ca_07823	R	MYB
Ca4_P1_20425177	Ca4	20425177	TTTATTATTATTATTATTATTATTATTATTATTATTATTATTATTATTATT/TTTATTATTATTATTATTATTATTATTATTATTATTATTATTATTATTATTATTATTATT	AATGAAAAACCGTGCAAAGC	ATTGGTGAATGGCAATTGGT	60.1	529	INTERGENIC	--	--	--
Ca4_P1_20510049	Ca4	20510049	GAAAA/GAAA	TCCTAGGGTCATTGTTTGACG	TGTCGCAGATGAGATCCAAG	60.0	567	INTERGENIC	--	--	--
Ca4_P1_2052943	Ca4	2052943	GATATATATATATATATATATATATAT/GATATATATATATATATATATAT	ACAAAATGGTCAGTGGTGGC	TCAAAATTGTGAGCATCAAACA	60.8	693	INTERGENIC	--	--	--
Ca4_P1_20650435	Ca4	20650435	TTATATATATATATATATATATATAT/TTATATATATATATATATATATAT	CGTGTGAGAAAACCTCGACA	CACCCTTACAAGTCGCCATT	59.9	733	INTERGENIC	--	--	--
Ca4_P1_20675480	Ca4	20675480	CATTATAA/CA	TTTCCGGCTAAAGTCCAATG	CGAATTGGATTAGGTGACCG	60.1	776	INTERGENIC	--	--	--
Ca4_P1_20709240	Ca4	20709240	ATTTTTTT/ATTTTTTTT	ACTTTGCCCATCAGTTTTGC	TCCGTTGGTGAAAGTTTGAA	60.1	251	INTERGENIC	--	--	--

INDEL marker IDs	Chromosomes/ unanchored scaffolds	Physical positions (bp)	InDels (*Kabuli* reference genome-CDC Frontier/PI)	Forward primers (5'-3')	Reverse primers (5'-3')	Anneling temperature (0C)	Expected amplified product size (bp)	Structural annotation		Functional annotation	
								Sequence components of *kabuli* genome	*Kabuli* gene accession IDs	NCBI-KOG	TFs
Ca4_P1_20714850	Ca4	20714850	AAAAATGATTTTT/AAAAATGATTTTTTAACAAAATGATTTTT	CATGTGGAAAGTAGGGGTGAA	ACAACGTCCGTCTGAGGTCT	59.8	930	INTERGENIC	--	--	--
Ca4_P1_20738577	Ca4	20738577	GGCTTATG/GG	CTCCCCTCCATGAGGTTACA	ACGCCACAAGGATTAACAGC	59.9	818	INTERGENIC	--	--	--
Ca4_P1_20773849	Ca4	20773849	CTTATTATTATTAT/CTTATTATTATTATTAT	TCTCGGCTGAAGGAACAGAT	AGCGGAACAACGACAAGATT	60.0	524	INTRON	Ca_17170	--	E2F/DP
Ca4_P1_20798644	Ca4	20798644	AAAAAAATA/AA	ACCTCGACTTAGGAGCCTCG	TTTGTCCTGGGAAAATGCTC	60.9	593	INTERGENIC	--	--	--
Ca4_P1_20798832	Ca4	20798832	TTTTATTTATTTATTTATT/TTTTATTTATTTATT	TAGTGCTTTTGCATGCCTCA	TTTGTCCTGGGAAAATGCTC	60.5	316	INTERGENIC	--	--	--
Ca4_P1_20845396	Ca4	20845396	CAAA/CAA	CCATAAGCATAAGGCGCAAT	TGCCACCCACTTTCAATGTA	60.1	590	INTERGENIC	--	--	--
Ca4_P1_20870966	Ca4	20870966	ATTTTTTTT/ATTTTTTT	ACTCATTGTGTTGGCTTTCG	GTGGATGAAGGGGGTAGGTT	58.8	776	DRR	Ca_19043	--	--
Ca4_P1_20884635	Ca4	20884635	T/TATATA	TCGGTTATAGAGATTAAACTCGGG	CAGAAAATAGAAACGGAAAATTCG	59.9	391	INTERGENIC	--	--	--
Ca4_P1_20954508	Ca4	20954508	CTATTATTATTATTATTATTATCATTATTATTATCATTAATATTATTATTATTATTATTATTATTATGATTATTATTATTATTATTATTATTATTATTATTATTATTATTATTATTATTATTATTATTATTATTATTATTATT[illegible]	TGAGAAAATGTTCCCTCTGATTC	TCGTTTATGTTAAATTACACCATGC	59.6	248	INTERGENIC	--	--	--
Ca4_P1_20962865	Ca4	20962865	TTATATATATATATATATATATATAT/TTATATATATATATATATATATAT	AAGAAAGGAGAATGATAAGATCTCAAA	GCCAACTGCTTCAGCTTTTC	59.3	827	INTERGENIC	--	--	--
Ca4_P1_21028273	Ca4	21028273	AATATATATATATATATATAT/AATATATATATATATAT	CCACCACTTGGTTCCAATCT	AGGCCATCTAGCTAAGCACAA	59.8	380	INTERGENIC	--	--	--
Ca4_P1_2103061	Ca4	2103061	A/ATAAT	TGTTTGCAAATGTTATGATGAATG	ACGGCAAAGATGGTTTTACG	59.8	834	INTERGENIC	--	--	--

INDEL marker IDs	Chromosomes/ unanchored scaffolds	Physical positions (bp)	InDels (*Kabuli* reference genome-CDC Frontier/PI)	Forward primers (5'-3')	Reverse primers (5'-3')	Anneling temperature (0C)	Expected amplified product size (bp)	Structural annotation		Functional annotation	
								Sequence components of *kabuli* genome	*Kabuli* gene accession IDs	NCBI-KOG	TFs
Ca4_P1_21047816	Ca4	21047816	TATAATAATAATAATAATAATAA/TATAATAATAATAATAATAATAA	CCTACCGGACCGTTTGTCTA	GCAGATCAATTTAACCGCATT	60.0	711	INTERGENIC	--	--	--
Ca4_P1_21057684	Ca4	21057684	AATATATATATATATA/AATATATATATATATATA	TTTAGAGGTCGCTTCACCGT	CCTTGGGGTAGACAAAGCAA	59.9	475	INTERGENIC	--	--	--
Ca4_P1_21060178	Ca4	21060178	GC/G	GGATGATAACTCCTTCAAAGCG	TGCTCATTTAATTTGACTGGGA	60.1	711	INTERGENIC	--	--	--
Ca4_P1_21071879	Ca4	21071879	T/TA	AATTGAGTGATCGAGTCGGC	ATGACGCGTGTCCAATTACA	60.2	837	INTERGENIC	--	--	--
Ca4_P1_21072127	Ca4	21072127	AG/A	TCTGTTTGGCAAGAAGAAGAAA	ATGACGCGTGTCCAATTACA	59.1	716	INTERGENIC	--	--	--
Ca4_P1_21076747	Ca4	21076747	CTGATGA/CTGA	GGCTTCAGTTAGGCAACAGC	GTCCAACAATGTTTTGTGCG	60.0	886	CDS (large-effect mutations)	Ca_19050	O	--
Ca4_P1_21098858	Ca4	21098858	GTTTTTTTTTT/GTTTTTTTTT	TGATCTTTTCCCCCACTTCA	AGCCATTTTCTCCCATTCCT	60.4	445	INTERGENIC	--	--	--
Ca4_P1_21122569	Ca4	21122569	CAAAAA/CAAAA	AGGTTTAAGTGCGAAGGTGC	CTATAAGCCCCTGACGGACA	59.4	701	INTERGENIC	--	--	--
Ca4_P1_21127401	Ca4	21127401	A/AAGTG	TGTTCACCGTTTGATCTTCG	GGTCGGTGAAAATGAGGCTA	59.7	510	DRR	Ca_19054	R	MYB
Ca4_P1_21175269	Ca4	21175269	C/CG	GACACCAGGAGGCATGTTTT	GGAAGGCTTGTCAGCTTGAG	60.0	386	DRR	Ca_19056	--	--
Ca4_P1_21181744	Ca4	21181744	A/ATGG	GAGGGAGATGATCGTGGAAA	GCTTCTTGGAAAAAGCGTTG	60.0	294	INTERGENIC	--	--	--
Ca4_P1_2121744	Ca4	2121744	TCCC/TCCCC	TGCCATAAGCAAATCACCAA	ACTGGAAAACCGTGCATAGC	60.1	708	DRR	Ca_07832	O	--

INDEL marker IDs	Chromosomes/ unanchored scaffolds	Physical positions (bp)	InDels (*Kabuli* reference genome-CDC Frontier/PI)	Forward primers (5'-3')	Reverse primers (5'-3')	Anneling temperature (0C)	Expected amplified product size (bp)	Structural annotation		Functional annotation	
								Sequence components of *kabuli* genome	*Kabuli* gene accession IDs	NCBI-KOG	TFs
Ca4_P1_21226048	Ca4	21226048	AAA/AAAAAGGAACCAA	CACGGCTCTACGAAGGAAAG	TGAACACCCCCAAGGTTAAG	60.0	287	INTERGENIC	--	--	--
Ca4_P1_21230858	Ca4	21230858	CATATATATATATATATA/CATATATATATATATA	GGTCTGCATTTTACACCGCT	AGTGCCCTCGTCAAACAAAT	60.1	920	DRR	Ca_19058	--	Trihelix
Ca4_P1_21248655	Ca4	21248655	TAAAAAAAAAAA/TAAAAAAAAAA	CAGGTTTAATTGTGGAGGTGC	TGCATTGCAGTGTGAAACG	59.5	102	INTERGENIC	--	--	--
Ca4_P1_21253893	Ca4	21253893	T/TA	CACAATTGCGAGATTTTCCA	CCCAACGACCAAATGAGTTT	59.7	598	INTERGENIC	--	--	--
Ca4_P1_21257917	Ca4	21257917	CAATAATAATAATAATAATAA/CAATAATAATAATAATAATAA	TATCGGCAAAAGATGAGGGA	CCGGAAATTTTGCCACTAAA	60.5	551	INTERGENIC	--	--	--
Ca4_P1_21261688	Ca4	21261688	AATATATATATATATATATATATATATATATATATA/AATATATATATATATATATATATATATATATATATATATATATATATA	GAAAAATTTGCTGAGCTGGTG	TGCGACAGAATCTTAATGTGC	59.9	485	INTERGENIC	--	--	--
Ca4_P1_21308758	Ca4	21308758	CAAAAAAA/CAAAAAA	TTGCTCCTCTGGTCCTCACT	CGTTCAACGTTCTCCATTTTT	60.0	854	INTERGENIC	--	--	--
Ca4_P1_21376315	Ca4	21376315	CATTATTATTATTATTATTATTATTATTATTATTATTATTATTATTATTA/CATTATTATTATTATTATTATTATTATTATTATTATTATTATTATTATTA	CCCCCTCTAAACCAACGTAA	CATTGGACTTGTGGCTTGTG	58.9	575	INTERGENIC	--	--	--
Ca4_P1_2139392	Ca4	2139392	TCCCCC/TCCCCCC	TATCTGGAACGACGACATGC	TCGTTCGCCATTAAAGAGGT	59.7	558	INTERGENIC	--	--	--
Ca4_P1_2140233	Ca4	2140233	AATATATATATATATATATATAT/AATATATATATATATATATATATAT	AACGTATTGGAAATTTGAATGG	TAGTACTCGTACCGGTGCCC	57.9	655	INTERGENIC	--	--	--
Ca4_P1_21404398	Ca4	21404398	ATTTTTTTTTT/ATTTTTTTTT	TCGCTCACATTCACGTTCTC	CAAGAAAATGACGCGTTTGA	60.0	860	INTERGENIC	--	--	--
Ca4_P1_2141158	Ca4	2141158	T/TATTACCGACACGG	CTATAGTACTCGCCCCGCAC	TGCGATTGAGCTGGTACTTG	59.8	956	INTERGENIC	--	--	--

INDEL marker IDs	Chromosomes/ unanchored scaffolds	Physical positions (bp)	InDels (*Kabuli* reference genome-CDC Frontier/PI)	Forward primers (5'-3')	Reverse primers (5'-3')	Anneling temperature (0C)	Expected amplified product size (bp)	Structural annotation		Functional annotation	
								Sequence components of *kabuli* genome	*Kabuli* gene accession IDs	NCBI-KOG	TFs
Ca4_P1_21426612	Ca4	21426612	ATTTTT/ATTTTTT	TGCAGATAAATGGCCAAACA	AGAACGGTTGCCAGAGAAGA	60.1	370	INTERGENIC	--	--	--
Ca4_P1_21514634	Ca4	21514634	ATGT/ATGTGT	TTGGAATCATGAGTTGATCTCG	AGAAGAACCACGACAAAGGC	60.1	656	INTERGENIC	--	--	--
Ca4_P1_21529168	Ca4	21529168	CAAAA/CAA	TGGGGGATTGTATTCCAAAA	TATGGAATGGACCTCCTGCT	60.0	499	INTERGENIC	--	--	--
Ca4_P1_21529241	Ca4	21529241	TATGAATGAA/TATGAA	TGGGGGATTGTATTCCAAAA	TATGGAATGGACCTCCTGCT	60.0	499	INTERGENIC	--	--	--
Ca4_P1_21529316	Ca4	21529316	T/TA	TGGGGGATTGTATTCCAAAA	TGAAATCGTCACCGTCTCAA	60.0	886	INTERGENIC	--	--	--
Ca4_P1_21581476	Ca4	21581476	TTTATTATTATTATTATTATTATTATTATTATTATTATT/TTTATTATTATTATTATTATTATTATTATTATTATTATTATTATTATT	CTGGGATGCCACCATCTACT	TTTGACTAAAATGCCGCACA	60.0	549	INTERGENIC	--	--	--
Ca4_P1_21622022	Ca4	21622022	T/TTGTG	GCCTTGAAATCCAAGCAGAA	TGATATATTGATTGGGTGTGCG	60.3	682	INTERGENIC	--	--	--
Ca4_P1_21622678	Ca4	21622678	ATGTG/ATGTGTG	CGTGTATATACGCACACCCAA	GTTTTGGCGTTGGTTTCACT	59.4	805	INTERGENIC	--	--	--
Ca4_P1_21651072	Ca4	21651072	TTATATATATATATATATATATATATATAT/TTATATATATATATATATATATATATATATATATATAT	TCCGTTCTCAAACGTGACAG	GTGCACACCGTCGATACAAT	59.9	584	INTERGENIC	--	--	--
Ca4_P1_21657242	Ca4	21657242	GTGAAAGAAAAT/GT	CGAGAGCTTTGAAGACTTTGC	CGGAGTTCCAATCTCACAGA	59.4	211	INTERGENIC	--	--	--
Ca4_P1_21657934	Ca4	21657934	TTATATATATATATATATATA/TTATATATATATATATATATATATATA	AAATGTGAAAGCCACAACCC	CCATGTAGCATGGCAACAAC	59.8	570	INTERGENIC	--	--	--
Ca4_P1_21703194	Ca4	21703194	AATATATATATATATATATATATAT/AATATATATATATATATATATATATAT	TAAAGGACCAAGTCGGGAAA	TGCACGTGTATACGAGACGG	59.5	373	INTERGENIC	--	--	--

INDEL marker IDs	Chromosomes/ unanchored scaffolds	Physical positions (bp)	InDels (*Kabuli* reference genome-CDC Frontier/PI)	Forward primers (5'-3')	Reverse primers (5'-3')	Anneling temperature (0C)	Expected amplified product size (bp)	Structural annotation		Functional annotation	
								Sequence components of *kabuli* genome	*Kabuli* gene accession IDs	NCBI-KOG	TFs
Ca4_P1_21703903	Ca4	21703903	GTTTTTTTTTTT/GTTTTTTTTTTTT	CGTATACACGTGCACACACAA	GGACACACAAGCGAAGATGA	59.1	943	INTERGENIC	--	--	--
Ca4_P1_21725823	Ca4	21725823	ATTTTTTT/ATTTTTT	TGACACTCATTGCTCCTCCA	CAACATTTGAATGAGGTGGC	60.4	531	INTERGENIC	--	--	--
Ca4_P1_21726235	Ca4	21726235	TAAAAAAA/TAAAAAAAA	CGACATTCATTGTTACCCCA	TGGAGGAAGAACCGTTATCAA	59.2	739	INTERGENIC	--	--	--
Ca4_P1_21731178	Ca4	21731178	TAAGAAGAAGAAGAAGAAGAAGAAGAAGAAGAAGAA/TAAGAAGAAGAAGAAGAAGAAGAAGAAGAA	TGCTGCACTGTAGAAAACCG	TGATGGACTTCGTGCTCTTG	60.1	273	INTERGENIC	--	--	--
Ca4_P1_21770743	Ca4	21770743	TTATATATATATATATATAT/TTATATATATATATATATATATATATATATATAT	TGCATTCTCCTTTGGCTTTT	TTGCATTTATTTCTTCTGGGG	59.8	536	INTERGENIC	--	--	--
Ca4_P1_21790580	Ca4	21790580	CAAAAAAAAA/CAAAAAAAAAAAA	GGGGAATTTAGAAAATCGTGC	CACCATCCTGCATGTGAATC	59.8	921	INTERGENIC	--	--	--
Ca4_P1_21804779	Ca4	21804779	CTGT/CTGTTATGT	CCGCATCATAAACAGCCTTT	CTAAAGCTGGTGCTTTTGGC	60.1	159	INTERGENIC	--	--	--
Ca4_P1_21907907	Ca4	21907907	AATATATAT/AATATAT	TGAGGATCCCTTTCAAATTCA	GAGCAATGGATGTTGGGTCT	59.5	781	INTERGENIC	--	--	--
Ca4_P1_21910161	Ca4	21910161	ATTTTTTTTTTT/ATTTTTTTTTT	TGCCCGTAGTTGTACCATGA	CGATGCAAACGTGGATTATG	60.0	383	INTERGENIC	--	--	--
Ca4_P1_21933188	Ca4	21933188	GAAAAAAAAAAA/GAAAAAAAAAA	GTTGGGCAATGGATCTGAAT	TGCTCACCGTTTGATCTTTG	59.8	862	DRR	Ca_20146	--	--
Ca4_P1_22042786	Ca4	22042786	ATTTTTTTTTTTTTTT/ATTTTTTTTTTTTTT	GCAGCTCTTCCGCTTAAAGA	GTTTCGGTTCGTGGTACGTT	59.9	508	INTERGENIC	--	--	--
Ca4_P1_22097668	Ca4	22097668	ATTTTTTTTTTTT/ATTTTTTTTTTT	TGAAGCACCTTTGATGAGGA	GACGTGATAACATGAAGGATGC	59.4	850	INTERGENIC	--	--	--

INDEL marker IDs	Chromosomes/ unanchored scaffolds	Physical positions (bp)	InDels (*Kabuli* reference genome-CDC Frontier/PI)	Forward primers (5'-3')	Reverse primers (5'-3')	Anneling temperature (0C)	Expected amplified product size (bp)	Structural annotation		Functional annotation	
								Sequence components of *kabuli* genome	*Kabuli* gene accession IDs	NCBI-KOG	TFs
Ca4_P1_22134995	Ca4	22134995	CATAT/CAT	GCACCTATGGTGGCAAAAAC	TTTGATGCCCACATTGAAGA	60.4	506	DRR	Ca_20134	J	--
Ca4_P1_22135412	Ca4	22135412	ATTT/ATTTTTT	TCTTCAATGTGGGCATCAAA	CCTCCTGTTCAGGTATCCACA	60.0	596	DRR	Ca_20134	J	--
Ca4_P1_22187580	Ca4	22187580	GCC/GCCTGTGTCC	GTCTCCCCTTGTGAGCTGAG	GGTGATGGTAGTACGGTGGC	60.0	179	INTERGENIC	--	--	--
Ca4_P1_22309344	Ca4	22309344	ATTTTTTTTT/ATTTTTTTTTT	TGCTTTGTTCAATTGCTTGC	AGTGCCAAGTAAGGTTGCTGA	60.0	566	INTERGENIC	--	--	--
Ca4_P1_22323554	Ca4	22323554	ATTTTTTTT/ATTTTTTT	ATGGAATTGGTGTCCGAGAG	GAGGTGATTTCGAACCAAAAA	59.9	363	INTERGENIC	--	--	--
Ca4_P1_22324512	Ca4	22324512	TAAAAAAAAAAA/TAAAAAAAAAAAA	CGCTGATTCATCCTTTTCGT	TTGAACTCCTTTCTGGGTGG	60.2	937	INTERGENIC	--	--	--
Ca4_P1_22381273	Ca4	22381273	AATATATATATATATATA/AATATATATATATATATATA	CAGTGGCGAAATCTGCATTA	TGGACGTAGGTTGTTGTGGA	59.8	702	INTERGENIC	--	--	--
Ca4_P1_22441886	Ca4	22441886	CTA/CTATA	TCCAATGGTGGAAAATAAACG	TCAATCACAAATTTCAAGGAGG	59.7	848	INTERGENIC	--	--	--
Ca4_P1_22476710	Ca4	22476710	CTTC/CTTCATTC	CCGGTGTCAAATGATAGGGT	AAGGAGACCAATGGGTTTGA	59.7	123	INTERGENIC	--	--	--
Ca4_P1_22499567	Ca4	22499567	A/ACACGACGCTAAAATACCC	ATGCAATGCGTATATGCCAA	TACATGGTGACCGCGATAGA	59.9	352	INTERGENIC	--	--	--
Ca4_P1_22508434	Ca4	22508434	CATATATATATATATATATATATAT/CATATATATATATATATATATATATATATATATATAT	TGGAAGGAAAACAATCGAGG	GCATTGCAATAATCCATCCA	60.0	647	INTERGENIC	--	--	--
Ca4_P1_22535036	Ca4	22535036	ATTTT/ATTTTT	TTTGAGTGTCGCATCCCATA	TCACGGCTTCATGGATGTTA	60.1	722	INTERGENIC	--	--	--

INDEL marker IDs	Chromosomes/ unanchored scaffolds	Physical positions (bp)	InDels (*Kabuli* reference genome-CDC Frontier/PI)	Forward primers (5'-3')	Reverse primers (5'-3')	Anneling temperature (0C)	Expected amplified product size (bp)	Structural annotation		Functional annotation	
								Sequence components of *kabuli* genome	*Kabuli* gene accession IDs	NCBI-KOG	TFs
Ca4_P1_22546535	Ca4	22546535	AATATATATATATA/AATATATATATATATA	CATTTTGGTTTCAAAGCCGT	CCAGGGGATGAGATCATAGG	60.0	714	INTERGENIC	--	--	--
Ca4_P1_22601747	Ca4	22601747	TAAAAAAAAA/TAAAAAAAAAAA	GTCGCGTCTTCCATTCATTT	GATTTTCCCTTGTCAACAATCA	60.1	229	INTERGENIC	--	--	--
Ca4_P1_22601909	Ca4	22601909	TG/T	TCGCGTCTTCCATTCATTTC	CGATTTCATGAATTGCAAATGT	61.1	626	INTERGENIC	--	--	--
Ca4_P1_22656603	Ca4	22656603	GAAGATTTCATTAAGATTT/GAAGATTT	TTCCTGGCTTCAAAGGAAAA	ACCTGACAACCCCAAATTGA	59.8	967	INTERGENIC	--	--	--
Ca4_P1_22656873	Ca4	22656873	TA/TAGAAAAGCAGA	GACACTCAAGGCTGAAGTAGAAAA	ACCTGACAACCCCAAATTGA	59.1	246	INTERGENIC	--	--	--
Ca4_P1_22663018	Ca4	22663018	ATTTTTTTT/ATTTTTTTTT	ACCCCCTCTTCTCGTGTTTT	AGGCTCAGGCAACTAGGGAT	60.0	536	INTERGENIC	--	--	--
Ca4_P1_22664879	Ca4	22664879	AA/AAGA	TCGCGTTCGATTTGTTCATA	GGTCATTCTTATTGATTGACCCA	60.2	570	INTERGENIC	--	--	--
Ca4_P1_22674188	Ca4	22674188	CAAAAAAAAAA/CAAAAAAAAA	AATGCATGTTGCACCACAGT	GCTGAAAAGGAAGGGAAAGG	60.0	292	INTERGENIC	--	--	--
Ca4_P1_22675724	Ca4	22675724	TTT/TTTCTT	CACAGAAAACTGAAACGCGA	ATGGCAATTAGGGTGTGCTC	60.0	325	INTERGENIC	--	--	--
Ca4_P1_22685908	Ca4	22685908	AC/ACC	AAGCTTGATTGTTGTTTCCGA	CTCAAGGACCCAAGACCAAA	59.7	195	INTERGENIC	--	--	--
Ca4_P1_22695676	Ca4	22695676	GATCGTTCATCGTTCATCGTTCATCGTTC/GATCGTTCATCGTTCATCGTTC	ATCAAGCATTTCCTCCAACG	AAGAACATGAATCGGGCAAG	60.1	411	INTERGENIC	--	--	--
Ca4_P1_22702418	Ca4	22702418	AAGTTATA/AA	GTCATTTGAAACGTGGGCTT	CATGTTGGAGGGGCTAAAAA	60.0	717	INTERGENIC	--	--	--

INDEL marker IDs	Chromosomes/ unanchored scaffolds	Physical positions (bp)	InDels (*Kabuli* reference genome-CDC Frontier/PI)	Forward primers (5'-3')	Reverse primers (5'-3')	Anneling temperature (0C)	Expected amplified product size (bp)	Structural annotation		Functional annotation	
								Sequence components of *kabuli* genome	*Kabuli* gene accession IDs	NCBI-KOG	TFs
Ca4_P1_22702676	Ca4	22702676	T/TAA	GGCTTAAGAGAGGGG GTTCA	TGCATTCACGTCAAAG AAGC	60.6	728	INTERGENIC	--	--	--
Ca4_P1_22702886	Ca4	22702886	AAATTGAATA/AA	CTGGGGCCAATTTAGA GGAT	TGCATTCACGTCAAAG AAGC	60.3	410	INTERGENIC	--	--	--
Ca4_P1_22703169	Ca4	22703169	ATTTTTT/ATTTTTTT	GTGCAAACAAGGAGCA ACAA	GCCTCCAATCCATGTC TGAT	59.9	322	INTERGENIC	--	--	--
Ca4_P1_22706706	Ca4	22706706	TCC/TC	CCAACAAATTGGGTGG AAAA	TCGCTGCAACCAATAA ATGA	60.6	776	INTERGENIC	--	--	--
Ca4_P1_22716804	Ca4	22716804	TC/T	ACGAACCCCTCTTGTG CTAA	TGCGATACGAATTTGG TGAA	59.7	677	INTERGENIC	--	--	--
Ca4_P1_22719475	Ca4	22719475	A/AT	ACACGTCATTTGTTTTG GCA	TGTTGCAATAGAAGGT CGCA	60.0	390	INTERGENIC	--	--	--
Ca4_P1_22719677	Ca4	22719677	TATAAA/TAA	TTTTGGCACGTCTTCT CAACT	CATCGACGATTGTACC AAAAA	59.9	548	INTERGENIC	--	--	--
Ca4_P1_22723833	Ca4	22723833	TGGGGG/TGGGGGG	TCTAACACCGGCAACA TCAA	AGGCCACGAAAGTGAA GAGA	60.1	527	DRR	Ca_14476	--	--
Ca4_P1_22727877	Ca4	22727877	TAAA/TAA	TTGGTTCCTTTATTAAC TAATTTTTGG	GGGACAAACGTTTTTG CTATG	59.3	809	INTERGENIC	--	--	--
Ca4_P1_22727990	Ca4	22727990	TAAAAAAAAA/TAAAAAAAA	AACCTATTTATCCAACT AACAAATGTC	GGGACAAACGTTTTTG CTATG	57.4	257	INTERGENIC	--	--	--
Ca4_P1_22735575	Ca4	22735575	AAAA/AAAATAAA	TTGAAGTTGTCGATCC CCTC	CATATTCAACGCAACA ACGC	60.1	553	DRR	Ca_14475	T	FAR1
Ca4_P1_22744609	Ca4	22744609	ACCC/ACC	GCCATCTCGAGTCAAC AACA	CATTGGTGGCTAGCAT GAGA	59.8	343	INTERGENIC	--	--	--

INDEL marker IDs	Chromosomes/ unanchored scaffolds	Physical positions (bp)	InDels (*Kabuli* reference genome-CDC Frontier/PI)	Forward primers (5'-3')	Reverse primers (5'-3')	Anneling temperature (0C)	Expected amplified product size (bp)	Structural annotation		Functional annotation	
								Sequence components of *kabuli* genome	*Kabuli* gene accession IDs	NCBI-KOG	TFs
Ca4_P1_22746108	Ca4	22746108	ACC/ACCC	GGAAGAAGCATGTGGGTTGT	TTGGCCTTGAGACCAGCTAT	60.0	721	INTERGENIC	--	--	--
Ca4_P1_22750911	Ca4	22750911	CTAATTAAT/CTAAT	CAAGTGTCTCATTGCAAATTCA	CCGAGGCCTCTTATTCTCAA	58.8	955	INTERGENIC	--	--	--
Ca4_P1_22755158	Ca4	22755158	ATTTTTTT/ATTTTTTTT	CTATGTGCGTCGTCAGGAAA	CCAAAAACGGTTTCACCAGT	59.9	595	INTERGENIC	--	--	--
Ca4_P1_22755286	Ca4	22755286	CT/CTTT	CTATGTGCGTCGTCAGGAAA	CCAAAAACGGTTTCACCAGT	59.9	595	INTERGENIC	--	--	--
Ca4_P1_22764594	Ca4	22764594	TAGA/TA	TCGATTCGGATTGGACTTTC	ATGTGGCCTTAGGAGGAGGT	60.0	670	INTERGENIC	--	--	--
Ca4_P1_22765355	Ca4	22765355	TAAAA/TAA	TGTTCGTAAATCAATGCCCA	TAGGCCCAAACCAAATCAAG	59.9	603	INTERGENIC	--	--	--
Ca4_P1_22765396	Ca4	22765396	GTTTTTTTTTTTTTTT/GTTTTTTTTTTTT	TGTTCGTAAATCAATGCCCA	TAGGCCCAAACCAAATCAAG	59.9	603	INTERGENIC	--	--	--
Ca4_P1_22765623	Ca4	22765623	CTT/CTTT	TGGGGACCTTCCCAATATAC	TATGGCCGATGCTACAACAA	58.6	219	INTERGENIC	--	--	--
Ca4_P1_22765942	Ca4	22765942	AGACG/AGACGACG	TAATTACTTTCCGCGGATGC	GGTGTAGCCTTTGCTTGGAG	60.1	424	INTERGENIC	--	--	--
Ca4_P1_22766072	Ca4	22766072	AATATA/AATATATATA	TAATTACTTTCCGCGGATGC	GGTGTAGCCTTTGCTTGGAG	60.1	424	INTERGENIC	--	--	--
Ca4_P1_22766863	Ca4	22766863	T/TGG	GCATAATCAAAAGCATTGGC	ATCGATTCTCAGTTGGACCG	58.3	756	INTERGENIC	--	--	--
Ca4_P1_22767329	Ca4	22767329	AACAC/AAC	CCAACTGAGAATCGATGGGT	TCTCCTTTTCAAATTCCTCCAA	59.9	274	INTERGENIC	--	--	--

INDEL marker IDs	Chromosomes/ unanchored scaffolds	Physical positions (bp)	InDels (*Kabuli* reference genome-CDC Frontier/PI)	Forward primers (5'-3')	Reverse primers (5'-3')	Anneling temperature (0C)	Expected amplified product size (bp)	Structural annotation		Functional annotation	
								Sequence components of *kabuli* genome	*Kabuli* gene accession IDs	NCBI-KOG	TFs
Ca4_P1_22780826	Ca4	22780826	TA/T	TCTATTTTTGATGATGTTGCGTT	TCCACACCGTTTTTGATATTG	58.7	495	INTERGENIC	--	--	--
Ca4_P1_22783764	Ca4	22783764	ATTTTT/ATTTTTT	CCTACAGAACAAGATAAAAACGG	ACTTTGATCCCGTTGCACAT	57.1	907	INTERGENIC	--	--	--
Ca4_P1_22790242	Ca4	22790242	TC/T	AGATAATCCCCCTGACACCC	AAAGGGGCAAACACTTGCTA	60.0	396	INTERGENIC	--	--	--
Ca4_P1_22792612	Ca4	22792612	ATTT/ATT	CCTTGAGTGAGTTTGACAGGAA	AAACCATAAAGCCCCAAACC	59.4	577	DRR	Ca_14472	--	--
Ca4_P1_22793993	Ca4	22793993	ATTT/ATT	CGAGTGGCAGAACTTCAAAA	GAATTATGCCGCAAGTACGA	59.0	318	DRR	Ca_14472	--	--
Ca4_P1_22799504	Ca4	22799504	AAATAA/AAA	CCAACACACCAAGACAGTGG	TCACACACAAATCACACCGA	60.0	213	INTERGENIC	--	--	--
Ca4_P1_22799819	Ca4	22799819	TAAAAAAA/TAAAAAAAA	CCAACACACCAAGACAGTGG	TTCGATCAATCGGTCACAAA	60.0	807	INTERGENIC	--	--	--
Ca4_P1_22799910	Ca4	22799910	AAAA/AAAAAATTAAA	TCGGTGTGATTTGTGTGTGA	GTGCAATCATGCAATCAAGG	59.5	672	INTERGENIC	--	--	--
Ca4_P1_22803782	Ca4	22803782	TA/T	CCAAGTTCCTCAAAAGCAGG	ATCCTGCCGTCATATGCTTC	59.8	682	INTRON	Ca_14471	Q	Nin-like
Ca4_P1_22809155	Ca4	22809155	AAAA/AAAATAAAATAAA	GCCGTTAAATTTCAGTGGGA	TTGGAGACAGTTGCTTCCCT	59.9	651	DRR	Ca_14471	Q	Nin-like
Ca4_P1_22818473	Ca4	22818473	GTATATATATAT/GTATATATATATAT	CAATTGCATGTTGAAATGGTG	TGGCCATATTGTTTTTGCAT	59.8	821	INTERGENIC	--	--	--
Ca4_P1_22819150	Ca4	22819150	TAAAAAA/TAAAAA	TGGCCAACTTCAAATAATCG	TCTAATAAAAATGGCGGTCCA	58.6	374	INTERGENIC	--	--	--

INDEL marker IDs	Chromosomes/ unanchored scaffolds	Physical positions (bp)	InDels (*Kabuli* reference genome-CDC Frontier/PI)	Forward primers (5'-3')	Reverse primers (5'-3')	Anneling temperature (0C)	Expected amplified product size (bp)	Structural annotation		Functional annotation	
								Sequence components of *kabuli* genome	*Kabuli* gene accession IDs	NCBI-KOG	TFs
Ca4_P1_22822736	Ca4	22822736	CTTTTTTT/CTTTTTT	CGAAAGTTTCACATTC ATCCAA	TTACCAAGTGTTGGAT TGGG	60.0	765	INTERGENIC	--	--	--
Ca4_P1_22824281	Ca4	22824281	AAA/AAAGTAA	ACATGCACCAGAGTTA ATGTTTT	GCTTCAACACCGGGAA TAGA	57.8	167	INTERGENIC	--	--	--
Ca4_P1_22824306	Ca4	22824306	TCCC/TCC	ACATGCACCAGAGTTA ATGTTTT	TGGGATTATTTTGATT GGACG	57.8	284	INTERGENIC	--	--	--
Ca4_P1_22824416	Ca4	22824416	CCGT/CCGTACGT	TCTATTCCCGGTGTTG AAGC	TGCACTCTAATGGGAT TATTTTGA	60.1	147	INTERGENIC	--	--	--
Ca4_P1_22824456	Ca4	22824456	TA/T	TCTATTCCCGGTGTTG AAGC	TTGACAACACAAATGT TTTAGAGGA	60.1	333	INTERGENIC	--	--	--
Ca4_P1_22825034	Ca4	22825034	TAAAAAA/TTAAAAAAA	TCCTCTAAAACATTTGT GTTGTCA	TCACTCTCTTACTATG GAAGCCAA	58.7	609	INTERGENIC	--	--	--
Ca4_P1_22837128	Ca4	22837128	ATTTT/ATT	TGTGTCGGGAAGTATG GTGA	TCACCATGGCAAGTTG AAAA	60.0	521	INTRON	Ca_14468	--	--
Ca4_P1_22837601	Ca4	22837601	CAAAAA/CAAAA	TTGAAATGGCAAAGAG GGTC	ATGCATGGGTTGAAAT ACCC	60.1	556	DRR	Ca_14468	--	--
Ca4_P1_22837669	Ca4	22837669	TAA/TAAAAA	TTGAAATGGCAAAGAG GGTC	ATGCATGGGTTGAAAT ACCC	60.1	556	DRR	Ca_14468	--	--
Ca4_P1_22837725	Ca4	22837725	AAATAATAA/AAATAA	TTGAAATGGCAAAGAG GGTC	ATGCATGGGTTGAAAT ACCC	60.1	556	DRR	Ca_14468	--	--
Ca4_P1_22848996	Ca4	22848996	TAAAAAAA/TAAAAAAAA	TTCATTATCTGTTCCCT CCCA	ACGACTGGCTAAGGT GCTGT	59.4	546	INTERGENIC	--	--	--
Ca4_P1_22849031	Ca4	22849031	TAAAAAAAAA/TAAAAAAAAAA	TTCATTATCTGTTCCCT CCCA	ACGACTGGCTAAGGT GCTGT	59.4	546	INTERGENIC	--	--	--

INDEL marker IDs	Chromosomes/ unanchored scaffolds	Physical positions (bp)	InDels (*Kabuli* reference genome-CDC Frontier/PI)	Forward primers (5'-3')	Reverse primers (5'-3')	Anneling temperature (0C)	Expected amplified product size (bp)	Structural annotation		Functional annotation	
								Sequence components of *kabuli* genome	*Kabuli* gene accession IDs	NCBI-KOG	TFs
Ca4_P1_22854055	Ca4	22854055	GT/G	TCAAAACACATTGGAGGCAC	AAAATGGCACAAGGCAAACT	59.5	858	INTERGENIC	--	--	--
Ca4_P1_22855998	Ca4	22855998	TATCCGAT/TAT	CCTGTCAACTCCAAGAATCTCC	AAAGCCATATTTTAACCCAAAAA	60.1	666	INTERGENIC	--	--	--
Ca4_P1_22856677	Ca4	22856677	TAAAAAA/TAAAAAAA	TTTTTGGGTTAAAATATGGCTTT	AAAAATGGAATTACGTCCAATGA	58.2	415	INTERGENIC	--	--	--
Ca4_P1_22864646	Ca4	22864646	TAGAGAGA/TAGAGA	TGAGACTGCAATTTGGCTTG	ACGACGATGTTCATTCCACA	60.0	914	DRR	Ca_14466	S	--
Ca4_P1_22867454	Ca4	22867454	GAAAAAAAAAA/GAAAAAAAAA	GATCTTCATCCCCTGCAGAC	CACCACCACCACAAAACACT	59.6	292	INTERGENIC	--	--	--
Ca4_P1_22869602	Ca4	22869602	TAAAAAAAAAA/TAAAAAAAAAAA	TTGCCGTGAAATAATCCTCA	TTGTGTTAGGACACACCCGA	59.1	294	INTERGENIC	--	--	--
Ca4_P1_22884702	Ca4	22884702	CAAAA/CAAA	TTTGGTACCCAATTTTCCCA	GGTGAAAGCGTTTGATGGAT	60.0	564	INTERGENIC	--	--	--
Ca4_P1_22889620	Ca4	22889620	TATAAAAATAAAAATAAAA/TATAAAAATAAAAATAAAAATAAAA	GGGTCAGGATACTTACAACCCA	TCCACCCCCAAATTTTATCA	60.1	717	INTERGENIC	--	--	--
Ca4_P1_22891439	Ca4	22891439	GAAAAAA/GAAAAAAA	AGTAAATGCAAGTTGCGCCT	AAGGTTGCTTGAGGTTTGCT	59.9	756	INTERGENIC	--	--	--
Ca4_P1_22896917	Ca4	22896917	GTTTTTTTTTTT/GTTTTTTTTT	CGATGCGCATGATAGAAAGA	AACGGAACCCCACTTAAAGG	59.9	590	INTERGENIC	--	--	--
Ca4_P1_22897920	Ca4	22897920	ATTTTTTTTT/ATTTTTTTTTT	GGTTGGCTTGTGATCTCCAT	TGCTTGTGGTCTGAAACCTG	59.9	463	INTERGENIC	--	--	--
Ca4_P1_22898673	Ca4	22898673	CAAAA/CAA	AACCCCACTTAAAGGGCAAC	AGGGTGAAGAAGCTCACACAA	60.2	684	INTERGENIC	--	--	--

INDEL marker IDs	Chromosomes/ unanchored scaffolds	Physical positions (bp)	InDels (*Kabuli* reference genome-CDC Frontier/PI)	Forward primers (5'-3')	Reverse primers (5'-3')	Anneling temperature (0C)	Expected amplified product size (bp)	Structural annotation		Functional annotation	
								Sequence components of *kabuli* genome	*Kabuli* gene accession IDs	NCBI-KOG	TFs
Ca4_P1_22901962	Ca4	22901962	CAAAA/CAAA	AGGGACTCAGTTTTCTGCGA	CAGACAAGCCTCTCGATTCC	60.0	593	INTERGENIC	--	--	--
Ca4_P1_22910353	Ca4	22910353	ATTT/ATT	TCGGCGAAGATTTGAGAACT	TTTTTGGGCTCCTATGCAAT	60.0	691	DRR	Ca_14463	R	NAC
Ca4_P1_22911497	Ca4	22911497	AC/A	TTTGTTGCATTTGTTTTCTTCG	GAAGACTCTCCACCGTCAGG	60.1	441	DRR	Ca_14462	J	--
Ca4_P1_22914105	Ca4	22914105	GTTTTTTTT/GTTTTTTTTTTT	AAAACCCTTTTGCTTCCGTT	TCCTACCAAAAACTTCATTCACAA	60.0	458	INTERGENIC	--	--	--
Ca4_P1_22914458	Ca4	22914458	CTTT/CT	ATTGCGCTTGCATCATCTTT	CCAAAGTTTCAACTTCGAGACA	60.8	671	INTERGENIC	--	--	--
Ca4_P1_22920774	Ca4	22920774	ACAA/ACAATGTTTCGGAACTCAA	CCGCAACTCAACCCAGTTAT	CACGTGAAGTTCGACAAGGA	60.0	509	INTERGENIC	--	--	--
Ca4_P1_22921547	Ca4	22921547	TAA/TAAAATAAATAAAAAA	TCGTCCGAACACAATCACAT	TGATCTCGTGCTTCTACGTGTT	60.0	311	INTERGENIC	--	--	--
Ca4_P1_22922451	Ca4	22922451	GA/GAA	CACCGTCCGAATACCAAAAC	AGTCATTTGCATGTGGGACA	60.2	391	INTERGENIC	--	--	--
Ca4_P1_22933272	Ca4	22933272	CAAATAAAAAATAAAAAAT/CAAATAAAAAAT	CTATAGTGACCGCACTCCGC	TTCCAAAATTCAGGAATCGC	60.8	774	INTRON	Ca_14461	--	--
Ca4_P1_22933918	Ca4	22933918	CTGAACAATT/CTGAACAATTGCGGTGAACAATT	AGAAACCCAAAAGGGGAAGA	TTTGAACCCAAGCTTCAACC	59.9	750	INTRON	Ca_14461	--	--
Ca4_P1_22938166	Ca4	22938166	ATTGTTG/ATTG	GATCTTTTTGGGCCAGTCAA	CCAAGATGGTTCTTGCCATT	60.1	343	INTRON	Ca_14461	--	--
Ca4_P1_22954544	Ca4	22954544	TG/TGG	GGTCCTATCCAGCTTCTCCC	CAAACCTTTTGTGGCCATTT	60.0	532	INTERGENIC	--	--	--

INDEL marker IDs	Chromosomes/ unanchored scaffolds	Physical positions (bp)	InDels (*Kabuli* reference genome-CDC Frontier/PI)	Forward primers (5'-3')	Reverse primers (5'-3')	Anneling temperature (0C)	Expected amplified product size (bp)	Structural annotation		Functional annotation	
								Sequence components of *kabuli* genome	*Kabuli* gene accession IDs	NCBI-KOG	TFs
Ca4_P1_22955278	Ca4	22955278	GAAAAAAAAA/GAAAAAAAAAA	CAGCCATTGCACCAATAAAA	TTTTCGCTGAAGCTTGGACT	59.6	772	INTERGENIC	--	--	--
Ca4_P1_22965696	Ca4	22965696	TAAAAAAAAAAA/TAAAAAAAAAA	CGGCTTTGTCACATTTGTTG	AATTCAAACCCCACTTCCTT	60.1	369	INTERGENIC	--	--	--
Ca4_P1_22970347	Ca4	22970347	CCTT/C	CAGCCTTTCATGTTTTGACG	CAACTGAAGCCTTCTTTGCC	59.3	787	INTRON	Ca_14460	--	--
Ca4_P1_22973152	Ca4	22973152	TTAAAATGTAAAATGTA/TTAAAATGTAAAATGTAAAATGTA	ATTTTATTGGCATCGGCATT	TGCGAATGTGCTTTCTGTCT	59.3	672	DRR	Ca_14460	--	--
Ca4_P1_22973209	Ca4	22973209	TAAAAAAAAA/TAAAAAAAAAAA	ATTTTATTGGCATCGGCATT	TGCGAATGTGCTTTCTGTCT	59.3	672	DRR	Ca_14460	--	--
Ca4_P1_22974231	Ca4	22974231	ATTTTTTTT/ATTTTTTT	TGATTGTATATCGGGGGTCG	ATTGTCAAAAGGATTTCGCC	60.5	537	DRR	Ca_14460	--	--
Ca4_P1_22974338	Ca4	22974338	A/AAT	ATTTTGGGCGAAATCCTTTT	GAACCACTCATCCTCCCAAA	59.8	442	DRR	Ca_14460	--	--
Ca4_P1_22978212	Ca4	22978212	TATGAT/TAT	GAGCAAATGAGGCACAATGA	TCAAGGGGCTTTGAGGTAGA	59.8	510	INTERGENIC	--	--	--
Ca4_P1_22980114	Ca4	22980114	CAAAAAAA/CAAAAAAAA	TCGCACAAGGCTACAATCAG	CTCGAAAAATCGAAGGGACA	60.0	749	INTERGENIC	--	--	--
Ca4_P1_22980181	Ca4	22980181	TAATAATAATAATAAT/TAATAATAATAATAATAAT	TCGCACAAGGCTACAATCAG	CTCGAAAAATCGAAGGGACA	60.0	749	INTERGENIC	--	--	--
Ca4_P1_22980878	Ca4	22980878	CTTTTTTT/CTTTTTTTTT	CGACGAAACGCCTTAAAAAG	AAAACGACGAAATCGCAAAT	59.9	332	INTERGENIC	--	--	--
Ca4_P1_22981123	Ca4	22981123	ATTTTTTTT/ATTTTTTTTTTT	CGACGAAACGCCTTAAAAAG	TTTTGTCATGTTTGCGAGGT	59.9	797	INTERGENIC	--	--	--

**INDEL marker IDs**	**Chromosomes/ unanchored scaffolds**	**Physical positions (bp)**	**InDels (*Kabuli* reference genome-CDC Frontier/PI)**	**Forward primers (5'-3')**	**Reverse primers (5'-3')**	**Anneling temperature (0C)**	**Expected amplified product size (bp)**	**Structural annotation**		**Functional annotation**	
								**Sequence components of *kabuli* genome**	***Kabuli* gene accession IDs**	**NCBI-KOG**	**TFs**
Ca4_P1_22981258	Ca4	22981258	TAGATTAAA/TA	TCCGCCGTTCTAAATT CTTG	CAAGCCCTCTCCCTCT CTTC	60.2	874	INTERGENIC	--	--	--
Ca4_P1_22981343	Ca4	22981343	ATTTTTTTT/ATTTTTTT	TCCGCCGTTCTAAATT CTTG	CAAGCCCTCTCCCTCT CTTC	60.2	874	INTERGENIC	--	--	--
Ca4_P1_22982428	Ca4	22982428	AGGGGG/AGGGG	GAGCTGACAAACGACC ATGA	AAAGAACGTCGGAACT CGTG	59.8	576	INTERGENIC	--	--	--
Ca4_P1_22984796	Ca4	22984796	GTTTA/GTTTAAACTTTA	CAAATTCCCTTTAGTG CCACA	TGCTTATGTGTTCCAT TTGCTC	60.0	802	INTERGENIC	--	--	--
Ca4_P1_22985522	Ca4	22985522	CAAATCAAAATCAAA/CAAATCAAAAT CAAAATCAAA	GTCATGAAATGGGAAG GCAT	CTTAGATGGGCCACCC AATA	59.8	729	INTERGENIC	--	--	--
Ca4_P1_23002548	Ca4	23002548	ATTATTTTATTTTATTTTATT/ATTATTT TATTTTATTTTATTTTATT	TCGATCGCTGATGTCA AACT	TGGGCTTGACCTATGT GTGT	59.4	147	INTERGENIC	--	--	--
Ca4_P1_23002623	Ca4	23002623	TTTACTATTACTATT/TTTACTATTACT ATTACTATT	TTTATGGCGGCTGGTT TTAC	ACGATGCATTTGATTG ATGTT	60.0	546	INTERGENIC	--	--	--
Ca4_P1_23012710	Ca4	23012710	AGGG/AGG	ATAACTCACACACACC CGCA	TCTTGTTGTTGGCGTT GTTT	60.0	359	INTERGENIC	--	--	--
Ca4_P1_23018299	Ca4	23018299	AATG/A	ATTTCGGTTTGTCCTT CGAG	TTTTAAAAAGATTCACA TGCACTAA	59.2	549	INTERGENIC	--	--	--
Ca4_P1_23018550	Ca4	23018550	GAAAAAAA/GAAAAAAAA	TCAGAGAGAATGCACG ACATTT	GGCCTGACGACACAG CTAAT	59.9	774	INTERGENIC	--	--	--
Ca4_P1_23018576	Ca4	23018576	A/AT	TCAGAGAGAATGCACG ACATTT	AGTTGGGCAGCACTTC ATCT	59.9	812	INTERGENIC	--	--	--
Ca4_P1_23018840	Ca4	23018840	AA/AATTA	CAAAATAAATCAAATGA TTGAAGTGA	GGCCTGACGACACAG CTAAT	58.6	651	INTERGENIC	--	--	--

INDEL marker IDs	Chromosomes/ unanchored scaffolds	Physical positions (bp)	InDels (*Kabuli* reference genome-CDC Frontier/PI)	Forward primers (5'-3')	Reverse primers (5'-3')	Anneling temperature (0C)	Expected amplified product size (bp)	Structural annotation		Functional annotation	
								Sequence components of *kabuli* genome	*Kabuli* gene accession IDs	NCBI-KOG	TFs
Ca4_P1_23019320	Ca4	23019320	CTTTTTTTTT/CTTTTTTT	AGATGAAGTGCTGCCCAACT	TTTGAATTTTCCGTCCAAAA	59.9	461	INTERGENIC	--	--	--
Ca4_P1_23020256	Ca4	23020256	TTAT/TT	TCATGACCAAGAAACAACGG	TCACATATCAAATGTTGGCACA	59.5	354	INTERGENIC	--	--	--
Ca4_P1_23020397	Ca4	23020397	TAGTAATAACA/TA	TCATGACCAAGAAACAACGG	TCGAAGGACAAAGGGAAGTG	59.5	946	INTERGENIC	--	--	--
Ca4_P1_23020630	Ca4	23020630	CAAAAAAAAAAA/CAAAAAAAAAA	CGCATCAGCAATTATGCGT	TCGAAGGACAAAGGGAAGTG	60.8	650	INTERGENIC	--	--	--
Ca4_P1_23027444	Ca4	23027444	T/TC	CCATTGGGAATTAGGCATTG	AAGGTTGTGGATCGATTCGT	60.1	868	INTERGENIC	--	--	--
Ca4_P1_23027613	Ca4	23027613	AGG/AGGG	GATGTTGGGAAGGGTGGTTA	AAGGTTGTGGATCGATTCGT	59.6	622	INTERGENIC	--	--	--
Ca4_P1_23027836	Ca4	23027836	CAAAAAAA/CAAAAAAAA	TGCGTCTTTGTTGATTCTCC	TGCCAATCACTATTCTCCCA	58.9	666	INTERGENIC	--	--	--
Ca4_P1_23030943	Ca4	23030943	CTTTTTTTTT/CTTTTTTTTTTT	TGGACGTGAGTGGTTCACAT	AACATGTGTGAAACATTAAATATCTGT	60.0	652	INTERGENIC	--	--	--
Ca4_P1_23034487	Ca4	23034487	CAAAAAAAA/CAAAAAAAAA	TGGGAATGACAACTTGGACA	GGTTGCCAAACAGTAGCCAT	59.9	758	INTERGENIC	--	--	--
Ca4_P1_23049719	Ca4	23049719	AAATAA/AAATAATAA	CCTCCCGATCGTTCAAAATA	CCTCATCAAGGGTTGCCTTA	59.9	564	INTERGENIC	--	--	--
Ca4_P1_23050771	Ca4	23050771	A/AAAT	TTTACCACGAACCACAACCA	TTAGCTCTGCCGTACGAGGT	59.9	430	INTERGENIC	--	--	--
Ca4_P1_23050902	Ca4	23050902	TAAAAAAAAAA/TAAAAAAAA	ACCTCGTACGGCAGAGCTAA	TGGATTTCGGTGTTCTCCTC	60.0	159	INTERGENIC	--	--	--

INDEL marker IDs	Chromosomes/ unanchored scaffolds	Physical positions (bp)	InDels (*Kabuli* reference genome-CDC Frontier/PI)	Forward primers (5'-3')	Reverse primers (5'-3')	Anneling temperature (0C)	Expected amplified product size (bp)	Structural annotation		Functional annotation	
								Sequence components of *kabuli* genome	*Kabuli* gene accession IDs	NCBI-KOG	TFs
Ca4_P1_23055017	Ca4	23055017	T/TG	GTGTGCTTGGAAAGTC GTTG	TTTGATGTTGTTGGAG ATGAAAA	59.3	485	DRR	Ca_14459	--	--
Ca4_P1_23055098	Ca4	23055098	CTTTATTTTATTTTATTTTATTTT/CTTT ATTTTATTTTATTTT	GTGTGCTTGGAAAGTC GTTG	TTTGATGTTGTTGGAG ATGAAAA	59.3	485	DRR	Ca_14459	--	--
Ca4_P1_23055622	Ca4	23055622	TAAGAA/TAAGAAAGAA	TGAATTAAAGGATTGC ATGAACA	CCGTTGAATGGAAAAT CACC	59.5	530	DRR	Ca_14459	--	--
Ca4_P1_23056430	Ca4	23056430	TAAAAAAAA/TAAAAAAAAA	ATGAATAGCATGCCAC GACA	TCATGTGGGCTCATTT GGTA	60.1	771	INTERGENIC	--	--	--
Ca4_P1_23056577	Ca4	23056577	TAAAAAAAAAA/TAAAAAAAAA	ATGAATAGCATGCCAC GACA	TCATGTGGGCTCATTT GGTA	60.1	771	INTERGENIC	--	--	--
Ca4_P1_23058519	Ca4	23058519	ATCT/AT	CGGATATGAGTTTTAT GGACGA	CTCCCTTTTTGCAACA CACA	59.0	434	INTERGENIC	--	--	--
Ca4_P1_23059712	Ca4	23059712	CTTTTT/CTTT	TCATTCGTTTGTGCAG TTTTG	TTTAATTTCCCTGCAC CCCT	59.8	612	INTERGENIC	--	--	--
Ca4_P1_23060171	Ca4	23060171	AATG/AATGCATG	TGCATTTGGCAATTTG AAGA	TCAACGCATGTGACTG CATA	60.2	254	INTERGENIC	--	--	--
Ca4_P1_23060568	Ca4	23060568	ATTTTTTTTT/ATTTTTTTTTT	ATGGGCATTATGCAGT CACA	TTTGCCAATTTATCAT GTTTGC	60.0	766	INTERGENIC	--	--	--
Ca4_P1_23067339	Ca4	23067339	GACAAAAACACAAAA/GACAAAA	GCAATATCGAAGCACG ACAA	ATTGCGGTCAGTTATT TCCG	59.8	148	INTRON	Ca_14458	R	--
Ca4_P1_23067690	Ca4	23067690	GTTTTTTTTT/GTTTTTTTTTT	CGGAAATAACTGACCG CAAT	GGAATCGAAAGTCGTG GAAA	60.0	373	INTERGENIC	--	--	--
Ca4_P1_23077402	Ca4	23077402	TAAAAAAAA/TAAAAAAAAA	ACCAAAGAATCCATCG GTTG	TGAATTCATGTGTATT GTCGGA	59.8	842	INTERGENIC	--	--	--

INDEL marker IDs	Chromosomes/ unanchored scaffolds	Physical positions (bp)	InDels (*Kabuli* reference genome-CDC Frontier/PI)	Forward primers (5'-3')	Reverse primers (5'-3')	Anneling temperature (0C)	Expected amplified product size (bp)	Structural annotation		Functional annotation	
								Sequence components of *kabuli* genome	*Kabuli* gene accession IDs	NCBI-KOG	TFs
Ca4_P1_23077586	Ca4	23077586	ATTTTTT/ATTTTTTT	AAGAAAAGGGTTGCGTTTGA	TGAATTCATGTGTATTGTCGGA	59.7	644	INTERGENIC	--	--	--
Ca4_P1_23078030	Ca4	23078030	GAAAAAA/GAAAAAAAA	TTTGAAGAACCAGAGTTTGAAGAA	TTCAATGCCCATAATCGTGA	59.5	656	INTERGENIC	--	--	--
Ca4_P1_23078082	Ca4	23078082	CTTTTTTT/CTTTTTT	TTTGAAGAACCAGAGTTTGAAGAA	TTCAATGCCCATAATCGTGA	59.5	656	INTERGENIC	--	--	--
Ca4_P1_23079294	Ca4	23079294	ATCTTCTT/ATCTT	TAAATAGCATTTGCGGTCCC	TTTTTCTTCCGATTTGGTCC	59.9	508	INTERGENIC	--	--	--
Ca4_P1_23079340	Ca4	23079340	CAAA/CAAAA	AATTGCAAGTGGACTGGACC	CCGAGCCAAAATCCAAAATA	60.0	787	INTERGENIC	--	--	--
Ca4_P1_23080112	Ca4	23080112	CAAAAAAA/CAAAAAA	CGGTGAATCCTTTACGGAGA	CACCGTAGTGTGGTGGTGAG	60.1	491	INTERGENIC	--	--	--
Ca4_P1_23098190	Ca4	23098190	TC/T	GGCGGTCGAGAGTTTATGTG	TGAGTGCCAACAAGCTGAAC	60.7	495	DRR	Ca_14457	R	MYB
Ca4_P1_23098234	Ca4	23098234	GTTTT/GTTTTT	GGCGGTCGAGAGTTTATGTG	TGAGTGCCAACAAGCTGAAC	60.7	495	DRR	Ca_14457	R	MYB
Ca4_P1_23098796	Ca4	23098796	GGA/GGAGA	GTTCAGCTTGTTGGCACTCA	TCATGAACCTCAACGAACGA	60.0	558	INTERGENIC	--	--	--
Ca4_P1_23103638	Ca4	23103638	ATTTTTT/ATTTTTTT	CATTTTGCATGCCATCTTTG	TGTCGGACCGAAAATGTAAA	60.1	680	INTERGENIC	--	--	--
Ca4_P1_23104080	Ca4	23104080	AGGGG/AGGGGG	TTTTGTTAAGCATTGTTGGGC	GCCGTTTAGTCGAAGTTTGG	60.0	343	INTERGENIC	--	--	--
Ca4_P1_23104118	Ca4	23104118	TA/T	TTTTGTTAAGCATTGTTGGGC	ATTCCAAACGTGAATCCGTC	60.0	594	INTERGENIC	--	--	--

INDEL marker IDs	Chromosomes/ unanchored scaffolds	Physical positions (bp)	InDels (*Kabuli* reference genome-CDC Frontier/PI)	Forward primers (5'-3')	Reverse primers (5'-3')	Anneling temperature (0C)	Expected amplified product size (bp)	Structural annotation		Functional annotation	
								Sequence components of *kabuli* genome	*Kabuli* gene accession IDs	NCBI-KOG	TFs
Ca4_P1_23134474	Ca4	23134474	ATTGTTGTTGT/ATTGTTGT	ATGCTGCGAAATTGTTGAAA	AAAAGCGTTGTAAATTGGATTGA	59.3	342	INTERGENIC	--	--	--
Ca4_P1_23135482	Ca4	23135482	GAAAAAA/GAAAAA	ATTCAGCAATGAGAGGTCCG	GATGATGCATGCCATTTGAG	60.2	752	INTERGENIC	--	--	--
Ca4_P1_23145891	Ca4	23145891	ATTTTTTTTTT/ATTTTTTTT	GCTTGAGGATTCAGTAGGCG	GAGTGGAGGACCACATGTCA	60.0	408	INTERGENIC	--	--	--
Ca4_P1_23217567	Ca4	23217567	AATATATATATATATATAT/AATATATATATATATATAT	CTCTAGGGAAGCGACCTCCT	CCAACAACCATTGTGGGAGT	60.0	633	INTERGENIC	--	--	--
Ca4_P1_23264893	Ca4	23264893	TAAAAAAAAAAAAA/TAAAAAAAAAAAAAAAA	TTAACCGGAAAACAATGCAA	TTTTAAGCATTGGGGTTTGG	59.1	742	INTERGENIC	--	--	--
Ca4_P1_23272983	Ca4	23272983	ATTTTTTTTT/ATTTTTTTT	ACCACATCGGAAGAAACGAC	GGACGAAGTAGAACGGACCA	60.0	596	INTERGENIC	--	--	--
Ca4_P1_23363366	Ca4	23363366	GAAAAAAAAAAAA/GAAAAAAAAAAAAA	GTTAACGTTGCCCTAACGGA	TCCAGGTAACGTTTTGGTCC	60.0	114	INTERGENIC	--	--	--
Ca4_P1_23366959	Ca4	23366959	GATATATATATATATATATATATATATATATATATA/GATATATATATATATATATATATATATATATATATATATATATATATATA	GGGAGTCATGTTGACCGTCT	TAGTGTGTAACCCGTGCGTC	60.0	227	INTERGENIC	--	--	--
Ca4_P1_23378919	Ca4	23378919	CATATATATATATATATA/CATATATATATATATATA	TGGGCCAAAGATCCATTAAC	AACAAGCGACCATTCGATTT	59.8	781	DRR	Ca_14444	--	ERF
Ca4_P1_23419838	Ca4	23419838	TA/TACA	AGGAAGAGTGGAGTTGCGAA	TTAACCTTGGAGTGATCGGC	60.0	623	INTERGENIC	--	--	--
Ca4_P1_23525491	Ca4	23525491	GTTTTTTTTTT/GTTTTTTTTT	TGAGATTATATGCACGCGGA	TGAATCGCGAAAGTAGTCCC	60.2	574	INTERGENIC	--	--	--
Ca4_P1_23606765	Ca4	23606765	TAAAAAAAAAAAA/TAAAAAAAAAAA	TTGCCCATTGCTCCTTTAAC	TGTGGAGGAAATGGAACACA	60.1	709	DRR	Ca_14435	--	--

INDEL marker IDs	Chromosomes/ unanchored scaffolds	Physical positions (bp)	InDels (*Kabuli* reference genome-CDC Frontier/PI)	Forward primers (5'-3')	Reverse primers (5'-3')	Anneling temperature (0C)	Expected amplified product size (bp)	Structural annotation		Functional annotation	
								Sequence components of *kabuli* genome	*Kabuli* gene accession IDs	NCBI-KOG	TFs
Ca4_P1_2366778	Ca4	2366778	AATATATATATATATATATATATATATATAT/AATATATATATATATATATATATATATATATATATATATATAT	ATCCGTCTCCACGCTAATTG	TATGGGACCTTCTGGCTCTG	60.1	482	INTRON	Ca_07858	Q	Nin-like
Ca4_P1_23721197	Ca4	23721197	TAAAACA/TAAAACAAAACA	ATTTGTGTGCATTTGCCTGA	TGGGCAATCGACTGACTAAA	60.1	353	INTERGENIC	--	--	--
Ca4_P1_23756124	Ca4	23756124	TGTAG/T	TTGGAACGTAATTTGATGCG	CACAATTCATTTGTTTGGCCT	59.6	611	INTERGENIC	--	--	--
Ca4_P1_23756363	Ca4	23756363	TTATATATATATATATATA/TTATATATATATATATATA	TTGGAACGTAATTTGATGCG	CCCGTTGCTTCTGTATGCTT	59.6	872	INTERGENIC	--	--	--
Ca4_P1_23828073	Ca4	23828073	G/GACATCGG	TGTTGAAAACGGTCCGTACA	CTTCATCCGAAGAGGCAGAG	60.0	744	INTERGENIC	--	--	--
Ca4_P1_2388178	Ca4	2388178	GTAATAATAATAATAATAATAATAATAATAATA/GTAATAATAATAATAATAATAATAATAATAATAATAATA	GCCATGGCATAAACCCTAGA	ATCAGGGAACACGGAACTTG	59.9	591	INTERGENIC	--	--	--
Ca4_P1_23960230	Ca4	23960230	AAATAATAATAATAATAATAATAAT/AAATAATAATAATAATAATAATAATAAT	GTCTGTGATTTGGTGCAACG	AGGCCAATGAGGCAAGAGTA	60.2	617	INTERGENIC	--	--	--
Ca4_P1_23977698	Ca4	23977698	AATTATTATTAT/AATTATTATTATTAT	CAAAGCAAGCAACGCATAAA	TGGATATTTGTTAATAATCACGGA	60.0	439	INTERGENIC	--	--	--
Ca4_P1_24073846	Ca4	24073846	ATTTTTTTTT/ATTTTTTTTTT	GCGTGTTTTAAATTTGGTGGA	ATTGATTGCAATGGCTTGAA	59.9	495	INTERGENIC	--	--	--
Ca4_P1_24100684	Ca4	24100684	AATATATATATATATATATATATATA/AATATATATATATATATATATATATATATA	AATGGTCCAAGGAAAAAGGG	ATCCCCAATTGGAACCTACC	60.2	753	INTERGENIC	--	--	--
Ca4_P1_24115337	Ca4	24115337	ATATAT/ATATATATCTATAT	CACACGTGCATGGATAGAGG	TTCACCGTGGATGAATTTGA	60.1	341	INTERGENIC	--	--	--
Ca4_P1_24140540	Ca4	24140540	AT/ATATT	TTATTTCACCGTCGTGTCCA	TTGTGTGTTGGCCGATTTTA	60.0	234	INTERGENIC	--	--	--

INDEL marker IDs	Chromosomes/ unanchored scaffolds	Physical positions (bp)	InDels (*Kabuli* reference genome-CDC Frontier/PI)	Forward primers (5'-3')	Reverse primers (5'-3')	Anneling temperature (0C)	Expected amplified product size (bp)	Structural annotation		Functional annotation	
								Sequence components of *kabuli* genome	*Kabuli* gene accession IDs	NCBI-KOG	TFs
Ca4_P1_24148555	Ca4	24148555	ATATTATTATTATTATTATTATTATTATTATTATTATTATTATTATTATTATTAT/ATATTATTATTATTATTATTATTATTATTATTATTATTATTAT	TTCATCTTTGTTCGCTGCAC	TTCGGGAGTCGATTACGAGT	60.0	597	INTERGENIC	--	--	--
Ca4_P1_24166868	Ca4	24166868	TA/TTTATTAA	GTCTGACCTGGCCTGACCTA	GAACGGCTACCGAATATGGA	60.3	296	INTERGENIC	--	--	--
Ca4_P1_24268223	Ca4	24268223	ATTTTTTTTTTTT/ATTTTTTTTTTT	CGAACATTCTCTCCATCCGT	GGCTCTTTCCGTAGACCCTT	60.1	565	INTERGENIC	--	--	--
Ca4_P1_24273906	Ca4	24273906	TTATATATATATATATATATATATATA/TTATATATATATATATATATATATATATA	GGGGGAGTTGTTATTCGGTT	GAAAAGTGTTGTCGTGTGTGC	60.1	646	INTERGENIC	--	--	--
Ca4_P1_24279827	Ca4	24279827	ATATTATTATTATTATTATTATTATTATTATTATTATTATTA/ATATTATTATTATTATTATTATTATTATTA	TGGTCCATATCCTGGGTTGT	GTACCCCGACTAAGCACCAA	60.1	720	INTERGENIC	--	--	--
Ca4_P1_24326964	Ca4	24326964	TAAAAAAAAAAA/TAAAAAAAAAA	CTGGGCAAGGGATCTGAATA	AATGCCCACCCATAATTTCA	60.0	646	INTERGENIC	--	--	--
Ca4_P1_24359275	Ca4	24359275	TAAAAAAAA/TAAAAAAAAAA	GGGAGGGTGGGAGTAAATGT	TGTGGCTTTCCAATGTACGA	60.1	571	INTERGENIC	--	--	--
Ca4_P1_24368839	Ca4	24368839	TAAAAAAA/TAAAAAA	TTTTTCCGACTTGGTCCTTT	GGACTTTGGTTCTCTTGGCA	58.7	381	INTERGENIC	--	--	--
Ca4_P1_24390102	Ca4	24390102	TAAAAAA/TAAAAAAA	AATCCTTCTGAGTGAGCGGA	CCTCCAACAGCTTCTTCACC	60.0	339	INTERGENIC	--	--	--
Ca4_P1_24397169	Ca4	24397169	GAAAAAAAAAA/GAAAAAAAAAAA	TGTTTCAATCCTCCCTCACA	TGGTCATTGTTGATGCGATT	59.1	853	INTERGENIC	--	--	--
Ca4_P1_24427573	Ca4	24427573	GTT/GTTT	CTGGAAAATCCTCCTCCTCC	TTCGTCTCAACAATCTCCCC	60.0	346	INTERGENIC	--	--	--
Ca4_P1_24438141	Ca4	24438141	CAAAAAA/CAAAAA	CCGCTCTGGAAATCATTCAT	AAGACTCGGGATAATGGCCT	60.0	551	DRR	Ca_20867	--	--

INDEL marker IDs	Chromosomes/ unanchored scaffolds	Physical positions (bp)	InDels (*Kabuli* reference genome-CDC Frontier/PI)	Forward primers (5'-3')	Reverse primers (5'-3')	Anneling temperature (0C)	Expected amplified product size (bp)	Structural annotation		Functional annotation	
								Sequence components of *kabuli* genome	*Kabuli* gene accession IDs	NCBI-KOG	TFs
Ca4_P1_24442934	Ca4	24442934	GCA/GCATTCA	TCGCCTTCAAAGAAAACCAC	GGCATGAAATCCATCACCTT	60.2	568	DRR	Ca_20866	R	NAC
Ca4_P1_24453845	Ca4	24453845	A/AT	TCCTTCGCAACCATATCACA	GTTTCCGGAAGCAATCGAAG	60.1	282	INTERGENIC	--	--	--
Ca4_P1_24455402	Ca4	24455402	TTATATATATATATAT/TTATATATATATATATAT	AGCACCAAAGCCACCATTAC	AGCCTCTTTTTGGGACCACT	60.0	236	INTERGENIC	--	--	--
Ca4_P1_2445623	Ca4	2445623	AATATATATATATATATATATATATATATATATATA/AATATATATATATATATATATATATATATATA	GGAGGTCACAACCACTCCAA	CGGACTTTCGACTTTCTTGC	61.0	654	INTERGENIC	--	--	--
Ca4_P1_24473624	Ca4	24473624	T/TA	GAGCTTAGACCGCCACAAAG	TTGTAGTTGAGTTCGCACAAAAA	60.0	743	INTERGENIC	--	--	--
Ca4_P1_24478956	Ca4	24478956	CAA/CAAA	CGGTTCTACACAAGTTGCGA	TCGATTATGCGTAGGGAAGG	59.9	746	INTERGENIC	--	--	--
Ca4_P1_24479035	Ca4	24479035	ATTTTTTT/ATTTTTTTTTT	CGGTTCTACACAAGTTGCGA	TCGATTATGCGTAGGGAAGG	59.9	746	INTERGENIC	--	--	--
Ca4_P1_24505723	Ca4	24505723	TAAAAAAAAAA/TAAAAAAAAAAA	GTTTCGTTCGCCAAACTTTC	TGCCCTGATTGATGACCATA	59.7	768	INTERGENIC	--	--	--
Ca4_P1_24510502	Ca4	24510502	CTGGAACATTGGAACATTG/CTGGAACATTG	CTGCAAAAGCCCTCACAAAT	TCCTCTTGCACACCACAAGA	60.2	969	INTERGENIC	--	--	--
Ca4_P1_24553735	Ca4	24553735	GATTAT/GAT	TGGTGCGATTGTGGAAAATA	GAGTAGATGTGCCAACAGCG	59.9	361	INTERGENIC	--	--	--
Ca4_P1_24553809	Ca4	24553809	ATTTTTTTT/ATTTTTTTTT	TGGTGCGATTGTGGAAAATA	TTCTTTGGTGGTTGAATTTTGTT	59.9	695	INTERGENIC	--	--	--
Ca4_P1_24554220	Ca4	24554220	T/TA	GTGCGGATTATGTCGGGTAT	ACTAGATGGTCGCATCCTGG	59.7	751	INTERGENIC	--	--	--

INDEL marker IDs	Chromosomes/ unanchored scaffolds	Physical positions (bp)	InDels (*Kabuli* reference genome-CDC Frontier/PI)	Forward primers (5'-3')	Reverse primers (5'-3')	Anneling temperature (0C)	Expected amplified product size (bp)	Structural annotation		Functional annotation	
								Sequence components of *kabuli* genome	*Kabuli* gene accession IDs	NCBI-KOG	TFs
Ca4_P1_24572568	Ca4	24572568	AA/AACA	ACAACACAAATCACCAGCCA	GTGGAAGAAAAGCGTCCAAA	60.0	935	INTERGENIC	--	--	--
Ca4_P1_24581547	Ca4	24581547	AATTA/AA	TTCCATTTCCTCCACAAAGC	TCATTCCAGAAGGAGATGGG	60.1	548	INTRON	Ca_20859	--	--
Ca4_P1_24597173	Ca4	24597173	TAAAAAAAAAAA/TAAAAAAAAAA	TGTCACGACAATATTGGATGATT	CCTTTAGAGGACCACCAATGTC	59.2	482	INTERGENIC	--	--	--
Ca4_P1_2460361	Ca4	2460361	CATGTTAGTTAATGTTAGTTAATG/CATGTTAGTTAATG	TTCAACCGACAAAATGCAAA	TGTTTTAAAGCATGCAAAGAGTTT	60.1	786	INTERGENIC	--	--	--
Ca4_P1_24624996	Ca4	24624996	CTTTTTTTTTT/CTTTTTTTTTTT	GTGTGCCTTGGAATTGTTCA	CATTTGCCAACTCACCAAAA	59.5	461	INTERGENIC	--	--	--
Ca4_P1_24626037	Ca4	24626037	AAATAATATAATAATAATATAATAATAATATAATAATAATATAATAATAATATAA/AAATAATATAATAATAATATAATAATAATATAATAATAATATAA	TGGAGGATGTGGCATGAATA	TGCTCTCCTCCTTTAACAACTTC	59.9	620	INTERGENIC	--	--	--
Ca4_P1_24628315	Ca4	24628315	AATATATATATATATATATATATATATATATATATATATAT/AATATATATATATATATATATATATATATATATATATATATATATATATATAT	TATTTTGGATTTTGGCTCGG	AGGAAGAGTGGAGTTGCGAA	59.9	540	INTERGENIC	--	--	--
Ca4_P1_24676114	Ca4	24676114	AAAAATGTT/A	ACCAGGGACTGTCCATCAAG	TTTTTGTCCGTCTTCCAAGT	60.0	624	INTERGENIC	--	--	--
Ca4_P1_24681027	Ca4	24681027	TTCTCTCTCTCTCTCTCTCTCT/TTCTCTCTCTCTCTCTCTCT	ATAAAAGGCGCGCTGTAAAA	TGGTTTGTAGATGTGCCGAA	59.9	383	INTERGENIC	--	--	--
Ca4_P1_24745546	Ca4	24745546	AATATATATATATATATATATATATATATATATATAT/AATATATATATATATATATATATAT	CGAGAAGCTTGATTGCTTCA	TTCAACCTAAGCCCATTACGA	59.3	787	INTERGENIC	--	--	--
Ca4_P1_24748380	Ca4	24748380	GTTATTATTATTATTATTATTATTATTATTATTAT/GTTATTATTATTATTATTATTATTATTATTATTAT	TGTTTTTAATGCCAATGCGA	TTTCTCACAGATGTGGTCGTG	60.1	546	INTERGENIC	--	--	--
Ca4_P1_2477995	Ca4	2477995	AATATATATATATATATATA/AATATATATATATATATATATATATATA	GCCATTTGGCTTGTAGGTTG	AGCGCTGTTGTATCCTTCGT	60.5	570	INTERGENIC	--	--	--

INDEL marker IDs	Chromosomes/ unanchored scaffolds	Physical positions (bp)	InDels (*Kabuli* reference genome-CDC Frontier/PI)	Forward primers (5'-3')	Reverse primers (5'-3')	Anneling temperature (0C)	Expected amplified product size (bp)	Structural annotation		Functional annotation	
								Sequence components of *kabuli* genome	*Kabuli* gene accession IDs	NCBI-KOG	TFs
Ca4_P1_24786287	Ca4	24786287	GTTAATTTAATTTAATTTAATTT/GTTAATTTAATTTAATTT	CTTCACTGCGTCCCTTCTTC	CTTTTGTCTTCTTGCCAGCC	60.0	749	DRR	Ca_20850	--	--
Ca4_P1_24828297	Ca4	24828297	ATTTTTTTTTT/ATTTTTTTTTTT	TGGAATTTCAGACTGCCTTG	GGGCACGAAGAAGCACTATC	58.8	716	INTERGENIC	--	--	--
Ca4_P1_24839816	Ca4	24839816	AATATATATATATATATATAT/AATATATATATATATATATATAT	GGAATTGGGAAAGATGGGAT	GCAACGTCACTTTGGGATTT	60.0	238	INTERGENIC	--	--	--
Ca4_P1_24897433	Ca4	24897433	GAAAAAAAAA/GAAAAAAAAAA	TGCACAAACCTTTCAATGCT	TTTTTGAATTTTGAACGAAACA	59.3	775	INTERGENIC	--	--	--
Ca4_P1_24905267	Ca4	24905267	CTATA/CTA	CCCATGACTGATTGGTATGCT	GGAAGATCACCCAAAAATGG	59.8	559	INTERGENIC	--	--	--
Ca4_P1_24905867	Ca4	24905867	ATTTTTTTTTTTTT/ATTTTTTTTTTTT	GCAATTTTGCAGAACATGGA	CCTTTCAGCCTTTTTGTTGG	59.7	140	INTERGENIC	--	--	--
Ca4_P1_24911942	Ca4	24911942	ATTTTTTTTTTT/ATTTTTTTTTT	ACACCGAGTAAACGGTGGAG	CCTTGTTCTCACAAAAACCCA	60.0	481	INTERGENIC	--	--	--
Ca4_P1_24940280	Ca4	24940280	GAAAAAAAAAAA/GAAAAAAAAAAAA	TTGTTGTCGCAATGGAATGT	TGCTTGGTCACTTTGGCATA	60.0	315	INTERGENIC	--	--	--
Ca4_P1_2495002	Ca4	2495002	ATCTCTCTCTCTCTCTCTCTCTCTCTCTCTCT/ATCTCTCTCTCTCTCTCTCTCTCTCTCTCTCTCTCTCTCT	GAGGTCCTTGATGTGCGAAT	AATTGGAGGCAACATTGAGG	60.1	812	INTRON	Ca_07866	S	--
Ca4_P1_24983663	Ca4	24983663	TTATATATATATATATATATATAT/TTATATATATATATATATATATATATATATAT	TTTTAGTGGGCAAGTGCAAA	GCTTTTGCATGCCATTTCTA	59.3	635	INTRON	Ca_20841	--	FAR1
Ca4_P1_25036918	Ca4	25036918	TAAAAAAAAA/TAAAAAAAAAAAAAAAA	ATCCAAGTTGAGCAAGCATT	CATTGAGACTTTGCCACGAA	57.4	605	INTERGENIC	--	--	--
Ca4_P1_25070539	Ca4	25070539	TTTTATTTATTTATTTATTTATTTATTT/TTTTATTTATTTATTTATTTATTT	TTTGCAAAAACCAATGAATACTT	GTTTTGAGCCCATTCACGAT	57.8	596	INTERGENIC	--	--	--

INDEL marker IDs	Chromosomes/ unanchored scaffolds	Physical positions (bp)	InDels (*Kabuli* reference genome-CDC Frontier/PI)	Forward primers (5'-3')	Reverse primers (5'-3')	Anneling temperature (0C)	Expected amplified product size (bp)	Structural annotation		Functional annotation	
								Sequence components of *kabuli* genome	*Kabuli* gene accession IDs	NCBI-KOG	TFs
Ca4_P1_25071336	Ca4	25071336	CA/C	TGAGACTGCTTGGCTACCCT	ATCTGAATCATCCCAAGCCA	60.0	488	INTERGENIC	--	--	--
Ca4_P1_25077515	Ca4	25077515	AATATATATATATATATAT/AATATATATATATAT	GTCAATTGAGTTGCGACGAA	TTGTTATGGCTGGGGCTAAG	59.8	308	INTERGENIC	--	--	--
Ca4_P1_25088043	Ca4	25088043	CCT/CCTCCTTCTTCT	AACCTTCGCAAGAACCCTTC	ATCAATCTCGTTTTCCTGGG	60.6	219	INTERGENIC	--	--	--
Ca4_P1_25097629	Ca4	25097629	CAAAAAA/CAAAAAAA	TCAATGATGGTGACTTGGGA	TACCCATTTGGTCTTGGGTC	59.9	820	INTERGENIC	--	--	--
Ca4_P1_25101214	Ca4	25101214	ATTTTTTTTTT/ATTTTTTTTT	TGTGTGTACGTGTGATTTGGC	ACAAGCAAGTGTGGCAAAAA	60.5	368	INTERGENIC	--	--	--
Ca4_P1_25109282	Ca4	25109282	ATT/ATTT	ATGCATCACAAATATGCACTCT	TGGTTTATTGAATTTGAAAAGAATGA	57.3	953	INTERGENIC	--	--	--
Ca4_P1_25137117	Ca4	25137117	TAGATGTCAAAAGA/TAGATGTCAAAAGATGTCAAAAGA	GCATTGTGGAACAAGGAACC	TAAGGCTGCCTAGTCCTTGC	60.4	114	INTERGENIC	--	--	--
Ca4_P1_25152424	Ca4	25152424	ATTTTTTTTTTTT/ATTTTTTTTTTT	GGTTTGGATGATTTTGGTGG	TGGCAACACATGAACCTGAT	60.0	361	INTRON	Ca_16561	Q	B3
Ca4_P1_25154078	Ca4	25154078	TA/T	AGAAAACAGTGGCTGCTCGT	GGATCTGTCCATGTGCTGTG	60.1	612	DRR	Ca_16561	Q	B3
Ca4_P1_25177814	Ca4	25177814	CAGAGAGA/CAGAGA	TCCTTGATCGGTTAAGTCGG	TAAAGGACCAAGTCGGGAAA	60.1	373	INTERGENIC	--	--	--
Ca4_P1_25186812	Ca4	25186812	TAAAAAAAAA/TAAAAAAAA	CCACAGGGTTAAGTTTCCGA	GCCAAACTCTCCTCCCTACC	60.0	657	INTRON	Ca_16562	--	--
Ca4_P1_25190584	Ca4	25190584	ATTTTTTTTTT/ATTTTTTTTTTTTT	TCACCTCCTTTGGTTCCACT	CAGTTTACTCAGGTGGCGGT	59.5	285	INTERGENIC	--	--	--

INDEL marker IDs	Chromosomes/ unanchored scaffolds	Physical positions (bp)	InDels (*Kabuli* reference genome-CDC Frontier/PI)	Forward primers (5'-3')	Reverse primers (5'-3')	Anneling temperature (0C)	Expected amplified product size (bp)	Structural annotation		Functional annotation	
								Sequence components of *kabuli* genome	*Kabuli* gene accession IDs	NCBI-KOG	TFs
Ca4_P1_25205443	Ca4	25205443	TAGATTTGGCACAAAAGATTTGG/TAGATTTGG	GATGGAGGATCAGCCACATT	ATGGTGGAATGCCTCAAAAG	59.9	648	INTERGENIC	--	--	--
Ca4_P1_25213751	Ca4	25213751	GAG/GAGATCATAG	TCATTCAACGTCATCGTATCAA	TGTGTTTGTATCAATATCGAAGGC	59.0	562	INTERGENIC	--	--	--
Ca4_P1_25228841	Ca4	25228841	AATATATATATATAT/AATATATATATATATAT	ATGATTACCTCGGCATGGAG	AGCACGAGATCAAAGCACAA	59.9	349	INTRON	Ca_16565	--	--
Ca4_P1_25235619	Ca4	25235619	G/GA	CAAAGGAAAACAATGGGGAG	TACTCTTGCTTTTGGCACCC	59.4	530	INTERGENIC	--	--	--
Ca4_P1_25237777	Ca4	25237777	ATTTTTTTTT/ATTTTTTTTTT	TGTCTCTCCGACCAAACCAT	GCAAAATTTTAAACTGGGCG	60.5	530	INTERGENIC	--	--	--
Ca4_P1_25237972	Ca4	25237972	ACC/AC	ATGAACAAACACGCCCAGTT	GGGTCGAACCCTATTTGTCA	60.4	567	INTERGENIC	--	--	--
Ca4_P1_25239132	Ca4	25239132	TTGG/TTGGATGG	TGCATCATGAGCTAGCCAAC	GGTGCTGGTCTTGGATTTGT	60.0	567	INTERGENIC	--	--	--
Ca4_P1_25275335	Ca4	25275335	T/TTCCAAA	TCTGCACCAATTGCCATAAA	TCTTCAAGGTGGGTGAATCC	60.1	577	INTERGENIC	--	--	--
Ca4_P1_25287465	Ca4	25287465	CAAAAAAAAA/CAAAAAAA	CTAGCGCCACTCCTGTAACC	TCCGATGGACACAAGTCTCA	59.9	330	INTERGENIC	--	--	--
Ca4_P1_25287739	Ca4	25287739	TC/T	CTAGCGCCACTCCTGTAACC	AAGCATGTAACTTCGCAAAATG	59.9	843	INTERGENIC	--	--	--
Ca4_P1_25290328	Ca4	25290328	GTTTTTTTT/GTTTTTTT	TCACACCGTGAGATTTTCCA	GAGGCTCCAGATCGAAGTTG	60.1	629	INTERGENIC	--	--	--
Ca4_P1_25291746	Ca4	25291746	CTT/CTTT	GCATGGGTGGGTGTCTAATC	TTGTGGAGCAAACATGTGGT	60.2	432	INTERGENIC	--	--	--

INDEL marker IDs	Chromosomes/ unanchored scaffolds	Physical positions (bp)	InDels (*Kabuli* reference genome-CDC Frontier/PI)	Forward primers (5'-3')	Reverse primers (5'-3')	Anneling temperature (0C)	Expected amplified product size (bp)	Structural annotation		Functional annotation	
								Sequence components of *kabuli* genome	*Kabuli* gene accession IDs	NCBI-KOG	TFs
Ca4_P1_25292705	Ca4	25292705	GTTAATTAA/GTTAA	GGCAGAAATTTGAACGTGGT	GGACAAAAGAAGAGCAAAAAGG	60.0	227	INTERGENIC	--	--	--
Ca4_P1_25301998	Ca4	25301998	TTTC/TTTCTTC	TTTTTCAATCTCCAATGGGC	AAAAATGTTGGGGCAAGATG	59.9	377	DRR	Ca_16570	--	Dof
Ca4_P1_25303619	Ca4	25303619	GTTTTT/GTTTTTT	TTGAGGCAGGAACCTCAACT	CATTTCCTTAAGCCCCATGA	59.8	601	INTERGENIC	--	--	--
Ca4_P1_25340691	Ca4	25340691	GAA/GA	GGTTGTAGATGGGTGGTTGG	TGACCACCACGATCATCATT	60.1	202	INTERGENIC	--	--	--
Ca4_P1_25347533	Ca4	25347533	GTTTTTTTTT/GTTTTTTTTTT	CTGTGGAAAAGGCACCTGAT	CGCCCTTTGCAAATAGTAAAA	60.1	354	INTERGENIC	--	--	--
Ca4_P1_25348751	Ca4	25348751	TCCCC/TCCC	GGCAAATGCTAACCCATGTT	TTGCTTACAATCCGAGACGA	59.8	224	INTERGENIC	--	--	--
Ca4_P1_25348827	Ca4	25348827	AGGGGGG/AGGGGG	GGCAAATGCTAACCCATGTT	TTGCTTACAATCCGAGACGA	59.8	224	INTERGENIC	--	--	--
Ca4_P1_25349261	Ca4	25349261	TCCC/TCC	TTCGTCTCGGATTGTAAGCA	CGTGTCAAACCAAAAGAGCA	59.4	716	INTERGENIC	--	--	--
Ca4_P1_25352179	Ca4	25352179	CCTCTC/CCTC	CACTGCTATAAGCCGCACAA	TGAATCAACGAGCTATCGAATG	60.0	382	INTERGENIC	--	--	--
Ca4_P1_25352495	Ca4	25352495	GGTT/G	GGAAATGGTTCTTTGAAAGCC	AGCCTTACGCATGTATTGCC	59.9	719	INTERGENIC	--	--	--
Ca4_P1_25352610	Ca4	25352610	T/TA	GGAAATGGTTCTTTGAAAGCC	AGCCTTACGCATGTATTGCC	59.9	719	INTERGENIC	--	--	--
Ca4_P1_25353133	Ca4	25353133	GAAAAAAAA/GAAAAAAA	CGTCGCACTAAGACGATGAA	GGGTAAGGGTACGTTGAGTCC	60.0	415	INTERGENIC	--	--	--

INDEL marker IDs	Chromosomes/ unanchored scaffolds	Physical positions (bp)	InDels (*Kabuli* reference genome-CDC Frontier/PI)	Forward primers (5'-3')	Reverse primers (5'-3')	Anneling temperature (0C)	Expected amplified product size (bp)	Structural annotation		Functional annotation	
								Sequence components of *kabuli* genome	*Kabuli* gene accession IDs	NCBI-KOG	TFs
Ca4_P1_25353195	Ca4	25353195	GT/GTT	CGTCGCACTAAGACGATGAA	GGGTAAGGGTACGTTGAGTCC	60.0	415	INTERGENIC	--	--	--
Ca4_P1_25353812	Ca4	25353812	A/AG	AAGTCCATAACGGTGTTTCCC	TTGCCCAAGAGGTCTCAAGT	60.1	404	INTERGENIC	--	--	--
Ca4_P1_25358015	Ca4	25358015	AG/AGATG	AAAATTCTGTTTGGGGGACC	ATTACACGCTTGCAGACGAA	60.0	886	INTERGENIC	--	--	--
Ca4_P1_25358106	Ca4	25358106	TTAAATAAATAAATAAATAAATAA/TTAAATAAATAAATAAATAA	GGGACTACTTTCGCGATTCA	ATTACACGCTTGCAGACGAA	60.2	842	INTERGENIC	--	--	--
Ca4_P1_25358567	Ca4	25358567	ATTTTTTTTT/ATTTTTTTT	TTTCGTCTGCAAGCGTGTAA	TGTTGTACGCCAAAATTTCA	60.6	396	INTERGENIC	--	--	--
Ca4_P1_25365917	Ca4	25365917	ATTTT/ATT	AATTTTGTTGCCCCTCCAAT	TGCTATGGCACAAGTCTCCA	60.5	547	INTERGENIC	--	--	--
Ca4_P1_25367189	Ca4	25367189	A/AT	TTTATTGTAGGTGGAGGCGG	GGCCAAATTGATGCAAAGTT	60.0	271	INTERGENIC	--	--	--
Ca4_P1_25369632	Ca4	25369632	TA/T	CTGGTGTGAGGTTGCGTTTA	CTCAGGTGCCTTTTCCACAT	59.8	474	INTERGENIC	--	--	--
Ca4_P1_25370682	Ca4	25370682	CAAA/CAAAAA	TTTTGCAACGACGACATTCT	CCCCACTTTGCACAAAGACT	59.3	766	INTERGENIC	--	--	--
Ca4_P1_25370722	Ca4	25370722	ATATTATTATTA/ATATTATTATTATTA	TTTTGCAACGACGACATTCT	CCCCACTTTGCACAAAGACT	59.3	766	INTERGENIC	--	--	--
Ca4_P1_25392312	Ca4	25392312	AGGG/AGG	TGACAAGCTCGATTGGACAC	ATAGCTTATTCCCGCCCAGT	59.8	892	INTERGENIC	--	--	--
Ca4_P1_25393702	Ca4	25393702	TACACA/TACACACA	TAAAAATGAGCCCTTGCACC	TTCCAGCACCAGAGGATAGC	60.1	679	INTERGENIC	--	--	--

INDEL marker IDs	Chromosomes/ unanchored scaffolds	Physical positions (bp)	InDels (*Kabuli* reference genome-CDC Frontier/PI)	Forward primers (5'-3')	Reverse primers (5'-3')	Anneling temperature (0C)	Expected amplified product size (bp)	Structural annotation		Functional annotation	
								Sequence components of *kabuli* genome	*Kabuli* gene accession IDs	NCBI-KOG	TFs
Ca4_P1_25434230	Ca4	25434230	AACACA/AACA	GATTATCCTCCTGCGGTCAA	TCCGCTCCTTACCATCACTC	60.0	797	DRR	Ca_16576	O	MIKC
Ca4_P1_25434376	Ca4	25434376	TAAAAAAAAAAA/TAAAAAAAA	TTTGTCCACCAAGAAAAGGC	TCCGCTCCTTACCATCACTC	60.1	732	DRR	Ca_16576	O	MIKC
Ca4_P1_25447723	Ca4	25447723	GAAAAAAAAA/GAAAAAAAA	GATACGTGGAAGCGCAATTT	TCACGCATCTGACTTTCGAC	60.1	535	INTERGENIC	--	--	--
Ca4_P1_25456544	Ca4	25456544	GA/G	TTTGCTTTCAAAACCGAAATC	TCGATGATAAAAATGATCGAGTG	59.2	723	INTERGENIC	--	--	--
Ca4_P1_25457334	Ca4	25457334	ATTTTTTT/ATTTTTTTT	CATTTTACTCATCGATCTTTGTGG	TGGACAAAATCATGTCTCTTTGA	59.9	336	DRR	Ca_16577	--	--
Ca4_P1_25457701	Ca4	25457701	AATATATATATATATATATATATATATA/AATATATATATATATATATATATATATATATA	TCAAAGAGACATGATTTTGTCCA	TCACTTTGCTTTGTGTGTCTCA	59.6	524	DRR	Ca_16577	--	--
Ca4_P1_25466972	Ca4	25466972	TAAAAAAA/TAAAAAAAA	TATAGGGAGCGGAACACACC	TCTAACTCCAGGTAACGCCG	60.0	840	INTERGENIC	--	--	--
Ca4_P1_25467705	Ca4	25467705	ATTTTTTTT/ATTTTTTT	CGGCGTTACCTGGAGTTAGA	TTTTCCATCACATTTGTATTTTGAA	60.3	436	INTERGENIC	--	--	--
Ca4_P1_25468253	Ca4	25468253	ATTTTTTT/ATTTTTTTT	CAAAATACAAATGTGATGGAAAAA	TTTTTGTTTCCTTATTGTTTGACA	58.0	648	INTERGENIC	--	--	--
Ca4_P1_25470351	Ca4	25470351	CTTTTTTTTT/CTTTTTTTTTT	TGCAATTATCCAAAGATGTATGAAA	TCCTTTTGTCCGTCGTCTCT	59.8	479	INTERGENIC	--	--	--
Ca4_P1_25472660	Ca4	25472660	CAAAAAAAAAAA/CAAAAAAAAAA	TGCACATAGCATGTCACTTTCA	GTTTGCCAATACATCGTCCC	60.3	619	INTERGENIC	--	--	--
Ca4_P1_25478172	Ca4	25478172	TAAAA/TAAAAA	TGTGATGCAGTTGCTCCATT	GACCTTGCTCTCTTGATGGG	60.3	703	INTERGENIC	--	--	--

INDEL marker IDs	Chromosomes/ unanchored scaffolds	Physical positions (bp)	InDels (*Kabuli* reference genome-CDC Frontier/PI)	Forward primers (5'-3')	Reverse primers (5'-3')	Anneling temperature (0C)	Expected amplified product size (bp)	Structural annotation		Functional annotation	
								Sequence components of *kabuli* genome	*Kabuli* gene accession IDs	NCBI-KOG	TFs
Ca4_P1_25501013	Ca4	25501013	TAAAAAAAA/TAAAAAAAAA	GGGCATTGGTAACTCCAAAA	CCAATTGTGCTCTCCTCTCC	59.8	909	INTERGENIC	--	--	--
Ca4_P1_2550484	Ca4	2550484	CT/CTGGAATTAGAATGTGTGTT	ATTGTTTGTCCAGCGATTCC	TCTGCAATGACCCTCTTGTG	59.9	433	INTERGENIC	--	--	--
Ca4_P1_25512047	Ca4	25512047	CTTTATT/CTTTATTTTATT	GCCACTGAATTGTCGGAGAT	ACCCCCAATGAATGAAAACA	60.1	324	INTRON	Ca_16579	S	--
Ca4_P1_25519864	Ca4	25519864	GAAAAAAAA/GAAAAAAAAA	GGGTCCTCGATCAAAGAGGT	TGTCAGTTGAGCTCCTGTCG	60.5	417	INTERGENIC	--	--	--
Ca4_P1_25523236	Ca4	25523236	TAAAA/TAAA	TGTGGTGGTGATCCTTGAAA	GATTCTCGCATCTGCTAGGC	59.9	786	INTERGENIC	--	--	--
Ca4_P1_25527044	Ca4	25527044	TAAAAAAAA/TAAAAAAAAA	ATTGAAGCGGCACCTATTGA	GCAAATTGTAAAAGTAGTCTTTGAGC	60.6	706	DRR	Ca_16580	--	--
Ca4_P1_25527454	Ca4	25527454	TAAAAAAAAAAAAAA/TAAAAAAAAAAAAAAAAAA	TCATGTGTAATTTTTATCTCAAGGAA	GGCTTGATTGATTCACATCCT	58.3	743	DRR	Ca_16580	--	--
Ca4_P1_25527696	Ca4	25527696	TAAAAAAAAAAAA/TAAAAAAAAAAAAA	TTGCAACTTGAATTGAAAACCTT	GACCCTCTAATGAGGGGAGAA	60.0	612	DRR	Ca_16580	--	--
Ca4_P1_25534530	Ca4	25534530	TTAT/TT	ATGATTTGGGACAATGTGGG	GATCGAGGAGCTAGGAGCAA	60.4	503	INTERGENIC	--	--	--
Ca4_P1_25535456	Ca4	25535456	ACACCTTGAGTTTGGTCCA/ACACCTTGAGTTTGGTCCACCTTGAGTTTGGTCCA	TGTGGGATGCATGTCCTTTA	TTGAAATTGAACCAATCCGA	59.9	682	INTERGENIC	--	--	--
Ca4_P1_25537627	Ca4	25537627	TAAAAAAAAAAA/TAAAAAAAAAAAA	AGTGAGCCGATCAAATGGAC	TGAAGCGGGATAAAAGGATG	60.1	647	INTERGENIC	--	--	--
Ca4_P1_25543797	Ca4	25543797	TTTTTTTT/TTTTTTTTTCTTCTTTTTTT	AGGACTTTCGACCACCACAC	TACCCTTTGCTAGCCCCTTT	60.0	415	INTERGENIC	--	--	--

INDEL marker IDs	Chromosomes/ unanchored scaffolds	Physical positions (bp)	InDels (*Kabuli* reference genome-CDC Frontier/PI)	Forward primers (5'-3')	Reverse primers (5'-3')	Anneling temperature (0C)	Expected amplified product size (bp)	Structural annotation		Functional annotation	
								Sequence components of *kabuli* genome	*Kabuli* gene accession IDs	NCBI-KOG	TFs
Ca4_P1_25565357	Ca4	25565357	TAAAAAAAAA/TAAAAAAAA	GGCGGCAACGGTAATACTAA	GCCACAATTGCATGTTTCAC	60.0	345	INTERGENIC	--	--	--
Ca4_P1_25579370	Ca4	25579370	TAAAAAAAAAA/TAAAAAAAAA	AAATCGGCTCCTTTTAAAACAATA	TCTTCTTCGACGTTTTTGGC	59.5	699	INTERGENIC	--	--	--
Ca4_P1_25596032	Ca4	25596032	TTAT/TTATAT	AGTGATGTGGGCAACAATGA	TGCATTTTACAGCGCTTTTG	60.0	515	INTERGENIC	--	--	--
Ca4_P1_25612150	Ca4	25612150	ATTTTTTTTT/ATTTTTTTT	TGAGTACGTGCTCGATTTGC	AAACTGCGGCATAATAACCG	60.0	672	INTERGENIC	--	--	--
Ca4_P1_25623331	Ca4	25623331	CATTATTATTATTATTATTATTATTATTATTATTATTATTATTATTATTATTATTATTAT/CATTATTATTATTATTATTATTATTATTATTATTATTATTATTATTATTATTATTATTATTAT	CACCGGAACCAAGCTTTTTA	GAGGAGGTTTGTTGGATGGA	60.1	376	INTERGENIC	--	--	--
Ca4_P1_25630011	Ca4	25630011	AT/A	AGTAGACTTGCCTCCCGGTT	CCTTGTTGTGGAGTGTGGTG	60.1	788	INTERGENIC	--	--	--
Ca4_P1_25651231	Ca4	25651231	CTTTTTTTTTT/CTTTTTTTTTTT	CGTTTGTCTGTGAAATGGGA	CAAGCGGTAAGCACTAGGGA	59.5	825	INTRON	Ca_16590	G	--
Ca4_P1_25711562	Ca4	25711562	GTTTATTTTATTTTATTTTATTT/GTTTATTTTATTTTATTT	CTAATCCCCAACACGTGACC	ACTCATTTCATGTTTCCGCC	60.2	274	INTERGENIC	--	--	--
Ca4_P1_25720958	Ca4	25720958	AAAGAAAGAGATA/AA	ACCACGCCATTCAAAATAGG	AGGACTTGACGTAACCCACG	59.8	738	INTERGENIC	--	--	--
Ca4_P1_25725898	Ca4	25725898	ATTTTTTTTT/ATTTTTTTTTT	ACTCTATGGTTCCGTGCGAT	GGTGCAGGGTAGAATCCTCA	59.6	312	INTERGENIC	--	--	--
Ca4_P1_25739354	Ca4	25739354	TCCACCACCACCACCACCACC/TCCACCACCACCACCACCACCACC	TATCATGTTTTTGACGCCGA	TTGAATCATGCTAGGAGGGC	60.1	457	DRR	Ca_16595	--	--
Ca4_P1_25758155	Ca4	25758155	CAAGTAAGT/CAAGT	TTGGTCCTCGTCATTTCCTC	TCCAAACTTCCATGTGATTCC	60.1	422	INTERGENIC	--	--	--

INDEL marker IDs	Chromosomes/ unanchored scaffolds	Physical positions (bp)	InDels (*Kabuli* reference genome-CDC Frontier/PI)	Forward primers (5'-3')	Reverse primers (5'-3')	Anneling temperature (0C)	Expected amplified product size (bp)	Structural annotation		Functional annotation	
								Sequence components of *kabuli* genome	*Kabuli* gene accession IDs	NCBI-KOG	TFs
Ca4_P1_25758636	Ca4	25758636	CTTTT/CTTTTTT	TGCACTTCAGTTAAATTGGGC	AGAACTTGCGTGGTGATTCC	60.1	530	INTERGENIC	--	--	--
Ca4_P1_25768326	Ca4	25768326	TAAAAAA/TAAAAAAA	ATGTTTCGTGCAATGGTCAA	CACATGGCTGAACGAGAAGA	60.0	382	INTERGENIC	--	--	--
Ca4_P1_25771593	Ca4	25771593	CTTTTTTTT/CTTTTTTT	GTGCAACAACTTGAAGCAGG	ATCTGAGGGAGGGACCAAAC	59.5	176	INTERGENIC	--	--	--
Ca4_P1_25774052	Ca4	25774052	TAAAAAAAAAAAA/TAAAAAAAAAAA	TGTCAAATCCAACGCAAAGA	TCGCAACCAAACAAAGCATA	60.2	661	INTERGENIC	--	--	--
Ca4_P1_25776089	Ca4	25776089	G/GAC	TGGAATGAGAGGGGAAGATG	TCCGAGACAGGTATTTTGGC	60.0	366	INTERGENIC	--	--	--
Ca4_P1_25780080	Ca4	25780080	A/AG	GCAGTGCTGAATTTTGCTGA	TCCCAACTTCCCAATAGTCG	60.1	342	INTERGENIC	--	--	--
Ca4_P1_25783388	Ca4	25783388	CTTTT/CTTTTT	GCGTGATGCATGATCTAGGA	TCTTTTGATTCCGAGTTGGG	59.8	658	INTERGENIC	--	--	--
Ca4_P1_25790471	Ca4	25790471	CAAACGAAACGAAACGAAAC/CAAACGAAAC	CCTCAAATTCCAATTGTGCC	TCATCCTCACCCCCAGTTAG	60.3	646	INTERGENIC	--	--	--
Ca4_P1_25802153	Ca4	25802153	GAAAAAAAA/GAAAAAAA	TGATGAATTTGATGTTGTTGGA	GCACACGTGGTCCCTTAACT	58.9	290	INTERGENIC	--	--	--
Ca4_P1_25812288	Ca4	25812288	CT/C	ATGGTTGTTGCAAGTTTCCC	CCTTGAAGACATATGGTGGCA	59.8	522	INTRON	Ca_16599	R	--
Ca4_P1_25819571	Ca4	25819571	TTATATATATATATATATA/TTATATATATATATATATATATATATATA	TACGGGATGCATTTTTGTGA	CATTGCAACTTGGAGCTGAA	59.9	504	DRR	Ca_16599	R	--
Ca4_P1_25822023	Ca4	25822023	ATTTTTTTTTT/ATTTTTTTTT	ACGCAAGAGCAACATCTACG	TGAAGTCTCGGGTTCAAACA	59.1	617	INTERGENIC	--	--	--

INDEL marker IDs	Chromosomes/ unanchored scaffolds	Physical positions (bp)	InDels (*Kabuli* reference genome-CDC Frontier/PI)	Forward primers (5'-3')	Reverse primers (5'-3')	Anneling temperature (0C)	Expected amplified product size (bp)	Structural annotation		Functional annotation	
								Sequence components of *kabuli* genome	*Kabuli* gene accession IDs	NCBI-KOG	TFs
Ca4_P1_25822369	Ca4	25822369	CAAAAAAA/CAAAAAA	TGTTTGAACCCGAGACTTCAC	CGTGGCATTTGAAAAAGCAT	60.1	611	INTERGENIC	--	--	--
Ca4_P1_25832753	Ca4	25832753	ATTTTTTTTTT/ATTTTTTTTTTT	ATTCAGGGGCGGATCTTAGT	CAAGTTTTATTGGGGGACCA	59.9	388	INTERGENIC	--	--	--
Ca4_P1_25834950	Ca4	25834950	AAGACGTTCCGTA/AA	TCGCTGCTTATTCATGTTCA	ACCACCAGTGAGGATTTTGC	58.0	336	INTERGENIC	--	--	--
Ca4_P1_25844453	Ca4	25844453	AATTATTATTATTATTATTATTATTATTAATGTTATTATTATTATTATTATTATTATT/AATTATTATTATTATTATTATTATTATTATTATTATTAATGTTATTATTATTATTATTATTATTATT	CACTTGGGTCACTGCTGAAG	GATGCTGCATGTGTTGATCC	59.5	662	DRR	Ca_16601	R	--
Ca4_P1_25847698	Ca4	25847698	T/TG	CATGCTACCAAAGCGATTGA	ATCGATTTCGCGAGTCTTGT	59.8	370	INTERGENIC	--	--	--
Ca4_P1_25854405	Ca4	25854405	ATATTATTATTATTATTATTATTATTATTATTATTATTATTATTATTA/ATATTATTATTATTATTATTATTATTATTATTATTATTATTATTATTATTATTATTATTA	GGGAAGTGAAGAACCACCAA	TGGGGACTAAACAGCAACTCA	59.9	927	DRR	Ca_16602	R	--
Ca4_P1_25854498	Ca4	25854498	T/TA	TGTTTACCCGGACATTTCTGT	TGGGGACTAAACAGCAACTCA	59.3	856	DRR	Ca_16602	R	--
Ca4_P1_25861719	Ca4	25861719	C/CAAATGTCTAGT	TCAAAAGGTCCACCCTCACT	TTTGGCCTCCAATAATCTCG	59.5	380	INTERGENIC	--	--	--
Ca4_P1_25863826	Ca4	25863826	GTTTTTTT/GTTTTTTTTTT	CATGGAGATTGAAGTCATCAAAA	GTTGGTTGTTGGAGTGATGC	59.1	481	INTERGENIC	--	--	--
Ca4_P1_25867798	Ca4	25867798	TG/T	TGCATTTCCCTCTTAATGCAA	TTTGGGTGCGTGGTAAAACT	60.6	397	INTERGENIC	--	--	--
Ca4_P1_25884525	Ca4	25884525	TA/T	AGCAACCAAATCGAAACACC	GGGAAATTTTGGAACCAATG	60.0	307	INTERGENIC	--	--	--
Ca4_P1_25890171	Ca4	25890171	AT/ATT	ACTGGTCCGTCTTGGTCAAC	CCACCCAACAAATACTTGCAT	60.0	872	INTERGENIC	--	--	--

INDEL marker IDs	Chromosomes/ unanchored scaffolds	Physical positions (bp)	InDels (*Kabuli* reference genome-CDC Frontier/PI)	Forward primers (5'-3')	Reverse primers (5'-3')	Anneling temperature (0C)	Expected amplified product size (bp)	Structural annotation		Functional annotation	
								Sequence components of *kabuli* genome	*Kabuli* gene accession IDs	NCBI-KOG	TFs
Ca4_P1_2589224	Ca4	2589224	CAGTTGCAAGTTGCAAG/CAGTTGCAAGTTGCAAGTTGCAAG	AAGATCGATCAGTTCCACCG	ATTGGGAACCAACTGACGAG	60.1	396	INTERGENIC	--	--	--
Ca4_P1_25897872	Ca4	25897872	GGTGTACCGTGTA/GGTGTA	AGTGCCCAACTTTCACCTTC	TCACGATGCACCCAAGAATA	59.2	493	INTERGENIC	--	--	--
Ca4_P1_25916811	Ca4	25916811	CTTTTTTTTTTT/CTTTTTTTTTTTTT	ATGGTAACAGATATCGCCGC	ATTGTAGCATTGCCCAAAGG	60.0	627	DRR	Ca_16606	I	--
Ca4_P1_25924178	Ca4	25924178	ATTTTTT/ATTTTTTT	TGATGATGTCATTGATGTGTTTG	CTTGTAAGTTGTAGGCGGGC	59.3	778	INTERGENIC	--	--	--
Ca4_P1_25925243	Ca4	25925243	GAAAAAAAAAAA/GAAAAAAAAAA	TTTCGCACAGCATAGGACAG	CAACGGCGCTGAGATTTATT	60.0	661	INTERGENIC	--	--	--
Ca4_P1_25927650	Ca4	25927650	AGG/AGGG	ATCACAACTGGGAACCCAAA	AAGCTCCAAGACCAGATGGA	60.2	470	INTERGENIC	--	--	--
Ca4_P1_25936338	Ca4	25936338	GTTTTTTTTTT/GTTTTTTTTT	GAGGTTTCGATTCCTCCACA	AAATTGCAATAGGTCGTCCG	60.1	705	INTERGENIC	--	--	--
Ca4_P1_25937535	Ca4	25937535	GC/GCTTTTGCTC	TCGACGTTGTCCATTTGTGT	ACACGAGCGTTTGAGCTTTT	60.0	621	INTERGENIC	--	--	--
Ca4_P1_25941878	Ca4	25941878	CAA/CAAAA	CGATCGTTGTTGGTATGTCG	CCGTTTTTCTTGCGATTCTC	60.0	899	INTERGENIC	--	--	--
Ca4_P1_25943779	Ca4	25943779	CAAAAA/CAAAA	CCTGGGGTATTCTCGACAGA	TGAAAACCCTTTCAAGGTGC	60.1	474	INTERGENIC	--	--	--
Ca4_P1_25945228	Ca4	25945228	CTTTTT/CTTTTTT	ATTCGCGCAAGGATAAGATG	AACGGAGAAAAACGGTGTTG	60.2	824	INTERGENIC	--	--	--
Ca4_P1_25947033	Ca4	25947033	CAAAAAAAAAAAAAAAA/CAAAAAAAAA	CACTAGAAGCGCGTGTCAGA	TTGTGAACGCCAATGGTAAA	60.3	924	INTERGENIC	--	--	--

INDEL marker IDs	Chromosomes/ unanchored scaffolds	Physical positions (bp)	InDels (*Kabuli* reference genome-CDC Frontier/PI)	Forward primers (5'-3')	Reverse primers (5'-3')	Anneling temperature (0C)	Expected amplified product size (bp)	Structural annotation		Functional annotation	
								Sequence components of *kabuli* genome	*Kabuli* gene accession IDs	NCBI-KOG	TFs
Ca4_P1_25954241	Ca4	25954241	A/ATTC	TTGCAAGAATCCATCCATCA	TTGAGCATTTCAGCAGAAGC	60.0	493	INTERGENIC	--	--	--
Ca4_P1_25956176	Ca4	25956176	ATTTTTTTTTTTTT/ATTTTTTTTTT	ATATTGGGACCGGTTAAGGG	TTTGGGTGTGTTGACTGCAT	59.9	784	INTERGENIC	--	--	--
Ca4_P1_25957806	Ca4	25957806	A/AGT	CCTGGCTTACGTCAATTCATC	TCTTTCGTTTGGGTGGAAGT	59.6	269	INTERGENIC	--	--	--
Ca4_P1_25958010	Ca4	25958010	TTATATATATAT/TTATATATATATAT	TCGGGGAAATCAATTCGTTA	TGGAAGTCATCGACCCATTT	60.3	673	INTERGENIC	--	--	--
Ca4_P1_25958150	Ca4	25958150	TTAAG/TTAAGTAAG	TCGGGGAAATCAATTCGTTA	TGGAAGTCATCGACCCATTT	60.3	673	INTERGENIC	--	--	--
Ca4_P1_25958849	Ca4	25958849	AACACA/AACA	CAAATGGGTCGATGACTTCC	TTGCCCTCTTCTTCATCTTCA	60.3	473	INTERGENIC	--	--	--
Ca4_P1_25959897	Ca4	25959897	AAACAA/AAA	CGTAATTTTGGCCCCCTTAT	CTGATTCGTAATTTTGGCCG	60.0	625	INTERGENIC	--	--	--
Ca4_P1_25962811	Ca4	25962811	GTATATATAT/GTATATATATAT	TGGTTGGAAGCTCCAGAAGT	GAAAAGGTGAGGAATCGCTG	59.8	275	DRR	Ca_16607	--	B3
Ca4_P1_25962938	Ca4	25962938	CTT/CT	TGGTTGGAAGCTCCAGAAGT	GTGGTAAGTTGTGTGGCCCT	59.8	577	DRR	Ca_16607	--	B3
Ca4_P1_25963240	Ca4	25963240	GAAAAAAA/GAAAAAAAA	AATCCAAGCACCAAAAGCAC	GTGGTAAGTTGTGTGGCCCT	60.1	353	DRR	Ca_16607	--	B3
Ca4_P1_25963279	Ca4	25963279	TTTTATTT/TTTT	AATCCAAGCACCAAAAGCAC	GTGGTAAGTTGTGTGGCCCT	60.1	353	DRR	Ca_16607	--	B3
Ca4_P1_25963788	Ca4	25963788	TAAA/TAAAA	GCCACACAACTTACCACCCT	TCAAGGCCGAAGAAGAAAAA	59.9	855	DRR	Ca_16607	--	B3

INDEL marker IDs	Chromosomes/ unanchored scaffolds	Physical positions (bp)	InDels (*Kabuli* reference genome-CDC Frontier/PI)	Forward primers (5'-3')	Reverse primers (5'-3')	Anneling temperature (0C)	Expected amplified product size (bp)	Structural annotation		Functional annotation	
								Sequence components of *kabuli* genome	*Kabuli* gene accession IDs	NCBI-KOG	TFs
Ca4_P1_25965378	Ca4	25965378	ACCCCCC/ACCCCC	GGTGTGCAAGAGGACTGGAT	GGTCCAAGAATATGCCCAAA	60.1	481	INTERGENIC	--	--	--
Ca4_P1_25966069	Ca4	25966069	ATTT/ATTTT	GGCATGGACAACTTCACCTT	CGAAATCCTCAAAATCGGAA	60.0	484	INTERGENIC	--	--	--
Ca4_P1_25967972	Ca4	25967972	ATTTTTTTT/ATTTTTTTTT	TTGTACCCTTGTGCAACCAA	TGGTTGGATACTTGCAATGG	60.0	180	INTERGENIC	--	--	--
Ca4_P1_25968820	Ca4	25968820	GTTT/GTTTT	TTCTTCTGCCCAAAAATGCT	CCTCAAGCAGACCTTTGGAG	59.8	412	INTERGENIC	--	--	--
Ca4_P1_25969511	Ca4	25969511	ACC/AC	TCCCTTGCCTCTAGCCAGTA	GCAGATGTTCTCAACAGCCA	60.0	350	INTERGENIC	--	--	--
Ca4_P1_25970159	Ca4	25970159	AC/A	TGGTTGGGTCTTGGTTTGTT	CACCTGCTATGGATGCAATG	60.2	709	INTERGENIC	--	--	--
Ca4_P1_25971096	Ca4	25971096	TAAAGTTCGAA/TAA	GGTTCACCCAAAGATGGCTA	ACGATCATCAAAGGGATTGG	59.9	374	INTERGENIC	--	--	--
Ca4_P1_25971278	Ca4	25971278	GATTGAAGAA/GA	GCTTCGCCAAGCTTACAATC	CCAACGATCATCAAAGGGAT	60.0	268	INTERGENIC	--	--	--
Ca4_P1_25975114	Ca4	25975114	TGGGG/TGGGGG	ACACATGTTGGCCAATGAGA	GAGTCCGATTTCCATGAGGA	60.0	621	CDS (FRAME SHIFT)	Ca_16608	--	--
Ca4_P1_25975332	Ca4	25975332	GTTTTTT/GTTTTTTT	GCCAATGAGAAAAGAGACGC	GAGTCCGATTTCCATGAGGA	60.0	611	CDS (FRAME SHIFT)	Ca_16608	--	--
Ca4_P1_25978445	Ca4	25978445	CT/CTTT	TGCCTCAAAGGCTTCATCTT	AGCACTCATCCTCGGGTAGA	60.0	435	INTERGENIC	--	--	--
Ca4_P1_25979689	Ca4	25979689	TG/TGG	GTCCTCGTCAGGAGGTTGAG	CCACTGTTGCTGGCAGATAA	59.8	707	INTERGENIC	--	--	--

INDEL marker IDs	Chromosomes/ unanchored scaffolds	Physical positions (bp)	InDels (*Kabuli* reference genome-CDC Frontier/PI)	Forward primers (5'-3')	Reverse primers (5'-3')	Anneling temperature (0C)	Expected amplified product size (bp)	Structural annotation		Functional annotation	
								Sequence components of *kabuli* genome	*Kabuli* gene accession IDs	NCBI-KOG	TFs
Ca4_P1_25981495	Ca4	25981495	GCT/G	CCTTTGATGACCGTTGGAAT	GGTCCAGCAAAGTTGAAAGC	59.8	464	INTERGENIC	--	--	--
Ca4_P1_25983794	Ca4	25983794	A/AC	TGACAACCCCAGATTGTTGA	TGGGTCCATGTTCCAATTTT	59.9	671	INTERGENIC	--	--	--
Ca4_P1_25984075	Ca4	25984075	TA/TAA	CGATTTTTAACAGCGTGGGT	TGGGTCCATGTTCCAATTTT	60.0	276	INTERGENIC	--	--	--
Ca4_P1_25984201	Ca4	25984201	AG/A	CGATTTTTAACAGCGTGGGT	TTTGGGCTTTGCTCTCAAAT	60.0	539	INTERGENIC	--	--	--
Ca4_P1_25984442	Ca4	25984442	TGAGAG/TGAGAGAG	AGATCCTGTTGACACGGCTT	CGGTTGTTGTTGTGATTTGC	59.7	524	INTERGENIC	--	--	--
Ca4_P1_25985391	Ca4	25985391	TCAC/TCACGATCACCAC	CCAAGGTGCAATCTCTCACA	TAGCGGTGGGGATAACTCTG	59.8	875	INTERGENIC	--	--	--
Ca4_P1_25986950	Ca4	25986950	CATATATATA/CATATATATATATA	TCAGTAGCATCGTAGCGTGG	TGAGCTGCGACGAGAGTAGA	60.0	379	INTERGENIC	--	--	--
Ca4_P1_25987244	Ca4	25987244	AATATATATAT/AATATATATATATATATAT	TCTACTCTCGTCGCAGCTCA	TGCTTAGGCATTTACCCCAG	60.0	250	INTERGENIC	--	--	--
Ca4_P1_25987622	Ca4	25987622	TCC/TC	TTCAACTGGGGTAAATGCCT	TGTTCTAGTCCAGATCAAAACGA	59.4	323	INTERGENIC	--	--	--
Ca4_P1_25988978	Ca4	25988978	AAAGAAGAAGAAG/AAAGAAGAAG	AAGCTGGATGTGGACTTTGC	AGTTCCAATTGGCCTCCTTT	60.3	404	INTERGENIC	--	--	--
Ca4_P1_25989281	Ca4	25989281	TCCCC/TCCC	AAGCTGGATGTGGACTTTGC	AGTTCCAATTGGCCTCCTTT	60.3	404	INTERGENIC	--	--	--
Ca4_P1_25989322	Ca4	25989322	GTTTTTT/GTTTTTTT	GTACGCGATCTAGTCCCCAA	CCGGGGAGAAAGTTCTAGTG	60.1	168	INTERGENIC	--	--	--

INDEL marker IDs	Chromosomes/ unanchored scaffolds	Physical positions (bp)	InDels (*Kabuli* reference genome-CDC Frontier/PI)	Forward primers (5'-3')	Reverse primers (5'-3')	Anneling temperature (0C)	Expected amplified product size (bp)	Structural annotation		Functional annotation	
								Sequence components of *kabuli* genome	*Kabuli* gene accession IDs	NCBI-KOG	TFs
Ca4_P1_25994447	Ca4	25994447	G/GT	ATCCACAACCGAGTTAGCGT	TACCCACACCCCTCAATCTT	59.6	389	INTERGENIC	--	--	--
Ca4_P1_25994543	Ca4	25994543	GTTTTTT/GTTTTTTT	TTAAGATTGAGGGGTGTGGG	CACCACTTCATTCGGGACTT	59.8	516	INTERGENIC	--	--	--
Ca4_P1_25994721	Ca4	25994721	AC/A	TTAAGATTGAGGGGTGTGGG	CACCACTTCATTCGGGACTT	59.8	516	INTERGENIC	--	--	--
Ca4_P1_25995802	Ca4	25995802	ATTTT/ATTT	CCCTAAATCAAAGCAGCGTC	TGCACTGTTGTTGTTGCTGA	59.8	470	INTERGENIC	--	--	--
Ca4_P1_25995977	Ca4	25995977	GTTTTTTTTT/GTTTTTTTTTT	CCCTAAATCAAAGCAGCGTC	TGCACTGTTGTTGTTGCTGA	59.8	470	INTERGENIC	--	--	--
Ca4_P1_25996238	Ca4	25996238	GAAAAAAAAAAA/GAAAAAAAAA	CCCTAAATCAAAGCAGCGTC	AACGTTGGAGGAACAAAACG	59.8	949	INTERGENIC	--	--	--
Ca4_P1_25998410	Ca4	25998410	TTA/TTATGTA	TATTGTTGCCCTGCATGTGT	TGGAGGCCAATTTGTTTCTC	60.0	531	DRR	Ca_16609	--	B3
Ca4_P1_26002044	Ca4	26002044	ATTTTTTT/ATTTTTT	TAGGGAGGATGCATTTTTGG	TGCCTGTGATCCAGGAATTT	59.9	809	INTRON	Ca_16610	C	--
Ca4_P1_26009631	Ca4	26009631	A/AC	GGAACTTCTCTTGTGGCAGC	TATTCCTCTGATTGGGTCGC	60.0	148	INTERGENIC	--	--	--
Ca4_P1_26011159	Ca4	26011159	AT/AACAGAACTCCT	GAAACCCCACAACAAAATGG	AGCATCATGGATTTCTTCCG	60.1	438	DRR	Ca_16611	C	--
Ca4_P1_26014657	Ca4	26014657	TAAAAAA/TAAAAAAA	CGATCGACCAAGGTTTTTGT	AGGGTTGGAATTTCAAGAATC	60.0	385	INTERGENIC	--	--	--
Ca4_P1_26015617	Ca4	26015617	AATAAATATA/AATA	TGCGAGGGCTAGCTTAAAAA	TGAGACAAAAGTTTGCGGTG	60.1	635	INTERGENIC	--	--	--

INDEL marker IDs	Chromosomes/ unanchored scaffolds	Physical positions (bp)	InDels (*Kabuli* reference genome-CDC Frontier/PI)	Forward primers (5'-3')	Reverse primers (5'-3')	Anneling temperature (0C)	Expected amplified product size (bp)	Structural annotation		Functional annotation	
								Sequence components of *kabuli* genome	*Kabuli* gene accession IDs	NCBI-KOG	TFs
Ca4_P1_26018351	Ca4	26018351	TAAAAAAAA/TAAAAAAAAA	AGAACATGGTGGATGCACAG	TCCAAGAGCTTGGTCTGACA	59.6	373	INTERGENIC	--	--	--
Ca4_P1_26020819	Ca4	26020819	GAAAAAAA/GAAAAAAAA	CGTTTGCAATTTCTTTTGGG	TGGCAATATGTCGTTGAGGA	60.5	716	INTERGENIC	--	--	--
Ca4_P1_26030952	Ca4	26030952	A/ACAAAC	CCGTACCGTTTACCAAGGTG	ACCGAATTTACTCACGGTGC	60.3	806	INTERGENIC	--	--	--
Ca4_P1_26032276	Ca4	26032276	TAAAAAAAAAAAA/TAAAAAAAAAAA	CCGAGCCAAAATCCAAAATA	AGAAAATGGACATGCAAGGG	59.9	357	INTERGENIC	--	--	--
Ca4_P1_26051855	Ca4	26051855	CTTTTTTTTTTTTT/CTTTTTTTTTT	TTTAACGGACGTTGTAGGGC	CTTCCACGGGATGTGTTTCT	60.0	448	INTERGENIC	--	--	--
Ca4_P1_26058753	Ca4	26058753	TAAA/TAAAA	AGGAACCAATCCTACCAGCC	TCTGGTCATGCATCCTTTGA	60.3	433	INTERGENIC	--	--	--
Ca4_P1_26065058	Ca4	26065058	TTAATAATAATAATAATAATAATA/TTAATAATAATAATAATAATAATAATAATA	CTCTCTGGAGGATTCATCGC	TTGTTCCGGAAAACTTGAGG	59.9	248	INTERGENIC	--	--	--
Ca4_P1_26074274	Ca4	26074274	CA/C	AGACCGCATTGTGAACTGTG	TTTGCTTGCTGAGTGATTGG	59.8	235	INTERGENIC	--	--	--
Ca4_P1_26074517	Ca4	26074517	GTTTA/GTTTAAATAAAATATTTTA	CCAATCACTCAGCAAGCAAA	CGAAGCATCCTGCATTTAAG	60.0	354	INTERGENIC	--	--	--
Ca4_P1_26075846	Ca4	26075846	TAAAAAA/TAAAAAAA	TTTAACAAAATCAAACAAACAGGA	AAGTGAAAAATATGTGTCCCGTACT	57.9	220	INTERGENIC	--	--	--
Ca4_P1_26076803	Ca4	26076803	TAAAAAAAAAA/TAAAAAAAAA	ACTTGGCCCTTATTTGTCATT	TGTGTCATTTGTTACGTGTTTTTG	57.6	480	INTERGENIC	--	--	--
Ca4_P1_26078815	Ca4	26078815	GTTTTTTTTT/GTTTTTTTTTTTT	TTATTGTGAGTCCGTGCAGC	GCTTTCAGACAAACCCTTGG	59.9	348	INTERGENIC	--	--	--

INDEL marker IDs	Chromosomes/ unanchored scaffolds	Physical positions (bp)	InDels (*Kabuli* reference genome-CDC Frontier/PI)	Forward primers (5'-3')	Reverse primers (5'-3')	Anneling temperature (0C)	Expected amplified product size (bp)	Structural annotation		Functional annotation	
								Sequence components of *kabuli* genome	*Kabuli* gene accession IDs	NCBI-KOG	TFs
Ca4_P1_26091418	Ca4	26091418	CATATATATATAT/CATATATATATATAT	AGGTGCATTTCTGAAGGTGG	GGGGCATTTTGTTGCATATT	60.1	729	INTERGENIC	--	--	--
Ca4_P1_26091461	Ca4	26091461	GAAAAAAAAAAA/GAAAAAAAAAA	AGGTGCATTTCTGAAGGTGG	GGGGCATTTTGTTGCATATT	60.1	729	INTERGENIC	--	--	--
Ca4_P1_2609181	Ca4	2609181	T/TGTGCTATA	GCTTCTGCTAGGCATCCATC	ATGCATGTGCTGATGCTTGT	59.9	907	DRR	Ca_12062	--	--
Ca4_P1_26091969	Ca4	26091969	CTTTTTTTTTTTTT/CTTTTTTTTTT	CACCCAATCAATTGTGATGC	TGATAGCCCCACATGCTACA	59.8	369	INTERGENIC	--	--	--
Ca4_P1_26094743	Ca4	26094743	AATATATATATATATATATATATATATA/AATATATATATATATATATATATATATATATA	AGGATCTTGATAGGGACGGG	ATCTTAAAAGCTCGGAGGGC	60.3	115	INTERGENIC	--	--	--
Ca4_P1_26096805	Ca4	26096805	ATTTTTTTT/ATTTTTTT	AATCACTTGACCACATGCCA	TGTTGGTGATGAACCAAGATAAA	60.0	700	INTERGENIC	--	--	--
Ca4_P1_26108181	Ca4	26108181	CTTTTTTTTTT/CTTTTTTTTTTT	TATGGATCTCTTTGGGCCAG	TGCACTGCTAATTGCCTCTG	60.0	785	INTERGENIC	--	--	--
Ca4_P1_26110333	Ca4	26110333	TCCC/TCCCC	CCAACAATCTCCCCCTTTTT	GCATCTGGTTGCAGTTCTCA	60.2	583	INTERGENIC	--	--	--
Ca4_P1_26118906	Ca4	26118906	CTTTTTTTTTT/CTTTTTTTTT	CTGGATTCCAACAATCTCCC	TTCTTTGCTTTTGCGCTTTT	59.3	851	INTERGENIC	--	--	--
Ca4_P1_26123905	Ca4	26123905	GCACACACACACACAC/GCACACACACACAC	TTGGATTAGCCTGAAATCGG	ATGGCTACACCAATGCATCC	60.0	170	INTERGENIC	--	--	--
Ca4_P1_26135507	Ca4	26135507	CAGCTAG/CAG	TCCACAAATAATGCAACACAAA	ATGCCATATCTCCCTGCTCA	59.0	700	INTERGENIC	--	--	--
Ca4_P1_26169276	Ca4	26169276	ATTTTTTTT/ATTTTTTT	TTTTTGTTTTGCCCTTTTGG	TTAAAGGCGTGAAAAACGGA	60.0	130	INTRON	Ca_16612	--	HD-ZIP

INDEL marker IDs	Chromosomes/ unanchored scaffolds	Physical positions (bp)	InDels (*Kabuli* reference genome-CDC Frontier/PI)	Forward primers (5'-3')	Reverse primers (5'-3')	Anneling temperature (0C)	Expected amplified product size (bp)	Structural annotation		Functional annotation	
								Sequence components of *kabuli* genome	*Kabuli* gene accession IDs	NCBI-KOG	TFs
Ca4_P1_26176783	Ca4	26176783	TAAAAAAAAA/TAAAAAAAA	CACAATCAATCACGCCCTTA	TGTCATTTTTGGCGTAGTGC	59.5	500	INTERGENIC	--	--	--
Ca4_P1_26176935	Ca4	26176935	GAAAAAAA/GAAAAAAAA	CACAATCAATCACGCCCTTA	TGTCATTTTTGGCGTAGTGC	59.5	500	INTERGENIC	--	--	--
Ca4_P1_26199378	Ca4	26199378	GAAAAAAAAA/GAAAAAAAA	TTTTCGGATTTAAAGGGCAA	CAATGCCATTCGACATCATC	59.5	166	INTERGENIC	--	--	--
Ca4_P1_26201717	Ca4	26201717	AATTATTATTATTATTATTATT/AATTATTATTATTATTATTATTATT	TCGCTAAGGAATTAGGGCAA	TCCCTTGGGAATCCATATCA	59.8	738	INTERGENIC	--	--	--
Ca4_P1_26203930	Ca4	26203930	TTATATATATATATATAT/TTATATATATATATATATATATAT	TTGGAAATAAGGACAAAACACC	TCCAAATTTGCTGGAATCCT	58.0	626	INTERGENIC	--	--	--
Ca4_P1_26204274	Ca4	26204274	CAAAAAAA/CAAAAAAAA	GAACATGAGAAGGCAATTTCG	AAGGCGTTTTACCCACAATG	59.7	432	INTERGENIC	--	--	--
Ca4_P1_26230345	Ca4	26230345	GTTTT/GTTT	CCTACATGCTTAGCTTCGCC	GAGTGTATGTCCGCAAGGGT	60.0	676	DRR	Ca_16615	F	--
Ca4_P1_26232772	Ca4	26232772	T/TG	TAGCAGCCATTTCCCAAATC	GAGCAGGACTTTAGGAGCCA	60.0	443	INTERGENIC	--	--	--
Ca4_P1_26236726	Ca4	26236726	TAAAAAAAAAAAA/TAAAAAAAAAA	GTTACGATTGTGTTCGGCCT	TAATCTCACTGCGCACCTTG	60.0	368	INTERGENIC	--	--	--
Ca4_P1_26251759	Ca4	26251759	CTTTTTTTTT/CTTTTTTTTTT	TAAAACGCAATCGGAAGACC	GAGAAACTTTCCGCTGCTTG	60.1	545	INTERGENIC	--	--	--
Ca4_P1_26251857	Ca4	26251857	ATTTTTTTTTTT/ATTTTTTTTTTTTT	AGCTTTCATGTAACACCCCG	GCCCTCAGATGAGAGTCAGG	60.0	453	INTERGENIC	--	--	--
Ca4_P1_26276734	Ca4	26276734	ATAATTAA/ATAA	GGTGCTACAGTGTCGAAGCA	TGCATTTTACAGCGCTTTTG	60.1	725	INTERGENIC	--	--	--

INDEL marker IDs	Chromosomes/ unanchored scaffolds	Physical positions (bp)	InDels (*Kabuli* reference genome-CDC Frontier/PI)	Forward primers (5'-3')	Reverse primers (5'-3')	Anneling temperature (0C)	Expected amplified product size (bp)	Structural annotation		Functional annotation	
								Sequence components of *kabuli* genome	*Kabuli* gene accession IDs	NCBI-KOG	TFs
Ca4_P1_2628635	Ca4	2628635	TTTTATTTATTTATTTATTT/TTTTATTTATTTATTTATTTATTT	TGGATTCGGATCAAGGAAAG	TTTATCCCCCAAAAAGAGGG	60.0	468	INTRON	Ca_12066	A	G2-like
Ca4_P1_26302087	Ca4	26302087	TAAAAAAAAA/TAAAAAAAAAA	AGCTTCCCCCAGTACTGTCA	TGACGCGTCCAAATTTATCA	59.7	494	INTERGENIC	--	--	--
Ca4_P1_26304368	Ca4	26304368	ATTTTTTTTT/ATTTTTTTTTT	CCGATTAACCTTAAACGCCA	TTTAAAGTCCCGACCCATGA	60.0	508	INTERGENIC	--	--	--
Ca4_P1_26304414	Ca4	26304414	GAAAAAAAAA/GAAAAAAAAAA	CCGATTAACCTTAAACGCCA	TTTAAAGTCCCGACCCATGA	60.0	508	INTERGENIC	--	--	--
Ca4_P1_26308021	Ca4	26308021	CTTTTTT/CTTTTT	ATAAAACATCATCGGGCGAC	TTCAGTGGCATTGAGACATTG	59.8	405	DRR	Ca_25307	S	--
Ca4_P1_26313553	Ca4	26313553	TAAAAAAAAAA/TAAAAAAAAAAA	TGAGGTATGCCCAAAGAAGG	TTTTGAAACATGAATTTCGGA	60.1	319	INTERGENIC	--	--	--
Ca4_P1_26326139	Ca4	26326139	GAAAAAAAA/GAAAAAAA	CTCCAACAGCATCAATTCCA	GATCCTGGGATTGTGAGCAT	59.6	724	DRR	Ca_25306	R	--
Ca4_P1_26343731	Ca4	26343731	CG/C	GCTGTGGAAAGTGTGAAGCA	GGCTTTTCATCAACAAAGCA	60.0	224	DRR	Ca_25304	S	--
Ca4_P1_26354768	Ca4	26354768	AGGG/AGGGG	TGCTGGCAGGTTTATGTTTG	TCACTGCCTTCCTTACGAGG	59.7	534	DRR	Ca_25303	I	--
Ca4_P1_26363618	Ca4	26363618	T/TTTAC	GCCAAAATTTCCTTCGTTGA	ATTCCCCAAGAACATCACCA	60.1	613	INTERGENIC	--	--	--
Ca4_P1_26363648	Ca4	26363648	CCTC/CCTCTC	GCCAAAATTTCCTTCGTTGA	ATTCCCCAAGAACATCACCA	60.1	613	INTERGENIC	--	--	--
Ca4_P1_26366710	Ca4	26366710	ACT/A	TCATTGGTCCATCCCATTTT	ACATCAGGCCTGTTATTGCC	60.0	365	INTERGENIC	--	--	--

INDEL marker IDs	Chromosomes/ unanchored scaffolds	Physical positions (bp)	InDels (*Kabuli* reference genome-CDC Frontier/PI)	Forward primers (5'-3')	Reverse primers (5'-3')	Anneling temperature (0C)	Expected amplified product size (bp)	Structural annotation		Functional annotation	
								Sequence components of *kabuli* genome	*Kabuli* gene accession IDs	NCBI-KOG	TFs
Ca4_P1_26368645	Ca4	26368645	ATTT/ATTTT	AACTTTGCAAATGGACGGAC	TCCCATTCAAATGCACAGAA	60.0	545	INTERGENIC	--	--	--
Ca4_P1_26371050	Ca4	26371050	T/TA	TTCATTAGAAGAAACGATGTGC	AGGCGCGTAAAAAGACAAAA	57.1	638	INTERGENIC	--	--	--
Ca4_P1_26381536	Ca4	26381536	TGGG/TGGGG	GGACATCTCCATCTCCCAGA	GCTGAAGAATTTTGCCGAAG	60.0	584	INTRON	Ca_25302	--	SAP
Ca4_P1_26389354	Ca4	26389354	TTATATATATATAT/TTATATATATATATAT	AGGAAGAGTGGAGTTGCGAA	TCATATTCGGTAGCCGTTCC	60.0	759	INTERGENIC	--	--	--
Ca4_P1_26410577	Ca4	26410577	TGGAGGAG/TGGAG	ATGGCATGTGATTGAGTGGA	GATGACTCTTCGTGGGGGTA	59.9	425	INTERGENIC	--	--	--
Ca4_P1_26417128	Ca4	26417128	CAAAA/CA	AGGAGGTAGGGACTGTCGGT	TTGCATGATAAGGGTTGCAG	60.0	538	DRR	Ca_25299	--	--
Ca4_P1_26461127	Ca4	26461127	CAAAAA/CAAAA	TTTGCCCCATGTTTTCTCTC	GGGCGTAAATTCCAACACAT	60.1	486	INTERGENIC	--	--	--
Ca4_P1_26468706	Ca4	26468706	CTCGATTCGA/CTCGATTCGATTCGA	ATCTTCCCGCCACTAAACCT	CTTCGCAGAAGGGCTAAATG	60.0	362	INTERGENIC	--	--	--
Ca4_P1_26483173	Ca4	26483173	TAAAAAAA/TAAAAAA	TTTTGAAAACCAATGCACCA	CGCTATATCACAGCTTGGCA	59.9	413	INTRON	Ca_25006	O	C2H2
Ca4_P1_26489826	Ca4	26489826	AC/A	TTGGTTGATCGGCTAATTGA	TCCGAGTAACCCGTTTTGAC	59.1	435	INTERGENIC	--	--	--
Ca4_P1_26493282	Ca4	26493282	TAAAAAA/TAAAAA	ATTAATGGCTTCGGCACAAC	CCTTAGCCGTTTTGCAATGT	60.0	327	INTERGENIC	--	--	--
Ca4_P1_26495554	Ca4	26495554	GAAAGTTGTTC/GAAAGTTGTTCAAAGTTGTTC	AACGATTTATCCTGGTCCCC	CCCGAATACAAACCCAAGTC	60.0	844	INTERGENIC	--	--	--

INDEL marker IDs	Chromosomes/ unanchored scaffolds	Physical positions (bp)	InDels (*Kabuli* reference genome-CDC Frontier/PI)	Forward primers (5'-3')	Reverse primers (5'-3')	Anneling temperature (0C)	Expected amplified product size (bp)	Structural annotation		Functional annotation	
								Sequence components of *kabuli* genome	*Kabuli* gene accession IDs	NCBI-KOG	TFs
Ca4_P1_26507557	Ca4	26507557	AATATATTTATA/AATATATTTATATATTTATA	AAAAACACTTCTCGCGTTGG	CGTATTCAGATGGCTCCGTT	60.3	762	INTERGENIC	--	--	--
Ca4_P1_26515466	Ca4	26515466	ATTTTTTTTTTT/ATTTTTTTTTT	AGGCTTCTAGACCGGACCAA	TGCAAAGCGTGTTAAGGATG	61.1	677	INTERGENIC	--	--	--
Ca4_P1_26519944	Ca4	26519944	CATTATTATTATTATT/CATTATTATTATT	GGCAGCGGGTACGTAAAATA	CATTTGTGCAACAATGAGGG	60.0	414	INTERGENIC	--	--	--
Ca4_P1_26523546	Ca4	26523546	GTTTTTTTTTT/GTTTTTTTTT	TTTTCTTTCGTTTCATGGCA	CAACCTGGCCCTCTATTTCA	59.3	806	DRR	Ca_25004	P	--
Ca4_P1_26529457	Ca4	26529457	ATCTCTCTCTCTCTC/ATCTCTCTCTCTCTCTC	ATAGCAGCAATTTCCATCCG	GTCCCACGAAGTAAGTCCCA	60.1	286	INTERGENIC	--	--	--
Ca4_P1_26542242	Ca4	26542242	TAAAAA/TAAAAAA	GCACGGAAGAAGACGAGTTT	GGCACACGTAAATGCACATC	59.5	185	INTERGENIC	--	--	--
Ca4_P1_26582216	Ca4	26582216	TAAAA/TAAA	GGAGAAGGAGGGAGAAATGG	TGAGGAGACACATGCTGAGG	60.0	881	DRR	Ca_25001	--	--
Ca4_P1_26597381	Ca4	26597381	CAAAAAAAAAA/CAAAAAAAAAAA	TCTCTGCAAAAAGGCAACCT	CCAGAATGTGCTCCTTCACA	60.0	718	INTERGENIC	--	--	--
Ca4_P1_26597868	Ca4	26597868	CTTTTTTTTTT/CTTTTTTTTT	TGTGAAGGAGCACATTCTGG	TTCCGTTAAGCACATGTTGG	59.8	425	INTERGENIC	--	--	--
Ca4_P1_26608127	Ca4	26608127	TTATATA/TTATATATA	TTTGTTTTGGTCTCGCAATG	CAAGATTTTGATGTTGCAGGC	59.7	629	DRR	Ca_25000	E	bHLH
Ca4_P1_26616894	Ca4	26616894	AACATTTTATACATTTTATACATTTTATACATTTTA/AACATTTTATACATTTTATACATTTTATACATTTTATACATTTTATACATTTTA	TTGCTGGATTGAAAACCCTC	CCAAAACCCATCATAATCCG	60.1	881	INTRON	Ca_25000	E	bHLH
Ca4_P1_26620467	Ca4	26620467	A/AGTAATT	CCGAAACCGAACAACAGATT	TGGGAACAAAACACAGCAAG	60.0	819	INTERGENIC	--	--	--

INDEL marker IDs	Chromosomes/ unanchored scaffolds	Physical positions (bp)	InDels (*Kabuli* reference genome-CDC Frontier/PI)	Forward primers (5'-3')	Reverse primers (5'-3')	Anneling temperature (0C)	Expected amplified product size (bp)	Structural annotation		Functional annotation	
								Sequence components of *kabuli* genome	*Kabuli* gene accession IDs	NCBI-KOG	TFs
Ca4_P1_26622087	Ca4	26622087	ATTTTTTTTTT/ATTTTTTTTTTT	TACCCCAATCAAAATGACCC	TGCACCAACAGTATCGAACAA	59.5	363	INTERGENIC	--	--	--
Ca4_P1_26623137	Ca4	26623137	TAAAAAA/TAAAAAAAA	TGGCTGTTGTCCATTGTGTT	CTTGAGGTTTTGTCCCTCCA	60.0	954	INTERGENIC	--	--	--
Ca4_P1_26624241	Ca4	26624241	C/CG	CCAAAAGTCCAACGATTGCT	TGTGCATGGTGCTTTTTGAT	60.1	447	INTERGENIC	--	--	--
Ca4_P1_26648113	Ca4	26648113	ATT/ATCTGAATTATCTT	TCGAACAGAAACCTGAGCCT	GATTCGGGAGTTGGTTGATG	60.0	440	INTRON	Ca_24999	L	--
Ca4_P1_26648267	Ca4	26648267	ATTTTTTTTT/ATTTTTTTT	TCGAACAGAAACCTGAGCCT	GATTCGGGAGTTGGTTGATG	60.0	440	INTRON	Ca_24999	L	--
Ca4_P1_26652549	Ca4	26652549	AATATATATATATA/AATATATATATATATATATA	AAGAGGTTGATGCGGCATAG	TGGTTCGGGTATTGGATTTT	60.2	222	INTERGENIC	--	--	--
Ca4_P1_26653534	Ca4	26653534	GAAAAAAAAAAA/GAAAAAAAAA	TGTGGCATTTGAGGTTGTGT	CCCACAAATTTTTAGGGGCT	60.0	497	INTERGENIC	--	--	--
Ca4_P1_26653751	Ca4	26653751	TTTTTTTTTAATTTTTTTTTA/TTTTTTTTTA	AGTCCACATGTTGGAGGGAC	AGATTCAGGCACAACCAACC	59.8	568	INTERGENIC	--	--	--
Ca4_P1_26662668	Ca4	26662668	ATTTTTTTTTT/ATTTTTTTTT	GTGACGTGGGGGAGTTAAGA	CAACGACCTTAACATTTGGTG	60.0	799	INTERGENIC	--	--	--
Ca4_P1_26664091	Ca4	26664091	TAAAAAAAAA/TAAAAAAAAAA	TGATACAATTGATTCGGCCA	TTTTTGTCACGCGAGCTCTA	59.9	371	INTERGENIC	--	--	--
Ca4_P1_26665824	Ca4	26665824	TA/T	TCGCGTGGCAACAATATTTA	TGAATTTGACATCATTTTGTTTGA	60.1	387	INTERGENIC	--	--	--
Ca4_P1_26666119	Ca4	26666119	CTTTTTTTT/CTTTTTTT	ACGCAAACACAAGAATCGGT	TTGGTCCAAATGGTAAGGGA	60.6	738	INTERGENIC	--	--	--

INDEL marker IDs	Chromosomes/ unanchored scaffolds	Physical positions (bp)	InDels (*Kabuli* reference genome-CDC Frontier/PI)	Forward primers (5'-3')	Reverse primers (5'-3')	Anneling temperature (0C)	Expected amplified product size (bp)	Structural annotation		Functional annotation	
								Sequence components of *kabuli* genome	*Kabuli* gene accession IDs	NCBI-KOG	TFs
Ca4_P1_26671228	Ca4	26671228	TAAAAAA/TAAAA	CGTTTATACACGTAAAATCGCA	CATTTTTACCCCACATTCGC	57.9	484	INTERGENIC	--	--	--
Ca4_P1_26672746	Ca4	26672746	ATTTTTTTTT/ATTTTTTTT	TGCTCAATTGAACCTGGAGA	CCATCCATCTGGGACAATTC	59.4	523	INTERGENIC	--	--	--
Ca4_P1_26672920	Ca4	26672920	CTTTT/CTTT	ACGGATGTCGTAGGGTGCTA	AAGACCCGAATCAGCATTTG	60.5	438	INTERGENIC	--	--	--
Ca4_P1_26739311	Ca4	26739311	TAAAAAAAAAA/TAAAAAAAAA	TCCATGATTTGGTGGTTCCT	TTCTTCAGCAATGGTCCTCC	60.2	492	INTERGENIC	--	--	--
Ca4_P1_26740649	Ca4	26740649	TAATTAATTAAT/TAATTAATTAATCAATTAATTAAT	TTGACCACAGGTCCACAAGA	TTTTAGGTGCGCATTTACCC	60.1	250	INTERGENIC	--	--	--
Ca4_P1_26741168	Ca4	26741168	GATAT/GATATGATATATATATAT	GGGTAAATGCGCACCTAAAA	ACTGAACGTGCAACAACTGC	60.0	660	INTERGENIC	--	--	--
Ca4_P1_26746302	Ca4	26746302	ACCCCCC/ACCCCC	GATCAATGAAGAGCCCCAAA	CGGACCATCCGTGATAAAGT	60.0	777	INTERGENIC	--	--	--
Ca4_P1_26748572	Ca4	26748572	GAAAAAAAA/GAAAAAAAAA	GGGTGTGAGTTGAAATGACG	TTTGTCGCAACAACACCATT	59.0	858	INTERGENIC	--	--	--
Ca4_P1_26751558	Ca4	26751558	CAAAA/CAAA	TGGGGGAGCTTGAAATAAAA	TGATATGCGGACAGTGTCAAA	59.5	235	INTERGENIC	--	--	--
Ca4_P1_26752184	Ca4	26752184	ATTTTTTTTTTT/ATTTTTTTTTT	TTGGGATCATAGAATCATTTGGT	TGATCACGGTCACTCCAAAG	59.6	475	INTERGENIC	--	--	--
Ca4_P1_26752542	Ca4	26752542	CATATATA/CATATATATA	TGAAGACTTTGGAGTGACCG	CCGAAGATTTTGCGAGTGTT	58.9	267	INTERGENIC	--	--	--
Ca4_P1_26753050	Ca4	26753050	GAA/GAAA	AACACTCGCAAAATCTTCGG	ATTGTTTGAGTTGCCGCTCT	60.2	623	INTERGENIC	--	--	--

INDEL marker IDs	Chromosomes/ unanchored scaffolds	Physical positions (bp)	InDels (*Kabuli* reference genome-CDC Frontier/PI)	Forward primers (5'-3')	Reverse primers (5'-3')	Anneling temperature (0C)	Expected amplified product size (bp)	Structural annotation		Functional annotation	
								Sequence components of *kabuli* genome	*Kabuli* gene accession IDs	NCBI-KOG	TFs
Ca4_P1_26753796	Ca4	26753796	G/GATTTA	AGAGCGGCAACTCAAACAAT	TGCAGGGGACATCTCTTACC	59.9	613	INTERGENIC	--	--	--
Ca4_P1_26763494	Ca4	26763494	TAAAAAAA/TAAAAAAAA	AAAAATACCGCGTCATTTCATT	TCGCTCAAAAACACAACGAG	59.8	572	INTERGENIC	--	--	--
Ca4_P1_26765991	Ca4	26765991	GAAAAAAAAA/GAAAAAAAA	AGTTTTGGCCCCTTTTGATT	AAAAACTCGCACACAACTGTTA	59.8	508	DRR	Ca_20437	K	MYB
Ca4_P1_26766647	Ca4	26766647	ATTTTTTTT/ATTTTTTTTT	AACAGTTGTGTGCGAGTTTTT	TGTAAGGGCAAAAGCAAACA	57.5	683	DRR	Ca_20437	K	MYB
Ca4_P1_26769049	Ca4	26769049	TC/T	TTGTGGTTGCCTAAACCCTC	ATACGGGTAGCGGGCTATTT	60.0	654	INTERGENIC	--	--	--
Ca4_P1_26774204	Ca4	26774204	ATTTTTTTTTTTTT/ATTTTTTTTTTTTTTTTT	CCGAAAATCTTTTGCGTACC	CGAATTCGAACTCAAAACCTC	59.6	441	INTERGENIC	--	--	--
Ca4_P1_26784175	Ca4	26784175	ATTTTTTT/ATTTTTTTT	CCCCCAATACTTAATTTTTCCA	CGTTTAGAATGGTCGTCGCT	59.1	450	INTERGENIC	--	--	--
Ca4_P1_26787145	Ca4	26787145	CTTTTTTTTTT/CTTTTTTTTT	GCGACTATGTCCGTCAACTG	TCGAAGCATGAAAGAAAAGGA	59.3	803	INTERGENIC	--	--	--
Ca4_P1_26795108	Ca4	26795108	TAAAAAAAAAAA/TAAAAAAAAAAAAA	AATTGAAAAGCTTGAGCCGA	GCTCCATCAAACAAGTCAAAGA	60.0	609	INTERGENIC	--	--	--
Ca4_P1_26805280	Ca4	26805280	TAAAAAAAAAAAAAAA/TAAAAAAAAAAAA	AAACATCATCCAAAATCATCCA	AAGCTAGCCATCCTCAACGA	59.1	389	DRR	Ca_20438	--	--
Ca4_P1_26816326	Ca4	26816326	CGGG/CGGGG	TTTCCCGACTTGGTCCTTTA	GCTTTTCGTTTCTTGTTCGC	59.5	639	INTERGENIC	--	--	--
Ca4_P1_26836955	Ca4	26836955	CAAAAAAA/CAAAAAA	AGAAGGGGTTTGGCTGTTTT	TCACTGCTCACTACCCACCA	60.0	713	INTERGENIC	--	--	--

INDEL marker IDs	Chromosomes/ unanchored scaffolds	Physical positions (bp)	InDels (*Kabuli* reference genome-CDC Frontier/PI)	Forward primers (5'-3')	Reverse primers (5'-3')	Anneling temperature (0C)	Expected amplified product size (bp)	Structural annotation		Functional annotation	
								Sequence components of *kabuli* genome	*Kabuli* gene accession IDs	NCBI-KOG	TFs
Ca4_P1_26847079	Ca4	26847079	AAAAATAAAATAAAATAAAATA/AAAAATAAAATA	GGAGAACGTTAAGGCAGCAG	GTGGTGTAGGTTTCGATCCG	60.0	689	INTERGENIC	--	--	--
Ca4_P1_26847495	Ca4	26847495	A/AC	AGCTTCAAGTCGGATCGAAA	AAAAATCTTGTGGGAATAATTCAA	60.0	216	INTERGENIC	--	--	--
Ca4_P1_26850722	Ca4	26850722	TAA/TAAA	GGAGAACGTTAAGGCAGCAG	GTGGTGTAGGTTTCGATCCG	60.0	726	INTERGENIC	--	--	--
Ca4_P1_26850744	Ca4	26850744	TTTATTATTATTATTATTATTATTATTATTATTATTATTATTATTATTATTATTATTATTATTATTATTATTATTA/TTTATTATTATTATTATTATTATTATTATTATTATTATTATTATTATTATTATTATTATTATTATTATTATTATTATTATTATTATTATTA	GGAGAACGTTAAGGCAGCAG	GTGGTGTAGGTTTCGATCCG	60.0	726	INTERGENIC	--	--	--
Ca4_P1_26853441	Ca4	26853441	ATTTTTTTT/ATTTTTTTTT	TGAGATTGTCAATGTTGGCG	TGGAAATGAAATCTTGAGGTATGA	60.7	393	INTERGENIC	--	--	--
Ca4_P1_26876873	Ca4	26876873	ATTTTTTTTTTT/ATTTTTTTTTTTT	CCACCGTTGTGCCTCTTATT	GCCAGAGAATGATCGAGAGG	60.0	439	INTERGENIC	--	--	--
Ca4_P1_2688293	Ca4	2688293	AT/A	AGCATGCTTCCACAAAGGAT	TTGGAGGTTGTGTTGTTGGA	59.7	532	DRR	Ca_12072	--	--
Ca4_P1_26898145	Ca4	26898145	CTTTTTTTTTT/CTTTTTTTTT	CGAAACTCAAAAACAACGGG	CGGCCCAATTCAAACTAAAA	60.5	588	INTERGENIC	--	--	--
Ca4_P1_26903304	Ca4	26903304	T/TG	ACTCGATTGGTCCATGCTTC	ATGTTTTGGACGCACACAGA	60.1	532	INTERGENIC	--	--	--
Ca4_P1_26905366	Ca4	26905366	TAAAAAAAAAA/TAAAAAAAAA	TGAAAGCCCAAAACTAATCAAGA	ATTGCTCGGTCGGTTTAGTC	60.1	471	DRR	Ca_20443	R	G2-like
Ca4_P1_26908402	Ca4	26908402	CATATATATATATATATATA/CATATATATATATATATATATA	CCTTATTGAGGCCGAAAACA	TGGTCGTATGGGTTTTGGAT	60.1	379	INTRON	Ca_20443	R	G2-like
Ca4_P1_26918578	Ca4	26918578	GA/G	TTGTGCTGGTTACCTTGCTG	TGCCTCCATCATTCAAACAA	59.9	370	INTERGENIC	--	--	--

INDEL marker IDs	Chromosomes/ unanchored scaffolds	Physical positions (bp)	InDels (*Kabuli* reference genome-CDC Frontier/PI)	Forward primers (5'-3')	Reverse primers (5'-3')	Anneling temperature (0C)	Expected amplified product size (bp)	Structural annotation		Functional annotation	
								Sequence components of *kabuli* genome	*Kabuli* gene accession IDs	NCBI-KOG	TFs
Ca4_P1_26942878	Ca4	26942878	CTTTTTTTTTT/CTTTTTTTTTTT	CCACATGGTTGAGACTTGAGG	TGGGAGGGAGCTCTAAGATG	60.6	559	INTERGENIC	--	--	--
Ca4_P1_26947230	Ca4	26947230	AATTATATTATATTATA/AATTATATTATA	CGTTTCTTCAAAAAGCGAGC	AGACGAGTTCGACGACATCC	60.1	513	DRR	Ca_20451	R	--
Ca4_P1_26966651	Ca4	26966651	TAAAAAAA/TAAAAAAAA	GCATAAGCGAATGTGAGAAGC	CCAACAATCACAATTCAACTCG	60.0	259	INTERGENIC	--	--	--
Ca4_P1_26967509	Ca4	26967509	TAAAAAAAA/TAAAAAAAAA	TTGGTGGGTTGTGTGTAATGA	TGAATGGGAAATGGAGGAAG	59.7	582	INTERGENIC	--	--	--
Ca4_P1_26991333	Ca4	26991333	ATTTTTTTTTT/ATTTTTTTTT	ACAAGGAAGCGCACCTCTAA	GTGCGACGAGCAAGAATGTA	60.0	762	INTERGENIC	--	--	--
Ca4_P1_27007761	Ca4	27007761	TAAAAAAAA/TAAAAAAAAA	TGTGATTCCTTGCTCGAGTG	TGCGCACATTTATCATTTAGTCA	60.0	445	INTERGENIC	--	--	--
Ca4_P1_27017251	Ca4	27017251	GTTTTTTTTT/GTTTTTTTTTT	CCTTTGCATCTCAGGGACAT	TGAGACCGGGTTTGAGTACC	60.1	453	INTERGENIC	--	--	--
Ca4_P1_27067931	Ca4	27067931	ATTTTTTTT/ATTTTTTTTT	AACCTCCATTAATTTTTGCCA	CATTTGCAATTCAAGCGAAC	58.5	438	INTERGENIC	--	--	--
Ca4_P1_27074666	Ca4	27074666	GAAAAAAAAA/GAAAAAAAAAA	ATTGGCATCAGAGCCAGTCT	TTTTCCAAGAGAGCGCATTT	59.8	566	INTERGENIC	--	--	--
Ca4_P1_27079794	Ca4	27079794	AAA/ACAA	AAACACTACCCGGCATCAAG	CAATTTTCTGCACTCTCGCA	60.0	516	INTERGENIC	--	--	--
Ca4_P1_27079917	Ca4	27079917	AA/AATATA	AAACACTACCCGGCATCAAG	CAATTTTCTGCACTCTCGCA	60.0	516	INTERGENIC	--	--	--
Ca4_P1_27087434	Ca4	27087434	TA/TAAAA	TTATTGCGATGCCGACTATG	AATGATGTGGCTGGGTTAGC	59.7	656	INTERGENIC	--	--	--

INDEL marker IDs	Chromosomes/ unanchored scaffolds	Physical positions (bp)	InDels (*Kabuli* reference genome-CDC Frontier/PI)	Forward primers (5'-3')	Reverse primers (5'-3')	Anneling temperature (0C)	Expected amplified product size (bp)	Structural annotation		Functional annotation	
								Sequence components of *kabuli* genome	*Kabuli* gene accession IDs	NCBI-KOG	TFs
Ca4_P1_27139983	Ca4	27139983	ACC/AC	CGTTCGTCTTTTCCTCTTGC	CATGATTGAGGGGCAAATCT	60.0	443	INTERGENIC	--	--	--
Ca4_P1_27149358	Ca4	27149358	ATTTTTTTT/ATTTTTTT	CGATGATGCTACCAGAGGGT	CGGGGATTCCCATATCACTA	60.1	611	INTERGENIC	--	--	--
Ca4_P1_27163385	Ca4	27163385	GTTTTTTTTTTT/GTTTTTTTTTT	TTGAACACACTAACCACCCAA	TCCTGGTAAGGAATTGTGGG	58.9	450	INTERGENIC	--	--	--
Ca4_P1_27168549	Ca4	27168549	CAAAAAA/CAAAAA	AGGGCCAAGGGACAATAAGT	TGGTTTGTGCAATGTCCCTA	59.8	523	INTERGENIC	--	--	--
Ca4_P1_27174775	Ca4	27174775	TTGTTTCAAA/TTGTTTCAAAAGGATGTTTCAAA	ACCAAAAAGCTGGAGAGCAA	CCAAGCTCTTGGTGCTTGTT	60.0	512	INTERGENIC	--	--	--
Ca4_P1_27184150	Ca4	27184150	AAATAATAATAATAATA/AAATAATAATAATAATAATA	TTTCTTGTTGCCTTGGTGTG	TAGAAACCGGTTGCCAAGAA	59.7	744	INTERGENIC	--	--	--
Ca4_P1_27184445	Ca4	27184445	GAAAAAAAAAA/GAAAAAAAAA	TGCAAAAATAACCCTTGTGC	TAGAAACCGGTTGCCAAGAA	58.7	189	INTERGENIC	--	--	--
Ca4_P1_27184800	Ca4	27184800	CATTAATTAATT/CATTAATT	TTATCGTTTCTTGGCAACCG	CAATTGCATGAAGGAGGGAT	61.0	502	INTERGENIC	--	--	--
Ca4_P1_27186691	Ca4	27186691	GTTTTTTTTTTT/GTTTTTTTTTT	TTATTGGGATTGGATGCAGA	TTCCTCACTTGACGTTTTTGAA	58.9	346	INTERGENIC	--	--	--
Ca4_P1_27193093	Ca4	27193093	GTTATTTTTTATTTTTTATTTTTT/GTTATTTTTTATTTTTTATTTTTTATTTTTT	GTAGAATGCCCCAACAAAGC	TTTCATCCTGCAATGTGTGA	59.6	463	INTERGENIC	--	--	--
Ca4_P1_27196099	Ca4	27196099	GAT/GATAT	CTGAAAACTCACAGGTGCCA	TCTTCCTCTAACTCGCGGTC	59.9	591	INTERGENIC	--	--	--
Ca4_P1_27197635	Ca4	27197635	TAAAAAAAAAAA/TAAAAAAAAAAAA	TTCAGGTTGCACCACAAAAC	TCAAATGCTACCGTTGGACA	59.6	691	DRR	Ca_20458	--	NF-YB

INDEL marker IDs	Chromosomes/ unanchored scaffolds	Physical positions (bp)	InDels (*Kabuli* reference genome-CDC Frontier/PI)	Forward primers (5'-3')	Reverse primers (5'-3')	Anneling temperature (0C)	Expected amplified product size (bp)	Structural annotation		Functional annotation	
								Sequence components of *kabuli* genome	*Kabuli* gene accession IDs	NCBI-KOG	TFs
Ca4_P1_27198549	Ca4	27198549	ACCCCCC/ACCCCCCC	TGCTTCCAAAAATCATCGTG	AAGGCTTCGAAGGTTTGACA	59.7	520	DRR	Ca_20458	--	NF-YB
Ca4_P1_27217904	Ca4	27217904	CAAAAAAAAAA/CAAAAAAAAAAA	TGTAGTGGCATGTTTGGCAT	TTTGGAGGACTTTTGGGTTG	60.0	662	INTERGENIC	--	--	--
Ca4_P1_27232925	Ca4	27232925	T/TG	CCAACGCTATTGGGTTTCAG	TTGATTCCTCTCATACCTTGTGAA	60.5	475	INTRON	Ca_20460	--	--
Ca4_P1_27234831	Ca4	27234831	AGTTTGAAATGTTTGAAATG/AGTTTGAAATG	CTGGGAAGCCAGAAAAATCA	ATGGTTGTGACCATGGGACT	60.2	384	INTRON	Ca_20460	--	--
Ca4_P1_27241081	Ca4	27241081	CTTTTTTTTTTT/CTTTTTTTTTTTTTT	AAGGACCAAGTCGGGAAAAA	AGGTTTTGATGTGTTTGACGG	60.8	705	INTRON	Ca_20460	--	--
Ca4_P1_27258927	Ca4	27258927	ATTTTTTTTTT/ATTTTTTTTT	AGCGCGGAGAGATAGTTGAA	TGGGTGACTTCATTTGGACA	60.1	393	INTERGENIC	--	--	--
Ca4_P1_27261165	Ca4	27261165	T/TC	CGTGGTTGCTCCAAATTTCT	AATTGAGCCCAGAAAACCCT	60.1	872	INTERGENIC	--	--	--
Ca4_P1_27267123	Ca4	27267123	TAAAAA/TAAAA	CCCTCAACTCAACTGAGATCC	TGCATTGGTTTGAATTTGGA	58.7	568	DRR	Ca_20461	R	--
Ca4_P1_27283830	Ca4	27283830	GTTT/GTTTT	TATTGGTCGTCAAGCAGCAG	TTTCCCAATCGATTTGCTTC	60.0	681	DRR	Ca_20463	--	--
Ca4_P1_27285679	Ca4	27285679	G/GAA	TATTTCGAGGGCGTACCAAC	CCAGCCTACCCCTTTTATCTG	60.0	549	INTERGENIC	--	--	--
Ca4_P1_2729716	Ca4	2729716	A/AGACATCAT	AGGGCTTGTAATGCCCTTTT	TTGGCTTCACAAGATACCCC	60.0	654	INTRON	Ca_12075	Z	--
Ca4_P1_27312714	Ca4	27312714	ATA/ATAATTA	AGTGGATTTTGTGGCTGCTT	TGATGGCGACTCATTTTTCA	59.7	546	INTERGENIC	--	--	--

**INDEL marker IDs**	**Chromosomes/ unanchored scaffolds**	**Physical positions (bp)**	**InDels (*Kabuli* reference genome-CDC Frontier/PI)**	**Forward primers (5'-3')**	**Reverse primers (5'-3')**	**Anneling temperature (0C)**	**Expected amplified product size (bp)**	**Structural annotation**		**Functional annotation**	
								**Sequence components of *kabuli* genome**	***Kabuli* gene accession IDs**	**NCBI-KOG**	**TFs**
Ca4_P1_27314909	Ca4	27314909	C/CG	TGCACCTTCAATTGGC ATTA	TGGCCTTTGCTTCTTG AACT	60.1	452	INTERGENIC	--	--	--
Ca4_P1_27317731	Ca4	27317731	CAAAAA/CAAAA	GACATTTGCTCCAGTG ACCA	TGATGTCAAGTCTGGA AGCG	59.7	686	INTERGENIC	--	--	--
Ca4_P1_27322564	Ca4	27322564	ATTTTTTT/ATTTTTTTT	ATCCTAATCGGTAGGG GTGG	AGGAAATCCTCATGGT GCAA	60.0	685	INTERGENIC	--	--	--
Ca4_P1_27331831	Ca4	27331831	A/AG	CAACAATCCCAAACCA ACAA	ATGGTGGTCAACGTCC CTTA	59.3	398	INTERGENIC	--	--	--
Ca4_P1_27332287	Ca4	27332287	TTGTGTGT/TTGTGT	TAAGGGACGTTGACCA CCAT	GGGTGTGATGGGTAA ACCTG	60.2	379	INTERGENIC	--	--	--
Ca4_P1_27354720	Ca4	27354720	C/CA	TAGACGCAACAACTGG CAAC	CCCGATTCAGGAGTC GATTA	59.9	710	INTERGENIC	--	--	--
Ca4_P1_27354842	Ca4	27354842	TAAAAAAA/TAAAAAA	TAGACGCAACAACTGG CAAC	GAACTTGTCGTGTCCG GTTT	59.9	821	INTERGENIC	--	--	--
Ca4_P1_27355192	Ca4	27355192	CGGG/CG	TGCATAATCATTGCATT CACC	GAACTTGTCGTGTCCG GTTT	59.4	242	INTERGENIC	--	--	--
Ca4_P1_27357133	Ca4	27357133	GAAAA/GAAA	TTGATTTGGTGCACAT CCAT	TAAAACTTGTCCCCAA CCCC	59.8	561	INTERGENIC	--	--	--
Ca4_P1_27365094	Ca4	27365094	GAAAAAAAAAAA/GAAAAAAAAAA	TTGCAATGGAAAAGGA GCTT	TAGCCATCTAACCATT CGGC	59.8	138	INTERGENIC	--	--	--
Ca4_P1_27365149	Ca4	27365149	AT/ATT	TTGCAATGGAAAAGGA GCTT	TAGCCATCTAACCATT CGGC	59.8	138	INTERGENIC	--	--	--
Ca4_P1_27367807	Ca4	27367807	CTTTT/CTTT	GCATCCTTGCTCACAA CTCA	TAAAGGCCCAAAGAAG CAAA	60.0	415	INTERGENIC	--	--	--

INDEL marker IDs	Chromosomes/ unanchored scaffolds	Physical positions (bp)	InDels (*Kabuli* reference genome-CDC Frontier/PI)	Forward primers (5'-3')	Reverse primers (5'-3')	Anneling temperature (0C)	Expected amplified product size (bp)	Structural annotation		Functional annotation	
								Sequence components of *kabuli* genome	*Kabuli* gene accession IDs	NCBI-KOG	TFs
Ca4_P1_27368649	Ca4	27368649	ATTTTTTTT/ATTTTTTT	GGGAAGACCATGTGCAAAAT	TTGTTCCAAGCCCTAAATGC	59.8	543	INTERGENIC	--	--	--
Ca4_P1_27369042	Ca4	27369042	ATGTGTG/ATGTGTGTGTG	GCATTTAGGGCTTGGAACAA	CTTTTGACAACACCAACCCA	60.1	378	INTERGENIC	--	--	--
Ca4_P1_27374248	Ca4	27374248	CTTTT/CTTTTT	GAGGCGGCAAAAATAATTGA	TGGAGAGTTTGTTGCGAGTG	60.0	847	INTERGENIC	--	--	--
Ca4_P1_27376069	Ca4	27376069	TAA/TAAA	ATCCTCTCCATTCCTTGCCT	TTGCTTTCACAGTCCAATGC	60.0	304	INTRON	Ca_21323	--	--
Ca4_P1_27377254	Ca4	27377254	CT/CTGT	TAAAGGACTTCCGTTCCGTG	TAGCAGACACCGGCATAGTG	60.1	587	DRR	Ca_21322	--	--
Ca4_P1_27379933	Ca4	27379933	CAAAAAAA/CAAAAAAAA	ATGGTACAAAGTGGCAAGGC	GAGAAAAGCAGGGTGAATGC	60.0	748	DRR	Ca_21323	--	--
Ca4_P1_27380010	Ca4	27380010	ATTT/ATT	ATGGTACAAAGTGGCAAGGC	GTGGACTGCAGTGGGTCTTT	60.0	909	DRR	Ca_21323	--	--
Ca4_P1_27383955	Ca4	27383955	GTTTTTTT/GTTTTTT	CGTCGATGTTGATTCCCTCT	TTTCGCATCTTGCTTCACTG	60.1	927	INTERGENIC	--	--	--
Ca4_P1_27384260	Ca4	27384260	ATTTT/ATTT	GGTGCCATCACTGTAAGCAA	AAAGAGCGCGTTGTTGTTCT	59.7	699	INTERGENIC	--	--	--
Ca4_P1_27388787	Ca4	27388787	T/TA	TGTATTGTCGGATCTGCTGC	AGGCAAAAACAGGGAGGAAT	59.8	520	INTERGENIC	--	--	--
Ca4_P1_27395838	Ca4	27395838	TT/TTGT	AAGTTTTGGGTTGCTCATGG	TTGTCCCCAAGCAACAATTA	60.0	627	INTERGENIC	--	--	--
Ca4_P1_27398676	Ca4	27398676	CTTTTTTTTTT/CTTTTTTTTT	GAGCGGGTCAATCATCCTAA	AATCCAGGGATCATGTTCGT	60.0	250	INTERGENIC	--	--	--

INDEL marker IDs	Chromosomes/ unanchored scaffolds	Physical positions (bp)	InDels (*Kabuli* reference genome-CDC Frontier/PI)	Forward primers (5'-3')	Reverse primers (5'-3')	Anneling temperature (0C)	Expected amplified product size (bp)	Structural annotation		Functional annotation	
								Sequence components of *kabuli* genome	*Kabuli* gene accession IDs	NCBI-KOG	TFs
Ca4_P1_27402844	Ca4	27402844	TAAAAAAAA/TAAAAAAA	CGAGTAGCGAAATCGACCAT	TATTTTGCTTCGTTGTTGCG	60.2	447	INTERGENIC	--	--	--
Ca4_P1_27403176	Ca4	27403176	GTTTTTT/GTTTTTTT	CGCAACAACGAAGCAAAATA	GCACGGTTGAGTGAAAAAGG	59.9	579	INTERGENIC	--	--	--
Ca4_P1_27403298	Ca4	27403298	CA/CAA	TAGATGGACGCAACAACGAA	TGGTTGCCTCTGTTTTCCTC	60.3	854	INTERGENIC	--	--	--
Ca4_P1_27405634	Ca4	27405634	TTATATATATATAT/TTATATATATAT	GAGTTGGTATTTCATTTGTTCGG	GTGGTTAAGATGGACCACCG	59.8	531	INTERGENIC	--	--	--
Ca4_P1_27405693	Ca4	27405693	TAAAA/TAAAAAA	GAGTTGGTATTTCATTTGTTCGG	GTGGTTAAGATGGACCACCG	59.8	531	INTERGENIC	--	--	--
Ca4_P1_27406421	Ca4	27406421	ACTCTC/ACTC	TTTGATGGTTCGAAATCGTT	CGCAAAATAAGAGAAAAGACGC	58.1	593	INTERGENIC	--	--	--
Ca4_P1_27409351	Ca4	27409351	TG/TGG	TGGATTTCAATGGGTTGGTT	AATTGGGCCCGGTAAGTATC	60.0	285	INTERGENIC	--	--	--
Ca4_P1_27409638	Ca4	27409638	TG/TGG	ATAGGGTTCAACGGGGTAGG	ATCGTGCTTATTGGGCAGTC	60.1	892	INTERGENIC	--	--	--
Ca4_P1_27474037	Ca4	27474037	TAAAA/TAA	GAACATTGAAACCCTATGCCA	CCCACTCTTAAGCGCACTTCT	59.8	413	INTERGENIC	--	--	--
Ca4_P1_27483001	Ca4	27483001	ATTTTTT/ATTTTTTT	GAAATCCAAGCCATTCTCCA	TGGTTGCGGAAATAAACACA	60.0	485	INTRON	Ca_21318	--	--
Ca4_P1_27491121	Ca4	27491121	ATTTTTTTT/ATTTTTTT	ATGCAGCACCTAGTTTTGGC	CATGTTCGTCGCTCTCATGT	60.3	531	INTERGENIC	--	--	--
Ca4_P1_27491848	Ca4	27491848	ATTTTTTT/ATTTTTT	TACGCGCTTTCTTTCATTCA	CGTCCCCTTCCTCAAACATA	59.6	503	INTERGENIC	--	--	--

INDEL marker IDs	Chromosomes/ unanchored scaffolds	Physical positions (bp)	InDels (*Kabuli* reference genome-CDC Frontier/PI)	Forward primers (5'-3')	Reverse primers (5'-3')	Anneling temperature (0C)	Expected amplified product size (bp)	Structural annotation		Functional annotation	
								Sequence components of *kabuli* genome	*Kabuli* gene accession IDs	NCBI-KOG	TFs
Ca4_P1_27496407	Ca4	27496407	CTG/CTGTG	TCGGAAACGAATTGGGAATA	GGAGTTTGAGAAAGGGGTCC	60.3	750	INTERGENIC	--	--	--
Ca4_P1_27499064	Ca4	27499064	CAAAAAAAA/CAAAAAAAAA	TTTGAGCATGGCTGTTGAAG	ACTGGTGGGCGCTAGATCAT	60.0	426	INTERGENIC	--	--	--
Ca4_P1_27503266	Ca4	27503266	AG/AGG	GGGGTTCTCCTTGAACCACT	CCTGCCATTTTCTTCCAAAA	60.3	343	INTERGENIC	--	--	--
Ca4_P1_27506038	Ca4	27506038	CAA/CAAA	GAGATCATTGCATCCCAGGT	CACCTGAAACCCCTTTTGAA	59.9	819	INTERGENIC	--	--	--
Ca4_P1_27507215	Ca4	27507215	ATTT/ATT	TGGCCTAGCTGAAAGGAGAA	CCACCTTGCCTTTGATAGGA	60.1	331	INTERGENIC	--	--	--
Ca4_P1_27512574	Ca4	27512574	CAAAAAAAAA/CAAAAAAAA	GTTGGCTGGGTGGAAATCTA	ATACGCGCTCCCTTAACTTG	59.9	806	INTERGENIC	--	--	--
Ca4_P1_27513599	Ca4	27513599	ATTTTTTT/ATTTTTTTT	TGATGGGGTTTTGTGTGAGA	ATGCATTACACGACCGCATA	59.9	612	INTERGENIC	--	--	--
Ca4_P1_27514350	Ca4	27514350	GTCTCT/GTCT	TGGTGCTCTCAACCATTGAA	TTATCCATCGCCTAAGTCGG	60.2	754	INTERGENIC	--	--	--
Ca4_P1_27523055	Ca4	27523055	TTA/T	TCATGTGCTTGCAGCTTTCT	CCTGCAAAATCAAAGGGAAA	59.7	552	INTERGENIC	--	--	--
Ca4_P1_27530914	Ca4	27530914	TCC/TC	TTCGAAGGTTGGGTTGAATC	CCATTAATTTCGGCCCATTA	59.9	635	INTERGENIC	--	--	--
Ca4_P1_27537181	Ca4	27537181	AGTGTGTGTGTGT/AGTGTGTGTGTGTGT	TTTCATCCTCTCTCACGGCT	TCTTCATGGCAACACTTCCA	60.0	478	INTERGENIC	--	--	--
Ca4_P1_27548804	Ca4	27548804	TAAAA/TAAA	TTCCCCATTCTTAACACCCA	TTCGCAACTCCACTCTTCCT	60.2	672	INTERGENIC	--	--	--

INDEL marker IDs	Chromosomes/ unanchored scaffolds	Physical positions (bp)	InDels (*Kabuli* reference genome-CDC Frontier/PI)	Forward primers (5'-3')	Reverse primers (5'-3')	Anneling temperature (0C)	Expected amplified product size (bp)	Structural annotation		Functional annotation	
								Sequence components of *kabuli* genome	*Kabuli* gene accession IDs	NCBI-KOG	TFs
Ca4_P1_2755654	Ca4	2755654	CAAAAAA/CAAAAAAA	AATTCGTTGCACAATTTGGTT	GCATGCTAAATGCCATCGTA	59.4	801	INTERGENIC	--	--	--
Ca4_P1_27562480	Ca4	27562480	GTTTTTTTTT/GTTTTTTTTTTT	TCAATCTGGGGTTGTTAGGC	CTGAAGGTGCAAACACGAGA	59.9	388	INTRON	Ca_21314	--	--
Ca4_P1_27574473	Ca4	27574473	ATTTTTTTTTTT/ATTTTTTTTT	CACCCTTACCTTGACCCCTT	GCTCGTATGCGTCAATTCAA	60.2	571	INTRON	Ca_21314	--	--
Ca4_P1_27576276	Ca4	27576276	GAA/GAAA	TCGCACAAAAGGTTAGGTCA	GCAACCAATTCGCTGAAAGT	59.3	698	INTRON	Ca_21314	--	--
Ca4_P1_27580274	Ca4	27580274	GAAAAAAAAA/GAAAAAAAAAA	TGGGGTCCATGTACCACTTT	AAGAAATGTGCCTCAGGTGC	60.1	305	INTRON	Ca_21314	--	--
Ca4_P1_27582209	Ca4	27582209	TGG/TG	AGGTGCAAACACAAGAAGGG	AATGTGTGGTGTGCCTTCAA	60.1	329	INTRON	Ca_21314	--	--
Ca4_P1_27593574	Ca4	27593574	GTTATTATTATTAT/GTTATTATTATTATTAT	TGCAACGCTTGATCGTTAAT	ACTCGAATCGTTTTGCAAGG	59.3	301	INTRON	Ca_21314	--	--
Ca4_P1_27594072	Ca4	27594072	CTTTTTTT/CTTTTTTTTT	ATCAGAGGTTGCGCAAAAAT	TTTCCTTCCAAATTTACGCC	59.7	668	INTRON	Ca_21314	--	--
Ca4_P1_27599972	Ca4	27599972	T/TG	ACGTCCAGTCGCTTCAAACT	AGAGCAGCCCTCATTGAAAA	59.9	343	INTRON	Ca_21314	--	--
Ca4_P1_27604352	Ca4	27604352	AAATGTTGATAAT/AAAT	ATTAGGAGTCTTGCTGCCCA	CCCTCCGAACTCTCAAACAG	59.8	320	INTRON	Ca_21314	--	--
Ca4_P1_27624221	Ca4	27624221	ATTTTTTTTT/ATTTTTTT	GGCGAAGGAAGAGAGAAAGG	GCAACATCAATGCTGCAACT	60.5	767	INTRON	Ca_21312	--	--
Ca4_P1_27634641	Ca4	27634641	CTTTTTT/CTTTTTTT	ACGAAGCAGAAGCGAACATT	CACGAGGTGTAGAATTGCCA	60.0	534	INTERGENIC	--	--	--

INDEL marker IDs	Chromosomes/ unanchored scaffolds	Physical positions (bp)	InDels (*Kabuli* reference genome-CDC Frontier/PI)	Forward primers (5'-3')	Reverse primers (5'-3')	Anneling temperature (0C)	Expected amplified product size (bp)	Structural annotation		Functional annotation	
								Sequence components of *kabuli* genome	*Kabuli* gene accession IDs	NCBI-KOG	TFs
Ca4_P1_27637430	Ca4	27637430	TG/TGG	TTTGGAGTGGTGGTTCTCAA	CCAATGATTTTCATGCCACA	59.1	482	DRR	Ca_21310	--	--
Ca4_P1_27649411	Ca4	27649411	TTTTATTT/TTTT	AAAATAAGGCATGGTGTTGGA	TAGTTTGTAACCCGTGCGTC	59.3	256	INTERGENIC	--	--	--
Ca4_P1_27649729	Ca4	27649729	TAAAAAAA/TAAAAAA	TTAAAATCGTTGTTTCCGTGC	ATTGGTGTGGGTTCGGTCTA	60.0	693	INTERGENIC	--	--	--
Ca4_P1_27656025	Ca4	27656025	TAAAAAAAAA/TAAAAAAAAAA	TTTTAATTGCGAGCATGAGAAG	AGACTCCCGATGTCACAACA	59.5	101	INTERGENIC	--	--	--
Ca4_P1_27656062	Ca4	27656062	TAAAAAAAAAAAAA/TAAAAAAAAAAAAAAA	GCGAGCATGAGAAGTTGTTG	TTTAAAATAGACTCCCGATGTCA	59.6	101	INTERGENIC	--	--	--
Ca4_P1_27657337	Ca4	27657337	ATTTTTT/ATTTTTTT	TGTTTGTGCAATTGAATTATAGTTATG	TCCATTTATGGTGAGTAATGACG	58.6	506	INTERGENIC	--	--	--
Ca4_P1_27682658	Ca4	27682658	T/TACTTAAG	AGACGGCGCAAGTAGAGAAC	TGTTTTCTTTGCGTTGCTTG	59.6	262	INTERGENIC	--	--	--
Ca4_P1_27698175	Ca4	27698175	CAAAAAAAAAAA/CAAAAAAAAAAAA	CTAACCTTGCATGGGGAAAA	ATTCATGGTGCTTTCTTGGC	59.9	274	DRR	Ca_21307	--	--
Ca4_P1_27746278	Ca4	27746278	CTTTTTTTTTTT/CTTTTTTTT	AGTTTTCTGTGGGTTCACCG	TGCCACATCACCCTAAATCA	60.0	546	INTERGENIC	--	--	--
Ca4_P1_27787881	Ca4	27787881	CAAAA/CAA	ACCTGCAAATAACACAGCCC	GTTACAAGGATCCTCCCACG	60.0	523	INTRON	Ca_21303	--	GATA
Ca4_P1_27814253	Ca4	27814253	T/TA	TGGGTCCCATGTACCATTTT	CACGCAAGAATCCTCAGTCA	59.9	510	INTERGENIC	--	--	--
Ca4_P1_27859134	Ca4	27859134	ATTTTTTTTT/ATTTTTTTTTT	ACCCTCCTTTTAGGTGTGCC	GGGAGATTTGGGGTTTTGAT	60.4	569	INTERGENIC	--	--	--

INDEL marker IDs	Chromosomes/ unanchored scaffolds	Physical positions (bp)	InDels (*Kabuli* reference genome-CDC Frontier/PI)	Forward primers (5'-3')	Reverse primers (5'-3')	Anneling temperature (0C)	Expected amplified product size (bp)	Structural annotation		Functional annotation	
								Sequence components of *kabuli* genome	*Kabuli* gene accession IDs	NCBI-KOG	TFs
Ca4_P1_27865338	Ca4	27865338	AAATAATAATAATAATAATAATAATAATAATAATAATAATAA/AAATAATAATAATAATAATAATAATAATAATAA	TCCTAAAACAAATGCACCCA	TTAGGCTTGATATGCCGACC	59.0	557	INTERGENIC	--	--	--
Ca4_P1_27873116	Ca4	27873116	TTTTATTTATTTATTTATTTATTTATTTATTT/TTTTATTTATTTATTTATTTATTTATTT	GCATGCGAACAAGAGTGAAG	GTTCAACTCCCGTTGGAAAA	59.6	693	INTERGENIC	--	--	--
Ca4_P1_27876725	Ca4	27876725	AGGGG/AG	TCTCCCTCCATGTCCAAAAC	AATGGACGAGGCAGATATGG	59.9	476	INTERGENIC	--	--	--
Ca4_P1_2787682	Ca4	2787682	TTTATTATTATTATTATTATTATTATTATT/TTTATTATTATTATTATTATTATTATTATT	ACCTGCCGAAACAAATAACG	TGCAATTAAATAAAAGGGAGTTG	60.0	512	INTERGENIC	--	--	--
Ca4_P1_2788113	Ca4	2788113	TTTATTATTATTATTATTATTATTATTATTATTATTATTATTATT/TTTATTATTATTATTATTATTATTATTATTATTATTATTATT	TGTGGCAAAACAAATAAGCG	ATATCCCGGGAACCAATTCT	59.7	734	INTERGENIC	--	--	--
Ca4_P1_27902426	Ca4	27902426	T/TC	CTGATTTGGCTCTGATGGCT	ATCATAGATCGCGAGCATCC	60.4	686	INTERGENIC	--	--	--
Ca4_P1_27920147	Ca4	27920147	GTATATATATATATAT/GTATATATATATATATAT	GCCCATCAAAACCCACATAC	CACAATCCTTTTCAACCGCT	60.1	466	INTERGENIC	--	--	--
Ca4_P1_27921168	Ca4	27921168	TTAT/TT	TTCCGTCTCATTTTCGGTTC	ACGGGCATGACCCTAATGTA	60.1	595	INTERGENIC	--	--	--
Ca4_P1_27923862	Ca4	27923862	CTATATATATATATATAT/CTATATATATATATATAT	CATTAATTTTGCGAGGAGGC	AATGGGTCTAAGTGCATGGC	59.7	783	DRR	Ca_21301	--	C2H2
Ca4_P1_27928820	Ca4	27928820	CACAAC/CACAACAAC	AGCGATGCATGTCTCAAGTG	AGCGCTTTGTGACCTCCTTA	60.0	754	INTERGENIC	--	--	--
Ca4_P1_27929931	Ca4	27929931	GTTTT/GTTTTT	CCTTCTCATTGCGAACCCTA	TGGTTGCACCACATTTGACT	60.2	427	INTERGENIC	--	--	--
Ca4_P1_27943723	Ca4	27943723	ATTTTTT/ATTTTT	CGATTGAATTAAGCCCAGGA	TCTCCATTTTCGTCCGTAGG	60.0	403	INTERGENIC	--	--	--

INDEL marker IDs	Chromosomes/ unanchored scaffolds	Physical positions (bp)	InDels (*Kabuli* reference genome-CDC Frontier/PI)	Forward primers (5'-3')	Reverse primers (5'-3')	Anneling temperature (0C)	Expected amplified product size (bp)	Structural annotation		Functional annotation	
								Sequence components of *kabuli* genome	*Kabuli* gene accession IDs	NCBI-KOG	TFs
Ca4_P1_27950194	Ca4	27950194	ATAATATAACTTTTAATATAACTTTTA/ ATAATATAACTTTTA	TTGGGATTTGCCAATT GTTT	ATCATGGGGGAGGTTA CACA	60.2	883	INTERGENIC	--	--	--
Ca4_P1_27950659	Ca4	27950659	ATTT/ATT	ACCTCAGTTGCAATAT CCGC	TATTTTTGGTCTTTTG GCCG	60.1	459	INTERGENIC	--	--	--
Ca4_P1_27951126	Ca4	27951126	CTT/CTTTCATTT	TGTGTAACCTCCCCCA TGAT	AAAGGGCACCAAATAC TCCA	60.1	858	INTERGENIC	--	--	--
Ca4_P1_27956988	Ca4	27956988	TCCCCCCC/TCCCCCCCC	ACTCCACTTCAAACAA CCGC	TTTTAAGCCACACCAC ACCA	60.2	603	INTERGENIC	--	--	--
Ca4_P1_27962102	Ca4	27962102	TA/T	TTTCCAAAACCGGTCA TTTT	AGTGGTGGAATCCCCA TAAA	59.3	756	INTERGENIC	--	--	--
Ca4_P1_27969810	Ca4	27969810	ATTTTTTTTTT/ATTTTTTTTTTTT	ATCGAGAAGAGGGTGT GTGG	GGGGCCCATAAGTCC AACTA	60.1	607	INTERGENIC	--	--	--
Ca4_P1_27971871	Ca4	27971871	GCTTCAC/G	TGTGTTTCGAGTTTTC GCTG	TGCCATGACAGAACCA ATGT	60.0	412	INTRON	Ca_21299	T	--
Ca4_P1_27993812	Ca4	27993812	TAAAAAAAAAAAAA/TAAAAAAAAAAAA	TTGTCTTGACAATTTTC ATTGTTG	TGGGGGTGGATTCAC ATATC	59.1	541	INTERGENIC	--	--	--
Ca4_P1_27994370	Ca4	27994370	ATTTTTTTTTT/ATTTTTTTTT	TTCCGGAATTTTTCTTG GTG	TCCCACGATTGAAGCT TACC	59.9	654	INTERGENIC	--	--	--
Ca4_P1_27998494	Ca4	27998494	CTTTTTTTTT/CTTTTTTTT	AAGGAGGAGGAGGTG AAGGA	GAAAAGGGAGCCACA AATGA	60.2	533	INTERGENIC	--	--	--
Ca4_P1_27999477	Ca4	27999477	AATATATATATATATATATATATATATA TATATA/AATATATATATATATATA	TCGTTTGCGTTTGACA ACAT	TTCCCTCCCTTTGTCC TCTT	60.2	772	INTERGENIC	--	--	--
Ca4_P1_28019097	Ca4	28019097	TAA/TAAA	TTCAACGTTTTTGAATC GGA	CCAATCCAACTTAATG CGGT	59.1	533	INTERGENIC	--	--	--

INDEL marker IDs	Chromosomes/ unanchored scaffolds	Physical positions (bp)	InDels (*Kabuli* reference genome-CDC Frontier/PI)	Forward primers (5'-3')	Reverse primers (5'-3')	Anneling temperature (0C)	Expected amplified product size (bp)	Structural annotation		Functional annotation	
								Sequence components of *kabuli* genome	*Kabuli* gene accession IDs	NCBI-KOG	TFs
Ca4_P1_28019581	Ca4	28019581	TTGT/TT	CGCATTAAGTTGGATTGGGT	TTGAGGTTGAACATGGTTGG	59.8	439	INTERGENIC	--	--	--
Ca4_P1_28020842	Ca4	28020842	GTTT/GTTTT	CCCGTGACGTAGGTCTCATT	CCAACCATTGTTGCTTCCTT	60.0	335	INTERGENIC	--	--	--
Ca4_P1_28022542	Ca4	28022542	GCC/GC	TGGAACATTGTGGCTGTGTT	GTTAGGTGCTGGGCTTTTTG	60.0	497	INTERGENIC	--	--	--
Ca4_P1_28023593	Ca4	28023593	GAAAAAAAAAA/GAAAAAAAA	CTCAAACCATTGAGGCCAGT	AATCTGCATCCCAAAATTGC	60.1	487	INTERGENIC	--	--	--
Ca4_P1_28027624	Ca4	28027624	CA/C	TGAAAAAGCACAAGCGACAC	GAGGGATGTATTTGAGGCCA	60.0	190	INTERGENIC	--	--	--
Ca4_P1_28042488	Ca4	28042488	T/TG	GTCCTTTTCCTTTGGCCTTC	CCTGAGGATAGCCTCGTGAA	60.1	159	INTERGENIC	--	--	--
Ca4_P1_28042758	Ca4	28042758	TC/T	GTCCTTTTCCTTTGGCCTTC	CACCCTCCTCCATATCCATT	60.1	361	INTERGENIC	--	--	--
Ca4_P1_28044154	Ca4	28044154	GAAAAAAA/GAAAAAAAA	GCCTTAGTCGTTGAAGCACC	CTTGGATTCCGACGAGGATA	59.9	521	INTERGENIC	--	--	--
Ca4_P1_28057637	Ca4	28057637	CTTTTTTTT/CTTTTTT	TTGGTGGTTCAAACTAGGGC	AATTTTCAAAACCGGTTGCTT	60.0	189	INTERGENIC	--	--	--
Ca4_P1_28057936	Ca4	28057936	GTTTTTTTTTT/GTTTTTTTTT	TTGGTGGTTCAAACTAGGGC	CATTTCGAACATTAACGGGG	60.0	654	INTERGENIC	--	--	--
Ca4_P1_28062057	Ca4	28062057	GT/GTT	TCAACAATTGTAATTGACTATCAACG	TATAGGCCGATCAGTCAGGC	59.4	771	INTERGENIC	--	--	--
Ca4_P1_28071470	Ca4	28071470	CAAAAAAAAAAA/CAAAAAAAAAAAA	GATGAGCAATTGGGGCTAAA	ACTTGATGGTCGATTCCTGG	60.0	171	INTERGENIC	--	--	--

INDEL marker IDs	Chromosomes/ unanchored scaffolds	Physical positions (bp)	InDels (*Kabuli* reference genome-CDC Frontier/PI)	Forward primers (5'-3')	Reverse primers (5'-3')	Anneling temperature (0C)	Expected amplified product size (bp)	Structural annotation		Functional annotation	
								Sequence components of *kabuli* genome	*Kabuli* gene accession IDs	NCBI-KOG	TFs
Ca4_P1_28071934	Ca4	28071934	CTTTTTTTTTT/CTTTTTTTTTTTT	CCAGGAATCGACCATCAAGT	CTATTTGACTTGGCTCGGGA	59.9	681	INTERGENIC	--	--	--
Ca4_P1_28086943	Ca4	28086943	GTAGAA/GTAGAATAGAA	TTTTGCATACGTCGGAATCA	ACGACGTTGATTGATCCAAA	60.1	577	INTERGENIC	--	--	--
Ca4_P1_28093890	Ca4	28093890	GAA/G	GAGAGTGCTCCTTTGGGAAA	GGGAAAAATTGCTTGGTGAA	59.4	300	DRR	Ca_23175	--	--
Ca4_P1_28108572	Ca4	28108572	TAAAAAAAA/TAAAAAAAAA	GCAAATGTTGGCGAAGTACA	AGTGTGTGCTTGTGGGATCA	59.7	301	INTERGENIC	--	--	--
Ca4_P1_28109349	Ca4	28109349	ATTTTTT/ATTTTTTT	CGGGGGTAGGGTTTTAGAGA	GCTGCTTGGTTGTGCTATGA	60.3	491	INTERGENIC	--	--	--
Ca4_P1_28112328	Ca4	28112328	ATTTTTTT/ATTTTTT	TTTTGTGGATGACAAAGACGA	CGAGACAAGACGTCCAACCT	59.2	461	INTERGENIC	--	--	--
Ca4_P1_28120280	Ca4	28120280	TAAAAAAAA/TAAAAAAAAA	TAGGGTGAAGGAGAACGGTG	TTTTTGTGCGTTTGACTGATG	60.1	822	INTERGENIC	--	--	--
Ca4_P1_28120543	Ca4	28120543	TAAAAAA/TAAAAAAA	CCAACTCCCTTCCCCTTTTA	CAATACGAGCACCTGGCTCT	60.3	994	INTERGENIC	--	--	--
Ca4_P1_28120712	Ca4	28120712	ATTTTTTTTTT/ATTTTTTTT	CATCAGTCAAACGCACAAAAA	CAATACGAGCACCTGGCTCT	59.8	433	INTERGENIC	--	--	--
Ca4_P1_28121722	Ca4	28121722	TAAAAAAAAAAAAAAA/TAAAAAAAAAAA	TGAACTCTTTCTTGGGTGGG	ATACGCATAATCGACTCCCG	60.1	643	INTERGENIC	--	--	--
Ca4_P1_28122657	Ca4	28122657	GAAAAAAAAAAAA/GAAAAAAAAAAA	CCCCGAGTTTGAGAAATTGA	CCTCCCGTCCTCTTTCTTTC	60.0	393	INTERGENIC	--	--	--
Ca4_P1_28122747	Ca4	28122747	GAAAAAAAAAA/GAAAAAAAAAAAAA	CCCCGAGTTTGAGAAATTGA	GCCACACGTCTTGTTCTTCC	60.0	474	INTERGENIC	--	--	--

INDEL marker IDs	Chromosomes/ unanchored scaffolds	Physical positions (bp)	InDels (*Kabuli* reference genome-CDC Frontier/PI)	Forward primers (5'-3')	Reverse primers (5'-3')	Anneling temperature (0C)	Expected amplified product size (bp)	Structural annotation		Functional annotation	
								Sequence components of *kabuli* genome	*Kabuli* gene accession IDs	NCBI-KOG	TFs
Ca4_P1_28125674	Ca4	28125674	CTTTTTTTTTTT/CTTTTTTTTTT	GAACAAGTTTCTCTTC GCGG	GTTTGCTGCAGGTTTT GTGA	60.0	799	INTRON	Ca_23174	--	bHLH
Ca4_P1_28131693	Ca4	28131693	TT/TTCGGTGGCT	TAGCAAGAACACCATC GCAA	TTAACCCTCAATTGCC GTTC	60.4	463	INTERGENIC	--	--	--
Ca4_P1_28143040	Ca4	28143040	ATTTTTTTTTTTTTTTT/ATTTTTTTTTT TTT	TCAAACAATGCCAAGT CCAA	TGGATATTGTGTGGGA ATGC	60.1	479	INTERGENIC	--	--	--
Ca4_P1_28146492	Ca4	28146492	TT/TTAT	TTGTGTGTGTGGGTTT TTGA	AATCCTGTATGGGGGA AAGG	58.4	430	INTERGENIC	--	--	--
Ca4_P1_28146874	Ca4	28146874	T/TACA	CCTTTCCCCCATACAG GATT	CCATTGAGGCACTGTA GGGT	60.0	186	INTERGENIC	--	--	--
Ca4_P1_28147858	Ca4	28147858	AT/ATTTAATAATTT	GGGAAAAGCCCCAAAC TAAG	TCCAACGTCGATTATG ACCA	59.9	424	INTERGENIC	--	--	--
Ca4_P1_28148756	Ca4	28148756	AT/ATCGCT	TGCATAAAAGTACGGA TCACCA	CTGCAAGCAGCAGAA GAACA	60.4	383	INTERGENIC	--	--	--
Ca4_P1_28153242	Ca4	28153242	CAA/C	TTGGCAGTGGTTCATT TTCA	AAAAGCTTGCGAACAT CACC	60.1	365	INTERGENIC	--	--	--
Ca4_P1_28159857	Ca4	28159857	TAAAAAAAAA/TAAAAAAAAAAA	TTTGGGGGTACAAGGG AAAT	TGCAACCTCATACTGG GAAA	60.4	645	INTERGENIC	--	--	--
Ca4_P1_28160415	Ca4	28160415	GAAAAAA/GAAAAAAA	CAAAATGCCCTTTGGC ACTA	TGACACGTTGTTTGTT TTGACA	61.0	457	INTERGENIC	--	--	--
Ca4_P1_28162271	Ca4	28162271	TAAAAAAAA/TAAAAAAA	AAACAAGTGGTTCTGG GCAC	CGAAACTTTGCATGCA TCTC	60.0	553	INTERGENIC	--	--	--
Ca4_P1_28167031	Ca4	28167031	CAAAAAA/CAAAAAAA	TTGAGATGGTTGCTTT GCAG	GCCCATTGGAGATTGA AAAA	60.0	661	DRR	Ca_23172	--	Dof

INDEL marker IDs	Chromosomes/ unanchored scaffolds	Physical positions (bp)	InDels (*Kabuli* reference genome-CDC Frontier/PI)	Forward primers (5'-3')	Reverse primers (5'-3')	Anneling temperature (0C)	Expected amplified product size (bp)	Structural annotation		Functional annotation	
								Sequence components of *kabuli* genome	*Kabuli* gene accession IDs	NCBI-KOG	TFs
Ca4_P1_28167305	Ca4	28167305	GGT/GGTGT	GCAACTTTGCCAACTCACAA	TTTTGAGCTAGTGTGTGCCG	59.9	552	DRR	Ca_23172	--	Dof
Ca4_P1_28174652	Ca4	28174652	TG/T	TTGCAACATTTGATGACGAA	GAAAAAGAAAATCACACCGCA	58.7	154	INTERGENIC	--	--	--
Ca4_P1_28179566	Ca4	28179566	AGGGG/AGGGGG	TGGGTAAAGTTCTGCCCACT	TAGCATGCTCGAAAACGAAA	59.6	129	INTERGENIC	--	--	--
Ca4_P1_28180097	Ca4	28180097	ATTTTTTTTT/ATTTTTTT	TCGAGCATGCTATAGTCCGA	ACCAATGTCCAACGGGAATA	59.5	869	INTERGENIC	--	--	--
Ca4_P1_28180264	Ca4	28180264	TAAAA/TAAA	GGAAGAGCGAGAAGGGAGAT	ACCAATGTCCAACGGGAATA	59.9	325	INTERGENIC	--	--	--
Ca4_P1_28180995	Ca4	28180995	CT/CTT	GTGATCATGGAGGCGAGTTT	GAGCAGACCAAACAGGGAAG	60.1	801	INTERGENIC	--	--	--
Ca4_P1_28183509	Ca4	28183509	GC/G	ACTGGCAGCCATTCACTCTT	TGACAACCCCAAACTTGTGA	59.9	745	INTERGENIC	--	--	--
Ca4_P1_28184807	Ca4	28184807	ATTT/ATTTT	GACCTTTGTTGACCTTGCGT	TGTTGAGTTGGAGATGAAGCA	60.2	595	INTERGENIC	--	--	--
Ca4_P1_2819881	Ca4	2819881	TAAAAAA/TAAAAAAA	TTGTTACCTCAATCGTGGGG	TTCGCCCCTACAACTACCAC	60.7	674	INTERGENIC	--	--	--
Ca4_P1_28201306	Ca4	28201306	C/CAAAT	AAAATCGAGAAAGGGGGAGA	TCCTTTTCCTGTTGTTTTTCG	60.0	179	INTERGENIC	--	--	--
Ca4_P1_28201530	Ca4	28201530	ATTTTTTTTTTT/ATTTTTTTTT	AAAATCGAGAAAGGGGGAGA	AAAATTTCACAATTCGAGAATCAA	60.0	562	INTERGENIC	--	--	--
Ca4_P1_28205048	Ca4	28205048	TA/T	GGCAACAATGTCTCAAAGCA	CACGTTGAATTCCACCTTCA	59.8	539	INTERGENIC	--	--	--

INDEL marker IDs	Chromosomes/ unanchored scaffolds	Physical positions (bp)	InDels (*Kabuli* reference genome-CDC Frontier/PI)	Forward primers (5'-3')	Reverse primers (5'-3')	Anneling temperature (0C)	Expected amplified product size (bp)	Structural annotation		Functional annotation	
								Sequence components of *kabuli* genome	*Kabuli* gene accession IDs	NCBI-KOG	TFs
Ca4_P1_28205282	Ca4	28205282	CAAAAAAA/CAAAAAA	GGCAACAATGTCTCAAAGCA	CACGTTGAATTCCACCTTCA	59.8	539	INTERGENIC	--	--	--
Ca4_P1_28207311	Ca4	28207311	TAAAAA/TAAAA	TTTTGTTTTGTATTTGACGATGC	TTAGCTCCGTTTCATGGACA	59.1	794	INTERGENIC	--	--	--
Ca4_P1_28208368	Ca4	28208368	TC/T	TGATGTGCGGTATGCTTTTC	TTCCCTCCAAAGTTGGACAC	59.7	670	INTERGENIC	--	--	--
Ca4_P1_28208396	Ca4	28208396	TAAAAAAA/TAAAAAAAA	TGATGTGCGGTATGCTTTTC	TTCCCTCCAAAGTTGGACAC	59.7	670	INTERGENIC	--	--	--
Ca4_P1_28208787	Ca4	28208787	CTTTTTTTTTTT/CTTTTTTTTTT	GTGTCCAACTTTGGAGGGAA	TTCCCATCGGACAACAAAAT	59.9	286	INTERGENIC	--	--	--
Ca4_P1_28209971	Ca4	28209971	T/TA	ATTGATGGAGCGAATCGAAC	AATTCCTCTTTTGGTGGGCT	60.0	891	INTERGENIC	--	--	--
Ca4_P1_28212107	Ca4	28212107	TCCC/TCC	TGCAGACATTTTCCAACGAG	TTTAATCTGCGTATCTCCCCA	59.8	232	INTERGENIC	--	--	--
Ca4_P1_28242856	Ca4	28242856	AGGGGGG/AGGGGGGG	ATTGGGAAGTGGGGAGACTT	CCATCTTTGGGTGAACCAGT	59.8	677	INTERGENIC	--	--	--
Ca4_P1_28254483	Ca4	28254483	CAACAA/CAACAAGACAAACAA	GGGGAACTCTCCAAGTCACA	CGTGGAAACCAACCTTGTCT	60.1	703	INTERGENIC	--	--	--
Ca4_P1_28274294	Ca4	28274294	GAAAAAAAAAA/GAAAAAAAAA	CCAGAAGCAGAAAAACCAGG	TTTGAAACTGTCGCGACTCA	59.8	631	INTERGENIC	--	--	--
Ca4_P1_28274340	Ca4	28274340	CAAAAAAAAAA/CAAAAAAAAA	CCAGAAGCAGAAAAACCAGG	TTTGAAACTGTCGCGACTCA	59.8	631	INTERGENIC	--	--	--
Ca4_P1_28277172	Ca4	28277172	GAAA/GAA	ATTTTCAGTGGCCTACACGC	TGTGGATCAGGTTTGGAGAA	60.1	210	INTERGENIC	--	--	--

INDEL marker IDs	Chromosomes/ unanchored scaffolds	Physical positions (bp)	InDels (*Kabuli* reference genome-CDC Frontier/PI)	Forward primers (5'-3')	Reverse primers (5'-3')	Anneling temperature (0C)	Expected amplified product size (bp)	Structural annotation		Functional annotation	
								Sequence components of *kabuli* genome	*Kabuli* gene accession IDs	NCBI-KOG	TFs
Ca4_P1_28281945	Ca4	28281945	CTTTTTTT/CTTTTTT	ATGCAAGTGGCCAAAAGAAC	GCTGCCACATGAATACAACC	60.1	673	INTRON	Ca_23166	TR	FAR1
Ca4_P1_28282247	Ca4	28282247	ATTTTTT/ATTTTTTT	TCCAGAAGGCATTTCTTGGT	AGGTTAGTTGGAGCGTTTGAA	59.7	430	DRR	Ca_23166	TR	FAR1
Ca4_P1_28283679	Ca4	28283679	TATTTAATTT/TATTT	ATGGGGATGTTGTGTGTGTG	TGAGAGTTGCTTGATCGGG	60.1	670	DRR	Ca_23166	TR	FAR1
Ca4_P1_28295619	Ca4	28295619	ATT/AT	CAACCATCCCCTTTCTTTGA	CCACTTGACCCAATTAAGCA	59.9	545	INTERGENIC	--	--	--
Ca4_P1_28296518	Ca4	28296518	ATC/A	TAGCTGCTCACACTGCCAAC	GGTTGTTGTGCCCAGAGATT	60.2	527	INTERGENIC	--	--	--
Ca4_P1_28297192	Ca4	28297192	ATTTTT/ATTTT	AATCTCTGGGCACAACAACC	TAGGTTGGACGTCTTGTCCC	60.0	764	INTERGENIC	--	--	--
Ca4_P1_28306538	Ca4	28306538	CTTTTTTTT/CTTTTTTTTT	GGGTTTGTCTTTTGCGAGAA	AATTGTCCCATCCCTTACCC	60.2	772	INTERGENIC	--	--	--
Ca4_P1_28306771	Ca4	28306771	CAAAAAAAAA/CAAAAAAAAAAA	TGATGGTCCATGAACTGACAT	CGAGCCAAAATCCAAAACAT	58.8	430	INTERGENIC	--	--	--
Ca4_P1_28321521	Ca4	28321521	CCGTACAGGCGTACA/CCGTACA	TCGAGTTGTGAAATTGAGCG	GCTGAAGAACCCGAGAAAGA	60.0	202	INTERGENIC	--	--	--
Ca4_P1_28331028	Ca4	28331028	CAAAAAAAA/CAAAAAAAAA	CTTGAAGGGACGGCTAGATG	TTGCCATAATGTTGAGAAGGG	59.8	458	INTERGENIC	--	--	--
Ca4_P1_28336605	Ca4	28336605	CAAAAAAAAAAAAAA/CAAAAAAAAAAAAA	CAAAGCAGGGAGTTTCCATC	TGGTTTAAGTCCTCGGCTGT	59.7	723	INTERGENIC	--	--	--
Ca4_P1_28337463	Ca4	28337463	AGTCTAT/A	ACAGCCGAGGACTTAAACCA	GACCATCTGGTCAACGCTTC	59.7	768	INTERGENIC	--	--	--

INDEL marker IDs	Chromosomes/ unanchored scaffolds	Physical positions (bp)	InDels (*Kabuli* reference genome-CDC Frontier/PI)	Forward primers (5'-3')	Reverse primers (5'-3')	Anneling temperature (0C)	Expected amplified product size (bp)	Structural annotation		Functional annotation	
								Sequence components of *kabuli* genome	*Kabuli* gene accession IDs	NCBI-KOG	TFs
Ca4_P1_28338388	Ca4	28338388	TAAAAA/TAAAAAA	TGACTCGGGCTGCAGATTAT	CACCGGTGGTCTTTCCTTTA	60.8	432	INTERGENIC	--	--	--
Ca4_P1_28338433	Ca4	28338433	GTTT/GTT	TGACTCGGGCTGCAGATTAT	GCTTTGAGCCCCATGAAATA	60.8	739	INTERGENIC	--	--	--
Ca4_P1_28342637	Ca4	28342637	GCCC/GCCCC	TCACAAATCCTGGTGGTTGA	GGAAGAAAGCAATGGTGGAA	59.9	359	INTERGENIC	--	--	--
Ca4_P1_28346696	Ca4	28346696	A/AT	ACCACAGGTGAAAGAAACCG	TTTTCAACTCTATCCGTAGCCC	60.0	340	INTERGENIC	--	--	--
Ca4_P1_28348918	Ca4	28348918	TATTTA/T	CTTTGCGATCAAATGGTCAA	TCAAGCTCAAAACGTGATGC	59.7	592	INTERGENIC	--	--	--
Ca4_P1_28351338	Ca4	28351338	TATA/T	GGCAGAAATTGTGTCCGTTT	CAACAAGTAGTCTCATGATTTCCC	60.0	346	INTERGENIC	--	--	--
Ca4_P1_2835298	Ca4	2835298	TATAATAATAATAATAATAATAAT/TATAATAATAATAATAATAAT	TTCGGTCAACCAAGTTTTAGG	TTGGGTTTAAGTGTGAGTGGC	59.1	562	INTERGENIC	--	--	--
Ca4_P1_28358498	Ca4	28358498	GAAAAAAA/GAAAAAAAA	GTGCACAAGCCTTGATTGAA	CGACCAAGACTCGTGTGAAA	59.8	488	INTERGENIC	--	--	--
Ca4_P1_28358687	Ca4	28358687	AAA/AAAGAA	GTGCACAAGCCTTGATTGAA	CATTGAGCAACAAAGCTCCA	59.8	751	INTERGENIC	--	--	--
Ca4_P1_28360925	Ca4	28360925	TTTATTTATCTTAT/TTTAT	CTCCGGTTCACCAAAATGAC	TGACCACCTGAAAACAAGGA	60.4	509	INTRON	Ca_23163	AYT	MYB
Ca4_P1_28362055	Ca4	28362055	GTTTTTTTTT/GTTTTTTTTTT	CAGCTGTTAGAGGGTGAGGG	TGTTCAAATATCATTCTTCCACG	59.9	149	INTRON	Ca_23163	AYT	MYB
Ca4_P1_28362288	Ca4	28362288	TA/T	CAGCTGTTAGAGGGTGAGGG	TTCCATAATTCCTGCTTCTTCC	59.9	742	INTRON	Ca_23163	AYT	MYB

INDEL marker IDs	Chromosomes/ unanchored scaffolds	Physical positions (bp)	InDels (*Kabuli* reference genome-CDC Frontier/PI)	Forward primers (5'-3')	Reverse primers (5'-3')	Anneling temperature (0C)	Expected amplified product size (bp)	Structural annotation		Functional annotation	
								Sequence components of *kabuli* genome	*Kabuli* gene accession IDs	NCBI-KOG	TFs
Ca4_P1_28364496	Ca4	28364496	CAAA/CAA	TCCCAATCGATTTGTTGGAT	TCAGGCAAACAAGCAGACAC	60.1	475	INTRON	Ca_23163	AYT	MYB
Ca4_P1_28369830	Ca4	28369830	ATTTTTTTTTT/ATTTTTTTTT	CCATCTTGCAATTGGTTCAA	TGTCATGCCAAATGAAAAATTC	59.5	526	INTERGENIC	--	--	--
Ca4_P1_28372180	Ca4	28372180	TATGTAATGT/TATGT	TTTGATAAACCCCGTGATCC	TGGAGCAGTTTTTCAACCAA	59.6	572	INTERGENIC	--	--	--
Ca4_P1_28372820	Ca4	28372820	ATTTT/ATTTTT	TAAGGGAAGGGGCTCTAGGA	CCCCTAAAATTTAAAAGGCCC	60.2	389	INTERGENIC	--	--	--
Ca4_P1_28374499	Ca4	28374499	GAAAA/GAAAAA	GAAGGAACACTGCAAAGCAA	ACACATCCTCAATTCACCGA	59.1	552	DRR	Ca_23162	C	--
Ca4_P1_28377332	Ca4	28377332	TAAA/TAA	CATGGAAAACAGGTGACACG	AACCCAGCAAATACCAATGC	60.0	364	INTRON	Ca_23162	C	--
Ca4_P1_28380287	Ca4	28380287	ATTTT/ATTTTT	ACTTTTCTCATTTTTAAGTCATCAAAT	CTTCAAAGTTGCCGGTGATT	57.3	919	INTERGENIC	--	--	--
Ca4_P1_28384863	Ca4	28384863	CTTTT/CTTTTTT	TCAAGCTGGAGAAGGTTCAGA	TTGCTTTGTATTCCCCAACC	60.1	601	INTERGENIC	--	--	--
Ca4_P1_28390462	Ca4	28390462	TAA/TAAA	GTCATCAAAAGCCGACGAAT	GGCCCACAATTTTGAAGAGA	60.1	786	INTERGENIC	--	--	--
Ca4_P1_28401988	Ca4	28401988	TAATTAA/TAATTAATTAAATAAAATTAA	ACTTGGATGAAGGGAAAGGG	GCATTTCGCAAAAATTTGAA	60.3	561	INTERGENIC	--	--	--
Ca4_P1_28402307	Ca4	28402307	GAAA/GAA	TGCTGACTATGCTTGTCGGA	TTGCCCACTTAATGAAGAAGAA	60.6	799	INTERGENIC	--	--	--
Ca4_P1_28404551	Ca4	28404551	AAG/AAGAG	GGAATTCAATTGATTGGGGA	CGTGTTGATGAACCATCATTG	59.6	460	INTERGENIC	--	--	--

INDEL marker IDs	Chromosomes/ unanchored scaffolds	Physical positions (bp)	InDels (*Kabuli* reference genome-CDC Frontier/PI)	Forward primers (5'-3')	Reverse primers (5'-3')	Anneling temperature (0C)	Expected amplified product size (bp)	Structural annotation		Functional annotation	
								Sequence components of *kabuli* genome	*Kabuli* gene accession IDs	NCBI-KOG	TFs
Ca4_P1_28406467	Ca4	28406467	TC/T	CGAGGATAAACTCGAGGACG	TGCAAAGATGAATGGTTCTCA	59.8	465	INTERGENIC	--	--	--
Ca4_P1_28407155	Ca4	28407155	ATTTTTTT/ATTTTTTTT	TTGGAACAATGGCCACCTAT	CCTGGTTCCCTTTATGACCA	60.2	456	INTERGENIC	--	--	--
Ca4_P1_28407636	Ca4	28407636	C/CTT	ACACCTAATTCACCCCCTCC	TCCCTTTTCCAACCACTTTG	60.1	517	INTERGENIC	--	--	--
Ca4_P1_28408105	Ca4	28408105	AAAAGAAA/AAAA	TCAAAGTGGTTGGAAAAGGG	CCCAACAATTTGAAGTGGCT	59.9	179	INTERGENIC	--	--	--
Ca4_P1_28410297	Ca4	28410297	GAAAAA/GAAAA	TTTTGGGATCTCAAGGTGAAA	ATCGTGGCAAAGATGACACA	59.5	613	INTERGENIC	--	--	--
Ca4_P1_28414370	Ca4	28414370	ATTTTTTTTTTTT/ATTTTTTTTTT	CCAGTAGTGGTTGCAGCCTT	ATTAAGCACAAACATGGGGC	60.3	377	INTERGENIC	--	--	--
Ca4_P1_28414876	Ca4	28414876	CAAT/CAATAAT	GCCCCATGTTTGTGCTTAAT	GGATTCAGCATCCGTCAAGT	59.8	741	INTERGENIC	--	--	--
Ca4_P1_28417839	Ca4	28417839	CAA/CAATAGAA	TGCCTTGGAAGAGGCTTTTA	TGAAAAATAGCCCAATGGTGA	60.0	715	INTERGENIC	--	--	--
Ca4_P1_28418179	Ca4	28418179	ATTTTTTTT/ATTTTTTT	TGCAAGGCCATTCTGTCTAA	GCAGGTTAGATGGGGAATCA	59.4	649	INTERGENIC	--	--	--
Ca4_P1_28420944	Ca4	28420944	TAAAGTTAGAAGAA/TAA	GTGTGGGCGGAAAAATCTAA	CACAGTGAAAAACCGATGGA	59.9	693	INTERGENIC	--	--	--
Ca4_P1_28421324	Ca4	28421324	AA/AAACA	CCAATCCATCGGTTTTTCAC	TGCATGGAAAGCTGTGGTAG	60.2	472	INTERGENIC	--	--	--
Ca4_P1_28422575	Ca4	28422575	ATGTGTTT/AT	TGAAGGAAACTTTGATGCCA	TTGGTTTAACGGCAAAGTGA	59.2	596	INTERGENIC	--	--	--

INDEL marker IDs	Chromosomes/ unanchored scaffolds	Physical positions (bp)	InDels (*Kabuli* reference genome-CDC Frontier/PI)	Forward primers (5'-3')	Reverse primers (5'-3')	Anneling temperature (0C)	Expected amplified product size (bp)	Structural annotation		Functional annotation	
								Sequence components of *kabuli* genome	*Kabuli* gene accession IDs	NCBI-KOG	TFs
Ca4_P1_28422969	Ca4	28422969	TA/T	TCACTTTGCCGTTAAACCAA	ACACCACATGGGGAAACCTA	59.2	213	INTERGENIC	--	--	--
Ca4_P1_28423269	Ca4	28423269	T/TA	TAGGTTTCCCCATGTGGTGT	AGCACTTGCCCCTTAGGAGT	60.1	697	INTERGENIC	--	--	--
Ca4_P1_28439809	Ca4	28439809	GTTTTTTT/GTTTTTTTT	TATGCCAATGCGAAATGAAA	GCGCAAACAAGAGAATAGGC	60.0	642	INTERGENIC	--	--	--
Ca4_P1_28440300	Ca4	28440300	TG/TGCAAATATTGATAG	TTGTTTGCGCTTCAGTATGG	TGCTACCCCTTTGTTGGAAG	59.9	257	INTERGENIC	--	--	--
Ca4_P1_28440649	Ca4	28440649	AT/ATT	CTTCCAACAAAGGGGTAGCA	CCCTCCATTCTCATGTTTGC	60.1	406	INTERGENIC	--	--	--
Ca4_P1_28447880	Ca4	28447880	ATTTTTTTT/ATTTTTTTTT	TCTTCTGTGTGAGCGGATTG	GCTCAACCTTGAGACCAAGC	60.0	252	INTERGENIC	--	--	--
Ca4_P1_28449436	Ca4	28449436	A/AT	CCAAATCTGGTGAAAGGGAA	CCATGTGGTTAGCATAGGGC	59.9	616	INTERGENIC	--	--	--
Ca4_P1_28454605	Ca4	28454605	TTGTCTG/T	GCCATTTGCCTAACCAAAAA	CTTGGTTCCAACAATCTCCC	59.9	493	INTERGENIC	--	--	--
Ca4_P1_28457671	Ca4	28457671	TCTCTA/TCTCTATTTAAACTCTA	TACAAAGCACCTGACCCTCC	CCCAAATCAAAGCAAGCAAT	60.1	765	INTERGENIC	--	--	--
Ca4_P1_28497094	Ca4	28497094	TAAAAAAA/TAAAAAA	GGTATCGGATCGAAACCAGA	CCCCAAGAATACATGAAAATTGA	59.9	841	INTERGENIC	--	--	--
Ca4_P1_28501292	Ca4	28501292	AAATATTTTATTATT/AAATATTTTATTATTAATATTTTATTATT	GCACGCACACACATATACCC	TTTGCAGGCACAAAAGACAG	59.9	747	INTERGENIC	--	--	--
Ca4_P1_28504169	Ca4	28504169	CAA/CA	GCTGGCGGGAATTAGTATGA	GCTTCCCCACTATCACTCCA	60.1	132	INTERGENIC	--	--	--

INDEL marker IDs	Chromosomes/ unanchored scaffolds	Physical positions (bp)	InDels (*Kabuli* reference genome-CDC Frontier/PI)	Forward primers (5'-3')	Reverse primers (5'-3')	Anneling temperature (0C)	Expected amplified product size (bp)	Structural annotation		Functional annotation	
								Sequence components of *kabuli* genome	*Kabuli* gene accession IDs	NCBI-KOG	TFs
Ca4_P1_28505491	Ca4	28505491	TAAAAAAAA/TAAAAAAAAA	GGATTTCACGGTGGTCTTTT	TCACACACCTCAAAACCCCT	58.9	433	INTERGENIC	--	--	--
Ca4_P1_28510451	Ca4	28510451	TAAAAAAAA/TAAAAAAAAAAA	AGGAAATTTCGCCCAAGAAC	GCACATTTTCCAAGGGCTTA	60.4	427	INTERGENIC	--	--	--
Ca4_P1_28519731	Ca4	28519731	AA/AAGA	GCCATGAAGTGGCCTAACAT	TCACGGTTTTGTAGAGCGGT	60.0	620	INTERGENIC	--	--	--
Ca4_P1_28522324	Ca4	28522324	CTTT/CTT	ATGCATTTACGTTGTTGCCA	TTTGTTGCATGCCAATTTGT	60.0	522	INTERGENIC	--	--	--
Ca4_P1_28534748	Ca4	28534748	ATTTTTTTTTTT/ATTTTTTTTTT	TGGTCAACATGCCAAAGAAA	TTGTTTTTCCTCCAAACAAATG	60.1	958	INTERGENIC	--	--	--
Ca4_P1_28535714	Ca4	28535714	TTT/TTTATT	GCCAGCTCCAATTACAATGC	CATGTCTTTTGAATTTTGATCAGC	60.6	816	INTERGENIC	--	--	--
Ca4_P1_28541959	Ca4	28541959	TAAAAAAAA/TAAAAAAA	TTGCATCGGCCTACTTTCTT	CGGATTCATCCAAAGTTTCAA	59.8	637	INTERGENIC	--	--	--
Ca4_P1_28543411	Ca4	28543411	ATTTTTTTTTT/ATTTTTTTTT	TCAAGTTTCGAACTCTCTCGC	GGAATGGAATGCGAAGAAAA	59.7	323	INTERGENIC	--	--	--
Ca4_P1_28555463	Ca4	28555463	GTTTTTTTTTTTT/GTTTTTTTTTTTTTT	GAGGGAGAGGAAGAGGCAGT	ATTAAAATTCGGGGACAGGG	60.0	433	INTERGENIC	--	--	--
Ca4_P1_28581947	Ca4	28581947	TAAAAAAAAAAA/TAAAAAAAAAA	CATTTCCCCACACCAATTTC	AAGGATGATGATTGTGCATGT	60.0	379	INTERGENIC	--	--	--
Ca4_P1_28581991	Ca4	28581991	CAAAAAAAAAA/CAAAAAAAAAAA	CATTTCCCCACACCAATTTC	AAGGATGATGATTGTGCATGT	60.0	379	INTERGENIC	--	--	--
Ca4_P1_28583861	Ca4	28583861	ATT/AT	AGAGGGAGGGACCAAAACTT	CTGGAGCAAGCTTTCCAAAC	59.0	524	INTERGENIC	--	--	--

INDEL marker IDs	Chromosomes/ unanchored scaffolds	Physical positions (bp)	InDels (*Kabuli* reference genome-CDC Frontier/PI)	Forward primers (5'-3')	Reverse primers (5'-3')	Anneling temperature (0C)	Expected amplified product size (bp)	Structural annotation		Functional annotation	
								Sequence components of *kabuli* genome	*Kabuli* gene accession IDs	NCBI-KOG	TFs
Ca4_P1_28586798	Ca4	28586798	ATTTTT/ATTTT	GCCCAAAGCATACGAGACAT	TTGGGGGATAATTGTTTGGA	60.1	516	INTERGENIC	--	--	--
Ca4_P1_28590176	Ca4	28590176	CTTTTT/CTTTTTT	CCACCTCCACCATTCCTCTA	CAGCACCACAAACATCCAAG	59.9	476	INTERGENIC	--	--	--
Ca4_P1_28616334	Ca4	28616334	GTTTTTTTTTT/GTTTTTTTTTTT	ATTGCATGTTGGAGGAAAGC	GGTGTACATGTGACAAGGCG	60.1	752	INTERGENIC	--	--	--
Ca4_P1_28623056	Ca4	28623056	A/AGAATTTGG	ATATGGTTGCGCACAAATGA	TGTCCGAAACATTAAACCCA	60.0	704	INTERGENIC	--	--	--
Ca4_P1_28714992	Ca4	28714992	GATAT/GAT	AGAGCCAACGCTCATAGGAA	TTAAAATTCCCTCCTCGGCT	60.0	531	INTERGENIC	--	--	--
Ca4_P1_28771989	Ca4	28771989	CTTTTTTTTT/CTTTTTTTTTT	AGAGGTCTATGGTGCCAGGA	ACCACCTTCTCGCACAAACT	59.7	658	DRR	Ca_23696	--	--
Ca4_P1_28783547	Ca4	28783547	CTTTTTTTTT/CTTTTTTTTTT	TGGAAAGCTTGCTCCTTCAT	CAAGTGATGTTGCCTATTCTGG	60.0	124	INTERGENIC	--	--	--
Ca4_P1_28791900	Ca4	28791900	TCACA/T	TGCCCCTCATGGTTATCCTA	CACAGAGTTCGACACCGCTA	60.3	580	INTERGENIC	--	--	--
Ca4_P1_28793395	Ca4	28793395	TAA/TAAAAACAA	CCAAAATTACACCAACGGTTC	TTGTTCGCATGCTCATTCTC	59.2	275	INTERGENIC	--	--	--
Ca4_P1_28802024	Ca4	28802024	GTTTT/GTTTTT	TGAGTGGGTGAATGAGATGTG	TTCCACAGTCCAAACCACAA	59.5	244	INTERGENIC	--	--	--
Ca4_P1_28802275	Ca4	28802275	AT/A	TTGTGGTTTGGACTGTGGAA	TTAGGTGCAAGTGTGGATGG	60.0	645	INTERGENIC	--	--	--
Ca4_P1_28806476	Ca4	28806476	T/TA	TGTTTGTTTTTGCAAGCAGG	GCAAGAAGCCATCCACAAGT	59.9	351	INTERGENIC	--	--	--

INDEL marker IDs	Chromosomes/ unanchored scaffolds	Physical positions (bp)	InDels (*Kabuli* reference genome-CDC Frontier/PI)	Forward primers (5'-3')	Reverse primers (5'-3')	Anneling temperature (0C)	Expected amplified product size (bp)	Structural annotation		Functional annotation	
								Sequence components of *kabuli* genome	*Kabuli* gene accession IDs	NCBI-KOG	TFs
Ca4_P1_28822607	Ca4	28822607	TAAAA/TAAA	CTTCTTCCGCAAATGGTGTT	TGTATTACCATGATTTTGTTGACTGA	60.1	448	INTERGENIC	--	--	--
Ca4_P1_28828876	Ca4	28828876	GTTTTTTTTTTT/GTTTTTTTTTTTTTT	GTGGCCTAAGCCCTCACAA	TCTTTATTTGTGGCGGTCAA	61.2	108	INTERGENIC	--	--	--
Ca4_P1_28834019	Ca4	28834019	AA/AAGA	TTTTGTTCCTCCATGTGATTTTT	ATAGTCTTCCATTGGCGTCG	59.7	795	INTERGENIC	--	--	--
Ca4_P1_28848955	Ca4	28848955	GTATAT/GTATATAT	AGAGGAAATGATGCCACAATG	AAACACTTGCGTAACCTGGC	59.9	794	INTERGENIC	--	--	--
Ca4_P1_28849010	Ca4	28849010	GAAAAAAAA/GAAAAAAAAA	AGAGGAAATGATGCCACAATG	AAACACTTGCGTAACCTGGC	59.9	794	INTERGENIC	--	--	--
Ca4_P1_28850439	Ca4	28850439	ATTATTTTATTTT/ATTATTTTTATTTTTATTTT	GTCAGAGGCAGTGAGCCTTC	TTTTAGCTTTCCAACGCCTG	60.1	500	INTERGENIC	--	--	--
Ca4_P1_28853222	Ca4	28853222	CAAAAAAAAA/CAAAAAAAAAA	TCCATTTTGGTCCATGAGAA	TTTTTCTTCTTCGTCCTCGC	58.9	435	INTERGENIC	--	--	--
Ca4_P1_28856354	Ca4	28856354	ATTTTTTT/ATTTTTTTT	GCGCGAATATCTCAACCAAT	TCCACCAACAACAAGATCCA	60.1	212	INTERGENIC	--	--	--
Ca4_P1_28856754	Ca4	28856754	ATT/AT	TGCCGTATTGATGGATCTGA	ACAAGGATGAGTTTGGCACC	60.0	923	INTRON	Ca_23701	T	E2F/DP
Ca4_P1_28870444	Ca4	28870444	A/AAT	TGAGGAGGCTTCTTGGAGTG	AAAACGACTTCGAGGCATGT	60.5	817	INTERGENIC	--	--	--
Ca4_P1_2888123	Ca4	2888123	AAGGAG/AAGGAGGAG	GCAAAGGGAAAATCATTGGA	GACACAGGTTCAAACTCGCA	59.9	400	DRR	Ca_12085	P	ERF
Ca4_P1_28887918	Ca4	28887918	TAAAAAAAAA/TAAAAAAAAAA	TGCCCTATTTTGTGTCCAGG	TGCGTTTGGCTGATGTTAAG	60.9	573	INTERGENIC	--	--	--

INDEL marker IDs	Chromosomes/ unanchored scaffolds	Physical positions (bp)	InDels (*Kabuli* reference genome-CDC Frontier/PI)	Forward primers (5'-3')	Reverse primers (5'-3')	Anneling temperature (0C)	Expected amplified product size (bp)	Structural annotation		Functional annotation	
								Sequence components of *kabuli* genome	*Kabuli* gene accession IDs	NCBI-KOG	TFs
Ca4_P1_28894593	Ca4	28894593	CCTTC/CCTTCTTC	AGGAGCATTTGGAGTTCTCG	AAAATGTAAACTCGTGCTCGC	59.4	645	INTERGENIC	--	--	--
Ca4_P1_28901728	Ca4	28901728	TC/T	GATGTTTCATCATGCGATCA	CCAAAGCTTTCAAGGTCCAA	58.0	145	INTERGENIC	--	--	--
Ca4_P1_2890812	Ca4	2890812	GA/GAATTA	ACTCAAACTCAAACGCGGAG	TAAAAGGTGGCACCACAACA	60.4	500	INTERGENIC	--	--	--
Ca4_P1_28908427	Ca4	28908427	ATCTT/ATCTTCTT	CGTGGGAAAAGGGGTAATTT	ATTTCCCGATTTGGACCTTT	60.0	531	INTERGENIC	--	--	--
Ca4_P1_28909780	Ca4	28909780	CAAAAA/CAAAAAA	TGAAAATTGTTTTTAACGCCA	CCCAGTTTGGAAGGAAACAG	58.3	781	INTERGENIC	--	--	--
Ca4_P1_28910424	Ca4	28910424	ATTTTCTTTTTCTTTTTCTT/ATTTTCTTTTTCTT	CTGTTTCCTTCCAAACTGGG	TGTAGAGGAATGTCTCGCCC	59.6	393	INTERGENIC	--	--	--
Ca4_P1_28910579	Ca4	28910579	A/AC	CTGTTTCCTTCCAAACTGGG	TGTAGAGGAATGTCTCGCCC	59.6	393	INTERGENIC	--	--	--
Ca4_P1_28913518	Ca4	28913518	TATATATATA/TATATATATAAATATATATA	TTCAGTGGATCCCTCAAACC	CAACGTCCTCCCATTTGTTT	59.9	951	INTERGENIC	--	--	--
Ca4_P1_28913641	Ca4	28913641	AAATAATAATAATAATAATAAT/AAATAATAATAATAATAAT	TACGTCGCTCCATGCAAATA	CAACGTCCTCCCATTTGTTT	60.2	822	INTERGENIC	--	--	--
Ca4_P1_28914437	Ca4	28914437	GA/GAAA	AAACAAATGGGAGGACGTTG	TGTTCCAGATATTTTGCCGA	59.8	508	INTERGENIC	--	--	--
Ca4_P1_28914633	Ca4	28914633	GTAATAATAATAATAATAATAATAA/GTAATAATAATAATAATAATAATAA	TCGGCAAAATATCTGGAACA	TGCGATCATGTGGAACATTT	59.1	573	INTERGENIC	--	--	--
Ca4_P1_28914767	Ca4	28914767	TTTATTATTATTATTATTA/TTTATTATTATTATTATTA	TCGGCAAAATATCTGGAACA	TGCGATCATGTGGAACATTT	59.1	573	INTERGENIC	--	--	--

INDEL marker IDs	Chromosomes/ unanchored scaffolds	Physical positions (bp)	InDels (*Kabuli* reference genome-CDC Frontier/PI)	Forward primers (5'-3')	Reverse primers (5'-3')	Anneling temperature (0C)	Expected amplified product size (bp)	Structural annotation		Functional annotation	
								Sequence components of *kabuli* genome	*Kabuli* gene accession IDs	NCBI-KOG	TFs
Ca4_P1_28915111	Ca4	28915111	TAAAAAAAA/TTAAAAAAAAAA	AAATGTTCCACATGATCGCA	TCCAAAAATAACGTTTTGCCA	59.9	400	INTERGENIC	--	--	--
Ca4_P1_28915187	Ca4	28915187	GTTTTTTTTT/GTTTTTTTT	AAATGTTCCACATGATCGCA	TCCAAAAATAACGTTTTGCCA	59.9	400	INTERGENIC	--	--	--
Ca4_P1_28916016	Ca4	28916016	TTAT/TTATAT	TTCTCCAGCCAAAAACACAA	GAGAGGATCAAAACTCCGCA	59.3	433	INTERGENIC	--	--	--
Ca4_P1_2891639	Ca4	2891639	A/AG	TGTTGTGGTGCCACCTTTTA	TAATACACACGGCCAAGCAA	60.0	823	INTERGENIC	--	--	--
Ca4_P1_28925061	Ca4	28925061	CAAAAAA/CAAAAAAAAA	GGGGTGGTTTTTAGCTTGGT	TAGCCCAGTTTGGAAGGAAA	60.2	773	INTERGENIC	--	--	--
Ca4_P1_28925610	Ca4	28925610	TAA/TA	TTTCCTTCCAAACTGGGCTA	AATGCGGTCTTCCCCTAAGT	59.7	622	INTERGENIC	--	--	--
Ca4_P1_28929142	Ca4	28929142	GGGG/GGGGAGGG	ATCTCTGTGATCCCCGTTTG	GAGGACTGGACGTACCCTTG	59.9	325	INTERGENIC	--	--	--
Ca4_P1_28932452	Ca4	28932452	GAAAAAAAAA/GAAAAAAAA	GAGCGGATTACCCCAAAAAT	TTGGAGTTGTGCCAATGTGT	60.2	742	INTERGENIC	--	--	--
Ca4_P1_28933902	Ca4	28933902	GTT/GT	GATGGACACTCATGTCAATGAAG	TGGCCAAACTGATGATTCAA	59.5	574	INTERGENIC	--	--	--
Ca4_P1_28936010	Ca4	28936010	TTTTATTTATTTATTTATTTAT/TTTTATTTATTTATTTATTTATTTATTTATTTAT	TTTGTCTTGTTGGGTGACCA	TGGCTGAATTGTGGATTCTTT	60.0	816	INTERGENIC	--	--	--
Ca4_P1_28936822	Ca4	28936822	CTT/CT	TAAAGACATTGGGTGGAGGC	TGGACTTTGATGACTGCTCG	59.9	415	INTERGENIC	--	--	--
Ca4_P1_2894996	Ca4	2894996	GAAAAAAAAA/GAAAAAAAAAA	AAAAGAAACGGAGGAAGTGTCA	TAAATACGGTGATCGGCCTC	60.1	127	DRR	Ca_12086	P	ERF

INDEL marker IDs	Chromosomes/ unanchored scaffolds	Physical positions (bp)	InDels (*Kabuli* reference genome-CDC Frontier/PI)	Forward primers (5'-3')	Reverse primers (5'-3')	Anneling temperature (0C)	Expected amplified product size (bp)	Structural annotation		Functional annotation	
								Sequence components of *kabuli* genome	*Kabuli* gene accession IDs	NCBI-KOG	TFs
Ca4_P1_28950587	Ca4	28950587	TTAT/TT	TCCCCAAGTGAAACCCACTA	TCTGGCAGCCATTCTCTTTT	60.3	168	INTERGENIC	--	--	--
Ca4_P1_28955300	Ca4	28955300	GAAAAAAAAA/GAAAAAAAAAA	CTTGCCTGTGTCGAAGATGA	ATTTCCTTCACGTGGGTCAA	60.0	209	INTERGENIC	--	--	--
Ca4_P1_2897791	Ca4	2897791	ATATTTATTTATTTATTTATTTATTT/ATATTTATTTATTTATTTATTTATTTATTT	AAAAAGACATGCTTTCAATTTGG	AAGCCTTGTGGAGCAGTTGT	59.5	948	INTERGENIC	--	--	--
Ca4_P1_28986690	Ca4	28986690	ATCGTCGT/ATCGT	AACAATGCAAAGTTGTCTCACA	AAAAATGGTGATGAGCAAAAA	58.3	423	INTERGENIC	--	--	--
Ca4_P1_28988768	Ca4	28988768	TAAAAAAAA/TAAAAAAAAA	AAAAGTCGACAGTTTTGGTTCA	ACTGGGTTGTCAATTGCCTC	58.8	681	INTERGENIC	--	--	--
Ca4_P1_28990312	Ca4	28990312	CAAAAA/CAAA	GTTTCGCAATCTGGGTTTGT	AACACGTGTAGCAAAGCACG	60.0	770	INTERGENIC	--	--	--
Ca4_P1_28992327	Ca4	28992327	TC/T	CAAGTCCATGCAGTAAGCGA	GGAACCTGGAACACCGTAGA	60.0	836	INTRON	Ca_23706	--	NF-YB
Ca4_P1_28993304	Ca4	28993304	ACAA/ACAATTTTCAA	GGATGGTTGTAAAAGGCGAA	TTTGATGCACATCATTGGGT	59.9	894	INTRON	Ca_23706	--	NF-YB
Ca4_P1_28994820	Ca4	28994820	CTGTT/CTGTTGTT	GCCACACTCTGCAAACGTAA	CTAAGCGCAGCACAACTGAG	59.9	725	INTRON	Ca_23706	--	NF-YB
Ca4_P1_28996493	Ca4	28996493	TTTGTTGTTGTT/TTTGTTGTTGTTGTT	GCACCCCTTTAGTGTTGGAA	ACAAGCAAACAAAGGGGACA	60.0	643	DRR	Ca_23706	--	NF-YB
Ca4_P1_28996676	Ca4	28996676	A/ATTT	TTTTTATGCTTGGATTGGGC	AACGGATACTCCCTCCGTCT	59.9	613	DRR	Ca_23706	--	NF-YB
Ca4_P1_29008246	Ca4	29008246	GTTTT/GTTT	CCTACAATCCCAAGACAAGGA	ATTAATGCCCATGAAGGCAC	59.0	252	INTERGENIC	--	--	--

INDEL marker IDs	Chromosomes/ unanchored scaffolds	Physical positions (bp)	InDels (*Kabuli* reference genome-CDC Frontier/PI)	Forward primers (5'-3')	Reverse primers (5'-3')	Anneling temperature (0C)	Expected amplified product size (bp)	Structural annotation		Functional annotation	
								Sequence components of *kabuli* genome	*Kabuli* gene accession IDs	NCBI-KOG	TFs
Ca4_P1_29008405	Ca4	29008405	CTTTTTTT/CTTTTTTTT	CCTACAATCCCAAGACAAGGA	ATTAATGCCCATGAAGGCAC	59.0	252	INTERGENIC	--	--	--
Ca4_P1_29031157	Ca4	29031157	A/AC	AAGGACCAAATAAAGGTTGGG	TTGATGCTCATGCACTGATTC	59.2	749	INTERGENIC	--	--	--
Ca4_P1_29034648	Ca4	29034648	AAAGTAAG/AAAG	CCTGAGAGTGGATGCTCACA	CCGGAAGAGTCGTCAACAAT	60.0	599	INTERGENIC	--	--	--
Ca4_P1_29037043	Ca4	29037043	GAAAA/GAAAAAAA	CCATTGAGCTACACTGTGCG	TTGGACAACGCTTCAAACTG	60.5	434	INTERGENIC	--	--	--
Ca4_P1_29037899	Ca4	29037899	TAA/TAAA	GAGCTGAAACATGCGAACAA	TGATACCTTGCATTGAACAAATTC	60.0	716	INTERGENIC	--	--	--
Ca4_P1_29049755	Ca4	29049755	TTA/TTAAAGCGAGAAATA	GGTTTTATAGGGCATGCTGG	TCCCAAGAAATCAATGCTCC	59.4	749	INTERGENIC	--	--	--
Ca4_P1_29059011	Ca4	29059011	TC/T	ATTCACCCCCACTCTTAGGC	ACCGAGAAATGGATGGACAG	60.3	788	INTERGENIC	--	--	--
Ca4_P1_29059821	Ca4	29059821	AG/AGG	CTGTCCATCCATTTCTCGGT	TGAAAGAAATTCCAGCGGAT	59.9	670	INTERGENIC	--	--	--
Ca4_P1_29060295	Ca4	29060295	ATTT/ATT	GCTGGAATTTCTTTCAGCCA	AACGTTAACATCGCAACCCT	60.3	647	INTERGENIC	--	--	--
Ca4_P1_29064946	Ca4	29064946	TA/T	GTTTCCCGAAGGTTTTGGAT	TCATCTTGGATCACACTCAACC	60.2	424	INTERGENIC	--	--	--
Ca4_P1_29065135	Ca4	29065135	TAAAAAAAAA/TAAAAAAAA	GTTTCCCGAAGGTTTTGGAT	TCATCTTGGATCACACTCAACC	60.2	424	INTERGENIC	--	--	--
Ca4_P1_29065949	Ca4	29065949	AATATATATATAT/AATATATATAT	GGATCAAGATGCTCTAGTTCCAA	CATAACATTTGCGCCACATC	59.7	547	INTERGENIC	--	--	--

INDEL marker IDs	Chromosomes/ unanchored scaffolds	Physical positions (bp)	InDels (*Kabuli* reference genome-CDC Frontier/PI)	Forward primers (5'-3')	Reverse primers (5'-3')	Anneling temperature (0C)	Expected amplified product size (bp)	Structural annotation		Functional annotation	
								Sequence components of *kabuli* genome	*Kabuli* gene accession IDs	NCBI-KOG	TFs
Ca4_P1_29068719	Ca4	29068719	GTTTTTTTTTTTTTTTT/GTTTTTTTTT	ATTCGTCGAACCAATGTTCC	AGGCAAGAGAATAACTGCCAA	59.8	587	INTERGENIC	--	--	--
Ca4_P1_29085024	Ca4	29085024	CT/CTT	AAGGAGAAAAGTGGGGGAGA	ACGTATCCCCAGGTGCAATA	60.0	602	INTERGENIC	--	--	--
Ca4_P1_29089519	Ca4	29089519	CTTTTTTTTTTT/CTTTTTTTTTTTT	TCGACGACAACAACGAGGTA	CACCGTATTACACCACAACCA	60.3	496	INTERGENIC	--	--	--
Ca4_P1_29091531	Ca4	29091531	AAAATAAATAAAT/AAAATAAATAAATAAAT	GCGTCAACAAAATAACACGC	TATTTGACGATGGCGATTCA	59.2	460	INTERGENIC	--	--	--
Ca4_P1_29093206	Ca4	29093206	TTTATTATTAT/TTTATTAT	GGAAGCTCGGTGATTCTGAC	CTGTGTTTTGCTCCCCAAAT	59.8	593	INTRON	Ca_23708	C	HD-ZIP
Ca4_P1_29098940	Ca4	29098940	TTTATGGATTTTGTGATAATTATGGATT/TTTATGGATT	TGTTTCTTCGCTTTACGCCT	CACTTTTTGTCCCCCATCAT	60.0	914	INTERGENIC	--	--	--
Ca4_P1_29100812	Ca4	29100812	GGTAGTA/GGTA	TCAAGAAATAGTGGGGGCAG	TGGAGAAACTCTTCCGCTGT	60.1	840	INTERGENIC	--	--	--
Ca4_P1_29109089	Ca4	29109089	GAA/GA	TTGTTGGTTGGTCTCGTAAAA	AAGGCATACACATGGCAACA	58.2	379	INTERGENIC	--	--	--
Ca4_P1_29109343	Ca4	29109343	CAAA/CAAAA	CGAAGTCACCGTTGTGTCTC	CGCAATTGGAATACTCCCTC	59.3	398	INTERGENIC	--	--	--
Ca4_P1_29110502	Ca4	29110502	ATTTTT/ATTTT	TGCAAACATGGATGGGAATA	CACGGTTTCTAACACCATTTGA	59.7	617	INTERGENIC	--	--	--
Ca4_P1_29110622	Ca4	29110622	TC/T	TGCAAACATGGATGGGAATA	GGAGATTTGTGCCCTAGCAG	59.7	651	INTERGENIC	--	--	--
Ca4_P1_29112901	Ca4	29112901	GAAAAA/GAAAA	AGCTTGACGCCTCAACTACTG	TCATGGTTGTGAGGACTGGA	59.7	338	INTERGENIC	--	--	--

INDEL marker IDs	Chromosomes/ unanchored scaffolds	Physical positions (bp)	InDels (*Kabuli* reference genome-CDC Frontier/PI)	Forward primers (5'-3')	Reverse primers (5'-3')	Anneling temperature (0C)	Expected amplified product size (bp)	Structural annotation		Functional annotation	
								Sequence components of *kabuli* genome	*Kabuli* gene accession IDs	NCBI-KOG	TFs
Ca4_P1_29122319	Ca4	29122319	TAAAAAAAA/TAAAAAAAAAAA	TTGTGTGCCACATCATCACA	TACCAGCGACATGCTTTCAG	60.6	832	INTERGENIC	--	--	--
Ca4_P1_29124250	Ca4	29124250	CTTTTT/CTTTT	GGTCAAGGTGTGATTTGCCT	TCCACCAAGTCCCAAAGTTC	60.0	539	INTERGENIC	--	--	--
Ca4_P1_29124667	Ca4	29124667	ATTTTTTTT/ATTTTTTT	GAACTTTGGGACTTGGTGGA	GAGCTCAAGTTATGGCCTGC	59.9	303	INTERGENIC	--	--	--
Ca4_P1_29136029	Ca4	29136029	ATTTT/ATTTTT	AGAAGAGGCAACCAACCGTA	GAGGGAGCAAAGTTAGGTTCAA	59.7	976	INTERGENIC	--	--	--
Ca4_P1_29156658	Ca4	29156658	CATGCAAATGCAAA/CATGCAAA	AGTTGGTTTGGTGGTGAAGG	GCATGGTAAAGGCTGTTGGT	59.9	841	DRR	Ca_23711	--	--
Ca4_P1_29157805	Ca4	29157805	GTTTTTTTTTTTTTTTTT/GTTTTTTTTTTTTT	TGCTGTTGTTGGTGGTTGTT	TCTCCACTCCTCTCTCCTCA	60.0	418	INTERGENIC	--	--	--
Ca4_P1_29159894	Ca4	29159894	CAT/CATAT	TGATTTTCAAAGAGAAACCCG	TTCCTGCGATGTCATTTGTT	59.2	450	INTERGENIC	--	--	--
Ca4_P1_29160022	Ca4	29160022	GAAAAAAA/GAAAAAAAA	AACAAATGACATCGCAGGAA	GGAAATGCATGAATTGAAACTG	59.1	461	INTERGENIC	--	--	--
Ca4_P1_29160471	Ca4	29160471	GATTATTATTATTATTATTATTATTATTAT/GATTATTATTATTATTATTATTATTATTATTATTATTAT	CAGTTTCAATTCATGCATTTCC	GTTTGAAATCGCACCCAAAT	59.5	334	INTERGENIC	--	--	--
Ca4_P1_2918158	Ca4	2918158	ATTTTTTTTT/ATTTTTTTT	GCATTGATGCTTACGTGCAT	GGTTTTTGGTTGAGTTGGGA	59.7	314	INTERGENIC	--	--	--
Ca4_P1_29188666	Ca4	29188666	TT/TTCT	CATTCTGCCTTGTTGAAGCA	TTTTTGCACCTTCAATTGGT	60.0	702	INTERGENIC	--	--	--
Ca4_P1_29188945	Ca4	29188945	T/TTA	ACAGAGTTTGAGCCTGCTGC	TTTTTGCACCTTCAATTGGT	60.7	567	INTERGENIC	--	--	--

INDEL marker IDs	Chromosomes/ unanchored scaffolds	Physical positions (bp)	InDels (*Kabuli* reference genome-CDC Frontier/PI)	Forward primers (5'-3')	Reverse primers (5'-3')	Anneling temperature (0C)	Expected amplified product size (bp)	Structural annotation		Functional annotation	
								Sequence components of *kabuli* genome	*Kabuli* gene accession IDs	NCBI-KOG	TFs
Ca4_P1_29190573	Ca4	29190573	GA/G	GCCAGTCTTCTCAAACAGCC	TTTGACCAAGTGGAAAAGAAGA	60.0	648	INTERGENIC	--	--	--
Ca4_P1_29191237	Ca4	29191237	CTTTTTTTT/CTTTTTTTTT	CCAACTTGGTTCCAACAATCT	GGAGAAAAGTTAGGGGGTGC	58.9	119	INTERGENIC	--	--	--
Ca4_P1_29195556	Ca4	29195556	TAAA/TAA	TGTCATCTTCAGATGCAAACG	ACTGCGGGAGTACTGCAAAG	59.9	485	INTERGENIC	--	--	--
Ca4_P1_29196980	Ca4	29196980	TTTTTTTATTTTTT/TTTTTTT	AATCAACGGCAAATCGTTTC	CCGTCTCTAAAATTCGTCGG	59.9	714	INTERGENIC	--	--	--
Ca4_P1_29202977	Ca4	29202977	AATGATAG/A	TCACCCCGATGTCACCTATT	ACATGCCATTGAATTACGCA	60.2	449	INTERGENIC	--	--	--
Ca4_P1_29203709	Ca4	29203709	TTATATA/TTATA	AGTGGTCTTGGTCAGCCCTA	TTAAACCTTGGTGTGGCTCC	59.7	523	INTERGENIC	--	--	--
Ca4_P1_29205093	Ca4	29205093	C/CCAAG	ACATATGGCTTCGCATGACA	TTAGGAGTGGCTTTGGATGG	60.1	492	INTERGENIC	--	--	--
Ca4_P1_29206292	Ca4	29206292	GAA/GA	TTTGGATTCGTTCCACCTTC	CGTCTTTGAAACTGACGCTG	59.9	938	INTERGENIC	--	--	--
Ca4_P1_29207358	Ca4	29207358	GT/GTT	TCCCCACCCATTTCTAATGA	ATCCTTCCAAGCAACACCAC	60.1	595	INTERGENIC	--	--	--
Ca4_P1_29213561	Ca4	29213561	GCCC/GCC	TGATTGCAAATAGCACCCAA	TCTCCGCATAAGCTTTCGAT	60.1	247	DRR	Ca_14152	--	Trihelix
Ca4_P1_29221170	Ca4	29221170	TAAAAAAAA/TAAAAAAAAA	TAACCATGGTCATCCCTCGT	ATGTTAACGTTCGGAGTGCC	60.2	374	INTERGENIC	--	--	--
Ca4_P1_29221975	Ca4	29221975	ATTTTTTTTTTTTTT/ATTTTTTTTTT	TTGTTCGTACATCCGGGAAT	CGCACATAGAGAGGTGCATT	60.2	735	INTERGENIC	--	--	--

INDEL marker IDs	Chromosomes/ unanchored scaffolds	Physical positions (bp)	InDels (*Kabuli* reference genome-CDC Frontier/PI)	Forward primers (5'-3')	Reverse primers (5'-3')	Anneling temperature (0C)	Expected amplified product size (bp)	Structural annotation		Functional annotation	
								Sequence components of *kabuli* genome	*Kabuli* gene accession IDs	NCBI-KOG	TFs
Ca4_P1_29222326	Ca4	29222326	ATTTTTTT/ATTTTTTTT	TGCACCTCTCTATGTGCGTG	TTCGTTCCGGACAAATCTTC	61.0	342	INTERGENIC	--	--	--
Ca4_P1_29228849	Ca4	29228849	T/TG	CATTTATTTCTTCCCCCGGT	CACGAAGACGTTGGAACAGA	60.0	362	INTERGENIC	--	--	--
Ca4_P1_29231819	Ca4	29231819	A/AT	TTCCTCCCATGAAACGATGT	CTGCAACTCTCTCTGACCCC	60.3	854	INTERGENIC	--	--	--
Ca4_P1_29234372	Ca4	29234372	AAACACACA/AA	TTTTCCGCCTTTGCTTCTAA	ATCCATGTTCCCCACCAATA	60.0	609	INTERGENIC	--	--	--
Ca4_P1_29238761	Ca4	29238761	A/AATT	CCATGAGATCCACATAATCCG	TTGCAAGAATCCATCCATCA	60.2	513	INTERGENIC	--	--	--
Ca4_P1_29238831	Ca4	29238831	A/AC	CCATGAGATCCACATAATCCG	TTGCAAGAATCCATCCATCA	60.2	513	INTERGENIC	--	--	--
Ca4_P1_29240532	Ca4	29240532	AA/AAGA	AGGGGGAGATTGTTGGAATC	TTCCTAGCTTGGTCAACAACAA	60.1	611	INTERGENIC	--	--	--
Ca4_P1_29243325	Ca4	29243325	CAAAA/CAAAAA	TGTGGCAGCTACGTTCAGTC	GTTGACCTCGCACAAGGAAT	60.1	553	INTERGENIC	--	--	--
Ca4_P1_29244811	Ca4	29244811	CAAAA/CAAA	CCGAGATGGTTTTGGAGAAA	GCTTCATAGCTTGCAAAGGG	60.0	488	INTERGENIC	--	--	--
Ca4_P1_29245084	Ca4	29245084	ATA/ATACTTA	GCCCTTTGCAAGCTATGAAG	TGGTGCAATTTGGTTGGTAA	60.0	418	INTERGENIC	--	--	--
Ca4_P1_29262137	Ca4	29262137	AAT/AATTAT	TTCAAAGGTGCATTGTCGAT	CACTGCGACGAAACTTACCA	59.1	619	INTERGENIC	--	--	--
Ca4_P1_29263303	Ca4	29263303	ATTTTTTTTTT/ATTTTTTTTTTT	ATGAAGACATCGGAACCCAA	TGCTAAGGCACAAGTCTCCA	60.3	262	INTERGENIC	--	--	--

INDEL marker IDs	Chromosomes/ unanchored scaffolds	Physical positions (bp)	InDels (*Kabuli* reference genome-CDC Frontier/PI)	Forward primers (5'-3')	Reverse primers (5'-3')	Anneling temperature (0C)	Expected amplified product size (bp)	Structural annotation		Functional annotation	
								Sequence components of *kabuli* genome	*Kabuli* gene accession IDs	NCBI-KOG	TFs
Ca4_P1_29270177	Ca4	29270177	CTTTTTTTT/CTTTTTTT	TTTCCCGACTTGGTCCTTTA	TCGACTCCCCACCTAATGAC	59.5	696	INTERGENIC	--	--	--
Ca4_P1_29274800	Ca4	29274800	GTTTTTTTTTT/GTTTTTTTTTTT	TTGGCGTGATAATTACATGAGG	GCCTACCGGAAATGAATGAA	59.9	864	INTERGENIC	--	--	--
Ca4_P1_29278808	Ca4	29278808	GTTTTTTTTTT/GTTTTTTTTTTT	CCACCGGATTTATGAAGGAA	GGGCATGAAATATGAGACTGC	59.8	415	INTERGENIC	--	--	--
Ca4_P1_29279654	Ca4	29279654	AATATATATATATATATA/AATATATATATATATATATATA	CCGGATTTTTGCACTTGAAT	CGTTTGATTTTGGACGACCT	59.9	444	INTERGENIC	--	--	--
Ca4_P1_29292121	Ca4	29292121	CTATTATTATTATTA/CTATTATTATTATTATTA	TGCAAATGGTTCTTTATGCG	CAAGTTGAAACATGTGTCTTGG	59.7	297	INTERGENIC	--	--	--
Ca4_P1_2929266	Ca4	2929266	CATATATATATATATATA/CATATATATATATATATATATA	AAATGGCAATGAAATCCCTG	TCACTCTATCAAATGTTCAAGCAA	59.8	628	INTERGENIC	--	--	--
Ca4_P1_29292913	Ca4	29292913	ATTTTTTTTT/ATTTTTTTTTT	ACACGCTAGCCATTAGAGCC	TCCTTTCTTTCCAAGAGGTTCA	59.5	306	INTERGENIC	--	--	--
Ca4_P1_29309376	Ca4	29309376	ATTT/AT	ACTCCCTAGGGCACTTGGAT	TAAAGGGCCAGCTGAAGAAA	60.0	258	INTERGENIC	--	--	--
Ca4_P1_29310722	Ca4	29310722	ATTTTTTTTTT/ATTTTTTTTT	GTGGTGTCCATCTCGGTCTT	TAAGCCAGCAACTCCAGGAT	60.0	658	INTERGENIC	--	--	--
Ca4_P1_29311174	Ca4	29311174	ATTTTTTTT/ATTTTTTTTT	CAACTCCTGGATTTCCCTCA	CTTGAGGTGGAGAAAGCTGG	60.0	556	INTERGENIC	--	--	--
Ca4_P1_29314549	Ca4	29314549	AA/AATA	AGTCTTGCTTGCTTTGGCTT	CGAAACCAATTCTCTCCCAA	59.3	569	INTERGENIC	--	--	--
Ca4_P1_29321184	Ca4	29321184	ATTTTTTT/ATTTTTTTT	AACACATCAGCAAATGGCAA	TGGGTTCGGGTACAAGCTAC	60.1	682	INTERGENIC	--	--	--

INDEL marker IDs	Chromosomes/ unanchored scaffolds	Physical positions (bp)	InDels (*Kabuli* reference genome-CDC Frontier/PI)	Forward primers (5'-3')	Reverse primers (5'-3')	Anneling temperature (0C)	Expected amplified product size (bp)	Structural annotation		Functional annotation	
								Sequence components of *kabuli* genome	*Kabuli* gene accession IDs	NCBI-KOG	TFs
Ca4_P1_29330235	Ca4	29330235	ATTTT/ATTT	TTTTGTTCAGATTGCG CTTG	GAGGGGATCCATTTTC CCTA	60.0	762	INTERGENIC	--	--	--
Ca4_P1_29335473	Ca4	29335473	TAAAAAAA/TAAAAAAAA	CCAACCACCTCCTTAA TCCA	GGATTTCATTTCGAAA AATTGG	59.8	384	INTERGENIC	--	--	--
Ca4_P1_29341821	Ca4	29341821	GA/GATATATATA	TTCTCCCCCTTTTGAC ATGA	CAACACTCGCTTCCAG AACA	60.4	379	DRR	Ca_14153	--	--
Ca4_P1_29348534	Ca4	29348534	AGGGGGG/AGGGGGGG	GTTGTGCGGAAGCCAT TTAT	GAGGACTGGACGTAC CCTTG	60.0	248	INTERGENIC	--	--	--
Ca4_P1_29350232	Ca4	29350232	AC/A	GTTAAATTTGCTGGAG CACG	ACCAAGATTGGGCATC TGAA	58.4	160	INTERGENIC	--	--	--
Ca4_P1_29356210	Ca4	29356210	ATTTTTT/ATTTTT	GCGGGTTTCCTTTTAT AGGC	TTTTCCAGCATGTGTG CATC	59.9	311	INTERGENIC	--	--	--
Ca4_P1_29356293	Ca4	29356293	CTTTTTTTTT/CTTTTTTTT	GCGGGTTTCCTTTTAT AGGC	TTTTCCAGCATGTGTG CATC	59.9	311	INTERGENIC	--	--	--
Ca4_P1_29360419	Ca4	29360419	AT/ATGT	TAGCCATGAAGCACGA CAAG	CGTGTTGCGCTCTTAC TTTG	60.0	585	INTERGENIC	--	--	--
Ca4_P1_29360677	Ca4	29360677	TAAAAAA/TAAAAAAA	CAAAGTAAGAGCGCAA CACG	TGCAAGTATCCACCAA AATGC	59.7	441	INTERGENIC	--	--	--
Ca4_P1_29361867	Ca4	29361867	TATTATTAATTATTAATT/TATTATTAAT TATTAATTATTAATT	TGCAAGGATTTATGGA CGGT	CACTTACACCGAGAAG TTCGTG	60.3	527	INTERGENIC	--	--	--
Ca4_P1_29363363	Ca4	29363363	TAA/TA	CTCGCGGTATCTTCAT GACT	AAAAACAGAGCAGGTA CGAGTG	57.9	313	INTERGENIC	--	--	--
Ca4_P1_29364474	Ca4	29364474	CAAA/CAA	GGGCATGAACTCCAAC TTGT	AACTTGATCAATCGCT GAGTGT	60.0	759	INTERGENIC	--	--	--

INDEL marker IDs	Chromosomes/ unanchored scaffolds	Physical positions (bp)	InDels (*Kabuli* reference genome-CDC Frontier/PI)	Forward primers (5'-3')	Reverse primers (5'-3')	Anneling temperature (0C)	Expected amplified product size (bp)	Structural annotation		Functional annotation	
								Sequence components of *kabuli* genome	*Kabuli* gene accession IDs	NCBI-KOG	TFs
Ca4_P1_29365710	Ca4	29365710	AT/ATT	GGGGTAAGAGGGGATACCAA	AGAAAGAGCCAAGACCACCA	60.0	469	INTERGENIC	--	--	--
Ca4_P1_29367849	Ca4	29367849	CTTTTTT/CTTTTT	ATTTTGGCAACACATCACCA	TGAATGTCACAACAATATGACCC	59.8	444	DRR	Ca_14154	--	--
Ca4_P1_29367942	Ca4	29367942	TTA/TTATATA	ATTTTGGCAACACATCACCA	TGGATCAAGTTGTTTGTCACG	59.8	634	DRR	Ca_14154	--	--
Ca4_P1_29368197	Ca4	29368197	TTA/TTAGTA	ATTTTGGCAACACATCACCA	CCTTGGCATATTTTTGCTGG	59.8	737	DRR	Ca_14154	--	--
Ca4_P1_29368326	Ca4	29368326	TC/T	TCGATTGTTTAGAGGCTATACGTG	TCGCTTGTGAGAGAGGACAA	59.7	511	DRR	Ca_14154	--	--
Ca4_P1_29377748	Ca4	29377748	GAAAAAAAAAA/GAAAAAAAAAAA	CATCCTTACGCGATCTCCAT	AATGGATACGACCCATTCCA	60.1	491	INTERGENIC	--	--	--
Ca4_P1_29388732	Ca4	29388732	ATTTT/ATTT	GGCATCCCAGATCTGAAAAA	AAGTCGGTACAGCACCATCC	60.0	931	INTERGENIC	--	--	--
Ca4_P1_29393253	Ca4	29393253	ATTATTTATTTATTTATTTATTTATTTATTT/ATTATTTATTTATTTATTTATTTATTTATTTATTTATTT	GCTTGCAGCTAGGAAGCAGT	TTTGTTGATCTACGCATCGG	59.9	404	INTERGENIC	--	--	--
Ca4_P1_29403004	Ca4	29403004	ATT/ATTATTGTT	GGGAAAAACAAAAACAAGGGA	TCATAACAACAACAAAAACAACAA	60.2	134	INTERGENIC	--	--	--
Ca4_P1_29415605	Ca4	29415605	AATAT/AAT	AATATGCATAACTCGACGGAGA	ACAGGTGTCGAAGACCGACT	58.7	481	INTERGENIC	--	--	--
Ca4_P1_29416570	Ca4	29416570	AGAGTTGAGTTG/AGAGTTG	CCAATGGAAAATGGTCATCC	GCATCAATGTCAACCAATGC	60.0	671	INTERGENIC	--	--	--
Ca4_P1_29418053	Ca4	29418053	GAATAAATAAATAAA/GAATAAATAAATAAATAAA	TTGCTCCTACGTATCTCCCG	AACTTCCACCGTACACCACC	60.2	836	INTERGENIC	--	--	--

INDEL marker IDs	Chromosomes/ unanchored scaffolds	Physical positions (bp)	InDels (*Kabuli* reference genome-CDC Frontier/PI)	Forward primers (5'-3')	Reverse primers (5'-3')	Anneling temperature (0C)	Expected amplified product size (bp)	Structural annotation		Functional annotation	
								Sequence components of *kabuli* genome	*Kabuli* gene accession IDs	NCBI-KOG	TFs
Ca4_P1_29418531	Ca4	29418531	TT/TTAT	GGTGGTGTACGGTGGAAGTT	GTCAAGGCAGACCAGAAAGC	59.7	383	INTERGENIC	--	--	--
Ca4_P1_29419703	Ca4	29419703	A/AC	TGATTTTACATCTGCACCATGA	GGCATAAGCTTGCATTGTGA	59.0	262	INTERGENIC	--	--	--
Ca4_P1_29420131	Ca4	29420131	CAAAAAAAAA/CAAAAAAAA	TCACAATGCAAGCTTATGCC	TTGAAACAAAAACCACGATGA	59.8	742	INTERGENIC	--	--	--
Ca4_P1_29420218	Ca4	29420218	ATTT/ATT	TCACAATGCAAGCTTATGCC	TTGAAACAAAAACCACGATGA	59.8	742	INTERGENIC	--	--	--
Ca4_P1_29420847	Ca4	29420847	T/TA	CTTCGCCGTTGCTTTTCTAC	GGCTCAAATGAAAAAGGCAT	60.0	526	INTERGENIC	--	--	--
Ca4_P1_29421150	Ca4	29421150	TAAAAAAAAAAAAAAAAAA/TAAAAAAAA	TCGCCGTTGCTTTTCTACTT	AGAGAGCCAAACGGCAGATA	60.0	728	INTERGENIC	--	--	--
Ca4_P1_29421598	Ca4	29421598	A/ATTCACTCTTCTG	CTATCTGCCGTTTGGCTCTC	TGGTTGGCTTATGCATTCAA	60.0	245	INTERGENIC	--	--	--
Ca4_P1_29426569	Ca4	29426569	ATTTTTTTTT/ATTTTTTTTTT	ATCCAGCGAATTTTTACCGA	TTTTGACACACACACACCCA	59.5	781	INTERGENIC	--	--	--
Ca4_P1_29426868	Ca4	29426868	TTATATATATATATATATATATATATATATATAT/TTATATATATATATATATATATATATATAT	TTCAAAGTAGTTAACGACACAAACA	TTCTTCTTGTGAGAAGTTGCCA	58.1	486	INTERGENIC	--	--	--
Ca4_P1_29427590	Ca4	29427590	ATTTTTT/ATTTTTTT	TGGCAACTTCTCACAAGAAGAA	CCATCGGATTTTGTTACCCT	60.0	460	INTERGENIC	--	--	--
Ca4_P1_29431394	Ca4	29431394	TAAAAAAAAAAAAA/TAAAAAAAAAA	TCGACCTTGTTAGGAGCGAT	CCATAGCCAATGGTTCCTTC	59.8	629	INTERGENIC	--	--	--
Ca4_P1_29443305	Ca4	29443305	CTTTTTTTTT/CTTTTTTTT	CCTGCCAACAAACCAACTTT	GGCTGGAGGAAAACAAACAA	60.0	643	INTERGENIC	--	--	--

INDEL marker IDs	Chromosomes/ unanchored scaffolds	Physical positions (bp)	InDels (*Kabuli* reference genome-CDC Frontier/PI)	Forward primers (5'-3')	Reverse primers (5'-3')	Anneling temperature (0C)	Expected amplified product size (bp)	Structural annotation		Functional annotation	
								Sequence components of *kabuli* genome	*Kabuli* gene accession IDs	NCBI-KOG	TFs
Ca4_P1_29443790	Ca4	29443790	GTTTTTTTTT/GTTTTTTTTTT	TTGTTTGTTTTCCTCCAGCC	TCGTGTCTCAAAATTCAAGGAG	60.1	667	INTERGENIC	--	--	--
Ca4_P1_29463257	Ca4	29463257	GAAAAAAA/GAAAAAA	TGCTTGGTTCTTGAGCCTCT	ATGTTCGTGTTTTTCCCCAA	60.1	297	INTERGENIC	--	--	--
Ca4_P1_29480362	Ca4	29480362	ACCCCCC/ACCCCC	GGAAACCCAATCTGAAGCAA	GCTTTGGAGCACAGAGGTTC	60.1	728	INTERGENIC	--	--	--
Ca4_P1_29498412	Ca4	29498412	ATTTTTTTT/ATTTTTTT	TTGGATTGATTTTTGGATTTGTC	AACTTGATTTGAATATTGCCATTG	60.0	779	INTERGENIC	--	--	--
Ca4_P1_29503488	Ca4	29503488	ATTTTTTTTTT/ATTTTTTTTTTTT	TGTATGTCCCTCCTCTTTGTCA	GGGGTACTCAACCCACAAAA	59.6	735	DRR	Ca_14160	--	--
Ca4_P1_29509804	Ca4	29509804	GTTTTTTTTTTT/GTTTTTTTTTT	GGCCGATAAGACAATCATTCA	ATCGGAAAAACCCTATGGGA	59.9	420	INTERGENIC	--	--	--
Ca4_P1_29511821	Ca4	29511821	CTTTTTTTTT/CTTTTTTTTTT	AAAGGGCTAAATAGCTCCCG	TCCCGATTTGGTCATTTGTT	59.7	506	INTERGENIC	--	--	--
Ca4_P1_29515377	Ca4	29515377	TAAAAAAAAA/TAAAAAAAA	CGATGAGAGATGGTTAGGGTG	GGGAAGAAGCTTGATTCACCT	59.6	436	INTERGENIC	--	--	--
Ca4_P1_29519692	Ca4	29519692	CAAAAAAAA/CAAAAAAAAA	CGAAAGTTTAAAATGCGATGTG	TTTCATAAAGCTTCTCGGTTTG	59.7	396	INTERGENIC	--	--	--
Ca4_P1_29522577	Ca4	29522577	C/CAT	CCATTTCATTCCATCCATCC	CAAAGCTGCTTCATTGTGGA	60.0	416	DRR	Ca_14161	--	--
Ca4_P1_29532946	Ca4	29532946	TAAAAAAAAAAA/TAAAAAAAAAAAAA	CCATATTATCCATGACTTGGCA	CCGTTAGAAATCAAATGGGC	59.7	562	INTERGENIC	--	--	--
Ca4_P1_29534339	Ca4	29534339	CAAAAAAA/CAAAAAAAA	GGTCCGTCAAAAGAGTGTGAG	CCTCGAGCTATGCAATATGATG	59.8	564	INTERGENIC	--	--	--

INDEL marker IDs	Chromosomes/ unanchored scaffolds	Physical positions (bp)	InDels (*Kabuli* reference genome-CDC Frontier/PI)	Forward primers (5'-3')	Reverse primers (5'-3')	Anneling temperature (0C)	Expected amplified product size (bp)	Structural annotation		Functional annotation	
								Sequence components of *kabuli* genome	*Kabuli* gene accession IDs	NCBI-KOG	TFs
Ca4_P1_29544316	Ca4	29544316	GATAT/GAT	CCATCAAACCACAACAAACA	CCCAATTTCCTTCCGTTTTT	57.8	706	INTERGENIC	--	--	--
Ca4_P1_29545135	Ca4	29545135	AATTATTTATTATTTAT/AATTATTTATTATTTATTATTTAT	CATACGTGATACGCTGGGTG	GTGAGGGAGGACCAAAACAA	60.0	659	INTERGENIC	--	--	--
Ca4_P1_29547360	Ca4	29547360	CTTTTTTTT/CTTTTTTT	TCCCACACATCCAAAACAAA	TTGGTCCCTATAAGTTGGCG	59.8	613	INTERGENIC	--	--	--
Ca4_P1_29564267	Ca4	29564267	TAAAAAAAAA/TAAAAAAAAAAAAA	AAGAAGGATTCTCGGGGAAA	CATGGACCGTTCTTCCAAGT	60.0	260	INTERGENIC	--	--	--
Ca4_P1_29564628	Ca4	29564628	GTTTT/GTTTTTT	GAACGGTCCATGCAAAAGTT	AGTTTCCCCGATCCTTATGC	60.0	535	INTERGENIC	--	--	--
Ca4_P1_29566926	Ca4	29566926	GTTTTTTTTTTTT/GTTTTTTTTTT	CTCTTTTGCCCTGCGATTAG	TGAAACCAGATTTTGAGTCCG	60.0	701	INTERGENIC	--	--	--
Ca4_P1_29570352	Ca4	29570352	CAAAAAAAAAAAAAAA/CAAAAAAAAAAAA	GAGATCCAAAAGGTCGTCCA	TTATTCTTCCTGCTCGCGTT	60.1	633	INTERGENIC	--	--	--
Ca4_P1_29582409	Ca4	29582409	TAAAA/TAAA	AGCTGTATTGGGCCTCCTTT	GCCGAATAAAACCCTCACAA	60.1	212	INTERGENIC	--	--	--
Ca4_P1_29595669	Ca4	29595669	T/TA	TGCACGTACTTCGCATTCTT	TAGCAACAGGCCAGAGAGGT	59.5	458	INTERGENIC	--	--	--
Ca4_P1_29618138	Ca4	29618138	C/CAATGTTGT	AGTGATTTCGGGACTGGATG	CATCATCAACAACAACAACAACA	59.9	443	INTERGENIC	--	--	--
Ca4_P1_29618955	Ca4	29618955	GAA/GAAA	ACCGTTGACACCGAATTAGC	CCTTTCTTACTCCTAACCCCC	60.0	727	INTERGENIC	--	--	--
Ca4_P1_29619182	Ca4	29619182	AG/A	TTGTCGTGGTGATTAATGAAAAA	GCCGACACCTTCAATCAAAT	59.4	751	INTERGENIC	--	--	--

INDEL marker IDs	Chromosomes/ unanchored scaffolds	Physical positions (bp)	InDels (*Kabuli* reference genome-CDC Frontier/PI)	Forward primers (5'-3')	Reverse primers (5'-3')	Anneling temperature (0C)	Expected amplified product size (bp)	Structural annotation		Functional annotation	
								Sequence components of *kabuli* genome	*Kabuli* gene accession IDs	NCBI-KOG	TFs
Ca4_P1_29622506	Ca4	29622506	ATTTTTTT/ATTTTTTTTT	CCCCACGATCCTGAAATAAA	AACGATGATCGCAACAACAA	59.8	281	INTERGENIC	--	--	--
Ca4_P1_29624503	Ca4	29624503	CCACA/CCA	TGCACAAGACCATGCTAACC	CCTTGATTGTGGCACCTTTT	59.7	619	INTERGENIC	--	--	--
Ca4_P1_29626397	Ca4	29626397	CTTTTTTTTTTT/CTTTTTTTTT	ACCCCATTTCATCCTCATCA	CTTAGTGAGTGGAGAGCGCC	60.1	398	INTERGENIC	--	--	--
Ca4_P1_29626809	Ca4	29626809	CTTTTTTTT/CTTTTTTT	CTAGGAGAAGGGGGATTTGG	TGTGTTTGAATTCTTGATTGGG	59.9	239	INTERGENIC	--	--	--
Ca4_P1_29628090	Ca4	29628090	TAA/TAAA	TGACACCATTACCTAGGCCC	CTTCGAGAGTTGTTCCTCCG	59.8	281	INTERGENIC	--	--	--
Ca4_P1_29640045	Ca4	29640045	TAAAGA/TAAAGAAAGA	GCCTGTCTGCATTGTTTCCT	CGGACAGCAACAATTTCCTT	60.3	753	INTERGENIC	--	--	--
Ca4_P1_29646991	Ca4	29646991	GGATCG/GG	AGGATTCGGTCTGTGTTTGG	TCAGGACTGCAAATAACCCA	60.0	555	DRR	Ca_14165	S	--
Ca4_P1_29647367	Ca4	29647367	ACCC/ACC	TGGGTTATTTGCAGTCCTGA	GGCAAATTGACCAATGCTCT	59.1	533	DRR	Ca_14165	S	--
Ca4_P1_29648026	Ca4	29648026	TC/T	CCACCGAACATTGCTATGAA	TCCAACAATAAACCGAAGGC	59.5	713	DRR	Ca_14165	S	--
Ca4_P1_29649360	Ca4	29649360	TAT/TATAAT	CTCGGTATGGCCTATTTCCA	TTGCTGGGCTAAATACCGAC	59.9	527	INTERGENIC	--	--	--
Ca4_P1_29650019	Ca4	29650019	T/TA	GTCGGTATTTAGCCCAGCAA	TAGCAAGCTTGGTGCATTTG	60.1	612	INTERGENIC	--	--	--
Ca4_P1_29654020	Ca4	29654020	CTAAGAATTA/CTA	CTCACACCCCCTCAACACTT	AGAATTCGTGTTTGCCTGGT	60.0	612	INTERGENIC	--	--	--

INDEL marker IDs	Chromosomes/ unanchored scaffolds	Physical positions (bp)	InDels (*Kabuli* reference genome-CDC Frontier/PI)	Forward primers (5'-3')	Reverse primers (5'-3')	Anneling temperature (0C)	Expected amplified product size (bp)	Structural annotation		Functional annotation	
								Sequence components of *kabuli* genome	*Kabuli* gene accession IDs	NCBI-KOG	TFs
Ca4_P1_29658881	Ca4	29658881	TGAGGAG/TGAG	CTTTTGAGGCATTCCACCAT	ATTTTGTTGGGGTGCTCTTG	59.9	176	INTERGENIC	--	--	--
Ca4_P1_29681493	Ca4	29681493	G/GT	ATCCCCCACACGATGAATAA	CCAAAGAGCTGAGCGAAAAT	60.0	404	INTRON	Ca_14166	Z	MYB_related
Ca4_P1_29684929	Ca4	29684929	ATTTTTTTTT/ATTTTTTTTTT	GCCCTGGCCTAAGAATAACC	CAAGCCAACAGAACCTGGAT	59.9	562	INTRON	Ca_14166	Z	MYB_related
Ca4_P1_29698366	Ca4	29698366	GAAAAAAAAA/GAAAAAAAAAAA	GAATGTGCATTGTCGGTGTC	CTCATACAATGCGTGGATCG	60.0	454	INTRON	Ca_14166	Z	MYB_related
Ca4_P1_29699888	Ca4	29699888	GTTTTT/GTTTTTT	TGGCCTTATTTGCTTTTTGG	CCAACATCATGCTCTCCAAC	60.1	446	INTERGENIC	--	--	--
Ca4_P1_29699958	Ca4	29699958	TTG/TTGGATG	TGGCCTTATTTGCTTTTTGG	CCAACATCATGCTCTCCAAC	60.1	446	INTERGENIC	--	--	--
Ca4_P1_29700063	Ca4	29700063	ATTTT/ATTT	TGGCCTTATTTGCTTTTTGG	CCTACACCCATACGTACCCG	60.1	716	INTERGENIC	--	--	--
Ca4_P1_29700916	Ca4	29700916	GTTTTATCTTTTTAT/GTTTTAT	CGGGTACGTATGGGTGTAGG	AAAGCCATGAGGGGAAAGAT	60.1	777	INTERGENIC	--	--	--
Ca4_P1_29701374	Ca4	29701374	G/GTTTAAATTTC	AGATCTTTCCCCTCATGGCT	CCTTGACAAGAAGCAACACG	60.0	474	INTERGENIC	--	--	--
Ca4_P1_29702135	Ca4	29702135	CTTTTTTT/CTTTTTTTTT	AATTAGGTGTGGAGGGCAAA	TTTCATTTTTAAAACCCCCTCA	59.4	421	INTERGENIC	--	--	--
Ca4_P1_29703578	Ca4	29703578	A/AG	TTTGTATGTACGCGTCATGTTT	TTGGTTGTGTTGGTCGAGAA	58.2	488	INTERGENIC	--	--	--
Ca4_P1_29717512	Ca4	29717512	GAAATAAATA/GAAATAAATAAATA	GTTGGCATTTCTCCACTCGT	GTGCCCAACAAACAAGGACT	60.1	458	INTERGENIC	--	--	--

INDEL marker IDs	Chromosomes/ unanchored scaffolds	Physical positions (bp)	InDels (*Kabuli* reference genome-CDC Frontier/PI)	Forward primers (5'-3')	Reverse primers (5'-3')	Anneling temperature (0C)	Expected amplified product size (bp)	Structural annotation		Functional annotation	
								Sequence components of *kabuli* genome	*Kabuli* gene accession IDs	NCBI-KOG	TFs
Ca4_P1_29717836	Ca4	29717836	CTCACATTTTACTC/CTCACATTTTACTCATCACATTTTACTC	AGTCCTTGTTTGTTGGGCAC	TAACAGCTCAACTGCCCTCC	60.0	373	INTERGENIC	--	--	--
Ca4_P1_29719863	Ca4	29719863	ATTTTTTT/ATTTTTTTT	GAGTTGCGCCCAAACTTTTA	AGGAACAGCAAGAACGGTGT	60.2	883	INTRON	Ca_14167	--	--
Ca4_P1_29722804	Ca4	29722804	TAAAAAAAAA/TAAAAAAAAAA	TCGCAGTGGAGTTTGAATGA	TGGCCTAAATTTCCAATCCA	60.4	739	DRR	Ca_14167	--	--
Ca4_P1_29764046	Ca4	29764046	CA/C	GGCAGCGACATGTGATAAGA	TGGAGAGACAGTCGATGACG	59.8	474	INTERGENIC	--	--	--
Ca4_P1_29774862	Ca4	29774862	T/TC	TGTGCTTTTCAAATCATGCC	CATTTGAGGAGCAGCAACAA	59.7	826	DRR	Ca_14170	K	HD-ZIP
Ca4_P1_29776445	Ca4	29776445	TAAAAAAAAA/TAAAAAAAAAA	TATTTTGGGCCTGGTGTTGT	TGGGCCACCCTATACCAATA	60.2	729	INTRON	Ca_14170	K	HD-ZIP
Ca4_P1_29781856	Ca4	29781856	TAAAA/TAAAAA	TGGTTGGTGAGGTTGATGTG	CCATCGAAAGGAGTGCAAAT	60.4	819	INTERGENIC	--	--	--
Ca4_P1_29782040	Ca4	29782040	GTTTTTTTTTTTT/GTTTTTTTTTTT	AAAAATACATCGGTCGAAAGAA	CCATCGAAAGGAGTGCAAAT	57.8	471	INTERGENIC	--	--	--
Ca4_P1_29793706	Ca4	29793706	TAAAAAAAAAA/TAAAAAAAAAAA	AGATGTTGCTTCCCCAAGAA	CAAAAGAACCTTAGCTTCCCC	59.7	718	INTERGENIC	--	--	--
Ca4_P1_29818124	Ca4	29818124	ATTTT/ATTTTT	TGAATTCACCTCCATGAGCA	TGGAGGGTCCGTAACTTTTG	60.2	719	INTERGENIC	--	--	--
Ca4_P1_29821605	Ca4	29821605	CTTTTTTTT/CTTTTTTT	GACTCATCCAAGGTTGCCAT	TTTGATGGCTCAGAAAGCCT	59.9	524	INTERGENIC	--	--	--
Ca4_P1_29862679	Ca4	29862679	ATGATCAAGATTTGATCAAGATTT/ATGATCAAGATTT	TCAAAACTTTTCCGCTGCTT	ACCTGACAACCCCAAATTCA	60.0	636	INTERGENIC	--	--	--

INDEL marker IDs	Chromosomes/ unanchored scaffolds	Physical positions (bp)	InDels (*Kabuli* reference genome-CDC Frontier/PI)	Forward primers (5'-3')	Reverse primers (5'-3')	Anneling temperature (0C)	Expected amplified product size (bp)	Structural annotation		Functional annotation	
								Sequence components of *kabuli* genome	*Kabuli* gene accession IDs	NCBI-KOG	TFs
Ca4_P1_29863037	Ca4	29863037	GTTATTATTATTAT/GTTATTATTATTATTAT	TCGGGGTGTTACAGTATGGA	ACATCCAAACCCCTCATTCA	58.8	605	INTERGENIC	--	--	--
Ca4_P1_29864644	Ca4	29864644	ATTT/ATT	TTTCTCCCGCTTTTGTCAAG	TATACAAGGCCTCTTGCGCT	60.4	725	INTERGENIC	--	--	--
Ca4_P1_29866382	Ca4	29866382	CAAA/CAA	TGGGGAGGAAGTTGACTCAG	CTTCGCACCTCTATCTTGCC	60.2	465	INTERGENIC	--	--	--
Ca4_P1_29872961	Ca4	29872961	GATATATATATATATA/GATATATATATATA	TTTGCCAAAATGTAAATGCG	GATAGAGGGATGCCGCATAA	59.6	315	INTERGENIC	--	--	--
Ca4_P1_29897854	Ca4	29897854	C/CTCAAGTTTCAT	GCCCACCCCATTTAGAATTT	GCCAAAGAAACACCATCGTT	60.0	367	INTERGENIC	--	--	--
Ca4_P1_29900525	Ca4	29900525	TTATA/TTA	AACAAAGAAGAACACGGGGT	CTTTCCTGCGCATAATCGAC	58.6	574	INTERGENIC	--	--	--
Ca4_P1_2990516	Ca4	2990516	ATTTTTT/ATTTTTTT	AAGGGTGAAATAGTTGGGGG	GACTGGTTTCGCATTTCCAT	60.0	441	DRR	Ca_12097	--	--
Ca4_P1_29911237	Ca4	29911237	TATGTTGA/TATGTTGATGTTGA	GTCAATGGCACAAAATGCTG	GCAGAAGGGTGTTGGAAAAA	60.1	738	INTERGENIC	--	--	--
Ca4_P1_29911958	Ca4	29911958	TAAAAAAAAAA/TAAAAAAAAA	TTTTTCCAACACCCTTCTGC	TAGATTTGAGTGCCCATCGC	60.1	684	INTERGENIC	--	--	--
Ca4_P1_29912693	Ca4	29912693	GATGTC/GATGTCATGTC	TGAGATCCCAACAGGCCTAA	TGGTCTTTGGAAAGCCTCAC	60.6	483	INTERGENIC	--	--	--
Ca4_P1_29925448	Ca4	29925448	G/GC	ATCGGACAGAGTTGACCCTC	GTTTTCCAAATCGTGGCCTA	59.1	686	INTERGENIC	--	--	--
Ca4_P1_29928651	Ca4	29928651	ATTT/ATT	TTCCCAGTTTTCCAACCAAA	TGCAACCCAAGGACACATAA	60.3	661	INTERGENIC	--	--	--

**INDEL marker IDs**	**Chromosomes/ unanchored scaffolds**	**Physical positions (bp)**	**InDels (*Kabuli* reference genome-CDC Frontier/PI)**	**Forward primers (5'-3')**	**Reverse primers (5'-3')**	**Anneling temperature (0C)**	**Expected amplified product size (bp)**	**Structural annotation**		**Functional annotation**	
								**Sequence components of *kabuli* genome**	***Kabuli* gene accession IDs**	**NCBI-KOG**	**TFs**
Ca4_P1_29931075	Ca4	29931075	GA/GAGTAA	TGTGCACCAATATCTGCCAT	GAAGAAAACCCGGGATCAGT	60.0	670	INTERGENIC	--	--	--
Ca4_P1_29932586	Ca4	29932586	TAAAAAAAAAA/TAAAAAAAAAAAA	TCTACGGCGTCTAGTGGACA	AGCCCGAAAGGATCTAAAGG	59.5	487	INTERGENIC	--	--	--
Ca4_P1_29933754	Ca4	29933754	ATTTTTTT/ATTTTTTTT	AGCCACACGATGTTGATTGA	TGATTAGGGAATCACCCGTT	60.1	845	INTERGENIC	--	--	--
Ca4_P1_29934874	Ca4	29934874	ATTTT/ATT	TCTTCTGACCATTCGGGTTT	ATAGACAGGGTTAGCGTGGC	59.5	370	INTERGENIC	--	--	--
Ca4_P1_29941572	Ca4	29941572	TAAAAAAA/TAAAAAA	CCGTGTAGGTATGATCTTTTGG	CCTTTCCTGTAAATCGGCAA	58.5	324	INTERGENIC	--	--	--
Ca4_P1_29946027	Ca4	29946027	AAAAATAAAAT/AAAAATAAAATAAAAT	GTCTAGCCGCCAAAAACTTG	GAATGAATTTCCAGGGCAGA	59.9	693	INTERGENIC	--	--	--
Ca4_P1_29974296	Ca4	29974296	A/ATTGAT	TTACTACAGCAGGGGCCAAG	TGTGGTGTGGTTACAAACCC	60.3	476	INTERGENIC	--	--	--
Ca4_P1_29979953	Ca4	29979953	TAAA/TAAAA	TCTCGATGTCGATTGTTGGA	GAGCTTTTCATTTTTCCAAGTG	60.2	628	INTERGENIC	--	--	--
Ca4_P1_29987923	Ca4	29987923	GATGGTAT/GATGGTATGGTAT	GAGCAGAGCTGCCTAATGCT	GGCGTTGTTGTAGAGTGCAA	59.9	419	INTERGENIC	--	--	--
Ca4_P1_30045432	Ca4	30045432	CATATAT/CATATATAT	GCGTGCATAAGTGGTTGATG	ACTCACGGTGATGAGTGTGG	60.1	345	DRR	Ca_14181	--	S1Fa-like
Ca4_P1_30046858	Ca4	30046858	TG/T	TGAATGAAATTTCTGGCAGG	CGTTTTGTTTGTGGCACATT	58.7	280	INTERGENIC	--	--	--
Ca4_P1_30046973	Ca4	30046973	GAAAAAAAAAA/GAAAAAAAAA	TGAATGAAATTTCTGGCAGG	CGTTTTGTTTGTGGCACATT	58.7	280	INTERGENIC	--	--	--

INDEL marker IDs	Chromosomes/ unanchored scaffolds	Physical positions (bp)	InDels (*Kabuli* reference genome-CDC Frontier/PI)	Forward primers (5'-3')	Reverse primers (5'-3')	Anneling temperature (0C)	Expected amplified product size (bp)	Structural annotation		Functional annotation	
								Sequence components of *kabuli* genome	*Kabuli* gene accession IDs	NCBI-KOG	TFs
Ca4_P1_30072648	Ca4	30072648	TAAAAAAA/TAAAAAAAA	TTTTCATGTTTCCGCATTCA	CGGGGTGAGTTAAATGTACGA	60.1	560	INTERGENIC	--	--	--
Ca4_P1_30075059	Ca4	30075059	CTAA/CTAATTGTTTTTATAA	TCGAAACCAACCACCTTTTC	GCAATTCGACAATGCACAAC	59.9	547	INTERGENIC	--	--	--
Ca4_P1_30076786	Ca4	30076786	GTTTTTTTT/GTTTTTTTTTT	GCACTTCCGCTAAAAGCATC	AAGCGCTCCCAAATATGCTA	60.0	432	INTERGENIC	--	--	--
Ca4_P1_30077264	Ca4	30077264	ATTTTTT/ATTTTTTT	GCATATTTGGGAGCGCTTTA	TGGATGATTGTGACAAAATTGC	60.2	508	INTERGENIC	--	--	--
Ca4_P1_30081112	Ca4	30081112	CCCTT/CCCTTCCTT	CACGGGACAAATCTTTGACAT	TCGTCCAAGTTCATCATCACA	59.8	941	INTERGENIC	--	--	--
Ca4_P1_30082739	Ca4	30082739	AAAAACAAAACAG/AAAAACAAAACAGTGGAAAACAAAACAG	TGCGTTGTATCATGTCGGTT	TCTGGTTTGTTCCTCCGTTT	60.0	389	INTERGENIC	--	--	--
Ca4_P1_30086302	Ca4	30086302	TTTTCATG/TTTTCATGCAAACTTTCATG	TTGATGTGGACTTTCTTGCG	TTTTCGGCGTAAAACCTTTG	59.8	238	INTERGENIC	--	--	--
Ca4_P1_30086898	Ca4	30086898	ACTTTCT/ACT	ACGCCGAAAAACACTTTCAC	TCCATTCGACTGTCCTTCAA	60.2	630	INTERGENIC	--	--	--
Ca4_P1_30087280	Ca4	30087280	A/AGTT	GGACAGTCGAATGGACATCA	GTTTGGCTGGGTGAGTTTGT	59.5	511	INTERGENIC	--	--	--
Ca4_P1_30091332	Ca4	30091332	GA/GAA	ACACAGTGGGGCATTTTAGC	AAAAAGGAAGAGGCATTTTGAA	60.0	380	INTERGENIC	--	--	--
Ca4_P1_30091400	Ca4	30091400	ATTTTTTTTTTTTTTTT/ATTTTTTTTTT	ACACAGTGGGGCATTTTAGC	AAAAAGGAAGAGGCATTTTGAA	60.0	380	INTERGENIC	--	--	--
Ca4_P1_30092712	Ca4	30092712	AT/ATTTT	GATGCGACCATCTACTGCAA	TGTCAAAACAAACGATGTGCT	59.8	347	INTERGENIC	--	--	--

INDEL marker IDs	Chromosomes/ unanchored scaffolds	Physical positions (bp)	InDels (*Kabuli* reference genome-CDC Frontier/PI)	Forward primers (5'-3')	Reverse primers (5'-3')	Anneling temperature (0C)	Expected amplified product size (bp)	Structural annotation		Functional annotation	
								Sequence components of *kabuli* genome	*Kabuli* gene accession IDs	NCBI-KOG	TFs
Ca4_P1_30092930	Ca4	30092930	GTTTTT/GTTTTTTT	GATGCGACCATCTACTGCAA	TGATGTGTCCCTAATATTTTCCG	59.8	529	INTERGENIC	--	--	--
Ca4_P1_30097465	Ca4	30097465	GAAA/GAA	GCAAAACGGGGACTAGAACA	CCAGGGGTTGCCTTAGATTT	60.1	307	INTERGENIC	--	--	--
Ca4_P1_30098406	Ca4	30098406	CTGCTTCATTGCTTC/CTGCTTC	CCTCATCCCTTGGAACTTCA	TTATGATCAACGATTCGCCA	60.0	372	INTERGENIC	--	--	--
Ca4_P1_30098435	Ca4	30098435	GTTT/GTT	CCTCATCCCTTGGAACTTCA	TTATGATCAACGATTCGCCA	60.0	372	INTERGENIC	--	--	--
Ca4_P1_30101633	Ca4	30101633	ATTTT/ATTT	CCAGTTGAGCTTCACCATCA	TGGTGGCAATTGAAGAATCA	59.8	394	INTERGENIC	--	--	--
Ca4_P1_30102999	Ca4	30102999	CC/CCCCTC	ATTTCCCAATCCCAAACACA	ATCCTTGCTAGCCACGAAAA	60.0	662	INTERGENIC	--	--	--
Ca4_P1_30107747	Ca4	30107747	ATTTTTTTTTTT/ATTTTTTTTTTTTTT	TGGTGAAGAAGCATGGTGAA	GTTGATTGCAGCCCAATCTT	60.2	608	DRR	Ca_14184	Z	Dof
Ca4_P1_30111644	Ca4	30111644	A/AAT	CGAATTTGGATGGATTGTTT	TTCAAACCCTAAGCCATAAACC	57.4	585	INTERGENIC	--	--	--
Ca4_P1_30112030	Ca4	30112030	TACGATTTAA/T	TTTTGCTCTTAATTTTCACCG	TCCAACAAAAGGTGCAACAA	57.2	884	INTERGENIC	--	--	--
Ca4_P1_30114713	Ca4	30114713	TAAAAAAAA/TAAAAAAA	GCTCAAGTTATAGCCTACAAAGGG	CAGTTTTGGTCAATCGCTCA	59.7	974	INTERGENIC	--	--	--
Ca4_P1_30115724	Ca4	30115724	TAAAAAAAAA/TAAAAAAA	ATCTTTCTTCTTGAGCGGCA	GGGGGCTTAAACCCTAACAA	60.1	337	INTERGENIC	--	--	--
Ca4_P1_30118694	Ca4	30118694	CATAGATA/CATAGATAGATA	CTCCTTCATTTCCTCCCTCC	AAGGTGGGTTAAAGAACCGC	60.0	778	INTERGENIC	--	--	--

INDEL marker IDs	Chromosomes/ unanchored scaffolds	Physical positions (bp)	InDels (*Kabuli* reference genome-CDC Frontier/PI)	Forward primers (5'-3')	Reverse primers (5'-3')	Anneling temperature (0C)	Expected amplified product size (bp)	Structural annotation		Functional annotation	
								Sequence components of *kabuli* genome	*Kabuli* gene accession IDs	NCBI-KOG	TFs
Ca4_P1_30118773	Ca4	30118773	TG/TGG	CTCCTTCATTTCCTCCCTCC	AAGGTGGGTTAAAGAACCGC	60.0	778	INTERGENIC	--	--	--
Ca4_P1_30120661	Ca4	30120661	TAAAA/TAAA	TTTGACATCCATCTTATGCAGC	CCCTTGGGTGCGTTAGTTAT	60.1	379	INTERGENIC	--	--	--
Ca4_P1_30122538	Ca4	30122538	ATT/AT	ATGTCAATGGGCCACTTAGC	TTGTTTGTTGTGCTTTGACCA	60.0	764	INTERGENIC	--	--	--
Ca4_P1_30122560	Ca4	30122560	GAA/GAAAA	ATGTCAATGGGCCACTTAGC	GTGGGGCCATTGATGAGTAA	60.0	962	INTERGENIC	--	--	--
Ca4_P1_30122790	Ca4	30122790	A/AG	TGTTGTGGGAGGTGATTTGA	ATCTTTTTGGGAGGTAGCGG	59.9	741	INTERGENIC	--	--	--
Ca4_P1_30122891	Ca4	30122891	CAA/CAAA	TGTTGTGGGAGGTGATTTGA	GGGTCAAACCTCAAAGACGA	59.9	862	INTERGENIC	--	--	--
Ca4_P1_30132561	Ca4	30132561	TTAGAT/TTAGATTAATAGAT	TTATTCTGGGGACTTCGTGG	TCATAACGTCAATTCGCTCAA	59.9	339	INTERGENIC	--	--	--
Ca4_P1_30133478	Ca4	30133478	TATAT/TATATAATACATAT	CGTCAATTGATGTCGTTCACTT	TCCAGTGCCCTTCATTTCTC	60.0	559	INTERGENIC	--	--	--
Ca4_P1_30134304	Ca4	30134304	GAATTAAT/GAAT	TCCTTTTGCATTACTATCCAAGC	GGGGGTTCCATGTAAAACATT	59.7	419	INTERGENIC	--	--	--
Ca4_P1_30134439	Ca4	30134439	CAAAAAA/CAAAA	TCCTTTTGCATTACTATCCAAGC	GGGGGTTCCATGTAAAACATT	59.7	419	INTERGENIC	--	--	--
Ca4_P1_30141754	Ca4	30141754	AATT/A	GAACAATGACCAAGTGAATTTTGA	TATGGGATGCGTTGAGATGA	60.3	428	INTERGENIC	--	--	--
Ca4_P1_30141789	Ca4	30141789	TTAT/TT	GAACAATGACCAAGTGAATTTTGA	TATGGGATGCGTTGAGATGA	60.3	428	INTERGENIC	--	--	--

**INDEL marker IDs**	**Chromosomes/ unanchored scaffolds**	**Physical positions (bp)**	**InDels (*Kabuli* reference genome-CDC Frontier/PI)**	**Forward primers (5'-3')**	**Reverse primers (5'-3')**	**Anneling temperature (0C)**	**Expected amplified product size (bp)**	**Structural annotation**		**Functional annotation**	
								**Sequence components of *kabuli* genome**	***Kabuli* gene accession IDs**	**NCBI-KOG**	**TFs**
Ca4_P1_30142165	Ca4	30142165	TAAAAAAAA/TAAAAAAA	GCCACCATTCATGCTCTACA	GGTGTCTGAGATGTGTGGGA	59.7	352	INTERGENIC	--	--	--
Ca4_P1_30147475	Ca4	30147475	ATCTTCTTCTTCTTCTTCTTCTTCTTCTTCTTC/ATCTTCTTCTTCTTCTTCTTC	CCCGTGGTTAGCAACACTTT	CATTCCAAATTTTCCCCCTT	60.0	471	CDS (large-effect mutations)	Ca_14185	--	--
Ca4_P1_30147920	Ca4	30147920	ATTTTTTTTTT/ATTTTTTTTTTTT	ATCACCACTTCTTGCATCCC	CATCTGCACGATTTTCCTTTT	59.9	536	INTRON	Ca_14185	--	--
Ca4_P1_30148542	Ca4	30148542	ATTTTTTTT/ATTTTTTTTT	GTTGTTGCTGTGAGTGCTCC	TTAGTCCCTTACGTCGTGGG	59.5	302	INTRON	Ca_14185	--	--
Ca4_P1_30150930	Ca4	30150930	TTCTCT/TTCT	GCCACCTTGTTTTCAGTGGT	AAAATGGCGGTCAATAGCTG	60.0	664	INTRON	Ca_14185	--	--
Ca4_P1_30170921	Ca4	30170921	AGGGG/AGGG	CACTCATTCGGGCTTCTAGC	CCTACCTTGAGCGAACCAAG	60.0	406	INTERGENIC	--	--	--
Ca4_P1_30172032	Ca4	30172032	G/GA	CTCAACAGCCTTATGTGCGA	GCATGGAGACTCAAAAATCCA	60.0	673	INTERGENIC	--	--	--
Ca4_P1_30172265	Ca4	30172265	TC/T	CTCAACAGCCTTATGTGCGA	TGCGATGGAATAATGTGTCG	60.0	752	INTERGENIC	--	--	--
Ca4_P1_3017713	Ca4	3017713	ATTATTTTAAATTTATTTTAAATTTATTTTAAATTTATTTTAAATTTATTTTAAATT/ATTATTTTAAATTTATTTTAAATTTATTTTAAATTTATTTTAAATTTATTTTAAATTTATTTTAAATT	GACAAATTAGTGGGGCAACC	TTGTATTGTTGGTGAGGCCA	59.3	902	INTERGENIC	--	--	--
Ca4_P1_30178438	Ca4	30178438	AAATTTCAATATTTAATTTCAATAT/AAATTTCAATATTTAATTTCAATATTTAATTTCAATAT	CAAGCAATTATGGTGCAACTG	AGTGAGGCAAATGGAAATGG	59.2	669	INTERGENIC	--	--	--
Ca4_P1_30180119	Ca4	30180119	C/CT	TCAGACCTACACTTCCGTCAAA	TGGTGAGTGAATTGTGTGGC	59.8	158	INTERGENIC	--	--	--
Ca4_P1_30180143	Ca4	30180143	ATTTTT/ATTTTTT	CTACCTCGGATCGATTCCTT	TGGTGAGTGAATTGTGTGGC	58.2	116	INTERGENIC	--	--	--

INDEL marker IDs	Chromosomes/ unanchored scaffolds	Physical positions (bp)	InDels (*Kabuli* reference genome-CDC Frontier/PI)	Forward primers (5'-3')	Reverse primers (5'-3')	Anneling temperature (0C)	Expected amplified product size (bp)	Structural annotation		Functional annotation	
								Sequence components of *kabuli* genome	*Kabuli* gene accession IDs	NCBI-KOG	TFs
Ca4_P1_30180525	Ca4	30180525	CAAAAAAAA/CAAAAAAA	GACATTCCAAAATGGCAAGG	ATTGTTCCCACTTCACCAGG	60.3	498	INTERGENIC	--	--	--
Ca4_P1_30184517	Ca4	30184517	TA/T	CGAGGGGCGTAAAAATCATA	GCGAGATTTGTTTCCGTTGT	59.9	579	INTERGENIC	--	--	--
Ca4_P1_30185691	Ca4	30185691	TAAAAAAAA/TAAAAAAAAAA	CATCGCTAACTCAGTGCCAA	AATGCGTTACGGGTGAAATG	60.0	876	INTERGENIC	--	--	--
Ca4_P1_30188012	Ca4	30188012	CTTT/CTTTT	CCTCGCTGCACAATCAAATA	TGAAAGTTGTTTCAAATGGCA	59.8	452	CDS (FRAME SHIFT)	Ca_14186	--	SAP
Ca4_P1_30194941	Ca4	30194941	AATTT/A	TTACATGGCATAGTGGGGGT	TGATCGTCAATGGGCATCTA	60.1	502	INTERGENIC	--	--	--
Ca4_P1_30198056	Ca4	30198056	TTTT/TTTTTGAGATTCTTT	GCCCATGTTTAATTAGGCCC	AGCCATTTCCATGTCTCTGC	60.5	860	INTERGENIC	--	--	--
Ca4_P1_30200799	Ca4	30200799	AGGGG/AGGG	GGGTCATTGTTCCTCAAACC	ATGGGGTCCTTCCAAAATTC	59.2	908	INTERGENIC	--	--	--
Ca4_P1_30201744	Ca4	30201744	A/ACCTT	CACAAGCTGGTTTTGCTTGA	GAAAGAATGCCCAGATTGGA	60.0	529	INTERGENIC	--	--	--
Ca4_P1_30206461	Ca4	30206461	TAAAAAAAAAAA/TAAAAAAAAA	TCCCTCGAGCTATAGGATGC	ATGCAATGGTTTATTCCCCA	59.4	643	DRR	Ca_14187	--	--
Ca4_P1_30207107	Ca4	30207107	TA/TACA	TGGGGAATAAACCATTGCAT	CCGGTAGTTTGGTGATTGGA	60.0	316	DRR	Ca_14187	--	--
Ca4_P1_30210048	Ca4	30210048	ATATCACTTT/AT	GGTTTGCTTATGGGGCAGTA	CACCCCGAATGAACTTTAGAA	60.0	631	INTERGENIC	--	--	--
Ca4_P1_30211443	Ca4	30211443	T/TA	ACATCAAACAAACAGCGCAC	CCGCTAAAATTGCCCAATAA	59.8	714	INTERGENIC	--	--	--

INDEL marker IDs	Chromosomes/ unanchored scaffolds	Physical positions (bp)	InDels (*Kabuli* reference genome-CDC Frontier/PI)	Forward primers (5'-3')	Reverse primers (5'-3')	Anneling temperature (0C)	Expected amplified product size (bp)	Structural annotation		Functional annotation	
								Sequence components of *kabuli* genome	*Kabuli* gene accession IDs	NCBI-KOG	TFs
Ca4_P1_30211511	Ca4	30211511	A/AT	ACATCAAACAAACAGCGCAC	CCGCTAAAATTGCCCAATAA	59.8	714	INTERGENIC	--	--	--
Ca4_P1_30213835	Ca4	30213835	T/TA	GCGACACAAAGGCCACTAAC	TTCGGTTCTTGTTTGCCTCT	60.7	888	INTERGENIC	--	--	--
Ca4_P1_30226816	Ca4	30226816	CAAAAAA/CAAAA	GCTTATGCAATGCACGAAAA	CATGGGTTGAAATGGAGACA	59.8	528	INTERGENIC	--	--	--
Ca4_P1_30237136	Ca4	30237136	GTTTTTTTTTTT/GTTTTTTTTTT	CGGGAGTATTTTGGTCACTTT	CCTCTTGTTGCTTCCTCTGG	58.1	589	INTERGENIC	--	--	--
Ca4_P1_30241127	Ca4	30241127	AT/ATT	ATCAGGCTCAATCGGTTGTC	GGAAAGTGAGGGAACCAATG	60.1	262	INTERGENIC	--	--	--
Ca4_P1_30252999	Ca4	30252999	AAAAAA/AAAAAAAATAAAAA	GGGCAGCGAACTTAGTCAAG	CGGTGGAAAAGTTTCGGATA	60.0	410	INTERGENIC	--	--	--
Ca4_P1_30258323	Ca4	30258323	GAAAAAAAAA/GAAAAAAAA	GTGTGCTGCGTTTGACACTT	TCACGTGCACTCATCCTCTC	60.0	454	DRR	Ca_14190	R	C3H
Ca4_P1_30316103	Ca4	30316103	AA/AATA	AACCATGCTACAGGGCATTC	TGCAGGCAACAAAGTGAAAG	60.0	412	INTERGENIC	--	--	--
Ca4_P1_30330570	Ca4	30330570	CTATATATATATATATATATA/CTATATATATATATATATATATA	TTCGATGGCTAGATTACATACTGC	CCCCTCAACTCAAGAATGATG	59.7	561	INTERGENIC	--	--	--
Ca4_P1_30332580	Ca4	30332580	ACC/AC	GTCCATCGACGAGACATGC	AATTGCAGTTTTGGTCTTCCT	60.2	517	INTERGENIC	--	--	--
Ca4_P1_30333302	Ca4	30333302	CTTTTTTTTTT/CTTTTTTTTT	AATCTATGTTCGGATGAGATTGA	AAGGGGAGAGAGATTTGGGA	57.7	749	INTERGENIC	--	--	--
Ca4_P1_30351459	Ca4	30351459	CTTTTTTT/CTTTTTTTT	TCTTTGAAACACATCACTTTCG	AAAATTTGATCGGTGGTGGA	57.9	314	INTERGENIC	--	--	--

INDEL marker IDs	Chromosomes/ unanchored scaffolds	Physical positions (bp)	InDels (*Kabuli* reference genome-CDC Frontier/PI)	Forward primers (5'-3')	Reverse primers (5'-3')	Anneling temperature (0C)	Expected amplified product size (bp)	Structural annotation		Functional annotation	
								Sequence components of *kabuli* genome	*Kabuli* gene accession IDs	NCBI-KOG	TFs
Ca4_P1_30390669	Ca4	30390669	T/TA	CTACTGAAGCCCAACGAAGG	CTTTCAGATTCGTGGCCATT	59.9	864	INTERGENIC	--	--	--
Ca4_P1_30402781	Ca4	30402781	GTTTTTTTT/GTTTTTTTTT	CCTAGATTGGGTGAACCAGG	GGAAATTCGACCCATACCCT	59.4	607	INTERGENIC	--	--	--
Ca4_P1_30404596	Ca4	30404596	C/CA	TTAACTGTTGGTGCGCAGTC	TGGGATAAAAGGACACCACTC	59.9	429	INTERGENIC	--	--	--
Ca4_P1_30404870	Ca4	30404870	AATAACAT/AAT	TTAACTGTTGGTGCGCAGTC	CGTCACATTTAACACGCCAT	59.9	798	INTERGENIC	--	--	--
Ca4_P1_30408872	Ca4	30408872	AA/AAAAGA	TTGCGTTACGGATTTGATGA	AGACTTTCGACCGACGCTAA	60.1	485	INTERGENIC	--	--	--
Ca4_P1_30415073	Ca4	30415073	GCATCATCATCATCA/GCATCATCATCATCATCA	TTGAACATATAGGGAGGCGG	CCATCCTTTTCAATGCGAGT	59.9	423	CDS (large-effect mutations)	Ca_14197	--	--
Ca4_P1_30422668	Ca4	30422668	TGTCAATTT/TGTCAATTTTAGTCAATTT	TCTGCCAAGTGACATTCCAA	CATGAACCAAACGAGTCGAA	60.2	686	INTERGENIC	--	--	--
Ca4_P1_30423088	Ca4	30423088	TAAAAAAA/TAAAAAAAA	AGGGGCAAAAATACTGCTTG	CTTGATCGGCTCATGTGTTG	59.2	938	INTERGENIC	--	--	--
Ca4_P1_30429374	Ca4	30429374	TATTCATT/TATT	TGATCGCCATATCCTTAGCC	ATTTGGTTTGGCATTGTTGC	60.0	811	INTERGENIC	--	--	--
Ca4_P1_30430504	Ca4	30430504	ATATATTTA/ATA	CCCCTTCTCGTTTTTCAACA	TTCATGCGTGTCCGATTTTA	60.1	643	INTERGENIC	--	--	--
Ca4_P1_30433017	Ca4	30433017	CAAAAAAA/CAAAAAA	GACTGACATTCCGATGAGCA	TGCAACACGATAAGGGAACA	59.8	721	INTERGENIC	--	--	--
Ca4_P1_30454501	Ca4	30454501	TAA/TAAA	CGTGCGCTGTGTGATAGAAT	CAATTCACTTTCGAGTTGGTG	59.9	761	INTERGENIC	--	--	--

INDEL marker IDs	Chromosomes/ unanchored scaffolds	Physical positions (bp)	InDels (*Kabuli* reference genome-CDC Frontier/PI)	Forward primers (5'-3')	Reverse primers (5'-3')	Anneling temperature (0C)	Expected amplified product size (bp)	Structural annotation		Functional annotation	
								Sequence components of *kabuli* genome	*Kabuli* gene accession IDs	NCBI-KOG	TFs
Ca4_P1_30456018	Ca4	30456018	AT/A	GGCGAGAAAAGTTTGTTGATG	TCGTTGATTTTCAACCACCA	59.7	377	INTERGENIC	--	--	--
Ca4_P1_30459024	Ca4	30459024	ATTTTTTT/ATTTTTT	GAGCTTGCATGCTACGTCCT	ATTTGGTGACTCAAAACGGC	60.6	758	INTERGENIC	--	--	--
Ca4_P1_30459877	Ca4	30459877	GTTTTTTTTTTTT/GTTTTTTTTTTT	AAACCCTTTGCAACCAATGA	TGCCTGTTGAAATTGTTGGA	60.3	446	INTERGENIC	--	--	--
Ca4_P1_30463711	Ca4	30463711	CAAAAA/CAAAA	TGGAAAGAAAATGCGTTTGA	ATGTGGGTCGCACTAACACA	59.3	635	INTERGENIC	--	--	--
Ca4_P1_30463898	Ca4	30463898	ATTTTTTTTT/ATTTTTTTT	GGGGGAAAAGTGATAATGGG	AATGGCATGCAAAAACATCA	60.4	572	INTERGENIC	--	--	--
Ca4_P1_30464438	Ca4	30464438	CTTTTTTT/CTTTTTT	TGTGTTAGTGCGACCCACAT	TGTGAAGGGAGTGAGTTGCTT	60.0	407	INTERGENIC	--	--	--
Ca4_P1_30464759	Ca4	30464759	GACAACAACAA/GACAACAA	TGATGTTTTTGCATGCCATT	TTGGATCATCACACTCCAACA	59.9	612	INTERGENIC	--	--	--
Ca4_P1_30468001	Ca4	30468001	TATGTTGTTATGAT/TAT	CCCCAATCACAATCATCGTT	TTTGACTGCAACAAAGCAGG	60.6	494	DRR	Ca_14199	--	G2-like
Ca4_P1_30468296	Ca4	30468296	AAT/AATAT	ATTGTGCATATTCCCTCCCA	CCTTAATCCACCAAAAGCCA	60.2	747	DRR	Ca_14199	--	G2-like
Ca4_P1_30468822	Ca4	30468822	AAA/AAAGAA	TGGCTTTTGGTGGATTAAGG	GGTCTCACGACCAAGTCACA	59.9	438	DRR	Ca_14199	--	G2-like
Ca4_P1_30477859	Ca4	30477859	CTTTTT/CTTTTTT	AAAATCAATCGGGGACATCA	GAATCAAACAAAGCCCCAAA	60.1	648	INTERGENIC	--	--	--
Ca4_P1_30501632	Ca4	30501632	AGT/A	TGGGGATTTATCCCTTCAAA	GCAAGTGTCAACTCACTCAATCA	59.2	595	INTERGENIC	--	--	--

INDEL marker IDs	Chromosomes/ unanchored scaffolds	Physical positions (bp)	InDels (*Kabuli* reference genome-CDC Frontier/PI)	Forward primers (5'-3')	Reverse primers (5'-3')	Anneling temperature (0C)	Expected amplified product size (bp)	Structural annotation		Functional annotation	
								Sequence components of *kabuli* genome	*Kabuli* gene accession IDs	NCBI-KOG	TFs
Ca4_P1_30502729	Ca4	30502729	CTATATATATATATAT/CTATATATATATATATAT	AATGTAGCATGTGCGTAGCG	CAATTCATTTGATTGTGTTGCAT	59.9	303	INTERGENIC	--	--	--
Ca4_P1_30503496	Ca4	30503496	A/AAAAAT	GAAAATATGTTAGCACACCACCA	TTGCTTTTGCATTTCACATACC	58.9	718	INTERGENIC	--	--	--
Ca4_P1_30528133	Ca4	30528133	ATTTTTTTTT/ATTTTTTTT	TTTTCCTCCGAATTGTGTGA	ACGCATCGTACTCTTTGCAC	59.1	429	INTERGENIC	--	--	--
Ca4_P1_30534956	Ca4	30534956	CTTTTTTTT/CTTTTTTTTT	CCATGCAAGCACAAATTCTTT	ATGCAATTAGCCTCTGGGAA	60.1	445	INTERGENIC	--	--	--
Ca4_P1_30535730	Ca4	30535730	GACCAAAGATA/G	CCATTCAGAATTAGAAAATCACCA	CCAAATTTCCCAAGGGTTCT	59.4	582	INTERGENIC	--	--	--
Ca4_P1_30535832	Ca4	30535832	TATAAATAAATAAATAAA/TATAAATAAATAAATAAATAAATAAATAAA	CCATTCAGAATTAGAAAATCACCA	CCAAATTTCCCAAGGGTTCT	59.4	582	INTERGENIC	--	--	--
Ca4_P1_30536932	Ca4	30536932	GTTTTTTTTT/GTTTTTTTTTTTTT	ATCATTGAACGACCGGAAAC	CGGATACAATGGCAATCAAA	59.8	431	INTRON	Ca_14204	--	NAC
Ca4_P1_30537523	Ca4	30537523	CA/CATA	TTTGATTGCCATTGTATCCG	CAAAATGGAAATGCAAGAACAA	59.4	803	INTRON	Ca_14204	--	NAC
Ca4_P1_30538166	Ca4	30538166	AAAGAAG/AAAGAAGAAG	TTGTTCTTGCATTTCCATTTTG	CGCCATCTTCCTCTTCTTTG	60.0	380	CDS (large-effect mutations)	Ca_14204	--	NAC
Ca4_P1_30542137	Ca4	30542137	TAAAAAA/TAAAAAAA	TAAGGCATGATAAGGCCGAC	GTCATGTCACCCGTGATGAG	60.1	299	DRR	Ca_14205	--	--
Ca4_P1_30543529	Ca4	30543529	T/TC	CACGAGATTCGTCGAGAACA	TGATGGGTCATTTAGTGCCA	60.0	196	DRR	Ca_14205	--	--
Ca4_P1_30552355	Ca4	30552355	TG/T	TGTCGCATGCAACCTCTTAG	ACCTGTGTGTTCCCTTCCAC	60.0	528	INTERGENIC	--	--	--

INDEL marker IDs	Chromosomes/ unanchored scaffolds	Physical positions (bp)	InDels (*Kabuli* reference genome-CDC Frontier/PI)	Forward primers (5'-3')	Reverse primers (5'-3')	Anneling temperature (0C)	Expected amplified product size (bp)	Structural annotation		Functional annotation	
								Sequence components of *kabuli* genome	*Kabuli* gene accession IDs	NCBI-KOG	TFs
Ca4_P1_30558963	Ca4	30558963	GC/G	CCCCTTTTGGCATCTATCAA	TCATCTCATTTTGCTTCCCT	59.9	510	INTERGENIC	--	--	--
Ca4_P1_30561689	Ca4	30561689	TAAAAA/TAAAA	GGAGGCACCATACTTTTCCA	AGAATGGCCACTTCAAGTCAA	59.9	198	INTERGENIC	--	--	--
Ca4_P1_30565218	Ca4	30565218	CTTTTTTTTTT/CTTTTTTTTT	ATGCAATCCATGTTCGTTCA	CAATTGCAAAAACATGTCGG	59.9	551	INTERGENIC	--	--	--
Ca4_P1_30566057	Ca4	30566057	CGGG/CGG	TTTTTGCAATTGGGCATACA	ACTGGGTTTGGGTTACCCTC	59.9	515	INTERGENIC	--	--	--
Ca4_P1_30566132	Ca4	30566132	ATTTTTTTT/ATTTTTTTTT	GAGGGTAACCCAAACCCAGT	CCACTTTATGTGCAACCCCT	60.1	440	INTERGENIC	--	--	--
Ca4_P1_30569960	Ca4	30569960	TAAAAAAAA/TAAAAAAAAA	AGAGAGTGTTGCAGGCGATT	AACTCGATTTTTGTGGAGGAAA	60.0	613	INTERGENIC	--	--	--
Ca4_P1_30570798	Ca4	30570798	TAAAAAAA/TAAAAAAAA	TGCTTCTCAATGACTAACACCAA	TGTTGGTGTGACTTTTCCTTCT	59.8	881	INTERGENIC	--	--	--
Ca4_P1_30573486	Ca4	30573486	GTT/GTTT	GGCAACAAATTAGTGTGCTTAGAA	TCCTCTTCGATCAAGCATCC	59.7	775	INTERGENIC	--	--	--
Ca4_P1_30573766	Ca4	30573766	T/TC	CATTATGATCACAGACGATGGTG	TCCTCTTCGATCAAGCATCC	60.3	259	DRR	Ca_14206	A	--
Ca4_P1_30582286	Ca4	30582286	GAAAAAAAAAA/GAAAAAAAAAAA	TTCCCTCCCTTCCTTTGAGT	TTTTCTGCAAGTTTGTCGTTG	60.0	615	INTERGENIC	--	--	--
Ca4_P1_3058285	Ca4	3058285	AGCCGCCGCCGCCGCC/AGCCGCCGCC	TTTCCAATTCCACCACCAAT	GTGTGGTTGCAAGGAATGTG	60.0	799	CDS (large-effect mutations)	Ca_12104	--	--
Ca4_P1_30583911	Ca4	30583911	GAAAAAA/GAAAAAAA	CCATTTTTGAAATTGACGAGC	GCAAATTGAGGTCACTCAACC	59.6	300	INTERGENIC	--	--	--

**INDEL marker IDs**	**Chromosomes/ unanchored scaffolds**	**Physical positions (bp)**	**InDels (*Kabuli* reference genome-CDC Frontier/PI)**	**Forward primers (5'-3')**	**Reverse primers (5'-3')**	**Anneling temperature (0C)**	**Expected amplified product size (bp)**	**Structural annotation**		**Functional annotation**	
								**Sequence components of *kabuli* genome**	***Kabuli* gene accession IDs**	**NCBI-KOG**	**TFs**
Ca4_P1_30585117	Ca4	30585117	CT/CTTT	GCAATGAATGTGTCCTCCAA	TGTTTGGGATGACTGACCTTT	59.5	524	INTERGENIC	--	--	--
Ca4_P1_30585901	Ca4	30585901	CTTTTTCTAATTTTTCTA/CTTTTTCTA	AAAGGTCAGTCATCCCAAACA	GAAACAGAAAACGGGAACCA	59.4	381	INTERGENIC	--	--	--
Ca4_P1_30605673	Ca4	30605673	TGGGGG/TGGGG	CTCCGAGACGAGAAAAGGTG	CGAGCAACTCTGGTTGATTT	60.0	670	INTERGENIC	--	--	--
Ca4_P1_30617600	Ca4	30617600	CAAAAAAAAAAAAAAAA/CAAAAAAAAAAAAAA	GCTATGTTGTCACGACCCAA	TGGTGGTTGTTGTCTCCTCA	59.6	466	INTERGENIC	--	--	--
Ca4_P1_30619694	Ca4	30619694	CAAAAA/CAAAA	ACTCACTCTGCCCCTGTGTT	CCAACTTGGTTCCAACAAAAA	59.8	566	INTERGENIC	--	--	--
Ca4_P1_30640898	Ca4	30640898	ATTTTT/ATTTTTT	TCGCGAAGAGATAAAAGGGA	ATGCACCAAAAATGCAACAA	59.9	831	INTERGENIC	--	--	--
Ca4_P1_30650156	Ca4	30650156	AT/ATT	AAGTTCCGTCGGGTAGGACT	CCAAGGCCCCATTTATTTTT	60.0	747	INTERGENIC	--	--	--
Ca4_P1_30650335	Ca4	30650335	ATTTTTTTTTT/ATTTTTTTTT	TAGGCACCCTATTGGTCCTG	CCAAGGCCCCATTTATTTTT	59.9	711	INTERGENIC	--	--	--
Ca4_P1_3065355	Ca4	3065355	CATA/CATATA	ACAAAGGTCACCCTCGTCAC	CCAAAACTGGGGAAAAACAA	60.0	622	INTERGENIC	--	--	--
Ca4_P1_30653740	Ca4	30653740	AC/ACC	TGCCCATTGATGCTAAACAA	GAATGATGTCGCGGTAGGAT	60.1	513	INTERGENIC	--	--	--
Ca4_P1_30685617	Ca4	30685617	TTA/TTATCAATA	GGGAAGAATGGATTTGGGAT	GGATGTTTCATTCCACCAAAG	60.0	192	INTERGENIC	--	--	--
Ca4_P1_30690853	Ca4	30690853	TAAAAAAAA/TAAAAAAAAA	GAAACGAGTTGTTGCACTTGA	TTGAGTTGCACGAGAATTGC	59.0	565	INTERGENIC	--	--	--

**INDEL marker IDs**	**Chromosomes/ unanchored scaffolds**	**Physical positions (bp)**	**InDels (*Kabuli* reference genome-CDC Frontier/PI)**	**Forward primers (5'-3')**	**Reverse primers (5'-3')**	**Anneling temperature (0C)**	**Expected amplified product size (bp)**	**Structural annotation**		**Functional annotation**	
								**Sequence components of *kabuli* genome**	***Kabuli* gene accession IDs**	**NCBI-KOG**	**TFs**
Ca4_P1_30692279	Ca4	30692279	GTTTTTTTTTTT/GTTTTTTTTTTTTT	CTCCTAACCCCCGTTGAGTT	TGCTAAAATTTGAGGTGGAAAAA	60.4	495	INTERGENIC	--	--	--
Ca4_P1_30693172	Ca4	30693172	CAAAAAAAAAAAAAA/CAAAAAAAAAAAAA	TTTTCACCGAAACTCCAAGG	GAAAATACCTAAACCCGCCC	60.1	768	INTERGENIC	--	--	--
Ca4_P1_30697175	Ca4	30697175	CTTTT/CTTT	AGATTCCATGGCACACACAA	CCCCAAGATTAACAAAGGCA	60.0	647	INTERGENIC	--	--	--
Ca4_P1_3069954	Ca4	3069954	TTTTATTTATTTATTTATTTAT/TTTTATTTATTTATTTATTTATTTAT	AAACCAAAACTGGCGAAAAA	TGAGAAAACATATTGGGAAGGC	59.6	686	DRR	Ca_12105	--	--
Ca4_P1_30700775	Ca4	30700775	TAAAAA/TAAAAAA	GGTGGAAAACTTTGCATTGG	CCCCTCATGCTTGAATTGTT	60.3	281	INTERGENIC	--	--	--
Ca4_P1_30718794	Ca4	30718794	AAACCT/A	TCCTAAGAGGAAGCCGATCA	TTTTGTGGTTTGGAAGGTCC	59.9	673	INTERGENIC	--	--	--
Ca4_P1_30737298	Ca4	30737298	ATTTTTTTTTTTTT/ATTTTTTTTTTT	AGGTGTGAGCCAGAATGTCC	GTTGTGGGATGAACTGACCC	60.1	613	INTERGENIC	--	--	--
Ca4_P1_30741967	Ca4	30741967	TCAATATTGAACA/T	ATCCGGTTCCTTTTTGGTTC	TAAGGACCGGTGATCTGACA	60.2	656	INTERGENIC	--	--	--
Ca4_P1_30752597	Ca4	30752597	TAAAAAAA/TAAAAAAAA	CGAGACACACATTTTTCACCA	GAGACACCCTTGTGCGTTTT	59.6	874	INTERGENIC	--	--	--
Ca4_P1_30755137	Ca4	30755137	ATTTTTTT/ATTTTTT	CAGTAGGAAGTCACATTCCCAGA	GCAACTGAGCTGAGAGCGTA	60.5	860	INTERGENIC	--	--	--
Ca4_P1_30755345	Ca4	30755345	TTTATTATTATTATTATTATTATTATTATTATTATTATTATTATTATTATTATTATTATTATTATT/TTTATTATTATTATTATTATTATTATTATTATTATTATTATTATTATTATTATTATTATTATTATTATT	CCAAACAAAACATATTGAATCGAA	GCAACTGAGCTGAGAGCGTA	60.1	498	INTERGENIC	--	--	--
Ca4_P1_30760524	Ca4	30760524	ATT/ATTT	TTTGGAAGCGGTCTCACTCT	AAACGAATACTGCAACGCAA	60.0	217	DRR	Ca_14211	--	ERF

INDEL marker IDs	Chromosomes/ unanchored scaffolds	Physical positions (bp)	InDels (*Kabuli* reference genome-CDC Frontier/PI)	Forward primers (5'-3')	Reverse primers (5'-3')	Anneling temperature (0C)	Expected amplified product size (bp)	Structural annotation		Functional annotation	
								Sequence components of *kabuli* genome	*Kabuli* gene accession IDs	NCBI-KOG	TFs
Ca4_P1_30773714	Ca4	30773714	CTTTTTTTTT/CTTTTTTTTTT	ATCCGATTGGACGGTTCATA	GCAAAATCAATTTATGCTGGAA	60.2	855	INTERGENIC	--	--	--
Ca4_P1_30775212	Ca4	30775212	CTTTTTTTTTT/CTTTTTTTTT	TGAACTCGTAACATAAGCCCAA	TGGGAATGCTTTCTTATGGG	59.6	931	INTERGENIC	--	--	--
Ca4_P1_30790861	Ca4	30790861	TAAAAAAAAA/TAAAAAAAAAA	GAATTTTGGCCATCACCACT	TGTCTCGCACACCCATTTTA	59.8	530	INTERGENIC	--	--	--
Ca4_P1_30792260	Ca4	30792260	TAAAAATAAAAA/TAAAAATAAAAAATAAAAA	TCAAATGAGTCGAAATTGAGC	TGTTGGTGGTGCATTCCTTA	58.0	740	INTERGENIC	--	--	--
Ca4_P1_30806217	Ca4	30806217	ATTTTTTTTTTTT/ATTTTTTTTTTT	TGCAATTGGAGCAAAAGTCA	GCCCTCAGATGAGAGTCAGG	60.4	616	INTERGENIC	--	--	--
Ca4_P1_3081231	Ca4	3081231	TAAAAAAAAAAA/TAAAAAAAAAA	TCGCCCCTTAATTGTTTGTC	TGTCTAGGTGGCCCTGTGAT	59.9	571	INTERGENIC	--	--	--
Ca4_P1_30823011	Ca4	30823011	GAAAAAAAAAAAAA/GAAAAAAAAAAAA	CGGAAAGAAACAGGACAAACA	CACCAGGAGGCGTGTTTTAT	60.1	165	INTERGENIC	--	--	--
Ca4_P1_30833853	Ca4	30833853	GC/G	GATCCTTCAAAAAGGAGGGC	TGTGAAAACGGCACAATGAT	60.0	434	INTERGENIC	--	--	--
Ca4_P1_30844654	Ca4	30844654	CTTTTTTTTTT/CTTTTTTTTT	CACCTGCTTTTGACCCATCT	GGCAATTGCTTTACGCTAGG	60.1	959	INTERGENIC	--	--	--
Ca4_P1_3085106	Ca4	3085106	AATATATATATATATATATATATATATATATATATATAT/AATATATATATATATATATATATATATATATATATATATATATATAT	GGGTGGAAAATTGGATGAAG	CGGGAAAGCATAAATATCACC	59.2	458	INTERGENIC	--	--	--
Ca4_P1_30856676	Ca4	30856676	A/AT	TGCACTGTATTGGAATTGTTGA	GAAATGACTGAAAAATTTTGGACC	59.1	482	DRR	Ca_14212	--	bZIP
Ca4_P1_30865017	Ca4	30865017	TAAA/TAA	AGGACGTGCAACCCTTATTG	TTGGAGCCTCATAGCCATCT	60.0	734	INTERGENIC	--	--	--

INDEL marker IDs	Chromosomes/ unanchored scaffolds	Physical positions (bp)	InDels (*Kabuli* reference genome-CDC Frontier/PI)	Forward primers (5'-3')	Reverse primers (5'-3')	Anneling temperature (0C)	Expected amplified product size (bp)	Structural annotation		Functional annotation	
								Sequence components of *kabuli* genome	*Kabuli* gene accession IDs	NCBI-KOG	TFs
Ca4_P1_30866796	Ca4	30866796	A/AC	AAGTTGCCTCTGCCTTTGAA	ATGTTTGCAACCCGTTTCTC	60.0	255	INTERGENIC	--	--	--
Ca4_P1_30867065	Ca4	30867065	GAAAAAAAAAAA/GAAAAAAA	AAGTTGCCTCTGCCTTTGAA	CACCCTTTTGGTTGCTCCTA	60.0	651	INTERGENIC	--	--	--
Ca4_P1_30871631	Ca4	30871631	A/AT	CACCCAATCTGCAATCTTGA	TGCTTGTTTTTCGACCATGA	59.6	362	INTERGENIC	--	--	--
Ca4_P1_30873864	Ca4	30873864	ACC/ACCC	CAAAGAGAGAAACTTGCCCG	GGAAAGCGAGAAGTTGAACG	60.0	566	INTERGENIC	--	--	--
Ca4_P1_30880709	Ca4	30880709	ATT/ATTTT	TGCAGAGTAAACCAGCAACG	TTGCAAGTTGTGACTGAGGC	60.1	557	INTERGENIC	--	--	--
Ca4_P1_30880741	Ca4	30880741	CAA/CAAA	TGCAGAGTAAACCAGCAACG	TTGCAAGTTGTGACTGAGGC	60.1	557	INTERGENIC	--	--	--
Ca4_P1_30883366	Ca4	30883366	ACTCT/ACT	TTGAAGCATGCATTTGAAGC	TGTGCTTTCTTCTCTGGCCT	60.0	897	INTERGENIC	--	--	--
Ca4_P1_30885134	Ca4	30885134	CAAAAAA/CAAAAAAA	GCATCACAAGTCCAGAACCA	TGCTCAAGTTCATTGCATCC	59.7	459	INTERGENIC	--	--	--
Ca4_P1_30887008	Ca4	30887008	TCTCCTC/TCTC	CCTGACCCTACAAGCCAATC	GTAGTCTCGCAAGGACTCCG	59.6	218	INTERGENIC	--	--	--
Ca4_P1_30888513	Ca4	30888513	T/TA	GGCATAGTTGGTATTCGCCA	TCGGCTCAAAGAGTCAAGGT	60.9	627	INTERGENIC	--	--	--
Ca4_P1_30928717	Ca4	30928717	TAAAAAAA/TAAAAAAAA	CAATGTCCATGTCGACCAAA	TCTGTCGCACCATCTCAGTC	60.4	949	INTERGENIC	--	--	--
Ca4_P1_30935136	Ca4	30935136	TG/T	CGGGACGTTTAATTTGCTGT	AGGCAAGAAGCTGATAGGCA	60.0	594	DRR	Ca_14214	R	HD-ZIP

INDEL marker IDs	Chromosomes/ unanchored scaffolds	Physical positions (bp)	InDels (*Kabuli* reference genome-CDC Frontier/PI)	Forward primers (5'-3')	Reverse primers (5'-3')	Anneling temperature (0C)	Expected amplified product size (bp)	Structural annotation		Functional annotation	
								Sequence components of *kabuli* genome	*Kabuli* gene accession IDs	NCBI-KOG	TFs
Ca4_P1_30954265	Ca4	30954265	CAAAAAAAA/CAAAAAAAAA	TTGCAAGAATCCATCCATCA	GCAGATGTAAACAAACCCGAA	60.0	299	INTERGENIC	--	--	--
Ca4_P1_30959142	Ca4	30959142	TCAAAA/T	TGTTTTGAAAGATTTATGATGGGA	CACCTCAGGCACAATAAATCG	59.7	464	INTERGENIC	--	--	--
Ca4_P1_30964064	Ca4	30964064	CTTTTTTTTTT/CTTTTTTTTTTT	ACTAGTTGGGGGACCAGCTT	CCCTTACGGCACACTTTGTT	60.0	569	INTERGENIC	--	--	--
Ca4_P1_30968151	Ca4	30968151	CTTTTTTTT/CTTTTTTT	CACATCCCTTGCCATTTCTT	CCCGCATTTGTAACCATCTT	59.9	827	INTERGENIC	--	--	--
Ca4_P1_31001917	Ca4	31001917	A/AGAGG	GCTGTTGAAAGATGCAACCA	CCTTTGCTCCTTGTGTCTCC	59.8	395	INTERGENIC	--	--	--
Ca4_P1_31002155	Ca4	31002155	GAGACA/GAGACAGACA	GCTGTTGAAAGATGCAACCA	TGACAAGCAAGTGATCGTCTG	59.8	607	INTERGENIC	--	--	--
Ca4_P1_31002850	Ca4	31002850	GATAT/GAT	CTTCAAAAGTTTTCACGGGC	CAAGATGAGGGGACAAGGAA	59.7	426	INTERGENIC	--	--	--
Ca4_P1_31011573	Ca4	31011573	CTT/CTTT	CAGTTCGGAAACAGTTCGGT	ATGGGGGAGCAGGAGTAGAT	60.1	928	INTERGENIC	--	--	--
Ca4_P1_31016017	Ca4	31016017	AG/A	TCAACCCTGATTCGACCTTC	TGAACCAAAACTCACGCAAG	60.1	551	INTERGENIC	--	--	--
Ca4_P1_31021850	Ca4	31021850	A/AC	ACGCACCAAACACATTGAGA	ATCCTATGCAGTGGATGCAA	60.2	781	INTERGENIC	--	--	--
Ca4_P1_31024618	Ca4	31024618	TAAAAAAAAA/TAAAAAAA	CGTCCTTGAAATCGTTTGGT	GCTCCCTTAACCAAAATCCC	60.0	724	INTERGENIC	--	--	--
Ca4_P1_31029219	Ca4	31029219	GA/GATAA	TGCATCGTGAGTTTGAGGAG	GCCATTTGGAACTGGAAGAA	60.0	714	DRR	Ca_14217	Q	--

INDEL marker IDs	Chromosomes/ unanchored scaffolds	Physical positions (bp)	InDels (*Kabuli* reference genome-CDC Frontier/PI)	Forward primers (5'-3')	Reverse primers (5'-3')	Anneling temperature (0C)	Expected amplified product size (bp)	Structural annotation		Functional annotation	
								Sequence components of *kabuli* genome	*Kabuli* gene accession IDs	NCBI-KOG	TFs
Ca4_P1_31049768	Ca4	31049768	A/AAT	AAGGCCAGATCATTGGACAC	TTCAGATTTGGCAAGCCTCT	59.9	378	INTERGENIC	--	--	--
Ca4_P1_31054236	Ca4	31054236	T/TCG	TCGAGAAAGGATTTTGGGTG	CAAGGCATTTCCATCACCTT	60.0	456	INTERGENIC	--	--	--
Ca4_P1_31055489	Ca4	31055489	TT/TTCAT	CATGCACAACTTGGTTAATGG	TGTAATTGGTGGCGTTTGAA	59.0	460	INTERGENIC	--	--	--
Ca4_P1_31062648	Ca4	31062648	TT/TTTTAT	GTTCGGGTGGTGCATAAACT	AAAACGACGCCTCCTATTCTAA	59.9	521	INTERGENIC	--	--	--
Ca4_P1_31062771	Ca4	31062771	CTTTTTTTTTT/CTTTTTTTTT	GTTCGGGTGGTGCATAAACT	AAAACGACGCCTCCTATTCTAA	59.9	521	INTERGENIC	--	--	--
Ca4_P1_31063154	Ca4	31063154	AATATATATATATATATATATAT/AATATATATATATATATATATAT	GAGGGGATGAATCATTAACTCG	GTAAGACCCCGCATTGTGTT	59.8	499	INTERGENIC	--	--	--
Ca4_P1_31071731	Ca4	31071731	TTGTGT/TTGTGTGT	TCAGTTTCGCAAACAATCCA	TGATGTGATCTAAAGGTGCGA	60.2	129	INTERGENIC	--	--	--
Ca4_P1_31072417	Ca4	31072417	TAA/TA	TGAATTCATAGTCGTGTCTCCAA	TTCCTTGTCTCCACATCCAA	59.6	438	INTERGENIC	--	--	--
Ca4_P1_31079661	Ca4	31079661	TGG/TG	GCTCGTGTCATTGCAGAAAA	CCAATTACCACAGATTCGGG	60.0	337	INTERGENIC	--	--	--
Ca4_P1_3109508	Ca4	3109508	AATATATATATATATATATATATAT/AATATATATATATATATATATATAT	AGTTCCATTGCCATTTCGAG	CAGAGTTGGGAAGCTGAAGG	60.1	751	DRR	Ca_12110	--	--
Ca4_P1_31102909	Ca4	31102909	ATTTTTT/ATTTTTTT	TTTTACATGGTAGCGTCCCC	TGAAATTGTAGGGATTGGGC	59.8	798	DRR	Ca_14221	U	bHLH
Ca4_P1_31106521	Ca4	31106521	CCTT/CCTTCTT	ATAAGAGTGTGGCGGTTTGG	TGGTTGAAATGGACGACAAA	60.0	584	INTERGENIC	--	--	--

INDEL marker IDs	Chromosomes/ unanchored scaffolds	Physical positions (bp)	InDels (*Kabuli* reference genome-CDC Frontier/PI)	Forward primers (5'-3')	Reverse primers (5'-3')	Anneling temperature (0C)	Expected amplified product size (bp)	Structural annotation		Functional annotation	
								Sequence components of *kabuli* genome	*Kabuli* gene accession IDs	NCBI-KOG	TFs
Ca4_P1_31107634	Ca4	31107634	GTTTT/GTTTTT	GCCTGATTTCTTGGTTGAGC	GGGACTGAATCCGACTTGAA	59.8	394	INTERGENIC	--	--	--
Ca4_P1_31108602	Ca4	31108602	GGAAGAAGAA/GGAAGAAGAAGAA	GGATCAGGCCCTATTGTTGA	AGTTGGCTTTGCTTGATGCT	59.9	476	INTERGENIC	--	--	--
Ca4_P1_31110947	Ca4	31110947	TAAAAAA/TAAAAA	GCTAAAGCAATCACCAAGGC	GCGTTTGGTGACATAGCTGA	59.9	412	INTERGENIC	--	--	--
Ca4_P1_31114996	Ca4	31114996	CT/CTGT	CTTCTTCAAGTCGGTGGCTC	TTTGCAGGTCTGGAATACCC	60.0	213	INTERGENIC	--	--	--
Ca4_P1_31121907	Ca4	31121907	CTA/C	ACCTTGGCAGAAACAGGATG	TGCTCACTGAAGATGTTGCC	60.1	737	INTERGENIC	--	--	--
Ca4_P1_31122661	Ca4	31122661	CGGGGG/CGGGG	CCAAAATCCACAAGCCACTT	CAAGGCAATGGGTATATGGG	60.0	597	INTERGENIC	--	--	--
Ca4_P1_31129446	Ca4	31129446	ATTTTTTTT/ATTTTTTTTT	TGGGTATCCAAACCATATCCA	TGATTCACGTGGATGGAGAA	59.9	741	INTERGENIC	--	--	--
Ca4_P1_31135621	Ca4	31135621	GCC/GC	AGCGGAGGAGAGTTGTGAAA	CCTTTGAGGATTAGAGCCCC	60.0	923	DRR	Ca_14222	R	MYB
Ca4_P1_31137565	Ca4	31137565	GAAAAAA/GAAAAAAA	TTTCCGAATCGGGAGTATGA	GTTCCTGCCATCCAACCTTA	60.4	113	DRR	Ca_14223	--	bHLH
Ca4_P1_31137943	Ca4	31137943	CAAA/CA	TAAGGTTGGATGGCAGGAAC	TTGAGACCATGCTGAAAAAGTT	59.9	798	DRR	Ca_14223	--	bHLH
Ca4_P1_31138748	Ca4	31138748	GTTAATT/GTT	TGCAGCTAGCTCGTTTTTCA	GGCAGCAAACTAACACAGCA	59.9	342	INTRON	Ca_14223	--	bHLH
Ca4_P1_31140324	Ca4	31140324	TAAAA/TAAAAA	ACTTCCATTCGCTTCAGCAT	AAATGATTCTGGTGGGGACA	59.8	536	INTRON	Ca_14223	--	bHLH

INDEL marker IDs	Chromosomes/ unanchored scaffolds	Physical positions (bp)	InDels (*Kabuli* reference genome-CDC Frontier/PI)	Forward primers (5'-3')	Reverse primers (5'-3')	Anneling temperature (0C)	Expected amplified product size (bp)	Structural annotation		Functional annotation	
								Sequence components of *kabuli* genome	*Kabuli* gene accession IDs	NCBI-KOG	TFs
Ca4_P1_31141569	Ca4	31141569	TTACTACTACTACTA/TTACTACTACTACTACTA	ATGTTCCATTCCCAATCCAA	TCTTATCCCTCCCCTGCTTT	60.0	497	INTERGENIC	--	--	--
Ca4_P1_31150025	Ca4	31150025	ATTTTTTTTTTTT/ATTTTTTTTTTT	TGAACGAAAAGCTTATGGATATGT	AATCGACCCAATCAAGAACG	59.1	412	INTERGENIC	--	--	--
Ca4_P1_31159995	Ca4	31159995	ATAAATTAAATT/ATAAATTAAATTAAATT	TTGAGCCAACAAATTGGAGA	AGTCCCCATGTAATTGTCACG	59.2	301	INTERGENIC	--	--	--
Ca4_P1_31172721	Ca4	31172721	TG/TGG	TCTTTGGATGGGTTCCATGT	TTACACCAATTCCCCCAAGA	60.2	663	INTERGENIC	--	--	--
Ca4_P1_31172943	Ca4	31172943	TA/T	TCGCCTAAGGTAAGGGCTTT	TTACACCAATTCCCCCAAGA	60.2	421	INTERGENIC	--	--	--
Ca4_P1_31173752	Ca4	31173752	TTTAATGTTA/TTTA	GCCTGTGTGGCTGGTTTAAT	TGCACCAATCAAACGTCATT	60.0	388	INTERGENIC	--	--	--
Ca4_P1_31182932	Ca4	31182932	T/TC	CGCATATTAGCATGGAGGGT	TCGCTCGAACCAGAGTTCTT	59.9	317	INTERGENIC	--	--	--
Ca4_P1_31192815	Ca4	31192815	TGG/TG	TTGAAGGAGAAGCCAGAGTCA	TGAGGCATGCAGAAACGTAG	60.1	238	INTERGENIC	--	--	--
Ca4_P1_31194912	Ca4	31194912	CTTTTTTTTTTT/CTTTTTTTTTT	TTCCATGTTTGTGTTGAAGCA	GCACATTGCAAGGGTATTGA	60.1	822	DRR	Ca_14224	--	S1Fa-like
Ca4_P1_31195163	Ca4	31195163	GAAAAAAAA/GAAAAAAAAA	TTTCCCGACTTGGTCCTTTA	TGCATGCAGATCTTGCTCTC	59.5	643	DRR	Ca_14224	--	S1Fa-like
Ca4_P1_31200114	Ca4	31200114	TA/TAGACCA	TTGTCTTAGGCAGCACTGGA	TGGTGAACGAAAGTGACGTG	59.6	903	INTERGENIC	--	--	--
Ca4_P1_3121760	Ca4	3121760	CATATATATATATATATATATATATATATATATATAT/CATATATATATATATATATATATATATATATATATATATATATAT	ATTCAATCAAACACACACAAAAA	ATTAATGACGACGCATGATCT	57.2	397	INTERGENIC	--	--	--

INDEL marker IDs	Chromosomes/ unanchored scaffolds	Physical positions (bp)	InDels (*Kabuli* reference genome-CDC Frontier/PI)	Forward primers (5'-3')	Reverse primers (5'-3')	Anneling temperature (0C)	Expected amplified product size (bp)	Structural annotation		Functional annotation	
								Sequence components of *kabuli* genome	*Kabuli* gene accession IDs	NCBI-KOG	TFs
Ca4_P1_31224726	Ca4	31224726	TAAAAAAAAAAAAAA/TAAAAAAAAAAAA	TGCACAAGTGAAAACCACAA	TTTGCAAATTGGTTGGTTCA	58.7	641	INTERGENIC	--	--	--
Ca4_P1_31226486	Ca4	31226486	GTTGGTGTTTCATATCCAT/GT	TTCCCAATGGTGGACTGTCT	CTGTAGGGCCACCTCCAATA	60.4	458	INTRON	Ca_14227	C	--
Ca4_P1_31229085	Ca4	31229085	CTTTTTTTT/CTTTTTTT	TGATGGTAAAAGTTTATATTGACGA	CAGTGCAACGGAAGGACATA	58.2	485	DRR	Ca_14227	C	--
Ca4_P1_31230420	Ca4	31230420	TTCTCTCTCTCT/TTCTCTCTCT	CTTTCCATCTCACTCCCTCG	TAGCCGACAGGGTCTCAGTT	59.8	435	INTERGENIC	--	--	--
Ca4_P1_31230662	Ca4	31230662	GAAAAA/GAAAA	CTTTCCATCTCACTCCCTCG	TAGCCGACAGGGTCTCAGTT	59.8	435	INTERGENIC	--	--	--
Ca4_P1_31235171	Ca4	31235171	GTATATATATAT/GTATATATATATATAT	GCCACCCTTAGGAGGAAAAG	ATTTAAATGTCGTGACCGGC	60.1	607	INTERGENIC	--	--	--
Ca4_P1_31235705	Ca4	31235705	ATTTTTTTTT/ATTTTTTTTTT	CAATTGCGGTTTCCACTTTT	TTCATGCATTTTCAATGGGA	60.0	434	INTERGENIC	--	--	--
Ca4_P1_31236090	Ca4	31236090	ATATTATTATTATTATT/ATATTATTATTATT	CAATTGCGGTTTCCACTTTT	TTTTTACCGTGGGTGAATCC	60.0	956	INTERGENIC	--	--	--
Ca4_P1_31237697	Ca4	31237697	TAAAAAAA/TAAAAAA	CGTACACGTGCATGGAAATC	TCGTTTGCGAGATGATTTTG	60.0	509	INTERGENIC	--	--	--
Ca4_P1_31250934	Ca4	31250934	ATT/ATTTT	AGTTTTCGCCATTTGTCACC	AACCACTTGAGCATGGGTTC	60.0	196	INTERGENIC	--	--	--
Ca4_P1_31258911	Ca4	31258911	GTT/GT	CAGGTCGTGCCAATTCTTTT	GGCTAGGATTTGCAACAGGT	60.1	264	INTERGENIC	--	--	--
Ca4_P1_31259145	Ca4	31259145	GATATATATATATATATATATATAT/GATATATCTATATATATATATATATATATAT	CAGGTCGTGCCAATTCTTTT	TTCATTGACGAATGTACCATCA	60.1	404	INTERGENIC	--	--	--

INDEL marker IDs	Chromosomes/ unanchored scaffolds	Physical positions (bp)	InDels (*Kabuli* reference genome-CDC Frontier/PI)	Forward primers (5'-3')	Reverse primers (5'-3')	Anneling temperature (0C)	Expected amplified product size (bp)	Structural annotation		Functional annotation	
								Sequence components of *kabuli* genome	*Kabuli* gene accession IDs	NCBI-KOG	TFs
Ca4_P1_31261697	Ca4	31261697	AGAAAC/AGAAACTATATGAAAC	GAGCGAGCGCTAGTAAATGC	TCGTCTCCTCTCATTTGTGAAA	60.3	617	DRR	Ca_14230	R	MYB_related
Ca4_P1_31274972	Ca4	31274972	TAAAAAAAAAA/TAAAAAAAA	CTATGGCACACACCAGCAAC	GATCAAGTAGGGCGGATTCA	60.2	699	INTERGENIC	--	--	--
Ca4_P1_31275360	Ca4	31275360	ATTTTTTTTT/ATTTTTTTT	TTCAATTAGGGTTTGGGTCG	ACAGTTTGTGCGTTGTTGGT	59.8	631	INTERGENIC	--	--	--
Ca4_P1_31275708	Ca4	31275708	ATT/ATTT	AACCACAAAACGTGCAACTTC	TTGTGCGTTGTTGGTATTTTG	60.1	533	INTERGENIC	--	--	--
Ca4_P1_31276483	Ca4	31276483	AAATA/AA	CACGTCGAATATCAACAAAACTT	TTCTCACAACCATACAACCATGT	58.2	415	INTERGENIC	--	--	--
Ca4_P1_31278515	Ca4	31278515	T/TA	CTCTCGCCATTCACAAACAC	TAGGAAGTTGGACAGGTGGC	59.3	773	INTERGENIC	--	--	--
Ca4_P1_31279281	Ca4	31279281	ACCC/ACC	GCCACCTGTCCAACTTCCTA	ATACCCAAGAGGGTATGGGC	60.1	454	INTERGENIC	--	--	--
Ca4_P1_31284485	Ca4	31284485	ATT/ATTT	ATGGCTACTCCAATGCATCC	ACATGGCTCCCCCTTACTCT	59.9	962	INTERGENIC	--	--	--
Ca4_P1_31284929	Ca4	31284929	ATTTT/ATTTTT	AACTGAACATCAATGCGCTG	ACATGGCTCCCCCTTACTCT	59.9	373	INTERGENIC	--	--	--
Ca4_P1_31290902	Ca4	31290902	GAAAAAAAAAA/GAAAAAAAAAAA	TCTGCAGGTCATGAAACCAA	TTCATCCCAAGCAGGAAAAC	60.2	847	INTERGENIC	--	--	--
Ca4_P1_31291193	Ca4	31291193	GAAAAAAAAAAAA/GAAAAAAAAAA	CCTTGAGTTCGACACTCCGT	ACTCCGACAATGGAACAAGG	60.3	803	INTERGENIC	--	--	--
Ca4_P1_31292377	Ca4	31292377	TCCCCCCCC/TCCCCCC	AAATAAGGATGGTCCCTGCC	AGGAACAACGAAAGGGGATT	60.2	476	INTERGENIC	--	--	--

INDEL marker IDs	Chromosomes/ unanchored scaffolds	Physical positions (bp)	InDels (*Kabuli* reference genome-CDC Frontier/PI)	Forward primers (5'-3')	Reverse primers (5'-3')	Anneling temperature (0C)	Expected amplified product size (bp)	Structural annotation		Functional annotation	
								Sequence components of *kabuli* genome	*Kabuli* gene accession IDs	NCBI-KOG	TFs
Ca4_P1_31302749	Ca4	31302749	GAAAAAAAAAAA/GAAAAAAAAAA	CACCCCACACCAAACT CTCT	GTTAGGTTGGACGGG ACCTC	60.0	680	INTRON	Ca_14231	--	bHLH
Ca4_P1_31303190	Ca4	31303190	CTTTTTTTTTTT/CTTTTTTTTTT	GAGGTCCCGTCCAACC TAAC	TCAGCACCGAGAGTGT CAAC	60.7	488	INTRON	Ca_14231	--	bHLH
Ca4_P1_31311462	Ca4	31311462	CAAA/CAAAA	ATCCATTGACAAGGCA CCAT	CAGAGAATGCAAATCT GGAAA	60.2	457	INTERGENIC	--	--	--
Ca4_P1_31316566	Ca4	31316566	GAAA/GAA	CATTGTTTTGCTGTTGT GGG	CCTCACGAGCTGCTTT AACC	60.0	664	INTERGENIC	--	--	--
Ca4_P1_31317473	Ca4	31317473	ATTTTTTTTT/ATTTTTTTT	TGAGGCGTCATGGTAG AACA	CAAATCGACATTGTTG CCAG	60.3	707	INTERGENIC	--	--	--
Ca4_P1_31317508	Ca4	31317508	TAAAAAAAA/TAAAAAAA	TTCGCATCAGCCATAT TTTG	CAAATCGACATTGTTG CCAG	59.7	509	INTERGENIC	--	--	--
Ca4_P1_31319641	Ca4	31319641	AG/A	CTTTGAGCACAAACAA GGCA	TCATCCGTGCAAAGTA TCCA	60.0	422	INTERGENIC	--	--	--
Ca4_P1_31322894	Ca4	31322894	GAAAAAAAA/GAAAAAAA	CTCCTAAGGGTGGCCT TTTC	GGGTTGTTCTTGGACC TTCA	60.1	623	INTERGENIC	--	--	--
Ca4_P1_31342614	Ca4	31342614	CTTTTTTTTTTTTT/CTTTTTTTTTTTT	CTTATCAAGAGCAGGC AGGG	GGAGAGAGGCAGAGA CGAGA	60.0	163	INTERGENIC	--	--	--
Ca4_P1_31348460	Ca4	31348460	TAAAAAAAAAA/TAAAAAAAAAAA	GTCACCTTTTGGCGAT GTCT	AAACGCAACCTTTGAT AATCG	60.1	700	INTERGENIC	--	--	--
Ca4_P1_31361662	Ca4	31361662	A/AC	CATGCGCACAAGCATA ACAT	GATTTGGATTTGGAGT GGGA	60.7	114	DRR	Ca_14232	--	Trihelix
Ca4_P1_31373366	Ca4	31373366	GAACTAACAAA/GAACTAACAAACTAA CAAA	GAGTGGCCAATCCCTC ATAA	CGGATGCATCACATTC AATC	59.9	250	INTERGENIC	--	--	--

INDEL marker IDs	Chromosomes/ unanchored scaffolds	Physical positions (bp)	InDels (*Kabuli* reference genome-CDC Frontier/PI)	Forward primers (5'-3')	Reverse primers (5'-3')	Anneling temperature (0C)	Expected amplified product size (bp)	Structural annotation		Functional annotation	
								Sequence components of *kabuli* genome	*Kabuli* gene accession IDs	NCBI-KOG	TFs
Ca4_P1_31406944	Ca4	31406944	GTTTTTTTTT/GTTTTTTTTTT	CAAATTATACTCGCAC AACTTGC	AAAGTGGACAAACACA AAACGA	58.9	782	INTERGENIC	--	--	--
Ca4_P1_31424088	Ca4	31424088	GAAAAA/GAAAA	TTTGAATCGAACATGG AAGAGA	TCCTTGTAAGCAATGG GAGAG	59.7	678	INTERGENIC	--	--	--
Ca4_P1_31424534	Ca4	31424534	TAAAAAAAAAAAA/TAAAAAAAAA	TCTCCCATTGCTTACA AGGAC	TTAAAGCATGACGTTG ACGG	59.2	601	INTERGENIC	--	--	--
Ca4_P1_31424757	Ca4	31424757	GTTTTTT/GTTTTTTT	AATTGGCGAAACCACT TTTT	TGCACTTGTTGGGGTT GATA	58.6	501	INTERGENIC	--	--	--
Ca4_P1_31445256	Ca4	31445256	ATT/ATTT	AGATCTATGCATCGGC AACC	AGGGTACTACAGCTGC CCCT	60.1	338	INTERGENIC	--	--	--
Ca4_P1_31448720	Ca4	31448720	ATTTTTTTTT/ATTTTTTTT	CAAACGGGAGAACGAG AGAG	CCATGCCTGTGATCTT GATG	60.0	864	DRR	Ca_15433	E	TALE
Ca4_P1_31451185	Ca4	31451185	CT/C	CAGCACCAATGGTTGA AATG	CACGTCGATCTTTGAC ACTCA	60.0	657	INTRON	Ca_15433	E	TALE
Ca4_P1_31452576	Ca4	31452576	CTTTTTTTT/CTTTTTTTTT	CCACTCGTGGTCCCTT AACT	TCGGCTAAATACCATT TGTGG	59.1	566	INTERGENIC	--	--	--
Ca4_P1_31452936	Ca4	31452936	GAAAAAAAAA/GAAAAAAAAAA	ACCACTCGTGGTCCCT TAAC	TGTCGTGCGACGAGA GTTAT	58.9	720	INTERGENIC	--	--	--
Ca4_P1_31454413	Ca4	31454413	TAA/TAAAA	TTCCTACCCCACACAC ACAA	TGCCCTCCATTGAATA TGAA	59.8	489	INTERGENIC	--	--	--
Ca4_P1_31463046	Ca4	31463046	AAGTAGTAGT/AAGTAGT	TAAAACGTGCGGAACG AAAT	GCTAACTGATGCAAGG AGCC	60.5	716	INTERGENIC	--	--	--
Ca4_P1_31463085	Ca4	31463085	CAAAAAAAAAAA/CAAAAAAAAA	TAAAACGTGCGGAACG AAAT	GCTAACTGATGCAAGG AGCC	60.5	716	INTERGENIC	--	--	--

INDEL marker IDs	Chromosomes/ unanchored scaffolds	Physical positions (bp)	InDels (*Kabuli* reference genome-CDC Frontier/PI)	Forward primers (5'-3')	Reverse primers (5'-3')	Anneling temperature (0C)	Expected amplified product size (bp)	Structural annotation		Functional annotation	
								Sequence components of *kabuli* genome	*Kabuli* gene accession IDs	NCBI-KOG	TFs
Ca4_P1_31529637	Ca4	31529637	CTTTTTTTTTT/CTTTTTTTTT	CCAAATCCGATGAGGAAAGA	TTTAAGACCCGCGATTTCAG	60.0	820	INTERGENIC	--	--	--
Ca4_P1_31531525	Ca4	31531525	G/GGT	AGGGATTGCTTGCAAATGAC	AATTCCCAAACCAATAGCCC	60.1	387	INTERGENIC	--	--	--
Ca4_P1_31532036	Ca4	31532036	CTTTTTATGG/C	ATGTACGTTTTGCCGCTTTC	TCCAAAGCGAAAGTTTGACC	60.1	520	INTERGENIC	--	--	--
Ca4_P1_31534458	Ca4	31534458	CAAAAAAA/CAAAAAAAAA	CAAGCCAACGTGTGTTTGTT	TGTGGGGGATCAAATTGTTT	59.7	371	INTERGENIC	--	--	--
Ca4_P1_31534512	Ca4	31534512	TCCCCC/TCCCC	CAAGCCAACGTGTGTTTGTT	GCAGGGATGTGAACCAAATAA	59.7	746	INTERGENIC	--	--	--
Ca4_P1_31550170	Ca4	31550170	ACAATAGCAATAGCAA/ACAATAGCAATAGCAATAGCAATAGCAA	TTGAGCTGGTACCGGTAGTAGTAG	CAAAGGCGGTTTCATCATCT	59.0	287	INTERGENIC	--	--	--
Ca4_P1_31553677	Ca4	31553677	CTTTTTTTTTTTT/CTTTTTTTTTT	GTGGAGATCGAGGATTTGGA	GGGCCGAAGTAAGGGAGTAG	60.0	414	DRR	Ca_15437	--	--
Ca4_P1_31553921	Ca4	31553921	G/GA	GTGGAGATCGAGGATTTGGA	GGGCCGAAGTAAGGGAGTAG	60.0	414	DRR	Ca_15437	--	--
Ca4_P1_31554097	Ca4	31554097	CGG/CG	GTGGAGATCGAGGATTTGGA	TGGCATGTTATCAGGGAACTC	60.0	859	DRR	Ca_15437	--	--
Ca4_P1_31554908	Ca4	31554908	ATGTTGT/ATGT	TTCAAGCTGGAGAGTGCAGA	CATGCATCGAAACTCGAAAA	59.9	453	DRR	Ca_15437	--	--
Ca4_P1_31559417	Ca4	31559417	AAAG/A	CTTGGATTTTCTCCAAGGTG	TGCAGTGGGATATCCTTTGA	57.2	696	INTERGENIC	--	--	--
Ca4_P1_31560414	Ca4	31560414	C/CG	CGCTTGTGGGAATACAGGTT	TTCCAAAATCATGCGTTGAA	60.0	315	INTERGENIC	--	--	--

INDEL marker IDs	Chromosomes/ unanchored scaffolds	Physical positions (bp)	InDels (*Kabuli* reference genome-CDC Frontier/PI)	Forward primers (5'-3')	Reverse primers (5'-3')	Anneling temperature (0C)	Expected amplified product size (bp)	Structural annotation		Functional annotation	
								Sequence components of *kabuli* genome	*Kabuli* gene accession IDs	NCBI-KOG	TFs
Ca4_P1_31568591	Ca4	31568591	TTCT/TT	ATGCTTTCCACACTCCTCAA	GTCTCCCCCTTGATCCATTT	58.3	745	INTERGENIC	--	--	--
Ca4_P1_31570022	Ca4	31570022	TGGGGGG/TGGGGGGG	TATTTTGGATTTTGGCTCGG	AGGAAGAGTGGAGTTGCGAA	59.9	505	INTERGENIC	--	--	--
Ca4_P1_31609144	Ca4	31609144	ATTTT/ATTT	TCGACGTTTATTTGATGGCA	TTGAGAAACTTAATTGTGGGCA	60.1	651	DRR	Ca_15441	A	--
Ca4_P1_31611840	Ca4	31611840	GTTTTTTTTTT/GTTTTTTTTTTTT	CGTTTTCAGCTGAGCAAATTC	TTGATCCATTGGGATTGTGA	60.0	392	INTERGENIC	--	--	--
Ca4_P1_31611867	Ca4	31611867	AAT/AATAT	CGTTTTCAGCTGAGCAAATTC	TTGATCCATTGGGATTGTGA	60.0	392	INTERGENIC	--	--	--
Ca4_P1_31612473	Ca4	31612473	TAAAAAAA/TAAAAAAAA	TAAAATCCAAAACCGCAAGC	CACTTAAGCAAACGGGGTGT	60.1	462	INTERGENIC	--	--	--
Ca4_P1_31615846	Ca4	31615846	ATAATTAA/ATAA	GATTGCATTTTTAGCAGGCA	ATCAAAATGGATTCCACCCA	58.9	328	INTERGENIC	--	--	--
Ca4_P1_31617249	Ca4	31617249	TAAAAAAAAAA/TAAAAAAAAA	CACGTTTATCGTGAGGCAAA	CTATGCAACATGTCACACGC	59.7	597	INTERGENIC	--	--	--
Ca4_P1_31619911	Ca4	31619911	CTTT/CTTTT	TATGAATTGGGTGGGGAATC	TGAGTCTACGTTCGGTTCAAA	59.4	377	INTERGENIC	--	--	--
Ca4_P1_31620795	Ca4	31620795	TT/TTAAT	TGAACGTGGCTTATTTGTCG	GATCGCATACAACAACCGTG	59.7	352	INTERGENIC	--	--	--
Ca4_P1_31620834	Ca4	31620834	AAAAAGAAAAGAAAAGAAA/AAAAAGAAAAGAAA	TGAACGTGGCTTATTTGTCG	GATCGCATACAACAACCGTG	59.7	352	INTERGENIC	--	--	--
Ca4_P1_31626130	Ca4	31626130	CTTTTTTTT/CTTTTTTTTT	TGATTCCAACAATCATCCCC	TGGCTGCAGTTCTCAACATC	60.5	624	INTERGENIC	--	--	--

INDEL marker IDs	Chromosomes/ unanchored scaffolds	Physical positions (bp)	InDels (*Kabuli* reference genome-CDC Frontier/PI)	Forward primers (5'-3')	Reverse primers (5'-3')	Anneling temperature (0C)	Expected amplified product size (bp)	Structural annotation		Functional annotation	
								Sequence components of *kabuli* genome	*Kabuli* gene accession IDs	NCBI-KOG	TFs
Ca4_P1_31626384	Ca4	31626384	ATG/ATGAAGTG	TGATTCCAACAATCATCCCC	TGGCTGCAGTTCTCAACATC	60.5	624	INTERGENIC	--	--	--
Ca4_P1_31630531	Ca4	31630531	AGG/AG	AGTTGAAGGTGCAAACACGA	AATTTACTTGGAGGGTCGGG	59.3	288	INTERGENIC	--	--	--
Ca4_P1_31630588	Ca4	31630588	TCA/TA	AAGGTGCAAACACGAGAAGG	AATTTACTTGGAGGGTCGGG	60.3	283	INTERGENIC	--	--	--
Ca4_P1_31630741	Ca4	31630741	TAA/TA	AAGGTGCAAACACGAGAAGG	AATTTACTTGGAGGGTCGGG	60.3	283	INTERGENIC	--	--	--
Ca4_P1_31631388	Ca4	31631388	CTT/CTTT	TTTCGATGCTTTTCTCCTTCA	GGCCATGCCTCTAGGTATCA	59.9	547	INTERGENIC	--	--	--
Ca4_P1_31631888	Ca4	31631888	AAAA/AAAATAAA	CCAACTTGGTTCCAACATCTG	TGGCTAATCATAGCGAAACG	60.4	832	INTERGENIC	--	--	--
Ca4_P1_31633774	Ca4	31633774	AG/AGG	AAATATTGTTCAATCCGCCG	CTCCTCCAAGCCAAGTGAAG	59.8	491	INTERGENIC	--	--	--
Ca4_P1_31635059	Ca4	31635059	AT/ATT	TCAATGTGAACGGGCATCTA	TTCACCTTAACCACGCACAA	60.1	466	INTERGENIC	--	--	--
Ca4_P1_31635242	Ca4	31635242	ATTTACCT/A	TCAATGTGAACGGGCATCTA	TGGTGCCTCCTACAATTTCC	60.1	778	INTERGENIC	--	--	--
Ca4_P1_31635868	Ca4	31635868	AAT/A	GGAAATTGTAGGAGGCACCA	GTTGCGCTACGAGATGTTGA	59.9	695	INTERGENIC	--	--	--
Ca4_P1_31636148	Ca4	31636148	AT/A	GGAAATTGTAGGAGGCACCA	GTTGCGCTACGAGATGTTGA	59.9	695	INTERGENIC	--	--	--
Ca4_P1_31636819	Ca4	31636819	CAA/CAAA	TCTCAACATCTCGTAGCGCA	TTCATGATTCATTGGGTCATCT	60.7	771	INTERGENIC	--	--	--

INDEL marker IDs	Chromosomes/ unanchored scaffolds	Physical positions (bp)	InDels (*Kabuli* reference genome-CDC Frontier/PI)	Forward primers (5'-3')	Reverse primers (5'-3')	Anneling temperature (0C)	Expected amplified product size (bp)	Structural annotation		Functional annotation	
								Sequence components of *kabuli* genome	*Kabuli* gene accession IDs	NCBI-KOG	TFs
Ca4_P1_31639620	Ca4	31639620	GTTTT/GTTTTT	ATTTGGAGAATGATTTGGCG	GTGTTTGGTTCAAGGGGAGA	59.9	412	INTERGENIC	--	--	--
Ca4_P1_31642398	Ca4	31642398	TAAAAAAAA/TAAAAAAAAA	TGTCACTGCACTGCCACATA	TTTCCGGCAAAGTTCTCATAA	59.9	482	INTRON	Ca_15442	U	EIL
Ca4_P1_31644451	Ca4	31644451	TA/T	AATGTCGGGAGTTTTCATCG	GCCATCTTAGGCTTTCCCTT	59.9	953	INTRON	Ca_15442	U	EIL
Ca4_P1_31644480	Ca4	31644480	TAAAA/TAAAAA	CGGGAGTTTTCATCGTTCTC	TGTGGTGGCAGGAATATCAA	59.7	976	INTRON	Ca_15442	U	EIL
Ca4_P1_31646633	Ca4	31646633	AA/AACA	ATGGAGAAGAGGGGGAGTGT	ACCAGTCTTTTACGGGCCTT	59.9	135	INTRON	Ca_15442	U	EIL
Ca4_P1_31646684	Ca4	31646684	C/CCAATCT	AAGGCCCGTAAAAGACTGGT	AAATTGTTCATTTGCCCCTG	60.0	141	INTRON	Ca_15442	U	EIL
Ca4_P1_31647747	Ca4	31647747	ATT/AT	CACGTGTGAAGGTCGAGAGA	AAGCTACCTGATCAATGCGG	60.0	863	INTRON	Ca_15442	U	EIL
Ca4_P1_31647770	Ca4	31647770	GTTTTTTTTT/GTTTTTTTT	CACGTGTGAAGGTCGAGAGA	AAGCTACCTGATCAATGCGG	60.0	863	INTRON	Ca_15442	U	EIL
Ca4_P1_31648071	Ca4	31648071	ATTTTTTTTT/ATTTTTTTT	AAAGCTCGTTTGTCACAGTTTTT	AAGCTACCTGATCAATGCGG	59.4	554	INTRON	Ca_15442	U	EIL
Ca4_P1_31649700	Ca4	31649700	ATTATTTTATTTTATTTTATTTT/ATTATTTTATTTTATTTT	TTGATGATCGAATTGACCGA	ATAAATACCTGCAGGGCACG	60.0	666	INTRON	Ca_15442	U	EIL
Ca4_P1_31649916	Ca4	31649916	AT/A	TGAGGCATTTGGAGACAACA	ATTGACCGGTGGAAGACATT	60.2	585	INTRON	Ca_15442	U	EIL
Ca4_P1_31649942	Ca4	31649942	CT/CTT	CGTGCCCTGCAGGTATTTAT	ATTGACCGGTGGAAGACATT	60.0	454	INTRON	Ca_15442	U	EIL

INDEL marker IDs	Chromosomes/ unanchored scaffolds	Physical positions (bp)	InDels (*Kabuli* reference genome-CDC Frontier/PI)	Forward primers (5'-3')	Reverse primers (5'-3')	Anneling temperature (0C)	Expected amplified product size (bp)	Structural annotation		Functional annotation	
								Sequence components of *kabuli* genome	*Kabuli* gene accession IDs	NCBI-KOG	TFs
Ca4_P1_31652250	Ca4	31652250	CTTTT/CTTTTT	CCATTACCCTTTCGTGATGC	TGAAAGCAAAGCCACGTATG	60.3	796	DRR	Ca_15442	U	EIL
Ca4_P1_31652588	Ca4	31652588	T/TG	CATACGTGGCTTTGCTTTCA	AACGTTTCGTAACACTGCCC	59.9	401	DRR	Ca_15442	U	EIL
Ca4_P1_31653241	Ca4	31653241	GATAAA/GATAAAAAATAAA	TTCAAACGATGGCAACAAAA	TCATGGGATTAAGCCTCCAC	60.1	268	DRR	Ca_15442	U	EIL
Ca4_P1_31653737	Ca4	31653737	T/TG	TTCTTCCGAATAAGGCATGG	CACGCTCGATGCTTCTATCA	60.0	553	DRR	Ca_15442	U	EIL
Ca4_P1_31657733	Ca4	31657733	TA/T	AATTCGTGTTCATCAAGCCA	CCAGCTATGTGGAGGCATCT	59.1	530	DRR	Ca_15443	--	--
Ca4_P1_31668617	Ca4	31668617	TAAA/TAAAA	ACTGAGCCTCCACCAAAGAA	ATCATGTCACCCGATCTCAA	59.8	249	INTERGENIC	--	--	--
Ca4_P1_31669067	Ca4	31669067	ATTTTTTT/ATTTTTTTT	ACTGAGCCTCCACCAAAGAA	CCTTTGACTCACCCGTTAGC	59.8	641	INTERGENIC	--	--	--
Ca4_P1_31676768	Ca4	31676768	AATATATATATATATATATATATATATATATATATATATAT/AATATATATATATATATATATATATATATATATATATATAT	TGCAATTACTGTTTTATTTTACCAAA	AACTTTATTGACGAGATTGCAAAA	58.3	225	DRR	Ca_15444	--	--
Ca4_P1_31712621	Ca4	31712621	CTATATATATATATATATATATA/CTATATATATATATATATATATATATATA	TTTTCACAAAAATCCAAAGGG	CAAAATGGACAAAATTGGGG	59.0	393	INTERGENIC	--	--	--
Ca4_P1_31713913	Ca4	31713913	ATTTTTTTTTTTT/ATTTTTTTTTT	GTTGTTGTTGTCGTTGTGGC	CCTTGCCCACTCTCTCTTTG	60.1	160	INTERGENIC	--	--	--
Ca4_P1_31714419	Ca4	31714419	ATTTTTTTTTTT/ATTTTTTTTTT	CAAAGAGAGAGTGGGCAAGG	TGAGTCCCAAAACTCAAGGAA	60.0	883	INTERGENIC	--	--	--
Ca4_P1_31714952	Ca4	31714952	ATTTTTTTTTTTTT/ATTTTTTTTTTT	TTGAGTTTTGGGACTCAAGTTGT	ATGCGCTTGACAAGAATGTG	60.1	228	INTERGENIC	--	--	--

**INDEL marker IDs**	**Chromosomes/ unanchored scaffolds**	**Physical positions (bp)**	**InDels (*Kabuli* reference genome-CDC Frontier/PI)**	**Forward primers (5'-3')**	**Reverse primers (5'-3')**	**Anneling temperature (0C)**	**Expected amplified product size (bp)**	**Structural annotation**		**Functional annotation**	
								**Sequence components of *kabuli* genome**	***Kabuli* gene accession IDs**	**NCBI-KOG**	**TFs**
Ca4_P1_31718401	Ca4	31718401	AT/ATT	ATTCCTCTAATGTCGGGGCT	GCCACCCTTGTGAGAGTTTC	59.9	501	INTERGENIC	--	--	--
Ca4_P1_31727083	Ca4	31727083	GTTTTTT/GTTTTT	TTGTTGGGTTGGGAGTTTTC	TTGAGATGATAAGCCATTGGG	59.8	581	INTRON	Ca_15445	G	--
Ca4_P1_31735357	Ca4	31735357	ATTTTTT/ATTTTTTTT	AAAAATGGAGGGCAAGGAGT	AAGGTTGAATCATGGGGATG	59.9	463	INTERGENIC	--	--	--
Ca4_P1_31735396	Ca4	31735396	TTCTCTC/TTCTCTCTC	AAAAATGGAGGGCAAGGAGT	AAGGTTGAATCATGGGGATG	59.9	463	INTERGENIC	--	--	--
Ca4_P1_31753116	Ca4	31753116	AAATAATAATAATAATAATAATAATAATAATAATAATAATA/AAATAATAATAATAATAATAATAATAATAATAATAATAATAATAATAATAATA	TTTCATCAAATTCGTCACGC	AGCCATATGTTGTGTGCCAA	59.7	673	INTERGENIC	--	--	--
Ca4_P1_31757461	Ca4	31757461	CTTTTTTTTT/CTTTTTTTT	TATGCTAAACCAACCGCCTC	TTTTTCCTGACTTGGTCCTTT	60.1	383	DRR	Ca_15447	R	bZIP
Ca4_P1_31761071	Ca4	31761071	ATTTTTTT/ATTTTTT	GTGGTTAGGGGGTGACAAGA	CATGCTCGCAATTAAAAATCA	59.8	529	INTERGENIC	--	--	--
Ca4_P1_31770186	Ca4	31770186	GTTTTTTTTT/GTTTTTTTTTT	TGGAGGGTCAGGTGCTTTAG	TTTGAAGGTGCAAACACGAG	60.2	276	INTERGENIC	--	--	--
Ca4_P1_31772554	Ca4	31772554	AC/A	TTATCCTCGCCAATGACCTC	ATTCCACCAACAATATGGGC	60.0	561	INTERGENIC	--	--	--
Ca4_P1_31797660	Ca4	31797660	GTTTTTTTTTTT/GTTTTTTTTTTTT	TTGACAACGGTGCCAATTTA	ATTGATCCGCTGTTTTGGAG	60.0	540	INTERGENIC	--	--	--
Ca4_P1_31806633	Ca4	31806633	ATGTGACTAATTTT/ATGTGACTAATTTTGTGACTAATTTT	ACAAAAGCCTGGATAGCGAA	TCACATATACCCTTAAAATCGCA	59.8	410	INTERGENIC	--	--	--
Ca4_P1_31816562	Ca4	31816562	ATTTTTTTTTTT/ATTTTTTTTTT	GGCTCCGATCAAAAGAAACA	GCCCTCAGATGAGAGTCAGG	60.2	545	INTERGENIC	--	--	--

INDEL marker IDs	Chromosomes/ unanchored scaffolds	Physical positions (bp)	InDels (*Kabuli* reference genome-CDC Frontier/PI)	Forward primers (5'-3')	Reverse primers (5'-3')	Anneling temperature (0C)	Expected amplified product size (bp)	Structural annotation		Functional annotation	
								Sequence components of *kabuli* genome	*Kabuli* gene accession IDs	NCBI-KOG	TFs
Ca4_P1_31822803	Ca4	31822803	ATTTTTTT/ATTTTTTTT	CACAGATTTCTCGAGGCACA	TCTAAAGCTCATGTCCCGCT	60.0	841	INTERGENIC	--	--	--
Ca4_P1_31825282	Ca4	31825282	GTTTTTT/GTTTTT	TCCCATCATGCTCATTTGAA	AAACAAGGCCAAATTGGTTG	60.0	496	INTERGENIC	--	--	--
Ca4_P1_31825775	Ca4	31825775	AC/A	CAACCAATTTGGCCTTGTTT	TGGACTGCCATTTTCACAAA	59.8	322	INTERGENIC	--	--	--
Ca4_P1_31837452	Ca4	31837452	ATTTTTTTT/ATTTTTTTTT	TGGAGACCATGGAAATGCTT	TTTTGTGAATAGTCCATGCTTCA	60.5	456	INTERGENIC	--	--	--
Ca4_P1_31852817	Ca4	31852817	TAAAAAAAA/TAAAAAAAAA	TTTGTCATGGCGAAACAAAC	GGTTTGTCTTTGCACCCATT	59.6	415	INTERGENIC	--	--	--
Ca4_P1_31859597	Ca4	31859597	TAAAAAAAAA/TAAAAAAAAAAA	GTTGGAGCCCCCACTTTATT	TGCAAGCAGCATCAGAGAAC	60.2	793	INTERGENIC	--	--	--
Ca4_P1_31871158	Ca4	31871158	AACACACA/AACACACACA	AAGGTGTGTTGCTAATCGGT	AAAAGGAATTTCTCAAATTGATGG	57.2	394	INTERGENIC	--	--	--
Ca4_P1_31873869	Ca4	31873869	TTATATATATATATATATATATATAT/TTATATATATATATATAT	CCCTGTTCATTTCATCCTTTTT	TCCCTTCAGTTCTTGGATGC	59.4	388	INTERGENIC	--	--	--
Ca4_P1_31885190	Ca4	31885190	CTATTTATTTATTTAT/CTATTTATTTATTTATTTAT	GGGTCCCTTATGTGATGCAG	CCCAATCCCAAATCAATCAC	60.3	352	INTERGENIC	--	--	--
Ca4_P1_31892250	Ca4	31892250	CTTTTTTTT/CTTTTTTTTT	AACCGTTAACCACCGAAACA	TGCGGTCAACACACTTAGGA	60.3	520	INTERGENIC	--	--	--
Ca4_P1_31892313	Ca4	31892313	TTTTTCTT/TTT	AACCGTTAACCACCGAAACA	TGCGGTCAACACACTTAGGA	60.3	520	INTERGENIC	--	--	--
Ca4_P1_31915422	Ca4	31915422	CTTTT/CTTT	AGCCACATGCTATTTCTGCC	AACCAAAACCAAATTGCAGG	60.2	446	INTERGENIC	--	--	--

INDEL marker IDs	Chromosomes/ unanchored scaffolds	Physical positions (bp)	InDels (*Kabuli* reference genome-CDC Frontier/PI)	Forward primers (5'-3')	Reverse primers (5'-3')	Anneling temperature (0C)	Expected amplified product size (bp)	Structural annotation		Functional annotation	
								Sequence components of *kabuli* genome	*Kabuli* gene accession IDs	NCBI-KOG	TFs
Ca4_P1_31917284	Ca4	31917284	AAACACGAACAC/AAACAC	ACCATGAAACCTTCGACCTG	GTGTTGGTGTTCGTCCTGTG	60.0	274	INTERGENIC	--	--	--
Ca4_P1_31918515	Ca4	31918515	ATTTTTTTTT/ATTTTTTTT	ATTCCACATGAAACTTCCGC	GAAAGTAGGGGGTGGGAAAC	59.9	568	INTERGENIC	--	--	--
Ca4_P1_31918660	Ca4	31918660	ATTTTTTTTTTTTT/ATTTTTTTTT	ATTCCACATGAAACTTCCGC	GAAAGTAGGGGGTGGGAAAC	59.9	568	INTERGENIC	--	--	--
Ca4_P1_31930111	Ca4	31930111	AATATATATATA/AATATATATATATATA	GAGGAGCATTGATGGAGGAA	TTTGGAAGACTTTTGGGCTC	60.2	574	INTERGENIC	--	--	--
Ca4_P1_31931258	Ca4	31931258	TAAAAAAAAA/TAAAAAAAA	TCAAACCCGAGAACAAGTGA	TAACAAGCGCGCAATGTATC	59.3	595	INTERGENIC	--	--	--
Ca4_P1_31934262	Ca4	31934262	GTT/GT	ACACTCTTTTCTCACCCCCA	GTCTTTTCTTCCCCTTTCCG	59.5	750	INTERGENIC	--	--	--
Ca4_P1_31937240	Ca4	31937240	TGG/TGGG	CTAAGGCCCTATGTGCAAGC	CGAGACTACTCGTGGGCTTC	59.9	835	CDS (FRAME SHIFT)	Ca_15455	--	Dof
Ca4_P1_31938648	Ca4	31938648	TTA/T	AATGCTCTACAGGAGCTGGC	GAGGAGGTCCAGGATGATGA	59.6	782	INTRON	Ca_15455	--	Dof
Ca4_P1_31948251	Ca4	31948251	AAAA/AAAATAAA	AAAATGCATATATTAATTCATCCCAA	CTGCATGTGCAATTGTGTCA	59.0	385	INTERGENIC	--	--	--
Ca4_P1_31953618	Ca4	31953618	AATA/AA	GTGGCTTCCTCTCTCCTTCC	ATTGCACTTCCTTGCACACA	60.3	313	INTERGENIC	--	--	--
Ca4_P1_31955209	Ca4	31955209	GCA/G	TGACGATGATTCTGGAACACA	TTGCATGTTCCTTTTCCACA	60.1	579	INTERGENIC	--	--	--
Ca4_P1_31963506	Ca4	31963506	AATATATA/AATATA	AAATGGCAGTGATGTGGTGA	TGTTGAGGCAAAATTCCAAA	60.0	572	INTERGENIC	--	--	--

**INDEL marker IDs**	**Chromosomes/ unanchored scaffolds**	**Physical positions (bp)**	**InDels (*Kabuli* reference genome-CDC Frontier/PI)**	**Forward primers (5'-3')**	**Reverse primers (5'-3')**	**Anneling temperature (0C)**	**Expected amplified product size (bp)**	**Structural annotation**		**Functional annotation**	
								**Sequence components of *kabuli* genome**	***Kabuli* gene accession IDs**	**NCBI-KOG**	**TFs**
Ca4_P1_31967535	Ca4	31967535	T/TC	TACCCTTAATGGCTGGGATG	AAAAACACTTGCGATGACCC	59.8	510	INTRON	Ca_15456	--	--
Ca4_P1_31967658	Ca4	31967658	GTTTT/GTT	GGGTCATCGCAAGTGTTTTT	TTGCATGTTCCTTTTCCACA	60.0	135	INTRON	Ca_15456	--	--
Ca4_P1_31972811	Ca4	31972811	CT/CTT	TTTGTGAAATTGGTTCCCTG	GGTCCAGCAGCATAGGACAT	58.4	523	INTERGENIC	--	--	--
Ca4_P1_31973044	Ca4	31973044	GTTTTTTTT/GTTTTTTT	ATGTCCTATGCTGCTGGACC	AAACAACCGTGCTATTTAGCCT	60.1	279	INTERGENIC	--	--	--
Ca4_P1_31973853	Ca4	31973853	CTTT/CTT	CGTAGTCTTCACCATTTTGTTTTG	ACCACACCCACTTTTTCAGC	60.0	262	INTERGENIC	--	--	--
Ca4_P1_31974107	Ca4	31974107	T/TATA	GCTGAAAAAGTGGGTGTGGT	GAGAGTGCTGTTGGCTGTGA	60.0	655	INTERGENIC	--	--	--
Ca4_P1_31974174	Ca4	31974174	AC/A	GCTGAAAAAGTGGGTGTGGT	GAGAGTGCTGTTGGCTGTGA	60.0	655	INTERGENIC	--	--	--
Ca4_P1_31974935	Ca4	31974935	ATTT/ATT	TCACAGCCAACAGCACTCTC	TCGTGCAATGTCCATTCATT	60.2	633	INTERGENIC	--	--	--
Ca4_P1_31977709	Ca4	31977709	CTTTTT/CTTTT	GCAGGTAGCGACATGAACAA	AAGTGCAACATGGCCATACA	59.9	419	INTERGENIC	--	--	--
Ca4_P1_31978850	Ca4	31978850	CAA/CAAA	ACATTCTTATGTCGGGGCAG	ACAGCCTTTTCTCCATCCCT	60.0	605	DRR	Ca_15457	S	--
Ca4_P1_31989643	Ca4	31989643	CTTTTTTTT/CTTTTTTTTTTT	GAAAGATTCCTCCCACTCCC	TGCACATTGGAAAATTGGTG	59.9	246	INTERGENIC	--	--	--
Ca4_P1_31991301	Ca4	31991301	GTTTTTTTT/GTTTTTTT	AGACAACGACTTTGGCGTTC	ATTGCTCCTTCAATCATGGG	60.3	534	INTERGENIC	--	--	--

INDEL marker IDs	Chromosomes/ unanchored scaffolds	Physical positions (bp)	InDels (*Kabuli* reference genome-CDC Frontier/PI)	Forward primers (5'-3')	Reverse primers (5'-3')	Anneling temperature (0C)	Expected amplified product size (bp)	Structural annotation		Functional annotation	
								Sequence components of *kabuli* genome	*Kabuli* gene accession IDs	NCBI-KOG	TFs
Ca4_P1_31994404	Ca4	31994404	TAAAA/TAAAAA	ATCAGCGAGGATGCAGAACT	CATCGCTCAGTTTGGACTCA	60.0	796	DRR	Ca_15458	--	--
Ca4_P1_32005440	Ca4	32005440	ATATTTATTTATTTATTTAT/ATATTTATTTATTTAT	GAGGTTGGAATGTTGGCACT	TGATGTGATGGGTGCATTTT	60.0	787	INTERGENIC	--	--	--
Ca4_P1_32008196	Ca4	32008196	TC/T	TCTGAGCACACTTGTGGGAC	TTGCGAAGTCAAGCAAACTG	59.9	418	INTERGENIC	--	--	--
Ca4_P1_32008454	Ca4	32008454	A/ATT	TCTGAGCACACTTGTGGGAC	CATTGGTCAATTAATTCCAAAACA	59.9	956	INTERGENIC	--	--	--
Ca4_P1_32008597	Ca4	32008597	TAAAAAA/TAAAAAAA	CAGTTTGCTTGACTTCGCAA	TTCGTACAATTCATACGCGG	60.2	659	INTERGENIC	--	--	--
Ca4_P1_32008875	Ca4	32008875	AATATATAT/AATAT	CAGTTTGCTTGACTTCGCAA	TTCGTACAATTCATACGCGG	60.2	659	INTERGENIC	--	--	--
Ca4_P1_32012239	Ca4	32012239	ATTTTTTTTTT/ATTTTTTTTTTT	TGTGATGTTTAAAGATGGGTGG	ATCGGCTTGCTATGTTTGCT	59.7	185	INTERGENIC	--	--	--
Ca4_P1_32021071	Ca4	32021071	ATT/ATTT	TCATGTGGCGTAAGTTCCAA	GAGTTGGGATGAAGGGACAA	60.1	744	INTERGENIC	--	--	--
Ca4_P1_32023198	Ca4	32023198	CAAATAAAATAAAATAAAA/CAAATAAAATAAAATAAAATAAAATAAAA	CAAGCTGCAACAAAACTCCA	CTAGGGGATGATGCGTGACT	60.0	535	INTERGENIC	--	--	--
Ca4_P1_32036080	Ca4	32036080	TAAAAAA/TAAAAAAA	ATTCAATTCCAGCATGCCTC	GCCTTGTATTGTGAGAATGGG	60.0	335	INTERGENIC	--	--	--
Ca4_P1_32042126	Ca4	32042126	TTTTATTTATTTATTTATTTATTTATTTATT/TTTTATTTATTTATTTATT	TGCTAATGGACCCCCTTATG	TCAACACACTGAGCCGCTAC	59.8	606	INTRON	Ca_15459	--	--
Ca4_P1_32044136	Ca4	32044136	CAA/CAAAA	CGGTGGCTTACGATGTTTTT	ATTTTCCAATGGAGTTCCCC	60.0	637	INTRON	Ca_15459	--	--

INDEL marker IDs	Chromosomes/ unanchored scaffolds	Physical positions (bp)	InDels (*Kabuli* reference genome-CDC Frontier/PI)	Forward primers (5'-3')	Reverse primers (5'-3')	Anneling temperature (0C)	Expected amplified product size (bp)	Structural annotation		Functional annotation	
								Sequence components of *kabuli* genome	*Kabuli* gene accession IDs	NCBI-KOG	TFs
Ca4_P1_32045859	Ca4	32045859	T/TA	TGGGGTTTTGATCCATTTGT	TGATGTCTTTCGATCCACCT	60.0	607	DRR	Ca_15459	--	--
Ca4_P1_32046470	Ca4	32046470	GTGT/GTGTTGT	ACTAGTGCCTTTGAAGCCGA	AATGACCCATGAAAACCAGC	60.0	258	DRR	Ca_15459	--	--
Ca4_P1_32046867	Ca4	32046867	TAA/T	ACTAGTGCCTTTGAAGCCGA	GTTCATGCAAAGAGCAGCAA	60.0	712	DRR	Ca_15459	--	--
Ca4_P1_32047003	Ca4	32047003	TTAATA/TTA	GCTGGTTTTCATGGGTCATT	GTTCATGCAAAGAGCAGCAA	59.8	474	DRR	Ca_15459	--	--
Ca4_P1_32047367	Ca4	32047367	TAAA/TAAAA	TTGCTGCTCTTTGCATGAAC	CAAATGGATCAAAACCCCAC	60.1	422	DRR	Ca_15459	--	--
Ca4_P1_32047491	Ca4	32047491	TTATATATATATATATATATATATA/TTATATATATATATATATATA	TTGCTGCTCTTTGCATGAAC	CAAATGGATCAAAACCCCAC	60.1	422	INTERGENIC	--	--	--
Ca4_P1_32047782	Ca4	32047782	G/GT	TGGGGTTTTGATCCATTTGT	AAAGCCCAAACCAACATTGA	60.0	329	INTERGENIC	--	--	--
Ca4_P1_32048513	Ca4	32048513	TAAAAAAAA/TAAAAAAAAA	CGATTTTGTCGGTCAATCTCT	TCACCGTAGGAAAGCTGGAT	59.2	355	INTERGENIC	--	--	--
Ca4_P1_32048942	Ca4	32048942	CTTAT/CTTATTAT	CGAAATCCAGCTTTCCTACG	TTTGGGCCTCCTTGAGATAA	59.8	670	INTERGENIC	--	--	--
Ca4_P1_32050169	Ca4	32050169	AT/ATT	TCACGTCATTAAAAATGGTGGA	CATTTGGAACTCTCCACTGCT	60.2	629	INTERGENIC	--	--	--
Ca4_P1_32057442	Ca4	32057442	TAAAAAAA/TAAAAAAAA	TATTGCTTCGCTTCGCTTTT	GGGAAACTGCCCCTAATTCT	60.1	451	INTERGENIC	--	--	--
Ca4_P1_32067769	Ca4	32067769	CTTTTTTT/CTTTTTT	TGTTGCATTTCTGTCGGAAG	GAGACACGGTGATTTTCGGT	59.8	802	INTERGENIC	--	--	--

INDEL marker IDs	Chromosomes/ unanchored scaffolds	Physical positions (bp)	InDels (*Kabuli* reference genome-CDC Frontier/PI)	Forward primers (5'-3')	Reverse primers (5'-3')	Anneling temperature (0C)	Expected amplified product size (bp)	Structural annotation		Functional annotation	
								Sequence components of *kabuli* genome	*Kabuli* gene accession IDs	NCBI-KOG	TFs
Ca4_P1_32069410	Ca4	32069410	A/AC	CCAGTGGTGTCCCTCTTGAT	GAGACGGAATTTGGGACAGA	60.0	767	DRR	Ca_15461	R	--
Ca4_P1_32101684	Ca4	32101684	TG/T	TCATTGCCTTGTCTGCAAAA	GCACTAACCCGACCCTAACA	60.4	632	INTRON	Ca_15462	--	--
Ca4_P1_32101908	Ca4	32101908	TG/T	TGTTAGGGTCGGGTTAGTGC	TTCTCTCGTTGTGTTGGTCG	60.0	178	INTRON	Ca_15462	--	--
Ca4_P1_32103137	Ca4	32103137	TAAA/TAAAA	TAGATCATGCGGCTGTTTCA	ACCACCATTCATCCGAGAAG	60.4	683	INTRON	Ca_15462	--	--
Ca4_P1_32103782	Ca4	32103782	TGTGGTG/TGTG	CTTCTCGGATGAATGGTGGT	TGGGATAGTGATGTAATGGGC	59.9	453	INTRON	Ca_15462	--	--
Ca4_P1_32105685	Ca4	32105685	T/TC	GAGGAAGGGATCAATAATGGC	TTGGATGATGGTGATTGATGA	59.8	650	INTRON	Ca_15462	--	--
Ca4_P1_32112436	Ca4	32112436	T/TA	TACATGTCGCGCAACTCTTC	AATTTGCACTTTTCATGGGG	60.0	842	INTRON	Ca_15462	--	--
Ca4_P1_32118643	Ca4	32118643	AAAT/A	GCCAAAGGTAACAAAGGCAA	TGATTTCTTCATTGCATCGG	60.1	569	INTERGENIC	--	--	--
Ca4_P1_32119212	Ca4	32119212	ATT/AT	TGTTTCGTTGTTGATAAGCATGT	TGTCCTCCTCCCTCCTTTTT	59.6	721	INTERGENIC	--	--	--
Ca4_P1_32121836	Ca4	32121836	AATA/AATATA	CCCCAAGCTTGTAAAGCAAA	AATGAAAGCGTTGATTGGGT	60.2	588	INTERGENIC	--	--	--
Ca4_P1_32122386	Ca4	32122386	CTTTTTTTTT/CTTTTTTTTTT	TGGGTTTTACCCAATCAACG	CACCAGCCAAGTTCTTCCTT	60.6	359	INTERGENIC	--	--	--
Ca4_P1_32123490	Ca4	32123490	GTTTTTTTT/GTTTTTTT	TGGATTTGGTTTCTCCTCAAA	AGTGCAAGTTGGTCCATTCC	59.5	716	INTERGENIC	--	--	--

INDEL marker IDs	Chromosomes/ unanchored scaffolds	Physical positions (bp)	InDels (*Kabuli* reference genome-CDC Frontier/PI)	Forward primers (5'-3')	Reverse primers (5'-3')	Anneling temperature (0C)	Expected amplified product size (bp)	Structural annotation		Functional annotation	
								Sequence components of *kabuli* genome	*Kabuli* gene accession IDs	NCBI-KOG	TFs
Ca4_P1_32124537	Ca4	32124537	AAGA/AAGAGA	CCAAGTCACCCATGTCAGTT	CGTGCAATGAACTTGCCTTA	58.4	717	INTERGENIC	--	--	--
Ca4_P1_32126593	Ca4	32126593	TAAAAAAAAAAAA/TAAAAAAAAAAAAAA	GGTGAAACTCCCAATCGAAA	GTCCGGCCTATTACCAGTCA	59.9	882	DRR	Ca_15463	J	GATA
Ca4_P1_32127096	Ca4	32127096	AGT/AGTGT	TTGACTGGTAATAGGCCGGA	CCGGACCGTGTATAGCACTT	60.5	362	DRR	Ca_15463	J	GATA
Ca4_P1_32128162	Ca4	32128162	ATTTT/ATTT	CCACATCACACATTTACGACG	CGGAGCCAGACCTACAAGAG	59.9	598	DRR	Ca_15463	J	GATA
Ca4_P1_32137662	Ca4	32137662	TAAAAAA/TAAAAAAA	TCTCTCTTGCAGGTTTCTGCT	CATGGACACAAATTTGCACAC	59.4	912	INTERGENIC	--	--	--
Ca4_P1_32137832	Ca4	32137832	CAAAAAAAA/CAAAAAAAAAA	CGACAGCACATTAGATAAACCG	ATGGATGATGGGCAGTAAGC	59.7	806	INTERGENIC	--	--	--
Ca4_P1_32137888	Ca4	32137888	GAAGAAA/GAA	CGACAGCACATTAGATAAACCG	ATGGATGATGGGCAGTAAGC	59.7	806	INTERGENIC	--	--	--
Ca4_P1_32141276	Ca4	32141276	GTG/GTGGCACGTTG	ACCAACCTGTTGAAGGAAGC	CCCTCATGATATCCACGACC	59.2	636	INTERGENIC	--	--	--
Ca4_P1_32143759	Ca4	32143759	GATATATA/GATATA	CGTTATCATTGCTTGAACGG	TTGAGCACTTACTGGTGGGA	59.2	941	INTERGENIC	--	--	--
Ca4_P1_32144375	Ca4	32144375	TAAAAAA/TAAAAAAA	TCCCACCAGTAAGTGCTCAA	GTCGTTGTTGGTCTGTGTGG	59.3	308	INTERGENIC	--	--	--
Ca4_P1_32147675	Ca4	32147675	AATATAT/AATAT	TTGCACAAAAGAAATCGAAAAA	ACAAATTGGCATTCACCCAT	59.7	471	INTERGENIC	--	--	--
Ca4_P1_32149191	Ca4	32149191	CTTTTTTT/CTTTTTT	CGGTGAAGGGACCACACTAT	TGCCCATAACTGCACAACAT	59.8	404	INTERGENIC	--	--	--

INDEL marker IDs	Chromosomes/ unanchored scaffolds	Physical positions (bp)	InDels (*Kabuli* reference genome-CDC Frontier/PI)	Forward primers (5'-3')	Reverse primers (5'-3')	Anneling temperature (0C)	Expected amplified product size (bp)	Structural annotation		Functional annotation	
								Sequence components of *kabuli* genome	*Kabuli* gene accession IDs	NCBI-KOG	TFs
Ca4_P1_32150610	Ca4	32150610	TTTGATTAC/T	GGCAAGGAATTTGTGAGAGC	AATGGTTAGGAATGAGCCTCC	59.8	953	INTERGENIC	--	--	--
Ca4_P1_32152120	Ca4	32152120	TC/T	GGCATTAAAAAGGAAATGGC	GGATATTTCGATGTGCATCAGT	58.6	618	INTERGENIC	--	--	--
Ca4_P1_32152405	Ca4	32152405	ATTAAT/ATTAATTTAAT	CTGATGCACATCGAAATATCC	TGTTCGAAATATGCAACAAAAA	58.1	445	INTERGENIC	--	--	--
Ca4_P1_32155362	Ca4	32155362	CGGG/CGG	ATGCGTGTTGTTTTGCCATA	GATGGGACTTGGTTGTACGG	60.0	716	INTERGENIC	--	--	--
Ca4_P1_32156632	Ca4	32156632	TAAAAAA/TAAAAAAA	TTCACTTGGTTGCTTTGCAG	CTTAAACCATAATCCCCGCA	60.0	692	INTERGENIC	--	--	--
Ca4_P1_32159010	Ca4	32159010	ATTTTTTTT/ATTTTTTTTTT	TCTCTTTTAATGGGCCATGC	ATGCATGGAATCCAGCTTTC	60.0	688	INTERGENIC	--	--	--
Ca4_P1_32159052	Ca4	32159052	TAAAA/TAAA	TCTCTTTTAATGGGCCATGC	ATGCATGGAATCCAGCTTTC	60.0	688	INTERGENIC	--	--	--
Ca4_P1_32159427	Ca4	32159427	ATTTTTTTTTT/ATTTTTTTTT	TTCCCTTGAAAGTGCATTGA	GCTTCTATGGGCAACGTACC	59.2	224	INTERGENIC	--	--	--
Ca4_P1_32167356	Ca4	32167356	CTATA/CTA	TCGAACTGGGGACCTTACAG	CATGCTACCTCCCATGGTGT	60.1	461	INTERGENIC	--	--	--
Ca4_P1_32169313	Ca4	32169313	ATTTTTTTT/ATTTTTTTTT	TACGTTAACGGTGACCACGA	GCACTTTGTAAACGTCCCGT	60.0	762	INTERGENIC	--	--	--
Ca4_P1_32173509	Ca4	32173509	AC/A	ACGCATGTGAGGTGTTTGAT	TACACATGGGTTAAGGGGGA	59.0	456	INTERGENIC	--	--	--
Ca4_P1_32173778	Ca4	32173778	GAAA/GAA	TGCAACACCGGAACTTCATA	TTTCAACCAATTGTTGCTCG	60.1	299	INTERGENIC	--	--	--

INDEL marker IDs	Chromosomes/ unanchored scaffolds	Physical positions (bp)	InDels (*Kabuli* reference genome-CDC Frontier/PI)	Forward primers (5'-3')	Reverse primers (5'-3')	Anneling temperature (0C)	Expected amplified product size (bp)	Structural annotation		Functional annotation	
								Sequence components of *kabuli* genome	*Kabuli* gene accession IDs	NCBI-KOG	TFs
Ca4_P1_32175950	Ca4	32175950	ATTTTTTTTT/ATTTTTTTTTT	AATGGTGGCTTTGAAGATGG	AAAATTGGAGGGAGGAAATG	59.9	535	INTERGENIC	--	--	--
Ca4_P1_32176267	Ca4	32176267	TAAAAAAAA/TAAAAAAA	GGATGGTTTCCCAATTCAAG	CATTTGTCGCATCGTTGAAG	59.2	834	INTERGENIC	--	--	--
Ca4_P1_32179388	Ca4	32179388	T/TA	TTCTTGCGCTTTGTCTCTCA	TGGAAGTGAACTAAACCCCTTT	59.9	744	INTERGENIC	--	--	--
Ca4_P1_32179682	Ca4	32179682	CAATAATCAAACAAA/CAATAATCAAACAAATAATCAAACAAA	TTGCATACCTCATACCATCCC	TTGATGCATTACCTCCCACA	59.7	731	INTERGENIC	--	--	--
Ca4_P1_32180768	Ca4	32180768	CAA/CA	TAAGCCAATGAAGAATCGCC	TTTTCATCCCACCCCAAATA	60.2	425	INTERGENIC	--	--	--
Ca4_P1_32186391	Ca4	32186391	CTTTT/CTTTTT	GGGTCAGGCAAAAATGAAAA	GGGGTGGTAGGGGAAAATTA	59.9	407	DRR	Ca_15464	R	ERF
Ca4_P1_32187077	Ca4	32187077	CTGTG/CTGTGTG	TTAAACATTCATTTCCGCCG	GCTTGGTTTGTGGCATGTTA	60.8	385	INTERGENIC	--	--	--
Ca4_P1_32187865	Ca4	32187865	TAAAAA/TAAAAAAAA	CACGCGAACGATAAATGATG	TGGGGGAATTGGAGTGATAA	60.1	185	INTERGENIC	--	--	--
Ca4_P1_32203955	Ca4	32203955	CTTTT/CTTTTT	GAAATGAATGGAGTCGCACA	TCGCGAAGTTGTTTTTGTTG	59.7	700	DRR	Ca_15465	--	--
Ca4_P1_32208900	Ca4	32208900	AG/A	CGTTGCGCGAGTATAATTTG	TCAATGTTTCAAACCAAACAAAA	59.4	370	INTERGENIC	--	--	--
Ca4_P1_32212035	Ca4	32212035	CTCA/CTCATTGATGCTTATGTCA	TGAATCAACAAATGAAAGCAGTG	TCCCGCTTCGATACAGAGAT	60.2	448	INTERGENIC	--	--	--
Ca4_P1_32212853	Ca4	32212853	CTTTTTT/CTTTTTTTTTT	GATCTCTGTATCGAAGCGGG	TTGCCTCCATTAAATCCTCG	59.8	700	DRR	Ca_15467	T	HB-other

INDEL marker IDs	Chromosomes/ unanchored scaffolds	Physical positions (bp)	InDels (*Kabuli* reference genome-CDC Frontier/PI)	Forward primers (5'-3')	Reverse primers (5'-3')	Anneling temperature (0C)	Expected amplified product size (bp)	Structural annotation		Functional annotation	
								Sequence components of *kabuli* genome	*Kabuli* gene accession IDs	NCBI-KOG	TFs
Ca4_P1_32213937	Ca4	32213937	AA/AAAGA	GGGTAGGGGGTCCTTGTTTA	CCACACCACTTGATTTGTTCA	60.0	738	DRR	Ca_15467	T	HB-other
Ca4_P1_32214848	Ca4	32214848	ATTTTTT/ATTTTTTT	CGCATGTGTATCCCTTTGTG	CGTTCTCGGAACCTTTCTTTC	60.0	471	INTRON	Ca_15467	T	HB-other
Ca4_P1_32215092	Ca4	32215092	AT/A	CGCATGTGTATCCCTTTGTG	ATGGGAATGGAACCATTGAA	60.0	918	INTRON	Ca_15467	T	HB-other
Ca4_P1_32215991	Ca4	32215991	TGTTTGAAGTTTGAAGTTTGAAGTTTGAAG/TGTTTGAAGTTTGAAGTTTGAAG	TTCAATGGTTCCATTCCCAT	ATAAGCGTGCGTTGACCTTT	60.0	599	INTERGENIC	--	--	--
Ca4_P1_32216251	Ca4	32216251	GTTTTTT/GTTTTTTTT	ATAAAGGTCAACGCACGCTT	GAAATCAACGGATCTCCATCA	59.8	384	INTERGENIC	--	--	--
Ca4_P1_32220024	Ca4	32220024	G/GCC	CGTGGGGGTATTTTTCATTG	GCCTCCCAGAATATGGATCA	60.0	749	INTERGENIC	--	--	--
Ca4_P1_32222918	Ca4	32222918	ACTCT/ACT	TCCATCATCGTTCTCATTGG	TCGAACAAAAGGCAACACAA	59.5	225	INTERGENIC	--	--	--
Ca4_P1_32224997	Ca4	32224997	T/TC	ATATTTGTTGCTTTTGGCCC	TGGTTGAATGATTGGAATTGA	58.9	692	INTERGENIC	--	--	--
Ca4_P1_32225876	Ca4	32225876	TTAT/TT	TCGTCGCATTGGTGTAATGT	TCCGTGAGGACACAAGTGAG	60.0	506	INTERGENIC	--	--	--
Ca4_P1_32227293	Ca4	32227293	AAAAA/AAAAATTAAAA	TCTTCACAACCCCTTGAACC	TTCTTCCAAACGAGCCTCTC	59.9	868	INTERGENIC	--	--	--
Ca4_P1_32227955	Ca4	32227955	TG/T	GAGAGGCTCGTTTGGAAGAA	GCTGTGCACTTGATGCTGAT	59.6	613	INTERGENIC	--	--	--
Ca4_P1_32239930	Ca4	32239930	TT/TTGGT	CAGGTAGAGTGCAGATTGCG	TAATGCTTGATGCCAAACCA	59.6	366	INTERGENIC	--	--	--

INDEL marker IDs	Chromosomes/ unanchored scaffolds	Physical positions (bp)	InDels (*Kabuli* reference genome-CDC Frontier/PI)	Forward primers (5'-3')	Reverse primers (5'-3')	Anneling temperature (0C)	Expected amplified product size (bp)	Structural annotation		Functional annotation	
								Sequence components of *kabuli* genome	*Kabuli* gene accession IDs	NCBI-KOG	TFs
Ca4_P1_32257454	Ca4	32257454	GTTTTTTTTTTT/GTTTTTTTTTT	TGCACTTGTTAACCACACAATG	TGTGGGAGACCGATTTCTCT	59.6	567	INTERGENIC	--	--	--
Ca4_P1_32263797	Ca4	32263797	CAAAAAAA/CAAAAAA	ACACATGTCCCAGTTGACGA	TGACGATGGAGGCTTTTAGG	60.0	682	INTERGENIC	--	--	--
Ca4_P1_32264452	Ca4	32264452	TAAAAAAAAAAA/TAAAAAAAAAAAAAAA	CCTAAAAGCCTCCATCGTCA	CCCAATTGAAGGGTAAGTGC	60.2	800	INTERGENIC	--	--	--
Ca4_P1_32264601	Ca4	32264601	TA/TAA	GATCAAATTCTCTATGCATGTGCT	CCAATTGAAGGGTAAGTGCC	59.7	608	INTERGENIC	--	--	--
Ca4_P1_32264980	Ca4	32264980	AAT/AATAT	GCACTTACCCTTCAATTGGG	AGGAGAGGTCCATTTCTCAGG	59.4	262	INTERGENIC	--	--	--
Ca4_P1_32290639	Ca4	32290639	ATT/ATTT	GGATCAGAGAAGAGACGGGA	TTACTTGTAGGGCAGGTCCG	59.3	703	INTERGENIC	--	--	--
Ca4_P1_32301138	Ca4	32301138	CAAAAAAAAAAAAAAAA/CAAAAAAAAAAAAA	TCAAAAGTCTTGTCCCCGAT	TGCACCAAATGTGTCCTTGT	59.5	116	DRR	Ca_15473	P	--
Ca4_P1_32301466	Ca4	32301466	CAAAAAAAA/CAAAAAAAAA	AACCATCCCACTTGATGGAA	TCAACCAGTTTTGCAATCCA	60.2	186	DRR	Ca_15472	DO	MYB_related
Ca4_P1_32301599	Ca4	32301599	TAAAAAAA/TAAAA	TGGATTGCAAAACTGGTTGA	CACGAGCTTACAAACCCACA	60.1	174	DRR	Ca_15472	DO	MYB_related
Ca4_P1_32315056	Ca4	32315056	AC/A	ATACCAACATCGACGAGGGA	GAACTTGTGTTGGGGGAGAA	60.3	333	INTERGENIC	--	--	--
Ca4_P1_32335179	Ca4	32335179	AAAGAAGAAGAAGAAGAAGAA/AAAGAAGAAGAAGAAGAA	AAAGTCCGTTTGTGAATCCG	TGGGCATTCAAATCAAACAA	60.0	507	INTERGENIC	--	--	--
Ca4_P1_32340514	Ca4	32340514	CAACAAAAAA/CAA	ATGGATTTGCATTTGCCTTC	TGCATCTCCTTGCTCAACAC	59.9	589	INTERGENIC	--	--	--

INDEL marker IDs	Chromosomes/ unanchored scaffolds	Physical positions (bp)	InDels (*Kabuli* reference genome-CDC Frontier/PI)	Forward primers (5'-3')	Reverse primers (5'-3')	Anneling temperature (0C)	Expected amplified product size (bp)	Structural annotation		Functional annotation	
								Sequence components of *kabuli* genome	*Kabuli* gene accession IDs	NCBI-KOG	TFs
Ca4_P1_32367636	Ca4	32367636	ATTTTTTTTTTTTT/ATTTTTTTTTTTT	TCCTCCTTGTGATCTTGGCT	CATTGCTCCCTCTTACTGCC	59.8	429	INTERGENIC	--	--	--
Ca4_P1_32369377	Ca4	32369377	TA/T	TTGGTTTCGTTTGCTTGTTG	AATAGTGCGCATGTGTGGTC	59.7	822	INTERGENIC	--	--	--
Ca4_P1_32370221	Ca4	32370221	GTTTTTTTT/GTTTTTTTTT	GACCACACATGCGCACTATT	TCATGCCCTTACGATCAACA	59.6	648	INTERGENIC	--	--	--
Ca4_P1_323717	Ca4	323717	ATTT/AT	CTGGGAATTGGACTTAAAAGAA	TTGTTGGGTTATCGTTGTCG	57.4	791	INTERGENIC	--	--	--
Ca4_P1_32384144	Ca4	32384144	AAAAAAATAAAA/AAAAAAATAAAAAATAAAA	GCTTGTGGTTGTCGGATCTT	ATGAGTTGCTTTTGGTTCCG	60.1	667	INTERGENIC	--	--	--
Ca4_P1_32384172	Ca4	32384172	TAAAAAA/TAAAAAAA	GCTTGTGGTTGTCGGATCTT	ATGAGTTGCTTTTGGTTCCG	60.1	667	INTERGENIC	--	--	--
Ca4_P1_32386770	Ca4	32386770	CAAAAA/CAAAAAA	TTCATTAGGGATTTTGGGCA	GGTTCCAACAATCTCCCCTT	60.3	618	INTERGENIC	--	--	--
Ca4_P1_32388402	Ca4	32388402	ATTTTTTTT/ATTTTTTTTT	TCAATTGCTTGTTGCCTCTG	AGAGACATAACAAAGGGCCG	60.0	606	INTERGENIC	--	--	--
Ca4_P1_32388627	Ca4	32388627	CTTTTTTTTTT/CTTTTTTTT	CGGCCCTTTGTTATGTCTCT	AGACCGTGAACGGCTTATAGA	59.2	611	INTERGENIC	--	--	--
Ca4_P1_32389433	Ca4	32389433	ATTTTT/ATTTT	TAAAATGGAATTCGCCCAAA	GCGTGTCTGCAATGATTGTT	60.3	732	INTERGENIC	--	--	--
Ca4_P1_32392989	Ca4	32392989	AT/A	ACAATGTTGTGGTCGCTTGA	CGAGAGTATTCATTGTCTTGTACCAT	60.2	620	DRR	Ca_15475	--	--
Ca4_P1_32395281	Ca4	32395281	TA/TAA	GGTTAACAAATCTCCTAAAGGCA	TTTTGTTAAAGAATTGCGACCTC	58.7	523	INTERGENIC	--	--	--

INDEL marker IDs	Chromosomes/ unanchored scaffolds	Physical positions (bp)	InDels (*Kabuli* reference genome-CDC Frontier/PI)	Forward primers (5'-3')	Reverse primers (5'-3')	Anneling temperature (0C)	Expected amplified product size (bp)	Structural annotation		Functional annotation	
								Sequence components of *kabuli* genome	*Kabuli* gene accession IDs	NCBI-KOG	TFs
Ca4_P1_32395719	Ca4	32395719	TAAAAAAAAAAA/TAAAAAAAAAA	GGTTAACAAATCTCCTAAAGGCA	CCAAACAAAGAAATGACGAACA	58.7	665	INTERGENIC	--	--	--
Ca4_P1_32396185	Ca4	32396185	TCCC/TCC	TCGTCATTTCTTTGTTTGGTTG	TCCAATTTTCCGTGTCATGT	60.0	346	INTERGENIC	--	--	--
Ca4_P1_32398144	Ca4	32398144	TAAAAAAAAAAA/TAAAAAAAAAA	ATTTTGAAAAATCTGCGGGG	GGACCACGAATGTAATTTAGCC	61.1	955	INTERGENIC	--	--	--
Ca4_P1_32399781	Ca4	32399781	GAAAAAAA/GAAAAAAAA	TTCTGTCCGAGTATGGGTCC	TTGATGGTTCGTTTAAAACAATTT	59.9	697	INTERGENIC	--	--	--
Ca4_P1_32400480	Ca4	32400480	ATTT/ATT	TCATAGAGCTTGCCCTAGCC	AACATTTGTGCCACACTCCA	59.6	375	INTERGENIC	--	--	--
Ca4_P1_32401010	Ca4	32401010	TAAA/TAAAA	TGGAGTGTGGCACAAATGTT	GAACACCACCGATTTCACCT	60.0	498	INTERGENIC	--	--	--
Ca4_P1_32410630	Ca4	32410630	TA/TAA	TTATTCCACCCCACCTCAAA	CTCAGCTCATTGGTTTGGTG	60.2	262	INTERGENIC	--	--	--
Ca4_P1_32413475	Ca4	32413475	AAT/AATAT	TTGTGCAACCCTTGAAATGA	CAATTTTCCCATTCTAAGCTTCTC	60.1	722	INTERGENIC	--	--	--
Ca4_P1_32415458	Ca4	32415458	CTTTT/CTTTTT	AACATGGAGGGTCATGGAAA	AACTTTGTCGATTAAGCACGG	60.2	435	INTERGENIC	--	--	--
Ca4_P1_32416231	Ca4	32416231	ATT/AT	CCCACCATTGAAATTTGGAG	GAAGGAGGGGAAATTTTGACA	60.2	680	INTERGENIC	--	--	--
Ca4_P1_32418479	Ca4	32418479	AAT/A	TGTTAGAACGAGTGGGTGAAAA	ACTTGCCTTGGTCCCTCTTT	59.6	501	INTERGENIC	--	--	--
Ca4_P1_32431643	Ca4	32431643	TTAATAATAA/TTAATAA	ACCCAACCTCATCTGCATTC	TTTCAGAAAAACTACGGAACAAA	59.9	362	DRR	Ca_15476	--	--

INDEL marker IDs	Chromosomes/ unanchored scaffolds	Physical positions (bp)	InDels (*Kabuli* reference genome-CDC Frontier/PI)	Forward primers (5'-3')	Reverse primers (5'-3')	Anneling temperature (0C)	Expected amplified product size (bp)	Structural annotation		Functional annotation	
								Sequence components of *kabuli* genome	*Kabuli* gene accession IDs	NCBI-KOG	TFs
Ca4_P1_32432521	Ca4	32432521	TC/TCC	CATGCATGTTTAATGTATTAGTGACAA	TTCATGACTTTGTGTCGGGA	59.3	625	INTERGENIC	--	--	--
Ca4_P1_32434494	Ca4	32434494	TACTT/TACTTATTTAACTT	GTTTTCCCGTTCTCTCCTCC	ACCAAGTCGAATTGTGGACC	60.1	400	INTERGENIC	--	--	--
Ca4_P1_32454180	Ca4	32454180	TAA/TAAA	GCGGATACATTTCTTTTGGG	AAACAAGCGGAAGAGCTTGA	59.4	310	INTERGENIC	--	--	--
Ca4_P1_32454199	Ca4	32454199	TAAAAAAAAAAA/TAAAAAAAAA	GCGGATACATTTCTTTTGGG	CTTCAGCTAATCGCTTTCCG	59.4	716	INTERGENIC	--	--	--
Ca4_P1_32457295	Ca4	32457295	AC/A	TGTGCTCAACCCAAATTGAA	ACAAGTTGTGGGAGTGGGAC	60.1	278	INTERGENIC	--	--	--
Ca4_P1_32460225	Ca4	32460225	TAAAAAAAAA/TAAAAAAAAAA	CGGAGATCCATAACACGGTC	TTTGAAAGAGAAATCCACCCA	60.3	242	INTERGENIC	--	--	--
Ca4_P1_32464896	Ca4	32464896	CTTTTTTTTT/CTTTTTTTT	CCTGTTTTGTGGGAACCTGT	TGGAGGAAGCAAGGAAAGAA	59.9	684	INTERGENIC	--	--	--
Ca4_P1_32467001	Ca4	32467001	GTTTTTTT/GTTTTTT	GCGTTTGTTCTTGTTTGGGT	CCATGCTACTTTGCATGTGG	60.0	688	INTERGENIC	--	--	--
Ca4_P1_32467491	Ca4	32467491	CTAATTAA/CTAA	AAATGCTAACGGTGCAAACA	GACGTAGAACGATTAACACCCA	59.2	465	INTERGENIC	--	--	--
Ca4_P1_32473583	Ca4	32473583	TTTTTTTATTTTTTATTTT/TTTTTTTATTTTTTATTTTTTATTTT	TGATCTTAGCGATGGTTCCC	TGAGCGTTCGATTTTGTTTG	60.0	687	INTERGENIC	--	--	--
Ca4_P1_32482372	Ca4	32482372	AGGGG/AGGGGG	TACCAAACCCCTTGGTCATC	TTTTGGGCGGAAATGTTTTA	59.6	583	INTERGENIC	--	--	--
Ca4_P1_32482909	Ca4	32482909	TG/T	GTTAAAACATTTCCGCCCAA	CAAGAACGAAGAAAAACGCC	59.8	485	INTERGENIC	--	--	--

INDEL marker IDs	Chromosomes/ unanchored scaffolds	Physical positions (bp)	InDels (*Kabuli* reference genome-CDC Frontier/PI)	Forward primers (5'-3')	Reverse primers (5'-3')	Anneling temperature (0C)	Expected amplified product size (bp)	Structural annotation		Functional annotation	
								Sequence components of *kabuli* genome	*Kabuli* gene accession IDs	NCBI-KOG	TFs
Ca4_P1_32484353	Ca4	32484353	CTTTTTT/CTTTTTTT	TTATCCTTTGCTGCCCAAAC	ATCATGTTGCCCCTAATCCA	60.1	753	INTERGENIC	--	--	--
Ca4_P1_32489291	Ca4	32489291	ATT/ATTGTT	CGACAAAGAGGTGGATGGTT	CCTCCATCAATAATAATAAAACCCC	60.0	423	INTERGENIC	--	--	--
Ca4_P1_32489332	Ca4	32489332	AA/AAATA	CGACAAAGAGGTGGATGGTT	CCTCCATCAATAATAATAAAACCCC	60.0	423	INTERGENIC	--	--	--
Ca4_P1_32492099	Ca4	32492099	ATTTTTT/ATTTTTTT	TGCTATTTTCGTGTGTGTTTGA	TGTCCGCTTCAGTGTAATGC	59.3	454	INTERGENIC	--	--	--
Ca4_P1_32492742	Ca4	32492742	AAGA/AA	AGTGGATTGGGTGGAAAATG	GCCAATTTCAATTAAGCCGA	59.6	296	INTERGENIC	--	--	--
Ca4_P1_32494646	Ca4	32494646	GAAAAAAA/GAAAAAAAA	CAATTCACTTGAAACGTCTCTCAT	CACAAAATCGACAAAGACATGC	59.7	386	INTERGENIC	--	--	--
Ca4_P1_32494672	Ca4	32494672	TA/T	CAATTCACTTGAAACGTCTCTCAT	CACAAAATCGACAAAGACATGC	59.7	386	INTERGENIC	--	--	--
Ca4_P1_32496585	Ca4	32496585	TATTTATTTTTA/TATTTATTTTTAAAATTTATTTTTA	CCACAATCCACCGTCTACCT	CCCAGACCCCTCATAATCAA	59.8	565	INTERGENIC	--	--	--
Ca4_P1_32496789	Ca4	32496789	TAAAAAAA/TAAAAAAAAAA	CCACAATCCACCGTCTACCT	CCCAGACCCCTCATAATCAA	59.8	565	INTERGENIC	--	--	--
Ca4_P1_32497649	Ca4	32497649	ATTTTTTTTTT/ATTTTTTTTT	TACTTCTGCATGAGGGGGAT	TCCAACCAAAGCGATTTTTC	59.5	505	INTERGENIC	--	--	--
Ca4_P1_32498847	Ca4	32498847	AG/A	TAGCACGGACCCCAATTAAG	TGAACCCCCATCAAAGGATA	60.0	897	INTERGENIC	--	--	--
Ca4_P1_32509855	Ca4	32509855	TAAAAAA/TAAAAA	GCATTCCCTCTGGACTTTGA	GAAACTTTGGCCCCTATGGT	60.2	454	INTERGENIC	--	--	--

INDEL marker IDs	Chromosomes/ unanchored scaffolds	Physical positions (bp)	InDels (*Kabuli* reference genome-CDC Frontier/PI)	Forward primers (5'-3')	Reverse primers (5'-3')	Anneling temperature (0C)	Expected amplified product size (bp)	Structural annotation		Functional annotation	
								Sequence components of *kabuli* genome	*Kabuli* gene accession IDs	NCBI-KOG	TFs
Ca4_P1_32511907	Ca4	32511907	T/TG	TTTCTCCCGTTATGTG TCGG	TGAGTGCTGGGAACTA ACCC	60.9	704	INTERGENIC	--	--	--
Ca4_P1_32512065	Ca4	32512065	AG/A	TTTCTCCCGTTATGTG TCGG	TGAGTGCTGGGAACTA ACCC	60.9	704	INTERGENIC	--	--	--
Ca4_P1_32514320	Ca4	32514320	GACATTGACTTTTT/GACATTGACTTT TTTCAACATTGACTTTTT	ATTACTGGACCAGAGG CACG	AATGTCCATTCTGGGA CGAG	60.1	510	INTERGENIC	--	--	--
Ca4_P1_32515759	Ca4	32515759	TG/T	CTATTGGCGGTTCTGT TGGT	AGGACCCCAAACATCA CAAG	60.0	586	INTERGENIC	--	--	--
Ca4_P1_32522290	Ca4	32522290	GAAAAAAAAA/GAAAAAAAAAA	ACTCATTCATTCCACA GCCC	TTGCTAACGGTAACAC AACGA	59.9	851	INTERGENIC	--	--	--
Ca4_P1_32523241	Ca4	32523241	TAAA/TAAAAAAA	GAAGGAGGAGGAGAG TGCAG	ATTGAACACGACGCAA ATCA	59.1	391	INTERGENIC	--	--	--
Ca4_P1_32523443	Ca4	32523443	TAAAAAAA/TAAAAAA	GAAGGAGGAGGAGAG TGCAG	ATTGAACACGACGCAA ATCA	59.1	391	INTERGENIC	--	--	--
Ca4_P1_32524102	Ca4	32524102	T/TG	CTTGGGCTCTCCTCCT CTCT	TTTCGGTACACAATTT CCCC	60.1	502	INTERGENIC	--	--	--
Ca4_P1_32524319	Ca4	32524319	ATTTTTTTTTT/ATTTTTTTT	CTTGGGCTCTCCTCCT CTCT	TTTCGGTACACAATTT CCCC	60.1	502	INTERGENIC	--	--	--
Ca4_P1_32525507	Ca4	32525507	CAAAA/CAAAAA	GTCATGCATATGTTGG ACGC	AAGTTTTCTTCCCGCT CACA	60.0	460	INTERGENIC	--	--	--
Ca4_P1_32529368	Ca4	32529368	GATATATATATAT/GATATATATATATA T	GGGTGTGATTAATGGG TTCG	TCAGGGGTTGCATGG TATTT	60.1	457	INTERGENIC	--	--	--
Ca4_P1_32531473	Ca4	32531473	TAA/T	TAAAATGGATGCATCG AGCC	ACAAATCAACCAGTGT CGCA	60.9	662	INTERGENIC	--	--	--

INDEL marker IDs	Chromosomes/ unanchored scaffolds	Physical positions (bp)	InDels (*Kabuli* reference genome-CDC Frontier/PI)	Forward primers (5'-3')	Reverse primers (5'-3')	Anneling temperature (0C)	Expected amplified product size (bp)	Structural annotation		Functional annotation	
								Sequence components of *kabuli* genome	*Kabuli* gene accession IDs	NCBI-KOG	TFs
Ca4_P1_32536057	Ca4	32536057	GTTTTTTTTTTTTT/GTTTTTTTTTT	TATCATTCGCATCAAACGGA	TGCGCTCAATTGTGATTCAT	60.0	578	INTERGENIC	--	--	--
Ca4_P1_32548891	Ca4	32548891	GT/GTTAT	CGAACCAAGTGAGGAAAGAGA	TGATTGGCATGAGTGCTGAC	59.5	603	INTERGENIC	--	--	--
Ca4_P1_32549547	Ca4	32549547	GTATA/GTA	TTGATGTCTATTGATGCAACG	ACATTGTGCCATCCCTTGAT	57.2	747	INTERGENIC	--	--	--
Ca4_P1_32550360	Ca4	32550360	ATTT/ATTTT	TGAATTAGCTTGGCCGTTTT	GAGAAAATCAACCCAACGTCA	59.7	530	INTERGENIC	--	--	--
Ca4_P1_32552051	Ca4	32552051	A/AT	GCATCGTGAGAATTCGATTG	TTGGTTGGAGGATTCTTTGG	59.2	398	INTERGENIC	--	--	--
Ca4_P1_32555810	Ca4	32555810	AAA/AAATTAA	TTTCGTCGTCTTAGTGTAGCGA	GGTGGTATGGGGAAACACAC	60.1	493	INTERGENIC	--	--	--
Ca4_P1_32555948	Ca4	32555948	AAAGAA/AAA	GTGTGTTTCCCCATACCACC	TGGGAATTTTGTTTGTGGGT	60.0	449	INTERGENIC	--	--	--
Ca4_P1_32557354	Ca4	32557354	TAAATAAAAT/TAAATAAAATAAAAT	AGGCAAAATACATGCAATCAT	TGCGTTTTGAGATTCGAAGA	57.1	497	INTERGENIC	--	--	--
Ca4_P1_32559742	Ca4	32559742	AAAGAAAGAAGAAAGAAGA/AAAGAAAGAAGA	TTCATTTCCAGTCTGTGGCA	ACGGCCGTTATAGGTTTCCT	60.2	669	INTERGENIC	--	--	--
Ca4_P1_32562355	Ca4	32562355	CTTTTT/CTTTTTT	AAATGGAGGAGAAGGAGGGA	CGGTGAATCCTTTACGGAGA	60.0	286	INTERGENIC	--	--	--
Ca4_P1_32578559	Ca4	32578559	TGTA/T	CTGTGCAGCAACAAAAGCAT	TCCTACTGGTCCGTACCCTG	60.1	749	INTERGENIC	--	--	--
Ca4_P1_3258516	Ca4	3258516	TTATATATATATATATATATAT/TTATATATATATATATATATAT	GTCAAATCTCCCTGCCAAAA	TGTAATTATAAAAATCATGCACGGA	60.1	712	INTERGENIC	--	--	--

INDEL marker IDs	Chromosomes/ unanchored scaffolds	Physical positions (bp)	InDels (*Kabuli* reference genome-CDC Frontier/PI)	Forward primers (5'-3')	Reverse primers (5'-3')	Anneling temperature (0C)	Expected amplified product size (bp)	Structural annotation		Functional annotation	
								Sequence components of *kabuli* genome	*Kabuli* gene accession IDs	NCBI-KOG	TFs
Ca4_P1_32585173	Ca4	32585173	TTTTC/TTTTCTCTCATGTTTATTTC	CACCCCTTCATTTGTTTGTG	GGCGCGTCTAGACTTACCAC	58.9	789	INTERGENIC	--	--	--
Ca4_P1_32586827	Ca4	32586827	GAAAAAAAA/GAAAAAAAAA	TATTCTTTGCCGCTCCAATC	AAGGACCAAAACCAATTGTCTC	60.2	750	INTERGENIC	--	--	--
Ca4_P1_32587039	Ca4	32587039	CTATTTATTTATTTATTTATTT/CTATTTATTTATTTATTTATTTATTTATTT	CGGGTTGTGTTATCATGCTC	AAGGACCAAAACCAATTGTCTC	59.0	389	INTERGENIC	--	--	--
Ca4_P1_32587822	Ca4	32587822	GTTTTTTTT/GTTTTTTTTT	AACGATAAGCCCCGAGAAAT	TAGCAATGCGGATTGAACAC	59.9	385	INTERGENIC	--	--	--
Ca4_P1_32592542	Ca4	32592542	ATTTTTTTTT/ATTTTTTTTTT	GGCGAGAGCTATGGAGAATG	GCGTTGTAAAAGGGTTGCAT	59.9	478	INTERGENIC	--	--	--
Ca4_P1_32595833	Ca4	32595833	TA/TATTGA	GTTGGGAATGACTCGTTCGT	CCAACTTATTGCATGGTTGGT	60.0	409	INTERGENIC	--	--	--
Ca4_P1_32602184	Ca4	32602184	CA/C	TGGGGATCAACAGACTCTAAAA	GTGAAAATCGAAGTGCGACA	58.7	456	DRR	Ca_15484	R	AP2
Ca4_P1_326188	Ca4	326188	AATATATATATATAT/AATATATATATATATAT	GCAATATCCAGTTTTTGAATAGGTT	ATGGCACATTGCACTGAACA	59.0	561	INTERGENIC	--	--	--
Ca4_P1_32622005	Ca4	32622005	T/TTAG	CATTGTTGCGCCTTAAATGA	GAATTTGAAATGCACCCGTT	59.7	525	DRR	Ca_15485	O	--
Ca4_P1_32622503	Ca4	32622503	ACC/AC	TCAATGCCATGGAAAGACAA	TTCCATCTCCAACAACATCG	60.0	867	DRR	Ca_15485	O	--
Ca4_P1_32622878	Ca4	32622878	GAAAA/GAAAAA	TTGATAGACTCGATGTTGTTGGA	TTGCATTATTTTGAGACGGAGA	59.6	343	DRR	Ca_15485	O	--
Ca4_P1_32623000	Ca4	32623000	TAAAAAAAAAA/TAAAAAAAAAAAA	TCGATGTTGTTGGAGATGGA	TCCATCTTTTGGAGGAATGG	60.0	566	DRR	Ca_15485	O	--

INDEL marker IDs	Chromosomes/ unanchored scaffolds	Physical positions (bp)	InDels (*Kabuli* reference genome-CDC Frontier/PI)	Forward primers (5'-3')	Reverse primers (5'-3')	Anneling temperature (0C)	Expected amplified product size (bp)	Structural annotation		Functional annotation	
								Sequence components of *kabuli* genome	*Kabuli* gene accession IDs	NCBI-KOG	TFs
Ca4_P1_32623906	Ca4	32623906	TAAAAA/TAAAA	TACTTGTTGAACCACG CAGC	TGGGCATACATCTGGT TTCA	59.9	530	INTRON	Ca_15485	O	--
Ca4_P1_32625448	Ca4	32625448	AAG/A	GGGATTCAAGCCCCAT TATT	GGTTCACATGGTGGCT TTCT	60.0	362	INTRON	Ca_15485	O	--
Ca4_P1_32634327	Ca4	32634327	TTTACTTACTTA/TTTACTTA	CAGACCCGTGTGAACA CAAA	GCCTTTGGAGTTGAAG ATTGA	60.6	591	INTERGENIC	--	--	--
Ca4_P1_32634885	Ca4	32634885	ATT/ATTT	GGCAACTAGAAAGTGC CACC	AGCAGATGCCTCTATC GCAT	59.7	258	INTERGENIC	--	--	--
Ca4_P1_32637847	Ca4	32637847	AT/ATGCT	TCCACCAAACCATGCT TGTA	GACCCCATAAGTCACA CGCT	60.0	811	INTERGENIC	--	--	--
Ca4_P1_32641631	Ca4	32641631	A/AAAG	ACACGGATTGAAACAT GCAA	TTTCAACTCACACACC ACCG	60.0	107	INTERGENIC	--	--	--
Ca4_P1_32647717	Ca4	32647717	TAAAAAAAAAAA/TAAAAAAAAAA	GGGTGCAATTAGGGAT GAGA	AGAATACCCCAGCAGC AATG	59.9	313	INTRON	Ca_15487	--	--
Ca4_P1_32650019	Ca4	32650019	ATTTTTT/ATTTTT	TTGTGTTTTGGTTGGG ATCA	TTTCATGGGTTTTGTT TGAAAT	59.8	451	DRR	Ca_15487	--	--
Ca4_P1_32650295	Ca4	32650295	TAAAAAAAAAA/TAAAAAAAAA	CTACTCGTGGTCCGTG AACTT	CACAACAACATTGAGG GTGC	59.3	776	DRR	Ca_15487	--	--
Ca4_P1_32662163	Ca4	32662163	CAAAAAAAA/CAAAAAAAAA	CTGGTGGTCTTGGATC TGGT	AAACTGCAAAAATTAG GGGG	60.0	568	INTERGENIC	--	--	--
Ca4_P1_32666526	Ca4	32666526	GTTTTTTTTT/GTTTTTTTTTT	GCCAATGAAACCATCC CTTA	GGTTCGAAGTTGTGAC ACACATA	59.8	375	INTERGENIC	--	--	--
Ca4_P1_32694376	Ca4	32694376	ATTTTTT/ATTTTTTT	AATCATTTTTGGAAGA CGCC	TGAAAACTGTGCAGCA TGAA	59.0	527	INTERGENIC	--	--	--

INDEL marker IDs	Chromosomes/ unanchored scaffolds	Physical positions (bp)	InDels (*Kabuli* reference genome-CDC Frontier/PI)	Forward primers (5'-3')	Reverse primers (5'-3')	Anneling temperature (0C)	Expected amplified product size (bp)	Structural annotation		Functional annotation	
								Sequence components of *kabuli* genome	*Kabuli* gene accession IDs	NCBI-KOG	TFs
Ca4_P1_32700117	Ca4	32700117	CTTTTTTT/CTTTTTTTT	TACGTGTCGCTTAGGCACTG	CGTCTTGTGCGGTGTATTTG	60.1	461	INTERGENIC	--	--	--
Ca4_P1_32714134	Ca4	32714134	GCTCTCTCTCTCTCTCTCTCTCTCTCTC/GCTCTCTCTCTCTCTCTCTCTCTCTCTCTCTC	AAGAACCCACGTTCACATCC	AACAATGATGACGAGAGGGG	59.8	150	INTERGENIC	--	--	--
Ca4_P1_32835028	Ca4	32835028	ATTTTT/ATTTT	TTGTGCAATGTTCGTTGGAT	CGCCATAACAAGCAATGGAT	60.0	463	INTERGENIC	--	--	--
Ca4_P1_32859234	Ca4	32859234	CNNNNNNNNNN/CNNNNN	TTGAATTTGCTGAAGGGGAC	GACAGACAGCCATACAGCCA	60.1	523	INTERGENIC	--	--	--
Ca4_P1_32873081	Ca4	32873081	ATTTT/ATTTTTT	GTCATTGACCGGGGATATTG	ACGGTTGCACCGAATAAAAA	60.0	162	INTERGENIC	--	--	--
Ca4_P1_32877122	Ca4	32877122	ATTTTTTTTTTTTT/ATTTTTTTTTTTTTT	CACAATCCAAAACAACGTGC	TACCGTTGGAGATGCTCTGA	60.0	523	INTRON	Ca_15496	--	ARF
Ca4_P1_32886390	Ca4	32886390	AC/ACC	AATCATCCCATGTTGCCATT	CGAGATTTCACACCAAGGGT	60.0	519	INTERGENIC	--	--	--
Ca4_P1_32913108	Ca4	32913108	TAAATTAAAATTAAAAT/TAAATTAAAATTAAAATTAAAAT	GGGTTTTTCAGAAAGAAACGG	AACGGTGAACTTAACACGGG	60.0	438	INTERGENIC	--	--	--
Ca4_P1_33050402	Ca4	33050402	ATTTTTTTTTTT/ATTTTTTTTTT	TTCCTCACACGCAAATTGTC	AAAAGAATTTGGGGTCTTTCG	59.7	531	DRR	Ca_24017	GC	HSF
Ca4_P1_33053978	Ca4	33053978	ATGTT/AT	TGCTTTCATCTTTGGAACCC	TTTGCATTACGGATTTGATGA	60.1	654	INTERGENIC	--	--	--
Ca4_P1_33054018	Ca4	33054018	TAA/TAAA	GGAATGGCAAACTTTCATGC	TTTGCATTACGGATTTGATGA	60.5	520	INTERGENIC	--	--	--
Ca4_P1_33054232	Ca4	33054232	TAAAAAAAA/TAAAAAAAAA	TCATCAAATCCGTAATGCAAA	GCACATGGGGTACCAGACTT	59.0	445	INTERGENIC	--	--	--

INDEL marker IDs	Chromosomes/ unanchored scaffolds	Physical positions (bp)	InDels (*Kabuli* reference genome-CDC Frontier/PI)	Forward primers (5'-3')	Reverse primers (5'-3')	Anneling temperature (0C)	Expected amplified product size (bp)	Structural annotation		Functional annotation	
								Sequence components of *kabuli* genome	*Kabuli* gene accession IDs	NCBI-KOG	TFs
Ca4_P1_33060640	Ca4	33060640	GAAAAAAAAA/GAAAAAAAAAAA	TGAAGAACGCTCTCCACATTT	TTTCTTAGCATGCCAGCCTT	59.9	239	INTERGENIC	--	--	--
Ca4_P1_33064079	Ca4	33064079	ATTGTTGTTGTTGTTGTTGTTG/ATTGTTGTTGTTGTTGTTGTTGTTG	TCGGGCCACTATCTGGATTA	GTGTGCCGTCACAAACAATC	60.4	810	INTERGENIC	--	--	--
Ca4_P1_33066353	Ca4	33066353	GTGATGATG/GTGATG	TGACGCGAACCATTGTTAAA	GGTCATAAAAGGTTCCGGG	60.1	435	INTERGENIC	--	--	--
Ca4_P1_33092824	Ca4	33092824	ATT/ATTTTT	TGAGAGGTGAGAATTGGAGGA	TGCGGATGTGTGTTTTCAAT	59.8	538	INTERGENIC	--	--	--
Ca4_P1_33100738	Ca4	33100738	GAAAAAAAAAA/GAAAAAAAAAAAA	TTGGGTTGTGAGGAATGGAT	TCATCTGTTAGGTACCCCGC	60.2	304	INTERGENIC	--	--	--
Ca4_P1_33102490	Ca4	33102490	ATTTTTT/ATTTTTTT	CCTTGAGTTCGACACTCGGT	GGGATTATTCTGCCTCCCAT	60.3	636	INTERGENIC	--	--	--
Ca4_P1_33111341	Ca4	33111341	AGTACCTCTT/AGTACCTCTTCAGGTACCTCTT	CCATGAGCAACCCAAAACTT	GTCTCAATTCTGCCCGAGAG	60.0	274	INTERGENIC	--	--	--
Ca4_P1_33113244	Ca4	33113244	GAAA/GAA	TGGGATTCCTCTCTCCCACT	GGCAAACTTGTCCACTTGGT	61.0	274	INTERGENIC	--	--	--
Ca4_P1_33137710	Ca4	33137710	ATTTTTTTTTTTTT/ATTTTTTTTTTTTTTT	TTGGGTTACGTCCAGTCCTC	TCTGTTTTCTTGTGCAACGG	60.0	726	INTERGENIC	--	--	--
Ca4_P1_33145619	Ca4	33145619	TAAAAAAA/TAAAAAA	AAACCGTCGTTCCAGTTGTC	AGGCAACAAACATAGAATCGC	60.0	550	INTERGENIC	--	--	--
Ca4_P1_33147211	Ca4	33147211	TAAAAAAAAAA/TAAAAAAAAAAA	TCACCATTTCTTTCCAAGGC	AGTGCAACCGAAAAATCCAC	60.1	333	INTERGENIC	--	--	--
Ca4_P1_33170728	Ca4	33170728	CAAAAAAAAA/CAAAAAAAA	TTTTTCTTGAAAATGTTGTCGTG	TGTCATATTCGTCGGCTTTG	59.2	774	INTERGENIC	--	--	--

INDEL marker IDs	Chromosomes/ unanchored scaffolds	Physical positions (bp)	InDels (*Kabuli* reference genome-CDC Frontier/PI)	Forward primers (5'-3')	Reverse primers (5'-3')	Anneling temperature (0C)	Expected amplified product size (bp)	Structural annotation		Functional annotation	
								Sequence components of *kabuli* genome	*Kabuli* gene accession IDs	NCBI-KOG	TFs
Ca4_P1_33176272	Ca4	33176272	ATTTTTTTTT/ATTTTTTTTTT	ATCCACGGACAATCCATTTG	TGGATGTCTTGTTCTGGGTTC	60.6	478	INTERGENIC	--	--	--
Ca4_P1_33185311	Ca4	33185311	GAAAAAAA/GAAAAAAAA	AAGTCGCCATTTGGGTGTAG	CCTCAAACACGAAACCCTGT	60.0	318	INTERGENIC	--	--	--
Ca4_P1_33186066	Ca4	33186066	A/AG	GCGCACAACAACAAGAAAAA	CGGGTTGTGAAACACCTTCT	59.9	620	INTERGENIC	--	--	--
Ca4_P1_33195041	Ca4	33195041	T/TAGAATTCGGTGA	TTGTCCGTGAGAGACTGCAC	GAATCCTGGGCTGAAATCAA	60.0	595	INTERGENIC	--	--	--
Ca4_P1_33212428	Ca4	33212428	TATAATAATAATAATAAT/TATAATAATAATAATAATAAT	TTTCGTTTCATTTTCATGTTATCAA	GAAATGCCACACTCCGAAAT	59.8	433	INTERGENIC	--	--	--
Ca4_P1_33256333	Ca4	33256333	CAAAAAAAAAA/CAAAAAAAAAAA	GCACGTGGGAAAATGAGATT	TGTTGCAAAACATTTCTGGG	59.9	540	INTERGENIC	--	--	--
Ca4_P1_33287086	Ca4	33287086	TA/T	CAACCACACCCTCATCCTCT	GGAGAAACCACTTCAAGGCA	60.0	434	INTERGENIC	--	--	--
Ca4_P1_33296074	Ca4	33296074	TAAAAAAAAAA/TAAAAAAAAAAA	TGACCTCCCACTGCATACAA	AAGAGACCGCATACGTGGAT	60.1	532	INTERGENIC	--	--	--
Ca4_P1_33308913	Ca4	33308913	GAAAA/GAAA	TCATTTTCCCCTCGTTTCTG	TTGGCCAAAACCTTTTGATG	60.0	183	DRR	Ca_24021	R	C2H2
Ca4_P1_33310326	Ca4	33310326	GAAAAAAAA/GAAAAAAA	TGCCTTGCTTTCTGGTTTTC	CGGTATATGTCACACTTCCGA	60.4	597	DRR	Ca_24021	R	C2H2
Ca4_P1_33314599	Ca4	33314599	AC/A	ATGTCCATAACGCATGCAGA	CATCATGCCACTTTGGATTG	60.1	661	INTERGENIC	--	--	--
Ca4_P1_33315743	Ca4	33315743	CTT/CTTT	ACATGAGTCTTTGTTGCCCC	TGGCACGTGATTTGTTTGAT	60.0	477	INTERGENIC	--	--	--

INDEL marker IDs	Chromosomes/ unanchored scaffolds	Physical positions (bp)	InDels (*Kabuli* reference genome-CDC Frontier/PI)	Forward primers (5'-3')	Reverse primers (5'-3')	Anneling temperature (0C)	Expected amplified product size (bp)	Structural annotation		Functional annotation	
								Sequence components of *kabuli* genome	*Kabuli* gene accession IDs	NCBI-KOG	TFs
Ca4_P1_33321015	Ca4	33321015	AATATA/AATATATA	TATTGTTGCACCCCCAATTT	ATGTCTGCCCCATTTTCTTG	60.1	229	INTERGENIC	--	--	--
Ca4_P1_33327000	Ca4	33327000	CA/C	AATATTCCCGTCGAATGTGC	TTTATTGCAACATCGCCTTTT	59.8	662	INTERGENIC	--	--	--
Ca4_P1_33327124	Ca4	33327124	ATTTTTTTTT/ATTTTTTTT	GCTACACGAAAAGCTCATTGC	TTTATTGCAACATCGCCTTTT	60.0	330	INTERGENIC	--	--	--
Ca4_P1_33327144	Ca4	33327144	CAAAAAAA/CAAAAAAAA	GCTACACGAAAAGCTCATTGC	CAATTCAAAAGGTTTTAGACGGA	60.0	379	INTERGENIC	--	--	--
Ca4_P1_33327822	Ca4	33327822	TAAAAAAAAAA/TAAAAAAAAAAAA	CGAAAATTCGAATGGGTAGC	GGTCAAGTGTTTGCCGAAAT	59.5	516	INTERGENIC	--	--	--
Ca4_P1_33327876	Ca4	33327876	TCCAAG/T	CGAAAATTCGAATGGGTAGC	AGTATGATGGGGTCATGGGA	59.5	631	INTERGENIC	--	--	--
Ca4_P1_33328529	Ca4	33328529	ATTTTTTTTTTTT/ATTTTTTTTT	TCCCATGACCCCATCATACT	TCTCACTGAATCGTCCACCTT	60.0	682	INTERGENIC	--	--	--
Ca4_P1_33329397	Ca4	33329397	TAAAAAAAA/TAAAAAAAAA	ACGTGGTCTCACGATAAGGC	CTCCATGTGAAAGTGCCAAA	60.1	552	INTERGENIC	--	--	--
Ca4_P1_33336961	Ca4	33336961	ATTTTTTTT/ATTTTTTT	CACGGAGTTTAACGATGTGAGA	CATCCCCTAACCCACTCAAA	60.2	911	INTERGENIC	--	--	--
Ca4_P1_33339958	Ca4	33339958	T/TA	TGGTGACAACAAGGTTTCCA	CCACATTTTCTCATCCACACA	60.0	437	INTERGENIC	--	--	--
Ca4_P1_33346959	Ca4	33346959	TT/TTAT	TTTTCGGTTTGTTCAAAGGC	GGAAAACATTGGGAAACCCT	60.1	766	INTERGENIC	--	--	--
Ca4_P1_33348179	Ca4	33348179	ATT/AT	AGGTTGAGCGATTGGAGAAA	TCCTAAAAGACTGCCCGCTA	59.8	378	INTERGENIC	--	--	--

INDEL marker IDs	Chromosomes/ unanchored scaffolds	Physical positions (bp)	InDels (*Kabuli* reference genome-CDC Frontier/PI)	Forward primers (5'-3')	Reverse primers (5'-3')	Anneling temperature (0C)	Expected amplified product size (bp)	Structural annotation		Functional annotation	
								Sequence components of *kabuli* genome	*Kabuli* gene accession IDs	NCBI-KOG	TFs
Ca4_P1_33353051	Ca4	33353051	TTG/TTGTG	ACCAATGCCGCTTAATTGAC	AGACGATCAATTGGTTTGCC	60.0	396	INTERGENIC	--	--	--
Ca4_P1_33357279	Ca4	33357279	CT/CTT	CATCCTTTGGGGAGAAGTGA	CCCCAATGACGAAAAATGAT	60.0	596	INTERGENIC	--	--	--
Ca4_P1_33376961	Ca4	33376961	ATTTTTTTTTTTTT/ATTTTTTTTTT	ACTCAAAGCCTGAAGAGGCA	AGAGTTTCTTGGAAACGCCA	60.1	595	INTERGENIC	--	--	--
Ca4_P1_33377078	Ca4	33377078	ACCCCC/ACCCC	ACTCAAAGCCTGAAGAGGCA	AGAGTTTCTTGGAAACGCCA	60.1	595	INTERGENIC	--	--	--
Ca4_P1_33386789	Ca4	33386789	CAAAATTAAAATTAAAATTAAA/CAAAATTAAAATTAAA	CCCTCCTTTGTGACGGTTTA	TTCGCACCTACACGATCAAG	60.0	763	INTERGENIC	--	--	--
Ca4_P1_33387360	Ca4	33387360	CAA/CAAA	TGTTGTGGCTACGCACTTTC	TGTTGTTGTTGTTTCCTTGTGA	59.9	103	INTERGENIC	--	--	--
Ca4_P1_33392985	Ca4	33392985	CAAAAAAA/CAAAAAA	TGCGCATGAATTTTACCACT	TTGTAGCTCGAAGGGCAACT	59.2	786	INTERGENIC	--	--	--
Ca4_P1_33393743	Ca4	33393743	AAGCTAGCTA/AAGCTA	AGGTGCTTTCATTGGCTGAG	TAATTTGTGGCGGTTGTGTG	60.4	439	INTERGENIC	--	--	--
Ca4_P1_33393816	Ca4	33393816	AT/ATT	AGGTGCTTTCATTGGCTGAG	TAATTTGTGGCGGTTGTGTG	60.4	439	INTERGENIC	--	--	--
Ca4_P1_33394479	Ca4	33394479	CATC/CATCGATC	CATTGGGGATTTTACAAATGG	GCCTCTCTTTTTCGCTTGTG	59.0	567	INTERGENIC	--	--	--
Ca4_P1_33397164	Ca4	33397164	TTATATATATATATATATA/TTATATATATATATATATATATA	ATAGGCTTTGCACTTCCCCT	CCCCAACAAAATGGAGCTAA	60.1	582	DRR	Ca_24022	--	--
Ca4_P1_33397720	Ca4	33397720	ATTTTTTTTTTT/ATTTTTTTTTT	CTCCATTTTGTTGGGGCTTA	GCCAACAAACAACCATGCTA	59.9	584	DRR	Ca_24022	--	--

INDEL marker IDs	Chromosomes/ unanchored scaffolds	Physical positions (bp)	InDels (*Kabuli* reference genome-CDC Frontier/PI)	Forward primers (5'-3')	Reverse primers (5'-3')	Anneling temperature (0C)	Expected amplified product size (bp)	Structural annotation		Functional annotation	
								Sequence components of *kabuli* genome	*Kabuli* gene accession IDs	NCBI-KOG	TFs
Ca4_P1_33413394	Ca4	33413394	ATTTTTTTTTT/ATTTTTTTTT	TAGAGCGGGGCTCACTTAAA	GCGGAGATTGCCATATCACT	60.0	447	INTERGENIC	--	--	--
Ca4_P1_33431052	Ca4	33431052	GAAAAAAAAAA/GAAAAAAAAA	TTTATGCTCCGTTTGTGCTG	CACACACCACCTCTCCCTTT	59.9	435	INTERGENIC	--	--	--
Ca4_P1_33431316	Ca4	33431316	GAAAAA/GAAAAATAAAAAAAA	TTTATGCTCCGTTTGTGCTG	CACACACCACCTCTCCCTTT	59.9	435	INTERGENIC	--	--	--
Ca4_P1_33467450	Ca4	33467450	CAAAAAAAAAA/CAAAAAAAAAAA	TGATGTAAAAATGATGATGTGAAA	AATTTGAGTTGCAATTTGATGA	57.1	558	INTERGENIC	--	--	--
Ca4_P1_33467899	Ca4	33467899	ATTTTTT/ATTTTTTT	TCATATCATCAAATTGCAACTCA	TGATGTTAAAAGCTACAGCAACAA	58.2	455	INTERGENIC	--	--	--
Ca4_P1_33493387	Ca4	33493387	TA/TATCCTAA	TGACCTTCTGCTGAGAAATGAA	TTGATCAGCTGCATCCAAAG	60.0	400	INTERGENIC	--	--	--
Ca4_P1_33500547	Ca4	33500547	TTAT/TT	TGCAATTCGAGTCAAAGTGATAA	GGAGCATAATTGTTGACGCA	59.8	391	INTERGENIC	--	--	--
Ca4_P1_33533176	Ca4	33533176	CAAAAAAAAAAA/CAAAAAAAAAA	GAACCAAGAGGCGATTGAAG	TCCATTTTGGGTGAACCATT	59.8	216	INTERGENIC	--	--	--
Ca4_P1_33546898	Ca4	33546898	GAAA/GAA	CTTGCTCTCCAGTTCCCAAG	AGCTTACCATCCACAAACGG	60.0	742	INTERGENIC	--	--	--
Ca4_P1_3355848	Ca4	3355848	A/AAAGGAT	GCCTTTACTCCAACAGCAGG	GAGGGTGATCAGCTTTCTCG	59.9	279	INTERGENIC	--	--	--
Ca4_P1_3356080	Ca4	3356080	AG/AAAGCCAAGCG	TCACCGGAAAGTAACTCGCT	ATCGATATTCTCCGCACCTG	59.9	622	INTERGENIC	--	--	--
Ca4_P1_33561407	Ca4	33561407	CAAA/CAA	TTTCATTTCCAATTGTGCCA	CAGCAGAGGAACCTAACCCA	59.9	704	DRR	Ca_24025	R	HD-ZIP

INDEL marker IDs	Chromosomes/ unanchored scaffolds	Physical positions (bp)	InDels (*Kabuli* reference genome-CDC Frontier/PI)	Forward primers (5'-3')	Reverse primers (5'-3')	Anneling temperature (0C)	Expected amplified product size (bp)	Structural annotation		Functional annotation	
								Sequence components of *kabuli* genome	*Kabuli* gene accession IDs	NCBI-KOG	TFs
Ca4_P1_33566311	Ca4	33566311	ATTTTTTTTTTT/ATTTTTTTTTTTTTT	TCCATCCTCCTCATCTGACC	TTGTCTCAACAATCTCCCCC	60.0	362	INTERGENIC	--	--	--
Ca4_P1_33572396	Ca4	33572396	ATTTTTTTTTTT/ATTTTTTTTTT	GTCCCTTCTCCATCCTCCTC	TTGTCTCAACAATCTCCCCC	60.0	370	INTERGENIC	--	--	--
Ca4_P1_33614721	Ca4	33614721	GAAAAAA/GAAAAAAA	GGTTGTTGATTTGATCGCAA	CAAGTTTGGAACGGTTTGTG	59.5	555	INTERGENIC	--	--	--
Ca4_P1_33615467	Ca4	33615467	ATT/ATTT	GGTCCTTGTTGAGTCCAACG	CTCAAAAACCTGAGCATGGC	60.5	646	INTERGENIC	--	--	--
Ca4_P1_33615658	Ca4	33615658	AG/A	GGTCCTTGTTGAGTCCAACG	AATGGAATAGCGGAGTGTGC	60.5	898	INTERGENIC	--	--	--
Ca4_P1_33617340	Ca4	33617340	CTTTTTTTTTT/CTTTTTTTTT	TTTTCCTAAATTTCGAGGACGA	TTTTCCCGATTTGGTCCTTT	60.1	798	INTERGENIC	--	--	--
Ca4_P1_33617796	Ca4	33617796	AAAAGAA/AAA	CCCCGATTTGGTCCTTTATT	TGCTAGTTGTTGCGACCAAA	60.0	765	INTERGENIC	--	--	--
Ca4_P1_33619158	Ca4	33619158	GTATATATATATATATATATATATATAT/GTATATATATATATATAT	GAACATGCTGAAAACGGGAT	TGAGGGCAATGACAAACAAA	59.9	122	INTERGENIC	--	--	--
Ca4_P1_33619321	Ca4	33619321	TAAAAAAAA/TAAAAA	GAACATGCTGAAAACGGGAT	CCAATTCCAAGGTCATCTGG	59.9	448	INTERGENIC	--	--	--
Ca4_P1_33620200	Ca4	33620200	TACACA/TACA	GCCATGCTACCATTTGGATT	TTACCGACGAACAAGCAGTG	59.8	603	INTERGENIC	--	--	--
Ca4_P1_33627666	Ca4	33627666	TATTTAAAATTTAAAATTTAAAATTTAAAAT/TATTTAAAATTTAAAATTTAAAAT	AGTGACATGACGGAAGTAGCA	TTGGTTCCAGTGTGCATGTT	58.4	432	INTERGENIC	--	--	--
Ca4_P1_33633372	Ca4	33633372	GTTTTTTT/GTTTTTT	ACGAGGAAACATATCGTCCG	ATGTCCACACATTGGGTGTC	60.0	726	INTERGENIC	--	--	--

INDEL marker IDs	Chromosomes/ unanchored scaffolds	Physical positions (bp)	InDels (*Kabuli* reference genome-CDC Frontier/PI)	Forward primers (5'-3')	Reverse primers (5'-3')	Anneling temperature (0C)	Expected amplified product size (bp)	Structural annotation		Functional annotation	
								Sequence components of *kabuli* genome	*Kabuli* gene accession IDs	NCBI-KOG	TFs
Ca4_P1_33714267	Ca4	33714267	T/TCTTAATC	GAACACGTTGTTAGACCGGC	TGACAAAATGGGGAGAAAGG	60.6	713	INTERGENIC	--	--	--
Ca4_P1_33714529	Ca4	33714529	ATT/ATTGTT	CGTTGTTAGACCGGCTCTTG	TGGGCTGACATAATCACCAA	60.8	940	INTERGENIC	--	--	--
Ca4_P1_33715942	Ca4	33715942	TAAAAAAAAAAAA/TAAAAAAAAAAAAAAAA	TTGGGTACGTTGGTGTAGCA	ATTGACTGGGCACCATGATT	60.0	634	INTERGENIC	--	--	--
Ca4_P1_33717821	Ca4	33717821	TAAAAAAAAAAAAAAAAAA/TAAAAAAAAAAA	CCAAAAACCCCTCTTTTTCC	CCACCAAAATTTGTCCAACC	59.8	730	INTERGENIC	--	--	--
Ca4_P1_33733806	Ca4	33733806	TT/TTTGTTTATACT	CGTTATTCCCATATGAGAGGC	TGTGATGTTGGACGTGTGTG	58.5	280	INTERGENIC	--	--	--
Ca4_P1_33736905	Ca4	33736905	ATTTTTTTT/ATTTTTTTTT	GGCTTTTCAAAGTTCATCGG	TTGAAGGATTTGAATTCCCC	59.7	789	INTERGENIC	--	--	--
Ca4_P1_33738599	Ca4	33738599	GAAAAAAAAA/GAAAAAAAAAAAAA	ATTGTTGCGCACAAATTGAA	CACCACCAAATCACAAGACG	60.1	734	INTERGENIC	--	--	--
Ca4_P1_33743516	Ca4	33743516	CAAAAAA/CAAAAA	TGTAGCTGGTGTCCACGAAG	AATGCAAGAATCCACCTTGG	59.9	435	INTERGENIC	--	--	--
Ca4_P1_33753503	Ca4	33753503	AATTATTATT/AATTATT	AGGATGTTTTTCAAGTGCCG	CTTAGCCATATGCAAGGGGA	60.1	760	INTERGENIC	--	--	--
Ca4_P1_33769585	Ca4	33769585	GGCT/GGCTGCT	TCTGTTTTTGCCATCAAGCA	TTTGGAGACCAATGGTGACA	60.4	502	INTERGENIC	--	--	--
Ca4_P1_33773434	Ca4	33773434	AC/ACC	GGATGTCTCCATGGGAATTG	GCATACAATTGCTTCTCGCA	60.1	528	INTERGENIC	--	--	--
Ca4_P1_33784625	Ca4	33784625	GTAT/GTATAT	CCGGTCTCAATTTTCCTCAA	TGTACGCTAGATTTCACTTTTGGA	60.0	440	INTERGENIC	--	--	--

**INDEL marker IDs**	**Chromosomes/ unanchored scaffolds**	**Physical positions (bp)**	**InDels (*Kabuli* reference genome-CDC Frontier/PI)**	**Forward primers (5'-3')**	**Reverse primers (5'-3')**	**Anneling temperature (0C)**	**Expected amplified product size (bp)**	**Structural annotation**		**Functional annotation**	
								**Sequence components of *kabuli* genome**	***Kabuli* gene accession IDs**	**NCBI-KOG**	**TFs**
Ca4_P1_33795008	Ca4	33795008	TTATATATATATATATAT/TTATATATATATAT	GTCGTGTCAATGCAACAACC	TCGACTAAGGACATTGGGCT	60.0	514	INTERGENIC	--	--	--
Ca4_P1_33807474	Ca4	33807474	TAAAAAAA/TAAAAAAAA	GTCATTTTGGCGGAACCTAA	TTGAGGTGGGATATGTTTGC	59.9	694	INTERGENIC	--	--	--
Ca4_P1_33813152	Ca4	33813152	TAA/TA	AGAGAAGTTGGCCGACAAAA	CTCCAATGCCCAACAAAAGT	59.9	734	INTERGENIC	--	--	--
Ca4_P1_33813355	Ca4	33813355	TAAAAAAAAAA/TAAAAAAAA	GACATATTTTGGACGCACCC	CTCCAATGCCCAACAAAAGT	60.2	200	INTERGENIC	--	--	--
Ca4_P1_33813940	Ca4	33813940	ATTT/ATTTTT	TGAAGCATGGACCAATCAAA	GTCCAATCACATGCACAAGG	60.0	483	INTERGENIC	--	--	--
Ca4_P1_33819598	Ca4	33819598	AAATAATAATAATAATAATA/AAATAATAATAATAATAATAATAATA	GCCATGTTGGCAAAATTACA	TGGAAATGGGGAGAATGGTA	59.4	389	INTRON	Ca_20371	O	--
Ca4_P1_33828542	Ca4	33828542	AATA/AATATA	TACATTGGTGCAACCTGCAT	TTGCCATGACACACCTGATT	60.0	703	INTRON	Ca_20369	Q	--
Ca4_P1_33833847	Ca4	33833847	TA/TAACTGCA	GCGTCATCGTCCCTACAAAT	TCCAACTCGCAAGGCTATCT	60.0	375	INTERGENIC	--	--	--
Ca4_P1_33835234	Ca4	33835234	TAAAAAAA/TAAAAAAAA	CCAATCAAATTGAGCATACCC	TGTGTTTGCTTTGCATGCTT	59.3	529	INTERGENIC	--	--	--
Ca4_P1_33844287	Ca4	33844287	CTAGAATA/CTAGAATAGAATA	GAGGGTTGTTTCCCAGCATA	GCCATGGTCAACGACCTACT	59.9	702	INTERGENIC	--	--	--
Ca4_P1_33844674	Ca4	33844674	TAAAAAAAAAAAA/TAAAAAAAAAAA	TCCAAAAACTGGAAAGGGAA	GCCATGGTCAACGACCTACT	59.5	211	INTERGENIC	--	--	--
Ca4_P1_33845547	Ca4	33845547	CTTTTTTTTTTTT/CTTTTTTTTTTT	CATTTAATGTTTCCCACACTTCA	TGTAGGGATGCTCAACTTGG	58.9	279	INTERGENIC	--	--	--

INDEL marker IDs	Chromosomes/ unanchored scaffolds	Physical positions (bp)	InDels (*Kabuli* reference genome-CDC Frontier/PI)	Forward primers (5'-3')	Reverse primers (5'-3')	Anneling temperature (0C)	Expected amplified product size (bp)	Structural annotation		Functional annotation	
								Sequence components of *kabuli* genome	*Kabuli* gene accession IDs	NCBI-KOG	TFs
Ca4_P1_33856380	Ca4	33856380	CTTTTTTTTTT/CTTTTTTTTTTTTT	CGGGAATTAAGAGGAG AGGG	AAAATTTAATGCCGTG ACCG	60.0	719	INTERGENIC	--	--	--
Ca4_P1_33857426	Ca4	33857426	AAAAATTAAAAT/AAAAATTAAAATTAA AAT	GTTTTGTGATGGGGGA TGAC	CAAATTTGGCGTTGTT CCTT	60.0	611	INTERGENIC	--	--	--
Ca4_P1_33865092	Ca4	33865092	AG/A	TTTGATGTTGTTGGAG ATGAAAA	CGTTTCAGAGGTGAAC CGTAA	59.5	407	INTERGENIC	--	--	--
Ca4_P1_33865441	Ca4	33865441	GTTTTTTTT/GTTTTTTTTT	TGTTTACGGTTCACCT CTGAAA	ATAACCCAGCTGAAAG GTGC	59.6	587	INTERGENIC	--	--	--
Ca4_P1_33878589	Ca4	33878589	CGTTTTCTGTTT/CGTTT	CTTTCAACAGCCTCCA AAGC	CGTGAATGGATCAGAT GTGC	60.0	748	INTERGENIC	--	--	--
Ca4_P1_33884406	Ca4	33884406	GTTTTTTTTT/GTTTTTTTTTT	AGGCTAGTGCATCATC CAGG	GTACCCCAATTTTTCC CGTT	60.2	303	INTERGENIC	--	--	--
Ca4_P1_33912297	Ca4	33912297	ATATCT/AT	AGAATCCAAATCGAAG CCCT	ACCCGCTCTAAGTCCA AAGC	60.0	606	INTERGENIC	--	--	--
Ca4_P1_33921285	Ca4	33921285	ATGCT/A	TGGGTGGTTGACACTT GAAA	CAGAGGAAAATGGGG TCAGA	60.0	560	INTRON	Ca_20365	O	GATA
Ca4_P1_33929002	Ca4	33929002	A/AC	GTGGGTTGTAAGGGCT GAAA	GCTTGCTGGAGTTTTC AAGG	60.0	664	INTRON	Ca_20365	O	GATA
Ca4_P1_33950107	Ca4	33950107	ACTCTCTCTCTCTCT/ACTCTCTCTCT CT	TATCCCTCCCACAGTT GCTT	ACAATGCCGAACATCT GTCA	59.6	721	INTRON	Ca_20365	O	GATA
Ca4_P1_33953368	Ca4	33953368	CAT/CATATCATAT	TATTTTGGGATTGTGG GCTC	AGATTAGGGCCTCAG GAACC	59.8	523	INTERGENIC	--	--	--
Ca4_P1_33960188	Ca4	33960188	TAA/TAAA	AAATAAAGGACCAGGT CGGG	TTTGGCCAGACCACCT TTAC	60.2	662	INTERGENIC	--	--	--

INDEL marker IDs	Chromosomes/ unanchored scaffolds	Physical positions (bp)	InDels (*Kabuli* reference genome-CDC Frontier/PI)	Forward primers (5'-3')	Reverse primers (5'-3')	Anneling temperature (0C)	Expected amplified product size (bp)	Structural annotation		Functional annotation	
								Sequence components of *kabuli* genome	*Kabuli* gene accession IDs	NCBI-KOG	TFs
Ca4_P1_34016739	Ca4	34016739	CTT/CT	TTGCTTTCTGAATGGCAGTG	TGGAAGGATGGGTTGAGAAG	60.0	492	INTERGENIC	--	--	--
Ca4_P1_34025530	Ca4	34025530	T/TC	ATAGCGTCGGATGGTCTTTG	GGGACATTTGAGAATTTGGG	60.1	453	DRR	Ca_20364	--	--
Ca4_P1_34026279	Ca4	34026279	TAAAAAAAAAA/TAAAAAAAAAAA	AAACAACCGCCATCCAACT	TTTCATGGACAAACAAGCCA	60.4	213	DRR	Ca_20364	--	--
Ca4_P1_34039084	Ca4	34039084	TAAAAAAAAAAA/TAAAAAAAAA	TACCAAAGTGTCGCCAACAA	AAAAAGGAAAAGCCAGCCTC	60.1	742	INTERGENIC	--	--	--
Ca4_P1_34072868	Ca4	34072868	CT/CTTT	ACCTTTAAACGGACCCTCGT	GAGGAACGAAAGCGTTTGAG	59.9	151	INTERGENIC	--	--	--
Ca4_P1_34128897	Ca4	34128897	ATTTTTTTTTT/ATTTTTTTTTTT	TAGACTTCCGATGCCACACC	ATTGCGAGCATGAGAAGTTG	61.1	100	INTERGENIC	--	--	--
Ca4_P1_34169598	Ca4	34169598	TTATATATATATATATATATATATATAT/TTATATATATATATAT	TCAAAGTGGCTCACAAGTGC	TCGACTCAAGAAGGCAAGTG	60.0	781	INTERGENIC	--	--	--
Ca4_P1_34169705	Ca4	34169705	CAATGA/CAATGATAATGA	TCAAAGTGGCTCACAAGTGC	TCGACTCAAGAAGGCAAGTG	60.0	781	INTERGENIC	--	--	--
Ca4_P1_34176341	Ca4	34176341	TT/TTAT	ATGAAACCTGGGATGCAAAG	TTCTGGTTTATTTGCCCTGC	59.9	644	INTERGENIC	--	--	--
Ca4_P1_34193644	Ca4	34193644	G/GC	GCAATCCCAATGCTAAAGGA	ACTCTTCCAAAGTAGGCGCA	60.0	249	INTERGENIC	--	--	--
Ca4_P1_34227066	Ca4	34227066	A/AG	ACGGCAAAAACCAGTGAAAT	TGAGGAAAATTGAGACCGGA	59.5	327	INTERGENIC	--	--	--
Ca4_P1_34239824	Ca4	34239824	GTTTTTTTT/GTTTTTTT	GGTTTTACGGCAACATCAGG	ACAAATCAGAGCTGGTCGCT	60.4	711	DRR	Ca_20356	L	--

INDEL marker IDs	Chromosomes/ unanchored scaffolds	Physical positions (bp)	InDels (*Kabuli* reference genome-CDC Frontier/PI)	Forward primers (5'-3')	Reverse primers (5'-3')	Anneling temperature (0C)	Expected amplified product size (bp)	Structural annotation		Functional annotation	
								Sequence components of *kabuli* genome	*Kabuli* gene accession IDs	NCBI-KOG	TFs
Ca4_P1_34240189	Ca4	34240189	CC/CCTGATGC	AGCGACCAGCTCTGATTTGT	GGGAGCTGCTTGATGAGAAG	60.0	168	DRR	Ca_20356	L	--
Ca4_P1_34246830	Ca4	34246830	ATT/AT	CGTCTTCCGTACAAAGCCTC	GATTGGGGACCATTCCATTA	59.9	320	INTERGENIC	--	--	--
Ca4_P1_34254143	Ca4	34254143	GTTTTTTTTTTT/GTTTTTTTTTTTT	TTTTGCGACATTTGATCGTT	TGTTCTCGCACACCAAAAAG	59.2	454	INTERGENIC	--	--	--
Ca4_P1_34257490	Ca4	34257490	CTTT/CT	TCCATTTCCGAGAAATCCAT	CACAAAGACCAAAGCCCAAT	59.3	490	INTERGENIC	--	--	--
Ca4_P1_34294129	Ca4	34294129	ATTTT/ATTT	CCAAGGTTGGATTCGAAGTG	TCCACTGTCTTTGCCATCAA	60.5	313	INTERGENIC	--	--	--
Ca4_P1_34319653	Ca4	34319653	ATTTTTTTT/ATTTTTTTTT	ATGTTTCCCCTTCCTTTTGG	GAAAAGGCGATAACAACCCA	60.2	762	DRR	Ca_20355	YU	--
Ca4_P1_34329441	Ca4	34329441	TAAAAAAAAAA/TAAAAAAAAA	CTCTCGAGGCGAGTTTTGTT	CTCTGGAATGACGCATTGTC	59.6	394	INTERGENIC	--	--	--
Ca4_P1_34330740	Ca4	34330740	TAAAAAAAAAAA/TAAAAAAAAAAAA	TGCACACATCTACCCCTGAA	GGCATGGCAACTTCACTTTT	60.1	502	INTERGENIC	--	--	--
Ca4_P1_34333563	Ca4	34333563	TAAAAAAAA/TAAAAAAA	ACGGTGACAAATTGGAGAGG	GGTCGCTCAACCTTTACGAG	60.0	744	INTERGENIC	--	--	--
Ca4_P1_34351448	Ca4	34351448	GAA/GA	TTTTTCCCAAGTTTGTCCTTT	AAAGTTTGTGGTCGGTCAGG	57.8	764	INTERGENIC	--	--	--
Ca4_P1_34367857	Ca4	34367857	TAAAAAAAAAAAA/TAAAAAAAAAAA	TTGGCCTTGTAAGTATCGCC	CACCTTCCTCAGGGTTTGAA	60.1	534	INTERGENIC	--	--	--
Ca4_P1_34375164	Ca4	34375164	ATTTTTTT/ATTTTTT	CAAATCTTCTTCAAATCTTGACCAT	CTATCGACCAAGCCACCATT	59.9	768	INTERGENIC	--	--	--

INDEL marker IDs	Chromosomes/ unanchored scaffolds	Physical positions (bp)	InDels (*Kabuli* reference genome-CDC Frontier/PI)	Forward primers (5'-3')	Reverse primers (5'-3')	Anneling temperature (0C)	Expected amplified product size (bp)	Structural annotation		Functional annotation	
								Sequence components of *kabuli* genome	*Kabuli* gene accession IDs	NCBI-KOG	TFs
Ca4_P1_34379819	Ca4	34379819	GAAAAACACTTAGAAAA/GA	CCCTGCAAGCCATACT TCTC	CCCAGATCAAAGCAAG CAAT	59.8	658	INTERGENIC	--	--	--
Ca4_P1_34382417	Ca4	34382417	GC/G	AAGGGGCTTTGAGGTA AGGA	TGGAAAATGGATGTTG ACGA	60.1	572	INTERGENIC	--	--	--
Ca4_P1_34388082	Ca4	34388082	CATATATATATATAT/CATATATATATA TATATATAT	GTTGGTTCCCTAGCAC CGTA	CTGTCAATTGAGTTGC GACG	60.0	484	INTERGENIC	--	--	--
Ca4_P1_34388181	Ca4	34388181	GAAA/GAAAA	GTTGGTTCCCTAGCAC CGTA	CTGTCAATTGAGTTGC GACG	60.0	484	INTERGENIC	--	--	--
Ca4_P1_34391197	Ca4	34391197	CTTTTTTTTTTT/CTTTTTTTTTTTT	TGTCATGGGGAAAATT GGTT	ATCACTTCTTTAAGCG CCGA	60.0	547	INTERGENIC	--	--	--
Ca4_P1_34393067	Ca4	34393067	ATTTTTTTTT/ATTTTTTTTTT	CATGATGCATGTCATT TGGTT	TCTATGGATTGACGGT CGTG	59.3	569	INTERGENIC	--	--	--
Ca4_P1_34413607	Ca4	34413607	TAAAAAAAAAAA/TAAAAAAAAAA	AGGCTCCCCTTGAATT TGAT	TTTCCCCCTTACTCGT TGTG	59.9	700	INTERGENIC	--	--	--
Ca4_P1_34416807	Ca4	34416807	CTTTTTTTTTTT/CTTTTTTTTTT	TGCCACTGAAAAAGCA AATG	GAATGCAACCACTATT GGGC	59.8	600	INTERGENIC	--	--	--
Ca4_P1_34458862	Ca4	34458862	CAAAA/CAAAAA	CCAAACGTTCTCCCAC AACT	GAGGATGCCAAAATGC CTAA	60.0	744	DRR	Ca_20351	--	--
Ca4_P1_34473954	Ca4	34473954	CTTTTTTT/CTTTTTTTT	ACCCAAATCCAGTGTT CTGC	GGCCTTGTCGAATGAT TGTT	60.0	839	INTERGENIC	--	--	--
Ca4_P1_34475378	Ca4	34475378	GAAAAAAA/GAAAAAAAA	CATTGTGGATGCAGTT TTGC	CGAAATAAGATGTCCA CCGC	60.1	535	INTERGENIC	--	--	--
Ca4_P1_34475759	Ca4	34475759	GAAAA/GAAA	GCGGTGGACATCTTAT TTCG	CGCATTGAAGCATAAA CCCT	60.5	446	INTERGENIC	--	--	--

INDEL marker IDs	Chromosomes/ unanchored scaffolds	Physical positions (bp)	InDels (*Kabuli* reference genome-CDC Frontier/PI)	Forward primers (5'-3')	Reverse primers (5'-3')	Anneling temperature (0C)	Expected amplified product size (bp)	Structural annotation		Functional annotation	
								Sequence components of *kabuli* genome	*Kabuli* gene accession IDs	NCBI-KOG	TFs
Ca4_P1_34482261	Ca4	34482261	GCCGCC/GCCGCCGTAAACCGCC	ACGCCATCTGCCATATGTTT	TTTGGGGTGATGGAAATGAT	60.4	497	INTRON	Ca_20350	R	G2-like
Ca4_P1_34492879	Ca4	34492879	TAAAAAAAAA/TAAAAAAAAAA	CTGATCAGTCGTTGGGAGGT	TGAGTTGATCGTTTCCACCA	60.1	538	DRR	Ca_20350	R	G2-like
Ca4_P1_34495119	Ca4	34495119	GTTTTTTTTTTT/GTTTTTTTTTTTT	TTTCTAGTGTGTGGCTCCCC	GCCTAAGAAGGGGAAGGTTG	60.1	107	INTERGENIC	--	--	--
Ca4_P1_34515993	Ca4	34515993	TC/T	CAGTATACCACCCAGGCCAT	TCACATGAATAAATTTACAAGCGAA	59.7	155	INTERGENIC	--	--	--
Ca4_P1_34517068	Ca4	34517068	TAAAAAAAAAA/TAAAAAAAAA	AAAACACATATTAAATTCACGCATC	CCACTTGCGCTCACTATGAA	58.5	253	INTERGENIC	--	--	--
Ca4_P1_34518791	Ca4	34518791	ATTTTTTT/ATTTTTT	CAGCTTCCCACCAAGGTAAA	TCGATGTGAACAATAGGCCA	60.1	463	DRR	Ca_20349	--	ERF
Ca4_P1_34525292	Ca4	34525292	TAAAA/TAAAAA	GGACAACCTGGGAAAAGTGA	CTTCGATCCGTGTAAGCCAT	59.9	212	INTERGENIC	--	--	--
Ca4_P1_34566172	Ca4	34566172	GTTTT/GTTTTT	CGCCTAAGGACTATGCAAGC	AAAATCCAACAATCTCCCCC	60.0	761	INTERGENIC	--	--	--
Ca4_P1_34566424	Ca4	34566424	ATT/AT	CCCTGAGACTCACGAAGAGG	AAAATCCAACAATCTCCCCC	60.0	612	INTERGENIC	--	--	--
Ca4_P1_34599013	Ca4	34599013	AGGGG/AGGG	TGTGCATCGAAAAGGTGTGT	AGCCTACGGAGGACCAAAAT	60.2	568	INTERGENIC	--	--	--
Ca4_P1_34599512	Ca4	34599512	TTA/T	TTGTGCCTCGAATAGGTGTG	ATTCTACGGAGGGCGAAAAT	59.7	669	INTERGENIC	--	--	--
Ca4_P1_34605005	Ca4	34605005	G/GT	AACCAATCTTGTGCATCCAA	TTGGACCAGAATAAGCCGAG	59.0	317	INTERGENIC	--	--	--

INDEL marker IDs	Chromosomes/ unanchored scaffolds	Physical positions (bp)	InDels (*Kabuli* reference genome-CDC Frontier/PI)	Forward primers (5'-3')	Reverse primers (5'-3')	Anneling temperature (0C)	Expected amplified product size (bp)	Structural annotation		Functional annotation	
								Sequence components of *kabuli* genome	*Kabuli* gene accession IDs	NCBI-KOG	TFs
Ca4_P1_34756	Ca4	34756	AATATATATATATATATATATA/AATATATATATATATATATA	ACACATGACTTCCTTTGCCC	TTGCAACAACAAAAACTGCC	60.0	673	INTERGENIC	--	--	--
Ca4_P1_34891617	Ca4	34891617	AGGGG/AGGG	GCAAATGTGTCGAAAGAGCA	CAATCAGATGGACCTTGGCT	60.0	483	INTERGENIC	--	--	--
Ca4_P1_3493500	Ca4	3493500	TGAGAGAGAGAGAGAGAGA/TGAGAGAGAGAGAGAGA	TTCACGAAATTGGTCCCTTT	TGTCCCCACTTGACAAAAAGA	59.4	529	INTERGENIC	--	--	--
Ca4_P1_34945613	Ca4	34945613	GTTTTTTTTT/GTTTTTTTT	CGCTCTTTTGGGTTTTCAAC	AAAAAGTTTGGCGGCCTTAT	59.7	384	INTERGENIC	--	--	--
Ca4_P1_34965051	Ca4	34965051	ATTTTTT/ATTTTTTT	TTGGGATTAGAGCAAGGTCG	CATGAGACCTTCATGTTGTCG	60.2	212	INTERGENIC	--	--	--
Ca4_P1_35106190	Ca4	35106190	TAAAAAAAAAAAA/TAAAAAAAAAAAAA	CGTCGCACTTCATCTTCCTT	CGAATGTTTTTCGTTTGCAG	60.4	229	INTERGENIC	--	--	--
Ca4_P1_35204590	Ca4	35204590	AAATAATAATAATAATAATAATAATAATAATAATAATAATAAT/AAATAATAATAATAATAATAATAATAATAATAATAAT	CGTGTTCCTGATATTTTGCG	TTTAATTCCAAAATGCCCCA	59.2	838	INTERGENIC	--	--	--
Ca4_P1_35237197	Ca4	35237197	TTTATTATTATTATTATTATTATTATTA/TTTATTATTATTATTATTATTATTATTATTATTATTATTATTA	TCACTGCTCACACACCAACA	TTGAAATGGGGTTGAAGGAC	59.9	276	INTERGENIC	--	--	--
Ca4_P1_35295864	Ca4	35295864	TAA/TAAA	CACGATGAATTAAATAGCATCCA	ATTCACATGGTTGTTCGCAA	59.0	239	INTERGENIC	--	--	--
Ca4_P1_35319446	Ca4	35319446	ATTTTTTTTT/ATTTTTTTTTT	GGTCAGTTCATCCTAGGGCA	TTTTTGGTTGCCTAAGTGGG	60.1	462	INTERGENIC	--	--	--
Ca4_P1_35343662	Ca4	35343662	TCCACAACC/TCCACAACCACAACC	CCACAACATGGTGCAAACTC	AACCGGTTCAGGTTCTGTTG	60.0	679	INTRON	Ca_20018	Z	NAC
Ca4_P1_35352556	Ca4	35352556	CTTTT/CTTTTT	CCATCAAAGTCCACGCCTAT	TCTCAGAGAACGACCTCCTATT	60.0	166	INTERGENIC	--	--	--

INDEL marker IDs	Chromosomes/ unanchored scaffolds	Physical positions (bp)	InDels (*Kabuli* reference genome-CDC Frontier/PI)	Forward primers (5'-3')	Reverse primers (5'-3')	Anneling temperature (0C)	Expected amplified product size (bp)	Structural annotation		Functional annotation	
								Sequence components of *kabuli* genome	*Kabuli* gene accession IDs	NCBI-KOG	TFs
Ca4_P1_35366554	Ca4	35366554	TTAGCTGATAG/TTAGCTGATAGCTGATAG	TCGAAAAACAAAAATAACCCG	GATTTACCATATACTCCTTCGCTT	59.0	498	INTERGENIC	--	--	--
Ca4_P1_35369859	Ca4	35369859	GATTAA/GATTAATTAA	TTTAGTGCGCATGAATTTTTACA	TTTCCACAAGCTAACAAAAATGA	59.7	494	INTERGENIC	--	--	--
Ca4_P1_35432013	Ca4	35432013	TTAT/TTATAT	TGTTCAACATCCACGAACAA	TGCCATACTCATGTCGGAAA	58.5	669	INTERGENIC	--	--	--
Ca4_P1_35432836	Ca4	35432836	TTATATATATATATATATATAT/TTATATATATATATATATATATAT	TTTCCGACATGAGTATGGCA	TTTGGAAAGTAACACTGCACG	60.1	785	INTERGENIC	--	--	--
Ca4_P1_35432970	Ca4	35432970	CATATATATATATATATATATATATATATA/CATATATATATATATATATATATATATATATATA	AAAAATGTGCATAAATTCGGTT	TTTGGAAAGTAACACTGCACG	57.7	428	INTERGENIC	--	--	--
Ca4_P1_35433291	Ca4	35433291	T/TATTATATATATATATA	CACGTGCAGTGTTACTTTCCA	TGACGAATTTCAAAAACCTGC	59.8	491	INTERGENIC	--	--	--
Ca4_P1_35440550	Ca4	35440550	AATATATATATATATATATATATATATATATAT/AATATATATATATATATATATATATATATAT	GGATTGTAAACGGTTTTAGCG	GGCTTGATGTGAATGTGCAA	58.7	766	INTERGENIC	--	--	--
Ca4_P1_35457008	Ca4	35457008	TTGC/T	AATTGCAGAAAAATGCGGTC	TTGTACCAGATATTGGCCCG	60.1	776	INTERGENIC	--	--	--
Ca4_P1_35620689	Ca4	35620689	TGA/TGAGA	ACAATCCCGACACAAAGTCA	GAAGCGAGAGGTAAGCGATG	59.0	499	INTERGENIC	--	--	--
Ca4_P1_35639160	Ca4	35639160	ATT/ATTT	GGAAAAGATGGTATTTGCCG	CTTCCCTGCTGGCAACTTAG	59.4	339	INTERGENIC	--	--	--
Ca4_P1_35695664	Ca4	35695664	GCC/GC	ACACGGGATGCTCAAAAGAC	CAGCCTGAAGAACCCATGTT	60.1	493	INTRON	Ca_22447	--	SAP
Ca4_P1_35708239	Ca4	35708239	GTTTTTT/GTTTTT	AAAGGACCAAGTCGGAAAAA	TGCTTCAAGATCAATCTGCG	58.7	578	INTERGENIC	--	--	--

INDEL marker IDs	Chromosomes/ unanchored scaffolds	Physical positions (bp)	InDels (*Kabuli* reference genome-CDC Frontier/PI)	Forward primers (5'-3')	Reverse primers (5'-3')	Anneling temperature (0C)	Expected amplified product size (bp)	Structural annotation		Functional annotation	
								Sequence components of *kabuli* genome	*Kabuli* gene accession IDs	NCBI-KOG	TFs
Ca4_P1_35720300	Ca4	35720300	TTATTATTATTATTATTATTATTATTATTATTATTATT/TTATTATTATTATTATTATTATTATTATTATTATTATT	GCTATAGCTGGCGACTCCAC	ACTCTTCCAAAGTAGGCGCA	60.0	688	INTERGENIC	--	--	--
Ca4_P1_35818039	Ca4	35818039	AAATAATAATAATAATA/AAATAATAATAATA	GTTCATAACGAAGATGACAATAACA	TGCAGTCAAACTTTAAAAGGAAG	57.4	728	INTERGENIC	--	--	--
Ca4_P1_35822043	Ca4	35822043	GTTT/GTTTT	CGGTCCATATTAATCGGGTG	GACTTTTCGAGCGATTTTCG	60.0	954	INTERGENIC	--	--	--
Ca4_P1_35822348	Ca4	35822348	TGG/TG	GATCAAATCGAGGACCGAAA	CGAACAAAACGATGGGAAGT	60.0	695	INTERGENIC	--	--	--
Ca4_P1_35910460	Ca4	35910460	TTATATATATATATATATATATATATATATATATATA/TTATATATATATATATATATATATATATATATATATATATATA	TGTCAACATTCAGGGAAGCA	GATGCAAGATGACATGGTGG	60.2	536	INTRON	Ca_14861	R	--
Ca4_P1_35924136	Ca4	35924136	AATATATATATATATATATATA/AATATATATATATATATATATATA	TTGTTGTTGCAATTTAAGCGA	ATGAAAACAACTTCACGCCC	59.4	224	INTERGENIC	--	--	--
Ca4_P1_35999977	Ca4	35999977	ATTTTTTTTTT/ATTTTTTTTTTT	GCTGGCAGCACAAATAAACA	TCGAACCAATGACCTCACAA	59.9	439	INTERGENIC	--	--	--
Ca4_P1_36079126	Ca4	36079126	TTGTG/TTGTGTG	ATTTTGATGCTCACGGTTCC	CGGAGATGATGGTTCAGGTT	59.9	595	INTERGENIC	--	--	--
Ca4_P1_36094744	Ca4	36094744	TAAAAAAAAAAA/TAAAAAAAAAA	CCTACCATTGCCTGACCTGT	TTCCGAGTTGTTTGAGAGGA	60.0	427	INTERGENIC	--	--	--
Ca4_P1_36130777	Ca4	36130777	AATATATATATATATATATATATATATATATATATATA/AATATATATATATATATATATATATATATATA	GAATTCAAAAATAAGGCATCATCA	AAATGGGTTAAAGGGACCGT	59.4	332	INTERGENIC	--	--	--
Ca4_P1_36245892	Ca4	36245892	TTATATATATATATATATATATATAT/TTATATATATATATATATATAT	TACATGCAAGCGAAATGGAA	AGGCATGGTGTTGGAGTCAT	60.2	287	DRR	Ca_14846	O	--
Ca4_P1_36256375	Ca4	36256375	ATTTTTTTTTT/ATTTTTTTTTTT	GATCATCGAGTTTCCCGTGT	AGTTCTGAGGGAGGCTCACA	59.9	350	INTERGENIC	--	--	--

INDEL marker IDs	Chromosomes/ unanchored scaffolds	Physical positions (bp)	InDels (*Kabuli* reference genome-CDC Frontier/PI)	Forward primers (5'-3')	Reverse primers (5'-3')	Anneling temperature (0C)	Expected amplified product size (bp)	Structural annotation		Functional annotation	
								Sequence components of *kabuli* genome	*Kabuli* gene accession IDs	NCBI-KOG	TFs
Ca4_P1_36299251	Ca4	36299251	ATTTT/ATT	AACCCAAGAGCGCACATATT	GAGCCAAACACTGCATCAGA	59.6	444	INTERGENIC	--	--	--
Ca4_P1_36301551	Ca4	36301551	ATTTTTTTTTTT/ATTTTTTTTTTTTT	CCGGGAGATATGTAGGAGCA	ATACTTTTTGTCGCGGCCTA	60.1	400	INTERGENIC	--	--	--
Ca4_P1_36310356	Ca4	36310356	ATTTT/ATTTTT	TTGATTCTTTTTAAAATTTTGCTCA	TTGGTGCGTTTCTCTAAACA	58.5	765	INTERGENIC	--	--	--
Ca4_P1_36318036	Ca4	36318036	T/TGTTG	TTTATGAGACAATTGTGTGGAGGT	ATGCATGCACGAATGTCATC	59.8	522	INTERGENIC	--	--	--
Ca4_P1_36335313	Ca4	36335313	ATTTTTT/ATTTTTTT	TTGCGACAAGATCAAGCAAC	TCAACACCAAACACAAGGGA	60.0	519	INTERGENIC	--	--	--
Ca4_P1_36336918	Ca4	36336918	TAAA/TATAAAA	ACAACAAGGGTGACCTCCAC	TGCTTGTCTATTCTGCGTCAA	59.9	736	INTERGENIC	--	--	--
Ca4_P1_36337139	Ca4	36337139	GC/G	TGCAATGTCAATATGAACCAAA	CCACAACCACTCATCACGAC	58.9	479	INTERGENIC	--	--	--
Ca4_P1_36337338	Ca4	36337338	TT/TTATTAT	TGACGCAGAATAGACAAGCAA	CCACAACCACTCATCACGAC	59.6	341	INTERGENIC	--	--	--
Ca4_P1_36392409	Ca4	36392409	T/TCTAC	TAAAGGACCAAATCGGGAGA	GGCCGAACCAAAGTCTACTG	59.5	438	INTERGENIC	--	--	--
Ca4_P1_36472761	Ca4	36472761	TAAAAAAAAA/TAAAAAAAAAA	CGTAGGACAGAAACCGTGGTA	TATGTAGCCCGTGTCAAGCA	60.0	679	INTERGENIC	--	--	--
Ca4_P1_36478044	Ca4	36478044	AATATA/AATA	AAAGTGATAACCGATGAGTGACAA	AAGGCTCCACATGAACAAGG	59.9	706	INTERGENIC	--	--	--
Ca4_P1_36503147	Ca4	36503147	CAAAAAAA/CAAAAAAAA	TCATTAAAGAAACACTAGACGGCA	AAGTTGGTCGGTTCATCCTG	60.2	463	INTERGENIC	--	--	--

INDEL marker IDs	Chromosomes/ unanchored scaffolds	Physical positions (bp)	InDels (*Kabuli* reference genome-CDC Frontier/PI)	Forward primers (5'-3')	Reverse primers (5'-3')	Anneling temperature (0C)	Expected amplified product size (bp)	Structural annotation		Functional annotation	
								Sequence components of *kabuli* genome	*Kabuli* gene accession IDs	NCBI-KOG	TFs
Ca4_P1_36529935	Ca4	36529935	TTATATATATATATATATATATATATATATA/TTATATATATATATATATATATATATATATA	TGGTGTAATTTGATTTCTTCCCT	TCTGGAAAAACTTGAACCACAA	58.9	523	INTERGENIC	--	--	--
Ca4_P1_36530142	Ca4	36530142	TAAAAAAAAA/TAAAAAAAA	TTGGTGTAATTTGATTTCTTCCC	TGAACTGTGTAGCCAAAAACG	59.3	778	INTERGENIC	--	--	--
Ca4_P1_36593028	Ca4	36593028	TAAAAAAAAAAA/TAAAAAAAAAAAA	CGCGGGGCATATTCTAGTTA	TGATTTGGCATTGTGTTTGC	60.1	527	INTERGENIC	--	--	--
Ca4_P1_36651037	Ca4	36651037	ACAAAATTGAGCTTCAAACCAAAATTG/ACAAAATTGAGCTTCAAACCAAAATTGAGCTTCAAACCAAAATTG	GCACATGGCTGGCTCTACTA	ATGGGACCAGTGATGTTGGT	59.1	675	INTERGENIC	--	--	--
Ca4_P1_36690853	Ca4	36690853	CTTTT/CTTT	TTGACAAGCGCTGTGAAATC	CAATGTGTATTCAACGACCGA	60.0	538	INTERGENIC	--	--	--
Ca4_P1_36731427	Ca4	36731427	CATATATATATATATATATATATATATATATAT/CATATATATATATATATATATATATATATATAT	AGGAAGAGTGGAGTTGCGAA	ATGTTTTGGATTTTGGCTCG	60.0	531	INTERGENIC	--	--	--
Ca4_P1_36779245	Ca4	36779245	TATAATAATAATAATAATAATAATAATAATAATAATAA/TATAATAATAATAATAATAATAATAATAATAATAA	AGCTTAGTTCAACATGGTCCG	CGGTCGACATATGGGTCAG	59.3	621	INTERGENIC	--	--	--
Ca4_P1_36854161	Ca4	36854161	GCA/GCATCA	TGTTCATGATGCTTCCTCTCC	CCAAACGATTATCCAACCCT	60.2	437	INTERGENIC	--	--	--
Ca4_P1_36906995	Ca4	36906995	CCTCCTCTCCTCT/CCTCCTCT	AACGGAGGATACAACAACGC	TTGGAAAATGGAAACCAAGG	60.0	629	INTERGENIC	--	--	--
Ca4_P1_36919128	Ca4	36919128	GTTTATTTATTTATTTATTTATTTATTTATT/GTTTATTTATTTATTTATTTATTTATTT	CCCAATCCACTTCCTTTTGA	CTTCAACTTGAGGCTGCACA	59.9	558	INTRON	Ca_14799	R	Trihelix
Ca4_P1_36928403	Ca4	36928403	TAAAAAAAAAA/TAAAAAAAAA	TTCAAAAATTGAAAACAATACCCA	TTGGAAAGCTTATTCTCGCA	59.7	548	DRR	Ca_14798	--	--
Ca4_P1_36952818	Ca4	36952818	TT/TTGAACT	GGGTTCATGTTTGAAGGGTG	ATGGGCCCTCTTTTGTCTTT	60.2	846	INTRON	Ca_14797	R	C2H2

INDEL marker IDs	Chromosomes/ unanchored scaffolds	Physical positions (bp)	InDels (*Kabuli* reference genome-CDC Frontier/PI)	Forward primers (5'-3')	Reverse primers (5'-3')	Anneling temperature (0C)	Expected amplified product size (bp)	Structural annotation		Functional annotation	
								Sequence components of *kabuli* genome	*Kabuli* gene accession IDs	NCBI-KOG	TFs
Ca4_P1_36953596	Ca4	36953596	AATATATATAT/AATATATATATATATAT	AAAAGAGGGCCCATGAGATT	TGTGCATTGGGAACAACACT	59.9	693	INTRON	Ca_14797	R	C2H2
Ca4_P1_36955326	Ca4	36955326	TTAAAATAAAA/TTAAAA	CACATGTTGCCTCTGGACAC	GGAGGAATCAACCTGGAACA	60.2	671	INTRON	Ca_14797	R	C2H2
Ca4_P1_36955749	Ca4	36955749	ATTTTTTTTTTTTTTT/ATTTTTTTTTTTTTT	TGTTCCAGGTTGATTCCTCC	TCTTCTTGGTGTTTTCTGGGA	59.9	432	INTERGENIC	--	--	--
Ca4_P1_36956074	Ca4	36956074	TAAAAAAAA/TAAAAAAAAAAAA	TGTTCCAGGTTGATTCCTCC	TGATGGCAGGGAAAATAAGG	59.9	963	INTERGENIC	--	--	--
Ca4_P1_36956595	Ca4	36956595	GTTTTTTTTT/GTTTTTTTTTTTT	CCTTATTTTCCCTGCCATCA	CTTGCATGCGTTCAATGTGT	59.9	467	INTERGENIC	--	--	--
Ca4_P1_36957693	Ca4	36957693	GTT/GTTT	GCCGAAAAAGTCCTCACAAA	GCGGCTAGAAGTATCAACGG	60.2	381	INTERGENIC	--	--	--
Ca4_P1_36959229	Ca4	36959229	TT/TTAT	GGACTTTTGTCGTCGGGTAA	AAAAGTCTGCCGCAAATGAT	60.0	412	INTERGENIC	--	--	--
Ca4_P1_36968194	Ca4	36968194	TAAAAAAAA/TAAAAAAAAA	GGCGAAAGTACCCAAACAAA	GACTTCTGCAAAGGACCTCG	60.0	426	INTERGENIC	--	--	--
Ca4_P1_36968275	Ca4	36968275	G/GTTAATAC	GGCGAAAGTACCCAAACAAA	GACTTCTGCAAAGGACCTCG	60.0	426	INTERGENIC	--	--	--
Ca4_P1_36968637	Ca4	36968637	TAAAAA/TAAAAAAA	TGGGGTACAAGAAAGAACCG	TTTTTGGCCCATGTAAATATCA	60.0	670	INTERGENIC	--	--	--
Ca4_P1_36969491	Ca4	36969491	TAAAAAAAAAAA/TAAAAAAAAAA	CATGGGCCAAAAATAAAAAGA	GAGCTGTGGAGAAGAAAAGCA	59.0	814	INTERGENIC	--	--	--
Ca4_P1_36969837	Ca4	36969837	CTTTTTTTTTT/CTTTTTTTTTTT	AGGTCATGCCATCAAAGACA	GAGCTGTGGAGAAGAAAAGCA	59.1	145	INTERGENIC	--	--	--

INDEL marker IDs	Chromosomes/ unanchored scaffolds	Physical positions (bp)	InDels (*Kabuli* reference genome-CDC Frontier/PI)	Forward primers (5'-3')	Reverse primers (5'-3')	Anneling temperature (0C)	Expected amplified product size (bp)	Structural annotation		Functional annotation	
								Sequence components of *kabuli* genome	*Kabuli* gene accession IDs	NCBI-KOG	TFs
Ca4_P1_36971752	Ca4	36971752	AAAAAGAAAA/AAAAA	TCTTTCAGTGGTCGCATCTCT	TGTCGCATTTTGTTGGTTGT	60.0	535	INTERGENIC	--	--	--
Ca4_P1_36972358	Ca4	36972358	CTTT/CTT	ACAACCAACAAAATGCGACA	AGCCTTGAGCGACCACTTTA	60.0	484	INTERGENIC	--	--	--
Ca4_P1_36972412	Ca4	36972412	GTT/GTTT	ACAACCAACAAAATGCGACA	TTGAAACATTCTCAGCCTTGTT	60.0	564	INTERGENIC	--	--	--
Ca4_P1_36972540	Ca4	36972540	AATATATATATATA/AATATATATATATATATATATA	TAAAGTGGTCGCTCAAGGCT	TCTACATTCAAATTGGGGGC	60.0	608	INTERGENIC	--	--	--
Ca4_P1_37007674	Ca4	37007674	AGGG/AGG	ATTGCAACTGAGGACAACCC	TGACCATTTAACGGTGACCA	60.0	643	INTERGENIC	--	--	--
Ca4_P1_37008544	Ca4	37008544	GAA/GA	GCCATTGAAGAATCCCAAGA	TGAGCTCATTGTTGCCAGAC	60.0	868	INTERGENIC	--	--	--
Ca4_P1_37033445	Ca4	37033445	CACAACAA/CACAACAACAA	CGTGGACTCCAAGTGATGAA	TCCAGCCATTGATAACCTCC	59.7	865	INTERGENIC	--	--	--
Ca4_P1_37034116	Ca4	37034116	TGGA/TGGAGGA	GGAGGTTATCAATGGCTGGA	TGGAGTGGAAAGGAATGGAG	59.9	536	INTERGENIC	--	--	--
Ca4_P1_37034691	Ca4	37034691	ATTTTT/ATTTT	CTCCATTCCTTTCCACTCCA	AGGGAAAACAAGGGTTTTGG	60.0	522	INTERGENIC	--	--	--
Ca4_P1_37035676	Ca4	37035676	GTAATAATAATAATAA/GTAATAATAATAATAATAA	TCAAACCCAATGAAAGGCTC	TCCCTTTACCTTGCCTCATTT	60.1	640	INTERGENIC	--	--	--
Ca4_P1_37035741	Ca4	37035741	TCC/TC	CCCAATGAAAGGCTCACAAT	TCCCTTTACCTTGCCTCATTT	59.9	635	INTERGENIC	--	--	--
Ca4_P1_37038560	Ca4	37038560	TGAGA/TGAGAGA	TGGTTTATAACACCGCTTATATTCA	AGGATTCAACCACAAAGGACA	59.0	242	INTERGENIC	--	--	--

INDEL marker IDs	Chromosomes/ unanchored scaffolds	Physical positions (bp)	InDels (*Kabuli* reference genome-CDC Frontier/PI)	Forward primers (5'-3')	Reverse primers (5'-3')	Anneling temperature (0C)	Expected amplified product size (bp)	Structural annotation		Functional annotation	
								Sequence components of *kabuli* genome	*Kabuli* gene accession IDs	NCBI-KOG	TFs
Ca4_P1_37038890	Ca4	37038890	GATATATATATATATATAT/GATATATA TATAT	TGTCCTTTGTGGTTGA ATCCT	TCGTACAATGTCCGGA TAAAA	59.4	724	INTERGENIC	--	--	--
Ca4_P1_37039202	Ca4	37039202	TAAAAA/TAAAA	CAATCTTTCTTTCAATT CTCTACAAA	TGATTCTTGACACGGT GGAA	57.5	644	INTERGENIC	--	--	--
Ca4_P1_37039222	Ca4	37039222	TGGG/TG	CAATCTTTCTTTCAATT CTCTACAAA	TGATTCTTGACACGGT GGAA	57.5	644	INTERGENIC	--	--	--
Ca4_P1_37039540	Ca4	37039540	TAAAAAAAAAAA/TAAAAAAAA	TTTATTGACGAGCATC GTTCC	TGGCAAGTGCAACCAA AATA	60.1	386	INTERGENIC	--	--	--
Ca4_P1_37040997	Ca4	37040997	GAAAAAAAAA/GAAAAAAAA	TTCACAAACTGGGTGG ACAA	TTGTTGGAGTATGCCC TTCC	60.0	143	INTERGENIC	--	--	--
Ca4_P1_37044142	Ca4	37044142	TAAAAAA/TAAAAAAA	TGATTGTTTTGATCCC CTCA	TCACGAAATTAGCCCT CCAA	58.9	328	INTERGENIC	--	--	--
Ca4_P1_37045211	Ca4	37045211	AAATACAA/AAA	ACCTCTTGTACCCAAC CCAA	CAGCTGGGACAAGGG AGTAT	59.3	503	INTERGENIC	--	--	--
Ca4_P1_37047512	Ca4	37047512	CAAA/CA	GAAGTGGGAAATGGGT TTGA	ATGATTGTGCCTCGTG AACA	59.8	662	INTERGENIC	--	--	--
Ca4_P1_37047776	Ca4	37047776	GC/GCC	ATACTCTGCACCACAC ACGG	ATGATTGTGCCTCGTG AACA	59.6	241	INTERGENIC	--	--	--
Ca4_P1_37053258	Ca4	37053258	T/TTTA	TTGAAGGAACTTTTCC GCTG	ATTCCCATCCCTACAG CCTT	60.4	827	INTERGENIC	--	--	--
Ca4_P1_37053592	Ca4	37053592	ATTTTTTTT/ATTTTTTT	AAATTTTTAAGGCAAAA GCTTACA	ATTCCCATCCCTACAG CCTT	57.9	221	INTERGENIC	--	--	--
Ca4_P1_37055356	Ca4	37055356	TAAAAAA/TAAAAAAAAA	TTCAAACACACTCCCT CCAA	AAACACAATACACTGT CCGGC	59.1	772	INTERGENIC	--	--	--

INDEL marker IDs	Chromosomes/ unanchored scaffolds	Physical positions (bp)	InDels (*Kabuli* reference genome-CDC Frontier/PI)	Forward primers (5'-3')	Reverse primers (5'-3')	Anneling temperature (0C)	Expected amplified product size (bp)	Structural annotation		Functional annotation	
								Sequence components of *kabuli* genome	*Kabuli* gene accession IDs	NCBI-KOG	TFs
Ca4_P1_37055534	Ca4	37055534	ATTTTT/ATTTT	TGCTTTAAAAGAGTGAGAGGGAA	AAACACAATACACTGTCCGGC	59.5	598	INTERGENIC	--	--	--
Ca4_P1_37055713	Ca4	37055713	A/AT	GCCGGACAGTGTATTGTGTTT	CCATATGCCACCATTTCCTT	59.9	552	INTERGENIC	--	--	--
Ca4_P1_37057248	Ca4	37057248	TAAA/TAA	TGCACAGTCAATCCTCTTCTG	TCATGTGTGTATGGAGCACAAA	59.0	474	INTERGENIC	--	--	--
Ca4_P1_37057959	Ca4	37057959	TA/T	TTTGTGCTCCATACACACATGA	CACCGTCTTCAAAGAATTCCA	60.0	771	INTERGENIC	--	--	--
Ca4_P1_37069003	Ca4	37069003	ATGTTGT/ATGT	TGAGCCATCCTTCAAAATCA	ATTGACCTCAGAATGTCGGG	59.2	267	INTERGENIC	--	--	--
Ca4_P1_37074535	Ca4	37074535	TCC/TCCC	TAAGAGAACCGTCGCAATCC	AAATCAATCGTTTGGGCTACA	60.2	946	INTERGENIC	--	--	--
Ca4_P1_37076395	Ca4	37076395	A/AGT	TTCCAAATTGCAAAACATGC	TGGTATTGTGGTGGCAAAGA	59.5	370	INTERGENIC	--	--	--
Ca4_P1_37082708	Ca4	37082708	GCT/G	GAAGTTGTTTGGGTTGGCAT	ATTGCAAATTGAGAGCCCAG	59.8	391	INTERGENIC	--	--	--
Ca4_P1_37082858	Ca4	37082858	GTTTTTTTTTTT/GTTTTTTTT	GAAGTTGTTTGGGTTGGCAT	ATTGCAAATTGAGAGCCCAG	59.8	391	INTERGENIC	--	--	--
Ca4_P1_37083111	Ca4	37083111	ATTTT/ATTT	GAAGTTGTTTGGGTTGGCAT	TTTGCCATCTGGAGTCTTGA	59.8	798	INTERGENIC	--	--	--
Ca4_P1_37087788	Ca4	37087788	TAAAAA/TAAAA	TTCGAATGTAGTTTGATCCCTTC	CAAAGGCCTAAACGGTGACT	59.5	588	INTERGENIC	--	--	--
Ca4_P1_37094096	Ca4	37094096	TA/T	CACTAATTGCCATGTAGGCTTTT	AGCTACGGCAATCAAGGAGA	59.6	755	INTERGENIC	--	--	--

INDEL marker IDs	Chromosomes/ unanchored scaffolds	Physical positions (bp)	InDels (*Kabuli* reference genome-CDC Frontier/PI)	Forward primers (5'-3')	Reverse primers (5'-3')	Anneling temperature (0C)	Expected amplified product size (bp)	Structural annotation		Functional annotation	
								Sequence components of *kabuli* genome	*Kabuli* gene accession IDs	NCBI-KOG	TFs
Ca4_P1_37098679	Ca4	37098679	GAAGATTCGATTAAGATT/GAAGATT	GTGCTATTGAGTGGTGCCAA	TCTTCTTCTTCTTCAGCCTTGA	59.7	734	INTERGENIC	--	--	--
Ca4_P1_37099831	Ca4	37099831	TAAAAAAA/TAAAAAA	TGAATAATTCGCGTACGGCT	TGTGAGGAGCTCTTTGTGGA	60.6	589	INTERGENIC	--	--	--
Ca4_P1_37101492	Ca4	37101492	GAAAAA/GAAAAAAA	TCAACTCAACCCGAGACACA	TCGACTCCCGAACTCAAAAT	60.3	171	INTERGENIC	--	--	--
Ca4_P1_37101687	Ca4	37101687	GAAAAAAAA/GAAAAAAAAAAA	TCAACTCAACCCGAGACACA	CACCGGGAGATACGTAGGAG	60.3	389	INTERGENIC	--	--	--
Ca4_P1_37105524	Ca4	37105524	AA/AACA	TTCAAATGGTCTCTCACCCC	ACGAAGGTCAAGCTCAAGGA	59.9	855	INTERGENIC	--	--	--
Ca4_P1_37108037	Ca4	37108037	ATTTTTTTTTT/ATTTTTTTTT	CAATCCTTCCAAGGGATGAA	TCATTTCTGCCTCAAACACAA	59.9	329	INTERGENIC	--	--	--
Ca4_P1_37108408	Ca4	37108408	A/AATAT	GATAGGATTTGAATGCGGGA	TCATGCTGGCATGTTATGCT	59.9	745	INTERGENIC	--	--	--
Ca4_P1_37117738	Ca4	37117738	CGG/CG	TACGGAAATCGGCTGGATAG	GCACGATAAATGCAACCCTT	60.1	496	INTERGENIC	--	--	--
Ca4_P1_37117919	Ca4	37117919	AT/A	TACGGAAATCGGCTGGATAG	GCACGATAAATGCAACCCTT	60.1	496	INTERGENIC	--	--	--
Ca4_P1_37118197	Ca4	37118197	ATTTTTTT/ATTTTTT	AAGGGTTGCATTTATCGTGC	CCAGCTCGTTTGTTTGAAGA	60.0	237	INTERGENIC	--	--	--
Ca4_P1_37118546	Ca4	37118546	ATT/AT	CCGATAAGCAGATGAAAGGG	TTTAGGAGCGATCAATGCAA	59.7	704	INTERGENIC	--	--	--
Ca4_P1_37118632	Ca4	37118632	ATT/ATTT	TCGTGCGCACTTAAAAATGA	TTTAGGAGCGATCAATGCAA	60.4	675	INTERGENIC	--	--	--

INDEL marker IDs	Chromosomes/ unanchored scaffolds	Physical positions (bp)	InDels (*Kabuli* reference genome-CDC Frontier/PI)	Forward primers (5'-3')	Reverse primers (5'-3')	Anneling temperature (0C)	Expected amplified product size (bp)	Structural annotation		Functional annotation	
								Sequence components of *kabuli* genome	*Kabuli* gene accession IDs	NCBI-KOG	TFs
Ca4_P1_37119880	Ca4	37119880	CTT/CTTCTTT	TGGGAAAAATTTGCACAACA	ACGAAAATTCATGGCTGTCC	59.9	718	INTERGENIC	--	--	--
Ca4_P1_37119914	Ca4	37119914	TTATA/TTATATA	TGGGAAAAATTTGCACAACA	ACGAAAATTCATGGCTGTCC	59.9	718	INTERGENIC	--	--	--
Ca4_P1_37122992	Ca4	37122992	ATTTTTTT/ATTTTTTTT	GATGGGACGGAGCTAAATGA	AATTGAGACCCTCTTGCGTG	60.0	195	INTERGENIC	--	--	--
Ca4_P1_37128543	Ca4	37128543	TAAAAA/TAAAA	TCGCACTTTTGGAGATTGAA	TGCATCCATCGTCAAATACAA	59.4	462	INTERGENIC	--	--	--
Ca4_P1_37130048	Ca4	37130048	ATTTTTTTTTT/ATTTTTTTTTTT	TTGCAGAAGGTTCCCAAATC	TGTTGGCCATCTTTTAGCTTT	60.1	841	INTERGENIC	--	--	--
Ca4_P1_37131217	Ca4	37131217	TC/T	TTGTGACCAAAGGTTGGAGG	AATTTGCGAGCATTACCTGC	60.9	710	INTERGENIC	--	--	--
Ca4_P1_37131327	Ca4	37131327	TAA/TAATAGTTTAAAAA	CAACTCAAACCACCTTCATTCA	AATTTGCGAGCATTACCTGC	60.0	633	INTERGENIC	--	--	--
Ca4_P1_37131533	Ca4	37131533	TAA/TAAA	GACTCGCAGGTAATGCTCG	TTCCACGATTTTCCCTTCAC	59.6	472	INTERGENIC	--	--	--
Ca4_P1_37131620	Ca4	37131620	A/AT	GACTCGCAGGTAATGCTCG	TTCCACGATTTTCCCTTCAC	59.6	472	INTERGENIC	--	--	--
Ca4_P1_37131804	Ca4	37131804	TA/T	GACTCGCAGGTAATGCTCG	TTCCACGATTTTCCCTTCAC	59.6	472	INTERGENIC	--	--	--
Ca4_P1_37131820	Ca4	37131820	TT/TTAT	GACTCGCAGGTAATGCTCG	TTCCACGATTTTCCCTTCAC	59.6	472	INTERGENIC	--	--	--
Ca4_P1_37311820	Ca4	37311820	GATATATATATATATATATATATA/GATATATATATATATATATATATATA	CGCTGTAAAATGCATGTGCT	TTGGGTGAACCATTAGGCAT	59.9	528	INTERGENIC	--	--	--

INDEL marker IDs	Chromosomes/ unanchored scaffolds	Physical positions (bp)	InDels (*Kabuli* reference genome-CDC Frontier/PI)	Forward primers (5'-3')	Reverse primers (5'-3')	Anneling temperature (0C)	Expected amplified product size (bp)	Structural annotation		Functional annotation	
								Sequence components of *kabuli* genome	*Kabuli* gene accession IDs	NCBI-KOG	TFs
Ca4_P1_37341946	Ca4	37341946	ATTTTTTTTTTTTT/ATTTTTTTTTTT	TTTCTCGCAGCTTTTTCCAT	TGTGGAGCATTTGCTTTCTG	60.0	703	INTRON	Ca_15142	--	--
Ca4_P1_37347347	Ca4	37347347	TTCTCTCTCTCTCTCTCTCTCTCTCTCTCT/TTCTCTCTCTCTCTCTCTCTCTCTCTCTCTCTCTCTCTCTCTCTCT	TTTGGCTTCCCAACAAAAAC	CAGTGTTGTGTCCGGTGTTC	60.0	311	INTRON	Ca_15141	S	--
Ca4_P1_37384193	Ca4	37384193	AATATATATATATATATATATATATATATATA/AATATATATATATATATATATATATATATATATATATA	GCACTGCAACAATCTTCCAA	ACCGTCCATCAGGGTCTATG	59.8	568	INTERGENIC	--	--	--
Ca4_P1_37397482	Ca4	37397482	AATATATATATATATATATATATA/AATATATATATATATATATATATATATA	TTTGTCTCCGCTTCAAATCA	GCTTATGCATGCCCTTCATT	59.4	429	DRR	Ca_15137	R	MYB
Ca4_P1_37443931	Ca4	37443931	CT/CTAT	CCGCATTTTCTTCATTAGGG	ACGTTAAGGCATGAGTTGGG	59.5	364	INTERGENIC	--	--	--
Ca4_P1_37444024	Ca4	37444024	TTAATAATAATAATAA/TTAATAATAATAATAATAA	CCGCATTTTCTTCATTAGGG	ACGTTAAGGCATGAGTTGGG	59.5	364	INTERGENIC	--	--	--
Ca4_P1_37515833	Ca4	37515833	ATTTTTTTTTT/ATTTTTTTTT	CGTGGATTGTTTTGTTGTCG	GGGCATGTGAGAGGTTTGTT	60.0	529	DRR	Ca_15127	--	--
Ca4_P1_37519504	Ca4	37519504	CTATTTATTTATTTATTTATTT/CTATTTATTTATTTATTT	GAAAAAGGAGTTGCCACCAA	TGGGGATACGTAGGAGCAAG	60.1	306	INTERGENIC	--	--	--
Ca4_P1_37659042	Ca4	37659042	TAAAAAAA/TAAAAAA	CTTTTATGTCAAACCGGCGT	TTTTTGAAGAGCGTCGATGA	60.0	486	INTRON	Ca_15114	T	TALE
Ca4_P1_37701400	Ca4	37701400	ATTTTTTTTTT/ATTTTTTTTTTTT	TGAACACCGCATTAATTGGA	GGGTTTTTGTTCGAAGATTTTG	59.9	262	DRR	Ca_15111	F	--
Ca4_P1_37704299	Ca4	37704299	T/TCAAA	AGGAGGGATCAAACCGAACT	TGTTGCTGCGTCTTGAAATC	59.9	472	INTERGENIC	--	--	--
Ca4_P1_37705943	Ca4	37705943	GA/GATAA	GCCGGGATGTTCTTTTTGTA	TTTACTGAATGCCATGCTGC	59.9	679	DRR	Ca_15110	C	MYB

INDEL marker IDs	Chromosomes/ unanchored scaffolds	Physical positions (bp)	InDels (*Kabuli* reference genome-CDC Frontier/PI)	Forward primers (5'-3')	Reverse primers (5'-3')	Anneling temperature (0C)	Expected amplified product size (bp)	Structural annotation		Functional annotation	
								Sequence components of *kabuli* genome	*Kabuli* gene accession IDs	NCBI-KOG	TFs
Ca4_P1_37706091	Ca4	37706091	TTCT/TT	GCCGGGATGTTCTTTTTGTA	GAGGGGAGGCCTTCTCATAC	59.9	818	DRR	Ca_15110	C	MYB
Ca4_P1_37706155	Ca4	37706155	AGGG/AGGGG	GCCGGGATGTTCTTTTTGTA	GAGGGGAGGCCTTCTCATAC	59.9	818	DRR	Ca_15110	C	MYB
Ca4_P1_37707068	Ca4	37707068	C/CTGGCAAGGAGCTACT	TGAGGCCTTAGCTTCGTGAT	CACAAGGACCTGACGGTTTT	60.0	555	INTERGENIC	--	--	--
Ca4_P1_37707135	Ca4	37707135	C/CGCCCCTCCCC	TGAGGCCTTAGCTTCGTGAT	CACAAGGACCTGACGGTTTT	60.0	555	INTERGENIC	--	--	--
Ca4_P1_37734970	Ca4	37734970	GTTATTATTATTATTATTATTATTATTATTATTATTATTATTATTATTATTATTATTAT/GTTATTATTATTATTATTATTATTATTATTATTATTATTATTATTATTAT	GCTTGAAGAAATGAGCGTGA	TGGGGAACGTATTGTTTTCA	59.2	512	INTERGENIC	--	--	--
Ca4_P1_37794166	Ca4	37794166	AGTGTT/AGTGTTGTGTT	GACAACCTTTCACCTTGTCCA	GGACACAATTTGGTTGGCTT	60.0	653	INTERGENIC	--	--	--
Ca4_P1_37817066	Ca4	37817066	AATCTCTCTCTAT/AATCTCTCTCTATCTCTCTCTAT	CCCTTCAAAAGGTGGTTCAA	ACAACACTCACCCTCAAGCC	59.9	816	INTERGENIC	--	--	--
Ca4_P1_37822334	Ca4	37822334	TT/TTATAGAACTCTAAT	TCAACCAAAATCCCCCAATA	CCCAATTTCGATTTACCCCT	60.0	329	INTERGENIC	--	--	--
Ca4_P1_37857649	Ca4	37857649	ATTTTTTTTTTT/ATTTTTTTTTT	ACCAATCGCACCTTAACACC	TAGCTGCTGATTCTCTGCGA	59.9	363	INTERGENIC	--	--	--
Ca4_P1_37872114	Ca4	37872114	CATATATATATATATATATATATATATATATATATATATAT/CATATATATATATATATATATATATATATATATATAT	TCAGCAAATCATAACCATCCA	CGCATAATACTCAAACCAAATCA	59.0	321	INTERGENIC	--	--	--
Ca4_P1_37913644	Ca4	37913644	ATTTTTTTTTTTTTTT/ATTTTTTTTTTTTTT	CCTTTTGTTCTTGTCGGGTC	TGAGACGAATCACACCGAAC	59.6	402	INTERGENIC	--	--	--
Ca4_P1_37993885	Ca4	37993885	CTATATATATATATATATATATATATATATA/CTATATATATATATATATATATATATA	GAAGGTCTTGCAAGTGAGTGC	CAAACGTGTGATGGATCTTCC	60.1	446	INTERGENIC	--	--	--

INDEL marker IDs	Chromosomes/ unanchored scaffolds	Physical positions (bp)	InDels (*Kabuli* reference genome-CDC Frontier/PI)	Forward primers (5'-3')	Reverse primers (5'-3')	Anneling temperature (0C)	Expected amplified product size (bp)	Structural annotation		Functional annotation	
								Sequence components of *kabuli* genome	*Kabuli* gene accession IDs	NCBI-KOG	TFs
Ca4_P1_38024785	Ca4	38024785	GTATATATATATATATATATATATATA/ GTATATATATATATATATATATATA	AAGTGAAAACCCCAAA GGCT	GCATGTGCCTCATTTT GTTG	60.0	230	INTERGENIC	--	--	--
Ca4_P1_38080710	Ca4	38080710	GTATATATATATATATATATATATATAT ATATATAT/GTATATATATATATATATA TATATATATATATATATATAT	GCTTTTGAGGCGTAAA ACCA	TTCCTCCAATAGAAAT GCCG	60.2	520	INTERGENIC	--	--	--
Ca4_P1_38231305	Ca4	38231305	TTTTATTTATTTATTTATTTATTTATTT ATTTA/TTTTATTTATTTATTTATTTATT TATTTATTTATTTA	GGCCATCAGAGAATCT TCCA	TGCACTTTTGGGAACA ACAA	60.2	580	INTRON	Ca_13131	--	--
Ca4_P1_3832898	Ca4	3832898	ATTT/ATTTTTT	CGGAGGGTACGAACCT GTAA	ACTGTTGTGGAAATCT GGGC	60.0	484	INTERGENIC	--	--	--
Ca4_P1_38439288	Ca4	38439288	CTTTTTTTTTTTT/CTTTTTTTTTTTTTT	TTTTGATGCTTTGTTG GCCT	GCGATTCAACTGAAAT AGGAGG	60.6	290	INTERGENIC	--	--	--
Ca4_P1_38614236	Ca4	38614236	GAATAAATAAATAAATAAATAAATAAA TAAATAAATAAATAAA/GAATAAATAAA TAAATAAATAAATAAATAAATAAATAAA TAAATAAA	GCTCTCCTCTGTACCG CATC	TGCTGCACAAAAATTT CAGG	60.0	203	INTRON	Ca_13104	O	NAC
Ca4_P1_38682630	Ca4	38682630	TTATATATATATATATATA/TTATATATA TATATATATATATA	ACGGTATCAATTAACA CAGGAAA	GTGGGGGCTTGTTGA GTTAC	57.6	707	INTERGENIC	--	--	--
Ca4_P1_38691909	Ca4	38691909	ATTTTTTTT/ATTTTTTT	GATGTCCCAAAGTGGA ATGG	TTTTTGTGTGCAAAAA TTGAAAA	60.2	725	INTERGENIC	--	--	--
Ca4_P1_3871430	Ca4	3871430	G/GGAGGAA	CACTGGGGCTGAACAT CTTT	ATTTAGGGTCTGCGTG GTTG	60.1	628	INTRON	Ca_03867	--	--
Ca4_P1_38822967	Ca4	38822967	TTATATATATATATATATATATATATAT/ TTATATATATATATATATATATATATAT ATAT	TCTATGGCAACCTAAG CCGT	CATGTGCAATGTACTT GGTTCC	59.7	622	INTERGENIC	--	--	--
Ca4_P1_38841033	Ca4	38841033	TAAAAAAAAAAA/TAAAAAAAAAAAA	TGGGCACATCAGACAA CATT	TGTGTGGAGATGGGTT TGAA	60.0	211	INTERGENIC	--	--	--
Ca4_P1_38884678	Ca4	38884678	CAAAAAAA/CAAAAAA	GGTGGTCATGGTGTAG TTTGG	TCACACGTCCTTTCAA AATCA	60.1	575	INTERGENIC	--	--	--

INDEL marker IDs	Chromosomes/ unanchored scaffolds	Physical positions (bp)	InDels (*Kabuli* reference genome-CDC Frontier/PI)	Forward primers (5'-3')	Reverse primers (5'-3')	Anneling temperature (0C)	Expected amplified product size (bp)	Structural annotation		Functional annotation	
								Sequence components of *kabuli* genome	*Kabuli* gene accession IDs	NCBI-KOG	TFs
Ca4_P1_38886301	Ca4	38886301	ATCAACTGCTCAA/ATCAACTGCTCAACTGCTCAA	ATTGGTTACGCGACCAACTC	TACACATGCATAGGTGGCCT	60.0	841	INTERGENIC	--	--	--
Ca4_P1_38936911	Ca4	38936911	ATTGGGAAAAATTT/ATTGGGAAAAATTTTGGGAAAAATTT	CCCTGAATCGATGAGTTTGAA	TGCTCAAAAGGCTGAGGAAT	60.1	272	INTERGENIC	--	--	--
Ca4_P1_3895254	Ca4	3895254	A/ATGGAAAG	AGGACAGCTCACGACCAAGT	AGGATGCGAATTCGAGGATA	59.9	603	INTERGENIC	--	--	--
Ca4_P1_39020626	Ca4	39020626	TAAAAAAAAA/TAAAAAAAAAA	TGGCATGACACTTAGCTCAAA	CCCCAATGTATTGGAACCAC	59.5	864	INTERGENIC	--	--	--
Ca4_P1_39022972	Ca4	39022972	TTTATTATTATTATTATTATTATTATTATTATTATTATTATTATT/TTTATTATTATTATTATTATTATTATTATTATTATTATTATTATTATTATTATTATT	TTGAAATGCACCGTAAATGG	AAATATTTGAGTTACGAATTTGATGA	59.4	499	INTERGENIC	--	--	--
Ca4_P1_39038460	Ca4	39038460	ACTTGCTTG/ACTTG	CCGGTCACATTCAATAGCCT	ATTGCGTTGTTTCTTCCAGG	60.0	411	DRR	Ca_13078	A	--
Ca4_P1_39070213	Ca4	39070213	TAATAAATAA/TAATAA	TATTTTGGATTTTGGCTCGG	AAGGAAGCAACGAAAACGAA	59.9	446	INTERGENIC	--	--	--
Ca4_P1_39081038	Ca4	39081038	CCACA/CCACACA	TGTTCAGGTTTCGGGATTCT	CTCGAGAAGAGGGAGCAATG	59.5	469	INTRON	Ca_13074	PT	FAR1
Ca4_P1_39147993	Ca4	39147993	AATATATATATATATATATATATAT/AATATATATATATATATATATATATATATAT	GGAAGGAGCCCTTATCTTCG	ATGTCAATTGATTTGCGACG	60.2	422	INTERGENIC	--	--	--
Ca4_P1_39165742	Ca4	39165742	TAAAAATAAAAAATAAAAAA/TAAAAATAAAAAATAAAAAATAAAAAA	CGTATACCGCTGCTCATCAA	TGATGTGATTTGTGCCATGA	59.9	259	INTERGENIC	--	--	--
Ca4_P1_39183756	Ca4	39183756	A/ATGAAGTGGG	AATTTGCAGGTGGAATCGTC	GCAAGCGAACAACATTGAGA	59.9	348	CDS (large-effect mutations)	Ca_13064	T	bHLH
Ca4_P1_39266620	Ca4	39266620	AT/ATT	TTTCACTGATGTGGCCTTCA	TGCCTCACTCCTAAGTTCTGC	60.2	222	DRR	Ca_13060	T	bHLH

INDEL marker IDs	Chromosomes/ unanchored scaffolds	Physical positions (bp)	InDels (*Kabuli* reference genome-CDC Frontier/PI)	Forward primers (5'-3')	Reverse primers (5'-3')	Anneling temperature (0C)	Expected amplified product size (bp)	Structural annotation		Functional annotation	
								Sequence components of *kabuli* genome	*Kabuli* gene accession IDs	NCBI-KOG	TFs
Ca4_P1_39270658	Ca4	39270658	AATATATATATATATATATATATATATATATATATATATATATATATAT/AATATATATATATATATATATATATATATATATAT	TGACATATGAACAAGACATGCG	ACATCACTTATCCCCCGACA	59.6	607	INTERGENIC	--	--	--
Ca4_P1_3949026	Ca4	3949026	AATATATATATATATATATATAT/AATATATATATATATATATAT	GGCAATTGGCTAAATTACTGTTC	TTGGCGATATTTCAGAAGGAA	59.1	522	INTERGENIC	--	--	--
Ca4_P1_3951582	Ca4	3951582	ATATGT/AT	TGGACATTTAATGGTTGCGA	TTTATCTCTCTCGTGCGTGC	59.9	563	INTERGENIC	--	--	--
Ca4_P1_39647050	Ca4	39647050	GAAAAA/GAAAAAA	GCATGGTGCAAGATGGTTTT	TTGGATTGCATGTGTTTGAGA	60.9	272	INTERGENIC	--	--	--
Ca4_P1_3966806	Ca4	3966806	GAAAAAAAAAAA/GAAAAAAAAAA	CCGTCGCTAAACACACTCAA	TGCACCAACAATCCTTGCTA	59.9	814	INTERGENIC	--	--	--
Ca4_P1_39668751	Ca4	39668751	AAATAATAATAATAATAATAA/AAATAATAATAATAATAATAATAA	CGACTTGAGAATTTTCACAAACA	TGCTTGCAACCCAATTATTTT	59.3	897	DRR	Ca_14933	T	bHLH
Ca4_P1_39689187	Ca4	39689187	AC/ACC	ATTACCGACATTGCAGAGGG	CTCCCATTCTGCCCTTGATA	60.0	329	INTERGENIC	--	--	--
Ca4_P1_39739876	Ca4	39739876	AATATATATATATATAT/AATATATATATATATATAT	TTTTGTGTGGGTGTCTTTTGA	CATGCAACTCATGAGCCCTA	59.1	848	INTERGENIC	--	--	--
Ca4_P1_39740716	Ca4	39740716	AG/A	TCCTAGGGCTCATGAGTTGC	GAGATGTGCAGTTTAATTCGTCC	60.4	882	INTERGENIC	--	--	--
Ca4_P1_3989437	Ca4	3989437	AATATATATATATATATATATAT/AATATATATATATATATATATATAT	TCCCGTTCCAAAACTTTTCA	TGATAGCACAAAATCGTGGA	60.5	594	DRR	Ca_03854	G	--
Ca4_P1_40019420	Ca4	40019420	ATTTTTTTTTT/ATTTTTTTTT	GCAAAAAGTTGAAATGGCGT	TTGCAACAATTCTTCCATCCT	60.1	891	INTERGENIC	--	--	--
Ca4_P1_40027152	Ca4	40027152	ATTTTTTTTT/ATTTTTTTTTT	AGCATTTCTCATGACACCTCG	CAGAGTGAATGGAGACATGGG	60.3	739	INTERGENIC	--	--	--

INDEL marker IDs	Chromosomes/ unanchored scaffolds	Physical positions (bp)	InDels (*Kabuli* reference genome-CDC Frontier/PI)	Forward primers (5'-3')	Reverse primers (5'-3')	Anneling temperature (0C)	Expected amplified product size (bp)	Structural annotation		Functional annotation	
								Sequence components of *kabuli* genome	*Kabuli* gene accession IDs	NCBI-KOG	TFs
Ca4_P1_40056699	Ca4	40056699	AATATATATATATATATATATATATATATATATATATATATATATATATAT/AATATATATATATATATATATATATATATATATATATATATATATATATATATATATAT	CAGGACAGGGATGGTGTTTT	TTGCAATTTCATGAATGTTACTCA	59.8	639	INTERGENIC	--	--	--
Ca4_P1_40080245	Ca4	40080245	CATATATATATATATATATATAT/CATATATATATATATATATATAT	AAAAGCGCAGTCGTAGGTGT	GGGTGCTATAGTGTCGAAGCA	59.9	886	INTERGENIC	--	--	--
Ca4_P1_40080777	Ca4	40080777	TTATATATATATATATATATATATATATA/TTATATATATATATATATATATATA	TGCTTCGACACTATAGCACCC	ACTTGCGCCACATGTATCAG	60.3	382	INTERGENIC	--	--	--
Ca4_P1_40102836	Ca4	40102836	ATGTTGTTGTTGTTGTTGTTGTTGTTGTTGTTGTTGTT/ATGTTGTTGTTGTTGTTGTTGTTGTTGTTGTTGTT	TTTAAAAAGGAAGCCAGCGA	CACTCAATACCAACACCTCCA	60.0	530	INTERGENIC	--	--	--
Ca4_P1_40112511	Ca4	40112511	AAATAATAATAATAATAATAATAATAA/AAATAATAATAATAATAATAATAATAATAA	TTTGTGATCCGTATTGCAAGTT	TTATTTTCCAACCAGCACCC	59.5	551	INTERGENIC	--	--	--
Ca4_P1_4029055	Ca4	4029055	GATCATCATCATCAT/GATCATCATCATCATCAT	ACATTCCTGTTGGTGGTGGT	ACATTCCAGTCCAAAACCCA	60.1	612	CDS (large-effect mutations)	Ca_03849	--	Dof
Ca4_P1_4029216	Ca4	4029216	AGG/AGGTGG	TTGGATGGAATTGGAGGAAG	AATGCAGTTCGCAACATCAA	59.9	289	CDS (large-effect mutations)	Ca_03849	--	Dof
Ca4_P1_4030815	Ca4	4030815	CAAAAA/CAAAA	TGGATCCACTAATAACATGGGA	CCTTTAAGGGGATTGAAGGA	59.2	516	DRR	Ca_03849	--	Dof
Ca4_P1_4031880	Ca4	4031880	GTTTTTTT/GTTTTTTTTT	GGGTTTGTTTTATTTGATTTTGA	GATCTGGAAAGGAGAAGGGC	57.2	238	INTERGENIC	--	--	--
Ca4_P1_4032141	Ca4	4032141	AACA/AA	GCGTCGTTTTAGTCGTGGTT	AGCTGGGGTTGCTTTACTGA	60.2	474	INTERGENIC	--	--	--
Ca4_P1_40323500	Ca4	40323500	TACA/TA	GTTTGCAAATGCGACTTCCT	TCGTTCATTCCACTCGAAAA	60.3	188	INTERGENIC	--	--	--
Ca4_P1_40324569	Ca4	40324569	AACTATGATA/AA	AAGTCGTTTTTGCAAATGGC	AGTTTGCATGATCCTCCGTC	60.1	874	INTERGENIC	--	--	--

INDEL marker IDs	Chromosomes/ unanchored scaffolds	Physical positions (bp)	InDels (*Kabuli* reference genome-CDC Frontier/PI)	Forward primers (5'-3')	Reverse primers (5'-3')	Anneling temperature (0C)	Expected amplified product size (bp)	Structural annotation		Functional annotation	
								Sequence components of *kabuli* genome	*Kabuli* gene accession IDs	NCBI-KOG	TFs
Ca4_P1_4032682	Ca4	4032682	T/TTTAATAAGAG	ATCAGTAAAGCAACCCCAGC	CAACGGTGGAGTTTGGATCT	59.2	346	INTERGENIC	--	--	--
Ca4_P1_4032754	Ca4	4032754	TG/T	ATCAGTAAAGCAACCCCAGC	CAACGGTGGAGTTTGGATCT	59.2	346	INTERGENIC	--	--	--
Ca4_P1_4037177	Ca4	4037177	GTTTTTTTTTTTT/GTTTTTTTTT	AAAGTGAAAGCCACTCCCAA	AATGTTCGCCACCACTTTTC	59.7	369	INTERGENIC	--	--	--
Ca4_P1_4037364	Ca4	4037364	AT/ATTT	AAAGTGAAAGCCACTCCCAA	AATGTTCGCCACCACTTTTC	59.7	369	INTERGENIC	--	--	--
Ca4_P1_4039327	Ca4	4039327	TACTC/T	TTTTGGTTTGGTAATTGTGGG	TGATGGAGAAATCCTCACCC	59.6	456	INTERGENIC	--	--	--
Ca4_P1_4039729	Ca4	4039729	TAAA/TAA	TGCAATTACACGAGGGTTCA	GTTCGTTGGCATTGGTGAC	60.1	380	INTERGENIC	--	--	--
Ca4_P1_4041834	Ca4	4041834	TA/T	TCATTCGCTGGTCGTCATTA	AACATGTGACCCAAGGACACT	60.2	387	INTERGENIC	--	--	--
Ca4_P1_4044595	Ca4	4044595	ATTTTTTTTTTTTT/ATTTTTTTTT	GGGTGCATTTGTTGGAACTT	TTTGTTTGGGTTAAAAAGAAGAGG	59.8	376	INTERGENIC	--	--	--
Ca4_P1_40449871	Ca4	40449871	TAAAAAAAAAA/TAAAAAAAAAAA	GCCCAATTGTTTTCTTTCCA	TGATGATGTGGGTTTGATCC	59.9	635	INTERGENIC	--	--	--
Ca4_P1_4044991	Ca4	4044991	A/AATTGGATC	GGGTGCATTTGTTGGAACTT	TTGGTACGTGTAGAACATCGTG	59.8	676	INTERGENIC	--	--	--
Ca4_P1_4045724	Ca4	4045724	ATTTTTTTTT/ATTTTTTT	TGTTGGGTTTGTTTCCTCCT	GCTTGATAATTTAAGGGCACCA	59.4	515	INTERGENIC	--	--	--
Ca4_P1_4046043	Ca4	4046043	TAAAA/TAAAAA	TGTTGGGTTTGTTTCCTCCT	GCTTGATAATTTAAGGGCACCA	59.4	515	INTERGENIC	--	--	--

INDEL marker IDs	Chromosomes/ unanchored scaffolds	Physical positions (bp)	InDels (*Kabuli* reference genome-CDC Frontier/PI)	Forward primers (5'-3')	Reverse primers (5'-3')	Anneling temperature (0C)	Expected amplified product size (bp)	Structural annotation		Functional annotation	
								Sequence components of *kabuli* genome	*Kabuli* gene accession IDs	NCBI-KOG	TFs
Ca4_P1_4046809	Ca4	4046809	TG/T	GAGTAAAACCAAACAC CCCC	GCCATTGCAATTTTCC ACTT	58.3	590	INTERGENIC	--	--	--
Ca4_P1_40483531	Ca4	40483531	ATT/ATTAGCGGTCTT	TCCCACATTGGTAGTT CCGT	AGCTGAACCCTTGCTG TGAT	60.2	500	DRR	Ca_14877	I	--
Ca4_P1_40484949	Ca4	40484949	AA/AATA	ATTGTGGTGAAGGTGG TGGT	CGGAGAACTCCAATG GAAAA	60.1	413	INTERGENIC	--	--	--
Ca4_P1_40484988	Ca4	40484988	GAAAAAAAAAAA/GAAAAAAAA	ATTGTGGTGAAGGTGG TGGT	CGGAGAACTCCAATG GAAAA	60.1	413	INTERGENIC	--	--	--
Ca4_P1_40485498	Ca4	40485498	GAAAAAAAA/GAAAAAAA	TTGGCTCCATCAATTG AACA	GGTCCCTTGAGCACAT TAGC	60.0	625	INTERGENIC	--	--	--
Ca4_P1_40491458	Ca4	40491458	AAG/A	TCCGTTAGATTGCTGA ATGC	CATCTCGAATGGCTTC CAAT	58.9	628	INTRON	Ca_14876	--	HD-ZIP
Ca4_P1_40498066	Ca4	40498066	AACATAACTAAATATTACA/AACATAAC TAAATATTACATAACTAAATATTACA	CCACCAAACATCATCA CAGC	TTGCATTCGAGTTGGA TTTTT	60.0	403	DRR	Ca_14875	--	--
Ca4_P1_40498650	Ca4	40498650	AAT/A	TCATTTGAAACATGGA CCACA	GCTTGACAATCATGGG GTTT	59.8	262	DRR	Ca_14875	--	--
Ca4_P1_40499161	Ca4	40499161	A/AAT	TCACGATTGTGGTGTC AAAAA	TTTTTGTTGGATGAAT TTGGC	60.0	761	DRR	Ca_14875	--	--
Ca4_P1_40499325	Ca4	40499325	T/TA	TGAAACAAACAATTAAG TCCGTG	TTTTTGTTGGATGAAT TTGGC	59.1	429	DRR	Ca_14875	--	--
Ca4_P1_40499362	Ca4	40499362	CA/CAA	TGAAACAAACAATTAAG TCCGTG	AAAGGGAAGTTTCTCG AGCC	59.1	802	DRR	Ca_14875	--	--
Ca4_P1_40499622	Ca4	40499622	ATTTTT/ATTTT	GGCCAAATTCATCCAA CAAA	GGGGTGGGTTGAAGA AAAAT	60.7	501	INTRON	Ca_14875	--	--

**INDEL marker IDs**	**Chromosomes/ unanchored scaffolds**	**Physical positions (bp)**	**InDels (*Kabuli* reference genome-CDC Frontier/PI)**	**Forward primers (5'-3')**	**Reverse primers (5'-3')**	**Anneling temperature (0C)**	**Expected amplified product size (bp)**	**Structural annotation**		**Functional annotation**	
								**Sequence components of *kabuli* genome**	***Kabuli* gene accession IDs**	**NCBI-KOG**	**TFs**
Ca4_P1_40501506	Ca4	40501506	ATTTTTTTTTT/ATTTTTTTT	TTTCAGCTAGCTTGACAACTCTC	TGGACCAAGTACCATGGGTT	58.1	501	INTERGENIC	--	--	--
Ca4_P1_40501605	Ca4	40501605	CTT/CTTT	TTTCAGCTAGCTTGACAACTCTC	CATGAGTTATTGATGGTGCGA	58.1	835	INTERGENIC	--	--	--
Ca4_P1_40501876	Ca4	40501876	ATTTTTTTTT/ATTTTTTTT	ACCCATGGTACTTGGTCCAC	CATGAGTTATTGATGGTGCGA	59.6	353	INTERGENIC	--	--	--
Ca4_P1_40503609	Ca4	40503609	TA/TACTCTCAA	GTGGTGTGTGCAAGAATTGG	CCAACAAGTAGGGTGGAGGA	60.0	800	INTERGENIC	--	--	--
Ca4_P1_40505698	Ca4	40505698	GATATATATATA/GATATATATATATA	TGGTTAAGGGGATGCTATCTTG	TGAAAGCCCAAACACAATGA	60.3	588	INTERGENIC	--	--	--
Ca4_P1_40506274	Ca4	40506274	CAGTATAATAGTA/CAGTATAATAGTATAATAGTA	GTGGCTGTTTATGGATTGGG	TGATCCCCTTCATTCCTTCA	60.2	239	INTERGENIC	--	--	--
Ca4_P1_40506520	Ca4	40506520	TTATTTGCT/TT	GGATCACAACCATAAACCCG	TTCAACAAACTTTCTGGCCTC	60.1	453	INTERGENIC	--	--	--
Ca4_P1_40506954	Ca4	40506954	TA/TAA	GAGGCCAGAAAGTTTGTTGAA	TTGCTTGGTGCATGTTTTTC	59.3	443	INTERGENIC	--	--	--
Ca4_P1_4050866	Ca4	4050866	TTA/TTATA	ATCATCCCTCATGGTGGAAA	CCTACTTCAAACGCCCATGT	60.1	519	INTRON	Ca_03848	--	bHLH
Ca4_P1_40508840	Ca4	40508840	GAAAAAAAA/GAAAAAAAAAA	GGGGAAAAAGAAAGGAAGGA	GTGGCTGCAAAAGCTAAAGG	59.5	294	DRR	Ca_14874	--	--
Ca4_P1_40509352	Ca4	40509352	TTTCT/TT	CTTTAGCTTTTGCAGCCACC	CATGGTTGGGCATAATTTGA	60.0	752	DRR	Ca_14874	--	--
Ca4_P1_40509479	Ca4	40509479	GTTTTTTTTTT/GTTTTTTTTT	CTTTAGCTTTTGCAGCCACC	GGTGGAACGGCTACTTACCA	60.0	881	DRR	Ca_14874	--	--

INDEL marker IDs	Chromosomes/ unanchored scaffolds	Physical positions (bp)	InDels (*Kabuli* reference genome-CDC Frontier/PI)	Forward primers (5'-3')	Reverse primers (5'-3')	Anneling temperature (0C)	Expected amplified product size (bp)	Structural annotation		Functional annotation	
								Sequence components of *kabuli* genome	*Kabuli* gene accession IDs	NCBI-KOG	TFs
Ca4_P1_40511968	Ca4	40511968	ATTTTTTT/ATTTTTT	TTTTTGTTGAAACGAAACACACTT	TGCCTACAATTCCTCCTTGC	60.0	417	INTRON	Ca_14874	--	--
Ca4_P1_40514146	Ca4	40514146	AAACATG/AAACATGAACATG	TTTTGATGTCTCTGCAACGC	GATGGTTAGACGCTGAACTCG	60.0	667	INTRON	Ca_14874	--	--
Ca4_P1_40514871	Ca4	40514871	AATA/AA	GGAGAACCAGCTTCCTCCTT	CGCTCTTGCTTCCTTTCACT	59.8	278	INTERGENIC	--	--	--
Ca4_P1_40515212	Ca4	40515212	CG/C	GGAGAACCAGCTTCCTCCTT	TTCTCGGTTCTCCTTTCTGC	59.8	916	INTERGENIC	--	--	--
Ca4_P1_40519165	Ca4	40519165	TAAA/TAAAA	TTCTTACAATTACCAAACACGCA	TGTCGACCATGTGAATTAGAGAA	59.6	488	INTERGENIC	--	--	--
Ca4_P1_40519317	Ca4	40519317	TC/T	ACAATTACCAAACACGCACA	TGTCGACCATGTGAATTAGAGAA	57.5	483	INTERGENIC	--	--	--
Ca4_P1_40519544	Ca4	40519544	CTTTATTTA/CTTTATTTATTTA	ACAATTACCAAACACGCACA	TGTCGACCATGTGAATTAGAGAA	57.5	483	INTERGENIC	--	--	--
Ca4_P1_40519957	Ca4	40519957	TAAAAAAA/TAAAAAAAAA	AATTCACATGGTCGACATTTTT	TTGTTTGTGCTGCCTTTGAG	58.4	532	INTERGENIC	--	--	--
Ca4_P1_40520777	Ca4	40520777	TAAAAAAAAAAAAA/TAAAAAAAAAAA	GCCTCCATCTCACGTGATTC	TGTGGAATTGTGTCGCATCT	60.6	922	INTERGENIC	--	--	--
Ca4_P1_40521264	Ca4	40521264	TTACTACTA/TTACTA	AGATGCGACACAATTCCACA	CATTAAAATTTCAAAGATTTTGCG	60.1	162	INTERGENIC	--	--	--
Ca4_P1_40521734	Ca4	40521734	ATTTTTTTTTTT/ATTTTTTTTTTTT	TCATCGGGACAACTTTGTATG	CGGCTCATCTCAATCCTTGT	58.5	655	INTERGENIC	--	--	--
Ca4_P1_40522888	Ca4	40522888	AAATATATATA/AAATATATATATATTTTTAGAATATATATA	GATGGGTTGGTTTGATTTGG	CGACAATTTTGGTCTCTCCA	60.0	946	INTERGENIC	--	--	--

INDEL marker IDs	Chromosomes/ unanchored scaffolds	Physical positions (bp)	InDels (*Kabuli* reference genome-CDC Frontier/PI)	Forward primers (5'-3')	Reverse primers (5'-3')	Anneling temperature (0C)	Expected amplified product size (bp)	Structural annotation		Functional annotation	
								Sequence components of *kabuli* genome	*Kabuli* gene accession IDs	NCBI-KOG	TFs
Ca4_P1_4052315	Ca4	4052315	AACA/AA	ATGCACCCAAAATGATTTCC	TTTTAACTTTGTCACGCCCC	59.6	568	DRR	Ca_03848	--	bHLH
Ca4_P1_40528670	Ca4	40528670	GTTTTTTTTT/GTTTTTTTT	AAATGCAACAGGAAGATGCC	TGGACTTGGAAGAGAAACGG	60.1	558	INTERGENIC	--	--	--
Ca4_P1_40529406	Ca4	40529406	CTTTTTTTTT/CTTTTTTTTTT	CCGTTTCTCTTCCAAGTCCA	CGCTAAGCACCAGTTTCCTC	60.2	608	INTERGENIC	--	--	--
Ca4_P1_40531599	Ca4	40531599	TTGT/TT	GGCGATGCAATTTTCAGTTT	TGCTTTCTCAAGCCAAACAA	60.1	546	INTRON	Ca_14873	--	--
Ca4_P1_4054903	Ca4	4054903	CAA/CA	AATTCGTTGTCATGCTCAGG	TCATCTAAATCGGTGCCAAA	58.7	857	INTERGENIC	--	--	--
Ca4_P1_40552426	Ca4	40552426	G/GA	CCATACAACATGGGGGAAAG	GAGCATGGATGATTCGACCT	60.0	412	INTRON	Ca_14870	--	--
Ca4_P1_40558899	Ca4	40558899	CTTTTTTTTTT/CTTTTTTT	GCGGTAAGGGCATTATTTCA	TCAGAAACCCTAATGGCCTG	59.9	681	DRR	Ca_14870	--	--
Ca4_P1_40559341	Ca4	40559341	TAA/TA	TCAGGCCATTAGGGTTTCTG	ATTGAATGCGGTCTCAGTGC	60.1	410	DRR	Ca_14870	--	--
Ca4_P1_40560588	Ca4	40560588	TAAAAAA/TAAAAAAA	TCATTCTCCCGATCTTCCAC	TTTCTGTGGACTCACGCATC	60.0	636	INTERGENIC	--	--	--
Ca4_P1_40560728	Ca4	40560728	CTT/CTTTT	TCATTCTCCCGATCTTCCAC	GGAGTGTGCTCCAAGGGTTA	60.0	943	INTERGENIC	--	--	--
Ca4_P1_40561270	Ca4	40561270	TTTCAA/T	ACTGATGCGTGAGTCCACAG	GGATGTTGGCTCCCAAGTTA	59.9	785	INTERGENIC	--	--	--
Ca4_P1_40573539	Ca4	40573539	AG/A	TATTGCACCGTTGCCTTACA	TCAACTTCCTTGGTTATATTTTTGC	60.1	721	INTERGENIC	--	--	--

INDEL marker IDs	Chromosomes/ unanchored scaffolds	Physical positions (bp)	InDels (*Kabuli* reference genome-CDC Frontier/PI)	Forward primers (5'-3')	Reverse primers (5'-3')	Anneling temperature (0C)	Expected amplified product size (bp)	Structural annotation		Functional annotation	
								Sequence components of *kabuli* genome	*Kabuli* gene accession IDs	NCBI-KOG	TFs
Ca4_P1_40573928	Ca4	40573928	CAAAA/CAA	TTCGTGGAATATCACAAAAGTTC	TGGACAAAAACTCATCTGCATT	58.2	895	INTERGENIC	--	--	--
Ca4_P1_40574863	Ca4	40574863	A/AC	TGCAGATGAGTTTTTGTCCA	AAAATTAAGACAAAAGGAAGTTCAACA	57.8	658	INTERGENIC	--	--	--
Ca4_P1_40575250	Ca4	40575250	TAA/TAAA	CAATGTTGAACTTCCTTTTGTCTT	AAAGGATGAGAATGAAACTTACGTG	58.8	687	INTERGENIC	--	--	--
Ca4_P1_40575649	Ca4	40575649	ATTTTTT/ATTTTT	TTTCTCATTTTCATGACTCCTATCA	TCACCAGTGACACGTTCGAT	59.2	375	INTERGENIC	--	--	--
Ca4_P1_40575944	Ca4	40575944	GAA/GAAA	AACATCGAACGTGTCACTGG	GACATGTACAGACATACGAGGCA	59.6	479	INTERGENIC	--	--	--
Ca4_P1_40586903	Ca4	40586903	CTTTTT/CTTTTTT	CTGATGTTGCTACCGCTGAA	GGATTCAAGTTCATGCACCA	60.0	513	DRR	Ca_14868	K	--
Ca4_P1_40593121	Ca4	40593121	CAAGT/CAAGTAAGT	CATTCCCACCATCCTTCACT	GGCCTATCGAACCACACAGT	59.8	932	INTRON	Ca_14867	R	BES1
Ca4_P1_40593400	Ca4	40593400	GTT/GTTTT	AAGGATTTGCTTGCTGATGC	GGCCTATCGAACCACACAGT	60.4	276	INTRON	Ca_14867	R	BES1
Ca4_P1_40602580	Ca4	40602580	ACC/AC	CCATTACGATGCACATTGCT	TGAATTGTTCTTACGAGCCG	59.6	257	INTERGENIC	--	--	--
Ca4_P1_40605387	Ca4	40605387	TAAAAAAAAAAA/TAAAAAAAAAA	AATCGGGCATGAAATATTGA	GTGTGTCCAAAAGTGCGAGA	57.9	808	INTERGENIC	--	--	--
Ca4_P1_40608651	Ca4	40608651	TAAAAAAAAAA/TAAAAAAAAA	TTTTTGCAAACGACAACTTTCT	TGTGAATACGTGACTTTTCTTTTCA	59.0	432	INTERGENIC	--	--	--
Ca4_P1_40614255	Ca4	40614255	ATTTTTTTT/ATTTTTTT	CGGTGTAGCAAGAACTTTACCA	ATTTGAACATCGTTGGGCTC	59.3	634	INTERGENIC	--	--	--

INDEL marker IDs	Chromosomes/ unanchored scaffolds	Physical positions (bp)	InDels (*Kabuli* reference genome-CDC Frontier/PI)	Forward primers (5'-3')	Reverse primers (5'-3')	Anneling temperature (0C)	Expected amplified product size (bp)	Structural annotation		Functional annotation	
								Sequence components of *kabuli* genome	*Kabuli* gene accession IDs	NCBI-KOG	TFs
Ca4_P1_40615032	Ca4	40615032	TAA/TA	GAGCCCAACGATGTTCAAAT	TGAACCCCACCAGCATTATT	59.9	793	INTERGENIC	--	--	--
Ca4_P1_40620475	Ca4	40620475	A/AT	TATGTGAAATTGCCACCGAA	ACCTCATCGAGCCATTGATT	59.9	509	INTERGENIC	--	--	--
Ca4_P1_40622124	Ca4	40622124	TAAAAAAAAAAAAA/TAAAAAAAAAAAA	ACATACACACGTCACGGGAA	TTTGCAAATGAGATTTCCTAAAGA	59.9	391	INTERGENIC	--	--	--
Ca4_P1_40625937	Ca4	40625937	GAAAAAAAA/GAAAAAAAAA	TTCCTCATGGAGCCATGTTA	GAAATTGCCACCGAAAAGAA	59.1	498	INTERGENIC	--	--	--
Ca4_P1_40626354	Ca4	40626354	TAGAAGAGAAGAGAAGA/TAGAAGAGAAGA	CTTTTCGGTGGCAATTTCAT	ACCCTTTCAGTGAGCACGTC	59.9	237	INTERGENIC	--	--	--
Ca4_P1_40626965	Ca4	40626965	TAA/TAAA	GACGTGCTCACTGAAAGGGT	CATCGAGGCCCATTATGATT	60.3	687	INTERGENIC	--	--	--
Ca4_P1_40633755	Ca4	40633755	GTTTTTTTTTTT/GTTTTTTTTTT	GTGTGTCCAAAAGTGCGAGA	CCCATAATATTTCTTGGGGC	59.9	726	INTERGENIC	--	--	--
Ca4_P1_40638727	Ca4	40638727	AATATATATAT/AATATATATATAT	TCCTTCTAGCACATGGTCCC	TTGGAAAAGCCGCAATCTAT	60.1	863	INTERGENIC	--	--	--
Ca4_P1_40639226	Ca4	40639226	AGG/AG	AATAGATTGCGGCTTTTCCA	CAAGAAACCGTTGTGGTCCT	59.7	158	INTERGENIC	--	--	--
Ca4_P1_40639757	Ca4	40639757	AAAATATTATAAATATTA/AAAATATTA	CCCCTGCCAAAATACTTTCA	TCCTATGGAACCACCAAAGC	59.9	752	INTERGENIC	--	--	--
Ca4_P1_40639974	Ca4	40639974	AATATATATAT/AATATATATATATAT	CAACGCTATTTACCCACCCA	TCCTATGGAACCACCAAAGC	60.7	200	INTERGENIC	--	--	--
Ca4_P1_40640542	Ca4	40640542	CA/CAAGTGA	TGCCATAATTGGTTGCAAAA	TGAATTTGATGTTGTTGAAGATGA	59.9	807	INTRON	Ca_14865	--	--

INDEL marker IDs	Chromosomes/ unanchored scaffolds	Physical positions (bp)	InDels (*Kabuli* reference genome-CDC Frontier/PI)	Forward primers (5'-3')	Reverse primers (5'-3')	Anneling temperature (0C)	Expected amplified product size (bp)	Structural annotation		Functional annotation	
								Sequence components of *kabuli* genome	*Kabuli* gene accession IDs	NCBI-KOG	TFs
Ca4_P1_40641097	Ca4	40641097	AATATA/AATA	GCAAATTTCATTAAATGCATGAC	CCCTCGCGAAAATTATTGAA	58.6	507	INTRON	Ca_14865	--	--
Ca4_P1_40641475	Ca4	40641475	ATTTTTTTT/ATTTTTTT	TTCAATAATTTTCGCGAGGG	TGAGTAATTGAGGGCTGCTG	60.0	515	INTRON	Ca_14865	--	--
Ca4_P1_40644205	Ca4	40644205	CTTTTTTTTTT/CTTTTTTT	TGGTGGATGTGTTCATGCTT	GATGCCCTCAAACGAATGTT	60.0	861	INTRON	Ca_14865	--	--
Ca4_P1_40649020	Ca4	40649020	ATTTTTT/ATTTTTTTT	TGTGATTATCCGGTACCACAAC	TGTATTCAGGACCTCGCAGA	59.6	207	INTERGENIC	--	--	--
Ca4_P1_40652676	Ca4	40652676	AAAAATAAAATAAAATAA/AAAAATAAAATAAAATAAAATAA	TGAGATGGACTTCTCGAGGG	AGGTCAACCCACCAACTGAA	60.3	622	DRR	Ca_14864	T	--
Ca4_P1_40653220	Ca4	40653220	TAA/TAAA	TTCAGTTGGTGGGTTGACCT	CAATGAGTGTGTTTATTGCGA	60.4	769	DRR	Ca_14864	T	--
Ca4_P1_40653582	Ca4	40653582	T/TC	TGTGCAATGGCAATAACTCAT	GGAATCGTTCTACTGGTCCAC	59.1	399	DRR	Ca_14864	T	--
Ca4_P1_40653790	Ca4	40653790	TAAAAAA/TAAAAA	TGTGCAATGGCAATAACTCAT	GGCAGTTCACCATAGCCACT	59.1	819	DRR	Ca_14864	T	--
Ca4_P1_4066971	Ca4	4066971	ATTTTTT/ATTTTTTT	TCCATCAATCGACGCAAATA	CGTGCATTGTACGGAGTTTT	60.0	609	INTERGENIC	--	--	--
Ca4_P1_4069280	Ca4	4069280	ATAGGGCTA/ATA	AATTCTGGAACTTTGCACCG	TCAATTCATAAAATTAGCCCCTT	60.1	551	INTERGENIC	--	--	--
Ca4_P1_40700886	Ca4	40700886	AGAC/A	TCTTGATGTAACGTTTTGTTTCAGA	TGAATATTTGAGTTACGAATTTGATGA	60.1	478	INTERGENIC	--	--	--
Ca4_P1_40702737	Ca4	40702737	ACTCT/ACT	CCCCCATCATAAAATTGTGC	AAGATTAGTGGGCGACCCTT	60.0	392	INTERGENIC	--	--	--

INDEL marker IDs	Chromosomes/ unanchored scaffolds	Physical positions (bp)	InDels (*Kabuli* reference genome-CDC Frontier/PI)	Forward primers (5'-3')	Reverse primers (5'-3')	Anneling temperature (0C)	Expected amplified product size (bp)	Structural annotation		Functional annotation	
								Sequence components of *kabuli* genome	*Kabuli* gene accession IDs	NCBI-KOG	TFs
Ca4_P1_40704105	Ca4	40704105	ATTTTTT/ATTTTT	GGATCTCCACTGCCAGGTTA	TTTCGTTTCCCATTCTACGC	60.1	648	INTERGENIC	--	--	--
Ca4_P1_40719941	Ca4	40719941	CTTTTTTTTTTT/CTTTTTTTTTT	TCCTCCCTTTTGTTTTGTGC	TCGTCATCACCCACACTCAT	60.1	410	INTRON	Ca_23911	BK	FAR1
Ca4_P1_40728044	Ca4	40728044	TATTTGTAATT/TATTTGTAATTTGTAATT	AGCATACATTGGCACCACAA	CATGCATTTCATGGTAACCG	60.0	502	INTERGENIC	--	--	--
Ca4_P1_40729597	Ca4	40729597	T/TG	TTGTGAAATTGAAAAGGTTCCA	CAACCATGTGTGAGGTCCAT	59.5	817	INTERGENIC	--	--	--
Ca4_P1_40731137	Ca4	40731137	TC/T	TCATGGATAAGGTGGAAGACG	TGAATTCGAAAATCCATTTCATC	59.9	471	INTERGENIC	--	--	--
Ca4_P1_40734477	Ca4	40734477	AGG/AGGG	ATTAATGGTCACGACCCTGC	CACTCGCAAAGACCAGATCA	59.8	293	INTRON	Ca_23909	--	--
Ca4_P1_40734940	Ca4	40734940	AGG/AGGG	TGCACATCTTTCTGGTGCTC	ACATATTGGGTGGGGTGAGA	60.0	272	DRR	Ca_23909	--	--
Ca4_P1_40743126	Ca4	40743126	AATATATATATATAT/AATATATATATATATATATATATATATATAT	CACAAACACAACATGCGACA	ACCGCAACACACAAAATTCA	60.2	373	INTERGENIC	--	--	--
Ca4_P1_40743537	Ca4	40743537	ATGTTG/ATGTTGTTG	GAATTTTGTGTGTTGCGGTG	GCGGTGTTTTTCAACACATTT	60.0	527	INTERGENIC	--	--	--
Ca4_P1_40743968	Ca4	40743968	A/AAAC	TGTTGAAAAACACCGCAAGA	GCAATGAAGTGTGTGCCAAT	60.3	575	INTERGENIC	--	--	--
Ca4_P1_40746698	Ca4	40746698	CAA/CA	CAGTGTTGTGTCCGATGTCC	GTACCAGCAGCATTTGCTCA	60.0	410	INTRON	Ca_23907	R	--
Ca4_P1_40747563	Ca4	40747563	TAATCAA/TAATCAAGCAAATCAA	ACTGGAAACGGGTGTCTACG	AATCAAAGCGGGGAGAAAGT	60.0	390	INTRON	Ca_23907	R	--

**INDEL marker IDs**	**Chromosomes/ unanchored scaffolds**	**Physical positions (bp)**	**InDels (*Kabuli* reference genome-CDC Frontier/PI)**	**Forward primers (5'-3')**	**Reverse primers (5'-3')**	**Anneling temperature (0C)**	**Expected amplified product size (bp)**	**Structural annotation**		**Functional annotation**	
								**Sequence components of *kabuli* genome**	***Kabuli* gene accession IDs**	**NCBI-KOG**	**TFs**
Ca4_P1_40748450	Ca4	40748450	T/TG	TGGTTTGCTTCCCTCATTTC	GCTTCTGCGCCATTAAGTTC	60.1	136	DRR	Ca_23907	R	--
Ca4_P1_40748583	Ca4	40748583	GAAAAAAA/GAAAAAAAA	GAACTTAATGGCGCAGAAGC	GGGGATTCATTCCCATCTTT	60.0	156	DRR	Ca_23907	R	--
Ca4_P1_40748781	Ca4	40748781	TAAAAAAAA/TAAAAAAAAAA	GAACTTAATGGCGCAGAAGC	TGCCATTTGTTGGATTACCA	60.0	809	DRR	Ca_23906	R	B3
Ca4_P1_40752302	Ca4	40752302	CGG/CG	TAACTGCACGCATTCACACA	CATCCCTTCCCACCTCACTA	59.9	794	INTRON	Ca_23906	R	B3
Ca4_P1_40753856	Ca4	40753856	GTATATATATATATA/GTATATATA	TTACGATATTGCCGCAACTG	GCCCTTTCTTTCAGGCTTCT	59.7	444	INTRON	Ca_23905	J	--
Ca4_P1_40759167	Ca4	40759167	GTTTATTTTT/GTTTATTTTTTATTTATTTTT	CAAAGCAACCCCCAAACTAA	TTTTGGGTGATCAAGTTTGC	60.0	761	DRR	Ca_23905	J	--
Ca4_P1_40759370	Ca4	40759370	AA/AACA	TGTCTGCAAAGAGGAGTTTCAA	CCGAATTTGAATAGGTTGGG	60.0	317	DRR	Ca_23905	J	--
Ca4_P1_40760167	Ca4	40760167	GAAAAAAAAAA/GAAAAAAAAA	TCCAAATTGTGAACACCCCT	CAAACAAACCTTAAAAGGGCA	60.2	274	DRR	Ca_23905	J	--
Ca4_P1_40761334	Ca4	40761334	A/ATATAT	GAACTTTGTTATTGCATTCGTTTT	TGGATACACCGTCCAAAGTG	58.7	714	INTERGENIC	--	--	--
Ca4_P1_40763607	Ca4	40763607	CAAAAA/CAAAA	GCTCTGCTTGATTGAAAATGC	CCAATGCATGAAATTTGTTCTT	60.0	696	INTERGENIC	--	--	--
Ca4_P1_40763691	Ca4	40763691	ATTTT/ATTT	GCTCTGCTTGATTGAAAATGC	GGGTCCCTCTAACTTGTGAGC	60.0	826	INTERGENIC	--	--	--
Ca4_P1_40767434	Ca4	40767434	GTTTT/GTTTTT	CCAAGAAGTCCACGTTTGGT	AGGTACGTCCAGGCATCAAG	60.0	644	INTRON	Ca_23904	Q	--

INDEL marker IDs	Chromosomes/ unanchored scaffolds	Physical positions (bp)	InDels (*Kabuli* reference genome-CDC Frontier/PI)	Forward primers (5'-3')	Reverse primers (5'-3')	Anneling temperature (0C)	Expected amplified product size (bp)	Structural annotation		Functional annotation	
								Sequence components of *kabuli* genome	*Kabuli* gene accession IDs	NCBI-KOG	TFs
Ca4_P1_40767882	Ca4	40767882	TGAGG/TGAGGAGG	CAAATTCCACTGTTTTGCGTT	TTGGACCGTGATTTTTACTTTTT	60.0	695	INTRON	Ca_23904	Q	--
Ca4_P1_40769867	Ca4	40769867	AACTTACTT/AACTT	TGCTACGCATGCTTGAGAAC	TAAAAGTTTGCATGCCCCTC	60.2	628	INTRON	Ca_23904	Q	--
Ca4_P1_40769926	Ca4	40769926	TCC/TCCC	CAAAATGAACCCAACAAGGC	GAGTTTGTTTGCCGATGGTT	60.3	633	INTRON	Ca_23904	Q	--
Ca4_P1_40772487	Ca4	40772487	GTTTTTTTTT/GTTTTTTTT	AGAGGGAGTTGGTTGCAAAA	GTATGAGGTCGCAAGGAGGA	59.7	387	INTERGENIC	--	--	--
Ca4_P1_40773874	Ca4	40773874	TAAAAAAAA/TAAAAAAAAA	GTGTGCACCATAGGGAAGGT	ATCCTTGCACATCCCAAAAA	59.9	405	INTERGENIC	--	--	--
Ca4_P1_40773929	Ca4	40773929	TTAT/TTATAT	GTGTGCACCATAGGGAAGGT	ATCCTTGCACATCCCAAAAA	59.9	405	INTERGENIC	--	--	--
Ca4_P1_40777832	Ca4	40777832	CTTTTTTTTT/CTTTTTTTT	TAAGGTCCAAAATCCAGGCA	CCCTTCTTCTCCAAACTCCC	60.4	905	INTERGENIC	--	--	--
Ca4_P1_40779355	Ca4	40779355	ATTTTTTT/ATTTTTT	TTAATTGGGGCTGTTGCTTT	TTTGGTCCAATGCTGACAGA	59.6	585	INTERGENIC	--	--	--
Ca4_P1_40783665	Ca4	40783665	TTTAT/TTTATTAT	CCTCTAGTGGGTTTCTTTCATCA	AATGATGCCTCCATGCTTTC	59.6	853	INTERGENIC	--	--	--
Ca4_P1_40783879	Ca4	40783879	TCTAATTG/T	TCTTTTTCTATCACATGGTCAACA	AATGATGCCTCCATGCTTTC	58.7	732	INTERGENIC	--	--	--
Ca4_P1_40784079	Ca4	40784079	AC/A	AAGCACTTTTCAATTCACCCA	AATGATGCCTCCATGCTTTC	59.6	199	INTERGENIC	--	--	--
Ca4_P1_40787317	Ca4	40787317	GTTTTTTT/GTTTTTT	CCTGAGTAGGTCGAACAGGC	TGTTGCTACCTGCGAAGTCA	59.9	976	INTERGENIC	--	--	--

INDEL marker IDs	Chromosomes/ unanchored scaffolds	Physical positions (bp)	InDels (*Kabuli* reference genome-CDC Frontier/PI)	Forward primers (5'-3')	Reverse primers (5'-3')	Anneling temperature (0C)	Expected amplified product size (bp)	Structural annotation		Functional annotation	
								Sequence components of *kabuli* genome	*Kabuli* gene accession IDs	NCBI-KOG	TFs
Ca4_P1_4078760	Ca4	4078760	CAAAAAAAAA/CAAAAAAAAAAA	TTCACTTTTTGTGGAAGGGG	TAATTATTGGCCCCAGCTCA	59.9	684	INTERGENIC	--	--	--
Ca4_P1_40787903	Ca4	40787903	TAA/TA	TGACTTCGCAGGTAGCAACA	GCTCCGATGCAATCCTTAAA	60.6	472	INTERGENIC	--	--	--
Ca4_P1_40788340	Ca4	40788340	ATTTTTTTTT/ATTTTTTTT	GCATCGGAGCATGAAATGTA	TTCGCACACGATGAAGACTAA	59.7	167	INTERGENIC	--	--	--
Ca4_P1_40790403	Ca4	40790403	AT/ATCT	TGTTGATTTTGTTTGTCACGA	TCATTGTCCAGTGAAGGCAA	57.7	472	INTERGENIC	--	--	--
Ca4_P1_40791133	Ca4	40791133	CA/C	TTTAGACCCTTTCCAAGGCA	CCTCAAGAAATCATGAAGGACA	59.7	543	INTERGENIC	--	--	--
Ca4_P1_40793165	Ca4	40793165	TA/T	ATCGTGGGATTTTGCTTGAC	GAAATAGGTGGCGACGGATA	59.9	946	INTRON	Ca_23903	--	--
Ca4_P1_40797470	Ca4	40797470	CAAAAAAA/CAAAAAAAAAA	CAAGGTCCCCAAACATCACT	ATGGACCATCGATTCTGGAG	59.8	610	INTERGENIC	--	--	--
Ca4_P1_40797521	Ca4	40797521	ATT/AT	CAAGGTCCCCAAACATCACT	ATGGACCATCGATTCTGGAG	59.8	610	INTERGENIC	--	--	--
Ca4_P1_40799151	Ca4	40799151	TTAGCATA/TTA	CCAGCAATCTAATGAACGCA	ATGTCACCAATGGGGTTTGT	59.8	323	DRR	Ca_23902	R	--
Ca4_P1_40799742	Ca4	40799742	TCCCCC/TCCCCCC	ACAAACCCCATTGGTGACAT	TGGCTTGTAAGGCTGTGTTG	60.0	740	DRR	Ca_23902	R	--
Ca4_P1_40801294	Ca4	40801294	AAAA/AAAATACCAGTAAA	AGCTTGCCTCTGAGCTCTTG	TTTATGGCATGGGACCAAAT	60.0	736	INTRON	Ca_23902	R	--
Ca4_P1_40802030	Ca4	40802030	A/AC	CATGAAGCCAAGAAGCAACA	TTGACACCTGTCACACATGC	60.0	518	INTRON	Ca_23902	R	--

INDEL marker IDs	Chromosomes/ unanchored scaffolds	Physical positions (bp)	InDels (*Kabuli* reference genome-CDC Frontier/PI)	Forward primers (5'-3')	Reverse primers (5'-3')	Anneling temperature (0C)	Expected amplified product size (bp)	Structural annotation		Functional annotation	
								Sequence components of *kabuli* genome	*Kabuli* gene accession IDs	NCBI-KOG	TFs
Ca4_P1_40807547	Ca4	40807547	TCAACAACAA/TCAACAACAACAA	CACCTGAAAGCTCCCTCAAG	CGACCTTGAAGGCTTAGTCG	60.0	505	INTRON	Ca_23902	R	--
Ca4_P1_40809750	Ca4	40809750	A/AC	TGCAAGAACAAATCCTTCCC	GCAGCTTTTGGCAGTTCTCT	60.1	278	INTRON	Ca_23902	R	--
Ca4_P1_40810384	Ca4	40810384	G/GAAAAAA	AAAAACGGCAGTAAAAGCCA	GGGACGAGGTACATGCAGAT	59.8	298	INTRON	Ca_23902	R	--
Ca4_P1_40811635	Ca4	40811635	GAAAA/GAA	TTACCACCAAGGTCAGCTCC	CGGGAAGATCATTCACGAAG	60.1	704	DRR	Ca_23901	R	MYB
Ca4_P1_40812283	Ca4	40812283	CTTT/CTT	TTGCAAATACATTGTGCCATC	GAAGTATCACGGCCCCACTA	59.4	322	DRR	Ca_23901	R	MYB
Ca4_P1_40819629	Ca4	40819629	TCAACAACAACAA/TCAACAACAACAACAA	GGCCTGGAACACTTTCCATA	CAAGGGGGTGAAAAATCAGA	59.9	407	INTRON	Ca_23900	R	--
Ca4_P1_40821058	Ca4	40821058	ACA/ACATGCCTCA	CCGTTAAAACGATGGTCGAT	TTAATTTTCCAGCCATGCAA	59.8	338	INTERGENIC	--	--	--
Ca4_P1_40826712	Ca4	40826712	GAAAAAAAA/GAAAAAAAAA	TGCCTGTGATGTAGGTCTCG	AATTGAGAACGACGACGAGG	59.9	850	INTERGENIC	--	--	--
Ca4_P1_40832080	Ca4	40832080	ATTTTTTTTTT/ATTTTTTTTT	AATGTGTCATTGTCCCAGAGC	TAACAAAATCACCGCACCAA	60.0	734	INTERGENIC	--	--	--
Ca4_P1_40833178	Ca4	40833178	TA/T	TTTTTATTTGTCAACCCGCA	CAATGATCGTCGTCTATTCACAA	59.1	582	INTERGENIC	--	--	--
Ca4_P1_40833473	Ca4	40833473	ATTTTTTTT/ATTTTTTTTTT	TTTTTATTTGTCAACCCGCA	TTCAAACCCCCATTTCTTGT	59.1	766	INTERGENIC	--	--	--
Ca4_P1_4083518	Ca4	4083518	ATATTAT/ATATTATTAT	TCTTATGAATGAGACAGAGGCA	CCAAGACCCCGAGTTAGAGA	57.6	491	INTERGENIC	--	--	--

INDEL marker IDs	Chromosomes/ unanchored scaffolds	Physical positions (bp)	InDels (*Kabuli* reference genome-CDC Frontier/PI)	Forward primers (5'-3')	Reverse primers (5'-3')	Anneling temperature (0C)	Expected amplified product size (bp)	Structural annotation		Functional annotation	
								Sequence components of *kabuli* genome	*Kabuli* gene accession IDs	NCBI-KOG	TFs
Ca4_P1_40836011	Ca4	40836011	ATTTTTTTTTT/ATTTTTTTTT	GTGTCGGTCACGACATTCAG	TCAAATTGAAATCGAACACATAAAA	60.2	253	INTERGENIC	--	--	--
Ca4_P1_40836419	Ca4	40836419	TAAAAAAAA/TAAAAAAA	GTGTCGGTCACGACATTCAG	AGATTGAAAAGTTGCACCCG	60.2	714	INTERGENIC	--	--	--
Ca4_P1_40837444	Ca4	40837444	TAAAAAAAAA/TAAAAAAAA	GGAAGCAGATGGAAAAGGAA	TCAACTTTGAGGACCCAACA	59.2	494	INTERGENIC	--	--	--
Ca4_P1_4083772	Ca4	4083772	CAT/CATAT	TCTCTAACTCGGGGTCTTGG	CCTTGCTGAAGAATCATCCC	59.3	489	INTERGENIC	--	--	--
Ca4_P1_40839647	Ca4	40839647	TA/TAA	AGGTGGGCTTCTCCATTTTT	CACAAAGTCAGCACTCCGAA	59.9	409	INTRON	Ca_23899	--	--
Ca4_P1_40839677	Ca4	40839677	GAT/G	AGGTGGGCTTCTCCATTTTT	CACAAAGTCAGCACTCCGAA	59.9	409	INTRON	Ca_23899	--	--
Ca4_P1_40842049	Ca4	40842049	A/AG	CATTTTCTTTCCATTTGGCG	CCTTTTGGACAACAGGCAGT	60.4	747	DRR	Ca_23898	--	--
Ca4_P1_40842793	Ca4	40842793	TC/T	CTGGATTGAGTTGAGGCCAT	TGGACCAAACCAAGCACATA	60.1	568	INTERGENIC	--	--	--
Ca4_P1_40842971	Ca4	40842971	AGTGTGT/AGTGTGTGT	AAAATTTGTGCATCAAGGGC	TCAAGATTGGGCCAAGAATC	59.9	551	INTERGENIC	--	--	--
Ca4_P1_40843717	Ca4	40843717	ATTTTTT/ATTTTT	GATTCTTGGCCCAATCTTGA	TTTGAGATGGTTGGTGTGTCA	60.0	624	INTERGENIC	--	--	--
Ca4_P1_40844159	Ca4	40844159	TAAAAAA/TAAAAAAA	AACATTAATGCAACACGCCA	GACTTCCGTCCCTTACAGAGAA	60.0	199	INTERGENIC	--	--	--
Ca4_P1_40846078	Ca4	40846078	TC/TCATGGTCC	CCATCAATTGGGTGGTTGTT	GGGAGAACATCATGCTCCAT	60.5	449	INTERGENIC	--	--	--

INDEL marker IDs	Chromosomes/ unanchored scaffolds	Physical positions (bp)	InDels (*Kabuli* reference genome-CDC Frontier/PI)	Forward primers (5'-3')	Reverse primers (5'-3')	Anneling temperature (0C)	Expected amplified product size (bp)	Structural annotation		Functional annotation	
								Sequence components of *kabuli* genome	*Kabuli* gene accession IDs	NCBI-KOG	TFs
Ca4_P1_40846133	Ca4	40846133	ATTT/ATTTT	CCATCAATTGGGTGGTTGTT	GGGAGAACATCATGCTCCAT	60.5	449	INTERGENIC	--	--	--
Ca4_P1_40852976	Ca4	40852976	T/TAA	ACCTTGAGATTGTTCGCACC	AAGTTAAGAGACCGCATAAGCAA	60.1	203	DRR	Ca_23897	--	--
Ca4_P1_40854093	Ca4	40854093	TAAAAAA/TAAAAA	ACACCCAGAAAGTACCCACG	TGAAATGCATTCGTCACACA	59.9	610	DRR	Ca_23897	--	--
Ca4_P1_4085722	Ca4	4085722	TAGAGA/TAGAGAGA	TCGGCCGATAGCAAAAGTTA	CCATTTTCTTCCACTCCCAA	60.7	685	INTERGENIC	--	--	--
Ca4_P1_4086337	Ca4	4086337	TAAAAAAAA/TAAAAAAAAAAA	GGGGCGTGTCAAAGTTAAAA	ACCATCCGTATAAAGCACGC	60.0	453	INTERGENIC	--	--	--
Ca4_P1_4086522	Ca4	4086522	GATATA/GATATATA	GCGTGCTTTATACGGATGGT	TGATGGAGGCCTAAACAAGG	60.0	499	INTERGENIC	--	--	--
Ca4_P1_40880303	Ca4	40880303	GA/GAA	GGAAGTTTCCAAAGCACGAG	AAGTTCCATTGCTTGCTGGT	59.9	411	INTERGENIC	--	--	--
Ca4_P1_40880423	Ca4	40880423	A/ATG	CTTTGGGAACCAAGCACAAT	CAAGGTACCATTGATTTGTGGA	60.0	392	INTERGENIC	--	--	--
Ca4_P1_40881164	Ca4	40881164	TTCA/TTCAATATATCTCA	AAGACCCACATTTGGTGAAAA	ATGAGTTCTTCATCGGTCGG	59.3	506	INTERGENIC	--	--	--
Ca4_P1_40881410	Ca4	40881410	CAAAA/CAAA	AAGACCCACATTTGGTGAAAA	ATGAGTTCTTCATCGGTCGG	59.3	506	INTERGENIC	--	--	--
Ca4_P1_40882947	Ca4	40882947	GGAA/G	GAAAACCAATTGGGTCATGC	GGTTAAATTGCATCTGTAGTCCC	60.2	489	INTRON	Ca_11021	--	NAC
Ca4_P1_40887107	Ca4	40887107	GAAAAAAAAA/GAAAAAAAA	TGAATCCAATCCATCCAACA	ACTATCGACCAACTGCCCAC	59.7	705	INTERGENIC	--	--	--

INDEL marker IDs	Chromosomes/ unanchored scaffolds	Physical positions (bp)	InDels (*Kabuli* reference genome-CDC Frontier/PI)	Forward primers (5'-3')	Reverse primers (5'-3')	Anneling temperature (0C)	Expected amplified product size (bp)	Structural annotation		Functional annotation	
								Sequence components of *kabuli* genome	*Kabuli* gene accession IDs	NCBI-KOG	TFs
Ca4_P1_40887198	Ca4	40887198	TAAAAAAA/TAAAAAA	TGAATCCAATCCATCCAACA	ACTATCGACCAACTGCCCAC	59.7	705	INTERGENIC	--	--	--
Ca4_P1_40887510	Ca4	40887510	ATGT/ATGTGT	GCCATTGCTAGCTCTTTCGT	ACTATCGACCAACTGCCCAC	59.6	296	INTERGENIC	--	--	--
Ca4_P1_40891579	Ca4	40891579	AATATATA/AATA	GCACAAAAGCAGTGTAGCCA	CCCGAACCTCTCCACAATAA	60.1	869	INTRON	Ca_11020	R	G2-like
Ca4_P1_40891676	Ca4	40891676	AA/AAATTA	GCACAAAAGCAGTGTAGCCA	CCCGAACCTCTCCACAATAA	60.1	869	INTRON	Ca_11020	R	G2-like
Ca4_P1_40891712	Ca4	40891712	TCC/TC	ATCGAATCCTCGTTGGAATG	CCCGAACCTCTCCACAATAA	59.9	725	INTRON	Ca_11020	R	G2-like
Ca4_P1_40892990	Ca4	40892990	AATT/AATTATT	TTTGTTGGCTTGCACTTTTG	TTTCCTCCTAGCCGAGTTCA	59.9	138	INTRON	Ca_11020	R	G2-like
Ca4_P1_40893226	Ca4	40893226	CTCTATCTAT/CTCTAT	TGAACTCGGCTAGGAGGAAA	TATATGGGGGTCCATTCCTG	59.9	294	DRR	Ca_11020	R	G2-like
Ca4_P1_40893320	Ca4	40893320	CATATA/CATA	TGAACTCGGCTAGGAGGAAA	CGCAAGTGTTGGACAATGAT	59.9	703	DRR	Ca_11020	R	G2-like
Ca4_P1_40896217	Ca4	40896217	GAAAAAAAA/GAAAAAAA	AGTTGCAGAAATGTGAAGAAAAA	TTGGATCACACCCATTTGAA	58.1	619	INTERGENIC	--	--	--
Ca4_P1_40896275	Ca4	40896275	ATGTGT/ATGT	CACAAATATTCATAAGTGTGACTTCAA	GAACCTGATGAACTGTGGCA	58.7	818	INTERGENIC	--	--	--
Ca4_P1_40901186	Ca4	40901186	ATTTTTATTATTTTTTATT/ATTTTTATT	GGGTATATTAATTTAACAACTCCCTCC	TTAGGAGGTCGCATCTTCGT	59.7	462	INTERGENIC	--	--	--
Ca4_P1_40901246	Ca4	40901246	AAA/AAATAA	GGGTATATTAATTTAACAACTCCCTCC	TTAGGAGGTCGCATCTTCGT	59.7	462	INTERGENIC	--	--	--

INDEL marker IDs	Chromosomes/ unanchored scaffolds	Physical positions (bp)	InDels (*Kabuli* reference genome-CDC Frontier/PI)	Forward primers (5'-3')	Reverse primers (5'-3')	Anneling temperature (0C)	Expected amplified product size (bp)	Structural annotation		Functional annotation	
								Sequence components of *kabuli* genome	*Kabuli* gene accession IDs	NCBI-KOG	TFs
Ca4_P1_40901551	Ca4	40901551	CTTTTTTTT/CTTTTTTTTT	ACGAAGATGCGACCTCCTAA	AACACAAAGGAAAGCGGAAA	59.8	561	INTERGENIC	--	--	--
Ca4_P1_40901611	Ca4	40901611	CTTTTTTT/CTTTTTTTT	ACGAAGATGCGACCTCCTAA	AACACAAAGGAAAGCGGAAA	59.8	561	INTERGENIC	--	--	--
Ca4_P1_40902003	Ca4	40902003	ACTCT/ACT	TTCCGCTTTCCTTTGTGTTT	CATGGAGTGATCGATTGTCG	59.7	476	INTERGENIC	--	--	--
Ca4_P1_40903317	Ca4	40903317	TAA/TA	GCCCAAGCACAAACCTACAT	GCTTTTGTCAACCCCTGGTA	60.0	310	INTERGENIC	--	--	--
Ca4_P1_40904392	Ca4	40904392	CAAA/CAA	AAAGGCAACCGACTAGGGTT	GAAATGGATCTAAGACGACGG	60.0	173	INTERGENIC	--	--	--
Ca4_P1_40907158	Ca4	40907158	TATACAT/TATACATAATACAT	TGTCGTTGAAATCAAAGCAAT	ATCGTTTGGGTGAAGAATCG	58.3	334	INTERGENIC	--	--	--
Ca4_P1_4090948	Ca4	4090948	T/TG	TAAGCACCCTTCCGCTTCTA	TGAAATAATGGGCTAGGGCTT	60.0	564	DRR	Ca_03847	R	SBP
Ca4_P1_4090976	Ca4	4090976	A/AT	TAAGCACCCTTCCGCTTCTA	TGAAATAATGGGCTAGGGCTT	60.0	564	DRR	Ca_03847	R	SBP
Ca4_P1_40912106	Ca4	40912106	CG/CGGCTGTTTGAACAAG	TGTGGGAAAATGAGACACCA	AAACAAATGGTGCAAGAGGG	59.9	484	DRR	Ca_11019	R	--
Ca4_P1_40922455	Ca4	40922455	CTTTTTTTTTT/CTTTTTTTTTTTT	CAGCATGCATCTGGAAAGAA	AATGAGGCGGTGGTATCTTG	59.9	534	INTRON	Ca_11016	R	--
Ca4_P1_40924286	Ca4	40924286	ACAATATG/ACAATATGTAGCTTCAATATG	GTGATCTACGTGGTGCCCTT	ACCCAAAATGAGGTGGTTCA	60.0	548	DRR	Ca_11015	--	bZIP
Ca4_P1_40926854	Ca4	40926854	TA/TAA	ACACACCGACACCGACAATA	GGTTGGAAAATGCAAGCCTA	59.9	311	INTRON	Ca_11015	--	bZIP

INDEL marker IDs	Chromosomes/ unanchored scaffolds	Physical positions (bp)	InDels (*Kabuli* reference genome-CDC Frontier/PI)	Forward primers (5'-3')	Reverse primers (5'-3')	Anneling temperature (0C)	Expected amplified product size (bp)	Structural annotation		Functional annotation	
								Sequence components of *kabuli* genome	*Kabuli* gene accession IDs	NCBI-KOG	TFs
Ca4_P1_40929080	Ca4	40929080	T/TC	ATGGTGGGGGTAAGCAATCT	AAGAAGCGCGTATTGGTGTT	60.6	813	INTRON	Ca_11015	--	bZIP
Ca4_P1_40929158	Ca4	40929158	C/CA	TCCATCATATTTGCAATGGTTC	AAGAAGCGCGTATTGGTGTT	59.7	506	INTRON	Ca_11015	--	bZIP
Ca4_P1_40929966	Ca4	40929966	TAAAAAAAAA/TAAAAAAA	TCATCGTTGGTAAAAATTTAGAGGA	TTAAGATGGTTGAGGCCTTATT	60.2	418	INTRON	Ca_11015	--	bZIP
Ca4_P1_40930508	Ca4	40930508	ATT/ATTTT	GGCCTCAACCATCTTAATAAAACA	AAGCATTCCACCATCACTCC	60.6	487	INTRON	Ca_11015	--	bZIP
Ca4_P1_40931865	Ca4	40931865	TCCCC/TCCC	TGCTTGGAAAACAAGCAATTT	AACTCGTGGGAACGCACTAC	59.8	779	INTRON	Ca_11015	--	bZIP
Ca4_P1_40934743	Ca4	40934743	A/AGC	GCTCGTCAACAACGTGAGAA	TGCAGATTGCAAAGACAAGG	60.0	689	INTERGENIC	--	--	--
Ca4_P1_40935936	Ca4	40935936	T/TC	TTTTCACGAATGATCCACCA	GTCGTTCAATCCAACCTCGT	59.9	350	INTERGENIC	--	--	--
Ca4_P1_40937505	Ca4	40937505	GTTTTTTTTT/GTTTTTTTTTT	TAATCCGATGGCAACATGAA	CGGTAGGAAATGATGAAACCA	59.9	615	INTERGENIC	--	--	--
Ca4_P1_40944823	Ca4	40944823	CAAAGAAA/CAAAGAAAGAAA	GCACATTGGATGTTTGATGG	TGGGTTTTGTTTGAAATTTTTG	59.8	698	INTERGENIC	--	--	--
Ca4_P1_40948499	Ca4	40948499	GAAAAAAAAA/GAAAAAAAAAA	AGGGTTGGGTTTGAGAGAAA	GGGAAGGGAAAAATGGATGT	58.6	702	DRR	Ca_11013	--	--
Ca4_P1_40948599	Ca4	40948599	CAAAAAAAA/CAAAAAAAAAAA	AGGGTTGGGTTTGAGAGAAA	GGGAAGGGAAAAATGGATGT	58.6	702	DRR	Ca_11013	--	--
Ca4_P1_40949429	Ca4	40949429	TT/TTAT	CCATGTGCACCATAAATTGC	CAGAATGTTCTTTCGGAATGG	59.8	612	INTRON	Ca_11013	--	--

INDEL marker IDs	Chromosomes/ unanchored scaffolds	Physical positions (bp)	InDels (*Kabuli* reference genome-CDC Frontier/PI)	Forward primers (5'-3')	Reverse primers (5'-3')	Anneling temperature (0C)	Expected amplified product size (bp)	Structural annotation		Functional annotation	
								Sequence components of *kabuli* genome	*Kabuli* gene accession IDs	NCBI-KOG	TFs
Ca4_P1_40950539	Ca4	40950539	TAAAAAAA/TAAAAAAAA	CCCAAAATAGAAACAC CAAAGG	AACGCAAAGATGGAAT GGAG	59.7	687	INTRON	Ca_11013	--	--
Ca4_P1_40950816	Ca4	40950816	CCTC/CCTCTC	TCCATCTTTGCGTTTCT CCT	CATGTGACAAAAAGGG TGGA	59.8	313	INTERGENIC	--	--	--
Ca4_P1_4095100	Ca4	4095100	TTCTTTTCT/TT	GGAGGACGCTTTGTTC TGAG	CCGGAATTCATTACCA CACC	60.0	313	INTRON	Ca_03846	T	--
Ca4_P1_40951362	Ca4	40951362	TAAAAAAAAA/TAAAAAAAA	TGTGTGCACATCACGA AAGA	ATCGTGAGTTTGAGGG GAGA	59.9	939	INTERGENIC	--	--	--
Ca4_P1_40956331	Ca4	40956331	CAA/CA	CCTCTTGGAAGTTTCC CTCC	GGGTACCCAACAAGG GATTT	60.0	682	INTERGENIC	--	--	--
Ca4_P1_40958047	Ca4	40958047	GTTT/GTTTT	CCGTGTTGGTTGTGTT GTTC	TTTGTGGGTGTTTGAG ACCA	59.9	558	INTERGENIC	--	--	--
Ca4_P1_40966159	Ca4	40966159	GCATTTG/GCATTTGATGCCATTGTCA TTTG	AACTATGGCTTTGTCG GGTG	CTCAAAAACGAGCCAA AAGC	60.0	399	INTERGENIC	--	--	--
Ca4_P1_40967580	Ca4	40967580	TAAAAAAAAAA/TAAAAAAAAAAA	AATGAAAAACTTGTTG CGGC	GTTTGGAATCGGGTAA CGAA	60.1	748	INTERGENIC	--	--	--
Ca4_P1_40968955	Ca4	40968955	TC/TCC	CACTGGAATGATTGCC AAAA	TTCTACCCAAGATCGG ATGG	59.5	398	INTERGENIC	--	--	--
Ca4_P1_40971058	Ca4	40971058	GAAAAAAAAA/GAAAAAAAAAA	GGACTCCCAACCCTTT TAGG	TGAGATGTGTCCTGTT TGGC	59.8	466	INTERGENIC	--	--	--
Ca4_P1_40996538	Ca4	40996538	GTTT/GTT	CGGTGTTAAAAATTGG GGTG	AAACGTGGAGGTGATT TTGC	60.1	445	DRR	Ca_11010	--	--
Ca4_P1_40996595	Ca4	40996595	GAAAAA/GAAAA	CGGTGTTAAAAATTGG GGTG	AAACGTGGAGGTGATT TTGC	60.1	445	DRR	Ca_11010	--	--

INDEL marker IDs	Chromosomes/ unanchored scaffolds	Physical positions (bp)	InDels (*Kabuli* reference genome-CDC Frontier/PI)	Forward primers (5'-3')	Reverse primers (5'-3')	Anneling temperature (0C)	Expected amplified product size (bp)	Structural annotation		Functional annotation	
								Sequence components of *kabuli* genome	*Kabuli* gene accession IDs	NCBI-KOG	TFs
Ca4_P1_40997097	Ca4	40997097	C/CT	GCAAAATCACCTCCACGTTT	AACGGTCGTCTCCATTCATC	60.0	762	DRR	Ca_11010	--	--
Ca4_P1_40998297	Ca4	40998297	TAAAAAA/TAAAAAAA	GCACAACTGCATCATTGGTT	TTTTGGCATAATTCTTATTTTGAA	59.6	820	INTERGENIC	--	--	--
Ca4_P1_40999557	Ca4	40999557	AAA/AAACAA	TTCTATCAAACAAACCCCGAA	TGCACGTCTAACTCAACTCACA	59.4	574	INTERGENIC	--	--	--
Ca4_P1_41000861	Ca4	41000861	GAAT/GAATTATTAACCAAT	TTTATTTGTGTGGCGGACTG	TAGGCTTCTTGTTGGATGGG	59.6	278	INTERGENIC	--	--	--
Ca4_P1_41000984	Ca4	41000984	CTAT/CTATAT	CCCATCCAACAAGAAGCCTA	CGAAAATATGTTTGTGGGGG	60.1	514	INTERGENIC	--	--	--
Ca4_P1_41003509	Ca4	41003509	A/AG	GCCAAATGAAATAATGATCGC	CGCTCTTTCGACACACTCAA	59.4	348	INTERGENIC	--	--	--
Ca4_P1_41007526	Ca4	41007526	CTTTT/CTTTTT	TATGCTGGCAAACAACCAAA	ATGCTCCTAACATGATCCGC	60.1	667	INTERGENIC	--	--	--
Ca4_P1_41007615	Ca4	41007615	AATATATATATA/AATATATATATATATA	TATGCTGGCAAACAACCAAA	ATGCTCCTAACATGATCCGC	60.1	667	INTERGENIC	--	--	--
Ca4_P1_41007675	Ca4	41007675	ATTTTT/ATTT	TATGCTGGCAAACAACCAAA	ATGCTCCTAACATGATCCGC	60.1	667	INTERGENIC	--	--	--
Ca4_P1_41007703	Ca4	41007703	CTTAATTAGTTAATTAGTTAA/CTTAATTAGTTAA	TATGCTGGCAAACAACCAAA	ATGCTCCTAACATGATCCGC	60.1	667	INTERGENIC	--	--	--
Ca4_P1_41010880	Ca4	41010880	CATTATATTATATT/CATTATATT	CTTCTCCCCTGTTAGTCCCC	AGGTCAAACCGTCAAAGTGC	59.9	730	DRR	Ca_11009	--	bHLH
Ca4_P1_41012180	Ca4	41012180	TAAAAA/TAAAA	GCTTGATGGGAGAGCAAAAA	TTTGGCAGCACTTCTATTGTG	60.3	738	DRR	Ca_11009	--	bHLH

INDEL marker IDs	Chromosomes/ unanchored scaffolds	Physical positions (bp)	InDels (*Kabuli* reference genome-CDC Frontier/PI)	Forward primers (5'-3')	Reverse primers (5'-3')	Anneling temperature (0C)	Expected amplified product size (bp)	Structural annotation		Functional annotation	
								Sequence components of *kabuli* genome	*Kabuli* gene accession IDs	NCBI-KOG	TFs
Ca4_P1_41012200	Ca4	41012200	CAAA/CAA	GCTTGATGGGAGAGCAAAAA	CCATTTGGGAATTATGAAGCA	60.3	807	DRR	Ca_11009	--	bHLH
Ca4_P1_41015733	Ca4	41015733	TAAAAAA/TAAAAAAA	CAAATTTTGATGATCTCACACGA	GCCCCATAAAAAGATGCTCA	60.0	504	INTERGENIC	--	--	--
Ca4_P1_41016384	Ca4	41016384	ATTTTTTT/ATTTTTTTT	AAACACGGGGCAACATAAAG	TGTTGGTGGTGAACGAGGTA	59.9	631	INTERGENIC	--	--	--
Ca4_P1_41027713	Ca4	41027713	AATATATATATATATATATATATATATAT/AATATATATATATATATATATATATATATATATAT	TGAATCCCAAAATTTGAACCA	TTTGATCGCAAGGGTTTAGG	60.2	637	INTERGENIC	--	--	--
Ca4_P1_41029836	Ca4	41029836	AATATATATATATATA/AATATATATATATATA	TTTCTCTCCATCGACCCATC	GAACCTTTGGGAAATCAGCA	60.0	760	INTERGENIC	--	--	--
Ca4_P1_4103235	Ca4	4103235	ACTC/ACTCCTC	AACATGGCAATTGATTGAAGG	AGTCCAACTTCATTGCCTGC	59.8	496	DRR	Ca_03844	--	--
Ca4_P1_4104877	Ca4	4104877	GAAAAAAAAAAAAAAA/GAAAAAAAAA	GAAGTGGGAATGGGCTGTTA	TCAAATTCAGTGCGTTTGCT	59.9	495	DRR	Ca_03843	T	BBR-BPC
Ca4_P1_4104993	Ca4	4104993	TTATAT/TTAT	GAAGTGGGAATGGGCTGTTA	AAGTTAGGGGGACACAACCC	59.9	677	DRR	Ca_03843	T	BBR-BPC
Ca4_P1_41159362	Ca4	41159362	ATTTTTTTTTTT/ATTTTTTTTTTTTT	ATGCTTGGACCACCATTCTC	CGTGTGAAACCTTTGCTGAA	59.9	891	INTERGENIC	--	--	--
Ca4_P1_41162022	Ca4	41162022	CATATATATATATATATATA/CATATATATATATATATATATA	TTTGATTGTCCTGCACCAAA	TGGTAGCCTTGAACTTGTGC	60.1	261	INTERGENIC	--	--	--
Ca4_P1_4116752	Ca4	4116752	TA/TACA	ACATGTTGGAAATCCGGGTA	TCGGCTACTCAAACAAAGGTG	60.1	516	INTERGENIC	--	--	--
Ca4_P1_4118588	Ca4	4118588	AATGCATTATG/AATG	CCCCTCGATAGATTGCAAAA	TAGGGTATAGCATGGGCAGG	60.0	301	INTERGENIC	--	--	--

INDEL marker IDs	Chromosomes/ unanchored scaffolds	Physical positions (bp)	InDels (*Kabuli* reference genome-CDC Frontier/PI)	Forward primers (5'-3')	Reverse primers (5'-3')	Anneling temperature (0C)	Expected amplified product size (bp)	Structural annotation		Functional annotation	
								Sequence components of *kabuli* genome	*Kabuli* gene accession IDs	NCBI-KOG	TFs
Ca4_P1_4118787	Ca4	4118787	CTTTTTTTTTTT/CTCTTTTTTTTTTTTT	CCCCTCGATAGATTGCAAAA	CATTCAGCACAAGCAAAAGC	60.0	410	INTERGENIC	--	--	--
Ca4_P1_41195132	Ca4	41195132	TTATATATATATATATATATATATATATATAT/TTATATATATATATATATATATATATATAT	TTCATGGGAATTTTGATAGATTTG	CAATTTAACAATTTGTGTTACGCC	59.3	862	DRR	Ca_10991	K	FAR1
Ca4_P1_41200706	Ca4	41200706	TAAAAAAAAAAAAA/TAAAAAAAAAAAA	CCCCCTGAAATATTGGACCT	TTTGGATGTTGAAGACGTGC	60.0	168	INTERGENIC	--	--	--
Ca4_P1_4120817	Ca4	4120817	GTTT/GTTTCATTTT	ATGGTCGAAACCAATTGAGC	TTTTGGAGGATTGAGATGGG	59.9	660	INTERGENIC	--	--	--
Ca4_P1_4120892	Ca4	4120892	G/GA	ATGGTCGAAACCAATTGAGC	TTTTGGAGGATTGAGATGGG	59.9	660	INTERGENIC	--	--	--
Ca4_P1_4121122	Ca4	4121122	AT/A	AAATGGCGAACTGAGTCCAT	TTTTGGAGGATTGAGATGGG	59.6	540	INTERGENIC	--	--	--
Ca4_P1_4121415	Ca4	4121415	ATT/AT	CTCATATGAACGGCCCATCT	TGTTTGTACGGACTCGACCA	59.9	403	INTERGENIC	--	--	--
Ca4_P1_4123332	Ca4	4123332	GAAAAA/GAAAAAAA	TGAATTGCTTTGCTCCCTTT	TGATTCTCGTTCTCGATCCC	59.8	652	INTERGENIC	--	--	--
Ca4_P1_41261010	Ca4	41261010	CTTATTATTATTATTATTATTATTATTATTATTATTATTATTATTATTATTATTATTATTATTATTATTATT/CTTATTATTATTATTATTATTATTATTATTATTATTATTATTATTATTATTATTATTATTATTATTATTATTATT	GCAATGTTGGACGACACAAT	CATTTAACAACCGTTTCGGG	59.4	844	INTERGENIC	--	--	--
Ca4_P1_4126408	Ca4	4126408	AATATATATATATATATATAT/AATATATATATATATATATATATAT	TTTGATCCATTTGCTCGTGA	TTTTTGGATATTGACCCGGA	60.2	947	INTERGENIC	--	--	--
Ca4_P1_41272417	Ca4	41272417	GATTATTATTATTATTATTATTATTATTATT/GATTATTATTATTATTATTATTATTATT	GGTTCACATGGAACTCCAACA	AGCCTTTTGCTTCCTTCCTC	60.8	737	INTERGENIC	--	--	--
Ca4_P1_41273196	Ca4	41273196	CTTTTTTT/CTTTTTT	AGAGGAAGGAAGCAAAAGGC	TCGCTTCCCCATAGAACAAC	60.0	530	INTERGENIC	--	--	--

INDEL marker IDs	Chromosomes/ unanchored scaffolds	Physical positions (bp)	InDels (*Kabuli* reference genome-CDC Frontier/PI)	Forward primers (5'-3')	Reverse primers (5'-3')	Anneling temperature (0C)	Expected amplified product size (bp)	Structural annotation		Functional annotation	
								Sequence components of *kabuli* genome	*Kabuli* gene accession IDs	NCBI-KOG	TFs
Ca4_P1_4127483	Ca4	4127483	AG/A	TGTGCATGACCAAATACTCCA	ATATACGGCCGCTCATCCTA	60.0	248	INTERGENIC	--	--	--
Ca4_P1_41283155	Ca4	41283155	AATATATATATATATATATAT/AATATATATATATATATATAT	TCTCTTGTGCCTCATCTATGACA	ATGCACTTAGCCATGTGTGC	59.9	636	DRR	Ca_10982	P	GRAS
Ca4_P1_4131889	Ca4	4131889	ATATTATTATTATTATTATTATTAT/ATATTATTATTATTATTATTAT	GCCTTCAATATTTATTTGGTCCAT	GATGAGAATGAAAATAGGAGGGA	59.5	817	INTERGENIC	--	--	--
Ca4_P1_41320371	Ca4	41320371	TGGGG/TGGG	TGGAGCATATGCGTAGCAAG	GCTTGCTTTTGCCCATACAT	60.0	761	DRR	Ca_10977	K	LBD
Ca4_P1_41327650	Ca4	41327650	TTTATTATTATTATTAT/TTTATTATTATTATTATTAT	AAGAGCTAGACGAGTTCGACG	GCCGCCAGACATTCATTATT	58.9	569	INTERGENIC	--	--	--
Ca4_P1_41328217	Ca4	41328217	GTCTCTCTCTCTCTCTCTCTCTCTCTCTCTCT/GTCTCTCTCTCTCTCTCTCTCTCTCTCTCTCT	AATAATGAATGTCTGGCGGC	GCCGCTTATTTGCTTCTGAC	59.9	669	INTERGENIC	--	--	--
Ca4_P1_41370951	Ca4	41370951	AATTATTATTATTATTATTATTATTATTATTATTATTATTATTATTATTATTATTATTATTATTATTATTATTAT/AATTATTATTATTATTATTATTATTATTATTATTATTATTATTATTATTATTATTATTATTATTATTATTATTAT	CATGAACGGGTCTAAAAATTCA	TTTGGGATTTTTGGTCTTGC	59.0	401	INTERGENIC	--	--	--
Ca4_P1_41376499	Ca4	41376499	TAAA/TAAAA	TATTCGCGAAATGGGTTTCT	CCTGCAGGTTCTTCGTCTTC	59.5	447	DRR	Ca_10970	--	NF-YB
Ca4_P1_41435167	Ca4	41435167	AA/AAATATTA	TAGGGGAACGATGTTTGCTT	TGGAGTTGATCATTTGTGTCA	59.6	476	INTERGENIC	--	--	--
Ca4_P1_41435407	Ca4	41435407	AATTATTATTATTATTATTATTATTATTATTATT/AATTATTATTATTATTATTATTATTATTATT	TAGGGGAACGATGTTTGCTT	CAGCGCATCAATCAAGTACA	59.6	698	INTERGENIC	--	--	--
Ca4_P1_4156202	Ca4	4156202	ACTT/ACTTCTT	TTGTGGTCCTTCCATTTTCC	AGCTGCAAGGAGCTATCCAA	59.8	316	DRR	Ca_03840	R	--
Ca4_P1_41713312	Ca4	41713312	TAAAAA/TAAAAAA	CCATCAAAACATGCATCCAC	CATGACAACACAACCAACCA	59.8	748	INTERGENIC	--	--	--

INDEL marker IDs	Chromosomes/ unanchored scaffolds	Physical positions (bp)	InDels (*Kabuli* reference genome-CDC Frontier/PI)	Forward primers (5'-3')	Reverse primers (5'-3')	Anneling temperature (0C)	Expected amplified product size (bp)	Structural annotation		Functional annotation	
								Sequence components of *kabuli* genome	*Kabuli* gene accession IDs	NCBI-KOG	TFs
Ca4_P1_41717468	Ca4	41717468	GAAAAAAAAAAAAAA/GAAAAAAAAAAAA	CCAGCGAAGACATAGTCCAAG	TGTCACACGCAAACATGAAA	59.9	315	INTERGENIC	--	--	--
Ca4_P1_41756748	Ca4	41756748	GAAAAAAAAAAA/GAAAAAAAAAAAA	GGGCTAAAGCAATCACCAAG	CACTTGTGTGCACCTTTGCT	59.7	896	INTERGENIC	--	--	--
Ca4_P1_41758727	Ca4	41758727	AATTATATTATATTATATTATATTATATTA/AATTATATTATATTATATTATATTATATATTATATTA	TTTGGAGATTGCATCACGTC	TGTGAAATATGTCAATGCCGA	59.7	505	INTERGENIC	--	--	--
Ca4_P1_41805048	Ca4	41805048	CGTGTGTGTGTGT/CGTGTGTGTGTGTGT	TCCAATAGCCAACCTTCACC	CAATTGAAAAGCACCCCACT	59.9	771	INTERGENIC	--	--	--
Ca4_P1_41806206	Ca4	41806206	CTA/CTAATA	TGCCTTGAATCTCTTCTTGGA	TGGAAAACCATAATTGAGCG	59.9	905	INTRON	Ca_10934	QR	B3
Ca4_P1_41808505	Ca4	41808505	ACC/AC	AATAAGTGTTTTCGGCGCAT	GGGTCAGTGCCATTATTGCT	59.6	430	DRR	Ca_10934	QR	B3
Ca4_P1_41810070	Ca4	41810070	AA/AAGCAAAAGAGGA	TCAACGAATGAGATCGTGATG	TCGTGAACATAGCTTGTGGC	59.7	345	INTERGENIC	--	--	--
Ca4_P1_41810677	Ca4	41810677	ATTTTT/ATTTT	CATGGCAAGTGGTGGAGTTT	TTTGTTGGTGGACGACAAAT	61.0	517	INTERGENIC	--	--	--
Ca4_P1_41823293	Ca4	41823293	ATTTTTTTT/ATTTTTTTTTT	TGGCAAAATATCTTTCATTTCG	TGGAACAATTGAACACCGTT	59.1	898	INTERGENIC	--	--	--
Ca4_P1_41823400	Ca4	41823400	A/AAT	GGAAATTTACTTGAATTCATGGCT	TGGAACAATTGAACACCGTT	59.8	420	INTERGENIC	--	--	--
Ca4_P1_41823592	Ca4	41823592	AT/A	GGAAATTTACTTGAATTCATGGCT	CAATTGTTCGTGTTGCGTCT	59.8	689	INTERGENIC	--	--	--
Ca4_P1_41823716	Ca4	41823716	TT/TTAAT	GGAAATTTACTTGAATTCATGGCT	CAATTGTTCGTGTTGCGTCT	59.8	689	INTERGENIC	--	--	--

INDEL marker IDs	Chromosomes/ unanchored scaffolds	Physical positions (bp)	InDels (*Kabuli* reference genome-CDC Frontier/PI)	Forward primers (5'-3')	Reverse primers (5'-3')	Anneling temperature (0C)	Expected amplified product size (bp)	Structural annotation		Functional annotation	
								Sequence components of *kabuli* genome	*Kabuli* gene accession IDs	NCBI-KOG	TFs
Ca4_P1_41823980	Ca4	41823980	CATCAGA/CA	CATCAAGACCATTTCCAGCA	CAATTGTTCGTGTTGCGTCT	59.6	114	INTERGENIC	--	--	--
Ca4_P1_41824482	Ca4	41824482	A/AAAAACTTT	CAAGTGGGTGCAGTTGATTG	GAGATTGGCCTACATTGGGA	60.2	654	INTERGENIC	--	--	--
Ca4_P1_41826986	Ca4	41826986	TC/T	TGCACCTGAAAATTCAGACC	AAACAAGCCCATGGAAAATG	58.7	508	INTERGENIC	--	--	--
Ca4_P1_41827335	Ca4	41827335	CATGA/CATGATGA	ATTTTCCATGGGCTTGTTTG	TACGATGAATCTCGGGCTCT	59.8	619	INTERGENIC	--	--	--
Ca4_P1_41832968	Ca4	41832968	AATATATATATATA/AATATATATATA	TTTCCATTGCACTCACTCCA	CCTATCATGGCCTGACCTGT	60.2	758	INTERGENIC	--	--	--
Ca4_P1_41892915	Ca4	41892915	GAAAAAAAA/GAAAAAAAAA	TTGAAAAGGCCAAAATCCAA	CCCGAAAATAACAAAGCGAA	60.4	298	INTERGENIC	--	--	--
Ca4_P1_41912186	Ca4	41912186	ATTTTTTTTTT/ATTTTTTTTTTT	ACATCACGCCTCCAATCTTT	TGAGCTTTTAGGTGGCACAA	59.6	883	INTERGENIC	--	--	--
Ca4_P1_419451	Ca4	419451	CAAA/CAAAAAA	CAAGATGCGATCCTGGAGTT	CGCCTCTTGCAAAAAGATGT	60.2	462	INTERGENIC	--	--	--
Ca4_P1_4194540	Ca4	4194540	TAATG/T	ATCTTCGGCAAAAGAAGCAA	AGAGAAGACTCACTGGGGCA	60.0	840	INTRON	Ca_03838	R	--
Ca4_P1_41954994	Ca4	41954994	TAAAAA/TAAA	GCGAAATTTCATGCAAACAA	TGTGTCAATTTTTATCGGTTGA	59.7	610	INTERGENIC	--	--	--
Ca4_P1_4196255	Ca4	4196255	CTATAAATT/CTATAAATTAATATAAATT	ATTGGCCGGAAATTGTACTG	TCAGGGGACCATAAAAGAAGG	59.8	378	DRR	Ca_03838	R	--
Ca4_P1_4196538	Ca4	4196538	ATTTT/ATTT	ATTGGCCGGAAATTGTACTG	TAACCCAAGGCCATATCCTG	59.8	841	DRR	Ca_03838	R	--

INDEL marker IDs	Chromosomes/ unanchored scaffolds	Physical positions (bp)	InDels (*Kabuli* reference genome-CDC Frontier/PI)	Forward primers (5'-3')	Reverse primers (5'-3')	Anneling temperature (0C)	Expected amplified product size (bp)	Structural annotation		Functional annotation	
								Sequence components of *kabuli* genome	*Kabuli* gene accession IDs	NCBI-KOG	TFs
Ca4_P1_41984896	Ca4	41984896	ACTCT/ACTCTCT	TCCGCTTTCTTTTGGGTATG	AATCCTCACACCCTACGTGC	60.1	845	DRR	Ca_10923	B	NF-YC
Ca4_P1_41993168	Ca4	41993168	CTGCAGTTGCAGTTGC/CTGCAGTTGC	GGGCTGGTTGGTATGTATGG	GTCTGCAACTGCGATTTCAA	60.1	381	INTRON	Ca_10922	P	--
Ca4_P1_42042301	Ca4	42042301	AATATATATATATATATATATATATATATATATATATATA/AATATATATATATATATATATATATATATATATATATATATATA	TCAACCCAATTGAACCGAAT	CGAAGCTGAAGGGCTAAATG	60.2	413	INTERGENIC	--	--	--
Ca4_P1_42078586	Ca4	42078586	AAGGCATGAAA/AAGGCATGAAACACAGGCATGAAA	TCTTTTGAACACTCCCCCTG	CCAAAGGAAACCACGACAAT	60.1	810	INTERGENIC	--	--	--
Ca4_P1_42083362	Ca4	42083362	CA/CAA	TTTCAAAGGTTTGCATTGTGA	AAAATTAACCGTATGGCCGA	59.2	327	INTERGENIC	--	--	--
Ca4_P1_42101261	Ca4	42101261	CGAGAGAGAGAGAGAGAGAGAGAGAGAGAGAGAGAGA/CGAGAGAGAGAGAGAGAGAGAGAGAGAGAGAGAGAGAGA	CCTCATTCCGTTGTTTCGTT	GCCGAACCGTTTCGTACTTA	60.0	565	INTERGENIC	--	--	--
Ca4_P1_42118929	Ca4	42118929	GTAT/GTATAT	GCAGAAGTTGGCTTCTTTGG	TCACGGCATAAGGTTTGTCA	60.0	677	DRR	Ca_10911	R	Nin-like
Ca4_P1_42120439	Ca4	42120439	TAAAAAAAAA/TAAAAAAAA	CACAAGCATTGGAGTCCCTT	AACTCGTGGGTTGGATTTTG	60.1	495	INTRON	Ca_10910	--	C2H2
Ca4_P1_42120489	Ca4	42120489	TAAAA/TAAA	CACAAGCATTGGAGTCCCTT	AACTCGTGGGTTGGATTTTG	60.1	495	INTRON	Ca_10910	--	C2H2
Ca4_P1_42332097	Ca4	42332097	GAAAAAAAAA/GAAAAAAAAAA	TCAGGGCCAACTGAAAGTCT	CTGGATGTGCATCCTGTTTG	59.8	628	INTERGENIC	--	--	--
Ca4_P1_42374619	Ca4	42374619	ATTTTTTTT/ATTTTTTT	TTTGATGTGTTCACGGCACT	CGGCATGCATGATTATTTTG	60.2	331	INTERGENIC	--	--	--
Ca4_P1_42407053	Ca4	42407053	GTTT/GTTTT	GCATAAGGATCGGGGAAACT	AATGATCGTCTTCGGATTGG	60.3	392	INTERGENIC	--	--	--

INDEL marker IDs	Chromosomes/ unanchored scaffolds	Physical positions (bp)	InDels (*Kabuli* reference genome-CDC Frontier/PI)	Forward primers (5'-3')	Reverse primers (5'-3')	Anneling temperature (0C)	Expected amplified product size (bp)	Structural annotation		Functional annotation	
								Sequence components of *kabuli* genome	*Kabuli* gene accession IDs	NCBI-KOG	TFs
Ca4_P1_42412637	Ca4	42412637	GA/GAA	CTTCTCAAGGGACGCATCAT	AATTTGGCAAGTTTGACACG	60.2	170	INTERGENIC	--	--	--
Ca4_P1_4242821	Ca4	4242821	A/ATCTG	TCCGTTTCAATCATCGTTCA	CACAAATAAGCACCTCCCAAA	60.0	765	DRR	Ca_03833	I	--
Ca4_P1_42433424	Ca4	42433424	AATATATATATATATATATATATATATATATATAT/AATATATATATATATATATATATATATAT	CCCAAGCAAGGTCGATTTTA	TTCTTTTCCCTCCAACCATT	60.1	381	INTERGENIC	--	--	--
Ca4_P1_42513890	Ca4	42513890	TTTATTATTATTATTATTATTATTATTATTATTATTATTATTATTA/TTTATTATTATTATTATTATTATTATTATTA	ACACGTTTTGTTTGTGGCAA	GTTGGCTTTTGTACCCCTCA	60.1	303	DRR	Ca_10898	--	WRKY
Ca4_P1_42516686	Ca4	42516686	CAAAA/CAAAAAA	GTCCTTTGCCATCTACCTCG	ATCTGCCAGAGCGATGTCTT	59.7	695	DRR	Ca_10897	--	Dof
Ca4_P1_42517427	Ca4	42517427	AATATATATATATATATATATATATATATATATA/AATATATATATATATATATATA	ATAATGTTCAGCACCCGAGG	GAGCTGTCCACGACACTCAA	60.0	466	INTRON	Ca_10897	--	Dof
Ca4_P1_42546801	Ca4	42546801	CAAAA/CAAAAA	GGTTGGTCAATATTAATTCCGC	CTAATATCATCGCCCCTTCG	59.6	686	INTERGENIC	--	--	--
Ca4_P1_42548370	Ca4	42548370	ATTTTTTTT/ATTTTTTTTT	CGTGCCTTTGTCGTCTCTTT	AGAGGCCCAATATCCTTGCT	60.4	528	INTERGENIC	--	--	--
Ca4_P1_42561342	Ca4	42561342	AATAGAGATAGAGATAGAGATAGAGA/AATAGAGATAGAGATAGAGA	CGGCAAAACAGAGACAATCA	TCTCACGGTTGTGAGGACTG	59.8	310	INTERGENIC	--	--	--
Ca4_P1_42588996	Ca4	42588996	TCC/TC	ACACGGCTGGTGTAAAGGAC	TATGGGAGAGGTTGAGACCG	60.0	766	INTERGENIC	--	--	--
Ca4_P1_42633986	Ca4	42633986	TATAATAATAATAATAATAATAATAATAATAATA/TATAATAATAATAATAATAATAATAATAATAATAATA	TTGCTTTAGATGAATCAAATCCC	CGACAGGCTTAGGTTAGACCA	59.5	362	INTERGENIC	--	--	--
Ca4_P1_42664491	Ca4	42664491	AATATATATATATATATATA/AATATATATATATATATATATA	TCGTTTGCTGACATGGGATA	TGCAATGGCGAATTACTCAA	60.1	800	INTERGENIC	--	--	--

INDEL marker IDs	Chromosomes/ unanchored scaffolds	Physical positions (bp)	InDels (*Kabuli* reference genome-CDC Frontier/PI)	Forward primers (5'-3')	Reverse primers (5'-3')	Anneling temperature (0C)	Expected amplified product size (bp)	Structural annotation		Functional annotation	
								Sequence components of *kabuli* genome	*Kabuli* gene accession IDs	NCBI-KOG	TFs
Ca4_P1_4269269	Ca4	4269269	TTATATATATATATATATATATAT/TTATATATATATATATATATATATATAT	ACACGGTGATCGTCAGTGAA	ACACGTGACACTTACCTAGCAA	60.2	596	INTERGENIC	--	--	--
Ca4_P1_42693293	Ca4	42693293	TC/TCC	AAATGCTCGAACGAAAATGC	TCGAAGTTGGATGCTTTTGA	60.2	503	INTERGENIC	--	--	--
Ca4_P1_4275930	Ca4	4275930	CAACGTA/CAACGTAACGTA	GTCTCCTTGGAGAAAAGGGC	AAAAGGCGAATGCAGAAGAA	60.2	652	DRR	Ca_03831	R	NAC
Ca4_P1_42776316	Ca4	42776316	TTTTTATTTTATTTTATTTTATTTTATT/TTTTTATTTTATTTTATTTTATT	AGGAAATGCTAGGTTGTGCTG	AAAACACCAAATCGGTGAGC	59.4	533	INTERGENIC	--	--	--
Ca4_P1_42945720	Ca4	42945720	CATATATATATAT/CATATATATATATAT	TGTCAATGTTACTTTATTTCAAAACTC	TTGGGTTATCTTCATTGCAAAA	57.2	739	INTERGENIC	--	--	--
Ca4_P1_4296365	Ca4	4296365	ATTTTTTTTTTT/ATTTTTTTTTTTT	CAAAGCCCCCACACTAAAAA	TATGGCGGTTGGACTGTGTA	60.0	525	INTERGENIC	--	--	--
Ca4_P1_43005724	Ca4	43005724	CTTTTTTTTTTT/CTTTTTTTTTT	TTGTAGTTGTATTCAATGACGAAGA	CAGAATTAATTTATTTGACAACTCCA	58.0	628	INTERGENIC	--	--	--
Ca4_P1_4302695	Ca4	4302695	ATATTATTATTATTATTATTATTATTATTATTATTATTATTATTATTAATATTATTATTATTATTA/ATATTATTATTATTATTATTATTATTATTATTATTATTATTATTATTATTATTATTAATATTATTATTATTATTA	GCATCTCCAAAATGCGATCT	TGAGTCCAAAACAAATGGTGTC	60.2	730	INTERGENIC	--	--	--
Ca4_P1_4305073	Ca4	4305073	ATTT/AT	TTGAATGGACGGGGTAAAAA	GAGGCGTTTGTTTGTGGATT	60.2	729	INTERGENIC	--	--	--
Ca4_P1_4315229	Ca4	4315229	CTTTT/CTTTTT	ATGCGACCTTCATCAAATGC	TCACTCGTTTTTACGTCTTTTG	61.0	813	DRR	Ca_03828	U	bHLH
Ca4_P1_43222555	Ca4	43222555	TTATATATATATATATATATATAT/TTATATATATATATATATAT	AAAGATTTAAAGGGCAAAAAGTG	CTGGCAGCTGGGAAAGATT	58.0	908	INTERGENIC	--	--	--
Ca4_P1_43273236	Ca4	43273236	G/GC	CGTCCCAAGACTCAATGGAT	AAAGGGTGATGAGCTCGCTA	59.9	633	INTERGENIC	--	--	--

INDEL marker IDs	Chromosomes/ unanchored scaffolds	Physical positions (bp)	InDels (*Kabuli* reference genome-CDC Frontier/PI)	Forward primers (5'-3')	Reverse primers (5'-3')	Anneling temperature (0C)	Expected amplified product size (bp)	Structural annotation		Functional annotation	
								Sequence components of *kabuli* genome	*Kabuli* gene accession IDs	NCBI-KOG	TFs
Ca4_P1_43278176	Ca4	43278176	GTTTTTTTT/GTTTTTTT	ACTGCGTCTCCATGGGTAAC	TTGGAATGGGCTTCTTTTTG	60.0	592	DRR	Ca_23681	--	--
Ca4_P1_43291624	Ca4	43291624	TAA/TAAAA	GAGCGATTACCGGTGTTGTT	GCCTTCAAATGTAACCCCCT	60.0	946	INTERGENIC	--	--	--
Ca4_P1_43301091	Ca4	43301091	ATAA/ATAATGTAA	ACCATAAAGGGAAACAGGGG	TCATGGTCTGCAATCTCGTC	60.0	714	INTERGENIC	--	--	--
Ca4_P1_43301725	Ca4	43301725	ATTTT/ATTT	GACGAGATTGCAGACCATGA	TGGGCCCTTAGTCTCTTTTT	59.8	753	INTERGENIC	--	--	--
Ca4_P1_43362642	Ca4	43362642	TG/T	AGAATCACACTGCCCCAAAC	TTGCAAGGGAATTTTTGTCA	60.0	590	DRR	Ca_23687	--	AP2
Ca4_P1_43385423	Ca4	43385423	ATTTTTTTTT/ATTTTTTTT	CACCATAAATTTCGAGGACGA	TGTGCACACTTAACATCAGTCAA	59.9	398	INTERGENIC	--	--	--
Ca4_P1_43420622	Ca4	43420622	T/TAA	ATGGCATACGATCCACCATT	GCCATTTGGAATGACGACTT	60.0	463	INTERGENIC	--	--	--
Ca4_P1_43421015	Ca4	43421015	GTATATATATATATATATATATATATATATATAT/GTATATATATATATATATATATATATATATAT	TTCTTGCAAAATCATATCCGC	CAGGCCCCATAGAAGAATGA	60.1	428	INTERGENIC	--	--	--
Ca4_P1_43445155	Ca4	43445155	TAAAAAAAAAAAA/TAAAAAAAAAAA	CAAAGGCTTGGTTGTCCATT	GTCAATGCCAAATGGTTGAA	60.0	458	INTERGENIC	--	--	--
Ca4_P1_43457820	Ca4	43457820	GTTTTTTTTTTTT/GTTTTTTTTTTT	TCAGAGTTCTTTCCACCGCT	TTTGTTGCTTTGGTGCTTTG	60.0	535	INTERGENIC	--	--	--
Ca4_P1_43633521	Ca4	43633521	TAAAAAAAAAAAAAA/TAAAAAAAAAAAAA	CAATTCCCACAGGTACGCTT	GACCAAGTCGGGAACAAAAA	60.0	423	INTERGENIC	--	--	--
Ca4_P1_43661821	Ca4	43661821	AA/AATA	AAAGGACAAATGGTCGCATC	TTCTTGTGGGCTGAGGCTAT	59.9	246	INTERGENIC	--	--	--

INDEL marker IDs	Chromosomes/ unanchored scaffolds	Physical positions (bp)	InDels (*Kabuli* reference genome-CDC Frontier/PI)	Forward primers (5'-3')	Reverse primers (5'-3')	Anneling temperature (0C)	Expected amplified product size (bp)	Structural annotation		Functional annotation	
								Sequence components of *kabuli* genome	*Kabuli* gene accession IDs	NCBI-KOG	TFs
Ca4_P1_43685747	Ca4	43685747	CAAAAAA/CAAAAAAA	CAACCATGATATATGG ACACCC	TTTGGTTCAATCGAAT TTAAAGG	58.9	706	INTERGENIC	--	--	--
Ca4_P1_43771826	Ca4	43771826	AAATAATAATAATAATAATAATAATAAT AATAATAATAATAATAATAATAA/AAAT AATAATAATAATAATAATAATAATAATA ATAATAATAATAATAATAA	TTCACATGCGGGTCAA ATTA	ATTCAAAGGAACCAAC CACG	59.9	478	INTERGENIC	--	--	--
Ca4_P1_4383126	Ca4	4383126	CG/CGTGGCAG	TAATTGCACTTTTGGTC CCC	GCGAAAGCATGATCAG TCAA	59.8	561	INTERGENIC	--	--	--
Ca4_P1_4383858	Ca4	4383858	TAAAAAAA/TAAAAAA	TTGACTGATCATGCTTT CGC	CTCTTCATCGCAGACA TCCA	60.0	828	INTERGENIC	--	--	--
Ca4_P1_43842884	Ca4	43842884	CTTTTTTTTTTT/CTTTTTTTTTTTTT	AACCGAACTTTGTTCA TCCC	GGCTAAATTACATATG TGGTCCC	58.9	646	INTERGENIC	--	--	--
Ca4_P1_43846525	Ca4	43846525	TAAAAAAAAAA/TAAAAAAAAAAA	GGCCGGTCTGTCATAC CTAA	TTTGAGATTTTGCCTC AACAA	60.0	541	INTERGENIC	--	--	--
Ca4_P1_43868196	Ca4	43868196	TTATATATATATATATATATATATAT/TT ATATATATATATATATATATATATATAT	TGCATTTTCTGCCAATT CTG	TTCTCCATTTGGGTTT CTGG	59.8	774	INTRON	Ca_09066	R	C2H2
Ca4_P1_43894968	Ca4	43894968	AATATATATATATATATATATATATATA TATAT/AATATATATATATATATATATATAT ATATATATATATATAT	TTGAAGCCCAATATGT CTCG	TTGATGGAGGACTACT CGGG	58.7	661	INTERGENIC	--	--	--
Ca4_P1_43902885	Ca4	43902885	TAAAAAAAAA/TAAAAAAAA	TGGTTTCGAACAATTT CTGG	TGTTGGTCGGGTTAAA CTCA	58.6	267	INTERGENIC	--	--	--
Ca4_P1_43964694	Ca4	43964694	TAAAAAAA/TAAAAAAAA	AGCGATTCACCAACCA AAAC	ACCAAACGAGCCTAGC TGAA	60.0	715	INTERGENIC	--	--	--
Ca4_P1_44040432	Ca4	44040432	TAAAAAAA/TAAAAAAAA	GATTGCGGAATTAGCC TTCTT	TGGTTAAGTGAAGCGC AAAC	59.7	774	INTERGENIC	--	--	--
Ca4_P1_44043408	Ca4	44043408	TAAAAAAAAA/TAAAAAAAAAA	TACAAGCCTGAGCCTG ACCT	ACTGGAGATGCCAATG GAAC	60.0	592	INTERGENIC	--	--	--

INDEL marker IDs	Chromosomes/ unanchored scaffolds	Physical positions (bp)	InDels (*Kabuli* reference genome-CDC Frontier/PI)	Forward primers (5'-3')	Reverse primers (5'-3')	Anneling temperature (0C)	Expected amplified product size (bp)	Structural annotation		Functional annotation	
								Sequence components of *kabuli* genome	*Kabuli* gene accession IDs	NCBI-KOG	TFs
Ca4_P1_44045635	Ca4	44045635	TAAAAAAAAAAAA/TAAAAAAAAAAA	TCGCTCCCGTCTTTTATCTT	TGACTCATTGATTTTGACTCGG	58.9	413	INTERGENIC	--	--	--
Ca4_P1_44057615	Ca4	44057615	TTTATTATTATTATTATTATTATTATT/TTTATTATTATTATTATTATTATT	AATGCGTGTTCTTGCTGTTG	CTTGGCCAAAGGAATCAAAA	59.9	198	INTRON	Ca_09080	K	ARR-B
Ca4_P1_4406006	Ca4	4406006	AATATATATATATATATATA/AATATATATATATA	AAGGAAATTCCAAATCTTGGTG	GATGACTTAGATTTGATGACAATGC	59.4	409	INTERGENIC	--	--	--
Ca4_P1_4406517	Ca4	4406517	TA/T	CAAGGTAATGCATTGTCATCAAA	CGATTGCTTGCGTGTATGAG	59.9	734	INTERGENIC	--	--	--
Ca4_P1_44083427	Ca4	44083427	TTATATATATATATATATA/TTATATATATATATATATA	TTGGCAGAGGTGAAGGTAGG	AAACCAACCATGCATAACCC	60.2	565	INTERGENIC	--	--	--
Ca4_P1_44098644	Ca4	44098644	ATTTTTTT/ATTTTTTTT	TCACCTTCATCCATCGTCAA	TGTGGTGTGCAAGATGACTG	60.0	408	INTERGENIC	--	--	--
Ca4_P1_44134460	Ca4	44134460	AAC/AACACAC	TACCAGATTGCCCCACTTTC	GCCAAAACGGATGAAGTTGT	59.9	554	INTERGENIC	--	--	--
Ca4_P1_44189746	Ca4	44189746	ATTTTTTT/ATTTTTTTT	TCCAAAATACAATTTTTCTAAACACG	TTTGAACCTTCCACACCACA	59.8	886	DRR	Ca_09095	--	GRAS
Ca4_P1_44285942	Ca4	44285942	AATATATATAT/AATATATAT	AATGTCCTCGATGCATACGTT	CGTTGCTGCGGATTTTATTT	59.5	547	INTRON	Ca_09102	--	C2H2
Ca4_P1_44350579	Ca4	44350579	TTATATATATATATATATATATATATATATATAT/TTATATATATATATATATATATATATATATATATATAT	GCTTGAGCTAACAACACGCA	GTGGCCAAAAGGTAGGTGAA	60.2	289	DRR	Ca_09105	S	--
Ca4_P1_44428431	Ca4	44428431	CATATATATATATATATAT/CATATATATATATATATAT	TGTTGCGGAAATGTTAGTTCA	TTCATCCATGCAATATTCAAAAA	59.2	699	INTERGENIC	--	--	--
Ca4_P1_44488143	Ca4	44488143	AAATAATAATAAT/AAATAATAATAATAAT	AAACCCAATTGTTGATGGGA	GCGACCATCTGCTTTCGTAT	60.0	336	INTERGENIC	--	--	--

INDEL marker IDs	Chromosomes/ unanchored scaffolds	Physical positions (bp)	InDels (*Kabuli* reference genome-CDC Frontier/PI)	Forward primers (5'-3')	Reverse primers (5'-3')	Anneling temperature (0C)	Expected amplified product size (bp)	Structural annotation		Functional annotation	
								Sequence components of *kabuli* genome	*Kabuli* gene accession IDs	NCBI-KOG	TFs
Ca4_P1_44488487	Ca4	44488487	T/TC	AAACCCAATTGTTGATGGGA	TTGACGGTTGTCAAAATCCA	60.0	715	INTERGENIC	--	--	--
Ca4_P1_44489358	Ca4	44489358	CTTTT/CTTT	TTTTGTTTCTCCACCCCAAA	TGACTGCACACAATAAACATTGA	60.3	400	INTERGENIC	--	--	--
Ca4_P1_44489529	Ca4	44489529	CATA/CATATA	TTTTGTTTCTCCACCCCAAA	TCAATCGATCAGTTGCTTTTT	60.3	785	INTERGENIC	--	--	--
Ca4_P1_44489710	Ca4	44489710	TTT/TTCTT	TTCTGGACTGCTCCAAAACA	TCAATCGATCAGTTGCTTTTT	59.4	611	INTERGENIC	--	--	--
Ca4_P1_44491674	Ca4	44491674	CAA/CA	GGCTGAATAATTGTGTCGGG	CACTGCCTTCCCATTTCCTA	60.3	314	INTERGENIC	--	--	--
Ca4_P1_44495959	Ca4	44495959	CTTTTTTTTT/CTTTTTTTTTTT	CTCAATCAAATGAAGGGGGA	ACCCATGTTGCATTGTGCTA	59.9	582	DRR	Ca_09122	--	--
Ca4_P1_44498939	Ca4	44498939	CTTT/CTT	ACCTTGGGCCTTAATTGAAA	CGGAACATCACTGTTCACAAA	58.6	410	INTERGENIC	--	--	--
Ca4_P1_44500579	Ca4	44500579	A/AT	TCTCAATCTAAAATCGGCTCAA	TTCATTATTGCAGTTTTGGTCC	59.0	391	INTERGENIC	--	--	--
Ca4_P1_44505236	Ca4	44505236	ATTT/AT	TCATTTCCTTCGGTTCATCC	CGCAAAATAGATTTTACAGCGTT	59.9	797	INTERGENIC	--	--	--
Ca4_P1_44507124	Ca4	44507124	TAA/TAAA	CCGGCTAGAAAACGTTGAAG	GGTCATTTTGAACAACCGCT	59.9	759	INTERGENIC	--	--	--
Ca4_P1_44507569	Ca4	44507569	CTTTT/CTTT	AGCGGTTGTTCAAAATGACC	AAAGCAAGTCCGACCAAAAA	60.0	421	INTERGENIC	--	--	--
Ca4_P1_44507737	Ca4	44507737	TTTCTTCTT/TTTCTTCTTCTT	AGCGGTTGTTCAAAATGACC	AAAGCAAGTCCGACCAAAAA	60.0	421	INTERGENIC	--	--	--

INDEL marker IDs	Chromosomes/ unanchored scaffolds	Physical positions (bp)	InDels (*Kabuli* reference genome-CDC Frontier/PI)	Forward primers (5'-3')	Reverse primers (5'-3')	Anneling temperature (0C)	Expected amplified product size (bp)	Structural annotation		Functional annotation	
								Sequence components of *kabuli* genome	*Kabuli* gene accession IDs	NCBI-KOG	TFs
Ca4_P1_44507825	Ca4	44507825	TGTA/TGTAACAGTAGTA	AGCGGTTGTTCAAAATGACC	TTAAGGGAGTGCATGAGCAA	60.0	835	INTERGENIC	--	--	--
Ca4_P1_44507935	Ca4	44507935	CT/CTT	AGATATTTGGCGGTGGTCAG	TTAAGGGAGTGCATGAGCAA	60.0	780	INTERGENIC	--	--	--
Ca4_P1_44508264	Ca4	44508264	TTCCCTG/T	GAAATGGATTTTTGGTCGGA	TGTGCATCCGTGATACATGA	59.7	961	INTERGENIC	--	--	--
Ca4_P1_44511339	Ca4	44511339	ATTTT/ATTTTT	GATGCAGGTCATGAAACCAA	GTCCTCAACGCTTCAAGAGG	59.5	494	INTERGENIC	--	--	--
Ca4_P1_44513152	Ca4	44513152	TAAAAAAAAA/TAAAAAAAA	AAGTTCAATGGGTCTCACGA	TGCTTTTTAAGGATTCGTTTGC	58.1	725	INTERGENIC	--	--	--
Ca4_P1_44513689	Ca4	44513689	ATTTTTTT/ATTTTTTTT	GCAAACGAATCCTTAAAAAGCA	TCTTGGGTTTGGCTCATCTC	60.6	569	INTERGENIC	--	--	--
Ca4_P1_44514418	Ca4	44514418	TC/TCC	GAGATGAGCCAAACCCAAGA	CGGAAGTATGCTGTGTACCG	60.2	889	INTERGENIC	--	--	--
Ca4_P1_44522624	Ca4	44522624	GAAAAAA/GAAAAAAA	GAAGTTGCTTGCTCCGATTC	CCCTAGCAAGTGCTAACCCA	60.0	675	DRR	Ca_09124	--	ERF
Ca4_P1_44522868	Ca4	44522868	A/AG	ACAATCCCTTCGTTCCCTCT	CCCTAGCAAGTGCTAACCCA	59.9	516	DRR	Ca_09124	--	ERF
Ca4_P1_44523921	Ca4	44523921	ATTTTTTTTTTT/ATTTTTTTTTTTT	GCAATGTGCTCACTTTTTGTCT	AACGAAGGAGGAGGGAAGAA	59.4	651	DRR	Ca_09124	--	ERF
Ca4_P1_44525079	Ca4	44525079	AGTATTTTTCTTA/AGTATTTTTCTTATGTATTTTTCTTA	ATGACGTTGGTGATTGGACA	TGGAGTAATCCCTAATCATTTCA	59.8	357	INTERGENIC	--	--	--
Ca4_P1_44525230	Ca4	44525230	TAAAAAAAAAAAA/TAAAAAAAA	ATGACGTTGGTGATTGGACA	GCGCTAGCAAAAATAGCCAC	59.8	884	INTERGENIC	--	--	--

INDEL marker IDs	Chromosomes/ unanchored scaffolds	Physical positions (bp)	InDels (*Kabuli* reference genome-CDC Frontier/PI)	Forward primers (5'-3')	Reverse primers (5'-3')	Anneling temperature (0C)	Expected amplified product size (bp)	Structural annotation		Functional annotation	
								Sequence components of *kabuli* genome	*Kabuli* gene accession IDs	NCBI-KOG	TFs
Ca4_P1_44526873	Ca4	44526873	ACTCCAAA/ACTCCAAATGCTCCTCCAAA	TTCCATTCTTTTTGCCAAGG	TTGGCTCAAGTGGGATAAGC	60.0	397	INTERGENIC	--	--	--
Ca4_P1_44531457	Ca4	44531457	TAAAAAAAA/TAAAAAAAAA	TTCGACAAAGACGAAAATCG	GTAAATGCAGTGGCGGATCT	58.9	282	INTERGENIC	--	--	--
Ca4_P1_44533305	Ca4	44533305	TAAAAAAAA/TAAAAAAA	CAAAATGCCGTCACAGAATG	CCTTGGTTTCGATGCCTAAA	60.1	368	DRR	Ca_09125	L	--
Ca4_P1_44540006	Ca4	44540006	CCTCTCTCTCTCTCTCT/CCTCTCTCTCTCTCT	TGGGAATTGGAACATGGAAC	CACACGATAGACCCCATTGTT	60.6	411	INTERGENIC	--	--	--
Ca4_P1_44540317	Ca4	44540317	TAAAAAAAAA/TAAAAAAAAAA	CACTCGATTTGACCCCTCTC	CCTACGATCTTAGACACGACCA	59.7	822	INTERGENIC	--	--	--
Ca4_P1_44540408	Ca4	44540408	TAA/TA	CACTCGATTTGACCCCTCTC	GGACCCATGGTCAAGAAAGA	59.7	896	INTERGENIC	--	--	--
Ca4_P1_44543641	Ca4	44543641	TAAAA/TAAAAA	CAAATATGTTGGGCCAATCC	GATCAAGTCCAGGGTTGTGG	60.0	473	INTERGENIC	--	--	--
Ca4_P1_44544825	Ca4	44544825	CCTACT/CCTACTACT	CAAATATGTTGGGCCAATCC	TTGACAAAATTTAACGTCGCA	60.0	417	INTERGENIC	--	--	--
Ca4_P1_44548432	Ca4	44548432	T/TC	TTTGCCGTAGTTTCTTCTCTGA	CTAGCTTGAGGGGGAGTGTG	59.2	816	INTERGENIC	--	--	--
Ca4_P1_44551365	Ca4	44551365	CAAAAAAAAAAA/CAAAAAAAAAA	CTTGAAAACCCTTGGGTCAA	ACACCCCCACAGCAGAATAG	59.9	294	INTERGENIC	--	--	--
Ca4_P1_44551458	Ca4	44551458	GT/G	CTTGAAAACCCTTGGGTCAA	GGACAGCCATTGGATCATTC	59.9	332	INTERGENIC	--	--	--
Ca4_P1_44557407	Ca4	44557407	ACC/AC	CAACCATCATTATGGACCCC	TTGATGGCCAAATCATTGAA	59.9	717	DRR	Ca_09126	--	--

INDEL marker IDs	Chromosomes/ unanchored scaffolds	Physical positions (bp)	InDels (*Kabuli* reference genome-CDC Frontier/PI)	Forward primers (5'-3')	Reverse primers (5'-3')	Anneling temperature (0C)	Expected amplified product size (bp)	Structural annotation		Functional annotation	
								Sequence components of *kabuli* genome	*Kabuli* gene accession IDs	NCBI-KOG	TFs
Ca4_P1_44559914	Ca4	44559914	TTATATATATAT/TTATATATATATAT	GCGGGGCATAAACTGATAGA	CATTTTCATATCACCAAATCACG	60.1	465	INTERGENIC	--	--	--
Ca4_P1_44560925	Ca4	44560925	ATTTTTTTT/ATTTTTTTTT	AACGTGATTTTGTTAACCATGC	TTTTGTTGTTGATTCTTTGATTTGA	58.9	682	INTERGENIC	--	--	--
Ca4_P1_44563494	Ca4	44563494	CTTCTTTTT/CTTT	GGCAAATCACATGCTAGGCT	TCGTCCAATCTTGTGGTTCA	60.2	513	INTERGENIC	--	--	--
Ca4_P1_44564443	Ca4	44564443	AGT/A	TTGTGGAAAACTACAGTCCCAA	CGTCAACTTCATGTAATCTTTCG	59.5	763	INTERGENIC	--	--	--
Ca4_P1_44570993	Ca4	44570993	TAAAAAAA/TAAAAAA	TGTCATGCAGTTCCCTCAAG	GGAATGGCTGCACTTATGGT	59.8	544	INTRON	Ca_09128	--	--
Ca4_P1_44581933	Ca4	44581933	GATATA/GATATATA	TGAGCAAGCACCTCATCATC	CTTTGGTGGGTGCAACATAA	60.0	473	INTRON	Ca_09130	--	--
Ca4_P1_4458327	Ca4	4458327	CAAAAAAAAAA/CAAAAAAAAA	GGAGTGATCGGATGTGTGTG	AATAGTGGGGAGGGAACCAG	60.0	709	DRR	Ca_03811	--	--
Ca4_P1_44591852	Ca4	44591852	TAAA/TAA	CTAGCCGAACCGAGAAGATG	AGCAAGCCCATGTAATCAGC	60.0	515	INTRON	Ca_09131	T	B3
Ca4_P1_44606474	Ca4	44606474	ATTTTTTTT/ATTTTTTT	CAACCGGTAACTAACAAAAATGC	TCATGACCACTTGTTTGATGTG	59.8	223	INTERGENIC	--	--	--
Ca4_P1_44606783	Ca4	44606783	ATTTT/ATTTTT	TTTCTTTCCTACATAGCCAACCA	TGCGTGAGAAATGATTCTTGA	60.0	926	INTERGENIC	--	--	--
Ca4_P1_44607011	Ca4	44607011	ACTCT/ACT	GGGGAATGTAAGGGTAGTATCG	AATGCCAAACCTAAAGCGTG	58.8	579	INTERGENIC	--	--	--
Ca4_P1_44607654	Ca4	44607654	TC/T	CACGCTTTAGGTTTGGCATT	AACAAAATTGTGCCGGAGAC	60.1	750	INTERGENIC	--	--	--

INDEL marker IDs	Chromosomes/ unanchored scaffolds	Physical positions (bp)	InDels (*Kabuli* reference genome-CDC Frontier/PI)	Forward primers (5'-3')	Reverse primers (5'-3')	Anneling temperature (0C)	Expected amplified product size (bp)	Structural annotation		Functional annotation	
								Sequence components of *kabuli* genome	*Kabuli* gene accession IDs	NCBI-KOG	TFs
Ca4_P1_44607935	Ca4	44607935	TGCAGC/TGCAGCAGC	GGACACGGAGACAGACTTGG	AACAAAATTGTGCCGGAGAC	60.7	689	INTERGENIC	--	--	--
Ca4_P1_44611506	Ca4	44611506	TAAA/TAA	TTTTTGCCCCTATCACCAAG	TCCAATGAAGACGACAACGA	59.9	807	INTERGENIC	--	--	--
Ca4_P1_44611929	Ca4	44611929	CTTTTTTT/CTTTTTT	TCGTTGTCGTCTTCATTGGA	CAATCTCAATGTGTTTGGCG	60.2	338	INTERGENIC	--	--	--
Ca4_P1_44613140	Ca4	44613140	T/TA	CCATCGTTGAAACGTTGTGAT	TGCGAGTTTCTTTATCATTTCC	60.8	428	INTERGENIC	--	--	--
Ca4_P1_44613867	Ca4	44613867	AACT/AACTGACT	TGATCAAGAAGTCACGAGTTCAA	TTAATGTTTGGGGAAGGCAA	59.9	312	INTERGENIC	--	--	--
Ca4_P1_44616332	Ca4	44616332	CTT/CTTT	AGGGGATGGCTTTCTTGTTT	CGGAGTTAATGTGGGGTTGT	59.9	500	INTERGENIC	--	--	--
Ca4_P1_44631148	Ca4	44631148	GTCT/GT	CCCTATTTGGGTGCGTCTTA	CCACTTGCACAAGACTTGGA	60.0	484	INTERGENIC	--	--	--
Ca4_P1_44632709	Ca4	44632709	CAAA/CAA	TCCTCCTCCTCCTCTCCTTC	TTGAAACTTTAACGGTGCCAT	59.9	418	INTERGENIC	--	--	--
Ca4_P1_44646219	Ca4	44646219	TA/TACA	ACTAGCCAACCATTCCATGC	ATTATGGCCAAAGGGTGGAT	60.0	634	INTRON	Ca_09136	O	--
Ca4_P1_44654366	Ca4	44654366	G/GGA	CCCGTACACACCATGTATTCC	ATGGTGCTGGCTTTGGTTAT	60.0	813	DRR	Ca_09137	R	NF-YA
Ca4_P1_44658102	Ca4	44658102	AG/A	GCATAGCCTGTCAATGCAAA	TGGCTATGTTCCACTCATTCC	59.8	745	INTRON	Ca_09137	R	NF-YA
Ca4_P1_44660873	Ca4	44660873	ATTTT/ATTT	CGGGAAGAAGTGGATTGTGT	CTCCTTGGTTGCCATTTGTT	60.0	965	INTERGENIC	--	--	--

INDEL marker IDs	Chromosomes/ unanchored scaffolds	Physical positions (bp)	InDels (*Kabuli* reference genome-CDC Frontier/PI)	Forward primers (5'-3')	Reverse primers (5'-3')	Anneling temperature (0C)	Expected amplified product size (bp)	Structural annotation		Functional annotation	
								Sequence components of *kabuli* genome	*Kabuli* gene accession IDs	NCBI-KOG	TFs
Ca4_P1_44661008	Ca4	44661008	TAAAAAA/TAAAAAAAAAA	AACTGTTGACCCAACT GACG	CTCCTTGGTTGCCATT TGTT	58.6	564	INTERGENIC	--	--	--
Ca4_P1_44661152	Ca4	44661152	CAAAAAA/CAAAAA	AACTGTTGACCCAACT GACG	CTCCTTGGTTGCCATT TGTT	58.6	564	INTERGENIC	--	--	--
Ca4_P1_44664978	Ca4	44664978	AGG/AG	CGAAGACAAACACTGT GAACG	TTGAGATTTGGGTGAG AGGG	59.4	482	INTERGENIC	--	--	--
Ca4_P1_44668585	Ca4	44668585	CA/CAA	CAAGGGTGGTGTGGC TAGTT	TGGCACACACCACTAG GAAA	60.0	396	INTERGENIC	--	--	--
Ca4_P1_44668816	Ca4	44668816	TTGGAGAAA/TTGGAGAAAATGGAGA AA	GTTCCTCATTTGGCAC ACCT	TCAAGTTTCAAAATGC AACCC	60.0	212	INTERGENIC	--	--	--
Ca4_P1_44669006	Ca4	44669006	T/TG	GTTCCTCATTTGGCAC ACCT	TCCGTTGGACCAATTT AACA	60.0	410	INTERGENIC	--	--	--
Ca4_P1_44676740	Ca4	44676740	T/TG	TAAGGGCCCCATCATG ACTA	GAACAACGGAGGAGG TGGTA	60.3	462	INTERGENIC	--	--	--
Ca4_P1_44678377	Ca4	44678377	TC/T	CATTTTGCCGATCAGA GTCA	GCAGGAACATGTGGG CTAGT	59.8	694	INTERGENIC	--	--	--
Ca4_P1_44679485	Ca4	44679485	TCATG/TCATGCATG	AATCCTCTATGGATGC CGTG	TTGTGGGATTAAAAGT CGAAAAA	59.9	707	INTERGENIC	--	--	--
Ca4_P1_44679620	Ca4	44679620	CTTT/CTT	AATCCTCTATGGATGC CGTG	AAAATGGAAAAAGCTC CAAAAA	59.9	868	INTERGENIC	--	--	--
Ca4_P1_44681210	Ca4	44681210	ATTTTT/ATTTTTT	TTTGCAATTCACCACC ACTC	CGGCACATTACCCAAA AGTT	59.5	827	INTERGENIC	--	--	--
Ca4_P1_44681450	Ca4	44681450	TA/T	CCGTCTGTACCCCTTA TCCA	CGGCACATTACCCAAA AGTT	59.8	329	INTERGENIC	--	--	--

INDEL marker IDs	Chromosomes/ unanchored scaffolds	Physical positions (bp)	InDels (*Kabuli* reference genome-CDC Frontier/PI)	Forward primers (5'-3')	Reverse primers (5'-3')	Anneling temperature (0C)	Expected amplified product size (bp)	Structural annotation		Functional annotation	
								Sequence components of *kabuli* genome	*Kabuli* gene accession IDs	NCBI-KOG	TFs
Ca4_P1_44682880	Ca4	44682880	TCAAAAACAAAA/TCAAAAACAAAAACAAAA	CGATTCGATTTTTACACCATAAA	CCACGCTCAACTCAACCATA	58.1	314	INTERGENIC	--	--	--
Ca4_P1_44691108	Ca4	44691108	TTAATAATAATAATAATAAT/TTAATAATAATAATAAT	CGAAAATGGAGCAATGAGGT	TGGGTTGAAAAATAAATGGAGG	60.1	539	INTERGENIC	--	--	--
Ca4_P1_44691557	Ca4	44691557	ATAA/ATAAAGTAATAGATTAGTAA	CCTCCATTTATTTTTCAACCCA	CCAAGTCGTTATGGAAAGCC	60.0	625	INTERGENIC	--	--	--
Ca4_P1_44693073	Ca4	44693073	C/CA	TCATGCCGTCCAAACTAACA	GCTGTCACCTGGATTTGGAT	60.1	665	INTERGENIC	--	--	--
Ca4_P1_44693618	Ca4	44693618	TTCT/TT	AGCTGTCTGCCCTTGAAAAA	TCATCAATGCAAGCTCGTTC	60.0	602	INTERGENIC	--	--	--
Ca4_P1_44694688	Ca4	44694688	TAGA/TAGAGA	GATTCGATCATCTGGGGAAA	AGGCTTCTGGTGCTGTTCTT	59.8	539	INTERGENIC	--	--	--
Ca4_P1_44694709	Ca4	44694709	TAGAGAG/TAGAG	GATTCGATCATCTGGGGAAA	AGGCTTCTGGTGCTGTTCTT	59.8	539	INTERGENIC	--	--	--
Ca4_P1_44695789	Ca4	44695789	CTTT/CTTTT	TGCCACTGCAACATAGACTTG	TTTCTCGTGTCGAATGGTGA	59.9	806	INTERGENIC	--	--	--
Ca4_P1_44697449	Ca4	44697449	AA/AATTA	TGCATGTGCCAATATTTGTG	GGGAAGAAGATGGCATGAAA	59.0	759	INTERGENIC	--	--	--
Ca4_P1_44697528	Ca4	44697528	A/AG	TGCATGTGCCAATATTTGTG	GGGAAGAAGATGGCATGAAA	59.0	759	INTERGENIC	--	--	--
Ca4_P1_44705100	Ca4	44705100	ATTT/ATT	ACCCAATTCTGATTGCGAAG	AAGCAAAATGGTTGAAACGG	60.1	293	INTRON	Ca_09141	O	--
Ca4_P1_44706655	Ca4	44706655	GAAA/GAA	AATAAACAAAGCACGCCTGG	GGTACTAGAGTTGCGGTGGC	60.1	576	INTRON	Ca_09141	O	--

INDEL marker IDs	Chromosomes/ unanchored scaffolds	Physical positions (bp)	InDels (*Kabuli* reference genome-CDC Frontier/PI)	Forward primers (5'-3')	Reverse primers (5'-3')	Anneling temperature (0C)	Expected amplified product size (bp)	Structural annotation		Functional annotation	
								Sequence components of *kabuli* genome	*Kabuli* gene accession IDs	NCBI-KOG	TFs
Ca4_P1_44708183	Ca4	44708183	G/GAAA	AGTCCAGTGGGCTGAAACAG	CAAGTTCATTGCACCGTTTTT	60.3	759	INTERGENIC	--	--	--
Ca4_P1_44709306	Ca4	44709306	GTTT/GTT	CCCATATCGCTTGGTGATTC	AGCATTCCACACCAACAACA	60.3	593	INTERGENIC	--	--	--
Ca4_P1_44709355	Ca4	44709355	ACTTTCAT/A	CCCATATCGCTTGGTGATTC	AGCATTCCACACCAACAACA	60.3	593	INTERGENIC	--	--	--
Ca4_P1_44709617	Ca4	44709617	TTTATAATTATTTTT/TTTATAATTATTTTTGTTATAATTATTTTT	TGTTGTTGGTGTGGAATGCT	TTGACGGTTCAAATGCAAAA	60.0	482	INTERGENIC	--	--	--
Ca4_P1_44709850	Ca4	44709850	TTATAT/TTAT	TGTTGTTGGTGTGGAATGCT	TTGACGGTTCAAATGCAAAA	60.0	482	INTERGENIC	--	--	--
Ca4_P1_44710249	Ca4	44710249	AAAGATAAGATAA/AAAGATAA	AGGAAGTGTCGCGGTTAAGA	TTTTTGTTGAATGGTGGAAAT	59.9	517	INTERGENIC	--	--	--
Ca4_P1_44710491	Ca4	44710491	GTA/GTATCATTTTTA	AGGAGTGGATGAGCCAAAAA	GGTGCTAAGTTCAAACACACAAA	59.7	822	INTERGENIC	--	--	--
Ca4_P1_4471743	Ca4	4471743	TAAGAAAAATACAAG/TAAGAAAAATACAAGAAAAATACAAG	ACATTTGATCACGGTCCTCC	AGGCATTGATTCTTCGTTCG	59.8	615	INTRON	Ca_03810	GOT	--
Ca4_P1_44718057	Ca4	44718057	ATTTTTTT/ATTTTTTTTTT	TCCTTTTTGTGCTCCACTTG	TTGACGCGATCTTTGATTTG	58.9	221	INTERGENIC	--	--	--
Ca4_P1_44718587	Ca4	44718587	GAAA/GAA	CAAAGATCGCGTCAATGAGA	ATCTTCCTTCCCAAAGCCAT	59.9	969	INTERGENIC	--	--	--
Ca4_P1_44725573	Ca4	44725573	AC/A	GGCTCAATTGGTCCAACCTA	ATTGGTTGTGATGCCATGAA	59.9	803	INTERGENIC	--	--	--
Ca4_P1_44726544	Ca4	44726544	TCACAACTCTGGCCACAA/TCACAA	TGGGACCGTGAGAGAGGATA	ATTCCACACGCCCACATATT	60.6	906	INTERGENIC	--	--	--

INDEL marker IDs	Chromosomes/ unanchored scaffolds	Physical positions (bp)	InDels (*Kabuli* reference genome-CDC Frontier/PI)	Forward primers (5'-3')	Reverse primers (5'-3')	Anneling temperature (0C)	Expected amplified product size (bp)	Structural annotation		Functional annotation	
								Sequence components of *kabuli* genome	*Kabuli* gene accession IDs	NCBI-KOG	TFs
Ca4_P1_44727621	Ca4	44727621	TA/TATTTTAATTTTTCCA	TCCTTTTAATGGGTGATCAGTAA	AAATGGACTGGCAAAATGAG	57.7	560	INTERGENIC	--	--	--
Ca4_P1_44727882	Ca4	44727882	AATTTT/AATTTTAAAATATTTT	CTCATTTTGCCAGTCCATTT	TTGATCTCGTGGTGAATACTCG	57.7	379	INTERGENIC	--	--	--
Ca4_P1_44728152	Ca4	44728152	AAAACAAA/AAAA	CTCATTTTGCCAGTCCATTT	AACCGCTAATTTTTCTGAGCA	57.7	793	INTERGENIC	--	--	--
Ca4_P1_44729278	Ca4	44729278	ATTTTTT/ATTTTTTT	GCAAGCTTGGCTCCATTAGA	TGGCCCTTTGTCCCTACATA	60.5	229	INTERGENIC	--	--	--
Ca4_P1_44735924	Ca4	44735924	CATAATAATAATAATAATAATAATAAT/ CATAATAATAATAATAATAATAAT	AACTGGATACGCCACTTTGG	GGTTGGGGAAAGAAACAACA	60.0	278	DRR	Ca_09142	C	bHLH
Ca4_P1_44737262	Ca4	44737262	CTTT/CTT	CGGTTGCTTTGATGATGTTG	CCAAACCCAAGTGGTTTTCTA	60.1	803	INTERGENIC	--	--	--
Ca4_P1_44738537	Ca4	44738537	ATTTTTTTTTT/ATTTTTTTT	TCATGCACCAACGTGCTTAT	AAGAAATTCCATGCAGGTGG	60.1	666	INTERGENIC	--	--	--
Ca4_P1_44738678	Ca4	44738678	AATA/AATATA	TCATGCACCAACGTGCTTAT	TCATGCATGGTTGGAAAAGA	60.1	906	INTERGENIC	--	--	--
Ca4_P1_44738779	Ca4	44738779	TT/TTAT	GGTTGCCTCTTTTACATGGG	TCATGCATGGTTGGAAAAGA	59.4	649	INTERGENIC	--	--	--
Ca4_P1_44739562	Ca4	44739562	TAAAAAAA/TAAAAAA	ACTCCTTTCCTTCCCACGAT	TCAAGGTCGTTTGTTGCTGT	59.9	490	INTERGENIC	--	--	--
Ca4_P1_44739765	Ca4	44739765	ATTT/ATTTT	ACTCCTTTCCTTCCCACGAT	TCAAGGTCGTTTGTTGCTGT	59.9	490	INTERGENIC	--	--	--
Ca4_P1_44741209	Ca4	44741209	GTTTTTTTTTT/GTTTTTTTT	TGGCCTTCAATGAAACTTCC	CTCGGGGACATCCTCATCTA	60.1	326	INTERGENIC	--	--	--

INDEL marker IDs	Chromosomes/ unanchored scaffolds	Physical positions (bp)	InDels (*Kabuli* reference genome-CDC Frontier/PI)	Forward primers (5'-3')	Reverse primers (5'-3')	Anneling temperature (0C)	Expected amplified product size (bp)	Structural annotation		Functional annotation	
								Sequence components of *kabuli* genome	*Kabuli* gene accession IDs	NCBI-KOG	TFs
Ca4_P1_44742903	Ca4	44742903	TCCC/TCCCC	TTTCGCATCCCAAAAT GAAT	TTTCCCTCTCGAGAAG CTGT	60.3	136	INTERGENIC	--	--	--
Ca4_P1_44744666	Ca4	44744666	GTTTTTTT/GTTTTTTTT	TTATTACACGCGTCAC GCTC	GAAGCCTTAATTGAAC CACAATG	59.9	749	INTERGENIC	--	--	--
Ca4_P1_44747786	Ca4	44747786	TAAAAA/TAAAA	TGGACTTGTGAAGCAA GCCT	TCCCCACTCCACAAAC CTAC	61.0	375	INTERGENIC	--	--	--
Ca4_P1_44747851	Ca4	44747851	ATTTTTTT/ATTTTTTTTT	TGGACTTGTGAAGCAA GCCT	CTTTCCCCGTGTGCTT ACAT	61.0	580	INTERGENIC	--	--	--
Ca4_P1_44748451	Ca4	44748451	TAAAA/TAA	ATGTAAGCACACGGGG AAAG	TCAGTCATTCATCAAC TCCCA	60.0	619	INTERGENIC	--	--	--
Ca4_P1_44748665	Ca4	44748665	AAATTA/AA	ATGTAAGCACACGGGG AAAG	TGTTGTGAACACCATT TTTGC	60.0	854	INTERGENIC	--	--	--
Ca4_P1_44770144	Ca4	44770144	GTAATCTATT/GT	CACATTCCCGACTTGT CTCA	CATTGGAACAGAAGCA AGCA	59.7	199	INTERGENIC	--	--	--
Ca4_P1_44772447	Ca4	44772447	AT/A	ACACCGCACGTAATGC TATG	CGAAATGTGTTTTGCT TTCG	59.6	149	INTERGENIC	--	--	--
Ca4_P1_44772492	Ca4	44772492	GTTTTTTTTTTTTTTTTTT/GTTTTTTTTTT	ACACCGCACGTAATGC TATG	CGAAATGTGTTTTGCT TTCG	59.6	149	INTERGENIC	--	--	--
Ca4_P1_44773439	Ca4	44773439	ATTTTTT/ATTTTTTTT	TTGTTTCACTTTTTCCA TTCCA	TAGGGTTCTTGATCCA TCGC	59.5	596	INTERGENIC	--	--	--
Ca4_P1_44773874	Ca4	44773874	G/GA	GCGATGGATCAAGAAC CCTA	GGATTTTGGCAACAGA TGCT	60.0	737	INTERGENIC	--	--	--
Ca4_P1_44775723	Ca4	44775723	CAAAA/CAAA	TGAAATTGGATGGATT GTGTG	ACTTGTGAGATGGAGT GGGG	59.2	476	INTERGENIC	--	--	--

INDEL marker IDs	Chromosomes/ unanchored scaffolds	Physical positions (bp)	InDels (*Kabuli* reference genome-CDC Frontier/PI)	Forward primers (5'-3')	Reverse primers (5'-3')	Anneling temperature (0C)	Expected amplified product size (bp)	Structural annotation		Functional annotation	
								Sequence components of *kabuli* genome	*Kabuli* gene accession IDs	NCBI-KOG	TFs
Ca4_P1_44775773	Ca4	44775773	CATA/CA	AGAGGGTTGTGGTCTTGTGG	ATTTACTGCAGGCCACCATC	60.0	219	INTERGENIC	--	--	--
Ca4_P1_44776173	Ca4	44776173	GA/G	AGAGGGTTGTGGTCTTGTGG	GAGGGGGATAAAAGTGAGGC	60.0	857	INTERGENIC	--	--	--
Ca4_P1_44780726	Ca4	44780726	TAAAA/TAAA	CAATTCCACGTGAGAGCCTT	TCATTTGCACCACATCAACA	60.3	303	INTERGENIC	--	--	--
Ca4_P1_44781972	Ca4	44781972	CTT/CTTT	AGCAGAAATGGTGGCAGATT	AATCCAAATGCCATAGCTGC	59.7	538	INTERGENIC	--	--	--
Ca4_P1_44784898	Ca4	44784898	A/AGTTGTC	GCAAGCACTTGCTGTGTTGT	GCCAGTCAGCTTGTTTCCAT	60.1	307	INTRON	Ca_09144	--	S1Fa-like
Ca4_P1_44785874	Ca4	44785874	GATGCACATATACGCATGCACAT/GATGCACATATACGCATGCACATATACGCATGCACAT	TAACAACAACCTTGAGGGGC	GTTCCAAGTCACCAAGGGAA	60.0	640	INTRON	Ca_09144	--	S1Fa-like
Ca4_P1_4478589	Ca4	4478589	TAAGTTAAAAGTTAAAAGTTAAAAGTTAAAA/TAAGTTAAAAGTTAAAAGTTAAAA	TGTAGACGCGCAGAGAGAGA	AATCATTTGCCTGGTGCTTT	60.0	593	INTERGENIC	--	--	--
Ca4_P1_44786400	Ca4	44786400	GTTTT/GTTTTT	TTCCCTTGGTGACTTGGAAC	AAGCGGCTTACAATGATGCT	59.9	364	INTRON	Ca_09144	--	S1Fa-like
Ca4_P1_44790743	Ca4	44790743	CCTATC/CCTATCTATC	GCCACAACCATCAACTTCAA	TTGGAGGGATCCAAGAGCTA	59.5	903	DRR	Ca_09144	--	S1Fa-like
Ca4_P1_44794120	Ca4	44794120	TAAAAA/TAAAA	TGTGATGATTTTGTGGAGGG	ATTTGCGGCCGATTTAATTT	59.3	624	INTRON	Ca_09145	--	--
Ca4_P1_44795219	Ca4	44795219	TAA/TA	CTTTGCAGCCAAAACTTTCC	GCAAAATCTTTTTGATTTTCCCT	59.9	394	INTRON	Ca_09145	--	--
Ca4_P1_44799502	Ca4	44799502	AATATAT/AATATATAT	CAACTTCATCTATTGTCACGCC	TTTCGCTGTTTCAGTGTTCG	59.6	730	INTRON	Ca_09145	--	--

INDEL marker IDs	Chromosomes/ unanchored scaffolds	Physical positions (bp)	InDels (*Kabuli* reference genome-CDC Frontier/PI)	Forward primers (5'-3')	Reverse primers (5'-3')	Anneling temperature (0C)	Expected amplified product size (bp)	Structural annotation		Functional annotation	
								Sequence components of *kabuli* genome	*Kabuli* gene accession IDs	NCBI-KOG	TFs
Ca4_P1_44799524	Ca4	44799524	GAAA/GAA	CAACTTCATCTATTGTCACGCC	TTTCGCTGTTTCAGTGTTCG	59.6	730	INTRON	Ca_09145	--	--
Ca4_P1_4480054	Ca4	4480054	TT/TTATTTGAAT	TCCTCCATTTTTCCACGCTA	TTTCATCCTCCATGTGACCA	60.6	409	INTERGENIC	--	--	--
Ca4_P1_4480380	Ca4	4480380	TC/TCC	TGGTCACATGGAGGATGAAA	TTTAATACCTCGTATTTCCTATCAAGA	59.9	609	DRR	Ca_03809	--	S1Fa-like
Ca4_P1_4481135	Ca4	4481135	TAA/TAAA	TGAAATCAAATTTTCTCAAGGC	AAACGACATTACGTTATTTCTTCATT	58.4	120	DRR	Ca_03809	--	S1Fa-like
Ca4_P1_4481516	Ca4	4481516	T/TTC	TGAAATCAAATTTTCTCAAGGC	GTTCCGCCTCTTCTACCTCC	58.4	760	DRR	Ca_03809	--	S1Fa-like
Ca4_P1_4481679	Ca4	4481679	TAAAAAAAA/TAAAAAAAAA	TTTTGCTTTTGATTACTTTCTTTTT	GTTCCGCCTCTTCTACCTCC	57.2	314	DRR	Ca_03809	--	S1Fa-like
Ca4_P1_4482067	Ca4	4482067	GAAAAA/GAAAAAA	GGAGGTAGAAGAGGCGGAAC	TCCAATCGTTGGATCAAGGT	60.2	744	DRR	Ca_03809	--	S1Fa-like
Ca4_P1_44831090	Ca4	44831090	TA/TAA	AAGACGCCAAAGTTCGAAGA	TTGGATTTTGTGGAGCATGA	60.0	754	DRR	Ca_09147	Z	NAC
Ca4_P1_44831235	Ca4	44831235	GAAAA/GAA	TTTGACCTTCAGCAGTGTGG	TTGGATTTTGTGGAGCATGA	59.9	463	DRR	Ca_09148	K	--
Ca4_P1_44831252	Ca4	44831252	AT/ATAAGTTT	TTTGACCTTCAGCAGTGTGG	TTGGATTTTGTGGAGCATGA	59.9	463	DRR	Ca_09148	K	--
Ca4_P1_44833120	Ca4	44833120	CACAACAA/CACAA	CCCTGAGTTTGGCTGAGAAG	TGGCATTTTTGTGGGGTATT	60.0	615	INTRON	Ca_09148	K	--
Ca4_P1_44833892	Ca4	44833892	TAAGAA/TAAGAAGAA	CGAATAAAACCGAAGTGCGT	TCGTTGGAACAACCACTGAA	60.1	474	INTERGENIC	--	--	--

INDEL marker IDs	Chromosomes/ unanchored scaffolds	Physical positions (bp)	InDels (*Kabuli* reference genome-CDC Frontier/PI)	Forward primers (5'-3')	Reverse primers (5'-3')	Anneling temperature (0C)	Expected amplified product size (bp)	Structural annotation		Functional annotation	
								Sequence components of *kabuli* genome	*Kabuli* gene accession IDs	NCBI-KOG	TFs
Ca4_P1_44834067	Ca4	44834067	CTTTTTTTTTTTTT/CTTTTTTTTTTT	CGAATAAAACCGAAGTGCGT	TCGTTGGAACAACCACTGAA	60.1	474	INTERGENIC	--	--	--
Ca4_P1_44837147	Ca4	44837147	G/GAGAT	CGGGGAGGCAAGAAAATAAC	TGCAATTTTACGTCCAAATCAT	60.8	815	INTERGENIC	--	--	--
Ca4_P1_44838015	Ca4	44838015	CTT/CTTT	GTCATTTGACGCATGGTTTG	TTTCGTTGATAATTTTTGGCA	60.0	499	INTERGENIC	--	--	--
Ca4_P1_44838837	Ca4	44838837	TC/T	TGAAGGAATGCTTCTTTGTTTG	GATGATTGCAACCAACATGG	59.4	304	INTERGENIC	--	--	--
Ca4_P1_44843289	Ca4	44843289	CTT/CT	CAATGGAATGTGGTGTGGAA	GAACATGGATCGCACACTTT	60.2	254	INTERGENIC	--	--	--
Ca4_P1_44843677	Ca4	44843677	TAAAAAA/TAAAAA	CAATGGAATGTGGTGTGGAA	TTGGGCTAGGTTGGATCTTG	60.2	634	INTERGENIC	--	--	--
Ca4_P1_44845646	Ca4	44845646	TCAAACAAAC/TCAAAC	AGTCCACACAAGCCTTCACC	GGAGCTTTTTCGTATGTGCC	60.2	797	INTERGENIC	--	--	--
Ca4_P1_44846286	Ca4	44846286	TTT/TTTCTT	CATGCAACAAGGGCACATAC	TGTGCATTCGTGAAGGTTTT	60.0	281	DRR	Ca_09149	--	--
Ca4_P1_44847649	Ca4	44847649	ATTT/ATTTT	TCCAGCTCAACACATGGAAA	ATGTTTTCAACTTGCAGGGG	60.2	510	DRR	Ca_09149	--	--
Ca4_P1_44854537	Ca4	44854537	ATTTTTTT/ATTTTTT	CCAACTTTTTCATACACAAACACA	TGTTGCACTCGAACAAACTCA	59.0	567	INTERGENIC	--	--	--
Ca4_P1_44864785	Ca4	44864785	AAA/AAAGAA	GGGACCACATCAATTGTCTCA	TCTCATTTTCACACAATGGTCA	60.8	542	INTERGENIC	--	--	--
Ca4_P1_44864908	Ca4	44864908	AATATATATATAT/AATATATATATATAT	GGGACCACATCAATTGTCTCA	TCTCATTTTCACACAATGGTCA	60.8	542	INTERGENIC	--	--	--

INDEL marker IDs	Chromosomes/ unanchored scaffolds	Physical positions (bp)	InDels (*Kabuli* reference genome-CDC Frontier/PI)	Forward primers (5'-3')	Reverse primers (5'-3')	Anneling temperature (0C)	Expected amplified product size (bp)	Structural annotation		Functional annotation	
								Sequence components of *kabuli* genome	*Kabuli* gene accession IDs	NCBI-KOG	TFs
Ca4_P1_44865492	Ca4	44865492	TA/TAGA	TGACCATTGTGTGAAAATGAGA	TGGTCGTAAAGTGCATTGGA	59.0	685	INTERGENIC	--	--	--
Ca4_P1_44866420	Ca4	44866420	ATTTTTTTT/ATTTTTTTTTT	CGAGAATCTATACACGGGAAGG	GGAAACCCCAGAATTTGGAT	60.0	840	INTERGENIC	--	--	--
Ca4_P1_44869625	Ca4	44869625	GTT/GTTGTACCTTT	TGTTATCTGCAGTGTCCCCA	GCGGTTCTCAACTTCTTTGC	60.1	580	INTRON	Ca_09151	R	--
Ca4_P1_44871777	Ca4	44871777	TTCACCACAAATAT/TT	TATTGGCGATTGGTTTGTGA	TTGCCAACCTTTGAACAGTG	59.9	627	DRR	Ca_09151	R	--
Ca4_P1_44872820	Ca4	44872820	T/TA	TTGAGGCTTGAAATCCTTTGA	TGTATTGCTCGTTGAGGGTG	59.8	174	DRR	Ca_09151	R	--
Ca4_P1_4487412	Ca4	4487412	AT/ATTAATAAAGT	TCAGACTCTCCCGAACGATT	CGTACGACGTCTTTCGTCAA	59.8	281	DRR	Ca_03808	--	--
Ca4_P1_44875930	Ca4	44875930	ATAAACATT/AT	CAACAGCACCAACCACAATC	TTGTTGGAAAGAAGAATGCAAA	60.0	365	INTERGENIC	--	--	--
Ca4_P1_44878187	Ca4	44878187	TG/TGG	CAATCGTGCAAACAATTTGG	ATGAAGCGATTGGTTGGAAG	60.0	738	INTERGENIC	--	--	--
Ca4_P1_44880910	Ca4	44880910	GTT/GTTT	TAAAGTCATGCCCATCACCA	TGTCCAAGCATGGGAGAAGT	59.9	647	INTERGENIC	--	--	--
Ca4_P1_44880954	Ca4	44880954	TTATATAT/TTATAT	TAAAGTCATGCCCATCACCA	TTGGCACTACAGCCATCTTG	59.9	745	INTERGENIC	--	--	--
Ca4_P1_44884987	Ca4	44884987	CAAAAAAAAA/CAAAAAAAAAAA	GCCTCCGTCAACATTTCATT	TGTGTTCTCCGTTGCCATAA	59.9	493	INTERGENIC	--	--	--
Ca4_P1_4488536	Ca4	4488536	TAAA/T	ACAGCACATTTCTGGTGGTG	TCTGCTAAACAGGGGATTGG	59.6	450	DRR	Ca_03808	--	--

INDEL marker IDs	Chromosomes/ unanchored scaffolds	Physical positions (bp)	InDels (*Kabuli* reference genome-CDC Frontier/PI)	Forward primers (5'-3')	Reverse primers (5'-3')	Anneling temperature (0C)	Expected amplified product size (bp)	Structural annotation		Functional annotation	
								Sequence components of *kabuli* genome	*Kabuli* gene accession IDs	NCBI-KOG	TFs
Ca4_P1_44886483	Ca4	44886483	TTCT/TT	TAATGGAGGGCAATTTCGAG	AAAACACCCAACCCCCTAAT	60.0	349	INTERGENIC	--	--	--
Ca4_P1_44889516	Ca4	44889516	ATT/ATTTGTT	AAACCGATATGTGTTGCCCT	TTCAAAATAGCCCTTCCAAAAA	59.3	711	INTERGENIC	--	--	--
Ca4_P1_4489645	Ca4	4489645	CA/C	TCACGGCACAATCTCCAATA	ATGCAAAATGCCTTGGTTTC	60.1	443	INTRON	Ca_03808	--	--
Ca4_P1_44897706	Ca4	44897706	ATTTTTTTT/ATTTTTTTTTTT	ATTTGTTGGAGGATGGGTCA	CACTCTTGTTCACGGCAAAA	60.2	623	INTERGENIC	--	--	--
Ca4_P1_44900157	Ca4	44900157	TAAAAAAA/TAAAAAAAA	CCCCATTCGACCAAGATTTA	AGAAAGTGGGAATGTGGGGT	59.8	526	INTERGENIC	--	--	--
Ca4_P1_44902733	Ca4	44902733	AAGAGAGAGAGAGAGAG/AAGAGAGAGAGAGAGAGAGAG	TGTGAAGCAACAAGGAACAGA	TCGATTCCCACATGACACTG	59.5	133	DRR	Ca_09152	--	C2H2
Ca4_P1_44904775	Ca4	44904775	AT/A	AGTATGGTGGCAGTCCCTTG	GACCAGTAAATGCCTTCCCA	60.0	767	INTRON	Ca_09153	--	--
Ca4_P1_44905028	Ca4	44905028	C/CT	TACAGTGTTGTGGTGCCGTT	GACCAGTAAATGCCTTCCCA	60.1	516	INTRON	Ca_09153	--	--
Ca4_P1_44905137	Ca4	44905137	ATTT/ATT	TACAGTGTTGTGGTGCCGTT	GACCAGTAAATGCCTTCCCA	60.1	516	INTRON	Ca_09153	--	--
Ca4_P1_44907347	Ca4	44907347	TTAAA/T	TGTGCGAGTCTTTCTGTTGG	CCGCTTGAATGACAGCACTA	60.0	356	INTRON	Ca_09153	--	--
Ca4_P1_44907961	Ca4	44907961	ATTTTTTTTT/ATTTTTTTTTT	TAGTGCTGTCATTCAAGCGG	AAAGGGCCATCTTCTGTTCA	60.0	833	DRR	Ca_09154	--	--
Ca4_P1_44909734	Ca4	44909734	TGA/T	AGCTCTCATGAGGGGCTACA	TCAATTTGAAGTGTCGGTGC	60.0	310	INTRON	Ca_09154	--	--

INDEL marker IDs	Chromosomes/ unanchored scaffolds	Physical positions (bp)	InDels (*Kabuli* reference genome-CDC Frontier/PI)	Forward primers (5'-3')	Reverse primers (5'-3')	Anneling temperature (0C)	Expected amplified product size (bp)	Structural annotation		Functional annotation	
								Sequence components of *kabuli* genome	*Kabuli* gene accession IDs	NCBI-KOG	TFs
Ca4_P1_44913191	Ca4	44913191	ATTTTTTTTTT/ATTTTTTTTTTT	TGGTTGTATATGCAATACTCCATTG	TTCACATTCCGACATAAACAAAA	60.0	382	INTERGENIC	--	--	--
Ca4_P1_44917486	Ca4	44917486	AATATATATATA/AATATATATATATA	TTCCACCTTCAATGCAACAA	TTTGGAATTGGTCCAAGAGC	60.1	899	DRR	Ca_09155	Q	--
Ca4_P1_44926400	Ca4	44926400	AG/A	AAAACGTTCTGCAACCAACC	TTGCTCCTACGTATCTCCCG	60.0	683	INTERGENIC	--	--	--
Ca4_P1_44932345	Ca4	44932345	AATA/AA	AATTTATGGGCCCAATCCTC	GCTGAAATCAAGAGAACGCC	60.0	616	INTERGENIC	--	--	--
Ca4_P1_44933428	Ca4	44933428	A/AG	TAACCATTTTGCACGGGTTT	AAAGTCTCAAAAGAGCCGCA	60.2	559	INTERGENIC	--	--	--
Ca4_P1_44933532	Ca4	44933532	GA/GAGAAAA	TGCGGCTCTTTTGAGACTTT	CATGTTTCAACGATTTGAATGAA	60.1	354	INTERGENIC	--	--	--
Ca4_P1_44935712	Ca4	44935712	TAATGTTAAC/T	ATCCTCAACGAAACCACGTC	CTTTGATGGGGCAAAGGTAA	60.0	266	INTERGENIC	--	--	--
Ca4_P1_44937033	Ca4	44937033	TAAAAAA/TAAAAA	TATCTCGCTCTTTCCTCCCA	GCTGTTTGATGGCTATGGGT	59.9	888	INTERGENIC	--	--	--
Ca4_P1_44938239	Ca4	44938239	GTTTTT/GTTTT	TTTGGTTACGTCACGGTTCA	GCAAATATATTGCATGGCTTGA	60.0	485	INTERGENIC	--	--	--
Ca4_P1_44939636	Ca4	44939636	TTA/TTATA	CTCGCTGAAATTGCCAAAGT	TTTTCCTAAAATAAACAACTCCCTG	60.4	460	INTERGENIC	--	--	--
Ca4_P1_44939813	Ca4	44939813	ATTTTT/ATTT	CTCGCTGAAATTGCCAAAGT	TGATAAAATGCTTTTGCACCAA	60.4	644	INTERGENIC	--	--	--
Ca4_P1_44944900	Ca4	44944900	TAAAA/TAAA	GGGAAACAAATTCGTGGAAA	ACATGCCAAATGCAATGTGT	59.8	388	DRR	Ca_09157	G	--

INDEL marker IDs	Chromosomes/ unanchored scaffolds	Physical positions (bp)	InDels (*Kabuli* reference genome-CDC Frontier/PI)	Forward primers (5'-3')	Reverse primers (5'-3')	Anneling temperature (0C)	Expected amplified product size (bp)	Structural annotation		Functional annotation	
								Sequence components of *kabuli* genome	*Kabuli* gene accession IDs	NCBI-KOG	TFs
Ca4_P1_44946195	Ca4	44946195	AC/A	CTGCTCTGGTCACTGCACAT	CAATTCCAAGGCAAGTTTGG	60.1	512	INTRON	Ca_09157	G	--
Ca4_P1_44946207	Ca4	44946207	TG/T	CTGCTCTGGTCACTGCACAT	CAATTCCAAGGCAAGTTTGG	60.1	512	INTRON	Ca_09157	G	--
Ca4_P1_44953374	Ca4	44953374	TACACACACA/TACACA	TCCCTCCATCACCAAAACTC	ATGTGCAGTGACCAGAGCAG	59.9	349	INTRON	Ca_09159	G	--
Ca4_P1_44965984	Ca4	44965984	TTCAACTT/TTCAACTTAATTTCTCAACTT	ATCTTTCCCCTCCACACACA	TGGTCGCATTCGCATATAAA	60.4	601	INTERGENIC	--	--	--
Ca4_P1_44967276	Ca4	44967276	AAAA/AAAATTAAA	GGAAGCACGGTGAGAAGAAG	TCACATTGCAATCACACAACA	60.0	412	INTRON	Ca_09160	R	C2H2
Ca4_P1_44968120	Ca4	44968120	ATTCTTCT/ATATTATTACTTCTTCTTCTTCT	ATTCTCCCAATCCACCATCA	GCATGTTTAAGTGGGGCCTA	60.1	184	CDS (large-effect mutations)	Ca_09160	R	C2H2
Ca4_P1_44970459	Ca4	44970459	CAAA/CA	CATTGCACGAGCACAACTG	TGGAACGTAATTCCTCCATT	60.0	661	DRR	Ca_09160	R	C2H2
Ca4_P1_44982193	Ca4	44982193	AC/A	CAGGGGGTAATGTGGTTCTG	TCAGGGATTGATAAGCAGGC	60.2	648	INTRON	Ca_09161	I	--
Ca4_P1_4498233	Ca4	4498233	ACC/AC	CAAAGACCAAACAAGCCCAT	AACACCAACCAACTCCTTGC	60.0	202	INTRON	Ca_03808	--	--
Ca4_P1_44987442	Ca4	44987442	TG/T	TCATGAAAACGTTTGGTTAATTG	AAAAGGGATACTGAATTTCATCCA	58.9	373	INTERGENIC	--	--	--
Ca4_P1_44987779	Ca4	44987779	GA/GAATA	ACTTTGGAACAAAAGGAGGAA	TGGTCAAGATGTGTTGACTTTG	57.8	570	INTERGENIC	--	--	--
Ca4_P1_44988091	Ca4	44988091	T/TTG	TGGATGAAATTCAGTATCCCTTTT	TGGGGACAGGTCAATCAAGT	60.1	856	INTERGENIC	--	--	--

INDEL marker IDs	Chromosomes/ unanchored scaffolds	Physical positions (bp)	InDels (*Kabuli* reference genome-CDC Frontier/PI)	Forward primers (5'-3')	Reverse primers (5'-3')	Anneling temperature (0C)	Expected amplified product size (bp)	Structural annotation		Functional annotation	
								Sequence components of *kabuli* genome	*Kabuli* gene accession IDs	NCBI-KOG	TFs
Ca4_P1_44988644	Ca4	44988644	ACACACCACACCACACCACAC/ACACACCACACCACACCACACCACACCACAC	CTTGATTGACCTGTCCCCAC	TAGGAAGGTTTTTGCAACGG	60.4	191	INTERGENIC	--	--	--
Ca4_P1_44994623	Ca4	44994623	TA/TACA	TGCATGCGAGGTATTCAAGA	ATTTGGCAACGAAGTTTTGG	60.4	827	DRR	Ca_09163	--	--
Ca4_P1_44994863	Ca4	44994863	TTATA/TTA	TTCCATCCCTTTTGTGAACC	ATTTGGCAACGAAGTTTTGG	59.8	413	INTRON	Ca_09163	--	--
Ca4_P1_44995144	Ca4	44995144	CAAAA/CAAAAA	CCAAAACTTCGTTGCCAAAT	AGGAAACTCTACCGCACGAA	60.0	528	INTRON	Ca_09163	--	--
Ca4_P1_44997151	Ca4	44997151	TAAA/TAAAA	AAATGCTAACCGGCAAAGTG	TTCTCTCCCAAAATTCCACG	60.1	505	INTRON	Ca_09163	--	--
Ca4_P1_44997568	Ca4	44997568	TCTCCAACTCCAACTCCAAC/TCTCCAACTCCAACTCCAACTCCAAC	CGTGGAATTTTGGGAGAGAA	AACAGGCGAGTTGAGAATTGA	60.0	189	CDS (large-effect mutations)	Ca_09163	--	--
Ca4_P1_44999560	Ca4	44999560	GTCTCTC/GTCTC	TTCATTTTCCCACCCCTACA	TCCCTACTCAAAGGGGTCAA	60.2	630	INTERGENIC	--	--	--
Ca4_P1_44999909	Ca4	44999909	CTGATT/CT	TGGCTAACAGCCACTTGATG	TTGGTTGTTCATCCACCTCA	59.9	709	INTERGENIC	--	--	--
Ca4_P1_45000855	Ca4	45000855	T/TCTCTCGAG	TGGTGAACTTTATGCTACACCG	TTCACGTTTTCAACGCATTC	60.1	318	INTERGENIC	--	--	--
Ca4_P1_45002591	Ca4	45002591	TAAAAAAAAA/TAAAAAAAAAA	TGTGACCGATAGCGATCTTG	AAGTTCACTCGTGTGTCCTGAA	59.8	944	INTERGENIC	--	--	--
Ca4_P1_4500375	Ca4	4500375	A/AT	CTCGAGTGCACTGTCGTCAT	CGGCCAAGTCTTCATATCGT	60.1	783	DRR	Ca_03807	--	FAR1
Ca4_P1_45005102	Ca4	45005102	A/AAT	CTCCGTTCAAGCCTCTTGTC	GAAAGTGTCGAGCAACACGA	60.0	225	INTERGENIC	--	--	--

INDEL marker IDs	Chromosomes/ unanchored scaffolds	Physical positions (bp)	InDels (*Kabuli* reference genome-CDC Frontier/PI)	Forward primers (5'-3')	Reverse primers (5'-3')	Anneling temperature (0C)	Expected amplified product size (bp)	Structural annotation		Functional annotation	
								Sequence components of *kabuli* genome	*Kabuli* gene accession IDs	NCBI-KOG	TFs
Ca4_P1_45006893	Ca4	45006893	ATCTCTCTCTCTCTCTCTCTCTCTCT/ ATCTCTCTCTCTCTCTCTCTCTCTCTC T	GGTCGGACAACATCCT CAGT	CCCCAACTTTTCATGC ATTT	60.0	604	DRR	Ca_09164	--	--
Ca4_P1_45007088	Ca4	45007088	ACC/AC	GGTCGGACAACATCCT CAGT	GAGTTGAACGCAGGTT GTCA	60.0	707	DRR	Ca_09164	--	--
Ca4_P1_45010579	Ca4	45010579	GAA/GAAA	AGTATCGAGGAACACG TGGG	TCAATGCCCATTCCTT CTCT	60.0	204	INTERGENIC	--	--	--
Ca4_P1_45011972	Ca4	45011972	CTTCAAC/CTTCAACATCATTTTCAAC	CATGCTCAATTGGACA ACGA	AAGACACCCATTTGTA GGCG	60.7	465	INTERGENIC	--	--	--
Ca4_P1_4501734	Ca4	4501734	TGAGAGAGA/TGAGA	TTCACCAATGTTTTCC GACA	ACGAAGGTCGAAGGC TTTTT	59.9	226	DRR	Ca_03807	--	FAR1
Ca4_P1_4502063	Ca4	4502063	C/CG	GCTGGATCCGTCGTTA CAAT	CCATCTGAGAAAGCTA GCCG	60.0	441	DRR	Ca_03807	--	FAR1
Ca4_P1_45030756	Ca4	45030756	CATTAATT/CATT	TGTTGACCAGCTTGGA GAGA	CCACCGTATGAAAGTT GGGT	59.5	331	INTERGENIC	--	--	--
Ca4_P1_4503851	Ca4	4503851	TAAAAAAAAA/TAAAAAAAA	TTCTCCCTCCGTCTCA CATC	TGGAGGCAATGATTTT GTCA	60.2	421	INTRON	Ca_03807	--	FAR1
Ca4_P1_45043891	Ca4	45043891	TGAAGA/TGAAGAAGA	TGCCACATGTGACTCC CTAA	CAGTGAGCAATGGCTT TTCA	60.1	470	INTERGENIC	--	--	--
Ca4_P1_45048444	Ca4	45048444	TAAAAAAAAAA/TAAAAAAA	TATTTAATGCGCGTCT GCAC	TTGGAGTGAATCTAAG GCGG	59.9	362	INTERGENIC	--	--	--
Ca4_P1_45057061	Ca4	45057061	GAAAAAA/GAAAAAAA	TTCCCATCTGTCTGGA AGAA	GAGCTGCGCTATCAAA GTCC	58.2	486	INTERGENIC	--	--	--
Ca4_P1_4505728	Ca4	4505728	ATAAATTTAAATT/ATAAATTTAAATTT AAATT	TCTTTTACACGCCATTC ATTTTT	TCATGTTGAAGGCTCT GTCG	59.9	685	INTERGENIC	--	--	--

INDEL marker IDs	Chromosomes/ unanchored scaffolds	Physical positions (bp)	InDels (*Kabuli* reference genome-CDC Frontier/PI)	Forward primers (5'-3')	Reverse primers (5'-3')	Anneling temperature (0C)	Expected amplified product size (bp)	Structural annotation		Functional annotation	
								Sequence components of *kabuli* genome	*Kabuli* gene accession IDs	NCBI-KOG	TFs
Ca4_P1_45057895	Ca4	45057895	CATGGA/CATGGATGGA	GGATTGCGTATCAGTCGTCA	CGCGAAATGCAAACAAACTA	59.7	344	INTERGENIC	--	--	--
Ca4_P1_45062190	Ca4	45062190	GAA/GAATAATAA	AAGGGACGTGGTGTTTGAAG	ACCCCGTTGGTAAAAACTCC	60.0	588	INTERGENIC	--	--	--
Ca4_P1_45063988	Ca4	45063988	CTT/CTTT	TTTTGAGCAATGGAGCAGTG	TTGGGAGGTGAAGTTGGTTC	60.0	940	INTERGENIC	--	--	--
Ca4_P1_45064783	Ca4	45064783	TAAA/TAA	GAACCAACTTCACCTCCCAA	ATCGAGTTGAGTCGTGGTCC	59.9	737	INTERGENIC	--	--	--
Ca4_P1_45065586	Ca4	45065586	TAAAA/TAAAAA	CGACTCAACTCGATCGTCAA	AAAACCTTAATCACCCGTGC	60.0	500	INTERGENIC	--	--	--
Ca4_P1_45066102	Ca4	45066102	C/CAAAACT	CCTCGAAGATCAAAGGGACA	CCCTAATGAGTGGAGCCAAA	60.2	353	INTERGENIC	--	--	--
Ca4_P1_45066949	Ca4	45066949	GATATATA/GATATATATA	GGAGGGACCATGTTTTTGTG	ATCCATGGTACAGGGGTGTC	60.2	867	INTERGENIC	--	--	--
Ca4_P1_45068051	Ca4	45068051	CAA/CAAA	CGTGGACTTCCCGACTACAT	ACAATTTACTCCCTCCGCCT	60.0	584	DRR	Ca_09168	T	WRKY
Ca4_P1_45076892	Ca4	45076892	CG/CGG	GGTCCAACATTCCTCCTTGA	TCCACAAATAACAGGTTTTCCC	59.9	606	INTERGENIC	--	--	--
Ca4_P1_45077297	Ca4	45077297	CTTTTTTT/CTTTTTT	GGGAAAACCTGTTATTTGTGGA	TGTTGGAATTTGGAAATATGGA	60.1	332	INTERGENIC	--	--	--
Ca4_P1_45079199	Ca4	45079199	TATTATTAAAAGATTATTAAAA/TATTATTAAAA	AAGGTATGAAAAATCCGAGTGC	TTGACTACTTTTTCGAGTGACATC	59.5	629	INTERGENIC	--	--	--
Ca4_P1_45081374	Ca4	45081374	ATTTT/ATTTTT	TCGTTTTCCTTCAAAGGTGG	TGCTGAGTTATGGCGTCTTG	60.1	917	INTERGENIC	--	--	--

**INDEL marker IDs**	**Chromosomes/ unanchored scaffolds**	**Physical positions (bp)**	**InDels (*Kabuli* reference genome-CDC Frontier/PI)**	**Forward primers (5'-3')**	**Reverse primers (5'-3')**	**Anneling temperature (0C)**	**Expected amplified product size (bp)**	**Structural annotation**		**Functional annotation**	
								**Sequence components of *kabuli* genome**	***Kabuli* gene accession IDs**	**NCBI-KOG**	**TFs**
Ca4_P1_4508477	Ca4	4508477	T/TA	GTCTCTTGGTTTACCGTGCC	ATGAGTGGTTTGCCACATGA	59.6	452	INTERGENIC	--	--	--
Ca4_P1_45088189	Ca4	45088189	A/AT	TGGCCTGTTCCTGGTACACT	TTACTCCCAACACACCCCTC	60.6	600	INTRON	Ca_09169	E	GATA
Ca4_P1_45092173	Ca4	45092173	GAAA/GAAAAAA	CAGCCACTTCACGTTCTTCA	TTTGTGAGAATCAAAAGTTTATTGG	60.0	527	INTERGENIC	--	--	--
Ca4_P1_4509348	Ca4	4509348	CAAAAAAAAA/CAAAAAAAAAA	GATGAAGGATTTTGCAACCAA	AGGTGAAACGAAGGAGGGAT	59.9	145	INTERGENIC	--	--	--
Ca4_P1_45098696	Ca4	45098696	ATTTTTT/ATTTTT	CATGACTCCAACACAATGCC	TTTTGCCAATTGATACAACAAAA	60.0	305	INTERGENIC	--	--	--
Ca4_P1_45102233	Ca4	45102233	TAAAAAAAAA/TAAAAAAAA	CAGCACATCACAATCATCCA	GGTCACAGTCACCCCAAAGT	59.0	877	INTERGENIC	--	--	--
Ca4_P1_45104586	Ca4	45104586	AGG/AGGG	CCATTTTATCTCATGATTCAGACC	GGTCAAGGAGAAAGCTGCAC	58.9	209	INTERGENIC	--	--	--
Ca4_P1_45107861	Ca4	45107861	CATAT/CAT	TGTGAGGGTGGGAAAAGAAC	TGCTTGGCTCACTGTTTCTG	59.9	512	DRR	Ca_09170	--	--
Ca4_P1_45108280	Ca4	45108280	A/ATT	CAGAAACAGTGAGCCAAGCA	AAAAACATTCCCGCCTTTTA	60.2	188	DRR	Ca_09170	--	--
Ca4_P1_45108878	Ca4	45108878	CAAAAA/CAAAAAA	AAACCCAAACTTTTTCATGGT	GCTTTGGAGGTTGGTTTTGA	57.5	393	INTERGENIC	--	--	--
Ca4_P1_45108939	Ca4	45108939	ATCTCTCTCTCTCTCT/ATCTCTCTCTCTCTCTCTCTCTCTCT	ATTTCCCTGTTTCCAGGCTC	GCTTTGGAGGTTGGTTTTGA	60.4	322	INTERGENIC	--	--	--
Ca4_P1_4510960	Ca4	4510960	ATT/ATTT	TGCATTCGTGTCTTTCTTGC	CGGCTTATTGCAAAGATGGT	60.0	338	INTERGENIC	--	--	--

INDEL marker IDs	Chromosomes/ unanchored scaffolds	Physical positions (bp)	InDels (*Kabuli* reference genome-CDC Frontier/PI)	Forward primers (5'-3')	Reverse primers (5'-3')	Anneling temperature (0C)	Expected amplified product size (bp)	Structural annotation		Functional annotation	
								Sequence components of *kabuli* genome	*Kabuli* gene accession IDs	NCBI-KOG	TFs
Ca4_P1_45114505	Ca4	45114505	AT/ATT	TGATTAATCTTTTTCGCCCG	AATGGTGCAAATGAATGCAA	60.0	388	DRR	Ca_09171	--	MYB_related
Ca4_P1_45115420	Ca4	45115420	TAAAAAAAAAAAAAAA/TAAAAAAAAAAAA	TCGCAATGAGTTGATTGTTCA	TCATCATCCGACTTTGCTTG	60.3	701	INTERGENIC	--	--	--
Ca4_P1_45115919	Ca4	45115919	CTT/CTTATT	TGCAGTGTTTGATTACGGGA	GGTGAAGATGATTCAATTTCCTTT	60.1	774	INTERGENIC	--	--	--
Ca4_P1_45116175	Ca4	45116175	A/AAAAT	GGAAACAGCAAGAAAGACGAA	TCCCTGATTTAAAAATATTGCCA	59.5	972	INTERGENIC	--	--	--
Ca4_P1_45116306	Ca4	45116306	TAAAAAAAA/TAAAAAAA	CGGAGTGGATTGAATTTATGA	GCAATTTCCGCTTTCTGTTT	57.6	889	INTERGENIC	--	--	--
Ca4_P1_45116369	Ca4	45116369	TA/T	AAAGGAAATTGAATCATCTTCACC	GCAATTTCCGCTTTCTGTTT	59.7	385	INTERGENIC	--	--	--
Ca4_P1_45118855	Ca4	45118855	AACTTACATA/AA	GCTTGAGGAAAAAGCCTGTG	ACAGACGGTGCACAATTGAA	60.0	441	INTRON	Ca_09172	T	--
Ca4_P1_4511887	Ca4	4511887	ATTTTT/ATTTTTT	CGTTGTTGGTGAAACTGCAT	CCGGCGAGAATTTTTCAATA	59.6	700	INTERGENIC	--	--	--
Ca4_P1_4512130	Ca4	4512130	GAAAAAAAAA/GAAAAAAAA	TAGACTCAACTCTGGCGGCT	CCGGCGAGAATTTTTCAATA	60.2	266	INTERGENIC	--	--	--
Ca4_P1_45126116	Ca4	45126116	CGTTGTTGTT/CGTTGTT	AACATTGGGACTGCACACAA	GGCGGATGACGAGAATATGT	60.0	913	DRR	Ca_09173	T	NAC
Ca4_P1_45126194	Ca4	45126194	ATTTTTTTTT/ATTTTTTTT	GCATTGCCAAAATAGTCGCT	GGCGGATGACGAGAATATGT	60.2	527	DRR	Ca_09173	T	NAC
Ca4_P1_45135433	Ca4	45135433	GACACTAACACT/GACACT	CATTCTGCGAGACACGAAAA	CATGCTGTGACTCGAAAGGA	60.0	661	DRR	Ca_09175	S	--

INDEL marker IDs	Chromosomes/ unanchored scaffolds	Physical positions (bp)	InDels (*Kabuli* reference genome-CDC Frontier/PI)	Forward primers (5'-3')	Reverse primers (5'-3')	Anneling temperature (0C)	Expected amplified product size (bp)	Structural annotation		Functional annotation	
								Sequence components of *kabuli* genome	*Kabuli* gene accession IDs	NCBI-KOG	TFs
Ca4_P1_45136981	Ca4	45136981	AG/AAAGG	ACTGAAACCAAGGTTGGCAC	TCCAATTCTCCTCCATCAGG	60.0	476	INTRON	Ca_09176	--	--
Ca4_P1_45138231	Ca4	45138231	CATTATATTATATTATATT/CATTATATTATATT	ACCACACTCATGCAACGAAA	AATAAATCGCAGGGCTTGTG	60.2	654	DRR	Ca_09177	T	--
Ca4_P1_45142192	Ca4	45142192	T/TAACAAGTTTTCC	CCAATTTCGATCCTCCTTGA	TGCACACAATGGAAGTGGTT	60.0	445	INTRON	Ca_09177	T	--
Ca4_P1_45145234	Ca4	45145234	ATT/ATTT	GCCTTGTCTGGATTGCATTT	TTCAAATTTTGCAGATAAATTCC	60.1	443	INTERGENIC	--	--	--
Ca4_P1_45145450	Ca4	45145450	GAAAAAAAAAA/GAAAAAAAA	GCCTTGTCTGGATTGCATTT	GAAAAACAATTTGGCTATTCACC	60.1	926	INTERGENIC	--	--	--
Ca4_P1_45151444	Ca4	45151444	AATAT/AAT	CGCTTCCTTGTTTCTTTTGG	CACTGAAAGCAAGGGTCACA	59.9	555	INTRON	Ca_09178	B	NAC
Ca4_P1_45153271	Ca4	45153271	CT/CTT	GACTGAGCCTTGCATCAACA	CATTGCGGTTTAGAAGGCAT	60.0	511	INTRON	Ca_09178	B	NAC
Ca4_P1_45153925	Ca4	45153925	ATGTGT/AT	ATGCCTTCTAAACCGCAATG	TGCTCTCCCTCGTTTTGATT	60.1	587	INTRON	Ca_09178	B	NAC
Ca4_P1_45155912	Ca4	45155912	GAATACAATACAATACAATA/GAATACAATA	AAGGGCACAGTGGTCAAAAC	GATGGAAAGGATCGGAGTGA	60.0	352	DRR	Ca_09178	B	NAC
Ca4_P1_45158654	Ca4	45158654	TA/TAA	TCAAGAGACGTGGATTTCCC	TTGCAGAATGACACAGCCTC	60.1	126	INTRON	Ca_09179	P	--
Ca4_P1_45159393	Ca4	45159393	GTTT/GTTTTT	TGGAGGCTTGCTTCAGATTT	GACTTGTCCATGAAGGGCAT	60.0	793	INTRON	Ca_09179	P	--
Ca4_P1_45163367	Ca4	45163367	ATTTTT/ATTTTTT	CAAGTTGCAGGGATACCGTT	TTCAGGCAAAGGAAATCACC	60.0	598	INTRON	Ca_09179	P	--

INDEL marker IDs	Chromosomes/ unanchored scaffolds	Physical positions (bp)	InDels (*Kabuli* reference genome-CDC Frontier/PI)	Forward primers (5'-3')	Reverse primers (5'-3')	Anneling temperature (0C)	Expected amplified product size (bp)	Structural annotation		Functional annotation	
								Sequence components of *kabuli* genome	*Kabuli* gene accession IDs	NCBI-KOG	TFs
Ca4_P1_45165066	Ca4	45165066	TA/TAA	TTGTTCTTCCTCCCCC TTCT	TTGCACTTCCAACATC CAAA	60.0	408	DRR	Ca_09179	P	--
Ca4_P1_45166587	Ca4	45166587	AATAT/AAT	TTGTGTGCTGCAAGGT TCTT	ACACCAAATTCGGCAA ACTC	59.5	518	INTERGENIC	--	--	--
Ca4_P1_4516701	Ca4	4516701	T/TGAC	TAACTTTTCCATGCTG GGCT	GCACACTTGTGCTTGG AAGA	59.7	868	INTERGENIC	--	--	--
Ca4_P1_45169402	Ca4	45169402	CTCCGACGATTTTC/CTC	TTGGTCTAGCTAATTTT CGAGGA	AAACGGTGCCAATGAG ATTT	59.4	532	INTERGENIC	--	--	--
Ca4_P1_4516975	Ca4	4516975	ATT/A	AAAATGCGTTTCACTC GGAC	GCACACTTGTGCTTGG AAGA	60.1	304	INTERGENIC	--	--	--
Ca4_P1_45172116	Ca4	45172116	ATTTTTTT/ATTTTTTTT	ATCGTTCGGTTACTTC GGTG	AGGGTGTAACCGCCA CAATA	60.0	893	INTERGENIC	--	--	--
Ca4_P1_4517245	Ca4	4517245	AAGA/AA	AAAATGCGTTTCACTC GGAC	CCGTTTTTCGTCACAA TTCA	60.1	550	INTERGENIC	--	--	--
Ca4_P1_45177041	Ca4	45177041	ACCCCCC/ACCCCC	CCAAGTTTTTGCAACC CAGT	TGTGTACCGGAAAACT TCAAAA	60.0	363	INTERGENIC	--	--	--
Ca4_P1_45177094	Ca4	45177094	ATT/AT	CCAAGTTTTTGCAACC CAGT	TGTGTACCGGAAAACT TCAAAA	60.0	363	INTERGENIC	--	--	--
Ca4_P1_45177173	Ca4	45177173	ATTT/ATT	CCAAGTTTTTGCAACC CAGT	TGTGTACCGGAAAACT TCAAAA	60.0	363	INTERGENIC	--	--	--
Ca4_P1_45177232	Ca4	45177232	A/AC	CCAAGTTTTTGCAACC CAGT	TCGTACGAAAAGTACA AACCCA	60.0	720	INTERGENIC	--	--	--
Ca4_P1_45178018	Ca4	45178018	GAAAAAA/GAAAAA	TGAAATGGGTTTGTAC TTTTCG	CCATTCGAGTGGTTGT GTTG	59.0	738	INTERGENIC	--	--	--

INDEL marker IDs	Chromosomes/ unanchored scaffolds	Physical positions (bp)	InDels (*Kabuli* reference genome-CDC Frontier/PI)	Forward primers (5'-3')	Reverse primers (5'-3')	Anneling temperature (0C)	Expected amplified product size (bp)	Structural annotation		Functional annotation	
								Sequence components of *kabuli* genome	*Kabuli* gene accession IDs	NCBI-KOG	TFs
Ca4_P1_45178267	Ca4	45178267	GTTTTTTTT/GTTTTTTTTT	TTTGTGAAATTGCATTCCGA	CCATTCGAGTGGTTGTGTTG	60.1	167	INTERGENIC	--	--	--
Ca4_P1_45182772	Ca4	45182772	AATATATATATATATAT/AATATATATAT	TGAAAAATTAAATAAAAAGCTGACGA	CAGTATTTATTCGGCGGGAA	59.6	629	INTERGENIC	--	--	--
Ca4_P1_45182830	Ca4	45182830	T/TATATGAA	AAAATTAAATAAAAAGCTGACGAAA	CAGTATTTATTCGGCGGGAA	57.1	626	INTERGENIC	--	--	--
Ca4_P1_45183778	Ca4	45183778	TTCTTTCTCTCT/TTCT	TGCAACGTTTGCTGAAAAAC	TGCATTGGTTCAATTTGCAT	59.9	674	INTERGENIC	--	--	--
Ca4_P1_45185940	Ca4	45185940	T/TG	TACTCGAGCACATGCAAACC	CTTTGAGGCCTCCATAGCTG	59.9	510	INTRON	Ca_09180	K	TALE
Ca4_P1_45189846	Ca4	45189846	CCAGCA/CCA	TTAATGGCTTCAAGGAACCG	CACGGTGGGGAAATCTCTTA	60.1	170	INTRON	Ca_09181	S	--
Ca4_P1_45193943	Ca4	45193943	TA/T	CACTGAATCGGTTGGTGTTG	GCAAGCAATCAAGTAAAAATGG	60.0	850	INTERGENIC	--	--	--
Ca4_P1_45195172	Ca4	45195172	TCTA/TCTAATTTTTTAGCTA	TCGACACTTTTGTGTCTCTCAA	AACGGCTTTTTATTAATCATGACA	58.6	904	INTERGENIC	--	--	--
Ca4_P1_45195206	Ca4	45195206	ATTT/ATTTTT	CACGTTATTTAAAACATCACACATCA	AACGGCTTTTTATTAATCATGACA	60.1	664	INTERGENIC	--	--	--
Ca4_P1_45197383	Ca4	45197383	GAAGAAAA/GAAGAAAAGAAAA	TTTCAAGTGACTGTTGCCAAA	GTCGGTGGCTATGACGAAAT	59.4	531	INTERGENIC	--	--	--
Ca4_P1_45197554	Ca4	45197554	TATTA/TATTATTA	ATTTCGTCATAGCCACCGAC	GTCGACATTGAGAGGGGAAA	60.0	395	INTERGENIC	--	--	--
Ca4_P1_4521076	Ca4	4521076	AATATATATATATATATATA/AATATATATATATATA	CCACAGAAAATGTTGGGCTT	TCGTAAAATAGTTGGCCGGA	60.0	568	INTERGENIC	--	--	--

INDEL marker IDs	Chromosomes/ unanchored scaffolds	Physical positions (bp)	InDels (*Kabuli* reference genome-CDC Frontier/PI)	Forward primers (5'-3')	Reverse primers (5'-3')	Anneling temperature (0C)	Expected amplified product size (bp)	Structural annotation		Functional annotation	
								Sequence components of *kabuli* genome	*Kabuli* gene accession IDs	NCBI-KOG	TFs
Ca4_P1_4521925	Ca4	4521925	CATATATAT/CATATATATAT	TGCTTGTATGGTTTTAATACGGG	AAACATAAAACAACACAGGACAAA	60.1	690	INTERGENIC	--	--	--
Ca4_P1_45222131	Ca4	45222131	TA/TAA	CCACCTTGTTCCCACTCCTA	TCCAATGAATTTGGAGAGCC	60.0	334	DRR	Ca_09184	A	C2H2
Ca4_P1_4522228	Ca4	4522228	ATTTTTTTTTT/ATTTTTTTT	TCAATGACAAATATTGATCCCA	CACGACACATAGTTTTCACCG	57.8	888	INTERGENIC	--	--	--
Ca4_P1_45222380	Ca4	45222380	TA/T	TTAGCCAATCACCCCAAAAC	ATACGAAGCAACAAGCACCC	59.8	353	DRR	Ca_09184	A	C2H2
Ca4_P1_45222705	Ca4	45222705	CTTTTTTTTT/CTTTTTTTTTTT	GGGTGCTTGTTGCTTCGTAT	TTTGCATATTTCCCCTCTTCA	60.1	489	DRR	Ca_09184	A	C2H2
Ca4_P1_45223122	Ca4	45223122	TAAAGTTGATATCTAAAAAGTTGATATCTAAAAA/TAAAGTTGATATCTAAAAA	GCCTTTTGCAGTGTGCCTAT	CATGTCCCCTCTTCATTTTTG	60.3	558	INTERGENIC	--	--	--
Ca4_P1_45226065	Ca4	45226065	CTTTTTTTTT/CTTTTTTTTTTT	GAAAGACGGCTTGTTGCTTC	GAGACCGGCCAGGAACTAAT	60.0	688	INTRON	Ca_09185	S	--
Ca4_P1_45228406	Ca4	45228406	ATTATT/ATTATTATTTTTATT	GAGAGGGTGGCTAGTTGCTG	TTGACCCATTTCGTATCACTG	60.0	672	DRR	Ca_09185	S	--
Ca4_P1_45228863	Ca4	45228863	ATATTATTATTATTATTATTATTATTATTA/ATATTATTATTATTATTATTATTATTATTATTATTATTA	TCAGTGATACGAAATGGGTCA	TTTGGTGATTGGCACAACAT	59.0	484	INTERGENIC	--	--	--
Ca4_P1_4522910	Ca4	4522910	ATTTTTTTT/ATTTTTTT	AATGACAAATTTTCGGCCC	TACCCTCCTTCGCATCAATC	59.8	775	INTERGENIC	--	--	--
Ca4_P1_4522975	Ca4	4522975	AATTTTAACATCAT/AAT	AATGACAAATTTTCGGCCC	TACCCTCCTTCGCATCAATC	59.8	775	INTERGENIC	--	--	--
Ca4_P1_4523208	Ca4	4523208	TATTAAATTAA/TATTAA	TGATAGAAAAATGATGCAAGAAGG	AATTTTCCATGCCAACTGGA	59.6	454	INTERGENIC	--	--	--

INDEL marker IDs	Chromosomes/ unanchored scaffolds	Physical positions (bp)	InDels (*Kabuli* reference genome-CDC Frontier/PI)	Forward primers (5'-3')	Reverse primers (5'-3')	Anneling temperature (0C)	Expected amplified product size (bp)	Structural annotation		Functional annotation	
								Sequence components of *kabuli* genome	*Kabuli* gene accession IDs	NCBI-KOG	TFs
Ca4_P1_4523289	Ca4	4523289	TT/TTATAT	TGATAGAAAAATGATGCAAGAAGG	AATTTTCCATGCCAACTGGA	59.6	454	INTERGENIC	--	--	--
Ca4_P1_4523557	Ca4	4523557	GAAAA/GAAAAA	GATTGATGCGAAGGAGGGTA	AACACACCAACCACCACTCA	60.0	490	INTERGENIC	--	--	--
Ca4_P1_45236013	Ca4	45236013	CATGTGCGAACAATG/CATGTGCGAACAATGTGCGAACAATG	CAAACCTGAAAAGGGCACAT	TAACGATGGGATTGGTCCAT	60.0	461	INTRON	Ca_09187	A	--
Ca4_P1_4523884	Ca4	4523884	CATATATATATATATATA/CATATATATATATATA	TGAGTGGTGGTTGGTGTGTT	TGCATTAATTGGGAGATGGC	59.9	198	INTERGENIC	--	--	--
Ca4_P1_4524273	Ca4	4524273	TA/TAAAATTAATAGA	TGAGTGGTGGTTGGTGTGTT	AACGGAGTGGGGTACCTTCT	59.9	690	INTERGENIC	--	--	--
Ca4_P1_45245680	Ca4	45245680	TAAAAAAAAA/TAAAAAAA	TGCTTGTTGCACAAGTTTCC	AAAAACGCAGTATCCATACAAAA	59.9	617	DRR	Ca_09188	--	B3
Ca4_P1_45247097	Ca4	45247097	ATCTTCTTCTTCTTCTTCTTCTT/ATCTTCTTCTTCTTCTTCTT	GCAAAACAAAATCCTCAGCC	AGTTCACCATGGCTTCCAAC	59.7	399	CDS (large-effect mutations)	Ca_09188	--	B3
Ca4_P1_45248759	Ca4	45248759	CTTT/CTTTTT	AAGTCTTGAAATCTTGCGGC	TTTGTCCCATCCGATTTTTC	59.5	334	INTERGENIC	--	--	--
Ca4_P1_45256690	Ca4	45256690	TAGAGAG/TAGAGAGAG	CCTGGGGTTCTTGGTGAGTA	CACAAACTCCACTTCGAGCA	60.0	303	INTRON	Ca_09189	O	--
Ca4_P1_45256888	Ca4	45256888	TCAGG/T	CCTGGGGTTCTTGGTGAGTA	CACAAACTCCACTTCGAGCA	60.0	303	INTRON	Ca_09189	O	--
Ca4_P1_45264987	Ca4	45264987	CATAATATCAA/CATAATATCAATAATATCAA	GCCGTTTGAGTCGAGCTATC	TCCAACCATCGAGACTTTCC	60.0	407	INTRON	Ca_09190	--	--
Ca4_P1_45267081	Ca4	45267081	CT/CTT	CTCTTCAATAGCCCCATCCA	TGGCCATGAAATCTTCTTCC	60.0	751	INTRON	Ca_09190	--	--

INDEL marker IDs	Chromosomes/ unanchored scaffolds	Physical positions (bp)	InDels (*Kabuli* reference genome-CDC Frontier/PI)	Forward primers (5'-3')	Reverse primers (5'-3')	Anneling temperature (0C)	Expected amplified product size (bp)	Structural annotation		Functional annotation	
								Sequence components of *kabuli* genome	*Kabuli* gene accession IDs	NCBI-KOG	TFs
Ca4_P1_45268948	Ca4	45268948	A/AAT	TGTTCATTTTGCACCGTGTT	AACATGGCGGTGAGAAAAAC	60.0	718	INTRON	Ca_09190	--	--
Ca4_P1_45269916	Ca4	45269916	TAAAAA/TAAAA	AAATACACAAGGCAATGGGG	TGACAGGCAACATTATCCGA	59.7	497	DRR	Ca_09190	--	--
Ca4_P1_45271723	Ca4	45271723	TATTTA/T	TTGTTGTGGTTTGGCTTCAA	CCCACCAATCTCTAAAGGCA	60.1	449	INTERGENIC	--	--	--
Ca4_P1_4527520	Ca4	4527520	TTATATATATATATATATATATATATATATATAT/TTATATATATATATATATATATATATATAT	AAGGTATGCTGTTTCAAGGGAA	AATGTGCATCTGCCACCATA	60.0	892	INTERGENIC	--	--	--
Ca4_P1_45289876	Ca4	45289876	GAAAAAAAAAA/GAAAAAAAA	AAGCGAGCATCAGAGAAAGC	CTTGATGGCAAAACCCAAGT	59.9	269	INTERGENIC	--	--	--
Ca4_P1_45290418	Ca4	45290418	GAAA/GAAAA	CTTGGGTTTTGCCATCAAGT	GAAGCAATCGTGTGTAGCCA	60.0	604	INTERGENIC	--	--	--
Ca4_P1_45297781	Ca4	45297781	ATTTTTTTTT/ATTTTTTTTTT	GTGTGTATCAAGGCGAGGGT	AGTTAAGAGAGCGCGTGTCC	60.0	579	INTERGENIC	--	--	--
Ca4_P1_45298506	Ca4	45298506	GA/GAA	GGACACGCGCTCTCTTAACT	GTTGTAGGTCAAGCTGGCAA	59.6	611	DRR	Ca_09193	--	SBP
Ca4_P1_45299425	Ca4	45299425	GATAAAATAAAATAAAA/GATAAAATAAAATAAAATAAAA	TCTCAATGGAGTGCTGAACG	TGAAGAAGAATTTTCCAGCACA	60.0	357	DRR	Ca_09193	--	SBP
Ca4_P1_45304688	Ca4	45304688	CATA/CATATA	TTGATGAAAAGGGTGGTATGG	TTTCTAGCAAGCACGCAAAA	59.7	428	INTERGENIC	--	--	--
Ca4_P1_45321052	Ca4	45321052	AC/A	TCATCGATTGCATCGACATT	CATTCCTTTGCTTCACCTCC	60.0	423	INTRON	Ca_09196	--	--
Ca4_P1_45323676	Ca4	45323676	CACGTA/CA	ATTGGTGGGTGCAATATGGT	AAACCAAACAAAAACCGCAG	59.9	837	DRR	Ca_09196	--	--

INDEL marker IDs	Chromosomes/ unanchored scaffolds	Physical positions (bp)	InDels (*Kabuli* reference genome-CDC Frontier/PI)	Forward primers (5'-3')	Reverse primers (5'-3')	Anneling temperature (0C)	Expected amplified product size (bp)	Structural annotation		Functional annotation	
								Sequence components of *kabuli* genome	*Kabuli* gene accession IDs	NCBI-KOG	TFs
Ca4_P1_4532626	Ca4	4532626	GTATTATTA/GTATTA	TGAACCCCAAAAATCACCAT	CAAACTCATAGCGGGCATTT	60.0	684	DRR	Ca_03806	--	--
Ca4_P1_4532939	Ca4	4532939	TAAAAAAA/TAAAAA	CGTGAATTGCATTCAACCTTT	CAAACTCATAGCGGGCATTT	60.0	503	DRR	Ca_03806	--	--
Ca4_P1_4533213	Ca4	4533213	CACAAA/CACAAATAACAAGGACAAA	AAATGCCCGCTATGAGTTTG	ACGAGGGGTTGAATTTTTCC	60.1	348	DRR	Ca_03805	P	--
Ca4_P1_45332248	Ca4	45332248	T/TCACAC	GGCCAATGTGCTCACTTTTT	TGCATTGGTTTGATGGCTAA	60.1	498	INTERGENIC	--	--	--
Ca4_P1_45332757	Ca4	45332757	CCCGT/CCCGTATCCGT	CTTTACGTGCCTCTCCCTTG	TCCAAAGCATGTGTATCCCA	59.9	479	INTERGENIC	--	--	--
Ca4_P1_4533290	Ca4	4533290	GAAAAA/GAAAA	AAATGCCCGCTATGAGTTTG	GGTGAAAAGAGTACGAGGGG	60.1	360	DRR	Ca_03805	P	--
Ca4_P1_45332940	Ca4	45332940	TAA/T	TGGGATACACATGCTTTGGA	TGACATTGTTTGGCACACTG	59.9	174	INTERGENIC	--	--	--
Ca4_P1_45333920	Ca4	45333920	TAAAA/TAAA	TTGTGGCATTATCTCCTCACC	TCATGTCTCTCTTTCCACATTAAAA	59.9	486	INTERGENIC	--	--	--
Ca4_P1_45334419	Ca4	45334419	ATT/ATTT	TTTAATGTGGAAAGAGAGACATGAA	AAACACATTTGACCGCCATT	59.2	357	INTERGENIC	--	--	--
Ca4_P1_45334532	Ca4	45334532	AG/A	TTTAATGTGGAAAGAGAGACATGAA	AAACACATTTGACCGCCATT	59.2	357	INTERGENIC	--	--	--
Ca4_P1_45334674	Ca4	45334674	TA/T	ATCATTCTTGAATGGCGGTC	CAAGACTTCGGGCACTTTTC	59.9	513	INTERGENIC	--	--	--
Ca4_P1_45334856	Ca4	45334856	AACG/AACGACG	ATCATTCTTGAATGGCGGTC	AGAAGCCATGTGATCGGAAC	59.9	684	INTERGENIC	--	--	--

INDEL marker IDs	Chromosomes/ unanchored scaffolds	Physical positions (bp)	InDels (*Kabuli* reference genome-CDC Frontier/PI)	Forward primers (5'-3')	Reverse primers (5'-3')	Anneling temperature (0C)	Expected amplified product size (bp)	Structural annotation		Functional annotation	
								Sequence components of *kabuli* genome	*Kabuli* gene accession IDs	NCBI-KOG	TFs
Ca4_P1_45334966	Ca4	45334966	ATT/ATTT	ATCATTCTTGAATGGCGGTC	AGAAGCCATGTGATCGGAAC	59.9	684	INTERGENIC	--	--	--
Ca4_P1_45335482	Ca4	45335482	ATTTT/ATTT	GTTCCGATCACATGGCTTCT	TGAGACGCTTTCGTCACAAC	60.1	406	INTRON	Ca_09198	L	--
Ca4_P1_45338666	Ca4	45338666	TA/TCA	GTTCTCCTTCGACAGGTTGG	AAGGAAACGGACGTCATCAG	59.7	433	INTRON	Ca_09198	L	--
Ca4_P1_45338691	Ca4	45338691	AT/A	GTTCTCCTTCGACAGGTTGG	AAGGAAACGGACGTCATCAG	59.7	433	INTRON	Ca_09198	L	--
Ca4_P1_45341287	Ca4	45341287	CATAT/CAT	TGCTCAAATGGATTGTTGTCA	TCCTCCTCTTTGTTTGTCCG	60.1	798	DRR	Ca_09199	F	--
Ca4_P1_45341374	Ca4	45341374	AAA/AAATAA	TTTGGCATATTTTAGGCTGGA	TCCTCCTCTTTGTTTGTCCG	59.6	536	DRR	Ca_09199	F	--
Ca4_P1_45341608	Ca4	45341608	AAGAGAATCGGA/AAGAGAATCGGAGAGAATCGGA	GCTGGACAAGTCTGCCAAG	CTAGTGTGCAGTGGTGCCAT	59.6	874	DRR	Ca_09198	L	--
Ca4_P1_45342973	Ca4	45342973	ATTTTTTTTTTT/ATTTTTTTTTTTTT	GGGCCTTTCTCCTTTTCATC	TCGCTTCTGTTCCGCTTAAT	60.0	762	INTRON	Ca_09199	F	--
Ca4_P1_45345425	Ca4	45345425	ATTTTTTT/ATTTTTT	CTTTTGCTGCAAGGCATACA	CTGCACATTCAACTCTGGGA	60.0	582	INTRON	Ca_09199	F	--
Ca4_P1_45346735	Ca4	45346735	GAAAAAAAAAA/GAAAAAAAAAAAAA	CTTCCTTACATTGAGCCCCA	GGTGTTGGATGCAACTGATG	60.1	670	INTRON	Ca_09199	F	--
Ca4_P1_45349934	Ca4	45349934	TAAAAAAAAA/TAAAAAAAA	AAAGGACTCTTGTTCTCCCCA	TTGACTTGGTCATCCAATTTTT	60.1	693	INTERGENIC	--	--	--
Ca4_P1_45350262	Ca4	45350262	TTA/TTATA	AGAATCGTTTTGTTGGGATCA	GTCCTTGGTAGCGAGTTGGA	59.4	778	INTERGENIC	--	--	--

INDEL marker IDs	Chromosomes/ unanchored scaffolds	Physical positions (bp)	InDels (*Kabuli* reference genome-CDC Frontier/PI)	Forward primers (5'-3')	Reverse primers (5'-3')	Anneling temperature (0C)	Expected amplified product size (bp)	Structural annotation		Functional annotation	
								Sequence components of *kabuli* genome	*Kabuli* gene accession IDs	NCBI-KOG	TFs
Ca4_P1_45350516	Ca4	45350516	A/AC	AAAAATTGGATGACCAAGTCAA	GTCCTTGGTAGCGAGTTGGA	58.5	517	INTERGENIC	--	--	--
Ca4_P1_45358850	Ca4	45358850	AATATTATATTAT/AATATTAT	TCATGTTATGGTTGCCGAAA	TTGGTCTTCACAATGATGCTTT	59.9	502	INTERGENIC	--	--	--
Ca4_P1_45363311	Ca4	45363311	AT/ATAATTT	GGCAAAACCAAAGAGAACCA	GTTGGAAAATGAGAGGTGCG	60.1	781	DRR	Ca_09200	K	MYB
Ca4_P1_4536669	Ca4	4536669	GTATA/GTA	GGTGGGTGAGCAAGAGAGAG	TTCGCAACCAACCACAATTA	60.0	410	INTERGENIC	--	--	--
Ca4_P1_45366808	Ca4	45366808	TT/TTAT	ACACCCCAATCCAAAATTGA	ATGCTCAAATGGGTAGCAGG	60.0	771	INTRON	Ca_09200	K	MYB
Ca4_P1_45368052	Ca4	45368052	AAAGAAGAA/AAAGAA	TGAAACAATGGGAAGAGCAA	AAAGGTGCTTGCTTTCAAGG	59.2	226	INTRON	Ca_09200	K	MYB
Ca4_P1_45368241	Ca4	45368241	CAAG/CAAGAAG	CCAACTTCCAGAAGTGCCAT	TCATCTGCTTTAGATTGTCACCA	60.1	209	INTERGENIC	--	--	--
Ca4_P1_45369151	Ca4	45369151	CTTTTTT/CTTTTT	TTGCATTCAATGTCTGCATTT	GGGCAGTCACCTCCACTAAA	59.2	575	INTERGENIC	--	--	--
Ca4_P1_45370800	Ca4	45370800	AG/AGCTTTCGG	GAGCTTCTTTTCCAACACGC	TCCGTCCTCTTGATTTTGCT	60.0	464	INTERGENIC	--	--	--
Ca4_P1_45371273	Ca4	45371273	AC/A	CGCAAATTTCGAAACCATTT	TGCGGATCACTTTTCTACCC	59.9	150	INTERGENIC	--	--	--
Ca4_P1_45372398	Ca4	45372398	AC/A	CGACACTCGAATGACACCAC	GGGTAATTCGGTGTGTGGAC	60.2	945	INTERGENIC	--	--	--
Ca4_P1_4537624	Ca4	4537624	TAATAAAATAAAATAAAAT/TAATAAAATAAAATAAAATAAAAT	TGCATCGCCCATATCAATAA	TATTGGACCGACCCTGAAAA	59.9	511	INTERGENIC	--	--	--

INDEL marker IDs	Chromosomes/ unanchored scaffolds	Physical positions (bp)	InDels (*Kabuli* reference genome-CDC Frontier/PI)	Forward primers (5'-3')	Reverse primers (5'-3')	Anneling temperature (0C)	Expected amplified product size (bp)	Structural annotation		Functional annotation	
								Sequence components of *kabuli* genome	*Kabuli* gene accession IDs	NCBI-KOG	TFs
Ca4_P1_45377418	Ca4	45377418	ATTGTCACTTTGTCACTTT/ATTGTCACTTT	CTCCATTGGTGGTGTCCTTT	GTCTGAGCGTTTGCATGGTA	59.8	821	DRR	Ca_09201	--	--
Ca4_P1_45377587	Ca4	45377587	ATTTTTTTT/ATTTTTTT	CTCCATTGGTGGTGTCCTTT	GTCTGAGCGTTTGCATGGTA	59.8	821	DRR	Ca_09201	--	--
Ca4_P1_45377807	Ca4	45377807	AC/A	TCCCATCATGTTTTGATAGCC	GTCTGAGCGTTTGCATGGTA	59.8	603	DRR	Ca_09201	--	--
Ca4_P1_45380400	Ca4	45380400	ACTACG/A	TTGCCGTTAATTCTTGGAGC	GTGGAGAAAACAACAACGGG	60.2	774	INTERGENIC	--	--	--
Ca4_P1_45383583	Ca4	45383583	TAAAAAAAAAA/TAAAAAAAAA	CAATCCCTAAAGACCGCTCA	TCGAATAATTATGACGCATGAC	60.2	399	INTERGENIC	--	--	--
Ca4_P1_4538533	Ca4	4538533	CTTT/CTTTT	CCGTACCAAAACAATCGTCC	TGCATCTATTCCAACCGTCA	60.2	597	INTERGENIC	--	--	--
Ca4_P1_45389098	Ca4	45389098	CA/CAA	CAATTTCATTTTGTTGGGGG	ATTTGAGCATGGGGAAGATG	60.0	623	INTERGENIC	--	--	--
Ca4_P1_4539131	Ca4	4539131	A/AG	TGTGCGCCATATTCTTTGAG	GGGTGGAAATAGGTTGGGTC	59.8	680	INTERGENIC	--	--	--
Ca4_P1_45391856	Ca4	45391856	CTTTTT/CTTTT	CCACACTGAGCTACTGCTCC	TCTCCGTGGGCTTAACAATC	58.6	634	INTERGENIC	--	--	--
Ca4_P1_45393727	Ca4	45393727	T/TG	CACATTCATGTTTCAACCGC	TCTGTGATTCAGACATTAGAGAATGA	60.0	372	INTERGENIC	--	--	--
Ca4_P1_45395670	Ca4	45395670	TC/T	TGATATGGGAGGTTAAGGACCA	CCCCACGAACTGTATCGTTT	60.6	604	INTERGENIC	--	--	--
Ca4_P1_4539713	Ca4	4539713	TTA/TTATA	TCGACCCAACCTATTTCCAC	GGTTAACCGTCGTAACACACTTT	59.8	340	INTERGENIC	--	--	--

INDEL marker IDs	Chromosomes/ unanchored scaffolds	Physical positions (bp)	InDels (*Kabuli* reference genome-CDC Frontier/PI)	Forward primers (5'-3')	Reverse primers (5'-3')	Anneling temperature (0C)	Expected amplified product size (bp)	Structural annotation		Functional annotation	
								Sequence components of *kabuli* genome	*Kabuli* gene accession IDs	NCBI-KOG	TFs
Ca4_P1_45397379	Ca4	45397379	TA/TAA	CACGTGAGTGTGACATGGTG	CAATGGCAAAAATATCCCAAA	59.6	626	INTERGENIC	--	--	--
Ca4_P1_4539900	Ca4	4539900	TTATATATATATATA/TTATATATATATATATATA	TCGACCCAACCTATTTCCAC	TCAATTAGGAGAATCACGTTTGAA	59.8	797	INTERGENIC	--	--	--
Ca4_P1_45399331	Ca4	45399331	ATTTTTTTTTTTTTTT/ATTTTTTTTTT	ACCACTTCACCACAAGAGGG	ATTCCGCTTTGCTGTCAAAC	60.0	445	INTERGENIC	--	--	--
Ca4_P1_45399782	Ca4	45399782	AG/A	GTTTGACAGCAAAGCGGAAT	ACAGCTTGCAACTGATGTGG	60.3	334	INTERGENIC	--	--	--
Ca4_P1_45402214	Ca4	45402214	ACAAAACCAAAACCAAAACC/ACAAAACCAAAACCAAAACCAAAACC	TGATAGTGGCTGGCACAAAA	GAAATGCGTTTATGCGTTCA	60.3	562	INTERGENIC	--	--	--
Ca4_P1_45404851	Ca4	45404851	GTTTCTTTTTTCT/GTTTCTTTTTTCTTTTTTCT	TTTCTTTTGTGTTTGCAGCG	GGGAGGAAAGTTGAAGTTGAAA	60.0	400	INTERGENIC	--	--	--
Ca4_P1_45404908	Ca4	45404908	CTTTTTTTT/CTTTTTTT	TTTCTTTTGTGTTTGCAGCG	TGCATATTTGCCACTACTCAAA	60.0	512	INTERGENIC	--	--	--
Ca4_P1_45414246	Ca4	45414246	AGCATTT/A	TGTTTTTCTCTCACCGCAAA	TGTGTGCATGCATATTCGTT	59.4	509	INTERGENIC	--	--	--
Ca4_P1_45414589	Ca4	45414589	CTT/CTTT	TGCATAAATGCACGATGAAAA	GTTCAAACCGGCAATGTCTT	60.1	740	INTERGENIC	--	--	--
Ca4_P1_45415143	Ca4	45415143	TTTTTATTTTATTTTAT/TTTTTATTTTATTTTATTTTAT	CTCCACCCTGTCAAGACCAT	GGCCTTGAACTTTGATTTGG	60.0	433	INTERGENIC	--	--	--
Ca4_P1_45415209	Ca4	45415209	AATTTATTTATTTATTTAT/AATTTATTTATTTATTTATTTAT	CTCCACCCTGTCAAGACCAT	GAACTTGGACTCCCTCCTCC	60.0	733	INTERGENIC	--	--	--
Ca4_P1_45415408	Ca4	45415408	ACA/ACACCA	CTCCACCCTGTCAAGACCAT	GAACTTGGACTCCCTCCTCC	60.0	733	INTERGENIC	--	--	--

INDEL marker IDs	Chromosomes/ unanchored scaffolds	Physical positions (bp)	InDels (*Kabuli* reference genome-CDC Frontier/PI)	Forward primers (5'-3')	Reverse primers (5'-3')	Anneling temperature (0C)	Expected amplified product size (bp)	Structural annotation		Functional annotation	
								Sequence components of *kabuli* genome	*Kabuli* gene accession IDs	NCBI-KOG	TFs
Ca4_P1_45416301	Ca4	45416301	T/TA	CAATTCATGGTACAAGGGGG	TCCCATTCCATTTTGAAAGC	60.0	741	INTRON	Ca_09202	K	TALE
Ca4_P1_45418187	Ca4	45418187	CAAA/CAAAA	CTTGACTTGGATGACATGATGAA	CTCGAGGATCAATCTCTGGC	60.0	508	INTRON	Ca_09202	K	TALE
Ca4_P1_4541961	Ca4	4541961	TAAAA/TAAA	AAGTTACCGCAGTGTGGTCC	TTTGGGCGCTTCTTGTAAAT	60.0	404	INTERGENIC	--	--	--
Ca4_P1_45431331	Ca4	45431331	GATATAT/GAT	GAGGGCAAGACCAAGATCAA	CCACCAAAAATCTCCCTATGA	60.2	248	INTERGENIC	--	--	--
Ca4_P1_45432100	Ca4	45432100	A/AATT	TTTGTTGTGTGCATTCATGTG	GGTAAACACATGACCCGTCC	59.0	746	INTERGENIC	--	--	--
Ca4_P1_45436331	Ca4	45436331	TT/TTCATAT	TGCTAATAACGTGTCACGCC	CTCTCCCAACATATGATGCCT	59.8	819	INTERGENIC	--	--	--
Ca4_P1_45436462	Ca4	45436462	AC/A	AACCATGCACATGACTCACAA	CGAGCGTGACGAAATAATCA	60.0	850	INTERGENIC	--	--	--
Ca4_P1_45437514	Ca4	45437514	GAGAAAGAAAGAAAGAAA/GAGAAAGAAAGAAAGAAAGAAAGAAAGAAAGAAA	TTTGGGTGAATGCAATTTGA	GGAGGATGAAGCCAAGTTGA	59.9	323	INTERGENIC	--	--	--
Ca4_P1_45437836	Ca4	45437836	AA/AATA	TCAACTTGGCTTCATCCTCC	TTCGAAACGCACAATTAATCA	60.2	435	INTERGENIC	--	--	--
Ca4_P1_4543916	Ca4	4543916	TAAAAAAAAA/TAAAAAAAAAA	TAGGTCTTTCCCTGGGTGTG	TTAGGCGTGCTTCCTTCACT	60.0	384	DRR	Ca_03804	--	--
Ca4_P1_45440447	Ca4	45440447	AT/A	GGAGGTCGAACTCGAGAATG	AAGTGGCGCTTAATCCAAAA	59.8	784	INTERGENIC	--	--	--
Ca4_P1_45505990	Ca4	45505990	AATTATTATTATTATTATTATTATTATTATTATTATTATTATTATTATTATTATTATTA/AATTATTATTATTATTATTATTATTATTATTATTATTATTATTATTATTATTATTATTATTATTA	GAATCACGCAGCATTCAAAA	CGGAGATTGCAAGTAAAAGAGTC	59.8	402	INTERGENIC	--	--	--

INDEL marker IDs	Chromosomes/ unanchored scaffolds	Physical positions (bp)	InDels (*Kabuli* reference genome-CDC Frontier/PI)	Forward primers (5'-3')	Reverse primers (5'-3')	Anneling temperature (0C)	Expected amplified product size (bp)	Structural annotation		Functional annotation	
								Sequence components of *kabuli* genome	*Kabuli* gene accession IDs	NCBI-KOG	TFs
Ca4_P1_45567618	Ca4	45567618	TTATATATATATATATATAT/TTATATAT ATATATATAT	TCACAAATGGCAGTTT TGTCTT	CCAATCCTAGCTTCTG AAAATG	59.7	635	INTERGENIC	--	--	--
Ca4_P1_45606495	Ca4	45606495	CATATATATATATATATATATATATAT/ CATATATATATATATATATATAT	AACCAACTTCGGCTAG GGTT	GTCATCCCGTGGAAAT ATGG	60.0	445	INTERGENIC	--	--	--
Ca4_P1_4561887	Ca4	4561887	GTAGCATCTA/GTA	GGAACATGGCAAGGAA GTGT	GCAGATCGATTCTTTA GCGG	60.0	912	INTRON	Ca_03802	UZ	--
Ca4_P1_45626888	Ca4	45626888	ATTT/ATTTT	TATCAGCAGCACCAAA TGGA	GCCTGATGTTTGTCAG GGTT	60.2	286	INTRON	Ca_09219	Z	MYB_related
Ca4_P1_4563881	Ca4	4563881	CTTTTTT/CTTTTTTT	TGCAGTTTGCTATGCC ACTT	GGCCAGTCATCAGATC CAGT	59.5	605	INTRON	Ca_03802	UZ	--
Ca4_P1_4563901	Ca4	4563901	ATT/ATTT	TGCAGTTTGCTATGCC ACTT	GGCCAGTCATCAGATC CAGT	59.5	605	INTRON	Ca_03802	UZ	--
Ca4_P1_4564033	Ca4	4564033	TAA/TAAAA	TTGCAAATTTTCGAATA CACTCA	GGCCAGTCATCAGATC CAGT	59.7	309	INTRON	Ca_03802	UZ	--
Ca4_P1_4564093	Ca4	4564093	CTG/CTGTG	TGGCATGGTAACAAAA GTCA	TGTGTTCCAAAGCCAG TGAG	57.6	539	INTRON	Ca_03802	UZ	--
Ca4_P1_45645932	Ca4	45645932	GT/G	AACTTGATGCCGAGTA GCGT	GCCGAATAAAACCCTC ACAA	59.9	713	INTERGENIC	--	--	--
Ca4_P1_4567851	Ca4	4567851	AAAAGTT/A	GTGGGGCATTACGATA CCAC	GGGGAAGCCATATCA CATCA	60.1	636	DRR	Ca_03802	UZ	--
Ca4_P1_4568058	Ca4	4568058	ATTTGATTTT/ATTTGATTTTTGATTTT	GTGGGGCATTACGATA CCAC	GGGGAAGCCATATCA CATCA	60.1	636	DRR	Ca_03802	UZ	--
Ca4_P1_45690560	Ca4	45690560	TAAAAAAAAAAAAAAA/TAAAAAAAAAA AAA	CCAATCCCGCAAAATT TCTA	TAAACCAACCCCCAAA TGAA	59.9	701	DRR	Ca_18393	--	--

INDEL marker IDs	Chromosomes/ unanchored scaffolds	Physical positions (bp)	InDels (*Kabuli* reference genome-CDC Frontier/PI)	Forward primers (5'-3')	Reverse primers (5'-3')	Anneling temperature (0C)	Expected amplified product size (bp)	Structural annotation		Functional annotation	
								Sequence components of *kabuli* genome	*Kabuli* gene accession IDs	NCBI-KOG	TFs
Ca4_P1_4569177	Ca4	4569177	TTATATATATATATATATATATATATA/TTATATATATATATATATATATATATATATATATATA	ATTTGAATTTGATTAAGTGTGGTTT	CTCGAACAGTGAGGTCGTGA	57.3	726	DRR	Ca_03802	UZ	--
Ca4_P1_4570487	Ca4	4570487	ATTTTT/ATTTTTT	ACTGTTGCTCCTGGTGAGGT	TCATGGGACTGGACAAAACA	59.8	559	INTRON	Ca_03801	--	--
Ca4_P1_4572666	Ca4	4572666	CTTTTTTTTT/CTTTTTTTTTT	ATGGTTTGTCTCCTGCATCC	TCAGAATTTTCGACTCCATGC	59.9	314	INTRON	Ca_03801	--	--
Ca4_P1_45730406	Ca4	45730406	AATATATATATATATATATATATATATATATATATATATATATATAT/AATATATATATATATATATATATATATATATATATAT	AATAAAAATCGCATTTTCGGA	TCGCTTCCCCAGATAACAAC	58.6	596	INTERGENIC	--	--	--
Ca4_P1_4574989	Ca4	4574989	AA/AAAGTA	TGCCACTAAATAGGGTTGGC	CGTTCTGAAAACGCGCTTAG	60.0	460	DRR	Ca_03801	--	--
Ca4_P1_4576559	Ca4	4576559	TAAAAAAA/TAAAAAAAAA	TCCATATGATTTGAACTCGATTTTT	GGTTGGTTTAACCGTTGGAA	60.1	729	INTERGENIC	--	--	--
Ca4_P1_4576739	Ca4	4576739	GAAAAA/GAAAAAA	TCCATATGATTTGAACTCGATTTTT	GGTTGGTTTAACCGTTGGAA	60.1	729	INTERGENIC	--	--	--
Ca4_P1_4582795	Ca4	4582795	CA/C	TGCAAAATATGGTTGATCCAAG	GCTGCTTCACCTTCACCTTC	59.8	444	DRR	Ca_03800	T	--
Ca4_P1_4584838	Ca4	4584838	AAAAGAA/AAA	GGCTGGACTTCAAATTGCTC	CATTCCTTGATGGACCTTGG	59.8	920	INTRON	Ca_03799	R	--
Ca4_P1_4587278	Ca4	4587278	TC/T	ACAGGAAGAGCTCCCTAGCC	GCAGTGGAGTACACGGCTCT	60.0	446	DRR	Ca_03799	R	--
Ca4_P1_45880223	Ca4	45880223	TACTAAATATAAAAAAAA/TACTAAATATAAAAAAAACTAAATATAAAAAAAA	CTTGGCCCATCGTCTTAATG	CGACAAAATTGTTTTCATTTTAGG	60.5	656	INTERGENIC	--	--	--
Ca4_P1_4588591	Ca4	4588591	TAAAAAAAAA/TAAAAAAAA	TTTTCCTCCATCGTTGATCC	GACCACGTGTACCGTTGAAA	59.9	785	INTERGENIC	--	--	--

INDEL marker IDs	Chromosomes/ unanchored scaffolds	Physical positions (bp)	InDels (*Kabuli* reference genome-CDC Frontier/PI)	Forward primers (5'-3')	Reverse primers (5'-3')	Anneling temperature (0C)	Expected amplified product size (bp)	Structural annotation		Functional annotation	
								Sequence components of *kabuli* genome	*Kabuli* gene accession IDs	NCBI-KOG	TFs
Ca4_P1_4589864	Ca4	4589864	CTTT/CTT	TTTCGCTCAATGTGAAAAGC	AACGGAGGCAGTAATTGTCG	59.0	322	INTERGENIC	--	--	--
Ca4_P1_4590717	Ca4	4590717	TC/T	GCATCACATCGTTATCGTGG	AAATTCACCATTGATATGCGAA	60.0	625	INTERGENIC	--	--	--
Ca4_P1_4592169	Ca4	4592169	GATATAT/GATATATAT	TCAGACCTGCGTGCATACTC	GAAATTGCCAACTACCATGTCA	60.0	777	DRR	Ca_03798	--	--
Ca4_P1_45922364	Ca4	45922364	GATTATTATTATTATTATTATTATTATTATTATTATTATTATTATTATT/GATTATTATTATTATTATTATTATTATTATTATTATTATT	TCGATTGACTCAACGCAACT	CAATCACCAGCCTTCGGTAT	59.4	750	INTERGENIC	--	--	--
Ca4_P1_4592350	Ca4	4592350	A/ATT	TCAGACCTGCGTGCATACTC	GAAATTGCCAACTACCATGTCA	60.0	777	DRR	Ca_03798	--	--
Ca4_P1_4593293	Ca4	4593293	A/ACTAAT	TGGATCAACACGTGGAAAAA	ACAATTGTTGCCAGGGGTAA	59.9	667	DRR	Ca_03798	--	--
Ca4_P1_45949230	Ca4	45949230	AATTATTATTATTATTATTATTATTATTATTATTATGATTATTATTATTAT/AATTATTATTATTATTATTATTATTATTATTATTATTATTATTATTATGATTATTATTATTAT	TCGAAAGAACCTCGACTCGT	TCAATATGAGGTCGCTTCCC	60.0	508	INTERGENIC	--	--	--
Ca4_P1_45959235	Ca4	45959235	TTTATTATTATTATTATTATTATTATTAT/TTTATTATTATTATTATTATTATTATTATTAT	GGCCTATGGCCTAGCCTACT	ACGGCCTACCATAGGCTTTT	59.7	555	INTERGENIC	--	--	--
Ca4_P1_46001807	Ca4	46001807	GATAATAATAATAATAATAATAATAATAATAATAATAATAATA/GATAATAATAATAATAATAATAATAATAATAATAATAATA	TTGCTCCTACGTATCCCCAG	CTCAATCGTGGGTGACCTTT	60.1	385	INTERGENIC	--	--	--
Ca4_P1_46003281	Ca4	46003281	TTTATTATTATTATTATTATTATTATTATTATTATT/TTTATTATTATTATTATTATTATTATTATTATTATTATT	ATTCTCTCCCCCTGGTGTCT	TGTTTGGCCTAAAATTGGGT	59.9	724	INTERGENIC	--	--	--
Ca4_P1_46004838	Ca4	46004838	TAAAAAA/TAAAAAAA	TGATTTCAATGAGAGGATTTTGTT	GCTCGATTGATGTTAAGGGC	59.0	425	INTERGENIC	--	--	--
Ca4_P1_46006002	Ca4	46006002	TATATAATATA/TATATA	TTTGTGTGCCGGAAAATATG	GGTGAAATGAAAGGACCCAA	59.4	793	INTERGENIC	--	--	--

INDEL marker IDs	Chromosomes/ unanchored scaffolds	Physical positions (bp)	InDels (*Kabuli* reference genome-CDC Frontier/PI)	Forward primers (5'-3')	Reverse primers (5'-3')	Anneling temperature (0C)	Expected amplified product size (bp)	Structural annotation		Functional annotation	
								Sequence components of *kabuli* genome	*Kabuli* gene accession IDs	NCBI-KOG	TFs
Ca4_P1_46040811	Ca4	46040811	T/TG	TGCGCTTCTCATTTACGTTG	AGCAAGTTCTCCCAAAAGCA	60.0	423	INTERGENIC	--	--	--
Ca4_P1_46058697	Ca4	46058697	TTATATATATATATATATA/TTATATATATATATATATATA	TCTGCCTTGGCTCTGTTTTT	TTCTTGCGACCTCCATCTCT	60.0	688	INTRON	Ca_18363	--	--
Ca4_P1_46137643	Ca4	46137643	AG/AGAAGG	TTATTCAATGGCTTAGGCGG	CTCCCGTGCAAAGGAAATAA	60.1	665	DRR	Ca_18358	--	G2-like
Ca4_P1_46221707	Ca4	46221707	TAAAAAAAAA/TAAAAAAAA	CAAACTATGGGAATGTGGGG	CGGAGATCAATAGAGAAACATGG	60.0	753	INTERGENIC	--	--	--
Ca4_P1_46229411	Ca4	46229411	AG/A	CAAATTCGTAATCCAAATCTTCAA	GCTAAATAGCGCTCGTGGTC	59.4	341	INTERGENIC	--	--	--
Ca4_P1_46253678	Ca4	46253678	TAAAAAAAAAAA/TAAAAAAAAAA	CTAATTTGCACCGGCTCATT	GATCCTGGCTCGGTTATGAA	60.1	530	INTERGENIC	--	--	--
Ca4_P1_46275521	Ca4	46275521	TATAATAATAATAATAATAATAATAATAATAATAATAATAATAATAATAATA/TATAATAATAATAATAATAATAATAATAATAATAATAATAATAATAATA	ACTCGGCAATGGTTGAGTTT	TGCTTTTGTTTGTGGCACTC	59.6	833	INTERGENIC	--	--	--
Ca4_P1_46301292	Ca4	46301292	TAAAAAAAAAA/TAAAAAAAAA	CGCTTTTGGTCTCTAATGATTT	ATATGTCGCAGTCTCCCACC	57.6	184	INTERGENIC	--	--	--
Ca4_P1_46376353	Ca4	46376353	TNNNNNNNNNN/TNNNNNN	ATTTCATGTGGGCCTTTCAC	TGAACCATGCTTTTCCACAA	59.8	622	INTERGENIC	--	--	--
Ca4_P1_46384693	Ca4	46384693	T/TCCCTATAAAAA	GAGGAGGGTGGAGAATGGAT	GGGTGCCTGGTACAAAACTC	60.3	708	INTERGENIC	--	--	--
Ca4_P1_46386159	Ca4	46386159	ATT/ATTT	GAGGAGGATGTCCCTAAGCC	AGAATCCAATGCTCCACAGG	60.0	779	INTRON	Ca_19274	--	Dof
Ca4_P1_46495777	Ca4	46495777	ATATTATTATTATTATTATTATTATTATTATTATTATTATTATTATTATTATTAT/ATATTATTATTATTATTATTATTATTATTATTATTATTATTATTATTATTATTAT	GAGGCTTCATTCCTCTCGTG	TGAATTGGTGGATCAATGAAAA	60.0	478	INTERGENIC	--	--	--

INDEL marker IDs	Chromosomes/ unanchored scaffolds	Physical positions (bp)	InDels (*Kabuli* reference genome-CDC Frontier/PI)	Forward primers (5'-3')	Reverse primers (5'-3')	Anneling temperature (0C)	Expected amplified product size (bp)	Structural annotation		Functional annotation	
								Sequence components of *kabuli* genome	*Kabuli* gene accession IDs	NCBI-KOG	TFs
Ca4_P1_46496090	Ca4	46496090	TAAAAAAAAA/TAAAAAAAAAA	AAAGTGGGGTCAACTTTTTGAA	TGTCAAGGAGACCCACCACT	59.9	481	INTERGENIC	--	--	--
Ca4_P1_46508599	Ca4	46508599	AATATATATATATATATATATATA/AATATATATATATATATATA	AAAGGGGGAGTATTGTTGGG	CAAAGCTCTTTCATAATTGTCTTGA	60.0	492	INTERGENIC	--	--	--
Ca4_P1_4651023	Ca4	4651023	GTTTTTTTTTTTTT/GTTTTTTTTTTTT	CCACGCACATCAATCATACC	TGTGTCGTTAAACTTGTTGCTTT	59.8	467	INTERGENIC	--	--	--
Ca4_P1_46524540	Ca4	46524540	AATATATATATATATATATAT/AATATATATATATATATAT	CCTAATTACCATTGACCGTGG	TGGCGATCAAGTAGGACCTC	59.2	479	INTERGENIC	--	--	--
Ca4_P1_46537753	Ca4	46537753	TATAATAATAATAATAATAATAATAATAATAATAATAATAATAATAATA/TATAATAATAATAATAATAATAATAATAATAATAATAATAATAATAATAATA	CAGCGAAATGTTAAGTGCTTTT	CGTTCTCTCCTCCCTTCCTC	58.6	610	INTERGENIC	--	--	--
Ca4_P1_46560817	Ca4	46560817	CAAAAAAAAAAA/CAAAAAAAAAA	ACCATTTCCTGATTTTTGCG	ATGAGGAGGATGTCAGCCC	59.9	838	INTERGENIC	--	--	--
Ca4_P1_46581335	Ca4	46581335	TAAAA/TAA	AGTCCCCATGTAATTGTCACG	TGAAAGGATCTATGATTTCCCTC	59.7	544	INTERGENIC	--	--	--
Ca4_P1_46581610	Ca4	46581610	CAATAATAATAATAATAATAATAATAATAATAATAATAATAATA/CAATAATAATAATAATAATAATAATAATAATAATAATAATAATAATAATA	CGTCCTCGAAACTAAGAGGAAA	TTTCGAAGAAGGCGATAACAA	59.9	993	INTERGENIC	--	--	--
Ca4_P1_46674801	Ca4	46674801	A/AG	GGATGACTCGAAACGGTGTT	TTACCCAAAACCCCAAATCA	60.0	667	INTERGENIC	--	--	--
Ca4_P1_47188403	Ca4	47188403	TTATATATATATATATATATATATAT/TTATATATATATATATATATATATATAT	ACGTCCACTGATTGGTCACA	ACAATAGCGCAATGCTCGTT	60.0	583	INTERGENIC	--	--	--
Ca4_P1_47193005	Ca4	47193005	TTTAT/TTTATTAT	CAATGTGGGTGAATTTGCTG	GATTCTGGCAAATTCGTCGT	60.0	287	INTRON	Ca_10862	--	--
Ca4_P1_47238699	Ca4	47238699	ATTT/ATT	TTTTGATCGAATCTGACCCA	TCGGCCATTTTTCTTCAATC	59.0	659	INTERGENIC	--	--	--

INDEL marker IDs	Chromosomes/ unanchored scaffolds	Physical positions (bp)	InDels (*Kabuli* reference genome-CDC Frontier/PI)	Forward primers (5'-3')	Reverse primers (5'-3')	Anneling temperature (0C)	Expected amplified product size (bp)	Structural annotation		Functional annotation	
								**Sequence components of *kabuli* genome**	***Kabuli* gene accession IDs**	**NCBI-KOG**	**TFs**
Ca4_P1_47240338	Ca4	47240338	AATATATATATA/AATATATATA	AAAGCTTAATTGTCGTGCATCA	TAGTTTGGAGCGAGGAGGAA	59.8	352	INTERGENIC	--	--	--
Ca4_P1_47245879	Ca4	47245879	TAATAAATAAATAAA/TAATAAATAAATAAATAAA	CAACGGGAGTTTGACCACTT	TAACACATCTCCCGACGACA	60.0	542	DRR	Ca_10857	--	B3
Ca4_P1_47247172	Ca4	47247172	CCTTTTTCTTTCTTTT/CCTTTT	GCGTTGGGAAATACCAAGAA	GCCAAACAACACAAGCATGA	59.9	663	DRR	Ca_10857	--	B3
Ca4_P1_47247298	Ca4	47247298	ATTTT/ATTT	GCGTTGGGAAATACCAAGAA	GCCAAACAACACAAGCATGA	59.9	663	DRR	Ca_10857	--	B3
Ca4_P1_47249263	Ca4	47249263	GAAA/GAA	TTCTCTTCCTTCTACGGCCA	TCAAAATTGATTTCCAACCTCA	59.9	663	DRR	Ca_10856	--	B3
Ca4_P1_47250438	Ca4	47250438	ATTTATTTTATTTTATTTTATTTTATTTTA/ATTTATTTTATTTTATTTTATTTTATTTTATTTTA	GCAGAAATTTCAGGATGAATGA	AGTGCCACTCGCTTGATTCT	59.2	725	INTERGENIC	--	--	--
Ca4_P1_47306163	Ca4	47306163	AATATATATATATATATATATAT/AATATATATATATATATATATATATATAT	GCAAATATCGGTCAATCATCA	GCATGGAGAAATTGGAAGGA	58.4	545	INTERGENIC	--	--	--
Ca4_P1_47410517	Ca4	47410517	CAAAA/CAAA	TTCTAACGCAAAGGCAGGTT	GAAGCTATGGTGGAGACCCA	59.9	304	INTERGENIC	--	--	--
Ca4_P1_47437962	Ca4	47437962	CATTGATTGATTGATTGA/CATTGATTGATTGA	CTTGACGTAAATTGCATGGC	TGACTTTGGAGCTTGGACCT	59.2	263	INTERGENIC	--	--	--
Ca4_P1_47441910	Ca4	47441910	ATTTTTTTT/ATTTTTTT	AAAGCATCAAACCCACGAAG	AGTGTGATTTTGCTCCCACA	60.1	557	DRR	Ca_10843	U	--
Ca4_P1_47442661	Ca4	47442661	AATATATATATATATATATATATATAT/AATATATATATATATATATATATATATATATAT	TGTGGGAGCAAAATCACACT	GACGATCAAATCGGAAGAAAA	59.1	688	DRR	Ca_10843	U	--
Ca4_P1_47470984	Ca4	47470984	CTATATATATATATATAT/CTATATATATATATAT	GACTTATTTAGTGTGGACCGTGG	ATGCATCCCAGCATATCAAA	59.8	301	INTERGENIC	--	--	--

INDEL marker IDs	Chromosomes/ unanchored scaffolds	Physical positions (bp)	InDels (*Kabuli* reference genome-CDC Frontier/PI)	Forward primers (5'-3')	Reverse primers (5'-3')	Anneling temperature (0C)	Expected amplified product size (bp)	Structural annotation		Functional annotation	
								Sequence components of *kabuli* genome	*Kabuli* gene accession IDs	NCBI-KOG	TFs
Ca4_P1_47533268	Ca4	47533268	ATTTTTTTT/ATTTTTTTTT	CAAAGGAGATTGAAGAGGCG	TTAAGCGGGTTTTGACCAAC	59.9	621	DRR	Ca_10834	R	WRKY
Ca4_P1_47565027	Ca4	47565027	AAGAGAGAGAGAGAGAGAGAGAGAGAGAGAGAGAG/AAGAGAGAGAGAGAGAGAGAGAGAGAGAGAG	AGAAAATTGGGGGAATTTGG	CGTGGACAAAGATCGTGATG	60.0	570	INTERGENIC	--	--	--
Ca4_P1_47604845	Ca4	47604845	GTTTTTTTTTT/GTTTTTTTTT	CGGGTGGACATTCTTTTCAT	GAGCGAATATAATTGTTTTGTGG	59.8	446	INTERGENIC	--	--	--
Ca4_P1_47669226	Ca4	47669226	AG/A	GATTCACCCAAAGTGGGCTA	AGCTGCTAAATCCTCCGTCA	59.9	611	INTERGENIC	--	--	--
Ca4_P1_47685774	Ca4	47685774	A/AGGTT	AGTCAGACGCTACTCGGCAT	CCGACGAAATAAGTCCCTCA	60.0	837	INTERGENIC	--	--	--
Ca4_P1_47705093	Ca4	47705093	C/CG	TTCGCATCCGTATCAAATCA	TCACACCCATGAAGATATGAGC	60.0	269	INTERGENIC	--	--	--
Ca4_P1_47723484	Ca4	47723484	ATTTTTTTTTT/ATTTTTTTTT	TGCGGATCGCTCAATTTTAC	CCCTTGCCACTACACCTTGT	61.1	715	INTERGENIC	--	--	--
Ca4_P1_47755689	Ca4	47755689	CTTTTTTTT/CTTTTTTT	TTTTCCTTTTGGTGGATTGG	TGTGCAACTTTCTCACCCAG	59.8	480	INTERGENIC	--	--	--
Ca4_P1_47767559	Ca4	47767559	TATAATAATAATAATAATAATAATAATAATAATAATAA/TATAATAATAATAATAATAATAATAATAATAATAATAATAA	GTCTGGTCTGGTCTGGGAAA	TTTCGGTTGGGTTTGCTTAC	60.1	636	INTERGENIC	--	--	--
Ca4_P1_47793461	Ca4	47793461	TATAAATAAATAAATAAATAAA/TATAAATAAATAAATAAA	TGTGCCAATGGATTGAGAAAT	CTGCAAGCACGTATGCCTTA	60.3	396	DRR	Ca_10815	C	--
Ca4_P1_4779910	Ca4	4779910	ATTTTTTTTTTT/ATTTTTTTTTT	AATTCGTGAATGGACATTGATT	TTCGTCATTCTTTGCCGAAT	58.3	695	INTERGENIC	--	--	--
Ca4_P1_4784534	Ca4	4784534	ATTTTTTTTT/ATTTTTTTT	CCCTTCGGAAGACCACATAA	TTCATTTCTTTACCCCGTTCA	59.9	657	DRR	Ca_03778	K	G2-like

INDEL marker IDs	Chromosomes/ unanchored scaffolds	Physical positions (bp)	InDels (*Kabuli* reference genome-CDC Frontier/PI)	Forward primers (5'-3')	Reverse primers (5'-3')	Anneling temperature (0C)	Expected amplified product size (bp)	Structural annotation		Functional annotation	
								Sequence components of *kabuli* genome	*Kabuli* gene accession IDs	NCBI-KOG	TFs
Ca4_P1_47848250	Ca4	47848250	TAAAAAAAAA/TAAAAAAAAAA	GACGGTGTTTGTAGCAGCAG	TTTCTTCCCCTTTTTGGGTT	59.5	273	INTERGENIC	--	--	--
Ca4_P1_47895561	Ca4	47895561	ATTT/ATTTT	AAAGGAGGCTAAATTCAAGAACC	AAAGAATGATCAAACGCAAGG	59.2	317	DRR	Ca_10803	S	--
Ca4_P1_47936613	Ca4	47936613	AT/A	TTTGGATACTATTTGCGAAGTTT	CCGTCACTGCAATTTCCTTT	57.2	457	INTERGENIC	--	--	--
Ca4_P1_47940203	Ca4	47940203	TATTAATTA/TATTA	TATGGTGGTCGGAGTGATGA	TAATTGCAAGTCATTGCCCA	59.9	115	INTRON	Ca_10795	--	ARF
Ca4_P1_47961030	Ca4	47961030	TG/T	CAAATTTATAACGATTCTCTCACAAA	GCAAATGCATAGAGCAGCAA	58.0	270	DRR	Ca_10794	--	ARF
Ca4_P1_47961387	Ca4	47961387	TG/T	GCTGCTCTATGCATTTGCTG	GAGCATGGACGTTTGGATTT	59.7	527	DRR	Ca_10794	--	ARF
Ca4_P1_47961570	Ca4	47961570	ATTTTTTTTT/ATTTTTTTT	TCCATGCATTCAAGAGCAAG	GAGCATGGACGTTTGGATTT	59.9	318	DRR	Ca_10794	--	ARF
Ca4_P1_47961712	Ca4	47961712	ATTT/ATT	TCCATGCATTCAAGAGCAAG	GAGCATGGACGTTTGGATTT	59.9	318	INTERGENIC	--	--	--
Ca4_P1_47962009	Ca4	47962009	ATTTTTTT/ATTTTTTTT	GAAATCCAAACGTCCATGCT	CCACCAATCACATCAACTCG	59.9	550	INTERGENIC	--	--	--
Ca4_P1_47966378	Ca4	47966378	CTTTTTTTTTT/CTTTTTTTT	AGCAAATTGGGAACCTTGTG	CGAGAAACCCATGAGGAAAA	60.0	361	DRR	Ca_10792	--	B3
Ca4_P1_47981505	Ca4	47981505	TCAATGGGA/T	TTCTGCATTGATGTGGAGGA	TGAGCCGTCATATTGTGTGA	60.2	381	INTRON	Ca_10790	U	ARF
Ca4_P1_47982618	Ca4	47982618	TTTTTATTTTATTTTATTTTATTTT/TTTTTATTTTATTTTATTTT	CCAAACTCCAATCAAATGGG	CAAATGAGAATTTTCTGGGCA	60.2	483	INTRON	Ca_10790	U	ARF

INDEL marker IDs	Chromosomes/ unanchored scaffolds	Physical positions (bp)	InDels (*Kabuli* reference genome-CDC Frontier/PI)	Forward primers (5'-3')	Reverse primers (5'-3')	Anneling temperature (0C)	Expected amplified product size (bp)	Structural annotation		Functional annotation	
								Sequence components of *kabuli* genome	*Kabuli* gene accession IDs	NCBI-KOG	TFs
Ca4_P1_47982672	Ca4	47982672	TC/TCC	CCAAACTCCAATCAAATGGG	CAAATGAGAATTTTCTGGGCA	60.2	483	INTRON	Ca_10790	U	ARF
Ca4_P1_47983864	Ca4	47983864	CTATATAT/C	GCTTTGTTTCCATAAGCCCA	GGACCTCTTTCTACCCCTGG	60.1	332	INTRON	Ca_10790	U	ARF
Ca4_P1_47985512	Ca4	47985512	G/GGTCAATATTGTTAA	ATGGGAAGGGAGCTTTTTGT	TTTGGATACTATTTGCGAAGTTT	59.9	383	INTRON	Ca_10790	U	ARF
Ca4_P1_47989970	Ca4	47989970	CTAA/CTAATTAA	TGGCTAAGTTGCCAAAGTTTA	TGAGTGTGCAAGCATTTCAA	57.7	194	INTERGENIC	--	--	--
Ca4_P1_47991117	Ca4	47991117	GTTTTTT/GTTTTTTTT	ATTAGCCACGACGATGGTTC	TGGTGTACATGTAGGCGTGC	60.0	564	DRR	Ca_10789	--	B3
Ca4_P1_48019545	Ca4	48019545	AATATTTATTTTATATTTATTTTATATTTATTT/AATATTTATTTTATATTTATTT	AAGCAACCCCGTTATTTGTG	TTCGTACCATACCGGCAGAT	59.9	588	INTERGENIC	--	--	--
Ca4_P1_48021995	Ca4	48021995	TAAAAAAAAAAAAA/TAAAAAAAAAAAA	AGACAAAAGGGGGAATTTGG	ATGCCACCAAGCCTAATCAC	60.2	896	INTRON	Ca_10786	V	NAC
Ca4_P1_48065584	Ca4	48065584	ATATCTTA/ATA	TTCAATTTGTTGCGTGCATT	CCAACTTGAGATGAGCCACC	60.1	312	INTERGENIC	--	--	--
Ca4_P1_48074355	Ca4	48074355	TG/T	GGTCGACATGACATCACTAAATG	AGTGTCAAAGGGGGAGGAAG	59.4	601	INTERGENIC	--	--	--
Ca4_P1_4808010	Ca4	4808010	GATTGCAGTCTC/GATTGCAGTCTCATTGCAGTCTC	CATGTTAAATTGGATGCCCC	GAGACTAAACCCACCCGTCA	60.0	774	INTERGENIC	--	--	--
Ca4_P1_48115647	Ca4	48115647	AATATATATATATATATATAT/AATATATATATATATATAT	TCCTTCATATCCCACGAACC	TGGTGTTGGAGTCATGCTTT	59.8	290	INTERGENIC	--	--	--
Ca4_P1_48116724	Ca4	48116724	AATATATATATATATA/AATATATATATATATATA	TTTGACGTTCGAAAAACCAC	ATGGAACAATCGGACACCAT	58.6	433	INTERGENIC	--	--	--

INDEL marker IDs	Chromosomes/ unanchored scaffolds	Physical positions (bp)	InDels (*Kabuli* reference genome-CDC Frontier/PI)	Forward primers (5'-3')	Reverse primers (5'-3')	Anneling temperature (0C)	Expected amplified product size (bp)	Structural annotation		Functional annotation	
								Sequence components of *kabuli* genome	*Kabuli* gene accession IDs	NCBI-KOG	TFs
Ca4_P1_48196964	Ca4	48196964	AATATATATATATATAT/AATATATATATATATATAT	GGGTTTTTACCTAGACGGTGA	TCATGACATGGCGTGGTAGT	58.1	709	INTERGENIC	--	--	--
Ca4_P1_4819702	Ca4	4819702	ATGTAGT/AT	AGCCAGCTGACACCTCATTT	TTAGCTGGCCATATGGTTCC	59.9	553	INTRON	Ca_03777	O	FAR1
Ca4_P1_48203210	Ca4	48203210	AAT/AATAT	TCCAACGGCTCTCATATCCT	GGTTATGGCCAAACATGGTAA	59.7	921	INTERGENIC	--	--	--
Ca4_P1_48204819	Ca4	48204819	T/TAA	AAAGAGGAAGAGGGCAAAGG	GCAAACACCGATAGAGAGGG	59.8	425	INTERGENIC	--	--	--
Ca4_P1_48206970	Ca4	48206970	CAGATAAAAG/CAG	ATCGAATGTGCATCGTGAAG	CAATAAGGATAAGGGGCGGT	59.7	631	INTERGENIC	--	--	--
Ca4_P1_48207508	Ca4	48207508	AGATATAGT/A	CCGCCCCTTATCCTTATTGT	TTTCAAGCAGCTAAAACGCA	60.2	568	INTERGENIC	--	--	--
Ca4_P1_48212976	Ca4	48212976	TAAAAAAAAAAA/TTAAAAAAAAAAAAA	AAGAGGCTAATTGTGGCATCA	AATCAATCCTCGGGTTTTCA	59.7	777	INTERGENIC	--	--	--
Ca4_P1_48214036	Ca4	48214036	AATATATATATATATATATATATAT/ AATATATATATATATATATATAT	CATAACCATCCTCACCCACC	TGAGTTCGAGGGGTCATTTC	60.1	616	INTERGENIC	--	--	--
Ca4_P1_48214303	Ca4	48214303	CAA/CA	TCAATTGATGATGACGGTGAA	TGAGTTCGAGGGGTCATTTC	59.9	245	INTERGENIC	--	--	--
Ca4_P1_48214982	Ca4	48214982	GAAT/GAATTAAT	ATGCTTTCAAACTCATCGCC	CCATCGGATAGGTCAGAACC	60.2	525	INTERGENIC	--	--	--
Ca4_P1_48216580	Ca4	48216580	GAAA/GAA	ATGGATGTGGGTATGGAGGA	CACGTCAATGTCATCTTGCC	60.0	518	INTERGENIC	--	--	--
Ca4_P1_48218291	Ca4	48218291	ATT/ATTT	GATTTAAGGAGCGCAACAAA	CGAGTGAGTGCAGCTGGTTA	58.0	929	INTERGENIC	--	--	--

INDEL marker IDs	Chromosomes/ unanchored scaffolds	Physical positions (bp)	InDels (*Kabuli* reference genome-CDC Frontier/PI)	Forward primers (5'-3')	Reverse primers (5'-3')	Anneling temperature (0C)	Expected amplified product size (bp)	Structural annotation		Functional annotation	
								Sequence components of *kabuli* genome	*Kabuli* gene accession IDs	NCBI-KOG	TFs
Ca4_P1_48222166	Ca4	48222166	AAAATAAATAAATAAATAAATAAATAAATA/AAAATAAATAAATAAATAAATA	GCCACCACTGGAGCATAGTT	TGGATTAAGGCAATTAAAAGGG	60.1	580	INTERGENIC	--	--	--
Ca4_P1_48222325	Ca4	48222325	T/TA	GCCACCACTGGAGCATAGTT	TGGATTAAGGCAATTAAAAGGG	60.1	580	INTERGENIC	--	--	--
Ca4_P1_48223068	Ca4	48223068	AC/A	CCCGAAGTAGAGTGATGACAAA	AACAAGGCGAGACAAGGCTA	59.2	479	INTERGENIC	--	--	--
Ca4_P1_48225858	Ca4	48225858	GTTGCAAA/GTTGCAAAAAATTGCAAA	TGCACAACTGCACCTTTCAC	CCATCACCCCCTTTTTCTTT	60.9	599	DRR	Ca_10766	--	DBB
Ca4_P1_48227983	Ca4	48227983	TTCTCT/TTCTCTCT	AAATTCGATCTCGGCAGCTA	AGACATGGATTCGCCACTTC	59.9	594	INTERGENIC	--	--	--
Ca4_P1_48230328	Ca4	48230328	A/AC	TCCTTGAGGGACCAGGATAA	ATTGTGGATTTTGTGTCGCA	59.5	431	INTERGENIC	--	--	--
Ca4_P1_48234625	Ca4	48234625	TAATATTCAGCTGTCAATATTCA/TAATATTCA	GACATGACAACCACGTTTGC	AATTCGGCCAATCCTCTTTT	60.0	569	DRR	Ca_10764	--	--
Ca4_P1_48243356	Ca4	48243356	CCTTCT/CCT	TGCATTTGTTGGACGCTTAG	AAATCGATAGTTTTGGCCCC	59.9	601	INTERGENIC	--	--	--
Ca4_P1_48246550	Ca4	48246550	GA/GAA	TCCAAGATGTTGTCGGTTGA	GAAATTTCGGCGATGTTGAT	60.1	884	INTERGENIC	--	--	--
Ca4_P1_48260072	Ca4	48260072	CATATA/CATA	GGTGATTCCCCAAGCTACAA	ATGCAAACAAAATGGCAACA	59.9	257	INTRON	Ca_10761	K	B3
Ca4_P1_4826016	Ca4	4826016	AT/A	GGATTGATTGCAGTTCCAAAA	GGCAAAATGCCTTTAGACCA	59.9	445	DRR	Ca_03777	O	FAR1
Ca4_P1_48260228	Ca4	48260228	GT/GTTTTAAGTT	TGTTGCCATTTTGTTTGCAT	ACCTGCACCACAAGAAAACC	60.0	635	INTRON	Ca_10761	K	B3

INDEL marker IDs	Chromosomes/ unanchored scaffolds	Physical positions (bp)	InDels (*Kabuli* reference genome-CDC Frontier/PI)	Forward primers (5'-3')	Reverse primers (5'-3')	Anneling temperature (0C)	Expected amplified product size (bp)	Structural annotation		Functional annotation	
								Sequence components of *kabuli* genome	*Kabuli* gene accession IDs	NCBI-KOG	TFs
Ca4_P1_48260377	Ca4	48260377	TGG/TGGG	TGTTGCCATTTTGTTTGCAT	ACCTGCACCACAAGAAAACC	60.0	635	INTRON	Ca_10761	K	B3
Ca4_P1_48265231	Ca4	48265231	ATTT/AT	TTTTTGGGACCCATGTATGTC	TGTTGCCGTTGTGTTGAAAT	59.5	910	DRR	Ca_10761	K	B3
Ca4_P1_48277169	Ca4	48277169	T/TG	ATGGCGTTTTCTGGTGATTC	TAAGACGACACGACCCATCA	59.9	637	INTERGENIC	--	--	--
Ca4_P1_48277827	Ca4	48277827	TCACACACACACA/TCACACACACACACA	TGATGGGTCGTGTCGTCTTA	CGAGTTCAAATCCGGAACAT	60.1	828	INTERGENIC	--	--	--
Ca4_P1_48278382	Ca4	48278382	CGAGAGA/CGAGA	ATGTTCCGGATTTGAACTCG	ACCAACGACGAGATAGGGTG	59.9	604	INTERGENIC	--	--	--
Ca4_P1_48278655	Ca4	48278655	ATTTTTTTT/ATTTTTTTTTTT	AATAGCAATGCCCAGGTTTG	ACCAACGACGAGATAGGGTG	60.0	297	INTERGENIC	--	--	--
Ca4_P1_48280011	Ca4	48280011	ATTT/ATT	TCCCAATAATCAAAATGAGCG	GCGTCGTTGCTAGTTCACAA	59.9	878	INTERGENIC	--	--	--
Ca4_P1_48292143	Ca4	48292143	ATTTTTTTTTT/ATTTTTTTTTTTTTT	AATAATTGGGGAGTCTGGGG	AACAATTGCTTGCACGACTG	60.0	660	INTERGENIC	--	--	--
Ca4_P1_48293271	Ca4	48293271	ATTC/ATTCTTC	CATGTCATGGGAACTCCAACT	GTTCGGTCGAAAATGACGAT	59.8	560	INTERGENIC	--	--	--
Ca4_P1_48293404	Ca4	48293404	CA/CAGTTA	TCCCATGAGGCATATCAAGA	GTTCGGTCGAAAATGACGAT	59.0	459	INTERGENIC	--	--	--
Ca4_P1_48297107	Ca4	48297107	AAATTTTAAT/AAATTTTAATTTTAAT	AACAAACACATTTGTGGCGA	TTCTGACTGTTTAGGCCGCT	60.0	678	DRR	Ca_10759	--	--
Ca4_P1_48300292	Ca4	48300292	AT/A	ACTTATTTTGGGGTCTGCCA	GCCATTGAGAAGCTCCTTTG	59.4	660	INTERGENIC	--	--	--

INDEL marker IDs	Chromosomes/ unanchored scaffolds	Physical positions (bp)	InDels (*Kabuli* reference genome-CDC Frontier/PI)	Forward primers (5'-3')	Reverse primers (5'-3')	Anneling temperature (0C)	Expected amplified product size (bp)	Structural annotation		Functional annotation	
								Sequence components of *kabuli* genome	*Kabuli* gene accession IDs	NCBI-KOG	TFs
Ca4_P1_48304435	Ca4	48304435	TTAC/TTACAGAATAAACTAC	CAGTGGTTTTGGAACTTCGC	GGCAACGGCTGTGTTTATTA	60.7	257	DRR	Ca_10757	B	C3H
Ca4_P1_4830820	Ca4	4830820	TTTATACATT/TTT	AAATGATGAAGGGGAAAGGG	CAACGGTTTACATGAATTGGG	60.1	815	INTERGENIC	--	--	--
Ca4_P1_48308452	Ca4	48308452	CTTTT/CTTT	ATCCGCCATTGATCCCTTAC	ATTGGGACGCGAATACAAAA	61.0	207	INTERGENIC	--	--	--
Ca4_P1_48309823	Ca4	48309823	CAAATTTAAATTT/CAAATTT	TGCCTCCACAAACGTCATTA	TGAAAACCCTAATCTCCGCA	60.1	832	INTERGENIC	--	--	--
Ca4_P1_48310613	Ca4	48310613	TGAGAGAGAGAGAGAGAGAG/TGAGAGAGAGAGAGAGAGAGAGAGAGAG	CAATGTTCAAACTGCACCGT	CAGATTCCACCCAAGGTCAA	59.6	706	INTERGENIC	--	--	--
Ca4_P1_4831068	Ca4	4831068	TGAAG/TG	CACCGATTCACCATTTAGCA	GGTTTTATTCGGCCACAAAAT	59.5	980	INTERGENIC	--	--	--
Ca4_P1_48314680	Ca4	48314680	ATTT/ATTTTT	GTAATGCGTTTGACCTGCAA	ATAGGCGCATCTCAATCACC	59.7	649	DRR	Ca_10756	--	BBR-BPC
Ca4_P1_48314877	Ca4	48314877	TA/T	TGCGCCTATCTCTCTGTGTG	TTCGATCCATTTTACCACCC	60.2	365	DRR	Ca_10756	--	BBR-BPC
Ca4_P1_48316349	Ca4	48316349	TAA/TA	ACGAGCACCAAAGAGGACAT	TTTTACCAGGTTGCATCCTAAA	59.7	493	INTERGENIC	--	--	--
Ca4_P1_48318482	Ca4	48318482	AATA/AA	CAACATATTTGCGGTTACCAA	CTCACCGTTTCACGTGATTG	58.5	920	INTERGENIC	--	--	--
Ca4_P1_48323095	Ca4	48323095	AACACACACACA/AACACACACACACA	GCAGCCAAACCAGCTTAGAC	AATTTGCAAAAATTCGGCTG	60.0	515	DRR	Ca_10754	Q	GeBP
Ca4_P1_48323352	Ca4	48323352	CCAAACACAAACACAAACAC/CCAAACACAAACAC	TTTTCTGCAGCCGAATTTTT	CATTTGAAATTGCTTTCCCC	59.8	625	DRR	Ca_10755	--	bZIP

INDEL marker IDs	Chromosomes/ unanchored scaffolds	Physical positions (bp)	InDels (*Kabuli* reference genome-CDC Frontier/PI)	Forward primers (5'-3')	Reverse primers (5'-3')	Anneling temperature (0C)	Expected amplified product size (bp)	Structural annotation		Functional annotation	
								Sequence components of *kabuli* genome	*Kabuli* gene accession IDs	NCBI-KOG	TFs
Ca4_P1_48327298	Ca4	48327298	G/GT	TCCCTGTATGCATGAACCAA	AAACCGGGAGGTGCTAGAAT	59.9	463	INTERGENIC	--	--	--
Ca4_P1_48330370	Ca4	48330370	AC/A	GCAAAGGAGCTTCTCAATGG	GCATCAGGAACAACCATCCT	60.0	617	INTRON	Ca_10753	J	--
Ca4_P1_48332704	Ca4	48332704	GTTTTT/GTTTTTT	GACCAATCCTTCGAGGAACA	AACTGGCAGATTCATCACACC	60.1	750	DRR	Ca_10753	J	--
Ca4_P1_48332874	Ca4	48332874	C/CAA	TTAGCTGTGGAAGGGCATTT	AACTGGCAGATTCATCACACC	59.7	590	DRR	Ca_10753	J	--
Ca4_P1_48335941	Ca4	48335941	TA/TAAA	CATGCACTCCATGTTTTTGG	CAGTCATGTGAACCACCCAC	60.0	493	INTERGENIC	--	--	--
Ca4_P1_48336369	Ca4	48336369	TTAT/TT	GTGGGTGGTTCACATGACTG	TCCTTAAAATTATAGGTGCCCG	59.8	374	INTERGENIC	--	--	--
Ca4_P1_48336651	Ca4	48336651	CTT/CTTTTT	GGAGAGCTTAGTTGGTTAATTGG	GTGCCTCTGTTGATGCTTGA	58.4	454	INTERGENIC	--	--	--
Ca4_P1_48340439	Ca4	48340439	ATTTTT/ATTT	TACGGCGGGCAATAAAAATA	AAAGGAAATCAAATGGCTCA	60.3	195	INTERGENIC	--	--	--
Ca4_P1_48341269	Ca4	48341269	TGG/TGGG	AACATTGATTTTGACGTGAGAAGA	TCGTTGTTGCACTATCCCAA	60.0	418	INTERGENIC	--	--	--
Ca4_P1_48342630	Ca4	48342630	TTA/TTACATA	GGGGCTTTTTGATACCCATT	TTTGCTTACAATATATTTGTGTCATTT	60.0	461	INTERGENIC	--	--	--
Ca4_P1_48345654	Ca4	48345654	T/TCA	GGTACGTTCGCTCTCTGAGG	AGCAGCTTGCAACTTCACAA	60.0	148	INTRON	Ca_10752	--	--
Ca4_P1_48351278	Ca4	48351278	TAATTCCAATTCCAATT/TAATTCCAATTCCAATTCCAATT	GACACGTGATGTCTCTGTAATGG	AATGTATGTATCGAGAGCGCC	59.5	651	INTERGENIC	--	--	--

INDEL marker IDs	Chromosomes/ unanchored scaffolds	Physical positions (bp)	InDels (*Kabuli* reference genome-CDC Frontier/PI)	Forward primers (5'-3')	Reverse primers (5'-3')	Anneling temperature (0C)	Expected amplified product size (bp)	Structural annotation		Functional annotation	
								Sequence components of *kabuli* genome	*Kabuli* gene accession IDs	NCBI-KOG	TFs
Ca4_P1_48366728	Ca4	48366728	A/AGAG	CAAGGGCTGGATTGTTTTGT	TTGGCTTTACACTTGGTCCC	60.0	673	DRR	Ca_10750	A	bHLH
Ca4_P1_48367765	Ca4	48367765	ATTT/ATTTT	CTGGTGCACTATCGCAAAAC	CCAACACTTGCAATAAAAGTGAA	59.3	418	INTERGENIC	--	--	--
Ca4_P1_48380924	Ca4	48380924	C/CCATACATACA	AATTGACCCCGTCTTTTGAA	GAAGAACCAAACACATGCCA	59.4	605	DRR	Ca_10748	--	ARF
Ca4_P1_48389863	Ca4	48389863	TC/T	CCGTTTTCCACATTTTCGAT	CCATCAAATTCATCCACCAA	59.8	522	INTERGENIC	--	--	--
Ca4_P1_48393205	Ca4	48393205	AAATTTCTAAATTT/AAATTTCTAAATTTAATTTCTAAATTT	TTGGGAATATGTCATGCGTC	GACCACGCCACACTTTAGGT	59.4	760	INTERGENIC	--	--	--
Ca4_P1_48395017	Ca4	48395017	ATTTTTTT/ATTTTTTTTATTTTTTTT	GCGACGTCTCAAGTTTGGAT	CAAAAAGTATGCACCGTATTTTGA	60.3	207	INTERGENIC	--	--	--
Ca4_P1_48395750	Ca4	48395750	ATTTTTTTT/ATTTTTTTTTT	AATTCGGACCTTTCCACTTTT	TGTATGATAAGGGCTTCGCC	59.0	479	INTERGENIC	--	--	--
Ca4_P1_4839788	Ca4	4839788	A/ATTTTT	ACGTAATACTTTGTGGCGGC	TCTCTCCTTTGTGACGGCTT	60.0	138	DRR	Ca_03775	--	--
Ca4_P1_48398628	Ca4	48398628	GTATA/GTATATA	ATGGCCCAATTAAGCTTTGA	CTGTTCTTGCGTGACCTTCA	59.5	565	INTERGENIC	--	--	--
Ca4_P1_4840884	Ca4	4840884	AC/A	TTGGGCCTTTGTGGAGTAAG	TACCAAAGTGGTGATGGTGG	60.1	496	DRR	Ca_03775	--	--
Ca4_P1_48410907	Ca4	48410907	ATTT/ATT	CACCGTCCAAATTATGCCTC	TGAGAACGTGCGACCTGTAG	60.3	518	INTERGENIC	--	--	--
Ca4_P1_48414216	Ca4	48414216	CATAT/CAT	ATGAGCATAGCATGTGCCAG	GCATGGTTATCGGCTGATTC	59.9	418	INTERGENIC	--	--	--

**INDEL marker IDs**	**Chromosomes/ unanchored scaffolds**	**Physical positions (bp)**	**InDels (*Kabuli* reference genome-CDC Frontier/PI)**	**Forward primers (5'-3')**	**Reverse primers (5'-3')**	**Anneling temperature (0C)**	**Expected amplified product size (bp)**	**Structural annotation**		**Functional annotation**	
								**Sequence components of *kabuli* genome**	***Kabuli* gene accession IDs**	**NCBI-KOG**	**TFs**
Ca4_P1_48414921	Ca4	48414921	GTTT/GTTTT	TTGAATGTGAATGGGTGCAT	AAACCGAACAAATCAATGCC	59.8	513	INTERGENIC	--	--	--
Ca4_P1_4842046	Ca4	4842046	AAATAATAATAATAATAAT/AAATAATAATAAT	TGAGAAGAAGGGGCTTAGCA	CGAGGAGGTTATTGGCGTAA	60.1	754	INTERGENIC	--	--	--
Ca4_P1_48430726	Ca4	48430726	TTTATTATTATTATTAT/TTTATTATTATTAT	GCTAAAACTGAGTCGTCCCG	ATGGGATGGGACAAAACAAA	59.9	639	INTERGENIC	--	--	--
Ca4_P1_48434508	Ca4	48434508	TGCGC/TGC	GGTGAGAGTAGGTGGGGACA	TCTGGCAAATGAAATGGAAA	60.0	112	INTERGENIC	--	--	--
Ca4_P1_48435268	Ca4	48435268	A/AG	TTGCAGTTGAATTTCGAAAGAA	TGATGTGATCCTAGGGTGAAGA	59.9	610	INTERGENIC	--	--	--
Ca4_P1_48440014	Ca4	48440014	AGGT/A	ATGTTGCGGGTTTGATCTTC	TGGCAAGAATACCTTGTAAACAAAT	59.9	412	INTERGENIC	--	--	--
Ca4_P1_48440263	Ca4	48440263	GTTTTTT/GTTTTTTT	ATGTTGCGGGTTTGATCTTC	TGGCAAGAATACCTTGTAAACAAAT	59.9	412	INTERGENIC	--	--	--
Ca4_P1_48440600	Ca4	48440600	GTT/GT	TTTGTTTACAAGGTATTCTTGCCA	GTCAATTGCACCTTTCCCAT	59.9	567	INTERGENIC	--	--	--
Ca4_P1_48442756	Ca4	48442756	TTATATATAT/TTATATAT	CTGATCCATTCATGGTGTGG	TCAATTCTCTCCATCCCTCAA	59.8	499	INTERGENIC	--	--	--
Ca4_P1_48443075	Ca4	48443075	ATTT/ATTTT	CCGGTTGTTAGCTTGTTTGC	CAATAGGATGGATGGTTGGG	60.7	609	INTERGENIC	--	--	--
Ca4_P1_48443839	Ca4	48443839	AT/A	TGCCATAACTTTGAAAAATTGC	GAGTTTGAGAAGTGCAGGGC	59.2	955	INTERGENIC	--	--	--
Ca4_P1_48444234	Ca4	48444234	TAAAA/TAAAAA	TGTCTTATGCAAAATAGAGAGACCA	GAGTTTGAGAAGTGCAGGGC	59.4	350	INTERGENIC	--	--	--

INDEL marker IDs	Chromosomes/ unanchored scaffolds	Physical positions (bp)	InDels (*Kabuli* reference genome-CDC Frontier/PI)	Forward primers (5'-3')	Reverse primers (5'-3')	Anneling temperature (0C)	Expected amplified product size (bp)	Structural annotation		Functional annotation	
								Sequence components of *kabuli* genome	*Kabuli* gene accession IDs	NCBI-KOG	TFs
Ca4_P1_48450705	Ca4	48450705	GTATATATATATATATATATATATAT/GTATATATATATATATATATATATATATATATAT	AATTGGTAACAAGCTCGTAATTG	CTGGACCAGAGGCATTCATT	57.5	619	INTERGENIC	--	--	--
Ca4_P1_48454259	Ca4	48454259	CTTTT/CTTT	TTGCTTTTCCTACCACCACC	CAGCAGAGTCCAATTCAGCA	60.0	376	INTERGENIC	--	--	--
Ca4_P1_48460211	Ca4	48460211	TATACAACAATACAA/TATACAA	TTGTGCTCGTCAAACAAACG	TTTCAGGGATTAAAATGACGG	60.9	744	DRR	Ca_10747	--	--
Ca4_P1_48461232	Ca4	48461232	TAGAG/TAG	TGCAAAGTTCGATCGGAATAA	AATTTCTCACCACCACCCAC	60.6	483	INTERGENIC	--	--	--
Ca4_P1_48462658	Ca4	48462658	GTATATATATAT/GTATATATATATAT	ACGTAACCGTTAATCGACGC	TTTCCCGTTACAAACAAAACAA	60.0	689	INTERGENIC	--	--	--
Ca4_P1_48465859	Ca4	48465859	GTTTTTTTTTTT/GTTTTTTTTTT	TGTGTGTCTCAGTGGTGGGT	GCCTTTCCTTGACAGTCTGC	60.0	619	INTRON	Ca_10746	LA	--
Ca4_P1_48467313	Ca4	48467313	TAG/TAGAG	CTATTTGCGTCTTTTCCCCA	ACGTGAACTTTTTGTTGCCC	60.1	202	INTRON	Ca_10746	LA	--
Ca4_P1_48483695	Ca4	48483695	ACT/A	CCTTGCTTATCCAACCCAGA	GCAAGAATCCCCGTGATAAA	60.1	311	INTERGENIC	--	--	--
Ca4_P1_48493736	Ca4	48493736	CAA/CAAA	AGGCACATGAGCAACATTGA	TTGGCAGTTTTTGTCAACCA	60.3	606	INTERGENIC	--	--	--
Ca4_P1_48494467	Ca4	48494467	TTTAATT/TTTAATTATGTTAATT	TGGTTGACAAAAACTGCCAA	ACTGGACCCCAAACCTCTCT	60.1	664	INTERGENIC	--	--	--
Ca4_P1_48501359	Ca4	48501359	GACA/GA	TACCTGGTATTCACGGAGCC	AAATGAATGTGTTAGGCGGC	60.0	915	INTRON	Ca_10741	J	E2F/DP
Ca4_P1_48502501	Ca4	48502501	GCATTAAGAC/GC	AGTGCAGCAGCAGCAATTTA	TTGATATGGTTGGTGGCCTT	59.8	478	INTRON	Ca_10741	J	E2F/DP

INDEL marker IDs	Chromosomes/ unanchored scaffolds	Physical positions (bp)	InDels (*Kabuli* reference genome-CDC Frontier/PI)	Forward primers (5'-3')	Reverse primers (5'-3')	Anneling temperature (0C)	Expected amplified product size (bp)	Structural annotation		Functional annotation	
								Sequence components of *kabuli* genome	*Kabuli* gene accession IDs	NCBI-KOG	TFs
Ca4_P1_48509401	Ca4	48509401	GAAAA/GAAAAA	ACTGTCGAGCATAAATGGGG	CGATCGACCCGAACTAAAAC	60.0	472	INTERGENIC	--	--	--
Ca4_P1_48515945	Ca4	48515945	GC/G	CAAATTCAATTCCAAGCTGACA	CAAAATAGTTTGACACATCTAGTGCAT	60.1	531	INTERGENIC	--	--	--
Ca4_P1_48516972	Ca4	48516972	CTCT/CTCTTCT	TGTCCCACAAGAAACATGACA	ACGTGTAGTTTCCTCCACCG	60.0	560	INTERGENIC	--	--	--
Ca4_P1_48517102	Ca4	48517102	AAGAAACA/AAGAAACAACAGAAACA	CTCACATCCTTGCCTCAACA	ACGTGTAGTTTCCTCCACCG	59.8	340	INTERGENIC	--	--	--
Ca4_P1_48518146	Ca4	48518146	CT/CTTT	TGGTGGTTGACCACAAAAGA	CCACACTGATCACAAGCACA	60.0	606	INTRON	Ca_10740	--	C2H2
Ca4_P1_48518404	Ca4	48518404	CTT/CTTT	TGGTGGTTGACCACAAAAGA	GCATGACGAACAGCTTGATG	60.0	660	INTRON	Ca_10740	--	C2H2
Ca4_P1_48520535	Ca4	48520535	GAGGGATAA/GAGGGATAAGGGATAA	ACCCACCAAAAGAACAGTGC	TCTCCACTATGTTCCGACCC	60.0	928	INTRON	Ca_10740	--	C2H2
Ca4_P1_48522137	Ca4	48522137	TTTATTATTATTATTATTATTATT/TTTATTATTATTATTATTATTATT	CATTGCAATCGGCCTCTATC	TCCCACTTTCACTTTGGGTC	60.6	657	DRR	Ca_10740	--	C2H2
Ca4_P1_48541135	Ca4	48541135	TAAAAAAAAAAAA/TAAAAAAAAA	AATTCCCACACGTACGCTTC	GATGTTCTTGGCTTGTGGCT	60.0	723	INTERGENIC	--	--	--
Ca4_P1_48547378	Ca4	48547378	TGAGAGAGAGAGAGAGAGAGAGAGA/TGAGAGAGAGAGAGAGAGAGAGAGAGA	TGTGTGGTCCCAAAAAGTCA	TCCAATTACCCACCACCCTA	60.0	533	INTERGENIC	--	--	--
Ca4_P1_4855498	Ca4	4855498	CTGCTTGCTTG/CTGCTTG	CAGGATCTCTCTGCCGAATC	ATGCTTTTGTTTTGGGTTGC	59.9	448	DRR	Ca_03773	--	--
Ca4_P1_48575054	Ca4	48575054	TGGGG/TGGGGG	GCAACAATTGAATCAACCCC	CTTCATGAATTGCACCATGC	60.2	705	CDS (FRAME SHIFT)	Ca_23007	P	--

INDEL marker IDs	Chromosomes/ unanchored scaffolds	Physical positions (bp)	InDels (*Kabuli* reference genome-CDC Frontier/PI)	Forward primers (5'-3')	Reverse primers (5'-3')	Anneling temperature (0C)	Expected amplified product size (bp)	Structural annotation		Functional annotation	
								Sequence components of *kabuli* genome	*Kabuli* gene accession IDs	NCBI-KOG	TFs
Ca4_P1_48576925	Ca4	48576925	AT/ATT	ATCTCACCCCAAGACAACCA	TCATTCCAGAAGGAGATGGG	60.4	211	INTRON	Ca_23007	P	--
Ca4_P1_48577228	Ca4	48577228	A/AC	AACACATCCGGGATCCATTA	TGATGACGAAGACGAAGACG	60.0	303	INTERGENIC	--	--	--
Ca4_P1_48651388	Ca4	48651388	AACTTTTAAGAGTACTTTTA/AACTTTTA	AAAACCACTCCAGACATCGG	CTTGCAATACCCGCAAAGTT	60.0	682	DRR	Ca_23016	O	LBD
Ca4_P1_48658941	Ca4	48658941	CTTTT/CTTTTT	CCATAATCAAAATGCAAGATGTG	TGATTTACGCTTGGGAACAA	59.4	636	INTERGENIC	--	--	--
Ca4_P1_48662498	Ca4	48662498	A/ATATATCAAACT	CGTTATAATATCGCGTTTTGATTAGA	AAATAAATAGCGCTGGCACC	59.8	609	INTERGENIC	--	--	--
Ca4_P1_48662786	Ca4	48662786	TAATAACAATAA/TAATAA	CAACATGTAGACCAAGTGGGG	AAATAAATAGCGCTGGCACC	60.3	216	INTERGENIC	--	--	--
Ca4_P1_48663015	Ca4	48663015	AAAA/AAAATTAAAAAATTGAAA	ATTAGACATTTCGGGTGCCA	TATGTTAGTGCCACTGCCCC	60.3	571	INTERGENIC	--	--	--
Ca4_P1_48664333	Ca4	48664333	G/GGGTTGGAGGTAT	GTGCCGCATTCATCCTTAGT	AAACTGGTTGACGGGATCTTT	60.1	504	INTERGENIC	--	--	--
Ca4_P1_48670108	Ca4	48670108	AAATATCT/A	TATCTCCATGGCGTCACAAA	CTGCGCCGACTAACAATACA	60.1	589	INTERGENIC	--	--	--
Ca4_P1_48670171	Ca4	48670171	AATTTA/AATTTATTTA	TATCTCCATGGCGTCACAAA	CTGCGCCGACTAACAATACA	60.1	589	INTERGENIC	--	--	--
Ca4_P1_48670468	Ca4	48670468	GATA/GATAAAATCTTATA	CTGTATTGTTAGTCGGCGCA	CCGACCCAACACGTTATGA	59.9	620	INTERGENIC	--	--	--
Ca4_P1_48670668	Ca4	48670668	CATAT/CAT	CTGTATTGTTAGTCGGCGCA	TGACAATGTGGAATCGGAAA	59.9	812	INTERGENIC	--	--	--

INDEL marker IDs	Chromosomes/ unanchored scaffolds	Physical positions (bp)	InDels (*Kabuli* reference genome-CDC Frontier/PI)	Forward primers (5'-3')	Reverse primers (5'-3')	Anneling temperature (0C)	Expected amplified product size (bp)	Structural annotation		Functional annotation	
								Sequence components of *kabuli* genome	*Kabuli* gene accession IDs	NCBI-KOG	TFs
Ca4_P1_48671006	Ca4	48671006	ATTTTTTTT/ATTTTTTTTTTT	GGTCGGCCCTGACTTTAGAT	TGACAATGTGGAATCGGAAA	60.5	198	INTERGENIC	--	--	--
Ca4_P1_48675486	Ca4	48675486	AT/ATT	AAAAGGAAATTGGCACGTTG	CACTTCATCATGCATCACCC	60.0	702	INTERGENIC	--	--	--
Ca4_P1_48676406	Ca4	48676406	TAAAAAAAAAA/TAAAAAAAA	GGGTGATGCATGATGAAGTG	TAAACTTGGTGGTGATGGCA	59.9	707	DRR	Ca_23018	--	LBD
Ca4_P1_4868200	Ca4	4868200	TAAA/TAAAA	ATTGGTTTTGCTTTGACCCA	CACCAATATTCCATTTCCCG	60.3	423	INTERGENIC	--	--	--
Ca4_P1_48683487	Ca4	48683487	TAAA/TAAAA	GTGTTTGTGCGATGATTTGG	TGGCATCATAAATTTGTGTGTGTA	60.0	200	INTERGENIC	--	--	--
Ca4_P1_48687183	Ca4	48687183	TTTACTTA/TTTACTTACTTA	TTCATAATTGCTGCACCCTG	ATGGAACCCAATGATCCTGA	59.7	677	INTRON	Ca_23019	R	--
Ca4_P1_48692254	Ca4	48692254	GTGATAT/GTGATATGATAT	TCCCGACATTGTGGGTTAAT	TTGTGAAAATGTTGATGGTCTTG	60.1	749	INTERGENIC	--	--	--
Ca4_P1_4869873	Ca4	4869873	CAA/CAAA	ATGGAAGCCTTGGTTGTGAC	AAATGGTCCCAAACTGCAAG	60.0	635	INTERGENIC	--	--	--
Ca4_P1_4870497	Ca4	4870497	AC/ACC	ACTGCCTAGGTCACCACCAC	AATGGCACACAATACCCCAT	60.0	142	INTERGENIC	--	--	--
Ca4_P1_4870551	Ca4	4870551	TC/TCGTGGCTGC	ACTGCCTAGGTCACCACCAC	AATGGCACACAATACCCCAT	60.0	142	INTERGENIC	--	--	--
Ca4_P1_48711156	Ca4	48711156	T/TC	CTCTGGGCTGATCAGAAAGG	ACTCTTTGGGCCCTCATTCT	59.9	229	INTRON	Ca_23021	U	--
Ca4_P1_48719311	Ca4	48719311	CAA/CAAA	TCAAAACCTTCTCCACCAGC	AGGTCCCAGTGTTCTGCAAC	60.2	550	DRR	Ca_23022	--	--

INDEL marker IDs	Chromosomes/ unanchored scaffolds	Physical positions (bp)	InDels (*Kabuli* reference genome-CDC Frontier/PI)	Forward primers (5'-3')	Reverse primers (5'-3')	Anneling temperature (0C)	Expected amplified product size (bp)	Structural annotation		Functional annotation	
								Sequence components of *kabuli* genome	*Kabuli* gene accession IDs	NCBI-KOG	TFs
Ca4_P1_48725994	Ca4	48725994	ATTTT/ATTT	AGGTGCGATTAGCAGCAAAA	TGTCGCAACTGGTTGATTGT	60.9	666	INTERGENIC	--	--	--
Ca4_P1_48740635	Ca4	48740635	CTTTTT/CTTTTTTT	TCAAGGAGGAATCCCAACAG	TTACACCTGTGGCCAATTCA	60.0	569	INTRON	Ca_23023	--	FAR1
Ca4_P1_48746264	Ca4	48746264	TA/TACCTCAAA	CGTACTTGCATCCATGAAACA	TCATCGCTGTCGTCAAACTC	59.6	546	INTRON	Ca_23023	--	FAR1
Ca4_P1_4875484	Ca4	4875484	GC/G	AGCCTATGGCTTGGCCTATT	GCCTAAGCTTGACTTGTGGC	60.1	687	INTERGENIC	--	--	--
Ca4_P1_48759040	Ca4	48759040	GCC/GC	CATGGAAAATCATGTTAGTCGC	TTATGCAGCATGTCTCACGTC	59.5	462	INTRON	Ca_23023	--	FAR1
Ca4_P1_48766746	Ca4	48766746	TAAAA/TAAAAA	ATATCCAGCCGTGTTTGCAT	TTCTCCCCACCACTAACTCG	60.4	261	INTRON	Ca_23023	--	FAR1
Ca4_P1_48767304	Ca4	48767304	TTGTGTGTGTGTGTGTG/TTGTGTGTGTGTG	CGAGTTAGTGGTGGGGAGAA	TTGGAAGGAGCCTTTACGTC	60.1	926	INTRON	Ca_23023	--	FAR1
Ca4_P1_48769666	Ca4	48769666	GTTTTTTTTT/GTTTTTTTTTT	TTTCAATGAAGCCTAGTCCG	TTAAGGGTCCGCATGATTGT	58.0	605	INTRON	Ca_23023	--	FAR1
Ca4_P1_48770331	Ca4	48770331	AC/A	TTTTGTTCACAATCATGCGG	TGTTTTAACGTGGCTGACCA	60.5	675	INTRON	Ca_23023	--	FAR1
Ca4_P1_48772701	Ca4	48772701	AT/A	GGTTGGAAAACGGAAAAACA	AAGAAAATCTAAACGCCAAGG	59.8	295	DRR	Ca_23023	--	FAR1
Ca4_P1_48773094	Ca4	48773094	AGATTCC/A	GGTAACCTTGGCGTTTAGATTTT	CATTGGTCGGACTAACAACTCA	59.8	427	DRR	Ca_23023	--	FAR1
Ca4_P1_48775290	Ca4	48775290	A/AAAATTTATT	GATGGAGAGGGAGACGAACA	CTTTCAAATCTGTAGGGCCG	60.2	313	INTERGENIC	--	--	--

INDEL marker IDs	Chromosomes/ unanchored scaffolds	Physical positions (bp)	InDels (*Kabuli* reference genome-CDC Frontier/PI)	Forward primers (5'-3')	Reverse primers (5'-3')	Anneling temperature (0C)	Expected amplified product size (bp)	Structural annotation		Functional annotation	
								Sequence components of *kabuli* genome	*Kabuli* gene accession IDs	NCBI-KOG	TFs
Ca4_P1_48776221	Ca4	48776221	AATATATATATATATATATA/AATATATATATATATATA	GCGACTTCCCACATGATTTT	TGTATCAAGGCGTCGTCAAA	59.9	753	INTERGENIC	--	--	--
Ca4_P1_48781102	Ca4	48781102	TAT/TATGAT	TCGGCATTTTCATTGTCTCA	TGTGTACCGACCGATGTTTG	60.2	190	INTERGENIC	--	--	--
Ca4_P1_48786645	Ca4	48786645	TTA/T	TCAAGGAGGAATCCCAACAG	TTACACCTGTGGCCAATTCA	60.0	641	INTRON	Ca_23024	--	FAR1
Ca4_P1_48791786	Ca4	48791786	TAAA/TA	ATTAAGCGGGTATGAGTGCG	TGATGGCAAATATGGTAGCG	60.1	674	INTERGENIC	--	--	--
Ca4_P1_48791939	Ca4	48791939	CAAA/CAA	AAACTGCGGGTATGAGGATG	CAAAAGACCAACTCGGGAAA	60.0	421	INTERGENIC	--	--	--
Ca4_P1_48792226	Ca4	48792226	TA/T	AAACTGCGGGTATGAGGATG	CAAAAGACCAACTCGGGAAA	60.0	421	INTERGENIC	--	--	--
Ca4_P1_48793555	Ca4	48793555	TTCTCTCTCTCTCTCTCTC/TTCTCTCTCTCTCTCTCTCTC	ACGGAGAGTTTGGGTGTGAC	GTTATTCCACCGCGAGAAAA	60.0	451	INTERGENIC	--	--	--
Ca4_P1_48809672	Ca4	48809672	GAAAAAAA/GAAAAAAAA	GCGAGCGAATTAAACTCACC	GGGAACATGACTTGGTTCGT	59.9	190	INTERGENIC	--	--	--
Ca4_P1_48815945	Ca4	48815945	T/TCAAAATA	AAAAATGGTGACGTTGCACA	CGGATGAAGACTTGGTCGAT	60.0	210	INTERGENIC	--	--	--
Ca4_P1_48825493	Ca4	48825493	AC/ACC	ACCCCACCATTGTTGTTCTT	TAAATCTTCCGGTCGCAATC	59.2	316	INTERGENIC	--	--	--
Ca4_P1_48826540	Ca4	48826540	TTCACAT/TT	ATATTGCATTGGCATGGGAT	TTGGAAATGTCATTGCTACCC	60.0	489	INTERGENIC	--	--	--
Ca4_P1_48829333	Ca4	48829333	GATCAT/GAT	TTTGTTGAGACTAAAAAGGTGGA	ATTGCACCGTAACTCTTGGG	58.0	340	DRR	Ca_23025	--	FAR1

INDEL marker IDs	Chromosomes/ unanchored scaffolds	Physical positions (bp)	InDels (*Kabuli* reference genome-CDC Frontier/PI)	Forward primers (5'-3')	Reverse primers (5'-3')	Anneling temperature (0C)	Expected amplified product size (bp)	Structural annotation		Functional annotation	
								Sequence components of *kabuli* genome	*Kabuli* gene accession IDs	NCBI-KOG	TFs
Ca4_P1_48831208	Ca4	48831208	GTATATA/GTATATATA	TATTTCCCAAGACCTCGTGC	ATGTGGTTGTTGTTGCCTGA	60.1	255	INTERGENIC	--	--	--
Ca4_P1_48831529	Ca4	48831529	TTATATATATATATATAT/TTTTATATATATATATATATATAT	TCAGGCAACAACAACCACAT	ATGGGTCCAAATATGCAGGT	60.0	627	INTRON	Ca_23026	--	FAR1
Ca4_P1_4883531	Ca4	4883531	AT/ATGTT	CACCTGCCTTTTTCTCAAGC	AGGAGCCAAACAGCAAGAAA	60.0	540	INTRON	Ca_03769	Z	NAC
Ca4_P1_48846764	Ca4	48846764	ACATCATCATCATCA/ACATCATCATCA	GGTAAACGTGTGTTGCATGG	ACAGCAGTAGCCCTTTGAGC	59.9	715	INTERGENIC	--	--	--
Ca4_P1_48850057	Ca4	48850057	GA/GATCA	GCATCAATATGAATCACCAATCA	TGACGATTTTATTTACGCCAA	59.7	857	INTERGENIC	--	--	--
Ca4_P1_48906072	Ca4	48906072	A/ATATT	TTTCGTGGCTAATTTTCGTT	GGCGTTGGTTTCACTCAAAT	57.5	864	INTERGENIC	--	--	--
Ca4_P1_4891737	Ca4	4891737	AATTATTATTATTATTATT/AATTATTATTATTATTATTATT	TGCCTTGTATCGTTCCATCA	TTCCTGCTTTTTCTTTCCGA	60.1	540	INTRON	Ca_03769	Z	NAC
Ca4_P1_4891996	Ca4	4891996	GAAAAAAAA/GAAAAAAAAA	TCGGAAAGAAAAAGCAGGAA	GGCAAATTGCTACTGAAGGG	59.9	265	INTRON	Ca_03769	Z	NAC
Ca4_P1_4893509	Ca4	4893509	CTTT/CTTTT	CAGCGTTTTGTCGCAAGTAA	GCCGAAGTTGAAGGACAGAG	60.1	480	INTERGENIC	--	--	--
Ca4_P1_48942067	Ca4	48942067	GTTTTTTTTTT/GTTTTTTTTTTT	TGATCTTTCTGCAATGAAACG	GATGGAAAATGAAGGTCCGA	58.9	528	INTERGENIC	--	--	--
Ca4_P1_4897344	Ca4	4897344	ATTT/ATTTT	CTCTGCAGCCTTGATTGGTT	ATGGAGTATGGGGCTGTGAC	60.4	278	INTRON	Ca_03768	K	--
Ca4_P1_48975362	Ca4	48975362	GAAAA/GAAAAA	TGGTTGGGATTTTTGGGTTA	GACCCTCTCCTCTCAATCCC	60.0	672	INTERGENIC	--	--	--

INDEL marker IDs	Chromosomes/ unanchored scaffolds	Physical positions (bp)	InDels (*Kabuli* reference genome-CDC Frontier/PI)	Forward primers (5'-3')	Reverse primers (5'-3')	Anneling temperature (0C)	Expected amplified product size (bp)	Structural annotation		Functional annotation	
								Sequence components of *kabuli* genome	*Kabuli* gene accession IDs	NCBI-KOG	TFs
Ca4_P1_48983285	Ca4	48983285	AACAC/AAC	ATAATGGACTTCCCCTCGCT	AACGTCTCAAGGTGGTGGTC	59.9	401	DRR	Ca_20769	O	--
Ca4_P1_49004237	Ca4	49004237	ATTAT/A	TGACATCAATTCCCAATCCTT	GGGGACAGAAACCTTATCCA	59.2	311	INTERGENIC	--	--	--
Ca4_P1_49073579	Ca4	49073579	GTT/GT	GTGACTTGGAGGGAGTGGAA	ATCCGCTAACCAATCACAGC	60.1	453	INTERGENIC	--	--	--
Ca4_P1_49074099	Ca4	49074099	ATTTTTTTTTT/ATTTTTTTTT	GCTGTGATTGGTTAGCGGAT	CGACGCGGGTTACACACTA	60.1	694	INTERGENIC	--	--	--
Ca4_P1_49074487	Ca4	49074487	AATATATATATATATATATATATATATATAT/AATATATATATATATATATATATATAT	CGTCGCACGGAAACTATCTT	TGAGTAGCACCGGAATGAAA	60.3	426	INTERGENIC	--	--	--
Ca4_P1_49135046	Ca4	49135046	ATATTATTATTATTATTATTATTATTATTATTATTATTATTATTATTATTATTATTATTATTATTATTAT/ATATTATTATTATTATTATTATTATTATTATTATTATTATTATTATTATTATTATTATTATTATTATTATTATTATTATTAT	TATAAAGCGGTTTGGTTCGG	ATCAACACAACATCCACCCA	60.0	938	INTERGENIC	--	--	--
Ca4_P1_49142216	Ca4	49142216	TTATATATATATATATATATATATATATATATATATATATATAT/TTATATATATATATATATATATATATATATATATATATATATAT	GATGGGATGGTTGGTAATGC	GGACCAAATCGAAGAGAAAAA	60.0	257	INTERGENIC	--	--	--
Ca4_P1_49149348	Ca4	49149348	TCC/TCCC	GCACCCTTGTATTTTGCTCC	GGGAAAACTTGTGTACCGGA	59.6	493	INTERGENIC	--	--	--
Ca4_P1_49160495	Ca4	49160495	TGGGTTTAGG/TGGGTTTAGGGTTTAGG	CCGGGGAGAATAGGAAGAAC	CCCCAAACCCTAAACCCTAA	59.9	459	INTERGENIC	--	--	--
Ca4_P1_4978107	Ca4	4978107	AAATAAATAA/AAA	GTCTAATCCCCCATTTTGGC	TCTTGTGGTCGTTGCTTGAG	60.5	557	INTRON	Ca_03758	R	--
Ca4_P1_5030136	Ca4	5030136	TAAAA/TAAAAA	AACAATTCCTTCGTATTTTTCCTC	CATTGCATGAGAACCAAACAA	59.0	778	INTERGENIC	--	--	--
Ca4_P1_5040391	Ca4	5040391	AATATATATATATATATATAT/AATATATATATATATATATATAT	GTGAAAAGAGACAACCGCCT	GGCCACACAAGACTTTGTCA	59.3	777	INTERGENIC	--	--	--

INDEL marker IDs	Chromosomes/ unanchored scaffolds	Physical positions (bp)	InDels (*Kabuli* reference genome-CDC Frontier/PI)	Forward primers (5'-3')	Reverse primers (5'-3')	Anneling temperature (0C)	Expected amplified product size (bp)	Structural annotation		Functional annotation	
								Sequence components of *kabuli* genome	*Kabuli* gene accession IDs	NCBI-KOG	TFs
Ca4_P1_5081255	Ca4	5081255	GATTATTATTATTATTATTATTATTATTAT/GATTATTATTATTATTATTATTATTATTATTAT	CACCAGCAATAGCTTGCTCA	GAAAAACAGGGCTAGCAACG	60.2	711	CDS (large-effect mutations)	Ca_03748	K	Trihelix
Ca4_P1_5097117	Ca4	5097117	TNNNNNNNNNN/TNNNNNNN	TGGAAGCAAGACACATCTGC	ATATTGGTGGGGTGCATGTT	60.0	550	INTRON	Ca_03746	S	--
Ca4_P1_5124845	Ca4	5124845	GATATATATATA/GATATATATATATA	CGAATAAGAGGGAGTATCCATTG	TATGGCCAACAACAGCACAT	59.0	162	INTERGENIC	--	--	--
Ca4_P1_5169385	Ca4	5169385	TAAAAAAA/TAAAAAAAA	CATTCAATTTGTCTGCCACG	CGCAGACCTTTGAGGTTGAT	60.1	768	INTERGENIC	--	--	--
Ca4_P1_5170510	Ca4	5170510	CTTT/CTT	CAAAGATAAAATGAACCCTGCC	GATTGAGGTGCTGTCACCCT	59.8	179	INTERGENIC	--	--	--
Ca4_P1_5222831	Ca4	5222831	T/TGAACCGAATC	CTTGGTGGAATAGCACGGAT	ACTGAAAAAGGTGGTGGTGC	60.0	660	INTRON	Ca_03733	S	LBD
Ca4_P1_5247724	Ca4	5247724	CAAA/CAAAA	GCCACTGCTGCTCATTCATA	ACCCAGAAAGCAACCACAAC	60.0	203	INTERGENIC	--	--	--
Ca4_P1_5248717	Ca4	5248717	ATTT/ATT	CCTCCAAAAAGCGATATCCA	TCACACCGAAATCGAAATCA	60.0	760	INTERGENIC	--	--	--
Ca4_P1_5300098	Ca4	5300098	TAAAAAAAAAA/TAAAAAAAAA	GGCTGGAGAAAGAATATGCG	AAAAGGAGTTTGGAAGGGGA	59.8	598	INTERGENIC	--	--	--
Ca4_P1_5335917	Ca4	5335917	TTCTCTCTCTCTCTCTCTCTCTCTC/TTCTCTCTCTCTCTCTCTCTCTC	AAGGAGCAGCATCCGACTAA	AAAGTGCTGACCAAAACGGT	60.0	444	INTERGENIC	--	--	--
Ca4_P1_5468294	Ca4	5468294	TTCT/TT	AGCTTTTGGCTCTCAGTGGA	GCAAAGGAGTGCCTGAAATC	60.1	389	INTRON	Ca_03705	P	--
Ca4_P1_5473420	Ca4	5473420	TTCTCTCTCTCTCTCTCTCTCTC/TTCTCTCTCTCTCTCTCTCTCTCTC	TATGGAGATGGAGGTCGAGG	ATCAAAAGCAGGGTCTCGAA	60.0	255	INTRON	Ca_03704	E	--

INDEL marker IDs	Chromosomes/ unanchored scaffolds	Physical positions (bp)	InDels (*Kabuli* reference genome-CDC Frontier/PI)	Forward primers (5'-3')	Reverse primers (5'-3')	Anneling temperature (0C)	Expected amplified product size (bp)	Structural annotation		Functional annotation	
								Sequence components of *kabuli* genome	*Kabuli* gene accession IDs	NCBI-KOG	TFs
Ca4_P1_5525661	Ca4	5525661	GAAAAAAAAAA/GAAAAAAAAA	TATTTAGCCAAAAGGGCAGC	TCCATGTGAATATGATGAACCAA	59.3	320	INTERGENIC	--	--	--
Ca4_P1_5564394	Ca4	5564394	GATATATATATATATATATATA/GATATATATATATATATATA	TTGCATCATTCCAAGGAACA	TCCACCTTCTTCTTAGAACCACA	60.0	591	INTERGENIC	--	--	--
Ca4_P1_5570234	Ca4	5570234	CTTTTTTTTTT/CTTTTTTTTTTT	TGATGCATAAGTACAGGTGGG	AAGTTTTTGGCTTTAAATAGTGTCA	58.5	581	INTERGENIC	--	--	--
Ca4_P1_5581102	Ca4	5581102	TAAAAAAAAAA/TAAAAAAAAAAA	TGGTGAACTAAAATGGCCAGA	TACGGGGAAAAATCTCCTGA	60.5	231	INTERGENIC	--	--	--
Ca4_P1_5618968	Ca4	5618968	GTTATTATTATTATTATTATTATTATTATTATTAT/GTTATTATTATTATTATTATTATTATTATTATTAT	GTGTTCCTGATGTTTTGCCA	GCAACTTCGAGATGCGACTT	59.5	752	INTERGENIC	--	--	--
Ca4_P1_5629484	Ca4	5629484	CATATATATATATATATATATATATATAT/CATATATATATATATATATATATATATATAT	TATCGCAAACCGGTCAATTT	GGGTCACGTGGATTTTGTCT	60.3	906	INTERGENIC	--	--	--
Ca4_P1_5655244	Ca4	5655244	TATA/TATAAATA	TGAACCCCCACCAAAATAAA	GCACCGCTCAAAATGAAATA	60.0	928	DRR	Ca_03683	R	--
Ca4_P1_568232	Ca4	568232	A/ACG	TTATCATTCCGCCAAGAAGG	TGTAACCACGACCATAGCCA	60.0	462	INTERGENIC	--	--	--
Ca4_P1_5707384	Ca4	5707384	AATATATATATATATATAT/AATATATATATATATATATAT	AAGCTAAGCACCGGTAACGA	TTCACGTCTTGAAATTTTCCG	59.9	711	DRR	Ca_03679	DKL	MYB_related
Ca4_P1_5712745	Ca4	5712745	CTTTTTTTT/CTTTTTTT	TGTTCCCTCAAAAGGAATGC	AACAATGTGAGAGCTTCCCC	60.1	714	INTERGENIC	--	--	--
Ca4_P1_5793201	Ca4	5793201	TAAAAA/TAAA	GACTGCCACCGACCACTTAT	GGTCACACCGTTCAGCTTTT	60.0	469	INTERGENIC	--	--	--
Ca4_P1_5824436	Ca4	5824436	ATATATTATATTA/ATATATTA	CCAAACACAAACCATCTCTCA	TGGAAAATCCAAATTCACCAA	58.6	452	DRR	Ca_03668	--	HSF

INDEL marker IDs	Chromosomes/ unanchored scaffolds	Physical positions (bp)	InDels (*Kabuli* reference genome-CDC Frontier/PI)	Forward primers (5'-3')	Reverse primers (5'-3')	Anneling temperature (0C)	Expected amplified product size (bp)	Structural annotation		Functional annotation	
								Sequence components of *kabuli* genome	*Kabuli* gene accession IDs	NCBI-KOG	TFs
Ca4_P1_582760	Ca4	582760	T/TATGGTGCTACTC	CAGGTGACTCATCCCGATTT	CGCAATTTCAGTGAAGCAAA	59.9	591	DRR	Ca_07688	O	FAR1
Ca4_P1_5846699	Ca4	5846699	TTATATATATATATATATATATATATATATAT/TTATATATATATATATATATATATATATATAT	CCACCCGTGATTGAGTTTTT	CCCTTGATCTCAGTCCAACC	59.8	503	INTERGENIC	--	--	--
Ca4_P1_5910055	Ca4	5910055	AATATATATATATATATA/AATATATATATATATA	AGGGGACAAATGGAGCTTTT	ACGGAACCCCTCTCTACCTC	59.9	829	INTRON	Ca_03658	--	--
Ca4_P1_5931200	Ca4	5931200	TT/TTATTAT	CCAGCCCAATTTACATGCTT	CCGCCCATTTCAACTTTTTA	60.0	527	INTERGENIC	--	--	--
Ca4_P1_5960166	Ca4	5960166	CTTGTGGTGATGTGT/CTTGTGGTGATGTGTTGTGGTGATGTGT	GAATCAGCCACTGAGTGCAA	AATGCCACCTACCTTCAACG	60.0	257	CDS (FRAME SHIFT)	Ca_03652	--	HD-ZIP
Ca4_P1_5961971	Ca4	5961971	T/TGTG	CCTCTAATATCGTCGCTCCAA	ATAATCGGTGCGGATTTCCT	59.3	668	INTERGENIC	--	--	--
Ca4_P1_6091215	Ca4	6091215	AATACAT/AAT	GTGGTTTGTTACGGCCACTT	GATTAATTGTTGGATGCGACG	59.9	551	INTERGENIC	--	--	--
Ca4_P1_6145049	Ca4	6145049	ATTTTTTTTTTTT/ATTTTTTTTTTTTT	ATCCCCTCAGAATCGGCTAT	TTCGTCTATGCATTTGTTCCTG	59.9	565	INTERGENIC	--	--	--
Ca4_P1_6146003	Ca4	6146003	ATTTTTTTTTT/ATTTTTTTTTTT	GCTCACATCTTCTTGCCACA	GGGAAAACGCACGTGTAACT	60.0	534	INTERGENIC	--	--	--
Ca4_P1_6167780	Ca4	6167780	TAAAAAAA/TAAAAAAAA	CATGAGAGGCATATTTAACAACTG	TGTGATAAATATTTGTTCCGTGC	57.9	381	INTERGENIC	--	--	--
Ca4_P1_6186297	Ca4	6186297	GTTTTTTTTT/GTTTTTTTTTT	TGTGTTTTCTCAATTTGACGATG	GCCATTTGAACCAAATTAAGAG	60.0	751	INTERGENIC	--	--	--
Ca4_P1_6209314	Ca4	6209314	TTATATATATATATATATATA/TTATATATATATATATATA	AAACTGTTAGACTTTGCAGGATGA	TCGCCCCTTAAATTTGACAC	59.4	205	INTERGENIC	--	--	--

INDEL marker IDs	Chromosomes/ unanchored scaffolds	Physical positions (bp)	InDels (*Kabuli* reference genome-CDC Frontier/PI)	Forward primers (5'-3')	Reverse primers (5'-3')	Anneling temperature (0C)	Expected amplified product size (bp)	Structural annotation		Functional annotation	
								Sequence components of *kabuli* genome	*Kabuli* gene accession IDs	NCBI-KOG	TFs
Ca4_P1_6271796	Ca4	6271796	GAAAAAAAAAA/GAAAAAAAAA	AAAAATTACGGGATGCATAAAAA	AAAGGAAAAATAATGTAAACTTTGAGG	58.9	684	INTERGENIC	--	--	--
Ca4_P1_6271908	Ca4	6271908	GTATATATATATATATATAT/GTATATATATATATATATATAT	AAAAATTACGGGATGCATAAAAA	TCCCCAAACAATGACAAAAA	58.9	775	INTERGENIC	--	--	--
Ca4_P1_6324755	Ca4	6324755	ATTAATAATTTAATAATTTAATAATT/ATTAATAATTTAATAATT	TGGGTGGAACATGACAGAGA	ATTTTGCCATTTGGGATTGA	60.1	588	INTERGENIC	--	--	--
Ca4_P1_6353994	Ca4	6353994	CAAAAAAAAAA/CAAAAAAAAAAA	TGGTCCTCAGAGTCGTTAACCT	ATCACAACTTCACAAGCCCC	60.2	968	INTERGENIC	--	--	--
Ca4_P1_6363964	Ca4	6363964	CAAAAA/CAAAAAA	ACAATATCCCCTAACGGCCT	TCCAAAGTTGCCCCTGTATT	59.7	704	INTRON	Ca_03603	T	bHLH
Ca4_P1_6389441	Ca4	6389441	TT/TTCCTTATGTGGAAAT	GCCCCACCTTCAGTGTTAAA	ATGCTGGCAAATGATGTCAC	60.0	728	DRR	Ca_03602	--	ERF
Ca4_P1_6479135	Ca4	6479135	AATATATATATATATATATATATATATATATATATATAT/AATATATATATATATATATATATATATATAT	TGACTCGTGCAACACAAGAG	GGATAAAACCGCATTTGAGC	58.6	675	INTERGENIC	--	--	--
Ca4_P1_6528150	Ca4	6528150	CTTTTTTTTTTTTT/CTTTTTTTTTTTT	TCCAAGTAAAGGATCGTGGG	AAAAATTCGAATGAAGAATCAAAA	59.9	690	INTERGENIC	--	--	--
Ca4_P1_6581955	Ca4	6581955	TAAAAAAAA/TAAAAAAA	GCACGTGTGTAATGGTGCTC	TCTTGCTCAACGAAAATCCC	60.2	612	INTERGENIC	--	--	--
Ca4_P1_6608507	Ca4	6608507	CGTGAAT/C	GGATGCACCATCTATTGCCT	GGATGCGACCATTTATTGCT	59.9	779	INTERGENIC	--	--	--
Ca4_P1_6609098	Ca4	6609098	TTTATTATTATTATTATTATTATTATTATTATT/TTTATTATTATTATTATTATTATTATT	AGCAATAAATGGTCGCATCC	CGTTCCACTCCCAACAACTT	59.9	594	INTERGENIC	--	--	--
Ca4_P1_6732925	Ca4	6732925	T/TC	AAAAACACTCGCCGTGAGAT	TCCAACCGTGAACTCATCAA	59.7	435	INTERGENIC	--	--	--

INDEL marker IDs	Chromosomes/ unanchored scaffolds	Physical positions (bp)	InDels (*Kabuli* reference genome-CDC Frontier/PI)	Forward primers (5'-3')	Reverse primers (5'-3')	Anneling temperature (0C)	Expected amplified product size (bp)	Structural annotation		Functional annotation	
								Sequence components of *kabuli* genome	*Kabuli* gene accession IDs	NCBI-KOG	TFs
Ca4_P1_6764856	Ca4	6764856	TAAAAAAAAAA/TAAAAAAAAAAA	AGCACGGTGTAGTCTTGGCT	GAGGGGTCATTTCGTTCTCA	59.9	326	DRR	Ca_03564	K	MYB
Ca4_P1_6869072	Ca4	6869072	ATTTTTTTTTT/ATTTTTTTTTTT	TGACACCAAGACAACTCCCA	AACGTTCTTGCTTTCACACG	60.1	552	INTERGENIC	--	--	--
Ca4_P1_6875620	Ca4	6875620	TGG/TG	GACAGTTTTGTGCAATCCGA	AACCTATACCCGCACAATGC	59.7	859	INTERGENIC	--	--	--
Ca4_P1_6907181	Ca4	6907181	TTATATATATATATATAT/TTATATATATATATAT	TTTGAAATGGCGACTTCCTT	CCAAATGGCAACTTCCAACT	59.7	569	INTERGENIC	--	--	--
Ca4_P1_7001379	Ca4	7001379	ATTTCTTTTTTTCTTTTTTTCTTTT/ATTTCTTTTTTTCTTTT	GGTACGATCTGCACAGGGTC	CAGGTCAAACTTCCTCCCAA	60.5	999	INTERGENIC	--	--	--
Ca4_P1_7009936	Ca4	7009936	TTATATATATATATATATATATATA/TTATATATATATATATATATATA	AATATGGGTCCCCAGACTCC	TCCTTTTTGTGCGGTAACAA	60.0	686	INTERGENIC	--	--	--
Ca4_P1_7031730	Ca4	7031730	GATATATATATATATATA/GATATATATATATATATA	GGATGTCGCTTGCAAGAAGT	TAAACTAACGTGTCGGCGTG	60.4	476	INTERGENIC	--	--	--
Ca4_P1_7081636	Ca4	7081636	AATATATATATATATATATATATATATA/AATATATATATATATATATATATATATATATATA	AATTGGCCACACTTCTTTCG	GGCTTTGTGAAAAACGAGGA	60.1	579	INTERGENIC	--	--	--
Ca4_P1_7089091	Ca4	7089091	GA/GAACAAAAGAGATTCACA	CTTTGCATACCCCTCTCGAC	TTGGGAGGATTGGTTTCAAG	59.7	644	INTERGENIC	--	--	--
Ca4_P1_7127718	Ca4	7127718	GAAAAAAAAAA/GAAAAAAAAA	AATTTGGACAATACCGCGAA	AGGGAAGGGGAAGAAGATCA	60.3	580	INTERGENIC	--	--	--
Ca4_P1_7134588	Ca4	7134588	ATTTTTTTTTTT/ATTTTTTTTTTTT	GAAAAGTCAAGCGCAACACA	TGCACCCTCAATAACAACCA	60.0	727	INTRON	Ca_03523	--	--
Ca4_P1_714706	Ca4	714706	AATATATATATATATATATATATATATATA/AATATATATATATATATATATATATATATATA	CATCAAATTGGCCCTTCAAT	GTTGGCCACCAAATCAAATC	59.8	640	INTERGENIC	--	--	--

INDEL marker IDs	Chromosomes/ unanchored scaffolds	Physical positions (bp)	InDels (*Kabuli* reference genome-CDC Frontier/PI)	Forward primers (5'-3')	Reverse primers (5'-3')	Anneling temperature (0C)	Expected amplified product size (bp)	Structural annotation		Functional annotation	
								Sequence components of *kabuli* genome	*Kabuli* gene accession IDs	NCBI-KOG	TFs
Ca4_P1_719535	Ca4	719535	TTTTTTTAAAATAATTTTTTTAAAAT/TTTTTTTAAAAT	GCAACAAGTCAGCAACGGTA	CATGTTTGATTCCATTTTCCA	59.9	675	INTERGENIC	--	--	--
Ca4_P1_7271578	Ca4	7271578	CAAAAAAAAAA/CAAAAAAAAAAA	GTTCGAAATTGCCCTTTTCA	ATCATAGGTTTCGGTGGACG	60.1	151	INTERGENIC	--	--	--
Ca4_P1_7282110	Ca4	7282110	TTATTATTA/TTATTATTAATATTATTA	CCCAACCCAAAAAGATGAGA	ACGGGTTATGCCTCATTTCA	59.9	764	DRR	Ca_03507	--	--
Ca4_P1_7299840	Ca4	7299840	T/TGTG	AGCTCTGTCCTTTGCCCATA	GCATTGATACCGATTCACCA	59.8	276	INTERGENIC	--	--	--
Ca4_P1_7319426	Ca4	7319426	TTATATATATATATATATATATATATATAT/TTATATATATATATATATATAT	TGGGAGTGAATGAAGGGTTC	TTTGAGTGGGCACAAATAAATG	59.9	677	INTERGENIC	--	--	--
Ca4_P1_7388463	Ca4	7388463	CAAAAAAAAAAA/CAAAAAAAAAA	TTTTCTCTAATCCCCGACTCA	TGTTGTTGTCAAACACCTCAAA	58.8	565	INTERGENIC	--	--	--
Ca4_P1_7415395	Ca4	7415395	TAA/TA	TTTCTGTCCTCACCCTCACC	TGTAGGTTTGGACGGTAGGC	60.1	535	INTRON	Ca_03496	F	YABBY
Ca4_P1_7452921	Ca4	7452921	C/CTTATTTG	TGCCAACTCGATTGAACAAG	GCACGCTAAAAGCCTGAAAC	59.8	364	INTERGENIC	--	--	--
Ca4_P1_7555397	Ca4	7555397	AATATG/A	TTAGGAATCGGTTGGGTTTG	GCAACATTTAACGTGTGGCA	59.8	183	INTERGENIC	--	--	--
Ca4_P1_7572773	Ca4	7572773	AC/ACGATTC	AAAATAGCAATGGAGCCGC	TGTTGTCCCGTCTGCAAATA	60.2	930	INTERGENIC	--	--	--
Ca4_P1_7607022	Ca4	7607022	TACACACACAC/TACACACACACAC	CGTGGTCGCAATGAAAGTTA	TCCACGAAGGATACACCACA	59.7	430	DRR	Ca_03474	--	WRKY
Ca4_P1_7620418	Ca4	7620418	GATATATATATAT/GATATATATATATAT	CGATTTTTGTGAAGTGCGTC	TCTCATCGCCGACAAAGTAA	59.3	837	INTERGENIC	--	--	--

INDEL marker IDs	Chromosomes/ unanchored scaffolds	Physical positions (bp)	InDels (*Kabuli* reference genome-CDC Frontier/PI)	Forward primers (5'-3')	Reverse primers (5'-3')	Anneling temperature (0C)	Expected amplified product size (bp)	Structural annotation		Functional annotation	
								Sequence components of *kabuli* genome	*Kabuli* gene accession IDs	NCBI-KOG	TFs
Ca4_P1_7638694	Ca4	7638694	AT/ATTT	TAGGCCATCATCATTTGGGT	TTCGAATCAGGGATAAGACGTT	60.2	896	INTERGENIC	--	--	--
Ca4_P1_7693662	Ca4	7693662	GTATATATATATATATATATATA/GTATATATATATATATATATATA	TCTTTTCTATTTTCATGCACGC	TTTTTGCCGCCACTAAAAGT	59.4	272	INTERGENIC	--	--	--
Ca4_P1_7710147	Ca4	7710147	AATATTATTATATATTA/AATATTATTATATATTATTATATATTA	TCGTTACCCCTCTCGTCATC	ACAGAATGCCTCATTGGTGG	60.1	927	DRR	Ca_03470	R	Nin-like
Ca4_P1_7713544	Ca4	7713544	ATTTTTTTTTT/ATTTTTTTTTTT	GTTCGACCTCGGTCCAATTT	TTTTTGAGATGTTAAAATTCCGATT	61.2	486	INTERGENIC	--	--	--
Ca4_P1_7752166	Ca4	7752166	A/ATTAT	AGCAGATAAGGGGAGGCAAT	TTGACAGTTTTAGTTCATTGTTTTGA	60.1	683	INTERGENIC	--	--	--
Ca4_P1_7783679	Ca4	7783679	GATATATATATATATATATATATATATATATA/GATATATATATATATATATATATATATATATATATATATATATA	GGAATATTCATTTGCATGTTGG	GACAAAACGTCGCACAAAAA	59.2	639	INTERGENIC	--	--	--
Ca4_P1_7938788	Ca4	7938788	CTTTTTTTTT/CTTTTTTTTTT	TGTGTGTCAGTTCTTCGCTACA	CCACATCTCACCACCTTCAA	59.6	235	INTERGENIC	--	--	--
Ca4_P1_8034485	Ca4	8034485	ATTTTTTTTTT/ATTTTTTTTTTT	TGTCGCTTTTTCTCAAAGCA	CGATCCGATAAAACACAGGAG	59.7	400	INTERGENIC	--	--	--
Ca4_P1_8039801	Ca4	8039801	ATAAAATTAAAAAACTAAAATTAAAAAACTAAAA/ATAAAATTAAAAAACTAAAATTAAAAAACTAAAATTAAAAAACTAAAA	TTCGAAAATTGGATTGCCA	TTTGTGCGAGATAGGTACGAAA	60.0	267	INTERGENIC	--	--	--
Ca4_P1_8060069	Ca4	8060069	TACA/TA	GGTGGAACCTTCTCGAATGA	CCACCACCAATGTGACTCTG	60.1	453	INTERGENIC	--	--	--
Ca4_P1_8083441	Ca4	8083441	ATTTTTTTTTTT/ATTTTTTTTTT	GCTAAATAGCACCCACGGTT	ACGAGCAGTAATGCATGGTG	59.1	579	INTERGENIC	--	--	--
Ca4_P1_8084283	Ca4	8084283	CTTATGACACTTGTTATGACACTTGTTA/CTTATGACACTTGTTA	TTAAAATTTCCCACCTCCCC	AATGATGTTTGGTCATGGCA	60.0	327	INTERGENIC	--	--	--

INDEL marker IDs	Chromosomes/ unanchored scaffolds	Physical positions (bp)	InDels (*Kabuli* reference genome-CDC Frontier/PI)	Forward primers (5'-3')	Reverse primers (5'-3')	Anneling temperature (0C)	Expected amplified product size (bp)	Structural annotation		Functional annotation	
								Sequence components of *kabuli* genome	*Kabuli* gene accession IDs	NCBI-KOG	TFs
Ca4_P1_8115754	Ca4	8115754	CATATATATATATATATATATATATA/CATATATATATATATATATATATA	GCTGCTAGGGATGCTGTTTC	GAATATTGGCCAAGGCTTCA	60.0	208	INTRON	Ca_03421	--	M-type
Ca4_P1_8169108	Ca4	8169108	T/TG	TAGCCCCCATTCAGTGTAGG	TCAACTGGAGGTGCCATAAA	59.9	413	INTERGENIC	--	--	--
Ca4_P1_8169426	Ca4	8169426	GTCGATC/GTC	GACATCCGAAATGGGCTAAA	ATTTCCAAGGTCACGGTTTG	59.9	907	INTERGENIC	--	--	--
Ca4_P1_8169626	Ca4	8169626	TAAATAAAAA/TAAATAAAAAATAAAAA	TATGGCACCTCCAGTTGAAA	TGTTAAATCCTCCAGACGGC	59.1	858	INTERGENIC	--	--	--
Ca4_P1_8170291	Ca4	8170291	G/GCTCA	GCCGTCTGGAGGATTTAACA	CACCGTGAGGTAGAAGAATTGA	60.1	604	INTERGENIC	--	--	--
Ca4_P1_8171890	Ca4	8171890	GT/GTTTAAT	TCAATTTGGTCTTTCCGCTC	ACCTACGTTTGTTGCCGAAC	60.2	572	INTERGENIC	--	--	--
Ca4_P1_8173142	Ca4	8173142	TAAAAAAAAA/TAAAAAAAA	TCCATCACACAATTGAAGGC	TGAATGTATCGCGTTGTTGG	59.5	569	INTERGENIC	--	--	--
Ca4_P1_8181721	Ca4	8181721	AATTTAATGAGTG/A	TTACCTTAGCCTCGGAACGA	ATCAAGGGCTTGAAGTCGAA	59.8	672	INTERGENIC	--	--	--
Ca4_P1_8181769	Ca4	8181769	CAAAAAA/CAAAAA	GTTACTGCCGGGAGAAATGA	ATCAAGGGCTTGAAGTCGAA	60.1	412	INTERGENIC	--	--	--
Ca4_P1_8183765	Ca4	8183765	TA/TAA	AACTCAATCCAACCAAGCCA	TCACCACACCAACCTCAAAA	60.5	548	INTERGENIC	--	--	--
Ca4_P1_8183910	Ca4	8183910	TAAA/TAA	AACTCAATCCAACCAAGCCA	TCACCACACCAACCTCAAAA	60.5	548	INTERGENIC	--	--	--
Ca4_P1_8183976	Ca4	8183976	TAAAAAAA/TAAAAAAAA	AACTCAATCCAACCAAGCCA	TCACCACACCAACCTCAAAA	60.5	548	INTERGENIC	--	--	--

INDEL marker IDs	Chromosomes/ unanchored scaffolds	Physical positions (bp)	InDels (*Kabuli* reference genome-CDC Frontier/PI)	Forward primers (5'-3')	Reverse primers (5'-3')	Anneling temperature (0C)	Expected amplified product size (bp)	Structural annotation		Functional annotation	
								Sequence components of *kabuli* genome	*Kabuli* gene accession IDs	NCBI-KOG	TFs
Ca4_P1_8186089	Ca4	8186089	ATTT/ATTTT	TCTCTATTCGAAGTGCGGAGA	CGTTTTGGTTTTTGCTGGAC	60.1	773	DRR	Ca_08308	T	--
Ca4_P1_8186328	Ca4	8186328	CTATATATATATATA/CTATATATATATA	TGCTTTTCATTTGCAGAATCC	TTTCACCTCCTGGTAGACGG	60.2	626	DRR	Ca_08308	T	--
Ca4_P1_8187438	Ca4	8187438	CATATAT/CATAT	CTAAGCCGAGCAGAAGCAAT	TCCCTTTAATGCAAAGGTGG	59.8	363	INTRON	Ca_08308	T	--
Ca4_P1_8187730	Ca4	8187730	TCC/TC	CTAAGCCGAGCAGAAGCAAT	TCCCTTTAATGCAAAGGTGG	59.8	499	INTRON	Ca_08308	T	--
Ca4_P1_8189663	Ca4	8189663	C/CGG	TGAGCATATCGGAACATGGA	TTATTTTCTCCGGTCAACGC	60.0	580	INTRON	Ca_08308	T	--
Ca4_P1_8192071	Ca4	8192071	GAAAAAAAAAAAAA/GAAAAAAAAAA	GAATGCGTATTCAAGGGTCTAA	TTTTTGACGTTGCGTTTTCC	58.3	240	DRR	Ca_08309	R	--
Ca4_P1_8193266	Ca4	8193266	TAAAAAAAAA/TAAAAAAAAAA	GCATCTCTCCAAGATGTCTGC	TGGGAATTGGGAGCTATCAG	60.0	620	DRR	Ca_08309	R	--
Ca4_P1_8201203	Ca4	8201203	GAAAAAAAAA/GAAAAAAAA	CATCGAAAATTATTTGGCTGAA	ATTGCAATCAACGTGGCTCT	59.1	332	INTERGENIC	--	--	--
Ca4_P1_8202902	Ca4	8202902	TC/T	CGGGGCTTTATGAAAAACAA	CCGATTTCTATCCTTCCACG	59.9	521	DRR	Ca_08310	T	MYB_related
Ca4_P1_8206232	Ca4	8206232	GTATA/GTATACTATA	CAAGGGGTTGAAGTTGCATT	TTGAAGCCACCCTCTGATCT	60.0	763	INTERGENIC	--	--	--
Ca4_P1_8207849	Ca4	8207849	CT/CTTAT	TTTGGTCAAATCAGATGGCTC	TCAGCTTTCCAAAGACATGC	60.1	954	INTERGENIC	--	--	--
Ca4_P1_8208745	Ca4	8208745	AAC/AACAC	TAGCGAAATTCAAGGATGGG	TGAAGCTCTTCACCTTCGGT	60.0	186	INTERGENIC	--	--	--

INDEL marker IDs	Chromosomes/ unanchored scaffolds	Physical positions (bp)	InDels (*Kabuli* reference genome-CDC Frontier/PI)	Forward primers (5'-3')	Reverse primers (5'-3')	Anneling temperature (0C)	Expected amplified product size (bp)	Structural annotation		Functional annotation	
								Sequence components of *kabuli* genome	*Kabuli* gene accession IDs	NCBI-KOG	TFs
Ca4_P1_8209835	Ca4	8209835	CTTTTTTTT/CTTTTTTT	AGACCAAGAGAGAGAG GCCC	GGGTTTGGTTTGTGC GTAGT	60.0	306	INTERGENIC	--	--	--
Ca4_P1_8210483	Ca4	8210483	GATAT/GATATAT	GGCCAGGAAATCAACT TCAA	ATGAATTGCATGCATG GTGA	60.1	439	INTERGENIC	--	--	--
Ca4_P1_8214967	Ca4	8214967	TT/TTGT	TCCTAACTCGTTGAGT AAGAAATCA	CAATTGACGCAAAGGA AACA	58.6	855	INTERGENIC	--	--	--
Ca4_P1_8216116	Ca4	8216116	ATTTTTTT/ATTTTTT	TCAAAATGCACGAGAT TAGCA	CGAAGCGGATTCGAA CTAAG	59.5	745	INTERGENIC	--	--	--
Ca4_P1_8216534	Ca4	8216534	GTT/GTTT	CGAATCCGCTTCGTGT TAAT	CGACCCGCTTAATATG TGGT	60.1	507	INTERGENIC	--	--	--
Ca4_P1_8216710	Ca4	8216710	T/TG	CGAATCCGCTTCGTGT TAAT	CGACCCGCTTAATATG TGGT	60.1	507	INTERGENIC	--	--	--
Ca4_P1_8216816	Ca4	8216816	CGG/CGGG	CGAATCCGCTTCGTGT TAAT	CGACCCGCTTAATATG TGGT	60.1	507	INTERGENIC	--	--	--
Ca4_P1_8220449	Ca4	8220449	GATTATTT/GATTATTTAATTATTT	CGGTGAAGCAACGTAG AACA	ACAGTTTTGGGTGCAA AAGG	59.9	688	DRR	Ca_08312	--	--
Ca4_P1_8220978	Ca4	8220978	ATTTT/ATTTTT	CCTTTTGCACCCAAAA CTGT	CGTGTTTCCCTCCTTG TGTT	60.0	404	DRR	Ca_08311	T	bHLH
Ca4_P1_8221070	Ca4	8221070	TTT/TTTATT	CCTTTTGCACCCAAAA CTGT	CGTGTTTCCCTCCTTG TGTT	60.0	404	DRR	Ca_08312	--	--
Ca4_P1_8221604	Ca4	8221604	TAAAAAA/TATTAAAAAAAA	AACACAAGGAGGGAAA CACG	CAAGAAACTGCCAATG CAAA	60.0	644	DRR	Ca_08311	T	bHLH
Ca4_P1_8221744	Ca4	8221744	AATATATAT/AATATATATAT	AGCCACCATGACTTTT CCAC	CAAGAAACTGCCAATG CAAA	60.0	533	DRR	Ca_08312	--	--

INDEL marker IDs	Chromosomes/ unanchored scaffolds	Physical positions (bp)	InDels (*Kabuli* reference genome-CDC Frontier/PI)	Forward primers (5'-3')	Reverse primers (5'-3')	Anneling temperature (0C)	Expected amplified product size (bp)	Structural annotation		Functional annotation	
								Sequence components of *kabuli* genome	*Kabuli* gene accession IDs	NCBI-KOG	TFs
Ca4_P1_8221810	Ca4	8221810	ATAT/A	CACCACAAAGATAGACGATCCA	CAAGAAACTGCCAATGCAAA	60.0	516	DRR	Ca_08312	--	--
Ca4_P1_8222692	Ca4	8222692	A/AC	TTCTGTCTTTGTTGTTGCGG	TGCTAAGGATTATGCAGGGG	59.9	483	INTRON	Ca_08312	--	--
Ca4_P1_8223032	Ca4	8223032	GAAAAAAAA/GAAAAAAA	CAACCTTTGGTGTTGGCTTT	ACGCGATAATCCAATCGAAC	60.0	252	INTRON	Ca_08312	--	--
Ca4_P1_8223617	Ca4	8223617	GAAAAAA/GAAAAAAA	GTTCGATTGGATTATCGCGT	GATACACTGCGTGCCAACTG	59.9	771	INTRON	Ca_08312	--	--
Ca4_P1_8224184	Ca4	8224184	AT/ATT	CAGTTGGCACGCAGTGTATC	TTGGGATTGCAAGATGTGAA	60.3	714	INTRON	Ca_08312	--	--
Ca4_P1_8226617	Ca4	8226617	TG/T	TATATGTGTTGCTGTGGGGG	GTACACCAAGGCGATTCGAT	59.3	969	INTERGENIC	--	--	--
Ca4_P1_8229365	Ca4	8229365	T/TTTAA	GCGAGAAGGTCGTGAGGTAG	TGCCTTTGAGCACATAGTGG	60.0	430	INTERGENIC	--	--	--
Ca4_P1_8232418	Ca4	8232418	TAT/TATAAT	CAGTATGGACGAATGGGAGG	GGTCTACTCTCGAACCACCG	60.3	563	INTERGENIC	--	--	--
Ca4_P1_8232626	Ca4	8232626	T/TC	CAGTATGGACGAATGGGAGG	GGTCTACTCTCGAACCACCG	60.3	563	INTERGENIC	--	--	--
Ca4_P1_8232812	Ca4	8232812	ATTT/ATTTT	CGGTGGTTCGAGAGTAGACC	CAATGATTGAGTCAGTTATGCCA	59.7	538	DRR	Ca_08313	--	--
Ca4_P1_8239700	Ca4	8239700	GTTTTTTT/GTTTTTTTT	TGTCCAATTTGAGGAGGTTG	CATGGTCATAACCTCACTAATGC	58.5	822	INTERGENIC	--	--	--
Ca4_P1_8240019	Ca4	8240019	TTTTTATTTAATTTTATTT/TTTTTATTT	CGTGGAATATGATAAAATAAATCGAA	CCAACCCATTAACATTTGCC	59.6	313	INTERGENIC	--	--	--

INDEL marker IDs	Chromosomes/ unanchored scaffolds	Physical positions (bp)	InDels (*Kabuli* reference genome-CDC Frontier/PI)	Forward primers (5'-3')	Reverse primers (5'-3')	Anneling temperature (0C)	Expected amplified product size (bp)	Structural annotation		Functional annotation	
								Sequence components of *kabuli* genome	*Kabuli* gene accession IDs	NCBI-KOG	TFs
Ca4_P1_8241602	Ca4	8241602	A/AAAG	TCGTCATATTTTTCTTCGCTCA	TAATAAGAGGTGGATGGCCG	59.9	268	INTERGENIC	--	--	--
Ca4_P1_8241700	Ca4	8241700	GTTTTT/GTTTT	TTCCTCATGTGATTTAAGCCC	TAATAAGAGGTGGATGGCCG	59.0	152	INTERGENIC	--	--	--
Ca4_P1_8266422	Ca4	8266422	CAAAA/CAAA	GCAAAGAGCAATTGGGTTGT	AACAATGCCTTCCTCAATGG	60.1	559	INTRON	Ca_08315	S	--
Ca4_P1_8280157	Ca4	8280157	AT/ATT	ACCATCAACTTGCGAAAACC	TCCAAATTAAAATGGCCCAA	60.0	517	INTERGENIC	--	--	--
Ca4_P1_8281041	Ca4	8281041	ATTTTTTT/ATTTTTTTT	TTTGGTGTCATTCGCCTTTT	TCGCAAGGATAATGGCTTTT	60.5	709	INTERGENIC	--	--	--
Ca4_P1_8281328	Ca4	8281328	CA/CCTAA	TTGGTGTCATTCGCCTTTTT	CAATCGCAAGGATAATGGCT	60.5	711	INTERGENIC	--	--	--
Ca4_P1_8283564	Ca4	8283564	GTTTTTTTTT/GTTTTTTTT	TTGGTTACAATAAACGAACACAAAA	GATTGTTCCACTCCGATGCT	59.8	757	INTERGENIC	--	--	--
Ca4_P1_8293335	Ca4	8293335	AT/AATT	CAATGGTTTTGCTTTTGGCT	TTCTCGAATGCGAAGAGTCA	60.1	492	INTERGENIC	--	--	--
Ca4_P1_8293495	Ca4	8293495	TA/TAAA	ACAATGGTTTTGCTTTTGGC	GGTCATTTCACTGCCAACCT	60.0	729	INTERGENIC	--	--	--
Ca4_P1_8295749	Ca4	8295749	ATTTTTATT/ATTTTTATTATTTTTTATT	TCAACCCTCATTCAACCACA	CCTGCGACAATCTGAAGGAT	59.9	840	INTERGENIC	--	--	--
Ca4_P1_8300206	Ca4	8300206	CTATTAACAATATTAACAAT/CTATTAACAAT	GCCAATTTGCCTGTGTTTTT	ACAAATGAAACGCAGCTTCC	60.0	979	DRR	Ca_08318	S	--
Ca4_P1_8300267	Ca4	8300267	TATAAAA/TATAAAATAAAA	GCTTCTTGGGCATTGATTGT	ACAAATGAAACGCAGCTTCC	60.1	605	DRR	Ca_08319	S	--

INDEL marker IDs	Chromosomes/ unanchored scaffolds	Physical positions (bp)	InDels (*Kabuli* reference genome-CDC Frontier/PI)	Forward primers (5'-3')	Reverse primers (5'-3')	Anneling temperature (0C)	Expected amplified product size (bp)	Structural annotation		Functional annotation	
								Sequence components of *kabuli* genome	*Kabuli* gene accession IDs	NCBI-KOG	TFs
Ca4_P1_8305739	Ca4	8305739	TAAA/TAA	CATCATGCAGTCGCCTCTAA	ACCACATCTGCCATCAATCA	60.0	510	INTRON	Ca_08319	S	--
Ca4_P1_8305796	Ca4	8305796	TTACTACCATACTACCAT/TTACTACCATACTACCATACTACCAT	CATCATGCAGTCGCCTCTAA	ACCACATCTGCCATCAATCA	60.0	510	INTRON	Ca_08319	S	--
Ca4_P1_8305998	Ca4	8305998	GAAA/GAA	CATCATGCAGTCGCCTCTAA	ACCACATCTGCCATCAATCA	60.0	510	INTRON	Ca_08319	S	--
Ca4_P1_8308551	Ca4	8308551	TAA/TAAA	TAACAGGTGAGGGCATGTCA	TATGCAAACCCAATTGACCA	60.1	321	INTERGENIC	--	--	--
Ca4_P1_8309234	Ca4	8309234	TCAACACAA/TCAACACAACACAA	GAATTTAAGAGGAAGGCGATGA	TTCACCCTAATTCGTAGGCG	59.7	450	INTERGENIC	--	--	--
Ca4_P1_8309328	Ca4	8309328	TTGGTGGT/TTGGT	CATCCCAGGATCAACACTCA	TTCACCCTAATTCGTAGGCG	59.5	272	INTERGENIC	--	--	--
Ca4_P1_8310925	Ca4	8310925	CAAAAA/CAAAAAA	CATGTGTGGGACTCTTTGGA	CTCAACACGGTTCTGCTCTG	59.5	764	INTERGENIC	--	--	--
Ca4_P1_8311787	Ca4	8311787	A/AG	TTACTCCCTCCTTCCCATGA	ATGGATGGGGGATAAAAAGG	59.5	433	INTERGENIC	--	--	--
Ca4_P1_8311844	Ca4	8311844	TG/T	TTACTCCCTCCTTCCCATGA	ATGGATGGGGGATAAAAAGG	59.5	433	INTERGENIC	--	--	--
Ca4_P1_8315320	Ca4	8315320	CCAAAGGCAAAGGCAAA/CCAAAGGCAAAGGCAAAGGCAAAGGCAAA	CCTTTACAAAAAGTGCCCCA	ATAAACAGCATCACGGGTCC	60.0	287	INTERGENIC	--	--	--
Ca4_P1_8315933	Ca4	8315933	CTT/CTTATT	GGACCCGTGATGCTGTTTAT	TCTCGGTTGATGTCATTATGCT	59.8	586	INTERGENIC	--	--	--
Ca4_P1_8316304	Ca4	8316304	GTTTTTTT/GTTTTTTTT	GCATAATGACATCAACCGAGAG	AAAAGAAATGGTTGTTTCTCGTTC	59.6	235	INTERGENIC	--	--	--

INDEL marker IDs	Chromosomes/ unanchored scaffolds	Physical positions (bp)	InDels (*Kabuli* reference genome-CDC Frontier/PI)	Forward primers (5'-3')	Reverse primers (5'-3')	Anneling temperature (0C)	Expected amplified product size (bp)	Structural annotation		Functional annotation	
								Sequence components of *kabuli* genome	*Kabuli* gene accession IDs	NCBI-KOG	TFs
Ca4_P1_8316423	Ca4	8316423	TAAAAAAA/TAAAAAA	GCATAATGACATCAACCGAGAG	CACGAGTGACACGCCATTTA	59.6	794	INTERGENIC	--	--	--
Ca4_P1_8316722	Ca4	8316722	ACTCT/ACT	GAACGAGAAACAACCATTTCTTTT	CACGAGTGACACGCCATTTA	59.9	583	INTERGENIC	--	--	--
Ca4_P1_8317987	Ca4	8317987	AAAATGACAA/AAA	GCATGGTAGCCAAAGAAAGC	TTCCGTTAAACTCAACTTCCG	59.9	758	INTERGENIC	--	--	--
Ca4_P1_8318067	Ca4	8318067	T/TA	GCATGGTAGCCAAAGAAAGC	TTCCGTTAAACTCAACTTCCG	59.9	758	INTERGENIC	--	--	--
Ca4_P1_8318141	Ca4	8318141	GTTTTTTT/GTTTTTTTTT	GCATGGTAGCCAAAGAAAGC	TTCCGTTAAACTCAACTTCCG	59.9	758	INTERGENIC	--	--	--
Ca4_P1_8318393	Ca4	8318393	TG/T	TGCTTTGAAAGTAAGGCACG	TAAAGGACCAAGTCGGGAAA	59.1	831	INTERGENIC	--	--	--
Ca4_P1_8319580	Ca4	8319580	CGTGTGT/CGT	TGTGACGTTTTTGTTGTGGTG	TTATATGGATGGGGGCAAAG	60.5	756	INTERGENIC	--	--	--
Ca4_P1_8320095	Ca4	8320095	AG/A	CTTTGCCCCCATCCATATAA	TGCACATCGGTGCTATCATT	59.6	333	INTERGENIC	--	--	--
Ca4_P1_8320682	Ca4	8320682	AAGAGAGA/AAGA	AAAATCTTCCGGTGTCTCCA	TTTTCCCAAGTTGGAAGTGG	59.5	668	INTERGENIC	--	--	--
Ca4_P1_8320715	Ca4	8320715	TAAAAAA/TAAAAA	GAATCATGGTTGAACCCAAAA	TTTTCCCAAGTTGGAAGTGG	59.7	200	INTERGENIC	--	--	--
Ca4_P1_8325707	Ca4	8325707	TA/T	ACGGGGCTGTTACTTTACCC	TTCTCAATGGGTGCGTACAA	60.2	739	INTERGENIC	--	--	--
Ca4_P1_8326843	Ca4	8326843	TTCT/TTCTCT	TAAAGAGCACGAATTGCACG	CCAATTCCAAAACCCATACG	60.0	601	INTERGENIC	--	--	--

INDEL marker IDs	Chromosomes/ unanchored scaffolds	Physical positions (bp)	InDels (*Kabuli* reference genome-CDC Frontier/PI)	Forward primers (5'-3')	Reverse primers (5'-3')	Anneling temperature (0C)	Expected amplified product size (bp)	Structural annotation		Functional annotation	
								Sequence components of *kabuli* genome	*Kabuli* gene accession IDs	NCBI-KOG	TFs
Ca4_P1_8329617	Ca4	8329617	T/TTTG	TGGTGATGTAAGCCAGGTGA	CTTCGTCGCCTTCAAGTTTC	60.1	476	INTERGENIC	--	--	--
Ca4_P1_8331477	Ca4	8331477	TTA/TTATA	AAGGGTATGCTGCCATGAAC	TGCTGCTACTGGCAAAAATG	60.0	666	DRR	Ca_08320	--	--
Ca4_P1_8333108	Ca4	8333108	GAAA/GAAAA	TCCCACTTTCATGTCTCCAA	TGATCACCTTTGAAAAGCCC	59.1	147	INTERGENIC	--	--	--
Ca4_P1_8333443	Ca4	8333443	GCTTCTTC/GCTTC	TCAAAGGTGATCATGGATGG	ACCCTTGCCAAAACACTACG	59.3	593	INTERGENIC	--	--	--
Ca4_P1_8333830	Ca4	8333830	ATTTTTTTTTT/ATTTTTTTTTTT	ACGTAGTGTTTTGGCAAGGG	TCACACATCACCGCAAACTT	60.0	342	INTERGENIC	--	--	--
Ca4_P1_8342865	Ca4	8342865	T/TTAAG	GAGCTTCAACCACAAGCACA	GCTTTTTGAGCAACTCGTCC	60.0	691	CDS (FRAME SHIFT)	Ca_08321	--	--
Ca4_P1_8345936	Ca4	8345936	AAAATCAC/AAAATCACTAAATCAC	CATACCTCGATGCACCTCCT	TGTGCGACCAAAGGTTACAA	60.1	213	DRR	Ca_08322	--	--
Ca4_P1_8349969	Ca4	8349969	CAAAAAAAAAAAAAA/CAAAAAAA	AGCCCAGAAAACAGTCCTCA	CTGATTCCTTCCATTTCGGA	59.8	839	DRR	Ca_08323	--	--
Ca4_P1_8350510	Ca4	8350510	ATTTTTTTTT/ATTTTTTTT	TCCGAAATGGAAGGAATCAG	CCCTTGATAACACCAACGCT	60.0	226	DRR	Ca_08323	--	--
Ca4_P1_8354913	Ca4	8354913	ATTTTTTTTTT/ATTTTTTTT	TTGATTTGGGGGCTTTGATA	GGAGCAGTTGTCCCATTTGT	60.3	765	DRR	Ca_08324	T	bZIP
Ca4_P1_8372871	Ca4	8372871	T/TG	TCTTTTTCTGCCCCACTAGC	CAGCTGAGTTGTGAGGTCCA	59.5	150	INTERGENIC	--	--	--
Ca4_P1_8373181	Ca4	8373181	TACA/TACACA	GGCAAAATGGACCTCACAAC	GGAGAGTCGGTCTTTGTGGA	60.4	706	INTERGENIC	--	--	--

INDEL marker IDs	Chromosomes/ unanchored scaffolds	Physical positions (bp)	InDels (*Kabuli* reference genome-CDC Frontier/PI)	Forward primers (5'-3')	Reverse primers (5'-3')	Anneling temperature (0C)	Expected amplified product size (bp)	Structural annotation		Functional annotation	
								Sequence components of *kabuli* genome	*Kabuli* gene accession IDs	NCBI-KOG	TFs
Ca4_P1_8373217	Ca4	8373217	TAAA/TAAAA	GGCAAAATGGACCTCACAAC	GGAGAGTCGGTCTTTGTGGA	60.4	706	INTERGENIC	--	--	--
Ca4_P1_8373461	Ca4	8373461	AAC/AACCAC	TGAGCATAAAGTGGCATTGG	GTCGGTCTTTGTGGAGAGGA	59.7	398	INTERGENIC	--	--	--
Ca4_P1_8373669	Ca4	8373669	TAGAAA/TAGAAAGAAA	TGAATTGGATCCTCTCCACC	AAGCCGAGTCTGGCCTATTT	59.9	680	INTERGENIC	--	--	--
Ca4_P1_8375280	Ca4	8375280	CTATA/CTATATA	AAGCCGTGTGGTACATACTCTTA	CCCTCACCAAGAGAAGTTCG	57.9	209	INTERGENIC	--	--	--
Ca4_P1_8376162	Ca4	8376162	CT/CTT	TCGGGCTTGAGCTCTATCAT	TGGCCGAAATTGAGAGAATC	59.9	541	INTERGENIC	--	--	--
Ca4_P1_8376866	Ca4	8376866	TAAAAAA/TAAAAAAA	GATTCTCTCAATTTCGGCCA	TGCCCCAAACACGTACACTA	60.2	575	DRR	Ca_08327	KL	--
Ca4_P1_8377254	Ca4	8377254	ATTTTTTTTTTT/ATTTTTTTTTTTTT	CCACATATATCTGCCCCACC	TGACTGTGTGCACTGAAACAA	60.0	563	DRR	Ca_08327	KL	--
Ca4_P1_8377411	Ca4	8377411	AT/ATATATATATT	TAGTGTACGTGTTTGGGGCA	GGGTGAGACTGAAGCTGGTC	60.0	949	DRR	Ca_08327	KL	--
Ca4_P1_8377585	Ca4	8377585	ATTT/ATT	TTCGTCCATTTGAATCCTGA	CCTGCAAGCACTTACGACAA	59.0	891	DRR	Ca_08327	KL	--
Ca4_P1_8379420	Ca4	8379420	TCGTGCG/T	GGAAGGAATGAATCCCACAA	CTGTTTTGCAGCATGGCTAA	59.7	376	INTRON	Ca_08327	KL	--
Ca4_P1_8381452	Ca4	8381452	TAAAAAAA/TAAAAAAAA	CAATCCGTTAAGGACCTGGA	GATGATTCCCTTGCTGGAAA	59.9	388	INTRON	Ca_08327	KL	--
Ca4_P1_8382072	Ca4	8382072	AT/ATTT	TTTCCAGCAAGGGAATCATC	GCATTCAAATCTGGGTTGGT	60.0	534	INTRON	Ca_08327	KL	--

INDEL marker IDs	Chromosomes/ unanchored scaffolds	Physical positions (bp)	InDels (*Kabuli* reference genome-CDC Frontier/PI)	Forward primers (5'-3')	Reverse primers (5'-3')	Anneling temperature (0C)	Expected amplified product size (bp)	Structural annotation		Functional annotation	
								Sequence components of *kabuli* genome	*Kabuli* gene accession IDs	NCBI-KOG	TFs
Ca4_P1_8384923	Ca4	8384923	AGGGGGG/AGGGGG	GCCGCAATAGTGTTTTTGGT	GCGTATCGGTTGGAATTGTT	60.0	807	INTRON	Ca_08327	KL	--
Ca4_P1_8387348	Ca4	8387348	TAAGAAGA/TAAGA	CATTGCCTGATTTTCCCAAT	GGGCTTTGAACCCGATAAAT	59.8	460	INTRON	Ca_08327	KL	--
Ca4_P1_8389681	Ca4	8389681	CATA/CA	CGGCAAACATGAAGATCTGA	CGTGGCTTAACCTTGGACAT	59.8	312	INTRON	Ca_08327	KL	--
Ca4_P1_8390760	Ca4	8390760	CG/CGG	TCCCTAATCAGAACCATCGC	CGTCGCTATTCTGACTCCCT	60.0	238	INTERGENIC	--	--	--
Ca4_P1_8391171	Ca4	8391171	TAC/TACAC	AGGGAGTCAGAATAGCGACG	CGGTGAAAATTTGTCTGACG	59.5	564	INTERGENIC	--	--	--
Ca4_P1_8396183	Ca4	8396183	T/TG	TTTACAACACATTGCGCACC	CGTTTATTGACGGAAGGCAT	60.6	681	DRR	Ca_08329	O	B3
Ca4_P1_8400811	Ca4	8400811	GAATTACTAAT/GAATTACTAATTACTAAT	CTTAAGCATCAAAGCCCGAG	CCTCAATTGCCACCAAAACT	60.0	469	INTRON	Ca_08329	O	B3
Ca4_P1_8400838	Ca4	8400838	CAAAAAAAA/CAAAAAAAAAA	CTTAAGCATCAAAGCCCGAG	CCTCAATTGCCACCAAAACT	60.0	469	INTRON	Ca_08329	O	B3
Ca4_P1_8401394	Ca4	8401394	GTT/GT	AGTTTTGGTGGCAATTGAGG	AACCACTACGGAACATTTGACA	60.0	742	INTERGENIC	--	--	--
Ca4_P1_8408536	Ca4	8408536	GCCC/GCCCC	CGCTGAAACAATTGCAGAAA	GGGAGGTCCACTCAAAGTGA	60.0	450	INTERGENIC	--	--	--
Ca4_P1_8408879	Ca4	8408879	TAAAA/TAAAAA	TCACTTTGAGTGGACCTCCC	TCCTACAAAATCACGGACACC	60.1	469	INTERGENIC	--	--	--
Ca4_P1_8409110	Ca4	8409110	GC/GCC	TCACTTTGAGTGGACCTCCC	TCCTACAAAATCACGGACACC	60.1	469	INTERGENIC	--	--	--

INDEL marker IDs	Chromosomes/ unanchored scaffolds	Physical positions (bp)	InDels (*Kabuli* reference genome-CDC Frontier/PI)	Forward primers (5'-3')	Reverse primers (5'-3')	Anneling temperature (0C)	Expected amplified product size (bp)	Structural annotation		Functional annotation	
								Sequence components of *kabuli* genome	*Kabuli* gene accession IDs	NCBI-KOG	TFs
Ca4_P1_8410839	Ca4	8410839	GATATA/GATA	AAGTACAGTGGCGGATCCAT	ATAGCGTAAGGGCACATGCT	59.4	206	INTERGENIC	--	--	--
Ca4_P1_8413582	Ca4	8413582	AGTTGT/AGT	AATGGTGAACAAGGAGCAGG	CAAGATGTGCCGAACGTGTA	60.1	279	INTERGENIC	--	--	--
Ca4_P1_8413764	Ca4	8413764	TA/T	AATGGTGAACAAGGAGCAGG	TCAAATATTAACTCCAAGCGCA	60.1	714	INTERGENIC	--	--	--
Ca4_P1_8414642	Ca4	8414642	GTTTTTTTTTT/GTTTTTTTTT	TCTTGGTCAGAACCATGTGC	AAATGGCCAAATTGCATGAT	59.7	616	INTERGENIC	--	--	--
Ca4_P1_8422191	Ca4	8422191	GAAA/GAA	AAGACGGATGCATTTTGCTC	GACACATGCCCAACTGAATG	60.2	841	INTERGENIC	--	--	--
Ca4_P1_8441953	Ca4	8441953	GTTTGTA/GTTTGTATTTTGTA	AGCAGGAGAGCTGAGATTGG	TACTGTGTATCGGGGCAACA	59.7	731	INTRON	Ca_08335	R	--
Ca4_P1_8450126	Ca4	8450126	TA/T	TTGAAGGGTGGAAATTCAGC	GCACTTATGTGGGCCAATCT	60.1	208	DRR	Ca_08336	--	--
Ca4_P1_8458330	Ca4	8458330	A/ATC	GAGTTCTTTGAAGGGCTCACA	TCCCCTACCCAAAATTGGAA	59.5	569	INTERGENIC	--	--	--
Ca4_P1_8458477	Ca4	8458477	ATTTTTTT/ATTTTTTTT	GAGTTCTTTGAAGGGCTCACA	TCCCCTACCCAAAATTGGAA	59.5	569	INTERGENIC	--	--	--
Ca4_P1_8458644	Ca4	8458644	ATTTT/ATTT	GCCACATTTGGATGTTCACC	TCCCCTACCCAAAATTGGAA	61.2	185	INTERGENIC	--	--	--
Ca4_P1_8459775	Ca4	8459775	G/GA	TGGACCACAGGTCTACTACCAA	TGTACCACTTTTGGTGACCG	59.5	260	INTERGENIC	--	--	--
Ca4_P1_8459845	Ca4	8459845	ATTTT/ATTTTT	TGGACCACAGGTCTACTACCAA	TGTACCACTTTTGGTGACCG	59.5	260	INTERGENIC	--	--	--

INDEL marker IDs	Chromosomes/ unanchored scaffolds	Physical positions (bp)	InDels (*Kabuli* reference genome-CDC Frontier/PI)	Forward primers (5'-3')	Reverse primers (5'-3')	Anneling temperature (0C)	Expected amplified product size (bp)	Structural annotation		Functional annotation	
								Sequence components of *kabuli* genome	*Kabuli* gene accession IDs	NCBI-KOG	TFs
Ca4_P1_8462210	Ca4	8462210	T/TTGTTTAGA	TCCGTGCACTTATTTTTCCC	ATTTCCAGCCAAGTGAGGTG	59.9	471	INTERGENIC	--	--	--
Ca4_P1_8467235	Ca4	8467235	CCATCATCATCATC/CCATCATCATC	GGCAAGCATTCAATTTCCAT	GGTTGGGATGTTATGTTCGG	59.9	449	INTERGENIC	--	--	--
Ca4_P1_8468674	Ca4	8468674	AGATTC/A	CCGAACATAACATCCCAACC	AGAACCCCTCAAATCGGAGT	60.1	851	INTERGENIC	--	--	--
Ca4_P1_8471441	Ca4	8471441	TA/TAA	TCTCCAACACCATTGGTTCA	TGTATTGGCGCAAGTTGAAG	59.9	727	DRR	Ca_08339	G	--
Ca4_P1_8475153	Ca4	8475153	ATTT/ATT	TGGACCGTACATTTTCGTGA	GTGGTAGCAGAAGGAGCAGG	60.0	829	INTRON	Ca_08339	G	--
Ca4_P1_8478290	Ca4	8478290	A/AT	TTTAGCTTGCCAAAACAGCA	CCTTTCTTCGTACGTCTCGC	59.6	607	INTRON	Ca_08339	G	--
Ca4_P1_8481188	Ca4	8481188	ATCTCTCTCTCTCTCT/ATCTCTCTCTCTCT	AGGGAAAAGTACCGGCAAAT	ATAGATCCAAACGGCACAGC	59.8	463	INTERGENIC	--	--	--
Ca4_P1_8482100	Ca4	8482100	T/TGTTGAA	GGTTTTGTGCGCTTTTCAAT	TGACTCTCCTCCTCTCCTCAA	60.1	403	DRR	Ca_08340	R	--
Ca4_P1_8482919	Ca4	8482919	TAAAAAAAAAAA/TAAAAAAAAAAAAAA	ATTTCGAGGAAAAGGGAGGA	TTTCAAGAGGGGCAATTGAG	60.0	515	DRR	Ca_08340	R	--
Ca4_P1_8483192	Ca4	8483192	A/ACT	ATTTCGAGGAAAAGGGAGGA	GACCCTATCAGAGTGGCAGC	60.0	886	DRR	Ca_08340	R	--
Ca4_P1_8483262	Ca4	8483262	TAAAAA/TAAA	CAATTGCCCCTCTTGAAAAA	TTTCTTGCAGGCATGTTGTC	60.0	518	DRR	Ca_08340	R	--
Ca4_P1_8483876	Ca4	8483876	TG/T	CAACAGAAGGCAATGAGCAA	AATTGAAGGCGTATTCGGTG	60.0	125	INTRON	Ca_08340	R	--

INDEL marker IDs	Chromosomes/ unanchored scaffolds	Physical positions (bp)	InDels (*Kabuli* reference genome-CDC Frontier/PI)	Forward primers (5'-3')	Reverse primers (5'-3')	Anneling temperature (0C)	Expected amplified product size (bp)	Structural annotation		Functional annotation	
								Sequence components of *kabuli* genome	*Kabuli* gene accession IDs	NCBI-KOG	TFs
Ca4_P1_8489479	Ca4	8489479	CACATACA/CACA	CCCAAATCACACACAT GAGC	AGAGTTGTTTCATCTC CAAACG	60.0	415	INTERGENIC	--	--	--
Ca4_P1_8490694	Ca4	8490694	TTATATATATATATA/TTATATATATATA TATATATA	TATGCCTTAGCGGTTT TTGG	GGTCCTTTGATCCCAC GTTA	60.1	498	INTERGENIC	--	--	--
Ca4_P1_8494464	Ca4	8494464	CAAAAAA/CAAAAAAA	TGGTGATGAAGAAGTG ATGGT	TCACAATGGGAACTGC AAAA	58.0	601	INTRON	Ca_08341	--	CPP
Ca4_P1_8497257	Ca4	8497257	TAAACTGAAGAAACT/TAAACTGAAGA AACTGAAGAAACT	AGCGTGAGAGGAACCT GTGT	GACTGGCAACAGCAAT TTGA	59.9	564	INTRON	Ca_08341	--	CPP
Ca4_P1_8502983	Ca4	8502983	AACGAC/AAC	CCAGTCAACTTTGCCC ATTT	ACCTCCAAATTTTCCC TTGG	60.0	533	INTERGENIC	--	--	--
Ca4_P1_8506656	Ca4	8506656	GACACACA/GACACA	GAGTGCCCAAGGACAA CCTA	CCTCTAGCAATTCCCA TCCA	60.1	414	INTRON	Ca_08343	YU	--
Ca4_P1_8509176	Ca4	8509176	CTTTT/CTT	GCCTTCTGGACAAAAC GAAG	TCTGCAGGTGAAGCAA CATC	59.9	578	INTRON	Ca_08343	YU	--
Ca4_P1_8509778	Ca4	8509778	TCTT/TCTTACTT	CCTTCGGCTTCAACTC AGTC	CCGCTGCTAAATTTCT ACGC	60.0	188	INTRON	Ca_08343	YU	--
Ca4_P1_8514326	Ca4	8514326	CTTTT/CTTTTT	TGTCGTGCGGTAAGTT TGAC	GAAGCAAAGCTAGGG GTGTG	59.8	449	DRR	Ca_08343	YU	--
Ca4_P1_8514977	Ca4	8514977	G/GT	CACACCCCTAGCTTTG CTTC	CACGAACGCCTGTGAA CTTA	59.9	547	DRR	Ca_08343	YU	--
Ca4_P1_8521020	Ca4	8521020	CATA/CATATATA	AATTCAACCAAATAAAT TCCCA	CACGCAAAATGAAAGG TTGA	57.4	308	INTERGENIC	--	--	--
Ca4_P1_8521823	Ca4	8521823	AACA/AA	AATTTGGCAAAAGGAG ATGC	AATAGGGGGACCAAAA CTGC	59.2	816	INTERGENIC	--	--	--

INDEL marker IDs	Chromosomes/ unanchored scaffolds	Physical positions (bp)	InDels (*Kabuli* reference genome-CDC Frontier/PI)	Forward primers (5'-3')	Reverse primers (5'-3')	Anneling temperature (0C)	Expected amplified product size (bp)	Structural annotation		Functional annotation	
								Sequence components of *kabuli* genome	*Kabuli* gene accession IDs	NCBI-KOG	TFs
Ca4_P1_8522151	Ca4	8522151	TG/T	TGTGATTGTTAGGATGATGACG	CCAAAACTGGGGAGAAACAA	58.5	288	INTERGENIC	--	--	--
Ca4_P1_8522296	Ca4	8522296	GT/GTT	GCAGTTTTGGTCCCCCTATT	GCAGTTTTGGTCCCCCTATT	60.2	328	INTERGENIC	--	--	--
Ca4_P1_8522801	Ca4	8522801	ATTTTTT/ATTTTTTT	TTGTTTCTCCCCAGTTTTGG	TATTCCACTCGCCCTTTCAC	59.9	896	INTERGENIC	--	--	--
Ca4_P1_8523011	Ca4	8523011	AAGA/AA	AATAGGGGGACCAAAACTGC	TATTCCACTCGCCCTTTCAC	60.2	638	INTERGENIC	--	--	--
Ca4_P1_8570714	Ca4	8570714	TAGAGAGAGAGAGAGAGAGAGAGAGA/TAGAGAGAGAGAGAGAGAGAGAGAGAGA	ACATTGATTGCACTGGGACA	CTGGAGGCGTAAAAAGCAAG	60.0	791	INTRON	Ca_08352	--	--
Ca4_P1_8573690	Ca4	8573690	CTTTTT/CTTTT	AAAAAGTGATGTGTGGCCCT	TCGATTTTCGGGTCTAATGTG	59.5	285	INTERGENIC	--	--	--
Ca4_P1_8576946	Ca4	8576946	GTTTT/GTTTTT	TGTCATAAGAAACAATTGGACG	TTGCAGACTAACCCAGATCG	57.7	440	INTERGENIC	--	--	--
Ca4_P1_8595450	Ca4	8595450	AAAGAAGAAGAAGAAGAAGAA/AAAGAAGAAGAAGAAGAA	CACAACACAGCTGCTACCAAA	GGTGGTCAAAGGAACCAAGA	60.0	267	INTERGENIC	--	--	--
Ca4_P1_8612545	Ca4	8612545	AA/AAATTAAAGGTGTA	TTTCTTTCTGCTGTCGGCTT	CATGCCATCCACTATGTTGC	60.1	393	INTERGENIC	--	--	--
Ca4_P1_8612629	Ca4	8612629	T/TTA	TTTCTTTCTGCTGTCGGCTT	CATGCCATCCACTATGTTGC	60.1	393	INTERGENIC	--	--	--
Ca4_P1_8612851	Ca4	8612851	CATT/CATTATTTGATATATTTTAATT	TTTGATGGGTACAGTGCTCG	CCTAAAGGGAAAACTAAAATGAAAGA	59.7	488	INTERGENIC	--	--	--
Ca4_P1_8613689	Ca4	8613689	TTTTTCT/TT	TGCATTTGGTTGAAAATGAAA	TGGTGGGCCTACGAGTAAAG	59.0	374	INTERGENIC	--	--	--

INDEL marker IDs	Chromosomes/ unanchored scaffolds	Physical positions (bp)	InDels (*Kabuli* reference genome-CDC Frontier/PI)	Forward primers (5'-3')	Reverse primers (5'-3')	Anneling temperature (0C)	Expected amplified product size (bp)	Structural annotation		Functional annotation	
								Sequence components of *kabuli* genome	*Kabuli* gene accession IDs	NCBI-KOG	TFs
Ca4_P1_8614446	Ca4	8614446	AAATTAAT/AAAT	GCAATTTTAACATAGACGACACG	TGCCAAAAACATTTTATCCAAA	58.8	383	INTERGENIC	--	--	--
Ca4_P1_8616583	Ca4	8616583	GATGT/GATGTATGT	GCGAGAATACAATTTCCAGCA	ACTCGATCGTGGAAGCAAGT	60.2	324	DRR	Ca_08355	O	--
Ca4_P1_8621480	Ca4	8621480	GAAAAAAAA/GAAAAAAAAA	GTGCATGACCCCTTCTTGTT	TGTCAGGCAACAGAAAACCA	60.0	889	INTERGENIC	--	--	--
Ca4_P1_8626887	Ca4	8626887	GTTTTTTT/GTTTTTTTT	CTCCCTCCCCTTCATTCTTC	CATCAGTAGCAGGTGGAGCA	60.0	378	INTERGENIC	--	--	--
Ca4_P1_8633518	Ca4	8633518	ATAAAAAAATT/AT	GCACGAAAAATTGTGAGCAA	ATGGGCATATGGGTCAATGT	59.9	171	INTERGENIC	--	--	--
Ca4_P1_8633576	Ca4	8633576	TAAAA/TAAA	TTGCACGAAAAATTGTGAGC	TAAATGGACGGAGTTGGGAA	59.9	527	INTERGENIC	--	--	--
Ca4_P1_8638452	Ca4	8638452	AACACACACACACACACAC/AACACACACACACACACACACACACAC	GGCAATGTCCAAACAACACA	ACCTTTTTAGGTAGCGGCGT	60.4	534	INTERGENIC	--	--	--
Ca4_P1_8639278	Ca4	8639278	AACCAC/AAC	TGAAGCCAGAGCAAAATGTG	GGCAATGCTTTCTTTTCTGC	60.0	243	CDS (large-effect mutations)	Ca_08357	--	--
Ca4_P1_8649594	Ca4	8649594	ATTTTTTTTTTT/ATTTTTTTTTT	CAGATTGACAAACTCGGGGT	CAAATCCCACTCAAAGGGAA	60.0	411	DRR	Ca_08358	--	G2-like
Ca4_P1_8686225	Ca4	8686225	TG/TGG	CAGAAATCAATGCTGAGGCA	GTTGAAGCGTGTTGGGTTTT	59.9	590	DRR	Ca_08364	T	C3H
Ca4_P1_8688868	Ca4	8688868	CTA/C	AGGACCCACAACATCCACAT	ACCAATCCATGCACGCTACT	60.1	505	INTERGENIC	--	--	--
Ca4_P1_8689007	Ca4	8689007	A/AT	AGGACCCACAACATCCACAT	ACCAATCCATGCACGCTACT	60.1	505	INTERGENIC	--	--	--

INDEL marker IDs	Chromosomes/ unanchored scaffolds	Physical positions (bp)	InDels (*Kabuli* reference genome-CDC Frontier/PI)	Forward primers (5'-3')	Reverse primers (5'-3')	Anneling temperature (0C)	Expected amplified product size (bp)	Structural annotation		Functional annotation	
								Sequence components of *kabuli* genome	*Kabuli* gene accession IDs	NCBI-KOG	TFs
Ca4_P1_8693633	Ca4	8693633	CTTTTTTTTTT/CTTTTTTTTTTT	ATTTCAGTTTGCTGGGGTTG	TTGCCCAAATATGACCCATT	60.0	162	INTRON	Ca_08365	G	--
Ca4_P1_8698891	Ca4	8698891	AAGAGTAGTAGAGTAGT/AAGAGTAGT	AAGCTGGCCACTGTAAGCAT	TCACAATCTCGACAACCCAA	59.9	246	INTRON	Ca_08365	G	--
Ca4_P1_8704241	Ca4	8704241	G/GC	TTTGTACCCAACAGGGCATT	ATGCATGCGTTGACAATGTT	60.2	569	DRR	Ca_08366	J	NAC
Ca4_P1_8705159	Ca4	8705159	GTT/GTTT	TCAATGGTGGAGCAAATTCA	GGTTCCCTTCCCCAATTTAG	60.0	399	DRR	Ca_08366	J	NAC
Ca4_P1_8706314	Ca4	8706314	CAAAAA/CAAAA	GATAGTCGCAGGTGCAGTCA	AAGTTGTCCTGCCTTTTTCG	60.0	150	INTERGENIC	--	--	--
Ca4_P1_8706854	Ca4	8706854	TAAAAA/TAAAAAA	CGAATTACGAAAAAGGCAGG	ATCTCTCCGACCATGTGGAA	59.7	935	INTERGENIC	--	--	--
Ca4_P1_8708208	Ca4	8708208	GTT/GTTTT	TCACTGCAATCAACCGCTTA	GCCAGTCGCTAATTTTTAGCTC	60.4	472	INTERGENIC	--	--	--
Ca4_P1_8708260	Ca4	8708260	AATT/A	TCACTGCAATCAACCGCTTA	TCAAACGATTTTATCATTTCTTTCAA	60.4	917	INTERGENIC	--	--	--
Ca4_P1_8708395	Ca4	8708395	GTTTTTTT/GTTTTTTTT	CCCATATGACCCCAAGTAGC	CCTAGCTTCCCACTCTTTCTTTT	59.3	807	INTERGENIC	--	--	--
Ca4_P1_8709451	Ca4	8709451	TAGATTCGAAAAGTTGTGAA/TA	AATTTGTGGGTGGGCATAAA	GGAGTATCCACACCTTACAGCA	60.1	608	INTERGENIC	--	--	--
Ca4_P1_8709656	Ca4	8709656	CAAAAAAA/CAAAAAAAA	AATTTGTGGGTGGGCATAAA	TGCATTGAGAGAATCACCAACT	60.1	701	INTERGENIC	--	--	--
Ca4_P1_8709676	Ca4	8709676	TG/T	AATTTGTGGGTGGGCATAAA	TGCATTGAGAGAATCACCAACT	60.1	701	INTERGENIC	--	--	--

INDEL marker IDs	Chromosomes/ unanchored scaffolds	Physical positions (bp)	InDels (*Kabuli* reference genome-CDC Frontier/PI)	Forward primers (5'-3')	Reverse primers (5'-3')	Anneling temperature (0C)	Expected amplified product size (bp)	Structural annotation		Functional annotation	
								Sequence components of *kabuli* genome	*Kabuli* gene accession IDs	NCBI-KOG	TFs
Ca4_P1_8711163	Ca4	8711163	CTTTTTTTTTTT/CTTTTTTTTT	CGCACCACTCTCATCACCTA	CTTTGCCAACCAAACCTTGT	59.9	826	INTERGENIC	--	--	--
Ca4_P1_8711907	Ca4	8711907	CTCTCTTTCTCTTTCT/CTCTCTTTCTCTTTCTCTTTCT	CAAGGTTTGGTTGGCAAAGT	GGGGGTGTTTCCATGTTATG	60.0	429	INTERGENIC	--	--	--
Ca4_P1_8712181	Ca4	8712181	TCAACAACAACAACAACAACAACAA/TCAACAACAACAACAACAACAACAACAA	ACTGCAGAGGGAGATGGAGA	CCCTTGATTGTCCTTGTGCT	59.9	190	CDS (large-effect mutations)	Ca_08367	R	C2H2
Ca4_P1_8720139	Ca4	8720139	AAAGT/AAAGTTTAAGT	GGCTGATGGAGAATTCCAAA	ACCAAAGACCAGATTGGCAC	60.0	860	INTRON	Ca_08368	Q	--
Ca4_P1_8723077	Ca4	8723077	T/TC	GCGTGTTTGGTTGAAAGGAT	GGTCGAGTTCCAAATTCCAA	60.0	619	INTERGENIC	--	--	--
Ca4_P1_8723541	Ca4	8723541	ATTTTTTTTTTTTTT/ATTTTTTTTTTT	TTGGAATTTGGAACTCGACC	AAGCAGGTGCCCTGAGACTA	59.9	429	INTERGENIC	--	--	--
Ca4_P1_8724004	Ca4	8724004	AT/A	TAGTCTCAGGGCACCTGCTT	TGCCTCTAAATACGTTGGACTG	60.0	546	INTERGENIC	--	--	--
Ca4_P1_8727978	Ca4	8727978	A/AG	TGAATGCCCAAAATAAGATGA	TGATTGAAAGAGCGTTGGATT	58.1	651	INTERGENIC	--	--	--
Ca4_P1_8730605	Ca4	8730605	CGTG/CTG	TTCACACCAACACCCTCAAA	ATTTTGGAAGAACAATCCGC	60.0	517	INTERGENIC	--	--	--
Ca4_P1_8730686	Ca4	8730686	TAA/TA	TTCACACCAACACCCTCAAA	ATTTTGGAAGAACAATCCGC	60.0	517	INTERGENIC	--	--	--
Ca4_P1_8733054	Ca4	8733054	TGGG/TGG	AAAAATCACCCAAAATTAACATCT	CACTTTCCTTGCTCCCTCTG	57.2	867	INTERGENIC	--	--	--
Ca4_P1_8734909	Ca4	8734909	TAAAAAAAAAAA/TAAAAAAAA	ACTTCCTTCCACATGACCCA	AATTGGGACCGTGAGTTCAG	60.4	410	DRR	Ca_08369	K	M-type

INDEL marker IDs	Chromosomes/ unanchored scaffolds	Physical positions (bp)	InDels (*Kabuli* reference genome-CDC Frontier/PI)	Forward primers (5'-3')	Reverse primers (5'-3')	Anneling temperature (0C)	Expected amplified product size (bp)	Structural annotation		Functional annotation	
								Sequence components of *kabuli* genome	*Kabuli* gene accession IDs	NCBI-KOG	TFs
Ca4_P1_8736441	Ca4	8736441	G/GATATTAGTATATC	TGTAAATGTCTCTCGCCGTG	CAGAGGTGCACATGGAATCA	59.9	598	INTRON	Ca_08370	R	WOX
Ca4_P1_8742911	Ca4	8742911	AAT/A	AGGTGCTCCACCAAAATCAG	CCACATCTTAATGTTTTGTTTGGT	60.1	547	INTERGENIC	--	--	--
Ca4_P1_8748201	Ca4	8748201	AG/A	CATTTCAATCATTCACCCCC	TCACAACATGAGAATGGGACA	60.0	218	INTERGENIC	--	--	--
Ca4_P1_8748360	Ca4	8748360	ATTTTTTT/ATTTTTT	CATTTCAATCATTCACCCCC	CAAAATTGCATTCAAAGTCCC	60.0	621	INTERGENIC	--	--	--
Ca4_P1_8748930	Ca4	8748930	TAAAAAAA/TAA	GGGACTTTGAATGCAATTTTGT	CGAGGATCTGTGACCAATCA	60.2	482	INTERGENIC	--	--	--
Ca4_P1_8749252	Ca4	8749252	T/TTA	ATACGGATTGGATGAGGTGC	GGCGTAATTACTTCAAGGCAA	59.8	376	INTERGENIC	--	--	--
Ca4_P1_8751297	Ca4	8751297	CAAAA/CAAA	ATGGATGGGAAGTATGCCAA	GTTTTCCACAAACCTTGCGT	60.2	591	INTERGENIC	--	--	--
Ca4_P1_8753314	Ca4	8753314	TGCTGGAACGCTGGAAC/TGCTGGAAC	TTCTTTCCCAGTCATTTCGG	CCAAATGGAGCTTCCACCTA	60.0	841	INTRON	Ca_08371	--	NAC
Ca4_P1_8753834	Ca4	8753834	TAA/TA	GGTGGAAGCTCCATTTGGTA	TTGCCACTTATTCACGGTCA	59.9	539	INTRON	Ca_08371	--	NAC
Ca4_P1_8754248	Ca4	8754248	T/TCGTAGATGATG	CATGGAAAATTTGCGATCCT	TAAGAAGGAACTGCGCCACT	59.9	139	INTRON	Ca_08371	--	NAC
Ca4_P1_8757256	Ca4	8757256	CATATA/CATATATA	TGGATATGGTGCAGATACGAA	AGGTGCATGAGTATCAATGTCAA	59.0	595	DRR	Ca_08371	--	NAC
Ca4_P1_8758495	Ca4	8758495	A/AG	TCACCATTCGTGAGTTGGAA	TGCTCATGTCTTCTAAAGATCCA	60.1	762	INTERGENIC	--	--	--

INDEL marker IDs	Chromosomes/ unanchored scaffolds	Physical positions (bp)	InDels (*Kabuli* reference genome-CDC Frontier/PI)	Forward primers (5'-3')	Reverse primers (5'-3')	Anneling temperature (0C)	Expected amplified product size (bp)	Structural annotation		Functional annotation	
								Sequence components of *kabuli* genome	*Kabuli* gene accession IDs	NCBI-KOG	TFs
Ca4_P1_8762153	Ca4	8762153	ATTTTT/ATTTT	TCATAAAGCTGTTGCCAAAAA	ATAGAGTGGGGTGTTGCCAG	58.5	546	INTERGENIC	--	--	--
Ca4_P1_8765633	Ca4	8765633	G/GATTCTAA	GGGTGAAATTGTAGCGGACT	ATGAATGGAGCTCCCTGATG	59.1	868	INTERGENIC	--	--	--
Ca4_P1_8774388	Ca4	8774388	TAAAAAA/TAAA	GACGATTGCGAATTTGAGGT	TGATTTATGTTTGTGGCGGA	60.1	295	DRR	Ca_08373	C	--
Ca4_P1_8775620	Ca4	8775620	CCTT/CCTTACTT	ACACATTGATCGAGACCCGT	GCAGATTTGGAAGATCACACC	60.4	702	DRR	Ca_08373	C	--
Ca4_P1_8787589	Ca4	8787589	TA/T	CCAAGGGTTTTCCTTCACAA	ATCAATCAAAATGGGTGGGA	59.9	436	INTRON	Ca_08375	R	NF-YA
Ca4_P1_8787727	Ca4	8787727	GTCCCACT/GTCCCACTTTCTTCCCACT	ATCCCACCCATTTTGATTGA	GGTACAGCAACAGCTGCAAA	60.0	107	INTRON	Ca_08375	R	NF-YA
Ca4_P1_8788652	Ca4	8788652	A/ATCTGCTTCC	CAGGGCAACCTGTTTCAAAT	TGGAATGGTCTCAACCACAA	60.0	624	INTRON	Ca_08375	R	NF-YA
Ca4_P1_8788897	Ca4	8788897	GAAAAAAA/GAAAAAA	TTGTGGTTGAGACCATTCCA	TTGACCAACATTCACCCTCA	59.9	219	INTRON	Ca_08375	R	NF-YA
Ca4_P1_8793043	Ca4	8793043	TAAAAAAAAAAA/TAAAAAAAAAAAAAA	GGAAATTCGAAATTGGCAGA	ACCCTCCATTCACTTGTTGC	60.0	632	INTERGENIC	--	--	--
Ca4_P1_8795970	Ca4	8795970	TAGAGAGAGAGAGAGAGA/TAGAGAGAGAGAGAGA	AACACGTGATATTCCAACGG	GTAGCCCATGCCCTCATAAA	58.4	756	DRR	Ca_08376	--	M-type
Ca4_P1_8797573	Ca4	8797573	TAAAAAA/TAAAAA	TTATAAAAGTGCGAGTGCGG	AAACGTCATTAGCTATAAGATTCCA	59.0	595	INTERGENIC	--	--	--
Ca4_P1_8797898	Ca4	8797898	ATTTT/ATTTTTTT	TTTTACGGTGGATTATCTATCAATG	CTTAAAACGCGATTGTTGGC	58.5	852	INTERGENIC	--	--	--

INDEL marker IDs	Chromosomes/ unanchored scaffolds	Physical positions (bp)	InDels (*Kabuli* reference genome-CDC Frontier/PI)	Forward primers (5'-3')	Reverse primers (5'-3')	Anneling temperature (0C)	Expected amplified product size (bp)	Structural annotation		Functional annotation	
								Sequence components of *kabuli* genome	*Kabuli* gene accession IDs	NCBI-KOG	TFs
Ca4_P1_8798276	Ca4	8798276	CATATATATATA/CATATATATATATATA	CGGTGGGAAACTAGGAGTGA	AACGAGTAGCCTTTAGCGGG	60.1	177	INTERGENIC	--	--	--
Ca4_P1_8799497	Ca4	8799497	TAATAGAGTTAAAAA/TAA	TCAAAGTGCGATACAGGAAGC	TGGGTTTAATACATGGAGATGG	60.4	922	INTERGENIC	--	--	--
Ca4_P1_8800004	Ca4	8800004	A/AT	TGATCGAGTTGCATCATACAAAA	TGTGATTTGATGGCTTTATTGG	60.5	377	INTERGENIC	--	--	--
Ca4_P1_8803000	Ca4	8803000	GTTT/GTT	TATACGACACACCTTGCCCA	AGGATATTGACATCCGCTGG	60.0	293	DRR	Ca_08377	--	--
Ca4_P1_8805450	Ca4	8805450	CTGT/CTGTGT	ATTATGATTCGCCGCAGAAC	CCGACCCCACTTATGAAGAA	60.1	324	INTRON	Ca_08378	AR	MYB
Ca4_P1_8810142	Ca4	8810142	ATTTTTTTT/ATTTTTTT	ACTCCTCCCTCCTGTCTCGT	TGAATATAAATAGACCAATACCGCAA	60.3	585	INTERGENIC	--	--	--
Ca4_P1_8812070	Ca4	8812070	AT/ATGTT	TGGTGCACTTTTTGTGTCCA	TCAAACACACAATAATGGCGA	61.2	648	INTERGENIC	--	--	--
Ca4_P1_8812862	Ca4	8812862	AC/A	GTTTTTAATTCGCACCCCAA	GCAGTTTAAGCAATAAAATGATGC	59.8	427	INTERGENIC	--	--	--
Ca4_P1_8816656	Ca4	8816656	AT/A	ATCGTTGTCGTAGATTGGGC	TTGGATCAAAATCTGAAGCG	60.0	789	INTERGENIC	--	--	--
Ca4_P1_8836055	Ca4	8836055	TATTTTTAATTTT/TATTTTTAATTTTTAATTTT	TGACCTGTAGGGCCGTAATC	TCACCAAATCGAAGCTCAAA	60.0	508	INTERGENIC	--	--	--
Ca4_P1_8836705	Ca4	8836705	TTAAT/TTAATAAT	AGCTTCGATTTGGTGAGGTG	TGATGTATACCACAATGATTCCG	60.3	444	INTERGENIC	--	--	--
Ca4_P1_8836902	Ca4	8836902	T/TG	AGCTTCGATTTGGTGAGGTG	TTGAGTTTCAATATGGTGATGTGA	60.3	854	INTERGENIC	--	--	--

INDEL marker IDs	Chromosomes/ unanchored scaffolds	Physical positions (bp)	InDels (*Kabuli* reference genome-CDC Frontier/PI)	Forward primers (5'-3')	Reverse primers (5'-3')	Anneling temperature (0C)	Expected amplified product size (bp)	Structural annotation		Functional annotation	
								Sequence components of *kabuli* genome	*Kabuli* gene accession IDs	NCBI-KOG	TFs
Ca4_P1_8837106	Ca4	8837106	AA/AAGA	AAATATTTCGGAATCATTGTGG	AATTTGGCCGATAAGCTCTC	57.9	627	INTERGENIC	--	--	--
Ca4_P1_8840379	Ca4	8840379	CTATA/CTA	CCAAAATCGGACAGGCTAAG	TTGTAAAATCATTGTTTGAAGAAAA	59.7	545	INTERGENIC	--	--	--
Ca4_P1_8841401	Ca4	8841401	ATTTTTTTT/ATTTTTTT	TTTGGTGACATTTTAGCAAAGAC	TCTTCTTCATTCACTTGTTTGTCA	58.4	229	INTERGENIC	--	--	--
Ca4_P1_8846872	Ca4	8846872	ATTTTT/ATTTTTT	ACAACGACACACGGTCTCTG	CGCAGAACATAGATGAGCCA	59.8	729	INTRON	Ca_08381	--	--
Ca4_P1_8858467	Ca4	8858467	TTACTAA/TTACTAATATTATAATATACTAA	TTGGTTTCCAAGTTCCCATC	TTCAACTTCGAGCAAAAGGG	59.8	602	INTRON	Ca_08382	--	MIKC
Ca4_P1_8861999	Ca4	8861999	CAAAAAAAA/CAAAAAAAAAAA	CGAGGGAGCATTATGCAACT	TCCTTCGCCTCTCAAGAAAA	60.2	432	DRR	Ca_08383	C	--
Ca4_P1_8864397	Ca4	8864397	A/ATT	TTCACCAAAGCCTCTTGCTT	GTAAAGCTCCCTTTCCCCAC	60.0	657	INTERGENIC	--	--	--
Ca4_P1_8867611	Ca4	8867611	AT/A	AGCTCCCATACCAGGACCTT	GTAGCCACCATTTTCCTCCA	60.0	374	INTERGENIC	--	--	--
Ca4_P1_8878798	Ca4	8878798	TAAAAAAAA/TAAAAAAA	ATGGACGGATTGGATGGATA	ATACGATGGTGGTTGGTGGT	60.0	581	INTERGENIC	--	--	--
Ca4_P1_8879630	Ca4	8879630	AATATATATATATATATA/AATATATATATATATATATATATA	ACCACCAACCACCATCGTAT	TGGATCGTCTCTGTCCCTTT	60.0	477	INTERGENIC	--	--	--
Ca4_P1_8882680	Ca4	8882680	CTCTTCTTCTTCTTCTT/CTCTTCTTCTTCTT	CCAACTGCATTCGACGACTA	TGTGGATCTCTTTATTGCCG	59.9	386	INTERGENIC	--	--	--
Ca4_P1_8882981	Ca4	8882981	TT/TTTAT	GCCTGGCTAAGCAGAAACAA	CAAATTTTACTGGGGGAATGAA	60.5	845	INTERGENIC	--	--	--

INDEL marker IDs	Chromosomes/ unanchored scaffolds	Physical positions (bp)	InDels (*Kabuli* reference genome-CDC Frontier/PI)	Forward primers (5'-3')	Reverse primers (5'-3')	Anneling temperature (0C)	Expected amplified product size (bp)	Structural annotation		Functional annotation	
								Sequence components of *kabuli* genome	*Kabuli* gene accession IDs	NCBI-KOG	TFs
Ca4_P1_8884777	Ca4	8884777	TAAT/TAATATATAAAT	ACACGTGGCTTGTTGGTGTA	GTCGATCTCGCTCACTCCAC	60.1	675	INTERGENIC	--	--	--
Ca4_P1_8884956	Ca4	8884956	ATTTT/ATT	GAGTGCCACAATGTGAACTGA	CGATCTCGCTCACTCCACTC	59.7	595	INTERGENIC	--	--	--
Ca4_P1_8885843	Ca4	8885843	CTGT/CTGTGT	TGAGACCCCGTAAGATCCAA	AAGAATGCAGATCAATCGCC	60.5	194	INTERGENIC	--	--	--
Ca4_P1_8885877	Ca4	8885877	AATATATATATAT/AATATATATAT	TGAGACCCCGTAAGATCCAA	AAGAATGCAGATCAATCGCC	60.5	194	INTERGENIC	--	--	--
Ca4_P1_8889120	Ca4	8889120	ATTT/ATT	TGAATCGAACAGGACATCCA	TTATGGCTGCAACGACTTGT	60.0	409	INTERGENIC	--	--	--
Ca4_P1_8889782	Ca4	8889782	A/AG	TTAAGAAGTATCACGCGGGC	TCCTTTCTCCGTGGTGAGTT	60.2	472	INTERGENIC	--	--	--
Ca4_P1_8890826	Ca4	8890826	CAGGTAG/CAG	TTTGAGGAGTGTGTGGTGGA	ACCCAACTCGCACTTTGAAT	60.1	360	INTERGENIC	--	--	--
Ca4_P1_8891264	Ca4	8891264	AAATTAATTAA/AAATTAA	AATTCAAAGTGCGAGTTGGG	CCCGATGTGTACCCTCTGAT	60.1	753	DRR	Ca_08386	--	--
Ca4_P1_8894308	Ca4	8894308	A/AG	TGATATCCGGTTTGGGTGTT	CTCCCGTTATCCGAAACAGA	60.1	569	INTERGENIC	--	--	--
Ca4_P1_8894416	Ca4	8894416	CTATATATATATAT/CTATATATATATATATATAT	TGATATCCGGTTTGGGTGTT	CTCCCGTTATCCGAAACAGA	60.1	569	INTERGENIC	--	--	--
Ca4_P1_8896251	Ca4	8896251	CAAAAA/CAAAA	GTCGCTAAACTGCAAAAGGC	TTTGTCCGGATGAGTTATCAAA	60.0	477	INTERGENIC	--	--	--
Ca4_P1_8897505	Ca4	8897505	TATT/TATTAATT	GCCATCCAGATGCAAAAGTT	TCATAGACGGTCCGATAGAGC	60.1	356	INTERGENIC	--	--	--

INDEL marker IDs	Chromosomes/ unanchored scaffolds	Physical positions (bp)	InDels (*Kabuli* reference genome-CDC Frontier/PI)	Forward primers (5'-3')	Reverse primers (5'-3')	Anneling temperature (0C)	Expected amplified product size (bp)	Structural annotation		Functional annotation	
								Sequence components of *kabuli* genome	*Kabuli* gene accession IDs	NCBI-KOG	TFs
Ca4_P1_8898244	Ca4	8898244	CA/C	TCGGACCGTCTATGATGTATG	CAAAATTTTCAAACAAAACCCA	58.5	680	INTERGENIC	--	--	--
Ca4_P1_8900032	Ca4	8900032	TCAAAA/TCAAAAATATAAACAAAA	AGACATGAAACATGTGGGCA	GCTTTTCGATCTGGCAACTC	60.0	751	INTERGENIC	--	--	--
Ca4_P1_8900762	Ca4	8900762	TC/T	GAGTTGCCAGATCGAAAAGC	AATCCCCTGTAATGGAGGGT	60.0	518	INTERGENIC	--	--	--
Ca4_P1_8901196	Ca4	8901196	GT/GTTT	CCCTCCATTACAGGGGATTT	AAGCTCATCACCGATCATCC	60.0	813	INTERGENIC	--	--	--
Ca4_P1_8901492	Ca4	8901492	TTTATATGTTATA/T	GCAACAAATTATCTTGCCGTC	AAGCTCATCACCGATCATCC	59.6	334	INTERGENIC	--	--	--
Ca4_P1_8903771	Ca4	8903771	GAATTAAT/GAAT	GGATCTCATTGGTTCTATGGG	CGAGTGTGGTGGGTTTTCTT	58.3	490	INTERGENIC	--	--	--
Ca4_P1_8903859	Ca4	8903859	CATAT/CAT	GGATCTCATTGGTTCTATGGG	CGAGTGTGGTGGGTTTTCTT	58.3	490	INTERGENIC	--	--	--
Ca4_P1_8904444	Ca4	8904444	C/CTCTTA	AAGAAAACCCACCACACTCG	ATGATTTCTGCATTCTCGGC	60.0	865	INTERGENIC	--	--	--
Ca4_P1_8943701	Ca4	8943701	GTTTTTTTTTT/GTTTTTTTTTTT	TTCGTTCATTTATCATTATTTCATTCA	TACAACGACGGACTGCAAAA	60.0	923	INTERGENIC	--	--	--
Ca4_P1_8993223	Ca4	8993223	CATAAATAAATAAATAAATAAATAAATAAA/CATAAATAAATAAATAAATAAATAAA	TCACAAATCACATTCACCCC	GGCGGTTTGACAGTAGGAAA	59.2	314	DRR	Ca_08394	GMW	--
Ca4_P1_9024370	Ca4	9024370	AAATAATAATAATAATAATAATAATAATAATAATAATAATAATAATAATAA/AAATAATAATAATAATAATAATAATAATAATAATAATAATAA	GAATGGGCTTAAGGGTGACA	TCCATTCCTTGAGTTTTCCG	59.9	885	INTERGENIC	--	--	--
Ca4_P1_9028821	Ca4	9028821	TTATATATATATATATATAT/TTATATATATATATATATATAT	ATGGAACAATCGGACACCAT	CTGTCAACTCACTCATCAAATCG	60.1	422	INTERGENIC	--	--	--

INDEL marker IDs	Chromosomes/ unanchored scaffolds	Physical positions (bp)	InDels (*Kabuli* reference genome-CDC Frontier/PI)	Forward primers (5'-3')	Reverse primers (5'-3')	Anneling temperature (0C)	Expected amplified product size (bp)	Structural annotation		Functional annotation	
								Sequence components of *kabuli* genome	*Kabuli* gene accession IDs	NCBI-KOG	TFs
Ca4_P1_9030140	Ca4	9030140	ATTTTTTTTTTT/ATTTTTTTTTTTT	TGGTGTTGGAGTCATGCTTT	AAATTTGCAATGACGGCTTC	59.1	656	INTERGENIC	--	--	--
Ca4_P1_9047358	Ca4	9047358	TTATATATATATATATATATATATATAT/TTATATATATATATATATAT	AGTTTATAACCCGTGCGTCG	TTTTCATCAATAACATTGAGCG	60.0	222	INTERGENIC	--	--	--
Ca4_P1_9047568	Ca4	9047568	CTTTTTTTTTTT/CTTTTTTTTTT	AGTTTATAACCCGTGCGTCG	TCACACAAATTCAAAGGATTCA	60.0	644	INTERGENIC	--	--	--
Ca4_P1_9066423	Ca4	9066423	ACC/AC	GCAACCCGTAGGTGTAGGAA	TGTGCGTTGGTAGTCAATGTG	60.0	546	INTERGENIC	--	--	--
Ca4_P1_9079145	Ca4	9079145	ATTTTTTTTTTT/ATTTTTTTTTT	AGTTGGAAGTCGCCAAAGAA	ATGGTGCAGTTCTTGCCATA	59.9	629	INTERGENIC	--	--	--
Ca4_P1_9130579	Ca4	9130579	CATATATATATATAT/CATATATATATAT	AATTCTGACATGCGAATCCC	TGTAAAGCGCTTCTAAGTCTCG	59.9	589	INTERGENIC	--	--	--
Ca4_P1_9224958	Ca4	9224958	CATATATATATATATATATATA/CATATATATATATATATATATATA	ACGCTGTAAAATGCAAACCC	CTGGGCAAGGGATCTGAATA	60.0	609	DRR	Ca_08413	--	Trihelix
Ca4_P1_9339969	Ca4	9339969	TAAAAAAAAAAAA/TAAAAAAAAAAA	TGAGGCCCAGTTTCTTTCTG	TACACCCACATAGTGGGCAA	60.4	856	INTRON	Ca_08423	Q	E2F/DP
Ca4_P1_9359201	Ca4	9359201	TAAAAAAAAAAA/TAAAAAAAAAA	TCTTTTCCACAATGTAAAGGAGATT	CGCATGCAACGAAATAAATG	59.5	658	INTERGENIC	--	--	--
Ca4_P1_9371255	Ca4	9371255	GAAAAAAAA/GAAAAAAAAA	GGAGCATATGCCTCGTTGTT	TGGAAATTCAACTTTTTGGACA	60.1	549	INTERGENIC	--	--	--
Ca4_P1_9385509	Ca4	9385509	ATTTTTTTTTTTT/ATTTTTTTTTTT	TCGTTCTCCAATAGATTGACCA	ATGTTGCGACATGAACCTCA	59.6	380	INTERGENIC	--	--	--
Ca4_P1_9388140	Ca4	9388140	C/CTTCAT	TCGTGTTGCGCCTAACAAT	AGTTTTTAATCCGTGCGTCG	60.3	976	INTERGENIC	--	--	--

INDEL marker IDs	Chromosomes/ unanchored scaffolds	Physical positions (bp)	InDels (*Kabuli* reference genome-CDC Frontier/PI)	Forward primers (5'-3')	Reverse primers (5'-3')	Anneling temperature (0C)	Expected amplified product size (bp)	Structural annotation		Functional annotation	
								Sequence components of *kabuli* genome	*Kabuli* gene accession IDs	NCBI-KOG	TFs
Ca4_P1_9388557	Ca4	9388557	GATATATATATATATATATA/GATATATATATATATATATATATA	CCACTTATACGGCAAAGGCT	AGTTTTTAATCCGTGCGTCG	59.2	423	INTERGENIC	--	--	--
Ca4_P1_9453892	Ca4	9453892	GTATATATATATATATATATATA/GTATATATATATATATATATATATA	TTGATGGTGCTAGGAGAAGACA	AAACCCACAATTCATCAGGTT	59.9	433	INTERGENIC	--	--	--
Ca4_P1_9460620	Ca4	9460620	ATTTTTTTTT/ATTTTTTTTTT	TTGGTGGAATAAATGCCAAA	TTGCGTGTTTTAAGAATTCGG	58.9	283	INTERGENIC	--	--	--
Ca4_P1_9569592	Ca4	9569592	CTT/C	CAAATGTCGAATTTAAGTATTGCG	TTGTCCTTCTCCCATTGTCC	59.9	355	INTERGENIC	--	--	--
Ca4_P1_9586182	Ca4	9586182	ATTTTTTTTTT/ATTTTTTTTTTT	CCACCTCCTTCTCTTCCTCC	TCACCAAAAATTGAATGCCC	60.2	597	INTERGENIC	--	--	--
Ca4_P1_9630289	Ca4	9630289	ATTAGTTAGTT/ATTAGTTAGTTAGTT	CCTCTTCCAAACAAATCTTCG	CCAGCTCATCAAAGCCATTT	58.8	550	INTERGENIC	--	--	--
Ca4_P1_9632653	Ca4	9632653	TG/T	TGGATTGTCGTGATCTCAAA	CACACCCGTACTCAGCATTG	57.6	630	INTERGENIC	--	--	--
Ca4_P1_9632938	Ca4	9632938	ATTTTTTTT/ATTTTTTT	CAATGCTGAGTACGGGTGTG	AACACAAGGAGGCCATGTTC	60.2	503	INTERGENIC	--	--	--
Ca4_P1_9653112	Ca4	9653112	TAAAAAAAAAA/TAAAAAAAAAAA	GGGTTGTACGATGACAGCAA	AAATCGTCGCAAATCAGTCC	59.6	208	INTERGENIC	--	--	--
Ca4_P1_9654621	Ca4	9654621	AATATATATATATATATATATATATATATAT/AATATATATATATATATATATATATATATATATATATATAT	GAATTAATCCGCACATCCCA	TGAATGCATACAAGGATCTCCA	60.7	483	INTERGENIC	--	--	--
Ca4_P1_9658674	Ca4	9658674	TAAAAAAA/TAAAA	TGCAATTTGATACCGAGCCT	TCCCCTTCTTTGAGGGATTT	60.6	914	INTERGENIC	--	--	--
Ca4_P1_9670676	Ca4	9670676	ATT/ATTT	AACACCCTCATTGCTCATCC	AATGGCAATCAGAAACAGCC	59.9	430	INTRON	Ca_08446	--	--

**INDEL marker IDs**	**Chromosomes/ unanchored scaffolds**	**Physical positions (bp)**	**InDels (*Kabuli* reference genome-CDC Frontier/PI)**	**Forward primers (5'-3')**	**Reverse primers (5'-3')**	**Anneling temperature (0C)**	**Expected amplified product size (bp)**	**Structural annotation**		**Functional annotation**	
								**Sequence components of *kabuli* genome**	***Kabuli* gene accession IDs**	**NCBI-KOG**	**TFs**
Ca4_P1_9722413	Ca4	9722413	TAAAAAAAAAAA/TAAAAAAAAAAAA	TTTTGTTTTTACATCCGCAAA	GGTCGCATGGGTTAAACACT	58.3	167	INTERGENIC	--	--	--
Ca4_P1_9722503	Ca4	9722503	AATATATATATATATATATATATATATATAT/AATATATATATATATATATATATATATATATATATATATATATAT	AGTGTTTAACCCATGCGACC	TGTTTTGCCATTAGATACACCAA	59.9	549	INTERGENIC	--	--	--
Ca4_P1_9732872	Ca4	9732872	A/AC	CGGTGGTTGATAAAGTCGTG	GAATTCAAATGGGGAAATGC	59.0	416	INTERGENIC	--	--	--
Ca4_P1_9754081	Ca4	9754081	ATGT/ATGTTGT	TCACCGTGAAAACGATGAGA	CAAAAGCAAATCCTTTGGGA	60.2	362	INTERGENIC	--	--	--
Ca4_P1_9764739	Ca4	9764739	GTTTTTTT/GTTTTTTTT	TCAGGCGGGTTTTATAGACA	CTCTGCTCACAACCCAACAA	58.2	701	INTERGENIC	--	--	--
Ca4_P1_978893	Ca4	978893	TAAAAAAAAA/TAAAAAAAA	TTTTGAGGTGCTTTCCCATC	CAATCCCCCAAGCTTAACAA	60.1	535	INTERGENIC	--	--	--
Ca4_P1_9827543	Ca4	9827543	GAAAAAAAAAA/GAAAAAAAAA	CGGTCACCGTGTACCACTTT	CAATCTGCCATGTCAATGAA	60.9	332	DRR	Ca_08460	--	--
Ca4_P1_9847676	Ca4	9847676	TAAAA/TAAA	GAGTTGCAGGGGATGTAGGA	CACAAAGAAAGGCCTGGAAG	60.1	240	INTRON	Ca_08462	Z	--
Ca4_P1_9857350	Ca4	9857350	TGTGCGAGTGCGAGTGCGAGT/TGTGCGAGTGCGAGT	TGAGACGTTTTTCACACCCA	CTGCAACAATGAATCTTGAAGT	60.1	469	INTERGENIC	--	--	--
Ca4_P1_988616	Ca4	988616	TGAG/TGAGAG	GCGTTGTCTTCCATCAAGGT	GAAGGATATTGGGAAACGCA	60.1	544	DRR	Ca_07731	R	S1Fa-like
Ca4_P1_988965	Ca4	988965	GA/GAGTGTAGGGAA	TGCGTTTCCCAATATCCTTC	TGGCAGGAAATATAGGGCTG	59.9	802	DRR	Ca_07731	R	S1Fa-like
Ca4_P1_992132	Ca4	992132	TTATATATATATATATATATAT/TTATATATATATATATATATATATAT	CAACCCCTTGAGATACACGA	GGGTTTGCCAAAACAAAGAA	58.6	420	INTERGENIC	--	--	--

INDEL marker IDs	Chromosomes/ unanchored scaffolds	Physical positions (bp)	InDels (*Kabuli* reference genome-CDC Frontier/PI)	Forward primers (5'-3')	Reverse primers (5'-3')	Anneling temperature (0C)	Expected amplified product size (bp)	Structural annotation		Functional annotation	
								Sequence components of *kabuli* genome	*Kabuli* gene accession IDs	NCBI-KOG	TFs
Ca4_P1_9923455	Ca4	9923455	AATATATATATATATATATA/AATATATATATATATATA	TGTGAACGTCGTCGTATGGT	CCGATAACAAAAGCTCTCGC	60.0	892	INTERGENIC	--	--	--
Ca4_P1_9937299	Ca4	9937299	AATATATATATATATATATATATAT/AATATATATATATATATAT	TTCAATGCTTCATCACGAGC	TGACAGCCGTTAGTCTCAAAA	60.0	767	INTERGENIC	--	--	--
Ca4_P1_9966088	Ca4	9966088	TA/T	TGCCCTTTATTAACTAATTCTTGTGA	TTCAAACTATTTGATGATGTGACACT	59.5	594	INTERGENIC	--	--	--
Ca5_P1_10001330	Ca5	10001330	ATG/A	CCAATTTTCCAAGAACACCC	TGCAGTTTTGATCTCCGTTG	59.3	415	INTERGENIC	--	--	--
Ca5_P1_10004188	Ca5	10004188	GTTGTTGTTATTGT/GTTGT	TCGCCATTTCCAACTACTCC	AATTTCCTCCTCCCACCAGT	60.1	703	INTERGENIC	--	--	--
Ca5_P1_10016204	Ca5	10016204	ATTTT/ATTT	TTTTGGAACCCAATTTTTAATTTT	ACATGTCACGCCTGATCAAA	59.1	773	INTERGENIC	--	--	--
Ca5_P1_10019391	Ca5	10019391	TTTT/TTTTCATTT	TGCATTGGTGGTGAAACTGT	GCATCAAAATGAAGGAGGGA	60.0	591	INTERGENIC	--	--	--
Ca5_P1_10021365	Ca5	10021365	A/AG	CGGCCACAATTTACATGACA	GTGGATGGACAAAAGGATGG	60.4	192	INTERGENIC	--	--	--
Ca5_P1_10023073	Ca5	10023073	A/AT	TGTGCCTAACGAGAATGGTG	CCACTTCCGTTCCCTGTTTA	59.7	386	INTERGENIC	--	--	--
Ca5_P1_10024689	Ca5	10024689	TAAA/TAAAA	GGGTCTTTAGGCCCTCTTGT	ACCTCGTGTGACTGATGATGA	59.6	565	INTERGENIC	--	--	--
Ca5_P1_10025602	Ca5	10025602	TAAAAAAAA/TAAAAAAA	TGAGTGCTCGACGACATTTT	AGACCTGTTGATTGTGTTTTGG	59.4	700	INTERGENIC	--	--	--
Ca5_P1_10028140	Ca5	10028140	ATTT/ATTTT	CAAAGATATTGCACATGCCG	AAAAGCGTTTCCTTTTAACCG	60.1	649	DRR	Ca_20513	--	--

INDEL marker IDs	Chromosomes/ unanchored scaffolds	Physical positions (bp)	InDels (*Kabuli* reference genome-CDC Frontier/PI)	Forward primers (5'-3')	Reverse primers (5'-3')	Anneling temperature (0C)	Expected amplified product size (bp)	Structural annotation		Functional annotation	
								Sequence components of *kabuli* genome	*Kabuli* gene accession IDs	NCBI-KOG	TFs
Ca5_P1_10028201	Ca5	10028201	TGAAG/T	TGGAACTTTCCATATT GGTTCA	TGGATAAAAGACGGAG TCACG	59.3	777	DRR	Ca_20513	--	--
Ca5_P1_10029431	Ca5	10029431	ATTT/ATTTT	AAAAATGCCCTTAGTTA CACACAA	GAGACATTGGCAAGGT TGGT	59.1	513	DRR	Ca_20513	--	--
Ca5_P1_10047959	Ca5	10047959	GAAAAA/GAAAA	TGGTAATCAACATGTA CCATCCA	TGCGATTATTTGTAAG GGGA	60.0	443	INTERGENIC	--	--	--
Ca5_P1_10048221	Ca5	10048221	AAAA/AAAATAAA	TCCCCTTACAAATAATC GCA	TTGCACTCTCACCATA TCACAA	58.1	493	INTERGENIC	--	--	--
Ca5_P1_10054215	Ca5	10054215	ACTCT/ACTCTCT	TTGCCCAACTTGATGA AACA	GCCATAGCACCTCTGT AGGC	60.1	531	DRR	Ca_20515	--	--
Ca5_P1_10054925	Ca5	10054925	AAT/AATAT	TATCCAGTTGGCGCTC TTTT	GGCACGATAGCAATGT AGCA	59.8	701	DRR	Ca_20515	--	--
Ca5_P1_10061262	Ca5	10061262	CAAAAAA/CAAAAAAA	TTGCTCAATTCCCAATA GAAAA	TACCTTTTAGCGCCAC CATT	58.7	650	INTERGENIC	--	--	--
Ca5_P1_10076657	Ca5	10076657	CT/CTAT	GGACGGGTTGAAACTT GAAA	GCTCCACCCACACTTT CATT	59.9	689	DRR	Ca_20517	I	--
Ca5_P1_10076985	Ca5	10076985	GATTTT/GATTTTAATATATTTT	TTGATATTTATCCGCC CTCG	ATTGCTCCACCCACAC TTTC	59.9	218	DRR	Ca_20517	I	--
Ca5_P1_10080488	Ca5	10080488	AAAGAAGAAGAAGAAGAAGAAGAAGA AGAA/AAAGAAGAAGAAGAAGAAGAA GAA	CAATGAAGGTGAAGAT GGCA	CCACGTTCATACACAC TCCG	59.6	801	CDS (large-effect mutations)	Ca_20518	K	MYB_related
Ca5_P1_10084501	Ca5	10084501	CTTGTTTTTGTTTTT/CTTGTTTTTGTT TTTGTTTTT	TTTTTGCCATTCCCATC ATT	TCATGAAAATCATAAG CCACG	60.1	603	INTERGENIC	--	--	--
Ca5_P1_10084594	Ca5	10084594	TGGTCTCTCAATTGAA/T	TGGCAAAGTCCTCTTG AAGG	TCATGAAAATCATAAG CCACG	60.4	386	INTERGENIC	--	--	--

INDEL marker IDs	Chromosomes/ unanchored scaffolds	Physical positions (bp)	InDels (*Kabuli* reference genome-CDC Frontier/PI)	Forward primers (5'-3')	Reverse primers (5'-3')	Anneling temperature (0C)	Expected amplified product size (bp)	Structural annotation		Functional annotation	
								Sequence components of *kabuli* genome	*Kabuli* gene accession IDs	NCBI-KOG	TFs
Ca5_P1_10121872	Ca5	10121872	TATT/TATTAATT	TTGCACTTTAGGCCATTTTTG	TTTATTGGCACAAAATAATCGTT	60.1	432	INTERGENIC	--	--	--
Ca5_P1_10124059	Ca5	10124059	TTTAATTAATTA/TTTAATTA	CCCACATTGACCTGACCTTT	TTTGTGGAGTTGTTTCAACCAT	59.8	309	INTERGENIC	--	--	--
Ca5_P1_10125443	Ca5	10125443	TTCTCTCTCTCTCTCTCTCTCTCTC/TTCTCTCTCTCTCTCTC	TTTTGGTTCTTGCTCAGCCT	CTTCAACCCAACCCCTTTTT	60.0	593	INTERGENIC	--	--	--
Ca5_P1_10125497	Ca5	10125497	AATTATTATTATTATTATT/AATTATTATTATTATTATTATT	TTTTGGTTCTTGCTCAGCCT	GCACTCGAATTGTGATCCCT	60.0	643	INTERGENIC	--	--	--
Ca5_P1_10127629	Ca5	10127629	AG/AGGG	CTAGTGAGCCGAAGGTGAGG	GCCGCTGAATTCTCTGGTAG	60.0	377	INTRON	Ca_20519	--	--
Ca5_P1_1012832	Ca5	1012832	GTTTTTTTTT/GTTTTTTTTTT	GATCCTAAGGCAGATGAGGG	TGCCATTCTTGAGTTGCTTG	58.7	871	INTERGENIC	--	--	--
Ca5_P1_10129472	Ca5	10129472	TAA/TAAA	TGAGGCAGACCAGGAAAACT	CTCACAAAGGGGGAATGAGA	59.8	265	INTRON	Ca_20519	--	--
Ca5_P1_10131612	Ca5	10131612	ATTTTGA/ATTTTGAAGAGTTTTGA	AATTAGACGACACGGGGTTG	GAGCCTCTTCATTTTTCACCTT	59.9	485	DRR	Ca_20519	--	--
Ca5_P1_10131669	Ca5	10131669	TAAAAAAAAAAA/TAAAAAAAAAAAAAA	AATTAGACGACACGGGGTTG	CGTATCAATTTCTTTTCAAATGG	59.9	635	DRR	Ca_20519	--	--
Ca5_P1_10132207	Ca5	10132207	CTTTTTT/CTTTTT	TCCATTTGAAAAGAAATTGATACG	TCATCAACGGTCATTCAACC	59.4	737	DRR	Ca_20519	--	--
Ca5_P1_10148305	Ca5	10148305	ATATAT/A	GAGGCGGAAAGAACACAAGA	GGAAAGGGCAACAATGAGAA	60.4	437	INTERGENIC	--	--	--
Ca5_P1_10150121	Ca5	10150121	CG/C	TTTGAAATTAGGTGCGATTCAA	AGCTAGTGCATTGACCTGCC	59.6	511	INTERGENIC	--	--	--

INDEL marker IDs	Chromosomes/ unanchored scaffolds	Physical positions (bp)	InDels (*Kabuli* reference genome-CDC Frontier/PI)	Forward primers (5'-3')	Reverse primers (5'-3')	Anneling temperature (0C)	Expected amplified product size (bp)	Structural annotation		Functional annotation	
								Sequence components of *kabuli* genome	*Kabuli* gene accession IDs	NCBI-KOG	TFs
Ca5_P1_10154398	Ca5	10154398	GGCAG/GGCAGCAG	CCTGCAATTGACAACATTGG	GGCTTCTTAAAAGGATCCCC	60.0	657	DRR	Ca_20521	K	--
Ca5_P1_10159630	Ca5	10159630	TG/T	CCAACAGGGCAAACTGAAAT	GCACAATGGGCTTATTCGAT	60.0	197	INTERGENIC	--	--	--
Ca5_P1_10160827	Ca5	10160827	GAT/GATAT	GGTGGTGACTTGGCTTGATT	GGTTGAGTTAGGGGTGTCAAA	60.0	328	DRR	Ca_20522	--	C3H
Ca5_P1_10161411	Ca5	10161411	ATTTTTTT/ATTTTTTTTTT	TTTGACACCCCTAACTCAACC	TCCTTACGATTTTGGTCGATG	58.9	769	DRR	Ca_20522	--	C3H
Ca5_P1_10164457	Ca5	10164457	CAAA/CAA	GCGACTAAAGCGAAAACCAG	TCCGGTTCTTACAGACCACC	60.0	923	INTRON	Ca_20522	--	C3H
Ca5_P1_10165334	Ca5	10165334	GATAAATAAATAAATAAAT/GATAAATAAATAAAT	GGTGGTCTGTAAGAACCGGA	AGCGAATAGTCCAACTGTCCA	60.0	564	INTRON	Ca_20522	--	C3H
Ca5_P1_10165940	Ca5	10165940	TATGACCAATGAC/T	TGGACAGTTGGACTATTCGCT	TTCCTCATTCAGGGGACAAG	59.7	587	INTRON	Ca_20522	--	C3H
Ca5_P1_10176653	Ca5	10176653	CA/C	CTCTCAAACGGTTGCATTGA	TGTTTCAGCTGTGATGCAAA	59.8	571	INTERGENIC	--	--	--
Ca5_P1_10185771	Ca5	10185771	CT/CTT	AAAAAGCAGTTTCGCCAGAA	GCCCTAGAATCCATGCAAAG	60.0	418	DRR	Ca_20524	--	--
Ca5_P1_10186000	Ca5	10186000	TTATC/T	ATTCTGCACCCTCCATGTTC	CCGTTGGCATGTTATTCAAA	59.9	784	DRR	Ca_20523	Z	--
Ca5_P1_10191175	Ca5	10191175	CTTAAATTAT/C	TCTTGTTCGTGTGTCATAGTTCG	CATTTTTGCGTGTGTGTGCT	60.2	407	INTERGENIC	--	--	--
Ca5_P1_10191320	Ca5	10191320	ATA/ATATTA	TCTTGTTCGTGTGTCATAGTTCG	CATTTTTGCGTGTGTGTGCT	60.2	407	INTERGENIC	--	--	--

INDEL marker IDs	Chromosomes/ unanchored scaffolds	Physical positions (bp)	InDels (*Kabuli* reference genome-CDC Frontier/PI)	Forward primers (5'-3')	Reverse primers (5'-3')	Anneling temperature (0C)	Expected amplified product size (bp)	Structural annotation		Functional annotation	
								Sequence components of *kabuli* genome	*Kabuli* gene accession IDs	NCBI-KOG	TFs
Ca5_P1_1020806	Ca5	1020806	TTTATTATTATTATTATTATTATTATTATTATTATTATTATTATTATT/TTTATTATTATTATTATTATTATTATTATTATTATTATTATTATTATTATT	AATTATGCTCCGACTTCCCC	GAAGCCGAGAAAACGACTTG	60.3	778	INTERGENIC	--	--	--
Ca5_P1_10222320	Ca5	10222320	ATTTTT/ATTTTTT	CCACCGAAAAACTTCATCCA	CTACCGGCAAACTTCATTCC	60.9	391	INTERGENIC	--	--	--
Ca5_P1_10223215	Ca5	10223215	CATAAAAA/C	AATGGGGGTACATGGGAAAT	TCAATTCCACTTGCAGCAATA	60.1	547	INTERGENIC	--	--	--
Ca5_P1_10224109	Ca5	10224109	TAAAAAAAA/TAAAAAAAAA	GAATTGGCCTGGTATGGTTG	TGGAAATTTTGACAATGGGG	60.2	182	INTERGENIC	--	--	--
Ca5_P1_10224537	Ca5	10224537	A/ATGATT	TCAAAAATCCGGTTGTGAAA	CATCTTTGTGGGTTGCATTG	59.0	537	INTERGENIC	--	--	--
Ca5_P1_10234428	Ca5	10234428	AATATATATATATAT/AATATATATATAT	ACGACTTGCCGTTTCAGACT	GGGAATGTTTTGAGAAACCG	59.9	599	INTERGENIC	--	--	--
Ca5_P1_10234470	Ca5	10234470	TAAA/TAAAA	ACGACTTGCCGTTTCAGACT	GGGAATGTTTTGAGAAACCG	59.9	599	INTERGENIC	--	--	--
Ca5_P1_10250599	Ca5	10250599	CCA/C	TCTTTGGGTCAACAACCTCC	TCCATTCTCATTCACTCGTCA	59.9	509	INTERGENIC	--	--	--
Ca5_P1_10251055	Ca5	10251055	TT/TTCT	ACCTGAATTGGATCAGCGAC	AAACAATTTTGATTTTTGGTCAAG	60.1	725	INTERGENIC	--	--	--
Ca5_P1_10251441	Ca5	10251441	A/AG	TGACGAGTGAATGAGAATGGA	ATAGTGCGCAATCATGCAAA	59.2	743	INTERGENIC	--	--	--
Ca5_P1_10262415	Ca5	10262415	AACACA/AACACACA	CATCAGGACCTCCCTTTGAA	AAGTCTGGTGGATTTGCTGC	60.0	635	INTERGENIC	--	--	--
Ca5_P1_10265605	Ca5	10265605	ATTTTTTTT/ATTTTTTTTTT	AGATGGAATGGATGCTTTGG	TCAAGATTAACGTCGTCACGTA	59.9	467	INTERGENIC	--	--	--

INDEL marker IDs	Chromosomes/ unanchored scaffolds	Physical positions (bp)	InDels (*Kabuli* reference genome-CDC Frontier/PI)	Forward primers (5'-3')	Reverse primers (5'-3')	Anneling temperature (0C)	Expected amplified product size (bp)	Structural annotation		Functional annotation	
								Sequence components of *kabuli* genome	*Kabuli* gene accession IDs	NCBI-KOG	TFs
Ca5_P1_10266065	Ca5	10266065	TAAAAAAAAA/TAAAAAAAAAA	CGTGACGACGTTAATCTTGA	CGAGGACAAGTCAAACGACA	57.3	271	INTERGENIC	--	--	--
Ca5_P1_10269975	Ca5	10269975	TTATATATATATAT/TTATATATATATATAT	TGCAATGTTTATTGGATGGC	AAAATGATATATGATGGGAACATGG	59.4	455	INTERGENIC	--	--	--
Ca5_P1_10270264	Ca5	10270264	A/ATT	TGCAATGTTTATTGGATGGC	AATGGAATGGTCAGAGAGTCAA	59.4	854	INTERGENIC	--	--	--
Ca5_P1_10270958	Ca5	10270958	AT/ATT	AGTAGCCAGTGGGTAATGCC	CCACCGACGAAAGGTATTGT	59.1	370	INTERGENIC	--	--	--
Ca5_P1_10275400	Ca5	10275400	A/AT	TGGATGGTTGTGATGTGCTAA	TGAATTGAAATATGGGTGATCAAA	60.0	295	INTERGENIC	--	--	--
Ca5_P1_10276357	Ca5	10276357	GC/GCC	TGCTCAAACAATTTTCAGTTTCA	GTTGGGAATTTCAAAAACGG	59.8	657	INTERGENIC	--	--	--
Ca5_P1_10276696	Ca5	10276696	A/AAAAAAAAGATAAAAT	TGCTAACAACAATGATTGGGC	GTTGGGAATTTCAAAAACGG	60.9	285	INTERGENIC	--	--	--
Ca5_P1_10278876	Ca5	10278876	TGA/T	TCTGATATCTGCACCCCTCC	CAGTGGTCGATTTTGTGTGG	60.0	174	INTERGENIC	--	--	--
Ca5_P1_10280837	Ca5	10280837	AATATATATA/AATATATATATA	AAGTTCGAAATGCCGATTTG	GCTGCATGTTCTTATGCTCG	60.1	689	INTERGENIC	--	--	--
Ca5_P1_10289518	Ca5	10289518	A/AT	TTGTGACACTTTTTACCCGTG	GTTACGGTGTGCGGTTTTCT	58.6	343	INTERGENIC	--	--	--
Ca5_P1_10299950	Ca5	10299950	CAA/CAAAA	CCTTTTCAGCATTCCAATTCA	GGTTAATTATTTTTAATGGGCGG	60.1	356	INTERGENIC	--	--	--
Ca5_P1_10300146	Ca5	10300146	GA/GATGATTAATAA	CCTTTTCAGCATTCCAATTCA	GGTTAATTATTTTTAATGGGCGG	60.1	356	INTERGENIC	--	--	--

INDEL marker IDs	Chromosomes/ unanchored scaffolds	Physical positions (bp)	InDels (*Kabuli* reference genome-CDC Frontier/PI)	Forward primers (5'-3')	Reverse primers (5'-3')	Anneling temperature (0C)	Expected amplified product size (bp)	Structural annotation		Functional annotation	
								Sequence components of *kabuli* genome	*Kabuli* gene accession IDs	NCBI-KOG	TFs
Ca5_P1_10300237	Ca5	10300237	TTCT/TT	CCTTTTCAGCATTCCAATTCA	TTGACTTAATTCAGAGCACCCTC	60.1	450	INTERGENIC	--	--	--
Ca5_P1_10313421	Ca5	10313421	CATATATATATATATATATATATATA/CATATATATATATATATATATATA	TTTGGAGACTCGTCAGGACA	ATAATTTAGATGCGCCACCG	59.4	452	INTERGENIC	--	--	--
Ca5_P1_10331676	Ca5	10331676	ATTTTTTTT/ATTTTTTTTT	AATGCCAAAATCTCAAACCG	GCCATGTTTGTCTTTGCACT	59.9	688	DRR	Ca_21237	O	E2F/DP
Ca5_P1_10331864	Ca5	10331864	TAAAAA/TAAAAAA	AATGCCAAAATCTCAAACCG	CAATGTTGTTGAGAACCCGA	59.9	847	DRR	Ca_21237	O	E2F/DP
Ca5_P1_10338471	Ca5	10338471	TTAAA/T	ACTGGCCATAAACTTGGTGC	AGAAAGATGGTGGTGCTGCT	60.0	703	INTRON	Ca_21237	O	E2F/DP
Ca5_P1_10346809	Ca5	10346809	GGTATATTGTATATTGTAT/GGTATATTGTATATTGTATATTGTAT	AACAACGGATTGCTTTGACC	CAACCTTCTGGTGCAACCTT	60.0	289	INTERGENIC	--	--	--
Ca5_P1_10348801	Ca5	10348801	ACC/ACCC	TGTGCCTGAAAATGATTGGA	CAGCTTCGTACACTTTGCCA	60.0	615	INTERGENIC	--	--	--
Ca5_P1_10363636	Ca5	10363636	T/TTAG	TTCCAGAGCGGACTCTTTGT	AGGAAATCCAGAGGAGCGAT	60.0	746	INTERGENIC	--	--	--
Ca5_P1_10363764	Ca5	10363764	TC/T	AACTGGCAAAAGAGCAAACC	TCCCAAGAAAAGGAGCTTGA	59.4	530	INTERGENIC	--	--	--
Ca5_P1_10370673	Ca5	10370673	TAAAAAAAAAA/TAAAAAAAAAAAA	AAGGATACGGTTGCCTTGTG	AGGATCACTCCTTGCGTGTC	60.0	675	DRR	Ca_21234	V	bHLH
Ca5_P1_10370717	Ca5	10370717	ATTTTTTTTTTT/ATTTTTTTTT	AAGGATACGGTTGCCTTGTG	TTTGTGCGTTGTGACGTGTA	60.0	696	DRR	Ca_21234	V	bHLH
Ca5_P1_10370791	Ca5	10370791	ATATTTATTTATTTA/ATATTTATTTA	AAGGATACGGTTGCCTTGTG	TTTGTGCGTTGTGACGTGTA	60.0	696	DRR	Ca_21234	V	bHLH

INDEL marker IDs	Chromosomes/ unanchored scaffolds	Physical positions (bp)	InDels (*Kabuli* reference genome-CDC Frontier/PI)	Forward primers (5'-3')	Reverse primers (5'-3')	Anneling temperature (0C)	Expected amplified product size (bp)	Structural annotation		Functional annotation	
								Sequence components of *kabuli* genome	*Kabuli* gene accession IDs	NCBI-KOG	TFs
Ca5_P1_10372230	Ca5	10372230	T/TGAATC	CGCGAACTAAGTCGCTCTCT	TGAGTGGCTTTAGGAAGCGT	59.9	296	INTERGENIC	--	--	--
Ca5_P1_10388504	Ca5	10388504	CTTT/CTTTT	TCCCATGTATTTGCCACATC	AAAATTGCAAGTGGAATGGC	59.2	464	INTERGENIC	--	--	--
Ca5_P1_10389384	Ca5	10389384	ATTTT/ATTTTT	CATCACTGCATCCAGAGCC	CCTTTGCCAAGATGCAAAAT	60.4	506	INTERGENIC	--	--	--
Ca5_P1_10389582	Ca5	10389582	CTACGTTA/CTA	TGGTTGCTTCATTGCTTGTG	GCGAAGGATATTCGACCAAG	60.8	216	INTERGENIC	--	--	--
Ca5_P1_10392503	Ca5	10392503	ATTTTTTTTT/ATTTTTTTTTT	TCGATTTTTCTTTCTTCGGG	GAGATAGATTTTGGTTGGTACGTT	59.3	533	INTERGENIC	--	--	--
Ca5_P1_10394462	Ca5	10394462	CATA/CA	GTTGTTTTGACGGGAAGGAA	TGAGCCACACATCACCATCT	59.9	614	INTERGENIC	--	--	--
Ca5_P1_10396682	Ca5	10396682	TAAAAAAAAAA/TAAAAAAAAA	TTGGAGGTCAAATCTGGGAG	AAAATTTTATGCCGCCACAC	60.0	310	INTERGENIC	--	--	--
Ca5_P1_10397846	Ca5	10397846	GAAAAAAA/GAAAAAAAA	TCAAATTGTTGCAACTCCGA	TTTTATCCCATGGATACCAAGA	60.2	617	INTERGENIC	--	--	--
Ca5_P1_10404196	Ca5	10404196	TAAA/TAA	CGACAAAAATTCATGGATGATAC	TGTGGGGGTGAATGTGTATG	58.4	526	INTERGENIC	--	--	--
Ca5_P1_10409173	Ca5	10409173	TTGTGT/TTGTGTGT	TGGCTCTTGAAAAGAAACGAA	TCAATGGTGCTTTTGTTGGA	60.0	805	DRR	Ca_21231	--	WRKY
Ca5_P1_10413807	Ca5	10413807	GTTTTTTTTTTTTT/GTTTTTTTTTTT	TTCTCATGCTCACTGGTTGC	TCGCATTTAACCATTCACACA	60.0	569	INTERGENIC	--	--	--
Ca5_P1_10414029	Ca5	10414029	TACCCAC/TAC	TTCTCATGCTCACTGGTTGC	TCGCATTTAACCATTCACACA	60.0	569	INTERGENIC	--	--	--

INDEL marker IDs	Chromosomes/ unanchored scaffolds	Physical positions (bp)	InDels (*Kabuli* reference genome-CDC Frontier/PI)	Forward primers (5'-3')	Reverse primers (5'-3')	Anneling temperature (0C)	Expected amplified product size (bp)	Structural annotation		Functional annotation	
								Sequence components of *kabuli* genome	*Kabuli* gene accession IDs	NCBI-KOG	TFs
Ca5_P1_10414356	Ca5	10414356	AG/A	TGTGTGAATGGTTAAATGCGA	TCGAGTACTCCAACGACACCT	60.0	291	INTERGENIC	--	--	--
Ca5_P1_10415934	Ca5	10415934	GTTTTTT/GTTTTTTT	TGCTCCAATGATCTGGTAAAAA	AGCCATGATGGGAGCATAAG	59.6	718	INTERGENIC	--	--	--
Ca5_P1_10417538	Ca5	10417538	A/AT	TGGGACCATAGCCCACATTA	GCATTCACCTCTCTTATACCCG	61.1	595	INTERGENIC	--	--	--
Ca5_P1_10420664	Ca5	10420664	AATATATATATATATATATATA/AATATATATATATATATATATATA	AAGGAAGGCCTACTCCCAAA	TACCAAGGCGTCGTCAAAAT	60.1	597	INTERGENIC	--	--	--
Ca5_P1_10456058	Ca5	10456058	TAAAAAAAA/TAAAAAAA	CACTAATCACAAACAAAAACCCA	CGATATGCTTGATGTAAATGTGC	59.0	609	INTERGENIC	--	--	--
Ca5_P1_10457260	Ca5	10457260	CACAATACTC/CACAATACTCTACAATACTC	GGGGCGAGATATTCAGACAA	TGATTGTTCTAGTGGTCGCTCT	60.0	108	INTERGENIC	--	--	--
Ca5_P1_10457939	Ca5	10457939	CAAAAAA/CAAAAAAA	CAGGTTGATGAAATAAGTATACACAAA	CAAGTTTGATACGCATGAAGAGA	57.9	480	INTERGENIC	--	--	--
Ca5_P1_10472256	Ca5	10472256	ATCTTCTTCTTCTTCTT/ATCTTCTTCTTCTT	CGGAATATTTTTCCACCACA	TGCGAAATCATTGAAGATTGA	58.3	795	INTERGENIC	--	--	--
Ca5_P1_10474049	Ca5	10474049	TTC/TTCTC	CCTTTGGTTCGTTGAAGCTC	GCTTCAGGTGTTGGCTTCTT	59.9	415	INTERGENIC	--	--	--
Ca5_P1_10474510	Ca5	10474510	T/TA	CCTTTGGTTCGTTGAAGCTC	AAGGGAATGTCACCATGGAA	59.9	695	INTERGENIC	--	--	--
Ca5_P1_10495487	Ca5	10495487	GTGAG/GTGAGATTGAG	GAACGGTTGTTGCACTTGATT	CAACAACATTGCGTTGAAGTG	60.0	741	INTERGENIC	--	--	--
Ca5_P1_10496835	Ca5	10496835	TTAAAATAAA/TTAAAATAAAATAAA	ATGGTTGCTCTTCGATGGTC	GGGTGCTCTTCGGTTGTAAA	60.1	410	INTERGENIC	--	--	--

INDEL marker IDs	Chromosomes/ unanchored scaffolds	Physical positions (bp)	InDels (*Kabuli* reference genome-CDC Frontier/PI)	Forward primers (5'-3')	Reverse primers (5'-3')	Anneling temperature (0C)	Expected amplified product size (bp)	Structural annotation		Functional annotation	
								Sequence components of *kabuli* genome	*Kabuli* gene accession IDs	NCBI-KOG	TFs
Ca5_P1_10497866	Ca5	10497866	AT/ATT	GCAAACGGGTTAATTGGAGA	GTCTTATGAGCCGAAGGTCG	59.9	764	INTERGENIC	--	--	--
Ca5_P1_10507484	Ca5	10507484	ATTTTTTTTT/ATTTTTTTTTT	TGCAAGTTAAAGAATAATTTAGAGCG	GGAAACGATTGGATTCGAGA	59.5	897	INTERGENIC	--	--	--
Ca5_P1_10508614	Ca5	10508614	GCA/GCACA	GCTTTGAAGCTCAACCTTGC	TGGGCCCTATTGCTCTAAAA	60.1	321	INTRON	Ca_21230	--	--
Ca5_P1_10513103	Ca5	10513103	AT/ATCTACACT	TTGACCGACACCAATCAGAA	CTTGCATCCCACAGGAAAAT	60.1	544	INTERGENIC	--	--	--
Ca5_P1_10513256	Ca5	10513256	AATATATATATATATATATA/AATATATATATATATATATA	TTGACCGACACCAATCAGAA	CTTGCATCCCACAGGAAAAT	60.1	544	INTERGENIC	--	--	--
Ca5_P1_10514189	Ca5	10514189	TAAAAA/TAAAAAA	TGGTTAAATTGCTCTGAAGTGA	GGGAGGATGGGACAATTTCT	57.6	501	INTERGENIC	--	--	--
Ca5_P1_10515370	Ca5	10515370	A/AT	CGATTAAAGTCAAAGTTTCACGA	AGCAAACCAGGTTGTCCTGT	58.5	560	INTERGENIC	--	--	--
Ca5_P1_10516286	Ca5	10516286	TTTTTATTTTATTTT/TTTTTATTTTATTTTATTTT	ATTTGGGTTGAAGTGTTGCC	ATTCAATGCAACTTCCCTGC	59.8	598	INTERGENIC	--	--	--
Ca5_P1_10531161	Ca5	10531161	G/GCTTGCA	ATGGTTTTCCAACGTTTTCG	CCTAGGTTCAAAACCACCCA	59.8	501	INTRON	Ca_21228	--	--
Ca5_P1_10549913	Ca5	10549913	A/AG	ATTTAGGCGGGTTAGGCACT	GGTTTTCACAAACAACGGCT	60.0	805	DRR	Ca_21226	--	--
Ca5_P1_10550017	Ca5	10550017	C/CATATTTTACCATTGAT	CTCCTCATATTGCAGGCGTT	CGGCTAAACAACAAGCCATT	60.2	703	DRR	Ca_21226	--	--
Ca5_P1_10559602	Ca5	10559602	AT/ATTTATCATTCATATGT	TGTCATTTCAAATTAGACCCAAAA	GCTTTTACGGAATGGCAAAA	59.8	250	INTERGENIC	--	--	--

INDEL marker IDs	Chromosomes/ unanchored scaffolds	Physical positions (bp)	InDels (*Kabuli* reference genome-CDC Frontier/PI)	Forward primers (5'-3')	Reverse primers (5'-3')	Anneling temperature (0C)	Expected amplified product size (bp)	Structural annotation		Functional annotation	
								Sequence components of *kabuli* genome	*Kabuli* gene accession IDs	NCBI-KOG	TFs
Ca5_P1_10561090	Ca5	10561090	TAGAGA/TAGAGAGA	CACGAGATCTTGAGGCATGA	TGTTGGTTTGTTGTGGGTTG	59.9	383	INTERGENIC	--	--	--
Ca5_P1_10564601	Ca5	10564601	ATTTT/ATT	TCCAAATCCCAAGCTACCAA	GGGCCTCATTTCCCTTTAAC	60.4	408	INTRON	Ca_21225	--	--
Ca5_P1_10569446	Ca5	10569446	TT/TTCTCT	GCATTGGGGGCATACATATC	AAAAGCTCACTGAAGCAGGG	60.0	362	INTERGENIC	--	--	--
Ca5_P1_1057278	Ca5	1057278	CCTC/CGGATTTACTC	TACGGTGCTAGGGAACCAAC	TTGGAAGGAGCCCTTACCTT	60.0	109	INTERGENIC	--	--	--
Ca5_P1_10574825	Ca5	10574825	A/AT	CGTGGACAAAGATCGTGATG	TTGTTTATTTAGCAGTGTCAATGG	60.1	479	INTERGENIC	--	--	--
Ca5_P1_10592850	Ca5	10592850	GAAAAAAAA/GAAAAAAAAAA	GATCAAATGTCAGACCTCACCA	ACCAGCCCCTAAACCAAGAT	60.0	770	INTERGENIC	--	--	--
Ca5_P1_10605600	Ca5	10605600	GT/GTT	AGTGAAACCAACGCCAAAAC	TCACTCAAAGGGCTACTGCC	60.0	272	DRR	Ca_21224	--	--
Ca5_P1_10625989	Ca5	10625989	TAAAAAAA/TAAAAAAAA	TGTAGGGGTACAGGGGAAAG	AACTTCCGTTGTTTTTGCCA	58.9	165	INTERGENIC	--	--	--
Ca5_P1_10634338	Ca5	10634338	AC/ACC	AATTTTTCAGTCCCGTGTCG	ATCCCTCAAAGGGCTACTGC	60.0	229	INTERGENIC	--	--	--
Ca5_P1_10638738	Ca5	10638738	GAAAAAAAA/GAAAAAAAAA	TTAAAACTGCAATGTCCCCC	TGTTTATTTGGTTGTGTCAATGG	59.8	541	INTERGENIC	--	--	--
Ca5_P1_1065182	Ca5	1065182	TAAAA/TAAAAA	AATGGCCACGAATCTGAAAG	ACCATTTTCAACTGCATCCC	60.1	634	INTERGENIC	--	--	--
Ca5_P1_10668282	Ca5	10668282	ATTT/ATTTT	GGGCGATTATATACCAGCGA	TGGCAACTATTTTGAGCTTTTTC	59.9	564	DRR	Ca_21221	--	--

INDEL marker IDs	Chromosomes/ unanchored scaffolds	Physical positions (bp)	InDels (*Kabuli* reference genome-CDC Frontier/PI)	Forward primers (5'-3')	Reverse primers (5'-3')	Anneling temperature (0C)	Expected amplified product size (bp)	Structural annotation		Functional annotation	
								Sequence components of *kabuli* genome	*Kabuli* gene accession IDs	NCBI-KOG	TFs
Ca5_P1_10679625	Ca5	10679625	TAAAAAAAA/TAAAAAAAAA	GGGATTGTTTTGAATATGAGGG	TCACACGATCATTTTGGCAT	59.6	596	INTERGENIC	--	--	--
Ca5_P1_10686981	Ca5	10686981	TAAAAAAAA/TAAAAAAAAAAA	GCTCCTTCTGCATCAACACA	TGCCCCACATTTTAGACCTC	60.0	509	INTERGENIC	--	--	--
Ca5_P1_10689004	Ca5	10689004	A/AAAC	GAGCCATCATCTAAACCCCA	TGCCAAAGAAAAAGGTTTCG	59.9	677	INTERGENIC	--	--	--
Ca5_P1_10689102	Ca5	10689102	TAAAA/TAA	ATGGGCCACCTATTGAGACA	TGCCAAAGAAAAAGGTTTCG	60.3	623	INTERGENIC	--	--	--
Ca5_P1_10689622	Ca5	10689622	AGAAGCATG/AG	CGAAACCTTTTTCTTTGGCA	AACGGATTGTCCCTTCATTG	60.2	418	INTERGENIC	--	--	--
Ca5_P1_10693789	Ca5	10693789	CTTGTGATTGTTTTGTTGTGATTG/CTTGTGATTG	CCAATTTGAATTGTCCGTCC	TTGCAAAAGCTGGGTCAAGT	60.2	406	INTERGENIC	--	--	--
Ca5_P1_10759502	Ca5	10759502	TA/T	GACCAGCAGCAAACTCTTCC	CAGCACGAATCTCTCTTCCC	60.0	856	INTERGENIC	--	--	--
Ca5_P1_10767937	Ca5	10767937	AATATATATATATATATATATATATATATATATATATATATATA/AATATATATATATATATATATATATATATATATATATATATATA	AGATGCAGACTTGCGTGTTG	CAAGTCATTCGTCAAAATCGG	60.1	202	INTERGENIC	--	--	--
Ca5_P1_10794967	Ca5	10794967	A/AC	GGTTTCAAAACCAAAATTCAAAA	TGCGTACTTAAAATTGTTTGGC	59.3	378	INTERGENIC	--	--	--
Ca5_P1_1083059	Ca5	1083059	ATTTTTTTTTTTTTTT/ATTTTTTTTTTTTT	TCAATCCGAACAAAACATGAA	CGATGATCTTCTTCTTCACCC	59.0	338	INTERGENIC	--	--	--
Ca5_P1_10852097	Ca5	10852097	AATATATATATAT/AATATATATAT	CACAGTTGTCCACGTACGAAA	AAATGGAGGTTTCAATTGCG	59.7	499	INTERGENIC	--	--	--
Ca5_P1_10854611	Ca5	10854611	ATTTTTT/ATTTTTTTTTT	GCGAGCATGAGAAGTTGTTG	CAATCACTCATTAATGTCAATCCA	59.6	599	INTERGENIC	--	--	--

INDEL marker IDs	Chromosomes/ unanchored scaffolds	Physical positions (bp)	InDels (*Kabuli* reference genome-CDC Frontier/PI)	Forward primers (5'-3')	Reverse primers (5'-3')	Anneling temperature (0C)	Expected amplified product size (bp)	Structural annotation		Functional annotation	
								Sequence components of *kabuli* genome	*Kabuli* gene accession IDs	NCBI-KOG	TFs
Ca5_P1_10931505	Ca5	10931505	AG/A	TTGATGAATTTGATGTCGTTGA	AAATACCTTACGCGGTCCCT	59.0	344	INTERGENIC	--	--	--
Ca5_P1_10995810	Ca5	10995810	AAATAATAATAATAATAATAAT/AAATAATAATAATAATAAT	ACTTCAGGTGGTCGATGTCC	GGGAGCGACCTCACACTAGA	60.0	923	INTERGENIC	--	--	--
Ca5_P1_11014094	Ca5	11014094	TTTATTATTATTATTATTATTATTAT/TTTATTATTATTATTATTATTAT	AGTGTGTCCGTTAGGGATGG	GGTCTGAGTCTGGCCTACGA	59.8	650	INTERGENIC	--	--	--
Ca5_P1_1105618	Ca5	1105618	TT/TTCCCT	GCACCAGTCATCTCATCACG	TTACCAATGCTTGAGATGCG	60.3	556	INTERGENIC	--	--	--
Ca5_P1_11058700	Ca5	11058700	AATATATATATATATATATATATATATAT/AATATATATATATATATATATATAT	AATTGCTCCCATAGTCCCTT	AATTGCTCATGCGGTCTCTT	57.6	469	INTERGENIC	--	--	--
Ca5_P1_11131180	Ca5	11131180	GT/GTT	CAAGTGCACAGCGGAATTTA	TCCGATTGGATTTTCAGGAC	59.9	148	INTERGENIC	--	--	--
Ca5_P1_11132723	Ca5	11132723	TTTATTATTATTATTATTA/TTTATTATTATTATTA	AAAAACCAACAATCTCCCCC	TGTTTTCTGCCTTTTTGCCT	60.0	262	INTERGENIC	--	--	--
Ca5_P1_11167847	Ca5	11167847	CAAAAAAAAAAAAA/CAAAAAAAA	GGTGGACTCGAGCAAGTAGC	AAAGCATCAGTTCCGTTTGAA	60.0	459	INTERGENIC	--	--	--
Ca5_P1_11168411	Ca5	11168411	AATA/AA	TTCAAACGGAACTGATGCTTT	TTGGTTGGGGGAGTTATTGA	59.7	954	INTERGENIC	--	--	--
Ca5_P1_11168737	Ca5	11168737	ATGT/A	TCGTTGAGGAAAAACAAACAAA	TTGGTTGGGGGAGTTATTGA	59.6	557	INTERGENIC	--	--	--
Ca5_P1_11168801	Ca5	11168801	ACTATTAGTCTATTA/ACTATTA	TCGTTGAGGAAAAACAAACAAA	TTGGTTGGGGGAGTTATTGA	59.6	557	INTERGENIC	--	--	--
Ca5_P1_11168904	Ca5	11168904	GTCTCTCT/GTCTCT	TCAATAACTCCCCCAACCAA	TCACAATTGGGTTTGGATCA	60.2	227	INTERGENIC	--	--	--

INDEL marker IDs	Chromosomes/ unanchored scaffolds	Physical positions (bp)	InDels (*Kabuli* reference genome-CDC Frontier/PI)	Forward primers (5'-3')	Reverse primers (5'-3')	Anneling temperature (0C)	Expected amplified product size (bp)	Structural annotation		Functional annotation	
								Sequence components of *kabuli* genome	*Kabuli* gene accession IDs	NCBI-KOG	TFs
Ca5_P1_11169035	Ca5	11169035	CG/C	TCAATAACTCCCCCAACCAA	TGACGCCGTGAAAAACATTA	60.2	626	INTERGENIC	--	--	--
Ca5_P1_11169613	Ca5	11169613	C/CG	TGAAGCAGTTGTTTGGGTGA	GAGCCCAAATCCAAAATTCA	60.3	225	CDS (FRAME SHIFT)	Ca_23744	A	--
Ca5_P1_11169708	Ca5	11169708	T/TGCAA	TGAAGCAGTTGTTTGGGTGA	GAGCCCAAATCCAAAATTCA	60.3	225	DRR	Ca_23744	A	--
Ca5_P1_11233017	Ca5	11233017	TAAAAA/TAAA	ATTGGACGTATCCCGAGTTG	GTTGTGGGATGAACTGACCC	59.8	633	INTERGENIC	--	--	--
Ca5_P1_11244127	Ca5	11244127	TAAAAAAA/TAAAAAA	AACTTGATGCCGAGTAGCGT	TCTCCCCTCCTCCTTCATTT	59.9	280	INTERGENIC	--	--	--
Ca5_P1_11272777	Ca5	11272777	A/ATTTATTTGTCGTGTAG	TTGTTAGTGATGGGCCACAA	ACGAATGTTGGTCAGGGAAG	60.0	519	INTERGENIC	--	--	--
Ca5_P1_11338251	Ca5	11338251	TAAAAAAAAA/TAAAAAAAA	CGAGGTAAAGCATGGTGGAT	TTTGCTTTTCAAAGTTGGCA	60.0	690	INTERGENIC	--	--	--
Ca5_P1_11340361	Ca5	11340361	GTTTTTTTTTT/GTTTTTTTTT	CATATTTGGGGGATGTCCAG	CACGTGTTGAATTAAGCGGA	60.0	341	INTERGENIC	--	--	--
Ca5_P1_11420785	Ca5	11420785	TTATTA/TTACTATTA	ATTAAGTGAATGGATCGCGG	AGTGTGGAAGGACGAGCAAG	59.9	485	INTERGENIC	--	--	--
Ca5_P1_11494201	Ca5	11494201	AGA/AGATATGA	CGTATGTTTATTTGAAATGTTTTGA	GGATGCCAATTGGTAGGTTT	57.4	295	INTERGENIC	--	--	--
Ca5_P1_11498601	Ca5	11498601	ACATCGAATAGTTGGATCATCGAATAGTTGGATCATCGAATAGTTGGATCATCGAA/ACATCGAATAGTTGGATCATCGAATAGTTGGATCATCGAA	CTGTTCAACCATCGAAATTGT	CTTCGTTGATCGAACGGTTT	57.6	417	INTERGENIC	--	--	--
Ca5_P1_11505972	Ca5	11505972	TTTCTTC/TTTC	GGACCTAAATTTTGCAACAACA	GAACGCGAGATATTGGGTGT	59.0	199	INTERGENIC	--	--	--

INDEL marker IDs	Chromosomes/ unanchored scaffolds	Physical positions (bp)	InDels (*Kabuli* reference genome-CDC Frontier/PI)	Forward primers (5'-3')	Reverse primers (5'-3')	Anneling temperature (0C)	Expected amplified product size (bp)	Structural annotation		Functional annotation	
								Sequence components of *kabuli* genome	*Kabuli* gene accession IDs	NCBI-KOG	TFs
Ca5_P1_115778	Ca5	115778	TTCACCGGTCA/TTCA	AAAGATTGCAATTTGCCTGC	TTTTTAGGGGGCGATTGTATT	60.2	511	INTERGENIC	--	--	--
Ca5_P1_11582454	Ca5	11582454	TT/TAAAAACCTTTTTAT	TGACTCATCAATTCGAACGC	GAATGCCACCAGGAAAAGAA	59.8	643	INTERGENIC	--	--	--
Ca5_P1_11582895	Ca5	11582895	AT/ATTGT	TTCTTTTCCTGGTGGCATTC	AGCGATGCATGTCTCAAGTG	60.1	490	INTERGENIC	--	--	--
Ca5_P1_11587595	Ca5	11587595	TTATATATATATATATATAT/TTATATATATATATATAT	TCGAACTACCTTCCCCCTTT	TTGTTGGAGTGGTTTTGACG	59.9	756	DRR	Ca_23350	--	Trihelix
Ca5_P1_11628665	Ca5	11628665	CNNNNNNNNNN/CNN	TATCCCCGTCCATTAACCAA	GGGAGTGGTTGAGACCTTGA	60.0	248	INTERGENIC	--	--	--
Ca5_P1_11729570	Ca5	11729570	AAATAATAATAA/AAATAATAA	CACGGTCCCTAGAGCCAATA	CGGGAGATACGTAGGAGCAA	60.1	672	INTERGENIC	--	--	--
Ca5_P1_11760778	Ca5	11760778	A/AC	ACAACTTCTCATGCTCGCAA	GGGAAAATCATGTTTATCACAATC	59.6	329	INTERGENIC	--	--	--
Ca5_P1_11861465	Ca5	11861465	GTATA/GTATACATATATATA	TGAACCAAAGACACAACGGA	TTTTTCAACAAAGCAATGGAAA	60.1	232	INTERGENIC	--	--	--
Ca5_P1_11878820	Ca5	11878820	CATATATATATATATATATATATATAT/CATATATATATATATATATATATAT	TATTTTGGATTTTGGCTCGG	AGAGGGGTTTGTGTTTGACG	59.9	599	INTERGENIC	--	--	--
Ca5_P1_11879108	Ca5	11879108	TAA/TAAA	AAGATTTCCTCCCTCCTCCA	CTTTAACATGCGTTTCCGGT	60.0	327	INTERGENIC	--	--	--
Ca5_P1_11886997	Ca5	11886997	GATAATAATAATAATAATAATAA/GATAATAATAATAATAATAATAA	TGCCTTCGGTTTTCCTATTG	AATGTTTGGAATTCCCCCTC	60.1	888	INTERGENIC	--	--	--
Ca5_P1_11930966	Ca5	11930966	TAAAAAAAAA/TAAAAAAAAAA	CATGCTGCAAGGGATTGTTA	GCGCGCTTGTTATACTCGTT	59.7	856	INTERGENIC	--	--	--

INDEL marker IDs	Chromosomes/ unanchored scaffolds	Physical positions (bp)	InDels (*Kabuli* reference genome-CDC Frontier/PI)	Forward primers (5'-3')	Reverse primers (5'-3')	Anneling temperature (0C)	Expected amplified product size (bp)	Structural annotation		Functional annotation	
								Sequence components of *kabuli* genome	*Kabuli* gene accession IDs	NCBI-KOG	TFs
Ca5_P1_11936712	Ca5	11936712	TTATATATATATATATATATAT/TTATATATATATATATATATATAT	TCTCTTCGAGTCCCTGTTTCA	GCTCTGGACCAATTACCACG	60.0	389	INTERGENIC	--	--	--
Ca5_P1_11973623	Ca5	11973623	AATATATATATATATATATATATATATATATATATATATATATATAT/AATATATATATATATATATATATATATATATATATAT	TCAATGCATATATTTACCGTGAGC	TTGCCAATGTTGGGATTTTT	60.2	520	INTERGENIC	--	--	--
Ca5_P1_11983861	Ca5	11983861	GTCT/GATCTTCT	CTGCTGTCAACTCCACTCCA	TCAAAATCCCCCAATAACCA	60.0	713	DRR	Ca_17106	--	--
Ca5_P1_1202538	Ca5	1202538	ATTTTTTTTTT/ATTTTTTTTTTT	AGAAAACCAAACAACTTCACACA	GATCCCCTGGGTCTCTTTTC	58.7	459	INTERGENIC	--	--	--
Ca5_P1_12040897	Ca5	12040897	T/TTTTAAATGAAG	TCGATGATGTTAACTCGCAA	GCGGTGTCATTTTGTTTCAA	57.9	636	INTERGENIC	--	--	--
Ca5_P1_12194821	Ca5	12194821	TTTATTATTATTATTATTATTATTATTATTATTATTATTATTATT/TTTATTATTATTATTATTATTATTATTATTATTATTATTATTATTATT	TATTTTGCTCCCCATCAAGC	GAGGAGTCACACCAATGCAA	60.0	560	INTERGENIC	--	--	--
Ca5_P1_12232596	Ca5	12232596	GTTTTTTTTTTT/GTTTTTTTTTT	CGGACACCATCTCTGCTGTT	AAAAATCAATTCAATCCATCCA	61.3	588	INTERGENIC	--	--	--
Ca5_P1_12272837	Ca5	12272837	CAAA/CAA	TGACGTTAACTGCATGTCCC	TTCATCTTCATCCTCCACCC	59.6	627	INTERGENIC	--	--	--
Ca5_P1_12292180	Ca5	12292180	AA/AATAATA	CAATGCAATCAGTGGAAGAAA	CCCCTCTCAAATTCATGACAA	58.8	663	INTERGENIC	--	--	--
Ca5_P1_1230644	Ca5	1230644	T/TCCGTA	ACGAAATCGAAGAGAAGCGA	TCCAGGACACAAACAATCCA	60.1	455	INTERGENIC	--	--	--
Ca5_P1_12371198	Ca5	12371198	TTATATATATATATATA/TTATATATATATATATATA	ACATGCGAGAAAACCTCGAT	GGTGATTGATGGAAGGAGACA	59.7	459	INTERGENIC	--	--	--
Ca5_P1_12448859	Ca5	12448859	ATTTT/ATTTTT	TTCTACCCGAAATTTGTGCC	GTCAGTCAGCCTCTCTTGCC	59.9	361	INTERGENIC	--	--	--

INDEL marker IDs	Chromosomes/ unanchored scaffolds	Physical positions (bp)	InDels (*Kabuli* reference genome-CDC Frontier/PI)	Forward primers (5'-3')	Reverse primers (5'-3')	Anneling temperature (0C)	Expected amplified product size (bp)	Structural annotation		Functional annotation	
								Sequence components of *kabuli* genome	*Kabuli* gene accession IDs	NCBI-KOG	TFs
Ca5_P1_12491196	Ca5	12491196	ATTTTTT/ATTTTT	CCAATAGGTGGTGGCAATCT	CACAAGCTGTTTTGTGCCAT	59.8	274	INTERGENIC	--	--	--
Ca5_P1_12619050	Ca5	12619050	AATATATATATATATATATATATA/AATATATATATATATATATATATATATATA	TCCTAACCTTTTGACTAATTTTTCTTC	TCAATCCTCTTAAGATATTTCCCTG	58.9	685	INTERGENIC	--	--	--
Ca5_P1_12621591	Ca5	12621591	ATATTATTATTATTATTATTATTATTATTATTATTATTATTAT/ATATTATTATTATTATTATTATTATTATTATTATTATTAT	GCGAATTGGTAAACGTAAAGG	TCAACTCTAGCACCATCCCC	58.7	410	INTERGENIC	--	--	--
Ca5_P1_12631984	Ca5	12631984	GCGTC/GC	GGTTATCACCGTGGTGCTCT	TGGCTTCTGGTTGGATTTTC	60.0	648	INTERGENIC	--	--	--
Ca5_P1_12635374	Ca5	12635374	T/TAA	TGGAACCAAACACACACCAT	AGCGAAAAGAAGATTGGTCG	59.7	642	INTERGENIC	--	--	--
Ca5_P1_1269046	Ca5	1269046	AAATT/A	TCCGGTACACAACTTTTCCC	ACCCATCATTTCCAACCAAC	59.8	376	DRR	Ca_26280	E	--
Ca5_P1_131876	Ca5	131876	GTTTT/GTTTTT	ATCCAAGTATCCAACGGCTG	CACCTCTGCCCACTTCATTT	60.0	493	INTERGENIC	--	--	--
Ca5_P1_132937	Ca5	132937	CAA/CAAA	CCAAAGACACAGCAAAGCAA	CACTTCCACCAGTTGCAAAA	60.0	569	INTERGENIC	--	--	--
Ca5_P1_133283	Ca5	133283	AATATAT/AATAT	ACCTTTACATGGTGCCAAGC	GCGTTGTGAAAACAGGACAA	60.0	433	INTERGENIC	--	--	--
Ca5_P1_133428	Ca5	133428	GAACA/GAACAATTTTTATAATAACA	ACCTTTACATGGTGCCAAGC	GCGTTGTGAAAACAGGACAA	60.0	433	INTERGENIC	--	--	--
Ca5_P1_133862	Ca5	133862	ATTTTTTTTTTTTT/ATTTTTTTTTTT	TTGTCCTGTTTTCACAACGC	TCAACAATTTGTGGGCTTCA	59.7	574	INTERGENIC	--	--	--
Ca5_P1_1338861	Ca5	1338861	TTTATTATTATTATTATTATTATTATTATTATTATTAT/TTTATTATTATTATTATTATTATTATTATTATTATTATTAT	AACAGGGAGCGGAATTTTCT	GATCGCAACTTGTGGATGTG	60.1	677	DRR	Ca_26443	O	--

**INDEL marker IDs**	**Chromosomes/ unanchored scaffolds**	**Physical positions (bp)**	**InDels (*Kabuli* reference genome-CDC Frontier/PI)**	**Forward primers (5'-3')**	**Reverse primers (5'-3')**	**Anneling temperature (0C)**	**Expected amplified product size (bp)**	**Structural annotation**		**Functional annotation**	
								**Sequence components of *kabuli* genome**	***Kabuli* gene accession IDs**	**NCBI-KOG**	**TFs**
Ca5_P1_133927	Ca5	133927	ATTT/ATTTT	TTGTCCTGTTTTCACAACGC	TCAACAATTTGTGGGCTTCA	59.7	574	INTERGENIC	--	--	--
Ca5_P1_133972	Ca5	133972	CAAAAAA/CAAAAA	TTGTCCTGTTTTCACAACGC	TCAACAATTTGTGGGCTTCA	59.7	574	INTERGENIC	--	--	--
Ca5_P1_134014	Ca5	134014	TA/T	CAGGACCGGTTTCTTACTGA	TCAACAATTTGTGGGCTTCA	57.8	265	INTERGENIC	--	--	--
Ca5_P1_135753	Ca5	135753	TTATATAT/TTAT	CTGCGCCATGATTAGGATTT	TGCTTGTTTGTTGACATTCG	60.1	866	INTERGENIC	--	--	--
Ca5_P1_13631028	Ca5	13631028	TAGAGAGAGAGAGAGAGAGAGAGAGAGAGAGA/TAGAGAGAGAGAGAGAGAGAGAGAGAGA	GTTGGTAGCGTGGAAGTGGT	TGAGCTTTGACTCCCGAACT	60.0	394	INTERGENIC	--	--	--
Ca5_P1_13636401	Ca5	13636401	A/AC	ATCGACAAACCAACATGCAA	GGGAAACTCACTTGCGGTTA	60.0	735	INTERGENIC	--	--	--
Ca5_P1_13636921	Ca5	13636921	CTGT/CT	TAACCGCAAGTGAGTTTCCC	GCGGTGGCTGAGTTTCTTAC	60.1	387	INTERGENIC	--	--	--
Ca5_P1_13714998	Ca5	13714998	TA/T	TGATGTGAGGTGACAGGCAT	GTAGCGTGGAGAGGAGCAAC	60.1	374	INTERGENIC	--	--	--
Ca5_P1_13730850	Ca5	13730850	AAAGGAAG/AAAG	CAATCCAACCCGCTTACACT	AAATGGAGGAGAAGGAGGGA	60.0	467	INTERGENIC	--	--	--
Ca5_P1_13764192	Ca5	13764192	C/CG	TTACAATTTACGCCCCTTCG	AAACATGACTCGTGTGCCAA	60.0	104	INTERGENIC	--	--	--
Ca5_P1_13822056	Ca5	13822056	TAACAAA/TAA	TCGGGAACTATCATGCAACA	ACGTAGGAGCAGCAAGGTGT	60.1	413	INTERGENIC	--	--	--
Ca5_P1_13825062	Ca5	13825062	ATATTATTATTATTATTATTATTATCATTATTATTATTAATATGATTATTATTATTATTATTATTATTATTATTATTATTATTATTATTATTATTATT/ATATTATTATTATTATTATTATTATTATTATCATTATTATTATTAATA[illegible]	TGGTTTCATAACGTTTGTCCA	TGATTTGGTCATTTGGTATTCA	58.9	823	INTERGENIC	--	--	--

INDEL marker IDs	Chromosomes/ unanchored scaffolds	Physical positions (bp)	InDels (*Kabuli* reference genome-CDC Frontier/PI)	Forward primers (5'-3')	Reverse primers (5'-3')	Anneling temperature (0C)	Expected amplified product size (bp)	Structural annotation		Functional annotation	
								Sequence components of *kabuli* genome	*Kabuli* gene accession IDs	NCBI-KOG	TFs
Ca5_P1_13837733	Ca5	13837733	TA/TAATCTTTGTAA	ATCATTTGCATGCCATCTCA	AAAGGGATACACCCTTTGGG	60.0	159	INTERGENIC	--	--	--
Ca5_P1_1384061	Ca5	1384061	TAAAA/TAAAAA	GAAGGGACTGCGGCATAATA	CCACTTCTCCCCCTTCTTTC	60.1	165	INTERGENIC	--	--	--
Ca5_P1_13855690	Ca5	13855690	TA/TAA	CCAGGTTTGCACTCAACTCA	GGGTGATGAGGACTCAGGAA	59.9	573	INTERGENIC	--	--	--
Ca5_P1_13858932	Ca5	13858932	A/AC	GATTCCCCATTCTTAACGCC	TCGCGAAGAGATAGAAGGGA	60.6	554	INTERGENIC	--	--	--
Ca5_P1_13970765	Ca5	13970765	TA/TGA	TATTTGGACAAACGAAGGGC	CCCCTTTTGCTTGTTGGTTA	59.9	282	INTERGENIC	--	--	--
Ca5_P1_13971864	Ca5	13971864	AATATATATATATATATATATATATA/AATATATATATATATATATATATATATA	ATGGAGACTTTCTGCAGCGT	TCCTCCGGCTGCTAAATAAA	60.0	691	INTERGENIC	--	--	--
Ca5_P1_14022502	Ca5	14022502	ATTT/ATTTT	GGAAATGGATGTCTGCCAAT	TTGCAATCTCATCAACTCGG	59.8	318	INTERGENIC	--	--	--
Ca5_P1_14022785	Ca5	14022785	GA/GATAATGAA	CGGACCGAGTTGATGAGATT	TGCTTTGAAGACCATGTGGA	60.1	618	INTERGENIC	--	--	--
Ca5_P1_14084083	Ca5	14084083	A/ACAAGGATG	TGTTGTTGTGATTGTGCCCT	TTAGCATGCACCTTGAAAGC	60.0	385	DRR	Ca_20825	K	--
Ca5_P1_140974	Ca5	140974	AT/ATTT	GCTGAAAGGGAAAATCACCA	CAGTTGTAGAACCCGCCAAT	60.1	417	INTRON	Ca_18170	--	--
Ca5_P1_14184624	Ca5	14184624	AATATATATATATATATATATA/AATATATATATATATATATATATA	GCGTTAATTTCCATCGCAGT	TTTAGGCCGCAACAAGTAGG	60.1	384	INTERGENIC	--	--	--
Ca5_P1_14221007	Ca5	14221007	CATATATATATATATATATATAT/CATATATATATATATATATAT	TCACTAGCATCGTAGCGTGG	TGAGGTGCCTTCTTTCCTGT	60.0	749	INTERGENIC	--	--	--

INDEL marker IDs	Chromosomes/ unanchored scaffolds	Physical positions (bp)	InDels (*Kabuli* reference genome-CDC Frontier/PI)	Forward primers (5'-3')	Reverse primers (5'-3')	Anneling temperature (0C)	Expected amplified product size (bp)	Structural annotation		Functional annotation	
								Sequence components of *kabuli* genome	*Kabuli* gene accession IDs	NCBI-KOG	TFs
Ca5_P1_14263455	Ca5	14263455	AATATATATATATATATATATATATA/AATATATATATATATATATATATATATA	ACTTGCAGCAATTTCCATCC	GGATGCTTCCCATAATCTGC	60.1	716	INTERGENIC	--	--	--
Ca5_P1_14307259	Ca5	14307259	TTTATTATTATTATTATTATTATTATTATTATTATTATTATTATTATTATTATTAT/TTTATTATTATTATTATTATTATTATTATTATTATTATTATTATTATTATTATTATTATTAT	GCTCTCCAACAACAAATCAACA	CACTTGAAATTTTAGGCCGC	60.2	798	INTERGENIC	--	--	--
Ca5_P1_14330970	Ca5	14330970	ATTTTTTTTTT/ATTTTTTTTTTT	TGATTAAGAAGTTTTGATTTGCG	GGTTTGGACAATCTGCCTTG	58.5	795	INTERGENIC	--	--	--
Ca5_P1_14344532	Ca5	14344532	GTCT/GT	CCCCAACAGTATCCGAAAAA	TGTTTGTAAATGCTCTCCTAATTGA	59.8	600	DRR	Ca_20838	R	--
Ca5_P1_14394852	Ca5	14394852	CTTTTT/CTTTT	TCTTCCACCCAGTTTTCACC	TTGAGGGCTATGTTTTTCGG	59.9	337	INTERGENIC	--	--	--
Ca5_P1_14419667	Ca5	14419667	TAAAAAAAAAAAAAA/TAAAAAAAAAAAA	ATTTGTCAAGCTTTGGCCTG	CTATTCTCCACGAGCGAAGC	60.2	624	INTERGENIC	--	--	--
Ca5_P1_144346	Ca5	144346	AATTATGACTTTATT/AATT	ATGGTTTAAGTCTGCGGTGG	TGCTGAAGTGGTTCCTTCCT	60.0	259	DRR	Ca_18170	--	--
Ca5_P1_146201	Ca5	146201	TAAAAAAAA/TAAAAAAAAA	GCCTCTATAAACACCGCACC	TTTTTCTCAAACATACCACCTATTAAA	59.6	833	INTERGENIC	--	--	--
Ca5_P1_146635	Ca5	146635	GAAAAAAAA/GAAAAAAAAA	TGCATTTATTACTTCTTTACCAACA	CGAGGAAGGTAATACCATTTTGA	57.2	712	INTERGENIC	--	--	--
Ca5_P1_150887	Ca5	150887	GTTTTTTTTTT/GTTTTTTT	CATTGTTGGTTTGAACCGTG	GAGTTTTGCCACCCAGTCAT	59.9	468	INTERGENIC	--	--	--
Ca5_P1_15176756	Ca5	15176756	AATATATATATATATATATATATATATAT/AATATATATATATATATATATATATATATATAT	ACATTTAGGGCCACTTGGTG	GTTGGTTCCCTAGCACCGTA	59.9	725	INTERGENIC	--	--	--
Ca5_P1_15184248	Ca5	15184248	AGGGGGGG/AGGGGGGGG	TCTAACCCGGAATCCTTTGA	TGAGGATTGAACGTAACCCA	59.5	300	DRR	Ca_21631	--	--

INDEL marker IDs	Chromosomes/ unanchored scaffolds	Physical positions (bp)	InDels (*Kabuli* reference genome-CDC Frontier/PI)	Forward primers (5'-3')	Reverse primers (5'-3')	Anneling temperature (0C)	Expected amplified product size (bp)	Structural annotation		Functional annotation	
								Sequence components of *kabuli* genome	*Kabuli* gene accession IDs	NCBI-KOG	TFs
Ca5_P1_15188475	Ca5	15188475	AA/AAAATA	TTCATTGGATTGTTTGTGCAT	GGTTCAAAAGAGGGGAGGAG	58.9	596	INTERGENIC	--	--	--
Ca5_P1_15219291	Ca5	15219291	AATCAAGTGATCAAGTGATCAA/AATCAAGTGATCAAGTGATCAAGTGATCAA	TGTGTTAGTCGTGTTTGGCA	CCTTTAGATTTTGCATCGCC	58.8	378	DRR	Ca_21632	--	Trihelix
Ca5_P1_15241568	Ca5	15241568	GAAAAAAA/GAAAAAA	CAAATGGAAAAGTTGCACACA	TTGCATATTAGTATTGCATTGTCAAA	59.6	393	INTERGENIC	--	--	--
Ca5_P1_15339602	Ca5	15339602	GAAAAAAAAAA/GAAAAAAAAA	AGGAGAGAAAAAGGGGGAAA	GGGAAACCCACCTCTTGATT	59.1	211	INTERGENIC	--	--	--
Ca5_P1_15341704	Ca5	15341704	GTTT/GTT	AAACACATCAGCTTCCTTCCA	CGAGTTGGGTGAACCAGAAT	59.7	388	INTERGENIC	--	--	--
Ca5_P1_15358764	Ca5	15358764	CTTTTTTTTTTT/CTTTTTTTTTT	AAAGGCAAGGCTCGTTACAA	TTGTGCTATGGTGCTTTTGC	59.9	392	INTERGENIC	--	--	--
Ca5_P1_155645	Ca5	155645	TAAAAAA/TAAAAAAA	GAGTGGTCACTCACGGGTTT	CCAATTTTCCCACTTGGTTG	60.0	773	INTERGENIC	--	--	--
Ca5_P1_1558914	Ca5	1558914	TAAAAAAAAA/TAAAAAAAAAAA	TCAAGATTGCAAACCTGCAT	TCTTCAGGTTCCAGCAAACC	59.3	452	INTERGENIC	--	--	--
Ca5_P1_15604604	Ca5	15604604	TAA/TAAA	ATGTTAACTGGGCGTTGAGG	TCACGGGTCAACACGATTTA	60.0	420	INTERGENIC	--	--	--
Ca5_P1_15634036	Ca5	15634036	CATATATATATATATATATATATAT/CATATATATATATATATATATAT	TTGGAAGGAGCCCTTACCTT	ATGCGTCTTGGAAATTGGAG	60.1	944	INTERGENIC	--	--	--
Ca5_P1_15704508	Ca5	15704508	TTATTATTATTATTAT/TTATTATTATTATTATTAT	CCCAAGTCGATCAAGGAAAA	TCCCAAACAGCCTAACAACC	60.0	421	INTERGENIC	--	--	--
Ca5_P1_15756736	Ca5	15756736	GACAC/GAC	GCGCGATTTACCAAAAATGT	TTTGAGTGGTGGGTTGTGAA	60.0	431	INTERGENIC	--	--	--

INDEL marker IDs	Chromosomes/ unanchored scaffolds	Physical positions (bp)	InDels (*Kabuli* reference genome-CDC Frontier/PI)	Forward primers (5'-3')	Reverse primers (5'-3')	Anneling temperature (0C)	Expected amplified product size (bp)	Structural annotation		Functional annotation	
								Sequence components of *kabuli* genome	*Kabuli* gene accession IDs	NCBI-KOG	TFs
Ca5_P1_15773178	Ca5	15773178	GATTATTATTATTATTATTATTATTATTATTATTATTATTATTATT/GATTATTATTATTATTATTATTATTATTATTATTATTATTATTATT	GCCTTAAAATTTGGCAATGG	TCTTCGCAAATCAAAAGAATGA	59.4	807	INTERGENIC	--	--	--
Ca5_P1_15835478	Ca5	15835478	CAAAA/CAAA	TGCATGATTCCAGAATTCCA	AGCGGTGAGGAGAACTCTGA	60.0	690	INTERGENIC	--	--	--
Ca5_P1_15877436	Ca5	15877436	GC/G	GGGCTACATCCAGTCCTCAC	TTTGGCTGTAGAGGTTCCAAA	59.5	474	INTERGENIC	--	--	--
Ca5_P1_158794	Ca5	158794	TAAAAAA/TAAAAA	TGATGCGTCATGGACATAAA	TTTTAAAAATGTCCATTTACACCG	58.5	498	INTERGENIC	--	--	--
Ca5_P1_15885113	Ca5	15885113	CTTT/CTT	CAACCTCCATGACATGCACT	TGTCGGAGGGATTATTCGAG	59.6	409	INTERGENIC	--	--	--
Ca5_P1_15918981	Ca5	15918981	TTTATTATTATTATTATTATTATTATTATTATTATTATTATTATTATT/TTTATTATTATTATTATTATTATTATTATTATTATTATTATTATTATTATTATTATT	CCTCCCCCATTTGTCTTCTT	GCCTGATGTTCGAATTTTTCA	60.3	462	INTERGENIC	--	--	--
Ca5_P1_15929247	Ca5	15929247	AACCAC/AAC	TGATGACGAAGACGAAGACG	AACACATCCGGGATCCATTA	60.0	416	INTERGENIC	--	--	--
Ca5_P1_15929348	Ca5	15929348	ACCC/ACC	TGATGACGAAGACGAAGACG	AACACATCCGGGATCCATTA	60.0	416	INTERGENIC	--	--	--
Ca5_P1_15984521	Ca5	15984521	TAAAAAAAAAA/TAAAAAAAAAAA	TTGTTTCTAGGATAAGTTTGTGATGC	CCATGCTTGTTGCACAATTT	60.0	811	INTERGENIC	--	--	--
Ca5_P1_160572	Ca5	160572	TAAAAAAAAA/TAAAAAAAA	AATCCAATGGCTTTCAATCAA	CACACGACAAAGTGGAGTATCAG	59.4	570	INTERGENIC	--	--	--
Ca5_P1_16097947	Ca5	16097947	TATAATAATAATAATAATAATAATAATAATAATAATAATAATAATAATAATAA/TATAATAATAATAATAATAATAATAATAATAATAATAATAATAATAATAATAATAA	AAAATCGATTATGGCACCCA	CAATTAGCTCTTTCGCCACA	60.2	862	INTERGENIC	--	--	--
Ca5_P1_16251480	Ca5	16251480	T/TAGTGTAGAAAGAGTG	TCAGAATCAACCGAGTTCCC	TAAACACACTCGTCCCCACA	60.1	249	INTERGENIC	--	--	--

INDEL marker IDs	Chromosomes/ unanchored scaffolds	Physical positions (bp)	InDels (*Kabuli* reference genome-CDC Frontier/PI)	Forward primers (5'-3')	Reverse primers (5'-3')	Anneling temperature (0C)	Expected amplified product size (bp)	Structural annotation		Functional annotation	
								Sequence components of *kabuli* genome	*Kabuli* gene accession IDs	NCBI-KOG	TFs
Ca5_P1_16319965	Ca5	16319965	TGGG/TGGGG	TTGTGTGCAGGATCCACATT	TTGACCATTGCACGAAACAT	60.0	502	INTERGENIC	--	--	--
Ca5_P1_16363340	Ca5	16363340	GTT/GTTT	AATCCTGGTGCACGATTTTC	ACATCGCCATTACCGAACTC	59.9	445	DRR	Ca_17921	R	MYB_related
Ca5_P1_16393546	Ca5	16393546	CTTTTTTTTTT/CTTTTTTTTT	CACAACTCCGCCATTACCTT	GTTGCAAGTTGCCGTGTCTA	60.0	713	INTERGENIC	--	--	--
Ca5_P1_164138	Ca5	164138	T/TGTAAATG	GGTAAAAGAGACTCGCGTGG	CTGATGTGCTCGTTGTTGCT	59.9	695	INTERGENIC	--	--	--
Ca5_P1_16462192	Ca5	16462192	CAAAA/CAAAAA	TCTGGTTTTGCTGATTCCTG	AGAGCTTGGCAAGGAACAAA	58.8	475	INTERGENIC	--	--	--
Ca5_P1_16469239	Ca5	16469239	GTATATATATATATATATATATATATATAT/GTATATATATATATATATATATATAT	TCGGTGTCAAATGAAGACCTC	CGATTTGACAACATCACGCT	60.1	561	INTERGENIC	--	--	--
Ca5_P1_16471569	Ca5	16471569	AATATATATATATATATATATAT/AATATATATATATATATAT	TACGAGCCAACAGTTGATGC	TTTAAGTCCATGCTCGCTGA	59.9	618	INTERGENIC	--	--	--
Ca5_P1_16487729	Ca5	16487729	TAAAAAAAAAAA/TAAAAAAAAAAAA	GTTTTTCAAAAGCGAGGGTG	CCTCAGATGAGAGCCAGGAC	59.7	443	INTERGENIC	--	--	--
Ca5_P1_16650628	Ca5	16650628	GTTTTTT/GTTTTTTT	CCCGAAAGTCCTCCAAAAAT	CCAAGTTCCCCAGAGTCAAA	60.3	583	INTERGENIC	--	--	--
Ca5_P1_16715155	Ca5	16715155	CTTTTTTTTTT/CTTTTTTTTTTT	TGGGTTCACCCCAAATTAAA	TCATCAAATCCGTAACGCAA	60.0	459	INTERGENIC	--	--	--
Ca5_P1_167611	Ca5	167611	CAAA/CA	TTTTGGGTGGAAGCGTATGT	TGTTGAACGAAGCCCTCTCT	60.4	492	DRR	Ca_18171	--	NAC
Ca5_P1_16765808	Ca5	16765808	TAGA/TA	CGATCGTGCTACGTCTTCAA	TGGTGGGAATGTTGTGATTG	60.0	677	DRR	Ca_17941	R	HSF

INDEL marker IDs	Chromosomes/ unanchored scaffolds	Physical positions (bp)	InDels (*Kabuli* reference genome-CDC Frontier/PI)	Forward primers (5'-3')	Reverse primers (5'-3')	Anneling temperature (0C)	Expected amplified product size (bp)	Structural annotation		Functional annotation	
								Sequence components of *kabuli* genome	*Kabuli* gene accession IDs	NCBI-KOG	TFs
Ca5_P1_1677289	Ca5	1677289	CTATATATATATATATATATATATATATAT/CTATATATATATATATATATATATATATATATAT	AAGCGCTGTAAAAGGTGCAT	ATGTGAATCCCACCAGGAAA	59.9	794	INTERGENIC	--	--	--
Ca5_P1_16780605	Ca5	16780605	AATATATATATATATATATATATATAT/AATATATATATATATATATATATATATATATATATATAT	AAAAGACCGCATCAACCATC	TTGAAATCATGGACGTGGTG	59.9	292	INTERGENIC	--	--	--
Ca5_P1_16781746	Ca5	16781746	ATTTTTTTTTTT/ATTTTTTTTTT	GCCATTTGGAATGACGACTT	TTTGGGAAATTGACCTTTCG	59.9	527	INTERGENIC	--	--	--
Ca5_P1_1678181	Ca5	1678181	TTATATATATAT/TTATATATATATATAT	CGGTGAATCCTTTACGGAGA	AGGAAGAGTGGAGTTGCGAA	60.1	384	INTERGENIC	--	--	--
Ca5_P1_16808865	Ca5	16808865	ATATGT/AT	GATCGCACTTTTGTCAAGCA	GCTTAAGGATGCCATTTTGA	60.0	777	INTERGENIC	--	--	--
Ca5_P1_16881811	Ca5	16881811	AT/ATATGT	AATCAATCACAACCGTTAAATCA	CACGTTGCTCTTGGTCTTGA	58.4	902	INTERGENIC	--	--	--
Ca5_P1_168831	Ca5	168831	GTGGTTGGTT/GTGGTT	CGAGATACGCGTCATTAAAAA	TCGTCGAGAACAAGTGCATC	58.0	358	INTERGENIC	--	--	--
Ca5_P1_16889364	Ca5	16889364	AATATATATATATATATATAT/AATATATATATATATATATATATATAT	GGAGAAAATTCATTTGGCATT	CCTTTGCATGGGACAGTTTT	58.0	985	INTERGENIC	--	--	--
Ca5_P1_16946068	Ca5	16946068	CAAAA/CAAAAA	ACATCCGTTCCCTCTGTGTC	TTTCGAAACCCATGATGTTG	60.0	786	INTERGENIC	--	--	--
[truncated: 715,188 more chars]
