# Supplementary material for: A High-Resolution InDel (Insertion–Deletion) Markers-Anchored Consensus Genetic Map Identifies Major QTLs Governing Pod Number and Seed Yield in Chickpea
Source: Front Plant Sci. 2016 Sep 16;7:1362. doi: 10.3389/fpls.2016.01362 (PMC5025440; doi:10.3389/fpls.2016.01362)
Supplement: Supplementary file 2 [file Table2.PDF]

Table S2. InDel markers (24166) differentiating low pod number-containing parental accession (ILWC 46) and homozygous bulk (LPNB) of a mapping population (Pusa 1103 x ILWC 46) from reference *kabuli* accession (CDC Frontier)

INDEL marker IDs	Chromosomes/unanchored scaffolds	Physical positions (bp)	InDels (*Kabuli* reference genome-CDC Frontier/PII)	Forward primers (5'-3')	Reverse primers (5'-3')	Anneling temperature (0C)	Expected amplified product size (bp)	Structural annotation		Functional annotation	
								Sequence components of *kabuli* genome	*Kabuli* gene accession IDs	NCBI-KOG	TFs
C11060010_P2_682	C11060010	682	AATTATT/AATTATTATT	TCCGATATGCACCAATGAAA	AGGCGTCGGTTTTTCTTTCT	60.2	413	INTERGENIC	--	--	--
C11108026_P2_1126	C11108026	1126	TTTAATTAATTAATTAATTAATTAATTAATT/TTTAATTAATTAATTAATTAATTAATT	TGGTCGCAAAAGCATATTCA	ATGGTGACCGTGCAATTTTT	60.2	529	INTERGENIC	--	--	--
C11108026_P2_1365	C11108026	1365	CAAAAAAAAAA/CAAAAAAAAA	CACGGTCACCATGTGCTATT	GGGTGAAATTGGTTTACAACG	59.2	544	INTERGENIC	--	--	--
C11119398_P2_787	C11119398	787	AATATA/AATA	AGCTCCAAATTTGATGGCAC	GTGGCGAGTGTTCCATTTCT	60.1	281	INTERGENIC	--	--	--
C11153802_P2_2086	C11153802	2086	CTT/CT	TGGAATTCCTAGCATAACCCAC	TGCGGTGTTTATTTGATTTCAG	60.0	512	INTERGENIC	--	--	--
C11154862_P2_1914	C11154862	1914	ATTTT/ATTTTT	GTCCAATCCCCAAACAACAC	TGAAATTATGGAAAGGAAGCTCA	60.1	521	INTERGENIC	--	--	--
C11154862_P2_4136	C11154862	4136	TAAAAAAA/TAAAAAA	GGGTTTCGGCCTCTTCTATC	CCTTTCAATTCAACATGGCA	59.5	856	INTERGENIC	--	--	--
C11155258_P2_5339	C11155258	5339	TTAAT/TT	CCCACATTTCATGATTTCCC	GCTTCAATGAAATCGCAATACA	60.1	456	INTERGENIC	--	--	--
C11155542_P2_2583	C11155542	2583	TAAAAAAAAAAAAA/TAAAAAAAAAAAAAA	TTGAACGCAGTTGATTTTGG	TTCAAATCATGCGGGTTTTT	60.3	512	INTERGENIC	--	--	--
C11159426_P2_3322	C11159426	3322	A/ATATAGAC	TCTCCCAAGAACTGTTGCAG	TTTGACTCGATGCACCTTCA	60.4	319	DRR	Ca_27806	--	--
C11159426_P2_3626	C11159426	3626	AAACGA/AA	CACATTGAAGGTGCATCGAG	TTGTGACGCTAACGACTTGG	59.9	462	DRR	Ca_27806	--	--

INDEL marker IDs	Chromosomes/unanchored scaffolds	Physical positions (bp)	InDels (*Kabuli* reference genome-CDC Frontier/PII)	Forward primers (5'-3')	Reverse primers (5'-3')	Anneling temperature (0C)	Expected amplified product size (bp)	Structural annotation		Functional annotation	
								Sequence components of *kabuli* genome	*Kabuli* gene accession IDs	NCBI-KOG	TFs
C11168930_P2_7035	C11168930	7035	ATTTTTT/ATTTTTTT	CGGCAACGTCCAAGATAGTT	CCATTTCAGACTGCGTGATG	60.3	131	INTERGENIC	--	--	--
C11169592_P2_2162	C11169592	2162	TAAAAAAAAA/TAAAAAAAAAA	TTATTTCAACCGTTTCTCTTTCTT	TGTGCACATCAAACATATGACA	58.5	911	INTERGENIC	--	--	--
C11169592_P2_3333	C11169592	3333	CTTTT/CTTTTT	TCTTTTTCCGATGTTTTCACG	GGGTGCCATCAACACATACA	60.2	125	INTERGENIC	--	--	--
C11174756_P2_4346	C11174756	4346	ATATTATTATTATTATTATTATTATTATTATTATTATTATTATTATTATTATTA/ATATTATTATTATTATTATTATTATTATTATTATTATTATTA	ATCTCGAACAATTATGCCGT	TGCCATAAATTTTACCGACG	58.6	619	INTERGENIC	--	--	--
C11177592_P2_10360	C11177592	10360	TCCC/TCC	GCTCCCAAAGAAGAAACACG	TTTCGATGGATACTTGAGCAGA	59.8	742	DRR	Ca_27747	--	--
C11178328_P2_12753	C11178328	12753	CAT/CATTAT	CCATCTTTTAGGCCTCCTCC	GAAGGCAACCATTCTAGGCA	60.2	925	DRR	Ca_28266	T	M-type
C11180336_P2_12584	C11180336	12584	CAAAAAAAAA/CAAAAAAAAAA	TCCAAGTTTTCTCCACGTGTT	GCATTTTTGAGTGATTTGCG	59.3	498	INTERGENIC		--	--
Ca1_P2_10034627	Ca1	10034627	ATTTTTTTTTTTTTT/ATTTTTTTTTTTTT	GAGGCGAAGGATTTGAATGA	ATTGAGATGTGTGGGTGGGT	60.1	302	DRR	Ca_02785	T	--
Ca1_P2_10040103	Ca1	10040103	ATTT/ATT	ATGCATCGAAAACCAACACA	TGCAATCCGGGCTTACTATC	60.1	378	INTRON	Ca_02784	LK	--
Ca1_P2_10057638	Ca1	10057638	CATATATATATATATATAT/CATATATATATATAT	TGATGGGAGCAATGAATGAA	TCAAATTCCGACTTGAGGGA	60.6	542	INTERGENIC	--	--	--
Ca1_P2_10064752	Ca1	10064752	TTATATATATATATATATA/TTATATATATATATATATATATATATA	ATGCAAATTCTCCCCCTCTT	TCATTTGATTTTAATTGGACGA	57.2	750	INTERGENIC	--	--	--
Ca1_P2_10072230	Ca1	10072230	TGG/TG	TGGAAATGGTTTTGATGGGT	TGATGCGTACCATTTTGCAT	60.0	154	INTERGENIC	--	--	--

INDEL marker IDs	Chromosomes/unanchored scaffolds	Physical positions (bp)	InDels (*Kabuli* reference genome-CDC Frontier/PII)	Forward primers (5'-3')	Reverse primers (5'-3')	Anneling temperature (0C)	Expected amplified product size (bp)	Structural annotation		Functional annotation	
								Sequence components of *kabuli* genome	*Kabuli* gene accession IDs	NCBI-KOG	TFs
Ca1_P2_10168587	Ca1	10168587	ATATTATTATTATTATTATTATTATTA/ATATTATTATTATTATTATTATTATTATTA	AGACAGGCATGGAGAAATGG	TTTAAGGCGTGGATGTCGTT	60.5	632	INTERGENIC	--	--	--
Ca1_P2_1027317	Ca1	1027317	C/CG	AAGAGGTCGGGGTTGAACTT	TCAACTTGAATTGGGTGCAA	60.1	745	INTERGENIC	--	--	--
Ca1_P2_10323177	Ca1	10323177	G/GAGCT	TTGAGTGCTTCACCATTTGC	GTTGTATTGGCTCTCCTCGC	59.8	463	INTRON	Ca_02746	B	C2H2
Ca1_P2_10414677	Ca1	10414677	CAGGAATNNNNNNNNNNAGGAAT/CAGGAAT	TTCTAAACGAGGCTTTTCAGTTG	GCATGTTGCTGATGATGGTC	60.1	489	DRR	Ca_02735	R	ERF
Ca1_P2_10418186	Ca1	10418186	TT/TTAT	CAACAAAAGCAAGCAACGAA	CTTGTGGGAAGCATGGAGTT	60.1	560	CDS (FRAME SHIFT)	Ca_02735	R	ERF
Ca1_P2_10433899	Ca1	10433899	TAAAAAAAAAAAAAAAAA/TAAAAAAAAAAAAAAA	TTTGGCACCTTGATTTCACA	TGTTTGGTAAGGCTCATTTGG	60.0	610	INTERGENIC	--	--	--
Ca1_P2_10458891	Ca1	10458891	TACACACA/TACACA	CCAATTTCAGTAGGCCCAAA	AGCATTGAAACCAAACCAGC	60.1	583	DRR	Ca_02729	--	HSF
Ca1_P2_10471260	Ca1	10471260	TTATATATATATATATATATA/TTATATATATATATATATATA	TTCTCCATTCGCACTCACAC	TCAAATTTGGAGGCTGGAAC	60.1	312	INTERGENIC	--	--	--
Ca1_P2_10513925	Ca1	10513925	CTTATTTATTTATTTATTTATTTATTTATTTATTTATTTATT/CTTATTTATTTATTTATTTATTTATTTATT	CCAGATTTCCTTTTCCCCTC	GGGTAGGCCACAGACTGTCTTA	60.5	634	INTERGENIC	--	--	--
Ca1_P2_10733080	Ca1	10733080	T/TG	CCGGTAACGAGTTGTGTTCA	AAAATGGCACGTTTTTCTGG	60.0	428	DRR	Ca_02689	--	DBB
Ca1_P2_10756482	Ca1	10756482	CTTTTTTTTTT/CTTTTTTTTTTT	GTTCGAATCTCTCAGGCGAC	CACTACCATGGTGAGCTGGA	59.7	608	INTERGENIC	--	--	--
Ca1_P2_1084300	Ca1	1084300	A/AC	TCCCAAATGACTTAATCCGC	ATCGTCGGAGAAATTTGACG	60.1	608	INTRON	Ca_00134	DO	LBD

INDEL marker IDs	Chromosomes/unanchored scaffolds	Physical positions (bp)	InDels (*Kabuli* reference genome-CDC Frontier/PII)	Forward primers (5'-3')	Reverse primers (5'-3')	Anneling temperature (0C)	Expected amplified product size (bp)	Structural annotation		Functional annotation	
								Sequence components of *kabuli* genome	*Kabuli* gene accession IDs	NCBI-KOG	TFs
Ca1_P2_10843023	Ca1	10843023	TT/TTATTATTATTATTAT	CCGATATCTTCCTGCGATGT	CACGGTTCAAAGTGACAACTTC	59.7	192	INTRON	Ca_02674	G	NAC
Ca1_P2_1084940	Ca1	1084940	CTTT/CTTTT	CGTCAAATTTCTCCGACGAT	CTGCGAGTGTTTTCTGGTCA	60.0	290	INTRON	Ca_00134	DO	LBD
Ca1_P2_10858035	Ca1	10858035	TTTCTTCTTCTTCTTCTTCTTCTTCTTCTT/TTTCTTCTTCTTCTTCTTCTTCTTCTTCTTCTT	TCATTTGGAAAAGCACACCA	CCACGAAGTCCCCAAGATAA	59.9	237	INTERGENIC	--	--	--
Ca1_P2_1085960	Ca1	1085960	CTGTGT/CTGT	GCCATCTTCACAAGCACTGA	CTTCTAATCCATTGCCCCAA	59.9	556	INTRON	Ca_00134	DO	LBD
Ca1_P2_1088478	Ca1	1088478	A/AC	GGCATCACATTTGTGTCGAA	TCACCCCACTATCCCGTTTA	60.2	717	DRR	Ca_00135	--	C2H2
Ca1_P2_1091564	Ca1	1091564	TCAGCAGCAGCAGCAG/TCAGCAGCAGCAGCAGCAG	CCAACATGACCATTGTAGCG	TTTGTTTGCTGTCAGCTTGG	60.0	708	INTRON	Ca_00135	--	C2H2
Ca1_P2_1092166	Ca1	1092166	TCC/TC	CCAAGCTGACAGCAAACAAA	TTATTGATCTCGCTGCGTTG	60.0	782	INTRON	Ca_00135	--	C2H2
Ca1_P2_10922594	Ca1	10922594	ATTTTTTTTTT/ATTTTTTTTTTT	TCATCATGGGTCTTTCTTTCG	CTGTTTCGCCAAAGCTTCTT	59.6	604	INTERGENIC	--	--	--
Ca1_P2_10944660	Ca1	10944660	TAAAAAAAAAA/TAAAAAAAAAAA	GCGACCTCGTGAATGAAAAA	GGCATAATTGTTCCCACAAA	58.3	534	INTERGENIC	--	--	--
Ca1_P2_1094498	Ca1	1094498	AC/A	TTGAGGAATGGTGCAAGATG	TGTACTTGCTATTCATTCTCGTTGA	60.2	254	INTERGENIC	--	--	--
Ca1_P2_1095096	Ca1	1095096	TTATA/TTATATA	TTTTGCAAAGATATTAAAGGGG	TTGTTGTGATGGATTGGGTG	60.2	957	INTERGENIC	--	--	--
Ca1_P2_1095596	Ca1	1095596	T/TCACAA	CACCCAATCCATCACAACAA	GCTTCTCCTTGCCATCAAAG	60.0	540	INTERGENIC	--	--	--

INDEL marker IDs	Chromosomes/unanchored scaffolds	Physical positions (bp)	InDels (*Kabuli* reference genome-CDC Frontier/PII)	Forward primers (5'-3')	Reverse primers (5'-3')	Anneling temperature (0C)	Expected amplified product size (bp)	Structural annotation		Functional annotation	
								Sequence components of *kabuli* genome	*Kabuli* gene accession IDs	NCBI-KOG	TFs
Ca1_P2_10960960	Ca1	10960960	TAAAAAAAAAAA/TAAAAAAAAAA	ACTTTGGAAGTTTGCCTCCC	TAAATCGAACGGGATACACG	58.5	602	INTERGENIC	--	--	--
Ca1_P2_1097354	Ca1	1097354	TACAACAACAA/TACAACAACAACAA	GGGACAAACTTGGAGTTGGA	TGCATGCCAATTAAATAAATCA	58.1	691	INTERGENIC	--	--	--
Ca1_P2_1100487	Ca1	1100487	ATTTTT/ATTTTTT	TTGCATTGAAAATAGCAACTATTGA	TAGACTGAACCAATGGCACG	59.7	531	INTERGENIC	--	--	--
Ca1_P2_1101385	Ca1	1101385	TAAAAAA/TAAAAAAA	TTCTTTTAATCGCGACTGGG	GAGCTTAGGTGAGGATGGCA	60.4	343	INTERGENIC	--	--	--
Ca1_P2_1102835	Ca1	1102835	A/AAT	TGGCATCTGATTGATGAAAGA	GGGACCCACATGGACTTCTA	59.8	858	INTERGENIC	--	--	--
Ca1_P2_11040099	Ca1	11040099	CAAA/CAA	AAATAAGGGGACCAATTCAATG	TGGAGGGAGGTGCAAAATAC	59.9	224	INTERGENIC	--	--	--
Ca1_P2_1104030	Ca1	1104030	G/GGTACAAAGA	CACCGACAATAATCCTTCCA	GCCATTTGCATTGAGTTCCT	60.1	441	INTERGENIC	--	--	--
Ca1_P2_11102279	Ca1	11102279	ATTTTTTTTTT/ATTTTTTTTT	TGCCGCGAATTTAGATCTTT	TCGGTGTTGGTGTCGTTATC	59.4	255	DRR	Ca_02649	C	--
Ca1_P2_1113940	Ca1	1113940	ATCAAAATCTCAAAATC/ATCAAAATCTCAAAATCTCAAAATC	GGACCCAAAAATCCAATTCC	TTGTCACTTGCGCCATAGAG	60.0	288	INTERGENIC	--	--	--
Ca1_P2_11145101	Ca1	11145101	TTATATATATATATATATATATATAT/TTATATATATATATATATATAT	TTTGAGTCCAAATTGAAGGCT	CGGATCAAAATTCGCACAAT	60.8	837	INTERGENIC	--	--	--
Ca1_P2_11148737	Ca1	11148737	CAATAATAATAAT/CAATAATAAT	CGTGTAATTGGTAATGTGGCA	GGCTAACTTGAAGTCGTGGC	59.9	693	INTERGENIC	--	--	--
Ca1_P2_11161526	Ca1	11161526	ATATATATTT/AT	TGGAACAATCCGATACCGTT	AAAAACATGATTCCAACGCC	59.8	676	DRR	Ca_02643	R	--

INDEL marker IDs	Chromosomes/unanchored scaffolds	Physical positions (bp)	InDels (*Kabuli* reference genome-CDC Frontier/PII)	Forward primers (5'-3')	Reverse primers (5'-3')	Anneling temperature (0C)	Expected amplified product size (bp)	Structural annotation		Functional annotation	
								Sequence components of *kabuli* genome	*Kabuli* gene accession IDs	NCBI-KOG	TFs
Ca1_P2_11162292	Ca1	11162292	CAAA/CA	GGCGTTGGAATCATGTTTTT	TGTAGCGGAAGCATCAACAG	60.0	556	INTERGENIC	--	--	--
Ca1_P2_11170203	Ca1	11170203	CATA/CATATATA	ATGAGGGTATCCGAGCCATA	CCACAAGAGTTTTGCCATTG	59.2	378	DRR	Ca_02641	--	--
Ca1_P2_11170701	Ca1	11170701	GGAGTTGTGTGTCGTGAGTTGTGTG/GGAGTTGTGTGTCGTGAGTTGTGTGTCGTGAGTTGTGTG	GCAATGGCAAAACTCTTGTG	ATCTCGAAAAGGGGTTCCAT	59.8	901	DRR	Ca_02642	U	--
Ca1_P2_11190216	Ca1	11190216	ATTACT/ATTACTTACT	TATGGCTAGCTGGTGAAGCA	TAGAATCCCACGCAGCTCTT	60.0	304	INTRON	Ca_02639	--	--
Ca1_P2_11241105	Ca1	11241105	ATTTTTTT/ATTTTTTTT	TTCAGTCAAAATATCTGCATGGTT	AGCCTCGTGGGATTAGGATT	59.9	348	INTERGENIC	--	--	--
Ca1_P2_11324975	Ca1	11324975	GAAGA/GAAGAAAGA	CTGCATCGGGGATACTTCAC	TTTTGACCGAGCCAGATACC	60.1	317	INTRON	Ca_02622	--	--
Ca1_P2_11400460	Ca1	11400460	GGAGGATTGTAGAAATTCTG/GGAGGATTGTAGAAATTCTGAGGATTGTAGAAATTCTG	CAAATGGCAAATCACGTGTC	CTGCACTACTTGCAGGTCCA	60.0	361	CDS (large-effect mutations)	Ca_02612	F	--
Ca1_P2_11413234	Ca1	11413234	ATTTT/ATTT	ACGCTTGGACTGAGCTTCAT	CGGACAACTCACCAAACCTT	60.0	517	INTRON	Ca_02610	Z	Dof
Ca1_P2_11456505	Ca1	11456505	TTATATATATATATATATATA/TTATATATATATATATATATATA	AACCGAGTGCCCTTTAACAA	TTGTTTTGCATTTTGGGACA	59.9	273	INTERGENIC	--	--	--
Ca1_P2_11521081	Ca1	11521081	GTTATTATT/GTTATTATTATT	GGTCATGCTGATAGGTGGATG	ACCACAACCTTGGAATGGAA	60.2	908	INTERGENIC	--	--	--
Ca1_P2_11558313	Ca1	11558313	TTATATATATATATATATATATATATATATATATATATATAT/TTATATATATATATATATATATATATATATATATATATATATATATATATATATAT	TGGGTCACTAAAACCTTGCC	GCTTCAACATATTTGGAATGTCA	59.0	679	INTERGENIC	--	--	--
Ca1_P2_1160199	Ca1	1160199	GATATATATATATATATATATATATA/GATATATATATATATATATATATATATATA	ATATTCGGGGAATCACCTCC	AGCCGACCTTCCCACTTAAT	60.0	578	DRR	Ca_00145	--	--

INDEL marker IDs	Chromosomes/unanchored scaffolds	Physical positions (bp)	InDels (*Kabuli* reference genome-CDC Frontier/PII)	Forward primers (5'-3')	Reverse primers (5'-3')	Anneling temperature (0C)	Expected amplified product size (bp)	Structural annotation		Functional annotation	
								Sequence components of *kabuli* genome	*Kabuli* gene accession IDs	NCBI-KOG	TFs
Ca1_P2_11728121	Ca1	11728121	TAAA/TAA	ACGTTGACCAATTCAGCTCC	CAAAATCGCCAGCACTGTAA	59.9	502	INTERGENIC	--	--	--
Ca1_P2_11735185	Ca1	11735185	GTATTATTATTATTATT/GTATTATTATTATTATTATTATT	TGACAGTCAGAGTGCGATCC	TCTCTGGCAACCGTTCTTCT	60.0	441	INTERGENIC	--	--	--
Ca1_P2_11786498	Ca1	11786498	TTATATATATATATATATATATATATATATAT/TTATATATATATATATATATATATATAT	TCACCTTTTACTCTTCAAAATGGA	CTACAACCGGAGGAGTTGGA	60.1	784	INTERGENIC	--	--	--
Ca1_P2_11811525	Ca1	11811525	AG/AGG	TCTTTGGCCTTTCATCCATC	AACCTGCGCATTTTGCTAAC	60.3	810	INTERGENIC	--	--	--
Ca1_P2_11868314	Ca1	11868314	TTATATATATATATATAT/TTATATATATATATATATATATAT	GCAAGTATGCAACAACCTTCC	TTGCATATATTGTCACACCGC	59.5	346	INTERGENIC	--	--	--
Ca1_P2_11912515	Ca1	11912515	CATATATATATATATAT/CATATATATATATATATAT	TGAGTCCGATTGATTGATGC	TGGCACAGTGAGTATCTCATACAA	59.7	665	INTERGENIC	--	--	--
Ca1_P2_11977187	Ca1	11977187	CAAAAAA/CAAAAAAA	GCAATTGGATGACATTGCATA	ACAGACAGGGCACAGGATCT	59.7	479	INTERGENIC	--	--	--
Ca1_P2_12036536	Ca1	12036536	A/AGG	GAGGAAGACGAAACGAACCA	CCTTGAGATCGAAAGACCCA	60.2	672	INTERGENIC	--	--	--
Ca1_P2_12044432	Ca1	12044432	GCTTCTTCTTCTTCTTCTTCTTCTTCTTCT/GCTTCTTCTTCTTCTTCTTCTTCTTCTTCTTCT	TCTCAACAAACTCTTCGCTTTG	TGATCGGAGAGAGAGGAGGA	60.0	185	INTERGENIC	--	--	--
Ca1_P2_1209194	Ca1	1209194	GAAAA/GAAAAA	TATGGAGTCGCCACCAAAAT	TCTCCTTGAGTTTTGAGACACG	59.5	548	INTERGENIC	--	--	--
Ca1_P2_12092762	Ca1	12092762	AT/ATATGT	CGGCAATTTTGAGTTGATTTG	CATCGGATGGTCCAGAAAGT	59.9	825	INTERGENIC	--	--	--
Ca1_P2_12110120	Ca1	12110120	A/ATT	TGAATTGGGCCAAGACATTT	TGGCAGACCACAGATAACCA	60.1	943	INTERGENIC	--	--	--

INDEL marker IDs	Chromosomes/unanchored scaffolds	Physical positions (bp)	InDels (*Kabuli* reference genome-CDC Frontier/PII)	Forward primers (5'-3')	Reverse primers (5'-3')	Anneling temperature (0C)	Expected amplified product size (bp)	Structural annotation		Functional annotation	
								Sequence components of *kabuli* genome	*Kabuli* gene accession IDs	NCBI-KOG	TFs
Ca1_P2_12112319	Ca1	12112319	C/CTTGG	GCGAAATTATTTGACGGAGTTT	CATTTCAGAAGTGAAGAGCAACA	59.5	874	INTERGENIC	--	--	--
Ca1_P2_12135832	Ca1	12135832	A/ATATATAT	GGTGTTGGAGTCATGCCTTT	GTCGCACGGAAACAACGTA	60.7	234	INTERGENIC	--	--	--
Ca1_P2_12180244	Ca1	12180244	GTTTTTTTTTTTT/GTTTTTTTTTTTTTT	CGCTTAAAGGCAACGCTATC	TGCAACAAGACGGAAAAACA	60.3	257	INTERGENIC	--	--	--
Ca1_P2_12195477	Ca1	12195477	CTTTTTTTTT/CTTTTTTTTTT	TTGTTTGTTTCCTTGTGGCA	AGAAAAATGCGATGGTTCTTT	57.9	250	INTERGENIC	--	--	--
Ca1_P2_12225439	Ca1	12225439	CTAGTTT/CTAGTTTTAGTTT	GCATAGGTGGCAGACGAAGT	GCTTTCGGTTTCCTGTTTCA	60.2	721	INTERGENIC	--	--	--
Ca1_P2_1228619	Ca1	1228619	TTATATATATATATATATATATATATATA/TTATATATATATATATATATATA	TTGTTAGTTTGACGAGGGCA	CCTTCTCATTGGCAACCCTA	60.1	499	INTERGENIC	--	--	--
Ca1_P2_12328187	Ca1	12328187	TTAAATTTAACAATAAAT/TTAAAT	ATTTGTCCAATGAGCCATCC	AGACCCAACATAGTGCCGTC	60.0	544	INTERGENIC	--	--	--
Ca1_P2_1237664	Ca1	1237664	GTC/G	CATCATCAATGAACGAGCCA	CGTAACTTGTGTTTTGCATGAGA	60.2	626	INTERGENIC	--	--	--
Ca1_P2_12392278	Ca1	12392278	TC/T	ATCTTAGGACTGGCCCTGGT	TGAACCATGACTGCAACACA	59.7	820	INTERGENIC	--	--	--
Ca1_P2_12420239	Ca1	12420239	CAAAAAAAAA/CAAAAAAAAAA	AAATGCAATCTTGGTTTCCA	TTTTGGTAATGTGCCCATGATA	60.1	595	INTERGENIC	--	--	--
Ca1_P2_12425587	Ca1	12425587	TAAAAAAAAAA/TAAAAAAAAAAA	TGTCTCCCGAAACTTTTGTTTT	AGCAATCATTTGAACTGGGC	60.1	419	INTERGENIC	--	--	--
Ca1_P2_12538333	Ca1	12538333	CTTTTTT/CTTTTT	ATTTTTCGTCGCTCTTCCAA	TTTTCCGACGTCGATGATAA	59.1	195	INTERGENIC	--	--	--

INDEL marker IDs	Chromosomes/unanchored scaffolds	Physical positions (bp)	InDels (*Kabuli* reference genome-CDC Frontier/PII)	Forward primers (5'-3')	Reverse primers (5'-3')	Anneling temperature (0C)	Expected amplified product size (bp)	Structural annotation		Functional annotation	
								Sequence components of *kabuli* genome	*Kabuli* gene accession IDs	NCBI-KOG	TFs
Ca1_P2_12550318	Ca1	12550318	ATTTTTTTTTTT/ATTTTTTTTTT	CGTCTTTATTTTATGCGAGCG	TAATGCCGATGGTGTCAAAA	59.9	252	INTERGENIC	--	--	--
Ca1_P2_12552821	Ca1	12552821	TAATAAATAAATAAATAAATAAA/TAATAAATAAATAAATAAATAAA	GCGTTTTTGTATCGTCCGTT	TTTTTGGAAAAAGCGCTGTAA	59.9	408	INTERGENIC	--	--	--
Ca1_P2_12571969	Ca1	12571969	ATT/AT	TTTTGCCTTGCTATTTTGATG	ATTCATACTCACTTGCGGGG	60.0	426	INTERGENIC	--	--	--
Ca1_P2_12580190	Ca1	12580190	TAAAAAAAAAA/TAAAAAAAAAAA	TAATTCACTCACCATCGCCA	TTCATGTTTGGTGTGGCACT	60.0	476	INTERGENIC	--	--	--
Ca1_P2_12620911	Ca1	12620911	T/TGCAATA	TTTATTTGGGTCATCCATTTGTT	CAATTTTAAAAGCGAGGAGGTG	60.1	615	INTERGENIC	--	--	--
Ca1_P2_12633621	Ca1	12633621	TT/TTAGATATCGAATGAT	TCTAAATTTGTCATATTGTCCCACTT	GACAAATACGGATAATGTCACCTTT	59.6	695	INTERGENIC	--	--	--
Ca1_P2_12644844	Ca1	12644844	CGG/CG	ACCCAACCGCAAAAACAATA	TGCCAGGATGTAACCCTTTC	59.9	440	INTERGENIC	--	--	--
Ca1_P2_12662540	Ca1	12662540	ATTTTTTTTTTT/ATTTTTTTTTT	AAGTGACACATGATGACCGTG	CCCGGTTTTGGAATAGGACT	60.2	560	INTERGENIC	--	--	--
Ca1_P2_12699932	Ca1	12699932	ACCCC/ACCC	ACCGAATGTCGAACTCAAGG	TTGGGAACTTGGGAGACTTG	60.1	768	INTERGENIC	--	--	--
Ca1_P2_12721902	Ca1	12721902	CATATATATATATATATATATATATAT/CATATATATATATATATATATAT	CGCATGATTCAAACAAATGG	CCAACTCAGGTTCAGCAAGA	59.0	746	DRR	Ca_02480	--	--
Ca1_P2_12727627	Ca1	12727627	TTATATATATATATATATATATATAT/TTATATATATATATATATAT	CCTTACGGACAACCAAGCAT	GCAAAAGAATTTGCAACAAAGA	59.4	419	INTERGENIC	--	--	--
Ca1_P2_12772623	Ca1	12772623	GTTTTTTTT/GGTTTTTTTTTT	GGAAGATGAGTGGGGATCAA	CAGATGAGAGAAGTTGAGGGG	58.9	497	INTERGENIC	--	--	--

INDEL marker IDs	Chromosomes/unanchored scaffolds	Physical positions (bp)	InDels (*Kabuli* reference genome-CDC Frontier/PII)	Forward primers (5'-3')	Reverse primers (5'-3')	Anneling temperature (0C)	Expected amplified product size (bp)	Structural annotation		Functional annotation	
								Sequence components of *kabuli* genome	*Kabuli* gene accession IDs	NCBI-KOG	TFs
Ca1_P2_12772687	Ca1	12772687	AT/A	GGAAGATGAGTGGGGATCAA	TGGTACCCCTGCAAGAATATG	59.8	888	INTERGENIC	--	--	--
Ca1_P2_12777387	Ca1	12777387	AC/ACC	CCGTCTGTGTAACAAGATTGC	TTTTGGTCGGAAACAGGAAG	60.1	736	INTERGENIC	--	--	--
Ca1_P2_1280971	Ca1	1280971	TTATATATATATATATATATA/TTATATATATATATATATATATATATA	GGGGAAAACCTTGCTCCTAC	AGGGGGAGCAAAACAAAAAT	59.8	379	INTRON	Ca_00158	--	--
Ca1_P2_12867966	Ca1	12867966	CATATATATATATATATATATATATATATATAT/CATATATATATATATATATATATATATATATATAT	AATCCTTTTTCTCAATGCATCA	AAACCCGATTCTCAATTCCA	59.4	475	INTERGENIC	--	--	--
Ca1_P2_12870844	Ca1	12870844	C/CATTTAT	TCACTGCCTTTGTCAGTTCG	CCCGCTTTTTATTGATCCTTC	59.9	549	INTERGENIC	--	--	--
Ca1_P2_12905675	Ca1	12905675	CAAA/CAA	TTGGGAGGATGCCAAATTAC	TTGAATAACCTCCACGCACA	60.1	941	INTERGENIC	--	--	--
Ca1_P2_12947634	Ca1	12947634	ATAAATTTAAA/ATAAATTTAAAATTTAAATTTAAA	AACTTTGTGGTCGTTTTGGC	TGCTTATTGTGGCTGCTTTG	60.0	647	INTERGENIC	--	--	--
Ca1_P2_12956191	Ca1	12956191	ATCTC/A	TGCTAACCCAACACAATTTCT	GTCAGATGTTTGGGGCATTT	59.8	469	INTERGENIC	--	--	--
Ca1_P2_12956423	Ca1	12956423	CAAAAAAA/CAAAAA	TTGTCGGATACCTGCTCAGT	TTGCAAATTCGGACAAACAA	60.1	455	INTERGENIC	--	--	--
Ca1_P2_12988934	Ca1	12988934	C/CGGG	ATCGGCATCCTTCCTTCTTT	GACGCTAAGCCCCTTTCTCT	60.0	354	INTERGENIC	--	--	--
Ca1_P2_13005887	Ca1	13005887	TAAAAAAAAAAAAA/TAAAAAAAAAAA	TCGTCATCTAGCACCAGCAC	TCCCTCGTAAAGACCCATCT	58.6	347	INTERGENIC	--	--	--
Ca1_P2_13007054	Ca1	13007054	ACT/A	ATGCAAAAAGGCTCCTGCTA	ACTAACCATCTGGACCCGTG	59.8	634	INTERGENIC	--	--	--

INDEL marker IDs	Chromosomes/unanchored scaffolds	Physical positions (bp)	InDels (*Kabuli* reference genome-CDC Frontier/PII)	Forward primers (5'-3')	Reverse primers (5'-3')	Anneling temperature (0C)	Expected amplified product size (bp)	Structural annotation		Functional annotation	
								Sequence components of *kabuli* genome	*Kabuli* gene accession IDs	NCBI-KOG	TFs
Ca1_P2_13007507	Ca1	13007507	TTAT/TTATATAT	CACGGGTCCAGATGGTTAGT	CCAAGCATCTTGGTGCATAA	59.7	322	INTERGENIC	--	--	--
Ca1_P2_13016584	Ca1	13016584	ATTTTTTTTTT/ATTTTTTTTTTT	TGTGGCAAGTCACATAAGCG	TTTGCAGGGGGAGTTATGTC	59.9	945	INTERGENIC	--	--	--
Ca1_P2_13018516	Ca1	13018516	AACCGCGTTGATCACC/AACCGCGTTGATCACCGCGTTGATCACC	CCATTGTTGACACCACTTCG	GCGTCATTTAAATCGGGAGA	60.0	467	DRR	Ca_14065	--	C2H2
Ca1_P2_13060064	Ca1	13060064	AAATAATAATAATAATAATAATAATAATAATAATAATAATAATAATAA/AAATAATAATAATAATAATAATAATAATAATAATAA	TCCTGAAACAAATGCACCAA	GGTCTGAAATAAGGCCGTGA	60.1	463	DRR	Ca_14070	O	--
Ca1_P2_13117590	Ca1	13117590	TAATAAAAATAAAAATAAAA/TAATAAAAATAAAA	TGTTGTTTACGGAATTTGGTTTT	TTTCGTTGTATATTAAAGCACGG	58.3	547	INTERGENIC	--	--	--
Ca1_P2_13134589	Ca1	13134589	TAAAAAAA/TAAAAAAAA	CGATGGATGACTTCCCGTAT	TTTACGTTGACGCACGAGAC	59.9	587	INTERGENIC	--	--	--
Ca1_P2_13264476	Ca1	13264476	AATATATATATATATATATATATATATATAT/AATATATATATATATATATATATATATATATATAT	GCTCGGGTCCTTTCGTACAT	TCATTTCGCAGGTCTGAGTCT	60.0	444	INTERGENIC	--	--	--
Ca1_P2_13392144	Ca1	13392144	ATTTTTTTTTT/ATTTTTTTTTTT	TCAGCCTGGCTTGATCTTTT	AATCAGTCAGCCGTCATTCC	60.1	205	INTERGENIC	--	--	--
Ca1_P2_13407952	Ca1	13407952	GAAAAAAAAAAA/GAAAAAAAAAA	GATCCGGTGATTTGATCTCG	TGTTTCATGGATGCAAGTACG	59.6	205	DRR	Ca_14105	--	--
Ca1_P2_13578342	Ca1	13578342	GAAAAAAAAAAA/GAAAAAAAAAA	CCGGCTTGTAGGACGAGTAG	TCTGTTTTCGAGGGCACTTT	59.9	519	INTERGENIC	--	--	--
Ca1_P2_1366043	Ca1	1366043	CTATATATATATATATATATATATATA/CTATATATATATATATATATATATA	TTTGGGACGAGACGGATAAC	CGAATTTTGTCAATCGAACCA	60.9	819	INTERGENIC	--	--	--
Ca1_P2_13674092	Ca1	13674092	ATTTT/ATTT	AACTGGTTCGATTCGGTTTTT	TGAAAATTTCGCATTCACCA	60.1	382	INTERGENIC	--	--	--

INDEL marker IDs	Chromosomes/unanchored scaffolds	Physical positions (bp)	InDels (*Kabuli* reference genome-CDC Frontier/PII)	Forward primers (5'-3')	Reverse primers (5'-3')	Anneling temperature (0C)	Expected amplified product size (bp)	Structural annotation		Functional annotation	
								Sequence components of *kabuli* genome	*Kabuli* gene accession IDs	NCBI-KOG	TFs
Ca1_P2_13674169	Ca1	13674169	AG/A	AACTGGTTCGATTCGGTTTTT	TGCATTTCGGAAGTTTCACA	60.2	757	INTERGENIC	--	--	--
Ca1_P2_13675228	Ca1	13675228	CTATAATTTTT/CTATAATTTTTATAATTTTT	ATGGTTCGCGACTGATTTTT	CCCAAAATTAAGCGTCGGTA	60.0	529	DRR	Ca_14132	--	--
Ca1_P2_13676036	Ca1	13676036	CTTTTTTT/CTTTTTT	CCGCTACAAGGCGATAGAAG	TTCTGATGAACGGGGGATTA	60.3	248	DRR	Ca_14132	--	--
Ca1_P2_13676261	Ca1	13676261	ACT/ACTCT	CCGCTACAAGGCGATAGAAG	GGGTGTCATCATAGGTGGCT	59.8	856	DRR	Ca_14132	--	--
Ca1_P2_13680810	Ca1	13680810	ATTTTT/ATTTT	TGGTTGAACTGTGAACAGGC	CCAATTTCCCCTACCCAAAT	59.9	559	INTERGENIC	--	--	--
Ca1_P2_13683980	Ca1	13683980	TAAA/TAAAA	ATCGAAATGGGTGCAAAGTC	GGTTGCCTTGGTATGGAGAA	59.9	621	DRR	Ca_14134	--	MYB_related
Ca1_P2_13686356	Ca1	13686356	T/TCTC	TTGTACGGGTGGTGTCAAAA	GAGGGTGACTCTGCTGGAAC	59.8	655	INTRON	Ca_14134	--	MYB_related
Ca1_P2_13686733	Ca1	13686733	CAAAAAAAAAA/CAAAAAAAAA	AGCATTTGCTACCGTGATCC	TTTACCTTGCCAAAAATGCC	59.9	698	INTRON	Ca_14134	--	MYB_related
Ca1_P2_13687868	Ca1	13687868	ATT/ATTT	CTTTTGCTGAGAGCATTCCC	CTCCGGAGCAAACTTGTGAT	60.3	514	INTERGENIC	--	--	--
Ca1_P2_13687994	Ca1	13687994	TC/T	CTTTTGCTGAGAGCATTCCC	CTCCGGAGCAAACTTGTGAT	60.3	514	INTERGENIC	--	--	--
Ca1_P2_13688125	Ca1	13688125	AATATATATATATA/AATATATATATA	CTTTTGCTGAGAGCATTCCC	CTCCGGAGCAAACTTGTGAT	60.3	514	INTERGENIC	--	--	--
Ca1_P2_13690477	Ca1	13690477	CAA/CAATCGGTTTTATAA	CCGGTTATTTAACCATAACCAA	TGCAAGGAACAAATGGCATA	60.1	200	INTERGENIC	--	--	--

INDEL marker IDs	Chromosomes/unanchored scaffolds	Physical positions (bp)	InDels (*Kabuli* reference genome-CDC Frontier/PII)	Forward primers (5'-3')	Reverse primers (5'-3')	Anneling temperature (0C)	Expected amplified product size (bp)	Structural annotation		Functional annotation	
								Sequence components of *kabuli* genome	*Kabuli* gene accession IDs	NCBI-KOG	TFs
Ca1_P2_13692764	Ca1	13692764	CTTTT/CTTTTT	CGCCAGTGGTTATGCTCTTA	AAGGAACCACACTTGAACCG	60.0	875	INTERGENIC	--	--	--
Ca1_P2_13694461	Ca1	13694461	GTA/GTAATA	CCACTTCGCCACGTTTTTAT	CGGTTTATCGTCAAAGCGAT	60.1	379	INTERGENIC	--	--	--
Ca1_P2_13696144	Ca1	13696144	TAAAAA/TAAAA	TTTGCGTTACGAATTTGATGA	AGAAGTTGAACTCACGAATCACA	58.9	757	INTERGENIC	--	--	--
Ca1_P2_13748153	Ca1	13748153	ATTTTT/ATTTT	ATTCGGGATAAGTGGAACCC	TGCAAATGAGATAACCAGCG	59.8	650	INTERGENIC	--	--	--
Ca1_P2_13831465	Ca1	13831465	TTAATAATAATAATAATAATAATAATAATAATAATAATAAT/TTAATAATAATAATAATAATAATAATAATAATAATAATAATAATAATAAT	AGTTTGCTTGTTGGGGTGTC	ACATTGAAAAATTGGTGGCG	60.7	380	INTERGENIC	--	--	--
Ca1_P2_13915344	Ca1	13915344	TTATATATATATATATATATA/TTATATATATATATATATATA	TGATGAATATTGAGGGGACAAA	ACGGCACACAATTGTACGAG	59.6	586	INTERGENIC	--	--	--
Ca1_P2_13916815	Ca1	13916815	TAA/TA	TGACCCTGGCTCCATTACTC	TTTCCCGACTTGGTCCTTTA	59.5	588	INTERGENIC	--	--	--
Ca1_P2_1397435	Ca1	1397435	CA/C	TGGAAACAGCAACGTGGTAG	CAAAGGGTTGGATTTTCCAC	59.3	555	INTERGENIC	--	--	--
Ca1_P2_13997509	Ca1	13997509	GGAAGAAGAAGAAGAAGAAGAAGAAGA/GGAAGAAGAAGAAGAAGAAGAAGAAGAAGA	TGGCCCAACAGAGAAGTACC	CGAGGTACTGGCCTTGTCTC	59.9	155	CDS (large-effect mutations)	Ca_07130	--	--
Ca1_P2_14210125	Ca1	14210125	G/GGTTATAAATTC	TCATATTTGCACACTTAAAATCCAA	AATTGGATCTGGCGATTGAG	60.0	367	INTERGENIC	--	--	--
Ca1_P2_1427442	Ca1	1427442	TTATATATATATATATATATA/TTATATATATATATATA	TTTGCGTAATTGTTTGTGTCAA	TCCACCCTCACCTCACTACC	60.0	554	DRR	Ca_00173	O	--
Ca1_P2_14370290	Ca1	14370290	A/AC	CTCCGTTTTCTGCACATCAA	TTTCCACCCAGAAAGCAATC	60.1	626	INTERGENIC	--	--	--

INDEL marker IDs	Chromosomes/unanchored scaffolds	Physical positions (bp)	InDels (*Kabuli* reference genome-CDC Frontier/PII)	Forward primers (5'-3')	Reverse primers (5'-3')	Anneling temperature (0C)	Expected amplified product size (bp)	Structural annotation		Functional annotation	
								Sequence components of *kabuli* genome	*Kabuli* gene accession IDs	NCBI-KOG	TFs
Ca1_P2_14385063	Ca1	14385063	CAA/CAAA	AATCGTCTCCGTCACACACA	GCGAATTGCAGACTGAAACA	60.0	662	DRR	Ca_07096	--	SAP
Ca1_P2_14649425	Ca1	14649425	CAATAATAATAATAATAATAATAATAATAATAATAA/CAATAATAATAATAATAATAATAATAATAATAATAA	ATGCATGACACATTCTCCGA	TTCCTTCAACCCATAACCCA	60.2	559	INTERGENIC	--	--	--
Ca1_P2_14669721	Ca1	14669721	ATTT/ATTTT	GGTGCTTGAAAAGGAAGCTG	ACCCAACACTGCTTGAATCC	60.0	226	DRR	Ca_07065	E	TCP
Ca1_P2_14682464	Ca1	14682464	CTTTT/CTTT	TCCATTGTTGAGCGAAAACA	TGGATGAAGGATCAACCACA	59.9	659	INTERGENIC	--	--	--
Ca1_P2_1470537	Ca1	1470537	T/TC	AAGTGGCGATAGGGTTTGTG	CAAAATGTGGCATATGAGCTG	59.2	834	INTERGENIC	--	--	--
Ca1_P2_14712075	Ca1	14712075	AATATATATATATAT/AATATATATATATATAT	TTCATGATTTTGGTGGTGAAA	CAGCCACAATAACTAGCGCA	60.0	459	DRR	Ca_07059	--	--
Ca1_P2_14777698	Ca1	14777698	TAAAAAAAA/TAAAAAAA	CAATGACCTTGGTCCTCCTG	TTAACTGCAGCCATGCATCT	59.4	799	DRR	Ca_07052	R	--
Ca1_P2_14796703	Ca1	14796703	AATAA/AATAAACATAA	TATTGGAAAGCTTGGCTGCT	GCAAACTTTTGCATGTCTCTCA	60.4	335	INTERGENIC	--	--	--
Ca1_P2_14850393	Ca1	14850393	CTTTTTTTTTT/CTTTTTTTTT	TGCAAGGAAGAACAACATGC	TGGTGGGTTATCATGAATGG	59.0	827	INTERGENIC	--	--	--
Ca1_P2_14855553	Ca1	14855553	AATATATATATATATATATATAT/AATATATATATATATATATATATAT	TTTCAAACTTGCACGCACTC	TGAAAATCCATGTGACAAGTG	57.0	778	INTRON	Ca_07046	I	--
Ca1_P2_14893455	Ca1	14893455	GTTTTTTTT/GTTTTTTT	AGGGACAAGGTTGGGTTTTT	TTAAAACTTTGCACGGAGGG	60.1	291	INTERGENIC	--	--	--
Ca1_P2_14906350	Ca1	14906350	TAAAAAAAAA/TAAAAAAAAAA	ATCTTGGGACATTGCGTTTC	ATGCATTCCTCCACCTTCAC	59.9	247	INTERGENIC	--	--	--

INDEL marker IDs	Chromosomes/unanchored scaffolds	Physical positions (bp)	InDels (*Kabuli* reference genome-CDC Frontier/PII)	Forward primers (5'-3')	Reverse primers (5'-3')	Anneling temperature (0C)	Expected amplified product size (bp)	Structural annotation		Functional annotation	
								Sequence components of *kabuli* genome	*Kabuli* gene accession IDs	NCBI-KOG	TFs
Ca1_P2_14995847	Ca1	14995847	ACATGCATGCA/ACATGCA	CAGCGCTTTTCCCAAATAAG	CCAAAAAGGGTGGAGATTGA	59.9	359	INTERGENIC	--	--	--
Ca1_P2_15025001	Ca1	15025001	TTATATATATATATATATATATATATATATA/TTATATATATATATATATATATATATATA	AAGCAATAAGATGAAACGAGACA	AGTTGTGATGGATATGGCCC	59.6	767	INTERGENIC	--	--	--
Ca1_P2_15186134	Ca1	15186134	AATATATATATATATATATAT/AATATATATATATATATATATAT	AGTGGCCCACGAGTAAGATG	CCACAGCACTCTTCTGTCCA	60.0	543	INTERGENIC	--	--	--
Ca1_P2_15257144	Ca1	15257144	AT/ATATATTT	ATTTTGGAATTTTGGCTCCC	TATGGGGCATAGTGCAACAA	60.0	522	INTRON	Ca_07008	OR	--
Ca1_P2_15262868	Ca1	15262868	ATTTTTTTTTTT/ATTTTTTTTTT	GAGAATGAGTTTTGGGTGCC	TCTCTGCAAACAATCACACCA	60.3	148	INTERGENIC	--	--	--
Ca1_P2_15303147	Ca1	15303147	ATTTTTTTTT/ATTTTTTTTTT	TTCTTCAACTGCAACAACGC	TTAAATGGCTCCAGGTGTCC	59.9	617	INTERGENIC	--	--	--
Ca1_P2_15326926	Ca1	15326926	CATATATATATAT/CATATATATAT	GTTAAGCATCCCAAGGTGGA	AGCCAAGAGTTTGTGGAGTTG	59.4	626	INTERGENIC	--	--	--
Ca1_P2_15341305	Ca1	15341305	TTATATATATATATATATATATATATATATATAT/TTATATATATATATATATATAT	AAATCAATGGACCGCCTCTT	ATCGAAGATGGTGGTTGAGG	59.9	414	INTERGENIC	--	--	--
Ca1_P2_1540452	Ca1	1540452	CTTTTTTT/CTTTTTTTTTTTTT	AGTCTAAAGCCCCCGATTGT	ATTTGGCGGGTCTTGTGTAG	60.0	476	INTERGENIC	--	--	--
Ca1_P2_15406728	Ca1	15406728	GTTTTTTTT/GTTTTTTTTT	ATGCTCAAATGTGCCAATCA	AACAAACCAATTCAATTCCGA	59.3	559	INTERGENIC	--	--	--
Ca1_P2_15570070	Ca1	15570070	AATATATATATATATATATATATATATA/AATATATATATATATATATATATA	TGGAATATCACCCCCTCAAA	ACATTGGACGTTCATCGACA	60.0	602	DRR	Ca_06985	S	--
Ca1_P2_15571799	Ca1	15571799	ATTTTTTT/ATTTTTT	CCCCATTTTGTTTCTCCTCA	TTGTTGAACGATGCTTCTGC	60.0	282	DRR	Ca_06985	S	--

INDEL marker IDs	Chromosomes/unanchored scaffolds	Physical positions (bp)	InDels (*Kabuli* reference genome-CDC Frontier/PII)	Forward primers (5'-3')	Reverse primers (5'-3')	Anneling temperature (0C)	Expected amplified product size (bp)	Structural annotation		Functional annotation	
								Sequence components of *kabuli* genome	*Kabuli* gene accession IDs	NCBI-KOG	TFs
Ca1_P2_15579150	Ca1	15579150	CATATATATATATATATATA/CATATATATATATATATATATA	CCAATTGCGTTCGTAACAGA	AAATCTTTCAAAAGAATCACAAAAA	57.6	587	INTRON	Ca_06985	S	--
Ca1_P2_15586273	Ca1	15586273	TTTATTATTATTATTATTATTATTATTATTATTATTATTATTATTATTATTATTATTATTAT/TTTATTATTATTATTATTATTATTATTATTATTATTATTATTATTATTATTATTATTAT	GGAGATTTGAAACCCGTGAA	GAGAGCCCCAAATAATGGGT	60.2	457	INTRON	Ca_06985	S	--
Ca1_P2_15650217	Ca1	15650217	CTTTTTTTTTTTT/CTTTTTTTTTTTTTT	GATATGGGATTAGGTGGCCC	TCATTGCTTTGTGTGCTTCC	59.8	521	INTERGENIC	--	--	--
Ca1_P2_15749342	Ca1	15749342	AATATATATATATATATATATAT/AATATATATATATATATATAT	CAGTTTTCTTAGGCCCTCCC	TTTTGCGTTTAAGACGACGAT	59.8	524	INTERGENIC	--	--	--
Ca1_P2_15773203	Ca1	15773203	A/ATATATT	CCTAAAGAAAGATGGGCCTG	TTTGTTCTTTGGGCCTTCTG	60.2	288	INTERGENIC	--	--	--
Ca1_P2_15811388	Ca1	15811388	TAAAAAAA/TAAAAAAAA	GCGAGATTTTCGACTTCTGC	TGCGGCAAACGTAATTGTAA	60.1	590	INTERGENIC	--	--	--
Ca1_P2_15821162	Ca1	15821162	ATT/AT	GGGGGAATTGGTGAAAAGTT	ATTAAACCAGCCACACAGGC	60.0	530	INTERGENIC	--	--	--
Ca1_P2_15821229	Ca1	15821229	A/ATT	GGGGGAATTGGTGAAAAGTT	ATTAAACCAGCCACACAGGC	60.0	530	INTERGENIC	--	--	--
Ca1_P2_15835234	Ca1	15835234	TTTATTATTATTATTATTATTATTATTATTATT/TTTATTATTATTATTATTATTATTATTATT	TCGTTTGCCGTTAACTAGGG	TTGGCCTAACCTGAAAGCAT	59.7	768	INTERGENIC	--	--	--
Ca1_P2_15848562	Ca1	15848562	AAA/AAATATGAATAA	TTTCAAAATATGGTCGTAACGG	TTCTTCCCAAAAACATTGCC	59.9	652	INTERGENIC	--	--	--
Ca1_P2_15849587	Ca1	15849587	AATATATATATATATATATATATATATATATATA/AATATATATATATATATATATATATATATATA	CCCCATTTTGTTTCTCTCCA	ATTCTTGGAGGAGCGGGTAT	59.9	872	INTERGENIC	--	--	--
Ca1_P2_15910790	Ca1	15910790	ATTTTTTTT/ATTTTTTT	TTCAGGCAACAATTTGTCCA	TCCCTCAAGTTCTTGCCACT	59.8	674	DRR	Ca_06960	S	--

INDEL marker IDs	Chromosomes/unanchored scaffolds	Physical positions (bp)	InDels (*Kabuli* reference genome-CDC Frontier/PII)	Forward primers (5'-3')	Reverse primers (5'-3')	Anneling temperature (0C)	Expected amplified product size (bp)	Structural annotation		Functional annotation	
								Sequence components of *kabuli* genome	*Kabuli* gene accession IDs	NCBI-KOG	TFs
Ca1_P2_15927565	Ca1	15927565	TAAAAAAAAAAA/TAAAAAAAAA	TGTTACGGTGGTAAAAGGCA	TATCGGCAACAGTTTGCTTG	59.9	656	INTERGENIC	--	--	--
Ca1_P2_15928284	Ca1	15928284	CAAAAAAAAAA/CAAAAAAAAAAA	AACTGTTGCCGATACTTGGG	GCACATTCAAAGAAATCTATCCC	59.0	651	INTERGENIC	--	--	--
Ca1_P2_15930994	Ca1	15930994	GAAAAAAAAAA/GAAAAAAAAAAA	CCAGAAAGAACATTGCACCA	AGCTTCCCCTCCCAAATAAA	59.9	243	INTERGENIC	--	--	--
Ca1_P2_15952106	Ca1	15952106	TTCTCTCTCTCTCTCTCTCTCTCTCTCTCTCTCTCTCTCTCTCTCTC/TTCTCTCTCTCTCTCTCTCTCTCTCTCTCTCTCTCTCTCTCTC	AGCGGCTCTTTCAATTTTCA	TGAGGTAGCCAAATCCAAGG	60.1	334	INTERGENIC	--	--	--
Ca1_P2_16062449	Ca1	16062449	CACAACAACAACAA/CACAACAACAACAACAACAA	GCAGCTCACAATCCAGTTCA	TGGTGTTGACGTTCAGTGGT	60.0	613	DRR	Ca_06944	J	--
Ca1_P2_16077599	Ca1	16077599	CAAAAAAA/CAAAAAA	GCAACATCGAACCCTCAAAT	TTGTCGTTTGAGCATTTAAAAAGT	59.3	494	INTERGENIC	--	--	--
Ca1_P2_16078538	Ca1	16078538	CTTTTTTTTT/CTTTTTTTT	TGCATGTTGTTGGTAGAGTCAA	GCACAAGGTATGCCAAATGA	59.5	632	INTERGENIC	--	--	--
Ca1_P2_16096244	Ca1	16096244	TG/TGG	ATGTAAGATTGGAAGCGGCA	CAATTGTTCCCCACTTGCTT	60.0	298	INTERGENIC	--	--	--
Ca1_P2_16097492	Ca1	16097492	TAAAAAAAA/TAAAAAAA	AAATGCCCATTGATCCGTTA	GGGAGCATTTGCAAACAATA	58.6	891	INTERGENIC	--	--	--
Ca1_P2_16110663	Ca1	16110663	TAAAAAAAAA/TAAAAAAAA	CGTAAGACTACACCCGTCCTTT	CTTCGCTTACCATCGTCAAA	58.9	640	INTRON	Ca_06940	R	--
Ca1_P2_16112834	Ca1	16112834	AATACTCATATA/AATA	CTTGCATGTGTGTCGATCAG	TTCAAATTAAGTTTTCACGTGGTT	59.0	518	INTRON	Ca_06940	R	--
Ca1_P2_16116426	Ca1	16116426	GA/GAA	AATGCAACGGTTGGTTTGAT	GCACAAATGAAAAGCAGAACA	59.0	826	DRR	Ca_06940	R	--

INDEL marker IDs	Chromosomes/unanchored scaffolds	Physical positions (bp)	InDels (*Kabuli* reference genome-CDC Frontier/PII)	Forward primers (5'-3')	Reverse primers (5'-3')	Anneling temperature (0C)	Expected amplified product size (bp)	Structural annotation		Functional annotation	
								Sequence components of *kabuli* genome	*Kabuli* gene accession IDs	NCBI-KOG	TFs
Ca1_P2_16118199	Ca1	16118199	AG/A	ATGCCATTTTGAAGACTGCC	TGGGGGCAGTTACCCTTATT	60.6	489	INTERGENIC	--	--	--
Ca1_P2_16119755	Ca1	16119755	T/TG	CTGCTCCAACCATCGGTACT	TTCCTAAAAGCGTCCCAAGA	59.8	547	INTERGENIC	--	--	--
Ca1_P2_16120380	Ca1	16120380	CTATA/CTA	GGACGCTTCCAGACAAAAAG	GCATTTGATCGAAACCGACT	60.1	815	INTERGENIC	--	--	--
Ca1_P2_16155793	Ca1	16155793	ATT/AT	GGCAACTCCATTGAATTCGT	AATCCCCACCCTAAATGACC	59.9	333	INTERGENIC	--	--	--
Ca1_P2_16156026	Ca1	16156026	A/ATTAAG	GGCAACTCCATTGAATTCGT	TGTCAAAACGGATATTCCACC	59.7	435	INTERGENIC	--	--	--
Ca1_P2_16156936	Ca1	16156936	CAA/CAAAA	AATGCCAATAGCACCTTTGC	GGTTTCGATTGATTTTTCGG	59.4	820	INTERGENIC	--	--	--
Ca1_P2_16157268	Ca1	16157268	GAAAAAAAAAA/GAAAAAAAAA	AGCCAAATTTGATGGCACTT	GGTTTCGATTGATTTTTCGG	59.4	231	INTERGENIC	--	--	--
Ca1_P2_16157361	Ca1	16157361	GAAAAAAAAAAA/GAAAAAAAAA	AGCCAAATTTGATGGCACTT	TCACCACAACAAAACAAACCA	59.9	541	INTERGENIC	--	--	--
Ca1_P2_16158999	Ca1	16158999	CAAAAAAAAA/CAAAAAAAA	AGTCCGCAACCCATTTAGTG	TTTGGGGAATGCTTAAAACC	58.9	619	INTERGENIC	--	--	--
Ca1_P2_16159274	Ca1	16159274	AAAAA/AAAAACAAAA	ATGGTTTTAAGCATTCCCCA	TTAAGTTGCGAGGTTGCTCA	59.6	457	INTERGENIC	--	--	--
Ca1_P2_16160389	Ca1	16160389	TT/TTCT	CATGTGGATTTGTCTTTGCG	GCACAAGATCCTCAAAACTGG	59.7	238	INTERGENIC	--	--	--
Ca1_P2_16161514	Ca1	16161514	TG/T	GGGGCTTTACGTATACAACCG	GGAATGAAAAGCCATGAGGA	60.0	253	INTERGENIC	--	--	--

INDEL marker IDs	Chromosomes/unanchored scaffolds	Physical positions (bp)	InDels (*Kabuli* reference genome-CDC Frontier/PII)	Forward primers (5'-3')	Reverse primers (5'-3')	Anneling temperature (0C)	Expected amplified product size (bp)	Structural annotation		Functional annotation	
								Sequence components of *kabuli* genome	*Kabuli* gene accession IDs	NCBI-KOG	TFs
Ca1_P2_16162054	Ca1	16162054	CTT/CTTT	TCCTCATGGCTTTTCATTCC	TCAATCTTCATAGGCTGAACGA	59.8	796	INTRON	Ca_06938	R	--
Ca1_P2_16165350	Ca1	16165350	T/TC	CCACTTGAAGAAGTGGGGAA	AAAATTGGGCCGAAAACTGT	60.7	216	DRR	Ca_06938	R	--
Ca1_P2_16167140	Ca1	16167140	TAAAA/TAAAAA	TTTTTAGCATGGCACGTTTT	CGGGTTGGATTAGATTTCAAG	58.6	378	INTERGENIC	--	--	--
Ca1_P2_16170875	Ca1	16170875	G/GA	CTAGATTCCCCCAACATGGA	TTTGCGATTTCAATAGAGAAAAGA	59.4	596	INTERGENIC	--	--	--
Ca1_P2_16171279	Ca1	16171279	TA/TATTTTTAAA	TGTCAAAATCAGTCCATTAAGCA	TGAACCGTGAATGTCCTCAA	60.1	707	INTERGENIC	--	--	--
Ca1_P2_16174129	Ca1	16174129	ATTTT/ATTTTTTTT	CCATGGGATCTCATTTCAAGA	AAAAACAATACCGCGTGGAG	60.0	928	INTERGENIC	--	--	--
Ca1_P2_16174383	Ca1	16174383	GT/G	ACGTGGGTTTTCGTATCTCG	AAAAACAATACCGCGTGGAG	60.0	297	INTERGENIC	--	--	--
Ca1_P2_1617458	Ca1	1617458	AC/ACC	TGAAATCAAGTTTGGAAGAGTCA	TAAAAAGGAAATTGCGTCCG	60.1	350	INTERGENIC	--	--	--
Ca1_P2_16174790	Ca1	16174790	TC/T	CCACGCGGTATTGTTTTTCT	TGGTTAGTTTATGTGGCAAAATG	59.0	715	INTERGENIC	--	--	--
Ca1_P2_16177614	Ca1	16177614	ATTTTTTTT/ATTTTTT	AGTCTCGTCGAGCCCACTAA	TCACGGCTGTGTACTTAAAACG	60.2	242	INTERGENIC	--	--	--
Ca1_P2_16183218	Ca1	16183218	AG/AGG	TGCACAAAAATTCCACCAGA	AGCATGCCAACTTTCTCCAT	59.7	900	DRR	Ca_06937	--	--
Ca1_P2_16183891	Ca1	16183891	AAATAATAATAATA/AAATAATAATA	ACTTGGCATGCAAGAGGACT	TCCTTTCACATACACACACATGA	59.0	268	DRR	Ca_06937	--	--

INDEL marker IDs	Chromosomes/unanchored scaffolds	Physical positions (bp)	InDels (*Kabuli* reference genome-CDC Frontier/PII)	Forward primers (5'-3')	Reverse primers (5'-3')	Anneling temperature (0C)	Expected amplified product size (bp)	Structural annotation		Functional annotation	
								Sequence components of *kabuli* genome	*Kabuli* gene accession IDs	NCBI-KOG	TFs
Ca1_P2_16187503	Ca1	16187503	AGTTTATGACG/AG	GTGTCACCATTGGCAATACG	TTCCTTCGACCTCACAATCC	60.1	382	CDS (large-effect mutations)	Ca_06937	--	--
Ca1_P2_16187739	Ca1	16187739	CA/CAATA	GGATTGTGAGGTCGAAGGAA	TGCATTTCTCTCCAACCATTT	59.6	484	INTERGENIC	--	--	--
Ca1_P2_16187793	Ca1	16187793	CTTTTTTTTTT/CTTTTTTTTTTTTTTT	GGATTGTGAGGTCGAAGGAA	TGCATTTCTCTCCAACCATTT	59.6	484	INTERGENIC	--	--	--
Ca1_P2_16196907	Ca1	16196907	CTTTTTTT/CTTTTTT	AATGGGATCCAACAAAAGCA	TACATGGCGAACATGAATGC	60.5	504	INTERGENIC	--	--	--
Ca1_P2_16201852	Ca1	16201852	GTTTTTTTTTT/GTTTTTTTTT	CCAACCGTGACCAAAAGACT	TTGCAATGTTTGGTGGAAGA	60.1	578	INTERGENIC	--	--	--
Ca1_P2_16202705	Ca1	16202705	TAAAAAAA/TAAAAAA	GCCGGATTCACAAAAACACT	AGTGACCTTCCCCACACAAG	60.0	478	INTERGENIC	--	--	--
Ca1_P2_16202757	Ca1	16202757	GTTTTTTTTTTTT/GTTTTTTTTTT	GCCGGATTCACAAAAACACT	AGTGACCTTCCCCACACAAG	60.0	478	INTERGENIC	--	--	--
Ca1_P2_16203089	Ca1	16203089	TC/T	CTTGTGTGGGGAAGGTCACT	TGTGTGACCCCTGCTGAATA	60.1	685	INTERGENIC	--	--	--
Ca1_P2_16204000	Ca1	16204000	TAAAAAA/TAAAAA	TGGGCAATGTGAAAATGAAA	TGAAGAAGTAGGACCCACGC	60.3	176	INTERGENIC	--	--	--
Ca1_P2_16206173	Ca1	16206173	TAAAA/TAAAAA	TTTTCCCTAAAATGTCACCAAA	GGGGCTTACGAGTACAGACTCA	60.7	644	INTERGENIC	--	--	--
Ca1_P2_16207593	Ca1	16207593	TTT/TTTTTTTCTT	CAACGCCCAACAACTAATCA	TTCATTTGGAGAATTTTTAGCAGA	59.3	125	INTERGENIC	--	--	--
Ca1_P2_16207830	Ca1	16207830	ATTTTT/ATTTTTT	CAACGCCCAACAACTAATCA	GAGAGGGTCTTGAGTGTCGC	60.0	657	INTERGENIC	--	--	--

INDEL marker IDs	Chromosomes/unanchored scaffolds	Physical positions (bp)	InDels (*Kabuli* reference genome-CDC Frontier/PII)	Forward primers (5'-3')	Reverse primers (5'-3')	Anneling temperature (0C)	Expected amplified product size (bp)	Structural annotation		Functional annotation	
								Sequence components of *kabuli* genome	*Kabuli* gene accession IDs	NCBI-KOG	TFs
Ca1_P2_16208087	Ca1	16208087	TTAATAATAA/TTAATAA	TCATGATATTTCAAAAGGCTTCA	GAGAGGGTCTTGAGTGTCGC	60.0	437	INTERGENIC	--	--	--
Ca1_P2_16208160	Ca1	16208160	T/TA	TTGCGACGACCACAAAATAA	GCAGCATTTCCAACTGTGGT	61.1	245	INTERGENIC	--	--	--
Ca1_P2_16209460	Ca1	16209460	CTTTTTT/CTTTTT	TGAATCCAATCGTCCATCAA	TTCCGTTGATGTAAAAGCGA	59.3	451	INTERGENIC	--	--	--
Ca1_P2_16209484	Ca1	16209484	GACAAC/GACAACAAC	TGAATCCAATCGTCCATCAA	TTCCGTTGATGTAAAAGCGA	59.3	451	INTERGENIC	--	--	--
Ca1_P2_16209624	Ca1	16209624	TT/TTAT	TGAATCCAATCGTCCATCAA	GTCCGGTGATCGGTTTCTAA	59.9	823	INTERGENIC	--	--	--
Ca1_P2_16209705	Ca1	16209705	ATT/AT	TCGCTTTTACATCAACGGAA	GTCCGGTGATCGGTTTCTAA	59.9	392	INTERGENIC	--	--	--
Ca1_P2_16209782	Ca1	16209782	TT/TTGAT	TCGCTTTTACATCAACGGAA	GTCCGGTGATCGGTTTCTAA	59.9	392	INTERGENIC	--	--	--
Ca1_P2_16213026	Ca1	16213026	CGAGTAGAGTA/CGAGTA	TTTGGAATGCTTCTGAGGCT	TTTCGTTACGGGTCCTTTTG	60.0	682	INTRON	Ca_06936	--	bHLH
Ca1_P2_16213262	Ca1	16213262	G/GAACCAAATC	TTTGCAACCACAATTCTCCA	TTTCGTTACGGGTCCTTTTG	60.0	621	INTRON	Ca_06936	--	bHLH
Ca1_P2_16213273	Ca1	16213273	T/TG	TTTGCAACCACAATTCTCCA	TTTCGTTACGGGTCCTTTTG	60.0	621	INTRON	Ca_06936	--	bHLH
Ca1_P2_16234731	Ca1	16234731	AATATATATATATATATATATATATA/AATATATATATATATATATATATATATA	GGAGGGGAAGTTGACACAAA	ATTGGCTCGTTAGAGGGGAT	59.9	647	INTERGENIC	--	--	--
Ca1_P2_1638459	Ca1	1638459	GAAAAAAAAAA/GAAAAAAAA	GTTCGCGACCCTTCTAACAA	AACAAAACCGTTCAATGCGT	60.4	446	INTERGENIC	--	--	--

INDEL marker IDs	Chromosomes/unanchored scaffolds	Physical positions (bp)	InDels (*Kabuli* reference genome-CDC Frontier/PII)	Forward primers (5'-3')	Reverse primers (5'-3')	Anneling temperature (0C)	Expected amplified product size (bp)	Structural annotation		Functional annotation	
								Sequence components of *kabuli* genome	*Kabuli* gene accession IDs	NCBI-KOG	TFs
Ca1_P2_16442402	Ca1	16442402	TTATATATATATATATATATATATATATATATATATATAT/TTATATATATATATATATATATATATATATATATATAT	AGTGTGTAACTCGTGCGTCG	AAAAAGTGGTGTTGGATGCC	59.8	259	INTERGENIC	--	--	--
Ca1_P2_16475900	Ca1	16475900	TNNNNNNNNNN/TNNNNNNN	ACCGGAGAAGAAGGAGGAAC	CCAGCTAGACCAACCTCTCG	60.0	281	CDS (large-effect mutations)	Ca_06916	Z	WRKY
Ca1_P2_16583164	Ca1	16583164	AATATATATATATATATATATATATA/AATATATATATATATATATATA	TTTGGCACTTTCTCATTCCC	GCCATGTATGGTCCAAGGTC	60.2	793	INTERGENIC	--	--	--
Ca1_P2_16620461	Ca1	16620461	TAAAAAAAA/TAAAAAAA	TGCGATTATGTGAAGCAAAA	TGAGGTTAATAGTTGGTCGAATCA	59.9	500	INTERGENIC	--	--	--
Ca1_P2_16629521	Ca1	16629521	TTTAATTAATTAATTAATT/TTTAATTAATTAATTAATTAATTAATT	AACGTTTGAGGATTCCGTTG	GAAACTCCAGCTCCAAAACG	59.9	370	INTERGENIC	--	--	--
Ca1_P2_16659024	Ca1	16659024	AATATATATATATATATATATATATA/AATATATATATATATATATATA	TTGCGCTCTTGAGTAGGGAT	TCATAGTGTAAAAGCTGCATTTGAT	59.3	702	INTERGENIC	--	--	--
Ca1_P2_1668876	Ca1	1668876	ATATTATTATTATTATTATTA/ATATTATTATTATTATTATTATTA	AGTGTTGGCAGACGACAATG	TTCCTATCAAGGGATCCACG	59.9	483	INTERGENIC	--	--	--
Ca1_P2_16707141	Ca1	16707141	TTATATATATATATATATATATATATATAT/TTATATATATATATATATATATATATATAT	CTTTACAACCGCCACAAACC	CGAACAAAGTGCTTGTGGAA	59.9	755	INTERGENIC	--	--	--
Ca1_P2_16780531	Ca1	16780531	CTTTTTTTTTTT/CTTTTTTTTTTTT	TTTCTTTTTGGTCCCCTGTG	TGGGCTAAATTACATTCGTGG	59.8	813	INTERGENIC	--	--	--
Ca1_P2_16808351	Ca1	16808351	AATATATATATATATATATATATATATATATATATATATATATATATATATA/AATATATATATATATATATATATATATATATATATATATATATATATATATATATATA	TCATGTCGAAACCTCCATCA	ACTCGCGTTCATCTCTTGCT	60.2	696	INTERGENIC	--	--	--
Ca1_P2_1687851	Ca1	1687851	AATATGATTTTATATACATGATTTTATA/AACATGATTTTATA	TTGTGCATAGTACCGCCAAG	CACGCGTGAAACTTACCCTT	60.2	370	INTERGENIC	--	--	--
Ca1_P2_1688068	Ca1	1688068	ATTTTTT/ATTTTT	TTGTGCATAGTACCGCCAAG	ACGACATGGGTTTACGGTTC	59.7	575	INTERGENIC	--	--	--

INDEL marker IDs	Chromosomes/unanchored scaffolds	Physical positions (bp)	InDels (*Kabuli* reference genome-CDC Frontier/PII)	Forward primers (5'-3')	Reverse primers (5'-3')	Anneling temperature (0C)	Expected amplified product size (bp)	Structural annotation		Functional annotation	
								Sequence components of *kabuli* genome	*Kabuli* gene accession IDs	NCBI-KOG	TFs
Ca1_P2_17051529	Ca1	17051529	ATTTTTTTTT/ATTTTTTTT	TGCAGTAGATCAGTTCGACAA	CTTTGAGCTTTTGGTCCTCC	58.9	464	INTERGENIC	--	--	--
Ca1_P2_17107416	Ca1	17107416	AATATATATATATATATATATATATATAT/AATATATATATATATATATATATATATAT	GCTTTTCTCACCTCTCCCCT	TCCAAACCATGCTGTAGAGG	58.7	495	INTERGENIC	--	--	--
Ca1_P2_17120418	Ca1	17120418	G/GA	ATGGGACCATAGGCATTTTG	TCAATGGTACTTTTTGTGGTTCA	59.4	888	INTERGENIC	--	--	--
Ca1_P2_17120609	Ca1	17120609	GAAA/GAA	TCTTCATGTTAAAAATGGATTGC	ACATTCAGGGGTGCAGGTAG	60.0	897	INTERGENIC	--	--	--
Ca1_P2_17120794	Ca1	17120794	A/AG	TGTCATGGTAGTATATTTTCGCAGA	ACATTCAGGGGTGCAGGTAG	60.0	596	INTERGENIC	--	--	--
Ca1_P2_1716073	Ca1	1716073	TTATATATATATATATATA/TTATATATATATATATA	GCCTGAATTTAGCAGATGGG	GATTATTGCACAAACTCCAAAAA	58.2	466	DRR	Ca_00214	--	bHLH
Ca1_P2_17185713	Ca1	17185713	TTTGATATTTGATTAT/TTTGATATTTGATTATTGATATTTGATTAT	GCGTTAAAGTTGGAAACCCA	TGACGTGACATCGAGAGTCTG	60.0	594	INTERGENIC	--	--	--
Ca1_P2_1723303	Ca1	1723303	TTGTAT/TT	ATGTCATCTGTGACCAAGCG	TGGATGAGTGAAAATTGATTGG	59.8	420	DRR	Ca_00216	R	--
Ca1_P2_17234878	Ca1	17234878	TAAAAAAAAAAAA/TAAAAAAAAAAA	CCCTATAGGCCCACTAAGCC	ACCCCAAAAGAGAAAGCACA	59.7	695	INTERGENIC	--	--	--
Ca1_P2_17236397	Ca1	17236397	T/TAGGGCGCTAATAC	CGGGAGATACGTAGGAGCAA	GCGGCCTAAATTTTCGAGTT	60.6	394	INTERGENIC	--	--	--
Ca1_P2_1729960	Ca1	1729960	AAATAATAATAATAATAATAATAATAATAATAATAATAATAATAATAATAATAATAA/AAATAATAATAATAATAATAATAATAATAATAATAATAATAATAATAATAATAATAATAATAATAA	GGAACGACCGTTGACTGATT	TTAACTCAGAGGAGCGCAGC	60.8	618	DRR	Ca_00217	--	TALE
Ca1_P2_17303519	Ca1	17303519	TTATATATATATATATAT/TTATATATATATATAT	TTTCAAACTCCACTGGTGTCA	ACCACGTATTAGCGGGTTTG	59.9	136	INTERGENIC	--	--	--

INDEL marker IDs	Chromosomes/unanchored scaffolds	Physical positions (bp)	InDels (*Kabuli* reference genome-CDC Frontier/PII)	Forward primers (5'-3')	Reverse primers (5'-3')	Anneling temperature (0C)	Expected amplified product size (bp)	Structural annotation		Functional annotation	
								Sequence components of *kabuli* genome	*Kabuli* gene accession IDs	NCBI-KOG	TFs
Ca1_P2_1733287	Ca1	1733287	CAAAA/CAAAAA	GACCAAATCCACCTTCTCCA	TGGCTTTTGTGGTGCTTATG	59.7	931	INTRON	Ca_00217	--	TALE
Ca1_P2_1736894	Ca1	1736894	ATT/AT	CGTCAATGAGTGCTGGAAAA	GGACCCAAAACTTCACCTCA	59.9	528	INTERGENIC	--	--	--
Ca1_P2_1738348	Ca1	1738348	CTTTTTTTTT/CTTTTTTTTTT	TTTCTTACGGAGGCAGCACT	ATTTGCGTTCTGTTTTGGGA	60.5	571	INTERGENIC	--	--	--
Ca1_P2_1745242	Ca1	1745242	AG/A	ATTGTGATTGCATTGCATGG	GAGTAGGTGCCTTCCCACAA	60.1	548	INTRON	Ca_00219	--	TALE
Ca1_P2_1745433	Ca1	1745433	ATGAATTGAATT/ATGAATTGAATTGAATT	TGTGTGATTTGATCGGTGCT	GAGTAGGTGCCTTCCCACAA	60.1	214	INTRON	Ca_00219	--	TALE
Ca1_P2_17462572	Ca1	17462572	GGA/GGAATGCGCACGA	AATCCGTCACAAAGGCAGAC	AACGCCAATGGCTATAATGC	60.0	173	DRR	Ca_14788	--	C2H2
Ca1_P2_17476559	Ca1	17476559	ATTTTTTTT/ATTTTTTTTT	ACCTTCTCACTCAAACCCCC	CAACATCATTGGCAGCAAAA	60.6	457	INTERGENIC	--	--	--
Ca1_P2_17477080	Ca1	17477080	ATTT/ATT	TTTTGCTGCCAATGATGTTG	CATTTCATCCGGGGAATAGA	59.7	275	INTRON	Ca_14787	--	--
Ca1_P2_17498653	Ca1	17498653	TTATATATATATATATATATATATAT/TTATATATATATATATATATATATATAT	TCATGCCCTAACACCCTCTC	GATGCGACCTCCTATGGTGT	60.0	268	INTERGENIC	--	--	--
Ca1_P2_17556793	Ca1	17556793	AATATATATATATATATATATATATATATATAT/AATATATATATATATATATATATATATAT	TTTTCAGTTGAATGGGAGGG	TGGGAAGCATAGGGCAAATA	60.4	529	DRR	Ca_14780	K	ARR-B
Ca1_P2_17642213	Ca1	17642213	TAA/TAAA	ACGCTGTGACACGATTTCAG	CCCTCAACACACTCATGTGG	60.0	243	INTRON	Ca_14766	--	--
Ca1_P2_1777586	Ca1	1777586	AGATGATGATGATGATGATGA/AGATGATGATGATGATGATGATGA	CGTCACAAAGCATCACAGGT	CTACGGTGCAAACAGAACCA	59.8	563	INTERGENIC	--	--	--

INDEL marker IDs	Chromosomes/unanchored scaffolds	Physical positions (bp)	InDels (*Kabuli* reference genome-CDC Frontier/PII)	Forward primers (5'-3')	Reverse primers (5'-3')	Anneling temperature (0C)	Expected amplified product size (bp)	Structural annotation		Functional annotation	
								Sequence components of *kabuli* genome	*Kabuli* gene accession IDs	NCBI-KOG	TFs
Ca1_P2_17776926	Ca1	17776926	AATATATATATATATATATATATATATATATATATAT/AATATATATATATATATATATATATATATATAT	TTTGAACGCAATTTGGATGA	TGCAGGGACTAAGACCACAA	59.3	281	INTRON	Ca_14759	I	--
Ca1_P2_1779935	Ca1	1779935	ATAAGTAAGTAA/ATAAGTAA	GGTGAATGTTTGCTTCCTCAA	AATAAATCACTTTTATTTTCCCGTT	57.8	637	INTERGENIC	--	--	--
Ca1_P2_17826942	Ca1	17826942	AACAGTA/AA	TTGTGGGGATCAATCCATTT	TGATGCTGGACCAAATCAAC	59.5	526	INTERGENIC	--	--	--
Ca1_P2_1786205	Ca1	1786205	CTTTT/CTTTTT	TGTCCAATGGTTAGTGTCCG	TTGGGTCATTTGTGCACTCT	59.1	686	DRR	Ca_00224	S	--
Ca1_P2_1786745	Ca1	1786745	AC/A	AGAGTGCACAAATGACCCAA	TGTTGGTGTGGCGTAGGTTA	60.0	607	INTERGENIC	--	--	--
Ca1_P2_1789018	Ca1	1789018	GTTTTTTTTT/GTTTTTTT	TCATAGACAGGTTTGCAGCG	CACAATCGTCCACCAAACTG	60.0	324	INTRON	Ca_00225	--	--
Ca1_P2_1790267	Ca1	1790267	TAA/TAAA	ATTTTAACCAGCCCCCGTAG	CCCGTCATATTTTCCTCCAA	59.8	389	DRR	Ca_00226	D	MYB_related
Ca1_P2_1793901	Ca1	1793901	C/CA	GAGAAGCCTCGCTTTCTGTG	TTAGCAGCGGTTTTCTCGTT	60.0	727	INTERGENIC	--	--	--
Ca1_P2_1794532	Ca1	1794532	TTATATAT/TTAT	TCTTGAACGAGAAAACCGCT	TATTATCACGGTGGGTGGGT	59.9	676	INTERGENIC	--	--	--
Ca1_P2_17970605	Ca1	17970605	TAATAATAATAATAATAATAATAATAATAATAATAATAATAATAATAATAATAATAATAATAATAATAATAATAATAAT/TAATAATAATAATAATAATAATAATAATAATAATAATAATAATAATAATAATAATAATAATAATAATAATAATA[illegible]	TTCGACATTTCCAACACATCA	ACTGCCCCCAATATACCGAC	61.0	333	INTERGENIC	--	--	--
Ca1_P2_1800007	Ca1	1800007	AGTT/A	TGTTGCACAAGGTCCAGAAG	TTTTGGCCAAGAGATGGATG	61.0	601	DRR	Ca_00227	P	--
Ca1_P2_1800171	Ca1	1800171	A/AG	TGTTGCACAAGGTCCAGAAG	TTTTGGCCAAGAGATGGATG	61.0	601	DRR	Ca_00227	P	--

INDEL marker IDs	Chromosomes/unanchored scaffolds	Physical positions (bp)	InDels (*Kabuli* reference genome-CDC Frontier/PII)	Forward primers (5'-3')	Reverse primers (5'-3')	Anneling temperature (0C)	Expected amplified product size (bp)	Structural annotation		Functional annotation	
								Sequence components of *kabuli* genome	*Kabuli* gene accession IDs	NCBI-KOG	TFs
Ca1_P2_1806013	Ca1	1806013	A/AG	TTGGGAAGCATGCACATTAG	TGAAGGAGCCAAATCACACA	60.2	779	INTERGENIC	--	--	--
Ca1_P2_18088	Ca1	18088	TA/TAA	CCGCTTGTTTTTCTTTGCAG	TTGATTGCTTGTTGCTCCAG	60.0	543	INTERGENIC	--	--	--
Ca1_P2_18181716	Ca1	18181716	AATATATATATATATATATATATATATA/AATATATATATATATATATATATATATATA	CGAATACTCATGGTTTTTGATCTG	ACCCAACGTAGCACGTTACC	59.9	626	INTERGENIC	--	--	--
Ca1_P2_1819590	Ca1	1819590	TA/TAA	AAAATTGATTCGTCACTCCATACA	CAGCACCAGCAACTCCTACA	60.0	434	INTERGENIC	--	--	--
Ca1_P2_1820515	Ca1	1820515	AT/ATT	TGTTGACATTAAGGAAGACCGTT	GCTGTTACTACATGCTCTTCCAA	58.6	823	INTERGENIC	--	--	--
Ca1_P2_1822426	Ca1	1822426	TG/T	TGTGCACGAGAGCAAAATTC	TCTTCATGGGCTGAAAAAGC	60.3	489	INTERGENIC	--	--	--
Ca1_P2_1822473	Ca1	1822473	AG/A	TGTGCACGAGAGCAAAATTC	TCTTCATGGGCTGAAAAAGC	60.3	489	INTERGENIC	--	--	--
Ca1_P2_18228329	Ca1	18228329	A/ATATATT	CCCTCCACAAGAGTGGAAAA	TGTGTGACCAACCTTAAAAGTCA	59.6	204	INTERGENIC	--	--	--
Ca1_P2_1824386	Ca1	1824386	ATGTGT/ATGTGTGTGT	ACGTGAAGCTTTGCGAGATT	TGAACCAAACACAGGGATGA	59.9	859	INTRON	Ca_00230	K	--
Ca1_P2_18298376	Ca1	18298376	CAAA/CAA	GGTAATGCCTCGTACAAAACAA	CATTCCTTATGCCTCCTACACC	59.9	751	INTERGENIC	--	--	--
Ca1_P2_18300620	Ca1	18300620	GTTTTTTTTT/GTTTTTTT	TTTCCATGAGTTATATTTGTCACGA	TGGCCTTCAATTTTCTTGCT	59.8	134	INTERGENIC	--	--	--
Ca1_P2_18300800	Ca1	18300800	ATTT/ATT	AGCAAGAAAATTGAAGGCCA	GGAAGCTTTAAAGGGAGCAAA	59.9	161	INTERGENIC	--	--	--

INDEL marker IDs	Chromosomes/unanchored scaffolds	Physical positions (bp)	InDels (*Kabuli* reference genome-CDC Frontier/PII)	Forward primers (5'-3')	Reverse primers (5'-3')	Anneling temperature (0C)	Expected amplified product size (bp)	Structural annotation		Functional annotation	
								Sequence components of *kabuli* genome	*Kabuli* gene accession IDs	NCBI-KOG	TFs
Ca1_P2_18301141	Ca1	18301141	AAAA/AAAAGAAA	AGCAAGAAAATTGAAGGCCA	GAGGTGTTAATTCAGCAAGACAAA	59.7	622	INTERGENIC	--	--	--
Ca1_P2_18301437	Ca1	18301437	AATTT/AT	TGATAAGCTAACAGGCCAAAAA	ACATGGGCTAGCAAGAAACC	59.2	417	INTERGENIC	--	--	--
Ca1_P2_18302283	Ca1	18302283	TTTATTATTATTATTATTATTATTATTATTATTATTATT/TTTATTATTATTATTATTATTATTATTATTATTATTATTATTATTATT	TTGTAAGGATGTGTTGACCG	AGGCCCGAGTATAGGGTCTC	59.6	643	INTERGENIC	--	--	--
Ca1_P2_18317638	Ca1	18317638	T/TG	TCATTTTCAACCCTTAGCAACA	TGGTAAGGTATACGCGGGAG	60.0	690	INTERGENIC	--	--	--
Ca1_P2_18325523	Ca1	18325523	AGG/AG	CGGATCATTACACACTTGCG	GCCAAAGCCATGTTTTCAAT	59.9	324	INTERGENIC	--	--	--
Ca1_P2_18328046	Ca1	18328046	CT/CTT	AAATTGACCAAAAGAAAGCCC	GCCCCATTTTGTGGTTTTAAT	59.9	151	INTERGENIC	--	--	--
Ca1_P2_18328170	Ca1	18328170	GTTTTTT/GTTTTTTT	TTAAAACCACAAAATGGGGC	GAGGCAAAGCTTCCAAATCA	60.3	475	INTERGENIC	--	--	--
Ca1_P2_18328227	Ca1	18328227	GTCTATCT/GTCT	TTAAAACCACAAAATGGGGC	GAGGCAAAGCTTCCAAATCA	60.3	475	INTERGENIC	--	--	--
Ca1_P2_1835247	Ca1	1835247	ATGAC/A	TCATAAGCCAGAACCCAAGC	GGTGGTGGAGATTCCAAGAA	59.9	448	INTERGENIC	--	--	--
Ca1_P2_18364053	Ca1	18364053	GAAA/GAAAA	CCCGTAGCAAGACTTGAAGC	TTGGAGAAACTTTTCCGCTG	60.4	627	INTERGENIC	--	--	--
Ca1_P2_1836845	Ca1	1836845	TATTTTAATT/TATTTTAATTTTAATT	CATTTGCATACCGTTTCACG	AGCACCACTTCTTCATTCAAAA	58.9	704	INTERGENIC	--	--	--
Ca1_P2_1837913	Ca1	1837913	TA/TAA	TCGTACTGATTAGTGAAGTGTGAGG	CAGTCAAATCGAAGCCATCA	59.8	617	INTERGENIC	--	--	--

**INDEL marker IDs**	**Chromosomes/unanchored scaffolds**	**Physical positions (bp)**	**InDels (*Kabuli* reference genome-CDC Frontier/PII)**	**Forward primers (5'-3')**	**Reverse primers (5'-3')**	**Anneling temperature (0C)**	**Expected amplified product size (bp)**	**Structural annotation**		**Functional annotation**	
								**Sequence components of *kabuli* genome**	***Kabuli* gene accession IDs**	**NCBI-KOG**	**TFs**
Ca1_P2_1841034	Ca1	1841034	TTTCTTCT/TTTCT	TCAAGGTGTTGATTCGGTAGG	TTTTGCAACCCGGTCTATTC	59.9	595	INTERGENIC	--	--	--
Ca1_P2_1841061	Ca1	1841061	TAAAA/TAAAAAAAA	TCAAGGTGTTGATTCGGTAGG	TTTTGCAACCCGGTCTATTC	59.9	595	INTERGENIC	--	--	--
Ca1_P2_1841424	Ca1	1841424	A/AT	GAATAGACCGGGTTGCAAAA	CCTCTTTCCGGACAACACAT	60.0	302	INTERGENIC	--	--	--
Ca1_P2_1841544	Ca1	1841544	A/AATGATTATTCGT	ATGTGTTGTCCGGAAAGAGG	TGAATCCGGAATAGAACCGA	60.4	147	INTERGENIC	--	--	--
Ca1_P2_1843254	Ca1	1843254	TAA/TA	AAGCCATAATACGCCCACAG	TGACGAGTCACCATCTTTGC	59.8	600	INTERGENIC	--	--	--
Ca1_P2_1843387	Ca1	1843387	ATATT/AT	CAACCAAAAATGCGACTTGA	GCCAAGCTAAGCTGACGAGT	59.8	526	INTERGENIC	--	--	--
Ca1_P2_18441371	Ca1	18441371	CACAAGAGAC/CAC	CCCTCTGTATGCCAGTCGAT	TGGCATTGATGATGGCTTTA	60.0	771	INTERGENIC	--	--	--
Ca1_P2_18472041	Ca1	18472041	CAAAAAAAAAA/CAAAAAAAAAAA	CCTACATTCGCACGAGTTCA	TTTTTCTCCAGTTTTGGTCCT	57.8	404	INTERGENIC	--	--	--
Ca1_P2_1847714	Ca1	1847714	GTTTTT/GTTTTTTT	AATGCCCCCTTCTTCTCCTA	AGTGAAGGCGATGGAATCAG	60.2	275	DRR	Ca_00232	K	MYB
Ca1_P2_1849075	Ca1	1849075	AATTATTATTATTATTATTATTATTAT/AATTATTATTATTATTATTAT	GAATTCAACGGTGTGGGACT	TGTTTGAGCCAAATGTGGAA	60.1	271	CDS (large-effect mutations)	Ca_00232	K	MYB
Ca1_P2_1850030	Ca1	1850030	CAATTAATTA/CAATTA	GTTGTCCGTCCTTCCTGGTA	GGACAACCGAAGAGGATCAA	60.1	563	INTRON	Ca_00232	K	MYB
Ca1_P2_1850089	Ca1	1850089	AT/ATT	GTTGTCCGTCCTTCCTGGTA	GGACAACCGAAGAGGATCAA	60.1	563	INTRON	Ca_00232	K	MYB

INDEL marker IDs	Chromosomes/unanchored scaffolds	Physical positions (bp)	InDels (*Kabuli* reference genome-CDC Frontier/PII)	Forward primers (5'-3')	Reverse primers (5'-3')	Anneling temperature (0C)	Expected amplified product size (bp)	Structural annotation		Functional annotation	
								Sequence components of *kabuli* genome	*Kabuli* gene accession IDs	NCBI-KOG	TFs
Ca1_P2_1858692	Ca1	1858692	CAGCTGC/CAGCTGCATAGTTAGCTGC	AGACAATGGCAGTGATTCCC	TGATCCAATTGATGGCACAC	60.3	949	INTRON	Ca_00233	FP	bHLH
Ca1_P2_1858938	Ca1	1858938	GA/GACA	CTCCTTCAACCACAAGAGCC	TGATCCAATTGATGGCACAC	60.3	677	INTRON	Ca_00233	FP	bHLH
Ca1_P2_18590568	Ca1	18590568	CAATAATAATAATAATAATAATAATAATAATAATAATAATAATAA/CAATAATAATAATAATAATAATAATAATAATAATAATAATAATAATAATAATAA	CCGCCACCTCCTATTTTACA	CTTCAGGAAGTCGCATCTCC	60.0	559	INTERGENIC	--	--	--
Ca1_P2_1859839	Ca1	1859839	ATT/ATTT	CTTGGAACCATGTTCGAGGT	CAACTCCGAATGTCCTGGTT	60.0	445	INTRON	Ca_00233	FP	bHLH
Ca1_P2_1862254	Ca1	1862254	TTAATAA/TTAATAATAA	TGCGTTGAAGAGGATGACTG	TCATAATTTTGAGCCGGTCC	59.9	466	INTERGENIC	--	--	--
Ca1_P2_18630512	Ca1	18630512	TTATATATATATATATATATATATATATATATATAT/TTATATATATATATATATATATATATATATATATATATATAT	ACAGCCAACAACCACATCAA	CCCATATTTCAAATGGCGAC	60.2	577	INTERGENIC	--	--	--
Ca1_P2_18630633	Ca1	18630633	C/CTTTA	ACCAAAGCGTCGTCAAAATC	TGGATGATGGCATTAATATTGG	59.5	625	INTERGENIC	--	--	--
Ca1_P2_186383	Ca1	186383	T/TC	TTCTGCATCACCATCTGAGG	TGATGAAGATCCCCCAAGAG	60.0	783	INTRON	Ca_00014	--	--
Ca1_P2_186468	Ca1	186468	AATC/AATCATC	CAGCAGAATCTTGGAAACCA	TGATGAAGATCCCCCAAGAG	60.0	502	INTRON	Ca_00014	--	--
Ca1_P2_186624	Ca1	186624	GG/GGCGATCG	CCACGCATGGGGACTAGATA	TGATGAAGATCCCCCAAGAG	60.0	212	CDS (large-effect mutations)	Ca_00014	--	--
Ca1_P2_18679893	Ca1	18679893	TTATATATATATATATATATATATATAT/TTATATATATATATATATATATATAT	GACAAAGGCATGTTGGTGTG	TGTTTTCCTCGTGCCTTTATG	60.1	651	INTERGENIC	--	--	--
Ca1_P2_18688865	Ca1	18688865	TTGT/TT	TAGCAGCTGGAGGAACATGA	GCACCCAGTGTAAGACCCAT	59.9	301	INTERGENIC	--	--	--

INDEL marker IDs	Chromosomes/unanchored scaffolds	Physical positions (bp)	InDels (*Kabuli* reference genome-CDC Frontier/PII)	Forward primers (5'-3')	Reverse primers (5'-3')	Anneling temperature (0C)	Expected amplified product size (bp)	Structural annotation		Functional annotation	
								Sequence components of *kabuli* genome	*Kabuli* gene accession IDs	NCBI-KOG	TFs
Ca1_P2_186939	Ca1	186939	TAA/TAAA	CTCTTGGGGGATCTTCATCA	GCATGCCATGTAGTGCATTT	59.6	329	DRR	Ca_00014	--	--
Ca1_P2_1874547	Ca1	1874547	TGGTAG/TGGTAGGTAG	GCTTCTGTTTTGATCTCGGG	TTTTAGTGGCCTATCGACGG	60.1	722	INTERGENIC	--	--	--
Ca1_P2_1874672	Ca1	1874672	TGGG/TG	GCTTCTGTTTTGATCTCGGG	TTTTAGTGGCCTATCGACGG	60.1	722	INTERGENIC	--	--	--
Ca1_P2_1876420	Ca1	1876420	AT/ATACGTAGTTCGGTTGAGT	CGGGTCTTCAAAATGGAATG	CAAAGGAAACGTGCTTCCAG	60.8	861	INTERGENIC	--	--	--
Ca1_P2_1881912	Ca1	1881912	AATAT/AAT	CGTGGACTCCTTCTTCAACC	GGTCATATTATGGGGATGCG	60.0	523	INTERGENIC	--	--	--
Ca1_P2_1882286	Ca1	1882286	CTAAT/CT	CGCATCCCCATAATATGACC	GGTGCAAATGCAAGAGTCAA	59.8	653	INTERGENIC	--	--	--
Ca1_P2_1882571	Ca1	1882571	TC/T	ATATCGTTTTTCACACCCGC	GGTGCAAATGCAAGAGTCAA	59.8	282	INTERGENIC	--	--	--
Ca1_P2_1882839	Ca1	1882839	GCATTA/GCATTAACAGTTCCATTA	TTGACTCTTGCATTTGCACC	ACATATGCGATTGGGACACA	59.8	581	DRR	Ca_00237	A	--
Ca1_P2_1882903	Ca1	1882903	GT/GTCAGCTTCTAT	TTGACTCTTGCATTTGCACC	ACATATGCGATTGGGACACA	59.8	581	DRR	Ca_00237	A	--
Ca1_P2_1883092	Ca1	1883092	C/CT	GCACGTGGCCCACTTATACT	ACATATGCGATTGGGACACA	59.8	244	DRR	Ca_00237	A	--
Ca1_P2_1883148	Ca1	1883148	G/GA	GCACGTGGCCCACTTATACT	ACATATGCGATTGGGACACA	59.8	244	DRR	Ca_00237	A	--
Ca1_P2_18864759	Ca1	18864759	TCCTGCC/TCC	GGGGGAATGTATCCTCTCCA	TGCCTCTCCTCCTTCTCTCA	60.2	431	INTERGENIC	--	--	--

INDEL marker IDs	Chromosomes/unanchored scaffolds	Physical positions (bp)	InDels (*Kabuli* reference genome-CDC Frontier/PII)	Forward primers (5'-3')	Reverse primers (5'-3')	Anneling temperature (0C)	Expected amplified product size (bp)	Structural annotation		Functional annotation	
								Sequence components of *kabuli* genome	*Kabuli* gene accession IDs	NCBI-KOG	TFs
Ca1_P2_18868658	Ca1	18868658	CGG/CGGGAATGG	AATCGATTGATTCAGCCCAG	CGTTTTTCAAAGCGAGGGTA	60.2	707	INTERGENIC	--	--	--
Ca1_P2_1888179	Ca1	1888179	TAAAAAAA/TAAAAAAAA	CCTCACCCCATTTTCCTTTT	ACCATGAACCATTGCCAAGT	60.2	455	INTERGENIC	--	--	--
Ca1_P2_1888548	Ca1	1888548	ATTTTTT/ATTTTTTT	CCAATGTGCCACTTGTTGAC	TAGTGTTGGGTGGGGTAAGG	59.7	262	INTERGENIC	--	--	--
Ca1_P2_18899804	Ca1	18899804	CTATATATATATATATATATAT/CTATATATATATATATATATATAT	GCTTCAATGTGCACGATACG	TTCTTTGTTGGTAGGGCACC	60.0	237	INTERGENIC	--	--	--
Ca1_P2_18928985	Ca1	18928985	CTTATTTATTTATTTATTTATTTAT/CTTATTTATTTATTTATTTATTTATTTAT	TTTACCCTATCCCACACCCC	TCCAATGATTCCTGTTTTTGC	59.9	613	INTERGENIC	--	--	--
Ca1_P2_19025184	Ca1	19025184	A/ATTG	CGGGATCCTTGAACTTGGTA	CTTGTGCAAAGGTTCCATCA	59.7	533	INTERGENIC	--	--	--
Ca1_P2_1907609	Ca1	1907609	ATTTCTTTT/ATTTCTTTTCTTTT	TGCAATGATGTCAATTGCTCT	AAATCAATTTTGGTCCGCTC	59.0	926	DRR	Ca_00239	--	--
Ca1_P2_1907983	Ca1	1907983	CTT/C	TTCCCGTGAATTTCATCCAT	GCATGGGAGGATAAAAGCAA	60.0	721	DRR	Ca_00239	--	--
Ca1_P2_19112991	Ca1	19112991	CTTTTTTTTTTTTTTTTTTTTT/CTTTTTTTTTTTTTTT	GAGAACATCCGGTTGCAGTT	GGATTTGGAATAAGCGGACA	59.9	926	INTERGENIC	--	--	--
Ca1_P2_19135325	Ca1	19135325	GC/GCC	ACTGCGCTCTACTTGAGGGA	ATGTTTCGTGCAATGGTCAA	60.0	404	INTERGENIC	--	--	--
Ca1_P2_19150621	Ca1	19150621	A/ATT	TTTAATATGTGGATCGGGGC	AACCCAAAGACGGGCTAGTT	60.0	648	INTERGENIC	--	--	--
Ca1_P2_19150973	Ca1	19150973	CTT/CT	AACTAGCCCGTCTTTGGGTT	GAAAACAAAAGCTTTACGGTCA	58.5	488	INTERGENIC	--	--	--

**INDEL marker IDs**	**Chromosomes/unanchored scaffolds**	**Physical positions (bp)**	**InDels (*Kabuli* reference genome-CDC Frontier/PII)**	**Forward primers (5'-3')**	**Reverse primers (5'-3')**	**Anneling temperature (0C)**	**Expected amplified product size (bp)**	**Structural annotation**		**Functional annotation**	
								**Sequence components of *kabuli* genome**	***Kabuli* gene accession IDs**	**NCBI-KOG**	**TFs**
Ca1_P2_1915229	Ca1	1915229	CTCAT/CTCATCTGTTTCAT	TGGATACACAAAAACCGAACA	CACCTTTTCCATCCATCGAC	60.3	709	INTERGENIC	--	--	--
Ca1_P2_19168	Ca1	19168	GCTCTCT/GCTCT	CAGAACCGAGGAATTAAGCG	AGGCTTGAAGCGTGGTAATG	60.3	637	INTERGENIC	--	--	--
Ca1_P2_1917237	Ca1	1917237	GAAAAA/GAAAA	TGGTTGATGAAAGAGAGATGGA	TGTAACAAGCAGATGGAATCTTG	59.3	247	INTERGENIC	--	--	--
Ca1_P2_1917413	Ca1	1917413	TTT/TTTATT	TGGTTGATGAAAGAGAGATGGA	TGTAACAAGCAGATGGAATCTTG	59.3	247	INTERGENIC	--	--	--
Ca1_P2_1920132	Ca1	1920132	AA/AACA	AAATGCCAATGAAACACTTGC	CCAAGTAGCCAACCAGCATT	60.1	305	DRR	Ca_00240	--	C3H
Ca1_P2_1927607	Ca1	1927607	G/GA	TCCGAAGGGAAAGTGAAGAA	TTCAATGGTTGATGCCATGT	59.8	626	INTERGENIC	--	--	--
Ca1_P2_1928153	Ca1	1928153	ATTTTT/ATTTT	ACATGGCATCAACCATTGAA	TGCATCAAATGAATCCTACATCA	60.3	576	INTERGENIC	--	--	--
Ca1_P2_1930262	Ca1	1930262	ATTTTTTTT/ATTTTTTTTTT	GGCAACATTTTACAAAAGTTAAGG	AGTTTGATTAATGTCGGGCG	60.0	318	INTERGENIC	--	--	--
Ca1_P2_19315964	Ca1	19315964	GTTTTTT/GTTTTTTT	GTCATTTGGAATGGCGACTT	GCTTTGATGGTTCAAACACG	59.2	364	INTERGENIC	--	--	--
Ca1_P2_19361983	Ca1	19361983	AATATATATATATATATATATATATA/AATATATATATATATATATATA	TCATTAACCACTAGAACAAAAGCAA	GGCCGTAGATACCAACAGGA	60.0	447	INTERGENIC	--	--	--
Ca1_P2_19422707	Ca1	19422707	TAAAAAAAAAA/TAAAAAAAA	ACCCGTTTTTGATTTGCTTG	TCGTGTCCCAAAACTCAATG	59.5	840	INTERGENIC	--	--	--
Ca1_P2_19422756	Ca1	19422756	TAAAAAA/TAAAAAAA	ACCCGTTTTTGATTTGCTTG	TCGTGTCCCAAAACTCAATG	59.5	840	INTERGENIC	--	--	--

INDEL marker IDs	Chromosomes/unanchored scaffolds	Physical positions (bp)	InDels (*Kabuli* reference genome-CDC Frontier/PII)	Forward primers (5'-3')	Reverse primers (5'-3')	Anneling temperature (0C)	Expected amplified product size (bp)	Structural annotation		Functional annotation	
								Sequence components of *kabuli* genome	*Kabuli* gene accession IDs	NCBI-KOG	TFs
Ca1_P2_19422886	Ca1	19422886	TTTTATTT/TTTTATTTATTT	GCCCATAACCTAGCCCATTT	TCGTGTCCCAAAACTCAATG	59.5	713	INTERGENIC	--	--	--
Ca1_P2_19460421	Ca1	19460421	T/TTCA	CGTTGCAATTGATGAGGATG	TTCGCTCCTTCTTCTTGTCC	59.6	748	INTERGENIC	--	--	--
Ca1_P2_19498678	Ca1	19498678	TTTTGATCCTTTGAT/TTTTGAT	GCCCGAGTTAACAACAAACG	ATCGATTTCCTCAGCACTGG	60.2	374	INTERGENIC	--	--	--
Ca1_P2_19506055	Ca1	19506055	GAA/GA	ACGAACGTTGAAAATCCAGC	TCGGTTCATGGAACGAGTTA	59.1	477	INTERGENIC	--	--	--
Ca1_P2_19523625	Ca1	19523625	AAATAATAATAATAATAATAATAATAATAATAATAATAATAATAATAATAATAATAA/AAATAATAATAATAATAATAATAATAATAATAATAATAATAATAATAATAATAATAATAA	CCTGATCAAACCCGATGAGT	TATTATCCATCCCGTCCCAA	60.0	364	INTERGENIC	--	--	--
Ca1_P2_1953853	Ca1	1953853	ACTCTCTCTCTCTCTCTC/ACTCTCTCTCTC	GTTGGAAATGCCAAAAAGGA	GGAGCAGAAGCCATTCAAAG	60.0	278	INTERGENIC	--	--	--
Ca1_P2_19567652	Ca1	19567652	T/TCCCATGCC	CGAAGACCGAAATGCTGATT	GGTCAAACCGTCAAAAAGGA	59.9	321	INTRON	Ca_08866	J	G2-like
Ca1_P2_19627166	Ca1	19627166	TTAATAATAATAATAATAATAATAATAATAATAATAATAATA/TTAATAATAATAATAATAATAATAATAATAATAATAATAATAATAATA	AACGCATCGGTAAACACTCC	TGAAGTTGAATGTTGGCAGG	59.7	327	INTERGENIC	--	--	--
Ca1_P2_19631803	Ca1	19631803	CATATATATATATATATATATAT/CATATATATATATATATATATATATAT	ATATTCGCAACGCGTATGTT	AAATGTGGCAAATTGGGACT	59.3	447	INTERGENIC	--	--	--
Ca1_P2_1963997	Ca1	1963997	ATATTTATTT/ATATTTATTTATTT	AGGCAAACAACTTTTGCACC	TTACAATGTGCATAGCGGGA	60.1	317	DRR	Ca_00244	--	B3
Ca1_P2_1965007	Ca1	1965007	TG/T	CCACCCCAAGAAGAAAAACA	TTTCCCAAGCCACTAACCAC	60.0	704	DRR	Ca_00244	--	B3
Ca1_P2_1966252	Ca1	1966252	TTCTCT/TTCTCTCT	GGCAAGAGAGCATCAAAAGG	CAAGTGAAGTTTTGATTCAAGGG	60.0	703	DRR	Ca_00245	U	bZIP

INDEL marker IDs	Chromosomes/unanchored scaffolds	Physical positions (bp)	InDels (*Kabuli* reference genome-CDC Frontier/PII)	Forward primers (5'-3')	Reverse primers (5'-3')	Anneling temperature (0C)	Expected amplified product size (bp)	Structural annotation		Functional annotation	
								Sequence components of *kabuli* genome	*Kabuli* gene accession IDs	NCBI-KOG	TFs
Ca1_P2_19662720	Ca1	19662720	TATAATAATAATAATAATAATAATAATAATAATAATAATAATAATAATAATAATAATAATAA/TATAATAATAATAATAATAATAATAATAATAATAATAATAATAATAATAATAATAATAATAATAATAA	TCCCTCAACCAACTCCACTC	TTCCAACCAACCACCGTTAT	60.1	684	INTERGENIC	--	--	--
Ca1_P2_19673430	Ca1	19673430	AAG/A	TGTAGGTGATCCTTCCACCG	AGGGACTTCGTCCCCTTAAA	59.9	508	INTERGENIC	--	--	--
Ca1_P2_1968368	Ca1	1968368	CAAAGAAAGAAAGAAAGA/CAAAGAAAGAAAGA	TCCAATCCATTCCACCATTT	TGCATTCCATTTAGCAGCAG	60.0	775	INTERGENIC	--	--	--
Ca1_P2_1969676	Ca1	1969676	TGG/TGGGG	TTTTGGCCGTGGTAGTTTTC	TTCGAATTTTCCCGTACCTG	59.9	724	INTERGENIC	--	--	--
Ca1_P2_1970052	Ca1	1970052	TTTAATTATATTAATTATA/TTTAATTATA	CGGATTTGGGCAGTGATATT	TTCGAATTTTCCCGTACCTG	59.9	266	INTERGENIC	--	--	--
Ca1_P2_1970410	Ca1	1970410	ATATTATTATTATTATTATTATTATTATTATTATTATTATTATTAT/ATATTATTATTATTATTATTATTATTATTATTATTATTATTAT	GGGAAAATTCGAACTCCGTT	TCGGTTAAATTCGAGGTGGT	59.4	626	INTERGENIC	--	--	--
Ca1_P2_1972044	Ca1	1972044	ACTTCTTCT/ACTTCTTCTTCT	GCCCTCAGCCTCACTTTATG	AGGGAGGCTACGAACGATTT	60.1	356	INTERGENIC	--	--	--
Ca1_P2_1973631	Ca1	1973631	TG/T	TTTTTGACAAGGCAGTGCAG	CAGCCACTGAAAGTAACGCA	60.1	290	INTRON	Ca_00246	A	--
Ca1_P2_1976496	Ca1	1976496	A/AATTTTCAC	TGATCAAGCAACCAAAGCTG	TGCCTTTCTAAAGCAGCCAT	60.0	574	DRR	Ca_00247	--	--
Ca1_P2_1979080	Ca1	1979080	CTTCTTA/CTTCTTAGTTCTTA	CGTAGGGAGGAGGTTGTTGA	TAGTGACGACTTGGGCACAG	59.9	481	INTERGENIC	--	--	--
Ca1_P2_1979133	Ca1	1979133	CA/C	CTGTGCCCAAGTCGTCACTA	TCTATGATCGGTTTCCACGA	59.1	160	INTERGENIC	--	--	--
Ca1_P2_1983802	Ca1	1983802	CTTA/CTTAATTA	GCCGTTAATTGTGATCTAACCA	TTCGAACTAAATGCAAGGGG	60.1	684	INTERGENIC	--	--	--

INDEL marker IDs	Chromosomes/unanchored scaffolds	Physical positions (bp)	InDels (*Kabuli* reference genome-CDC Frontier/PII)	Forward primers (5'-3')	Reverse primers (5'-3')	Anneling temperature (0C)	Expected amplified product size (bp)	Structural annotation		Functional annotation	
								Sequence components of *kabuli* genome	*Kabuli* gene accession IDs	NCBI-KOG	TFs
Ca1_P2_1986085	Ca1	1986085	TAAAAA/TAAAAAA	TGGATAAATTGTAGGCCCCA	GAGAAAAGTTGAAACCATGACCA	60.4	679	DRR	Ca_00248	T	--
Ca1_P2_19864874	Ca1	19864874	AATTATTATTATTATTATTATTATTATTATTATTATTATTATTATTATTATTATTATTATTATTATTATTATTATTATTATTAT/AATTATTATTATTATTATTATTATTATTATTATTATTATTATTATTATTATTATTATTATTATTATTATTATT[illegible]	AAATATTAATCATGCGGGATCG	GGATGCTTGTTTTTGGAACC	59.4	582	INTERGENIC	--	--	--
Ca1_P2_1987558	Ca1	1987558	ATAGTAG/ATAG	GTGGCATAGGCTCCAATCAT	GGAATGGTGCTGGACATTTT	59.8	922	DRR	Ca_00248	T	--
Ca1_P2_1988980	Ca1	1988980	TC/TCCC	ACCTGCCAAAAGCAGAAGAA	CACTGAGTTTTCGGGGAGAA	60.2	776	INTRON	Ca_00248	T	--
Ca1_P2_1989852	Ca1	1989852	AAA/AAATAA	TTGGGCTTATTGGGTCACTC	TATCCAATCCCAACCCAAAA	60.0	115	INTERGENIC	--	--	--
Ca1_P2_19903627	Ca1	19903627	TA/TAATTGGCAGCA	TGGGCTGAAAAGCAACTTCT	ACCAGTGCTGTTCCCAAATC	60.0	233	INTRON	Ca_08847	K	--
Ca1_P2_1990497	Ca1	1990497	TAA/TA	TGGGTCAACGTTGGTGTCTA	CCACTCAAAGGCTCACACAA	59.9	769	INTERGENIC	--	--	--
Ca1_P2_19909063	Ca1	19909063	TTATATATATATATATATATATATATATATATAT/TTATATATATATATATATATATATATATATATATATATATATATATATAT	CCATAATTTTTGGATCCTCCT	TTTGCTTATGAGAAAAAGGAGATGT	59.7	235	INTERGENIC	--	--	--
Ca1_P2_1991447	Ca1	1991447	GATATATATATATATATATATATATATAT/GATATATATATATATATATATATAT	CCAACCCTTAGGGGACAAAT	CATGTGCCAGAAAGCTCAAA	60.0	505	DRR	Ca_00249	CR	HB-other
Ca1_P2_1995431	Ca1	1995431	GATAT/GAT	AACCATGCACCATGTTTTCA	GTTGATGACGTGTAGGGGCT	60.0	406	DRR	Ca_00250	CR	HB-other
Ca1_P2_1996315	Ca1	1996315	T/TTTTTTTCATAA	AAAATGAACTTTGCGGTTGG	AGGGCTTGTTTACTTTGCGA	59.9	538	INTRON	Ca_00250	CR	HB-other
Ca1_P2_1997441	Ca1	1997441	AC/A	AAGCAATACTGAACCTGCGG	ATGAGCCGGTTATCTCATGG	59.9	473	INTRON	Ca_00250	CR	HB-other

INDEL marker IDs	Chromosomes/unanchored scaffolds	Physical positions (bp)	InDels (*Kabuli* reference genome-CDC Frontier/PII)	Forward primers (5'-3')	Reverse primers (5'-3')	Anneling temperature (0C)	Expected amplified product size (bp)	Structural annotation		Functional annotation	
								Sequence components of *kabuli* genome	*Kabuli* gene accession IDs	NCBI-KOG	TFs
Ca1_P2_1998915	Ca1	1998915	T/TTATAAAAATGA	TGCTTGTTTCACCACTTTGC	CGACCGATATTCGTCCACTC	60.5	481	INTERGENIC	--	--	--
Ca1_P2_1999792	Ca1	1999792	CTTTTTTTTTTT/CTTTTTTTTT	TCCTTTTCCGATGTTTGGAC	AATGGGAGACAAAGGGAGAGA	60.1	548	INTERGENIC	--	--	--
Ca1_P2_2000025	Ca1	2000025	TAAAAAAA/TAAAAAA	TCCTTTTCCGATGTTTGGAC	TTAAGGACGTCGTTGGAAGG	60.1	778	INTERGENIC	--	--	--
Ca1_P2_2000345	Ca1	2000345	TAAGA/TAAGAAGA	TCTCTCCCTTTGTCTCCCATT	TGTTAGGATTGTGCTGAGCG	60.0	405	INTERGENIC	--	--	--
Ca1_P2_2000540	Ca1	2000540	AT/ATT	CGACGTCCTTAAAAGGTCCA	TGTTAGGATTGTGCTGAGCG	60.0	166	DRR	Ca_00251	J	--
Ca1_P2_2001789	Ca1	2001789	ATGT/AT	AGCACTGGGCAGAAAGAAAA	CACACGTCATGATCCCCATA	60.2	751	DRR	Ca_00251	J	--
Ca1_P2_20097334	Ca1	20097334	CNNNNNNNNNNNNNN/CNNN	TTTCGTTTGCGATAGTGATCC	TCATTTCAAGATCCTGGTTCC	59.0	250	INTERGENIC	--	--	--
Ca1_P2_2015582	Ca1	2015582	TTGT/TT	CTCTAGAACCCCCAACTCCC	CTCCAATGTTGGGAGACTACG	59.6	499	DRR	Ca_00253	E	--
Ca1_P2_2018076	Ca1	2018076	A/AGTGAGACTCT	TTGTCTTGCAGCAACTTTGG	GCCGCAATGATGTGTTTATG	60.0	831	INTRON	Ca_00254	FP	bHLH
Ca1_P2_2019732	Ca1	2019732	ATTTTTT/ATTTTTTT	AACTCAACCGGAGGTCCTTT	GCACATTTAGGTGTGGAGCA	59.7	745	INTRON	Ca_00254	FP	bHLH
Ca1_P2_2019873	Ca1	2019873	AT/ATT	CCATCTTCGGAAAGTTGGAA	GATATCAACACCGCCAAACC	60.2	848	INTRON	Ca_00254	FP	bHLH
Ca1_P2_2023719	Ca1	2023719	ATTTT/ATTTTT	CGGTCAAAGTAAATCTCGTCG	TCCGTTGTTGAGGATGATGA	60.0	608	DRR	Ca_00255	--	--

INDEL marker IDs	Chromosomes/unanchored scaffolds	Physical positions (bp)	InDels (*Kabuli* reference genome-CDC Frontier/PII)	Forward primers (5'-3')	Reverse primers (5'-3')	Anneling temperature (0C)	Expected amplified product size (bp)	Structural annotation		Functional annotation	
								Sequence components of *kabuli* genome	*Kabuli* gene accession IDs	NCBI-KOG	TFs
Ca1_P2_2023898	Ca1	2023898	TAA/TAAA	TAATGAGGGCTTGAATTCGC	TCCGTTGTTGAGGATGATGA	60.0	386	DRR	Ca_00255	--	--
Ca1_P2_2044788	Ca1	2044788	T/TATCGTAAA	GAGCTTCTCCACCTCACGTC	TGGCATGTTTCCACTAGGCT	60.7	633	DRR	Ca_00259	U	M-type
Ca1_P2_2057962	Ca1	2057962	GAAAAGTTA/GA	TGTGCATGAAAGCAAAATTGA	GGCCGTGAAAGATATTTGGA	59.9	712	DRR	Ca_00261	--	--
Ca1_P2_2073961	Ca1	2073961	CTTTT/CTTT	AGAGGCACGAGATTTCCAGA	AAATATCCCCGCAATAAGGG	60.0	336	CDS (FRAME SHIFT)	Ca_00263	T	M-type
Ca1_P2_2083881	Ca1	2083881	TCCCC/TCCCCC	AGCACCTAGGAACACGGAAA	GAGAAAGCGTGTGTGTGCAT	59.9	103	INTERGENIC	--	--	--
Ca1_P2_2085633	Ca1	2085633	CTTT/CTT	TGGGTTTGTAAAATGAAAAGTGA	TTGAGATTTTCCAAGCTCAAAG	58.6	737	INTERGENIC	--	--	--
Ca1_P2_2091229	Ca1	2091229	AGGGGG/AGGGGGGG	TTCAGTTCATCAATTGCCCA	TCGAACTGTTGTTCTGGCTG	60.0	618	DRR	Ca_00266	--	MYB_related
Ca1_P2_2094556	Ca1	2094556	ATTTT/ATTTTT	CAAACCCTGCCCCCTATAAT	GGGAGCACGTGAGTTGATTT	60.1	947	INTRON	Ca_00266	--	MYB_related
Ca1_P2_2095011	Ca1	2095011	AT/A	CCAAGCCCTGCTATTGTCAT	GCAAAAACGGTATTCTGGGA	59.9	196	INTRON	Ca_00266	--	MYB_related
Ca1_P2_2099948	Ca1	2099948	ATTTTT/ATTTT	TTGGTATATGTATGAATGTTGGCA	TGGTGTTAATTAATCTCGTCACTT	57.0	574	INTERGENIC	--	--	--
Ca1_P2_2106022	Ca1	2106022	CTCTTTNNNNNNNNNNNTCTTT/CTCTTT	CTCAAATATCATGGTGGGGG	GTCAATGGAGGTTTGCCATC	60.3	276	CDS (FRAME SHIFT)	Ca_00267	D	NAC
Ca1_P2_2108793	Ca1	2108793	GTT/GTTTT	GTTTTATCTGCCGAACCTGC	CAATGGCAGTGATCTGCTCT	59.0	678	INTRON	Ca_00267	D	NAC

INDEL marker IDs	Chromosomes/unanchored scaffolds	Physical positions (bp)	InDels (*Kabuli* reference genome-CDC Frontier/PII)	Forward primers (5'-3')	Reverse primers (5'-3')	Anneling temperature (0C)	Expected amplified product size (bp)	Structural annotation		Functional annotation	
								Sequence components of *kabuli* genome	*Kabuli* gene accession IDs	NCBI-KOG	TFs
Ca1_P2_2113146	Ca1	2113146	TAAAAAAAAAA/TAAAAAAAA	TGTGGACACGTTAAGTTGTGG	TGATCTCGCCCTATTATTCAAAA	59.9	409	INTERGENIC	--	--	--
Ca1_P2_2117774	Ca1	2117774	T/TTATTG	TGCATGATACCCATCAAACG	TGCCTTTGCCTCTGACTACC	60.4	633	INTERGENIC	--	--	--
Ca1_P2_2120405	Ca1	2120405	TTTAATTTAATTAA/TTTAATTTAATTAATTTAATTAA	TGTATGGAAAGAGGTAAAATGGAA	AACGACAAATGTTGCCATGA	60.0	532	INTERGENIC	--	--	--
Ca1_P2_2121527	Ca1	2121527	ATT/AT	AACGCAAAATCAAACACCAA	TCTCGAAAGCAACTCACTCG	59.3	398	INTERGENIC	--	--	--
Ca1_P2_2131072	Ca1	2131072	A/AAAG	TGCTATTCATCCCACAAGCA	CTTGGATTCGATGCATTCCT	60.0	640	INTERGENIC	--	--	--
Ca1_P2_2131592	Ca1	2131592	TTAGG/TTAGGCTAGG	AGGAATGCATCGAATCCAAG	CCACAAGTGCCATTCAGCTA	59.9	516	INTERGENIC	--	--	--
Ca1_P2_2131832	Ca1	2131832	CTTT/CTTTT	AGGAATGCATCGAATCCAAG	CCACAAGTGCCATTCAGCTA	59.9	516	INTERGENIC	--	--	--
Ca1_P2_2132014	Ca1	2132014	G/GTGC	CATGGATGGTTAGCCCAAAG	AATGACCAACCCACAAGTGC	60.8	107	INTERGENIC	--	--	--
Ca1_P2_2135161	Ca1	2135161	AGTGTG/AGTGTGTG	AGAATTTACTCCACGTCCCG	TGAACCTCGTGCATGTAATTCT	59.6	437	INTERGENIC	--	--	--
Ca1_P2_21595611	Ca1	21595611	TTATATATATATATATATATATATATATATATATATATATATATATA/TTATATATATATATATATATATATATATATATATATATATATATATATATA	GGCATTATGGTCACCCACTT	TGTCAAGTCCGTAGGACGAA	59.3	512	INTERGENIC	--	--	--
Ca1_P2_21653919	Ca1	21653919	TTATATATATATATATATATATATAT/TTATATATATATATATATATATATATATAT	GCTTGCAAAACAGATGCAGA	ACGAAACGAAAGCGAGAAAA	60.0	536	INTERGENIC	--	--	--
Ca1_P2_2169507	Ca1	2169507	ATT/ATTT	TCGCTGAAAAAGAAAGGTGA	ATGTAGGTGATGGGATGGGA	60.0	509	DRR	Ca_00273	Z	--

INDEL marker IDs	Chromosomes/unanchored scaffolds	Physical positions (bp)	InDels (*Kabuli* reference genome-CDC Frontier/PII)	Forward primers (5'-3')	Reverse primers (5'-3')	Anneling temperature (0C)	Expected amplified product size (bp)	Structural annotation		Functional annotation	
								Sequence components of *kabuli* genome	*Kabuli* gene accession IDs	NCBI-KOG	TFs
Ca1_P2_21725211	Ca1	21725211	AATATATATATATATATATATATATA/AATATATATATATATATATATATATATA	TTGCTTTTTGGGTACTTGGG	TTTCCATTCAAATTTACTCCTCAA	59.1	387	INTERGENIC	--	--	--
Ca1_P2_21746056	Ca1	21746056	AC/ACACATATCAGCTTTC	CTATTGCCCCCTCCTCCTAC	TACCGATACCTCCAAGTGCC	60.0	585	INTRON	Ca_22116	S	--
Ca1_P2_21772275	Ca1	21772275	CATATATATATATATAT/CATATATATATATATATAT	CTTTTGTCTCCCCATCCTCA	AAAACTGAGACGAAAAAGTTGTCC	60.1	342	DRR	Ca_22115	--	MYB
Ca1_P2_21801995	Ca1	21801995	T/TTTCCTACTACATCTTA	CTGGAACCACCGATTTCACT	TTGGTTTGGAGCTAGCTTGG	60.4	694	INTERGENIC	--	--	--
Ca1_P2_21852841	Ca1	21852841	CAAAAAA/CAAAAAAA	CTTTTGCGTTTCCGTGATTT	GGACCCAATGGTCAATGAAT	59.5	589	INTERGENIC	--	--	--
Ca1_P2_21862084	Ca1	21862084	ATT/ATTT	CGTATGTTCTCCACGACCTG	TGTTTTTGAATTGCATCGAGA	59.3	941	INTERGENIC	--	--	--
Ca1_P2_21918405	Ca1	21918405	AAAGAAGAAGAAGAAGAAGAAGAAGAAGAAGAAGAAGAAGAAGAAGAAGAAGAAGAAGAAGAAGAAGAAGAAGAA/AAAGAAGAAGAAGAAGAAGAAGAAGAAGAAGAAGAAGAAGAAGAAGAAGAAGAAGAAGA[illegible]	TGTCAGTCACAGATAGCGGC	GCTGCGAATAGCACCCTAAC	59.9	682	INTRON	Ca_22107	P	GRAS
Ca1_P2_21974667	Ca1	21974667	T/TCCTTA	GTTGGGGATTTGAGGTGATG	TGGAAATCCCGCTAGTACAAA	59.6	340	INTERGENIC	--	--	--
Ca1_P2_21999732	Ca1	21999732	AGGGGG/AGGG	CGCCTTTGGCTATTCATCAT	TCCCTTATTGCCCAAACATC	59.8	492	DRR	Ca_22101	R	MYB
Ca1_P2_22086077	Ca1	22086077	ATATTTATTTATTTATTTATTTATTTATTTAT/ATATTTATTTATTTATTTATTTATTTATTTATTTAT	TCGCACCCCTACTTATACCG	CCGTGGGTAATTTTTCCGTA	59.7	581	INTERGENIC	--	--	--
Ca1_P2_22100599	Ca1	22100599	ATATATATATAT/A	ATGTGTGAGAAAACCTCGGC	TGTCCAGCTTCATTCAACCA	60.2	574	INTERGENIC	--	--	--
Ca1_P2_22131328	Ca1	22131328	AATATATAT/AATATATATATAT	GTGTGGACATGCCTAAAACG	GTTGCAATTGAGGTGCTGAA	59.8	403	INTERGENIC	--	--	--

INDEL marker IDs	Chromosomes/unanchored scaffolds	Physical positions (bp)	InDels (*Kabuli* reference genome-CDC Frontier/PII)	Forward primers (5'-3')	Reverse primers (5'-3')	Anneling temperature (0C)	Expected amplified product size (bp)	Structural annotation		Functional annotation	
								Sequence components of *kabuli* genome	*Kabuli* gene accession IDs	NCBI-KOG	TFs
Ca1_P2_22131513	Ca1	22131513	GAAAAA/GAAAA	GTGTGGACATGCCTAAAACG	GTTGCAATTGAGGTGCTGAA	59.8	403	INTERGENIC	--	--	--
Ca1_P2_22132039	Ca1	22132039	G/GC	GGATGGTAGTTCAGCACCTCA	GAGCATTAGTGCTTGGCACA	60.0	774	INTERGENIC	--	--	--
Ca1_P2_22133403	Ca1	22133403	CTTT/CTT	GCTGTAGCCAAGGAATAGCG	CATCCCTTGAGTGGGTTTTG	60.3	731	INTERGENIC	--	--	--
Ca1_P2_22159776	Ca1	22159776	ATTTTTTTTTTT/ATTTTTTTTTTTT	TGTTTCGAAAGTTTGGCCTT	TAAGGGAAAGCCTCCTCCAT	60.0	333	INTERGENIC	--	--	--
Ca1_P2_22188648	Ca1	22188648	AATATATATATATATATATATATATATATATAT/AATATATATATATATATATATATATATATAT	TTTTGGTGCTTTACATTTTCAA	CAATAGCCGGCTCTAAATCG	59.8	128	INTERGENIC	--	--	--
Ca1_P2_22204494	Ca1	22204494	TTATATATAT/TTATATATATAT	CTCACATGCCATAGCTGCAT	TTTAGTAAATCCAAATGGGCA	57.2	952	DRR	Ca_20635	--	TALE
Ca1_P2_2223979	Ca1	2223979	TATAATAATAATAATAATAATAATAATAATAATAA/TATAATAATAATAATAATAATAATAATAATAA	AATAGGTCAGGCCAGGCTTT	TATGGATGGGAATAGGCTCG	59.9	659	INTERGENIC	--	--	--
Ca1_P2_22270085	Ca1	22270085	TAAA/TAAAA	CAAGGACCAACTCGAGGAAA	TATGCAATTGGGATGAATGC	59.3	659	INTERGENIC	--	--	--
Ca1_P2_22287070	Ca1	22287070	TA/TAA	ACTCGACTGGTGGCTGAACT	GTGTTCGATCGGATAACGGT	59.8	675	INTERGENIC	--	--	--
Ca1_P2_22384776	Ca1	22384776	G/GAACAAGTGTGT	CTTCAGATCCTTGAGCTGCC	TGAATCTTTGTGTCCCACCC	60.8	539	INTERGENIC	--	--	--
Ca1_P2_2239989	Ca1	2239989	AATATATATATATATATATATAT/AATATATATATATATATAT	ATGGTGGCAACACAACTTGA	TGGTTTTGATTTGAGCTTTCC	59.2	775	DRR	Ca_00280	I	--
Ca1_P2_22401909	Ca1	22401909	GTATTATTATTATTATTATTATTATTATTATTATTATTATTATTATTATT/GTATTATTATTATTATTATTATTATTATTATTATTATT	TAGCGGAAATTTTGTGCCTT	GTTCAACGGTGTTTTTCGCT	60.2	494	INTERGENIC	--	--	--

INDEL marker IDs	Chromosomes/unanchored scaffolds	Physical positions (bp)	InDels (*Kabuli* reference genome-CDC Frontier/PII)	Forward primers (5'-3')	Reverse primers (5'-3')	Anneling temperature (0C)	Expected amplified product size (bp)	Structural annotation		Functional annotation	
								Sequence components of *kabuli* genome	*Kabuli* gene accession IDs	NCBI-KOG	TFs
Ca1_P2_22404929	Ca1	22404929	AATATATATATATATATATATATATATATATATATA/AATATATATATATATATATATATATATATATA	TGTTTTATGTGAACGACGGG	TTCCTGCTTCAAGAACGATT	57.5	249	INTERGENIC	--	--	--
Ca1_P2_22512128	Ca1	22512128	AATATATATATATATATATATATATATATATA/AATATATATATATATATATATATATATATATATATA	AACACATCCGGGATCCATTA	AACGATGATGACGATGACGA	60.1	533	DRR	Ca_20620	--	--
Ca1_P2_2254515	Ca1	2254515	ATTTTTTTT/ATTTTTTT	TTGAGTTGGTTCGTGGATGA	CCAACAATCCTCTTCACCCT	59.0	620	INTERGENIC	--	--	--
Ca1_P2_22580970	Ca1	22580970	TATAATAATAATAATAATAATAATAATAATAATAATAATAAT/TATAATAATAATAATAATAATAATAATAATAATAATAATAATAAT	CGAAGACATGCGAATTTCAA	ATTTATCTGCACAGGTGGGG	59.8	847	INTERGENIC	--	--	--
Ca1_P2_22745256	Ca1	22745256	AATATATATATATATATATATATATATA/AAAATATATATATATATATATATATATATATA	CTCCAGAAATTTTGCCTCCA	GTTAGGCCGAGGCTCTCTTT	60.0	480	INTRON	Ca_20613	BD	bHLH
Ca1_P2_22847792	Ca1	22847792	AATATATATATATA/AATATATATATATATA	TCCAATTCTTCTTGGATGCC	TTGTGTATTGTGTAACACCCCAA	60.1	916	INTERGENIC	--	--	--
Ca1_P2_22853183	Ca1	22853183	C/CG	TACATTCCCATCCACAGGGT	TCGGGTGTAAGTGTGATGGA	60.0	780	INTERGENIC	--	--	--
Ca1_P2_22877135	Ca1	22877135	TTATATATATATATAT/TTATATATATATAT	AAGGGGAGAATTGTAACACCC	TCCCTCCAATTTTCCAATTTT	59.6	746	INTERGENIC	--	--	--
Ca1_P2_22885854	Ca1	22885854	TTA/TTATA	TGATTTGGGGTTTTGGGTAA	GGGTGAGTTCTTTGAGGGTG	59.5	531	INTERGENIC	--	--	--
Ca1_P2_22886112	Ca1	22886112	GAA/GAAA	TGATTTGGGGTTTTGGGTAA	GGGTGAGTTCTTTGAGGGTG	59.5	531	INTERGENIC	--	--	--
Ca1_P2_22896758	Ca1	22896758	CTTTTTTTTTTTTTTT/CTTTTTTTTTTT	GCACCGTTTACAACGCTTTT	GATGAAACCGAGGACCAGAA	60.1	400	INTERGENIC	--	--	--
Ca1_P2_22906568	Ca1	22906568	T/TG	CCCAAAAAGCGCTGTAAAAG	GCATGCATGTTGAGCTTGTT	59.9	469	INTERGENIC	--	--	--

INDEL marker IDs	Chromosomes/unanchored scaffolds	Physical positions (bp)	InDels (*Kabuli* reference genome-CDC Frontier/PII)	Forward primers (5'-3')	Reverse primers (5'-3')	Anneling temperature (0C)	Expected amplified product size (bp)	Structural annotation		Functional annotation	
								Sequence components of *kabuli* genome	*Kabuli* gene accession IDs	NCBI-KOG	TFs
Ca1_P2_22965842	Ca1	22965842	TCACA/TCA	TAACAAGAGCTCGGCAACCT	TTGTCGGATTAGTGATGCCA	60.1	456	INTERGENIC	--	--	--
Ca1_P2_2299530	Ca1	2299530	AATATATATATATATATATATATATATATA/AATATATATATATATATATATATATATATATATATA	CAGCAACTGCGATGAGATGT	TCACGGCGAGTCAATAAAAA	59.3	835	DRR	Ca_00288	R	--
Ca1_P2_23003159	Ca1	23003159	GAAAAAAAAAAA/GAAAAAAAAAA	TGCGCGCTTGTTATACTTGT	ATGACATGCTGCAAGCGATA	60.4	510	INTERGENIC	--	--	--
Ca1_P2_23075722	Ca1	23075722	CAATAATAATAATAATAATAATAATAATAATAATAATAATAATAATAATAATAATAAT/CAATAATAATAATAATAATAATAATAATAATAATAATAATAATAATAATAATAATAATAATAAT	TCATTCATACGACGAATGTGC	GGCCATAAACATTGGATTGC	60.2	414	INTERGENIC	--	--	--
Ca1_P2_23116761	Ca1	23116761	AATATATATATATATATATATATATATATATATATA/AATATATATATATATATATATATATATATATA	TTGATGATCCAACGGTTCAA	ATCATCCGCAAAACTGGTTC	59.9	863	INTERGENIC	--	--	--
Ca1_P2_23192284	Ca1	23192284	TTAATAATAATAATAATAATAATAATAATAATAATAATAATAATAATAATAATAATAA/TTAATAATAATAATAATAATAATAATAATAATAATAATAATAATAATAATAATAATAATAATAA	TTCGAAACTTTTGGCCACTT	TTGGATTTTGGCCACATTTT	60.2	527	INTERGENIC	--	--	--
Ca1_P2_23226713	Ca1	23226713	AATATATATATATATATATATATATATATATA/AATATATATATATATATATATATATATATATA	AAAAGTACGCGACCGTTTTG	GCCTTTGTGCTTCCCATAAA	60.1	710	INTERGENIC	--	--	--
Ca1_P2_23250478	Ca1	23250478	CAAGAAG/CAAG	TTGGTTTCATGACCTGCATC	CGGCCGTTTCTAACATCATT	60.0	351	INTERGENIC	--	--	--
Ca1_P2_23304825	Ca1	23304825	CATATATATATATATAT/CATATATATATATATATATATAT	CCATTACGTGCAACCAGAAA	TGACCTGCGTCCATAAATCA	60.1	928	INTERGENIC	--	--	--
Ca1_P2_2336589	Ca1	2336589	TCCTGCNNNNNNNNNNNCCTGC/TCCTGC	GATGCCTTTCCATTGTGCTT	TTTTCCATCAAAATCCCCAA	60.1	662	INTRON	Ca_00294	A	--
Ca1_P2_23386171	Ca1	23386171	GTTATTATTATTATTATTATTATTATTATTATTATTATTAT/GTTATTATTATTATTATTATTATTATTATTATTATTAT	ACAAGAATGGCAAATGGAGC	TTTGCCCCACTAACTCTTGG	60.1	537	INTERGENIC	--	--	--
Ca1_P2_23422424	Ca1	23422424	ATTTTTTTT/ATTTTTT	AACATCACTCAAGCGCACAT	TAGCGTTGATGGAATCGTGA	60.2	548	INTERGENIC	--	--	--

INDEL marker IDs	Chromosomes/unanchored scaffolds	Physical positions (bp)	InDels (*Kabuli* reference genome-CDC Frontier/PII)	Forward primers (5'-3')	Reverse primers (5'-3')	Anneling temperature (0C)	Expected amplified product size (bp)	Structural annotation		Functional annotation	
								Sequence components of *kabuli* genome	*Kabuli* gene accession IDs	NCBI-KOG	TFs
Ca1_P2_23426237	Ca1	23426237	TTATATATATATATATATATATATATATATAT/TTATATATATATATATATATATATATATATAT	AAGTTAAGGAACCACGAGTGC	AAACATGTTGTTGATGCGTACA	59.0	256	DRR	Ca_19317	--	--
Ca1_P2_23492453	Ca1	23492453	AATATATATATATATATATATATATAT/AATATATATATATATATATATATATATATATAT	TGTGTAGCGCCCCTTAACTT	ACGGTAGCTTTAACGAGCGA	60.0	935	INTERGENIC	--	--	--
Ca1_P2_23496241	Ca1	23496241	CT/CTTT	AGGGTGGATCAGTTCGATTG	TTATATTAATTGGGTGCGCGT	59.4	376	DRR	Ca_19314	--	--
Ca1_P2_23516838	Ca1	23516838	CAAAAAA/CAAAAA	GGAATCCAAACCAAAACCCT	TTGGTGTGGTTTTTGTGGAA	59.8	634	INTERGENIC	--	--	--
Ca1_P2_23544752	Ca1	23544752	CATATATATATATATATATA/CATATATATATATATATA	TGGGTGCTATTTGCAATCAA	GCTTCGACACTGTAGCACCTC	60.1	247	DRR	Ca_19313	--	Trihelix
Ca1_P2_23569704	Ca1	23569704	ATATTATTATTATTATTATTATTATTATTATTATTATTATTATTATTATTATTAT/ATATTATTATTATTATTATTATTATTATTATTATTATTATTATTATTATTAT	GCTATTGAATGCGTCTTTCG	TTCAAATTGTGGCGACTTTG	59.7	876	INTERGENIC	--	--	--
Ca1_P2_23571644	Ca1	23571644	AA/AAGTCTTCCGATCTTTTGA	TGTGTTGGCTTAAAACATTTGAT	GGGCAATGTTGGTGGTACTC	60.2	810	INTERGENIC	--	--	--
Ca1_P2_236262	Ca1	236262	TAAAAAAAAA/TAAAAAAAA	TGAAAGAACAATATTCCACTAAACCA	GCAGCAATTGATGGTGTGAT	59.5	129	INTERGENIC	--	--	--
Ca1_P2_23639295	Ca1	23639295	AATATATATATATATATATATA/AATATATATATATATATATATATATA	ATAACATGGGATGAACGGGA	ATTCCCCACCAGAGATACCC	60.0	695	INTERGENIC	--	--	--
Ca1_P2_23650187	Ca1	23650187	GTTTTTTTTTT/GTTTTTTTTT	CGCTAGTTTGGGTTGACTGG	TTGTGCCTTAACAACTCCAAGA	59.8	759	INTERGENIC	--	--	--
Ca1_P2_23683623	Ca1	23683623	CATATATATATATATATATATATATATATAT/CATATATATATATATATATATATATATATAT	TATTGTGTTGCTTTGCCAGC	TGACCAATGCAAAAGAGACG	59.8	783	DRR	Ca_19303	R	MYB
Ca1_P2_23683980	Ca1	23683980	A/AAAGGAAGGAGCGTAGCAC	AAAATGAGGAAAGAGGTCATAAAAA	CGTTGACCAATGCAAAAGAG	59.3	101	DRR	Ca_19303	R	MYB

INDEL marker IDs	Chromosomes/unanchored scaffolds	Physical positions (bp)	InDels (*Kabuli* reference genome-CDC Frontier/PII)	Forward primers (5'-3')	Reverse primers (5'-3')	Anneling temperature (0C)	Expected amplified product size (bp)	Structural annotation		Functional annotation	
								Sequence components of *kabuli* genome	*Kabuli* gene accession IDs	NCBI-KOG	TFs
Ca1_P2_23777095	Ca1	23777095	TTATATATATATATATATATATATATATAT/TTATATATATATATATATATATAT	ACCTCACCCGTTTTGAGATG	CAAACACGAGCAATTGTGTCA	60.7	157	INTERGENIC	--	--	--
Ca1_P2_23777727	Ca1	23777727	AATATATATATATATATATATATATA/AATATATATATATATATATATATATATA	ACCAACATCTTCGATGTAAGAGA	AAGAAGCCCGGAATTTGTTT	59.9	367	INTERGENIC	--	--	--
Ca1_P2_23784964	Ca1	23784964	ATTTTTTTT/ATTTTTT	GTCAACCAATTGACCCCAAC	TGCCAAGAAAACACCATGAA	60.1	663	INTERGENIC	--	--	--
Ca1_P2_23819907	Ca1	23819907	CTT/CT	GCAGCTCTTTTGAGGTTTTCA	AACCAAACAGGGAGAAGGTT	57.6	409	INTERGENIC	--	--	--
Ca1_P2_23824032	Ca1	23824032	AGT/AGCCTAGGT	CCAAGGAAGGACGAAAATGA	TTTTCGGTGATTCAAAAGCC	60.1	390	INTERGENIC	--	--	--
Ca1_P2_23838777	Ca1	23838777	ATGTGTGTGTGT/ATGTGTGT	AATGCCCACCCATAATTTCA	TGTACGAGAAGGTCGTGTGC	59.9	593	INTERGENIC	--	--	--
Ca1_P2_23966814	Ca1	23966814	GA/GAA	AACTGTGGAGGAATCCATGC	GCTTCAAGTCTTGCTACGGG	60.0	490	INTERGENIC	--	--	--
Ca1_P2_24013951	Ca1	24013951	TAAAAAAAAAAA/TAAAAAAAAAA	TCCATCCTCTTACTTTGGTTCC	TGGTCTGACCTATTCCCACC	59.8	788	INTERGENIC	--	--	--
Ca1_P2_24030518	Ca1	24030518	C/CATGTT	CCAATTCAAAAGCTCCCAAA	CAGATCCTCCTTTCCCAACA	60.0	424	DRR	Ca_19291	OK	--
Ca1_P2_24058877	Ca1	24058877	GAAAAAAAAAAA/GAAAAAAAAA	GGGCTTCCAATGTGATTGTT	AAGAGGGACCATTTCCCTGA	60.8	801	INTERGENIC	--	--	--
Ca1_P2_24065596	Ca1	24065596	AATATATATATATATATATATATATATATATATAT/AATATATATATATATATATATATATATATATATATAT	ACTAATGGTCAAGCACACAAAA	TGAATTTGATGTTGTTGGAGATG	59.9	635	INTERGENIC	--	--	--
Ca1_P2_24098971	Ca1	24098971	GATATATATATATATATATATATATATATATA/GATATATATATATATATATATATATATATA	AGGCATGGTGTTGGAGTCAT	TGCATTTTTCCACGTTCAAG	59.7	526	INTERGENIC	--	--	--

INDEL marker IDs	Chromosomes/unanchored scaffolds	Physical positions (bp)	InDels (*Kabuli* reference genome-CDC Frontier/PII)	Forward primers (5'-3')	Reverse primers (5'-3')	Anneling temperature (0C)	Expected amplified product size (bp)	Structural annotation		Functional annotation	
								Sequence components of *kabuli* genome	*Kabuli* gene accession IDs	NCBI-KOG	TFs
Ca1_P2_24099811	Ca1	24099811	ATTTTTTTTTT/ATTTTTTTTT	TCGCAAAAGTAGTCCCCTCA	TCAAGCCACACCACATCATT	60.0	177	INTERGENIC	--	--	--
Ca1_P2_24154735	Ca1	24154735	ATTTTT/ATTTTTT	GGGTGCCATCATAAGCAAGT	CAGATACTGCAGCCTCCACA	60.0	516	INTERGENIC	--	--	--
Ca1_P2_24220141	Ca1	24220141	TTAATTAATAATTAATAATTAATAATTAATAAT/TTAATTAATAATTAATAATTAATAATTAATAATTAATAAT	TGATTCAGTGCGAAAAGACG	GCAGTCGATTGGGAGAAGAG	60.0	473	INTERGENIC	--	--	--
Ca1_P2_24242300	Ca1	24242300	TAAAAAAAAAAA/TAAAAAAAAAA	ACCAACTCATCCCTTTCCCT	ATGATTGCAGCATTTCACGA	60.2	638	INTERGENIC	--	--	--
Ca1_P2_24277831	Ca1	24277831	CATATATATATATATATATATATAT/CATATATATATATATATATATATATATAT	AGCAAACCAATTGACCATCTG	ACGCCTCTCTAGGTTGTTGC	59.5	695	INTERGENIC	--	--	--
Ca1_P2_24290063	Ca1	24290063	CAGAGAGAGAGAGAGAGAGA/CAGAGAGAGAGAGAGAGAGAGAGAGAGAGAGAGA	AAATGCTTCAGCCAGACACC	CGAACCCTAAAGACGCAAAG	59.9	378	INTERGENIC	--	--	--
Ca1_P2_24392808	Ca1	24392808	AAATTGAA/AAA	CCGAGCATCGATTTGGTAAT	TGATGGTGAAAGTGGGTTGA	59.9	376	INTERGENIC	--	--	--
Ca1_P2_24471395	Ca1	24471395	AA/ATATAATA	GCAATGCATACATGGACCCT	GGCATTCATCCAAAATCACC	60.1	340	INTERGENIC	--	--	--
Ca1_P2_24514649	Ca1	24514649	TAAAAAAAAAA/TAAAAAAAAAAA	TACTCGAGCAACAACGCATC	GTAGAGCATTGGATTCGGGA	60.0	848	INTRON	Ca_18341	--	SAP
Ca1_P2_24611579	Ca1	24611579	AATATATATATATATA/AATATATATATATATA	TGCCCACTTGATACAACAAAA	CATCGCCTTTGACGAATACA	59.7	330	INTERGENIC	--	--	--
Ca1_P2_24626336	Ca1	24626336	TAA/TAAA	TCTCCCCTCCTCCTTCATTT	AACTTGATGCCGAGTAGCGT	59.9	284	INTERGENIC	--	--	--
Ca1_P2_24648370	Ca1	24648370	ATTTTTTTTTT/ATTTTTTTTTTT	TGCAGGGATGAAATCCAAAT	CATGCAATGCTTTTTCTGGA	59.8	629	INTERGENIC	--	--	--

INDEL marker IDs	Chromosomes/unanchored scaffolds	Physical positions (bp)	InDels (*Kabuli* reference genome-CDC Frontier/PII)	Forward primers (5'-3')	Reverse primers (5'-3')	Anneling temperature (0C)	Expected amplified product size (bp)	Structural annotation		Functional annotation	
								Sequence components of *kabuli* genome	*Kabuli* gene accession IDs	NCBI-KOG	TFs
Ca1_P2_24723595	Ca1	24723595	TTTAATTAATTAATT/TTTAATTAATT	CGGAGGCTATGAGAAGGAGA	TTGTGCTGCCAACTAAATGC	59.9	890	INTRON	Ca_18333	--	Dof
Ca1_P2_24778446	Ca1	24778446	TATAA/T	ATGCCGTGCACTGACTTTTT	GGGCTTTCTGATTATGCCAA	60.0	826	INTERGENIC	--	--	--
Ca1_P2_24975919	Ca1	24975919	ACC/AC	ATGGCAAATAATTGGTGGGA	GGAAAAGGGCCCATAAAGTC	59.8	759	INTERGENIC	--	--	--
Ca1_P2_24998307	Ca1	24998307	ATTTTTTTTTTT/ATTTTTTTTTT	AAACAGCGTTGAAATTTGGG	GCTAAATAACACATGTGGTCCC	58.4	632	INTERGENIC	--	--	--
Ca1_P2_25058286	Ca1	25058286	AATATATATATATATATATATATATATAT/AATATATATATATATATATATATATAT	CATCCTGCAACACATGCTCT	TCATGCAAATTCAAAAGAAATGA	59.6	794	DRR	Ca_18316	--	--
Ca1_P2_2529049	Ca1	2529049	AATATATATATATATATA/AATATATATATATATA	GTCCACTATGGTCGAAGCCT	AACGCAAGACGAAGCTGTTT	60.1	657	DRR	Ca_00315	R	WRKY
Ca1_P2_25303242	Ca1	25303242	CATATATATATATATATATATATATATATAT/CATATATATATATATATATATATATATATATAT	TAGGCAACCTTGGGTGTTGT	TCAGCATGTCAATTTCCCAA	60.0	813	INTERGENIC	--	--	--
Ca1_P2_25382145	Ca1	25382145	AC/A	GAATCACTAGGCTTGCCAGG	GGGGTGAGAGAACGTTTGAA	60.1	714	INTERGENIC	--	--	--
Ca1_P2_25382333	Ca1	25382333	AAC/ATGGCTAC	CAAATGCCACACATTTAACGA	GGGGTGAGAGAACGTTTGAA	60.1	255	INTERGENIC	--	--	--
Ca1_P2_25451336	Ca1	25451336	ATTTTTTTTTTT/ATTTTTTTTTT	CTGACCCACCAAACCAATCT	ATGCAGGGTTTTTGTTTCCA	60.3	466	INTERGENIC	--	--	--
Ca1_P2_25458751	Ca1	25458751	AATATATATATATATATATATATATATATATATA/AATATATATATATATATATATATATA	GACGTCGCTTCCGAAGTTAG	TCACTCAGGTTGCCAAAACTT	59.8	538	INTERGENIC	--	--	--
Ca1_P2_25507843	Ca1	25507843	CAATAATAATAATAATAATAATAATAATAATAATAATA/CAATAATAATAATAATAATAATAATAATAATAATA	TCTCTGGCAACCGTTCTTCT	GAATGAAGTCCAAGGGTCCA	59.9	341	INTERGENIC	--	--	--

INDEL marker IDs	Chromosomes/unanchored scaffolds	Physical positions (bp)	InDels (*Kabuli* reference genome-CDC Frontier/PII)	Forward primers (5'-3')	Reverse primers (5'-3')	Anneling temperature (0C)	Expected amplified product size (bp)	Structural annotation		Functional annotation	
								Sequence components of *kabuli* genome	*Kabuli* gene accession IDs	NCBI-KOG	TFs
Ca1_P2_25509778	Ca1	25509778	AATATATATATATATATATATATATATATATATATATCTATATATATAT/AATATATATATATATATATATATATATATATATATATATCTATATATATAT	ATTTTGGCTCGGAGGTTTTT	GTTTTCGAATCAACGAGGGA	60.1	550	INTERGENIC	--	--	--
Ca1_P2_25519824	Ca1	25519824	CAGACCCA/CAGACCCAAACATAAGACCCA	TCAGGGACAAAATTGCATGA	CGTTCCTCTGAAGGTTACGAA	59.4	382	INTERGENIC	--	--	--
Ca1_P2_25526955	Ca1	25526955	A/ACG	TTCGTAATGGACGGGATGAT	GGGTTTTGCATGTTGAGCTT	60.1	510	INTERGENIC	--	--	--
Ca1_P2_25572037	Ca1	25572037	GTTTT/GTTTTT	GTCAGGCATTCCCCTCTACA	CCCGAAACGCATTGAATTAT	59.8	781	INTERGENIC	--	--	--
Ca1_P2_25578670	Ca1	25578670	CAAAAA/CAAA	GTTGGTTCCCTAGCACCGTA	CGAGTTCGCCTTTGTTCTTC	60.0	273	INTERGENIC	--	--	--
Ca1_P2_25630122	Ca1	25630122	ATTT/AT	AGATCACGCTCCACTCCTTG	ATTTCACTCATGCCTCCACC	59.9	776	INTERGENIC	--	--	--
Ca1_P2_25688981	Ca1	25688981	TAAAAAAA/TAAAAAA	AATGTCCCCAACAGAGTTGC	TCGTATCGAACTTGCCCATT	60.5	542	INTERGENIC	--	--	--
Ca1_P2_25781138	Ca1	25781138	GGA/GGGAAATACTTGA	GCTTCAAGAGACGAAATCCG	ACACGACCGATAGGGATCAG	60.0	709	DRR	Ca_18582	--	--
Ca1_P2_25948887	Ca1	25948887	TTATATATATATATATATATATATA/TTATATATATATATATATATATATATA	TTTCTATAAAGCAAATCCGCA	GTCGCCGACAGTTTTAAGGA	60.2	855	INTERGENIC	--	--	--
Ca1_P2_26142349	Ca1	26142349	TTAC/T	CTTCGCACAACCATTGACAC	TTCCTGCTGTTGCTGATTTG	60.0	206	CDS (large-effect mutations)	Ca_18596	R	ZF-HD
Ca1_P2_26181400	Ca1	26181400	GATATATATATATA/GATATATATATATATA	ACCCAAGCAAAGTTCAAGGA	AACAACATGCAAAGTGTGCC	59.6	614	INTERGENIC	--	--	--
Ca1_P2_26208589	Ca1	26208589	CATATATATATATATATATATATATATATATA/CATATATATATATATATATATATATA	CAGCCACCCCAAACTTTTTA	TCACCACCAGAGACAAACCA	60.1	419	INTERGENIC	--	--	--

INDEL marker IDs	Chromosomes/unanchored scaffolds	Physical positions (bp)	InDels (*Kabuli* reference genome-CDC Frontier/PII)	Forward primers (5'-3')	Reverse primers (5'-3')	Anneling temperature (0C)	Expected amplified product size (bp)	Structural annotation		Functional annotation	
								Sequence components of *kabuli* genome	*Kabuli* gene accession IDs	NCBI-KOG	TFs
Ca1_P2_26219980	Ca1	26219980	TGGGGG/TGGGGGG	CCATGGTGTTGAAGGTGCTA	AAACACGAGAAGGGGGAGTT	60.0	561	INTERGENIC	--	--	--
Ca1_P2_26230776	Ca1	26230776	CAAA/CAA	ATCAACGCACATAACACCCA	CCCTCCTTTCTCCCATTCTC	60.0	517	INTERGENIC	--	--	--
Ca1_P2_26263650	Ca1	26263650	ATATTATTATTATTATTATTATTATTA/ATATTATTATTATTATTATTATTA	TCACCCAACAAGATCGTGAA	CCAAGCCCAATATCCAACAT	59.6	780	INTERGENIC	--	--	--
Ca1_P2_26267110	Ca1	26267110	TA/T	CCGAGCATATGTGTCGGATA	TTACAGAAGTTTCGTCCCCG	60.1	318	INTERGENIC	--	--	--
Ca1_P2_26358637	Ca1	26358637	TTTTAGT/TTTTAGTTTAGT	TGTGTTCATCCTTTCCACCA	GGGTTCAACTTCAAAAGCCA	60.1	754	INTRON	Ca_18604	U	LSD
Ca1_P2_2655089	Ca1	2655089	TG/T	CGGACTAGTTTATTTCCACGC	TCCAAAACGCGAAAGGTTAC	60.1	574	INTERGENIC	--	--	--
Ca1_P2_2655633	Ca1	2655633	AGG/AG	TCCGTGGAAATGAACCTAGC	CATGGACTCAAAAACAGCCA	59.7	812	INTERGENIC	--	--	--
Ca1_P2_26577309	Ca1	26577309	TTATATATATATATATATATATATA/TTATATATATATATATATATATATATA	TACCATTTGATGCCAAACCA	GCTGGAAGTTGACCACCAAT	60.0	508	INTERGENIC	--	--	--
Ca1_P2_2658146	Ca1	2658146	T/TCAATTTC	GTGGGAAAGTTTGGTGATGG	TTCCGAACAAATAAATGGCA	59.0	684	INTERGENIC	--	--	--
Ca1_P2_26589205	Ca1	26589205	ATTTTTTTTT/ATTTTTTTT	TTCCTAACATTCTCCGCTGG	TGATGTTGCATCAACCATTTT	58.9	168	INTERGENIC	--	--	--
Ca1_P2_26592081	Ca1	26592081	C/CTGACAGTGA	ACACACAAATTTTGACCGCA	GCAATTTTCTCCAATTGCGT	60.1	801	INTERGENIC	--	--	--
Ca1_P2_2660856	Ca1	2660856	ATTTCTTT/A	TCGTCGTTGTCCATGTTGTT	GAGTTTGAAGAAAAACGGCG	59.9	183	INTERGENIC	--	--	--

INDEL marker IDs	Chromosomes/unanchored scaffolds	Physical positions (bp)	InDels (*Kabuli* reference genome-CDC Frontier/PII)	Forward primers (5'-3')	Reverse primers (5'-3')	Anneling temperature (0C)	Expected amplified product size (bp)	Structural annotation		Functional annotation	
								Sequence components of *kabuli* genome	*Kabuli* gene accession IDs	NCBI-KOG	TFs
Ca1_P2_26617808	Ca1	26617808	TTATATATATATATATATATATATAT/TTATATATATATATATATATATATATAT	TCAATTGAGTTGCGACGAAG	GAAGAATGAGTTTCGCGAGG	60.0	278	INTERGENIC	--	--	--
Ca1_P2_2662211	Ca1	2662211	AATATA/AATA	CTCTGCACTCGAACACGCTA	TTTGTCTCTCCCACTCCCAC	60.1	345	INTERGENIC	--	--	--
Ca1_P2_2663715	Ca1	2663715	TGAAAAGAAAA/TGAAAA	GTTGCCATGCCTATAACGGT	TGAAGACCATGGACACCAAC	59.4	547	INTERGENIC	--	--	--
Ca1_P2_2664699	Ca1	2664699	TGG/TG	CCAAAAATTGATGAATTGACGTT	CCCATTTCTCAAGTTGCCTC	59.7	648	INTERGENIC	--	--	--
Ca1_P2_26683611	Ca1	26683611	TG/T	CAATTGTGTAAAACGTGCGG	TTGCATTTGGTTTGACTCCA	60.1	538	INTERGENIC	--	--	--
Ca1_P2_2669959	Ca1	2669959	TAAAAAAAAAAAAAA/TAAAAAAAAAA	GAGGCACATAGCAGCACAAA	AGCCAGATATCAGACCGCAC	60.2	818	DRR	Ca_00327	--	ERF
Ca1_P2_26720249	Ca1	26720249	A/AAT	ACATGCTTTTGGCCTTTTTG	TATTGTGACCGCAAATCCAA	59.9	833	INTERGENIC	--	--	--
Ca1_P2_26725325	Ca1	26725325	CTATATATATATATATATATAT/CTATATATATATATATAT	AATTAGCAGCGGAAGGAAAG	GCATCACAGTTAAATCATCGC	58.3	352	INTERGENIC	--	--	--
Ca1_P2_2674516	Ca1	2674516	CTAGATTGGAT/CTAGATTGGATTAGATTGGAT	TGTCGGAATATGGGAAGCTC	GCCGTTGTTTTCAGATCCAT	59.9	934	INTERGENIC	--	--	--
Ca1_P2_2675683	Ca1	2675683	AG/AGG	TCAAAACATCACACACACAAACA	ACCAACCGGATGCACATAAC	60.6	309	INTERGENIC	--	--	--
Ca1_P2_26761299	Ca1	26761299	AT/ATTATTATTTT	CCGATACTGGTTCATTTTCCA	GACGTGGTTAGAAGGGGGAT	60.2	458	INTERGENIC	--	--	--
Ca1_P2_2676429	Ca1	2676429	CAAATA/CAAATAAATA	TGGAATTCACCAATTAACCCA	TGTCAAACGAATCCGAAATG	59.5	347	INTERGENIC	--	--	--

INDEL marker IDs	Chromosomes/unanchored scaffolds	Physical positions (bp)	InDels (*Kabuli* reference genome-CDC Frontier/PII)	Forward primers (5'-3')	Reverse primers (5'-3')	Anneling temperature (0C)	Expected amplified product size (bp)	Structural annotation		Functional annotation	
								Sequence components of *kabuli* genome	*Kabuli* gene accession IDs	NCBI-KOG	TFs
Ca1_P2_26774824	Ca1	26774824	TCTTTCCNNNNNNNNNNCTTTCC/TCTTTCC	TTGCACCCTTCTTAGCACCT	ACAATGAGTCGTCGCACAAA	60.3	212	INTERGENIC	--	--	--
Ca1_P2_26810976	Ca1	26810976	ATTTTTTTT/ATTTTTTTTT	TTGTCGGGTTGTGACAAAAA	CCAAAATTCTTTGGGGATCA	59.7	425	INTERGENIC	--	--	--
Ca1_P2_26866750	Ca1	26866750	ATTTTTTTTTT/ATTTTTTTTTTT	TCTCAGTGGAAAACGAACCC	TCTCCACCATCTTCCTCTTCA	59.8	679	INTERGENIC	--	--	--
Ca1_P2_2686857	Ca1	2686857	TG/T	AATGTGATGCACGTTTTGGA	CAAATTTCACGCAACAATCG	60.1	444	DRR	Ca_00328	--	MIKC
Ca1_P2_2686910	Ca1	2686910	AGTATTTGGGT/AGT	AATGTGATGCACGTTTTGGA	CAAATTTCACGCAACAATCG	60.1	444	DRR	Ca_00328	--	MIKC
Ca1_P2_26876468	Ca1	26876468	C/CG	TTGTGCATCCGTCTATCCAC	TCGGTGGAAATTGTAATGAGAG	59.1	937	INTERGENIC	--	--	--
Ca1_P2_2692135	Ca1	2692135	AC/ACCTC	ACAATGCCATTTTCAAGGCT	CGTGAAGACTAAAGGTCAGAAAAA	59.0	532	DRR	Ca_00330	--	--
Ca1_P2_2693052	Ca1	2693052	CT/CTT	CAACCAGTAATAATGCGCCA	CGTGACAGAGGACGCTTGTA	60.0	468	DRR	Ca_00330	--	--
Ca1_P2_2694120	Ca1	2694120	AATAT/AAT	TTTTGGTTGCACATGTCGAT	TCACCAATTGTCAAAAGGCA	60.1	190	INTRON	Ca_00330	--	--
Ca1_P2_26958726	Ca1	26958726	GTTT/GTTTT	TGAGCGTGAAAAATTGTGGT	CCGACCAATAGACTTTTGTTGA	59.1	204	INTERGENIC	--	--	--
Ca1_P2_26982272	Ca1	26982272	ATTTTTTTTTT/ATTTTTTTTTTT	GGTCCATCAAATGTTGTGGTT	TTACCCCCGAATGTTTGTTT	59.2	792	INTERGENIC	--	--	--
Ca1_P2_26997237	Ca1	26997237	TAAA/TAA	TCTTGGGGGAATTGGTGTAA	ATTAAACCAGCCACACAGGC	60.0	392	INTERGENIC	--	--	--

INDEL marker IDs	Chromosomes/unanchored scaffolds	Physical positions (bp)	InDels (*Kabuli* reference genome-CDC Frontier/PII)	Forward primers (5'-3')	Reverse primers (5'-3')	Anneling temperature (0C)	Expected amplified product size (bp)	Structural annotation		Functional annotation	
								Sequence components of *kabuli* genome	*Kabuli* gene accession IDs	NCBI-KOG	TFs
Ca1_P2_2702474	Ca1	2702474	CTT/CTTATT	CTGGCAGCATTGTTGTTGTT	ACTTCCTTCCACCACCACTG	60.0	228	CDS (large-effect mutations)	Ca_00331	--	--
Ca1_P2_2703175	Ca1	2703175	AA/AAGA	AATTGGCTGTCTCTGGCAGT	TGGCTTTCCACAAAGAGTCC	60.2	555	INTERGENIC	--	--	--
Ca1_P2_27113847	Ca1	27113847	ACCT/ACCTCCT	CACGAGAAACGGCAAAAGAT	ACGCGAATTAAGATTGCGTA	58.5	580	INTERGENIC	--	--	--
Ca1_P2_27118318	Ca1	27118318	CAAA/CAA	CGAACATGGTTCGTGAACTG	TTTTGCTACCAACACCCCTT	59.5	537	INTERGENIC	--	--	--
Ca1_P2_27132468	Ca1	27132468	TGAA/T	AAAAAGTGGCGGTTCACAAC	GATTGTGATGGGAGCTGGTT	59.9	928	DRR	Ca_20310	KR	--
Ca1_P2_27139492	Ca1	27139492	ATTTTTTTTTTT/ATTTTTTTTTTTTTT	GTGCAGCATAGGTTGCTTGA	GAAATTAAGTTAAGGGACCGCA	59.5	190	INTERGENIC	--	--	--
Ca1_P2_27143610	Ca1	27143610	AATATATATATATATATATATATATATATA/AATATATATATATATATATATATATATATATATATATATATA	GTTCAATCAATACTCGGCGG	GGCATCTTTGATCAATGGTCTT	60.3	681	INTERGENIC	--	--	--
Ca1_P2_27196096	Ca1	27196096	GAA/GAAA	TCGTGTGCTCCAAATCCATA	CGGGGGTAACTTGGAATTTT	60.0	886	INTRON	Ca_26748	S	--
Ca1_P2_27204677	Ca1	27204677	C/CT	GCCGAAGTACCAATTACCGA	TACGGTGCTAGGGAACCAAC	60.0	705	INTERGENIC	--	--	--
Ca1_P2_27219220	Ca1	27219220	GTTATTATTATTATTATTATTATTATTATTATTATTA/GTTATTATTATTATTATTATTATTATTATTATTA	TGAGGGAGATATTTGGTGGG	TACGGCGCTGTTCTTTTCTT	60.0	277	INTERGENIC	--	--	--
Ca1_P2_27282873	Ca1	27282873	GTATA/GTATATA	AAAATCCAACAATCTCCCCC	CCCTGAGACTCACGAAGAGG	60.0	561	INTERGENIC	--	--	--
Ca1_P2_2729433	Ca1	2729433	ATTTTTTT/ATTTTTT	ACAGAATCAAAAGCATGGGG	TTCATTCCACTTCCACCTCC	59.9	740	INTRON	Ca_00335	O	MYB

INDEL marker IDs	Chromosomes/unanchored scaffolds	Physical positions (bp)	InDels (*Kabuli* reference genome-CDC Frontier/PII)	Forward primers (5'-3')	Reverse primers (5'-3')	Anneling temperature (0C)	Expected amplified product size (bp)	Structural annotation		Functional annotation	
								Sequence components of *kabuli* genome	*Kabuli* gene accession IDs	NCBI-KOG	TFs
Ca1_P2_27320145	Ca1	27320145	GAAAAAAAAAAAA/GAAAAAAAAAAA	GAAACTTCGGTGCCATCATT	CGGTCACAGAGAGGAAGAGG	60.0	616	INTERGENIC	--	--	--
Ca1_P2_27335479	Ca1	27335479	CATATATATATATATATATATATATAT/CATATATATATATATATATATATAT	TGAAGAAGCTGAAGGGGAAA	TGAGTGATGTGATGTGCGAA	59.8	138	INTERGENIC	--	--	--
Ca1_P2_27335704	Ca1	27335704	AATATATATATATATATATATATATATATATATATATATAT/AATATATATATATATATATATATATATATATATATATATAT	TGAAGAAGCTGAAGGGGAAA	TTGATCAAATCTGGAAGACCC	59.0	453	INTERGENIC	--	--	--
Ca1_P2_27344175	Ca1	27344175	GTATATATATATATATATATATATATATATATATATA/GTATATATATATATATATATATATATATATATATATATATATAT	CTCATTGCCCATTGTTCCTT	AGGGAGGTCACTCAAGCAGA	60.0	528	INTERGENIC	--	--	--
Ca1_P2_27431047	Ca1	27431047	CCACA/CCACACA	TCCCAACTACATCCACCCAT	CTGATTGCTGTTGGCTTGAA	60.0	823	INTRON	Ca_25064	D	--
Ca1_P2_27597497	Ca1	27597497	G/GTGT	AAAATCCAACAATCTCCCCC	CCCTGAGACTCACGAAGAGG	60.0	559	DRR	Ca_25069	--	--
Ca1_P2_2762433	Ca1	2762433	T/TC	TATTTGGACAAACGAAGGGC	TGGAAGGATGGATGCTTAGG	60.0	126	INTERGENIC	--	--	--
Ca1_P2_2766077	Ca1	2766077	CTTATGTT/CTTATGTTATGTT	AAAACAATTGATTGGGCTCC	CCAATCATACGCAGGTGAAA	59.5	561	INTERGENIC	--	--	--
Ca1_P2_2767121	Ca1	2767121	GTTTAA/GTTTAATTTAA	TGATGAGGTGCATGGTTTGT	CAGAAAGCGGGACCAATTTA	60.1	177	INTERGENIC	--	--	--
Ca1_P2_27715858	Ca1	27715858	AATATATATATATATATATATATATATATATATAT/AATATATATATATATATATATATATATATATAT	TGATTGAAGATCTCAAACGGT	TTGAGAGGATTTTTCGCTCAT	58.9	686	INTERGENIC	--	--	--
Ca1_P2_2789005	Ca1	2789005	TTATTTTAT/TT	AACGAAGCGCTTTGACCTAA	GCACTATGCTCGCCCTAAAT	59.3	189	INTERGENIC	--	--	--
Ca1_P2_27894626	Ca1	27894626	CATATATATATATATATATATATAT/CATATATATATATATATATATATATAT	TCGGACAACAACGACGATAA	ACGACATTTATACCGTGGGC	59.7	592	INTERGENIC	--	--	--

INDEL marker IDs	Chromosomes/unanchored scaffolds	Physical positions (bp)	InDels (*Kabuli* reference genome-CDC Frontier/PII)	Forward primers (5'-3')	Reverse primers (5'-3')	Anneling temperature (0C)	Expected amplified product size (bp)	Structural annotation		Functional annotation	
								Sequence components of *kabuli* genome	*Kabuli* gene accession IDs	NCBI-KOG	TFs
Ca1_P2_2790553	Ca1	2790553	AATA/AA	TCCCAAATAAAAGAGAAACCCA	TGCACATCAATGAAACACCC	60.4	759	INTERGENIC	--	--	--
Ca1_P2_2790615	Ca1	2790615	TAAAAAAAA/TAAAAAAAAA	ATTTTGAAGCTGTTGTTCAATTT	TGCACATCAATGAAACACCC	60.4	628	INTERGENIC	--	--	--
Ca1_P2_2791646	Ca1	2791646	TT/TTTTAAT	ATCGAAAGCAACCATTTTGG	TTGTGGTGGTTCTTTGTTGG	59.4	869	INTERGENIC	--	--	--
Ca1_P2_2796096	Ca1	2796096	ATAAATTAA/ATAA	TACTATCCAATTCGCGGTGG	TCCCAAATCAAACTCCATCC	59.7	111	INTERGENIC	--	--	--
Ca1_P2_2796283	Ca1	2796283	T/TG	GGTGGACAGAGGCATCAACT	AAAAAGCGAGTTTCAGGGTG	59.4	530	INTERGENIC	--	--	--
Ca1_P2_2796306	Ca1	2796306	AATATATATATATATATATATATATATATATAT/AATATATATATATATATATATATATATATATATATATAT	GGTGGACAGAGGCATCAACT	AAAAAGCGAGTTTCAGGGTG	59.4	530	INTERGENIC	--	--	--
Ca1_P2_2797326	Ca1	2797326	A/AC	CCCTGTAAATTCAGACACAATCG	AAATAGTTGACAAACCGCCG	60.0	272	INTERGENIC	--	--	--
Ca1_P2_2800448	Ca1	2800448	ATTTTTTTTT/ATTTTTTT	GAGAGCAAGAGCGAGCAAGT	ACCACATGAGGGGCATTTTA	60.2	439	INTERGENIC	--	--	--
Ca1_P2_2800724	Ca1	2800724	TTTT/TTTTTTTATTT	GAGAGCAAGAGCGAGCAAGT	TCCATTTTCTCCTTCAAGCG	60.3	754	INTRON	Ca_00344	--	NAC
Ca1_P2_28010764	Ca1	28010764	A/ACTAG	TGAGACCGAGCTTTTGTGTG	GCAAAACTTGGTGGTCCAGT	60.0	557	INTERGENIC	--	--	--
Ca1_P2_28011021	Ca1	28011021	CAAAAAA/CAAAAAAA	TGAGACCGAGCTTTTGTGTG	GCAAAACTTGGTGGTCCAGT	60.0	557	INTERGENIC	--	--	--
Ca1_P2_2802455	Ca1	2802455	ATTTTT/ATTTT	GGGTTACAGACCGACCTTGA	GCGGATTTGTCCATCTTTGT	59.9	765	DRR	Ca_00344	--	NAC

INDEL marker IDs	Chromosomes/unanchored scaffolds	Physical positions (bp)	InDels (*Kabuli* reference genome-CDC Frontier/PII)	Forward primers (5'-3')	Reverse primers (5'-3')	Anneling temperature (0C)	Expected amplified product size (bp)	Structural annotation		Functional annotation	
								Sequence components of *kabuli* genome	*Kabuli* gene accession IDs	NCBI-KOG	TFs
Ca1_P2_28074634	Ca1	28074634	CAATAATAATAATAATAATAACAATAATAATAATAATAATAATAATAATAA/CAATAATAATAATAATAATAATAACAATAATAATAATAATAATAATAATAA	TTGCAAAGCCCAAAAAGAGT	ACCCGTTCCTGTGTTTTCTG	60.0	489	INTRON	Ca_18523	U	LSD
Ca1_P2_28170518	Ca1	28170518	AATATATATATATATATA/AATATATATATATATA	CGCGTGTTAACAAGACCTTT	ATACTTGTGGTGCGATCCGT	60.4	437	INTERGENIC	--	--	--
Ca1_P2_28239060	Ca1	28239060	TTATATATATATATATA/TTATATATATATATATA	GTCTGGGAAAAATGAGTCGC	CCACCGTTGGCTGTAATTTT	59.9	568	INTERGENIC	--	--	--
Ca1_P2_2827577	Ca1	2827577	TTC/T	TCGAATTCATAACTCAAATCTTCA	AGATCTGTTGGAGGTGGTGG	60.0	505	INTERGENIC	--	--	--
Ca1_P2_2828962	Ca1	2828962	TAATAACAATAA/TAATAA	ACCACGTTTTACATGCGTCA	TTTAGCCGTGTCAATGAATTT	57.3	483	INTERGENIC	--	--	--
Ca1_P2_2831499	Ca1	2831499	GTTTTTTTTTTTTTT/GTTTTTTTTTTTT	CCTCTGACCACATGGGACTT	TCAAAAACAAAAGCGCACTG	60.0	625	DRR	Ca_00347	--	ARF
Ca1_P2_28322869	Ca1	28322869	GAAAAAAAAAA/GAAAAAAAAA	GAGAACAACAACTTGACCCAAA	GTTACCAGCCCATCAATGCT	60.0	376	INTERGENIC	--	--	--
Ca1_P2_2837552	Ca1	2837552	TTCTCTCTCTCTCTCTCTCTCTCT/TTCTCTCTCTCTCTCTCTCT	CAGCACAAGCATGCCATAGT	TGCATCTGTTCCTGCTTCAC	60.0	671	INTERGENIC	--	--	--
Ca1_P2_28398850	Ca1	28398850	GTTTTTTT/GTTTTTTTT	TTTTGCCCTATTGGTGGAAG	TCCTTCTCATTTTCCCCCTT	59.9	667	INTRON	Ca_18514	A	--
Ca1_P2_28449282	Ca1	28449282	ACGAATATTTTCCGAATATTTTCCGAATATTT/ACGAATATTTTCCGAATATTT	TGGATTGGTTGAAGGAGGTC	CTCTGTCACTGGCGTCACAT	59.9	695	DRR	Ca_18508	--	--
Ca1_P2_2850106	Ca1	2850106	ATTTT/ATT	TAGGTGGGGCTCTCATTGTC	GTTTCTCCATGCGCAAAAAT	60.1	902	INTERGENIC	--	--	--
Ca1_P2_2851127	Ca1	2851127	TAT/TATAAT	TGTCGTTTTCAATGGACTGG	AGAGAGTATGGGGCAGTCAAA	58.8	226	INTERGENIC	--	--	--

INDEL marker IDs	Chromosomes/unanchored scaffolds	Physical positions (bp)	InDels (*Kabuli* reference genome-CDC Frontier/PII)	Forward primers (5'-3')	Reverse primers (5'-3')	Anneling temperature (0C)	Expected amplified product size (bp)	Structural annotation		Functional annotation	
								Sequence components of *kabuli* genome	*Kabuli* gene accession IDs	NCBI-KOG	TFs
Ca1_P2_28550132	Ca1	28550132	ATTTTTTTTTT/ATTTTTTT	TGGAAGCACGCTTAAGGACT	TTTCCCAATCGATTTGCTTC	60.0	664	INTERGENIC	--	--	--
Ca1_P2_28550236	Ca1	28550236	T/TG	CTGACACAGGGGGAGAAAAA	TTTCCCAATCGATTTGCTTC	60.0	159	INTERGENIC	--	--	--
Ca1_P2_2862848	Ca1	2862848	A/AG	TAATGGCCAAATTCTCTCCG	GCTTTCACCAATTCCACGTT	60.0	691	INTRON	Ca_00348	R	--
Ca1_P2_2862989	Ca1	2862989	CAA/CA	TAATGGCCAAATTCTCTCCG	GCTTTCACCAATTCCACGTT	60.0	691	INTRON	Ca_00348	R	--
Ca1_P2_28638034	Ca1	28638034	ATATTATTATTATTATTATTATTATTATTATTATTATTATTATT/ATATTATTATTATTATTATTATTATTATTATTATTATTATTATTATTATTATTATT	TGGCAGGAAATGTTGTGTGT	TTCGTTTTGCCAATCATCAA	60.1	597	INTERGENIC	--	--	--
Ca1_P2_2866655	Ca1	2866655	CTTT/CTTTT	ATTGATTGCAAGCACAACCA	CCTCCAAAGAAACGAATGGA	60.0	806	DRR	Ca_00348	R	--
Ca1_P2_2867577	Ca1	2867577	AGGG/AGGGG	AATGGGAAATGAGAAAGGGG	CGAATGTTATGGATCCTCTGC	59.5	541	DRR	Ca_00348	R	--
Ca1_P2_2867984	Ca1	2867984	AAT/AATGAT	ATGAGAAAGGGGAAGAGGGA	AAGGGGTGAACAGTGAGGTG	60.0	646	INTERGENIC	--	--	--
Ca1_P2_28703262	Ca1	28703262	CATATATATATATATATAT/CATATATATATATATATATAT	CCGAGCCAAAATCCAAAATA	TACTGGAACGAACACCACCA	60.0	609	INTERGENIC	--	--	--
Ca1_P2_28713213	Ca1	28713213	TTA/TTAGAAAGAGTGTA	TTCATTTCTCTGATTCCCCG	TCCGCATTATAACCTCTGCC	60.1	425	INTERGENIC	--	--	--
Ca1_P2_28826704	Ca1	28826704	CATTATTATTATTATTATTATTATTATTATTATTATTATTATTATTATTATTATTATTATTATTAT/CATTATTATTATTATTATTATTATTATTATTATTATTATTATTATTATTATTATTATTAT	TGCAGAGCACGCTGTATATTG	TAATTTCATGCGACCACCAA	59.9	944	INTERGENIC	--	--	--
Ca1_P2_2885377	Ca1	2885377	ATTTTTTT/ATTTTTTTT	GCCATTGTTAACTCCGCAAT	CATTTGGAGAGGGCATAGGA	60.0	360	DRR	Ca_00351	T	M-type

INDEL marker IDs	Chromosomes/unanchored scaffolds	Physical positions (bp)	InDels (*Kabuli* reference genome-CDC Frontier/PII)	Forward primers (5'-3')	Reverse primers (5'-3')	Anneling temperature (0C)	Expected amplified product size (bp)	Structural annotation		Functional annotation	
								Sequence components of *kabuli* genome	*Kabuli* gene accession IDs	NCBI-KOG	TFs
Ca1_P2_28905061	Ca1	28905061	CAAA/CAAAA	GGCAGCTTTGAAACCAATGT	TAAGAAACGCCAATGCAACA	60.3	165	INTERGENIC	--	--	--
Ca1_P2_2890664	Ca1	2890664	TGG/TG	TGCATGTGTCATGTTGATGG	CAAAGCACTCACAGGGTTGA	59.9	501	INTRON	Ca_00353	R	C2H2
Ca1_P2_28906702	Ca1	28906702	TAAAAAAA/TAAAAAA	TATGGAGAGCAGCGACTCAA	TGTTGGGTTGCACAACATTC	60.4	184	INTERGENIC	--	--	--
Ca1_P2_28910299	Ca1	28910299	CATT/CATTAATT	GAAAATGATCGGTGGAGGAA	CTTGCGTGTTGCACGAGTAT	59.9	620	DRR	Ca_18486	R	--
Ca1_P2_28910424	Ca1	28910424	TA/T	GAGTCATTGGTGCCTTGGTT	CTTGCGTGTTGCACGAGTAT	59.9	385	DRR	Ca_18486	R	--
Ca1_P2_28910492	Ca1	28910492	GATTAA/GATTAATTAA	GAGTCATTGGTGCCTTGGTT	CTTGCGTGTTGCACGAGTAT	59.9	385	DRR	Ca_18486	R	--
Ca1_P2_28911150	Ca1	28911150	TCAC/TC	ATACTCGTGCAACACGCAAG	CGAACAATATTTGTGTCCCA	58.4	525	DRR	Ca_18486	R	--
Ca1_P2_28911391	Ca1	28911391	T/TGA	TGGGACACAAAATATTGTTCGAT	GCAAAACCGATCTAAGGAAGC	60.2	595	DRR	Ca_18486	R	--
Ca1_P2_28929600	Ca1	28929600	GTTTTTTT/GTTTTTT	AAATTCGAACTGGGCATTTG	TGAAGGCACCATGTCTTTGA	60.2	750	INTERGENIC	--	--	--
Ca1_P2_28930954	Ca1	28930954	A/ATT	TGAAAAGTTTCTTTGTCCCTTTG	TGGTGTTGCCTTCAAATTCA	60.1	349	INTERGENIC	--	--	--
Ca1_P2_28935215	Ca1	28935215	ATTTTTTTT/ATTTTTTTTT	TTTTGTTGGCAGTTTGTTTG	CAGTCAACATTGATGTGACGTTAT	59.0	490	INTERGENIC	--	--	--
Ca1_P2_28936099	Ca1	28936099	ATTTTTTTT/ATTTTTTTTTTT	GAAATTTGACCAGAGCTGCC	TCGGTCACCGTTTTTAGTTT	57.3	465	INTERGENIC	--	--	--

INDEL marker IDs	Chromosomes/unanchored scaffolds	Physical positions (bp)	InDels (*Kabuli* reference genome-CDC Frontier/PII)	Forward primers (5'-3')	Reverse primers (5'-3')	Anneling temperature (0C)	Expected amplified product size (bp)	Structural annotation		Functional annotation	
								Sequence components of *kabuli* genome	*Kabuli* gene accession IDs	NCBI-KOG	TFs
Ca1_P2_28936233	Ca1	28936233	AT/ATGTAATT	GAAATTTGACCAGAGCTGCC	GGACCAATTACTCTGGTGGAA	58.9	650	INTERGENIC	--	--	--
Ca1_P2_28939471	Ca1	28939471	AAAATAAAACAAA/AAAA	CCATGTGTGAACCCTAATTGTT	GTTTTGGCGTTGGTTTCACT	60.0	226	INTERGENIC	--	--	--
Ca1_P2_2893987	Ca1	2893987	CT/CTT	TGATCCATGTTTCAATCCCA	AGCCATACACCTCCACAAGC	60.1	894	DRR	Ca_00353	R	C2H2
Ca1_P2_28941503	Ca1	28941503	CAA/CAAAA	TGGGGTTTAGGGTGTCACAT	CTTTTGTAAAGGCAGCTCCG	60.0	207	INTERGENIC	--	--	--
Ca1_P2_28941877	Ca1	28941877	AA/AATA	TGGGGTTTAGGGTGTCACAT	TCAATTTGGATTTCTTGGTGG	59.8	847	INTERGENIC	--	--	--
Ca1_P2_28943560	Ca1	28943560	GG/GGTG	ACTTGGGACAAATCACATGG	TGATCACCAGCTTTCCAATG	59.6	252	INTERGENIC	--	--	--
Ca1_P2_28944496	Ca1	28944496	TTATATATAT/TTATATAT	TTGGCACCAGACTTCATATTG	AAATGTACTTTGCGGCACAT	58.2	775	INTERGENIC	--	--	--
Ca1_P2_28944803	Ca1	28944803	AACTA/AA	TTTCAATTGCATTAATCCGTTC	CAATAAAAATTGCGGTCCGA	60.8	963	INTERGENIC	--	--	--
Ca1_P2_2897109	Ca1	2897109	AA/AAATA	TGCTGCTTCTTTTCCAGGTT	TTCAGGGTCTTGTAGGGATGA	59.5	467	INTRON	Ca_00354	--	bHLH
Ca1_P2_2901135	Ca1	2901135	TTCTGAGAAAGTTATCT/TTCTGAGAAAGTTATCTGAGAAAGTTATCT	AGCTTTCCACGAAAATCAGC	GCACATTTACAGTTGGGGCT	60.0	565	INTRON	Ca_00354	--	bHLH
Ca1_P2_2904188	Ca1	2904188	TG/T	AGGAAGCAGGTGTAGGGGAT	ACACGGAAGTTGGTTTCCTG	60.0	602	DRR	Ca_00354	--	bHLH
Ca1_P2_29061700	Ca1	29061700	TTATATATATATATATATATATATATATATATATATA/TTATATATATATATATATATATATATATATATATATATATA	AGATGATCGACCACTTTGGG	GCTTTAGCAACGGAATGACC	59.7	477	INTERGENIC	--	--	--

INDEL marker IDs	Chromosomes/unanchored scaffolds	Physical positions (bp)	InDels (*Kabuli* reference genome-CDC Frontier/PII)	Forward primers (5'-3')	Reverse primers (5'-3')	Anneling temperature (0C)	Expected amplified product size (bp)	Structural annotation		Functional annotation	
								Sequence components of *kabuli* genome	*Kabuli* gene accession IDs	NCBI-KOG	TFs
Ca1_P2_2907520	Ca1	2907520	T/TTC	CTGAAGCCTGAAGTGAAGGG	ATTGCATGAATGTGGAAGCA	60.1	727	DRR	Ca_00356	U	WOX
Ca1_P2_2911194	Ca1	2911194	CTATGAGTGGTTA/CTA	TGGCTACACTGACAAATCCCT	GTTTTACTTTGGTTCGGGCA	60.0	908	INTERGENIC	--	--	--
Ca1_P2_29124803	Ca1	29124803	ATTT/ATTTT	AGTGGCATTGGGCAAATTAC	ACCAAATATACGCGTCTGCC	60.0	350	INTERGENIC	--	--	--
Ca1_P2_2914658	Ca1	2914658	TTTTATTTATTTATT/TTTTATTTATT	TGTGATGGTTCATGCAGAAAG	CAGAAAGGTCTGACCCGAAA	60.2	722	DRR	Ca_00357	--	WOX
Ca1_P2_2914725	Ca1	2914725	ATT/AT	TGTGATGGTTCATGCAGAAAG	CAGAAAGGTCTGACCCGAAA	60.2	722	DRR	Ca_00357	--	WOX
Ca1_P2_2915564	Ca1	2915564	GATATATAT/GATAT	TGCAAAAATGGTGTTTTGTG	CATATGGTGAAAAGCGGTGA	59.5	523	DRR	Ca_00357	--	WOX
Ca1_P2_29158614	Ca1	29158614	AGG/AG	TGAGCTTTTAGGTGGCACAA	TCTCCATCTCTTCCTCCATCA	59.7	145	INTERGENIC	--	--	--
Ca1_P2_2918789	Ca1	2918789	GTTATTATTAT/GTTATTATTATTAT	TTGCAGGCAGGCTTAGACTT	TTCCATGTTCCCTTTGCTTC	60.1	770	INTERGENIC	--	--	--
Ca1_P2_2920371	Ca1	2920371	TTGATAT/TTGATATATATGGAGGTGATAT	TCACGTGAGCTATAGCCATAAA	TGACAGCTGAAAGCACAACC	60.0	245	INTERGENIC	--	--	--
Ca1_P2_2921755	Ca1	2921755	A/ATATG	CAACTGGGCCAAATGCTTAT	TGATGATCACACGAGGGAGA	60.2	570	INTERGENIC	--	--	--
Ca1_P2_2923437	Ca1	2923437	GTCTCTCTCT/GTCTCTCTCTCTCTCTCTCT	TGACATATGAAAAGGGGACCA	TGATGCGATTGTAGTCCCAA	60.1	134	INTERGENIC	--	--	--
Ca1_P2_2927297	Ca1	2927297	A/AG	TCTCTTCCATGGACCACACA	GCAAATTCACACAAGAACATTCA	60.0	794	INTERGENIC	--	--	--

INDEL marker IDs	Chromosomes/unanchored scaffolds	Physical positions (bp)	InDels (*Kabuli* reference genome-CDC Frontier/PII)	Forward primers (5'-3')	Reverse primers (5'-3')	Anneling temperature (0C)	Expected amplified product size (bp)	Structural annotation		Functional annotation	
								Sequence components of *kabuli* genome	*Kabuli* gene accession IDs	NCBI-KOG	TFs
Ca1_P2_2927323	Ca1	2927323	TG/T	TCATCAATGAGTTAATGCCCC	GCAAATTCACACAAGAACATTCA	60.0	737	INTERGENIC	--	--	--
Ca1_P2_2932731	Ca1	2932731	AA/AATAGGTTGA	GAATCTGGTTCCGCACTCAT	GATTCCGGTATTTGCCTCAA	59.9	771	DRR	Ca_00358	E	--
Ca1_P2_29343809	Ca1	29343809	AATA/AATATA	AACTTTCGGTGCAAACAACC	TGGAATAAACCACATGCGAG	59.5	392	INTERGENIC	--	--	--
Ca1_P2_2937855	Ca1	2937855	G/GT	CGGATGGAACAAGTGGAAGT	CGAACTCAATCTTCTATTCGCC	60.2	933	INTERGENIC	--	--	--
Ca1_P2_2938785	Ca1	2938785	T/TTG	GGCGGATAGACAAAATCGAA	GTCGCGAAAAGATTTGCACT	60.4	640	INTERGENIC	--	--	--
Ca1_P2_2939062	Ca1	2939062	TGTGA/TGTGATCGGAAGTGA	GGCGGATAGACAAAATCGAA	GCAACCGTGTGGATATGTTG	59.8	703	INTERGENIC	--	--	--
Ca1_P2_2943241	Ca1	2943241	TTATATATATATATATAT/TTATATATATAT	GGTATTGCATGAGAGGACCAA	GGTTGGAAGCTCTAACGTGG	59.7	689	INTERGENIC	--	--	--
Ca1_P2_2944510	Ca1	2944510	CTTT/CTTTT	AATCGCTTACGTCACCCAAC	TGCATATATCATTGCGGTGC	60.5	568	INTERGENIC	--	--	--
Ca1_P2_2945700	Ca1	2945700	GATATA/GATATATA	ATCAAATTGCGTTGGTAGCC	TGGCGATGGATACACGATAA	59.9	885	INTERGENIC	--	--	--
Ca1_P2_2945955	Ca1	2945955	AACT/A	TACTTGATGATACGCACGGC	TGGCGATGGATACACGATAA	59.9	670	INTERGENIC	--	--	--
Ca1_P2_2946069	Ca1	2946069	TA/TAA	ATTCCATCGCCATAGCATTC	GGGACCAACAAATTCAAACAA	59.7	553	INTERGENIC	--	--	--
Ca1_P2_2950691	Ca1	2950691	CCAAG/C	TCACGTGTCTCCTTGCATTC	CATTCTGCCCAATCCAAATC	60.3	660	DRR	Ca_00360	--	ERF

INDEL marker IDs	Chromosomes/unanchored scaffolds	Physical positions (bp)	InDels (*Kabuli* reference genome-CDC Frontier/PII)	Forward primers (5'-3')	Reverse primers (5'-3')	Anneling temperature (0C)	Expected amplified product size (bp)	Structural annotation		Functional annotation	
								Sequence components of *kabuli* genome	*Kabuli* gene accession IDs	NCBI-KOG	TFs
Ca1_P2_29587419	Ca1	29587419	AGG/AGGG	TGATGATGCTCCTCAAGCAC	AACAAAAGCGAGAAAAGCCA	60.0	617	INTERGENIC	--	--	--
Ca1_P2_29613616	Ca1	29613616	TT/TTATTATTAT	AGGTGCAAAAAGGAATGTGG	ACACAATTGGTGCAAGGACA	60.0	681	INTERGENIC	--	--	--
Ca1_P2_29636835	Ca1	29636835	AA/AAGA	TCACAAATCTCAACCCTCCC	ATCAGTTGGAGCCTGGTTGT	59.6	497	INTRON	Ca_24382	AR	--
Ca1_P2_29637072	Ca1	29637072	GAAAAAAAAA/GAAAAAAAAAAA	ACAACCAGGCTCCAACTGAT	ATGCTGCCAGCCTACAAAAT	59.7	503	INTRON	Ca_24382	AR	--
Ca1_P2_2964067	Ca1	2964067	AATTTATTTATT/AATTTATTTATTTATTTATT	TGGACCAAGTGTGGTATTTAACC	CTGCTAGAACACCGCATGAA	60.0	368	INTERGENIC	--	--	--
Ca1_P2_2965477	Ca1	2965477	AG/A	ACATGCTGTGATTTGGGTCA	AAAGCGAATTTACAGACGTGG	59.3	873	INTERGENIC	--	--	--
Ca1_P2_2965539	Ca1	2965539	CAAAA/CAAA	ACATGCTGTGATTTGGGTCA	AAAGCGAATTTACAGACGTGG	59.3	873	INTERGENIC	--	--	--
Ca1_P2_2970188	Ca1	2970188	TAAAAAAA/TAAAAAAAAA	GCCTGTTTTCGTGCATTTCT	GTGAGGGGATGGTGCTCTAA	60.1	608	INTERGENIC	--	--	--
Ca1_P2_29709028	Ca1	29709028	ACC/AC	AAAGATTTGTTGAGGCGTGG	GACGAGAATGGAGATGGGAA	60.0	580	INTERGENIC	--	--	--
Ca1_P2_2973113	Ca1	2973113	TATA/TATAATA	TTTGATGTGGCGGCATAGTA	TGTTTGCCAATCACAGCTTC	59.8	281	INTRON	Ca_00361	--	--
Ca1_P2_29738380	Ca1	29738380	GTATATATATATATATATATAT/GTATATATATATATATATATATAT	TATTTTGGATTTTGGCTCGG	AAACCGGTAAAATCTCCGCT	60.0	699	INTERGENIC	--	--	--
Ca1_P2_29753388	Ca1	29753388	TTATATATATATATATAT/TTATATATATATATATATAT	CAACACCGTTTTTCTCCGTT	TTTGGGAGGTTATGGGTGAA	60.2	321	INTERGENIC	--	--	--

INDEL marker IDs	Chromosomes/unanchored scaffolds	Physical positions (bp)	InDels (*Kabuli* reference genome-CDC Frontier/PII)	Forward primers (5'-3')	Reverse primers (5'-3')	Anneling temperature (0C)	Expected amplified product size (bp)	Structural annotation		Functional annotation	
								Sequence components of *kabuli* genome	*Kabuli* gene accession IDs	NCBI-KOG	TFs
Ca1_P2_29763169	Ca1	29763169	AATATATATATATATATATATATATATATATATATATATA/AATATATATATATATATATATATATATATATATATATATATATA	TATTTTGGATTTTGGCTCGG	AAATGGAGGAAGGAGGGAGA	60.0	429	INTERGENIC	--	--	--
Ca1_P2_2976854	Ca1	2976854	TA/TAA	CCTAATCCCAAAGCTGGAAA	AAAATTTCCTCGTCCAAGCA	59.7	747	INTRON	Ca_00361	--	--
Ca1_P2_2979061	Ca1	2979061	CATAT/CATATATAT	CATCCTTTCATTCTTCTCCCC	TTCAAACCAAGCCTCAAAGTT	58.9	290	INTRON	Ca_00361	--	--
Ca1_P2_29791416	Ca1	29791416	GAAAA/GAAAAA	TCCGATAAAATCTCCGTTCG	AAAAACACGTTCCCCTCCTT	59.8	323	INTERGENIC	--	--	--
Ca1_P2_2979509	Ca1	2979509	A/ATTATTT	GGAATTCCAAAACTGATCCAA	TGCCAACAAATCCTGAACAA	60.1	653	INTRON	Ca_00361	--	--
Ca1_P2_29801275	Ca1	29801275	TAAAAAAAAAAA/TAAAAAAAAAA	GTTTTTCCATCTTGGTGCGT	TCACCCAAATTTATGGCATGT	60.1	573	INTERGENIC	--	--	--
Ca1_P2_29806954	Ca1	29806954	ATT/ATTT	GCATGCTGCAAGGGATAGTT	CGCGCTGTTAAAAATCAAGG	60.7	423	INTERGENIC	--	--	--
Ca1_P2_29825613	Ca1	29825613	GCTTT/G	CAAGACTTAAGGCTCCCCCT	CCCATTTGCCTAACCAAGAA	59.9	500	INTERGENIC	--	--	--
Ca1_P2_29842423	Ca1	29842423	AC/A	TCATCAAAGGCATGCAAATC	GACCAAAACACAACGCAATG	60.0	359	INTERGENIC	--	--	--
Ca1_P2_29843804	Ca1	29843804	G/GA	ATGGATCAAGAGAAGGGGCT	CCTATGCGAAGTGGTGGTTT	60.0	535	INTERGENIC	--	--	--
Ca1_P2_29844165	Ca1	29844165	TTTATT/TTT	ATGGATCAAGAGAAGGGGCT	CCTATGCGAAGTGGTGGTTT	60.0	535	INTERGENIC	--	--	--
Ca1_P2_29846530	Ca1	29846530	CTTTT/CTTTTT	TGGATTGGGGATCATTCATT	TCAACATTTGGAAGCACAAGA	59.3	897	DRR	Ca_24388	--	--

INDEL marker IDs	Chromosomes/unanchored scaffolds	Physical positions (bp)	InDels (*Kabuli* reference genome-CDC Frontier/PII)	Forward primers (5'-3')	Reverse primers (5'-3')	Anneling temperature (0C)	Expected amplified product size (bp)	Structural annotation		Functional annotation	
								Sequence components of *kabuli* genome	*Kabuli* gene accession IDs	NCBI-KOG	TFs
Ca1_P2_29846735	Ca1	29846735	ACT/AATATATATATATCT	AGAACGGTTTTCTGTCCGTG	TCAACATTTGGAAGCACAAGA	59.3	689	DRR	Ca_24388	--	--
Ca1_P2_29846941	Ca1	29846941	TAAAAAAAAAA/TAAAAAAAA	TGTTGCCAACTGAAAATCGT	ATACAGACACCGAATGGGGA	60.2	828	DRR	Ca_24388	--	--
Ca1_P2_29847994	Ca1	29847994	TAAAAAA/TAAAAAAAA	TCTTTTCATCAGGCCCAATC	GGAAAGTGAGGGAACCAATG	59.4	228	INTRON	Ca_24388	--	--
Ca1_P2_29848026	Ca1	29848026	ATGT/AT	TCTTTTCATCAGGCCCAATC	GGAAAGTGAGGGAACCAATG	59.4	228	INTRON	Ca_24388	--	--
Ca1_P2_29853119	Ca1	29853119	TAA/TA	TCCACGAGATTTTCCTTTGG	TGTCATCATTCTCTGCTGGC	60.0	357	INTERGENIC	--	--	--
Ca1_P2_29853730	Ca1	29853730	GTGGAAGTCT/GTGGAAGTCTGGAAGTCT	TTGCATTCCTAGGTTCAGCC	TATCGCTCGACCTCTGCTTT	60.1	602	INTERGENIC	--	--	--
Ca1_P2_29856647	Ca1	29856647	CTTT/CTTTT	GCATTCCCTGCTCAAGACTC	AGACAAGGGAGAGTCCCACA	59.7	812	INTERGENIC	--	--	--
Ca1_P2_29857046	Ca1	29857046	GTTTTTTTT/GTTTTTTTTTT	TGGTAAACACCCTCTTGGGA	CCTCCCGAGCAATACACATT	60.0	496	INTERGENIC	--	--	--
Ca1_P2_2993052	Ca1	2993052	ATTTTTTTT/ATTTTTTTTTTT	AGCTGCTTCTTGCCATGTTT	GGAAGCAACAGAGAACAGCC	60.0	534	INTRON	Ca_00363	PET	NAC
Ca1_P2_2993988	Ca1	2993988	AATTATCTAT/AATTATCTATTATCTAT	TATGGGGTAGGGAATGGTCA	CCAAGATAGATCCGAGGAGG	58.7	623	INTERGENIC	--	--	--
Ca1_P2_3001318	Ca1	3001318	CTTT/CTTTT	ATGTGACTTTTGCCAGGACC	AAACCCATGCCAACATGAAT	60.1	770	INTRON	Ca_00364	A	bHLH
Ca1_P2_3002623	Ca1	3002623	TCA/TCACA	TCTTCATTGATTTCCCTCGG	CCAAGCAAGGGAACGTAGAA	60.2	523	DRR	Ca_00364	A	bHLH

INDEL marker IDs	Chromosomes/unanchored scaffolds	Physical positions (bp)	InDels (*Kabuli* reference genome-CDC Frontier/PII)	Forward primers (5'-3')	Reverse primers (5'-3')	Anneling temperature (0C)	Expected amplified product size (bp)	Structural annotation		Functional annotation	
								Sequence components of *kabuli* genome	*Kabuli* gene accession IDs	NCBI-KOG	TFs
Ca1_P2_3003710	Ca1	3003710	AATATATATATATATATA/AATATATATATATATATATATATATATA	GAAAGGCATGCAATATGGGT	AATCTGCCGACGCAAACTAT	59.7	440	DRR	Ca_00364	A	bHLH
Ca1_P2_30097998	Ca1	30097998	ATTTTTTTTTT/ATTTTTTTTTTTT	TCACCGGTCGTGATACAAAA	CGTCTCTGTAGGTTCTCGCC	60.0	520	INTERGENIC	--	--	--
Ca1_P2_3016499	Ca1	3016499	ATAA/ATAATTAA	AGTCAATGCATGCCACGTAA	GCGACTTACCTCCATTTCCA	60.1	574	INTERGENIC	--	--	--
Ca1_P2_3016722	Ca1	3016722	ATAAAATTA/ATAAAATTACTAAAATTA	TGGAAATGGAGGTAAGTCGC	TTTGATTATGAAGCATTGTCAACAT	59.8	535	INTERGENIC	--	--	--
Ca1_P2_3016741	Ca1	3016741	AATATATATATATATATA/AATATATATATATATATATA	TGGAAATGGAGGTAAGTCGC	TTTGATTATGAAGCATTGTCAACAT	59.8	535	INTERGENIC	--	--	--
Ca1_P2_3017821	Ca1	3017821	ATTTT/ATTT	TATGAAGCACAATGTGGCCC	CAAATTTTGACAAGGCGATTT	59.1	551	INTERGENIC	--	--	--
Ca1_P2_3020023	Ca1	3020023	ATAATATTAATAT/ATAATATTAATATTAATAT	CAAGCAACATGCTGAAGGAA	TTTAAGCACATCTGGTCAATCA	58.3	237	INTERGENIC	--	--	--
Ca1_P2_3020354	Ca1	3020354	AGG/AGGG	CAAGCAACATGCTGAAGGAA	ATGTCCTTAGTGTCACGGGG	59.8	808	INTERGENIC	--	--	--
Ca1_P2_30264384	Ca1	30264384	TAAAAA/TAAAA	TGTGCAAATAACGCAGATTC	GGGTTTTATTGCAATCTCATGG	60.5	511	INTERGENIC	--	--	--
Ca1_P2_3032265	Ca1	3032265	TATTTGGAATTTGGA/TATTTGGA	AATTCTCACGCAGCGTTCTT	AACTTCAGCGGAATCCAAAA	59.7	706	DRR	Ca_00369	--	--
Ca1_P2_3033238	Ca1	3033238	CT/CTTT	CCGCTGAAGTTGAAGCAGTA	TCAGTTGCAAAGCCAAACAC	59.9	758	DRR	Ca_00369	--	--
Ca1_P2_3035970	Ca1	3035970	TTCT/TT	AAGGAAGAAGCGCAATTGAA	ATCCATCCCCCTCATTCTCT	59.7	463	DRR	Ca_00370	--	ARF

INDEL marker IDs	Chromosomes/unanchored scaffolds	Physical positions (bp)	InDels (*Kabuli* reference genome-CDC Frontier/PII)	Forward primers (5'-3')	Reverse primers (5'-3')	Anneling temperature (0C)	Expected amplified product size (bp)	Structural annotation		Functional annotation	
								Sequence components of *kabuli* genome	*Kabuli* gene accession IDs	NCBI-KOG	TFs
Ca1_P2_30399037	Ca1	30399037	CATATATATATATATATATATAT/CATATATATATATATATATATATATAT	TAACGTGGCAAAGGTAAGGG	GGGGTGCTCAACTTGTTTGT	60.0	232	INTERGENIC	--	--	--
Ca1_P2_3040746	Ca1	3040746	C/CAT	TTTATGAAAGTGTCCCCCTTTT	TGCACGGAACCTGTTTGTTA	60.1	734	INTERGENIC	--	--	--
Ca1_P2_3042228	Ca1	3042228	ATTT/ATTTT	GAGCCAAGATCGATGGGAT	TTTGTATGAACACAACCCCAAA	60.1	502	INTERGENIC	--	--	--
Ca1_P2_30432201	Ca1	30432201	CTTTTTTT/CTTTTTT	CCACAATATCAAAGGGCCAC	AGAGAAAACAAAAGGCGCAA	60.0	471	INTERGENIC	--	--	--
Ca1_P2_3043596	Ca1	3043596	GTATATATATATATAT/GTATATATATATATATAT	GGACCGAAATGTTGCCTAAA	GACTTGGTCAATTTGCCGAT	59.9	531	INTERGENIC	--	--	--
Ca1_P2_3045718	Ca1	3045718	TCAC/TCACTACAC	CGTAATCACATACATAGGGCTCA	CATTGCACGGTCAATTCTTG	60.1	479	DRR	Ca_00371	I	--
Ca1_P2_3046144	Ca1	3046144	TAAAAAAAAAAA/TAAAAAAAAA	ACCGTGCAATGCAGATGTAA	TGATGCAGCCTAAGTGGATG	59.8	398	DRR	Ca_00371	I	--
Ca1_P2_30475415	Ca1	30475415	A/AC	ACGGCAAGACCCTAAAACCT	AAACTTCTACTCGGGCCCAT	60.0	906	INTERGENIC	--	--	--
Ca1_P2_30475519	Ca1	30475519	ATTT/ATTTT	AGGATGCATGGGATGAAAAG	AAACTTCTACTCGGGCCCAT	60.0	766	INTERGENIC	--	--	--
Ca1_P2_3049357	Ca1	3049357	ATT/AT	ATCCTTAAAGAGAGGGGCCA	AAACCCAGGAAAGGGTCACT	59.8	888	INTRON	Ca_00371	I	--
Ca1_P2_3055427	Ca1	3055427	AA/AATACA	TTCTCATTTTGGCTCACGGT	TTGAGGATGTGGGAATGGAT	60.1	553	DRR	Ca_00372	S	--
Ca1_P2_30568244	Ca1	30568244	TA/TAA	CAGCGGAAAAGTTTCTCCAA	GTCACGTTAGGAGGATCCCA	59.9	247	INTERGENIC	--	--	--

INDEL marker IDs	Chromosomes/unanchored scaffolds	Physical positions (bp)	InDels (*Kabuli* reference genome-CDC Frontier/PII)	Forward primers (5'-3')	Reverse primers (5'-3')	Anneling temperature (0C)	Expected amplified product size (bp)	Structural annotation		Functional annotation	
								Sequence components of *kabuli* genome	*Kabuli* gene accession IDs	NCBI-KOG	TFs
Ca1_P2_30627831	Ca1	30627831	AATATATATATATATATATAT/AATATATATATATATATATATATATATAT	CTTGGGAACTGGAGAGCAAG	CCAACGTCACTTCCAAGGTT	60.0	797	INTERGENIC	--	--	--
Ca1_P2_30661908	Ca1	30661908	CTTTTTTTT/CTTTTTTT	TAAAATTTTGGCACGCAACA	CGTTGGAAAGGGTTCAGCTA	60.2	534	INTERGENIC	--	--	--
Ca1_P2_3066310	Ca1	3066310	GAAAAAAAAA/GAAAAAAAAAAAA	ACTATTTGACGGGTTCACGC	GCATGGTGTGGAATGTTCTG	60.0	373	INTERGENIC	--	--	--
Ca1_P2_30682856	Ca1	30682856	CATATATATATATATATATATAT/CATATATATATATATATATAT	GTTGGTTCCCTAGCACCGTA	TCCGCCTAGGAGATAGCAGA	60.1	672	INTERGENIC	--	--	--
Ca1_P2_3068457	Ca1	3068457	TTA/TTAAGTA	TCATTGGGTTGGGTTTTCAT	TTATCAGTTGCAGCCTGTGC	60.0	626	INTRON	Ca_00374	EH	bHLH
Ca1_P2_3070486	Ca1	3070486	AC/ACCATCACTTAGC	AGAATGGGGATCTCGTGTTG	GATTTGCTCCATTTGTGCCT	60.1	807	DRR	Ca_00374	EH	bHLH
Ca1_P2_3070792	Ca1	3070792	CTTT/CTT	TTCCAACAGTGGTTGGTTCA	GATTTGCTCCATTTGTGCCT	60.1	400	DRR	Ca_00374	EH	bHLH
Ca1_P2_30709542	Ca1	30709542	A/AAGAAGAGCG	TCACACAGTCGTTTTGGCAT	TCTCTCCGTTTTCATCCAGG	60.2	287	INTERGENIC	--	--	--
Ca1_P2_3073093	Ca1	3073093	CTTTTT/CTTTTTT	CCTCCTTACGGCGAAACATA	CGTTGGAACTCCCAATGACT	60.0	234	INTRON	Ca_00375	--	bHLH
Ca1_P2_30734904	Ca1	30734904	AATATATATATATATATATATATATATATATATATATATATA/AATATATATATATATATATATATATATATATATA	CGATTTATGCAATCAAGCACA	TCAAATTTGGGAGGAACCAC	59.8	723	INTERGENIC	--	--	--
Ca1_P2_30738786	Ca1	30738786	AATATATATATATATATATATATAT/AATATATATATATATATATATATATAT	AGCGGAAAACATGTTGGACT	GGAAAGATGATGGGTGACAGA	59.9	508	DRR	Ca_22454	OE	WRKY
Ca1_P2_30740000	Ca1	30740000	TTATATATATATATATATATATATATA/TTATATATATATATATATATATATA	TTCGGAGCACAACCTACACA	GAATGGTGGCCATTATTAAGGT	59.1	382	DRR	Ca_22454	OE	WRKY

INDEL marker IDs	Chromosomes/unanchored scaffolds	Physical positions (bp)	InDels (*Kabuli* reference genome-CDC Frontier/PII)	Forward primers (5'-3')	Reverse primers (5'-3')	Anneling temperature (0C)	Expected amplified product size (bp)	Structural annotation		Functional annotation	
								Sequence components of *kabuli* genome	*Kabuli* gene accession IDs	NCBI-KOG	TFs
Ca1_P2_30740126	Ca1	30740126	CTGT/CT	TCGGAGCACAACCTACACAA	ACATTTGCCACCGTTAGAGG	60.0	901	DRR	Ca_22454	OE	WRKY
Ca1_P2_30745547	Ca1	30745547	C/CT	TTTTTGGGTTTCCTTTTCCC	TGCAATATGTATCGTGGATGG	59.3	817	INTERGENIC	--	--	--
Ca1_P2_3074672	Ca1	3074672	GCAC/GC	CACGAGGTTTGACCTCTTCC	CGTGTGAACATTCATACACCTCA	60.9	578	INTRON	Ca_00375	--	bHLH
Ca1_P2_3075180	Ca1	3075180	CT/CTTT	GAGGTGTATGAATGTTCACACGTA	CGATCGTCGCATTTTTCATA	59.7	285	INTRON	Ca_00375	--	bHLH
Ca1_P2_3076592	Ca1	3076592	CATA/CATATA	TCGGTCAAGCTACAGAACCA	ATTTCCAGGGACGATGAGTG	59.9	173	INTRON	Ca_00375	--	bHLH
Ca1_P2_30768111	Ca1	30768111	GAAAAA/GAAAAAA	TCCGGTATAAAAGGTTTCCG	CCGTTGAAAACAACACATGAA	59.5	482	INTERGENIC	--	--	--
Ca1_P2_30769862	Ca1	30769862	TAAAAAA/TAAAAAAAA	ATGGGTCTTGGAAACACTCG	ACCAACTCCTCCAAGCCACT	61.1	354	INTERGENIC	--	--	--
Ca1_P2_30770184	Ca1	30770184	TAAA/TAAAA	AGTGGCTTGGAGGAGTTGGT	CGTTCGGCTTTCTTCTTCTG	60.1	535	INTERGENIC	--	--	--
Ca1_P2_3078920	Ca1	3078920	TACACACA/TACACA	CTGCGATTGCATCCCTTAAT	TTGAAGATTTGAGTTACGAATTTGA	59.2	905	DRR	Ca_00375	--	bHLH
Ca1_P2_30790839	Ca1	30790839	TGTAAC/T	TTTTTGATGGTTCAACGAGAA	GGTTGAATAATTGAAAAGAGGTGG	60.1	250	INTERGENIC	--	--	--
Ca1_P2_3079517	Ca1	3079517	A/AAATTTTTTG	TCAAATTCGTAACTCAAATCTTCAA	ATTGCCCCAAGACATTATCG	59.8	478	DRR	Ca_00375	--	bHLH
Ca1_P2_3081375	Ca1	3081375	TG/T	AGATGGTGACCAGATTGACG	TGCCTCCTATCTCTCCCACA	60.8	683	INTRON	Ca_00376	--	bHLH

INDEL marker IDs	Chromosomes/unanchored scaffolds	Physical positions (bp)	InDels (*Kabuli* reference genome-CDC Frontier/PII)	Forward primers (5'-3')	Reverse primers (5'-3')	Anneling temperature (0C)	Expected amplified product size (bp)	Structural annotation		Functional annotation	
								Sequence components of *kabuli* genome	*Kabuli* gene accession IDs	NCBI-KOG	TFs
Ca1_P2_30828577	Ca1	30828577	AATATATATATATATATATATATATATAT/AATATATATATATATATATATATATATATATATATATAT	CCTCACTGAGCCAAAAGCTC	TGCGATTGAAAGAGAAAATGA	58.5	778	INTERGENIC	--	--	--
Ca1_P2_3083516	Ca1	3083516	AT/ATT	TCATTTGACTGAGGCTGCAT	GGACGAGGAGTTGGATTTGA	60.1	884	INTRON	Ca_00376	--	bHLH
Ca1_P2_3085008	Ca1	3085008	GTAGT/G	TTCATCCAAGAGGGACTTGC	CAACCATTTCAAACATGACCC	60.1	491	INTRON	Ca_00376	--	bHLH
Ca1_P2_3085305	Ca1	3085305	CA/CATA	GATCTCCAAGCCACTGTTCC	AAAAGTTCCACTGCCCCTTC	60.5	927	INTRON	Ca_00376	--	bHLH
Ca1_P2_30870705	Ca1	30870705	CAA/CA	TGAAATGTCCATGATGGGAG	CTTTCCTTGGGTAGGGCTTC	60.1	806	INTERGENIC	--	--	--
Ca1_P2_30874605	Ca1	30874605	A/AT	TGTGATGCAGTTGCTCCATT	CAAGCGTCATGCCAAACTTA	59.9	624	INTERGENIC	--	--	--
Ca1_P2_3087515	Ca1	3087515	CAAA/CAAAAA	GGGCAATTCTATCGCTCAAA	ACTGGTGGACGAGCAAGACT	59.9	681	DRR	Ca_00376	--	bHLH
Ca1_P2_3088550	Ca1	3088550	A/AG	CAAGTCGTTTGAGCTTGTGC	TTCCCGATAAGACGTTTTGC	60.1	349	INTRON	Ca_00377	J	--
Ca1_P2_3089287	Ca1	3089287	TTGTAAT/TT	GCAAAACGTCTTATCGGGAA	TGCAAACTATTTGACATTGACG	58.7	591	DRR	Ca_00378	I	--
Ca1_P2_3089369	Ca1	3089369	AT/ATT	AAAATCGGTCTTGGGCTCTT	CCGTGTTAGTCGTCTATTTTTACCA	60.6	713	DRR	Ca_00378	I	--
Ca1_P2_3093464	Ca1	3093464	CATATATATATAT/CATATATATATATATAT	ATATATAGGCAGGGGCGGAC	AATTGAACCCAAGTGCAGGT	59.5	481	INTERGENIC	--	--	--
Ca1_P2_3094552	Ca1	3094552	TA/T	GGTGCTCTTAGAGGCAAAGG	TTGGAACAATGACGGTGTTG	60.4	633	INTERGENIC	--	--	--

INDEL marker IDs	Chromosomes/unanchored scaffolds	Physical positions (bp)	InDels (*Kabuli* reference genome-CDC Frontier/PII)	Forward primers (5'-3')	Reverse primers (5'-3')	Anneling temperature (0C)	Expected amplified product size (bp)	Structural annotation		Functional annotation	
								Sequence components of *kabuli* genome	*Kabuli* gene accession IDs	NCBI-KOG	TFs
Ca1_P2_3096580	Ca1	3096580	TAAAAAA/TAAAAAAA	AACATAGCGGAGCATTTTGG	CATGGTAGCCCAAATCCAAT	59.6	511	INTERGENIC	--	--	--
Ca1_P2_30968052	Ca1	30968052	AATTATTATTTATTA/AATTATTATTTATTATTATTTATTA	TGCGATTGTGGGAAATATCA	ACCAAAATGTCACACAGCGA	60.2	670	INTERGENIC	--	--	--
Ca1_P2_30993040	Ca1	30993040	AAAAATTAAAATTAAAATTAA/AAAAATTAAAATTAAAATTAAAATTAA	TCCTTCCGATCGAGTAGTGG	AAACATGAAATGGACAATACAAAAA	58.8	398	INTERGENIC	--	--	--
Ca1_P2_3103121	Ca1	3103121	AT/ATT	GGGTGCAATGCTGGATTATT	GTTAAGTGAGGTCCGTCCCA	60.0	462	DRR	Ca_00380	--	--
Ca1_P2_31086522	Ca1	31086522	TAAAA/TAAA	TATAGGTTGGCACCCAAAGC	GGTTCGCCATATACCAATGC	60.2	706	DRR	Ca_25826	S	--
Ca1_P2_31090559	Ca1	31090559	CAA/CA	CGCTCGCTCATTGAATAACA	CTCCACGACCATCCTGAAAT	59.9	417	INTRON	Ca_25826	S	--
Ca1_P2_31095231	Ca1	31095231	TTTATTAT/TTTAT	GATACATTATGCACGCCTCG	TGACGATGGTTTTGGGTTTT	60.2	506	INTERGENIC	--	--	--
Ca1_P2_31112808	Ca1	31112808	AC/A	AGGTCAAATCGGGACAATCA	TGCCATTATCGAAGGCTTGT	60.6	583	INTERGENIC	--	--	--
Ca1_P2_31118424	Ca1	31118424	AC/A	TCGTGTGTCGCACATAAACA	TGTTACACCACGATTTGACGA	60.0	801	INTERGENIC	--	--	--
Ca1_P2_31130464	Ca1	31130464	AAG/AAGAG	GCAGATTCTCCACAATGGGT	TCCACCCACGATACTGATGA	59.9	503	INTERGENIC	--	--	--
Ca1_P2_3113687	Ca1	3113687	TTATATATATATATATATATATATATATA/TTATATATATATATATATATATA	TTTTGGGACAGATGAATGGA	GGAATCGAACTCGAGACTGC	60.0	971	DRR	Ca_00381	--	--
Ca1_P2_3113743	Ca1	3113743	GATATATATATAT/GATATATATAT	TGATTACAATCCTTTAAGCACG	GGAATCGAACTCGAGACTGC	60.0	564	DRR	Ca_00381	--	--

INDEL marker IDs	Chromosomes/unanchored scaffolds	Physical positions (bp)	InDels (*Kabuli* reference genome-CDC Frontier/PII)	Forward primers (5'-3')	Reverse primers (5'-3')	Anneling temperature (0C)	Expected amplified product size (bp)	Structural annotation		Functional annotation	
								Sequence components of *kabuli* genome	*Kabuli* gene accession IDs	NCBI-KOG	TFs
Ca1_P2_31141128	Ca1	31141128	C/CA	ATTATTGAGGTTGGCCCCTC	TCCACTTTTTGTGCTTTAGGC	59.4	376	INTERGENIC	--	--	--
Ca1_P2_31162887	Ca1	31162887	T/TG	CCGGGGATTGCCATATAACT	CTAATAGGCTGCATCGGAGG	59.8	342	INTERGENIC	--	--	--
Ca1_P2_31163466	Ca1	31163466	T/TAGA	ACTTTTGCGGAGAAGCACAT	CGTTCGTCTTTTCCTCTTGC	60.0	360	INTERGENIC	--	--	--
Ca1_P2_31212342	Ca1	31212342	CTTTTTTTTTTTT/CTTTTTTTTTT	GCCAAAAATGCCATTAAGGA	TGCCTCCAAATATCCTCCAA	60.4	514	INTERGENIC	--	--	--
Ca1_P2_31238518	Ca1	31238518	ATT/AT	CATCGTAAGCATGTGGTTGG	TAATGGCCCCATAGCAGAAG	60.1	861	DRR	Ca_21848	R	FAR1
Ca1_P2_3125906	Ca1	3125906	GAAAAA/GAAAAAA	GAAATCCACACCATCGAACC	TGATTCATGTGCCTTACCCA	59.9	595	INTRON	Ca_00382	--	--
Ca1_P2_31262303	Ca1	31262303	TTA/TTAATA	ATTCCTAACCCTCTGCCCAT	TTGAGAATGTGAGGGTGCAA	60.2	788	INTERGENIC	--	--	--
Ca1_P2_3126822	Ca1	3126822	AATAT/AATATATTATTGATAT	TGTTGCTTGGTGTAGGGACA	CCTTTAGCCCGTGAGTCTTG	59.9	987	INTERGENIC	--	--	--
Ca1_P2_3130963	Ca1	3130963	G/GTCAACACGGATA	GGTTTTCAAGCCAGACCAGA	CCCATTTTTCTGACAACCGT	59.8	601	INTERGENIC	--	--	--
Ca1_P2_3131301	Ca1	3131301	TATA/TATACTCATA	GGCCAGACTCAGACCTTTCA	ACCCATTTTTCTGACAACCG	59.8	506	INTERGENIC	--	--	--
Ca1_P2_3133490	Ca1	3133490	AATTATATATTATA/AATTATA	CCACATTTTGCCACTTTTCA	TCGCCTTTTAAACCAACCAG	60.1	853	INTERGENIC	--	--	--
Ca1_P2_31354820	Ca1	31354820	AAAG/A	CGGTCTTCTCCACAAAGAGC	TTAAAGGCATCTACCCGTGC	60.1	718	INTERGENIC	--	--	--

INDEL marker IDs	Chromosomes/unanchored scaffolds	Physical positions (bp)	InDels (*Kabuli* reference genome-CDC Frontier/PII)	Forward primers (5'-3')	Reverse primers (5'-3')	Anneling temperature (0C)	Expected amplified product size (bp)	Structural annotation		Functional annotation	
								Sequence components of *kabuli* genome	*Kabuli* gene accession IDs	NCBI-KOG	TFs
Ca1_P2_31367687	Ca1	31367687	CTCTTCTTCTTCTTCTTCTTCTTCTTC/CTCTTCTTCTTCTTCTTCTTCTTCTTCTTC	TGGTTGGGTTGCATGTAAGA	AATTAAGCCCGACGTTGTTG	60.0	379	INTRON	Ca_21855	O	--
Ca1_P2_3138107	Ca1	3138107	A/ATT	ATCGGGGTGGACAAAACATA	GGGCTTTAAATTGAGTGAGGC	60.1	683	INTERGENIC	--	--	--
Ca1_P2_31413565	Ca1	31413565	AG/AGG	TAATTCAAACCCCGTTCCTT	CCGAAGGATGAAAGATCCAA	60.0	466	DRR	Ca_21859	--	--
Ca1_P2_31428294	Ca1	31428294	ATATTTTTATTTTTATTTTTATTTTTAT/ATATTTTTATTTTTATTTTTAT	TCAACAGGTTGATGGGTGAA	TGAGATGGAGGTGATGGTGA	60.0	370	INTERGENIC	--	--	--
Ca1_P2_3145431	Ca1	3145431	AAAAGAAAGAAAGAAAGAAAGAAAGAAAGAA/AAAAGAAAGAAAGAA	TTGCAGCCATATGTGGTCAT	AATGCTTGTCCTGTTGGACC	60.0	933	INTRON	Ca_00385	--	--
Ca1_P2_3147144	Ca1	3147144	AATATATATATATATATATATATA/AATATATATATATATA	TCCCAATGCTGCATGTTTTA	TGGGTTGAGTTGTGAGTTCG	59.7	650	INTRON	Ca_00385	--	--
Ca1_P2_31498685	Ca1	31498685	AGG/AG	TTGGAGCTTCAGCATTTGTG	CCTCTTTTCTTCGCATTTCG	60.0	747	DRR	Ca_21863	--	M-type
Ca1_P2_3155613	Ca1	3155613	AAACTGCAA/AAA	TGTCCCATAGACACCCTTTTG	GTGGCGGAGAAAGACTCAAC	59.9	610	INTERGENIC	--	--	--
Ca1_P2_3157589	Ca1	3157589	AAAT/A	ATTTTTCATGAGGTGGCTGC	TGGTGATACGCAAGTTCTGG	59.7	468	INTERGENIC	--	--	--
Ca1_P2_31592114	Ca1	31592114	CT/CTTT	TCAGCTGCAGTTCCAACATC	GCACAAGTCCTTGAGGAAGC	60.0	375	INTRON	Ca_21868	F	--
Ca1_P2_31600651	Ca1	31600651	AC/A	GGATGGAAGAGAAATTGGGA	AGGATCATGTTTGTGGTTGC	58.4	899	INTERGENIC	--	--	--
Ca1_P2_31600847	Ca1	31600847	TG/T	AAGGTAGTGAAAGAGGGGCTG	AGGATCATGTTTGTGGTTGC	58.4	669	INTERGENIC	--	--	--

INDEL marker IDs	Chromosomes/unanchored scaffolds	Physical positions (bp)	InDels (*Kabuli* reference genome-CDC Frontier/PII)	Forward primers (5'-3')	Reverse primers (5'-3')	Anneling temperature (0C)	Expected amplified product size (bp)	Structural annotation		Functional annotation	
								Sequence components of *kabuli* genome	*Kabuli* gene accession IDs	NCBI-KOG	TFs
Ca1_P2_31627142	Ca1	31627142	TAAAAAAAA/TAAAAAAA	CTTCGAATCCAACCTTGGTG	CCGGAACCACATTCAGACTT	60.0	892	INTERGENIC	--	--	--
Ca1_P2_31665837	Ca1	31665837	G/GA	TAAAGGGTCTTGCTGTTGGG	GGCAAAAGAAATATAGTGCCAAA	59.5	538	INTERGENIC	--	--	--
Ca1_P2_31716302	Ca1	31716302	A/AC	TCGGTATTTGCCATGAGACA	AAACAACCTTCATGCCTTGG	60.0	482	INTERGENIC	--	--	--
Ca1_P2_31768486	Ca1	31768486	TTATA/TTTTTTATATA	ACACATTATATTAAATGCGCGA	TGCCAAACGATAGGTTGTTG	59.6	646	INTERGENIC	--	--	--
Ca1_P2_31783442	Ca1	31783442	TGGG/TGG	TGGATTGGGCTGGAATTAAA	TATAACCGACGAGGGACGAG	60.1	466	INTERGENIC	--	--	--
Ca1_P2_31783488	Ca1	31783488	AATATATATATATATATATATATAT/AATATATATATATATAT	TGGATTGGGCTGGAATTAAA	TATAACCGACGAGGGACGAG	60.1	466	INTERGENIC	--	--	--
Ca1_P2_31783811	Ca1	31783811	TTAATAATAATAATAATAATAATAATAATAATAAT/TTAATAATAATAATAATAATAATAATAATAAT	ATACTGCCTTCGATCCGTTG	CGGGAAAAAGGGTGGTAAAT	60.0	477	INTERGENIC	--	--	--
Ca1_P2_31785672	Ca1	31785672	ATTTTTTT/ATTTTTT	GTTTTGGCGTTGGTTTCACT	TTTTACGTTCATACGTGAGTTGG	59.1	765	INTERGENIC	--	--	--
Ca1_P2_31803627	Ca1	31803627	TAAA/TAAAA	CTTACGGTGAAGGAGAACGG	ATCATTCTTTTATGCGTTTGACT	57.1	793	INTERGENIC	--	--	--
Ca1_P2_3194081	Ca1	3194081	ACGAATGGTCAG/A	GCAAGGCTTGATTCCAAAAA	GGTTGCACCTTCCATGAACT	60.0	799	DRR	Ca_00390	O	E2F/DP
Ca1_P2_31956113	Ca1	31956113	ATTATATAT/A	TGCTACACGTGCATCAAGGT	TGTTGGATGGCATGTGAGTT	60.0	554	INTERGENIC	--	--	--
Ca1_P2_31956151	Ca1	31956151	TATATATAA/T	TGCTACACGTGCATCAAGGT	TGTTGGATGGCATGTGAGTT	60.0	554	INTERGENIC	--	--	--

INDEL marker IDs	Chromosomes/unanchored scaffolds	Physical positions (bp)	InDels (*Kabuli* reference genome-CDC Frontier/PII)	Forward primers (5'-3')	Reverse primers (5'-3')	Anneling temperature (0C)	Expected amplified product size (bp)	Structural annotation		Functional annotation	
								Sequence components of *kabuli* genome	*Kabuli* gene accession IDs	NCBI-KOG	TFs
Ca1_P2_31995451	Ca1	31995451	T/TCAC	TGCTTTGAAGACCATGTGGA	AAAAGGATTCATGCGTGTCC	59.9	354	INTERGENIC	--	--	--
Ca1_P2_32030892	Ca1	32030892	AATATATATATATATATATATATATATATATATATAT/AATATATATATATATATATATATATATATATAT	CATGGATGGGTTTTGTACCC	TTGGAAGGAGCCCTTACCTT	60.1	771	INTERGENIC	--	--	--
Ca1_P2_32037255	Ca1	32037255	CA/CTTGGTCAA	GGGAAAACTTTTGTGTGGGA	TTGGCAAATCGATTAGACTGG	60.1	802	INTERGENIC	--	--	--
Ca1_P2_32037334	Ca1	32037334	CTTTTT/CTTTT	TCCAGTCATGTGGCTTCTCA	CTGAGCTTGTGACCAAATCG	59.4	559	INTERGENIC	--	--	--
Ca1_P2_32037356	Ca1	32037356	CTTTTTT/CTTTTT	TCCAGTCATGTGGCTTCTCA	CTGAGCTTGTGACCAAATCG	59.4	559	INTERGENIC	--	--	--
Ca1_P2_32078246	Ca1	32078246	TA/TAAAAA	CTCGGTTCGAACAGGAGAAG	TATGTCAACAACCCCCTGGT	60.1	702	INTRON	Ca_26405	--	--
Ca1_P2_32080106	Ca1	32080106	T/TC	TTCCCCATGTCGATCTAAGC	AACCCTGAACAGATTGTCGG	60.0	612	INTRON	Ca_26405	--	--
Ca1_P2_32080183	Ca1	32080183	C/CGGCGATTACT	TTCCCCATGTCGATCTAAGC	AACCCTGAACAGATTGTCGG	60.0	612	INTRON	Ca_26405	--	--
Ca1_P2_3211182	Ca1	3211182	ATTTTTTTTT/ATTTTTTTT	TCCAAGCTGTATCTGGCTCA	CCAAAATCGGATGGAATATGA	59.6	624	INTERGENIC	--	--	--
Ca1_P2_3213987	Ca1	3213987	TTATATATATATATATATATATATA/TTATATATATATATATATATATATATATA	TCGGCAATGTGGTACTGAAA	TTGCAAATGCGACTTTCTTG	60.0	344	INTERGENIC	--	--	--
Ca1_P2_32147043	Ca1	32147043	GTTTTTTTTTT/GTTTTTTTTTTT	CGGCCTCCAAATCTTTAACA	TTGTCATGAAAGTCTCGCCT	58.4	670	INTERGENIC	--	--	--
Ca1_P2_32150556	Ca1	32150556	CTTTTT/CTTTTTT	GTTGGTTCCCTAGCACCGTA	TTTTGTTGACAAATCGGACG	59.6	746	INTERGENIC	--	--	--

INDEL marker IDs	Chromosomes/unanchored scaffolds	Physical positions (bp)	InDels (*Kabuli* reference genome-CDC Frontier/PII)	Forward primers (5'-3')	Reverse primers (5'-3')	Anneling temperature (0C)	Expected amplified product size (bp)	Structural annotation		Functional annotation	
								Sequence components of *kabuli* genome	*Kabuli* gene accession IDs	NCBI-KOG	TFs
Ca1_P2_32150572	Ca1	32150572	TCC/TC	GTTGGTTCCCTAGCACCGTA	TTTTGTTGACAAATCGGACG	59.6	746	INTERGENIC	--	--	--
Ca1_P2_32151710	Ca1	32151710	TAAAAAAAAAAA/TAAAAAAAAAAAAAAAA	TGAAGGTGTGAGCAGATTCG	TCGGGTGGAGAATTCCTATG	59.9	593	INTERGENIC	--	--	--
Ca1_P2_32153452	Ca1	32153452	TTTATTTTAT/TTTATTTTATATTATTTTAT	GTTTTGGCGTTGGTTTCACT	TGGGTTTGACCCTATGTGTG	59.3	353	INTERGENIC	--	--	--
Ca1_P2_32153737	Ca1	32153737	TTATATATATATAT/TTATATATATATATAT	CACACATAGGGTCAAACCCA	ATACATGGATTGGTTGGGGA	59.9	585	INTERGENIC	--	--	--
Ca1_P2_32154060	Ca1	32154060	TGTATTGTAAGTATTGTAAG/TGTATTGTAAG	ATGGAATACTCCCCAACCAA	ACTTGCAAAATCCAAATGCC	59.9	446	INTERGENIC	--	--	--
Ca1_P2_32191667	Ca1	32191667	ATTTTTTTTT/ATTTTTTT	CATCATGATCAAGGGACCAA	AAGTTAGCGGTCGGGAGAAT	60.1	413	INTERGENIC	--	--	--
Ca1_P2_32203410	Ca1	32203410	TGGGG/TGGGGG	CAAGGAGGTTGCAAAAGAGG	CCTTTCGTGGACCAGATGTT	60.0	511	INTERGENIC	--	--	--
Ca1_P2_32222272	Ca1	32222272	ATTTT/ATTTTT	ACAACTTCTCATGCTCGCAA	GGTGGCATACCTCAACGAGT	60.0	620	INTRON	Ca_26127	--	Trihelix
Ca1_P2_32235747	Ca1	32235747	GNNNNNNNNNNNN/GNN	AAACGCAATCCTTGAGTTCG	GCACGTAACAACTCGACCCT	60.2	755	INTRON	Ca_26127	--	Trihelix
Ca1_P2_32263460	Ca1	32263460	TAAAAAAAA/TAAAAAAAAA	CGGAAATTTCGAAAAACGAA	TAGGACGAATGTTTGTGGCA	60.1	487	INTERGENIC	--	--	--
Ca1_P2_32267954	Ca1	32267954	T/TG	AAGCTATGTCAAGGAATGTGATTT	CCGTGTTTTTGAAAATTGACAC	59.4	378	INTERGENIC	--	--	--
Ca1_P2_32284188	Ca1	32284188	CATC/CATCGATC	ATCCAGTGACCCAACCAAAA	AATGGTGCACTCAAGAACCC	60.0	642	DRR	Ca_26129	--	--

INDEL marker IDs	Chromosomes/unanchored scaffolds	Physical positions (bp)	InDels (*Kabuli* reference genome-CDC Frontier/PII)	Forward primers (5'-3')	Reverse primers (5'-3')	Anneling temperature (0C)	Expected amplified product size (bp)	Structural annotation		Functional annotation	
								Sequence components of *kabuli* genome	*Kabuli* gene accession IDs	NCBI-KOG	TFs
Ca1_P2_32312793	Ca1	32312793	GTT/GT	GATTGGCTCAGCAGAAGTCC	TTTCAAGCAGCGGAAAAAGT	60.0	561	INTERGENIC	--	--	--
Ca1_P2_3244914	Ca1	3244914	CATATATATATATATAT/CATATATATATATATATAT	CTTGATCACCACCACTCCCT	TCAATTAAAAACCGGGTCAA	58.0	797	INTERGENIC	--	--	--
Ca1_P2_32454365	Ca1	32454365	A/AG	AGGAAACACATGAAGGCACC	TCTTCGTTGTCACTCCATGC	59.8	572	INTERGENIC	--	--	--
Ca1_P2_3246500	Ca1	3246500	AATATATATATATATATATATAT/AATATATATATATATATATATATAT	TGTTTGGACGAGAGAATTTTATGA	GGCAAATTTGGAGATTGTGG	60.3	855	INTERGENIC	--	--	--
Ca1_P2_32524824	Ca1	32524824	TATACA/TA	CCGGTGCATACCTTGTTCTT	AAATTTTGGGATTAATTTCCG	57.1	819	INTERGENIC	--	--	--
Ca1_P2_32760818	Ca1	32760818	TTATATATATATATA/TTATATATATATATATA	AATGGAGATGGGAACTCGTG	ATTGGACTGCGGATTAGTGG	60.0	539	INTERGENIC	--	--	--
Ca1_P2_32790193	Ca1	32790193	TGGG/TGGGG	CATTTTGGATTCAACTCGGG	AGGGCAACTGTTTTGGATTG	60.0	133	INTERGENIC	--	--	--
Ca1_P2_32796156	Ca1	32796156	CAAA/CAAAA	TTGAGCCCCTGACTTAATGG	ACGTTCCTGAGGGAATTGTG	60.0	410	INTERGENIC	--	--	--
Ca1_P2_3281135	Ca1	3281135	GTATATATATATATATATATATATATATAAATACATATATATATATATATATATATATATATATATA/GTATATATATATATATATATATATATATATAAATACATATATATATATATATATATATATATATA	TAATTTTTGCTAATAAGTGCCTTG	TTTTTATAAGCAATTATGCATTCAA	57.3	182	INTRON	Ca_00398	--	LBD
Ca1_P2_32817874	Ca1	32817874	T/TGATTTATTTA	GCCTGTGTGGCTGGTTTAAT	AAAGGTCTCATTTTCCCACG	59.0	223	INTERGENIC	--	--	--
Ca1_P2_32848422	Ca1	32848422	AC/A	CAAGGCTCAAAGGGGCTAGT	GGAGGCAACCCACAAGTAGA	60.1	547	INTERGENIC	--	--	--
Ca1_P2_32851240	Ca1	32851240	TATAATAATAATAATAATAATAATAATAATAATAATAA/TATAATAATAATAATAATAATAATAATAA	CAAGCTTTGAAAGGCCTGAG	ATAGACTAGGCCGTCCGTCA	59.7	618	INTERGENIC	--	--	--

INDEL marker IDs	Chromosomes/unanchored scaffolds	Physical positions (bp)	InDels (*Kabuli* reference genome-CDC Frontier/PII)	Forward primers (5'-3')	Reverse primers (5'-3')	Anneling temperature (0C)	Expected amplified product size (bp)	Structural annotation		Functional annotation	
								Sequence components of *kabuli* genome	*Kabuli* gene accession IDs	NCBI-KOG	TFs
Ca1_P2_32862144	Ca1	32862144	AATATATATATATATATATATATA/AATATATATATATATATATATATATATATATATATA	AAAAGCCTCCGAACCAAAAT	ATTTCTGCACGAATTGGACC	59.9	734	INTERGENIC	--	--	--
Ca1_P2_32905999	Ca1	32905999	AATATAT/AATAT	TTGCTTCGTCAGCACAATTC	TCGTATGTCATAGCGGTGCT	59.3	920	INTERGENIC	--	--	--
Ca1_P2_32906329	Ca1	32906329	TTATATATATATATATAT/TTATATATATATAT	TCATTAAATCAAAGGCACACAAA	TCGTATGTCATAGCGGTGCT	59.3	488	INTERGENIC	--	--	--
Ca1_P2_32911601	Ca1	32911601	AAATAATAATAATAA/AAATAATAATAATAATAA	CAAAATGGAGCAAGAAACAGG	CAAGTTCAACGAGCGCCTAT	60.4	892	INTERGENIC	--	--	--
Ca1_P2_32933974	Ca1	32933974	TTTCGTTC/TTTC	TAACCATCGTGGGGTTGTTT	TTTTAGCTTTCCAACGCCTG	60.4	851	INTERGENIC	--	--	--
Ca1_P2_33018948	Ca1	33018948	CTT/CT	TTCGCAACTCCACTCTTCCT	CACATCATCACAACATGCCA	60.0	481	INTERGENIC	--	--	--
Ca1_P2_33066887	Ca1	33066887	TT/TTAT	TATTGCCTCTCCCTCTCCCT	TTCCATGACGGTGAACACAT	59.8	517	DRR	Ca_19437	J	GATA
Ca1_P2_33084283	Ca1	33084283	T/TC	CCGCACTTGGAGAAAAGAAA	AGGATTCCACGAGCAAACAG	60.3	443	INTERGENIC	--	--	--
Ca1_P2_33105635	Ca1	33105635	TC/T	TGCCAAGAACGATAAAAGCA	TTCTGCATTTTCCTTGAGCA	59.5	101	DRR	Ca_19438	--	--
Ca1_P2_33129418	Ca1	33129418	ATATAAGCAGAAAATAAGTATAAGCAGAAAAT/ATATAAGCAGAAAATAAGTATAAGCAGAAAATAAGTATAAGCAGAAAAT	TTTCATAGGGCTTTTCCACG	CGGATTGATGAATCAGGGTT	59.8	878	INTERGENIC	--	--	--
Ca1_P2_33154204	Ca1	33154204	AGCGCTGTAAAATGGGCGCTGTAAAATGGGCGCTGT/AGCGCTGTAAAATGGGCGCTGTAAAATGGGCGCTGTAAAATGGGCGCTGT	TTCAATGAAAGCGCATGTGT	GGGATTGACCAAAACTGTCG	60.4	344	INTERGENIC	--	--	--
Ca1_P2_33193839	Ca1	33193839	GATATATATATAT/GATATATATATATAT	CGCAATGAAGGTGGTAGGTT	TAATCCCCCTCACTCCCTTT	59.8	758	INTRON	Ca_19440	A	--

INDEL marker IDs	Chromosomes/unanchored scaffolds	Physical positions (bp)	InDels (*Kabuli* reference genome-CDC Frontier/PII)	Forward primers (5'-3')	Reverse primers (5'-3')	Anneling temperature (0C)	Expected amplified product size (bp)	Structural annotation		Functional annotation	
								Sequence components of *kabuli* genome	*Kabuli* gene accession IDs	NCBI-KOG	TFs
Ca1_P2_33240386	Ca1	33240386	T/TG	AAACGCAATCCTTGAGTTCG	CTATCTAGCGTCGGAGTCGG	60.0	280	INTERGENIC	--	--	--
Ca1_P2_33247360	Ca1	33247360	GTTTTTATTT/GTTTTTATTTTTTATTT	TCCCCCTTTTGTCATCAGAC	CATGCCAACAACAGAAGTTGA	59.7	499	INTERGENIC	--	--	--
Ca1_P2_33329257	Ca1	33329257	AAA/AAAGAA	TTTGTGAGTTGCGAAGTAGAATTT	CTCCAAGGCACACACAAATG	60.2	599	INTRON	Ca_19441	--	MIKC
Ca1_P2_33336930	Ca1	33336930	GAAAAA/GAAAA	CAATGGCAACAAGGTCAATG	TCACATCTGTTGGTAACGGG	59.4	856	INTRON	Ca_19441	--	MIKC
Ca1_P2_33344607	Ca1	33344607	AG/AGG	CCACCCAGAAAGGAGTTCAA	TAAAAGGAAGGCCAAGCTCA	60.0	321	INTRON	Ca_19441	--	MIKC
Ca1_P2_33365804	Ca1	33365804	AC/A	CAATTTTGGTCCCTCTCTCTTTT	TTTTTCTCCCGATTTGGTCA	60.4	552	INTRON	Ca_19441	--	MIKC
Ca1_P2_33407768	Ca1	33407768	TA/TAA	TGGCCATCAATTAATCCACTC	GATCTGCAACTGGGGGTAAA	59.9	230	INTERGENIC	--	--	--
Ca1_P2_33445892	Ca1	33445892	TTATATATATATATAT/TTATATATATATAT	GGGCGGATCTTTTCTAGGAC	ATTCACTTCTCGAGCGCATT	60.0	378	INTERGENIC	--	--	--
Ca1_P2_33476619	Ca1	33476619	CTTT/CTT	ACCAGCAACCATAAAAACGC	AATTGGTTTCATGACCTGCAC	59.9	450	INTERGENIC	--	--	--
Ca1_P2_33590290	Ca1	33590290	T/TTCA	GCAAATGTCTGGTTGCAAAA	TCAGTTGCCTTTGAAATCCC	60.1	940	INTERGENIC	--	--	--
Ca1_P2_33670245	Ca1	33670245	TAAAAAAAAAAA/TAAAAAAAAAA	AATCACACTCGATCCAACCC	GCATCTTTGATTCCTCCCAA	60.0	665	INTERGENIC	--	--	--
Ca1_P2_33722309	Ca1	33722309	GAAAAAAAA/GAAAAAAAAA	GGGACGTACACAAGAGGGAA	ATGAGCGAGGTAGAAGCCAA	60.0	359	DRR	Ca_19456	--	--

INDEL marker IDs	Chromosomes/unanchored scaffolds	Physical positions (bp)	InDels (*Kabuli* reference genome-CDC Frontier/PII)	Forward primers (5'-3')	Reverse primers (5'-3')	Anneling temperature (0C)	Expected amplified product size (bp)	Structural annotation		Functional annotation	
								Sequence components of *kabuli* genome	*Kabuli* gene accession IDs	NCBI-KOG	TFs
Ca1_P2_33746473	Ca1	33746473	GTATATATATATATATAT/GTATATATATAT	CCAATCGATTTCCTCCAAAA	TTTCATCAAAAACCCCCTTG	59.8	779	INTERGENIC	--	--	--
Ca1_P2_33765021	Ca1	33765021	AAATAAAATTAATAAATAATAAAAT/AAATAAAAT	GCGACCGAATATGGAACAGT	TAGGCTAGGTCGTCCGTTTG	60.3	301	INTERGENIC	--	--	--
Ca1_P2_33777775	Ca1	33777775	ATTT/ATT	CATGCTCAATACACGGATGG	AACGTTCTTGAGTGGCTTGG	60.3	870	INTERGENIC	--	--	--
Ca1_P2_33781062	Ca1	33781062	CTTTTTTT/CTTTTTTTTT	AATTTCCCAATCACCCACAA	TCCACAATGTCTCCCAACAA	59.9	517	CDS (FRAME SHIFT)	Ca_19460	--	--
Ca1_P2_3391465	Ca1	3391465	GATAATAATAATAATAATAATAATAATAAT/GATAATAATAATAATAATAATAATAAT	CAGGAGCGACACAAGACAAA	TCGTTCCTTCAGGAAGTCGT	59.8	389	INTRON	Ca_00410	--	S1Fa-like
Ca1_P2_34003692	Ca1	34003692	TTATATATATATATATATATATATATATA/TTATATATATATATATATATATATATATATA	CGGAGGGTAACCAAAGATGA	TCTGAACGAAACCACTGCAC	59.9	852	INTRON	Ca_27634	--	--
Ca1_P2_34057382	Ca1	34057382	C/CA	GGGGATTAAGTGCTTGGACA	AAAGAAAGGCACTAAGGCCC	59.7	156	INTERGENIC	--	--	--
Ca1_P2_34136220	Ca1	34136220	CTTTGGT/CT	GGGCATGTTTCCTCTGATGT	TGATGTGGGTCACCAAGAAA	59.9	803	DRR	Ca_25472	QI	bHLH
Ca1_P2_34137295	Ca1	34137295	CTTT/CTT	GCAAGCATTGTCCTCGACTT	TGTTACCAGGCTGAACCACA	60.2	845	DRR	Ca_25472	QI	bHLH
Ca1_P2_34143628	Ca1	34143628	TTA/TTAAAATGTTCTATA	CCCCCTAAACAAATTCGACA	TTTATGATTTACGCCCCTCG	59.9	714	INTERGENIC	--	--	--
Ca1_P2_34147315	Ca1	34147315	C/CTG	TCAAATCGATTGGGACATCA	TACTCGCCAAACTCTCACGA	59.6	900	INTERGENIC	--	--	--
Ca1_P2_34167302	Ca1	34167302	CTTT/CTT	CATCATGCCAATGCATCATAA	GTACCTCAAACGGAGGGGAT	60.2	804	INTERGENIC	--	--	--

INDEL marker IDs	Chromosomes/unanchored scaffolds	Physical positions (bp)	InDels (*Kabuli* reference genome-CDC Frontier/PII)	Forward primers (5'-3')	Reverse primers (5'-3')	Anneling temperature (0C)	Expected amplified product size (bp)	Structural annotation		Functional annotation	
								Sequence components of *kabuli* genome	*Kabuli* gene accession IDs	NCBI-KOG	TFs
Ca1_P2_34182031	Ca1	34182031	TC/T	CGTGTCTCCACCTCTCAACA	CAAAATCACGACCACCACTG	60.0	217	INTERGENIC	--	--	--
Ca1_P2_34200799	Ca1	34200799	CTTTTTTTTTTT/CTTTTTTTTTTTT	AGGTTGGTGCCATTATGAGG	AGAGGAAGCCCTACGTGGAT	60.1	425	INTERGENIC	--	--	--
Ca1_P2_34286273	Ca1	34286273	ATATTTTTTATTTTTTATTTTTT/ATATTTTTTATTTTTT	TATGCATGCGGACAAGAGTC	TTGGAAAAGCCCACCTACAC	60.0	448	INTERGENIC	--	--	--
Ca1_P2_34313358	Ca1	34313358	GTG/GTGATG	GGGGGTAATAGGATGTCACG	TTATCGCAGATTGTCCCACA	60.1	590	INTRON	Ca_25478	R	ARR-B
Ca1_P2_34320054	Ca1	34320054	CGACCTCCAGA/CGA	GCATGGAGGGGGACTTTTAT	GTTTCTTTGGTTCAAGGGCA	60.1	565	DRR	Ca_25479	C	MYB_related
Ca1_P2_34328412	Ca1	34328412	AA/AATTA	AACGCTTCTAAAGAACGCCA	TGGATCCGTGATCCTTCTTC	60.0	313	INTERGENIC	--	--	--
Ca1_P2_34332252	Ca1	34332252	GA/G	AATTGGAAGCAGGGGTTTTT	GGATTGTACCCTTGGGGTTT	59.9	849	INTERGENIC	--	--	--
Ca1_P2_34348577	Ca1	34348577	TAAA/TAA	TTTGTGGACACGACTACCGA	TGGGCAAAAGGAATTTCAAG	60.0	252	INTERGENIC	--	--	--
Ca1_P2_34351954	Ca1	34351954	GAAAA/GAAAATAAAAAA	AGAGCTACCACCGCAAGAAA	CAGACGCAAGTGTGGAAGAA	60.0	743	INTERGENIC	--	--	--
Ca1_P2_34352370	Ca1	34352370	GTATATATATATATATATATATATATATATATATATATATA/GTATATATATATATATATATATATATATATATATATATATA	TTCTTCCACACTTGCGTCTG	TTTGACTAGTTTGTCCCCGC	60.1	463	INTERGENIC	--	--	--
Ca1_P2_34364559	Ca1	34364559	T/TCTC	TGGGAAAACCTTGACCAATC	TCATGAGTGGGAGTGAACCA	60.1	865	INTERGENIC	--	--	--
Ca1_P2_34371294	Ca1	34371294	AAAGAAG/AAAGAAGAAG	AAACGGGTGCAAGAAGATTG	TCTTTACGACCTTTGTGGGC	60.1	233	INTERGENIC	--	--	--

INDEL marker IDs	Chromosomes/unanchored scaffolds	Physical positions (bp)	InDels (*Kabuli* reference genome-CDC Frontier/PII)	Forward primers (5'-3')	Reverse primers (5'-3')	Anneling temperature (0C)	Expected amplified product size (bp)	Structural annotation		Functional annotation	
								Sequence components of *kabuli* genome	*Kabuli* gene accession IDs	NCBI-KOG	TFs
Ca1_P2_34386272	Ca1	34386272	GTTT/GTTTT	AATCTGGAGGGCGGTAAGTT	ATATCGGTCAAACGCACACA	60.0	413	INTERGENIC	--	--	--
Ca1_P2_34428919	Ca1	34428919	TCACA/TCA	GGGAGATTTTTGTGGTAAGCC	GCATTTCCGCAGTTTCACTT	60.3	661	INTERGENIC	--	--	--
Ca1_P2_34431310	Ca1	34431310	TCC/TC	AAAAGACCAAATCGAGAAGAAAAA	TTTGCATCACTCGACAATGAG	59.9	388	INTERGENIC	--	--	--
Ca1_P2_34647162	Ca1	34647162	TGG/TG	TGGATGAATGCTTGGATGAA	TAGTCCAACAATGGCCAACA	60.0	978	INTERGENIC	--	--	--
Ca1_P2_34647577	Ca1	34647577	CCA/CCATTAACA	GGGAGCAAGAAGTTGGTTGA	TAGTCCAACAATGGCCAACA	60.0	132	INTERGENIC	--	--	--
Ca1_P2_34666828	Ca1	34666828	TAAAAAAAAA/TAAAAAAAA	TGTTTCAGGATGTCGACCAA	GTTACCGCAGAGGGACGTAA	60.1	640	INTERGENIC	--	--	--
Ca1_P2_34667053	Ca1	34667053	ATTTTTTTTTT/ATTTTTTTTT	AGACTGCTCGGTCCAAAGAA	GTTACCGCAGAGGGACGTAA	60.1	264	INTERGENIC	--	--	--
Ca1_P2_34747960	Ca1	34747960	ACTCTCTCTCTCTCTCTCTCTC/ACTCTCTCTCTCTCTCTC	GTGGTTGCATTGGAGAAGGT	TTTCATCAGCAAAAGCAACG	60.0	371	INTERGENIC	--	--	--
Ca1_P2_34812970	Ca1	34812970	ATTTTTTTTTTT/ATTTTTTTTTT	CACAACAATGTGGGTTGAGG	ACCAAGAACAAAATGGTGCC	59.8	428	INTERGENIC	--	--	--
Ca1_P2_34818334	Ca1	34818334	C/CA	GTGGTAATCGCGTTGAAGGT	TCTGACTTAAGGCCCAATCG	60.2	314	INTERGENIC	--	--	--
Ca1_P2_34838110	Ca1	34838110	AATATA/AATATATATA	TGAGTTTATTGGGGGTGGAG	CAAAATTCATCCCATTCGCT	59.9	455	INTERGENIC	--	--	--
Ca1_P2_34870195	Ca1	34870195	TCAGCA/TCAGCAGCA	ACGTTGCATGGAAAAGAACC	TGTCCAAATAAAGGGGACCA	60.2	575	INTRON	Ca_21411	--	MYB_related

INDEL marker IDs	Chromosomes/unanchored scaffolds	Physical positions (bp)	InDels (*Kabuli* reference genome-CDC Frontier/PII)	Forward primers (5'-3')	Reverse primers (5'-3')	Anneling temperature (0C)	Expected amplified product size (bp)	Structural annotation		Functional annotation	
								Sequence components of *kabuli* genome	*Kabuli* gene accession IDs	NCBI-KOG	TFs
Ca1_P2_34881849	Ca1	34881849	T/TTTA	GAATGGTTTTGGGCTGAGAA	AAAAATTTTGAAAACGGACAAA	57.8	654	INTERGENIC	--	--	--
Ca1_P2_34952469	Ca1	34952469	AAATAATAATAATAATAA/AAATAATAATAATAA	CTTCAACCAATGCCTCCACT	AAAAAGGAGGTGCGACAAAA	59.7	603	INTERGENIC	--	--	--
Ca1_P2_34954237	Ca1	34954237	ATACT/AT	TGCTTATTGTGGCTGCTTTG	TGTGTGCATGCATTTGAAGA	59.8	669	INTERGENIC	--	--	--
Ca1_P2_34962921	Ca1	34962921	CAAAAAAA/CAAAAAA	CCATCTTGCCCTTTTCTTGA	CGTGGGAAAAAGGGGTATTT	60.0	725	INTERGENIC	--	--	--
Ca1_P2_34963287	Ca1	34963287	AGG/AG	CCAACAACATGCGTTGTCAT	CGTGGGAAAAAGGGGTATTT	60.0	606	INTERGENIC	--	--	--
Ca1_P2_34971020	Ca1	34971020	CAAAAAAA/CAAAAA	AAATACCCCTTTTTCCCACG	TGAAAACGCTCAATGCCATA	60.2	689	INTERGENIC	--	--	--
Ca1_P2_34978712	Ca1	34978712	GT/GTT	ATGGCATGGTGGATTTTGTT	CTAGAAAGTTGCAGCCCAGG	60.0	525	INTRON	Ca_21407	--	--
Ca1_P2_34986077	Ca1	34986077	ATTT/ATT	TGGCAATGGGCTAATTTCTC	TGCATAATCGATTGGCACAT	59.9	120	DRR	Ca_21406	--	--
Ca1_P2_35042644	Ca1	35042644	TC/T	ATAGTAGTTGCGGTGGTCGG	GGACCAGTTTTGGGTGATTG	60.2	386	INTERGENIC	--	--	--
Ca1_P2_35044743	Ca1	35044743	AG/A	GTACCGAATAGCCCGCATAA	CCAGTCCATATCGGGTCAGT	59.8	654	INTERGENIC	--	--	--
Ca1_P2_35096364	Ca1	35096364	AT/A	TTGATGTGGTCGGAGATTGA	ACAATTTTCAGCAACCCGAG	60.1	588	INTERGENIC	--	--	--
Ca1_P2_35153435	Ca1	35153435	GTTTTTTTTTTTTT/GTTTTTT	TTTGGTTTCCGATTGCTAGG	GGTTGTATGGCTCCCAAGAA	59.9	409	INTERGENIC	--	--	--

INDEL marker IDs	Chromosomes/unanchored scaffolds	Physical positions (bp)	InDels (*Kabuli* reference genome-CDC Frontier/PII)	Forward primers (5'-3')	Reverse primers (5'-3')	Anneling temperature (0C)	Expected amplified product size (bp)	Structural annotation		Functional annotation	
								Sequence components of *kabuli* genome	*Kabuli* gene accession IDs	NCBI-KOG	TFs
Ca1_P2_35158359	Ca1	35158359	C/CT	ATCTTGGTTGGCTTTTGTCG	GGATTCGAAGTGAAACTCATCC	59.9	471	INTERGENIC	--	--	--
Ca1_P2_35160383	Ca1	35160383	GT/G	CATTGCTCCCAAGTGCTTTT	CAATTCCCACAGGTACGCTT	60.0	542	INTERGENIC	--	--	--
Ca1_P2_35165919	Ca1	35165919	GTTTTTT/GTTTTTTTTTTTT	TCATCGCAGGTGTACCATGT	CACCTAAGGGAATCGGTCAA	59.9	377	INTERGENIC	--	--	--
Ca1_P2_35166209	Ca1	35166209	TAAAAAAAAAA/TAAAAAAAA	AAGAGAGGTCACTTTGGCGA	CAATTCCCACAGGTACGCTT	60.0	852	INTERGENIC	--	--	--
Ca1_P2_35222825	Ca1	35222825	TAAAAAAAA/TAAAAAAAAAA	TTTGCATGCATCAAGTTCCT	AGGTGGTTTCCTTCCTTGCT	60.1	492	INTERGENIC	--	--	--
Ca1_P2_35235628	Ca1	35235628	TATAATAATAATAATAATAATAATAATAATAATAA/TATAATAATAATAATAATAATAATAATAATAATAA	GGGATGCGACCTTGTACTGT	TCCTCCTCTTTTGGCACATT	59.7	463	INTERGENIC	--	--	--
Ca1_P2_35258820	Ca1	35258820	A/AC	AGCAGAGATCCTAGGGTGGG	CCACCGACACCTCTCAATTT	60.0	870	INTERGENIC	--	--	--
Ca1_P2_35261821	Ca1	35261821	ATT/AT	TGGATTGTCATGGTCTGGAA	AATGGTCTTGAATTTTGCCG	59.9	640	INTERGENIC	--	--	--
Ca1_P2_35266220	Ca1	35266220	ATAT/ATATTAT	CGCGGTAGAGTTATTGGAAAA	TGGGGATACGTAGGAGCAAG	60.1	682	INTERGENIC	--	--	--
Ca1_P2_35281570	Ca1	35281570	TC/T	ATGACACGTCATTCGTTTCG	GGTTCGAGACATCCATAATTCA	58.9	733	INTRON	Ca_26099	QI	bHLH
Ca1_P2_35281940	Ca1	35281940	TA/TAA	AAACGTGATCTATACAGTTTCCAAAA	TTCACATCTGGAAACAACGC	59.7	529	INTRON	Ca_26099	QI	bHLH
Ca1_P2_35283797	Ca1	35283797	TGGGGG/TGGGG	GGGTGTGTTTGATAGGAGGG	CTCCCCTTCCATCTCCTCTT	59.6	139	INTERGENIC	--	--	--

INDEL marker IDs	Chromosomes/unanchored scaffolds	Physical positions (bp)	InDels (*Kabuli* reference genome-CDC Frontier/PII)	Forward primers (5'-3')	Reverse primers (5'-3')	Anneling temperature (0C)	Expected amplified product size (bp)	Structural annotation		Functional annotation	
								Sequence components of *kabuli* genome	*Kabuli* gene accession IDs	NCBI-KOG	TFs
Ca1_P2_35284017	Ca1	35284017	ATTT/ATT	GAGATGGAAGGGGAGGAAAG	GCACAAAAGAAAAATACAAGTCCA	59.6	273	INTERGENIC	--	--	--
Ca1_P2_35284253	Ca1	35284253	TT/TTGT	GAGATGGAAGGGGAGGAAAG	TCTGATCAAATATGTGGCAGAAA	59.6	657	INTERGENIC	--	--	--
Ca1_P2_35321165	Ca1	35321165	GTTT/GTTTT	CATCATTCGTTTCAAATTTGTTG	ACGAAGGTTTGAGACATCCA	58.1	586	INTRON	Ca_26101	QI	bHLH
Ca1_P2_35325462	Ca1	35325462	CTTT/CTTTT	CACACTCAAGTGCCTCGAAA	AACACGGGTTACGTCCAGTC	59.9	411	INTERGENIC	--	--	--
Ca1_P2_35353239	Ca1	35353239	GAA/GAAA	CCAACAACTTCCTTTCGAGC	AGCCCATTCAGATTTTGTGG	59.9	147	INTERGENIC	--	--	--
Ca1_P2_35540299	Ca1	35540299	AA/AATA	GGGGTGCTCAACTTGTTTGT	GGCCAAGGTAAGGGTGAGAT	60.3	254	INTERGENIC	--	--	--
Ca1_P2_3556309	Ca1	3556309	TTATATATATACATATATATAT/TTATATATAT	TGACCTCATGGATACCCACA	AAGGTCGCATCTTCATCCTG	60.2	371	INTERGENIC	--	--	--
Ca1_P2_35564952	Ca1	35564952	CACAA/CACAACAA	AGCGATGCATGTCTCAAGTG	GCCCTTCTTCTTTTCCTGGT	59.7	427	INTERGENIC	--	--	--
Ca1_P2_35585435	Ca1	35585435	ATGTGTGTGTGTGTGTGTGTGTGTGT/ATGTGTGTGTGTGTGTGTGTGTGT	ATGTTGAGGGTCATGCTTCC	GCAAATGAGATGAACAGCGA	60.0	690	INTERGENIC	--	--	--
Ca1_P2_35598879	Ca1	35598879	CTTT/CTT	ATTCCCCATTCTTAATGCCC	CGGTGCTAGTGGACCAATTT	60.0	949	INTERGENIC	--	--	--
Ca1_P2_35610504	Ca1	35610504	ATGT/ATATATATGTGT	TATTTTGGATTTTGGCTCGG	TTTTCCTTTCTTTTTCCCCC	59.4	261	INTERGENIC	--	--	--
Ca1_P2_35663461	Ca1	35663461	AG/AGG	CACTTTCTTCGAGTAGGCCG	TGGAGGTGAACAACTTTCCC	59.9	855	INTERGENIC	--	--	--

INDEL marker IDs	Chromosomes/unanchored scaffolds	Physical positions (bp)	InDels (*Kabuli* reference genome-CDC Frontier/PII)	Forward primers (5'-3')	Reverse primers (5'-3')	Anneling temperature (0C)	Expected amplified product size (bp)	Structural annotation		Functional annotation	
								Sequence components of *kabuli* genome	*Kabuli* gene accession IDs	NCBI-KOG	TFs
Ca1_P2_35679606	Ca1	35679606	CAAAAAAAAAAA/CAAAAAAAAAA	GTGCACAACCTTGTGGTGTC	TAGCCAACATGCCATACGTC	59.6	604	INTERGENIC	--	--	--
Ca1_P2_35711382	Ca1	35711382	GCTTACTTA/GCTTA	CAAATGGAGACGTGAGGGTT	CGCACCAAATAAACCGAAGA	61.0	222	DRR	Ca_24110	K	--
Ca1_P2_35743539	Ca1	35743539	GAAAAAAAAAA/GAAAAAAAAAAA	TCACATCACCCTAAATCAACTCA	TTGGAGTTGTGCCAATGTGT	60.0	511	INTERGENIC	--	--	--
Ca1_P2_35767551	Ca1	35767551	GTGAT/GT	TGGGAGATGCGATATGTTTT	ACAACTTGTGATTTTCGGGG	59.8	867	INTERGENIC	--	--	--
Ca1_P2_35888969	Ca1	35888969	AAA/AAATATAA	CATTCATTGTGCCTTTGGTG	GACGTCTGACCCATTCGTTT	60.0	792	INTERGENIC	--	--	--
Ca1_P2_35896608	Ca1	35896608	TTATATATATATATATATATATATATATATATA/TTATATATATATATATATATATATATATATATATATA	TTTGACCGATGCTTAGTCCC	TTTTGTGCACGACAATCACA	59.7	592	INTERGENIC	--	--	--
Ca1_P2_35899211	Ca1	35899211	TTTTAGACAC/TTTTAGACACATTTAGACAC	CACCTCCCCTGAGTTCGTTA	GCAAGCTCCAAATGGATTACA	60.1	404	INTERGENIC	--	--	--
Ca1_P2_35902931	Ca1	35902931	AATATATATATA/AATATATATA	TTGTTTGTTCTCTCACTCGATCA	TCCATCATACCCAGAGCAAA	59.1	595	INTERGENIC	--	--	--
Ca1_P2_35946848	Ca1	35946848	AT/ATTTT	GTTTCGTAGCGCCGTTTATC	ATTTCTTGCCCTTTGTCCCT	59.9	531	INTERGENIC	--	--	--
Ca1_P2_35947069	Ca1	35947069	G/GTGTCTCC	AGGGACAAAGGGCAAGAAAT	CCAAATTGACCTTCTTGGGA	59.9	269	INTERGENIC	--	--	--
Ca1_P2_35977319	Ca1	35977319	TA/TAA	AACTCGTTGGAACTTCCCTCT	GCAAGCAGCCTTGTTGACTA	59.2	540	INTERGENIC	--	--	--
Ca1_P2_36017731	Ca1	36017731	CATATATATATATATATATATATATATATA/CATATATATATATATATATATATA	TCTAAGTCATTGCATTTGTGCT	CGAAGTTGTTTTTGGGGCTA	60.1	256	INTERGENIC	--	--	--

INDEL marker IDs	Chromosomes/unanchored scaffolds	Physical positions (bp)	InDels (*Kabuli* reference genome-CDC Frontier/PII)	Forward primers (5'-3')	Reverse primers (5'-3')	Anneling temperature (0C)	Expected amplified product size (bp)	Structural annotation		Functional annotation	
								Sequence components of *kabuli* genome	*Kabuli* gene accession IDs	NCBI-KOG	TFs
Ca1_P2_36044248	Ca1	36044248	CGG/CG	CGATTTCCCTGCATGTTTTT	GCCTGTGATAGGCGGTACAT	60.0	540	INTERGENIC	--	--	--
Ca1_P2_36063199	Ca1	36063199	TCC/TCCCC	CGTTCGTGGTCCCTTAACTT	TTGCGTTACGGATTTGATGA	60.1	227	INTERGENIC	--	--	--
Ca1_P2_36146895	Ca1	36146895	AATTATTATTATTATTATTATTATTATTATTATTATTATTATTATTA/AATTATTATTATTATTATTATTATTATTATTATTATTATTATTATTATTA	TTCTAAGGTCCAGCAACCTCT	GATTGATAGGCCTTGCCCTT	60.4	185	INTERGENIC	--	--	--
Ca1_P2_36166710	Ca1	36166710	CATTATTATTATTATTATTATTATTATTA/CATTATTATTATTATTATTATTATTA	TCGTTGGCCTATGGTACTCC	CCAACGATTCATACAGGGCT	60.0	574	INTERGENIC	--	--	--
Ca1_P2_36171485	Ca1	36171485	TA/T	TTCGGTTGTTGTTCACTCCA	TAAAACCCATTGGTCCCTTT	58.3	749	INTERGENIC	--	--	--
Ca1_P2_3622177	Ca1	3622177	ATT/A	TTAGCTACGTTTTGGGTGGG	CAGCCAGCTAGATGGTGACA	60.0	488	INTRON	Ca_00444	T	bZIP
Ca1_P2_36271104	Ca1	36271104	TTATATATATATATATATATATATATAT/TTATATATATATATATATATATAT	TGTTATGGGTGAAATGGCAA	AGGGAGGTTTGTGTTTGACG	60.0	387	INTERGENIC	--	--	--
Ca1_P2_36283002	Ca1	36283002	ACC/ACCC	TGAGTTTGTTTGAGAGGATTTGTT	CAAGCGCACTCACTATGCTC	59.8	520	INTERGENIC	--	--	--
Ca1_P2_3636721	Ca1	3636721	TAA/T	TTCATGGAACATTCTGCCAA	CGACAAGTTTGAGGGGTTGT	60.0	350	INTERGENIC	--	--	--
Ca1_P2_36415628	Ca1	36415628	CATATATATATATATATATATATATAT/CATATATATATATATATATATATAT	AACCAAGGCAAACAAGAGGA	TTGTTTAAGCCCTTTGAGCC	59.3	592	INTERGENIC	--	--	--
Ca1_P2_36467494	Ca1	36467494	AGGGG/AGGGGG	AGAGCACGGAGAAGGAACAA	CCCGAGTTGGGTGAACTATG	60.4	571	INTERGENIC	--	--	--
Ca1_P2_36473784	Ca1	36473784	AA/AATA	TTTTGGATTTCGGTTTTGAA	TTGACCAAAATTCAAGGAGACA	59.6	284	DRR	Ca_21691	--	--

INDEL marker IDs	Chromosomes/unanchored scaffolds	Physical positions (bp)	InDels (*Kabuli* reference genome-CDC Frontier/PII)	Forward primers (5'-3')	Reverse primers (5'-3')	Anneling temperature (0C)	Expected amplified product size (bp)	Structural annotation		Functional annotation	
								Sequence components of *kabuli* genome	*Kabuli* gene accession IDs	NCBI-KOG	TFs
Ca1_P2_36720036	Ca1	36720036	AATATATATATATATATATATATATATATATATATATATATATAT/AATATATATATATATATATATATATATATATATATATATATATATATATAT	TACCAATCGGATGCAAATGA	CTGCAAGTACCGCTTCCTTC	60.0	726	INTERGENIC	--	--	--
Ca1_P2_36724754	Ca1	36724754	CTTTTTTTT/CTTTTTTTTTT	TTGCACCACACTTCTTCACC	CTTATTTCGACGCTTTTCGC	60.0	279	INTERGENIC	--	--	--
Ca1_P2_36726460	Ca1	36726460	GAAA/GAAAA	ACAGGAACGACCGAGAGAAA	ATAGTTTTTGGGCGGGCTAC	60.3	502	INTERGENIC	--	--	--
Ca1_P2_36727739	Ca1	36727739	TCC/TC	AGGCTGCATTTTAGTCCACG	ACGACCACTGCAAACACAAA	60.2	218	INTERGENIC	--	--	--
Ca1_P2_3677073	Ca1	3677073	ATTTTTT/ATTTTTTT	GCACTTTCCGTTCTCGTGAT	TCGATTGTTTGGACGATGTG	60.5	741	INTERGENIC	--	--	--
Ca1_P2_36958396	Ca1	36958396	TAAAAAAAAAA/TAAAAAAAAAAA	TTCAGTTGCACGAGAATTGC	GTGGCTAGGACGGTTGAAAA	60.1	784	DRR	Ca_21823	--	--
Ca1_P2_37050284	Ca1	37050284	GAAAAAAAAAA/GAAAAAAAAAAA	TAGCTGATGATCTTTGGGGG	TTGAACCGTTCTCCTTCACC	60.1	379	INTERGENIC	--	--	--
Ca1_P2_37075088	Ca1	37075088	TTAAA/T	GCCCTCAGATGAGAGTCAGG	ATTCTGCAGCACGGATCTCT	60.0	830	INTERGENIC	--	--	--
Ca1_P2_37078511	Ca1	37078511	TTAT/TTATAT	CCATTCCAAAAAGGAGTTGG	TGAAAATGTTGGAGTGATGTGA	59.0	938	INTERGENIC	--	--	--
Ca1_P2_37078904	Ca1	37078904	TTAAATAAATAAATA/TTAAATAAATAAATAAATAAATA	TGTGACACCCTAAACCCTAAGAA	TTGATTGCTTGCGTGTTCTC	60.0	499	INTERGENIC	--	--	--
Ca1_P2_37122051	Ca1	37122051	CAAAAAAAA/CAAAAAAA	CGTATCTCGTATCACCGACG	AGTTGCGCTCCTCCTAATCA	60.0	873	INTERGENIC	--	--	--
Ca1_P2_37122104	Ca1	37122104	CAAAAAAAAAA/CAAAAAAAAAAAAA	CGTATCTCGTATCACCGACG	AGTTGCGCTCCTCCTAATCA	60.0	873	INTERGENIC	--	--	--

INDEL marker IDs	Chromosomes/unanchored scaffolds	Physical positions (bp)	InDels (*Kabuli* reference genome-CDC Frontier/PII)	Forward primers (5'-3')	Reverse primers (5'-3')	Anneling temperature (0C)	Expected amplified product size (bp)	Structural annotation		Functional annotation	
								Sequence components of *kabuli* genome	*Kabuli* gene accession IDs	NCBI-KOG	TFs
Ca1_P2_37122520	Ca1	37122520	AGG/AG	TGCAATCATTGGTGCTTAGG	AAAAAGCGCCGTAAAATGAG	59.4	710	INTERGENIC	--	--	--
Ca1_P2_37122699	Ca1	37122699	GTTT/GTT	GGAGGAGCGCAACTTAATCA	AAAAAGCGCCGTAAAATGAG	59.4	372	INTERGENIC	--	--	--
Ca1_P2_37122810	Ca1	37122810	TACAAC/TACAACAAC	GGAGGAGCGCAACTTAATCA	AAAAAGCGCCGTAAAATGAG	59.4	372	INTERGENIC	--	--	--
Ca1_P2_37125192	Ca1	37125192	GATGGAGATGGTTTGCAATGGAGATG/GATGGAGATG	AGCGGTTTAGGGTTTAGGGA	CCAAATGTGCATACGAACCA	60.4	113	INTERGENIC	--	--	--
Ca1_P2_37128515	Ca1	37128515	ATTTTTTT/ATTTT	CAATTCCCACAGGTACGCTT	TTGGTCTCATCAATGATTGTAAAAA	59.8	568	INTERGENIC	--	--	--
Ca1_P2_37131203	Ca1	37131203	ATTTTT/ATTTT	TTGAAAGGTGCGTAAATAAAGGA	CAATTGAGAATTTATTTGATGACGA	59.4	898	INTERGENIC	--	--	--
Ca1_P2_37159439	Ca1	37159439	CTTTTTTTTTT/CTTTTTTTTT	TCACAACCAAATTGCTCTGC	GGGTGCCACCTGAAGAGATA	60.1	126	INTERGENIC	--	--	--
Ca1_P2_37212808	Ca1	37212808	TTCTCTCTCTCTCTCTCT/TTCTCTCTCTCTCTCT	CAACAGCAGGCTGTCAGAAA	AGGGGGTGAGTTTGAATGTG	59.8	392	INTERGENIC	--	--	--
Ca1_P2_37391477	Ca1	37391477	TTATATATATATATATATATATAT/TTATATATATATATATATATATAT	CTGTCAATTGAGATGCGACG	GCGATTACGGTACCACGAGT	60.0	600	INTERGENIC	--	--	--
Ca1_P2_37410251	Ca1	37410251	GAAAAAAAAAAA/GAAAAAAAAA	TGAATGAGGAGCACGTCTTG	CTGGGCAAAGGGGTATTTTT	60.2	401	INTERGENIC	--	--	--
Ca1_P2_37410363	Ca1	37410363	GAAAAAA/GAAAAAAA	TGAATGAGGAGCACGTCTTG	CTGTTGTTGCCAACGGTTTT	61.0	445	INTERGENIC	--	--	--
Ca1_P2_37412282	Ca1	37412282	CCATG/CCATGCATG	CGTGGATGATGCTTTTCTCA	TGCTTCTCATCTGCTTGGTG	60.1	580	DRR	Ca_21842	R	GRAS

INDEL marker IDs	Chromosomes/unanchored scaffolds	Physical positions (bp)	InDels (*Kabuli* reference genome-CDC Frontier/PII)	Forward primers (5'-3')	Reverse primers (5'-3')	Anneling temperature (0C)	Expected amplified product size (bp)	Structural annotation		Functional annotation	
								Sequence components of *kabuli* genome	*Kabuli* gene accession IDs	NCBI-KOG	TFs
Ca1_P2_37420598	Ca1	37420598	ATTTT/ATTT	GGGAAAAGGAAACCCAAAAA	TGAGTGAGGGAGACAAAGGG	60.2	950	INTERGENIC	--	--	--
Ca1_P2_37421082	Ca1	37421082	CCTCACTCACTCACTCACTCAC/CCTCACTCACTCACTCAC	ACTTGACGGGGACTCCACTA	ACATGTTCCTTGTGCTCGTG	59.8	637	INTERGENIC	--	--	--
Ca1_P2_37424329	Ca1	37424329	CATATATATATATATATATAT/CATATATATATATATAT	AAAAAGTATTGAACAAACACGCT	TGAAATCCAAATTAAATTGCTGAA	59.9	549	INTERGENIC	--	--	--
Ca1_P2_37424731	Ca1	37424731	ATTTTTT/ATTTTTTT	GCAATTTAATTTGGATTTCATGG	AGTTTGGTTCAATTGTTTCGAT	57.7	104	INTERGENIC	--	--	--
Ca1_P2_37424983	Ca1	37424983	AC/A	GCAATTTAATTTGGATTTCATGG	TCACATCCATCCACGGTACA	60.8	761	INTERGENIC	--	--	--
Ca1_P2_37425133	Ca1	37425133	TTATATATA/TTATATA	GCAATTTAATTTGGATTTCATGG	TCACATCCATCCACGGTACA	60.8	761	INTERGENIC	--	--	--
Ca1_P2_37425153	Ca1	37425153	GCA/GATCA	GCAATTTAATTTGGATTTCATGG	TCACATCCATCCACGGTACA	60.8	761	INTERGENIC	--	--	--
Ca1_P2_37425439	Ca1	37425439	TG/T	TGTACCGTGGATGGATGTGA	TGACACGTATCAATTTAGGGAGAA	59.9	222	INTERGENIC	--	--	--
Ca1_P2_37425620	Ca1	37425620	ACG/A	TGTACCGTGGATGGATGTGA	TATGCATCCTCACGGTTTTG	59.5	675	INTERGENIC	--	--	--
Ca1_P2_37425673	Ca1	37425673	ATTTTTTTTTT/ATTTTTTTTTTTT	TGTACCGTGGATGGATGTGA	TATGCATCCTCACGGTTTTG	59.5	675	INTERGENIC	--	--	--
Ca1_P2_37426537	Ca1	37426537	ATT/AT	CTTCAAAACCGTGAGGATGC	TCTGAGCTTCTGATGCTTGC	59.4	916	INTERGENIC	--	--	--
Ca1_P2_37433380	Ca1	37433380	CAA/CAACGTAAA	AATAAATTAGTGCAGCAAACCAA	GGCAAAGAGAGGAAAGTGGA	59.4	756	INTERGENIC	--	--	--

INDEL marker IDs	Chromosomes/unanchored scaffolds	Physical positions (bp)	InDels (*Kabuli* reference genome-CDC Frontier/PII)	Forward primers (5'-3')	Reverse primers (5'-3')	Anneling temperature (0C)	Expected amplified product size (bp)	Structural annotation		Functional annotation	
								Sequence components of *kabuli* genome	*Kabuli* gene accession IDs	NCBI-KOG	TFs
Ca1_P2_37433540	Ca1	37433540	TATAATAGATAATAGATAATA/TATAATAGATAATA	TGAAAAACATCTCTCTAATAGGCG	ACCACTGCACCAAGGGTAAG	60.0	532	INTERGENIC	--	--	--
Ca1_P2_37450942	Ca1	37450942	GT/G	ACACGCTTATTGGATGCACA	CAGTGATTTTCGTCTGCCCT	60.3	377	INTERGENIC	--	--	--
Ca1_P2_37489959	Ca1	37489959	AT/ATT	ATTGTCCAGTTTCCTCACCG	CGATTGGTAAATCGATTGGG	60.1	594	INTERGENIC	--	--	--
Ca1_P2_37490066	Ca1	37490066	A/AATGTAT	ATTGTCCAGTTTCCTCACCG	TGAACTCTGAGCGTGTGACC	60.0	775	INTERGENIC	--	--	--
Ca1_P2_37501680	Ca1	37501680	TAAAAA/TAAAA	GGGAGGACGACAACGTCTTA	ACCAATCTTGTGCATCCCAT	60.2	369	INTERGENIC	--	--	--
Ca1_P2_37516914	Ca1	37516914	TAAAAAA/TAAAAA	AGGGAGGACGAAAATGACCT	ACCAATCTCGTGCATCCAAT	60.3	567	INTERGENIC	--	--	--
Ca1_P2_37597011	Ca1	37597011	GAATTTAAATTTAA/GAATTTAAATTTAAATTTAA	TGCGTTCACAGTTTTCAAGTT	ATTATGCTGATTCGATCGCC	60.0	648	INTERGENIC	--	--	--
Ca1_P2_3767709	Ca1	3767709	AATATATATATATATA/AATATATATATATATA	AAACCAACCAAAAGGTTCCA	TGGCAGCAAATTCAAATGAC	59.7	963	INTERGENIC	--	--	--
Ca1_P2_37693180	Ca1	37693180	AATTAT/AAT	TGTGGAACATTCTCTGCGTC	TTCCCGAGAGATTTGTCTTCA	59.8	593	DRR	Ca_23533	--	G2-like
Ca1_P2_37698093	Ca1	37698093	CAT/CATAT	CAAGTTTTCCAGTACACCCCA	TCACGTCGTCGAGACTATGG	59.9	709	INTERGENIC	--	--	--
Ca1_P2_37712787	Ca1	37712787	A/ACATGTG	GGTGTTGGAGGTGTAATGGG	CATGTCCCGTATTCGCTTTT	60.0	639	INTERGENIC	--	--	--
Ca1_P2_37716852	Ca1	37716852	T/TG	TGATGTTGTTGTTGTTGTTGGA	GTTTTGGGTACACCCCATTG	59.9	473	INTERGENIC	--	--	--

INDEL marker IDs	Chromosomes/unanchored scaffolds	Physical positions (bp)	InDels (*Kabuli* reference genome-CDC Frontier/PII)	Forward primers (5'-3')	Reverse primers (5'-3')	Anneling temperature (0C)	Expected amplified product size (bp)	Structural annotation		Functional annotation	
								Sequence components of *kabuli* genome	*Kabuli* gene accession IDs	NCBI-KOG	TFs
Ca1_P2_37724930	Ca1	37724930	CCACACACAC/CCACACAC	ACCCTGTTGCTGCCAAATAC	GCTCCAATTCCCTCAGATCA	60.2	664	INTRON	Ca_23534	--	--
Ca1_P2_37758491	Ca1	37758491	TA/TAA	TGACGAGGTTCGTGATGATG	AGCGAATGATTTCAACCACC	59.9	489	INTERGENIC	--	--	--
Ca1_P2_37763281	Ca1	37763281	TGTG/TGTGTATTGTCGGAGTG	GAATTGATAGACCCGCCAAA	AGAACGTCTCATTGGTTGCC	60.1	375	INTERGENIC	--	--	--
Ca1_P2_37771437	Ca1	37771437	CTTTTT/CTTTT	CTGTGGCCCGACAAAGTATT	TCGTATATGCAGCAAGTGGC	59.9	627	INTERGENIC	--	--	--
Ca1_P2_37779617	Ca1	37779617	AT/ATTATTATTATTATTTT	TCACGAACCAATTTGAGATG	TGTTCGTGACCCAGAGATGA	60.2	515	INTERGENIC	--	--	--
Ca1_P2_37780594	Ca1	37780594	TAAAAAAAAA/TAAAAAAAAAAAA	CCGGAAGTCCTACTGGTTCA	CCACTAGAAACCTGGTTGGA	57.6	501	INTERGENIC	--	--	--
Ca1_P2_37800107	Ca1	37800107	TA/TATTTAAA	TGGGTCCTAAACCGCAATAC	CAAAAATGATGCAACCATCG	59.9	371	INTERGENIC	--	--	--
Ca1_P2_37803540	Ca1	37803540	TTATATATATATATATATATATATATATATATATATATATAT/TTATATATATATATATATATATATATATATATATATATATAT	ATCGATCACATCAACGTCCA	AGCAAATGCGGAAGAAAGAA	60.0	656	INTERGENIC	--	--	--
Ca1_P2_37809250	Ca1	37809250	ACTTTTCCTTTTCCT/ACTTTTCCT	TGCCTTTGGGGATTTTATCA	TTTTGCATTAAAAATGGATGAA	57.3	579	INTERGENIC	--	--	--
Ca1_P2_3783481	Ca1	3783481	AATA/AA	AATGATGATAATTCATTTGCCA	GCGACTATCCAGGAAATCCA	60.0	522	INTERGENIC	--	--	--
Ca1_P2_3783626	Ca1	3783626	TATTTGCATTTG/TATTTG	TTCACAATTACCACAAAGATCG	GCGACTATCCAGGAAATCCA	60.0	433	INTERGENIC	--	--	--
Ca1_P2_37849378	Ca1	37849378	G/GA	CCTAAGGGCCGTAGTAACCTG	AGGATCCACCAAGCAAATCA	60.5	154	INTRON	Ca_23538	T	E2F/DP

INDEL marker IDs	Chromosomes/unanchored scaffolds	Physical positions (bp)	InDels (*Kabuli* reference genome-CDC Frontier/PII)	Forward primers (5'-3')	Reverse primers (5'-3')	Anneling temperature (0C)	Expected amplified product size (bp)	Structural annotation		Functional annotation	
								Sequence components of *kabuli* genome	*Kabuli* gene accession IDs	NCBI-KOG	TFs
Ca1_P2_37913733	Ca1	37913733	TAAA/TAA	ACGTTCGCCACCTATCTCAT	CCTTATGCGGTAATCCCTGA	59.9	563	INTERGENIC	--	--	--
Ca1_P2_37917967	Ca1	37917967	ATATGACAGTTA/ATA	TCCACAAGTTATTCGACGAAAC	TTCCATTGATCTACGTTAATGTTTG	59.4	558	INTERGENIC	--	--	--
Ca1_P2_37918071	Ca1	37918071	ATTTTTTTTT/ATTTTTTT	TCCACAAGTTATTCGACGAAAC	AAAATAGGGGACCAAACCTG	57.9	757	INTERGENIC	--	--	--
Ca1_P2_37926604	Ca1	37926604	TATAATAATAATAATAAT/TATAATAATAATAAT	TGAATGTGACGTCTTGAACCTT	CAAACTTCCGCGATCAAAAT	60.1	685	INTERGENIC	--	--	--
Ca1_P2_37927331	Ca1	37927331	TAAA/TAAATTGTATCAAA	ATTTTGATCGCGGAAGTTTG	TCAATCAACCAGATTTTGCATT	59.5	553	INTERGENIC	--	--	--
Ca1_P2_37927862	Ca1	37927862	G/GTA	TGCAAAATCTGGTTGATTGAG	TCCCAGATCAAAATTGGGTT	59.2	745	INTERGENIC	--	--	--
Ca1_P2_37928039	Ca1	37928039	G/GTTACATT	TCAAAATTGGAATTGTTCAGGA	GAATTGACATTGTTGCACGG	60.0	675	INTERGENIC	--	--	--
Ca1_P2_37928765	Ca1	37928765	T/TTTTGATG	CCGTGCAACAATGTCAATTC	TGGTTGCATGTTTGTAACTTGA	59.1	176	INTERGENIC	--	--	--
Ca1_P2_37928853	Ca1	37928853	CT/CTGAAT	CCGTGCAACAATGTCAATTC	GACCACCATATTAACCGTCACA	59.6	571	INTERGENIC	--	--	--
Ca1_P2_37929646	Ca1	37929646	TAAAAAAAA/TAAAAAAAAAA	TGTGACGGTTAATATGGTGGTC	TTGACTTGTTTTGCGGATTG	59.7	817	INTERGENIC	--	--	--
Ca1_P2_37929753	Ca1	37929753	ATTTTTTTTT/ATTTTTTTTTTTT	TGTGACGGTTAATATGGTGGTC	TTGACTTGTTTTGCGGATTG	59.7	817	INTERGENIC	--	--	--
Ca1_P2_37954337	Ca1	37954337	GTTTTT/GTTT	TCTTTTCTCCGATCCCATTG	CGACTCCCGAACTCAAAATC	59.7	868	INTERGENIC	--	--	--

INDEL marker IDs	Chromosomes/unanchored scaffolds	Physical positions (bp)	InDels (*Kabuli* reference genome-CDC Frontier/PII)	Forward primers (5'-3')	Reverse primers (5'-3')	Anneling temperature (0C)	Expected amplified product size (bp)	Structural annotation		Functional annotation	
								Sequence components of *kabuli* genome	*Kabuli* gene accession IDs	NCBI-KOG	TFs
Ca1_P2_37954669	Ca1	37954669	TAAAAAAATAAAAAAAAAATAAAAA/TAAAAAAATAAAAA	AGAAGGCTACCACTACGCGA	CGGGAGATACGTAGGAGCAA	60.2	716	INTERGENIC	--	--	--
Ca1_P2_37993308	Ca1	37993308	CAAAAAAAAAAAAAAA/CAAAAAAAAA	TCATCGCAGGTGTACCATGT	GGATTGGTAAAGGGCTCACA	59.9	482	INTERGENIC	--	--	--
Ca1_P2_37993665	Ca1	37993665	CAAAAAA/CAAAAA	GTGAGCCCTTTACCAATCCA	CAATTCCCACAGGTACGCTT	60.0	654	INTERGENIC	--	--	--
Ca1_P2_38024874	Ca1	38024874	TATGAATGAAT/TATGAATGAATGAAT	GCAAGCTCAGTTCCAGTTCC	TGCATCAATTATGGCTCCAA	60.0	655	INTRON	Ca_27443	S	--
Ca1_P2_38030335	Ca1	38030335	TTAAAATAAAATAAAATAAAA/TTAAAATAAAATAAAATAAAATAAAATAAAA	AGCTCGAAACATGGTATCCAA	CTAGCCATCCTCAGCGAGAG	60.3	662	INTERGENIC	--	--	--
Ca1_P2_38060326	Ca1	38060326	CGG/CG	GGTTTGTCCGTTCTTCTCCA	CTTGTTGCGACGATGAAAAA	59.8	310	INTERGENIC	--	--	--
Ca1_P2_38069381	Ca1	38069381	AT/ATGGGGCTT	GCAATTTTGGTCCCCCTATT	GACAAATAGTCGGGTTCGGA	59.9	559	INTERGENIC	--	--	--
Ca1_P2_38070666	Ca1	38070666	T/TA	AACCATTGGGGGAGAATAGAA	TTGTTAGAACCATTGGGGGA	60.2	362	INTERGENIC	--	--	--
Ca1_P2_38070895	Ca1	38070895	ATGTGTGT/ATGTGT	CTCCCCCAATGGTTCTAACA	CACTGGAATCCGTTTCTTTTG	59.6	587	INTERGENIC	--	--	--
Ca1_P2_38071534	Ca1	38071534	TACACACAC/TACACAC	CTCCTCCTCATTCACCCAAC	ACACACCCACTCTCCTTTGC	60.2	189	INTERGENIC	--	--	--
Ca1_P2_38091918	Ca1	38091918	GAAAAAAAAAAA/GAAAAAAAAAA	TATGGCAATTTTGGCATCCT	AAACCCTTTGAATAGACCCCA	59.7	521	INTERGENIC	--	--	--
Ca1_P2_38110776	Ca1	38110776	AT/ATTTT	GGGAGCATCCATAACATTGC	GCCATTTCAAACACATCACG	60.0	188	INTERGENIC	--	--	--

INDEL marker IDs	Chromosomes/unanchored scaffolds	Physical positions (bp)	InDels (*Kabuli* reference genome-CDC Frontier/PII)	Forward primers (5'-3')	Reverse primers (5'-3')	Anneling temperature (0C)	Expected amplified product size (bp)	Structural annotation		Functional annotation	
								Sequence components of *kabuli* genome	*Kabuli* gene accession IDs	NCBI-KOG	TFs
Ca1_P2_38131605	Ca1	38131605	C/CT	CGTAACTCGTGTTGGGTGAA	GAAGCAAGAGGCAACTCCAC	60.0	379	INTERGENIC	--	--	--
Ca1_P2_38141538	Ca1	38141538	GGAA/GGAAGAA	TCATCAAATCCGTAATGCAAA	TGTTTTATAAGCATCATGTGTCACTC	59.5	166	INTERGENIC	--	--	--
Ca1_P2_38164426	Ca1	38164426	ATTT/ATTTT	TTCGAGAATTCCCGCTCTAA	GCCATGGCATCTATGTTGTG	60.0	815	INTERGENIC	--	--	--
Ca1_P2_38180047	Ca1	38180047	GGTAATA/G	AAACGCAATCCTTGAGTTCG	TCACTACGACCCCAACATGA	60.0	844	INTERGENIC	--	--	--
Ca1_P2_38184317	Ca1	38184317	ATTTT/ATTTTT	TGACTCGTGATCCTCACTGC	TTAAGCGTGCTCTCAACGTG	60.2	572	DRR	Ca_13975	--	--
Ca1_P2_38187305	Ca1	38187305	AT/A	ATCACTCCACCGACTTCCAC	GAGTAGGGCTGTGCATGGTT	60.1	602	INTERGENIC	--	--	--
Ca1_P2_38193154	Ca1	38193154	TAAAAAAAAAAA/TAAAAAAAAA	CGAACAAACTCACCGGATCT	CCAATGGAGTCCCAAAAAGA	59.9	724	INTERGENIC	--	--	--
Ca1_P2_38195545	Ca1	38195545	CATA/CATATA	CAGACCAACTGCAAGTCCAA	TCACGAATTCCCATGTTTGA	59.9	539	INTRON	Ca_13974	L	C2H2
Ca1_P2_38227247	Ca1	38227247	GT/GTT	CTGAGAATGGCAGCACAAAA	TTGATGTATTTTGGCGATTCA	59.0	348	INTERGENIC	--	--	--
Ca1_P2_38227299	Ca1	38227299	CAAAATAAAATAAAATAAAATA/CAAAATAAAATAAAATA	CTGAGAATGGCAGCACAAAA	TGCTTAGGGAAATAAGGCATGT	60.0	770	INTERGENIC	--	--	--
Ca1_P2_38244423	Ca1	38244423	ATTTTTT/ATTTTTTT	GCGGAAATATTTTTGCAGGA	AACCAATTGCATCACCCTTC	59.8	523	INTERGENIC	--	--	--
Ca1_P2_38273940	Ca1	38273940	CAAAAAAAAAAA/CAAAAAAAAAA	TATTTGGGGACCAAAACTGC	CATGGTTTGAATGCGAGTTG	60.1	637	INTERGENIC	--	--	--

INDEL marker IDs	Chromosomes/unanchored scaffolds	Physical positions (bp)	InDels (*Kabuli* reference genome-CDC Frontier/PII)	Forward primers (5'-3')	Reverse primers (5'-3')	Anneling temperature (0C)	Expected amplified product size (bp)	Structural annotation		Functional annotation	
								Sequence components of *kabuli* genome	*Kabuli* gene accession IDs	NCBI-KOG	TFs
Ca1_P2_38281902	Ca1	38281902	TTCT/TT	GAAACACGCTCGAACTCCTC	TGGAAGAAAGAACCTTTGGC	59.3	888	INTERGENIC	--	--	--
Ca1_P2_38284193	Ca1	38284193	TC/T	GCCAACACATGGTGAAAGTG	AACAAAGTTTGCATTTGGGC	60.0	939	INTERGENIC	--	--	--
Ca1_P2_38335336	Ca1	38335336	GTTTTT/GTTTTTT	TGCATCTCTAGGCTGCCTTT	ACCTTCTGGCCATGAATTTG	59.9	852	INTERGENIC	--	--	--
Ca1_P2_38335567	Ca1	38335567	CTATA/CTA	AGCAGGTTGCCAACATCTTC	CGCGAAACCGTTACCTACAT	60.0	676	INTRON	Ca_13967	--	--
Ca1_P2_38338321	Ca1	38338321	CAAAA/CAAAAA	TCCTCTCTCATCCCAACACC	AATCCCCAATTCGAAAAACC	60.0	244	DRR	Ca_13967	--	--
Ca1_P2_38349813	Ca1	38349813	A/AC	CGTGGGTTCCAACAACTTTT	TTTCATGGTTAGCACACCGA	60.1	674	INTERGENIC	--	--	--
Ca1_P2_38353011	Ca1	38353011	ATT/ATTT	GTGGTGATTGGAGTGTGTCG	CAAATTCCCCTCCATGTTTG	60.2	710	INTERGENIC	--	--	--
Ca1_P2_38365522	Ca1	38365522	TTATATATATAT/TTATATATATATAT	CAACAATGGAGGAAGGAGGA	TTAACTGCGGCGAATCTTTT	59.9	263	INTERGENIC	--	--	--
Ca1_P2_38476810	Ca1	38476810	TTAT/TTATAT	TTGCAAGTACGTTCTCCAAAA	GTGTTACACGCCCTTCATCC	60.4	280	INTERGENIC	--	--	--
Ca1_P2_38479463	Ca1	38479463	AATATATATATATATATATATATATAT A/AATATATATATATATATATATATATA	AGCGTGAATGTGACGTCTTG	TTAAAGAGATTCGCAGCGGT	60.0	100	INTERGENIC	--	--	--
Ca1_P2_38487463	Ca1	38487463	ATTTTTTTTTT/ATTTTTTTTT	TGCTACCTGCAACCAGTGAG	GTCAACTTTAAGGGGGCGTC	60.9	872	DRR	Ca_13962	--	--
Ca1_P2_38495552	Ca1	38495552	AAT/AATAATTATTAT	AACACCTTCCTGATCCTCCC	TGGCGAACTTTTAAGAATGGA	59.7	659	INTERGENIC	--	--	--

INDEL marker IDs	Chromosomes/unanchored scaffolds	Physical positions (bp)	InDels (*Kabuli* reference genome-CDC Frontier/PII)	Forward primers (5'-3')	Reverse primers (5'-3')	Anneling temperature (0C)	Expected amplified product size (bp)	Structural annotation		Functional annotation	
								Sequence components of *kabuli* genome	*Kabuli* gene accession IDs	NCBI-KOG	TFs
Ca1_P2_38499923	Ca1	38499923	TTATATATATATATATATATATATATATATATATAT/TTATATATATATATATATATATATATATATATATAT	GAACAATGTCAATTTGCCCC	GGCAATTAAAAGAATGAAACCAA	59.4	911	INTERGENIC	--	--	--
Ca1_P2_38544271	Ca1	38544271	ATT/ATTT	GGCAATGAGCTGAATCCTTT	CGATTGGCACATTGATTTTG	59.9	164	INTERGENIC	--	--	--
Ca1_P2_38559526	Ca1	38559526	ATTT/ATTTT	GTGCAAAACAGACGTTCAGC	GCATCGCTGGGTAATTTGTT	60.0	363	DRR	Ca_13957	--	--
Ca1_P2_38571843	Ca1	38571843	CAAA/CAAAA	CACCTGACACTGCAAACCAG	TGGTCAAAGATTTTCATCAATCA	59.4	617	INTERGENIC	--	--	--
Ca1_P2_38577185	Ca1	38577185	GTTTTT/GTTTT	CCATTTTCTGTCCCCTTTCA	GCATTGGCTAAGCATGGATT	60.1	760	INTERGENIC	--	--	--
Ca1_P2_38597715	Ca1	38597715	ATAATTTTAATTTTAATTT/ATAATTTTAATTT	CATTTGCCTCCCTTGTTGTT	TGTAGTTGCTGCAAACGAGG	60.1	551	DRR	Ca_13956	--	--
Ca1_P2_38614804	Ca1	38614804	TTAAGATATAGCAGTAA/TTAA	TGATGGATTTGGGTTCCATT	GGATGATGCATTTGATGCTTT	59.9	756	INTERGENIC	--	--	--
Ca1_P2_38658433	Ca1	38658433	CA/C	GAAATGGCAGGAAGACCAGA	CTTTGTTAAACGCACTCGCA	60.1	282	INTERGENIC	--	--	--
Ca1_P2_38683280	Ca1	38683280	AA/AAGTA	AGGCACTCCAAGCTTTGCTA	TTTTGAAATGCCCCTCTTTG	60.0	778	INTERGENIC	--	--	--
Ca1_P2_3872384	Ca1	3872384	TTATATATATATATATATATATATA/TTATATATATATATATATATATATATA	GGGCAGAGCAAGAAAACAAG	CTGACCGTTTTTCGATTTCC	59.5	393	INTERGENIC	--	--	--
Ca1_P2_38733678	Ca1	38733678	AATATATATATATATA/AATATATATATATATATA	CCAAACCCCTTTGGTTGTAA	GTGTCTGGGAAATCGCAGTT	60.1	720	INTERGENIC	--	--	--
Ca1_P2_38740282	Ca1	38740282	GAATTAA/GAA	AAACCGTTACCTGCATTTCG	TGAGAGCTATTTTTCGGACACA	59.9	548	INTRON	Ca_13954	--	--

INDEL marker IDs	Chromosomes/unanchored scaffolds	Physical positions (bp)	InDels (*Kabuli* reference genome-CDC Frontier/PII)	Forward primers (5'-3')	Reverse primers (5'-3')	Anneling temperature (0C)	Expected amplified product size (bp)	Structural annotation		Functional annotation	
								Sequence components of *kabuli* genome	*Kabuli* gene accession IDs	NCBI-KOG	TFs
Ca1_P2_38759787	Ca1	38759787	AGTGT/AGT	GCCATAAACCCGAAATTTGA	TGTGCTTGTCCCACACACTT	60.2	328	INTERGENIC	--	--	--
Ca1_P2_38766832	Ca1	38766832	ACT/ACTCT	GAGCTTCTTCAAGTCGGTGG	GTTTGGAATACCCTGAGCCA	59.9	222	INTERGENIC	--	--	--
Ca1_P2_38790509	Ca1	38790509	AC/A	AGAGCTCTGAAGTCAAGCGG	GAAATCCTTGTTGCCTTGGA	60.1	163	DRR	Ca_13953	--	--
Ca1_P2_3881904	Ca1	3881904	TTATATATATATATATATATATATATATATATATATATATATATATATA/TTATATATATATATATATATATATATATATATATATATATATATATATATATATATA	GACGGTACCGGAGAGTGAAA	GTCCACGTTGGCTTTGAAAT	60.0	580	INTERGENIC	--	--	--
Ca1_P2_38885222	Ca1	38885222	T/TG	CTGGTGAGGGAAGGTTGTGT	AACTGCCCAAATGTTTCTGG	60.0	427	INTERGENIC	--	--	--
Ca1_P2_38886459	Ca1	38886459	AAAATAAA/AAAA	GCATGTTGTATGCTTTCTTCCA	ATTTATCCATCCATGTCGCC	59.6	863	INTERGENIC	--	--	--
Ca1_P2_38895765	Ca1	38895765	GAAA/GAA	GAGGGCAATTTGGGATTTTC	TATTGGGTGCGTTTGTTTCA	60.0	242	INTERGENIC	--	--	--
Ca1_P2_38963161	Ca1	38963161	TAAAAAAAAAAAA/TAAAAAAAAA	TCTGTACCGGAAAACTTGGG	TTTCTTCGAGGCTTGTGCTT	60.1	619	INTERGENIC	--	--	--
Ca1_P2_38983134	Ca1	38983134	CATATATATATATATATATATATATATATATA/CATATATATATATATATATATATATATATATATA	TAAATTCTTGCGGTGGGTTG	ATCAATGCAGGGAAGAGGAA	59.6	689	INTERGENIC	--	--	--
Ca1_P2_39028142	Ca1	39028142	AATATATATATAT/AATATATATAT	TCATGGAAGGAGCAATCACA	TAAGGTTTAGGATGCCCCAA	59.4	764	INTRON	Ca_13946	--	--
Ca1_P2_39038586	Ca1	39038586	GTATATATATATATATATATATATATATATATATATA/GTATATATATATATATATATATATATATATATATATATATATATA	CGATTTTGAATTTGGGTCTCA	TTAAATAGGCTTCCAGGCCA	59.7	708	INTRON	Ca_13946	--	--
Ca1_P2_39144961	Ca1	39144961	CACGTAC/CAC	CATCCTTTGCAGGATTGGTT	TTGTGCTTTGGAAAATGCAG	59.8	494	INTRON	Ca_13942	--	ARF

INDEL marker IDs	Chromosomes/unanchored scaffolds	Physical positions (bp)	InDels (*Kabuli* reference genome-CDC Frontier/PII)	Forward primers (5'-3')	Reverse primers (5'-3')	Anneling temperature (0C)	Expected amplified product size (bp)	Structural annotation		Functional annotation	
								Sequence components of *kabuli* genome	*Kabuli* gene accession IDs	NCBI-KOG	TFs
Ca1_P2_39147028	Ca1	39147028	AT/ATT	AAAATGATCAGGGAAAATCATGT	ACAACTTCTCATGCTCGCAA	59.6	339	DRR	Ca_13942	--	ARF
Ca1_P2_39163701	Ca1	39163701	C/CCTG	AAGTCTGCCACAAAGCCACT	GCTTGAAAATGGAAGGGACA	60.1	473	INTRON	Ca_13941	--	Trihelix
Ca1_P2_39183702	Ca1	39183702	ATTT/ATTTT	GGGTGCTTTCTATGATGTGC	GGGCTGCTATTCAGCTTTTG	60.0	758	INTERGENIC	--	--	--
Ca1_P2_39220811	Ca1	39220811	T/TCTTA	TGGGAGGCTCTCCAGTTCTA	AGGCACTTGGTTTTCTTCCA	59.7	478	CDS (FRAME SHIFT)	Ca_13939	T	M-type
Ca1_P2_39261977	Ca1	39261977	A/AT	GGTTTGCTGTCGAGCCTTAG	AAACACAAGTCCAATCAAAAGG	58.2	421	INTERGENIC	--	--	--
Ca1_P2_39265068	Ca1	39265068	GTTTTT/GTTTTTT	TTGGAAGCTAAGAAGCCGAA	ATAATGGCCAAGGAACATGC	59.8	566	INTERGENIC	--	--	--
Ca1_P2_39303782	Ca1	39303782	ATTTT/ATTTTT	CCATCCACATTTTCAAGTGATTT	TGAATCGTGAGGGTGTTATGA	59.0	533	INTERGENIC	--	--	--
Ca1_P2_39366651	Ca1	39366651	CTT/CTTT	CCTTTGGAGGACGAAAATGA	AATGGCCACGAATCTGAAAG	60.1	621	DRR	Ca_13933	--	--
Ca1_P2_39384808	Ca1	39384808	T/TA	GGGCCAAGAAACAACAAAAA	TTCCACCAATCAATTAGGCA	59.0	105	INTERGENIC	--	--	--
Ca1_P2_39388747	Ca1	39388747	T/TGG	GAAGGTGAAGCTCCAAGCAC	TTGTGACCTGTCATGTGGCT	60.2	716	INTERGENIC	--	--	--
Ca1_P2_39392030	Ca1	39392030	ATT/ATTT	AGCTACCCCATGGTAAGCCT	GCACCCATGCACTTAGTCCT	60.1	729	INTERGENIC	--	--	--
Ca1_P2_39419858	Ca1	39419858	CTTTTTTTTTTT/CTTTTTTTTTTTTT	TGGGGAAGTTTTGATACAAGG	CTTTGTTGCTGCATTTTTCG	59.5	688	INTERGENIC	--	--	--

INDEL marker IDs	Chromosomes/unanchored scaffolds	Physical positions (bp)	InDels (*Kabuli* reference genome-CDC Frontier/PII)	Forward primers (5'-3')	Reverse primers (5'-3')	Anneling temperature (0C)	Expected amplified product size (bp)	Structural annotation		Functional annotation	
								Sequence components of *kabuli* genome	*Kabuli* gene accession IDs	NCBI-KOG	TFs
Ca1_P2_39428398	Ca1	39428398	TAAATCGATTGGGAAATCGATT/TAAATCGATT	CGGAACCAATTGTTGGACTT	GTGCCAGTCGATTGCCTAAT	60.1	284	INTERGENIC	--	--	--
Ca1_P2_39442566	Ca1	39442566	A/AT	GCCTCAAGGATCGGATAACA	GCCAGTGTTCGTGCTTCATA	59.9	917	INTRON	Ca_13931	Z	NAC
Ca1_P2_39442792	Ca1	39442792	A/ACT	TGAATGGCATGCAAGAAAAG	GCCAGTGTTCGTGCTTCATA	59.9	631	INTRON	Ca_13931	Z	NAC
Ca1_P2_39444043	Ca1	39444043	TAGTTAA/TA	AATGATGGAAAAGCCGAATG	GGAGGGACCTGAAGTCACAA	60.1	838	INTRON	Ca_13931	Z	NAC
Ca1_P2_39444170	Ca1	39444170	TAAAA/TAA	AATGATGGAAAAGCCGAATG	GGAGGGACCTGAAGTCACAA	60.1	838	INTRON	Ca_13931	Z	NAC
Ca1_P2_39445206	Ca1	39445206	ATTTT/ATTT	TTTACAGGGGTCATCTTGGG	TCAAATTGAAGGCGTGTCAG	59.8	448	INTRON	Ca_13931	Z	NAC
Ca1_P2_39524520	Ca1	39524520	GA/G	CTTGGAGTTAGGGATGGCAA	TGAAACAAACGCACCCAATA	60.0	405	INTERGENIC	--	--	--
Ca1_P2_39575023	Ca1	39575023	ATTTTTTTT/ATTTTTTTTTTT	TCAGACCTCCATGAAAAGCA	TGACCGTTTTTCTCCTTCGT	59.7	599	INTERGENIC	--	--	--
Ca1_P2_39598601	Ca1	39598601	TC/TCCC	TTTCGTTATTTAAGCTACAACGG	GCGAAGATGGTGCCAATAAT	59.9	930	INTERGENIC	--	--	--
Ca1_P2_39609113	Ca1	39609113	AATAT/AAT	ATGGCCTTTCATTTTCAACG	TTGTCTCAAGCGTTCAATTTTT	58.9	725	INTERGENIC	--	--	--
Ca1_P2_39641416	Ca1	39641416	CTCT/CTCTTTCT	TAATTGCCCCAATCTGAAGC	TGGAATGGAAGCGGTACTTC	60.1	537	INTERGENIC	--	--	--
Ca1_P2_39675431	Ca1	39675431	CATATATATATATATAT/CATATATATATATATATATATATAT	CACCAAAGACAGGACCACCT	CCTAGGGTTTTTGGTTGGGT	60.1	784	DRR	Ca_13920	--	--

INDEL marker IDs	Chromosomes/unanchored scaffolds	Physical positions (bp)	InDels (*Kabuli* reference genome-CDC Frontier/PII)	Forward primers (5'-3')	Reverse primers (5'-3')	Anneling temperature (0C)	Expected amplified product size (bp)	Structural annotation		Functional annotation	
								Sequence components of *kabuli* genome	*Kabuli* gene accession IDs	NCBI-KOG	TFs
Ca1_P2_39733036	Ca1	39733036	CTGAT/C	TGATTGAGAGGGGAGAAAGG	AAACAGACCCCGAATGTCAC	59.8	117	DRR	Ca_13916	R	bZIP
Ca1_P2_39850133	Ca1	39850133	GTTAGTAATTCAAAAATTA/GTTA	CACAGCACCAGGTTATCCCT	CTCCTTGTGGAGGGACCATA	59.9	563	INTERGENIC	--	--	--
Ca1_P2_39871055	Ca1	39871055	TAAA/TAAAA	CATTGGCCACCTTTAGGAAA	TGCATTGCTTCAGTCATGGT	60.3	329	INTERGENIC	--	--	--
Ca1_P2_39976327	Ca1	39976327	CA/CATA	TTTAAGAGAAGCGTTTGAGCG	CTTTGACTACGTTTCCCCGA	60.1	211	INTERGENIC	--	--	--
Ca1_P2_3999056	Ca1	3999056	ATCTCTCTCTCTCTCT/ATCTCTCTCTCTCT	CAAGCAGAAGCTACAAGGGG	TGAAGAACCATTTCCAAGCC	60.1	577	INTERGENIC	--	--	--
Ca1_P2_40089909	Ca1	40089909	TGG/TG	AGTTGGGTTTGGTTGCTTTG	CCCGTCAATTGCAGTCTCTT	60.3	390	INTRON	Ca_13902	J	NAC
Ca1_P2_4009762	Ca1	4009762	AT/ATT	CGCACACACTTTCTACGCAT	TTGATCTGGTCAAATAAGCCA	58.2	841	INTERGENIC	--	--	--
Ca1_P2_40142062	Ca1	40142062	AAAAATAAAATAAAATA/AAAAATAAAATAAAATAAAATA	GACAGAATTGGAGTGGGGAA	GGTTTGAGAACGATGACACG	59.1	746	INTERGENIC	--	--	--
Ca1_P2_40224219	Ca1	40224219	TAAAAAAAAAA/TAAAAAAAA	CATTTGCATCTGATTGGTGG	TCTTTGCCTCATAGGAGCGT	60.0	664	INTERGENIC	--	--	--
Ca1_P2_40225071	Ca1	40225071	ATTATTT/ATTATTTATTTATTT	GTTGGAACTGTCCATTGGCT	AAGTCCGTTTCAGGGTTGTG	60.0	355	INTERGENIC	--	--	--
Ca1_P2_4024954	Ca1	4024954	AAATAATAATAATAATAATAATAATAATAATAATAATAATAATAATAATAATAATA/AAATAATAATAATAATAATAATAATAATAATAATAATAATAATAATAATAATAATAATA	TGGTTTTGATTGGAAAGTCG	GGGTTTGTCTCGGAGATTGA	60.1	361	INTERGENIC	--	--	--
Ca1_P2_40277677	Ca1	40277677	AA/AACA	TTCCCTTTGCATTAGATGGC	GAATCCATCGGGATGCTCTA	60.0	524	INTERGENIC	--	--	--

INDEL marker IDs	Chromosomes/unanchored scaffolds	Physical positions (bp)	InDels (*Kabuli* reference genome-CDC Frontier/PII)	Forward primers (5'-3')	Reverse primers (5'-3')	Anneling temperature (0C)	Expected amplified product size (bp)	Structural annotation		Functional annotation	
								Sequence components of *kabuli* genome	*Kabuli* gene accession IDs	NCBI-KOG	TFs
Ca1_P2_40279256	Ca1	40279256	AATATAT/AATATATAT	AACGCATCGGTAAACACTCC	CCATGCCAAATCAGAAACACT	60.0	273	INTERGENIC	--	--	--
Ca1_P2_40370849	Ca1	40370849	GATAGACTTAAT/GAT	AAACTCAAATCCATCGGTCG	TTGTGTGAGAAGCTGCCCTA	59.6	724	INTERGENIC	--	--	--
Ca1_P2_40371176	Ca1	40371176	CTATTATTA/CTATTA	TCGTCATCCTCTGCATCTCA	TTGTGTGAGAAGCTGCCCTA	59.6	559	INTERGENIC	--	--	--
Ca1_P2_40380273	Ca1	40380273	CTATA/CTATATATA	CCTGGTCCCACTTTTACGAA	GAAAAAGCGCTGTAAATGGC	59.9	487	INTRON	Ca_13892	--	Trihelix
Ca1_P2_40391433	Ca1	40391433	ACCCCC/ACCCCCC	TCCTGAAGGGGATTCATCAG	GTTAGGCGATCGCGAATACT	59.3	610	INTERGENIC	--	--	--
Ca1_P2_40424994	Ca1	40424994	C/CCAAAATATTATAA	TGCAAAACTTTGTTCATCGAG	GCATCATTCTCCTTCGAGACA	60.4	748	INTERGENIC	--	--	--
Ca1_P2_40426155	Ca1	40426155	ATTTTTTTT/ATTTTTTTTTT	CTTTACTGCGTTGGACCACA	ATAAATTGGATTTGCACCGC	59.8	544	INTERGENIC	--	--	--
Ca1_P2_40461964	Ca1	40461964	CATATATATATATATATATATATATA/CATATATATATATATATATATATATATA	GGGTCCCACACGTAGAAGAA	TGGGTTCATGTTAGAAATGGG	59.7	621	INTERGENIC	--	--	--
Ca1_P2_40534315	Ca1	40534315	GAAA/GAAAA	CAATTTTGACCATGGAACCC	TGAATTGACCTCGCAGATTG	59.8	730	INTERGENIC	--	--	--
Ca1_P2_40535465	Ca1	40535465	TTAATAATAATAATAATAATA/TTAATAATAATAATAATAATAATA	CATGCGTTCCTTGTGGTAGA	ATCACAGTTTGGCCAAGTCC	60.0	599	INTERGENIC	--	--	--
Ca1_P2_40535882	Ca1	40535882	AATATATATATATATATATATAT/AATATATATATATATATATATATAT	GATGATGATGATGTCGTGCC	ATCACAGTTTGGCCAAGTCC	60.0	311	INTERGENIC	--	--	--
Ca1_P2_40663043	Ca1	40663043	TAGAAAGAA/TAGAA	AATCCCCTTTCGGAACAAAC	GTTTCGCACTTCCCAAACAT	60.0	487	INTERGENIC	--	--	--

INDEL marker IDs	Chromosomes/unanchored scaffolds	Physical positions (bp)	InDels (*Kabuli* reference genome-CDC Frontier/PII)	Forward primers (5'-3')	Reverse primers (5'-3')	Anneling temperature (0C)	Expected amplified product size (bp)	Structural annotation		Functional annotation	
								Sequence components of *kabuli* genome	*Kabuli* gene accession IDs	NCBI-KOG	TFs
Ca1_P2_40665843	Ca1	40665843	TTATATATATATATATATAT/TTATATATATATATATATATAT	CCACGTCAATTTTGACATACATC	ACCAAGATCCGCTTACATGG	60.0	290	INTERGENIC	--	--	--
Ca1_P2_40688107	Ca1	40688107	TGG/TG	CACAACTGAATCAATCCACCA	TGAGGGTTTCGAAGGAAAGA	59.8	587	INTERGENIC	--	--	--
Ca1_P2_4075360	Ca1	4075360	CTTTTTTTTTTT/CTTTTTTTTTTTT	TTCTTTCACAAATTAGCTTGGATT	CGAATGACTTGTCTCGTCCC	60.7	710	INTERGENIC	--	--	--
Ca1_P2_40756087	Ca1	40756087	CAAAAAAAA/CAAAAAAAAA	AAGCGGCTTATGTGCTTGTT	ACGCTTTGTTGGGTGTAAGG	60.0	506	INTERGENIC	--	--	--
Ca1_P2_40795125	Ca1	40795125	AATATATATATATATATATATATAT/AATATATATATATATATATAT	TGAAATCAATTGAGGACATTCG	ACGTAGCGAGCCTCGAATTA	60.0	547	INTERGENIC	--	--	--
Ca1_P2_40814689	Ca1	40814689	G/GCTT	TGAATGTTTTGTCTTGAAAATCG	CTATCACAAGCCCGTACCGT	60.0	146	INTERGENIC	--	--	--
Ca1_P2_40861492	Ca1	40861492	GTTTTTTTT/GTTTTTTTTTTTTT	ATTTCTAAACCCGCAAACCC	TCCACCCACGATACTGATGA	59.9	941	INTERGENIC	--	--	--
Ca1_P2_40889769	Ca1	40889769	TTATATA/TTA	GCATCTCTTGATGCCCTTTT	AACATACGCCATACCCAAGC	59.9	289	INTERGENIC	--	--	--
Ca1_P2_40890558	Ca1	40890558	A/AC	CGGATTTGCAAGTGTTCGTA	TGGGGTTTAGGATATCACATCA	59.2	707	INTERGENIC	--	--	--
Ca1_P2_40890662	Ca1	40890662	CAAA/CA	CAAGAAACAAATTGCAATGGA	TCAAGTTTGGGAGGAACCAC	59.9	704	INTERGENIC	--	--	--
Ca1_P2_40953274	Ca1	40953274	G/GACT	CTTAACAAATGAGGCGGAGG	TAACCGATTTAGCCACGGAG	60.1	187	INTERGENIC	--	--	--
Ca1_P2_41043357	Ca1	41043357	AATATATATATATATATATATATATATAT/AATATATATATATATATATATATATATATATATATATAT	TAGTGGCACCCCTACACCTT	TTTCATTCGAAATCCATCCTC	59.0	604	INTERGENIC	--	--	--

INDEL marker IDs	Chromosomes/unanchored scaffolds	Physical positions (bp)	InDels (*Kabuli* reference genome-CDC Frontier/PII)	Forward primers (5'-3')	Reverse primers (5'-3')	Anneling temperature (0C)	Expected amplified product size (bp)	Structural annotation		Functional annotation	
								Sequence components of *kabuli* genome	*Kabuli* gene accession IDs	NCBI-KOG	TFs
Ca1_P2_4105789	Ca1	4105789	A/AGCACT	AAAAGTTTGCTGAGGAGCCA	AAAGGGCAAGGATGTGTTTG	60.0	163	INTRON	Ca_00493	D	TCP
Ca1_P2_41058981	Ca1	41058981	CTTTTTTTTTTT/CTTTTTTTTTT	GCATGGGAAATGGATTCTCT	TGGCTCATACACAGTTTTCTGG	60.2	679	INTERGENIC	--	--	--
Ca1_P2_41076452	Ca1	41076452	T/TAAC	AAATGGAACAGAAGCAACCG	TGTGCACATGTCTCGATTGAC	60.8	650	INTERGENIC	--	--	--
Ca1_P2_41103616	Ca1	41103616	T/TGAGAGAATTTA	TTATTAGGACGGACAACCGC	TTGTTCGTGTTGCGTTTAGC	59.9	284	INTERGENIC	--	--	--
Ca1_P2_41103687	Ca1	41103687	TACACACA/TACACA	GGACGGACAACCGCTATTTA	TTGTTCGTGTTGCGTTTAGC	59.9	278	INTERGENIC	--	--	--
Ca1_P2_41167792	Ca1	41167792	AATATATATATATATATATATA/AATATATATATATATATATA	TTTTGGTCGGTATTCCAATTT	GGGTCAAAATGTATGGTCGG	60.1	802	DRR	Ca_18480	--	--
Ca1_P2_41208187	Ca1	41208187	GATATATATATATATATATATA/GATATATATATATATATATATATATATATA	CATGTGTGCATTGAAAATAGGA	GAAAGGAAGCTCGGGCTAGT	60.0	174	DRR	Ca_18484	QR	B3
Ca1_P2_4131582	Ca1	4131582	AAAATAAATAAATAAATAAATAAATAAA/AAAATAAATAAATAAATAAATAAATAAATAAATAAATAAATAAA	TCCGAATAAATCGCATACCC	CCAGAGAGACAGATGCACCA	60.0	631	INTERGENIC	--	--	--
Ca1_P2_41503661	Ca1	41503661	CAAAAAA/CAAAAAAA	TCGATTATGCGTAGGGAAGG	AGCTTTGATTTCTCCATCGC	59.4	272	DRR	Ca_23123	--	--
Ca1_P2_41503912	Ca1	41503912	TCC/TC	TCGATTATGCGTAGGGAAGG	CTTGGTGGTTTTGAAACTCAAT	58.2	540	INTERGENIC	--	--	--
Ca1_P2_41504479	Ca1	41504479	CTT/CTTT	AAATTGAGTTTCAAAACCACCAA	TGCTCAGCTACGGTTCCTTT	60.0	883	INTERGENIC	--	--	--
Ca1_P2_4157854	Ca1	4157854	TA/T	GTGGACACGCGTATGATTTG	TAACAGGCGATGCAATTGAG	59.8	307	INTERGENIC	--	--	--

INDEL marker IDs	Chromosomes/unanchored scaffolds	Physical positions (bp)	InDels (*Kabuli* reference genome-CDC Frontier/PII)	Forward primers (5'-3')	Reverse primers (5'-3')	Anneling temperature (0C)	Expected amplified product size (bp)	Structural annotation		Functional annotation	
								Sequence components of *kabuli* genome	*Kabuli* gene accession IDs	NCBI-KOG	TFs
Ca1_P2_41639098	Ca1	41639098	GAAAAAAAAA/GAAAAAAAAAA	ACCCCTATTGATGTCCCCTC	CAAAAGTCCCTTATTGCCTTTG	60.0	592	INTERGENIC	--	--	--
Ca1_P2_4165803	Ca1	4165803	CTATATATATATATATATATAT/CTATATATATATATATATAT	CTCTTTGCCCTTTGAACTGC	CACAATATTGGCCACACAGC	60.0	405	INTERGENIC	--	--	--
Ca1_P2_41683229	Ca1	41683229	AATATATATATATATATA/AATATATATATATATA	GCTGAGCACACGATCCTTTT	GGATTTCTCGAAATGCTCCA	60.2	634	INTERGENIC	--	--	--
Ca1_P2_41695402	Ca1	41695402	CTA/CTATA	TACTTCCATGCAAACCAGCA	CCAACTCTGAAATACAAAAGCCA	60.5	486	INTERGENIC	--	--	--
Ca1_P2_41695561	Ca1	41695561	ATTTTTTTTTT/ATTTTTTTTTTTT	TTCCAAAGTATTGTGGGACACTT	TTGTTGGAGATAATTGAAGCTGA	58.9	964	INTERGENIC	--	--	--
Ca1_P2_41704419	Ca1	41704419	CATAATAAT/CATAAT	GGGGAATTTTTCATTTTGTGG	TGTTTTGGTTTCAAGGGACA	59.0	311	INTERGENIC	--	--	--
Ca1_P2_41704503	Ca1	41704503	CTTTTTTT/CTTTTTT	GGGGAATTTTTCATTTTGTGG	TGTTTTGGTTTCAAGGGACA	59.0	311	INTERGENIC	--	--	--
Ca1_P2_41705538	Ca1	41705538	AATTAT/AAT	CATCACCGTAACATGACTTACCA	ACATTGCACATCGAACGAAA	60.1	263	INTERGENIC	--	--	--
Ca1_P2_41705653	Ca1	41705653	ATTTTTTTT/ATTTTTTT	CATCACCGTAACATGACTTACCA	TCCCAATAATTTTTCCTTTTCG	59.4	723	INTERGENIC	--	--	--
Ca1_P2_41718814	Ca1	41718814	T/TG	GGGTCGAATGTCGATTCTGT	TCATCTCTCAGCCCCACTCT	59.9	156	INTERGENIC	--	--	--
Ca1_P2_41719424	Ca1	41719424	AATATATATATATATATAT/AATATATATATATATATATAT	TCCGCTCATCATCACAAAAA	ATTCTTTACAAGCTCTTAAACCTCA	57.1	478	INTERGENIC	--	--	--
Ca1_P2_41748047	Ca1	41748047	CCGTATT/CCGTATTCGTATT	ATTGGAGGATTGTTGATGCC	GAAAAATTAAACCCCCGGAA	60.0	303	INTERGENIC	--	--	--

INDEL marker IDs	Chromosomes/unanchored scaffolds	Physical positions (bp)	InDels (*Kabuli* reference genome-CDC Frontier/PII)	Forward primers (5'-3')	Reverse primers (5'-3')	Anneling temperature (0C)	Expected amplified product size (bp)	Structural annotation		Functional annotation	
								Sequence components of *kabuli* genome	*Kabuli* gene accession IDs	NCBI-KOG	TFs
Ca1_P2_41774189	Ca1	41774189	CAAAA/CAA	TCCATTCACACTTGTTGGGA	GGCACATTCATGTTCAGACG	60.1	237	DRR	Ca_22517	G	--
Ca1_P2_41817802	Ca1	41817802	TAT/TATTATTATAAT	TTTTTGTTGATGTGGTTCATCC	TCACCTTGCTTATCGTGGTTT	59.6	498	INTERGENIC	--	--	--
Ca1_P2_41863807	Ca1	41863807	GTTTTTTT/GTTTTTTTT	GGGAGGGTTACACATTGGAG	AAGGGTGCAATCACTACCTCA	59.6	962	INTERGENIC	--	--	--
Ca1_P2_41864496	Ca1	41864496	GAAAAAAAA/GAAAAAAAAA	TTATGGATGACCCTTCCCAA	AATTTACAATGTATGTGGGGGT	57.2	231	INTERGENIC	--	--	--
Ca1_P2_41912357	Ca1	41912357	AATATATATATATATATATATAT/AATATATATATATATATATAT	TATGGTTCAAATTGCGACGA	TGTTATATTTTGTCATGTCTTCGG	58.1	689	INTERGENIC	--	--	--
Ca1_P2_419222	Ca1	419222	TAAAAATTGAAAAATTGAAAA/TAAAAATTGAAAA	TCTGAGCTCGTTCAAATGGA	GTTGGAAGGGACAAAAGGGT	60.2	593	INTERGENIC	--	--	--
Ca1_P2_41978699	Ca1	41978699	TAAAAAAAA/TAAAAAAA	GAGAGTCGATGAGCGACAAC	AATTCTTTTCCACGCAATGG	59.9	782	INTERGENIC	--	--	--
Ca1_P2_41998443	Ca1	41998443	CG/CGG	CCATTTTTCTCACGTGCTTTC	ATCCCTCTATTGGTGGGTCC	60.0	339	INTERGENIC	--	--	--
Ca1_P2_42021612	Ca1	42021612	TAAA/TAAAA	CTCCTTCAGTTTTGAGACACGA	TCGATTATGCGTAGGGAAGG	60.1	442	INTERGENIC	--	--	--
Ca1_P2_42026239	Ca1	42026239	AAACAACGACAACAACGACAACAAC/AAACAACGACAACAAC	TCCTTCATATGGACCAAGCC	TCGTCGGTGTTGTCATTGTT	60.0	321	INTERGENIC	--	--	--
Ca1_P2_42026909	Ca1	42026909	AACAACAACGAC/AACAACAACGACAACAACGAC	GACGACGACAACATGACGAC	TGTTGTTGCCGTTGTTGTTT	60.1	374	INTERGENIC	--	--	--
Ca1_P2_42081964	Ca1	42081964	AT/ATT	TCAAAATCAAGCAGTGATTCG	TGCAAATAAATGGCCAAACA	59.9	858	INTERGENIC	--	--	--

INDEL marker IDs	Chromosomes/unanchored scaffolds	Physical positions (bp)	InDels (*Kabuli* reference genome-CDC Frontier/PII)	Forward primers (5'-3')	Reverse primers (5'-3')	Anneling temperature (0C)	Expected amplified product size (bp)	Structural annotation		Functional annotation	
								Sequence components of *kabuli* genome	*Kabuli* gene accession IDs	NCBI-KOG	TFs
Ca1_P2_4211915	Ca1	4211915	TTATATATATATATATATATA/TTATATATATATATATATATATA	ATCTCTCACGCGACGAAGTT	CCAAATCCGTTCCGCTAGTA	60.1	276	INTERGENIC	--	--	--
Ca1_P2_42209181	Ca1	42209181	GAAA/GAAAA	TTTTTCCCCCTTATGGTGAG	GCGAGCATGAGAAGTTGTTG	59.6	319	INTERGENIC	--	--	--
Ca1_P2_4233360	Ca1	4233360	TTTAAAATTAAAATTAAAATT/TTTAAAATTAAAATT	TAATGGGCTTCGTGGGTTAG	GTGCAGCACATTCGAGAGAA	60.1	683	DRR	Ca_00504	--	--
Ca1_P2_42363852	Ca1	42363852	GAAAAAAAA/GAAAAAAA	TGAGGATGTCCATGTTCCTTT	TTGACACAATATCCAGCGTGA	60.1	323	INTERGENIC	--	--	--
Ca1_P2_42374683	Ca1	42374683	ATA/ATATATATTAAATTA	CAGAGAGATGAGAGCCAGGG	AAGTTAAGGAACCGTTGATGC	58.3	302	INTERGENIC	--	--	--
Ca1_P2_42426294	Ca1	42426294	A/AC	CTTCCGGGATTCTAACCACA	TGGGGAGAGGAATTGATACG	59.9	698	INTERGENIC	--	--	--
Ca1_P2_42432936	Ca1	42432936	AATATATATATATATATATATAT/AATATATATATATATATAT	CAAATTCCTCTTGCTCCACC	TGAAAGCTGATGAAAATGGTC	57.8	695	INTERGENIC	--	--	--
Ca1_P2_42526018	Ca1	42526018	GAA/GA	TGCTCGACTACAAAAATCCCA	ATGGATGATGGGTCGACATT	60.0	507	INTERGENIC	--	--	--
Ca1_P2_42572678	Ca1	42572678	TTATATATATATATATATATA/TTATATATATATATATATATATA	TTCCTCGTATTTCCACCGTC	ACGATGATGACGATGACGAA	60.1	351	INTERGENIC	--	--	--
Ca1_P2_4260907	Ca1	4260907	A/AC	TTCAGCCTCGTCAGCTTTTT	TGTAAAATGACACCGTTCATCA	58.9	294	DRR	Ca_00507	--	--
Ca1_P2_42712797	Ca1	42712797	TCCA/T	GCTCGTCGAACTTTTTCAGG	AAAGCATCTCACACAGCACG	60.1	429	INTERGENIC	--	--	--
Ca1_P2_42712903	Ca1	42712903	CGTGT/CGT	AACAACAATTTCCCTTTGCG	TCTCCAGCAAAAGTCGTGTG	60.0	711	INTERGENIC	--	--	--

INDEL marker IDs	Chromosomes/unanchored scaffolds	Physical positions (bp)	InDels (*Kabuli* reference genome-CDC Frontier/PII)	Forward primers (5'-3')	Reverse primers (5'-3')	Anneling temperature (0C)	Expected amplified product size (bp)	Structural annotation		Functional annotation	
								Sequence components of *kabuli* genome	*Kabuli* gene accession IDs	NCBI-KOG	TFs
Ca1_P2_42737936	Ca1	42737936	AAATAATAATAATAATAATAATAATAATAATAATAATAATAAT/AAATAATAATAATAATAATAATAATAATAATAATAATAATAATAAT	CAGCGCATTTTTAGAAGGGA	TTTGCATGAACAACAATGTCAA	60.0	985	INTERGENIC	--	--	--
Ca1_P2_4279698	Ca1	4279698	TAA/TAAA	CTCAAAGGGTTCCTTCCACA	CAAAGTTTGGACAAACGTGAA	58.7	706	INTERGENIC	--	--	--
Ca1_P2_42862144	Ca1	42862144	AATATATATATATATATATATATATATATATATATATAT/AATATATATATATATATATATATATATATAT	CCCCAACTATTTTGGATGGA	CCTCTCCTTGTGATTGGCAT	60.1	724	DRR	Ca_13596	O	--
Ca1_P2_42928186	Ca1	42928186	CNNNNNNNNNN/CNNNNNN	TGAGCGCGTACGAAGAAATA	TTGTAACATTTTGCCCCCTC	59.8	497	INTERGENIC	--	--	--
Ca1_P2_43010316	Ca1	43010316	TT/TTAT	GGCGGAGGAGGGAAGTAATA	CTCATATTTTATGCCGCACG	59.2	300	INTERGENIC	--	--	--
Ca1_P2_43066988	Ca1	43066988	ATTT/ATT	AGCGCTGTAAAATGCAGACC	TGCATCACTTGGTTCGTGTT	60.2	312	DRR	Ca_13587	--	--
Ca1_P2_43119967	Ca1	43119967	AATATATATATATATATATATA/AATATATATATATATATATATATA	TTTCCGGGAATAAATCCCTC	GAAGGAGAGTTCGCGTTGAC	60.0	361	INTERGENIC	--	--	--
Ca1_P2_43335241	Ca1	43335241	AATATATATATATATATATATATATATATATATATATAT/AATATATATATATATATATATATATATATAT	CGTGTATATATGCGCACCCA	CACAAACGGAAAAGTCGGAT	60.0	788	INTERGENIC	--	--	--
Ca1_P2_43401371	Ca1	43401371	TAAAAAAAAAA/TAAAAAAAAA	TGACCGAAAATTAAATCCGC	TGTTGCCTTTAAAATTTCCTTTT	58.4	668	INTERGENIC	--	--	--
Ca1_P2_43441438	Ca1	43441438	CTA/CTAATTTA	TTCATGGTGTTTTCTTGGCA	CGAGAAATAGCTCACGAGGC	60.1	376	INTERGENIC	--	--	--
Ca1_P2_43469250	Ca1	43469250	A/ATATATTATT	GAGGGTTTGCAGCTCTTGTC	AACGCATCGGTAAACACTCC	60.0	547	INTERGENIC	--	--	--
Ca1_P2_43491966	Ca1	43491966	ATATTATTATTATTATTATTATTATTATTATTATTATTA/ATATTATTATTATTATTATTATTATTATTATTATTATTATTA	GAGGGTTTGCAGCTCTTGTC	AAAGCTGATCGTGGAGAAA	59.8	340	INTERGENIC	--	--	--

INDEL marker IDs	Chromosomes/unanchored scaffolds	Physical positions (bp)	InDels (*Kabuli* reference genome-CDC Frontier/PII)	Forward primers (5'-3')	Reverse primers (5'-3')	Anneling temperature (0C)	Expected amplified product size (bp)	Structural annotation		Functional annotation	
								Sequence components of *kabuli* genome	*Kabuli* gene accession IDs	NCBI-KOG	TFs
Ca1_P2_4359134	Ca1	4359134	AATATATATATATATATATATAT/AATATATATATATATATATATAT	GTTGGGGTTTTGCTGACCTA	GGGCAAGCCCAACCTATTTA	61.2	379	INTERGENIC	--	--	--
Ca1_P2_4360786	Ca1	4360786	TTA/TATCGTA	CGTCTGGTATCCGTCATGTG	TTGACTTTGTAAGTTGGAGTTTATTCA	59.6	492	INTERGENIC	--	--	--
Ca1_P2_43638401	Ca1	43638401	A/ATATGTC	CGCTTCATGTGTGTTGCTTT	ACCCCTCCTAAGTGGGTTGA	60.7	857	INTERGENIC	--	--	--
Ca1_P2_43704336	Ca1	43704336	T/TGACA	GGCCACTAAAAAGTGTCCCA	CCTTCTCCTGTACGCGAGTC	60.0	358	INTERGENIC	--	--	--
Ca1_P2_43781549	Ca1	43781549	CG/CGTGGTGGTTAG	ATCGGTGAACGAGGATCAAG	CTTTCCTTTCCGGTGAACAA	60.1	747	INTRON	Ca_13549	--	--
Ca1_P2_43834719	Ca1	43834719	CTTATTATTATTATTATTATTATTATTATTATTAT/CTTATTATTATTATTATTATTATTATTAT	AACCCTTACCTTGGCCACTT	AAAGGCCCTCGTCATGTTTA	59.6	867	INTERGENIC	--	--	--
Ca1_P2_43867975	Ca1	43867975	AATATATATATATATATATATATATATA/AATATATATATATATATATATATATA	TTGCTGGCATAAGTGAATCG	CCGTGAATTTTCATGGAATAAA	58.8	711	INTERGENIC	--	--	--
Ca1_P2_44045336	Ca1	44045336	CAAAAAAAAAAA/CAAAAAAAAAA	GAAGTGTGGGAACAACACGA	AAGTGGAACCAATGTTTGGC	59.8	474	INTERGENIC	--	--	--
Ca1_P2_44093089	Ca1	44093089	G/GAA	AAAGAGCCAAGAGCCAAACA	TATTTTGGAGCCCAGTGGTG	60.9	522	DRR	Ca_13529	S	--
Ca1_P2_44103543	Ca1	44103543	CTATTATTATTATTATTATTATTATT/CTATTATTATTATTATTATTATTATTATT	GCCACATTTATTCAATCGCA	GACCAGTAGGAAGTCGCTGG	59.9	904	INTERGENIC	--	--	--
Ca1_P2_44103725	Ca1	44103725	TAA/TA	TTTTCCAATCATACGAATCATTTTT	GACCAGTAGGAAGTCGCTGG	59.9	667	INTERGENIC	--	--	--
Ca1_P2_44103753	Ca1	44103753	T/TC	TTTTCCAATCATACGAATCATTTTT	GACCAGTAGGAAGTCGCTGG	59.9	667	INTERGENIC	--	--	--

INDEL marker IDs	Chromosomes/unanchored scaffolds	Physical positions (bp)	InDels (*Kabuli* reference genome-CDC Frontier/PII)	Forward primers (5'-3')	Reverse primers (5'-3')	Anneling temperature (0C)	Expected amplified product size (bp)	Structural annotation		Functional annotation	
								Sequence components of *kabuli* genome	*Kabuli* gene accession IDs	NCBI-KOG	TFs
Ca1_P2_44172001	Ca1	44172001	GTTTTT/GTTTTTT	ACCTATGCCCACTGATGAGG	GCAATTGCCAATGACGACTA	59.7	750	INTERGENIC	--	--	--
Ca1_P2_44201341	Ca1	44201341	TAAAAAAAAAAA/TAAAAAAAAAA	CAACGCATCAAGCTATTCCA	TATGCATGCGGACAAGAGTC	59.8	695	INTERGENIC	--	--	--
Ca1_P2_44247311	Ca1	44247311	GTTTTTTTTTT/GTTTTTTTTT	TTCTCCAGACTCTAGCCCGA	AGCTTCTCAGTAGGGGGCTC	60.0	592	INTERGENIC	--	--	--
Ca1_P2_44325935	Ca1	44325935	TAA/TA	TCTAGGTGTACACATCGCCG	AACCCAATAGTGGTGGTGGA	60.1	297	INTERGENIC	--	--	--
Ca1_P2_44351850	Ca1	44351850	TTATATATATATATATA/TTATATATATATATATATA	TGAAATTCGGAAGAACCAGG	CGCATTCCCTTCACCTCTAA	60.2	111	INTERGENIC	--	--	--
Ca1_P2_44376568	Ca1	44376568	C/CCAGAAA	CAGGGAACACGAAATGGAGT	TGACATTTGCCATCAAAGGA	60.0	757	INTERGENIC	--	--	--
Ca1_P2_44442119	Ca1	44442119	GACTTCTTTATTAACCAA/GA	ATGGCATGTGGTTTTCGTTT	GTCGCACCGTAATTATGCCT	60.0	433	DRR	Ca_23499	--	--
Ca1_P2_4444544	Ca1	4444544	AATATATATATATATATATATA/AATATATATATATATA	AATCGATTGGCTGAAATCCA	GACATTTAACAGCGCCCATT	60.0	663	INTERGENIC	--	--	--
Ca1_P2_44467058	Ca1	44467058	T/TG	ATGCAGCTCAAAAACAGCCT	GGTTCACCCAAAGATGGCTA	59.9	537	INTERGENIC	--	--	--
Ca1_P2_44467119	Ca1	44467119	GTT/GTTT	ATGCAGCTCAAAAACAGCCT	GGTTCACCCAAAGATGGCTA	59.9	537	INTERGENIC	--	--	--
Ca1_P2_44589434	Ca1	44589434	TA/TATAAAACA	TTGGTTTATTTGGGTTGGGA	AATAATGGCGTTCGGATTGA	60.3	797	INTERGENIC	--	--	--
Ca1_P2_44592980	Ca1	44592980	TAATGA/TAATGAATGA	TATTTTGGGGTTATCGAGCG	TTCCTGGTGGGATTCACATT	60.2	437	INTERGENIC	--	--	--

INDEL marker IDs	Chromosomes/unanchored scaffolds	Physical positions (bp)	InDels (*Kabuli* reference genome-CDC Frontier/PII)	Forward primers (5'-3')	Reverse primers (5'-3')	Anneling temperature (0C)	Expected amplified product size (bp)	Structural annotation		Functional annotation	
								Sequence components of *kabuli* genome	*Kabuli* gene accession IDs	NCBI-KOG	TFs
Ca1_P2_44650256	Ca1	44650256	T/TC	TCGTTTGCGTCATTGGATTA	AGGGAGGACGAAAATGACCT	59.9	444	INTERGENIC	--	--	--
Ca1_P2_44776081	Ca1	44776081	AATATATATATATATATATATATATATATATATATATATATA/AATATATATATATATATATATATATATATATATATATATATATATATATA	TGGAATACCATATATTTTACGTTTTCA	TGACGGATCCTTTCTTGACA	59.2	795	INTERGENIC	--	--	--
Ca1_P2_44795508	Ca1	44795508	ATTTTTTTTTTTTTTT/ATTTTTTTTTTTTTT	AAACGAACACCAAAGTTCCG	GGCTTTGTGGTTGTTGTCCT	60.0	525	INTERGENIC	--	--	--
Ca1_P2_44808505	Ca1	44808505	GAAAAAAAAA/GAAAAAAAAAA	GAAATGAGCTGCACACCAGA	TAAGGCATGCAAAGGTTGGT	60.5	383	INTERGENIC	--	--	--
Ca1_P2_44846998	Ca1	44846998	CTGTTTGTTTG/CTGTTTG	TACTGCCATACTACATCTGTTGC	AAAATCCAACAATCTCCCCC	60.0	459	INTERGENIC	--	--	--
Ca1_P2_44847079	Ca1	44847079	ATTTTTTTTTTT/ATATTTTTTTTTTTTTT	GACATGCTCAGTAATTGCCC	AAAATCCAACAATCTCCCCC	60.0	373	INTERGENIC	--	--	--
Ca1_P2_44847252	Ca1	44847252	ATCTGATGTTCTGATG/ATCTGATGTTCTGATGTTCTGATG	TGTTTTCTGCCTTTTTGCCT	AAAATCCAACAATCTCCCCC	60.0	274	INTERGENIC	--	--	--
Ca1_P2_44847313	Ca1	44847313	TATAATAATAATAATAATAATA/TATAATAATAATAATA	TGTTTTCTGCCTTTTTGCCT	AAAATCCAACAATCTCCCCC	60.0	274	INTERGENIC	--	--	--
Ca1_P2_44851320	Ca1	44851320	GCT/GCTGGACTGGGTTCT	CTCAGTTTTCATTGCTGCCA	TATGAGCCCCTCACTCCATC	60.0	440	INTERGENIC	--	--	--
Ca1_P2_44859136	Ca1	44859136	AAAATAAATAAATAAATAAATAAATA/AAAATAAATAAATAAATAAATA	TCATTTCAATGTCTGTAAGAATGATAC	AACCTTTTTGCAATGTTGGC	60.0	774	INTERGENIC	--	--	--
Ca1_P2_44921329	Ca1	44921329	ACTT/A	CCTCAAACGATTTTCTGGGA	CTTTCCCAATGCGTGAAGAT	60.1	478	INTERGENIC	--	--	--
Ca1_P2_45003074	Ca1	45003074	AA/ATAAAATTCCGAATCA	TCTTCCACATTGATTTTGTTGC	TGCTTATTCCCCAGATACCG	59.9	988	DRR	Ca_25714	--	--

INDEL marker IDs	Chromosomes/unanchored scaffolds	Physical positions (bp)	InDels (*Kabuli* reference genome-CDC Frontier/PII)	Forward primers (5'-3')	Reverse primers (5'-3')	Anneling temperature (0C)	Expected amplified product size (bp)	Structural annotation		Functional annotation	
								Sequence components of *kabuli* genome	*Kabuli* gene accession IDs	NCBI-KOG	TFs
Ca1_P2_45033030	Ca1	45033030	ATTTTTTTT/ATTTTTTTTTT	TTTCCCCATGCAGAGTTAGG	TAAACCAGCCACTAAGGCGT	59.8	894	INTERGENIC	--	--	--
Ca1_P2_45057964	Ca1	45057964	CAAA/CAA	AAACTAGATTATGGCCTGCG	TGCTCGCAATTAAAACATGC	59.8	426	INTERGENIC	--	--	--
Ca1_P2_45058076	Ca1	45058076	TAAAAAA/TAAAAAAA	AAACTAGATTATGGCCTGCG	TGCTCGCAATTAAAACATGC	59.8	426	INTERGENIC	--	--	--
Ca1_P2_45082109	Ca1	45082109	CATATATATATATATATATATATATATATATATAT/CATATATATATATATATATATATATATATATATATATATATATATATAT	GTGGCAGCTTCACTTGTTCA	CTCGTCGAATTCGGGTATGT	60.0	899	INTERGENIC	--	--	--
Ca1_P2_45104438	Ca1	45104438	TTAATAATAATAATAATAATAATAATAATAATAATAATAATAATAATAATAATAATAATAATAATAATAA/TTAATAATAATAATAATAATAATAATAATAATAATAATAATAATAATAATAATAATAATAATAATAATAATAATAATAA	TTTCAGGCGATTTTCGTACC	AAGTGGGGAACGTTGACTTG	60.0	744	INTERGENIC	--	--	--
Ca1_P2_45231841	Ca1	45231841	T/TATTGTGGCAG	TGCTATCAATTGTGCTCCCA	TTCATTGATCGGGGCTTAAC	59.9	619	INTERGENIC	--	--	--
Ca1_P2_45232358	Ca1	45232358	T/TAGCAATAATG	GTTAAGCCCCGATCAATGAA	CATCCCCCTCTTTAACCCAT	60.0	515	INTERGENIC	--	--	--
Ca1_P2_45243785	Ca1	45243785	TA/TAGACA	TGTGCCAAGGTTTCAGAACA	CTTTTATTGCAAGACCCCGA	60.1	605	DRR	Ca_26020	--	--
Ca1_P2_45246034	Ca1	45246034	C/CGTCTTGTT	CGATTTCGCTTTTAGCATCC	CAATTGCGATATTATGGGGG	60.0	253	INTERGENIC	--	--	--
Ca1_P2_45253870	Ca1	45253870	TA/T	TGCCAATAAGGCAATGTGAA	TGGATTAGGAGCGTGAAAACA	60.6	478	INTERGENIC	--	--	--
Ca1_P2_45339273	Ca1	45339273	TA/TAA	TGATTGGGAAATCGATTGGT	TTGATCCTTGCAGTCACTCG	60.0	566	DRR	Ca_24431	--	--
Ca1_P2_45354235	Ca1	45354235	AATATATATATATATATATATATATATATAT/AATATATATATATATATATATATATATATATATATATAT	TGACTTGACTCTTGTCCGCA	CAAAGTGTTTGGGCTCCTGT	60.1	596	INTERGENIC	--	--	--

INDEL marker IDs	Chromosomes/unanchored scaffolds	Physical positions (bp)	InDels (*Kabuli* reference genome-CDC Frontier/PII)	Forward primers (5'-3')	Reverse primers (5'-3')	Anneling temperature (0C)	Expected amplified product size (bp)	Structural annotation		Functional annotation	
								Sequence components of *kabuli* genome	*Kabuli* gene accession IDs	NCBI-KOG	TFs
Ca1_P2_45392704	Ca1	45392704	ATTT/ATTTT	GGGGGCTCTTCATTTTTCTC	CACAGTTTCATCGCTCCTCA	60.0	476	INTERGENIC	--	--	--
Ca1_P2_45394591	Ca1	45394591	CAAAAAAAAA/CAAAAAAAAAA	GGCCCTGATCCAGAAACATA	TGGTTCTTCTGTCGTTCGTG	59.9	847	INTERGENIC	--	--	--
Ca1_P2_45441724	Ca1	45441724	CAAAAAAAAAAA/CAAAAAAAAAAAA	GCCCTGAGATGAGAGTCAGG	CTGCTGTAACACCCCGTTTT	60.0	459	INTERGENIC	--	--	--
Ca1_P2_4547442	Ca1	4547442	TA/TAGAGAGA	TCGAACACACCAACGCTATC	CATTTTCCCCACCAAACAAC	60.1	356	INTERGENIC	--	--	--
Ca1_P2_45497100	Ca1	45497100	CTTTTTTTTTTT/CTTTTTTTTTTTTT	AGTATAAGTGGGGCCGTGTG	TGGATTGTGCCAACTTTTCA	60.1	237	INTERGENIC	--	--	--
Ca1_P2_45512409	Ca1	45512409	AAATAATAATAATAATAATAATAATAATAATAATAATAATAATAATAATAATAATA/AAATAATAATAATAATAATAATAATAATAATAATAATAATAATAATAATAATAATAATAATAATA	TCTCAATTCTGCAACGCATC	GCTTAGCACCGCTTTTCATC	60.0	881	INTERGENIC	--	--	--
Ca1_P2_4551372	Ca1	4551372	GTGAATTT/GT	AACCACGGACTAATTGTCTCA	AGTGTTCAACGTGGCTGTTG	59.8	187	INTERGENIC	--	--	--
Ca1_P2_45566205	Ca1	45566205	GT/GTT	ATTTACCGACGGACAAGCTG	AAGAGGATGAGCAACGCACT	60.0	635	DRR	Ca_24438	--	--
Ca1_P2_45609121	Ca1	45609121	C/CT	TCGCGTGAGAAAATAATCCC	CGAACCCTCGGTAAACAAAA	60.0	295	INTERGENIC	--	--	--
Ca1_P2_45635524	Ca1	45635524	AATATATATATATATATATATATATATATAT/AATATATATATATATATATATATATATATATATATAT	ATGTTTTGGATTTTGGCTCG	GGAGAAGGAGGGAGAAATGG	60.0	433	INTERGENIC	--	--	--
Ca1_P2_45737919	Ca1	45737919	TAAAAAAAAAAA/TAAAAAAAAAA	TGAGCATGGCTAAGTTGCAT	TGAGTGTTGTTGATCGACTGG	59.7	391	INTERGENIC	--	--	--
Ca1_P2_45749239	Ca1	45749239	GAAAAAAAAAA/GAAAAAAAAA	TTGTGCGCTTGACTGATGTT	GTTTTGGCGTTGGTTTCACT	60.0	857	INTERGENIC	--	--	--

INDEL marker IDs	Chromosomes/unanchored scaffolds	Physical positions (bp)	InDels (*Kabuli* reference genome-CDC Frontier/PII)	Forward primers (5'-3')	Reverse primers (5'-3')	Anneling temperature (0C)	Expected amplified product size (bp)	Structural annotation		Functional annotation	
								Sequence components of *kabuli* genome	*Kabuli* gene accession IDs	NCBI-KOG	TFs
Ca1_P2_45812093	Ca1	45812093	TA/TAA	ATAACCCGACAATCTCCACG	TGTTGTTGTTATCGTGCCGT	60.0	473	INTERGENIC	--	--	--
Ca1_P2_45901008	Ca1	45901008	CTTTTTTTTTTTTT/CTTTTTTTTTTT	GAGAAAAAGGTGGAAGCCGT	TTTTTCCGAGTTGGTCCTTT	58.7	673	INTERGENIC	--	--	--
Ca1_P2_45929718	Ca1	45929718	AATATATATATATATATATATATATATATATATATATATAT/AATATATATATATATATATATATATATATATATATATATATAT	TTCCTAAAACGAATGCACCC	GGCTAGGCCCGAAATAAAAG	60.1	506	INTERGENIC	--	--	--
Ca1_P2_45941271	Ca1	45941271	AATATATATATATA/AATATATATATATATA	GAAAGGAGATCACGAGCAGC	GTTGAATGGAACAGGCGAGT	60.1	723	INTERGENIC	--	--	--
Ca1_P2_45941295	Ca1	45941295	GATATATATATATATATATATATAT/GATATATATATATATATATATATATAT	GAAAGGAGATCACGAGCAGC	CAAGTCGTGGGGAGCATTAT	60.0	846	INTERGENIC	--	--	--
Ca1_P2_46023584	Ca1	46023584	CACTAGTTACTAGTTACTAGTTACTAGTTACT/CACTAGTTACTAGTTACTAGTTACT	TTTTCCACCAACAAACCTCA	AGTTGTGGAATCTGCCTGCT	59.9	330	INTERGENIC	--	--	--
Ca1_P2_46083317	Ca1	46083317	T/TATCCTTTTAAC	TGGGTATGTGGTTATGGCCT	TAACTTGGGAGTTGGCTTGG	60.1	415	INTERGENIC	--	--	--
Ca1_P2_46088799	Ca1	46088799	CTATATATATATATATATATATATATATATAT/CTATATATATATATATATATATATATATATATATAT	TAAGTCCGTAAGTGGTGCCC	GGGCTTTCCAAAGTGTTATCC	59.8	586	INTERGENIC	--	--	--
Ca1_P2_46186559	Ca1	46186559	AGTGATTGGTTTGTTTGTGATTGGTTTGTTTG/AGTGATTGGTTTGTTTGTGATTGGTTTGTTTGTGATTGGTTTGTTTG	AAAGCACACGATTTTCACCC	TTCGGCATCTAGCACTTCCT	60.0	775	INTERGENIC	--	--	--
Ca1_P2_46278771	Ca1	46278771	ATGGCCTGGCCTGGCCTGGCCTG/ATGGCCTGGCCTG	ACCTTCAGTCAGGGGCCTAT	AAGTCTGGTCTGGCCTGAAA	59.8	538	INTERGENIC	--	--	--
Ca1_P2_46285366	Ca1	46285366	GTTTTTTTTTT/GTTTTTTTTTTTT	AAATCGAACCGAACTGGTTTT	ATGCGGAAGTTTCATATGGG	59.8	379	INTERGENIC	--	--	--
Ca1_P2_46286614	Ca1	46286614	CTTTT/CTTT	CCAAGTTATGCTTGATCGCA	CGAGAACCATCCTGCATTTT	60.1	820	INTERGENIC	--	--	--

INDEL marker IDs	Chromosomes/unanchored scaffolds	Physical positions (bp)	InDels (*Kabuli* reference genome-CDC Frontier/PII)	Forward primers (5'-3')	Reverse primers (5'-3')	Anneling temperature (0C)	Expected amplified product size (bp)	Structural annotation		Functional annotation	
								Sequence components of *kabuli* genome	*Kabuli* gene accession IDs	NCBI-KOG	TFs
Ca1_P2_46286661	Ca1	46286661	GTAGGGATA/GTA	CCAAGTTATGCTTGATCGCA	CGAGAACCATCCTGCATTTT	60.1	820	INTERGENIC	--	--	--
Ca1_P2_46352138	Ca1	46352138	CACAAC/CACAACAAC	AGCGATGCATGTCTCAAGTG	TGCCCTTCTTCTTTTCCTAGTG	59.9	396	INTERGENIC	--	--	--
Ca1_P2_46352231	Ca1	46352231	TTAATAAT/TTAAT	AGCGATGCATGTCTCAAGTG	TCAATGGCTCTTTTACAGCG	59.1	827	INTERGENIC	--	--	--
Ca1_P2_46380105	Ca1	46380105	TTATATATATATATATATATATATATATATATATATATATATATAT/TTATATATATATATATATATATATATATATATATATATATATATATAT	TGTGTGGGGCATTGACTTTA	ACTTTCACACCCATTGCACA	60.0	494	INTERGENIC	--	--	--
Ca1_P2_46401991	Ca1	46401991	GGAGAGAGA/GGAGAGAGAGAGAGAGA	TCCCCTACCCATTGGTTACA	ACCTCTTCGGGAAGAACGAT	60.1	408	DRR	Ca_21546	--	--
Ca1_P2_46417437	Ca1	46417437	AGATTGA/AGA	TCGAATGGTGTTTTAACTCCTACA	CTGTTTTGGCCTCCACAAAT	60.0	541	INTERGENIC	--	--	--
Ca1_P2_46460221	Ca1	46460221	CTATATATATATATATATATATAT/CTATATATATATATATATATATATAT	TGCAATCCTACTCACCCCTC	AAACCGTGCACATTTTGTGA	60.0	574	DRR	Ca_21548	R	--
Ca1_P2_46491737	Ca1	46491737	CAAG/CAAGAAG	GATGCATCTCACCAAGGGTT	CGGTGCAAACTCTACGTTCA	59.9	420	INTERGENIC	--	--	--
Ca1_P2_46500141	Ca1	46500141	GCC/GC	GTAGCCAAAGGATTGCCAAA	ATGTGCAAGGAACCCGTTAG	60.0	586	INTERGENIC	--	--	--
Ca1_P2_46523548	Ca1	46523548	CAAAAAAAAAAAAAAAA/CAAAAAAAAAAAAA	GTGGACAAACCTGCATTCCT	ACCATGCACAGTTGCTGAGA	60.5	675	INTERGENIC	--	--	--
Ca1_P2_46524349	Ca1	46524349	TAAAAAAAAA/TAAAAAAAAAAA	TGAAGCATGCACAAATCCTC	TTGATGTAGCAACGGCTGAG	60.0	843	INTERGENIC	--	--	--
Ca1_P2_46525859	Ca1	46525859	GAAAAAA/GAAAAAAA	CCCACCTGGATACAAGGCTA	AGTAGAGGGAGTTCGGGTGC	60.6	711	INTERGENIC	--	--	--

INDEL marker IDs	Chromosomes/unanchored scaffolds	Physical positions (bp)	InDels (*Kabuli* reference genome-CDC Frontier/PII)	Forward primers (5'-3')	Reverse primers (5'-3')	Anneling temperature (0C)	Expected amplified product size (bp)	Structural annotation		Functional annotation	
								Sequence components of *kabuli* genome	*Kabuli* gene accession IDs	NCBI-KOG	TFs
Ca1_P2_46526676	Ca1	46526676	TAAAA/TAAA	TTAAAATTCCGAGGCGAAAA	ACATGGTTGTGGTGGTGATG	60.1	698	INTERGENIC	--	--	--
Ca1_P2_46528558	Ca1	46528558	CT/CTT	TTAAAATTCCCTCCTCGGCT	AACTCTGGGAACCCTCCTGT	60.0	398	INTERGENIC	--	--	--
Ca1_P2_46528763	Ca1	46528763	G/GC	TTAAAATTCCCTCCTCGGCT	GGGGAAAACATCCCGTTATT	59.9	865	INTERGENIC	--	--	--
Ca1_P2_46530950	Ca1	46530950	CAAATTGAAAATTGAAAATT/CAAATTGAAAATT	ATTTGAGAATGTTCGGGCAA	GTCTGCGGATAATTTCGCTT	59.3	665	DRR	Ca_21551	M	--
Ca1_P2_46531041	Ca1	46531041	T/TG	GCGAAAATAATGTTGATGCAGA	GTCTGCGGATAATTTCGCTT	59.3	139	DRR	Ca_21551	M	--
Ca1_P2_46539303	Ca1	46539303	TTCAAATCTTCATCAAATCTTC/TTCAAATCTTC	TCAAGCCAATCTTCCCAAAC	TCTGATGACAAAAGGGGGAG	60.0	757	INTERGENIC	--	--	--
Ca1_P2_46539360	Ca1	46539360	CAAA/CAAAA	TCCCAAACAGCCTGATAACC	CTGATGACAAAAGGGGGAGA	60.0	744	INTERGENIC	--	--	--
Ca1_P2_46598729	Ca1	46598729	GATATATATATAT/GATATATATATATATAT	TGATTTGTTAGCTTGTGGCAA	TGGAAGCATGATCCTCAACA	60.2	477	INTERGENIC	--	--	--
Ca1_P2_46779104	Ca1	46779104	AATATATATATATATAT/AATATATATATATATATAT	TGCCAACCCAACATCACTAA	GCAGCTACGGACTCGTTTTC	60.0	617	INTRON	Ca_25114	--	C2H2
Ca1_P2_46797747	Ca1	46797747	CCCTAAACCTGAAATCCTAAACCTGAAA/CCCTAAACCTGAAATCCTAAACCTGAAATCCTAAACCTGAAA	AAACCCGAAACGCTTAACCT	AGGGTTTCTGGTGTAGGGCT	60.0	385	INTERGENIC	--	--	--
Ca1_P2_46831943	Ca1	46831943	CTTTT/CTTT	GTGGTTGGTTTGCAAGAGGT	AACTGGGTGAGAGCCTGAGA	60.0	725	INTRON	Ca_25116	U	--
Ca1_P2_46856347	Ca1	46856347	GCT/GCTGAACT	ATGCAATCCGATAAGCAACC	ACGATTTTAGAAGAGGCGCA	60.0	980	INTERGENIC	--	--	--

INDEL marker IDs	Chromosomes/unanchored scaffolds	Physical positions (bp)	InDels (*Kabuli* reference genome-CDC Frontier/PII)	Forward primers (5'-3')	Reverse primers (5'-3')	Anneling temperature (0C)	Expected amplified product size (bp)	Structural annotation		Functional annotation	
								Sequence components of *kabuli* genome	*Kabuli* gene accession IDs	NCBI-KOG	TFs
Ca1_P2_46916401	Ca1	46916401	GATATATATATATATATATATA/GATATATATATATATATATA	CAGCAAGCACCAACCACTTA	TTGCCAGTTATTTTTGCAAGG	60.1	363	DRR	Ca_12949	--	--
Ca1_P2_46978141	Ca1	46978141	TTATATATATATATAT/TTATATATATATATATAT	GAATGCAGTTGGATCCTCGT	ATTACCCCTAACCACTCCCG	60.1	764	INTERGENIC	--	--	--
Ca1_P2_46999543	Ca1	46999543	AAT/AATAATTATTAT	AAAATTCTGTTTGGGGGACC	TGGCTCATGAGAGTTTGTGG	59.8	651	INTERGENIC	--	--	--
Ca1_P2_47016631	Ca1	47016631	ACTCTCTCTCTCTCT/ACTCTCTCTCTCTCTCT	ATTGCCTCTCAGTCGCGTTA	AAAATTCGACATCGTGAGGG	59.9	531	INTERGENIC	--	--	--
Ca1_P2_47029872	Ca1	47029872	CAAA/CAAAA	CGGATTTTTCAATTTTCCGA	CAAGGGTTTTGGTCATGCTT	60.0	749	INTERGENIC	--	--	--
Ca1_P2_47059529	Ca1	47059529	AG/AGTGGG	GGAGAACGTTAAGGCAGCAG	GAAACCAGAGGCAACCAAAA	60.1	585	INTERGENIC	--	--	--
Ca1_P2_47097781	Ca1	47097781	TAAAAAAA/TAAAAAAAA	TTTCCCACCATCAAACCTGT	TTGAAATGGTGCTAGGTCCC	59.9	460	INTRON	Ca_12937	--	--
Ca1_P2_47108136	Ca1	47108136	ATTTTTTTTTTT/ATTTTTTTTTT	TTTCTTTGGTCCATAAACTTTTGTT	CATACTATACCGCACCCCCA	60.6	620	INTERGENIC	--	--	--
Ca1_P2_47116478	Ca1	47116478	AATATATATATATATATATATATAT/AATATATATATATATATATAT	ACACGCTTCCCTGTTGACAT	TAGGCCTGAACTGGGATGAC	60.1	772	INTRON	Ca_12935	--	ERF
Ca1_P2_47148955	Ca1	47148955	C/CT	GCTTGTTGGTCATGCAGCTA	AGGCAGGAAGATGATGCAGT	59.8	807	DRR	Ca_12932	--	S1Fa-like
Ca1_P2_4720237	Ca1	4720237	CTTA/CTTAATTA	TCAGAGTTCATTGCAAACGG	GGGATTCCCATTTTCCATCT	60.0	691	INTRON	Ca_00555	T	MYB_related
Ca1_P2_47230123	Ca1	47230123	AATATATATATATATATATATATATATAT/AATATATATATATATATATATATATATATATATATATAT	CAAAAGTCCCCTGTCATCGT	CAACCTAAACCATGCAGCAA	59.7	745	INTERGENIC	--	--	--

INDEL marker IDs	Chromosomes/unanchored scaffolds	Physical positions (bp)	InDels (*Kabuli* reference genome-CDC Frontier/PII)	Forward primers (5'-3')	Reverse primers (5'-3')	Anneling temperature (0C)	Expected amplified product size (bp)	Structural annotation		Functional annotation	
								Sequence components of *kabuli* genome	*Kabuli* gene accession IDs	NCBI-KOG	TFs
Ca1_P2_47321461	Ca1	47321461	AACA/AACATGCGACAAAAACACA	GGTTGGTAGAGGAATAGCGG	ATTGGGATTTTGTTCGTTGC	59.8	827	DRR	Ca_12921	--	--
Ca1_P2_4732444	Ca1	4732444	AATATATATATATATATATATATATATATATA/AATATATATATATATATATATATATATATATATATATATA	GGTTTAGGCAACGGACTGAA	TGCGTTAATTAAATCCAAAGGTT	59.0	527	INTERGENIC	--	--	--
Ca1_P2_47336854	Ca1	47336854	TC/TCATAACAC	GCGAGTTACAGTCGGTGGTT	CCGATTCACGGAATTTGTCT	59.9	670	INTERGENIC	--	--	--
Ca1_P2_47337298	Ca1	47337298	C/CAAATATATACT	ATTGGGCATAGGAAGCACTG	AGTTGACCACGGACCCATAG	59.8	533	DRR	Ca_12919	--	--
Ca1_P2_47350834	Ca1	47350834	A/ATT	CGCGTAAGTAGTTTGGTTGATG	TTTTTCCCGAACAAAAATGA	58.1	388	INTERGENIC	--	--	--
Ca1_P2_4736299	Ca1	4736299	ATTTTTTTTT/ATTTTTTTT	TGTTTTGGCACCATTTAGCA	GGAGCAACAGCTAAGGCAAC	60.0	901	DRR	Ca_00557	--	--
Ca1_P2_47373127	Ca1	47373127	T/TTGTTGC	CAGCAGCAAGAGCCATGTTA	GCCATTCCTCTACCAACCAA	59.9	475	INTERGENIC	--	--	--
Ca1_P2_47385431	Ca1	47385431	ANNNNNNNNNN/ANNNNNNNN	ACCTCGGTTTCGGTTTCTTT	TGACTTTCTCACCCCATTCC	59.9	196	INTERGENIC	--	--	--
Ca1_P2_4742142	Ca1	4742142	TAAA/TAA	GACCGCGTAGATGTGGAACT	GGATGAGACCTCCTACTTGCC	60.1	677	INTERGENIC	--	--	--
Ca1_P2_47455552	Ca1	47455552	TTAATAATAATAATAATAA/TTAATAATAATAATAA	TTGGGGTCAGTCAAACACAA	CGGTGAAATAGGTATGCCAAA	59.8	600	INTERGENIC	--	--	--
Ca1_P2_47456048	Ca1	47456048	AAATAATAATAATAATAATAATAATAATAA/AAATAATAATAATAATAATAATAATAA	TTTGGCATACCTATTTCACCG	TCCAAAAGAAAAAGGAAGAAGTTG	60.1	681	INTERGENIC	--	--	--
Ca1_P2_47489764	Ca1	47489764	AATTATTATTATTATTATTATTATTATTATTATT/AATTATTATTATTATTATTATTATTATTATTATTATTATT	GAGCGGAGAGAAGATGATTGA	TAAAAACCAATTCGGTTCGG	59.8	581	INTERGENIC	--	--	--

INDEL marker IDs	Chromosomes/unanchored scaffolds	Physical positions (bp)	InDels (*Kabuli* reference genome-CDC Frontier/PII)	Forward primers (5'-3')	Reverse primers (5'-3')	Anneling temperature (0C)	Expected amplified product size (bp)	Structural annotation		Functional annotation	
								Sequence components of *kabuli* genome	*Kabuli* gene accession IDs	NCBI-KOG	TFs
Ca1_P2_47508107	Ca1	47508107	TTATATATATATATATATATATATATATATATATATATATA/TTATATATATATATATATATATATATATATATATATATATATATA	CCTTCTCATTGCGAACCCTA	CTTCGACACTGTAGCACCCA	59.9	380	INTERGENIC	--	--	--
Ca1_P2_47511261	Ca1	47511261	ATTTTTTTT/ATTTTTTTTT	TCGCGTGTTAGAGCTATCGTT	TCTTTTTGCCCAAACTCTTGA	59.8	644	INTERGENIC	--	--	--
Ca1_P2_47573948	Ca1	47573948	GAAA/GAAAA	GCATCAAGTGCGTCTGTGTT	ACGCAGCCAGGAACAATAAT	59.6	492	INTERGENIC	--	--	--
Ca1_P2_47630703	Ca1	47630703	TAAAAAGAAAAAGAAAAAGAAA/TAAAAAGAAAAAGAAAAAGAAAAAGAAA	CGTGTCCGGCAAAGTAAAAT	GTGATGCACGCAGTAATTGG	60.1	517	INTERGENIC	--	--	--
Ca1_P2_47630977	Ca1	47630977	AC/ACC	GGCCTTCCATCGTGAATAAA	TTTCGAATTCCAGAACTGCC	60.2	916	INTERGENIC	--	--	--
Ca1_P2_47654204	Ca1	47654204	ATTTTTTTTTTT/ATTTTTTTTTT	AGGAGCACTTTCGGAGTTTG	CCTCTTGTTGTTGAGGCAGC	61.0	394	INTERGENIC	--	--	--
Ca1_P2_47664148	Ca1	47664148	ATTTTTTTTTT/ATTTTTTTTTTT	CGAATGAACGACGAGTCTCA	TCCGGATATATTTGGCCTTG	59.8	952	INTERGENIC	--	--	--
Ca1_P2_47676057	Ca1	47676057	ATTTTTTTTTTT/ATTTTTTTTTTTT	AATCCCTCCCTGCCTAAAAA	ATGTGGTGGTCCAATCAACA	59.7	619	INTRON	Ca_12903	--	--
Ca1_P2_47698915	Ca1	47698915	AAGAGAGAGAGAGAGAGAGAGAGAGAGAGAGA/AAGAGAGAGAGAGAGAGAGAGAGAGAGAGAGAGAGA	TCATGTGACCACCAAGTGCT	GAGGGCGGTTATAGGTAGGG	59.8	861	INTERGENIC	--	--	--
Ca1_P2_47712293	Ca1	47712293	ACCCCCCC/ACCCCCCCC	CCCTCTATCCACCCAGTGAA	ACCAAATTTCCGGTGAACAA	60.2	758	INTERGENIC	--	--	--
Ca1_P2_47822692	Ca1	47822692	TTATATATATATATATATAT/TTATATATATATATATATATAT	TGTTATGCATCACCTTTTCCA	TGTCCAGTGCCATGATTTCT	59.1	426	INTERGENIC	--	--	--
Ca1_P2_47875198	Ca1	47875198	AT/ATT	GAAAAACAAGACCCGCGATA	TTCCTATTGCTCACCCCTTG	60.1	466	INTERGENIC	--	--	--

INDEL marker IDs	Chromosomes/unanchored scaffolds	Physical positions (bp)	InDels (*Kabuli* reference genome-CDC Frontier/PII)	Forward primers (5'-3')	Reverse primers (5'-3')	Anneling temperature (0C)	Expected amplified product size (bp)	Structural annotation		Functional annotation	
								Sequence components of *kabuli* genome	*Kabuli* gene accession IDs	NCBI-KOG	TFs
Ca1_P2_47883205	Ca1	47883205	GAAAAAAAAAAAAA/GAAAAAAAAAAAA	GTCGGCAAACATATTGCCTT	ACCGCTTAATCGTGACCAAC	60.0	629	INTERGENIC	--	--	--
Ca1_P2_47906772	Ca1	47906772	AATATATATATATATATATATATAT/AATATATATATATATATATATATAT	CAACTTGGGTTTTGGTTTCC	AATGGCTGAGACAGCTACCC	59.3	520	INTERGENIC	--	--	--
Ca1_P2_47913934	Ca1	47913934	CTTTA/CTTTATTTA	ATACCCAGGGACGGTTTTTC	ATGTACGTGGCTTTGCATGA	60.1	383	DRR	Ca_12886	O	E2F/DP
Ca1_P2_47916750	Ca1	47916750	ATT/ATTTCAGGTTT	GTTGGTGATGCCACTGAATG	GAGAGAAAGGCAAGTGTGGG	59.8	979	DRR	Ca_12885	O	M-type
Ca1_P2_47920789	Ca1	47920789	TTTATTATTATTATTATTATTATTATTATTATTATT/TTTATTATTATTATTATTATTATTATTATTATTATTATT	AGTTGTTCATCGCAACATCG	CAACTTCATTCGTTTTGGCA	59.7	629	INTERGENIC	--	--	--
Ca1_P2_47945221	Ca1	47945221	TCAAGCCA/TCA	AGGACAGTGCCAAAAGATGG	GTCATGTATGCATTGGGCAG	60.0	226	DRR	Ca_12882	R	--
Ca1_P2_48010052	Ca1	48010052	TTATATATATATATATATATATATATA/TTATATATATATATATATATATATATATATA	TGCTCACGTGTCTTTCCATC	TTTTTCACGAACTTCTCCGC	60.4	677	INTERGENIC	--	--	--
Ca1_P2_48064288	Ca1	48064288	TTATATATATATATATATATATATATA/TTATATATATATATATATA	TGGGTGGATAGATGAGAGGC	TCTGAATAGGTTTGGTCGGC	60.1	404	INTERGENIC	--	--	--
Ca1_P2_48091761	Ca1	48091761	TAAAAAAAA/TAAAAAAA	TCTGGAGATGGGACCTCTTG	CCAAATCGGAGGAAGTTGTT	59.0	700	INTERGENIC	--	--	--
Ca1_P2_48099930	Ca1	48099930	TAAAAAAAAAA/TAAAAAAAAAAAA	ACAAACGCCAATGTTGATGA	GGTAACAACCGATTCCCCTT	60.1	244	INTERGENIC	--	--	--
Ca1_P2_48114146	Ca1	48114146	TTATATATATATATATATATATATAT/TTATATATATATATATATATATAT	TGATTGTTGTTGGGCCACTA	TTTGTTCTCCATTTTGCTTTTG	59.3	807	INTERGENIC	--	--	--
Ca1_P2_48172069	Ca1	48172069	AAATAATAATAATAATAATAATAATAATAATAATAATAATA/AAATAATAATAATAATAATAATAATAATAATAATAATAATAATAATAATA	TAAATGGCAGGTAAAGGCGA	AGACTTTGCTGCCACCTCAT	59.9	315	DRR	Ca_12862	R	WRKY

INDEL marker IDs	Chromosomes/unanchored scaffolds	Physical positions (bp)	InDels (*Kabuli* reference genome-CDC Frontier/PII)	Forward primers (5'-3')	Reverse primers (5'-3')	Anneling temperature (0C)	Expected amplified product size (bp)	Structural annotation		Functional annotation	
								Sequence components of *kabuli* genome	*Kabuli* gene accession IDs	NCBI-KOG	TFs
Ca1_P2_48210401	Ca1	48210401	ATATATATATATT/A	TGACATTGCCGGTGAAATAA	CACTGTGTTGTGTGTGCTGC	60.0	489	INTERGENIC	--	--	--
Ca1_P2_48236817	Ca1	48236817	TAA/TA	TGGGTGTTGCCCATATATCA	TTTGGATCCATTTCATTTGATT	58.3	626	INTERGENIC	--	--	--
Ca1_P2_48261489	Ca1	48261489	TAAAAAAAAA/TAAAAAAAA	GGCAACATGGTGAAGAGACA	TCTTCTTTATGGACCAGCCC	59.1	611	INTERGENIC	--	--	--
Ca1_P2_4900100	Ca1	4900100	TATTCAAT/TATTCAATTCAAT	TCTTTGCAGCATGCTTTTTG	TTTCTGAAGCGACCAATTCC	60.2	561	DRR	Ca_00575	--	--
Ca1_P2_4900555	Ca1	4900555	AGAAGTAGACTCTGAAGTA/AGAAGTA	GGAATTGGTCGCTTCAGAAA	TCCATAAGCTGATCCCCAAG	60.0	428	DRR	Ca_00576	R	M-type
Ca1_P2_4907874	Ca1	4907874	AGGGAAANNNNNNNNNNGGGAAA/AGGGAAA	CGTGGGAGAGAAAAAGCAAG	GTTTGAAGCATCACAAGCGA	60.0	592	CDS (FRAME SHIFT)	Ca_00577	T	M-type
Ca1_P2_49600	Ca1	49600	CTT/CT	TGGGTTCAAATTTCTCGACC	GAACCATATTGCCACCCATC	60.0	916	INTERGENIC	--	--	--
Ca1_P2_5002157	Ca1	5002157	AATATATATATATATATATATAT/AATATATATATATATATATATATATATAT	AAAGGGACGTGTATCGTTGG	TTGGCGGCTTCGTATAATTC	60.1	488	INTERGENIC	--	--	--
Ca1_P2_5040464	Ca1	5040464	ACC/AC	CAAAATGCTCGCAATGAGAA	GAACTCCCCATGTGGTTGAT	59.6	635	INTERGENIC	--	--	--
Ca1_P2_5106336	Ca1	5106336	CT/CTCTAT	CGAGTGATTTGCTTCATTGG	TGTTACGCATTGTTTGGGAA	60.0	651	INTERGENIC	--	--	--
Ca1_P2_5132478	Ca1	5132478	GT/GATCCCGCTCATTCT	GAGTCCGATCCACCGTAGAA	AGTTCCAGCTTGCTTCCAAA	60.0	722	INTERGENIC	--	--	--
Ca1_P2_5189106	Ca1	5189106	TTAATAATAATAATAATAATAATAATAATAATAATAATAATAATAATAATAA/TTAATAATAATAATAATAATAATAATAATAATAATAATAATAATAA	TGGTGGCTACCACATGAAGA	GGAGAACGTTAAGGCAGCAG	60.0	716	INTERGENIC	--	--	--

INDEL marker IDs	Chromosomes/unanchored scaffolds	Physical positions (bp)	InDels (*Kabuli* reference genome-CDC Frontier/PII)	Forward primers (5'-3')	Reverse primers (5'-3')	Anneling temperature (0C)	Expected amplified product size (bp)	Structural annotation		Functional annotation	
								Sequence components of *kabuli* genome	*Kabuli* gene accession IDs	NCBI-KOG	TFs
Ca1_P2_5254524	Ca1	5254524	CATTCATTATTCATTATT/CATTCATTATT	AATTCACCCAAACGACCAAC	ATAATTGGTTGGAGCCCACA	60.2	521	INTERGENIC	--	--	--
Ca1_P2_5255796	Ca1	5255796	TTTATTATTATTATTATTATTATTATTATTATTATTATTATTATTA/TTTATTATTATTATTATTATTATTATTATTATTATTATTATTA	CCGTTGAAAACGTGGATCTT	GTGTGTCGGTGCTTTTTGAA	59.7	990	INTERGENIC	--	--	--
Ca1_P2_5273611	Ca1	5273611	TTATATATATATATATATATATATAT/TTATATATATATATATATATATATATATATAT	TGCATTAATTTTGAAAACGTCAA	TGTTTGCCACGGTTGTTAAA	60.0	593	INTERGENIC	--	--	--
Ca1_P2_5341655	Ca1	5341655	TTATATATATATATATATATATATAT/TTATATATATATATATATATATAT	GGAAGGTTATCTCCACACGAA	CATCGGTAACCATGTGCATC	59.8	562	INTERGENIC	--	--	--
Ca1_P2_5376097	Ca1	5376097	ATGGTGGTG/ATGGTG	AAAAGATTAGTGGCTGCCGA	TTTGTGGTGGTCAAAGTCCA	60.0	219	INTERGENIC	--	--	--
Ca1_P2_5403862	Ca1	5403862	C/CTTTATTATT	ACGGTTGACTAATTGTGCCA	TCGCATCCCTACGATACGAT	60.5	629	DRR	Ca_00627	--	--
Ca1_P2_5421383	Ca1	5421383	AAA/AAATAA	GCTGGGTTTCAGGTTTGAGA	CAGGGTGGCTCTTGCTAAAC	59.9	579	INTERGENIC	--	--	--
Ca1_P2_5423307	Ca1	5423307	CTTTT/CTTTTT	CGGGGTAGTTTTTGTATTCCC	GGTGACGCCTTTTAATTTGC	59.6	640	INTERGENIC	--	--	--
Ca1_P2_5425033	Ca1	5425033	TTTTATTT/TTTTATTTATTTATTT	TCACGGTGAAAGTTCTGTGC	AAATCCCAATAAGCAAGGCA	59.5	427	INTERGENIC	--	--	--
Ca1_P2_5433441	Ca1	5433441	TC/T	TCGGGATTTGTTTGTTTACCA	GGGCCCTATGCCTTTTTATG	60.6	511	INTERGENIC	--	--	--
Ca1_P2_5441585	Ca1	5441585	TGACTCCAGA/TGA	TGGATGAATTTGGTTGGATTT	ACTTGGAATTCAATGCAGGC	60.1	688	DRR	Ca_00629	G	--
Ca1_P2_5445910	Ca1	5445910	GTAATAATAATAATAA/GTAATAATAATAA	GAACGCAATTAGGTCAGGGA	CACAAGGCAACACAACGATT	59.6	364	DRR	Ca_00630	--	--

INDEL marker IDs	Chromosomes/unanchored scaffolds	Physical positions (bp)	InDels (*Kabuli* reference genome-CDC Frontier/PII)	Forward primers (5'-3')	Reverse primers (5'-3')	Anneling temperature (0C)	Expected amplified product size (bp)	Structural annotation		Functional annotation	
								Sequence components of *kabuli* genome	*Kabuli* gene accession IDs	NCBI-KOG	TFs
Ca1_P2_5447363	Ca1	5447363	AAGAGAATGAGAGA/AAGAGA	AACAATTTGGGGTCATTATTGC	ATTTTGATTGGGTAGCGCAG	60.1	580	DRR	Ca_00630	--	--
Ca1_P2_5464246	Ca1	5464246	T/TC	TGGTAAATGGCCATGAGGAT	CCCTCAACACACTCATGTGG	60.0	468	INTRON	Ca_00631	--	--
Ca1_P2_5482579	Ca1	5482579	TAAA/TAAAA	ACAAATCCGGATCGAAACAC	GATTTGCTGCCACTCCTCTT	59.4	929	DRR	Ca_00633	--	S1Fa-like
Ca1_P2_5484796	Ca1	5484796	TAA/TAAA	CGCGCGTTGTGTTTATAAGT	TTACCAAGATTGCCCCTCTG	60.1	444	INTERGENIC	--	--	--
Ca1_P2_5488076	Ca1	5488076	ATGTGT/ATGTGTGT	ATCCCATAGGTTCCCTCCAC	ATGGCCTCCACTTTGATTTG	59.9	300	INTRON	Ca_00634	--	--
Ca1_P2_5500920	Ca1	5500920	CTAT/CTATAAACGTCATAT	GGAAGGGAATCGATGAGAAA	TATTCATTGGTTGGGGGAAA	60.0	456	INTERGENIC	--	--	--
Ca1_P2_5501358	Ca1	5501358	CAA/CA	TTTCCCCCAACCAATGAATA	CTTGCTTTGTGCCATGAAGA	60.0	523	INTERGENIC	--	--	--
Ca1_P2_5521185	Ca1	5521185	ATTTTT/ATTTTTT	TCGGGAACATCTATCCAATCT	GTGCCACCGTTATGATGCTA	59.6	138	INTERGENIC	--	--	--
Ca1_P2_5529079	Ca1	5529079	ATTTTTTTT/ATTTTTT	AGCCCGTGAGCATACAGTTC	ATCCATCCCACAACTCAAGC	59.9	599	INTERGENIC	--	--	--
Ca1_P2_5531171	Ca1	5531171	AATATATATATATATATATATATA/AATATATATATATATATATA	TGCTTCTCTTGGCTTTTTCC	CCAAACTTCCCACATTGGAG	60.3	265	INTERGENIC	--	--	--
Ca1_P2_5600652	Ca1	5600652	AGG/A	TTGGAATTGTGCCAATGTGT	TCAATATGGGTCCACTGTGC	59.4	269	DRR	Ca_00644	--	ERF
Ca1_P2_5611256	Ca1	5611256	ATTTTTTTTT/ATTTTTTTT	TCTCAGAACCGACACTGTTCA	TGAATTCAGGTGAGCGTGAG	60.0	886	INTRON	Ca_00645	--	G2-like

INDEL marker IDs	Chromosomes/unanchored scaffolds	Physical positions (bp)	InDels (*Kabuli* reference genome-CDC Frontier/PII)	Forward primers (5'-3')	Reverse primers (5'-3')	Anneling temperature (0C)	Expected amplified product size (bp)	Structural annotation		Functional annotation	
								Sequence components of *kabuli* genome	*Kabuli* gene accession IDs	NCBI-KOG	TFs
Ca1_P2_5623602	Ca1	5623602	GTATATATATATATATATA/GTATATATATATATATATATA	CAATAAAATATCAATTCCAATCGC	CGTATTACACATATGCGCGG	60.0	448	INTERGENIC	--	--	--
Ca1_P2_5688442	Ca1	5688442	TTATATATATATATATATATATATATATATATATATATA/TTATATATATATATATATATATATATATATATATATATATATATATATA	TGTTTTCCCCATAAAAATCCA	GGCAAAGTTCCCACTCAAAA	60.1	550	INTERGENIC	--	--	--
Ca1_P2_5754591	Ca1	5754591	CATA/CA	AACCTCCAGATCCTCGAACA	CTCCACAAAGAAAAGGGCAA	60.2	344	DRR	Ca_00667	J	TALE
Ca1_P2_5803481	Ca1	5803481	TTAAGATAAGAT/TTAAGAT	GCTCATGAGTTCCCTACCCA	GTCAGGGGCTCGTGTATGAT	60.0	560	INTRON	Ca_00671	O	FAR1
Ca1_P2_5804081	Ca1	5804081	AT/ATT	AATAACACGGGCACAAGAGG	AGGTCCACCAGGTTTGTCAG	60.0	457	INTRON	Ca_00671	O	FAR1
Ca1_P2_5816176	Ca1	5816176	GATTTTTATTTTTATTTTTATTTTTATT/GATTTTTATTTTTATT	CGATGAAAAATTGGACACGA	TTTAGTGCATGAGGTGCCAA	60.3	825	INTERGENIC	--	--	--
Ca1_P2_5872810	Ca1	5872810	TTATATATATATATATATATAT/TTATATATATATATATATAT	GTAAGCTTCTTGCGACCACC	TGAAATGACTATAATTTTGATCGAATG	59.7	454	INTERGENIC	--	--	--
Ca1_P2_5874435	Ca1	5874435	TAAAAAAAAA/TAAAAAAAA	TCTTCTACAAAAGCGAGGACAA	GCAGTTCACGGACTCTCACA	60.0	597	INTERGENIC	--	--	--
Ca1_P2_5907254	Ca1	5907254	ATCTTCTTCTTCTTCTTCTTCTTC/ATCTTCTTCTTCTTCTTC	TTGCCCAAGAGCCAATAATC	CTACCGTTGAAACCGCAAAT	60.0	905	CDS (large-effect mutations)	Ca_00678	ODR	--
Ca1_P2_5955465	Ca1	5955465	AATATATATATATATATATATATATATA/AATATATATATATATATATATATATATA	TGCAGTGGAAGTGTTGTTGA	GAACGAGGTCGGCAAAAGTA	60.2	686	INTERGENIC	--	--	--
Ca1_P2_6015678	Ca1	6015678	ATATAT/ATATATTTATAT	AAATTTGAAGTCGCTGTTTGC	TGACCTCAAGTTTGCAGCAC	60.0	562	DRR	Ca_00689	I	--
Ca1_P2_6023010	Ca1	6023010	TTATATATATATATATATATAT/TTATATATATATATATATAT	GCGGCAAATACGAAGAAGAG	TCCAATTTTTAGGTTTTGTTTTTCA	60.1	545	INTERGENIC	--	--	--

INDEL marker IDs	Chromosomes/unanchored scaffolds	Physical positions (bp)	InDels (*Kabuli* reference genome-CDC Frontier/PII)	Forward primers (5'-3')	Reverse primers (5'-3')	Anneling temperature (0C)	Expected amplified product size (bp)	Structural annotation		Functional annotation	
								Sequence components of *kabuli* genome	*Kabuli* gene accession IDs	NCBI-KOG	TFs
Ca1_P2_6130097	Ca1	6130097	T/TG	GAAATAATTAACGCCGGCAA	CGTGTGGGTCTGTGATGTTC	60.0	456	INTERGENIC	--	--	--
Ca1_P2_6137600	Ca1	6137600	T/TC	TCTCATAGAAGGCGACGGTT	CATTTCCGGAGCCAGTAAAA	60.1	412	INTERGENIC	--	--	--
Ca1_P2_6142050	Ca1	6142050	T/TTAA	AAGATTCAACCTCCCCAACC	TTGGATTAGGGTGAGGAACG	59.9	416	INTERGENIC	--	--	--
Ca1_P2_6156071	Ca1	6156071	AACACAC/AACAC	TTCGTGAAAGCTTCCGTCTT	TTTAACCGCTGGTTTTACCG	60.0	315	DRR	Ca_00700	G	--
Ca1_P2_6156406	Ca1	6156406	TTA/TTATA	TTGGCAAAAATGCGATAACA	TCCATCAAACCAAATGAACA	57.4	847	DRR	Ca_00700	G	--
Ca1_P2_6156764	Ca1	6156764	AACGTA/AACGTACGTA	AAGGGAGTGGGATTTTTGTTTT	TGGTCCTCAATGACACTCCA	60.1	836	DRR	Ca_00700	G	--
Ca1_P2_6158170	Ca1	6158170	ATTTT/ATTT	TGGTTTGTCATTCCGGTCAT	ATGTCTCATTCATGCTCCCC	59.9	359	INTERGENIC	--	--	--
Ca1_P2_6174612	Ca1	6174612	TTAT/TTATAT	CCTCTCATCCACATCCATCC	GATATCCATCGGCTTACGACA	59.9	538	INTERGENIC	--	--	--
Ca1_P2_6176716	Ca1	6176716	GGAAT/G	GCACATAGATAGGTGCCTGC	CGGGCTGAACTATTACCACC	59.5	162	INTERGENIC	--	--	--
Ca1_P2_6176982	Ca1	6176982	A/AAT	GGTGGTAATAGTTCAGCCCG	AATCTTTGCCAATGGTTTGT	57.1	564	INTERGENIC	--	--	--
Ca1_P2_6202713	Ca1	6202713	CTTTTT/CTTTTTT	CGCTTCCTTCTTCACACACA	AAAAGGCAATACATCCAATCAA	58.5	640	INTERGENIC	--	--	--
Ca1_P2_6203281	Ca1	6203281	GTTTTTTTTT/GTTTTTTTTTT	CTCCTTGAGTTTTGAGACACGA	TCGATTATGCGTAGGGAAGG	60.1	453	INTERGENIC	--	--	--

INDEL marker IDs	Chromosomes/unanchored scaffolds	Physical positions (bp)	InDels (*Kabuli* reference genome-CDC Frontier/PII)	Forward primers (5'-3')	Reverse primers (5'-3')	Anneling temperature (0C)	Expected amplified product size (bp)	Structural annotation		Functional annotation	
								Sequence components of *kabuli* genome	*Kabuli* gene accession IDs	NCBI-KOG	TFs
Ca1_P2_6204822	Ca1	6204822	AAGA/AA	TGGTTTGGTTGCATCACTTT	TCGAAATTTGTGTTCCAAAGA	59.6	543	DRR	Ca_07880	T	--
Ca1_P2_6205233	Ca1	6205233	CAAAAAAAAAAA/CAAAAAAAAAAAA	GGGTATTGGATAAAAATTTCAAAAA	TGAAAATTAAGGGACGGGTTT	59.7	604	DRR	Ca_07880	T	--
Ca1_P2_6206568	Ca1	6206568	CTTTTTT/CTTTTTTTT	TTATGCTTTCAACCAGTGCG	TGTCGGATAAGTGTGATATATTCG	58.1	747	INTERGENIC	--	--	--
Ca1_P2_6212978	Ca1	6212978	AATATATATATAT/AATATATATATATAT	GTGCGCAGTACAATTCTCCA	CAACCGCTCCCAAATAGAAA	60.1	573	INTERGENIC	--	--	--
Ca1_P2_6219100	Ca1	6219100	TTACGGTAT/TT	TGGGAAAACTTGTTCCATGAG	TTGATCTCCACATCACCAAGA	59.1	587	INTERGENIC	--	--	--
Ca1_P2_6219483	Ca1	6219483	TTTGTTGTTGTTGTTG/TTTGTTGTTGTTG	CTTGGTGATGTGGAGATCAAA	TGGAGTTATGATATTTGTGCCA	58.0	543	INTERGENIC	--	--	--
Ca1_P2_6245829	Ca1	6245829	TAAAAAAAAAAA/TAAAAAAAAAA	TCATATTCCCGCACTTGTGA	GCAAGTGGTTGAGTGCTTGA	60.0	500	INTERGENIC	--	--	--
Ca1_P2_6259182	Ca1	6259182	AATGGTCATGGTCATGGTCA/AATGGTCATGGTCA	TGATGATTTTGCTCTTCCCC	GTCCACTCCACCGATCAACT	60.0	575	INTRON	Ca_07885	R	bHLH
Ca1_P2_6259902	Ca1	6259902	AAAATAAATA/AAAATAAATAAATAAATA	AGTTGATCGGTGGAGTGGAC	AAGCGGGAGGTCTTGAGTTC	60.8	621	DRR	Ca_07885	R	bHLH
Ca1_P2_6267078	Ca1	6267078	GAAA/GA	TTTCTTGCACTGGTTTGTGC	TGGGTCCCTCATTGTACTCC	59.8	560	INTERGENIC	--	--	--
Ca1_P2_6267684	Ca1	6267684	GT/GTTT	GGAGTACAATGAGGGACCCA	GCCGCGGTATTATTCCTACA	60.0	384	INTERGENIC	--	--	--
Ca1_P2_6269792	Ca1	6269792	GAAGAAAAAGAAAAAGAAAAAG/GAAGAAAAAGAAAAAGAAAAAGAAAAAG	AACCACTATTATGGCGGTGC	CAGGAATCTCTGCCACACAA	59.8	415	INTERGENIC	--	--	--

INDEL marker IDs	Chromosomes/unanchored scaffolds	Physical positions (bp)	InDels (*Kabuli* reference genome-CDC Frontier/PII)	Forward primers (5'-3')	Reverse primers (5'-3')	Anneling temperature (0C)	Expected amplified product size (bp)	Structural annotation		Functional annotation	
								Sequence components of *kabuli* genome	*Kabuli* gene accession IDs	NCBI-KOG	TFs
Ca1_P2_6288427	Ca1	6288427	TA/TAA	TGGCTTCTGATAGATTGCGA	CCAAGCCATCTAACCTGGAA	60.1	591	INTERGENIC	--	--	--
Ca1_P2_6293780	Ca1	6293780	TAAA/TAAAA	ACACACTGATCGATGATCCC	TATCTTTTTGGTGGGCCAAG	59.9	716	INTERGENIC	--	--	--
Ca1_P2_6298845	Ca1	6298845	T/TG	GAGGCAAAATTGGCTCTTGT	TAATTGACGACGACGACGAG	59.9	124	DRR	Ca_07890	--	--
Ca1_P2_6299112	Ca1	6299112	TGTGCATCTCATAAG/TG	CTCGTCGTCGTCGTCAATTA	TGAAAGCTCATTTTTCGTATGTTG	60.5	439	DRR	Ca_07890	--	--
Ca1_P2_6299712	Ca1	6299712	T/TGTTG	CAACATACGAAAAATGAGCTTTCA	GTGACATCAAGCCGGATTCT	60.1	895	DRR	Ca_07890	--	--
Ca1_P2_6301080	Ca1	6301080	ATTTTTTT/ATTTTTTTTTT	TGCCAGTTTATGGTCAACCTC	CACAATTCCACAATTGACGC	60.0	850	INTERGENIC	--	--	--
Ca1_P2_6303467	Ca1	6303467	CTGTGTG/CTGTG	GGTGTGGCCTAAAGCAAGAG	GGTGCACATGAGCAATGAAT	59.5	573	DRR	Ca_07891	--	--
Ca1_P2_6311323	Ca1	6311323	T/TCACGA	CGGAGAACCAATCACTCACA	GTTGAGCCGAGGCAACTATG	60.8	601	INTERGENIC	--	--	--
Ca1_P2_6314613	Ca1	6314613	CAAA/CAA	CACAAAATTAAAAAGCACGTGAA	GCGACATACGAAACGAGGAT	60.1	592	INTERGENIC	--	--	--
Ca1_P2_6317430	Ca1	6317430	TTAAAG/TTAAAGCCATAAATAATAAAG	GCTGAGCATGTTGGAACAAA	AAGATCCCCCATGGAAACTC	60.1	606	INTRON	Ca_07892	B	MYB_related
Ca1_P2_6326554	Ca1	6326554	ATCACT/ATCACTCACT	CAAGATGAAACCCGCTTTGT	TTTGTTTCACATTCCTGCAAA	59.2	566	INTERGENIC	--	--	--
Ca1_P2_6337132	Ca1	6337132	AAA/AAATAA	TCGGCCCACATTATTGTTTT	ATAAATCCCACAAGTGCCCA	60.2	258	INTERGENIC	--	--	--

INDEL marker IDs	Chromosomes/unanchored scaffolds	Physical positions (bp)	InDels (*Kabuli* reference genome-CDC Frontier/PII)	Forward primers (5'-3')	Reverse primers (5'-3')	Anneling temperature (0C)	Expected amplified product size (bp)	Structural annotation		Functional annotation	
								Sequence components of *kabuli* genome	*Kabuli* gene accession IDs	NCBI-KOG	TFs
Ca1_P2_6338411	Ca1	6338411	AAATTAA/AAA	CAACGTTGATTGGGTCAAAA	CCCTTCATACACGGACCATC	60.2	662	INTERGENIC	--	--	--
Ca1_P2_6342773	Ca1	6342773	TG/T	AGGGTCATGGGTGTCACAAT	AGCACATCCACCTTCCATTC	59.9	693	INTERGENIC	--	--	--
Ca1_P2_6365021	Ca1	6365021	CT/CTT	TACCGCACCACATTCACCTA	CGAGTTTATTTCGCTTGGGA	60.2	280	INTERGENIC	--	--	--
Ca1_P2_6375372	Ca1	6375372	GTATATATATATA/GTATATATATATATA	GGCAATTGAGGGCTTTATCA	ACGGTTTATGCACTCCAAGC	60.1	483	INTERGENIC	--	--	--
Ca1_P2_6383460	Ca1	6383460	ATT/ATTT	TACCGACATCGTTGTTTCCA	TTGACAACGAATGAAGCAGC	60.0	600	INTERGENIC	--	--	--
Ca1_P2_6400773	Ca1	6400773	TCAAACCAAAC/TCAAACCAAACCAAAC	TGGATAATTTTTCGGATTCAAGA	AATTGAGGGACGTCTTGGTG	60.0	444	INTERGENIC	--	--	--
Ca1_P2_6404378	Ca1	6404378	ATTTTTTTT/ATTTTTTTTT	AATTGCATTTTATGTCCGCC	TGTTGCAGGTTCTGAGATCG	60.0	858	INTERGENIC	--	--	--
Ca1_P2_6415075	Ca1	6415075	GTAATAATAATAATAATAAT/GTAATAATAATAATAATAATAATAAT	TCCCTCCTTTTCTCCCTTGT	CCCTCGTTACATGTGCCTTT	60.0	971	INTRON	Ca_07905	P	MIKC
Ca1_P2_6415669	Ca1	6415669	CTATTATTAT/CTATTATTATTAT	AAAGGCACATGTAACGAGGG	CATGTGGATGTGGGGTTTCT	60.6	589	INTRON	Ca_07905	P	MIKC
Ca1_P2_6425270	Ca1	6425270	GTT/GTTT	TCTTCTCTGGCAACCGTTCT	AGCCACCGTTGATGAAAATC	59.9	479	INTERGENIC	--	--	--
Ca1_P2_6430790	Ca1	6430790	GTAAT/GT	CCACCTCTGCCACCAACTAT	ATCGGTCGTATTTTGAACCG	59.8	689	INTERGENIC	--	--	--
Ca1_P2_6436385	Ca1	6436385	GTACTTGTTTTACTTGT/GTACTTGT	ATCCAAACTCCCTTCTTGCC	TCGAAAGGAGGCAAAATCAA	60.7	409	INTRON	Ca_07907	T	C3H

INDEL marker IDs	Chromosomes/unanchored scaffolds	Physical positions (bp)	InDels (*Kabuli* reference genome-CDC Frontier/PII)	Forward primers (5'-3')	Reverse primers (5'-3')	Anneling temperature (0C)	Expected amplified product size (bp)	Structural annotation		Functional annotation	
								Sequence components of *kabuli* genome	*Kabuli* gene accession IDs	NCBI-KOG	TFs
Ca1_P2_6443801	Ca1	6443801	TAAAA/TAAA	CTCCTTACACTTCTTCGCCG	GCCAACACTTTTGCAACCAT	60.9	387	DRR	Ca_07908	K	Trihelix
Ca1_P2_6453816	Ca1	6453816	AT/ATT	GGTCTTGCTGACCTTGAAGC	CACGTTAAAATCTCTAACGATGAA	57.6	644	DRR	Ca_07909	--	Trihelix
Ca1_P2_6460044	Ca1	6460044	GAAAAAAAAAA/GAAAAAAAAAAA	AGCTTTCTCAAGGCACGGTA	GAATGGCCTTTCCTCAACAA	60.1	742	INTERGENIC	--	--	--
Ca1_P2_6463116	Ca1	6463116	TAAAAAAAAAA/TAAAAAAA	AACCATGAGGTCATAACGCC	TCATAACAAAATGCTTTCTTCAACA	60.1	332	INTERGENIC	--	--	--
Ca1_P2_6463783	Ca1	6463783	TGATTATAGATTATAAATTATAGATTA/TGATTATAGATTATAGATTATAAATTATAGATTA	TGCTTATTTTGAATGGCCTAA	TGTGGGTAGACATTTCAGGCT	59.6	166	INTERGENIC	--	--	--
Ca1_P2_6467793	Ca1	6467793	GGAG/GGAGAG	AAAGGGCAAGATTTGTCCAC	AAGGGATAATGTGTTCCCCC	59.9	197	DRR	Ca_07910	S	--
Ca1_P2_6479424	Ca1	6479424	AATA/AA	CAGGAGATTGAAAGTGGCGT	GGCAAGCAAGTGTCACAAAA	59.9	540	INTERGENIC	--	--	--
Ca1_P2_6481070	Ca1	6481070	TA/T	TTTCACACCCACTTAAGCCC	AATGGGATTGTGGGATCGTA	60.0	433	INTRON	Ca_07911	D	B3
Ca1_P2_6489856	Ca1	6489856	AAAGAGCAAGAGCAAGAGCAAGAGCAAGAGCA/AAAGAGCAAGAGCAAGAGCAAGAGCA	CCTGGTTGGCTGATGAAAGT	CTCCGTTTAGGTGCTGGAAC	59.7	513	CDS (large-effect mutations)	Ca_07911	D	B3
Ca1_P2_6523042	Ca1	6523042	GAACAAACA/GAACA	GGAGCAGTTGTTCCCAAAAA	CGAGGAAGAAGCGGATATTG	59.8	444	DRR	Ca_07917	I	Trihelix
Ca1_P2_6534234	Ca1	6534234	TATAAATAAA/TATAAA	AAATTTGAGGGGCACAACTG	TACAACTTTTCTGGGCCGAC	60.1	669	INTERGENIC	--	--	--
Ca1_P2_6534439	Ca1	6534439	CA/CAA	AAATTTGAGGGGCACAACTG	TACAACTTTTCTGGGCCGAC	60.1	669	INTERGENIC	--	--	--

INDEL marker IDs	Chromosomes/unanchored scaffolds	Physical positions (bp)	InDels (*Kabuli* reference genome-CDC Frontier/PII)	Forward primers (5'-3')	Reverse primers (5'-3')	Anneling temperature (0C)	Expected amplified product size (bp)	Structural annotation		Functional annotation	
								Sequence components of *kabuli* genome	*Kabuli* gene accession IDs	NCBI-KOG	TFs
Ca1_P2_6534633	Ca1	6534633	A/AC	TTACTGGGCCACGTTTACAA	TGGTTCCAAAGGAAAAGCAT	59.5	328	INTERGENIC	--	--	--
Ca1_P2_6535430	Ca1	6535430	GTTATAGTTTTTATAGTT/GTTATAGTT	GCGATGATCGATGACACTCTT	CAAAGTCGAGTATCACCGCC	60.7	236	INTERGENIC	--	--	--
Ca1_P2_6535745	Ca1	6535745	TAAAAAAAA/TAAAAAAAAAAA	CACTCAATGGTGGCACACTC	TCTTTTCATATTTCGACGATGC	59.2	702	INTERGENIC	--	--	--
Ca1_P2_6543436	Ca1	6543436	AAA/AAATAAATAA	GTTTGACATGATCGTCGTGG	TATGGTAATCCCTCACCCCA	60.0	655	INTERGENIC	--	--	--
Ca1_P2_6543932	Ca1	6543932	A/AGAAGAAGAGAGAG	TGGGGTGAGGGATTACCATA	AGCATGAGAGACAGGTGCAA	59.6	492	INTERGENIC	--	--	--
Ca1_P2_6559705	Ca1	6559705	CATATATATATATATATATA/CATATATATATATATATATA	GGGTCAAAACCCTTGGAAAT	TCCTCGTGTTCACTTTCGC	60.0	910	INTERGENIC	--	--	--
Ca1_P2_6593836	Ca1	6593836	TT/TTATAT	AAGTGGGTTGTCGCTACCTC	CGTTCTTCTTCTTCCACATTCC	60.1	590	DRR	Ca_07923	R	--
Ca1_P2_6697194	Ca1	6697194	TTTATTTTTAAATGAAC/T	TTAAAAGTGGGGCAATCTGG	TGTTAGCATTTGACAAAAAGTAATAGC	59.3	793	DRR	Ca_07933	--	--
Ca1_P2_6712717	Ca1	6712717	ATTTTTT/ATTTTT	ACAAGGAACCTTTCCCCTTT	TTGTCCGAATAAACCTATTTTTCA	59.0	923	DRR	Ca_07935	R	Nin-like
Ca1_P2_6806099	Ca1	6806099	CA/C	TCATGCCTTCAAATTCCACA	ATGCGTGCTTCAAAACTCAA	59.5	970	DRR	Ca_07947	S	--
Ca1_P2_6965841	Ca1	6965841	CTTTTTT/CTTTTTTT	GTTTTCGTTCTCTCCTCCCC	TGTCATCAGGGAAACCACAA	59.9	759	INTERGENIC	--	--	--
Ca1_P2_6999458	Ca1	6999458	TTATATATATATATATATATATATAT/TTATATATATATATATATATATATATAT	AACAACGGCTTTCATTGACTTT	TTTTTCCTGAAACAGGCAGT	57.5	795	INTERGENIC	--	--	--

INDEL marker IDs	Chromosomes/unanchored scaffolds	Physical positions (bp)	InDels (*Kabuli* reference genome-CDC Frontier/PII)	Forward primers (5'-3')	Reverse primers (5'-3')	Anneling temperature (0C)	Expected amplified product size (bp)	Structural annotation		Functional annotation	
								Sequence components of *kabuli* genome	*Kabuli* gene accession IDs	NCBI-KOG	TFs
Ca1_P2_7014540	Ca1	7014540	CTATTATTATTATTATTATTATTATTATTATTA/CTATTATTATTATTATTATTATTATTATTATTATTATTA	CCGTAAGTCCACCACCATCT	GACCGCAAATGTAATTTAGCCT	59.5	639	INTERGENIC	--	--	--
Ca1_P2_7215414	Ca1	7215414	AA/AACA	AACCCCCAACTACAAGGTCC	CAATTTGACAAGCCCCCTAA	59.9	639	INTERGENIC	--	--	--
Ca1_P2_7215436	Ca1	7215436	GAAAAAAAA/GAAAAAAAAAAAA	AACCCCCAACTACAAGGTCC	CAATTTGACAAGCCCCCTAA	59.9	639	INTERGENIC	--	--	--
Ca1_P2_7274484	Ca1	7274484	TAAAAAAAAA/TAAAAAAAAAA	TTTTGAAGTCGTTCACGCTG	CCCTAACACACCACACATGC	59.9	613	INTERGENIC	--	--	--
Ca1_P2_7423449	Ca1	7423449	CTTTTTTTTTTTT/CTTTTTTTTTTT	CCATTTGGTTGCTTGGTTTT	TTTTGCTTATATGTTTGGTTCTTGA	59.2	118	INTERGENIC	--	--	--
Ca1_P2_7582813	Ca1	7582813	CATATATATATATATATATATATATATATATATATATATATATAT/CATATATATATATATATATATATATATATATATATATATATAT	CTCCAGTCGATGAGATTCAAA	TGTTGAAATTCCATTCATTAGCA	59.5	769	INTERGENIC	--	--	--
Ca1_P2_7600007	Ca1	7600007	CAAAAAAAAAAAA/CAAAAAAAAAAAAA	ATCAAACGGTTGGTGAAAGC	CGAACCGGTTTCAAACACTT	60.0	815	INTRON	Ca_08014	--	HD-ZIP
Ca1_P2_7634571	Ca1	7634571	GT/GTT	AAAGTGAATTGCTCAATTTCGTC	AATTGACACGTGAAGATTTGTTT	57.7	777	INTERGENIC	--	--	--
Ca1_P2_7634633	Ca1	7634633	ATTTTTTTT/ATTTTTTTTTT	TCCAGCACAAATACCTTTTTACC	TGAATTCGCGTTGTTTGTGT	60.2	666	INTERGENIC	--	--	--
Ca1_P2_7634819	Ca1	7634819	AA/AAGAAAGA	TCCAGCACAAATACCTTTTTACC	TGAATTCGCGTTGTTTGTGT	60.2	666	INTERGENIC	--	--	--
Ca1_P2_7675315	Ca1	7675315	AA/AAGA	TGGTATTGAATTTTATGGATGTTTG	TCGGATTTAACTTGGGTCTCA	59.6	505	INTERGENIC	--	--	--
Ca1_P2_7675331	Ca1	7675331	GAAA/GAA	TGGTATTGAATTTTATGGATGTTTG	TCGGATTTAACTTGGGTCTCA	59.6	505	INTERGENIC	--	--	--

INDEL marker IDs	Chromosomes/unanchored scaffolds	Physical positions (bp)	InDels (*Kabuli* reference genome-CDC Frontier/PII)	Forward primers (5'-3')	Reverse primers (5'-3')	Anneling temperature (0C)	Expected amplified product size (bp)	Structural annotation		Functional annotation	
								Sequence components of *kabuli* genome	*Kabuli* gene accession IDs	NCBI-KOG	TFs
Ca1_P2_7677905	Ca1	7677905	CTATTATTATTATTATTATTATT/CTATTATTATTATTATTATTATT	ATCGGAATCAAACCACTTCG	TGAGCCGGAATATTGAGATTG	60.0	567	INTERGENIC	--	--	--
Ca1_P2_7678204	Ca1	7678204	C/CATTT	TCACGCACTTCATACCAATCA	CATGAGCAATCACGGTTTTG	60.1	776	INTERGENIC	--	--	--
Ca1_P2_7687191	Ca1	7687191	AC/A	CACGACACAAAATTTCACTTATGA	GGATGGACTTCATGCTTCCT	59.1	369	INTERGENIC	--	--	--
Ca1_P2_7688348	Ca1	7688348	AAAAA/AAAAAGAAAA	AAGCCAAAATAATAAGGCTCAAGA	AGCACAACAGGGATTTACGG	60.0	895	INTERGENIC	--	--	--
Ca1_P2_7706750	Ca1	7706750	AAACAACAACAA/AAACAACAA	TTTGGTGTCGGTTTGACAAT	AAATTTCCAGACACATGCCC	59.8	685	INTERGENIC	--	--	--
Ca1_P2_7711941	Ca1	7711941	AGTT/A	CGCATCAGCCAACTACTCAA	GCCTATGACGAAGCTTCCAA	60.4	314	DRR	Ca_08023	K	bHLH
Ca1_P2_7727269	Ca1	7727269	T/TC	GAAAGCAAATCTTCCATCCAA	CGCGTGAATAGCGACAATAA	59.9	690	INTERGENIC	--	--	--
Ca1_P2_7775058	Ca1	7775058	C/CCTTAAT	AGCCCAACCCTTGACCTAAT	GCACGGAAACAACATGTGAC	60.0	838	INTERGENIC	--	--	--
Ca1_P2_7777758	Ca1	7777758	GATATATATATATATATATATATATATATATATAT/GATATATATATATATATATATATATATAT	TTCCATGACGATTTGACGAG	CGTCGCACGGAAATAATGTA	59.6	552	INTERGENIC	--	--	--
Ca1_P2_7778630	Ca1	7778630	CTTT/C	CCACACACATCAACCACCAC	GGGCGATTGACTTGAGACAT	60.1	444	INTERGENIC	--	--	--
Ca1_P2_7783469	Ca1	7783469	CGACCTGA/CGA	ACAAAATGCTGAAATTGCCA	CTGTTGTTTTTGCCTCAACG	59.4	334	INTERGENIC	--	--	--
Ca1_P2_7788046	Ca1	7788046	T/TCAA	ATCCAAAATACATTGCGCTT	TGAATTGGCGATGAATTTGA	60.0	482	INTERGENIC	--	--	--

INDEL marker IDs	Chromosomes/unanchored scaffolds	Physical positions (bp)	InDels (*Kabuli* reference genome-CDC Frontier/PII)	Forward primers (5'-3')	Reverse primers (5'-3')	Anneling temperature (0C)	Expected amplified product size (bp)	Structural annotation		Functional annotation	
								Sequence components of *kabuli* genome	*Kabuli* gene accession IDs	NCBI-KOG	TFs
Ca1_P2_7789078	Ca1	7789078	TAAAAAAAAAAAAA/TAAAAAAAAAA	AGGGAAGAAGCACGATCTTT	AGATGTTAACCCGCGTTTTG	60.0	728	INTERGENIC	--	--	--
Ca1_P2_7789677	Ca1	7789677	ATTTTT/ATTT	CAAAACGCGGGTTAACATCT	TGAAGGGAGGAGATTTGGTG	60.0	779	INTERGENIC	--	--	--
Ca1_P2_7790700	Ca1	7790700	AAAA/AAAACTGCAAA	TCCATAATGTCAAGTTTTGAGCC	ACATCCACAGCACTTGCATC	59.7	949	INTERGENIC	--	--	--
Ca1_P2_7790927	Ca1	7790927	TAAGAG/T	AATGTGCGCATTTCATGTTC	TGCAACAAGAGTGAGCCAAG	60.2	642	INTERGENIC	--	--	--
Ca1_P2_7802950	Ca1	7802950	CAA/CAAA	TTGCTGTTTTGTGAGAGTTATCC	ACCTCGCAACAGGTCCATAC	60.0	278	INTERGENIC	--	--	--
Ca1_P2_7810637	Ca1	7810637	G/GAA	AACGATTGGTTGAGACCCAG	ACCTTGTGTCTATCTGGCCG	60.1	505	DRR	Ca_08031	--	FAR1
Ca1_P2_7813090	Ca1	7813090	GTT/GTTT	ACCCATTCCGCATACCAATA	CACTTGCTGAGGTGCAAAAA	60.0	580	INTRON	Ca_08032	--	--
Ca1_P2_7820020	Ca1	7820020	CTTTTTTTT/CTTTT	AGGTATAGCTCTTGCCGGGT	CAGACACTGCGCGATTCTTA	60.2	501	INTRON	Ca_08033	G	FAR1
Ca1_P2_7824017	Ca1	7824017	AT/ATT	AAGAATGGTGGACACTTGCC	ACCCATGTGGCTACTGGAAG	60.0	433	INTERGENIC	--	--	--
Ca1_P2_7884700	Ca1	7884700	CAAAAA/CAAA	TTCACTCCCGAGGAAAACAC	TCCAATTGAGGAGATCCCAG	60.0	438	INTRON	Ca_08037	I	bHLH
Ca1_P2_7895366	Ca1	7895366	GAAA/GAA	TTGCCACAAGCTAAAAGGCT	TGGTCAGTTTGGCCTATTCC	59.9	514	DRR	Ca_08038	--	--
Ca1_P2_7902635	Ca1	7902635	TAAAA/TA	CGCGCCTATTTTATGCATTT	GCCTTTCAAAATCAATTGCC	59.5	611	INTERGENIC	--	--	--

INDEL marker IDs	Chromosomes/unanchored scaffolds	Physical positions (bp)	InDels (*Kabuli* reference genome-CDC Frontier/PII)	Forward primers (5'-3')	Reverse primers (5'-3')	Anneling temperature (0C)	Expected amplified product size (bp)	Structural annotation		Functional annotation	
								Sequence components of *kabuli* genome	*Kabuli* gene accession IDs	NCBI-KOG	TFs
Ca1_P2_7913956	Ca1	7913956	GTGACTTGTTGAC/GTGAC	ACACTTGCAAAAATGAGGGG	TGTGTGTGGACCAAGAATTGA	60.0	696	INTERGENIC	--	--	--
Ca1_P2_7923910	Ca1	7923910	TATTGTG/TATTGTGATAAATTGTG	AATGGTGTCTTCGAAGGGTG	CTACTCACACCAAACCCCGT	59.9	553	INTRON	Ca_08042	--	HB-other
Ca1_P2_7928927	Ca1	7928927	TAAAATAAA/TAA	CCCTTTATTTGCATGGAGGA	TACGATTCAGCTTGCGGATA	59.4	684	INTERGENIC	--	--	--
Ca1_P2_7945191	Ca1	7945191	AACA/AA	TTTTGTGGGCAAATTGGAGT	AGGTACTCATTGCCACCAGG	60.0	593	INTRON	Ca_08044	QI	B3
Ca1_P2_7947587	Ca1	7947587	AC/ACC	TCTTCCTCCAAATTCCAATCA	TTTTTATTCTCGTGGGAGTGAG	58.4	578	INTERGENIC	--	--	--
Ca1_P2_7948210	Ca1	7948210	TTATATATATATATATAT/TTATATATATATATAT	CTCACTCCCACGAGAATAAAAA	TGGAAGAGGAGAGATGCAAAA	59.9	661	DRR	Ca_08045	QI	B3
Ca1_P2_7954074	Ca1	7954074	TAAAAAAAAA/TAAAAAAAA	TGACATGGGACAGTGACACC	AAAATGAAAGTTCCGAGGGG	60.3	705	INTRON	Ca_08045	QI	B3
Ca1_P2_7973178	Ca1	7973178	AGTT/AGTTGTT	GAGCTATTGAGCTTGGTGCC	GAGCTTCTGGGTGTAAAGCG	60.0	591	INTRON	Ca_08047	U	MYB
Ca1_P2_7990610	Ca1	7990610	AATATATATATA/AATATATA	AGTTGGCCATGGATGATTGT	GCAAATGTGTTGTCAATGGG	59.8	349	INTRON	Ca_08049	--	WRKY
Ca1_P2_7995324	Ca1	7995324	ATAGTAGT/ATAGTAGTAGT	CGGATTGTTTTCGATATGGG	AAGGGATTTTTATTCACAAAACAA	58.1	527	INTERGENIC	--	--	--
Ca1_P2_8000857	Ca1	8000857	ATGT/AT	CGCCATCTTCACGTCACTTA	TCCAACCCAAATTTCAAACA	58.8	699	INTERGENIC	--	--	--
Ca1_P2_8006710	Ca1	8006710	TG/TGG	TCTTCCCTCCTTCCTTGGAT	TAACACAAGTAGTCGCCCCC	60.0	748	INTERGENIC	--	--	--

INDEL marker IDs	Chromosomes/unanchored scaffolds	Physical positions (bp)	InDels (*Kabuli* reference genome-CDC Frontier/PII)	Forward primers (5'-3')	Reverse primers (5'-3')	Anneling temperature (0C)	Expected amplified product size (bp)	Structural annotation		Functional annotation	
								Sequence components of *kabuli* genome	*Kabuli* gene accession IDs	NCBI-KOG	TFs
Ca1_P2_8008581	Ca1	8008581	TG/T	ATAACAGCGCCCAAAACGTA	CGATAGAGGTACCACATTCATGC	60.8	782	DRR	Ca_08050	R	MYB
Ca1_P2_8028400	Ca1	8028400	TTA/TTATATA	TTCGTAAGTACCCACCCGTC	GCACATGAGCCATTTGACAT	59.5	776	INTERGENIC	--	--	--
Ca1_P2_8035534	Ca1	8035534	T/TTA	CAAATGCAAATGCAATGACC	AGGCATCTTTTCAGGTTCCC	60.4	782	INTRON	Ca_08051	M	FAR1
Ca1_P2_8041662	Ca1	8041662	TAAC/TAACATTAAAC	CCCTTCATTGGAGATGTTCC	TTGGGGACACTAAGAGGTGG	60.0	866	INTERGENIC	--	--	--
Ca1_P2_8101006	Ca1	8101006	GT/GTT	CCTCACCCGTGCATTCTTAT	GGCGGAGACAAAGAGTCAAG	60.0	587	DRR	Ca_08056	C	--
Ca1_P2_8105908	Ca1	8105908	TTTGATTGATTGA/TTTGATTGA	TCGTTGATTGATTCTCAAAGAAAA	TGGGTGGAAATATGTGTCTTTA	57.1	528	INTERGENIC	--	--	--
Ca1_P2_8116795	Ca1	8116795	AAG/A	CCAAAAGCCCAAAAGTACCA	CAATCGAACGAGACGAGTCA	60.0	979	INTERGENIC	--	--	--
Ca1_P2_8125888	Ca1	8125888	G/GACATATTAAAA	TGTTGGGTTACGAGGGAGTC	TTGCGGGTTTTTAACCTGAG	60.1	526	INTERGENIC	--	--	--
Ca1_P2_8126149	Ca1	8126149	TAAAAAAAA/TAAAA	TCAAAATTTGTCCACGTCCA	AATTTACAAACACGGCGACC	59.9	478	INTERGENIC	--	--	--
Ca1_P2_8127338	Ca1	8127338	CTT/CTTT	CCCTCCTTCCAGAAAACCTT	AATCCTGCAGTGAAACCACC	60.0	708	INTERGENIC	--	--	--
Ca1_P2_8248772	Ca1	8248772	ATTTTTTTTTT/ATTTTTTTTTTT	TGCAAACATGAATTGAAAGGA	ATAGTCGGGAATGCAACCTG	60.0	486	INTERGENIC	--	--	--
Ca1_P2_8270887	Ca1	8270887	T/TG	GCCGAATAAAACCCTCACAA	ATGAAGGAGGAGGGGAGAAA	60.0	561	INTERGENIC	--	--	--

INDEL marker IDs	Chromosomes/unanchored scaffolds	Physical positions (bp)	InDels (*Kabuli* reference genome-CDC Frontier/PII)	Forward primers (5'-3')	Reverse primers (5'-3')	Anneling temperature (0C)	Expected amplified product size (bp)	Structural annotation		Functional annotation	
								Sequence components of *kabuli* genome	*Kabuli* gene accession IDs	NCBI-KOG	TFs
Ca1_P2_831015	Ca1	831015	T/TCTCTTATAAATGA	GCCGGATTAAATATAAATCCACA	TGACCTAATAAGCCTGTTTGTTT	57.1	595	DRR	Ca_00101	--	--
Ca1_P2_8327656	Ca1	8327656	A/ATATT	AATAGCTCCCGCAAGTTTGA	CATGACATCTTTCAATCCAATCA	59.8	425	INTERGENIC	--	--	--
Ca1_P2_8329444	Ca1	8329444	TAAAAAAAAAAA/TAAAAAAAAAA	CAAAAGCGATAGTGCCAACA	GGTTTCCCAAGGCCATATTC	60.5	399	INTERGENIC	--	--	--
Ca1_P2_8372194	Ca1	8372194	CAATAATAATAATAATAATAATAATAATAATAA/CAATAATAATAATAATAATAATAATAATAATAA	AAACGCGTTAAATTAAAGGAAAAA	GGGGAGAAAGAGTGCAAAAA	59.3	893	INTERGENIC	--	--	--
Ca1_P2_8403563	Ca1	8403563	TTAATAATAATAATAATAATAATAATAATAATAATAATAATAATAATAATAATA/TTAATAATAATAATAATAATAATAATAATAATAATAATAATA	CCCCTAAAAATTAGTCGAACTCC	GAGTCTTTGTCCCCAGCAAG	59.8	474	INTRON	Ca_08076	--	--
Ca1_P2_8415420	Ca1	8415420	AG/A	ATGGGTGAAACGAGCAACTC	TTTTCCTTTTGCATCATCTTTG	59.2	910	INTERGENIC	--	--	--
Ca1_P2_8430467	Ca1	8430467	TT/TTATTATTATTAT	AGGTCATGGGCTGGATGTAG	ACCCTTTCACTGCGCTCTTA	60.0	612	INTERGENIC	--	--	--
Ca1_P2_8434947	Ca1	8434947	G/GTT	TGGCTTTTCCTGGGTGATAG	CCTTGCCACACATTGATGAC	60.0	799	INTERGENIC	--	--	--
Ca1_P2_84627	Ca1	84627	TTAATAATAATAATAATAATAATAATAATAA/TTAATAATAATAATAATAATAATAATAATAATAA	TCACAAATGGTTTGGTGTGG	GTGGGAACGTTAAGCCAAAA	60.0	657	INTERGENIC	--	--	--
Ca1_P2_8522654	Ca1	8522654	AATAAATA/AA	TGCCACGACCAAATATGAAA	CCACCGATTTCATTGCTTTT	59.9	718	INTRON	Ca_08089	--	NAC
Ca1_P2_8576194	Ca1	8576194	TTATATATATATATATATATATATATATAT/TTATATATATATATATATATATATATATAT	TCCCATCATGCCTTATTCTTG	AAAATCAGAGGGAGAGACCAAA	59.2	571	INTERGENIC	--	--	--
Ca1_P2_8596087	Ca1	8596087	ATTTTTTT/ATTTTTT	GGGCTGTTTATGGTTGGTTTT	CCCAGGGAGAGTTTTCCTTC	60.0	655	INTERGENIC	--	--	--

INDEL marker IDs	Chromosomes/unanchored scaffolds	Physical positions (bp)	InDels (*Kabuli* reference genome-CDC Frontier/PII)	Forward primers (5'-3')	Reverse primers (5'-3')	Anneling temperature (0C)	Expected amplified product size (bp)	Structural annotation		Functional annotation	
								Sequence components of *kabuli* genome	*Kabuli* gene accession IDs	NCBI-KOG	TFs
Ca1_P2_8621700	Ca1	8621700	TTATATATATATATATATATATAT/TTATATATATATATATATATATATATATAT	TGAACTTCATCTTCCCCACA	CACCAAATTACAAGTCGCCA	59.6	459	INTERGENIC	--	--	--
Ca1_P2_876057	Ca1	876057	AATATATATATATATATATATATA/AATATATATATATATATATA	GGTAGCCAGCAATGCAAGAT	GAATGGAACCATCACATCCC	60.0	818	INTERGENIC	--	--	--
Ca1_P2_8830262	Ca1	8830262	TTATATATATATATATATATATATATA/TTATATATATATATATATATATATATA	GCTACCTTAGCCTCTCCATGC	GGAATGCCATAGAAACACCAA	59.8	811	INTERGENIC	--	--	--
Ca1_P2_8848542	Ca1	8848542	TTATATATATATATATATATATATAT/TTATATATATATATATATATAT	CGCCCCTACCGATAATCATA	CAAGTGAAACCGGTGTGAAA	59.6	875	INTERGENIC	--	--	--
Ca1_P2_8875092	Ca1	8875092	CATATATATATATATATATATATATATATATAT/CATATATATATATATATATATATATATATATATATATATATATATAT	TGGCGTGAGAAAAAGAAAAA	CAACAGTGTCATTTCCAGCC	59.1	620	INTERGENIC	--	--	--
Ca1_P2_8882846	Ca1	8882846	CATATATATATATATATATATATATA/CATATATATATATATATATATATATATATA	GCGCACGATGGTTAATTTTT	TTTTCCACTCAATTCCCTGC	60.1	621	DRR	Ca_02920	U	--
Ca1_P2_8909387	Ca1	8909387	T/TCC	AGGCAAAAGGTTTTTGGGTT	GTGAGGCGTGAAGTGGTTTT	60.2	806	DRR	Ca_02914	--	--
Ca1_P2_8952246	Ca1	8952246	ATATTATTATTATTATTATTATTATTATTATTATTATTATT/ATATTATTATTATTATTATTATTATTATTATTATTATT	CGTTTTTCATCAATCAACGC	ATAAACCCCTGCACCAAATG	59.7	872	INTERGENIC	--	--	--
Ca1_P2_8968123	Ca1	8968123	AT/A	CAAGCTCCCGAATCAAACAC	CACAAAACCGAAAAGGGAGA	60.1	305	INTERGENIC	--	--	--
Ca1_P2_8992280	Ca1	8992280	TAAAAAAAA/TAAAAAAAAA	TGCAAGAACCATTGAGTTAGGTT	AGGTGCAAGTTCAAATTGGG	60.0	654	DRR	Ca_02903	--	--
Ca1_P2_9233850	Ca1	9233850	GTTTTTTTTTTTTT/GTTTTTTTTTT	TGAGTTCTTTTGAATTTGCGTG	GTTGCGTCTAGCAACTTTCTG	57.9	637	INTERGENIC	--	--	--
Ca1_P2_928963	Ca1	928963	AAATAATAATAATAAT/AAATAATAATAAT	CACCGTCACAAGCTTCTTCA	ATGAGGGAAATGCAACTTGG	59.9	333	INTRON	Ca_00116	--	B3

INDEL marker IDs	Chromosomes/unanchored scaffolds	Physical positions (bp)	InDels (*Kabuli* reference genome-CDC Frontier/PII)	Forward primers (5'-3')	Reverse primers (5'-3')	Anneling temperature (0C)	Expected amplified product size (bp)	Structural annotation		Functional annotation	
								Sequence components of *kabuli* genome	*Kabuli* gene accession IDs	NCBI-KOG	TFs
Ca1_P2_9297941	Ca1	9297941	AATATATATATATATATATATATATATATATA/AATATATATATATATATATATATATATATATATATATATATATATA	TTTTTCTTTCCTTCGTTTACTTTTC	TTTTTCATGGCATGTGTAGAGA	58.3	732	INTERGENIC	--	--	--
Ca1_P2_9314844	Ca1	9314844	TAAAAAAA/TAAAAAAAA	TTCAATTTTGTAAATCAATCCGA	TTTTGTTCTTCCATGGAGCC	60.1	768	INTERGENIC	--	--	--
Ca1_P2_9511687	Ca1	9511687	GA/GAAA	CAACCCGCTTATGTCGTTTT	TGATGCTACAAATGGCCAAA	60.1	510	INTRON	Ca_02846	QR	B3
Ca1_P2_953140	Ca1	953140	GATATATATATATATATATATATATAT/GATATATATATATATATATATATATATATAT	GTCCATTTTCCTGGCTCAAA	TCCCTAAACACCACTCACCC	59.8	636	DRR	Ca_00118	E	--
Ca1_P2_9554397	Ca1	9554397	ATTTTTTT/ATTTTTTTT	GAAATGCAGGAAGCCATGAT	GGGAATTGAAGATGGGATGA	59.7	771	INTRON	Ca_02842	R	--
Ca1_P2_9561666	Ca1	9561666	CTT/CTTCTTATTATTATTATT	ACCATTCACGGGTTCACATT	CTAACGCCTTTGTTGGTGGT	60.0	502	INTERGENIC	--	--	--
Ca1_P2_9686293	Ca1	9686293	TTTATTATTATTATTATTATTATTATTATTATTATTATTATTATTA/TTTATTATTATTATTATTATTATTATTATTATTATTATTATTATTATTATTA	TCAAATGCTTTAATGGCTCAGA	TTTTTAGTTTGAAGACCTTTGTCA	57.3	446	INTERGENIC	--	--	--
Ca1_P2_9867132	Ca1	9867132	AAATAATAATAATAATAATAATAATAATAATAATAATAATAATAAT/AAATAATAATAATAATAATAATAATAATAATAATAAT	GGGTCTATGTTTGTCTTGATGC	CAGGGCAAAATTGAACTTTAATAGA	59.9	482	INTERGENIC	--	--	--
Ca1_P2_9871519	Ca1	9871519	ATT/A	CTTAGTCCACGTGCAAACGA	AAAGTGCGGGCCAATATCTA	59.6	600	INTERGENIC	--	--	--
Ca1_P2_9874160	Ca1	9874160	GTTTTTT/GTT	TGCAGCATTCAACACAATCA	TTCGGGGAAAAGGGTTAGTT	59.8	493	DRR	Ca_02802	G	--
Ca1_P2_9944669	Ca1	9944669	TTAATAATAAATAATAATAAAT/TTAATAATAAAT	GTACCGTGCATCCCTTCACT	GTACCGTGCACTCTTCACCA	59.8	963	INTERGENIC	--	--	--
Ca1_P2_9945105	Ca1	9945105	CAAAAAAAAAAA/CAAAAAAAAAA	CGAGCATTACGTTTCGGAAG	CTTTGGGCCCATTGTCTTTA	59.9	319	INTERGENIC	--	--	--

INDEL marker IDs	Chromosomes/unanchored scaffolds	Physical positions (bp)	InDels (*Kabuli* reference genome-CDC Frontier/PII)	Forward primers (5'-3')	Reverse primers (5'-3')	Anneling temperature (0C)	Expected amplified product size (bp)	Structural annotation		Functional annotation	
								Sequence components of *kabuli* genome	*Kabuli* gene accession IDs	NCBI-KOG	TFs
Ca2_P2_1000852	Ca2	1000852	ATTTTTTTTTTTT/ATTTTTTTTTTTTT	TGTTGAACGTGGGAGTTTGT	TATCCCAGCTCTGGATCACC	60.0	716	INTERGENIC	--	--	--
Ca2_P2_10013835	Ca2	10013835	ATTTTTTTTT/ATTTTTTTT	TACTCACCCAAATCCGCTTC	TAGACCCTCATCCTCATCCG	60.0	393	INTERGENIC	--	--	--
Ca2_P2_10032701	Ca2	10032701	AATATATATATATATA/AATATATATATATA	TTCTTCAGAGAAAAGAGTGGTTTTT	CCGTGTTTACGCTCTGTTGA	59.9	392	INTERGENIC	--	--	--
Ca2_P2_10095157	Ca2	10095157	TTATATATATATATATATATATATATATATA/TTATATATATATATATATATATATATATATATATA	TAGCAACACACATCCCGAAA	TTGTTTGGCAGTGGATTGAA	60.1	864	DRR	Ca_20378	S	--
Ca2_P2_10097433	Ca2	10097433	CG/C	GCTAAATTCGTCTTCGACGC	CATTCAAATGGCAATGCAAC	59.9	139	DRR	Ca_20377	J	--
Ca2_P2_10175437	Ca2	10175437	TTGTGT/TTGT	TGGAACACAACGCCAAAATA	CCCCTTTGTCAATTAAGGCA	59.9	630	DRR	Ca_20376	--	--
Ca2_P2_10183002	Ca2	10183002	AATATATATATATATATATATATATAT/AATATATATATATATATATATATATATAT	TTGACGGACCAACCTTTTTC	TTCGATTGGACTTGTGTCGT	59.1	583	INTERGENIC	--	--	--
Ca2_P2_10187103	Ca2	10187103	AATATATATATATATATATATATA/AATATATATATATATATATATATATATATATA	AAGGCGAGAACAAGCCATTA	CCTGAATATGCGGCGTTATC	60.4	314	INTERGENIC	--	--	--
Ca2_P2_10207704	Ca2	10207704	AAGAGAGAGAGAGAGAGAGAGAG/AAGAGAGAGAGAGAGAGAGAG	TGATTGATGGGAAAGGTTCA	CCATCACCTCATAAGTTTCAACA	59.0	411	INTERGENIC	--	--	--
Ca2_P2_10240968	Ca2	10240968	AATATATATATATATATATATATA/AATATATATATATA	AAGATGCGACCATGTGTTGA	GGTTTGTGGTTGGCACTTCT	60.0	450	DRR	Ca_17599	--	--
Ca2_P2_1024227	Ca2	1024227	CAATAAATAAATAAATAAATAAATAAATAAATAAATAAAT/CAATAAATAAATAAATAAATAAATAAATAAATAAATAAATAAATAAAT	TTGTCAAAAGGGTCTGTCTTG	TGACTGCCGCACATTTAGAG	60.0	411	INTERGENIC	--	--	--
Ca2_P2_10284789	Ca2	10284789	CTGTG/CTG	GGTGACAAATGCAGGTGTTG	AGAATTTGTTGGGTTGGCTG	60.0	662	DRR	Ca_17594	R	--

INDEL marker IDs	Chromosomes/unanchored scaffolds	Physical positions (bp)	InDels (*Kabuli* reference genome-CDC Frontier/PII)	Forward primers (5'-3')	Reverse primers (5'-3')	Anneling temperature (0C)	Expected amplified product size (bp)	Structural annotation		Functional annotation	
								Sequence components of *kabuli* genome	*Kabuli* gene accession IDs	NCBI-KOG	TFs
Ca2_P2_10315365	Ca2	10315365	ATTTTTTTTTTTTTTT/ATTTTTTTTTTTTTTTTT	AATTTCCCTTGTACCCCCAC	TCCGGTACACAACTTTTCCC	59.8	324	INTERGENIC	--	--	--
Ca2_P2_1040699	Ca2	1040699	CTAGATAGATA/CTAGATA	TTTCTCGAGCTTCCAATGGT	TGATTCCATGAAAATTGCGA	60.0	351	INTRON	Ca_17010	A	C3H
Ca2_P2_1047594	Ca2	1047594	CAGAAGAAGAAGAAG/CAGAAGAAGAAG	AAATCTCCTCTTGCCACACG	GGTCGCTGAAGAGAAAGTCG	60.1	364	CDS (large-effect mutations)	Ca_17010	A	C3H
Ca2_P2_10498057	Ca2	10498057	CTTTTTTTTTTT/CTTTTTTTTTTTT	TGACCAAATCGGAAAGAAAAA	AGGGAATGAGAGCAATGACG	60.2	454	INTERGENIC	--	--	--
Ca2_P2_10567636	Ca2	10567636	CTCTTCTTCTTCTTCTT/CTCTTCTTCTTCTTCTTCTT	TACCCACACCCCTCAAATGT	TGATGATTGATGGGGATTGA	59.7	241	INTERGENIC	--	--	--
Ca2_P2_10611337	Ca2	10611337	ATT/ATTT	ATGGAACCTTGTGAGTTGGC	GCCAGATGTGTTTGCAAGTG	60.3	804	INTRON	Ca_17572	--	SBP
Ca2_P2_10612588	Ca2	10612588	CAAA/CA	AACATTCTACCCCCTCGTCC	TGCTGCTGCTGGAAATTATG	60.0	508	INTRON	Ca_17572	--	SBP
Ca2_P2_10658848	Ca2	10658848	GTTTTTTTTTT/GTTTTTTTTT	TCAAGATGGTGAGGGTTGGT	TGTTGTTGTTGGAGGTGAAAA	59.1	351	INTERGENIC	--	--	--
Ca2_P2_1074408	Ca2	1074408	TTTTATTTATTTATT/TTTTATTTATTTATTTATT	CCTCCTCGGTCTCTTCTCCT	AAATTGAGTTTCAAAACCACCAA	59.8	397	INTERGENIC	--	--	--
Ca2_P2_1075762	Ca2	1075762	ATTTTTTTTTTT/ATTTTTTTTTT	TTAGGCCGCGACAATAATTC	CTGTACTCACGTCAGGCTGC	59.6	707	INTERGENIC	--	--	--
Ca2_P2_10778432	Ca2	10778432	TTTTTCTTTT/TTTTT	TTTCGACGATTGAACAATGTAA	TGCGGTGCATAAGAAAAGTG	59.9	757	INTERGENIC	--	--	--
Ca2_P2_10779124	Ca2	10779124	TTA/TTACATA	CACTTTTCTTATGCACCGCA	GCAGCGAGTAGGAGAAATGC	60.1	663	INTERGENIC	--	--	--

INDEL marker IDs	Chromosomes/unanchored scaffolds	Physical positions (bp)	InDels (*Kabuli* reference genome-CDC Frontier/PII)	Forward primers (5'-3')	Reverse primers (5'-3')	Anneling temperature (0C)	Expected amplified product size (bp)	Structural annotation		Functional annotation	
								Sequence components of *kabuli* genome	*Kabuli* gene accession IDs	NCBI-KOG	TFs
Ca2_P2_10789942	Ca2	10789942	ATTTTTTT/ATTTTTTTT	TTGAATCTGCACCCAAATGA	GTGGCTTCATAAGGCAGAGG	59.8	888	INTERGENIC	--	--	--
Ca2_P2_10853311	Ca2	10853311	ATTTTTTTTTT/ATTTTTTTTT	CAACTCAACCCCTAAACCGA	TGAGAAATTTTTCCGCTGCT	60.0	261	INTERGENIC	--	--	--
Ca2_P2_10913431	Ca2	10913431	GAAAAAAAAAAA/GAAAAAAAAAA	TGCATAATCGATTGGCACAT	TCACTTACCGATTCCAGCCT	59.7	838	DRR	Ca_17557	--	--
Ca2_P2_10928556	Ca2	10928556	ATTTTTTTT/ATTTTTTTTT	CATCCGTGGTCCCTTAACTT	GCTAAATTACATTCGTGGTCCC	59.7	370	INTERGENIC	--	--	--
Ca2_P2_11082192	Ca2	11082192	CTATATATATATATATATAT/CTATATATATATATATATATATATATATATAT	AAGTTCCAGGAGAGTGGCAA	CCGGGTGTCCAAAATCTCTA	59.9	942	INTERGENIC	--	--	--
Ca2_P2_11085781	Ca2	11085781	AATATATATATATATATATATATATATATATATATATTTATATATATATATAT/AATATATATATATATATATATATATATATATATATATATATTTATATATATATAT	TTTGGTTTTGAACTCGGAGG	CACCGTAGTGTGGTGGTGAG	60.1	660	INTERGENIC	--	--	--
Ca2_P2_1110782	Ca2	1110782	TA/T	TTGAAAAACCCGAATCGAAC	TTGTTCATCAGTGGCTGCTC	60.0	586	INTRON	Ca_20673	--	--
Ca2_P2_11136560	Ca2	11136560	GAAAAAAAAA/GAAAAAAAAAA	TGACCAATGGAAAAAGGGAG	TGAAAATTTTGGGGTGTTACAA	59.2	300	INTERGENIC	--	--	--
Ca2_P2_11157229	Ca2	11157229	AC/A	ATTCGGCATACTTCACCTGC	TCCTACTTCACCGTTCCACC	60.0	673	INTERGENIC	--	--	--
Ca2_P2_11169617	Ca2	11169617	GTTATTATTATTATTATTATTATTAT/GTTATTATTATTATTATTATTAT	CTGGCCTCATCTATTGGGAA	TTTTTCAATATTTTTCCCTCCA	57.7	537	INTRON	Ca_17548	--	C2H2
Ca2_P2_11220118	Ca2	11220118	ANNNNNNNNNN/ANNNNNNNN	TTGCACTTGGTTTGACTCCA	CTCTCAACCCTCTCGTTTGC	60.0	939	INTERGENIC	--	--	--
Ca2_P2_11226197	Ca2	11226197	GATATATATATATATATATATA/GATATATATATATATATATATA	CGCGATCAAAACAAAACGTA	CCAACATGGCATGAGTTGAC	60.0	757	INTERGENIC	--	--	--

INDEL marker IDs	Chromosomes/unanchored scaffolds	Physical positions (bp)	InDels (*Kabuli* reference genome-CDC Frontier/PII)	Forward primers (5'-3')	Reverse primers (5'-3')	Anneling temperature (0C)	Expected amplified product size (bp)	Structural annotation		Functional annotation	
								Sequence components of *kabuli* genome	*Kabuli* gene accession IDs	NCBI-KOG	TFs
Ca2_P2_11240445	Ca2	11240445	ATTTTTT/ATTTTTTT	TCGACCGAATCAAACAATGA	ACGTTTCCTCACGTTTCCAC	60.0	622	INTERGENIC	--	--	--
Ca2_P2_1147777	Ca2	1147777	CAATTTCAAAAATTTCAAAA/CAATTTCAAAA	AAAGCCTTGGTTCTTGGACA	TGCATGGAATGCATCATCTT	60.0	774	INTERGENIC	--	--	--
Ca2_P2_11546825	Ca2	11546825	ATTTTTTTTT/ATTTTTTTTTT	CACCCAGCCGTAAAACAGAT	CCCCCTTTGTCACAACCTAA	59.8	574	INTERGENIC	--	--	--
Ca2_P2_11623009	Ca2	11623009	TATTTGCATTTGCATTTGCATTTGCAT/TATTTGCATTTGCATTTGCAT	GCCCAAGTTCCCAAAGAGAT	AAAAGAGTGGGACACAACGC	60.2	169	INTERGENIC	--	--	--
Ca2_P2_11625278	Ca2	11625278	AATATATATATATATATATATATATATATA/AATATATATATATATATATATATATATATATATATA	GTTTAGCGCTCCTCCATCAT	CTGCCCGTAAAAATCTTCCA	60.1	839	INTERGENIC	--	--	--
Ca2_P2_11648268	Ca2	11648268	AATATATATATATATATATATA/AATATATATATATATATATATATATATATA	TGAGTGCAAGCCTAGTGTGG	TGCACGGAAAACTGACTCTG	60.0	521	INTERGENIC	--	--	--
Ca2_P2_11711787	Ca2	11711787	ATATTATTATTATTATTATTATTATTATTATTATTA/ATATTATTATTATTATTATTATTATTATTATTATTATTA	TTCAAAATTGCTGGTTTGGTC	TGAAAGAAGCGCGGTAAAAC	60.4	596	INTERGENIC	--	--	--
Ca2_P2_11716435	Ca2	11716435	TTATATATATATATATATATATATATAT/TTATATATATATATATATATATATATATATATATAT	CACAGTTCCGCATGTGCTAT	TGCACTGACAGGGAAATCAA	60.2	857	INTERGENIC	--	--	--
Ca2_P2_12040246	Ca2	12040246	TTATATATATATATATATAT/TTATATATATATATAT	TGTCAATCCATTTATAATTTCTTTCAA	CAGCTTTTTGGTTGATGTGG	59.2	490	INTERGENIC	--	--	--
Ca2_P2_12040519	Ca2	12040519	ATT/AT	CCACATCAACCAAAAAGCTG	GTGAGGTGGCATTCGAGAGT	60.3	665	INTERGENIC	--	--	--
Ca2_P2_12048675	Ca2	12048675	AATAATTTATAATTTATA/AATAATTTATA	CAAACATGCTGATGTGGACC	CCATTGGTAAATGGCTAACAA	57.6	278	INTERGENIC	--	--	--
Ca2_P2_12146761	Ca2	12146761	TTATATATATATATATATATATATATATATAT/TTATATATATATATATATATATATATATATATATATATATAT	TGCACTCACTCCAACAAACA	AAGTCTGGCCTACGGCCTAT	60.1	828	INTERGENIC	--	--	--

INDEL marker IDs	Chromosomes/unanchored scaffolds	Physical positions (bp)	InDels (*Kabuli* reference genome-CDC Frontier/PII)	Forward primers (5'-3')	Reverse primers (5'-3')	Anneling temperature (0C)	Expected amplified product size (bp)	Structural annotation		Functional annotation	
								Sequence components of *kabuli* genome	*Kabuli* gene accession IDs	NCBI-KOG	TFs
Ca2_P2_12222534	Ca2	12222534	T/TC	GATTGTTCGTACCCTCCCCT	TTGTTGACGTTGGGTTTTGA	60.0	784	INTERGENIC	--	--	--
Ca2_P2_12307967	Ca2	12307967	GAAA/GAAAA	TGTCTTTGGTCCCTGCAAAT	TCAAACCCCCATTTTTGTGT	60.1	755	INTERGENIC	--	--	--
Ca2_P2_1233900	Ca2	1233900	C/CG	CGTTTTTGGAAGCCATTTGT	CTGCCATAAAGGCTGCAGAT	60.4	830	INTERGENIC	--	--	--
Ca2_P2_12360564	Ca2	12360564	TAAAAAAAAAAAA/TAAAAAAAAAAA	TCGCACTCACACAACAAAATC	CCTCTTCACTTTTTGGAAGGG	60.1	155	INTERGENIC	--	--	--
Ca2_P2_12384702	Ca2	12384702	TAAAA/TAAA	CCTACGTATCTCCTGGTGCG	TTAATCGTTGCGTTGCTGAG	60.0	649	INTERGENIC	--	--	--
Ca2_P2_12553140	Ca2	12553140	ATTTTTTTTTT/ATTTTTTTTTTT	CCCCCTCCAACCTTTCATAC	ATCCAAATTCAATGCATCCC	59.6	811	INTERGENIC	--	--	--
Ca2_P2_12707442	Ca2	12707442	GAATTTCAATTTCAAT/GAATTTCAATTTCAATTTCAAT	AGCAGCTGACCGAGCATATT	GGATGGGTGATTGAAAATCG	60.1	902	INTRON	Ca_18086	R	--
Ca2_P2_12755246	Ca2	12755246	AATATATATATATATATATATATATATATATATATAT/AATATATATATATATATATATATATATATATATATATATATATAT	AAAATTGAACGGGTCACGAT	AAATGTCCCCCATGATGTTC	59.5	696	INTERGENIC	--	--	--
Ca2_P2_1293074	Ca2	1293074	CTTTTTTT/CTTTTTT	AAAAAGTGAAAAATTGTTTGGCA	CATTCATGAGTGGTGCTTCG	60.3	674	INTERGENIC	--	--	--
Ca2_P2_12933106	Ca2	12933106	A/AT	AGGTTGAAAGAAGCCCCACT	ATCGGTTGGTTGGTGAAATG	60.6	834	INTERGENIC	--	--	--
Ca2_P2_12933331	Ca2	12933331	TTATATATATATATATATATATAT/TTATATATATATATATATATATATATATATATAT	TGGAGTTTTGGTCTCCATATTT	AGGGACATGCAATGGGTTAG	59.8	618	INTERGENIC	--	--	--
Ca2_P2_12933573	Ca2	12933573	CACCAACCAACC/CACCAACC	TCATTGACAAGGAAAGAACATGA	CGAATTGGTTTTGGGATTTG	60.2	413	INTERGENIC	--	--	--

INDEL marker IDs	Chromosomes/unanchored scaffolds	Physical positions (bp)	InDels (*Kabuli* reference genome-CDC Frontier/PII)	Forward primers (5'-3')	Reverse primers (5'-3')	Anneling temperature (0C)	Expected amplified product size (bp)	Structural annotation		Functional annotation	
								Sequence components of *kabuli* genome	*Kabuli* gene accession IDs	NCBI-KOG	TFs
Ca2_P2_12934202	Ca2	12934202	AAA/AAACAA	AGAAAGCAAGCAAATCCCAA	TTCCAACACCTCACGATTCA	60.1	488	INTERGENIC	--	--	--
Ca2_P2_12934976	Ca2	12934976	TATCAATC/TATC	TGCCTCAAAAATCTGGTGTG	CTGCTCCAAACTGATCACGA	60.0	516	INTERGENIC	--	--	--
Ca2_P2_12937651	Ca2	12937651	ATTTT/ATTTTT	GATTGCTTTCCTCTATGCCG	GTTTTGGCGTTGGTTTCACT	60.0	353	INTERGENIC	--	--	--
Ca2_P2_12939688	Ca2	12939688	AATATATA/AATATA	TTTTGGTGTTGAGGGTGTCA	TTAATCAAAGCGGCAAAAGAA	59.8	564	INTERGENIC	--	--	--
Ca2_P2_12941200	Ca2	12941200	CAAAA/CAAA	TGTGCTTCTTCCTGATTTTGG	GGCCAAAGACGGATATGGTA	59.8	888	INTERGENIC	--	--	--
Ca2_P2_12941800	Ca2	12941800	T/TA	CATATCCGTCTTTGGCCATT	CCTTGAACAATTTGGGCAGT	60.0	643	INTERGENIC	--	--	--
Ca2_P2_12947970	Ca2	12947970	G/GT	TGCTTTTATGAGCATTACGGG	CGACTTCAAATCCCTTCGAG	59.8	777	INTERGENIC	--	--	--
Ca2_P2_12949902	Ca2	12949902	CTAAAAGAAATAAAAGAAATAA/CTAAAAGAAATAA	CCTTCGATTCCCCATTTTCT	TCATGATGGAATTTACGGCA	59.9	484	INTERGENIC	--	--	--
Ca2_P2_12952557	Ca2	12952557	ACATATCATATCA/ACATATCA	AAAGCATTTTATCCCGACCA	TTTTTCATCTCACTCTCTCCCA	58.9	787	INTERGENIC	--	--	--
Ca2_P2_12952944	Ca2	12952944	TGTCATGATT/TGTCATGATTGTAGTCATGATT	TGGGAGAGAGTGAGATGAAAAA	ACGGATGTCTAATCCGTTGG	59.8	308	INTERGENIC	--	--	--
Ca2_P2_13148618	Ca2	13148618	G/GGAAT	CTTTCTCCATCCCAACAGGA	TTTTGGAGTCATGCTACCCC	59.9	776	INTERGENIC	--	--	--
Ca2_P2_13247575	Ca2	13247575	ATTTTTTTT/ATTTTTTT	TGAAAAGCTAAATGCATGGAA	TCATGACCATGGACCCACTA	59.8	937	INTERGENIC	--	--	--

INDEL marker IDs	Chromosomes/unanchored scaffolds	Physical positions (bp)	InDels (*Kabuli* reference genome-CDC Frontier/PII)	Forward primers (5'-3')	Reverse primers (5'-3')	Anneling temperature (0C)	Expected amplified product size (bp)	Structural annotation		Functional annotation	
								Sequence components of *kabuli* genome	*Kabuli* gene accession IDs	NCBI-KOG	TFs
Ca2_P2_13334478	Ca2	13334478	GTTTTTTTTTTTT/GTTTTTTTTTTTTT	TTTTAATCGCTGCACAGCAC	TTTTTCCGACTTGGTCCTTT	58.7	805	INTERGENIC	--	--	--
Ca2_P2_1350612	Ca2	1350612	TAAAAA/TAAAA	GCGTTGTAACTGATTTGCGA	ATCAAACGGGTTTAAGTGCG	60.0	272	INTERGENIC	--	--	--
Ca2_P2_13622321	Ca2	13622321	TTATATATATATATATATATATATATAT/TTATATATATATATATATATATATATATATAT	TTGTCTTTTGTTTAGCACATCTCA	GAAGCTCACTAAGCACTTGCC	59.3	491	INTERGENIC	--	--	--
Ca2_P2_13639596	Ca2	13639596	TAATTAAATAAAAT/TAAT	AAGCCAGCCGCATGTAAATA	TTCACTTCCTCTGCATCTTTCA	60.0	788	INTERGENIC	--	--	--
Ca2_P2_13808036	Ca2	13808036	GTTTT/GTTT	AAAGGGAATGGGAAACACAA	TCCTACGTATCCCCAGATGC	59.9	688	INTERGENIC	--	--	--
Ca2_P2_13853423	Ca2	13853423	CAAAAAAAA/CAAAAAAAAA	CATTGCCAGTATGCGACATC	AAGGTCCACAAAGAGCCTCC	60.6	529	INTERGENIC	--	--	--
Ca2_P2_13858331	Ca2	13858331	AATTATTATTATTATTATTATTATTATTATTATTATTAT/AATTATTATTATTATTATTATTATTATTATTATTAT	TGAAACCAGTTGGGTCTGAA	GATTTATAAGCGAGCGTGGC	59.8	812	INTERGENIC	--	--	--
Ca2_P2_13955227	Ca2	13955227	ACCCCC/ACCCC	GCTCCACCACCAACTTGTTT	TGAGCTCTTGTTGTGGTTGG	59.9	719	INTERGENIC	--	--	--
Ca2_P2_13956600	Ca2	13956600	AATTATTATT/AATTATTATTATT	TGAAATTTGTGATTTAGTTGCTTAAAA	AAACAAGTTGGTGGTGGAGC	60.0	686	INTERGENIC	--	--	--
Ca2_P2_13957739	Ca2	13957739	TGG/TG	ATTATCCCCACCCCCACTAC	TTTCAAAAACGGTGGTGTGA	60.0	755	INTERGENIC	--	--	--
Ca2_P2_13957974	Ca2	13957974	A/AG	TTAGCCATTGTGCGTAGCTG	GTGGGGATAGTAGTGGGGGT	59.9	218	INTERGENIC	--	--	--
Ca2_P2_13959549	Ca2	13959549	ATTTTTTTTTT/ATTTTTTTTTTT	TGAAATTTGTGATTTAGTTGCTTAAAA	CATAGATTCCCCACCCAATG	60.0	752	INTERGENIC	--	--	--

INDEL marker IDs	Chromosomes/unanchored scaffolds	Physical positions (bp)	InDels (*Kabuli* reference genome-CDC Frontier/PII)	Forward primers (5'-3')	Reverse primers (5'-3')	Anneling temperature (0C)	Expected amplified product size (bp)	Structural annotation		Functional annotation	
								Sequence components of *kabuli* genome	*Kabuli* gene accession IDs	NCBI-KOG	TFs
Ca2_P2_13983591	Ca2	13983591	ATTTTTTTTTT/ATTTTTTTTTTT	ACACGAGGCGGAGAAATAAA	TGCAATTGTGGTCCATTTGT	59.8	893	INTERGENIC	--	--	--
Ca2_P2_13995550	Ca2	13995550	CAT/CATAT	TATTGCTTTGTTTCCTGGGG	TGTGTGCACATGCCAAGATA	59.7	734	CDS (FRAME SHIFT)	Ca_11631	GE	HB-other
Ca2_P2_14086143	Ca2	14086143	C/CT	GCTTCTGATGGTGCACTTGA	TGGCCTAAAGCAAGCTCCTA	60.1	371	INTERGENIC	--	--	--
Ca2_P2_14117374	Ca2	14117374	AATATATATATATATATATATATATATATAT/AATATATATATATATATATATATATATATATATATATAT	ACCTACGGATGTTAGCCGTG	GGTCCATGCAAAATCATTCA	59.3	768	INTERGENIC	--	--	--
Ca2_P2_14192522	Ca2	14192522	GAAAAAAAAAA/GAAAAAAAAAAA	TACAAATTGGATGCGGTTGA	CCCTCTGCAATGTCGGTAAT	60.0	755	INTERGENIC	--	--	--
Ca2_P2_14200236	Ca2	14200236	A/AT	GCGCTGTAAAATGTGCAAAA	TCGGCTATATGATTGTCCCA	58.9	258	DRR	Ca_11619	--	Trihelix
Ca2_P2_1481432	Ca2	1481432	AAATAATAATAATAATAATAATA/AAATAATAATAATAATAATAATAATA	TTGGTTTCAAGAAGGATGGC	TTGAAATGTTGATGTGTTGGG	59.3	750	INTERGENIC	--	--	--
Ca2_P2_15151368	Ca2	15151368	CTAATAA/CTAATAATAA	TCTCCATGTCCGTTACCTCC	TCCTGAAGATGAGATGCGTG	59.9	873	INTRON	Ca_18572	S	SAP
Ca2_P2_15255186	Ca2	15255186	ATTTTTT/ATTTTT	TGGATGCAGTGGAGCTAATG	GGCGGTCACAAAATTGAGTT	60.0	611	INTERGENIC	--	--	--
Ca2_P2_15278206	Ca2	15278206	TTATATATATATATATATATATATATATATATATATATAT/TTATATATATATATATATATATATATATATATATATATATATAT	ATAACGTGGCAAGGAGCAAC	GACGAAGCTCCTTTTAGGGG	60.2	680	INTERGENIC	--	--	--
Ca2_P2_15280624	Ca2	15280624	AATATATATATATATATATATATATATATATATATA/AATATATATATATATATATATATATATATATATATATATA	AGAGGCAGAAATGGTGGTGT	AAGATTTTAGGCGATTCTACGTG	58.9	659	INTERGENIC	--	--	--
Ca2_P2_15339865	Ca2	15339865	TAAAAA/TAAAA	TGGCTTTTATTTTGGATACACG	CCAGTTTTTGGGTCCTTTTT	58.1	421	INTERGENIC	--	--	--

INDEL marker IDs	Chromosomes/unanchored scaffolds	Physical positions (bp)	InDels (*Kabuli* reference genome-CDC Frontier/PII)	Forward primers (5'-3')	Reverse primers (5'-3')	Anneling temperature (0C)	Expected amplified product size (bp)	Structural annotation		Functional annotation	
								Sequence components of *kabuli* genome	*Kabuli* gene accession IDs	NCBI-KOG	TFs
Ca2_P2_15363207	Ca2	15363207	TGCAGAACCAGAACAAGCAG/TGCAGAACCAGAACAAGCAGAACCAGAACAAGCAG	CTCTTCTCATGATTGGGGGA	CTGCATTTTGATGTGCTGCT	60.0	325	INTERGENIC	--	--	--
Ca2_P2_15400009	Ca2	15400009	ATTTTTTTTT/ATTTTTTTT	TCGGTAACACCAAAAGGAGG	GGGACTACTTTCGCGATTCA	60.2	565	INTERGENIC	--	--	--
Ca2_P2_15479768	Ca2	15479768	ATTTTTTTTTTT/ATTTTTTTTTTTT	GGGGTTCTTCAAAATTGGGT	CAAACTTGGGGATGAAAACG	60.3	461	INTERGENIC	--	--	--
Ca2_P2_15497081	Ca2	15497081	TTAATAATAATAATAATAATAATAATAATAATAATAATAATAATAATAATAATAATAAT/TTAATAATAATAATAATAATAATAATAATAATAATAATAATAATAATAATAATAATAATAAT	CAAATGGAAAAGTTGCACACA	TTTTTGCCTTTGTGGGATTC	59.9	902	DRR	Ca_18548	C	--
Ca2_P2_15511402	Ca2	15511402	TGG/T	GGGCTGTTGTTCGTCAAAAT	TTTCGCGATTTGCCTTACTT	59.9	496	INTRON	Ca_18547	--	--
Ca2_P2_15512215	Ca2	15512215	TAAAAA/TAAAAAA	ATTGTGGAAGGAAGCTCGTG	CCCCCTAACCTGACCAAAAT	60.0	617	INTRON	Ca_18547	--	--
Ca2_P2_15535688	Ca2	15535688	TAAATCGATT/TAAATCGATTGAGAAATCGATT	GGGACCAAAACTGTCGACTT	CGTATTGCTCACCCCTTGAT	60.0	618	INTERGENIC	--	--	--
Ca2_P2_15557649	Ca2	15557649	AATATATATATATATATATATATATATA/AATATATATATATATATATATATATATATA	GAAAGCAATTGGGCTACTGG	CCAAAAACGTCTTTTCAATGG	59.5	251	INTERGENIC	--	--	--
Ca2_P2_1558409	Ca2	1558409	TCGGAAGNNNNNNNNNNCGGAAG/TCGGAAG	TCGAAATTTTAGGCCACGAC	TGAGGTTGTTGCTTTGACCA	60.3	631	INTERGENIC	--	--	--
Ca2_P2_15589036	Ca2	15589036	ATTT/ATTTT	ATACGCCTCAACAAAATGGC	CTGCTTTGCTTGATTCCGTT	60.4	376	INTERGENIC	--	--	--
Ca2_P2_15598813	Ca2	15598813	TA/TAA	GGAGGCGCATTAGATATGGA	GCCTCGTGAGTTGCATCATA	59.8	518	INTERGENIC	--	--	--
Ca2_P2_15629838	Ca2	15629838	AATATATATATATATATATATATATATATATATATATATATATATATATATATATATAT/AATATATATATATATATATATATATATATATATATATATATATATAT	GGGGACATTTGTAAGACCCA	GTGAGAAGGAGGAGGAAGGG	60.2	540	INTERGENIC	--	--	--

INDEL marker IDs	Chromosomes/unanchored scaffolds	Physical positions (bp)	InDels (*Kabuli* reference genome-CDC Frontier/PII)	Forward primers (5'-3')	Reverse primers (5'-3')	Anneling temperature (0C)	Expected amplified product size (bp)	Structural annotation		Functional annotation	
								Sequence components of *kabuli* genome	*Kabuli* gene accession IDs	NCBI-KOG	TFs
Ca2_P2_15691178	Ca2	15691178	TTATATATATATATATATATATATAT/TTATATATATATATATATATATATATAT	GACTGTCCAAATTTGTGGGG	TTTTTGGCATCAAGTTGTCTTTT	60.0	581	DRR	Ca_18540	--	--
Ca2_P2_15700651	Ca2	15700651	ATTTTTTTTTTT/ATTTTTTTTTT	GTGACTCCAACATGACCCCT	CCAATGCATTCCATTCAACA	60.3	743	INTERGENIC	--	--	--
Ca2_P2_15722479	Ca2	15722479	AATATATATATATATATATATATATAT/AATATATATATATATATATATATAT	TCCATTGAGTTGCGACGATA	CGCCTTCTCCTTTCTTCCTT	60.0	195	INTERGENIC	--	--	--
Ca2_P2_16203896	Ca2	16203896	GTATATATATATATATATATATATATAT/GTATATATATATATATATATATAT	GTGCCAGTCGATTGCCTAAT	CCTCCTACTCCCGACATTGA	60.1	491	INTERGENIC	--	--	--
Ca2_P2_16296988	Ca2	16296988	TAAAAAAAAAA/TAAAAAAAAAAAA	GAGGGAGGTGTGTAGTCCCA	GCGTTGTTGTATCCTCCGTT	60.0	623	INTERGENIC	--	--	--
Ca2_P2_16309679	Ca2	16309679	TACTC/T	GCGTTGCTCAGTTGAATGAA	CTCAACTCACGACCACCAGA	59.9	596	INTERGENIC	--	--	--
Ca2_P2_16375278	Ca2	16375278	AT/ATATATTT	TTTTTCCCATTTTTCGTTTTTC	TTTCGATTTGAGCTTGGCTT	60.0	591	INTERGENIC	--	--	--
Ca2_P2_1640062	Ca2	1640062	ATTTTTTTTT/ATTTTTT	AGATCATGTGCAAAAAGGGG	AGTAACCGTGAGGGAACGTG	60.0	509	INTERGENIC	--	--	--
Ca2_P2_16496636	Ca2	16496636	GTCAAAC/GTCAAACTAAAAGGACTATTCAAAC	TATGGACTTTGTGGTGGCAA	TTGGCACGTACTCCCATACA	60.0	441	INTERGENIC	--	--	--
Ca2_P2_16695716	Ca2	16695716	CTAATAATAATAATAATAATAATAATAATAATAATA/CTAATAATAATAATAATAATAATAATAATAATA	ATTTTGCCAAATTCACCGTC	CCAGCAGTAACGTTGTTGGA	59.8	449	INTERGENIC	--	--	--
Ca2_P2_16712405	Ca2	16712405	CATATATATATATATATATAT/CATATATATATATATATATATATAT	AAATTCGCACACCACAAAAA	TGTTTAATTCGGCAAGCACA	60.3	607	INTERGENIC	--	--	--
Ca2_P2_16841574	Ca2	16841574	TTATATATATATATATATATATATATAT/TTATATATATATATATATATATATAT	GCCATTAATTTTCCTTCATTCG	CCTCAAAGGAATGTCAAACCA	60.0	529	INTERGENIC	--	--	--

INDEL marker IDs	Chromosomes/unanchored scaffolds	Physical positions (bp)	InDels (*Kabuli* reference genome-CDC Frontier/PII)	Forward primers (5'-3')	Reverse primers (5'-3')	Anneling temperature (0C)	Expected amplified product size (bp)	Structural annotation		Functional annotation	
								Sequence components of *kabuli* genome	*Kabuli* gene accession IDs	NCBI-KOG	TFs
Ca2_P2_16921710	Ca2	16921710	TTTATTATTATTATTATTATTATTATTATTATTATTATTATTAT/TTTATTATTATTATTATTATTATTATTATTATTATTATTAT	TGCTTGTTGCCTAACACGAC	CCGACAAAACATGATGGTCA	60.4	502	DRR	Ca_24772	--	NAC
Ca2_P2_16958740	Ca2	16958740	TG/T	GCCACCCTTAGGAGGAAAAG	GGTCGAAGTATTTGTTCCACG	59.5	872	INTERGENIC	--	--	--
Ca2_P2_16998327	Ca2	16998327	ACTCT/ACT	TCATGCCGAACAAAAATTCA	CAAGGGTGTGCAAGAGGTTT	60.1	345	INTERGENIC	--	--	--
Ca2_P2_17046294	Ca2	17046294	AA/AAAATTTA	CTATTCCATCCACGAATGCC	TCCATGGAGGATATTTATGTCG	58.8	523	DRR	Ca_24768	--	G2-like
Ca2_P2_17061321	Ca2	17061321	TAATATATA/TAATATATAAATATATA	TTTGGCTCGGAAGCTTTTTA	AGGAAGAGTGGAGTTGCGAA	60.0	498	INTERGENIC	--	--	--
Ca2_P2_17061679	Ca2	17061679	T/TCCA	TTCGCAACTCCACTCTTCCT	CGGTAAAAATCTCTGCTCGC	60.0	190	INTERGENIC	--	--	--
Ca2_P2_17196393	Ca2	17196393	CTTTTTTTT/CTTTTTTT	CCGAGTGGAGCTCCTTATTG	ACTTGCAATCGTCTCAAGGC	60.4	382	INTERGENIC	--	--	--
Ca2_P2_1728113	Ca2	1728113	AATTATTATTATTATTATTATTATTATTATTATT/AATTATTATTATTATTATTATTATTATTATT	GAAAAGACACACATATTTGGCA	GTTTGTTGTGGTTGCAGTGG	60.0	491	INTERGENIC	--	--	--
Ca2_P2_17333975	Ca2	17333975	GCAACAACAACAACAACAACA/GCAACAACAACAACAACAACAACA	TACTCCGACTCCGAAGAAGC	TGCAGCAACCCTCTATTGTG	59.9	382	DRR	Ca_16008	--	--
Ca2_P2_17491578	Ca2	17491578	CATATATATATATATATATATATATATA/CATATATATATATATATATATATATA	AGTTGGTCCCCTAGAACCGT	CGGTGAATCCTTTACGGAGA	60.1	541	INTERGENIC	--	--	--
Ca2_P2_17512742	Ca2	17512742	GTATATATATATATATATATATATATATATATATAT/GTATATATATATATATATATATATAT	AGCTTATTCTGGCTCCCACA	GTGGCCCTTCATCAAAAAGA	60.1	680	DRR	Ca_16004	R	Nin-like
Ca2_P2_17551687	Ca2	17551687	G/GC	ATGTGGTTGATAAGGCCTCG	AGCAAATGCAATGCTCTGAA	59.6	398	CDS (FRAME SHIFT)	Ca_16000	OE	--

INDEL marker IDs	Chromosomes/unanchored scaffolds	Physical positions (bp)	InDels (*Kabuli* reference genome-CDC Frontier/PII)	Forward primers (5'-3')	Reverse primers (5'-3')	Anneling temperature (0C)	Expected amplified product size (bp)	Structural annotation		Functional annotation	
								Sequence components of *kabuli* genome	*Kabuli* gene accession IDs	NCBI-KOG	TFs
Ca2_P2_17799529	Ca2	17799529	ATGTTTGTTTGTTTGTTTGTTTGTTTGTTTGTTTGTTTGTTTGTTT/ATGTTTGTTTGTTTGTTTGTTTGTTTGTTTGTTTGTTTGTTTGTTTGTTT	CATGCATGCAAAATGGAAAG	AAATCCCTGAACTCGTGGTG	60.0	778	INTERGENIC	--	--	--
Ca2_P2_17994863	Ca2	17994863	T/TATATA	TGTGGCCCTATGTACTGCTG	TTGGAAGGAGCCCCTTACCTT	60.1	799	INTERGENIC	--	--	--
Ca2_P2_17994954	Ca2	17994954	GACA/GGCACA	AAAATACCTCGGGTTGCTGA	TTGGAAGGAGCCCCTTACCTT	60.1	544	INTERGENIC	--	--	--
Ca2_P2_18101343	Ca2	18101343	A/AT	ACTGCGGTGAATCCTTTACG	AATTCCTTCCCACACAACCA	60.2	469	INTERGENIC	--	--	--
Ca2_P2_18124367	Ca2	18124367	ATTTTTTTTTTTTTT/ATTTTTTTTTTTTTTTT	TTCAAATGCACGTGAGAACC	CCTCAGATGAGAGCCAGGAC	59.9	765	INTERGENIC	--	--	--
Ca2_P2_18225059	Ca2	18225059	GTTTTTTTTT/GTTTTTTTTTT	TTGTTTTGATGGATGATTTGGA	AAGCAAACATTTGGAATGGC	59.9	460	INTERGENIC	--	--	--
Ca2_P2_18225951	Ca2	18225951	TTTAATTTAATTAATTTAATT/TTTAATTTAATTAATTTAATTAATTTAATT	CGGGAAATCGTTATTAGCCA	AACAACGCAGAAGAGAAGCC	59.6	127	INTERGENIC	--	--	--
Ca2_P2_18237555	Ca2	18237555	ATGGT/AT	TTGGTTGTTTGGGGATCTTC	AATTTTGCTCCTCCCAAGGT	59.9	479	DRR	Ca_15980	TZ	--
Ca2_P2_18323354	Ca2	18323354	ATTT/ATTTTTT	CGGGAGATACGTAGGAGCAA	TCGATTATGCGTAGGGAAGG	60.1	253	INTERGENIC	--	--	--
Ca2_P2_18343132	Ca2	18343132	T/TGGCGACAACATA	TCAAATGAATGGTTTGAGCG	TCCCTCCTTCACAAAATTGG	59.9	657	INTERGENIC	--	--	--
Ca2_P2_1835747	Ca2	1835747	TTATATATATATATATATATATATATAT/TTATATATATATATATATAT	CACAATGCACCATGTGACAA	TGGAACTGAAGCCATATCCC	59.9	831	INTRON	Ca_12601	--	--
Ca2_P2_18391802	Ca2	18391802	AATATATATATATATATA/AATATATATATATATA	CAAAGCGGGTAGCAAGGATA	GCCGCTCCTCTTCAAGTAAA	59.6	524	INTERGENIC	--	--	--

INDEL marker IDs	Chromosomes/unanchored scaffolds	Physical positions (bp)	InDels (*Kabuli* reference genome-CDC Frontier/PII)	Forward primers (5'-3')	Reverse primers (5'-3')	Anneling temperature (0C)	Expected amplified product size (bp)	Structural annotation		Functional annotation	
								Sequence components of *kabuli* genome	*Kabuli* gene accession IDs	NCBI-KOG	TFs
Ca2_P2_18394672	Ca2	18394672	CTCTGCTTC/CTCTGCTTCTGCTTC	TAGTGATGGGAGCTGCAGTG	TGGTCCATCCAGACTCAGTG	59.7	483	INTERGENIC	--	--	--
Ca2_P2_18523267	Ca2	18523267	TAAAAA/TTAAAAAA	CAAACATGCCACCACAAAAG	GGAGGCAACCCACAAGTAGA	60.1	304	INTERGENIC	--	--	--
Ca2_P2_18547380	Ca2	18547380	ATTTTTTTT/ATTTTTTTTT	TCGGGCCTAAAAACAACAAC	ATGGAAGCGGAAAAACAAGA	59.7	678	INTRON	Ca_15969	--	--
Ca2_P2_18584340	Ca2	18584340	GAAAAAAAAAA/GAAAAAAAAAAA	ATGTACCTCCGCCTCATTTG	TCTCTGGCAACCGTTCTTCT	60.0	257	INTERGENIC	--	--	--
Ca2_P2_18610007	Ca2	18610007	TAA/TAACAA	TTGATCCCTTATTTCCTGCG	TTATTTCACCGTCGTGTCCA	60.0	492	INTERGENIC	--	--	--
Ca2_P2_186251	Ca2	186251	ATATTATTATTATTATTATTATTATTATTATTATTATTATTAT/ATATTATTATTATTATTATTATTATTATTATTATTATTATTAT	GATGCACTTAGGGGCAATTT	TGGATCAAGCCAAACCTTTC	60.1	610	INTERGENIC	--	--	--
Ca2_P2_18764616	Ca2	18764616	GAATAATAATAATAATAATAATA/GAATAATAATAATAATAATA	ATTTGCCTCCCTCAACCTTT	TGTCTTCATGGGTGCATTGT	60.0	841	INTERGENIC	--	--	--
Ca2_P2_1879905	Ca2	1879905	CAAAAAAA/CAAAAAAAA	AACATCTCTAGCGTGTGCCA	CACACCCACTCCCTTGTCTT	60.0	711	DRR	Ca_12598	YU	--
Ca2_P2_18833380	Ca2	18833380	TAAAAAAAAAAA/TAAAAAAAAAAAA	TCGAAAAATCCCTTGAAAAAGA	GGACCTATCAAGTGAGCTTGTG	58.9	551	INTERGENIC	--	--	--
Ca2_P2_18870465	Ca2	18870465	GAAAAAAAAA/GAAAAAAAAAA	TTGCTCCGATTCTATCCTTCA	TTGATCCTGCCATCTTGTCA	60.2	766	INTERGENIC	--	--	--
Ca2_P2_18905246	Ca2	18905246	GTTTTTTTTTTT/GTTTTTTTTTTTT	TGTGAGCTCAATTATTGGAACAG	TACTCAACCACCACCGTCAA	60.0	595	INTERGENIC	--	--	--
Ca2_P2_19008979	Ca2	19008979	TAAAAAA/TAAAAAAAA	GGCTATATCTCCCATGCCAA	GCAAGAAACGAGGAAAGAAGG	60.4	663	INTRON	Ca_15954	--	bHLH

INDEL marker IDs	Chromosomes/unanchored scaffolds	Physical positions (bp)	InDels (*Kabuli* reference genome-CDC Frontier/PII)	Forward primers (5'-3')	Reverse primers (5'-3')	Anneling temperature (0C)	Expected amplified product size (bp)	Structural annotation		Functional annotation	
								Sequence components of *kabuli* genome	*Kabuli* gene accession IDs	NCBI-KOG	TFs
Ca2_P2_19081800	Ca2	19081800	ATTT/ATT	TCACGGGTCAGCACAATTTA	CGATTGTTGTGGATGGAGTC	58.9	152	INTERGENIC	--	--	--
Ca2_P2_19154584	Ca2	19154584	ATTTTTTT/ATTTTTTTT	TCACTGCAAATTTCGTGGAT	CGTGCATAATCAACATCAGTCA	59.6	385	INTERGENIC	--	--	--
Ca2_P2_1919646	Ca2	1919646	TC/TCTCC	TTGAAGCGATGTGGTTGAAG	CCAAAACAACTATGCCCGTT	59.9	339	INTERGENIC	--	--	--
Ca2_P2_19207562	Ca2	19207562	TAAAAAAA/TAAAAAAAA	ACAAACCAAAACCCAACCAA	TTTCACCGACCCAAACAAAT	60.2	663	INTERGENIC	--	--	--
Ca2_P2_19219016	Ca2	19219016	ATTTT/ATTT	CCCGTGAGATCCTCCTAACA	TGAATCTACGGCTTTCTGGG	60.2	498	INTERGENIC	--	--	--
Ca2_P2_19276814	Ca2	19276814	ATTTTTT/ATTTTTTT	TTTTCACATGTGCTCGTTTACC	CAATAGCAAGACATTGCCCA	59.7	810	INTERGENIC	--	--	--
Ca2_P2_19363197	Ca2	19363197	A/ACTT	GCAGCGAAACTTAGAGACGG	ACCGAGTGTCGAACTCAAGG	60.3	606	INTERGENIC	--	--	--
Ca2_P2_19387244	Ca2	19387244	TTATATATATATATATAT/TTATATATATATATATAT	CGACATTGTAGCACCCCTTT	TTTAGATCCCTTGCACGACC	60.1	191	INTERGENIC	--	--	--
Ca2_P2_19513567	Ca2	19513567	TG/T	GCCCCTGAGTCAATACCAAA	ATCGCCAAACATTCAACAAA	59.0	615	INTERGENIC	--	--	--
Ca2_P2_19517696	Ca2	19517696	AAAGAA/AAA	CCCAAATCACCTAACTTGTGAA	TGTAAAAGCTGCATCGGACA	60.4	213	INTERGENIC	--	--	--
Ca2_P2_19594396	Ca2	19594396	ATT/ATTTAATCTCTT	GATGCAATTTTAGAGGAGAAGCA	TTTTTGGCCTTTGAGAGGAA	59.8	221	DRR	Ca_24535	--	--
Ca2_P2_19705772	Ca2	19705772	GAAAA/GAAAAA	TATTTTGGATTTTGGCTCGG	AGGAAGAGTGGAGTTGCGAA	60.0	475	INTERGENIC	--	--	--

INDEL marker IDs	Chromosomes/unanchored scaffolds	Physical positions (bp)	InDels (*Kabuli* reference genome-CDC Frontier/PII)	Forward primers (5'-3')	Reverse primers (5'-3')	Anneling temperature (0C)	Expected amplified product size (bp)	Structural annotation		Functional annotation	
								Sequence components of *kabuli* genome	*Kabuli* gene accession IDs	NCBI-KOG	TFs
Ca2_P2_19791685	Ca2	19791685	C/CG	TAGGCCATTTTCGTCCTCAC	AATCCAATGACACAAACGCA	60.0	392	INTERGENIC	--	--	--
Ca2_P2_19976429	Ca2	19976429	TATATATATATAT/TATATATATATATATATACATATATATATAT	ATGTTTTGGATTTTGGCTCG	AGGAAGAGTGGAGTTGCGAA	60.0	526	INTERGENIC	--	--	--
Ca2_P2_20086670	Ca2	20086670	GAAAAAAAAA/GAAAAAAAAAA	GCTGGCTGACATCTTCACAA	GAATGGGAACCCTTTTGGTT	60.0	710	INTERGENIC	--	--	--
Ca2_P2_20166723	Ca2	20166723	TTATATATATATATATATATATAT/TTATATATATATATATATATATATATATAT	CAAATGCCGTCACAAACATC	GCAAATGCTCCCCTAATTGA	60.0	541	INTERGENIC	--	--	--
Ca2_P2_20196984	Ca2	20196984	AAATAA/AAAATAATAA	GTTTGGGGTGGTGATTCTCA	TCCTAGAACATGCATCACGC	59.8	345	INTERGENIC	--	--	--
Ca2_P2_20232350	Ca2	20232350	TNNNNNNNNNN/TNNNNNNNNN	TTACGTATCTCTCGGTGCGA	AACGTCAACTTCGACCCTTG	60.1	541	INTERGENIC	--	--	--
Ca2_P2_20275069	Ca2	20275069	CATATATATATATATATATATATATATATATATATATATA/CATATATATATATATATATATATATATATA	GAGTATAACCACGCCGGAAA	GTTCCCCAGTTGCTTGTGTT	60.0	625	INTERGENIC	--	--	--
Ca2_P2_20280019	Ca2	20280019	TAAAAAAAAAAA/TAAAAAAAAAA	GCAAGGTTTGACAGTGAGCA	CATCGACAACCAATCCACAG	60.0	554	INTERGENIC	--	--	--
Ca2_P2_20321211	Ca2	20321211	GTTTTTTTT/GTTTTTTT	TCCAACAATCAACATAAGACATGA	CCGCAAGTAAATCCGGTAAA	60.0	786	INTERGENIC	--	--	--
Ca2_P2_20353586	Ca2	20353586	T/TG	TCTCCCTCCTTCCTCCATTT	CATTAAGAGGTTCCCCCTCC	59.8	844	INTERGENIC	--	--	--
Ca2_P2_20380428	Ca2	20380428	CTATATATATATATATATAT/CTATATATATATATATAT	CTTTCCCCATCAAACAATGG	CTAAGCCCGATAACGGATGA	60.1	538	INTERGENIC	--	--	--
Ca2_P2_20451589	Ca2	20451589	CAAAA/CA	ACCAAATTGACTCGGTGCTC	TGTGGGATGACCCTTTTTGT	60.2	460	INTERGENIC	--	--	--

INDEL marker IDs	Chromosomes/unanchored scaffolds	Physical positions (bp)	InDels (*Kabuli* reference genome-CDC Frontier/PII)	Forward primers (5'-3')	Reverse primers (5'-3')	Anneling temperature (0C)	Expected amplified product size (bp)	Structural annotation		Functional annotation	
								Sequence components of *kabuli* genome	*Kabuli* gene accession IDs	NCBI-KOG	TFs
Ca2_P2_20474785	Ca2	20474785	GAAAA/GAAAAA	ATACTGGCTCGGAGGCTTTT	CGTAGCGTGGTAGTGAGCAA	60.1	614	INTERGENIC	--	--	--
Ca2_P2_20490707	Ca2	20490707	T/TCAAAACATTA	AGTAGACCCACCGATGTTGC	TTTCAGAGCTTGCGGATTTT	60.0	437	INTERGENIC	--	--	--
Ca2_P2_20586221	Ca2	20586221	A/AC	ATTCCAAACCAAAACCCTCC	TAAATCGTGTTGACCCGTGA	60.0	197	INTERGENIC	--	--	--
Ca2_P2_20588077	Ca2	20588077	ATTTTTTTTTTT/ATTTTTTTTTT	CCGAGCCAAAATCCAAAATA	CTCCCCCTTTTTGATGATGA	59.9	814	INTERGENIC	--	--	--
Ca2_P2_20647114	Ca2	20647114	AATATATATATATATATATAT/AATATATATATATATATATATAT	ATCACTCGCACAACAAACCA	TTGGAAGGAGCCCTTACCTT	60.1	920	INTERGENIC	--	--	--
Ca2_P2_20687157	Ca2	20687157	ATT/ATTT	GATTGGTCGGTTGACTCGTT	AAGCATTCCCATCTGCATTC	60.0	253	INTERGENIC	--	--	--
Ca2_P2_20745579	Ca2	20745579	TC/TCC	CTCGTGCAAATCACGAGTCT	TTGTGTTTTGGTTGTGACCG	60.4	323	INTERGENIC	--	--	--
Ca2_P2_20761850	Ca2	20761850	CATA/CATATA	AAAATCCAACAATCTCCCCC	ACTCCTCCACAAGCCTCTGA	60.0	939	DRR	Ca_23762	--	--
Ca2_P2_20970910	Ca2	20970910	ATTCCCTCTT/ACTT	AACAAAGGAACAAAATGGCG	AGGGACAAAGGGCAAGAAAT	59.9	853	INTERGENIC	--	--	--
Ca2_P2_21088001	Ca2	21088001	TA/TAAAAGCATCA	AGAACGATGACACGTTGGGT	CTTCTGTCCAAAAAGCAGCC	60.0	812	INTERGENIC	--	--	--
Ca2_P2_21308559	Ca2	21308559	CTTTTCCTTTGTTTCTTTTTCCTT/CTTTTCCTTTGTTTCTTTTTCCTTTGTTTCTTTTTCCTT	TTTCGAAGCAACGAGGAAGT	TTGGTATTGAACTCGGAGGC	60.1	474	INTERGENIC	--	--	--
Ca2_P2_21312312	Ca2	21312312	GTTTTTT/GTTTTTTTT	TATCAGAGTTCTCCCCACCG	AGAAACCACAACGCAACACA	60.2	356	INTERGENIC	--	--	--

INDEL marker IDs	Chromosomes/unanchored scaffolds	Physical positions (bp)	InDels (*Kabuli* reference genome-CDC Frontier/PII)	Forward primers (5'-3')	Reverse primers (5'-3')	Anneling temperature (0C)	Expected amplified product size (bp)	Structural annotation		Functional annotation	
								Sequence components of *kabuli* genome	*Kabuli* gene accession IDs	NCBI-KOG	TFs
Ca2_P2_21314611	Ca2	21314611	AG/AGG	CAAACCCTTTCAAGGTGCAT	GCACTGCCCCAAGAATTTTA	60.1	443	INTERGENIC	--	--	--
Ca2_P2_21553694	Ca2	21553694	TTTATTATTATTATTATTATTATTATTATTATTATTATTATT/TTTATTATTATTATTATTATTATTATTATTATTATTATTATTATTATTATTA TTATTATT	TGTGCTCTCAATTGTGCTCC	AATTACCCGCATGTTGAAGC	60.0	792	INTERGENIC	--	--	--
Ca2_P2_21596317	Ca2	21596317	TTTATTATTATTATTATTATTATTATTATTATT/TTTATTATTATTATTATTATTATTATTATTATTATTATT	AATCCCTCCCTTCGTCACTT	TCCCTTTCTACAAGCGTTCA	58.5	210	INTERGENIC	--	--	--
Ca2_P2_21672716	Ca2	21672716	ATT/AT	TGATGCCGTGCCATAAATAG	TAACCTTGCCTTTGGGTGAG	60.1	595	INTERGENIC	--	--	--
Ca2_P2_2168744	Ca2	2168744	TTTTT/TTTTTT	AGCTTGCCAACTTCTGGGTA	GTAAAACCGTAGGGGGCATT	60.1	928	INTERGENIC	--	--	--
Ca2_P2_2169056	Ca2	2169056	C/CG	AACCTCTCCAAGAGGATCGG	GGAGAGTGAAACGGATGGAA	60.1	648	INTERGENIC	--	--	--
Ca2_P2_2169072	Ca2	2169072	TTCTCTCTC/TTCTCTCTCTC	AACCTCTCCAAGAGGATCGG	GGAGAGTGAAACGGATGGAA	60.1	648	INTERGENIC	--	--	--
Ca2_P2_2169184	Ca2	2169184	GTTTT/GTTT	TCTCACGGTTTCAACTTCCC	GTCTGATGGAGGAGGCAGAG	59.9	447	INTERGENIC	--	--	--
Ca2_P2_21699071	Ca2	21699071	AAATAATAATAATAATAATAATAA/AAATAATAATAATAATAATAATAATAATAATAA	GGCCTAGGCAAAAATTAGGG	AGGTTGCCCTTTCAGGTTTT	60.0	210	INTERGENIC	--	--	--
Ca2_P2_21717164	Ca2	21717164	TA/T	GGTAGAGGCAGGCCATATCA	GATTTGTGAGGGATTTTTCAGC	60.0	841	INTERGENIC	--	--	--
Ca2_P2_21735219	Ca2	21735219	AATATATATATATATATATATATATATATATATATATATATATATAT/AATATATATATATATATATATATATATATATATATATATATATAT	GCACACAAAATCAAAACACACA	TCCAAAGGCTTGAGCTTGAT	60.0	418	INTERGENIC	--	--	--
Ca2_P2_21757860	Ca2	21757860	CTTTT/CTTT	TTGACCGAAGAAAAATTGGG	CCGAAATGAGTCCCATCTGT	59.9	569	INTERGENIC	--	--	--

INDEL marker IDs	Chromosomes/unanchored scaffolds	Physical positions (bp)	InDels (*Kabuli* reference genome-CDC Frontier/PII)	Forward primers (5'-3')	Reverse primers (5'-3')	Anneling temperature (0C)	Expected amplified product size (bp)	Structural annotation		Functional annotation	
								Sequence components of *kabuli* genome	*Kabuli* gene accession IDs	NCBI-KOG	TFs
Ca2_P2_21783996	Ca2	21783996	ATTTTTT/ATTTTTTTT	ATCTCGTGGGTGGATCTGAC	GCTATGGGACCTATCAGGCA	60.1	381	DRR	Ca_23057	--	--
Ca2_P2_21798248	Ca2	21798248	TAAAAAAAAAAAA/TAAAAAAAAAAAAA	AGCTTTGATTTCTCCATCGC	TCAATCGAGTCACCACCAAA	60.1	370	INTERGENIC	--	--	--
Ca2_P2_21889862	Ca2	21889862	G/GGT	CGATGTTAGTGGTGACCGTG	CTGCTTGAGATGGCGTTGTA	60.0	606	INTERGENIC	--	--	--
Ca2_P2_21943275	Ca2	21943275	AGCGCG/AGCG	GCAGATTTGGAAGGTGTGGT	ACAATCAGCACGCAAGAATG	59.9	461	INTERGENIC	--	--	--
Ca2_P2_22103225	Ca2	22103225	AAGAGAGAGAG/AAGAGAGAG	AAGAGAAGGCACTGCCACAT	TGTAGGAATCCCTTGGCAAC	59.9	804	INTERGENIC	--	--	--
Ca2_P2_22173952	Ca2	22173952	GAAAAAAAAAAA/GAAAAAAAAAA	TGAATGCACAACCGCTAAAG	TGGACCAACCTTCTCACCAT	60.4	521	INTRON	Ca_15007	--	Trihelix
Ca2_P2_22219214	Ca2	22219214	GTGGTGGTCG/GTGGTGGTCGTTGGTGGTCG	CAAAGCGGTCGAAGTAGAGG	GTTGTTCGGGGTTGTGAGTT	59.9	391	INTERGENIC	--	--	--
Ca2_P2_22220209	Ca2	22220209	CATATATATATATATATATATATATATATATATATA/CATATATATATATATATATATATATATATATATATATATATATATA	AACTCACAACCCCGAACAAC	CTGCTTGGGGATAACTTGGA	60.1	911	INTERGENIC	--	--	--
Ca2_P2_2222122	Ca2	2222122	TAAAAAAAAAAAAAAA/TAAAAAAAAAAAA	TGTCCTTGTTGTTAGTGGCTG	TGTTTGAATTTGAGTCCCCA	58.9	179	DRR	Ca_12559	--	FAR1
Ca2_P2_22286038	Ca2	22286038	GAAGCGC/GAAGCGCACCCAAGCGC	GGGAGGCAACCCAATTTTAT	GCTTAGATCATCCGCTCCTG	59.9	368	INTERGENIC	--	--	--
Ca2_P2_2232996	Ca2	2232996	CTTTTTTTTTTTTTTTTTTTT/CTTTTTTTTTTTTTTTTTTTTTTTTTTTT	GCAAACACCGTCTTAATTGGA	GGAATTGTTTGCAAGTCCGT	60.0	685	INTERGENIC	--	--	--
Ca2_P2_22337782	Ca2	22337782	TTATATATATATATATATATATATA/TTATATATATATATATATATATA	GGGTTAGAGGACACCCCAAT	CCTTTTTGGAAATGCAGGAA	60.0	654	INTERGENIC	--	--	--

INDEL marker IDs	Chromosomes/unanchored scaffolds	Physical positions (bp)	InDels (*Kabuli* reference genome-CDC Frontier/PII)	Forward primers (5'-3')	Reverse primers (5'-3')	Anneling temperature (0C)	Expected amplified product size (bp)	Structural annotation		Functional annotation	
								Sequence components of *kabuli* genome	*Kabuli* gene accession IDs	NCBI-KOG	TFs
Ca2_P2_2235889	Ca2	2235889	TTATTA/TTATTACTATTA	TCTTGCACAACTCATTTCGG	AAAAAGCAAAGAAATTGTCAGAA	57.3	330	INTERGENIC	--	--	--
Ca2_P2_22483232	Ca2	22483232	ATAATTAAT/ATAAT	CGTGCCACTTTTCTCTAGGC	AGATCATGTGCAAAAAGGGG	59.9	369	INTERGENIC	--	--	--
Ca2_P2_22489205	Ca2	22489205	CTTTT/CTTT	GATCAGATCTGGCAGCAACA	AAGTGATTTTGGCCTGAGGA	59.7	401	INTERGENIC	--	--	--
Ca2_P2_22527944	Ca2	22527944	TAAAAAAA/TAAAAAAAA	TGCTCCCTCATTCTTCCATT	ATCTTGGAGCCCCTGATCTT	60.0	713	INTERGENIC	--	--	--
Ca2_P2_22650634	Ca2	22650634	TG/T	GAGAGGAGGGTTCCCAGAGT	GTGCATCCTCCATCTTTGGT	59.9	550	DRR	Ca_14991	--	--
Ca2_P2_22652275	Ca2	22652275	TAATCGATAGACAAATCGAT/TAATCGAT	CGGAGCAAATCGATTAGGAA	GCACTGGACGTAACCCAGAT	60.0	500	INTERGENIC	--	--	--
Ca2_P2_2278595	Ca2	2278595	CTATTTATTTAT/CTATTTAT	GTTTGGAAAATGTTGCATCG	CGAACCATCAGGCCAATAAA	60.8	718	INTERGENIC	--	--	--
Ca2_P2_228183	Ca2	228183	GTTTTTTTTTT/GTTTTTTTTT	TTCGAAACGTTGGATGAATG	GCAACCATCTGACCAAGTGA	59.7	418	INTERGENIC	--	--	--
Ca2_P2_22825489	Ca2	22825489	ATATTATTATTATTATTATTATTATTATTATTATTATTATTATTATTATTATTATTA/ATATTATTATTATTATTATTATTATTATTATTATTATTATTATTATTATTATTATTATTA	TTGGTAATTCAGGACGAGGC	ACGAGGTAACGAAATGGCAG	60.1	828	DRR	Ca_14985	O	GeBP
Ca2_P2_22846943	Ca2	22846943	ATTTTTTTTTT/ATTTTTTTTTTT	GGTTCCAAACTCTCGGTTGA	GCACGTGGCTAGGGTTATGT	60.0	569	DRR	Ca_14984	--	--
Ca2_P2_22890617	Ca2	22890617	TTATATATATATATATATATAT/TTATATATATATATATATATATAT	GCCACCCTTGTGAGAGTTTC	CACGAGTCCGATAAGGGTGT	60.0	554	INTERGENIC	--	--	--
Ca2_P2_23029103	Ca2	23029103	TATA/TATAGTAATA	GGTTCCTGTTGTGGCTTTTT	TGCTCATGTGTTGGAAGTGC	60.9	586	INTERGENIC	--	--	--

INDEL marker IDs	Chromosomes/unanchored scaffolds	Physical positions (bp)	InDels (*Kabuli* reference genome-CDC Frontier/PII)	Forward primers (5'-3')	Reverse primers (5'-3')	Anneling temperature (0C)	Expected amplified product size (bp)	Structural annotation		Functional annotation	
								Sequence components of *kabuli* genome	*Kabuli* gene accession IDs	NCBI-KOG	TFs
Ca2_P2_23050911	Ca2	23050911	AATG/AATGGTCTATTGGCTTATG	TATGGGGATCGGAAATCAAA	ACAATCGAAATTTTAGGCCG	59.1	907	INTERGENIC	--	--	--
Ca2_P2_23058191	Ca2	23058191	GTTTTTTTTTTT/GTTTTTTTTTTTT	GAAACTGTGTCACGAGGCAA	AGAGCCGTCCAGAAAAATCA	59.8	121	INTERGENIC	--	--	--
Ca2_P2_23183113	Ca2	23183113	TAAAAAA/TAAAAAAA	TGGATACATATGCCGCTCAA	ACCGTGGACCACCTTTGATA	60.2	565	DRR	Ca_14971	--	--
Ca2_P2_23193293	Ca2	23193293	GTT/GTTT	CCACAAATGCTCATCGTGAC	TTTGCAGCCACAAAAGACAG	60.0	561	INTERGENIC	--	--	--
Ca2_P2_23193466	Ca2	23193466	TA/T	CCACAAATGCTCATCGTGAC	TTTGCAGCCACAAAAGACAG	60.0	561	INTERGENIC	--	--	--
Ca2_P2_23243854	Ca2	23243854	AAATAATAATAATAATAATAATAATAATAATAAT/AAATAATAATAATAATAATAATAATAATAATAATAATAAT	TTTCATGATAATTTTGGAGGGA	CGGCTTTACACCACCTGTTT	60.0	252	INTERGENIC	--	--	--
Ca2_P2_23294259	Ca2	23294259	GGATGCA/G	TCTAACACTCCCCCTCAAGC	TTTGTCTCAGATGCACTGGC	60.0	342	INTRON	Ca_14967	S	ERF
Ca2_P2_23365942	Ca2	23365942	TAAAAAAAAA/TAAAAAAAAAA	GAGCTGTGTCTTGGCAATCA	TCTCTGGCAACCGTTCTTCT	60.0	597	INTERGENIC	--	--	--
Ca2_P2_23401826	Ca2	23401826	AT/ATTT	TTCGTAATCGATTGCAGGTG	TTTTCGTCAAACCCAAAAGC	60.1	920	INTERGENIC	--	--	--
Ca2_P2_23408235	Ca2	23408235	AATATATATATATATATATATATAT/AATATATATATATATATATATATATAT	GGTAGGAATTGGGTGTGGTG	AGGAAGAGTGGAGTTGCGAA	60.0	710	INTERGENIC	--	--	--
Ca2_P2_23409125	Ca2	23409125	T/TA	CGATTTGTGTCCGTTGTTTG	AGATTCCCCCTTCTTAACGC	59.6	335	INTERGENIC	--	--	--
Ca2_P2_2344236	Ca2	2344236	ACATTCATTCATTCATTC/ACATTCATTCATTCATTCATTCATTCATTC	GAAGGATAGGTCGCATTGGA	TGCAGCTGTGATGTTGTTCA	60.0	384	INTRON	Ca_12544	S	BBR-BPC

INDEL marker IDs	Chromosomes/unanchored scaffolds	Physical positions (bp)	InDels (*Kabuli* reference genome-CDC Frontier/PII)	Forward primers (5'-3')	Reverse primers (5'-3')	Anneling temperature (0C)	Expected amplified product size (bp)	Structural annotation		Functional annotation	
								Sequence components of *kabuli* genome	*Kabuli* gene accession IDs	NCBI-KOG	TFs
Ca2_P2_23472039	Ca2	23472039	CTTTATTTATTTATTTATTTATTTATTTATTTATTTAT/CTTTATTTATTTATTTATTTATTTATTTATTTATTTATTTATTTATTTAT	TTTTGCTAACCACACCCCTC	TGACCACGTGTTTTTGGGTA	59.9	434	INTRON	Ca_14958	--	--
Ca2_P2_23495114	Ca2	23495114	ATTTTTTT/ATTTTTT	GCTCCCCATGAGCACTAGAA	AGGCTCTCAATGCCAACCTA	59.8	710	INTERGENIC	--	--	--
Ca2_P2_23496145	Ca2	23496145	TTATATATATATATATATAT/TTATATATATATATATATATAT	GTTTTGTAGTGGCCCCAAAG	CTTTTCAAACGGCGACTTTC	59.9	557	INTERGENIC	--	--	--
Ca2_P2_23509535	Ca2	23509535	AAAAACTTAAAACTTAAAACTTA/AAAAACTTAAAACTTA	CCCTCTTGAATTTTTGTTTTCC	CCCACACCCTCGACTAAAGA	60.1	542	INTERGENIC	--	--	--
Ca2_P2_2354894	Ca2	2354894	ATT/ATTT	CCTTCCCCAGATCCTTAACC	TGTAGTGGGTAATTTGAGCCG	60.0	866	INTERGENIC	--	--	--
Ca2_P2_2373367	Ca2	2373367	AATATATATATATATATATATATA/AATATATATATATATATATATATATATATATATA	AAAATTGTCAGGTTCACCGC	GGTGGTGATTTGGTGCTCTT	60.0	896	DRR	Ca_12541	R	DBB
Ca2_P2_23792320	Ca2	23792320	GGAGTAAGAGAGTAA/GGAGTAAGAGAGTAAGAGAGTAA	TCAGAATCCCTGATCCCAAG	GCAACCAGGAGGCAAGTCTA	60.4	490	INTERGENIC	--	--	--
Ca2_P2_23820380	Ca2	23820380	TTATATATATATATAT/TTATATATATATATATAT	GTGCTCTCGTCAAACGAACA	AAGCGCTGTAAATGGTGCAT	60.7	716	DRR	Ca_14945	--	--
Ca2_P2_2382639	Ca2	2382639	T/TCATA	TTGGAAATGCCAGAGGTATTAGA	TGTGATTTGGTTTCGCAAAG	59.7	658	DRR	Ca_12540	R	--
Ca2_P2_23829520	Ca2	23829520	GTT/GT	AAATGGTTTGCCCATCAAAA	CTTGCCTTTCCTCACCTCAG	60.0	791	DRR	Ca_14944	T	YABBY
Ca2_P2_23838319	Ca2	23838319	GTTTTTTTTTTTTT/GTTTTTTTTTTTT	CAACAATCTCTCCCGCTCTC	TGGCGACTAGGAGGTTTTGA	60.8	665	INTERGENIC	--	--	--
Ca2_P2_23849485	Ca2	23849485	A/ATATAT	TTGTGTGTGGGAAGAACCAA	AGACACCCCAAGAGATGTGG	60.0	521	INTERGENIC	--	--	--

INDEL marker IDs	Chromosomes/unanchored scaffolds	Physical positions (bp)	InDels (*Kabuli* reference genome-CDC Frontier/PII)	Forward primers (5'-3')	Reverse primers (5'-3')	Anneling temperature (0C)	Expected amplified product size (bp)	Structural annotation		Functional annotation	
								Sequence components of *kabuli* genome	*Kabuli* gene accession IDs	NCBI-KOG	TFs
Ca2_P2_23851284	Ca2	23851284	GATATATATATATATATATATATATATA/GATATATATATATATATATATATATATATA	TGCCTTCATCATGTGGTTGT	ATGGCAATTTCTCAGGATGG	59.9	501	INTERGENIC	--	--	--
Ca2_P2_23932608	Ca2	23932608	TAAATTAATGAATAAAAATTAAT/TAAATTAAT	AAGGCATCCAATCAAAGCAC	CTCAAGATTTTAGCGCCGTC	60.0	460	INTERGENIC	--	--	--
Ca2_P2_23946313	Ca2	23946313	ACA/ACAGCTGCCA	GAGCCAAACACTGCATCAGA	GGTAGCGGCTTGTGCTAGTT	59.5	662	INTERGENIC	--	--	--
Ca2_P2_23986096	Ca2	23986096	TTATATATATATATATATATA/TTATATATATATATATATATATA	CAAAATTTTCAAAACAAACCCG	GCACGACAAACACCTCAAAA	59.7	574	INTERGENIC	--	--	--
Ca2_P2_23989802	Ca2	23989802	AAAAACAAAACAAAA/AAAAACAAAACAAAACAAAA	AATTCGTTGAGCCGGTCTAGT	AGCACGGGGAGTATGAGAAA	59.7	421	INTERGENIC	--	--	--
Ca2_P2_23990182	Ca2	23990182	AAAAACAAAACAAAA/AAAAACAAAACAAAACAAAA	TCTCATACTCCCCGTGCTTT	ATCCCCTACACCAGATGCAC	59.8	565	INTERGENIC	--	--	--
Ca2_P2_24102909	Ca2	24102909	GT/GTT	TGGGGGATCCAAGTTAAAAA	GAAAACTCTCCGGCATTCAA	60.2	428	CDS (FRAME SHIFT)	Ca_21670	S	--
Ca2_P2_24157441	Ca2	24157441	ATTTTTTT/ATTTTTTTT	CAATATTCCGGAGGAGACGA	CTTCAGCATGCACCCCTAAT	60.1	727	INTERGENIC	--	--	--
Ca2_P2_24190689	Ca2	24190689	ATTT/ATTTT	AACACTTTCATCCAATAAAAATTGA	TTGTTGAAAATAATCGCGTGAC	60.0	526	INTERGENIC	--	--	--
Ca2_P2_24251584	Ca2	24251584	ACCCCC/ACCCCCC	GATTGCTTTCTGGGTGGAAA	TGATCAACCCTTGGTGTGAA	59.9	652	INTERGENIC	--	--	--
Ca2_P2_24251932	Ca2	24251932	A/AC	GATCCTCAAATCGACCTCCA	GCACAGTGTAAGCTTTGCCA	60.1	410	INTERGENIC	--	--	--
Ca2_P2_24252139	Ca2	24252139	TAA/TAAGAAA	GATCCTCAAATCGACCTCCA	TGTCACCATTGGTCTCCAAA	59.9	465	INTERGENIC	--	--	--

INDEL marker IDs	Chromosomes/unanchored scaffolds	Physical positions (bp)	InDels (*Kabuli* reference genome-CDC Frontier/PII)	Forward primers (5'-3')	Reverse primers (5'-3')	Anneling temperature (0C)	Expected amplified product size (bp)	Structural annotation		Functional annotation	
								Sequence components of *kabuli* genome	*Kabuli* gene accession IDs	NCBI-KOG	TFs
Ca2_P2_24319865	Ca2	24319865	GAAAAAAAAA/GAAAAAAAAAA	CCGAGCCAAAATCCAAAATA	ACCGACATCTGGCTGAGTCT	59.9	972	INTERGENIC	--	--	--
Ca2_P2_24393200	Ca2	24393200	G/GAAGATA	TCCTAGAACATGCATCACGC	TACCCTTTGCTAGCCCCTTT	60.1	505	INTERGENIC	--	--	--
Ca2_P2_24475160	Ca2	24475160	AATATATATATATATATATATATATA/AATATATATATATATATATATA	TTCATGCCTCGTAAGCTTCAT	TGGTGACCACGCTCATATTT	59.0	460	INTERGENIC	--	--	--
Ca2_P2_24538889	Ca2	24538889	ATATTATTATTATTATTATTATTATTAT/ATATTATTATTATTATTATTATTATTATTATTATTAT	GGAGGACATGCAAATGAGGT	GTGGTTTGCAGGCTATGGAT	60.0	829	INTERGENIC	--	--	--
Ca2_P2_24563119	Ca2	24563119	TCCCC/TCCC	CCATCTACTGCCCTTGGAAA	GATCCAGGCAAGGTCAATGT	59.9	260	INTERGENIC	--	--	--
Ca2_P2_24571099	Ca2	24571099	TA/T	CGGTTAGGTGGTAAAAGAGCA	CCGCATCGTTAAAAATCTCA	58.8	469	INTERGENIC	--	--	--
Ca2_P2_24597881	Ca2	24597881	T/TTGGAGATCATC	TTATTCGGGCCTGTCAAAAC	ATGGCGTTTACCCAAGACTG	60.0	564	INTERGENIC	--	--	--
Ca2_P2_24650875	Ca2	24650875	TATAATAATAATAATAATAATAATAATAATAATAATAATAATAATAAT/TATAATAATAATAATAATAATAATAATAATAATAATAATAATAATAATAAT	TGTTGTTTCCATACCCTCCC	ATGAGGTCCGACTTAACATCA	57.1	413	INTERGENIC	--	--	--
Ca2_P2_24660741	Ca2	24660741	AAATAATAATAATAATAATAATAATAATAATAATAATAATAATA/AAATAATAATAATAATAATAATAATAATAATAATAATAATAATAATAATA	CTTCGAAGGCCTACACCAAA	TTGGTGAAAACCCCTAAACG	59.8	569	INTERGENIC	--	--	--
Ca2_P2_24943252	Ca2	24943252	CTTT/CTT	AACCCCTCCAAAGAACGACT	AGGGCCCCACTAACTCCTTA	60.0	680	INTERGENIC	--	--	--
Ca2_P2_24948166	Ca2	24948166	CTTTTT/CTTTT	GGTGAGACTTTCGAGGGCTA	GGCATTGAACGTTTTCACAA	59.6	295	INTERGENIC	--	--	--
Ca2_P2_25072393	Ca2	25072393	CTATATATATATATATATATATATATATATAT/CTATATATATATATATATATATATATATAT	GAAGGAAGGAGGTCGGATTT	GGAGTTGCAGGCAGAAAAAG	60.0	561	DRR	Ca_14240	R	MYB

INDEL marker IDs	Chromosomes/unanchored scaffolds	Physical positions (bp)	InDels (*Kabuli* reference genome-CDC Frontier/PII)	Forward primers (5'-3')	Reverse primers (5'-3')	Anneling temperature (0C)	Expected amplified product size (bp)	Structural annotation		Functional annotation	
								Sequence components of *kabuli* genome	*Kabuli* gene accession IDs	NCBI-KOG	TFs
Ca2_P2_25135786	Ca2	25135786	GTT/GTTT	GGAAGCTGACTTTTTGGCAT	TGGCTTGATTCCTTTTGCTT	59.8	160	INTERGENIC	--	--	--
Ca2_P2_25171520	Ca2	25171520	TCAACAA/TCAA	GGAAATGAGCTGAATGGGAA	CCAGAGGGGATGTTGAGAAA	60.0	551	INTRON	Ca_14250	T	M-type
Ca2_P2_25228216	Ca2	25228216	T/TTGG	TGTCCTTTTCAAACCCCATC	ACAAGCAAAACCTCACCACC	60.0	351	CDS (large-effect mutations)	Ca_14254	R	--
Ca2_P2_25241576	Ca2	25241576	GATATATATATATATA/GATATATATATATATATATA	TATTTTGGATTTTGGCTCGG	AAATGGAGGAAGGAGGGAGA	60.0	433	INTERGENIC	--	--	--
Ca2_P2_25352886	Ca2	25352886	ATTTTTTTTTT/ATTTTTTTTTTT	AAAGAGACACATGACATAACTCCTC	ATGGGGGATGGACCTAGAAC	60.0	685	DRR	Ca_14268	--	--
Ca2_P2_25411955	Ca2	25411955	TAATTATAAA/TAATTATAAAATTATAAA	CAAAATCATTACTCACCATAAGTGG	TCACATCAGCCAAAAAGCTG	60.0	405	INTERGENIC	--	--	--
Ca2_P2_25427657	Ca2	25427657	TTATATATATATATATATATATATATATATAT/TTATATATATATATATATATATATATATAT	TGTTGTTGAAGATGAAATATTGGC	TTTGGCGTAGTGAAGTTGACA	59.4	723	INTERGENIC	--	--	--
Ca2_P2_25449852	Ca2	25449852	CGTAGATA/CGTAGATAAAATAGTAGATA	TATTTACCGACGGACAAGCC	TCGCATGGCTATGAAAAGTG	59.8	522	DRR	Ca_14275	K	G2-like
Ca2_P2_25460200	Ca2	25460200	TTATATATATATATATATATATATATATATATATATAT/TTATATATATATATATATATATATATATATATATATATATAT	AAAGCGTTGGCTTTTTCTGA	GTGCAACTTCATCCCCACTT	60.0	774	INTRON	Ca_14276	Q	GeBP
Ca2_P2_2546989	Ca2	2546989	AT/ATCATAGTTT	CCCGTCGATCAAAAGAAAAA	TTGAATTGCTTTCCAGTGTCC	60.1	705	INTRON	Ca_10592	R	--
Ca2_P2_25529098	Ca2	25529098	A/ATC	CCATTGAATTTACCGACGAAA	TTTACCGGGATTCTGCAAAG	60.1	663	INTERGENIC	--	--	--
Ca2_P2_25529864	Ca2	25529864	GTTTTTT/GTTTTTTTTT	AGCAGCCCAAGCAAGACTTA	TCATGCCGATTGATAGAGGTT	59.5	553	INTERGENIC	--	--	--

INDEL marker IDs	Chromosomes/unanchored scaffolds	Physical positions (bp)	InDels (*Kabuli* reference genome-CDC Frontier/PII)	Forward primers (5'-3')	Reverse primers (5'-3')	Anneling temperature (0C)	Expected amplified product size (bp)	Structural annotation		Functional annotation	
								Sequence components of *kabuli* genome	*Kabuli* gene accession IDs	NCBI-KOG	TFs
Ca2_P2_25530031	Ca2	25530031	A/ATACCCTTG	AGCAGCCCAAGCAAGACTTA	ATCCGACCGTTGAAGATTTG	59.9	724	INTERGENIC	--	--	--
Ca2_P2_25535050	Ca2	25535050	CTTTTTTTTT/CTTCTTTTTTTTTTTTTTT	CCAGATTTGAACTGGGGAAA	AATCAAGTGGGTTCGAGTGG	60.0	551	DRR	Ca_14283	R	MYB
Ca2_P2_25554310	Ca2	25554310	TTGTGTGTGTGTGT/TTGTGTGTGTGT	ATGCGGCAGGTATGAGAACA	ATATCAACGCCCTTTTGCAT	59.4	473	INTERGENIC	--	--	--
Ca2_P2_25593755	Ca2	25593755	TTATATATATATATATATATATATATATAT/TTATATATATATATATATATATATATATATATATATAT	AACCCGTTTTGATTGAGTGC	TGCGAAATTAGGCTTCCATC	60.2	466	INTERGENIC	--	--	--
Ca2_P2_25619256	Ca2	25619256	CTTTTTT/CTTTTTTT	TTGAGAGACCAAAGTGTCCAAA	AAGTGACTATTTCCTCGGTCCTT	59.6	829	INTERGENIC	--	--	--
Ca2_P2_25642637	Ca2	25642637	TAAAAAAAAA/TAAAAAAAA	AGTAGCCACTCCCAACAAGG	TGGTTGTTCCGATCCTTCTC	60.1	651	INTERGENIC	--	--	--
Ca2_P2_25733189	Ca2	25733189	TAAAAAA/TAAAAAAA	CTCAGAAAGCGGATCAAAGG	CTCAACCAACCACCCACTCT	60.0	477	INTERGENIC	--	--	--
Ca2_P2_25767704	Ca2	25767704	GAAGTTAAGTT/GAAGTT	ATGAATTTTGGCATCCAACC	CGTGGACAAAGATCGTGATG	60.1	654	INTRON	Ca_14294	G	NAC
Ca2_P2_25806540	Ca2	25806540	TAAAAAAAAAAAA/TAAAAAAAAAAAAA	AATGTGATTGAGGAGTTGCG	TCTATGCGTAGCCACAATGG	59.7	871	INTERGENIC	--	--	--
Ca2_P2_25911445	Ca2	25911445	GAAAAAAAAAAA/GAAAAAAAAAA	TTTCCCGACTTGGTCCTTTA	TGCGCTTAAAGACAGGGAGT	60.0	417	INTERGENIC	--	--	--
Ca2_P2_2610952	Ca2	2610952	CATATATATATATATATATATATATATATATATAT/CATATATATATATATATATATATATATAT	TACCCTCGCTCCTGTCGTAT	GGGAATCGGTTTTGAGTGAA	59.9	392	INTERGENIC	--	--	--
Ca2_P2_26109919	Ca2	26109919	CTATATATATATATATATATATATATATATATATA/CTATATATATATATATATATATATATATATATATATATATA	TTGTGCCTATTGTTGTGGGA	CAACCACTACTCCACGCTCA	59.9	573	INTERGENIC	--	--	--

INDEL marker IDs	Chromosomes/unanchored scaffolds	Physical positions (bp)	InDels (*Kabuli* reference genome-CDC Frontier/PII)	Forward primers (5'-3')	Reverse primers (5'-3')	Anneling temperature (0C)	Expected amplified product size (bp)	Structural annotation		Functional annotation	
								Sequence components of *kabuli* genome	*Kabuli* gene accession IDs	NCBI-KOG	TFs
Ca2_P2_26111863	Ca2	26111863	AATATATATATATATATATATATATAT/AATATATATATATATATATATATATAT	ATGCGTACATCAAGGGTGGT	GGCTCATATATTTGCATTCACG	59.5	560	INTERGENIC	--	--	--
Ca2_P2_2624920	Ca2	2624920	CGGCTGGCT/CGGCT	GGCCAAGTTGCTGAAGGATA	CGCTAAGAGGCCGTTATCAA	60.4	610	INTRON	Ca_10586	--	--
Ca2_P2_263972	Ca2	263972	TAAAAAAAAAAAAA/TAAAAAAAAAAAA	TTGGGATTAAAAACCCATTCC	TGCACGTTCTTCTCCTCCTT	60.0	467	INTERGENIC	--	--	--
Ca2_P2_26492795	Ca2	26492795	GAAAAAAAAAAA/GAAAAAAAAAA	TGACGACATCAACCAACCAC	GTTGAATTATTGGCGGGAAA	59.8	538	INTRON	Ca_17249	Q	GeBP
Ca2_P2_26504194	Ca2	26504194	AGG/AG	CCGACGTCCAGTCAGAATTT	GGATTGGAGTTGGTTGGATG	60.2	319	INTERGENIC	--	--	--
Ca2_P2_26504489	Ca2	26504489	CAA/CAAAA	CCGACGTCCAGTCAGAATTT	CTCAATTGGACACCGTTCCT	60.0	772	INTERGENIC	--	--	--
Ca2_P2_2671437	Ca2	2671437	TAAAAAAAAAA/TAAAAAAAAAAA	TAATTCAGCCAAGGAAGCCA	GGAAAACGCAAAAGGAATCA	60.1	235	INTERGENIC	--	--	--
Ca2_P2_26811414	Ca2	26811414	TAAAAAAA/TAAAAAAAA	GGGCTTGTTTGATTGTGTTTT	GGCTCCATTCTTTGCTCTTG	60.0	584	INTERGENIC	--	--	--
Ca2_P2_26830567	Ca2	26830567	AAAATATAAATA/AAAATATAAATATAAATA	TCGTAGGGAATCACTCCAAA	GACCTTGCATCTCTTGAGCC	60.0	543	DRR	Ca_17273	--	WRKY
Ca2_P2_2692293	Ca2	2692293	ATTTTTTTT/ATTTTTTT	AAAGGTGATGAAGCAGATTTTGA	TGGTGTCCTCTCTTCTCTAATCG	59.9	478	INTERGENIC	--	--	--
Ca2_P2_2703935	Ca2	2703935	TAAAAAAAAA/TAAAAAAAA	CCCAGCCATAGTTTGGAAAA	TTCACCCAGTTGGTGTTCAA	60.0	656	INTRON	Ca_10573	Q	B3
Ca2_P2_27068285	Ca2	27068285	TCC/TCCC	CGTGCTGAATTTTGGTGAGA	TTGGTTTCATGACCTGCATC	59.5	713	INTERGENIC	--	--	--

INDEL marker IDs	Chromosomes/unanchored scaffolds	Physical positions (bp)	InDels (*Kabuli* reference genome-CDC Frontier/PII)	Forward primers (5'-3')	Reverse primers (5'-3')	Anneling temperature (0C)	Expected amplified product size (bp)	Structural annotation		Functional annotation	
								Sequence components of *kabuli* genome	*Kabuli* gene accession IDs	NCBI-KOG	TFs
Ca2_P2_27153936	Ca2	27153936	TTATATATATATATATATATATAT/TTATATATATATATATATATAT	TCCAAACTAGGTGGCAACAA	TTGCCCACTCATTTCCTTTC	60.1	478	INTERGENIC	--	--	--
Ca2_P2_27401550	Ca2	27401550	TAAAAAAAAAAAA/TAAAAAAAAAAAAA	GCCGTTGGGTTTGAGTTAGA	CCAAATCACCTCCAGTCACC	60.4	678	INTERGENIC	--	--	--
Ca2_P2_27432353	Ca2	27432353	G/GT	TCATGATGGTAACCCGACCT	CATCAAACCCACACACAACC	59.7	319	INTERGENIC	--	--	--
Ca2_P2_27436271	Ca2	27436271	CAAA/CAA	TCCTACGTATCTTTTGATGCGA	CGTGCCCAATCTCTCAATCT	60.2	382	INTERGENIC	--	--	--
Ca2_P2_27468383	Ca2	27468383	TTATATATATATATATATATATATATATATATATATAT/TTATATATATATATATATATATATATATATAT	TTAACCGAGAAAATAAACGCA	ATTGCAAGTCAAACATGGGA	59.0	670	INTERGENIC	--	--	--
Ca2_P2_2749945	Ca2	2749945	CTTT/CTT	ATATGGATTCATGCCCCAAA	TTGCATTTTTCAGAGGGAGG	60.2	580	INTRON	Ca_10569	GMO	E2F/DP
Ca2_P2_27560889	Ca2	27560889	TTCCCTCCTATCCCTC/TTCCCTC	GGTGCATTTGGGAACCTTTA	CTGCAGCATCCCTACACTCA	60.0	767	INTERGENIC	--	--	--
Ca2_P2_27614728	Ca2	27614728	AAATAATAATAATAATAATAATAATAATAATAATAATAATAATGCTAATAATAATAATAATAATTGTAATAATAATAATAATAATAACAATAATAATAATAATAA/AAATAATAATAATAATAATAATAATAATAATAATAATGC[illegible]	CGCGCGAGCCATAATCTAGT	CGACGCCAATCTGCTTATTT	60.2	625	INTERGENIC	--	--	--
Ca2_P2_27692325	Ca2	27692325	TG/T	AGACAAAACGTTCAAGTTGTCG	TCTTTAATTGCATGCGCAAA	60.3	762	DRR	Ca_15671	--	ERF
Ca2_P2_27804750	Ca2	27804750	TCC/TC	CAGGAAACTCTGCCTTTTCG	TAGCAGCTGTATGGTGCAGG	60.0	137	INTERGENIC	--	--	--
Ca2_P2_2794197	Ca2	2794197	ACTCTCTCTCTCTCTCTCTCTCTCTCTC/ACTCTCTCTCTCTCTCTCTCTCTCTCTCTC	GACACCCCTTCACCTCAAAA	CGAGTTCTATTGCCTCTCGC	60.1	680	INTRON	Ca_10562	B	WRKY
Ca2_P2_27944905	Ca2	27944905	AATATATATATATATATATATATATATATATATATATATATATATATATATAT/AATATATATATATATATATATATATATATATATATATATATATATATATATATATATATATAT	TTGACCCAATCTTGGTGGAT	ATTGCCTTATGCCTTGGTTG	60.0	600	INTERGENIC	--	--	--

INDEL marker IDs	Chromosomes/unanchored scaffolds	Physical positions (bp)	InDels (*Kabuli* reference genome-CDC Frontier/PII)	Forward primers (5'-3')	Reverse primers (5'-3')	Anneling temperature (0C)	Expected amplified product size (bp)	Structural annotation		Functional annotation	
								Sequence components of *kabuli* genome	*Kabuli* gene accession IDs	NCBI-KOG	TFs
Ca2_P2_2798707	Ca2	2798707	TAAAAATAAAAAAAA/TAAAAATAAAAAAAAATAAAAAAAAA	CAGCACGTGAAGAACCAAAA	TTTGCTCCGCTCTCTCTCTC	60.0	444	INTRON	Ca_10562	B	WRKY
Ca2_P2_27989435	Ca2	27989435	CAAAAAAAAAA/CAAAAAAAAA	CCACGCTTCCTTTTGTCAAT	TCAAGCCAAATCTGAATGTCC	60.1	451	INTERGENIC	--	--	--
Ca2_P2_28009782	Ca2	28009782	TTATATATATATATATATAT/TTATATATATATATATATATAT	TGGCAATGTGGTACTGGAGA	GGAAGTCCGAATTTGCAATG	60.4	206	INTERGENIC	--	--	--
Ca2_P2_28118930	Ca2	28118930	TAAAAAAAAA/TAAAAAAAAAA	ACTGTGGTGGGATTGACCAT	AACGAGAGAGGTTGGAGGGT	60.1	688	DRR	Ca_14391	M	HB-other
Ca2_P2_28157707	Ca2	28157707	GATATATATATATATATATATATATATATATATAT/GATATATATATATATATATATATATAT	TGCAGTGCAGTTAGTTCAGACC	AAGCACGACCAATCCTCAAC	60.1	741	DRR	Ca_14385	O	MYB_related
Ca2_P2_28157957	Ca2	28157957	TTATATATATATATATATATATATAT/TTATATATATATATATATATATATATATATAT	GCATGGACATTCAAAAATGAGA	CCATGTCTAAGTGGCAGGGT	60.0	583	DRR	Ca_14385	O	MYB_related
Ca2_P2_28171942	Ca2	28171942	ANNNNNNNNNN/ANNNNNN	TTAACGGCAATTTTGCACAC	CGGACACATTGGCCTTTATT	59.8	386	INTERGENIC	--	--	--
Ca2_P2_28235432	Ca2	28235432	AATATATATATATATATATATA/AATATATATATATATATATATATA	TCCAAATAGCGACTTCCCAC	GCTGCATGTTCTTATGCTCG	59.6	849	INTERGENIC	--	--	--
Ca2_P2_28464989	Ca2	28464989	AAAAA/AAAAATAAAA	TCATCGCAGGTGTACCATGT	AACATTGCACCGGTTTGATT	60.2	414	INTERGENIC	--	--	--
Ca2_P2_28465098	Ca2	28465098	CAAAAAAAAAAAA/CAAAAAAAAAAAAAA	TCATCGCAGGTGTACCATGT	AACATTGCACCGGTTTGATT	60.2	414	INTERGENIC	--	--	--
Ca2_P2_28465231	Ca2	28465231	CAAAAA/CAAAA	TCATCGCAGGTGTACCATGT	GGTATCAGAGCCCACCATGT	59.8	908	INTERGENIC	--	--	--
Ca2_P2_28465336	Ca2	28465336	AAT/A	AAGAGAGGTCACTTTGGCGA	GCAATTCCCACAGGTATGCT	60.0	886	INTERGENIC	--	--	--

INDEL marker IDs	Chromosomes/unanchored scaffolds	Physical positions (bp)	InDels (*Kabuli* reference genome-CDC Frontier/PII)	Forward primers (5'-3')	Reverse primers (5'-3')	Anneling temperature (0C)	Expected amplified product size (bp)	Structural annotation		Functional annotation	
								Sequence components of *kabuli* genome	*Kabuli* gene accession IDs	NCBI-KOG	TFs
Ca2_P2_28465620	Ca2	28465620	A/AT	AGCTCCATTGCAAAAACACC	GCAATTCCCACAGGTATGCT	60.0	553	INTERGENIC	--	--	--
Ca2_P2_28562582	Ca2	28562582	ATT/ATTGGTGGGAGGTCGTCTT	GGTGTGGGGATATGTTCTGG	GTGACCACTGGTATTCGGCT	60.0	600	INTERGENIC	--	--	--
Ca2_P2_285679	Ca2	285679	AAATAATAATAATAATAATAATAATAATAATAATAATAATACTAATAATAATAATAATAATAA/AAATAATAATAATAATAATAATAATAATAATAATAATAATAATACTAATAATAATAATAATAATAA	AAACGACTCACACCCGAAAC	GCCATTGATTCATTTCTTCG	58.2	614	INTERGENIC	--	--	--
Ca2_P2_28569036	Ca2	28569036	TGG/TGGG	TCTCCAAATGATTGGAAGGC	GACTGGACGTAACCCACGTT	59.9	913	INTERGENIC	--	--	--
Ca2_P2_28569281	Ca2	28569281	T/TC	CATCTGTAATGATGCGCCAC	GACTGGACGTAACCCACGTT	59.9	503	INTERGENIC	--	--	--
Ca2_P2_28573863	Ca2	28573863	AATATATATATATATATATAT/AATATATATATATATATAT	GTAGCACGTTTTTGGGCATT	CAGGATTTAAAGAAATAATTTGGTGC	60.4	707	INTERGENIC	--	--	--
Ca2_P2_28583450	Ca2	28583450	A/AT	GGCTTCGGTGAAGTGGTAAA	TGATTCTGGAGCGACACAAG	60.0	370	INTERGENIC	--	--	--
Ca2_P2_28595017	Ca2	28595017	TATAATAATAATAATAATAATAATAATAATAATAATAA/TATAATAATAATAATAATAATAATAATAATAATAATAATAA	AATAGGCCAGGCCAGACTTT	ACGGACATGATCAATCACCA	59.8	912	INTERGENIC	--	--	--
Ca2_P2_28614815	Ca2	28614815	AATATATATATATATATA/AATATATATATATATATA	ATTCCACATCCACCAAGGAA	GCAGTGCAGCAACAGAAAAA	60.2	441	INTERGENIC	--	--	--
Ca2_P2_28617666	Ca2	28617666	TTTTATTTATTTATTTATT/TTTTATTTATTTATTTATTTATTTATT	ATCGACTCCCGAACTCAAAA	CCACGGTTCTTAGAGCCATTA	59.2	621	INTERGENIC	--	--	--
Ca2_P2_28644589	Ca2	28644589	ATTTTTTTTTT/ATTTTTTTTTTT	AAATGCCAGAACCATTGTGA	TTTGAAGGAGCAGAAGTCGG	60.5	463	INTERGENIC	--	--	--
Ca2_P2_286502	Ca2	286502	TAAAAAAA/TAAAAAAAA	AATAGGCTGCCACAAGAGGA	TGGACACGACGGTGAAATAA	60.0	610	INTERGENIC	--	--	--

INDEL marker IDs	Chromosomes/unanchored scaffolds	Physical positions (bp)	InDels (*Kabuli* reference genome-CDC Frontier/PII)	Forward primers (5'-3')	Reverse primers (5'-3')	Anneling temperature (0C)	Expected amplified product size (bp)	Structural annotation		Functional annotation	
								Sequence components of *kabuli* genome	*Kabuli* gene accession IDs	NCBI-KOG	TFs
Ca2_P2_28723461	Ca2	28723461	GTTTAGGTT/GGTATTTAGGTT	GCATGATGCATGTTGAGCTT	GAAAGCGCTGTAAAAGGTGC	60.0	811	DRR	Ca_14359	R	--
Ca2_P2_28723746	Ca2	28723746	AAACACACA/AA	AAAAAGCGCTGTTCATTTCAA	AAAAAGCCTCCACCAAAGGT	60.0	511	DRR	Ca_14359	R	--
Ca2_P2_28765007	Ca2	28765007	TC/T	CTTCATTTTTCAAAGGCGGA	ACCACTTGTGGAACACCTCA	59.0	398	INTERGENIC	--	--	--
Ca2_P2_28930173	Ca2	28930173	GTTATTATTATTATTATTATTATTATTATTATTATTATTATT/GTTATTATTATTATTATTATTATTATTATT	TGCCTTCAGCTTTTGCTTTT	TGAATGCTGCACATTTGGAT	60.1	685	DRR	Ca_14345	--	--
Ca2_P2_29089904	Ca2	29089904	CTAAATTTTTTAAATTTT/CTAAATTTTTTAAATTTTTTAAATTTT	TCATAAATCACCCCCTCCTTT	TGGTGAAACAATTCAAACGC	59.6	644	INTERGENIC	--	--	--
Ca2_P2_29220253	Ca2	29220253	GATATATATATATATATATATATA/GATATATATATATATATATATATATATATA	AGGCATGGTGTTGGAGTCAT	CTAGTTCGTAACCCGTGCGT	60.2	253	INTERGENIC	--	--	--
Ca2_P2_29220546	Ca2	29220546	GATAATAATAATAATAATAATAATAATAATAAT/GATAATAATAATAATAATAATAATAATAATAATAATAAT	CACGTGTCAAACTTGCCAAT	GTAACCGTTGGAGCACCACT	60.0	534	INTERGENIC	--	--	--
Ca2_P2_29226326	Ca2	29226326	GC/GCC	AATTAAAGAACCGCGGGAAC	AACAACCATGACCAAGAGGC	60.0	917	INTERGENIC	--	--	--
Ca2_P2_29238503	Ca2	29238503	A/AGTT	TGTGGAACACCAACGAATGT	ACCTCCCGCTAATGTCAATG	60.0	746	DRR	Ca_14317	--	--
Ca2_P2_29238544	Ca2	29238544	AAATAATAATAATAATAATAATAATAATAATAATAATAATAATAATAA/AAATAATAATAATAATAATAATAATAATAATAATAATAATAATAATAATAA	TGTGGAACACCAACGAATGT	ACCTCCCGCTAATGTCAATG	60.0	746	DRR	Ca_14317	--	--
Ca2_P2_29239999	Ca2	29239999	AATT/AT	GGCTGCTGATCCTGTTCTTC	CTGACGGACGGACTCATTTT	60.1	482	INTERGENIC	--	--	--
Ca2_P2_29240150	Ca2	29240150	TC/TCACC	CTTAGAGCATCTCCAACGGC	GAAGCATCGTTCCTTCCTGA	60.3	134	INTERGENIC	--	--	--

INDEL marker IDs	Chromosomes/unanchored scaffolds	Physical positions (bp)	InDels (*Kabuli* reference genome-CDC Frontier/PII)	Forward primers (5'-3')	Reverse primers (5'-3')	Anneling temperature (0C)	Expected amplified product size (bp)	Structural annotation		Functional annotation	
								Sequence components of *kabuli* genome	*Kabuli* gene accession IDs	NCBI-KOG	TFs
Ca2_P2_29240273	Ca2	29240273	ATTTTTTTT/ATTTTTTT	CTTAGAGCATCTCCAACGGC	CAACGGTGAACTCAATATGGG	60.2	671	INTERGENIC	--	--	--
Ca2_P2_29240382	Ca2	29240382	TCCC/TCC	CTTAGAGCATCTCCAACGGC	CCTTGCACTATCATTTTCGGA	60.1	803	INTERGENIC	--	--	--
Ca2_P2_29240881	Ca2	29240881	G/GGAC	CCATATTGAGTTCACCGTTGG	TGTCCTTGTTCCCTTGCACT	60.7	187	INTERGENIC	--	--	--
Ca2_P2_29252004	Ca2	29252004	GTT/GT	TCAAAGGTGCACTCTTGCTATT	TGGCTTCCGGAAAGACTAAA	59.8	502	INTERGENIC	--	--	--
Ca2_P2_29253047	Ca2	29253047	TTTATTATTATTATTATTATTATTATTATTATTATTATTATTATTATTATTATTATTATT/TTTATTATTATTATTATTATTATTATTATTATTATTATTATTATTATTATTATTATTATTATTATT	GACACCCAAATATAGTGTCAACGA	CCCGGGTATAGGGTCTCACT	60.2	583	INTERGENIC	--	--	--
Ca2_P2_29256743	Ca2	29256743	TTCCCACAA/T	TCAGACTCGAGGTCTTTTTCC	CCAAATTTTGAGTTCGGGAG	59.5	332	INTERGENIC	--	--	--
Ca2_P2_29257012	Ca2	29257012	TA/T	ACCTTCCCACAAACCCTACA	AAATAAAAGGGGTGGGATCG	60.0	714	INTERGENIC	--	--	--
Ca2_P2_29270627	Ca2	29270627	TCACAC/TCAC	TGGTTTTCCATTTGCAATGTT	AGTATCGTTGCGTGCACTGT	59.4	513	INTERGENIC	--	--	--
Ca2_P2_29285559	Ca2	29285559	GC/GCC	TCCATCAACAGCAAAACAGC	AGGACAATCGGAAATCAACG	59.9	211	INTERGENIC	--	--	--
Ca2_P2_29339791	Ca2	29339791	CAAA/CAAAA	CCACAGCCCTACCTGAACAT	CCCAAGGTATGCAGTGGATT	59.8	502	DRR	Ca_25183	TZ	--
Ca2_P2_29417303	Ca2	29417303	TACA/TAACA	CCCAGAAATCGATTAGGCAA	TCAACGTTCGTTTTGGTTCA	60.1	522	INTERGENIC	--	--	--
Ca2_P2_29427514	Ca2	29427514	GAAAA/GAAAAA	TTCAAACCCCCTTTCTTGTG	TGGAAATCGTTTTGCTTTGA	59.3	735	INTERGENIC	--	--	--

INDEL marker IDs	Chromosomes/unanchored scaffolds	Physical positions (bp)	InDels (*Kabuli* reference genome-CDC Frontier/PII)	Forward primers (5'-3')	Reverse primers (5'-3')	Anneling temperature (0C)	Expected amplified product size (bp)	Structural annotation		Functional annotation	
								Sequence components of *kabuli* genome	*Kabuli* gene accession IDs	NCBI-KOG	TFs
Ca2_P2_29444244	Ca2	29444244	ATTTT/ATTTTT	AAATCCGACTGTCTAACCCG	TTTTCTCCTCCAATTTAGTCCAA	59.2	826	INTERGENIC	--	--	--
Ca2_P2_29445927	Ca2	29445927	AACCCGAACCC/AACCCGAACCCACCCGAACCC	TGTCTAACCCGTCTAACCCG	CCCTACCTAGGGGTGGACAT	60.1	138	INTERGENIC	--	--	--
Ca2_P2_29482178	Ca2	29482178	CAAAAAAAAAAAAAAAAA/CAAAAAAAAAAAAAAA	AGGACAAAGGCAAGCAAGAA	CAGAACACGGGATCACAATG	60.0	407	INTERGENIC	--	--	--
Ca2_P2_29483509	Ca2	29483509	CTATATATATATATATATATATATA/CTATATATATATATATATATATATATA	TCAGGTGACCATTGTATTTGGA	GGAAATTTGAGGCGTTCAGA	60.2	272	INTERGENIC	--	--	--
Ca2_P2_29511065	Ca2	29511065	AAGAGAGAGAGAGAGAGAGAGAGAGAGAGAGAGA/AAGAGAGAGAGAGAGAGAGAGAGAGAGAGAGAGAGAGAGAGAGAGAGA	GGTTAGGCCACCTGCACTTA	CTGAGATGCAAAAACGCAAA	60.0	623	DRR	Ca_16135	--	--
Ca2_P2_29534597	Ca2	29534597	ATTTTTTTT/ATTTTTTT	AGAGTCGCTCCCAAGCATTA	GGAGTGGTTGACGTTGGATT	59.8	472	INTERGENIC	--	--	--
Ca2_P2_2960458	Ca2	2960458	GAAA/GAA	TATGGGTGGAAGTCAGGGAG	CAAGCACCTTGAAAGCATGA	60.0	832	INTRON	Ca_10547	O	--
Ca2_P2_29608982	Ca2	29608982	TAAAAAAA/TAAAAAA	TTTGCCACAAACAAAACGTG	TGAAATTTCCCTACGATGCC	59.9	504	INTERGENIC	--	--	--
Ca2_P2_29666169	Ca2	29666169	ATTTTTT/ATTTTTTT	TGATGGATTTGATGTTGTTGG	TTTCACCTCAACGCCCTAGT	59.7	571	INTERGENIC	--	--	--
Ca2_P2_29677109	Ca2	29677109	ATTTTTT/ATTTTTTT	TGCAACGAGTTAAGTGTCCAA	CAAATTTAACGACCAGATGTGTG	59.4	362	INTERGENIC	--	--	--
Ca2_P2_29739972	Ca2	29739972	GA/GACA	GGGCGCAGTCTTGATAGAAG	TGAGCCTAAGAGTCTGGGGA	59.9	249	INTERGENIC	--	--	--
Ca2_P2_2974815	Ca2	2974815	ATATT/AT	ATCTACGAAGACGTGGTGGG	GGTCCTTTCCAAGTCGTGAA	60.1	237	INTERGENIC	--	--	--

INDEL marker IDs	Chromosomes/unanchored scaffolds	Physical positions (bp)	InDels (*Kabuli* reference genome-CDC Frontier/PII)	Forward primers (5'-3')	Reverse primers (5'-3')	Anneling temperature (0C)	Expected amplified product size (bp)	Structural annotation		Functional annotation	
								Sequence components of *kabuli* genome	*Kabuli* gene accession IDs	NCBI-KOG	TFs
Ca2_P2_2975267	Ca2	2975267	GAAAAAAAA/GAAAAAAA	TTCACGACTTGGAAAGGACC	TGATCGCCATTGATTGAGAA	60.2	750	INTERGENIC	--	--	--
Ca2_P2_2977761	Ca2	2977761	A/ACTT	CAACTTGCCAAACAGCTTCA	TGGTGCAAGGATAGTACGACA	59.2	620	INTERGENIC	--	--	--
Ca2_P2_2978014	Ca2	2978014	CTAT/CTATAT	TTGCTGTAATTGTTGCACATGA	CCTCCCCAACTTGTAATTTCTG	59.9	542	INTERGENIC	--	--	--
Ca2_P2_2981462	Ca2	2981462	TCACCA/TCACCACCA	CATTTGTTAAAAGAATGATGGAAGAA	ATTTTAGGTGGCCCATGTGT	59.2	372	INTERGENIC	--	--	--
Ca2_P2_29829940	Ca2	29829940	TT/TTTCGAAAAAAT	CTTTTCGTCGGAGTTTGTCC	TCGTCCTCTGGTTTTCTCGT	59.8	476	INTERGENIC	--	--	--
Ca2_P2_2984317	Ca2	2984317	AT/ATT	GCTCACAACAGCAAAAACCA	TCTCTTTCTTAGACCGGAGCA	59.2	443	INTERGENIC	--	--	--
Ca2_P2_2984359	Ca2	2984359	CTTTTTTTTTTTT/CTTTTTTTTTT	GCTCACAACAGCAAAAACCA	TCTCTTTCTTAGACCGGAGCA	59.2	443	INTERGENIC	--	--	--
Ca2_P2_2984403	Ca2	2984403	GAT/GATATAT	GCTCACAACAGCAAAAACCA	TCTCTTTCTTAGACCGGAGCA	59.2	443	INTERGENIC	--	--	--
Ca2_P2_2984949	Ca2	2984949	AG/A	TGCTCCGGTCTAAGAAAGAGA	GGGAAATTGTTGGAGGGAAT	60.0	786	INTERGENIC	--	--	--
Ca2_P2_2985774	Ca2	2985774	ATGT/AT	GTGAAGCTGTATGGCCCAAT	AGATCTGGGTCGGGAAAAGT	59.9	540	INTERGENIC	--	--	--
Ca2_P2_2987297	Ca2	2987297	ATT/ATTGTT	GCCAAATGATTCGGTTGCTA	GCAAATGGAGCTTTGAAACA	58.9	262	INTERGENIC	--	--	--
Ca2_P2_2987500	Ca2	2987500	TAAAAAAAAA/TAAAAAAAAAAA	GCCAAATGATTCGGTTGCTA	GCCGACCATGGAGTAATTGT	59.8	700	INTERGENIC	--	--	--

INDEL marker IDs	Chromosomes/unanchored scaffolds	Physical positions (bp)	InDels (*Kabuli* reference genome-CDC Frontier/PII)	Forward primers (5'-3')	Reverse primers (5'-3')	Anneling temperature (0C)	Expected amplified product size (bp)	Structural annotation		Functional annotation	
								Sequence components of *kabuli* genome	*Kabuli* gene accession IDs	NCBI-KOG	TFs
Ca2_P2_2987706	Ca2	2987706	TAAAAAAA/TAAAAAAAAAA	TGTTTCAAAGCTCCATTTGC	GCCGACCATGGAGTAATTGT	59.8	458	INTERGENIC	--	--	--
Ca2_P2_29887425	Ca2	29887425	AATATATATATATATATATATATATATA/AATATATATATATATATATATATATATATA	TTCGTTTGAAAACTCCTAGCAA	TACGACCTACCGAAGCAACA	59.3	708	INTERGENIC	--	--	--
Ca2_P2_29892926	Ca2	29892926	CAAAAAAAAA/CAAAAAAAA	CAAAAAGGTCATTTCAGCGA	TTGAATTCGAGAATTTGGGC	60.0	689	INTERGENIC	--	--	--
Ca2_P2_2996172	Ca2	2996172	ATTT/ATT	TTCTAAGGACCAACGCAACA	GTATGATCGATTATGCGCGA	59.7	434	INTERGENIC	--	--	--
Ca2_P2_29962779	Ca2	29962779	G/GC	AGCCAACGGACCACTAACAC	TTCGCCATTCTTCTTCGTCT	60.0	237	INTERGENIC	--	--	--
Ca2_P2_29962875	Ca2	29962875	CAAGAAGAAGAAGAAGAAGAAGAA/CAAGAAGAAGAAGAAGAAGAA	TGGAAGCCATGATTAGGAGG	TCTCCCATTCGTTGTTGATG	59.5	182	INTERGENIC	--	--	--
Ca2_P2_29963169	Ca2	29963169	TGG/TGGG	TGGAAGCCATGATTAGGAGG	TGGAATCAGAGAGGGACGTT	59.7	876	INTERGENIC	--	--	--
Ca2_P2_29996298	Ca2	29996298	CAAAAAA/CAAAAAAA	CCACGAATCTCTTGTGGATG	GCTTTAGTGAGGAGGGGAGA	58.5	681	DRR	Ca_16090	--	--
Ca2_P2_3000644	Ca2	3000644	ATTTTTT/ATTTT	TGATTCAATTGTGAAGTTTTAATGTG	GGAGAAACATCACGTTGACC	58.0	949	INTERGENIC	--	--	--
Ca2_P2_3001687	Ca2	3001687	CAAA/CAAAAA	TTCCAATTTTCGGCAACTTC	GCTGATGAAGGAGAGGAACG	60.0	915	INTERGENIC	--	--	--
Ca2_P2_3003019	Ca2	3003019	GTTTTTT/GTTTTTTTT	AGGCAGACCCAAAAGACAAA	GAGGGCTAATTAAACTTGACCAAA	59.9	230	INTERGENIC	--	--	--
Ca2_P2_3003272	Ca2	3003272	CTTT/CTT	AGGCAGACCCAAAAGACAAA	GGAAAATGCAGAAGCAAATCA	60.2	696	INTERGENIC	--	--	--

INDEL marker IDs	Chromosomes/unanchored scaffolds	Physical positions (bp)	InDels (*Kabuli* reference genome-CDC Frontier/PII)	Forward primers (5'-3')	Reverse primers (5'-3')	Anneling temperature (0C)	Expected amplified product size (bp)	Structural annotation		Functional annotation	
								Sequence components of *kabuli* genome	*Kabuli* gene accession IDs	NCBI-KOG	TFs
Ca2_P2_3003635	Ca2	3003635	CAAT/CAATTAAT	TTTGGTCAAGTTTAATTAGCCCTC	AGAATTGGGCCTAAAACCGT	59.8	602	INTERGENIC	--	--	--
Ca2_P2_3007459	Ca2	3007459	CAAAAAAAAAAAA/CAAAAAAAAAAA	TGGTTTAAAAGTTCGTGCCA	TTGTGAGGTTGTGGTGTTGG	60.5	302	DRR	Ca_10544	G	--
Ca2_P2_3009059	Ca2	3009059	ACCCCC/ACCCC	ATTCGCTTGCCTCTTTGGTA	CCCGAAACTTTCTGTGCATT	60.1	658	INTRON	Ca_10544	G	--
Ca2_P2_30095786	Ca2	30095786	TAAAAAAA/TAAAAAA	AAATGATGAACGATGGAGCC	CCAAAATGTAGCGGGAATGT	59.8	257	INTERGENIC	--	--	--
Ca2_P2_30095996	Ca2	30095996	TAAAAAAA/TAAAAAAAAA	CATTCCCGCTACATTTTGGT	GGAGAGAGGCCAATTTTTCA	59.2	226	INTERGENIC	--	--	--
Ca2_P2_30096276	Ca2	30096276	TA/TAAA	CATTCCCGCTACATTTTGGT	GAAGCTGCTTGTGTGCCATA	60.0	876	INTERGENIC	--	--	--
Ca2_P2_30096870	Ca2	30096870	ATATATATATATATATATATATATATATATATATAT/ATATATATATATATATATATATATATATATATATAT	CATTGCAGGTGCTATGGTTG	TTCTCTTATGAAGCGGAGGA	57.6	759	INTERGENIC	--	--	--
Ca2_P2_3011662	Ca2	3011662	TAAAAAAAA/TAAAAAAAAAAA	TGATTTGGGGTGTTCAGAAA	TCCGCACACCAACATTAAAA	60.0	657	INTERGENIC	--	--	--
Ca2_P2_3011716	Ca2	3011716	G/GA	TGATTTGGGGTGTTCAGAAA	TCCGCACACCAACATTAAAA	60.0	657	INTERGENIC	--	--	--
Ca2_P2_30125989	Ca2	30125989	TAAAAAA/TAAAAAAA	TTCCACTTATGGTGAGTAATGATTTT	TTGTGAGCATGAGAAGTTGTTG	59.0	641	INTERGENIC	--	--	--
Ca2_P2_30146454	Ca2	30146454	CAAAAAA/CAAAAAAAA	GCCCAAAACCAAATTTAGCA	AAGACCCAGAAACTTCCCGT	60.0	452	INTERGENIC	--	--	--
Ca2_P2_3016524	Ca2	3016524	GAAAAAAAA/GAAAAAAAAA	TGGGAGAGATAAAACGCTGC	TCTTTCACAACCTCAAAAGTGC	59.4	353	INTERGENIC	--	--	--

INDEL marker IDs	Chromosomes/unanchored scaffolds	Physical positions (bp)	InDels (*Kabuli* reference genome-CDC Frontier/PII)	Forward primers (5'-3')	Reverse primers (5'-3')	Anneling temperature (0C)	Expected amplified product size (bp)	Structural annotation		Functional annotation	
								Sequence components of *kabuli* genome	*Kabuli* gene accession IDs	NCBI-KOG	TFs
Ca2_P2_30174020	Ca2	30174020	CTT/CTTT	TTATCCCCGCATATTGGACT	AGTCCTTGGGACCGAAAAGT	60.0	227	INTERGENIC	--	--	--
Ca2_P2_30174230	Ca2	30174230	ATTT/ATTTCTTTTTTTTT	ACTTTTCGGTCCCAAGGACT	AAATTCTGTATGGTGATGGTTGG	60.0	157	INTERGENIC	--	--	--
Ca2_P2_3022835	Ca2	3022835	TTAA/TTAATAA	CCCGACTCGAAAGTTTCTCA	AAAATTGACACGGCTTCGTC	60.1	544	INTERGENIC	--	--	--
Ca2_P2_3023942	Ca2	3023942	GAAAAAAA/GAAAAAA	TGTTGATGTCATCGCCAACT	CCTCCTTGTTATCCTATGCCA	59.1	513	INTERGENIC	--	--	--
Ca2_P2_30243585	Ca2	30243585	CTT/CT	TTCCTACTCCCACATTTTAACGA	GCCTGTATCCAGCCATTTGT	60.0	467	INTERGENIC	--	--	--
Ca2_P2_3024878	Ca2	3024878	ATTTTTTTT/ATTTTTTTTTT	CTGGTTTTCAAACGCGCTAC	CGGGGTCGGTATTATTTTGA	59.7	799	INTERGENIC	--	--	--
Ca2_P2_3025182	Ca2	3025182	CTATATATATAT/CTATATATATATAT	TCAGCCGACTTAACAACGAA	CGGGGTCGGTATTATTTTGA	59.7	413	INTERGENIC	--	--	--
Ca2_P2_3025547	Ca2	3025547	GAAAAAAAAAA/GAAAAAAAAA	TCAAAATAATACCGACCCCG	AAAAACGAGGCGATGACAAT	59.6	589	INTERGENIC	--	--	--
Ca2_P2_3028297	Ca2	3028297	TAAAAA/TAAAA	TCAGTTAATCCCAAACTGCAAA	ATGCACCAAGTGCAACTGAG	59.9	565	INTERGENIC	--	--	--
Ca2_P2_3029449	Ca2	3029449	TTTAATAATTATT/TTTAATAATTATTAATAATTATT	ACCGCTCTATTAAGGGCGAG	TCCATCGCAATTGAATAATGA	59.0	765	INTERGENIC	--	--	--
Ca2_P2_3030942	Ca2	3030942	CAAGA/C	TTTTCATTTTCTCCCTTCCCT	TCCATCGCAATTGAATAATGA	59.0	592	INTERGENIC	--	--	--
Ca2_P2_3031259	Ca2	3031259	ATTTTT/ATTTTTT	TCAATTGCGATGGATAGAGAAA	AATTGGTGTACCGATTGCGT	60.3	647	INTERGENIC	--	--	--

INDEL marker IDs	Chromosomes/unanchored scaffolds	Physical positions (bp)	InDels (*Kabuli* reference genome-CDC Frontier/PII)	Forward primers (5'-3')	Reverse primers (5'-3')	Anneling temperature (0C)	Expected amplified product size (bp)	Structural annotation		Functional annotation	
								Sequence components of *kabuli* genome	*Kabuli* gene accession IDs	NCBI-KOG	TFs
Ca2_P2_3032104	Ca2	3032104	AT/ATAATT	TTGGATACGCAATCGGTACA	ATGCACTTGCAAGACCTTTT	57.5	440	INTERGENIC	--	--	--
Ca2_P2_3032802	Ca2	3032802	ATT/ATTTCTTTT	AACGCAACAAGCTCCAAAAG	TGCATGACTGAGCATTGGAT	60.2	417	INTERGENIC	--	--	--
Ca2_P2_3033081	Ca2	3033081	TA/TAA	AACGCAACAAGCTCCAAAAG	CGTTAGGCCTTATCCTAGCC	58.0	902	INTERGENIC	--	--	--
Ca2_P2_3033174	Ca2	3033174	CTTTTTTT/CTTTTTT	GATCCAATGCTCAGTCATGC	GCACCATCATTTGACCCTTC	60.3	619	INTERGENIC	--	--	--
Ca2_P2_3033295	Ca2	3033295	CAAAAAAAAA/CAAAAAAAAAAA	GATCCAATGCTCAGTCATGC	GCACCATCATTTGACCCTTC	60.3	619	INTERGENIC	--	--	--
Ca2_P2_3033478	Ca2	3033478	CAAAAAAAA/CAAAAAAA	GATCCAATGCTCAGTCATGC	CCTCCATTCCTGGTCAATGT	59.8	927	INTERGENIC	--	--	--
Ca2_P2_30335425	Ca2	30335425	CTT/CTTT	AAAGGTCGACAAGGTGAACG	GGTTGGCTACAGGGATCAAA	59.9	696	INTRON	Ca_12519	J	--
Ca2_P2_30341034	Ca2	30341034	TAA/TAAA	TGTCTTATCCTGAACTCAAACTCG	ATTTGGTCAACCACCCAAAA	60.1	537	INTERGENIC	--	--	--
Ca2_P2_30342769	Ca2	30342769	ATTTTTT/ATTTTTTTTT	TGCCTAATTTTGACTTCCCC	TCTCTAATCTGCTACGCATCCA	60.0	439	INTERGENIC	--	--	--
Ca2_P2_30342844	Ca2	30342844	ATTTTT/ATTT	TGCCTAATTTTGACTTCCCC	TCTCTAATCTGCTACGCATCCA	60.0	439	INTERGENIC	--	--	--
Ca2_P2_30342955	Ca2	30342955	TAAA/TAAAA	TGCCTAATTTTGACTTCCCC	TCTCTAATCTGCTACGCATCCA	60.0	439	INTERGENIC	--	--	--
Ca2_P2_30343145	Ca2	30343145	TAAA/TAA	TGGATGCGTAGCAGATTAGAGA	CGTCACTTGGTCACTTGGTG	60.2	581	INTERGENIC	--	--	--

INDEL marker IDs	Chromosomes/unanchored scaffolds	Physical positions (bp)	InDels (*Kabuli* reference genome-CDC Frontier/PII)	Forward primers (5'-3')	Reverse primers (5'-3')	Anneling temperature (0C)	Expected amplified product size (bp)	Structural annotation		Functional annotation	
								Sequence components of *kabuli* genome	*Kabuli* gene accession IDs	NCBI-KOG	TFs
Ca2_P2_30343224	Ca2	30343224	CT/CTT	TGGATGCGTAGCAGATTAGAGA	CGTCACTTGGTCACTTGGTG	60.2	581	INTERGENIC	--	--	--
Ca2_P2_30343408	Ca2	30343408	TTAATAAA/T	TGGATGCGTAGCAGATTAGAGA	CGTCACTTGGTCACTTGGTG	60.2	581	INTERGENIC	--	--	--
Ca2_P2_30343585	Ca2	30343585	CACCAAGTGACCAAGTGAC/CACCAAGTGAC	CAATCAATAATTTTGACGTCGG	CATTTCACGGGGCTCTTAAA	60.1	658	INTERGENIC	--	--	--
Ca2_P2_30344848	Ca2	30344848	TAAAAAAAAAAAAA/TAAAAAAAAAA	TTCATACTTGGTTGGGGACC	GGATGATTTCGCCCAACTTA	59.9	408	INTERGENIC	--	--	--
Ca2_P2_30345200	Ca2	30345200	TG/T	TAAGTTGGGCGAAATCATCC	TGGGGTGGGTGTTTAGAAAA	60.2	524	INTERGENIC	--	--	--
Ca2_P2_30345472	Ca2	30345472	C/CG	TAAGTTGGGCGAAATCATCC	CCCATTTTGAAGAGTCCGAA	60.0	603	INTERGENIC	--	--	--
Ca2_P2_30346932	Ca2	30346932	A/ACAT	GCGGTATCTTCCTGTGTCGT	GGTCCAACTTCGGAATACCA	59.8	853	INTRON	Ca_12518	GE	bZIP
Ca2_P2_30347185	Ca2	30347185	A/AC	TCATCAATTGTCGGTGATGG	AGTTAACAACCGCGATTTGG	60.0	732	INTRON	Ca_12518	GE	bZIP
Ca2_P2_30347838	Ca2	30347838	T/TG	TGGTATTCCGAAGTTGGACC	CAAACAAATGGAAGCTCGGT	60.1	793	INTRON	Ca_12518	GE	bZIP
Ca2_P2_30352008	Ca2	30352008	T/TG	TTTTAACCGAACCGAACTGG	TAAGAAGCAATTGAAGCGCA	59.7	572	INTERGENIC	--	--	--
Ca2_P2_30355244	Ca2	30355244	CAAAAAAAAA/CAAAAAAAAAA	TGTAATTGGGTTCCTTTCGC	TCATTGAAGAAATTGCAAATAATAA	57.2	648	DRR	Ca_12517	--	ERF
Ca2_P2_30359195	Ca2	30359195	GC/GCC	TCACAAGGTTCCCTCTCCAG	AATTTGGATTTTCCCCCATC	59.8	171	INTERGENIC	--	--	--

INDEL marker IDs	Chromosomes/unanchored scaffolds	Physical positions (bp)	InDels (*Kabuli* reference genome-CDC Frontier/PII)	Forward primers (5'-3')	Reverse primers (5'-3')	Anneling temperature (0C)	Expected amplified product size (bp)	Structural annotation		Functional annotation	
								Sequence components of *kabuli* genome	*Kabuli* gene accession IDs	NCBI-KOG	TFs
Ca2_P2_30359357	Ca2	30359357	A/ATTC	TCACAAGGTTCCCTCTCCAG	TCCTTCATCTTCAACCAAAACA	59.6	401	INTERGENIC	--	--	--
Ca2_P2_30361475	Ca2	30361475	TATC/TATCGATC	TCCGCTAATAGTGGCAGATAAA	ACCAATTTGTGAAAGCTGGC	60.1	617	INTERGENIC	--	--	--
Ca2_P2_30361673	Ca2	30361673	CAAA/CAA	TCCGCTAATAGTGGCAGATAAA	ACCAATTTGTGAAAGCTGGC	60.1	617	INTERGENIC	--	--	--
Ca2_P2_30362499	Ca2	30362499	CAAA/CAAAA	TCCTTAGACGTTTACGGCCT	CATGCCATTTACCATTTCCC	60.0	502	INTERGENIC	--	--	--
Ca2_P2_3036254	Ca2	3036254	CTAT/CTATAT	TTCGGAGTCGGATAATGGAC	CACCATATTTCCCCCTCAAA	59.6	555	INTERGENIC	--	--	--
Ca2_P2_30364268	Ca2	30364268	CTTTTGAA/C	GCAGCAAGTTGTGACAAGGA	AAAAATATTGCCACAAGGCG	60.0	575	DRR	Ca_12516	--	--
Ca2_P2_30364332	Ca2	30364332	GAAAAAAAAAAAA/GAAAAAAAAAA	GCAGCAAGTTGTGACAAGGA	AAAAATATTGCCACAAGGCG	60.0	575	DRR	Ca_12516	--	--
Ca2_P2_30365777	Ca2	30365777	TTTGTTGTT/TTTGTT	TGCCATTGTCATTTTGCCTA	GGTGGCAGACCAGAATTTGT	60.0	278	INTRON	Ca_12515	Q	--
Ca2_P2_30368280	Ca2	30368280	ATTTTTT/ATTTTTTT	TAAAATAAAACGCGCCAGGT	GCAATTGCAAGCATTGTGTT	59.7	688	DRR	Ca_12515	Q	--
Ca2_P2_30369374	Ca2	30369374	TAAAA/TAAAAA	TGTACTTCCTCCGATTCCAAA	TGAATGACGACGACGGTAAA	60.1	632	DRR	Ca_12515	Q	--
Ca2_P2_30369585	Ca2	30369585	TAAAA/TAAA	TTCCCGAACCCAAGTTAGTG	TATTAGGAAAATCGTGCCGC	60.1	553	INTERGENIC	--	--	--
Ca2_P2_30371355	Ca2	30371355	TAAA/TAAAA	ACCTTGCTGCTTTCATTGCT	GAATTGGGTTTCCAAAGGGT	60.0	806	INTERGENIC	--	--	--

INDEL marker IDs	Chromosomes/unanchored scaffolds	Physical positions (bp)	InDels (*Kabuli* reference genome-CDC Frontier/PII)	Forward primers (5'-3')	Reverse primers (5'-3')	Anneling temperature (0C)	Expected amplified product size (bp)	Structural annotation		Functional annotation	
								Sequence components of *kabuli* genome	*Kabuli* gene accession IDs	NCBI-KOG	TFs
Ca2_P2_30373739	Ca2	30373739	TAAAAAAAAAA/TAAAAAAAAA	CGGGAATGAAAAGTGGAAGA	CCAGGGGCAAAACAAAGTAA	60.0	518	DRR	Ca_12514	S	--
Ca2_P2_30375213	Ca2	30375213	TAAAAAAA/TAAAAAA	AATTGAATCAACCCCCTTCC	GGTTTGAAGCTAATGTCGTGC	59.8	737	DRR	Ca_12514	S	--
Ca2_P2_30375265	Ca2	30375265	CT/C	AACCCCCTTCCTTTTGTGTC	TTGATTTTTGGGGTGGCTAA	60.3	963	DRR	Ca_12514	S	--
Ca2_P2_30375925	Ca2	30375925	GTTT/GTT	TAGCCACCCCAAAAATCAAA	AAGAGGGTGAAAGAAAGGGTG	59.6	288	INTERGENIC	--	--	--
Ca2_P2_30378672	Ca2	30378672	CTTTTTTTTTT/CTTTTTTTTTTT	ATCTTGAGCAGCCAGGTCAT	CTGCCTATATTCTGGCCCAA	60.1	252	DRR	Ca_12513	O	MYB
Ca2_P2_30379917	Ca2	30379917	TTATATATATA/TATATATATATA	AAGCGTGAGGTAGCCAAGTG	TGCTCGCAAGCAAGAAAGTA	59.9	493	INTERGENIC	--	--	--
Ca2_P2_30380142	Ca2	30380142	CTTT/CTTTTT	AAGCGTGAGGTAGCCAAGTG	TGCTCGCAAGCAAGAAAGTA	59.9	493	INTERGENIC	--	--	--
Ca2_P2_3038248	Ca2	3038248	GAAAAAAA/GAAAAAAAA	ACTTGCACTTTGGAGTGCCT	CCCTTGTGCCTTATGGTTTG	60.4	784	INTERGENIC	--	--	--
Ca2_P2_30383976	Ca2	30383976	T/TC	TGGCAAGCCATCACTCATAA	TTCTCCGATTAACCCGTTTG	59.9	706	INTERGENIC	--	--	--
Ca2_P2_3038524	Ca2	3038524	CA/CATGA	AACCAAAGGATGAATTTGAGAGA	CCCTTGTGCCTTATGGTTTG	60.4	269	INTERGENIC	--	--	--
Ca2_P2_30385483	Ca2	30385483	ATTT/ATTTCTTTT	TGATTTGGCCCAACACCTAT	ACAGAAAATGTCGTCCCTGC	60.1	744	INTERGENIC	--	--	--
Ca2_P2_30385637	Ca2	30385637	TTAATA/TTAATAATA	TTGACGGTCCTTTGTATTTGC	ACAGAAAATGTCGTCCCTGC	60.1	384	INTERGENIC	--	--	--

**INDEL marker IDs**	**Chromosomes/unanchored scaffolds**	**Physical positions (bp)**	**InDels (*Kabuli* reference genome-CDC Frontier/PII)**	**Forward primers (5'-3')**	**Reverse primers (5'-3')**	**Anneling temperature (0C)**	**Expected amplified product size (bp)**	**Structural annotation**		**Functional annotation**	
								**Sequence components of *kabuli* genome**	***Kabuli* gene accession IDs**	**NCBI-KOG**	**TFs**
Ca2_P2_30387130	Ca2	30387130	ATTTTTTT/ATTTTTTTT	TAACCGCACTGATGCCAATA	CAGCAGCATATGTTGACACG	58.9	640	DRR	Ca_12512	R	--
Ca2_P2_30387330	Ca2	30387330	TTA/TTATTAATA	TAACCGCACTGATGCCAATA	CACTTGCTGGTCCAAGATCA	59.8	814	DRR	Ca_12512	R	--
Ca2_P2_30387634	Ca2	30387634	GATCA/GATCATCA	TGGTCCACCACACTAAGCAA	TGCTGTTGAACATCCAGCTC	60.0	649	DRR	Ca_12512	R	--
Ca2_P2_30388874	Ca2	30388874	AT/ATGGGT	TGCCTTCTTCGTCTTCCATT	CCCAACTTGGAAGTCCTTGA	60.1	623	INTRON	Ca_12512	R	--
Ca2_P2_30389928	Ca2	30389928	GAAAAAAAAAAA/GAAAAAAAAAAAAA	CGACAACTGAGCTTTCCTCC	CCACCAAACACTTTTCTGCC	60.5	586	INTRON	Ca_12512	R	--
Ca2_P2_30391104	Ca2	30391104	AT/ATT	TTGCATTCTCAAGGCTCAGA	TTTTGGGAGTGCTTGGTTTC	60.1	461	INTRON	Ca_12512	R	--
Ca2_P2_30394924	Ca2	30394924	CATATA/CATATATATA	GAGAGAGGGGCAAGGTCAC	AGGCTTTTGTTGTTGTTGGG	60.0	392	INTERGENIC	--	--	--
Ca2_P2_30395203	Ca2	30395203	CATATATATA/CATATATATATA	CCCAACAACAACAAAAGCCT	AAACCAGCATGACAAAAGCA	59.3	289	INTERGENIC	--	--	--
Ca2_P2_30395506	Ca2	30395506	TTCGAT/TT	CCCAACAACAACAAAAGCCT	CCAAATGAAACAATCGAGGAA	59.9	780	INTERGENIC	--	--	--
Ca2_P2_30395881	Ca2	30395881	AATA/AATATA	TGCTTTTGTCATGCTGGTTT	GCGCGTTCATAAACTTCGTA	59.0	702	INTERGENIC	--	--	--
Ca2_P2_30400839	Ca2	30400839	ATTT/ATT	TGGTGGTTGTTTGGTCGTTA	CCAAAAACCCTTTGTGATGC	60.3	514	INTERGENIC	--	--	--
Ca2_P2_30401762	Ca2	30401762	TAAAAAAA/TAAAAAAAA	GGTCGAGTTTGCAATGATGA	TGACCACAAGTTGCCAAAAA	60.1	761	INTERGENIC	--	--	--

INDEL marker IDs	Chromosomes/unanchored scaffolds	Physical positions (bp)	InDels (*Kabuli* reference genome-CDC Frontier/PII)	Forward primers (5'-3')	Reverse primers (5'-3')	Anneling temperature (0C)	Expected amplified product size (bp)	Structural annotation		Functional annotation	
								Sequence components of *kabuli* genome	*Kabuli* gene accession IDs	NCBI-KOG	TFs
Ca2_P2_30408247	Ca2	30408247	AA/AAATATGTTA	TCCCTATTTGGCTGAAGGTG	GCTGTCCGAGGTTGGTATGT	60.0	490	INTRON	Ca_12511	--	--
Ca2_P2_30410480	Ca2	30410480	TAGGAG/TAGGAGGAG	CGCAAAACATTTGTTTATCCTA	TAAGGGGACGTCGAAAGTTG	60.1	884	INTERGENIC	--	--	--
Ca2_P2_30414684	Ca2	30414684	T/TTAA	AACATATTCTGGCACAGCCAC	ACTTGTGTGTTAAACGGGGC	59.9	611	INTRON	Ca_12510	--	--
Ca2_P2_30415101	Ca2	30415101	CCTCTCTCTCTCTC/CCTCTCTCTCTCTCTCTC	GCCCCGTTTAACACACAAGT	TTTTACAGCCCCCTGTTTTG	60.0	399	INTRON	Ca_12510	--	--
Ca2_P2_30415294	Ca2	30415294	ATTTTTTTT/ATTTTTTTTT	CAAAACAGGGGGCTGTAAAA	AATCTTCAACGGGTTGTTGC	60.0	506	INTRON	Ca_12510	--	--
Ca2_P2_30415621	Ca2	30415621	CTTTTTTTT/CTTTTTTTTT	CAAAACAGGGGGCTGTAAAA	AATCTTCAACGGGTTGTTGC	60.0	506	INTRON	Ca_12510	--	--
Ca2_P2_30419368	Ca2	30419368	TTCATCAT/TTCAT	GTTGCAATCATCACCACACC	TGAGTGTTCTTGGTGATTTGG	58.6	568	DRR	Ca_12509	C	M-type
Ca2_P2_3042033	Ca2	3042033	TTTTGTTTGCTT/TTT	TGCCAAATCATGCGTTTTTA	CTCTGATGCGAAGGTTGACA	60.0	100	INTRON	Ca_10543	--	--
Ca2_P2_30423136	Ca2	30423136	CTATATATATATATATATATATATA/CTATATATATATATATATATATATATATATATA	CACCATGGCCTAGCATTTTC	TCCCCATTGAATTCCCATAA	60.0	303	INTRON	Ca_12509	C	M-type
Ca2_P2_3042791	Ca2	3042791	G/GT	GCAAAGGGGAGGTATGTTGA	ACCGCAGAGAAAGCTGAAAA	60.1	415	INTRON	Ca_10543	--	--
Ca2_P2_30433045	Ca2	30433045	AT/A	ATGAATCAAACGAACACCGA	TTACAGTTAAGGGCCAACCG	60.0	778	INTERGENIC	--	--	--
Ca2_P2_30433778	Ca2	30433778	TA/TAA	CGGTTGGCCCTTAACTGTAA	TCCTCAAACCCAAAATGGTC	59.8	674	INTERGENIC	--	--	--

INDEL marker IDs	Chromosomes/unanchored scaffolds	Physical positions (bp)	InDels (*Kabuli* reference genome-CDC Frontier/PII)	Forward primers (5'-3')	Reverse primers (5'-3')	Anneling temperature (0C)	Expected amplified product size (bp)	Structural annotation		Functional annotation	
								Sequence components of *kabuli* genome	*Kabuli* gene accession IDs	NCBI-KOG	TFs
Ca2_P2_30434159	Ca2	30434159	AATTAATTATATTAATTATATTAAT/AATTAATTATATTAAT	TATCCCAAACGCAAGTCACA	TGGTCTTGACAATCTTGCACA	60.3	909	INTERGENIC	--	--	--
Ca2_P2_30439226	Ca2	30439226	GTAATT/GT	CGAGAGATCAAAACTGTCGAA	ATGGAGAATTCAGCCCAACA	60.5	664	INTERGENIC	--	--	--
Ca2_P2_30439297	Ca2	30439297	AGGG/AG	CCAAGTTCAACACTCATATGCC	ATGGAGAATTCAGCCCAACA	60.5	406	INTERGENIC	--	--	--
Ca2_P2_3044354	Ca2	3044354	AT/A	TTGACGACTTGCGTTTGAAG	GGGACCATGAAAACAATCCA	60.6	874	INTRON	Ca_10543	--	--
Ca2_P2_30445916	Ca2	30445916	TAAAAAA/TAAAAAAA	CAAAAATATATCGATCGCAACAA	AATTGAAAGCTGCATTGGCT	59.9	406	INTERGENIC	--	--	--
Ca2_P2_30447321	Ca2	30447321	TAAAAAA/TAAAAAAA	CTTTTGTCTTCTCGGCCTTG	AGCTTTCCAACCCCTTTTGT	60.0	155	INTERGENIC	--	--	--
Ca2_P2_30447611	Ca2	30447611	A/AT	CTTTTGTCTTCTCGGCCTTG	CGTGCTCATCTCCATTGTTG	60.3	891	INTERGENIC	--	--	--
Ca2_P2_30447804	Ca2	30447804	TTG/TTGTG	AACAAAAGGGGTTGGAAAGC	ATCTCCTCAGCATTGTCGCT	60.0	809	INTERGENIC	--	--	--
Ca2_P2_30449164	Ca2	30449164	TAAAAAAA/TAAAAAAAA	TGTTGCAAAGAGAAAGAACTTCA	TGACGGTGGCAATCGATATT	61.3	557	DRR	Ca_12507	--	--
Ca2_P2_30456719	Ca2	30456719	TAAAAAAAA/TAAAAAAA	TTCCTGATTCCGATCTCACC	CATACCTTGGGGATGCAAAG	60.3	601	DRR	Ca_12504	--	WOX
Ca2_P2_30457303	Ca2	30457303	A/AT	TGCATCCCCAAGGTATGACT	CCACCATTAGATTTGCGGTT	59.8	649	DRR	Ca_12504	--	WOX
Ca2_P2_30457888	Ca2	30457888	GAAAAAA/GAAAAAAA	CATCTCACCAAGTCCTGCAA	CGTGAAAGTGTTTTGGAATCAA	60.0	180	INTRON	Ca_12504	--	WOX

INDEL marker IDs	Chromosomes/unanchored scaffolds	Physical positions (bp)	InDels (*Kabuli* reference genome-CDC Frontier/PII)	Forward primers (5'-3')	Reverse primers (5'-3')	Anneling temperature (0C)	Expected amplified product size (bp)	Structural annotation		Functional annotation	
								Sequence components of *kabuli* genome	*Kabuli* gene accession IDs	NCBI-KOG	TFs
Ca2_P2_30461396	Ca2	30461396	CTTTTTTT/CTTTTTTTTT	AAAAAGAAGTGGTTGTCCGC	AAAACGATGCTTATCCGTGAA	59.6	929	INTERGENIC	--	--	--
Ca2_P2_30461477	Ca2	30461477	A/AC	CGTTTACAAATAATATGAACACATCG	AAAACGATGCTTATCCGTGAA	59.6	407	INTERGENIC	--	--	--
Ca2_P2_30462203	Ca2	30462203	GTTTTT/GTTTT	TTCACGGATAAGCATCGTTTT	GGATAACCGCACGCTTTTTA	60.1	478	INTERGENIC	--	--	--
Ca2_P2_30464291	Ca2	30464291	CA/CAA	CCACCTCCCAAACAAACACT	GGGTGCATGGCTTCTACATT	60.0	792	INTERGENIC	--	--	--
Ca2_P2_30465481	Ca2	30465481	TAAAAAA/TAAAAA	ACAGTCAAACATTCCGTCCA	GCAGTCAACCAGCGACAATA	59.9	918	DRR	Ca_12503	J	--
Ca2_P2_3047152	Ca2	3047152	CTTT/CTTTT	TGAAATTTAACGGGCCTTCA	GTGGAAAAACTGGACCCTCA	59.9	643	INTRON	Ca_10543	--	--
Ca2_P2_3047767	Ca2	3047767	TGG/TGGG	TGAGGGTCCAGTTTTTCCAC	AACCAAGTTGGCCCAAAAA	60.3	266	DRR	Ca_10543	--	--
Ca2_P2_30480817	Ca2	30480817	TAAA/TAAAA	CTGCGTCCCATTTCAAGAAT	ATGTGTCTTTGGGAGCCAAC	60.0	880	INTRON	Ca_12502	G	--
Ca2_P2_30481523	Ca2	30481523	TTTAAAAAAT/TTTAAAAAATATGTTAAAAAAT	GTTGGCTCCCAAAGACACAT	TCGATTTTCATTAAAGTGTTGGA	58.7	631	INTERGENIC	--	--	--
Ca2_P2_30481983	Ca2	30481983	ATATTATTT/ATATTATTTAAATTATTATTT	TCAATGCATGGAAAATAATCTCT	TGGTCCTCATCCATCAAACA	59.9	401	INTERGENIC	--	--	--
Ca2_P2_30484697	Ca2	30484697	TGTAGTA/TGTA	ACATGAGACCGTCAATGCAA	CGAAAAATCAAAATAACATCGC	58.7	386	INTERGENIC	--	--	--
Ca2_P2_30484794	Ca2	30484794	ATTTTTTT/ATTTTTTTTT	ACATGAGACCGTCAATGCAA	CGAAAAATCAAAATAACATCGC	58.7	386	INTERGENIC	--	--	--

INDEL marker IDs	Chromosomes/unanchored scaffolds	Physical positions (bp)	InDels (*Kabuli* reference genome-CDC Frontier/PII)	Forward primers (5'-3')	Reverse primers (5'-3')	Anneling temperature (0C)	Expected amplified product size (bp)	Structural annotation		Functional annotation	
								Sequence components of *kabuli* genome	*Kabuli* gene accession IDs	NCBI-KOG	TFs
Ca2_P2_30487680	Ca2	30487680	TCGAAAAGG/T	GCGTCTCCCAAATTTTTGTT	AAATTTTGGTGGCGACTTCA	60.5	451	INTERGENIC	--	--	--
Ca2_P2_30489361	Ca2	30489361	TTAT/TT	TGCAAAGTTTGATGGAGTGC	TAGAACCCCAATTTTGTCCG	59.8	363	INTERGENIC	--	--	--
Ca2_P2_30489892	Ca2	30489892	GTTTTTTTTTTTT/GTTTTTTT	CGGACAAAATTGGGGTTCTA	CCCAAATGCAATTTTTCCAA	60.7	558	INTERGENIC	--	--	--
Ca2_P2_30490349	Ca2	30490349	T/TTA	GGAAAAATTGCATTTGGGAA	GTTGGCTTACACATTGGGCT	60.0	578	INTERGENIC	--	--	--
Ca2_P2_30490798	Ca2	30490798	AA/AACA	AGCCCAATGTGTAAGCCAAC	AAGCTTTTGCAAATGGGAGA	59.8	410	INTERGENIC	--	--	--
Ca2_P2_30494831	Ca2	30494831	GAAAAAAAAA/GAAAAAAAAAA	TGAGAACGCCCATGTCAATA	TCTCTGGCAACCGTTCTTCT	60.0	404	INTERGENIC	--	--	--
Ca2_P2_30504150	Ca2	30504150	TG/T	TGCGATTCGGGCTAATTACT	GGTGAGACTTTGATCGGATTG	59.5	625	INTERGENIC	--	--	--
Ca2_P2_30505914	Ca2	30505914	ATTTTTTTTTT/ATTTTTTTTTTT	TTTAACGCTTCTCGCGTTTT	CGTGCTCCATAGTTCCCAAC	60.5	730	INTRON	Ca_12501	GMW	--
Ca2_P2_30510478	Ca2	30510478	TAAAA/TAAA	ACTCCCCCTTGTAAGGTGCT	ATTGACAAAGCGTGTGCAAG	59.9	524	DRR	Ca_12501	GMW	--
Ca2_P2_3051243	Ca2	3051243	ATT/AT	TTTACGACTCGGTTTTCCCA	CCGATTTTGATTTTCTCGGA	60.0	363	INTERGENIC	--	--	--
Ca2_P2_30514265	Ca2	30514265	AAGA/AA	CACCAAATCACCCACAATCA	ATGGGCTGGTATATCATGGG	59.5	972	INTERGENIC	--	--	--
Ca2_P2_30514306	Ca2	30514306	ATTTTTTTTT/ATTTTTTTT	TGTGAAGTGAATGCTTCCAA	AAACTTTCAAATGCATGGGC	59.9	598	INTERGENIC	--	--	--

INDEL marker IDs	Chromosomes/unanchored scaffolds	Physical positions (bp)	InDels (*Kabuli* reference genome-CDC Frontier/PII)	Forward primers (5'-3')	Reverse primers (5'-3')	Anneling temperature (0C)	Expected amplified product size (bp)	Structural annotation		Functional annotation	
								Sequence components of *kabuli* genome	*Kabuli* gene accession IDs	NCBI-KOG	TFs
Ca2_P2_30521356	Ca2	30521356	TTC/TTCGCTC	TAACAATTCCTTCCGCCAAG	GCAGCCGTCTATGCTATTCA	59.0	361	INTERGENIC	--	--	--
Ca2_P2_30522267	Ca2	30522267	AATTAT/AAT	GAACAGTGGCAATTCATCCA	TTTTGGTGCCAAATTCTTCTC	59.2	556	INTERGENIC	--	--	--
Ca2_P2_30523404	Ca2	30523404	AAGTTTAGTTT/AAGTTTAGTTTAGTTT	TCATCGTTTGCAAGGTTTTG	CGGAAGAGGCTACTGGAGAG	59.2	794	INTERGENIC	--	--	--
Ca2_P2_30524430	Ca2	30524430	GCGAG/GCGAGCGAG	TGGAACGATCCAATGAAACA	AAGCCCAAAGCAGCATCTAA	60.0	632	INTERGENIC	--	--	--
Ca2_P2_30526615	Ca2	30526615	TTATATATATA/TTATATATA	GCTGAATTTTGTTAAAAACTACCACA	AATTCCGACCGACCTAATCC	60.2	712	INTERGENIC	--	--	--
Ca2_P2_30527100	Ca2	30527100	TAAAAAAAAAA/TAAAAAAAA	CTCGTGAAGGATTAGGTCGG	TTGCAACGCATCTCAATCTC	60.0	353	INTERGENIC	--	--	--
Ca2_P2_30527508	Ca2	30527508	TAAAAAAAAAAAA/TAAAAAAAAAA	TTGAGATGCGTTGCAATAGG	CGTGCCACTTTCTTCCTTTC	59.9	681	INTERGENIC	--	--	--
Ca2_P2_30537225	Ca2	30537225	TAAAA/TAAAAA	GTACAACGATAGATGCCGCC	GTAGCTGCAGTTGCTACCCC	59.9	512	DRR	Ca_12497	J	--
Ca2_P2_30550219	Ca2	30550219	TCC/TC	TTTCTTTATTCTCGGTGGCG	TGCATTCGATCAAGCTCAAC	60.0	640	INTERGENIC	--	--	--
Ca2_P2_30550405	Ca2	30550405	CTATATA/CTATA	TTTCTTTATTCTCGGTGGCG	TGCATTCGATCAAGCTCAAC	60.0	640	INTERGENIC	--	--	--
Ca2_P2_30551575	Ca2	30551575	ATCTC/ATC	GTGTGTGCTCTGGCCCTAGT	TTTCCAGGAAGATTCCAACG	60.0	806	INTERGENIC	--	--	--
Ca2_P2_30553379	Ca2	30553379	GAAAA/GAAAAA	TTTTTATGAGGCGTGGAAGG	CCATGAACACGCCAAACATA	60.4	874	INTERGENIC	--	--	--

INDEL marker IDs	Chromosomes/unanchored scaffolds	Physical positions (bp)	InDels (*Kabuli* reference genome-CDC Frontier/PII)	Forward primers (5'-3')	Reverse primers (5'-3')	Anneling temperature (0C)	Expected amplified product size (bp)	Structural annotation		Functional annotation	
								Sequence components of *kabuli* genome	*Kabuli* gene accession IDs	NCBI-KOG	TFs
Ca2_P2_30555764	Ca2	30555764	AAG/AAGACACGTGTAG	AAAACATAACCTTTTGCCCG	TCACTCCAAGCTTCCCAAAT	59.7	257	INTERGENIC	--	--	--
Ca2_P2_30558242	Ca2	30558242	CTATATAT/CTATATATAT	TGAAGAAGCAAAATGAATGGG	TGATTGCCTCGACATTGCTA	60.4	823	INTERGENIC	--	--	--
Ca2_P2_30558299	Ca2	30558299	TAAAAAAAAA/TAAAAAAAAAA	TGAAGAAGCAAAATGAATGGG	TGATTGCCTCGACATTGCTA	60.4	823	INTERGENIC	--	--	--
Ca2_P2_30558600	Ca2	30558600	CTAT/CTATATAT	GATGCATACAAAAGTCACCCC	GGATTGATGAAGTGGTTGGG	60.2	254	INTERGENIC	--	--	--
Ca2_P2_30565181	Ca2	30565181	CTTTTT/CTTTTTT	TGGTCCCCTCAACCACTAAG	TGTCTCTGTTTGGTTGCAAAA	59.4	708	INTERGENIC	--	--	--
Ca2_P2_30565683	Ca2	30565683	GAAAAAAAAA/GAAAAAAAA	TGCAACCAAACAGAGACAAAA	CCGAATGTTACAACCAATTTCTC	59.8	376	INTERGENIC	--	--	--
Ca2_P2_30567133	Ca2	30567133	TAAAAAAAA/TAAAAAAA	AAATTACCGCAACAATTGGC	GAAGCGCAACTAAACCAAGC	60.0	539	INTERGENIC	--	--	--
Ca2_P2_30568142	Ca2	30568142	AT/ATGT	CTTGTTTGTCGCAGTCGCTA	GGTCATACCAAGGCCAAGAA	59.9	453	INTRON	Ca_12495	E	--
Ca2_P2_30569147	Ca2	30569147	TCCCCC/TCCCC	GAAAATGTGGTTCACTCGGG	CCGTCCAATTTTCTCTTGGA	60.0	875	INTRON	Ca_12495	E	--
Ca2_P2_30573602	Ca2	30573602	CGGG/CGGGG	TGGCTCATGACCCCTTTATC	GGCCCCTTTTCATTTTCATT	60.1	709	DRR	Ca_12495	E	--
Ca2_P2_30574328	Ca2	30574328	TAAAAA/TAAAAAAA	CCATTGAGGAGTGGAGAACTTT	CTTGTGTGTTCCCAATGCAA	60.6	783	INTERGENIC	--	--	--
Ca2_P2_30575054	Ca2	30575054	AT/A	TGCAGCAACAATTATCCAGC	GACACACACCTCGTTTGCAT	59.6	858	INTERGENIC	--	--	--

**INDEL marker IDs**	**Chromosomes/unanchored scaffolds**	**Physical positions (bp)**	**InDels (*Kabuli* reference genome-CDC Frontier/PII)**	**Forward primers (5'-3')**	**Reverse primers (5'-3')**	**Anneling temperature (0C)**	**Expected amplified product size (bp)**	**Structural annotation**		**Functional annotation**	
								**Sequence components of *kabuli* genome**	***Kabuli* gene accession IDs**	**NCBI-KOG**	**TFs**
Ca2_P2_30575439	Ca2	30575439	ATCT/ATCTCT	GGTTGATTATGGTTTTGGCG	ATCCAAAGCAGCATCCATTC	60.0	862	INTERGENIC	--	--	--
Ca2_P2_30580411	Ca2	30580411	TAAAAAAAA/TAAAAAAA	ACATGCATCAACAACGGAAA	TGCATTTTGAGGTTTCACCA	60.1	408	INTRON	Ca_12493	--	--
Ca2_P2_30593141	Ca2	30593141	CTAT/CTATAT	TTCCTTGGCTAAGACGGATG	GAACACGTGGTTTGATTTTTGA	59.9	389	INTERGENIC	--	--	--
Ca2_P2_30622722	Ca2	30622722	A/ACCGTAATCC	CTTGGTCAGAATCACGGAGC	CACGTTGACCCTTTTTGTCA	59.6	380	INTRON	Ca_12490	R	--
Ca2_P2_30623473	Ca2	30623473	TAAA/TGAAAAA	TGACAAAAAGGGTCAACGTG	CCGCAATCAATTTAGCGAAT	60.1	686	INTERGENIC	--	--	--
Ca2_P2_30623593	Ca2	30623593	TTATGTTTTAT/TTATGTTTTATATGTTTTAT	AGTAGAGCCCGACAAGATGC	CCGCAATCAATTTAGCGAAT	60.1	483	INTERGENIC	--	--	--
Ca2_P2_30624454	Ca2	30624454	C/CT	TCATGAATGCAACGTGGTTT	TTTTGCTGTTTGTTTTACGTCAT	58.8	646	INTERGENIC	--	--	--
Ca2_P2_30624742	Ca2	30624742	GTTTTTT/GTTTTTTT	TTGCTTGCAACACAAAACTTC	TGAACCATTCATCACAAAAAGAG	59.1	837	INTERGENIC	--	--	--
Ca2_P2_30624786	Ca2	30624786	ATAATTAAT/ATAATTAATTAAT	TGAACGTATAAGTGCGTCAAAAA	TGAACCATTCATCACAAAAAGAG	59.1	701	INTERGENIC	--	--	--
Ca2_P2_30626028	Ca2	30626028	AC/A	TTGTCATTTTGATCATTGATTCG	GGTGGTTTGTAACAACAGCAAA	59.9	515	INTERGENIC	--	--	--
Ca2_P2_30630152	Ca2	30630152	A/AG	CCAAAAACTAGGGAGAAGAGCA	TGAAATCGAGACTTTACGGTTG	59.3	763	INTERGENIC	--	--	--
Ca2_P2_30630331	Ca2	30630331	GTTTATTTATTTAT/GTTTATTTATTTATTTAT	TGCGTGTATCATTTTCAACTCA	GGAGATCCAATAGATGGCGA	60.0	841	INTERGENIC	--	--	--

INDEL marker IDs	Chromosomes/unanchored scaffolds	Physical positions (bp)	InDels (*Kabuli* reference genome-CDC Frontier/PII)	Forward primers (5'-3')	Reverse primers (5'-3')	Anneling temperature (0C)	Expected amplified product size (bp)	Structural annotation		Functional annotation	
								Sequence components of *kabuli* genome	*Kabuli* gene accession IDs	NCBI-KOG	TFs
Ca2_P2_30632787	Ca2	30632787	TAAAAAA/TAAAAAAA	CCAACAATGCTCACACATCC	ATCTTGCAAACCACCACACC	60.8	783	INTERGENIC	--	--	--
Ca2_P2_30639636	Ca2	30639636	AG/A	TCTCATCCAACCTTTGCAAGT	TCTCAAACTCGTCCTCACCC	60.2	882	INTERGENIC	--	--	--
Ca2_P2_30641972	Ca2	30641972	GTATA/GTA	TGGGAGAGAAAAGATGTTGGA	AAAATTGTCGGCAACTGCAC	61.1	774	INTERGENIC	--	--	--
Ca2_P2_30642500	Ca2	30642500	GAAAAAAAAA/GAAAAAAAAAAA	TGCAGTTGCCGACAATTTTAT	TCCTCGGTAACCAAACAAGC	60.1	128	INTERGENIC	--	--	--
Ca2_P2_30645883	Ca2	30645883	TAAAAAAAAAA/TAAAAAAAAAAA	TGCTGGGTTGATGGATGTAA	TGCATGCATGGATCCCTAA	61.0	816	INTERGENIC	--	--	--
Ca2_P2_30647034	Ca2	30647034	CAT/CATAT	TTGACGTACCCAACCATTGA	CCCTCACTTAAGCCCACAAA	60.1	443	INTERGENIC	--	--	--
Ca2_P2_30652935	Ca2	30652935	TAA/TA	TTTCTTCAATCTTAATGGTTTTTGG	CATTCGACCAAGGAAAGTTCA	60.1	432	INTERGENIC	--	--	--
Ca2_P2_30664626	Ca2	30664626	GTTTTTTT/GTTTTTTTTT	AAAGTGACAACCCAGAACCG	CTAAACCCCGAACCATGAGA	59.9	728	INTRON	Ca_12489	QI	bHLH
Ca2_P2_30664649	Ca2	30664649	CTTTTTTTTTTT/CTTTTTTTTTT	AAAGTGACAACCCAGAACCG	CTAAACCCCGAACCATGAGA	59.9	728	INTRON	Ca_12489	QI	bHLH
Ca2_P2_30665443	Ca2	30665443	ATTTTTTTTTT/ATTTTTTTT	TCATGGTGGACCGTAATCTG	CTACTGCCTTGGATTCCTTTT	57.5	403	INTRON	Ca_12489	QI	bHLH
Ca2_P2_30665911	Ca2	30665911	ATTTTTTTTTTT/ATTTTTTTTTTTT	AAAAGGAATCCAAGGCAGTAG	TCCATTACCATCATACGCCA	59.8	788	INTRON	Ca_12489	QI	bHLH
Ca2_P2_30668742	Ca2	30668742	TAAAAAAA/TAAAAAA	TGTGGTGTGGTCTCCTTTCA	TATTTCAAATTTCCGCCGAC	59.9	197	INTRON	Ca_12489	QI	bHLH

INDEL marker IDs	Chromosomes/unanchored scaffolds	Physical positions (bp)	InDels (*Kabuli* reference genome-CDC Frontier/PII)	Forward primers (5'-3')	Reverse primers (5'-3')	Anneling temperature (0C)	Expected amplified product size (bp)	Structural annotation		Functional annotation	
								Sequence components of *kabuli* genome	*Kabuli* gene accession IDs	NCBI-KOG	TFs
Ca2_P2_30670677	Ca2	30670677	CTATATAT/CTAT	CCGTGTTTCTTCACCGTTTT	TCTTTGGCACCCTCATTTTC	60.1	435	DRR	Ca_12489	QI	bHLH
Ca2_P2_30671540	Ca2	30671540	TAAAAAA/TAAAAA	TTCGATTGATGGTTAAGAGACA	ATAACGTGTTGGATCAGCCC	59.8	697	DRR	Ca_12489	QI	bHLH
Ca2_P2_30672058	Ca2	30672058	ATTTT/ATT	GGGCTGATCCAACACGTTAT	CCATGGGAAACGTTGGTAAC	60.1	396	DRR	Ca_12489	QI	bHLH
Ca2_P2_30672151	Ca2	30672151	TGGGG/TGGG	GGGCTGATCCAACACGTTAT	CCATGGGAAACGTTGGTAAC	60.1	396	DRR	Ca_12489	QI	bHLH
Ca2_P2_30675995	Ca2	30675995	A/AC	TTAAGTGGGTTGCGCTTTTT	AAGAACTTGGACGTTGGTGG	60.0	536	INTERGENIC	--	--	--
Ca2_P2_30676386	Ca2	30676386	GAAAAAAAAAA/GAAAAAAAAAAA	ATCCACCAACGTCCAAGTTC	CCTTGGTATGACCCTAATTCCTT	59.6	532	INTERGENIC	--	--	--
Ca2_P2_30676782	Ca2	30676782	ACTAGC/ACTAGCTAGC	GCTCCAGCAAAAATAGAAATGG	TCATCAATCAAACTACTTACGCAGA	60.2	504	INTERGENIC	--	--	--
Ca2_P2_30678202	Ca2	30678202	TA/T	AGCTAATTGTGGGCGAAAAA	TGGAAAAGGTTTTGGGTTTG	59.8	922	INTERGENIC	--	--	--
Ca2_P2_30678362	Ca2	30678362	TTC/T	AGCATGGAGAGAGGGGAAGT	TGGAAAAGGTTTTGGGTTTG	59.8	433	INTERGENIC	--	--	--
Ca2_P2_30678630	Ca2	30678630	CTTTT/CTTT	AGCATGGAGAGAGGGGAAGT	CTCACGCATCTCCAAACTCA	60.0	782	INTERGENIC	--	--	--
Ca2_P2_30678822	Ca2	30678822	ATT/ATTTTT	CAAACCCAAAACCTTTTCCA	CTCACGCATCTCCAAACTCA	60.0	369	INTERGENIC	--	--	--
Ca2_P2_30690091	Ca2	30690091	CT/CTTT	GCGGTGTTTGTTTGGGTAGT	GGAGCACACAGATGCGAATA	59.8	335	INTERGENIC	--	--	--

INDEL marker IDs	Chromosomes/unanchored scaffolds	Physical positions (bp)	InDels (*Kabuli* reference genome-CDC Frontier/PII)	Forward primers (5'-3')	Reverse primers (5'-3')	Anneling temperature (0C)	Expected amplified product size (bp)	Structural annotation		Functional annotation	
								Sequence components of *kabuli* genome	*Kabuli* gene accession IDs	NCBI-KOG	TFs
Ca2_P2_30690274	Ca2	30690274	A/AT	TTAGGGGAACGTGAAATTGG	CGGTCATACTGCACACACAA	59.2	444	INTERGENIC	--	--	--
Ca2_P2_30690304	Ca2	30690304	ACC/AC	GCGGTGTTTGTTTGGGTAGT	TGTACATTCAAATCAAACAAAATTCA	59.7	825	INTERGENIC	--	--	--
Ca2_P2_30690585	Ca2	30690585	AAATAATAATAATAATAA/AAATAATAATAATAATAATAATAATAA	TATTCGCATCTGTGTGCTCC	CGTGGTTGAGTAGATGTGCC	59.2	670	INTERGENIC	--	--	--
Ca2_P2_30690967	Ca2	30690967	CAAAAAAAAA/CAAAAAAA	GGCACATCTACTCAACCACG	GGAATTGTGTGATAAATTGCTTGA	60.2	580	INTERGENIC	--	--	--
Ca2_P2_30694795	Ca2	30694795	TTATATATATATATATATATATATAT/TTATATATATATATATAT	CACGTGTCAAACTTGCCAAT	CACTTGCCTTGACCATTGACT	60.2	984	DRR	Ca_12487	KLB	YABBY
Ca2_P2_30695617	Ca2	30695617	CTTTTTTTTTT/CTTTTTTTTTTT	CCCATGCATATCACGTTTTG	ACGCAGGGCCATGATACTAC	60.0	361	DRR	Ca_12487	KLB	YABBY
Ca2_P2_30700575	Ca2	30700575	CAAGTAAG/CAAG	CGCTAAAGGCACCTCAAAAG	GCAGCTTTCTGTTAGTGGGC	60.0	583	INTRON	Ca_12487	KLB	YABBY
Ca2_P2_30700832	Ca2	30700832	ATCCAAGTCCAAGTC/ATCCAAGTC	GCCCACTAACAGAAAGCTGC	GCAGAATCCAGGGCAGATAA	60.2	460	INTRON	Ca_12487	KLB	YABBY
Ca2_P2_30701503	Ca2	30701503	T/TAATC	TTATCTGCCCTGGATTCTGC	TTGGAAGAGGCCAAATATGC	60.0	753	INTRON	Ca_12487	KLB	YABBY
Ca2_P2_30701983	Ca2	30701983	TCCC/TCC	GCATATTTGGCCTCTTCCAA	TGACCCTTGCCAAAGAAATC	60.1	223	CDS (FRAME SHIFT)	Ca_12487	KLB	YABBY
Ca2_P2_30702625	Ca2	30702625	GAAAAA/GAAAAAA	CTTCCGCCATCAATGACAC	AATCTCTTCAATCCCCCGTT	59.8	566	INTRON	Ca_12487	KLB	YABBY
Ca2_P2_30702934	Ca2	30702934	ATTTTTTTTTT/ATTTTTTTTT	AACGGGGGATTGAAGAGATT	GCAAATTCTGCGGGTGTTAT	60.0	508	INTERGENIC	--	--	--

INDEL marker IDs	Chromosomes/unanchored scaffolds	Physical positions (bp)	InDels (*Kabuli* reference genome-CDC Frontier/PII)	Forward primers (5'-3')	Reverse primers (5'-3')	Anneling temperature (0C)	Expected amplified product size (bp)	Structural annotation		Functional annotation	
								Sequence components of *kabuli* genome	*Kabuli* gene accession IDs	NCBI-KOG	TFs
Ca2_P2_30702988	Ca2	30702988	TT/TTTTAT	AACGGGGGATTGAAGAGATT	GCAAATTCTGCGGGTGTTAT	60.0	508	INTERGENIC	--	--	--
Ca2_P2_30703095	Ca2	30703095	TT/TTATAAACT	AACGGGGGATTGAAGAGATT	CGCATACATACGGTGATTGC	60.0	882	INTERGENIC	--	--	--
Ca2_P2_30703181	Ca2	30703181	TAAAAAAA/TAAAAAA	AACGGGGGATTGAAGAGATT	CGCATACATACGGTGATTGC	60.0	882	INTERGENIC	--	--	--
Ca2_P2_30704814	Ca2	30704814	CAA/CAAA	AAATTCAGTGCAACGCACAT	CCCACTTTGGATAACAATTCC	58.3	445	INTERGENIC	--	--	--
Ca2_P2_30705130	Ca2	30705130	CTTTTTTT/CTTTTTT	GCAATTCAAGTACACAACGTGC	CAAGAAAGGAAAATGTGGCA	58.7	239	INTERGENIC	--	--	--
Ca2_P2_30710524	Ca2	30710524	ATCTCTCTCTCT/ATCTCTCTCTCTCT	GGACCATAAAGTTGGCAAGG	TCATCGCCACATGCAGTATT	60.1	767	DRR	Ca_12486	T	--
Ca2_P2_30710563	Ca2	30710563	TA/T	GGACCATAAAGTTGGCAAGG	TCATCGCCACATGCAGTATT	60.1	767	DRR	Ca_12486	T	--
Ca2_P2_30719829	Ca2	30719829	A/AT	CAACGGACAAGAAAATCTCACA	TCACAAATTAACGGCCACAA	60.0	804	INTERGENIC	--	--	--
Ca2_P2_30720315	Ca2	30720315	TATTA/T	TTGTGGCCGTTAATTTGTGA	GTTGATGTTATCAAATAAACAATTTCA	57.7	344	INTERGENIC	--	--	--
Ca2_P2_30720521	Ca2	30720521	CAAAAAAAA/CAAAAAAA	TTGTGGCCGTTAATTTGTGA	CGAGTTTCTTTTTCCCGTCA	60.2	787	INTERGENIC	--	--	--
Ca2_P2_30720601	Ca2	30720601	TAAAAA/TAAAAAA	TTGTGGCCGTTAATTTGTGA	TTGTTGTGGATGCGGTTAAA	60.0	917	INTERGENIC	--	--	--
Ca2_P2_30730862	Ca2	30730862	TAAAAAATATAATGAGTAAAAAAATATAATGAGTAAAAAAATATA/TAAAAAATATAATGAGTAAAAAAATATA	ATGGGCATGAAAGAAAATGG	TAGAGCTGTCAAAACGGGCT	60.0	335	INTERGENIC	--	--	--

INDEL marker IDs	Chromosomes/unanchored scaffolds	Physical positions (bp)	InDels (*Kabuli* reference genome-CDC Frontier/PII)	Forward primers (5'-3')	Reverse primers (5'-3')	Anneling temperature (0C)	Expected amplified product size (bp)	Structural annotation		Functional annotation	
								Sequence components of *kabuli* genome	*Kabuli* gene accession IDs	NCBI-KOG	TFs
Ca2_P2_30731584	Ca2	30731584	C/CG	TATACTCATGCTTGCCGTGC	CCCACCATTGGGATACACTC	60.1	758	INTERGENIC	--	--	--
Ca2_P2_30732861	Ca2	30732861	A/AG	TCACAGAAGGGGTTTTGGTC	AAAATTGTGAACGGAGACCG	60.0	394	INTRON	Ca_12484	T	RAV
Ca2_P2_30737308	Ca2	30737308	TAAAAAAAAA/TAAAAAAAAAAA	TTTTGCCATGGTCTTGGATT	TCAAGAAAATTGCCAACCCT	59.5	618	DRR	Ca_12484	T	RAV
Ca2_P2_30737463	Ca2	30737463	TAAAAAA/TAAAAAAA	TTTTGCCATGGTCTTGGATT	TGGGCCCTCATGATATTGAT	60.1	720	DRR	Ca_12484	T	RAV
Ca2_P2_30766209	Ca2	30766209	CAAAAAAAA/CAAAAAAAAAA	TGTCGTGCGAGAAAAATGAG	GTTGCTTTCGGATAGCTTGC	60.0	770	DRR	Ca_12479	K	ERF
Ca2_P2_30773455	Ca2	30773455	GAAAAAAAAA/GAAAAAAAAAA	TGACTCCATCCACTTATCCCA	CCCATGAGTTTATGTTGGGG	60.0	158	INTERGENIC	--	--	--
Ca2_P2_30774109	Ca2	30774109	CT/CTT	TCGATCAATTGGCATAACCA	TCATCAACGCAAAACTGACTG	59.9	310	INTERGENIC	--	--	--
Ca2_P2_30776002	Ca2	30776002	CATAAGTATAAGTAT/CATAAGTATAAGTATAAGTAT	GACTCAAAGCCACTAAGGCAA	CTTTTGGTGCACAAACACTCT	57.9	131	INTERGENIC	--	--	--
Ca2_P2_30778514	Ca2	30778514	AG/A	AAATTGGTGCATGTGTGATTG	CTTGAATTTCGCTTCTTGGG	59.8	767	INTERGENIC	--	--	--
Ca2_P2_30783813	Ca2	30783813	CTTTTTTTT/CTTTTTTT	AAGGACATGGTACCACCAGC	TGTGAACTATGCCGATTAACTGA	59.7	710	INTERGENIC	--	--	--
Ca2_P2_30788694	Ca2	30788694	TTTTATTTATTTATT/TTTTATTTATTTATTTATT	TCTATCCTCACCACCTCAAGC	TGCATGTCAAGTTGGATTCAT	59.0	774	INTERGENIC	--	--	--
Ca2_P2_30790808	Ca2	30790808	GAAAAAAAA/GAAAAAAAAA	CACTCAGGGGATGAATGTACG	CGAAGTTTGGACATGAGCTG	59.4	480	INTERGENIC	--	--	--

INDEL marker IDs	Chromosomes/unanchored scaffolds	Physical positions (bp)	InDels (*Kabuli* reference genome-CDC Frontier/PII)	Forward primers (5'-3')	Reverse primers (5'-3')	Anneling temperature (0C)	Expected amplified product size (bp)	Structural annotation		Functional annotation	
								Sequence components of *kabuli* genome	*Kabuli* gene accession IDs	NCBI-KOG	TFs
Ca2_P2_30794649	Ca2	30794649	TAAAAAAAAA/TAAAAAAAA	TGATTCTCGACTGAAAACATGAG	TTGCATTTCACTGGAGACGTT	60.7	641	INTERGENIC	--	--	--
Ca2_P2_30795227	Ca2	30795227	TTTATTATTA/TTTATTATTATTA	ACGTCTCCAGTGAAATGCAA	AGCCTTGATGTTGTTTGCG	59.9	506	INTERGENIC	--	--	--
Ca2_P2_30795438	Ca2	30795438	TAAA/TAA	GCAAACAACATCAAGGCTCA	TGAAAATAGTTTTTGGTCCCTG	58.2	558	INTERGENIC	--	--	--
Ca2_P2_30795526	Ca2	30795526	TTAATATAATAT/TTAATAT	GCAAACAACATCAAGGCTCA	TGAAAATAGTTTTTGGTCCCTG	58.2	558	INTERGENIC	--	--	--
Ca2_P2_30795679	Ca2	30795679	GAAAAAA/GAAAAA	GCAAACAACATCAAGGCTCA	TGAAAATAGTTTTTGGTCCCTG	58.2	558	INTERGENIC	--	--	--
Ca2_P2_30795952	Ca2	30795952	CAAAAAAAA/CAAAAAAAAA	CAGGGACCAAAAACTATTTTCA	CCAAGCCCATTTGGACTCTA	60.1	522	INTERGENIC	--	--	--
Ca2_P2_30796973	Ca2	30796973	CAAA/CAAAA	CGTGTCCCTGATATTTTGCC	GGGACAGTTTTGTTTGTGGC	60.4	108	INTERGENIC	--	--	--
Ca2_P2_30799488	Ca2	30799488	TATAAT/TAT	TCGATTTACCCAGGAGCATC	TGAATTGGAAGGATTGAGCA	59.2	561	DRR	Ca_12478	R	MYB
Ca2_P2_30799752	Ca2	30799752	T/TGAC	CCATGAAGTTGAAGGCAAGA	TCTCTGGTGGGAAGGTTCAG	60.2	556	DRR	Ca_12478	R	MYB
Ca2_P2_30803822	Ca2	30803822	TAAAAAAAAAA/TAAAAAAAAAAA	CGGATTCCGTATGGATTGAT	GGTTCATCAAATTCTCGTGTTTC	59.9	723	INTRON	Ca_12478	R	MYB
Ca2_P2_30804787	Ca2	30804787	GAGAGAGAGA/GAGAGAGAGAAAGAGAGAGA	TGCTGAATTGGATAGAGGGG	AGACAGAGACATTGTTTGGCTT	58.0	247	INTERGENIC	--	--	--
Ca2_P2_30805135	Ca2	30805135	CATTAATTA/CATTA	CCTCATGGTGTGTGATTTGC	GCGAAAGCAAAATAGAGTGGA	59.5	880	INTERGENIC	--	--	--

INDEL marker IDs	Chromosomes/unanchored scaffolds	Physical positions (bp)	InDels (*Kabuli* reference genome-CDC Frontier/PII)	Forward primers (5'-3')	Reverse primers (5'-3')	Anneling temperature (0C)	Expected amplified product size (bp)	Structural annotation		Functional annotation	
								Sequence components of *kabuli* genome	*Kabuli* gene accession IDs	NCBI-KOG	TFs
Ca2_P2_30805765	Ca2	30805765	TAAAAAAAAA/TAAAAAAAAAA	TCCACTCTATTTTGCTTTCGC	TCTTTGACCCCACCAAAAC	59.8	667	INTERGENIC	--	--	--
Ca2_P2_30808989	Ca2	30808989	ATTTTTT/ATTTTT	TTCTGAATCAGTGCCCTTCC	TGGGTAGGAGAGCCTCATGT	59.7	392	INTERGENIC	--	--	--
Ca2_P2_30809026	Ca2	30809026	ATTTTTTTT/ATTTTTTT	TTCTGAATCAGTGCCCTTCC	TGGGTAGGAGAGCCTCATGT	59.7	392	INTERGENIC	--	--	--
Ca2_P2_30809205	Ca2	30809205	AG/AGG	ACATGAGGCTCTCCTACCCA	TGTTCACCAACAAAATTGACAAA	60.3	592	INTERGENIC	--	--	--
Ca2_P2_30820733	Ca2	30820733	ATGCAATGTCTTT/AT	TTGCCACGTGTTATCCAAAA	TTCACAGCTGCCACATATCC	59.7	530	DRR	Ca_12476	--	--
Ca2_P2_30826487	Ca2	30826487	TAAA/TAA	TTCCAGCATCCTGTCATAACC	ACGGAGTGACCAGGAAACAC	60.0	640	INTERGENIC	--	--	--
Ca2_P2_30843280	Ca2	30843280	AAATTAA/AAA	CCACAATCATTGGGAAGTGA	TGGATGAATAGGGGATTGCT	59.3	305	INTERGENIC	--	--	--
Ca2_P2_30844033	Ca2	30844033	T/TAA	TTTTCCACATGTTCCCTTCA	TTCATCTAACGCAGTCGTGG	59.9	686	INTERGENIC	--	--	--
Ca2_P2_30844075	Ca2	30844075	ATTTTTTTT/ATTTTTTT	TCATCAAAGGTCATAGGAGGG	TTCATCTAACGCAGTCGTGG	59.9	646	INTERGENIC	--	--	--
Ca2_P2_30844365	Ca2	30844365	TTATATATATATATATA/TTATATATATATA	CCACGACTGCGTTAGATGAA	CAGAATCAGCACGCAAACAT	59.9	372	INTERGENIC	--	--	--
Ca2_P2_30846096	Ca2	30846096	GAAAA/GAAAAA	CCTTTGCGAACTTGAAGACC	TCCTCCCTCTTGCAGGTTTA	59.8	815	INTERGENIC	--	--	--
Ca2_P2_30848508	Ca2	30848508	TTGATAAT/TTGATAATAATGATAAT	TTGAAGGACCAAAATCATGAAC	CATACAACGCAAGCTAGGCA	60.0	533	INTERGENIC	--	--	--

INDEL marker IDs	Chromosomes/unanchored scaffolds	Physical positions (bp)	InDels (*Kabuli* reference genome-CDC Frontier/PII)	Forward primers (5'-3')	Reverse primers (5'-3')	Anneling temperature (0C)	Expected amplified product size (bp)	Structural annotation		Functional annotation	
								Sequence components of *kabuli* genome	*Kabuli* gene accession IDs	NCBI-KOG	TFs
Ca2_P2_30860320	Ca2	30860320	TAA/TAAA	TGAACAAAAAGGTTGGGTGA	TGCTCAACTTTGACCCATTG	59.7	487	INTERGENIC	--	--	--
Ca2_P2_30860644	Ca2	30860644	CAAA/CA	TGGGTCAAAGTTGAGCATGA	CACCTTTGGAAAGAGCTGGA	60.4	557	INTERGENIC	--	--	--
Ca2_P2_30860811	Ca2	30860811	CTTT/CTT	TGGGTCAAAGTTGAGCATGA	CACCTTTGGAAAGAGCTGGA	60.4	557	INTERGENIC	--	--	--
Ca2_P2_30865173	Ca2	30865173	GAAAAAAAA/GAAAAAAA	GCTGAGGGATTTCGAAAGTAA	ATAATACCCCTCAGGGTGGC	60.0	576	INTERGENIC	--	--	--
Ca2_P2_30871113	Ca2	30871113	TGGG/TGG	CAGCAGAACCTGACGATGAA	GTTTGCCTTGCAGAAAAAGC	60.0	827	INTRON	Ca_12473	U	--
Ca2_P2_30871455	Ca2	30871455	A/AG	ATTTGTCTTTTGGCGGTGAG	GTTTGCCTTGCAGAAAAAGC	60.0	340	INTRON	Ca_12473	U	--
Ca2_P2_30872234	Ca2	30872234	CAT/CATAT	CAAAAGCACAACAAGCCAGA	AATTTAAAGAAAGGCCGGGA	59.9	268	INTRON	Ca_12473	U	--
Ca2_P2_30876227	Ca2	30876227	AT/ATTT	GCAGCCAAATTTTCTTGAGC	CAAAGCAGCATTCAGGACAA	60.0	678	INTRON	Ca_12473	U	--
Ca2_P2_30880301	Ca2	30880301	C/CTTGT	GCATTTTTAAGTTGCGCCAT	CAACGGTCTCTGGTGGTTTT	60.0	126	INTRON	Ca_12473	U	--
Ca2_P2_30883523	Ca2	30883523	TGCG/TG	CACATCAACAGGGCAAAATG	GTGTGTGCAATCTCTGGCAT	59.7	682	INTRON	Ca_12473	U	--
Ca2_P2_30887300	Ca2	30887300	TTAATAA/TTAATAATAA	TCAAGGGAGGATTACATGGG	TATTAGAATCCGTGCGATGC	58.7	250	INTERGENIC	--	--	--
Ca2_P2_30887613	Ca2	30887613	AT/A	TCAAGGGAGGATTACATGGG	AAAGGGAAAGTTGCATCACG	60.1	826	INTERGENIC	--	--	--

INDEL marker IDs	Chromosomes/unanchored scaffolds	Physical positions (bp)	InDels (*Kabuli* reference genome-CDC Frontier/PII)	Forward primers (5'-3')	Reverse primers (5'-3')	Anneling temperature (0C)	Expected amplified product size (bp)	Structural annotation		Functional annotation	
								Sequence components of *kabuli* genome	*Kabuli* gene accession IDs	NCBI-KOG	TFs
Ca2_P2_3088792	Ca2	3088792	TATTTTTA/TATTTTTACATTTTTA	AGACATCCATTCAAGGGCAC	ACCGGATGATAGAGCATACAA	57.2	950	INTERGENIC	--	--	--
Ca2_P2_30888121	Ca2	30888121	TAAAAAA/TAAAAAAA	TTAAGGGAAGGTCGTGATGC	GGGCTAGTGGCAGTCTTCAG	60.0	232	INTERGENIC	--	--	--
Ca2_P2_30888708	Ca2	30888708	TG/T	CTGAAGACTGCCACTAGCCC	AATGGGAGCCAATGAAGATG	59.9	892	INTERGENIC	--	--	--
Ca2_P2_30888970	Ca2	30888970	ATCTTT/AT	CAGTCCATAGGCCATTTTGAA	GCAGGCTGAGTTCAGGTAGG	60.0	567	INTERGENIC	--	--	--
Ca2_P2_30890128	Ca2	30890128	AAAATAAATAAATAAATAAA/AAAATAAATAAATAAA	GATGATGTAAATGGGAGCCAA	CATTTGTTGAATGTTGCCCA	60.4	697	INTERGENIC	--	--	--
Ca2_P2_30890182	Ca2	30890182	AAA/AAAGAA	GATGATGTAAATGGGAGCCAA	GGAATTGATCCACAACTCGG	60.3	958	INTERGENIC	--	--	--
Ca2_P2_30890429	Ca2	30890429	A/ACGAC	TGGGCAACATTCAACAAATG	GCGGTGAGACTCGAAAAGAG	60.1	321	INTERGENIC	--	--	--
Ca2_P2_30890896	Ca2	30890896	ATTTTTT/ATTTTTTT	CTCTTTTCGAGTCTCACCGC	ATGCCATGACGTGAACAAAA	60.0	566	INTERGENIC	--	--	--
Ca2_P2_30892153	Ca2	30892153	A/ATGTAC	AGGATGGGTTTCTGTGTTGC	CTAAGGGGCAAAAGCGTACA	60.3	617	INTRON	Ca_12472	--	--
Ca2_P2_30892524	Ca2	30892524	GTTT/GTT	AGTGGCTTGGTTTGTGGAAA	TAAACAAAATGACGCCACCA	60.0	342	INTRON	Ca_12472	--	--
Ca2_P2_30896118	Ca2	30896118	ATT/AT	TGCCAAAAAGAAAAAGGAATG	TGTTAAAGCCAAAAACCCCA	60.3	742	DRR	Ca_12472	--	--
Ca2_P2_30899363	Ca2	30899363	ATTTT/ATTT	GAATTGGAAATGGCAGCCTA	TTGCACAAGCTGGATACTCG	60.0	459	INTRON	Ca_12471	--	--

INDEL marker IDs	Chromosomes/unanchored scaffolds	Physical positions (bp)	InDels (*Kabuli* reference genome-CDC Frontier/PII)	Forward primers (5'-3')	Reverse primers (5'-3')	Anneling temperature (0C)	Expected amplified product size (bp)	Structural annotation		Functional annotation	
								Sequence components of *kabuli* genome	*Kabuli* gene accession IDs	NCBI-KOG	TFs
Ca2_P2_30901376	Ca2	30901376	CTTT/CTTTTTTCTTTTTT	TCATTGGTGTTTACCGGGAT	TGCAGTGCTTCCATTCTCAG	60.1	323	DRR	Ca_12470	DO	--
Ca2_P2_30901717	Ca2	30901717	TG/T	TCATTGGTGTTTACCGGGAT	TAGCCTTTTGCAATATGGGG	59.9	886	DRR	Ca_12470	DO	--
Ca2_P2_3090420	Ca2	3090420	CCTCTCTCTCTCTCTCTCTCTCTCTC/CCTCTCTCTCTCTCTCTCTCTCTCTC	TACAAGCACTCTCTCCCGCT	TTTCCATGCCCAAAAGTCTC	60.1	257	INTERGENIC	--	--	--
Ca2_P2_30904965	Ca2	30904965	CTATAT/CTAT	TTGGACCCACCCTATATTGC	GCCTGATTGAGCACTTTCCT	59.4	419	INTRON	Ca_12470	DO	--
Ca2_P2_3090922	Ca2	3090922	TA/T	GAGACTTTTGGGCATGGAAA	TGGTCAGTCAAATTCTCCGA	59.2	368	INTRON	Ca_10541	T	HD-ZIP
Ca2_P2_30912621	Ca2	30912621	T/TCCCAC	GCACACGTGCAAAACAAAAC	TTGGCCCAAGATAGCAGATT	59.7	515	INTRON	Ca_12470	DO	--
Ca2_P2_30912940	Ca2	30912940	TGGG/TGGGG	GATGCCTCTGCTTCTGTTCC	CAATGAGCTTGGGGACAAAT	59.9	381	INTRON	Ca_12470	DO	--
Ca2_P2_30916172	Ca2	30916172	TCC/TCCC	TGAATTGCCACAGGTCTTGA	TAAAGAGTGGCACCCCTCAC	60.1	411	INTRON	Ca_12470	DO	--
Ca2_P2_30945167	Ca2	30945167	GT/GTCT	GACCATGGAATCGGACAAGT	CTTCTTGCATCCCCTTGTGT	60.1	684	INTRON	Ca_12469	U	bHLH
Ca2_P2_3096067	Ca2	3096067	CAAGTAA/CAA	AAAGACCACACCCAAGCATC	TCGGCGGATACTTTTAAACAA	59.6	622	DRR	Ca_10541	T	HD-ZIP
Ca2_P2_3096143	Ca2	3096143	ATT/AT	AAAGACCACACCCAAGCATC	CTTTATCTTCATTTCGGCGG	59.7	635	DRR	Ca_10541	T	HD-ZIP
Ca2_P2_30963140	Ca2	30963140	GT/G	TAAGCCCATTGAACTGCTCC	CACAATTGCCTACACCATCG	60.0	780	INTERGENIC	--	--	--

INDEL marker IDs	Chromosomes/unanchored scaffolds	Physical positions (bp)	InDels (*Kabuli* reference genome-CDC Frontier/PII)	Forward primers (5'-3')	Reverse primers (5'-3')	Anneling temperature (0C)	Expected amplified product size (bp)	Structural annotation		Functional annotation	
								Sequence components of *kabuli* genome	*Kabuli* gene accession IDs	NCBI-KOG	TFs
Ca2_P2_3096490	Ca2	3096490	ATTTT/ATTTTT	TGGTTTTCCTTAAACATGCAA	CTTTATCTTCATTTCGGCGG	59.7	134	DRR	Ca_10541	T	HD-ZIP
Ca2_P2_30968434	Ca2	30968434	ATT/ATTT	TTCTTGATCGGGTAGGTTCG	ATTTTGCTCCCGTCTCCTTT	60.1	507	INTERGENIC	--	--	--
Ca2_P2_30983014	Ca2	30983014	AGTGT/AGT	ACAAAATGGGATGGATGGAA	TAATCCCAAGGCGATGCTAC	60.1	517	INTERGENIC	--	--	--
Ca2_P2_3098929	Ca2	3098929	TTTTCTT/TTT	CAAATGCTTCACGAAAGGGT	AGCCACAAAACCACAACACA	60.0	361	INTERGENIC	--	--	--
Ca2_P2_30992771	Ca2	30992771	ACCC/ACC	TGACCGATTCAACCAGTCCT	CCCGAGTTCCAAATGACAAC	60.4	333	INTERGENIC	--	--	--
Ca2_P2_3099296	Ca2	3099296	CACATGACATG/CACATGACATGACATG	GTTGTGGTTTTGTGGCTGTG	CCAATGCTTGACCCATCTTT	59.9	649	INTERGENIC	--	--	--
Ca2_P2_3099476	Ca2	3099476	ATTT/ATTTT	GTTGTGGTTTTGTGGCTGTG	CCAATGCTTGACCCATCTTT	59.9	649	INTERGENIC	--	--	--
Ca2_P2_30997225	Ca2	30997225	A/AT	TCACCCTCGTATCCACAACA	ATGTGGCCATTGACAAGTGA	60.0	245	DRR	Ca_12465	--	ERF
Ca2_P2_30998171	Ca2	30998171	ATGTTGTTGTTGTTGTT/ATGTTGTTGTTGTT	ACGCAGCGTAAGGAAGAAAA	CACTTCCACAGCAATCCCTT	60.1	818	CDS (large-effect mutations)	Ca_12465	--	ERF
Ca2_P2_3100168	Ca2	3100168	AGTGT/AGT	AAAGATGGGTCAAGCATTGG	CGGTTTGTTAGAAGCGAAAAA	59.4	823	INTRON	Ca_10540	C	--
Ca2_P2_31003034	Ca2	31003034	TTAAT/TTAATAAT	CGACATATTATTTGGGACAAACAT	GCAATACTTCCTCTTTGGCG	59.8	368	INTERGENIC	--	--	--
Ca2_P2_31003144	Ca2	31003144	TA/TAGA	CGACATATTATTTGGGACAAACAT	GCAATACTTCCTCTTTGGCG	59.8	368	INTERGENIC	--	--	--

INDEL marker IDs	Chromosomes/unanchored scaffolds	Physical positions (bp)	InDels (*Kabuli* reference genome-CDC Frontier/PII)	Forward primers (5'-3')	Reverse primers (5'-3')	Anneling temperature (0C)	Expected amplified product size (bp)	Structural annotation		Functional annotation	
								Sequence components of *kabuli* genome	*Kabuli* gene accession IDs	NCBI-KOG	TFs
Ca2_P2_31013894	Ca2	31013894	TAAAAAAAAA/TAAAAAAAAAAAA	GTGGTAGAGGGAAGGCACAA	TCATGGATTTGTGGTGTGCT	60.0	615	INTERGENIC	--	--	--
Ca2_P2_31017880	Ca2	31017880	A/ATTG	TCCTTGATGCTAAGGTTGCC	GACCCATCTCCAATAGCCCT	60.3	584	DRR	Ca_12462	R	G2-like
Ca2_P2_31029004	Ca2	31029004	TAAAAAAAAAA/TAAAAAAAAAAAA	AAGAAACAATTCCTTTGTTTTCAA	TCCTCCCTCCAAATTTCCTC	60.4	570	DRR	Ca_12461	--	LBD
Ca2_P2_31029414	Ca2	31029414	CG/C	GGAAATTTGGAGGGAGGAGA	CCATATGATGTTCGGCAGTG	59.9	461	DRR	Ca_12461	--	LBD
Ca2_P2_31030970	Ca2	31030970	AAACAA/AAACAACAA	AGTCGCTCCAACATGAGCTT	TGCCCCAAATGGAACACTAT	60.2	277	INTRON	Ca_12461	--	LBD
Ca2_P2_31039004	Ca2	31039004	AT/A	TCGAGTTTCCATCCTCTCTGA	GGTTGAGTTGAGGGGATCAA	59.9	476	INTERGENIC	--	--	--
Ca2_P2_31040581	Ca2	31040581	CTTTTTTT/CTTTTTTTT	CCGGACTTACGGTGAAGAAA	GTTAAGGAACCGCCGATGTA	60.0	567	INTERGENIC	--	--	--
Ca2_P2_31041300	Ca2	31041300	GGAA/GGAAGAAGAA	TACATCGGCGGTTCCTTAAC	GGCTAAATTACATCTGTGGTCCTT	59.8	553	INTERGENIC	--	--	--
Ca2_P2_3104148	Ca2	3104148	TAAAAAAA/TAAAAAAAA	GGAAACGCCGAGACATACAT	GCACAACCTTTTTGGCGTAT	60.0	360	DRR	Ca_10539	P	WOX
Ca2_P2_31041815	Ca2	31041815	TAAAA/TAA	AAGGACCACAGATGTAATTTAGCC	ATATCAAGTGGGGGATGCAA	60.2	965	INTERGENIC	--	--	--
Ca2_P2_31047686	Ca2	31047686	CTATTAACAATATTAACAAT/CTATTAACAAT	AACTTTGGAATCATCACCGC	ATCAAAGAGGTGATTTGGCG	60.1	577	INTERGENIC	--	--	--
Ca2_P2_31049093	Ca2	31049093	GTTT/GTT	CTTTGGGCCAAGGGATTTAT	CTTTGAGGCATGGCCATATT	59.9	412	DRR	Ca_12459	D	--

INDEL marker IDs	Chromosomes/unanchored scaffolds	Physical positions (bp)	InDels (*Kabuli* reference genome-CDC Frontier/PII)	Forward primers (5'-3')	Reverse primers (5'-3')	Anneling temperature (0C)	Expected amplified product size (bp)	Structural annotation		Functional annotation	
								Sequence components of *kabuli* genome	*Kabuli* gene accession IDs	NCBI-KOG	TFs
Ca2_P2_31051310	Ca2	31051310	A/AC	CCAAACGGCCACTAATGTCT	TTCTTGTGGAGACAGGACCC	60.1	195	INTRON	Ca_12459	D	--
Ca2_P2_31051512	Ca2	31051512	GTATATATATATATAT/GTATATATATATATATATATAT	CCAAACGGCCACTAATGTCT	CTGCATTTTCCATCCCAAGT	59.9	614	INTRON	Ca_12459	D	--
Ca2_P2_3106218	Ca2	3106218	TTAA/T	CCAGGAGCTGGCTCATTAAC	TGGGGGATGGAGTAGAAGTG	59.9	372	INTRON	Ca_10539	P	WOX
Ca2_P2_31066601	Ca2	31066601	TTGTGTGTGTGTGTGTGTGTGT/TTGTGTGTGTGTGTGTGTGT	GATTCGTGAGACCGTTGGAT	AAGACATGTCCATAACGAACCA	59.4	640	INTRON	Ca_12458	M	--
Ca2_P2_31067008	Ca2	31067008	ACTAGAGTCTAGAG/ACTAGAG	TTTGTGTGTGTGTGTGTGTGTG	AAGGGCACATGACCTTCAAC	60.0	733	INTRON	Ca_12458	M	--
Ca2_P2_31071398	Ca2	31071398	T/TTTA	GCGGTTTCATTTTCTTCTCG	CGACTATATGTATCCGCCCG	60.3	197	INTERGENIC	--	--	--
Ca2_P2_31080419	Ca2	31080419	AAAAAATA/AAAAAATATGAAAAATA	GACTTCGGGAAAAGGAAAGG	ATGCTTTGGGTTTTCACTGG	60.0	601	INTERGENIC	--	--	--
Ca2_P2_3108123	Ca2	3108123	TAAAAAAA/TAAAAAA	GCATCGTCATCGAGCAACTA	ATCCCTTCCATTTTCCCATC	60.0	773	INTRON	Ca_10539	P	WOX
Ca2_P2_31084768	Ca2	31084768	AAAA/AAAATAAA	TTGGCAACAAGTTCAAATGG	AATGATGCAACAAGGTTCCC	59.8	866	DRR	Ca_12455	--	--
Ca2_P2_31085057	Ca2	31085057	T/TTAAA	CACCAACATAAACAATGTCGAA	ACCACAAATGGAGAAGGTGC	60.0	942	DRR	Ca_12455	--	--
Ca2_P2_31088737	Ca2	31088737	GTTTTTTT/GTTTTTT	GGACCGACAAGACCTATCCA	TCATAAAGCAGGCGGAAGTT	59.8	628	INTRON	Ca_12454	J	--
Ca2_P2_31090173	Ca2	31090173	TTAAT/TTAATAAT	TCACTATTGGCAAGGGAACC	AGATTGCTTCTTGCAACCGT	59.9	565	DRR	Ca_12453	P	--

INDEL marker IDs	Chromosomes/unanchored scaffolds	Physical positions (bp)	InDels (*Kabuli* reference genome-CDC Frontier/PII)	Forward primers (5'-3')	Reverse primers (5'-3')	Anneling temperature (0C)	Expected amplified product size (bp)	Structural annotation		Functional annotation	
								Sequence components of *kabuli* genome	*Kabuli* gene accession IDs	NCBI-KOG	TFs
Ca2_P2_31099158	Ca2	31099158	ACTCTC/ACTC	TGCTGGCCAATCTAGCATAA	GGATGTTGTAGTGTGCCACG	60.0	554	DRR	Ca_12452	F	--
Ca2_P2_31105226	Ca2	31105226	CTTTTTTTTTTTTTTTT/CTTTTTTTTTTT	AACACACCAAACCTCTTCCG	CAGCATTTTTCTCATTCTCCG	59.8	759	INTERGENIC	--	--	--
Ca2_P2_31105310	Ca2	31105310	G/GGTAAAATTTAAAAT	CCACCCATGGCTTATGTCTC	CAGCATTTTTCTCATTCTCCG	59.8	331	INTERGENIC	--	--	--
Ca2_P2_31105800	Ca2	31105800	AT/AATCTTTTTTCTGT	CGGAGAATGAGAAAAATGCTG	TTTTTCCCGCTTAAAATGTAGC	59.7	782	INTERGENIC	--	--	--
Ca2_P2_31105983	Ca2	31105983	TAAAAAA/TAAAAAAA	TGTGGATTATCACCACTCCAA	TTTTTCCCGCTTAAAATGTAGC	59.7	557	INTERGENIC	--	--	--
Ca2_P2_31106191	Ca2	31106191	ATTTTTTTTT/ATTTTTTT	TGTGGATTATCACCACTCCAA	GGGCAATGTGCTCACTTTTA	58.8	950	INTERGENIC	--	--	--
Ca2_P2_31106302	Ca2	31106302	GTA/GTATA	GCTACATTTTAAGCGGGAAAA	GGGCAATGTGCTCACTTTTA	58.8	415	INTERGENIC	--	--	--
Ca2_P2_31106498	Ca2	31106498	CAAAAA/CAAAAAA	GCTACATTTTAAGCGGGAAAA	AATATATGTCCAGGGCCGGT	60.4	763	INTERGENIC	--	--	--
Ca2_P2_31107340	Ca2	31107340	TAAAAAA/TAAAAAAA	ATAGATTGCCGCTGAGTTGG	GTGGCAATTTGGTCTCCCTA	59.9	126	INTERGENIC	--	--	--
Ca2_P2_31108593	Ca2	31108593	ATTTTT/ATTTTTT	TCCAACCAAGTGTCAATCCA	TGCTAGATTGGCCAGCATAA	59.4	792	INTERGENIC	--	--	--
Ca2_P2_3111346	Ca2	3111346	TA/TAA	TGGCAAAGCAATTAACATTCC	CATAGGCAGCTCTACCCCAA	60.2	390	INTRON	Ca_10538	--	--
Ca2_P2_31114173	Ca2	31114173	TA/TAAA	ATCACAAATGGCAACCTTCC	TGTTTTGGCCACCAGGTAAT	60.2	559	INTRON	Ca_12451	F	--

INDEL marker IDs	Chromosomes/unanchored scaffolds	Physical positions (bp)	InDels (*Kabuli* reference genome-CDC Frontier/PII)	Forward primers (5'-3')	Reverse primers (5'-3')	Anneling temperature (0C)	Expected amplified product size (bp)	Structural annotation		Functional annotation	
								Sequence components of *kabuli* genome	*Kabuli* gene accession IDs	NCBI-KOG	TFs
Ca2_P2_31115561	Ca2	31115561	ATTT/ATTTT	CGGTGTGCTTGTATTGGCTA	TCATGGTCGCATTTCCATTA	59.9	360	INTERGENIC	--	--	--
Ca2_P2_31117267	Ca2	31117267	TTATAT/TTATATAT	TGGGTTTTTCATGTCATGCT	ATGGTCGCTGTGGATAAAGG	60.0	248	INTERGENIC	--	--	--
Ca2_P2_31118846	Ca2	31118846	ATATTATTATTATTATTAT/ATATTATTATTATTAT	AACGCCAAGTCAAGACGAGT	AAGTATGTTGCATTGCGATTTTT	59.9	352	INTERGENIC	--	--	--
Ca2_P2_31125141	Ca2	31125141	TTCT/TT	TCTCTTCCGTCTGCCATTTT	CCACATTGGGTATTTTTGGG	59.9	581	INTERGENIC	--	--	--
Ca2_P2_31125327	Ca2	31125327	TTTTTATGTTTTAT/TTTTTATGTTTTATGTTTTATGTTTTAT	TCTCTTCCGTCTGCCATTTT	CCACATTGGGTATTTTTGGG	59.9	581	INTERGENIC	--	--	--
Ca2_P2_31125648	Ca2	31125648	CTTT/CTT	CCACTTCCCAAAAATACCCA	ACCGGGAGAACGAAGTTTTT	60.0	593	INTERGENIC	--	--	--
Ca2_P2_31127493	Ca2	31127493	TT/TTGAT	TGGGTGGAAATTCTCCATGT	TGCTCACTGCATCTGCTCTT	59.9	880	INTRON	Ca_12450	--	LBD
Ca2_P2_31128616	Ca2	31128616	TAAAAAAAAA/TAAAAAAAAAAA	TCTTAGACCATGGCCCTCAC	TGGCCTCAAATTACGATCAA	59.1	726	DRR	Ca_12450	--	LBD
Ca2_P2_31131673	Ca2	31131673	GAATAATAA/GAATAATAATAA	TTTGGACAGCAAGAAGATGG	CATGCAAGAAATGCAAGGAA	59.8	473	INTERGENIC	--	--	--
Ca2_P2_31133111	Ca2	31133111	CAA/CAAA	GGTGATCAAACACATCGTCG	TTAGGTGGAGAGGGTGAGGA	59.6	807	INTERGENIC	--	--	--
Ca2_P2_3113326	Ca2	3113326	AACTTATACTT/AACTTATACTTATACTT	ATTTGTTCTGGCCCACCTTT	AGCTTGATGCTGCAATTCCT	60.0	535	INTRON	Ca_10538	--	--
Ca2_P2_31138425	Ca2	31138425	AA/AATA	TGATGAGACGTTGGTCAAACA	TTTCCTCCTTGTTCACCTCC	59.1	464	INTRON	Ca_12449	--	--

INDEL marker IDs	Chromosomes/unanchored scaffolds	Physical positions (bp)	InDels (*Kabuli* reference genome-CDC Frontier/PII)	Forward primers (5'-3')	Reverse primers (5'-3')	Anneling temperature (0C)	Expected amplified product size (bp)	Structural annotation		Functional annotation	
								Sequence components of *kabuli* genome	*Kabuli* gene accession IDs	NCBI-KOG	TFs
Ca2_P2_31138891	Ca2	31138891	ATTTTTTT/ATTTTTT	GGAGGTGAACAAGGAGGAAA	GGCCCAAAAGGATGAATTTT	60.1	820	DRR	Ca_12449	--	--
Ca2_P2_3114349	Ca2	3114349	TTAT/TT	ATGCCAACACATTGATCAGG	AACCAAGCACTTTGCCACTT	59.8	870	INTERGENIC	--	--	--
Ca2_P2_31143846	Ca2	31143846	TCC/TCCC	GCAAATCAATTTCACACCGA	CACGGCTAGGAGTTGGTCAT	60.1	641	DRR	Ca_12448	--	--
Ca2_P2_31146520	Ca2	31146520	CA/CACTAATTTTAGA	CTAGAAAGTCCGGCGTATCG	TTGGCCTGAAGAATGACCTC	60.2	557	INTERGENIC	--	--	--
Ca2_P2_31148192	Ca2	31148192	ACC/AC	ACCGACGACACAACAAATGA	CAGCAACAAAACAGAGCCAA	60.0	333	INTERGENIC	--	--	--
Ca2_P2_31150853	Ca2	31150853	CCGG/CCGGACCAAGCGG	ACCGTTTTTAACGGATGTCG	TCCACTGACCAGTACCACCA	60.0	381	INTERGENIC	--	--	--
Ca2_P2_31151652	Ca2	31151652	A/AG	ATGGCTTCCTGAATGGTACG	CCCAACTTGAGGGAGTGTGT	60.0	561	INTERGENIC	--	--	--
Ca2_P2_31151914	Ca2	31151914	AGG/AGGG	AGCCAAAGTGAGAAAGGCAA	AAGGATCCAAGCCCACTTCT	60.1	740	INTERGENIC	--	--	--
Ca2_P2_31151939	Ca2	31151939	A/AT	AGCCAAAGTGAGAAAGGCAA	AAGGATCCAAGCCCACTTCT	60.1	740	INTERGENIC	--	--	--
Ca2_P2_31152319	Ca2	31152319	TAAAAAAAA/TAAAAAAAAAA	TCCAACCTTCCAATTTTTCG	AAGGATCCAAGCCCACTTCT	60.1	256	DRR	Ca_12447	L	ERF
Ca2_P2_31158685	Ca2	31158685	GAAAA/GAAA	GGGAACCTGGATTACCACCT	TCCATTCAATGCTCACCGTA	60.1	179	INTRON	Ca_12447	L	ERF
Ca2_P2_31159922	Ca2	31159922	TGAGAGAGAGAGAGAGAGAGAGAGAGAGAGAGA/TGAGAGAGAGAGAGAGAGAGAGAGAGAGAGAGA	AGCAGTGGCATGATGATGAA	CCCATTAACCGCGAATATCA	60.7	232	INTERGENIC	--	--	--

INDEL marker IDs	Chromosomes/unanchored scaffolds	Physical positions (bp)	InDels (*Kabuli* reference genome-CDC Frontier/PII)	Forward primers (5'-3')	Reverse primers (5'-3')	Anneling temperature (0C)	Expected amplified product size (bp)	Structural annotation		Functional annotation	
								Sequence components of *kabuli* genome	*Kabuli* gene accession IDs	NCBI-KOG	TFs
Ca2_P2_31160544	Ca2	31160544	T/TGG	AGTTTGAGAGGAAAACACGCA	TTTTGAACGTCACTTTATGATCG	59.2	426	INTERGENIC	--	--	--
Ca2_P2_31160632	Ca2	31160632	ATTTTTTTTTTT/ATTTTTT	AGTTTGAGAGGAAAACACGCA	TTTTGAACGTCACTTTATGATCG	59.2	426	INTERGENIC	--	--	--
Ca2_P2_31163345	Ca2	31163345	CTGAAATGAAATG/CTGAAATGAAATGAAATG	CACGAGTTTGTCAACTCCTCC	ATTTCGTCGTGATTCGCATT	60.5	495	INTERGENIC	--	--	--
Ca2_P2_31165144	Ca2	31165144	TTCTTTAATTCTCTT/TTCTTTAATTCTCTTTAATTCTCTT	CTGAAGATGGTGATTGAACCAA	TTGGGCCTATCTGAAAATGG	59.9	141	DRR	Ca_12446	--	ERF
Ca2_P2_31165732	Ca2	31165732	C/CTT	AAGTTGCAGTCAAGGCCAAC	CTCTGTGGCCACTTTTGTCA	59.9	397	DRR	Ca_12446	--	ERF
Ca2_P2_31169525	Ca2	31169525	AATA/AATATATA	CAAACCTTTTTGACGGAACA	GCACATGCGTCTTTTAAGTTTG	59.8	506	INTERGENIC	--	--	--
Ca2_P2_31170607	Ca2	31170607	GT/G	GATTGGGCAAGGGAAACTTA	TGGGCCCTATAAAATGGTGA	60.1	436	INTERGENIC	--	--	--
Ca2_P2_31170706	Ca2	31170706	TA/TATATACA	GATTGGGCAAGGGAAACTTA	TCGGATATGCGAATGCAATA	60.0	617	INTERGENIC	--	--	--
Ca2_P2_31172482	Ca2	31172482	TATTTAAT/TAT	CCTTGAATAAAAATATGGGGCA	TCAATAACTTTTAACAGTCCGACAA	59.2	526	INTERGENIC	--	--	--
Ca2_P2_31172925	Ca2	31172925	AATATATATATA/AATATATATATATA	TTGTCGGACTGTTAAAAGTTATTGA	TAGTGCGCGACATTTTATGC	59.9	265	INTERGENIC	--	--	--
Ca2_P2_31175907	Ca2	31175907	T/TG	TCAGCTGAATGCAATCTCTTGT	TCCACGAGGAATATTTGGAA	58.0	581	INTERGENIC	--	--	--
Ca2_P2_31177108	Ca2	31177108	ATT/ATTT	TCCAATTTGATATCGGTCTCTTG	AAACATAAAACAACGTGAGACAAA	57.9	970	INTERGENIC	--	--	--

INDEL marker IDs	Chromosomes/unanchored scaffolds	Physical positions (bp)	InDels (*Kabuli* reference genome-CDC Frontier/PII)	Forward primers (5'-3')	Reverse primers (5'-3')	Anneling temperature (0C)	Expected amplified product size (bp)	Structural annotation		Functional annotation	
								Sequence components of *kabuli* genome	*Kabuli* gene accession IDs	NCBI-KOG	TFs
Ca2_P2_31178815	Ca2	31178815	CTTTTTTTTT/CTTTTTTT	TGTAACAAAAAGGGAGGAAAAA	ACGCAGATGCCAAAGTTGTA	59.3	691	INTERGENIC	--	--	--
Ca2_P2_31180716	Ca2	31180716	TAA/TAAA	GGCCAATTGCTAAGTGGATG	GCTTTGATTCTTGCAATGTCC	59.7	364	INTERGENIC	--	--	--
Ca2_P2_31189040	Ca2	31189040	GATCA/GATCATCA	CAATAGGGTTTGCATCCCAC	TTCATGGTTGAGAGCCACTG	59.8	532	INTRON	Ca_12445	Q	--
Ca2_P2_3118907	Ca2	3118907	TAAAAA/TAAAAAAA	TGCACCAGAATGCCTTGTTA	TGGCCTTCACATTTCAAACA	60.1	752	INTRON	Ca_10537	--	AP2
Ca2_P2_31189468	Ca2	31189468	ATAAT/ATAATTAAT	CAGCCCATCCTGTTCTGATT	ATGGACAATTTCCAGGTCCA	60.2	403	INTRON	Ca_12445	Q	--
Ca2_P2_31190112	Ca2	31190112	GAAAAAA/GAAAAAAA	CTTCCAAAGTTGGACCTGGA	CTACCGGGTGCAACAACTTT	60.0	745	INTRON	Ca_12445	Q	--
Ca2_P2_31190584	Ca2	31190584	TAAAAAAAAAAAAA/TAAAAAAAAA	AAAGTTGTTGCACCCGGTAG	CCAAATTGCGGACACTTTTT	60.0	631	INTRON	Ca_12445	Q	--
Ca2_P2_31191888	Ca2	31191888	TATTTA/T	CGTAGTGCTCTCACCCACAA	CCAAGCGTTAGCTTCCATGT	60.3	743	INTERGENIC	--	--	--
Ca2_P2_31195552	Ca2	31195552	TAAAAAAAAAAA/TAAAAAAAA	CTGGTCGTCCATCTCAACCT	ATTGGTGGTGTGCAAATTCA	59.8	695	INTERGENIC	--	--	--
Ca2_P2_31195882	Ca2	31195882	GTT/GTTT	TGTTCATTGACGCATTTGTTT	ATTGGTGGTGTGCAAATTCA	59.8	248	INTERGENIC	--	--	--
Ca2_P2_31203736	Ca2	31203736	CTTTTTTT/CTTTTTT	GACTAGTTGGGTTCGCCTCA	TTTTATGTTCCTTGCTCCCG	60.1	697	DRR	Ca_12444	U	--
Ca2_P2_31210081	Ca2	31210081	CTTTAATTTA/CTTTA	ACAATGCCGTTTATTCTCCG	TTTTGTTGGTAAGAAAAACTAATTCAA	58.8	599	INTERGENIC	--	--	--

**INDEL marker IDs**	**Chromosomes/unanchored scaffolds**	**Physical positions (bp)**	**InDels (*Kabuli* reference genome-CDC Frontier/PII)**	**Forward primers (5'-3')**	**Reverse primers (5'-3')**	**Anneling temperature (0C)**	**Expected amplified product size (bp)**	**Structural annotation**		**Functional annotation**	
								**Sequence components of *kabuli* genome**	***Kabuli* gene accession IDs**	**NCBI-KOG**	**TFs**
Ca2_P2_3121485	Ca2	3121485	TAAA/TAA	TTCTTATGTCTTGGACCGGC	CTAGAGGCTCCCCACATTCA	60.2	286	DRR	Ca_10536	--	--
Ca2_P2_31232054	Ca2	31232054	CATCTATTTAAT/CATCTATTTAATCTATTTAAT	AGGCAACAAGGAAGAAGTCG	CTTTGACGCATCTTGATGACA	59.9	474	INTERGENIC	--	--	--
Ca2_P2_31232370	Ca2	31232370	ATAT/A	TGTCATCAAGATGCGTCAAAG	TCATTCTTTCCATCACGGGT	60.3	527	INTERGENIC	--	--	--
Ca2_P2_31232642	Ca2	31232642	GTTAATTAATTA/GTTAATTA	TGTCATCAAGATGCGTCAAAG	CCATTGTGATGTTGGTGGAA	60.2	830	INTERGENIC	--	--	--
Ca2_P2_3123340	Ca2	3123340	ACC/AC	CCCAAGGTTGAAAAGCACAT	TGCTTCTGTCACCACCAAAC	59.7	534	INTRON	Ca_10536	--	--
Ca2_P2_31233960	Ca2	31233960	AG/A	GGGTGGAAATCATGGTGAAC	TTGGTTGGAAGGAATGGAAG	59.9	436	INTRON	Ca_12440	--	--
Ca2_P2_31240228	Ca2	31240228	TCAA/T	CTGCCCCCACTATTTCTCAA	GAAACACTTGGCATGTTAAGGA	59.1	479	INTERGENIC	--	--	--
Ca2_P2_31242575	Ca2	31242575	TAC/T	GGGAGTTCACCATTTTGTGG	TTGGGTGTGTACAGGTTCTAGC	59.2	496	INTERGENIC	--	--	--
Ca2_P2_31244339	Ca2	31244339	AGG/AG	CAAGACTCAGGGTGGGTTGT	AAATCCATTGGGAAATGCAG	59.8	547	INTRON	Ca_12439	--	C3H
Ca2_P2_31245171	Ca2	31245171	AAAGGCAA/AAAGGCAAGGCAA	GGGCAATCATATTGGCACTT	AGGAGCTGTTGCTACTCCCA	60.0	597	INTRON	Ca_12439	--	C3H
Ca2_P2_3124790	Ca2	3124790	ATT/A	GGGAAAATGTGGAAGCAAAA	CTTCAAGGTACCCCTCTCCC	59.9	348	INTERGENIC	--	--	--
Ca2_P2_3125216	Ca2	3125216	GAAAAAAA/GAAAAAAAA	AGGGAGAGGGGTACCTTGAA	CCCTTCTTTTCTCCTCCACC	60.0	288	INTERGENIC	--	--	--

INDEL marker IDs	Chromosomes/unanchored scaffolds	Physical positions (bp)	InDels (*Kabuli* reference genome-CDC Frontier/PII)	Forward primers (5'-3')	Reverse primers (5'-3')	Anneling temperature (0C)	Expected amplified product size (bp)	Structural annotation		Functional annotation	
								Sequence components of *kabuli* genome	*Kabuli* gene accession IDs	NCBI-KOG	TFs
Ca2_P2_3126083	Ca2	3126083	CTATATATAT/CTATATATATAT	AACGATTCCATGACTAATTTGAA	TTGAACGGTTTAAATGTGGCT	59.5	518	INTERGENIC	--	--	--
Ca2_P2_3126150	Ca2	3126150	ATT/AT	AACGATTCCATGACTAATTTGAA	TTGAACGGTTTAAATGTGGCT	59.5	518	INTERGENIC	--	--	--
Ca2_P2_3128127	Ca2	3128127	AATTA/AA	GAAGCGCAAAAGTTCCTTAAA	TGTCTCCATTCCAAAGCACA	60.2	615	INTERGENIC	--	--	--
Ca2_P2_31286944	Ca2	31286944	A/ATAATG	TGGTATATTTGCACGGACCA	AAATGCGAGTCTAAGAGGATGC	59.9	330	INTERGENIC	--	--	--
Ca2_P2_31294019	Ca2	31294019	AG/AGAGG	AGACCGTCGGTTTCAATGAC	AAAAATCTTTAACAAGCGGGT	57.0	494	INTERGENIC	--	--	--
Ca2_P2_31298498	Ca2	31298498	CAAAAAAAAAAAA/CAAAAAAAAAA	TCGCATCACCATTCTCACAT	ATCGTTGACACCTCTGCTCA	59.4	972	INTERGENIC	--	--	--
Ca2_P2_31299649	Ca2	31299649	TAAAAAAAAAA/TAAAAAAAA	GCTGCAATGGAAATGCATAA	GCGAATTTCACAATCCCACT	59.9	972	INTERGENIC	--	--	--
Ca2_P2_31300005	Ca2	31300005	A/AG	CATTCCAATGTGCAACCTCT	GCGAATTTCACAATCCCACT	59.9	535	INTERGENIC	--	--	--
Ca2_P2_3130104	Ca2	3130104	TTCTAATTTCTAAT/TTCTAATTTCTAATTTCTAAT	TTGTTTTCTTTAACCAAAGCTCG	GAATGAACGGGATCCTGAGA	60.0	838	INTERGENIC	--	--	--
Ca2_P2_31302251	Ca2	31302251	AT/A	TCTCACCAAACCAACCAACA	GCAACGTCACCACTGAAGAA	59.9	746	DRR	Ca_12435	--	--
Ca2_P2_31303509	Ca2	31303509	T/TAA	AAGAAAAGGGCAGTGCAAGA	TTTGACAATGGAATGGAACG	59.4	933	INTERGENIC	--	--	--
Ca2_P2_31304211	Ca2	31304211	C/CCT	CCCACCATTTACGTTATGCC	GAGCGTTTCCCATTGTTGTT	60.0	232	INTERGENIC	--	--	--

INDEL marker IDs	Chromosomes/unanchored scaffolds	Physical positions (bp)	InDels (*Kabuli* reference genome-CDC Frontier/PII)	Forward primers (5'-3')	Reverse primers (5'-3')	Anneling temperature (0C)	Expected amplified product size (bp)	Structural annotation		Functional annotation	
								Sequence components of *kabuli* genome	*Kabuli* gene accession IDs	NCBI-KOG	TFs
Ca2_P2_31310762	Ca2	31310762	TAATAATAA/TAATAATAAAAAATAATAA	CGCATGAAACATGAAATGCT	TCATGCCAGCTTCAACCATA	60.2	461	INTERGENIC	--	--	--
Ca2_P2_31310921	Ca2	31310921	GTGTATGTA/GTGTA	CGCATGAAACATGAAATGCT	TCATGCCAGCTTCAACCATA	60.2	461	INTERGENIC	--	--	--
Ca2_P2_31311015	Ca2	31311015	T/TA	CGCATGAAACATGAAATGCT	TCATGCCAGCTTCAACCATA	60.2	461	INTERGENIC	--	--	--
Ca2_P2_31312308	Ca2	31312308	ATTTTTTTT/ATTTTTTTTTT	GTTGCATGTGCATTCGTCTC	TGCATGACAGCATGAGCATA	60.0	843	INTERGENIC	--	--	--
Ca2_P2_31316288	Ca2	31316288	AG/A	GGTGACATTCGGGAGTCTGT	CGAGTTGCGCGGTAGAAT	60.0	931	INTERGENIC	--	--	--
Ca2_P2_31316352	Ca2	31316352	GAAAAAA/GAAAAAAA	AGGCACACACGAATGAATTG	TGGGTTTGCATATGTCTTTGA	59.0	647	DRR	Ca_12431	U	--
Ca2_P2_31316763	Ca2	31316763	TTATA/TTA	TGCATGCTTAGATGGACAATG	CTTGGCCATAAGTTTGCCAT	60.0	879	DRR	Ca_12431	U	--
Ca2_P2_31317319	Ca2	31317319	AACT/A	ATGGCAAACTTATGGCCAAG	ACCAAAATTGGAAAGTCGCA	60.5	664	DRR	Ca_12431	U	--
Ca2_P2_31317559	Ca2	31317559	GTT/GTTT	ATGGCAAACTTATGGCCAAG	GAGTGGGTTGGTTGTTTGGT	59.7	831	DRR	Ca_12431	U	--
Ca2_P2_31317661	Ca2	31317661	T/TC	TTGAGCAAAAGTGGCAATGA	GAGTGGGTTGGTTGTTTGGT	59.7	614	DRR	Ca_12431	U	--
Ca2_P2_31333849	Ca2	31333849	T/TG	ATATCCGTTGGACACGCTTC	CTGCTCAAATCGTTGGTGAA	59.8	328	INTERGENIC	--	--	--
Ca2_P2_31335738	Ca2	31335738	A/AAAT	ATATAAACGACGCACCACCC	CGACTGAAACGAATAAAAAGTGAA	59.7	381	INTERGENIC	--	--	--

INDEL marker IDs	Chromosomes/unanchored scaffolds	Physical positions (bp)	InDels (*Kabuli* reference genome-CDC Frontier/PII)	Forward primers (5'-3')	Reverse primers (5'-3')	Anneling temperature (0C)	Expected amplified product size (bp)	Structural annotation		Functional annotation	
								Sequence components of *kabuli* genome	*Kabuli* gene accession IDs	NCBI-KOG	TFs
Ca2_P2_31342497	Ca2	31342497	AATATATATATATATATA/AATATATATATATATATATA	GACGTGGTCTTACAACCATTTTT	TGTGTGACAGGTTGGATTGG	60.4	597	INTERGENIC	--	--	--
Ca2_P2_31345875	Ca2	31345875	CT/C	GGGACAAAATTATAGCACTGGC	TTCGTCAAAACAGATGACACG	59.7	551	DRR	Ca_12427	R	--
Ca2_P2_31346609	Ca2	31346609	TAAAAAAA/TAAAAAA	CGTGTCATCTGTTTTGACGAA	TTGGCACATTATCACCTTGC	59.5	333	DRR	Ca_12428	E	--
Ca2_P2_31352669	Ca2	31352669	CTTTT/CTTT	CCCTTGCTGTGGATGGTAGT	TCCAAAATCTGCAAGAGGCT	60.0	569	INTERGENIC	--	--	--
Ca2_P2_31352863	Ca2	31352863	TTG/T	CCCTTGCTGTGGATGGTAGT	TCCAAAATCTGCAAGAGGCT	60.0	569	INTERGENIC	--	--	--
Ca2_P2_31355084	Ca2	31355084	AT/ATT	GCATGGAAAAATGAAGGCAT	GAATGCATTGCTCATTTGGA	59.6	674	INTERGENIC	--	--	--
Ca2_P2_31355163	Ca2	31355163	TT/TTAT	GCATGGAAAAATGAAGGCAT	GAATGCATTGCTCATTTGGA	59.6	674	INTERGENIC	--	--	--
Ca2_P2_31355232	Ca2	31355232	CTTTTTTTT/CTTTT	GCATGGAAAAATGAAGGCAT	TCCAGAGGGGCAAAAACTTA	59.7	899	INTERGENIC	--	--	--
Ca2_P2_31361796	Ca2	31361796	CA/CAA	GAGGAGTGTGATGACGAGCA	CGTGAATTTTTCGTTAGCCA	58.8	796	INTERGENIC	--	--	--
Ca2_P2_31362383	Ca2	31362383	TAAAAAAAAA/TAAAAAAA	TGGCTAACGAAAAATTCACG	TTGCGAGAGAGCAAAACTCA	59.9	514	INTERGENIC	--	--	--
Ca2_P2_31363352	Ca2	31363352	TGTAT/TGTATGATGTGGTAT	TGGATAAATACTGGCAGGCA	GTTCCACGATGTCGATGATG	59.9	556	INTERGENIC	--	--	--
Ca2_P2_31364749	Ca2	31364749	TATTA/TATTAATTA	AAGCATTGTGCTCGAGGAAT	TGGTCAAATTCTGCCGTAAA	59.2	846	INTERGENIC	--	--	--

INDEL marker IDs	Chromosomes/unanchored scaffolds	Physical positions (bp)	InDels (*Kabuli* reference genome-CDC Frontier/PII)	Forward primers (5'-3')	Reverse primers (5'-3')	Anneling temperature (0C)	Expected amplified product size (bp)	Structural annotation		Functional annotation	
								Sequence components of *kabuli* genome	*Kabuli* gene accession IDs	NCBI-KOG	TFs
Ca2_P2_31365275	Ca2	31365275	CTATATA/CTATA	TTTACGGCAGAATTTGACCA	TGGCTTTTTCCCTACTTCCA	59.7	361	INTERGENIC	--	--	--
Ca2_P2_31365474	Ca2	31365474	CT/C	TTTACGGCAGAATTTGACCA	TGGCTTTTTCCCTACTTCCA	59.7	361	INTERGENIC	--	--	--
Ca2_P2_31367352	Ca2	31367352	AT/ATT	ATTGTAATGCGGTTCCGAAG	CTCCACACGTGCCATCATAC	60.0	485	INTERGENIC	--	--	--
Ca2_P2_31367430	Ca2	31367430	TA/TAGGCAA	ATTGTAATGCGGTTCCGAAG	CTCCACACGTGCCATCATAC	60.0	485	INTERGENIC	--	--	--
Ca2_P2_31367840	Ca2	31367840	CTATA/CTA	GTATGATGGCACGTGTGGAG	CCTTCCCTTTCCCTTGAAAA	60.4	399	INTERGENIC	--	--	--
Ca2_P2_31368405	Ca2	31368405	ATTT/ATTTT	TTTTCAAGGGAAAGGGAAGG	CCGTTCCATTATCGGAGAGA	60.0	797	INTERGENIC	--	--	--
Ca2_P2_31368994	Ca2	31368994	AT/ATTAGT	TCTCTCCGATAATGGAACGG	CCCTCCGCTCGAATCTATATC	60.0	658	INTERGENIC	--	--	--
Ca2_P2_31372461	Ca2	31372461	ATTTTTTT/ATTTTTTTTT	GCACAAGCTGTGAAACAAGC	TGGCGGGTTACTACTTGGTC	60.0	538	DRR	Ca_12425	--	bHLH
Ca2_P2_31381351	Ca2	31381351	AGTATTTCATT/AGTATTTCATTGTATTTCATT	TTCATTTCAGGCAGCCTCTT	TCAGAGATGTGGCACTGAGG	60.0	816	INTRON	Ca_12423	--	--
Ca2_P2_31386815	Ca2	31386815	TAAA/TAA	CAGTCGAATCCGCCATATCT	TTGGGAATCGCATTTTTCTC	60.0	587	INTRON	Ca_12422	--	MYB_related
Ca2_P2_31388110	Ca2	31388110	TAAAA/TAAA	TCAGGGGACAATAACTTGGC	TCCATTTACCCACTAGACCCA	59.3	919	DRR	Ca_12422	--	MYB_related
Ca2_P2_31388227	Ca2	31388227	TAAAA/TAAAAA	CATCAGTCCCCTCACTGGAT	TCCATTTACCCACTAGACCCA	59.3	707	DRR	Ca_12422	--	MYB_related

INDEL marker IDs	Chromosomes/unanchored scaffolds	Physical positions (bp)	InDels (*Kabuli* reference genome-CDC Frontier/PII)	Forward primers (5'-3')	Reverse primers (5'-3')	Anneling temperature (0C)	Expected amplified product size (bp)	Structural annotation		Functional annotation	
								Sequence components of *kabuli* genome	*Kabuli* gene accession IDs	NCBI-KOG	TFs
Ca2_P2_31388368	Ca2	31388368	TTT/TTTTCTT	CATCAGTCCCCTCACTGGAT	CGAAACAGTTCCCGAAAGAA	60.2	866	DRR	Ca_12422	--	MYB_related
Ca2_P2_31388637	Ca2	31388637	CTTTT/CTTT	TGGGTCTAGTGGGTAAATGGA	CGAAACAGTTCCCGAAAGAA	60.2	180	DRR	Ca_12422	--	MYB_related
Ca2_P2_31392467	Ca2	31392467	TAAAAAAAAA/TAAAAAAAA	ATGTTCCGGCTATTCGAGTG	CTTCAAAATGCAGGGACGTT	60.1	708	INTERGENIC	--	--	--
Ca2_P2_31407170	Ca2	31407170	GAAAAAAA/GAAAAAAAA	TGGAACCCAGCCGTATAAAG	TCTTGCTCAAGGGTCATTCA	59.4	223	INTRON	Ca_12421	--	ERF
Ca2_P2_31407415	Ca2	31407415	AG/A	CCCTTGAGCAAGAGACTTGG	TCTTTTGCTCTTGTATTCCGAAC	59.8	199	INTRON	Ca_12421	--	ERF
Ca2_P2_31408666	Ca2	31408666	TATATATCAAA/TATATATCAAATATATCAAA	TCCCAATCTTTCTTCCCAAG	TGAGTGTCACGACCCATGAT	60.0	708	INTERGENIC	--	--	--
Ca2_P2_31410939	Ca2	31410939	CAAAA/CAAA	CGTTCGGAATGGGTAGAAAA	GGTGCAATGAAATGCCTCTT	60.1	536	DRR	Ca_12420	--	LBD
Ca2_P2_31419374	Ca2	31419374	TTCT/TT	GGCTTTCCATTTTGGGTTTT	TCCATTCACATGGGTGTGTC	60.2	202	INTERGENIC	--	--	--
Ca2_P2_31419525	Ca2	31419525	T/TGG	GGCTTTCCATTTTGGGTTTT	ATTCCAATGTGGAAGGACAA	57.8	258	INTERGENIC	--	--	--
Ca2_P2_31423587	Ca2	31423587	ATTTTTTTTTT/ATTTTTTTTTTTT	TTATTCCCCTTCCCCAAAAC	CTTTGCAAACCAATCAACCC	60.3	432	INTERGENIC	--	--	--
Ca2_P2_31423787	Ca2	31423787	GTTTTTTT/GTTTTTTTT	TTATTCCCCTTCCCCAAAAC	TCCAAAAATCCTTGGGAATG	59.7	800	INTERGENIC	--	--	--
Ca2_P2_31439958	Ca2	31439958	GAAAAAAA/GAAAAAAAA	CAAATGTCAAGGTGAAAATCCA	TGCTCCTTATGCCAACTCCT	59.8	492	INTERGENIC	--	--	--

INDEL marker IDs	Chromosomes/unanchored scaffolds	Physical positions (bp)	InDels (*Kabuli* reference genome-CDC Frontier/PII)	Forward primers (5'-3')	Reverse primers (5'-3')	Anneling temperature (0C)	Expected amplified product size (bp)	Structural annotation		Functional annotation	
								Sequence components of *kabuli* genome	*Kabuli* gene accession IDs	NCBI-KOG	TFs
Ca2_P2_31441363	Ca2	31441363	A/AAT	GGGTTTTCGGCCTTCTTAAA	GCTAAAACAAGTGTGCCGAA	59.0	470	INTERGENIC	--	--	--
Ca2_P2_31446059	Ca2	31446059	A/AT	GGCATAAGTCTGGCCTACGA	CAGAGGCCTATGTCCTGACC	59.7	624	INTERGENIC	--	--	--
Ca2_P2_31448144	Ca2	31448144	TAAAAAAAAA/TAAAAAAAAAA	TTATTGTGGGATTGCGTGAA	TTGTGCGATGGAATATTTGG	59.4	875	INTERGENIC	--	--	--
Ca2_P2_31449619	Ca2	31449619	GAAAAAAAAAAAAA/GAAAAAAAAA	TATGGACCGGAAATGGATGT	TCGTAGGATCGATTGGGAAG	60.0	559	INTERGENIC	--	--	--
Ca2_P2_31450111	Ca2	31450111	TCC/TC	TTCCCAATCGATCCTACGAG	ATTGTGGAGAATCTGCAGGG	60.1	313	INTERGENIC	--	--	--
Ca2_P2_31453758	Ca2	31453758	CACGTTGAT/CACGTTGATACGTTGAT	GTGCATTCTCGGTGTTCCTT	CAATCGTCAATTGTGATGGC	59.9	501	INTERGENIC	--	--	--
Ca2_P2_31456735	Ca2	31456735	ATTTTT/ATTTTTT	AGCGCGCAATATGTCTACCT	CCACTCTAGCTCGGTCCTTG	60.0	300	DRR	Ca_12418	--	Trihelix
Ca2_P2_3145944	Ca2	3145944	TTCT/TT	TCGAACTTCGCTTTGTTGTG	GCATTGCTGTGTGAAAATGC	60.3	907	INTERGENIC	--	--	--
Ca2_P2_31459966	Ca2	31459966	GTAATAATAATAATAATAA/GTAATAATAATAATAATAATAATAATAA	TGTTGGGGGTTTGAGGATAA	CCTATGCCTACGACATGCCT	60.1	519	INTERGENIC	--	--	--
Ca2_P2_3146125	Ca2	3146125	TAAAAA/TAAA	TCGATTACGGGGAATTCAAG	GCATTGCTGTGTGAAAATGC	60.3	543	INTERGENIC	--	--	--
Ca2_P2_31511966	Ca2	31511966	T/TAATATATA	CAATTGAGTTGCGACGAGAG	GCGACAGAGGAAGAAGATGG	60.0	276	INTERGENIC	--	--	--
Ca2_P2_31534378	Ca2	31534378	CG/CGG	AGGAAAGCAACGTGAACAGC	CCAAGACAGGCAAAGAAAGC	60.0	519	INTERGENIC	--	--	--

INDEL marker IDs	Chromosomes/unanchored scaffolds	Physical positions (bp)	InDels (*Kabuli* reference genome-CDC Frontier/PII)	Forward primers (5'-3')	Reverse primers (5'-3')	Anneling temperature (0C)	Expected amplified product size (bp)	Structural annotation		Functional annotation	
								Sequence components of *kabuli* genome	*Kabuli* gene accession IDs	NCBI-KOG	TFs
Ca2_P2_31534854	Ca2	31534854	TT/TTCT	GCTTTCTTTGCCTGTCTTGG	ACTTTGTCCACTGCCAAACC	60.0	331	INTERGENIC	--	--	--
Ca2_P2_3153753	Ca2	3153753	TATTAA/T	AGATGCGAGGGTGGACATAG	TCCAAGTTTTGAGGCTAGTGG	59.4	660	INTERGENIC	--	--	--
Ca2_P2_3154111	Ca2	3154111	A/AT	TTCGTCCTGTCATCCAAAAT	TTTGACGGTGAAAAAGTGATATG	59.0	975	INTERGENIC	--	--	--
Ca2_P2_31545554	Ca2	31545554	TC/T	AGAGGGACTAGCTACCCCGA	TGAGTGAAGCGAAATTATTCCA	59.7	709	INTERGENIC	--	--	--
Ca2_P2_31550288	Ca2	31550288	CTTTTT/CTTTTTT	ACAAATATCCCATCCCACGA	TGCACGTAAAATTGCCTAAAA	58.4	721	INTERGENIC	--	--	--
Ca2_P2_31552083	Ca2	31552083	A/AT	AATTTTTCAATCGCGTCTCG	TCACCCAACTCAACCTGAGA	59.2	928	INTERGENIC	--	--	--
Ca2_P2_31556890	Ca2	31556890	TTGGCCTGGCCTGGCCT/TTGGCCTGGCCTGGCCTGGCCT	TTAAATAGGCTTCCAGGCCA	GACCTTGGAGGTATTCGGGT	60.2	207	INTERGENIC	--	--	--
Ca2_P2_31559832	Ca2	31559832	CTATATATATATA/CTATATATATATATA	TTGCTCCAAATTGATCACGA	TTGGACCAATTTTGTGGCTT	60.3	678	INTERGENIC	--	--	--
Ca2_P2_31562756	Ca2	31562756	ACC/ACCC	CACTTAGGAATTCCCCACCA	AGCTTGAAACCCTTGACCCT	60.1	476	INTERGENIC	--	--	--
Ca2_P2_3156648	Ca2	3156648	GTCT/GTCTCT	GGTCAGAACAAAGGGTTGGA	GTTTGTTCCCTTCTAGGCCC	59.9	488	INTERGENIC	--	--	--
Ca2_P2_3158645	Ca2	3158645	AT/ATT	TTGCGACCCTATTTCCAAAC	TTCTTCATGCAACTTTTGCG	60.0	619	INTERGENIC	--	--	--
Ca2_P2_3158875	Ca2	3158875	GTATTATTATTATTATTAT/GTATTATTATTATTATTATTAT	CGCAAAAGTTGCATGAAGAA	TCTAAGAAATTTGCCCCAATG	59.1	295	INTERGENIC	--	--	--

INDEL marker IDs	Chromosomes/unanchored scaffolds	Physical positions (bp)	InDels (*Kabuli* reference genome-CDC Frontier/PII)	Forward primers (5'-3')	Reverse primers (5'-3')	Anneling temperature (0C)	Expected amplified product size (bp)	Structural annotation		Functional annotation	
								Sequence components of *kabuli* genome	*Kabuli* gene accession IDs	NCBI-KOG	TFs
Ca2_P2_3159298	Ca2	3159298	ATTTTTTTT/ATTTTTTTTTT	CGCAAAAGTTGCATGAAGAA	TGCAGGACGGTCTAACAATG	59.7	600	INTERGENIC	--	--	--
Ca2_P2_31593028	Ca2	31593028	AAACTTCAACTTCAACT/AAACTTCAACT	TCCTTCTGCCTCATTCTGCT	AGAAGCAGTTGCTGCATTGA	59.7	535	DRR	Ca_17855	U	bHLH
Ca2_P2_31593870	Ca2	31593870	CAAAAAA/CAAAAA	GCAGCAACTGCTTCTTGATG	CAATCGGTTTCGTTGTGATG	60.0	748	INTRON	Ca_17855	U	bHLH
Ca2_P2_31597025	Ca2	31597025	AATATATATATATATATATATATATATATATATATATAT/AATATATATATATATATATATATATATATATATATATAT	TCCTGTCCTTTGATCGGTTC	GCGCAACATGCAAGAAGTTA	60.0	251	INTERGENIC	--	--	--
Ca2_P2_31602924	Ca2	31602924	TATAATAATAATAAT/TATAATAATAAT	GGAACTCAGCAGCGGAATAG	TCCACACGCGTATGTGTTTT	60.0	404	INTRON	Ca_17854	--	SAP
Ca2_P2_31613310	Ca2	31613310	GAAAAAAAAAAA/GAAAAAAA	TACTGGCAATCTCAAACCCC	TGAAAACAACATCAACGGGA	59.9	557	INTERGENIC	--	--	--
Ca2_P2_31614676	Ca2	31614676	ATTTTTTTTT/ATTTTTTTT	TGCAAATTTAGATTTAGGGAGGA	TTGCATGGATGTAGTTGGGA	59.9	286	INTERGENIC	--	--	--
Ca2_P2_31616756	Ca2	31616756	AATATATATATATAT/AATATATATATAT	GGCCCTTCTTTTCTCGAACT	GAAGCCCTATTTCCATGTGGT	60.2	524	INTERGENIC	--	--	--
Ca2_P2_3161786	Ca2	3161786	TTCTCTCTC/TTCTCTCTCTC	GCATTGCATGCATGTGTTTT	CGTGCGCAGTGTGTATGAAT	60.8	342	INTRON	Ca_10531	S	--
Ca2_P2_31619110	Ca2	31619110	AT/ATT	AAGACTCCCTCTCCTCTGGC	ACACCATTCTGGCATTCTCC	59.9	667	INTERGENIC	--	--	--
Ca2_P2_3162145	Ca2	3162145	CAAAAAA/CAAAAA	CTCTTGCTTCTCTCTCCCCC	GGCCTTCTGTGAGAAGCATT	59.4	788	INTRON	Ca_10531	S	--
Ca2_P2_31622713	Ca2	31622713	ATTT/ATTTT	TGGATCCAATAGCTTTCCTCA	GGACCTGGTGGTAATTTGGA	59.6	976	DRR	Ca_17853	--	--

INDEL marker IDs	Chromosomes/unanchored scaffolds	Physical positions (bp)	InDels (*Kabuli* reference genome-CDC Frontier/PII)	Forward primers (5'-3')	Reverse primers (5'-3')	Anneling temperature (0C)	Expected amplified product size (bp)	Structural annotation		Functional annotation	
								Sequence components of *kabuli* genome	*Kabuli* gene accession IDs	NCBI-KOG	TFs
Ca2_P2_31624008	Ca2	31624008	GATTAATTAATTAATTA/GATTAATTAATTA	AAAGGGCACCACTTTTGTTG	TTGACATGTGATTGTACTTTTTAATTG	58.6	645	INTERGENIC	--	--	--
Ca2_P2_31634009	Ca2	31634009	CAAA/CAA	GGGTGAAGGTGAAGAAGTGC	TTGGGCTTGATAAAAGTGGG	59.9	524	DRR	Ca_17852	--	--
Ca2_P2_31635582	Ca2	31635582	TAAAAAAA/TAAAAAA	TTTCCATCTTCGTCTCAAATTAGT	TGAGAGATTTAATTTGGTCTTTGAA	58.4	527	INTRON	Ca_17852	--	--
Ca2_P2_31635915	Ca2	31635915	TCC/TCCC	AAAATATTTCCATCTTCGTCTCAAA	TCGACCATCTCATGATGGAA	60.0	848	INTRON	Ca_17852	--	--
Ca2_P2_31637112	Ca2	31637112	TAA/TA	GGGGTGTTGATGTGTCCTTT	GTGCCACAACAAGAAAACCC	60.4	596	INTERGENIC	--	--	--
Ca2_P2_31648369	Ca2	31648369	AATAT/AATATCTCATATAT	TTCACGTTGGAACTTGCTTG	ACCCGTTGGAAGATGAAATG	59.8	897	INTERGENIC	--	--	--
Ca2_P2_31657254	Ca2	31657254	CTTTTTTTT/CTTTTTTTTT	GAACGTGGAAAACTTCCATCA	GTTGGGAGGGTTCACTTCTG	59.5	570	INTERGENIC	--	--	--
Ca2_P2_3166305	Ca2	3166305	CTT/CTTT	CATGTTTTCAGCCCAACAAA	TGTCGGGTATAAAGATTGGTCA	59.3	190	INTERGENIC	--	--	--
Ca2_P2_3166543	Ca2	3166543	T/TA	CATGTTTTCAGCCCAACAAA	TTCCGCACATCAAACACAAT	60.0	849	INTERGENIC	--	--	--
Ca2_P2_31670858	Ca2	31670858	CGTCGCTGTCGCTGTCGCTGTCGCTG/CGTCGCTGTCGCTG	ACCGCCACCATTGTAGTCTC	CCGAGCCATCAAAAGATGAT	60.0	434	CDS (large-effect mutations)	Ca_17848	--	--
Ca2_P2_31677553	Ca2	31677553	GAAAAAAAA/GAAAAAAAAAA	TTGAAAAGGCATCTTGCACA	GCGCTATAAAAGACGCGCTA	60.6	688	INTERGENIC	--	--	--
Ca2_P2_31677730	Ca2	31677730	TAAAAAAAAAA/TAAAAAAAAAAAA	TTGAAAAGGCATCTTGCACA	GAGGGCGCTTCAGTTTACAG	60.0	859	INTERGENIC	--	--	--

INDEL marker IDs	Chromosomes/unanchored scaffolds	Physical positions (bp)	InDels (*Kabuli* reference genome-CDC Frontier/PII)	Forward primers (5'-3')	Reverse primers (5'-3')	Anneling temperature (0C)	Expected amplified product size (bp)	Structural annotation		Functional annotation	
								Sequence components of *kabuli* genome	*Kabuli* gene accession IDs	NCBI-KOG	TFs
Ca2_P2_31678452	Ca2	31678452	AAAAACAAATTTAA/AAAAACAAATTTAAAACAAATTTAA	CTGTAAACTGAAGCGCCCTC	TGGAAACCAAGGTCCAAGTC	59.9	560	INTERGENIC	--	--	--
Ca2_P2_31678680	Ca2	31678680	TC/T	GGAGCGCTTTAAACACAAGC	TGGAAACCAAGGTCCAAGTC	59.9	517	INTERGENIC	--	--	--
Ca2_P2_3169411	Ca2	3169411	TTCT/TACTCT	TTGTCGGTTTAAACTTGTGTGC	TGACATCGATATGTATGGACTCG	59.9	536	INTERGENIC	--	--	--
Ca2_P2_31694754	Ca2	31694754	A/AAT	GCTTTTCATAAGAAGCGTTGTAA	AATTGAGCTTGTTGATGTGGA	58.2	305	INTERGENIC	--	--	--
Ca2_P2_3169671	Ca2	3169671	ATTTTTTT/ATTTTTT	TTGTCGGTTTAAACTTGTGTGC	TTTTCCAAGGGATTTCAACTCT	59.1	892	INTERGENIC	--	--	--
Ca2_P2_31697056	Ca2	31697056	TTTT/TTTTTATTT	GTGTGCAGGTAAGGCTGACA	ATTTGGCATTGGGTTGGATA	60.0	759	INTERGENIC	--	--	--
Ca2_P2_31697285	Ca2	31697285	TCC/TCCCC	TGCATTGGTAAAAGTGTGCC	ATTTGGCATTGGGTTGGATA	60.0	380	INTERGENIC	--	--	--
Ca2_P2_31698036	Ca2	31698036	AATTATT/AATTATTATTATT	AAAATGGAAGGGCTTGGAGT	GGAAGGTTGAAAGAAGAGGGA	59.7	195	INTERGENIC	--	--	--
Ca2_P2_31713656	Ca2	31713656	TAAG/TAAGAAG	TCGGTTTCTCCTTCACCATC	TTGTCAAACACGCAATCCAT	60.0	595	INTERGENIC	--	--	--
Ca2_P2_31713823	Ca2	31713823	TAAAAAA/TAAAAA	TCGGTTTCTCCTTCACCATC	TTGTCAAACACGCAATCCAT	60.0	595	INTERGENIC	--	--	--
Ca2_P2_31728640	Ca2	31728640	AT/ATT	GGAGGGTATAAATGAAGCAGCA	AACTTGCGGCAAACAAAAAG	60.3	486	INTERGENIC	--	--	--
Ca2_P2_31728994	Ca2	31728994	AATTTATATTTATATTTA/AATTTATATTTA	GCTATTCACCGAAAGGGACA	AACTTGCGGCAAACAAAAAG	60.3	429	INTERGENIC	--	--	--

INDEL marker IDs	Chromosomes/unanchored scaffolds	Physical positions (bp)	InDels (*Kabuli* reference genome-CDC Frontier/PII)	Forward primers (5'-3')	Reverse primers (5'-3')	Anneling temperature (0C)	Expected amplified product size (bp)	Structural annotation		Functional annotation	
								Sequence components of *kabuli* genome	*Kabuli* gene accession IDs	NCBI-KOG	TFs
Ca2_P2_31736227	Ca2	31736227	T/TA	ACGTGAATCGTTGTGTCCA	CCGGAAAAGGTTTGTCACAT	59.8	381	DRR	Ca_17845	--	--
Ca2_P2_31743852	Ca2	31743852	TAAAAAAAA/TAAAAAAA	GAGGGCGCTTCAGTTTACAG	TGTCTCGTTGCACCCCTAAT	60.5	695	DRR	Ca_17844	--	--
Ca2_P2_31745106	Ca2	31745106	TCTTCGTCTCCGTCACCTTCGTCTCCGTCACC/TCTTCGTCTCCGTCACCTTCGTCTCCGTCACCTTCGTCTCCGTCACC	TGTGTTCCTCAACCCTGTCA	ATTTGGCATTGGGTTGGTTA	60.1	580	CDS (large-effect mutations)	Ca_17844	--	--
Ca2_P2_31746177	Ca2	31746177	AATAT/AATATAT	CGAGTTGTAAAATTTGGCTTCC	GGGGATGTGTATTAAAGGGGA	59.9	914	INTERGENIC	--	--	--
Ca2_P2_31780888	Ca2	31780888	CTTTTTTTTT/CTTTTTTTTTTT	TATTCCATGGCCAATTGTGA	CCCCCAACAGCTGATTAGAA	60.1	879	INTERGENIC	--	--	--
Ca2_P2_31781012	Ca2	31781012	TAAAAAAAA/TAAAAAAAAA	CATGTTTGCCTTTTGGATCA	CCCCCAACAGCTGATTAGAA	60.1	805	INTERGENIC	--	--	--
Ca2_P2_31794099	Ca2	31794099	TAA/TAAA	TGTGTACGTGTGCATGGAAG	ATCCTTGGCCTTTTGGCTAT	59.9	247	INTRON	Ca_17840	--	--
Ca2_P2_31794560	Ca2	31794560	AATATA/AATA	ATAGCCAAAAGGCCAAGGAT	TGCTGCTTCATTTCATAGCC	59.0	753	INTERGENIC	--	--	--
Ca2_P2_31794610	Ca2	31794610	GAAAAA/GAAAAAAA	ATAGCCAAAAGGCCAAGGAT	CTGGTCTGTCACTTTCAGCG	59.6	804	INTERGENIC	--	--	--
Ca2_P2_31795221	Ca2	31795221	T/TC	CGCTGAAAGTGACAGACCAG	TTCTTGTGCTTAGAACGTTTCTTG	60.0	627	INTERGENIC	--	--	--
Ca2_P2_31797437	Ca2	31797437	TAA/TA	ACAAAATCAAAAACGCCGAA	CATTTGGTGGCAAAATTGAA	59.4	108	INTERGENIC	--	--	--
Ca2_P2_31797941	Ca2	31797941	CATATATATATATATATATA/CATATATATATATATATATATA	TTTGCCACCAAATGTTCTTG	AAAAACGCAAACACCACTCC	60.0	579	INTERGENIC	--	--	--

INDEL marker IDs	Chromosomes/unanchored scaffolds	Physical positions (bp)	InDels (*Kabuli* reference genome-CDC Frontier/PII)	Forward primers (5'-3')	Reverse primers (5'-3')	Anneling temperature (0C)	Expected amplified product size (bp)	Structural annotation		Functional annotation	
								Sequence components of *kabuli* genome	*Kabuli* gene accession IDs	NCBI-KOG	TFs
Ca2_P2_31799043	Ca2	31799043	CTTTTTT/CTTTTTTT	AAATGTCAACGTGGCGTACA	GGTCCGTAAACGAGTTGTCC	59.5	320	INTERGENIC	--	--	--
Ca2_P2_31799253	Ca2	31799253	TAAAAAA/TAAAAA	AAATGTCAACGTGGCGTACA	GCACGAGGATTGCTATGTGA	59.8	832	INTERGENIC	--	--	--
Ca2_P2_31799508	Ca2	31799508	TATAATA/TATA	GACAACTCGTTTACGGACCC	GCACGAGGATTGCTATGTGA	59.8	531	INTERGENIC	--	--	--
Ca2_P2_31805812	Ca2	31805812	CATTATT/CATT	GTTACGCGCAAGATGTTTGA	AAGGCTGCAGCAATTTTCAT	59.9	344	INTERGENIC	--	--	--
Ca2_P2_31814386	Ca2	31814386	CAAAAAAA/CAAAAAA	GGAGGCAACAACAAGGAGAG	CCATCTTTCAAGCCAACTCC	59.7	685	INTRON	Ca_17839	--	--
Ca2_P2_31814697	Ca2	31814697	GAA/GCTGATAA	GGAGGCAACAACAAGGAGAG	GACGAGTTGCCATCCATCTT	60.1	698	INTRON	Ca_17839	--	--
Ca2_P2_31828138	Ca2	31828138	AA/AACGGAACTATAGCGTA	TTCGATTGGTGGACATGAGA	GAAAGGGTCATCCATTGTGG	60.2	892	INTRON	Ca_17836	--	MYB_related
Ca2_P2_31828278	Ca2	31828278	ATTGTT/ATTGTTGTT	AAAAATCGCCATTGTTTTGG	AAAAATAGGGAATCCGGTGC	60.2	510	INTRON	Ca_17836	--	MYB_related
Ca2_P2_31828392	Ca2	31828392	CTTTTTTT/CTTTTTT	AAAAATCGCCATTGTTTTGG	AAAAATAGGGAATCCGGTGC	60.2	510	INTRON	Ca_17836	--	MYB_related
Ca2_P2_31828525	Ca2	31828525	A/AG	AAAAATCGCCATTGTTTTGG	AAAAATAGGGAATCCGGTGC	60.2	510	INTRON	Ca_17836	--	MYB_related
Ca2_P2_3183388	Ca2	3183388	ATTT/ATTTT	CTAGAAGAACCACGCCGAAG	TGGCTGTCTCTAACCTCCAGA	60.0	588	INTRON	Ca_10528	T	HD-ZIP
Ca2_P2_31834171	Ca2	31834171	CGG/CGGG	TCACGCTCTGAGCTTCTCAA	CGTATCGTTTAGCGGTCGTT	60.2	882	INTRON	Ca_17835	V	bHLH

INDEL marker IDs	Chromosomes/unanchored scaffolds	Physical positions (bp)	InDels (*Kabuli* reference genome-CDC Frontier/PII)	Forward primers (5'-3')	Reverse primers (5'-3')	Anneling temperature (0C)	Expected amplified product size (bp)	Structural annotation		Functional annotation	
								Sequence components of *kabuli* genome	*Kabuli* gene accession IDs	NCBI-KOG	TFs
Ca2_P2_31838256	Ca2	31838256	GTGCGGTG/GTGCGGTGGAAATATGCGGTG	ACTTGACGATGGTTTTTGGC	CTAACCGCGAAACCAAACAT	60.0	393	DRR	Ca_17834	--	--
Ca2_P2_31842704	Ca2	31842704	ATTTTTT/ATTTTTTT	GCAGTATCTGAAACAAACGACAA	TTTTTCGGGGTTTGAACAAC	59.8	661	INTERGENIC	--	--	--
Ca2_P2_3184404	Ca2	3184404	AAAA/AAAATAAA	TGCATATCCTTGTGTTCCCA	CTTCCATGGGCTGTGAGAAT	60.1	486	INTRON	Ca_10528	T	HD-ZIP
Ca2_P2_31846061	Ca2	31846061	TTTATTATTATTATTATTATTATTATTATTATTATT/TTTATTATTATTATTATTATTATTATTATTATTATTATT	GATTGGAGCACCATCCTCTC	TTCGAGACAAAACGTTCAACC	60.1	805	INTERGENIC	--	--	--
Ca2_P2_31846722	Ca2	31846722	GAA/GAAGAAATTAA	TGGTTGGTTAGCCAAGTTTTG	GACAAGATCAGATGCAGCCA	60.0	472	INTERGENIC	--	--	--
Ca2_P2_31849042	Ca2	31849042	AAACAAATGAAC/AAAC	GACAAAATGGCACCGAACTT	TCCTTGATCTTTTCCATGGTATT	58.9	526	DRR	Ca_17833	--	--
Ca2_P2_31849105	Ca2	31849105	CTTTTTTT/CTTTTTTTTT	GACAAAATGGCACCGAACTT	GAGATGCCACCTCTTGGTGT	60.1	709	DRR	Ca_17833	--	--
Ca2_P2_31851053	Ca2	31851053	CACTTCTACTTCTAC/CACTTCTAC	ATCCCTCTCTCTCAATGGCA	TCCATAGGTCCCACTGAACC	59.8	201	INTERGENIC	--	--	--
Ca2_P2_31851416	Ca2	31851416	A/AG	AAAATTGGCCCCCAAACTAC	GAAGCAAGCATGCGAATTTA	59.1	775	INTERGENIC	--	--	--
Ca2_P2_3185239	Ca2	3185239	AAGA/AA	GATGTTGGAGGGCACCTAGA	GTGAAGGAGGGTTTGGAACA	59.9	661	INTRON	Ca_10528	T	HD-ZIP
Ca2_P2_31853436	Ca2	31853436	CTTTTTT/CTTTTT	GGCCGATGCTAAAATCAGTT	TTTTAGGTGGGCTACGGTTG	60.0	503	INTERGENIC	--	--	--
Ca2_P2_31853745	Ca2	31853745	ATTT/ATTATATTTT	CAACCGTAGCCCACCTAAAA	AATGCGCAAAGAAAGTTTGG	60.2	610	INTERGENIC	--	--	--

INDEL marker IDs	Chromosomes/unanchored scaffolds	Physical positions (bp)	InDels (*Kabuli* reference genome-CDC Frontier/PII)	Forward primers (5'-3')	Reverse primers (5'-3')	Anneling temperature (0C)	Expected amplified product size (bp)	Structural annotation		Functional annotation	
								Sequence components of *kabuli* genome	*Kabuli* gene accession IDs	NCBI-KOG	TFs
Ca2_P2_31853792	Ca2	31853792	AA/AAGA	CAACCGTAGCCCACCTAAAA	AATGCGCAAAGAAAGTTTGG	60.2	610	INTERGENIC	--	--	--
Ca2_P2_3185412	Ca2	3185412	TT/TTGT	CTTCAGCCTGCACAAATGAC	GTGAAGGAGGGTTTGGAACA	59.9	634	INTRON	Ca_10528	T	HD-ZIP
Ca2_P2_31854609	Ca2	31854609	ATTTTAAAAT/A	GTGTTCCGCCTGATTTCATT	TCAAGGAGAAAACACAACCAAA	59.6	349	INTERGENIC	--	--	--
Ca2_P2_31857740	Ca2	31857740	TAAA/TAA	CAAACTTGTTACACTGAGAGTGCAT	CATGCTTTCTCTACCACCTCAA	59.4	630	INTERGENIC	--	--	--
Ca2_P2_3185815	Ca2	3185815	GA/G	TGTTCCAAACCCTCCTTCAC	CTTTGTGGAGACAAGAGCCC	59.8	360	INTRON	Ca_10528	T	HD-ZIP
Ca2_P2_31859055	Ca2	31859055	C/CCGGCTT	TCCTCCCAAATTTGAAAAACA	AAATGAACCAAGCCAGACTTTC	59.6	407	INTERGENIC	--	--	--
Ca2_P2_31861522	Ca2	31861522	ATTTT/ATTT	TTGCAAAATGCTTGAGAGAAAA	ATGGCCTATGCCAATGAAAC	59.8	459	INTERGENIC	--	--	--
Ca2_P2_31871335	Ca2	31871335	CTATATATATATA/CTATATATATA	ATGGGTCAAAATGGGTGTGT	AGGCTGATTCAAACCCTGTG	60.1	290	INTRON	Ca_17832	--	FAR1
Ca2_P2_31871724	Ca2	31871724	GTATATATATATATATATA/GTATATATATATATATATA	CACAGGGTTTGAATCAGCCT	AAGCACAACTTGGTGGAAGG	60.1	554	DRR	Ca_17832	--	FAR1
Ca2_P2_3188198	Ca2	3188198	TTAAT/TT	GAGGGATGCATTTTCTGAGC	AAGTGACTTTCGTGAGACGGA	59.9	313	INTERGENIC	--	--	--
Ca2_P2_31882952	Ca2	31882952	CTTTATTTATTTAT/CTTTATTTATTTATTTAT	TTTCTGGTGGGATCAGCTCT	GACATCCTGCGCCAGTTATT	60.1	591	INTRON	Ca_17830	D	LBD
Ca2_P2_31883010	Ca2	31883010	AAGAG/AAG	TTTCTGGTGGGATCAGCTCT	GACATCCTGCGCCAGTTATT	60.1	591	INTRON	Ca_17830	D	LBD

INDEL marker IDs	Chromosomes/unanchored scaffolds	Physical positions (bp)	InDels (*Kabuli* reference genome-CDC Frontier/PII)	Forward primers (5'-3')	Reverse primers (5'-3')	Anneling temperature (0C)	Expected amplified product size (bp)	Structural annotation		Functional annotation	
								Sequence components of *kabuli* genome	*Kabuli* gene accession IDs	NCBI-KOG	TFs
Ca2_P2_31883642	Ca2	31883642	TAA/TAAA	AATAACTGGCGCAGGATGTC	TAAAGATGGCACCAGGAAGG	60.1	632	INTRON	Ca_17830	D	LBD
Ca2_P2_31884266	Ca2	31884266	GATAT/GAT	TAGCGCCAAGATTTCAGCTT	TGAAGGAAGCAAAAGTGGCT	60.0	506	INTRON	Ca_17830	D	LBD
Ca2_P2_3189167	Ca2	3189167	A/AATT	GAAGTTTACGGATCTCCGGTC	TCTCTTCCCCAAAAATTCAGA	58.7	652	INTERGENIC	--	--	--
Ca2_P2_31892669	Ca2	31892669	GAAAAAA/GAAAAAAA	TTTTGGTCTGTGAATCTGCG	TTCTACCCAAGAAAACATGCG	60.1	781	INTERGENIC	--	--	--
Ca2_P2_31892832	Ca2	31892832	TC/T	CATTTGGTCGGTCAAAAGAAA	TTCTACCCAAGAAAACATGCG	60.1	473	INTERGENIC	--	--	--
Ca2_P2_31892912	Ca2	31892912	GAAAAAAA/GAAAAAAAA	CATTTGGTCGGTCAAAAGAAA	TTCTACCCAAGAAAACATGCG	60.1	473	DRR	Ca_17828	I	C3H
Ca2_P2_31893581	Ca2	31893581	ATTTTTTTT/ATTTTTTT	CCTTCCCTTAAGTGGGGGTA	TTTATATTGGGTCGGTCGGA	60.1	308	DRR	Ca_17828	I	C3H
Ca2_P2_3189476	Ca2	3189476	GTTAAAAA/GTTAAAAATTAAAAA	TGTGTAGTGTTCGAATTTTTGC	CGGGGGATGTAGAATCTTTG	59.4	498	INTERGENIC	--	--	--
Ca2_P2_31896861	Ca2	31896861	GAAAAAAA/GAAAAAAAA	TGCAACCAATGTTCCCTACA	CTTAGAAGAGTGCATCCGCC	60.0	326	INTRON	Ca_17828	I	C3H
Ca2_P2_31901464	Ca2	31901464	TAA/TAAA	TTCCAACTACAGACCCGTCC	TGGAAGCTGGTACACAACCA	60.2	689	INTERGENIC	--	--	--
Ca2_P2_31901515	Ca2	31901515	TAA/TAAAA	TCCGCAAGCACACAGAATTA	TGGAAGCTGGTACACAACCA	60.2	566	INTERGENIC	--	--	--
Ca2_P2_31902363	Ca2	31902363	GCATGT/GCATGTTACATGT	AGGCTCATTTTCCTCCCACT	CCAACCACTACGACTCACCA	59.6	536	INTERGENIC	--	--	--

INDEL marker IDs	Chromosomes/unanchored scaffolds	Physical positions (bp)	InDels (*Kabuli* reference genome-CDC Frontier/PII)	Forward primers (5'-3')	Reverse primers (5'-3')	Anneling temperature (0C)	Expected amplified product size (bp)	Structural annotation		Functional annotation	
								Sequence components of *kabuli* genome	*Kabuli* gene accession IDs	NCBI-KOG	TFs
Ca2_P2_31902708	Ca2	31902708	TACTGA/TACTGACTGA	TGGTGAGTCGTAGTGGTTGG	CTGGTAAGAAGGGGTGACCA	60.0	268	DRR	Ca_17827	--	C3H
Ca2_P2_31903942	Ca2	31903942	GGTAT/GGTATTAGTAT	CATCAGTCTTTGGGGCAAGT	AAAGGCTTTGGTGGTTTTCA	59.6	445	DRR	Ca_17827	--	C3H
Ca2_P2_31905149	Ca2	31905149	CTAATTAAT/CTAAT	AATAAGGTGGTTTGGGGGAG	GGTATTCGTTGCGTCATGTG	60.0	450	INTRON	Ca_17827	--	C3H
Ca2_P2_31905190	Ca2	31905190	ATTTT/ATTTTT	AATAAGGTGGTTTGGGGGAG	GGTATTCGTTGCGTCATGTG	60.0	450	INTRON	Ca_17827	--	C3H
Ca2_P2_31905735	Ca2	31905735	CTACTATACT/CTACTATACTATACTATACT	TTCCAACGGTGAAAACAACA	CGATATAGGGTTGGTTTGCG	60.3	207	DRR	Ca_17826	O	--
Ca2_P2_31905894	Ca2	31905894	GAAAAAA/GAAAAA	CACATGACGCAACGAATACC	TTTGAAGGCCACGATTTATTT	58.6	512	DRR	Ca_17826	O	--
Ca2_P2_31905921	Ca2	31905921	TAAAAAAAAA/TAAAAAAAA	CACATGACGCAACGAATACC	TGATAGTATTTGAAGGCCACGA	59.6	520	DRR	Ca_17826	O	--
Ca2_P2_31907449	Ca2	31907449	CTT/CTTT	CGGTGACGGAGTTCAAGATT	CTGACCGTCCAAAGGAGGTA	60.1	531	INTRON	Ca_17826	O	--
Ca2_P2_31912393	Ca2	31912393	TTATCTA/TTATCTATCTA	TGAGAAATCTCCATCCCCAG	CGCATCCTTGACAACTCTGA	60.0	650	INTRON	Ca_17826	O	--
Ca2_P2_31916232	Ca2	31916232	C/CG	TTTGCCCAAGAACAAGAACC	AAAAGGGGAAACCCTTTGTG	60.2	463	DRR	Ca_17825	O	--
Ca2_P2_31920016	Ca2	31920016	AATTATAATCAATTAT/AATTATAATCAATTATTATAATCAATTAT	TTCAGAAGAAGGAAACAGCACA	GATCCACAAAATTGGTTTTTCA	58.8	877	INTERGENIC	--	--	--
Ca2_P2_31921460	Ca2	31921460	TA/TAA	GGAAAGTCAAACTCTTAAATGGGA	TCTGTATCGCATGGTTGCTT	59.3	533	INTERGENIC	--	--	--

INDEL marker IDs	Chromosomes/unanchored scaffolds	Physical positions (bp)	InDels (*Kabuli* reference genome-CDC Frontier/PII)	Forward primers (5'-3')	Reverse primers (5'-3')	Anneling temperature (0C)	Expected amplified product size (bp)	Structural annotation		Functional annotation	
								Sequence components of *kabuli* genome	*Kabuli* gene accession IDs	NCBI-KOG	TFs
Ca2_P2_31922902	Ca2	31922902	CATA/CATATATA	AAAGGTGTTGGGATTGTCATT	GCTTGTTATGGCTTGATCACC	59.6	394	INTERGENIC	--	--	--
Ca2_P2_31924149	Ca2	31924149	TAA/TA	TGCAAGCATTCTTCGTTCTG	TCGGTATCGATTCCAACCAT	60.2	515	INTERGENIC	--	--	--
Ca2_P2_31924611	Ca2	31924611	ATAATTTTGCT/AT	TACCTTGCGGTTTGGATGTT	GAAGGTTAGGGAGGCTGGTC	60.1	523	INTERGENIC	--	--	--
Ca2_P2_31925279	Ca2	31925279	GTTTTTTTTT/GTTTTTTTT	GACCAGCCTCCCTAACCTTC	TGTTGCCGATAGACCATGAA	60.1	538	INTERGENIC	--	--	--
Ca2_P2_31926359	Ca2	31926359	CTTTTTTTTTTTTTTT/CTTTTTTTTTTT	GTGGGAGATGCAATCCAAGT	TTGCTGAGTTTCGTTTGGTT	58.4	848	DRR	Ca_17824	--	ERF
Ca2_P2_31930201	Ca2	31930201	AATTATTATTATTATTGTTATTATTATTA/AATTATTATTATTATTATTGTTATTATTATTA	CGACCTCCCAAAGGATTTTT	TGTCGTTATGAGGAATTTACCG	59.0	444	INTERGENIC	--	--	--
Ca2_P2_31931300	Ca2	31931300	T/TAA	TCGTAACGCAAATTTTCCAA	TCCATCTCCAATTTTGTTTTCA	59.4	377	INTERGENIC	--	--	--
Ca2_P2_31937123	Ca2	31937123	T/TG	TTGAACCCAAGACTTTGTAATGAA	GGTTAAAGACGTAACAAGAAAAACG	59.6	614	INTERGENIC	--	--	--
Ca2_P2_31937481	Ca2	31937481	TTTC/T	AATGCCATATTGGATTTTGTTAC	TCCGATCGTTCAAATTTTCC	59.9	661	INTERGENIC	--	--	--
Ca2_P2_31939044	Ca2	31939044	ATTTTTTTT/ATTTTTTT	ATCACATTGCAAAGCAACGA	TGTATTCACGGTTTGGGTTTT	59.2	586	DRR	Ca_17823	R	C3H
Ca2_P2_31939650	Ca2	31939650	CAAAAAAAA/CAAAAAAAAA	GACCCCAAGAGAGAAAAGGG	TTTTGGCCATTCCTCAGAAC	60.1	482	DRR	Ca_17823	R	C3H
Ca2_P2_31943060	Ca2	31943060	TTTGT/TT	AGGCAGAGATTGAGCCGTAA	TATGCTTGATCGCACATGGT	60.1	494	INTRON	Ca_17823	R	C3H

INDEL marker IDs	Chromosomes/unanchored scaffolds	Physical positions (bp)	InDels (*Kabuli* reference genome-CDC Frontier/PII)	Forward primers (5'-3')	Reverse primers (5'-3')	Anneling temperature (0C)	Expected amplified product size (bp)	Structural annotation		Functional annotation	
								Sequence components of *kabuli* genome	*Kabuli* gene accession IDs	NCBI-KOG	TFs
Ca2_P2_31944652	Ca2	31944652	T/TC	TCCACGTGGCAACTCAAATA	TGAAATTTGGAGTCGTACCCT	58.6	834	INTERGENIC	--	--	--
Ca2_P2_31945382	Ca2	31945382	AAT/A	AGGGTACGACTCCAAATTTCA	TTCCTTTAGCCTTCCATGTG	57.8	600	INTERGENIC	--	--	--
Ca2_P2_31945753	Ca2	31945753	TAT/TATTTAAT	CACATGGAAGGCTAAAGGAA	TTGAGTACCAAGGCATTGACC	60.0	614	INTERGENIC	--	--	--
Ca2_P2_31945989	Ca2	31945989	ATTTTTTTTT/ATTTTTTT	CACATGGAAGGCTAAAGGAA	CCACAAGTGTGGATGACAGG	60.0	838	INTERGENIC	--	--	--
Ca2_P2_31950497	Ca2	31950497	CTTT/CTTTT	AACAAAAAGGCCATAGCCAA	CACTAGCGTGCGAGTTTATCTG	60.1	821	INTERGENIC	--	--	--
Ca2_P2_31950818	Ca2	31950818	GTTATAC/GTTATACGAAAATAAATTATAC	CCAATTAATAAGTTAGCGGGTGA	TGTGTGAGGAGGACAACAAAA	59.2	870	INTERGENIC	--	--	--
Ca2_P2_31958606	Ca2	31958606	AT/ATGTT	ACGTTGTCGGACATTCATCA	TTGCAACTGACTTGAATCCG	59.8	606	INTERGENIC	--	--	--
Ca2_P2_31963928	Ca2	31963928	GTACTT/GTACTTACTT	CCTCGAGAAACCCAGCTAGA	GCATTCATGAGTTTGCCCTT	60.1	187	INTERGENIC	--	--	--
Ca2_P2_31964265	Ca2	31964265	TG/T	AAGGGCAAACTCATGAATGC	GGTGTGACAAAGGAAACGTG	59.0	674	INTERGENIC	--	--	--
Ca2_P2_31964495	Ca2	31964495	G/GA	AAGGGCAAACTCATGAATGC	TTGATGGTCACCTTTGTTGC	59.5	792	INTERGENIC	--	--	--
Ca2_P2_31973893	Ca2	31973893	TGTTTCTACAGCTACAACAGTTTCTACAGCTACAACA/TGTTTCTACAGCTACAACA	TTTCAAAGACCGAGTTGGAGA	TGTCCATTCAAAATACGGCA	59.9	327	INTERGENIC	--	--	--
Ca2_P2_31979574	Ca2	31979574	ACC/A	TATGGGGAATAGGTGGTGGA	GGGGAAGGGAAATTTGAGAG	59.9	323	DRR	Ca_17820	--	--

INDEL marker IDs	Chromosomes/unanchored scaffolds	Physical positions (bp)	InDels (*Kabuli* reference genome-CDC Frontier/PII)	Forward primers (5'-3')	Reverse primers (5'-3')	Anneling temperature (0C)	Expected amplified product size (bp)	Structural annotation		Functional annotation	
								Sequence components of *kabuli* genome	*Kabuli* gene accession IDs	NCBI-KOG	TFs
Ca2_P2_31981326	Ca2	31981326	AT/ATTT	ACGTTGTGTGGACAATGAGG	TCCGGAGGGTTTTTCTACCT	59.9	353	INTERGENIC	--	--	--
Ca2_P2_31991643	Ca2	31991643	ATTTTTTTTTTT/ATTTTTTTTTTTTT	TGGTCAATGAAATTTCCGGT	GGTTGTGTGTTTTCCATCCC	60.1	230	INTERGENIC	--	--	--
Ca2_P2_3199733	Ca2	3199733	T/TATGG	GAGCATGTGTTGGTGAATGG	TCACCGGAGGTTAGATCTCG	60.2	309	INTERGENIC	--	--	--
Ca2_P2_3199849	Ca2	3199849	TT/TTATTATTAGT	GAGCATGTGTTGGTGAATGG	TCACCGGAGGTTAGATCTCG	60.2	309	INTERGENIC	--	--	--
Ca2_P2_3199930	Ca2	3199930	TCCGG/TCCGGCCGG	GAGCATGTGTTGGTGAATGG	AATTTTATTTGAGACACGTCGAA	57.9	691	INTERGENIC	--	--	--
Ca2_P2_32000117	Ca2	32000117	TC/T	TAAGTTGGGATTGAGCCACA	TCAATGAAGGTGCATTTGGT	59.0	596	INTERGENIC	--	--	--
Ca2_P2_3200285	Ca2	3200285	TCA/T	CGAGATCTAACCTCCGGTGA	TTGATGCATGCTGCTACAAAT	59.3	811	DRR	Ca_10527	K	HD-ZIP
Ca2_P2_3200442	Ca2	3200442	CAT/CATAT	GGAGTTGAATTAGCCTCTTTATCC	TTGATGCATGCTGCTACAAAT	59.3	503	DRR	Ca_10527	K	HD-ZIP
Ca2_P2_3200504	Ca2	3200504	AA/AAAAAGATA	CATTTCCCGATTTTAGATTATGC	AATTGAAGGAAACATTTGCCA	59.4	511	DRR	Ca_10527	K	HD-ZIP
Ca2_P2_3200632	Ca2	3200632	CAATT/CAATTAATT	AAGGAGTATCAACCAAAACAGAGG	AATTGAAGGAAACATTTGCCA	59.4	423	DRR	Ca_10527	K	HD-ZIP
Ca2_P2_32008641	Ca2	32008641	GAATTAATTTT/GAATTAATTTTATAAAATTAATTTT	TTTTGTTTCAGAGACGCTTTTG	CACCAACGCAACTTGACATC	60.2	731	INTERGENIC	--	--	--
Ca2_P2_3201185	Ca2	3201185	ATTTTTTTTT/ATTTTTTTTTT	TTTGGCAAATGTTTCCTTCA	GAGTGGGGGTTGGAGGTAGT	60.2	405	DRR	Ca_10527	K	HD-ZIP

INDEL marker IDs	Chromosomes/unanchored scaffolds	Physical positions (bp)	InDels (*Kabuli* reference genome-CDC Frontier/PII)	Forward primers (5'-3')	Reverse primers (5'-3')	Anneling temperature (0C)	Expected amplified product size (bp)	Structural annotation		Functional annotation	
								Sequence components of *kabuli* genome	*Kabuli* gene accession IDs	NCBI-KOG	TFs
Ca2_P2_3201946	Ca2	3201946	ATTTTTT/ATTTTTTT	ATGATTCCAAAGCACCAACA	GGGATTGCTTTGAAAATGGA	59.9	908	DRR	Ca_10527	K	HD-ZIP
Ca2_P2_32024632	Ca2	32024632	AC/ACC	GCATCAAACCAACCTTAATCG	CACATCACTCGCTCAATGCT	60.0	516	INTERGENIC	--	--	--
Ca2_P2_3203039	Ca2	3203039	AT/A	TGGATTTCTGACACTGCCAC	ATGCTCTTCCCTGTCTCCAA	59.8	348	INTRON	Ca_10527	K	HD-ZIP
Ca2_P2_32034535	Ca2	32034535	AATATATA/AATATATATATATATA	CGGTGTTGAAGATGGGAAAT	GCACTTTTGAAGGCTGAAGG	60.0	874	INTRON	Ca_17818	R	--
Ca2_P2_3204387	Ca2	3204387	T/TATATATA	AAAGGTGCACCACTACCACC	ACTCCTTCCCTTCCTTTCCA	60.0	502	INTRON	Ca_10527	K	HD-ZIP
Ca2_P2_32046007	Ca2	32046007	AATAACATTATAACATTAT/AATAACATTAT	CAATTGGCGGACATTAAAAA	AGAATGTGTCTCGTGCGTTG	59.9	803	INTERGENIC	--	--	--
Ca2_P2_32048288	Ca2	32048288	AC/A	CCAAATCGAACTCTCTCCTCA	CGTGATGCACCGATAAATTG	60.0	634	DRR	Ca_17817	R	--
Ca2_P2_32054569	Ca2	32054569	CATCATTAT/CATCATTATCATTAT	TGATGACATGTCGTAATGGGA	GGGCATTCAAATTTTTCTATCG	59.8	809	INTERGENIC	--	--	--
Ca2_P2_3205849	Ca2	3205849	TC/T	ATTGTGGAGACATAACCGCC	ACATCGAGACGTACGGGAAA	60.5	284	INTERGENIC	--	--	--
Ca2_P2_32060578	Ca2	32060578	CATATATAT/CATATAT	TTGACGCTGAAAATCCATCA	CCATCTCGTTTCCATTGAGG	60.5	498	INTRON	Ca_17816	R	--
Ca2_P2_32061716	Ca2	32061716	A/AG	GGTTCTGCCTTGCAAATGAT	TGCCATTCTTGTTGCTTCTG	60.0	640	INTRON	Ca_17816	R	--
Ca2_P2_32080788	Ca2	32080788	TAAAAAAAAAAAAA/TAAAAAAAAA	TGTGCATTAAAGATGCTCTTGG	AAGCGTTGAAACCACTGTGA	59.3	441	INTERGENIC	--	--	--

INDEL marker IDs	Chromosomes/unanchored scaffolds	Physical positions (bp)	InDels (*Kabuli* reference genome-CDC Frontier/PII)	Forward primers (5'-3')	Reverse primers (5'-3')	Anneling temperature (0C)	Expected amplified product size (bp)	Structural annotation		Functional annotation	
								Sequence components of *kabuli* genome	*Kabuli* gene accession IDs	NCBI-KOG	TFs
Ca2_P2_32081128	Ca2	32081128	GTTT/GTT	TCAGCTGGTGCAAAACAAAG	CCCTTCATCTCTACTCACCTGG	60.1	226	INTERGENIC	--	--	--
Ca2_P2_32088009	Ca2	32088009	TTAATAATAATAATAATAATAATAATA/TTAATAATAATAATAATAATAATAATAATA	TCAGCAAGCAAGCATTATGG	ATGGGGAACGTTGACAGAAG	60.0	605	INTERGENIC	--	--	--
Ca2_P2_32095647	Ca2	32095647	T/TCACACGTGAC	TTGCTTAAACCACATGTTTTCATA	GCCATAAAACGTGTCAAGCA	59.7	623	INTERGENIC	--	--	--
Ca2_P2_32102387	Ca2	32102387	TAAA/TAAAAAAA	TTCCAAACGGAATCCATCAT	TGTGGTCTTCCTTTGGCACT	60.7	308	INTERGENIC	--	--	--
Ca2_P2_32109642	Ca2	32109642	TGAG/TGAGAG	TGGTTGCCTCCTTAGTTTGG	GTTTGCTTCTTGAAGGGTGC	59.9	504	INTERGENIC	--	--	--
Ca2_P2_32112721	Ca2	32112721	T/TAAA	TTGTCTTTCCTATGCCACCC	TAAAGGACATGTGCAAACGC	59.7	508	INTERGENIC	--	--	--
Ca2_P2_32117375	Ca2	32117375	CGG/CTGAGGAAGG	GGACGTGGGTAACATAAACCAT	TTCTAGAGGCGAGTAATGTAGAAAAA	59.1	644	INTERGENIC	--	--	--
Ca2_P2_32127033	Ca2	32127033	TGC/TGCGC	TGCAAGTTGTGATGGGTTGT	TGTCTGTTCTGCACGGAGTC	60.0	650	INTRON	Ca_17812	O	--
Ca2_P2_32130215	Ca2	32130215	ATTGAA/ATTGAATTTGAATTTGAA	CCGGGTCAGTTTCCTTCTTT	ACTCCACCAACTTCCAATGC	60.0	388	INTRON	Ca_17811	J	--
Ca2_P2_32137242	Ca2	32137242	ATT/ATTT	GTGTGAAAGGTCCTGCCTGT	CCCCTGCTACTCCTCTGACA	60.4	390	INTRON	Ca_17809	J	--
Ca2_P2_32145004	Ca2	32145004	TA/TACA	GGCCTTCAAAATTCAATGAGA	TGGATACCTAGCACCTTAGCAA	58.9	598	INTRON	Ca_17808	--	G2-like
Ca2_P2_32146442	Ca2	32146442	AT/ATT	ATTCCCACGCCACTTAGATG	CACTGTCACAGAGATAAGATGCAA	59.4	807	INTRON	Ca_17808	--	G2-like

INDEL marker IDs	Chromosomes/unanchored scaffolds	Physical positions (bp)	InDels (*Kabuli* reference genome-CDC Frontier/PII)	Forward primers (5'-3')	Reverse primers (5'-3')	Anneling temperature (0C)	Expected amplified product size (bp)	Structural annotation		Functional annotation	
								Sequence components of *kabuli* genome	*Kabuli* gene accession IDs	NCBI-KOG	TFs
Ca2_P2_32154593	Ca2	32154593	GGT/GGTTATATGT	ATTCGAAAAAGGGCATTTGA	GAAGCTTGGCTTTGGTTGTC	59.9	691	INTERGENIC	--	--	--
Ca2_P2_32161753	Ca2	32161753	TCCCC/TCCC	AGGCCATTAAGCATGACACC	TTCTTGGAACCCTGCATACC	59.9	867	INTERGENIC	--	--	--
Ca2_P2_32161954	Ca2	32161954	ATTTT/ATT	GTCCCCAGACTTGGCTGTAA	AAACATTTGCGGTTTCTTGG	60.0	638	INTERGENIC	--	--	--
Ca2_P2_32163428	Ca2	32163428	CATA/CATATATATATA	TCAAAGAGACATGATTTTGTCCA	GGTCAATCAGCTGTGAGCAA	60.0	415	INTERGENIC	--	--	--
Ca2_P2_32168741	Ca2	32168741	ATA/ATAGTAAATTA	CCTGGCCATTGTTCCTTAGA	AAATGAGTAGATGGTGCAACGA	59.6	687	INTERGENIC	--	--	--
Ca2_P2_32173774	Ca2	32173774	TC/T	ATGTTTTGATTGGGGCTTTG	TAAATCAGCCCTTTTGGTGG	59.9	631	DRR	Ca_17806	--	WRKY
Ca2_P2_32179090	Ca2	32179090	TATTATAAAATAAATTATAAAATAA/TATTATAAAATAA	CGACATTTATGAGTGATTAGGTGG	CAACAATTTCTCATGCTCGC	59.4	292	INTERGENIC	--	--	--
Ca2_P2_32182829	Ca2	32182829	GATATATATATATATATATATATATATATATATATA/GATATATATATATATATATATATATATATATATA	TGAAATTCTGAATGGTGGGAG	TGACTAACTTTGTGCGGTCG	59.9	886	INTERGENIC	--	--	--
Ca2_P2_32203826	Ca2	32203826	GATATATATATATATATATATA/GATATATATATATATATATATA	GGGCAATTCCTGCAATTTTA	GATGCACCGAATAAGGAAGC	59.7	390	INTRON	Ca_10295	--	G2-like
Ca2_P2_32209131	Ca2	32209131	T/TG	GCAAAGATAGACACCACCCC	AACCATTCCCATGGAACAAA	60.0	577	INTERGENIC	--	--	--
Ca2_P2_32209334	Ca2	32209334	CTATATATA/CTATA	TCCATGGGAATGGTTTTGAT	TTGAAGAGGAAGTTTTGGCA	58.5	342	INTERGENIC	--	--	--
Ca2_P2_32209671	Ca2	32209671	TC/TCC	TCCATGGGAATGGTTTTGAT	TTTTAGTGTGCACCAATGTCAA	59.1	899	INTERGENIC	--	--	--

INDEL marker IDs	Chromosomes/unanchored scaffolds	Physical positions (bp)	InDels (*Kabuli* reference genome-CDC Frontier/PII)	Forward primers (5'-3')	Reverse primers (5'-3')	Anneling temperature (0C)	Expected amplified product size (bp)	Structural annotation		Functional annotation	
								Sequence components of *kabuli* genome	*Kabuli* gene accession IDs	NCBI-KOG	TFs
Ca2_P2_32210099	Ca2	32210099	CA/CAAATA	TCAGCTTCAATTATCTCCAACAA	TCGGCGACAAAAATCACATA	60.1	644	INTERGENIC	--	--	--
Ca2_P2_32210241	Ca2	32210241	TA/T	TGACATTGGTGCACACTAAAAA	CCACGTCATCTTCCTTTGGT	60.0	505	INTERGENIC	--	--	--
Ca2_P2_32211970	Ca2	32211970	A/AG	ATGGAATTCTGAATGGTGGG	AAAACCACAAAATGGGGTCA	60.1	763	INTERGENIC	--	--	--
Ca2_P2_32213171	Ca2	32213171	GTGAT/GTGATGAT	CAACATGACAAGGACTTTCCAA	TTACCGGTATCGCAAGGAAC	60.0	529	INTERGENIC	--	--	--
Ca2_P2_32213424	Ca2	32213424	CAAA/CAAAA	AGGGATAGATAACGGCACGA	AAGGCCCTGCTAGAATGATG	59.3	504	INTERGENIC	--	--	--
Ca2_P2_32234513	Ca2	32234513	AAAATTTGA/AAAATTTGAATTAAATTTGA	TCGCATCCGTATTCGTTTTT	TAACCCGGTTTATCCAATCG	59.7	778	INTERGENIC	--	--	--
Ca2_P2_32236823	Ca2	32236823	TATTTGA/TATTTGAATTTGA	TTCGGGTTAAACAAAGTGCC	CCCCAGCATCAAGAAAAAGA	60.2	482	INTRON	Ca_10293	--	--
Ca2_P2_32240359	Ca2	32240359	TAAGAAA/TAA	GGCGTCTTAAGCAAGGTGTC	CTGAGGGTGACGTCTCACAA	59.9	525	DRR	Ca_10293	--	--
Ca2_P2_32241009	Ca2	32241009	GAAAAAAAAAAAA/GAAAAAAAAA	TGAGACGTCACCCTCAGTTG	GAGGCCAAAGGTCCAATATG	59.4	568	DRR	Ca_10293	--	--
Ca2_P2_32241156	Ca2	32241156	TA/TAA	TTCATCGAATAACGAGATGGG	TGCTTGCCAATTCATGTCTC	59.8	711	DRR	Ca_10293	--	--
Ca2_P2_32241234	Ca2	32241234	AATATATATATATATAT/AATATATATATATAT	CATATTGGACCTTTGGCCTC	TGCTTGCCAATTCATGTCTC	59.8	434	DRR	Ca_10293	--	--
Ca2_P2_32243132	Ca2	32243132	TCAA/TCAACAA	TCTGAGGGGATTCGTTATGC	TCCATAACTCTTGCCAACCC	59.9	524	CDS (large-effect mutations)	Ca_10292	--	bHLH

INDEL marker IDs	Chromosomes/unanchored scaffolds	Physical positions (bp)	InDels (*Kabuli* reference genome-CDC Frontier/PII)	Forward primers (5'-3')	Reverse primers (5'-3')	Anneling temperature (0C)	Expected amplified product size (bp)	Structural annotation		Functional annotation	
								Sequence components of *kabuli* genome	*Kabuli* gene accession IDs	NCBI-KOG	TFs
Ca2_P2_32244010	Ca2	32244010	GTTTTT/GTTTT	CCTTGGTCATTGTGTTGCTG	CGGAAAAACATGAAGGGAAA	59.9	397	DRR	Ca_10292	--	bHLH
Ca2_P2_32245556	Ca2	32245556	TTAAGTGGGTAAT/TT	GCCAAAATTAAACATTGGAAACA	CATGCAATCTTTTGTCGGTTT	60.0	298	DRR	Ca_10292	--	bHLH
Ca2_P2_32245950	Ca2	32245950	TA/TAGA	AACCGACAAAAGATTGCATGT	GCAGTTGCACAAGGTGGTAA	59.8	586	INTERGENIC	--	--	--
Ca2_P2_32246591	Ca2	32246591	AAACCAACCA/AAACCA	TACCACCTTGTGCAACTGCT	TGTGGTATGTGCCAAATGGT	59.7	604	INTERGENIC	--	--	--
Ca2_P2_32249149	Ca2	32249149	ATGTG/ATG	GCTTTGGTCACTTGCTGACA	TTGTTGAAACCAGCAATGGA	60.1	472	INTRON	Ca_10291	E	C3H
Ca2_P2_32252298	Ca2	32252298	CATTAAATAA/CATTAAATAAATTAAATAA	CCGAGGAGGGATTTTTCTTC	TGTATGTCCGTTGGGTTTTT	57.9	695	INTERGENIC	--	--	--
Ca2_P2_32255815	Ca2	32255815	T/TTA	TTGATTATTTTGGCCCCGTA	CGTTGCTCTTGTGGTAAACG	59.4	379	DRR	Ca_10289	K	TALE
Ca2_P2_32260922	Ca2	32260922	ATACACATGTA/ATA	TGTTGTTTCCTCCAAACCCT	TTTTACCTTCTTTTCCGGGG	60.3	157	INTERGENIC	--	--	--
Ca2_P2_32268747	Ca2	32268747	GAAAA/GAAAAA	CATGCTGCCTCCTTAGGTGT	TGGGGAGCTTTTCATCAATC	60.0	775	INTERGENIC	--	--	--
Ca2_P2_32282292	Ca2	32282292	CATTAGTGATT/CATTAGTGATTAGTGATT	ACCCTCCAGGGTGCTAAGAT	GCCAACATTTTCTGGTTGCT	60.1	726	INTRON	Ca_10287	S	--
Ca2_P2_32291118	Ca2	32291118	AG/A	TGTGCCAATAATTTGGTTGC	AAAGCTTTGGAGGCATGCTA	60.0	532	INTERGENIC	--	--	--
Ca2_P2_32293632	Ca2	32293632	AAGAAATTCAGAAATTCA/AAGAAATTCAGAAATTCAGAAATTCA	AAACCCTACGAAGTTTGGCA	TTGTCACCCGTCCAAATACA	59.8	237	INTERGENIC	--	--	--

INDEL marker IDs	Chromosomes/unanchored scaffolds	Physical positions (bp)	InDels (*Kabuli* reference genome-CDC Frontier/PII)	Forward primers (5'-3')	Reverse primers (5'-3')	Anneling temperature (0C)	Expected amplified product size (bp)	Structural annotation		Functional annotation	
								Sequence components of *kabuli* genome	*Kabuli* gene accession IDs	NCBI-KOG	TFs
Ca2_P2_32324833	Ca2	32324833	TAAAAAAAAAAA/TAAAAAAAAAAAA	CCGAGAAGTTTACCAATTGTTTG	TGAATGGCCGTTTTCTATGG	60.8	694	INTERGENIC	--	--	--
Ca2_P2_32324896	Ca2	32324896	ATTTTTTTTTTTT/ATTTTTTTTT	CCGAGAAGTTTACCAATTGTTTG	TATTACCACGCGAAACACGA	60.1	896	INTERGENIC	--	--	--
Ca2_P2_32327172	Ca2	32327172	CT/CTAT	GTCACGCCACCGGTTAGTAT	GCACCGATAGTGAAGGGAAA	60.1	358	INTRON	Ca_10285	R	--
Ca2_P2_32327707	Ca2	32327707	GT/GTAATGATAT	TTTCCCTTCACTATCGGTGC	CAAATTTGCAATTGTGCCTG	60.1	660	DRR	Ca_10285	R	--
Ca2_P2_32332253	Ca2	32332253	CTTTTTTTTT/CTTTTTTTTTTT	TTGCAAATTTGTTGAAGAAAGG	TCGTCGTTAAATTGCGACAC	59.7	480	INTERGENIC	--	--	--
Ca2_P2_32332565	Ca2	32332565	AAAATAAATAAATAAATA/AAAATAAATAAATAAATAAATA	CGCCAAAGTCATTGATTGAA	CATCTTGTCAGCCATAGCCA	59.8	338	INTERGENIC	--	--	--
Ca2_P2_32340350	Ca2	32340350	TGG/TG	TCCATTCAATGCTTTCAAATTC	TTGTGGTCATTTAAGGTGGTTG	59.8	530	INTERGENIC	--	--	--
Ca2_P2_32341023	Ca2	32341023	GAAT/GAATGTTAAAAT	CAACCACCTTAAATGACCACAA	AAAAACAATGTCATGCAGAGG	57.7	682	INTERGENIC	--	--	--
Ca2_P2_32348102	Ca2	32348102	GTTTTTTTTTT/GTTTTTTTT	GCAGTTGTGCAAGAACCTCA	TCGCTGACCCAAATATCTCC	60.0	629	INTRON	Ca_10283	--	--
Ca2_P2_32349769	Ca2	32349769	GTTTTTTTT/GTTTTTTTTTTTTTTTTTT	AAGTTGGAGGCTCAAAAGCA	AAAATTGCAAATGCACACCA	60.0	661	INTRON	Ca_10283	--	--
Ca2_P2_32349885	Ca2	32349885	ATGT/ATGTGT	AAGTTGGAGGCTCAAAAGCA	AAAATTGCAAATGCACACCA	60.0	661	INTRON	Ca_10283	--	--
Ca2_P2_32349934	Ca2	32349934	CAAAA/CAA	ATTCTGACGCCTCTTGCACT	AAAATTGCAAATGCACACCA	60.0	390	INTRON	Ca_10283	--	--

INDEL marker IDs	Chromosomes/unanchored scaffolds	Physical positions (bp)	InDels (*Kabuli* reference genome-CDC Frontier/PII)	Forward primers (5'-3')	Reverse primers (5'-3')	Anneling temperature (0C)	Expected amplified product size (bp)	Structural annotation		Functional annotation	
								Sequence components of *kabuli* genome	*Kabuli* gene accession IDs	NCBI-KOG	TFs
Ca2_P2_32353326	Ca2	32353326	G/GA	GAACGACAACAATTTCGCCT	CAATGGATTTCTTTGGCCTT	59.0	513	DRR	Ca_10283	--	--
Ca2_P2_32353407	Ca2	32353407	TGAAC/T	GAACGACAACAATTTCGCCT	CAATGGATTTCTTTGGCCTT	59.0	513	DRR	Ca_10283	--	--
Ca2_P2_32357617	Ca2	32357617	CTTT/CTTTT	TCCCAACTCTTCGAAATCGT	TTGGATGATAGACATTGAAATTTTG	59.3	367	INTERGENIC	--	--	--
Ca2_P2_32358261	Ca2	32358261	CAAAAAAA/CAAAAAA	TCAAAATTTCAATGTCTATCATCCA	GCGAGGATGAGAAGTTGTTGT	59.3	658	INTERGENIC	--	--	--
Ca2_P2_32359800	Ca2	32359800	TAGA/T	TTGATGAGTTCGGTCATGGA	AGCCGCTTAATTGATCGTTG	60.2	195	INTERGENIC	--	--	--
Ca2_P2_32362657	Ca2	32362657	TAAAAAAA/TAAAAAAAA	GCAAAAGCACAACCAAATCA	AATGACCACAATTTGCATTGA	58.9	260	INTERGENIC	--	--	--
Ca2_P2_32366586	Ca2	32366586	TATTA/T	GCCAAAATTCAAAGAGTCGTG	GCTTGGCAACATTCTTCACA	59.8	385	INTERGENIC	--	--	--
Ca2_P2_32369826	Ca2	32369826	CT/CTCGT	TGGTTTCAAGGTCTTCCACA	GAAAAGTTAAAATCCAAAATAACATTC	57.2	794	INTERGENIC	--	--	--
Ca2_P2_32369843	Ca2	32369843	ATTTTTTTT/ATTTTTTTTT	TGGTTTCAAGGTCTTCCACA	TGTTTGTGCAATTGAATTATAGTTATG	58.6	933	INTERGENIC	--	--	--
Ca2_P2_32370223	Ca2	32370223	AAAAAATTAATGAAAAATTAATGAAAAATTAA/AAAAAATTAATGAAAAATTAA	TCAAGCAAGTCACAACATTTCA	TGCTATTTTAATTGTGAGCATGAGA	60.2	637	INTERGENIC	--	--	--
Ca2_P2_32373466	Ca2	32373466	TAAGA/TAAGAAGA	TTCGGTTATTGGTTATCGCC	TTCCACGTCTGCAGTTGAAT	59.3	619	DRR	Ca_10282	--	--
Ca2_P2_32374333	Ca2	32374333	AAC/AACAC	TCAAGGTTTTGTGATTGCCA	TCAACTTTGCCACTGAACCA	60.3	286	DRR	Ca_10282	--	--

INDEL marker IDs	Chromosomes/unanchored scaffolds	Physical positions (bp)	InDels (*Kabuli* reference genome-CDC Frontier/PII)	Forward primers (5'-3')	Reverse primers (5'-3')	Anneling temperature (0C)	Expected amplified product size (bp)	Structural annotation		Functional annotation	
								Sequence components of *kabuli* genome	*Kabuli* gene accession IDs	NCBI-KOG	TFs
Ca2_P2_32378599	Ca2	32378599	AACACA/AACA	GTGGAAGGTCGGTGGTCTTA	ACCACACCCAATCCTTTCAA	60.2	144	INTERGENIC	--	--	--
Ca2_P2_32379585	Ca2	32379585	AATTAATTTT/AATTAATTTTGATTAATTTT	TGTGCGAGTTTATTGCGGTA	CATTTGGTTCAAAATGTCATGG	60.1	624	INTERGENIC	--	--	--
Ca2_P2_32380306	Ca2	32380306	GTTTGAATTTAATTA/GTTTGAATTTAATTATTTGAATTTAATTA	AACTAACTTTTCGCAGCCCA	GGCAACTTGGCCAACACTAT	60.0	587	INTERGENIC	--	--	--
Ca2_P2_32393042	Ca2	32393042	ATTTTTTTT/ATTTTTTTTT	TGCCCATCCTTATGCTCTTC	ATCACGTGCAACCACAAGTC	59.6	626	INTERGENIC	--	--	--
Ca2_P2_32393092	Ca2	32393092	TAAA/TTAAAA	TGCCCATCCTTATGCTCTTC	ATCACGTGCAACCACAAGTC	59.6	626	INTERGENIC	--	--	--
Ca2_P2_32393117	Ca2	32393117	T/TC	TGCCCATCCTTATGCTCTTC	ATCACGTGCAACCACAAGTC	59.6	626	INTERGENIC	--	--	--
Ca2_P2_32393187	Ca2	32393187	AATAT/A	TGCCCATCCTTATGCTCTTC	ATCACGTGCAACCACAAGTC	59.6	626	INTERGENIC	--	--	--
Ca2_P2_32393441	Ca2	32393441	TGG/TGTGGG	TCGCACTTTTTCGTGTAGGA	GGGCTTGTTTGACGCATTAT	60.0	588	INTERGENIC	--	--	--
Ca2_P2_32393633	Ca2	32393633	TAACATAATAA/TA	GACTTGTGGTTGCACGTGAT	GGGCTTGTTTGACGCATTAT	60.0	445	INTERGENIC	--	--	--
Ca2_P2_32397852	Ca2	32397852	G/GA	AAAAGTCAAACATCGCCGAC	TAAAGGGGCACACAACACAA	60.0	312	INTERGENIC	--	--	--
Ca2_P2_32399194	Ca2	32399194	T/TATTAATGTCTAA	TCTTGCACGGGTGAGAACTA	CCTATTTGAAGAGTGCCCCA	60.1	587	INTERGENIC	--	--	--
Ca2_P2_32399302	Ca2	32399302	ATTTTTTTTT/ATTTTTTTTTT	AATTTGGGGCACTCTTCAAA	GCCTCTTCAAAAGTCGGTCA	60.4	219	INTERGENIC	--	--	--

INDEL marker IDs	Chromosomes/unanchored scaffolds	Physical positions (bp)	InDels (*Kabuli* reference genome-CDC Frontier/PII)	Forward primers (5'-3')	Reverse primers (5'-3')	Anneling temperature (0C)	Expected amplified product size (bp)	Structural annotation		Functional annotation	
								Sequence components of *kabuli* genome	*Kabuli* gene accession IDs	NCBI-KOG	TFs
Ca2_P2_32400495	Ca2	32400495	GATATAT/GATATATATAT	TATGTCTCCTTTTGGGCGTC	CAAAATCGATCCAAGCAACA	59.7	868	INTERGENIC	--	--	--
Ca2_P2_32406094	Ca2	32406094	AGG/AG	GGTGGAAAAGGGAGGAGAAG	ACGTGTAACGGGTGATTTGG	60.7	197	INTERGENIC	--	--	--
Ca2_P2_32406123	Ca2	32406123	CAAA/CAAAA	GGTGGAAAAGGGAGGAGAAG	ACGTGTAACGGGTGATTTGG	60.7	197	INTERGENIC	--	--	--
Ca2_P2_32406444	Ca2	32406444	CATTT/CATTTAATATTAATATTT	GGTGGAAAAGGGAGGAGAAG	GTCAGCTTGGAAAGGGTCTG	59.8	832	INTERGENIC	--	--	--
Ca2_P2_32407126	Ca2	32407126	T/TC	CAGACCCTTTCCAAGCTGAC	TCTCCATTGAGGCAATCACA	60.2	729	INTERGENIC	--	--	--
Ca2_P2_32408863	Ca2	32408863	CATAT/CAT	GGACTGAGGGAGGTGCATTA	AGAAGCATGCAAATTCCACC	60.1	606	INTRON	Ca_10279	--	bHLH
Ca2_P2_32417284	Ca2	32417284	ATTTTTTTT/ATTTTTTTTT	AAATTTGCGTGGAAAGGTTG	CTCTGGTGGCATGTATCCAA	59.5	207	INTRON	Ca_10278	--	bHLH
Ca2_P2_32423562	Ca2	32423562	AGG/AG	GTGAGAGCATTCGTCGAACA	TGTCGCGTGTTCATCAACTT	60.3	520	DRR	Ca_10277	--	--
Ca2_P2_32423881	Ca2	32423881	CAAAA/CAAAAA	TGAGGCATGATAATTGAGCG	TGTTGTCATCAATCACGCAG	59.2	536	DRR	Ca_10277	--	--
Ca2_P2_32427812	Ca2	32427812	ATT/AGAGTT	CCACATGAGTGTGTTGAGGG	GGCTATCGCTTATGCAGGAC	59.8	718	DRR	Ca_10276	--	SAP
Ca2_P2_32427863	Ca2	32427863	TTTATTATTATT/TTTATTATT	CCACATGAGTGTGTTGAGGG	GGCTATCGCTTATGCAGGAC	59.8	718	DRR	Ca_10276	--	SAP
Ca2_P2_32427895	Ca2	32427895	GTTATTA/GTTATTATTA	CCACATGAGTGTGTTGAGGG	GGCTATCGCTTATGCAGGAC	59.8	718	DRR	Ca_10276	--	SAP

INDEL marker IDs	Chromosomes/unanchored scaffolds	Physical positions (bp)	InDels (*Kabuli* reference genome-CDC Frontier/PII)	Forward primers (5'-3')	Reverse primers (5'-3')	Anneling temperature (0C)	Expected amplified product size (bp)	Structural annotation		Functional annotation	
								Sequence components of *kabuli* genome	*Kabuli* gene accession IDs	NCBI-KOG	TFs
Ca2_P2_32432077	Ca2	32432077	AC/ACTC	GTCGGTGGAATTCGAAGAAA	TAAGTTGGGGGCATAAACCA	60.2	782	INTERGENIC	--	--	--
Ca2_P2_32435485	Ca2	32435485	TT/TTGT	TTTTTATTACCGACATGGCTCA	GCAACTCTCCCTATTTTGTCG	58.8	832	DRR	Ca_10275	O	C2H2
Ca2_P2_32453109	Ca2	32453109	ATTTTTTT/ATTTTTTTTTT	TCTTCACACAATCCCACCAA	CTCAAGTCGACAAGCCTTCC	60.0	659	INTERGENIC	--	--	--
Ca2_P2_32476919	Ca2	32476919	ATGT/AT	CATGGATGGTGCTTCAAATG	CAACTGCAATGTTCCATATTCG	60.4	885	DRR	Ca_10273	R	C3H
Ca2_P2_32480670	Ca2	32480670	T/TA	TCCACATGCTTCCAATCAAA	ACCACTCCAACCAGTCGTTT	59.5	584	INTERGENIC	--	--	--
Ca2_P2_32480880	Ca2	32480880	AC/A	TCCACATGCTTCCAATCAAA	CAACGCCTCAAGTAGGGGTA	60.1	744	INTERGENIC	--	--	--
Ca2_P2_32480992	Ca2	32480992	GTTT/GTT	CATGCTTCCAATCAAACACG	CAACGCCTCAAGTAGGGGTA	60.1	740	INTERGENIC	--	--	--
Ca2_P2_32481466	Ca2	32481466	GTTTTTTTTTT/GTTTTTTTTTTTTT	AACGACTGGTTGGAGTGGTC	TTTCTTCTGCATTTCCACCC	60.1	538	INTERGENIC	--	--	--
Ca2_P2_32481790	Ca2	32481790	ATATAT/A	GGGTGGAAATGCAGAAGAAA	AAGCTTTCGGTGATTGCTGT	59.9	535	INTERGENIC	--	--	--
Ca2_P2_3250898	Ca2	3250898	CATATATATATATATATATAT/CATATATATATATATATATATATATATAT	TTCTCTTTTTCCCCACATGC	GCTCTCGGTGGAGTACTTGC	60.0	464	INTERGENIC	--	--	--
Ca2_P2_3264088	Ca2	3264088	CATGCTTATGC/CATGCTTATGCTTATGC	GGGCCCCTCAAAGAATAAAG	CATAAGCAGGCCAGAAGGAG	60.0	459	DRR	Ca_10525	Q	Nin-like
Ca2_P2_32642955	Ca2	32642955	ATTTTTTTTTT/ATTTTTTTTT	CAAAGTTGAGGAAAGGCCAG	GGAATCACCCATCCACATTC	60.0	869	DRR	Ca_10258	--	--

INDEL marker IDs	Chromosomes/unanchored scaffolds	Physical positions (bp)	InDels (*Kabuli* reference genome-CDC Frontier/PII)	Forward primers (5'-3')	Reverse primers (5'-3')	Anneling temperature (0C)	Expected amplified product size (bp)	Structural annotation		Functional annotation	
								Sequence components of *kabuli* genome	*Kabuli* gene accession IDs	NCBI-KOG	TFs
Ca2_P2_32648297	Ca2	32648297	AAAGAA/AAAGAAGAA	AAGCGCTAAATGATGGTGAA	ACCTTTTGATCCATGCTTGC	60.1	519	INTERGENIC	--	--	--
Ca2_P2_32672427	Ca2	32672427	TC/T	ATTCAACGTTTCTGGATGGC	ATAAGGCAGTGGCGTAGAGC	59.5	452	DRR	Ca_10254	DR	--
Ca2_P2_32673561	Ca2	32673561	ATTTTTTTTTTT/ATTTTTTTTTT	TTCAACCAAGCCTACCCTTC	TGGCCAACAAAATTTAACCA	58.9	449	DRR	Ca_10254	DR	--
Ca2_P2_32678344	Ca2	32678344	CATTATTATTATTATTATTATTATTATTATTATTATTA/CATTATTATTATTATTATTATTATTATTATTATTATTA	AAGCGTGAAGGGTTTCCTTT	ATTGCATTCCGCAATCTAGG	60.1	846	INTERGENIC	--	--	--
Ca2_P2_32696355	Ca2	32696355	CTTTT/CTTT	TGTCATCAAATTTAATATGGGTCG	TTCACCTGAAAAACATGCCA	60.1	775	INTERGENIC	--	--	--
Ca2_P2_32713700	Ca2	32713700	TTTTTTTTT/TTTTTTTTTTT	AACGCATCGGTAAACACTCC	GAGGTACTACAGCTTGCCCC	58.8	234	INTERGENIC	--	--	--
Ca2_P2_32713895	Ca2	32713895	CAAAAAAA/CAAAACAAAAAAAA	AACGCATCGGTAAACACTCC	TGTTTTGCAGCAAGATGTCC	59.8	695	INTERGENIC	--	--	--
Ca2_P2_32756102	Ca2	32756102	TAAAAAAAAAAA/TAAAAAAAAAAAA	TCATTTCCCAATTGAAAGGC	AGCTTCGAACTTGCCATGAT	59.8	543	INTERGENIC	--	--	--
Ca2_P2_32777553	Ca2	32777553	CTC/CTCATC	TGCTGAGGCATAGTGACTGG	GAGCTTACGCCGTCTCATTC	60.0	629	CDS (large-effect mutations)	Ca_10245	R	C3H
Ca2_P2_32817790	Ca2	32817790	CGAGAGAGAGAGAGAGAGAGAGAGAGAGAGAGAGAGAG/CGAGAGAGAGAGAGAGAGAGAGAGAGAGAGAGAGAGAGAGAGAGAG	CGGGAATGGAGTTAGTGGTG	GGCAAATCTGATCCCTCAAA	60.0	593	INTERGENIC	--	--	--
Ca2_P2_32914739	Ca2	32914739	TTAATAA/TTAA	GTGCGATTTGAGGGAATACG	GGGAGCTGAATGACCATTTG	60.5	718	INTERGENIC	--	--	--
Ca2_P2_32934778	Ca2	32934778	TTAAATAAATAAATAAATAAA/TTAAATAAATAAATAAATAAATAAA	ACAGCGACACGAAGGACTCT	GGAACAAGTTTCCGTGAAGG	59.6	396	INTRON	Ca_10230	OJ	MYB_related

INDEL marker IDs	Chromosomes/unanchored scaffolds	Physical positions (bp)	InDels (*Kabuli* reference genome-CDC Frontier/PII)	Forward primers (5'-3')	Reverse primers (5'-3')	Anneling temperature (0C)	Expected amplified product size (bp)	Structural annotation		Functional annotation	
								Sequence components of *kabuli* genome	*Kabuli* gene accession IDs	NCBI-KOG	TFs
Ca2_P2_32970157	Ca2	32970157	TAATCAAAGTGGTATAAATCAA/TAATCAA	GGTCAATTTCGCGAATTAGTTA	TCAGATTCAGCTGGACAAAGA	58.6	609	INTERGENIC	--	--	--
Ca2_P2_33007830	Ca2	33007830	AAGAGAGAGAGAGAGAGAGAGAGAGAGAGAGAGAGAGAGAGAGAGAGAGA/AAGAGAGAGAGAGAGAGAGAGAGAGAGAGAGAGAGAGAGAGAGA	AAAGCGGTCCAATTTGAGAA	TTTTCCAATCCTAAGCCGTG	60.1	276	INTERGENIC	--	--	--
Ca2_P2_33030318	Ca2	33030318	AATATATATATATATATATATATATAT/AATATATATATATATATATATAT	CAGCATGGGTTCATGCACTA	GCAAATTGGAATGAGTTGGTC	59.4	601	INTERGENIC	--	--	--
Ca2_P2_33048104	Ca2	33048104	AATATATATATATATATATATATATATAT/AATATATATATATATATATATATATAT	GAGGGATGGGTGTTGTTGAC	CAGAGTTGGATGGGGTGATT	59.8	532	INTERGENIC	--	--	--
Ca2_P2_33049370	Ca2	33049370	TATATAA/T	AGCTAAGTAACACCGCCACG	CAGACCCATTTACCCTTGGA	59.8	419	INTERGENIC	--	--	--
Ca2_P2_33079848	Ca2	33079848	GAAAAAAAA/GAAAAAAA	AGACGCCACGTAATGGAAAC	TCAACACACATTGCCCTTGT	60.0	440	INTERGENIC	--	--	--
Ca2_P2_33257195	Ca2	33257195	ATTTTTTTTT/ATTTTTTTTTT	ATCGCATTTTGCCTTTGTTT	GCTGTCGTTTGCAGCATAAA	60.0	801	INTERGENIC	--	--	--
Ca2_P2_33279130	Ca2	33279130	AATATATATATA/AATATATATATATA	GCAACCACCTTCTTGAAACC	ATCAGTGCCTGCTCCATTCT	59.8	720	INTERGENIC	--	--	--
Ca2_P2_33306373	Ca2	33306373	TTATATATATATATATATA/TTATATATATATATA	GAATCCGTTTCCGAGTTTGA	TCAATGGAAATCAAAGTTGAAGG	60.3	644	INTERGENIC	--	--	--
Ca2_P2_33345639	Ca2	33345639	GTT/GT	CTCCATTTTGGGCAGGTTTA	GGATGATCTGTTTTTCGCGT	60.1	573	INTRON	Ca_10191	T	FAR1
Ca2_P2_33346809	Ca2	33346809	C/CA	ATGCACATGAACCGATTGAA	AAACAAGGTTGAATCACGCC	60.0	718	INTRON	Ca_10191	T	FAR1
Ca2_P2_33397405	Ca2	33397405	CAGACAAG/CAGACAAGACAAG	CAATTTGCCTGAACCAGGAT	CCAATTTTCAAGGCACGTTT	60.0	580	DRR	Ca_10186	T	M-type

INDEL marker IDs	Chromosomes/unanchored scaffolds	Physical positions (bp)	InDels (*Kabuli* reference genome-CDC Frontier/PII)	Forward primers (5'-3')	Reverse primers (5'-3')	Anneling temperature (0C)	Expected amplified product size (bp)	Structural annotation		Functional annotation	
								Sequence components of *kabuli* genome	*Kabuli* gene accession IDs	NCBI-KOG	TFs
Ca2_P2_33398330	Ca2	33398330	TCACA/TCA	AGTCAATGGCATGAGAAGGC	AAATGCAGGGTCACAACACA	60.0	513	DRR	Ca_10186	T	M-type
Ca2_P2_33398441	Ca2	33398441	TACACAC/TACAC	AGAGCCTACAGCAAGCCAAA	AAATGCAGGGTCACAACACA	60.0	205	DRR	Ca_10186	T	M-type
Ca2_P2_33405227	Ca2	33405227	AATATATATATA/AATATATATA	AAAGAGACATGACTTTGTCCATCA	TTTCGCTACCCATCATACCC	59.8	586	INTERGENIC	--	--	--
Ca2_P2_33498318	Ca2	33498318	AATATATATATATATATATATATATAT/AATATATATATATATATATATATATAT	TCATTGAAGCCATCAAATTAGTGT	TCCTTCATTCCTTTTGGTGC	60.1	342	INTERGENIC	--	--	--
Ca2_P2_33585937	Ca2	33585937	T/TC	CTCAACATGCATGCAAAACC	TCCCAACCCAAATAAACCAA	60.0	536	INTERGENIC	--	--	--
Ca2_P2_33586099	Ca2	33586099	GTTA/GTTAATTA	CTCAACATGCATGCAAAACC	TCCCAACCCAAATAAACCAA	60.0	536	INTERGENIC	--	--	--
Ca2_P2_33639777	Ca2	33639777	TAAAAAAAA/TAAAAAAA	CGACGGAATAGTTTGCTCTCT	CGGAAAACCGTAGCTTAATACG	60.0	892	INTERGENIC	--	--	--
Ca2_P2_33642680	Ca2	33642680	TATAATAATAATAA/TATAATAATAATAATAA	TGGATAGCCAGTGACAGGAA	TTTTTGCCGATAATGGGAAC	59.8	290	INTERGENIC	--	--	--
Ca2_P2_3367372	Ca2	3367372	AATATATATATATATATATATATATATATATATATATA/AATATATATATATATATATATATATATATATATA	CATTTGACCAAAATGCCACA	ACCAATACAAGGTCGCATCC	59.8	182	INTERGENIC	--	--	--
Ca2_P2_33715359	Ca2	33715359	ANNNNNNNNNN/ANNNNNNNN	CAAAAGGCGTGCATGAATTA	CTTGCCTTATTTTTCCTGCG	59.8	271	DRR	Ca_10165	K	G2-like
Ca2_P2_33955296	Ca2	33955296	TTAAAATAAA/TTAAA	AATTTCGACTATTCTCTAATGATGG	TCGTTTTGGTGTTGGTTTCA	60.0	520	INTERGENIC	--	--	--
Ca2_P2_33959397	Ca2	33959397	ATATTTATTTATTT/ATATTTATTT	GGCTAATTTTCGTTATATGGATGC	ACTAGTGATGGCCGAAATGG	60.0	764	INTERGENIC	--	--	--

INDEL marker IDs	Chromosomes/unanchored scaffolds	Physical positions (bp)	InDels (*Kabuli* reference genome-CDC Frontier/PII)	Forward primers (5'-3')	Reverse primers (5'-3')	Anneling temperature (0C)	Expected amplified product size (bp)	Structural annotation		Functional annotation	
								Sequence components of *kabuli* genome	*Kabuli* gene accession IDs	NCBI-KOG	TFs
Ca2_P2_33978678	Ca2	33978678	CATATATATATATATAT/CATATATATATATATATAT	AGGAAGAGTGGAGTTGCGAA	TGCGTTACGGTGAAATGGTA	60.0	364	INTERGENIC	--	--	--
Ca2_P2_33984310	Ca2	33984310	C/CCATA	TGGCTATGATCACAATTGGC	CCCTTTGCAAGAGACTCCAA	60.4	869	INTERGENIC	--	--	--
Ca2_P2_33986265	Ca2	33986265	TT/TTATAT	AACTTTGGATCTACACGCGA	ACTCCAAGATCATCATCGGC	60.0	623	INTERGENIC	--	--	--
Ca2_P2_34025051	Ca2	34025051	TAG/TAGAG	CCTATTCGTGGTCGTGGAAT	TTTTGTTAGGCTGCAAGAATTG	59.4	528	INTERGENIC	--	--	--
Ca2_P2_34028835	Ca2	34028835	AC/A	AAAGGGGTTTTGGCTATGCT	CATGATCATAATTGCATGGAAAA	59.7	229	INTERGENIC	--	--	--
Ca2_P2_34054495	Ca2	34054495	AATATATATATATAT/AATATATATATAT	AGAATTGAATGCGTTACGGG	ATTCCGGTAAAATCTCCGCT	59.9	513	INTERGENIC	--	--	--
Ca2_P2_34054996	Ca2	34054996	GTT/GTTT	GGTAAGGGCTCCTTCCAAAC	ATCACGCAACATAACACCCA	59.8	207	INTERGENIC	--	--	--
Ca2_P2_34073061	Ca2	34073061	TTATATATATATATATATATATATATATAT/TTATATATATATATATATATATATATATATATAT	TGAATGTTTTCGATGATGTGC	GATAAGTCCATTGGTCCCCTT	59.2	758	INTERGENIC	--	--	--
Ca2_P2_34097693	Ca2	34097693	G/GCTAGGCT	TGCATGCATCATCACAACAT	AGAAGCGCCTACCATTCTCA	60.0	447	DRR	Ca_15231	I	--
Ca2_P2_3415073	Ca2	3415073	CTTATTATTATTATTATTATTATTATTATTATTATTATTATTATTATTATTATTATTATTATTATTATTATTATTATTATTATTATTAT/CTTATTATTATTATTATTATTATTATTATTATTATTATTATTATTATTATTATTATTATTATTAT[illegible]	TGCTCAATTCTGGCAGATCA	AGTTTCATCACGGCAAAACC	60.0	447	INTERGENIC	--	--	--
Ca2_P2_34353805	Ca2	34353805	GTTTTTTTT/GTTTTTTTTT	AGTTGAAGGAGTGCATGAGGA	TAGGAGGCATATGTGAGGGG	59.9	633	INTERGENIC	--	--	--
Ca2_P2_34355206	Ca2	34355206	CTTGGGCT/CTTGGGCTTTGTTGGGCT	TCTTTGATGCAATGCCAGAG	ATCATTCGAGGTGCCATCAT	60.3	975	INTERGENIC	--	--	--

INDEL marker IDs	Chromosomes/unanchored scaffolds	Physical positions (bp)	InDels (*Kabuli* reference genome-CDC Frontier/PII)	Forward primers (5'-3')	Reverse primers (5'-3')	Anneling temperature (0C)	Expected amplified product size (bp)	Structural annotation		Functional annotation	
								Sequence components of *kabuli* genome	*Kabuli* gene accession IDs	NCBI-KOG	TFs
Ca2_P2_34355457	Ca2	34355457	TAAAAAA/TAAAAAAA	ATTCTTGGGCTGTGTTTTGG	GGGTGATGAGGACTGGTTGT	59.8	725	INTERGENIC	--	--	--
Ca2_P2_34355554	Ca2	34355554	CAAAAAAAAAA/CAAAAAAAAAAAA	ATTCTTGGGCTGTGTTTTGG	GGGTGATGAGGACTGGTTGT	59.8	725	INTERGENIC	--	--	--
Ca2_P2_34357732	Ca2	34357732	CATATATATATATATATATATATATATATAT/CATATATATATATATATATATATATATATATATATATATATATATATAT	CATGAGCTTGAGTCGCAAAA	GCAACAAGATGGGGACAAGT	60.0	654	INTERGENIC	--	--	--
Ca2_P2_34515091	Ca2	34515091	TTTATTATTATTATTATTATTATTATTATTATTATTATTATTATTATTATTATTATTA/TTTATTATTATTATTATTATTATTATTATTATTATTATTATTATTATTATTATTATTATTATTATTATTATTA	TGCCAATGTAATGATTCAGGA	AGCGTAAGTTTCGAGGACCA	59.9	766	INTERGENIC	--	--	--
Ca2_P2_34654101	Ca2	34654101	GTATATATATATATATATATATATATAT/GTATATATATATATATATATATATAT	AGGACGAGACAAAATGAGCC	TGGAAACCAAGGTCCAAGTC	59.9	219	INTERGENIC	--	--	--
Ca2_P2_34683764	Ca2	34683764	TC/TCC	GTTTTTCGCAACCACCTTTC	CGCTTTTTGCACTAACCTCC	59.9	682	INTERGENIC	--	--	--
Ca2_P2_34684386	Ca2	34684386	CAACGTTAAC/CAAC	ACAATACTGCGGCCTGTTTC	GATTCCGATCGTTTTTCACG	60.4	830	INTERGENIC	--	--	--
Ca2_P2_34684594	Ca2	34684594	TA/TACACACA	TTCAGTAGACGTTGAACCATCG	GATTCCGATCGTTTTTCACG	60.4	651	INTERGENIC	--	--	--
Ca2_P2_34684923	Ca2	34684923	ATTTTTTT/ATTTTTTTT	TCGTGAAAAACGATCGGAAT	TGCTTGATTTTAATGGATGCC	59.9	550	INTERGENIC	--	--	--
Ca2_P2_34685252	Ca2	34685252	CATAAATAAATAAATAAA/CATAAATAAA	TCGTGAAAAACGATCGGAAT	TGGAGAGAAACAAAATGGGG	59.9	816	INTERGENIC	--	--	--
Ca2_P2_34685921	Ca2	34685921	TAAAAAAA/TAAAAA	CCAAAACTGGGGAGAAACAA	TACGATAGGGGGATCTCACG	59.9	503	INTERGENIC	--	--	--
Ca2_P2_34686878	Ca2	34686878	TTGACAGGGTAG/T	GAATCCTGGGCTGAAATCAA	ACGTTCCTGAGGGAATTGTG	60.0	612	INTERGENIC	--	--	--

INDEL marker IDs	Chromosomes/unanchored scaffolds	Physical positions (bp)	InDels (*Kabuli* reference genome-CDC Frontier/PII)	Forward primers (5'-3')	Reverse primers (5'-3')	Anneling temperature (0C)	Expected amplified product size (bp)	Structural annotation		Functional annotation	
								Sequence components of *kabuli* genome	*Kabuli* gene accession IDs	NCBI-KOG	TFs
Ca2_P2_34706742	Ca2	34706742	GAAAAA/GAAAA	CTCTCTCTCCATCTCGGCAC	AAAGTAGCCGGCAAGGTCTC	60.8	459	INTERGENIC	--	--	--
Ca2_P2_34775151	Ca2	34775151	TATAATAATAATAATAATAATAATAATAATAATAATAATAATAATAATA/TATAATAATAATAATAATAATAATAATAATAATAATAATAATAATAATAATAATAATAATA	CTCGTATCTAGTGTTTGTGTTTGAA	ACTCAGAACCACCACTTGGG	60.0	553	INTERGENIC	--	--	--
Ca2_P2_34775299	Ca2	34775299	AATATATATATATATATATATA/AATATATATATATATATATATATA	CCCAAGTGGTGGTTCTGAGT	CATGCTTCACTCTTAGGGCA	59.0	473	INTERGENIC	--	--	--
Ca2_P2_34809034	Ca2	34809034	GAAAAAAAAAAAAAAAAAAAAAAAA/GAAAAAAAAAAAAAAAAAA	TTGAAATAAATGGCCAAATCG	CAATAAAAGGGCAAAACCCA	59.8	262	INTERGENIC	--	--	--
Ca2_P2_34983521	Ca2	34983521	AATATATATATATATATATATATATATATATATATATA/AATATATATATATATATATATATATATATATATATATATATA	CAAATGCATGCTCCCCTAAT	CTAAAAGCCCAAAGCTGCAC	60.0	450	INTERGENIC	--	--	--
Ca2_P2_35074492	Ca2	35074492	TTATA/TTATATA	GGTTTAACATCCCCACAAGC	CGGACGACCTCTCTCGTAAG	60.0	718	INTRON	Ca_16865	TU	C3H
Ca2_P2_35180722	Ca2	35180722	GTATATATATATATATATAT/GTATATATATATATATATATATAT	TTTAACCGCAATTTAATTCATCA	GGTCTCCCTATATCACGCCA	59.9	301	INTERGENIC	--	--	--
Ca2_P2_35228331	Ca2	35228331	TAT/TATTATTATCAT	TTATTTCACCGTCGTGTCCA	GGAAAAGGAGTCACCACCAA	59.9	624	INTERGENIC	--	--	--
Ca2_P2_35258590	Ca2	35258590	A/ATT	AAAAAGTTTTAACACGGAGGTTTTT	TGTTGTTGGATTGGACATGG	60.2	487	INTERGENIC	--	--	--
Ca2_P2_35274069	Ca2	35274069	GAAAA/GAAA	TTAAAAATATCGCGGGTTGC	TCAAATATCAAATGGGTTCGC	59.8	279	INTERGENIC	--	--	--
Ca2_P2_35288109	Ca2	35288109	ATTTTTT/ATTTTTTT	TGCTTTATAGTGCTATTATGTGCCA	CGTCATTAAAAGGGAAACCAA	59.0	757	INTERGENIC	--	--	--
Ca2_P2_35288455	Ca2	35288455	TT/TTAT	TTCGATGGTATGCAGTAATTGG	TTCCCATGAAAGAGAGGTGG	60.0	433	INTERGENIC	--	--	--

INDEL marker IDs	Chromosomes/unanchored scaffolds	Physical positions (bp)	InDels (*Kabuli* reference genome-CDC Frontier/PII)	Forward primers (5'-3')	Reverse primers (5'-3')	Anneling temperature (0C)	Expected amplified product size (bp)	Structural annotation		Functional annotation	
								Sequence components of *kabuli* genome	*Kabuli* gene accession IDs	NCBI-KOG	TFs
Ca2_P2_35289402	Ca2	35289402	ACCCCC/ACCCC	ACCCACCTCTTTTTCTTGGG	TGGTCCTCGAAATTTGTAATGA	59.5	609	INTERGENIC	--	--	--
Ca2_P2_35297774	Ca2	35297774	G/GAACTGAAC	ACCAGGAAAAGAAGAAGGGC	TTTTGGGTTGGGATGACATT	60.0	535	INTERGENIC	--	--	--
Ca2_P2_35297846	Ca2	35297846	TTGA/TTGATTCAACTGAACTGA	ACCAGGAAAAGAAGAAGGGC	TTTTGGGTTGGGATGACATT	60.0	535	INTERGENIC	--	--	--
Ca2_P2_35298866	Ca2	35298866	AA/AACA	TGGGGGCTTAGAGCTTTGTA	CGATGTTGTTGATCTGATATCGT	59.0	527	INTERGENIC	--	--	--
Ca2_P2_35303488	Ca2	35303488	ATTTTTT/ATTTTTTT	TTCCGAATAAGGATTTCTTGG	ATGGTTTGGGGAAAAGAACC	60.0	137	INTERGENIC	--	--	--
Ca2_P2_35304698	Ca2	35304698	C/CG	GCTTGGAAACAGCCTCAGAC	CAGGGTGGTACTGCAATGTG	60.0	536	INTERGENIC	--	--	--
Ca2_P2_3531881	Ca2	3531881	AATATATATATATATATATATATATATATATAT/AATATATATATATATATATATATATATATATATATATATATATATAT	CTTGTGTGGGGGACTGCTAT	TTTCTCCTTTTCACTTGTCACG	59.4	689	DRR	Ca_10506	--	--
Ca2_P2_35329169	Ca2	35329169	AATATATATATATATATATATATATAT/AATATATATATATATATATATATAT	CAATTTTGGAAGACGATTTTGA	TGTCCCGAAAAAGTTCATCC	59.9	330	INTERGENIC	--	--	--
Ca2_P2_35354742	Ca2	35354742	TAAAAAAAAAAA/TAAAAAAAAAAAAAA	AATGTCAACGACCCATTTGTT	TTGTCCCTTCCTCCATCTTG	60.0	581	INTERGENIC	--	--	--
Ca2_P2_35359085	Ca2	35359085	TTATATATATATATATATATATATATATATAT/TTATATATATATATATATATATATATATAT	CGTCGAATCCTAGAGCGAAT	TGGTGTTGGAGTCATACTTTTCA	59.5	306	INTERGENIC	--	--	--
Ca2_P2_354010	Ca2	354010	TTCTCTCTCTCTCTCTCTCTCTCTCTCTC/TTCTCTCTCTCTCTCTCTCTCTCTCTC	GAGGAATTGGAACGCGTAAA	TGATTCAGGGCTTTTGATCC	60.0	675	INTERGENIC	--	--	--
Ca2_P2_35481606	Ca2	35481606	T/TTCTGGTTGA	TGAAAATGTGCATGCTTGGT	GTCAGAGGAAGGAGGCACTG	60.0	410	CDS (large-effect mutations)	Ca_09709	L	--

INDEL marker IDs	Chromosomes/unanchored scaffolds	Physical positions (bp)	InDels (*Kabuli* reference genome-CDC Frontier/PII)	Forward primers (5'-3')	Reverse primers (5'-3')	Anneling temperature (0C)	Expected amplified product size (bp)	Structural annotation		Functional annotation	
								Sequence components of *kabuli* genome	*Kabuli* gene accession IDs	NCBI-KOG	TFs
Ca2_P2_35581740	Ca2	35581740	AATATATATATATATATATATATATATA/AATATATATATATATATATATATATA	TTGCCACTTGATACAGCAAAA	TTGAAAGGATAACATGGCCG	60.8	690	INTERGENIC	--	--	--
Ca2_P2_35599561	Ca2	35599561	ATTTTCTTTTCTTTTCTTTTCTTTTC/ATTTTCTTTTCTTTTCTTTTCTTTTCTTTTCTTTTC	TTTAAATGAGGCGGGTATGG	ATGATAGGGACAGACCGTCG	60.0	405	INTERGENIC	--	--	--
Ca2_P2_35797971	Ca2	35797971	CTATATATATATATATATATATATAT/CTATATATATATATATATATATATATAT	AATGTGAAAACCAAGGCACC	TCTCGTCATCTTTTTCCTCCA	59.8	486	DRR	Ca_09755	--	--
Ca2_P2_3593906	Ca2	3593906	CTTATTATTATTATTATTATTATTATTATT/CTTATTATTATTATTATTATTATTATTATT	GTTCCAACCCATACCAAACG	GGAGATGCGACTTGGTGAAT	60.1	621	INTERGENIC	--	--	--
Ca2_P2_35944964	Ca2	35944964	CAAAA/CA	TCCAACCATACCTGAAAGGC	CTCGTGCGAACTTGTGTGTT	59.9	313	CDS (large-effect mutations)	Ca_09769	--	--
Ca2_P2_35961062	Ca2	35961062	TT/TTATTATTATTAGT	CATTTTCATTCACACTCCACTCA	GGCACGAATGTCTTTGATGA	59.7	557	DRR	Ca_09770	--	--
Ca2_P2_35976572	Ca2	35976572	AATATATATATATATATATATATATATATATATATATATATA/AATATATATATATATATATATATATATATATATATATATATATA	AGGACATGATGTTGCTGCTG	AACAAAAATGAAGGAGGGGG	60.2	286	INTRON	Ca_09772	T	LBD
Ca2_P2_36146623	Ca2	36146623	GAAAAAA/GAAAAA	AAACAGCAACAAAAATCCCAA	TTTGGAAATCAAGAGGTGGG	59.9	201	INTERGENIC	--	--	--
Ca2_P2_36146917	Ca2	36146917	TCCCCC/TCCCCCC	AGGGAAACCCACCTCTTGAT	AAATCCAAAGTGCTTGCGTT	59.8	623	INTERGENIC	--	--	--
Ca2_P2_36174197	Ca2	36174197	AATATATATATATATATA/AATATATATATATATATATA	CCAGAACCCGGACTTTATCA	TGCATGCAGTACCACAACCT	60.2	301	INTERGENIC	--	--	--
Ca2_P2_36181406	Ca2	36181406	GTTTTTTTTTTTTTTTTTT/GTTTTTTTTTTTTTT	GGCAGCATGTCGAGTGTTAG	TGCATTATCTTGTGTTCTCCG	58.8	285	DRR	Ca_09798	R	bHLH
Ca2_P2_36223052	Ca2	36223052	TATAAATAAATAAATAAATAAATAA/TATAAATAAATAAATAAATAA	TTGTTAAACGCAATTTACCGAG	TTGATTTGAAAACCCCAACA	58.8	366	INTERGENIC	--	--	--

**INDEL marker IDs**	**Chromosomes/unanchored scaffolds**	**Physical positions (bp)**	**InDels (*Kabuli* reference genome-CDC Frontier/PII)**	**Forward primers (5'-3')**	**Reverse primers (5'-3')**	**Anneling temperature (0C)**	**Expected amplified product size (bp)**	**Structural annotation**		**Functional annotation**	
								**Sequence components of *kabuli* genome**	***Kabuli* gene accession IDs**	**NCBI-KOG**	**TFs**
Ca2_P2_36224008	Ca2	36224008	CTTTTTTTTTT/CTTTTTTTTTTT	TTTTCACCACGACACACAAA	TGCACGTTTATAAGGAATCGG	60.0	354	INTERGENIC	--	--	--
Ca2_P2_36245487	Ca2	36245487	ATTTTTTTT/ATTTTTTTTT	TCCCAAAAAGGAATTTTGTTTC	GGTGAGATACTGCATCACTTCAA	59.3	519	INTERGENIC	--	--	--
Ca2_P2_36290906	Ca2	36290906	ACCCCCCCCCCC/ACCCCCCCCCC	TGCGATGTGCTGACAGTGTA	CGTGCGTCCACTGAAAATTA	59.7	884	INTERGENIC	--	--	--
Ca2_P2_3630204	Ca2	3630204	CATATATATATATATATATATATATATAT/CATATATATATATATATATATATATAT	TGAAACAAGCATATGCGTCAA	TTTGCCTTCAATAATTGCACA	59.2	158	INTRON	Ca_10502	P	ERF
Ca2_P2_3644500	Ca2	3644500	AT/AATCT	GTCCCAACCTGCTTTTGTTT	AGCAAAGTTTTTGCACCCAT	59.6	759	INTERGENIC	--	--	--
Ca2_P2_3705697	Ca2	3705697	TAAA/TAAAA	CGTGAGGGGTTGAAAATAGG	GTGTTTCAAGCACCACCAAA	59.6	650	DRR	Ca_10491	D	WRKY
Ca2_P2_3708826	Ca2	3708826	G/GATACATT	ATGATACCGCGAACATGACA	GGGAGGATGTTTGCTTTGAA	60.1	457	INTRON	Ca_10491	D	WRKY
Ca2_P2_3710197	Ca2	3710197	GAACAAACAAACAAACA/GAACAAACAAACAAACAAACA	AGCTGCAGAAGCTGTAAGCC	AACGGATCTGACAAACCCAA	60.4	625	INTERGENIC	--	--	--
Ca2_P2_3710445	Ca2	3710445	CTTTTTTT/CTTTTTTTTTTTTT	TTGGGTTTGTCAGATCCGTT	CACACTCCAAAATGTCACATCA	59.5	558	INTERGENIC	--	--	--
Ca2_P2_3711910	Ca2	3711910	A/AT	TGTTGAGATTCAACGATTTGATTT	GCTTGGCACGAACTACACAC	59.4	715	INTERGENIC	--	--	--
Ca2_P2_3711927	Ca2	3711927	TA/T	TGTTGAGATTCAACGATTTGATTT	GCTTGGCACGAACTACACAC	59.4	715	INTERGENIC	--	--	--
Ca2_P2_3723233	Ca2	3723233	TATTATCAATTATCAATTA/TATTATCAATTA	ATTTCGTTGCGTTTTGGACT	TGGTCAATTTTTGTTTGCCA	59.9	234	INTERGENIC	--	--	--

INDEL marker IDs	Chromosomes/unanchored scaffolds	Physical positions (bp)	InDels (*Kabuli* reference genome-CDC Frontier/PII)	Forward primers (5'-3')	Reverse primers (5'-3')	Anneling temperature (0C)	Expected amplified product size (bp)	Structural annotation		Functional annotation	
								Sequence components of *kabuli* genome	*Kabuli* gene accession IDs	NCBI-KOG	TFs
Ca2_P2_3723695	Ca2	3723695	TAAAAAA/TAAAAAAA	TGGCAAACAAAAATTGACCA	TTCCTCAAATTGCAACACCA	60.1	752	INTERGENIC	--	--	--
Ca2_P2_3733191	Ca2	3733191	TTAGAGACTAA/T	ACCTGCAACATGTGCGATAA	TGCATGAGATTGGAAATGGA	60.0	349	DRR	Ca_10489	G	C3H
Ca2_P2_3733907	Ca2	3733907	AGG/AG	CCAAGCAAATTGTGCACCTA	GGTGTTGGTGCTAACGAGGT	60.0	554	DRR	Ca_10489	G	C3H
Ca2_P2_3738112	Ca2	3738112	CAAAA/CAAAAA	GATGAGACAGCCAAGCCTTC	CCCACCCTTTTGTGTTTTGT	59.7	563	INTRON	Ca_10488	--	WRKY
Ca2_P2_3739763	Ca2	3739763	AATATATATATATATAT/AATATATATATAT	GTTTCGTCCACAAAATGGCT	AGGTTTTCTCACCATCACCG	60.0	689	INTRON	Ca_10488	--	WRKY
Ca2_P2_3741294	Ca2	3741294	CT/CTTT	GGTGGAGAGGGATGATGAGA	GCAGAGCACCAAACACAAGA	60.0	605	DRR	Ca_10487	--	--
Ca2_P2_3743015	Ca2	3743015	A/AATACAAATGGTG	CACCACTGGATTTAGCCGTT	TCCTTTTCGCTGATGAAACA	59.4	722	INTRON	Ca_10487	--	--
Ca2_P2_3743829	Ca2	3743829	CTTTTTT/CTTTTT	TTGGCCAATACTCCAACCAT	CAGCAAGTCCCACGAAAGAT	60.3	311	INTRON	Ca_10487	--	--
Ca2_P2_3745143	Ca2	3745143	TAAAA/TAAA	GAAGCACTGGACCTCCCATA	TGACTTGGAATAGATTGGACCAT	59.7	630	INTERGENIC	--	--	--
Ca2_P2_3746503	Ca2	3746503	TTA/TTAAACATA	TCCACATTGCACCAACACTT	TTGCTCCTCTGGTCCTCACT	60.0	925	INTERGENIC	--	--	--
Ca2_P2_3752919	Ca2	3752919	ATTT/ATTTT	GGTACCACATGGGTTGGAAT	CACTCAGCAACTCTTTCTGCC	60.2	344	INTRON	Ca_10486	DL	--
Ca2_P2_3754191	Ca2	3754191	TCC/TC	TCCCTGGTTCTCGTGAGACT	CATGTTGAAAGCACACCACC	60.0	500	DRR	Ca_10486	DL	--

INDEL marker IDs	Chromosomes/unanchored scaffolds	Physical positions (bp)	InDels (*Kabuli* reference genome-CDC Frontier/PII)	Forward primers (5'-3')	Reverse primers (5'-3')	Anneling temperature (0C)	Expected amplified product size (bp)	Structural annotation		Functional annotation	
								Sequence components of *kabuli* genome	*Kabuli* gene accession IDs	NCBI-KOG	TFs
Ca2_P2_3754940	Ca2	3754940	GA/GATA	TCCTTCAACTGTGGAACCAA	GCTTCTTGTGTGGGCTTAGG	59.9	751	DRR	Ca_10486	DL	--
Ca2_P2_3755215	Ca2	3755215	CTTTT/CTTT	AGGCAACACAACAAGCACAA	TCTTGTGTGGGCTTAGGCAT	60.7	326	DRR	Ca_10486	DL	--
Ca2_P2_3755748	Ca2	3755748	GTTTTTT/GTTTTTTT	GCCTAAGCCCACACAAGAAG	AGCTGATCCGACTGATTTTTG	59.3	742	DRR	Ca_10486	DL	--
Ca2_P2_3755819	Ca2	3755819	CTTTTTTTT/CTTTTTTT	CCTAAGCCCACACAAGAAGC	ACCTATTTGTGTTCGTGGGC	59.9	869	DRR	Ca_10486	DL	--
Ca2_P2_3756233	Ca2	3756233	ATTTT/ATTTTT	CAAAAATCAGTCGGATCAGC	TTTAGAATGCGAAAATCGGG	60.0	287	INTERGENIC	--	--	--
Ca2_P2_3758161	Ca2	3758161	CTCTCTCTCTCTCTCTCTCTCTCTCTCT/CTCTCTCTCTCTCTCTCTCTCTCTCTCTCTCTCT	GGTGTTTGTGCTCCATAGGC	GAAACAGCACTGCCCAATTT	60.1	200	DRR	Ca_10485	J	--
Ca2_P2_3758374	Ca2	3758374	G/GTTATAT	AAATTGGGCAGTGCTGTTTC	GGAGGTTGGAGGAGACACAA	60.1	428	DRR	Ca_10485	J	--
Ca2_P2_3760103	Ca2	3760103	CTTTTTTT/CTTTTTTTT	GTGAAGCTTTTGGTGGTGGT	TTCCAATCCAACCAATGAAA	58.8	636	DRR	Ca_10484	--	--
Ca2_P2_3761698	Ca2	3761698	TAA/TAAA	ACCCTCCATTGCTGTTTTTG	CTCGGAACATGCATAGAATGA	58.7	262	INTERGENIC	--	--	--
Ca2_P2_3766566	Ca2	3766566	ATTTTT/ATTTTTT	GAGGTGCTGAGGTCCCTTTA	ATACCAGGACCACCCTACCC	59.9	831	INTERGENIC	--	--	--
Ca2_P2_3766913	Ca2	3766913	ATTTTTT/ATTTTTTT	AGAACCATGTGCTTCATCCC	CAACACAATTGAATGGCAGG	60.0	478	INTERGENIC	--	--	--
Ca2_P2_3768714	Ca2	3768714	CAAAAAAAAAA/CAAAAAAAAAAAA	ACCTCAAAATTAGTCGGTCGT	TATTTTTCCAGGTTCGCAGG	60.1	430	INTERGENIC	--	--	--

INDEL marker IDs	Chromosomes/unanchored scaffolds	Physical positions (bp)	InDels (*Kabuli* reference genome-CDC Frontier/PII)	Forward primers (5'-3')	Reverse primers (5'-3')	Anneling temperature (0C)	Expected amplified product size (bp)	Structural annotation		Functional annotation	
								Sequence components of *kabuli* genome	*Kabuli* gene accession IDs	NCBI-KOG	TFs
Ca2_P2_3771310	Ca2	3771310	TTATATATATATATATA/TTATATATATATA	GCTCGACGGAATAGCATTTT	TGGCCAAGCATGATCTAACA	60.2	612	DRR	Ca_10483	E	--
Ca2_P2_3771545	Ca2	3771545	AT/A	GCTCGACGGAATAGCATTTT	TGGCCAAGCATGATCTAACA	60.2	612	DRR	Ca_10483	E	--
Ca2_P2_3771652	Ca2	3771652	TTGA/TTGAGCTATTATATATGA	GGACACGGTCCTTTAAACAAA	TGGCCAAGCATGATCTAACA	60.2	223	DRR	Ca_10483	E	--
Ca2_P2_3777166	Ca2	3777166	AA/AACTGATTA	AATTTTTGGGGGAAAAATGC	CTCTGATAGACCCTCCCACCT	59.6	646	INTERGENIC	--	--	--
Ca2_P2_3777372	Ca2	3777372	AATATA/AATATATA	AATTTTTGGGGGAAAAATGC	CTCTGATAGACCCTCCCACCT	59.6	646	INTERGENIC	--	--	--
Ca2_P2_3782950	Ca2	3782950	TAAAAAAA/TAAAAAAAA	TGCCATGGGAGCTCTATGAT	CGCACTTGGAGTTGATGTGT	59.8	554	INTERGENIC	--	--	--
Ca2_P2_3783594	Ca2	3783594	ATTTTTTT/ATTTTTT	ACACATCAACTCCAAGTGCG	CAAACACTGAAACACACCCG	60.0	756	INTERGENIC	--	--	--
Ca2_P2_3783648	Ca2	3783648	A/AT	ACACATCAACTCCAAGTGCG	CAAACACTGAAACACACCCG	60.0	756	INTERGENIC	--	--	--
Ca2_P2_3785084	Ca2	3785084	ATT/A	CATGCACTAAAACAATATTGACCA	CCCAAATTAGGGTTTGGGTT	59.9	870	INTERGENIC	--	--	--
Ca2_P2_3785150	Ca2	3785150	GTTT/GTTTT	AAACTCTGTGACGACTCAAAAAT	CCCAAATTAGGGTTTGGGTT	59.9	352	INTERGENIC	--	--	--
Ca2_P2_3786172	Ca2	3786172	TGGG/TGG	ATTGAGGAGGGATGCACAAG	TGCTCAGCTGAAGAGATCGT	58.9	614	INTRON	Ca_10482	S	--
Ca2_P2_3786408	Ca2	3786408	GA/GAAA	ATTGAGGAGGGATGCACAAG	TTAGGAAGAGGCCCTGGAAT	60.0	860	INTRON	Ca_10482	S	--

INDEL marker IDs	Chromosomes/unanchored scaffolds	Physical positions (bp)	InDels (*Kabuli* reference genome-CDC Frontier/PII)	Forward primers (5'-3')	Reverse primers (5'-3')	Anneling temperature (0C)	Expected amplified product size (bp)	Structural annotation		Functional annotation	
								Sequence components of *kabuli* genome	*Kabuli* gene accession IDs	NCBI-KOG	TFs
Ca2_P2_3788353	Ca2	3788353	TAAAAAA/TAAAA	GAAAGAGGTGGATTGTTAAAGGG	CCCCAGACCATGCTTTTCTA	60.1	696	DRR	Ca_10481	O	--
Ca2_P2_3800163	Ca2	3800163	TA/TAA	TTCCTCATTTTTCTCTTGGTTTG	CCACACTGCGCTTCAGATAA	60.0	377	INTERGENIC	--	--	--
Ca2_P2_3805996	Ca2	3805996	TAAAAA/TAAAAAA	GAACAAGAGCAGGCGTTAGG	CCACCCCTTGAATCAAAAGA	59.9	567	INTRON	Ca_10479	--	--
Ca2_P2_3806132	Ca2	3806132	GTTCGATTCGA/GTTCGA	GAACAAGAGCAGGCGTTAGG	CCACCCCTTGAATCAAAAGA	59.9	567	INTRON	Ca_10479	--	--
Ca2_P2_3809090	Ca2	3809090	TTT/TTTAAACAAAAGAGTT	TGACGACGGCAAGAGTGTAG	CTAAAATAAGGCACGCCGAA	60.2	862	DRR	Ca_10479	--	--
Ca2_P2_3809184	Ca2	3809184	TTATATATATATATATATATATATATATATAT/TTATATATATATATATATATATATATATAT	TCTTCGAGCAATTGTGTGCT	CTAAAATAAGGCACGCCGAA	60.2	538	DRR	Ca_10479	--	--
Ca2_P2_3810432	Ca2	3810432	ATTTT/ATTTTT	TGAGATGAGGGAATCCAAGG	TCCTCTATTGTTTGGTGCCTG	60.1	431	DRR	Ca_10479	--	--
Ca2_P2_3812277	Ca2	3812277	TATTAATTAATTAATTA/TATTAATTAATTA	TGTTCTGCAAAATCGCAAAG	TGGTGACATGACTAATTTTGGA	58.0	414	INTERGENIC	--	--	--
Ca2_P2_3813617	Ca2	3813617	CAA/CAAA	TTGAAGCATACCTTGAGTGGTG	TTAATCTGGTTTTGGTGGGC	59.8	570	INTERGENIC	--	--	--
Ca2_P2_3817971	Ca2	3817971	ACTCTCTCTCTCTCTC/ACTCTCTCTCTCTC	TCCTCACCAACCAAACCAAC	AGAGAACGTGGAAGCAAGGA	60.0	540	INTERGENIC	--	--	--
Ca2_P2_3857450	Ca2	3857450	TTTTATTTATTTATTTATTTATTTATTT/TTTTATTTATTTATTTATTTATTTATTTATTTATTTATTTATTT	GGGAAATTGTGGTGTGAGGT	TTCGTGCAACGCTATAATTCC	60.1	513	INTERGENIC	--	--	--
Ca2_P2_3874478	Ca2	3874478	CCTTCTTC/CCTTCTTCTTC	TTTGCATCCTAAGGACCACAC	AGAGGTGGAGAAGCTGGTCA	60.0	515	CDS (large-effect mutations)	Ca_10470	GC	HSF

INDEL marker IDs	Chromosomes/unanchored scaffolds	Physical positions (bp)	InDels (*Kabuli* reference genome-CDC Frontier/PII)	Forward primers (5'-3')	Reverse primers (5'-3')	Anneling temperature (0C)	Expected amplified product size (bp)	Structural annotation		Functional annotation	
								Sequence components of *kabuli* genome	*Kabuli* gene accession IDs	NCBI-KOG	TFs
Ca2_P2_3901584	Ca2	3901584	C/CGAG	TTCAAGTTCAGCAGTGACCG	ACAGCAAACTTATGGGCACC	60.0	565	DRR	Ca_10467	G	--
Ca2_P2_3901844	Ca2	3901844	AC/ACTTCACGAGC	GTCGCTATCTCCCCAGTGAA	CATTCTGTGCCGCTATCTGA	60.0	248	DRR	Ca_10466	GT	--
Ca2_P2_3944863	Ca2	3944863	AATATATATATATATATATATATATATATATATAT/AATATATATATATATATATATATATATATATATATATATATAT	AAGGCAAAAGCAACAAAAGC	GATGCACAATCAATGTGTTCG	60.0	417	INTERGENIC	--	--	--
Ca2_P2_4013903	Ca2	4013903	ATTTTTTT/ATTTTTTTT	CGGTCACAAGCATCTCATGT	TGATGCTTAAAAACTATGGGACAA	59.9	669	INTERGENIC	--	--	--
Ca2_P2_4014195	Ca2	4014195	AATATATATATATATATATAT/AATATATATATATATATATATATAT	TGGAAATTATGAGTCCCTTGA	TGAATACCAACGACACACCG	60.4	642	INTERGENIC	--	--	--
Ca2_P2_4033531	Ca2	4033531	CTTTTTTTTTTT/CTTTTTTTTTT	TGCGGTCCAATCCAAATTAT	TTCTCATCCGTGCTCACAAG	60.0	703	INTERGENIC	--	--	--
Ca2_P2_4051277	Ca2	4051277	CAAAAAAAAAAA/CAAAAAAAAAAAA	TTCTTGGACGTTTCACCCAT	TAACGTGCACATGAAAAGGG	59.6	223	INTERGENIC	--	--	--
Ca2_P2_4060085	Ca2	4060085	AATATATATATATATATATATATATATA/AATATATATATATATATATATATA	GACCTTGCATGGAGTGTTGA	GGCATAGCCATGAAGAACTTG	59.7	690	INTERGENIC	--	--	--
Ca2_P2_410847	Ca2	410847	AATATATATATATATATATATATATATATATATATATATATATATATATATATA/AATATATATATATATATATATATATATATATATATATATATA	AATCTTTGCAAGGTTATCCGA	TGTTTCAACGAGGAAACGTG	59.7	551	INTERGENIC	--	--	--
Ca2_P2_4116151	Ca2	4116151	GTTTTTTTTTT/GTTTTTTTTTTT	TTTTTATGACGCAGATGCCC	TGGATCCTTACATGGTCGGT	60.2	665	INTERGENIC	--	--	--
Ca2_P2_4118911	Ca2	4118911	TAAAAAAAAAAA/TAAAAAAAAAA	ATCAACGAAACGCAACACAA	ATTGGGGTTTGTCATCTTGC	59.8	233	INTERGENIC	--	--	--
Ca2_P2_4123258	Ca2	4123258	AATATATATATATA/AATATATATATATA	GCCCAATCATTTTCAAGGAA	CGTTCAACTCCCACAGGATT	60.0	667	INTERGENIC	--	--	--

INDEL marker IDs	Chromosomes/unanchored scaffolds	Physical positions (bp)	InDels (*Kabuli* reference genome-CDC Frontier/PII)	Forward primers (5'-3')	Reverse primers (5'-3')	Anneling temperature (0C)	Expected amplified product size (bp)	Structural annotation		Functional annotation	
								Sequence components of *kabuli* genome	*Kabuli* gene accession IDs	NCBI-KOG	TFs
Ca2_P2_4204531	Ca2	4204531	GTATATATATATATATATATATATATA/GTATATATATATATATATATATA	GGGTTTGAACCGTCCCTACT	CTTTCTCCATCTCCTTCCCC	60.0	633	INTRON	Ca_21114	--	--
Ca2_P2_42172	Ca2	42172	TTATATATATATATATATATATATA/TTATATATATATATATA	TGGCAGTCTGTTTCACGTTT	CAAGGGACCAGATTCTGCAT	60.1	553	INTERGENIC	--	--	--
Ca2_P2_4217766	Ca2	4217766	TTACTA/TTA	TTCATCCCCTCAAGTACAAGC	TTTTCAGCTTTCAACTCATTCA	57.7	608	INTERGENIC	--	--	--
Ca2_P2_422540	Ca2	422540	G/GTCTTCCT	AGAGTTGGAGGGTGCAGCTA	CTTCGAATCCAACCTTGGTG	60.5	829	INTERGENIC	--	--	--
Ca2_P2_4253061	Ca2	4253061	A/AC	CACGTGTCAATGTTGTGTCG	TGTGCCTGAATCCACAAAAA	60.1	272	INTRON	Ca_21112	--	--
Ca2_P2_4285697	Ca2	4285697	TAAAA/TAAA	CCAGTCCCGAAATTAAACGA	ACTTGCGATAATCGATTGGG	59.9	613	INTERGENIC	--	--	--
Ca2_P2_4353642	Ca2	4353642	CAAAAAAAAAA/CAAAAAAAAAAA	AGAATGTTCTCCACAACGCC	ATGGGGTTCCTTTTATTGGC	60.0	500	INTRON	Ca_21108	GC	HSF
Ca2_P2_4412680	Ca2	4412680	CAAAAAAAA/CAAAAAAA	TTGAGGTAGCCCAATCCAAG	TGCACCCACTAACGATCAAA	60.1	539	INTERGENIC	--	--	--
Ca2_P2_4418355	Ca2	4418355	CTTTTTTTTTT/CTTTTTTTTTTT	TGTGGCGTTTTGATTTTTCA	GGTTGTTTTTGCATTTGGGT	59.7	371	INTERGENIC	--	--	--
Ca2_P2_4513905	Ca2	4513905	GAAAAAAAAAAA/GAAAAAAAAAAAA	ACGCGTTGAAGTGTTGTTTG	GTGGTTGCGGAGATAGTGGT	60.0	944	INTERGENIC	--	--	--
Ca2_P2_4527479	Ca2	4527479	GAAGACAAAAAAAACATAAGACAAAAAAAACAT/GAAGACAAAAAAAACATAAGACAAAAAAAACATAAGACAAAAAAAACAT	GCAATTTCACCTCTTCTGCC	TGACGAAGCCCACAATAACA	60.1	523	INTERGENIC	--	--	--
Ca2_P2_4538239	Ca2	4538239	CATATATATATATATATAT/CATATATATATATATATATAT	TGATTTGTCGATTGGGTTGA	TGATTTGAGGCACCGGATA	60.0	266	INTERGENIC	--	--	--

INDEL marker IDs	Chromosomes/unanchored scaffolds	Physical positions (bp)	InDels (*Kabuli* reference genome-CDC Frontier/PII)	Forward primers (5'-3')	Reverse primers (5'-3')	Anneling temperature (0C)	Expected amplified product size (bp)	Structural annotation		Functional annotation	
								Sequence components of *kabuli* genome	*Kabuli* gene accession IDs	NCBI-KOG	TFs
Ca2_P2_4557579	Ca2	4557579	CAAAAAA/CAAAAAAA	ACTGACATTCGCGAGCTTTT	AATTATTAGCACGGGCAGCA	60.6	401	INTERGENIC	--	--	--
Ca2_P2_469349	Ca2	469349	TA/TAA	CCCAAGACAACCTTGCAGAT	ATGAAGTGCAAACCATGCTG	59.7	674	DRR	Ca_16986	--	--
Ca2_P2_4700407	Ca2	4700407	ATTATTTATTTATTTATTTATTTATT/ATTATTTATTTATTTATTTATT	TCTCACGGTTGTGAGGACTG	CCTACCTCTGGAGTGTCCGT	59.2	926	INTERGENIC	--	--	--
Ca2_P2_4713992	Ca2	4713992	AC/ACCC	TTAAGTCTGGCCCATCAAGG	TGCTTTGTCAACCTTGCTACA	59.5	239	INTERGENIC	--	--	--
Ca2_P2_4793170	Ca2	4793170	GATAATAATAATAATAATAATAATAAT/GATAATAATAATAATAATAATAATAATAAT	GCCATGTGGAAGAACAAGGT	CCAAAACTGGGGAGAAACAA	59.9	669	INTERGENIC	--	--	--
Ca2_P2_4812881	Ca2	4812881	ATTTTTTT/ATTTTTTTTT	AACCAGTTCCGTTCCAAGTG	TCCCCCAAACAGAATTTGAG	59.9	731	DRR	Ca_21090	--	MYB
Ca2_P2_4824675	Ca2	4824675	TAAAAAAAAAA/TAAAAAAAAA	CCCCAAGTTCACAATATGGC	TCATTGATTCATTTCTCCGGT	59.4	524	INTERGENIC	--	--	--
Ca2_P2_4850252	Ca2	4850252	GTTATTATTATTATTATTATTATTATTATTATTATTATTATTATTATT/GTTATTATTATTATTATTATTATTATTATTATTATTATTATT	AAATTCATCCTCGCTCGCTA	TTTCCCGACTTGGTCCTTTA	59.5	658	INTERGENIC	--	--	--
Ca2_P2_4892794	Ca2	4892794	CATATATATATATATATATATATATATATATAT/CATATATATATATATATATATATATATATATATATATATATAT	GCGGACATAAGGAAGATGTGA	AGGTCTGCGAAGATGGAAAA	59.8	606	INTERGENIC	--	--	--
Ca2_P2_4894086	Ca2	4894086	TA/TAGACAACTCAA	TTGAACTACAACCAGCGCAC	TTTTGTTCAATTTCTTGCTCCA	59.7	493	INTERGENIC	--	--	--
Ca2_P2_4895677	Ca2	4895677	AA/AATATGA	CCAGGATAAAACAGCCCAGA	TGGATGCTTTCCCAACTTTC	60.1	467	INTERGENIC	--	--	--
Ca2_P2_4913166	Ca2	4913166	AAATAATAATAATAATAATAATAATAATAATAATAATAATAATAATAATAATAATAA/AAATAATAATAATAATAATAATAATAATAATAATAATAATAATAA	CCTTGATGTCGCTGCTTACA	AGAGGCAAACAAGAACCGAA	59.9	241	INTERGENIC	--	--	--

INDEL marker IDs	Chromosomes/unanchored scaffolds	Physical positions (bp)	InDels (*Kabuli* reference genome-CDC Frontier/PII)	Forward primers (5'-3')	Reverse primers (5'-3')	Anneling temperature (0C)	Expected amplified product size (bp)	Structural annotation		Functional annotation	
								Sequence components of *kabuli* genome	*Kabuli* gene accession IDs	NCBI-KOG	TFs
Ca2_P2_5003705	Ca2	5003705	G/GATT	TGTAGCATGCAGGTTGAAGG	GAAAGGACTTTGTCCAGCCA	60.2	605	INTRON	Ca_14709	I	--
Ca2_P2_5027666	Ca2	5027666	TAAAAAAAAA/TAAAAAAAA	AAATGCAGGAACTTGGAGGA	TGAATTTTCACATTTGCTTACCA	59.5	658	INTERGENIC	--	--	--
Ca2_P2_5062465	Ca2	5062465	TTATATATATATATATATATATATATATATATATATATAT/TTATATATATATATATATATATATATATATAT	TGGTTTGTCGTACGTTCTCCT	AGTGTGGCAAAGATCCAAAAA	59.6	536	INTERGENIC	--	--	--
Ca2_P2_5096452	Ca2	5096452	AATATATATATATATATATATATATAT/AATATATATATATATATATATATATAT	GTTTTGTGGTGCAGTTTGGA	TTTGAAAGAGTCCGAATTCAAATA	59.2	570	INTERGENIC	--	--	--
Ca2_P2_5108884	Ca2	5108884	TAAAAAAAAAAAA/TAAAAAAAAAAA	CGTTCTCCGTTTTCTGTTTTC	TACCGTGTGCGAAATACCAA	60.0	925	INTERGENIC	--	--	--
Ca2_P2_5135372	Ca2	5135372	AATATATATATATATATATATATATATATATATATATATATATATAT/AATATATATATATATATATATATATATATATATATATATAT	TGATTTATTTCCACGCCTACG	TTGTTTCTCCCCAGTTTTGG	59.9	952	INTERGENIC	--	--	--
Ca2_P2_5163788	Ca2	5163788	CAAAAAAAAA/CAAAAAAAAAA	TGCGAGAAAGTGAATTCCAG	GGAATGATGATGGGGTTTTG	60.0	658	DRR	Ca_14696	--	--
Ca2_P2_5175283	Ca2	5175283	TAAAAAAAAAAA/TAAAAAAAAAA	AATGCCAAAATCCACCACAT	GAGCAAAGACATCACCAGCA	60.0	769	CDS (FRAME SHIFT)	Ca_14694	--	--
Ca2_P2_5286155	Ca2	5286155	AATATATATATATATATATATATATATATA/AATATATATATATATATATATATATATATATATA	TTCATGCCTCCCATTTCTTC	CGCTCTGTCAACATGTCAGC	60.6	752	INTRON	Ca_14679	T	--
Ca2_P2_5466464	Ca2	5466464	GGAGAGAGAGAGAGAGAGAGAGAGAGAGAGAGAGAGAGAGA/GGAGAGAGAGAGAGAGAGAGAGAGAGAGAGAGAGAGAGAGAGAGAGAGAGAGA	GAGGCGGCAAGTAAAGTGTC	TCCAACTTCAACTCCAACCC	59.9	444	INTERGENIC	--	--	--
Ca2_P2_5502541	Ca2	5502541	TTCTCTCTCTCTCTCTCTCTCTCT/TTCTCTCTCTCTCTCTCTCTCTCT	CCAACCCTATTAATGGCAGC	TGGAATGAACAAACACCATGA	59.8	633	INTERGENIC	--	--	--
Ca2_P2_5518338	Ca2	5518338	AATATATATATATATAT/AATATATATATATATATAT	AAGATGGAGCTGTCCACGAC	GTACTGGCTTCTGAATCGCC	59.8	418	INTERGENIC	--	--	--

INDEL marker IDs	Chromosomes/unanchored scaffolds	Physical positions (bp)	InDels (*Kabuli* reference genome-CDC Frontier/PII)	Forward primers (5'-3')	Reverse primers (5'-3')	Anneling temperature (0C)	Expected amplified product size (bp)	Structural annotation		Functional annotation	
								Sequence components of *kabuli* genome	*Kabuli* gene accession IDs	NCBI-KOG	TFs
Ca2_P2_5524326	Ca2	5524326	A/ACAG	AATGAAAAACTTGTTGCGGC	CGTTGGGATTTGTTTATCGG	60.2	397	INTERGENIC	--	--	--
Ca2_P2_55390	Ca2	55390	GACACACACACACACACA/GACACACACACACACACA	GCACCGGGAGATACGTAAGA	TCACGGCTTCACAAAACAAG	59.9	798	INTERGENIC	--	--	--
Ca2_P2_5566169	Ca2	5566169	GAAAAAAAAAAA/GAAAAAAAAAAAA	TGGGAGTATCCTTCACTGCC	TTCGGATTTTCCTTCCCTTT	59.9	836	INTERGENIC	--	--	--
Ca2_P2_5626731	Ca2	5626731	ATTTTTTTTTTT/ATTTTTTTTTT	GAACCCAATATAGCCGCAAA	TTTGCTGCAACAACTCCAAG	60.0	782	INTRON	Ca_14650	I	C3H
Ca2_P2_5674299	Ca2	5674299	TCTTTACTTTACTT/TCTTTACTTTACTTTACTT	ATTAGTCCACTGTGCTGGGG	GCTTCCAAATTGCTTGTTCC	59.7	815	INTRON	Ca_14646	O	FAR1
Ca2_P2_5743663	Ca2	5743663	TAA/TA	TCAAAATTTGCGGTGAGTGA	CGGCCTACTTGGAATGTGTT	60.0	345	INTERGENIC	--	--	--
Ca2_P2_5751248	Ca2	5751248	TC/TCC	CCCAACTTCCCAAGAGTCAA	TCGTGTAGCACATTTGGAGC	59.9	722	INTERGENIC	--	--	--
Ca2_P2_5752201	Ca2	5752201	ATGTTGT/ATGT	TAACGGTTTCCGTTTCATGC	GCAACCATTGAAGAGAAATGG	59.6	699	INTERGENIC	--	--	--
Ca2_P2_5767970	Ca2	5767970	GAA/GAGTAAAAACAA	AAAATCCAACAATCTCCCCC	CGCCTAAGGACTATGCAAGC	60.0	589	DRR	Ca_14639	--	--
Ca2_P2_5859673	Ca2	5859673	ATATAAT/ATATAATTATAAT	CGGACATTTCAATCATTCACC	TTGGCATTGTACGGTTTTTG	59.5	405	INTERGENIC	--	--	--
Ca2_P2_6075442	Ca2	6075442	CAAAA/CAAA	CGGAGTCTAATGTGGACGGT	TGCGTATGCACTCTTATCGC	60.0	711	INTRON	Ca_19709	--	--
Ca2_P2_6317566	Ca2	6317566	GTATATATATATATATATATATATATATAT/GTATATATATATATATATATATATATAT	CACCAAATGATGACGACGAC	ATTCACGTCGTTCCTGTTCC	60.0	324	INTERGENIC	--	--	--

INDEL marker IDs	Chromosomes/unanchored scaffolds	Physical positions (bp)	InDels (*Kabuli* reference genome-CDC Frontier/PII)	Forward primers (5'-3')	Reverse primers (5'-3')	Anneling temperature (0C)	Expected amplified product size (bp)	Structural annotation		Functional annotation	
								Sequence components of *kabuli* genome	*Kabuli* gene accession IDs	NCBI-KOG	TFs
Ca2_P2_6505178	Ca2	6505178	TA/TAA	CGTAATAACGGCGAGGAAAA	TGAGCAGGGGGAAGAGTAGA	59.9	548	INTERGENIC	--	--	--
Ca2_P2_6522206	Ca2	6522206	AT/ATT	GGAAGTGAAGGTCACGGTGT	TGAGCACGAATAATTGCAGC	60.0	593	DRR	Ca_20947	--	--
Ca2_P2_6640316	Ca2	6640316	TAAAAAA/TAAAAAAA	ATGGCCACGAATCTGAAGAG	ACGGTTGTTTTGGCATTGAT	60.2	890	INTERGENIC	--	--	--
Ca2_P2_6640452	Ca2	6640452	GAAAA/GAAAAA	AAAAAGGTCCTAGGGTGGGA	ACGGTTGTTTTGGCATTGAT	60.2	473	INTERGENIC	--	--	--
Ca2_P2_6641737	Ca2	6641737	CATATAT/CATATATATAT	GTATTCAGGGGCGAAGGAAT	AATGCAAACGACAAAGGAGG	60.1	500	INTERGENIC	--	--	--
Ca2_P2_6641916	Ca2	6641916	TG/T	GTTCTATGGGGACAGGGTCA	TATCAACTACCAGAGCGGGG	60.1	476	INTERGENIC	--	--	--
Ca2_P2_6642487	Ca2	6642487	ATTTT/ATTTTTTTTT	CCCCGCTCTGGTAGTTGATA	ACCCACTTTGAGAAACGTGC	60.2	479	INTERGENIC	--	--	--
Ca2_P2_6642515	Ca2	6642515	TTATATATATATATATA/TTATATATATATATATA	CCCCGCTCTGGTAGTTGATA	ACCCACTTTGAGAAACGTGC	60.2	479	INTERGENIC	--	--	--
Ca2_P2_6643765	Ca2	6643765	AT/ATT	ATGGAACAAGCAGCGAAGTT	GTTGATACGAGGGGGTTGAA	59.8	537	INTERGENIC	--	--	--
Ca2_P2_6643843	Ca2	6643843	CTTTTTTTTTTT/CTTTTTTTT	ATGGAACAAGCAGCGAAGTT	GTTGATACGAGGGGGTTGAA	59.8	537	INTERGENIC	--	--	--
Ca2_P2_6648530	Ca2	6648530	TAAAAAA/TAAAAAAAA	TTATCGATCGTCGGTGTCAA	TTCCATATGCACCTCCCATT	60.2	612	INTERGENIC	--	--	--
Ca2_P2_6648980	Ca2	6648980	ATTTTTTTTTT/ATTTTTTTTTTT	AGCCTAATGGGAGGTGCATA	TATGAGGGGGTTGAATTGGA	60.1	563	INTERGENIC	--	--	--

INDEL marker IDs	Chromosomes/unanchored scaffolds	Physical positions (bp)	InDels (*Kabuli* reference genome-CDC Frontier/PII)	Forward primers (5'-3')	Reverse primers (5'-3')	Anneling temperature (0C)	Expected amplified product size (bp)	Structural annotation		Functional annotation	
								Sequence components of *kabuli* genome	*Kabuli* gene accession IDs	NCBI-KOG	TFs
Ca2_P2_6690462	Ca2	6690462	CAAAAAAAAAA/CAAAAAAAAAAA	TCCAATTCAACCCCCTCATA	GGGAACCAAAACAAAAAGCA	60.0	299	INTERGENIC	--	--	--
Ca2_P2_6742633	Ca2	6742633	AATTATTATTATTATTATTATTATTATTATTATTATTATTATTATT/AATTATTATTATTATTATTATTATTATTATTATTATTATT	CAGGCGTACTGTGGGTTTTT	CTTAATCGACCGGCGTATGT	60.0	520	INTRON	Ca_19156	C	FAR1
Ca2_P2_6761366	Ca2	6761366	CTT/CTTT	TTAAACTTGAGCAGGGCTCG	TGGAGCTTTACTGGGTGAAGA	59.9	622	DRR	Ca_19154	O	ERF
Ca2_P2_6815005	Ca2	6815005	ATTATTTATTTATTTATTTATTTA/ATTATTTATTTATTTATTTA	GAAATTTTAGGCGCAACAGC	TCCAATCCCATTGTCCACTT	60.2	703	INTERGENIC	--	--	--
Ca2_P2_6831436	Ca2	6831436	AC/A	CCTGTGACATGTTGCCTTTG	AAATGGGCTGAGTTTGAACA	58.2	360	INTERGENIC	--	--	--
Ca2_P2_6867609	Ca2	6867609	CAAAAAAAAAAA/CAAAAAAAAAA	TAACCACATTGCAAGCCAAA	CAATCAAAATGCAACAGACACA	59.6	451	INTERGENIC	--	--	--
Ca2_P2_6998417	Ca2	6998417	TATGATGATGATGATGATGATGATGATGATG/TATGATGATGATGATGATGATGATGATGATGATG	TAAGGCATCATTCTCACCCC	CCAACTTTGCAATTTGCCTT	60.1	420	DRR	Ca_19137	S	--
Ca2_P2_7043443	Ca2	7043443	AATATATATATATATATATATATAT/AATATATATATATATATATATATATATAT	ATGGGTCGTGTTCGATTGTT	AACCAACTATTGACCCCAAAAA	59.6	267	INTERGENIC	--	--	--
Ca2_P2_7144237	Ca2	7144237	A/AG	GGACCGGAATCATAATGCAA	TTTTTCCGTAAAAGTCCCCC	60.2	743	INTERGENIC	--	--	--
Ca2_P2_7171869	Ca2	7171869	TTAT/TT	CGGCCATTAAATTTCTGACA	TTGCATTATGTTTCTGGCAAA	59.2	320	INTERGENIC	--	--	--
Ca2_P2_7171912	Ca2	7171912	TATTTAATTTAATTTAATT/TATTTAATTTAATT	CGGCCATTAAATTTCTGACA	TCATTTCATAACGAGTGACCCA	60.4	584	INTERGENIC	--	--	--
Ca2_P2_7172121	Ca2	7172121	CTATATATATATAT/CTATATATATAT	TTTGCCAGAAACATAATGCAA	TCATTTCATAACGAGTGACCCA	60.4	285	INTERGENIC	--	--	--

INDEL marker IDs	Chromosomes/unanchored scaffolds	Physical positions (bp)	InDels (*Kabuli* reference genome-CDC Frontier/PII)	Forward primers (5'-3')	Reverse primers (5'-3')	Anneling temperature (0C)	Expected amplified product size (bp)	Structural annotation		Functional annotation	
								Sequence components of *kabuli* genome	*Kabuli* gene accession IDs	NCBI-KOG	TFs
Ca2_P2_7172172	Ca2	7172172	ATTTTTTTTT/ATTTTTTTTTT	TTTGCCAGAAACATAATGCAA	TTTTGGAAAAATGAAGGACCA	59.4	774	INTERGENIC	--	--	--
Ca2_P2_7173102	Ca2	7173102	GAAAAAA/GAAAAA	CTCAATCCAAACACACGCTT	AATGACCACCTCTTGTTGCC	60.0	787	INTERGENIC	--	--	--
Ca2_P2_7206191	Ca2	7206191	CGG/CG	TATTGTACATGGCGACCGAA	TCACGGGTCAACACGATTTA	60.0	165	INTERGENIC	--	--	--
Ca2_P2_7249042	Ca2	7249042	CATATATATATATATATATATATATATATATA/CATATATATATATATATATATATATATATATATATATA	CGCAACGTGGAGATGAGTAA	TTTGAGAAGTGATGGGAGGG	60.0	359	INTERGENIC	--	--	--
Ca2_P2_7276294	Ca2	7276294	CAAAA/CAAAAA	TCCACCACAATCGTTAAGCA	AGCGGAGATTTTACCGGATT	59.9	676	INTERGENIC	--	--	--
Ca2_P2_7302005	Ca2	7302005	TTATATATATATATATATATA/TTATATATATATATATATATATA	TGTAGCAAAACGTGCAGACC	GGATATTTTGCTCTCTCGCAA	59.4	159	INTERGENIC	--	--	--
Ca2_P2_735929	Ca2	735929	TCACACACACACACACA/TCACACACACACA	TCAATCCTTTCTTTTCCCTCA	GGGAATACCATATTCTCCACCTT	59.5	232	INTERGENIC	--	--	--
Ca2_P2_7407914	Ca2	7407914	TTCTAGTTTTGGGATCCTCTAGTTTT/TTCTAGTTTTGGGATCCTCTAGTTTTGGGATCCTCTAGTTTT	TCCCTTTGGTTCAGGTTGTC	GGAAAAGCAAGGATGCTGAA	60.3	372	INTERGENIC	--	--	--
Ca2_P2_7535934	Ca2	7535934	TAAAAAAAAAA/TAAAAAAAAAAA	AACCGGACATTTTGTTGGAG	TGCACAAATTGGTGGAAAAA	59.9	626	INTERGENIC	--	--	--
Ca2_P2_753916	Ca2	753916	CTTTTTTTTTT/CTTTTTTTTT	TTGCCCATTAACTTTACCCG	ACTTGTGCATTTGCATTAGTTC	57.0	555	DRR	Ca_17000	--	ARF
Ca2_P2_7540916	Ca2	7540916	TATAGTAATAAATAGTAATAAAT/TATAGTAATAAAT	AATATTCGAACCTCCCACCC	TTAATCGTCCAACTCCCACC	59.8	890	INTERGENIC	--	--	--
Ca2_P2_7546466	Ca2	7546466	TATAAATAAATAAATAAA/TATAAATAAATAAATAAATAAA	TGCTCTTCGCAACACATTTC	TCCCAATTCATTCGCTTTTC	60.0	432	INTRON	Ca_18113	--	--

INDEL marker IDs	Chromosomes/unanchored scaffolds	Physical positions (bp)	InDels (*Kabuli* reference genome-CDC Frontier/PII)	Forward primers (5'-3')	Reverse primers (5'-3')	Anneling temperature (0C)	Expected amplified product size (bp)	Structural annotation		Functional annotation	
								Sequence components of *kabuli* genome	*Kabuli* gene accession IDs	NCBI-KOG	TFs
Ca2_P2_7555681	Ca2	7555681	GTTTTTTTTTTT/GTTTTTTTTTT	AATTTTGCGTTCATCGGAGA	AATCACACACGACACCGAGA	60.2	178	INTERGENIC	--	--	--
Ca2_P2_7557882	Ca2	7557882	TAAA/TAA	AAATTTGAATTCCGTCGCAG	TGTTAGAACTTCCCGCCACT	59.7	690	INTERGENIC	--	--	--
Ca2_P2_7590008	Ca2	7590008	TAAATT/TAAATTAAAATT	TTTTGTGATGGCTTTTGCAG	TTGATTTTAATTTATTTCGGTTGG	58.5	640	INTERGENIC	--	--	--
Ca2_P2_7617731	Ca2	7617731	CTAAAATAAAATAAAA/CTAAAATAAAATAAAATAAAATAAAA	CCAAGATCTCGTAGTGCAACA	TGGGTTTAGTCCGTTTTTCTTC	59.5	468	INTERGENIC	--	--	--
Ca2_P2_7626491	Ca2	7626491	AAAAATTAAAATTAAAATTAAA/AAAAATTAAAATTAAA	AACAAGCATTTTGTGAGGGC	ATTCGACTAGTGGTGGTGCC	60.0	931	DRR	Ca_18119	--	--
Ca2_P2_7644680	Ca2	7644680	AATATATATATATATATATATATAT/AATATATATATATATATATATAT	AAAGGACCGCATGTGCTATT	TGTTGCGGATGAAGAAGATG	59.8	475	INTERGENIC	--	--	--
Ca2_P2_7655016	Ca2	7655016	T/TAAA	CCGTTCCTTCCTGACATACC	GCAAAATTGGCTTCTTACGG	59.7	516	INTERGENIC	--	--	--
Ca2_P2_7655778	Ca2	7655778	TGAG/TG	CCCACAATAGCACCACTCTTG	AGGAAAAGCCAAAAAGCACA	59.9	793	INTERGENIC	--	--	--
Ca2_P2_7657147	Ca2	7657147	CTTTTT/CTTTTTT	TTATGTGGGGGATTTGGATG	TTCCACGTCCTTTTCATTCC	59.9	705	INTERGENIC	--	--	--
Ca2_P2_7657698	Ca2	7657698	CTTTTTTTT/CTTTTTTT	CCTCTTAGGATGTGCCCAAA	CGTTTGAATTGATGGAAAGTGA	60.0	573	DRR	Ca_18120	--	--
Ca2_P2_7657952	Ca2	7657952	CTTTTTTTTTTTTT/CTTTTTTTTTTTT	TTGAACGTCATGCACAACAA	ATGTGACGTGGCAGAGTCAA	60.3	591	DRR	Ca_18120	--	--
Ca2_P2_7668122	Ca2	7668122	TCCCCC/TCCCCCC	GGCCAGAGAGTAAGTCCACG	GATGTTGGTGCTTGTGTTGG	60.0	625	INTERGENIC	--	--	--

INDEL marker IDs	Chromosomes/unanchored scaffolds	Physical positions (bp)	InDels (*Kabuli* reference genome-CDC Frontier/PII)	Forward primers (5'-3')	Reverse primers (5'-3')	Anneling temperature (0C)	Expected amplified product size (bp)	Structural annotation		Functional annotation	
								Sequence components of *kabuli* genome	*Kabuli* gene accession IDs	NCBI-KOG	TFs
Ca2_P2_7668484	Ca2	7668484	CC/CCCAC	CCAACACAAGCACCAACATC	GGAGGGGTAGAAGTGGAAGG	59.9	188	INTERGENIC	--	--	--
Ca2_P2_7669241	Ca2	7669241	TAA/TAAA	TGTTCGCCTCTTCATCAGTG	TGTGTTTTGGGTTCGACTAGG	60.0	475	INTERGENIC	--	--	--
Ca2_P2_7669371	Ca2	7669371	TAAAAAAAA/TAAAAAAAAAAA	TGTTCGCCTCTTCATCAGTG	TGTGTTTTGGGTTCGACTAGG	60.0	475	INTERGENIC	--	--	--
Ca2_P2_7669611	Ca2	7669611	A/ACACTCATT	CCTAGTCGAACCCAAAACACA	TTGCTCACGAAGGAAATGACT	59.9	450	INTERGENIC	--	--	--
Ca2_P2_7670497	Ca2	7670497	C/CT	TGAGATTTTGAAATCCATTGACTC	GCATCTTTTTGGCTACTGGC	59.9	762	INTERGENIC	--	--	--
Ca2_P2_7675590	Ca2	7675590	TAAAAAAA/TAAAAAAAA	ACTCCGGATTTGGAGAAAGG	AAAATGAGTTTACATTCACGGTACA	59.0	882	INTERGENIC	--	--	--
Ca2_P2_7678375	Ca2	7678375	AAATAA/AAA	GGTGAGGATGCATGAGATGA	ACCAAGATGACCAGCAAAGG	60.1	949	INTERGENIC	--	--	--
Ca2_P2_7678453	Ca2	7678453	GAAAAAAAAA/GAAAAAAAA	TTTTCGCATTTGATTAGAGAGATT	ACCAAGATGACCAGCAAAGG	60.1	515	INTERGENIC	--	--	--
Ca2_P2_7681881	Ca2	7681881	TAAAAA/TAAAA	GAAAAAGAGCCACGGTGGTA	GCGAGAAAAGATTTGTTGAGAA	58.6	383	INTRON	Ca_18121	R	ERF
Ca2_P2_7681954	Ca2	7681954	TAAAA/TAAA	GAAAAAGAGCCACGGTGGTA	TTTCCAGCTCCAAGCTCATT	60.0	643	INTRON	Ca_18121	R	ERF
Ca2_P2_7682063	Ca2	7682063	CTTTTTTT/CTTTTTT	GAAAAAGAGCCACGGTGGTA	TTTCCAGCTCCAAGCTCATT	60.0	643	INTRON	Ca_18121	R	ERF
Ca2_P2_7682985	Ca2	7682985	AT/ATT	AACGGTTCAAATGAAAATCCC	ACCAACCCATCAAAAATCCA	60.0	335	INTRON	Ca_18121	R	ERF

INDEL marker IDs	Chromosomes/unanchored scaffolds	Physical positions (bp)	InDels (*Kabuli* reference genome-CDC Frontier/PII)	Forward primers (5'-3')	Reverse primers (5'-3')	Anneling temperature (0C)	Expected amplified product size (bp)	Structural annotation		Functional annotation	
								Sequence components of *kabuli* genome	*Kabuli* gene accession IDs	NCBI-KOG	TFs
Ca2_P2_7683046	Ca2	7683046	TT/TTAT	AACGGTTCAAATGAAAATCCC	ACCAACCCATCAAAAATCCA	60.0	335	INTRON	Ca_18121	R	ERF
Ca2_P2_7683059	Ca2	7683059	TAAAAAAAA/TAGAAAAAAAAAAA	AACGGTTCAAATGAAAATCCC	ACCAACCCATCAAAAATCCA	60.0	335	INTRON	Ca_18121	R	ERF
Ca2_P2_7695766	Ca2	7695766	TG/T	GGCGGGACAATTATTTTGAA	CCATATAGGAAGTACGAGTACGTGA	58.7	401	INTERGENIC	--	--	--
Ca2_P2_7699107	Ca2	7699107	TAAAAAAA/TAAAAAAAA	CCAAAAATTGTGGAGATTTGG	GCTGATGATGCCTCCATTTT	60.0	846	INTERGENIC	--	--	--
Ca2_P2_7699126	Ca2	7699126	TAAAAAAAA/TAAAAAAA	CCAAAAATTGTGGAGATTTGG	GCTGATGATGCCTCCATTTT	60.0	846	INTERGENIC	--	--	--
Ca2_P2_7703845	Ca2	7703845	ATTTTTTTT/ATTTTTTT	TCTCTTTTATTGGGGCATGG	GATGTAAGGGGTGTGGAAAA	57.4	909	DRR	Ca_18123	R	ERF
Ca2_P2_7703919	Ca2	7703919	AAATAAAATAATAAAATA/AAATAAAATA	TCTCTTTTATTGGGGCATGG	AGATGTAAGGGGTGTGGAAAA	58.4	910	DRR	Ca_18123	R	ERF
Ca2_P2_7704113	Ca2	7704113	TTAATAAT/TTAATAATAATAAT	TACTTCCAAAGTCGGGCAAG	AGATGTAAGGGGTGTGGAAAA	58.4	660	DRR	Ca_18123	R	ERF
Ca2_P2_7704404	Ca2	7704404	T/TTA	TTTTCCACACCCCTTACATCT	GAGGTTTTTGGTATCGTGCC	59.4	507	DRR	Ca_18123	R	ERF
Ca2_P2_7704992	Ca2	7704992	ATTTTTTT/ATTTTTTTT	TGGCACGATACCAAAAACCT	AGGAGATGGTTGTACCGCAG	60.1	282	DRR	Ca_18123	R	ERF
Ca2_P2_7705341	Ca2	7705341	ATAGTA/ATA	CTGCGGTACAACCATCTCCT	CGCCGTCGTTACTGTTCATA	59.8	355	INTERGENIC	--	--	--
Ca2_P2_7706488	Ca2	7706488	AAAATAATTAAATAATTAAAT/AAAATAATTAAATAATTAAATAATTAAAT	GCCTATTTTGCTTTTCCTGC	TCATCCACTTTCTTGCCTGT	58.3	689	INTERGENIC	--	--	--

INDEL marker IDs	Chromosomes/unanchored scaffolds	Physical positions (bp)	InDels (*Kabuli* reference genome-CDC Frontier/PII)	Forward primers (5'-3')	Reverse primers (5'-3')	Anneling temperature (0C)	Expected amplified product size (bp)	Structural annotation		Functional annotation	
								Sequence components of *kabuli* genome	*Kabuli* gene accession IDs	NCBI-KOG	TFs
Ca2_P2_7707720	Ca2	7707720	ATTTTTTTT/ATTTTTTTTT	TTTAAAATCGGTTATGCGGG	CTTTTAGGAGGTCCCAAGGC	60.1	469	INTERGENIC	--	--	--
Ca2_P2_7709881	Ca2	7709881	TAAAAAAAA/TAAAAAAA	TGACACATGCAAAACACCCT	AGAAGCACGAAGATATGCACTT	58.2	619	INTERGENIC	--	--	--
Ca2_P2_7714112	Ca2	7714112	TG/TTGG	CTCACTCGATCGATGGTTCA	TGACAATGCTGGCTGTTAGC	60.0	588	INTERGENIC	--	--	--
Ca2_P2_7716515	Ca2	7716515	TA/TACCTGA	ATGGGCTTTAAATGTGGCAG	ACCCCGTTTGGCTTCTATTT	59.8	105	DRR	Ca_18124	--	--
Ca2_P2_7717172	Ca2	7717172	ATTGGTTGG/ATTGG	TCATTCTTGTGGGTGTTGGA	TGAGACATGCAATGACGGAT	60.1	410	DRR	Ca_18124	--	--
Ca2_P2_7718496	Ca2	7718496	C/CT	CCTGCCACCACTCTTAGCTT	TGGGATGCTACCTCCTATGG	59.9	511	INTERGENIC	--	--	--
Ca2_P2_7718955	Ca2	7718955	TGAAA/T	GAGCTTCATTGAAATCGGGA	TCATATAGGAGGTCGCACCC	59.9	821	INTERGENIC	--	--	--
Ca2_P2_7720145	Ca2	7720145	GAAAAAAAA/GAAAAAAAAAAA	ATCACCGGAAGAGGTAAGGG	GCATCCATTTAGGCGGATTA	59.9	591	INTERGENIC	--	--	--
Ca2_P2_7720508	Ca2	7720508	TAAA/TAA	TAATCCGCCTAAATGGATGC	CTCCCATAGGATGAAGGGGT	60.1	530	INTERGENIC	--	--	--
Ca2_P2_7720908	Ca2	7720908	AAT/A	TTCACCCCTTCATCCTATGG	TCCAAATGTTTGTGATTGTGC	59.4	490	INTERGENIC	--	--	--
Ca2_P2_7722018	Ca2	7722018	ACT/ACTCT	TATGCACGCGAATCTTTGAC	AAACGGAAGTTTCTGCAAGC	59.5	784	INTERGENIC	--	--	--
Ca2_P2_7724864	Ca2	7724864	TA/TAAAGAGATTGAGA	ACCAGAGACACTGCAGCAAA	AACAAGATTCTCGAATGCTCC	58.4	464	INTERGENIC	--	--	--

INDEL marker IDs	Chromosomes/unanchored scaffolds	Physical positions (bp)	InDels (*Kabuli* reference genome-CDC Frontier/PII)	Forward primers (5'-3')	Reverse primers (5'-3')	Anneling temperature (0C)	Expected amplified product size (bp)	Structural annotation		Functional annotation	
								Sequence components of *kabuli* genome	*Kabuli* gene accession IDs	NCBI-KOG	TFs
Ca2_P2_7725038	Ca2	7725038	ATTTT/ATTT	ACCAGAGACACTGCAGCAAA	AGCCATTTGAGACTGTGCAA	59.4	789	INTERGENIC	--	--	--
Ca2_P2_7729483	Ca2	7729483	TAAA/TAAAA	AAAGGAAGAAGGGGTGCATT	ATGTTCTAAAGCCCATGCCA	60.5	843	INTERGENIC	--	--	--
Ca2_P2_7730325	Ca2	7730325	ATTTTTTTTTT/ATTTTTTTTT	GTCACGGCGCAAGTTATACA	CCACAAAAAGCACACAATGG	60.0	555	INTERGENIC	--	--	--
Ca2_P2_7731377	Ca2	7731377	GAAAAA/GAA	GTGGTTGTGATGTTGAACGC	CTCCCGCTAACTCCTCTTCC	60.3	697	INTERGENIC	--	--	--
Ca2_P2_7739105	Ca2	7739105	ACTTTGT/A	GGTTCTACGGCAACGTCAAT	TTACGCGTGTCATTTTCTCG	59.9	790	INTERGENIC	--	--	--
Ca2_P2_7743342	Ca2	7743342	TG/T	AGCTCCCCTTCCGTACATTT	ATCTCCACCCGTTCTCTTGA	59.7	728	INTERGENIC	--	--	--
Ca2_P2_7743405	Ca2	7743405	CG/C	ATCCATCATGTTCATGCCCT	ATCTCCACCCGTTCTCTTGA	59.7	579	INTERGENIC	--	--	--
Ca2_P2_7743532	Ca2	7743532	ATTTT/ATTT	ATCCATCATGTTCATGCCCT	ATCTCCACCCGTTCTCTTGA	59.7	579	INTERGENIC	--	--	--
Ca2_P2_7743551	Ca2	7743551	AAACCAAACTTAA/AAA	TCAAGCTTTATAGCGGAGCC	ATCTCCACCCGTTCTCTTGA	59.7	526	INTERGENIC	--	--	--
Ca2_P2_7743661	Ca2	7743661	AATC/AATCGATC	TCAAGAGAACGGGTGGAGAT	GATTCAAAATGTGAGCGCAA	59.8	530	INTERGENIC	--	--	--
Ca2_P2_7747929	Ca2	7747929	TAAAAAAAA/TAAAAAAA	TGGTGAAATGCTGCAGAAAA	CTCAACTTGTCGATGCCTCA	60.0	147	INTERGENIC	--	--	--
Ca2_P2_7749318	Ca2	7749318	ATTTTTTTTT/ATTTTTTTT	TACCGACTCAACCCATGACA	GCCCTTGTCACAATCACAAC	59.0	585	INTERGENIC	--	--	--

INDEL marker IDs	Chromosomes/unanchored scaffolds	Physical positions (bp)	InDels (*Kabuli* reference genome-CDC Frontier/PII)	Forward primers (5'-3')	Reverse primers (5'-3')	Anneling temperature (0C)	Expected amplified product size (bp)	Structural annotation		Functional annotation	
								Sequence components of *kabuli* genome	*Kabuli* gene accession IDs	NCBI-KOG	TFs
Ca2_P2_7749605	Ca2	7749605	AAAATAAATAAATAAATAAATAAA/AAAATAAATAAATAAATAAATAAATAAATAAA	CAACCCTTATGGATTCCGTC	GGAGAAGATGGTTGCAGCTC	60.0	613	INTERGENIC	--	--	--
Ca2_P2_7753358	Ca2	7753358	C/CAAT	CGGAGGTTTGGTGTAAACTGA	AGCTTGCCATTGCAGCTACT	60.2	447	INTRON	Ca_18125	--	MYB_related
Ca2_P2_7771490	Ca2	7771490	ATTTTTTTTT/ATTTTTTTTTTT	ACCATTTATTATTTTCGTGTTAATGA	GGTACCATGGATGATATTTAGCC	58.7	188	INTERGENIC	--	--	--
Ca2_P2_7771633	Ca2	7771633	TTAT/TT	CATCCATGGTACCTTAATTTAATTTTT	TAAATAACACCCGTGGTCCC	59.5	512	INTERGENIC	--	--	--
Ca2_P2_7771838	Ca2	7771838	ATTTTTTTT/ATTTTTTTTTT	CATCAAAATTTTTAAACAAAACCG	TAAATAACACCCGTGGTCCC	59.5	371	INTERGENIC	--	--	--
Ca2_P2_7772671	Ca2	7772671	AAAAAAATAAAAA/AAAAAA	CAATTGGTGGCCGTTATTTT	TGCCCTAAGGGGAATAAGCTA	60.1	750	INTERGENIC	--	--	--
Ca2_P2_7773617	Ca2	7773617	TTAAATAAAAT/TTAAATAAAATAAATAAAAT	GCTGAGCCCAAACAAGCTAA	CCACTGTGGAAAGGTAAATGC	59.5	845	INTERGENIC	--	--	--
Ca2_P2_7774796	Ca2	7774796	TAAAAAAAAAA/TAAAAAAAA	TTGGATCATTTTGAGTTTGAGG	TCCTTACGTTCCACGGTAATTT	59.8	358	INTERGENIC	--	--	--
Ca2_P2_7778320	Ca2	7778320	ATT/ATTT	AGACCTCTTACTGTGCGCGT	TGCACTTTTGGTCCCTCAAT	60.5	321	INTERGENIC	--	--	--
Ca2_P2_7780730	Ca2	7780730	ATTTTTTTTTTT/ATTTTTTTTTT	TTGTCTCCATCTCCAGCCTT	TGTTGTTTGAGTTGGGACACA	60.0	995	INTERGENIC	--	--	--
Ca2_P2_7782164	Ca2	7782164	AG/A	TCATTTCAATCCCCCATCAT	TTTGATGCTATGCGAAGGAG	59.0	388	INTERGENIC	--	--	--
Ca2_P2_7786133	Ca2	7786133	AC/A	GCTCCTTTGGTGTATCGCTC	CATTCCATTCCATGTTGCAC	59.8	369	INTERGENIC	--	--	--

INDEL marker IDs	Chromosomes/unanchored scaffolds	Physical positions (bp)	InDels (*Kabuli* reference genome-CDC Frontier/PII)	Forward primers (5'-3')	Reverse primers (5'-3')	Anneling temperature (0C)	Expected amplified product size (bp)	Structural annotation		Functional annotation	
								Sequence components of *kabuli* genome	*Kabuli* gene accession IDs	NCBI-KOG	TFs
Ca2_P2_7786190	Ca2	7786190	GAAAAAAAAAA/GAAAAAAAAA	GCTCCTTTGGTGTATCGCTC	CATTCCATTCCATGTTGCAC	59.8	369	INTERGENIC	--	--	--
Ca2_P2_7788894	Ca2	7788894	TAC/TACAC	GCTCTTTTACGTTTGCCAGC	CCGATTCCATTATGACCTCAA	59.8	204	INTERGENIC	--	--	--
Ca2_P2_7788989	Ca2	7788989	TC/T	GCTCTTTTACGTTTGCCAGC	TGTTTTAGATTGTCTTCTATTTTACGG	58.5	566	INTERGENIC	--	--	--
Ca2_P2_7789072	Ca2	7789072	TAAAAAAA/TAAAAAAAA	GCTCTTTTACGTTTGCCAGC	TGTTTTAGATTGTCTTCTATTTTACGG	58.5	566	INTERGENIC	--	--	--
Ca2_P2_7790064	Ca2	7790064	CA/CATGTTA	AGAGTTGGCCAATTGGTTTG	GAATGTCAAGCATTGTGAAGGA	60.1	467	INTERGENIC	--	--	--
Ca2_P2_7812840	Ca2	7812840	TTA/T	TAGAAGGTCGCTTCTTCGGA	CGACATCGTAGATGTGGGACT	60.0	453	INTERGENIC	--	--	--
Ca2_P2_7815016	Ca2	7815016	TAATAACAAATAACAAA/TAATAACAAATAACAAATAACAAA	TTTTCACCAACACCGTCAAA	AAATAATCGTGGCCTTGCAC	60.0	901	INTERGENIC	--	--	--
Ca2_P2_7816294	Ca2	7816294	CAAAAAA/CAAAAAAA	TTCGATCCACCATCTCACCT	CCTTTGTGCTTTTCAATCTTTT	57.6	507	INTERGENIC	--	--	--
Ca2_P2_7824116	Ca2	7824116	T/TG	TGTCTCGTGGATCTGGTTTG	TGACTTTGTGTCGGAATTTG	57.2	258	INTERGENIC	--	--	--
Ca2_P2_7844283	Ca2	7844283	TAAAAAAAA/TAAAAAAA	GTCCACTGTGCCACATCATC	AACTGCTTTGAAGCACCGTT	59.9	611	INTERGENIC	--	--	--
Ca2_P2_7881274	Ca2	7881274	GTAT/GTATCGTTAT	GCCACTCCAACACCAATTTC	TGGAGACCACATGGAGATGA	60.0	423	INTERGENIC	--	--	--
Ca2_P2_7881305	Ca2	7881305	TTAT/TT	GCCACTCCAACACCAATTTC	TGGAGACCACATGGAGATGA	60.0	423	DRR	Ca_18136	--	--

INDEL marker IDs	Chromosomes/unanchored scaffolds	Physical positions (bp)	InDels (*Kabuli* reference genome-CDC Frontier/PII)	Forward primers (5'-3')	Reverse primers (5'-3')	Anneling temperature (0C)	Expected amplified product size (bp)	Structural annotation		Functional annotation	
								Sequence components of *kabuli* genome	*Kabuli* gene accession IDs	NCBI-KOG	TFs
Ca2_P2_7881740	Ca2	7881740	ATATTCC/ATATTCCTATTCC	TCATCTCCATGTGGTCTCCA	TGCAAATGACCACCCACTTA	60.0	642	DRR	Ca_18136	--	--
Ca2_P2_7882645	Ca2	7882645	ATTTT/ATTT	CTTCTCACGTTGAACCACCA	TGACCTTGTCTGGTCCATGT	58.9	733	DRR	Ca_18136	--	--
Ca2_P2_7883781	Ca2	7883781	GAAAAAAAAAAA/GAAAAAAAAA	TCCTTGTTCCTTCCCCTCTT	TGTCCCCTCACCTTTTCAAC	59.9	440	INTERGENIC	--	--	--
Ca2_P2_7884349	Ca2	7884349	TT/TTAT	GTTGAAAAGGTGAGGGGACA	GGCCTTCTCCTTAGAATGCAA	60.7	546	INTERGENIC	--	--	--
Ca2_P2_7886656	Ca2	7886656	T/TATA	AAAAGGCAGACAACATGGAT	GTCCTTTTGCTCCTCTGCAC	60.0	461	DRR	Ca_18137	E	--
Ca2_P2_7887346	Ca2	7887346	AATAT/AATATAT	GTGCAGAGGAGCAAAAGGAC	TGGAATTTGAGACATGTCAACC	59.8	889	DRR	Ca_18137	E	--
Ca2_P2_7887518	Ca2	7887518	ATTTTTTT/ATTTTTTTT	AAGCAAAATACATCTCGAAGTTCT	TCCCAAGAAAAGCTTCAACC	59.3	550	DRR	Ca_18137	E	--
Ca2_P2_7887647	Ca2	7887647	CAACATA/C	AAGCAAAATACATCTCGAAGTTCT	TCCCAAGAAAAGCTTCAACC	59.3	550	DRR	Ca_18137	E	--
Ca2_P2_7894638	Ca2	7894638	ATTTTT/ATTTTTT	GTCATGGTTGTAGCATCCCC	GCCAAATGAGGTTAATGAAGGA	60.3	278	INTERGENIC	--	--	--
Ca2_P2_7895896	Ca2	7895896	CG/C	CGATCAAATAAAGCAACCGAA	TACAATCAGGATGCGTCCAA	60.1	482	DRR	Ca_18138	--	Dof
Ca2_P2_7913902	Ca2	7913902	GAAAAAAAAAA/GAAAAAAAAA	TCAAATCTACCTCTAACGATTCACA	AAGGACAGTTGTCCCAATGC	60.0	326	DRR	Ca_18139	FE	--
Ca2_P2_7916006	Ca2	7916006	A/AC	CTCATTTGCATCCGATTGGT	TATCGAAAGTTTCCGCGTTT	59.7	525	INTERGENIC	--	--	--

INDEL marker IDs	Chromosomes/unanchored scaffolds	Physical positions (bp)	InDels (*Kabuli* reference genome-CDC Frontier/PII)	Forward primers (5'-3')	Reverse primers (5'-3')	Anneling temperature (0C)	Expected amplified product size (bp)	Structural annotation		Functional annotation	
								Sequence components of *kabuli* genome	*Kabuli* gene accession IDs	NCBI-KOG	TFs
Ca2_P2_7922625	Ca2	7922625	TAA/TA	GAAGCATCACTAGGTGGGGA	CAACCAAAACCATTTCAAAACT	58.1	369	DRR	Ca_18141	--	bHLH
Ca2_P2_7925840	Ca2	7925840	ATTTTTTT/ATTTTTTTTT	CTCGACATCAAAGGCCGTAT	CTTATCCCTTCACTTCGCCA	60.2	788	INTRON	Ca_18142	U	--
Ca2_P2_792603	Ca2	792603	ATTTTTTTTTT/ATTTTTTTTTTT	TCAATGATTTGCACGGAGAA	AACCAACACTTCCATGCCTC	60.0	772	INTERGENIC	--	--	--
Ca2_P2_7928503	Ca2	7928503	CTTTT/CTTT	TGAGATTGAGTTGCCTGTGC	GCAAAAGGTGAGAGAGACGG	60.0	521	DRR	Ca_18143	--	--
Ca2_P2_7929259	Ca2	7929259	CTTTT/CTTT	GTGGGTATCAGGCTCTTCCA	CACATGAAGTACAGGCTCGC	59.5	289	DRR	Ca_18142	U	--
Ca2_P2_7930077	Ca2	7930077	GA/GACA	AACCGGCGACAATATTCAAG	AGGGGAGTATCCAGCGTCTT	60.1	200	DRR	Ca_18143	--	--
Ca2_P2_7933051	Ca2	7933051	AC/A	CCCCAAAAAGTGCTTCAAAA	TGACAAGCACGCTTAAGGAA	59.6	694	INTERGENIC	--	--	--
Ca2_P2_7933926	Ca2	7933926	ATT/AT	TTCCTTAAGCGTGCTTGTCA	CCAAAATAACCCACAATCGG	60.0	539	INTERGENIC	--	--	--
Ca2_P2_7936147	Ca2	7936147	TA/TAA	TATGGCAATGCAAGTTCGAC	TCGCAAAACTTCAAGAGTGG	59.0	443	DRR	Ca_18144	--	--
Ca2_P2_7936230	Ca2	7936230	AT/ATT	TATGGCAATGCAAGTTCGAC	TCGCAAAACTTCAAGAGTGG	59.0	443	DRR	Ca_18144	--	--
Ca2_P2_7937530	Ca2	7937530	AAAA/AAAAGAAA	AATCGTTCCCCTCCTCTTGT	AATTCATGCGGTTGAGTTCC	59.9	524	INTRON	Ca_18144	--	--
Ca2_P2_7937938	Ca2	7937938	ATTTTTTTTTTT/ATTTTTTTTT	GCCCACCTTCATCCAAAATA	CAAATCCACAGGTCATGCTG	60.1	162	INTRON	Ca_18144	--	--

INDEL marker IDs	Chromosomes/unanchored scaffolds	Physical positions (bp)	InDels (*Kabuli* reference genome-CDC Frontier/PII)	Forward primers (5'-3')	Reverse primers (5'-3')	Anneling temperature (0C)	Expected amplified product size (bp)	Structural annotation		Functional annotation	
								Sequence components of *kabuli* genome	*Kabuli* gene accession IDs	NCBI-KOG	TFs
Ca2_P2_7940035	Ca2	7940035	TA/TAA	CAACATTGACAACGGAATCG	AAAATGGACAAAATTGGAGCA	59.4	927	INTERGENIC	--	--	--
Ca2_P2_7940136	Ca2	7940136	ATTTTTTT/ATTTTTTTT	TCTTGACCGTTAGATTGAACCA	CAAAATGGACAAAATTGGAGC	59.4	714	INTERGENIC	--	--	--
Ca2_P2_7940384	Ca2	7940384	CTTAATTTAAATATT/C	TGACCAAAATCTTCTCTTTCCA	GTGCCTCAACCATGACACTG	60.2	818	INTERGENIC	--	--	--
Ca2_P2_7963268	Ca2	7963268	ATTTTTTTTTT/ATTTTTTTTTTT	TTTCGGAGAGGCTAAAGAAGAA	TTTGCCTCACTCACAATTCAA	59.3	532	INTERGENIC	--	--	--
Ca2_P2_7966683	Ca2	7966683	ATTTTTTTTTTT/ATTTTTTTTTTTT	GATGTCACTCCCTGGCTCAT	TTTTCACATTTTCATGTCAAATACA	58.5	397	INTERGENIC	--	--	--
Ca2_P2_797060	Ca2	797060	TAAAAAAAAAA/TAAAAAAAAAAA	CTTCTCATTGGTGGGGAAAA	CGGGTCCGTAATTTTTAGCA	60.0	438	INTERGENIC	--	--	--
Ca2_P2_7982128	Ca2	7982128	CTTTTTTTTTTT/CTTTTTTTTT	GCCCGAATCAAGTAGTGGAA	ATCAATGGAGGATAGCGACG	60.1	644	INTERGENIC	--	--	--
Ca2_P2_8016754	Ca2	8016754	ATTTTTTTTTTT/ATTTTTTTTTTTT	TTTCCCCTGTACCTCCACAG	CCCCCTTTGTCACAACCTAA	59.8	671	INTERGENIC	--	--	--
Ca2_P2_8027702	Ca2	8027702	ATTTTTTTTTTTTTT/ATTTTTTTTTTTT	CATTGGGCAACTTAGTGCAA	ACCTCATCAACTCCACCGAC	60.0	694	INTERGENIC	--	--	--
Ca2_P2_8117986	Ca2	8117986	AATATATATATATATATATAT/AATATATATATATATATATATAT	TGAAGTGGACGTCAAAGAAAAA	ACGTTTACAAATGCGGCTTC	60.1	232	INTERGENIC	--	--	--
Ca2_P2_8163022	Ca2	8163022	TACTT/TACTTTTGACTT	TCACGGGTCAACACGATTTA	GGGAAGGAATTTGTCAGGGT	60.2	886	INTERGENIC	--	--	--
Ca2_P2_8166930	Ca2	8166930	AAATAATAATAATAATAATAATAATAAT/AAATAATAATAATAATAATAATAATAATAATAAT	CCTAGGGATGCGACCATCTA	TCCTCCTCTTTTGGCACATT	59.7	385	INTERGENIC	--	--	--

INDEL marker IDs	Chromosomes/unanchored scaffolds	Physical positions (bp)	InDels (*Kabuli* reference genome-CDC Frontier/PII)	Forward primers (5'-3')	Reverse primers (5'-3')	Anneling temperature (0C)	Expected amplified product size (bp)	Structural annotation		Functional annotation	
								Sequence components of *kabuli* genome	*Kabuli* gene accession IDs	NCBI-KOG	TFs
Ca2_P2_8208107	Ca2	8208107	TAAAAAAAAA/TAAAAAAAAAA	TCCAACCAATTGACGTCGTA	ACATAGGGGTTGTACACGGG	59.6	409	INTERGENIC	--	--	--
Ca2_P2_8216990	Ca2	8216990	AATATATATATATATATATATATATATATATATAT/AATATATATATATATATATATATATATATATATATATATAT	GATAATGGACGTCCTTGCGT	AAACACACGTTCGAACCAAA	59.1	602	INTERGENIC	--	--	--
Ca2_P2_8269566	Ca2	8269566	GTATATATATATATATAT/GTATATATATATATATAT	AGCCTGCAAGTTCAAGTGGT	CACCCCACACACAACAAAGA	60.5	301	INTERGENIC	--	--	--
Ca2_P2_8308316	Ca2	8308316	TAAAAAAAA/TAAAAAAA	AATTGGGACCATTTGATAGGC	GCCTCTACCATCTTTGACAGC	59.0	171	INTERGENIC	--	--	--
Ca2_P2_8328118	Ca2	8328118	TATTTATTTATGCATTT/TATTTATTTATGCATTTATTTATGCATTT	AGGTTCAATTTGGTGGCAAG	TTGAATTGTTATGCCCGTGA	59.9	570	INTRON	Ca_21469	--	C2H2
Ca2_P2_8332435	Ca2	8332435	GTTATTATTATTATTATTATTATTATTATTATTATTATTATT/GTTATTATTATTATTATTATTATTATTATTATTATTATTATTATTATT	CTACGGAGGATGGAACTGGA	TTTGGTGATCTGGGGAGAAG	60.0	734	INTRON	Ca_21468	TU	--
Ca2_P2_833993	Ca2	833993	TAATAAAATAAAATAAAATAAAATA/TAATAAAATAAAATA	TTGCATCATGGTTATGCGAC	CATAGCCTCGTACATGCAACA	59.8	490	INTERGENIC	--	--	--
Ca2_P2_8343398	Ca2	8343398	AATATATATATATAT/AATATATATATATATAT	CCAGTGTTTGACCTCCAAGC	TTTGCAACAACGCGAAATAG	59.9	743	INTERGENIC	--	--	--
Ca2_P2_8362914	Ca2	8362914	GAATGAAATGAAA/GAATGAAATGAAATGAAATGAAA	GTGGGTATCCGGAGGTATCA	TCCAAACACTCCCAAAAAGC	60.1	307	DRR	Ca_21465	--	--
Ca2_P2_8387275	Ca2	8387275	ATTTTTTTT/ATTTTTTTTT	ATGAGAACCCCATACCCCTC	AACAATAAAGGGACGCGTTG	60.0	739	INTERGENIC	--	--	--
Ca2_P2_8420270	Ca2	8420270	AACA/AACACA	CCAAGTTTGAAATGCCGATT	GCTGCATGATCTTATGCTCG	59.6	451	INTERGENIC	--	--	--
Ca2_P2_8478800	Ca2	8478800	AATTT/AT	CCCGTATGAATGAACGGAAC	CTACCCCCACATTTCAATCG	60.2	519	INTERGENIC	--	--	--

INDEL marker IDs	Chromosomes/unanchored scaffolds	Physical positions (bp)	InDels (*Kabuli* reference genome-CDC Frontier/PII)	Forward primers (5'-3')	Reverse primers (5'-3')	Anneling temperature (0C)	Expected amplified product size (bp)	Structural annotation		Functional annotation	
								Sequence components of *kabuli* genome	*Kabuli* gene accession IDs	NCBI-KOG	TFs
Ca2_P2_8478910	Ca2	8478910	ACT/A	CCCGTATGAATGAACGGAAC	CTACCCCCACATTTCAATCG	60.2	519	INTERGENIC	--	--	--
Ca2_P2_8479884	Ca2	8479884	ATTTT/ATTTTT	AAGAAAGCACTCAAAGCCCA	ATTCTTCTGGGGGCAAGAGT	60.1	305	INTERGENIC	--	--	--
Ca2_P2_8483756	Ca2	8483756	A/ATCACAAATATC	TTTGGTTGCCAGGAAATAGG	GTATTTTGATGCGTGAAGCG	59.3	775	DRR	Ca_21454	--	--
Ca2_P2_8539885	Ca2	8539885	TAAA/TAAACAAA	CTATGAACACCACATTGCCG	TCCATGGTCAAGACGTGATT	58.9	582	INTERGENIC	--	--	--
Ca2_P2_8541841	Ca2	8541841	TAAAAAAAAAA/TAAAAAAAAAAA	TTCATATCATTCAAATGCCCA	ACACCTAGTTCACCCCCTCC	60.2	643	INTERGENIC	--	--	--
Ca2_P2_8640240	Ca2	8640240	AATATATATATATATATATATATATATAT/AATATATATATATATATATATATATATATATAT	CATAAGACCACCTCCAATCCA	TTGATTCTACGGTAACAGCAAAAA	60.1	344	INTERGENIC	--	--	--
Ca2_P2_8647024	Ca2	8647024	TACA/TA	CGGGGAGCAGATCTAAAAAG	GAACCCCTCCCTCTCTCTTG	60.2	415	INTERGENIC	--	--	--
Ca2_P2_8647425	Ca2	8647425	CAGG/CAGGAGG	TCAAGAGAGAGGGAGGGGTT	TTTGCGTTACGAATTTGATGA	59.2	553	INTERGENIC	--	--	--
Ca2_P2_8708914	Ca2	8708914	ATTTTTTTT/ATTTTTTTTT	GCCCACACCGAAGAAATAAA	TTCATTTTGCTCCATCACTTTC	59.2	714	INTERGENIC	--	--	--
Ca2_P2_8718137	Ca2	8718137	TTTCATCA/T	GGGACTTGCACTTTGTGCTT	GGGAGCCTTATACCTCTGGG	59.9	590	INTRON	Ca_22308	U	--
Ca2_P2_8777880	Ca2	8777880	ATTTTTTTTT/ATTTTTTTTTT	CATGTCCTCAGGTGGTTGTG	TTTGTCGTGGGATGAACTGA	60.1	807	INTERGENIC	--	--	--
Ca2_P2_8794654	Ca2	8794654	C/CAA	GAGTTGAATGCATGTGTGGG	ACCCACGACAATTCAAAAGC	60.0	539	DRR	Ca_22304	J	--

INDEL marker IDs	Chromosomes/unanchored scaffolds	Physical positions (bp)	InDels (*Kabuli* reference genome-CDC Frontier/PII)	Forward primers (5'-3')	Reverse primers (5'-3')	Anneling temperature (0C)	Expected amplified product size (bp)	Structural annotation		Functional annotation	
								Sequence components of *kabuli* genome	*Kabuli* gene accession IDs	NCBI-KOG	TFs
Ca2_P2_8820736	Ca2	8820736	ATTTTTTTTTTT/ATTTTTTTTTT	TTACCATGATGTCGGTGGTG	GTGAGCAAATGTTTCGGGAT	59.9	690	INTERGENIC	--	--	--
Ca2_P2_8839893	Ca2	8839893	AG/A	ATGGGCCCCTCATTTATACC	CGTGGATTGAACCACATCAC	59.8	257	INTRON	Ca_22301	--	GRAS
Ca2_P2_88585	Ca2	88585	TG/T	TGAATTTGACGAAATTTGCATT	GGCTATGAGTGCTCCATTTGA	60.2	752	INTERGENIC	--	--	--
Ca2_P2_903067	Ca2	903067	TTATATTAAAATATATTAAAATATA/TTATATTAAAATATA	GCACATCACTTATGATTGCGAG	TAGGAGGATAGACACCGCCA	60.6	516	INTERGENIC	--	--	--
Ca2_P2_904938	Ca2	904938	GGTGT/GGTGTGT	TGAGCAGGTTGTTGCAATTC	CAGGGTTCTGTGGCATTTCT	60.1	575	INTERGENIC	--	--	--
Ca2_P2_9233338	Ca2	9233338	TAGAGAGAGAGAGAGAGAGAGAGAGA/TAGAGAGAGAGAGAGAGAGAGAGA	TCGACCGAATCGTAGGAAAC	TCATTCCATCTTTCATGGCA	60.0	557	INTERGENIC	--	--	--
Ca2_P2_9269046	Ca2	9269046	AATATATATATATATATATATATATA/AATATATATATATATATATATA	GGTTGATGTGTTTGCGTTTG	AAAAATCAAATGTGTATTGATGGA	57.5	521	INTERGENIC	--	--	--
Ca2_P2_9277232	Ca2	9277232	AGG/AG	ATCGTGCAATCAACCTGTCA	GTTTCATGCAAATTGGGTCC	60.2	439	DRR	Ca_20035	--	--
Ca2_P2_9287043	Ca2	9287043	ATTTTTTTTTT/ATTTTTTTTT	CTCTTTCTCCTCCCGAAACA	AAGGGTTTTGTTGAAAGGGG	60.2	706	INTERGENIC	--	--	--
Ca2_P2_9298699	Ca2	9298699	AAATAATAATAATAATAATAATAATAATAATAATAA/AAATAATAATAATAATAATAATAATAATAATAATAATAATAATAA	TTGGTAGGAAGGTTGATCTGTTC	TTTGCAGAGATGTGAACCAAA	59.3	632	INTERGENIC	--	--	--
Ca2_P2_9353417	Ca2	9353417	CATATATATATATATATATATATATAT/CATATATATATATATATATATATATATATATAT	CAACAATGGAGGAAGGAGGA	ATTTTGGCTCGGAGGTTTTT	59.9	441	INTERGENIC	--	--	--
Ca2_P2_9408358	Ca2	9408358	CTT/CTTTCTATTATTATT	CATCAAAAACAGCAGCGAAA	TCGTGTCTTCTTTCCATCCA	59.2	934	INTERGENIC	--	--	--

INDEL marker IDs	Chromosomes/unanchored scaffolds	Physical positions (bp)	InDels (*Kabuli* reference genome-CDC Frontier/PII)	Forward primers (5'-3')	Reverse primers (5'-3')	Anneling temperature (0C)	Expected amplified product size (bp)	Structural annotation		Functional annotation	
								Sequence components of *kabuli* genome	*Kabuli* gene accession IDs	NCBI-KOG	TFs
Ca2_P2_9428131	Ca2	9428131	GTATATATATATATAT/GTATATATATATAT	GAAAATGCTAATGGTTGCGG	TTCCAAAACACTTACCCCCA	60.2	657	INTERGENIC	--	--	--
Ca2_P2_9485330	Ca2	9485330	TTTTTATTTTATTTTATTTT/TTTTTATTTTATTTT	TTATGCGCCACATCACATTT	CGCCGAAAAGGTTACACAAT	60.0	604	INTRON	Ca_20024	T	M-type
Ca2_P2_9494071	Ca2	9494071	T/TA	GAACTGCAACAACAACGACAA	GGTCTCGAAATCCCTGATGA	60.0	609	INTERGENIC	--	--	--
Ca2_P2_9494155	Ca2	9494155	CAAA/CAAAA	GAACTGCAACAACAACGACAA	GGTCTCGAAATCCCTGATGA	60.0	609	INTERGENIC	--	--	--
Ca2_P2_9500229	Ca2	9500229	CGTATGCTAGACCTGG/CGTATGCTAGACCTGGGTATGCTAGACCTGG	AGAGGTTGGGGGAGAAGAAA	GCAACAACAGCAGAGACAATG	59.5	213	INTERGENIC	--	--	--
Ca2_P2_9650270	Ca2	9650270	CATATATATATATATATA/CATATATATATATATA	GAAGGGGTATGAACGAAGCA	TGAGTAGGGATGGAGGACCA	60.5	590	INTERGENIC	--	--	--
Ca2_P2_9653674	Ca2	9653674	ATT/ATTTT	GCAACCACAAAGGCAAATTA	CTGGGAGGAAAGCACTTGAG	60.0	748	INTERGENIC	--	--	--
Ca2_P2_9715169	Ca2	9715169	AATTATTATTATTATTATTATTATTATTATTATT/AATAATTATTATTATTATTATTATTATTATTATTATTATT	TTTGAACGATGATGTGAGACG	ATGAGCAAGAATCTCGCGTT	60.0	762	INTERGENIC	--	--	--
Ca2_P2_975081	Ca2	975081	CTTTTTTTTTT/CTTTTTTTTTTT	CACCCAACATTAAGCGTGTG	CATTCGTCCCACAATGAGTG	60.0	618	INTERGENIC	--	--	--
Ca2_P2_9779226	Ca2	9779226	ATTTT/ATTT	GCTAGTGGGGACAACTCAGG	CCCAAACACATATGGAAGCC	60.2	324	INTERGENIC	--	--	--
Ca2_P2_9884034	Ca2	9884034	AATATATATAT/AATATATAT	TAAGTTGTTGTGGCATCGGA	CCGACCTAATCACTCATAAATATCG	60.1	322	INTERGENIC	--	--	--
Ca2_P2_9981127	Ca2	9981127	TTATATATATATATATATATATATATAT/TTATATATATATATATATATATATATATATATATAT	GGTATTTTCGCATGCAGATG	AGCCAGCATACCAGCAAAAG	60.4	819	INTERGENIC	--	--	--

INDEL marker IDs	Chromosomes/unanchored scaffolds	Physical positions (bp)	InDels (*Kabuli* reference genome-CDC Frontier/PII)	Forward primers (5'-3')	Reverse primers (5'-3')	Anneling temperature (0C)	Expected amplified product size (bp)	Structural annotation		Functional annotation	
								Sequence components of *kabuli* genome	*Kabuli* gene accession IDs	NCBI-KOG	TFs
Ca2_P2_9997900	Ca2	9997900	AAATAATAATAATAATAATAATAATAATAATA/AAATAATAATAATAATAATAATAATAATAATAATA	TAAATACGCGACGACCAACA	TTTCTGTCATAAAAAGTGGCACA	59.7	407	INTERGENIC	--	--	--
Ca3_P2_10067227	Ca3	10067227	CTTTTTTTTTTT/CTTTTTTTTTT	CCCTGGTGCAACAAAAACAT	TCGTCGTCCACATTACTCCA	60.1	719	INTERGENIC	--	--	--
Ca3_P2_10082918	Ca3	10082918	GTTTTTTTTTTTTTT/GTTTTTTTTTTTTTTT	AAGTTGTTACAATTCGATCGTCC	CAGAAAATGTGGATGCGTTG	60.1	153	INTERGENIC	--	--	--
Ca3_P2_100899	Ca3	100899	AATATATATATATATATATATATATATATAT/AATATATATATATATATATATATATAT	AACAAACGACGACGTGACAT	ACGGTAAATGCATAGCCACC	59.9	746	INTERGENIC	--	--	--
Ca3_P2_1009100	Ca3	1009100	CAATATAAATATAAA/CAATATAAA	CAGCAGCATCATCGTGAACT	TGGCAAGGATAAAACTTGGG	59.9	390	INTERGENIC	--	--	--
Ca3_P2_10159368	Ca3	10159368	GA/G	CTAGGCTCACCAACCGGATA	CTAAGGGCGCTACAACAAGC	60.0	354	INTERGENIC	--	--	--
Ca3_P2_10159468	Ca3	10159468	T/TA	TGGGGCAAACAAAGCTATTC	CTAAGGGCGCTACAACAAGC	60.0	107	INTERGENIC	--	--	--
Ca3_P2_10244842	Ca3	10244842	GGAGAGAGAGAGAGAGA/GGAGAGAGAGAGAGAGAGA	AAAACGTGTGTGGGAGAAGG	CAATTTGATGGCACTTGGTG	60.0	203	INTERGENIC	--	--	--
Ca3_P2_10299801	Ca3	10299801	CTATATATATATATATATATATATATATATATATA/CTATATATATATATATATATATATATATA	AGCACCGGTAGAAATAGCCA	CACCTTCGACTTTTGTCAGG	58.3	997	INTERGENIC	--	--	--
Ca3_P2_10303005	Ca3	10303005	AATATATATATATATATATATATATATATATATATATAT/AATATATATATATATATATATATATAT	GGGATATTTTGGAGGGGAGA	TGAGCTTTGCTCTTGCATTC	59.3	262	INTERGENIC	--	--	--
Ca3_P2_10312456	Ca3	10312456	TAAAA/TAAAAA	TGACACGCAATATAGAACCG	CATCTTGGTGTGTGAAAGCG	60.3	598	INTERGENIC	--	--	--
Ca3_P2_10350276	Ca3	10350276	CAAGAAG/CAAGAAGAAG	ATTGGCAAATCGGTTCAGTC	GCCAAACCCTTACGAGATCA	60.1	459	DRR	Ca_18818	--	--

INDEL marker IDs	Chromosomes/unanchored scaffolds	Physical positions (bp)	InDels (*Kabuli* reference genome-CDC Frontier/PII)	Forward primers (5'-3')	Reverse primers (5'-3')	Anneling temperature (0C)	Expected amplified product size (bp)	Structural annotation		Functional annotation	
								Sequence components of *kabuli* genome	*Kabuli* gene accession IDs	NCBI-KOG	TFs
Ca3_P2_10381884	Ca3	10381884	AAATAATAATAATAATAATAATAATAATAATAATAATAATAATAATAATAATAATA/AAATAATAATAATAATAATAATAATAATAATAATAATAATAATAATAATAATAATA	TTCATGGTTGGATCCCATTT	TTATAGAACCCGCCCAACAA	60.3	382	INTERGENIC	--	--	--
Ca3_P2_10398321	Ca3	10398321	G/GATTGATCTTTCCC	CGGATTGCCAAGTACAAGGT	CAAACCACAACCAGCACAAC	60.0	770	DRR	Ca_18819	J	--
Ca3_P2_10570421	Ca3	10570421	GATATATATATATA/GATATATATATATATA	ACCCGAATACCTCCAAGGTC	GATCTGCAGTTGCTGATGGA	60.0	816	INTERGENIC	--	--	--
Ca3_P2_10623857	Ca3	10623857	ATTTTTTTTTTT/ATTTTTTTTTT	TTTTGGCTCGAAGCTCATTT	AGGTGCGATGGAGAAATCAA	60.6	372	DRR	Ca_18826	O	--
Ca3_P2_10636458	Ca3	10636458	TTTATTATTATTATTATTATTATTATTATTATTATTATTATT/TTTATTATTATTATTATTATTATTATTATTATTATTATTATTATT	ATCGACTCCCGAACTCAAAA	CTCCAGAGCCACGTACTCGT	60.5	657	INTERGENIC	--	--	--
Ca3_P2_10657869	Ca3	10657869	TAAAAAAAAAAA/TAAAAAAAAAAAA	GCTCCAAAACCAAGGATTGA	GATCCCATTTCTGCTGCATT	60.0	520	INTERGENIC	--	--	--
Ca3_P2_10684192	Ca3	10684192	CTATTATTATTATTATTATTATTATTATTATTATTA/CTATTATTATTATTATTATTATTATTATTA	TTTCATCCAATGCTTCAGGA	TTGTGGTTGCATCCCTAACA	60.0	822	DRR	Ca_18828	O	--
Ca3_P2_10724964	Ca3	10724964	GTTATTATTATTATTATTATTATTAT/GTTATTATTATTATTATTATTATTATTAT	TTGAAGCCCAAATTTTCACC	CAGGAGCAGCAAACAACAAA	60.0	588	INTRON	Ca_18831	--	--
Ca3_P2_10846962	Ca3	10846962	AATATATATATATATATATATATATATATA/AATATATATATATATATATATATATATATATATATA	CCAAATGACGACTTCCCACT	CAAGACGTCGTCAAAATCCC	60.5	683	INTERGENIC	--	--	--
Ca3_P2_10860554	Ca3	10860554	TG/T	GCACACTGGTGTTGGATTTG	TCAATGGTAACTTCCCCAGC	59.9	851	INTRON	Ca_19358	G	--
Ca3_P2_10923237	Ca3	10923237	T/TCC	GCTGATTGAAATCGAGCACA	TTAAAACAAGCACCAAGGGC	60.1	347	INTERGENIC	--	--	--
Ca3_P2_10994316	Ca3	10994316	AG/A	CCCGTGAGATCCTCCTAACA	TTCGGTTGGTGACCCTTTAC	59.8	146	INTERGENIC	--	--	--

INDEL marker IDs	Chromosomes/unanchored scaffolds	Physical positions (bp)	InDels (*Kabuli* reference genome-CDC Frontier/PII)	Forward primers (5'-3')	Reverse primers (5'-3')	Anneling temperature (0C)	Expected amplified product size (bp)	Structural annotation		Functional annotation	
								Sequence components of *kabuli* genome	*Kabuli* gene accession IDs	NCBI-KOG	TFs
Ca3_P2_11044475	Ca3	11044475	CAAAAAAAAAA/CAAAAAAAAA	GCATCCATCCAAACGACTTT	CATTACATCTTATTTGAGGGAGAGC	59.6	286	INTERGENIC	--	--	--
Ca3_P2_11182328	Ca3	11182328	GAAAAAAAAAA/GAAAAAAAAAAA	AGGTGCCCTTCTTTTTGGTT	CCATCGTCGACCTTGAGAAT	60.1	527	INTERGENIC	--	--	--
Ca3_P2_11247051	Ca3	11247051	AAAATAAATAAATAAATAAATA/AAAATAAATAAATAAATAAATAAATA	CTGGGGATACGTAGGAGCAA	TCCCTTAAAACGGCTACCCT	60.0	180	INTERGENIC	--	--	--
Ca3_P2_11273271	Ca3	11273271	ATTTTTTTTT/ATTTTTTTT	GCGCGCGGATAAAATAAATA	TCATGACGCAGCACTTTTTC	60.0	738	INTERGENIC	--	--	--
Ca3_P2_11279419	Ca3	11279419	CTATTATTATTATTATTATTA/CTATTATTATTATTATTATTATTA	TTCCAGGGCAGACTTCTTGT	GGCCCATTATGACCAAAACTT	60.1	641	INTERGENIC	--	--	--
Ca3_P2_11307149	Ca3	11307149	TTATATATATATATATAT/TTATATATATATATATAT	GTGGTACTGGAGGCCACATT	CCAAAATGCCCCCTTTTATT	60.0	792	INTERGENIC	--	--	--
Ca3_P2_11315222	Ca3	11315222	TTATATATATATATATATATATATATATATATAT/TTATATATATATATATATATATATATATATATATATATAT	CTCATGATCGGGAAAATGGT	GTTGAGAGGCATGAAGAGGC	60.0	217	INTERGENIC	--	--	--
Ca3_P2_11442489	Ca3	11442489	AAATAAATAA/AAATAAATAATAAATAA	AATGTTTGGATGATTCCCCA	ATACGCATAATCGACTCCCG	59.9	543	INTERGENIC	--	--	--
Ca3_P2_11465111	Ca3	11465111	GA/GAA	GTGCTGAACCCGTGATAGGT	ATTCCAAACCAAAACCCTCC	60.0	276	INTERGENIC	--	--	--
Ca3_P2_11475230	Ca3	11475230	TTGT/TT	GCATAAGCAGCGCTTTCATT	TCGATTATGCGTAGGGAAGG	60.1	809	INTERGENIC	--	--	--
Ca3_P2_1149110	Ca3	1149110	TAAAAAAAAAAAA/TAAAAAAAAAAA	TGTGCAATCGTTTTAACCCA	TGAGGGGTAATTTGAACCGA	60.3	272	INTERGENIC	--	--	--
Ca3_P2_1149320	Ca3	1149320	ATTTTTTTTT/ATTTTTTTTTT	TGTGCAATCGTTTTAACCCA	CCCTTACCACAAAATCAACTCA	59.0	719	INTERGENIC	--	--	--

INDEL marker IDs	Chromosomes/unanchored scaffolds	Physical positions (bp)	InDels (*Kabuli* reference genome-CDC Frontier/PII)	Forward primers (5'-3')	Reverse primers (5'-3')	Anneling temperature (0C)	Expected amplified product size (bp)	Structural annotation		Functional annotation	
								Sequence components of *kabuli* genome	*Kabuli* gene accession IDs	NCBI-KOG	TFs
Ca3_P2_11497481	Ca3	11497481	ATTATTTATTTATTTATTTATTTATTTATGTATTTATGTATTTATGTATTTATTTATTCATTTATTTATTTATT/ATTATTTATTTATTTATTTATTTATTTATGTATTTATGTATTTATGTATTTATTTATTCATTTATTTATTTATT	GGGTTTGCACTTGAAGGTGT	CCTCCGAGCCAAAATACAAA	60.1	617	INTERGENIC	--	--	--
Ca3_P2_11521142	Ca3	11521142	TAAA/TAA	TCCATCGTACCGGGAGATAC	TCGATTATGCGTAGGGAAGG	60.1	235	INTERGENIC	--	--	--
Ca3_P2_11523659	Ca3	11523659	GT/G	TTGGAGGAGAAAATCGGTTG	GGTTGACGACTAAGGGTGGA	60.0	596	INTERGENIC	--	--	--
Ca3_P2_11531328	Ca3	11531328	CAAAAAAAA/CAAAAAAAAA	TTTCCTTTCAACCCAGCATC	CGATGCAGTCGTCTCTTTGA	60.1	482	INTERGENIC	--	--	--
Ca3_P2_11587128	Ca3	11587128	CAAAA/CAAAAA	TTTGGGCTTACTTTCCATGC	GGTGTCAATTTCTTTGCGGT	60.0	543	DRR	Ca_19379	--	--
Ca3_P2_11611655	Ca3	11611655	AATATATATATATATATATATATATATATATATATATATATATATATAT/AATATATATATATATATATATATATATATATATATATATATATATATATATATATATATAT	AAAACCATCATGCCACAAAA	GCCTCACAAGTAACAAGAAGGG	60.2	368	INTERGENIC	--	--	--
Ca3_P2_11661143	Ca3	11661143	TTTATTATTATTATTATTATTATTATTATTATTATTATTATTATTA/TTTATTATTATTATTATTATTATTATTATTATTATTATTA	GCTTCTTGAAGGTTTCGACG	AATGCTTAATTTGAATATGAGAATCTT	57.3	744	INTERGENIC	--	--	--
Ca3_P2_11720789	Ca3	11720789	ATTATTTATTTATTTATT/ATTATTTATTTATTTATTTATTTATT	CCTTCTCGTGTTTGCACCTT	TGCCATGTTTTAATGGCTGT	59.0	620	INTERGENIC	--	--	--
Ca3_P2_11765428	Ca3	11765428	ATTT/ATTTT	GATCAGATCTGGCAGCAACA	TGGCATACATCTAGGGGGAG	59.9	574	DRR	Ca_19390	--	--
Ca3_P2_11830341	Ca3	11830341	AC/A	AACCCAAGGTTTGGTGAGTG	TTGGAAGGAGCCCTTACCTT	60.1	297	INTERGENIC	--	--	--
Ca3_P2_11877050	Ca3	11877050	TAAAAAAAAA/TAAAAAAAA	TTCAAACCTTTCAATTTTGCTC	GCGATTTTGGTGAAATCCAT	59.8	594	INTERGENIC	--	--	--
Ca3_P2_11919605	Ca3	11919605	GAA/GA	CGGAAATTGAAATCGGAAAA	ATCGCGAAAGTAGTCTCCCA	59.8	633	INTERGENIC	--	--	--

INDEL marker IDs	Chromosomes/unanchored scaffolds	Physical positions (bp)	InDels (*Kabuli* reference genome-CDC Frontier/PII)	Forward primers (5'-3')	Reverse primers (5'-3')	Anneling temperature (0C)	Expected amplified product size (bp)	Structural annotation		Functional annotation	
								Sequence components of *kabuli* genome	*Kabuli* gene accession IDs	NCBI-KOG	TFs
Ca3_P2_12006600	Ca3	12006600	TTATATATATATATATATATATATATATATAT/TTATATATATATATATATATATATATATATATATATATATAT	TTCACCTCCTCAATTCGTTT	GTTGGGCCTAGCCTTCTTTT	59.7	156	INTERGENIC	--	--	--
Ca3_P2_12092540	Ca3	12092540	TAAAAAAA/TAAAAAAAA	GGGTTCCACTTGGAGAGTGA	AGATGTTCGTCCGGATTGTT	59.4	668	INTERGENIC	--	--	--
Ca3_P2_12140676	Ca3	12140676	A/AT	TTCCTATTGCTCACCCCTTG	GAAAAACAAGACCCGCGATA	60.1	525	INTERGENIC	--	--	--
Ca3_P2_12238343	Ca3	12238343	TAAAAAAAAAA/TAAAAAAAAAAA	GTGAATCGGTTCGCAAAAGT	TCTCTTTTGGTTTGTTGGGG	59.9	266	INTERGENIC	--	--	--
Ca3_P2_12253700	Ca3	12253700	AATATATATATATATATATATATATATATATATATA/AATATATATATATATATATATATATATATATATATATA	TGCAAAAGTAGGAGCAGTGA	ATTGGCAGAACTTTGTTGGG	60.0	773	INTERGENIC	--	--	--
Ca3_P2_12263072	Ca3	12263072	A/AGAATCACAT	CAAAGAAGGGGGTTTGAATTG	TCCACTTTGGGTTAACCAGG	59.8	230	INTERGENIC	--	--	--
Ca3_P2_12402301	Ca3	12402301	T/TG	GAAAAGGTTGAGCAACGAGC	GAATGATTTTGAAGTGGGGG	59.2	880	DRR	Ca_21030	--	--
Ca3_P2_12549942	Ca3	12549942	TTATATATATATATAT/TTATATATATATAT	AGCATGCTGCAAGGGATAGT	TGATACAATATGCCCCCTCA	58.8	864	INTERGENIC	--	--	--
Ca3_P2_12594254	Ca3	12594254	AATATATATATATATATATATATAT/AATATATATATATATATATATATAT	TGGCATTGCTTACCATAGGA	GTCAGCCGGTCAAATACCTT	59.1	342	INTERGENIC	--	--	--
Ca3_P2_12601166	Ca3	12601166	TCCTGCTNNNNNNNNNNCCTGCT/TCCTGCT	CTTCGGTGGAAGTGTCACAA	TGGTTTGGTTTAAATATGCGG	59.7	732	INTERGENIC	--	--	--
Ca3_P2_12606196	Ca3	12606196	A/AC	GGGATTGTCAGCTTGATGGT	AAGAGAGGTCACTTTGGCGA	60.0	194	INTERGENIC	--	--	--
Ca3_P2_12619453	Ca3	12619453	TAAAAAAAA/TAAAAAAA	TTTGGCCTAGTGAAAATGCC	TAAGGGTGTTCATGGTGCAA	60.0	816	INTERGENIC	--	--	--

INDEL marker IDs	Chromosomes/unanchored scaffolds	Physical positions (bp)	InDels (*Kabuli* reference genome-CDC Frontier/PII)	Forward primers (5'-3')	Reverse primers (5'-3')	Anneling temperature (0C)	Expected amplified product size (bp)	Structural annotation		Functional annotation	
								Sequence components of *kabuli* genome	*Kabuli* gene accession IDs	NCBI-KOG	TFs
Ca3_P2_12659757	Ca3	12659757	CATATATATATATATATATATATATATAT/CATATATATATATATATATATATATATATAT	TATTTTGGATTTTGGCTCGG	CACCGTAGTGTGGTGGTGAG	60.1	672	INTERGENIC	--	--	--
Ca3_P2_12697436	Ca3	12697436	GTTTTTATTTTTATTTTTATTTTTATTTTTATTTT/GTTTTTATTTTTATTTTTATTTTTATTTTTATTTTTATTTT	AGCTTGCCAACTTCTGGGTA	TGCAAAACGTGTCATCCATT	60.0	670	INTERGENIC	--	--	--
Ca3_P2_12715466	Ca3	12715466	T/TACACATTAAAG	TATTCCCGATTAATGCAGCC	GTCCCGAATGCAATGTCTCT	60.1	667	INTERGENIC	--	--	--
Ca3_P2_12747032	Ca3	12747032	TGGG/TGG	CCTACGGACGGGAATGTAGA	CGTTTTTCTAAGCGAGGGTG	59.9	398	INTERGENIC	--	--	--
Ca3_P2_1288242	Ca3	1288242	GTTTTTTTTTTT/GTTTTTTTTTT	TGGTGAGATCAACGATACGC	TGGTAACAAATCTCCGAAGGA	59.6	371	INTRON	Ca_19421	R	--
Ca3_P2_12911294	Ca3	12911294	C/CCT	TATCGAGTGTTTCCGGTTCC	CAGGTTCACGTGAGCAAGAA	60.0	337	INTERGENIC	--	--	--
Ca3_P2_12969818	Ca3	12969818	TAAAAAAA/TAAAAAAAA	TTACACCAATTCCCCCAAGA	CATGCAAGGTTGGGCTAGAT	60.1	400	INTERGENIC	--	--	--
Ca3_P2_12971127	Ca3	12971127	CAAAAAAAAAAA/CAAAAAAAAAAAA	TGCACATTATGCCAAGAAAA	CGGAGGAAAAATAGGAGGGA	60.4	841	INTERGENIC	--	--	--
Ca3_P2_12987107	Ca3	12987107	CTTTTTTTTTTTT/CTTTTTTTTTTT	TCACAGATTCACCACCTCCA	ATGCAAAACAGATCCTTGGC	60.1	409	INTERGENIC	--	--	--
Ca3_P2_12997438	Ca3	12997438	GTATATATATATATATATATATATATATATATAT/GTATATATATATATATATATATATATATATATATATATAT	GAAGGAAGGAGGGAGAATGG	TGCGCTTTTATTCCAACACA	60.3	648	INTERGENIC	--	--	--
Ca3_P2_1304633	Ca3	1304633	CAAAAAA/CAAAAAAA	GACCGCCTTCTTTTGTTTGA	TTGAGAAATGTGCCACCAAA	60.1	670	INTERGENIC	--	--	--
Ca3_P2_13068182	Ca3	13068182	AG/A	CAACTCGGGTTACGTCCAGT	TTCTGCAATTCGCAAAACAG	60.0	351	INTERGENIC	--	--	--

INDEL marker IDs	Chromosomes/unanchored scaffolds	Physical positions (bp)	InDels (*Kabuli* reference genome-CDC Frontier/PII)	Forward primers (5'-3')	Reverse primers (5'-3')	Anneling temperature (0C)	Expected amplified product size (bp)	Structural annotation		Functional annotation	
								Sequence components of *kabuli* genome	*Kabuli* gene accession IDs	NCBI-KOG	TFs
Ca3_P2_13097036	Ca3	13097036	A/AT	CCTTCTTTACGGGACCCAAT	TCATCAGTATCGTGGGTGGA	59.9	364	INTERGENIC	--	--	--
Ca3_P2_13163446	Ca3	13163446	AATATATATATATATATATATATATATAT/AATATATATATATATATATATATATATAT	TGCAAAGTGAGGATTGACCA	TTTTGGGTCTTGGATTTTGG	59.8	420	INTERGENIC	--	--	--
Ca3_P2_1317155	Ca3	1317155	GTTCTTCTTCTTCTTCTTC/GTTCTTCTTCTTCTTCTTCTTC	ATTCGTTAACCGTTGGATCG	ACCAAGAACAGAATGACGCC	60.1	517	INTERGENIC	--	--	--
Ca3_P2_13171985	Ca3	13171985	GATATATATATATATATATATATA/GATATATATATATATATATATATATA	TCCCAACTACTGGGAATCCA	TAGGGGAATGACTCCACCAC	59.8	414	INTERGENIC	--	--	--
Ca3_P2_13236644	Ca3	13236644	AATATATATATATATATATATAT/AATATATATATATATATATATATAT	AAGACCTGTGGGATCATTGC	TTTTGTGATAGGTTCCTGATGC	59.1	928	INTERGENIC	--	--	--
Ca3_P2_13265127	Ca3	13265127	TAAA/TAA	CGTTTCTCTTCGAGCACACA	TTGAGCTTGCAACTTGATCACT	60.1	693	INTERGENIC	--	--	--
Ca3_P2_13265149	Ca3	13265149	TAA/TAACAA	TTTTTGAATGGTTCCAAGGG	TTGAGCTTGCAACTTGATCACT	60.1	655	INTERGENIC	--	--	--
Ca3_P2_13313491	Ca3	13313491	CATTATTATTATTATTATTATTATTATTATTATTAATATTATTATTATTAT/CATTATTATTATTATTATTATTATTATTATTAATATTATTATTAT	AACCCACCTCTTGATTTCCA	CGACAATTTTGTTGGTGACG	60.0	637	INTERGENIC	--	--	--
Ca3_P2_13417490	Ca3	13417490	TAAAAAAAA/TAAAAAAAAA	TGGGAATAGGTCAGGCTTTG	TGCACGAGAGGATTTGTTTG	59.8	339	INTERGENIC	--	--	--
Ca3_P2_13449893	Ca3	13449893	GATAAATAAATAAATAAATAAA/GATAAATAAATAAATAAATAAATAAA	ACTTATCTTCCGGTGCGATG	TTTGTCTCAACAAGGGTTTCA	58.2	822	INTERGENIC	--	--	--
Ca3_P2_13462560	Ca3	13462560	GATATATATATATATATATAT/GATATATATATATATATATATAT	TGGAAAAAGGGCAATTGAAG	GCATGCAATGGATGATGAAG	60.0	316	INTERGENIC	--	--	--
Ca3_P2_135372	Ca3	135372	CA/C	GTTGTAATGGGGCTTAGGCA	TTGGTCGTTTGAGCCTTTCT	59.9	478	INTERGENIC	--	--	--

INDEL marker IDs	Chromosomes/unanchored scaffolds	Physical positions (bp)	InDels (*Kabuli* reference genome-CDC Frontier/PII)	Forward primers (5'-3')	Reverse primers (5'-3')	Anneling temperature (0C)	Expected amplified product size (bp)	Structural annotation		Functional annotation	
								Sequence components of *kabuli* genome	*Kabuli* gene accession IDs	NCBI-KOG	TFs
Ca3_P2_1371048	Ca3	1371048	CAAAAAAAAAAAA/CAAAAAAAAAAA	CGGATCTCAGTTCGTGGGTA	CAGAGAGAGCGAGAGGGAGA	60.0	567	INTERGENIC	--	--	--
Ca3_P2_13796912	Ca3	13796912	CAAAAAAAAAAA/CAAAAAAAAAAAA	TATCATGCATTTGCGGTTGT	CCTGAGAGAGAAAGGGGCTT	60.0	650	INTERGENIC	--	--	--
Ca3_P2_13827207	Ca3	13827207	TTATATATATATATATATAT/TTATATATATATATATATAT	CTACTCCCGACACTGAAGCC	TTTTCCAGGAAGGAATCGTG	60.0	527	DRR	Ca_25325	--	--
Ca3_P2_13827457	Ca3	13827457	AAC/AACAACATGTAC	CTACTCCCGACACTGAAGCC	TTTTCCAGGAAGGAATCGTG	60.0	527	INTERGENIC	--	--	--
Ca3_P2_13836046	Ca3	13836046	TAAAAAAAAA/TAAAAAAAAAA	TGAGCTGATGGTGAAGTGATTT	TGGTTGAGCTTCAGACATGC	60.0	530	INTERGENIC	--	--	--
Ca3_P2_13845318	Ca3	13845318	ATTTTTTTTT/ATTTTTTTTTT	CCTCAGATGAGAGCCAGGAC	GAGCTGAACCTGCTTGCTTT	59.8	805	INTERGENIC	--	--	--
Ca3_P2_1386021	Ca3	1386021	T/TTTA	AAAGAACATTTCCAATGCGG	ACGTATCCCCAGGTGCAATA	60.2	704	INTERGENIC	--	--	--
Ca3_P2_13868652	Ca3	13868652	TT/TTCCCTTAAT	CCTTTTGCACAAGCTTCACA	TGGTTCGATCACATTTGGAA	59.9	266	INTERGENIC	--	--	--
Ca3_P2_13899750	Ca3	13899750	CTTTT/CTTTTT	CTAGCCCAAGCACGCTTAAC	GATCCCATTAGAACTCCGCA	60.0	345	INTERGENIC	--	--	--
Ca3_P2_13952343	Ca3	13952343	TTTATTATTATTATTATTATTATTATTATTAT/TTTATTATTATTATTATTATTATTATTATTATTAT	TACGGGTTGAGGACCAAAAG	TGCAGAGGACAATTCTTGCT	58.6	702	INTERGENIC	--	--	--
Ca3_P2_14018560	Ca3	14018560	CTTTTTTTTTTT/CTTTTTTTTTTTT	AGGGCAATCACGAGATTTTG	CACGACTTGAGTGGTCCAAG	59.3	635	INTERGENIC	--	--	--
Ca3_P2_1403982	Ca3	1403982	GAAAAAAA/GAAGAAAAAAAA	TCCCCCGTAATAGGTGCATA	TCTTTGTTTCTGCGAACCCT	59.9	521	INTERGENIC	--	--	--

INDEL marker IDs	Chromosomes/unanchored scaffolds	Physical positions (bp)	InDels (*Kabuli* reference genome-CDC Frontier/PII)	Forward primers (5'-3')	Reverse primers (5'-3')	Anneling temperature (0C)	Expected amplified product size (bp)	Structural annotation		Functional annotation	
								Sequence components of *kabuli* genome	*Kabuli* gene accession IDs	NCBI-KOG	TFs
Ca3_P2_14130012	Ca3	14130012	AATATATATATATATAT/AATATATATATATATATAT	GGGCACATATGCTTGACTCC	GGGGAATAACAAAGGGCAAT	60.0	601	INTERGENIC	--	--	--
Ca3_P2_14197660	Ca3	14197660	ATTTTTTTTTT/ATTTTTTTTTTT	TTCCTGTCCCCAATTCAAAC	AACGTTGGGTGGGTAGAGTT	58.4	536	INTERGENIC	--	--	--
Ca3_P2_14205152	Ca3	14205152	AATATATATATATATATA/AATATATATATATATATATA	ACGGAACACAACCTTTGGAG	TGCTGCCTCTCATTCTCACA	60.7	452	INTERGENIC	--	--	--
Ca3_P2_14454312	Ca3	14454312	GTTTTTTTTTTT/GTTTTTTTTTTTT	ACCAAGCACAGCGACTTTCT	CGTTGCAATTGAACAAACGA	60.7	634	INTRON	Ca_22513	K	G2-like
Ca3_P2_14503491	Ca3	14503491	AATATATATATATATATATATATATAT/AATATATATATATATATATATATAT	TGCGTTACGGTGAAATGGTA	AGGAAGAGTGGAGTTGCGAA	60.0	374	INTERGENIC	--	--	--
Ca3_P2_14565992	Ca3	14565992	CAAAAAA/CAAAAAAA	CGTGGAATCGTATTCGTGTG	TGTCAAAATCATGATCAAGAGGA	59.6	757	INTERGENIC	--	--	--
Ca3_P2_14600005	Ca3	14600005	ATTTTATTAATTTTTATTAATTTTTATTAATTTTTATTAATTTT/ATTTTATTAATTTTTATTAATTTTTATTAATTTTTATTAATTTTTATTAATTTT	AGAATGGGGGTTAGGATTGG	GTGTGACAAGAAAAGGGGCA	61.1	743	DRR	Ca_22507	--	C2H2
Ca3_P2_14615841	Ca3	14615841	CAAAAAACAAA/CAAAAAACAAAAAAACAAA	AAGACAACCATGCCTTTTGG	GGGTTGGGCTATCAAATCAA	59.8	334	INTERGENIC	--	--	--
Ca3_P2_14653349	Ca3	14653349	A/AG	AGGAGCTTCTGCCATTGAAA	TAAAAGGAGCAAAGGCCAGA	60.0	408	INTRON	Ca_22505	O	--
Ca3_P2_14686306	Ca3	14686306	T/TC	ATTGCATTAGGGTGTTTGGC	CCCCGTGAGATCTTCCTACA	60.1	835	INTERGENIC	--	--	--
Ca3_P2_14801451	Ca3	14801451	GATATATATATATATATATATAT/GATATATATATATATATATATATAT	ATGAGCCACAAGCTCCAAGT	TGCCATTTGGTGAACGATTA	59.9	357	INTERGENIC	--	--	--
Ca3_P2_1484259	Ca3	1484259	TAC/T	TTGCGGTAACCGGATAGAAC	CAGAGAGGTGTGCGTACCAA	59.9	628	INTERGENIC	--	--	--

INDEL marker IDs	Chromosomes/unanchored scaffolds	Physical positions (bp)	InDels (*Kabuli* reference genome-CDC Frontier/PII)	Forward primers (5'-3')	Reverse primers (5'-3')	Anneling temperature (0C)	Expected amplified product size (bp)	Structural annotation		Functional annotation	
								Sequence components of *kabuli* genome	*Kabuli* gene accession IDs	NCBI-KOG	TFs
Ca3_P2_14843003	Ca3	14843003	AATATATATATATATATATATATATATATA/AATATATATATATATATATATATATATATA	GAGAAAGAAGATATCGGATTCTCAA	AAAAATCAACGCAGTGGGAG	60.1	356	INTERGENIC	--	--	--
Ca3_P2_14846195	Ca3	14846195	A/AAG	GACCTCTTGAAGAAGCCGTG	TCCTCAGGGAACGATTTCAC	60.1	143	INTERGENIC	--	--	--
Ca3_P2_1486345	Ca3	1486345	ATCTCTCTCTCTCTCTCTCTCTCTCTC/ATCTCTCTCTCTCTCTCTCTCTCTCTCTC	TTGAAACCTTTGTTAGGAAGCA	TCCTCCCACCTCACAATAGAA	59.5	371	DRR	Ca_19415	K	MYB
Ca3_P2_14874833	Ca3	14874833	GCC/GCCC	AGGTCATTTTCGTCCTCCCT	CAAACACACCTGTTGGATGC	60.0	679	INTERGENIC	--	--	--
Ca3_P2_14880871	Ca3	14880871	TG/TGTCG	TTTCGGTCATTTTCGTCCTC	ACACACCTATCGGATGCACA	60.0	616	INTERGENIC	--	--	--
Ca3_P2_14902851	Ca3	14902851	GAA/GAAA	CGGTGGGGAGAACTCTGATA	TTCACATGACATCATCCCGT	59.8	474	INTERGENIC	--	--	--
Ca3_P2_14919264	Ca3	14919264	GTTT/GTTTT	CGATTGTGTTTGTCACAGGG	GAAGCAAATCGATTGGGAAA	60.0	303	INTERGENIC	--	--	--
Ca3_P2_15039270	Ca3	15039270	TC/T	CAACACGATCATGACAAGCA	GCATTTTGCATTTGTCAACG	60.1	677	INTERGENIC	--	--	--
Ca3_P2_15039370	Ca3	15039370	ATTTTTTTTT/ATTTTTTTT	TGGTATTGGTAGGGATCTTTGG	GCATTTTGCATTTGTCAACG	60.1	568	INTERGENIC	--	--	--
Ca3_P2_15047295	Ca3	15047295	TA/T	TACTTTGTGCCATGCTCTGC	ACACAAGCCAACACATCCAA	60.0	117	INTERGENIC	--	--	--
Ca3_P2_15047748	Ca3	15047748	ATT/AATTT	TTGGATGTGTTGGCTTGTGT	TGCGGATGTGTTGATTGTTT	60.0	531	INTERGENIC	--	--	--
Ca3_P2_15072147	Ca3	15072147	AATATATATATATATATATATATATATA/AATATATATATATATATATATATATA	GAGTGGTGTTGGAGTCATGTTT	AGACATGTGTTTCCCTTCCG	60.0	424	DRR	Ca_17011	--	--

INDEL marker IDs	Chromosomes/unanchored scaffolds	Physical positions (bp)	InDels (*Kabuli* reference genome-CDC Frontier/PII)	Forward primers (5'-3')	Reverse primers (5'-3')	Anneling temperature (0C)	Expected amplified product size (bp)	Structural annotation		Functional annotation	
								Sequence components of *kabuli* genome	*Kabuli* gene accession IDs	NCBI-KOG	TFs
Ca3_P2_15073326	Ca3	15073326	GAAAAAAAAAAA/GAAAAAAAAAA	TGTGTGTGCATCCAAAAGGT	TTTGGGAGTAGGCCTTCCTT	60.1	205	DRR	Ca_17012	R	--
Ca3_P2_15080816	Ca3	15080816	AATATATATATATATATATATATATATATATATATATATATATA/AATATATATATATATATATATATATATATATATATATA	TGGAGCTACGAAGACACACG	GTGCTTCGCACGGAAACTAT	60.3	550	INTERGENIC	--	--	--
Ca3_P2_15095814	Ca3	15095814	GAAAAAAAAAAAA/GAAAAAAAAAAA	TTGGTGCAAGCCACAAATAA	ATGATGGTGGTGATGGAGGT	60.1	547	INTERGENIC	--	--	--
Ca3_P2_15231571	Ca3	15231571	ATGAACTGAACTGAACTGAA/ATGAACTGAACTGAA	ACCAGGAAAAGAAGAAGGGC	TTGGTTTATTTGGGTTGGGA	60.0	462	INTERGENIC	--	--	--
Ca3_P2_15232166	Ca3	15232166	A/ATTTAA	TCCCAACCCAAATAAACCAA	CCCAAAAAGCGCTGTAAAAG	59.9	777	INTERGENIC	--	--	--
Ca3_P2_15232531	Ca3	15232531	TAAAAA/TAAAA	CTTTTACAGCGCTTTTTGGG	TTCGTAATGGACGGGATGAT	60.2	245	INTERGENIC	--	--	--
Ca3_P2_15241800	Ca3	15241800	GTAATAATAATAATAATAATAATAATAATAATAATAAT/GTAATAATAATAATAATAATAATAATAATAATAATAAT	GAATGAATTCCGTGGCAAAT	CCTCAAGGGCAGATCCATAA	60.0	325	INTERGENIC	--	--	--
Ca3_P2_15299542	Ca3	15299542	GAAAAAAAAAAA/GAAAAAAAAAA	CTAGTTTGCCCGGTGATTGT	GGGTTTGCGATTTGTTCTGT	60.0	868	INTERGENIC	--	--	--
Ca3_P2_15318264	Ca3	15318264	TAAAAAAA/TAAAAAA	CGAAGGAGCTTTTCTTGTGG	CGCATTTTGCATTTGTCAAC	60.1	651	INTERGENIC	--	--	--
Ca3_P2_1533357	Ca3	1533357	TCAAAA/TCAAAACTCCAAAA	ACCCAAAAGCTAACTTGCCA	GCATCAACAAAGCAGCAGAA	60.1	533	INTERGENIC	--	--	--
Ca3_P2_1534003	Ca3	1534003	AAACTGCAACTAACT/AAACTGCAACTAACTGCAACTAACT	TTCTGCTGCTTTGTTGATGC	GTGGATGGGTGTTTTCCATC	60.0	774	INTERGENIC	--	--	--
Ca3_P2_15388201	Ca3	15388201	CATATATATAT/CATATATAT	AAACACATGCGCTTTCATTG	AATCGATTGGCTGAAATCCA	60.4	479	INTERGENIC	--	--	--

INDEL marker IDs	Chromosomes/unanchored scaffolds	Physical positions (bp)	InDels (*Kabuli* reference genome-CDC Frontier/PII)	Forward primers (5'-3')	Reverse primers (5'-3')	Anneling temperature (0C)	Expected amplified product size (bp)	Structural annotation		Functional annotation	
								Sequence components of *kabuli* genome	*Kabuli* gene accession IDs	NCBI-KOG	TFs
Ca3_P2_15424721	Ca3	15424721	ATTTTTTTTTTTT/ATTTTTTTTTTT	TTCGTGCCTATATGTAAGATCCA	GTCGCATTTTCAGACAACCA	59.7	295	INTRON	Ca_17030	OE	WRKY
Ca3_P2_15463471	Ca3	15463471	CATATATATATATATATATATA/CATATATATATATATATATA	TTTGTGGATTTGCATTGATGA	TGAATTCACCGTAAACGGAA	59.0	783	INTERGENIC	--	--	--
Ca3_P2_15503247	Ca3	15503247	C/CTAAAATATTAGGGAA	ATTGGGAAGTGGGGAAACTC	CGGGGTACATTGATAGCGTT	59.8	800	INTERGENIC	--	--	--
Ca3_P2_15524418	Ca3	15524418	GTTTTTT/GTTTTTTT	GACGTGTGTGTGGAAAGAGC	CGGCCAAAGTAATGGATTGT	59.8	720	INTERGENIC	--	--	--
Ca3_P2_15658491	Ca3	15658491	A/AC	AAAGCATTCCCACTACGGTG	TTGTCACAGCCTAAAACCCC	60.0	343	DRR	Ca_17043	--	--
Ca3_P2_15658525	Ca3	15658525	TC/TCATTCC	AAAGCATTCCCACTACGGTG	TTGTCACAGCCTAAAACCCC	60.0	343	DRR	Ca_17043	--	--
Ca3_P2_15660475	Ca3	15660475	TA/TAA	AAGTGAGCTAACGGGGGTTT	TGGACAGAGCAAGTGAGGTG	60.0	140	INTERGENIC	--	--	--
Ca3_P2_1573297	Ca3	1573297	TAAAAAAAAAAA/TAAAAAAAAAA	ATAAGACCAAAGAAGCGGCA	GCATGCAATTGGTTTGACAC	60.0	607	INTERGENIC	--	--	--
Ca3_P2_15824653	Ca3	15824653	C/CCTGCTT	CCCTCAAGACTCCCACGATA	CATTGGCTTGAAGTGGGTCT	60.1	268	INTERGENIC	--	--	--
Ca3_P2_15838572	Ca3	15838572	AATATATATATATATATATATAT/AATATATATATATATATATATATAT	GCGTTTGTTCTAACCACTCG	CCCACCGCAATATGTAGGTC	60.2	661	INTRON	Ca_17052	Q	--
Ca3_P2_15868825	Ca3	15868825	ATATTATTATTATTATTATTATTATTATTATTATTATTATTATTATTATTATTATTATTAT/ATATTATTATTATTATTATTATTATTATTATTATTATTATTATTATTATTATTATTATTATTATTATTATTAT	AATCGTGCGTTTTTGCTTTT	AGGATTCTCAGTGCAAAGCG	60.5	472	INTERGENIC	--	--	--
Ca3_P2_15912164	Ca3	15912164	ATTTTTTTT/ATTTTTTT	ATCCCATTTTTCCCATTTCC	CCCAAAAATCCCAAACAAAA	59.6	213	INTERGENIC	--	--	--

INDEL marker IDs	Chromosomes/unanchored scaffolds	Physical positions (bp)	InDels (*Kabuli* reference genome-CDC Frontier/PII)	Forward primers (5'-3')	Reverse primers (5'-3')	Anneling temperature (0C)	Expected amplified product size (bp)	Structural annotation		Functional annotation	
								Sequence components of *kabuli* genome	*Kabuli* gene accession IDs	NCBI-KOG	TFs
Ca3_P2_16108101	Ca3	16108101	TAAAAAAAAAAA/TAAAAAAAAA	AAAACAGGGCAAGAAAGCAA	AACAGCAAAAGAGAAGGCGA	60.1	292	INTERGENIC	--	--	--
Ca3_P2_16133157	Ca3	16133157	GTTTTATTTTATTTTATTT/GTTTTATTTTATTTTATTTTATTT	CGTCTCTTGAAGCGATGAAA	CATTGTTGGTCACTAGCAAATCA	60.0	698	INTERGENIC	--	--	--
Ca3_P2_16133384	Ca3	16133384	CAATAATAATAATAATAATAATAATAATAATAATAATA/CAATAATAATAATAATAATAATAATAATAATA	CGTCTCTTGAAGCGATGAAA	ATAAGCCGTCAATCTGCCAC	60.1	807	INTERGENIC	--	--	--
Ca3_P2_16133499	Ca3	16133499	TA/TACACAAAGAA	TTTGCCCCATTATAAACAAAA	ATAAGCCGTCAATCTGCCAC	60.1	625	INTERGENIC	--	--	--
Ca3_P2_16137267	Ca3	16137267	CATATATATATATATATATA/CATATATATATATATATATATA	TGGCACATCTAAAAATAGGAGGA	AGAAAGTCGCATCCCAGAGA	60.0	544	INTERGENIC	--	--	--
Ca3_P2_16138352	Ca3	16138352	TTATATATATATATATATATATATATATA/TTATATATATATATATATATATATATATATATATATATATATATATA	GCCACAATCTGTCCCCAATA	TAGTGAAGGCATTGGCAGAA	59.4	855	INTERGENIC	--	--	--
Ca3_P2_16163642	Ca3	16163642	TAAAAAAAAAAA/TAAAAAAAAAA	TTCAAAATCGCAAACACTGC	ACCCACAAAACGCAAAGATT	59.5	795	INTERGENIC	--	--	--
Ca3_P2_16166714	Ca3	16166714	TTTTG/TTTTGCTTTG	TCAACCATCTGGGTTGGAAT	CCCCTAGCAAAGGAAACAAA	59.2	674	INTERGENIC	--	--	--
Ca3_P2_16168531	Ca3	16168531	AG/A	TGGAGGAGGATTTCCATCTG	TGCTTTCTTGCCCTGTTTTT	59.9	547	INTERGENIC	--	--	--
Ca3_P2_16171083	Ca3	16171083	T/TG	TACAGCGCTTCATTCCACAG	CGTGCATGAGCAAAAGAAAA	60.0	468	INTERGENIC	--	--	--
Ca3_P2_16182804	Ca3	16182804	ATTTTTTTT/ATTTTTTTTTT	CCGGCATCACCTATCAGAGT	TCCGACCTCCTTTGAGAAGA	59.9	583	INTERGENIC	--	--	--
Ca3_P2_16190775	Ca3	16190775	CA/C	TCGCGGTTCTTGATGATGTA	AGCTCGACTTATCCTTCGCA	60.1	423	INTERGENIC	--	--	--

INDEL marker IDs	Chromosomes/unanchored scaffolds	Physical positions (bp)	InDels (*Kabuli* reference genome-CDC Frontier/PII)	Forward primers (5'-3')	Reverse primers (5'-3')	Anneling temperature (0C)	Expected amplified product size (bp)	Structural annotation		Functional annotation	
								Sequence components of *kabuli* genome	*Kabuli* gene accession IDs	NCBI-KOG	TFs
Ca3_P2_16195131	Ca3	16195131	AT/ATT	TGATAAAAGACTAACGATGCAAACA	TAAACCATCAATCTGCCACG	59.5	655	INTERGENIC	--	--	--
Ca3_P2_16195368	Ca3	16195368	CATATAT/CATATATAT	ACGTGGCAGATTGATGGTTT	TTTGATGTTGGCGTAGAGGA	59.3	350	INTERGENIC	--	--	--
Ca3_P2_16195709	Ca3	16195709	AC/A	ACGTGGCAGATTGATGGTTT	GAACGTACGGAAGAGAAGCG	60.0	754	INTERGENIC	--	--	--
Ca3_P2_16197261	Ca3	16197261	TCTTAA/T	CTGCATCGAATTAATGCAACA	AAGGGGGATCATGGAAAACT	59.6	316	DRR	Ca_18251	--	--
Ca3_P2_16212378	Ca3	16212378	A/AACATTC	ATGCACTTTGCCACTGTTGT	GAGGGGATGATGCTTCAAAA	60.0	879	INTERGENIC	--	--	--
Ca3_P2_16235197	Ca3	16235197	ATAATAGTAATAGTAATAGTAATAGTAATAGTAATAGTA/ATAATAGTAATAGTAATAGTAATAGTAATAGTAATAGTAATAGTA	TGCTCCTCTTTATGTTGGCA	GCCAGAGATGGCTATCCAAG	59.8	463	INTERGENIC	--	--	--
Ca3_P2_16253024	Ca3	16253024	TTTTTTT/TTTTTTTATTTTTT	AACACAGTGGCTGCTTCCTC	AAGAGAGGTCACTTTGGCGA	60.0	491	INTERGENIC	--	--	--
Ca3_P2_16347875	Ca3	16347875	CAAAAAA/CAAAAAAA	GCGTTTTTATGGTTGCTGGT	TATGGAAGGTGAGACAGGGG	59.9	690	INTERGENIC	--	--	--
Ca3_P2_16423935	Ca3	16423935	AATATATATATATATATATATAT/AATATATATATATATATATATAT	TCCAGAAAATAAATCTTCTTCACG	CAAGGATGGGTTGATCGTCT	59.9	702	INTERGENIC	--	--	--
Ca3_P2_1643316	Ca3	1643316	ATCTTTTTTCTTTTTTCT/ATCTTTTTTCTTTTTTCTTTTTTTCT	TCCAATTCCTGATTTCTGCC	TGCCATCAACCTTACACCAA	60.0	579	INTERGENIC	--	--	--
Ca3_P2_16476513	Ca3	16476513	TNNNNNNNNNN/TNNN	GGCTCACATGTGGAGGCTAT	CCAATGCCATACCATAGAGGA	59.8	344	INTERGENIC	--	--	--
Ca3_P2_16518599	Ca3	16518599	A/AACC	CTTTGGAGCAAGCTGGGTAG	TTTTGCGATGTTTGATCGTT	59.2	544	INTERGENIC	--	--	--

INDEL marker IDs	Chromosomes/unanchored scaffolds	Physical positions (bp)	InDels (*Kabuli* reference genome-CDC Frontier/PII)	Forward primers (5'-3')	Reverse primers (5'-3')	Anneling temperature (0C)	Expected amplified product size (bp)	Structural annotation		Functional annotation	
								Sequence components of *kabuli* genome	*Kabuli* gene accession IDs	NCBI-KOG	TFs
Ca3_P2_1653851	Ca3	1653851	TTTATTATTATTATTATTATTATTATTATTATTATT/TTTATTATTATTATTATTATTATTATTATTATTATTATT	TTTCTAGGGTGCGACCATGT	ACGCGTTTGGGTAGATATGC	60.0	627	INTERGENIC	--	--	--
Ca3_P2_16544552	Ca3	16544552	GTTATTATTATTATTATTATTATTATTATTATTATTATTATTA/GTTATTATTATTATTATTATTATTATTATTATTATTA	TGCTCGACATGTTTCTCCTT	CCACACAAACTGACAACTCCA	59.6	605	INTERGENIC	--	--	--
Ca3_P2_16568096	Ca3	16568096	TTATATATATATATATATATATATATA/TTATATATATATATATATATATATATATATATA	CTTGAATCGCAGCATTGTGT	TTCTTGGCATTGTTCAGCAG	60.0	602	INTRON	Ca_18273	G	C2H2
Ca3_P2_16643955	Ca3	16643955	CTATTATTATTATTATTATTATTATTATTATTATTATT/CTATTATTATTATTATTATTATTATTATTATTATTATTATT	GTCGTTCTCTCCTCCCCTTC	AGTCAAATCGACCGTGTTTGT	59.5	257	INTERGENIC	--	--	--
Ca3_P2_16778238	Ca3	16778238	GAAAAAAA/GAAAAAA	TCCTATGGTGGCTCATCTCC	TTTTGCCTCAACAATCTCCC	60.1	359	INTERGENIC	--	--	--
Ca3_P2_16793468	Ca3	16793468	TTATA/TTATATA	TAATCAAATGCCCCCAATGT	TTGAGTTCAAGCGTGAGTGG	60.0	851	INTERGENIC	--	--	--
Ca3_P2_16820332	Ca3	16820332	AATATATATATATATATA/AATATATATATATATATA	AGAGGACATCGGTTAGCTGC	AAGAAAGAAGGGGTCCCAAA	59.9	633	INTERGENIC	--	--	--
Ca3_P2_1685924	Ca3	1685924	TTATATATATATATATATATATATATATA/TTATATATATATATATATATATATATATATATATA	CTCGCTCTCCTATATCCCCC	GGGTGAAAAATTAACCAAACCA	60.0	508	INTERGENIC	--	--	--
Ca3_P2_16879724	Ca3	16879724	CT/CTCCCACTGGT	AAGCGTACCTGTGGGAATTG	GCGGATCCACTCACTAAAGG	59.7	818	DRR	Ca_18286	R	MYB
Ca3_P2_16885390	Ca3	16885390	CTATATATATATATATATATA/CTATATATATATATATA	CATTTTCATCAAAGGTCGCA	CAAATTTGCCACTGTCTTGC	59.3	244	INTERGENIC	--	--	--
Ca3_P2_16954725	Ca3	16954725	CATATATATATATATATATATATATA/CATATATATATATATATATATATATATA	TAAATGACCAAGCCGGAAAA	CGTTTGATCGATTGCAGTTC	59.3	573	INTERGENIC	--	--	--
Ca3_P2_17011730	Ca3	17011730	AT/ATTATT	AGATCGGTGTTGGAAACTGG	TTGAGCCCAACAGTTCAATG	59.7	663	INTERGENIC	--	--	--

INDEL marker IDs	Chromosomes/unanchored scaffolds	Physical positions (bp)	InDels (*Kabuli* reference genome-CDC Frontier/PII)	Forward primers (5'-3')	Reverse primers (5'-3')	Anneling temperature (0C)	Expected amplified product size (bp)	Structural annotation		Functional annotation	
								Sequence components of *kabuli* genome	*Kabuli* gene accession IDs	NCBI-KOG	TFs
Ca3_P2_17033772	Ca3	17033772	AAATAATAATAATAATAATAATAATAATAATAATAATAATAAT/AAATAATAATAATAATAATAATAATAATAATAATAATAATAATAATAATAAT	ATGCGATCATGTGGATGAGA	TACCCCTTGTTGGTCCTGAG	60.0	840	INTERGENIC	--	--	--
Ca3_P2_17039245	Ca3	17039245	CAAAAAA/CAAAAAAAA	TTCTAACTTTGCTGTCCCATTTT	GGGGTAAAACCAGGAGGAAG	59.8	750	INTERGENIC	--	--	--
Ca3_P2_17039469	Ca3	17039469	CTTTT/CTTTTT	TGTTTTGAGGACAAATGTTTTGA	GGGGTAAAACCAGGAGGAAG	59.8	301	INTERGENIC	--	--	--
Ca3_P2_17122676	Ca3	17122676	ATTTTTTTTTT/ATTTTTTTTTTT	AATGCACCACTTTATGCACG	CCAGTTTTCATTCAGGCACA	59.7	358	INTERGENIC	--	--	--
Ca3_P2_1719355	Ca3	1719355	ATATATCTATAT/ATATAT	TTTCCCTCCAAACACACACA	CTTTTGATGCCCCGATTAAA	59.9	351	INTERGENIC	--	--	--
Ca3_P2_17201946	Ca3	17201946	TTATATATATATATATATATATATATATATATATAT/TTATATATATATATATATATATATATATATAT	AAAATCAAACATGGTGGGGA	AAGGCATGATGTTGGAGTCA	59.1	398	DRR	Ca_24619	S	--
Ca3_P2_17217773	Ca3	17217773	TCC/TC	ACAATCGGACACCGTTTCAT	TCATGTGGCAACACTGTTCA	59.7	253	INTERGENIC	--	--	--
Ca3_P2_17261676	Ca3	17261676	AATATATATATATATATATATA/AATATATATATATATATATA	TGTGCATTTTGGAGAAACCA	GTCAGGGCATCAACCACTTT	60.0	395	INTRON	Ca_23345	T	WRKY
Ca3_P2_17312408	Ca3	17312408	AG/A	AGGTTTTCTGCTCGCTTGAA	TGTGTGAAACGCTAAGCGAC	60.1	178	INTERGENIC	--	--	--
Ca3_P2_17328838	Ca3	17328838	ATAGATTA/ATA	CATTTCCTAAACGAAAGCAAGG	TCCAAAGTTCCGAACCAAAG	60.1	582	INTERGENIC	--	--	--
Ca3_P2_17392321	Ca3	17392321	AGCT/A	GCAACACTTGTTCGAATGTGA	CGGATTGAAAAAGGAACGAA	60.0	289	INTERGENIC	--	--	--
Ca3_P2_17428991	Ca3	17428991	CAAAAAA/CAAAAA	CCAACGCCTATTTTGATGGT	TGTGGTTGAGTTGGTGATGAA	60.0	263	INTERGENIC	--	--	--

INDEL marker IDs	Chromosomes/unanchored scaffolds	Physical positions (bp)	InDels (*Kabuli* reference genome-CDC Frontier/PII)	Forward primers (5'-3')	Reverse primers (5'-3')	Anneling temperature (0C)	Expected amplified product size (bp)	Structural annotation		Functional annotation	
								Sequence components of *kabuli* genome	*Kabuli* gene accession IDs	NCBI-KOG	TFs
Ca3_P2_17474697	Ca3	17474697	AATATATATATATATATATATATATATATAT/AATATATATATATATATATATATATATATATATAT	CCAACATGCCGACAAACTTA	TACCATTCACATGCAATGCC	60.4	765	INTERGENIC	--	--	--
Ca3_P2_17491839	Ca3	17491839	AATATATATATATATA/AATATATATATATA	TGAATTCATGGCTAAAAAGCCT	GCAAAGATGATAATTCGGCAA	60.1	654	INTERGENIC	--	--	--
Ca3_P2_17542001	Ca3	17542001	TAAAAAAAAAAA/TAAAAAAAAAAAA	TTCATGAGGTTGTTGTTGCC	GAATCGACAAACGGAGGAAA	60.1	540	INTERGENIC	--	--	--
Ca3_P2_17579559	Ca3	17579559	TAAAA/TAAATAAAAA	TGCATTTTCACCAACATCGT	AATCACCGAGCTGAAATCAAA	59.7	912	INTERGENIC	--	--	--
Ca3_P2_17706970	Ca3	17706970	AATTATTATTATTATTATTATTATTATTATTATTA/AATTATTATTATTATTATTATTATTATTATTA	ACGGAAGCAGATGGTAGTGG	ATTTTAACAGATGGCCGCAT	59.4	517	INTERGENIC	--	--	--
Ca3_P2_17725458	Ca3	17725458	CATATATATATATATATATAT/CATATATATATATATATATATAT	AGAAGAAGGAGGATTCGCGT	GCGATTTGGGAGAGAAACAG	59.8	572	INTERGENIC	--	--	--
Ca3_P2_17791032	Ca3	17791032	GAAAAAA/GAAAAA	GGACGGAAAACAAATATGGG	CCTCATACGCGATTTGGATT	59.9	283	INTERGENIC	--	--	--
Ca3_P2_17819480	Ca3	17819480	AATATATATATATATATATATATATATATATATA/AATATATATATATATATATATATATATA	AACGATCTGATTGTAATGTGTTTCTT	GTCGTGAACGGTGCTACATC	59.2	884	INTERGENIC	--	--	--
Ca3_P2_1786985	Ca3	1786985	AAAAATTAAAATTAAAATTAAAATT/AAAAATTAAAATTAAAATT	TTTTAATTGTAGCTTTGGTCCCTC	CTCACGAAACCGGTCTTTCTA	59.4	404	INTERGENIC	--	--	--
Ca3_P2_17940721	Ca3	17940721	GAAAAAAAAAAA/GAAAAAAAAAA	GGCTAAATTACATTTGTGGTCCC	GGCTAAATTATATTCGTGGTCCC	60.0	382	INTERGENIC	--	--	--
Ca3_P2_18015978	Ca3	18015978	TTATATATATATATATATATATATATATATAT/TTATATATATATATATATATATATATATAT	TCAGCAATAGAAGCCAAATGC	CGAACTTTGCCGTACGTCTT	60.3	470	INTERGENIC	--	--	--
Ca3_P2_18065169	Ca3	18065169	TTATATATATATATATATATAT/TTATATATATATATATATATAT	TCTTGGAACAAACGCACTCA	TGCCCTATTGCACATCGTAA	60.1	760	INTERGENIC	--	--	--

INDEL marker IDs	Chromosomes/unanchored scaffolds	Physical positions (bp)	InDels (*Kabuli* reference genome-CDC Frontier/PII)	Forward primers (5'-3')	Reverse primers (5'-3')	Anneling temperature (0C)	Expected amplified product size (bp)	Structural annotation		Functional annotation	
								Sequence components of *kabuli* genome	*Kabuli* gene accession IDs	NCBI-KOG	TFs
Ca3_P2_18153020	Ca3	18153020	CTTTTTTTTTTTT/CTTTTTTTTTTTTT	TACCAATGCGTTGATCGAAA	TTGAAGTTGAAATCCCCTCG	60.0	782	INTERGENIC	--	--	--
Ca3_P2_18178087	Ca3	18178087	T/TA	AACAGTGGTTGTCCACGTCA	CTTCGCTGCTGCATATACCA	60.0	148	INTERGENIC	--	--	--
Ca3_P2_18192577	Ca3	18192577	GTTTTTTTTTTT/GTTTTTTTTTT	GCTGTAAAATGCACCCCATT	TTGTTGTGTGCCCTGATTGT	60.0	451	DRR	Ca_21141	--	--
Ca3_P2_18241779	Ca3	18241779	CTATATATATATATA/CTATATATATATATATA	CTCTACCACGCGATTACGGT	GGAAGTGATGGGTGAAATGG	60.2	390	INTERGENIC	--	--	--
Ca3_P2_18268210	Ca3	18268210	GAAAAAAAAAA/GAAAAAAAAAAA	AACGAAGAAGTTGAGGTGGC	CCGAATTGCTCTTCATTGGT	60.1	940	DRR	Ca_19613	O	--
Ca3_P2_18272727	Ca3	18272727	A/AC	TTTGCCAATCGATTATCGTG	GGGCAATCGACTGACTGAAT	60.1	683	INTERGENIC	--	--	--
Ca3_P2_18291883	Ca3	18291883	CAAAAAAAAAAA/CAAAAAAAAAAAA	GAAAGAGATCAAACTCGCGG	TGCAATATTTCAGGCTAATCGTT	60.0	879	INTERGENIC	--	--	--
Ca3_P2_18345308	Ca3	18345308	TAAAAAAA/TAAAAAAAA	GGTTTCCCTCCTAAGTTAAGGGT	GCGGTGCAATCAGTAAACCT	60.1	271	DRR	Ca_19619	O	--
Ca3_P2_18454468	Ca3	18454468	ATT/A	GCGCGTGGAATAATGAGACT	TACACGATGCATCTACCCCA	59.9	518	INTERGENIC	--	--	--
Ca3_P2_18536264	Ca3	18536264	GAAAAAAAAAA/GAAAAAAAAAAA	TTACGGTGCACCAATTCAAA	TTCATTTGCAATTCTTTTCACAA	59.6	574	INTERGENIC	--	--	--
Ca3_P2_18632022	Ca3	18632022	AATATATATATATATATAT/AATATATATATATATATATATAT	TGGGGCTGGTCCAATATAAA	AGATGTGGGGACAACGACTC	60.0	399	INTERGENIC	--	--	--
Ca3_P2_18647131	Ca3	18647131	ATTTTTTTTT/ATTTTTTTTTT	AATTGATTTAAGCCCAAAAATAGA	AGAAGTGGCAAGATGGATGG	60.1	576	DRR	Ca_19638	Q	--

INDEL marker IDs	Chromosomes/unanchored scaffolds	Physical positions (bp)	InDels (*Kabuli* reference genome-CDC Frontier/PII)	Forward primers (5'-3')	Reverse primers (5'-3')	Anneling temperature (0C)	Expected amplified product size (bp)	Structural annotation		Functional annotation	
								Sequence components of *kabuli* genome	*Kabuli* gene accession IDs	NCBI-KOG	TFs
Ca3_P2_18663228	Ca3	18663228	AATT/AGAGTTTAAACAAATTATT	TGTGCACAACTCATCGGATT	AAAAGCGTGATTTTTGTGGG	60.0	260	INTERGENIC	--	--	--
Ca3_P2_18695410	Ca3	18695410	AAATAATAATAATAATAATAATA/AAATAATAATAATAATAATAATA	CTCTAGGTTTGCGCTTGTCC	TCGACTGTGGGATTGACAAA	60.1	776	INTERGENIC	--	--	--
Ca3_P2_18835973	Ca3	18835973	TAAAA/TAAAAA	ATTTTCCGATTCTGCATTCG	CCGACAATCTCCCAATCAAT	59.8	627	INTERGENIC	--	--	--
Ca3_P2_18862499	Ca3	18862499	TGG/TG	GGAATCGGGTAACAAATCCA	CCGATAAACAAATCCCAACG	60.2	241	INTERGENIC	--	--	--
Ca3_P2_18943181	Ca3	18943181	AAT/A	TGGAGTGTTGATGTCGTTGTC	AAATCGAAGATCGCAACAGT	57.4	428	INTERGENIC	--	--	--
Ca3_P2_19056367	Ca3	19056367	ATATTATTATTATTATTATTATTATTATTATTATTATTGTTATTATTATTATTATTATTATTAT/ATATTATTATTATTATTATTATTATTATTATTATTATTATTGTTATTATTATTATTATTATTATTAT	CAACACAACGATAATGGGCA	GACATTGGTTGGGGTTTGAC	60.1	717	INTERGENIC	--	--	--
Ca3_P2_19062819	Ca3	19062819	AATATATATATATATATATATATATATATATATATATATA/AATATATATATATATATATATATATATATATATATATATA	CATGCTAAATAACATGAAGAATGC	GCAGGTTTGACAGTGAGCAA	60.0	637	INTERGENIC	--	--	--
Ca3_P2_19062993	Ca3	19062993	AG/AGG	TTGTTTCATCAATTTACAATGCG	GCAGGTTTGACAGTGAGCAA	60.0	185	INTERGENIC	--	--	--
Ca3_P2_19089322	Ca3	19089322	T/TATC	CACCGTTGACCAGTTTGATG	CACGGGAAACTCGATGATCT	60.1	713	INTERGENIC	--	--	--
Ca3_P2_19138250	Ca3	19138250	CTTTTTTTTTT/CTTTTTTTTT	TGCCAGCCTAGTCTGTTGTG	GAGATTTAGAACCATCAAATTGTCA	58.6	556	INTERGENIC	--	--	--
Ca3_P2_19141746	Ca3	19141746	TTTTATTTATTTATTTATTTATTTATTTATTTATTTATTTATTTATTT/TTTTATTTATTTATTTATTTATTTATTTATTTATTT	GGAGCTCCTGGTTACAAAACA	GGGTGTACATTGGTTGGGTC	60.0	484	INTERGENIC	--	--	--
Ca3_P2_19148213	Ca3	19148213	CATATATATATATATA/CATATATATATATATATA	GCACAAGACTTTACGTCCATCTC	TATTTTGTGTCGTGCGCATT	60.1	691	INTERGENIC	--	--	--

INDEL marker IDs	Chromosomes/unanchored scaffolds	Physical positions (bp)	InDels (*Kabuli* reference genome-CDC Frontier/PII)	Forward primers (5'-3')	Reverse primers (5'-3')	Anneling temperature (0C)	Expected amplified product size (bp)	Structural annotation		Functional annotation	
								Sequence components of *kabuli* genome	*Kabuli* gene accession IDs	NCBI-KOG	TFs
Ca3_P2_19163962	Ca3	19163962	GGTGTGTGTGTGT/GGTGTGTGTGT	AGTTAACCGACGAAACCCCT	TTAGCAATCAAACCAACGCA	60.3	321	INTERGENIC	--	--	--
Ca3_P2_19181508	Ca3	19181508	GCC/GC	GAAGGCTTGTTTTTGTCCGA	TGAGATGCTTCGTCCAGAGA	59.7	896	INTERGENIC	--	--	--
Ca3_P2_19198715	Ca3	19198715	A/AC	TCTTCCACCCAGTTTTCACC	ACCGAGGAATGATATCGACG	59.9	385	INTERGENIC	--	--	--
Ca3_P2_19358625	Ca3	19358625	GTTTTTTTTTTTTT/GTTTTTTTT	AAGATTTGGTCCGTTATGCG	GTGGGAATGAACAAAGGACG	60.4	517	INTERGENIC	--	--	--
Ca3_P2_19359038	Ca3	19359038	TAAAA/TAAA	CGTCCTTTGTTCATTCCCAC	TGAGGGCCTTTAGAATCGTG	60.2	670	INTERGENIC	--	--	--
Ca3_P2_19359334	Ca3	19359334	CCA/C	AGGGAAATCAACGACAGAGC	TGAGGGCCTTTAGAATCGTG	60.2	365	INTERGENIC	--	--	--
Ca3_P2_19362553	Ca3	19362553	TAAAAA/TAAAA	CCAAAATGATGTTGCAGGGT	TCATCATCATTGAAAATCCGTT	59.3	361	INTERGENIC	--	--	--
Ca3_P2_19368426	Ca3	19368426	GTTTTTTTT/GTTTTTTTTT	GGCACTCAAAGTGTTGGGTT	TGGGTTAGCTTTTGGGATTG	59.9	920	DRR	Ca_19336	--	--
Ca3_P2_19368565	Ca3	19368565	GTTTTT/GTTTTTT	GGTGATGCATGATGATTTGC	TGGGTTAGCTTTTGGGATTG	59.9	630	DRR	Ca_19336	--	--
Ca3_P2_19369038	Ca3	19369038	CCAACAAC/CCAAC	CAATCCCAAAAGCTAACCCA	AAGAATCTGCGCCAACATCT	59.8	173	INTERGENIC	--	--	--
Ca3_P2_19371062	Ca3	19371062	TAAAAAAAA/TAAAAAAAAA	TTCATTGGATTGTTTGTGGG	GAATTCTGCGTTTTTCCTGC	59.8	845	INTERGENIC	--	--	--
Ca3_P2_19373696	Ca3	19373696	TAA/TA	GCTGAATGCGTGAAGAACAA	GGGTAGAAAACACGCGGAAT	61.2	339	INTERGENIC	--	--	--

INDEL marker IDs	Chromosomes/unanchored scaffolds	Physical positions (bp)	InDels (*Kabuli* reference genome-CDC Frontier/PII)	Forward primers (5'-3')	Reverse primers (5'-3')	Anneling temperature (0C)	Expected amplified product size (bp)	Structural annotation		Functional annotation	
								Sequence components of *kabuli* genome	*Kabuli* gene accession IDs	NCBI-KOG	TFs
Ca3_P2_19375049	Ca3	19375049	AAAAGAAA/AAAAGAAAGAAA	TCTCCGTTCCATTTACACGA	AACTCCAAATCCCTGCTCAA	59.7	365	INTERGENIC	--	--	--
Ca3_P2_19375109	Ca3	19375109	GAGA/GAGAGAGAGAGAGAGAAGA	TCTCCGTTCCATTTACACGA	AACTCCAAATCCCTGCTCAA	59.7	365	INTERGENIC	--	--	--
Ca3_P2_19376077	Ca3	19376077	A/ATTT	TGACCTTGTGCATTGTTTTGA	GCAAACTCAACCTTGCCAAT	60.1	333	INTERGENIC	--	--	--
Ca3_P2_19380131	Ca3	19380131	TG/T	TTGGAGAGAATACTCCCCGTT	TGACGATCAGGAACACCAAA	60.1	413	INTERGENIC	--	--	--
Ca3_P2_19381558	Ca3	19381558	TTATTATTAT/TTATTATTATTAT	GGAGAAAAGCATCGAAAAGC	ACCTAACCCTCCAAGCCAGT	60.0	338	INTERGENIC	--	--	--
Ca3_P2_19382845	Ca3	19382845	TTT/TTTATT	TAGGGGCATATGGTCTCGTC	ACCACATGATGCTCTCCACA	60.1	782	INTERGENIC	--	--	--
Ca3_P2_19383912	Ca3	19383912	CAAA/CAA	GTCAGGTCAGGCTGGAAAAA	TCGTCTTAATCCACTTGGTGC	60.1	436	DRR	Ca_19337	--	--
Ca3_P2_19384308	Ca3	19384308	CTTTTTTTT/CTTTTTTTTT	GCACCAAGTGGATTAAGACGA	TCAAGATAACCACCGCAACA	60.1	745	DRR	Ca_19337	--	--
Ca3_P2_19384571	Ca3	19384571	TTTATTATTATTATTATTATTATTATTATTATTATTATTATTATTATTATTATTA/TTTATTATTATTATTATTATTATTATTATTATTATTATTATTATTATTATTATTATTATTATTATTA	GGGATTGGACTATTGGATGG	ACGAGGCAGTTGCTCATCTT	60.0	659	DRR	Ca_19337	--	--
Ca3_P2_19402891	Ca3	19402891	AGA/AAGATGA	ACCCAATCCAAGTTTGTCCA	GAAGGCGACCTCAAAGACTG	60.0	632	INTERGENIC	--	--	--
Ca3_P2_19403408	Ca3	19403408	CC/CCTTTC	AGGGTAATCCGCCAGTCTTT	GCAACATTCGTCCTCTCCAT	60.1	674	INTERGENIC	--	--	--
Ca3_P2_19471860	Ca3	19471860	TAAC/TAACAAC	TGATGGTTTCTTTATTCCGTGA	GGTCAAAACCCAAGGAAACA	59.8	223	INTRON	Ca_19344	Q	--

INDEL marker IDs	Chromosomes/unanchored scaffolds	Physical positions (bp)	InDels (*Kabuli* reference genome-CDC Frontier/PII)	Forward primers (5'-3')	Reverse primers (5'-3')	Anneling temperature (0C)	Expected amplified product size (bp)	Structural annotation		Functional annotation	
								Sequence components of *kabuli* genome	*Kabuli* gene accession IDs	NCBI-KOG	TFs
Ca3_P2_19513000	Ca3	19513000	TACACACACACACA/TACACACACACACACA	AAGCTTCCTCCTTTGCCTTC	GACATTGTGTGTCAGGTGCC	60.0	443	INTERGENIC	--	--	--
Ca3_P2_19532606	Ca3	19532606	TTATATATATATATATATATATATATATATATATATATATA/TTATATATATATATATATATATATATATATATATATATATATATA	ACAAAATATCGAGGGGAGCC	GGAAATTGACCGAAGACGAA	60.1	828	INTERGENIC	--	--	--
Ca3_P2_19560633	Ca3	19560633	TAAAAAAAAA/TAAAAAAAAAA	TCAGTGCGATGGAGAAATCA	TGACATTTTAAACCTTGAGTTGC	58.4	453	INTERGENIC	--	--	--
Ca3_P2_19626677	Ca3	19626677	GATATATATATATATATATATATA/GATATATATATATATATATATATA	TTCCCCATGCAAGGTTAGAC	TCATCGCAGGTGTACCATGT	60.0	797	INTERGENIC	--	--	--
Ca3_P2_19639547	Ca3	19639547	CT/CTTATAT	TGCAATCAAGAGAGGACACAA	CGAGAAATAGCTCACGAGGC	60.1	521	INTERGENIC	--	--	--
Ca3_P2_1964709	Ca3	1964709	A/AT	TCTCTTTTCTCGAACCGCAT	AAACGCAATCCTTGAGTTCG	60.2	317	INTERGENIC	--	--	--
Ca3_P2_19676099	Ca3	19676099	GTTTTTTTTTTT/GTTTTTTTTTTTTT	CAAACCCAAGCCATCTTCAT	TAAACTTTTTGGCGGTTTGG	60.0	380	INTERGENIC	--	--	--
Ca3_P2_19716338	Ca3	19716338	TTATATATATATATATATATATATATATATATA/TTATATATATATATATATATATATATATATATATATA	TGTTGTTGGAGACGAAAACG	TTTGTTGGTTGGTGGGTAAA	58.8	283	INTERGENIC	--	--	--
Ca3_P2_19753066	Ca3	19753066	ATTTTTTTT/ATTTTTTTTT	TTCTCACCAGTTTTGGTCCC	GCACATGAGATCTTGAGGCA	60.0	107	INTERGENIC	--	--	--
Ca3_P2_19899505	Ca3	19899505	CGGG/CGG	ACCCGCACTTGAAAAATCTG	ATTTCAAATTTCCGGGAAGG	60.1	218	DRR	Ca_09393	--	--
Ca3_P2_19960902	Ca3	19960902	TAAAAAAAAAAA/TAAAAAAAAAA	AGCTTTGATTTCTCCATCGC	GGTATTAGCACCCTACGGCA	60.0	243	INTERGENIC	--	--	--
Ca3_P2_19969942	Ca3	19969942	GATCAACGCATC/GATCAACGCATCAACGCATC	CATAAGGATCGGGGAACAAA	AGGATCATCCATCGTCTTCG	60.0	541	INTERGENIC	--	--	--

INDEL marker IDs	Chromosomes/unanchored scaffolds	Physical positions (bp)	InDels (*Kabuli* reference genome-CDC Frontier/PII)	Forward primers (5'-3')	Reverse primers (5'-3')	Anneling temperature (0C)	Expected amplified product size (bp)	Structural annotation		Functional annotation	
								Sequence components of *kabuli* genome	*Kabuli* gene accession IDs	NCBI-KOG	TFs
Ca3_P2_20005707	Ca3	20005707	AATATATATATATATATA/AATATATATATATATA	CCACAACAGAACGATGTTGG	TTCCCAAAACTATTTATGCATGG	60.1	312	INTERGENIC	--	--	--
Ca3_P2_20011560	Ca3	20011560	GTTGACACTT/GTTGACACTTGACACTT	GCAATTGCAAGAGCGTGATA	TTTGGCATGCAATCAAACAT	59.9	277	DRR	Ca_09404	--	--
Ca3_P2_20128194	Ca3	20128194	A/AATAATT	GGGGCCAATGTAAAAAGGTT	CCCAATTAATGGGAAGGGTAA	59.9	111	INTERGENIC	--	--	--
Ca3_P2_20159047	Ca3	20159047	AATATATATATATATATATATATATATA/AATATATATATATATATATATATATATATATATATATA	GATTGTTTATGCGCAAGGGT	TGCTTATTCCCCTCCTTCCT	60.0	489	INTERGENIC	--	--	--
Ca3_P2_20221448	Ca3	20221448	TATAATAATAATAATAATAATAATAATAATAATAATAATAATAATAATAATAATATTAATAATAATAATAA/TATAATAATAATAATAATAATAATAATAATAATAATAATAATAATAATAATAATATTAATAATAATAATAA	CCTGAGTGTTTTCGAGAGGC	GGGAAACCCACCTCTTGATT	60.2	772	INTERGENIC	--	--	--
Ca3_P2_20221557	Ca3	20221557	AT/ATT	TGGTTGGATGTGTTGAAGTTG	GGGAAACCCACCTCTTGATT	60.2	727	INTERGENIC	--	--	--
Ca3_P2_2023417	Ca3	2023417	CTTTTTTTTT/CTTTTTTTTTT	TCTCTGGCAACCGTTCTTCT	TGACAGTCAGAGTGCGATCC	60.0	436	INTERGENIC	--	--	--
Ca3_P2_20313249	Ca3	20313249	TTATATATATATATATATAT/TTATATATATATATAT	TGATGCTCTTTTTCCCCTTTT	AACAATAAAAGTCCGATTGCC	58.1	532	INTERGENIC	--	--	--
Ca3_P2_20329423	Ca3	20329423	CTTTTTTTTT/CTTTTTTTT	AGTTGTGGTTGTCCTGCTCC	ATGGTAGAAGGGGCCAAACT	59.8	316	DRR	Ca_09430	--	--
Ca3_P2_20343217	Ca3	20343217	TAAAAAAAAAA/TAAAAAAAAA	TTCACCCACTTCATTCGAAAC	GTCTCTTTTTCACCCCCACA	59.9	887	INTERGENIC	--	--	--
Ca3_P2_20347422	Ca3	20347422	AATATATATATATATATATATATATATATATAGATATATATATAT/AATATATATATATATATATATATATATATATATATATAGATATATATATAT	TTGTATGATGTCTCCTTGACCA	CTTTTCCAATCGACCTGCAT	60.1	777	INTERGENIC	--	--	--
Ca3_P2_20349202	Ca3	20349202	TTATATATATATATATATATAT/TTATATATATATATATATATAT	ACTGCCTAGCTCACTTCCCA	GGCGATTTTCATCGTGAGTT	60.1	417	INTERGENIC	--	--	--

INDEL marker IDs	Chromosomes/unanchored scaffolds	Physical positions (bp)	InDels (*Kabuli* reference genome-CDC Frontier/PII)	Forward primers (5'-3')	Reverse primers (5'-3')	Anneling temperature (0C)	Expected amplified product size (bp)	Structural annotation		Functional annotation	
								Sequence components of *kabuli* genome	*Kabuli* gene accession IDs	NCBI-KOG	TFs
Ca3_P2_20439188	Ca3	20439188	TGAAG/TG	CCGGTTTCATTCAAAAATGG	TGTTCAGCCTTGTGTTCAGC	60.0	565	INTERGENIC	--	--	--
Ca3_P2_20445164	Ca3	20445164	TTATATATATATATATATATATATAT/TTATATATATATATATATATAT	AGTGCCACATGTAACACCCC	GTTTTGGCGTTGGTTTCACT	60.0	815	INTERGENIC	--	--	--
Ca3_P2_20473105	Ca3	20473105	TAAA/TAA	AAAGCGGAAAAAGCACTCAC	CCCTCACCCTCTCTGCTCTA	59.5	807	DRR	Ca_09437	GC	HSF
Ca3_P2_20526351	Ca3	20526351	TTATATATATATATATATATATATATATATAT/TTATATATATATATATATATATAT	TGCATGTTCTTACGCTCGTC	AATTCACAAGACGCCATTCC	59.9	504	INTERGENIC	--	--	--
Ca3_P2_20559803	Ca3	20559803	ATTTTTTT/ATTTTTTTT	GCGCTTCGAATGTTGTACCT	TGAATTTTGTTTGAAGATTTTAACG	58.7	765	INTERGENIC	--	--	--
Ca3_P2_20617847	Ca3	20617847	AATATATATATATATATATATATA/AATATATATATATATATATATATA	AATCGATTGGCTGAAATCCA	TCGAGTTTGAGCATGTGAGG	60.0	809	INTERGENIC	--	--	--
Ca3_P2_2065051	Ca3	2065051	AATTATTATTATTATTATTATTATTATTATTA/AATTATTATTATTATTATTATTATTATTA	CGACCATGTAGTGAGGCAAA	AATAATTGCCCTACACCAACAA	58.4	685	INTERGENIC	--	--	--
Ca3_P2_20672094	Ca3	20672094	CAAAAAAAAAAAA/CAAAAAAAAAAA	TGTGCGGAAGAATTGATGAG	TTCCCCCTTCTCTTTCACCT	60.0	718	INTRON	Ca_09450	--	--
Ca3_P2_20728954	Ca3	20728954	A/AAAT	ATCGTCACATCTCCCACCTC	GATTGAGTTCCGGTTGTCGT	60.0	229	INTERGENIC	--	--	--
Ca3_P2_20750487	Ca3	20750487	CTTTATTTATTTTATTTATTTTATTTATTTTATTTATTTTATTTATTTTATTT/CTTTATTTATTTTATTTATTTTATTTATTTTATTTATTTTATTTATTTTATTT	TGTTATGTTGGGTTTGGGCT	TGCAGATGGGTTGAGTGTGT	60.2	875	INTERGENIC	--	--	--
Ca3_P2_20811683	Ca3	20811683	CATAATAATAATAATAATAATAATAATAATAATAATAATAATA/CATAATAATAATAATAATAATAATAATAATAATAATAATAATAATAATA	ATTCCACATAAGCGTTTGCC	CGATTGTGGACAATGGAAGA	59.5	361	INTERGENIC	--	--	--
Ca3_P2_20956913	Ca3	20956913	ATTTTTTTTTT/ATTTTTTTTT	TTGCAAGGCCCAATAAAAAC	TTTTCCGGAAGCCACTTATG	60.1	445	DRR	Ca_09466	--	--

INDEL marker IDs	Chromosomes/unanchored scaffolds	Physical positions (bp)	InDels (*Kabuli* reference genome-CDC Frontier/PII)	Forward primers (5'-3')	Reverse primers (5'-3')	Anneling temperature (0C)	Expected amplified product size (bp)	Structural annotation		Functional annotation	
								Sequence components of *kabuli* genome	*Kabuli* gene accession IDs	NCBI-KOG	TFs
Ca3_P2_20988635	Ca3	20988635	AATATATATATATATATATATATATATATATATATATATATAT/AATATATATATATATATATATATATATATATATATATATATATATATAT	CAAGCTGAAGGACTTCCACA	ATTATGTGGCGAGTGACGGT	60.4	477	INTERGENIC	--	--	--
Ca3_P2_21003278	Ca3	21003278	G/GCT	GCCTTTGGGGTACATTGTTG	GAGTGAAGGGGCAGAACAAA	60.2	413	INTERGENIC	--	--	--
Ca3_P2_21068865	Ca3	21068865	A/AG	ACAGGAGGTCCTGCTCTTCC	ATCCACCAGGGTCAATTCAA	60.2	483	INTRON	Ca_09474	J	--
Ca3_P2_21097843	Ca3	21097843	ATTTTTTTTT/ATTTTTTTTTT	GGCATTTTGATTTGATTCGC	CAAATTTTGTGTTTCAAAAACCATT	60.4	653	INTERGENIC	--	--	--
Ca3_P2_21311568	Ca3	21311568	GAAAAAAAAA/GAAAAAAAAAA	CACGTCACATCGCAATTTTT	CATGTCATTTCAACCCACGA	60.4	542	INTERGENIC	--	--	--
Ca3_P2_21319728	Ca3	21319728	AACTTACTTA/AACTTA	TGCTGTTCTAGTGTTCTAATTGCTC	GCCTGCCAGACGTTTTACAT	60.1	562	INTERGENIC	--	--	--
Ca3_P2_213670	Ca3	213670	AATATATATATATATATATAT/AATATATATATATATATATATAT	TTCCTTTTCCAATTTGCAGG	TCACGTAGAATGCTATCACTTTGA	59.0	544	DRR	Ca_22935	M	MYB_related
Ca3_P2_21376415	Ca3	21376415	TTAAAATAAAATAAAATAAAATAAAA/TTAAAATAAAATAAAATAAAA	TGGTATCATGACTTTCGGAGG	GCAATTGCCGAATGAAGATT	60.0	559	INTERGENIC	--	--	--
Ca3_P2_21396927	Ca3	21396927	ATGTTGTTGTTGT/ATGTTGTTGTTGTTGT	TATTGCATTGCTTCAACCCA	TTATGGCAAACCAGAAAGTCG	60.1	530	INTERGENIC	--	--	--
Ca3_P2_21424576	Ca3	21424576	CTTTTTT/CTTTTT	ACGTAGTTGGGTCTTGGCAC	CAAATTCTGTCCCGTCTGGT	60.0	385	INTERGENIC	--	--	--
Ca3_P2_214694	Ca3	214694	CG/CGAG	ATGTGGGGAGAACGTGAGTC	TGATTTCGTCAGGTCCCATT	60.3	472	DRR	Ca_22935	M	MYB_related
Ca3_P2_21563174	Ca3	21563174	CAAGAAG/CAAG	TCCCTTCTTCTCTCCCTTCC	GTTCCTGATCCGCTTGTTGT	60.1	158	INTERGENIC	--	--	--

INDEL marker IDs	Chromosomes/unanchored scaffolds	Physical positions (bp)	InDels (*Kabuli* reference genome-CDC Frontier/PII)	Forward primers (5'-3')	Reverse primers (5'-3')	Anneling temperature (0C)	Expected amplified product size (bp)	Structural annotation		Functional annotation	
								Sequence components of *kabuli* genome	*Kabuli* gene accession IDs	NCBI-KOG	TFs
Ca3_P2_21583634	Ca3	21583634	CTTTTTTTTTTTTTT/CTTTTTTTTT	CAATTCCCACAGGTACGCTT	AAGAGAGGTCACTTTGGCGA	60.0	812	INTERGENIC	--	--	--
Ca3_P2_21599415	Ca3	21599415	CAAAAAA/CAAAAAAA	GCAGCAGTTGTTTGCAGAAG	TCTCTTGCATGCCTAACACG	60.0	941	INTERGENIC	--	--	--
Ca3_P2_21603413	Ca3	21603413	GATATATATATA/GATATATATATATA	TGAGTCAAACAAACATCACAACA	GCCTCTCTAATCCACTCCCC	60.0	324	INTERGENIC	--	--	--
Ca3_P2_21635589	Ca3	21635589	GCATCATCA/GCATCA	AAGCATGCCAAACCAAAAAC	TGAACATCAAAGATCTGCGG	59.8	521	INTRON	Ca_09516	--	--
Ca3_P2_21635884	Ca3	21635884	CGAAGACAAAGAAGAAGA/CGAAGA	TGTCATGTTGTGGAGGAGGA	GGAAGCAATAGAAAATGGCG	59.7	573	CDS (large-effect mutations)	Ca_09516	--	--
Ca3_P2_2176981	Ca3	2176981	GATTATTATTATTATTATTATTATTATTATTATTATTATTATTATGATTATTATTATTA/GATTATTATTATTATTATTATTATTATTATTATTATTATTATGATTATTATTATTA	ACGGATTGTGTTTTGCTTCT	CCTCTTCTTCGGTGATCTTTTG	60.2	906	INTERGENIC	--	--	--
Ca3_P2_21874469	Ca3	21874469	ATTTTTCTTT/ATT	CAATGCACAAAAGTGTGGGT	ATTGTTCGCGTTTTGCTTGT	60.7	887	INTERGENIC	--	--	--
Ca3_P2_21897869	Ca3	21897869	ATATTATTATTATTATTATTATTATTA/ATATTATTATTATTATTATTATTA	GAGTTGAGTGTTGCCAGGGT	AGCTACCAAGCTGATGGTGAA	59.9	296	INTERGENIC	--	--	--
Ca3_P2_21989858	Ca3	21989858	TTATATATATATATATATATATATATATATATAT/TTATATATATATATATATATATATATAT	TGAAGCAAACCAAATAAATCCA	GACCGGTGGTAGACCTTTGA	60.0	592	INTERGENIC	--	--	--
Ca3_P2_22042563	Ca3	22042563	CTTTTTT/CTTTTTTT	AGGAAAGTGCCATGTGAAAAA	TCGTAGGTCAAGTTCAGACCG	60.3	764	INTERGENIC	--	--	--
Ca3_P2_22124545	Ca3	22124545	A/AGT	TCAATGTAAGGACCAGATGAATG	CGGTTTGGTTTGGTTGAGTT	59.9	296	INTERGENIC	--	--	--
Ca3_P2_22125727	Ca3	22125727	CAAAA/CAAAAA	TAACCCCTTGATCCTTGCAG	AGGGTTCAGCTAAGTGCCAA	59.9	647	DRR	Ca_20414	--	--

INDEL marker IDs	Chromosomes/unanchored scaffolds	Physical positions (bp)	InDels (*Kabuli* reference genome-CDC Frontier/PII)	Forward primers (5'-3')	Reverse primers (5'-3')	Anneling temperature (0C)	Expected amplified product size (bp)	Structural annotation		Functional annotation	
								Sequence components of *kabuli* genome	*Kabuli* gene accession IDs	NCBI-KOG	TFs
Ca3_P2_22160995	Ca3	22160995	CAAAAAAA/CAAAAAA	CCTTTTCCTTCCCTTTCCTG	CAATGTGCTCACTTTTGTCTCA	59.0	527	INTERGENIC	--	--	--
Ca3_P2_22165237	Ca3	22165237	AG/A	TTTGAAATGCCAATAAAGTTTGAA	CATGCAATGCACGAGATCAT	60.7	502	DRR	Ca_20412	U	--
Ca3_P2_22287300	Ca3	22287300	GATATATATATATATATATATATAT/GATATATATATATATATATATAT	TCACCAAAATGGTCACTGGA	TGTTCTTCCTTCCCCAGATG	60.0	913	INTRON	Ca_06280	K	MIKC
Ca3_P2_22377503	Ca3	22377503	TGTT/TGTTAGTT	TTCATTAACGCTTATTCCCTCA	TGCAACTCTAACAGCGACAA	58.2	694	INTERGENIC	--	--	--
Ca3_P2_22439659	Ca3	22439659	AATATATATATATATATATATATATA/AATATATATATATATATATATATATATA	CGGGTGCAGGTGTAGATTTT	GGACCACGGATGTAATTTGG	60.1	335	INTERGENIC	--	--	--
Ca3_P2_22478435	Ca3	22478435	AATATATATATATATATATATATATATATATATAT/AATATATATATATATATATATATATATAT	AATTATTGCGTGCATGTGGA	CAAAGAAACAGAAATGTGGAGG	58.7	638	INTERGENIC	--	--	--
Ca3_P2_22482988	Ca3	22482988	CAA/CA	TTGGATGGAGAAGAATGCAA	TGGATCAAACCCCTCAATTT	59.2	551	INTERGENIC	--	--	--
Ca3_P2_22534501	Ca3	22534501	TTTATTATTATTATTATTAT/TTTATTATTATTATTAT	CAACGAGGGATGGGAATAGA	GTGCACGGTACAAACCTCCT	60.0	594	INTERGENIC	--	--	--
Ca3_P2_22565599	Ca3	22565599	AATATATATATATATATATATATATATATA/AATATATATATATATATATA	CAATTTCCTTCTCTTTTGGCA	ACTGCGAGTTAGCGGTGAGT	60.1	690	INTERGENIC	--	--	--
Ca3_P2_22597436	Ca3	22597436	TTAAGTTGATATAAGTTGATAT/TTAAGTTGATAT	ATAGGTTGGGTTATTCGGGC	GAAAATGAGGGTGGGGAAAT	60.0	374	INTERGENIC	--	--	--
Ca3_P2_22661182	Ca3	22661182	AATTATTATTATTATTATTATTATTATTATTATTATTATTAT/AATTATTATTATTATTATTATTATTATTATTATTATTAT	AGAGAAGGTGAGGGGGTGTT	AGCCTGATAGGTGGTGGAAA	59.6	763	INTRON	Ca_06245	R	--
Ca3_P2_22676886	Ca3	22676886	CTTTTTTTTTTT/CTTTTT	TTTCTCCATGTAATCAATGTCG	ATATTTTATCCGCGGTCCCT	59.7	888	INTERGENIC	--	--	--

INDEL marker IDs	Chromosomes/unanchored scaffolds	Physical positions (bp)	InDels (*Kabuli* reference genome-CDC Frontier/PII)	Forward primers (5'-3')	Reverse primers (5'-3')	Anneling temperature (0C)	Expected amplified product size (bp)	Structural annotation		Functional annotation	
								Sequence components of *kabuli* genome	*Kabuli* gene accession IDs	NCBI-KOG	TFs
Ca3_P2_2273008	Ca3	2273008	CAAAAAAAAAAA/CAAAAAAAAAAAA	TAAAGGATCGGACCCACTTG	GGCGATCCAAGAGTTCAATC	59.6	822	INTERGENIC	--	--	--
Ca3_P2_22892590	Ca3	22892590	CTTTTTTTTTTT/CTTTTTTTTTTTT	TCTTTGTCAAGCACACATTTGA	GAGTTGATATCCGACACCCAA	59.8	793	DRR	Ca_06218	--	FAR1
Ca3_P2_22934054	Ca3	22934054	G/GCCGATACAATTTGT	CCACATTATGCCTTCGGTTT	GGTTTTCGCGATATTTTCCA	59.9	210	INTERGENIC	--	--	--
Ca3_P2_22936637	Ca3	22936637	TTAATAATAATAATAATAATAATAATAATAATAAAAATAAAAATAATAATAATAATAATAATATTAATATTAATAATAATAATAATAATAATAATAATAATAATAATAATAATAATAATAATAATAATAATAATAATAATAATAATAATAA/TTAATAATAATAATAATAATAATAATAAT[illegible]	ACCACAAATTTCGAGGACGA	TGAAAGATGTGGGTAGCAATAA	57.3	778	INTERGENIC	--	--	--
Ca3_P2_22951232	Ca3	22951232	GTTTTTTTT/GTTTTTTT	CAAACTGTGCAATTACGGATT	CCAACTTCATCCTTCTCACCA	60.1	538	INTERGENIC	--	--	--
Ca3_P2_23014028	Ca3	23014028	CAAAAAAAAAAAAAAA/CAAAAAAAAAAAAA	GGATGTTGCACAATCAAGGA	GGCAGTGGTAGTGAAGCACA	59.9	370	INTERGENIC	--	--	--
Ca3_P2_23016776	Ca3	23016776	TCCTACCT/TCCT	GCACACCTATGTGGTCTTGC	CCATTTTGTCGAAGTTTGGTT	59.0	615	DRR	Ca_06208	QR	B3
Ca3_P2_23102761	Ca3	23102761	CTATATATAT/CTATATATATAT	CACCTTGTCACATTCACAGACA	CCTTGAAGGTTAACAATTTCGT	57.4	543	INTERGENIC	--	--	--
Ca3_P2_23111581	Ca3	23111581	CATTATTATTATTATTATTATTATTATTATTATTATTATTATTATTATTATTATTATTATT/CATTATTATTATTATTATTATTATTATTATTATTATTATTATTATTATTATTATTATTATTATTATTATTATTATT	CCAATGCCACCACTAACCAT	AATATTGGTCTCTGCCACCTT	57.7	759	INTERGENIC	--	--	--
Ca3_P2_23112762	Ca3	23112762	TT/TTTCCTTGATACTTCGT	TGATGCCAATTTGGTTCTGA	TTCGAATCCGTCACAAATGA	60.0	628	INTERGENIC	--	--	--
Ca3_P2_23121593	Ca3	23121593	AGG/AG	TCCGCTTATATTTGGTTCGG	ACGGATGTACCTGGGTTGAG	59.8	769	INTERGENIC	--	--	--
Ca3_P2_23136591	Ca3	23136591	ATTT/ATTTT	GGGTGAGTTTCTGGGTCTCA	CTTCAAACAATGGTCCCTCAA	60.0	723	INTERGENIC	--	--	--

INDEL marker IDs	Chromosomes/unanchored scaffolds	Physical positions (bp)	InDels (*Kabuli* reference genome-CDC Frontier/PII)	Forward primers (5'-3')	Reverse primers (5'-3')	Anneling temperature (0C)	Expected amplified product size (bp)	Structural annotation		Functional annotation	
								Sequence components of *kabuli* genome	*Kabuli* gene accession IDs	NCBI-KOG	TFs
Ca3_P2_23201611	Ca3	23201611	TAGGTCGATCAG/TAG	AAAAAGGAGAGTTCTCCGGC	ATCGAGCGAACACATTGTCA	60.3	677	INTERGENIC	--	--	--
Ca3_P2_23219721	Ca3	23219721	CATTATTATTATTATTATTATTATTAT/CATTATTATTATTATTATTATTATTATTATTATTAT	GATTAGAGCGGCCAAAATGA	TTTTCAATTAGTCGGGTGGC	59.9	593	INTERGENIC	--	--	--
Ca3_P2_23252453	Ca3	23252453	TAA/TA	TCGGAAAATGAAAATGCTGA	TCCAAACATTGTTGACACATCA	59.9	208	INTERGENIC	--	--	--
Ca3_P2_2327052	Ca3	2327052	CAAAAAAAAAAAAAA/CAAAAAAAAAAAAA	GATACGGAACCATCGCACTT	TTGGTTTCGTTCTCCATTCA	59.1	343	DRR	Ca_19393	K	MYB
Ca3_P2_23308159	Ca3	23308159	CTTTTTTTTTT/CTTTTTTTTTTT	TTGCCTTCATGCTTTCTCAA	CATGTTCCGATATGCCACTG	59.9	461	INTERGENIC	--	--	--
Ca3_P2_23322269	Ca3	23322269	TAAAAAAAA/TAAAAAAAAA	GAGTGTGATGTCCAGGCTCA	CCCCTCTTTCGAGACTTTGA	59.4	735	INTERGENIC	--	--	--
Ca3_P2_23356020	Ca3	23356020	GA/G	TCGTTTCCGGAGTCAAGTTC	TGGGCTGGTATGTGACTTTG	59.6	718	DRR	Ca_06185	O	--
Ca3_P2_23370537	Ca3	23370537	TAACCCTAAACCCTAAACCCTAA/TAACCCTAAACCCTAA	TCAAACCAAAATTGTTCTCCG	GGGTTTAAGGTTCGGGGTTA	60.0	778	INTERGENIC	--	--	--
Ca3_P2_2339879	Ca3	2339879	ATTTTTTTTTT/ATTTTTTTTT	TTATGCAATGGCGATGAAGA	CGTTGAGACGTGGCTGATAA	59.9	660	INTERGENIC	--	--	--
Ca3_P2_23424382	Ca3	23424382	TATTAAAAAAT/TATTAAAAAATTAAAAAAT	CATTGTCTGGACTACATTGTGTGA	GAAGGGATCAATTTCTACCCA	58.0	674	INTERGENIC	--	--	--
Ca3_P2_23530917	Ca3	23530917	AATATATATATATATATATATATAT/AATATATATATATATATATATATATAT	ACCCATTAGCGTAGGAGTGC	AACGTAAATGCACTTGAACCG	60.0	786	INTERGENIC	--	--	--
Ca3_P2_23541848	Ca3	23541848	TTATATATATATATATATATATATATAT/TTATATATATATATATATATATATATATATATATATATATAT	ATAAGTTGGCGTTGCCTCTG	CGTCATATGGCGAAATAGATTG	59.5	518	INTERGENIC	--	--	--

INDEL marker IDs	Chromosomes/unanchored scaffolds	Physical positions (bp)	InDels (*Kabuli* reference genome-CDC Frontier/PII)	Forward primers (5'-3')	Reverse primers (5'-3')	Anneling temperature (0C)	Expected amplified product size (bp)	Structural annotation		Functional annotation	
								Sequence components of *kabuli* genome	*Kabuli* gene accession IDs	NCBI-KOG	TFs
Ca3_P2_23551096	Ca3	23551096	AACAATTTACAATTTACGATTTACAATTT/AACAATTTACAATTTACAATTTACGATTTACAATTT	TCCCATTTTGATTCCCTTCA	TCCCTAAATCGCTGAATCTGA	59.8	363	INTERGENIC	--	--	--
Ca3_P2_23572160	Ca3	23572160	CATATATATATATATATAT/CATATATATATATATATATAT	TGTGAACACCTCACACATCAAA	TTTGAATTTTTCAATTAACGCAA	58.8	560	INTERGENIC	--	--	--
Ca3_P2_23642178	Ca3	23642178	CCTAG/CCTAGCTAG	TGTCAAACTTTGGAGCTTCG	GGTGTCCTATCCGAGCAAAA	60.1	737	INTERGENIC	--	--	--
Ca3_P2_23651690	Ca3	23651690	TTAATAATAATAATAATAATAATAATAATAATAAT/TTAATAATAATAATAATAATAATAATAATAATAATAAT	GGTACGTTTGGTCTTGCAGC	TGAGCGCTTATGATCCATTTT	59.7	529	DRR	Ca_06162	--	NAC
Ca3_P2_236571	Ca3	236571	CGAGAGAGAGAGAGA/CGAGAGAGAGAGA	CAATTCACAGCAAAAGCGAA	CCTAGGGTTTCTTCCCCTCA	60.4	191	INTERGENIC	--	--	--
Ca3_P2_23830553	Ca3	23830553	ACTCTCTCTCTCTCTCTCTCTCTCTCTC/ACTCTCTCTCTCTCTCTCTCTCTCTCTCTCTC	TCACGAAATTGGTCCCCTTA	GGGACCAAAACTGGAGAGAA	59.1	878	INTERGENIC	--	--	--
Ca3_P2_23863549	Ca3	23863549	ATT/ATTT	TTAACCGCCAACGATGTGTA	TCATTACTCACCAGAATTGGAA	57.2	644	INTERGENIC	--	--	--
Ca3_P2_23876465	Ca3	23876465	GTATATA/GTATATATATA	CATAATCCCATCGCTCTCGT	TCGGTTAACTGGCCAAACTC	60.1	568	DRR	Ca_06137	--	--
Ca3_P2_23993355	Ca3	23993355	AATAAATTATAAATTATAAATTA/AATAAATTATAAATTA	CCGAGCCAAAATCCAAAATA	GTTTGGGGCACAATTGAGAT	59.8	599	INTERGENIC	--	--	--
Ca3_P2_24011224	Ca3	24011224	ATTT/ATT	TCATGAAGATTTGGAAGAGAAAGA	TGTTGCTACGCTGAAACTGG	60.1	272	INTERGENIC	--	--	--
Ca3_P2_24014416	Ca3	24014416	ATTTTTTTTT/ATTTTTTTT	CGCATTGTTAGTTTCGTCTTTG	AGGCTTCCATGTCAGGTCAG	60.3	653	INTERGENIC	--	--	--
Ca3_P2_24033300	Ca3	24033300	TTAATAATAATAATAATAATAATAATA/TTAATAATAATAATAATAATAATA	TCCAAGAAAAGTGTTGGAAATG	CCTTCCTACGCCTCAAATCA	60.2	376	INTERGENIC	--	--	--

INDEL marker IDs	Chromosomes/unanchored scaffolds	Physical positions (bp)	InDels (*Kabuli* reference genome-CDC Frontier/PII)	Forward primers (5'-3')	Reverse primers (5'-3')	Anneling temperature (0C)	Expected amplified product size (bp)	Structural annotation		Functional annotation	
								Sequence components of *kabuli* genome	*Kabuli* gene accession IDs	NCBI-KOG	TFs
Ca3_P2_240424	Ca3	240424	GGTTCAATGGGTAATAACA/GGTTCAATGGGTAATAACAGTTCAATGGGTAATAACA	AAACTGCCATCCACAAGGTC	CATCATTTCCAACAATCCCC	60.0	671	INTERGENIC	--	--	--
Ca3_P2_24069078	Ca3	24069078	GAAAAAAAAA/GAAAAAAAAAA	CCAATCAACATACACACGCC	GAGGTGCCGAAGCTCATAAC	59.8	511	INTERGENIC	--	--	--
Ca3_P2_24117383	Ca3	24117383	TTACATACATACATAC/TTACATACATAC	GAAGAGGTGCTTGGTGGAGA	TACACCAGAATTTGCTCCCC	59.9	342	DRR	Ca_06107	O	--
Ca3_P2_2414966	Ca3	2414966	A/AC	TTAATGAGGGGCTGAATTGG	AAGCGTACCTGTGGGAATTG	60.0	259	INTERGENIC	--	--	--
Ca3_P2_24184057	Ca3	24184057	TC/TCC	CCAGCCCACACTAGGTGTTT	AAAATCCAACAATCTCCCCC	60.0	549	INTERGENIC	--	--	--
Ca3_P2_24193117	Ca3	24193117	ATTT/ATT	TAAGGTTCAGGTTATGGCCG	CGCCACTATGTCGGCTATTT	60.1	695	INTRON	Ca_06100	J	--
Ca3_P2_24272959	Ca3	24272959	GAATAATAA/GAATAATAATAA	GCATGTCCGTTACCTCCACT	GCGATTTATTATTCAGAGTTATTAGCG	60.0	646	INTERGENIC	--	--	--
Ca3_P2_2441504	Ca3	2441504	AGGG/AGG	CATGCATAGCTTCGTAGGCA	CCTTGCCTGATAATTGGGAA	59.9	810	INTERGENIC	--	--	--
Ca3_P2_24470050	Ca3	24470050	AAATAATAATAATAATAATAATAATAATAATAATAATAA/AAATAATAATAATAATAATAATAATAATAATAATAATAATAATAA	CCTCCTCTTTTGGCACGTTA	ACCAAAATGCCACACATTGA	59.8	481	INTERGENIC	--	--	--
Ca3_P2_24509560	Ca3	24509560	ATTT/ATT	GTTGGCTTTTGTACCCCTCA	CCGAAATTTCAGGATGAATGA	59.9	551	INTERGENIC	--	--	--
Ca3_P2_24701158	Ca3	24701158	ATTTTTTTTTT/ATTTTTTTTT	TTTGGCACATCTATTCAACTGC	CGCATTCTTGGGATGCTTAT	60.1	136	INTERGENIC	--	--	--
Ca3_P2_24785287	Ca3	24785287	CTTT/CT	ACTCCGGCCTTCTCCATATT	TGGGAATAAGCTTGGGATTG	59.9	242	INTERGENIC	--	--	--

**INDEL marker IDs**	**Chromosomes/unanchored scaffolds**	**Physical positions (bp)**	**InDels (*Kabuli* reference genome-CDC Frontier/PII)**	**Forward primers (5'-3')**	**Reverse primers (5'-3')**	**Anneling temperature (0C)**	**Expected amplified product size (bp)**	**Structural annotation**		**Functional annotation**	
								**Sequence components of *kabuli* genome**	***Kabuli* gene accession IDs**	**NCBI-KOG**	**TFs**
Ca3_P2_24864498	Ca3	24864498	TAA/TAAA	CACGAGGTCCATTTGATGTG	TAAATCTCGGATGCATGGAA	59.1	239	DRR	Ca_06041	JT	C3H
Ca3_P2_24957469	Ca3	24957469	TAA/TA	TTGAGGGATAATCATTTTCTCG	GAAAAAGCGCTGTAAATGGC	59.9	820	DRR	Ca_06033	--	Trihelix
Ca3_P2_24969495	Ca3	24969495	CATATATATATATATATATATATATATATATA/CATATATATATATATATA	TTGAAAATGTGATTCGATTGC	TTGAGGATTATTGCCTTGGG	59.9	469	DRR	Ca_06032	--	ERF
Ca3_P2_24984768	Ca3	24984768	CTTTTTTTTT/CTTTTTTTT	TATATGGGCTGCTCTCCCTG	TTTTGCTTTTGAAGCCAACC	60.2	663	INTRON	Ca_06031	--	SAP
Ca3_P2_25079344	Ca3	25079344	AATTATTATTATTATTATTATTATTATTATTATTATTAT/AATTATTATTATTATTATTATTATTATTATTATTATTATTATTATTATTAT	GCGAGCAGGTTTTGTCTCTC	TTAAAATTCGGAGGCGAGAA	59.8	379	INTERGENIC	--	--	--
Ca3_P2_25164347	Ca3	25164347	TTAT/TT	ATTATCGAGCAGGAGGCTGA	TCAGCCATCATATCCTTCCC	59.9	897	INTRON	Ca_06013	I	--
Ca3_P2_25199115	Ca3	25199115	TTTATTATTATTATTATTATTATTATTATTATTATTATT/TTTATTATTATTATTATTATTATTATTATT	AATGCACGAAGAGAGAGGGA	GAGGGAGTTGTGAAGAGACAAAA	59.8	562	INTERGENIC	--	--	--
Ca3_P2_25316453	Ca3	25316453	TTATATATATATATATATATATAT/TTATATATATATATATATAT	ATGCGATGCAAGCCTAAAGT	TGACTTTTCAAAACACATCGAAA	59.7	680	INTERGENIC	--	--	--
Ca3_P2_25341114	Ca3	25341114	TTTCAAAAA/TTTCAAAAATTCAAAAA	TGTGAAAAAGCTGCGATGTC	GAGGAAAGTGTGCCAGGAAA	60.2	118	INTERGENIC	--	--	--
Ca3_P2_25413762	Ca3	25413762	GATATAT/GATAT	TCTAAAAGGTACCGGTGCTCA	TCTGCAACCAAAAACATCCA	60.1	316	INTERGENIC	--	--	--
Ca3_P2_25585812	Ca3	25585812	TAAAAAAAA/TAAAAAAA	TGTTTCGTTTTTGTTCCCTCT	TCCATTGCAATCGTACTTAATGA	59.5	735	DRR	Ca_05976	--	B3
Ca3_P2_25659301	Ca3	25659301	TAGAGAGAGAGAGAG/TAGAGAGAGAGAG	TCAAGTATTCCACCGCAATCT	CGCTTGTTGCTTTTCATTTG	59.5	704	INTRON	Ca_23893	R	bHLH

INDEL marker IDs	Chromosomes/unanchored scaffolds	Physical positions (bp)	InDels (*Kabuli* reference genome-CDC Frontier/PII)	Forward primers (5'-3')	Reverse primers (5'-3')	Anneling temperature (0C)	Expected amplified product size (bp)	Structural annotation		Functional annotation	
								Sequence components of *kabuli* genome	*Kabuli* gene accession IDs	NCBI-KOG	TFs
Ca3_P2_25719339	Ca3	25719339	TAGAGAGAGAGAGAGAGAGAGAGAGAGA/TAGAGAGAGAGAGAGAGAGAGAGAGAGA	AGGTCCTCCTCTCACCACCT	GCAGGATTTTGGAAATGGAA	59.9	833	INTRON	Ca_23884	G	--
Ca3_P2_25758872	Ca3	25758872	TAAAAAAAAA/TAAAAAAAA	GGATCCGGTGCTACTCCATA	ATTTGAGGTTGTGATTATGCAA	57.2	713	INTERGENIC	--	--	--
Ca3_P2_2580419	Ca3	2580419	GGAGAGAGAGAGAGAGAGAGAGAGAGAGAGAGAGAGAGA/GGAGAGAGAGAGAGAGAGAGAGAGAGAGAGAGA	GTGGAAAAAGGCAAGAGTGC	CGCATTTCATCCTCACCATA	59.5	266	DRR	Ca_22145	--	--
Ca3_P2_25864422	Ca3	25864422	TAAAAAAAAAAA/TAAAAAAAAAAAA	TTTGTTTGTGCGGTCAAGAG	TGGGCAGATATGACCAACAA	59.9	321	INTERGENIC	--	--	--
Ca3_P2_25870977	Ca3	25870977	ATTTTT/ATTTT	AATCGCAGGAACCCTAACCT	GGTACGCGACTGCAACTGTA	59.9	571	INTERGENIC	--	--	--
Ca3_P2_25872845	Ca3	25872845	ACTAAATTGGTCGCTAAAT/ACTAAAT	TGTGTACAAGATTGTGAAGGCA	CACGTAAGATCGCCAAAATC	58.2	680	INTERGENIC	--	--	--
Ca3_P2_25888060	Ca3	25888060	AT/ATT	TGTGGAAGACATCCTTTACTGC	ACCTGACCTATTTCCACCCC	60.1	551	INTERGENIC	--	--	--
Ca3_P2_25888907	Ca3	25888907	GTTTTTTT/GTTTTTTTT	CAGGTCATGCCAGACTTTGA	TCTGACCCGACCTGGTCTAC	60.1	578	INTERGENIC	--	--	--
Ca3_P2_25889145	Ca3	25889145	ATTTTTTTT/ATTTTTTTTT	GTAGACCAGGTCGGGTCAGA	TGATATGTCACATTGTCGCAC	58.0	403	INTERGENIC	--	--	--
Ca3_P2_25891751	Ca3	25891751	TAAA/TAAAA	CGGTTCCTAATTGAAAAGGC	CAACGCTTTGTTCTCTCAACA	59.1	341	INTERGENIC	--	--	--
Ca3_P2_25916145	Ca3	25916145	AATATATATATATATATATATATATATATATATATATAT/AATATATATATATATATATATATATATATATATATATATATATATATATAT	CCGCTCACAATATGCAAGAA	AAATGGGTGGGACATGTGAT	59.9	765	INTERGENIC	--	--	--
Ca3_P2_25937655	Ca3	25937655	TTATATATATATATATATATATAT/TTATATATATATATATATAT	TCGAGTATGACCAATTAACCGA	ATAAACTTTTAGGCGCCGGT	60.0	785	INTERGENIC	--	--	--

INDEL marker IDs	Chromosomes/unanchored scaffolds	Physical positions (bp)	InDels (*Kabuli* reference genome-CDC Frontier/PII)	Forward primers (5'-3')	Reverse primers (5'-3')	Anneling temperature (0C)	Expected amplified product size (bp)	Structural annotation		Functional annotation	
								Sequence components of *kabuli* genome	*Kabuli* gene accession IDs	NCBI-KOG	TFs
Ca3_P2_26018200	Ca3	26018200	AATATATATATATATATATATATA/AATATATATATATATATATATATATA	TGTTAATATCCGCCCCATGT	CCCAATGGATCTCTCTCCAA	60.0	536	INTERGENIC	--	--	--
Ca3_P2_26020458	Ca3	26020458	CATATATATATATATATATAT/CATATATATATATATATATATATATAT	CCCACTAAAGGCCCATATCA	TTCAAATTCACGCCCCTAAC	59.9	789	INTERGENIC	--	--	--
Ca3_P2_26069971	Ca3	26069971	TT/TTTTAT	TCAAATGGCGACTTACCACA	GGAAGCCCAATTTTCAAACA	59.9	747	INTRON	Ca_08292	--	MYB_related
Ca3_P2_26070506	Ca3	26070506	GT/GTAT	TGTTTGAAAATTGGGCTTCC	TGCAAATCTTGAAAACAATTCG	60.1	375	INTRON	Ca_08292	--	MYB_related
Ca3_P2_26071275	Ca3	26071275	ATTTTT/ATTTTTTT	TGTGTTTGTTTGGTTTCATCTTG	TGTCAACGTGATTGGAGCAT	60.1	563	INTRON	Ca_08292	--	MYB_related
Ca3_P2_26071877	Ca3	26071877	TAAA/TAAAA	TGCTCCAATCACGTTGACAT	AGACGCTGCAACATCAAAGC	61.5	397	INTRON	Ca_08292	--	MYB_related
Ca3_P2_26074434	Ca3	26074434	TTATATATA/TTATATATATA	TTTTCAAAATGCTCAAATTTTCTT	GCTTTGATGGAAATCAGGGA	60.0	779	INTRON	Ca_08292	--	MYB_related
Ca3_P2_26114065	Ca3	26114065	AG/A	TTTATCCTGGGGACTTCGTG	AGGAAAATACACGGGCAAGA	59.6	364	INTRON	Ca_08292	--	MYB_related
Ca3_P2_26114282	Ca3	26114282	ATTATAGTTTTTATT/AT	TTTATCCTGGGGACTTCGTG	ATACACCGATGGAGGGCTTT	60.7	838	INTRON	Ca_08292	--	MYB_related
Ca3_P2_26114943	Ca3	26114943	GTTTT/GTTT	TGGCATGGTACTCAACCAGA	TTCACATGAATGATTGTTGGGT	60.1	545	INTRON	Ca_08292	--	MYB_related
Ca3_P2_26115029	Ca3	26115029	AACG/A	TGGCATGGTACTCAACCAGA	TTCACATGAATGATTGTTGGGT	60.1	545	INTRON	Ca_08292	--	MYB_related
Ca3_P2_26125921	Ca3	26125921	CA/CAAGCCAGACTA	CCGAATATGAAAGGACTATCGG	ACATGGGCAATGTGTGTGTT	59.7	327	INTERGENIC	--	--	--

INDEL marker IDs	Chromosomes/unanchored scaffolds	Physical positions (bp)	InDels (*Kabuli* reference genome-CDC Frontier/PII)	Forward primers (5'-3')	Reverse primers (5'-3')	Anneling temperature (0C)	Expected amplified product size (bp)	Structural annotation		Functional annotation	
								Sequence components of *kabuli* genome	*Kabuli* gene accession IDs	NCBI-KOG	TFs
Ca3_P2_26134563	Ca3	26134563	AAATAATAATAATAATAATAATAATAATAATAATAATA/AAATAATAATAATAATAATAATAATAATAATA	TCCTGTGCAATACGAGTTGG	AAAGCAGCACCATTGGTTTC	60.1	516	DRR	Ca_08290	T	--
Ca3_P2_26159882	Ca3	26159882	CATATATAT/CATATAT	TTGACCAGACCGAGATCACA	GGGGCATTATGGACTTGAAA	59.8	779	INTERGENIC	--	--	--
Ca3_P2_26245714	Ca3	26245714	C/CA	CGCCTGTACCGGTTGATACT	ACCCATTCCCAGTGTTTTCA	60.2	678	INTRON	Ca_08279	QI	B3
Ca3_P2_26328372	Ca3	26328372	AAGAGAGAGAGAGAGAGAGAGAGA/AAGAGAGAGAGAGAGAGAGAGA	TTGTTGAATTTGATGTCGTTGA	TCCCTGATTTTGTAAGACCAA	57.2	327	INTERGENIC	--	--	--
Ca3_P2_26336721	Ca3	26336721	GATATATATATATATATATATATATATA/GATATATATATATATATATATATATATATA	AGAAAGGCCGTTTTCTGGAT	ACCACCGACCACCTACTTCA	60.4	574	INTRON	Ca_08268	YU	bHLH
Ca3_P2_26349764	Ca3	26349764	GTTAACA/G	TCTGCATTGTCCAAAACCAA	TTATCCATGATTTGGGAGGG	59.6	737	INTERGENIC	--	--	--
Ca3_P2_26389085	Ca3	26389085	TTATATATATATATATATATATATA/TTATATATATATATATATATATATATATA	AATCGACTTGCTTTGTGGCT	CATCTAGGACCCGTGCAATC	60.5	375	INTERGENIC	--	--	--
Ca3_P2_2653586	Ca3	2653586	GTTTTTTT/GTTTTTTTT	TGTTTTGCAGCAAGATGTCC	TGGAGATGGTTAGCAATGGA	59.1	536	INTERGENIC	--	--	--
Ca3_P2_26628740	Ca3	26628740	AATATATATATATATATATATATATATATATAT/AATATATATATATATATATATATATATATAT	TGTGATTTTGAGTTTCCTCTCG	CTTGCACGTATGCAAGAACA	58.5	342	INTERGENIC	--	--	--
Ca3_P2_26777304	Ca3	26777304	GTATATATATATATATAT/GTATATATATATATATAT	CACTCATCACAACGGGACAC	CAATGAATCGATGAAATGAATTG	59.3	754	DRR	Ca_08228	P	--
Ca3_P2_26826640	Ca3	26826640	GAATAATAATAATAATAATAATAATAATAATAATAATA/GAATAATAATAATAATAATAATAATAATAATAATAATAATAATA	TGAAGTTGCCACTTGTTGAA	TTTTGTGTCAAAAGTCAAAACCA	59.6	603	DRR	Ca_08225	--	ARF
Ca3_P2_26872317	Ca3	26872317	CAGAGAGAGAGAGAGAGAGAGAGAGAG/CAGAGAGAGAGAGAGAGAGAGAGAGAGAG	CAATGTGCTCGATTTCTCCA	TTACATTCCTTGCCTCGTCA	59.3	389	INTERGENIC	--	--	--

INDEL marker IDs	Chromosomes/unanchored scaffolds	Physical positions (bp)	InDels (*Kabuli* reference genome-CDC Frontier/PII)	Forward primers (5'-3')	Reverse primers (5'-3')	Anneling temperature (0C)	Expected amplified product size (bp)	Structural annotation		Functional annotation	
								Sequence components of *kabuli* genome	*Kabuli* gene accession IDs	NCBI-KOG	TFs
Ca3_P2_26961684	Ca3	26961684	ATTTTTTTTTTTT/ATTTTTTTTTTT	CACGGTTGTGATGACTCGAC	GTCGCGTCAACGTTTGTTTA	59.8	538	INTERGENIC	--	--	--
Ca3_P2_26998517	Ca3	26998517	AATATATATATATATATATATATATATATAT/AATATATATATATATATATATATATATATATATATATATAT	TGGGATGTTTCAAATTTCTGC	AAAATGGCATTGGTGTGGTT	60.1	576	INTERGENIC	--	--	--
Ca3_P2_27067574	Ca3	27067574	GTTTTTGGTTTTTTT/GTTTTT	TGATCGGATGGTCGTTGATA	CCACCAAAAAGTGGAGCCTA	60.1	466	INTRON	Ca_08197	E	--
Ca3_P2_27142432	Ca3	27142432	CATATATATATATATATA/CATATATATATATATATATA	GCTTCTTTCACACCCACGTT	GCCGTAACGTAACCGTCAGT	60.1	547	INTERGENIC	--	--	--
Ca3_P2_27221901	Ca3	27221901	AATTAT/AAT	TGGCCTAAAAATGACCGACT	TGTGCGCTGATAGGCATTAG	60.0	929	INTERGENIC	--	--	--
Ca3_P2_27328014	Ca3	27328014	TAAAAAAAAAA/TAAAAAAAAA	TTCTCCTTGAAGCTGTCGGT	GACGTTTTTGCCCTCGTTTA	60.1	547	DRR	Ca_08178	--	--
Ca3_P2_27340222	Ca3	27340222	AA/AAGGA	TGATGAAATTTGCATTAAATCG	CGGGGTTATACGCTGACACT	60.0	402	INTERGENIC	--	--	--
Ca3_P2_27382854	Ca3	27382854	GTTTTTTTTTTTT/GTTTTTTTTTTT	GCGGTAAAAGAAGGCAAGTG	TGTGGGTACCTTAAACCCTAAAAA	60.0	591	INTERGENIC	--	--	--
Ca3_P2_27571944	Ca3	27571944	TTATATATATATATATATATATATATATAT/TTATATATATATATATATATATATATATATAT	GCTTTGATAGGGTGTTGCGT	AGTAGGCATGTCAACGGGAC	60.0	474	INTERGENIC	--	--	--
Ca3_P2_27572261	Ca3	27572261	TTATATATATATATATATATAT/TTATATATATATATATATATATATATATATAT	ACATGCCTACTCGTGTCGTG	TTTTCCTTTTTATTTTCTTTTGTGA	57.7	604	INTERGENIC	--	--	--
Ca3_P2_27613485	Ca3	27613485	TGGG/TGG	GAATGATGCTGCTCGGGTAT	GTTTCGTCTGGTTCACCGTT	60.0	428	INTRON	Ca_08146	--	bHLH
Ca3_P2_2762503	Ca3	2762503	GTTTTT/GTTTTTT	CTCTCACCCAAGAAGATCGC	TTGGAACAAAACACCTGCAA	60.1	590	INTERGENIC	--	--	--

INDEL marker IDs	Chromosomes/unanchored scaffolds	Physical positions (bp)	InDels (*Kabuli* reference genome-CDC Frontier/PII)	Forward primers (5'-3')	Reverse primers (5'-3')	Anneling temperature (0C)	Expected amplified product size (bp)	Structural annotation		Functional annotation	
								Sequence components of *kabuli* genome	*Kabuli* gene accession IDs	NCBI-KOG	TFs
Ca3_P2_27648871	Ca3	27648871	AATATATATATATATATATATATAT/AATATATATATATATATATATATAT	AATCCCATGGCACTGAGAAC	AGCGTGATCACATTCACAGC	59.9	670	INTERGENIC	--	--	--
Ca3_P2_27854087	Ca3	27854087	ATAAATAATTGTAAA/ATAAA	TCGATAATGAGTGATTAGGTGGG	ACCTCACCCTCGAATGTCAC	60.0	459	INTERGENIC	--	--	--
Ca3_P2_27854154	Ca3	27854154	TGG/TG	TCGATAATGAGTGATTAGGTGGG	ACCTCACCCTCGAATGTCAC	60.0	459	INTERGENIC	--	--	--
Ca3_P2_27854804	Ca3	27854804	T/TA	GGATTGAGAGGGGAGAAAGG	TTTGAAAGCAAGAGCGTCAA	59.7	516	INTERGENIC	--	--	--
Ca3_P2_27856755	Ca3	27856755	TAAAAAAAAA/TAAAAAAAA	CGAAGATGAAGAGGCTTGAAA	CAACAACTTCTGATGCTCGC	59.6	593	INTERGENIC	--	--	--
Ca3_P2_27866552	Ca3	27866552	TTAATAATAATAATAATAATAATAATAATAATAATAATAATAATAGTAATAATAATAATA/TTAATAATAATAATAATAATAATAATAATAATAATAGTAATAATAATAATA	TCAGAATTTTGGTGAGTTGGC	GGGATTCGCTGGTTATCTCA	60.0	552	INTERGENIC	--	--	--
Ca3_P2_27867074	Ca3	27867074	TCAC/TCACAC	TGAGATAACCAGCGAATCCC	ATTCGGGATAAGTGGAACCC	60.0	550	INTERGENIC	--	--	--
Ca3_P2_27867135	Ca3	27867135	AT/ATT	TGAGATAACCAGCGAATCCC	ATTCGGGATAAGTGGAACCC	60.0	550	INTERGENIC	--	--	--
Ca3_P2_27873354	Ca3	27873354	AA/AACTGA	TGGGGAGAAATTACCAAAAGAA	CCCCTATAATCTTGAGGGAGG	58.9	487	INTERGENIC	--	--	--
Ca3_P2_2791398	Ca3	2791398	GTTTTTTTTTTTTTT/GTTTTTTTTTTTTT	GTGAATGGAAAAAGGCTCCA	TGAGCGTACGGAACATCAAG	59.9	242	INTERGENIC	--	--	--
Ca3_P2_2798836	Ca3	2798836	ATTTTTTTTTT/ATTTTTTTTTTT	TGAGGTTTCTTGGGTTGAGG	GCCCTCAGATGAGAGTCAGG	59.9	561	INTERGENIC	--	--	--
Ca3_P2_28016436	Ca3	28016436	AATATATATATATATATATATATATATATATATA/AATATATATATATATATATATATATATA	TCGAAGTAACTTGATCCCTCTTG	GGATGGTTGCGGTATGATGT	60.6	526	DRR	Ca_08108	O	GeBP

INDEL marker IDs	Chromosomes/unanchored scaffolds	Physical positions (bp)	InDels (*Kabuli* reference genome-CDC Frontier/PII)	Forward primers (5'-3')	Reverse primers (5'-3')	Anneling temperature (0C)	Expected amplified product size (bp)	Structural annotation		Functional annotation	
								Sequence components of *kabuli* genome	*Kabuli* gene accession IDs	NCBI-KOG	TFs
Ca3_P2_28052758	Ca3	28052758	CACAGAC/CAC	ACTTCCGTTCACACGTTTCC	CGATGCAGGTGAAGATCAAA	59.8	481	INTERGENIC	--	--	--
Ca3_P2_28084076	Ca3	28084076	TT/TTTTATAT	TGTTTGACCGTTAGAATGTGC	CGTTCCCATAGCCAAAGTCT	59.2	378	INTERGENIC	--	--	--
Ca3_P2_28113252	Ca3	28113252	ATTTTTTTT/ATTTTTTT	TCCTTCTCCTCTTCCTTCCA	TGTTTGTGCAATTGAATTATAGTTATG	58.6	849	INTERGENIC	--	--	--
Ca3_P2_28188438	Ca3	28188438	CAAAA/CAAAAA	ACCCATAACGCACTCGTTTC	TCCTCCATTTTTCGTTTTCG	60.0	434	INTERGENIC	--	--	--
Ca3_P2_28191077	Ca3	28191077	CTTTTTTTTT/CTTTTTTTTTT	TAAGATGCGTGGGTTTAGGG	GCTTGTGTTTAAAGCGCTCC	60.0	659	INTERGENIC	--	--	--
Ca3_P2_28262424	Ca3	28262424	A/AAAGTCG	TACTTTTACCCCCATTGCCA	AAACACCCATCCCACGTATG	60.5	414	INTERGENIC	--	--	--
Ca3_P2_28262509	Ca3	28262509	GTTTTTTTT/GTTTTTTTTTT	TACTTTTACCCCCATTGCCA	AAACACCCATCCCACGTATG	60.5	414	INTERGENIC	--	--	--
Ca3_P2_28289088	Ca3	28289088	TAGAGAGAGAGAGAGAGAGAG/TAGAGAGAGAGAGAGAGAG	CTTCCCATCTTGAAAACCGA	CGAACGTCTCTCCACTCTCC	60.0	369	INTERGENIC	--	--	--
Ca3_P2_28293830	Ca3	28293830	TAAAAAAAAAA/TAAAAAAAAA	GATTACTGCTAGGGCCCAAAT	ATCCCACTTGCAACACTTCC	60.0	147	INTERGENIC	--	--	--
Ca3_P2_28294648	Ca3	28294648	AATATATATATATATATATAT/AATATATATATATATATATATAT	TACCAGTACCAGGAGGCAGG	TGGTGGGTAATCCGATGAAT	60.0	670	INTERGENIC	--	--	--
Ca3_P2_28334138	Ca3	28334138	TTATATATATATATATATATATA/TTATATATATATATATATATATATATATA	CGACCTCCTGAAGATCCAAA	TCGATGCTCCATGTTAATGC	59.7	853	DRR	Ca_07149	R	NAC
Ca3_P2_28428889	Ca3	28428889	TTATATATATATATATATATA/TTATATATATATATATATATA	AGGCAACTCGATTCAAGCAT	AAACAAACAAGCCCACCAAC	59.9	923	INTERGENIC	--	--	--

INDEL marker IDs	Chromosomes/unanchored scaffolds	Physical positions (bp)	InDels (*Kabuli* reference genome-CDC Frontier/PII)	Forward primers (5'-3')	Reverse primers (5'-3')	Anneling temperature (0C)	Expected amplified product size (bp)	Structural annotation		Functional annotation	
								Sequence components of *kabuli* genome	*Kabuli* gene accession IDs	NCBI-KOG	TFs
Ca3_P2_28511565	Ca3	28511565	AATATATATATATATAT/AATATATATATATAT	TGGGATAGAGTTTGCTTGCC	CCCTCCCATGTTTTCCACTA	59.8	642	INTERGENIC	--	--	--
Ca3_P2_28514570	Ca3	28514570	ATATTATTATTATTATTATTATTATTATTATT/ATATTATTATTATTATTATTATTATTATT	CAAAGAAAATGGTGGGGATG	AAATTGGACTATGCCACCGA	60.3	841	INTERGENIC	--	--	--
Ca3_P2_28533888	Ca3	28533888	ATTTTTTTT/ATTTTTTTTT	ATCCAATAATGGGTGTGGGA	TGGAGATAGCTGGGGAAGAA	59.8	477	INTERGENIC	--	--	--
Ca3_P2_28535222	Ca3	28535222	AATTCATAT/AAT	AAGCGCCAATCTAGTTTCATTT	AAAATGAGTCGTTGGATTCAAAA	59.9	411	INTERGENIC	--	--	--
Ca3_P2_2855993	Ca3	2855993	TAAA/TAA	CTTGGTTGGAATTGCATGTG	CGTTTTGGAAGGTAGGGTGT	59.0	514	INTERGENIC	--	--	--
Ca3_P2_28589622	Ca3	28589622	GAAAAAAAAAAA/GAAAAAAAAAAAA	TTTCCCGACTTGGTCCTTTA	GGAATCGCTACCGAATATGG	59.4	447	INTERGENIC	--	--	--
Ca3_P2_28636768	Ca3	28636768	AATATATATATATATATATATAT/AATATATATATATATATATAT	CGCGGACACCTATCCAACT	CGCATGTTGATATGCGATTT	59.5	870	INTERGENIC	--	--	--
Ca3_P2_28680371	Ca3	28680371	AATATATATATATATATATATATATATATATAT/AATATATATATATATATATATATATATATATATATAT	TGTGTAATGGGTGCATGCTT	GCTTTCAAGGACTTTGCTGG	60.0	642	INTRON	Ca_07175	E	C3H
Ca3_P2_28797139	Ca3	28797139	CAA/CAATTCGATAA	CAAGGAGCTGTTTTGCTTCA	GTAAAATGGCACGCGGTAGT	60.0	609	INTERGENIC	--	--	--
Ca3_P2_28797825	Ca3	28797825	A/AG	CATAGTCTGCGAAACCAGCA	GGTGGAATTGATCGGAGAGA	60.0	454	INTERGENIC	--	--	--
Ca3_P2_28799476	Ca3	28799476	G/GTC	TCTTGGACCTCACTGGGTTT	TGTCCTTGATCTTGTTTCCG	58.7	836	INTERGENIC	--	--	--
Ca3_P2_28799571	Ca3	28799571	AG/A	TCTTGGACCTCACTGGGTTT	TGTCCTTGATCTTGTTTCCG	58.7	836	INTERGENIC	--	--	--

INDEL marker IDs	Chromosomes/unanchored scaffolds	Physical positions (bp)	InDels (*Kabuli* reference genome-CDC Frontier/PII)	Forward primers (5'-3')	Reverse primers (5'-3')	Anneling temperature (0C)	Expected amplified product size (bp)	Structural annotation		Functional annotation	
								Sequence components of *kabuli* genome	*Kabuli* gene accession IDs	NCBI-KOG	TFs
Ca3_P2_28799969	Ca3	28799969	GTA/GTATA	TGCAGCAGCAAAACTTGTGT	CGAATTAAAAACTCAAGGGTTCA	59.5	302	INTERGENIC	--	--	--
Ca3_P2_28859122	Ca3	28859122	CAAAAAAAAA/CAAAAAAAAAA	TTTCCCGACTTGGTCCTTTA	ATGACATTTCGCCAAACACA	60.0	432	INTERGENIC	--	--	--
Ca3_P2_28932040	Ca3	28932040	GTATATATATATATATATATATATATATAT/GTATATATATATATATATATATATAT	ATTCCACCCATTAACCCACA	CTTTTCTGGCATTTGGCTGT	60.2	711	INTERGENIC	--	--	--
Ca3_P2_28966806	Ca3	28966806	TTATATATATATATATATATATAT/TTATATATATATATATATATATATATAT	TCGATATTTAGAATTTACGAACGAGA	ATTCAAGAAAGTTCGCCGTG	60.2	850	INTERGENIC	--	--	--
Ca3_P2_28984656	Ca3	28984656	GTCTCTCT/GTCTCT	GAGAACGCAAGCTAACGGAC	GGATCAGAGAAAACGTGGGA	60.1	326	INTERGENIC	--	--	--
Ca3_P2_28994933	Ca3	28994933	AC/A	CAAATTAAGCAGAGCCCGTC	TTTTGGACAATGTCAACCCA	59.8	165	INTRON	Ca_07207	KTDL	--
Ca3_P2_28995118	Ca3	28995118	CAA/CAAA	CAAATTAAGCAGAGCCCGTC	CGGAATTTGAACAAAACGCT	60.1	769	INTRON	Ca_07207	KTDL	--
Ca3_P2_28999636	Ca3	28999636	CT/CTT	TCTGAATTTCTGTACCGCCC	GGGATAGGGCATGTGTGAGT	59.8	536	INTRON	Ca_07207	KTDL	--
Ca3_P2_29000078	Ca3	29000078	TG/T	GCCCTCTGTTCAGGGTAGGT	GATGAGACCACCCAAGCACT	60.1	606	INTRON	Ca_07207	KTDL	--
Ca3_P2_29030044	Ca3	29030044	CG/CGATCTTTCCCCTAAG	CCTTCCTTCCCTTACTTCCG	ATACATTAGGCGATGCAGCC	60.1	417	INTRON	Ca_07209	--	--
Ca3_P2_29051833	Ca3	29051833	AATTATTATTATTATTATTATTAT/AATTATTATTATTATTATTAT	CCCACCTCTACCACCGACTA	TCTGAACCGTCTTGATTCGAT	59.7	387	INTERGENIC	--	--	--
Ca3_P2_29053872	Ca3	29053872	TTATATATATATATATATATA/TTATATATATATATATATATATA	TCCTCCGTCTTTTCATTTGG	GGTTGGGGGAGTGTTAGCTT	60.4	456	INTERGENIC	--	--	--

INDEL marker IDs	Chromosomes/unanchored scaffolds	Physical positions (bp)	InDels (*Kabuli* reference genome-CDC Frontier/PII)	Forward primers (5'-3')	Reverse primers (5'-3')	Anneling temperature (0C)	Expected amplified product size (bp)	Structural annotation		Functional annotation	
								Sequence components of *kabuli* genome	*Kabuli* gene accession IDs	NCBI-KOG	TFs
Ca3_P2_29097133	Ca3	29097133	CTACTTATTTA/C	AAAATGCTGCACTCAAACGA	CACGGGTTGCGTATATCCAT	60.6	233	INTERGENIC	--	--	--
Ca3_P2_29174780	Ca3	29174780	TTATATATATATATAT/TTATATATATATATATAT	ATCCACCCCACATTCAAATC	GGAGAGAAATACGCGTGGAG	59.8	439	INTERGENIC	--	--	--
Ca3_P2_29228366	Ca3	29228366	TTATATATATATATATATAT/TTATATATATATATATAT	TCGGAGGCAGAGACAAGTTT	TTAATTGGGGGAAATCCACA	60.0	364	INTERGENIC	--	--	--
Ca3_P2_2930472	Ca3	2930472	ATCTC/ATCTCTC	AGGCGATGGAGGATAAAACC	CGGAAACGAGTGAAACCAAT	60.0	502	INTRON	Ca_22158	D	--
Ca3_P2_29334875	Ca3	29334875	TTATATATATATATATATAT/TTATATATATATATATATATATAT	GGGTGGCTCATGAAGCTTAG	TCTCAACCGTTTTGGTGACA	60.1	576	INTERGENIC	--	--	--
Ca3_P2_29407345	Ca3	29407345	T/TGTTG	AAAAGCCAAAATGGAACGTG	GAGCTGGGACACTTTGGAAG	59.8	509	INTRON	Ca_07245	O	--
Ca3_P2_29421952	Ca3	29421952	TAATTTTAATAAATTTTAAT/TAATTTTAAT	GTCGTTGTAGAGAGCGAGGG	TGTGAATGAGGGATAAAAGCAA	59.6	627	INTERGENIC	--	--	--
Ca3_P2_29446926	Ca3	29446926	GCA/GCACA	CAGCAGGGGCATACGTTAAT	GATTCCCCTCCCTGATTCTC	59.8	306	INTERGENIC	--	--	--
Ca3_P2_29506460	Ca3	29506460	CGTT/C	TGCCCCTGTCATCAATGTTA	TCAATCCCCATCAATCATCA	59.7	598	DRR	Ca_07256	T	FAR1
Ca3_P2_29507017	Ca3	29507017	AAAGAAGAAGAAGAAGAAGAAGAAGAAGAAGAAGA/AAAGAAGAAGAAGAAGAAGAAGAAGAAGAAGAAGAAGAAGAAGAAGAAGAAGA	TGATGATTGATGGGGATTGA	TACCCACACCCCTCAAATGT	60.1	259	DRR	Ca_07256	T	FAR1
Ca3_P2_29512683	Ca3	29512683	AATATATATATATATATAT/AATATATATATATATATAT	TGCAATGAATGGAATCTTGG	TCCAAAACAACAAGGACACG	59.6	380	INTERGENIC	--	--	--
Ca3_P2_29563741	Ca3	29563741	TATTCAAATAAATT/TATTCAAATAAATTCAAATAAATT	GCACATGCGGTCCTTTAACT	TTCCCCTCTCTCCCTCTCTC	59.9	648	INTERGENIC	--	--	--

INDEL marker IDs	Chromosomes/unanchored scaffolds	Physical positions (bp)	InDels (*Kabuli* reference genome-CDC Frontier/PII)	Forward primers (5'-3')	Reverse primers (5'-3')	Anneling temperature (0C)	Expected amplified product size (bp)	Structural annotation		Functional annotation	
								Sequence components of *kabuli* genome	*Kabuli* gene accession IDs	NCBI-KOG	TFs
Ca3_P2_29572508	Ca3	29572508	TTTA/TTTAGCCTAAAAAGTATTA	GGACCAATTCGGAAAAAGAA	TTGGCACCCCAAATTTTAAG	59.8	359	DRR	Ca_07262	R	bHLH
Ca3_P2_2962592	Ca3	2962592	TTTATTATTATTATTATTATTATTATTATT/TTTATTATTATTATTATTATTATTATT	AGAGGCAAACAAGAACCGAA	AACTCGGTGCCAACAGTCTT	59.8	596	INTERGENIC	--	--	--
Ca3_P2_29722160	Ca3	29722160	TTATATATATATATATATATATATATAT/TTATATATATATATATATATATATATATAT	CGAAGGAGTGGACAAGAAGC	TTTCGTCGATGTTTGCTGAG	60.0	610	DRR	Ca_07279	--	--
Ca3_P2_29748543	Ca3	29748543	CATTATTATTATTATTATTATTATTATTATTATTATTATTATTATTATTATTATTATTATTATTATTATTAT/CATTATTATTATTATTATTATTATTATTATTATTATTATTATTATTATTATTATTATTATTATTATTATTAT	TGCACCAAGTCACTAACAAAAA	GGTGGCCCTCAACTTTCTTT	60.5	404	INTERGENIC	--	--	--
Ca3_P2_29752847	Ca3	29752847	TAAAAAAAAAA/TAAAAAAAAAAA	AATCGAGATGCCTTAGTGCG	CCTATTCCCACCCCCTTTTA	60.0	868	INTERGENIC	--	--	--
Ca3_P2_29767581	Ca3	29767581	AATATATATATATATATATATATATATAT/AATATATATATATATAT	AGCAAGGTCGACTTTCTCCA	TGGCACAACTCTAACAACCAA	59.2	694	DRR	Ca_07284	--	bHLH
Ca3_P2_29771272	Ca3	29771272	CTTTTTTTTTTT/CTTTTTTTTTT	GGATTGCATTATTGCCAAGTT	GCGAGACATTTTGGGTGAAT	59.9	264	INTERGENIC	--	--	--
Ca3_P2_29772076	Ca3	29772076	ATTTTTTTTTTTT/ATTTTTTTTTTTTT	CCAGCTCCACAACCATCTTT	GCAAATGTTCTGGTCGCATA	59.7	391	INTERGENIC	--	--	--
Ca3_P2_29779379	Ca3	29779379	TTATATATATATATATATAT/TTATATATATATATATATATAT	GAGGAAAGCTTGTTGCTTGG	CCGAATATAAATCATTTGTGGAAA	59.2	323	DRR	Ca_07285	--	--
Ca3_P2_29794446	Ca3	29794446	TG/T	TTGGACCTTTGGGAAAGTTG	TCGACAGTGAAGTTGGGTTG	59.7	463	CDS (FRAME SHIFT)	Ca_07286	Q	bHLH
Ca3_P2_29794859	Ca3	29794859	AATATATATATATATATAT/AATATATATATATATATATAT	TCGAAAGTTCCAACCCAACT	ATTTGCATTCTCTTCGCCAT	59.7	212	INTRON	Ca_07286	Q	bHLH
Ca3_P2_29809144	Ca3	29809144	ATT/ATAATTTTT	GCTCATGCCTCCTTCAAAAA	AAGCAGCCATAATGACAGGG	60.1	856	INTERGENIC	--	--	--

INDEL marker IDs	Chromosomes/unanchored scaffolds	Physical positions (bp)	InDels (*Kabuli* reference genome-CDC Frontier/PII)	Forward primers (5'-3')	Reverse primers (5'-3')	Anneling temperature (0C)	Expected amplified product size (bp)	Structural annotation		Functional annotation	
								Sequence components of *kabuli* genome	*Kabuli* gene accession IDs	NCBI-KOG	TFs
Ca3_P2_29846757	Ca3	29846757	ATTTTTTTTT/ATTTTTTTTTT	TGACTGAAATGCCATCGTTT	TCTAGCTGCCTCTCTCCCTT	58.4	743	INTERGENIC	--	--	--
Ca3_P2_29849306	Ca3	29849306	AATTATTATTATTATTATTATTATTATTATTATTATTATTATTATTATTATTATTATT/AATTATTATTATTATTATTATTATTATTATTATTATTATTATTATTATTATTATTATTATT	TGGCAATCATTTGCTTTTTG	AGAGGCATTGACTTTGGTGG	60.1	228	INTERGENIC	--	--	--
Ca3_P2_29922532	Ca3	29922532	AAATAATAATAATAATAATAATAATAATAATAA/AAATAATAATAATAATAATAATAATAATAATAATAA	CCTGTTTAATTTGGTTCGCC	AATGACGTGCATGACAAAGC	59.7	699	INTERGENIC	--	--	--
Ca3_P2_29962865	Ca3	29962865	TAAAAAAAAAA/TAAAAAAAAA	GGTCGCATCACGAACTTTCT	GGTGCTGGTATCCTAAGACAAAA	59.6	283	INTRON	Ca_07306	S	C3H
Ca3_P2_29970428	Ca3	29970428	CATATAATATAATATAATAT/CATATAATATAATAT	GCAAATAACATCGAGGTTCAGG	AACGACTCAGATGAAATGCAA	58.4	454	INTERGENIC	--	--	--
Ca3_P2_30049696	Ca3	30049696	CATATATATATATATATATATATATATAT/CATATATATATATATATATATATATATATAT	TCCCCTTTTCCTCATCCTCT	TGCACTTTGTTCCCTCAACA	60.3	546	INTRON	Ca_07320	--	--
Ca3_P2_30071057	Ca3	30071057	TAAAAAAAAAA/TAAAAAAAAA	TAAAAGCCAATGGAACTGGG	ATCGCTCTCCTCCTCAAACA	60.0	247	INTERGENIC	--	--	--
Ca3_P2_30100381	Ca3	30100381	ATATTATTATTATTATTATTATT/ATATTATTATTATTATT	TTGCACATTTACTCAACCGC	ATTCCAGAAATGCGACCTTG	60.1	319	INTERGENIC	--	--	--
Ca3_P2_3013494	Ca3	3013494	GATATATATATATATATATATATATATATAT/GATATATATATATATATATATATATATATATATAT	TCACATGAAGCCAATTTCCA	GCTCAGGGGATGTTGTTAGC	59.7	582	INTERGENIC	--	--	--
Ca3_P2_3017046	Ca3	3017046	TGA/TGAGA	TTTGAAAAGAAATGCAATGTTGA	TGGATGTGAGCACATGAAAA	58.6	653	INTERGENIC	--	--	--
Ca3_P2_3022233	Ca3	3022233	A/ATTTTTC	AGTTTTGGTAATTGTTGGTTTTATTT	CTCTAAGCCCCACAGAGTCG	60.0	328	INTERGENIC	--	--	--
Ca3_P2_30339041	Ca3	30339041	AAA/AAATAA	CCTCCTCCAACCATGAAATG	CCTGTGTGGTCAGAAAGGGT	60.0	496	INTERGENIC	--	--	--

INDEL marker IDs	Chromosomes/unanchored scaffolds	Physical positions (bp)	InDels (*Kabuli* reference genome-CDC Frontier/PII)	Forward primers (5'-3')	Reverse primers (5'-3')	Anneling temperature (0C)	Expected amplified product size (bp)	Structural annotation		Functional annotation	
								Sequence components of *kabuli* genome	*Kabuli* gene accession IDs	NCBI-KOG	TFs
Ca3_P2_30380300	Ca3	30380300	ATTTTTTTTTTT/ATTTTTTTTTTTT	GTCATGGGCAATAGGGTCAC	CACATGTGTTTAAATGAAATCGAA	59.0	656	INTERGENIC	--	--	--
Ca3_P2_30450479	Ca3	30450479	ATCTTCTTCTTCTTCTTCTTCT/ATCTTCTTCTTCTTCT	TCATGGGCCACAGCATATAA	GTCGTGCCAGCACAAGAGTA	60.1	366	INTERGENIC	--	--	--
Ca3_P2_30536672	Ca3	30536672	AATATATATATATATATATATATATAT/AATATATATATATATATATATATATAT	TCGCCTTGTGAATATAGGGC	GCCTAACGACAAGGCTTTTG	59.9	376	INTERGENIC	--	--	--
Ca3_P2_3054800	Ca3	3054800	TAAAAAAAAAA/TAAAAAAAAA	TCTACGTATCTCTCGGTGCG	TGTCTTCTTCGTTGTTGCCA	60.4	639	INTERGENIC	--	--	--
Ca3_P2_30602335	Ca3	30602335	TTG/T	GCCCTCAGATGAGAGTCAGG	AAATTAAACCCTCATCCCCG	60.0	842	INTERGENIC	--	--	--
Ca3_P2_30619779	Ca3	30619779	ATTTTTTTTTT/ATTTTTTTTTTT	AAGTTGGTCCATGATAGCAAAAA	CCCTCTGTTCATTTGCCTGT	60.1	732	INTERGENIC	--	--	--
Ca3_P2_30629336	Ca3	30629336	ATTTTTTTTTT/ATTTTTTTTTTT	GATTGATTGCGCATCGACTA	GGGCAAAAGTACAAGTGGGA	60.0	625	INTERGENIC	--	--	--
Ca3_P2_30646834	Ca3	30646834	ATTTTTTTTTTT/ATTTTTTTTTT	TTTCCGGTACACAATTTCCA	CGCCTTCTACGACATTCACA	59.9	260	INTERGENIC	--	--	--
Ca3_P2_30706994	Ca3	30706994	AT/A	TCCACTAATGCGGAGAGTCA	ATAGGAGGTCGCTTCTCCGT	60.2	445	INTERGENIC	--	--	--
Ca3_P2_30729039	Ca3	30729039	TTATATATATATATAT/TTATATATATATAT	CGAAGTTTTATGGTCCCCAA	CCTCGCGCGATAAAAAGTTA	60.3	763	INTERGENIC	--	--	--
Ca3_P2_30747282	Ca3	30747282	ATCTCTCTCTCTCTCTCTCTCTCTCTCTCTCTCTCT/ATCTCTCTCTCTCTCTCTCTCTCTCTCTCTCTCTCT	AAACCACCGTTTTAGTCCCC	CTCGTTTGGGTTGTGGATCT	60.0	764	INTRON	Ca_07395	S	--
Ca3_P2_30930504	Ca3	30930504	GTTTTTATTTTTATTTTTA/GTTTTTATTTTTA	GCATGGACCGTTTAAAATTG	TTTAGGTGGAAAATTCATTAGAAAA	57.6	818	INTERGENIC	--	--	--

INDEL marker IDs	Chromosomes/unanchored scaffolds	Physical positions (bp)	InDels (*Kabuli* reference genome-CDC Frontier/PII)	Forward primers (5'-3')	Reverse primers (5'-3')	Anneling temperature (0C)	Expected amplified product size (bp)	Structural annotation		Functional annotation	
								Sequence components of *kabuli* genome	*Kabuli* gene accession IDs	NCBI-KOG	TFs
Ca3_P2_31101603	Ca3	31101603	ATTTTTTTTTTT/ATTTTTTTTTTTT	ATGGTCCCAATGAAATCCAA	TGTGTTGTCTCTGGCCTTTG	59.9	843	INTRON	Ca_12207	--	--
Ca3_P2_31172417	Ca3	31172417	GTTTTTTTTTTT/GTTTTTTTTTT	CAATGGGAATGGGACTTGTT	TTGTACTGCCTTGAGATGCG	60.0	720	DRR	Ca_12214	--	LBD
Ca3_P2_31205524	Ca3	31205524	GTTTTTTTTT/GTTTTTTTT	TGTGATGAATTTTTCAACCAAT	CACGCTTACATCTCTGCCCT	60.4	512	DRR	Ca_12217	O	TCP
Ca3_P2_31211125	Ca3	31211125	GTTTTTTTTTT/GTTTTTTTTT	TGGAGAGAAGCAACAAAGCA	AATTTTAGGGGGCCATCATC	60.0	342	INTERGENIC	--	--	--
Ca3_P2_31264246	Ca3	31264246	TTATATATATA/TTATATATATATA	AAAAACGAGTGGATGCGTTC	AGAAGACCATCTCTCGTTAGGC	59.0	479	INTERGENIC	--	--	--
Ca3_P2_31274302	Ca3	31274302	TA/T	GCGTTGAGAGAACGCTAACC	GGGATTTGTTCGAAGATTTTG	58.5	732	INTERGENIC	--	--	--
Ca3_P2_31274347	Ca3	31274347	TTATATATAT/TTATATAT	GCGTTGAGAGAACGCTAACC	GGGATTTGTTCGAAGATTTTG	58.5	732	INTERGENIC	--	--	--
Ca3_P2_31279080	Ca3	31279080	ATTTTTTTTTT/ATTTTTTTTT	TACACGCAATTTCGAGCAGT	AAGATGTGGGCATAACGGAG	60.0	920	INTRON	Ca_12224	S	--
Ca3_P2_31285016	Ca3	31285016	A/AT	CGTCGTTTTCAACTGGGAAT	GGATACGAAGTCGGTTGCAT	60.0	328	INTERGENIC	--	--	--
Ca3_P2_31285044	Ca3	31285044	TATAATAATA/TATAATA	CGTCGTTTTCAACTGGGAAT	GGATACGAAGTCGGTTGCAT	60.0	328	INTERGENIC	--	--	--
Ca3_P2_31290021	Ca3	31290021	T/TTGA	GGTTCAAACGTGTTCCCTTG	ACGCTCTCAAATCGACCATC	60.2	929	DRR	Ca_12225	P	ERF
Ca3_P2_31290271	Ca3	31290271	AATATATA/AATATA	TTCGAAAGAAAACATTGTGGG	ACGCTCTCAAATCGACCATC	60.2	681	DRR	Ca_12225	P	ERF

INDEL marker IDs	Chromosomes/unanchored scaffolds	Physical positions (bp)	InDels (*Kabuli* reference genome-CDC Frontier/PII)	Forward primers (5'-3')	Reverse primers (5'-3')	Anneling temperature (0C)	Expected amplified product size (bp)	Structural annotation		Functional annotation	
								Sequence components of *kabuli* genome	*Kabuli* gene accession IDs	NCBI-KOG	TFs
Ca3_P2_31290446	Ca3	31290446	A/AGGTCTAAGCT	TTTAAAGAAGGGAGGTCGCTC	TTGAAGCAGTGTTGTGGGAA	60.3	632	DRR	Ca_12225	P	ERF
Ca3_P2_31290884	Ca3	31290884	GAAAAAAA/GAAAAAA	GATGGTCGATTTGAGAGCGT	TTCATAGGGTCACGGCAAAT	60.3	812	DRR	Ca_12225	P	ERF
Ca3_P2_31293397	Ca3	31293397	T/TC	TCCATAATTGTAGAATTCGAAGAGTC	TGCTTTTCTCAAACATCCCA	59.2	222	INTERGENIC	--	--	--
Ca3_P2_31293455	Ca3	31293455	TG/TGG	TGGGATGTTTGAGAAAAGCA	ATCCCATCCCACCAACAATA	59.9	522	INTERGENIC	--	--	--
Ca3_P2_31293673	Ca3	31293673	G/GA	TGGGATGTTTGAGAAAAGCA	CCCACATCGTTTATCCCATC	60.0	534	INTERGENIC	--	--	--
Ca3_P2_31295937	Ca3	31295937	AAAAAA/AAAAAACAAAAA	GGACCATGGACTCCAGAAGA	TCGTCCTCCATCAGTTCGAT	60.6	688	INTRON	Ca_12226	K	MYB
Ca3_P2_31296813	Ca3	31296813	CGTGTGT/CGTGT	TCGGTTTTAAGGGAAGCAAA	TGCTGCAATAGCTGACCATC	60.0	806	INTRON	Ca_12226	K	MYB
Ca3_P2_31298126	Ca3	31298126	GTTATTATTATTAT/GTTATTATTATTATTAT	GGACTTTTGGCACGATGTTT	TTTGCAACTTTTGACGGACA	60.3	716	DRR	Ca_12226	K	MYB
Ca3_P2_31298291	Ca3	31298291	CT/CTTTT	TGAGAGGTCGATTTTGGAGG	TTTGCAACTTTTGACGGACA	60.3	506	DRR	Ca_12226	K	MYB
Ca3_P2_31298325	Ca3	31298325	TCC/TC	TGAGAGGTCGATTTTGGAGG	TTTGCAACTTTTGACGGACA	60.3	506	DRR	Ca_12226	K	MYB
Ca3_P2_31313566	Ca3	31313566	TTCTTCATCTTCATCTTCATCTT/TTCTTCATCTTCATCTT	TCGAACACATTGTTGCCATT	GCCACAGCAGACAAGATGAA	60.0	493	CDS (large-effect mutations)	Ca_12230	--	Dof
Ca3_P2_31319681	Ca3	31319681	GAAAA/GAAAAA	CGTGGCTCCTCAAAAGTCTC	ATGCACAAGGCTTCCATACC	60.0	599	DRR	Ca_12232	U	bHLH

INDEL marker IDs	Chromosomes/unanchored scaffolds	Physical positions (bp)	InDels (*Kabuli* reference genome-CDC Frontier/PII)	Forward primers (5'-3')	Reverse primers (5'-3')	Anneling temperature (0C)	Expected amplified product size (bp)	Structural annotation		Functional annotation	
								Sequence components of *kabuli* genome	*Kabuli* gene accession IDs	NCBI-KOG	TFs
Ca3_P2_31322105	Ca3	31322105	TAAAAAAAAAA/TAAAAAAAAA	GTTTCAACATGACCATCCCC	CCCCCTGAAAGATACCCAAT	60.0	868	INTRON	Ca_12232	U	bHLH
Ca3_P2_31323281	Ca3	31323281	CTTTT/CTTTTT	AATGCTTCAACAGCCTTCGT	TGGGATCGGTTACATGGATT	60.0	415	INTRON	Ca_12232	U	bHLH
Ca3_P2_31325736	Ca3	31325736	ATTT/ATTTT	ATTAATTGGGCTGACGTGGA	GTCCACATTAGGGACACGCT	60.0	426	INTERGENIC	--	--	--
Ca3_P2_31326387	Ca3	31326387	GTTTT/GTTT	CGGGTACAGCCGGTAAGTAA	GGGTGCCTTTAAAGTGTGGA	60.0	639	INTERGENIC	--	--	--
Ca3_P2_31326762	Ca3	31326762	CTTTTTTTTTTT/CTTTTTTTTTT	TCCACACTTTAAAGGCACCC	CCAGTTTGTCCATGCATTTG	60.0	560	INTERGENIC	--	--	--
Ca3_P2_31327189	Ca3	31327189	TAAAAAAAAA/TAAAAAAAAAA	CAAATGCATGGACAAACTGG	GGATCATTCGCGACCATAAT	59.8	426	INTERGENIC	--	--	--
Ca3_P2_31327483	Ca3	31327483	AT/ATT	CAAATGCATGGACAAACTGG	TTGGATGCCTCCATATCTAACTT	59.0	922	INTERGENIC	--	--	--
Ca3_P2_31327768	Ca3	31327768	GTTT/GTT	ATTATGGTCGCGAATGATCC	AATTGCGGTTAGTTGGATGC	60.0	528	INTERGENIC	--	--	--
Ca3_P2_31328317	Ca3	31328317	ATTTTTT/ATTTTTTT	TCCAACTAACCGCAATTCAA	TCATGGCAAATTTAGGATGTTG	59.8	447	INTERGENIC	--	--	--
Ca3_P2_31328367	Ca3	31328367	AA/AATA	TCCAACTAACCGCAATTCAA	TCATGGCAAATTTAGGATGTTG	59.8	447	INTERGENIC	--	--	--
Ca3_P2_31330534	Ca3	31330534	ATTTTTTTTT/ATTTTTTTT	GTGTCAAATGTTCGTGTCGG	GAAAGGCAACGGTGATTCAT	59.9	825	INTERGENIC	--	--	--
Ca3_P2_31333274	Ca3	31333274	ATTTTTTTT/ATTTTTTT	TTCTCCGTGAAGGAGCTGTT	GACAACCAATGTTTCTGCCC	60.4	703	INTRON	Ca_12233	S	bZIP

INDEL marker IDs	Chromosomes/unanchored scaffolds	Physical positions (bp)	InDels (*Kabuli* reference genome-CDC Frontier/PII)	Forward primers (5'-3')	Reverse primers (5'-3')	Anneling temperature (0C)	Expected amplified product size (bp)	Structural annotation		Functional annotation	
								Sequence components of *kabuli* genome	*Kabuli* gene accession IDs	NCBI-KOG	TFs
Ca3_P2_31334839	Ca3	31334839	ACCCCC/ACCCC	GCATCAAATCTTCACCCACC	GCAAAAGCAGGGTCTCAAAC	59.9	622	DRR	Ca_12233	S	bZIP
Ca3_P2_31336446	Ca3	31336446	ACTCT/ACT	GAAACGTGCGGTGTATCCTT	TGCATTTGTATTGAAAATTGTTTG	59.0	754	INTERGENIC	--	--	--
Ca3_P2_31336604	Ca3	31336604	T/TC	AGCAGGCCTGGACCTTTACT	GGAATGCCAACGAATGAAAA	60.8	555	INTERGENIC	--	--	--
Ca3_P2_31336842	Ca3	31336842	AAAAAAAATAATAAAAA/AAAAAA	AGCAGGCCTGGACCTTTACT	GGAATGCCAACGAATGAAAA	60.8	555	INTERGENIC	--	--	--
Ca3_P2_31339236	Ca3	31339236	T/TC	CCAACCCATGTACCGACATT	AGAGACCGTTTCATGAGATTTG	58.3	525	INTERGENIC	--	--	--
Ca3_P2_31340782	Ca3	31340782	GAAAAAAAAAA/GAAAAAAAAAAAAA	GGGTAAATTGGTAGGGGCAT	GTGTTGGTGGTGATGGTTGA	60.3	948	INTERGENIC	--	--	--
Ca3_P2_31342905	Ca3	31342905	GATA/GATATATA	TGGCAAGTTTTCACTTCCATT	TGCATTGCACACAAGAATGA	59.8	315	DRR	Ca_12234	--	--
Ca3_P2_31343075	Ca3	31343075	CAATAAAAATAAAAATAAAAAT/CAATAAAAATAAAAAT	TGGCAAGTTTTCACTTCCATT	GATCACGGGAGGATCGACTA	60.0	792	DRR	Ca_12234	--	--
Ca3_P2_31343206	Ca3	31343206	AG/A	TGGTTTGAAATACTGGTCATGC	GATCACGGGAGGATCGACTA	60.0	257	DRR	Ca_12234	--	--
Ca3_P2_31343763	Ca3	31343763	TAAA/TAAAA	AATAGTCGATCCTCCCGTGAT	CCATGCTCAAGTCTAGGTTCG	59.9	510	DRR	Ca_12234	--	--
Ca3_P2_31348024	Ca3	31348024	AC/A	GGTCAACAACATCAATTGCG	TAGGGGACCAAAACTGAGGA	59.5	924	INTERGENIC	--	--	--
Ca3_P2_31348057	Ca3	31348057	TAAAA/TAAAAA	GTTTGGATTCCACCGAAGAA	TAGGGGACCAAAACTGAGGA	59.5	895	INTERGENIC	--	--	--

INDEL marker IDs	Chromosomes/unanchored scaffolds	Physical positions (bp)	InDels (*Kabuli* reference genome-CDC Frontier/PII)	Forward primers (5'-3')	Reverse primers (5'-3')	Anneling temperature (0C)	Expected amplified product size (bp)	Structural annotation		Functional annotation	
								Sequence components of *kabuli* genome	*Kabuli* gene accession IDs	NCBI-KOG	TFs
Ca3_P2_31348114	Ca3	31348114	T/TCATAA	TCTCCGTTGGCACTACAAGA	TAGGGGACCAAAACTGAGGA	59.5	844	INTERGENIC	--	--	--
Ca3_P2_31350700	Ca3	31350700	TCATG/TCATGCATG	TCTTAGCGTAGATTCGCCGT	AACATCTGGGCTAATCGGTG	60.0	280	INTERGENIC	--	--	--
Ca3_P2_31351028	Ca3	31351028	A/ATAT	CACCGATTAGCCCAGATGTT	TGTCATTGAGGTGGGTGTTG	60.4	521	INTERGENIC	--	--	--
Ca3_P2_31353219	Ca3	31353219	CAAAAA/CAAAAAA	TCCCTAAAAGTTTCTTCATCCAA	GAATGACATATTGTGGCAGCA	59.6	225	INTERGENIC	--	--	--
Ca3_P2_31355710	Ca3	31355710	CT/C	ATTTACCGACGGTGATCTGC	ATCACAACCCAGCTGAAACC	60.0	781	DRR	Ca_12236	R	NAC
Ca3_P2_31359527	Ca3	31359527	AA/AAGGTTTAATTTATCTTA	GCTTCAGCAACGACAAACAA	CCACTCAAATGGGTAATGCC	60.2	804	INTRON	Ca_12236	R	NAC
Ca3_P2_31361081	Ca3	31361081	CTATATATATATA/CTATATATATATATA	CCTCTTCGGTTGGATGAAAA	CAGGCTAACAGCTGCAATGA	60.2	585	INTRON	Ca_12236	R	NAC
Ca3_P2_31364505	Ca3	31364505	CTTTTTTTT/CTTTTTTTTT	ACCAATGTCTCGCAAGCTCT	GGTAAACATTTGCATCGTTGG	60.2	458	INTERGENIC	--	--	--
Ca3_P2_31365247	Ca3	31365247	TAAAAAAAAA/TAAAAAAAA	GGAGCGGACAATTCAAACAT	AGTGAAAAATACGTGTCCCG	57.6	223	INTERGENIC	--	--	--
Ca3_P2_31365869	Ca3	31365869	GTTTT/GTTT	AATTAAACCCGTGCAAATTCC	AAATGACATGCGATCCAACA	59.9	209	INTERGENIC	--	--	--
Ca3_P2_31366021	Ca3	31366021	A/ATT	CCCTCTTGTGCCTGTCTTTC	CAACGGGAATCTTCCGTCTA	60.1	469	INTERGENIC	--	--	--
Ca3_P2_31371532	Ca3	31371532	TATCAT/TATCATCAT	GGATCAGCCCAATTGGTAAA	TAGACCATAAGCCCCTGACG	60.1	322	INTERGENIC	--	--	--

INDEL marker IDs	Chromosomes/unanchored scaffolds	Physical positions (bp)	InDels (*Kabuli* reference genome-CDC Frontier/PII)	Forward primers (5'-3')	Reverse primers (5'-3')	Anneling temperature (0C)	Expected amplified product size (bp)	Structural annotation		Functional annotation	
								Sequence components of *kabuli* genome	*Kabuli* gene accession IDs	NCBI-KOG	TFs
Ca3_P2_31375441	Ca3	31375441	ATTTTTT/ATTTTT	TTGATTCGTTGCTAGTGCAAA	CATATGTGAACCATGGCAGC	60.0	871	INTERGENIC	--	--	--
Ca3_P2_31376677	Ca3	31376677	TT/TAGTATTTTTCAT	AGGGGCATGCTTGTGTAAAT	GACCAACTTTATGCGTGCAA	59.7	581	INTERGENIC	--	--	--
Ca3_P2_31376995	Ca3	31376995	GT/GTT	TCCTTGCACGCATAAAGTTG	GAAATCTCACTGCCCCATTC	59.5	563	INTERGENIC	--	--	--
Ca3_P2_31378790	Ca3	31378790	TAA/TGATTATTTTTAAA	TCACAATAGACAAGTGGCCG	CGCCAACCCAATCTAACACT	60.0	508	INTERGENIC	--	--	--
Ca3_P2_31380098	Ca3	31380098	ATTTTTTTTT/ATTTTTTT	CATCGTCTTTGATTTTTATGAATTG	TTATTGCCCTGCTTTCATCC	60.0	683	INTERGENIC	--	--	--
Ca3_P2_31380874	Ca3	31380874	ATTTTTTTT/ATTTTTTT	CTTTTTGTTTGTGGCTGGGT	ATTGGTCCCAATGGTTGGT	59.9	752	INTERGENIC	--	--	--
Ca3_P2_31381715	Ca3	31381715	GATATA/GATA	TAAACCTTGGCTGGGTCAAC	TTGTTTATTTTGTTCGTCGTGTT	58.6	403	INTERGENIC	--	--	--
Ca3_P2_31381813	Ca3	31381813	AT/A	TAAACCTTGGCTGGGTCAAC	TTGCTACCATTGTAAAGCTTGTTT	59.3	611	INTERGENIC	--	--	--
Ca3_P2_31384239	Ca3	31384239	TCTAGAGATCTGCTAGA/TCTAGA	TTCGACTCGGGTCCATAAAC	TGTCTACACCGTCAACAGCC	59.8	564	INTERGENIC	--	--	--
Ca3_P2_31391479	Ca3	31391479	CTTT/CTTTT	GTGCCGCTTCATCCAAATTA	TTGTTGATAATGTGGGCACG	60.4	247	INTERGENIC	--	--	--
Ca3_P2_31391982	Ca3	31391982	ATTTT/ATTT	CGTGCCCACATTATCAACAA	TCTTGCTCATGGTGCTTCTG	60.1	793	INTERGENIC	--	--	--
Ca3_P2_31392191	Ca3	31392191	A/ATAAAT	CACCCCATTTTGTATCGAGTT	TCTTGCTCATGGTGCTTCTG	60.1	351	INTERGENIC	--	--	--

INDEL marker IDs	Chromosomes/unanchored scaffolds	Physical positions (bp)	InDels (*Kabuli* reference genome-CDC Frontier/PII)	Forward primers (5'-3')	Reverse primers (5'-3')	Anneling temperature (0C)	Expected amplified product size (bp)	Structural annotation		Functional annotation	
								Sequence components of *kabuli* genome	*Kabuli* gene accession IDs	NCBI-KOG	TFs
Ca3_P2_31394583	Ca3	31394583	AA/AAGA	TCACACATAACCCGTGATCC	AGCACGAGGACAAGTTCACC	60.3	610	INTERGENIC	--	--	--
Ca3_P2_31396962	Ca3	31396962	A/AG	GCATTTTATTCAAATTTCATGGG	ATCGTGAGTTTGAGGGGAGA	59.7	840	INTERGENIC	--	--	--
Ca3_P2_31402077	Ca3	31402077	TAAAA/TAAAAA	TGTACACGTCGGTGAACAACT	TGTGGCGACAACTAACTTTGA	59.4	634	INTERGENIC	--	--	--
Ca3_P2_31402737	Ca3	31402737	ATTTTTTTTTTT/ATTTTTTTTTT	CACCGAAATAAATATTGGTCCC	CTTTTTCCATCATTTTTCCATT	57.2	826	DRR	Ca_12237	O	--
Ca3_P2_31413249	Ca3	31413249	AA/AATA	TGTTGCACGTTGGACAAGTT	GACTGCTACCAACCCAGCAT	60.1	374	DRR	Ca_12238	A	--
Ca3_P2_31415275	Ca3	31415275	T/TG	GTAGCTAACCCAACCGGTGA	GGGCCCGTTATATATTGGCT	60.0	565	INTERGENIC	--	--	--
Ca3_P2_31415533	Ca3	31415533	TAAAAAAA/TAAAAAAAA	CAAGGGTGTGGGAGAGAAAA	TTTTGAAATTTCCATGTTCAGG	59.0	386	INTERGENIC	--	--	--
Ca3_P2_31418574	Ca3	31418574	ATTTTTTTT/ATTTTTTTTT	TGCACTCCTCTGCATGATTC	GTCTATCTCGCGGCTGAAAC	60.0	497	INTERGENIC	--	--	--
Ca3_P2_31418976	Ca3	31418976	CATTATTATTATTAT/CATTATTATTATTATTAT	GTTTCAGCCGCGAGATAGAC	CCATTTAATCCTTCAAATTGCC	59.7	341	INTRON	Ca_12239	R	YABBY
Ca3_P2_31419172	Ca3	31419172	T/TA	GTTTCAGCCGCGAGATAGAC	TGCAGCAAAGGAAAAACACA	60.4	736	INTRON	Ca_12239	R	YABBY
Ca3_P2_31426177	Ca3	31426177	C/CCCAACAAGTGT	TGAGTCCTCATCGGTCACAA	TCATCAATGACTAAAATTTGTTTTGTA	58.3	573	DRR	Ca_12239	R	YABBY
Ca3_P2_31441706	Ca3	31441706	AT/ATGAAATT	AACAATTTTTGGAACCCCTAA	TTTGCAAGTGCAACTTCTGC	60.2	669	DRR	Ca_12242	--	--

INDEL marker IDs	Chromosomes/unanchored scaffolds	Physical positions (bp)	InDels (*Kabuli* reference genome-CDC Frontier/PII)	Forward primers (5'-3')	Reverse primers (5'-3')	Anneling temperature (0C)	Expected amplified product size (bp)	Structural annotation		Functional annotation	
								Sequence components of *kabuli* genome	*Kabuli* gene accession IDs	NCBI-KOG	TFs
Ca3_P2_31442973	Ca3	31442973	CTTTTT/CTTTTTT	TAGATGATTTTGGCAAGGGC	CTGGACTTTCGTCATCAGCA	60.0	457	INTRON	Ca_12242	--	--
Ca3_P2_31443022	Ca3	31443022	CGT/C	TAGATGATTTTGGCAAGGGC	CTGGACTTTCGTCATCAGCA	60.0	457	INTRON	Ca_12242	--	--
Ca3_P2_31443328	Ca3	31443328	ATTTTT/ATTTTTT	TGCTGATGACGAAAGTCCAG	CATATTGGATGGGATGGTCC	59.8	302	INTRON	Ca_12242	--	--
Ca3_P2_31444469	Ca3	31444469	GTATATAT/GTATAT	TGGTCGAAATTTCAGGTGTG	AACTCAACTAATCCGGCCAA	59.6	544	DRR	Ca_12243	G	--
Ca3_P2_31446074	Ca3	31446074	CTTTTT/CTTTT	CATCCCAATTTGCATTGGTT	TGGAGGCTATTGGTACAGGG	59.9	531	DRR	Ca_12243	G	--
Ca3_P2_31448937	Ca3	31448937	ATTT/ATTTAGAAAGTTT	TGGCCACCACTAACACAAAA	ACAGGTGATCCTCTTTCCCC	60.3	757	INTERGENIC	--	--	--
Ca3_P2_31453959	Ca3	31453959	CTCATTTTTCATTTTTCAT/CTCATTTTTCATTTTTCATTTTTCAT	CTCCAATGGCAAGGTCACTT	GGATCAATGGCAAGAGCATT	60.0	310	INTRON	Ca_12244	P	--
Ca3_P2_31457627	Ca3	31457627	ATTTTTTTTTT/ATTTTTTTTT	AACATTAGGATGCACCCAGC	GAGGCACGAAATTCAGGTGT	60.1	760	INTRON	Ca_12244	P	--
Ca3_P2_31463027	Ca3	31463027	TAG/TAGAATTTAAG	TCTTCCGAGTCCTTTTGGTG	AAAGTATTGACGGTGCCCTG	60.0	416	INTRON	Ca_12245	P	--
Ca3_P2_31468994	Ca3	31468994	TAGAGAGAGAG/TAGAGAGAG	ATTTTGCCTCCAAACACACC	ACTTGCAACGCAAGCCTAAT	59.9	352	INTERGENIC	--	--	--
Ca3_P2_31471455	Ca3	31471455	CTTATTTATTTATTT/CTTATTTATTTATTTATTT	AAAACCCAAAAGCTGAATCG	CCCAAAGTTGGCATGAAGTT	60.0	624	INTERGENIC	--	--	--
Ca3_P2_31475126	Ca3	31475126	TAAAAAAAAA/TAAAAAAAA	AACACCGCAACAAGTCACTG	AAATGACGGCACGACAAGAT	60.5	497	INTERGENIC	--	--	--

INDEL marker IDs	Chromosomes/unanchored scaffolds	Physical positions (bp)	InDels (*Kabuli* reference genome-CDC Frontier/PII)	Forward primers (5'-3')	Reverse primers (5'-3')	Anneling temperature (0C)	Expected amplified product size (bp)	Structural annotation		Functional annotation	
								Sequence components of *kabuli* genome	*Kabuli* gene accession IDs	NCBI-KOG	TFs
Ca3_P2_31476304	Ca3	31476304	ATTTTT/ATTTT	TGGTTCAGATTTGTTTGGTGA	CAAACGTGAGTGAAACAACCA	59.6	568	INTERGENIC	--	--	--
Ca3_P2_31476572	Ca3	31476572	AAATA/AA	TGGTTGTTTCACTCACGTTTG	GGATGGCAATCCTCCATTAC	59.2	519	INTERGENIC	--	--	--
Ca3_P2_31478334	Ca3	31478334	TCC/TCCC	TGGTGTGTGTTAGCCATAAAGG	GGGGCCTCTTCTTTTGTTTC	60.1	614	INTRON	Ca_12246	--	--
Ca3_P2_31480211	Ca3	31480211	TTAATAATAATAATAATAATAATAATAA/TTAATAATAATAATAATAATAATAA	TTCTGCACATCAAATGGGAA	TTTGAGCTGCAAAACCATTT	58.4	683	DRR	Ca_12246	--	--
Ca3_P2_31482581	Ca3	31482581	CTAAT/CTAATTAAT	TGCTCATGTGCCAAGAGTTG	TAGGGCCCACAATTCAAAAG	59.9	425	INTERGENIC	--	--	--
Ca3_P2_31483041	Ca3	31483041	ATAA/ATAAGTCTAA	CTTCCTTGACACGCTAAGGG	TGGAGAATTTGAGTGGGGAG	60.0	714	INTERGENIC	--	--	--
Ca3_P2_31489433	Ca3	31489433	ATTT/ATTTTT	GGTGCCAAGAGACCAGAGAG	AGTGGAGCTAGCAGGAACCA	60.0	383	INTRON	Ca_12247	T	--
Ca3_P2_31491087	Ca3	31491087	CTTTTT/CTTTT	CCCCCAAACCTTCAAAAAGT	CGTTTGATAATTTGAGGGGC	59.4	421	DRR	Ca_12247	T	--
Ca3_P2_31495232	Ca3	31495232	CTTT/CTT	CCAAAATGGTGAGAGACTTCG	TTCGGTGCTTTGTCTCTTCC	60.4	809	INTERGENIC	--	--	--
Ca3_P2_31496027	Ca3	31496027	GTTTTTTTT/GTTTTTTT	ATGGTGTCTTTGTTCCTGGC	ACTTTCATCCTCCTGCAACG	60.3	522	INTRON	Ca_12248	T	--
Ca3_P2_31496131	Ca3	31496131	ATTTTTTTTTTTTTT/ATTTTTTTTTTT	ATGGTGTCTTTGTTCCTGGC	ACTTTCATCCTCCTGCAACG	60.3	522	INTRON	Ca_12248	T	--
Ca3_P2_31498407	Ca3	31498407	ATTT/ATTTT	CTAGCTCGGGTTTTCTGACG	TTGGCCACAGTTTAACCACA	60.0	531	DRR	Ca_12248	T	--

INDEL marker IDs	Chromosomes/unanchored scaffolds	Physical positions (bp)	InDels (*Kabuli* reference genome-CDC Frontier/PII)	Forward primers (5'-3')	Reverse primers (5'-3')	Anneling temperature (0C)	Expected amplified product size (bp)	Structural annotation		Functional annotation	
								Sequence components of *kabuli* genome	*Kabuli* gene accession IDs	NCBI-KOG	TFs
Ca3_P2_31501132	Ca3	31501132	TAA/TAAA	TCCCAAGTGAACATCACCAA	TTGGATTGTATGGGTTCGGT	60.1	496	INTRON	Ca_12249	I	--
Ca3_P2_31509699	Ca3	31509699	TTTATATT/TTT	ACTTGCTCCCCATGAAATTG	TGAGGGAAGAAAGCAGAAGG	59.5	170	INTRON	Ca_12249	I	--
Ca3_P2_31512169	Ca3	31512169	TAAAAAAAAAA/TAAAAAAAAA	AAGGTTGATGTTTCCGTTGC	TGAATTTGATCGCAGAGACG	59.9	837	INTERGENIC	--	--	--
Ca3_P2_31518374	Ca3	31518374	ATTTTTTTTT/ATTTTTTTT	TTGTTAAGGCAGCAAAGGCT	TTCAGGGAAATGCCTCAAAG	60.2	625	INTERGENIC	--	--	--
Ca3_P2_31519164	Ca3	31519164	C/CT	TACACGTGGCCTCAAACAAA	TCAAGCATGGTTCCAATCAG	59.6	364	INTERGENIC	--	--	--
Ca3_P2_31530560	Ca3	31530560	TTGGGT/TTGGGTGGGT	AACTCCTTCTGCACATTGGG	TGGGAAATAGATCGGTCTCG	60.0	449	INTRON	Ca_12252	E	TALE
Ca3_P2_31536384	Ca3	31536384	GTTTTTTT/GTTTTTT	AAAACGTCATCGTTCCAAGG	ACAATCCACCAAGCAGGAAC	60.0	590	INTRON	Ca_12253	LK	--
Ca3_P2_31540731	Ca3	31540731	CTATATATATATATATATATATATA/CTATATATATATATATATATATATATA	CTGTTCCATTGTCCAACACG	CACATGGGTGACATCTGAGG	60.0	566	INTRON	Ca_12253	LK	--
Ca3_P2_31544066	Ca3	31544066	TACAAGCAAAACAAGCAAA/TACAAGCAAA	TGTGCATTCCAAAAGTGCAT	CCTCCAGGGAGCCCTATAAC	59.9	561	DRR	Ca_12253	LK	--
Ca3_P2_31544146	Ca3	31544146	TAAAAAAA/TAAAAAAAA	TGTGCATTCCAAAAGTGCAT	CCTCCAGGGAGCCCTATAAC	59.9	561	DRR	Ca_12253	LK	--
Ca3_P2_31560705	Ca3	31560705	A/AAG	TGTTCACTGCCAAACTCTGC	GCAGTGCACAACTGATGGTT	59.8	585	INTERGENIC	--	--	--
Ca3_P2_31563003	Ca3	31563003	CATA/CATATA	GAATGGTGTTCCTTTGTCCC	AATTTCACCCAAAGACACGG	59.8	274	INTERGENIC	--	--	--

INDEL marker IDs	Chromosomes/unanchored scaffolds	Physical positions (bp)	InDels (*Kabuli* reference genome-CDC Frontier/PII)	Forward primers (5'-3')	Reverse primers (5'-3')	Anneling temperature (0C)	Expected amplified product size (bp)	Structural annotation		Functional annotation	
								Sequence components of *kabuli* genome	*Kabuli* gene accession IDs	NCBI-KOG	TFs
Ca3_P2_31566099	Ca3	31566099	CG/C	AAAGGGCATGGGGAAAATAG	TTTTCATGCATTTGAGACGC	59.8	338	DRR	Ca_12255	--	G2-like
Ca3_P2_31568511	Ca3	31568511	CTA/CTATA	TGCAATGTGCATCACTCTCA	GCAGATGGAGGTCCAGAAAC	59.7	548	INTRON	Ca_12255	--	G2-like
Ca3_P2_31569781	Ca3	31569781	GAAAA/GAAA	TGTCAGTGCTGTGTGCGTTA	TTTGTTGAATACCGTTGGCA	60.0	635	INTRON	Ca_12255	--	G2-like
Ca3_P2_31576995	Ca3	31576995	TAAAAAAAAAAA/TAAAAAAAAAA	AAACGATAAAATCAGACCAAATCA	TAACCCGACTAGCACAACCC	60.0	451	INTERGENIC	--	--	--
Ca3_P2_31578728	Ca3	31578728	CATAT/CAT	TCCATGAAATTGAAATAAAACGC	GCCCGTTAGGATGTGGAGTA	60.0	715	DRR	Ca_12256	--	--
Ca3_P2_31589916	Ca3	31589916	CCT/CCTCT	TGCTTGTTCAAAATTGTCGC	TTTCACCCATCACTGCTTCA	60.2	399	INTRON	Ca_12258	BK	G2-like
Ca3_P2_31595214	Ca3	31595214	CATTTATAAACAT/C	TTGATGTTATTTATTGGTGCAAAA	AGTAACGACAACCTCCGTGG	60.0	623	INTERGENIC	--	--	--
Ca3_P2_31602376	Ca3	31602376	GT/GTGTTTCTTT	GCACGAGCACAACTTCAAAA	AAAGAACCATCATCGCAGGT	59.6	618	INTERGENIC	--	--	--
Ca3_P2_31602428	Ca3	31602428	GAGTGCTAGTGCTA/GAGTGCTAGTGCTAGTGCTA	GCACGAGCACAACTTCAAAA	AAAGAACCATCATCGCAGGT	59.6	618	INTERGENIC	--	--	--
Ca3_P2_31602554	Ca3	31602554	ATGT/AT	GCACGAGCACAACTTCAAAA	AAAGAACCATCATCGCAGGT	59.6	618	INTERGENIC	--	--	--
Ca3_P2_31602911	Ca3	31602911	ATGTT/ATGTTGTT	ACCGAATATTCCATGCTCCA	CCAAGTGATGTGTCATGGCT	59.6	565	INTERGENIC	--	--	--
Ca3_P2_31603278	Ca3	31603278	CA/C	ACCGAATATTCCATGCTCCA	CCAAGTGATGTGTCATGGCT	59.6	565	INTERGENIC	--	--	--

INDEL marker IDs	Chromosomes/unanchored scaffolds	Physical positions (bp)	InDels (*Kabuli* reference genome-CDC Frontier/PII)	Forward primers (5'-3')	Reverse primers (5'-3')	Anneling temperature (0C)	Expected amplified product size (bp)	Structural annotation		Functional annotation	
								Sequence components of *kabuli* genome	*Kabuli* gene accession IDs	NCBI-KOG	TFs
Ca3_P2_31606915	Ca3	31606915	ATCTTCTTCTTCTTC/ATCTTCTTCTTCTTCTTC	GTGGCAGCTGCAAGAATACA	ATGCCATTTGGCCCAATAAT	61.2	894	CDS (large-effect mutations)	Ca_12260	--	--
Ca3_P2_31607270	Ca3	31607270	ATTTTTTTT/ATTTTTTT	TACCCTAACGGTGCTCCATC	AAGATCATTCCCCAAAACCC	60.0	567	INTRON	Ca_12260	--	--
Ca3_P2_31607443	Ca3	31607443	G/GT	TTTGACATTTGAGAAATGGGG	CCAATTGCTGCTACTCCTCC	59.8	209	INTRON	Ca_12260	--	--
Ca3_P2_31607764	Ca3	31607764	CA/C	GCAGTTGGAGGAGTAGCAGC	TGATATGGTGGGGACAAGAA	58.8	311	DRR	Ca_12260	--	--
Ca3_P2_31608359	Ca3	31608359	ATA/ATAGTA	TGCCATCCTCTTCCAATTTC	TCTTTCCCAGTTTCCCCTTT	59.9	168	DRR	Ca_12260	--	--
Ca3_P2_31609545	Ca3	31609545	TAAAAAAAAA/TTAAAAAAAAAA	GACAAAGCCAGAACCTTTGC	ACCCAAGCAGGCATACAAAC	60.0	538	DRR	Ca_12260	--	--
Ca3_P2_31611126	Ca3	31611126	GAAAAGTAAAAGTAAAA/GAAAAGTAAAA	TTTGGATTAGCCATAACATCCA	CTTCCACCTTCGTGGAAAGA	60.2	617	INTERGENIC	--	--	--
Ca3_P2_31628087	Ca3	31628087	CT/CTT	TCAAAATTGCGTATTTGGTCC	TGATAGATCGGAGAGGGGTG	60.0	792	DRR	Ca_12261	A	--
Ca3_P2_31629115	Ca3	31629115	ATTTTTT/ATTTTT	GTTTGTTTCGCAGGTTGGTT	ATTTTGGAGGGATTTGGAGG	60.1	498	INTRON	Ca_12262	T	--
Ca3_P2_31629285	Ca3	31629285	GGGGGAGGGGAGGGGAG/GGGGGAGGGGAG	GTTTGTTTCGCAGGTTGGTT	TCACTCCACTCCGGTACTCC	60.1	711	INTRON	Ca_12262	T	--
Ca3_P2_31633339	Ca3	31633339	GTATAT/GTAT	TGGACCCAAGAACAGTGTCA	GCAATTGCAGAAATGCAAGA	60.0	496	INTRON	Ca_12262	T	--
Ca3_P2_31634280	Ca3	31634280	AGTT/AGTTGTT	TCAGCGTGGTTCACGATAAG	GACGATCAGCACAACTTCCA	59.8	339	DRR	Ca_12262	T	--

INDEL marker IDs	Chromosomes/unanchored scaffolds	Physical positions (bp)	InDels (*Kabuli* reference genome-CDC Frontier/PII)	Forward primers (5'-3')	Reverse primers (5'-3')	Anneling temperature (0C)	Expected amplified product size (bp)	Structural annotation		Functional annotation	
								Sequence components of *kabuli* genome	*Kabuli* gene accession IDs	NCBI-KOG	TFs
Ca3_P2_31634878	Ca3	31634878	ATTT/ATT	TGGAAGTTGTGCTGATCGTC	AAGTTGGGTTTCTTTCGCAG	59.4	839	DRR	Ca_12262	T	--
Ca3_P2_31635608	Ca3	31635608	GTTTTTTTTTTT/GTTTTTTT	AATGGGTTCCCTGGATTTTC	CTTTGGTGGGATCAGAGAGC	59.8	222	DRR	Ca_12262	T	--
Ca3_P2_31636736	Ca3	31636736	TTATA/TTA	TGCGTAAAAGCCAAACAAAA	GGCCTAAAATTCTACTTTGCAC	57.2	374	DRR	Ca_12264	--	NF-YB
Ca3_P2_31637117	Ca3	31637117	TGA/TGAGGA	TGCGTAAAAGCCAAACAAAA	GCACAAGCAGAGCAAAATGA	60.1	943	DRR	Ca_12264	--	NF-YB
Ca3_P2_31637556	Ca3	31637556	TTTTGCT/TTTTGCTTTGCCTTTGCT	TAAACATCACCCGCATTTCG	GAATGCATCAGGCTTTTCAA	58.8	102	DRR	Ca_12264	--	NF-YB
Ca3_P2_31672357	Ca3	31672357	CATATATATATATATATATATATATATAT/CATATATATATATATATATATATATAT	AAGGCCGATTAGCCAAAAAT	TAGGAGGTCGCTTCTCCAAA	59.9	211	INTERGENIC	--	--	--
Ca3_P2_31682159	Ca3	31682159	GAAAAAAAAA/GAAAAAAAA	GAAACCTGTCGAAGCCAATG	TGTATGATGTGTGCGAGGGT	60.0	289	DRR	Ca_12270	--	G2-like
Ca3_P2_31755384	Ca3	31755384	CTATATATATATATATATATATATAT/CTATATATATATATATATATATATAT	GCAGCTAAAGAAGATTGGCCT	TTTGCGATATGAACCAAATCA	59.0	467	INTERGENIC	--	--	--
Ca3_P2_31778792	Ca3	31778792	CAT/C	TGCAAAATTCACATAGGCCA	ATTTGGGGATTTAGGGTTCG	60.0	541	INTERGENIC	--	--	--
Ca3_P2_31810634	Ca3	31810634	AAC/AACCTTTGAC	TGTGTGATTGCACAGGAACA	TTAGCGTGGGCAAATAGTCA	59.3	253	INTERGENIC	--	--	--
Ca3_P2_31852962	Ca3	31852962	TTTATTATTATTATTATTATTATTATTATTATTATTATTATTATTATTATTATTATT/TTTATTATTATTATTATTATTATTATTATTATTATTATTATTATTATTATTATTATTATT	TTTAGGGAAATGGGTCATGG	TCAATCTGTCTCGTTTTGATTGA	59.7	576	DRR	Ca_12289	G	STAT
Ca3_P2_3185857	Ca3	3185857	TAAAAAAAAAA/TAAAAAAAA	CGCCTCTCTTTTTCTATGCG	TGTTGCTGTTGTTGCCTCTC	60.0	468	INTERGENIC	--	--	--

INDEL marker IDs	Chromosomes/unanchored scaffolds	Physical positions (bp)	InDels (*Kabuli* reference genome-CDC Frontier/PII)	Forward primers (5'-3')	Reverse primers (5'-3')	Anneling temperature (0C)	Expected amplified product size (bp)	Structural annotation		Functional annotation	
								Sequence components of *kabuli* genome	*Kabuli* gene accession IDs	NCBI-KOG	TFs
Ca3_P2_31901383	Ca3	31901383	CCTCTCTCTCTCTCTCTCTCTCTCT/CCTCTCTCTCTCTCT	GGAGCTGCTTCACCTTCAAC	ATCCAAAGGAACGCGATATG	59.9	426	INTERGENIC	--	--	--
Ca3_P2_31901719	Ca3	31901719	ATATTATTATTATTATTATTATTATTATTATTATTATTATTATTATTATTATTATT/ATATTATTATTATTATTATTATTATTATTATTATTATTATTATTATTATTATTATTATTATTATTATTATT	CGCGTTCCTTTGGATAATGT	TTCCCTTTCGTATTGCTTGG	60.1	616	INTERGENIC	--	--	--
Ca3_P2_31908295	Ca3	31908295	TAAAAAAAAAAAA/TAAAAAAAAAA	GAGGTGAAAATGGTGTAGATTAGAG	TCGTTCAACACCAAATGGAA	59.9	863	INTERGENIC	--	--	--
Ca3_P2_31912229	Ca3	31912229	GAAAA/GAAA	CTCGGATACTGCCGATTGAT	TGCCTGTTCTCTGTGATTGC	60.0	958	INTRON	Ca_12292	--	--
Ca3_P2_31939527	Ca3	31939527	TTATATATATATATATATATATATATAT/TTATATATATATATATATATATATATATATATAT	GCACGGAAACAGCGATTTTA	CAAAAATAAGACATGGTGTTGGA	58.9	246	INTERGENIC	--	--	--
Ca3_P2_31939882	Ca3	31939882	TAAAAA/TAAAA	GCACGGAAACAGCGATTTTA	GCGGTTGTCCGTCCTAATAA	60.0	896	INTERGENIC	--	--	--
Ca3_P2_3194968	Ca3	3194968	CA/CAA	CATCCAGGAAGGCAGAAAAC	GGAGGCAAACCAAGTGAAAA	60.1	201	INTERGENIC	--	--	--
Ca3_P2_3235341	Ca3	3235341	AGCTTCAGTCGCT/AGCT	TGGAGTGAGCTGTGGTTGAG	TGGTCACCACTTGGTGTTTG	60.5	840	INTERGENIC	--	--	--
Ca3_P2_3235471	Ca3	3235471	TAAA/TAA	ATAGGCCAAGCAACCAAAGA	GACGATGGTGAAGATGACGA	59.6	663	INTERGENIC	--	--	--
Ca3_P2_3235549	Ca3	3235549	GCACACA/GCACACACA	ATAGGCCAAGCAACCAAAGA	GACGATGGTGAAGATGACGA	59.6	663	INTERGENIC	--	--	--
Ca3_P2_3235914	Ca3	3235914	ATCTTCTTCATCCTCTTCTTCATCCTC/ATCTTCTTCATCCTC	TCGTCATCTTCACCATCGTC	CGCCAAAAAGTTGATTGGTT	60.0	251	INTERGENIC	--	--	--
Ca3_P2_32376435	Ca3	32376435	AACACA/AACACACA	TTTACGCAATCGAGATGCAC	TCACCGTGGCAAAACTGATA	60.1	443	INTERGENIC	--	--	--

INDEL marker IDs	Chromosomes/unanchored scaffolds	Physical positions (bp)	InDels (*Kabuli* reference genome-CDC Frontier/PII)	Forward primers (5'-3')	Reverse primers (5'-3')	Anneling temperature (0C)	Expected amplified product size (bp)	Structural annotation		Functional annotation	
								Sequence components of *kabuli* genome	*Kabuli* gene accession IDs	NCBI-KOG	TFs
Ca3_P2_32392706	Ca3	32392706	ATTTTTTTT/ATTTTTTTTT	CCAGATTAAGGAAAGGACACTTG	GCAAAGAAACCACACATGGA	59.5	927	INTERGENIC	--	--	--
Ca3_P2_32399895	Ca3	32399895	CATATATATATATATATATATATATATATATATAT/CATATATATATATATATATATATATATATATATATATATATAT	TTTCTTTCCGAAAATGGAGA	TGTGAATCCCGCTTTAACAA	59.2	523	INTERGENIC	--	--	--
Ca3_P2_32400390	Ca3	32400390	GATATATATATATATATATATATATATATATAT/GATATATATATATATATATATATATATATATAT	TGTTAAAGCGGGATTCACAA	ATTGCATTTCTGCACAACGA	60.3	196	INTERGENIC	--	--	--
Ca3_P2_32498219	Ca3	32498219	TA/TACA	GTTCAAAGGTGGAGTGGGAA	TGTGTGAATAAGTGAGACCTTTTGA	60.1	720	INTERGENIC	--	--	--
Ca3_P2_32563568	Ca3	32563568	TTATATATATATATATATAT/TTATATATATATATATATATATAT	CGAAAGAAAAAGAAAACATCGC	GAAGGTGGCTCGGCATATAA	60.1	574	INTERGENIC	--	--	--
Ca3_P2_32582344	Ca3	32582344	CGTGT/CGT	CAGAACTGATTCTTCCCCCA	TCTCGGGTAAGTAAAGGGCA	59.7	408	DRR	Ca_11984	P	--
Ca3_P2_32589900	Ca3	32589900	CTTTTTT/CTTTTTTTT	TCGATGCAATGATGATCAAA	TGGACACGACGGTGAAATAA	60.0	774	INTERGENIC	--	--	--
Ca3_P2_32604694	Ca3	32604694	CTTCTTGATTTATTTATTTCTTGAT/CTTCTTGATTTATTTATTTCTTGATTTATTTATTTCTTGAT	TCGGCACATCACAATCATTT	TTGGCTTGCTTCACACATTC	59.8	677	DRR	Ca_11988	NI	--
Ca3_P2_32629755	Ca3	32629755	TAAAAAAAA/TAAAAAAA	TTTTTGCGGTCCTTTGAATTA	GCGGTATTCACTCCAAACTGA	60.1	819	INTERGENIC	--	--	--
Ca3_P2_32683395	Ca3	32683395	ACTTTC/ACTTTCAATGCATCTTTATCTTTC	GGTGCATAGTATCACGTGCG	TTTGGGTAAATCAGGACCCA	60.2	550	INTERGENIC	--	--	--
Ca3_P2_32691639	Ca3	32691639	A/ACCT	AATACCCACATGGCCAAAAA	CCGACAACTATGTGCACCTTT	60.0	498	INTERGENIC	--	--	--
Ca3_P2_32697535	Ca3	32697535	CAAAAAAAAAAAAA/CAAAAAAAAAAAAAA	GGCCTGAGTCTGACCTACGA	TAGGCCTGACAGACCCATTT	59.6	701	INTERGENIC	--	--	--

INDEL marker IDs	Chromosomes/unanchored scaffolds	Physical positions (bp)	InDels (*Kabuli* reference genome-CDC Frontier/PII)	Forward primers (5'-3')	Reverse primers (5'-3')	Anneling temperature (0C)	Expected amplified product size (bp)	Structural annotation		Functional annotation	
								Sequence components of *kabuli* genome	*Kabuli* gene accession IDs	NCBI-KOG	TFs
Ca3_P2_3272993	Ca3	3272993	TTATATATATATATATATATATATATATATA/TTATATATATATATATATATATATATATA	TTGTAGTATCCCGACAATGTAAAA	TGATTCAACAATGATCCCCA	59.7	687	INTERGENIC	--	--	--
Ca3_P2_32789037	Ca3	32789037	ATTTTTTTTT/ATTTTTTTTTT	TGGAGAAAATGAAAGTAACGATTC	ACGTCGTTTGCTCCCTCTAA	59.9	710	INTERGENIC	--	--	--
Ca3_P2_32836823	Ca3	32836823	AATATATATATATATATATATATATATATATATATAT/AATATATATATATATATATATATATAT	TATGGATGAAGCTTTCGCCT	TGGTTATTTAATTTGGGCCATT	59.5	610	INTERGENIC	--	--	--
Ca3_P2_32853814	Ca3	32853814	CTTAATATGA/CTTAATATGATTAATATGA	GAAGGAATCATCATGTGGCA	TTGCTCAAAACCAAGCTTCA	59.6	891	INTRON	Ca_12021	O	--
Ca3_P2_32961119	Ca3	32961119	ATTTTT/ATTTT	TGAGAGGGGTTCATAAGCCT	TCCTACGCTTTCGCTTTCAT	60.0	409	INTERGENIC	--	--	--
Ca3_P2_33196113	Ca3	33196113	TAAAAAAA/TAAAAAA	CAGGGGCACAAATACATCAA	TTGCAATTCCTTGTGAACCA	60.1	334	INTERGENIC	--	--	--
Ca3_P2_33243720	Ca3	33243720	CTT/CT	GATGTGGCTGCAACAGCTTA	TGACAGACTCGTCGTTCAGG	60.0	462	CDS (FRAME SHIFT)	Ca_12059	--	--
Ca3_P2_33346296	Ca3	33346296	GATATATATATATATATATATATA/GATATATATATATATATATATATATA	ATTTTGCCACATGATACGCA	GCTTCACACGGAAACAACAA	59.7	531	DRR	Ca_23675	--	--
Ca3_P2_33351580	Ca3	33351580	ATATTTTATTTTATTTTATTTTATTTTAT/ATATTTTATTTTATTTTATTTTAT	CTGGTTTTCTTGGCCATTGT	CAAAGAAAATTGCGTTAGAAAGG	59.4	689	INTERGENIC	--	--	--
Ca3_P2_33387153	Ca3	33387153	AATATATATATATATATATATATATATATAT/AATATATATATATATATATATATATATATATATATATATATAT	ATACCTACGGATTTGTGCCG	TTTGTTGTTGAAGATGGCAA	57.7	553	INTERGENIC	--	--	--
Ca3_P2_33398638	Ca3	33398638	GAAAAAAAAA/GAAAAAAAAAA	AGATGATTGAGACGGTTGGG	GGGTAAATGCTTTGCCACAT	59.8	731	INTERGENIC	--	--	--
Ca3_P2_33423898	Ca3	33423898	T/TGAATAA	TTCAAAATTGTCCCTCCACC	TGAGGGAAGGACTTAAACTGTCA	60.2	382	DRR	Ca_23672	R	C2H2

INDEL marker IDs	Chromosomes/unanchored scaffolds	Physical positions (bp)	InDels (*Kabuli* reference genome-CDC Frontier/PII)	Forward primers (5'-3')	Reverse primers (5'-3')	Anneling temperature (0C)	Expected amplified product size (bp)	Structural annotation		Functional annotation	
								Sequence components of *kabuli* genome	*Kabuli* gene accession IDs	NCBI-KOG	TFs
Ca3_P2_33424399	Ca3	33424399	AA/AATA	TGACAGTTTAAGTCCTTCCCTCA	CATGATGGCAAGTTTGTTGG	60.0	684	DRR	Ca_23672	R	C2H2
Ca3_P2_33531472	Ca3	33531472	CAAA/CAAAA	TTGCCTTTGTATCACTGCCA	CTTGCCACTTGGTTGGAAAT	60.0	228	INTRON	Ca_23669	S	--
Ca3_P2_33545135	Ca3	33545135	C/CTATCAGTAAACATT	ACGTCATCAGTCGAAGTCCC	AGTTTGCGTAGGTTGTTGGG	60.0	329	INTERGENIC	--	--	--
Ca3_P2_33581359	Ca3	33581359	TAAAAAAAAAAA/TAAAAAAAAAA	TGGGTCAAGATTAGAGCCGT	CAATTTGACAAGCCCCCTAA	59.9	610	INTERGENIC	--	--	--
Ca3_P2_33646476	Ca3	33646476	GTTTT/GTTT	AAGGAAGCAACGAAAACGAA	ATTTTGGCTCGGAGGTTTTT	59.9	437	INTERGENIC	--	--	--
Ca3_P2_33662476	Ca3	33662476	TAAA/TA	ATGCCGTTTGGAATTACTCG	TTGGAAGGAGCCCTTACCTT	60.1	664	INTERGENIC	--	--	--
Ca3_P2_33748722	Ca3	33748722	CTTTTTTTTTTT/CTTTTTTTTTTTT	AGGTGCCAAAATTGATGAGG	CAAGTTGCGATGAACACCTG	60.3	399	INTERGENIC	--	--	--
Ca3_P2_33748791	Ca3	33748791	TAGTTTCAG/TAGTTTCAGTTTCAG	AGGTGCCAAAATTGATGAGG	CCTAGAAGGTCGCATCTCCA	60.4	417	INTERGENIC	--	--	--
Ca3_P2_33752607	Ca3	33752607	TAAAAA/TAAAAAA	TGCGATTCATCTCCTTCCTT	GAGTTCCAGGCGATTGTCTC	59.8	832	INTERGENIC	--	--	--
Ca3_P2_33828272	Ca3	33828272	TTTATTATTATTATTATTATTATTATTATTATTATTATTATTATTATTATTATT/TTTATTATTATTATTATTATTATTATTATTATTATTATTATTATTATTATTATTATT	AGAGGCAAACAAGAACCGAA	TGCATATAAGGAATTGACGCC	59.9	318	INTERGENIC	--	--	--
Ca3_P2_33830576	Ca3	33830576	GAAA/GAA	CTTTTATTCCGACCGAACCA	TTTGAAATGCAAAAACCTTCG	60.1	850	INTRON	Ca_20879	R	--
Ca3_P2_33859343	Ca3	33859343	TT/TTATTATTAT	CAATTGAATTCATTCTTCATACAGG	TTGGAAAATGTTGCAAGTTCA	59.2	978	INTERGENIC	--	--	--

INDEL marker IDs	Chromosomes/unanchored scaffolds	Physical positions (bp)	InDels (*Kabuli* reference genome-CDC Frontier/PII)	Forward primers (5'-3')	Reverse primers (5'-3')	Anneling temperature (0C)	Expected amplified product size (bp)	Structural annotation		Functional annotation	
								Sequence components of *kabuli* genome	*Kabuli* gene accession IDs	NCBI-KOG	TFs
Ca3_P2_33883185	Ca3	33883185	ATTTTTTTTTTTTTT/ATTTTTTTTTTTTT	CATGCCAGACCAGACTTTGA	AGGGGCCTATGGTCTGACTT	60.0	620	INTERGENIC	--	--	--
Ca3_P2_33929125	Ca3	33929125	ATTTTTTTTTTT/ATTTTTTTTTTTT	TGACGGGTTGTGGTTGTAGA	CCATCATTTACGTGGGAACC	60.1	830	INTERGENIC	--	--	--
Ca3_P2_3394940	Ca3	3394940	GAAAAA/GAAAA	TTTCAATTGCATTAAGTGGGA	CCCGGGAGTAGGAATTTGAT	60.1	498	INTERGENIC	--	--	--
Ca3_P2_33954528	Ca3	33954528	ATATTATTATTATTATTATTATTATTATTATTATTATTATTATTATTATTATTATTATTATT/ATATTATTATTATTATTATTATTATTATTATTATTATTATTATTATTATTATTATTATTATTATTATT	GGCTTCAAAGCCAACATCAT	TCATACAAAAACCAAACGGG	58.4	622	INTERGENIC	--	--	--
Ca3_P2_33983043	Ca3	33983043	TA/TAGACACTGA	GGTGTTTTACGAATGGGGTG	GGGACTCATTTATGCCGATG	60.3	353	INTERGENIC	--	--	--
Ca3_P2_34032860	Ca3	34032860	CCAAAGC/CC	TAAATGTTGCGAACCTGCTG	CGTCGCGGTTATCACTTACA	59.8	678	INTERGENIC	--	--	--
Ca3_P2_34057106	Ca3	34057106	GAAAA/GAAA	TCTTTGCGGCAGGTTTATTT	TCACGATTTCGTAAACGCAT	59.2	402	INTERGENIC	--	--	--
Ca3_P2_3411224	Ca3	3411224	CATATATATATATATATAT/CATATATATATATATATATATATATATAT	TGAATTTTTAAATGATTTTCTGCAA	ATAGTCGGCAAAACCCTCCT	60.0	785	INTERGENIC	--	--	--
Ca3_P2_34161169	Ca3	34161169	GAAAA/GAAAAA	GAAGCAAATCGATTGGGAAA	CTGGGGTTCCACTGTGACTT	60.0	239	INTERGENIC	--	--	--
Ca3_P2_34161241	Ca3	34161241	AG/A	GAAGCAAATCGATTGGGAAA	CTGGGGTTCCACTGTGACTT	60.0	239	INTERGENIC	--	--	--
Ca3_P2_34212501	Ca3	34212501	AATTATCTCTATTATCTC/AATTATCTC	TCCGAAAACGTTCGTACACA	AACGTTGTCACTTTCTTGGAGA	58.9	371	INTERGENIC	--	--	--
Ca3_P2_34229674	Ca3	34229674	GATATATATATATATATATATATATATAT/GATATATATATATATATATATATATATATATAT	GCATGATATGGATGTGCGAG	GGGGGCTTGTTAAACTGACA	60.0	856	INTERGENIC	--	--	--

INDEL marker IDs	Chromosomes/unanchored scaffolds	Physical positions (bp)	InDels (*Kabuli* reference genome-CDC Frontier/PII)	Forward primers (5'-3')	Reverse primers (5'-3')	Anneling temperature (0C)	Expected amplified product size (bp)	Structural annotation		Functional annotation	
								Sequence components of *kabuli* genome	*Kabuli* gene accession IDs	NCBI-KOG	TFs
Ca3_P2_3424341	Ca3	3424341	CAAAAAAAAA/CAAAAAAAAAA	TTGCTCATGCGGTCTATCAA	CGTTGACAAAGGGATCGAGT	60.1	600	INTERGENIC	--	--	--
Ca3_P2_34264150	Ca3	34264150	TAAAAAAAAA/TAAAAAAAA	AATGGAAAGAGCATTGGGTG	GCAAACAAACAAAAACACATTGA	59.9	709	INTERGENIC	--	--	--
Ca3_P2_34323944	Ca3	34323944	ATTTTTTTTT/ATTTTTTTTTT	TGGGTTTGTCCAATGGAGTT	AACTTCATGGTGTGGCCTCT	59.6	682	INTERGENIC	--	--	--
Ca3_P2_34365238	Ca3	34365238	T/TC	TGACCCAAAATGTGACTTGC	ATGCCAAGAATCCAACTCGT	59.6	865	INTERGENIC	--	--	--
Ca3_P2_34371009	Ca3	34371009	ATATGTAT/ATAT	TTTGACCATTGCTTTGCTTG	TGGAGCAAATCGGTAAGGTT	59.6	795	INTERGENIC	--	--	--
Ca3_P2_3438296	Ca3	3438296	CATATATATATATATATATAT/CATATATATATATATATATAT	ACGTTATTACACCTTGGCGG	TGGTCTTCCATCCTCAGACC	60.0	686	INTERGENIC	--	--	--
Ca3_P2_34456382	Ca3	34456382	AGG/AG	TCCCCTGTTTGGTTGTTGAT	GTAGTGTTGGTTTGTGGGGG	60.1	418	INTERGENIC	--	--	--
Ca3_P2_34474422	Ca3	34474422	TAAAAAAAA/TAAAAAAA	AAAAATCAATTTATGCCGTTGT	TTATGCCGGATTGACACTCTT	59.6	478	INTERGENIC	--	--	--
Ca3_P2_34520648	Ca3	34520648	GAA/GAAA	AAAGACGAAAGGGAACACCA	GAAATTGGAAACAAAGTCCGA	59.1	411	INTERGENIC	--	--	--
Ca3_P2_34520742	Ca3	34520742	ATTT/ATT	AAAGACGAAAGGGAACACCA	TCGAAGGACGTGATGTCATAG	58.7	775	INTERGENIC	--	--	--
Ca3_P2_34521509	Ca3	34521509	CTTTTTT/CTTTTTTT	ACCCATCTTGCGTTTTTCAG	TTAAATGGCGGCAAATTGAT	60.3	310	INTERGENIC	--	--	--
Ca3_P2_34525463	Ca3	34525463	CTTTTTTTTTTTTT/CTTTTTTTTTT	TTATCGGGGACAAATTCAGC	GCTTGGACAAATCAGCACAA	59.8	375	INTRON	Ca_00726	S	--

INDEL marker IDs	Chromosomes/unanchored scaffolds	Physical positions (bp)	InDels (*Kabuli* reference genome-CDC Frontier/PII)	Forward primers (5'-3')	Reverse primers (5'-3')	Anneling temperature (0C)	Expected amplified product size (bp)	Structural annotation		Functional annotation	
								Sequence components of *kabuli* genome	*Kabuli* gene accession IDs	NCBI-KOG	TFs
Ca3_P2_34531723	Ca3	34531723	A/AG	CTGAAATGGCACAGGTTCAA	ACAAGACAAGCAGCAGCTCA	59.9	605	INTRON	Ca_00726	S	--
Ca3_P2_34537757	Ca3	34537757	GTTA/GTTATTTA	TCATCAATGCCCTTTCCTTC	GGGCATTTATTCTGGCTTCA	60.0	550	INTRON	Ca_00726	S	--
Ca3_P2_34538569	Ca3	34538569	ACTCT/ACT	AGTAGCCTTTTCTGGCCCAT	TTGCAGCATTTTGTTACCCA	60.1	587	INTRON	Ca_00726	S	--
Ca3_P2_34540545	Ca3	34540545	GAAAA/GAAAAAAA	GCCGATATGATCATCAGCCT	ATCGATCATGGCTTCGTTTC	60.0	411	INTRON	Ca_00726	S	--
Ca3_P2_34542316	Ca3	34542316	ATTTT/ATTT	TGGGCGTTAGCAGCTAGTTT	TTTGTATCGCAAAGAACCCC	59.9	543	INTRON	Ca_00726	S	--
Ca3_P2_34545193	Ca3	34545193	TCCCCC/TCCCCCC	AGTCCTGGCTACTGCTGGAA	AACGATCTGCAACTAACCCG	60.1	413	INTRON	Ca_00726	S	--
Ca3_P2_34546901	Ca3	34546901	A/AC	GAGATCCACCCTCAACGTGT	GAGCAAAAATTGCAGCTTCC	60.0	406	INTRON	Ca_00726	S	--
Ca3_P2_34546963	Ca3	34546963	ATTCTTCTT/ATTCTT	GAGATCCACCCTCAACGTGT	GAGCAAAAATTGCAGCTTCC	60.0	406	INTRON	Ca_00726	S	--
Ca3_P2_34549339	Ca3	34549339	ATT/ATTT	TGACCTTGAGATGGGAAAGG	CACCAGATGAAGGAAGCACA	59.8	365	INTRON	Ca_00726	S	--
Ca3_P2_34557105	Ca3	34557105	CAAAAAAAA/CAAAAAAAAA	AAACTCGCACATCACATTCG	TGGAACTCGATTGTTCTTCG	58.8	850	DRR	Ca_00726	S	--
Ca3_P2_34557367	Ca3	34557367	GAAAAAAAAAA/GAAAAAAAAA	TGTTCAATTATTTTGGGATGGA	TCCTCCAATTTTCTTTCCCC	60.2	582	DRR	Ca_00726	S	--
Ca3_P2_34560809	Ca3	34560809	GAAAAAAA/GAAAAAAAA	GGCTCAGATCTTCCCACGTA	CATGCGATCGGAGATAATTG	59.1	307	INTERGENIC	--	--	--

INDEL marker IDs	Chromosomes/unanchored scaffolds	Physical positions (bp)	InDels (*Kabuli* reference genome-CDC Frontier/PII)	Forward primers (5'-3')	Reverse primers (5'-3')	Anneling temperature (0C)	Expected amplified product size (bp)	Structural annotation		Functional annotation	
								Sequence components of *kabuli* genome	*Kabuli* gene accession IDs	NCBI-KOG	TFs
Ca3_P2_34562955	Ca3	34562955	GTTTTATATTTTATAT/GTTTTATAT	GCTCCAAAAACAAGCAGAGG	AACTGGTCCAACACACCACA	59.9	652	INTERGENIC	--	--	--
Ca3_P2_34565884	Ca3	34565884	AG/AGG	TGCTTTGGGTTTCATTGGAT	CGAATTATATCTCCACCTAATTGTTTG	60.3	735	DRR	Ca_00727	GC	HSF
Ca3_P2_34565949	Ca3	34565949	AA/AAGA	TGCTTTAGTTTGGCTTTGGC	TGATAGGATTTGTTGGAAATCG	58.9	876	DRR	Ca_00727	GC	HSF
Ca3_P2_34568477	Ca3	34568477	CCTCCGA/CCTCCGACTCCGA	GTTTGTGCCCAGTCCAAAGT	ACAAACACAACGCCAAACAA	60.1	549	INTERGENIC	--	--	--
Ca3_P2_34571269	Ca3	34571269	GCGGTTC/GC	ATGAAAAGACCTTGCCATGC	TGGTGGTGTAATCGTGATGG	60.2	921	DRR	Ca_00728	GC	HSF
Ca3_P2_34582473	Ca3	34582473	TG/T	GGGGTGTTGAAGAATCGAGA	GGGAAATAGCGGTTTGTTCA	59.9	614	INTERGENIC	--	--	--
Ca3_P2_34584603	Ca3	34584603	CTTT/CT	TTGCATCCAATGGTGTCACT	TTTCCAGCTCTGAAAGCCAT	60.0	232	INTERGENIC	--	--	--
Ca3_P2_34591879	Ca3	34591879	GAAAAAAAAAAAAA/GAAAAAAAAAAA	TGGTAGTGATGCATGATTGGA	TGTTGGAATAGCGGTGGATT	60.3	754	INTERGENIC	--	--	--
Ca3_P2_34593590	Ca3	34593590	CTTT/CTTTT	CGACCAGTTTATCGGGCTTA	GGTCAATAGTCAGTGGCCGT	60.0	493	DRR	Ca_00732	--	B3
Ca3_P2_34601431	Ca3	34601431	TAAAAAAAAAAA/TAAAAAAAAAA	TCAGCGAAAAGCAACAAATG	TTGCCTTGGGTGTGTATTGA	60.0	528	INTRON	Ca_00732	--	B3
Ca3_P2_34605719	Ca3	34605719	CTTTTTAATTTTTTAATTTTTTAAT/CTTTTTAATTTTTTAAT	ATGTCTTTGGTTGGGCAGAG	GGGTTTGGAGGAGAGGAGAA	60.6	404	INTERGENIC	--	--	--
Ca3_P2_34605969	Ca3	34605969	TAAAAAAA/TAAAAAAAA	TTCTCCTCTCCTCCAAACCC	TTTGCCCTATAGGCACGTATT	58.7	456	INTERGENIC	--	--	--

INDEL marker IDs	Chromosomes/unanchored scaffolds	Physical positions (bp)	InDels (*Kabuli* reference genome-CDC Frontier/PII)	Forward primers (5'-3')	Reverse primers (5'-3')	Anneling temperature (0C)	Expected amplified product size (bp)	Structural annotation		Functional annotation	
								Sequence components of *kabuli* genome	*Kabuli* gene accession IDs	NCBI-KOG	TFs
Ca3_P2_34608015	Ca3	34608015	CTTTTTTTTT/CTTTTTTTTTTT	TGGAGAAACAAAATACGGGA	TTGGGTAGACATTTTCCCTTG	58.9	575	INTERGENIC	--	--	--
Ca3_P2_34608178	Ca3	34608178	ATTG/A	TGGAGAAACAAAATACGGGA	TTGGGTAGACATTTTCCCTTG	58.9	575	INTERGENIC	--	--	--
Ca3_P2_34608223	Ca3	34608223	CTTTTTTT/CTTTTTT	TGGAGAAACAAAATACGGGA	TTGGGTAGACATTTTCCCTTG	58.9	575	INTERGENIC	--	--	--
Ca3_P2_34617440	Ca3	34617440	ATTTT/ATT	TGGATGGTTAAAAATGGGGA	TTGCGGTAACAAACGTCAAA	60.1	525	INTERGENIC	--	--	--
Ca3_P2_34619372	Ca3	34619372	CAAA/CAA	CGGCTCAGTGATCCTCTTTC	CCAAGGGAAAAGATAACCAACA	60.2	334	INTERGENIC	--	--	--
Ca3_P2_34623607	Ca3	34623607	AATATATTAT/AAT	CCAAGCAAGTGGCATTATGA	ACGATTAGATTTGACCGAAAAA	57.8	457	INTERGENIC	--	--	--
Ca3_P2_34623825	Ca3	34623825	TC/T	CCAAGCAAGTGGCATTATGA	GGAGCCAAGCTCCAGTTATT	58.4	778	INTERGENIC	--	--	--
Ca3_P2_34623936	Ca3	34623936	ATTTTT/ATTTT	CAGATAATTTGGGCAAGAGAA	GGAGCCAAGCTCCAGTTATT	58.4	716	INTERGENIC	--	--	--
Ca3_P2_34624335	Ca3	34624335	TAAAAAAA/TAAAAAAAA	GAGCTTGGCTCCGAAAAA	TGGACTCAACAAATGAATCG	57.1	356	INTERGENIC	--	--	--
Ca3_P2_34626479	Ca3	34626479	CTTTT/CTTTTT	TTTGCAAACCAAATTCTTCAAA	CTTGTGCAATATCCGAAACG	59.2	562	DRR	Ca_00734	FH	--
Ca3_P2_34626639	Ca3	34626639	C/CAAATGATT	TTTGCAAACCAAATTCTTCAAA	GATGGGTCCTATTGGTGGAA	59.6	938	DRR	Ca_00734	FH	--
Ca3_P2_34632668	Ca3	34632668	TTA/TTATA	TGTTTTTGGGATTGTGAATTTCT	TTTGAGTGGGGTCGACGTAT	60.4	479	INTERGENIC	--	--	--

INDEL marker IDs	Chromosomes/unanchored scaffolds	Physical positions (bp)	InDels (*Kabuli* reference genome-CDC Frontier/PII)	Forward primers (5'-3')	Reverse primers (5'-3')	Anneling temperature (0C)	Expected amplified product size (bp)	Structural annotation		Functional annotation	
								Sequence components of *kabuli* genome	*Kabuli* gene accession IDs	NCBI-KOG	TFs
Ca3_P2_34636018	Ca3	34636018	TAACAACAACAACAA/TAACAACAACAACAACAA	TGATGATGTATGGGTGGTGG	CGTTTTTCAATGTGTGTCGG	60.0	749	INTRON	Ca_00735	--	--
Ca3_P2_34636156	Ca3	34636156	A/ATATTTG	TTCCAGATTTTGGTTGAGGC	CGTTTTTCAATGTGTGTCGG	60.0	476	INTRON	Ca_00735	--	--
Ca3_P2_34637631	Ca3	34637631	GAAAAAAAA/GAAAAAA	AAAAATTTGAAATGCTTGCTAAAG	TCTCACATCAAAAGGGTGGTT	59.4	656	INTERGENIC	--	--	--
Ca3_P2_34639121	Ca3	34639121	AC/A	CGTTAATCATCCCAAAACCG	GAGCGAAGAAACAACGGAAC	59.9	726	INTERGENIC	--	--	--
Ca3_P2_34640762	Ca3	34640762	T/TC	GCAATGAGCACTTCAGGGAT	AACAGTTCCATCACCAAGGC	60.0	223	INTRON	Ca_00736	S	--
Ca3_P2_34644909	Ca3	34644909	T/TTA	TTGAAGCCAGATTTTCCACC	AAATGGATCGCCACTCTGTC	60.1	478	DRR	Ca_00736	S	--
Ca3_P2_34645157	Ca3	34645157	TACAACAACAACAACAACAA/TACAACAACAACAACAA	GACAGAGTGGCGATCCATTT	GTGTTGCACCAATAACACGG	59.9	651	INTRON	Ca_00737	--	--
Ca3_P2_34648656	Ca3	34648656	TTTTA/TTTTATTAATTTA	CAAACCATTTTCTCTCCATTCC	TCTTCCACGGCCAATAGTTC	60.1	684	DRR	Ca_00738	J	--
Ca3_P2_34657286	Ca3	34657286	AGGG/AGGGG	CAACTGTGAGCTTATCCGCA	AGAGAGAGTGGCGCGTGTAT	60.0	618	INTERGENIC	--	--	--
Ca3_P2_34657306	Ca3	34657306	GTTTT/GTTTTT	CAACTGTGAGCTTATCCGCA	AGAGAGAGTGGCGCGTGTAT	60.0	618	INTERGENIC	--	--	--
Ca3_P2_34657606	Ca3	34657606	CTTTTTTTTTT/CTTTTTTTTT	ATACACGCGCCACTCTCTCT	ACTCACTTTTGGCGATGTCA	59.3	258	INTERGENIC	--	--	--
Ca3_P2_34658972	Ca3	34658972	GTTCTTTCTTTCTTTCTTT/GTTCTTTCTTTCTTTCTTTCTTT	CACTCCGGCTTCTCTTTACG	AGCAAACACCGGTCAAAAAC	60.0	683	INTRON	Ca_00740	AJ	NAC

INDEL marker IDs	Chromosomes/unanchored scaffolds	Physical positions (bp)	InDels (*Kabuli* reference genome-CDC Frontier/PII)	Forward primers (5'-3')	Reverse primers (5'-3')	Anneling temperature (0C)	Expected amplified product size (bp)	Structural annotation		Functional annotation	
								Sequence components of *kabuli* genome	*Kabuli* gene accession IDs	NCBI-KOG	TFs
Ca3_P2_34660669	Ca3	34660669	GAA/GAAA	TCTTGCACCAGCAATTTGAG	TAAACCCAATGGATAGCCGA	60.3	824	INTRON	Ca_00740	AJ	NAC
Ca3_P2_34665269	Ca3	34665269	TGGGGG/TGGGG	CAAGAAGGTGGGAGGATTCA	TTGGTACAAGCCCAGACAAA	59.2	593	INTERGENIC	--	--	--
Ca3_P2_34669424	Ca3	34669424	GTTTTTTT/GTTTTTTTT	AAAGGGCACTTGAGAAAGCA	GCAGCCTTAAATTTTTCCCC	59.9	454	CDS (FRAME SHIFT)	Ca_00741	T	--
Ca3_P2_34674867	Ca3	34674867	TAAAAAA/TAAAAAAA	GGAGGAACTCAAACCCACAA	GTTTCGACTGGTGCAGATCA	59.8	659	INTERGENIC	--	--	--
Ca3_P2_34676663	Ca3	34676663	GTTTTTTTTTTT/GTTTTTTTTTT	TCAACTTTGCAGGCCCTATT	TGAAAATCACAGTTATTGGCG	58.7	586	DRR	Ca_00742	T	bHLH
Ca3_P2_34680558	Ca3	34680558	T/TAAAA	GCTGAAGCGATTGTGTTTGA	AGAAGATTAGCATGGCCCCT	60.1	595	INTERGENIC	--	--	--
Ca3_P2_34681160	Ca3	34681160	ATAAAATTAAAAT/ATAAAATTAAAATTAAAAT	AGGGGCCATGCTAATCTTCT	TTTGAAGGGAGAAATCCATCA	59.5	552	DRR	Ca_00743	V	GRAS
Ca3_P2_34681675	Ca3	34681675	GTTTTTTT/GTTTTTT	TGATGGATTTCTCCCTTCAAA	TTGGTTGTGGACCTGTGGTA	59.8	475	DRR	Ca_00743	V	GRAS
Ca3_P2_34685118	Ca3	34685118	TAAAAAAA/TAAAAAAAA	TGGATAGAGGATTGAACCAGTAA	TGTCCACCAAACGAGACATAA	59.0	512	INTERGENIC	--	--	--
Ca3_P2_34685742	Ca3	34685742	TAAAAAAA/TAAAAAA	TTATGTCTCGTTTGGTGGACA	TTAGTGACATGCGTGGCTTC	59.9	586	INTERGENIC	--	--	--
Ca3_P2_34686333	Ca3	34686333	ATTTTT/ATTTT	GAAGCCACGCATGTCACTAA	GGGAGTGTGGAGAGAATGGA	60.0	431	INTERGENIC	--	--	--
Ca3_P2_34689105	Ca3	34689105	AG/AGG	TCGATCCGACACTTTCCTTC	ATGCGCAAAAGAACTCACAA	59.5	588	INTERGENIC	--	--	--

INDEL marker IDs	Chromosomes/unanchored scaffolds	Physical positions (bp)	InDels (*Kabuli* reference genome-CDC Frontier/PII)	Forward primers (5'-3')	Reverse primers (5'-3')	Anneling temperature (0C)	Expected amplified product size (bp)	Structural annotation		Functional annotation	
								Sequence components of *kabuli* genome	*Kabuli* gene accession IDs	NCBI-KOG	TFs
Ca3_P2_34693848	Ca3	34693848	CAAAA/CAAAAA	GCCCTTCACATGCTGGTATT	CCGGATTTGAACTTTCCTGA	60.0	493	DRR	Ca_00745	O	--
Ca3_P2_34717035	Ca3	34717035	CAAAATTAAAATT/CAAAATTAAAATTAAAATT	AAAACATGTAGATCCATTTGAGTGA	AACTTTTTGGCTTTCCGGTT	60.0	640	INTERGENIC	--	--	--
Ca3_P2_34718760	Ca3	34718760	ATTTT/ATTT	TTTGGAAGATTTCCCTCGTG	TTCAAAGGGAAAACGACACC	59.9	757	INTRON	Ca_00746	GMW	ERF
Ca3_P2_34721625	Ca3	34721625	ACTATCATTCTATCA/ACTATCA	CAATCCGTTTAGAACTCCCG	TGTTGCACGTAGCCTAAGAAA	58.6	625	DRR	Ca_00746	GMW	ERF
Ca3_P2_34722333	Ca3	34722333	ATT/AT	CTCAAACACGGACACCCTTT	CCTTCTTCTCGTTCGTTTGC	60.0	364	DRR	Ca_00746	GMW	ERF
Ca3_P2_34723546	Ca3	34723546	ATTTTTTTTTTTTT/ATTTTTTTTTTTTTTT	TGGAGGACATTTGGAGGAAG	AGCCCAAGTTCCAAACAATG	60.0	382	DRR	Ca_00747	--	--
Ca3_P2_34726598	Ca3	34726598	ATTTTTTTT/ATTTTTTTTT	AATCGGTAGTTGTCATCCGC	AGGGGTGCCTGTAGTCTCAA	59.7	948	INTERGENIC	--	--	--
Ca3_P2_34727897	Ca3	34727897	CGT/CGTGT	TTCCACCAAAGCTTTCATCC	AAGTGTGGCCAAACCAAGTC	60.0	294	INTRON	Ca_00748	--	--
Ca3_P2_34730315	Ca3	34730315	GTATA/GTATATA	ATTTTGGGCCCTCTTCTCAT	AGCCAGCCAAAACAGACACT	59.9	647	INTRON	Ca_00748	--	--
Ca3_P2_34731798	Ca3	34731798	TAAAAAAAA/TAAAAAAAAAA	TTTTCTGCCAAAATCATCCC	AAGAAAGAGTACCGTCGCCA	59.9	671	DRR	Ca_00748	--	--
Ca3_P2_34732162	Ca3	34732162	TAAA/TAAAAA	TGGCGACGGTACTCTTTCTT	CACACTTTTAGGCCACCCAT	59.9	389	DRR	Ca_00748	--	--
Ca3_P2_34735063	Ca3	34735063	C/CAGAT	TGGTTGTTGGAGGAATGTGA	TCGGTCCGTTTTCATATTGG	60.7	871	INTERGENIC	--	--	--

INDEL marker IDs	Chromosomes/unanchored scaffolds	Physical positions (bp)	InDels (*Kabuli* reference genome-CDC Frontier/PII)	Forward primers (5'-3')	Reverse primers (5'-3')	Anneling temperature (0C)	Expected amplified product size (bp)	Structural annotation		Functional annotation	
								Sequence components of *kabuli* genome	*Kabuli* gene accession IDs	NCBI-KOG	TFs
Ca3_P2_34735388	Ca3	34735388	ATT/ATTT	GCGACGAGTTGAATGTAAATG	GTTGATTGTTCCGCGAATTT	59.9	278	INTERGENIC	--	--	--
Ca3_P2_34735825	Ca3	34735825	CTTTTTTTTTT/CTTTTTTTTT	AAATTCGCGGAACAATCAAC	CTTCACAAAATTGCAAGCGT	59.0	556	INTRON	Ca_00749	J	--
Ca3_P2_34737485	Ca3	34737485	CTTTTTTT/CTTTTTT	TGCTCTTCGGTTCGTTTCTT	ACCCAAGGATACAATGCCAA	60.2	650	DRR	Ca_00749	J	--
Ca3_P2_34739285	Ca3	34739285	TT/TTGGT	AGGTAGAGCCAGTGAGGTGG	TCTTTGGATGTGGTGGAACA	59.9	841	DRR	Ca_00749	J	--
Ca3_P2_34739482	Ca3	34739482	ATTTT/ATTT	AGGCAATTCAAAGTTAAAGGTG	TCTTTGGATGTGGTGGAACA	59.9	508	INTERGENIC	--	--	--
Ca3_P2_34739896	Ca3	34739896	AATT/A	ATCGGATTGTTCCACCACAT	TTCTACTTGACACCCCCACC	59.8	384	INTERGENIC	--	--	--
Ca3_P2_34743651	Ca3	34743651	TAAA/TAA	TGCGAGGGAATTCAAATAGG	GAGGAAGTAATGGACAAACACAAA	59.5	201	INTRON	Ca_00750	K	HD-ZIP
Ca3_P2_34745881	Ca3	34745881	TGGG/TGGGG	CAGAAAATGGTTTAAAGACCCC	AAAAACAAACGTGTAGACTAAAAAGC	58.6	340	INTERGENIC	--	--	--
Ca3_P2_34745952	Ca3	34745952	CTTTTTTT/CTTTTTTTT	CAGAAAATGGTTTAAAGACCCC	AAAAACAAACGTGTAGACTAAAAAGC	58.6	340	INTERGENIC	--	--	--
Ca3_P2_34746354	Ca3	34746354	GAT/GATCAT	TGGTGTATAACTAAGAGACACGTCAA	GGGGAGGAGGAAGAAGCTC	60.3	810	INTERGENIC	--	--	--
Ca3_P2_34746515	Ca3	34746515	CAA/CA	GCTTTTTAGTCTACACGTTTGTTTTT	ATTTGCATGAGGTAGCCAGC	60.2	746	INTERGENIC	--	--	--
Ca3_P2_34749435	Ca3	34749435	TC/T	TGGGGGAGAGACGTCATAAT	TGTCCTCGACATTTCTCTCCA	60.8	834	INTERGENIC	--	--	--

INDEL marker IDs	Chromosomes/unanchored scaffolds	Physical positions (bp)	InDels (*Kabuli* reference genome-CDC Frontier/PII)	Forward primers (5'-3')	Reverse primers (5'-3')	Anneling temperature (0C)	Expected amplified product size (bp)	Structural annotation		Functional annotation	
								Sequence components of *kabuli* genome	*Kabuli* gene accession IDs	NCBI-KOG	TFs
Ca3_P2_34749745	Ca3	34749745	A/ACCTTAT	CGTCAATATTTGTTGCAGGC	TATGGGTGGTTCCATTCCAC	60.4	332	INTERGENIC	--	--	--
Ca3_P2_34751724	Ca3	34751724	TAA/TAAAATAAA	TCCCTCTCTGCAGGAAAGTC	ATGAGACTCCAACCCACGTC	60.0	260	INTERGENIC	--	--	--
Ca3_P2_34753443	Ca3	34753443	TAA/TAACAA	TCCAAAACCACAACAACCAA	CTAAAATGTCCCCCGTCTCA	59.9	689	INTERGENIC	--	--	--
Ca3_P2_34755544	Ca3	34755544	T/TA	TTTTTCCATTTTACCGGTCG	TTTTCAAGTGGCATCAACCA	60.1	587	DRR	Ca_00751	--	HD-ZIP
Ca3_P2_34759483	Ca3	34759483	AACACA/AACACACA	CTGCAAAACCCTAACACCGT	CTCCGTTTAATGCCTACGGA	60.1	176	INTRON	Ca_00751	--	HD-ZIP
Ca3_P2_34760720	Ca3	34760720	T/TG	AACGATCTAACGGTTGTAATTGA	TGCACTTTTTGTTCTCTTATTTTGTC	60.1	579	INTERGENIC	--	--	--
Ca3_P2_34762413	Ca3	34762413	T/TA	GGTGCTCAAAATGGTCGAGT	GCTCCTGGTTTTGTCTTTGC	59.9	360	INTERGENIC	--	--	--
Ca3_P2_34763162	Ca3	34763162	CAAATAAAAATAAAA/CAAATAAAAATAAAAATAAAAATAAAA	TTCTCATGGTTGACGACTGC	GCAAATCCTCCTCTCACACA	58.8	937	INTERGENIC	--	--	--
Ca3_P2_34764284	Ca3	34764284	ATTTTTTTTTTTTT/ATTTTTTTTTTT	ATTTGGTGCAAGCACATGAA	TCGACAAAATGACCAAATTGA	59.0	294	INTERGENIC	--	--	--
Ca3_P2_34764651	Ca3	34764651	AATATAT/AATATATAT	TGAGGATGAATTTGGTGCAA	TGAGGGTAAAATGTGGAGGG	59.8	821	INTERGENIC	--	--	--
Ca3_P2_34770011	Ca3	34770011	CTTTTTTTTTT/CTTTTTTTT	TGCTTGTGAGACCTATTGCG	TATGGCGGGCAATTAAAAAG	59.9	483	INTRON	Ca_00753	G	MYB_related
Ca3_P2_34777528	Ca3	34777528	T/TG	AGAGACCGAAGGAACGAACA	CCCAAATCTTATTGGGAGCA	59.9	514	INTERGENIC	--	--	--

INDEL marker IDs	Chromosomes/unanchored scaffolds	Physical positions (bp)	InDels (*Kabuli* reference genome-CDC Frontier/PII)	Forward primers (5'-3')	Reverse primers (5'-3')	Anneling temperature (0C)	Expected amplified product size (bp)	Structural annotation		Functional annotation	
								Sequence components of *kabuli* genome	*Kabuli* gene accession IDs	NCBI-KOG	TFs
Ca3_P2_34784573	Ca3	34784573	T/TGG	TTGTGTTCAAATATCACCGCTC	ACGTGTTTGGTGAAAACGAA	59.1	380	INTERGENIC	--	--	--
Ca3_P2_34784668	Ca3	34784668	T/TA	TTGTGTTCAAATATCACCGCTC	ACGTGTTTGGTGAAAACGAA	59.1	380	INTERGENIC	--	--	--
Ca3_P2_34786765	Ca3	34786765	ACTTTTTTTT/ACTTTTTTTTTATCTTTTTTTT	CCAAGCGATAAAACAAAAGCA	GAACTTTCCGAAGTTGAGCG	60.0	355	INTERGENIC	--	--	--
Ca3_P2_34788229	Ca3	34788229	CATATATAT/CATAT	GAGCGAGCCTGTTCTGCTAT	TGGAGACGAGACACCAAATC	58.6	663	INTERGENIC	--	--	--
Ca3_P2_34789290	Ca3	34789290	TTACAAAAT/TT	GCAATGTTGGGACACTGATG	ATGATGCACAATTTCCTGCC	60.9	302	INTERGENIC	--	--	--
Ca3_P2_34789632	Ca3	34789632	AACTA/AACTAGTACTA	ATTGTGGCAGGAAATTGTGC	CCATCCCAAAAGATCCTCAA	59.9	676	INTERGENIC	--	--	--
Ca3_P2_34789772	Ca3	34789772	TA/TAACAA	ATTGTGGCAGGAAATTGTGC	CCATCCCAAAAGATCCTCAA	59.9	676	INTERGENIC	--	--	--
Ca3_P2_34790104	Ca3	34790104	TTATA/TTATATA	TTGAGGATCTTTTGGGATGG	GGGTTGATGCCTCACTCTAAA	59.2	333	INTERGENIC	--	--	--
Ca3_P2_34790577	Ca3	34790577	CATATTAATATTAAT/CATATTAATATTAATATTAAT	CCTCACAGCTGCCCCTATTA	AAAACACACAGGGCATGATAA	58.0	692	INTERGENIC	--	--	--
Ca3_P2_34791845	Ca3	34791845	TTCTCTCTCTCTCTCTCTCTCTCTC/TTCTCTCTCTCTCTCTCTCTCTCTC	AGCAAGAGAATGACACCCCA	AGGAAGAGGAATACGGGGAA	59.9	683	INTERGENIC	--	--	--
Ca3_P2_34793167	Ca3	34793167	TAATAATGAAT/TAAT	CTAACGTGGCGGATTTGTTT	CGTGGGACAAGAAGTGGAAT	60.0	660	INTRON	Ca_00755	Q	B3
Ca3_P2_34795848	Ca3	34795848	TAAAAAAAAAAAAA/TAAAAAAAAAA	CAAGATCTCGAAGCCAAAGG	CCAAACCAAAAAGATTCCCA	59.8	868	INTERGENIC	--	--	--

INDEL marker IDs	Chromosomes/unanchored scaffolds	Physical positions (bp)	InDels (*Kabuli* reference genome-CDC Frontier/PII)	Forward primers (5'-3')	Reverse primers (5'-3')	Anneling temperature (0C)	Expected amplified product size (bp)	Structural annotation		Functional annotation	
								Sequence components of *kabuli* genome	*Kabuli* gene accession IDs	NCBI-KOG	TFs
Ca3_P2_34796964	Ca3	34796964	GA/GTGTTATTTCA	ATGCACTCAAACCAACCACA	GGCCGCTCTATTTAAGGCAT	60.6	410	INTERGENIC	--	--	--
Ca3_P2_34797791	Ca3	34797791	GAAAA/GAAAAA	TTGTCAAACATTGCAACAAGAA	CACACTGAAGGTCTCCGGTT	60.2	902	INTERGENIC	--	--	--
Ca3_P2_34797856	Ca3	34797856	GTTTTTTTT/GTTTTTT	TTGTCAAACATTGCAACAAGAA	CACACTGAAGGTCTCCGGTT	60.2	902	INTERGENIC	--	--	--
Ca3_P2_34799142	Ca3	34799142	CAAAAAAA/CAAAAAA	CAATTGAACCTACGTGGGACT	AAGTGTGGCAAGCTCTTGGT	59.9	344	DRR	Ca_00756	--	--
Ca3_P2_34806753	Ca3	34806753	CT/CTTT	GTTTCGATTTCTGGAGCTGG	CCAAAATTGAATCAAACCACA	58.4	351	DRR	Ca_00757	--	--
Ca3_P2_34811902	Ca3	34811902	AAGCTA/AAGCTAGCTA	CACACTCGTGTTTGCCTCTT	TGCATGTGAAAAGAATGGGA	60.0	623	INTERGENIC	--	--	--
Ca3_P2_34815185	Ca3	34815185	TG/T	CACACTTGTGGTTGAGGCTG	GGAAGCAGAGCTGGAATGTC	60.0	473	INTERGENIC	--	--	--
Ca3_P2_34817190	Ca3	34817190	GTT/GT	CTCAAAACTGTCGGTGGGTT	CCATGCCTTTTACCTCCAAA	59.9	605	INTRON	Ca_00758	--	--
Ca3_P2_34818238	Ca3	34818238	TAAAAAAAAA/TAAAAAAAA	TGGGGAAAAGTTCAATTTCTACA	ATTGCGATCCGAAAATCAAC	59.9	320	INTRON	Ca_00758	--	--
Ca3_P2_34818325	Ca3	34818325	TTATATATATATAT/TTATATATATAT	TGGGGAAAAGTTCAATTTCTACA	ATTGCGATCCGAAAATCAAC	59.9	320	INTRON	Ca_00758	--	--
Ca3_P2_34818875	Ca3	34818875	AAATAATAATAAT/AAATAATAATAATAATAAT	TTGTTGTCGCCATCATTGTT	ACTCCCGCAGTCCCTATTCT	60.1	455	INTRON	Ca_00758	--	--
Ca3_P2_34821783	Ca3	34821783	CAAAAAAAAAAA/CAAAAAAAAAA	ATTGTCACGTGAGTTGCTGG	ACTAACCCTCACCACCCTCC	60.2	498	DRR	Ca_00758	--	--

INDEL marker IDs	Chromosomes/unanchored scaffolds	Physical positions (bp)	InDels (*Kabuli* reference genome-CDC Frontier/PII)	Forward primers (5'-3')	Reverse primers (5'-3')	Anneling temperature (0C)	Expected amplified product size (bp)	Structural annotation		Functional annotation	
								Sequence components of *kabuli* genome	*Kabuli* gene accession IDs	NCBI-KOG	TFs
Ca3_P2_34832562	Ca3	34832562	TAAAAAAAAAAA/TAAAAAAAAAAAAAAA	TCCGTTAGGCAAAGAGCATT	GAAGGGAGGTGAGGTGATGA	60.0	458	INTERGENIC	--	--	--
Ca3_P2_34835748	Ca3	34835748	TC/T	ATGCATTCACCAACCCATTT	TGTTATTGGCCTTCGCTTTC	60.2	504	INTERGENIC	--	--	--
Ca3_P2_34835888	Ca3	34835888	GAAAAAAAAAAA/GAAAAAAAAA	ATGCATTCACCAACCCATTT	TGTTATTGGCCTTCGCTTTC	60.2	504	INTERGENIC	--	--	--
Ca3_P2_34836958	Ca3	34836958	TTTTTTTAAATTTTTTA/TTTTTTTA	GTGGTAATCCAACCGTCGAG	GAACCGATCCAACATGCTTT	59.9	443	INTRON	Ca_00761	G	--
Ca3_P2_34837304	Ca3	34837304	AATACACACATACACA/AATACACACATACACACATACACA	AAAGCATGTTGGATCGGTTC	GCGATGATGCTGTTCACATT	59.7	507	INTRON	Ca_00761	G	--
Ca3_P2_34840194	Ca3	34840194	TGG/TG	GAAGCCATGAAGATGGGTGT	AAGCCACCTTCATCACCAAC	60.0	402	INTRON	Ca_00761	G	--
Ca3_P2_34840396	Ca3	34840396	CTTTTTTTTTTTT/CTTTTTTTTT	GTTGGTGATGAAGGTGGCTT	ATCCGGTCCCCATAACTTTC	60.0	457	INTRON	Ca_00761	G	--
Ca3_P2_34840506	Ca3	34840506	GTTTTTTTTTT/GTTTTTTTT	GTTGGTGATGAAGGTGGCTT	ATCCGGTCCCCATAACTTTC	60.0	457	INTRON	Ca_00761	G	--
Ca3_P2_34844133	Ca3	34844133	TAAAAAAAAAA/TAAAAAAAAAAAA	AGGATGAAACAATTGCCAAA	TAAAAGCCCACAGAGCAACC	60.2	546	DRR	Ca_00761	G	--
Ca3_P2_34845314	Ca3	34845314	GCAAA/G	ATGGGTACACACGAAAAGCA	ACACCCCCGTACTTCAAACA	60.3	563	INTERGENIC	--	--	--
Ca3_P2_34845361	Ca3	34845361	ATTTT/ATTT	ATGGGTACACACGAAAAGCA	ACACCCCCGTACTTCAAACA	60.3	563	INTERGENIC	--	--	--
Ca3_P2_34850302	Ca3	34850302	AA/AACTCTA	CTATGGGGCGAGGTTAACAA	CCCATTGGAAGAGAACCTCA	60.0	587	DRR	Ca_00762	A	C3H

INDEL marker IDs	Chromosomes/unanchored scaffolds	Physical positions (bp)	InDels (*Kabuli* reference genome-CDC Frontier/PII)	Forward primers (5'-3')	Reverse primers (5'-3')	Anneling temperature (0C)	Expected amplified product size (bp)	Structural annotation		Functional annotation	
								Sequence components of *kabuli* genome	*Kabuli* gene accession IDs	NCBI-KOG	TFs
Ca3_P2_34851745	Ca3	34851745	GAAAAAAAA/GAAAAAAA	ACAAACCCTTCACTTGGCAC	GGTGGCCATGTTGATACTCC	60.2	375	INTRON	Ca_00763	--	C2H2
Ca3_P2_34851854	Ca3	34851854	TATAA/TATAATAA	ACAAACCCTTCACTTGGCAC	ACCGCAAACTCTATGTTGGG	60.0	648	INTRON	Ca_00763	--	C2H2
Ca3_P2_34852048	Ca3	34852048	CTACTTTA/CTACTTTACTTTA	ACAAACCCTTCACTTGGCAC	ACCGCAAACTCTATGTTGGG	60.0	648	INTRON	Ca_00763	--	C2H2
Ca3_P2_34855207	Ca3	34855207	GAAA/GAAAACAGCGATAAA	ACCGTTGAAAGGTGGACAAG	TTGAAATCATCTCGAAGGGG	60.0	824	INTRON	Ca_00763	--	C2H2
Ca3_P2_34856152	Ca3	34856152	TAAAAAAAAAA/TAAAAAAAAAAAAAA	AAAAGCGTTATCACATCCGC	GTGGAGCCAAGCATTTCTTC	59.8	192	INTRON	Ca_00763	--	C2H2
Ca3_P2_34857328	Ca3	34857328	T/TG	AAAAGAGGGCAAAGTTCGATT	TGATCCCCTTTCCATGTGAT	60.1	667	INTERGENIC	--	--	--
Ca3_P2_34861205	Ca3	34861205	GT/GTGTGTCT	GATTTGTATGCGAAGCGGAT	GAAGGGATGAAAGCCAATGA	60.0	387	INTRON	Ca_00764	A	C2H2
Ca3_P2_34866347	Ca3	34866347	CTATT/CTATTATT	TACCATGATGATGAAGCGGA	TCAACCGAATCAACAACCAA	59.9	308	DRR	Ca_00764	A	C2H2
Ca3_P2_34866807	Ca3	34866807	AC/A	TTGGTTGTTGATTCGGTTGA	GTCGTCGGCAAAATTAAAGG	59.6	487	DRR	Ca_00764	A	C2H2
Ca3_P2_34867338	Ca3	34867338	T/TA	TTTTGCCGACGACTCTTATTC	AAGAGGTTGGAATGTGACCG	60.0	677	DRR	Ca_00764	A	C2H2
Ca3_P2_34869900	Ca3	34869900	T/TAA	TGGCCCTAGCAAAAGAAAAA	TGAGCTGATAACCGATGAAAAA	59.7	631	INTERGENIC	--	--	--
Ca3_P2_34870723	Ca3	34870723	ATTTTTTT/ATTTTTT	CTGGGACACAATCCCAAACT	CGGTGAGTCACTATACTCCCTCTT	60.1	666	INTERGENIC	--	--	--

INDEL marker IDs	Chromosomes/unanchored scaffolds	Physical positions (bp)	InDels (*Kabuli* reference genome-CDC Frontier/PII)	Forward primers (5'-3')	Reverse primers (5'-3')	Anneling temperature (0C)	Expected amplified product size (bp)	Structural annotation		Functional annotation	
								Sequence components of *kabuli* genome	*Kabuli* gene accession IDs	NCBI-KOG	TFs
Ca3_P2_34872921	Ca3	34872921	ATTTTTT/ATTTTTTT	CACAAATGCGATTAAGGGCT	CATGTTGAATGGAAGCACACA	60.6	685	INTERGENIC	--	--	--
Ca3_P2_34873119	Ca3	34873119	CATAATT/C	TACGACTCAACGAAGGGACC	AAACATAATCCCTGCCATCG	59.8	835	INTERGENIC	--	--	--
Ca3_P2_34873728	Ca3	34873728	TA/T	AACACATTTTTGCAAGTGCG	GGCTTCTGAAAAACACAGCC	59.9	728	INTERGENIC	--	--	--
Ca3_P2_34873766	Ca3	34873766	CAAAAAAA/CAAAAAA	AACACATTTTTGCAAGTGCG	GGCTTCTGAAAAACACAGCC	59.9	728	INTERGENIC	--	--	--
Ca3_P2_34877612	Ca3	34877612	AATATATATAT/AATATATAT	GACGCATTCTCACAACGAGA	TTGTTACGGTTGTGAAGCAAA	59.3	823	DRR	Ca_00765	--	--
Ca3_P2_34877774	Ca3	34877774	ATTTTTTTTT/ATTTTTTTT	ACCAACTGGGGAAACAAGTG	TTGTTACGGTTGTGAAGCAAA	59.3	550	DRR	Ca_00765	--	--
Ca3_P2_34880048	Ca3	34880048	ACCC/ACC	TCTTCGAGGATGGTGATGTG	TGCCCCCGTCTATCATAATC	59.7	107	INTERGENIC	--	--	--
Ca3_P2_34884069	Ca3	34884069	GTT/GAAACTT	TGGTGCAATAAGCTCACCAC	CAGCAAGGAGTTCAAATCCAA	60.2	379	DRR	Ca_00766	--	CPP
Ca3_P2_34884996	Ca3	34884996	CAAAAAAAAAA/CAAAAAAAAA	TTTCGCTCATTCTCCTCCAT	TGAAAACAAAATTGCCCAAA	59.0	531	DRR	Ca_00766	--	CPP
Ca3_P2_34885342	Ca3	34885342	TAAAAAAAAAAA/TAAAAAAAAAA	TTTGGGCAATTTTGTTTTCA	GGAACTTGCACGGTGACTTT	60.2	459	DRR	Ca_00766	--	CPP
Ca3_P2_34887394	Ca3	34887394	GT/GTT	TCTTCCACACAAAACCCTCC	ACGTGCATCACAAAACCAAA	60.0	571	INTRON	Ca_00767	G	ERF
Ca3_P2_34887847	Ca3	34887847	C/CG	CCATCCCTTAAGGTTGGGAT	GGACGAGCCTTACAGCAAAC	59.9	443	INTRON	Ca_00767	G	ERF

INDEL marker IDs	Chromosomes/unanchored scaffolds	Physical positions (bp)	InDels (*Kabuli* reference genome-CDC Frontier/PII)	Forward primers (5'-3')	Reverse primers (5'-3')	Anneling temperature (0C)	Expected amplified product size (bp)	Structural annotation		Functional annotation	
								Sequence components of *kabuli* genome	*Kabuli* gene accession IDs	NCBI-KOG	TFs
Ca3_P2_34890320	Ca3	34890320	CTACTTA/CTA	ATCGGCCTTCTTCCACTTTT	TTGTAACCAAAAAGCCCAGC	60.1	748	DRR	Ca_00767	G	ERF
Ca3_P2_34890533	Ca3	34890533	TAAAAAAAAA/TAAAAAAAAAA	GTGCTACTGAAGCAAACGCA	TTGTAACCAAAAAGCCCAGC	60.1	252	DRR	Ca_00767	G	ERF
Ca3_P2_34897093	Ca3	34897093	CA/CAA	CGCGTGTATAGTGCCTTTGA	AAGTGGTGGGTGGAGATTTG	59.8	935	INTERGENIC	--	--	--
Ca3_P2_34897314	Ca3	34897314	TA/TAAA	AGAACAAATTTTGGCGCAAC	AGTTTTGTCGGTCCATGAGC	60.1	537	INTERGENIC	--	--	--
Ca3_P2_34897346	Ca3	34897346	TAAAAAAA/TAAAAAAAA	AGAACAAATTTTGGCGCAAC	AGTTTTGTCGGTCCATGAGC	60.1	537	INTERGENIC	--	--	--
Ca3_P2_34901143	Ca3	34901143	ATAAATTTAAAT/ATAAATTTAAATTTAAAT	CCAAGTTCCAACACCCATCT	TGGTGGATAACCCCTTTAACA	59.2	692	INTERGENIC	--	--	--
Ca3_P2_34901385	Ca3	34901385	AATCTTA/AA	AAATGACCACCAACAAAAGAAA	TGGTGGATAACCCCTTTAACA	59.2	372	INTERGENIC	--	--	--
Ca3_P2_34906102	Ca3	34906102	CTTTTTTT/CTTTTTTTTT	TGGATGGTGATTATCCGACTC	TCCAAGTCTTTCAATACAAAACACA	60.0	605	INTERGENIC	--	--	--
Ca3_P2_34910960	Ca3	34910960	GTTTTTT/GTTTTTTT	TCAAGGTCAAGTGTGGCAAA	CTTTGGTCGACGATGAGGAT	60.1	819	INTERGENIC	--	--	--
Ca3_P2_34913784	Ca3	34913784	GTATATATATATA/GTATATATATATATATA	ACCCGTTTCTTTGTTGTTGG	CTATCCAAGAAGCATCCCCA	60.0	427	DRR	Ca_00768	--	ERF
Ca3_P2_34913867	Ca3	34913867	AA/AACACTTGA	TGGGGATGCTTCTTGGATAG	AAAATTTCGAGGCCCATACC	60.2	154	DRR	Ca_00768	--	ERF
Ca3_P2_34914599	Ca3	34914599	T/TCGGG	TGGGAAGATGGAGGAAGAAA	GCCGGTTTCACACTTTTGAT	60.0	387	INTERGENIC	--	--	--

INDEL marker IDs	Chromosomes/unanchored scaffolds	Physical positions (bp)	InDels (*Kabuli* reference genome-CDC Frontier/PII)	Forward primers (5'-3')	Reverse primers (5'-3')	Anneling temperature (0C)	Expected amplified product size (bp)	Structural annotation		Functional annotation	
								Sequence components of *kabuli* genome	*Kabuli* gene accession IDs	NCBI-KOG	TFs
Ca3_P2_34915693	Ca3	34915693	CGA/CGAGA	GAGAGAGTGAGGCGGATGAC	TGAGTTATTGCTTCCGGTCC	60.1	885	INTERGENIC	--	--	--
Ca3_P2_34916105	Ca3	34916105	GTTTT/GTTTTTT	TCGACCATCGTCATACTCCA	TCTCCCCTTCATATGACCCTT	59.8	671	INTERGENIC	--	--	--
Ca3_P2_34916279	Ca3	34916279	GAAAAAA/GAAAAAAA	TCGACCATCGTCATACTCCA	TGCCAAAGTTCGAAAATCAA	59.3	799	INTERGENIC	--	--	--
Ca3_P2_34923351	Ca3	34923351	TT/TTAT	GGGGTTTTATTAGCTGGGGA	TCTCTGCCCTTGCTTTCCTA	60.1	513	INTERGENIC	--	--	--
Ca3_P2_34923380	Ca3	34923380	TAGAGAGAGAG/TAGAGAGAGAGAG	GGGGTTTTATTAGCTGGGGA	TCTCTGCCCTTGCTTTCCTA	60.1	513	INTERGENIC	--	--	--
Ca3_P2_34923971	Ca3	34923971	CAAAA/CAAA	TGGTAGCAAAATGTTGGTGAA	AAGGCACATTCCTACATCCG	60.0	545	INTERGENIC	--	--	--
Ca3_P2_34925950	Ca3	34925950	GAAAAAA/GAAAAAAA	ATCTTTCAATTCGCCCCTTT	CCATCACTCTTACATGCCCA	59.5	875	INTERGENIC	--	--	--
Ca3_P2_34927968	Ca3	34927968	AGG/AGGG	AGTCAGCAACAAAGTGGCAA	GCATAAAACTTGGAGCCTCG	59.8	322	INTERGENIC	--	--	--
Ca3_P2_34929792	Ca3	34929792	TA/T	TCAATGCATGTCCAAGTGGT	ACGTCACCGACAAACAAACA	60.0	935	INTERGENIC	--	--	--
Ca3_P2_34930417	Ca3	34930417	CG/C	TTTTCGTTTGCGATGATTGA	CCGTTAAAACAACGTTGGGA	60.8	217	INTERGENIC	--	--	--
Ca3_P2_34931259	Ca3	34931259	AAAA/AAAAGAAA	TGCACCATCGTAGTTGATTACA	TGTCCTTAGTGGTGGAAGGG	60.0	307	INTERGENIC	--	--	--
Ca3_P2_34932929	Ca3	34932929	TAAAAAAA/TAAAAAAAA	ACTTGCTTTGCACCTCGTTT	TTGAAAGATGACTCTTCACCGA	59.9	631	INTERGENIC	--	--	--

INDEL marker IDs	Chromosomes/unanchored scaffolds	Physical positions (bp)	InDels (*Kabuli* reference genome-CDC Frontier/PII)	Forward primers (5'-3')	Reverse primers (5'-3')	Anneling temperature (0C)	Expected amplified product size (bp)	Structural annotation		Functional annotation	
								Sequence components of *kabuli* genome	*Kabuli* gene accession IDs	NCBI-KOG	TFs
Ca3_P2_34933144	Ca3	34933144	AT/A	TCGGTGAAGAGTCATCTTTCAA	GCTGCATTGTGAGAAAATGTG	59.3	517	INTERGENIC	--	--	--
Ca3_P2_34933338	Ca3	34933338	AGTAAATATAG/AGTAAATATAGAATATGTAAATATAG	TCGGTGAAGAGTCATCTTTCAA	GGGCCTTCACGTGTCTAAAA	60.1	687	INTERGENIC	--	--	--
Ca3_P2_34942332	Ca3	34942332	TAAAAAAAA/TAAAAAAA	CCAAATCAAGCTATTTGGGTAG	TCGGCAATTTATCACCTTGA	59.1	425	INTERGENIC	--	--	--
Ca3_P2_34943152	Ca3	34943152	TTATATATATATATATATA/TTATATATATATATATATA	TGCATTTTTGTGATTGTGTGTG	GCTGGCTCTTTGGGTATGAG	59.8	454	INTERGENIC	--	--	--
Ca3_P2_34943202	Ca3	34943202	ATTT/ATTTT	TGCATTTTTGTGATTGTGTGTG	GCTGGCTCTTTGGGTATGAG	59.8	454	INTERGENIC	--	--	--
Ca3_P2_34943765	Ca3	34943765	ATTTTTTTTT/ATTTTTTTT	GTGTTTGCTTGCTTTGACCA	CGGAAACTACTCCATCGTGAA	60.1	384	DRR	Ca_00771	--	S1Fa-like
Ca3_P2_34946762	Ca3	34946762	AAAAATAAAATAAAA/AAAAATAAAA	GAACAAAACCACAGATCGGG	TTCAGTTGGGTCGTGTTGAA	60.1	461	INTRON	Ca_00771	--	S1Fa-like
Ca3_P2_34947756	Ca3	34947756	TAG/T	CCTTTGCCTTTAGGGACCAT	AGTTGGGCCAGATCACATTC	59.9	597	INTERGENIC	--	--	--
Ca3_P2_34952227	Ca3	34952227	AT/A	ACACGTGTGGATGTGGAAGA	TGAAAGACATAGACTCATTTTTACCAA	59.5	679	INTERGENIC	--	--	--
Ca3_P2_34952792	Ca3	34952792	AATATATATATATA/AATATATATATATATATATATATATATATA	AAGTTTTTCAATGACAACCCTTAAA	ACCAAAACCAAATTGCTTGC	60.0	349	INTERGENIC	--	--	--
Ca3_P2_34952880	Ca3	34952880	TAAAAAAA/TAAAAAAAAA	TCGTTATTTAGTTGGTTGGCA	ACCAAAACCAAATTGCTTGC	60.0	239	INTERGENIC	--	--	--
Ca3_P2_34953359	Ca3	34953359	AATATATATAT/AATATATAT	GCAAGCAATTTGGTTTTGGT	TGTTGTAGAGGAATCACGGG	58.6	644	INTERGENIC	--	--	--

INDEL marker IDs	Chromosomes/unanchored scaffolds	Physical positions (bp)	InDels (*Kabuli* reference genome-CDC Frontier/PII)	Forward primers (5'-3')	Reverse primers (5'-3')	Anneling temperature (0C)	Expected amplified product size (bp)	Structural annotation		Functional annotation	
								Sequence components of *kabuli* genome	*Kabuli* gene accession IDs	NCBI-KOG	TFs
Ca3_P2_34953729	Ca3	34953729	ATTTTTT/ATTTTT	CCCGTGATTCCTCTACAACA	GAACTCGCTTGAGGACGAAG	60.1	395	INTERGENIC	--	--	--
Ca3_P2_34953897	Ca3	34953897	TAAAAAA/TAAAAAAA	CCCGTGATTCCTCTACAACA	GAACTCGCTTGAGGACGAAG	60.1	395	INTERGENIC	--	--	--
Ca3_P2_34954280	Ca3	34954280	TC/T	CTTCGTCCTCAAGCGAGTTC	TGCATGCAGGAGAAGTATTTATT	58.0	681	INTERGENIC	--	--	--
Ca3_P2_34954400	Ca3	34954400	ATTTTTTTTTT/ATTTTTTTTT	CTTCGTCCTCAAGCGAGTTC	TTGTTGTCGAGGAAATGATCC	59.9	857	INTERGENIC	--	--	--
Ca3_P2_34955034	Ca3	34955034	AATG/AATGATG	GGCACCTTTGTCGTATGGTT	CAGACTCAATCATGGGCATT	58.5	522	INTERGENIC	--	--	--
Ca3_P2_34958868	Ca3	34958868	ACCCC/ACCC	GCAACACCATGATAACGTGC	GATGAGATGTTCGCCCTGTT	60.1	220	INTERGENIC	--	--	--
Ca3_P2_34964095	Ca3	34964095	GCC/GCCCC	GGTGACCATGAAGCTCGTTT	GCAATCAACAAACAAGTGTGC	59.2	488	INTERGENIC	--	--	--
Ca3_P2_34967501	Ca3	34967501	TAAAAA/TAAAAAA	TGGTGGTTTTGGACTTGATG	TTTCTTCTTCTGCCTTCCACA	60.0	626	INTERGENIC	--	--	--
Ca3_P2_34967661	Ca3	34967661	GTATTTTTATTTGATATATTTT/GTATTTT	TGGTGGTTTTGGACTTGATG	ATCTGTAATTGGCAGGCAGG	60.1	773	INTERGENIC	--	--	--
Ca3_P2_34970200	Ca3	34970200	CTTTTTTTT/CTTTTTTT	AACACCAACAGATTCCCCAG	CAAGCTAAAATGGCTTCCCA	60.2	234	INTRON	Ca_00772	--	GRF
Ca3_P2_34970367	Ca3	34970367	TTTCTT/TTTCTTCTT	AACACCAACAGATTCCCCAG	CAAGCTAAAATGGCTTCCCA	60.2	234	INTRON	Ca_00772	--	GRF
Ca3_P2_34972470	Ca3	34972470	TA/T	ATGAAGCTTTGGATTGTGGG	TCCACAGCGTACCAACAAAA	60.1	491	INTRON	Ca_00772	--	GRF

INDEL marker IDs	Chromosomes/unanchored scaffolds	Physical positions (bp)	InDels (*Kabuli* reference genome-CDC Frontier/PII)	Forward primers (5'-3')	Reverse primers (5'-3')	Anneling temperature (0C)	Expected amplified product size (bp)	Structural annotation		Functional annotation	
								Sequence components of *kabuli* genome	*Kabuli* gene accession IDs	NCBI-KOG	TFs
Ca3_P2_34976263	Ca3	34976263	T/TC	GTTCTACGTTTGGAGCAGCC	CAATTCAGCAAAGCGCAATA	60.0	358	INTRON	Ca_00773	G	--
Ca3_P2_34981658	Ca3	34981658	CAAAAAAAAAAA/CAAAAAAAAAAAAA	TTGCCTGTGGGTAGGAGAAC	TCCTTACGCTCTTTCGTCGT	60.0	334	INTRON	Ca_00773	G	--
Ca3_P2_34993883	Ca3	34993883	GATCTATTGTGAATCTATTGT/GATCTATTGT	GCACGTTGATTGAAGCAGAA	TTGTTTGCAGTGCTACAGGC	60.1	717	INTRON	Ca_00773	G	--
Ca3_P2_35017950	Ca3	35017950	AC/A	TGACCGAAAAGTGGTCAAAA	GCCTAGAAAGTAGGGGTGTTACAA	60.0	760	INTERGENIC	--	--	--
Ca3_P2_35018016	Ca3	35018016	AGTTT/A	CAGGCGCATATGTTTTTACC	GCCTAGAAAGTAGGGGTGTTACAA	60.0	535	INTERGENIC	--	--	--
Ca3_P2_35019374	Ca3	35019374	A/AG	CCCCTGAAAATCGTATCACAA	GCTGAGTTTAATGACCCGGA	60.1	765	DRR	Ca_00776	Q	E2F/DP
Ca3_P2_35030161	Ca3	35030161	GC/GCTACTAAAGTTC	GATGTCCAACTCCGACGACT	GAAACCGGATTTTCCCACTT	60.2	370	INTERGENIC	--	--	--
Ca3_P2_35031349	Ca3	35031349	CAA/C	ACTCATCTGTTCCCACAGCC	ATTCATGATGGTCCCTCAGC	59.9	584	INTERGENIC	--	--	--
Ca3_P2_35033028	Ca3	35033028	GATATA/GATA	TTCAAACTAGTCACACCAACGG	TTTCATGTTGTTCCCCCAAT	60.0	477	DRR	Ca_00777	R	--
Ca3_P2_35033116	Ca3	35033116	TGGGGG/TGGGGGG	TTCAAACTAGTCACACCAACGG	CACGTTCATCTATTTTGGGGA	59.8	853	DRR	Ca_00777	R	--
Ca3_P2_35035644	Ca3	35035644	TA/TAA	GCTGAACTCCGGATTACCAA	TCATGCTGCTTCAAAGGTTG	60.0	701	INTRON	Ca_00777	R	--
Ca3_P2_35040031	Ca3	35040031	GA/GAA	CATTCCAACCACACTTCCCT	GAACAACCACCATCTTTGGG	60.2	521	INTRON	Ca_00777	R	--

INDEL marker IDs	Chromosomes/unanchored scaffolds	Physical positions (bp)	InDels (*Kabuli* reference genome-CDC Frontier/PII)	Forward primers (5'-3')	Reverse primers (5'-3')	Anneling temperature (0C)	Expected amplified product size (bp)	Structural annotation		Functional annotation	
								Sequence components of *kabuli* genome	*Kabuli* gene accession IDs	NCBI-KOG	TFs
Ca3_P2_35040555	Ca3	35040555	A/AAAAAAAAG	AGCAAGGCCATCTTTCTTCA	TCAAGCTCTGCATATGGGTG	59.8	259	INTRON	Ca_00777	R	--
Ca3_P2_35040626	Ca3	35040626	CTT/CTTT	AGCAAGGCCATCTTTCTTCA	AATATCAAGCCGTGCAAACC	60.0	722	INTRON	Ca_00777	R	--
Ca3_P2_35048375	Ca3	35048375	TA/T	AGCTGATCGCAACTTAGGGA	TTATGGGCAAAATAGCAGGG	59.9	536	INTERGENIC	--	--	--
Ca3_P2_35049078	Ca3	35049078	GAAAAAAAA/GAAAAAAA	TCCCTGCTATTTTGCCCATA	GGGAAGCGTAGTGACATCGT	60.1	687	INTERGENIC	--	--	--
Ca3_P2_35053156	Ca3	35053156	TAAAAAAA/TAAAAAA	GACACCCAGTCCATAGTGCC	ACACGGGACAAATGTTTTGA	58.9	698	INTRON	Ca_00778	Q	E2F/DP
Ca3_P2_35053556	Ca3	35053556	CATATATA/CATATA	TTGTCAAAACATTTGTCCCG	GATTTGATCTTCCTATTGTCAACG	59.1	280	INTRON	Ca_00778	Q	E2F/DP
Ca3_P2_35053715	Ca3	35053715	ATTTTTTTT/ATTTTTT	TTGTCAAAACATTTGTCCCG	AACCATTATCAAATTTTAGGCACA	58.9	534	INTRON	Ca_00778	Q	E2F/DP
Ca3_P2_35061749	Ca3	35061749	A/AACTGT	TCAATACCCCAGCACCAAAT	TTCTGCCTTCCATTCAAACC	60.1	710	CDS (FRAME SHIFT)	Ca_00778	Q	E2F/DP
Ca3_P2_35063966	Ca3	35063966	TTTTACATCTT/TTT	CGCTCGCAATTAAAACATCA	CGCGTTTCGCGGATATTA	60.7	452	DRR	Ca_00778	Q	E2F/DP
Ca3_P2_35075809	Ca3	35075809	AATTATTATTAT/AATTATTATTATTATTAT	ATCCAATCCAATGTTGCCAT	TGCCGACCTCTTTTCTCCTA	59.9	506	INTERGENIC	--	--	--
Ca3_P2_35076089	Ca3	35076089	TG/T	TAGGAGAAAAGAGGTCGGCA	AAAGGACCAAGTCGGAAAAA	58.7	332	INTERGENIC	--	--	--
Ca3_P2_35077901	Ca3	35077901	ATTTTT/ATTTTTTT	GTCCATCACGACTTGGACCT	TGCAGTCCAGCATTGGTAAC	59.7	281	INTERGENIC	--	--	--

INDEL marker IDs	Chromosomes/unanchored scaffolds	Physical positions (bp)	InDels (*Kabuli* reference genome-CDC Frontier/PII)	Forward primers (5'-3')	Reverse primers (5'-3')	Anneling temperature (0C)	Expected amplified product size (bp)	Structural annotation		Functional annotation	
								Sequence components of *kabuli* genome	*Kabuli* gene accession IDs	NCBI-KOG	TFs
Ca3_P2_35080677	Ca3	35080677	TC/T	TTTTGCTTCAATTCCTTGGG	CTAAGGTGGCAGCTTTCCTG	60.0	649	INTRON	Ca_00780	--	bZIP
Ca3_P2_35086395	Ca3	35086395	TAAAAAAAAAA/TAAAAAAAAA	TGCCTTTCCATCTACTTGGG	GGGCAATCGACCATTCATAG	60.3	690	INTRON	Ca_00781	--	G2-like
Ca3_P2_35086678	Ca3	35086678	ATTTTTT/ATTTTT	TCTGCTTCATGTGGCAAAAG	CATGTTGGAGACTGCGATTG	60.3	487	INTRON	Ca_00781	--	G2-like
Ca3_P2_35086999	Ca3	35086999	TGTTGCAGTTGCAGTTG/TGTTGCAGTTGCAGTTGCAGTTG	TGTTGTGGTGGTGTTGTTCC	TCACTCATTTCGCGTTCTTG	60.0	458	CDS (large-effect mutations)	Ca_00781	--	G2-like
Ca3_P2_35087553	Ca3	35087553	CATAATA/CATA	CAAGAACGCGAAATGAGTGA	AGCTTGGGCCAAACCATAG	60.1	239	INTRON	Ca_00781	--	G2-like
Ca3_P2_35087732	Ca3	35087732	GTTTTTTTTTT/GTTTTTTTTT	CAAGAACGCGAAATGAGTGA	TGACAAACCGAAATGGGAAT	60.2	819	INTRON	Ca_00781	--	G2-like
Ca3_P2_35090168	Ca3	35090168	CTTTTTTTTT/CTTTTTTTT	GAGTTGTCATGTTTGGGCCT	TTGTCAGTGCCAGTCAAAAA	57.9	513	INTERGENIC	--	--	--
Ca3_P2_35090812	Ca3	35090812	GTTTTTTTTTTT/GTTTTTTTTTT	TTTGTGCAAAAATTTCAAGTCA	AGCTTTGGTCGCAGACACTT	60.1	613	INTERGENIC	--	--	--
Ca3_P2_35092745	Ca3	35092745	GTGATGATGATGATGATG/GTGATGATG	TTTTAATGTTGCCGGTGTCA	TCACAAAGCTTCACTTCCCA	59.4	390	INTERGENIC	--	--	--
Ca3_P2_35092956	Ca3	35092956	TG/TGGTGTCTATAG	TTTTAATGTTGCCGGTGTCA	TCACAAAGCTTCACTTCCCA	59.4	390	INTERGENIC	--	--	--
Ca3_P2_35093080	Ca3	35093080	GTTT/GTTTTATTTTTT	TTTTAATGTTGCCGGTGTCA	TGGAGGCAACTTGATTTCACT	59.7	445	INTERGENIC	--	--	--
Ca3_P2_35094993	Ca3	35094993	TAAAA/TAAAAA	TGAACCTAACTAAGGGTCTCAACA	AATAGCAATGGCGATGAAGG	60.1	681	DRR	Ca_00782	F	--

INDEL marker IDs	Chromosomes/unanchored scaffolds	Physical positions (bp)	InDels (*Kabuli* reference genome-CDC Frontier/PII)	Forward primers (5'-3')	Reverse primers (5'-3')	Anneling temperature (0C)	Expected amplified product size (bp)	Structural annotation		Functional annotation	
								Sequence components of *kabuli* genome	*Kabuli* gene accession IDs	NCBI-KOG	TFs
Ca3_P2_35095475	Ca3	35095475	AATATATATATATATATATA/AATATATATATATATA	CTTGATAACAAGGCCAGGGA	ACTCCATCCACCAACGAAAG	60.0	483	DRR	Ca_00782	F	--
Ca3_P2_35098773	Ca3	35098773	TAAGTA/TAAGTAAAACTCAAGTA	AGCACGGTTATTTCACCAGG	GCAGAAGGAAGCACTGAACC	60.0	372	INTRON	Ca_00782	F	--
Ca3_P2_35099657	Ca3	35099657	GATA/GATATA	TGGGTACCGGACACACTATG	AAACGCCGAGAAGCTCATTA	60.0	180	INTRON	Ca_00782	F	--
Ca3_P2_35100602	Ca3	35100602	TAAAAAAA/TAAAAAAAA	TTATAAAGTTTGGGGCACCG	CAACAGCGGTACAATCATCG	60.1	701	INTERGENIC	--	--	--
Ca3_P2_35101262	Ca3	35101262	TTAAAAAGTAAAA/TTAAAAAGTAAAAAGTAAAA	CGATGATTGTACCGCTGTTG	CGAAGGAAGTGTTATCCGAA	57.8	885	INTERGENIC	--	--	--
Ca3_P2_35102203	Ca3	35102203	TAAAAAAAA/TAAAAAAA	TTCGTGGAAGAATATAGCGACA	TGTGTAACCCTAACAATGGCA	59.0	118	INTERGENIC	--	--	--
Ca3_P2_35102977	Ca3	35102977	GAGATCAAAA/GA	ATGAATTACGGTTGTTGCGG	TGGAAAAAGATTGTGACGTTTG	60.0	254	INTERGENIC	--	--	--
Ca3_P2_35103080	Ca3	35103080	ATTTTTTTT/ATTTTTTTTT	ATGAATTACGGTTGTTGCGG	TGGAAAAAGATTGTGACGTTTG	60.0	254	INTERGENIC	--	--	--
Ca3_P2_35103163	Ca3	35103163	CAAAAAAAAAA/CAAAAAAAAAAA	ATGAATTACGGTTGTTGCGG	GCACATGTTGGATGCACATAA	60.4	708	INTERGENIC	--	--	--
Ca3_P2_35106644	Ca3	35106644	AATATATATATATA/AATATATATATA	TGTCAAATGGGAAAGGGAAG	TTTGCTGCAATACATTTTCAGG	60.1	608	INTRON	Ca_00783	S	--
Ca3_P2_35111987	Ca3	35111987	T/TG	GATAAGAACGACCCCACCAA	TGGTTGTGGTGGAATTGAGA	59.9	586	INTERGENIC	--	--	--
Ca3_P2_35112555	Ca3	35112555	CTTTTTTTT/CTTTTTTT	CCACCACAACCACTCCTTCT	TGCGTCAGAAACGATAGACG	60.0	588	INTERGENIC	--	--	--

INDEL marker IDs	Chromosomes/unanchored scaffolds	Physical positions (bp)	InDels (*Kabuli* reference genome-CDC Frontier/PII)	Forward primers (5'-3')	Reverse primers (5'-3')	Anneling temperature (0C)	Expected amplified product size (bp)	Structural annotation		Functional annotation	
								Sequence components of *kabuli* genome	*Kabuli* gene accession IDs	NCBI-KOG	TFs
Ca3_P2_35117240	Ca3	35117240	CTTT/CTT	GGTGCACCATACAATAACAACAA	CATGGCATCGTAACAACGAC	60.0	580	DRR	Ca_00785	G	C3H
Ca3_P2_35118983	Ca3	35118983	AC/A	AGTTGGACCCACTTTGTTGG	AAAATGGATGGGTCTGTTGC	59.8	545	INTRON	Ca_00785	G	C3H
Ca3_P2_35119008	Ca3	35119008	G/GCACTTTTA	AGTTGGACCCACTTTGTTGG	AAAATGGATGGGTCTGTTGC	59.8	545	INTRON	Ca_00785	G	C3H
Ca3_P2_35126936	Ca3	35126936	CAAAA/CAAAAA	ACCTCCGCCACACAATACTC	TTTCTTCACATCCACCACCA	59.9	245	INTERGENIC	--	--	--
Ca3_P2_35126954	Ca3	35126954	AC/ACC	ACCTCCGCCACACAATACTC	TTTCTTCACATCCACCACCA	59.9	245	INTERGENIC	--	--	--
Ca3_P2_35136413	Ca3	35136413	CTAT/CTATATAT	ATACCACTAGCACGCACACG	TTTATGCATATCACATCAACAAAAA	58.5	660	INTERGENIC	--	--	--
Ca3_P2_35138675	Ca3	35138675	TAA/TAAA	AGGGCCAGATTCTCTTGGTT	GTCAGATGAATTCCCGCATC	60.4	563	INTRON	Ca_00787	G	C3H
Ca3_P2_35143342	Ca3	35143342	ATTT/ATTTT	CACATGCACTTTGGAGTTGG	TCTTCGTCTCCCTCCATTTC	59.2	428	DRR	Ca_00788	G	C3H
Ca3_P2_35147082	Ca3	35147082	T/TAAAGA	GTCGGAGCCAAAAATGAAAA	TGTGTTTCTTTCCAGCAGAGAC	59.5	317	INTERGENIC	--	--	--
Ca3_P2_35147709	Ca3	35147709	AG/A	TCTCTGCTGGAAAGAAACACA	ACCCGTATTTTGAACAAGCG	60.0	848	INTERGENIC	--	--	--
Ca3_P2_35152845	Ca3	35152845	A/AC	TCATTGAGCATGTAACGGGA	TGGAGGGGGAAGGGTAATAG	60.1	746	INTERGENIC	--	--	--
Ca3_P2_35155451	Ca3	35155451	GTA/GTATA	CCCTTTTGTTTCCTCAACCA	TTGAGTCTTTTGGACCACCC	59.9	711	INTERGENIC	--	--	--

INDEL marker IDs	Chromosomes/unanchored scaffolds	Physical positions (bp)	InDels (*Kabuli* reference genome-CDC Frontier/PII)	Forward primers (5'-3')	Reverse primers (5'-3')	Anneling temperature (0C)	Expected amplified product size (bp)	Structural annotation		Functional annotation	
								Sequence components of *kabuli* genome	*Kabuli* gene accession IDs	NCBI-KOG	TFs
Ca3_P2_35155696	Ca3	35155696	ATGTG/ATGTGTG	GGACAGCCGAAAGAAACAAT	TTTGGACCACCCTGTCAAAT	60.2	127	INTERGENIC	--	--	--
Ca3_P2_35156142	Ca3	35156142	ATTTTT/ATTTTTT	ATTTGACAGGGTGGTCCAAA	AAAGTCTCCTGGTTGGGTCA	59.5	714	INTERGENIC	--	--	--
Ca3_P2_35157017	Ca3	35157017	TGG/TGGG	TTGCATTGCCTCTGTTTCTG	AACGATGATTTCAAGCTCGG	60.2	718	INTERGENIC	--	--	--
Ca3_P2_35166785	Ca3	35166785	TAGAAAG/TAG	TGGCATGTGGTTTGTCATTT	AATGTGACCAACTTCCTCGG	60.0	859	INTERGENIC	--	--	--
Ca3_P2_35172864	Ca3	35172864	TTGTCGAAATGT/TTGT	TCGTTTCTATTGCCTATTGGAGA	TCATCTTTCGAATTAAGGTTTGC	59.6	886	DRR	Ca_00790	R	--
Ca3_P2_35174879	Ca3	35174879	CATATATATATATATA/CATATATATATATATATA	CCAGGAACATCAGGGTCTGT	CCATGAGTTCTTCCCTTCCA	60.0	203	INTRON	Ca_00790	R	--
Ca3_P2_35178313	Ca3	35178313	TTTGAATCATGTTGAATCA/TTTGAATCA	GATTTCGAAGAACCCAACCA	TCTACCTTTGAACCAGATCCA	57.3	487	INTERGENIC	--	--	--
Ca3_P2_35178777	Ca3	35178777	ATTTTTTTTTT/ATTTTTTTTT	TGAATCATGTTGAATCAGCCA	CGCTCCTAATCTCTTTGGAACA	60.7	730	INTERGENIC	--	--	--
Ca3_P2_35180181	Ca3	35180181	AT/A	ATTTTCCCAAATTGAACCCC	GGATCAGCCGATGATTATTTTT	59.3	607	INTERGENIC	--	--	--
Ca3_P2_35185977	Ca3	35185977	CTTTTTTTTT/CTTTTTTTT	TTGGCAGGCTTACACAGTTG	TTTGCCTTCTGAGCTTTGGT	60.0	373	DRR	Ca_00792	--	--
Ca3_P2_35187164	Ca3	35187164	GTCT/GTCTCT	GGGCACTAAAACAAAAGGCA	TCAAATCCTGTGGTTGTGGA	59.9	588	DRR	Ca_00791	--	bZIP
Ca3_P2_35187474	Ca3	35187474	CAGA/CAGAGA	CCCCATTTTCTCCTTTGACA	TATAGCTCCACCACCCTTGG	59.9	326	DRR	Ca_00791	--	bZIP

INDEL marker IDs	Chromosomes/unanchored scaffolds	Physical positions (bp)	InDels (*Kabuli* reference genome-CDC Frontier/PII)	Forward primers (5'-3')	Reverse primers (5'-3')	Anneling temperature (0C)	Expected amplified product size (bp)	Structural annotation		Functional annotation	
								Sequence components of *kabuli* genome	*Kabuli* gene accession IDs	NCBI-KOG	TFs
Ca3_P2_35191007	Ca3	35191007	TAAA/TAAAA	GGAGAATACTGGCCATAGCG	AACAAAAATCTTAATGTGACGCAA	59.9	881	INTERGENIC	--	--	--
Ca3_P2_35192465	Ca3	35192465	AGG/AG	ATAAGGCCTCCCACAAGAGG	AACCTTTTAGATCAGTCCGAACC	59.9	663	INTERGENIC	--	--	--
Ca3_P2_35195471	Ca3	35195471	TAAAAAAAAAAAAAA/TAAAAAAAAAAAAAAAA	TGTTTGGGAGGAGGATCTTG	GGCTGCAATTCCTTCTTGAG	60.0	857	INTRON	Ca_00793	--	GATA
Ca3_P2_35204214	Ca3	35204214	AT/ATTT	GGGGGTGCACAATACAATTC	CAATTTGCATGAGTTGTCGG	60.1	741	INTERGENIC	--	--	--
Ca3_P2_35204680	Ca3	35204680	AT/ATCT	TGGCCAAATCACAAACCTATT	AATACCAATTTGGCGAGTGC	60.0	560	INTERGENIC	--	--	--
Ca3_P2_35208857	Ca3	35208857	GAAAAAAAAAA/GAAAAAAAAA	CTCGCAAAAAGCCGTATAGC	ACAGGGTATCATGAGCGGAC	60.0	588	INTRON	Ca_00795	R	B3
Ca3_P2_35212793	Ca3	35212793	TAAA/TAA	TTTCCAAAACGCAACAAACA	TTTTGCACGTGAATGAAGTTAAA	59.7	704	INTERGENIC	--	--	--
Ca3_P2_35213093	Ca3	35213093	CTTTT/CTTT	GCCATCACATTCATGACAAAA	TGATATTGCAAGAAACCCCA	59.0	758	INTERGENIC	--	--	--
Ca3_P2_35214818	Ca3	35214818	TCCAGCCCAGCCCA/TCCAGCCCAGCCCAGCCCA	AAAAGTGCGGTGCAAAAAGT	AACGCCTCCTCCTCTTCTTC	60.0	335	INTERGENIC	--	--	--
Ca3_P2_35215168	Ca3	35215168	TTTTTCCTTTCATTTTCCTTT/TTTTTCCTTTCATTTTCCTTTCATTTTCCTTT	GCGAAGAAAAAGAGGTCGTG	GTAATCGACAGCCTGCAACA	59.9	316	INTRON	Ca_00796	C	--
Ca3_P2_35216734	Ca3	35216734	AA/AATA	AACGTCGTTGTTGGTTAGGC	ACAGCCAAATGTGGTGCATA	60.0	474	DRR	Ca_00796	C	--
Ca3_P2_35216771	Ca3	35216771	ATTTTTTTTTTT/ATTTTTTTTT	GATTTGACTACTCCGCTCGC	ACAGCCAAATGTGGTGCATA	60.0	168	DRR	Ca_00796	C	--

INDEL marker IDs	Chromosomes/unanchored scaffolds	Physical positions (bp)	InDels (*Kabuli* reference genome-CDC Frontier/PII)	Forward primers (5'-3')	Reverse primers (5'-3')	Anneling temperature (0C)	Expected amplified product size (bp)	Structural annotation		Functional annotation	
								Sequence components of *kabuli* genome	*Kabuli* gene accession IDs	NCBI-KOG	TFs
Ca3_P2_35219260	Ca3	35219260	CTTTTTTTTTTT/CTTTTTTTTTTTTTTTT	TGAGGCCATTTTTAACCCAC	CCCAAGATTTTCCCTACCTTG	59.8	551	INTERGENIC	--	--	--
Ca3_P2_35220172	Ca3	35220172	AAAACA/AA	TCACTAGCGGAGATCGAACC	TTCTTCTTCCTTTCCCAGCA	59.9	435	INTERGENIC	--	--	--
Ca3_P2_35224402	Ca3	35224402	TAA/TA	AACCTGCCAATGCAAAATTC	ATTCAATCCACATCCCTCCA	60.1	519	INTRON	Ca_00797	I	--
Ca3_P2_35226113	Ca3	35226113	TG/TGG	TTCTCGATGGACGATCAATG	GACACAGCATGTCATCCCAC	60.0	504	INTRON	Ca_00797	I	--
Ca3_P2_35227900	Ca3	35227900	GTTA/GTTAGTTGCCTCCATGTTATTA	TTCAAATTCTTCGGGCTTTG	TTACCATTGAGAGCAGCGTG	60.0	278	INTRON	Ca_00797	I	--
Ca3_P2_35230228	Ca3	35230228	CAA/CAAA	ACTCGCCTAAATTCTGCACG	GAAACTCCACGCTGAAAAGC	60.0	502	INTERGENIC	--	--	--
Ca3_P2_35234401	Ca3	35234401	GAAAAAA/GAAAAAAA	CGTCGTTATCAAACCGTTCC	CACTCCCCGTTGCAATTATC	60.3	490	INTERGENIC	--	--	--
Ca3_P2_35239414	Ca3	35239414	ATGTGT/ATGT	TTCGAACCTACGGGACATTC	TGGTCCACTGGTCGTTATGA	60.0	416	INTERGENIC	--	--	--
Ca3_P2_3524015	Ca3	3524015	AATATATATATATAT/AATATATATATATATATATAT	GTTTGCAGGAAGAGGCAAAG	GCCAGCAGCCTTCAAGATAG	60.1	712	DRR	Ca_21350	J	--
Ca3_P2_35241583	Ca3	35241583	TTTTATTTATTTATTTATT/TTTTATTTATTTATT	ACCAAGTCACAATGGTGCAA	AACCATTGTATGAGCGTCCC	59.8	658	INTRON	Ca_00801	--	--
Ca3_P2_35241980	Ca3	35241980	CTT/CTTAGTT	GGGACGCTCATACAATGGTT	CGCACAGTGAAGTGCAAAGT	60.1	331	INTRON	Ca_00801	--	--
Ca3_P2_35246038	Ca3	35246038	GTTTT/GTTTTT	CATAACAATCCCTTGTGCCA	ACGCTAAAGCGTTATGCACC	60.3	732	DRR	Ca_00802	--	--

INDEL marker IDs	Chromosomes/unanchored scaffolds	Physical positions (bp)	InDels (*Kabuli* reference genome-CDC Frontier/PII)	Forward primers (5'-3')	Reverse primers (5'-3')	Anneling temperature (0C)	Expected amplified product size (bp)	Structural annotation		Functional annotation	
								Sequence components of *kabuli* genome	*Kabuli* gene accession IDs	NCBI-KOG	TFs
Ca3_P2_35247904	Ca3	35247904	CATATATAT/CATATAT	CACTATCTTTGGGCGGTTGT	TTCTCTGACCGTGAAAGTGG	58.9	382	INTERGENIC	--	--	--
Ca3_P2_35248313	Ca3	35248313	ATTTTTTTT/ATTTTTTT	ACCACTTTCACGGTCAGAGA	CTCCACCCAATTGCATCTTT	59.9	859	INTERGENIC	--	--	--
Ca3_P2_35248340	Ca3	35248340	AATTATATT/AATT	ACCACTTTCACGGTCAGAGA	TACAATTTGCACTCCACCCA	60.0	870	INTERGENIC	--	--	--
Ca3_P2_35249900	Ca3	35249900	CAA/CAAA	TGGAACTCATGCATCTCTCG	GAAAAATTTTGTGGACCCGA	59.8	809	INTERGENIC	--	--	--
Ca3_P2_35250196	Ca3	35250196	TAAAAAAAA/TAAAAAA	AATCAAAAGCAACGACCAAAA	GAAATCTATTGGCCACCGAA	59.9	636	INTERGENIC	--	--	--
Ca3_P2_35254278	Ca3	35254278	CAAAAA/CAAAAAA	TGGAAACGTTCGAATGTGAG	TCATGAGTGGCAATTCACAG	58.2	779	INTERGENIC	--	--	--
Ca3_P2_35254794	Ca3	35254794	CTT/CTTT	TGCCACTCATGACTACCAAAA	GTACTCGCGCGGTAGGTAAG	59.9	356	INTERGENIC	--	--	--
Ca3_P2_35260978	Ca3	35260978	TACT/TACTGTAACTGCACT	ATGGGAACCAATTTGTTGGA	GCCTTGTGTTTGGGCTATGT	60.0	537	DRR	Ca_00803	O	--
Ca3_P2_35261471	Ca3	35261471	AG/A	ACATAGCCCAAACACAAGGC	GAACAGTTTCACCCCCAAAA	59.8	584	DRR	Ca_00803	O	--
Ca3_P2_35261676	Ca3	35261676	TTG/T	ACATAGCCCAAACACAAGGC	GAACAGTTTCACCCCCAAAA	59.8	584	DRR	Ca_00803	O	--
Ca3_P2_35261808	Ca3	35261808	CGTCATAATGTCATAATGT/CGTCATAATGT	ACATAGCCCAAACACAAGGC	GAACAGTTTCACCCCCAAAA	59.8	584	DRR	Ca_00803	O	--
Ca3_P2_35262067	Ca3	35262067	TAAAAAAAAAAA/TAAAAAAAAAA	TTTTGGGGGTGAAACTGTTC	AGTGAACGGGTCAAAAATCG	60.0	479	DRR	Ca_00803	O	--

INDEL marker IDs	Chromosomes/unanchored scaffolds	Physical positions (bp)	InDels (*Kabuli* reference genome-CDC Frontier/PII)	Forward primers (5'-3')	Reverse primers (5'-3')	Anneling temperature (0C)	Expected amplified product size (bp)	Structural annotation		Functional annotation	
								Sequence components of *kabuli* genome	*Kabuli* gene accession IDs	NCBI-KOG	TFs
Ca3_P2_35263394	Ca3	35263394	AAAT/A	TTTTCTTTCAATTTTCCCTTTCA	GCTCCCCTAAAGTTCCCAAC	59.9	498	INTERGENIC	--	--	--
Ca3_P2_35264243	Ca3	35264243	A/AG	ATGTCAACGCACCTCCATTT	TGAGTGAGTGTTCTATGGTTTGC	59.3	402	INTERGENIC	--	--	--
Ca3_P2_35264670	Ca3	35264670	AATAACTTCTA/AATAACTTCTACTATAACTTCTA	TCTCAAATCCAGCATGCCTA	TGGGATATTTCCTGACTCCAA	59.4	676	INTERGENIC	--	--	--
Ca3_P2_35265195	Ca3	35265195	TC/TCC	TTGGAGTCAGGAAATATCCCA	GCAACGAGGATTGACCGTAT	60.0	820	INTERGENIC	--	--	--
Ca3_P2_35267022	Ca3	35267022	ATTCGAAGT/AT	ATTTTTACCTCCCACCCCCT	TGCAGAAACCTTCGGAACTT	59.9	779	DRR	Ca_00804	--	--
Ca3_P2_35267134	Ca3	35267134	AC/A	CTTTTCTCACCACCACTTTCG	TGCAGAAACCTTCGGAACTT	59.9	713	DRR	Ca_00804	--	--
Ca3_P2_35267597	Ca3	35267597	TAAAAAAAAA/TAAAAAAAA	TCCGAAGGTTTCTGCATTTC	GGCAGTGCTCAAATATCGGT	60.1	391	DRR	Ca_00804	--	--
Ca3_P2_35270131	Ca3	35270131	ATTTTTTTT/ATTTTTTTTT	TGCATGCTGTGAAGTGTGAA	TGTGACCAAAACCATGTACCA	59.7	343	INTERGENIC	--	--	--
Ca3_P2_35278837	Ca3	35278837	GAA/GAAA	TATCATGCGATCGTGGAAAA	TCCTTCACCAGTATCCCGAG	60.1	647	DRR	Ca_00806	A	bZIP
Ca3_P2_35284220	Ca3	35284220	ACT/A	TCACGTTCAACGATTGGCTA	CCTCGCTGATGCTTGTTGTA	60.0	151	INTERGENIC	--	--	--
Ca3_P2_35285983	Ca3	35285983	G/GC	GAAGGGGTTCATGTTGAGGA	GCTAACATGGTGAGGGAGGA	60.1	643	DRR	Ca_00807	--	--
Ca3_P2_35287641	Ca3	35287641	AC/A	GGATCAATCCTGGGGCTAAT	TATGTTGGAAGGAGGGTTCG	59.9	686	DRR	Ca_00808	U	--

INDEL marker IDs	Chromosomes/unanchored scaffolds	Physical positions (bp)	InDels (*Kabuli* reference genome-CDC Frontier/PII)	Forward primers (5'-3')	Reverse primers (5'-3')	Anneling temperature (0C)	Expected amplified product size (bp)	Structural annotation		Functional annotation	
								Sequence components of *kabuli* genome	*Kabuli* gene accession IDs	NCBI-KOG	TFs
Ca3_P2_35288170	Ca3	35288170	ATTTTTTTTTTT/ATTTTTTTTTT	GTGGGGCTGATCCTTTTACA	ACGCAAACGAAATGCCATAC	60.9	592	DRR	Ca_00808	U	--
Ca3_P2_35291358	Ca3	35291358	GAAAAA/GAAAA	ATGCATGCAGATCAGACGAC	TGCACAAATGGGTTCTGTGT	60.0	283	INTRON	Ca_00808	U	--
Ca3_P2_35293542	Ca3	35293542	CAAAAAAAAAAA/CAAAAAAAAAAAA	GCTTCTTGCCCTGTCATAGC	TTGACACTGCTGGTAGGCTG	60.0	325	INTRON	Ca_00808	U	--
Ca3_P2_35299387	Ca3	35299387	TAAA/TAAAA	CCTCTGGCATCGAAAGAAAG	ATTGAAGGCCCTCTACAGCA	59.8	639	INTRON	Ca_00809	T	--
Ca3_P2_35300047	Ca3	35300047	CAAAAAAA/CAAAAAA	AAAAGGAATGTTTGGCCTGA	GGCTGGCCTTCTGATGTTAG	59.8	773	INTRON	Ca_00809	T	--
Ca3_P2_35303218	Ca3	35303218	ATTTT/ATTTTT	AATCCATGTCCCAAATCCAA	CACCACCACCTCATTGTTTG	59.8	889	INTERGENIC	--	--	--
Ca3_P2_35303259	Ca3	35303259	AAAG/A	ACTAACCATTCGCTGCCATC	CACCACCACCTCATTGTTTG	59.8	821	INTERGENIC	--	--	--
Ca3_P2_35303338	Ca3	35303338	TAAAAAAAAAA/TAAAAAAAA	AGTAAGGAATTGGGGAAGCC	CACCACCACCTCATTGTTTG	59.8	683	INTERGENIC	--	--	--
Ca3_P2_35304616	Ca3	35304616	AA/AATCTGTTA	AAATCAAATTGGAGGGAGGG	AACCACCACATGTACGCATC	59.3	896	INTERGENIC	--	--	--
Ca3_P2_35305963	Ca3	35305963	A/AGTTTTGTAGGTAGTT	CCCCGCAAAGAAACTACTCA	ACTGGCGGTACGAATCAAAC	60.0	666	DRR	Ca_00810	A	--
Ca3_P2_35306134	Ca3	35306134	TAAA/TAAAA	TTACCCACCAGCATTGAACC	TGTGCCACCTGGAATCATAA	59.9	214	DRR	Ca_00810	A	--
Ca3_P2_35307396	Ca3	35307396	TAAA/TAA	CAATCATGCGATTTGCATTC	ATTTGGTTTTGCGATGAAGC	60.1	636	INTRON	Ca_00810	A	--

**INDEL marker IDs**	**Chromosomes/unanchored scaffolds**	**Physical positions (bp)**	**InDels (*Kabuli* reference genome-CDC Frontier/PII)**	**Forward primers (5'-3')**	**Reverse primers (5'-3')**	**Anneling temperature (0C)**	**Expected amplified product size (bp)**	**Structural annotation**		**Functional annotation**	
								**Sequence components of *kabuli* genome**	***Kabuli* gene accession IDs**	**NCBI-KOG**	**TFs**
Ca3_P2_35307472	Ca3	35307472	AA/ACATTATGAATA	CAATCATGCGATTTGCATTC	ATTTGGTTTTGCGATGAAGC	60.1	636	INTRON	Ca_00810	A	--
Ca3_P2_35307977	Ca3	35307977	AT/ATT	GCTTCATCGCAAAACCAAAT	TATAATCGCAAAAATGCCGC	60.9	212	INTRON	Ca_00810	A	--
Ca3_P2_35314687	Ca3	35314687	TAAAAAA/TAAAAAAAA	CACGTACACGCGTCACATAA	GCAATGGGACCCACTTAGAC	59.4	677	DRR	Ca_00811	--	--
Ca3_P2_35316586	Ca3	35316586	A/AT	CACGTGGTTGTTGAGGATTG	AAATTAAAGGACCGCGAATG	59.1	633	INTRON	Ca_00811	--	--
Ca3_P2_35317314	Ca3	35317314	TATATAATATAATAT/TATATAATAT	TGACGTTGTTGGAGATGAAAA	TGTCGGTCCACTCTCTACCC	60.1	590	INTRON	Ca_00811	--	--
Ca3_P2_35320060	Ca3	35320060	TTA/TTACATA	AACCACCATGTGCACTTCAG	TGTCACAATTGGTGGAACAAA	59.8	855	INTERGENIC	--	--	--
Ca3_P2_35320939	Ca3	35320939	ATTTTT/ATTTTTT	GAGCAAAATTCTGCATGCCT	TTTTCTCCGTCCATGAACTTT	58.7	171	INTERGENIC	--	--	--
Ca3_P2_35323484	Ca3	35323484	ATT/ATTT	TTGTTCATTTGCCACGTTGT	AAGAATCATAATTGAGCCCAAAA	59.0	305	INTERGENIC	--	--	--
Ca3_P2_35329050	Ca3	35329050	TTG/TTGGTG	CGCCAATGTTTTTCATGTTG	CATCCATCAACAGATGGCAC	59.9	600	INTRON	Ca_00812	Z	MYB_related
Ca3_P2_35329937	Ca3	35329937	TA/TAA	ATAATATGCGCCTGCTTGCT	TCCAAATCGGCTCGTAAAAC	60.1	473	INTRON	Ca_00812	Z	MYB_related
Ca3_P2_35333838	Ca3	35333838	CAAAAA/CAAAAAA	TGTGGATTTTCCTTGATGCT	TTGCATTGAGCGATTCTTTG	60.0	693	DRR	Ca_00812	Z	MYB_related
Ca3_P2_35336928	Ca3	35336928	GT/GTT	TCTAAAACTCTCCGCCCTTG	ACCGAACCTTTTGGGTTGTA	59.3	607	INTRON	Ca_00813	G	--

INDEL marker IDs	Chromosomes/unanchored scaffolds	Physical positions (bp)	InDels (*Kabuli* reference genome-CDC Frontier/PII)	Forward primers (5'-3')	Reverse primers (5'-3')	Anneling temperature (0C)	Expected amplified product size (bp)	Structural annotation		Functional annotation	
								Sequence components of *kabuli* genome	*Kabuli* gene accession IDs	NCBI-KOG	TFs
Ca3_P2_35339243	Ca3	35339243	ATTTTTTTTTT/ATTTTTTTT	TTTTGGGCTTTTGACTTTGG	GCGGTTGAACTTGGGATTTA	59.9	539	INTRON	Ca_00813	G	--
Ca3_P2_35339486	Ca3	35339486	ATAAGTAAGTA/ATAAGTA	TAAATCCCAAGTTCAACCGC	CCAGCAAATGATGGCTTGTA	59.7	434	INTRON	Ca_00813	G	--
Ca3_P2_35339957	Ca3	35339957	T/TTAACTCC	AGCCATCATTTGCTGGATTT	GTAACGGCTAGCACCGGATA	60.1	261	INTRON	Ca_00813	G	--
Ca3_P2_35342556	Ca3	35342556	TAAAAAAAA/TAAAAAAAAA	GCGGTTTCAAACACAACCTT	GTTATTTCCCCACTCCAGCA	59.9	410	INTERGENIC	--	--	--
Ca3_P2_35347211	Ca3	35347211	T/TA	CGACGCTGTAATCGTTGAGA	TGTCGATTTACCATCACCCA	59.8	176	DRR	Ca_00814	QR	B3
Ca3_P2_35347629	Ca3	35347629	CTTTTTTTT/CTTTTTTT	TGGGTGATGGTAAATCGACA	TCACTGGGTCATCAAAAATCA	59.0	449	DRR	Ca_00814	QR	B3
Ca3_P2_35349843	Ca3	35349843	AG/AGG	CCAAAACAAGCAAGGGGTTA	TGGGAGCTTACTGGTCTCGT	59.9	237	DRR	Ca_00815	J	YABBY
Ca3_P2_35356175	Ca3	35356175	CTT/CTTT	CATCTTGAGTCGAGGTGCAA	TGGTGATTCTGTTCAGTGGC	59.7	808	INTRON	Ca_00816	R	--
Ca3_P2_35356389	Ca3	35356389	ATTTTTT/ATTTTTTT	CCCACTCACCTACCCAAAAA	ATCATGGAAAATCAGCGGAG	60.0	692	INTRON	Ca_00816	R	--
Ca3_P2_35361036	Ca3	35361036	TAAAAAAAAAAAA/TAAAAAAAAAA	TCACGTTACCATGCCTACCA	CCAAAATTTCACACCCCTTC	59.3	507	INTERGENIC	--	--	--
Ca3_P2_35361083	Ca3	35361083	TGG/TG	CTTCCCCCTCTAAAGTTGGC	AACCAATTCGTTATTCCCCA	59.1	552	INTERGENIC	--	--	--
Ca3_P2_35361414	Ca3	35361414	AAATAATAATAATAATAA/AAATAATAATAATAA	GGAAGAAGAAGCTACGCCAA	AGAACCAAACCAACCACTCG	60.0	696	INTERGENIC	--	--	--

INDEL marker IDs	Chromosomes/unanchored scaffolds	Physical positions (bp)	InDels (*Kabuli* reference genome-CDC Frontier/PII)	Forward primers (5'-3')	Reverse primers (5'-3')	Anneling temperature (0C)	Expected amplified product size (bp)	Structural annotation		Functional annotation	
								Sequence components of *kabuli* genome	*Kabuli* gene accession IDs	NCBI-KOG	TFs
Ca3_P2_35362095	Ca3	35362095	ATATTATT/ATATTATTATT	CGAGTGGTTGGTTTGGTTCT	TGCTTAATTTTCAACAAAGTTGC	59.7	789	INTERGENIC	--	--	--
Ca3_P2_35365941	Ca3	35365941	ATTTTTTTTT/ATTTTTTTTTTT	AACAACGGGGTGTCACCTAC	TTGGTGGGTATCTTGTGAAGC	60.0	681	DRR	Ca_00817	--	--
Ca3_P2_35366034	Ca3	35366034	A/ATTTTTT	AACAACGGGGTGTCACCTAC	CATTGCATTTGCAGTGAGAGA	60.0	713	DRR	Ca_00817	--	--
Ca3_P2_35368592	Ca3	35368592	TA/T	TCAACTCACCACCAATCCAA	GCCGATTCTGAATATGTGTAAGG	59.9	617	INTERGENIC	--	--	--
Ca3_P2_35368688	Ca3	35368688	AATATATATATATATATATATATATATA/AATATATATATATATATATATATA	TCAACTCACCACCAATCCAA	GAAGTGTTGACATTGACCTCCA	60.0	861	INTERGENIC	--	--	--
Ca3_P2_35370511	Ca3	35370511	CT/CTT	TTCTTCAAATTTGTTTTACTCCGA	CCAAATCTTTGGCACTCGAT	60.1	829	INTERGENIC	--	--	--
Ca3_P2_35370619	Ca3	35370619	GAAAAAAAAA/GAAAAAAAA	AAAATGTTTTACAGTCCCGTTG	CCAAATCTTTGGCACTCGAT	60.1	388	INTERGENIC	--	--	--
Ca3_P2_35372617	Ca3	35372617	TAAA/TAAAA	GGTGTGGGCAAAGTTCAGTT	GCGCGGTTTGGTTAAAAATA	60.0	683	INTERGENIC	--	--	--
Ca3_P2_35372925	Ca3	35372925	CAAAAAAAAA/CAAAAAAAA	AACATCCTTGTCTCCAACGG	GAAACGATAACCCGGTAGCA	60.0	424	INTERGENIC	--	--	--
Ca3_P2_35373136	Ca3	35373136	ATTTTGTTTTTTGTTTTTTGTTTTT/ATTTTGTTTTTTGTTTTT	TGCTACCGGGTTATCGTTTC	CCATGATGCCAAAATGAAGA	59.5	480	INTRON	Ca_00818	--	NAC
Ca3_P2_35376259	Ca3	35376259	CTTT/CTTTT	GGTCTGCATTTAGCCTTTGC	GGGTTCCACTTGAGGAGTGA	60.1	598	DRR	Ca_00818	--	NAC
Ca3_P2_35379481	Ca3	35379481	TAAG/TAAGAGGACTACAAG	CGACTCAATCTCCGTCTGCT	TTACTTTGTGCAGCCTCGTG	60.1	795	INTERGENIC	--	--	--

INDEL marker IDs	Chromosomes/unanchored scaffolds	Physical positions (bp)	InDels (*Kabuli* reference genome-CDC Frontier/PII)	Forward primers (5'-3')	Reverse primers (5'-3')	Anneling temperature (0C)	Expected amplified product size (bp)	Structural annotation		Functional annotation	
								Sequence components of *kabuli* genome	*Kabuli* gene accession IDs	NCBI-KOG	TFs
Ca3_P2_35382356	Ca3	35382356	AATCATCA/AATCATCATCA	GAGCTTTTGGAAACTGCTGG	GGACAAAGTTTGAGGTGGGA	59.9	353	DRR	Ca_00819	O	--
Ca3_P2_35390580	Ca3	35390580	CG/C	TAGATCGGACGGTCGAAATC	CACGCACCTAGAACTTGCAG	59.7	704	INTRON	Ca_00819	O	--
Ca3_P2_35393263	Ca3	35393263	CTTTTT/CTTTTTT	AAGGAGATGCATGCCCTATG	CACCATCCCATATTTGGACC	59.9	715	INTRON	Ca_00820	--	--
Ca3_P2_35393421	Ca3	35393421	CAA/CA	AAGGAGATGCATGCCCTATG	CACCATCCCATATTTGGACC	59.9	715	INTRON	Ca_00820	--	--
Ca3_P2_35395949	Ca3	35395949	AG/A	AAGTGCACCACCTCCAAACT	TGAAGACATGCAAATGTTTACCA	60.4	575	DRR	Ca_00820	--	--
Ca3_P2_35396014	Ca3	35396014	CAAAAA/CAAAA	AAGTGCACCACCTCCAAACT	ATCTGTGTCGACCGAAAAGG	60.1	721	DRR	Ca_00820	--	--
Ca3_P2_35396573	Ca3	35396573	CTT/CTGATTTCTTGTT	CCTTTTCGGTCGACACAGAT	TGAGATTGAGCACCTTCGTG	60.0	273	DRR	Ca_00820	--	--
Ca3_P2_35397271	Ca3	35397271	TGTC/T	GATTCTCTTCACCGCTGCTC	ACAAACCGTTATTTTCCGCA	60.4	663	INTRON	Ca_00821	KA	--
Ca3_P2_35397297	Ca3	35397297	ATTAATTATTGTTAATTATTGTTA/ATTAATTATTGTTA	CGACTTCAACATCGAAACCA	ACAAACCGTTATTTTCCGCA	60.4	630	INTRON	Ca_00821	KA	--
Ca3_P2_35399079	Ca3	35399079	TA/T	ACTGCTTCAACCATTCCCAC	AACAGCTTCGCAACGAAAGT	60.1	374	INTRON	Ca_00821	KA	--
Ca3_P2_35399243	Ca3	35399243	C/CCGTCTCATTCA	ACTGCTTCAACCATTCCCAC	TAAGCATCCCACAGAAAGGG	60.1	793	INTRON	Ca_00821	KA	--
Ca3_P2_35399432	Ca3	35399432	ACC/AC	ACTTTCGTTGCGAAGCTGTT	TCCCACAGAAAGGGTTCAAG	60.1	433	INTRON	Ca_00821	KA	--

INDEL marker IDs	Chromosomes/unanchored scaffolds	Physical positions (bp)	InDels (*Kabuli* reference genome-CDC Frontier/PII)	Forward primers (5'-3')	Reverse primers (5'-3')	Anneling temperature (0C)	Expected amplified product size (bp)	Structural annotation		Functional annotation	
								Sequence components of *kabuli* genome	*Kabuli* gene accession IDs	NCBI-KOG	TFs
Ca3_P2_35403982	Ca3	35403982	ATCT/AT	TTCGCCTGCTCTCTTTTTGT	TTGGTCAAATCATTCAATCAGA	58.1	730	INTERGENIC	--	--	--
Ca3_P2_35405211	Ca3	35405211	AATATATATA/AATATATA	TGGGTATGTGAATGGAAAACA	TGTCTGGTGATTCGCTTTTT	58.4	670	INTERGENIC	--	--	--
Ca3_P2_35405768	Ca3	35405768	AAAAAGCAAAA/AAAAAGCAAAAGCAAAA	AAAAAGCGAATCACCAGACA	CCACAGACGGCGAAAATAAT	60.0	172	DRR	Ca_00823	--	--
Ca3_P2_35407498	Ca3	35407498	GT/G	ATTTTGAGTGTCTGGGCACC	AATGGGAGGGATAGTTTGGG	60.0	544	DRR	Ca_00823	--	--
Ca3_P2_35409813	Ca3	35409813	GTTTTTTTTT/GTTTTTTTTTT	TTTAGTCTGTGCGCATCTGG	TGCATCTTCTATGGTCCCAA	59.1	666	INTERGENIC	--	--	--
Ca3_P2_35410009	Ca3	35410009	CAAAAAAAAAAA/CAAAAAAAAA	CCCTTCATCGTTGAATAGGTTG	TGCATCTTCTATGGTCCCAA	59.1	561	INTERGENIC	--	--	--
Ca3_P2_35410044	Ca3	35410044	ATTTTTTT/ATTTTTTTTT	TTGTGACCGAATAGATGAGTAGTTG	TTTATTGCATCTTCTATGGTCCC	59.4	445	INTERGENIC	--	--	--
Ca3_P2_35410157	Ca3	35410157	GTTTTTTTT/GTTTTTTTTT	TGGGACCATAGAAGATGCAA	ACTCGATTTGGCACCATTTT	59.4	602	INTERGENIC	--	--	--
Ca3_P2_35413716	Ca3	35413716	TGG/TG	CCAGCTTTCCCATTTGTCAT	GTTTTGCCGGACCAACTTTA	60.0	704	INTRON	Ca_00824	S	--
Ca3_P2_35421003	Ca3	35421003	GCACA/GCA	ATTGAGAGATTCCAGCCCCT	ACCTTTCGGGTGCATAAGTG	60.0	509	INTRON	Ca_00824	S	--
Ca3_P2_35423816	Ca3	35423816	ATCTT/ATCTTCTT	AAGAAATGCCAACGGTTCTG	CATAAAATTCGACGGCGATAA	59.9	528	INTERGENIC	--	--	--
Ca3_P2_35425621	Ca3	35425621	GTTTTTTT/GTTTTTTTT	GTCGCCCCTGTGTCAGTTAT	TTCGCAATGTTTGCAGTAGC	60.0	508	INTERGENIC	--	--	--

INDEL marker IDs	Chromosomes/unanchored scaffolds	Physical positions (bp)	InDels (*Kabuli* reference genome-CDC Frontier/PII)	Forward primers (5'-3')	Reverse primers (5'-3')	Anneling temperature (0C)	Expected amplified product size (bp)	Structural annotation		Functional annotation	
								Sequence components of *kabuli* genome	*Kabuli* gene accession IDs	NCBI-KOG	TFs
Ca3_P2_35434798	Ca3	35434798	TATAAATAAATAAATAAATAA/TATAAATAAATAAATAA	CAGCCTTAGCACCCTGAAAG	AGGCTGATGATCCGAATGAG	60.2	475	INTRON	Ca_00826	--	B3
Ca3_P2_35435217	Ca3	35435217	GACA/GACACA	TGCAAATGCCCATACTTTCA	TATCCTGCGCATCCTTATCC	60.0	747	INTRON	Ca_00826	--	B3
Ca3_P2_35436004	Ca3	35436004	ATTTTTTTTT/ATTTTTTTT	GAAATAACGTTCCGCGTGTT	CATCATTGCCAAGTGCAAGT	59.7	454	INTERGENIC	--	--	--
Ca3_P2_35437574	Ca3	35437574	TT/TTCT	TTTCAATTCAACCTGGCCTC	CCACTGTTTGTGGCAAAATG	60.0	475	INTERGENIC	--	--	--
Ca3_P2_35438606	Ca3	35438606	GAAAAAAA/GAAAAAA	TTCGTTGAGACACCCTTTCC	TGTGTGTGTCATGTTTATGATTTTG	59.7	545	INTERGENIC	--	--	--
Ca3_P2_35439933	Ca3	35439933	TC/TCC	CATGAGCATGACTTTTTAGATGG	TTTATGGATATGCTGCACAGTT	57.4	564	INTERGENIC	--	--	--
Ca3_P2_35440050	Ca3	35440050	ATTT/ATT	CATGAGCATGACTTTTTAGATGG	CAAGGGCGGGTCTAGTATGT	59.1	952	INTERGENIC	--	--	--
Ca3_P2_35440193	Ca3	35440193	T/TCA	AACTGTGCAGCATATCCATAAA	CAAGGGCGGGTCTAGTATGT	59.1	410	INTERGENIC	--	--	--
Ca3_P2_35441304	Ca3	35441304	ATTTTTTTT/ATTTTTTT	CCCAAATAAAAACTTGTCCCAA	TCAAACATGGAAAAACTCAAGC	59.2	570	INTERGENIC	--	--	--
Ca3_P2_35447848	Ca3	35447848	TAA/TAAAA	AAAGACTGCACCCTTGTGCT	TGGAGTGGTGGAGGGTAAAG	60.0	517	INTRON	Ca_00827	OR	--
Ca3_P2_35449751	Ca3	35449751	CTATATATATATATATATATAT/CTATATATATATATAT	TGAGGTGCACGTGAAAACAT	CATCTGCTCCAATCCTCCAT	60.0	400	INTRON	Ca_00827	OR	--
Ca3_P2_35450073	Ca3	35450073	GAAAAAA/GAAAAAAA	ATGGAGGATTGGAGCAGATG	GGCATCATGCAAAATGATTG	59.9	248	INTRON	Ca_00827	OR	--

INDEL marker IDs	Chromosomes/unanchored scaffolds	Physical positions (bp)	InDels (*Kabuli* reference genome-CDC Frontier/PII)	Forward primers (5'-3')	Reverse primers (5'-3')	Anneling temperature (0C)	Expected amplified product size (bp)	Structural annotation		Functional annotation	
								Sequence components of *kabuli* genome	*Kabuli* gene accession IDs	NCBI-KOG	TFs
Ca3_P2_35456526	Ca3	35456526	CATATATATAT/CATATATAT	TGCGGTCGTATGAAGTAGCA	TCAAAAGTCCTTGAAAGAACAACA	60.2	820	INTERGENIC	--	--	--
Ca3_P2_35458777	Ca3	35458777	TAA/TA	TGCGGCTTTTTACGAGTTTC	ATGAAGAAAGGGAAAGCGGT	60.1	747	INTERGENIC	--	--	--
Ca3_P2_35468557	Ca3	35468557	TG/T	CGAACCATGCTTTGGAGATT	GCAGATGGTCCTTTTTCAGC	59.8	472	INTERGENIC	--	--	--
Ca3_P2_35469378	Ca3	35469378	TC/TCC	CATGACCCTCACTTCCACCT	GCACAAGAAGTCTGGGAAGC	60.0	577	INTERGENIC	--	--	--
Ca3_P2_35470778	Ca3	35470778	ATTTTTTTT/ATTTTTTT	CGTTGAACCCTAGTCCCAAA	ACGTAGCTTGTGGCGAGATT	59.9	736	INTERGENIC	--	--	--
Ca3_P2_35484198	Ca3	35484198	CA/CAGTTAA	TTTATGCAGATGTGCCGTGT	TCTAGCAGTGGAGGTTGGCT	60.0	608	INTRON	Ca_00831	R	--
Ca3_P2_35488328	Ca3	35488328	GAAA/GAA	CTCGCTGGAATTTGCTAACC	AGGACGTTGCTGCAAGATTT	59.9	502	INTRON	Ca_00832	S	--
Ca3_P2_35492143	Ca3	35492143	GAAAAAAAAAAA/GAAAAAAAAAA	CCTTGCGCTCTTGAGTAACC	TTTTCATTGCCAAATGGTGA	59.9	189	INTRON	Ca_00832	S	--
Ca3_P2_35494118	Ca3	35494118	TTATATATATATATATATATATATATA/TTATATATATATATATATATATA	GTCTCGGCCCAAAAGTAACA	TGCCAAATTTTACCCCTTTG	59.8	720	INTERGENIC	--	--	--
Ca3_P2_35495999	Ca3	35495999	GAAA/GAAAA	TGGGTTTTGTTTGAAGATTTTG	TGAGAGTTGTGGAGGAGAGGA	60.0	447	INTERGENIC	--	--	--
Ca3_P2_35496087	Ca3	35496087	AAAAGA/AA	TGGGTTTTGTTTGAAGATTTTG	TGAGAGTTGTGGAGGAGAGGA	60.0	447	INTERGENIC	--	--	--
Ca3_P2_35498417	Ca3	35498417	ATTCATATATTTTA/ATTCATATATTTTATCACTTCATATATTTTA	GCTGTCTTGACTTTGACGGA	TCGGTGCCATCATAAGTGTC	59.5	604	INTERGENIC	--	--	--

INDEL marker IDs	Chromosomes/unanchored scaffolds	Physical positions (bp)	InDels (*Kabuli* reference genome-CDC Frontier/PII)	Forward primers (5'-3')	Reverse primers (5'-3')	Anneling temperature (0C)	Expected amplified product size (bp)	Structural annotation		Functional annotation	
								Sequence components of *kabuli* genome	*Kabuli* gene accession IDs	NCBI-KOG	TFs
Ca3_P2_35505847	Ca3	35505847	TAAAA/TAAA	CCGTCCTCCAATATAACCCA	TGTTTGGAGGGAAAACAAGG	59.9	334	INTERGENIC	--	--	--
Ca3_P2_35508699	Ca3	35508699	TAAA/TAAAA	GTGCGCTCCATTTTTACCAT	CGTCGCATGAGTGGAGTCTA	60.0	549	DRR	Ca_00833	--	--
Ca3_P2_35515650	Ca3	35515650	AGTGTG/AGTG	ATGACATATAAGAGCCGCCG	GCGCCGATTTCAGAATCTAC	59.8	337	INTERGENIC	--	--	--
Ca3_P2_35527763	Ca3	35527763	ATTTT/ATTTTT	TGCCAATCACATGCAAAAAT	ATTGGATGAACGATGACACG	59.4	324	INTERGENIC	--	--	--
Ca3_P2_35528890	Ca3	35528890	ATTAT/ATTATTTTTAT	AACGTCGACCAGCCTACACT	ATCCACACAAAACTCGGCAT	60.4	422	INTERGENIC	--	--	--
Ca3_P2_35532266	Ca3	35532266	GTT/GT	TTCGAGATGTCAGATTCCCC	CCAGTTTGCTTCTCTGGACC	59.8	348	DRR	Ca_00835	--	--
Ca3_P2_35532624	Ca3	35532624	TG/T	CACATAATGTTGGCGTGGTC	ACCGTATGTGGACTCTCGCT	59.8	797	INTERGENIC	--	--	--
Ca3_P2_35536371	Ca3	35536371	TAAAT/TAAATATCTAAATCAAAT	GCCCTTTTGTGTTTTTGGAG	TGCAAACTCTCATTCGCATC	60.0	568	INTERGENIC	--	--	--
Ca3_P2_35539553	Ca3	35539553	AAA/AAAGAA	TTAATGGAATAATGGCCGCT	AGCCAACGATACACCTTCGT	59.6	859	INTERGENIC	--	--	--
Ca3_P2_35540047	Ca3	35540047	TATATATA/T	ACGAAGGTGTATCGTTGGCT	TTGCGATTGAAGCATTGAAG	60.0	340	INTERGENIC	--	--	--
Ca3_P2_35553013	Ca3	35553013	CAAAAA/CAAAAAA	GCCACTGTTCCCATACCATT	GCCATGTCTCTCACTCACGA	60.0	399	INTRON	Ca_00837	--	--
Ca3_P2_35558006	Ca3	35558006	TAA/TA	ATCCCGCGTAATTCCTCTCT	ATCCCCTAACATTTCCGACC	60.0	403	INTERGENIC	--	--	--

INDEL marker IDs	Chromosomes/unanchored scaffolds	Physical positions (bp)	InDels (*Kabuli* reference genome-CDC Frontier/PII)	Forward primers (5'-3')	Reverse primers (5'-3')	Anneling temperature (0C)	Expected amplified product size (bp)	Structural annotation		Functional annotation	
								Sequence components of *kabuli* genome	*Kabuli* gene accession IDs	NCBI-KOG	TFs
Ca3_P2_35560442	Ca3	35560442	GAAAAAAAAAAA/GAAAAAAAAAA	TCTGAGAGCCTGGACCAGAT	CTGCTTGGGCTTACCTTCAG	60.0	709	DRR	Ca_00839	--	--
Ca3_P2_35562376	Ca3	35562376	ATG/AATCTATTGTG	TTTTCTTCCAGACACCCGTT	CAGCTATATTACCTCATATCCACCAA	59.7	494	INTERGENIC	--	--	--
Ca3_P2_35562625	Ca3	35562625	TAAAAAAA/TAAAAAAAA	TGATCTATTTGGTGGATATGAGG	CAAACATTCATTCGCACTATTCA	60.0	566	INTERGENIC	--	--	--
Ca3_P2_35562721	Ca3	35562721	A/AT	TGATCTATTTGGTGGATATGAGG	TGCAATAGGTAAAGCCACCT	57.4	795	INTERGENIC	--	--	--
Ca3_P2_35564542	Ca3	35564542	CTTTTTTT/CTTTTTT	ACCCATGCATACGACACTGA	GGCTTCAAAATTGGGTGCTA	60.1	336	INTRON	Ca_00840	O	--
Ca3_P2_35566663	Ca3	35566663	GAA/GAAA	CCAGACCCCAAACTTAGCAA	ATGGCCAGTGCTATATTCCG	59.9	550	DRR	Ca_00840	O	--
Ca3_P2_35568618	Ca3	35568618	AACGTACGT/AACGT	TGTGGCATGCAAATGCTTAT	TCCTGTGTTGTTGTGTCCGT	60.0	572	DRR	Ca_00841	--	--
Ca3_P2_35574829	Ca3	35574829	AG/A	TTCAAGATTGAAAATGCTTTGTG	AATCGCATCGGCTAAACAAT	59.6	412	INTERGENIC	--	--	--
Ca3_P2_35575076	Ca3	35575076	G/GAGTA	TTCAAGATTGAAAATGCTTTGTG	AATCGCATCGGCTAAACAAT	59.6	412	INTERGENIC	--	--	--
Ca3_P2_35575589	Ca3	35575589	ATA/ATAAAATTTA	ATTGTTTAGCCGATGCGATT	TGGGTTTAGCAACCACCTTC	60.0	729	INTERGENIC	--	--	--
Ca3_P2_35584142	Ca3	35584142	A/AGC	TATCACGTGGTGAATTGGCA	AGGGGAAAATGAAAGGGATG	60.1	761	INTERGENIC	--	--	--
Ca3_P2_35584493	Ca3	35584493	CTTT/CTT	CATCCCTTTCATTTTCCCCT	TTGTGTTTGGGCAATGTGTT	59.9	136	INTERGENIC	--	--	--

**INDEL marker IDs**	**Chromosomes/unanchored scaffolds**	**Physical positions (bp)**	**InDels (*Kabuli* reference genome-CDC Frontier/PII)**	**Forward primers (5'-3')**	**Reverse primers (5'-3')**	**Anneling temperature (0C)**	**Expected amplified product size (bp)**	**Structural annotation**		**Functional annotation**	
								**Sequence components of *kabuli* genome**	***Kabuli* gene accession IDs**	**NCBI-KOG**	**TFs**
Ca3_P2_35591231	Ca3	35591231	TAAAA/T	CACACCTTTGAAGATGGGTCT	TGTCTCCGGATAGATGACATTG	60.0	287	DRR	Ca_00843	--	bZIP
Ca3_P2_35593827	Ca3	35593827	GTT/GT	ACGTTGGGACGGACTATGAG	ACGCGCTTTTTCACTTGTTT	59.9	391	INTERGENIC	--	--	--
Ca3_P2_35596803	Ca3	35596803	ATTTTTTTT/ATTTTTTTTT	GTCAACGCAAGCGAAAACTA	CGAAAAATCATCCGGAGAAA	60.0	524	DRR	Ca_00844	--	bZIP
Ca3_P2_35600296	Ca3	35600296	TTG/T	GGCCTTAAAATCTGAAACCCA	CAGCTCTAGCAATGCGACTG	59.9	861	DRR	Ca_00845	--	--
Ca3_P2_35600374	Ca3	35600374	TAAAAAAAAA/TAAAAAA	GGCCTTAAAATCTGAAACCCA	CAGCTCTAGCAATGCGACTG	59.9	861	DRR	Ca_00845	--	--
Ca3_P2_35600454	Ca3	35600454	TAA/TAAA	AATTTCACAGTCCCGTCACA	CAATGCGACTGGGTTTACCT	60.0	334	DRR	Ca_00845	--	--
Ca3_P2_35602083	Ca3	35602083	CATGTTTTAT/CAT	GGGTGCGTTAGTGCATTTTT	GCCATGCTTTAACGAAGAGG	59.8	940	INTERGENIC	--	--	--
Ca3_P2_35602156	Ca3	35602156	A/ACTATCAAATT	TCCTCTTCGTTAATGGCTGC	CGCGTAGATCCCACAAAATC	60.5	750	INTERGENIC	--	--	--
Ca3_P2_35602730	Ca3	35602730	G/GA	CCTCTTCGTTAAAGCATGGC	TGGGCCTAGTTTTGTTGACC	60.0	653	INTERGENIC	--	--	--
Ca3_P2_35605337	Ca3	35605337	TC/TCC	CAAAGTGGCATCCATCACAT	CAATTGGATGGCCCTTAGAA	59.9	232	DRR	Ca_00846	--	--
Ca3_P2_35605669	Ca3	35605669	GAAAA/GAAAACAAAAA	TGCTAGAAAGGACCACCACC	TATGCTCGCGTGCAATTTTA	60.4	307	DRR	Ca_00846	--	--
Ca3_P2_35605855	Ca3	35605855	CATATATATA/CATATATATATATATATATATATA	TGCTAGAAAGGACCACCACC	TGAATCATCTCCCCTTTTGG	59.9	661	DRR	Ca_00846	--	--

INDEL marker IDs	Chromosomes/unanchored scaffolds	Physical positions (bp)	InDels (*Kabuli* reference genome-CDC Frontier/PII)	Forward primers (5'-3')	Reverse primers (5'-3')	Anneling temperature (0C)	Expected amplified product size (bp)	Structural annotation		Functional annotation	
								Sequence components of *kabuli* genome	*Kabuli* gene accession IDs	NCBI-KOG	TFs
Ca3_P2_35614019	Ca3	35614019	ATTTTTTTTT/ATTTTTTTTTT	TGATCAAGATTTGAATTTTTGTTTTC	ATCCCCGTGTGTTGTTGTTT	60.1	256	INTERGENIC	--	--	--
Ca3_P2_35615874	Ca3	35615874	AG/A	TTGCACTGACCATTTTTCTTAAA	CACTACGGATGCTCCAAACA	59.7	323	DRR	Ca_00847	--	--
Ca3_P2_35615927	Ca3	35615927	TAAAAAAAA/TAAAAAAA	TCATGCATCCCTACAAATTCA	CACTACGGATGCTCCAAACA	59.7	140	DRR	Ca_00847	--	--
Ca3_P2_35618022	Ca3	35618022	GTTTTTTTT/GTTTTTTT	TGCGTACCTTAATCAGGAACAA	TTTACGCGGGCGTATTTTAC	60.0	882	INTERGENIC	--	--	--
Ca3_P2_35618119	Ca3	35618119	T/TC	TGTGAAACGAACAAAGCTTAAA	TTTACGCGGGCGTATTTTAC	60.0	555	INTERGENIC	--	--	--
Ca3_P2_35621774	Ca3	35621774	TAAAAAAAA/TAAAAAAA	TGTCGATTAAGAAACATGCGA	ATACTGGGGGTAATCCCGTC	59.9	591	DRR	Ca_00848	--	--
Ca3_P2_35624827	Ca3	35624827	CTTTTTT/CTTTTTTT	CCTTGCGTAACAATTCACCA	TTGGGACAAAGAATGCATGA	60.0	248	DRR	Ca_00849	--	--
Ca3_P2_35625961	Ca3	35625961	GTTTTTTTT/GTTTTTTTTT	AGGGGGAAGGAGAAAAGACA	CCCAACGATCCTAGCTTCAA	60.2	230	DRR	Ca_00850	--	--
Ca3_P2_35625993	Ca3	35625993	TGAA/TGAAGAA	AGGGGGAAGGAGAAAAGACA	CCCAACGATCCTAGCTTCAA	60.2	230	DRR	Ca_00850	--	--
Ca3_P2_35626462	Ca3	35626462	TCTAC/TC	TGCAGTGGAAATGGATTTGA	GCGGTGATTCAGATTCAAAAG	59.7	513	DRR	Ca_00850	--	--
Ca3_P2_35626631	Ca3	35626631	ATTTTTT/ATTTTTTT	TTGAATCTGAATCACCGCTTT	CCAAAAGGAGTATGGATCAGTAA	57.3	459	DRR	Ca_00850	--	--
Ca3_P2_35626825	Ca3	35626825	CTTTTTTTT/CTTTTTT	TTGAATCTGAATCACCGCTTT	TGTTTGTGTCGGAGTTTTCAG	58.8	707	DRR	Ca_00850	--	--

INDEL marker IDs	Chromosomes/unanchored scaffolds	Physical positions (bp)	InDels (*Kabuli* reference genome-CDC Frontier/PII)	Forward primers (5'-3')	Reverse primers (5'-3')	Anneling temperature (0C)	Expected amplified product size (bp)	Structural annotation		Functional annotation	
								Sequence components of *kabuli* genome	*Kabuli* gene accession IDs	NCBI-KOG	TFs
Ca3_P2_35631172	Ca3	35631172	TAAA/TAAAA	CACCCCTAAAATCCCTTTCT	CTGCAGCAATATCAAAGGTCA	58.9	642	DRR	Ca_00851	--	--
Ca3_P2_35631361	Ca3	35631361	ATAAATTT/AT	CACCCCTAAAATCCCTTTCT	CCAGCTGCAGCAATATCAAA	60.0	646	DRR	Ca_00851	--	--
Ca3_P2_35631433	Ca3	35631433	TAAAAA/TAAAA	CACCCCTAAAATCCCTTTCT	CCAGCTGCAGCAATATCAAA	60.0	646	DRR	Ca_00851	--	--
Ca3_P2_35632027	Ca3	35632027	ATTTTT/ATTTTTT	TACACCTCCATTTTGCACGA	TTCCACTCACCCTATTTGGC	59.9	624	DRR	Ca_00851	--	--
Ca3_P2_35633984	Ca3	35633984	AATATATATATATATA/AATATATATATATATATATATATATA	TCACGAGGAAGTATGGGGAG	GCAATGAATGGTGCATTTTT	58.5	864	INTERGENIC	--	--	--
Ca3_P2_35635776	Ca3	35635776	CTTTT/CTTT	GTAATTTTGGCGCTGGTGTT	GCGAATGCATAGAGCCTACC	59.8	635	INTERGENIC	--	--	--
Ca3_P2_35643455	Ca3	35643455	T/TA	TGTGTTTCGTCGGCTGATTA	GCACCTGGAACTCAGGAAAC	59.7	518	INTRON	Ca_00852	S	--
Ca3_P2_35644013	Ca3	35644013	ATACCT/ATACCTACCT	GTTTCCTGAGTTCCAGGTGC	TGGTTCGCTGTTTGTCACTC	59.9	249	CDS (FRAME SHIFT)	Ca_00852	S	--
Ca3_P2_35644626	Ca3	35644626	TTCTC/TTCTCTCTC	CTCCCAAGAGTGAGAGCACC	CAGTTGGAGGTACCACCGTT	59.9	269	INTRON	Ca_00852	S	--
Ca3_P2_35649725	Ca3	35649725	A/ACTC	TGAAAGAAAATGGGTATGGCA	GACTGGACACCATACAGGGG	60.2	526	INTERGENIC	--	--	--
Ca3_P2_35651872	Ca3	35651872	ATTT/AT	ATTCTTCTCCCGCTCACTCA	AGACTGGTGGTATGGGCAAA	60.4	532	INTRON	Ca_00853	--	--
Ca3_P2_35652779	Ca3	35652779	GTT/GTTTT	AAGTGTTTGGTGGTGGTGGT	CCACTGTCCAGTCTCTGCAA	60.0	499	INTRON	Ca_00853	--	--

INDEL marker IDs	Chromosomes/unanchored scaffolds	Physical positions (bp)	InDels (*Kabuli* reference genome-CDC Frontier/PII)	Forward primers (5'-3')	Reverse primers (5'-3')	Anneling temperature (0C)	Expected amplified product size (bp)	Structural annotation		Functional annotation	
								Sequence components of *kabuli* genome	*Kabuli* gene accession IDs	NCBI-KOG	TFs
Ca3_P2_35655203	Ca3	35655203	ATTTTTGTTGTTGTTTTTG/ATTTTTG	GGTCGTTCTTCAGGAATGGA	TGAATCATCCAAGTGCCAAA	60.0	717	DRR	Ca_00853	--	--
Ca3_P2_35656833	Ca3	35656833	TAAAAAAAAA/TAAAAAAAA	TCGAGCCATTTTTCTTCTTCA	TTCCATGTGTTGTGCCAAGT	60.0	970	DRR	Ca_00854	--	WRKY
Ca3_P2_35662531	Ca3	35662531	GGTCCT/G	TTCGATTATAAATGGGACCACA	CGTGTCTAGAACACACACGGA	59.8	399	INTERGENIC	--	--	--
Ca3_P2_35664034	Ca3	35664034	ATTTTT/ATT	GCATTTGGCCACATTTCTTT	TGTAACACGTCGACCAATCAA	60.0	711	INTERGENIC	--	--	--
Ca3_P2_35678085	Ca3	35678085	TTCT/TT	TTGAGCACGACAGAATCACC	ACAATGGCCCAACTCTGTTC	60.0	441	DRR	Ca_00855	--	--
Ca3_P2_35678189	Ca3	35678189	GTT/GT	GAACAGAGTTGGGCCATTGT	ACATGCCATAACAACTCGCA	60.1	191	DRR	Ca_00855	--	--
Ca3_P2_35679181	Ca3	35679181	ATTTTTTTT/ATTTTTTT	TTCATTCTTTGGCTTGGGAC	CGTCATGGTCGTGGCTTTAT	60.9	959	DRR	Ca_00856	R	SBP
Ca3_P2_35679729	Ca3	35679729	TTCTCTCTCTCT/TTCTCTCTCT	GCCACGACCATGACGTACTA	GCCAATATGGGGTTTTCCTT	60.0	252	DRR	Ca_00856	R	SBP
Ca3_P2_35683554	Ca3	35683554	TTAAT/TT	TGAAGACAAGGATCCCAAGG	CTGGAATGTGGGCAGTTCTT	60.1	555	INTRON	Ca_00856	R	SBP
Ca3_P2_35685531	Ca3	35685531	ATTTTTTTT/ATTTTTTTTTT	GAAATAGGCAGAGACAGGTTCAA	CCTCTGCTCTATGCATTGCC	60.9	985	INTERGENIC	--	--	--
Ca3_P2_35685757	Ca3	35685757	TAA/TA	TGCAAAATCGATCTCTCCTTC	GGCCCCATAGTTTTTCCAAT	60.0	580	INTERGENIC	--	--	--
Ca3_P2_35685874	Ca3	35685874	AA/AATA	TGCAAAATCGATCTCTCCTTC	GGCCCCATAGTTTTTCCAAT	60.0	580	INTERGENIC	--	--	--

INDEL marker IDs	Chromosomes/unanchored scaffolds	Physical positions (bp)	InDels (*Kabuli* reference genome-CDC Frontier/PII)	Forward primers (5'-3')	Reverse primers (5'-3')	Anneling temperature (0C)	Expected amplified product size (bp)	Structural annotation		Functional annotation	
								Sequence components of *kabuli* genome	*Kabuli* gene accession IDs	NCBI-KOG	TFs
Ca3_P2_35686882	Ca3	35686882	AATATATATATATATATA/AATATATATATATA	GCATCAGCATTTCCGAATCT	CACTCGATAGTGGCTGCTGA	60.2	393	INTERGENIC	--	--	--
Ca3_P2_35692305	Ca3	35692305	C/CAAA	CCTCGTGACCCGACACTTAT	CACTCTATTTGGGCCGTGTT	60.0	816	DRR	Ca_00858	R	ZF-HD
Ca3_P2_35694757	Ca3	35694757	TAAAAAAAAAA/TAAAAAAAAA	CATTAAAGGGCGGAACAAAA	TGTGCCAATTAGGGATTAAACT	57.7	164	INTERGENIC	--	--	--
Ca3_P2_35697688	Ca3	35697688	TAAAAAAA/TAAAAAAAAA	TTTCATATCCACCCGTCAAA	TGAATTTGCCTAATTTGGTCC	58.9	628	INTERGENIC	--	--	--
Ca3_P2_35699071	Ca3	35699071	CTGTAATGTAAT/CTGTAAT	ACGAATCTCTCAACCATCGG	TGAGGCTCTCTTTGTGACTTTG	59.7	711	INTERGENIC	--	--	--
Ca3_P2_35699306	Ca3	35699306	TAAAAA/TAAAAAA	GCTTCCTGGTAGGACAAACAA	TGAGGCTCTCTTTGTGACTTTG	59.7	455	INTERGENIC	--	--	--
Ca3_P2_35703336	Ca3	35703336	CAAAAAA/CAAAAAAA	CGGATATGCGGACAAAAACT	TTAGCTTTGGGGTTTGATGG	59.9	600	INTERGENIC	--	--	--
Ca3_P2_35703384	Ca3	35703384	A/ATATATAT	CGGATATGCGGACAAAAACT	TTAGCTTTGGGGTTTGATGG	59.9	600	INTERGENIC	--	--	--
Ca3_P2_35703988	Ca3	35703988	CA/CATAATAA	CCATCAAACCCCAAAGCTAA	CACTGTTTCTTTCACAACTCTCG	59.1	942	INTERGENIC	--	--	--
Ca3_P2_35706702	Ca3	35706702	AAAATTAAATT/AAAATT	TCGGCCACTATTTCTTCCAC	CGTTGTGCCTAAATTGGGAT	59.8	985	INTERGENIC	--	--	--
Ca3_P2_35708125	Ca3	35708125	CTATATAGTATATA/CTATATA	ATTTGGGAGCTAGCAAAGCA	CATATCCAACTCAAAGTCGCAA	60.1	593	INTERGENIC	--	--	--
Ca3_P2_35709940	Ca3	35709940	AT/ATCGT	TGTGTGATGTGATGTGTGCC	CCCTCTCCACTGTTGCTCTC	60.0	524	INTERGENIC	--	--	--

INDEL marker IDs	Chromosomes/unanchored scaffolds	Physical positions (bp)	InDels (*Kabuli* reference genome-CDC Frontier/PII)	Forward primers (5'-3')	Reverse primers (5'-3')	Anneling temperature (0C)	Expected amplified product size (bp)	Structural annotation		Functional annotation	
								Sequence components of *kabuli* genome	*Kabuli* gene accession IDs	NCBI-KOG	TFs
Ca3_P2_35711338	Ca3	35711338	AT/A	AAACCACCGTCCCTAAATCC	TGCGGTTTGATTCCATTTTT	60.3	388	INTERGENIC	--	--	--
Ca3_P2_35712326	Ca3	35712326	AATATATATATA/AATATATATATATA	TCGAAATTACACCGCAAACA	TGGCAAAATGAGGGACAAAT	60.3	519	INTERGENIC	--	--	--
Ca3_P2_35725765	Ca3	35725765	A/AT	TGACACGATTAATATCGCTTTGA	GCACCCCTCCTTTAGAGTCC	60.1	647	INTERGENIC	--	--	--
Ca3_P2_35725827	Ca3	35725827	TAAAAAA/TAAAAAAA	AAATCATAAGAACAAGGAGGATCG	GCACCCCTCCTTTAGAGTCC	60.1	549	INTERGENIC	--	--	--
Ca3_P2_35725900	Ca3	35725900	CTAAA/CTAAAAGTAAA	TGTGTGTGTCTACCAAAATACTCG	TTTCCACACTCTAATCGGGG	59.9	743	INTERGENIC	--	--	--
Ca3_P2_35726187	Ca3	35726187	TG/T	GGACTCTAAAGGAGGGGTGC	TTTCCACACTCTAATCGGGG	59.9	326	INTERGENIC	--	--	--
Ca3_P2_35726503	Ca3	35726503	A/AC	CCCCGATTAGAGTGTGGAAA	GCACTTTCACATGGTGTGGT	59.4	410	INTERGENIC	--	--	--
Ca3_P2_35726533	Ca3	35726533	TTATATA/TTATATATATA	CCCCGATTAGAGTGTGGAAA	GCACTTTCACATGGTGTGGT	59.4	410	INTERGENIC	--	--	--
Ca3_P2_35726898	Ca3	35726898	CAAAAAAAAA/CAAAAAAAAAAAA	CAACCACACCATGTGAAAGTG	ACGCATACGGCATTTTTCTC	60.1	803	INTERGENIC	--	--	--
Ca3_P2_35730551	Ca3	35730551	CATT/CATTAATT	TTAAAAGGCCATGGGCATAA	TGGTGGTGCTGGACAATTAG	59.6	125	INTERGENIC	--	--	--
Ca3_P2_35733605	Ca3	35733605	CTTT/CTT	TCTCCCCTTTCCTTTCATTT	AAAAACGTTAATTTGAGACAGCAA	59.3	360	INTERGENIC	--	--	--
Ca3_P2_35733690	Ca3	35733690	AATTTTATTTT/AATTTT	TCTCCCCTTTCCTTTCATTT	TGGGGAATTCATACTTATCCAAA	59.6	767	INTERGENIC	--	--	--

INDEL marker IDs	Chromosomes/unanchored scaffolds	Physical positions (bp)	InDels (*Kabuli* reference genome-CDC Frontier/PII)	Forward primers (5'-3')	Reverse primers (5'-3')	Anneling temperature (0C)	Expected amplified product size (bp)	Structural annotation		Functional annotation	
								Sequence components of *kabuli* genome	*Kabuli* gene accession IDs	NCBI-KOG	TFs
Ca3_P2_35734649	Ca3	35734649	A/AAT	CTGCGTGCGTCATTGTATTC	TGTTCATCCCAAATTGAAAATG	59.7	860	INTERGENIC	--	--	--
Ca3_P2_35740511	Ca3	35740511	TCC/TC	GTCGTGAATTTCCCCTTTCA	TGCCGCATTGATTCATTTTA	60.0	547	DRR	Ca_00861	--	CPP
Ca3_P2_35740818	Ca3	35740818	TAAAAAAA/TAAAAAA	GTCGTGAATTTCCCCTTTCA	TGCCGCATTGATTCATTTTA	60.0	547	DRR	Ca_00861	--	CPP
Ca3_P2_35741226	Ca3	35741226	TGG/TG	CATCCGAAAAATAATGGCGT	CGATGACTAACGGTCGACAA	59.7	588	DRR	Ca_00861	--	CPP
Ca3_P2_35741530	Ca3	35741530	C/CG	TGCAGGTTCACTTTCCAACA	CATTTCATCCATCCACCCTC	60.1	683	DRR	Ca_00861	--	CPP
Ca3_P2_35743869	Ca3	35743869	TCC/T	AATTTGGTCTTTGTCACAGTCA	TGTTTTTGTTCATTTCGGTCA	59.0	384	INTERGENIC	--	--	--
Ca3_P2_35746621	Ca3	35746621	CTT/CTTT	TTGTTAGGGCTCGATCTGAAA	ATCGTTCAAGCACGTATCCC	60.0	538	INTERGENIC	--	--	--
Ca3_P2_35746767	Ca3	35746767	ATTTTTTTTTTT/ATTTTTTTT	AAAGCCTCGTATATGCACCG	TGCGAACAAGTTCATAGCATTT	59.8	510	INTERGENIC	--	--	--
Ca3_P2_35746875	Ca3	35746875	ATATTATTATT/ATATTATTATTATTATT	AAAGCCTCGTATATGCACCG	TGCGAACAAGTTCATAGCATTT	59.8	510	INTERGENIC	--	--	--
Ca3_P2_35749541	Ca3	35749541	ATTG/ATTGTTTTG	TGAAGATTGGTTAGGCCTGG	GTTGCGGAAAACAACTGTCA	59.7	495	INTRON	Ca_00862	D	--
Ca3_P2_35751166	Ca3	35751166	GCTTCTT/GCTT	TTGATAAAGCCTGGAAGCGT	CTTTCACACCACTTCCCGAT	60.0	209	INTRON	Ca_00862	D	--
Ca3_P2_35751286	Ca3	35751286	TCCCCCCC/TCCCCCC	ATCGGGAAGTGGTGTGAAAG	ACCCTTGGTCTGATTCGATG	59.9	464	INTRON	Ca_00862	D	--

INDEL marker IDs	Chromosomes/unanchored scaffolds	Physical positions (bp)	InDels (*Kabuli* reference genome-CDC Frontier/PII)	Forward primers (5'-3')	Reverse primers (5'-3')	Anneling temperature (0C)	Expected amplified product size (bp)	Structural annotation		Functional annotation	
								Sequence components of *kabuli* genome	*Kabuli* gene accession IDs	NCBI-KOG	TFs
Ca3_P2_35765376	Ca3	35765376	GTATATATATATA/GTATATATATATATA	ACATTTGGTCTGGGTCAAGC	CATGGTTCCTCGTTCCTGTT	60.0	327	DRR	Ca_00862	D	--
Ca3_P2_35766292	Ca3	35766292	AGGGTGGG/AGGG	CTATTCGTGTGCGCTGAAAA	AAAGGCATTGGAGTAGGACG	59.2	612	DRR	Ca_00862	D	--
Ca3_P2_35767362	Ca3	35767362	AAAAA/AAAAAATAAAA	GGGTGCTTCCTCAATCTTCA	GACTTTGGAAGTCGGCAAAA	60.2	798	INTERGENIC	--	--	--
Ca3_P2_35768780	Ca3	35768780	T/TC	CCTTATCCCCGAAATTTGAA	GCGGATAGCAAATCGTTCAT	60.1	271	INTERGENIC	--	--	--
Ca3_P2_35768860	Ca3	35768860	TAAAAAA/TAAAAAAA	CCTTATCCCCGAAATTTGAA	GCGGATAGCAAATCGTTCAT	60.1	271	INTERGENIC	--	--	--
Ca3_P2_35770921	Ca3	35770921	GTAAT/GT	GCATGGTGCAGTGAAAGAGA	AATTGAGGTCCCGTCAGTTG	60.0	448	DRR	Ca_00863	U	bZIP
Ca3_P2_35776584	Ca3	35776584	TTATATAT/TTATATATAT	GGTGGTTTTGCACTTTTGCT	TCACATACTCGCCAGACTGC	60.0	712	DRR	Ca_00865	R	G2-like
Ca3_P2_35779416	Ca3	35779416	CTTTTTTTTTTTTT/CTTTTTTTTTTT	GCAAATCCTCTGCTCCTGTC	TTCCATTTCACTCACCACCA	59.9	548	DRR	Ca_00866	K	NF-YA
Ca3_P2_35779467	Ca3	35779467	TA/TTATAA	GCAAATCCTCTGCTCCTGTC	TTCCATTTCACTCACCACCA	59.9	548	DRR	Ca_00866	K	NF-YA
Ca3_P2_35779513	Ca3	35779513	GAAAAAAA/GAAAAAAAA	GCAAATCCTCTGCTCCTGTC	TTCCATTTCACTCACCACCA	59.9	548	DRR	Ca_00866	K	NF-YA
Ca3_P2_35779977	Ca3	35779977	TACAACAA/TACAA	TGGTGGTGAGTGAAATGGAA	GTGAAATGCAAAGCCCAAAT	59.9	176	DRR	Ca_00866	K	NF-YA
Ca3_P2_35783291	Ca3	35783291	TAAAAAAA/TAAAAAA	TGGAAAGTCAACTCAATGCG	TGCTTCGGAAAATCTAGATGAA	59.0	303	INTRON	Ca_00866	K	NF-YA

INDEL marker IDs	Chromosomes/unanchored scaffolds	Physical positions (bp)	InDels (*Kabuli* reference genome-CDC Frontier/PII)	Forward primers (5'-3')	Reverse primers (5'-3')	Anneling temperature (0C)	Expected amplified product size (bp)	Structural annotation		Functional annotation	
								Sequence components of *kabuli* genome	*Kabuli* gene accession IDs	NCBI-KOG	TFs
Ca3_P2_35783476	Ca3	35783476	ACTATTC/AC	TGGAAAGTCAACTCAATGCG	CTTACATCGAGCCGACACAA	59.9	745	INTRON	Ca_00866	K	NF-YA
Ca3_P2_35783609	Ca3	35783609	ATGT/ATGTGT	TGGAAAGTCAACTCAATGCG	CTTACATCGAGCCGACACAA	59.9	745	INTRON	Ca_00866	K	NF-YA
Ca3_P2_35784516	Ca3	35784516	A/AG	TGGTATGCTCTTTCCCCAAC	TCCAGCTTCTGTTTCAAGACC	59.5	486	INTERGENIC	--	--	--
Ca3_P2_35784715	Ca3	35784715	TAAAAAA/TAAAAA	GGTGCTGCTCCTGAATTAGC	GGCTCCAATCTAATGGGGTT	60.2	798	INTERGENIC	--	--	--
Ca3_P2_35784745	Ca3	35784745	TAAAAAAAA/TAAAAAAAAA	GGTGCTGCTCCTGAATTAGC	GGCTCCAATCTAATGGGGTT	60.2	798	INTERGENIC	--	--	--
Ca3_P2_35787094	Ca3	35787094	TAAAAAAAA/TAAAAAAAAA	AGCATTAGGGTGAATCGTGC	AACTTGAAGCGTGAAGGCAT	59.9	732	INTERGENIC	--	--	--
Ca3_P2_35787147	Ca3	35787147	ATTTTTTTT/ATTTTTTTTTT	AGCATTAGGGTGAATCGTGC	AACTTGAAGCGTGAAGGCAT	59.9	732	INTERGENIC	--	--	--
Ca3_P2_35788163	Ca3	35788163	GATAAATATTTTATAAA/GATAAATATTTTATAAATATTTTATAAA	CCTTCACCTTAGGCCATTGA	CACCTTGATTTTACCACACTCG	59.5	645	INTERGENIC	--	--	--
Ca3_P2_35789381	Ca3	35789381	TATAAAATAAAATAAAA/TATAAAATAAAA	ATGGCTACTGCTCGTTTGTT	AAACGTGGGGCACATTTTAG	59.9	766	INTERGENIC	--	--	--
Ca3_P2_35791544	Ca3	35791544	TAGCCA/TAGCCAGCCA	AAGTGATACACCGACCGTCA	TGAATGACGCCTTCTTCCTC	60.3	455	INTERGENIC	--	--	--
Ca3_P2_35795684	Ca3	35795684	ATTTTTTT/ATTTTTTTT	GACCATGGCATTGAGTTCCT	GGTCTCCAGCTCAAGATTGC	60.0	676	INTERGENIC	--	--	--
Ca3_P2_35796430	Ca3	35796430	CTTTT/CTTT	GCAATCTTGAGCTGGAGACC	GGCACAAGTCAAATGGTCAA	59.5	909	INTERGENIC	--	--	--

INDEL marker IDs	Chromosomes/unanchored scaffolds	Physical positions (bp)	InDels (*Kabuli* reference genome-CDC Frontier/PII)	Forward primers (5'-3')	Reverse primers (5'-3')	Anneling temperature (0C)	Expected amplified product size (bp)	Structural annotation		Functional annotation	
								Sequence components of *kabuli* genome	*Kabuli* gene accession IDs	NCBI-KOG	TFs
Ca3_P2_35798935	Ca3	35798935	CATAGAATAGAATAGAATAGAAT/CATAGAATAGAATAGAAT	AGCTCAGTGCAACGATTGTG	AGTGCATCATTTGTCCACCA	60.0	355	INTERGENIC	--	--	--
Ca3_P2_35799328	Ca3	35799328	CTTT/CT	TGGTGGACAAATGATGCACT	TCACCCGAATATTCCAAAGA	59.4	293	INTERGENIC	--	--	--
Ca3_P2_35799677	Ca3	35799677	ATTTTT/ATTTT	TAACTGCATTTGATTGCGGA	TGCTTTTGCAAGTGTTTTTGA	59.5	610	INTERGENIC	--	--	--
Ca3_P2_35800877	Ca3	35800877	TGAG/TGAGAG	GCGTTCCAAGAACAACCAAT	TTTGATGCAATTCAACTCGG	59.7	535	INTERGENIC	--	--	--
Ca3_P2_35801766	Ca3	35801766	TAA/TA	TTACCATGATTGAGGCACCA	CCTTGTTTGGTTTTGGTTGG	60.2	395	INTERGENIC	--	--	--
Ca3_P2_35802689	Ca3	35802689	TGGTCGG/TGGTCGGTCGG	TTGGTTGTGCTAACATGGGA	CAACATCCACACATTCACACG	60.9	217	DRR	Ca_00867	O	--
Ca3_P2_35805111	Ca3	35805111	CATATATATAT/CATATAT	TTTAGCATCGTGGTTTCCTTT	TGCTAAAATAAACTCCGCCAA	59.7	521	INTERGENIC	--	--	--
Ca3_P2_35806267	Ca3	35806267	CAAAAAAAAA/CAAAAAAAA	CCATGAAACTCTTCCAAGCA	CGAAAATCCATGGCTCTTTTTA	60.4	976	DRR	Ca_00868	--	--
Ca3_P2_35809465	Ca3	35809465	TAAAAAAAAAA/TAAAAAAAAAAAAAA	CTGTGTTCCCGTGAGAACCT	TGCGGTTTGTTTTGTGTGTT	60.1	795	INTRON	Ca_00868	--	--
Ca3_P2_35809496	Ca3	35809496	CAAA/CAAAA	CTGTGTTCCCGTGAGAACCT	TGCGGTTTGTTTTGTGTGTT	60.1	795	INTRON	Ca_00868	--	--
Ca3_P2_35809695	Ca3	35809695	CAAAAAAA/CAAAAAAAA	CCGGAAGGAAAATTCCAAAT	TGCGGTTTGTTTTGTGTGTT	60.1	297	INTRON	Ca_00868	--	--
Ca3_P2_35809791	Ca3	35809791	CTTTTTT/CTTTTTTT	CCGGAAGGAAAATTCCAAAT	TGCGGTTTGTTTTGTGTGTT	60.1	297	INTRON	Ca_00868	--	--

INDEL marker IDs	Chromosomes/unanchored scaffolds	Physical positions (bp)	InDels (*Kabuli* reference genome-CDC Frontier/PII)	Forward primers (5'-3')	Reverse primers (5'-3')	Anneling temperature (0C)	Expected amplified product size (bp)	Structural annotation		Functional annotation	
								Sequence components of *kabuli* genome	*Kabuli* gene accession IDs	NCBI-KOG	TFs
Ca3_P2_35811026	Ca3	35811026	TCC/TC	TGTGTGCGTGTGTTTGAGAA	TGGTAGTAAATCACTCGCAGTTG	59.3	743	INTERGENIC	--	--	--
Ca3_P2_35811115	Ca3	35811115	TTTTTATTTTATTTTATTTTATTTTATTTTA/TTTTTATTTTATTTTATTTTATTTTA	TTTTGGGTTTCTCACTTGCC	TGGTAGTAAATCACTCGCAGTTG	59.3	455	INTERGENIC	--	--	--
Ca3_P2_35811467	Ca3	35811467	CTATAT/CTAT	TGAACAAATAAAATTCAACTGCG	TCTGAATTTCTCCCGTGGAT	59.5	450	INTERGENIC	--	--	--
Ca3_P2_35811730	Ca3	35811730	CTATAT/CTATATAT	TGAACAAATAAAATTCAACTGCG	TCTGAATTTCTCCCGTGGAT	59.5	450	INTERGENIC	--	--	--
Ca3_P2_35812032	Ca3	35812032	ATTTTTTTTTTTTTT/ATTTTTTTTT	TCCACGGGAGAAATTCAGAC	GCTCATGTGCGAAATGCTAA	60.0	727	INTERGENIC	--	--	--
Ca3_P2_35813539	Ca3	35813539	TAAAAA/TAA	GATCATATGGCAAACCACCC	ACACACTTCCCAATGTGCAA	60.0	168	INTERGENIC	--	--	--
Ca3_P2_35828389	Ca3	35828389	ATTT/ATT	ATCCGCGATTGAGATAGGTG	CACACCGAAAGAACAAATGC	59.2	791	INTRON	Ca_00870	--	--
Ca3_P2_35832602	Ca3	35832602	CTATAT/CTAT	TTGGACACCACAGACCAAAA	GGTGTTGAATTTGAGGGCAG	60.5	697	DRR	Ca_00871	--	--
Ca3_P2_35832906	Ca3	35832906	GCA/GA	TGGGGCTTCAAAGGTATGAC	TTTGCATTCTTTTCGTTATTTTT	57.2	930	DRR	Ca_00871	--	--
Ca3_P2_35833491	Ca3	35833491	TTA/T	TGTGAGAAAGCATCAAAACCT	CAAACTACGGTCGGTGTCCT	60.0	238	DRR	Ca_00871	--	--
Ca3_P2_35838680	Ca3	35838680	ACCTTTTG/ACCTTTTGCCTTTTG	AGGACCAAGCGGTAATGTTG	GGATCTGTTCCTGTGTGCCT	60.1	338	INTRON	Ca_00872	--	--
Ca3_P2_35839159	Ca3	35839159	ATTTTTTTTTTTTTT/ATTTTTTTTTT	GCACACAGGAACAGATCCAA	CCGGATAAATGCATGGAAAT	59.6	504	DRR	Ca_00872	--	--

INDEL marker IDs	Chromosomes/unanchored scaffolds	Physical positions (bp)	InDels (*Kabuli* reference genome-CDC Frontier/PII)	Forward primers (5'-3')	Reverse primers (5'-3')	Anneling temperature (0C)	Expected amplified product size (bp)	Structural annotation		Functional annotation	
								Sequence components of *kabuli* genome	*Kabuli* gene accession IDs	NCBI-KOG	TFs
Ca3_P2_35839497	Ca3	35839497	GT/GTCCTCTTAAACT	ATGGAATTGCATGATTGGGT	GGTTGAATTTTGTCCGCTACA	60.0	514	DRR	Ca_00872	--	--
Ca3_P2_35848406	Ca3	35848406	TCTAACT/TCTAACTAACT	TAATCGCAACCTCCCATCTC	GAATGTTCAAGAAGGCTCGC	60.0	645	DRR	Ca_00873	S	--
Ca3_P2_35848490	Ca3	35848490	A/AC	TAATCGCAACCTCCCATCTC	GAATGTTCAAGAAGGCTCGC	60.0	645	DRR	Ca_00873	S	--
Ca3_P2_35852219	Ca3	35852219	AAGGAATTGACTAG/AAG	CCGGCCTGTCAGACCTAATA	TGGCCAACAAGACAATTTCA	60.1	639	INTERGENIC	--	--	--
Ca3_P2_35853994	Ca3	35853994	ATTTTTTTTT/ATTTTTTTT	GTCATGGAACGCAACCTCTT	AGTGGCGTCGCACTAACTTT	59.9	814	DRR	Ca_00875	--	--
Ca3_P2_35856021	Ca3	35856021	ATTTTTTTTTTTT/ATTTTTTTTTTTTTTT	CTTTGACCGAAGCATTAGGC	ACCCCAAAAAGGAGGTGTTC	60.2	435	INTERGENIC	--	--	--
Ca3_P2_35856163	Ca3	35856163	T/TATA	CTTTGACCGAAGCATTAGGC	ACCCCAAAAAGGAGGTGTTC	60.2	435	INTERGENIC	--	--	--
Ca3_P2_35859165	Ca3	35859165	TTATATATATATATATA/TTATATATATATATA	AATGCGGAAACGAATTTGAG	AATCACTCACCACCACCACA	59.8	375	INTERGENIC	--	--	--
Ca3_P2_35859927	Ca3	35859927	TGGG/TGGGG	GAAATCAATTGAAAGAAAAGCCA	TGTCAACAATGTTATCTTCACAAAAA	59.9	808	INTERGENIC	--	--	--
Ca3_P2_35859979	Ca3	35859979	ATCTTCTT/ATCTT	AAACGTCATAATTGCTTATGGG	TGGCTTTTGTTTGAAGATTTTG	59.3	205	INTERGENIC	--	--	--
Ca3_P2_35862589	Ca3	35862589	ACCC/ACC	TTCAACTGTTCTACGGCAACA	TGGGGTTTCCAAAAGTTCAG	59.9	654	INTERGENIC	--	--	--
Ca3_P2_3586328	Ca3	3586328	GTTTTTTTT/GTTTTTTTTT	TGTCCCCAACTAAACCGTTC	TACGACTACATGACCGTGCC	59.6	850	DRR	Ca_21349	--	ZF-HD

INDEL marker IDs	Chromosomes/unanchored scaffolds	Physical positions (bp)	InDels (*Kabuli* reference genome-CDC Frontier/PII)	Forward primers (5'-3')	Reverse primers (5'-3')	Anneling temperature (0C)	Expected amplified product size (bp)	Structural annotation		Functional annotation	
								Sequence components of *kabuli* genome	*Kabuli* gene accession IDs	NCBI-KOG	TFs
Ca3_P2_35873093	Ca3	35873093	TC/T	TTCAATGGGAATGGGAATGT	GTCTCCGACACCGACACTTT	60.2	663	INTERGENIC	--	--	--
Ca3_P2_35873417	Ca3	35873417	TATAAATAAATAAATAAA/TATAAATAAA	GCCATGCCATGTTACCATTT	GTCTCCGACACCGACACTTT	60.2	470	DRR	Ca_00878	O	--
Ca3_P2_35874357	Ca3	35874357	CTT/C	AGCTACCCCCACCACTTCTT	ACACGTAGGCCACGATGATT	60.4	243	DRR	Ca_00878	O	--
Ca3_P2_35874648	Ca3	35874648	ATTTTT/ATTT	AGCTACCCCCACCACTTCTT	TGGATCTTGCCTTCCAGTTT	59.7	701	DRR	Ca_00878	O	--
Ca3_P2_35874800	Ca3	35874800	TTAT/TT	AGCTACCCCCACCACTTCTT	TGGATCTTGCCTTCCAGTTT	59.7	701	DRR	Ca_00878	O	--
Ca3_P2_35880580	Ca3	35880580	A/AC	GAAGTGGCTGAAACAGAGGC	CTCGTATAGGGGTGGCAAAA	60.0	461	INTERGENIC	--	--	--
Ca3_P2_35887062	Ca3	35887062	CT/CTAATAATT	GCAACATGAAAGCACGTTTG	CGTTGGAACTTGGAACCATT	59.8	507	INTERGENIC	--	--	--
Ca3_P2_35889852	Ca3	35889852	TT/TTAT	GACCCGTCGGAACTTTGTAA	TTAATCGAAGGAGGGTGTGG	59.9	415	INTERGENIC	--	--	--
Ca3_P2_35894033	Ca3	35894033	AACCTTTTACC/AACC	CACACAAATTGGTTGGTGCT	TCAAAAGTTTGACTCCTAAACGG	59.7	508	INTERGENIC	--	--	--
Ca3_P2_35894525	Ca3	35894525	GTATATATATATATA/GTATATATATATATATATATATA	TGAAACCTTTTACCAGACTCCAA	GTTAATCAAAGGGCCGACAA	59.9	952	INTERGENIC	--	--	--
Ca3_P2_35895341	Ca3	35895341	CAAAAAA/CAAAAAAAA	TTTCCTTAGGACGCGAAGAA	CGGAAGTTCCAATGGAGAAA	60.0	553	INTERGENIC	--	--	--
Ca3_P2_35895922	Ca3	35895922	TTT/TTTTTTTCTT	ATACGGCAAAAACAGCCATC	TAGATAAACCCACCGGCTCA	60.5	464	DRR	Ca_00881	DO	WRKY

INDEL marker IDs	Chromosomes/unanchored scaffolds	Physical positions (bp)	InDels (*Kabuli* reference genome-CDC Frontier/PII)	Forward primers (5'-3')	Reverse primers (5'-3')	Anneling temperature (0C)	Expected amplified product size (bp)	Structural annotation		Functional annotation	
								Sequence components of *kabuli* genome	*Kabuli* gene accession IDs	NCBI-KOG	TFs
Ca3_P2_35897194	Ca3	35897194	GTTATTTT/GTTATTTTATTTT	TAGGAAAGGGCCAATGTCAG	AGTAGGAGGAGGGGTGTGGT	59.8	266	DRR	Ca_00881	DO	WRKY
Ca3_P2_35900336	Ca3	35900336	CTTTTTTT/CTTTTTT	GTGACGTTGTATTGCGATGG	CCAAAACTGGGGAGAAACAA	59.9	656	DRR	Ca_00882	Q	Nin-like
Ca3_P2_35901183	Ca3	35901183	ATTTTTTTTTT/ATTTTTTT	TTGTTTCTCCCCAGTTTTGG	TTTTTCTTCAATCCACGGGT	59.4	607	DRR	Ca_00882	Q	Nin-like
Ca3_P2_35910653	Ca3	35910653	TAAGAAG/TAAG	TAATTTTCGTCCTCCATGCC	AGCCAAATCCAACCATTCAC	59.8	521	INTERGENIC	--	--	--
Ca3_P2_35911655	Ca3	35911655	AG/A	GCATGCGACTCATTTACCAA	TCGAATTATTTTTGAGTTGGTTTG	59.5	599	INTERGENIC	--	--	--
Ca3_P2_35914804	Ca3	35914804	TTGTGTGTGTGTGTGTGTGT/TTGTGTGTGTGTGTGTGT	CCTTGAAGTCCCGTGATGAT	GGGATAGGAAGGACGAGGAC	59.9	617	INTERGENIC	--	--	--
Ca3_P2_35917238	Ca3	35917238	AC/A	ATCTTAGCCCACTCAATCCG	GTGTCGTAAGCCTCCTCGAA	60.4	522	INTERGENIC	--	--	--
Ca3_P2_35918984	Ca3	35918984	GAATT/GAATTTAAATT	TGGAAGGAAGGAGGAGGATT	GAGATTCGATTTGAGGGCAA	60.2	825	INTERGENIC	--	--	--
Ca3_P2_35919061	Ca3	35919061	T/TGAATAAA	TTCAATCCCGTAAAAAGGCA	TTGCAAAAACGAAGAACACG	59.9	936	INTERGENIC	--	--	--
Ca3_P2_35919080	Ca3	35919080	AATAAAGA/AATAAAGATAAAGA	TGAGAGGGTCTGCAAAGAAAA	TTGCAAAAACGAAGAACACG	59.9	788	INTERGENIC	--	--	--
Ca3_P2_35919142	Ca3	35919142	GC/GCAC	AGGGATGGTGATCAGTTTGG	GAAACTCCATGAGAGCTCGG	60.0	520	INTERGENIC	--	--	--
Ca3_P2_35919349	Ca3	35919349	CTTTTTT/CTTTTT	TTGCCCTCAAATCGAATCTC	GAAACTCCATGAGAGCTCGG	60.0	315	INTERGENIC	--	--	--

INDEL marker IDs	Chromosomes/unanchored scaffolds	Physical positions (bp)	InDels (*Kabuli* reference genome-CDC Frontier/PII)	Forward primers (5'-3')	Reverse primers (5'-3')	Anneling temperature (0C)	Expected amplified product size (bp)	Structural annotation		Functional annotation	
								Sequence components of *kabuli* genome	*Kabuli* gene accession IDs	NCBI-KOG	TFs
Ca3_P2_35922731	Ca3	35922731	ACCCC/ACC	ACTTCAGAAGGAGGTTGGCA	CGACCAGAGCTCCCATCTTA	60.4	646	DRR	Ca_00884	--	--
Ca3_P2_35925530	Ca3	35925530	TTAAT/TTAATAAT	CGGTGAACAACTCCCAACTT	GAGTCTGCGTTGGTCCAAAT	60.1	458	INTRON	Ca_00885	--	--
Ca3_P2_35925572	Ca3	35925572	CTTTTTT/CTTTTTTT	CGGTGAACAACTCCCAACTT	GAGTCTGCGTTGGTCCAAAT	60.1	458	INTRON	Ca_00885	--	--
Ca3_P2_35928406	Ca3	35928406	TTGCCTG/TTG	CGAAAGGTAGCGCATAGGTC	TCAAACACTAATGATGCCCA	57.5	647	DRR	Ca_00886	R	NAC
Ca3_P2_35928795	Ca3	35928795	ATTTTTTT/ATTTTTT	GATGGGCATCATTAGTGTTTGA	TTCAGTTGCACCTCAACGTC	59.9	336	DRR	Ca_00885	--	--
Ca3_P2_35929392	Ca3	35929392	T/TG	AAGGAAATGCAAGTCGTCGT	CGTTCACATCAACGTTTTCA	58.2	425	DRR	Ca_00885	--	--
Ca3_P2_35932284	Ca3	35932284	AAG/A	GGTTCTTAGCATGTGGGCAT	ATTGCATTTGGGGGTTGTAA	60.1	845	DRR	Ca_00887	BT	GATA
Ca3_P2_35935534	Ca3	35935534	CAA/CAAA	TGGAACCTGGGTTGAGGTAG	GAAAATTGACCCAAAAGCCA	59.9	633	DRR	Ca_00888	--	--
Ca3_P2_35935554	Ca3	35935554	AA/AATA	TGGAACCTGGGTTGAGGTAG	GAAAATTGACCCAAAAGCCA	59.9	633	DRR	Ca_00888	--	--
Ca3_P2_35936442	Ca3	35936442	GAAAAAAAAAA/GAAAAAAAAA	CCACATCCATGTTTCACCAA	CGGTGTAAGGTCGTGGAGAT	60.0	254	DRR	Ca_00888	--	--
Ca3_P2_35941651	Ca3	35941651	CATATATAT/CATATAT	CAAGTCCAACCGTGGAAGAT	TCGGGACGTTACACATAGCA	60.1	251	INTERGENIC	--	--	--
Ca3_P2_35947189	Ca3	35947189	C/CTATGTCTCTG	GGTTACAAGGCCTCACGGTA	ATTCAGAAATGCACCCCATC	59.8	300	DRR	Ca_00889	J	--

INDEL marker IDs	Chromosomes/unanchored scaffolds	Physical positions (bp)	InDels (*Kabuli* reference genome-CDC Frontier/PII)	Forward primers (5'-3')	Reverse primers (5'-3')	Anneling temperature (0C)	Expected amplified product size (bp)	Structural annotation		Functional annotation	
								Sequence components of *kabuli* genome	*Kabuli* gene accession IDs	NCBI-KOG	TFs
Ca3_P2_35947619	Ca3	35947619	CAA/CA	GATGGGGTGCATTTCTGAAT	TTGAAATTTTGACCGCATCA	60.1	830	DRR	Ca_00889	J	--
Ca3_P2_35947883	Ca3	35947883	CTTT/CT	TCCTGTCTAGCCGTAGTCCTG	TTGAAATTTTGACCGCATCA	60.1	434	DRR	Ca_00889	J	--
Ca3_P2_35950711	Ca3	35950711	ATTTA/ATTTAAAATTACATGCAATTTTA	CATGCGATGGAACTGAATTG	GTAAAGTTGGCACAGGGGAA	60.0	479	INTERGENIC	--	--	--
Ca3_P2_35960562	Ca3	35960562	AGTTTGATT/AGTTTGATTGTTTGATT	TCCGACTTGGTAAAACAGCA	TTTGGACGAAATAAATTTGAAAAA	59.0	398	DRR	Ca_00891	V	GRAS
Ca3_P2_35960868	Ca3	35960868	TAA/TAAA	GGATGCTACATTTCTTGGTGG	TGAAGGAGGGAGAAACATGG	60.0	835	DRR	Ca_00891	V	GRAS
Ca3_P2_35963982	Ca3	35963982	CAAAAAAAAA/CAAAAAAAA	CACAGAACTGTGATGTCTTTAGTTTG	ACGCTAGTACAGCCACCGAT	59.8	819	INTERGENIC	--	--	--
Ca3_P2_35966241	Ca3	35966241	GTATCCAATAATAT/GTAT	CCCTATCGAGTTTAAGGGCA	TTTGAGTACAACGAATTTGAAAGTG	59.6	494	INTERGENIC	--	--	--
Ca3_P2_35967842	Ca3	35967842	TAAAAAAAAAA/TAAAAAAAAAAA	TTGATTATGAGCACCAAGCG	TGTATTAAACGTCATTTCCCCC	60.0	716	DRR	Ca_00892	C	--
Ca3_P2_35973469	Ca3	35973469	AC/A	TGCCAGTGATCATCGAAAAA	AGGGGCAAAGGTAACAAAGC	60.5	374	INTRON	Ca_00892	C	--
Ca3_P2_35973692	Ca3	35973692	CG/C	TGCCAGTGATCATCGAAAAA	TTCTGTGAAGGATGCAGCAG	60.1	778	INTRON	Ca_00892	C	--
Ca3_P2_35974404	Ca3	35974404	G/GGA	TAGCAACATCTGCTGCATCC	ATCACACGGGAAATCTGAGC	60.1	721	INTRON	Ca_00892	C	--
Ca3_P2_35981540	Ca3	35981540	ATTTTTTTT/ATTTTTTTTT	ATCAAACCAAATGGACCCAA	TCATAAAACCTGCATTTGTGAAG	59.2	527	DRR	Ca_00893	--	--

INDEL marker IDs	Chromosomes/unanchored scaffolds	Physical positions (bp)	InDels (*Kabuli* reference genome-CDC Frontier/PII)	Forward primers (5'-3')	Reverse primers (5'-3')	Anneling temperature (0C)	Expected amplified product size (bp)	Structural annotation		Functional annotation	
								Sequence components of *kabuli* genome	*Kabuli* gene accession IDs	NCBI-KOG	TFs
Ca3_P2_35981806	Ca3	35981806	GAAAAAA/GAAAAAAA	ATCAAACCAAATGGACCCAA	TCGAAACTTTTGAGAGATGGC	59.4	770	DRR	Ca_00893	--	--
Ca3_P2_35982476	Ca3	35982476	TGG/TG	TCGAAAAGAACTTTATCCGCA	TGAATTGCTGTAGCCTGTGG	59.9	408	DRR	Ca_00893	--	--
Ca3_P2_35982788	Ca3	35982788	ATTT/ATT	CGGAGCCGTAGGATTGATTA	CGTGCAGGGTGGAGTTTATT	60.0	324	DRR	Ca_00893	--	--
Ca3_P2_35986617	Ca3	35986617	CCA/CCACA	GGTGGATTCTTTGCTTCCAA	AGGTGCAAGTGCAAGTTCCT	59.9	479	INTRON	Ca_00893	--	--
Ca3_P2_35990406	Ca3	35990406	ACTG/A	TGAATTTACACCCTGCCCTC	GCTCGAGATGAAGGGACTTG	60.0	331	INTERGENIC	--	--	--
Ca3_P2_35994489	Ca3	35994489	CT/CTAAATATT	TGAATGATGTTTCATTCCATCA	GAATTCGGAACAAAACGTTCA	60.0	311	INTERGENIC	--	--	--
Ca3_P2_35996017	Ca3	35996017	T/TTA	GCTTTGTGGCAAACCATTTT	GATCCAGATTGCCTGCATTT	60.0	766	INTRON	Ca_00895	E	--
Ca3_P2_35997682	Ca3	35997682	GTT/GT	GTGCTTTTTGTTCGGTCCAT	ACAAATGAGGCAAATACGGC	60.0	768	INTRON	Ca_00895	E	--
Ca3_P2_35998171	Ca3	35998171	CAAA/CAA	TGTAGCTTCATTCGTGCCAG	ATGCTGATTGGTGTGTCCAA	60.0	727	DRR	Ca_00895	E	--
Ca3_P2_35998366	Ca3	35998366	CAAAAAAAAAAAAAA/CAAAAAAAAAAA	TGTAGCTTCATTCGTGCCAG	ATGCTGATTGGTGTGTCCAA	60.0	727	DRR	Ca_00895	E	--
Ca3_P2_36003419	Ca3	36003419	TGGG/TGGGG	TCAATTGTCACCGCATTTGT	CAAAAGTTGTTGGCTGCTCA	60.0	370	INTRON	Ca_00896	OPR	--
Ca3_P2_36006229	Ca3	36006229	CAA/CAAAA	GGTTGCTGAGCCAACTCTTC	ATGTTGAATTTCTGCCAGCC	60.1	549	INTRON	Ca_00896	OPR	--

INDEL marker IDs	Chromosomes/unanchored scaffolds	Physical positions (bp)	InDels (*Kabuli* reference genome-CDC Frontier/PII)	Forward primers (5'-3')	Reverse primers (5'-3')	Anneling temperature (0C)	Expected amplified product size (bp)	Structural annotation		Functional annotation	
								Sequence components of *kabuli* genome	*Kabuli* gene accession IDs	NCBI-KOG	TFs
Ca3_P2_36012310	Ca3	36012310	ATTTTT/ATTTTTT	ACACGAAAGCATCAAAACCC	TTTATCAATGGGGTGGCAAT	60.0	444	DRR	Ca_00897	--	--
Ca3_P2_36016163	Ca3	36016163	TAAAAAAAAAA/TAAAAAAAAA	CAACTGCACAGATGGATTGG	AATTTGCAATTTGATCCCCA	60.1	650	DRR	Ca_00900	O	CAMTA
Ca3_P2_36016763	Ca3	36016763	TAA/TA	CGCGGAACAAGAATCAAAAT	ATCTCGCGAAGGTGAGAGAA	60.1	384	DRR	Ca_00900	O	CAMTA
Ca3_P2_36016803	Ca3	36016803	GTTTTTTTTTTTT/GTTTTTTTTTTTTTTTTTT	CGCGGAACAAGAATCAAAAT	ATCTCGCGAAGGTGAGAGAA	60.1	384	DRR	Ca_00900	O	CAMTA
Ca3_P2_36016832	Ca3	36016832	TAAAAAAA/TAAAAAA	CGCGGAACAAGAATCAAAAT	ATCTCGCGAAGGTGAGAGAA	60.1	384	DRR	Ca_00900	O	CAMTA
Ca3_P2_36018756	Ca3	36018756	TAAAA/TAA	GGGCTTCAACTTCCACTTCA	TGTATCCAAGGGGGCATAAA	60.1	498	INTRON	Ca_00900	O	CAMTA
Ca3_P2_36019541	Ca3	36019541	AT/ATGT	ACCTTTGGATTCATGGTTGG	CCGATAGCCATTTGGACACT	60.0	614	INTERGENIC	--	--	--
Ca3_P2_36020478	Ca3	36020478	ATT/ATTT	AGGTGAAGGTTGCGTTAGTGA	TCAAATTGTGTGGGAGAAACA	59.0	536	INTERGENIC	--	--	--
Ca3_P2_36021252	Ca3	36021252	GAT/G	GTTTGCGGATTTGGTTGAGT	TATTTTAGGACCGCCTGACG	60.1	442	DRR	Ca_00901	O	CAMTA
Ca3_P2_36025976	Ca3	36025976	ATTTTTTT/ATTTTTTTT	CAACGGAACTAAACCGTCTTTC	AACGGCGAATCTAAGTGCAT	59.7	579	INTERGENIC	--	--	--
Ca3_P2_36027369	Ca3	36027369	AATATATATAT/AATATATAT	GCTGATTCAAAAAGCCCTCA	TGTGCATTTTATTTTTGTTGGAA	59.4	375	INTERGENIC	--	--	--
Ca3_P2_36027559	Ca3	36027559	CGA/C	GCTGATTCAAAAAGCCCTCA	CATGAACACCCTATCTAGCACAA	59.2	467	INTERGENIC	--	--	--

INDEL marker IDs	Chromosomes/unanchored scaffolds	Physical positions (bp)	InDels (*Kabuli* reference genome-CDC Frontier/PII)	Forward primers (5'-3')	Reverse primers (5'-3')	Anneling temperature (0C)	Expected amplified product size (bp)	Structural annotation		Functional annotation	
								Sequence components of *kabuli* genome	*Kabuli* gene accession IDs	NCBI-KOG	TFs
Ca3_P2_36032758	Ca3	36032758	ATTTTTT/ATTTTTTT	GGTCATTTTGGTTCAGCGTT	TCGATTGGATAGTGCCATCA	60.0	705	DRR	Ca_00902	S	bZIP
Ca3_P2_36040095	Ca3	36040095	TAAAAAAAAAA/TAAAAAAAAA	TGTTGCACTATCGGCGTAAA	TTTAAAACAGGCAGGGTTGC	60.1	782	INTRON	Ca_00902	S	bZIP
Ca3_P2_36040732	Ca3	36040732	ATTTT/ATTT	CCACCACTGCAGAAGTGAGA	TCCGAACTGGAGAGTGTGAA	59.4	461	INTRON	Ca_00902	S	bZIP
Ca3_P2_36040968	Ca3	36040968	CAAA/CAAAA	CCACCACTGCAGAAGTGAGA	TGTTGGTAAGATTTTGTGAAAATTG	59.4	510	INTERGENIC	--	--	--
Ca3_P2_36041204	Ca3	36041204	ATT/AT	TTCACACTCTCCAGTTCGGA	AAGTCGACGTTTGTGAAGAGTTC	59.8	699	INTERGENIC	--	--	--
Ca3_P2_36042265	Ca3	36042265	ATTTTTTTT/ATTTTTTTTTTT	CTCCAGTTTTGGACCCTCAA	GTTCCGCCATTTTGTTTCTC	59.6	210	INTERGENIC	--	--	--
Ca3_P2_36043188	Ca3	36043188	A/AT	TGGAATGAATTAAACCATCACG	GCCACAAAATCATGCTGAAA	59.7	245	INTERGENIC	--	--	--
Ca3_P2_36044685	Ca3	36044685	TAA/TAAAAAA	TGTCAAATATCAAGGGGGATG	TTTGTGGCTGTATTTGGCAG	59.7	722	INTERGENIC	--	--	--
Ca3_P2_36045650	Ca3	36045650	ATT/ATTT	TGTGATTGAAAAGATGTGTCAAAA	GGGGTACGGAGTGGAGGTAT	60.1	622	INTERGENIC	--	--	--
Ca3_P2_36046088	Ca3	36046088	GTTTTTTT/GTTTTTTTT	ATACCTCCACTCCGTACCCC	GGCCTTGCATGATTTGAAAC	60.5	416	INTERGENIC	--	--	--
Ca3_P2_36048215	Ca3	36048215	CTTTTTTTTTTT/CTTTTTTTTT	AACCGCCCATCTATCCCTAC	GGACGTTGAAAAGCAGAAGC	60.0	468	INTRON	Ca_00903	A	--
Ca3_P2_36053633	Ca3	36053633	TAAAA/TAAAAA	AAAAAGGGTTGTTTGCCAGA	AACGGGATTAGGGATTTTGG	60.0	581	INTERGENIC	--	--	--

INDEL marker IDs	Chromosomes/unanchored scaffolds	Physical positions (bp)	InDels (*Kabuli* reference genome-CDC Frontier/PII)	Forward primers (5'-3')	Reverse primers (5'-3')	Anneling temperature (0C)	Expected amplified product size (bp)	Structural annotation		Functional annotation	
								Sequence components of *kabuli* genome	*Kabuli* gene accession IDs	NCBI-KOG	TFs
Ca3_P2_36055468	Ca3	36055468	TATA/TATAATA	GCTCAATCCTATCCCCACAA	ATGATGGTCAATGCAAAGCA	60.1	591	INTERGENIC	--	--	--
Ca3_P2_36059105	Ca3	36059105	ATTTTTTTTTT/ATTTTTTTT	GACCTACTTCCATGGGCAAA	TCTAAACCCGAACACGAAGG	60.1	562	INTRON	Ca_00904	A	--
Ca3_P2_36067685	Ca3	36067685	GTTTTTTTT/GTTTTTTTTT	GCATTTACTGCATTCTGCCC	GGCCAAACTCCGAAGTACAA	60.1	529	INTRON	Ca_00904	A	--
Ca3_P2_36076083	Ca3	36076083	ATAAAA/ATAAAAATTAAAA	TGTCAAAATTTGGATTGCCA	CCTTGTTGAAACAAATGCAAGA	60.1	539	INTERGENIC	--	--	--
Ca3_P2_36086627	Ca3	36086627	AC/ACC	ATTCCAACGGAAAACATCCA	CTCCCAAACAAAGCCTACGA	60.2	605	INTRON	Ca_00907	I	--
Ca3_P2_36087882	Ca3	36087882	CTTTTTTTTTTTTTTTT/CTTTTTTTTTTTTTT	GCAAAGGGAGAAAAGCAGTG	CACTATGGCAAGGTTCGGTT	60.0	348	INTRON	Ca_00907	I	--
Ca3_P2_36088258	Ca3	36088258	AT/ATT	AACCGAACCTTGCCATAGTG	AGACAGATTACCCACGGCTG	60.1	355	INTERGENIC	--	--	--
Ca3_P2_36089072	Ca3	36089072	GAAAAAAAAAAA/GAAAAAAAAAA	CACCTACTAAAAAGCGTGCTGA	TCCAAAACAAAACAAAGCCC	60.0	146	INTERGENIC	--	--	--
Ca3_P2_36089210	Ca3	36089210	CAAAAAAAAA/CAAAAAAAA	GGGCTTTGTTTTGTTTTGGA	AATAAAGGGGAAAAGGCCAA	59.8	185	INTERGENIC	--	--	--
Ca3_P2_36089981	Ca3	36089981	TAAAAGTCAA/TAA	AATACGAATCATTGGAGCTTTTT	AAACTGGTTTGAGTGACGGC	60.2	167	INTERGENIC	--	--	--
Ca3_P2_36092729	Ca3	36092729	ATTTTTTT/ATTTTTTTT	TGGAATAGGCTAGACCGTCG	TTTCCGATTTGGTCTAACTTGAA	60.0	691	INTERGENIC	--	--	--
Ca3_P2_36096942	Ca3	36096942	GAAAAAAA/GAAAAAAAAA	AAGGGAGGAACACAAAAATTGA	TTTCGAACAGGGGCTTCTAA	59.8	390	INTERGENIC	--	--	--

INDEL marker IDs	Chromosomes/unanchored scaffolds	Physical positions (bp)	InDels (*Kabuli* reference genome-CDC Frontier/PII)	Forward primers (5'-3')	Reverse primers (5'-3')	Anneling temperature (0C)	Expected amplified product size (bp)	Structural annotation		Functional annotation	
								Sequence components of *kabuli* genome	*Kabuli* gene accession IDs	NCBI-KOG	TFs
Ca3_P2_36097056	Ca3	36097056	TGG/TG	GCCATTTCTTAGAAGCCCCT	TTTTGCCTGGATATTCCCTG	59.9	485	INTERGENIC	--	--	--
Ca3_P2_36097089	Ca3	36097089	TATAA/T	GCCATTTCTTAGAAGCCCCT	TTTTGCCTGGATATTCCCTG	59.9	485	INTERGENIC	--	--	--
Ca3_P2_36098483	Ca3	36098483	TATGCT/TATGCTTCTTTGGCATGCT	TGTTAACGCGCCTCAAATAA	GGCAATTGCTATAATCCCCA	59.8	521	INTERGENIC	--	--	--
Ca3_P2_36101762	Ca3	36101762	AACATATATA/AA	TTCCCGGAAGTGAATTGAAG	GAAGCAACCCAAAGTTTCCA	60.1	492	INTRON	Ca_00908	K	--
Ca3_P2_36107876	Ca3	36107876	ATTTT/ATTT	CAATCCCTTTAACTTCGCCA	GGTGGGTGACAATGATTTCC	60.0	658	INTERGENIC	--	--	--
Ca3_P2_36109027	Ca3	36109027	CTTTTT/CTTTTTT	TCCATGCCATCAAAATCAAA	TTTCGTGAAGGCATACTCCA	59.3	316	DRR	Ca_00909	J	--
Ca3_P2_36109234	Ca3	36109234	AAAATA/AA	TCCATGCCATCAAAATCAAA	GACCCATTCACTCAACCGAT	59.8	813	DRR	Ca_00909	J	--
Ca3_P2_36109369	Ca3	36109369	CTTG/CTTGGTTG	TGGAGTATGCCTTCACGAAA	TGCCGTGGTTAAGGGAATAG	60.0	652	DRR	Ca_00909	J	--
Ca3_P2_36110040	Ca3	36110040	CATA/CATATA	TCCCTTAACCACGGCATTAG	GGCAAAGGGTGAGGTCATTA	59.9	643	DRR	Ca_00909	J	--
Ca3_P2_36114057	Ca3	36114057	ATTT/ATTTT	TATGTGCAGAAGCAAGTGGC	TTTGGGAGGAGTCGAAAATG	60.0	421	INTRON	Ca_00910	--	--
Ca3_P2_36117243	Ca3	36117243	GAAAAAAAA/GAAAAAAA	TGAATGATTGAGCCAGCAAG	AGAAGGATGTCGAAAAGCGA	60.0	393	DRR	Ca_00911	D	--
Ca3_P2_36118869	Ca3	36118869	TA/T	CATCGTCCTCTTCTGCTTCA	TCAAATCTGGAAAGGTTGGG	59.9	579	INTRON	Ca_00911	D	--

INDEL marker IDs	Chromosomes/unanchored scaffolds	Physical positions (bp)	InDels (*Kabuli* reference genome-CDC Frontier/PII)	Forward primers (5'-3')	Reverse primers (5'-3')	Anneling temperature (0C)	Expected amplified product size (bp)	Structural annotation		Functional annotation	
								Sequence components of *kabuli* genome	*Kabuli* gene accession IDs	NCBI-KOG	TFs
Ca3_P2_36120455	Ca3	36120455	TGGG/TGG	AAAGCACCTGCAAGAAGGAA	GGCTTGGTCTCTTCCAATCA	60.2	479	INTRON	Ca_00911	D	--
Ca3_P2_36120818	Ca3	36120818	TA/TAA	TGATTGGAAGAGACCAAGCC	CAATGGTGCAACAAACTTGG	60.0	680	INTRON	Ca_00911	D	--
Ca3_P2_36123850	Ca3	36123850	TAA/TA	CCCCACAAAATCGGCTAGTA	TGTTTGACATGCAATTCGGT	60.0	876	INTERGENIC	--	--	--
Ca3_P2_36124148	Ca3	36124148	GAAAAAAA/GAAAAAAAA	TTGGACAAAGATAGGGCCAC	TGTTTGACATGCAATTCGGT	60.0	174	INTERGENIC	--	--	--
Ca3_P2_36128642	Ca3	36128642	ATTTTTTT/ATTTTTT	ACTAACGGAACGACGAATGG	AAAGTCGAGGTGCTTTTTGC	59.5	657	INTERGENIC	--	--	--
Ca3_P2_36129655	Ca3	36129655	GATATA/GATA	TTGCTCGTTTGATTGGTGTG	TTTTTGTGATTTTACGTAAGGGTA	57.1	589	DRR	Ca_00912	YU	--
Ca3_P2_36129790	Ca3	36129790	T/TC	TTGCTCGTTTGATTGGTGTG	TTTTTGTGATTTTACGTAAGGGTA	57.1	589	DRR	Ca_00912	YU	--
Ca3_P2_36131712	Ca3	36131712	CAA/CAAA	GGCGTGAAAAGTGACTGGAT	TGTTTCCTAGTACCGTGCCC	60.0	471	INTRON	Ca_00912	YU	--
Ca3_P2_36135574	Ca3	36135574	ATT/AT	CAAATTAATTGGTTTGGATTTGA	TTTAGCACCGTTCTGGTGTG	59.8	873	DRR	Ca_00913	--	M-type
Ca3_P2_36135942	Ca3	36135942	A/AC	ATAAAAATGCATGCACGCTG	TGAAGCAAACGTGTCAAAGC	60.0	525	DRR	Ca_00913	--	M-type
Ca3_P2_36136545	Ca3	36136545	CA/CATACATACATA	GCTTTGACACGTTTGCTTCA	ATTGTGGGAAGTGCTTACGG	60.0	368	INTRON	Ca_00913	--	M-type
Ca3_P2_36138195	Ca3	36138195	TAAAAAAAA/TAAAAAAAAA	AGCTAATGAGCGGAAGCAAA	AATCTCCAAATTGGTGGTGG	59.6	558	INTERGENIC	--	--	--

INDEL marker IDs	Chromosomes/unanchored scaffolds	Physical positions (bp)	InDels (*Kabuli* reference genome-CDC Frontier/PII)	Forward primers (5'-3')	Reverse primers (5'-3')	Anneling temperature (0C)	Expected amplified product size (bp)	Structural annotation		Functional annotation	
								Sequence components of *kabuli* genome	*Kabuli* gene accession IDs	NCBI-KOG	TFs
Ca3_P2_36141214	Ca3	36141214	TGCCACC/T	AAAGAGCAAGGCTGATCCAA	AAAAACGTTTCGGGCTTACA	59.6	570	INTERGENIC	--	--	--
Ca3_P2_36148820	Ca3	36148820	TAAAA/TAAA	TTGCTTATGTCTCACGGCTG	CACCTTTGGAAGCAAACAGAG	59.9	901	INTERGENIC	--	--	--
Ca3_P2_36154002	Ca3	36154002	AA/AACA	TTATTTTTCGGGCTTTTGTCA	CTTCCATTTGCAGGGTTTGT	60.0	839	DRR	Ca_00916	A	GeBP
Ca3_P2_36157100	Ca3	36157100	G/GCTCCCTTG	TGAATCAATCATCGCCAAAA	CATGACTTAATGGCGTGGTG	60.0	435	INTRON	Ca_00916	A	GeBP
Ca3_P2_36157685	Ca3	36157685	CT/CTAT	ACAAAGGGATGGGGAAAGAC	TACCGAAACACATGACGGAA	60.0	644	INTRON	Ca_00916	A	GeBP
Ca3_P2_36161068	Ca3	36161068	ATTT/ATTTT	TTGCATCTTCCCATGATTGA	TTTGGGGTCGCTTTTATTTTT	59.8	443	INTERGENIC	--	--	--
Ca3_P2_36167042	Ca3	36167042	GTTTTTTTTT/GTTTTTTTTTT	TTTTGACCCATCCAAGAAGG	CCCCGTCTAATTAGCCAACA	60.0	459	DRR	Ca_00918	KTDL	--
Ca3_P2_36168799	Ca3	36168799	CTCATAAGTCA/CTCA	GCCTGCGAAAGACAAAGTTC	ATTTGCAGGAGCTGCTGTTT	60.0	610	INTRON	Ca_00918	KTDL	--
Ca3_P2_36173279	Ca3	36173279	CTTTTTTT/CTTTTTTTT	CATTGGAATGGGAACTGCTT	ATGCTGCAAGGGTTATTTGG	60.0	596	INTERGENIC	--	--	--
Ca3_P2_36176812	Ca3	36176812	GAAAAAAAAAA/GAAAAAAAAAAA	TAATTCGCCACATCACTCCA	TTCTCATCTGCAAATGCTGC	60.1	515	DRR	Ca_00919	R	bHLH
Ca3_P2_36177769	Ca3	36177769	A/AC	TCAATGAAGCAATGAGGGCT	CCAATGCCCTCCAGGTACTA	59.9	534	INTRON	Ca_00920	--	--
Ca3_P2_36177820	Ca3	36177820	GTCTCT/GTCT	TCAATGAAGCAATGAGGGCT	CCAATGCCCTCCAGGTACTA	59.9	534	INTRON	Ca_00920	--	--

**INDEL marker IDs**	**Chromosomes/unanchored scaffolds**	**Physical positions (bp)**	**InDels (*Kabuli* reference genome-CDC Frontier/PII)**	**Forward primers (5'-3')**	**Reverse primers (5'-3')**	**Anneling temperature (0C)**	**Expected amplified product size (bp)**	**Structural annotation**		**Functional annotation**	
								**Sequence components of *kabuli* genome**	***Kabuli* gene accession IDs**	**NCBI-KOG**	**TFs**
Ca3_P2_36178035	Ca3	36178035	TC/T	CTCAATGAAGCAATGAGGGC	GCATTCTCCGGTCTTATCCA	60.0	898	INTRON	Ca_00920	--	--
Ca3_P2_36178743	Ca3	36178743	TAA/TAAA	TGGATAAGACCGGAGAATGC	TCCTTATAAAATTATCATGCACTCAA	58.3	690	DRR	Ca_00921	Q	B3
Ca3_P2_36178911	Ca3	36178911	ATTT/ATT	TGGATAAGACCGGAGAATGC	TCTCGAAACTAGAAAAGAACAAAAA	58.0	812	DRR	Ca_00921	Q	B3
Ca3_P2_36179397	Ca3	36179397	CTTTTTTTTTT/CTTTTTTTTT	AAAGACTACGCATATATTTACCCC	TGCATTTGAGTGATGACGTG	59.2	527	DRR	Ca_00921	Q	B3
Ca3_P2_36182833	Ca3	36182833	GTAA/GTAATTTAA	AGCATGCAGCAACAGAGAGA	TTGCCAAAGGATTGATGTGA	60.0	415	INTERGENIC	--	--	--
Ca3_P2_36184874	Ca3	36184874	AAAA/AAAATAAA	AACTGTCAACACGTTGAGTATCG	GGCGTACACATGTCTTTGGA	59.6	395	INTERGENIC	--	--	--
Ca3_P2_36186889	Ca3	36186889	GT/GTT	ATGCCTTGTTTGGTAGGGTG	TGCATTGCCAACATAGATTCA	60.1	453	INTERGENIC	--	--	--
Ca3_P2_36195467	Ca3	36195467	TTTTATTTATTTATTTATTTATTTATTTATTTATTT/TTTTATTTATTTATTTATTTATTT	TATTGCCGACGTGAGTCTGA	TTCCCTTTCTTTTCGTCTGC	59.4	283	DRR	Ca_00923	--	bHLH
Ca3_P2_36199467	Ca3	36199467	TAA/T	CACTCAAGGGACTAGGAGCG	ACAGCCGAGACAGCACCTAT	59.9	646	DRR	Ca_00924	--	--
Ca3_P2_36199876	Ca3	36199876	CTTT/CTTTT	ATAGGTGCTGTCTCGGCTGT	CATATTACCCATCGATCCGC	60.1	109	DRR	Ca_00924	--	--
Ca3_P2_36200602	Ca3	36200602	CCCAGGT/CCCAGGTCCAGGT	TGCATTTCATTCATTCCCCT	ACTTTGTGCAATCCTTTGGC	60.1	489	CDS (large-effect mutations)	Ca_00924	--	--
Ca3_P2_36200954	Ca3	36200954	GGCATGC/GGCATGCATGC	GCCAAAGGATTGCACAAAGT	TTCCCGGCTTCAACAATTAG	60.1	338	INTERGENIC	--	--	--

INDEL marker IDs	Chromosomes/unanchored scaffolds	Physical positions (bp)	InDels (*Kabuli* reference genome-CDC Frontier/PII)	Forward primers (5'-3')	Reverse primers (5'-3')	Anneling temperature (0C)	Expected amplified product size (bp)	Structural annotation		Functional annotation	
								Sequence components of *kabuli* genome	*Kabuli* gene accession IDs	NCBI-KOG	TFs
Ca3_P2_36203025	Ca3	36203025	CACTTAC/CAC	ATCGAAGCCGGTTAGGTTTT	TACATCTTAATTGGGCCGGA	60.3	168	INTERGENIC	--	--	--
Ca3_P2_36204918	Ca3	36204918	ATTT/ATTTATTAAGTTT	GTCGTTGGAGTCTTTGCGAT	TTCACCAAATGTTGACCGAA	59.9	452	DRR	Ca_00925	--	--
Ca3_P2_36205967	Ca3	36205967	GT/GTCT	GCATGGCATCAACAAGAATG	CATCAGAAGTCTGTTGCCGA	60.0	467	DRR	Ca_00925	--	--
Ca3_P2_36207336	Ca3	36207336	T/TGTTGGCAATTC	TACCCCCATAGTCATCCCAA	CAACTCCAGTGGTTTCGTCA	59.7	532	INTRON	Ca_00925	--	--
Ca3_P2_36207565	Ca3	36207565	CTTT/CTTTT	TCCCTAGAGGCTATGTCCCA	GTTCAAACTTCGTTGCCTCC	59.7	836	INTRON	Ca_00925	--	--
Ca3_P2_36207784	Ca3	36207784	GA/GAA	TGGGTTCATTAGCCACATCA	GTTCAAACTTCGTTGCCTCC	59.7	237	INTRON	Ca_00925	--	--
Ca3_P2_36208259	Ca3	36208259	CTT/CTTACATATAATTTTT	GAGGCAACGAAGTTTGAACC	GGTTTCGTCGTCGTTTGAGT	60.2	675	INTRON	Ca_00925	--	--
Ca3_P2_36218021	Ca3	36218021	TG/T	TTGATGTCTCCCACAAGCTG	TAATCAGATGATCGCGCAAG	59.9	480	INTRON	Ca_00927	--	CO-like
Ca3_P2_36218063	Ca3	36218063	CAAAAAAAA/CAAAAAAAAAA	TTGATGTCTCCCACAAGCTG	TAATCAGATGATCGCGCAAG	59.9	480	INTRON	Ca_00927	--	CO-like
Ca3_P2_36219342	Ca3	36219342	GTTT/GTT	CATGTGGCGAAGAGAGTGAA	GTATTTCCTTTGGGCGTGAA	59.9	531	INTERGENIC	--	--	--
Ca3_P2_36219993	Ca3	36219993	TCA/T	TTCACGCCCAAAGGAAATAC	ACCGGTGATGACTCCAGTTC	60.0	757	DRR	Ca_00928	--	--
Ca3_P2_36222692	Ca3	36222692	AA/AAGGTGGTCTCA	AGGAAATTCGATTGGCTGTG	ACGCTTGCTGATTCCAGAGT	60.0	761	INTRON	Ca_00928	--	--

INDEL marker IDs	Chromosomes/unanchored scaffolds	Physical positions (bp)	InDels (*Kabuli* reference genome-CDC Frontier/PII)	Forward primers (5'-3')	Reverse primers (5'-3')	Anneling temperature (0C)	Expected amplified product size (bp)	Structural annotation		Functional annotation	
								Sequence components of *kabuli* genome	*Kabuli* gene accession IDs	NCBI-KOG	TFs
Ca3_P2_36226234	Ca3	36226234	AATAGAT/AATAGATAGAT	TGGAGCCCATCGAAATAAAG	CCACATGGTGAAGAGCAGAA	59.8	729	INTERGENIC	--	--	--
Ca3_P2_36226493	Ca3	36226493	ATTTTTT/ATTTTTTT	GTTGAGTTTGTGGGAATGGG	GTTCCATTTCGGATTTCGTG	60.3	532	INTERGENIC	--	--	--
Ca3_P2_36226833	Ca3	36226833	CGAGGAGG/CGAGG	CACGAAATCCGAAATGGAAC	TTGTTTGCTCTGATTTTGGAGA	59.9	537	INTERGENIC	--	--	--
Ca3_P2_36228493	Ca3	36228493	AA/AACA	CAGAAGTTGCAGCACAAGGA	ATCATCACAGCACATCCTGC	59.7	588	INTRON	Ca_00929	J	--
Ca3_P2_36230517	Ca3	36230517	T/TG	TGGGACAATTGTTAGCACGA	TCCAAAAGGAAAGGACTGGA	59.6	372	DRR	Ca_00929	J	--
Ca3_P2_36230990	Ca3	36230990	T/TA	TCCAGTCCTTTCCTTTTGGA	TGAGTTAAAGGACCACATGCC	60.0	353	DRR	Ca_00929	J	--
Ca3_P2_36231579	Ca3	36231579	ACTCTCT/ACTCT	TCGAATTTTTAAAACAAAACCCA	TTAAAACCAGTTCGGTTCGG	60.0	717	DRR	Ca_00929	J	--
Ca3_P2_36234537	Ca3	36234537	GAAAAAA/GAAAAAAA	ACCACACCCTTTTCAGATGC	TCTGCTGGTTTGGGAGTTCT	59.8	157	INTERGENIC	--	--	--
Ca3_P2_36236307	Ca3	36236307	CTAAA/CTAAATAAA	GCCGTTGAAAAATTGTGGTT	ATGTTGATTCCCAGCCACTC	59.9	239	DRR	Ca_00931	O	--
Ca3_P2_36238941	Ca3	36238941	CTTTTTT/CTTTTT	CCGCTTCAGAATCATGGAAT	GTGGAAACGCATTTCATTCA	59.5	292	INTERGENIC	--	--	--
Ca3_P2_36240433	Ca3	36240433	TAAAAAAAAA/TAAAAAAAA	TTGTTTTGAATTTGGGCCAG	TTCCAAAATCTCACCATTCATT	58.4	859	INTERGENIC	--	--	--
Ca3_P2_36241653	Ca3	36241653	CC/CCAC	TTCGCAGAAAAACTATGCCC	TCCGTATTCGAATTTGAGACAA	59.6	808	INTERGENIC	--	--	--

INDEL marker IDs	Chromosomes/unanchored scaffolds	Physical positions (bp)	InDels (*Kabuli* reference genome-CDC Frontier/PII)	Forward primers (5'-3')	Reverse primers (5'-3')	Anneling temperature (0C)	Expected amplified product size (bp)	Structural annotation		Functional annotation	
								Sequence components of *kabuli* genome	*Kabuli* gene accession IDs	NCBI-KOG	TFs
Ca3_P2_36246782	Ca3	36246782	ATTTTTTTTT/ATTTTTTTTTTT	TCATTTTTGAGCGTTTTGACA	CATGCTGTATGCTTAGGCCA	59.9	577	DRR	Ca_00932	TO	--
Ca3_P2_36249313	Ca3	36249313	A/AT	TTCCATCTATTTGGTTCGCC	GGATAGCGGATTTTTCGGAT	60.2	578	DRR	Ca_00933	O	--
Ca3_P2_36250776	Ca3	36250776	AAATAATAATAATAATAATAA/AAATAATAATAATAATAA	GCAAAGCCAAACCGCTATAA	AACGCTAACGTTGGTTCCAC	60.0	935	DRR	Ca_00933	O	--
Ca3_P2_36252617	Ca3	36252617	T/TTGCTAGTATAA	TCAATTTGGGAATCCCTTGA	CCATCCAAACACCATTTCCT	59.6	420	INTERGENIC	--	--	--
Ca3_P2_36254231	Ca3	36254231	GTTTTTTTTT/GTTTTTTTTTT	TGCCGATGTAATCTACCGAA	CGCCATTAGAAGTGGGAAAA	60.1	652	DRR	Ca_00934	O	--
Ca3_P2_36255251	Ca3	36255251	TAAAAAAAAAA/TAAAAAAAAAAA	CCTAACGCATATGCCAAGGT	TATTCGTCAACAAGGGAGGG	59.9	631	DRR	Ca_00935	--	--
Ca3_P2_36255389	Ca3	36255389	GAAA/GAA	CCTAACGCATATGCCAAGGT	TATTCGTCAACAAGGGAGGG	59.9	631	DRR	Ca_00934	O	--
Ca3_P2_36257643	Ca3	36257643	GTTTTTTTTTTTTTT/GTTTTTTTTTTTT	ACAGACACACACCATTCGGA	ATGGTGCCATGAAGAAGGAA	60.5	721	DRR	Ca_00936	--	MYB_related
Ca3_P2_36274255	Ca3	36274255	G/GCAACAT	TGGGTTTTCATTGGTTTGGT	AAGCCGACTTCAAGAAGGAC	58.5	419	DRR	Ca_00937	J	Dof
Ca3_P2_36274277	Ca3	36274277	T/TA	TGGGTTTTCATTGGTTTGGT	AAGCCGACTTCAAGAAGGAC	58.5	419	DRR	Ca_00937	J	Dof
Ca3_P2_36279811	Ca3	36279811	CTTTTTTTT/CTTTTTTTTT	GCTTAAGGTCTGACACCCGA	TGGAAACATTCTAGGCCGTC	60.1	607	INTRON	Ca_00938	R	--
Ca3_P2_36282822	Ca3	36282822	TGCT/TGCTGTTTGGCT	AGGTCCATGCCAGTGAAAAC	AAGTTGCTCATGGATCGGTT	59.6	363	DRR	Ca_00938	R	--

INDEL marker IDs	Chromosomes/unanchored scaffolds	Physical positions (bp)	InDels (*Kabuli* reference genome-CDC Frontier/PII)	Forward primers (5'-3')	Reverse primers (5'-3')	Anneling temperature (0C)	Expected amplified product size (bp)	Structural annotation		Functional annotation	
								Sequence components of *kabuli* genome	*Kabuli* gene accession IDs	NCBI-KOG	TFs
Ca3_P2_36283526	Ca3	36283526	ATTTTTTTTT/ATTTTTTTTTTTT	GCGGTCGTGTGACAGGTACT	TCTTATGCCGTTTTCCTTCG	60.2	654	DRR	Ca_00938	R	--
Ca3_P2_36286597	Ca3	36286597	ATTTTTTT/ATTTTTTTT	TCATTTTGGCAACTTACCACC	TGCCTGAATTTATTGGGAATG	59.8	754	INTERGENIC	--	--	--
Ca3_P2_36286847	Ca3	36286847	AACA/AA	TGTGGGATAAGAAAAAGACACG	CGGGCATTAGAGATCCACAT	59.9	581	INTERGENIC	--	--	--
Ca3_P2_36288728	Ca3	36288728	TAAAAAAAAA/TAAAAAAAA	TCGTCGAAAAACGTGAAAAA	TCTTGGTCGTGTCCATGAAA	60.1	707	INTERGENIC	--	--	--
Ca3_P2_36290289	Ca3	36290289	TAAAAA/TAAAA	CACCAAGACGGAAACACAAA	ATCAAAGGCGAATTTTGAGA	57.4	408	INTERGENIC	--	--	--
Ca3_P2_36291284	Ca3	36291284	GTT/GT	TCAGAGTTTATTTACCCCACTCAA	GGTTTGGTTCCTTCGGTTTT	60.2	562	INTERGENIC	--	--	--
Ca3_P2_36295270	Ca3	36295270	TTGTTTTCATGTTT/TTGTTT	TGTAGAGAACGGAAATGGGG	TAAGGTGGCGAGGATTGAAC	60.1	593	INTRON	Ca_00939	--	--
Ca3_P2_36299818	Ca3	36299818	AT/ATTT	ACCATGTTATTGCTGCACGA	TGGCAAAATTCACATCCTGA	60.0	492	INTRON	Ca_00939	--	--
Ca3_P2_36303469	Ca3	36303469	GTTTTTTTTTT/GTTTTTTTTT	GCAAACATGGTAAATTGGCA	CAAAAATTGTCACGTCGGTG	60.0	469	INTERGENIC	--	--	--
Ca3_P2_36307893	Ca3	36307893	T/TCTACTAATC	CGTAAATAGCAGTGGTTGTTCG	TCTGCTGTATCAAGTGGCAAA	59.5	573	INTERGENIC	--	--	--
Ca3_P2_36308195	Ca3	36308195	T/TC	TTTGCCACTTGATACAGCAGA	TTGAACCTAAACGATGACACCTT	59.9	309	INTERGENIC	--	--	--
Ca3_P2_36308619	Ca3	36308619	GTTTTTT/GTTTTTTT	TTTGCCACTTGATACAGCAGA	GAGACCCGAGTTTGGTTTCA	60.1	835	INTERGENIC	--	--	--

INDEL marker IDs	Chromosomes/unanchored scaffolds	Physical positions (bp)	InDels (*Kabuli* reference genome-CDC Frontier/PII)	Forward primers (5'-3')	Reverse primers (5'-3')	Anneling temperature (0C)	Expected amplified product size (bp)	Structural annotation		Functional annotation	
								Sequence components of *kabuli* genome	*Kabuli* gene accession IDs	NCBI-KOG	TFs
Ca3_P2_36312854	Ca3	36312854	AAA/AAATTAAGTAA	CTATGCACGAGTTTGGGGTT	CAATCAAGAAGCGCATTGAA	60.0	586	DRR	Ca_00941	D	TCP
Ca3_P2_36318491	Ca3	36318491	AATAT/AAT	TTGATTTGCACGGTTTACCA	TATTCAAATAGCGGCCATCC	59.9	607	INTRON	Ca_00941	D	TCP
Ca3_P2_36320561	Ca3	36320561	CTTTTTTTT/CTTTTTTTTTTT	GTCTCAAGCAATCCTCAGCC	CAACGAGAGAGTGGATGGGT	60.1	442	INTRON	Ca_00941	D	TCP
Ca3_P2_36320888	Ca3	36320888	CAAAAAAAAAA/CAAAAAAAAAAA	ACCCATCCACTCTCTCGTTG	TTTTCTTATGCAAGGCCAGC	60.3	417	INTRON	Ca_00941	D	TCP
Ca3_P2_36324338	Ca3	36324338	TTTCT/TT	CAACAGGCCAACATAAAATGA	GGAATGTTTCATGGACACCC	60.0	330	INTERGENIC	--	--	--
Ca3_P2_3632559	Ca3	3632559	ATTTTTTTTTTTTTT/ATTTTTTTTTTTTT	TTTGAGTCTAACCCGAACCAA	GTGGGGTAGGCAGAGACAAA	60.1	765	INTERGENIC	--	--	--
Ca3_P2_36327376	Ca3	36327376	ATTTTTTTT/ATTTTTTT	TCAAAATTTGCAAGTTGGTCC	TTGTTTTCCCCCAATTCTTG	59.8	504	INTERGENIC	--	--	--
Ca3_P2_36327903	Ca3	36327903	ATTTTTTTTT/ATTTTTTTTTTTTTTT	CAAGAATTGGGGGAAAACAA	ATACATCTCGCCACAATGGG	60.7	296	INTERGENIC	--	--	--
Ca3_P2_36328591	Ca3	36328591	GAAA/GAA	CACGATGAAATCACTATGAAAAGC	CGTTCCAGACCTCCATTGTT	60.0	440	INTERGENIC	--	--	--
Ca3_P2_36340133	Ca3	36340133	A/AGTAGAGATATAATTG	TAGCCCGTCACAATTTAGGG	CTCTTTCTTTCCCTCCGCTC	60.5	910	DRR	Ca_00942	--	HB-other
Ca3_P2_36344189	Ca3	36344189	GCCC/GCC	GGCCTTTTGATCCTCTAGGC	TGCAATTTTTATCACATCCCC	59.7	777	DRR	Ca_00943	R	C2H2
Ca3_P2_36344245	Ca3	36344245	CATAT/CAT	GGCCTTTTGATCCTCTAGGC	TGCAATTTTTATCACATCCCC	59.7	777	DRR	Ca_00943	R	C2H2

INDEL marker IDs	Chromosomes/unanchored scaffolds	Physical positions (bp)	InDels (*Kabuli* reference genome-CDC Frontier/PII)	Forward primers (5'-3')	Reverse primers (5'-3')	Anneling temperature (0C)	Expected amplified product size (bp)	Structural annotation		Functional annotation	
								Sequence components of *kabuli* genome	*Kabuli* gene accession IDs	NCBI-KOG	TFs
Ca3_P2_36351449	Ca3	36351449	TA/TAA	AAGACCAACATTGGGGACAA	CGATTCAAGTGTCGCATGTC	60.3	473	INTERGENIC	--	--	--
Ca3_P2_36351563	Ca3	36351563	TAAAAAAA/TAAAAAA	AAGACCAACATTGGGGACAA	GCCTGCAATGAACCGTAAGT	60.1	873	INTERGENIC	--	--	--
Ca3_P2_36352882	Ca3	36352882	AT/ATT	TCACCTCGAGCAAACAAGTG	AAGCACCATTTGTAGTTTTCATGT	59.0	380	INTERGENIC	--	--	--
Ca3_P2_36355454	Ca3	36355454	GTT/GT	TTTTGGCACTGGGATCTTTT	CCGGCCTCCTTAACAAACTA	59.2	795	DRR	Ca_00944	--	--
Ca3_P2_36356666	Ca3	36356666	CAAAAAAAAAAAA/CAAAAAAAAAAA	ATTTAAAAGTCACGCGGAGC	TCCAGGTATGCGGTAGTTCC	60.0	373	DRR	Ca_00944	--	--
Ca3_P2_36362238	Ca3	36362238	CATTAATTAATTAATTAATT/CATTAATTAATTAATTAATTAATT	GAAAATCGACCTGGGGAAAT	GATCGCTCGTTTTCTTGCTC	60.1	289	INTERGENIC	--	--	--
Ca3_P2_36363400	Ca3	36363400	ATTTTTTTT/ATTTTTTT	CAATGCATATGGACGGAATG	GACGACAAAGGATATTCATGAGC	60.0	352	DRR	Ca_00945	--	NF-X1
Ca3_P2_36369683	Ca3	36369683	AATA/AA	TTTTCTGTATCGGCCATTCC	GGAATCTCGCATTTTCTCCA	60.2	660	INTRON	Ca_00945	--	NF-X1
Ca3_P2_36371742	Ca3	36371742	ATTTTTTTT/ATTTTTTT	GGGTTTTCTAGTCCCCCAAG	TGTTTTTCATTATGTGCCCG	59.4	483	INTERGENIC	--	--	--
Ca3_P2_36371828	Ca3	36371828	GT/GTTAT	GGGTTTTCTAGTCCCCCAAG	TGTTTTTCATTATGTGCCCG	59.4	483	INTERGENIC	--	--	--
Ca3_P2_36372036	Ca3	36372036	TAAAAAAAA/TAAAAAAAAA	GGGTTTTCTAGTCCCCCAAG	TGTTTTTCATTATGTGCCCG	59.4	483	INTERGENIC	--	--	--
Ca3_P2_36377150	Ca3	36377150	GAAAAAAAAA/GAAAAAAAAAA	TGAAAAACAATCGTGGTGGA	AGGCAAACACATGAAAAGGG	60.0	565	INTRON	Ca_00946	D	WRKY

INDEL marker IDs	Chromosomes/unanchored scaffolds	Physical positions (bp)	InDels (*Kabuli* reference genome-CDC Frontier/PII)	Forward primers (5'-3')	Reverse primers (5'-3')	Anneling temperature (0C)	Expected amplified product size (bp)	Structural annotation		Functional annotation	
								Sequence components of *kabuli* genome	*Kabuli* gene accession IDs	NCBI-KOG	TFs
Ca3_P2_36381407	Ca3	36381407	ATTTTTT/ATTTTTTT	TTTTGAGAGCGCATTTTGTG	CCATGCTTCCCACGTCTAAT	60.0	352	INTERGENIC	--	--	--
Ca3_P2_36383257	Ca3	36383257	A/AC	CACAATGACCACCAAAATCG	GCACCCATCATACGTGTCAG	60.0	436	INTERGENIC	--	--	--
Ca3_P2_36383999	Ca3	36383999	AT/ATGTTT	TGGCACTGTTTTGGTCTTTG	TGGACCCAAAATTTCCCATA	60.0	605	INTERGENIC	--	--	--
Ca3_P2_36393062	Ca3	36393062	TC/TTTTTTCC	TTCAAGAATTTACGGGCTGG	CCACCAGTCAGGCCAATAAT	59.8	585	INTERGENIC	--	--	--
Ca3_P2_36396966	Ca3	36396966	AAAATT/AAAATTTCAAATT	CTCACATCTGATCGAAGGCA	ACTGTAACCTTGTGCTCCCG	60.2	332	INTRON	Ca_00950	P	--
Ca3_P2_36397163	Ca3	36397163	TTATATATAT/TTATATATATATAT	CTCACATCTGATCGAAGGCA	TGCAGCAGCAGAACCATTAC	60.0	708	INTRON	Ca_00950	P	--
Ca3_P2_36398787	Ca3	36398787	T/TC	GTTTGGCCATTTGCAACTTT	ACGATGTGTGGCCCAATTAT	60.1	545	DRR	Ca_00950	P	--
Ca3_P2_36399143	Ca3	36399143	ATTTTTT/ATTTTTTT	ATAATTGGGCCACACATCGT	TGCAATGTGGAAATCAGAGAA	59.3	208	DRR	Ca_00950	P	--
Ca3_P2_36399524	Ca3	36399524	T/TG	ATAATTGGGCCACACATCGT	AACAAGCAAGCGCAACACTA	59.7	712	DRR	Ca_00950	P	--
Ca3_P2_36400923	Ca3	36400923	TAAA/TAAAA	TCCTGAACAAGCTTCCGAGT	CAAGCTTGCCAATGTTCAAA	59.8	434	INTRON	Ca_00951	H	--
Ca3_P2_36400979	Ca3	36400979	TTATA/TTATATA	TCCTGAACAAGCTTCCGAGT	CAAGCTTGCCAATGTTCAAA	59.8	434	INTRON	Ca_00951	H	--
Ca3_P2_36408046	Ca3	36408046	AATATATATATA/AATATATATATATA	TTAGGAATGCCTTGCTTGCT	CTGTGTCTGGGCCCTTAAAA	60.1	727	DRR	Ca_00952	--	--

INDEL marker IDs	Chromosomes/unanchored scaffolds	Physical positions (bp)	InDels (*Kabuli* reference genome-CDC Frontier/PII)	Forward primers (5'-3')	Reverse primers (5'-3')	Anneling temperature (0C)	Expected amplified product size (bp)	Structural annotation		Functional annotation	
								Sequence components of *kabuli* genome	*Kabuli* gene accession IDs	NCBI-KOG	TFs
Ca3_P2_36410216	Ca3	36410216	ACCCCCCC/ACCCCCC	CGGAAATGTCATGTGTGGTC	ATGTTTGTCCACTTGCTCCC	60.0	458	DRR	Ca_00953	--	HSF
Ca3_P2_36410249	Ca3	36410249	GAAAAAAAAAA/GAAAAAAAAAAAA	CGGAAATGTCATGTGTGGTC	ATGTTTGTCCACTTGCTCCC	60.0	458	DRR	Ca_00953	--	HSF
Ca3_P2_36410296	Ca3	36410296	GTTTTTTT/GTTTTTTTTT	ATGGTTGTTCATCGTCCCAT	ATGTTTGTCCACTTGCTCCC	60.0	126	DRR	Ca_00953	--	HSF
Ca3_P2_36414062	Ca3	36414062	TGG/TGGG	GATGCGCTATCCAACCTCAC	GCCGCCCACATATCATTTAT	59.6	659	INTRON	Ca_00954	G	--
Ca3_P2_36416411	Ca3	36416411	CAAAAAAAAAAAA/CAAAAAAAAAA	AGGAGTTTTCGGTGACATGG	CATCGAGGTTTTCTGCCATT	60.1	623	INTRON	Ca_00954	G	--
Ca3_P2_36418781	Ca3	36418781	GAAAA/GAAA	TCCTCCTTACCACATCGGTC	ACGTAGAAACAATGGCCGTC	60.0	929	INTRON	Ca_00954	G	--
Ca3_P2_36420292	Ca3	36420292	GGAGC/G	TGTTTTTCGCTCATCCATTG	TGGTAGAAATCACGATGCCA	60.1	710	INTERGENIC	--	--	--
Ca3_P2_36420971	Ca3	36420971	AATATATATATATAT/AATATATATAT	TGGCATCGTGATTTCTACCA	GGCCATGGTGATTGTAAAGG	60.2	746	INTERGENIC	--	--	--
Ca3_P2_36422241	Ca3	36422241	ATTTT/ATTT	GTGGACCTTCCAAGGATGAA	CGTTGTTTGAGGTTTGCTGA	59.9	895	INTERGENIC	--	--	--
Ca3_P2_36426774	Ca3	36426774	TAGATGAAGATGAAGATGAAGATGA/TAGATGAAGATGAAGATGAAGATGAAGATGA	TCAAAGGAAGCTTAGCCCAA	GCAAACGCTTCTCTGGTTTC	60.0	861	CDS (large-effect mutations)	Ca_00956	GM	WRKY
Ca3_P2_36427924	Ca3	36427924	CAAATATAAAT/CAAATATAAATATAAAT	TGGCCCTTTATCACAACTCA	TTTTGGTTGTTTCAATCGCA	60.1	493	INTERGENIC	--	--	--
Ca3_P2_36429101	Ca3	36429101	TAAAAAA/TAAAAAAAAAA	CCATCTTGCGAAATGTGAAG	AAATTTAACGACGCTCCACG	60.1	397	INTERGENIC	--	--	--

INDEL marker IDs	Chromosomes/unanchored scaffolds	Physical positions (bp)	InDels (*Kabuli* reference genome-CDC Frontier/PII)	Forward primers (5'-3')	Reverse primers (5'-3')	Anneling temperature (0C)	Expected amplified product size (bp)	Structural annotation		Functional annotation	
								Sequence components of *kabuli* genome	*Kabuli* gene accession IDs	NCBI-KOG	TFs
Ca3_P2_36429786	Ca3	36429786	G/GA	AGGGATGCACCAGCTGTAAC	AGAGAAGAAACCTGGTGCGA	60.0	593	INTERGENIC	--	--	--
Ca3_P2_36429931	Ca3	36429931	GCTTCTTCTTCTTCTTCTTCT/GCTTCTTCTTCTTCTTCTTCTTCTTCTTCT	AGGGATGCACCAGCTGTAAC	AGAGAAGAAACCTGGTGCGA	60.0	593	CDS (large-effect mutations)	Ca_00957	H	--
Ca3_P2_36431158	Ca3	36431158	CT/CTGTT	CCCATTTGCTGTATTTGCCT	TAGTCCAACCCAATCCTCCA	60.3	530	INTRON	Ca_00957	H	--
Ca3_P2_36431342	Ca3	36431342	TTG/TGAACTG	CCCATTTGCTGTATTTGCCT	TTGCATCAGGCTTGTACCTG	59.9	694	INTRON	Ca_00957	H	--
Ca3_P2_36435267	Ca3	36435267	T/TAACACAG	CGAAAAATTCCTGAACCATCC	AAGGTGGAGGAGGGAAAAGA	60.0	787	DRR	Ca_00957	H	--
Ca3_P2_36439660	Ca3	36439660	TACCAACCAACCAACC/TACCAACCAACCAACCAACC	TAATGGGGCCAGTTTGCTAC	TAAGGAAAATACCGGACGCA	60.4	441	DRR	Ca_00959	R	--
Ca3_P2_36443024	Ca3	36443024	TGAGAGA/TGAGA	TTGCAATTGAAGTTCCCACA	GGCAAGCTCATTTCTGCTTC	60.1	427	INTRON	Ca_00959	R	--
Ca3_P2_36443050	Ca3	36443050	ATG/A	TTGCAATTGAAGTTCCCACA	GGCAAGCTCATTTCTGCTTC	60.1	427	INTRON	Ca_00959	R	--
Ca3_P2_36443105	Ca3	36443105	GCATCAT/GCAT	TTGCAATTGAAGTTCCCACA	GGCAAGCTCATTTCTGCTTC	60.1	427	INTRON	Ca_00959	R	--
Ca3_P2_36446380	Ca3	36446380	CAAAAAA/CAAAA	TTTCCGCATAGAAATCTGGG	ATCCCAAATTGCACTCATCC	59.8	407	DRR	Ca_00960	O	--
Ca3_P2_36447416	Ca3	36447416	AGCTGC/AGCTGCTGC	GGACACCAACGTCTCCACTT	GAGCAAGAAGTGTCGAAGCC	60.1	210	CDS (large-effect mutations)	Ca_00960	O	--
Ca3_P2_36454087	Ca3	36454087	CGAT/CGATCATATTGGAT	TGATTCGGTTTGTGCTGTGT	CGTGACAGCCCATAGTTGAA	59.7	931	INTERGENIC	--	--	--

INDEL marker IDs	Chromosomes/unanchored scaffolds	Physical positions (bp)	InDels (*Kabuli* reference genome-CDC Frontier/PII)	Forward primers (5'-3')	Reverse primers (5'-3')	Anneling temperature (0C)	Expected amplified product size (bp)	Structural annotation		Functional annotation	
								Sequence components of *kabuli* genome	*Kabuli* gene accession IDs	NCBI-KOG	TFs
Ca3_P2_36455748	Ca3	36455748	AC/AGTTTTACTCTTAATACATC	TCAAATTACAACAAGGGCGA	CGTAAGGGAGCAAAGTGGTC	59.7	792	INTERGENIC	--	--	--
Ca3_P2_36455997	Ca3	36455997	GTAAATTT/GTAAATTTGTTTATAAATTT	ATTAACCACTTTGCCGACGA	GACGCAACAACGTAAGGGAG	60.7	532	INTERGENIC	--	--	--
Ca3_P2_36456355	Ca3	36456355	AATTTTTTT/AATTTTTTTAATATTTTTTT	GACCACTTTGCTCCCTTACG	AAGTCTCAAACAAACCACCGA	59.6	585	INTERGENIC	--	--	--
Ca3_P2_36458050	Ca3	36458050	AG/A	TGGGTCCCAAAAGTGGTATC	CAATTTGGCCACTTGAATGA	59.5	772	INTERGENIC	--	--	--
Ca3_P2_36458200	Ca3	36458200	A/ATTCTCAT	TGGGTCCCAAAAGTGGTATC	TGTTTTGCAAGGTGAATTGG	59.6	910	INTERGENIC	--	--	--
Ca3_P2_36458326	Ca3	36458326	ATTTTTT/ATTTTTTT	TTTGCAAAGTGGCGGTAGTT	TGTTTTGCAAGGTGAATTGG	59.6	525	INTERGENIC	--	--	--
Ca3_P2_36458434	Ca3	36458434	GATCAT/GAT	TTTGCAAAGTGGCGGTAGTT	ATCAATAATTGGGCAGACGC	59.9	753	INTERGENIC	--	--	--
Ca3_P2_36458751	Ca3	36458751	TAAAAAAAAA/TAAAAAAAA	CCAATTCACCTTGCAAAACA	ATCAATAATTGGGCAGACGC	59.9	248	INTERGENIC	--	--	--
Ca3_P2_36460868	Ca3	36460868	ATT/ATTTT	TACAGGAGCCGCTGAGATTT	TGAAGCGTGGTGCTTCTCTA	59.7	523	INTRON	Ca_00962	--	--
Ca3_P2_36465593	Ca3	36465593	A/AAGTT	AAGCGCGTGGTAATAAATGG	AATTCCTCCTGCTCACAATGA	59.7	664	INTERGENIC	--	--	--
Ca3_P2_36468870	Ca3	36468870	CAAAAAA/CAAAA	TAACCAAAAGGAGGACAGCG	ATCTTGAAAGATTGGCCGTG	60.1	542	INTERGENIC	--	--	--
Ca3_P2_36469879	Ca3	36469879	GAAAAAAAAA/GAAAAAAAAAAA	CGTTAAGAACCAAAAGGGCA	TTGGGAGCAACTTTACCCAC	60.0	534	DRR	Ca_00964	A	B3

INDEL marker IDs	Chromosomes/unanchored scaffolds	Physical positions (bp)	InDels (*Kabuli* reference genome-CDC Frontier/PII)	Forward primers (5'-3')	Reverse primers (5'-3')	Anneling temperature (0C)	Expected amplified product size (bp)	Structural annotation		Functional annotation	
								Sequence components of *kabuli* genome	*Kabuli* gene accession IDs	NCBI-KOG	TFs
Ca3_P2_36472177	Ca3	36472177	GAAAAAAA/GAAAAAAA	GAGTCCCGCCTTGAATCATA	GCAGGTACCACTGGGATTGT	59.9	370	INTRON	Ca_00964	A	B3
Ca3_P2_36472539	Ca3	36472539	TAAAAAAAAAA/TAAAAAAAAA	GAGTCCCGCCTTGAATCATA	AGGGGATGTTTGTTTTTCCC	60.0	627	INTRON	Ca_00964	A	B3
Ca3_P2_36477906	Ca3	36477906	CAAAAAAAA/CAAAAAAA	TGGCATTGCAAAATAGATGG	ATGTCGTCGGCAATAACACA	60.0	465	INTERGENIC	--	--	--
Ca3_P2_36480664	Ca3	36480664	T/TG	AAATAGAAGGGGGTGTTGGG	AAAGAGTGGATCGCCTCAAA	59.8	164	INTERGENIC	--	--	--
Ca3_P2_36486572	Ca3	36486572	TAAAA/TAAAAA	AATTTCGTGATTTTGTGGGG	CCTTGCAACTTGGAAAAGAGA	59.5	713	INTERGENIC	--	--	--
Ca3_P2_36489025	Ca3	36489025	GTT/GT	CGGGAACATGAAAGGAAGAA	ATCCAAATTCCAACCCACAA	60.0	720	DRR	Ca_00965	--	--
Ca3_P2_36490446	Ca3	36490446	TA/TCACA	CAGGGTCACCTGCTCAAAAT	TCCAAGATCAGTTGACTGCG	60.0	333	DRR	Ca_00965	--	--
Ca3_P2_36497213	Ca3	36497213	C/CTTGTTTTTGTATATGG	CGCAGATTCCCCATACTTTC	TGTTGAGCTCCCCCAATTTA	60.4	871	INTERGENIC	--	--	--
Ca3_P2_36497594	Ca3	36497594	CAAA/CAA	TTATTCTGTCAATGAACCGCC	TGTTGAGCTCCCCCAATTTA	60.4	178	INTERGENIC	--	--	--
Ca3_P2_36498592	Ca3	36498592	TATAGATA/TATA	TTCTTGCACGGGCTGATTAT	TCAATAATTTTCCCATCTCCAA	58.4	577	INTERGENIC	--	--	--
Ca3_P2_36498648	Ca3	36498648	TAC/TACAC	TTCTTGCACGGGCTGATTAT	TTCACGAAATTCTCTCAAATGG	59.2	678	INTERGENIC	--	--	--
Ca3_P2_36498881	Ca3	36498881	TC/T	TTCTTGCACGGGCTGATTAT	CATGGGAATAATGCTTGGCT	59.9	709	INTERGENIC	--	--	--

INDEL marker IDs	Chromosomes/unanchored scaffolds	Physical positions (bp)	InDels (*Kabuli* reference genome-CDC Frontier/PII)	Forward primers (5'-3')	Reverse primers (5'-3')	Anneling temperature (0C)	Expected amplified product size (bp)	Structural annotation		Functional annotation	
								Sequence components of *kabuli* genome	*Kabuli* gene accession IDs	NCBI-KOG	TFs
Ca3_P2_36500472	Ca3	36500472	GAT/G	TGGTAGGAAAGCCAACGAGT	TTTTGCAGACGGAAGCTTTT	60.0	708	DRR	Ca_00967	S	--
Ca3_P2_36500819	Ca3	36500819	TAAAAAAAAAAA/TAAAAAAAAAA	ATTTGTGTTTTGAAACGGGG	TTTTGCAGACGGAAGCTTTT	60.0	596	DRR	Ca_00967	S	--
Ca3_P2_36502102	Ca3	36502102	GACTGCAA/GACTGCAACTGCAA	AACCAAAGCAACCACACACA	TTGCTGCGGATGATGTAGAG	60.0	762	INTRON	Ca_00967	S	--
Ca3_P2_36508250	Ca3	36508250	T/TC	TTGATTCGTGTGATTGGTATGA	GGCCAACGACTTCCTATTGA	60.1	130	INTERGENIC	--	--	--
Ca3_P2_36512527	Ca3	36512527	TA/TATACTTAA	TTGCACACTATGCCTTCGAC	CCCCACAAAACCCTATGTTG	60.1	662	DRR	Ca_00968	A	--
Ca3_P2_36516587	Ca3	36516587	GAAAAAAAA/GAAAAAAA	CTTCTTCTTCTCCACGACCG	TGCCAAATGAAAGATGACCT	58.1	656	INTERGENIC	--	--	--
Ca3_P2_36518859	Ca3	36518859	TAAAAAAA/TAAAAAA	TGATCAAGGAGTATCACGGG	GGAGTCACCTTTTTGTGAAGC	58.8	525	INTERGENIC	--	--	--
Ca3_P2_36521786	Ca3	36521786	CTT/CTTGTTATT	TTTGTGGCATTGACACCTTT	TGCGTTCAACTACTATGCCG	59.9	628	INTERGENIC	--	--	--
Ca3_P2_36522315	Ca3	36522315	CAA/CAAA	CGGCATAGTAGTTGAACGCA	TTGTTCTTGGAGGATAGCAAAAA	60.1	381	INTERGENIC	--	--	--
Ca3_P2_36522650	Ca3	36522650	ACCC/ACCCC	TTTTTGCTATCCTCCAAGAACAA	CGTCATTCGTTTAATTTCTTGTG	58.7	465	INTERGENIC	--	--	--
Ca3_P2_36525538	Ca3	36525538	AAT/AAAATCTTTAT	TCCAAGGACTAAAACGTCCAA	TCCAAAGAGACTCATCCCAAA	59.7	519	DRR	Ca_00969	--	--
Ca3_P2_36526742	Ca3	36526742	AAA/AAATTAA	AAGTCGCCAACGCAATAAAC	CTCTTTGTGCAAACAGGCAA	60.0	827	DRR	Ca_00970	--	--

INDEL marker IDs	Chromosomes/unanchored scaffolds	Physical positions (bp)	InDels (*Kabuli* reference genome-CDC Frontier/PII)	Forward primers (5'-3')	Reverse primers (5'-3')	Anneling temperature (0C)	Expected amplified product size (bp)	Structural annotation		Functional annotation	
								Sequence components of *kabuli* genome	*Kabuli* gene accession IDs	NCBI-KOG	TFs
Ca3_P2_36527391	Ca3	36527391	CT/C	GCCATAAATTGTGTTTGGGG	AAATTTTGCCGTTTGGTGAG	60.0	387	DRR	Ca_00970	--	--
Ca3_P2_36529799	Ca3	36529799	AAAGA/AA	CCCTATCCAACATTCATGGG	ACCAAACTGGTGGAACTTGC	60.0	722	DRR	Ca_00971	U	--
Ca3_P2_36530182	Ca3	36530182	T/TC	GATTTAGCAGGAGTGCCGAG	TCGGATCGACTCTTTGGAAC	60.2	686	DRR	Ca_00971	U	--
Ca3_P2_36533542	Ca3	36533542	CTTTTTT/CTTTTTTT	ACTATTCTACATTTTGTTGGGCA	TGCGAACGATGGATGAGTAA	60.2	953	INTERGENIC	--	--	--
Ca3_P2_36550076	Ca3	36550076	CAAAAAAA/CAAAAAAAAA	TGGGGATCAAACACTCTTCC	CCCAAGTTCCAAGTCCAAAA	59.9	579	INTRON	Ca_00973	I	B3
Ca3_P2_36560979	Ca3	36560979	TAAAAAAAAAAAA/TTCAAAAAAAAAAAAAA	ATCTGATTTGAGCAATGGGC	GGTAAGGCCATTTTGGGTAA	58.8	452	INTERGENIC	--	--	--
Ca3_P2_36565372	Ca3	36565372	T/TATTTA	CGGTACAAGACGTCCAACCT	TGTATGCAACCACGAGGAAA	60.1	600	INTERGENIC	--	--	--
Ca3_P2_36571020	Ca3	36571020	ATTTTTTT/ATTTTT	TACCGAATCCTTTGTCCTGC	GAGTTTGCGCGTTTAAGAGG	60.0	772	DRR	Ca_00977	--	NAC
Ca3_P2_36573606	Ca3	36573606	GATAT/GAT	CGGATTGTCATGTTCACTGC	TGATGATGGTTGGAGATGGA	59.9	598	INTRON	Ca_00977	--	NAC
Ca3_P2_36576249	Ca3	36576249	TAAAAAAAAAAA/TAAAAAAAAAA	CCATACTCAATGCAAAATCGTT	AGAAAAACATGCGTTGTCCC	60.0	590	INTERGENIC	--	--	--
Ca3_P2_36577180	Ca3	36577180	GAAAAAAAAAA/GAAAAAAAAA	AAGGAATTCATGCCTGCAAC	CTTCAATGCTCGACTGCAAG	59.7	309	INTERGENIC	--	--	--
Ca3_P2_36593212	Ca3	36593212	GAAAAAAAA/GAAAAAAAAA	CAGCAGTATCACCCCAACCT	TGGAACCCTTGCTTGATTTC	60.1	448	INTRON	Ca_00979	M	HB-other

INDEL marker IDs	Chromosomes/unanchored scaffolds	Physical positions (bp)	InDels (*Kabuli* reference genome-CDC Frontier/PII)	Forward primers (5'-3')	Reverse primers (5'-3')	Anneling temperature (0C)	Expected amplified product size (bp)	Structural annotation		Functional annotation	
								Sequence components of *kabuli* genome	*Kabuli* gene accession IDs	NCBI-KOG	TFs
Ca3_P2_36596139	Ca3	36596139	GAAAAAA/GAAAAAAA	AAAGTTTTTGGCAAGTCGGA	AGAGCAAGGAAACCGAATCA	59.8	596	INTERGENIC	--	--	--
Ca3_P2_36597752	Ca3	36597752	ATTTTTTT/ATTTTTTTT	TTGTTTTCGTAGGCGAGACT	TTTCATGCGGTTGTTGGTTA	60.0	380	INTERGENIC	--	--	--
Ca3_P2_36597966	Ca3	36597966	TAAAAAAAA/TAAAAAAA	TAACCAACAACCGCATGAAA	TCAATTTCTTAAAAGAGGTGGCA	60.1	242	INTERGENIC	--	--	--
Ca3_P2_36608541	Ca3	36608541	T/TA	TGGCTCAAAGATAGGATGGG	CCCTTCCTCCATCAATCTCA	60.0	315	DRR	Ca_00981	--	--
Ca3_P2_36609637	Ca3	36609637	CATATAT/CATAT	ATTGAAATGCGGGTTCTCAG	TTGATCACAACGAGGAGCTG	60.0	519	DRR	Ca_00981	--	--
Ca3_P2_36609660	Ca3	36609660	CTT/CTTT	ATTGAAATGCGGGTTCTCAG	TTGATCACAACGAGGAGCTG	60.0	519	DRR	Ca_00981	--	--
Ca3_P2_36613694	Ca3	36613694	AT/A	CAGGGGTAAAATCCACGAGA	GGATGAATGCGAAACTTTTGA	60.1	792	INTERGENIC	--	--	--
Ca3_P2_36614124	Ca3	36614124	TGTA/T	TTGCATTTCAAAAGTTTCGC	TGTGGCTCAACGAGAGATTT	58.4	277	INTERGENIC	--	--	--
Ca3_P2_36616040	Ca3	36616040	AAATTGATAATTGAT/AAATTGATAATTGATAATTGAT	CACCAAGATTCACTCCCGTT	GCATTCTCCTCATCCAAAGG	59.6	785	INTRON	Ca_00982	J	bHLH
Ca3_P2_36616270	Ca3	36616270	TT/TTAT	CCTGTGCTTGGTTCCACTTT	CAAACTTCCTTTCTCGGCTG	60.0	725	INTRON	Ca_00982	J	bHLH
Ca3_P2_36618059	Ca3	36618059	CAAA/CAAAA	TCCTCTGCCGCTTGATATTC	ATACTCAGGCCGCCATCTTT	61.0	587	DRR	Ca_00983	T	--
Ca3_P2_36618106	Ca3	36618106	CAAGAAG/CAAG	TCCTCTGCCGCTTGATATTC	CCATTTGTTGAACGACACCA	60.4	772	DRR	Ca_00982	J	bHLH

INDEL marker IDs	Chromosomes/unanchored scaffolds	Physical positions (bp)	InDels (*Kabuli* reference genome-CDC Frontier/PII)	Forward primers (5'-3')	Reverse primers (5'-3')	Anneling temperature (0C)	Expected amplified product size (bp)	Structural annotation		Functional annotation	
								Sequence components of *kabuli* genome	*Kabuli* gene accession IDs	NCBI-KOG	TFs
Ca3_P2_36624066	Ca3	36624066	ATTTTTTT/ATTTTTT	TCACTCTCCCGTCATTTTCC	TGTGGTGCAGTGGAAATCAT	60.0	563	INTERGENIC	--	--	--
Ca3_P2_36624385	Ca3	36624385	CAAAAAAAAAAA/CAAAAAAAA	TGATTTCCACTGCACCACAT	TGCAACACAAACTCCTTTGC	59.9	517	INTERGENIC	--	--	--
Ca3_P2_36624455	Ca3	36624455	ATTTTTTT/ATTTTTTTT	TGATTTCCACTGCACCACAT	TGCAACACAAACTCCTTTGC	59.9	517	INTERGENIC	--	--	--
Ca3_P2_36624904	Ca3	36624904	GTTT/GTTTCTTTTTT	TGCAAAGGAGTTTGTGTTGC	AACATAAAAGAACAGAGCGGAGA	59.4	211	INTERGENIC	--	--	--
Ca3_P2_36625899	Ca3	36625899	TTATTTATTTA/T	ATTTCACTGACTGTCGCCCT	CAACAGGAAGGGGATTAGAAAA	59.5	356	INTERGENIC	--	--	--
Ca3_P2_36627027	Ca3	36627027	CTTTTTTTTTTTTTT/CTTTTTTTTTTTT	CATGGTGAGTGCCATGTGTT	TTGCGATTACCTCAAAATTCG	60.1	502	INTERGENIC	--	--	--
Ca3_P2_36636712	Ca3	36636712	CTTTTTT/CTTTTT	CGGATAACACTTGTTTGTGAGTTT	GTCTTTTCTTGATCCCGACG	59.7	776	INTERGENIC	--	--	--
Ca3_P2_36638153	Ca3	36638153	TG/T	TCGTCAACTCGCAACTCATC	AAAAAGCCAAACAATTCTAGAAACA	59.6	487	INTERGENIC	--	--	--
Ca3_P2_36638232	Ca3	36638232	AATTTT/A	TCGTCAACTCGCAACTCATC	AAAAAGCCAAACAATTCTAGAAACA	59.6	487	INTERGENIC	--	--	--
Ca3_P2_36639234	Ca3	36639234	TG/T	CAAACAACGCGTGAAAGAAA	TAGTTCGGTTCGGTTCGATT	59.6	735	INTERGENIC	--	--	--
Ca3_P2_36647114	Ca3	36647114	GAAAAAAA/GAAAAAA	TGACTTTCGTCCCTCTGCTT	TGTACCAGAATGGATGGCAA	59.9	447	DRR	Ca_00985	U	--
Ca3_P2_36647346	Ca3	36647346	CCTTCTT/CCTT	TGACTTTCGTCCCTCTGCTT	AACGGTCGATAAACGTGGAC	59.9	821	DRR	Ca_00985	U	--

INDEL marker IDs	Chromosomes/unanchored scaffolds	Physical positions (bp)	InDels (*Kabuli* reference genome-CDC Frontier/PII)	Forward primers (5'-3')	Reverse primers (5'-3')	Anneling temperature (0C)	Expected amplified product size (bp)	Structural annotation		Functional annotation	
								Sequence components of *kabuli* genome	*Kabuli* gene accession IDs	NCBI-KOG	TFs
Ca3_P2_36652791	Ca3	36652791	TGGG/TG	TCACAAAGAAAGCCAGCTCA	TCCAGAGCTTTTCATGGCTT	60.0	451	DRR	Ca_00986	--	bZIP
Ca3_P2_36654188	Ca3	36654188	TT/TTAT	TGGCTTCATTATGGATTCA	GGCTCCAAAAATAGCCAACTT	59.6	499	INTERGENIC	--	--	--
Ca3_P2_36673352	Ca3	36673352	CAAAAAAA/CAAAAAA	GGGGAACAATGAAAAAGACAA	CGTCTTCGAACACTGTGCAT	59.9	855	DRR	Ca_00988	P	GRAS
Ca3_P2_36673542	Ca3	36673542	TATAC/T	TGTTGTTAGCATTCACGCTATTC	TTTTTCGACCCACAAGAGGT	59.6	408	DRR	Ca_00988	P	GRAS
Ca3_P2_36677699	Ca3	36677699	ATTTT/ATTTTT	TCAGACCGTGTAACCAACCA	TAAGTATGGGCCTCTTGCCC	61.3	716	DRR	Ca_00989	--	--
Ca3_P2_36678142	Ca3	36678142	CA/CAA	GGGCAAGAGGCCCATACTTA	AAGAAAAGAAAAATGTTTGCGG	59.7	424	DRR	Ca_00989	--	--
Ca3_P2_36678243	Ca3	36678243	GAA/GA	GGGCAAGAGGCCCATACTTA	AAGAAAAGAAAAATGTTTGCGG	59.7	424	DRR	Ca_00989	--	--
Ca3_P2_36681076	Ca3	36681076	A/AT	ATGGAATGCAATTAGGCTGG	AACAACCAACCGAAAGATGG	59.8	475	INTERGENIC	--	--	--
Ca3_P2_36681303	Ca3	36681303	ATTTTTTTT/ATTTTTTTTT	CCATCTTTCGGTTGGTTGTT	TAATCCAGCTGTTTGGCCTC	60.2	182	INTERGENIC	--	--	--
Ca3_P2_36682375	Ca3	36682375	GTTTTTTTTTT/GTTTTTTTTT	ACAAGGATCATTCCTCTGCC	TTGTCCGAACATCGATGAAG	59.6	194	INTERGENIC	--	--	--
Ca3_P2_36682594	Ca3	36682594	GTTTTTTTTT/GTTTTTTTTTT	TTCATCGATGTTCGGACAAA	CCAACTCGGTTCATTTACTCG	59.6	536	INTERGENIC	--	--	--
Ca3_P2_36688981	Ca3	36688981	TTCTTTATCTTTATCTTTATC/TTCTTTATCTTTATCTTTATCTTTATC	AGGGGAGACAACCATAGGCT	GTGATGTGATTGGCTGGTTG	60.0	862	DRR	Ca_00990	O	C3H

INDEL marker IDs	Chromosomes/unanchored scaffolds	Physical positions (bp)	InDels (*Kabuli* reference genome-CDC Frontier/PII)	Forward primers (5'-3')	Reverse primers (5'-3')	Anneling temperature (0C)	Expected amplified product size (bp)	Structural annotation		Functional annotation	
								Sequence components of *kabuli* genome	*Kabuli* gene accession IDs	NCBI-KOG	TFs
Ca3_P2_36690698	Ca3	36690698	TAAAA/TAAA	TTGTTGTATTGGGCTGGACA	TTGGGGAAAAGAGCTAAATCC	59.6	355	DRR	Ca_00991	--	--
Ca3_P2_36690863	Ca3	36690863	TAAAAAAAA/TAAAAAAA	TTGTTGTATTGGGCTGGACA	AAGCATGGAAACGTGTGTTG	59.6	769	DRR	Ca_00991	--	--
Ca3_P2_36694628	Ca3	36694628	AATTATTATTATTA/AATTATTATTATTATTA	ATGTGGGGCGATATATGAGG	ATTGGGACTGGGAGAGTGTG	60.0	790	INTERGENIC	--	--	--
Ca3_P2_36695647	Ca3	36695647	A/AAAC	TTTGTTTTTCTTCATGGCCC	CAAAATCCTACATTTTGGGATATACA	59.5	649	INTERGENIC	--	--	--
Ca3_P2_36695663	Ca3	36695663	GAAAA/GAAA	TTTGTTTTTCTTCATGGCCC	CAAAATCCTACATTTTGGGATATACA	59.5	649	INTERGENIC	--	--	--
Ca3_P2_36697642	Ca3	36697642	ATT/AT	CGACGCATATTTCAAATGACTT	TCTTTTGGCAACAGGTTGGT	60.5	634	INTERGENIC	--	--	--
Ca3_P2_36697708	Ca3	36697708	AC/A	CGACGCATATTTCAAATGACTT	AGTTGCATGCTTCTTTTGGC	60.4	645	INTERGENIC	--	--	--
Ca3_P2_36699656	Ca3	36699656	TTAT/TTATAT	TGCCGTATAATCATGCAACC	TGTGTGTTTGTGAATCTTAGCG	58.9	759	INTERGENIC	--	--	--
Ca3_P2_36700703	Ca3	36700703	T/TTG	AATTGGCTATGGTTGCTTCG	CCATTGACCCGAAACATATTC	59.1	462	INTERGENIC	--	--	--
Ca3_P2_36702095	Ca3	36702095	GTTTTTTTTT/GTTTTTTTTTT	ACGTCAATGGCAGTGTGTGT	CGGAATTTGAATCCACGACT	59.9	634	INTERGENIC	--	--	--
Ca3_P2_36706321	Ca3	36706321	GTATATATATA/GTATATATA	TAAATTGTGGGTTAGGCGGA	CGTCCAACTCGCTAACTGGT	60.3	513	INTERGENIC	--	--	--
Ca3_P2_36707043	Ca3	36707043	CT/C	CTCCTCCCAGTGGTAACAAA	CAGTGAAAAATTGAATCCCCA	59.8	595	INTERGENIC	--	--	--

INDEL marker IDs	Chromosomes/unanchored scaffolds	Physical positions (bp)	InDels (*Kabuli* reference genome-CDC Frontier/PII)	Forward primers (5'-3')	Reverse primers (5'-3')	Anneling temperature (0C)	Expected amplified product size (bp)	Structural annotation		Functional annotation	
								Sequence components of *kabuli* genome	*Kabuli* gene accession IDs	NCBI-KOG	TFs
Ca3_P2_36707318	Ca3	36707318	TA/TAAA	CAAAAACAGTTTGGGGATTCA	CATTTGACCTTACGTCCATCC	59.3	502	INTERGENIC	--	--	--
Ca3_P2_36707636	Ca3	36707636	TTTTATTTATT/TTTTATTTATTTATTTATT	CAAAAACAGTTTGGGGATTCA	GATGATGGAAGGGGATGAGA	59.8	868	INTERGENIC	--	--	--
Ca3_P2_36707907	Ca3	36707907	ATTTTTTTT/ATTTTTT	TGGACGTAAGGTCAAATGGA	GATGATGGAAGGGGATGAGA	59.8	384	INTERGENIC	--	--	--
Ca3_P2_36710235	Ca3	36710235	TAT/TATCAT	GCCAACACAAAACTCGATCC	CTGTTCTCCACGTCGGATTT	60.1	370	INTERGENIC	--	--	--
Ca3_P2_36712698	Ca3	36712698	CA/C	AGTGCTGGAGGCTGTGTTTT	TCTCTTCCCAGGTGCTGACT	60.0	329	INTRON	Ca_00995	O	MYB
Ca3_P2_36713875	Ca3	36713875	CAAAA/CAAAAAA	TGAGAAAGGCATGCAAGATG	AACGCATCCAGTGATCAACA	60.1	568	INTRON	Ca_00995	O	MYB
Ca3_P2_36716843	Ca3	36716843	GC/G	TTTTATTTGAGGGGTGACCG	GATGGATTGGTCGTGAGGTT	59.8	554	DRR	Ca_00995	O	MYB
Ca3_P2_36723949	Ca3	36723949	C/CTA	CCTCCCGTCCTTGTGATCTA	AAGAACGTGGGTTTGGTTTC	58.9	385	DRR	Ca_00996	--	--
Ca3_P2_36725525	Ca3	36725525	TA/T	CTGTGGCAGATCCCAAACTT	GGGAATCCGATTTACCCTTT	59.1	329	INTERGENIC	--	--	--
Ca3_P2_36730803	Ca3	36730803	ATGT/A	CGGTAACGTGCAAAGGAAAT	GATTAATTGAGTTTGAAAGGCTTG	58.5	584	DRR	Ca_00997	--	--
Ca3_P2_36732284	Ca3	36732284	TGCAGCAGCAGCAGCA/TGCAGCAGCAGCAGCAGCAGCA	CCCTTTCGCATTTTGTTTGT	ACAGAGGTTGGGTTCTGGTG	60.0	624	CDS (large-effect mutations)	Ca_00997	--	--
Ca3_P2_36735770	Ca3	36735770	AATAAATAAATATAAATAAATATAAATA/AATAAATAAATATAAATA	TACGTGGCCTATTAGGTGGC	GCATATGGTGATTGCTGACTCT	59.2	648	INTERGENIC	--	--	--

INDEL marker IDs	Chromosomes/unanchored scaffolds	Physical positions (bp)	InDels (*Kabuli* reference genome-CDC Frontier/PII)	Forward primers (5'-3')	Reverse primers (5'-3')	Anneling temperature (0C)	Expected amplified product size (bp)	Structural annotation		Functional annotation	
								Sequence components of *kabuli* genome	*Kabuli* gene accession IDs	NCBI-KOG	TFs
Ca3_P2_36735874	Ca3	36735874	TAAAAAAA/TAAAAAA	TACGTGGCCTATTAGGTGGC	CGGCGGAGTCAGGTAATAAA	60.1	834	INTERGENIC	--	--	--
Ca3_P2_36735945	Ca3	36735945	AT/ATT	TACGTGGCCTATTAGGTGGC	ATGGGTAACCGGGTGTATCA	59.9	874	INTERGENIC	--	--	--
Ca3_P2_36737503	Ca3	36737503	GTTTT/GTTTTT	TTGATGAAGCAAATTGCGAG	AATTGTGGGGTCAATGGAAA	60.0	237	INTERGENIC	--	--	--
Ca3_P2_36751775	Ca3	36751775	C/CCTATTAACAAGGAAA	TAACATCCATGGCCTCACAA	CGGTTTGTCTTTGGATAATGG	59.3	835	INTRON	Ca_00998	--	C2H2
Ca3_P2_36752313	Ca3	36752313	AT/ATT	ACGAATCAACTTGGCCTACG	GTTGAAGGTGGCTGCTTGTT	60.3	429	INTRON	Ca_00998	--	C2H2
Ca3_P2_36752520	Ca3	36752520	AAGTAGTAGTAGTAGTA/AAGTAGTAGTAGTAGTAGTA	ACGAATCAACTTGGCCTACG	AGGGCAATGTACAATGGAGG	59.8	602	INTRON	Ca_00998	--	C2H2
Ca3_P2_36752957	Ca3	36752957	GTTTTTTTT/GTTTTTTTTTT	ACAACCTGCGCAATTTTACC	TCCTCAGCAACTTGCACAAC	60.0	305	INTRON	Ca_00998	--	C2H2
Ca3_P2_36753275	Ca3	36753275	TTTTTTAATGAATTTTTAATG/TTTTTTAATG	ACAACCTGCGCAATTTTACC	TGCGAACCAACCAATTATGA	59.9	632	INTRON	Ca_00998	--	C2H2
Ca3_P2_36756795	Ca3	36756795	TAAAAAA/TAAAAAAA	AAGTGCTTGTTGCTGGAGTG	TCGTCGCATCTACAAACAAAA	59.4	529	INTERGENIC	--	--	--
Ca3_P2_36756835	Ca3	36756835	ATTATATTTTTATTTTATATTTT/ATTATATTTT	AAGTGCTTGTTGCTGGAGTG	TCGTCGCATCTACAAACAAAA	59.4	529	INTERGENIC	--	--	--
Ca3_P2_36761612	Ca3	36761612	CAAAAA/CAAAAAA	CAATTTTTGTTGGGTGGGAC	CCGAATTTAAGCATTTGGGA	59.9	918	INTERGENIC	--	--	--
Ca3_P2_36763028	Ca3	36763028	CTTT/CTTTT	ATGCAATGGGAAATGGTGTT	TACGGGTGGAAATGTGTTCA	59.8	589	INTERGENIC	--	--	--

INDEL marker IDs	Chromosomes/unanchored scaffolds	Physical positions (bp)	InDels (*Kabuli* reference genome-CDC Frontier/PII)	Forward primers (5'-3')	Reverse primers (5'-3')	Anneling temperature (0C)	Expected amplified product size (bp)	Structural annotation		Functional annotation	
								Sequence components of *kabuli* genome	*Kabuli* gene accession IDs	NCBI-KOG	TFs
Ca3_P2_36763413	Ca3	36763413	T/TA	TGGTTTGGTTGTTGGATCTG	TCAAAACAACCTCTACCGGG	60.0	864	INTERGENIC	--	--	--
Ca3_P2_36768622	Ca3	36768622	GAAAAAAAAA/GAAAAAAAAAA	CCACATGCAAACAAAATTCA	CAAGGTTTTCCACAATTTTCC	58.5	843	INTERGENIC	--	--	--
Ca3_P2_36772130	Ca3	36772130	ATTTTTT/ATTTT	GCTTTGCACCCTCTTTTATCC	GAGGCATTTGGTGATGGTTT	59.8	128	INTERGENIC	--	--	--
Ca3_P2_36773167	Ca3	36773167	TATATAAATATA/TATATA	ATTTGCTTTCCTTCACACGG	GGCACGGTTGAAATTAACGA	60.9	752	INTERGENIC	--	--	--
Ca3_P2_36834412	Ca3	36834412	GTTTTTT/GTTTTTTT	GTACCTTCCACCCCCATTTT	GGAAAGTTCCGGAATACATTTTT	59.6	597	DRR	Ca_01008	K	GRAS
Ca3_P2_36888821	Ca3	36888821	CATATATATATATATATATATATAT/CATATATATATATATATATATATAT	TGATGTCACTGGTGCTGAAA	TTTCAAGGCTTGCACCGTAT	60.6	499	INTRON	Ca_01015	O	--
Ca3_P2_36932559	Ca3	36932559	GAAAAAAAAAAAAA/GAAAAAAAAAAAA	GTTACGATTTTCATTGGCGA	CTTTGGTATGTTGGGCCTGT	59.9	917	DRR	Ca_01018	--	WRKY
Ca3_P2_37017817	Ca3	37017817	AATATATATATATATATATATATATATATATATATA/AATATATATATATATATATATATATATA	CAGAAGGCCAGGAGAAGAAA	TGTGCGTGAAGTTTCATGGT	60.2	707	INTERGENIC	--	--	--
Ca3_P2_37030222	Ca3	37030222	AATATATATATATATATATATATATATA/AATATATATATATATATATATATATA	GAAAAGCCCATTTTGTCTCG	AAAGGTTTGAAAATGGATGGTC	59.2	593	DRR	Ca_01031	G	C3H
Ca3_P2_37033553	Ca3	37033553	AATATATATATATATATATATATATATATATAT/AATATATATATATATATATATATATATATAT	TGGGCATTTGACAGTTGAAA	TGGTGAAAGGATTTATTGGCA	60.3	454	INTRON	Ca_01031	G	C3H
Ca3_P2_37082941	Ca3	37082941	TAAAAAA/TAAAAA	GACTCCACCCTCCCTTTCTT	GAAAATTTGTTGGACGGCAT	59.8	465	DRR	Ca_01034	R	--
Ca3_P2_3712839	Ca3	3712839	TCC/T	TGACACACCCACCCACTAAA	GACTGAGATGATGATTCACTTTGC	60.1	520	INTERGENIC	--	--	--

INDEL marker IDs	Chromosomes/unanchored scaffolds	Physical positions (bp)	InDels (*Kabuli* reference genome-CDC Frontier/PII)	Forward primers (5'-3')	Reverse primers (5'-3')	Anneling temperature (0C)	Expected amplified product size (bp)	Structural annotation		Functional annotation	
								Sequence components of *kabuli* genome	*Kabuli* gene accession IDs	NCBI-KOG	TFs
Ca3_P2_37150967	Ca3	37150967	ATTTTTTTT/ATTTTTTTTT	TTTTGCTTGCGTGAAAATTG	CCAAATGATAATCCATGGGC	60.0	604	INTERGENIC	--	--	--
Ca3_P2_37183401	Ca3	37183401	CTATATATATATATATATATAT/CTATATATATATATATATATATATAT	GCAGGTTTAGTCTCCGACGA	GCGGCGATATCTTCAATCAT	60.0	839	DRR	Ca_01050	--	WRKY
Ca3_P2_37191129	Ca3	37191129	TAAAAAAAAAA/TAAAAAAAAA	TGGTTTCATTCATCTTTTGCAG	GCCCAAATCAACCCATTTTA	59.6	445	INTERGENIC	--	--	--
Ca3_P2_37257790	Ca3	37257790	GAAAAAAAAAA/GAAAAAAAAAAA	GTTGCATGAGCTTGTAGCCA	TCATGCAGTGACCACAACAA	59.7	469	INTERGENIC	--	--	--
Ca3_P2_37258845	Ca3	37258845	TC/T	CGTAGGATCCGTGGTACGTT	GCGTCAAAAATCGCATAGGT	60.1	645	DRR	Ca_01060	M	--
Ca3_P2_37414792	Ca3	37414792	GAAAAAAAAAA/GAAAAAAAAAAA	TGTCACCGTGTTATGTGGCT	CAGTCACGAGTCGCGATTTA	60.0	488	INTERGENIC	--	--	--
Ca3_P2_37428682	Ca3	37428682	T/TGGA	TTGAGGATCAGCTACACCCC	TGGAACAATTTTGGTGGTGA	59.8	546	INTERGENIC	--	--	--
Ca3_P2_37464289	Ca3	37464289	TAAAAAAAAA/TAAAAAAAAAA	ATGTGCAGCCCTGCATACTA	GCTGCTGCCTGCATGTATAA	60.0	824	DRR	Ca_01080	--	--
Ca3_P2_37513649	Ca3	37513649	ATTTTTTTTTTT/ATTTTTTTTTT	TGCAACTTATTTATGGGCCAC	ATGGCAATGATTCCCCTACA	60.2	769	DRR	Ca_01085	--	SAP
Ca3_P2_37532293	Ca3	37532293	TAGAGA/TAGA	CCTCCTTGGCTGAGACTTTG	ACCAAAATTTGAGGAAGCCA	59.5	902	DRR	Ca_01088	G	FAR1
Ca3_P2_37560956	Ca3	37560956	AATATATATATATATATATATATA/AATATATATATATATATA	TGGCACCTACATCACCTCAC	GGAGACACGCAAACGATTTT	60.1	513	INTERGENIC	--	--	--
Ca3_P2_37577755	Ca3	37577755	CAATAATAATAATAATAATAATAATAATAATAATAATA/CAATAATAATAATAATAATAATAATAATAATAATA	TGTCACGGGAGGAGAAAGAC	AAAACGCAATTAGCGGAGAT	58.9	687	DRR	Ca_01095	--	--

INDEL marker IDs	Chromosomes/unanchored scaffolds	Physical positions (bp)	InDels (*Kabuli* reference genome-CDC Frontier/PII)	Forward primers (5'-3')	Reverse primers (5'-3')	Anneling temperature (0C)	Expected amplified product size (bp)	Structural annotation		Functional annotation	
								Sequence components of *kabuli* genome	*Kabuli* gene accession IDs	NCBI-KOG	TFs
Ca3_P2_37594905	Ca3	37594905	TTATATATATATATATATATATATA/TTATATATATATATATATATATATATA	GTCCGCCCTAAAAAGGTAGC	TCAAAACACCACATCAAAACG	59.5	188	INTERGENIC	--	--	--
Ca3_P2_3777233	Ca3	3777233	CAATAATAATAATAATA/CAATAATAATAATAATAATA	TCTCTGGCAACCGTTCTTCT	GAGCTGTGTCTTGGCAATCA	60.0	594	DRR	Ca_21336	--	--
Ca3_P2_37875029	Ca3	37875029	GT/GTT	ATGGCGTGACTGAACATGAA	AAGGGCAAGATCCCAAAAAC	60.3	672	INTERGENIC	--	--	--
Ca3_P2_3798409	Ca3	3798409	ATAGTA/ATA	AATTTGAAGCCACTGCTGCT	AGCATCTCCCTTCAAAAGTCA	58.9	753	INTERGENIC	--	--	--
Ca3_P2_37989883	Ca3	37989883	GTATATATATATATATATATA/GTATATATATATATATATATATATATA	TGTCACGGGTGGACAAATTA	TAGGCGATTGTAGGGCTTTG	60.2	749	INTERGENIC	--	--	--
Ca3_P2_37991623	Ca3	37991623	TAAAAAAAAA/TAAAAAAAAAA	TAGATTCCCCACCAGTCCAG	TGGTACGCGACAATTTTTGTT	60.4	793	DRR	Ca_01140	--	--
Ca3_P2_38009510	Ca3	38009510	GTTTTTTTTTTT/GTTTTTTTTTT	TTCATGGATCAATAAAATTAGATAGGC	TAACCGAAAATCGGAAGTCG	60.1	577	INTERGENIC	--	--	--
Ca3_P2_3803273	Ca3	3803273	CTATTATTATTATTATTATT/CTATTATTATTATTATTATTATT	TGACAGTCAGAGTGCGATCC	TCTCTGGCAACCGTTCTTCT	60.0	436	INTERGENIC	--	--	--
Ca3_P2_38064707	Ca3	38064707	TTATATATATATATATATATATATATATATATAT/TTATATATATATATATATATATATATATATATATAT	ATTTTGCACCCACCACATTT	CGCCATATTCAAACAAAATTCA	59.8	944	INTERGENIC	--	--	--
Ca3_P2_38102155	Ca3	38102155	TTAATTATATATA/TTA	AGAACGCCATCGCAACTACT	GCGGAGATTTCACGGTGTAT	60.0	227	INTERGENIC	--	--	--
Ca3_P2_38129457	Ca3	38129457	CATATATATATATATA/CATATATATATATATATA	CAAAAGCCGGACCAAAATTA	GGGCATGGGCAATAATCTAA	59.8	398	INTERGENIC	--	--	--
Ca3_P2_38136282	Ca3	38136282	GTTTTTTT/GTTTTTT	CGAGGTGAAATTGTGAAGCA	AACCATGTGATCCAAACACG	59.3	381	INTERGENIC	--	--	--

INDEL marker IDs	Chromosomes/unanchored scaffolds	Physical positions (bp)	InDels (*Kabuli* reference genome-CDC Frontier/PII)	Forward primers (5'-3')	Reverse primers (5'-3')	Anneling temperature (0C)	Expected amplified product size (bp)	Structural annotation		Functional annotation	
								Sequence components of *kabuli* genome	*Kabuli* gene accession IDs	NCBI-KOG	TFs
Ca3_P2_38138552	Ca3	38138552	CTAT/CTATAT	CGAGGCATCCTTTGTTTAGG	AAAAACGTTCAAGTTTAATGCAA	58.0	506	INTERGENIC	--	--	--
Ca3_P2_38139076	Ca3	38139076	A/AC	TTGCATTAAACTTGAACGTTTTT	TCAGGCCAACCCAGTACATT	60.4	416	INTERGENIC	--	--	--
Ca3_P2_38141259	Ca3	38141259	TTA/T	GGAAAGTCATGATGTCCTTTTT	GTCATTTGTAAATGGCGGCT	60.0	436	INTERGENIC	--	--	--
Ca3_P2_38141514	Ca3	38141514	ATTTTTT/ATTTTTTT	CCGCCATTTACAAATGACACT	TCGTCAAAATTATTTAGCGCAT	58.8	536	INTERGENIC	--	--	--
Ca3_P2_38144557	Ca3	38144557	ATTTGTT/ATT	GCACAAGAGCTGACTTTCCC	CCCACTTACAAATGGCTGCT	60.1	791	INTRON	Ca_01158	C	--
Ca3_P2_38146150	Ca3	38146150	TAAAAAAAA/TAAAAAAAAA	CAACCCTTCTGTGCCAATTT	ATTTCGCAATGCTCGTCTTT	59.9	545	INTRON	Ca_01158	C	--
Ca3_P2_38149794	Ca3	38149794	T/TA	AGTTATTACATGCGGCAGGG	TTAACCATGTGTCAAGGTCG	57.0	287	DRR	Ca_01158	C	--
Ca3_P2_38149993	Ca3	38149993	ATTA/ATTAACTTA	AGTTATTACATGCGGCAGGG	GCCAAACAGTGTTGTAATTCG	58.2	752	DRR	Ca_01158	C	--
Ca3_P2_38154708	Ca3	38154708	CGGAGGA/CGGAGGAGGA	AGAGCGGAAAAGTTATCGCA	TTCATGTTGTCCCTTTTGACA	59.0	477	DRR	Ca_01160	L	MYB_related
Ca3_P2_38155393	Ca3	38155393	ATTTT/ATTT	GTTTTGCGCCAATTTTCATT	AGGGTGTACATTCCGTGCAT	60.3	484	DRR	Ca_01160	L	MYB_related
Ca3_P2_38156627	Ca3	38156627	ACTCT/ACT	CTGCAAGAGGGGTAAAACCA	ATGTTCTGAAAATGAGGCGG	60.1	560	INTRON	Ca_01160	L	MYB_related
Ca3_P2_38162427	Ca3	38162427	GTACTACTA/GTACTA	TCCAGGCGAGATAGCAAGAT	GTGGTCCAGGAAAAGCAAGA	60.2	811	INTRON	Ca_01160	L	MYB_related

INDEL marker IDs	Chromosomes/unanchored scaffolds	Physical positions (bp)	InDels (*Kabuli* reference genome-CDC Frontier/PII)	Forward primers (5'-3')	Reverse primers (5'-3')	Anneling temperature (0C)	Expected amplified product size (bp)	Structural annotation		Functional annotation	
								Sequence components of *kabuli* genome	*Kabuli* gene accession IDs	NCBI-KOG	TFs
Ca3_P2_38164329	Ca3	38164329	TG/TGAGAGAGAG	TATTCCCCAATTCCTAGCCC	CAAGATGCTGGCACAGAAAA	60.0	740	INTRON	Ca_01160	L	MYB_related
Ca3_P2_38170265	Ca3	38170265	TAA/TA	CCCAGATCTGCCTAATGGAA	GATGGGTTTGGAACATCCAG	60.2	594	INTRON	Ca_01160	L	MYB_related
Ca3_P2_38174447	Ca3	38174447	A/AT	TTCACATCCACACTCATCGG	CTGGCACTGGCATTCCTAAT	60.1	628	DRR	Ca_01161	--	NF-YA
Ca3_P2_38174640	Ca3	38174640	GTTTTTTTTTT/GTTTTTTTTT	ATTTCACCCTACAGCCATGC	CTGGCACTGGCATTCCTAAT	60.1	145	DRR	Ca_01161	--	NF-YA
Ca3_P2_38178356	Ca3	38178356	GTTTTTT/GTTTTTTT	GATTCGCCTGCTATCAAAGG	TCCGTCTGATCAATCGTCTG	59.8	374	INTRON	Ca_01161	--	NF-YA
Ca3_P2_3818047	Ca3	3818047	G/GGA	ATGCTCTGCACTTGCAGCTA	TTCATCGCCTCAAAGTGTTG	59.8	707	INTERGENIC	--	--	--
Ca3_P2_38183694	Ca3	38183694	TAAA/TAAAA	CGACACGCACAAACCTTTTA	AACCTTCTTCGTTTCGTGGA	59.7	856	INTERGENIC	--	--	--
Ca3_P2_38189173	Ca3	38189173	TAAAAA/TAAAA	TGGGTTAGGGGGACATCATA	TGAAAATGGCCATCAAAACA	59.9	499	INTERGENIC	--	--	--
Ca3_P2_38197547	Ca3	38197547	TAAAA/TAAA	GTAACAGGCAGGACCTCAGC	TTTCTCTCCCTGTCGGCTAA	59.9	444	INTERGENIC	--	--	--
Ca3_P2_38201387	Ca3	38201387	CTTTTTTTTT/CTTTTTT	ATTGTGGAGATTGGTGGAGC	GTCGCAGGCAGCTTTATAGG	60.0	427	INTRON	Ca_01163	E	--
Ca3_P2_38205894	Ca3	38205894	TAAAAAAAAA/TAAAAAAAA	ACAACACTCGGCCTATCACC	CTCCCCCTTCTCCAGGATAG	60.0	768	DRR	Ca_01163	E	--
Ca3_P2_38205985	Ca3	38205985	GATA/G	ACAACACTCGGCCTATCACC	CTCCCCCTTCTCCAGGATAG	60.0	768	DRR	Ca_01163	E	--

INDEL marker IDs	Chromosomes/unanchored scaffolds	Physical positions (bp)	InDels (*Kabuli* reference genome-CDC Frontier/PII)	Forward primers (5'-3')	Reverse primers (5'-3')	Anneling temperature (0C)	Expected amplified product size (bp)	Structural annotation		Functional annotation	
								Sequence components of *kabuli* genome	*Kabuli* gene accession IDs	NCBI-KOG	TFs
Ca3_P2_38206711	Ca3	38206711	GAAAAAAAAAAA/GAAAAAAAAAAAAA	CTATCCTGGAGAAGGGGGAG	GCTATTGCATTGGAGTTGCTC	59.9	706	DRR	Ca_01163	E	--
Ca3_P2_38206767	Ca3	38206767	AAGAG/AAG	CTATCCTGGAGAAGGGGGAG	GCTATTGCATTGGAGTTGCTC	59.9	706	DRR	Ca_01163	E	--
Ca3_P2_38208971	Ca3	38208971	T/TCTC	CTGGGAAGAATGAAGGGTCA	CTTTCCTCCATCGGATTTGA	60.0	537	INTERGENIC	--	--	--
Ca3_P2_38208982	Ca3	38208982	ACTTTCTTTCTTTCTTT/ACTTTCTTTCTTTCTTTCTTT	CTGGGAAGAATGAAGGGTCA	CTTTCCTCCATCGGATTTGA	60.0	537	INTERGENIC	--	--	--
Ca3_P2_38212511	Ca3	38212511	GTTTTTTT/GTTTTTT	ATTCAGCTCCCCCTGAGATT	TCAGAGGGATCGGACATAGG	60.0	665	INTRON	Ca_01164	T	WRKY
Ca3_P2_38215897	Ca3	38215897	CTT/CTTCTCTTCATT	TGTTGCCTGGAAATGAGTTG	TGCTGCATTGAAATCGAGTC	60.0	688	DRR	Ca_01165	R	--
Ca3_P2_38219046	Ca3	38219046	TCC/TCCC	GGTGAGGTTCTTGCAACCAT	TATGCATGGTGAAAAAGCGA	60.2	655	DRR	Ca_01166	Z	B3
Ca3_P2_38219080	Ca3	38219080	TAAAAA/TAAAAAA	GGTGAGGTTCTTGCAACCAT	TATGCATGGTGAAAAAGCGA	60.2	655	DRR	Ca_01166	Z	B3
Ca3_P2_38219148	Ca3	38219148	A/AC	CTACCGGCTACTTTCTTGCG	TATGCATGGTGAAAAAGCGA	60.2	463	DRR	Ca_01166	Z	B3
Ca3_P2_38219165	Ca3	38219165	TAAAAAAA/TAAAAAA	CTACCGGCTACTTTCTTGCG	TATGCATGGTGAAAAAGCGA	60.2	463	DRR	Ca_01166	Z	B3
Ca3_P2_38219214	Ca3	38219214	TTCT/TT	CTACCGGCTACTTTCTTGCG	TATGCATGGTGAAAAAGCGA	60.2	463	DRR	Ca_01166	Z	B3
Ca3_P2_38221107	Ca3	38221107	TCTCCTC/TCTC	CCACTGGTGGATTCAGAGGT	GAGAACTCATGTGCCGGAAT	60.1	566	CDS (large-effect mutations)	Ca_01166	Z	B3

INDEL marker IDs	Chromosomes/unanchored scaffolds	Physical positions (bp)	InDels (*Kabuli* reference genome-CDC Frontier/PII)	Forward primers (5'-3')	Reverse primers (5'-3')	Anneling temperature (0C)	Expected amplified product size (bp)	Structural annotation		Functional annotation	
								Sequence components of *kabuli* genome	*Kabuli* gene accession IDs	NCBI-KOG	TFs
Ca3_P2_38225612	Ca3	38225612	G/GC	CCACCATCCTTGTGCTTTTT	CCCCTCTCCCACGTTTAGTT	60.4	444	DRR	Ca_01167	R	--
Ca3_P2_38228425	Ca3	38228425	A/AGTGTGAATAC	ATGATCCACTGAGATTCCGC	CCAGAGTGTGACGCTTTTGA	60.0	472	DRR	Ca_01168	R	bHLH
Ca3_P2_38228566	Ca3	38228566	GAA/GAAA	AAATGTGAAAGCATGAGGGG	TGTCCACTTCCTCGGCTTAC	60.3	796	DRR	Ca_01168	R	bHLH
Ca3_P2_38233215	Ca3	38233215	ATA/ATAGTA	TTCGCGATTGCCAATATAGC	TCGCATGCTTATTTGTTAGTCAA	59.8	137	DRR	Ca_01169	G	--
Ca3_P2_38237845	Ca3	38237845	GTATA/GTA	GAGATTGCTTTCCCATTCCA	ACATGAGCTCCTGGACTGCT	60.0	733	INTRON	Ca_01169	G	--
Ca3_P2_38240356	Ca3	38240356	TCCCC/TCCC	GTGGTGAGTTCAGTTCCGGT	ACCGTTAGAGGAGAGGAGCC	59.8	475	INTERGENIC	--	--	--
Ca3_P2_38241149	Ca3	38241149	A/AAT	GGCTCCTCTCCTCTAACGGT	GACAAGTGCAACAATTTTCATCA	60.0	527	INTERGENIC	--	--	--
Ca3_P2_38241254	Ca3	38241254	AAA/AAAAAAATAA	TTGATGAAAATTGTTGCACTTG	TTGCTAACACAAAAGTATTTGAATGTC	59.9	189	INTERGENIC	--	--	--
Ca3_P2_38241297	Ca3	38241297	TTTATT/TTT	TTGATGAAAATTGTTGCACTTG	TTGCTAACACAAAAGTATTTGAATGTC	59.9	189	INTERGENIC	--	--	--
Ca3_P2_38326832	Ca3	38326832	ATTTTTTTTTTTT/ATTTTTTTTTTT	TGCGGCTTTCACTTGTATCA	GCAGCCGCTGTCGTATATTT	60.3	533	INTERGENIC	--	--	--
Ca3_P2_38417598	Ca3	38417598	CTATATATATATATATATATATATATATA/CTATATATATATATATATATATATATATATATATA	GTTGGTGGCGTTTTCAACTT	TGTAGCCCAAAAGAGACCCA	60.6	226	DRR	Ca_01193	T	--
Ca3_P2_3847338	Ca3	3847338	CATATATATATAT/CATATATATATATATAT	AGGAAGAGTGGAGTTGCGAA	ATTTTGGTTCGGAGGCTTTT	59.9	496	INTERGENIC	--	--	--

INDEL marker IDs	Chromosomes/unanchored scaffolds	Physical positions (bp)	InDels (*Kabuli* reference genome-CDC Frontier/PII)	Forward primers (5'-3')	Reverse primers (5'-3')	Anneling temperature (0C)	Expected amplified product size (bp)	Structural annotation		Functional annotation	
								Sequence components of *kabuli* genome	*Kabuli* gene accession IDs	NCBI-KOG	TFs
Ca3_P2_38612369	Ca3	38612369	TAAAAA/TAAAA	TTTTGAAAGCTCTCGCTTGA	TGCGACCTCATGAAGACTAAAA	59.9	423	DRR	Ca_01216	--	GeBP
Ca3_P2_38665334	Ca3	38665334	CTT/CTTCATTT	CCCCTTCATCAGTGGTGAGT	TCTTCTCCTCCCAAAAGCAA	59.9	257	INTERGENIC	--	--	--
Ca3_P2_38690322	Ca3	38690322	AATATATATATATATATATATA/AATATATATATATATATATATATATATATATATA	GAAGCAGGGAGGAGGAAAAC	TGTCAATGAGGATTATGCCAA	59.0	910	DRR	Ca_01224	K	Trihelix
Ca3_P2_38690500	Ca3	38690500	TTAA/T	GGCAGCTAGCTTGAGGGATA	TGTCAATGAGGATTATGCCAA	59.0	559	DRR	Ca_01224	K	Trihelix
Ca3_P2_38690878	Ca3	38690878	GAAAAATAAAAATAAAAA/GAAAAATAAAAA	TTGGCATAATCCTCATTGACA	CCAAAACAAAGACCTCCGAA	60.1	469	DRR	Ca_01225	--	NAC
Ca3_P2_38697951	Ca3	38697951	T/TA	GTACGACACCGCAATTCCTT	CTATTGAGGTCTTTGGACCG	57.3	434	DRR	Ca_01227	O	--
Ca3_P2_38729060	Ca3	38729060	GATATATATATATA/GATATATATATATATA	CAGATCATGTCGGAGCTTCA	CCTTTTCCTTCTTGCCCTTC	60.2	535	INTERGENIC	--	--	--
Ca3_P2_38735210	Ca3	38735210	GTGTTG/GTG	CACCTGCACAAACTTCCAAA	TGGTGTTACTCCCTTCCTTCA	59.6	220	INTERGENIC	--	--	--
Ca3_P2_3873650	Ca3	3873650	GAAAAAAAAAA/GAAAAAAAAAAA	GCTGTTGGTTAGTCGGGGTA	GCAGTAATGCATCCGTTGAA	59.7	631	INTERGENIC	--	--	--
Ca3_P2_38764047	Ca3	38764047	TAAAAATAAAA/TAAAAATAAAAAATAAAA	TGGGCTCCGAGAGTCTTATG	CGTGGATTAATCGCTACCGT	60.0	253	DRR	Ca_01234	--	bZIP
Ca3_P2_38783791	Ca3	38783791	ATTTTTTTTT/ATTTTTTTT	TCCCACCCCTAATGACAGTT	AACCATTGTCAAATCTACTCTCAA	57.0	690	INTERGENIC	--	--	--
Ca3_P2_38788784	Ca3	38788784	CTGCTT/CTGCTTTGCTT	GGCTAAACAAAGGGAACGGT	TTTATTGCCAACAGCAACCA	60.1	581	DRR	Ca_01237	R	--

INDEL marker IDs	Chromosomes/unanchored scaffolds	Physical positions (bp)	InDels (*Kabuli* reference genome-CDC Frontier/PII)	Forward primers (5'-3')	Reverse primers (5'-3')	Anneling temperature (0C)	Expected amplified product size (bp)	Structural annotation		Functional annotation	
								Sequence components of *kabuli* genome	*Kabuli* gene accession IDs	NCBI-KOG	TFs
Ca3_P2_3881329	Ca3	3881329	AAAAATATATAAAATATATAAAATA/AAAAATATATAAAATATATAAAATATATAAAATA	AGGTAGGCAAGTCTGAGGCA	CAAACGCATGACCAAGACAG	60.3	773	INTERGENIC	--	--	--
Ca3_P2_38870959	Ca3	38870959	AAATAATAATAATAATAATAATAATAATAATAATAATAATAATAA/AAATAATAATAATAATAATAATAATAATAATAATAATAA	TTTGCATATGACATTGAAAGCA	TGAAACAGGCAATCTTTCCC	60.1	292	INTERGENIC	--	--	--
Ca3_P2_38906965	Ca3	38906965	TTT/TTTCTT	AATTTTGGGTCACCGAATCA	CGTAAATGATGTGGCATTCG	60.0	496	INTERGENIC	--	--	--
Ca3_P2_38988784	Ca3	38988784	TGAGAGAGAGAGAGAGAGAGAG/TGAGAGAGAGAGAGAGAGAG	CTTCAACCGGCAAAAACAAT	GGAATGGGTTTATGGGCTTT	60.0	966	INTERGENIC	--	--	--
Ca3_P2_38998818	Ca3	38998818	TAAAAAAAAAAAAA/TAAAAAAAAAAAAAA	GTATTGGGGCGTCACTTTTC	AGGTGGGGGTAAAAACAAGC	60.2	213	INTERGENIC	--	--	--
Ca3_P2_39051137	Ca3	39051137	AG/AGCG	AGTTTCATTTTTGCCCGTTG	TTCCAACCGTGTGATTTTGA	59.9	467	DRR	Ca_01267	--	bHLH
Ca3_P2_39106800	Ca3	39106800	ATTTTTTTTTTTT/ATTTTTTTTTTT	AAAGTTGTTGGAATGGCTGG	AGACAACCATTCTAGGGTTTCA	57.7	400	INTERGENIC	--	--	--
Ca3_P2_39317786	Ca3	39317786	CATATATATATATATATATATAT/CATATATATATATATATAT	AGGACCATTCCATCCTGTCA	TGCATCCAAACGAGCACTAA	60.4	664	INTERGENIC	--	--	--
Ca3_P2_39355870	Ca3	39355870	TAAAAAAAAAA/TAAAAAAAAAAA	TGCGAGATCGAAGATTTGG	CGACGCGACTGGATTGTAA	60.8	725	INTERGENIC	--	--	--
Ca3_P2_39367471	Ca3	39367471	ATTTTTTTTTTTT/ATTTTTTTTTTT	GGAAAAGCCATCAAACCAGA	GAGTTGATTTTGACCCTTCCA	59.0	296	INTERGENIC	--	--	--
Ca3_P2_39374463	Ca3	39374463	TAAAAAAAAAA/TAAAAAAAAAAA	GGGGACTTCACTTTCGGATA	TCCCATAACCTTTACCACATCC	59.9	754	INTRON	Ca_01301	--	--
Ca3_P2_39401096	Ca3	39401096	ATTTTTTTTTT/ATTTTTTTTT	ATTTGGGTCAAGGATGATGC	GCTTTGCTTGCCCATTTTTA	60.2	940	INTERGENIC	--	--	--

INDEL marker IDs	Chromosomes/unanchored scaffolds	Physical positions (bp)	InDels (*Kabuli* reference genome-CDC Frontier/PII)	Forward primers (5'-3')	Reverse primers (5'-3')	Anneling temperature (0C)	Expected amplified product size (bp)	Structural annotation		Functional annotation	
								Sequence components of *kabuli* genome	*Kabuli* gene accession IDs	NCBI-KOG	TFs
Ca3_P2_39417852	Ca3	39417852	AAT/A	TGAGTTGTGAGATGCTTCCCT	CCTTTGCAATTTTCTTTGCC	59.7	692	INTERGENIC	--	--	--
Ca3_P2_39465898	Ca3	39465898	GTTATTATTATTATTATTATTATTATTATTATTATTATTATTATTATTAT/GTTATTATTATTATTATTATTATTATTATTATTATTATTATTAT	GAAGTTCGCTTCCTCCAAAA	TTGGCAGTACCAACGAATGA	60.1	358	DRR	Ca_01313	--	--
Ca3_P2_39488222	Ca3	39488222	GATATATATATATA/GATATATATATA	CCACTCAAACTGTCGAGCAA	CAATCCACCACATGTACCCA	60.1	634	INTERGENIC	--	--	--
Ca3_P2_39571556	Ca3	39571556	GAAAAAAAAA/GAAAAAAAA	AATTTGTTTCGGCAATTCCA	GCAAAAGTGGCTGATGACAA	59.8	553	INTERGENIC	--	--	--
Ca3_P2_39611074	Ca3	39611074	T/TC	ACCGGACACGATATCAGGAG	TGCTTCAACAAAGGCAAACA	60.4	474	INTRON	Ca_01338	--	--
Ca3_P2_39611327	Ca3	39611327	CAAA/CAA	TGTTTGCCTTTGTTGAAGCA	CCCAATTTCGAAGCTCTCTG	59.9	198	INTRON	Ca_01338	--	--
Ca3_P2_3962696	Ca3	3962696	AAAAATATTT/AAAAATATTTAATATAAAATATTT	AGTTCCATGGGCTTTTCCTT	AGACTGTGGCACACAATGGA	60.2	552	INTERGENIC	--	--	--
Ca3_P2_3963300	Ca3	3963300	CAAAAAAAAA/CAAAAAAAAAAAAA	TCCATTGTGTGCCACAGTCT	TCTAGTCCGCGACTCAACCT	60.0	505	INTERGENIC	--	--	--
Ca3_P2_39663553	Ca3	39663553	TAAAAAAAAA/TAAAAAAAA	GTGGCCACGTATCGAGGTAG	TCCAAAAGAAAATCATGGGG	59.7	516	INTERGENIC	--	--	--
Ca3_P2_39670282	Ca3	39670282	TTATATATATATATATATATAT/TTATATATATATATATATATAT	TGGTGCGACAATCATTAACC	TGGATATCGAACCCTCACCT	59.4	720	INTERGENIC	--	--	--
Ca3_P2_39763068	Ca3	39763068	CAGAGAGAGAGAGAGAGAGA/CAGAGAGAGAGAGAGAGA	GGTCGCACATTGTCATCATC	AAAGGTTTCGACCACGTCAC	60.0	522	INTRON	Ca_01363	R	--
Ca3_P2_39810656	Ca3	39810656	TTA/TTAAATATA	ATGGGGATGTTGATCCAGAA	TCAACGTCCCAAAATGATGA	59.9	744	INTERGENIC	--	--	--

INDEL marker IDs	Chromosomes/unanchored scaffolds	Physical positions (bp)	InDels (*Kabuli* reference genome-CDC Frontier/PII)	Forward primers (5'-3')	Reverse primers (5'-3')	Anneling temperature (0C)	Expected amplified product size (bp)	Structural annotation		Functional annotation	
								Sequence components of *kabuli* genome	*Kabuli* gene accession IDs	NCBI-KOG	TFs
Ca3_P2_39866735	Ca3	39866735	C/CT	ACAGTCTTTGGTTCGATGGG	CTGCCCCAAAAGACACAAAT	60.0	288	INTRON	Ca_01372	--	WRKY
Ca3_P2_39926798	Ca3	39926798	CT/CTGTAT	CATCTGCAGCCAAAAACTCA	GTGTGGGCTTTTCCTTTGAA	60.1	418	INTERGENIC	--	--	--
Ca3_P2_39933774	Ca3	39933774	TT/TTTTAT	ATGTCATGCACTTTGTTGCC	TCCGTGTTGTCAAGATTAGTCG	60.2	493	INTERGENIC	--	--	--
Ca3_P2_39988476	Ca3	39988476	GTTTT/GTTT	AGAACAGAGCTGGCCTCAAA	ATTGCAAGAAAACCAGCACC	60.1	148	INTERGENIC	--	--	--
Ca3_P2_4017573	Ca3	4017573	TAA/TAAA	ACCACAGATCACCAGTCGCT	ACGTCCAAGGTAAGGGCTTT	60.0	683	INTERGENIC	--	--	--
Ca3_P2_4042829	Ca3	4042829	GATATATATATATATATATATATATAT/GATATATATATATATATATATATATATAT	ACACGAAGAGCAGGATCGTT	AAACAAAAATAAAAACTCGGAGACA	59.5	743	DRR	Ca_21325	QR	--
Ca3_P2_4043334	Ca3	4043334	AATATATATATATATATATATATATATATA/AATATATATATATATATATATATATATATATATATATA	TTCAAAATTCAAAACTTCGATTG	TTGCATTGGAAATAGACAAGTCA	59.6	522	DRR	Ca_21325	QR	--
Ca3_P2_4073796	Ca3	4073796	CT/CTT	CTCAACATCCACCATGCAAT	TGCAAATTTCATCAAATGCATAA	60.3	746	INTERGENIC	--	--	--
Ca3_P2_4076583	Ca3	4076583	ATATTATTATTATTATTATTATTATTATTATTATTATTATTATTATTATT/ATATTATTATTATTATTATTATTATTATTATTATTATTATTATTATT	ATAGTCCTCACCCACGCATC	AAGCGTACCTGTGGGAATTG	60.0	607	INTERGENIC	--	--	--
Ca3_P2_4121386	Ca3	4121386	CTTT/CTTTT	GACTGGACGTAACCCACGTT	AGCATTGTGCTTACGACGAA	59.5	379	DRR	Ca_22386	--	--
Ca3_P2_4132295	Ca3	4132295	TAAAAAAAAAAAAAA/TAAAAAAAAAAAAAAAAA	CGTGTGAGAAAACCTCGACA	CAAACCCAAGTTCGAAATGC	60.5	261	INTERGENIC	--	--	--
Ca3_P2_4132392	Ca3	4132392	TTATATATATATATATATAT/TTATATATATATATATATATATATAT	CGTGTGAGAAAACCTCGACA	GAAGGCCAACTTCCAAATGA	60.1	738	INTERGENIC	--	--	--

INDEL marker IDs	Chromosomes/unanchored scaffolds	Physical positions (bp)	InDels (*Kabuli* reference genome-CDC Frontier/PII)	Forward primers (5'-3')	Reverse primers (5'-3')	Anneling temperature (0C)	Expected amplified product size (bp)	Structural annotation		Functional annotation	
								Sequence components of *kabuli* genome	*Kabuli* gene accession IDs	NCBI-KOG	TFs
Ca3_P2_41406	Ca3	41406	AATATATATATATATATATATATATATATATATATATATATATATAT/AATATATATATATATATATATATATATATATATATATATATATATAT	GCAAATATGCAATAACCTTCCA	GGGTTTAGCCGCCCATATAA	61.0	418	INTERGENIC	--	--	--
Ca3_P2_4231908	Ca3	4231908	AGG/AGGTGG	CCCGTGATAGGCAGTACGTT	AATTCCTACGCGATCACCAC	60.0	517	INTERGENIC	--	--	--
Ca3_P2_4305334	Ca3	4305334	AATA/AA	CGTCATCACTTTCTACGCGA	GTTTGCAAGGTTGGGTGAAT	59.8	614	INTERGENIC	--	--	--
Ca3_P2_4378055	Ca3	4378055	AATATATATATATATATATATATATA/AATATATATATATATATATATATA	TATTTTGGATTTTGGCTCGG	GTTTTCGATTCAACGAGGGA	60.1	502	INTERGENIC	--	--	--
Ca3_P2_4428002	Ca3	4428002	GAAAAAAAAAAAA/GAAAAAAAAAAA	TGGAGCATTTAATTGTGGGA	TGCGTTGCCTAATAATAAAAGGA	60.0	643	INTERGENIC	--	--	--
Ca3_P2_4513626	Ca3	4513626	T/TCATGA	AGATCGTGAATCAATTCGTGG	AAGGAGGGGAGGATCATTGT	59.8	720	DRR	Ca_22078	K	MYB
Ca3_P2_451560	Ca3	451560	ATAACGTGATCTTA/ATA	AGCAGAAAGGGAGAATGGGT	ACGGTGAAGCTCCGAAAATA	59.7	734	INTERGENIC	--	--	--
Ca3_P2_4527615	Ca3	4527615	AATATATATATATATATATA/AATATATATATATATATA	TTTGGTATTGACTCAGGGGC	TTGGAAGGAGCCCTTACCTT	60.1	711	INTERGENIC	--	--	--
Ca3_P2_454494	Ca3	454494	AGGG/AGG	GGATTGGAGTGTTCAGCCAT	TTGTCATTAGTTTCAATCTCACCAA	59.9	460	INTERGENIC	--	--	--
Ca3_P2_454633	Ca3	454633	GATATATATATATAT/GATATATATATAT	GGATTGGAGTGTTCAGCCAT	TTGTCATTAGTTTCAATCTCACCAA	59.9	460	INTERGENIC	--	--	--
Ca3_P2_458081	Ca3	458081	TTATATATATATATAT/TTATATATATATAT	GCGGTTTCCTTTAATTTTGGA	GAGGGAACGCAGAAAGTTCA	60.4	543	INTERGENIC	--	--	--
Ca3_P2_4608979	Ca3	4608979	TTATATATATATATATATATATATAT/TTATATATATATATATATATATATATATAT	GAAATTGAGCGTGCAACTGA	TTGCCACTTGATACAGCAAAA	59.4	823	INTERGENIC	--	--	--

INDEL marker IDs	Chromosomes/unanchored scaffolds	Physical positions (bp)	InDels (*Kabuli* reference genome-CDC Frontier/PII)	Forward primers (5'-3')	Reverse primers (5'-3')	Anneling temperature (0C)	Expected amplified product size (bp)	Structural annotation		Functional annotation	
								Sequence components of *kabuli* genome	*Kabuli* gene accession IDs	NCBI-KOG	TFs
Ca3_P2_4622794	Ca3	4622794	ATATTATTATTATTATTATTATTATTATTATTATTATTATTATTA/ATATTATTATTATTATTATTATTATTATTATTATTATTATTATTATTATTATTA	TTGTCAAGTTAGGGCACATTTTT	TCTTGAAGTGTGTGGCCATT	59.1	627	INTERGENIC	--	--	--
Ca3_P2_4655846	Ca3	4655846	CATTTAGGA/CATTTAGGATTTAGGA	GGGGATTGCTTGAGTCCATA	CTACCAAAGGGGAAGAAGGC	60.1	356	INTERGENIC	--	--	--
Ca3_P2_4689138	Ca3	4689138	TAAAAAA/TAAAAAAA	CAGCTTGCATTTGCTTTTGA	TTGCTCCTACGTATCTCCCG	60.2	888	INTERGENIC	--	--	--
Ca3_P2_474304	Ca3	474304	GTTTTTT/GTTTTTTT	ACGTTGCGAGCAGAGATTTT	TTGGCTTCTGAATTTGGTCC	60.1	655	INTERGENIC	--	--	--
Ca3_P2_4766178	Ca3	4766178	CTATATATATATATATATAT/CTATATATATATATATATAT	AGGCGAAAGCGCACTATTTA	GCAAGTCAGGAAAATGGGAA	60.1	753	INTRON	Ca_22087	T	YABBY
Ca3_P2_4787200	Ca3	4787200	CA/CATA	GTGTCGGATGTCTGACGCTA	GACGACTTGTCACTGGACGA	59.9	450	INTERGENIC	--	--	--
Ca3_P2_4869501	Ca3	4869501	GAAAAAAAAAAA/GAAAAAAAAAA	CCCGGTCGTAGAGAGAAAGA	TTGCTTGGACTTTGAAGCCT	60.0	665	INTERGENIC	--	--	--
Ca3_P2_488374	Ca3	488374	ATTTTTTTTTTT/ATTTTTTTTTTTT	TTTGCGTTACGAATTTGATGA	ACAAAATACCAAGTGCCCCA	60.2	361	INTERGENIC	--	--	--
Ca3_P2_4898542	Ca3	4898542	AATATATATATATATATATATATATATAT/AATATATATATATATATATATATATATATATATAT	GCCAAAAACGAACCACAAAC	GCCCGCTTTGATAGCTGATA	60.3	491	INTERGENIC	--	--	--
Ca3_P2_4943052	Ca3	4943052	GTTTTTTTTTTT/GTTTTTTTTTT	GCCACAAAATTGGTCCAAAA	TATGTTGGTTTAGGGGGCTG	59.8	582	INTERGENIC	--	--	--
Ca3_P2_4944486	Ca3	4944486	AATATATATATATATATATATATATATATA/AATATATATATATATATATATATATATATATATATATA	AATCGACATTCGACCCACTC	CCCCGCTCTGATAGGTGATA	60.1	398	INTERGENIC	--	--	--
Ca3_P2_4965548	Ca3	4965548	ATTTT/ATTTTT	CGGGGAATGTGATTGATAGG	GTTGGAGAGATCGGTGGAAA	60.1	948	INTERGENIC	--	--	--

INDEL marker IDs	Chromosomes/unanchored scaffolds	Physical positions (bp)	InDels (*Kabuli* reference genome-CDC Frontier/PII)	Forward primers (5'-3')	Reverse primers (5'-3')	Anneling temperature (0C)	Expected amplified product size (bp)	Structural annotation		Functional annotation	
								Sequence components of *kabuli* genome	*Kabuli* gene accession IDs	NCBI-KOG	TFs
Ca3_P2_4965627	Ca3	4965627	T/TGAG	TGCAAATCTGGAGCAGAGAA	GTTGGAGAGATCGGTGGAAA	60.1	510	INTERGENIC	--	--	--
Ca3_P2_4966098	Ca3	4966098	CTTTTTTTT/CTTTTTTT	TTTCCACCGATCTCTCCAAC	AAACCCGCTCCTTTCAAAAT	59.9	514	INTERGENIC	--	--	--
Ca3_P2_4975195	Ca3	4975195	AATATATATATATATA/AACATATATATATATATATA	CAAAGTTCAATACGCCAGCA	AGGTTTTGGCGTAACTCCCT	60.0	455	INTERGENIC	--	--	--
Ca3_P2_5031216	Ca3	5031216	TA/T	AAAAACAGGAGGGGCTGAGT	TTGTCGGAAGAACCCTTGTC	60.1	742	INTERGENIC	--	--	--
Ca3_P2_5089285	Ca3	5089285	CAAAAAAAAAA/CAAAAAAAAAAA	TGCCAAGAAAACACCATGAA	GCCAACCAACATTGTGTCAG	60.0	700	INTERGENIC	--	--	--
Ca3_P2_5160337	Ca3	5160337	GAAAAAAAA/GAAAAAAAAA	TTTTTCCCGACTTTGTCCTTT	GGCTAAATTACATTTGTGGTCCC	60.8	315	INTERGENIC	--	--	--
Ca3_P2_5182639	Ca3	5182639	ATTTTTTTTT/ATTTTTTTTTT	TCTCTTTCTAAAACTAGGCAACCA	TTGAGGTGGCCAAAGCTACT	59.9	492	INTERGENIC	--	--	--
Ca3_P2_5239462	Ca3	5239462	AATATATATATATATATATATATATATAT/AATATATATATATATATATATATATATATAT	TGAGATCAGTTTTTGTGGAGGA	CAGTCACCACCCTTGCTTTT	60.1	482	INTERGENIC	--	--	--
Ca3_P2_5268526	Ca3	5268526	T/TATTTACAAGCAG	GGTTTGCTTGCTCCAATGTT	GGGTCCTTGGACACTTTTGA	59.9	657	INTERGENIC	--	--	--
Ca3_P2_5269970	Ca3	5269970	CAAACAATAA/CAA	GATTGGCACGTCGATTAACA	TTCTCGCTTTCGTTTCGTTT	60.0	563	INTERGENIC	--	--	--
Ca3_P2_5270478	Ca3	5270478	TATCAATC/TATC	ACGAAACGAAAGCGAGAAAA	CGACCTCGAAATAATCGGAA	60.0	521	INTERGENIC	--	--	--
Ca3_P2_5278342	Ca3	5278342	ATT/AT	CACCCCAGTTTTGAGGGTAA	TTTGCCAATCGATTATCGTG	59.5	549	DRR	Ca_20669	--	--

INDEL marker IDs	Chromosomes/unanchored scaffolds	Physical positions (bp)	InDels (*Kabuli* reference genome-CDC Frontier/PII)	Forward primers (5'-3')	Reverse primers (5'-3')	Anneling temperature (0C)	Expected amplified product size (bp)	Structural annotation		Functional annotation	
								Sequence components of *kabuli* genome	*Kabuli* gene accession IDs	NCBI-KOG	TFs
Ca3_P2_5286221	Ca3	5286221	ATTT/ATT	CACCCCTCCCAAAACATCT	TTGTGAGTGCATTTGATTTTGTT	59.5	876	INTERGENIC	--	--	--
Ca3_P2_5288032	Ca3	5288032	AATATATATATATATATATATATAT/AATATATATATATATATAT	AGAAGGTCGCATCTCCAAGA	TGGCACTTGTATTCACTCGC	59.9	456	INTERGENIC	--	--	--
Ca3_P2_5347423	Ca3	5347423	CAATAATAATAATAATAATAATAATAATAATAA/CAATAATAATAATAATAATAATAATAA	TTGCTCCTACGTATCTCCCG	TTGTGACAATTTGATGGTTCTAAA	58.6	563	INTERGENIC	--	--	--
Ca3_P2_5389213	Ca3	5389213	AATTATTATTATTATTATT/AATTATTATTATTATTATT	AAGAGACTTCGCGTCCAAAA	CAAGTTTCAACAAATAAATGCACA	59.1	229	INTERGENIC	--	--	--
Ca3_P2_5403691	Ca3	5403691	ACTCTCTCTCTCTCTCTCT/ACTCTCTCTCTCTCTCT	GAGAGGAGCCAACTGGAACA	TGAACCTGTTGAAGTGAAGCA	59.5	326	INTERGENIC	--	--	--
Ca3_P2_5447948	Ca3	5447948	TAAAAAAAAAAA/TAAAAAAAAAA	GCATGAAGACGAATGAGCAA	CATCCCTCTAACCCCACAAA	59.8	667	DRR	Ca_20658	--	--
Ca3_P2_5448109	Ca3	5448109	AAAAATTTAAAATTTAAAATTT/AAAAATTTAAAATTT	GCATGAAGACGAATGAGCAA	GTTTCAAGGGCTTCTTTCCC	60.1	918	DRR	Ca_20658	--	--
Ca3_P2_5502243	Ca3	5502243	TG/T	TGCGTTTTTCTGCATTTCTG	ATTCTGCGCGACTATCAACC	60.2	661	INTERGENIC	--	--	--
Ca3_P2_5502450	Ca3	5502450	GTATATATATATATATA/GTATATATATATATA	TGCGTTTTTCTGCATTTCTG	ATTCTGCGCGACTATCAACC	60.2	661	INTERGENIC	--	--	--
Ca3_P2_5507746	Ca3	5507746	AATATATATATATATATATATATATATATATAT/AATATATATATATATATATATATATATATATATATATATAT	TATTTTGGATTTTGGGCTCG	GGAGGGATAAGGTTTCTCGC	60.0	427	INTERGENIC	--	--	--
Ca3_P2_5520403	Ca3	5520403	GTATATATATATATATATATATATATA/GTATATATATATATATATATATATA	CAAATGACGACGACGATGAC	GAATGATGACAATGCGGTTG	59.9	503	INTERGENIC	--	--	--
Ca3_P2_5689239	Ca3	5689239	GA/GATTATGTTA	TTGGTCAATGTGGCTTCTCA	TGAACTCTGAGCGTGTGACC	60.0	439	INTERGENIC	--	--	--

INDEL marker IDs	Chromosomes/unanchored scaffolds	Physical positions (bp)	InDels (*Kabuli* reference genome-CDC Frontier/PII)	Forward primers (5'-3')	Reverse primers (5'-3')	Anneling temperature (0C)	Expected amplified product size (bp)	Structural annotation		Functional annotation	
								Sequence components of *kabuli* genome	*Kabuli* gene accession IDs	NCBI-KOG	TFs
Ca3_P2_5689325	Ca3	5689325	ATTT/ATT	TTGGTCAATGTGGCTTCTCA	TGAACTCTGAGCGTGTGACC	60.0	439	INTERGENIC	--	--	--
Ca3_P2_571479	Ca3	571479	GTT/GTTGATT	GTGAGTGGTGAGGGCCTAAT	TTGCATACCTCGTATCATCCC	59.8	407	INTERGENIC	--	--	--
Ca3_P2_5864065	Ca3	5864065	AAGAG/AAG	TGAGTGGTGGGCTGTGATTA	ATGCAATGCGTATATGCCAA	59.9	731	INTERGENIC	--	--	--
Ca3_P2_6057834	Ca3	6057834	TTATATATATATATAT/TTATATATATATATATAT	AATTCAATAGAAAGCAGGGGG	TCTTTGCCTGGTCTGACCTT	59.8	734	INTERGENIC	--	--	--
Ca3_P2_613347	Ca3	613347	ATTTTTTTTTTT/ATTTTTTTTTT	TGGAGCTTTGTTTTGGACCT	AGTAAGTGTCGTCGTTGGGC	60.2	409	INTERGENIC	--	--	--
Ca3_P2_615331	Ca3	615331	CAAAAAAAA/CAAAAAAA	CAAGTTTTGGTTCTGGCGTT	TGAAATTATGGGTGGGCATT	60.0	513	DRR	Ca_22653	--	Trihelix
Ca3_P2_6243942	Ca3	6243942	CTTATT/CTT	TGATGGACTTCGTGCTCTTG	CCTCAGGATAAAACAGCCCA	60.1	298	INTERGENIC	--	--	--
Ca3_P2_6289426	Ca3	6289426	CATATATATATAT/CATATATATATATATAT	CGGTTCAGAACTGCATTGAA	CCTTACAAGGCCCACCAATA	59.8	457	INTERGENIC	--	--	--
Ca3_P2_62942	Ca3	62942	AATATATATATATATATATATATA/AATATATATATATATATATATATATATATA	TTCTCTCGAGGATAGTCCGA	AGTTAAACGAGGGGTTTGCC	60.4	936	INTERGENIC	--	--	--
Ca3_P2_6353465	Ca3	6353465	CATTATTATTATTATTATTATTATTATTATTATTATTATT/CATTATTATTATTATTATTATTATTATTATTATTATTATTATTATTATTATTATTATTATT	ATTTTAGGCCGCGACAACTA	TGGACATCCAGAAGGCTACC	60.1	427	INTERGENIC	--	--	--
Ca3_P2_6386934	Ca3	6386934	TGG/TGGG	TGTACGAGAAGGTCGTGTGC	GCCCAACAGCAAGCTATTTC	59.9	574	INTRON	Ca_24834	--	Trihelix
Ca3_P2_6587330	Ca3	6587330	T/TA	TTGAAGGGTTGCATGAATGA	GGAGGTCGAAAATGACCAAA	59.9	741	INTERGENIC	--	--	--

INDEL marker IDs	Chromosomes/unanchored scaffolds	Physical positions (bp)	InDels (*Kabuli* reference genome-CDC Frontier/PII)	Forward primers (5'-3')	Reverse primers (5'-3')	Anneling temperature (0C)	Expected amplified product size (bp)	Structural annotation		Functional annotation	
								Sequence components of *kabuli* genome	*Kabuli* gene accession IDs	NCBI-KOG	TFs
Ca3_P2_6622230	Ca3	6622230	AG/AGG	ATGATGGCCCCCTAAAATTC	AATCCAATGACACAAACGCA	60.0	573	INTERGENIC	--	--	--
Ca3_P2_6627284	Ca3	6627284	ATTTTTTTTTTT/ATTTTTTTTTT	CAATGCAGCCTTGAAAATGA	CAGACTTGGTCCCTCTCTCAA	59.4	805	INTRON	Ca_25776	G	MYB_related
Ca3_P2_6632125	Ca3	6632125	T/TC	ACGATAACCCTTTTGGGACC	CATTTGGGGTCCTTCTCGTA	59.9	394	INTERGENIC	--	--	--
Ca3_P2_6632855	Ca3	6632855	GAAAAAAAAAA/GAAAAAAAAAAA	CTATGGTCTGGCCTGGAATG	TTCTGGACAGACGATTGACG	59.8	704	INTERGENIC	--	--	--
Ca3_P2_6636800	Ca3	6636800	TG/T	ATTCCAAATGAGTCATCGGC	GTTGATCGGGCCACAATATC	60.2	632	INTRON	Ca_25775	G	MYB_related
Ca3_P2_6646283	Ca3	6646283	AATATATATATATATATATATATATATATATATATATATAT/AATATATATATATATATATATATATATATATATATATATATATATAT	TTTGTTGGTTGAGGTTGTGG	AAGTCGGACGGTTGGTACTG	60.0	380	INTERGENIC	--	--	--
Ca3_P2_6664320	Ca3	6664320	TAAAAAA/TAAAAAAA	TGAAATTATGGGTGGGCATT	GCTGAAAAAGTGCCGAAGAA	60.5	569	INTERGENIC	--	--	--
Ca3_P2_6672422	Ca3	6672422	TTTTATTTATTTATTTATTTATTTATTTAT/TTTTATTTATTTATTTATTTATTTATTTATTTAT	GCGCCGCAATAGAATAGAAG	CAACAAAGGGAGGCAAATGT	60.0	672	INTRON	Ca_25772	A	--
Ca3_P2_6687388	Ca3	6687388	ATTTTTTTT/ATTTTTTT	GAACAATGTGGGCAGGAAGT	GTGGTTGCCAGGCTTCTTTA	60.2	563	DRR	Ca_25772	A	--
Ca3_P2_6708682	Ca3	6708682	GTTTTTTTTTT/GTTTTTTTTTTT	GGCCCTTCACCTTGTGATTA	AAAGTGAGGCAGGAAATGGA	59.7	641	INTERGENIC	--	--	--
Ca3_P2_6772905	Ca3	6772905	GTTT/GTT	TCCCTTTGTCTTTTCGCACT	CACTGCCGTTCTTCACTTCA	60.0	383	DRR	Ca_16559	R	NAC
Ca3_P2_6774064	Ca3	6774064	T/TTAA	TTTGCTGGACTTTGAAGCCT	CCCGGTCCTAGAGAGAAAGA	58.5	640	DRR	Ca_16559	R	NAC

INDEL marker IDs	Chromosomes/unanchored scaffolds	Physical positions (bp)	InDels (*Kabuli* reference genome-CDC Frontier/PII)	Forward primers (5'-3')	Reverse primers (5'-3')	Anneling temperature (0C)	Expected amplified product size (bp)	Structural annotation		Functional annotation	
								Sequence components of *kabuli* genome	*Kabuli* gene accession IDs	NCBI-KOG	TFs
Ca3_P2_6774329	Ca3	6774329	ATTTTTTTT/ATTTTTTT	TCTTTCTCTCTAGGACCGGG	GGGTTAGTCTTTGGAGAACACA	58.2	138	DRR	Ca_16559	R	NAC
Ca3_P2_6780457	Ca3	6780457	A/ACAAT	GGATGCTTCGTTTGGTGTTT	TTTGAACCAAGATGCAGCAG	60.0	832	INTRON	Ca_16557	R	NAC
Ca3_P2_6788233	Ca3	6788233	AATTTATTAT/AAT	TTTTTCACTATTGTGTGAAATCAGT	TTTCCTACCATATTCCCGCT	58.5	826	DRR	Ca_16556	R	NAC
Ca3_P2_6790470	Ca3	6790470	T/TA	GGCTGGCTACTTAGGGATCA	GACCTCGTCCAGCTTTTCAG	60.0	582	INTERGENIC	--	--	--
Ca3_P2_6821985	Ca3	6821985	TTATATATATATATATATATATATATA/TTATATATATATATATATATATATATATATATATA	GCGTGATCCCAAGCTACAAT	CATCTTTTAAGGTGCGGGTG	60.5	139	INTERGENIC	--	--	--
Ca3_P2_6838667	Ca3	6838667	AGCA/AGCAGGATCCAATGCAATGGCA	GGAACCCCACAATCATCATC	GCAGCCAATTTTGTTGCATA	59.7	547	CDS (large-effect mutations)	Ca_16550	R	bHLH
Ca3_P2_6844921	Ca3	6844921	GACACACACACACA/GACACACACACACACA	CATCTCCCATCCTGGTAAGC	TCAGCCACTCCAAGCTTTTT	60.0	734	INTERGENIC	--	--	--
Ca3_P2_6909242	Ca3	6909242	ATCTCTCTCTCTCTCTC/ATCTCTCTCTCTCTCTCTC	GCCTCTTTAGGACTTCGGCT	TCAAACTGAACTGCATTGAAAAA	59.8	656	INTERGENIC	--	--	--
Ca3_P2_70555	Ca3	70555	TATGAGTTATGA/TATGAGTTATGATGAATGAGTTATGA	CAAGTAAACGCACGTGAACC	TACCCACACCCCCATTTTTA	59.9	582	INTERGENIC	--	--	--
Ca3_P2_7076805	Ca3	7076805	C/CCCGAATGT	TCTTCGATTCCATTCCAACC	TTGGGGGAGACGATATTCTG	59.9	701	INTERGENIC	--	--	--
Ca3_P2_7223058	Ca3	7223058	TA/TATACA	AGGTTATGTCGTCCGTCAGG	GCGTAGTGGGAGCTTGTAGG	59.9	767	INTERGENIC	--	--	--
Ca3_P2_7228177	Ca3	7228177	GAGTTACATAGTTACATA/GAGTTACATA	CTGTTGGGCAAGGGATCTAA	ACAGCGCTTTTGTGCTTTTT	60.1	463	DRR	Ca_16534	--	--

INDEL marker IDs	Chromosomes/unanchored scaffolds	Physical positions (bp)	InDels (*Kabuli* reference genome-CDC Frontier/PII)	Forward primers (5'-3')	Reverse primers (5'-3')	Anneling temperature (0C)	Expected amplified product size (bp)	Structural annotation		Functional annotation	
								Sequence components of *kabuli* genome	*Kabuli* gene accession IDs	NCBI-KOG	TFs
Ca3_P2_726429	Ca3	726429	ATTTTTTT/ATTTTTTTT	TCCTGTATCTTACCAAACAAGGC	GTTTGTTGTCGGCTTACGGT	60.0	715	INTERGENIC	--	--	--
Ca3_P2_7312652	Ca3	7312652	TAAAAAAAAA/TAAAAAAAAAA	CGCAAGATTTTACCTACCTGTG	TGATCAAGCTTCCAGTCCAA	59.4	720	DRR	Ca_16529	--	LFY
Ca3_P2_7330454	Ca3	7330454	AG/A	CCAACAAAAGGACGGAAGAA	TTTTGTCCCAACAATCTCCC	59.8	605	INTERGENIC	--	--	--
Ca3_P2_7340219	Ca3	7340219	T/TTA	GCCATCTTATGAAATCTTTGGG	TGTGCGTGTGTGAAAGTGAA	59.9	865	INTERGENIC	--	--	--
Ca3_P2_7367436	Ca3	7367436	CGAAGAAGA/CGAAGA	TGATTGTTTGTCTGGTGGGA	CACTCTCTTCCTTGGCCTTG	60.0	331	INTERGENIC	--	--	--
Ca3_P2_7368189	Ca3	7368189	AAATAATAATAATAATAATAATAATAA/AAATAATAATAATAATAATAA	ATTGGGGCAATAACCAACAA	TGGTGCGATTGTGGAAAATA	59.9	854	INTERGENIC	--	--	--
Ca3_P2_7385240	Ca3	7385240	TAAAA/TAAAAA	AATCGGTGAGGAAAATGCAG	AGCAAAGTGTTTTGCCCTTG	60.3	567	INTERGENIC	--	--	--
Ca3_P2_7440399	Ca3	7440399	TGGTGGTGGTGGCGGTGGT/TGGTGGTGGTGGCGGTGGTGGTGGCGGTGGT	ATCACAGAGATCCAATCGCC	TTCATCACCTCCTCCGAATC	60.0	384	CDS (large-effect mutations)	Ca_16525	R	--
Ca3_P2_7467677	Ca3	7467677	AACATTAGA/AACATTAGACATTAGA	CCACACCCCTCACACTTTCT	AGCAAAGGGGATGAAAAGGT	59.9	461	INTERGENIC	--	--	--
Ca3_P2_7494196	Ca3	7494196	T/TC	TTGCTTTGGTTTGATGTGGA	GAAGTTGCCTTAGTGGCAGG	59.9	694	INTERGENIC	--	--	--
Ca3_P2_7535269	Ca3	7535269	TAAAAAAAAAA/TAAAAAAAAAAA	TATCCCTCACCCCTCCTTTT	GCATCTTTATATCTGGCGACC	58.7	343	INTERGENIC	--	--	--
Ca3_P2_7543380	Ca3	7543380	TAAAAAAAAAAAA/TAAAAAAAAAAAAA	ACAAAAGGCCACCAAGAAAA	CGGACCAAAGTGGTCAATGT	60.8	619	INTERGENIC	--	--	--

INDEL marker IDs	Chromosomes/unanchored scaffolds	Physical positions (bp)	InDels (*Kabuli* reference genome-CDC Frontier/PII)	Forward primers (5'-3')	Reverse primers (5'-3')	Anneling temperature (0C)	Expected amplified product size (bp)	Structural annotation		Functional annotation	
								Sequence components of *kabuli* genome	*Kabuli* gene accession IDs	NCBI-KOG	TFs
Ca3_P2_7549569	Ca3	7549569	T/TACTCAAGTTTAGCAC	TGTCTACGCTAGCACGAACAA	TGTGGCGAGATATTTTGTAGTGT	58.7	618	INTERGENIC	--	--	--
Ca3_P2_7580880	Ca3	7580880	TAAAAAAAAA/TAAAAAAAAAA	GTGGTTCCGGAAAAGATGAA	CCCCATCCATTTCCCTATCT	60.0	373	INTERGENIC	--	--	--
Ca3_P2_7636412	Ca3	7636412	CTTATTATTATTATTATTATTATTATTATTATTATTATTATTATT/CTTATTATTATTATTATTATTATTATTATTATTATTATTATTATTATTATT	TGGCCTTGTGTGTATTGCAT	CTGAATTGCGTGTGTCTGGT	59.8	566	INTERGENIC	--	--	--
Ca3_P2_7749695	Ca3	7749695	G/GACT	GCTTGGTTTTCTGGGTTTGA	ACCTTGAATCGCTGCCTCTA	60.0	870	INTERGENIC	--	--	--
Ca3_P2_7788612	Ca3	7788612	ATTT/ATT	CCTCAGATGAGAGCCAGGAC	TGGGTAACTAATGCCCCAAC	59.7	582	INTERGENIC	--	--	--
Ca3_P2_7792015	Ca3	7792015	TAT/TATTATTCTTTCCCTAAT	TAGGTAAAGGTGGTTTGCCG	TGAGGGATAGATGCAGGTCA	59.2	611	INTERGENIC	--	--	--
Ca3_P2_781722	Ca3	781722	CAA/CAAAA	AAAACGCGCCACTAATGACT	AGCAAGAGTGCAAAGTCGGT	60.1	546	DRR	Ca_22643	V	B3
Ca3_P2_7880432	Ca3	7880432	TCTAG/T	AGGAAGAGTGGAGTTGCGAA	TATTTTGGATTTTGGCTCGG	59.9	503	INTERGENIC	--	--	--
Ca3_P2_7922530	Ca3	7922530	TTATATATATATATATATAT/TTATATATATATATATAT	AGGAAGAGTGGAGTTGCGAA	TATTTTGGATTTTGGCTCGG	59.9	504	INTERGENIC	--	--	--
Ca3_P2_794982	Ca3	794982	GTTTTTTTTTTT/GTTTTTTTTTT	TAGAGGGAGGAGGTGCAAGA	GGTGATATAGACGGGGCAGA	59.9	686	INTERGENIC	--	--	--
Ca3_P2_7959750	Ca3	7959750	TTAATTTGATTATGTTAAGTAATTTGATTATGTTAA/TTAATTTGATTATGTTAAGTAATTTGATTATGTTAAGTAATTTGATTATGTTAA	TTGGCATAGGTCATGGAAGTT	TTTGCCAATGTTGCTAGCTG	60.0	848	INTERGENIC	--	--	--
Ca3_P2_8059631	Ca3	8059631	GTTT/GTT	TGTGCGACCATCTACTGCTC	GCAATCTCCTCAACTCGGTC	59.8	813	INTERGENIC	--	--	--

INDEL marker IDs	Chromosomes/unanchored scaffolds	Physical positions (bp)	InDels (*Kabuli* reference genome-CDC Frontier/PII)	Forward primers (5'-3')	Reverse primers (5'-3')	Anneling temperature (0C)	Expected amplified product size (bp)	Structural annotation		Functional annotation	
								Sequence components of *kabuli* genome	*Kabuli* gene accession IDs	NCBI-KOG	TFs
Ca3_P2_8081601	Ca3	8081601	CATATATATATATATATATA/CATATATATATATATATA	AAAGCGCTGTAAAATGCACA	GAAATAGCTTGCTGTTGGGC	59.9	580	DRR	Ca_24371	--	Trihelix
Ca3_P2_8146397	Ca3	8146397	AT/A	TCAAGAAGGATGGAGGATCG	TCCTTTTGCACATGCTTCAC	59.8	448	INTERGENIC	--	--	--
Ca3_P2_8241639	Ca3	8241639	ATTTT/ATTTTATGATTTT	TTTGTTTCAATCGATTCCCC	TGTGTGTTTGTTTGGAGGGA	60.0	627	INTERGENIC	--	--	--
Ca3_P2_8245494	Ca3	8245494	TCT/TCTTCTGCT	TGCGCCTCTTCTAAAATCGT	TATCGCAATCAGAGGAACCC	60.0	670	INTERGENIC	--	--	--
Ca3_P2_8290056	Ca3	8290056	TT/TTCT	AACGATGAAGAATGGGTTCG	CGTGGATTAGCTTCGAGGAA	60.3	426	INTRON	Ca_24378	--	--
Ca3_P2_8317445	Ca3	8317445	GTTTTTTTTTT/GTTTTTTTTTTT	GGTTTTTGGTTGTTGTCCCA	AACACGCCTATGGTGACTCC	60.0	451	INTERGENIC	--	--	--
Ca3_P2_8393314	Ca3	8393314	GTATATATATATATATATAT/GTATATATATATATATATATATAT	GTTGGTTCCCTAGCACCGTA	TATTTTGGATTTTGGCTCGG	59.9	602	INTERGENIC	--	--	--
Ca3_P2_8437173	Ca3	8437173	T/TAA	TGCCAAGAAAACACCATGAA	GTTTAGAACGTGACCCCGAA	60.0	662	INTERGENIC	--	--	--
Ca3_P2_8523296	Ca3	8523296	ATTTTTTTTTT/ATTTTTTTT	CACATGACAAAATTGGTGGAA	CGTTAAGGCACATCTCACTCC	59.7	508	INTERGENIC	--	--	--
Ca3_P2_8527625	Ca3	8527625	GAAGGAAAAGGAAAAGGAAAAG/GAAGGAAAAGGAAAAG	ACCCAATTACTGGAGATGCG	CCGTAGCGTGATAGTGAGCA	60.0	570	INTERGENIC	--	--	--
Ca3_P2_8537399	Ca3	8537399	AATATATATATATATATATATATATATATATA/AATATATATATATATATATATATATATATA	ATCACCACTTCACCAAAGGC	CATGTCCAGTCCAGTAACTCATGT	60.3	583	INTERGENIC	--	--	--
Ca3_P2_8611742	Ca3	8611742	A/ATTGTTTT	TTTAACGGACGTTGTAGGGC	AGCCCGGGTATAGAGTCCAC	60.3	422	INTERGENIC	--	--	--

INDEL marker IDs	Chromosomes/unanchored scaffolds	Physical positions (bp)	InDels (*Kabuli* reference genome-CDC Frontier/PII)	Forward primers (5'-3')	Reverse primers (5'-3')	Anneling temperature (0C)	Expected amplified product size (bp)	Structural annotation		Functional annotation	
								Sequence components of *kabuli* genome	*Kabuli* gene accession IDs	NCBI-KOG	TFs
Ca3_P2_8656594	Ca3	8656594	AATATATATATATATATATATATATATATATATATATA/AATATATATATATATATATATATATATATATATATA	GCACATTCACTGCCAAGAGA	GCGTAGTGGTAGCCTTCTGG	59.9	588	INTERGENIC	--	--	--
Ca3_P2_8682830	Ca3	8682830	CATATATATATATATATATATATAT/CATATATATATATATATATATATATAT	TAATACCCGCTTACGTTCGG	CAAAGCGTGGAAATGAGACA	59.8	484	INTERGENIC	--	--	--
Ca3_P2_8754025	Ca3	8754025	GTATATATATATATATATATATATATATAT/GTATATATATATATATATATATATATATATATAT	AAAATCCTTGGCCTTTCGAT	GATGAGGTATGTGCGTGTGG	60.0	863	INTRON	Ca_23720	--	B3
Ca3_P2_8775439	Ca3	8775439	GTATATATATATATATATATATATATATATATAT/GTATATATATATATATATATATATATATATATATATATATATATATATAT	AGTCGCATCTTCGTCGTTTC	GGCATCGGGAGTCTGTTTTA	60.1	537	INTERGENIC	--	--	--
Ca3_P2_8809963	Ca3	8809963	TTTATTATTATTATTATTATTATTATTATTATTATTATTATTATTATTATTATTATTATTATTATTATTATTATTATTATTATTATTATT/TTTATTATTATTATTATTATTATTATTATTATTATTATTATTATTATTATTATTATTATTATTATTATTATTATTATTATTATT	CCATGTTTCAAAATGTTGCG	TCATGTTCTTCGCCTTTGTG	59.8	773	DRR	Ca_23725	--	--
Ca3_P2_8828007	Ca3	8828007	GTTATTATTATTATTATTATTATTATTAT/GTTATTATTATTATTATTATTAT	AGAAAATGCGGGTTATGTCG	TTGATAACTTGAAAACGAAATGAAA	59.1	331	INTERGENIC	--	--	--
Ca3_P2_8830310	Ca3	8830310	AATATATATATATATATATATATATATATATATATA/AATATATATATATATATATATATATATATATATATA	AAAGGCTTTTGCCTCTCACA	TGAATCGCCTAGCTGCTTTT	60.1	934	INTERGENIC	--	--	--
Ca3_P2_8854221	Ca3	8854221	TTATATATATATATATATATATATATATATATATATAT/TTATATATATATATATATATATATATATATATATATATATATATAT	TCACGTGCTCCATGGTTTTA	TGGATAAGGGGTGTGTGGTT	60.1	619	DRR	Ca_23727	--	--
Ca3_P2_8887696	Ca3	8887696	AATATATATATATATATATATATATATATATA/AATATATATATATATATATA	GCGCTTTTAAGCGTTGTTGT	GGTGCTACAGTGTCGAAGCA	60.1	778	INTERGENIC	--	--	--
Ca3_P2_8942193	Ca3	8942193	AT/ATT	TGGGGAAAAACCCTTACCTT	CACATGATTTCAATTGGCTTTG	60.4	326	INTERGENIC	--	--	--
Ca3_P2_9042314	Ca3	9042314	CAAAA/CAAA	TCATCGCAGGTGTACCATGT	CGTGTTTGAAAAATTAGGGTTTG	59.8	811	INTERGENIC	--	--	--
Ca3_P2_9048134	Ca3	9048134	TAAAAAAAA/TAAAAAAA	TTGATTTTGCCATCACTCCA	GGGCCCTTAAGCCTACAAAA	60.4	220	INTERGENIC	--	--	--

INDEL marker IDs	Chromosomes/unanchored scaffolds	Physical positions (bp)	InDels (*Kabuli* reference genome-CDC Frontier/PII)	Forward primers (5'-3')	Reverse primers (5'-3')	Anneling temperature (0C)	Expected amplified product size (bp)	Structural annotation		Functional annotation	
								Sequence components of *kabuli* genome	*Kabuli* gene accession IDs	NCBI-KOG	TFs
Ca3_P2_9092559	Ca3	9092559	AA/AACA	CATGTCCCAAAAATATGCCC	TCTTTCAAGTGGGTTGGGTC	59.9	304	INTERGENIC	--	--	--
Ca3_P2_9186815	Ca3	9186815	CTATATATATATATATATATATATA/CTATATATATATATATATATATATA	TCCACAAACACGATAACATTGA	TGTAACAAGCATTTGTCGGG	59.6	693	INTERGENIC	--	--	--
Ca3_P2_9189477	Ca3	9189477	AATATATATATATATATATATATATATAT ATATAT/AATATATATATATATATATATATAT ATATATATATATATAT	AAAGGTTTGGTTGGAGATACG	TTGGCTGCAAAATATCCCTC	60.0	856	INTERGENIC	--	--	--
Ca3_P2_9241046	Ca3	9241046	TTTTATTTATTTATTTATTTATTTAT/TTT TATTTATTTATTTATTTATTTATTTATTTA T	TTTGATGTTGTTGGAAATGAAAA	GCACATGTTCACCGACATTC	60.0	445	DRR	Ca_20955	--	--
Ca3_P2_9289555	Ca3	9289555	TTATATATATATATATATATATAT/TTATATATATATATATATATATATAT	GAACCAAGTTGCATGGGAAT	CCTAGGGACTTCGTGGTTGA	60.1	651	INTERGENIC	--	--	--
Ca3_P2_9306348	Ca3	9306348	AATATATATATATATATATATATA/AATATATATATATATATATA	TCCAGGTGAAAAGTAACGCC	TGCTGAGCAAAACACTCTGG	60.2	432	DRR	Ca_20961	--	Trihelix
Ca3_P2_9327176	Ca3	9327176	GTTTTTTTTTT/GTTTTTTTTTTT	TTGGAACTTTCTCCGTTTGG	TTAATGGCAAAAACAGGGGA	60.3	647	INTERGENIC	--	--	--
Ca3_P2_939073	Ca3	939073	GTT/GTTTT	ATTGGTTCTGCTCTGGGTTG	GGGACCACAGCACCTATGTT	59.9	296	INTERGENIC	--	--	--
Ca3_P2_9426071	Ca3	9426071	CAAAAAAAAA/CAAAAAAAAAA	CCATAAAACCATTTCCATTGC	TACTCTAGCAACCACCGCCT	59.9	262	INTERGENIC	--	--	--
Ca3_P2_943806	Ca3	943806	ATCT/ATCTCT	GTGAATTCATCCCATGTCCC	AGCCCTGGTAAGCCTTTTGT	60.1	515	INTERGENIC	--	--	--
Ca3_P2_9577976	Ca3	9577976	AGG/AGGG	TTGCGTTCAAAGACTCGATG	TGAATTGCAGAATCCCATGA	60.0	575	INTRON	Ca_20977	--	--
Ca3_P2_959453	Ca3	959453	CAAA/CAA	CCTATCACGGATCAGCACAA	TACGGTGCTAGGGAACCAAC	60.0	924	INTERGENIC	--	--	--

INDEL marker IDs	Chromosomes/unanchored scaffolds	Physical positions (bp)	InDels (*Kabuli* reference genome-CDC Frontier/PII)	Forward primers (5'-3')	Reverse primers (5'-3')	Anneling temperature (0C)	Expected amplified product size (bp)	Structural annotation		Functional annotation	
								Sequence components of *kabuli* genome	*Kabuli* gene accession IDs	NCBI-KOG	TFs
Ca3_P2_960261	Ca3	960261	GTATATATATATATATATATATATATATATA/GTATATATATATATATATATATATATATATATATA	GTTGGTTCCCTAGCACCGTA	CTTGGGAACTGGAGAGCAAG	60.0	755	INTERGENIC	--	--	--
Ca3_P2_962679	Ca3	962679	T/TTCGATAC	TGTGTCAATGGAGCCACAAT	CCTCCAAAGTTCAAGGGTGA	60.1	748	INTERGENIC	--	--	--
Ca3_P2_9649838	Ca3	9649838	ATTT/ATT	TAAGGCAGATTGCCCAAAAC	TTTCGGGAATGAAACTGGAC	59.9	462	DRR	Ca_20979	--	--
Ca3_P2_9650661	Ca3	9650661	AG/ACG	ACCAATCGATTTCCACAAGC	CCCTGTGACAAACACAATCG	60.0	257	DRR	Ca_20979	--	--
Ca3_P2_9651257	Ca3	9651257	CTTTTTT/CTTTTT	CTCCAAACTGGGGTGTCACT	CGGTCTCAACCTCTCCCATA	60.1	882	DRR	Ca_20979	--	--
Ca3_P2_9940793	Ca3	9940793	ATTCCAATTTCCAATT/ATTCCAATTTCCAATTTCCAATT	TCAGGGATTACCGCATAAGG	CATTCTTCCACTAGGCACGC	60.8	293	INTERGENIC	--	--	--
Ca3_P2_9953160	Ca3	9953160	GAAAAAA/GAAAAAAA	TGCCACGTTTCTATTTCCCT	ATCACGCCATAAGCACCTTC	60.1	511	INTERGENIC	--	--	--
Ca3_P2_9979893	Ca3	9979893	AT/A	GACGGCAGTCCTCCTTTGTA	GAGCACAAGCAGTTCATCCA	60.0	519	INTRON	Ca_18805	C	bHLH
Ca3_P2_9998089	Ca3	9998089	TTAATAATAATAATAATAATAATAATAATAATAAT/TTAATAATAATAATAATAATAATAATAATAATAATAATAAT	GCAACCTGTTGAAGCCAAAT	ATGTGCAAAATGGAGGAAGC	60.1	907	INTERGENIC	--	--	--
Ca4_P2_10033685	Ca4	10033685	TTATATATATATATATATAT/TTATATATATATATATATATAT	CTAGTTCGTAACCCGTGCGT	AGGCATGGTGTTGGAGTCAT	60.4	248	INTERGENIC	--	--	--
Ca4_P2_10057169	Ca4	10057169	AAAATTCAAATTCAAATTCAAATTCAAA/AAAATTCAAATTCAAATTCAAA	AACGTGCATCGACACTTTGA	AACGGAAGAATACGCCCTTTA	60.0	542	INTERGENIC	--	--	--
Ca4_P2_10072492	Ca4	10072492	GTTTTTTT/GTTTTTTTT	AAGTGTGGCAATTTTGGAGG	CCTTTGAAATTTGGTGCGTT	60.0	711	INTERGENIC	--	--	--

INDEL marker IDs	Chromosomes/unanchored scaffolds	Physical positions (bp)	InDels (*Kabuli* reference genome-CDC Frontier/PII)	Forward primers (5'-3')	Reverse primers (5'-3')	Anneling temperature (0C)	Expected amplified product size (bp)	Structural annotation		Functional annotation	
								Sequence components of *kabuli* genome	*Kabuli* gene accession IDs	NCBI-KOG	TFs
Ca4_P2_10092984	Ca4	10092984	GTTTTTTTT/GTTTTTTTTT	CGCACGAGTAACACGTCATT	ACGCGGAACAAATATTGTCA	59.0	432	INTERGENIC	--	--	--
Ca4_P2_10108026	Ca4	10108026	GTAATAATAATAATAATAATAATAATAAT/GTAATAATAATAATAATAATAATAATAATAAT	AACCGAATGAAATGAAACGG	AAGCGCACAGGAAAACCTTA	59.9	551	INTERGENIC	--	--	--
Ca4_P2_10173137	Ca4	10173137	ATTTTTTTT/ATTTTTTTTT	CCCGTTATTTTTGACGAAACA	TGTGTTGAGTGGTGGCATTT	60.0	791	INTERGENIC	--	--	--
Ca4_P2_10180518	Ca4	10180518	AT/A	AGGGCTGGAATTAGTTGGCT	CAGATGACAACCGAAAGCAA	59.8	321	DRR	Ca_08489	G	--
Ca4_P2_10233239	Ca4	10233239	GACAATTATTTGGTACAATTAT/GACAATTATTTGGTACAATTATTTGGTACAATTAT	TGTCGGTTGTTGCTTGAAGA	ATACAATCATTTTCCCGCCA	60.2	403	INTERGENIC	--	--	--
Ca4_P2_10253630	Ca4	10253630	GAAAAAAAA/GAAAAAAA	TGTGTTTGAATGGGCAAAAA	CTTCATTTAAACGCCCCAAA	59.9	312	INTERGENIC	--	--	--
Ca4_P2_10310413	Ca4	10310413	CAAAAAAA/CAAAAAAAA	GAGGAGGAGGTGGAGGGTAG	TTGTGCACCATCGATCATTT	59.9	732	INTERGENIC	--	--	--
Ca4_P2_10460957	Ca4	10460957	CATATATATATATATATATATATAT/CATATATATATATATATATATATATATATAT	TTAATTCCAATGGCGGGTAG	TCACATTCAGCTTTTGGTCG	59.8	428	INTERGENIC	--	--	--
Ca4_P2_10624424	Ca4	10624424	TTAATAATAATAATAATAATA/TTAATAATAATAATAATA	GGATGTCCCAAAGGGTTTTT	GGATGATTCTGGAGCGAGAC	59.8	303	INTERGENIC	--	--	--
Ca4_P2_10657750	Ca4	10657750	T/TCTAACACTCTTTCC	ATACAAAAACATTTGGGCCG	TTCACATGAAGTGGCGAGAC	59.8	417	DRR	Ca_04301	--	--
Ca4_P2_10661685	Ca4	10661685	CTTTTTTTTTTT/CTTTTTTTTTT	GGAGAAATGAGTGAAGGTGGA	AAATACCACTCGAGGTCCCA	59.4	801	INTERGENIC	--	--	--
Ca4_P2_10758002	Ca4	10758002	AATATATATATATATATATATATATATATATATATATATATATATATAT/AATATATATATATATATATATATATATATATATATATATATATATATATATATAT	ACATAGCCAAAAACCGATGC	ATCCAACGGTTAACGAATGC	59.8	579	INTERGENIC	--	--	--

INDEL marker IDs	Chromosomes/unanchored scaffolds	Physical positions (bp)	InDels (*Kabuli* reference genome-CDC Frontier/PII)	Forward primers (5'-3')	Reverse primers (5'-3')	Anneling temperature (0C)	Expected amplified product size (bp)	Structural annotation		Functional annotation	
								Sequence components of *kabuli* genome	*Kabuli* gene accession IDs	NCBI-KOG	TFs
Ca4_P2_10793822	Ca4	10793822	AT/ATGGT	TGGTGAAAATAGATTAGGACGAGA	CTGAACATTTGCATGCTCTCA	60.0	943	INTERGENIC	--	--	--
Ca4_P2_10939792	Ca4	10939792	G/GAGTGTTGGTC	GCAGCAAAAAGGGATAACCA	AAGAAACCTCGTGTGCCATC	60.1	231	DRR	Ca_04328	GMW	NAC
Ca4_P2_10954430	Ca4	10954430	AATATATATATATATATATATA/AATATATATATATATATATATATA	TTCGTGAATTAGTTTTCCGTGA	TAACGTAACATGCCATTGCC	59.4	554	INTERGENIC	--	--	--
Ca4_P2_10993124	Ca4	10993124	CAAAAAAA/CAAAAAAAA	CCTAAAACCCTAACCCCCAA	AGCACCCCCAAAAAGAAAGT	60.0	613	INTERGENIC	--	--	--
Ca4_P2_1104791	Ca4	1104791	GATAATAATAATAATAATAATAATAATAATAATAATAATA/GATAATAATAATAATAATAATAATAATAATAATAATAATAATAATAATA	AAATTCCATCAGCTTCTTTGAA	AAGAGAGGCAATGTTCCACG	60.3	579	DRR	Ca_07743	--	NAC
Ca4_P2_11070438	Ca4	11070438	T/TATTACG	TTTGCCCCAATAAAGAGCAC	AACAACTTTTGCGAACCACC	60.0	701	DRR	Ca_04340	J	--
Ca4_P2_11077353	Ca4	11077353	TTCTCTCTCTCTCTCTCTCTCTCTCTCTCTCTCTCTCTCTC/TTCTCTCTCTCTCTCTCTCTCTCTCTCTCTCTCTCTCTCTCTCTC	TAGTTCAGACGTGCATTCGC	TTGCTTCAGAGGCACAAATG	60.0	701	INTRON	Ca_04340	J	--
Ca4_P2_11203626	Ca4	11203626	TTATATATATATATATATATATATATATATATATATATA/TTATATATATATATATATATATATATATATATATATATATATATA	TCGGGGCTGTTTGTTTAAGA	GCGATAATGAGTCCGTTTGC	60.6	487	INTERGENIC	--	--	--
Ca4_P2_11206085	Ca4	11206085	TTATATATATATATATATATAT/TTATATATATATATATATATAT	GATTGCCACATGCTCACATC	CTCGTGCAAGTGCAAGTGAT	60.1	467	INTERGENIC	--	--	--
Ca4_P2_11209323	Ca4	11209323	TAAAAAAA/TAAAAAA	TTAGTGTTGAATTGACGGCG	CAATGCCTAGAATCACGGGT	60.0	960	INTERGENIC	--	--	--
Ca4_P2_11220000	Ca4	11220000	AAATAATAATAA/AAATAATAATAATAA	ATTTCTTCGTGTACCGCACC	CTGATAGACCGACACACACACA	59.7	465	INTERGENIC	--	--	--
Ca4_P2_11228979	Ca4	11228979	ATGATATT/ATGATATTGATATT	CCCATGTAAGTGAGTGAAATCG	GGCTTGCATGATGCTAAATTC	59.7	694	INTRON	Ca_04355	G	--

INDEL marker IDs	Chromosomes/unanchored scaffolds	Physical positions (bp)	InDels (*Kabuli* reference genome-CDC Frontier/PII)	Forward primers (5'-3')	Reverse primers (5'-3')	Anneling temperature (0C)	Expected amplified product size (bp)	Structural annotation		Functional annotation	
								Sequence components of *kabuli* genome	*Kabuli* gene accession IDs	NCBI-KOG	TFs
Ca4_P2_11259655	Ca4	11259655	CATTATTATTATTAT/CATTATTATTATTATTATTAT	TGTTTGGTCGTTGAAATGGA	AACCATCATGAAAGGGTGGA	60.2	629	INTERGENIC	--	--	--
Ca4_P2_11268024	Ca4	11268024	AAAGAA/AAA	TAGTGCCCTCCAAAAGGATG	GCGTTGAGGTGTCAACTGTG	60.4	733	INTERGENIC	--	--	--
Ca4_P2_11285447	Ca4	11285447	C/CAA	TCCACATGCACCTGTGAACT	AGTAGTGCCTCAAACCACGG	60.2	382	DRR	Ca_04361	GE	GATA
Ca4_P2_11291621	Ca4	11291621	ATTT/ATTTT	GATGAAAGTTTTTGGTGTGGC	AGCGCAGTGTTAACGTTCCT	59.9	946	INTERGENIC	--	--	--
Ca4_P2_11300403	Ca4	11300403	ATATTATT/ATATT	GTGTTACTTCCGCCGTTGAT	TGTCAAGTTGTTTCATGCTGC	59.9	185	INTERGENIC	--	--	--
Ca4_P2_11309352	Ca4	11309352	AATGAT/AATGATGAT	AAGAGCTACCTACGGAGGGC	ACCCAACAGCAAAACCAAAC	59.9	354	INTRON	Ca_04363	--	--
Ca4_P2_11311504	Ca4	11311504	A/ACAC	TGATGGTTGGCCTAAATGGT	TTGCTGAAGACTGATGCCAC	60.0	463	DRR	Ca_04363	--	--
Ca4_P2_11326193	Ca4	11326193	CATATATA/CATA	CACCTGGATTGTTTGTGTGC	ATATTGGTCAAGCTGCTGGC	60.2	272	INTRON	Ca_04365	--	M-type
Ca4_P2_11337922	Ca4	11337922	TAAA/TAAAA	ATCGAGGAATAACACGGACG	GGCCATCATTTGTCCCTCTA	59.9	770	INTERGENIC	--	--	--
Ca4_P2_11340703	Ca4	11340703	CG/C	TCATCACCGTCCAATTTCAA	CTCCTTGCCCAATTTGTTGT	60.0	333	INTERGENIC	--	--	--
Ca4_P2_11368276	Ca4	11368276	CG/C	TGATGAATTTTCATTCACCAATC	TCCCAAGGTCAATTAGGCAG	60.1	116	INTERGENIC	--	--	--
Ca4_P2_11404509	Ca4	11404509	TTTCTTC/TTTCTTCTTC	GATGTGGGTAAGCTTGGAGG	TGATGGTTCATGTGGTGGTC	60.2	508	DRR	Ca_04374	--	bZIP

INDEL marker IDs	Chromosomes/unanchored scaffolds	Physical positions (bp)	InDels (*Kabuli* reference genome-CDC Frontier/PII)	Forward primers (5'-3')	Reverse primers (5'-3')	Anneling temperature (0C)	Expected amplified product size (bp)	Structural annotation		Functional annotation	
								Sequence components of *kabuli* genome	*Kabuli* gene accession IDs	NCBI-KOG	TFs
Ca4_P2_11427452	Ca4	11427452	AATATATATATATATATAT/AATATATATATATATATATATAT	TATCCGCATGTGGACAAAAA	TTGGGTTGAATTTTTCTGGTG	59.8	555	INTERGENIC	--	--	--
Ca4_P2_11433750	Ca4	11433750	TTTGATT/TTT	TATGCCATGATTCTCGGACA	AATTGGAGCCGGAAGTTTCT	60.1	515	INTRON	Ca_04378	S	--
Ca4_P2_11435815	Ca4	11435815	AAC/A	CGGAGGTTTCAGTTCAGCTC	GTGAATTGGTCGAGCGGTAT	60.0	906	DRR	Ca_04378	S	--
Ca4_P2_11448454	Ca4	11448454	AATATATATATATATATATATATATATAT/AATATATATATATATATATATAT	ATCACCGCCCATATTATCCA	TGAAGGTGATTGACACAAGGA	59.1	882	INTERGENIC	--	--	--
Ca4_P2_1144979	Ca4	1144979	CATATATATATATATATATATATATATATA/CATATATATATATATATATATATATATA	AACGCATGCAATCTATTCCC	ATTGTTGGCGTCTTCCAAAC	60.0	813	INTERGENIC	--	--	--
Ca4_P2_11461111	Ca4	11461111	AG/AGATATG	TGCAACTTTCATCCTCACCA	CCCCCTTCTTCCTTCACTTC	60.0	703	INTERGENIC	--	--	--
Ca4_P2_11472631	Ca4	11472631	AC/A	TTTCTTGTCTCCTCGGTTGG	TTTGTCATGTTCACGCACTG	59.3	632	INTERGENIC	--	--	--
Ca4_P2_11477897	Ca4	11477897	GATATATATATA/GATATATATATATATATA	CCGCTTCTCTTCAATGCTCT	AACACACCCCAATTTGCATT	60.1	463	INTERGENIC	--	--	--
Ca4_P2_11484974	Ca4	11484974	ATTTTTTTT/ATTTTTTTTTTT	TTTATCACTCCTCAATTCGGGT	TGTACTGCAGACAAAATTGTGATA	57.5	202	INTERGENIC	--	--	--
Ca4_P2_11505808	Ca4	11505808	GACA/GACAAAAACA	GCAATGATGACGGTGTATCG	CATGAACCATGATGTCGAGG	59.9	470	INTERGENIC	--	--	--
Ca4_P2_11506583	Ca4	11506583	CTTA/CTTAATTA	CTCGACATCATGGTTCATGG	CGGATCTATGCATGGGATAA	58.4	507	INTERGENIC	--	--	--
Ca4_P2_11514114	Ca4	11514114	AAAACA/AAAACAAACA	TGGATTTATGTAATGGGCTGG	TCGCCGAGTTTCAACTCTTT	60.0	509	INTERGENIC	--	--	--

INDEL marker IDs	Chromosomes/unanchored scaffolds	Physical positions (bp)	InDels (*Kabuli* reference genome-CDC Frontier/PII)	Forward primers (5'-3')	Reverse primers (5'-3')	Anneling temperature (0C)	Expected amplified product size (bp)	Structural annotation		Functional annotation	
								Sequence components of *kabuli* genome	*Kabuli* gene accession IDs	NCBI-KOG	TFs
Ca4_P2_11514646	Ca4	11514646	AT/ATT	AAGAGTTGAAACTCGGCGAA	AAGCCACATTTTGGGTCAAG	60.0	700	INTERGENIC	--	--	--
Ca4_P2_11529329	Ca4	11529329	TTGTGTGTGT/TTGTGTGT	TTCAAGATGTCGCTGCAATC	CCAATCTTCCCCTTTTGTCA	59.9	368	INTERGENIC	--	--	--
Ca4_P2_11549653	Ca4	11549653	TAA/TAAAA	AAATTCGTAAAGGTCCACCG	CCAACGGTGTGCATAATGAG	60.0	729	INTERGENIC	--	--	--
Ca4_P2_11551467	Ca4	11551467	TTTTTTTTAATTTTTTTA/TTTTTTTTA	ATCCAAAAGCCCCCAATAAC	TCACTGTTGCATCTCCAAGC	60.0	844	INTERGENIC	--	--	--
Ca4_P2_11567821	Ca4	11567821	CA/CACGAATA	TTTCTTTTGCCCTCATTTCAA	TTCCACCTGATCATCATTTCA	58.9	463	INTERGENIC	--	--	--
Ca4_P2_11635720	Ca4	11635720	CTTCCCCATTCCCCA/CTTCCCCA	TCTGCATTTGTTTGCTGAGG	AAAGCCGCATTTTAGAAGCA	60.0	500	INTERGENIC	--	--	--
Ca4_P2_11648302	Ca4	11648302	G/GAGAACAT	TGTGGATACTGGCAAAACCA	GCTGCAACATTGCTTTACGA	60.0	455	CDS (FRAME SHIFT)	Ca_04392	J	--
Ca4_P2_11850311	Ca4	11850311	AATATATATATATATATATATATATAT/AATATATATATATATATATATAT	AAATGGGAGCTCCATGATGA	TTGGATCTTGTTCACAATGTTCTT	59.9	253	INTERGENIC	--	--	--
Ca4_P2_11866901	Ca4	11866901	TATAATAATAATAATAA/TATAATAATAA	GAATAGACCAGGCCAGACCA	CTATTAGGCCTGACAAGGCG	59.9	493	INTERGENIC	--	--	--
Ca4_P2_11926596	Ca4	11926596	GATATATATATATATATATATATATATAT/GATATATATATATATATATATATATAT	TGCCTTGTCTTCTTCCTGCT	TTCAGCATTTCCCATTCTCC	60.0	526	DRR	Ca_04414	O	Trihelix
Ca4_P2_11931582	Ca4	11931582	TGAAAAACAAGAAGAAAAACAA/TGAAAAACAAGAAGAAAAACAAGAAGAAAAACAA	CAAAGAACCTGTGACGAGCA	TTTAGTTAACGCAGGCACCC	60.1	480	INTRON	Ca_04415	O	Trihelix
Ca4_P2_11973466	Ca4	11973466	GTT/GTTT	AGCATGGGGATGTAGCTCAA	CTTGGAGGACAAAACGGTGT	60.0	338	INTERGENIC	--	--	--

INDEL marker IDs	Chromosomes/unanchored scaffolds	Physical positions (bp)	InDels (*Kabuli* reference genome-CDC Frontier/PII)	Forward primers (5'-3')	Reverse primers (5'-3')	Anneling temperature (0C)	Expected amplified product size (bp)	Structural annotation		Functional annotation	
								Sequence components of *kabuli* genome	*Kabuli* gene accession IDs	NCBI-KOG	TFs
Ca4_P2_11976075	Ca4	11976075	GTTTTTTTTTTTT/GTTTTTTTTTTT	AGCAACCCTTCGAATCTTCA	ATCCAAAATTGGCATCCTTG	59.8	777	INTERGENIC	--	--	--
Ca4_P2_12027795	Ca4	12027795	AAAAAAGAAATAAAAAGAAATAAA/AAAAAAGAAATAAA	GATGTCGTCATGGCTGTTTG	TCATTGCGTTCTTTAGTGCG	60.0	510	INTERGENIC	--	--	--
Ca4_P2_12032031	Ca4	12032031	AGGGG/AGGGGG	CCGCCCGACTTAATAAATGA	AAACGGCAACGGTTGTAAAG	60.0	813	DRR	Ca_04429	--	S1Fa-like
Ca4_P2_1203846	Ca4	1203846	AT/A	TGACCAAGATTTTTCCCCTG	ACATCCCATCCAACCACAGT	60.1	585	INTRON	Ca_07750	I	--
Ca4_P2_12063275	Ca4	12063275	TTTTA/T	TTTTGTGTGCGTTTGACTGA	AACCACCAAAATACCTTTCAAGA	59.0	694	INTERGENIC	--	--	--
Ca4_P2_12135685	Ca4	12135685	ATTTTTTTTT/ATTTTTTTT	TTTCCAAATCTTGGACTCGG	AGTGGTCGGTGAGTGCTCTT	59.9	383	INTERGENIC	--	--	--
Ca4_P2_12140267	Ca4	12140267	TTCTCTCTCTCTCTCTCTCTCTCTCT/TTCTCTCTCTCTCTCTCTCTCTCTCTCTCTCTCT	AAAATATGCCCGATCACTCG	AGAGAAAGGGGGAGCAAAAC	59.7	441	INTERGENIC	--	--	--
Ca4_P2_12197695	Ca4	12197695	AATGAT/AAT	CGGTGGTGGAGGAAGTAAAA	TGCATCTGCTTTACCCATGA	60.2	329	INTERGENIC	--	--	--
Ca4_P2_12298586	Ca4	12298586	TACCA/TACCAACACCA	TGGCAAGTTTGTTTCAATGTG	TTTTTGGGGTGATATTGTCG	58.3	454	INTERGENIC	--	--	--
Ca4_P2_12313260	Ca4	12313260	G/GA	ATATTTTCGATGTGCAGGGC	TGAAGGAGGCTTCTAGGACG	59.6	545	DRR	Ca_04455	J	--
Ca4_P2_12334767	Ca4	12334767	C/CTATT	GTCCTCCAACTGACCCAAAA	AGGTTAGGTCGTCCGTCAAG	59.2	377	INTERGENIC	--	--	--
Ca4_P2_12342974	Ca4	12342974	AAAGGAAGCCCTG/A	TACCCACGACTGGTAAAGGC	AGGGGTGTTCCAGTCAACAG	60.0	374	INTERGENIC	--	--	--

INDEL marker IDs	Chromosomes/unanchored scaffolds	Physical positions (bp)	InDels (*Kabuli* reference genome-CDC Frontier/PII)	Forward primers (5'-3')	Reverse primers (5'-3')	Anneling temperature (0C)	Expected amplified product size (bp)	Structural annotation		Functional annotation	
								Sequence components of *kabuli* genome	*Kabuli* gene accession IDs	NCBI-KOG	TFs
Ca4_P2_1235200	Ca4	1235200	TAAAAAAAAA/TAAAAAAAAAA	AAGGATGCATTTAGCCGATG	CTTTGTTCCACTTGTTGGGC	60.5	493	INTERGENIC	--	--	--
Ca4_P2_12471352	Ca4	12471352	TG/TTCAATACAAG	CCGTACAGCTACCTTTTGCAG	TCAAACCTCCATGCTTGACT	58.3	253	DRR	Ca_04469	Z	bHLH
Ca4_P2_12480535	Ca4	12480535	CAT/CATACAAAAT	CAAGTTTTGCTTTACATGGTCG	AAGTTGTGCTTTTTGTGCCC	60.2	438	INTERGENIC	--	--	--
Ca4_P2_12480728	Ca4	12480728	GTTTT/GTTT	GGGCACAAAAAGCACAACTT	TTCCATTGGTGAATTAAAAACG	58.9	258	INTERGENIC	--	--	--
Ca4_P2_12481811	Ca4	12481811	TAAA/TAAAA	CGAATCAAGGGTGAATCTCAA	AATGCGTGTTTTTAGTGCCC	60.0	781	INTERGENIC	--	--	--
Ca4_P2_12487827	Ca4	12487827	ATTTTTTTTT/ATTTTTTTT	GGGTGGAAGATGTTGCTGAT	AGGTAAGGCGTGAGGTTGTG	60.2	682	DRR	Ca_04471	R	C3H
Ca4_P2_12490666	Ca4	12490666	TGTAGTAGTAGTAGTAGTAGTAGTAGTA/TGTAGTAGTAGTAGTAGTAGTAGTAGTAGTAGTAGTAGTAGTAGTA	GGAATATGGCAGCAGACGAT	GCCTGCATATGGCTAAGAGC	60.0	734	DRR	Ca_04472	R	NAC
Ca4_P2_12493248	Ca4	12493248	ATATTTATTATTTATTAT/ATATTTATTATTTATTATTTATTAT	GTTGAGGGGAACAAGGTTGA	TCTGAAAACGGACGAGGAAT	59.7	462	DRR	Ca_04473	--	--
Ca4_P2_12504806	Ca4	12504806	CAAA/CAAAAA	TGGCACCAAAGATACCATCA	TGCAACCGCAATATTTACCA	60.0	328	INTRON	Ca_04474	--	MIKC
Ca4_P2_12505639	Ca4	12505639	GTCTTAAA/GTCTTAAATATCTTAAA	GCAAGGACAAGGGATGTAGG	TCAACCGCAATTGAGATTTTC	60.1	677	INTERGENIC	--	--	--
Ca4_P2_12506017	Ca4	12506017	ATTTTTTT/ATTTTTTTT	TCAATTGCGGTTGAATTATCA	AAGCTGCTCTCAACACCCAT	59.9	485	INTERGENIC	--	--	--
Ca4_P2_12506512	Ca4	12506512	TAAA/TAA	ATGGGTGTTGAGAGCAGCTT	AGACAGCCCAGAAAGCACAT	59.9	227	INTERGENIC	--	--	--

INDEL marker IDs	Chromosomes/unanchored scaffolds	Physical positions (bp)	InDels (*Kabuli* reference genome-CDC Frontier/PII)	Forward primers (5'-3')	Reverse primers (5'-3')	Anneling temperature (0C)	Expected amplified product size (bp)	Structural annotation		Functional annotation	
								Sequence components of *kabuli* genome	*Kabuli* gene accession IDs	NCBI-KOG	TFs
Ca4_P2_12518595	Ca4	12518595	ATTTTTTTT/ATTTTTTTTT	GGAGCCTTACACATGTGGCT	TCATTTGTGAGATTGTCACGTT	58.1	364	INTERGENIC	--	--	--
Ca4_P2_12519771	Ca4	12519771	A/AT	AGCATAGCCTTGCTTTCCAA	GATCGTCCCAATAATGCACC	60.2	227	INTERGENIC	--	--	--
Ca4_P2_12522756	Ca4	12522756	ATTTTTTT/ATTTTTT	TTGTTGAACGCCAGAACTTG	TAGAGGAGGGGTAGGAGGGA	60.0	317	INTRON	Ca_04475	C	bZIP
Ca4_P2_12523831	Ca4	12523831	TTGCT/TT	GCTGCTGCCACTTGTAATCA	GGTACGGAATGGGACAAAAA	59.7	482	INTRON	Ca_04475	C	bZIP
Ca4_P2_12524149	Ca4	12524149	AC/ACC	GCTGCTGCCACTTGTAATCA	GAAAATTTGGACCCCATCAA	59.6	523	INTRON	Ca_04475	C	bZIP
Ca4_P2_12524698	Ca4	12524698	GAAAAAAAA/GAAAAAAAAA	TGGCATCAGAGATATGGTCCT	TTTGGGTCTCCAAACTCAGG	60.1	284	INTRON	Ca_04475	C	bZIP
Ca4_P2_12528293	Ca4	12528293	AT/ATT	TTCCTTCGACCAACAAAACC	CATGGGACCCTAGCGTTAAA	60.0	573	DRR	Ca_04475	C	bZIP
Ca4_P2_12528630	Ca4	12528630	TAAAAAA/TAAAAA	TAACGCTAGGGTCCCATGTC	AGCTTCTTGGAGCCATGTCA	60.9	211	DRR	Ca_04475	C	bZIP
Ca4_P2_12529769	Ca4	12529769	AATA/AATATA	AACGTGTTCAAACTCGGAGC	GCAAAGCAAATGAAGTAGGCA	60.4	111	INTERGENIC	--	--	--
Ca4_P2_12529895	Ca4	12529895	TAA/TAAA	AACGTGTTCAAACTCGGAGC	ACAACACCTTATTCTGCGCC	60.1	630	INTERGENIC	--	--	--
Ca4_P2_12529954	Ca4	12529954	CTTTT/CTTTTT	AACGTGTTCAAACTCGGAGC	AGCTTAGCTGGGTGGGTTTT	60.1	707	INTERGENIC	--	--	--
Ca4_P2_12529978	Ca4	12529978	T/TC	AACGTGTTCAAACTCGGAGC	AGCTTAGCTGGGTGGGTTTT	60.1	707	INTERGENIC	--	--	--

INDEL marker IDs	Chromosomes/unanchored scaffolds	Physical positions (bp)	InDels (*Kabuli* reference genome-CDC Frontier/PII)	Forward primers (5'-3')	Reverse primers (5'-3')	Anneling temperature (0C)	Expected amplified product size (bp)	Structural annotation		Functional annotation	
								Sequence components of *kabuli* genome	*Kabuli* gene accession IDs	NCBI-KOG	TFs
Ca4_P2_12531515	Ca4	12531515	ATT/ATTT	TTTGCTTCCAATTGGGTGAT	AATTTCAAGGCCATGTGAGG	59.9	712	INTRON	Ca_04476	--	--
Ca4_P2_12532996	Ca4	12532996	C/CA	ATGAATTTGATGAGCAGGGC	GTTCCAGTTGCCCTGTGTTT	60.0	349	DRR	Ca_04477	K	MYB
Ca4_P2_12542560	Ca4	12542560	TAAAAAAA/TAAAAAAAA	TGCCAAATTGTGTTTGTCGT	TATGACGGGAATGCATTTGA	59.9	631	INTERGENIC	--	--	--
Ca4_P2_12551844	Ca4	12551844	TGAAGAAGAAGAAGAAGAA/TGATGAAGAAGAAGAAGAAGAAGAA	CCATTCCTTTGCAAACCTGT	CCAATCAAAGCAGGTTGGTT	60.0	472	INTERGENIC	--	--	--
Ca4_P2_12552603	Ca4	12552603	TTA/TTATA	TGCAGACCCAAAGAGTGACA	TCCAAATTTGCTGTTGTTGC	59.7	708	INTERGENIC	--	--	--
Ca4_P2_12555453	Ca4	12555453	AAT/AATTTAT	TAAAACTGCAGCGCAATCAC	AGATCATCTCCTTCCTGCGA	59.9	335	DRR	Ca_04478	A	EIL
Ca4_P2_12559174	Ca4	12559174	TAG/TAGAG	AGTTCAAGAAACCCCCGAGT	GGCACGTGGAGAAGAGACAT	60.3	496	INTRON	Ca_04478	A	EIL
Ca4_P2_12561730	Ca4	12561730	CTT/CTTT	ATTTTTGGGAGTTCCATCGG	AAATTGTCCGATGTTGAAAGC	59.1	655	INTERGENIC	--	--	--
Ca4_P2_12561884	Ca4	12561884	AATAT/AAT	TCGTCTATGCGAGTCGTAACA	TTCAAACCTAAGCCACTGCAT	59.8	727	INTERGENIC	--	--	--
Ca4_P2_12561951	Ca4	12561951	TAAAA/TAAAAA	TCGTCTATGCGAGTCGTAACA	TTCAAACCTAAGCCACTGCAT	59.8	727	INTERGENIC	--	--	--
Ca4_P2_12561994	Ca4	12561994	ATTTTTT/ATTTTTTT	TCGTCTATGCGAGTCGTAACA	TTCAAACCTAAGCCACTGCAT	59.8	727	INTERGENIC	--	--	--
Ca4_P2_12562229	Ca4	12562229	TAAAAAA/TAAAAA	TCGAAAGTTTTGATGTCTCCC	TTCAAACCTAAGCCACTGCAT	59.8	472	INTERGENIC	--	--	--

INDEL marker IDs	Chromosomes/unanchored scaffolds	Physical positions (bp)	InDels (*Kabuli* reference genome-CDC Frontier/PII)	Forward primers (5'-3')	Reverse primers (5'-3')	Anneling temperature (0C)	Expected amplified product size (bp)	Structural annotation		Functional annotation	
								Sequence components of *kabuli* genome	*Kabuli* gene accession IDs	NCBI-KOG	TFs
Ca4_P2_12562479	Ca4	12562479	CAAAA/CAA	TGCAGTGGCTTAGGTTTGAA	TGTGGTGGGTCAGTCCAGTA	60.0	642	INTERGENIC	--	--	--
Ca4_P2_12563798	Ca4	12563798	GTTTTT/GTTTT	TGTGATATTGGGTCCATTGGT	TCACAAATCGAAATGCGGTA	60.1	672	INTERGENIC	--	--	--
Ca4_P2_12566101	Ca4	12566101	TAAAAAA/TAAAAA	GTTGTGAATCAACTTGGCCC	CGGAGAGAATATGGAAAGACTGA	59.7	558	INTERGENIC	--	--	--
Ca4_P2_12566654	Ca4	12566654	AG/A	TCAGTCTTTCCATATTCTCTCCG	AAAACCCAGGAGGTCAGCTT	60.1	619	INTERGENIC	--	--	--
Ca4_P2_12567928	Ca4	12567928	AATATATATAT/AATATATAT	ATTCGGCAATTCTACCCCTT	GAGGTAGAGCTCGACGATGG	60.0	446	INTERGENIC	--	--	--
Ca4_P2_12570942	Ca4	12570942	T/TC	GGCTCACATCAGGAATGGAT	CACCAGATCCCAATTTGCTT	59.9	654	DRR	Ca_04479	E	--
Ca4_P2_12571013	Ca4	12571013	GTATATA/GTATA	GGCTCACATCAGGAATGGAT	GGCTCTTGGTCATTGGATGT	59.9	968	DRR	Ca_04479	E	--
Ca4_P2_12572622	Ca4	12572622	TAAAAAAAAA/TAAAAAAAAAA	TGGAAGAGCATGCAAAACAA	TGAGATCTTGAGGCATGGTG	59.8	644	INTERGENIC	--	--	--
Ca4_P2_12573367	Ca4	12573367	AAT/A	GGGACTACTTTCGCGATTCA	TGTTAATTGAAACATGCGACA	57.7	402	INTERGENIC	--	--	--
Ca4_P2_12573605	Ca4	12573605	CAATTAAAT/CAATTAAATAATTAAAT	GGAGGCAGAGAATATGATACGG	AGATTTGGCGCGCTTTACTA	60.0	884	INTERGENIC	--	--	--
Ca4_P2_12577207	Ca4	12577207	A/AC	TGGAAAAGCATGGGTGTGTA	GAAGTGCATTGGCTGACAGA	60.0	577	INTRON	Ca_04480	L	--
Ca4_P2_12577562	Ca4	12577562	T/TC	TCTGTCAGCCAATGCACTTC	CAGGAATGCCAAAACCAAAT	59.8	409	INTRON	Ca_04480	L	--

INDEL marker IDs	Chromosomes/unanchored scaffolds	Physical positions (bp)	InDels (*Kabuli* reference genome-CDC Frontier/PII)	Forward primers (5'-3')	Reverse primers (5'-3')	Anneling temperature (0C)	Expected amplified product size (bp)	Structural annotation		Functional annotation	
								Sequence components of *kabuli* genome	*Kabuli* gene accession IDs	NCBI-KOG	TFs
Ca4_P2_12577604	Ca4	12577604	CTTTTTTTTTT/CTTTTTTTT	TCTGTCAGCCAATGCACTTC	CTGTCACCAGCCAACAAAGA	59.9	561	INTRON	Ca_04480	L	--
Ca4_P2_12578159	Ca4	12578159	CTTTTTTT/CTTTTTTTT	TCTTTGTTGGCTGGTGACAG	ACCCAATGCCTAAGAAGCCT	60.1	289	INTRON	Ca_04480	L	--
Ca4_P2_12578358	Ca4	12578358	TAT/TATACCAGCAT	AGGCTTCTTAGGCATTGGGT	ATTCTTGTAATCCCGCGTTG	60.0	346	INTRON	Ca_04480	L	--
Ca4_P2_12580791	Ca4	12580791	T/TTA	AAATTGCGCTCTGATTTGCT	CTTAACCCAATTTGGAGGCA	59.9	946	INTRON	Ca_04480	L	--
Ca4_P2_12581069	Ca4	12581069	TAA/TA	TTTTAGTTGCCGCCTTTTTG	CTTCAGGAGGGTGATACCGA	60.1	468	INTRON	Ca_04480	L	--
Ca4_P2_12585143	Ca4	12585143	A/ATCTACAT	TGACGGGACAATTCACAAAA	AAAAAGACCATGTCAAGCCG	60.1	144	DRR	Ca_04480	L	--
Ca4_P2_12586799	Ca4	12586799	AC/A	AACCTAATAAGCCCGGAAGC	ATGACCCGTACCTTGTTTGC	59.9	833	INTERGENIC	--	--	--
Ca4_P2_12588156	Ca4	12588156	TAAA/TAA	ACAATTCCGAAGATACCCCC	GATGTGGTCGTTGTCGAGTG	60.2	506	INTERGENIC	--	--	--
Ca4_P2_12588357	Ca4	12588357	TAAAAAAAAAA/TAAAAAAAAAAAAA	ACAATTCCGAAGATACCCCC	GATGTGGTCGTTGTCGAGTG	60.2	506	INTERGENIC	--	--	--
Ca4_P2_12589173	Ca4	12589173	ATTTTTTTTTT/ATTTTTTTTT	CTTTCGAGATCTGATGCGTG	ACAACGTCTTCCATTACGCC	60.0	753	INTERGENIC	--	--	--
Ca4_P2_12589362	Ca4	12589362	CTTTTTTTTT/CTTTTTT	CAATGGTTCTTTTGGGGTTC	ACAACGTCTTCCATTACGCC	60.0	615	INTERGENIC	--	--	--
Ca4_P2_12589473	Ca4	12589473	G/GA	TGTGGCATTTTGGTAATGCT	ACAACGTCTTCCATTACGCC	60.0	200	INTERGENIC	--	--	--

INDEL marker IDs	Chromosomes/unanchored scaffolds	Physical positions (bp)	InDels (*Kabuli* reference genome-CDC Frontier/PII)	Forward primers (5'-3')	Reverse primers (5'-3')	Anneling temperature (0C)	Expected amplified product size (bp)	Structural annotation		Functional annotation	
								Sequence components of *kabuli* genome	*Kabuli* gene accession IDs	NCBI-KOG	TFs
Ca4_P2_12592291	Ca4	12592291	ATA/ATACAAATACTTGATGTA	TGACTGCCGCTTTGTCATTA	TTTCGATTCAAACCCAAACC	59.8	588	DRR	Ca_04481	R	--
Ca4_P2_12592831	Ca4	12592831	AATATATATATATAT/AATATATATAT	TGCAAAATGTGCGATAGAGG	AGCCCATTATGATGGACGAT	59.2	732	DRR	Ca_04481	R	--
Ca4_P2_12593132	Ca4	12593132	AT/ATGTTGT	TCACTGTAAGTCCATGCCTCC	GCCCTTGTCAAAACAAGTTGA	60.1	454	INTERGENIC	--	--	--
Ca4_P2_12593182	Ca4	12593182	TAAAA/TAAA	TCACTGTAAGTCCATGCCTCC	GCCCTTGTCAAAACAAGTTGA	60.1	454	INTERGENIC	--	--	--
Ca4_P2_12594768	Ca4	12594768	T/TCTG	AGGAAGACCTGCCTCCTCTC	GCACAACCAAACAGGTCAAA	59.6	570	INTRON	Ca_04482	R	--
Ca4_P2_12597000	Ca4	12597000	AT/ATCTAATGGT	CACATGGTTCCAGGTTCCTT	CTTATGGCGTGGCCTTAAAC	59.6	820	DRR	Ca_04482	R	--
Ca4_P2_12598611	Ca4	12598611	CAA/CA	GGACCAAACCCTCTGCATAA	TACGATGCAGTGAAACTCCG	59.9	466	INTERGENIC	--	--	--
Ca4_P2_12599330	Ca4	12599330	TAAAAAAA/TAAAAAA	CGGAGTTTCACTGCATCGTA	TTTGAGCGGTTGTTATGTCG	59.7	374	INTERGENIC	--	--	--
Ca4_P2_12600801	Ca4	12600801	TAAAAAA/TAAAAA	ACAGCGTTGTGCTTACGATG	GGGGACAACGAACCTAGTCA	60.0	870	INTERGENIC	--	--	--
Ca4_P2_12601482	Ca4	12601482	CAAA/CAA	TTAATTCGCTTGGGATCAGG	AGCGCTACATCTCATTGGTG	58.9	185	INTERGENIC	--	--	--
Ca4_P2_12604165	Ca4	12604165	CTTTTTTTTTTTTT/CTTTTTTTTTTT	CGATTTCACTTCCCCAACTT	AGCAGCAGAAAGCATGGACT	60.2	209	INTERGENIC	--	--	--
Ca4_P2_12637168	Ca4	12637168	TAAAAAAAAA/TAAAAAAAAAA	GTGGCTGCATTGATATGGTG	CAAAATCACTTCAGGTGCCA	59.7	697	DRR	Ca_04486	--	--

INDEL marker IDs	Chromosomes/unanchored scaffolds	Physical positions (bp)	InDels (*Kabuli* reference genome-CDC Frontier/PII)	Forward primers (5'-3')	Reverse primers (5'-3')	Anneling temperature (0C)	Expected amplified product size (bp)	Structural annotation		Functional annotation	
								Sequence components of *kabuli* genome	*Kabuli* gene accession IDs	NCBI-KOG	TFs
Ca4_P2_12639362	Ca4	12639362	TTTATTATTATTATTATTATTATTATTATTATTATTATTATTA/TTTATTATTATTATTATTATTATTATTATTATTA	TGCTTAAATGGCCCAGACTT	CGAAATTGATGTCGGTTACA	57.1	336	INTERGENIC	--	--	--
Ca4_P2_12644877	Ca4	12644877	CATATATATATATATATAT/CATATATATATATATATATATAT	TCACTTGTTAGTATGACCACAACA	TTTGAGGCCATTAGTTTGGG	59.9	877	INTERGENIC	--	--	--
Ca4_P2_12665662	Ca4	12665662	AATATATATATAT/AATATATATATATAT	TTACCCCAATCCCCTACACA	GCTTTTGAATGAAAGGCAAA	58.0	142	INTERGENIC	--	--	--
Ca4_P2_12676299	Ca4	12676299	CATATGTGATATGTGATA/CATATGTGATATGTGATATGTGATA	GATCTCGGGTTAGATGGTCG	TCCACTCTCGCTCTCAATCC	60.5	740	INTERGENIC	--	--	--
Ca4_P2_12700504	Ca4	12700504	CAAAAAAAAAAAA/CAAAAAAAAAAA	ACATGCACGACTCATACCCA	TTTTTCGTGTTTCGGTTGTAA	58.3	570	INTERGENIC	--	--	--
Ca4_P2_12702239	Ca4	12702239	ATTTTTTTTTTTTT/ATTTTTTTTTTTT	TTTTGGACTCATATGTTACCAATTT	TTTTCGACTCAAAACACCCC	59.9	483	INTERGENIC	--	--	--
Ca4_P2_12715012	Ca4	12715012	TA/TATAGAAAGA	AGAAGCGATGCAGACAACTTT	AGGGGCGAATATCGCTAACT	60.1	373	INTERGENIC	--	--	--
Ca4_P2_12724267	Ca4	12724267	GAAAAAAAAAAA/GAAAAAAAAAAAAA	CCAAGTGCAATGACGAGCTA	CAAGGATATGGATCCAATGTGA	59.6	610	INTERGENIC	--	--	--
Ca4_P2_12738952	Ca4	12738952	GATATATATATATATATATATATA/GATATATATATATATATATATATATATA	CCTCGCAGCTGTAAAATGGT	TCTGGGTGAGACGACAATCA	60.2	631	INTRON	Ca_04493	--	FAR1
Ca4_P2_12784480	Ca4	12784480	TATAATAATAATAA/TATAATAATAATAATAA	TAAAGCCATCATCAATGCCA	GACAAACCATGCACAAGTGG	60.0	524	INTERGENIC	--	--	--
Ca4_P2_12788740	Ca4	12788740	TTATATATATATATATATATATA/TTATATATATATATATATATATATA	TCACATGCGTCCAATTTTTG	AAAACGTGTGGCCAGCTAAC	60.2	845	INTERGENIC	--	--	--
Ca4_P2_12852660	Ca4	12852660	AATATATATAT/AATATATATATAT	CATTCTCTCTATGCCACGCA	AACTTGTGTCCTTCAAGGGG	59.0	593	INTERGENIC	--	--	--

INDEL marker IDs	Chromosomes/unanchored scaffolds	Physical positions (bp)	InDels (*Kabuli* reference genome-CDC Frontier/PII)	Forward primers (5'-3')	Reverse primers (5'-3')	Anneling temperature (0C)	Expected amplified product size (bp)	Structural annotation		Functional annotation	
								Sequence components of *kabuli* genome	*Kabuli* gene accession IDs	NCBI-KOG	TFs
Ca4_P2_12866217	Ca4	12866217	GGG/GGGAGCTTTATTTTTTGG	TAGACTCCACTAAGCCCCCA	CCTTCCAACGCAACATTTCT	60.1	294	DRR	Ca_04506	T	FAR1
Ca4_P2_12885868	Ca4	12885868	ATTTTTTTTTT/ATTTTTTTTT	AGGTCATTGGTGTGCAAGGT	TGAAGCTGACTGCACTTTGG	60.2	666	DRR	Ca_04508	R	--
Ca4_P2_12892488	Ca4	12892488	CTTTTTTTTTTTTTTTTT/CTTTTTTTTTTTTTT	TACCGACCATTCTGTCCTCC	TGGCATGAAGTGTCACCAAT	60.0	296	INTERGENIC	--	--	--
Ca4_P2_12904478	Ca4	12904478	ATTTTTTTT/ATTTTTTTTT	GCTTCCGCCCCTAAAAATAC	ACCGTATTGTTTCTCCGTCG	60.0	519	INTERGENIC	--	--	--
Ca4_P2_1297403	Ca4	1297403	TAAAAAAAAAA/TAAAAAAAAA	TGTTATAGCAAATTGCCACTGTC	CTCTCAAATAATGATGTGACAAAAA	57.5	542	INTERGENIC	--	--	--
Ca4_P2_12999105	Ca4	12999105	TTATATATATATATATATATATATAT/TTATATATATATATATATATATATATATAT	TTTTCATGATTGTTGCGGTT	AGATACGAAAGCCAAATGCG	60.2	971	INTERGENIC	--	--	--
Ca4_P2_13028368	Ca4	13028368	CTTGTTG/CTTGTTGTTG	TGCACGCTCCACATTATTTC	CTTTTCTGGGGCAAATTGAA	60.0	367	INTRON	Ca_04525	T	bHLH
Ca4_P2_13068436	Ca4	13068436	AATATATATATATATATATATATATAT/AATATATATATATATATATATATATATATATATATATAT	GCTCAATTTTTCTTAGGGCG	TGTCGCAACAATTTGTCTCTT	58.4	432	INTERGENIC	--	--	--
Ca4_P2_13068683	Ca4	13068683	CTATATATATATATATATATATATATATATATATAT/CTATATATATATATATATATATATATATATATATAT	TGCGACATTTTGTAGAGTTTCAA	TGAAAAACATAATAGGTCCCCTT	58.0	292	DRR	Ca_04530	--	G2-like
Ca4_P2_1309061	Ca4	1309061	GAAAAAA/GAAAAAAA	AGCTACAAAATGGGCAATGG	CCACGGTACCGAAAAATGAT	59.7	237	INTERGENIC	--	--	--
Ca4_P2_13096912	Ca4	13096912	TTATATATATATATATAT/TTATATATATATATAT	TTACCTTAAACACCCCGCAC	TTCCGCACTAACGACTTCCT	59.9	316	INTERGENIC	--	--	--
Ca4_P2_1310245	Ca4	1310245	ATTTTTTTTTTT/ATTTTTTTTTT	TGAAATTGCTTGATTTTGCTTT	CGACCGATTTTATCCGTCTC	59.5	825	INTERGENIC	--	--	--

INDEL marker IDs	Chromosomes/unanchored scaffolds	Physical positions (bp)	InDels (*Kabuli* reference genome-CDC Frontier/PII)	Forward primers (5'-3')	Reverse primers (5'-3')	Anneling temperature (0C)	Expected amplified product size (bp)	Structural annotation		Functional annotation	
								Sequence components of *kabuli* genome	*Kabuli* gene accession IDs	NCBI-KOG	TFs
Ca4_P2_13133292	Ca4	13133292	ATTTT/ATTTTTT	CAGCCGCCTCCAACTATTAC	CCATCCATGCAATTGAACAC	59.8	408	INTERGENIC	--	--	--
Ca4_P2_13162283	Ca4	13162283	T/TAATAA	TCGTGCGAGTATTCAAAACG	ATGGCAATGCATCAAATCAA	59.9	524	INTERGENIC	--	--	--
Ca4_P2_13325790	Ca4	13325790	ATTTTTT/ATTT	TTGATCATCCTATTTCAAAAGTTCA	ATTTTCGACTCCACACACGG	61.0	640	INTERGENIC	--	--	--
Ca4_P2_13386456	Ca4	13386456	AATTATTATTATTATTATTATTATTATTATTATTATTATTATTATTATT/AATTATTATTATTATTATTATTATTATTATTATTATTATTATTATTATT	CGGTAGGTGCTAAAATTGCC	GCGTCCTTTTCCAACACATT	60.0	346	INTERGENIC	--	--	--
Ca4_P2_13387249	Ca4	13387249	AATATATATATATATATATATATATATATATATATA/AATATATATATATATATATATATATATATATA	ATCAGCTCATGCTTTGATCG	CCTTTTGGTCTCTCAACCGA	60.2	615	INTERGENIC	--	--	--
Ca4_P2_13433311	Ca4	13433311	TA/T	CGAGCAAACACAATGAATGG	TTGGTTGGTTCATTGGTTGA	59.8	569	INTERGENIC	--	--	--
Ca4_P2_13467814	Ca4	13467814	GATATATATATATATATATATATATATAT/GATATATATATATATATATATATATATATATATATAT	AAGTTGATTGTCCACACAGGC	ACGGTGGATCCCTTCTCAAT	60.7	703	INTERGENIC	--	--	--
Ca4_P2_13509527	Ca4	13509527	ATTTT/ATTTTATTAAAAAATTTATTTTT	CCTCTTCACGGTGCTCATTT	CCTCGTTTTAAGCCCAATCA	60.1	158	INTERGENIC	--	--	--
Ca4_P2_13601854	Ca4	13601854	T/TC	GAAAACTGCCACTTACCGGA	TTCCGACAATAATTTAAACGGT	57.7	625	INTERGENIC	--	--	--
Ca4_P2_13604124	Ca4	13604124	GA/G	TTCAGACCCCAGTTTCATCA	GACATGTATCGTGTGCGGTC	60.0	631	INTERGENIC	--	--	--
Ca4_P2_13608641	Ca4	13608641	ATT/ATTT	GGTGACGTGGCTTACTCTCC	CCCTTTCTGCATGGACAAGT	60.1	413	INTERGENIC	--	--	--
Ca4_P2_13617448	Ca4	13617448	TG/TGG	GGGCCTTTTCTTCAGCTTTT	TGCATGTTATCCGCAATCTC	59.7	713	DRR	Ca_04579	--	--

INDEL marker IDs	Chromosomes/unanchored scaffolds	Physical positions (bp)	InDels (*Kabuli* reference genome-CDC Frontier/PII)	Forward primers (5'-3')	Reverse primers (5'-3')	Anneling temperature (0C)	Expected amplified product size (bp)	Structural annotation		Functional annotation	
								Sequence components of *kabuli* genome	*Kabuli* gene accession IDs	NCBI-KOG	TFs
Ca4_P2_13620272	Ca4	13620272	A/ATAT	TCAACATGTGGTTGAAATTGG	ATTGGTGGGAGCAAGTTAGC	59.2	930	INTERGENIC	--	--	--
Ca4_P2_13643715	Ca4	13643715	GTT/GTTT	ATAGTTGTTGAAGGGGCACG	GGGTGAAGAATGTATGCCAAA	59.8	798	INTERGENIC	--	--	--
Ca4_P2_13643938	Ca4	13643938	AA/AATATA	GCTTCCGACAAAGGTCAGAG	GGGTGAAGAATGTATGCCAAA	59.8	641	INTERGENIC	--	--	--
Ca4_P2_13650120	Ca4	13650120	GAT/GATCAATATAT	GAACTGGTATCGGGCTTGAA	CGTGCATTGAGCAACTGTCT	60.1	635	INTERGENIC	--	--	--
Ca4_P2_13655349	Ca4	13655349	AG/A	AATTCACAAGGTCCAGTGGC	GGGCATGATTTTTCCCCTAT	60.0	790	INTERGENIC	--	--	--
Ca4_P2_13657824	Ca4	13657824	ATATT/AT	TGCATGCATGAATAGTGTTTGA	TCTCCCAATGTGATTGCTCA	60.2	395	INTERGENIC	--	--	--
Ca4_P2_13666161	Ca4	13666161	ACTG/ACTGCTG	CCCATTTCACCTCTTTGGAA	TTCCCGCAACAAATAGATCC	59.9	840	CDS (large-effect mutations)	Ca_04586	Q	B3
Ca4_P2_13691259	Ca4	13691259	GTT/GT	CCATTTATTTCCGGCATTTC	AGCCGACCAAACACTATTGG	60.0	271	INTERGENIC	--	--	--
Ca4_P2_13692964	Ca4	13692964	TGTATCAGTAT/TGTAT	TGTATCAAGTTCGTCCACCG	TTGATTTACGCCTGTTTCCC	59.9	247	DRR	Ca_04590	--	--
Ca4_P2_13700589	Ca4	13700589	GATATATATATATA/GATATATATATATATA	CCAAATCCCAAATCAGCATC	GCCCATACGCCTCTTAACAA	60.1	825	INTRON	Ca_04591	--	--
Ca4_P2_1371815	Ca4	1371815	TATAATAATAATAATAATAA/TATAATAATAATAATAATAATAA	ATGAAGTTCCCCATGAGTGC	AGGCAAAAGGGAAAGACCAC	60.5	463	INTERGENIC	--	--	--
Ca4_P2_13730284	Ca4	13730284	TTATATATATATATATATATATAT/TTATATATATATATATATATATATATAT	AACACATAACCACTTCGCCC	TGCAGCGAAATTATTCGAGA	59.5	458	INTERGENIC	--	--	--

INDEL marker IDs	Chromosomes/unanchored scaffolds	Physical positions (bp)	InDels (*Kabuli* reference genome-CDC Frontier/PII)	Forward primers (5'-3')	Reverse primers (5'-3')	Anneling temperature (0C)	Expected amplified product size (bp)	Structural annotation		Functional annotation	
								Sequence components of *kabuli* genome	*Kabuli* gene accession IDs	NCBI-KOG	TFs
Ca4_P2_13739989	Ca4	13739989	TA/T	TTTCTCCGTCTTAGTGCGGT	AAATGATCAAATCGAAAAGAAAAA	58.3	432	INTERGENIC	--	--	--
Ca4_P2_13743876	Ca4	13743876	GAA/GAAGCATATGCAA	CTGGTTCATGCCCCTACAAT	TCAAAGCACTAGCCACATGC	60.0	740	DRR	Ca_04598	R	MYB_related
Ca4_P2_13757687	Ca4	13757687	ACTTGC/AC	TAACCTGTTGCCATGGTTCA	AAGATGGGGAGAGGGAAAGA	60.0	649	INTRON	Ca_04600	--	NF-YB
Ca4_P2_13757858	Ca4	13757858	ATTTTT/ATTTT	GCTCTCCATTTCATCCAAGC	AGATGGGGAGAGGGAAAGAA	60.0	327	INTRON	Ca_04600	--	NF-YB
Ca4_P2_13776936	Ca4	13776936	GTATATATATATA/GTATATATATA	TGTGTCTGCCCATGAGTGTT	ATATGCGGTTTCATCCTTGC	59.9	301	INTERGENIC	--	--	--
Ca4_P2_13784894	Ca4	13784894	T/TATA	ACCCAACAAGACTGAGGCAC	CCACTCCAAAAACTCATGTTAGC	60.0	440	INTERGENIC	--	--	--
Ca4_P2_13800813	Ca4	13800813	CGGTTG/CG	CCGCACGTGGAATTAAAAAT	TTCTGCTTCGATGTATCCCC	60.0	645	DRR	Ca_04606	--	NF-YA
Ca4_P2_13808256	Ca4	13808256	T/TATATTC	TCGAGTTGAAACCCGAGAAA	CGGAGAAGTGAACGTGGAAT	60.1	525	INTERGENIC	--	--	--
Ca4_P2_13810887	Ca4	13810887	TTGA/TTGAATGA	GATTAGGTGTTGCCTGACGC	GCTGAGATGAAGGCAAAAATG	59.8	281	INTERGENIC	--	--	--
Ca4_P2_13815340	Ca4	13815340	TTTTTTTATTTTTT/TTTTTTT	CAACCCTTTGTGTTGTGGTG	TGTCAAAGGGAAGTGCAAGA	59.4	265	INTERGENIC	--	--	--
Ca4_P2_13826688	Ca4	13826688	ATT/AT	TGGAACTATGTGAAGCGGAA	GTTGAGGAAAACGCAGGAAG	59.9	898	INTERGENIC	--	--	--
Ca4_P2_13864543	Ca4	13864543	CTATATAT/CTATATATATATAT	AATGCAGCTGCTCAAAACCT	CCGGTCACTGATATATCCACAA	59.7	283	INTRON	Ca_04610	R	--

INDEL marker IDs	Chromosomes/unanchored scaffolds	Physical positions (bp)	InDels (*Kabuli* reference genome-CDC Frontier/PII)	Forward primers (5'-3')	Reverse primers (5'-3')	Anneling temperature (0C)	Expected amplified product size (bp)	Structural annotation		Functional annotation	
								Sequence components of *kabuli* genome	*Kabuli* gene accession IDs	NCBI-KOG	TFs
Ca4_P2_13896280	Ca4	13896280	CGAAGAAGAAGAAGAA/CGAAGAAGAAGAA	TACACTGGCCCATCATCTTG	GCCTCGTTGGAATGATTTGT	59.9	482	INTERGENIC	--	--	--
Ca4_P2_13908536	Ca4	13908536	TC/T	CTCCTCCGATGTCTGGAAAA	CGCGGTTGCTTCAATTCTAT	60.2	232	INTERGENIC	--	--	--
Ca4_P2_13909254	Ca4	13909254	CA/CAA	CATCACGATTTGACGAGTTGA	AAAAACATGACTCCAAACACCA	59.4	636	INTERGENIC	--	--	--
Ca4_P2_14011652	Ca4	14011652	TAA/TAACAA	GCACCGACTTGCTGATGTAA	GCCTCATGCTCCAATTTTGT	60.1	755	INTERGENIC	--	--	--
Ca4_P2_1405750	Ca4	1405750	CATATATATATATATATATA/CATATATATATATATATA	GGCCACGTGGAAGTAACTGT	ATTTGGTGCCAAAGGAATTG	59.8	432	INTERGENIC	--	--	--
Ca4_P2_14196867	Ca4	14196867	CTATATATATATATATAT/CTATATATATATATAT	CACTCGTGTTTCCCAAGGTT	TTGCAAGTGCATCCAAATTC	59.7	584	INTERGENIC	--	--	--
Ca4_P2_14223211	Ca4	14223211	T/TATATATATA	AAGTCCCAAGTTCTCATTTGGA	TAGCCATGGTTGGTCTGTCA	60.1	763	DRR	Ca_04655	--	B3
Ca4_P2_14273982	Ca4	14273982	TAAAAAAAAAA/TAAAAAAAAAAA	TGCATTTTACAGCGCTTTTG	TCATGCACTACGCGAAAAAC	59.9	227	DRR	Ca_04664	--	--
Ca4_P2_14278885	Ca4	14278885	AATATATATATATATATATATATAT/AATATATATATATATATATATATATAT	TGTCTCAAATGGTCTGACTTGAA	TGATCAACGTTGTTGCCTTT	59.2	706	INTERGENIC	--	--	--
Ca4_P2_14299282	Ca4	14299282	GTTTTTTTT/GTTTTTTTTT	GAGCTTTCTTTTTGGGGGAC	CGAGATCTTGAGGCATTGTG	59.4	416	INTERGENIC	--	--	--
Ca4_P2_14322937	Ca4	14322937	TNNNNNNNNNN/TNNNNNNNNN	TGAATGGCAATTTGGTTTTT	GCGTGCATATGCGAGTAGAA	60.0	685	INTERGENIC	--	--	--
Ca4_P2_14361079	Ca4	14361079	ATTTTTTTTTT/ATTTTTTTTT	TGAAGATGGTGTGTGGTCGT	TACAAACCCACTGCAAGCAA	60.3	628	INTERGENIC	--	--	--

INDEL marker IDs	Chromosomes/unanchored scaffolds	Physical positions (bp)	InDels (*Kabuli* reference genome-CDC Frontier/PII)	Forward primers (5'-3')	Reverse primers (5'-3')	Anneling temperature (0C)	Expected amplified product size (bp)	Structural annotation: Sequence components of *kabuli* genome	Structural annotation: *Kabuli* gene accession IDs	Functional annotation: NCBI-KOG	Functional annotation: TFs
Ca4_P2_14397300	Ca4	14397300	GATATATATATATATATATATGTATATATATATATA/GATATATATATATATATATATATATGTATATATATATATA	TAAATGCATGCTCCCCTGAT	AACACATCCGGGATCCATTA	60.0	273	INTERGENIC	--	--	--
Ca4_P2_14405624	Ca4	14405624	ATGTTGTT/ATGTTGTTGTT	CGGTGGTTGCTCTCTCTTTC	GGGGTACAATGTGGAACCAA	60.5	440	DRR	Ca_05663	E	MYB
Ca4_P2_14405755	Ca4	14405755	ATCTCTCTCT/ATCTCTCT	ATGTGCATGCCAAGTTGTGT	ACCTATAAGCCCTTTGCGCT	60.2	963	DRR	Ca_05663	E	MYB
Ca4_P2_14406037	Ca4	14406037	GTCTTT/GT	TTGGTTCCACATTGTACCCC	TTGTTGCTGGAGTTGGATCA	60.2	639	DRR	Ca_05663	E	MYB
Ca4_P2_14406133	Ca4	14406133	CAAA/CAAAA	TTGGTTCCACATTGTACCCC	CCATTGGTTGGGATATGGAG	60.0	892	DRR	Ca_05663	E	MYB
Ca4_P2_14416755	Ca4	14416755	TTATATATATATA/TTATATATATATATATA	TCATTTCAAACCCTTAAACTCCA	TTGCGGTCAATTGTATGAAGAC	60.0	392	INTERGENIC	--	--	--
Ca4_P2_14543102	Ca4	14543102	CTATTATTATTATTATTATTATTATTATTATTATTATTATTATTATTATTATTATTATTATTATTATTATT/CTATTATTATTATTATTATTATTATTATTATTATTATTATTATTATTATTATTATTATTATTATTATTATTATTATT	AGCATTGCCAAGCCTTAAAA	AAAAAGCAAGTGTGAGTCAAAAA	58.2	338	INTERGENIC	--	--	--
Ca4_P2_14569481	Ca4	14569481	TTT/TTTCATATTATTATT	CAAAACTCCAAAAGTAAGAAGGAA	TTTCAGCAATTTGACCGTGA	60.2	570	INTERGENIC	--	--	--
Ca4_P2_14677950	Ca4	14677950	CAAAAAAA/CAAAAAAAA	TGTTGGGCAAAATAAATTGAGA	CAAACCTTACCACCGTCTTG	58.1	424	INTERGENIC	--	--	--
Ca4_P2_14812398	Ca4	14812398	CNNNNNNNNNNNNNNN/CNNN	ATGCAGTAGCTGCCCTTGTT	CTTCTTCTTCCCACGTTTCG	59.8	761	CDS (large-effect mutations)	Ca_05621	R	HB-PHD
Ca4_P2_1491046	Ca4	1491046	AATATATATATATATATATAT/AATATATATATATATATATATATATATATATATATAT	GGCTGAATTGATTAAATTTGTCG	GAGTTGCAAGGGGACCAATA	59.9	584	DRR	Ca_07779	--	WRKY
Ca4_P2_14967549	Ca4	14967549	GTAATAATAATAATAATAATAATAATAATAA/GTAATAATAATAATAATAATAATAATAATAATAA	CAAAGTGCTTTTGTACCCCC	CCGTTTGTTTTGGGACATCT	59.8	899	INTERGENIC	--	--	--

INDEL marker IDs	Chromosomes/unanchored scaffolds	Physical positions (bp)	InDels (*Kabuli* reference genome-CDC Frontier/PII)	Forward primers (5'-3')	Reverse primers (5'-3')	Anneling temperature (0C)	Expected amplified product size (bp)	Structural annotation		Functional annotation	
								Sequence components of *kabuli* genome	*Kabuli* gene accession IDs	NCBI-KOG	TFs
Ca4_P2_15012755	Ca4	15012755	AATATATATATATATATA/AATATATATATATATATATA	TACTCCCAAATGGCGACTTC	GACATTTCGAACTTGAATTTCCT	58.7	322	INTERGENIC	--	--	--
Ca4_P2_15016370	Ca4	15016370	CTAGT/CT	CTACAAATCGCCTTTCCCAA	CATCATTCCATAACAATGCCA	59.2	437	INTERGENIC	--	--	--
Ca4_P2_15045995	Ca4	15045995	CTATATATATATATATATATATATATATATATATA/CTATATATATATATATATATATATATATATATATATATATA	GAAAATTATATTCTTTCCCAAATGC	GGAGGAGAGAGACGAAGGCT	60.1	402	INTERGENIC	--	--	--
Ca4_P2_15048181	Ca4	15048181	TA/TAA	CAAAAGCTGTAACACCCCGT	AATCGATTGATTCAGCCCAG	60.0	528	INTERGENIC	--	--	--
Ca4_P2_15081049	Ca4	15081049	ATTT/ATTTT	TTTGCAACTCCATTCTTCCC	GATTCCCCATTCTTAACGCC	60.6	688	INTERGENIC	--	--	--
Ca4_P2_15095206	Ca4	15095206	TTATATATATATATATATATATAT/TTATATATATATATATATATATATAT	TGACCGCTAAGTTGTTGTGC	TTTTACCACTGCACACGCAT	60.2	359	DRR	Ca_05591	D	LBD
Ca4_P2_15111425	Ca4	15111425	A/ATAAT	TTGCACTTTTGAGAAGTCACG	TCAAGAGGTAGCATTCCGAGA	60.0	949	INTERGENIC	--	--	--
Ca4_P2_15216075	Ca4	15216075	TAAAAAAAAAA/TAAAAAAAAAAA	TGAACCAAAAATCATCGCAA	AGGCTCTTGTTAAGCGACCA	60.0	402	INTERGENIC	--	--	--
Ca4_P2_15221737	Ca4	15221737	ATTTTTT/ATTTTTTT	AGGGTGCGATCAGGTAAATG	AACAAGTGGTGCGATTGTGA	60.2	430	INTERGENIC	--	--	--
Ca4_P2_15234278	Ca4	15234278	AA/AATTAATAATAATAATA	AGGTTGTTCACACCAACCAA	TTGGGGTTATGTGTCGTCG	60.4	439	INTERGENIC	--	--	--
Ca4_P2_15251363	Ca4	15251363	AAAATAAATAAATAAATAAATAAATAAATAA/AAAATAAATAAATAAATAAATAAATAAATAAATAA	TCCTGAAACAAACGCATTCA	AAATAGGTTAGGCCGTCCGT	59.9	685	INTERGENIC	--	--	--
Ca4_P2_15252487	Ca4	15252487	GTA/GTACATATATA	TTTTTGAGTGAAACCAGTTTGG	CCAACCATGTTGGTGTGCTA	60.4	533	INTERGENIC	--	--	--

INDEL marker IDs	Chromosomes/unanchored scaffolds	Physical positions (bp)	InDels (*Kabuli* reference genome-CDC Frontier/PII)	Forward primers (5'-3')	Reverse primers (5'-3')	Anneling temperature (0C)	Expected amplified product size (bp)	Structural annotation		Functional annotation	
								Sequence components of *kabuli* genome	*Kabuli* gene accession IDs	NCBI-KOG	TFs
Ca4_P2_15272177	Ca4	15272177	AG/A	GTTTCGGCTAATGGGTTGAA	TTGCTTGTAATTTTGGGTTTTG	59.1	878	DRR	Ca_05572	A	--
Ca4_P2_15291109	Ca4	15291109	TAAAAAAAAAAAAAAAA/TAAAAAAAAAAAAAA	TGCACTCCATCAAAAAGCAG	AAACAGCCACAAAAAGGTGG	60.0	557	INTERGENIC	--	--	--
Ca4_P2_15333395	Ca4	15333395	GACACACA/GACACA	ACGATAGATGAGCAAGGCGT	AATGAAAAATGGGCTCAACG	59.9	463	INTRON	Ca_05564	--	MYB_related
Ca4_P2_15347067	Ca4	15347067	TA/TAA	GAACATGGCCAAGGAGGTAA	TGTGGCGTACTTTGGTGTGT	60.1	792	DRR	Ca_05564	--	MYB_related
Ca4_P2_15375076	Ca4	15375076	TAAAAAAA/TAAAAAAAA	CTGAAATCGCCTTTCTCGTC	TTTTGCAAGCATGGATGATG	60.6	192	INTRON	Ca_05559	C	--
Ca4_P2_1541443	Ca4	1541443	CATTGTTTTTGGGTAGAGA/CATTGTTTTTGGGTAGAGAATTGTTTTTGGGTAGAGA	TCATGTTCCGCTATGAAGTTTG	AAGCTATTGTGCAAATCGGG	60.1	274	DRR	Ca_07783	S	--
Ca4_P2_15465052	Ca4	15465052	T/TG	GCAACTCATTCGTCCCATTT	GGAAACGAAAAATTTGCAGC	59.7	712	INTERGENIC	--	--	--
Ca4_P2_15473254	Ca4	15473254	TTATATATATATATATAT/TTATATATATATATAT	TTGGACCGAAGAAATTGTCA	CTCCGTTAATCCTCATGCGT	60.1	702	INTERGENIC	--	--	--
Ca4_P2_15481101	Ca4	15481101	AATA/AA	TAGTAGGGCTCTTGCTCCCA	CCAATTGTACCGGGATCTTC	59.2	933	INTERGENIC	--	--	--
Ca4_P2_15609414	Ca4	15609414	GAACGTT/G	TTGCATTTGCTTGACTTGGA	CCATGACAAAGGGGGATATG	60.0	688	DRR	Ca_05541	--	--
Ca4_P2_15637043	Ca4	15637043	TA/T	CGGATGAACGTGGCTTTATT	GAGGTGTTTTGTGTGAGCGA	59.9	639	INTERGENIC	--	--	--
Ca4_P2_15643039	Ca4	15643039	TA/TTACTGTAGGGAA	TGCACCGACAAACTGAAGTC	ACGCGTGAGTTTGCTCACTA	59.7	592	INTERGENIC	--	--	--

INDEL marker IDs	Chromosomes/unanchored scaffolds	Physical positions (bp)	InDels (*Kabuli* reference genome-CDC Frontier/PII)	Forward primers (5'-3')	Reverse primers (5'-3')	Anneling temperature (0C)	Expected amplified product size (bp)	Structural annotation		Functional annotation	
								Sequence components of *kabuli* genome	*Kabuli* gene accession IDs	NCBI-KOG	TFs
Ca4_P2_15724429	Ca4	15724429	CTTCTTCCTCTTTCTTCCTCTTT/CTTCTTCCTCTTT	TCGTTACCCCTCTCGTCATC	GCCTGCCATCAATATTCACA	59.5	390	DRR	Ca_05532	R	ERF
Ca4_P2_1575145	Ca4	1575145	GAAATAAATAAATAAATAAATAAA/GAAATAAATAAATAAATAAATAAATAAA	TGGAAAGCAATTAATCACCG	AGTTCGCGCGCTTTTATTTA	60.0	335	INTERGENIC	--	--	--
Ca4_P2_15764982	Ca4	15764982	AATATATATATATATATATATATATATAT/AATATATATATATATATATATATATATATAT	GGGGCTAAGGAGTTTTGTGA	CGTCGCTGTGGCATTAAATA	59.7	250	INTERGENIC	--	--	--
Ca4_P2_15786147	Ca4	15786147	ATTTTTTTTT/ATTTTTTTTTT	AAAGAAGGAAGGGAAATCGG	CTGGTGATGGCGTTAATTTG	59.0	533	INTERGENIC	--	--	--
Ca4_P2_15867938	Ca4	15867938	CGCACTCGCGGATAT/C	TGGAGCAGGGATGTCATGTA	AGATTTTCCCCACCCAAATC	60.0	527	DRR	Ca_05521	T	bHLH
Ca4_P2_15877801	Ca4	15877801	CA/CAA	TCAAGCAACGACATTTCTGC	AGCCAATGCAGAAGCAGTTT	60.0	785	INTRON	Ca_05520	T	bHLH
Ca4_P2_15882468	Ca4	15882468	AA/AAATATTTA	TGCATTGGGAGTTGTTTTTG	TGTCGCGAGTTCTATCTGTGA	59.6	317	DRR	Ca_05519	--	--
Ca4_P2_15882875	Ca4	15882875	CTA/CTACTGCTATTA	TCACAGATAGAACTCGCGACA	GTTAAAGTGTGGGGGTGCAT	59.7	730	DRR	Ca_05519	--	--
Ca4_P2_15885406	Ca4	15885406	A/AT	CCTCAGTGAGATCAAGGTTGG	TTTTTCCCAATACATGCACA	57.5	676	INTERGENIC	--	--	--
Ca4_P2_15885901	Ca4	15885901	TA/T	TGTGCATGTATTGGGAAAAA	GAGATGATGCACCAAATCCA	59.5	493	INTERGENIC	--	--	--
Ca4_P2_15891601	Ca4	15891601	CTTTTTTTT/CTTTTTTT	AATCGTATCCCCTTGTGGGT	CCGATGCTCCCTAGTTGCTA	60.4	965	INTERGENIC	--	--	--
Ca4_P2_15891918	Ca4	15891918	T/TA	CGACGTCAACACAAATTGC	AGATGCGCGGTGATTAGAGT	59.9	802	INTERGENIC	--	--	--

INDEL marker IDs	Chromosomes/unanchored scaffolds	Physical positions (bp)	InDels (*Kabuli* reference genome-CDC Frontier/PII)	Forward primers (5'-3')	Reverse primers (5'-3')	Anneling temperature (0C)	Expected amplified product size (bp)	Structural annotation		Functional annotation	
								Sequence components of *kabuli* genome	*Kabuli* gene accession IDs	NCBI-KOG	TFs
Ca4_P2_15891944	Ca4	15891944	CTTT/CTTTT	CGACGTCAACACAAATTGC	AGATGCGCGGTGATTAGAGT	59.9	802	INTERGENIC	--	--	--
Ca4_P2_15892004	Ca4	15892004	AC/ACCGACTC	CGACGTCAACACAAATTGC	AGATGCGCGGTGATTAGAGT	59.9	802	INTERGENIC	--	--	--
Ca4_P2_15892103	Ca4	15892103	AG/A	TAGCAACTAGGGAGCATCGG	AGATGCGCGGTGATTAGAGT	59.9	292	INTERGENIC	--	--	--
Ca4_P2_15898293	Ca4	15898293	T/TA	CCTTCAAAATTTGTGAGTCCG	ATGGGAATTGGTGAATGGAA	60.0	941	INTERGENIC	--	--	--
Ca4_P2_15903673	Ca4	15903673	CTTTTTTTTTT/CTTTTTTTTT	GAAGGATGCAATGGAAGGAA	TGTGCGAGACCAATTCAAAA	60.2	822	DRR	Ca_05518	T	B3
Ca4_P2_15906376	Ca4	15906376	ATT/AT	TCACCAATTGGATTGCAAAA	GCTCCCTTCCAACATTTCAA	60.1	553	CDS (FRAME SHIFT)	Ca_05517	T	S1Fa-like
Ca4_P2_15908188	Ca4	15908188	CTA/CTAATTA	ATGTCGATTGAAGTTGCTGC	CGAAGGTGCATGTCAAAGAA	59.8	827	DRR	Ca_05517	T	S1Fa-like
Ca4_P2_15917692	Ca4	15917692	TAA/TAAA	CCAGACAGGGAAGGAAGACA	GCCTTCATTTTGGGTCACAT	59.8	952	DRR	Ca_05516	--	bHLH
Ca4_P2_15923050	Ca4	15923050	GAAAAAAAAA/GAAAAAAAA	CAACGGCTCTTATTTGCTTTC	GTCCTGTGTTTTTGGCACCT	60.0	419	DRR	Ca_05515	--	ERF
Ca4_P2_15926036	Ca4	15926036	AAAGA/AAAGAAGA	GTTTCCCTTACGCCAGATGA	GCATTGCTGTATTGGTGCAT	59.6	532	DRR	Ca_05513	R	--
Ca4_P2_15926090	Ca4	15926090	ACT/ACTCT	AGCAGCACCCACCAAATATC	TGCTGGTGTTCTAGTTGGCA	60.4	674	DRR	Ca_05513	R	--
Ca4_P2_15930383	Ca4	15930383	CAAAAAAAAA/CAAAAAAAAAA	ACCCCACACAATGATCACCT	CTGTGCTTTGTCCGTGCTTA	60.1	438	INTRON	Ca_05513	R	--

INDEL marker IDs	Chromosomes/unanchored scaffolds	Physical positions (bp)	InDels (*Kabuli* reference genome-CDC Frontier/PII)	Forward primers (5'-3')	Reverse primers (5'-3')	Anneling temperature (0C)	Expected amplified product size (bp)	Structural annotation		Functional annotation	
								Sequence components of *kabuli* genome	*Kabuli* gene accession IDs	NCBI-KOG	TFs
Ca4_P2_15932632	Ca4	15932632	GAAAAAAAAA/GAAAAAAAAAAA	TGCTGCTTTGTGGTTCTGAC	CAGGAGTACCCGACTGGTGT	60.0	811	INTRON	Ca_05513	R	--
Ca4_P2_15934332	Ca4	15934332	ACACACATTTCTAGCACACAT/ACACACAT	CCCAATGCATACCAAAAGGT	CTGAGGACAGTGACTTGCCA	60.0	189	INTRON	Ca_05513	R	--
Ca4_P2_15937811	Ca4	15937811	TC/T	TCAGTTGATGTTCTGCCGAC	AACTATGTGCACCTACCGCC	60.0	401	INTERGENIC	--	--	--
Ca4_P2_15939075	Ca4	15939075	AT/ATT	GTGCACCGGTAACGAAGTTT	TTCGTTGGAGGTTTGGATTC	59.9	452	INTERGENIC	--	--	--
Ca4_P2_15945430	Ca4	15945430	AAAA/AAAAATCAAA	AGTCCAGCCTCTGCACAAAT	GGAGCAAGATGGAACAATCAA	60.1	212	DRR	Ca_05512	O	C2H2
Ca4_P2_15950381	Ca4	15950381	ATTTTTTTTT/ATTTTTTTT	AAGCACGCTGCAATTGATTA	AGATCCAGTCTCACGTCGCT	60.0	707	INTERGENIC	--	--	--
Ca4_P2_15950510	Ca4	15950510	GTTATTTTTATT/GTTATTTTTATTATTATTTTTATT	AAAAGGGATTGTCTTCCCAGA	CAAAGGTAGCCCTGCTTCAG	60.0	601	INTERGENIC	--	--	--
Ca4_P2_15952722	Ca4	15952722	AG/A	ATTTCAACGTCAATCACGCA	ACCCACCATAGCTACCACCA	60.3	822	INTERGENIC	--	--	--
Ca4_P2_15956008	Ca4	15956008	AATTATTATTA/AATTATTATTATTA	CAATTCCAACCATTTGGGTC	TGGGTAGTGGGGTTTGTTGT	60.1	540	INTERGENIC	--	--	--
Ca4_P2_15958115	Ca4	15958115	ATTAAA/ATTAAAGTTAAA	TTTGGTTCACATGATCGCAT	CCCCTATTCACCGCATAAGA	59.9	743	DRR	Ca_05511	--	C2H2
Ca4_P2_15958358	Ca4	15958358	CT/C	ACAAAGCTCAAACTCGCCAT	CCCCTATTCACCGCATAAGA	59.9	342	DRR	Ca_05511	--	C2H2
Ca4_P2_15962161	Ca4	15962161	TAAAAA/TAAAA	TTCAACCGAACCCATCTAGG	CCATGATGCACAATTTCCTG	59.9	474	INTERGENIC	--	--	--

INDEL marker IDs	Chromosomes/unanchored scaffolds	Physical positions (bp)	InDels (*Kabuli* reference genome-CDC Frontier/PII)	Forward primers (5'-3')	Reverse primers (5'-3')	Anneling temperature (0C)	Expected amplified product size (bp)	Structural annotation		Functional annotation	
								Sequence components of *kabuli* genome	*Kabuli* gene accession IDs	NCBI-KOG	TFs
Ca4_P2_15962571	Ca4	15962571	ACTT/ACTTCTCTT	CAGGAAATTGTGCATCATGG	TGGTGCATGACTGAATGGTT	60.0	723	INTERGENIC	--	--	--
Ca4_P2_15966850	Ca4	15966850	TTATATAT/TTATAT	TTTGCTCGTAGAGGTGCTCA	TTGCCCCCTACGACACTATC	60.0	846	INTERGENIC	--	--	--
Ca4_P2_15969051	Ca4	15969051	CAA/CA	TAAGGCGTTAACTTGCACCC	TGGTTGTGGCGATTACAAGA	60.1	494	DRR	Ca_05510	J	NF-YA
Ca4_P2_15982123	Ca4	15982123	AT/ATT	GGAAATAACGCAAAAAGGCA	TCAAAGTGTCCATGAATCCG	59.5	567	INTERGENIC	--	--	--
Ca4_P2_15983115	Ca4	15983115	AATT/AATTATT	TGATGTTGATGATATGACGTGG	ATTGTTCTGCAAATGAGGGG	59.9	729	INTERGENIC	--	--	--
Ca4_P2_15987672	Ca4	15987672	GTTACAA/GTTACAAATTACAA	TGAATGCATTGTTGCTGGTT	CTGGACTAAAACCACCCGAA	60.0	224	INTERGENIC	--	--	--
Ca4_P2_15989208	Ca4	15989208	T/TC	AACAGCGCACAAGTGTTTCA	TCTCATCCATATATTGCGAATCA	59.4	238	INTERGENIC	--	--	--
Ca4_P2_15990207	Ca4	15990207	ATA/ATATTA	GATTGTCCAACAAACGAGCTT	AATGGTAGTCCGTTTTGCCA	60.4	305	INTERGENIC	--	--	--
Ca4_P2_15998419	Ca4	15998419	G/GCTTAC	TTTTTCCAAACCAGGAAACG	CCAATTACCACAAGCTTCCAA	60.0	300	INTERGENIC	--	--	--
Ca4_P2_16000633	Ca4	16000633	TACA/TA	CGATGTGATTTTTGTAGTCAAGC	TATTTTCCGCACACCAATGA	59.9	392	INTERGENIC	--	--	--
Ca4_P2_16025562	Ca4	16025562	TAAAA/TAAA	TTGAACGATCAAAATTGGCA	CGTAGGGTTCACCAAGCAAT	60.0	744	DRR	Ca_05507	T	--
Ca4_P2_16027847	Ca4	16027847	A/AG	AATTTTGGCTTTTTGCGTTG	ATCATATCCATCAAACGGGC	59.6	536	INTERGENIC	--	--	--

INDEL marker IDs	Chromosomes/unanchored scaffolds	Physical positions (bp)	InDels (*Kabuli* reference genome-CDC Frontier/PII)	Forward primers (5'-3')	Reverse primers (5'-3')	Anneling temperature (0C)	Expected amplified product size (bp)	Structural annotation		Functional annotation	
								Sequence components of *kabuli* genome	*Kabuli* gene accession IDs	NCBI-KOG	TFs
Ca4_P2_16028033	Ca4	16028033	CT/C	ATGCAACCTCCATTAGCCTG	TTGGTTTCTAACCCAACTCATATTC	59.7	322	INTERGENIC	--	--	--
Ca4_P2_16028385	Ca4	16028385	GTTTTTTTT/GTTTTTTT	CCCTCGTGCACAAGTTCTTT	CATTTTGCATTTGATACACACG	59.0	885	INTERGENIC	--	--	--
Ca4_P2_16028817	Ca4	16028817	CGTGTGT/CGTGTGTGT	TGATTTTACACAAGGGTAATTTTG	CGCCGCATACTGTAATGGTA	59.6	951	INTERGENIC	--	--	--
Ca4_P2_16043366	Ca4	16043366	A/AG	CGCAAACGCTTTAGAACTCC	ATGGACACGAGGGTTTTTCA	60.4	484	INTERGENIC	--	--	--
Ca4_P2_16046439	Ca4	16046439	GTTT/GTTTT	GCTTGTGGTGTTGTTTGGTG	AGCAACAGGTGCTGCCTTAT	59.9	853	INTRON	Ca_05504	J	bHLH
Ca4_P2_16046471	Ca4	16046471	TATCATCAT/TATCAT	GCTTGTGGTGTTGTTTGGTG	ATCTTCTTCGGGTGCCTTTT	60.1	880	INTRON	Ca_05504	J	bHLH
Ca4_P2_16048595	Ca4	16048595	TG/T	GACCATTTTTGGTGATTGGG	AGGTACCACCAACCAAGTGC	59.9	258	INTRON	Ca_05504	J	bHLH
Ca4_P2_16054222	Ca4	16054222	CTTAATTAA/CTTAA	CACAGGACAGGACGGTAGGT	GGATCCTTGAGGTTGTTCGT	59.0	626	DRR	Ca_05504	J	bHLH
Ca4_P2_16055262	Ca4	16055262	GCTT/G	TTCCCATTTTAATTCCCATCA	CCAGTGGCGGAGAAAAATAA	60.1	803	DRR	Ca_05504	J	bHLH
Ca4_P2_16055486	Ca4	16055486	CAAAAAAA/CAAAAAA	TGCTGGTAGTCACGGGGTAT	CCAGTGGCGGAGAAAAATAA	60.1	229	DRR	Ca_05504	J	bHLH
Ca4_P2_16055970	Ca4	16055970	TAAAAAAA/TAAAAAAAA	AGCAACCTCGGTAGCATCAT	ATTGAGTGTGTGGTACGGGG	60.7	509	INTERGENIC	--	--	--
Ca4_P2_16056262	Ca4	16056262	TAAAAAAAAAAA/TAAAAAAAAAA	AAGGGATGTATCCCCGTACC	GGCGTATCCAAATGGTTGAG	60.3	574	INTERGENIC	--	--	--

**INDEL marker IDs**	**Chromosomes/unanchored scaffolds**	**Physical positions (bp)**	**InDels (*Kabuli* reference genome-CDC Frontier/PII)**	**Forward primers (5'-3')**	**Reverse primers (5'-3')**	**Anneling temperature (0C)**	**Expected amplified product size (bp)**	**Structural annotation**		**Functional annotation**	
								**Sequence components of *kabuli* genome**	***Kabuli* gene accession IDs**	**NCBI-KOG**	**TFs**
Ca4_P2_16057308	Ca4	16057308	CG/C	CAGGTCGGGATTCTTTTTGA	CATACTTACCCGTGCAGCAA	59.8	705	INTERGENIC	--	--	--
Ca4_P2_16058692	Ca4	16058692	ATTTTTTTTT/ATTTTTTTTTT	TGCGCAAGAATTGAAATCAG	AACTCACCAACCAGACATTGA	58.0	828	INTERGENIC	--	--	--
Ca4_P2_16059109	Ca4	16059109	TAA/TA	TTGACGTGTCAAAATGTGACTG	CGTCACTGACAGCGTAGCAC	60.7	346	INTERGENIC	--	--	--
Ca4_P2_16060314	Ca4	16060314	AAAAGAAA/AAAA	TTGGATGATACTTCTTCCGTCTC	AGAACACCCTGCAGGTTCAC	60.2	559	INTERGENIC	--	--	--
Ca4_P2_16063311	Ca4	16063311	A/ACATGCGCAAGAAATTC	AAGTTGGCGTGCTTGTTTCT	CATCATGGTGAAGGTCGTTG	60.0	666	INTRON	Ca_05503	K	MYB
Ca4_P2_16064554	Ca4	16064554	T/TTA	TTGCAAGTAGCTTCCCCATT	AGGTGAGGTGAGGTGATTGA	58.1	820	INTERGENIC	--	--	--
Ca4_P2_16064877	Ca4	16064877	GT/G	CACTTTCTCTTATTCCACTTTCCC	GGCGACTTCCATTGGACTAA	60.1	497	INTERGENIC	--	--	--
Ca4_P2_16065224	Ca4	16065224	CCTCTC/CCTC	TCAATCACCTCACCTCACCT	GGCGACTTCCATTGGACTAA	60.1	310	INTERGENIC	--	--	--
Ca4_P2_16066680	Ca4	16066680	CAAAA/CAAA	TCTCGATTGAAGTGCATTGG	CATTAGAGTTCACTTGGCGG	58.4	514	INTERGENIC	--	--	--
Ca4_P2_16066890	Ca4	16066890	ATTTTT/ATTTT	AGAACCGATTTTGGTGATGC	GGGACCAGTGATTTAATTTAGCC	60.1	705	INTERGENIC	--	--	--
Ca4_P2_16067505	Ca4	16067505	CAAAAAAAA/CAAAAAAA	GGCTAAATTAAATCACTGGTCCC	TACATCTGCGGCCCTTTAAC	60.1	572	INTERGENIC	--	--	--
Ca4_P2_16072660	Ca4	16072660	ATATTAT/ATATTATTAT	GTCACAGCTCTGATGCCAAA	GCGATACGGAGTGGACATCT	60.1	787	INTERGENIC	--	--	--

INDEL marker IDs	Chromosomes/unanchored scaffolds	Physical positions (bp)	InDels (*Kabuli* reference genome-CDC Frontier/PII)	Forward primers (5'-3')	Reverse primers (5'-3')	Anneling temperature (0C)	Expected amplified product size (bp)	Structural annotation		Functional annotation	
								Sequence components of *kabuli* genome	*Kabuli* gene accession IDs	NCBI-KOG	TFs
Ca4_P2_16080327	Ca4	16080327	CTTTTTTTTTTTTT/CTTTTTTTTTTT	TTCCTCACGTGATCCCTTTC	ACAATTGCTCCAACGGTCAT	60.4	567	INTERGENIC	--	--	--
Ca4_P2_16080441	Ca4	16080441	TAAAAAAAAAAAA/TAAAAAAAAAA	TTCCTCACGTGATCCCTTTC	ACAATTGCTCCAACGGTCAT	60.4	567	INTERGENIC	--	--	--
Ca4_P2_16081516	Ca4	16081516	TA/TAA	CATGCAATAAATTTAGAGCGAAGA	TGCAACAGATTGCCTTTCAC	59.8	776	INTERGENIC	--	--	--
Ca4_P2_16086955	Ca4	16086955	GTATA/GTATATATA	CCTCCAATCCGGTATTTTCA	CGTGCAACCTGCAGATAATG	60.3	721	INTERGENIC	--	--	--
Ca4_P2_16089933	Ca4	16089933	A/AC	CCAAAACAGAACTGGCAACA	TTTTTAAAACATTGCACCAGTGA	59.6	467	INTERGENIC	--	--	--
Ca4_P2_16095391	Ca4	16095391	GCAACAAC/GCAAC	TCGACGCGAGACATGTTAAA	GTGGAGGGAGCGATTCTGTA	60.2	437	INTERGENIC	--	--	--
Ca4_P2_16095440	Ca4	16095440	TA/TAA	AGCCCAAGAAAGAAAACGCT	AAGGCAAAAGTCGTCCAATC	59.2	726	INTERGENIC	--	--	--
Ca4_P2_16095927	Ca4	16095927	GATAATATAATATAATATA/GATAATATAATATAATATAATATA	TACAGAATCGCTCCCTCCAC	TACATGGTTCGGTTGGGTTT	60.1	665	INTERGENIC	--	--	--
Ca4_P2_16097658	Ca4	16097658	ATTTTTTTTTT/ATTTTTTTTTTTTT	GTGGAATGTAAGATCGCCGT	TCTTTGAACAATTGGCCCTC	60.1	307	DRR	Ca_05500	--	--
Ca4_P2_16097693	Ca4	16097693	GAAAAAAA/GAAAAAA	GTGGAATGTAAGATCGCCGT	GCAAACAGAGGAAGAGAAGGAA	60.0	535	DRR	Ca_05500	--	--
Ca4_P2_16098339	Ca4	16098339	TAAAAAAAAA/TAAAAAAA	CCTTCTCTTCCTCTGTTTGCTC	CTCATACATGAGGGGAAAGCA	60.1	674	DRR	Ca_05500	--	--
Ca4_P2_16099117	Ca4	16099117	TAAAAAAA/TAAAAAA	TTCAATTCAAAATATAAACGGCA	AGGTTGACGATTGGTGTTCC	59.8	398	DRR	Ca_05500	--	--

INDEL marker IDs	Chromosomes/unanchored scaffolds	Physical positions (bp)	InDels (*Kabuli* reference genome-CDC Frontier/PII)	Forward primers (5'-3')	Reverse primers (5'-3')	Anneling temperature (0C)	Expected amplified product size (bp)	Structural annotation		Functional annotation	
								Sequence components of *kabuli* genome	*Kabuli* gene accession IDs	NCBI-KOG	TFs
Ca4_P2_16099943	Ca4	16099943	T/TGG	GGAAAACAAAGTTCCCAACC	TTTTGTTTGATGGCTTGCAG	59.8	748	INTERGENIC	--	--	--
Ca4_P2_16102523	Ca4	16102523	ATTTTTTT/ATTTTTTTT	GTTGAGTGGTGGCATGAAAT	TTCGAGTTGAGGTCCTTCTCA	60.0	820	DRR	Ca_05499	--	--
Ca4_P2_16103095	Ca4	16103095	TAAAAAAAAAAA/TAAAAAAAA	TGAGAAGGACCTCAACTCGAA	CAAATTCAAATAAAGCATGGATCA	60.2	628	DRR	Ca_05499	--	--
Ca4_P2_16114063	Ca4	16114063	TCTCAAACACT/TCTCAAACACTCAAACACT	AATATCGTCAGCGACCTGCT	TTTTGCCAATGAAGTTGTGC	59.7	544	INTERGENIC	--	--	--
Ca4_P2_16120236	Ca4	16120236	AATATATATATATATATATA/AATATATATATATATATA	TCGAAAGCATTCTTCTCCAAA	TGGTTTGACCTATTTATGGTTGG	60.0	550	INTERGENIC	--	--	--
Ca4_P2_16123203	Ca4	16123203	GATATATATATATATATATATATA/GATATATATATATATATATATA	CTCTGCTTGTTGGCACTTGA	CCCCCAGCCCTCTATATTTC	59.8	831	INTERGENIC	--	--	--
Ca4_P2_16138106	Ca4	16138106	TTTATTATTATTATTATTATTATTATTATT/TTTATTATTATTATTATTATTATTATTATTATTATT	CAATGACATTACTTGGGCCTT	GGGAGAAGAAAAGAATGGGAA	59.5	391	INTERGENIC	--	--	--
Ca4_P2_16175560	Ca4	16175560	CATATATATATATA/CATATATATATA	TGTTTGCTTGCTTTTGATGC	GGTGTGTGTAGGGGAAGGAA	59.8	269	INTERGENIC	--	--	--
Ca4_P2_16186698	Ca4	16186698	T/TC	TTGTTTACAAGAGATGTTTTGTCATTT	AAACCACGACTCCAAGCAAT	59.6	372	DRR	Ca_05493	Z	--
Ca4_P2_16197589	Ca4	16197589	ATTACATTTACA/ATTACATTTACATTTACA	CCTAAGTGAAGGGCAGCTTG	AGCTTGCGGTTTTCCTGTTA	59.9	513	INTRON	Ca_05492	--	C2H2
Ca4_P2_16237710	Ca4	16237710	ATCTCTCTCTCTCTCTCTCTC/ATCTCTCTCTCTCTCTCTCTCTC	ATTGATGGCTCCCAGCATAC	TAGCTAGGAGCGCTGTGTGA	59.9	468	INTERGENIC	--	--	--
Ca4_P2_16288880	Ca4	16288880	TAAAAAAAAAA/TAAAAAAAAAAA	GGCATTTTCAAACAATCCCT	TGATTGAATTCCCACCCAAT	60.0	514	INTERGENIC	--	--	--

INDEL marker IDs	Chromosomes/unanchored scaffolds	Physical positions (bp)	InDels (*Kabuli* reference genome-CDC Frontier/PII)	Forward primers (5'-3')	Reverse primers (5'-3')	Anneling temperature (0C)	Expected amplified product size (bp)	Structural annotation		Functional annotation	
								Sequence components of *kabuli* genome	*Kabuli* gene accession IDs	NCBI-KOG	TFs
Ca4_P2_16313772	Ca4	16313772	TAAAAAAAAAAAAA/TAAAAAAAAAAAAAA	TTTGGCATTTTGGACTAGGG	GCCCAATTTCAGGCTTATGA	60.0	283	INTERGENIC	--	--	--
Ca4_P2_16329274	Ca4	16329274	A/AAGCATAC	TTGGACGATAGTACCGTATTCAA	CGAAGCAAATAGCAAGACTGC	60.2	815	INTERGENIC	--	--	--
Ca4_P2_16444215	Ca4	16444215	TAGAGAGAGAGAAGAGAGAGAGAGAAGAGAGAGAGAG/TAGAGAGAGAGAAGAGAGAGAGAGAAGAGAGAGAGAGAGAGAGAAGAGAGAGAG	CGCTCCACTTTCAACACTCA	AATTCGTAACGGCACGGTAG	60.0	322	INTERGENIC	--	--	--
Ca4_P2_16523278	Ca4	16523278	AATTATTATTATTATTATTATTATTATTATTATTATTATTATTATTATTAT/AATTATTATTATTATTATTATTATTATTATTATTATTATTATTATTATTATTATTATTATTATTATTATTAT	TTTCCCAGCCCTCTTAGACA	TTGGTTGTTCTTCCTGCACA	60.3	451	DRR	Ca_05473	V	NAC
Ca4_P2_16545258	Ca4	16545258	AATATATATATATATATATAT/AATATATATATATATATAT	TTTGGGCTTACTTTTGTGAGAA	CAAAGAGTTACAACGATGCGA	59.0	313	INTERGENIC	--	--	--
Ca4_P2_16626685	Ca4	16626685	TCCCCCCCC/TCCCCCCCCC	TGTTTTGGGCACAACGATTA	TCTGAAACGGCGACTTCTTT	60.0	580	INTERGENIC	--	--	--
Ca4_P2_1665800	Ca4	1665800	CATATATATATATATATATA/CATATATATATATATATATATATA	CATTGAAAGCGCGTTGATAA	GGTGAATTGATTTATGCTGGAA	58.9	394	INTERGENIC	--	--	--
Ca4_P2_16713373	Ca4	16713373	AATATATATATATATATATATAT/AATATATATATATATATATATATATATATATATAT	GCTTTGGCTTTCCTCTCCTT	GTGGAAAAAGATGTGGACGG	60.4	790	INTERGENIC	--	--	--
Ca4_P2_16728462	Ca4	16728462	TAAA/TAAAA	CATGGGGATGCTGCTATTTT	CAACTTCCTCTTTTGGCACA	58.9	388	DRR	Ca_05454	O	MYB
Ca4_P2_16728592	Ca4	16728592	TAAAAAAAAAA/TAAAAAAAAA	CATGGGGATGCTGCTATTTT	CAACTTCCTCTTTTGGCACA	58.9	388	DRR	Ca_05454	O	MYB
Ca4_P2_1677374	Ca4	1677374	TAAAAAAA/TAAAAA	TCCAAACTAGTCCGACGTGA	AGCTGCTGGAGGAGTTGGTA	60.0	450	INTRON	Ca_07795	--	--
Ca4_P2_16784125	Ca4	16784125	ATTTTTTTTTTT/ATTTTTTTTTTTTT	ACCTTGACACAACGGAAAGG	TCTTGCAATGCACCACAAAT	60.1	614	INTERGENIC	--	--	--

INDEL marker IDs	Chromosomes/unanchored scaffolds	Physical positions (bp)	InDels (*Kabuli* reference genome-CDC Frontier/PII)	Forward primers (5'-3')	Reverse primers (5'-3')	Anneling temperature (0C)	Expected amplified product size (bp)	Structural annotation		Functional annotation	
								Sequence components of *kabuli* genome	*Kabuli* gene accession IDs	NCBI-KOG	TFs
Ca4_P2_16787893	Ca4	16787893	CTTTTTTTT/CTTTTTTT	CCCTTTAAAATGAGAGAATTGAAG	ATCGTCACGTGCAAACAATC	59.6	628	INTERGENIC	--	--	--
Ca4_P2_16793556	Ca4	16793556	AG/AGG	ACACTTGCTATGAAACGGGG	ACGCTTTGCAAGGTCTGAAT	59.9	590	INTERGENIC	--	--	--
Ca4_P2_16810468	Ca4	16810468	TAAAAAAAAAAA/TAAAAAAAAAA	TTTTGGATGTGCAGTTCAGC	GGTTGCATGGTGTTTGTCTG	60.0	418	INTRON	Ca_05443	--	--
Ca4_P2_16813063	Ca4	16813063	CTAGAAGCTTTAGAAGCTTTA/CTAGAAGCTTTA	GTAAGTGATGGGTGCGTGTG	TGTGAACCCAGCTGCTCTAA	59.6	700	DRR	Ca_05442	--	--
Ca4_P2_16827901	Ca4	16827901	AATATTGATTATA/AATATTGATTATATTGATTATA	ACGCCAACATTTGTGAGTGA	TCGGATTCACTTAGTTGCACA	59.3	853	INTERGENIC	--	--	--
Ca4_P2_16846897	Ca4	16846897	C/CTT	ACATGTGCCTTGGGACTAGC	GCGAAAAGTCGAAGATCCTG	60.0	571	INTRON	Ca_05437	--	--
Ca4_P2_16862733	Ca4	16862733	GTTTTTTTTTT/GTTTTTTTTTTT	TTTGATGCAATTATGAACCGA	TTGGAGGGTTGAAATTGAAAA	59.4	689	INTERGENIC	--	--	--
Ca4_P2_16878770	Ca4	16878770	GCACACACACACACACACACACACA/GCACACACACACACACACACACA	TTTTGCATCTCACGCATCAT	CACGAAAATGGTTCCCTCAT	59.8	612	DRR	Ca_05436	--	--
Ca4_P2_16925907	Ca4	16925907	TAAAAAAAAAAAA/TAAAAAAAAAAA	TGCAAAACGTCGCTGTCTAC	TGGTCACTGGTCACTCTTGG	59.7	566	DRR	Ca_05430	R	C2H2
Ca4_P2_16933042	Ca4	16933042	TAAAAAAAAAA/TAAAAAAAAA	CCAAATAAACTACTTTTGTGAGACGA	CACACTCTGCCTCCACTGAA	60.0	388	INTERGENIC	--	--	--
Ca4_P2_16943222	Ca4	16943222	TATGAATGAAT/TATGAATGAATGAAT	CTCAAGCTCAGTCCCAGTCC	TGCGTTTTGTTTGGTGTAGC	59.8	668	INTERGENIC	--	--	--
Ca4_P2_16983681	Ca4	16983681	A/AT	TGGTCAGCCTCAGTCTCCTT	CACACATGGCACCCTTGTTA	60.4	381	INTRON	Ca_05426	U	--

INDEL marker IDs	Chromosomes/unanchored scaffolds	Physical positions (bp)	InDels (*Kabuli* reference genome-CDC Frontier/PII)	Forward primers (5'-3')	Reverse primers (5'-3')	Anneling temperature (0C)	Expected amplified product size (bp)	Structural annotation		Functional annotation	
								Sequence components of *kabuli* genome	*Kabuli* gene accession IDs	NCBI-KOG	TFs
Ca4_P2_16984107	Ca4	16984107	ATTTTTTTT/ATTTTTTT	TAACAAGGGTGCCATGTGTG	ATTGATTTGGCAATGGTGGT	60.1	822	INTRON	Ca_05426	U	--
Ca4_P2_16985095	Ca4	16985095	ATTTTT/ATTTTTT	CATGAGACTCGTCAAGGCAA	GTGTGGAAGTGCCAAAGGTT	60.0	914	INTRON	Ca_05426	U	--
Ca4_P2_17010261	Ca4	17010261	GAGACA/GAGACAGACA	GTTAGCCTCACAGCACCCAT	GCTTCTAACTCGGCAAGTGG	60.0	342	DRR	Ca_05422	--	G2-like
Ca4_P2_17017376	Ca4	17017376	TAAAATAT/TAAAATATCAAAAAAATAT	AATCGTTCAATACCCTTCCA	ATCGGCACAAGTCAACTTCC	60.1	392	INTERGENIC	--	--	--
Ca4_P2_17017613	Ca4	17017613	ATTTTT/ATTTTTT	GGAAGTTGACTTGTGCCGAT	TTGTTCTGAAAGAAACGGGG	60.1	527	INTERGENIC	--	--	--
Ca4_P2_17018846	Ca4	17018846	ATT/AT	TTCTCCCCAGAATCAATTTGT	ATTCCTTTTGCCTTTGCCTT	60.1	598	INTERGENIC	--	--	--
Ca4_P2_17029753	Ca4	17029753	ATTTTTTTTT/ATTTTTTTT	ACCATTTCCTCCTGTGTTGC	CAAAAATTTGAGGGTTCAAACA	59.0	728	INTERGENIC	--	--	--
Ca4_P2_17030182	Ca4	17030182	TAA/TAAAA	AAGATTTTGTGTTTGAACCCTCA	TCTCGCCTTTTGTATCGTCA	59.4	426	DRR	Ca_05420	R	bHLH
Ca4_P2_17038197	Ca4	17038197	T/TCGA	AGCCTTTGATCTTCCCCTGT	AGTGGACTCGTGAGGGAATG	60.1	326	INTRON	Ca_05419	C	bHLH
Ca4_P2_17044386	Ca4	17044386	CT/CTT	AAACAATTTACGCGAATGCC	TATTAAAAGGCCCGCACAAC	60.0	896	INTERGENIC	--	--	--
Ca4_P2_17047766	Ca4	17047766	ATTTTTTTTTTT/ATTTTTTTTTT	GAACCCAGTGCACAACTCCT	AAGCGACCAGTTGAAGGAAA	59.9	662	DRR	Ca_05418	DA	--
Ca4_P2_17047788	Ca4	17047788	ATTTTTTTTT/ATTTTTTTTTT	GAACCCAGTGCACAACTCCT	AAGCGACCAGTTGAAGGAAA	59.9	662	DRR	Ca_05418	DA	--

INDEL marker IDs	Chromosomes/unanchored scaffolds	Physical positions (bp)	InDels (*Kabuli* reference genome-CDC Frontier/PII)	Forward primers (5'-3')	Reverse primers (5'-3')	Anneling temperature (0C)	Expected amplified product size (bp)	Structural annotation		Functional annotation	
								Sequence components of *kabuli* genome	*Kabuli* gene accession IDs	NCBI-KOG	TFs
Ca4_P2_17047964	Ca4	17047964	GAAAAAAAAAAA/GAAAAAAAAA	AGAGGTCATTTGGGTGGACA	AAGCGACCAGTTGAAGGAAA	59.9	453	DRR	Ca_05418	DA	--
Ca4_P2_17048363	Ca4	17048363	TAAAAAAAA/TAAAAAAAAA	TTTCCTTCAACTGGTCGCTT	TCATCATCTTGCTCTCCACG	59.9	527	DRR	Ca_05418	DA	--
Ca4_P2_17050258	Ca4	17050258	AGG/AGGG	GCGAAATGTTCTTCTCCTGC	TTTGGATAACCACCCCTACG	59.7	887	INTRON	Ca_05418	DA	--
Ca4_P2_17053264	Ca4	17053264	TAGTGATTAAGTGATT/TAGTGATTAAGTGATTAAGTGATT	TAGACGCGGAAAACAGTGTG	GTCAGTGGAAAAGGGTGGAA	59.9	277	INTRON	Ca_05418	DA	--
Ca4_P2_17057323	Ca4	17057323	AAAATAAATAAATAAATAAA/AAAATAAATAAATAAA	AAAAATCCTCGATTGGATAAAAGA	TGTGTTAATTGAGGACGTTGTTG	59.9	837	DRR	Ca_05417	--	--
Ca4_P2_17063810	Ca4	17063810	TAAAAAAAA/TAAAAAAAAAA	CAAAAGAAAAGCTTTATACGGTGA	TTCGGTTTATGTAGCAGCCC	60.1	558	INTERGENIC	--	--	--
Ca4_P2_17066705	Ca4	17066705	ATTTTTT/ATTTTTTT	GGTGATGGAAGAAGGTGAGG	TTTCCATCTAACGGTGGAGC	60.1	873	INTERGENIC	--	--	--
Ca4_P2_17067831	Ca4	17067831	TAAAAAAAA/TAAAAAAAAA	TTCACCAAACAGCCAGTATCC	GTGAGTCATGGGACGGTTCT	60.0	402	INTERGENIC	--	--	--
Ca4_P2_17071722	Ca4	17071722	ATTTTTTTTT/ATTTTTTTTTT	GGGCTGCTACATAAACCGAA	TCCGACATGCCATTTTGTTA	59.9	189	INTERGENIC	--	--	--
Ca4_P2_17074453	Ca4	17074453	TCTC/T	TTGTTGTGGTGGGAGTGAAA	AAGGACGGTGTGGCTCATAG	60.1	332	CDS (large-effect mutations)	Ca_05416	--	bHLH
Ca4_P2_17081756	Ca4	17081756	TTATA/TTATATA	TACCGGAAGATTCGGAAATG	CTGCAGGTAAGCATCCCAAT	60.1	694	INTRON	Ca_05415	--	--
Ca4_P2_17081772	Ca4	17081772	TAATA/T	TACCGGAAGATTCGGAAATG	CTGCAGGTAAGCATCCCAAT	60.1	694	INTRON	Ca_05415	--	--

INDEL marker IDs	Chromosomes/unanchored scaffolds	Physical positions (bp)	InDels (*Kabuli* reference genome-CDC Frontier/PII)	Forward primers (5'-3')	Reverse primers (5'-3')	Anneling temperature (0C)	Expected amplified product size (bp)	Structural annotation		Functional annotation	
								Sequence components of *kabuli* genome	*Kabuli* gene accession IDs	NCBI-KOG	TFs
Ca4_P2_17081881	Ca4	17081881	T/TTTCC	TTTTGACAATGGGCACTCAA	CTGCAGGTAAGCATCCCAAT	60.1	295	CDS (FRAME SHIFT)	Ca_05415	--	--
Ca4_P2_17082915	Ca4	17082915	CTTTTT/CTTTTTT	TCAGCTCACTCTCGCTTTGA	TGATGGAAGAATAAGGGGTCC	60.1	503	DRR	Ca_05415	--	--
Ca4_P2_17089846	Ca4	17089846	T/TC	CCAACATTAAAACGGCCCTA	GACTTAAGGGAGGGGGCTC	60.0	397	INTERGENIC	--	--	--
Ca4_P2_17089902	Ca4	17089902	ATTTTTTT/ATTTTTTTT	CCAACATTAAAACGGCCCTA	TGTGCTAGGATTGCACGGTA	60.3	574	INTERGENIC	--	--	--
Ca4_P2_17090015	Ca4	17090015	ATATTTATTTATTTATTTATTT/ATATTTATTTATTTATTT	CCAACATTAAAACGGCCCTA	GCATTTTCACAATGTGGTGC	60.0	698	INTERGENIC	--	--	--
Ca4_P2_17101140	Ca4	17101140	TAACAAC/TAACAACAAC	TGGTTTGATTTATTGTGGTGC	CATTGGTTTTAACAAGTGTTGTTGT	59.3	472	DRR	Ca_05414	--	--
Ca4_P2_17101199	Ca4	17101199	A/AC	TGGTTTGATTTATTGTGGTGC	GTTCTTTGAAATTGCACGAATA	57.0	812	DRR	Ca_05414	--	--
Ca4_P2_17101268	Ca4	17101268	TAAAAAA/TAAAAAAA	TGGTTTGATTTATTGTGGTGC	TCGAGAGTTGGGTCATGGTC	61.1	881	DRR	Ca_05414	--	--
Ca4_P2_17101583	Ca4	17101583	AG/A	TGATGTTAACTGACAAATGCTGTTT	GTCGAGAGTTGGGTCATGGT	60.0	322	DRR	Ca_05414	--	--
Ca4_P2_17102148	Ca4	17102148	AA/AAGA	ATGACCCAACTCTCGACACC	TCAGGTGATTGTTGACCCAA	59.9	733	DRR	Ca_05414	--	--
Ca4_P2_17104717	Ca4	17104717	TAAAAAAAA/TAAAAAAA	ATGATGTTTTCCGTTTTGCC	CGAAACGGATTTTAACGGAA	59.9	455	INTERGENIC	--	--	--
Ca4_P2_17105095	Ca4	17105095	G/GGATAA	TTCCGTTAAAATCCGTTTCG	TGAATGGGACATGAATCCTC	58.3	253	INTERGENIC	--	--	--

INDEL marker IDs	Chromosomes/unanchored scaffolds	Physical positions (bp)	InDels (*Kabuli* reference genome-CDC Frontier/PII)	Forward primers (5'-3')	Reverse primers (5'-3')	Anneling temperature (0C)	Expected amplified product size (bp)	Structural annotation		Functional annotation	
								Sequence components of *kabuli* genome	*Kabuli* gene accession IDs	NCBI-KOG	TFs
Ca4_P2_17114726	Ca4	17114726	GAAAA/GAAAAA	CAAAAACAAGGGCACCAACT	TATTTGAAACGAACGGTCCC	59.8	736	DRR	Ca_05412	CR	Nin-like
Ca4_P2_17116278	Ca4	17116278	ATTT/ATTTTT	ACCGACCGAACACAAAAGAC	TTCAGCAAAGTGGTTCCAAA	59.3	623	DRR	Ca_05412	CR	Nin-like
Ca4_P2_17130703	Ca4	17130703	TAAAAAA/TAAAAA	CCATGAAGGGGACAAAGATG	GCATGTAATGTTGGAAGCCC	60.3	931	INTRON	Ca_05411	CR	Nin-like
Ca4_P2_17133672	Ca4	17133672	AATATTTG/A	TCGGGTATTTTTCCTTCATCA	TTTTGAAAAATCCTTTGCGG	60.0	522	INTRON	Ca_05411	CR	Nin-like
Ca4_P2_17133934	Ca4	17133934	A/AAAAGACTTAGTT	GAATTCGGGAAACACTCGAA	GATGTCATTCACACCATGCC	59.8	237	INTRON	Ca_05411	CR	Nin-like
Ca4_P2_17134051	Ca4	17134051	CAAAAA/CAAAA	GAATTCGGGAAACACTCGAA	GATGTCATTCACACCATGCC	59.8	237	INTRON	Ca_05411	CR	Nin-like
Ca4_P2_17135091	Ca4	17135091	CATA/CATATA	TGATATGGCAAAATACACGGA	AGACATCCTCCGTCACCAAC	60.0	755	INTRON	Ca_05411	CR	Nin-like
Ca4_P2_17136748	Ca4	17136748	T/TC	GGTTGAATATGTCAATCGCGT	TCAATTAAGCCCCTAAAGTCCA	60.0	590	INTERGENIC	--	--	--
Ca4_P2_17140168	Ca4	17140168	ACTTCTTCT/ACTTCT	ATCATTGGCCACTCCTTCAT	AAGCAACAACTCGTAGCCGT	59.9	675	INTERGENIC	--	--	--
Ca4_P2_17141313	Ca4	17141313	T/TC	TTTCAATCGTATCAAGTTTTTGC	AAACTGAAAGGCCTAATTCCAA	59.2	682	DRR	Ca_05410	CR	Nin-like
Ca4_P2_17141537	Ca4	17141537	ATT/AT	TGCAAAATCAAACATAAAGGTTGT	AAACTGAAAGGCCTAATTCCAA	59.2	233	DRR	Ca_05410	CR	Nin-like
Ca4_P2_17141571	Ca4	17141571	TAAAAAA/TAAAAAAA	TGCAAAATCAAACATAAAGGTTGT	AAACTGAAAGGCCTAATTCCAA	59.2	233	DRR	Ca_05410	CR	Nin-like

INDEL marker IDs	Chromosomes/unanchored scaffolds	Physical positions (bp)	InDels (*Kabuli* reference genome-CDC Frontier/PII)	Forward primers (5'-3')	Reverse primers (5'-3')	Anneling temperature (0C)	Expected amplified product size (bp)	Structural annotation		Functional annotation	
								Sequence components of *kabuli* genome	*Kabuli* gene accession IDs	NCBI-KOG	TFs
Ca4_P2_17157304	Ca4	17157304	CAAAAAAAAAA/CAAAAAAAAAAAA	ATCTCCATCTCTGGACCGTG	ACGACGCATAGCAAGGTTCT	59.9	691	INTERGENIC	--	--	--
Ca4_P2_17157766	Ca4	17157766	TTAAT/TTAATAAT	AGAACCTTGCTATGCGTCGT	CATGGTGTGGACAAGATTGG	59.8	501	DRR	Ca_05407	--	--
Ca4_P2_17159336	Ca4	17159336	A/AC	GCCACGCTCTAGGTTGAGAT	CCTCAGGCCTGCAACTGTAT	60.3	222	DRR	Ca_05407	--	--
Ca4_P2_17166544	Ca4	17166544	ATTTTTTTT/ATTTTTTTTT	GCCAGACCATAAGTAGGCCA	TCATGTGGACACTTGGATGAA	60.0	721	INTERGENIC	--	--	--
Ca4_P2_17167099	Ca4	17167099	AT/ATAGTT	TTCCACTCTCCACCATTTCC	CACCAACAAGGATCGGATTT	59.8	429	INTERGENIC	--	--	--
Ca4_P2_17167273	Ca4	17167273	T/TC	TTCCACTCTCCACCATTTCC	CACCAACAAGGATCGGATTT	59.8	429	INTERGENIC	--	--	--
Ca4_P2_17168225	Ca4	17168225	GAAAAAAA/GAAAAAAAAA	CTCTACCCTTGAGAGTGTGTGG	TGGACGGAGTATGCATTATTGA	60.3	581	INTERGENIC	--	--	--
Ca4_P2_17172228	Ca4	17172228	TAAAAAAAAAA/TAAAAAAAAAAAA	TCTCCAGCATGTAATTACGGTTA	AAACGAAACATCCTCGTTGC	60.1	368	INTERGENIC	--	--	--
Ca4_P2_17172307	Ca4	17172307	ATT/AT	TCTCCAGCATGTAATTACGGTTA	AAACGAAACATCCTCGTTGC	60.1	368	INTERGENIC	--	--	--
Ca4_P2_17185190	Ca4	17185190	TATAATAATAAT/TATAATAATAATAAT	GTTGGCTTATGCTGTGCTTG	GGAAGTCGCTTTCCCATAAA	59.2	651	INTERGENIC	--	--	--
Ca4_P2_17187280	Ca4	17187280	TTTACTTTATTACTTTAT/TTTACTTTAT	TTTGACACATACCTCTGATCAAATC	CACAAAACTTTCTGCTGCCA	60.0	659	INTERGENIC	--	--	--
Ca4_P2_17193639	Ca4	17193639	ATTTT/ATTT	AAGCAGCAAGGATCACCAAT	CCCCATTAGTATTGCCGCTA	59.9	532	DRR	Ca_05405	T	--

INDEL marker IDs	Chromosomes/unanchored scaffolds	Physical positions (bp)	InDels (*Kabuli* reference genome-CDC Frontier/PII)	Forward primers (5'-3')	Reverse primers (5'-3')	Anneling temperature (0C)	Expected amplified product size (bp)	Structural annotation		Functional annotation	
								Sequence components of *kabuli* genome	*Kabuli* gene accession IDs	NCBI-KOG	TFs
Ca4_P2_17193665	Ca4	17193665	TAAAAAAA/TAAAAAAAA	AAGCAGCAAGGATCACCAAT	GGTGGTTGGGTTTAGCAGAA	60.0	612	DRR	Ca_05405	T	--
Ca4_P2_17194134	Ca4	17194134	TAAA/TAAAA	TAGCGGCAATACTAATGGGG	AAACCGTTTCACTCCACCAG	60.0	410	DRR	Ca_05405	T	--
Ca4_P2_17196085	Ca4	17196085	TTCTCTCTCTCTCTCTCTCTCTCTCTCT/TTCTCTCTCTCTCTCTCTCTCTCTCT	CCTCAACCATGGCCTGTACT	AGTTGCATTGGAGACAAGGG	60.1	118	INTERGENIC	--	--	--
Ca4_P2_17196504	Ca4	17196504	GTTTTTTTTT/GTTTTTTTTTT	CCTCAACCATGGCCTGTACT	AGGAGAATCAGGAGGAGGGA	60.1	889	INTERGENIC	--	--	--
Ca4_P2_17203357	Ca4	17203357	TAAGA/TAAGAAGA	TGTGCTCCATGTCTCTTTGC	CTCTTGGCTGGTAGCTTTGG	60.0	611	INTRON	Ca_05403	--	--
Ca4_P2_17206399	Ca4	17206399	ATTTTTT/ATTTTT	AACCGTTTGATGCGAAAAAT	ATCTATTGCCATCTGCCAGG	60.1	925	INTERGENIC	--	--	--
Ca4_P2_17206661	Ca4	17206661	T/TA	AATCAATCAACCAAGAACGGA	ATCTATTGCCATCTGCCAGG	60.1	598	INTERGENIC	--	--	--
Ca4_P2_17206969	Ca4	17206969	TAAAAAAAA/TAAAAAAA	CCTGGCAGATGGCAATAGAT	GAAAATGGATCCGACGAAGA	60.0	593	INTERGENIC	--	--	--
Ca4_P2_17209722	Ca4	17209722	CT/CTT	CCATTCAGAAAAGGGCAAGA	AAGCCTCGGGTAAAATTGGT	59.8	442	INTRON	Ca_05402	C	--
Ca4_P2_17210187	Ca4	17210187	ATTTTTTTTTTT/ATTTTTTTTT	GTGTCACTGGACATGCTTGG	TAGCCTTCTGAATTCCCCCT	60.0	626	INTRON	Ca_05402	C	--
Ca4_P2_17210535	Ca4	17210535	CTT/CT	TTCAGGGAGTTCCATCAACC	TAGCCTTCTGAATTCCCCCT	60.0	448	INTRON	Ca_05402	C	--
Ca4_P2_17210903	Ca4	17210903	TC/T	AGGGGGAATTCAGAAGGCTA	GTCACTCTCTCCATCGAGCC	60.0	681	INTRON	Ca_05402	C	--

INDEL marker IDs	Chromosomes/unanchored scaffolds	Physical positions (bp)	InDels (*Kabuli* reference genome-CDC Frontier/PII)	Forward primers (5'-3')	Reverse primers (5'-3')	Anneling temperature (0C)	Expected amplified product size (bp)	Structural annotation		Functional annotation	
								Sequence components of *kabuli* genome	*Kabuli* gene accession IDs	NCBI-KOG	TFs
Ca4_P2_17214428	Ca4	17214428	T/TG	GCTTTTTAGGCTGGGGAATC	TTGGTCAATGCGGAAGTGTA	60.1	627	DRR	Ca_05402	C	--
Ca4_P2_17219620	Ca4	17219620	GTTTTTT/GTTTTT	GGAATGAGAGAGAGCGTTGG	TTATGCGATGCCAATTCAAA	60.0	563	INTRON	Ca_05401	O	--
Ca4_P2_17223825	Ca4	17223825	GAAAAGAAAAA/GAAAA	TTTTCTGCATATCACTGCGA	GGCCTGGTTCTCAAAATTCA	60.1	246	DRR	Ca_05401	O	--
Ca4_P2_17227245	Ca4	17227245	ATTTT/ATTT	CATTGACGACCAGGTTTGTG	TCAGACACCGTTGTGGGATA	60.0	732	INTRON	Ca_05400	YU	--
Ca4_P2_17228265	Ca4	17228265	GTTTTTT/GTTTTT	ATGAATCCGTGATTGAAGCC	CAGCACCTCAACAATAGGCA	59.9	564	INTRON	Ca_05400	YU	--
Ca4_P2_17243952	Ca4	17243952	A/AT	AGCTTTGCGATTCTATTGGG	CAACCGTTTGAAAATGACGA	59.6	501	INTERGENIC	--	--	--
Ca4_P2_17247653	Ca4	17247653	GAAAAAAAA/GAAAAAAAAAA	TTTATGTTTGGCCAACTCCC	TTTTTAGGGACGCAATTCAAA	59.6	539	INTERGENIC	--	--	--
Ca4_P2_17247848	Ca4	17247848	TAAAAAAAAA/TAAAAAAAAAA	TTTATGTTTGGCCAACTCCC	TTTTTAGGGACGCAATTCAAA	59.6	539	INTERGENIC	--	--	--
Ca4_P2_17247936	Ca4	17247936	TAAAAA/TAAAA	CAAGACATTGGGGACAAACA	TCTGGCCAAAAATTAGCTTCA	59.8	851	INTERGENIC	--	--	--
Ca4_P2_17248915	Ca4	17248915	T/TC	ATCCCAAATTCATGCCAAAA	GCCCTCTTTTGAGTTGGAAA	59.3	396	INTERGENIC	--	--	--
Ca4_P2_17250466	Ca4	17250466	GATC/GATCCGATAGTATC	GGCCAACTTGACCCAAAATA	ACACACTTTGGTTTTTCGGC	60.0	402	INTERGENIC	--	--	--
Ca4_P2_17250760	Ca4	17250760	A/AC	GCCGAAAAACCAAAGTGTGT	CGTTGGATTCCTTTCCTCTC	58.7	514	INTERGENIC	--	--	--

INDEL marker IDs	Chromosomes/unanchored scaffolds	Physical positions (bp)	InDels (*Kabuli* reference genome-CDC Frontier/PII)	Forward primers (5'-3')	Reverse primers (5'-3')	Anneling temperature (0C)	Expected amplified product size (bp)	Structural annotation		Functional annotation	
								Sequence components of *kabuli* genome	*Kabuli* gene accession IDs	NCBI-KOG	TFs
Ca4_P2_17250847	Ca4	17250847	ATTTT/ATTT	GCCGAAAAACCAAAGTGTGT	CGTTGGATTCCTTTCCTCTC	58.7	514	INTERGENIC	--	--	--
Ca4_P2_17253834	Ca4	17253834	AAATAA/AAA	TGCAAATATCATTTCATGTTCTTG	TGATGAAGTGAGTCGCTTGG	60.0	843	INTERGENIC	--	--	--
Ca4_P2_17253939	Ca4	17253939	T/TA	TCAACATCTAGTTAGAGGTGCAAAA	TGATGAAGTGAGTCGCTTGG	60.0	780	INTERGENIC	--	--	--
Ca4_P2_17254087	Ca4	17254087	G/GTATTTATC	AAACTCCCTTACTTTGAAACTGG	TGATGAAGTGAGTCGCTTGG	60.0	212	INTERGENIC	--	--	--
Ca4_P2_17254750	Ca4	17254750	GTAATAA/GTAA	CAAATATTGTGCCAGCCAAA	TCTTAATCTTGACCAGCCCG	60.2	898	INTERGENIC	--	--	--
Ca4_P2_17257056	Ca4	17257056	ATTTT/ATTT	CCAACAAGCATTACATTGCG	ACGTTGACATTCCAGCATGA	60.1	423	INTERGENIC	--	--	--
Ca4_P2_17257099	Ca4	17257099	ATTTTTT/ATTTTTTT	CCAACAAGCATTACATTGCG	ACGTTGACATTCCAGCATGA	60.1	423	INTERGENIC	--	--	--
Ca4_P2_17257163	Ca4	17257163	ATTTTTTT/ATTTTTT	TGTACGCCAAAGTAATGCCA	TCCAATGAAATTAAACGCAAA	58.2	897	INTERGENIC	--	--	--
Ca4_P2_17257948	Ca4	17257948	AATTACTATTACTAT/AATTACTAT	ACATGGCAGGTTGGATTCAT	TGGGTACCCCACCTTAGTTT	58.3	602	INTERGENIC	--	--	--
Ca4_P2_17257974	Ca4	17257974	AATA/AATATA	ACATGGCAGGTTGGATTCAT	TCATGGGTACCCCACCTTAG	59.7	605	INTERGENIC	--	--	--
Ca4_P2_17259822	Ca4	17259822	TGA/T	ATCGTATGCCAAACTCAGGC	CGTGTGTGCATTGGAATTGT	60.4	422	INTERGENIC	--	--	--
Ca4_P2_17263202	Ca4	17263202	CAA/CA	TAAAACCAGTTTGGTTCGGC	GGTTCAAAATGGCGTTGAGT	60.0	729	INTRON	Ca_05398	R	--

INDEL marker IDs	Chromosomes/unanchored scaffolds	Physical positions (bp)	InDels (*Kabuli* reference genome-CDC Frontier/PII)	Forward primers (5'-3')	Reverse primers (5'-3')	Anneling temperature (0C)	Expected amplified product size (bp)	Structural annotation		Functional annotation	
								Sequence components of *kabuli* genome	*Kabuli* gene accession IDs	NCBI-KOG	TFs
Ca4_P2_17263445	Ca4	17263445	CT/CTT	CGGTTCAATTTTCAAACGGT	GGTTCAAAATGGCGTTGAGT	60.0	658	INTRON	Ca_05398	R	--
Ca4_P2_17264416	Ca4	17264416	TAAAAAAAAAAAAA/TAAAAAAAAA	CCATTGCCTAGCTCTTGCAT	TTCCCAACTGGAACCTTGTC	59.9	156	INTRON	Ca_05398	R	--
Ca4_P2_17278734	Ca4	17278734	CTTTTTTTT/CTTTTTTTTT	CCCGAGCGAGTAATCAAGAG	AGCTTCACGAGGCTTATCCA	60.0	710	DRR	Ca_05394	R	--
Ca4_P2_17280110	Ca4	17280110	ATT/AT	CATTGGCTGCTTTGTTGCTA	GCAATATGGAGCCACGGTAT	59.8	596	DRR	Ca_05395	R	--
Ca4_P2_17284874	Ca4	17284874	GATA/GA	TTGAATATGAGCCTCCAAGAGA	CTTGTTGAGGCTTTGGGTGT	60.1	794	DRR	Ca_05393	R	--
Ca4_P2_17285160	Ca4	17285160	ACT/A	CATTTCCTTTGAGTCATGAGAGG	CTTGTTGAGGCTTTGGGTGT	60.1	310	DRR	Ca_05393	R	--
Ca4_P2_17286686	Ca4	17286686	CATTGGATTGGATTG/CATTGGATTG	TGCATTTCATGCCTTTGCTA	TGATTAGCCCGTATTTTGGG	59.8	648	INTRON	Ca_05393	R	--
Ca4_P2_17290871	Ca4	17290871	GTTTAATT/GTTTAATTTTAATT	CCAAGTATGGCAGGTGGAAT	TCATGAGCTAGAATGGTTGTAAAAA	59.3	615	INTERGENIC	--	--	--
Ca4_P2_17291312	Ca4	17291312	AAT/A	AAAATTATCGCGTGACGCTT	TAGATCACCCACACCAACGA	60.0	590	INTERGENIC	--	--	--
Ca4_P2_17291561	Ca4	17291561	T/TA	TCGTTGGTGTGGGTGATCTA	TGTCGGTCACGTCATCATTT	60.0	450	INTERGENIC	--	--	--
Ca4_P2_17292884	Ca4	17292884	ATCTTCTT/ATCTT	GGGACTATTTTCGCGATTCA	GCGGATGCGGATTATGTTAT	59.8	233	DRR	Ca_05392	R	--
Ca4_P2_17293698	Ca4	17293698	TAAAAAAAAA/TAAAAAAAA	TCACATCCGTTGAACGAAGA	TTTGTTTTATTAGTTTTGGTGTGG	57.4	652	DRR	Ca_05392	R	--

INDEL marker IDs	Chromosomes/unanchored scaffolds	Physical positions (bp)	InDels (*Kabuli* reference genome-CDC Frontier/PII)	Forward primers (5'-3')	Reverse primers (5'-3')	Anneling temperature (0C)	Expected amplified product size (bp)	Structural annotation		Functional annotation	
								Sequence components of *kabuli* genome	*Kabuli* gene accession IDs	NCBI-KOG	TFs
Ca4_P2_17297028	Ca4	17297028	TA/TAA	TTTGCCTCAGCTGTGTTGAC	TCATCAGCTGGACAACTTCG	60.0	607	INTRON	Ca_05392	R	--
Ca4_P2_17297165	Ca4	17297165	TATATATATAAAT/TAT	TTTGCCTCAGCTGTGTTGAC	TCATCAGCTGGACAACTTCG	60.0	607	INTRON	Ca_05392	R	--
Ca4_P2_17297693	Ca4	17297693	TATA/TATAATA	CGAAGTTGTCCAGCTGATGA	CCACGAGGATCGGACAGTAT	60.0	528	INTRON	Ca_05392	R	--
Ca4_P2_17299468	Ca4	17299468	CTTTTTTTT/CTTTTTTTTT	GACCAACGCAAACAAGGATT	TCGAGGCAAGGTTAGAAGGA	59.9	671	DRR	Ca_05391	TBLD	--
Ca4_P2_17312805	Ca4	17312805	TATTATTGATTATTGATTATTGATGATTGATTATTGAT/TATTATTGATTATTGATTATTGATTATTGATGATTGATTATTGAT	AGCGAACCGATAGCTTCAAA	ACGCATAGCTGCATCCTCTT	60.0	509	INTRON	Ca_05391	TBLD	--
Ca4_P2_17313521	Ca4	17313521	TAAA/TAA	GCTAGACAGGTTTGCCTTCG	AAGGCTCCTGCAAGCATTTA	60.0	966	INTERGENIC	--	--	--
Ca4_P2_17314621	Ca4	17314621	ATTTTTT/ATTTTT	ACGCGATTGAGAACAAAAGG	CTTCCCCACTCACAGTCCAT	60.0	604	DRR	Ca_05390	M	--
Ca4_P2_17314963	Ca4	17314963	TGGGG/TGGGGG	ATGGACTGTGAGTGGGGAAG	CAATGGAGAGGTTCGTGACA	59.7	156	DRR	Ca_05390	M	--
Ca4_P2_17315192	Ca4	17315192	TAAA/TAAAA	ATGGACTGTGAGTGGGGAAG	TTGGTCCAACAGCTTGATGA	60.2	716	DRR	Ca_05390	M	--
Ca4_P2_17315979	Ca4	17315979	ATT/ATTT	GTGACGGGCACTCCATAACT	ATACCGTCTCATGCCTCTCG	60.2	704	DRR	Ca_05390	M	--
Ca4_P2_17316090	Ca4	17316090	A/AG	GTGACGGGCACTCCATAACT	AGGTCCGCTCTGATTGAAGA	60.0	757	DRR	Ca_05390	M	--
Ca4_P2_17317909	Ca4	17317909	ATTTTT/ATTTTTT	CTCAAAAGTGGGATAGGGCA	ACAGCGTGAAACAAGATCCC	60.1	429	INTRON	Ca_05390	M	--

INDEL marker IDs	Chromosomes/unanchored scaffolds	Physical positions (bp)	InDels (*Kabuli* reference genome-CDC Frontier/PII)	Forward primers (5'-3')	Reverse primers (5'-3')	Anneling temperature (0C)	Expected amplified product size (bp)	Structural annotation		Functional annotation	
								Sequence components of *kabuli* genome	*Kabuli* gene accession IDs	NCBI-KOG	TFs
Ca4_P2_17318086	Ca4	17318086	A/AG	CTCAAAAGTGGGATAGGGCA	ACAGCGTGAAACAAGATCCC	60.1	429	INTRON	Ca_05390	M	--
Ca4_P2_17318109	Ca4	17318109	AA/AATATTA	CTCAAAAGTGGGATAGGGCA	ACAGCGTGAAACAAGATCCC	60.1	429	INTRON	Ca_05390	M	--
Ca4_P2_17321092	Ca4	17321092	CGATGATGATGATGATGATG/CGATGATGATGATGATGATG	CATCGGCAAAGACTGAGTGA	CGATGGTGCAGATAGCAGAA	60.0	582	INTERGENIC	--	--	--
Ca4_P2_17322906	Ca4	17322906	AATATATATATATATATATATATATA/AATATATATATATATATATATA	TCATCCATAGAGCAGTGGCA	CGATCACGATTGTGTTTTGG	60.0	225	INTERGENIC	--	--	--
Ca4_P2_17323787	Ca4	17323787	TAA/TAAA	CTTGGTACAGGTTTGGCGAT	TCTTGTGGTTGTGGGTTTCA	60.0	490	INTERGENIC	--	--	--
Ca4_P2_17323828	Ca4	17323828	CATATA/CATA	CTTGGTACAGGTTTGGCGAT	TCTTGTGGTTGTGGGTTTCA	60.0	490	INTERGENIC	--	--	--
Ca4_P2_17327734	Ca4	17327734	ATTTT/ATTT	CGAACACAACGATAAGATCTCG	TGCACTTAATTTGCCAACGA	60.3	876	INTERGENIC	--	--	--
Ca4_P2_17328369	Ca4	17328369	GAAAA/GAAAAA	ACACGTGCAATATCGGTGAA	TTCACAAGTTTTTCGATACGGA	59.6	429	INTERGENIC	--	--	--
Ca4_P2_17328665	Ca4	17328665	GAA/GAAAA	ACACGTGCAATATCGGTGAA	TCCTCCCTCTTTATCATGCC	59.1	750	INTERGENIC	--	--	--
Ca4_P2_17328879	Ca4	17328879	GTTTTTT/GTTTTTTT	CGTGATAAAACGTGCATCAA	TCCTCCCTCTTTATCATGCC	59.1	244	INTERGENIC	--	--	--
Ca4_P2_17329245	Ca4	17329245	TCACA/TCA	GGCATGATAAAGAGGGAGGA	TAACCTCTCACGTGCAATCG	59.9	386	INTERGENIC	--	--	--
Ca4_P2_17329310	Ca4	17329310	AC/A	GGCATGATAAAGAGGGAGGA	TAACCTCTCACGTGCAATCG	59.9	386	INTERGENIC	--	--	--

INDEL marker IDs	Chromosomes/unanchored scaffolds	Physical positions (bp)	InDels (*Kabuli* reference genome-CDC Frontier/PII)	Forward primers (5'-3')	Reverse primers (5'-3')	Anneling temperature (0C)	Expected amplified product size (bp)	Structural annotation		Functional annotation	
								Sequence components of *kabuli* genome	*Kabuli* gene accession IDs	NCBI-KOG	TFs
Ca4_P2_17337403	Ca4	17337403	CAA/CAAA	AATGGCTGCCGTTATTTTTG	AACAACATTTGCCTTCCCTG	60.0	198	INTERGENIC	--	--	--
Ca4_P2_17339262	Ca4	17339262	CTTTTTTTTT/CTTTTTTTT	ACTTGGGAACCCGAAGAAGT	AGAGTAGCCGGCAAGGTCTC	60.9	707	INTERGENIC	--	--	--
Ca4_P2_17342951	Ca4	17342951	ATTTTTTT/ATTTTTT	TTTAGCTTAGCTCCTATGCGG	GGCTTAGCTTGGTCTCATGC	60.0	918	DRR	Ca_05389	A	--
Ca4_P2_17353180	Ca4	17353180	TAAAAAAAAAA/TAAAAAAAAA	GCTTAGGTGGACTTATGGGC	CGATCCATTATTTCAGTGGGA	59.8	379	INTERGENIC	--	--	--
Ca4_P2_17354406	Ca4	17354406	TA/T	TTGTTCTTCTGTGGACACCAA	AATATGTCATAATTGACCGAATCA	57.5	464	INTERGENIC	--	--	--
Ca4_P2_17354782	Ca4	17354782	TAAAAAAAA/TAAAAAAA	TATTGATTCGGTCAATTATGACA	TGGATTAGGCCTTGAAGCAT	59.7	728	INTERGENIC	--	--	--
Ca4_P2_17355433	Ca4	17355433	ATTTTTTTTTT/ATTTTTTTTTTTT	AAAGGACCCCGAAAAAGAAA	TGAGGCTTATAGGCCCAATG	60.1	334	INTERGENIC	--	--	--
Ca4_P2_17360464	Ca4	17360464	TTATATATATATATATAT/TTATATATATATATATATATATATATAT	GTGTGCGATTGAGTTGAACG	CTTGCTGGGCTTTTCAAGAC	60.0	579	INTRON	Ca_05388	T	NAC
Ca4_P2_17361143	Ca4	17361143	TGAGAGAGAGA/TGAGAGAGAGAGAGAGAGA	GTCTTGAAAAGCCCAGCAAG	CCTACCCTGCTCAAAGTTGC	59.9	469	INTRON	Ca_05388	T	NAC
Ca4_P2_17365284	Ca4	17365284	TA/TAA	CCCCGGCTTACACATTCTTA	TATTGGTGTAACCGAACGCA	60.0	557	INTRON	Ca_05388	T	NAC
Ca4_P2_17365456	Ca4	17365456	TAAAAAAAAAAA/TAAAAAAAAA	TGCGTTCGGTTACACCAATA	ACGTAGAAAGGGCGGAAGAT	60.1	397	INTRON	Ca_05388	T	NAC
Ca4_P2_17366027	Ca4	17366027	GAAAAAAAAA/GAAAAAAAAAAA	AAGAACATCTTCCGCCCTTT	GGCAGTGGATTCATTTCGTT	59.9	325	INTRON	Ca_05388	T	NAC

INDEL marker IDs	Chromosomes/unanchored scaffolds	Physical positions (bp)	InDels (*Kabuli* reference genome-CDC Frontier/PII)	Forward primers (5'-3')	Reverse primers (5'-3')	Anneling temperature (0C)	Expected amplified product size (bp)	Structural annotation		Functional annotation	
								Sequence components of *kabuli* genome	*Kabuli* gene accession IDs	NCBI-KOG	TFs
Ca4_P2_17367667	Ca4	17367667	AC/ACC	TAAATGTGATTTGCGAGCGA	ATGCATGGAGGTGATGATGA	59.9	284	INTERGENIC	--	--	--
Ca4_P2_17372359	Ca4	17372359	CC/CCCTCACTAC	TGCATAAACAAAGATCGGGT	CGTGTTTCCAAGGGTCATCT	60.0	691	INTERGENIC	--	--	--
Ca4_P2_17372607	Ca4	17372607	TAAAAAAAA/TAAAAAAA	TCTGTCCAAACCACATTTCAA	CGTGTTTCCAAGGGTCATCT	60.0	455	INTERGENIC	--	--	--
Ca4_P2_17375359	Ca4	17375359	ATAGGATT/ATAGGATTAGGATT	TTTCACCGAGTAGCTTGAGGA	TCTCCTTGAAGTGTGCAACAA	59.5	649	INTERGENIC	--	--	--
Ca4_P2_17375692	Ca4	17375692	TAAAAAAAAAAA/TAAAAAAAAAAAA	TTGTTGCACACTTCAAGGAGA	TCGTAGTTGGTCACCGATCA	60.1	435	INTERGENIC	--	--	--
Ca4_P2_17375846	Ca4	17375846	CTTTTTTTT/CTTTTTTT	TTGTTGCACACTTCAAGGAGA	TCGTAGTTGGTCACCGATCA	60.1	435	INTERGENIC	--	--	--
Ca4_P2_17376469	Ca4	17376469	CAAAAAAAAAAAA/CAAAAAAAAAAA	TGATCGGTGACCAACTACGA	AACACATTTGAAGATGGGGG	59.6	536	DRR	Ca_05387	R	--
Ca4_P2_17376716	Ca4	17376716	ATTTTTTTTT/ATTTTTTTTTTT	CCCCCATCTTCAAATGTGTT	AGATGTAGCAACGCAAACCA	59.3	698	DRR	Ca_05387	R	--
Ca4_P2_17379518	Ca4	17379518	TG/T	AACAAATGGTGCATCACGAA	TCTTCATTGGTCTCGTGCTT	58.4	740	INTRON	Ca_05387	R	--
Ca4_P2_17380152	Ca4	17380152	AT/ATT	GCAACCGAACCCAAGTAAAG	GGAATCAATGCAAAGCCCTA	60.0	200	INTRON	Ca_05387	R	--
Ca4_P2_17380447	Ca4	17380447	CG/C	TAGGGCTTTGCATTGATTCC	CAACCTCATCCACTTTCCGT	60.0	352	INTRON	Ca_05387	R	--
Ca4_P2_17388357	Ca4	17388357	TAAAAAAAAAAAAA/TAAAAAAAAA	AAATCGCCATTTGGAACTTG	CACGATCATGTGTTCCACAA	58.9	308	INTERGENIC	--	--	--

INDEL marker IDs	Chromosomes/unanchored scaffolds	Physical positions (bp)	InDels (*Kabuli* reference genome-CDC Frontier/PII)	Forward primers (5'-3')	Reverse primers (5'-3')	Anneling temperature (0C)	Expected amplified product size (bp)	Structural annotation		Functional annotation	
								Sequence components of *kabuli* genome	*Kabuli* gene accession IDs	NCBI-KOG	TFs
Ca4_P2_17408958	Ca4	17408958	ATGTTGT/ATGT	CCAAAGAATTTTCCGACCAA	CTCCTCCCCTCAAAATCTCC	60.0	567	INTERGENIC	--	--	--
Ca4_P2_17420097	Ca4	17420097	TA/TAA	TCGTTCAACCATTGCATCAT	TCCGAATGAGTGACCAAAATC	59.9	612	INTERGENIC	--	--	--
Ca4_P2_17421248	Ca4	17421248	G/GTTTAA	CCCGTGAGTTGTTGGAAGTT	TCGATAATTTGAAACCGCCT	59.5	569	INTERGENIC	--	--	--
Ca4_P2_17423293	Ca4	17423293	TTATATA/TTA	GTGTGGGTTGGCTTTCATCT	GAAACGCTATGATGCCGTAAA	60.1	492	INTERGENIC	--	--	--
Ca4_P2_17431572	Ca4	17431572	GC/G	TCTGTCATTCCTGCCATTTTT	TCGCAATGTAGCTCCCTCTT	60.0	858	INTRON	Ca_05384	K	--
Ca4_P2_17431624	Ca4	17431624	T/TG	TGTCCCTCATTATAAGCACCC	TCGCAATGTAGCTCCCTCTT	60.0	606	INTRON	Ca_05384	K	--
Ca4_P2_17435255	Ca4	17435255	GTACTACTA/GTACTA	TCAAGCTGCTTTCAATGGTG	GAGGCCAATGGAGTGGTTTA	59.9	241	INTRON	Ca_05384	K	--
Ca4_P2_17436350	Ca4	17436350	CTTTTT/CTTT	AAGATCCTGCCCCTCAAGAT	TGCTCCAACTGAGTGGTGAC	59.9	459	INTRON	Ca_05384	K	--
Ca4_P2_17437453	Ca4	17437453	T/TG	CTTGCTTTTGGAGACCTTGC	CATCCAAGGCAAGGAACAAT	59.9	463	INTRON	Ca_05384	K	--
Ca4_P2_17437950	Ca4	17437950	TTGT/TT	ATGGGTTCCCATGAAAATCA	CAATGCCGCACTCATTACAC	60.1	585	INTRON	Ca_05384	K	--
Ca4_P2_17438191	Ca4	17438191	ATTTTTTTTT/ATTTTTTTT	ATGGGTTCCCATGAAAATCA	ACCGAGTGAATGGCCATAAG	60.0	757	INTRON	Ca_05384	K	--
Ca4_P2_17439449	Ca4	17439449	AC/A	CATCAACTATCCCCACACCC	ATGAGGCCCAAGAAACCTCT	60.1	571	INTRON	Ca_05384	K	--

INDEL marker IDs	Chromosomes/unanchored scaffolds	Physical positions (bp)	InDels (*Kabuli* reference genome-CDC Frontier/PII)	Forward primers (5'-3')	Reverse primers (5'-3')	Anneling temperature (0C)	Expected amplified product size (bp)	Structural annotation		Functional annotation	
								Sequence components of *kabuli* genome	*Kabuli* gene accession IDs	NCBI-KOG	TFs
Ca4_P2_17443644	Ca4	17443644	GAAAAAAAAA/GAAAAAAAA	GTTGGTGTTGGCTTTTGCTT	CGTTGGTTTACAGAGGGCAT	60.0	768	DRR	Ca_05384	K	--
Ca4_P2_17448021	Ca4	17448021	TATTTTCATTTTC/TATTTTCATTTTCATTTTC	GAGTTGAGGAAACAATTATGTCAAA	TTCTTTTTCTGCGATTTCTCG	59.6	654	INTERGENIC	--	--	--
Ca4_P2_17449171	Ca4	17449171	A/AG	TTCTTCACCGAGCTTTCGTT	AATGCATGCTTGATGGTTCA	60.1	654	INTERGENIC	--	--	--
Ca4_P2_17457595	Ca4	17457595	ATT/ATTT	CTGCACGAAGGCAACATAAA	TTGGACCACCTAACCTGCTC	60.1	795	DRR	Ca_05382	--	ERF
Ca4_P2_17463034	Ca4	17463034	A/AT	AGAAATAAAATCACGAGTAACCAAA	TTGCGGATTCCCAGTTACTC	60.1	734	INTERGENIC	--	--	--
Ca4_P2_17463437	Ca4	17463437	ATTTT/ATTTTTGATTTTT	GAGTAACTGGGAATCCGCAA	GATACTTTGGCCGTGACGTT	60.0	385	INTERGENIC	--	--	--
Ca4_P2_17463503	Ca4	17463503	TTTTATTTATTTATTTATT/TTTTATTTATTTATTTATTTATTTATT	GAGTAACTGGGAATCCGCAA	GATACTTTGGCCGTGACGTT	60.0	385	INTERGENIC	--	--	--
Ca4_P2_17464247	Ca4	17464247	C/CGAGG	ACCACTCACCAACCTTCCAG	AACCCGGAGACCCAATTTAG	60.2	635	INTRON	Ca_05381	O	GRAS
Ca4_P2_17470566	Ca4	17470566	TGGG/TGG	TGGTGCAACCACTCTGCTAT	TCCACTTGGAAGCAGCTTTT	60.0	425	DRR	Ca_05380	AB	--
Ca4_P2_17470593	Ca4	17470593	GTTTTTTTTTTTTTTT/GTTTTTTTTTTTTT	TCCTTGCAACATTTGTTTGG	TCCACTTGGAAGCAGCTTTT	60.0	157	DRR	Ca_05380	AB	--
Ca4_P2_17470871	Ca4	17470871	GTGGCTA/GTGGCTAATGGCTA	GTCCCACCAAAGAAAGCAAA	CACTTGAAGAGGCTCCATCC	59.8	343	DRR	Ca_05380	AB	--
Ca4_P2_17472385	Ca4	17472385	CACATCAAA/CACATCAAAACATCAAA	TAATGATCCCTAGGGGAGGG	CGCTTTAAGCCTGACAAAGG	60.0	376	DRR	Ca_05380	AB	--

INDEL marker IDs	Chromosomes/unanchored scaffolds	Physical positions (bp)	InDels (*Kabuli* reference genome-CDC Frontier/PII)	Forward primers (5'-3')	Reverse primers (5'-3')	Anneling temperature (0C)	Expected amplified product size (bp)	Structural annotation		Functional annotation	
								Sequence components of *kabuli* genome	*Kabuli* gene accession IDs	NCBI-KOG	TFs
Ca4_P2_17475440	Ca4	17475440	GT/GTTGTTATT	CGGGCAGTAGCATTAGATGG	GGATGAATGGGAGGTGGTTA	59.6	210	INTERGENIC	--	--	--
Ca4_P2_17480298	Ca4	17480298	ATATTATTATTATTATTAT/ATATTATTATTAT	TTGTTGGCCGTCATATGCTA	GACCAATTACAAGCGAGGGA	60.1	617	INTRON	Ca_05379	O	--
Ca4_P2_17482178	Ca4	17482178	ATTTTTTT/ATTTTTTTT	TCCATCTGAACCCTGACTCC	TGCCCTAAGATGGTTTGGTC	59.9	445	INTRON	Ca_05379	O	--
Ca4_P2_17482484	Ca4	17482484	CGTTG/CGTTGTTG	TCCATCTGAACCCTGACTCC	AAGTTGGGTGATGAAGTGCC	60.0	628	INTRON	Ca_05379	O	--
Ca4_P2_17484481	Ca4	17484481	ATTTTTTTTT/ATTTTTTTTTT	TCGCCTGATGCTTGTACTTG	AGGAGGCTTTTCCACAGGTT	60.1	361	INTRON	Ca_05379	O	--
Ca4_P2_17489115	Ca4	17489115	GAAAAAAA/GAAAAAA	CATCCACCTCAGGGAAGAAA	CGGCGGCATCTACATTTTAT	60.0	575	DRR	Ca_05379	O	--
Ca4_P2_17489726	Ca4	17489726	AC/ACC	ATAAAATGTAGATGCCGCCG	CCAGCTTGTGACAAAAAGCA	60.0	349	DRR	Ca_05379	O	--
Ca4_P2_17494020	Ca4	17494020	GAAA/GAAAA	GGGTGTGGAAAAATGAAAGC	AGAATTGCTGCTGCTCAGGT	60.2	734	INTRON	Ca_05378	--	--
Ca4_P2_17494426	Ca4	17494426	AGG/AG	AAATCACACCAAAGCAAAAGC	CCGAAGCCGTTAACATTCAT	60.0	674	DRR	Ca_05377	R	B3
Ca4_P2_17495202	Ca4	17495202	GT/G	GTGTGAAGTGGGGTTTGCTT	CGAAATTCATGCATTCAACG	60.1	476	DRR	Ca_05377	R	B3
Ca4_P2_17495441	Ca4	17495441	AG/A	GTGTGAAGTGGGGTTTGCTT	CGAAATTCATGCATTCAACG	60.1	476	DRR	Ca_05377	R	B3
Ca4_P2_17495808	Ca4	17495808	A/AT	TCATTGCTGAAGAGCACGAT	TGCTCAACAACAGGAAGTGG	59.9	728	DRR	Ca_05377	R	B3

INDEL marker IDs	Chromosomes/unanchored scaffolds	Physical positions (bp)	InDels (*Kabuli* reference genome-CDC Frontier/PII)	Forward primers (5'-3')	Reverse primers (5'-3')	Anneling temperature (0C)	Expected amplified product size (bp)	Structural annotation		Functional annotation	
								Sequence components of *kabuli* genome	*Kabuli* gene accession IDs	NCBI-KOG	TFs
Ca4_P2_17528276	Ca4	17528276	TGG/TGGG	AAGCTTTCCATAAGCCGTGA	GACAACGCAGAAGCAGAACA	60.2	911	INTRON	Ca_05374	R	NAC
Ca4_P2_17532742	Ca4	17532742	TAAAA/TAAAAA	CCCATCTTGTTTGGTTCACA	CTGAAAGGGCATAAAGGCAA	60.2	954	INTERGENIC	--	--	--
Ca4_P2_17533392	Ca4	17533392	CTT/CTTT	CTTTTTGCTGGTGCCATTTT	GGCATTGTGCTGAGTGATTTT	60.1	679	INTERGENIC	--	--	--
Ca4_P2_17533461	Ca4	17533461	AA/AATTA	CTTTTTGCTGGTGCCATTTT	TGCAAGAAGAAGGAAGCCAT	60.0	713	INTERGENIC	--	--	--
Ca4_P2_17533529	Ca4	17533529	GT/G	CTTTTTGCTGGTGCCATTTT	TGCAAGAAGAAGGAAGCCAT	60.0	713	INTERGENIC	--	--	--
Ca4_P2_17534928	Ca4	17534928	TAAAAAAAAA/TAAAAAAAA	TTTTTGTGTGTGTGCGGTTT	TGTGTCAAAGATGGTATTTCAATG	58.9	410	INTERGENIC	--	--	--
Ca4_P2_17534970	Ca4	17534970	AAAA/AAAAACATATTTCAAA	TTTTTGTGTGTGTGCGGTTT	TGTGTCAAAGATGGTATTTCAATG	58.9	410	INTERGENIC	--	--	--
Ca4_P2_17535175	Ca4	17535175	ACT/ACTCT	TTTTTGTGTGTGTGCGGTTT	GGCTGGAAGTGAGCTAGTGG	60.0	811	INTERGENIC	--	--	--
Ca4_P2_17545144	Ca4	17545144	TATTAAAATAATAATTA/TATTAAAATAATAATTAAAATAATAATTA	ATCCAAAAGGTCGTCCACAG	AAATCGGTTTCCCCAGTACC	60.1	652	INTERGENIC	--	--	--
Ca4_P2_17545339	Ca4	17545339	TAAAAAAAAAA/TAAAAAAAAA	ATGCCCTTATCGTCCTGATG	AAATCGGTTTCCCCAGTACC	60.1	537	INTERGENIC	--	--	--
Ca4_P2_17545627	Ca4	17545627	TAAAAAAAA/TAAAAAAA	AGTTTAGGGGCAAGCACTGA	TGCATCGGAGATCACATCTT	59.2	363	INTERGENIC	--	--	--
Ca4_P2_17547225	Ca4	17547225	TTTATT/TTT	CAACATCAGATGCACATGGA	TTCGTGATCGTTTGGTGAAA	60.1	845	INTERGENIC	--	--	--

INDEL marker IDs	Chromosomes/unanchored scaffolds	Physical positions (bp)	InDels (*Kabuli* reference genome-CDC Frontier/PII)	Forward primers (5'-3')	Reverse primers (5'-3')	Anneling temperature (0C)	Expected amplified product size (bp)	Structural annotation		Functional annotation	
								Sequence components of *kabuli* genome	*Kabuli* gene accession IDs	NCBI-KOG	TFs
Ca4_P2_17552174	Ca4	17552174	GTT/GTTT	AAGTGAGGCCACTGGAAAGA	ATGTGTTGGAACTTTTCCGC	60.0	204	INTERGENIC	--	--	--
Ca4_P2_17552666	Ca4	17552666	TAAAAAAA/TAAAAAAAA	GCGGAAAAGTTCCAACACAT	ATTCGATTTTTGAGCGGTTG	60.1	536	INTERGENIC	--	--	--
Ca4_P2_17600797	Ca4	17600797	TC/T	AAGGGTGATGGACGAAAAAC	TTAAATGGTCGCATAGAGCAA	58.5	682	INTERGENIC	--	--	--
Ca4_P2_17600811	Ca4	17600811	TAAAAAA/TAAAAAAA	AAGGGTGATGGACGAAAAAC	TCCAACAACATCAAATTCATCC	59.7	951	INTERGENIC	--	--	--
Ca4_P2_17601510	Ca4	17601510	TC/T	GGATGAATTTGATGTTGTTGGA	CCGTGTCTATCCCCATTTTG	60.2	515	INTERGENIC	--	--	--
Ca4_P2_17604443	Ca4	17604443	TA/T	TATTGGGTAGCTTGGCCACT	TTCACACCTCACAGCAGGAC	59.9	977	INTRON	Ca_05371	S	--
Ca4_P2_17606592	Ca4	17606592	ATTTTTTTTTT/ATTTTTTTTT	GCATTGTGAACAAATTATCGTAGC	GCGGAAGTAGCGATCTCAAG	60.1	767	DRR	Ca_05371	S	--
Ca4_P2_17616508	Ca4	17616508	CAAAAAAAAA/CAAAAAAAAAA	ACCAAACACCGGTAAGCAAG	AACAAAACGAACAACCCAGC	60.0	472	INTRON	Ca_05369	E	--
Ca4_P2_17617805	Ca4	17617805	GAAAAAAAA/GAAAAAAA	CATCTAGTGTTGGGGAATGTGA	ACGTTTCGCCCTACATCATT	59.5	838	INTERGENIC	--	--	--
Ca4_P2_17617845	Ca4	17617845	AATATATATATATATATATATATATATAT/AATATATATATATATATATATATAT	CATCTAGTGTTGGGGAATGTGA	ACGTTTCGCCCTACATCATT	59.5	838	INTERGENIC	--	--	--
Ca4_P2_17628873	Ca4	17628873	TA/TACTTAAAATAA	TACAAGCAATCGGTGATCCA	CAGCGGCTTCAAACCTTTAG	60.0	559	INTERGENIC	--	--	--
Ca4_P2_17629803	Ca4	17629803	ATTTTTTTT/ATTTTTTTTTTT	CTTTTCCGCGAACACATTTT	GTACATACATGGACGCGACG	60.0	587	INTERGENIC	--	--	--

INDEL marker IDs	Chromosomes/unanchored scaffolds	Physical positions (bp)	InDels (*Kabuli* reference genome-CDC Frontier/PII)	Forward primers (5'-3')	Reverse primers (5'-3')	Anneling temperature (0C)	Expected amplified product size (bp)	Structural annotation		Functional annotation	
								Sequence components of *kabuli* genome	*Kabuli* gene accession IDs	NCBI-KOG	TFs
Ca4_P2_1763030	Ca4	1763030	ATTT/ATTTT	AAAGGCATCATTCACGTTCA	ACATTTTGGAAGGGCATCTG	59.9	757	INTERGENIC	--	--	--
Ca4_P2_17631583	Ca4	17631583	TT/TTAGAAAT	TGCACTTTTCATCGCTTTTG	ACATGGATGGGATCTGTGT	60.1	347	INTRON	Ca_05367	Z	WRKY
Ca4_P2_17633397	Ca4	17633397	ACCC/ACC	GTGAGAGTGGAGCAGGGAAG	GTGCTCCAGCACATAGCTGA	60.2	649	INTRON	Ca_05367	Z	WRKY
Ca4_P2_17636026	Ca4	17636026	C/CCAAGATATA	GGCCTCATCTCTAGCCAATG	CCAAGGAAGTTTCACCAGGA	60.1	297	INTRON	Ca_05367	Z	WRKY
Ca4_P2_17636087	Ca4	17636087	GTT/GTTT	GGCCTCATCTCTAGCCAATG	CCAAGGAAGTTTCACCAGGA	60.1	297	INTRON	Ca_05367	Z	WRKY
Ca4_P2_17637226	Ca4	17637226	CTATT/C	ACACAAGTTAGGTGGGGCAG	CTGCATCTGTAGTGAGGCCA	60.0	346	INTRON	Ca_05367	Z	WRKY
Ca4_P2_17637458	Ca4	17637458	GAC/G	ACACAAGTTAGGTGGGGCAG	GCATGGCTTGATGTGAGAGA	60.0	475	INTRON	Ca_05367	Z	WRKY
Ca4_P2_1763837	Ca4	1763837	TTTTT/TTTTTGTTTT	AGAAAAATAGCGGTGGTGGA	GGTTGATGAGCAATGCCTCT	60.2	390	INTERGENIC	--	--	--
Ca4_P2_17638808	Ca4	17638808	CAA/CA	GTTGCTTCAGGGGTTACCAA	GACAACTGCAGCATCAATGG	60.3	301	INTRON	Ca_05367	Z	WRKY
Ca4_P2_17640423	Ca4	17640423	CTTTTTTT/CTTTTTT	CAAGAAGAAGTGGTGGGGAA	GAACCCTTTCCATCGAGTCA	60.1	524	DRR	Ca_05367	Z	WRKY
Ca4_P2_17649020	Ca4	17649020	TTTTAAA/TTTTAAATTTAAA	CCAAAGAGCTGAAATGCACA	GGAAGCCGAAAAACTAAGGC	60.2	399	DRR	Ca_05365	H	--
Ca4_P2_17651073	Ca4	17651073	CTTG/CTTTTGTTTTTTGTTG	ACGTTGACAGCCCAATTCAT	AGAAAAAGAAGCAACGGCAA	60.0	467	INTERGENIC	--	--	--

INDEL marker IDs	Chromosomes/unanchored scaffolds	Physical positions (bp)	InDels (*Kabuli* reference genome-CDC Frontier/PII)	Forward primers (5'-3')	Reverse primers (5'-3')	Anneling temperature (0C)	Expected amplified product size (bp)	Structural annotation		Functional annotation	
								Sequence components of *kabuli* genome	*Kabuli* gene accession IDs	NCBI-KOG	TFs
Ca4_P2_17653817	Ca4	17653817	AATATA/AATA	GATGTGAGGGCTTGCAATTT	GGTGCAACTTTATTTGGTCG	58.1	870	DRR	Ca_05364	S	--
Ca4_P2_17654502	Ca4	17654502	CTTT/CTTTT	TTGTTTTCGATCATTTGACCA	AAACGAGAGCAGTGGTGTCA	59.5	262	DRR	Ca_05364	S	--
Ca4_P2_17656569	Ca4	17656569	GATA/GATATA	AATTTGGGGCGTTACAAACA	GTGCAAGATCCGCTACCAGT	60.3	427	INTERGENIC	--	--	--
Ca4_P2_17658487	Ca4	17658487	TAA/TA	GGCCCATTGGTGGTTTATTA	CAAACCGAACGTTTCCTTGT	60.0	526	INTERGENIC	--	--	--
Ca4_P2_1765972	Ca4	1765972	ATTAACTTT/ATT	TATTGGGTGTGAATGCGAAA	TCTCAGCATGCAACTGTATGAA	59.5	205	INTERGENIC	--	--	--
Ca4_P2_17661499	Ca4	17661499	TAAAAAAA/TAAAAAAAA	TGGTAGCATCAATGAGCCTG	TCTTTGCTATGTGACCGAACA	59.3	690	DRR	Ca_05363	R	WRKY
Ca4_P2_17662013	Ca4	17662013	TAAAAAAAAAA/TAAAAAAAAA	TGTTCGGTCACATAGCAAAGA	TTAACGGTTCGTTTTCCAGG	60.0	719	DRR	Ca_05363	R	WRKY
Ca4_P2_17666631	Ca4	17666631	GAAAAAA/GAAAAA	GTGGGGGCAATATGGTAAGA	AAACATGGCTTGCATGTGAA	60.1	106	DRR	Ca_05361	--	--
Ca4_P2_17672684	Ca4	17672684	GATA/GA	TGTAGTCCCTCTGTCCCAAAA	CGGAAGATACCAAGTCGCAT	60.1	433	DRR	Ca_05360	--	--
Ca4_P2_17677636	Ca4	17677636	CT/CTT	GGGGGCCAAAAATTAAGAGA	ATATTGGGGATGATGTGGGA	59.8	528	INTERGENIC	--	--	--
Ca4_P2_17689554	Ca4	17689554	ATTTT/ATTT	GGGGATACAAAGGATGAGGAA	AGGGGAGAGTGGGAAGAGAG	59.8	557	INTERGENIC	--	--	--
Ca4_P2_17692749	Ca4	17692749	ATTTT/ATT	GGGAGAAGTTGAACCCATGA	TCCAACCACGGGTAGATAGC	60.0	568	INTRON	Ca_05356	TU	--

INDEL marker IDs	Chromosomes/unanchored scaffolds	Physical positions (bp)	InDels (*Kabuli* reference genome-CDC Frontier/PII)	Forward primers (5'-3')	Reverse primers (5'-3')	Anneling temperature (0C)	Expected amplified product size (bp)	Structural annotation		Functional annotation	
								Sequence components of *kabuli* genome	*Kabuli* gene accession IDs	NCBI-KOG	TFs
Ca4_P2_17697196	Ca4	17697196	TAAAAAAAAA/TAAAAAAAAAA	GGAAATTTGGAAAGTTGGCA	ATAAGGTTCCCCCTGCAATC	60.2	564	INTERGENIC	--	--	--
Ca4_P2_17698774	Ca4	17698774	CAAAAAAAA/CAAAAAAAAA	TGCTTGCAGTCTGAAATTCG	ATCACGGATTGACCTTGGAG	59.9	532	INTERGENIC	--	--	--
Ca4_P2_1771183	Ca4	1771183	TATATATA/TATATATAACATATATA	GATCAATATCTCCATGCGGG	GTTGGGCACCGTCATAAAGT	59.9	423	INTERGENIC	--	--	--
Ca4_P2_17713409	Ca4	17713409	ATT/AT	TCCGTCGTTTATTCTTTGGC	TGAATCGTTTTGGAAAATTCG	59.9	673	INTERGENIC	--	--	--
Ca4_P2_17715218	Ca4	17715218	GAAAAAAAAAA/GAAAAAAAA	CTGTTCCTCACATCACGCAT	GGAGTCACCCTCCAAATTGA	59.9	143	INTERGENIC	--	--	--
Ca4_P2_17715291	Ca4	17715291	CTT/CT	TCAATTTGGAGGGTGACTCC	TACCAGCTTTGGTTTCTGCC	60.2	250	INTERGENIC	--	--	--
Ca4_P2_17715589	Ca4	17715589	TAAAAAA/TAAAAAAAA	TCAATTTGGAGGGTGACTCC	AAAAATTGTTGCACCATCCC	59.7	372	INTERGENIC	--	--	--
Ca4_P2_17715629	Ca4	17715629	ATTTTTTTTTT/ATATATTTTTTTTTTTT	TCAATTTGGAGGGTGACTCC	GCGCTGATGCAATACATACAC	59.2	532	INTERGENIC	--	--	--
Ca4_P2_17716034	Ca4	17716034	ATAA/ATAATTAA	TTTAGTGTGACGGGATGGTG	GGTTTGGAGAAACAAAACTTGG	59.9	624	INTERGENIC	--	--	--
Ca4_P2_17717044	Ca4	17717044	TGG/TG	TCTGTCAAAGTCGGTGCAAG	ATAATGGCGCCACAGAAAAG	60.1	821	INTERGENIC	--	--	--
Ca4_P2_17720367	Ca4	17720367	CTGATCTTGATC/CTGATCTTGATCTTGATC	ACCCTCAGCATGGTCAAAAG	TAGTGGTGGCTCCCCTAATG	59.9	480	DRR	Ca_05352	KL	bHLH
Ca4_P2_17721034	Ca4	17721034	AT/A	CAAGACTTTGTTGGGGGAAA	TCAGTTCTGTCACAGTCGGC	60.0	413	DRR	Ca_05353	O	SRS

INDEL marker IDs	Chromosomes/unanchored scaffolds	Physical positions (bp)	InDels (*Kabuli* reference genome-CDC Frontier/PII)	Forward primers (5'-3')	Reverse primers (5'-3')	Anneling temperature (0C)	Expected amplified product size (bp)	Structural annotation		Functional annotation	
								Sequence components of *kabuli* genome	*Kabuli* gene accession IDs	NCBI-KOG	TFs
Ca4_P2_17725651	Ca4	17725651	TGTGTGAGAG/TG	CCCACTCCAGCACCTCTTAG	CTTGATGGCAAGCCGATAAT	60.1	786	INTRON	Ca_05352	KL	bHLH
Ca4_P2_17727392	Ca4	17727392	AAAGTGGAGA/AA	GGGTGCCTCCTGTACATCAT	GCGTTGACCGGATAGATTGT	60.0	474	INTRON	Ca_05352	KL	bHLH
Ca4_P2_17735131	Ca4	17735131	GATATATATATA/GATATATATATATATA	TTTTTAAAGGGCCCGATTG	TGGACGGTCAAAAATAGAAGAAA	60.0	345	INTERGENIC	--	--	--
Ca4_P2_17735379	Ca4	17735379	ATATT/AT	TTTTTAAAGGGCCCGATTG	TGATTGTCACGTCATCAAAATTC	59.9	554	INTERGENIC	--	--	--
Ca4_P2_17735574	Ca4	17735574	T/TA	TCACGATCGAAAAACCTATTTG	AAACATGACAAAAGTTGATGTTGAA	59.8	916	INTERGENIC	--	--	--
Ca4_P2_17738283	Ca4	17738283	ATTTTTTTTTT/ATTTTTTTTTTTT	TTTTCCTTCATAATTCGGCG	AACGTTGGAGGATGGAGATG	59.9	395	INTERGENIC	--	--	--
Ca4_P2_17740594	Ca4	17740594	GTTTTTTTT/GTTTTTTT	ATATGGCAAGGAACAACGGA	TACCTTGGAATTGGCTGGTC	59.9	664	INTERGENIC	--	--	--
Ca4_P2_17742712	Ca4	17742712	TG/TGG	CCCAAAATCAATTCCGTGAT	GCACCAAAGCCCTGTAACAT	60.0	576	INTERGENIC	--	--	--
Ca4_P2_1774381	Ca4	1774381	CCTTCTT/CCTTCTTCTT	CACACGAGTGTCGCCTAAGA	TGGTATCCAGAGCCATCACA	60.1	437	INTERGENIC	--	--	--
Ca4_P2_17744706	Ca4	17744706	A/ATAAAT	CAAAAGGTCAAAGTCTCCGC	TCAAAAACAGATTCAACTTCAACAA	60.1	466	INTERGENIC	--	--	--
Ca4_P2_17746874	Ca4	17746874	G/GA	CGATGGCAATTTTGGTTTCT	ACTCGTCGCTGTCTTGGACT	60.1	543	INTERGENIC	--	--	--
Ca4_P2_17755784	Ca4	17755784	CTAAGTA/CTAAGTAAGTA	TGTTTTTCTGAAAGCAGGATGA	TGAAAAGAAACCAACTGCCA	59.3	279	INTERGENIC	--	--	--

INDEL marker IDs	Chromosomes/unanchored scaffolds	Physical positions (bp)	InDels (*Kabuli* reference genome-CDC Frontier/PII)	Forward primers (5'-3')	Reverse primers (5'-3')	Anneling temperature (0C)	Expected amplified product size (bp)	Structural annotation: Sequence components of *kabuli* genome	Structural annotation: *Kabuli* gene accession IDs	Functional annotation: NCBI-KOG	Functional annotation: TFs
Ca4_P2_1775612	Ca4	1775612	TT/TTTCAATTTTTGCT	GAGAAATGGAGAGGGAAGGG	TCGTCTGACACCACCAACAT	60.0	718	DRR	Ca_07800	K	HSF
Ca4_P2_17756932	Ca4	17756932	ATT/ATTT	AGTGGGAGAGTGGTGTTTGG	AATGGTGTGGGGTTGGTTTA	59.9	618	INTERGENIC	--	--	--
Ca4_P2_17757375	Ca4	17757375	T/TC	TAAACCAACCCCACACCATT	ACAATGCCTTTTTGCCACTC	60.1	252	DRR	Ca_05349	O	--
Ca4_P2_17757630	Ca4	17757630	CTT/CT	TAAACCAACCCCACACCATT	GGACCAATGTCTTTGATGGC	60.3	689	DRR	Ca_05349	O	--
Ca4_P2_17758892	Ca4	17758892	GTTTTTTTT/GTTTTTTT	TTTGATGCAAACAAGTTCCTG	CGATGAAGGAGTTCTCAGCC	60.0	499	DRR	Ca_05349	O	--
Ca4_P2_17759108	Ca4	17759108	TAAA/TAAAA	GGCTGAGAACTCCTTCATCG	CGCACCACCTTTACATTCCT	60.0	431	DRR	Ca_05349	O	--
Ca4_P2_1776360	Ca4	1776360	TAA/TA	TGAGACCCTTAATCCCACCA	TGGCCAAAAAGAGAAGGCTA	60.0	874	DRR	Ca_07800	K	HSF
Ca4_P2_17765663	Ca4	17765663	TAAA/TAA	TAAACCGTGGTAAAGGCTGG	GGCTTAAACATTGGTCGCAT	60.0	839	INTRON	Ca_05349	O	--
Ca4_P2_17766593	Ca4	17766593	TAAAA/TAAA	ATTGCATTGTGCTCCACTTG	AGGGGTTGGTATAGTAGAAGGAA	57.4	512	DRR	Ca_05348	--	--
Ca4_P2_17769321	Ca4	17769321	GAACAAACAAACAAACAAACAA/GAACAAACAAACAAACAA	GAGCCCATTCCAAAAGATGA	TTCAGCCTCGACCAGTTTTT	59.9	814	INTRON	Ca_05348	--	--
Ca4_P2_17769598	Ca4	17769598	TCATCATCAACATCATCA/TCATCATCAACATCATCAACATCATCA	GTCTTTGATGGGACGGATGT	TTCAGCCTCGACCAGTTTTT	59.9	126	CDS (large-effect mutations)	Ca_05348	--	--
Ca4_P2_17771969	Ca4	17771969	GTCTCTCT/GTCTCT	CATGGAAGAGATGCTGGGTT	CTTTTGGAATCGGGATGAGA	60.0	790	INTRON	Ca_05347	R	--

INDEL marker IDs	Chromosomes/unanchored scaffolds	Physical positions (bp)	InDels (*Kabuli* reference genome-CDC Frontier/PII)	Forward primers (5'-3')	Reverse primers (5'-3')	Anneling temperature (0C)	Expected amplified product size (bp)	Structural annotation		Functional annotation	
								Sequence components of *kabuli* genome	*Kabuli* gene accession IDs	NCBI-KOG	TFs
Ca4_P2_17772365	Ca4	17772365	AACTT/AACTTACTT	TCTCATCCCGATTCCAAAAG	CGTAAGTTTCCCGTTCCAAA	60.0	387	INTRON	Ca_05347	R	--
Ca4_P2_17775890	Ca4	17775890	TAAA/TA	TGCATTCAGCAAAAAGTACCA	GGCACTGCCGTCAAAAGTAT	60.1	525	DRR	Ca_05347	R	--
Ca4_P2_17777647	Ca4	17777647	CATCTGAATCTGAA/CATCTGAA	CCTTAGGGGAGATTTGCATTC	TGCAGATAAATGGCCAAACA	60.1	852	INTERGENIC	--	--	--
Ca4_P2_1778100	Ca4	1778100	CAAAAAAAA/CAAAAAAA	AGGGGACGTCTCCCATTTAT	AGATAGGACCCCCACCATTC	60.0	705	INTRON	Ca_07800	K	HSF
Ca4_P2_17793841	Ca4	17793841	ATTTTTTTTT/ATTTTTTT	CCATCCCACCTACTAGCCAA	TTGACTTGGCCAAACAACAC	59.6	472	INTERGENIC	--	--	--
Ca4_P2_17821019	Ca4	17821019	AAGA/AA	GGAACTGTCGTATTGTTCCCA	AGATGTGGGCATGTGTTTCA	60.0	805	INTERGENIC	--	--	--
Ca4_P2_17821086	Ca4	17821086	AGGGGG/AGGGG	GGAACTGTCGTATTGTTCCCA	AGATGTGGGCATGTGTTTCA	60.0	805	INTERGENIC	--	--	--
Ca4_P2_1782351	Ca4	1782351	TAA/TA	TGGTTTGGATTTGGAGCTGT	TAGATCCGGAATGAAGACCG	60.0	361	INTRON	Ca_07801	T	--
Ca4_P2_17823791	Ca4	17823791	TAAAAAAAAAAAA/TAAAAAAAAAA	TCTCATGTCTTGCAAAATTCTTCT	TTTGTGATTGATATTTGCCGTT	59.4	354	INTERGENIC	--	--	--
Ca4_P2_17823999	Ca4	17823999	GTTTT/GTTT	TTGACCAATTTCACTTCGTG	AATAGGGGGACCAAAACTGC	60.2	571	INTERGENIC	--	--	--
Ca4_P2_17825906	Ca4	17825906	ATTTTTTT/ATTTTTT	AAAAGGCCAACCAAATAGCA	CCTCAATTCTCGATTTCCCA	60.0	555	INTERGENIC	--	--	--
Ca4_P2_17827610	Ca4	17827610	A/AT	TCAACTTCGGTGCTTGACAG	TTGGGGTACACGAACAATGA	59.8	141	INTERGENIC	--	--	--

INDEL marker IDs	Chromosomes/unanchored scaffolds	Physical positions (bp)	InDels (*Kabuli* reference genome-CDC Frontier/PII)	Forward primers (5'-3')	Reverse primers (5'-3')	Anneling temperature (0C)	Expected amplified product size (bp)	Structural annotation		Functional annotation	
								Sequence components of *kabuli* genome	*Kabuli* gene accession IDs	NCBI-KOG	TFs
Ca4_P2_17830486	Ca4	17830486	ATTTTTTTTT/ATTTTTTTTTT	TGGCTTGCATTCTCATTCAG	TTTGGATGCATAGGCCATTT	60.3	296	INTERGENIC	--	--	--
Ca4_P2_17837344	Ca4	17837344	CTTTTTTTTTT/CTTTTTTT	TAGCCCCTTGTCATGATGGT	GAATGCCCTAAATGGGACAA	59.8	403	INTERGENIC	--	--	--
Ca4_P2_17838219	Ca4	17838219	ATTTTTTTT/ATTTTTTT	CATGGAAACCCTGATTTGCT	GGTGTGGGATTGGTTGATTC	60.0	634	INTERGENIC	--	--	--
Ca4_P2_17838269	Ca4	17838269	TTGACGAG/T	CATGGAAACCCTGATTTGCT	GGTGTGGGATTGGTTGATTC	60.0	634	INTERGENIC	--	--	--
Ca4_P2_17839861	Ca4	17839861	TGTGGTGGTGGTGGTGGTG/TGTGGTGGTGGTGGTG	AAACACTGTTCCCCGAAGTG	GAGTCGGAAACCCGATAACA	59.9	325	INTRON	Ca_18620	--	--
Ca4_P2_17843324	Ca4	17843324	TA/T	GGGGGAGACCTACCGTATCT	AGAAACGGGTTGTGCCTATG	60.0	224	INTERGENIC	--	--	--
Ca4_P2_17847500	Ca4	17847500	CTCCTTTTCATC/CTCCTTTTCATCCTTTTCATC	TGAACGATGGTGGAAAGACA	GCAGAGCTCTTTGGCAGAGT	59.9	391	INTRON	Ca_18621	J	--
Ca4_P2_17848856	Ca4	17848856	AATAAATTATTATAA/AATAA	TCTGCTGAGACCCGTCTTTT	AAGCCAGGGCCAGAAATTAT	59.9	602	INTRON	Ca_18621	J	--
Ca4_P2_17849606	Ca4	17849606	TGAGAGAGAGAGAGAGAGAGAGAGA/TGAGAGAGAGAGAGAGAGAGA	AGTGTAAGTGCCCAAGGTGG	TTTCCTCGGCTCTGTTCTGT	60.0	609	INTRON	Ca_18621	J	--
Ca4_P2_17851237	Ca4	17851237	AC/A	TTTCCTTGAAGCACAAGGCT	GGCGATATTTACCGAGCTGA	60.2	308	INTRON	Ca_18621	J	--
Ca4_P2_17854136	Ca4	17854136	CCTCTCTCTCTCTCTCTCTC/CCTCTCTCTCTCTCTCTC	GCGAAGGGTTTTAGGGTTTC	TATAGCGAGAAGTTCGCCGT	60.0	521	INTRON	Ca_18622	K	WRKY
Ca4_P2_17862487	Ca4	17862487	CTTT/CTT	TTTTCTTCGGATGGAAATGC	ACAAAAATGGCAATGCACAA	60.0	571	INTRON	Ca_18623	--	WOX

INDEL marker IDs	Chromosomes/unanchored scaffolds	Physical positions (bp)	InDels (*Kabuli* reference genome-CDC Frontier/PII)	Forward primers (5'-3')	Reverse primers (5'-3')	Anneling temperature (0C)	Expected amplified product size (bp)	Structural annotation		Functional annotation	
								Sequence components of *kabuli* genome	*Kabuli* gene accession IDs	NCBI-KOG	TFs
Ca4_P2_17862773	Ca4	17862773	AGT/AGTGT	TTTTCTTCGGATGGAAATGC	ACAAAAATGGCAATGCACAA	60.0	571	INTRON	Ca_18623	--	WOX
Ca4_P2_17863620	Ca4	17863620	CTTATTTGGAATTATTTGGAATTATT/CTTATTTGGAATTATT	TTTGATTGTGGCTTTTGCAG	ACGAGCTAAAGGATGGGTTG	59.2	540	DRR	Ca_18623	--	WOX
Ca4_P2_17865279	Ca4	17865279	CTTTTTTTTTTTT/CTTTTTTTTTT	ATATGGAAAGTCGCCGTTTG	CCGAGGCAATAAAACACCTC	59.6	494	INTERGENIC	--	--	--
Ca4_P2_17873426	Ca4	17873426	AAGGA/AAGGAGGA	CGAATGGAAAAAGCGAGTTC	CAATAATCCCCGCTCTTGAA	60.0	303	INTERGENIC	--	--	--
Ca4_P2_17882499	Ca4	17882499	ATTTTTTTTTTT/ATTTTTTTTTT	TTTCCAAATATCGTTGCCGT	TGGGTGGGGATTTAATTCTAGTT	60.0	756	DRR	Ca_18626	--	WRKY
Ca4_P2_17891	Ca4	17891	AATATATATATATATATATATATAT/AATATATATATATATATATAT	TTTGGTTGCTTTGTGTCGAA	TGCCCTCGAAGGATTTTAAG	59.3	877	INTERGENIC	--	--	--
Ca4_P2_1791916	Ca4	1791916	AGTTAGTTTGTTA/AGTTAGTTTGTTAGTTTGTTA	CGATATTGTCAAATGGCGTG	CAATGATCACACCAAGGTCG	60.0	741	INTRON	Ca_07801	T	--
Ca4_P2_17942733	Ca4	17942733	CTATATATATATAT/CTATATATATATATATAT	CATCGTCAGCACATCACAAA	CTTAGGAGGTCTTGCCCCTC	60.2	447	INTERGENIC	--	--	--
Ca4_P2_17989441	Ca4	17989441	TAAAAAAAAAAAA/TAAAAAAAAAAA	TTGGTGATCGCAAAGACATC	CCCTATTTTGCTTTGCTTGC	59.9	950	INTERGENIC	--	--	--
Ca4_P2_17999604	Ca4	17999604	ATATTTATTTATTTATTTAT/ATATTTATTTATTTAT	CAAATCCACCCTTTGCAAGT	TCATGGTCTTGCTCTCTCCC	60.3	472	INTRON	Ca_18641	I	bHLH
Ca4_P2_1800090	Ca4	1800090	C/CG	TCGATCCAAGGAAATATGGC	AGAATGGGGGAAGCAAGAAT	59.9	616	INTRON	Ca_07801	T	--
Ca4_P2_18001317	Ca4	18001317	AAATAATAATAATAATAATAATAATAATAATAATAATAAT/AAATAATAATAATAATAATAATAATAATAATAATAATAATAATAATAATAAT	TGGACCAATAGAGATTGAGCG	AAAACGTTCACACTCCGGTC	60.0	364	INTERGENIC	--	--	--

INDEL marker IDs	Chromosomes/unanchored scaffolds	Physical positions (bp)	InDels (*Kabuli* reference genome-CDC Frontier/PII)	Forward primers (5'-3')	Reverse primers (5'-3')	Anneling temperature (0C)	Expected amplified product size (bp)	Structural annotation		Functional annotation	
								Sequence components of *kabuli* genome	*Kabuli* gene accession IDs	NCBI-KOG	TFs
Ca4_P2_18033819	Ca4	18033819	A/AT	AGCACACGCCCTTTGTACTT	TCATTCCAGAAGGAGATGGG	60.0	746	INTRON	Ca_18645	--	--
Ca4_P2_18033913	Ca4	18033913	CT/CTT	CTGAGTGACCTCCCTTCCAA	TCATTCCAGAAGGAGATGGG	60.0	433	INTRON	Ca_18645	--	--
Ca4_P2_1805860	Ca4	1805860	CAAAA/CAA	AAAGAACATGACAAACCGGC	CTTACGTGCCTCCACCAAAT	60.0	657	INTRON	Ca_07801	T	--
Ca4_P2_18061059	Ca4	18061059	TATAATAATAATAATAATAATAATAA/TATAATAATAATAATAATAATAATAATAATAATAA	GAAGCGACCACCTGAAGAAC	CAGAACAAATGACGTGTCCC	59.0	536	INTERGENIC	--	--	--
Ca4_P2_18091373	Ca4	18091373	TCATCAC/T	CCACGGATGTGATTTAGCCT	GAAGCACCGTTCCTTCTTGA	60.4	401	INTERGENIC	--	--	--
Ca4_P2_18121565	Ca4	18121565	TAAGAAG/TAAG	GCTGCACCATGAGATCTTGA	ATGGTGCTGGAAGTTGCTTT	59.7	440	INTRON	Ca_18652	--	--
Ca4_P2_18196212	Ca4	18196212	ATTT/ATTTAATCTTTTT	GTGGTCCAGATTCTCCCAAA	TGTCGCTTCAGCAAAAAGAA	59.7	549	INTERGENIC	--	--	--
Ca4_P2_1820931	Ca4	1820931	AC/A	ATTTGCAGGGATTTGAACCA	TGACTGATTGTCGCTGATGA	58.9	578	INTERGENIC	--	--	--
Ca4_P2_1822745	Ca4	1822745	TAAA/TA	TTTCTTTTCAGCAAAAGTACAACC	TTGAAAAGGGGTTCCTCAAA	59.5	320	INTERGENIC	--	--	--
Ca4_P2_1823869	Ca4	1823869	CTC/CTCTAATC	GCATCAACATGCTTACACACG	GTGAAAAGCGTTTTGACCCT	59.2	444	INTERGENIC	--	--	--
Ca4_P2_1827582	Ca4	1827582	TATAATAATAATAAT/TATAATAATAAT	TTCAACCCGCATATCTCTCC	TGTGTCTCACCGCATTCATT	60.1	603	INTERGENIC	--	--	--
Ca4_P2_1828432	Ca4	1828432	TTTTTTTTA/T	TCGTTCTCGTCTTCGTTCCT	CAACGCTGCAATTCACATTC	60.3	138	INTRON	Ca_07803	T	--

INDEL marker IDs	Chromosomes/unanchored scaffolds	Physical positions (bp)	InDels (*Kabuli* reference genome-CDC Frontier/PII)	Forward primers (5'-3')	Reverse primers (5'-3')	Anneling temperature (0C)	Expected amplified product size (bp)	Structural annotation		Functional annotation	
								Sequence components of *kabuli* genome	*Kabuli* gene accession IDs	NCBI-KOG	TFs
Ca4_P2_1829151	Ca4	1829151	ATT/AT	AGGGGGCAGTGTAGATTGTG	TGCAGCCCAAAATGTGTAAA	60.1	596	INTRON	Ca_07803	T	--
Ca4_P2_1831102	Ca4	1831102	TAAAAAAAAA/TAAAAAAAA	TCCTGAGGAAAAATGTTGGC	GAGATTAAGCCGGAAGACCC	60.0	768	DRR	Ca_07803	T	--
Ca4_P2_18314259	Ca4	18314259	CAAAAAAAA/CAAAAAAAAA	TCACTGAAGGCTCAAGCTCA	TTCATCATTGTCGTTCCAGG	59.5	348	INTERGENIC	--	--	--
Ca4_P2_1831456	Ca4	1831456	TTA/TTATA	TCCAATAAGGTTGCCTAAGGA	GAGATTAAGCCGGAAGACCC	60.0	256	DRR	Ca_07804	--	--
Ca4_P2_1831678	Ca4	1831678	GTTTTTTTT/GTTTTTTTTT	GGGTCTTCCGGCTTAATCTC	TCAACCTTTCCTTCCCCTTT	59.9	357	DRR	Ca_07804	--	--
Ca4_P2_1834191	Ca4	1834191	CTTTTTT/CTTTTT	ATTCTCGGGTTCTCCACCTT	TGCTGATTGTTAGACCGCAG	60.0	333	INTRON	Ca_07804	--	--
Ca4_P2_1835541	Ca4	1835541	CTTTTTTTT/CTTTTTTTTT	ACCATTCAAGGGTTCACTGC	GCAATGCGGTTATGGAGACT	60.1	583	INTRON	Ca_07804	--	--
Ca4_P2_1837868	Ca4	1837868	ATGTGT/ATGTGTGT	GGACAATGTCTGGTCAGCAA	ATCCGTACTTGGACCCACCT	60.6	523	INTERGENIC	--	--	--
Ca4_P2_1840597	Ca4	1840597	ATTCATTCAAAATTAATTTCATTCAAAATTAATTTCATT/ATTCATTCAAAATTAATTTCATT	ACTCATAATTGCGTCTGCCC	TTCTCTTCCATCTGCTGGCT	60.1	506	DRR	Ca_07805	--	--
Ca4_P2_18407838	Ca4	18407838	AATATATATATATATATATATATATATA/AATATATATATATATATATATATATATA	TAATAGGAACCCACACCCCA	ATGCTCCGGTGAACAATCTC	60.1	360	INTERGENIC	--	--	--
Ca4_P2_18446227	Ca4	18446227	GTTTTTT/GTTTTTTT	TCTCACGGTTGTGAGGACTG	GGTGGAGGTGAAGGTAAACAA	58.9	304	INTERGENIC	--	--	--
Ca4_P2_18462391	Ca4	18462391	GTTTTTTTTTTT/GTTTTTTTTTT	AGGAAGGTGGATGCAGATTG	TGGAAGAAAAGGAATCCGAA	59.6	455	INTERGENIC	--	--	--

INDEL marker IDs	Chromosomes/unanchored scaffolds	Physical positions (bp)	InDels (*Kabuli* reference genome-CDC Frontier/PII)	Forward primers (5'-3')	Reverse primers (5'-3')	Anneling temperature (0C)	Expected amplified product size (bp)	Structural annotation		Functional annotation	
								Sequence components of *kabuli* genome	*Kabuli* gene accession IDs	NCBI-KOG	TFs
Ca4_P2_1848187	Ca4	1848187	TATAATAATAA/TATAATAATAATAA	AGAAGCAAAAGAGCCCTTCC	ATAGGAATATAGGCGCGGGT	59.8	323	INTERGENIC	--	--	--
Ca4_P2_1848385	Ca4	1848385	CA/CAATA	AGAAGCAAAAGAGCCCTTCC	TTTTGAAATTGTAAATCGCGG	60.0	805	INTERGENIC	--	--	--
Ca4_P2_1848879	Ca4	1848879	ATTTTTTTT/ATTTTTTTTTT	AACCGCGATTTACAATTTCA	ATCGCGTGAGAACAAAGAGG	60.4	130	INTERGENIC	--	--	--
Ca4_P2_1849730	Ca4	1849730	ATTTT/ATTTTT	TTTGGGAGCAATAAATTTTCAA	ATCAACTGAAACGACCTGGC	60.1	228	INTERGENIC	--	--	--
Ca4_P2_18515805	Ca4	18515805	TATAAATAAATAAATAAATAAATAAAT/TATAAATAAATAAATAAATAAATAAATAAAT	TTTTTGAGATTGGTACGGGA	TCAGCGTTTGTTTGTTGACTT	58.5	738	INTERGENIC	--	--	--
Ca4_P2_18518061	Ca4	18518061	GTTTTTTTT/GTTTTTTTTT	AGCTAGACGAGTTCAGACACCA	GTCGCAAGATGCGAATGTAA	59.8	187	INTERGENIC	--	--	--
Ca4_P2_1855105	Ca4	1855105	TTTAT/TTTATTAT	CACGACCCTTTCGATTGTTT	AAACCAGCTGCCAATTCTGT	59.7	430	INTERGENIC	--	--	--
Ca4_P2_18582757	Ca4	18582757	GTATATATATATATAT/GTATATATATAT	CGAAGATTTTATGACACGGGA	CGGTTAATTTCCACACCGAC	60.2	806	INTERGENIC	--	--	--
Ca4_P2_1858416	Ca4	1858416	GAAAAA/GAAAA	ATGGTGCACACAAAACAGGA	AAACCGATATGCCCTTTGAA	59.4	788	INTERGENIC	--	--	--
Ca4_P2_1860625	Ca4	1860625	C/CTA	TGAACTGTTCCAATGCCAAA	TAAAAGGTGTTTTGTCCGCC	60.0	532	INTERGENIC	--	--	--
Ca4_P2_1861853	Ca4	1861853	AT/ATGAACCCT	TGAGGACAAAAGATCCCCTG	AGGGTCCACAGAAAATCGTG	60.0	870	INTERGENIC	--	--	--
Ca4_P2_1861881	Ca4	1861881	GTTTATTTATTTATTTATTTATT/GTTTATTTATTTATTTATTTATTTATT	TGAGGACAAAAGATCCCCTG	AGGGTCCACAGAAAATCGTG	60.0	870	INTERGENIC	--	--	--

INDEL marker IDs	Chromosomes/unanchored scaffolds	Physical positions (bp)	InDels (*Kabuli* reference genome-CDC Frontier/PII)	Forward primers (5'-3')	Reverse primers (5'-3')	Anneling temperature (0C)	Expected amplified product size (bp)	Structural annotation		Functional annotation	
								Sequence components of *kabuli* genome	*Kabuli* gene accession IDs	NCBI-KOG	TFs
Ca4_P2_1864820	Ca4	1864820	GATA/GATATATA	CTTCTGGGTGTCAGCAATGA	AATCAAATCAACGTGTGCCA	60.0	432	INTRON	Ca_07807	P	MIKC
Ca4_P2_18649901	Ca4	18649901	AATATATATATATATATATATATATATATATAT/AATATATATATATATATATATATATATAT	CTCTTTGGTTCTACGGCTGC	TATGGCTTACGAGGGACTGG	60.1	911	INTERGENIC	--	--	--
Ca4_P2_18651098	Ca4	18651098	AATATATATATATATATATAT/AATATATATATATATATATATAT	TGCTTTCTTTGATGTGCTGG	TTCCCCCAAACATCATTTAGTT	59.6	385	INTERGENIC	--	--	--
Ca4_P2_1865918	Ca4	1865918	CATATATATATATATAT/CATATATATATATAT	TAAATGCCCACCAGATCACA	AAGGATGTGAGTAGCAGAAGTGC	60.0	848	INTRON	Ca_07807	P	MIKC
Ca4_P2_1867347	Ca4	1867347	ATTTTTTT/ATTTTTTTTT	GCAAACCGTGTTTCTGGAAT	ATTCCCAACAGCTGCCAATA	60.5	560	DRR	Ca_07808	--	MYB_related
Ca4_P2_1867692	Ca4	1867692	ATTTTTTTTT/ATTTTTTTTTT	GCAAACCGTGTTTCTGGAAT	TTGACCAAAACAAGCTGCAC	59.9	765	DRR	Ca_07808	--	MYB_related
Ca4_P2_1875458	Ca4	1875458	A/AC	GGCGCCATAGCATTATCAGT	ATGGTTGGAGGTTGAGTTGC	60.0	265	INTRON	Ca_07808	--	MYB_related
Ca4_P2_18781017	Ca4	18781017	TAAAAAAAAAAA/TAAAAAAAAAA	GAGAAACAACGCAGGGAAAC	ATTATTGCCGCCAATTCAAA	60.3	350	INTERGENIC	--	--	--
Ca4_P2_18819263	Ca4	18819263	ATTTTTTTTTTT/ATTTTTTTTTTTT	TGTCGGTGCTTGAGTGATTC	CCGGATGAAAACAACATTGA	59.4	804	INTERGENIC	--	--	--
Ca4_P2_18819341	Ca4	18819341	ATTTTTTTTTT/ATTTTTTTTTTT	GAGAAAAGTTCTGCGCGATT	CCGGATGAAAACAACATTGA	59.4	771	INTERGENIC	--	--	--
Ca4_P2_18821207	Ca4	18821207	CATTATTATTATTATTATTATTATTATT/CATTATTATTATTATTATTATTATTATTATTATT	AAAGTCGCATTCTGGGGATA	ATGTGCCAACTTCGACAATG	59.6	722	INTERGENIC	--	--	--
Ca4_P2_18836993	Ca4	18836993	TGG/TGGG	AGTGGAAAAAGCAGAAGGCA	CAACTGCTAGACGTTGGGC	59.5	137	INTERGENIC	--	--	--

INDEL marker IDs	Chromosomes/unanchored scaffolds	Physical positions (bp)	InDels (*Kabuli* reference genome-CDC Frontier/PII)	Forward primers (5'-3')	Reverse primers (5'-3')	Anneling temperature (0C)	Expected amplified product size (bp)	Structural annotation		Functional annotation	
								Sequence components of *kabuli* genome	*Kabuli* gene accession IDs	NCBI-KOG	TFs
Ca4_P2_18860078	Ca4	18860078	TTATATATATATATATATATATATAT/TTATATATATATATATATATATATATATATAT	GAGAAACTCCGCTCTTGTGG	GAACTCCCCGTCCAACACTA	60.0	700	INTERGENIC	--	--	--
Ca4_P2_18977694	Ca4	18977694	GAAAA/GAAAAA	TCGTGTCTCAAAATTCAAGGAG	AGACGCTAGTTCGGCTTGAC	59.6	883	DRR	Ca_13997	A	--
Ca4_P2_18993059	Ca4	18993059	GTTTTTTT/GTTTTTT	CGTTAAGAATGCGTTGCAGA	TCGCAACTTTTGTTGGTTTTT	59.7	302	INTERGENIC	--	--	--
Ca4_P2_19009000	Ca4	19009000	TAAAAAAA/TAAAAAAAA	ATGGTATGCGAATTGCGAG	TGGGTTTAGTGGGACCTCTG	60.0	564	INTERGENIC	--	--	--
Ca4_P2_1904018	Ca4	1904018	AATATATATATATATATA/AATATATATATATATA	ACCCACTGAAACCCATTTGA	GGAGGAAAGGGGTAACGAAG	59.9	665	INTERGENIC	--	--	--
Ca4_P2_19045820	Ca4	19045820	A/AT	GGGCTTGCTTGGTTTGAATA	TCAACTGCACGTGTTAAATGC	59.8	825	INTERGENIC	--	--	--
Ca4_P2_19046060	Ca4	19046060	CAAAAAAA/CAAAAAAAA	TGAAAATGAGATTAGAACCACCAA	TCTTCACCTGCATCGAAATG	59.8	677	INTERGENIC	--	--	--
Ca4_P2_1906081	Ca4	1906081	GAAAAAA/GAAAAAAA	GTCATTGTCCGACACCTCCT	GGCAAACATCCATGTGACAA	60.4	587	INTERGENIC	--	--	--
Ca4_P2_19106756	Ca4	19106756	CTATATATATATATATATA/CTATATATATATATATATATA	TTTTAAAGCGCTCCCATCAG	GCACTGTAAAAGCCCTCCTT	58.5	367	INTERGENIC	--	--	--
Ca4_P2_19198535	Ca4	19198535	CTTTTTTTTTTT/CTTTTTTTTTTTTT	AGAGGCGGGTGAGAAATTGT	CGTGCTATTCGGCAATCATA	59.7	707	INTRON	Ca_14019	O	--
Ca4_P2_19294536	Ca4	19294536	TTATATATATATATATATATATATATAT/TTATATATATATATATATATATATATATATATATAT	GCATACTCTGTCCTTTCGTCC	GGCTGCCATCACTTCATCTC	60.8	586	INTERGENIC	--	--	--
Ca4_P2_19322386	Ca4	19322386	AATATATATATATATATATATATA/AATATATATATATATATATATATATATA	AAAAACGGCCTTAAACCACA	ATGTTGTCCACATTGGGTGA	59.7	600	INTERGENIC	--	--	--

INDEL marker IDs	Chromosomes/unanchored scaffolds	Physical positions (bp)	InDels (*Kabuli* reference genome-CDC Frontier/PII)	Forward primers (5'-3')	Reverse primers (5'-3')	Anneling temperature (0C)	Expected amplified product size (bp)	Structural annotation		Functional annotation	
								Sequence components of *kabuli* genome	*Kabuli* gene accession IDs	NCBI-KOG	TFs
Ca4_P2_19387051	Ca4	19387051	CAAAA/CAA	GGAGTACTCTGACAACGTCCAA	ACGGTCTTCCAATCGAGATG	60.1	256	DRR	Ca_14033	O	--
Ca4_P2_19400429	Ca4	19400429	TGG/TG	CAACTGAGGCCGAATACGTT	GGAGAGGCAGAAGAGGTGTG	60.0	814	INTERGENIC	--	--	--
Ca4_P2_19405152	Ca4	19405152	TAA/TAAA	GACTGGACGTAACCCACGTT	GATTTTTGGGATGTGCTCGT	59.9	709	INTERGENIC	--	--	--
Ca4_P2_19412758	Ca4	19412758	TTATATATATATATATATATATATATATATA/TTATATATATATATATATATATATATATATATATATA	TGGCTTTATCTATCGGTGCC	CGACCTGTTGGGTGATTCTT	60.0	255	INTERGENIC	--	--	--
Ca4_P2_19424767	Ca4	19424767	ATT/AT	TTTATGGTTTGGGCCCTGTA	TTGCGTTCAAAGTGTTTGGT	59.2	705	INTERGENIC	--	--	--
Ca4_P2_1943645	Ca4	1943645	CATATATATATATATATATATATATAT/CATATATATATATATATATATAT	GCACATGGTGTGAAACTGCT	TTGGGGAGATGGAAGAAAAA	59.5	662	INTERGENIC	--	--	--
Ca4_P2_19498296	Ca4	19498296	TC/T	TTCATGCGGTCCCTTAACTT	TTTAAATCCCGTGCAAAACG	60.8	433	INTERGENIC	--	--	--
Ca4_P2_19534272	Ca4	19534272	TAAAAAAAAAAAAAAA/TAAAAAAAAAAAAAA	AATTGCACACAGATGGATTGA	GGGTTTGATTTTCTCCCTCC	59.7	715	INTERGENIC	--	--	--
Ca4_P2_19643688	Ca4	19643688	CTTTTT/CTTTTTT	GGTGCAGTTGTTTGATGCAC	ATGCGATTTACATGGTTGGC	60.7	255	INTERGENIC	--	--	--
Ca4_P2_19678061	Ca4	19678061	C/CGGCTGGTACAA	ATTTTGGCAACGGCTGATAC	CCACAATCCTCCGTTGTCTT	60.0	492	INTERGENIC	--	--	--
Ca4_P2_19742155	Ca4	19742155	AATATATATATATATATATATATATATATATA/AATATATATATATATATATATATATATATATATATATATATA	GGATGTTGATGTTTGGTCCC	TTAATACAGGCTGTGCCCCT	59.6	701	INTERGENIC	--	--	--
Ca4_P2_1975483	Ca4	1975483	C/CACCACAACACTCT	TGTGTTTGGTTCTACGGTGA	CGGCACCGGAATCTTAGTTA	60.1	796	INTERGENIC	--	--	--

INDEL marker IDs	Chromosomes/unanchored scaffolds	Physical positions (bp)	InDels (*Kabuli* reference genome-CDC Frontier/PII)	Forward primers (5'-3')	Reverse primers (5'-3')	Anneling temperature (0C)	Expected amplified product size (bp)	Structural annotation		Functional annotation	
								Sequence components of *kabuli* genome	*Kabuli* gene accession IDs	NCBI-KOG	TFs
Ca4_P2_19757541	Ca4	19757541	GAAAAAAAAAAAA/GAAAAAAAAAAAAAA	TATGAGGAAAAATGGGCCAG	AACCTATTGCGAACGTCGTC	60.1	163	DRR	Ca_14064	U	GATA
Ca4_P2_19780699	Ca4	19780699	CTTGTAATTTGTAATTTGT/CTTGTAATTTGTAATTTGTAATTTGT	AGCAAACCACACAACCACAA	TTTGTGACCACCACTTCCAA	60.0	484	INTERGENIC	--	--	--
Ca4_P2_19911140	Ca4	19911140	TAAAAAAA/TAAAAAAAA	CCAACAACCAAATTGCCTCT	GTTGCACTGGGACGAAAAAT	60.0	632	INTERGENIC	--	--	--
Ca4_P2_20030982	Ca4	20030982	CA/C	ACAACTTCTCATGCTCGCAA	TGAGGAAGAAGCTTCAAGGC	59.7	512	INTERGENIC	--	--	--
Ca4_P2_20035757	Ca4	20035757	AATATATATATATATATATATATATATATATATAT/AATATATATATATATATATATATATATATATATATATATATAT	TCTCAAACGATTCGTCCAAA	CCTCCATTTAAGCCATTCCA	59.9	342	INTERGENIC	--	--	--
Ca4_P2_20061713	Ca4	20061713	GATATATATA/GATATATATATA	TGAATCCTAGTTGGTGACTTGG	AACGTTTGATGAAAACAACATGA	59.4	627	INTERGENIC	--	--	--
Ca4_P2_20097233	Ca4	20097233	ATTT/ATTTT	TTGTGAAAACGTTCAATGCC	CAAACGTCATTCATGCAACC	60.0	580	INTERGENIC	--	--	--
Ca4_P2_20097310	Ca4	20097310	CTA/CTAAATCTTTTATTA	ACGCCTTAGTGGCTGGTTTA	AGTCGTTCTTTGGAGGGGTT	60.0	497	INTERGENIC	--	--	--
Ca4_P2_20108947	Ca4	20108947	TTATATATATA/TTATATATA	GGCTAACATATGGACTCGCC	TGGTCATTTTGACCTCCACA	59.9	572	INTERGENIC	--	--	--
Ca4_P2_20141078	Ca4	20141078	CTAATAATAATAATAATAATAATAATAATAATAATAATAATAATAATAATAATAATAATAATAA/CTAATAATAATAATAATAATAATAATAATAATAATAATAATAATAATAATAATAATAATAATAATAATAATAATAA	TGACGAGTTGCCATGAGAAG	CGATTTTCAAACCCGTGAGT	60.0	790	INTERGENIC	--	--	--
Ca4_P2_20162535	Ca4	20162535	ATTTTTTTTTT/ATTTTTTTTTTT	TCCCACACACAAATTGGAAA	TGCAGCCCAATCTAAACCTC	60.2	819	DRR	Ca_17138	--	MYB_related
Ca4_P2_20186434	Ca4	20186434	AATA/AA	GAGCTGTCTCGGTCAGGAGT	CAATTCCCACAGGTACGCTT	60.0	419	INTERGENIC	--	--	--

INDEL marker IDs	Chromosomes/unanchored scaffolds	Physical positions (bp)	InDels (*Kabuli* reference genome-CDC Frontier/PII)	Forward primers (5'-3')	Reverse primers (5'-3')	Anneling temperature (0C)	Expected amplified product size (bp)	Structural annotation		Functional annotation	
								Sequence components of *kabuli* genome	*Kabuli* gene accession IDs	NCBI-KOG	TFs
Ca4_P2_20189946	Ca4	20189946	TTTATTATTATTATTATTATTATTATTATTATTATTATTATTATTATTATTATTATTATTATTATTTTATTATTATTATTATTATTATTATTATTATTATTATTATTATTATTATTATTATTATTATTATTATTATTATTATT	ATTTCGGAGGAAGTATGCCC	TGTCAAAAATTGATGGAGATGAG	59.1	503	INTERGENIC	--	--	--
Ca4_P2_20198875	Ca4	20198875	ATTT/AT	AAAATTTTCAACCAAAACCCA	CGTTCGCGGTCTTTTAATTT	59.3	391	INTERGENIC	--	--	--
Ca4_P2_2022623	Ca4	2022623	AAATAATAATAATAATAATAATAATAATAATAATAA/AAATAATAATAATAATAATAATAATAATAATAA	AACGTGTTTGAGTGCGAAAA	TCGAGATGCGATTATGTGGA	60.2	819	INTERGENIC	--	--	--
Ca4_P2_20266418	Ca4	20266418	T/TGC	CTGCAGAATGTTTGCAGAATG	ACCATCAAGGACCCAAACAA	60.2	508	INTERGENIC	--	--	--
Ca4_P2_2032207	Ca4	2032207	TC/T	TGCTGAGTTCGAGGAAAGGT	CACATCTCTTGTTCGCTCCA	60.0	517	CDS (FRAME SHIFT)	Ca_07822	D	--
Ca4_P2_2032547	Ca4	2032547	CTGTGTGTGT/CTGTGTGT	TGCTGAGTTCGAGGAAAGGT	CACATCTCTTGTTCGCTCCA	60.0	517	INTRON	Ca_07822	D	--
Ca4_P2_20327473	Ca4	20327473	CATATATATATATATATATATATATATATATATATATATATA/CATATATATATATATATATATATATATATATATATA	TAAGGTCATGAGGGATTGCC	CACCCCCTTTCCTTTTATGC	60.7	521	INTERGENIC	--	--	--
Ca4_P2_2034424	Ca4	2034424	CTTTT/CTTTTT	ACCAGATACCGCACCAAAAG	TTCTGCATACTTGCTCCACG	60.0	352	INTRON	Ca_07822	D	--
Ca4_P2_20345579	Ca4	20345579	GTTTTTTT/GTTTTTTTT	TTGGTGAAAACTTTGTGTCATGT	TGTGGCCCATATTTGTCACTT	60.2	640	INTERGENIC	--	--	--
Ca4_P2_2034663	Ca4	2034663	TACA/TACACA	ACCAGATACCGCACCAAAAG	AGTTGGCAAGTTTGTCAGGC	60.3	570	INTRON	Ca_07822	D	--
Ca4_P2_2035160	Ca4	2035160	CAA/CA	TAATGCATGGCCTGACAAAC	TGGGCAGCTTAGACTGGACT	60.0	692	CDS (FRAME SHIFT)	Ca_07822	D	--
Ca4_P2_2035225	Ca4	2035225	GGTTTAA/G	CGCGCCAGAAGTATGTGATA	TGGGCAGCTTAGACTGGACT	60.0	275	INTRON	Ca_07822	D	--

INDEL marker IDs	Chromosomes/unanchored scaffolds	Physical positions (bp)	InDels (*Kabuli* reference genome-CDC Frontier/PII)	Forward primers (5'-3')	Reverse primers (5'-3')	Anneling temperature (0C)	Expected amplified product size (bp)	Structural annotation		Functional annotation	
								Sequence components of *kabuli* genome	*Kabuli* gene accession IDs	NCBI-KOG	TFs
Ca4_P2_2035282	Ca4	2035282	GTTTTTT/GTTTTTTT	CGCGCCAGAAGTATGTGATA	TGGGCAGCTTAGACTGGACT	60.0	275	INTRON	Ca_07822	D	--
Ca4_P2_2035697	Ca4	2035697	ATTT/AT	AGTCCAGTCTAAGCTGCCCA	TGGAATCATGCCATGCTAGA	60.2	436	DRR	Ca_07823	R	MYB
Ca4_P2_2035798	Ca4	2035798	A/AGTGT	AGTCCAGTCTAAGCTGCCCA	TGGAATCATGCCATGCTAGA	60.2	436	DRR	Ca_07823	R	MYB
Ca4_P2_20425177	Ca4	20425177	TTTATTATTATTATTATTATTATTATTATTATTATTATTATTATTATT/TTTATTATTATTATTATTATTATTATTATTATTATTATTATTATTATTATTATT	AATGAAAAACCGTGCAAAGC	ATTGGTGAATGGCAATTGGT	60.1	529	INTERGENIC	--	--	--
Ca4_P2_20452006	Ca4	20452006	ATTTTTTTTTT/ATTTTTTTTT	TAGCAATGCTTCTGTGCTGG	CCAGCTGCTGCATATTACCA	59.9	564	INTERGENIC	--	--	--
Ca4_P2_20464790	Ca4	20464790	AATATATATATATATATATAT/AATATATATATATATATATAT	AACGTCTCTATGGCTCTATGCTTT	TGGATTTTGAATAATCGCCA	59.0	707	INTERGENIC	--	--	--
Ca4_P2_20471816	Ca4	20471816	GNNNNNNNNNN/GNNN	ATTGGTAGCTGGAAACACGG	AAAAACCCGGACATGCATTA	60.2	575	DRR	Ca_17155	O	FAR1
Ca4_P2_20538748	Ca4	20538748	CAAAAAAAAAAAAA/CAAAAAAAAAAAA	GGGCTTATGGACAACAGCAT	CCATTGTCGTTGTGTTTTGC	60.0	728	INTERGENIC	--	--	--
Ca4_P2_20545384	Ca4	20545384	CAAAAAAAAAAAA/CAAAAAAAAAAA	GCAGTCGTTCCATATTCGGT	ATATGGCACGGTAGATCCCA	60.2	681	INTERGENIC	--	--	--
Ca4_P2_20548671	Ca4	20548671	GATATATATATATATATATATATA/GATATATATATATATATATATA	AAGTGGTGTTGGAATGAGCC	CACGGAAATAATGCGGAAAT	59.8	236	INTERGENIC	--	--	--
Ca4_P2_20650435	Ca4	20650435	TTATATATATATATATATATATAT/TTATATATATATATATATATAT	CGTGTGAGAAAACCTCGACA	CACCCTTACAAGTCGCCATT	60.0	733	INTERGENIC	--	--	--
Ca4_P2_20709240	Ca4	20709240	ATTTTTTT/ATTTTTTTT	ACTTTGCCCATCAGTTTTGC	TCCGTTGGTGAAAGTTTGAA	59.1	251	INTERGENIC	--	--	--

INDEL marker IDs	Chromosomes/unanchored scaffolds	Physical positions (bp)	InDels (*Kabuli* reference genome-CDC Frontier/PII)	Forward primers (5'-3')	Reverse primers (5'-3')	Anneling temperature (0C)	Expected amplified product size (bp)	Structural annotation		Functional annotation	
								Sequence components of *kabuli* genome	*Kabuli* gene accession IDs	NCBI-KOG	TFs
Ca4_P2_20714850	Ca4	20714850	AAAAATGATTTTT/AAAAATGATTTTTTTAACAAAATGATTTTT	CATGTGGAAAGTAGGGGTGAA	ACAACGTCCGTCTGAGGTCT	59.8	930	INTERGENIC	--	--	--
Ca4_P2_20798632	Ca4	20798632	CAAAAAAAAAAAAAAAAAA/CAAAAAAAAAA	ACCTCGACTTAGGAGCCTCG	TTTGTCCTGGGAAAATGCTC	60.1	593	INTERGENIC	--	--	--
Ca4_P2_20798832	Ca4	20798832	TTTTATTTATTTATTTATT/TTTTATTTATTTATT	TAGTGCTTTTGCATGCCTCA	TTTGTCCTGGGAAAATGCTC	60.1	316	INTERGENIC	--	--	--
Ca4_P2_20870966	Ca4	20870966	ATTTTTTTT/ATTTTTTT	ACTCATTGTGTTGGCTTTCG	GTGGATGAAGGGGGTAGGTT	60.1	776	DRR	Ca_19043	--	--
Ca4_P2_20904738	Ca4	20904738	TG/T	AGCGCCTGCTGGTTCTAATA	GGAGAGAGAGTTGGCAGTGG	60.0	530	INTRON	Ca_19045	E	--
Ca4_P2_20912258	Ca4	20912258	CAA/CAAA	AGTGGGACAGCATTGGCTTA	ACTACACATTTGCAGCGCAC	59.9	611	INTERGENIC	--	--	--
Ca4_P2_20919644	Ca4	20919644	ATTTTTTTTT/ATTTTTTTTTT	TCAGAAATTTGTCATTTGTCCA	TTCGTTGAAATCGGTTATTTGA	59.5	719	INTERGENIC	--	--	--
Ca4_P2_21028015	Ca4	21028015	TAAAAAA/TAAAAAAA	TCCATGAGACCCAACTCTCC	AGATTGGAACCAAGTGGTGG	59.8	499	INTERGENIC	--	--	--
Ca4_P2_21028273	Ca4	21028273	AATATATATATATATATATAT/AATATATATATATATATAT	CCACCACTTGGTTCCAATCT	AGGCCATCTAGCTAAGCACAA	59.1	380	INTERGENIC	--	--	--
Ca4_P2_21060178	Ca4	21060178	GC/G	GGATGATAACTCCTTCAAAGCG	TGCTCATTTAATTTGACTGGGA	59.6	711	INTERGENIC	--	--	--
Ca4_P2_21071879	Ca4	21071879	T/TA	AATTGAGTGATCGAGTCGGC	ATGACGCGTGTCCAATTACA	60.0	837	INTERGENIC	--	--	--
Ca4_P2_21082826	Ca4	21082826	AAATAATAATAATAATAATAATAA/AAATAATAATAATAATAATAA	GTGCGTGCTTTGGGATTTTA	CGAACTGAAGAACGATTAGGTG	58.9	332	INTERGENIC	--	--	--

INDEL marker IDs	Chromosomes/unanchored scaffolds	Physical positions (bp)	InDels (*Kabuli* reference genome-CDC Frontier/PII)	Forward primers (5'-3')	Reverse primers (5'-3')	Anneling temperature (0C)	Expected amplified product size (bp)	Structural annotation		Functional annotation	
								Sequence components of *kabuli* genome	*Kabuli* gene accession IDs	NCBI-KOG	TFs
Ca4_P2_21134198	Ca4	21134198	AATATATATATATATATATA/AATATATATATATATATA	GCCATTCCTCACTTGTCCAT	TCACTCCCAAATCTCGAACA	59.2	871	INTERGENIC	--	--	--
Ca4_P2_21134274	Ca4	21134274	TTATATATATATATATATATAT/TTATATATATATATATAT	ACAAGTCGCCTTTTGGAATG	TCACTCCCAAATCTCGAACA	59.2	521	INTERGENIC	--	--	--
Ca4_P2_21181744	Ca4	21181744	A/ATGG	GAGGGAGATGATCGTGGAAA	GCTTCTTGGAAAAAGCGTTG	60.0	294	INTERGENIC	--	--	--
Ca4_P2_21226048	Ca4	21226048	AAA/AAAAAGGAACCAA	CACGGCTCTACGAAGGAAAG	TGAACACCCCCAAGGTTAAG	59.8	287	INTERGENIC	--	--	--
Ca4_P2_2123443	Ca4	2123443	GAAAAAAAAAAAAAA/GAAAAAAAAAAAA	TATTTGTCGGGGCAGTTTTC	TAAACCAAGTGGCACACCAA	60.0	695	INTERGENIC	--	--	--
Ca4_P2_21271302	Ca4	21271302	AATATATATATATATATATATATATATATATATATATATAT/AATATATATATATATATATATATATATATATATATAT	AAGAAAGGAAGAATGAAAGGGG	ATTAAATTGGGCCTTTTGCC	60.1	563	INTERGENIC	--	--	--
Ca4_P2_21317275	Ca4	21317275	CCAAAATCAAAATCAAAATCAAAATCAAAATCAAA/CCAAAATCAAAATCAAAATCAAAATCAAA	AATCGCGAAAGTAGTCCCCT	TGCTGTTGCTTTTGAGGATG	60.0	689	DRR	Ca_19065	R	C2H2
Ca4_P2_21376315	Ca4	21376315	CATTATTATTATTATTATTATTATTATTATTATTATTATTATTATTATTA/CATTATTATTATTATTATTATTATTATTATTATTATTATTATTATTATTA	CCCCCTCTAAACCAACGTAA	CATTGGACTTGTGGCTTGTG	60.2	575	INTERGENIC	--	--	--
Ca4_P2_2139392	Ca4	2139392	TCCCCC/TCCCCCC	TATCTGGAACGACGACATGC	TCGTTCGCCATTAAAGAGGT	59.7	558	INTERGENIC	--	--	--
Ca4_P2_21426612	Ca4	21426612	ATTTTT/ATTTTTT	TGCAGATAAATGGCCAAACA	AGAACGGTTGCCAGAGAAGA	60.0	370	INTERGENIC	--	--	--
Ca4_P2_21426745	Ca4	21426745	CTCA/C	TGTGCAGATAAATGGCCAAA	CCACGAGGTTTGTGTTTCCT	60.0	620	INTERGENIC	--	--	--
Ca4_P2_2145836	Ca4	2145836	GAAAAAAAAA/GAAAAAAAAAA	TAGGTGGCACCACCATGATA	TATGCACCAGGACAACCTCA	60.1	669	INTERGENIC	--	--	--

INDEL marker IDs	Chromosomes/unanchored scaffolds	Physical positions (bp)	InDels (*Kabuli* reference genome-CDC Frontier/PII)	Forward primers (5'-3')	Reverse primers (5'-3')	Anneling temperature (0C)	Expected amplified product size (bp)	Structural annotation		Functional annotation	
								Sequence components of *kabuli* genome	*Kabuli* gene accession IDs	NCBI-KOG	TFs
Ca4_P2_21514634	Ca4	21514634	ATGT/ATGTGT	TTGGAATCATGAGTTGATCTCG	AGAAGAACCACGACAAAGGC	59.3	656	INTERGENIC	--	--	--
Ca4_P2_21519679	Ca4	21519679	TAAAAAA/TAAAAAAA	CCAATTTGTAGCTTCCACGG	TTTTGAACAGATACGTAATCCCC	59.3	189	INTERGENIC	--	--	--
Ca4_P2_21529168	Ca4	21529168	CAAAA/CAA	TGGGGGATTGTATTCCAAAA	TATGGAATGGACCTCCTGCT	59.5	499	INTERGENIC	--	--	--
Ca4_P2_21529316	Ca4	21529316	T/TA	TGGGGGATTGTATTCCAAAA	TGAAATCGTCACCGTCTCAA	60.2	886	INTERGENIC	--	--	--
Ca4_P2_21570950	Ca4	21570950	CATATATATATATATATATATATATATATATATAT/CATATATATATATATATATATATATATATATATATATATATAT	GCTACGTGGGAAGTCGAAAG	TGGTGCGATTGTGGAAAATA	59.9	419	INTERGENIC	--	--	--
Ca4_P2_21601808	Ca4	21601808	TAAA/TAA	TTGATCCTTGCAGTCACTCG	ACCAAATCGATTGGCACTTC	59.9	643	DRR	Ca_19071	--	--
Ca4_P2_21622139	Ca4	21622139	AG/A	TCCAACAGAGGGAATGATGA	TGATATATTGATTGGGTGTGCG	60.6	517	INTERGENIC	--	--	--
Ca4_P2_2162453	Ca4	2162453	TAATAA/TAATAATAGAAATAA	TTGCAAGGCTCAAGTCTTCTC	CTCACAAAAGCACCGTTGAA	59.9	728	INTERGENIC	--	--	--
Ca4_P2_21657934	Ca4	21657934	TTATATATATATATATATATA/TTATATATATATATATATATATATA	AAATGTGAAAGCCACAACCC	CCATGTAGCATGGCAACAAC	60.0	570	INTERGENIC	--	--	--
Ca4_P2_21676549	Ca4	21676549	TTATATATATATATATATATATATATATATATATATATA/TTATATATATATATATATATATATATATATATA	GTTGGGGTATCCGTTCTCAA	GCAAGCCAACAACAACAATG	60.2	604	INTERGENIC	--	--	--
Ca4_P2_21717914	Ca4	21717914	ATTTTTTTTTTTTT/ATTTTTTTTTTTTTT	ATTTCACAATCGTGCCAACA	TTACCCGTCCCATTTAACCA	60.0	514	DRR	Ca_19077	E	--
Ca4_P2_21728459	Ca4	21728459	TAA/TAATAATAATAAAAA	CCATTTCGTTGCTCCATCTT	TGGTTTTTGTTGTTTGGTTTCT	58.6	267	INTERGENIC	--	--	--

INDEL marker IDs	Chromosomes/unanchored scaffolds	Physical positions (bp)	InDels (*Kabuli* reference genome-CDC Frontier/PII)	Forward primers (5'-3')	Reverse primers (5'-3')	Anneling temperature (0C)	Expected amplified product size (bp)	Structural annotation		Functional annotation	
								Sequence components of *kabuli* genome	*Kabuli* gene accession IDs	NCBI-KOG	TFs
Ca4_P2_21731786	Ca4	21731786	TAAA/TAAAA	CTCCTTCACAAAATTGCGGT	GGGGATTGTTGGAAATGATG	60.0	171	INTERGENIC	--	--	--
Ca4_P2_21767863	Ca4	21767863	ATATTATTATTATTATTATTATTATTATTATTAT/ATATTATTATTATTATTATTATTATTATTAT	AATCACCCGAAACGAGATTG	TCGTTTTTCACATAAGCGTTTT	58.9	632	INTERGENIC	--	--	--
Ca4_P2_21779923	Ca4	21779923	TATAATAATAATAATAATAATAATAATAATAATAATAATAATAATAAAAATAATAATAATAATTATTATAATAATAACAATAATAATAATAATAATAATAATAATAATAATAATAATAATAATAATAATAATAACAATAATAATAATAATA/TATAATAATAATAATAATAATAATA	ACAATGCACCCTCCAATTTC	TGTTTCTTGCCTCTGTTTTGAA	59.9	754	INTERGENIC	--	--	--
Ca4_P2_21804779	Ca4	21804779	CTGT/CTGTTATGT	CCGCATCATAAACAGCCTTT	CTAAAGCTGGTGCTTTTGGC	60.0	159	INTERGENIC	--	--	--
Ca4_P2_21840503	Ca4	21840503	CTTTTTTTT/CTTTTTTTTT	TCCTTTTTACCGCGATGTTC	AATTGGCACTGGTTTATGGG	59.7	415	INTERGENIC	--	--	--
Ca4_P2_21896851	Ca4	21896851	ATTTTTTTTTTTTTTTT/ATTTTTTTTTTTTT	CCGGAAATTTCAAAAACGAA	GTTGCTGCTGCTGATCATGT	60.0	424	INTERGENIC	--	--	--
Ca4_P2_21910161	Ca4	21910161	ATTTTTTTTTTT/ATTTTTTTTTT	TGCCCGTAGTTGTACCATGA	CGATGCAAACGTGGATTATG	60.0	383	INTERGENIC	--	--	--
Ca4_P2_22035290	Ca4	22035290	ATTTTTTTTTT/ATTTTTTTTT	TCGCGATTCAGCTAAAATAGG	TTTGGTTCCTACCTTGTCGG	60.0	800	INTRON	Ca_20139	--	--
Ca4_P2_22052600	Ca4	22052600	AATTATTATTATTATTATTA/AATTATTATTATTATTA	GGATGCGACCTGCTACTGAT	GGATGCGACCTCCTACTACG	59.7	801	INTERGENIC	--	--	--
Ca4_P2_22097668	Ca4	22097668	ATTTTTTTTTTTT/ATTTTTTTTTTTT	TGAAGCACCTTTGATGAGGA	GACGTGATAACATGAAGGATGC	59.5	850	INTERGENIC	--	--	--
Ca4_P2_22134995	Ca4	22134995	CATAT/CAT	GCACCTATGGTGGCAAAAAC	TTTGATGCCCACATTGAAGA	60.0	506	DRR	Ca_20134	J	--
Ca4_P2_22181064	Ca4	22181064	C/CTACTTG	GGTTGAACTTGTTTTGCGGT	CGAACAACACGACACACACA	60.3	426	INTERGENIC	--	--	--

INDEL marker IDs	Chromosomes/unanchored scaffolds	Physical positions (bp)	InDels (*Kabuli* reference genome-CDC Frontier/PII)	Forward primers (5'-3')	Reverse primers (5'-3')	Anneling temperature (0C)	Expected amplified product size (bp)	Structural annotation		Functional annotation	
								Sequence components of *kabuli* genome	*Kabuli* gene accession IDs	NCBI-KOG	TFs
Ca4_P2_22233819	Ca4	22233819	TTATATATATATATATATATATATAT/TTATATATATATATATATATATAT	GTGAAATTGAGGCATGAGCA	CGAAGCTCCCTACTTCGTTG	60.0	843	INTERGENIC	--	--	--
Ca4_P2_22234165	Ca4	22234165	CG/C	AATTTGCCCCGACACTCTTA	TTGCTGGAAAATGAAGAAAGC	59.5	209	INTERGENIC	--	--	--
Ca4_P2_22237363	Ca4	22237363	TAG/T	CCCTCTTGATTTGGCATGAT	AATAAACAGCATGGCGGAAG	60.1	568	DRR	Ca_20127	--	ERF
Ca4_P2_22309344	Ca4	22309344	ATTTTTTTTT/ATTTTTTTTTT	TGCTTTGTTCAATTGCTTGC	AGTGCCAAGTAAGGTTGCTGA	59.9	566	INTERGENIC	--	--	--
Ca4_P2_22324512	Ca4	22324512	TAAAAAAAAAAA/TAAAAAAAAAAAA	CGCTGATTCATCCTTTTCGT	TTGAACTCCTTTCTGGGTGG	60.1	937	INTERGENIC	--	--	--
Ca4_P2_22327394	Ca4	22327394	TG/T	ACCTCTAGGAACGATGGCCT	GCGTGAAATTTCCGGTAGAA	60.1	807	INTERGENIC	--	--	--
Ca4_P2_22334594	Ca4	22334594	ATTTTTTTTT/ATTTTTTTTTT	CAACATCCCTTGAACCAACC	GGGGATTGCCAATTCACTATT	60.0	291	INTERGENIC	--	--	--
Ca4_P2_22344704	Ca4	22344704	AATATATATATATATATATATATATATAT/AATATATATATATATATATATATATATATATATAT	AAGGGATCAAAATCGCACAA	TGTCATGTGAAATGCGACAA	59.7	461	INTERGENIC	--	--	--
Ca4_P2_22361520	Ca4	22361520	ATTTTTTTTT/ATTTTTTTTTT	GAGTGCGTGGGATATGCTTT	GAGTTGGAACTTCGGATGGA	60.1	676	DRR	Ca_20123	R	WRKY
Ca4_P2_22381273	Ca4	22381273	AATATATATATATATATA/AATATATATATATATATATA	CAGTGGCGAAATCTGCATTA	TGGACGTAGGTTGTTGTGGA	60.0	702	INTERGENIC	--	--	--
Ca4_P2_22412136	Ca4	22412136	TGGG/TGG	TAGGGGGTTGAGTTGTCAGG	GTTATTCATAGCCTCCGCCA	60.1	197	INTERGENIC	--	--	--
Ca4_P2_22441886	Ca4	22441886	CTA/CTATA	TCCAATGGTGGAAAATAAACG	TCAATCACAAATTTCAAGGAGG	59.1	848	INTERGENIC	--	--	--

INDEL marker IDs	Chromosomes/unanchored scaffolds	Physical positions (bp)	InDels (*Kabuli* reference genome-CDC Frontier/PII)	Forward primers (5'-3')	Reverse primers (5'-3')	Anneling temperature (0C)	Expected amplified product size (bp)	Structural annotation		Functional annotation	
								Sequence components of *kabuli* genome	*Kabuli* gene accession IDs	NCBI-KOG	TFs
Ca4_P2_22460898	Ca4	22460898	GTT/GTTT	TTAATGCCCTCGTCATGTTT	TAATTTCCGCTGTGCACTTG	59.9	480	INTERGENIC	--	--	--
Ca4_P2_22476709	Ca4	22476709	ACTTC/ACTTCCTTC	CCGGTGTCAAATGATAGGGT	AAGGAGACCAATGGGTTTGA	59.4	123	INTERGENIC	--	--	--
Ca4_P2_22499567	Ca4	22499567	A/ACACGACGCTAAAATACCC	ATGCAATGCGTATATGCCAA	TACATGGTGACCGCGATAGA	60.1	352	INTERGENIC	--	--	--
Ca4_P2_22503071	Ca4	22503071	AACTGCGGAAAATACTGCGG/AACTGCGG	CGGGATGGTGGCATAGTATC	ATGGAGTTGCGAAAACCTTG	60.1	804	INTERGENIC	--	--	--
Ca4_P2_22508434	Ca4	22508434	CATATATATATATATATATATATAT/CATATATATATATATATATATATATATATATATAT	TGGAAGGAAAACAATCGAGG	GCATTGCAATAATCCATCCA	59.3	647	INTERGENIC	--	--	--
Ca4_P2_22546535	Ca4	22546535	AATATATATATATA/AATATATATATATATA	CATTTTGGTTTCAAAGCCGT	CCAGGGGATGAGATCATAGG	59.3	714	INTERGENIC	--	--	--
Ca4_P2_22555367	Ca4	22555367	CTAT/CT	CAGTTGTCCTTTCTGTGGCA	GCATGCATAACCTGGTTCCT	60.0	569	INTERGENIC	--	--	--
Ca4_P2_22601747	Ca4	22601747	TAAAAAAAAA/TAAAAAAAAAAA	GTCGCGTCTTCCATTCATTT	GATTTTCCCTTGTCAACAATCA	58.9	229	INTERGENIC	--	--	--
Ca4_P2_22601946	Ca4	22601946	AAATAATAATAATAATAATAATAATAATAATAATA/AAATAATAATAATAATAATAATAATAATAATAATAATAATA	TCGCGTCTTCCATTCATTTC	CGATTTCATGAATTGCAAATGT	59.8	626	INTERGENIC	--	--	--
Ca4_P2_22652216	Ca4	22652216	T/TG	TCATGTATTTGGCGCGTTTA	CGGCGTCTTATTTTCTTTCG	59.8	242	INTERGENIC	--	--	--
Ca4_P2_22656873	Ca4	22656873	TA/TAGAAAAGCAGA	GACACTCAAGGCTGAAGTAGAAAA	ACCTGACAACCCCAAATTGA	60.2	246	INTERGENIC	--	--	--
Ca4_P2_22657313	Ca4	22657313	ATTTTTTTT/ATTTTTTT	CTTGTAGGGTCAAGGGGGTT	CCATCATCCACATGAAACCA	60.2	347	INTERGENIC	--	--	--

INDEL marker IDs	Chromosomes/unanchored scaffolds	Physical positions (bp)	InDels (*Kabuli* reference genome-CDC Frontier/PII)	Forward primers (5'-3')	Reverse primers (5'-3')	Anneling temperature (0C)	Expected amplified product size (bp)	Structural annotation		Functional annotation	
								Sequence components of *kabuli* genome	*Kabuli* gene accession IDs	NCBI-KOG	TFs
Ca4_P2_22664879	Ca4	22664879	AA/AAGA	TCGCGTTCGATTTGTTCATA	GGTCATTCTTATTGATTGACCCA	60.1	570	INTERGENIC	--	--	--
Ca4_P2_22665150	Ca4	22665150	ATTTTTTTTTT/ATTTTTTTTTTT	TCGCGTTCGATTTGTTCATA	GGTCATTCTTATTGATTGACCCA	60.1	570	INTERGENIC	--	--	--
Ca4_P2_22665456	Ca4	22665456	GTTTTT/GTTTTTT	TTCCTTCAAATCCATACAAAAACA	TCAAACATTTGGCATACAACAA	59.0	300	INTERGENIC	--	--	--
Ca4_P2_22666443	Ca4	22666443	ATTTCTACTTT/ATTT	CATCCAGTGGTTGCGAGTTA	GTGTACAAACCCATGTTGCG	59.9	632	INTERGENIC	--	--	--
Ca4_P2_22667821	Ca4	22667821	GGA/G	GTGAAGCTAGCAAGCCGAAG	TGAATGAAAGGACGGGAAAC	59.9	656	INTERGENIC	--	--	--
Ca4_P2_22668695	Ca4	22668695	TGG/TG	GTTTCCCGTCCTTTCATTCA	CAGTGACAAAAGGCCAATCA	59.7	712	INTERGENIC	--	--	--
Ca4_P2_22674188	Ca4	22674188	CAAAAAAAAAA/CAAAAAAAAA	AATGCATGTTGCACCACAGT	GCTGAAAAGGAAGGGAAAGG	60.2	292	INTERGENIC	--	--	--
Ca4_P2_22674455	Ca4	22674455	AATATATATATATATATATATATATA/AATATATATATATATATATATA	AATGCATGTTGCACCACAGT	ATGGACCGCTCAAAGAGAGA	60.0	805	INTERGENIC	--	--	--
Ca4_P2_22675513	Ca4	22675513	C/CCG	ATCAAATTGGATCCACGGAA	ATGGCAATTAGGGTGTGCTC	60.0	400	INTERGENIC	--	--	--
Ca4_P2_22675724	Ca4	22675724	TTT/TTTCTT	CACAGAAAACTGAAACGCGA	ATGGCAATTAGGGTGTGCTC	60.0	325	INTERGENIC	--	--	--
Ca4_P2_22677817	Ca4	22677817	AC/ACC	TCTCCCCCTTTTTGATGATG	TCCTAAGGCTGAACCCTCAA	59.8	546	INTERGENIC	--	--	--
Ca4_P2_22679339	Ca4	22679339	TAAAAAAA/TAAAAAA	GTGGGAAGGTTGAGGATTGA	ACATCCCCAACTCAAGAACG	60.0	405	DRR	Ca_14477	--	--

INDEL marker IDs	Chromosomes/unanchored scaffolds	Physical positions (bp)	InDels (*Kabuli* reference genome-CDC Frontier/PII)	Forward primers (5'-3')	Reverse primers (5'-3')	Anneling temperature (0C)	Expected amplified product size (bp)	Structural annotation		Functional annotation	
								Sequence components of *kabuli* genome	*Kabuli* gene accession IDs	NCBI-KOG	TFs
Ca4_P2_22699364	Ca4	22699364	A/AAAG	GCAGAATGGGTCCTTGATGT	GCTCGATAGAACCGAGTTGC	60.0	342	INTERGENIC	--	--	--
Ca4_P2_22701237	Ca4	22701237	GACAACA/GACAACAACA	ACCGCAATGAAATCAGGAAG	GTCTGCATTTTCTTGGCACA	59.8	726	INTERGENIC	--	--	--
Ca4_P2_22702092	Ca4	22702092	AAAT/A	GTCATTTGAAACGTGGGCTT	TATAAGCCCTCTGAACCCCC	60.3	434	INTERGENIC	--	--	--
Ca4_P2_22702413	Ca4	22702413	CTTGAAA/CTTGAAATTTTTTTGAAA	GTCATTTGAAACGTGGGCTT	CATGTTGGAGGGGCTAAAAA	59.9	717	INTERGENIC	--	--	--
Ca4_P2_22702639	Ca4	22702639	ATTTTTTT/ATTTTTTTT	GGCTTAAGAGAGGGGGTTCA	TGCATTCACGTCAAAGAAGC	60.0	728	INTERGENIC	--	--	--
Ca4_P2_22703169	Ca4	22703169	ATTTTTT/ATTTTTTT	GTGCAAACAAGGAGCAACAA	GCCTCCAATCCATGTCTGAT	59.9	322	INTERGENIC	--	--	--
Ca4_P2_22703292	Ca4	22703292	AT/ATGT	GTGCAAACAAGGAGCAACAA	AATATCGGACCAAGACGACG	60.0	717	INTERGENIC	--	--	--
Ca4_P2_22704154	Ca4	22704154	CT/CTTT	CGTCGTCTTGGTCCGATATT	TTTGTGCTTATTGACGATTTGAA	59.7	498	INTERGENIC	--	--	--
Ca4_P2_22718798	Ca4	22718798	ATTTTTTTT/ATTTTTTTTT	TCAATAGAAGGTCGCATCCC	TGCCAAAACAAATGACGTGT	60.0	412	INTERGENIC	--	--	--
Ca4_P2_22719677	Ca4	22719677	TATAA/TA	TTTTGGCACGTCTTCTCAACT	CATCGACGATTGTACCAAAAA	58.5	548	INTERGENIC	--	--	--
Ca4_P2_22720347	Ca4	22720347	GAAAAAAAAA/GAAAAAAAAAA	ATGCCAAAACAAATGACTGTT	TGTTTGGTTTTGATTGCGAA	60.1	587	INTERGENIC	--	--	--
Ca4_P2_22723629	Ca4	22723629	CTTTTT/CTTTT	TCTAACACCGGCAACATCAA	AGGCCACGAAAGTGAAGAGA	60.0	527	DRR	Ca_14476	--	--

INDEL marker IDs	Chromosomes/unanchored scaffolds	Physical positions (bp)	InDels (*Kabuli* reference genome-CDC Frontier/PII)	Forward primers (5'-3')	Reverse primers (5'-3')	Anneling temperature (0C)	Expected amplified product size (bp)	Structural annotation		Functional annotation	
								Sequence components of *kabuli* genome	*Kabuli* gene accession IDs	NCBI-KOG	TFs
Ca4_P2_22723833	Ca4	22723833	TGGGGG/TGGGGGG	TCTAACACCGGCAACATCAA	AGGCCACGAAAGTGAAGAGA	60.0	527	DRR	Ca_14476	--	--
Ca4_P2_22727877	Ca4	22727877	TAAA/TAA	TTGGTTCCTTTATTAACTAATTTTTGG	GGGACAAACGTTTTTGCTATG	59.5	809	INTERGENIC	--	--	--
Ca4_P2_22735575	Ca4	22735575	AAAA/AAAATAAA	TTGAAGTTGTCGATCCCCTC	CATATTCAACGCAACAACGC	60.1	553	DRR	Ca_14475	T	FAR1
Ca4_P2_22746108	Ca4	22746108	ACC/ACCC	GGAAGAAGCATGTGGGTTGT	TTGGCCTTGAGACCAGCTAT	59.8	721	INTERGENIC	--	--	--
Ca4_P2_22754464	Ca4	22754464	GTTT/GTTTT	TGAATTTTGGTCGATGTGAAA	ATGTTGCGGAAATTGAAAGG	59.9	705	INTERGENIC	--	--	--
Ca4_P2_22754706	Ca4	22754706	TCCC/TCC	CTTGATTCGGGTAACCATCG	ATGTTGCGGAAATTGAAAGG	59.9	205	INTERGENIC	--	--	--
Ca4_P2_22755158	Ca4	22755158	ATTTTTTT/ATTTTTTTT	CTATGTGCGTCGTCAGGAAA	CCAAAAACGGTTTCACCAGT	59.9	595	INTERGENIC	--	--	--
Ca4_P2_22765430	Ca4	22765430	ATT/ATTT	TGTTCGTAAATCAATGCCCA	TAGGCCCAAACCAAATCAAG	59.9	603	INTERGENIC	--	--	--
Ca4_P2_22765623	Ca4	22765623	CTT/CTTT	TGGGGACCTTCCCAATATAC	TATGGCCGATGCTACAACAA	60.1	219	INTERGENIC	--	--	--
Ca4_P2_22765775	Ca4	22765775	CATTAATT/CATT	TGTTGTAGCATCGGCCATAA	GGTGTAGCCTTTGCTTGGAG	59.9	518	INTERGENIC	--	--	--
Ca4_P2_22766072	Ca4	22766072	AATATA/AATATATATA	TAATTACTTTCCGCGGATGC	GGTGTAGCCTTTGCTTGGAG	59.9	424	INTERGENIC	--	--	--
Ca4_P2_22766863	Ca4	22766863	T/TGG	GCATAATCAAAAGCATTGGC	ATCGATTCTCAGTTGGACCG	60.1	756	INTERGENIC	--	--	--

INDEL marker IDs	Chromosomes/unanchored scaffolds	Physical positions (bp)	InDels (*Kabuli* reference genome-CDC Frontier/PII)	Forward primers (5'-3')	Reverse primers (5'-3')	Anneling temperature (0C)	Expected amplified product size (bp)	Structural annotation		Functional annotation	
								Sequence components of *kabuli* genome	*Kabuli* gene accession IDs	NCBI-KOG	TFs
Ca4_P2_22767329	Ca4	22767329	AACAC/AAC	CCAACTGAGAATCGATGGGT	TCTCCTTTTCAAATTCCTCCAA	60.0	274	INTERGENIC	--	--	--
Ca4_P2_22780960	Ca4	22780960	TAAAAAAAAA/TAAAAAAAAAA	TCTATTTTTGATGATGTTGCGTT	TGAGCTCTTAATCCCTTCATTTC	58.9	565	INTERGENIC	--	--	--
Ca4_P2_22783139	Ca4	22783139	AATATATA/AATATATATA	TCCAATTCATCAATGCCAAA	CCGTTTTTATCTTGTTCTGTAGG	57.1	270	INTERGENIC	--	--	--
Ca4_P2_22789395	Ca4	22789395	GCCCC/GCCCCC	ACACCATTCCATGCTTCACA	GTGGGGGTTGTCTAATGTGG	60.1	463	INTERGENIC	--	--	--
Ca4_P2_22791089	Ca4	22791089	CAAAAAAAAAAAAA/CAAAAAAAAAAA	AAATGCGCAAATGCATACAA	GTGACTTTAGAGCCGCCATC	59.8	691	INTERGENIC	--	--	--
Ca4_P2_22791236	Ca4	22791236	TTTAAT/TTTAATTAAT	AAATGCGCAAATGCATACAA	GTGACTTTAGAGCCGCCATC	59.8	691	INTERGENIC	--	--	--
Ca4_P2_22791929	Ca4	22791929	ATTTTTTTTTT/ATTTTTTTT	CTCATTTTCATTCACCGGCT	CATGACAAAATCCATCCCTTC	59.2	276	INTRON	Ca_14472	--	--
Ca4_P2_22792612	Ca4	22792612	ATTT/ATT	CCTTGAGTGAGTTTGACAGGAA	AAACCATAAAGCCCCAAACC	60.1	577	DRR	Ca_14472	--	--
Ca4_P2_22793825	Ca4	22793825	GTTTTTTTTTTT/GTTTTTTTTT	AGGAGGCAAGACGAAAACAG	TGAAGTTCTGCCACTCGTCA	60.6	572	DRR	Ca_14472	--	--
Ca4_P2_22793993	Ca4	22793993	ATTT/ATT	CGAGTGGCAGAACTTCAAAA	GAATTATGCCGCAAGTACGA	58.8	318	DRR	Ca_14472	--	--
Ca4_P2_22798315	Ca4	22798315	CATAA/C	GCTTTATGAGCCCCATTTCA	CAGGGGAAATTTTTGTGGTG	60.2	562	INTERGENIC	--	--	--
Ca4_P2_22799511	Ca4	22799511	AAATAA/AAA	CCAACACACCAAGACAGTGG	TCACACACAAATCACACCGA	59.5	213	INTERGENIC	--	--	--

INDEL marker IDs	Chromosomes/unanchored scaffolds	Physical positions (bp)	InDels (*Kabuli* reference genome-CDC Frontier/PII)	Forward primers (5'-3')	Reverse primers (5'-3')	Anneling temperature (0C)	Expected amplified product size (bp)	Structural annotation		Functional annotation	
								Sequence components of *kabuli* genome	*Kabuli* gene accession IDs	NCBI-KOG	TFs
Ca4_P2_22799910	Ca4	22799910	AAAA/AAAAAATTAAA	TCGGTGTGATTTGTGTGTGA	GTGCAATCATGCAATCAAGG	60.1	672	INTERGENIC	--	--	--
Ca4_P2_22800253	Ca4	22800253	CTAAAACTCAAT/C	CCTTGATTGCATGATTGCAC	CCTAAATTTTTAAGGGCTAAAATGG	59.7	165	INTERGENIC	--	--	--
Ca4_P2_22808161	Ca4	22808161	A/AC	CATGGTTTTCGCTTACCGTT	TTGGGTGATGTGGCAAAATA	59.8	559	DRR	Ca_14470	H	--
Ca4_P2_22808307	Ca4	22808307	ATT/AT	CATGGTTTTCGCTTACCGTT	TTGGGTGATGTGGCAAAATA	59.8	559	DRR	Ca_14471	Q	Nin-like
Ca4_P2_22808419	Ca4	22808419	TG/T	CATGGTTTTCGCTTACCGTT	TTGGGTGATGTGGCAAAATA	59.8	559	DRR	Ca_14471	Q	Nin-like
Ca4_P2_22809160	Ca4	22809160	AAAA/AAAATAAAATAAA	GCCGTTAAATTTCAGTGGGA	TTGGAGACAGTTGCTTCCCT	59.8	651	DRR	Ca_14471	Q	Nin-like
Ca4_P2_22815726	Ca4	22815726	ATTTTTTTT/ATTTTTTT	GGAAGGAGTCCCTTTAACCC	CAACTCATTTGGGAGCCATT	59.9	840	INTERGENIC	--	--	--
Ca4_P2_22816244	Ca4	22816244	AGGGG/AGG	TCCCAAATGAGTTGGTGGAT	CAATTCCAAATCAAAGCAAAC	57.3	358	INTERGENIC	--	--	--
Ca4_P2_22818481	Ca4	22818481	AT/ATCT	CAATTGCATGTTGAAATGGTG	TGGCCATATTGTTTTTGCAT	58.9	821	INTERGENIC	--	--	--
Ca4_P2_22821558	Ca4	22821558	ACTAGCTAGC/ACTAGC	CCTGGGTGAAGTCTTCTGCT	GAAGTGGCGGTGTTTTTCAT	60.0	569	INTERGENIC	--	--	--
Ca4_P2_22824416	Ca4	22824416	CCGT/CCGTACGT	TCTATTCCCGGTGTTGAAGC	TGCACTCTAATGGGATTATTTTGA	59.9	147	INTERGENIC	--	--	--
Ca4_P2_22825034	Ca4	22825034	T/TTA	TCCTCTAAAACATTTGTGTTGTCA	TCACTCTCTTACTATGGAAGCCAA	59.5	609	INTERGENIC	--	--	--

INDEL marker IDs	Chromosomes/unanchored scaffolds	Physical positions (bp)	InDels (*Kabuli* reference genome-CDC Frontier/PII)	Forward primers (5'-3')	Reverse primers (5'-3')	Anneling temperature (0C)	Expected amplified product size (bp)	Structural annotation		Functional annotation	
								Sequence components of *kabuli* genome	*Kabuli* gene accession IDs	NCBI-KOG	TFs
Ca4_P2_22825336	Ca4	22825336	TTT/TTTGTT	TGGCTTCCATAGTAAGAGAGTGAA	AAATAACGTGTCACGGGGAT	59.2	546	INTERGENIC	--	--	--
Ca4_P2_22827669	Ca4	22827669	GAAAAAA/GAAAAA	CATGCAGAAAATGCGAAAGA	AAGGGGAATAACCCAACACC	59.9	881	INTRON	Ca_14469	--	EIL
Ca4_P2_22836441	Ca4	22836441	TAAAAAAAAA/TAAAAAAAAAA	ATCTCCCAATCACCACCCTT	TCACCATACTTCCCGACACA	60.0	901	INTERGENIC	--	--	--
Ca4_P2_22837601	Ca4	22837601	CAAAAA/CAAAA	TTGAAATGGCAAAGAGGGTC	ATGCATGGGTTGAAATACCC	59.5	556	DRR	Ca_14468	--	--
Ca4_P2_22837725	Ca4	22837725	AAATAATAA/AAATAA	TTGAAATGGCAAAGAGGGTC	ATGCATGGGTTGAAATACCC	59.5	556	DRR	Ca_14468	--	--
Ca4_P2_22848263	Ca4	22848263	GTTTTTTTT/GTTTTTTTTT	TTGTCAAGCCTGATAAGCCA	TCCAATGGGAAGGATAGTGG	59.7	219	INTERGENIC	--	--	--
Ca4_P2_22848996	Ca4	22848996	TAAAAAAA/TAAAAAAAA	TTCATTATCTGTTCCCTCCCA	ACGACTGGCTAAGGTGCTGT	59.9	546	INTERGENIC	--	--	--
Ca4_P2_22850231	Ca4	22850231	GAA/GAAAATAAA	TACGCCCAATTTACGTCACA	GGCTCTGTTGTAGTAGCGGC	60.0	402	INTERGENIC	--	--	--
Ca4_P2_22851617	Ca4	22851617	CTAATA/CTAATAATA	GCGGTTGGAGTTGCATTAGT	TGCAGATCATGCCAAATTGT	60.1	325	DRR	Ca_14467	--	--
Ca4_P2_22853847	Ca4	22853847	ATTTTTTTTTT/ATTTTTTTTTTT	TCAAAACACATTGGAGGCAC	AAGCGTAAATGGCAGATGTGT	59.7	344	INTERGENIC	--	--	--
Ca4_P2_22854209	Ca4	22854209	T/TC	CACATCTGCCATTTACGCTT	AAAATGGCACAAGGCAAACT	59.6	534	INTERGENIC	--	--	--
Ca4_P2_22854290	Ca4	22854290	GAAAAAAAAA/GAAAAAAAA	CACATCTGCCATTTACGCTT	AAAATGGCACAAGGCAAACT	59.6	534	INTERGENIC	--	--	--

INDEL marker IDs	Chromosomes/unanchored scaffolds	Physical positions (bp)	InDels (*Kabuli* reference genome-CDC Frontier/PII)	Forward primers (5'-3')	Reverse primers (5'-3')	Anneling temperature (0C)	Expected amplified product size (bp)	Structural annotation		Functional annotation	
								Sequence components of *kabuli* genome	*Kabuli* gene accession IDs	NCBI-KOG	TFs
Ca4_P2_22856126	Ca4	22856126	GTTTATTT/GTTT	CCTGTCAACTCCAAGAATCTCC	AAAGCCATATTTTAACCCAAAAA	58.2	666	INTERGENIC	--	--	--
Ca4_P2_22864646	Ca4	22864646	TAGAGAGA/TAGAGA	TGAGACTGCAATTTGGCTTG	ACGACGATGTTCATTCCACA	60.0	914	DRR	Ca_14466	S	--
Ca4_P2_22867224	Ca4	22867224	TTATTC/TTATTCATCTATTC	TCACCACTGACCAACCCTTT	CACCACCACCACAAAACACT	59.3	485	INTERGENIC	--	--	--
Ca4_P2_22867292	Ca4	22867292	TAAAAAAAAA/TAAAAAAAAAA	TCACCACTGACCAACCCTTT	CACCACCACCACAAAACACT	59.3	485	INTERGENIC	--	--	--
Ca4_P2_22867454	Ca4	22867454	GAAAAAAAAAA/GAAAAAAAAA	GATCTTCATCCCCTGCAGAC	CACCACCACCACAAAACACT	59.3	292	INTERGENIC	--	--	--
Ca4_P2_22867805	Ca4	22867805	ATTTTTTTTTTTTT/ATTTTTTTTTTTT	GATCTTCATCCCCTGCAGAC	TTCCAAAAAGATAGTTTCACCTCA	59.2	555	INTERGENIC	--	--	--
Ca4_P2_22870716	Ca4	22870716	A/AC	GGAATCGGTGAAAACCAAAA	AGGTAACGCGATTTTATGCG	60.1	786	INTERGENIC	--	--	--
Ca4_P2_22877567	Ca4	22877567	A/ATT	GCAATCTTGCTCCTGCGTAT	TTGCGACAACTTGGTGATTC	59.7	582	INTERGENIC	--	--	--
Ca4_P2_22879215	Ca4	22879215	A/AAT	TCATCTGCGTTTTTCTGCAC	ACCAATCCGAAGACGATCAC	59.9	828	INTERGENIC	--	--	--
Ca4_P2_22884702	Ca4	22884702	CAAAA/CAAA	TTTGGTACCCAATTTTCCCA	GGTGAAAGCGTTTGATGGAT	59.9	564	INTERGENIC	--	--	--
Ca4_P2_22889518	Ca4	22889518	ATTTTTTTT/ATTTTTTTTTT	TGGAGACTGTGGGTCTACTGG	TCCACCCCCAAATTTTATCA	60.0	901	INTERGENIC	--	--	--
Ca4_P2_22889580	Ca4	22889580	AC/A	GGGTCAGGATACTTACAACCCA	TCCACCCCCAAATTTTATCA	60.0	717	INTERGENIC	--	--	--

INDEL marker IDs	Chromosomes/unanchored scaffolds	Physical positions (bp)	InDels (*Kabuli* reference genome-CDC Frontier/PII)	Forward primers (5'-3')	Reverse primers (5'-3')	Anneling temperature (0C)	Expected amplified product size (bp)	Structural annotation		Functional annotation	
								Sequence components of *kabuli* genome	*Kabuli* gene accession IDs	NCBI-KOG	TFs
Ca4_P2_22889620	Ca4	22889620	TATAAAAATAAAAATAAAA/TATAAAAATAAAAATAAAAATAAAA	GGGTCAGGATACTTACAACCCA	TCCACCCCCAAATTTTATCA	60.0	717	INTERGENIC	--	--	--
Ca4_P2_22890323	Ca4	22890323	GG/GGAGTCCATTATG	TGATAAAATTTGGGGGTGGA	AGGTAGGAGCAAAATCGCAA	59.8	622	INTERGENIC	--	--	--
Ca4_P2_22890995	Ca4	22890995	AGCAAGATTTT/AGCAAGATTTTATTTTTGGCAAGATTTT	TTGCGATTTTGCTCCTACCT	AGGCGCAACTTGCATTTACT	59.9	526	INTERGENIC	--	--	--
Ca4_P2_22892881	Ca4	22892881	TAAAAAAAAA/TAAAAAAAA	CACGTGAGTTGATTTAAAAGTGA	TATGTTTGGGTGTGGGACCT	60.1	186	INTERGENIC	--	--	--
Ca4_P2_22896917	Ca4	22896917	GTTTTTTTTTTT/GTTTTTTTTT	CGATGCGCATGATAGAAAGA	AACGGAACCCCACTTAAAGG	60.2	590	INTERGENIC	--	--	--
Ca4_P2_22897920	Ca4	22897920	ATTTTTTTTT/ATTTTTTTTTT	GGTTGGCTTGTGATCTCCAT	TGCTTGTGGTCTGAAACCTG	59.9	463	INTERGENIC	--	--	--
Ca4_P2_22898104	Ca4	22898104	ATTTTTTT/ATTTTTTTT	GGTTGGCTTGTGATCTCCAT	CCCTTTAAGTGGGGTTCCAT	60.0	797	INTERGENIC	--	--	--
Ca4_P2_22898673	Ca4	22898673	CAAAA/CAA	AACCCCACTTAAAGGGCAAC	AGGGTGAAGAAGCTCACACAA	59.9	684	INTERGENIC	--	--	--
Ca4_P2_22901962	Ca4	22901962	CAAAA/CAAA	AGGGACTCAGTTTTCTGCGA	CAGACAAGCCTCTCGATTCC	60.0	593	INTERGENIC	--	--	--
Ca4_P2_22902727	Ca4	22902727	ATT/ATTT	CACTATGCAAAATGGCCTCA	TCTCTTATGTGGGGTGCCTC	60.1	186	INTERGENIC	--	--	--
Ca4_P2_22914105	Ca4	22914105	GTTTTTTTT/GTTTTTTTTTTT	AAAACCCTTTTGCTTCCGTT	TCCTACCAAAAACTTCATTCACAA	59.9	458	INTERGENIC	--	--	--
Ca4_P2_22914458	Ca4	22914458	CTTT/CT	ATTGCGCTTGCATCATCTTT	CCAAAGTTTCAACTTCGAGACA	59.4	671	INTERGENIC	--	--	--

INDEL marker IDs	Chromosomes/unanchored scaffolds	Physical positions (bp)	InDels (*Kabuli* reference genome-CDC Frontier/PII)	Forward primers (5'-3')	Reverse primers (5'-3')	Anneling temperature (0C)	Expected amplified product size (bp)	Structural annotation		Functional annotation	
								Sequence components of *kabuli* genome	*Kabuli* gene accession IDs	NCBI-KOG	TFs
Ca4_P2_22915979	Ca4	22915979	GTTTTT/GCGTTTTTTT	GGTGGAAGGCTTACGAAACA	ACTCGGTGGATGGAAGACAC	60.0	333	INTERGENIC	--	--	--
Ca4_P2_22920453	Ca4	22920453	TTA/T	GTGCCCCAAGAGGTACTCAA	ATAACTGGGTTGAGTTGCGG	60.0	429	INTERGENIC	--	--	--
Ca4_P2_22921547	Ca4	22921547	TAA/TAAAATAAATAAAAAA	TCGTCCGAACACAATCACAT	TGATCTCGTGCTTCTACGTGTT	59.9	311	INTERGENIC	--	--	--
Ca4_P2_22933272	Ca4	22933272	CAAATAAAAAATAAAAAT/CAAATAAAAAAT	CTATAGTGACCGCACTCCGC	TTCCAAAATTCAGGAATCGC	60.0	774	INTRON	Ca_14461	--	--
Ca4_P2_22933918	Ca4	22933918	CTGAACAATT/CTGAACAATTGCGGTGAACAATT	AGAAACCCAAAAGGGGAAGA	TTTGAACCCAAGCTTCAACC	60.1	750	INTRON	Ca_14461	--	--
Ca4_P2_22934181	Ca4	22934181	GCACACACA/GCACACA	AAGGGAATAGGGTCCGAAGA	TTTGAACCCAAGCTTCAACC	60.1	321	INTRON	Ca_14461	--	--
Ca4_P2_22943635	Ca4	22943635	GTT/GTTT	TGCATTAATTGAAGGGGAGC	TTTGCTTCAACAATTTCCCC	59.9	112	INTRON	Ca_14461	--	--
Ca4_P2_22945533	Ca4	22945533	TAAAAAAAA/TAAAAAAA	TGTTTTCACCACCAGGATCA	TTGGCATTTTTAGTTGAAGTCG	59.3	758	INTRON	Ca_14461	--	--
Ca4_P2_22947061	Ca4	22947061	TAAAAAAAA/TAAAAAAAAA	AACCACCACGGATCAAAGAG	GGAAGGTAACTATGGGGCGT	60.2	455	INTRON	Ca_14461	--	--
Ca4_P2_22947175	Ca4	22947175	CAAAA/CAAA	AACCACCACGGATCAAAGAG	TTAAATGGGGGATTGTTGGA	60.0	966	INTRON	Ca_14461	--	--
Ca4_P2_22947623	Ca4	22947623	TTGTTCCAACCCTGTTCCAACCC/TTGTTCCAACCC	ACGCCCCATAGTTACCTTCC	TTAAATGGGGGATTGTTGGA	60.0	531	INTRON	Ca_14461	--	--
Ca4_P2_22947673	Ca4	22947673	TAAAAAAAAA/TAAAAAAAAAA	TTCCAACAATCCCCCATTTA	AATGCAATAATGGACGGAGC	59.9	412	INTRON	Ca_14461	--	--

INDEL marker IDs	Chromosomes/unanchored scaffolds	Physical positions (bp)	InDels (*Kabuli* reference genome-CDC Frontier/PII)	Forward primers (5'-3')	Reverse primers (5'-3')	Anneling temperature (0C)	Expected amplified product size (bp)	Structural annotation		Functional annotation	
								Sequence components of *kabuli* genome	*Kabuli* gene accession IDs	NCBI-KOG	TFs
Ca4_P2_22969338	Ca4	22969338	CAG/C	GCATGTATGGTGTCCTGCAC	CATCCGCCATTTCATTACCT	59.8	269	INTERGENIC	--	--	--
Ca4_P2_22970209	Ca4	22970209	ATTAAGACTTAAGA/ATTAAGA	TTCAAGTGGGATGGGACACT	TACGCACCTGGTCCAACATA	60.0	852	INTRON	Ca_14460	--	--
Ca4_P2_22973209	Ca4	22973209	TAAAAAAAAA/TAAAAAAAAAAA	ATTTTATTGGCATCGGCATT	TGCGAATGTGCTTTCTGTCT	59.6	672	DRR	Ca_14460	--	--
Ca4_P2_22974338	Ca4	22974338	A/AAT	ATTTTGGGCGAAATCCTTTT	GAACCACTCATCCTCCCAAA	59.9	442	DRR	Ca_14460	--	--
Ca4_P2_22974622	Ca4	22974622	TGG/TGGG	ATTTTGGGCGAAATCCTTTT	GAACCACTCATCCTCCCAAA	59.9	442	INTERGENIC	--	--	--
Ca4_P2_22981343	Ca4	22981343	ATTTTTTTT/ATTTTTTT	TCCGCCGTTCTAAATTCTTG	CAAGCCCTCTCCCTCTCTTC	60.5	874	INTERGENIC	--	--	--
Ca4_P2_22984796	Ca4	22984796	GTTTA/GTTTAAACTTTA	CAAATTCCCTTTAGTGCCACA	TGCTTATGTGTTCCATTTGCTC	60.1	802	INTERGENIC	--	--	--
Ca4_P2_22986372	Ca4	22986372	A/AT	GCCCATCTAAGCAACGAGAA	TTTACACATCAAACGCATTAGAGA	58.9	468	INTERGENIC	--	--	--
Ca4_P2_22988646	Ca4	22988646	ATTTTTTT/ATTTTTTTT	ATGTGACACCCTAAACCCCA	TCGAAACTCACCTCCTTCGT	59.8	373	INTERGENIC	--	--	--
Ca4_P2_23002548	Ca4	23002548	ATTATTTTATTTTATTTTATT/ATTATTTTATTTTATTTTATTTTATTTTATT	TCGATCGCTGATGTCAAACT	TGGGCTTGACCTATGTGTGT	59.0	147	INTERGENIC	--	--	--
Ca4_P2_23002623	Ca4	23002623	TTTACTATTACTATT/TTTACTATTACTATTACTATT	TTTATGGCGGCTGGTTTTAC	ACGATGCATTTGATTGATGTT	58.0	546	INTERGENIC	--	--	--
Ca4_P2_23013499	Ca4	23013499	CTA/CTATA	TCGGTTGGTACACACCAATTT	AATTGGAGATAGCATCCCCTC	59.4	506	INTERGENIC	--	--	--

INDEL marker IDs	Chromosomes/unanchored scaffolds	Physical positions (bp)	InDels (*Kabuli* reference genome-CDC Frontier/PII)	Forward primers (5'-3')	Reverse primers (5'-3')	Anneling temperature (0C)	Expected amplified product size (bp)	Structural annotation		Functional annotation	
								Sequence components of *kabuli* genome	*Kabuli* gene accession IDs	NCBI-KOG	TFs
Ca4_P2_23018840	Ca4	23018840	AA/AATTA	CAAAATAAATCAAATGATTGAAGTGA	GGCCTGACGACACAGCTAAT	60.3	651	INTERGENIC	--	--	--
Ca4_P2_23019641	Ca4	23019641	G/GT	CCAATTGGCCCTCATTAGAC	GGAACCGTTGTTTCTTGGTC	59.4	445	INTERGENIC	--	--	--
Ca4_P2_23020256	Ca4	23020256	TTAT/TT	TCATGACCAAGAAACAACGG	TCACATATCAAATGTTGGCACA	59.9	354	INTERGENIC	--	--	--
Ca4_P2_23027358	Ca4	23027358	GTTTTT/GTTTTTT	CCATTGGGAATTAGGCATTG	ACAAACTTGTGGGTGGGTTT	59.2	696	INTERGENIC	--	--	--
Ca4_P2_23027383	Ca4	23027383	TTTAT/TTTATTAT	CCATTGGGAATTAGGCATTG	AAGGTTGTGGATCGATTCGT	59.4	868	INTERGENIC	--	--	--
Ca4_P2_23027444	Ca4	23027444	T/TC	CCATTGGGAATTAGGCATTG	AAGGTTGTGGATCGATTCGT	59.4	868	INTERGENIC	--	--	--
Ca4_P2_23031555	Ca4	23031555	CGTGTGT/CGTGT	AAACATTCAGACCATGTATCGG	CAACTCGATCACCATCATGC	60.1	163	INTERGENIC	--	--	--
Ca4_P2_23037799	Ca4	23037799	AC/ACC	TCGACAAAAGCAAAGCAATG	GCGTTCTTGGTTTTCCAGTC	59.7	349	INTERGENIC	--	--	--
Ca4_P2_23049316	Ca4	23049316	GAAAAAAA/GAAAAAA	AGTGTGGGCTGCTTCAAAGT	TATTTTGAACGATCGGGAGG	59.9	327	INTERGENIC	--	--	--
Ca4_P2_23055622	Ca4	23055622	TAAGAA/TAAGAAAGAA	TGAATTAAAGGATTGCATGAACA	CCGTTGAATGGAAAATCACC	60.2	530	DRR	Ca_14459	--	--
Ca4_P2_23056577	Ca4	23056577	TAAAAAAAAAA/TAAAAAAAAA	ATGAATAGCATGCCACGACA	TCATGTGGGCTCATTTGGTA	59.9	771	INTERGENIC	--	--	--
Ca4_P2_23057259	Ca4	23057259	TAA/TA	TACCAAATGAGCCCACATGA	GTGTGATCCAAAATCGTCCC	60.2	883	INTERGENIC	--	--	--

INDEL marker IDs	Chromosomes/unanchored scaffolds	Physical positions (bp)	InDels (*Kabuli* reference genome-CDC Frontier/PII)	Forward primers (5'-3')	Reverse primers (5'-3')	Anneling temperature (0C)	Expected amplified product size (bp)	Structural annotation		Functional annotation	
								Sequence components of *kabuli* genome	*Kabuli* gene accession IDs	NCBI-KOG	TFs
Ca4_P2_23058698	Ca4	23058698	ATTTT/ATTTTT	CGGATATGAGTTTTATGGACGA	CTCCCTTTTTGCAACACACA	59.7	434	INTERGENIC	--	--	--
Ca4_P2_23060077	Ca4	23060077	GAAAAAAAAAAA/GAAAAAAAAAA	TGCATTTGGCAATTTGAAGA	TCAACGCATGTGACTGCATA	59.9	254	INTERGENIC	--	--	--
Ca4_P2_23060568	Ca4	23060568	ATTTTTTTTT/ATTTTTTTTTT	ATGGGCATTATGCAGTCACA	TTTGCCAATTTATCATGTTTGC	59.8	766	INTERGENIC	--	--	--
Ca4_P2_23064176	Ca4	23064176	CAAGTAAG/CAAG	CTTTGAAAATTGGATGGCGT	CAGGTGCCAGAATCCTGTTT	60.1	562	DRR	Ca_14458	R	--
Ca4_P2_23067690	Ca4	23067690	GTTTTTTTTT/GTTTTTTTTTT	CGGAAATAACTGACCGCAAT	GGAATCGAAAGTCGTGGAAA	60.1	373	INTERGENIC	--	--	--
Ca4_P2_23077402	Ca4	23077402	TAAAAAAAA/TAAAAAAAAA	ACCAAAGAATCCATCGGTTG	TGAATTCATGTGTATTGTCGGA	58.9	842	INTERGENIC	--	--	--
Ca4_P2_23077444	Ca4	23077444	GTTTTTT/GTTTTTTT	ACCAAAGAATCCATCGGTTG	TGAATTCATGTGTATTGTCGGA	58.9	842	INTERGENIC	--	--	--
Ca4_P2_23077586	Ca4	23077586	ATTTTTT/ATTTTTTT	AAGAAAAGGGTTGCGTTTGA	TGAATTCATGTGTATTGTCGGA	58.9	644	INTERGENIC	--	--	--
Ca4_P2_23077647	Ca4	23077647	ATT/AT	AAGAAAAGGGTTGCGTTTGA	TGAATTCATGTGTATTGTCGGA	58.9	644	INTERGENIC	--	--	--
Ca4_P2_23078030	Ca4	23078030	GAAAAAA/GAAAAAAAA	TTTGAAGAACCAGAGTTTGAAGAA	TTCAATGCCCATAATCGTGA	59.9	656	INTERGENIC	--	--	--
Ca4_P2_23078167	Ca4	23078167	TC/T	TTTGAAGAACCAGAGTTTGAAGAA	TTCAATGCCCATAATCGTGA	59.9	656	INTERGENIC	--	--	--
Ca4_P2_23078243	Ca4	23078243	TAAAA/TAAA	AAAAATTCCGACAATACACATGAA	GAATGCGTACACCTTGACGA	59.7	897	INTERGENIC	--	--	--

INDEL marker IDs	Chromosomes/unanchored scaffolds	Physical positions (bp)	InDels (*Kabuli* reference genome-CDC Frontier/PII)	Forward primers (5'-3')	Reverse primers (5'-3')	Anneling temperature (0C)	Expected amplified product size (bp)	Structural annotation		Functional annotation	
								Sequence components of *kabuli* genome	*Kabuli* gene accession IDs	NCBI-KOG	TFs
Ca4_P2_23078933	Ca4	23078933	AA/AAATGA	CCTGTTTTGGTTCAAGCCAT	ATGTTAAGGGACCGCAAATG	59.8	446	INTERGENIC	--	--	--
Ca4_P2_23078978	Ca4	23078978	AAGT/A	CCTGTTTTGGTTCAAGCCAT	ATGTTAAGGGACCGCAAATG	59.8	446	INTERGENIC	--	--	--
Ca4_P2_23079340	Ca4	23079340	CAAA/CAAAA	AATTGCAAGTGGACTGGACC	CCGAGCCAAAATCCAAAATA	59.9	787	INTERGENIC	--	--	--
Ca4_P2_23098064	Ca4	23098064	AGT/A	GGCGGTCGAGAGTTTATGTG	TGAGTGCCAACAAGCTGAAC	60.0	495	DRR	Ca_14457	R	MYB
Ca4_P2_23101001	Ca4	23101001	GAT/GATCAAT	GGTAGCTAAAACCACTTTTGCACT	CACCCACTAGGGGATGGATA	59.6	283	INTERGENIC	--	--	--
Ca4_P2_23104007	Ca4	23104007	AT/A	GAGGTAAAAGTGAGGGGTGGA	GCCGTTTAGTCGAAGTTTGG	59.7	383	INTERGENIC	--	--	--
Ca4_P2_23105473	Ca4	23105473	TGG/TG	ATGGGAGTTGAAGCATTTGG	TTGTGCTTCACTTCTGACCG	60.0	693	INTRON	Ca_14456	--	--
Ca4_P2_23108687	Ca4	23108687	C/CT	GTGGGTGGAATGGAAATACG	GCATCGGGAGATACGTAGGA	60.1	659	DRR	Ca_14456	--	--
Ca4_P2_23111205	Ca4	23111205	ATTTTTTTT/ATTTTTTT	GCCGCCTTTTTAGGTTTTTC	CTCCGAATTACGAGGGATCA	60.0	184	INTERGENIC	--	--	--
Ca4_P2_23121191	Ca4	23121191	GTATA/GTA	TAGTTGGATGTTGGGGAACC	AAAGTCTCCCAACAGAGCGA	60.0	898	INTERGENIC	--	--	--
Ca4_P2_23130145	Ca4	23130145	ATTTTTT/A	TGATGGAAGAAGTGGAGCAA	GGCCATTCTCCTCGTGTAAA	60.1	718	INTERGENIC	--	--	--
Ca4_P2_23130325	Ca4	23130325	TGGCCCTCG/TG	TGATGGAAGAAGTGGAGCAA	GGCCATTCTCCTCGTGTAAA	60.1	718	INTERGENIC	--	--	--

INDEL marker IDs	Chromosomes/unanchored scaffolds	Physical positions (bp)	InDels (*Kabuli* reference genome-CDC Frontier/PII)	Forward primers (5'-3')	Reverse primers (5'-3')	Anneling temperature (0C)	Expected amplified product size (bp)	Structural annotation		Functional annotation	
								Sequence components of *kabuli* genome	*Kabuli* gene accession IDs	NCBI-KOG	TFs
Ca4_P2_23134474	Ca4	23134474	ATTGTTGTTGT/ATTGTTGT	ATGCTGCGAAATTGTTGAAA	AAAAGCGTTGTAAATTGGATTGA	59.9	342	INTERGENIC	--	--	--
Ca4_P2_23135482	Ca4	23135482	GAAAAAA/GAAAAA	ATTCAGCAATGAGAGGTCCG	GATGATGCATGCCATTTGAG	60.0	752	INTERGENIC	--	--	--
Ca4_P2_23135678	Ca4	23135678	CGG/CG	GGGATGAACACAGAAGGGTG	GATGATGCATGCCATTTGAG	60.0	504	INTERGENIC	--	--	--
Ca4_P2_23136851	Ca4	23136851	TAAAAAAAAAAAA/TAAAAAA	CCACCATTGCAACTTCCTTT	ACCACCTTATTTGTGGCTGC	60.0	727	INTERGENIC	--	--	--
Ca4_P2_23192116	Ca4	23192116	CTA/CTATA	TTCGAACAAAATGATTCCCA	TAAACCAATGCCACACATGG	60.2	640	INTERGENIC	--	--	--
Ca4_P2_23207027	Ca4	23207027	ATTGTTGTTGTTGTTGTTGTTGTTGTTGTTGTTGTTGTT/ATTGTTGTTGTTGTTGTTGTTGTTGTTGTT	TCCTATTTGATTTGCATGCCT	GGTTCACCTTCGTCGTTCAT	60.0	569	INTERGENIC	--	--	--
Ca4_P2_23217567	Ca4	23217567	AATATATATATATATATAT/AATATATATATATATATATATATAT	CTCTAGGGAAGCGACCTCCT	CCAACAACCATTGTGGGAGT	60.7	633	INTERGENIC	--	--	--
Ca4_P2_23243883	Ca4	23243883	TTATATATATATATATATATATATATAT/TTATATATATATATATATATATATATATATATATATATATAT	GTTCGGAGTGCCAATTTTTC	ATGCATGGTTGGGAAGAAAG	59.9	675	INTERGENIC	--	--	--
Ca4_P2_23312749	Ca4	23312749	AGACACACA/AGACACACACGACACACA	TATGTGTCTTGTGGCGGTGT	GGTATTCGACCCTTTGACCA	59.8	604	INTERGENIC	--	--	--
Ca4_P2_23313149	Ca4	23313149	CAAAAAAAAAAA/CAAAAAAAAAA	AATCCTTGGATGTTTGGCAG	TCACCCAAGGGTTTTGTTGT	60.2	600	INTERGENIC	--	--	--
Ca4_P2_23352912	Ca4	23352912	G/GT	AAGAAGGGAAAAGGCAAAGC	TTTGCCATCATTGCTTTTGA	60.2	450	INTERGENIC	--	--	--
Ca4_P2_23421612	Ca4	23421612	TT/TTGT	GATGCCAGATGGGTATGCTT	CGTTGCAATTGATGAGGATG	60.1	703	INTERGENIC	--	--	--

INDEL marker IDs	Chromosomes/unanchored scaffolds	Physical positions (bp)	InDels (*Kabuli* reference genome-CDC Frontier/PII)	Forward primers (5'-3')	Reverse primers (5'-3')	Anneling temperature (0C)	Expected amplified product size (bp)	Structural annotation		Functional annotation	
								Sequence components of *kabuli* genome	*Kabuli* gene accession IDs	NCBI-KOG	TFs
Ca4_P2_23474599	Ca4	23474599	ATTTTTT/ATTTTTTT	GCATTATTGATCCCGCAGTT	GATGAGATGAGATTTCAGAGAGCA	60.0	599	DRR	Ca_14438	--	CPP
Ca4_P2_23494359	Ca4	23494359	AATATATATATATATATATATATA/AATATATATATATATATATA	CTCAAGGAACTCGCTTCACC	ATTGCGAGTCAGATTCCAGC	60.4	664	INTERGENIC	--	--	--
Ca4_P2_23500297	Ca4	23500297	AG/A	GAACCCCAAGCAAGAATCAA	TGCACAAAAGCACTTCTGAAA	59.6	379	INTERGENIC	--	--	--
Ca4_P2_23525491	Ca4	23525491	GTTTTTTTTTT/GTTTTTTTTT	TGAGATTATATGCACGCGGA	TGAATCGCGAAAGTAGTCCC	60.2	574	INTERGENIC	--	--	--
Ca4_P2_23575550	Ca4	23575550	TAAAAAAAAAAAAA/TAAAAAAAAAAAA	CATTGGTAAAAGTGAAAAATCCG	AACCGAAACAAATCGCACAT	60.4	363	INTERGENIC	--	--	--
Ca4_P2_23583040	Ca4	23583040	TTATATATATATATATATATATAT/TTATATATATATATATATATATATATAT	AAGGAAGTGGTTGTTGACGG	TTATGGGGCGAGTTTAGCAG	60.2	688	INTERGENIC	--	--	--
Ca4_P2_23721198	Ca4	23721198	AAAACA/AAAACAAAACA	ATTTGTGTGCATTTGCCTGA	TGGGCAATCGACTGACTAAA	59.3	353	INTERGENIC	--	--	--
Ca4_P2_23756363	Ca4	23756363	TTATATATATATATATATA/TTATATATATATATATATA	TTGGAACGTAATTTGATGCG	CCCGTTGCTTCTGTATGCTT	60.3	872	INTERGENIC	--	--	--
Ca4_P2_2379618	Ca4	2379618	CATATATATATATATAT/CATATATATATATATAT	CGTTGCGTGGTTAAAAGTGA	CCATTGCGGTTCTTTTCATT	59.9	604	DRR	Ca_07859	--	MYB
Ca4_P2_23828072	Ca4	23828072	TG/TCGG	TGTTGAAAACGGTCCGTACA	CTTCATCCGAAGAGGCAGAG	60.1	744	INTERGENIC	--	--	--
Ca4_P2_23881120	Ca4	23881120	GATATATATATATATATATATATATATATATAT/GATATATATATATATATATATATATATATATATATATATATATAT	AAAAGGTCGCATCCTGAAGA	ATTCTCCAAACCCACCATGA	60.2	801	INTERGENIC	--	--	--
Ca4_P2_2390421	Ca4	2390421	ATTTTTT/ATTTTTTT	GCACTTTTGGTCCCCCTATT	TATTCCACGTATGCGTCTGC	59.7	168	INTERGENIC	--	--	--

INDEL marker IDs	Chromosomes/unanchored scaffolds	Physical positions (bp)	InDels (*Kabuli* reference genome-CDC Frontier/PII)	Forward primers (5'-3')	Reverse primers (5'-3')	Anneling temperature (0C)	Expected amplified product size (bp)	Structural annotation		Functional annotation	
								Sequence components of *kabuli* genome	*Kabuli* gene accession IDs	NCBI-KOG	TFs
Ca4_P2_23914195	Ca4	23914195	TAAA/TAA	ATCTTCGACCTCTACGTGGC	TCCTCCCCATTCTAGCCTTT	60.0	549	INTERGENIC	--	--	--
Ca4_P2_23957142	Ca4	23957142	GAAAAAAAAAA/GAAAAAAAAA	TTTATAGTCGGTCGTTGGGC	ACCGACCCGCAAATAAGTAA	59.5	390	INTERGENIC	--	--	--
Ca4_P2_23960230	Ca4	23960230	AAATAATAATAATAATAATAATAAT/AAATAATAATAATAATAATAATAAT	GTCTGTGATTTGGTGCAACG	AGGCCAATGAGGCAAGAGTA	59.8	617	INTERGENIC	--	--	--
Ca4_P2_23984716	Ca4	23984716	ATTT/ATTTACTAGTTT	GCATCAACCTTAGCTACCCG	AAAACTTGGCCAAACCATTG	59.8	605	INTERGENIC	--	--	--
Ca4_P2_24003800	Ca4	24003800	CATATATATATATATATA/CATATATATATATATA	AGGTCATTTTTCGAGTCCGA	TTTGCATTGCATTTGGTGAT	59.9	502	INTERGENIC	--	--	--
Ca4_P2_24100684	Ca4	24100684	AATATATATATATATATATATATA/AATATATATATATATATATATATATATATATATA	AATGGTCCAAGGAAAAAGGG	ATCCCCAATTGGAACCTACC	59.9	753	INTERGENIC	--	--	--
Ca4_P2_24115337	Ca4	24115337	ATATATC/ATCTATATC	CACACGTGCATGGATAGAGG	TTCACCGTGGATGAATTTGA	59.9	341	INTERGENIC	--	--	--
Ca4_P2_24140540	Ca4	24140540	ATTATT/ATATTTATT	TTATTTCACCGTCGTGTCCA	TTGTGTGTTGGCCGATTTTA	60.0	234	INTERGENIC	--	--	--
Ca4_P2_24140557	Ca4	24140557	CATTATTATTATTATTATTA/CATTATTATTATTATTATTATTA	TTATTTCACCGTCGTGTCCA	TTGTGTGTTGGCCGATTTTA	60.0	234	INTERGENIC	--	--	--
Ca4_P2_24147447	Ca4	24147447	TAA/TAAAAA	GGAAAACGACTTACACCCGA	TCCCCTTTGTCTTCTTGTGG	60.1	813	INTERGENIC	--	--	--
Ca4_P2_24148613	Ca4	24148613	CATTATTATTATTATTATTA/CATTATTATTATTATTATTA	TTCATCTTTGTTCGCTGCAC	TTCGGGAGTCGATTACGAGT	59.7	597	INTERGENIC	--	--	--
Ca4_P2_24174892	Ca4	24174892	GAAAAAAAAAAA/GAAAAAAAAAA	ACAAGCGCAGTGGAAAAAGT	TATCCTCAACTCGGTCTGGG	60.1	524	INTERGENIC	--	--	--

INDEL marker IDs	Chromosomes/unanchored scaffolds	Physical positions (bp)	InDels (*Kabuli* reference genome-CDC Frontier/PII)	Forward primers (5'-3')	Reverse primers (5'-3')	Anneling temperature (0C)	Expected amplified product size (bp)	Structural annotation		Functional annotation	
								Sequence components of *kabuli* genome	*Kabuli* gene accession IDs	NCBI-KOG	TFs
Ca4_P2_24228039	Ca4	24228039	AATATTGATATATTG/AATATTGATATATTGATATATTG	TGATCCTCCCATCCTACCAA	TTGCGTCTTCTGTTTGATGC	60.0	560	INTERGENIC	--	--	--
Ca4_P2_24273906	Ca4	24273906	TTATATATATATATATATATATATATA/TTATATATATATATATATATATATA	GGGGGAGTTGTTATTCGGTT	GAAAAGTGTTGTCGTGTGTGC	59.3	646	INTERGENIC	--	--	--
Ca4_P2_24346747	Ca4	24346747	GCATATAACATATAAC/GCATATAAC	TGGTTTGTTTCATTGGGCTT	GAAGCTTTGAAAAACGGGTG	59.7	818	INTERGENIC	--	--	--
Ca4_P2_24355412	Ca4	24355412	AATATATATATAT/AATATATATATATAT	TGCCATTTTCAAATTTGTGG	AGGCACACCTTGTAGCCAAC	60.2	376	INTERGENIC	--	--	--
Ca4_P2_24359275	Ca4	24359275	TAAAAAAAA/TAAAAAAAAAA	GGGAGGGTGGGAGTAAATGT	TGTGGCTTTCCAATGTACGA	60.1	571	INTERGENIC	--	--	--
Ca4_P2_24362931	Ca4	24362931	TAA/TA	GGTTTAGATGGGGGATCGTT	CCCCGAGTTGGTCCTTTATT	60.2	947	INTERGENIC	--	--	--
Ca4_P2_24368839	Ca4	24368839	TAAAAAAA/TAAAAAA	TTTTTCCGACTTGGTCCTTT	GGACTTTGGTTCTCTTGGCA	60.2	381	INTERGENIC	--	--	--
Ca4_P2_24378768	Ca4	24378768	GTTTTTTTTTTT/GTTTTTTTTTTTT	AGTCAATGTTGGCGATCCTC	TCCACCATCTGATCTGGACA	60.0	627	INTERGENIC	--	--	--
Ca4_P2_24427573	Ca4	24427573	GTT/GTTT	CTGGAAAATCCTCCTCCTCC	TTCGTCTCAACAATCTCCCC	60.1	346	INTERGENIC	--	--	--
Ca4_P2_24442934	Ca4	24442934	GCA/GCATTCA	TCGCCTTCAAAGAAAACCAC	GGCATGAAATCCATCACCTT	59.8	568	DRR	Ca_20866	R	NAC
Ca4_P2_24445284	Ca4	24445284	GC/GCC	CTCGCCAAATCCATACACCT	CACAGCACCAGGTTATCCCT	60.0	383	INTERGENIC	--	--	--
Ca4_P2_2445413	Ca4	2445413	AATATATATATATATATA/AATATATATATATATATATA	GGAGGTCACAACCACTCCAA	ATTTGTGACGTTCCCCTCAA	60.4	504	INTERGENIC	--	--	--

INDEL marker IDs	Chromosomes/unanchored scaffolds	Physical positions (bp)	InDels (*Kabuli* reference genome-CDC Frontier/PII)	Forward primers (5'-3')	Reverse primers (5'-3')	Anneling temperature (0C)	Expected amplified product size (bp)	Structural annotation		Functional annotation	
								Sequence components of *kabuli* genome	*Kabuli* gene accession IDs	NCBI-KOG	TFs
Ca4_P2_24455402	Ca4	24455402	TTATATATATATATAT/TTATATATATATATATAT	AGCACCAAAGCCACCATTAC	AGCCTCTTTTTGGGACCACT	60.1	236	INTERGENIC	--	--	--
Ca4_P2_2445623	Ca4	2445623	AATATATATATATATATATATATATATATATATATA/AATATATATATATATATATATATATATATATATA	GGAGGTCACAACCACTCCAA	CGGACTTTCGACTTTCTTGC	60.0	654	INTERGENIC	--	--	--
Ca4_P2_24478956	Ca4	24478956	CAA/CAAA	CGGTTCTACACAAGTTGCGA	TCGATTATGCGTAGGGAAGG	60.1	746	INTERGENIC	--	--	--
Ca4_P2_24479035	Ca4	24479035	ATTTTTTT/ATTTTTTTTTT	CGGTTCTACACAAGTTGCGA	TCGATTATGCGTAGGGAAGG	60.1	746	INTERGENIC	--	--	--
Ca4_P2_24488859	Ca4	24488859	TAAA/TAAAA	TTGGAACTCATGCAAATGGA	CGATACTCACCCCTCGTTGT	60.0	557	INTERGENIC	--	--	--
Ca4_P2_24498031	Ca4	24498031	AT/ATT	TAGGCCGCGACAATTTATCT	GAAGGAGGAAAAAGGCCAAC	60.1	798	INTERGENIC	--	--	--
Ca4_P2_24510502	Ca4	24510502	CTGGAACATTGGAACATTG/CTGGAACATTG	CTGCAAAAGCCCTCACAAAT	TCCTCTTGCACACCACAAGA	60.4	969	INTERGENIC	--	--	--
Ca4_P2_24540030	Ca4	24540030	TAAAAAAAAA/TAAAAAAAA	CCGGTCAAATATTAAACGTCAA	TTCATGGTGAAAGCATTGGA	60.0	308	INTERGENIC	--	--	--
Ca4_P2_24549935	Ca4	24549935	TAG/TAGAG	CGCATAAAGTGATCCCCAAT	TGATCGTTCAATTTCCACTCA	59.1	563	DRR	Ca_20863	--	--
Ca4_P2_24553735	Ca4	24553735	GATTAT/GAT	TGGTGCGATTGTGGAAAATA	GAGTAGATGTGCCAACAGCG	59.5	361	INTERGENIC	--	--	--
Ca4_P2_24587937	Ca4	24587937	TNNNNNNNNNN/TNNNNNN	CTCTCATTCTCGTGCATTCG	AATGGAGAGGAGGGGAGAAA	60.0	290	DRR	Ca_20858	R	ERF
Ca4_P2_24647459	Ca4	24647459	CAAAAAAAAAAA/CAAAAAAAAAA	TTGAAAATGATGGATGGTTGAA	CTTGGTTTTGGGAGGCATTA	59.9	810	INTERGENIC	--	--	--

INDEL marker IDs	Chromosomes/unanchored scaffolds	Physical positions (bp)	InDels (*Kabuli* reference genome-CDC Frontier/PII)	Forward primers (5'-3')	Reverse primers (5'-3')	Anneling temperature (0C)	Expected amplified product size (bp)	Structural annotation		Functional annotation	
								Sequence components of *kabuli* genome	*Kabuli* gene accession IDs	NCBI-KOG	TFs
Ca4_P2_24648777	Ca4	24648777	CTTTTTTTTTTT/CTTTTTTTTTTTT	TTCCTTGTTGTTGACCCTCC	GGTGTGTGGATGATTGGTGT	59.1	754	INTERGENIC	--	--	--
Ca4_P2_2470923	Ca4	2470923	TTATATATATATATATATATAT/TTATATATATATATATATATATAT	CGGCCCTGATTTTAGTTGAA	TCGCTGCCACCTCTAAATCT	60.0	298	INTERGENIC	--	--	--
Ca4_P2_24748227	Ca4	24748227	CATATATATATATATATATATATATATATA/CATATATATATATATATA	TGTTTTTAATGCCAATGCGA	CGCACGGAGACAAATTATGA	59.7	127	INTERGENIC	--	--	--
Ca4_P2_24786286	Ca4	24786286	TGTTAA/T	CTTCACTGCGTCCCTTCTTC	CTTTTGTCTTCTTGCCAGCC	60.0	749	DRR	Ca_20850	--	--
Ca4_P2_24828297	Ca4	24828297	ATTTTTTTTTT/ATTTTTTTTTTT	TGGAATTTCAGACTGCCTTG	GGGCACGAAGAAGCACTATC	59.8	716	INTERGENIC	--	--	--
Ca4_P2_24837534	Ca4	24837534	ATTTTT/ATTTT	CCAATGGACCGTTGCTAGTT	GCTTAGGCAGCCACAAAATC	59.9	905	INTERGENIC	--	--	--
Ca4_P2_24897433	Ca4	24897433	GAAAAAAAAA/GAAAAAAAAAA	TGCACAAACCTTTCAATGCT	TTTTTGAATTTTGAACGAAACA	57.4	775	INTERGENIC	--	--	--
Ca4_P2_24905867	Ca4	24905867	ATTTTTTTTTTTTT/ATTTTTTTTTTTT	GCAATTTTGCAGAACATGGA	CCTTTCAGCCTTTTTGTTGG	59.7	140	INTERGENIC	--	--	--
Ca4_P2_24925584	Ca4	24925584	CAAAAAAAAAA/CAAAAAAAAAAA	TCGATTCTTTGAACCATCTCTTC	GACCATTTGAAAATAAAGTTAGGCA	59.8	464	INTERGENIC	--	--	--
Ca4_P2_24940280	Ca4	24940280	GAAAAAAAAAAA/GAAAAAAAAAAAA	TTGTTGTCGCAATGGAATGT	TGCTTGGTCACTTTGGCATA	60.3	315	INTERGENIC	--	--	--
Ca4_P2_24956663	Ca4	24956663	GAAAAAAAAAA/GAAAAAAAAAAA	AAATTGGTGCTATTGCTGGG	GATTCTAACCGTCGTTTCCG	59.6	159	INTERGENIC	--	--	--
Ca4_P2_24997166	Ca4	24997166	T/TG	TGTTGAATGCTGAAACAGCC	CATCGGGGCTATGAGACCTA	60.1	626	INTERGENIC	--	--	--

INDEL marker IDs	Chromosomes/unanchored scaffolds	Physical positions (bp)	InDels (*Kabuli* reference genome-CDC Frontier/PII)	Forward primers (5'-3')	Reverse primers (5'-3')	Anneling temperature (0C)	Expected amplified product size (bp)	Structural annotation		Functional annotation	
								Sequence components of *kabuli* genome	*Kabuli* gene accession IDs	NCBI-KOG	TFs
Ca4_P2_25011140	Ca4	25011140	GAAAAA/GAAAA	GGCTGAAAATGGGAAATGAA	AGGTTTGATTTTGTGCCTGG	60.0	778	INTERGENIC	--	--	--
Ca4_P2_25036917	Ca4	25036917	T/TA	ATCCAAGTTGAGCAAGCATT	CATTGAGACTTTGCCACGAA	59.8	605	INTERGENIC	--	--	--
Ca4_P2_25046968	Ca4	25046968	TGCTGAGCTGA/TGCTGA	ATGTTTCGTGCAATGGTCAA	AAGGTCAAGGGCCAAAAACT	60.0	601	INTERGENIC	--	--	--
Ca4_P2_25049378	Ca4	25049378	TAAAAAAAAA/TAAAAAAAAAAA	GGACACTACCTTAACATTTGAAAGC	CATTGAGACTTTGCCACGAA	59.8	476	INTERGENIC	--	--	--
Ca4_P2_25053449	Ca4	25053449	ACTCT/ACT	ACGCATCCGTTGAATACTCC	TGGGGAATCATAAGCCTCAG	60.0	256	INTERGENIC	--	--	--
Ca4_P2_25053641	Ca4	25053641	TAAAAAAAA/TAAAAAAAAA	CTGAGGCTTATGATTCCCCA	CGATAAGGGTGTCGAGGAAA	60.1	154	INTERGENIC	--	--	--
Ca4_P2_25062584	Ca4	25062584	TAAAAAAAAAAA/TAAAAAAAAA	AATCGACTCCCGAACTCAAA	ACGTTCTTCGTTTCCCTGAA	59.7	524	INTERGENIC	--	--	--
Ca4_P2_25064267	Ca4	25064267	AATATATATATATATATA/AATATATATATATATATA	AATTCCATCCCCTCTCAACC	ACCGTTTTGGTGTTTCTTGC	60.0	561	INTERGENIC	--	--	--
Ca4_P2_25070539	Ca4	25070539	TTTTATTTATTTATTTATTTATTTATTT/TTTTATTTATTTATTTATTTATTT	TTTGCAAAAACCAATGAATACTT	GTTTTGAGCCCATTCACGAT	59.9	596	INTERGENIC	--	--	--
Ca4_P2_25071336	Ca4	25071336	CA/C	TGAGACTGCTTGGCTACCCT	ATCTGAATCATCCCAAGCCA	60.4	488	INTERGENIC	--	--	--
Ca4_P2_25097629	Ca4	25097629	CAAAAAA/CAAAAAAA	TCAATGATGGTGACTTGGGA	TACCCATTTGGTCTTGGGTC	59.6	820	INTERGENIC	--	--	--
Ca4_P2_25098043	Ca4	25098043	CAAA/CAAAA	ACCCCAAGAACGTTATGCAA	GTTTGGCATTCTGACTTCGC	60.8	525	INTERGENIC	--	--	--

INDEL marker IDs	Chromosomes/unanchored scaffolds	Physical positions (bp)	InDels (*Kabuli* reference genome-CDC Frontier/PII)	Forward primers (5'-3')	Reverse primers (5'-3')	Anneling temperature (0C)	Expected amplified product size (bp)	Structural annotation		Functional annotation	
								Sequence components of *kabuli* genome	*Kabuli* gene accession IDs	NCBI-KOG	TFs
Ca4_P2_25106094	Ca4	25106094	TAAAAAA/TAAAAA	GTTGGTCATGCAGAAAGCAA	GAGGGGAGTGGAGATGATGA	60.0	590	DRR	Ca_16560	--	--
Ca4_P2_25139153	Ca4	25139153	TCC/TC	TTGTGTTGCAATAGTGCTTGC	TGCTCCACATAAAGCGAAGA	59.6	642	INTERGENIC	--	--	--
Ca4_P2_25142345	Ca4	25142345	TTATATATATATATATATATATATATATATA/TTATATATATATATATATA	AGCTGCTGCTACTGTTGGATT	CCCTTTTGAACGCAGAAAAC	59.7	439	INTERGENIC	--	--	--
Ca4_P2_25144894	Ca4	25144894	GTATATTTATATTTATATTTATATTTATATTTATATTTATAT/GTATATTTATATTTATATTTATATTTATAT	GAACCAAACACGGGCATAAT	TCTTCGTTCGATCATGTGAAA	59.3	346	INTERGENIC	--	--	--
Ca4_P2_25145575	Ca4	25145575	TAAAAAAAA/TAAAAAAAAA	TCACATGATCGAACGAAGACA	CCCATGTGCATGAGACTTTG	60.1	845	INTERGENIC	--	--	--
Ca4_P2_25150451	Ca4	25150451	TAAATTTTAAAATTTTAAAATT/TAAATTTTAAAATT	TAAATGCACATGCAAAAGCG	TCGCCAAAAACAATCAATCA	60.1	370	INTERGENIC	--	--	--
Ca4_P2_25156464	Ca4	25156464	TTA/TTAAGTA	CGATGAATGACATGTGTTGG	GACGTGCTTAACAACTACTTTGC	58.2	477	INTERGENIC	--	--	--
Ca4_P2_25164375	Ca4	25164375	A/AAT	AAATGAAGAAGGGTCGGTCC	TGCCACCATTATTTGGGATT	60.0	444	INTERGENIC	--	--	--
Ca4_P2_25177814	Ca4	25177814	CAGAGAGA/CAGAGA	TCCTTGATCGGTTAAGTCGG	TAAAGGACCAAGTCGGGAAA	59.5	373	INTERGENIC	--	--	--
Ca4_P2_25178138	Ca4	25178138	GAAAAAAAAA/GAAAAAAAAAAAA	TTTCCCGACTTGGTCCTTTA	TGACTAACGCATTGCCAAAA	60.3	619	INTERGENIC	--	--	--
Ca4_P2_25180205	Ca4	25180205	GAAAAAAAA/GAAAAAAAAAA	AATCCTAAGCACAACGAGGG	ATGACAACAATCATTCGGGA	58.8	592	DRR	Ca_16562	--	--
Ca4_P2_25182596	Ca4	25182596	TATATATATATA/TATATATATATAAATATATATATA	TGGTAACTTGCATTCACCCA	CTTTTGGTCCCCGACATAGA	59.9	252	INTRON	Ca_16562	--	--

INDEL marker IDs	Chromosomes/unanchored scaffolds	Physical positions (bp)	InDels (*Kabuli* reference genome-CDC Frontier/PII)	Forward primers (5'-3')	Reverse primers (5'-3')	Anneling temperature (0C)	Expected amplified product size (bp)	Structural annotation		Functional annotation	
								Sequence components of *kabuli* genome	*Kabuli* gene accession IDs	NCBI-KOG	TFs
Ca4_P2_25186812	Ca4	25186812	TAAAAAAAAA/TAAAAAAAA	CCACAGGGTTAAGTTTCCGA	GCCAAACTCTCCTCCCTACC	60.1	657	INTRON	Ca_16562	--	--
Ca4_P2_25191410	Ca4	25191410	GTTTTTTTTTT/GTTTTTTTTT	CCTGACCATTTAGCATCAATCA	CGGAATTTGGCATTTAAGGA	59.9	567	INTERGENIC	--	--	--
Ca4_P2_25193095	Ca4	25193095	GAAAAAAAAAA/GAAAAAAAAA	CATCACCCACCATTGACAAG	CATCCACGAAACACTCATGG	60.0	587	INTERGENIC	--	--	--
Ca4_P2_25205443	Ca4	25205443	TAGATTTGGCACAAAAGATTTGG/TAGATTTGG	GATGGAGGATCAGCCACATT	ATGGTGGAATGCCTCAAAAG	59.9	648	INTERGENIC	--	--	--
Ca4_P2_25213751	Ca4	25213751	GAG/GAGATCATAG	TCATTCAACGTCATCGTATCAA	TGTGTTTGTATCAATATCGAAGGC	60.3	562	INTERGENIC	--	--	--
Ca4_P2_25214544	Ca4	25214544	CAAA/CAA	TCTCCATTTGCATAAAGCCC	GGTGTGACCCTTCCTTTTCA	59.9	347	INTERGENIC	--	--	--
Ca4_P2_25215378	Ca4	25215378	TA/TAA	GTCAATTGAGTTGCGACGAG	TGGGAGTTTCCAACATCTACG	60.0	886	INTERGENIC	--	--	--
Ca4_P2_25216110	Ca4	25216110	G/GC	AATTGGCAAGTTATCGCAGC	AAATGGCAGATCCAGAATTTTA	57.8	235	INTERGENIC	--	--	--
Ca4_P2_25220713	Ca4	25220713	C/CT	TGAATTCATCACCGATCCAA	TGAATCCAAATTTTGTGCGA	60.1	400	DRR	Ca_16564	--	--
Ca4_P2_25228841	Ca4	25228841	AATATATATATATAT/AATATATATATATATAT	ATGATTACCTCGGCATGGAG	AGCACGAGATCAAAGCACAA	59.6	349	INTRON	Ca_16565	--	--
Ca4_P2_25235418	Ca4	25235418	TAA/TA	TCGATATTTGAGTTTGGTTTGG	TTGCAGCACATGTTTATCCC	59.5	606	INTERGENIC	--	--	--
Ca4_P2_25241552	Ca4	25241552	GTTTTTTT/GTTTTTT	CACTGCGTTTGCATATGACC	AAGGAAGAGGCGAACATGAA	59.8	434	INTERGENIC	--	--	--

INDEL marker IDs	Chromosomes/unanchored scaffolds	Physical positions (bp)	InDels (*Kabuli* reference genome-CDC Frontier/PII)	Forward primers (5'-3')	Reverse primers (5'-3')	Anneling temperature (0C)	Expected amplified product size (bp)	Structural annotation		Functional annotation	
								Sequence components of *kabuli* genome	*Kabuli* gene accession IDs	NCBI-KOG	TFs
Ca4_P2_25248594	Ca4	25248594	CTGG/CTGGAAACTTCCTATGG	TCCTTCAGGCTTTAGAGGCA	TGGACGAAGAAAATCGAAGG	60.2	346	INTERGENIC	--	--	--
Ca4_P2_25254264	Ca4	25254264	CTAA/CTAATTAA	GTGGCCCTAACTTCCACTCA	CCAGGGTAACAAGATCCGAA	59.9	717	INTRON	Ca_16567	CD	--
Ca4_P2_25259085	Ca4	25259085	TAAA/TAA	AAACACTTTTGCTCCCCACA	TGGAGGTTGATTGGTCAAGA	59.1	918	INTERGENIC	--	--	--
Ca4_P2_25275223	Ca4	25275223	CAAAAAA/CAAAAAAA	TCTGCACCAATTGCCATAAA	TCTTCAAGGTGGGTGAATCC	59.9	577	INTERGENIC	--	--	--
Ca4_P2_25290328	Ca4	25290328	GTTTTTTTT/GTTTTTTT	TCACACCGTGAGATTTTCCA	GAGGCTCCAGATCGAAGTTG	60.0	629	INTERGENIC	--	--	--
Ca4_P2_25292705	Ca4	25292705	GTTAATTAA/GTTAA	GGCAGAAATTTGAACGTGGT	GGACAAAAGAAGAGCAAAAAGG	59.4	227	INTERGENIC	--	--	--
Ca4_P2_25300340	Ca4	25300340	AGATATATTCT/A	TGCACATGCTGGTAGAGACC	ATCAGGATTGGGTGCTCAAG	60.1	786	DRR	Ca_16570	--	Dof
Ca4_P2_25342530	Ca4	25342530	ATTTTTTTTT/ATTTTTTTTTT	TCACCACAAACGGACTCAAA	GGATGCATATGTGATGGGTG	59.6	497	DRR	Ca_16572	J	--
Ca4_P2_25346612	Ca4	25346612	GTTTTTTTTT/GTTTTTTTT	TTGCATTTGCTGCTGATTTC	TTTAAACCCTTTGGGTGCAG	60.0	404	INTERGENIC	--	--	--
Ca4_P2_25348226	Ca4	25348226	T/TCTAC	CGTCATTTACAAATGCGACTT	AACATGGGTTAGCATTTGCC	59.8	896	INTERGENIC	--	--	--
Ca4_P2_25349410	Ca4	25349410	TA/TAA	TCGTTTGTTTTGGCACATCT	TCAAACCAAAAGAGCAGGAAG	59.5	349	INTERGENIC	--	--	--
Ca4_P2_25352495	Ca4	25352495	GGTT/G	GGAAATGGTTCTTTGAAAGCC	AGCCTTACGCATGTATTGCC	60.1	719	INTERGENIC	--	--	--

INDEL marker IDs	Chromosomes/unanchored scaffolds	Physical positions (bp)	InDels (*Kabuli* reference genome-CDC Frontier/PII)	Forward primers (5'-3')	Reverse primers (5'-3')	Anneling temperature (0C)	Expected amplified product size (bp)	Structural annotation		Functional annotation	
								Sequence components of *kabuli* genome	*Kabuli* gene accession IDs	NCBI-KOG	TFs
Ca4_P2_25352610	Ca4	25352610	T/TA	GGAAATGGTTCTTTGAAAGCC	AGCCTTACGCATGTATTGCC	60.1	719	INTERGENIC	--	--	--
Ca4_P2_25353098	Ca4	25353098	GA/GAAA	CGTCGCACTAAGACGATGAA	GGGTAAGGGTACGTTGAGTCC	59.7	415	INTERGENIC	--	--	--
Ca4_P2_25353133	Ca4	25353133	GAAAAAAAA/GAAAAAAA	CGTCGCACTAAGACGATGAA	GGGTAAGGGTACGTTGAGTCC	59.7	415	INTERGENIC	--	--	--
Ca4_P2_25357817	Ca4	25357817	ATTTTTTTT/AATTTTTTTTT	AAAATTCTGTTTGGGGGACC	ACATGCATGCAATTCAATAGAG	57.8	677	INTERGENIC	--	--	--
Ca4_P2_25358106	Ca4	25358106	TTAAATAAATAAATAAATAAATAA/TTAAATAAATAAATAAATAA	GGGACTACTTTCGCGATTCA	ATTACACGCTTGCAGACGAA	59.5	842	INTERGENIC	--	--	--
Ca4_P2_25359522	Ca4	25359522	GATATAT/GATATATATAT	CGACAAAGCCTCACGAGTTA	GGAGCCACAAACGATTGACT	60.1	660	INTERGENIC	--	--	--
Ca4_P2_25365917	Ca4	25365917	ATTTT/ATT	AATTTTGTTGCCCCTCCAAT	TGCTATGGCACAAGTCTCCA	60.4	547	INTERGENIC	--	--	--
Ca4_P2_25367189	Ca4	25367189	A/AT	TTTATTGTAGGTGGAGGCGG	GGCCAAATTGATGCAAAGTT	59.9	271	INTERGENIC	--	--	--
Ca4_P2_25393862	Ca4	25393862	TATTAAAATT/TATTAAAATTAAAATT	CAATTGGATTGTGGCTTCCT	CAGGCGTCGAACAATTCATA	59.7	909	INTERGENIC	--	--	--
Ca4_P2_25400137	Ca4	25400137	GAAAAAA/GAAAAA	ACTCGCATACGAGACACACG	TTTGCTTCAATTGTTTTTCTTTTG	59.7	286	INTERGENIC	--	--	--
Ca4_P2_25401216	Ca4	25401216	TCTGACTG/TCTG	CTTGTGATGGAAAAGCAGCA	TATTCCCCGACTGGAAGTTG	59.9	628	INTERGENIC	--	--	--
Ca4_P2_25402747	Ca4	25402747	AAATAAAAAT/AAATAAAAATAATGAATAAAAAT	TTTGATATGTGGAAGCGCAA	CATTCCAACTCGTTTGATGC	59.1	405	INTERGENIC	--	--	--

INDEL marker IDs	Chromosomes/unanchored scaffolds	Physical positions (bp)	InDels (*Kabuli* reference genome-CDC Frontier/PII)	Forward primers (5'-3')	Reverse primers (5'-3')	Anneling temperature (0C)	Expected amplified product size (bp)	Structural annotation		Functional annotation	
								Sequence components of *kabuli* genome	*Kabuli* gene accession IDs	NCBI-KOG	TFs
Ca4_P2_25434234	Ca4	25434234	CATA/CA	GATTATCCTCCTGCGGTCAA	TCCGCTCCTTACCATCACTC	60.2	797	DRR	Ca_16576	O	MIKC
Ca4_P2_25439114	Ca4	25439114	TAA/TA	GAGGTGGAGAATGATTGGTCA	TCGAGACTTGGCTCATGTTG	60.0	674	INTERGENIC	--	--	--
Ca4_P2_25456545	Ca4	25456545	ATTTTTTTTTT/ATTTTTTTT	TTTGCTTTCAAAACCGAAATC	TCGATGATAAAAATGATCGAGTG	59.1	723	INTERGENIC	--	--	--
Ca4_P2_25457334	Ca4	25457334	ATTTTTTT/ATTTTTTTT	CATTTTACTCATCGATCTTTGTGG	TGGACAAAATCATGTCTCTTTGA	59.6	336	DRR	Ca_16577	--	--
Ca4_P2_25466972	Ca4	25466972	TAAAAAAA/TAAAAAAAA	TATAGGGAGCGGAACACACC	TCTAACTCCAGGTAACGCCG	60.3	840	INTERGENIC	--	--	--
Ca4_P2_25468010	Ca4	25468010	TTATATATATATATATATATATAT/TTATATATATATATATATATATATATATATAT	TTCAAAATACAAATGTGATGGAAAA	TTTTTGTTTCCTTATTGTTTGACA	57.9	650	INTERGENIC	--	--	--
Ca4_P2_25468253	Ca4	25468253	ATTTTTTT/ATTTTTTTT	CAAAATACAAATGTGATGGAAAAA	TTTTTGTTTCCTTATTGTTTGACA	57.9	648	INTERGENIC	--	--	--
Ca4_P2_25470351	Ca4	25470351	CTTTTTTTTT/CTTTTTTTTTT	TGCAATTATCCAAAGATGTATGAAA	TCCTTTTGTCCGTCGTCTCT	59.8	479	INTERGENIC	--	--	--
Ca4_P2_25478172	Ca4	25478172	TAAAA/TAAAAA	TGTGATGCAGTTGCTCCATT	GACCTTGCTCTCTTGATGGG	59.8	703	INTERGENIC	--	--	--
Ca4_P2_25492419	Ca4	25492419	CAAAAAAAAAAAA/CAAAAAAAAAAAAA	ACTTGCTTCAATTGTGCGTG	GTAGGCACTACCGCAACCAT	60.0	291	INTERGENIC	--	--	--
Ca4_P2_25494077	Ca4	25494077	TGGGGGG/TGGGGG	ACATGGATTGTTGGTGCAGA	TCTGAAATAACCGCCCTCAC	60.1	647	INTERGENIC	--	--	--
Ca4_P2_25496001	Ca4	25496001	TA/TAGCTCATAGA	CAATGGTTATGCGATTGCTG	TCGTAGCTCAATTGGCAGTG	60.0	215	INTERGENIC	--	--	--

INDEL marker IDs	Chromosomes/unanchored scaffolds	Physical positions (bp)	InDels (*Kabuli* reference genome-CDC Frontier/PII)	Forward primers (5'-3')	Reverse primers (5'-3')	Anneling temperature (0C)	Expected amplified product size (bp)	Structural annotation		Functional annotation	
								Sequence components of *kabuli* genome	*Kabuli* gene accession IDs	NCBI-KOG	TFs
Ca4_P2_2550001	Ca4	2550001	CTTTTTTT/CTTTTTTTT	GGTGACCGAAAACCATCTTT	GGAATCGCTGGACAAACAAT	59.9	705	INTERGENIC	--	--	--
Ca4_P2_25501013	Ca4	25501013	TAAAAAAAA/TAAAAAAAAA	GGGCATTGGTAACTCCAAAA	CCAATTGTGCTCTCCTCTCC	59.8	909	INTERGENIC	--	--	--
Ca4_P2_25501416	Ca4	25501416	CTTT/CTTTT	TTGACACTTGTTGGCACCTAA	GAGTAGATCCGCGAGTCAGC	60.1	950	INTERGENIC	--	--	--
Ca4_P2_25502917	Ca4	25502917	T/TA	CCAGACGCGACAACACTAGA	GTTGCTAGAGTGAGGGGCAG	60.0	694	INTERGENIC	--	--	--
Ca4_P2_25512047	Ca4	25512047	CTTTATT/CTTTATTTTATT	GCCACTGAATTGTCGGAGAT	ACCCCCAATGAATGAAAACA	60.0	324	INTRON	Ca_16579	S	--
Ca4_P2_25523236	Ca4	25523236	TAAAA/TAAA	TGTGGTGGTGATCCTTGAAA	GATTCTCGCATCTGCTAGGC	60.1	786	INTERGENIC	--	--	--
Ca4_P2_25527454	Ca4	25527454	TAAAAAAAAAAAAAA/TAAAAAAAAAAAAAAAAAA	TCATGTGTAATTTTTATCTCAAGGAA	GGCTTGATTGATTCACATCCT	59.0	743	DRR	Ca_16580	--	--
Ca4_P2_25579370	Ca4	25579370	TAAAAAAAAAA/TAAAAAAAAA	AAATCGGCTCCTTTTAAAACAATA	TCTTCTTCGACGTTTTTGGC	60.4	699	INTERGENIC	--	--	--
Ca4_P2_25580470	Ca4	25580470	GTTTTTTTT/GTTTTTTTTT	TTCTTTCTTTCTTTTCCTTCCG	GCAAATCCATTGGCAATTCT	59.9	453	INTERGENIC	--	--	--
Ca4_P2_25590282	Ca4	25590282	TAAAAAAAA/TAAAAAAAAA	GCATGGATGCAACCTCCTAT	TGACCCCACACTGCAAATTA	60.0	400	INTERGENIC	--	--	--
Ca4_P2_25596032	Ca4	25596032	TTAT/TTATAT	AGTGATGTGGGCAACAATGA	TGCATTTTACAGCGCTTTTG	60.0	515	INTERGENIC	--	--	--
Ca4_P2_2560005	Ca4	2560005	AAAAAATAA/AAA	TGGTGTCACTCATCTCACCAA	TTCGTGTTCCGTTGATGTGT	60.0	675	INTERGENIC	--	--	--

INDEL marker IDs	Chromosomes/unanchored scaffolds	Physical positions (bp)	InDels (*Kabuli* reference genome-CDC Frontier/PII)	Forward primers (5'-3')	Reverse primers (5'-3')	Anneling temperature (0C)	Expected amplified product size (bp)	Structural annotation		Functional annotation	
								Sequence components of *kabuli* genome	*Kabuli* gene accession IDs	NCBI-KOG	TFs
Ca4_P2_2560053	Ca4	2560053	TAATAATAAATAA/TAATAA	TGGTGTCACTCATCTCACCAA	TTCGTGTTCCGTTGATGTGT	60.0	675	INTERGENIC	--	--	--
Ca4_P2_25605220	Ca4	25605220	GATTATTATTATTATTATTATTATTATTATTATTATTATTATTATTATT/GATTATTATTATTATTATTATTATTATTATTATTATTATTATTATTATTATTATT	GTGGAAATCACCATTCCCTG	GTATGTTGGGCGCTTTGTTT	60.0	705	INTERGENIC	--	--	--
Ca4_P2_25607880	Ca4	25607880	CATATATATATATATATATATATAT/CATATATATATATATATATATATATATATATAT	TTGGTCAAGAGGTTGTGACG	TGGTACCCACCTTCTTCCAA	60.3	676	INTERGENIC	--	--	--
Ca4_P2_25618351	Ca4	25618351	CTTTTTTTTTTTT/CTTTTTTTTTTT	TCACCCACAACTACGAAATCA	TCCAACCACACCTTGCATAA	60.0	457	INTERGENIC	--	--	--
Ca4_P2_25623331	Ca4	25623331	CATTATTATTATTATTATTATTATTATTATTATTATTATTATTATTATTATTATTAT/CATTATTATTATTATTATTATTATTATTATTATTATTATTATTATTATTATTATTATTAT	CACCGGAACCAAGCTTTTTA	GAGGAGGTTTGTTGGATGGA	59.9	376	INTERGENIC	--	--	--
Ca4_P2_25642263	Ca4	25642263	CAAAAAA/CAAAAA	TTTGTTGTGGTGTTCTGGGA	TACAACCAAACCCCTTGAGC	60.0	318	INTERGENIC	--	--	--
Ca4_P2_25651231	Ca4	25651231	CTTTTTTTTTT/CTTTTTTTTTTT	CGTTTGTCTGTGAAATGGGA	CAAGCGGTAAGCACTAGGGA	60.4	825	INTRON	Ca_16590	G	--
Ca4_P2_25671775	Ca4	25671775	TTTTCTTTCTTTC/TTTTCTTTCTTTCTTTC	CCAGGATGCTTCCCTGATAA	AGAGCGCGAACTGTCAAAAT	60.0	809	INTERGENIC	--	--	--
Ca4_P2_25694157	Ca4	25694157	GTTTTTTTTTTTTT/GTTTTTTTTTT	ATTTGCTTCATTTTGGGTGC	TTGATCCCTAAGCGAAGTGG	60.2	823	INTERGENIC	--	--	--
Ca4_P2_25694552	Ca4	25694552	CAAAAAAAAAAA/CAAAAAAAAAA	CCACTTCGCTTAGGGATCAA	TTTGCCATTTTTGCAAATCC	60.8	440	INTERGENIC	--	--	--
Ca4_P2_25699421	Ca4	25699421	GAAAAAAAAA/GAAAAAAAAAA	ATTGTGGAAATCTGGGCTTG	CAAGTTGAGCCTCATGGACA	59.8	531	INTERGENIC	--	--	--
Ca4_P2_25709629	Ca4	25709629	ATTTTTTTT/ATTTTTTT	CGCATTGCACATATTTATCG	AAAATTTGATGTGTGCGTCA	57.6	175	INTERGENIC	--	--	--

INDEL marker IDs	Chromosomes/unanchored scaffolds	Physical positions (bp)	InDels (*Kabuli* reference genome-CDC Frontier/PII)	Forward primers (5'-3')	Reverse primers (5'-3')	Anneling temperature (0C)	Expected amplified product size (bp)	Structural annotation		Functional annotation	
								Sequence components of *kabuli* genome	*Kabuli* gene accession IDs	NCBI-KOG	TFs
Ca4_P2_25711685	Ca4	25711685	ATTTTTTTTT/ATTTTTTTTTT	GGCGGAAACATGAAATGAGT	CCACTCACCAAGCGAAGAAT	60.3	228	INTERGENIC	--	--	--
Ca4_P2_25715947	Ca4	25715947	ATTTTTTTTTTTTT/ATTTTTTTTTTT	GGTTCCCCTTCAACTCACCT	TGAACTGAGTCCGCGTATGT	59.3	820	DRR	Ca_16593	K	GATA
Ca4_P2_25719010	Ca4	25719010	TA/T	GTCCACTTGTTGCCAAGGTT	GTTGCAGAACCTGCTGATGA	60.0	884	DRR	Ca_16594	--	--
Ca4_P2_25758636	Ca4	25758636	CTTTT/CTTTTTT	TGCACTTCAGTTAAATTGGGC	AGAACTTGCGTGGTGATTCC	60.1	530	INTERGENIC	--	--	--
Ca4_P2_25761284	Ca4	25761284	ATTTTTTTTT/ATTTTTTTTTT	TTGTTGTGTGCAAGAGGATTG	GGAGATCATGGACGAAGGTG	60.5	366	INTERGENIC	--	--	--
Ca4_P2_25763103	Ca4	25763103	CTT/CT	ATCCTTGCGGGGATTTAGAG	ATTGCCTCATCCAAACGAAC	59.9	295	INTERGENIC	--	--	--
Ca4_P2_25768326	Ca4	25768326	TAAAAAA/TAAAAAAA	ATGTTTCGTGCAATGGTCAA	CACATGGCTGAACGAGAAGA	60.0	382	INTERGENIC	--	--	--
Ca4_P2_25776089	Ca4	25776089	G/GAC	TGGAATGAGAGGGGAAGATG	TCCGAGACAGGTATTTTGGC	60.1	366	INTERGENIC	--	--	--
Ca4_P2_2577945	Ca4	2577945	GTTTTTTTTTT/GTTTTTTTTTTT	CCAAAGTTGGAGGCTTTGAA	TCTTCCAGTCCGAAGTGGTC	60.2	813	DRR	Ca_07877	R	bHLH
Ca4_P2_25781212	Ca4	25781212	TAAAAAAAAAAA/TAAAAAAAAAA	TCAAGTTTTTGCAGCCATTG	CACTTAAGAGGCCCAATCCA	60.1	376	INTERGENIC	--	--	--
Ca4_P2_25783388	Ca4	25783388	CTTTT/CTTTTT	GCGTGATGCATGATCTAGGA	TCTTTTGATTCCGAGTTGGG	60.0	658	INTERGENIC	--	--	--
Ca4_P2_25790471	Ca4	25790471	CAAACGAAACGAAACGAAAC/CAAACGAAAC	CCTCAAATTCCAATTGTGCC	TCATCCTCACCCCCAGTTAG	59.9	646	INTERGENIC	--	--	--

INDEL marker IDs	Chromosomes/unanchored scaffolds	Physical positions (bp)	InDels (*Kabuli* reference genome-CDC Frontier/PII)	Forward primers (5'-3')	Reverse primers (5'-3')	Anneling temperature (0C)	Expected amplified product size (bp)	Structural annotation		Functional annotation	
								Sequence components of *kabuli* genome	*Kabuli* gene accession IDs	NCBI-KOG	TFs
Ca4_P2_25799557	Ca4	25799557	GAAAA/GAAA	TCAAATTCGCATTTCAACATATAAA	AACACCCTTCTCGTCACACC	60.0	657	INTERGENIC	--	--	--
Ca4_P2_25812288	Ca4	25812288	CT/C	ATGGTTGTTGCAAGTTTCCC	CCTTGAAGACATATGGTGGCA	60.9	522	INTRON	Ca_16599	R	--
Ca4_P2_25822023	Ca4	25822023	ATTTTTTTTTT/ATTTTTTTTT	ACGCAAGAGCAACATCTACG	TGAAGTCTCGGGTTCAAACA	59.3	617	INTERGENIC	--	--	--
Ca4_P2_25822369	Ca4	25822369	CAAAAAAA/CAAAAAA	TGTTTGAACCCGAGACTTCAC	CGTGGCATTTGAAAAAGCAT	61.0	611	INTERGENIC	--	--	--
Ca4_P2_25830554	Ca4	25830554	CAAAAAAAAAA/CAAAAAAAAA	ATCGTGCACATTCCCATTTT	TTCAAATTGCTCCTTCTGGG	60.2	621	INTERGENIC	--	--	--
Ca4_P2_25830993	Ca4	25830993	CTTTTTTTTTTTT/CTTTTTTTTTT	CCCAGAAGGAGCAATTTGAA	TTGCGGTTTCCTTAATGGTC	59.9	315	INTERGENIC	--	--	--
Ca4_P2_25844453	Ca4	25844453	AATTATTATTATTATTATTATTATTATTAATGTTATTATTATTATTATTATTATTATT/AATTATTATTATTATTATTATTATTATTATTATTATTAATGTTATTATTATTATTATTATTATTATT	CACTTGGGTCACTGCTGAAG	GATGCTGCATGTGTTGATCC	60.1	662	DRR	Ca_16601	R	--
Ca4_P2_25847698	Ca4	25847698	T/TG	CATGCTACCAAAGCGATTGA	ATCGATTTCGCGAGTCTTGT	59.8	370	INTERGENIC	--	--	--
Ca4_P2_25854591	Ca4	25854591	TAAAAAAAAAAAAA/TAAAAAAAAAAAA	TCACCCATGATATAAGGCTTTTG	TGGGGACTAAACAGCAACTCA	60.7	401	DRR	Ca_16602	R	--
Ca4_P2_25869082	Ca4	25869082	ATTTTT/ATTTT	TATTTGTCGATTTGCGTGGA	TTATAAGTGCGGTTAGCGGG	60.1	693	INTERGENIC	--	--	--
Ca4_P2_25884525	Ca4	25884525	TA/T	AGCAACCAAATCGAAACACC	GGGAAATTTTGGAACCAATG	59.1	307	INTERGENIC	--	--	--
Ca4_P2_25887792	Ca4	25887792	TTATTATTATTATTATTATTATTATTATTAGTATTATTATTATCATCATTATTATTATTATTATTATTATTATTATTATTATTATTATTATTATTATTAT/TTATTATTATTATTATTATTATTATTATTATTATTAGTATTATTATTATCATCA[illegible]	GGCTGAAGAAGAAAATCATCAAA	CACAGCACTTGACCTTCCAA	59.9	401	INTERGENIC	--	--	--

INDEL marker IDs	Chromosomes/unanchored scaffolds	Physical positions (bp)	InDels (*Kabuli* reference genome-CDC Frontier/PII)	Forward primers (5'-3')	Reverse primers (5'-3')	Anneling temperature (0C)	Expected amplified product size (bp)	Structural annotation		Functional annotation	
								Sequence components of *kabuli* genome	*Kabuli* gene accession IDs	NCBI-KOG	TFs
Ca4_P2_25890750	Ca4	25890750	TGGGG/TGGGGG	TGCAAGTATTTGTTGGGTGG	TCTCGGGATGGATGTAGTATATGA	59.7	460	INTERGENIC	--	--	--
Ca4_P2_25891004	Ca4	25891004	AGGG/AGGGG	TGCAAGTATTTGTTGGGTGG	CGTCAGTGACAAGTGATGGTG	60.2	823	INTERGENIC	--	--	--
Ca4_P2_2589224	Ca4	2589224	CAGTTGCAAGTTGCAAG/CAGTTGCAAGTTGCAAGTTGCAAG	AAGATCGATCAGTTCCACCG	ATTGGGAACCAACTGACGAG	60.0	396	INTERGENIC	--	--	--
Ca4_P2_25906423	Ca4	25906423	TC/T	TAATGGCATTGGTACAGGCA	AAAAATCCTGAATGTCCCCC	60.0	472	INTERGENIC	--	--	--
Ca4_P2_25916811	Ca4	25916811	CTTTTTTTTTTT/CTTTTTTTTTTTTT	ATGGTAACAGATATCGCCGC	ATTGTAGCATTGCCCAAAGG	60.0	627	DRR	Ca_16606	I	--
Ca4_P2_25924178	Ca4	25924178	ATTTTTT/ATTTTTTT	TGATGATGTCATTGATGTGTTTG	CTTGTAAGTTGTAGGCGGGC	59.8	778	INTERGENIC	--	--	--
Ca4_P2_25933783	Ca4	25933783	CTCGGTTCGGTTCGG/CTCGGTTCGGTTCGGTTCGG	TGGCATTGATGATGGCTTTA	ACCTTGTGGATGGCAGTTTC	60.0	340	INTERGENIC	--	--	--
Ca4_P2_25937535	Ca4	25937535	GC/GCTTTTGCTC	TCGACGTTGTCCATTTGTGT	ACACGAGCGTTTGAGCTTTT	60.1	621	INTERGENIC	--	--	--
Ca4_P2_25949029	Ca4	25949029	CTTTTT/CTTTTTT	TTCATTCAGATTGATTATGCTCG	GCTAAACACATGGAGATCAAACA	59.1	754	INTERGENIC	--	--	--
Ca4_P2_25954241	Ca4	25954241	A/ATTC	TTGCAAGAATCCATCCATCA	TTGAGCATTTCAGCAGAAGC	59.3	493	INTERGENIC	--	--	--
Ca4_P2_25956176	Ca4	25956176	ATTTTTTTTTTTTT/ATTTTTTTTTT	ATATTGGGACCGGTTAAGGG	TTTGGGTGTGTTGACTGCAT	60.0	784	INTERGENIC	--	--	--
Ca4_P2_25957374	Ca4	25957374	ATTTTTTTTTTTT/ATTTTTTTTTTT	TCATACCAGATCCACGGTGA	TAACGAATTGATTTCCCCGA	60.3	505	INTERGENIC	--	--	--

INDEL marker IDs	Chromosomes/unanchored scaffolds	Physical positions (bp)	InDels (*Kabuli* reference genome-CDC Frontier/PII)	Forward primers (5'-3')	Reverse primers (5'-3')	Anneling temperature (0C)	Expected amplified product size (bp)	Structural annotation		Functional annotation	
								Sequence components of *kabuli* genome	*Kabuli* gene accession IDs	NCBI-KOG	TFs
Ca4_P2_25957805	Ca4	25957805	AA/AAGTTATAAATCTA	CCTGGCTTACGTCAATTCATC	TCTTTCGTTTGGGTGGAAGT	59.6	269	INTERGENIC	--	--	--
Ca4_P2_25958357	Ca4	25958357	TTC/T	TTGTGCACTTCATTTTCAGACC	TGGAAGTCATCGACCCATTT	60.3	173	INTERGENIC	--	--	--
Ca4_P2_25959074	Ca4	25959074	GCC/GC	TGAAGATGAAGAAGAGGGCAA	TGTGGCATAAGGAGGAGTTTC	59.2	376	INTERGENIC	--	--	--
Ca4_P2_25962811	Ca4	25962811	GTATATATAT/GTATATATATAT	TGGTTGGAAGCTCCAGAAGT	GAAAAGGTGAGGAATCGCTG	59.8	275	DRR	Ca_16607	--	B3
Ca4_P2_25962938	Ca4	25962938	CTT/CT	TGGTTGGAAGCTCCAGAAGT	GTGGTAAGTTGTGTGGCCCT	59.9	577	DRR	Ca_16607	--	B3
Ca4_P2_25963240	Ca4	25963240	GAAAAAAA/GAAAAAAAA	AATCCAAGCACCAAAAGCAC	GTGGTAAGTTGTGTGGCCCT	59.9	353	DRR	Ca_16607	--	B3
Ca4_P2_25963279	Ca4	25963279	TTTTATTT/TTTT	AATCCAAGCACCAAAAGCAC	GTGGTAAGTTGTGTGGCCCT	59.9	353	DRR	Ca_16607	--	B3
Ca4_P2_25965378	Ca4	25965378	ACCCCCC/ACCCCC	GGTGTGCAAGAGGACTGGAT	GGTCCAAGAATATGCCCAAA	59.8	481	INTERGENIC	--	--	--
Ca4_P2_25966069	Ca4	25966069	ATTT/ATTTT	GGCATGGACAACTTCACCTT	CGAAATCCTCAAAATCGGAA	60.0	484	INTERGENIC	--	--	--
Ca4_P2_25967268	Ca4	25967268	T/TA	TTCAAACTCTTTCTGCCCAA	AAAGCACCAGTCGAAACTGC	60.4	670	INTERGENIC	--	--	--
Ca4_P2_25967972	Ca4	25967972	ATTTTTTTT/ATTTTTTTTT	TTGTACCCTTGTGCAACCAA	TGGTTGGATACTTGCAATGG	59.4	180	INTERGENIC	--	--	--
Ca4_P2_25968820	Ca4	25968820	GTTT/GTTTT	TTCTTCTGCCCAAAAATGCT	CCTCAAGCAGACCTTTGGAG	60.0	412	INTERGENIC	--	--	--

INDEL marker IDs	Chromosomes/unanchored scaffolds	Physical positions (bp)	InDels (*Kabuli* reference genome-CDC Frontier/PII)	Forward primers (5'-3')	Reverse primers (5'-3')	Anneling temperature (0C)	Expected amplified product size (bp)	Structural annotation		Functional annotation	
								Sequence components of *kabuli* genome	*Kabuli* gene accession IDs	NCBI-KOG	TFs
Ca4_P2_25969511	Ca4	25969511	ACC/AC	TCCCTTGCCTCTAGCCAGTA	GCAGATGTTCTCAACAGCCA	60.0	350	INTERGENIC	--	--	--
Ca4_P2_25970159	Ca4	25970159	AC/A	TGGTTGGGTCTTGGTTTGTT	CACCTGCTATGGATGCAATG	60.1	709	INTERGENIC	--	--	--
Ca4_P2_25972652	Ca4	25972652	TGGG/TGG	TTGTCTCAACAATCTCCCCC	CTCAGTTCCTAGCACGAGGG	60.0	676	INTERGENIC	--	--	--
Ca4_P2_25975114	Ca4	25975114	TGGGG/TGGGGG	ACACATGTTGGCCAATGAGA	GAGTCCGATTTCCATGAGGA	60.0	621	CDS (FRAME SHIFT)	Ca_16608	--	--
Ca4_P2_25976449	Ca4	25976449	CTTTTTT/CTTTTT	CCCCTTTTTGCACATGATCT	GGGAACTCCTATCCACCCAT	60.0	510	INTERGENIC	--	--	--
Ca4_P2_25978445	Ca4	25978445	CT/CTTT	TGCCTCAAAGGCTTCATCTT	AGCACTCATCCTCGGGTAGA	59.8	435	INTERGENIC	--	--	--
Ca4_P2_25979689	Ca4	25979689	TG/TGG	GTCCTCGTCAGGAGGTTGAG	CCACTGTTGCTGGCAGATAA	59.9	707	INTERGENIC	--	--	--
Ca4_P2_25982514	Ca4	25982514	CAATAATA/CAATAATAATA	GCGCAAAAACAAGAAGGAAG	GGTCCAGGTTGTTTGGAGAA	59.9	290	INTERGENIC	--	--	--
Ca4_P2_25983794	Ca4	25983794	A/AC	TGACAACCCCAGATTGTTGA	TGGGTCCATGTTCCAATTTT	60.0	671	INTERGENIC	--	--	--
Ca4_P2_25984075	Ca4	25984075	TA/TAA	CGATTTTTAACAGCGTGGGT	TGGGTCCATGTTCCAATTTT	60.0	276	INTERGENIC	--	--	--
Ca4_P2_25984442	Ca4	25984442	TGAGAG/TGAGAGAG	AGATCCTGTTGACACGGCTT	CGGTTGTTGTTGTGATTTGC	60.0	524	INTERGENIC	--	--	--
Ca4_P2_25987001	Ca4	25987001	T/TTAAA	TCAGTAGCATCGTAGCGTGG	TGAGCTGCGACGAGAGTAGA	60.0	379	INTERGENIC	--	--	--

INDEL marker IDs	Chromosomes/unanchored scaffolds	Physical positions (bp)	InDels (*Kabuli* reference genome-CDC Frontier/PII)	Forward primers (5'-3')	Reverse primers (5'-3')	Anneling temperature (0C)	Expected amplified product size (bp)	Structural annotation		Functional annotation	
								Sequence components of *kabuli* genome	*Kabuli* gene accession IDs	NCBI-KOG	TFs
Ca4_P2_25987244	Ca4	25987244	AATATATATAT/AATATATATATATATATAT	TCTACTCTCGTCGCAGCTCA	TGCTTAGGCATTTACCCCAG	60.1	250	INTERGENIC	--	--	--
Ca4_P2_25987622	Ca4	25987622	TCC/TC	TTCAACTGGGGTAAATGCCT	TGTTCTAGTCCAGATCAAAACGA	58.9	323	INTERGENIC	--	--	--
Ca4_P2_25987851	Ca4	25987851	CTTT/CT	TTCCTTCCTAATGTTCGTTTTGA	CGTGTCTCATCTTTGTGCTTT	58.0	732	INTERGENIC	--	--	--
Ca4_P2_25988978	Ca4	25988978	AAAGAAGAAGAAG/AAAGAAGAAG	AAGCTGGATGTGGACTTTGC	AGTTCCAATTGGCCTCCTTT	59.9	404	INTERGENIC	--	--	--
Ca4_P2_25989281	Ca4	25989281	TCCCC/TCCC	AAGCTGGATGTGGACTTTGC	AGTTCCAATTGGCCTCCTTT	59.9	404	INTERGENIC	--	--	--
Ca4_P2_25989322	Ca4	25989322	GTTTTT/GTTTTTT	GTACGCGATCTAGTCCCCAA	CCGGGGAGAAAGTTCTAGTG	58.8	168	INTERGENIC	--	--	--
Ca4_P2_25994447	Ca4	25994447	G/GT	ATCCACAACCGAGTTAGCGT	TACCCACACCCCTCAATCTT	59.3	389	INTERGENIC	--	--	--
Ca4_P2_25994721	Ca4	25994721	AC/A	TTAAGATTGAGGGGTGTGGG	CACCACTTCATTCGGGACTT	60.0	516	INTERGENIC	--	--	--
Ca4_P2_25995802	Ca4	25995802	ATTTT/ATTT	CCCTAAATCAAAGCAGCGTC	TGCACTGTTGTTGTTGCTGA	60.1	470	INTERGENIC	--	--	--
Ca4_P2_25996238	Ca4	25996238	GAAAAAAAAAAA/GAAAAAAAAA	CCCTAAATCAAAGCAGCGTC	AACGTTGGAGGAACAAAACG	60.0	949	INTERGENIC	--	--	--
Ca4_P2_25997913	Ca4	25997913	GATACTTTCA/G	TCTGCTTCTTGAGGTCTTGC	CACATGCAGGGCAACAATAG	60.1	581	DRR	Ca_16610	C	--
Ca4_P2_25998410	Ca4	25998410	TTA/TTATGTA	TATTGTTGCCCTGCATGTGT	TGGAGGCCAATTTGTTTCTC	60.1	531	DRR	Ca_16609	--	B3

INDEL marker IDs	Chromosomes/unanchored scaffolds	Physical positions (bp)	InDels (*Kabuli* reference genome-CDC Frontier/PII)	Forward primers (5'-3')	Reverse primers (5'-3')	Anneling temperature (0C)	Expected amplified product size (bp)	Structural annotation		Functional annotation	
								Sequence components of *kabuli* genome	*Kabuli* gene accession IDs	NCBI-KOG	TFs
Ca4_P2_26009737	Ca4	26009737	TC/T	GGAACTTCTCTTGTGGCAGC	ACCCTTTGCATCCCTTTCTT	59.9	617	INTERGENIC	--	--	--
Ca4_P2_26011159	Ca4	26011159	A/AACAGAACTCC	GAAACCCCACAACAAAATGG	AGCATCATGGATTTCTTCCG	60.0	438	DRR	Ca_16611	C	--
Ca4_P2_26015617	Ca4	26015617	AATAAATATA/AATA	TGCGAGGGCTAGCTTAAAAA	TGAGACAAAAGTTTGCGGTG	59.9	635	INTERGENIC	--	--	--
Ca4_P2_26030877	Ca4	26030877	CAAAA/CAAAAA	AACGTACTGCCTATCACGGG	ACCGAATTTACTCACGGTGC	60.0	827	INTERGENIC	--	--	--
Ca4_P2_26058753	Ca4	26058753	TAAA/TAAAA	AGGAACCAATCCTACCAGCC	TCTGGTCATGCATCCTTTGA	60.2	433	INTERGENIC	--	--	--
Ca4_P2_26067428	Ca4	26067428	CTTTTTTTTT/CTTTTTTTTTT	AGGTTGTTGCAGTGCAGACTT	TTGCGTTACGGATTTGATGA	60.1	604	INTERGENIC	--	--	--
Ca4_P2_26067529	Ca4	26067529	TAAAAAAAAAAA/TAAAAAAAAAAAAA	TAAAAAGGTCAGGCCGGAAA	TTGCGTTACGGATTTGATGA	60.1	428	INTERGENIC	--	--	--
Ca4_P2_26072163	Ca4	26072163	AAATAGTACTGACATT/A	TTACACCCACCACGACGTAA	CCAGAAAGTTGTTAGAAGCACG	59.1	523	INTERGENIC	--	--	--
Ca4_P2_26076271	Ca4	26076271	AAATA/AA	GCCGATAAAATAAAAATATACTGGACA	AATGACAAATAAGGGCCAAGT	57.6	424	INTERGENIC	--	--	--
Ca4_P2_26079571	Ca4	26079571	TA/TAA	TGCACAAACTCAAAATGCCT	GGCATTGGAAAAATCACGTT	59.8	194	INTERGENIC	--	--	--
Ca4_P2_26091108	Ca4	26091108	ATTTTTTTTT/ATTTTTTTTTTT	AGGTGCATTTCTGAAGGTGG	TGTTCAAGATCATTCAAATGCC	59.9	564	INTERGENIC	--	--	--
Ca4_P2_26095258	Ca4	26095258	CTGATTTCATGTGTTGATTT/CTGATTT	TATTAAAATTGGCCCTCCGA	GCAGTAGGCGACAAAACACA	59.9	849	INTERGENIC	--	--	--

INDEL marker IDs	Chromosomes/unanchored scaffolds	Physical positions (bp)	InDels (*Kabuli* reference genome-CDC Frontier/PII)	Forward primers (5'-3')	Reverse primers (5'-3')	Anneling temperature (0C)	Expected amplified product size (bp)	Structural annotation		Functional annotation	
								Sequence components of *kabuli* genome	*Kabuli* gene accession IDs	NCBI-KOG	TFs
Ca4_P2_26109434	Ca4	26109434	CTTTTT/CTTTTTT	GGAAAGGAATCATGTCAGGG	AAGACTGGCTTGGAAGGTCA	59.8	445	INTERGENIC	--	--	--
Ca4_P2_26110090	Ca4	26110090	AG/A	AGAACCTGACCTTCCAAGCC	AAAAAGGGGGAGATTGTTGG	60.2	606	INTERGENIC	--	--	--
Ca4_P2_26110333	Ca4	26110333	TCCC/TCCCC	CCAACAATCTCCCCCTTTTT	GCATCTGGTTGCAGTTCTCA	60.0	583	INTERGENIC	--	--	--
Ca4_P2_26110396	Ca4	26110396	CTTTTTTTTTT/CTTTTTTTTTTT	CCAACAATCTCCCCCTTTTT	GCATCTGGTTGCAGTTCTCA	60.0	583	INTERGENIC	--	--	--
Ca4_P2_26118906	Ca4	26118906	CTTTTTTTTTT/CTTTTTTTTT	CTGGATTCCAACAATCTCCC	TTCTTTGCTTTTGCGCTTTT	60.1	851	INTERGENIC	--	--	--
Ca4_P2_26121900	Ca4	26121900	CT/CTTGT	AGGGGTTAAAAAGGAAGGCA	CACGTCCCCTTTATTTTTGC	59.5	217	INTERGENIC	--	--	--
Ca4_P2_26153740	Ca4	26153740	TAAAAAA/TAAAAA	TTGGGTTGACCCGTATATTCT	CCGTGACAGTAGCCCGTATT	60.0	743	INTERGENIC	--	--	--
Ca4_P2_26155943	Ca4	26155943	ATTTTTTT/ATTTTTT	GCAATGCCTTACCGTGAAAT	CGACAGCACATTACCCAAAA	59.6	384	INTERGENIC	--	--	--
Ca4_P2_26176935	Ca4	26176935	GAAAAAAA/GAAAAAAAA	CACAATCAATCACGCCCTTA	TGTCATTTTTGGCGTAGTGC	59.7	500	INTERGENIC	--	--	--
Ca4_P2_2617790	Ca4	2617790	GTTTTTTTTTTTT/GTTTTTTTTTTTTTT	GATTGGAGCAAAGGGAAACA	ACGCCAACACCAAAATCAA	60.0	327	INTERGENIC	--	--	--
Ca4_P2_26194244	Ca4	26194244	ATTTTTTTTTT/ATTTTTTTTTTTT	TTCCTCAGCCTTCGTTGTCT	TCTCCCCCTTTTTGATGATG	59.9	358	DRR	Ca_16613	--	--
Ca4_P2_26199834	Ca4	26199834	A/AT	GGGCAAGGTGAACACAAGAT	GCATCGTCCTTCCATGATTT	59.9	383	INTERGENIC	--	--	--

INDEL marker IDs	Chromosomes/unanchored scaffolds	Physical positions (bp)	InDels (*Kabuli* reference genome-CDC Frontier/PII)	Forward primers (5'-3')	Reverse primers (5'-3')	Anneling temperature (0C)	Expected amplified product size (bp)	Structural annotation		Functional annotation	
								Sequence components of *kabuli* genome	*Kabuli* gene accession IDs	NCBI-KOG	TFs
Ca4_P2_26203930	Ca4	26203930	TTATATATATATATATAT/TTATATATATATATATATATATAT	TTGGAAATAAGGACAAAACACC	TCCAAATTTGCTGGAATCCT	59.5	626	INTERGENIC	--	--	--
Ca4_P2_26219618	Ca4	26219618	CA/C	ACGGTTTCTTTCCTTCCGAT	CGAGAACCAACCCTATCCAA	59.9	571	INTERGENIC	--	--	--
Ca4_P2_26236726	Ca4	26236726	TAAAAAAAAAAAA/TAAAAAAAAAA	GTTACGATTGTGTTCGGCCT	TAATCTCACTGCGCACCTTG	60.0	368	INTERGENIC	--	--	--
Ca4_P2_26251759	Ca4	26251759	CTTTTTTTTT/CTTTTTTTTTT	TAAAACGCAATCGGAAGACC	GAGAAACTTTCCGCTGCTTG	60.1	545	INTERGENIC	--	--	--
Ca4_P2_26251857	Ca4	26251857	ATTTTTTTTTTT/ATTTTTTTTTTTTT	AGCTTTCATGTAACACCCCG	GCCCTCAGATGAGAGTCAGG	59.9	453	INTERGENIC	--	--	--
Ca4_P2_26258704	Ca4	26258704	AT/ATAAACTTTTT	TTCTCGAATCAATTCCGTCC	TGGGTGTGTTTGTTTCAGGA	60.0	332	INTERGENIC	--	--	--
Ca4_P2_26259040	Ca4	26259040	ATTTTTTTTTT/ATTTTTTTTT	TTCTCGAATCAATTCCGTCC	CTCAGAGGCCGACCTATACA	57.9	888	INTERGENIC	--	--	--
Ca4_P2_26276734	Ca4	26276734	ATAATTAA/ATAA	GGTGCTACAGTGTCGAAGCA	TGCATTTTACAGCGCTTTTG	60.0	725	INTERGENIC	--	--	--
Ca4_P2_26302275	Ca4	26302275	TAAAAAAAAAA/TAAAAAAAAAAA	AGCTTCCCCCAGTACTGTCA	TGACGCGTCCAAATTTATCA	60.1	494	INTERGENIC	--	--	--
Ca4_P2_26308021	Ca4	26308021	CTTTTTT/CTTTTT	ATAAAACATCATCGGGCGAC	TTCAGTGGCATTGAGACATTG	59.7	405	DRR	Ca_25307	S	--
Ca4_P2_26326139	Ca4	26326139	GAAAAAAAA/GAAAAAAA	CTCCAACAGCATCAATTCCA	GATCCTGGGATTGTGAGCAT	59.9	724	DRR	Ca_25306	R	--
Ca4_P2_26330439	Ca4	26330439	CCTAAGCTCTAAGCTCT/CCTAAGCTCT	ATGAGCCCCGAATTCTAACA	GAACAATGGGGATGTGGTTC	60.0	662	INTERGENIC	--	--	--

INDEL marker IDs	Chromosomes/unanchored scaffolds	Physical positions (bp)	InDels (*Kabuli* reference genome-CDC Frontier/PII)	Forward primers (5'-3')	Reverse primers (5'-3')	Anneling temperature (0C)	Expected amplified product size (bp)	Structural annotation		Functional annotation	
								Sequence components of *kabuli* genome	*Kabuli* gene accession IDs	NCBI-KOG	TFs
Ca4_P2_26337485	Ca4	26337485	GTTTTTT/GTTTTTTT	CCTTTCCACCCAGAAAGGAG	TGGCAGAGCACTACCAACAA	60.4	362	DRR	Ca_25305	--	FAR1
Ca4_P2_26339363	Ca4	26339363	CAAGAAGAAGAAGAAGAAGAAGAA/CAAGAAGAAGAAGAAGAAGAAGAAGAAGAA	TCAAGACGTTGATCTCGTGC	CATCTTCATTGCCTCCCATT	59.9	397	INTERGENIC	--	--	--
Ca4_P2_26341643	Ca4	26341643	ATTTTTTTTTTTTTTTT/ATTTTTTTTTTTT	CACAGTCTTATTTGAGGCACCA	TTTGCGCTATGATTTGAGTGA	59.5	651	INTERGENIC	--	--	--
Ca4_P2_26344119	Ca4	26344119	CAAAAA/CAAAA	GGGATGGACAAAAACCAAAA	TCAAACAATTTCGACATTTACCA	59.4	589	DRR	Ca_25304	S	--
Ca4_P2_26345302	Ca4	26345302	TTTCTACTTCTACTTCTACTT/TTTCTACTTCTACTTCTACTTCTACTT	ACCGTCCAGTGACAGAAACC	TTAATATCAAACGGAGGCCG	59.9	504	DRR	Ca_25304	S	--
Ca4_P2_26350658	Ca4	26350658	TGGGG/TGGG	CTCTGACATGCGACAATGCT	GTTTGGTCGGGTTAAGGGAT	60.1	555	INTRON	Ca_25303	I	--
Ca4_P2_26361460	Ca4	26361460	CTTTTTTT/CTTTTTT	GAAACTCGCAGACTTCGGAC	TATTGGGCGAATCAAGGAAG	60.0	520	INTERGENIC	--	--	--
Ca4_P2_26363618	Ca4	26363618	T/TTTAC	GCCAAAATTTCCTTCGTTGA	ATTCCCCAAGAACATCACCA	60.2	613	INTERGENIC	--	--	--
Ca4_P2_26366195	Ca4	26366195	AATATATATATATATATATATATA/AATATATATATATATATATA	ACGACACCAACAACAACCAA	AAAATGGGATGGACCAATGA	60.0	614	INTERGENIC	--	--	--
Ca4_P2_26369375	Ca4	26369375	ATTTTTTTT/ATTTTTTT	TTCTGTGCATTTGAATGGGA	AAACCCCAAACCCTACGAAC	60.1	721	INTERGENIC	--	--	--
Ca4_P2_26389354	Ca4	26389354	TTATATATATATAT/TTATATATATATATAT	AGGAAGAGTGGAGTTGCGAA	TCATATTCGGTAGCCGTTCC	59.9	759	INTERGENIC	--	--	--
Ca4_P2_26398945	Ca4	26398945	AATTTAATTT/AATTTAATTTATCTATTTAATTT	GTGGCGAATTGATTTGTCCT	CGACGTAATCTGGGTTGGTT	59.9	413	INTERGENIC	--	--	--

INDEL marker IDs	Chromosomes/unanchored scaffolds	Physical positions (bp)	InDels (*Kabuli* reference genome-CDC Frontier/PII)	Forward primers (5'-3')	Reverse primers (5'-3')	Anneling temperature (0C)	Expected amplified product size (bp)	Structural annotation		Functional annotation	
								Sequence components of *kabuli* genome	*Kabuli* gene accession IDs	NCBI-KOG	TFs
Ca4_P2_26404295	Ca4	26404295	ATTTTTTTTTT/ATTTTTTTTT	TTTGGGTCCTTGGTGGTTAG	CCCACTTGGTAAAGACCACTG	59.5	430	INTERGENIC	--	--	--
Ca4_P2_26411287	Ca4	26411287	GTTT/GTTTT	TGTTCTGCTTAAGGGGCCTA	GATCAAGAGGTCACCGGTTC	59.5	841	INTERGENIC	--	--	--
Ca4_P2_26489000	Ca4	26489000	ATTTTTTTTTTTT/ATTTTTTTTTTTTT	CATCGCTCACACGTGTCTTT	CAGCGTGTCATATCAAGCCT	58.9	651	INTERGENIC	--	--	--
Ca4_P2_26492251	Ca4	26492251	ACTC/AC	CAAATGCACATGAGAACCGT	AATGATCCGGAACCATTTCA	60.1	519	INTERGENIC	--	--	--
Ca4_P2_26495554	Ca4	26495554	GAAAGTTGTTC/GAAAGTTGTTCAAAGTTGTTC	AACGATTTATCCTGGTCCCC	CCCGAATACAAACCCAAGTC	59.3	844	INTERGENIC	--	--	--
Ca4_P2_26504817	Ca4	26504817	GAAAAAAA/GAAAAAAAA	TGGTTCGAGAAGTACGACCA	TTGCTACGTCCAATCCCTTC	60.1	710	INTERGENIC	--	--	--
Ca4_P2_26514235	Ca4	26514235	GAAAAAAAAA/GAAAAAAAAAA	CTAGCCTGGCAAGCCTATCA	GGACGACCTAGCCTATTCCC	59.9	644	INTERGENIC	--	--	--
Ca4_P2_26514316	Ca4	26514316	CAAAAAAAAAAA/CAAAAAAAAAA	CTAGCCTGGCAAGCCTATCA	GGACGACCTAGCCTATTCCC	59.9	644	INTERGENIC	--	--	--
Ca4_P2_26514892	Ca4	26514892	GAAAAAAAA/GAAAAAAA	TGCTCTTGGGGATGTCAAAT	TGGTCCGGTCTAGAAGCCTA	59.8	687	INTERGENIC	--	--	--
Ca4_P2_26519771	Ca4	26519771	CAAA/CAAAAAA	AAACCTCTTCGCCTTTTTGC	TATTTTACGTACCCGCTGCC	60.0	351	DRR	Ca_25005	R	NAC
Ca4_P2_26523067	Ca4	26523067	ATTTTTTTT/ATTTTTTTTT	TATCTGCCAGGGAGAAAACG	TGCCATGAAACGAAAGAAAA	59.3	362	DRR	Ca_25004	P	--
Ca4_P2_26523126	Ca4	26523126	TCCC/TCC	TATCTGCCAGGGAGAAAACG	TGCAAGAGAGGAAACGAAAA	58.6	436	DRR	Ca_25004	P	--

INDEL marker IDs	Chromosomes/unanchored scaffolds	Physical positions (bp)	InDels (*Kabuli* reference genome-CDC Frontier/PII)	Forward primers (5'-3')	Reverse primers (5'-3')	Anneling temperature (0C)	Expected amplified product size (bp)	Structural annotation		Functional annotation	
								Sequence components of *kabuli* genome	*Kabuli* gene accession IDs	NCBI-KOG	TFs
Ca4_P2_26524092	Ca4	26524092	GAA/GAAAA	TGAAATAGAGGGCCAGGTTG	TTCGTCCTTTGTCATCAAACA	59.2	317	DRR	Ca_25004	P	--
Ca4_P2_26524408	Ca4	26524408	C/CCTTAA	TGTTTGATGACAAAGGACGAA	AGAGGCACATTGAGAGCGTA	58.6	421	DRR	Ca_25004	P	--
Ca4_P2_26529457	Ca4	26529457	ATCTCTCTCTCTCTC/ATCTCTCTCTCTCTCTC	ATAGCAGCAATTTCCATCCG	GTCCCACGAAGTAAGTCCCA	60.0	286	INTERGENIC	--	--	--
Ca4_P2_26532976	Ca4	26532976	TCCCC/TCCC	TCAGATGCAGTTCCTTCGTG	CTGTGTACTGCAAGGGGGTT	60.0	477	INTRON	Ca_25003	T	--
Ca4_P2_26581837	Ca4	26581837	TTTCCTTCCTTCCTT/TTTCCTTCCTTCCTTCCTT	GGAGAAGGAGGGAGAAATGG	ATGCATTACGGGTGAAATGG	60.6	263	DRR	Ca_25001	--	--
Ca4_P2_26620467	Ca4	26620467	A/AGTAATT	CCGAAACCGAACAACAGATT	TGGGAACAAAACACAGCAAG	59.7	819	INTERGENIC	--	--	--
Ca4_P2_26622922	Ca4	26622922	GAAAAAAAAA/GAAAAAAAA	TGGCTGTTGTCCATTGTGTT	TCAGACACAGTTGGTCGGTT	59.2	604	INTERGENIC	--	--	--
Ca4_P2_26624241	Ca4	26624241	C/CG	CCAAAAGTCCAACGATTGCT	TGTGCATGGTGCTTTTTGAT	60.1	447	INTERGENIC	--	--	--
Ca4_P2_26626709	Ca4	26626709	A/AT	TATATGGGATGAAGCGCACA	AGCACATGATTTGCAAGCCT	60.8	887	INTERGENIC	--	--	--
Ca4_P2_26648267	Ca4	26648267	ATTTTTTTTT/ATTTTTTTT	TCGAACAGAAACCTGAGCCT	GATTCGGGAGTTGGTTGATG	60.3	440	INTRON	Ca_24999	L	--
Ca4_P2_26664091	Ca4	26664091	TAAAAAAAAA/TAAAAAAAAAA	TGATACAATTGATTCGGCCA	TTTTTGTCACGCGAGCTCTA	59.8	371	INTERGENIC	--	--	--
Ca4_P2_26672419	Ca4	26672419	TAAAAAAAAAA/TAAAAAAAAA	GGATTCAAAAATTTGGAGCGT	TAGCACCCTACGACATCCGT	60.5	981	INTERGENIC	--	--	--

INDEL marker IDs	Chromosomes/unanchored scaffolds	Physical positions (bp)	InDels (*Kabuli* reference genome-CDC Frontier/PII)	Forward primers (5'-3')	Reverse primers (5'-3')	Anneling temperature (0C)	Expected amplified product size (bp)	Structural annotation		Functional annotation	
								Sequence components of *kabuli* genome	*Kabuli* gene accession IDs	NCBI-KOG	TFs
Ca4_P2_26672920	Ca4	26672920	CTTTT/CTTT	ACGGATGTCGTAGGGTGCTA	AAGACCCGAATCAGCATTTG	60.1	438	INTERGENIC	--	--	--
Ca4_P2_26673641	Ca4	26673641	ATTTTTTTTT/ATTTTTTTTTT	CAAATGCTGATTCGGGTCTT	AAAATGCACGCCCATAACTC	60.0	653	DRR	Ca_24998	--	Trihelix
Ca4_P2_26726880	Ca4	26726880	ACTTCTT/ACTT	CATTGTTCGAATCCACGTTTT	CCCCTCATTAGCCACTTAGC	58.8	422	INTERGENIC	--	--	--
Ca4_P2_26727123	Ca4	26727123	GTTTTTTTTTTTTTTTTTTT/GTTTTTTTTTTTTTTTT	CATTGTTCGAATCCACGTTTT	TGTGGGAACACGACCATAAA	59.8	690	INTERGENIC	--	--	--
Ca4_P2_26733398	Ca4	26733398	ATTTTTTT/ATTTTTT	AGTACCATCCAAGTGCGGTC	TCTTTTTCACCATCCCGACT	59.5	302	INTERGENIC	--	--	--
Ca4_P2_26733428	Ca4	26733428	TAAAAAAAAA/TAAAAAAAA	AGTACCATCCAAGTGCGGTC	TCTTTTTCACCATCCCGACT	59.5	302	INTERGENIC	--	--	--
Ca4_P2_26735683	Ca4	26735683	CTTTTTTTT/CTTTTTTT	TTTACCCATGCTTCCGTAGG	TCCAGCCAGAAATCCATAGG	60.0	751	DRR	Ca_20436	--	--
Ca4_P2_26748572	Ca4	26748572	GAAAAAAAA/GAAAAAAAAA	GGGTGTGAGTTGAAATGACG	TTTGTCGCAACAACACCATT	60.0	858	INTERGENIC	--	--	--
Ca4_P2_26750453	Ca4	26750453	GTATA/GTATATA	AAAAAGCAAATGATCCCATCC	TGGATCCTCTCAATTCAAATCTC	59.5	672	INTERGENIC	--	--	--
Ca4_P2_26752184	Ca4	26752184	ATTTTTTTTTTT/ATTTTTTTTTT	TTGGGATCATAGAATCATTTGGT	TGATCACGGTCACTCCAAAG	59.7	475	INTERGENIC	--	--	--
Ca4_P2_26753050	Ca4	26753050	GAA/GAAA	AACACTCGCAAAATCTTCGG	ATTGTTTGAGTTGCCGCTCT	59.9	623	INTERGENIC	--	--	--
Ca4_P2_26761814	Ca4	26761814	TCC/TC	ATCATTTTAGCGCAGCCACT	ACACTCACACTCCCCAGAGG	60.2	643	INTERGENIC	--	--	--

INDEL marker IDs	Chromosomes/unanchored scaffolds	Physical positions (bp)	InDels (*Kabuli* reference genome-CDC Frontier/PII)	Forward primers (5'-3')	Reverse primers (5'-3')	Anneling temperature (0C)	Expected amplified product size (bp)	Structural annotation		Functional annotation	
								Sequence components of *kabuli* genome	*Kabuli* gene accession IDs	NCBI-KOG	TFs
Ca4_P2_26763494	Ca4	26763494	TAAAAAAA/TAAAAAAAA	AAAAATACCGCGTCATTTCATT	TCGCTCAAAAACACAACGAG	60.0	572	INTERGENIC	--	--	--
Ca4_P2_26765991	Ca4	26765991	GAAAAAAAAA/GAAAAAAAA	AGTTTTGGCCCCTTTTGATT	AAAAACTCGCACACAACTGTTA	57.6	508	DRR	Ca_20437	K	MYB
Ca4_P2_26775300	Ca4	26775300	TAAAAAAAAAAA/TAAAAAAAAAAAA	ACGGCGCTAAACCAACATAG	TTGTGTGCTTAACCTTTGCG	59.9	320	INTERGENIC	--	--	--
Ca4_P2_26777289	Ca4	26777289	TAAAAAAAAAAA/TAAAAAAAAA	TCAAAAATAGAGGCAGCCAAA	TCGTGTTCGACACACATCAA	59.7	417	INTERGENIC	--	--	--
Ca4_P2_26778550	Ca4	26778550	CTTT/CTTTT	GCCCATCCAAGTGATGAGAT	TTCCTTTCGGCAAAAATCAC	60.1	811	INTERGENIC	--	--	--
Ca4_P2_26787145	Ca4	26787145	CTTTTTTTTTT/CTTTTTTTTT	GCGACTATGTCCGTCAACTG	TCGAAGCATGAAAGAAAAGGA	59.9	803	INTERGENIC	--	--	--
Ca4_P2_26791133	Ca4	26791133	CTTTTTTT/CTTTTTTTT	GACGACAGAGTTGATGCCATT	TCAAGTCAACGCTCTGGTTG	60.0	603	INTERGENIC	--	--	--
Ca4_P2_26801659	Ca4	26801659	ATTTTTTTTTTTT/ATTTTTTTTTT	TTTGCGTTACGAATTTGATGA	TTTCCCGATTTGGTCCTTTA	59.4	178	INTERGENIC	--	--	--
Ca4_P2_26805293	Ca4	26805293	AAAATA/AA	TTCCATTGATACATTCCAGCA	AAGCTAGCCATCCTCAACGA	60.0	234	DRR	Ca_20438	--	--
Ca4_P2_26831033	Ca4	26831033	CAAAAAAAAAAA/CAAAAAAAAAA	TCTTCCCGTATATTGCTCGG	CCAGCGTGACGTAGCTGATA	60.0	677	INTERGENIC	--	--	--
Ca4_P2_26842237	Ca4	26842237	GAAAAAAAAAA/GAAAAAAAAA	TAGCATGCCTCAAATCCCTC	AGCAACACCGTATTGACATCC	59.9	726	INTERGENIC	--	--	--
Ca4_P2_26844220	Ca4	26844220	TAAAAAAAAAA/TAAAAAAAAA	ATCAACCCACAGGTGACACA	CACAAAGCCACATCCACAAC	60.0	363	INTERGENIC	--	--	--

INDEL marker IDs	Chromosomes/unanchored scaffolds	Physical positions (bp)	InDels (*Kabuli* reference genome-CDC Frontier/PII)	Forward primers (5'-3')	Reverse primers (5'-3')	Anneling temperature (0C)	Expected amplified product size (bp)	Structural annotation		Functional annotation	
								Sequence components of *kabuli* genome	*Kabuli* gene accession IDs	NCBI-KOG	TFs
Ca4_P2_26847079	Ca4	26847079	AAAAATAAAATAAAATAAAATA/AAAAATAAAATA	GGAGAACGTTAAGGCAGCAG	GTGGTGTAGGTTTCGATCCG	60.4	689	INTERGENIC	--	--	--
Ca4_P2_26847495	Ca4	26847495	A/AC	AGCTTCAAGTCGGATCGAAA	AAAAATCTTGTGGGAATAATTCAA	58.1	216	INTERGENIC	--	--	--
Ca4_P2_26867246	Ca4	26867246	CTTTTTTTTTT/CTTTTTTTTTTT	AAGTATGCATCGCGTCCTTC	ATTGACATTGGCACATCTCG	59.5	140	INTERGENIC	--	--	--
Ca4_P2_26876873	Ca4	26876873	ATTTTTTTTTTT/ATTTTTTTTTTTT	CCACCGTTGTGCCTCTTATT	GCCAGAGAATGATCGAGAGG	59.9	439	INTERGENIC	--	--	--
Ca4_P2_2688293	Ca4	2688293	AT/A	AGCATGCTTCCACAAAGGAT	TTGGAGGTTGTGTTGTTGGA	60.0	532	DRR	Ca_12072	--	--
Ca4_P2_26898145	Ca4	26898145	CTTTTTTTTTT/CTTTTTTTTT	CGAAACTCAAAAACAACGGG	CGGCCCAATTCAAACTAAAA	59.9	588	INTERGENIC	--	--	--
Ca4_P2_26903304	Ca4	26903304	T/TG	ACTCGATTGGTCCATGCTTC	ATGTTTTGGACGCACACAGA	60.2	532	INTERGENIC	--	--	--
Ca4_P2_26905366	Ca4	26905366	TAAAAAAAAAA/TAAAAAAAAA	TGAAAGCCCAAAACTAATCAAGA	ATTGCTCGGTCGGTTTAGTC	59.2	471	DRR	Ca_20443	R	G2-like
Ca4_P2_26936665	Ca4	26936665	AGTTAATATGTTAATATGTTAAT/AGTTAATATGTTAAT	TCAAGGCTTTAGGCGTGAGT	GTAAATGCGTTGTGCGATCA	60.7	615	INTERGENIC	--	--	--
Ca4_P2_26948125	Ca4	26948125	GTTATTATTATTATTATTATTATT/GTTATTATTATTATTATTATT	GATGAACCGGAAGAACCTCA	ATAGGAGATCGCTTTTCGGG	60.5	547	DRR	Ca_20451	R	--
Ca4_P2_26954877	Ca4	26954877	GTTTTTTTTTT/GTTTTTTTTTTT	TCAATTTTGATGATGTGGCAA	CGGTCAAACAATCAGCTTCA	59.8	405	INTERGENIC	--	--	--
Ca4_P2_26955373	Ca4	26955373	CTTTTTTTTTT/CTTTTTTTTTTTT	TGAAGCTGATTGTTTGACCG	CGTGTTTTGAAGCACCGATA	59.7	604	INTERGENIC	--	--	--

INDEL marker IDs	Chromosomes/unanchored scaffolds	Physical positions (bp)	InDels (*Kabuli* reference genome-CDC Frontier/PII)	Forward primers (5'-3')	Reverse primers (5'-3')	Anneling temperature (0C)	Expected amplified product size (bp)	Structural annotation		Functional annotation	
								Sequence components of *kabuli* genome	*Kabuli* gene accession IDs	NCBI-KOG	TFs
Ca4_P2_26957417	Ca4	26957417	GTTTTGATTTTTTTTT/GTTTTGATTTTTTTTTATTTTTGATTTTTTTTT	GTTTGTCCCCAAGCAACAAT	CTAGCCCATTTGAGCTTTGC	60.0	642	INTERGENIC	--	--	--
Ca4_P2_26966437	Ca4	26966437	CAAAAAA/CAAAAAAA	GGGTGCGTTTGTTTGAGTTT	TGCACAAATAGCATGTCACCT	59.2	845	INTERGENIC	--	--	--
Ca4_P2_26970810	Ca4	26970810	TAAAAAAAA/TAAAAAAA	GAAAAGCTGAAACTGTATTTTGGAA	AACAGTGCCGATGAATAGTTTT	57.8	306	DRR	Ca_20452	A	--
Ca4_P2_26991333	Ca4	26991333	ATTTTTTTTTT/ATTTTTTTTT	ACAAGGAAGCGCACCTCTAA	GTGCGACGAGCAAGAATGTA	60.0	762	INTERGENIC	--	--	--
Ca4_P2_27017251	Ca4	27017251	GTTTTTTTTT/GTTTTTTTTTT	CCTTTGCATCTCAGGGACAT	TGAGACCGGGTTTGAGTACC	60.0	453	INTERGENIC	--	--	--
Ca4_P2_27037667	Ca4	27037667	TG/T	CAATTGCAAAGGACATCCAG	AACGTGGGTTACGTCCAGTC	59.9	678	INTERGENIC	--	--	--
Ca4_P2_27052245	Ca4	27052245	ATTTTTTTTTTT/ATTTTTTTTTTTT	TGAAGGACCAAATCGAAAAGA	CGTGTCAAGTTAAACCATGCC	60.4	651	INTERGENIC	--	--	--
Ca4_P2_27054869	Ca4	27054869	TAAAAAAAAAAAAA/TAAAAAAAAAAA	GCTCAACTAAACGGTCCTGC	AATCAAGCAACCCACCAAAC	59.8	394	INTRON	Ca_20455	L	--
Ca4_P2_27054943	Ca4	27054943	ATTTTTTTTT/ATTTTTTTTTT	GCTCAACTAAACGGTCCTGC	AATCAAGCAACCCACCAAAC	59.8	394	INTRON	Ca_20455	L	--
Ca4_P2_27063954	Ca4	27063954	TAAAAA/TAAAAAA	CGCAAAAGGGCATTGATTAT	TGGGGTGTTACAAGTAGGGTG	59.8	571	INTERGENIC	--	--	--
Ca4_P2_27072516	Ca4	27072516	CAAAAAAAAAA/CAAAAAAAAA	GGAATGTGGAAGTGCGATTT	GTCGCAGAAGATGGGAATGT	60.1	839	INTERGENIC	--	--	--
Ca4_P2_27074666	Ca4	27074666	GAAAAAAAAA/GAAAAAAAAAA	ATTGGCATCAGAGCCAGTCT	TTTTCCAAGAGAGCGCATTT	60.0	566	INTERGENIC	--	--	--

INDEL marker IDs	Chromosomes/unanchored scaffolds	Physical positions (bp)	InDels (*Kabuli* reference genome-CDC Frontier/PII)	Forward primers (5'-3')	Reverse primers (5'-3')	Anneling temperature (0C)	Expected amplified product size (bp)	Structural annotation		Functional annotation	
								Sequence components of *kabuli* genome	*Kabuli* gene accession IDs	NCBI-KOG	TFs
Ca4_P2_27079794	Ca4	27079794	AAA/ACAA	AAACACTACCCGGCATCAAG	CAATTTTCTGCACTCTCGCA	60.1	516	INTERGENIC	--	--	--
Ca4_P2_27079864	Ca4	27079864	GATATATATATATATA/GATATATATATATATATA	AAACACTACCCGGCATCAAG	CAATTTTCTGCACTCTCGCA	60.1	516	INTERGENIC	--	--	--
Ca4_P2_27082135	Ca4	27082135	GATATATATATATATATATA/GATATATATATATA	GACGCCTGACGGTTATTGTT	TTAGCCAGAAAAGCGGAAAA	60.0	578	INTERGENIC	--	--	--
Ca4_P2_27087434	Ca4	27087434	TA/TAAAA	TTATTGCGATGCCGACTATG	AATGATGTGGCTGGGTTAGC	60.0	656	INTERGENIC	--	--	--
Ca4_P2_27090201	Ca4	27090201	ATTTTTTTTTTTTTTT/ATTTTTTTTTTTTT	ATGGCAAATCATCTTGCTCC	TCTCCCCCTTTTTGATGATG	59.9	433	INTERGENIC	--	--	--
Ca4_P2_27094849	Ca4	27094849	TAT/TATCAAT	GCCAAAACGACCACAAAGTT	CCGCCACAAAGGAATAAAGA	60.1	716	INTERGENIC	--	--	--
Ca4_P2_27096186	Ca4	27096186	GAAAAAAAAA/GAAAAAAAAAA	GGTTAGGTGAGCGACTGAGC	GCTCTTTTCTTTTTCCCGGT	59.7	122	INTERGENIC	--	--	--
Ca4_P2_27115538	Ca4	27115538	TTCTCTCTCTCTCTCTCTCTCTCTCTC/TTCTCTCTCTCTCTCTCTCTCTC	TATTAGCGCTCGCTGGTTTT	ACCTCCAATGCTCAAAGCAC	60.3	477	INTRON	Ca_20456	R	--
Ca4_P2_27138018	Ca4	27138018	ATTTTGTTTTTGTTTTTGTTTTTGTTTTT/ATTTTGTTTTTGTTTTT	TCCTAGAACATGCATCACGC	TACCCTTTGCTAGCCCCTTT	60.1	729	INTERGENIC	--	--	--
Ca4_P2_27149358	Ca4	27149358	ATTTTTTTT/ATTTTTTT	CGATGATGCTACCAGAGGGT	CGGGGATTCCCATATCACTA	59.6	611	INTERGENIC	--	--	--
Ca4_P2_27161752	Ca4	27161752	ATTTTTT/ATTTTTTT	TTTCAATGCAGTTCGATTTTTG	TTTTCAAAATCCCGACGTTA	58.1	504	INTERGENIC	--	--	--
Ca4_P2_27165248	Ca4	27165248	A/AG	AGGGGCAGAAATGACATGAA	TTCAAAATCAGCATTATCCACG	60.0	582	INTERGENIC	--	--	--

INDEL marker IDs	Chromosomes/unanchored scaffolds	Physical positions (bp)	InDels (*Kabuli* reference genome-CDC Frontier/PII)	Forward primers (5'-3')	Reverse primers (5'-3')	Anneling temperature (0C)	Expected amplified product size (bp)	Structural annotation		Functional annotation	
								Sequence components of *kabuli* genome	*Kabuli* gene accession IDs	NCBI-KOG	TFs
Ca4_P2_27168549	Ca4	27168549	CAAAAAA/CAAAAAA	AGGGCCAAGGGACAATAAGT	TGGTTTGTGCAATGTCCCTA	60.0	523	INTERGENIC	--	--	--
Ca4_P2_27168934	Ca4	27168934	AAC/A	TCCTACATGCCCCAAGAAAC	TGGTTTGTGCAATGTCCCTA	60.0	339	INTERGENIC	--	--	--
Ca4_P2_27171043	Ca4	27171043	ATTG/AGCAAAATACATTGTTG	ATCAGCGCTCGGTCTCTAAA	CTTTCTTCGCAGCAGAGGTT	59.8	253	INTERGENIC	--	--	--
Ca4_P2_27184150	Ca4	27184150	AAATAATAATAATAATA/AAATAATAATAATAATAATA	TTTCTTGTTGCCTTGGTGTG	TAGAAACCGGTTGCCAAGAA	60.6	744	INTERGENIC	--	--	--
Ca4_P2_27185776	Ca4	27185776	TAAAAAAAAA/TAAAAAAAAAA	GCCCAAAACGGGAATAATTT	GGGCAAAAGATGGAACAAGA	60.1	712	INTERGENIC	--	--	--
Ca4_P2_27196717	Ca4	27196717	A/ACGCTGACACGCAC	TTGGCACCAGAAATCTCACA	TTTGGGACCCCAATTATTCA	60.0	892	INTRON	Ca_20458	--	NF-YB
Ca4_P2_27206500	Ca4	27206500	ATTTTTTTTTTTTTTTT/ATTTTTTTTTT	GCACTTCATTCAGCTGTGCT	TGTTGATGATGCTGATTGGG	60.5	345	INTERGENIC	--	--	--
Ca4_P2_27213395	Ca4	27213395	ATTTTTTTTT/ATTTTTTTTTT	TTTTTCCAGACACCAGGAGG	AGAAAGGCTCAAACGACCAA	59.9	648	INTERGENIC	--	--	--
Ca4_P2_27217904	Ca4	27217904	CAAAAAAAAAA/CAAAAAAAAAAA	TGTAGTGGCATGTTTGGCAT	TTTGGAGGACTTTTGGGTTG	59.9	662	INTERGENIC	--	--	--
Ca4_P2_27231581	Ca4	27231581	CTTTTTTTTTTT/CTTTTTTTTT	GGGTTGGTTTGATTTGGATG	TTGTTTCTCCCCAGTTTTGG	59.9	622	INTRON	Ca_20460	--	--
Ca4_P2_27231813	Ca4	27231813	CAA/CAAA	CCCCATTTTGTTTCTCTCCA	TTGTTTCTCCCCAGTTTTGG	59.9	236	INTRON	Ca_20460	--	--
Ca4_P2_27240444	Ca4	27240444	CTTTTTTTTTT/CTTTTTTTT	ATTGTTCTCCTTGTGCTGCC	TTTCCCGACTTGGTCCTTTT	60.8	373	INTRON	Ca_20460	--	--

INDEL marker IDs	Chromosomes/unanchored scaffolds	Physical positions (bp)	InDels (*Kabuli* reference genome-CDC Frontier/PII)	Forward primers (5'-3')	Reverse primers (5'-3')	Anneling temperature (0C)	Expected amplified product size (bp)	Structural annotation		Functional annotation	
								Sequence components of *kabuli* genome	*Kabuli* gene accession IDs	NCBI-KOG	TFs
Ca4_P2_27241081	Ca4	27241081	CTTTTTTTTTTT/CTTTTTTTTTTTTTT	AAGGACCAAGTCGGGAAAAA	AGGTTTTGATGTGTTTGACGG	59.9	705	INTRON	Ca_20460	--	--
Ca4_P2_27242609	Ca4	27242609	GTTTTTTTTTTTT/GTTTTTTTTT	CGATGTCGAATATGGAACGA	CGGAAATTAGATTGAACGGC	59.5	561	INTRON	Ca_20460	--	--
Ca4_P2_27249645	Ca4	27249645	GTTTTTTTTTTT/GTTTTTTTTTTTTTTTT	GAAATAATGGGTCGGGGTCT	CCATCATGTGCTTGTCAAAA	58.1	872	DRR	Ca_20460	--	--
Ca4_P2_27258927	Ca4	27258927	ATTTTTTTTTT/ATTTTTTTTT	AGCGCGGAGAGATAGTTGAA	TGGGTGACTTCATTTGGACA	59.9	393	INTERGENIC	--	--	--
Ca4_P2_27259252	Ca4	27259252	TAAAAAAAAA/TAAAAAAAA	AATTTATGCGCTTGGGACAC	CAGGGGTGAGTGTCACTTTG	59.1	551	INTERGENIC	--	--	--
Ca4_P2_27260077	Ca4	27260077	GAAA/GAAAA	GCGTCCAAGTCTTTGAGGAA	TTTGGGGTTGCATAATTTGA	58.9	498	INTERGENIC	--	--	--
Ca4_P2_27261340	Ca4	27261340	CAAAAAAA/CAAAAAAAA	ACTCCAACACACAACCCCTC	AATTGAGCCCAGAAAACCCT	59.9	366	INTERGENIC	--	--	--
Ca4_P2_27267123	Ca4	27267123	TAAAAA/TAAAA	CCCTCAACTCAACTGAGATCC	TGCATTGGTTTGAATTTGGA	59.9	568	DRR	Ca_20461	R	--
Ca4_P2_27270866	Ca4	27270866	TAAAAAAAAAAAAAAAAAA/TAAAAAAAAAAAAAAA	CGGAACCAGCTTTGAGTAGC	GTTGGAGCCATTTCGGATAA	59.9	299	DRR	Ca_20462	R	--
Ca4_P2_27273452	Ca4	27273452	GTTT/GTT	TACATGAGGGTTTTCTGGGC	AACGTTCAGAGATGGTTGGG	60.0	272	INTERGENIC	--	--	--
Ca4_P2_27273711	Ca4	27273711	CT/CTT	TACATGAGGGTTTTCTGGGC	ATCAGAGGAAGGGTGGGACT	59.9	757	INTERGENIC	--	--	--
Ca4_P2_27274392	Ca4	27274392	GAA/GA	AGTCCCACCCTTCCTCTGAT	TTGCCAACCGATTGTGTTTA	60.0	805	INTERGENIC	--	--	--

INDEL marker IDs	Chromosomes/unanchored scaffolds	Physical positions (bp)	InDels (*Kabuli* reference genome-CDC Frontier/PII)	Forward primers (5'-3')	Reverse primers (5'-3')	Anneling temperature (0C)	Expected amplified product size (bp)	Structural annotation		Functional annotation	
								Sequence components of *kabuli* genome	*Kabuli* gene accession IDs	NCBI-KOG	TFs
Ca4_P2_27283830	Ca4	27283830	GTTT/GTTTT	TATTGGTCGTCAAGCAGCAG	TTTCCCAATCGATTTGCTTC	60.0	681	DRR	Ca_20463	--	--
Ca4_P2_27285679	Ca4	27285679	G/GAA	TATTTCGAGGGCGTACCAAC	CCAGCCTACCCCTTTTATCTG	60.0	549	INTERGENIC	--	--	--
Ca4_P2_2729716	Ca4	2729716	A/AGACATCAT	AGGGCTTGTAATGCCCTTTT	TTGGCTTCACAAGATACCCC	59.9	654	INTRON	Ca_12075	Z	--
Ca4_P2_27326152	Ca4	27326152	TAA/TA	TCACCCAATATGGGTTACGTC	TACAACTCCCAACGGACACA	60.0	530	INTERGENIC	--	--	--
Ca4_P2_27330002	Ca4	27330002	GAAAAAAAAAAA/GAAAAAAAAAA	GATGCACAATGCTGAAGGAA	ATTTTTGGTGTTGGGTTGGA	60.1	947	INTERGENIC	--	--	--
Ca4_P2_27330072	Ca4	27330072	TAAAAA/TAAAAAA	AAATCTGGAAAATGCAGGGA	ATTTTTGGTGTTGGGTTGGA	60.1	762	INTERGENIC	--	--	--
Ca4_P2_27331930	Ca4	27331930	GAAAAAAAA/GAAAAAAA	CAACAATCCCAAACCAACAA	ATGGTGGTCAACGTCCCTTA	60.2	398	INTERGENIC	--	--	--
Ca4_P2_27332406	Ca4	27332406	ACT/ACTTCT	TAAGGGACGTTGACCACCAT	GGGTGTGATGGGTAAACCTG	60.1	379	INTERGENIC	--	--	--
Ca4_P2_27336275	Ca4	27336275	CTTTTTTTTTTTTTTTTTTTTTT/CTTTTTTTTTTTTTT	AACCATGTTGGGCCATGTAT	CCATGATTTAGCCCGAGAA	60.0	329	INTERGENIC	--	--	--
Ca4_P2_27354234	Ca4	27354234	CTTTTTTTTTT/CTTTTTTTT	TGAGCTTTGTGCCGTAACAG	CGAGTACGGAAATCGTCCAT	60.0	550	INTERGENIC	--	--	--
Ca4_P2_27357133	Ca4	27357133	GAAAA/GAAA	TTGATTTGGTGCACATCCAT	TAAAACTTGTCCCCAACCCC	60.9	561	INTERGENIC	--	--	--
Ca4_P2_27366853	Ca4	27366853	TG/T	TGAGACAATGAGGCTTGTGG	TGCACTTGGTCAATGTGTGA	59.7	460	INTERGENIC	--	--	--

INDEL marker IDs	Chromosomes/unanchored scaffolds	Physical positions (bp)	InDels (*Kabuli* reference genome-CDC Frontier/PII)	Forward primers (5'-3')	Reverse primers (5'-3')	Anneling temperature (0C)	Expected amplified product size (bp)	Structural annotation		Functional annotation	
								Sequence components of *kabuli* genome	*Kabuli* gene accession IDs	NCBI-KOG	TFs
Ca4_P2_27367807	Ca4	27367807	CTTTT/CTTT	GCATCCTTGCTCACAACTCA	TAAAGGCCCAAAGAAGCAAA	59.8	415	INTERGENIC	--	--	--
Ca4_P2_27368144	Ca4	27368144	GAAAAAAAA/GAAAAAAA	AGGCATTCAGCTCGACAACT	ATTTTGCACATGGTCTTCCC	59.8	800	INTERGENIC	--	--	--
Ca4_P2_27368524	Ca4	27368524	T/TG	GGGAAGACCATGTGCAAAAT	TTGTTCCAAGCCCTAAATGC	60.1	543	INTERGENIC	--	--	--
Ca4_P2_27374369	Ca4	27374369	CTT/CTTT	TGATGCAATTCTCATTTGGG	TGGAGAGTTTGTTGCGAGTG	60.0	687	INTERGENIC	--	--	--
Ca4_P2_27376069	Ca4	27376069	TAA/TAAA	ATCCTCTCCATTCCTTGCCT	TTGCTTTCACAGTCCAATGC	59.8	304	INTRON	Ca_21323	--	--
Ca4_P2_27378491	Ca4	27378491	TAAAAAAAAAA/TAAAAAAAAA	GGATCCGTCTCCTGCATAAA	TTTGCACAGGTTGTTTGTTG	58.2	585	DRR	Ca_21323	--	--
Ca4_P2_27379933	Ca4	27379933	CAAAAAAA/CAAAAAAAA	ATGGTACAAAGTGGCAAGGC	GAGAAAAGCAGGGTGAATGC	59.8	748	DRR	Ca_21323	--	--
Ca4_P2_27380010	Ca4	27380010	ATTT/ATT	ATGGTACAAAGTGGCAAGGC	GTGGACTGCAGTGGGTCTTT	60.2	909	DRR	Ca_21323	--	--
Ca4_P2_27381951	Ca4	27381951	AG/AGAAAGTGGTAGCG	GAAAAGATCGAATCGGGTGA	GTGGAGACACCGGAAGATGT	60.0	525	INTERGENIC	--	--	--
Ca4_P2_27382900	Ca4	27382900	AAC/ACACAAATCAC	TGGAATGAAAGGCACACAAA	GTGGTGGCTTTGAAACAGGT	60.0	649	INTERGENIC	--	--	--
Ca4_P2_27405693	Ca4	27405693	TAAAA/TAAAAAA	GAGTTGGTATTTCATTTGTTCGG	GTGGTTAAGATGGACCACCG	60.2	531	INTERGENIC	--	--	--
Ca4_P2_27419983	Ca4	27419983	A/AC	TTGTTGGGAAATTGACATGC	CTCACATTATCGCTACGCCA	59.9	879	INTERGENIC	--	--	--

INDEL marker IDs	Chromosomes/unanchored scaffolds	Physical positions (bp)	InDels (*Kabuli* reference genome-CDC Frontier/PII)	Forward primers (5'-3')	Reverse primers (5'-3')	Anneling temperature (0C)	Expected amplified product size (bp)	Structural annotation		Functional annotation	
								Sequence components of *kabuli* genome	*Kabuli* gene accession IDs	NCBI-KOG	TFs
Ca4_P2_27421262	Ca4	27421262	AT/A	AATGATGGAAGGGATGCTTG	TCGGCCAATAAATTCGTTAGTT	59.9	518	INTERGENIC	--	--	--
Ca4_P2_27422554	Ca4	27422554	TGA/T	CATCAATGAGGTCACCATCC	CCAAATGCGATAACCCTCAT	59.8	471	INTERGENIC	--	--	--
Ca4_P2_27424528	Ca4	27424528	ATTTTTTTT/ATTTTT	CGAACCAAAAGTGGATTTGA	AAAGTTTTGTGGCCGTCTTT	58.7	733	INTERGENIC	--	--	--
Ca4_P2_27434008	Ca4	27434008	CTT/CT	GAGGAAGACAAACCTGCCAC	TTAGTGGATTGTGCGGGAAT	60.3	101	INTERGENIC	--	--	--
Ca4_P2_27480414	Ca4	27480414	ATG/A	GGCCACTACCTGGTGGACTA	GTATCGAGCCTTTGCACCTC	59.8	283	INTRON	Ca_21319	J	--
Ca4_P2_27483001	Ca4	27483001	ATTTTTT/ATTTTTTT	GAAATCCAAGCCATTCTCCA	TGGTTGCGGAAATAAACACA	60.0	485	INTRON	Ca_21318	--	--
Ca4_P2_27484490	Ca4	27484490	AC/A	ACACATGTTGGTGGCTTTGA	CCAATGCTTGAGTTATGCCA	59.7	679	INTERGENIC	--	--	--
Ca4_P2_27491121	Ca4	27491121	ATTTTTTTT/ATTTTTTT	ATGCAGCACCTAGTTTTGGC	CATGTTCGTCGCTCTCATGT	59.9	531	INTERGENIC	--	--	--
Ca4_P2_27491616	Ca4	27491616	TATAA/TATAAGAATAA	TACGCGCTTTCTTTCATTCA	CGTCCCCTTCCTCAAACATA	59.9	503	INTERGENIC	--	--	--
Ca4_P2_27491848	Ca4	27491848	ATTTTTTT/ATTTTTT	TACGCGCTTTCTTTCATTCA	CGTCCCCTTCCTCAAACATA	59.9	503	INTERGENIC	--	--	--
Ca4_P2_27491894	Ca4	27491894	TA/T	TACGCGCTTTCTTTCATTCA	CGTCCCCTTCCTCAAACATA	59.9	503	INTERGENIC	--	--	--
Ca4_P2_27496407	Ca4	27496407	CTG/CTGTG	TCGGAAACGAATTGGGAATA	GGAGTTTGAGAAAGGGGTCC	59.9	750	INTERGENIC	--	--	--

**INDEL marker IDs**	**Chromosomes/unanchored scaffolds**	**Physical positions (bp)**	**InDels (*Kabuli* reference genome-CDC Frontier/PII)**	**Forward primers (5'-3')**	**Reverse primers (5'-3')**	**Anneling temperature (0C)**	**Expected amplified product size (bp)**	**Structural annotation**		**Functional annotation**	
								**Sequence components of *kabuli* genome**	***Kabuli* gene accession IDs**	**NCBI-KOG**	**TFs**
Ca4_P2_27499638	Ca4	27499638	TA/T	ATATGATCTAGCGCCCACCA	ATTTTGGCAGACCAAAGGTG	60.0	763	INTERGENIC	--	--	--
Ca4_P2_27500422	Ca4	27500422	ATTTTT/ATTTTTTT	CACCTTTGGTCTGCCAAAAT	CATGCATGCACACATAATTCA	59.0	533	INTERGENIC	--	--	--
Ca4_P2_27503266	Ca4	27503266	AG/AGG	GGGGTTCTCCTTGAACCACT	CCTGCCATTTTCTTCCAAAA	60.0	343	INTERGENIC	--	--	--
Ca4_P2_27504292	Ca4	27504292	AG/A	CATTCATGCCACAGGTATCG	AACACCAACACACCAAAGCA	60.0	353	INTERGENIC	--	--	--
Ca4_P2_27511083	Ca4	27511083	AATATATATAT/AATATATATATATATATATATAT	TTCATTACGCGGGATACGTT	CCACAAAAAGTGTGGGGTTC	60.2	337	INTERGENIC	--	--	--
Ca4_P2_27512475	Ca4	27512475	CTTTTTTTTTTT/CTTTTTTTTTT	GTTGGCTGGGTGGAAATCTA	ACGCGCTCCCTTAACTTGAT	61.1	804	INTERGENIC	--	--	--
Ca4_P2_27512574	Ca4	27512574	CAAAAAAAAA/CAAAAAAAA	GTTGGCTGGGTGGAAATCTA	ATACGCGCTCCCTTAACTTG	59.4	806	INTERGENIC	--	--	--
Ca4_P2_27512817	Ca4	27512817	ATTTTTTTTT/ATTTTTTTT	TGCCTTTAGAGAAGGGTGGA	TCTCACACAAAACCCCATCA	59.9	898	INTERGENIC	--	--	--
Ca4_P2_27523736	Ca4	27523736	ATTTTTTTT/ATTTTTTT	TCCTCGAGACGTTGTGTTTG	GCCTCCACGAATTTGTCATT	59.9	603	INTERGENIC	--	--	--
Ca4_P2_27530785	Ca4	27530785	CTTTTTTT/CTTTTTTTT	TTCGAAGGTTGGGTTGAATC	CCATTAATTTCGGCCCATTA	59.6	635	INTERGENIC	--	--	--
Ca4_P2_27536967	Ca4	27536967	TCCCCCCCC/TCCCCCCC	GATTTGGAAATGGCTGTCGT	AGCCGTGAGAGAGGATGAAA	60.0	492	INTERGENIC	--	--	--
Ca4_P2_27538878	Ca4	27538878	A/ATATGTTATT	AAACGGTCGTTGAGAATGTG	AGGCTTTGAGGCTTTCACAA	60.0	529	INTERGENIC	--	--	--

INDEL marker IDs	Chromosomes/unanchored scaffolds	Physical positions (bp)	InDels (*Kabuli* reference genome-CDC Frontier/PII)	Forward primers (5'-3')	Reverse primers (5'-3')	Anneling temperature (0C)	Expected amplified product size (bp)	Structural annotation		Functional annotation	
								Sequence components of *kabuli* genome	*Kabuli* gene accession IDs	NCBI-KOG	TFs
Ca4_P2_27539521	Ca4	27539521	TAA/TAACATAGTGAA	CTTGTGAAAGCCTCAAAGCC	TTGATTTCGTGAGTTCATTTCAA	59.6	776	INTERGENIC	--	--	--
Ca4_P2_27539986	Ca4	27539986	ATA/ATAGATTA	TTGAAATGAACTCACGAAATCAA	CACCCTCGCTTAGAAAAACG	59.9	542	INTERGENIC	--	--	--
Ca4_P2_27548804	Ca4	27548804	TAAAA/TAAA	TTCCCCATTCTTAACACCCA	TTCGCAACTCCACTCTTCCT	60.0	672	INTERGENIC	--	--	--
Ca4_P2_27551058	Ca4	27551058	TGG/TG	CATGTGATGTGTTTTTCCGC	TAACCGGCATTCCAATTGTT	60.2	580	INTERGENIC	--	--	--
Ca4_P2_27555037	Ca4	27555037	GCTCC/GCTCATCCTCC	CCAACAACACAGATGCAACC	TGCGCTTTCCTTTGTTATGA	59.4	527	INTERGENIC	--	--	--
Ca4_P2_2755654	Ca4	2755654	CAAAAAA/CAAAAAAA	AATTCGTTGCACAATTTGGTT	GCATGCTAAATGCCATCGTA	59.7	801	INTERGENIC	--	--	--
Ca4_P2_27564213	Ca4	27564213	ATTTTTTT/ATTTTTTTT	TAGCTTGACAACCCCAAACC	CCAATGTCCAGGTTAGAAGCA	60.1	900	INTRON	Ca_21314	--	--
Ca4_P2_27574074	Ca4	27574074	AAGAGAG/AAGAG	CGTGTGGGGATGCTTAACTT	AACCACTCAAGTGTTTGGGC	60.0	453	INTRON	Ca_21314	--	--
Ca4_P2_27574473	Ca4	27574473	ATTTTTTTTTTT/ATTTTTTTTT	CACCCTTACCTTGACCCCTT	GCTCGTATGCGTCAATTCAA	59.8	571	INTRON	Ca_21314	--	--
Ca4_P2_27580274	Ca4	27580274	GAAAAAAAAA/GAAAAAAAAAA	TGGGGTCCATGTACCACTTT	AAGAAATGTGCCTCAGGTGC	60.3	305	INTRON	Ca_21314	--	--
Ca4_P2_27586048	Ca4	27586048	T/TC	AAAACCCAAGCAGTGGTCTG	CCAGGATCTCGTTGTCCCTA	60.1	651	INTRON	Ca_21314	--	--
Ca4_P2_27594072	Ca4	27594072	CTTTTTTT/CTTTTTTTTT	ATCAGAGGTTGCGCAAAAAT	TTTCCTTCCAAATTTACGCC	59.1	668	INTRON	Ca_21314	--	--

INDEL marker IDs	Chromosomes/unanchored scaffolds	Physical positions (bp)	InDels (*Kabuli* reference genome-CDC Frontier/PII)	Forward primers (5'-3')	Reverse primers (5'-3')	Anneling temperature (0C)	Expected amplified product size (bp)	Structural annotation		Functional annotation	
								Sequence components of *kabuli* genome	*Kabuli* gene accession IDs	NCBI-KOG	TFs
Ca4_P2_27603513	Ca4	27603513	TTTATTATT/TTTATT	TGGGATAGGGACGTTGGTTA	ACGCCCTGGAAACAATACAG	60.0	701	INTRON	Ca_21314	--	--
Ca4_P2_27604352	Ca4	27604352	AAATGTTGATAAT/AAAT	ATTAGGAGTCTTGCTGCCCA	CCCTCCGAACTCTCAAACAG	59.8	320	INTRON	Ca_21314	--	--
Ca4_P2_27629377	Ca4	27629377	GAA/GA	ATTCATGCATTGCTCCAACA	TCTCCAGTTTTGGTCTCCCA	60.6	448	DRR	Ca_21311	--	--
Ca4_P2_27631111	Ca4	27631111	GTTTTTTT/GTTTTTT	ATCAACGCGATAATTGGAGC	TGCATGCAACAATCAAGAAA	58.8	813	INTERGENIC	--	--	--
Ca4_P2_27637430	Ca4	27637430	TG/TGG	TTTGGAGTGGTGGTTCTCAA	CCAATGATTTTCATGCCACA	60.3	482	DRR	Ca_21310	--	--
Ca4_P2_27639809	Ca4	27639809	TCC/TC	TTTTGCAAGTGATTTCCACG	GCAAGTTGTTTGTTGCGTGT	59.8	626	INTERGENIC	--	--	--
Ca4_P2_27639986	Ca4	27639986	CTTTTTTTTTTTTTTT/CTTTTTTTTTTT	ACGGTGTTTACGATCGTTCC	TAATCAGTCCCCAAACGGAG	59.9	585	INTERGENIC	--	--	--
Ca4_P2_27647933	Ca4	27647933	CC/CCACTAC	TTGTCCACGATACATGCGTT	GCGGGTGGTTGCTAAAGTAG	59.8	542	INTERGENIC	--	--	--
Ca4_P2_27649411	Ca4	27649411	TTTTATTT/TTTT	AAAATAAGGCATGGTGTTGGA	TAGTTTGTAACCCGTGCGTC	58.7	256	INTERGENIC	--	--	--
Ca4_P2_27656062	Ca4	27656062	TAAAAAAAAAAAAA/TAAAAAAAAAAAAAAA	GCGAGCATGAGAAGTTGTTG	TTTAAAATAGACTCCCGATGTCA	57.8	101	INTERGENIC	--	--	--
Ca4_P2_27673654	Ca4	27673654	TAGAGAGAGAGA/TAGAGAGAGAGAGA	TCGAAGAAATGGCGAAGAGT	GTATGCCTTTGCACTCCGTT	60.1	250	INTERGENIC	--	--	--
Ca4_P2_27689404	Ca4	27689404	GATAT/GAT	TGCGTCATAAAATGGCTTGA	GGATGAGCTGACCCCTTACA	60.1	419	INTERGENIC	--	--	--

INDEL marker IDs	Chromosomes/unanchored scaffolds	Physical positions (bp)	InDels (*Kabuli* reference genome-CDC Frontier/PII)	Forward primers (5'-3')	Reverse primers (5'-3')	Anneling temperature (0C)	Expected amplified product size (bp)	Structural annotation		Functional annotation	
								Sequence components of *kabuli* genome	*Kabuli* gene accession IDs	NCBI-KOG	TFs
Ca4_P2_27692193	Ca4	27692193	TAAAAAAAA/TAAAAAAA	TTGGTCGCAAATAAAACTAACAAA	TGACAATTACATGGGGACGA	59.8	247	INTERGENIC	--	--	--
Ca4_P2_27702742	Ca4	27702742	GAAAAAAAA/GAAAAAAA	TCATGAAGACACCGAAACCA	AACTGCTCCAATGGCGTTAG	60.3	282	INTERGENIC	--	--	--
Ca4_P2_2773308	Ca4	2773308	GAAA/GAA	ATATAAGGCCCCATATCGCC	TGCACTTTCAATCCCAAAGA	59.2	564	INTERGENIC	--	--	--
Ca4_P2_27744353	Ca4	27744353	ATTCAACCCCCTCTTC/ATTCAACCCCCTCTTCAACCCCCTCTTC	CCATCTTTGGGTGAACCAGT	CCTCACCGCAACCACTATTT	60.0	481	DRR	Ca_21304	--	--
Ca4_P2_27764770	Ca4	27764770	TTAT/TATAT	AACCTCAACGAAATCTTTCCAA	GCGTGCGATAGGTTGGTATT	60.0	452	INTERGENIC	--	--	--
Ca4_P2_27785713	Ca4	27785713	TC/TTCGACAC	GGCAGGAGAACCAAACAAAA	ACTCCGGCACAACTATGGAC	60.0	815	INTRON	Ca_21303	--	GATA
Ca4_P2_27790734	Ca4	27790734	CAAAAA/CAAAAAAA	TTCCTGTTTCAGTCTCGGCT	CGATCTTTGAGAGCCTTGCT	59.7	499	INTERGENIC	--	--	--
Ca4_P2_27809345	Ca4	27809345	CTTTTTTT/CTTTTTTTT	TCATGGTTCACCAAAAACGA	TCCATTGTGTGCATCCCTAA	59.9	983	INTERGENIC	--	--	--
Ca4_P2_27822472	Ca4	27822472	TA/T	TTGATGCTTGCTCAACTTGG	TATCGCGGGTCTTGTTTTTC	60.1	470	INTERGENIC	--	--	--
Ca4_P2_2782813	Ca4	2782813	ATTTTTTTTTTT/ATTTTTTTTTT	CACATGCGCTTGTATTTTGG	TTCTCGATATTCCTTCGGACT	57.9	626	INTERGENIC	--	--	--
Ca4_P2_27859134	Ca4	27859134	ATTTTTTTTT/ATTTTTTTTTT	ACCCTCCTTTTAGGTGTGCC	GGGAGATTTGGGGTTTTGAT	60.0	569	INTERGENIC	--	--	--
Ca4_P2_27865338	Ca4	27865338	AAATAATAATAATAATAATAATAATAATAATAATAATAATAA/AAATAATAATAATAATAATAATAATAATAATAATAATAA	TCCTAAAACAAATGCACCCA	TTAGGCTTGATATGCCGACC	60.1	557	INTERGENIC	--	--	--

INDEL marker IDs	Chromosomes/unanchored scaffolds	Physical positions (bp)	InDels (*Kabuli* reference genome-CDC Frontier/PII)	Forward primers (5'-3')	Reverse primers (5'-3')	Anneling temperature (0C)	Expected amplified product size (bp)	Structural annotation		Functional annotation	
								Sequence components of *kabuli* genome	*Kabuli* gene accession IDs	NCBI-KOG	TFs
Ca4_P2_2787061	Ca4	2787061	GAAAAAAAA/GAAAAAAAAA	CCTTTGCAAAATTCGGTCAT	CGTTATTTGTTTCGGCAGGT	60.0	724	INTERGENIC	--	--	--
Ca4_P2_27908754	Ca4	27908754	ATTTTTTTTTTT/ATTTTTTTTTT	AGAAACCAACCTTGGCATTG	TGTGTCAGAAGGATCTTGCG	60.0	537	INTERGENIC	--	--	--
Ca4_P2_27918921	Ca4	27918921	CAAAAAA/CAAAAA	GCCTCAAGCAATTTGTGGAT	CCTTAAAGTGCGCAAACACA	59.9	507	INTERGENIC	--	--	--
Ca4_P2_27919596	Ca4	27919596	TAAAAAAAAAAAAAA/TAAAAAAAAAAAA	CATGATGGCGATGTAGGATG	ACCCAACACAATCCGTTGAT	60.1	552	INTERGENIC	--	--	--
Ca4_P2_27919988	Ca4	27919988	ATTTTTTTTTTTT/ATTTTTTTTTTT	ATCAACGGATTGTGTTGGGT	GTATGTGGGTTTTGATGGGC	60.1	210	INTERGENIC	--	--	--
Ca4_P2_27920147	Ca4	27920147	GTATATATATATATAT/GTATATATATATATATATAT	GCCCATCAAAACCCACATAC	CACAATCCTTTTCAACCGCT	60.1	466	INTERGENIC	--	--	--
Ca4_P2_27928820	Ca4	27928820	CACAAC/CACAACAAC	AGCGATGCATGTCTCAAGTG	AGCGCTTTGTGACCTCCTTA	60.0	754	INTERGENIC	--	--	--
Ca4_P2_27929931	Ca4	27929931	GTTTT/GTTTTT	CCTTCTCATTGCGAACCCTA	TGGTTGCACCACATTTGACT	60.0	427	INTERGENIC	--	--	--
Ca4_P2_27932993	Ca4	27932993	CATATATATATATATATA/CATATATATATATATA	TGGGTGCTATTTGCAATCAA	CTTGCAGCATGCAATTTCTC	59.6	469	DRR	Ca_21300	--	--
Ca4_P2_27943723	Ca4	27943723	ATTTTTT/ATTTTT	CGATTGAATTAAGCCCAGGA	TCTCCATTTTCGTCCGTAGG	60.1	403	INTERGENIC	--	--	--
Ca4_P2_27951126	Ca4	27951126	CTT/CTTTCATTT	TGTGTAACCTCCCCCATGAT	AAAGGGCACCAAATACTCCA	59.4	858	INTERGENIC	--	--	--
Ca4_P2_27954238	Ca4	27954238	A/AT	ACTTCTTGACCATGCCAACC	CCATTAGTGGTTGTTGCCCT	59.9	466	INTERGENIC	--	--	--

INDEL marker IDs	Chromosomes/unanchored scaffolds	Physical positions (bp)	InDels (*Kabuli* reference genome-CDC Frontier/PII)	Forward primers (5'-3')	Reverse primers (5'-3')	Anneling temperature (0C)	Expected amplified product size (bp)	Structural annotation		Functional annotation	
								Sequence components of *kabuli* genome	*Kabuli* gene accession IDs	NCBI-KOG	TFs
Ca4_P2_27960253	Ca4	27960253	GTTTTTTTTTT/GTTTTTTTTTTTT	CACCATACCAATGTTTGCCA	TGCACGCTCAAATGATTTTT	59.3	757	INTERGENIC	--	--	--
Ca4_P2_27981509	Ca4	27981509	GTTTTTTTTTTT/GTTTTTTTTTTTTTT	ATGCTCGTCTGGAACCAAAG	TACCCCAAATTCTAAGCCCC	60.1	433	INTERGENIC	--	--	--
Ca4_P2_28022155	Ca4	28022155	CGT/C	ATTGTCTTGTTTGCTTGCCC	AACACAGCCACAATGTTCCA	60.0	354	INTERGENIC	--	--	--
Ca4_P2_28025126	Ca4	28025126	TGTGGTG/TGTGGTGGTG	TTCATCAAACCTCTACGAACGA	TTGTCGATGACTGCTTTTGG	59.8	892	INTERGENIC	--	--	--
Ca4_P2_28026871	Ca4	28026871	TAAGAA/TAAGAAGAA	GACATCACACCAATTCGACG	TTTTGAAGGAGATGGGGTTG	59.9	885	INTERGENIC	--	--	--
Ca4_P2_28042488	Ca4	28042488	T/TG	GTCCTTTTCCTTTGGCCTTC	CCTGAGGATAGCCTCGTGAA	60.4	159	INTERGENIC	--	--	--
Ca4_P2_28045732	Ca4	28045732	TGAAGAAG/TGAAG	AAGAAACGGTAAGGTTCGGG	CGCATTGTCCCATCTCTCTT	60.2	220	INTERGENIC	--	--	--
Ca4_P2_28053081	Ca4	28053081	G/GT	GCGATTGATTTGGTGGACTT	AAAATATTGGGGGCCTATGG	59.9	218	INTERGENIC	--	--	--
Ca4_P2_28062048	Ca4	28062048	GTTTTTTTT/GTTTTTTTTT	TCAACAATTGTAATTGACTATCAACG	TATAGGCCGATCAGTCAGGC	60.2	771	INTERGENIC	--	--	--
Ca4_P2_28087494	Ca4	28087494	CAAAAAAAAA/CAAAAAAAA	TTTGGATCAATCAACGTCGTA	TCAAAAACCACAACCACCAC	59.3	738	INTERGENIC	--	--	--
Ca4_P2_28093890	Ca4	28093890	GAA/G	GAGAGTGCTCCTTTGGGAAA	GGGAAAAATTGCTTGGTGAA	59.9	300	DRR	Ca_23175	--	--
Ca4_P2_28108572	Ca4	28108572	TAAAAAAAA/TAAAAAAAAA	GCAAATGTTGGCGAAGTACA	AGTGTGTGCTTGTGGGATCA	60.2	301	INTERGENIC	--	--	--

INDEL marker IDs	Chromosomes/unanchored scaffolds	Physical positions (bp)	InDels (*Kabuli* reference genome-CDC Frontier/PII)	Forward primers (5'-3')	Reverse primers (5'-3')	Anneling temperature (0C)	Expected amplified product size (bp)	Structural annotation		Functional annotation	
								Sequence components of *kabuli* genome	*Kabuli* gene accession IDs	NCBI-KOG	TFs
Ca4_P2_28109349	Ca4	28109349	ATTTTTT/ATTTTTTT	CGGGGGTAGGGTTTTAGAGA	GCTGCTTGGTTGTGCTATGA	60.0	491	INTERGENIC	--	--	--
Ca4_P2_28112014	Ca4	28112014	GTA/GTATA	TGTCAATGACGAACATTTATTTCA	CAAGAATCGTCTTTGTCATCCA	60.1	399	INTERGENIC	--	--	--
Ca4_P2_28120543	Ca4	28120543	TAAAAAA/TAAAAAAA	CCAACTCCCTTCCCCTTTTA	CAATACGAGCACCTGGCTCT	60.4	994	INTERGENIC	--	--	--
Ca4_P2_28120712	Ca4	28120712	ATTTTTTTTTT/ATTTTTTTT	CATCAGTCAAACGCACAAAAA	CAATACGAGCACCTGGCTCT	60.4	433	INTERGENIC	--	--	--
Ca4_P2_28122657	Ca4	28122657	GAAAAAAAAAAAA/GAAAAAAAAAAA	CCCCGAGTTTGAGAAATTGA	CCTCCCGTCCTCTTTCTTTC	60.2	393	INTERGENIC	--	--	--
Ca4_P2_28131945	Ca4	28131945	TAGAA/TA	CGGCAATTGAGGGTTAAAGA	GAGGCCTTTCAAGTACACGC	59.9	344	INTERGENIC	--	--	--
Ca4_P2_28145617	Ca4	28145617	AA/AAATAATAGA	TGGAAATGAATCACCGGAAT	TTTTTCAAAATGGCATGGGT	60.2	703	INTERGENIC	--	--	--
Ca4_P2_28146035	Ca4	28146035	CTA/CTATA	TTTTTGGCTGCAAAATCCAT	TCAAAAACCCACACACACAA	58.4	881	INTERGENIC	--	--	--
Ca4_P2_28146419	Ca4	28146419	TG/T	CCTTGTAATTGTGTGTGTGGG	AATCCTGTATGGGGGAAAGG	60.0	438	INTERGENIC	--	--	--
Ca4_P2_28147858	Ca4	28147858	AT/ATTTAATAATTT	GGGAAAAGCCCCAAACTAAG	TCCAACGTCGATTATGACCA	59.9	424	INTERGENIC	--	--	--
Ca4_P2_28148545	Ca4	28148545	ATACTTACT/ATACT	TGGTCATAATCGACGTTGGA	CTGCAAGCAGCAGAAGAACA	60.5	956	INTERGENIC	--	--	--
Ca4_P2_28159857	Ca4	28159857	TAAAAAAAAA/TAAAAAAAAAAA	TTTGGGGGTACAAGGGAAAT	TGCAACCTCATACTGGGAAA	59.1	645	INTERGENIC	--	--	--

INDEL marker IDs	Chromosomes/unanchored scaffolds	Physical positions (bp)	InDels (*Kabuli* reference genome-CDC Frontier/PII)	Forward primers (5'-3')	Reverse primers (5'-3')	Anneling temperature (0C)	Expected amplified product size (bp)	Structural annotation		Functional annotation	
								Sequence components of *kabuli* genome	*Kabuli* gene accession IDs	NCBI-KOG	TFs
Ca4_P2_28159950	Ca4	28159950	ATTTTTT/ATTTTTTT	AGTCAATGGTTGTGGGCTCT	TGCAACCTCATACTGGGAAA	59.1	584	INTERGENIC	--	--	--
Ca4_P2_28160415	Ca4	28160415	GAAAAAA/GAAAAAAA	CAAAATGCCCTTTGGCACTA	TGACACGTTGTTTGTTTTGACA	60.1	457	INTERGENIC	--	--	--
Ca4_P2_28161200	Ca4	28161200	AACGGAC/AAC	CCCCGTAATGCGATATCAAG	TTAAACACCCCTCCTCAAGC	59.2	585	INTERGENIC	--	--	--
Ca4_P2_28169092	Ca4	28169092	GC/G	TTTTTGCCCATTCAAACACA	AAATGCGTCAGCCCAGTAAG	60.3	829	INTERGENIC	--	--	--
Ca4_P2_28169343	Ca4	28169343	TAAAAAAAA/TAAAAAAA	GGGGTGAGAAAAGAGTGTGG	AAATGCGTCAGCCCAGTAAG	60.3	180	INTERGENIC	--	--	--
Ca4_P2_28173222	Ca4	28173222	CTTTTTTTTTTTT/CTTTTTTTTT	ACCTAACCCTCCAAGCCAGT	TTTTCCCTCTTCACACCACC	59.9	910	INTERGENIC	--	--	--
Ca4_P2_28179172	Ca4	28179172	TAAAAAAAAA/TAAAAAAAAAA	CGGCTCACGTTTATTTGTTG	TTCCACATTTTTCCCCTCAA	60.3	485	INTERGENIC	--	--	--
Ca4_P2_28179566	Ca4	28179566	AGGGG/AGGGGG	TGGGTAAAGTTCTGCCCACT	TAGCATGCTCGAAAACGAAA	59.6	129	INTERGENIC	--	--	--
Ca4_P2_28179824	Ca4	28179824	ATT/ATTT	TGAGGGGAAAAATGTGGAAA	ATCTCCCTTCTCGCTCTTCC	59.9	623	INTERGENIC	--	--	--
Ca4_P2_28180097	Ca4	28180097	ATTTTTTTTT/ATTTTTTT	TCGAGCATGCTATAGTCCGA	ACCAATGTCCAACGGGAATA	60.1	869	INTERGENIC	--	--	--
Ca4_P2_28184015	Ca4	28184015	A/ACG	TCACAAGTTTGGGGTTGTCA	ACGCAAGGTCAACAAAGGTC	60.2	641	INTERGENIC	--	--	--
Ca4_P2_28184807	Ca4	28184807	ATTT/ATTTT	GACCTTTGTTGACCTTGCGT	TGTTGAGTTGGAGATGAAGCA	59.4	595	INTERGENIC	--	--	--

**INDEL marker IDs**	**Chromosomes/unanchored scaffolds**	**Physical positions (bp)**	**InDels (*Kabuli* reference genome-CDC Frontier/PII)**	**Forward primers (5'-3')**	**Reverse primers (5'-3')**	**Anneling temperature (0C)**	**Expected amplified product size (bp)**	**Structural annotation**		**Functional annotation**	
								**Sequence components of *kabuli* genome**	***Kabuli* gene accession IDs**	**NCBI-KOG**	**TFs**
Ca4_P2_2819881	Ca4	2819881	TAAAAAA/TAAAAAAA	TTGTTACCTCAATCGTGGGG	TTCGCCCCTACAACTACCAC	60.0	674	INTERGENIC	--	--	--
Ca4_P2_28206465	Ca4	28206465	AACA/AACACA	CCAAATGCCTCCAGAATGTT	TCCTGTCCAGAAGCTATGCC	60.4	643	INTERGENIC	--	--	--
Ca4_P2_28206696	Ca4	28206696	CATC/CATCGATC	GGGATGATAATCGGCATAGC	TGCATCGTCAAATACAAAACAA	59.1	300	INTERGENIC	--	--	--
Ca4_P2_28209971	Ca4	28209971	T/TA	ATTGATGGAGCGAATCGAAC	AATTCCTCTTTTGGTGGGCT	59.9	891	INTERGENIC	--	--	--
Ca4_P2_28213499	Ca4	28213499	CTTTTTTTT/CTTTTTTTTT	ACTGACCTGACCTAGGCGTG	GTTGCAAAATTGGTGTCTGC	59.2	646	INTERGENIC	--	--	--
Ca4_P2_28213716	Ca4	28213716	T/TCAA	AATTGCAGACACCAATTTTGC	GTGTGGACGCGCACATATTA	60.6	390	INTERGENIC	--	--	--
Ca4_P2_28214061	Ca4	28214061	CA/CATA	AATTGCAGACACCAATTTTGC	CAGACATGGGCCATCAAATA	59.4	739	INTERGENIC	--	--	--
Ca4_P2_28214278	Ca4	28214278	ATATAATT/AT	TCCGAGTCTAATCGGGCTAA	TGGAAAAGGGAGCCAATATG	59.9	539	INTERGENIC	--	--	--
Ca4_P2_28214353	Ca4	28214353	GACAA/GACAACAA	TCCGAGTCTAATCGGGCTAA	TGGAAAAGGGAGCCAATATG	59.9	539	INTERGENIC	--	--	--
Ca4_P2_28237673	Ca4	28237673	CCCTTTA/C	TTCTGCAAGATTTTGTTGCG	GCAGTTTCGTGCTAAGGAGG	60.0	675	INTERGENIC	--	--	--
Ca4_P2_28238860	Ca4	28238860	GTTTTTTTT/GTTTTTTTTTTTTT	CTGTCCCTTAGAAGGGGTCC	TTTGGAGCAAAAGGAACCTG	60.2	729	INTERGENIC	--	--	--
Ca4_P2_28246910	Ca4	28246910	TAAAAAA/TAAAA	AGGCGTGCCACTTTTCTCTA	TCAGCCACTCAATGTTGCTC	60.0	518	INTERGENIC	--	--	--

INDEL marker IDs	Chromosomes/unanchored scaffolds	Physical positions (bp)	InDels (*Kabuli* reference genome-CDC Frontier/PII)	Forward primers (5'-3')	Reverse primers (5'-3')	Anneling temperature (0C)	Expected amplified product size (bp)	Structural annotation		Functional annotation	
								Sequence components of *kabuli* genome	*Kabuli* gene accession IDs	NCBI-KOG	TFs
Ca4_P2_28254483	Ca4	28254483	CAACAA/CAACAAGACAAACAA	GGGGAACTCTCCAAGTCACA	CGTGGAAACCAACCTTGTCT	60.0	703	INTERGENIC	--	--	--
Ca4_P2_28274340	Ca4	28274340	CAAAAAAAAAA/CAAAAAAAAA	CCAGAAGCAGAAAAACCAGG	TTTGAAACTGTCGCGACTCA	60.6	631	INTERGENIC	--	--	--
Ca4_P2_28275123	Ca4	28275123	ACAAATAAGTGGCAAATAAGT/ACAAATAAGT	AAACATTGCCGATCGATTTC	TTTGTTCTGGTAACACCCCA	58.9	336	INTERGENIC	--	--	--
Ca4_P2_28277260	Ca4	28277260	CT/CTACAAAT	GCCTACACGCAACAATGAGA	TGTGGATCAGGTTTGGAGAA	59.1	200	INTERGENIC	--	--	--
Ca4_P2_28277388	Ca4	28277388	AATATATATATA/AATATATATA	GCCTACACGCAACAATGAGA	AAGTTTTTCTAAACCGCAATTTTT	58.8	423	INTERGENIC	--	--	--
Ca4_P2_28277547	Ca4	28277547	CAAAAAAAAAA/CAAAAAAAAAAA	GCCTACACGCAACAATGAGA	TTGTGGTCATTCAAATTCCTTTT	59.7	818	INTERGENIC	--	--	--
Ca4_P2_28280754	Ca4	28280754	TTGAAATATGAAATATGAAATA/TTGAAATATGAAATA	GGGCGTCTGATCCTAATTGA	TGCACTAAATCACCATCCCA	59.9	707	INTRON	Ca_23166	TR	FAR1
Ca4_P2_28281644	Ca4	28281644	CTAT/CTATATAT	ATGCAAGTGGCCAAAAGAAC	ACCAAGAAATGCCTTCTGGA	59.7	405	INTRON	Ca_23166	TR	FAR1
Ca4_P2_28282850	Ca4	28282850	GTTTTTTTT/GTTTTTTTTTT	AAAAACAAAAGACTCGGGGAA	CACACACACAACATCCCCAT	60.1	434	DRR	Ca_23166	TR	FAR1
Ca4_P2_28284800	Ca4	28284800	AAAGAAGAAGAAGAAGAAGAAG/AAAGAAGAAGAAGAAGAAG	TTGAATCCTCATTTCTGCCC	CCTTCCACCGAGTCAACATT	60.0	813	INTERGENIC	--	--	--
Ca4_P2_28287770	Ca4	28287770	T/TC	TTCACAGGTTTGGGACATCA	CCTCCATTGGCTAGTTTGGA	60.1	574	INTERGENIC	--	--	--
Ca4_P2_28289460	Ca4	28289460	TAAAAAAAAAAAA/TAAAAAAAAA	TTGCATTGCACTTCTTTTGC	CGGTGATGCCTCTGTTTCTT	60.3	585	INTERGENIC	--	--	--

INDEL marker IDs	Chromosomes/unanchored scaffolds	Physical positions (bp)	InDels (*Kabuli* reference genome-CDC Frontier/PII)	Forward primers (5'-3')	Reverse primers (5'-3')	Anneling temperature (0C)	Expected amplified product size (bp)	Structural annotation		Functional annotation	
								Sequence components of *kabuli* genome	*Kabuli* gene accession IDs	NCBI-KOG	TFs
Ca4_P2_28296294	Ca4	28296294	TNNNNNNNNNN/TNNN	TTGCTTAATTGGGTCAAGTGG	GTTGGCAGTGTGAGCAGCTA	60.2	469	INTERGENIC	--	--	--
Ca4_P2_28298251	Ca4	28298251	ATTTTTTT/ATTTTTTTTT	CGGATACAATGCTTGCTTGA	CAAACCGACGCCAACTAAAT	60.0	589	INTERGENIC	--	--	--
Ca4_P2_28306771	Ca4	28306771	CAAAAAAAAA/CAAAAAAAAAAA	TGATGGTCCATGAACTGACAT	CGAGCCAAAATCCAAAACAT	59.9	430	INTERGENIC	--	--	--
Ca4_P2_28320855	Ca4	28320855	ATTTTTTTT/ATTTTTTT	TGAGAAACGGCAAAACACAA	AAATATTGGGCATGTGAGCC	59.8	450	INTERGENIC	--	--	--
Ca4_P2_28321240	Ca4	28321240	TAAAAAAAA/TAAAAAAA	AGGTCCCGACCCATTATTTT	ACCAACCAACGCTCAATTTC	60.0	486	INTERGENIC	--	--	--
Ca4_P2_28321521	Ca4	28321521	CCGTACAGGCGTACA/CCGTACA	TCGAGTTGTGAAATTGAGCG	GCTGAAGAACCCGAGAAAGA	59.6	202	INTERGENIC	--	--	--
Ca4_P2_28330076	Ca4	28330076	AT/ATT	CACAGGCAGCTGAGTAGCAA	TTGTTGTTCGACATGGACAAT	58.9	704	INTERGENIC	--	--	--
Ca4_P2_28331028	Ca4	28331028	CAAAAAAAA/CAAAAAAAAA	CTTGAAGGGACGGCTAGATG	TTGCCATAATGTTGAGAAGGG	59.9	458	INTERGENIC	--	--	--
Ca4_P2_28335781	Ca4	28335781	A/AT	CCCAGTTCTTGAGCATGACA	TGGACAAAACTTTGGTGCTTC	60.1	783	INTERGENIC	--	--	--
Ca4_P2_28336543	Ca4	28336543	TAAAAAAAAAAA/TAAAAAAAAA	CAAAGCAGGGAGTTTCCATC	TGGTTTAAGTCCTCGGCTGT	59.7	723	INTERGENIC	--	--	--
Ca4_P2_28336605	Ca4	28336605	CAAAAAAAAAAAAAA/CAAAAAAAAAAAAA	CAAAGCAGGGAGTTTCCATC	TGGTTTAAGTCCTCGGCTGT	59.7	723	INTERGENIC	--	--	--
Ca4_P2_28338433	Ca4	28338433	GTTT/GTT	TGACTCGGGCTGCAGATTAT	GCTTTGAGCCCCATGAAATA	60.0	739	INTERGENIC	--	--	--

INDEL marker IDs	Chromosomes/unanchored scaffolds	Physical positions (bp)	InDels (*Kabuli* reference genome-CDC Frontier/PII)	Forward primers (5'-3')	Reverse primers (5'-3')	Anneling temperature (0C)	Expected amplified product size (bp)	Structural annotation		Functional annotation	
								Sequence components of *kabuli* genome	*Kabuli* gene accession IDs	NCBI-KOG	TFs
Ca4_P2_28338753	Ca4	28338753	TGGGGCT/TGGGGCTTAAGAGAGGGGGCT	AAGACCACCGGTGTTTATGG	TCAATTTGACAAGCCCCTTA	58.2	724	INTERGENIC	--	--	--
Ca4_P2_28342637	Ca4	28342637	GCCC/GCCCC	TCACAAATCCTGGTGGTTGA	GGAAGAAAGCAATGGTGGAA	60.1	359	INTERGENIC	--	--	--
Ca4_P2_28346696	Ca4	28346696	A/AT	ACCACAGGTGAAAGAAACCG	TTTTCAACTCTATCCGTAGCCC	59.6	340	INTERGENIC	--	--	--
Ca4_P2_28351841	Ca4	28351841	CTTAAT/CTTAATTAAT	GAGTTCGATTTCTATCATCACCA	TGCATTGCAAACTCAGAGG	58.5	572	INTERGENIC	--	--	--
Ca4_P2_28360547	Ca4	28360547	ATTTTT/ATTTT	ATGGACATAGCAATTGGCGT	GTCATTTTGGTGAACCGGAG	60.4	520	INTRON	Ca_23163	AYT	MYB
Ca4_P2_28367927	Ca4	28367927	GGA/GGAGA	GAAAAGACGGCGATGAAGAA	ACTATGGAGCTGGATGGCTG	60.2	803	DRR	Ca_23163	AYT	MYB
Ca4_P2_28368172	Ca4	28368172	G/GA	CCAATAATGGCTTTGGCAAT	AGCCCCTCTCTTGACACTCA	60.0	453	DRR	Ca_23163	AYT	MYB
Ca4_P2_28372178	Ca4	28372178	TTTATGT/TT	TTTGATAAACCCCGTGATCC	TGGAGCAGTTTTTCAACCAA	59.3	572	INTERGENIC	--	--	--
Ca4_P2_28372820	Ca4	28372820	ATTTT/ATTTTT	TAAGGGAAGGGGCTCTAGGA	CCCCTAAAATTTAAAAGGCCC	60.0	389	INTERGENIC	--	--	--
Ca4_P2_28372972	Ca4	28372972	A/AT	TAAGGGAAGGGGCTCTAGGA	CCCCTAAAATTTAAAAGGCCC	60.0	389	INTERGENIC	--	--	--
Ca4_P2_28373073	Ca4	28373073	AC/A	TAAGGGAAGGGGCTCTAGGA	CCCCTAAAATTTAAAAGGCCC	60.0	389	INTERGENIC	--	--	--
Ca4_P2_28375729	Ca4	28375729	CAAAAAAAAA/CAAAAAAAA	GTGTTTGACTGCACGCTTGT	GATGGGGGTTTGTGGTTAGA	59.6	630	DRR	Ca_23162	C	--

INDEL marker IDs	Chromosomes/unanchored scaffolds	Physical positions (bp)	InDels (*Kabuli* reference genome-CDC Frontier/PII)	Forward primers (5'-3')	Reverse primers (5'-3')	Anneling temperature (0C)	Expected amplified product size (bp)	Structural annotation		Functional annotation	
								Sequence components of *kabuli* genome	*Kabuli* gene accession IDs	NCBI-KOG	TFs
Ca4_P2_28380287	Ca4	28380287	ATTTT/ATTTTT	ACTTTTCTCATTTTTAAGTCATCAAAT	CTTCAAAGTTGCCGGTGATT	60.1	919	INTERGENIC	--	--	--
Ca4_P2_28380372	Ca4	28380372	CGT/C	AAATTGTCTTTTTGGACAATCTTTA	CTTCAAAGTTGCCGGTGATT	60.1	489	INTERGENIC	--	--	--
Ca4_P2_28382490	Ca4	28382490	CTTAATAG/CTTAATAGCTAATAGGTTAATAG	TCAAACGAACTCGAACCCTT	TGTTGGATTTTGGAGGTTCA	58.9	489	INTERGENIC	--	--	--
Ca4_P2_28385530	Ca4	28385530	TAAAAAAA/TAAAAAAAA	AGCGCATATAGGGAAGCAAA	TGACGCCTCCTCTCATTACA	59.4	608	INTERGENIC	--	--	--
Ca4_P2_28389709	Ca4	28389709	T/TA	GTTCCTTGCCAAGTGCTGAT	CACCCCGAATCCATTATTTG	60.0	520	INTERGENIC	--	--	--
Ca4_P2_28390462	Ca4	28390462	TAA/TAAA	GTCATCAAAAGCCGACGAAT	GGCCCACAATTTTGAAGAGA	60.1	786	INTERGENIC	--	--	--
Ca4_P2_28401904	Ca4	28401904	AT/ATT	ACTTGGATGAAGGGAAAGGG	GCATTTCGCAAAAATTTGAA	58.8	561	INTERGENIC	--	--	--
Ca4_P2_28401988	Ca4	28401988	TAATTAA/TAATTAATTAAATAAAATTAA	ACTTGGATGAAGGGAAAGGG	GCATTTCGCAAAAATTTGAA	58.8	561	INTERGENIC	--	--	--
Ca4_P2_28403960	Ca4	28403960	TCTCCT/TCTCCTCCT	CGTTTAAGTCGACGTGTCCAT	TGCACTTGTTCACGTGAGTG	59.5	721	INTERGENIC	--	--	--
Ca4_P2_28414876	Ca4	28414876	CAAT/CAATAAT	GCCCCATGTTTGTGCTTAAT	GGATTCAGCATCCGTCAAGT	60.1	741	INTERGENIC	--	--	--
Ca4_P2_28420944	Ca4	28420944	TAAAGTTAGAAGAA/TAA	GTGTGGGCGGAAAAATCTAA	CACAGTGAAAAACCGATGGA	59.5	693	INTERGENIC	--	--	--
Ca4_P2_28421267	Ca4	28421267	TAAAAAAAAAAAAA/TAAAAAAAAAAA	GACAAAAGAACGTGATTGGGA	TGCATGGAAAGCTGTGGTAG	59.9	639	INTERGENIC	--	--	--

INDEL marker IDs	Chromosomes/unanchored scaffolds	Physical positions (bp)	InDels (*Kabuli* reference genome-CDC Frontier/PII)	Forward primers (5'-3')	Reverse primers (5'-3')	Anneling temperature (0C)	Expected amplified product size (bp)	Structural annotation		Functional annotation	
								Sequence components of *kabuli* genome	*Kabuli* gene accession IDs	NCBI-KOG	TFs
Ca4_P2_28421518	Ca4	28421518	T/TA	CCAATCCATCGGTTTTTCAC	TGCATGGAAAGCTGTGGTAG	59.9	472	INTERGENIC	--	--	--
Ca4_P2_28423269	Ca4	28423269	T/TA	TAGGTTTCCCCATGTGGTGT	AGCACTTGCCCCTTAGGAGT	60.3	697	INTERGENIC	--	--	--
Ca4_P2_28424700	Ca4	28424700	TC/T	TTAGGCTATGTTTGGCGGTC	ATTGTCTCCATCGGAGCATC	60.0	280	INTERGENIC	--	--	--
Ca4_P2_28443084	Ca4	28443084	AAT/AATAT	CGATGAACTTGCGGTTTCTA	CCACATTGTTTCCAACTCCA	59.4	658	INTERGENIC	--	--	--
Ca4_P2_28447880	Ca4	28447880	ATTTTTTTT/ATTTTTTTTT	TCTTCTGTGTGAGCGGATTG	GCTCAACCTTGAGACCAAGC	60.0	252	INTERGENIC	--	--	--
Ca4_P2_28452206	Ca4	28452206	CAAAAAAAAA/CAAAAAAAA	CAAACTTCCGCACTGCACTA	ACAAATGGAACCCAACCGTA	60.1	473	INTERGENIC	--	--	--
Ca4_P2_28457671	Ca4	28457671	TCTCTA/TCTCTATTTAAACTCTA	TACAAAGCACCTGACCCTCC	CCCAAATCAAAGCAAGCAAT	60.1	765	INTERGENIC	--	--	--
Ca4_P2_28458251	Ca4	28458251	CAAAA/CAAA	ATTGCTTGCTTTGATTTGGG	GAAGAAAATCTTTGACCAAGTTGAA	60.0	486	INTERGENIC	--	--	--
Ca4_P2_28497621	Ca4	28497621	TAAAAAAAAA/TAAAAAAAAAAA	TCAATTTTCATGTATTCTTGGGG	TTGTCTGATTGATGTCAATGTCAG	60.0	577	INTERGENIC	--	--	--
Ca4_P2_28497882	Ca4	28497882	TC/T	TCAATTTTCATGTATTCTTGGGG	GGTTTTTGCGAGGAAAATTG	59.6	823	INTERGENIC	--	--	--
Ca4_P2_28501292	Ca4	28501292	AAATATTTTATTATT/AAATATTTTATTATTAATATTTTATTATT	GCACGCACACACATATACCC	TTTGCAGGCACAAAAGACAG	60.0	747	INTERGENIC	--	--	--
Ca4_P2_28505491	Ca4	28505491	TAAAAAAAA/TAAAAAAAAA	GGATTTCACGGTGGTCTTTT	TCACACACCTCAAAACCCCT	60.4	433	INTERGENIC	--	--	--

INDEL marker IDs	Chromosomes/unanchored scaffolds	Physical positions (bp)	InDels (*Kabuli* reference genome-CDC Frontier/PII)	Forward primers (5'-3')	Reverse primers (5'-3')	Anneling temperature (0C)	Expected amplified product size (bp)	Structural annotation		Functional annotation	
								Sequence components of *kabuli* genome	*Kabuli* gene accession IDs	NCBI-KOG	TFs
Ca4_P2_28511873	Ca4	28511873	CAA/CA	CCGAGTGCTTTGACCCTTAG	TCTTCACTCAAGCAAAACTCCA	60.0	687	INTERGENIC	--	--	--
Ca4_P2_28513752	Ca4	28513752	ATTTTT/ATTTTTT	CTATTCCGTGAACAACCCGT	TCACTATGGTGTAAAACAATTGGTAA	58.9	738	INTERGENIC	--	--	--
Ca4_P2_28516643	Ca4	28516643	TCTAA/TCTAACTCTAA	TAATCTTCGGCGAAACTGCT	TAACAATCTGGTGCTGCTCG	60.0	897	CDS (large-effect mutations)	Ca_26231	--	--
Ca4_P2_28529463	Ca4	28529463	TC/T	CATGGAAGCGTATGTCGATG	ACCTGCCCCTAGACCAATTC	60.3	311	INTERGENIC	--	--	--
Ca4_P2_28534748	Ca4	28534748	ATTTTTTTTTTT/ATTTTTTTTTT	TGGTCAACATGCCAAAGAAA	TTGTTTTTCCTCCAAACAAATG	59.0	958	INTERGENIC	--	--	--
Ca4_P2_28535714	Ca4	28535714	TTT/TTTATT	GCCAGCTCCAATTACAATGC	CATGTCTTTTGAATTTTGATCAGC	60.0	816	INTERGENIC	--	--	--
Ca4_P2_28552997	Ca4	28552997	CTTTTTTTTTTT/CTTTTTTTTTT	TTTGCATGGAGATGGTTTGA	TCATGCGGTTAAAATGTGGA	59.9	437	INTERGENIC	--	--	--
Ca4_P2_28554798	Ca4	28554798	TAGAG/TAG	TGGTACCCAATGCCAATTCT	AACCGCCCCCATTTTATATC	59.9	732	INTERGENIC	--	--	--
Ca4_P2_28559705	Ca4	28559705	AT/ATT	TGATCATCGAGGAGCAAACA	CATCTCTTGTTTCGGGTGGT	60.0	722	INTERGENIC	--	--	--
Ca4_P2_28563243	Ca4	28563243	TG/T	GCAGACTTTGGCAGTGAACA	AGATCTATGCATCGGCAACC	60.1	575	INTERGENIC	--	--	--
Ca4_P2_28581706	Ca4	28581706	CAAAAAAA/CAAAAAAAAA	ATGGGAGAGGGGAAGAAAGA	AATTGGTGTGGGGAAATGAA	60.0	585	INTERGENIC	--	--	--
Ca4_P2_28581947	Ca4	28581947	TAAAAAAAAAAA/TAAAAAAAAAA	CATTTCCCCACACCAATTTC	AAGGATGATGATTGTGCATGT	57.9	379	INTERGENIC	--	--	--

INDEL marker IDs	Chromosomes/unanchored scaffolds	Physical positions (bp)	InDels (*Kabuli* reference genome-CDC Frontier/PII)	Forward primers (5'-3')	Reverse primers (5'-3')	Anneling temperature (0C)	Expected amplified product size (bp)	Structural annotation		Functional annotation	
								Sequence components of *kabuli* genome	*Kabuli* gene accession IDs	NCBI-KOG	TFs
Ca4_P2_28581991	Ca4	28581991	CAAAAAAAAAA/CAAAAAAAAAAA	CATTTCCCCACACCAATTTC	AAGGATGATGATTGTGCATGT	57.9	379	INTERGENIC	--	--	--
Ca4_P2_28591975	Ca4	28591975	TTATATATATATATATA/TTATATATATATATATATATATA	ATCGATGCGACCACATGTAA	TGGGGTTCAAAGAAAGGTCA	60.5	245	INTERGENIC	--	--	--
Ca4_P2_28592937	Ca4	28592937	CTTCTCATTTTCTCATTTTCT/CTTCTCATTTTCT	TTTGGTAGTTTTTGCCCAGC	CGTCGTCGATGTCTCTTTCA	60.0	478	INTERGENIC	--	--	--
Ca4_P2_28623056	Ca4	28623056	A/AGAATTTGG	ATATGGTTGCGCACAAATGA	TGTCCGAAACATTAAACCCA	58.9	704	INTERGENIC	--	--	--
Ca4_P2_28623236	Ca4	28623236	ATTTTTTTT/ATTTTTTT	GAAATGTTGAATGTGTGCCG	TGTCCGAAACATTAAACCCA	58.9	249	INTERGENIC	--	--	--
Ca4_P2_28714992	Ca4	28714992	GATAT/GAT	AGAGCCAACGCTCATAGGAA	TTAAAATTCCCTCCTCGGCT	60.0	531	INTERGENIC	--	--	--
Ca4_P2_28770606	Ca4	28770606	ATTT/ATTTT	AACACCTGTGGTCCCTTAACTT	CCGACAAGTTTGGATGTCTG	59.1	970	INTERGENIC	--	--	--
Ca4_P2_28783066	Ca4	28783066	TAAA/TAA	AGGAGAGAAGTGGTTTGGCA	ATGAAGGAGCAAGCTTTCCA	60.0	711	INTERGENIC	--	--	--
Ca4_P2_28783547	Ca4	28783547	CTTTTTTTTT/CTTTTTTTTTT	TGGAAAGCTTGCTCCTTCAT	CAAGTGATGTTGCCTATTCTGG	59.6	124	INTERGENIC	--	--	--
Ca4_P2_28791900	Ca4	28791900	TCACA/T	TGCCCCTCATGGTTATCCTA	CACAGAGTTCGACACCGCTA	60.0	580	INTERGENIC	--	--	--
Ca4_P2_28793395	Ca4	28793395	TAA/TAAAAACAA	CCAAAATTACACCAACGGTTC	TTGTTCGCATGCTCATTCTC	60.0	275	INTERGENIC	--	--	--
Ca4_P2_28806476	Ca4	28806476	TT/TAT	TGTTTGTTTTTGCAAGCAGG	GCAAGAAGCCATCCACAAGT	60.3	351	INTERGENIC	--	--	--

INDEL marker IDs	Chromosomes/unanchored scaffolds	Physical positions (bp)	InDels (*Kabuli* reference genome-CDC Frontier/PII)	Forward primers (5'-3')	Reverse primers (5'-3')	Anneling temperature (0C)	Expected amplified product size (bp)	Structural annotation		Functional annotation	
								Sequence components of *kabuli* genome	*Kabuli* gene accession IDs	NCBI-KOG	TFs
Ca4_P2_28822073	Ca4	28822073	TA/TAA	ATTCAGAATGGCGGATCTTG	AACACCATTTGCGGAAGAAG	60.1	646	INTERGENIC	--	--	--
Ca4_P2_28822156	Ca4	28822156	TC/T	AAGCGCAAAAGTGCATGTTA	AACACCATTTGCGGAAGAAG	60.1	361	INTERGENIC	--	--	--
Ca4_P2_28823076	Ca4	28823076	TCC/T	TCAGTCAACAAAATCATGGTAATACA	TTCTCAAGATGGTCAACAACTCA	59.8	733	INTERGENIC	--	--	--
Ca4_P2_28823148	Ca4	28823148	TAAAAAAAA/TAAAAAAAAA	TCAGTCAACAAAATCATGGTAATACA	TTCTCAAGATGGTCAACAACTCA	59.8	733	INTERGENIC	--	--	--
Ca4_P2_28823207	Ca4	28823207	ACC/AC	TGGTTTTAATGTGTGCATTTTATG	TTCTCAAGATGGTCAACAACTCA	59.8	266	INTERGENIC	--	--	--
Ca4_P2_28823256	Ca4	28823256	CAAAAAA/CAAAAAAA	TGGTTTTAATGTGTGCATTTTATG	TTCTCAAGATGGTCAACAACTCA	59.8	266	INTERGENIC	--	--	--
Ca4_P2_28823535	Ca4	28823535	AAATAATAATAATAATAATAATAATAATA/AAATAATAATAATAATAATAATA	TGAGTTGTTGACCATCTTGAGAA	ACCAAGGGCAGATCTTAGGG	60.5	399	INTERGENIC	--	--	--
Ca4_P2_28830966	Ca4	28830966	CTA/CTATA	TCGCCTATGTGAATGTGGAA	CCGCTCTAAAAATTGAAGGG	58.8	706	INTERGENIC	--	--	--
Ca4_P2_2884124	Ca4	2884124	T/TA	TGCAACAACCAATGCAGTTT	ATATCATCAAACTTCCGCCG	59.9	580	INTERGENIC	--	--	--
Ca4_P2_28848503	Ca4	28848503	A/AG	CGAAAAACCGTCCTCTTTCA	CATTGTGGCATCATTTCCTCT	59.9	151	INTERGENIC	--	--	--
Ca4_P2_28849010	Ca4	28849010	GAAAAAAAA/GAAAAAAAAA	AGAGGAAATGATGCCACAATG	AAACACTTGCGTAACCTGGC	60.2	794	INTERGENIC	--	--	--
Ca4_P2_28850439	Ca4	28850439	ATTATTTTTATTTT/ATTATTTTTATTTTTATTTT	GTCAGAGGCAGTGAGCCTTC	TTTTAGCTTTCCAACGCCTG	60.4	500	INTERGENIC	--	--	--

INDEL marker IDs	Chromosomes/unanchored scaffolds	Physical positions (bp)	InDels (*Kabuli* reference genome-CDC Frontier/PII)	Forward primers (5'-3')	Reverse primers (5'-3')	Anneling temperature (0C)	Expected amplified product size (bp)	Structural annotation		Functional annotation	
								Sequence components of *kabuli* genome	*Kabuli* gene accession IDs	NCBI-KOG	TFs
Ca4_P2_28856354	Ca4	28856354	ATTTTTTT/ATTTTTTTT	GCGCGAATATCTCAACCAAT	TCCACCAACAACAAGATCCA	59.9	212	INTERGENIC	--	--	--
Ca4_P2_28856666	Ca4	28856666	TA/T	TGCCGTATTGATGGATCTGA	GAAGGTGCGAGTGATGTGAA	59.8	729	INTRON	Ca_23701	T	E2F/DP
Ca4_P2_28871484	Ca4	28871484	AAA/AAATTCATGAA	CTCCTACTTGCGCACACAAA	ACAGTGCCTTCCATAAGCCT	58.8	233	DRR	Ca_23703	R	YABBY
Ca4_P2_28872061	Ca4	28872061	GAAAAAAAAAA/GAAAAAAAAAAAA	TGATAAAGGGAGAAAAGGGC	AAACCAAGGCCCTGAAAACT	60.0	782	DRR	Ca_23703	R	YABBY
Ca4_P2_28872106	Ca4	28872106	ATTTTTTTTT/ATTTTTTTT	TGATAAAGGGAGAAAAGGGC	AAACCAAGGCCCTGAAAACT	60.0	782	DRR	Ca_23703	R	YABBY
Ca4_P2_28879341	Ca4	28879341	AGT/AGTGTGATGT	TCTCACCACAATGGGATTCA	AAAAGCAAAACCAGCGAATG	60.2	518	INTERGENIC	--	--	--
Ca4_P2_28883001	Ca4	28883001	T/TC	CAAACGACGTTCTTTGGACA	ACACCATCGCAATGTTTTCA	60.0	405	INTERGENIC	--	--	--
Ca4_P2_28887918	Ca4	28887918	TAAAAAAAAA/TAAAAAAAAAA	TGCCCTATTTTGTGTCCAGG	TGCGTTTGGCTGATGTTAAG	59.9	573	INTERGENIC	--	--	--
Ca4_P2_28898361	Ca4	28898361	TAAAAAAAAAA/TAAAAAAAAAAA	AACACAAACCAAATTAACATCAACA	CGGAAGTCTTGGAAAAGGTG	59.7	934	INTRON	Ca_23704	--	C2H2
Ca4_P2_2890459	Ca4	2890459	CA/CAAA	CGTAGGCTAAACTAACATGCCC	CTCCGCGTTTGAGTTTGAGT	60.4	385	INTERGENIC	--	--	--
Ca4_P2_28910424	Ca4	28910424	ATTTTCTTTTTCTTTTTCTT/ATTTTCTTTTTCTT	CTGTTTCCTTCCAAACTGGG	TGTAGAGGAATGTCTCGCCC	60.2	393	INTERGENIC	--	--	--
Ca4_P2_28910579	Ca4	28910579	A/AC	CTGTTTCCTTCCAAACTGGG	TGTAGAGGAATGTCTCGCCC	60.2	393	INTERGENIC	--	--	--

INDEL marker IDs	Chromosomes/unanchored scaffolds	Physical positions (bp)	InDels (*Kabuli* reference genome-CDC Frontier/PII)	Forward primers (5'-3')	Reverse primers (5'-3')	Anneling temperature (0C)	Expected amplified product size (bp)	Structural annotation		Functional annotation	
								Sequence components of *kabuli* genome	*Kabuli* gene accession IDs	NCBI-KOG	TFs
Ca4_P2_28913641	Ca4	28913641	AAATAATAATAATAATAATAAT/AAATAATAATAATAATAAT	TACGTCGCTCCATGCAAATA	CAACGTCCTCCCATTTGTTT	59.8	822	INTERGENIC	--	--	--
Ca4_P2_28913802	Ca4	28913802	TTTATTATTATTATTATTATTATTATTATTATTATTATT/TTTATTATTATTATTATTATTATTATT	AAATAGAAAGTCGCATCCCG	CAACGTCCTCCCATTTGTTT	59.8	510	INTERGENIC	--	--	--
Ca4_P2_28914437	Ca4	28914437	GA/GAAA	AAACAAATGGGAGGACGTTG	TGTTCCAGATATTTTGCCGA	59.1	508	INTERGENIC	--	--	--
Ca4_P2_2891639	Ca4	2891639	A/AG	TGTTGTGGTGCCACCTTTTA	TAATACACACGGCCAAGCAA	60.1	823	INTERGENIC	--	--	--
Ca4_P2_28923563	Ca4	28923563	AGATAC/A	CCGAGTGTAGGGTTTGAGGA	AGGGGCTATGTCCAGAAGGT	60.0	523	INTERGENIC	--	--	--
Ca4_P2_28925061	Ca4	28925061	CAAAAAA/CAAAAAAAAA	GGGGTGGTTTTTAGCTTGGT	TAGCCCAGTTTGGAAGGAAA	59.7	773	INTERGENIC	--	--	--
Ca4_P2_28925445	Ca4	28925445	AC/ACCTCTC	TCCAATTTTATCTTTATGCTGCC	GTGGCACAAGAATTGGAACC	60.4	189	INTERGENIC	--	--	--
Ca4_P2_28925610	Ca4	28925610	TAA/TA	TTTCCTTCCAAACTGGGCTA	AATGCGGTCTTCCCCTAAGT	60.0	622	INTERGENIC	--	--	--
Ca4_P2_28926033	Ca4	28926033	AC/ACGCACGC	AGGTTCCAATTCTTGTGCCA	CTGATGAAAGTTTGCGACGA	60.0	858	INTERGENIC	--	--	--
Ca4_P2_28930811	Ca4	28930811	CATTATTATTATTATT/CATTATTATTATTATTATTATTATT	CTCGTTCAAAGGGCTAGACG	TAATTTGAAATGGCGCACAA	60.1	722	INTERGENIC	--	--	--
Ca4_P2_2894996	Ca4	2894996	GAAAAAAAAA/GAAAAAAAAAA	AAAAGAAACGGAGGAAGTGTCA	TAAATACGGTGATCGGCCTC	59.9	127	DRR	Ca_12086	P	ERF
Ca4_P2_2895172	Ca4	2895172	TCCC/TCC	GAGGCCGATCACCGTATTTA	TTTTTCCCCTTTGAAATAACACA	59.8	545	DRR	Ca_12086	P	ERF

INDEL marker IDs	Chromosomes/unanchored scaffolds	Physical positions (bp)	InDels (*Kabuli* reference genome-CDC Frontier/PII)	Forward primers (5'-3')	Reverse primers (5'-3')	Anneling temperature (0C)	Expected amplified product size (bp)	Structural annotation		Functional annotation	
								Sequence components of *kabuli* genome	*Kabuli* gene accession IDs	NCBI-KOG	TFs
Ca4_P2_28955741	Ca4	28955741	CAT/CATAT	TTTTAATGGGCTTGACCCAC	TTTCAAAATGGGTAGAGGCG	60.1	858	INTERGENIC	--	--	--
Ca4_P2_28965010	Ca4	28965010	GTTTTTTTT/GTTTTTTTTT	CTTTGGTCATACGGCATGTG	TTGAGTTGGGGGAGAAAAGA	59.6	168	INTERGENIC	--	--	--
Ca4_P2_28965039	Ca4	28965039	ATTTTTTTT/ATTTTTTTTT	CTTTGGTCATACGGCATGTG	TTGAGTTGGGGGAGAAAAGA	59.6	168	INTERGENIC	--	--	--
Ca4_P2_28970044	Ca4	28970044	ATTTTTTTT/ATTTTTTTTT	TTGTTTCTCCCCAGTTTTGG	TCCATTTTGTTTCTCCCCAG	59.9	233	INTERGENIC	--	--	--
Ca4_P2_2897791	Ca4	2897791	ATATTTATTTATTTATTTATTTATTT/ATATTTATTTATTTATTTATTTATTTATTT	AAAAAGACATGCTTTCAATTTGG	AAGCCTTGTGGAGCAGTTGT	59.9	948	INTERGENIC	--	--	--
Ca4_P2_28981567	Ca4	28981567	GAAAAAAAA/GAAAAAAAAA	GCCCTCAGATGAGAGTCAGG	AACTTTACCGCGATTGTTGG	60.0	778	INTERGENIC	--	--	--
Ca4_P2_28984089	Ca4	28984089	GAAAA/GAAA	ATGGAATGGGATATGGGTCA	TAATCATGGCTTGCCACAAA	60.1	601	INTERGENIC	--	--	--
Ca4_P2_28986695	Ca4	28986695	CGTA/C	AACAATGCAAAGTTGTCTCACA	TCAGTTCAAAAAGAAAATCAACAGA	59.4	808	INTERGENIC	--	--	--
Ca4_P2_28987257	Ca4	28987257	CTAT/CTATAT	TCTGTTGATTTTCTTTTTGAACTGA	TTTTCCTCAAGCTACAGCCTTT	59.6	293	INTERGENIC	--	--	--
Ca4_P2_28988082	Ca4	28988082	GAAAAAAAAA/GAAAAAAAAAAA	TCCACATAGACACGAACCCA	CCAAAATGGAATAAGCTGACC	58.6	261	INTERGENIC	--	--	--
Ca4_P2_28988768	Ca4	28988768	TAAAAAAAA/TAAAAAAAAA	AAAAGTCGACAGTTTTGGTTCA	ACTGGGTTGTCAATTGCCTC	60.0	681	INTERGENIC	--	--	--
Ca4_P2_28990312	Ca4	28990312	CAAAAA/CAAA	GTTTCGCAATCTGGGTTTGT	AACACGTGTAGCAAAGCACG	60.0	770	INTERGENIC	--	--	--

INDEL marker IDs	Chromosomes/unanchored scaffolds	Physical positions (bp)	InDels (*Kabuli* reference genome-CDC Frontier/PII)	Forward primers (5'-3')	Reverse primers (5'-3')	Anneling temperature (0C)	Expected amplified product size (bp)	Structural annotation		Functional annotation	
								Sequence components of *kabuli* genome	*Kabuli* gene accession IDs	NCBI-KOG	TFs
Ca4_P2_28991379	Ca4	28991379	CCTGCTGC/CCTGC	GAAGCGTTTAGTTGCTTCCG	GTTGTCAATTGCGGGATTTC	60.3	751	INTRON	Ca_23706	--	NF-YB
Ca4_P2_28992492	Ca4	28992492	CA/CAAA	GGCGGTACAATTGCTTGATT	GGAACCTGGAACACCGTAGA	60.0	592	INTRON	Ca_23706	--	NF-YB
Ca4_P2_28993058	Ca4	28993058	CTTT/CT	TCTACGGTGTTCCAGGTTCC	AATGGATGGTTAACGAGTTGC	59.0	500	INTRON	Ca_23706	--	NF-YB
Ca4_P2_28996068	Ca4	28996068	CTTTTTTTTTTTT/CTTTTTTTTTT	TAACAGCATTGCATTGGGAA	TTCCAACACTAAAGGGGTGC	60.0	243	DRR	Ca_23706	--	NF-YB
Ca4_P2_29030081	Ca4	29030081	CGG/CGGG	ACCATTGCCGAATTGAAAAA	GTGCCCAAATTGTCCTCACT	60.0	501	INTERGENIC	--	--	--
Ca4_P2_29030327	Ca4	29030327	CAA/CAACTAAAAA	GCGAGTCGGATGGACATAAT	GTGCCCAAATTGTCCTCACT	60.0	177	INTERGENIC	--	--	--
Ca4_P2_29030631	Ca4	29030631	GAA/GA	AGTGAGGACAATTTGGGCAC	CCCAACCTTTATTTGGTCCTT	59.2	480	INTERGENIC	--	--	--
Ca4_P2_29031991	Ca4	29031991	ATTTTTTT/ATTTTTT	TCACAAGGAGTGCGAATCAA	CACGAAATGAACCACAACGA	60.6	726	INTERGENIC	--	--	--
Ca4_P2_29032247	Ca4	29032247	T/TG	CGGAGTTTTGTGGCATTTTT	CACGAAATGAACCACAACGA	60.6	482	INTERGENIC	--	--	--
Ca4_P2_29034169	Ca4	29034169	TA/TAA	TGAATGGAAAGAACCTGGACA	TGTGAGCATCCACTCTCAGG	60.0	758	INTERGENIC	--	--	--
Ca4_P2_29035883	Ca4	29035883	ATTTTTTT/ATTTTTTTT	TTGGACAGAGGCATGCAATA	GGACCAACTCTGGAAGAAAAA	58.3	735	INTERGENIC	--	--	--
Ca4_P2_29036852	Ca4	29036852	CAAA/CA	TTGAGCCATGGATTTGATGA	TCCATTCATTCCTTGCCATT	60.3	806	INTERGENIC	--	--	--

INDEL marker IDs	Chromosomes/unanchored scaffolds	Physical positions (bp)	InDels (*Kabuli* reference genome-CDC Frontier/PII)	Forward primers (5'-3')	Reverse primers (5'-3')	Anneling temperature (0C)	Expected amplified product size (bp)	Structural annotation		Functional annotation	
								Sequence components of *kabuli* genome	*Kabuli* gene accession IDs	NCBI-KOG	TFs
Ca4_P2_29037043	Ca4	29037043	GAAAA/GAAAAAAA	CCATTGAGCTACACTGTGCG	TTGGACAACGCTTCAAACTG	59.9	434	INTERGENIC	--	--	--
Ca4_P2_29038018	Ca4	29038018	TC/T	GAGCTGAAACATGCGAACAA	TGATACCTTGCATTGAACAAATTC	60.2	716	DRR	Ca_23707	--	--
Ca4_P2_29046225	Ca4	29046225	TAAAAAAA/TAAAAAA	TTTCAACGCGCTTTAGACAAT	TGACCAGGCTTGTTACTCAAA	58.4	740	INTERGENIC	--	--	--
Ca4_P2_29046983	Ca4	29046983	CTTTTTTTT/CTTTTTTTTTT	AGCACCGTTGTCGAGAATTT	CCACCTGCATGAATTTGTTG	60.0	349	INTERGENIC	--	--	--
Ca4_P2_29049755	Ca4	29049755	TTA/TTAAAGCGAGAAATA	GGTTTTATAGGGCATGCTGG	TCCCAAGAAATCAATGCTCC	60.0	749	INTERGENIC	--	--	--
Ca4_P2_29052614	Ca4	29052614	CTTTTTTT/CTTTTTT	GGTTCCAACACGTGTCCTCT	CAATCAACATTGCAACAGGG	60.0	536	INTERGENIC	--	--	--
Ca4_P2_29053469	Ca4	29053469	ACCCC/ACCC	CCTGTTGCAATGTTGATTGG	ACATCAGAGAAATCACCGCC	60.1	855	INTERGENIC	--	--	--
Ca4_P2_29055386	Ca4	29055386	CG/CGG	AACAAAGGTCCTAGGGTGGG	CTTGCATGCGTATCTTCCAA	59.8	398	INTERGENIC	--	--	--
Ca4_P2_29061010	Ca4	29061010	ATTTTTTT/ATTTTTTTT	AACGCGATGTGGAGGTTTAG	TTATATTGGCAACCGGCCTA	60.3	287	INTERGENIC	--	--	--
Ca4_P2_29064946	Ca4	29064946	TA/T	GTTTCCCGAAGGTTTTGGAT	TCATCTTGGATCACACTCAACC	60.0	424	INTERGENIC	--	--	--
Ca4_P2_29065135	Ca4	29065135	TAAAAAAAAA/TAAAAAAAA	GTTTCCCGAAGGTTTTGGAT	TCATCTTGGATCACACTCAACC	60.0	424	INTERGENIC	--	--	--
Ca4_P2_29068157	Ca4	29068157	C/CTCAAATAG	CTTGGCTACCACCACGAAAT	ATCCAAGGGAGAAAGAAGGG	59.5	767	INTERGENIC	--	--	--

INDEL marker IDs	Chromosomes/unanchored scaffolds	Physical positions (bp)	InDels (*Kabuli* reference genome-CDC Frontier/PII)	Forward primers (5'-3')	Reverse primers (5'-3')	Anneling temperature (0C)	Expected amplified product size (bp)	Structural annotation		Functional annotation	
								Sequence components of *kabuli* genome	*Kabuli* gene accession IDs	NCBI-KOG	TFs
Ca4_P2_29070763	Ca4	29070763	TAAAAAAAAA/TAAAAAAAAAAA	ATACCAGACGCGATAGGTGG	TCATCCATAATTTTAGCCCGA	59.4	819	INTERGENIC	--	--	--
Ca4_P2_29073548	Ca4	29073548	ATTTTTTTTT/ATTTTTTTTTT	TGTCAAAAATGAGGGTGCAA	CACCTATTTTGTGCCCCAAC	60.2	546	INTERGENIC	--	--	--
Ca4_P2_29085024	Ca4	29085024	CT/CTT	AAGGAGAAAAGTGGGGGAGA	ACGTATCCCCAGGTGCAATA	60.2	602	INTERGENIC	--	--	--
Ca4_P2_29085344	Ca4	29085344	AATCGATC/AATC	TCGCGAATCTGCTCTTGAAT	CGCCGCCAATTTTATTTTTA	59.9	556	INTERGENIC	--	--	--
Ca4_P2_29085408	Ca4	29085408	CTGG/CTGGAGATGG	TCGCGAATCTGCTCTTGAAT	CGCCGCCAATTTTATTTTTA	59.9	556	INTERGENIC	--	--	--
Ca4_P2_29085860	Ca4	29085860	TAAAAAA/TAAAAAAA	TAAAAATAAAATTGGCGGCG	TCCAATTCAATTCATGGGGT	60.0	348	INTERGENIC	--	--	--
Ca4_P2_29086174	Ca4	29086174	GAAAAAA/GAAAAAAA	ACCCCATGAATTGAATTGGA	GCCTCCAAAAGATCAAGCAG	60.0	374	INTERGENIC	--	--	--
Ca4_P2_29089032	Ca4	29089032	AT/A	GGAAGGGATGTTCGTAGCAA	ACGGGAGGAATGATACATGG	59.6	457	INTERGENIC	--	--	--
Ca4_P2_29094195	Ca4	29094195	TCTA/T	TCGGTAGCTGGCTGAAATCT	TGCGTTGTCGCGTAAGTAAC	59.9	470	INTRON	Ca_23708	C	HD-ZIP
Ca4_P2_29094516	Ca4	29094516	ATTTTTTT/ATTTTTTTT	TCGGTAGCTGGCTGAAATCT	TGCGTTGTCGCGTAAGTAAC	59.9	470	INTRON	Ca_23708	C	HD-ZIP
Ca4_P2_29107247	Ca4	29107247	TTTAT/TTTATTAT	TGCGTCCAAAAAGAATTCAA	TTGCTTCCTCTCCAAGCATT	60.0	747	INTERGENIC	--	--	--
Ca4_P2_29110502	Ca4	29110502	ATTTTT/ATTTT	TGCAAACATGGATGGGAATA	CACGGTTTCTAACACCATTTGA	59.9	617	INTERGENIC	--	--	--

INDEL marker IDs	Chromosomes/unanchored scaffolds	Physical positions (bp)	InDels (*Kabuli* reference genome-CDC Frontier/PII)	Forward primers (5'-3')	Reverse primers (5'-3')	Anneling temperature (0C)	Expected amplified product size (bp)	Structural annotation		Functional annotation	
								Sequence components of *kabuli* genome	*Kabuli* gene accession IDs	NCBI-KOG	TFs
Ca4_P2_29110940	Ca4	29110940	AGATTC/AGATTCATGAATGATTC	CGTAAAGTCCACTTCCATTTCA	GGAGATTTGTGCCCTAGCAG	59.8	300	INTERGENIC	--	--	--
Ca4_P2_29118229	Ca4	29118229	GAGAAG/GAG	TGGTCTGCTCTTCTCAATCG	TGATTGAACGTTGGCCATAA	59.9	307	INTERGENIC	--	--	--
Ca4_P2_29119825	Ca4	29119825	AT/ATAAATTGT	TTCACACCTGTTTTTATGGGC	TTGTTTTCTTCCCCTTTGTCA	59.6	431	INTERGENIC	--	--	--
Ca4_P2_29122267	Ca4	29122267	TAA/TAAAA	TTGTGTGCCACATCATCACA	TACCAGCGACATGCTTTCAG	60.0	832	INTERGENIC	--	--	--
Ca4_P2_29133293	Ca4	29133293	CAGAGGA/CAGAGGAAACAACTAGAGGA	GCCAAACCAATTCCAATGTC	TTCGGATTTTGAGGATTTCG	60.0	209	INTERGENIC	--	--	--
Ca4_P2_29137078	Ca4	29137078	TC/T	GCAACAGAAGTGCCAGATGA	GCCTTCGGAACCAGAATGTA	60.1	526	INTERGENIC	--	--	--
Ca4_P2_29137332	Ca4	29137332	TAAAAAAAA/TAAAAAAA	GCAACAGAAGTGCCAGATGA	TCATCAGAGAAGTCCACCCC	60.0	712	INTERGENIC	--	--	--
Ca4_P2_29155494	Ca4	29155494	GCC/GC	AGTCGTTAGTCGTCGGTCGT	AACCAACCGATTTGATCGAG	59.9	436	DRR	Ca_23711	--	--
Ca4_P2_29155695	Ca4	29155695	ATTTTTT/ATTTTTTT	CAAATCGGTTGGTTTGATCC	AAAAGATCTCGACCCTTTTGG	59.6	505	DRR	Ca_23711	--	--
Ca4_P2_29156546	Ca4	29156546	CTATA/CTA	AGTTGGTTTGGTGGTGAAGG	GCATGGTAAAGGCTGTTGGT	60.0	841	DRR	Ca_23711	--	--
Ca4_P2_29156658	Ca4	29156658	CATGCAAATGCAAA/CATGCAAA	AGTTGGTTTGGTGGTGAAGG	GCATGGTAAAGGCTGTTGGT	60.0	841	DRR	Ca_23711	--	--
Ca4_P2_29158297	Ca4	29158297	ATT/ATTT	TGAGGAGAGAGGAGTGGAGA	TGTCCATTTAACGTTGACGA	57.6	433	INTERGENIC	--	--	--

INDEL marker IDs	Chromosomes/unanchored scaffolds	Physical positions (bp)	InDels (*Kabuli* reference genome-CDC Frontier/PII)	Forward primers (5'-3')	Reverse primers (5'-3')	Anneling temperature (0C)	Expected amplified product size (bp)	Structural annotation		Functional annotation	
								Sequence components of *kabuli* genome	*Kabuli* gene accession IDs	NCBI-KOG	TFs
Ca4_P2_29159894	Ca4	29159894	CAT/CATAT	TGATTTTCAAAGAGAAACCCG	TTCCTGCGATGTCATTTGTT	59.1	450	INTERGENIC	--	--	--
Ca4_P2_29162382	Ca4	29162382	AATA/AA	CTTTCCCGATTTCGTCCTTT	GGCCAGCGAAAATTACTACC	58.7	804	INTERGENIC	--	--	--
Ca4_P2_2918070	Ca4	2918070	AG/A	GCATTGATGCTTACGTGCAT	GGTTTTTGGTTGAGTTGGGA	59.8	314	INTERGENIC	--	--	--
Ca4_P2_29187399	Ca4	29187399	TAGAGAGAGAGAGAGAGA/TAGAGAGAGAGAGAGAGAGAGAGA	TCCAAAGACGACGACACTTG	GCTACATCAAGTCCTCCCCA	60.1	752	INTERGENIC	--	--	--
Ca4_P2_29190573	Ca4	29190573	GA/G	GCCAGTCTTCTCAAACAGCC	TTTGACCAAGTGGAAAAGAAGA	58.9	648	INTERGENIC	--	--	--
Ca4_P2_29191237	Ca4	29191237	CTTTTTTTT/CTTTTTTTTT	CCAACTTGGTTCCAACAATCT	GGAGAAAAGTTAGGGGGTGC	59.9	119	INTERGENIC	--	--	--
Ca4_P2_29194603	Ca4	29194603	GTTTTTTTT/GTTTTTTTTTT	TCCTCAAAAGTTTGGGGTTG	GCCAATCGAGTGTTTGAGTG	59.3	631	INTERGENIC	--	--	--
Ca4_P2_29196580	Ca4	29196580	TTCT/TT	AATCAACGGCAAATCGTTTC	ATGGTTTGATCCATCGGGTA	60.0	466	INTERGENIC	--	--	--
Ca4_P2_29197206	Ca4	29197206	CAT/CATCACGAAT	CCGACGAATTTTAGAGACGG	TGAATTTGGAGGGTTGGAGA	60.4	312	INTERGENIC	--	--	--
Ca4_P2_29202977	Ca4	29202977	AATGATAG/A	TCACCCCGATGTCACCTATT	ACATGCCATTGAATTACGCA	60.0	449	INTERGENIC	--	--	--
Ca4_P2_29203316	Ca4	29203316	CAAAA/CAAA	TGCGTAATTCAATGGCATGT	TAGGGCTGACCAAGACCACT	59.7	621	INTERGENIC	--	--	--
Ca4_P2_29205093	Ca4	29205093	C/CCAAG	ACATATGGCTTCGCATGACA	TTAGGAGTGGCTTTGGATGG	60.1	492	INTERGENIC	--	--	--

INDEL marker IDs	Chromosomes/unanchored scaffolds	Physical positions (bp)	InDels (*Kabuli* reference genome-CDC Frontier/PII)	Forward primers (5'-3')	Reverse primers (5'-3')	Anneling temperature (0C)	Expected amplified product size (bp)	Structural annotation		Functional annotation	
								Sequence components of *kabuli* genome	*Kabuli* gene accession IDs	NCBI-KOG	TFs
Ca4_P2_29210343	Ca4	29210343	G/GT	TCAAGCATAGCGTCAAATGG	GGAGAACAACCACTATTGGGC	60.7	355	INTERGENIC	--	--	--
Ca4_P2_29211121	Ca4	29211121	AT/A	GATGTTTAAGGTCTGTTTGGCA	AACTTCAGCCGTCATTGGTT	59.6	641	DRR	Ca_14151	--	--
Ca4_P2_29215455	Ca4	29215455	ATTT/ATT	ACGAGCTTCCTTCCACAAGA	TCGTTTGCAGACTGCTTTTG	60.2	555	INTRON	Ca_14152	--	Trihelix
Ca4_P2_29221169	Ca4	29221169	A/AG	TAACCATGGTCATCCCTCGT	ATGTTAACGTTCGGAGTGCC	60.0	374	INTERGENIC	--	--	--
Ca4_P2_29221849	Ca4	29221849	CTTTTTTT/CTTTTTT	TTGTTCGTACATCCGGGAAT	CGCACATAGAGAGGTGCATT	58.9	735	INTERGENIC	--	--	--
Ca4_P2_29238761	Ca4	29238761	A/AATT	CCATGAGATCCACATAATCCG	TTGCAAGAATCCATCCATCA	60.0	513	INTERGENIC	--	--	--
Ca4_P2_29244811	Ca4	29244811	CAAAA/CAAA	CCGAGATGGTTTTGGAGAAA	GCTTCATAGCTTGCAAAGGG	60.0	488	INTERGENIC	--	--	--
Ca4_P2_29245084	Ca4	29245084	ATA/ATACTTA	GCCCTTTGCAAGCTATGAAG	TGGTGCAATTTGGTTGGTAA	59.8	418	INTERGENIC	--	--	--
Ca4_P2_29245578	Ca4	29245578	T/TA	TTACCAACCAAATTGCACCA	CCCTAATGGGCCAAATTACA	59.7	583	INTERGENIC	--	--	--
Ca4_P2_29249082	Ca4	29249082	TTAT/TTATAT	AGTCAGGACAATGGCCAAAA	CGCCAAACACAAACATCAAC	60.0	681	INTERGENIC	--	--	--
Ca4_P2_29266562	Ca4	29266562	AAAATAGATAAAT/AAAAT	TGGTTTCCAGCACCTTCAAT	TGCGAGTTTTGTCCAGTTTG	59.9	687	INTERGENIC	--	--	--
Ca4_P2_29270177	Ca4	29270177	CTTTTTTTT/CTTTTTTT	TTTCCCGACTTGGTCCTTTA	TCGACTCCCCACCTAATGAC	59.9	696	INTERGENIC	--	--	--

INDEL marker IDs	Chromosomes/unanchored scaffolds	Physical positions (bp)	InDels (*Kabuli* reference genome-CDC Frontier/PII)	Forward primers (5'-3')	Reverse primers (5'-3')	Anneling temperature (0C)	Expected amplified product size (bp)	Structural annotation		Functional annotation	
								Sequence components of *kabuli* genome	*Kabuli* gene accession IDs	NCBI-KOG	TFs
Ca4_P2_29270431	Ca4	29270431	TAAAAAA/TAAAAAAA	CACGTCATTAGGTGGGGAGT	TTCCTTGAATTTGTCCCTGC	60.1	427	INTERGENIC	--	--	--
Ca4_P2_29274800	Ca4	29274800	GTTTTTTTTTT/GTTTTTTTTTTT	TTGGCGTGATAATTACATGAGG	GCCTACCGGAAATGAATGAA	59.9	864	INTERGENIC	--	--	--
Ca4_P2_29278808	Ca4	29278808	GTTTTTTTTTT/GTTTTTTTTTTT	CCACCGGATTTATGAAGGAA	GGGCATGAAATATGAGACTGC	59.5	415	INTERGENIC	--	--	--
Ca4_P2_29279766	Ca4	29279766	ATTTTTTTTTT/ATTTTTTTTTTT	CCGGATTTTTGCACTTGAAT	CGTTTGATTTTGGACGACCT	60.0	444	INTERGENIC	--	--	--
Ca4_P2_29280125	Ca4	29280125	ATTTTTTTTTTTTT/ATTTTTTTTTTTT	AGGTCGTCCAAAATCAAACG	TTCATCGCTCGCATTCATAC	59.8	328	INTERGENIC	--	--	--
Ca4_P2_29292512	Ca4	29292512	TCC/TC	CACATGTTTCAACTTGGTTGC	GATCCTCATGGGAACGTGAT	59.7	631	INTERGENIC	--	--	--
Ca4_P2_2929266	Ca4	2929266	CATATATATATATATATA/CATATATATATATATATATA	AAATGGCAATGAAATCCCTG	TCACTCTATCAAATGTTCAAGCAA	58.9	628	INTERGENIC	--	--	--
Ca4_P2_29292814	Ca4	29292814	CTTTTTTTTTT/CTTTTTTTTT	ACACGCTAGCCATTAGAGCC	ACGAGTGCAATCAGTCGAGA	59.6	176	INTERGENIC	--	--	--
Ca4_P2_29292913	Ca4	29292913	ATTTTTTTTT/ATTTTTTTTTT	ACACGCTAGCCATTAGAGCC	TCCTTTCTTTCCAAGAGGTTCA	60.2	306	INTERGENIC	--	--	--
Ca4_P2_29310722	Ca4	29310722	ATTTTTTTTTT/ATTTTTTTTT	GTGGTGTCCATCTCGGTCTT	TAAGCCAGCAACTCCAGGAT	59.8	658	INTERGENIC	--	--	--
Ca4_P2_29310770	Ca4	29310770	TAAAAAAAAA/TAAAAAAAA	GTGGTGTCCATCTCGGTCTT	TAAGCCAGCAACTCCAGGAT	59.8	658	INTERGENIC	--	--	--
Ca4_P2_29312136	Ca4	29312136	CAAAAAAA/CAAAAAAAAA	CATGCCCGGTCTACATCTCT	GGTTTTGGACAGGCAAGGTA	60.0	524	INTERGENIC	--	--	--

INDEL marker IDs	Chromosomes/unanchored scaffolds	Physical positions (bp)	InDels (*Kabuli* reference genome-CDC Frontier/PII)	Forward primers (5'-3')	Reverse primers (5'-3')	Anneling temperature (0C)	Expected amplified product size (bp)	Structural annotation		Functional annotation	
								Sequence components of *kabuli* genome	*Kabuli* gene accession IDs	NCBI-KOG	TFs
Ca4_P2_29315886	Ca4	29315886	CTTTTTTTTT/CTTTTTTTTTT	CGTGGCAACCCAAGACTTAT	TCAATGTTCAAGAATCGGTCC	59.9	733	INTERGENIC	--	--	--
Ca4_P2_29316470	Ca4	29316470	ATCTTCTT/ATCTT	GGACCGATTCTTGAACATTGA	GAAAATCTTCGAACAAGTGGAAA	59.6	413	INTERGENIC	--	--	--
Ca4_P2_29319511	Ca4	29319511	A/AC	GAATCTTCGATGCCTTGGTC	TTGGGAAATGATTGCTTTTTG	59.9	531	INTERGENIC	--	--	--
Ca4_P2_29321184	Ca4	29321184	ATTTTTTT/ATTTTTTTT	AACACATCAGCAAATGGCAA	TGGGTTCGGGTACAAGCTAC	60.0	682	INTERGENIC	--	--	--
Ca4_P2_29330235	Ca4	29330235	ATTTT/ATTT	TTTTGTTCAGATTGCGCTTG	GAGGGGATCCATTTTCCCTA	60.1	762	INTERGENIC	--	--	--
Ca4_P2_29330289	Ca4	29330289	CAAATTAAAATTAAA/CAAATTAAA	TTTTGTTCAGATTGCGCTTG	GAGGGGATCCATTTTCCCTA	60.1	762	INTERGENIC	--	--	--
Ca4_P2_29348534	Ca4	29348534	AGGGGGG/AGGGGGGG	GTTGTGCGGAAGCCATTTAT	GAGGACTGGACGTACCCTTG	59.6	248	INTERGENIC	--	--	--
Ca4_P2_29356210	Ca4	29356210	ATTTTTT/ATTTTT	GCGGGTTTCCTTTTATAGGC	TTTTCCAGCATGTGTGCATC	60.7	311	INTERGENIC	--	--	--
Ca4_P2_29356293	Ca4	29356293	CTTTTTTTTT/CTTTTTTTT	GCGGGTTTCCTTTTATAGGC	TTTTCCAGCATGTGTGCATC	60.7	311	INTERGENIC	--	--	--
Ca4_P2_29359586	Ca4	29359586	AATAT/AAT	GGGCTCGATATCTCATCCAA	CTTGTCGTGCTTCATGGCTA	60.0	541	INTERGENIC	--	--	--
Ca4_P2_29363363	Ca4	29363363	TAA/TA	CTCGCGGTATCTTCATGACT	AAAAACAGAGCAGGTACGAGTG	58.6	313	INTERGENIC	--	--	--
Ca4_P2_29366628	Ca4	29366628	ATTT/ATTTACCTTT	AAGGAAGAGCTCGACCATCA	TCTGCCATCACTCCCATACA	60.1	584	CDS (large-effect mutations)	Ca_14154	--	--

**INDEL marker IDs**	**Chromosomes/unanchored scaffolds**	**Physical positions (bp)**	**InDels (*Kabuli* reference genome-CDC Frontier/PII)**	**Forward primers (5'-3')**	**Reverse primers (5'-3')**	**Anneling temperature (0C)**	**Expected amplified product size (bp)**	**Structural annotation**		**Functional annotation**	
								**Sequence components of *kabuli* genome**	***Kabuli* gene accession IDs**	**NCBI-KOG**	**TFs**
Ca4_P2_29368326	Ca4	29368326	TC/T	TCGATTGTTTAGAGGCTATACGTG	TCGCTTGTGAGAGAGGACAA	59.7	511	DRR	Ca_14154	--	--
Ca4_P2_29371338	Ca4	29371338	GAA/GA	TTTTAAATTGGCAAGTCACCA	TCCACATTATCGTGTCCAAAA	58.9	526	INTERGENIC	--	--	--
Ca4_P2_29377597	Ca4	29377597	CTTTTTTTTT/CTTTTTTTTTT	CATCCTTACGCGATCTCCAT	AATGGATACGACCCATTCCA	60.0	491	INTERGENIC	--	--	--
Ca4_P2_29379238	Ca4	29379238	CAAAAAAA/CAAAAAA	ATGCAAGTGGGCCTAGACAG	CACTTCGTTTTGGAATCGGT	60.0	630	INTERGENIC	--	--	--
Ca4_P2_29388732	Ca4	29388732	ATTTT/ATTT	GGCATCCCAGATCTGAAAAA	AAGTCGGTACAGCACCATCC	60.0	931	INTERGENIC	--	--	--
Ca4_P2_29401976	Ca4	29401976	GTTTTT/GTTTTTT	CCCCGAGTTTGAGAATTTGA	TCTCTATTGCCACCACTCCC	60.1	575	INTERGENIC	--	--	--
Ca4_P2_29416514	Ca4	29416514	ATTTGTTT/ATTTGTTTGTTT	CCAATGGAAAATGGTCATCC	GCATCAATGTCAACCAATGC	59.9	671	INTERGENIC	--	--	--
Ca4_P2_29418053	Ca4	29418053	GAATAAATAAATAAA/GAATAAATAAATAAATAAA	TTGCTCCTACGTATCTCCCG	AACTTCCACCGTACACCACC	59.7	836	INTERGENIC	--	--	--
Ca4_P2_29418531	Ca4	29418531	TT/TTAT	GGTGGTGTACGGTGGAAGTT	GTCAAGGCAGACCAGAAAGC	60.0	383	INTERGENIC	--	--	--
Ca4_P2_29418584	Ca4	29418584	C/CT	GGTGGTGTACGGTGGAAGTT	GTCAAGGCAGACCAGAAAGC	60.0	383	INTERGENIC	--	--	--
Ca4_P2_29419385	Ca4	29419385	AA/AATA	GCCCCCAAAAAGAAAGTCTC	GGCATAAGCTTGCATTGTGA	59.8	682	INTERGENIC	--	--	--
Ca4_P2_29419619	Ca4	29419619	ATTTTT/ATTTT	GCCCCCAAAAAGAAAGTCTC	GGCATAAGCTTGCATTGTGA	59.8	682	INTERGENIC	--	--	--

INDEL marker IDs	Chromosomes/unanchored scaffolds	Physical positions (bp)	InDels (*Kabuli* reference genome-CDC Frontier/PII)	Forward primers (5'-3')	Reverse primers (5'-3')	Anneling temperature (0C)	Expected amplified product size (bp)	Structural annotation		Functional annotation	
								Sequence components of *kabuli* genome	*Kabuli* gene accession IDs	NCBI-KOG	TFs
Ca4_P2_29419673	Ca4	29419673	TAAAAAA/TAAAAAAA	TGATTTTACATCTGCACCATGA	GGCATAAGCTTGCATTGTGA	59.8	262	INTERGENIC	--	--	--
Ca4_P2_29419703	Ca4	29419703	AA/ACA	TGATTTTACATCTGCACCATGA	GGCATAAGCTTGCATTGTGA	59.8	262	INTERGENIC	--	--	--
Ca4_P2_29421150	Ca4	29421150	TAAAAAAAAAAAAAAAAAA/TAAAAAAAA	TCGCCGTTGCTTTTCTACTT	AGAGAGCCAAACGGCAGATA	60.0	728	INTERGENIC	--	--	--
Ca4_P2_29426569	Ca4	29426569	ATTTTTTTTT/ATTTTTTTTTT	ATCCAGCGAATTTTTACCGA	TTTTGACACACACACACCCA	59.4	781	INTERGENIC	--	--	--
Ca4_P2_29428218	Ca4	29428218	ATTTTTTTTTT/ATTTTTTTTT	TCCATCTTCTTCATTGATCACG	TGCCAAAAATACTCTTGTAAACAAAT	59.5	539	INTERGENIC	--	--	--
Ca4_P2_29439380	Ca4	29439380	AT/ATT	ATGGGAAACGTGAGGTATGC	CAAGTGGAGCTTGCCAATTT	60.2	604	INTERGENIC	--	--	--
Ca4_P2_29444899	Ca4	29444899	GTTTTTTTTTT/GTTTTTTTTT	TCGAAATTTTAGGCCACGAC	CCAGAAGGCTACCACTACGC	59.9	415	INTERGENIC	--	--	--
Ca4_P2_29457394	Ca4	29457394	ATTTTT/ATTTT	CCAATCCGAAGACGATCATT	TGGTGATTGGCAAAAACAAA	59.9	319	INTERGENIC	--	--	--
Ca4_P2_29471358	Ca4	29471358	GTTTTTTTTT/GTTTTTTTTTT	TACGGAAAAATTACCCACGG	CAATCTGGAGGAGGTTGGAA	60.0	472	INTERGENIC	--	--	--
Ca4_P2_29498412	Ca4	29498412	ATTTTTTTTT/ATTTTTTTT	TTGGATTGATTTTTGGATTTGTC	AACTTGATTTGAATATTGCCATTG	59.3	779	INTERGENIC	--	--	--
Ca4_P2_29503488	Ca4	29503488	ATTTTTTTTTT/ATTTTTTTTTTTT	TGTATGTCCCTCCTCTTTGTCA	GGGGTACTCAACCCACAAAA	59.7	735	DRR	Ca_14160	--	--
Ca4_P2_29508624	Ca4	29508624	CTTTTTTTTTTTT/CTTTTTTTTTTTTT	AGGGGGAATGAGAGGAAGAG	AGTGGTTGGGTCCTTGATGA	60.4	307	INTERGENIC	--	--	--

INDEL marker IDs	Chromosomes/unanchored scaffolds	Physical positions (bp)	InDels (*Kabuli* reference genome-CDC Frontier/PII)	Forward primers (5'-3')	Reverse primers (5'-3')	Anneling temperature (0C)	Expected amplified product size (bp)	Structural annotation		Functional annotation	
								Sequence components of *kabuli* genome	*Kabuli* gene accession IDs	NCBI-KOG	TFs
Ca4_P2_29515377	Ca4	29515377	TAAAAAAAAA/TAAAAAAAA	CGATGAGAGATGGTTAGGGTG	GGGAAGAAGCTTGATTCACCT	59.7	436	INTERGENIC	--	--	--
Ca4_P2_29519322	Ca4	29519322	TAAAAA/TAAAA	TGATCGCATTCTTATCAAACACA	CAAATTTTGAGTTCAGAGGTCG	58.9	239	INTERGENIC	--	--	--
Ca4_P2_29520331	Ca4	29520331	TATATATATAAAG/T	AAAAATCGAAACACATGGGG	TTTCCGAAACTCAAAAAGGG	59.2	320	INTERGENIC	--	--	--
Ca4_P2_29529676	Ca4	29529676	AAGA/AAGAGA	CCTGCTCCTCCTTCTTGATG	TGCATGTTGCCATTTTGTTT	60.0	273	INTRON	Ca_14161	--	--
Ca4_P2_29531556	Ca4	29531556	GT/GAT	CAAGGTCGGGGATTTGACTA	GTTATGCGCAAAATAGCGGT	60.1	578	INTERGENIC	--	--	--
Ca4_P2_29532848	Ca4	29532848	TAAAAAAA/TAAAAAAAA	TCCTTGCAAAGGACATTAAACA	TGCCAAGTCATGGATAATATGGT	60.5	544	INTERGENIC	--	--	--
Ca4_P2_29541970	Ca4	29541970	TTCATCATCATCATCATCATCA/TTCATCATCATCATCA	ATTCCCCACCCGTATACCTC	TGAGTGTTGAATGAATTTTGTGC	60.0	582	INTERGENIC	--	--	--
Ca4_P2_29544316	Ca4	29544316	GATAT/GAT	CCATCAAACCACAACAAACA	CCCAATTTCCTTCCGTTTTT	60.2	706	INTERGENIC	--	--	--
Ca4_P2_29545135	Ca4	29545135	AATTATTTATTATTTAT/AATTATTTATTATTTATTATTTAT	CATACGTGATACGCTGGGTG	GTGAGGGAGGACCAAAACAA	59.9	659	INTERGENIC	--	--	--
Ca4_P2_29545575	Ca4	29545575	GTTTTTTTTTT/GTTTTTTTTTTTT	TTGTTTTGGTCCTCCCTCAC	TTGAGCATTCATTGTAGTTCTTGA	58.9	322	INTERGENIC	--	--	--
Ca4_P2_29547360	Ca4	29547360	CTTTTTTTT/CTTTTTTT	TCCCACACATCCAAAACAAA	TTGGTCCCTATAAGTTGGCG	60.0	613	INTERGENIC	--	--	--
Ca4_P2_29564628	Ca4	29564628	GTTTT/GTTTTTT	GAACGGTCCATGCAAAAGTT	AGTTTCCCCGATCCTTATGC	60.3	535	INTERGENIC	--	--	--

INDEL marker IDs	Chromosomes/unanchored scaffolds	Physical positions (bp)	InDels (*Kabuli* reference genome-CDC Frontier/PII)	Forward primers (5'-3')	Reverse primers (5'-3')	Anneling temperature (0C)	Expected amplified product size (bp)	Structural annotation		Functional annotation	
								Sequence components of *kabuli* genome	*Kabuli* gene accession IDs	NCBI-KOG	TFs
Ca4_P2_29566926	Ca4	29566926	GTTTTTTTTTTTT/GTTTTTTTTTT	CTCTTTTGCCCTGCGATTAG	TGAAACCAGATTTTGAGTCCG	60.1	701	INTERGENIC	--	--	--
Ca4_P2_29577146	Ca4	29577146	TAAAATAAAAATAAAAATAAAAATAAAAATAAAAAT/TAAAATAAAAATAAAAATAAAAATAAAAAATAAAAATAAAAAT	CCTTGTTGAGGAATCGGTGT	TTTTTCACATCGCAAATCCA	60.1	279	INTERGENIC	--	--	--
Ca4_P2_29581998	Ca4	29581998	TTATAT/TTATATATAT	AACTTGATGCCGAGTAGCGT	AGGCCGTCACAAATGGATAG	60.0	366	INTERGENIC	--	--	--
Ca4_P2_29582409	Ca4	29582409	TAAAA/TAAA	AGCTGTATTGGGCCTCCTTT	GCCGAATAAAACCCTCACAA	59.9	212	INTERGENIC	--	--	--
Ca4_P2_29583344	Ca4	29583344	A/AAACATG	TTGGCAAGATTGAAAAACGA	TAAGAAGGGAGCAATCACGC	60.4	780	INTERGENIC	--	--	--
Ca4_P2_29585533	Ca4	29585533	ATTTTTTTTTT/ATTTTTTTTTTT	CGAAAGTTTGGGAAACGAAA	AAAGCTTGCCATCATTCCAC	60.1	915	INTERGENIC	--	--	--
Ca4_P2_29596763	Ca4	29596763	GAACAACAACAACAA/GAACAACAACAACAACAACAA	TATACGTGCCACAACGATGC	CGGTCACCATATGTCACTTCA	59.4	816	INTERGENIC	--	--	--
Ca4_P2_29596950	Ca4	29596950	TTTTATTTATTTATTTATTTATTTATTTATTTAT/TTTTATTTATTTATTTATTTATTTATTTATTTATTTATTTATTTAT	CCTGTTTTTGAACAAGCCAAA	CGGTCACCATATGTCACTTCA	59.4	399	INTERGENIC	--	--	--
Ca4_P2_29619182	Ca4	29619182	AG/A	TTGTCGTGGTGATTAATGAAAAA	GCCGACACCTTCAATCAAAT	59.9	751	INTERGENIC	--	--	--
Ca4_P2_29622506	Ca4	29622506	ATTTTTTT/ATTTTTTTTT	CCCCACGATCCTGAAATAAA	AACGATGATCGCAACAACAA	60.1	281	INTERGENIC	--	--	--
Ca4_P2_29624385	Ca4	29624385	ATT/ATTTT	ACCGAACCTGTTTGGATTTG	GGGGAACCAAAACCTTGAAT	60.0	537	INTERGENIC	--	--	--
Ca4_P2_29626397	Ca4	29626397	CTTTTTTTTTTT/CTTTTTTTTT	ACCCCATTTCATCCTCATCA	CTTAGTGAGTGGAGAGCGCC	60.2	398	INTERGENIC	--	--	--

INDEL marker IDs	Chromosomes/unanchored scaffolds	Physical positions (bp)	InDels (*Kabuli* reference genome-CDC Frontier/PII)	Forward primers (5'-3')	Reverse primers (5'-3')	Anneling temperature (0C)	Expected amplified product size (bp)	Structural annotation		Functional annotation	
								Sequence components of *kabuli* genome	*Kabuli* gene accession IDs	NCBI-KOG	TFs
Ca4_P2_29626809	Ca4	29626809	CTTTTTTTT/CTTTTTTT	CTAGGAGAAGGGGGATTTGG	TGTGTTTGAATTCTTGATTGGG	59.8	239	INTERGENIC	--	--	--
Ca4_P2_29640045	Ca4	29640045	TAAAGA/TAAAGAAAGA	GCCTGTCTGCATTGTTTCCT	CGGACAGCAACAATTTCCTT	60.1	753	INTERGENIC	--	--	--
Ca4_P2_29640624	Ca4	29640624	GATCAATGAAAATCAATGA/GATCAATGA	AAGGAAATTGTTGCTGTCCG	CAATTGAGTTAGGGCCCATT	58.9	355	INTERGENIC	--	--	--
Ca4_P2_29641443	Ca4	29641443	ATTT/ATTTT	TGGAGCAATGATCTTCCCTT	TCAAGCAAGAATCCAAGTCAAA	59.9	608	INTERGENIC	--	--	--
Ca4_P2_29648827	Ca4	29648827	CTTTT/CTTT	GCCTTCGGTTTATTGTTGGA	TGGAAATAGGCCATACCGAG	59.9	714	INTERGENIC	--	--	--
Ca4_P2_29650151	Ca4	29650151	CATA/CA	TCACCACAATCTAGCGATGC	TAGCAAGCTTGGTGCATTTG	60.0	558	INTERGENIC	--	--	--
Ca4_P2_29650782	Ca4	29650782	A/AT	ATTTTCCAGGTGCATTTTCG	CCTAGAGCTTGGTTTGGCAG	60.0	492	INTERGENIC	--	--	--
Ca4_P2_29654133	Ca4	29654133	CA/CAAAA	TGGCGTTGCAACACTAATTC	GATGAGGATGTGGAAGGCTG	60.6	983	INTERGENIC	--	--	--
Ca4_P2_29656227	Ca4	29656227	ACCCC/ACCC	GGACGATAATGCCCTTTGAA	TGGGAATTTATCCGCAAAAG	59.9	308	INTERGENIC	--	--	--
Ca4_P2_29658881	Ca4	29658881	TGAGGAG/TGAG	CTTTTGAGGCATTCCACCAT	ATTTTGTTGGGGTGCTCTTG	60.0	176	INTERGENIC	--	--	--
Ca4_P2_29661405	Ca4	29661405	CT/CTAT	TGCCTTATCAACGGCTCTCT	GGAGACAACCCCAGACAGAA	60.1	495	INTERGENIC	--	--	--
Ca4_P2_29661513	Ca4	29661513	CTTT/CTT	TGCCTTATCAACGGCTCTCT	GGAGACAACCCCAGACAGAA	60.1	495	INTERGENIC	--	--	--

INDEL marker IDs	Chromosomes/unanchored scaffolds	Physical positions (bp)	InDels (*Kabuli* reference genome-CDC Frontier/PII)	Forward primers (5'-3')	Reverse primers (5'-3')	Anneling temperature (0C)	Expected amplified product size (bp)	Structural annotation		Functional annotation	
								Sequence components of *kabuli* genome	*Kabuli* gene accession IDs	NCBI-KOG	TFs
Ca4_P2_29670240	Ca4	29670240	TAAAAAA/TAAAAAAA	TGGGGGATACCATTCTTTGA	TGCCCTTGAGCTTTGAGTTT	60.0	479	INTERGENIC	--	--	--
Ca4_P2_29671539	Ca4	29671539	GTTATGG/GTTATGGACTTATGG	CACTTGTGTTGGAACGTTGG	TGCATGTCAGCCATACCAAT	60.0	158	DRR	Ca_14166	Z	MYB_related
Ca4_P2_29681493	Ca4	29681493	G/GT	ATCCCCCACACGATGAATAA	CCAAAGAGCTGAGCGAAAAT	59.6	404	INTRON	Ca_14166	Z	MYB_related
Ca4_P2_29684929	Ca4	29684929	ATTTTTTTTT/ATTTTTTTTTT	GCCCTGGCCTAAGAATAACC	CAAGCCAACAGAACCTGGAT	60.1	562	INTRON	Ca_14166	Z	MYB_related
Ca4_P2_29688505	Ca4	29688505	C/CA	TGGATGCCAGTATCAACCTG	TGGTGGTTGTTTGTGATGCT	60.0	604	INTRON	Ca_14166	Z	MYB_related
Ca4_P2_29690022	Ca4	29690022	GAAAAAAAA/GAAAAAAAAAA	GAGGATAAGCCATGGATGTCA	AGCAAGCCAAGAGAATCGAA	60.1	524	INTRON	Ca_14166	Z	MYB_related
Ca4_P2_29696963	Ca4	29696963	AGGTGGT/AGGTGGTTTGGTGGT	TCTGCAGTTTTCGATGTTCG	GCTACCGGAGTTGATTGGAA	60.1	482	INTRON	Ca_14166	Z	MYB_related
Ca4_P2_29697671	Ca4	29697671	GAATCGGCCT/GAATCGGCCTCTAAATCGGCCT	AACGTATTCATCACCAGCCA	ACCGACAATGCACATTCCTT	60.4	458	INTRON	Ca_14166	Z	MYB_related
Ca4_P2_29699958	Ca4	29699958	TTG/TTGGATG	TGGCCTTATTTGCTTTTTGG	CCAACATCATGCTCTCCAAC	59.1	446	INTERGENIC	--	--	--
Ca4_P2_29701374	Ca4	29701374	G/GTTTAAATTTC	AGATCTTTCCCCTCATGGCT	CCTTGACAAGAAGCAACACG	59.5	474	INTERGENIC	--	--	--
Ca4_P2_29702135	Ca4	29702135	CTTTTTTT/CTTTTTTTTT	AATTAGGTGTGGAGGGCAAA	TTTCATTTTTAAAACCCCCTCA	59.7	421	INTERGENIC	--	--	--
Ca4_P2_29702690	Ca4	29702690	AG/A	TGAGGGGGTTTTAAAAATGAAA	CTGCTACACTCTCCTTGCCC	60.0	609	INTERGENIC	--	--	--

INDEL marker IDs	Chromosomes/unanchored scaffolds	Physical positions (bp)	InDels (*Kabuli* reference genome-CDC Frontier/PII)	Forward primers (5'-3')	Reverse primers (5'-3')	Anneling temperature (0C)	Expected amplified product size (bp)	Structural annotation		Functional annotation	
								Sequence components of *kabuli* genome	*Kabuli* gene accession IDs	NCBI-KOG	TFs
Ca4_P2_29712850	Ca4	29712850	TAAAAAAAA/TAAAAAAA	TCATGTTGAGCAAAGCCAAA	TCAGTTCGGTAGTGGCTTCC	60.3	406	INTERGENIC	--	--	--
Ca4_P2_29717512	Ca4	29717512	GAAATAAATA/GAAATAAATAAATA	GTTGGCATTTCTCCACTCGT	GTGCCCAACAAACAAGGACT	60.0	458	INTERGENIC	--	--	--
Ca4_P2_29719863	Ca4	29719863	ATTTTTTT/ATTTTTTTT	GAGTTGCGCCCAAACTTTTA	AGGAACAGCAAGAACGGTGT	59.8	883	INTRON	Ca_14167	--	--
Ca4_P2_29721814	Ca4	29721814	TC/T	CCTCACCTTCCCTTTCAACA	TGTTCTTCTTTGGATGAGTGGA	59.7	649	DRR	Ca_14167	--	--
Ca4_P2_29722401	Ca4	29722401	AAT/AATAT	TTCGCAGTGGAGTTTGAATG	CGATAAAAACAAAATTGTACACCG	59.7	550	DRR	Ca_14167	--	--
Ca4_P2_29769152	Ca4	29769152	AT/ATT	CCGATGAAGAAGAAAAAGCAA	TTGCAGTTGGATTTTGTAGGG	60.0	479	INTERGENIC	--	--	--
Ca4_P2_29776445	Ca4	29776445	TAAAAAAAAA/TAAAAAAAAAA	TATTTTGGGCCTGGTGTTGT	TGGGCCACCCTATACCAATA	60.0	729	INTRON	Ca_14170	K	HD-ZIP
Ca4_P2_29793706	Ca4	29793706	TAAAAAAAAAA/TAAAAAAAAAAA	AGATGTTGCTTCCCCAAGAA	CAAAAGAACCTTAGCTTCCCC	59.3	718	INTERGENIC	--	--	--
Ca4_P2_2979984	Ca4	2979984	ATTTTTTTTTTT/ATTTTTTTTTT	TGCCATTCCACCACTAACAA	GCTTTTGGCGTATGTTTTGG	60.5	512	INTERGENIC	--	--	--
Ca4_P2_29811877	Ca4	29811877	CCAAGAGT/C	TAGGGCACAAGTCTCCCAAG	ACTTTCATGAAGACACCGGG	60.0	212	INTERGENIC	--	--	--
Ca4_P2_29821605	Ca4	29821605	CTTTTTTTT/CTTTTTTT	GACTCATCCAAGGTTGCCAT	TTTGATGGCTCAGAAAGCCT	60.0	524	INTERGENIC	--	--	--
Ca4_P2_29826827	Ca4	29826827	GTGTTGTT/GTGTT	CGAACCTACGAGGCTAAACG	GGGTGTCGAAACATTCCATC	60.2	290	INTERGENIC	--	--	--

INDEL marker IDs	Chromosomes/unanchored scaffolds	Physical positions (bp)	InDels (*Kabuli* reference genome-CDC Frontier/PII)	Forward primers (5'-3')	Reverse primers (5'-3')	Anneling temperature (0C)	Expected amplified product size (bp)	Structural annotation		Functional annotation	
								Sequence components of *kabuli* genome	*Kabuli* gene accession IDs	NCBI-KOG	TFs
Ca4_P2_29864003	Ca4	29864003	ATTTTTTTTTTT/ATTTTTTTTTT	CAAAGTGATCCAACTTCTGCTG	CTTCTTGACAAAAGCGGGAG	60.0	861	INTERGENIC	--	--	--
Ca4_P2_29866382	Ca4	29866382	CAAA/CAA	TGGGGAGGAAGTTGACTCAG	CTTCGCACCTCTATCTTGCC	60.0	465	INTERGENIC	--	--	--
Ca4_P2_2986997	Ca4	2986997	ATTTTTTTTTTT/ATTTTTTTTTT	TTTGCGTTACGAATTTGATGA	GTAATGTTCCCACGTTGCTG	59.1	261	INTERGENIC	--	--	--
Ca4_P2_29876041	Ca4	29876041	CTT/CTTT	TCGGTCAATGATGACGTTGT	TAATTGCAGTTTTGGTCCCC	59.8	359	INTERGENIC	--	--	--
Ca4_P2_29877079	Ca4	29877079	TAAAAAAAAAA/TAAAAAAAAA	ATGATTCGGTCTGCGTTTTC	TTGGGAGGTGGCAGATTTAG	60.1	551	INTERGENIC	--	--	--
Ca4_P2_29900525	Ca4	29900525	TTATA/TTA	AACAAAGAAGAACACGGGGT	CTTTCCTGCGCATAATCGAC	60.7	574	INTERGENIC	--	--	--
Ca4_P2_29901176	Ca4	29901176	TAAAAAAAAAA/TAAAAAAAAA	CGAATTCAATGGAGTTGCCT	TCGATCCCACCGAGAGATAC	60.0	363	INTERGENIC	--	--	--
Ca4_P2_29906707	Ca4	29906707	GAAAAAAAAAA/GAAAAAAAAAAA	ATTTGCTCCACCTTGGTTTG	AATTGGCGCAAGCTTAACTC	59.5	582	INTRON	Ca_14176	--	M-type
Ca4_P2_29907949	Ca4	29907949	AATATATATATATATATAT/AATATATATATATATAT	CGAAGCTTTGGGATTATTGC	TGGTTAAAATTAAAACAAAAGGTCA	58.2	736	INTRON	Ca_14176	--	M-type
Ca4_P2_29916991	Ca4	29916991	TC/T	GAGCAACGTCTGGTTTGGTT	GGAGGAGCAGAAGCAACAAC	60.0	363	INTERGENIC	--	--	--
Ca4_P2_29924754	Ca4	29924754	TTATATATATATA/TTATATATATATATA	GGGAATCCCCTGAAGGTAAA	AGAGGGTCAACTCTGTCCGA	59.8	915	INTERGENIC	--	--	--
Ca4_P2_29925363	Ca4	29925363	CAAAAAAA/CAAAAAAAA	ATCGGACAGAGTTGACCCTC	GAATGGGCTGGCTTGACTAA	60.2	394	INTERGENIC	--	--	--

INDEL marker IDs	Chromosomes/unanchored scaffolds	Physical positions (bp)	InDels (*Kabuli* reference genome-CDC Frontier/PII)	Forward primers (5'-3')	Reverse primers (5'-3')	Anneling temperature (0C)	Expected amplified product size (bp)	Structural annotation		Functional annotation	
								Sequence components of *kabuli* genome	*Kabuli* gene accession IDs	NCBI-KOG	TFs
Ca4_P2_29925448	Ca4	29925448	G/GC	ATCGGACAGAGTTGACCCTC	GTTTTCCAAATCGTGGCCTA	59.9	686	INTERGENIC	--	--	--
Ca4_P2_29925949	Ca4	29925949	CATATATATATATATATATATATAT/CATATATATATAT	GGCCACGATTTGGAAAACTA	TTATGGCAATATGCGATGGA	59.9	534	INTERGENIC	--	--	--
Ca4_P2_29930444	Ca4	29930444	CAAAAAAAAAA/CAAAAAAAAAAAA	CCGACACGGTCTTTTTCCTA	AATGACCCGCAATTATGAGC	59.9	392	INTERGENIC	--	--	--
Ca4_P2_29930687	Ca4	29930687	TTTGATCTT/TTT	GCTCATAATTGCGGGTCATT	AGGTATGGACAAAATTGCGG	59.8	547	INTERGENIC	--	--	--
Ca4_P2_29931076	Ca4	29931076	ATG/AGTAACAATAAGTTG	TGTGCACCAATATCTGCCAT	GAAGAAAACCCGGGATCAGT	60.3	670	INTERGENIC	--	--	--
Ca4_P2_29932586	Ca4	29932586	TAAAAAAAAAA/TAAAAAAAAAAAA	TCTACGGCGTCTAGTGGACA	AGCCCGAAAGGATCTAAAGG	59.7	487	INTERGENIC	--	--	--
Ca4_P2_29933754	Ca4	29933754	ATTTTTTT/ATTTTTTTT	AGCCACACGATGTTGATTGA	TGATTAGGGAATCACCCGTT	59.2	845	INTERGENIC	--	--	--
Ca4_P2_29934671	Ca4	29934671	ATTTT/ATTT	TTTGGCAATTCCTTGTGAGA	TTTCAAAAACCCGAATGGTC	59.8	104	INTERGENIC	--	--	--
Ca4_P2_29941572	Ca4	29941572	TAAAAAAA/TAAAAAA	CCGTGTAGGTATGATCTTTTGG	CCTTTCCTGTAAATCGGCAA	60.1	324	INTERGENIC	--	--	--
Ca4_P2_29942701	Ca4	29942701	AG/A	TCATCCCATGTAGCCATCAA	CAAAGGGTTCGTTAAAGGCA	60.1	822	INTERGENIC	--	--	--
Ca4_P2_29945039	Ca4	29945039	A/AAT	AAATGCAATAGCAGGTTGGC	CGATGAGGATAAAAGGAGCG	59.8	904	INTERGENIC	--	--	--
Ca4_P2_29961872	Ca4	29961872	TAAAAAAA/TAAAAAAAAA	ACCTGGAGAGGCATAAGCAA	CTTGGGGGTACGTAGGAGTG	59.5	577	INTERGENIC	--	--	--

INDEL marker IDs	Chromosomes/unanchored scaffolds	Physical positions (bp)	InDels (*Kabuli* reference genome-CDC Frontier/PII)	Forward primers (5'-3')	Reverse primers (5'-3')	Anneling temperature (0C)	Expected amplified product size (bp)	Structural annotation		Functional annotation	
								Sequence components of *kabuli* genome	*Kabuli* gene accession IDs	NCBI-KOG	TFs
Ca4_P2_29962434	Ca4	29962434	GTAT/GTATATAT	CTAGTCGAAGTAGCCGGTGG	TTTGGAAGCATTGTGGTTGA	60.1	486	INTERGENIC	--	--	--
Ca4_P2_29974650	Ca4	29974650	A/AACTCTTAATATTGT	GGGTTTGTAACCACACCACA	GTTGAGTTGGCCGCATTATT	60.0	416	INTERGENIC	--	--	--
Ca4_P2_29974969	Ca4	29974969	ATGAGTTCTCACCCTGAGTTCTCACCC/ATGAGTTCTCACCC	AATAATGCGGCCAACTCAAC	CCAAGAACAATCTTGGAATGC	59.6	491	INTERGENIC	--	--	--
Ca4_P2_29979575	Ca4	29979575	GAA/GAAAA	TCTCGATGTCGATTGTTGGA	TTTGGGCACCAATATCTCAA	59.0	355	INTERGENIC	--	--	--
Ca4_P2_29986196	Ca4	29986196	TA/T	AAATTATTTTTAATTGAATGGCGA	GCATGTATTCGCACTGGTTG	60.1	701	INTERGENIC	--	--	--
Ca4_P2_29986284	Ca4	29986284	A/AT	AAATTATTTTTAATTGAATGGCGA	GCATGTATTCGCACTGGTTG	60.1	701	INTERGENIC	--	--	--
Ca4_P2_29986497	Ca4	29986497	GTGCGAATACATGCG/GTGCG	AAAAAGGATATAAATACCCGCAA	AAAAATCTTCTCCACAAGATGAA	57.1	449	INTERGENIC	--	--	--
Ca4_P2_29986830	Ca4	29986830	AAATAATAATA/AAATAATA	CAACCAGTGCGAATACATGC	TGTGCTTATCCCTTTGTGACC	60.0	755	INTERGENIC	--	--	--
Ca4_P2_29987923	Ca4	29987923	GATGGTAT/GATGGTATGGTAT	GAGCAGAGCTGCCTAATGCT	GGCGTTGTTGTAGAGTGCAA	59.9	419	INTERGENIC	--	--	--
Ca4_P2_29989392	Ca4	29989392	GTATATA/GTATA	TTTTACTGCGTTGGTTGCAG	CAGAGCTGTTGTACAGGGCA	60.0	525	INTERGENIC	--	--	--
Ca4_P2_29989748	Ca4	29989748	TATACA/TA	ACAACAGCTCTGTCCCTGCT	GGGGGTTTTGGGAGATTTTA	60.0	468	INTERGENIC	--	--	--
Ca4_P2_30009279	Ca4	30009279	ATT/AT	CCCGAACCCCCTAAAAATAA	GTGCACCACTTTCGGAGAAT	60.1	463	INTERGENIC	--	--	--

INDEL marker IDs	Chromosomes/unanchored scaffolds	Physical positions (bp)	InDels (*Kabuli* reference genome-CDC Frontier/PII)	Forward primers (5'-3')	Reverse primers (5'-3')	Anneling temperature (0C)	Expected amplified product size (bp)	Structural annotation		Functional annotation	
								Sequence components of *kabuli* genome	*Kabuli* gene accession IDs	NCBI-KOG	TFs
Ca4_P2_30045432	Ca4	30045432	CATATAT/CATATATAT	GCGTGCATAAGTGGTTGATG	ACTCACGGTGATGAGTGTGG	59.6	345	DRR	Ca_14181	--	S1Fa-like
Ca4_P2_30046973	Ca4	30046973	GAAAAAAAAAA/GAAAAAAAAA	TGAATGAAATTTCTGGCAGG	CGTTTTGTTTGTGGCACATT	59.5	280	INTERGENIC	--	--	--
Ca4_P2_30075059	Ca4	30075059	CTAA/CTAATTGTTTTTATAA	TCGAAACCAACCACCTTTTC	GCAATTCGACAATGCACAAC	60.1	547	INTERGENIC	--	--	--
Ca4_P2_30076682	Ca4	30076682	ATTTTTTTTT/ATTTTTTTTTTT	GCACTTCCGCTAAAAGCATC	AAGCGCTCCCAAATATGCTA	59.8	432	INTERGENIC	--	--	--
Ca4_P2_30077034	Ca4	30077034	TAAATTAAAATTAAAAT/TAAATTAAAATTAAAATTAAAAT	GCATATTTGGGAGCGCTTTA	TGGATGATTGTGACAAAATTGC	60.7	508	INTERGENIC	--	--	--
Ca4_P2_30077264	Ca4	30077264	ATTTTTT/ATTTTTTT	GCATATTTGGGAGCGCTTTA	TGGATGATTGTGACAAAATTGC	60.7	508	INTERGENIC	--	--	--
Ca4_P2_30077287	Ca4	30077287	CTTTTTTTT/CTTTTTTTTTT	GCATATTTGGGAGCGCTTTA	TGGATGATTGTGACAAAATTGC	60.7	508	INTERGENIC	--	--	--
Ca4_P2_30078781	Ca4	30078781	AATATA/AATA	GATCCCTCATTGCGAACATT	TGAGGGATGCACATGAAAAA	60.0	507	INTERGENIC	--	--	--
Ca4_P2_30081791	Ca4	30081791	CCTT/CCTTTCTT	TTGTCGTTGCAATGGTTCAT	TTCACAAAAGGGGGTGAAAG	59.9	467	INTERGENIC	--	--	--
Ca4_P2_30082739	Ca4	30082739	AAAAACAAAACAG/AAAAACAAAACAGTGGAAAACAAAACAG	TGCGTTGTATCATGTCGGTT	TCTGGTTTGTTCCTCCGTTT	59.6	389	INTERGENIC	--	--	--
Ca4_P2_30083166	Ca4	30083166	ATTTTTTTTTTTT/ATTTTTTTTT	AACGGAGGAACAAACCAGAA	CAACCTCGATCTCGACCAAT	60.1	790	INTERGENIC	--	--	--
Ca4_P2_30085065	Ca4	30085065	A/AAATATC	TGACCACCACAATGCAACTT	ATTGGTTGCATATCGGCTTC	59.9	480	INTERGENIC	--	--	--

INDEL marker IDs	Chromosomes/unanchored scaffolds	Physical positions (bp)	InDels (*Kabuli* reference genome-CDC Frontier/PII)	Forward primers (5'-3')	Reverse primers (5'-3')	Anneling temperature (0C)	Expected amplified product size (bp)	Structural annotation		Functional annotation	
								Sequence components of *kabuli* genome	*Kabuli* gene accession IDs	NCBI-KOG	TFs
Ca4_P2_30085385	Ca4	30085385	CAA/C	TCTCATGAGGTGCATTGGAA	TCGTTGCAACTGTTGTTTCAT	59.2	471	INTERGENIC	--	--	--
Ca4_P2_30085998	Ca4	30085998	AATTTAGTTGTATTTAGTTGTAT/AATTTAGTTGTAT	TTCGAAAATTTTGGTTCCTCA	CGCAAGAAAGTCCACATCAA	59.8	632	INTERGENIC	--	--	--
Ca4_P2_30086302	Ca4	30086302	TTTTCATG/TTTTCATGCAAACTTTCATG	TTGATGTGGACTTTCTTGCG	TTTTCGGCGTAAAACCTTTG	60.1	238	INTERGENIC	--	--	--
Ca4_P2_30086898	Ca4	30086898	ACTTTCT/ACT	ACGCCGAAAAACACTTTCAC	TCCATTCGACTGTCCTTCAA	59.2	630	INTERGENIC	--	--	--
Ca4_P2_30087680	Ca4	30087680	CTTTTTTTTT/CTTTTTTTTTT	AACTCACCCAGCCAAACAAC	TGCAAACCCAACCTAACAAA	59.1	116	INTERGENIC	--	--	--
Ca4_P2_30089605	Ca4	30089605	TA/TAA	ACGCACTTTGGACGTTTTTC	ATGTGAATCAGGGGCAGTGT	60.4	915	INTERGENIC	--	--	--
Ca4_P2_30090101	Ca4	30090101	TA/TAA	TCGAGCGCGTATCCTCTAAT	TTGGTCAAACCCCTATCAGC	59.9	524	INTERGENIC	--	--	--
Ca4_P2_30094188	Ca4	30094188	ATTTTTTTTT/ATTTTTTTTTT	TCCCATGCCAACATGTCTAA	GTTTGAATTTGCAGCACCCT	60.1	370	INTERGENIC	--	--	--
Ca4_P2_30097465	Ca4	30097465	GAAA/GAA	GCAAAACGGGGACTAGAACA	CCAGGGGTTGCCTTAGATTT	60.3	307	INTERGENIC	--	--	--
Ca4_P2_30098960	Ca4	30098960	ATTTTTTTT/ATTTTTTTTT	TGGCGAATCGTTGATCATAA	ATATGGCTTGAAGCAAACGC	60.2	697	INTERGENIC	--	--	--
Ca4_P2_3010101	Ca4	3010101	ATTT/ATTTT	AGCATGGTTAAGCCCTGTGT	CGTAGGGGAAAAATGCAAAA	59.9	527	INTRON	Ca_12099	--	--
Ca4_P2_30102122	Ca4	30102122	CTCTTCCAAT/CTCTTCCAATTCTTCCAAT	TGATTCTTCAATTGCCACCA	ACGAGAGACAAGTGAGGGGA	59.8	580	INTERGENIC	--	--	--

INDEL marker IDs	Chromosomes/unanchored scaffolds	Physical positions (bp)	InDels (*Kabuli* reference genome-CDC Frontier/PII)	Forward primers (5'-3')	Reverse primers (5'-3')	Anneling temperature (0C)	Expected amplified product size (bp)	Structural annotation		Functional annotation	
								Sequence components of *kabuli* genome	*Kabuli* gene accession IDs	NCBI-KOG	TFs
Ca4_P2_30102440	Ca4	30102440	TG/T	TCCCCTCACTTGTCTCTCGT	GTGTTTGGGATTGGGAAATG	60.0	609	INTERGENIC	--	--	--
Ca4_P2_30118361	Ca4	30118361	AAAA/AAAATAAA	TCCTTCATTTCCTCCCTCCT	GTGGATAAGAGACGCAAGGG	59.7	114	INTERGENIC	--	--	--
Ca4_P2_30118491	Ca4	30118491	CGG/CG	TCCTTCATTTCCTCCCTCCT	TGCGTGAATTTTGACTTTTGA	59.3	282	INTERGENIC	--	--	--
Ca4_P2_30118694	Ca4	30118694	CATAGATA/CATAGATAGATA	CTCCTTCATTTCCTCCCTCC	AAGGTGGGTTAAAGAACCGC	60.4	778	INTERGENIC	--	--	--
Ca4_P2_30120661	Ca4	30120661	TAAAA/TAAA	TTTGACATCCATCTTATGCAGC	CCCTTGGGTGCGTTAGTTAT	59.0	379	INTERGENIC	--	--	--
Ca4_P2_30122560	Ca4	30122560	GAA/GAAAA	ATGTCAATGGGCCACTTAGC	GTGGGGCCATTGATGAGTAA	60.7	962	INTERGENIC	--	--	--
Ca4_P2_30122953	Ca4	30122953	AC/A	TGTTGTGGGAGGTGATTTGA	GGGTCAAACCTCAAAGACGA	60.1	862	INTERGENIC	--	--	--
Ca4_P2_30125286	Ca4	30125286	ATT/ATTT	ATCTGGCAACGAGGTTCATC	ACCGCCAATCTACCATCATC	59.8	666	INTERGENIC	--	--	--
Ca4_P2_30125897	Ca4	30125897	TAAAA/TAAAAAAA	ATCGAAAACACCATAAGCCG	TTATGCAAGTGTCTGCCGAG	60.0	330	INTERGENIC	--	--	--
Ca4_P2_30134439	Ca4	30134439	CAAAAAA/CAAAA	TCCTTTTGCATTACTATCCAAGC	GGGGGTTCCATGTAAAACATT	59.8	419	INTERGENIC	--	--	--
Ca4_P2_30139802	Ca4	30139802	GAAAAAAAAA/GAAAAAAAA	TCTTCAGGAAATGGCACACA	CACAGAGCAACCCAAACAAA	59.7	619	INTERGENIC	--	--	--
Ca4_P2_30141273	Ca4	30141273	T/TG	TGAACCTGGACCAAAACCTC	TCAAAATTCACTTGGTCATTGTTC	60.3	871	INTERGENIC	--	--	--

INDEL marker IDs	Chromosomes/unanchored scaffolds	Physical positions (bp)	InDels (*Kabuli* reference genome-CDC Frontier/PII)	Forward primers (5'-3')	Reverse primers (5'-3')	Anneling temperature (0C)	Expected amplified product size (bp)	Structural annotation		Functional annotation	
								Sequence components of *kabuli* genome	*Kabuli* gene accession IDs	NCBI-KOG	TFs
Ca4_P2_30141789	Ca4	30141789	TTAT/TT	GAACAATGACCAAGTGAATTTTGA	TATGGGATGCGTTGAGATGA	60.0	428	INTERGENIC	--	--	--
Ca4_P2_30142421	Ca4	30142421	AGATACTTATATGAT/AGAT	TCATCTCAACGCATCCCATA	CGTTGCAACTGAACACATACA	58.3	827	INTERGENIC	--	--	--
Ca4_P2_30142701	Ca4	30142701	ATT/ATTTAAAGTTTCAATTTTT	TTTCCCATGCAAAGTTAGGC	AAGGAAAGGGGACGTGTCTT	60.0	755	INTERGENIC	--	--	--
Ca4_P2_30143750	Ca4	30143750	GTTT/GTT	TAAGAAACCCCCAATGATGC	GGGATGGATCAAACACAAGG	60.2	170	INTERGENIC	--	--	--
Ca4_P2_30144088	Ca4	30144088	ATTTTTT/ATTTTT	AGGTTTGGGATGCCTTTCTT	GAGGATGCAGCGTAAAGTCA	59.0	726	INTERGENIC	--	--	--
Ca4_P2_30148542	Ca4	30148542	ATTTTTTTT/ATTTTTTTTT	GTTGTTGCTGTGAGTGCTCC	TTAGTCCCTTACGTCGTGGG	60.0	302	INTRON	Ca_14185	--	--
Ca4_P2_30170493	Ca4	30170493	TCTTTA/T	TCTCTCCGGCAAGAAACACT	CATGGTTCAGATCATGCTGC	60.2	719	INTERGENIC	--	--	--
Ca4_P2_30172004	Ca4	30172004	CG/CGG	CTCAACAGCCTTATGTGCGA	GCCTTGCAAGACAAAAATCC	59.7	182	INTERGENIC	--	--	--
Ca4_P2_3017713	Ca4	3017713	ATTATTTTAAATTTATTTTAAATTTATTTTAAATTTATTTTAAATTTATTTTAAATT/ATTATTTTAAATTTATTTTAAATTTATTTTAAATTTATTTTAAATTTATTTTAAATTTATTTTAAATT	GACAAATTAGTGGGGCAACC	TTGTATTGTTGGTGAGGCCA	60.0	902	INTERGENIC	--	--	--
Ca4_P2_30178438	Ca4	30178438	AAATTTCAATATTTAATTTCAATAT/AAATTTCAATATTTAATTTCAATATTTAATTTCAATAT	CAAGCAATTATGGTGCAACTG	AGTGAGGCAAATGGAAATGG	59.9	669	INTERGENIC	--	--	--
Ca4_P2_30178760	Ca4	30178760	TAAAAAAA/TAAAAAA	CCATTTCCATTTGCCTCACT	GAGCCACTTCTCCCCTTTCT	59.8	559	INTERGENIC	--	--	--
Ca4_P2_30184517	Ca4	30184517	TA/T	CGAGGGGCGTAAAAATCATA	GCGAGATTTGTTTCCGTTGT	60.1	579	INTERGENIC	--	--	--

INDEL marker IDs	Chromosomes/unanchored scaffolds	Physical positions (bp)	InDels (*Kabuli* reference genome-CDC Frontier/PII)	Forward primers (5'-3')	Reverse primers (5'-3')	Anneling temperature (0C)	Expected amplified product size (bp)	Structural annotation		Functional annotation	
								Sequence components of *kabuli* genome	*Kabuli* gene accession IDs	NCBI-KOG	TFs
Ca4_P2_30184775	Ca4	30184775	TG/T	ACAACGGAAACAAATCTCGC	GGGGGTTAGGAGTAAGAAAGGA	59.8	116	INTERGENIC	--	--	--
Ca4_P2_30185691	Ca4	30185691	TAAAAAAAA/TAAAAAAAAAA	CATCGCTAACTCAGTGCCAA	AATGCGTTACGGGTGAAATG	60.8	876	INTERGENIC	--	--	--
Ca4_P2_30187625	Ca4	30187625	GCC/GCCC	CCGTTGTGTAAGAGGGGTGT	TATTTGATTGTGCAGCGAGG	59.8	628	DRR	Ca_14186	--	SAP
Ca4_P2_30189123	Ca4	30189123	AAGAG/AAG	TTCGTTCCTGAATTTTTCGG	TGTTCCGAATGGAAGACACA	60.1	391	INTERGENIC	--	--	--
Ca4_P2_30191058	Ca4	30191058	ATTAAATTT/ATT	TTAAGCATCGTGCTGTTTGG	TGTGTGGTGGCTCTAGCGTA	60.5	742	INTERGENIC	--	--	--
Ca4_P2_30192150	Ca4	30192150	GCATTTT/G	AACGCATTTTGTTGATATCCG	CGATTGACCAATGCTAACCC	60.3	835	INTERGENIC	--	--	--
Ca4_P2_30196284	Ca4	30196284	AAT/A	TCAAACCATGATTTTGGTGC	TGTCAAACAACCTCTTGGCT	58.3	112	INTERGENIC	--	--	--
Ca4_P2_30197320	Ca4	30197320	CAAAAA/CAAAAAA	CTTGTCCTTTTTCCCATCCA	GGCCTAATTAAACATGGGCA	59.8	793	INTERGENIC	--	--	--
Ca4_P2_30202940	Ca4	30202940	G/GC	GTGCCATGTCCTTGTCTCCT	GTCTAATGTTGGTTGCGTGC	59.2	546	INTERGENIC	--	--	--
Ca4_P2_30205470	Ca4	30205470	CTAGAACT/C	CGTTTGAAATGTCACCCCTT	TCAATGCACTTCTGAGCCAC	60.0	385	INTERGENIC	--	--	--
Ca4_P2_30206114	Ca4	30206114	ATTTTTTT/ATTTTTT	GTGGCTCAGAAGTGCATTGA	TTGCAAGCATCCATATCCAA	60.0	832	DRR	Ca_14187	--	--
Ca4_P2_30206517	Ca4	30206517	ACCCCCC/ACCCC	TCCCTCGAGCTATAGGATGC	ATGCAATGGTTTATTCCCCA	60.0	643	DRR	Ca_14187	--	--

INDEL marker IDs	Chromosomes/unanchored scaffolds	Physical positions (bp)	InDels (*Kabuli* reference genome-CDC Frontier/PII)	Forward primers (5'-3')	Reverse primers (5'-3')	Anneling temperature (0C)	Expected amplified product size (bp)	Structural annotation		Functional annotation	
								Sequence components of *kabuli* genome	*Kabuli* gene accession IDs	NCBI-KOG	TFs
Ca4_P2_30209458	Ca4	30209458	CTTTTTTTTT/CTTTTTTTTTT	TGGATGTGGCTTTCTGTTGA	TACTGCCCCATAAGCAAACC	60.0	566	INTERGENIC	--	--	--
Ca4_P2_30210048	Ca4	30210048	ATATCACTTT/AT	GGTTTGCTTATGGGGCAGTA	CACCCCGAATGAACTTTAGAA	59.1	631	INTERGENIC	--	--	--
Ca4_P2_30211443	Ca4	30211443	T/TA	ACATCAAACAAACAGCGCAC	CCGCTAAAATTGCCCAATAA	59.9	714	INTERGENIC	--	--	--
Ca4_P2_30213725	Ca4	30213725	GAAAAAAA/GAAAAAAAA	CGGCGGTGAAATAATCTGTT	TGCCACAAGAAGACAAACGA	60.4	571	INTERGENIC	--	--	--
Ca4_P2_30225198	Ca4	30225198	T/TC	AGGCAAACAAGAACCGAAAA	ATGTGCTTTTAGCACCAGGG	60.1	424	INTERGENIC	--	--	--
Ca4_P2_30226816	Ca4	30226816	CAAAAAA/CAAAA	GCTTATGCAATGCACGAAAA	CATGGGTTGAAATGGAGACA	59.3	528	INTERGENIC	--	--	--
Ca4_P2_30227136	Ca4	30227136	T/TC	TCTCCATTTCAACCCATGAA	GATGCCCTTTTCCCCTATGT	60.2	399	INTERGENIC	--	--	--
Ca4_P2_30230201	Ca4	30230201	TAAAAAAAAAAA/TAAAAAAAAA	GGTATGCTGTTTCGGTTCGT	TTCGCTAATTTTGGCAAACC	60.1	406	DRR	Ca_14188	--	--
Ca4_P2_30230521	Ca4	30230521	A/AAAG	GGTATGCTGTTTCGGTTCGT	TTCGCTAATTTTGGCAAACC	60.1	406	DRR	Ca_14188	--	--
Ca4_P2_30240512	Ca4	30240512	ATTTTTTTTTT/ATTTTTTTTT	TTGGGCATTTTGATGATTGA	GACAACCGATTGAGCCTGAT	60.1	539	INTERGENIC	--	--	--
Ca4_P2_30252991	Ca4	30252991	TAAAAAAAAAAAA/TAAAAATAAAAAAAAAAAAAA	GGGCAGCGAACTTAGTCAAG	CGGTGGAAAAGTTTCGGATA	59.9	410	INTERGENIC	--	--	--
Ca4_P2_30329942	Ca4	30329942	AACACACACACACACACA/AACACACACACACACA	GCTAGCTTCCGACACTCCAG	GCAGTATGTAATCTAGCCATCGAA	59.7	514	INTERGENIC	--	--	--

INDEL marker IDs	Chromosomes/unanchored scaffolds	Physical positions (bp)	InDels (*Kabuli* reference genome-CDC Frontier/PII)	Forward primers (5'-3')	Reverse primers (5'-3')	Anneling temperature (0C)	Expected amplified product size (bp)	Structural annotation		Functional annotation	
								Sequence components of *kabuli* genome	*Kabuli* gene accession IDs	NCBI-KOG	TFs
Ca4_P2_30330570	Ca4	30330570	CTATATATATATATATATATA/CTATATATATATATATATATATATA	TTCGATGGCTAGATTACATACTGC	CCCCTCAACTCAAGAATGATG	59.5	561	INTERGENIC	--	--	--
Ca4_P2_30351459	Ca4	30351459	CTTTTTTT/CTTTTTTTT	TCTTTGAAACACATCACTTTCG	AAAATTTGATCGGTGGTGGA	60.2	314	INTERGENIC	--	--	--
Ca4_P2_30363670	Ca4	30363670	ATTTTTTTTTT/ATTTTTTTTTTTT	AATCTTCCTGGTGGTGCAAG	AGGAGTGTCAAAATGCACCC	60.0	542	INTERGENIC	--	--	--
Ca4_P2_30371345	Ca4	30371345	GAAAAAAAAA/GAAAAAAAAAA	TCACGTGCTTGCATTTCTTC	TCACCGGAAATTTCATTCGT	60.3	224	INTERGENIC	--	--	--
Ca4_P2_30390669	Ca4	30390669	T/TA	CTACTGAAGCCCAACGAAGG	CTTTCAGATTCGTGGCCATT	60.1	864	INTERGENIC	--	--	--
Ca4_P2_30404870	Ca4	30404870	AATAACAT/AAT	TTAACTGTTGGTGCGCAGTC	CGTCACATTTAACACGCCAT	59.5	798	INTERGENIC	--	--	--
Ca4_P2_30414673	Ca4	30414673	ATTT/ATT	TTTGGAAGACCATTGCTTGA	CTCCCGCCTCCCTATATGTT	60.3	234	INTERGENIC	--	--	--
Ca4_P2_30415073	Ca4	30415073	GCATCATCATCATCA/GCATCATCATCATCATCA	TTGAACATATAGGGAGGCGG	CCATCCTTTTCAATGCGAGT	60.1	423	CDS (large-effect mutations)	Ca_14197	--	--
Ca4_P2_30416417	Ca4	30416417	TCC/TCCC	GCAGGGATGTGCTTGTGTTA	CGACAAAGCACCCCAATAAA	60.9	593	INTRON	Ca_14197	--	--
Ca4_P2_30421550	Ca4	30421550	ATTTTTTTT/ATTTTTTT	AGAGCGACAAATGTGTTAGGG	GGACCGTCAAAAGTACAATGTT	58.0	952	INTERGENIC	--	--	--
Ca4_P2_30423088	Ca4	30423088	TAAAAAAA/TAAAAAAAA	AGGGGCAAAAATACTGCTTG	CTTGATCGGCTCATGTGTTG	60.3	938	INTERGENIC	--	--	--
Ca4_P2_30429188	Ca4	30429188	T/TGA	TGATCGCCATATCCTTAGCC	GTACCGCATGAGCAATTGTG	60.1	315	INTERGENIC	--	--	--

INDEL marker IDs	Chromosomes/unanchored scaffolds	Physical positions (bp)	InDels (*Kabuli* reference genome-CDC Frontier/PII)	Forward primers (5'-3')	Reverse primers (5'-3')	Anneling temperature (0C)	Expected amplified product size (bp)	Structural annotation		Functional annotation	
								Sequence components of *kabuli* genome	*Kabuli* gene accession IDs	NCBI-KOG	TFs
Ca4_P2_30429374	Ca4	30429374	TATTCATT/TATT	TGATCGCCATATCCTTAGCC	ATTTGGTTTGGCATTGTTGC	60.7	811	INTERGENIC	--	--	--
Ca4_P2_30430477	Ca4	30430477	AATATATATATATATATATATATATATATAT/AATATATATATATATATATATATAT	CCCCTTCTCGTTTTTCAACA	TTCATGCGTGTCCGATTTTA	60.1	643	INTERGENIC	--	--	--
Ca4_P2_30451525	Ca4	30451525	GTTTTTTTTT/GTTTTTTTTTT	GGATGTGAGTCCAGTCCAAAA	ATTCATGCGCACAATCTGAA	60.2	427	INTERGENIC	--	--	--
Ca4_P2_30451648	Ca4	30451648	TCC/TC	TCAGCAATTCAGATTGTGCG	AGGGTGGAAAGCAAGATTCC	60.4	167	INTERGENIC	--	--	--
Ca4_P2_30452448	Ca4	30452448	AAT/AATACCTTATATAT	TGAAGCAGATACCCAGACGA	TGCTATTGTTGTTGATGATGGA	59.0	705	INTERGENIC	--	--	--
Ca4_P2_30452527	Ca4	30452527	ATAAATATTAAATAT/ATAAATAT	TTCATTGGTTGGACGAATTTT	CACATGGTCACGTTGCAGTT	60.6	831	INTERGENIC	--	--	--
Ca4_P2_30453156	Ca4	30453156	TAAA/TAAAA	CAACGTGACCATGTGGATTC	CAATAGTCACCGCCAACCTT	60.0	541	INTERGENIC	--	--	--
Ca4_P2_30453969	Ca4	30453969	AT/A	GTGTTCGCGATATCTGGGTT	ATTCTATCACACAGCGCACG	59.9	545	INTERGENIC	--	--	--
Ca4_P2_30458112	Ca4	30458112	C/CCTT	CAGGCAATGGGACATGACTT	AAGATTCGCTTTGATACGTGG	59.2	830	INTERGENIC	--	--	--
Ca4_P2_30462755	Ca4	30462755	GGAA/G	GAATTTTATTGCGATTTCATGG	TCGGCTAAATTACATTCGTGG	60.0	513	DRR	Ca_14198	--	G2-like
Ca4_P2_30465071	Ca4	30465071	TAAA/TAA	CAAGCAAATTTGTGAGCACC	TCAAGCGACTCAAATCGTGA	60.5	399	INTERGENIC	--	--	--
Ca4_P2_30466296	Ca4	30466296	AAATAATAA/AAATAATAATAA	TGATGGGTTTGGATGTGCTA	GTTGTAGGGGCAACTGAGGA	60.1	575	INTRON	Ca_14199	--	G2-like

INDEL marker IDs	Chromosomes/unanchored scaffolds	Physical positions (bp)	InDels (*Kabuli* reference genome-CDC Frontier/PII)	Forward primers (5'-3')	Reverse primers (5'-3')	Anneling temperature (0C)	Expected amplified product size (bp)	Structural annotation		Functional annotation	
								Sequence components of *kabuli* genome	*Kabuli* gene accession IDs	NCBI-KOG	TFs
Ca4_P2_30468296	Ca4	30468296	AAT/AATAT	ATTGTGCATATTCCCTCCCA	CCTTAATCCACCAAAAGCCA	59.9	747	DRR	Ca_14199	--	G2-like
Ca4_P2_30468822	Ca4	30468822	AAA/AAAGAA	TGGCTTTTGGTGGATTAAGG	GGTCTCACGACCAAGTCACA	59.7	438	DRR	Ca_14199	--	G2-like
Ca4_P2_30472727	Ca4	30472727	ATT/AT	ATCAGGAACGAGGTTGGATG	GAGGGTTGTTGTCTCCCAAA	59.9	653	DRR	Ca_14200	--	--
Ca4_P2_30477859	Ca4	30477859	CTTTTT/CTTTTTT	AAAATCAATCGGGGACATCA	GAATCAAACAAAGCCCCAAA	59.9	648	INTERGENIC	--	--	--
Ca4_P2_30482085	Ca4	30482085	TGAA/TGAAGTTTAGAA	TTTGGAACAGAGGTTTTGCC	GGGTATGGGGACCAATTTTT	59.8	720	DRR	Ca_14201	--	SBP
Ca4_P2_3049939	Ca4	3049939	CATATATATATATATA/CATATATATATATATATA	GCCGAATGGACCAAACATAC	GGCAACAAAATAGAATCGGG	59.4	516	INTERGENIC	--	--	--
Ca4_P2_30501632	Ca4	30501632	AGT/A	TGGGGATTTATCCCTTCAAA	GCAAGTGTCAACTCACTCAATCA	60.3	595	INTERGENIC	--	--	--
Ca4_P2_30502836	Ca4	30502836	ATTTTTTTT/ATTTTTTT	AATGTAGCATGTGCGTAGCG	CAATTCATTTGATTGTGTTGCAT	59.8	303	INTERGENIC	--	--	--
Ca4_P2_30524382	Ca4	30524382	GAAAAAAAAAA/GAAAAAAAAAAA	ATCCCCTCTCTACACCCACC	TTGAACGCCTCCTCTGAGTT	60.0	908	INTERGENIC	--	--	--
Ca4_P2_30535832	Ca4	30535832	TATAAATAAATAAATAAA/TATAAATAAATAAATAAATAAATAAATAAA	CCATTCAGAATTAGAAAATCACCA	CCAAATTTCCCAAGGGTTCT	60.2	582	INTERGENIC	--	--	--
Ca4_P2_30536932	Ca4	30536932	GTTTTTTTTT/GTTTTTTTTTTTTT	ATCATTGAACGACCGGAAAC	CGGATACAATGGCAATCAAA	59.4	431	INTRON	Ca_14204	--	NAC
Ca4_P2_30537523	Ca4	30537523	CA/CATA	TTTGATTGCCATTGTATCCG	CAAAATGGAAATGCAAGAACAA	60.0	803	INTRON	Ca_14204	--	NAC

INDEL marker IDs	Chromosomes/unanchored scaffolds	Physical positions (bp)	InDels (*Kabuli* reference genome-CDC Frontier/PII)	Forward primers (5'-3')	Reverse primers (5'-3')	Anneling temperature (0C)	Expected amplified product size (bp)	Structural annotation		Functional annotation	
								Sequence components of *kabuli* genome	*Kabuli* gene accession IDs	NCBI-KOG	TFs
Ca4_P2_30538166	Ca4	30538166	AAAGAAG/AAAGAAGAAG	TTGTTCTTGCATTTCCATTTTG	CGCCATCTTCCTCTTCTTTG	59.9	380	CDS (large-effect mutations)	Ca_14204	--	NAC
Ca4_P2_30539496	Ca4	30539496	G/GCAA	CCGTGTAGCCAATTTCACCT	TACTCCAGGTTCTGCCCTTG	60.2	517	DRR	Ca_14204	--	NAC
Ca4_P2_30539573	Ca4	30539573	A/AATTG	CCGTGTAGCCAATTTCACCT	TACTCCAGGTTCTGCCCTTG	60.2	517	DRR	Ca_14204	--	NAC
Ca4_P2_30540068	Ca4	30540068	ATTTTTTT/ATTTTTTTT	GATTTATCGTCGCTCTTGCC	CAACTGGATCTGCCACGTTA	59.7	268	DRR	Ca_14204	--	NAC
Ca4_P2_30542137	Ca4	30542137	TAAAAAA/TAAAAAAA	TAAGGCATGATAAGGCCGAC	GTCATGTCACCCGTGATGAG	60.0	299	DRR	Ca_14205	--	--
Ca4_P2_30542725	Ca4	30542725	AT/A	ACTCATCACGGGTGACATGA	CCGAGTATGATGGTTGGCTT	60.0	767	DRR	Ca_14205	--	--
Ca4_P2_30542760	Ca4	30542760	AT/A	TGCCCAAGCTCAATTAATCC	CCGAGTATGATGGTTGGCTT	60.0	362	DRR	Ca_14205	--	--
Ca4_P2_30545976	Ca4	30545976	C/CTTACA	CCAAAGACGAGGTTGACCAT	TGGCATTCACAATTCGAAAA	60.1	423	INTERGENIC	--	--	--
Ca4_P2_30551689	Ca4	30551689	ATTT/ATTTT	CGGAAATGGACTTTACGAATG	TGATATGGCAATCCCTGACA	59.9	548	INTERGENIC	--	--	--
Ca4_P2_30552249	Ca4	30552249	GTTTTTTT/GTTTTTTTT	TGTCGCATGCAACCTCTTAG	ACCTGTGTGTTCCCTTCCAC	59.9	528	INTERGENIC	--	--	--
Ca4_P2_30552355	Ca4	30552355	TG/T	TGTCGCATGCAACCTCTTAG	ACCTGTGTGTTCCCTTCCAC	59.9	528	INTERGENIC	--	--	--
Ca4_P2_30552695	Ca4	30552695	ATTTTTTT/ATTTTTTTT	CCAATTGAGAAGGGAATGGA	AATGACCCAACAAAATTGCC	59.7	646	INTERGENIC	--	--	--

INDEL marker IDs	Chromosomes/unanchored scaffolds	Physical positions (bp)	InDels (*Kabuli* reference genome-CDC Frontier/PII)	Forward primers (5'-3')	Reverse primers (5'-3')	Anneling temperature (0C)	Expected amplified product size (bp)	Structural annotation		Functional annotation	
								Sequence components of *kabuli* genome	*Kabuli* gene accession IDs	NCBI-KOG	TFs
Ca4_P2_30558234	Ca4	30558234	GTTTTTTT/GTTTTTTTT	GCGAAATCTAGGGGCAACTT	TTGATAGATGCCAAAAGGGG	59.9	725	INTERGENIC	--	--	--
Ca4_P2_30558963	Ca4	30558963	GC/G	CCCCTTTTGGCATCTATCAA	TCATCTCATTTTGCTTCCCT	57.3	510	INTERGENIC	--	--	--
Ca4_P2_30560828	Ca4	30560828	T/TC	CAGATTGTTCTTCCTCCCCA	CCACATCATCTCCAACCAAA	59.3	704	INTERGENIC	--	--	--
Ca4_P2_30560848	Ca4	30560848	GAAAAAAAAAAAAAAAA/GAAAAAAAAAA	CAGATTGTTCTTCCTCCCCA	CCACATCATCTCCAACCAAA	59.3	704	INTERGENIC	--	--	--
Ca4_P2_30561391	Ca4	30561391	TAAAAAAAAA/TAAAAAAAA	TTTGGTTGGAGATGATGTGG	TGGAAAAGTATGGTGCCTCC	59.9	431	INTERGENIC	--	--	--
Ca4_P2_30561689	Ca4	30561689	TAAAAA/TAAAA	GGAGGCACCATACTTTTCCA	AGAATGGCCACTTCAAGTCAA	59.7	198	INTERGENIC	--	--	--
Ca4_P2_30566057	Ca4	30566057	CGGG/CGG	TTTTTGCAATTGGGCATACA	ACTGGGTTTGGGTTACCCTC	60.1	515	INTERGENIC	--	--	--
Ca4_P2_30570798	Ca4	30570798	TAAAAAAA/TAAAAAAAA	TGCTTCTCAATGACTAACACCAA	TGTTGGTGTGACTTTTCCTTCT	58.7	881	INTERGENIC	--	--	--
Ca4_P2_30573766	Ca4	30573766	T/TC	CATTATGATCACAGACGATGGTG	TCCTCTTCGATCAAGCATCC	60.3	259	DRR	Ca_14206	A	--
Ca4_P2_30575118	Ca4	30575118	ATT/ATTT	TGTTCAACCATCAATTGTTTCC	GGCGGAATTTTTCATAGCAA	60.0	222	DRR	Ca_14206	A	--
Ca4_P2_30581429	Ca4	30581429	CA/CAA	GCAAACAAACTTCTGCGACA	TCGGCTCCTTCTTTTCTTCA	60.1	732	INTERGENIC	--	--	--
Ca4_P2_30582286	Ca4	30582286	GAAAAAAAAAA/GAAAAAAAAAAA	TTCCCTCCCTTCCTTTGAGT	TTTTCTGCAAGTTTGTCGTTG	59.0	615	INTERGENIC	--	--	--

**INDEL marker IDs**	**Chromosomes/unanchored scaffolds**	**Physical positions (bp)**	**InDels (*Kabuli* reference genome-CDC Frontier/PII)**	**Forward primers (5'-3')**	**Reverse primers (5'-3')**	**Anneling temperature (0C)**	**Expected amplified product size (bp)**	**Structural annotation**		**Functional annotation**	
								**Sequence components of *kabuli* genome**	***Kabuli* gene accession IDs**	**NCBI-KOG**	**TFs**
Ca4_P2_30582999	Ca4	30582999	AC/ACC	GGAGGGATGCAATATTAAGCA	ATTTCATAGACGCACCCGAG	60.1	436	INTERGENIC	--	--	--
Ca4_P2_30585901	Ca4	30585901	CTTTTTCTAATTTTTCTA/CTTTTTCTA	AAAGGTCAGTCATCCCAAACA	GAAACAGAAAACGGGAACCA	59.9	381	INTERGENIC	--	--	--
Ca4_P2_30587522	Ca4	30587522	TCC/TCCC	AAGCCAACCATCATACTCGG	GAATGATGTCGGTGTTGTCG	60.0	400	INTERGENIC	--	--	--
Ca4_P2_30587570	Ca4	30587570	G/GT	CGACAACACCGACATCATTC	CCTTGTCGTGAATGGTCCTT	60.0	113	INTERGENIC	--	--	--
Ca4_P2_30587846	Ca4	30587846	ACCCC/ACCC	CGACAACACCGACATCATTC	CCTTGTCGTCCTTGTTTGGT	60.0	678	INTERGENIC	--	--	--
Ca4_P2_30595068	Ca4	30595068	GAAAAAAAAA/GAAAAAAAAAA	ATCTCACAGTGTGCGTCTGC	CCGTCAAGTGGATCAGTGAA	59.7	305	INTERGENIC	--	--	--
Ca4_P2_30605971	Ca4	30605971	AATATATATATATATATATAT/AATATATATATATATATATATATATATATAT	TCCGACATCACACACCAAAC	TCGGTGTTAAAAATCGGAGTG	60.0	850	INTERGENIC	--	--	--
Ca4_P2_30606887	Ca4	30606887	TAAAAAAAAA/TAAAAAAAAAA	GCAATTTCAGCACGACTCAA	ATGTGCAGTTTTTGCCACTG	59.8	294	INTERGENIC	--	--	--
Ca4_P2_30608454	Ca4	30608454	AC/A	TTGCCTCACGTGAAGAATGA	CATTATGATGTTTGGCACGC	60.0	631	INTERGENIC	--	--	--
Ca4_P2_30619694	Ca4	30619694	CAAAAA/CAAAA	ACTCACTCTGCCCCTGTGTT	CCAACTTGGTTCCAACAAAAA	59.9	566	INTERGENIC	--	--	--
Ca4_P2_30621604	Ca4	30621604	ATTTTTTTTTTT/ATTTTTTTTTTTT	TGGGCTTCAATTTAGTCCTTG	CTGATGACAAAAGGGGGAGA	60.0	516	INTERGENIC	--	--	--
Ca4_P2_30628637	Ca4	30628637	ATTCTGGTTCTG/ATTCTG	ATCGGGTTTGTCTGATGGTG	ACAAACCAAGACCCAACCAG	59.9	363	INTERGENIC	--	--	--

INDEL marker IDs	Chromosomes/unanchored scaffolds	Physical positions (bp)	InDels (*Kabuli* reference genome-CDC Frontier/PII)	Forward primers (5'-3')	Reverse primers (5'-3')	Anneling temperature (0C)	Expected amplified product size (bp)	Structural annotation		Functional annotation	
								Sequence components of *kabuli* genome	*Kabuli* gene accession IDs	NCBI-KOG	TFs
Ca4_P2_30645520	Ca4	30645520	ATTTTTTTTT/ATTTTTTTT	GTTTGTGTTACGCTTTGGCA	GTTGTCCCTGCCTTACGTGT	60.0	377	INTERGENIC	--	--	--
Ca4_P2_30653740	Ca4	30653740	AC/ACC	TGCCCATTGATGCTAAACAA	GAATGATGTCGCGGTAGGAT	59.9	513	INTERGENIC	--	--	--
Ca4_P2_30655579	Ca4	30655579	TAAAAAAAAAA/TAAAAAAAAA	TTTTCTGGCCTAGCTCCTTG	TGCTCAAAGCATGTTGTTTCT	58.6	488	INTERGENIC	--	--	--
Ca4_P2_30668648	Ca4	30668648	ATGT/ATGTTGT	GAACAATTGAGAATGCGGGT	TCTTTCTTTGCTGGCGTTTT	60.0	479	DRR	Ca_14210	S	bZIP
Ca4_P2_30675981	Ca4	30675981	TCCCCCCC/TCCCCCC	ATCGGGCTCATTTGTTTGAG	AGTTGGGAATGGATAGCACG	60.0	427	INTRON	Ca_14210	S	bZIP
Ca4_P2_30684801	Ca4	30684801	GGT/GGTGT	TGTTAGGGATTGTGTGGGGT	GAAGTTGGGGATGAGGACAA	59.9	605	INTERGENIC	--	--	--
Ca4_P2_30685617	Ca4	30685617	TTA/TTATCAATA	GGGAAGAATGGATTTGGGAT	GGATGTTTCATTCCACCAAAG	59.3	192	INTERGENIC	--	--	--
Ca4_P2_30685756	Ca4	30685756	GTTTTT/GTTTTTT	GGGAAGAATGGATTTGGGAT	TTTCTCCTATGTGGCAACGC	61.2	649	INTERGENIC	--	--	--
Ca4_P2_30687259	Ca4	30687259	TTTA/TTTATTA	TCATCATTCATAGCTTGGGCT	AAATTTCCCAAGCAAAAGGG	60.3	393	INTERGENIC	--	--	--
Ca4_P2_30690397	Ca4	30690397	GTTTTTTTTTT/GTTTTTTTTT	AAGTGAAACACGGAACACCA	TGCAACAACTCGTTTCTCAA	58.0	662	INTERGENIC	--	--	--
Ca4_P2_30697175	Ca4	30697175	CTTTT/CTTT	AGATTCCATGGCACACACAA	CCCCAAGATTAACAAAGGCA	59.9	647	INTERGENIC	--	--	--
Ca4_P2_3069954	Ca4	3069954	TTTTATTTATTTATTTATTTAT/TTTTATTTATTTATTTATTTATTTAT	AAACCAAAACTGGCGAAAAA	TGAGAAAACATATTGGGAAGGC	60.3	686	DRR	Ca_12105	--	--

INDEL marker IDs	Chromosomes/unanchored scaffolds	Physical positions (bp)	InDels (*Kabuli* reference genome-CDC Frontier/PII)	Forward primers (5'-3')	Reverse primers (5'-3')	Anneling temperature (0C)	Expected amplified product size (bp)	Structural annotation		Functional annotation	
								Sequence components of *kabuli* genome	*Kabuli* gene accession IDs	NCBI-KOG	TFs
Ca4_P2_30723844	Ca4	30723844	CTAATTAA/CTAA	TGCTCATGAAGTAGCAAAATTGA	TCATCTTTGGCATGAGAATCA	59.2	837	INTERGENIC	--	--	--
Ca4_P2_30730238	Ca4	30730238	ATTGTGAAA/ATTGTGAAACTTTTTGTGAAA	TGTGGATTTTCAAGGTTCTCG	TCGAAAAAGCGGGATGTTAC	60.1	667	INTERGENIC	--	--	--
Ca4_P2_30731659	Ca4	30731659	CAAA/CAAAA	TATGGACGCACCACACAACT	GGCAACCACCAAAAGAGAAA	60.1	606	INTERGENIC	--	--	--
Ca4_P2_30741967	Ca4	30741967	TCAATATTGAACA/T	ATCCGGTTCCTTTTTGGTTC	TAAGGACCGGTGATCTGACA	59.1	656	INTERGENIC	--	--	--
Ca4_P2_30748634	Ca4	30748634	GTTTTTTTTTTTT/GTTTTTTTTTTTTTTT	TTTAGTGAGCCACAAAGGGG	AAGGGGATCCAAAATGGTGT	60.4	822	INTERGENIC	--	--	--
Ca4_P2_30753677	Ca4	30753677	ATTTTTTTT/ATTTTTTT	TTGAATGACAAACATTGAACTACAGA	TGGGAATTTATTTCGCGAGT	59.5	347	INTERGENIC	--	--	--
Ca4_P2_30755137	Ca4	30755137	ATTTTTTT/ATTTTTT	CAGTAGGAAGTCACATTCCCAGA	GCAACTGAGCTGAGAGCGTA	59.5	860	INTERGENIC	--	--	--
Ca4_P2_30760524	Ca4	30760524	ATT/ATTT	TTTGGAAGCGGTCTCACTCT	AAACGAATACTGCAACGCAA	59.4	217	DRR	Ca_14211	--	ERF
Ca4_P2_30772757	Ca4	30772757	ATTTTTTTTTT/ATTTTTTTTTTT	TCGTACTTTGTTTTGATTGCG	GCACCTATGAACCGTCCAAT	59.8	907	INTERGENIC	--	--	--
Ca4_P2_30774640	Ca4	30774640	TAAAAAAAAAA/TAAAAAAAAAAA	CGAATCTGTAGCCGAAAAGC	TTGGGCTTATGTTACGAGTTCA	59.6	249	INTERGENIC	--	--	--
Ca4_P2_30774863	Ca4	30774863	TA/T	CGAATCTGTAGCCGAAAAGC	GGAAAGACCCCGAATAATGA	58.8	770	INTERGENIC	--	--	--
Ca4_P2_30782871	Ca4	30782871	GAAAAAAAAA/GAAAAAAAA	CCGTGCTCTCTTGATTTGGT	CAATTTGACAAGCCCCCTAA	59.9	789	INTERGENIC	--	--	--

INDEL marker IDs	Chromosomes/unanchored scaffolds	Physical positions (bp)	InDels (*Kabuli* reference genome-CDC Frontier/PII)	Forward primers (5'-3')	Reverse primers (5'-3')	Anneling temperature (0C)	Expected amplified product size (bp)	Structural annotation		Functional annotation	
								Sequence components of *kabuli* genome	*Kabuli* gene accession IDs	NCBI-KOG	TFs
Ca4_P2_30790861	Ca4	30790861	TAAAAAAAAA/TAAAAAAAAAA	GAATTTTGGCCATCACCACT	TGTCTCGCACACCCATTTTA	60.1	530	INTERGENIC	--	--	--
Ca4_P2_30791078	Ca4	30791078	AATATATATATATATATATA/AATATATATATATATA	TAAAATGGGTGTGCGAGACA	ATAGGGCCCCAATTTAGCAG	60.3	199	INTERGENIC	--	--	--
Ca4_P2_30791534	Ca4	30791534	TTTTATTTATTTATTTATT/TTTTATTTATTTATT	AATTGGGGCCCTATCATTTC	TGAGCTCAATTTCGACTCATTT	59.0	639	INTERGENIC	--	--	--
Ca4_P2_30796000	Ca4	30796000	ATTTTTTTTT/ATTTTTTTTTTTTT	AGAAAACAAGGGGTCCAGGT	TGAATGGGAGAACACAAGCA	60.2	791	INTERGENIC	--	--	--
Ca4_P2_30797389	Ca4	30797389	CAAAAAA/CAAAAAAA	GGGAAACCTGTTGTCGTCAT	CTTCATGTTGTTCCACACGC	60.2	518	INTERGENIC	--	--	--
Ca4_P2_30801791	Ca4	30801791	ACTC/ACTCTC	GGAATCCCTACATGCCTTTG	ATTACATGCCATAGTGGGGG	59.5	404	INTERGENIC	--	--	--
Ca4_P2_3081231	Ca4	3081231	TAAAAAAAAAAA/TAAAAAAAAAA	TCGCCCCTTAATTGTTTGTC	TGTCTAGGTGGCCCTGTGAT	60.5	571	INTERGENIC	--	--	--
Ca4_P2_30821800	Ca4	30821800	TTTCTTCTT/TTTCTT	TTTTGAGGACTTTCGGGTTG	GCTCATGGGATTGCAACTTT	60.1	307	INTERGENIC	--	--	--
Ca4_P2_30841561	Ca4	30841561	GTCTTCTTCTTC/GTCTTCTTCTTCTTCTTCTTCTTC	TTGGTTGCTTCTTCTTGGCT	TGACGTCTCCCATTCACAAA	60.1	617	INTERGENIC	--	--	--
Ca4_P2_30844654	Ca4	30844654	CTTTTTTTTTT/CTTTTTTTTT	CACCTGCTTTTGACCCATCT	GGCAATTGCTTTACGCTAGG	59.9	959	INTERGENIC	--	--	--
Ca4_P2_30857682	Ca4	30857682	ATTTTTTTT/ATTTTTTTTT	ATTTTCCACCGCATATCCAA	GTGAACCGGCCTATTTAGCA	60.1	220	INTERGENIC	--	--	--
Ca4_P2_30866796	Ca4	30866796	A/AC	AAGTTGCCTCTGCCTTTGAA	ATGTTTGCAACCCGTTTCTC	60.0	255	INTERGENIC	--	--	--

INDEL marker IDs	Chromosomes/unanchored scaffolds	Physical positions (bp)	InDels (*Kabuli* reference genome-CDC Frontier/PII)	Forward primers (5'-3')	Reverse primers (5'-3')	Anneling temperature (0C)	Expected amplified product size (bp)	Structural annotation		Functional annotation	
								Sequence components of *kabuli* genome	*Kabuli* gene accession IDs	NCBI-KOG	TFs
Ca4_P2_30867064	Ca4	30867064	TGAAA/T	AAGTTGCCTCTGCCTTTGAA	CACCCTTTTGGTTGCTCCTA	60.1	651	INTERGENIC	--	--	--
Ca4_P2_30868286	Ca4	30868286	A/AG	CAAAGAATCGTGCAAGAGCA	CGATAGTGCCATGTTTGCAT	59.6	730	INTERGENIC	--	--	--
Ca4_P2_30868944	Ca4	30868944	ATTTTTTTT/ATTTTTTTTTTT	CATGGCACTATCGGCTAACA	TTTCTGGCTTCGTCCAAATC	60.2	711	INTERGENIC	--	--	--
Ca4_P2_30870230	Ca4	30870230	GAAAAAA/GAAAAA	TCAAGAAATGGCATGAACCA	TTGAGTTCCTTTTGGGATGC	60.1	434	INTERGENIC	--	--	--
Ca4_P2_30871301	Ca4	30871301	ATTTTTTTTT/ATTTTTTT	GCAACAAGAGCTTGGTACGAG	TGCAGATTGGGTGTATTGGA	59.9	369	INTERGENIC	--	--	--
Ca4_P2_30872091	Ca4	30872091	ATTTTTTT/ATTTTTTTTT	CCAAAAATCCAATTTTGCGT	TGGTTCCAACAATCTCCCTC	59.9	563	INTERGENIC	--	--	--
Ca4_P2_30873753	Ca4	30873753	GAAA/GAAAAA	CAAGCTCAAACTTCCAAATTCA	ACCCCAAATTGTTAAAGGCA	59.3	190	INTERGENIC	--	--	--
Ca4_P2_30873864	Ca4	30873864	ACC/ACCC	CAAAGAGAGAAACTTGCCCG	GGAAAGCGAGAAGTTGAACG	60.0	566	INTERGENIC	--	--	--
Ca4_P2_30880709	Ca4	30880709	ATT/ATTTT	TGCAGAGTAAACCAGCAACG	TTGCAAGTTGTGACTGAGGC	60.0	557	INTERGENIC	--	--	--
Ca4_P2_30880741	Ca4	30880741	CAA/CAAA	TGCAGAGTAAACCAGCAACG	TTGCAAGTTGTGACTGAGGC	60.0	557	INTERGENIC	--	--	--
Ca4_P2_30881744	Ca4	30881744	CAAAAAAAAAAA/CAAAAAAAAAAAAA	CTTTTGCGGTTGTTCGGTAT	CATTAAAGACAACGCCTAATCG	58.8	363	INTERGENIC	--	--	--
Ca4_P2_30883608	Ca4	30883608	GAAA/GAA	TGAAATTCATTCACGCTCCA	TGTGCTTTCTTCTCTGGCCT	60.1	495	INTERGENIC	--	--	--

INDEL marker IDs	Chromosomes/unanchored scaffolds	Physical positions (bp)	InDels (*Kabuli* reference genome-CDC Frontier/PII)	Forward primers (5'-3')	Reverse primers (5'-3')	Anneling temperature (0C)	Expected amplified product size (bp)	Structural annotation		Functional annotation	
								Sequence components of *kabuli* genome	*Kabuli* gene accession IDs	NCBI-KOG	TFs
Ca4_P2_30887008	Ca4	30887008	TCTCCTC/TCTC	CCTGACCCTACAAGCCAATC	GTAGTCTCGCAAGGACTCCG	60.0	218	INTERGENIC	--	--	--
Ca4_P2_30928717	Ca4	30928717	TAAAAAAA/TAAAAAAAA	CAATGTCCATGTCGACCAAA	TCTGTCGCACCATCTCAGTC	60.0	949	INTERGENIC	--	--	--
Ca4_P2_30942574	Ca4	30942574	GTTTTTTTT/GTTTTTTTTT	TTTGTGTGCCTTGATTGCTC	GTGCCCAGAGGCAGAATAAG	59.8	511	INTERGENIC	--	--	--
Ca4_P2_30955630	Ca4	30955630	TAAAAAAAAA/TAAAAAAAA	CAAAGCCATGTTTCTTTCCA	TGAGCAAATGAGGCAAAATG	59.8	579	INTERGENIC	--	--	--
Ca4_P2_30956638	Ca4	30956638	ATTTTTTTT/ATTTTTTTTT	TTGTTGGTGAAATTTCCGGT	GATGGATTTCCTACCCCCTC	59.6	236	INTERGENIC	--	--	--
Ca4_P2_30956675	Ca4	30956675	CAATTTT/CAATTTTAAATTTT	TTGTTGGTGAAATTTCCGGT	AGTGCATCGTTTCCTTGCTT	59.9	661	INTERGENIC	--	--	--
Ca4_P2_30958639	Ca4	30958639	GAAAAAAA/GAAAAAA	GCCAAACCAACTCATTCTTACA	CCCATCATAAATCTTTCAAAACAA	59.3	664	INTERGENIC	--	--	--
Ca4_P2_30967907	Ca4	30967907	T/TTCTCTAAACCATG	CACATCCCTTGCCATTTCTT	AGTCGAATCTCGTTGCACAAT	59.8	237	INTERGENIC	--	--	--
Ca4_P2_30968151	Ca4	30968151	CTTTTTTTT/CTTTTTTT	CACATCCCTTGCCATTTCTT	CCCGCATTTGTAACCATCTT	59.8	827	INTERGENIC	--	--	--
Ca4_P2_30976868	Ca4	30976868	GAAAA/GAAAAA	CACGGGAGTTAATTTTAGGACAA	TGTGAAGAGGCAAATGAAAAA	58.4	598	INTERGENIC	--	--	--
Ca4_P2_31022687	Ca4	31022687	CTT/CTTT	TATGGCACATGCGAGGAATA	GGCCTTGGTAGAAGGGAGAC	60.1	444	INTERGENIC	--	--	--
Ca4_P2_31024986	Ca4	31024986	CTTTTTTTTT/CTTTTTTTTTT	GGGATTTTGGTTAAGGGAGC	TTGAACTCACTCATCCACGG	59.7	176	INTERGENIC	--	--	--

INDEL marker IDs	Chromosomes/unanchored scaffolds	Physical positions (bp)	InDels (*Kabuli* reference genome-CDC Frontier/PII)	Forward primers (5'-3')	Reverse primers (5'-3')	Anneling temperature (0C)	Expected amplified product size (bp)	Structural annotation		Functional annotation	
								Sequence components of *kabuli* genome	*Kabuli* gene accession IDs	NCBI-KOG	TFs
Ca4_P2_31041871	Ca4	31041871	TGATG/T	AGCATGCCTCCAATGAGTCT	TTTTGGGCTTGTTCTTGGAC	60.1	499	INTERGENIC	--	--	--
Ca4_P2_31054804	Ca4	31054804	CAA/CAAA	AAGGAATGTGTTTTGGGCTG	GCCTTGCATTGGTAGTCACA	59.7	269	INTERGENIC	--	--	--
Ca4_P2_31055592	Ca4	31055592	TAAAAACAAAAACAAAAA/TAAAAACAAAAACAAAAACAAAAA	CATGCACAACTTGGTTAATGG	TGTAATTGGTGGCGTTTGAA	60.0	460	INTERGENIC	--	--	--
Ca4_P2_3105955	Ca4	3105955	GTTTTTTTTTTTTT/GTTTTTTTTTTTT	GGGGTCACAAACCTTACCCT	AATGGCCTAGCCTACCCTGT	60.0	643	INTERGENIC	--	--	--
Ca4_P2_31063154	Ca4	31063154	AATATATATATATATATATATAT/AATATATATATATATATATATAT	GAGGGGATGAATCATTAACTCG	GTAAGACCCCGCATTGTGTT	59.9	499	INTERGENIC	--	--	--
Ca4_P2_31063577	Ca4	31063577	CATGA/CA	AACACAATGCGGGGTCTTAC	TTTCGCATGAACAACAAGAAA	59.3	674	INTERGENIC	--	--	--
Ca4_P2_31063707	Ca4	31063707	GGTT/GGTTGTT	AACACAATGCGGGGTCTTAC	CCAACATACCCATTCCTTGAA	59.7	738	INTERGENIC	--	--	--
Ca4_P2_31064264	Ca4	31064264	CTTTTGTTTT/CTTTTGTTTTGTTCTTTTTTTGTTTT	TTAGTTCTCCTCCGTGGCTC	TGCACATTGGTGATTTTTCG	60.5	186	INTERGENIC	--	--	--
Ca4_P2_31079661	Ca4	31079661	TGG/TG	GCTCGTGTCATTGCAGAAAA	CCAATTACCACAGATTCGGG	60.2	337	INTERGENIC	--	--	--
Ca4_P2_31103766	Ca4	31103766	GAAAAAAAAAA/GAAAAAAAAAAA	CCCTACAATTTCAGAGGGCA	TTTTGCGATTTCCCGTAGTC	60.1	575	DRR	Ca_14221	U	bHLH
Ca4_P2_31104781	Ca4	31104781	TG/T	ACTGACGCTTTGTCATGTCG	GTGCTTGGGGTTTTGTCTGT	60.0	610	INTRON	Ca_14221	U	bHLH
Ca4_P2_31106521	Ca4	31106521	CCTT/CCTTCTT	ATAAGAGTGTGGCGGTTTGG	TGGTTGAAATGGACGACAAA	59.9	584	INTERGENIC	--	--	--

INDEL marker IDs	Chromosomes/unanchored scaffolds	Physical positions (bp)	InDels (*Kabuli* reference genome-CDC Frontier/PII)	Forward primers (5'-3')	Reverse primers (5'-3')	Anneling temperature (0C)	Expected amplified product size (bp)	Structural annotation		Functional annotation	
								Sequence components of *kabuli* genome	*Kabuli* gene accession IDs	NCBI-KOG	TFs
Ca4_P2_31106765	Ca4	31106765	GAAAAAAAAT/GAAAAAAAATAAAAAAAAT	TTGTCGTCCATTTCAACCAA	CTTTGATTCCCTTTCCCACA	59.9	334	INTERGENIC	--	--	--
Ca4_P2_31108602	Ca4	31108602	GGAAGAAGAA/GGAAGAAGAAGAA	GGATCAGGCCCTATTGTTGA	AGTTGGCTTTGCTTGATGCT	60.0	476	INTERGENIC	--	--	--
Ca4_P2_31108864	Ca4	31108864	ACAAGCATCAAGCA/ACAAGCA	GGATCAGGCCCTATTGTTGA	CCAGAACTTGCATCCCCTTA	60.1	527	INTERGENIC	--	--	--
Ca4_P2_31109191	Ca4	31109191	ATTTT/ATTT	CTTGCAAGGATTGCTTCACA	GAAGGCCATGTCCACATCTT	59.9	456	INTERGENIC	--	--	--
Ca4_P2_31110947	Ca4	31110947	TAAAAAA/TAAAAA	GCTAAAGCAATCACCAAGGC	GCGTTTGGTGACATAGCTGA	59.9	412	INTERGENIC	--	--	--
Ca4_P2_31111668	Ca4	31111668	GAAAAAAAAA/GAAAAAAAAAA	ATTGAAAAGGGCGAGCTACA	AAATGTTTATCCACGCTGCC	60.0	434	INTERGENIC	--	--	--
Ca4_P2_31118703	Ca4	31118703	GA/G	CAGGCAATTTGTTTGACCCT	TTGGATGCTACTAGTGGGGG	59.9	634	INTERGENIC	--	--	--
Ca4_P2_31123070	Ca4	31123070	TAAAA/TAAA	CCCATATACCCATTGCCTTG	TTGGAATTCATCCCTGCAAT	60.3	300	INTERGENIC	--	--	--
Ca4_P2_31128051	Ca4	31128051	CT/CTCAT	CGGGATCGTCAGACTGTTTT	GCAACGTTTGTCCCTTCATT	60.0	110	INTERGENIC	--	--	--
Ca4_P2_31128772	Ca4	31128772	GATA/GATACATA	CAACCTACGTTCTCGTGTTTGT	TCTTTAAATGCGGGGATGTT	59.4	619	INTERGENIC	--	--	--
Ca4_P2_31137263	Ca4	31137263	TAA/T	CTTTTATTTAAGGTGCGCCG	TCCTGCCATCCAACCTTATC	59.9	660	DRR	Ca_14223	--	bHLH
Ca4_P2_31137294	Ca4	31137294	AG/A	CTTTTATTTAAGGTGCGCCG	TCCTGCCATCCAACCTTATC	59.9	660	DRR	Ca_14223	--	bHLH

INDEL marker IDs	Chromosomes/unanchored scaffolds	Physical positions (bp)	InDels (*Kabuli* reference genome-CDC Frontier/PII)	Forward primers (5'-3')	Reverse primers (5'-3')	Anneling temperature (0C)	Expected amplified product size (bp)	Structural annotation		Functional annotation	
								Sequence components of *kabuli* genome	*Kabuli* gene accession IDs	NCBI-KOG	TFs
Ca4_P2_31137565	Ca4	31137565	GAAAAAA/GAAAAAAA	TTTCCGAATCGGGAGTATGA	GTTCCTGCCATCCAACCTTA	59.9	113	DRR	Ca_14223	--	bHLH
Ca4_P2_31138420	Ca4	31138420	TAAAAAA/TAAAAAAA	CAAAGTGTTTTGACTTAGCCCA	ATGAGGATGCAGAAGGCAGA	60.9	762	DRR	Ca_14223	--	bHLH
Ca4_P2_31140324	Ca4	31140324	TAAAA/TAAAAA	ACTTCCATTCGCTTCAGCAT	AAATGATTCTGGTGGGGACA	60.2	536	INTRON	Ca_14223	--	bHLH
Ca4_P2_31172727	Ca4	31172727	A/AC	TCTTTGGATGGGTTCCATGT	TTACACCAATTCCCCCAAGA	60.2	663	INTERGENIC	--	--	--
Ca4_P2_31172943	Ca4	31172943	TA/T	TCGCCTAAGGTAAGGGCTTT	TTACACCAATTCCCCCAAGA	60.2	421	INTERGENIC	--	--	--
Ca4_P2_31182932	Ca4	31182932	T/TC	CGCATATTAGCATGGAGGGT	TCGCTCGAACCAGAGTTCTT	60.1	317	INTERGENIC	--	--	--
Ca4_P2_31192815	Ca4	31192815	TGG/TG	TTGAAGGAGAAGCCAGAGTCA	TGAGGCATGCAGAAACGTAG	60.0	238	INTERGENIC	--	--	--
Ca4_P2_31194912	Ca4	31194912	CTTTTTTTTTTT/CTTTTTTTTTT	TTCCATGTTTGTGTTGAAGCA	GCACATTGCAAGGGTATTGA	59.5	822	DRR	Ca_14224	--	S1Fa-like
Ca4_P2_31195163	Ca4	31195163	GAAAAAAAA/GAAAAAAAAA	TTTCCCGACTTGGTCCTTTA	TGCATGCAGATCTTGCTCTC	60.3	643	DRR	Ca_14224	--	S1Fa-like
Ca4_P2_31200114	Ca4	31200114	TA/TAGACCA	TTGTCTTAGGCAGCACTGGA	TGGTGAACGAAAGTGACGTG	60.8	903	INTERGENIC	--	--	--
Ca4_P2_31222344	Ca4	31222344	ATTTTTTTTTT/ATTTTTTTTTTTT	TTGAACGCTACATTCACGTTG	TTTGAAAATCCCAAATTGCC	59.8	748	INTERGENIC	--	--	--
Ca4_P2_31222733	Ca4	31222733	GAC/GACAC	GAGGGCAATTTGGGATTTTC	TGACAAGTTTATGTGTGGGGG	60.7	523	INTERGENIC	--	--	--

INDEL marker IDs	Chromosomes/unanchored scaffolds	Physical positions (bp)	InDels (*Kabuli* reference genome-CDC Frontier/PII)	Forward primers (5'-3')	Reverse primers (5'-3')	Anneling temperature (0C)	Expected amplified product size (bp)	Structural annotation		Functional annotation	
								Sequence components of *kabuli* genome	*Kabuli* gene accession IDs	NCBI-KOG	TFs
Ca4_P2_31228747	Ca4	31228747	ATTTT/ATTT	GCAGCAAAGTTACAAAACCCA	TCCATCTCAAAATATCAGCAAAAA	60.0	975	DRR	Ca_14227	C	--
Ca4_P2_31230420	Ca4	31230420	TTCTCTCTCTCT/TTCTCTCTCT	CTTTCCATCTCACTCCCTCG	TAGCCGACAGGGTCTCAGTT	59.9	435	INTERGENIC	--	--	--
Ca4_P2_31235705	Ca4	31235705	ATTTTTTTTT/ATTTTTTTTTT	CAATTGCGGTTTCCACTTTT	TTCATGCATTTTCAATGGGA	59.9	434	INTERGENIC	--	--	--
Ca4_P2_31236090	Ca4	31236090	ATATTATTATTATTATT/ATATTATTATTATT	CAATTGCGGTTTCCACTTTT	TTTTTACCGTGGGTGAATCC	59.7	956	INTERGENIC	--	--	--
Ca4_P2_31248260	Ca4	31248260	CAA/C	AAAAGCGCTGTAAAATGCGT	GACACGTTTGCATACCAATGA	59.5	629	DRR	Ca_14228	--	Trihelix
Ca4_P2_31248427	Ca4	31248427	TAAAAAAA/TAAAAAA	AAAAGCGCTGTAAAATGCGT	GACACGTTTGCATACCAATGA	59.5	629	DRR	Ca_14228	--	Trihelix
Ca4_P2_31248634	Ca4	31248634	ACC/AC	TGGGTCATTTCTCATTATGTCG	TGCTATGCCTTTTGATCGC	59.9	645	DRR	Ca_14228	--	Trihelix
Ca4_P2_31251511	Ca4	31251511	GTTTTTTT/GTTTTTTTT	AACCCATGCTCAAGTGGTTC	CAAGCGCATTTGTCCCTTAT	60.1	894	INTERGENIC	--	--	--
Ca4_P2_31254902	Ca4	31254902	TAAAAAAAAAAA/TAAAAAAAAAAAAA	CCCTCTTCTCTTGTTTGGTTTG	GGAAACTGCTCTGGTAAGCG	60.0	338	DRR	Ca_14229	R	bHLH
Ca4_P2_31255173	Ca4	31255173	ACAG/ACAGGCAG	CGCTTACCAGAGCAGTTTCC	GACCCAATAACCCCAACCTT	59.9	285	DRR	Ca_14229	R	bHLH
Ca4_P2_31255343	Ca4	31255343	GAAAA/GAAAAA	CGCTTACCAGAGCAGTTTCC	GAGAGAAGTGTCTTTGCGGG	60.0	681	DRR	Ca_14229	R	bHLH
Ca4_P2_31258911	Ca4	31258911	GTT/GT	CAGGTCGTGCCAATTCTTTT	GGCTAGGATTTGCAACAGGT	59.2	264	INTERGENIC	--	--	--

INDEL marker IDs	Chromosomes/unanchored scaffolds	Physical positions (bp)	InDels (*Kabuli* reference genome-CDC Frontier/PII)	Forward primers (5'-3')	Reverse primers (5'-3')	Anneling temperature (0C)	Expected amplified product size (bp)	Structural annotation		Functional annotation	
								Sequence components of *kabuli* genome	*Kabuli* gene accession IDs	NCBI-KOG	TFs
Ca4_P2_31259145	Ca4	31259145	GATATATATATATATATATATATAT/GATATATCTATATATATATATATATATATAT	CAGGTCGTGCCAATTCTTTT	TTCATTGACGAATGTACCATCA	58.9	404	INTERGENIC	--	--	--
Ca4_P2_31262288	Ca4	31262288	AATA/AATATA	TCACAAATGAGAGGAGACGAGA	AATTTTCCATCCATTGCTGC	59.9	793	DRR	Ca_14230	R	MYB_related
Ca4_P2_31268063	Ca4	31268063	ATTTTTTTTTTTTT/ATTTTTTTTTTT	CGATCAACGTCCCTTCTTTC	CACAACCCTAATCATGCAAAAA	59.9	746	INTERGENIC	--	--	--
Ca4_P2_31268618	Ca4	31268618	TAAAAAAAA/TAAAAAAAAA	TTTTTGCATGATTAGGGTTGTG	CGAGTGCGAGATAGATTGGA	59.0	249	INTERGENIC	--	--	--
Ca4_P2_31268857	Ca4	31268857	ATTTTTTTTTTT/ATTTTTTTTTTTT	TCCAATCTATCTCGCACTCG	GAAAACCCAAAACACGGAAA	59.8	491	INTERGENIC	--	--	--
Ca4_P2_31268906	Ca4	31268906	TAA/TA	TCCAATCTATCTCGCACTCG	GAAAACCCAAAACACGGAAA	59.8	491	INTERGENIC	--	--	--
Ca4_P2_31271135	Ca4	31271135	TAAAA/TAAAAA	TGTTTCCTTTGTATGCATTCCTT	GGCTACAATTCAACAGTCTTGTG	58.8	753	INTERGENIC	--	--	--
Ca4_P2_31274077	Ca4	31274077	GTTTTTTTTTTTTTTTTTTT/GTTTTTTTTTT	TTGTTTATCTGCCCGTTTGA	GTTGCTGGTGTGTGCCATAG	60.2	751	INTERGENIC	--	--	--
Ca4_P2_31274891	Ca4	31274891	TTTATT/TTT	CTATGGCACACACCAGCAAC	GATCAAGTAGGGCGGATTCA	60.0	699	INTERGENIC	--	--	--
Ca4_P2_31274972	Ca4	31274972	TAAAAAAAAAA/TAAAAAAAA	CTATGGCACACACCAGCAAC	GATCAAGTAGGGCGGATTCA	60.0	699	INTERGENIC	--	--	--
Ca4_P2_31275360	Ca4	31275360	ATTTTTTTTT/ATTTTTTTT	TTCAATTAGGGTTTGGGTCG	ACAGTTTGTGCGTTGTTGGT	59.1	631	INTERGENIC	--	--	--
Ca4_P2_31279281	Ca4	31279281	ACCC/ACC	GCCACCTGTCCAACTTCCTA	ATACCCAAGAGGGTATGGGC	60.0	454	INTERGENIC	--	--	--

INDEL marker IDs	Chromosomes/unanchored scaffolds	Physical positions (bp)	InDels (*Kabuli* reference genome-CDC Frontier/PII)	Forward primers (5'-3')	Reverse primers (5'-3')	Anneling temperature (0C)	Expected amplified product size (bp)	Structural annotation		Functional annotation	
								Sequence components of *kabuli* genome	*Kabuli* gene accession IDs	NCBI-KOG	TFs
Ca4_P2_31281553	Ca4	31281553	CAA/CA	TAGTACCTGTACCCGCACCC	ACAAAACGCCAATGACACAA	60.0	471	INTERGENIC	--	--	--
Ca4_P2_31284485	Ca4	31284485	ATT/ATTT	ATGGCTACTCCAATGCATCC	ACATGGCTCCCCCTTACTCT	60.0	962	INTERGENIC	--	--	--
Ca4_P2_31288200	Ca4	31288200	AGAGGAAAAGAAGAACGAGGAAAAGAAGAA/AGAGGAAAAGAAGAA	TATCACCCTCAGATCCAGCC	TAGCTTGGCGCCTCAACTAT	60.0	594	INTERGENIC	--	--	--
Ca4_P2_31291385	Ca4	31291385	A/ATTT	GTTTTCCTGCTTGGGATGAA	ACTCCGACAATGGAACAAGG	60.0	339	INTERGENIC	--	--	--
Ca4_P2_31297319	Ca4	31297319	TCAAC/TCAACAAC	GCACCAAAATAAACCAAACCA	TGCAGTTCTGAACCGATTTG	59.8	390	INTERGENIC	--	--	--
Ca4_P2_31298440	Ca4	31298440	CTTTTTTTTTTT/CTTTTTTTTTTTTT	GTTTGGAAGGGAGGGGAGTA	AACAGTACAGCAACCCCACC	59.9	628	DRR	Ca_14231	--	bHLH
Ca4_P2_31302749	Ca4	31302749	GAAAAAAAAAAA/GAAAAAAAAAA	CACCCCACACCAAACTCTCT	GTTAGGTTGGACGGGACCTC	60.7	680	INTRON	Ca_14231	--	bHLH
Ca4_P2_31302962	Ca4	31302962	AATTGT/A	AGAAAAATGTCGCTGGAACG	TCAGCACCGAGAGTGTCAAC	60.0	903	INTRON	Ca_14231	--	bHLH
Ca4_P2_31303190	Ca4	31303190	CTTTTTTTTTTT/CTTTTTTTTTT	GAGGTCCCGTCCAACCTAAC	TCAGCACCGAGAGTGTCAAC	60.0	488	INTRON	Ca_14231	--	bHLH
Ca4_P2_31307742	Ca4	31307742	CAAAAA/CAAAA	GGTCGCGTAGACCTTCAGAG	AAGCATTTGATCGTCTGGCT	59.8	166	INTERGENIC	--	--	--
Ca4_P2_31312729	Ca4	31312729	CTTTTTTTTTTTTT/CTTTTTTTTT	GATGCTTTTCTCCTTCAGCC	TCTTTGACCAAGTTGAAAAGAAGAT	59.7	341	INTERGENIC	--	--	--
Ca4_P2_31317473	Ca4	31317473	ATTTTTTTTT/ATTTTTTTT	TGAGGCGTCATGGTAGAACA	CAAATCGACATTGTTGCCAG	60.1	707	INTERGENIC	--	--	--

INDEL marker IDs	Chromosomes/unanchored scaffolds	Physical positions (bp)	InDels (*Kabuli* reference genome-CDC Frontier/PII)	Forward primers (5'-3')	Reverse primers (5'-3')	Anneling temperature (0C)	Expected amplified product size (bp)	Structural annotation		Functional annotation	
								Sequence components of *kabuli* genome	*Kabuli* gene accession IDs	NCBI-KOG	TFs
Ca4_P2_31317508	Ca4	31317508	TAAAAAAAA/TAAAAAAA	TTCGCATCAGCCATATTTTG	CAAATCGACATTGTTGCCAG	60.1	509	INTERGENIC	--	--	--
Ca4_P2_31322872	Ca4	31322872	GAAAAAAAAA/GAAAAAAAA	CTCCTAAGGGTGGCCTTTTC	GGGTTGTTCTTGGACCTTCA	59.9	623	INTERGENIC	--	--	--
Ca4_P2_31322894	Ca4	31322894	GAAAAAAAA/GAAAAAAA	CTCCTAAGGGTGGCCTTTTC	GGGTTGTTCTTGGACCTTCA	59.9	623	INTERGENIC	--	--	--
Ca4_P2_31327989	Ca4	31327989	ATTTT/ATTTTT	TTGAAATGCGTATGATGGGA	ACGCCACTTGTGCTTTTTCT	59.9	465	INTERGENIC	--	--	--
Ca4_P2_31332105	Ca4	31332105	ATTTT/ATTT	GCACATGTGTGGGTTGACTC	TTGAACCAACATGATCCAATTC	59.7	557	INTERGENIC	--	--	--
Ca4_P2_31346807	Ca4	31346807	ATTTTTTTTTT/ATTTTTTTTTTT	TGGACGAAAGAGTTGTTGTCA	TATGCATCTGACGATCTGCG	60.9	468	INTERGENIC	--	--	--
Ca4_P2_31349174	Ca4	31349174	TAAAAAAAAAA/TAAAAAAAAAAA	TCGATTATCAAAGGTTGCGTT	TCCAACTACAATCCTTCCGC	60.1	779	INTERGENIC	--	--	--
Ca4_P2_31350277	Ca4	31350277	CCAA/CCAAAACAA	CAAAATGCAAGAACCCGTTT	GCTCTTTGCTCCTCATCCAC	60.0	134	INTERGENIC	--	--	--
Ca4_P2_31360317	Ca4	31360317	TAAAAAAA/TAAAAAAAA	ACTCATATGACGGGGTGCAT	GGCAGACGTTGGAAAGCTAA	60.4	715	DRR	Ca_14232	--	Trihelix
Ca4_P2_31384014	Ca4	31384014	CTTTTTTTTTT/CTTTTTTTTT	TCGACTAAAAGATTTTTGGAAGATTT	GAGCCAACTGCATTTGTTGA	59.8	391	INTERGENIC	--	--	--
Ca4_P2_31387262	Ca4	31387262	CTTTTTT/CTTTTTTT	CGAAACCGTAACCCTACGAA	GCCACTTACAGCGCTTTTTC	60.0	580	INTERGENIC	--	--	--
Ca4_P2_31387318	Ca4	31387318	CTTTTTTTT/CTTTTTTT	TGCCCTCGTCAAACTAACAA	AGCCACTTACAGCGCTTTTT	59.2	277	INTERGENIC	--	--	--

INDEL marker IDs	Chromosomes/unanchored scaffolds	Physical positions (bp)	InDels (*Kabuli* reference genome-CDC Frontier/PII)	Forward primers (5'-3')	Reverse primers (5'-3')	Anneling temperature (0C)	Expected amplified product size (bp)	Structural annotation		Functional annotation	
								Sequence components of *kabuli* genome	*Kabuli* gene accession IDs	NCBI-KOG	TFs
Ca4_P2_31392404	Ca4	31392404	AT/A	CTCAAATCGAACCCTCCAAA	AAGCCAGGTGGTTGTTTGAC	60.0	403	INTERGENIC	--	--	--
Ca4_P2_31406944	Ca4	31406944	GTTTTTTTTT/GTTTTTTTTTT	CAAATTATACTCGCACAACTTGC	AAAGTGGACAAACACAAAACGA	59.6	782	INTERGENIC	--	--	--
Ca4_P2_31414937	Ca4	31414937	GTTTTTTT/GTTTTTTTT	GGAATACCGCATGCAAATCT	TGGTATCGGGAGAGAGGAGA	59.8	847	INTERGENIC	--	--	--
Ca4_P2_31424757	Ca4	31424757	GTTTTTT/GTTTTTTT	AATTGGCGAAACCACTTTTT	TGCACTTGTTGGGGTTGATA	60.0	501	INTERGENIC	--	--	--
Ca4_P2_31445256	Ca4	31445256	ATT/ATTT	AGATCTATGCATCGGCAACC	AGGGTACTACAGCTGCCCCT	60.2	338	INTERGENIC	--	--	--
Ca4_P2_31449673	Ca4	31449673	TA/T	TTCCGCCATTCCCATAAGTA	CCCTTTTTGTGAAGCAAGGA	60.2	353	CDS (FRAME SHIFT)	Ca_15433	E	TALE
Ca4_P2_31451186	Ca4	31451186	TCC/TC	CAGCACCAATGGTTGAAATG	CACGTCGATCTTTGACACTCA	59.9	657	INTRON	Ca_15433	E	TALE
Ca4_P2_31452576	Ca4	31452576	CTTTTTTTT/CTTTTTTTTT	CCACTCGTGGTCCCTTAACT	TCGGCTAAATACCATTTGTGG	59.8	566	INTERGENIC	--	--	--
Ca4_P2_31463756	Ca4	31463756	CTTTTTTTT/CTTTTTTTTT	GGCTCCTTGCATCAGTTAGC	GAAAACCGATTAGGGATTCG	58.5	683	INTERGENIC	--	--	--
Ca4_P2_31509721	Ca4	31509721	CATATATATATATATATATATAT/CATATATATATATATATATAT	TGTTCACTAGCACCGTAGCG	TTATGGAGCCCCGATTAAAA	59.4	570	INTERGENIC	--	--	--
Ca4_P2_31511106	Ca4	31511106	TTATATATATATATATATATATA/TTATATATATATATATATATATATA	TGTGGGGTGATTAGTGGGTT	TTGAATGCTGAAATTTGGGTT	59.4	454	INTERGENIC	--	--	--
Ca4_P2_31529637	Ca4	31529637	CTTTTTTTTTT/CTTTTTTTTT	CCAAATCCGATGAGGAAAGA	TTTAAGACCCGCGATTTCAG	60.2	820	INTERGENIC	--	--	--

INDEL marker IDs	Chromosomes/unanchored scaffolds	Physical positions (bp)	InDels (*Kabuli* reference genome-CDC Frontier/PII)	Forward primers (5'-3')	Reverse primers (5'-3')	Anneling temperature (0C)	Expected amplified product size (bp)	Structural annotation		Functional annotation	
								Sequence components of *kabuli* genome	*Kabuli* gene accession IDs	NCBI-KOG	TFs
Ca4_P2_31531525	Ca4	31531525	G/GGT	AGGGATTGCTTGCAAATGAC	AATTCCCAAACCAATAGCCC	60.0	387	INTERGENIC	--	--	--
Ca4_P2_31532942	Ca4	31532942	CTTATTTATTTATT/CTTATTTATTTATTTATT	CAGGCTGTGAAGTGAGACGA	GCTACGAAATCGCAATGAAA	58.9	644	INTERGENIC	--	--	--
Ca4_P2_31534458	Ca4	31534458	CAAAAAAA/CAAAAAAAAA	CAAGCCAACGTGTGTTTGTT	TGTGGGGGATCAAATTGTTT	60.0	371	INTERGENIC	--	--	--
Ca4_P2_31546446	Ca4	31546446	TAAA/TAAAAA	CAGCATTTTCCCCTTGACAT	TTTATGCCATTTTGCGTTCA	60.1	499	INTERGENIC	--	--	--
Ca4_P2_31549537	Ca4	31549537	A/AT	AAATCCAAACTTTGCAATCTTGA	CCAAAACTGCAATTGAAAGG	58.3	218	INTERGENIC	--	--	--
Ca4_P2_31549926	Ca4	31549926	TAAAAAAA/TAAAAAAAAA	TCCTTTCAATTGCAGTTTTGG	CAAAGGCGGTTTCATCATCT	60.1	801	INTERGENIC	--	--	--
Ca4_P2_31549950	Ca4	31549950	AT/A	TCCTTTCAATTGCAGTTTTGG	CAAAGGCGGTTTCATCATCT	60.1	801	INTERGENIC	--	--	--
Ca4_P2_31560045	Ca4	31560045	ATTTTTTTTT/ATTTTTTTT	GGATATCCCACTGCAAGGAA	AACCTGTATTCCCACAAGCG	60.0	608	INTERGENIC	--	--	--
Ca4_P2_31561636	Ca4	31561636	CTTT/CTT	TGCCAAAGTTCCTTTGTTGA	CGTGTCCCACTAACACCACA	60.5	502	INTERGENIC	--	--	--
Ca4_P2_31561946	Ca4	31561946	GAAAA/GAAA	TGTGGTGTTAGTGGGACACG	TGGGACAACCATCTTTTTCAG	60.0	532	INTERGENIC	--	--	--
Ca4_P2_31568988	Ca4	31568988	CTTTTTTTTT/CTTTTTTTTTTT	TGCGTACATCAATGGAGGTTT	CGTGTATCGAACATCATGCC	60.0	552	INTERGENIC	--	--	--
Ca4_P2_31570022	Ca4	31570022	TGGGGGG/TGGGGGGG	TATTTTGGATTTTGGCTCGG	AGGAAGAGTGGAGTTGCGAA	60.0	505	INTERGENIC	--	--	--

INDEL marker IDs	Chromosomes/unanchored scaffolds	Physical positions (bp)	InDels (*Kabuli* reference genome-CDC Frontier/PII)	Forward primers (5'-3')	Reverse primers (5'-3')	Anneling temperature (0C)	Expected amplified product size (bp)	Structural annotation		Functional annotation	
								Sequence components of *kabuli* genome	*Kabuli* gene accession IDs	NCBI-KOG	TFs
Ca4_P2_31609144	Ca4	31609144	ATTTT/ATTT	TCGACGTTTATTTGATGGCA	TTGAGAAACTTAATTGTGGGCA	59.6	651	DRR	Ca_15441	A	--
Ca4_P2_31611840	Ca4	31611840	GTTTTTTTTTT/GTTTTTTTTTTTT	CGTTTTCAGCTGAGCAAATTC	TTGATCCATTGGGATTGTGA	59.7	392	INTERGENIC	--	--	--
Ca4_P2_31611867	Ca4	31611867	AAT/AATAT	CGTTTTCAGCTGAGCAAATTC	TTGATCCATTGGGATTGTGA	59.7	392	INTERGENIC	--	--	--
Ca4_P2_31612473	Ca4	31612473	TAAAAAAA/TAAAAAAAA	TAAAATCCAAAACCGCAAGC	CACTTAAGCAAACGGGGTGT	60.0	462	INTERGENIC	--	--	--
Ca4_P2_31616497	Ca4	31616497	ATTTTTTTTTTTT/ATTTTTTTTTT	TTTGTCCATCCCACACAATG	AACTCGAAAGAGGGCAACCT	60.2	202	INTERGENIC	--	--	--
Ca4_P2_31622995	Ca4	31622995	GA/GAA	TTCCAAAAGAAAGGCACAAGA	TATGGCCTTTGGTGAAGACC	59.9	323	INTERGENIC	--	--	--
Ca4_P2_31626932	Ca4	31626932	ATTTTTT/ATTTTT	GATGTTGAGAACTGCAGCCA	AACCCCCAGGTTTTGAAAGT	59.7	862	INTERGENIC	--	--	--
Ca4_P2_31628781	Ca4	31628781	ATTT/ATTTT	ATTGCAAGCACCCATTTACC	CGAGCACGCATCTATGATTT	58.9	305	INTERGENIC	--	--	--
Ca4_P2_31630531	Ca4	31630531	AGG/AG	AGTTGAAGGTGCAAACACGA	AATTTACTTGGAGGGTCGGG	60.2	288	INTERGENIC	--	--	--
Ca4_P2_31630588	Ca4	31630588	TC/T	AAGGTGCAAACACGAGAAGG	AATTTACTTGGAGGGTCGGG	60.2	283	INTERGENIC	--	--	--
Ca4_P2_31631071	Ca4	31631071	TAAAAAAAA/TAAAAAAAAA	ACCCGACCCTCCAAGTAAAT	TGATGAAACTCACCAAGTTGAAA	59.6	511	INTERGENIC	--	--	--
Ca4_P2_31631888	Ca4	31631888	AAAA/AAAATAAA	CCAACTTGGTTCCAACATCTG	TGGCTAATCATAGCGAAACG	58.9	832	INTERGENIC	--	--	--

INDEL marker IDs	Chromosomes/unanchored scaffolds	Physical positions (bp)	InDels (*Kabuli* reference genome-CDC Frontier/PII)	Forward primers (5'-3')	Reverse primers (5'-3')	Anneling temperature (0C)	Expected amplified product size (bp)	Structural annotation		Functional annotation	
								Sequence components of *kabuli* genome	*Kabuli* gene accession IDs	NCBI-KOG	TFs
Ca4_P2_31633774	Ca4	31633774	AG/AGG	AAATATTGTTCAATCCGCCG	CTCCTCCAAGCCAAGTGAAG	60.0	491	INTERGENIC	--	--	--
Ca4_P2_31634027	Ca4	31634027	CAAA/CAA	CACTTGGCTTGGAGGAGAAG	TTCCCACCAAGTGTGAGTGA	60.1	315	INTERGENIC	--	--	--
Ca4_P2_31635242	Ca4	31635242	ATTTACCT/A	TCAATGTGAACGGGCATCTA	TGGTGCCTCCTACAATTTCC	59.9	778	INTERGENIC	--	--	--
Ca4_P2_31635868	Ca4	31635868	AAT/A	GGAAATTGTAGGAGGCACCA	GTTGCGCTACGAGATGTTGA	60.0	695	INTERGENIC	--	--	--
Ca4_P2_31639620	Ca4	31639620	GTTTT/GTTTTT	ATTTGGAGAATGATTTGGCG	GTGTTTGGTTCAAGGGGAGA	59.9	412	INTERGENIC	--	--	--
Ca4_P2_31640017	Ca4	31640017	AATCACTCATATAT/AATCACTCATATATATCACTCATATAT	TGATTTGGCGATTGTTTTGA	CTAGATGTCTCCAGACGCCC	59.8	617	INTERGENIC	--	--	--
Ca4_P2_31642398	Ca4	31642398	TAAAAAAAA/TAAAAAAAAA	TGTCACTGCACTGCCACATA	TTTCCGGCAAAGTTCTCATAA	59.7	482	INTRON	Ca_15442	U	EIL
Ca4_P2_31643009	Ca4	31643009	GTAGTGA/GTAGTGATAGTGA	AAGAATCTATTGTTCGGGGGA	CGCCAAACTCGTTTTCATTT	60.1	385	INTRON	Ca_15442	U	EIL
Ca4_P2_31644451	Ca4	31644451	TA/T	AATGTCGGGAGTTTTCATCG	GCCATCTTAGGCTTTCCCTT	59.7	953	INTRON	Ca_15442	U	EIL
Ca4_P2_31646633	Ca4	31646633	AA/AACA	ATGGAGAAGAGGGGGAGTGT	ACCAGTCTTTTACGGGCCTT	60.0	135	INTRON	Ca_15442	U	EIL
Ca4_P2_31647747	Ca4	31647747	ATT/AT	CACGTGTGAAGGTCGAGAGA	AAGCTACCTGATCAATGCGG	60.2	863	INTRON	Ca_15442	U	EIL
Ca4_P2_31647770	Ca4	31647770	GTTTTTTTTT/GTTTTTTTT	CACGTGTGAAGGTCGAGAGA	AAGCTACCTGATCAATGCGG	60.2	863	INTRON	Ca_15442	U	EIL

INDEL marker IDs	Chromosomes/unanchored scaffolds	Physical positions (bp)	InDels (*Kabuli* reference genome-CDC Frontier/PII)	Forward primers (5'-3')	Reverse primers (5'-3')	Anneling temperature (0C)	Expected amplified product size (bp)	Structural annotation		Functional annotation	
								Sequence components of *kabuli* genome	*Kabuli* gene accession IDs	NCBI-KOG	TFs
Ca4_P2_31649175	Ca4	31649175	GTTTTTTT/GTTTTTT	CATCCTTTATGTGACGCCCT	TTTGTCGGTCAATTCGATCA	60.0	165	INTRON	Ca_15442	U	EIL
Ca4_P2_31649916	Ca4	31649916	AT/A	TGAGGCATTTGGAGACAACA	ATTGACCGGTGGAAGACATT	59.3	585	INTRON	Ca_15442	U	EIL
Ca4_P2_31649942	Ca4	31649942	CT/CTT	CGTGCCCTGCAGGTATTTAT	ATTGACCGGTGGAAGACATT	59.3	454	INTRON	Ca_15442	U	EIL
Ca4_P2_31649993	Ca4	31649993	GAAAAA/GAAAA	CGTGCCCTGCAGGTATTTAT	ATTGACCGGTGGAAGACATT	59.3	454	INTRON	Ca_15442	U	EIL
Ca4_P2_31650019	Ca4	31650019	TAAA/TAAAA	CGTGCCCTGCAGGTATTTAT	ATTGACCGGTGGAAGACATT	59.3	454	INTRON	Ca_15442	U	EIL
Ca4_P2_31652250	Ca4	31652250	CTTTT/CTTTTT	CCATTACCCTTTCGTGATGC	TGAAAGCAAAGCCACGTATG	59.9	796	DRR	Ca_15442	U	EIL
Ca4_P2_31653241	Ca4	31653241	GATAAA/GATAAAAAATAAA	TTCAAACGATGGCAACAAAA	TCATGGGATTAAGCCTCCAC	59.9	268	DRR	Ca_15442	U	EIL
Ca4_P2_31653364	Ca4	31653364	AC/ATGACC	TTCAAACGATGGCAACAAAA	TCATGGGATTAAGCCTCCAC	59.9	268	DRR	Ca_15442	U	EIL
Ca4_P2_31703062	Ca4	31703062	AT/A	TGTGGTTGTGAGTTGGATGG	TGGTTTGCACCGTTAGTCCT	60.6	562	INTERGENIC	--	--	--
Ca4_P2_31718401	Ca4	31718401	AT/ATT	ATTCCTCTAATGTCGGGGCT	GCCACCCTTGTGAGAGTTTC	59.7	501	INTERGENIC	--	--	--
Ca4_P2_31720164	Ca4	31720164	ATTTTTTTTT/ATTTTTTTTTT	AGGGAGGGGAAGAAACAGAG	TACCTCAGCCATTGGACACA	60.1	712	INTERGENIC	--	--	--
Ca4_P2_31724488	Ca4	31724488	GAAAAAAAAAA/GAAAAAAAAAAA	CCTGAGGGGTTTGTTCCTTT	TCCATCAGCAAATGCACCTA	60.2	324	INTRON	Ca_15445	G	--

INDEL marker IDs	Chromosomes/unanchored scaffolds	Physical positions (bp)	InDels (*Kabuli* reference genome-CDC Frontier/PII)	Forward primers (5'-3')	Reverse primers (5'-3')	Anneling temperature (0C)	Expected amplified product size (bp)	Structural annotation		Functional annotation	
								Sequence components of *kabuli* genome	*Kabuli* gene accession IDs	NCBI-KOG	TFs
Ca4_P2_31731129	Ca4	31731129	CAAAAAAAAAAAAA/CAAAAAAAAAAA	AGGGTCATAGCAGCTCCAAA	TCGCAAAGGAAAAATCCAAT	59.5	527	INTRON	Ca_15445	G	--
Ca4_P2_31734174	Ca4	31734174	TGAGAGAGA/TGAGAGA	CGGTCCCTCCTTCTGATTTT	CCACAGAATCGGTCTCCCTA	60.1	303	INTERGENIC	--	--	--
Ca4_P2_31751264	Ca4	31751264	CAAAAAA/CAAAAA	TCTCGTGGAAAATGGACCTC	ATCGGGATGCCCTAATTCTT	59.8	232	INTERGENIC	--	--	--
Ca4_P2_31754106	Ca4	31754106	TAAAAAAAAA/TAAAAAAAAAAA	GAACTGCCAAGTTATCGCAA	AGCAAAAATCTGCACCAACC	60.1	325	DRR	Ca_15446	--	--
Ca4_P2_31754266	Ca4	31754266	CTTTTTTTTTTT/CTTTTTTTTTTTTT	GGTTGGTGCAGATTTTTGCT	AGCACAAAGGCAGAGAAGGA	60.1	266	DRR	Ca_15446	--	--
Ca4_P2_31754313	Ca4	31754313	CTTTTTTTTTTT/CTTTTTTTTTT	GGTTGGTGCAGATTTTTGCT	AGCACAAAGGCAGAGAAGGA	60.1	266	DRR	Ca_15446	--	--
Ca4_P2_31757461	Ca4	31757461	CTTTTTTTTT/CTTTTTTTT	TATGCTAAACCAACCGCCTC	TTTTTCCTGACTTGGTCCTTT	57.8	383	DRR	Ca_15447	R	bZIP
Ca4_P2_31759099	Ca4	31759099	CTTTTTTTTTTT/CTTTTTTTTTT	TCTCCCCACCATCCACTTAG	CTCCACCTCCCCCTATTCAT	60.1	847	INTRON	Ca_15447	R	bZIP
Ca4_P2_31806633	Ca4	31806633	ATGTGACTAATTTT/ATGTGACTAATTTTGTGACTAATTTT	ACAAAAGCCTGGATAGCGAA	TCACATATACCCTTAAAATCGCA	58.6	410	INTERGENIC	--	--	--
Ca4_P2_31816562	Ca4	31816562	ATTTTTTTTTTT/ATTTTTTTTTT	GGCTCCGATCAAAAGAAACA	GCCCTCAGATGAGAGTCAGG	59.9	545	INTERGENIC	--	--	--
Ca4_P2_31833854	Ca4	31833854	ATTTTTT/ATTTTTTT	ACCAACCTCTGTTTGTGGGT	CTCGCCGTTCTCTCCACTAC	60.0	121	INTERGENIC	--	--	--
Ca4_P2_31837048	Ca4	31837048	ATTTTTTTTTTTTT/ATTTTTTTTTTTTTTT	TTCTGGATGGACATTTTCTTTTT	GCATTTCCATGGTCTCCATT	59.8	578	INTERGENIC	--	--	--

INDEL marker IDs	Chromosomes/unanchored scaffolds	Physical positions (bp)	InDels (*Kabuli* reference genome-CDC Frontier/PII)	Forward primers (5'-3')	Reverse primers (5'-3')	Anneling temperature (0C)	Expected amplified product size (bp)	Structural annotation		Functional annotation	
								Sequence components of *kabuli* genome	*Kabuli* gene accession IDs	NCBI-KOG	TFs
Ca4_P2_31837452	Ca4	31837452	ATTTTTTTT/ATTTTTTTTT	TGGAGACCATGGAAATGCTT	TTTTGTGAATAGTCCATGCTTCA	59.6	456	INTERGENIC	--	--	--
Ca4_P2_31859170	Ca4	31859170	GATAATA/GATA	CCTCGATGGATAAGGGTGAA	AAAATTGTCGACCAATCACTTG	59.0	418	INTERGENIC	--	--	--
Ca4_P2_31859597	Ca4	31859597	TAAAAAAAAA/TAAAAAAAAAAA	GTTGGAGCCCCCACTTTATT	TGCAAGCAGCATCAGAGAAC	60.3	793	INTERGENIC	--	--	--
Ca4_P2_31861646	Ca4	31861646	CTTTTTTTTTT/CTTTTTTTTTTT	TTTTGATGATTCACCTGCTCA	TATCCGCTCAATGATGTCCA	60.0	473	INTERGENIC	--	--	--
Ca4_P2_31885833	Ca4	31885833	AAGTTAGTTA/AAGTTA	TTCCATTTGCATTTCCAACA	AACGCGGTAGTTCTTGGATG	60.1	128	INTERGENIC	--	--	--
Ca4_P2_31892250	Ca4	31892250	CTTTTTTTT/CTTTTTTTTT	AACCGTTAACCACCGAAACA	TGCGGTCAACACACTTAGGA	60.3	520	INTERGENIC	--	--	--
Ca4_P2_31894163	Ca4	31894163	AAGAGAGA/AAGAGAGAGA	CCCCACCAAACCAATCATAG	AAACCCCGCATATCTTTTCC	60.2	928	INTERGENIC	--	--	--
Ca4_P2_31917120	Ca4	31917120	AG/A	ACCATGAAACCTTCGACCTG	TTCGTATTGGTGTTCGTCCA	60.0	235	INTERGENIC	--	--	--
Ca4_P2_31921150	Ca4	31921150	CAAAAAAAAAAAA/CAAAAAAAAAA	GGGATAACTTGCTTTTGCGA	TTGAACACATTCAGCAAGCC	59.8	540	DRR	Ca_15453	--	--
Ca4_P2_31929869	Ca4	31929869	TAATAAATAAATAAATAAA/TAATAAATAA	CCATGCTTTCAAAAATGCCT	TTTGGAAGACTTTTGGGCTC	59.3	961	INTERGENIC	--	--	--
Ca4_P2_31930111	Ca4	31930111	AATATATATATA/AATATATATATATATA	GAGGAGCATTGATGGAGGAA	TTTGGAAGACTTTTGGGCTC	59.3	574	INTERGENIC	--	--	--
Ca4_P2_31931258	Ca4	31931258	TAAAAAAAAA/TAAAAAAAA	TCAAACCCGAGAACAAGTGA	TAACAAGCGCGCAATGTATC	59.9	595	INTERGENIC	--	--	--

INDEL marker IDs	Chromosomes/unanchored scaffolds	Physical positions (bp)	InDels (*Kabuli* reference genome-CDC Frontier/PII)	Forward primers (5'-3')	Reverse primers (5'-3')	Anneling temperature (0C)	Expected amplified product size (bp)	Structural annotation		Functional annotation	
								Sequence components of *kabuli* genome	*Kabuli* gene accession IDs	NCBI-KOG	TFs
Ca4_P2_31931370	Ca4	31931370	TAAAAAAAAA/TAAAAAAAAAA	TACGTGTGGATTTGCCTACG	TGTTTGCACCTTTTACAGCG	59.9	430	INTERGENIC	--	--	--
Ca4_P2_31934155	Ca4	31934155	ATTTTTTTTT/ATTTTTTTT	ACAGTTTTACCTCCCACCCC	GTCTTTTCTTCCCCTTTCCG	60.0	835	INTERGENIC	--	--	--
Ca4_P2_31934185	Ca4	31934185	ATATTATTATTATTATT/ATATTATTATTATTATTATT	ACACTCTTTTCTCACCCCCA	GTCTTTTCTTCCCCTTTCCG	60.0	750	INTERGENIC	--	--	--
Ca4_P2_31937240	Ca4	31937240	TGG/TGGG	CTAAGGCCCTATGTGCAAGC	CGAGACTACTCGTGGGCTTC	60.0	835	CDS (FRAME SHIFT)	Ca_15455	--	Dof
Ca4_P2_31948252	Ca4	31948252	AAA/AAATAA	AAAATGCATATATTAATTCATCCCAA	CTGCATGTGCAATTGTGTCA	60.3	385	INTERGENIC	--	--	--
Ca4_P2_31950817	Ca4	31950817	ATTTTTTTTTTT/ATTTTTTTTTT	GAGCAACAGTCAGTGGCGTA	CAACGCGAGCAAAATATCAA	59.8	742	INTERGENIC	--	--	--
Ca4_P2_31954873	Ca4	31954873	TAAAAAAAA/TAAAAAAAAA	CACCCCTCACATACTCGGAT	TGTGTTCCAGAATCATCGTCA	60.1	586	INTERGENIC	--	--	--
Ca4_P2_31966507	Ca4	31966507	TC/T	AGGAAAAGGAAGGGCTTGAA	TCGTTTCAATGTTAGTGACGG	58.7	537	INTERGENIC	--	--	--
Ca4_P2_31967496	Ca4	31967496	ATTTTTT/ATTTTTTT	TACCCTTAATGGCTGGGATG	AAAAACACTTGCGATGACCC	60.0	510	INTRON	Ca_15456	--	--
Ca4_P2_31967535	Ca4	31967535	T/TC	TACCCTTAATGGCTGGGATG	AAAAACACTTGCGATGACCC	60.0	510	INTRON	Ca_15456	--	--
Ca4_P2_31967596	Ca4	31967596	GT/GTTTT	ATCATCAAAAGGGTCATCGC	AGGAACGATGCAAGGAAGAA	59.8	257	INTRON	Ca_15456	--	--
Ca4_P2_31972811	Ca4	31972811	CT/CTT	TTTGTGAAATTGGTTCCCTG	GGTCCAGCAGCATAGGACAT	60.1	523	INTERGENIC	--	--	--

INDEL marker IDs	Chromosomes/unanchored scaffolds	Physical positions (bp)	InDels (*Kabuli* reference genome-CDC Frontier/PII)	Forward primers (5'-3')	Reverse primers (5'-3')	Anneling temperature (0C)	Expected amplified product size (bp)	Structural annotation		Functional annotation	
								Sequence components of *kabuli* genome	*Kabuli* gene accession IDs	NCBI-KOG	TFs
Ca4_P2_31974174	Ca4	31974174	AC/A	GCTGAAAAAGTGGGTGTGGT	GAGAGTGCTGTTGGCTGTGA	60.2	655	INTERGENIC	--	--	--
Ca4_P2_31978699	Ca4	31978699	CATAAACAAATA/CA	ACATTCTTATGTCGGGGCAG	TGGACAGTGATTGATGTTGGA	60.0	159	DRR	Ca_15457	S	--
Ca4_P2_31985613	Ca4	31985613	ATT/ATTT	AAGACTGGACGCCTGAAAAA	GATTTGACATCCATGCTTGC	59.1	344	INTRON	Ca_15457	S	--
Ca4_P2_31985970	Ca4	31985970	TAAAAAAA/TAAAAAAAA	ATGTGCAATAACACCGCAAA	CTGTCTTTTGGACTCGGAGC	60.0	682	INTRON	Ca_15457	S	--
Ca4_P2_31989643	Ca4	31989643	CTTTTTTTT/CTTTTTTTTTTT	GAAAGATTCCTCCCACTCCC	TGCACATTGGAAAATTGGTG	60.4	246	INTERGENIC	--	--	--
Ca4_P2_31991301	Ca4	31991301	GTTTTTTTT/GTTTTTT	AGACAACGACTTTGGCGTTC	ATTGCTCCTTCAATCATGGG	59.9	534	INTERGENIC	--	--	--
Ca4_P2_31992393	Ca4	31992393	ATGACACATTGA/ATGA	AATCAGGATGGTGAGAACCG	TTTTGAAAAACCCATCCTCG	59.9	540	INTERGENIC	--	--	--
Ca4_P2_31992893	Ca4	31992893	CTGAGTTAA/CTGAGTTAATGAGTTAA	TGACACAATTGCTTATGCGG	TTCCAACGGTCATAAAAGGC	59.9	869	DRR	Ca_15458	--	--
Ca4_P2_31994404	Ca4	31994404	TAAAA/TAAAAA	ATCAGCGAGGATGCAGAACT	CATCGCTCAGTTTGGACTCA	60.0	796	DRR	Ca_15458	--	--
Ca4_P2_32000683	Ca4	32000683	ATTTTTT/ATTTTTTT	TGCCCATGTTTACCACAAAA	GCAATGAGGAACTTCAAGCC	59.8	445	INTERGENIC	--	--	--
Ca4_P2_32001064	Ca4	32001064	GTTTTTTT/GTTTTTT	CTTGTTGCATCCAAAACACG	AAAAAGGAAAGCTCGGGGTA	60.1	163	INTERGENIC	--	--	--
Ca4_P2_32001207	Ca4	32001207	TC/T	CTTGTTGCATCCAAAACACG	GCCAAGCAGAAAGTTCATCC	59.8	207	INTERGENIC	--	--	--

INDEL marker IDs	Chromosomes/unanchored scaffolds	Physical positions (bp)	InDels (*Kabuli* reference genome-CDC Frontier/PII)	Forward primers (5'-3')	Reverse primers (5'-3')	Anneling temperature (0C)	Expected amplified product size (bp)	Structural annotation		Functional annotation	
								Sequence components of *kabuli* genome	*Kabuli* gene accession IDs	NCBI-KOG	TFs
Ca4_P2_32003550	Ca4	32003550	TAAAAAAAAA/TAAAAAAAAAA	ATCAGCGAGGATGCAGAACT	TTGAAATTTTTGTCAGGCCA	59.1	731	INTERGENIC	--	--	--
Ca4_P2_32005767	Ca4	32005767	TAAAAAAAAA/TAAAAAAAA	CATTGGAGGCTTGTTGTCAC	AGGTGCACGCTTCAGCTATT	60.0	674	INTERGENIC	--	--	--
Ca4_P2_32006778	Ca4	32006778	ATTTTTT/ATTTTT	CAAAAGAAGGGGGAATGACC	GCAAAGGGCAAGACTAATGG	59.7	760	INTERGENIC	--	--	--
Ca4_P2_32007020	Ca4	32007020	TCCCCCC/TCCCCCCC	TTGGAAAAAGTTGGAGAAGGA	GCAAAGGGCAAGACTAATGG	59.7	348	INTERGENIC	--	--	--
Ca4_P2_32007324	Ca4	32007324	A/ACCAAT	CCATTAGTCTTGCCCTTTGC	CAAATAAAATCATGGCTGCAC	58.2	450	INTERGENIC	--	--	--
Ca4_P2_32008597	Ca4	32008597	TAAAAAA/TAAAAAAA	CAGTTTGCTTGACTTCGCAA	TTCGTACAATTCATACGCGG	59.6	659	INTERGENIC	--	--	--
Ca4_P2_32012018	Ca4	32012018	AT/ATT	GGACAAGAGAGAAGAGGGAAAA	ATCGGCTTGCTATGTTTGCT	59.9	380	INTERGENIC	--	--	--
Ca4_P2_32019443	Ca4	32019443	AAAATTAA/AAA	TCAACGTGTTCCCTTCATTG	CGCTGAATCTCATCAAAAGTTG	59.9	896	INTERGENIC	--	--	--
Ca4_P2_32022692	Ca4	32022692	A/AAT	CCGTTTTATTTTCATCGAACAAG	TGCAGCTTGTCTCTTTAGCG	59.5	648	INTERGENIC	--	--	--
Ca4_P2_32022731	Ca4	32022731	AC/A	CCGTTTTATTTTCATCGAACAAG	TGCAGCTTGTCTCTTTAGCG	59.5	648	INTERGENIC	--	--	--
Ca4_P2_32031978	Ca4	32031978	T/TTTTCTGGAAATC	TTCTTGCTCCGAGGATGACT	ACATGTGGTCTGTGGTGGAA	59.8	626	INTERGENIC	--	--	--
Ca4_P2_32034803	Ca4	32034803	TAAAAAAAAAAA/TAAAAAAAA	CGTCCACTTCCACTACCGAT	ATTAAAATTTGGAGCGGGGA	60.6	553	INTERGENIC	--	--	--

INDEL marker IDs	Chromosomes/unanchored scaffolds	Physical positions (bp)	InDels (*Kabuli* reference genome-CDC Frontier/PII)	Forward primers (5'-3')	Reverse primers (5'-3')	Anneling temperature (0C)	Expected amplified product size (bp)	Structural annotation		Functional annotation	
								Sequence components of *kabuli* genome	*Kabuli* gene accession IDs	NCBI-KOG	TFs
Ca4_P2_32035302	Ca4	32035302	CCTTTAA/CCTTTAATTCAAACTTTAA	TTGGCCTATGTCCATTTCGT	TTCTGTTGAGGCTGAGCGTA	59.7	704	INTERGENIC	--	--	--
Ca4_P2_32035344	Ca4	32035344	TAAAAAAAA/TAAAAAAAAA	TTGGCCTATGTCCATTTCGT	TTCTGTTGAGGCTGAGCGTA	59.7	704	INTERGENIC	--	--	--
Ca4_P2_32035468	Ca4	32035468	ATTTTTTTTT/AATTTTTTTTTT	TTGGCCTATGTCCATTTCGT	TTCTGTTGAGGCTGAGCGTA	59.7	704	INTERGENIC	--	--	--
Ca4_P2_32035530	Ca4	32035530	ATTTTTTTT/ATTTTTTTTTTT	TTGGCCTATGTCCATTTCGT	TTCTGTTGAGGCTGAGCGTA	59.7	704	INTERGENIC	--	--	--
Ca4_P2_32035805	Ca4	32035805	GTC/GTCTC	ATTCAATTCCAGCATGCCTC	TTGATGGATCAAATCTCCCA	58.8	108	INTERGENIC	--	--	--
Ca4_P2_32045859	Ca4	32045859	T/TA	TGGGGTTTTGATCCATTTGT	TGATGTCTTTCGATCCACCT	58.1	607	DRR	Ca_15459	--	--
Ca4_P2_32045999	Ca4	32045999	T/TGATACACTC	TTTGGGCTTTTCTTGATTGG	AGTCGGCTTCAAAGGCACTA	60.0	646	DRR	Ca_15459	--	--
Ca4_P2_32046303	Ca4	32046303	GTTTTTT/GTTTTTTT	TTTGGGCTTTTCTTGATTGG	AGTCGGCTTCAAAGGCACTA	60.0	646	DRR	Ca_15459	--	--
Ca4_P2_32046367	Ca4	32046367	ATTTTTTT/ATTTTTTTTTTTT	AGGTGGATCGAAAGACATCA	AGTCGGCTTCAAAGGCACTA	60.0	394	DRR	Ca_15459	--	--
Ca4_P2_32047782	Ca4	32047782	G/GT	TGGGGTTTTGATCCATTTGT	AAAGCCCAAACCAACATTGA	60.3	329	INTERGENIC	--	--	--
Ca4_P2_32048513	Ca4	32048513	TAAAAAAAA/TAAAAAAAAA	CGATTTTGTCGGTCAATCTCT	TCACCGTAGGAAAGCTGGAT	59.7	355	INTERGENIC	--	--	--
Ca4_P2_32049087	Ca4	32049087	ATTTTTTTT/ATTTTTTTTTT	CGAAATCCAGCTTTCCTACG	TTTGGGCCTCCTTGAGATAA	59.6	670	INTERGENIC	--	--	--

INDEL marker IDs	Chromosomes/unanchored scaffolds	Physical positions (bp)	InDels (*Kabuli* reference genome-CDC Frontier/PII)	Forward primers (5'-3')	Reverse primers (5'-3')	Anneling temperature (0C)	Expected amplified product size (bp)	Structural annotation		Functional annotation	
								Sequence components of *kabuli* genome	*Kabuli* gene accession IDs	NCBI-KOG	TFs
Ca4_P2_32049749	Ca4	32049749	AAA/AAAGAA	CCAAATCATTGGCATTCACA	TTCCTATCACATTGCATATCAAAAA	59.8	693	INTERGENIC	--	--	--
Ca4_P2_32050483	Ca4	32050483	TAAAAAA/TAAAAA	AGCAGTGGAGAGTTCCAAATG	GGCCTTCTTGTGACAATTCAA	60.1	547	INTERGENIC	--	--	--
Ca4_P2_32051212	Ca4	32051212	ATTTTTTTTTT/ATTTTTTTTT	TTGGATCAAACCTCATCATCA	TCGAATTTTACCCACCACAA	58.9	658	INTERGENIC	--	--	--
Ca4_P2_32051935	Ca4	32051935	ATTTTTTTTTTT/ATTTTTTTTT	TTGAAGATGGTGAATGTGGAA	TATGACCCGTTGGATTCGAT	60.2	797	INTERGENIC	--	--	--
Ca4_P2_32057338	Ca4	32057338	TG/T	TATTGCTTCGCTTCGCTTTT	GGGAAACTGCCCCTAATTCT	59.4	451	INTERGENIC	--	--	--
Ca4_P2_32059894	Ca4	32059894	TTCTCTCTCT/TTCTCTCT	TTTCCCGACTTGGTCCTTTA	TTTTTATGACCGGACTTCGT	57.2	580	INTERGENIC	--	--	--
Ca4_P2_32061418	Ca4	32061418	T/TGTTTTTTTA	ATCTGTGGCGAAGAGTCCAG	TGTTCAAGGGATCAAAAGGG	59.9	359	INTRON	Ca_15460	--	--
Ca4_P2_32062669	Ca4	32062669	CTTTTT/CTTTTTT	GCCAGGGTTTAAATTTGGGT	GGCCTTGAAACAAGCAGAAG	60.0	287	INTRON	Ca_15460	--	--
Ca4_P2_32063511	Ca4	32063511	CGG/CG	TTGCCTATGCCAATCAATCA	TCGGAATAGGCTTCAATTCG	60.2	390	INTRON	Ca_15460	--	--
Ca4_P2_32063547	Ca4	32063547	TCC/TC	TTGCCTATGCCAATCAATCA	TCGGAATAGGCTTCAATTCG	60.2	390	INTRON	Ca_15460	--	--
Ca4_P2_32069410	Ca4	32069410	A/AC	CCAGTGGTGTCCCTCTTGAT	GAGACGGAATTTGGGACAGA	60.1	767	DRR	Ca_15461	R	--
Ca4_P2_32073125	Ca4	32073125	TAAAA/TAAAAA	CGGGTCCTTGAAGTGATGAT	ACATACTCATAGCGGCCACC	60.0	864	INTERGENIC	--	--	--

INDEL marker IDs	Chromosomes/unanchored scaffolds	Physical positions (bp)	InDels (*Kabuli* reference genome-CDC Frontier/PII)	Forward primers (5'-3')	Reverse primers (5'-3')	Anneling temperature (0C)	Expected amplified product size (bp)	Structural annotation		Functional annotation	
								Sequence components of *kabuli* genome	*Kabuli* gene accession IDs	NCBI-KOG	TFs
Ca4_P2_32101684	Ca4	32101684	TG/T	TCATTGCCTTGTCTGCAAAA	GCACTAACCCGACCCTAACA	60.0	632	INTRON	Ca_15462	--	--
Ca4_P2_32103781	Ca4	32103781	GTGT/G	CTTCTCGGATGAATGGTGGT	TGGGATAGTGATGTAATGGGC	59.7	453	INTRON	Ca_15462	--	--
Ca4_P2_32113890	Ca4	32113890	ATTTT/ATTTTT	AAATGGGTAAGCGTTGTTGC	AGCTGCTAGGGTGCATTTGT	59.9	796	CDS (FRAME SHIFT)	Ca_15462	--	--
Ca4_P2_32118643	Ca4	32118643	AAAT/A	GCCAAAGGTAACAAAGGCAA	TGATTTCTTCATTGCATCGG	59.6	569	INTERGENIC	--	--	--
Ca4_P2_32119212	Ca4	32119212	ATT/AT	TGTTTCGTTGTTGATAAGCATGT	TGTCCTCCTCCCTCCTTTTT	60.0	721	INTERGENIC	--	--	--
Ca4_P2_32119266	Ca4	32119266	A/AC	TGTTTCGTTGTTGATAAGCATGT	TGTCCTCCTCCCTCCTTTTT	60.0	721	INTERGENIC	--	--	--
Ca4_P2_32121497	Ca4	32121497	TAAAAAAAA/TAAAAAAAAAA	TGGCATACGACCAACTCAAA	TTTGCTTTACAAGCTTGGGG	60.2	440	INTERGENIC	--	--	--
Ca4_P2_32121752	Ca4	32121752	ACCC/ACC	CCCCAAGCTTGTAAAGCAAA	AATGAAAGCGTTGATTGGGT	59.4	588	INTERGENIC	--	--	--
Ca4_P2_32121836	Ca4	32121836	AATA/AATATA	CCCCAAGCTTGTAAAGCAAA	AATGAAAGCGTTGATTGGGT	59.4	588	INTERGENIC	--	--	--
Ca4_P2_32121863	Ca4	32121863	AC/A	CCCCAAGCTTGTAAAGCAAA	AATGAAAGCGTTGATTGGGT	59.4	588	INTERGENIC	--	--	--
Ca4_P2_32122386	Ca4	32122386	CTTTTTTTTT/CTTTTTTTTTT	TGGGTTTTACCCAATCAACG	CACCAGCCAAGTTCTTCCTT	59.3	359	INTERGENIC	--	--	--
Ca4_P2_32137227	Ca4	32137227	TTTTATTTATTTATT/TTTTATTTATTTATTTATT	TTTAACTCTGCCCCCTCCTT	TTCATCGGTCAAACAATTTCA	59.0	449	INTERGENIC	--	--	--

INDEL marker IDs	Chromosomes/unanchored scaffolds	Physical positions (bp)	InDels (*Kabuli* reference genome-CDC Frontier/PII)	Forward primers (5'-3')	Reverse primers (5'-3')	Anneling temperature (0C)	Expected amplified product size (bp)	Structural annotation		Functional annotation	
								Sequence components of *kabuli* genome	*Kabuli* gene accession IDs	NCBI-KOG	TFs
Ca4_P2_32139471	Ca4	32139471	TAAAAAA/TAAAAA	TGTGGTTTGGATTGGGTTTT	TCCGTGTGACTATTTGGTGG	59.4	169	INTERGENIC	--	--	--
Ca4_P2_32139759	Ca4	32139759	AAAAGCTTT/AAAAGCTTTAAAGCTTT	TGTGGTTTGGATTGGGTTTT	ACTTCAAAAATTGAGGGGCA	59.5	736	INTERGENIC	--	--	--
Ca4_P2_32141015	Ca4	32141015	T/TTTAGC	GTGTGTTGCGCTTGATCCTA	TGCATTCTTTTGCCATGAAC	59.7	730	INTERGENIC	--	--	--
Ca4_P2_32143759	Ca4	32143759	GATATATA/GATATA	CGTTATCATTGCTTGAACGG	TTGAGCACTTACTGGTGGGA	59.3	941	INTERGENIC	--	--	--
Ca4_P2_32143997	Ca4	32143997	TC/T	TTTGCCTGTTGTTCTTGTACG	TTGAGCACTTACTGGTGGGA	59.3	381	INTERGENIC	--	--	--
Ca4_P2_32144375	Ca4	32144375	TAAAAAA/TAAAAAAA	TCCCACCAGTAAGTGCTCAA	GTCGTTGTTGGTCTGTGTGG	60.0	308	INTERGENIC	--	--	--
Ca4_P2_32148430	Ca4	32148430	AC/ACC	TGAGTTCAAGTGTTGGGTGG	TTGCCTTTTCTTCCACCTTTT	60.1	655	INTERGENIC	--	--	--
Ca4_P2_32148558	Ca4	32148558	GTAAT/GT	TGAGTTCAAGTGTTGGGTGG	AAGCACCAAAGCCACAACTT	59.8	878	INTERGENIC	--	--	--
Ca4_P2_32151280	Ca4	32151280	GAAAAAA/GAAAAAAA	TCGAACCATTGTGCTTGAAA	GCCATTTCCTTTTTAATGCC	58.6	531	INTERGENIC	--	--	--
Ca4_P2_32156632	Ca4	32156632	TAAAAAA/TAAAAAAA	TTCACTTGGTTGCTTTGCAG	CTTAAACCATAATCCCCGCA	59.8	692	INTERGENIC	--	--	--
Ca4_P2_32159052	Ca4	32159052	TAAAA/TAAA	TCTCTTTTAATGGGCCATGC	ATGCATGGAATCCAGCTTTC	60.0	688	INTERGENIC	--	--	--
Ca4_P2_32159336	Ca4	32159336	TAAAAA/TAAAA	TGTCCCTATCGTAAATGGGC	ATGCATGGAATCCAGCTTTC	60.0	662	INTERGENIC	--	--	--

INDEL marker IDs	Chromosomes/unanchored scaffolds	Physical positions (bp)	InDels (*Kabuli* reference genome-CDC Frontier/PII)	Forward primers (5'-3')	Reverse primers (5'-3')	Anneling temperature (0C)	Expected amplified product size (bp)	Structural annotation		Functional annotation	
								Sequence components of *kabuli* genome	*Kabuli* gene accession IDs	NCBI-KOG	TFs
Ca4_P2_32159427	Ca4	32159427	ATTTTTTTTTT/ATTTTTTTTT	TTCCCTTGAAAGTGCATTGA	GCTTCTATGGGCAACGTACC	59.6	224	INTERGENIC	--	--	--
Ca4_P2_32159712	Ca4	32159712	T/TC	GTACGTTGCCCATAGAAGCC	TTTTGGTGTTTTCCCACCTC	59.8	634	INTERGENIC	--	--	--
Ca4_P2_32165683	Ca4	32165683	ATTTTTTTTT/ATTTTTTTTTT	TTTGCTGGCATTTAGGCTCT	AACCATTAGCTCCACCTACCAA	59.9	843	INTERGENIC	--	--	--
Ca4_P2_32167202	Ca4	32167202	TTATATAT/TTATATATAT	TCGAACTGGGGACCTTACAG	CATGCTACCTCCCATGGTGT	60.8	461	INTERGENIC	--	--	--
Ca4_P2_32173778	Ca4	32173778	GAAA/GAA	TGCAACACCGGAACTTCATA	TTTCAACCAATTGTTGCTCG	59.7	299	INTERGENIC	--	--	--
Ca4_P2_32176859	Ca4	32176859	ATTTTTT/ATTTT	TGGTCTTGCAATCTCTGTCG	TTTGTTTCCATCTTTGGTTGG	59.8	445	INTERGENIC	--	--	--
Ca4_P2_32179388	Ca4	32179388	T/TA	TTCTTGCGCTTTGTCTCTCA	TGGAAGTGAACTAAACCCCTTT	59.0	744	INTERGENIC	--	--	--
Ca4_P2_32179682	Ca4	32179682	CAATAATCAAACAAA/CAATAATCAAACAAATAATCAAACAAA	TTGCATACCTCATACCATCCC	TTGATGCATTACCTCCCACA	59.9	731	INTERGENIC	--	--	--
Ca4_P2_32180017	Ca4	32180017	AATGTTTGAT/AATGTTTGATGTTTGAT	TGGGAGGTAATGCATCAAAA	TGACCCAAAATTTCGTTTGC	60.8	304	INTERGENIC	--	--	--
Ca4_P2_32180074	Ca4	32180074	CTTTTTTTT/CTTTTTTTTTT	TGGGAGGTAATGCATCAAAA	TGACCCAAAATTTCGTTTGC	60.8	304	INTERGENIC	--	--	--
Ca4_P2_32180768	Ca4	32180768	CAA/CA	TAAGCCAATGAAGAATCGCC	TTTTCATCCCACCCCAAATA	60.0	425	INTERGENIC	--	--	--
Ca4_P2_32188268	Ca4	32188268	TGG/TG	TTATCACTCCAATTCCCCCA	CCTCCATCGAATTTCTCCAA	60.0	605	INTERGENIC	--	--	--

INDEL marker IDs	Chromosomes/unanchored scaffolds	Physical positions (bp)	InDels (*Kabuli* reference genome-CDC Frontier/PII)	Forward primers (5'-3')	Reverse primers (5'-3')	Anneling temperature (0C)	Expected amplified product size (bp)	Structural annotation		Functional annotation	
								Sequence components of *kabuli* genome	*Kabuli* gene accession IDs	NCBI-KOG	TFs
Ca4_P2_32188418	Ca4	32188418	CAGCAAT/C	TTATCACTCCAATTCCCCCA	CCTCCATCGAATTTCTCCAA	60.0	605	INTERGENIC	--	--	--
Ca4_P2_32202833	Ca4	32202833	TGA/TGAAGA	GATGAAGCCAATGATGCTGA	ACCCTGCAGCTACTTCCTGA	60.0	499	CDS (large-effect mutations)	Ca_15465	--	--
Ca4_P2_32212035	Ca4	32212035	CTCA/CTCATTGATGCTTATGTCA	TGAATCAACAAATGAAAGCAGTG	TCCCGCTTCGATACAGAGAT	59.8	448	INTERGENIC	--	--	--
Ca4_P2_32214848	Ca4	32214848	ATTTTTT/ATTTTTTT	CGCATGTGTATCCCTTTGTG	CGTTCTCGGAACCTTTCTTTC	60.2	471	INTRON	Ca_15467	T	HB-other
Ca4_P2_32215092	Ca4	32215092	AT/A	CGCATGTGTATCCCTTTGTG	ATGGGAATGGAACCATTGAA	60.0	918	INTRON	Ca_15467	T	HB-other
Ca4_P2_32216067	Ca4	32216067	GATTT/G	TGTTGGGTTCTGATCCAATG	ATAAGCGTGCGTTGACCTTT	59.8	539	INTERGENIC	--	--	--
Ca4_P2_32216199	Ca4	32216199	ATTTTTTTT/ATTTTTTT	ATAAAGGTCAACGCACGCTT	GAAATCAACGGATCTCCATCA	59.9	384	INTERGENIC	--	--	--
Ca4_P2_32218753	Ca4	32218753	AT/ATT	GCATTGAAGGGGTCAACACT	GAGGGAGCAAATCGGTATCA	60.0	390	INTERGENIC	--	--	--
Ca4_P2_32218807	Ca4	32218807	T/TA	GCATTGAAGGGGTCAACACT	GAGGGAGCAAATCGGTATCA	60.0	390	INTERGENIC	--	--	--
Ca4_P2_32218844	Ca4	32218844	CT/CTAT	GCATTGAAGGGGTCAACACT	GAGGGAGCAAATCGGTATCA	60.0	390	INTERGENIC	--	--	--
Ca4_P2_32222918	Ca4	32222918	ACTCT/ACT	TCCATCATCGTTCTCATTGG	TCGAACAAAAGGCAACACAA	60.3	225	INTERGENIC	--	--	--
Ca4_P2_32224229	Ca4	32224229	A/AT	TCTTCTTCCACAGGGGATTG	ATATGGGCCAAAAGCAACAA	60.3	519	INTERGENIC	--	--	--

INDEL marker IDs	Chromosomes/unanchored scaffolds	Physical positions (bp)	InDels (*Kabuli* reference genome-CDC Frontier/PII)	Forward primers (5'-3')	Reverse primers (5'-3')	Anneling temperature (0C)	Expected amplified product size (bp)	Structural annotation		Functional annotation	
								Sequence components of *kabuli* genome	*Kabuli* gene accession IDs	NCBI-KOG	TFs
Ca4_P2_32225442	Ca4	32225442	TTATATAT/TTAT	TCAATTCCAATCATTCAACCA	ACATTACACCAATGCGACGA	60.0	349	INTERGENIC	--	--	--
Ca4_P2_32225876	Ca4	32225876	TTAT/TT	TCGTCGCATTGGTGTAATGT	TCCGTGAGGACACAAGTGAG	59.9	506	INTERGENIC	--	--	--
Ca4_P2_32225930	Ca4	32225930	GAAAAAAA/GAAAAAAAA	TCGTCGCATTGGTGTAATGT	TCCGTGAGGACACAAGTGAG	59.9	506	INTERGENIC	--	--	--
Ca4_P2_32227293	Ca4	32227293	AAAAA/AAAAATTAAAA	TCTTCACAACCCCTTGAACC	TTCTTCCAAACGAGCCTCTC	59.6	868	INTERGENIC	--	--	--
Ca4_P2_32227770	Ca4	32227770	GT/GTGATATATGTT	GAAGAGAGGCTCGTTTGGAA	CTAATGGCTCCCGAATTCAA	60.0	474	INTERGENIC	--	--	--
Ca4_P2_32239930	Ca4	32239930	TT/TTGGT	CAGGTAGAGTGCAGATTGCG	TAATGCTTGATGCCAAACCA	60.1	366	INTERGENIC	--	--	--
Ca4_P2_32240927	Ca4	32240927	CTTTTTT/CTTTTTTT	CCCTTTTATTCAGCCATCCA	CTCAAGATCTCGTGGTGCAA	60.0	895	INTERGENIC	--	--	--
Ca4_P2_3224587	Ca4	3224587	AAGAGAGAGAGAGAGAGAGAGAGAGA/AAGAGAGAGAGAGAGAGAGAGAGAGAGA	TCGTTTCATTCGAGCACTTG	TTAATTGCGCACACACACAA	59.8	362	DRR	Ca_12123	T	E2F/DP
Ca4_P2_32255817	Ca4	32255817	TAAAAAAAAAA/TAAAAAAAA	TTCTCAATGCATCATTCAAAAAT	TTGGGAAGGATGAGTTTTGTT	58.5	784	INTERGENIC	--	--	--
Ca4_P2_32263496	Ca4	32263496	A/AC	ACACATGTCCCAGTTGACGA	AAGCACATACATAGGGGATTTGA	59.8	516	INTERGENIC	--	--	--
Ca4_P2_32263960	Ca4	32263960	TAAAAAAA/TAAAAAAAAAA	GATCAAATCCCCTATGTATGTGC	TGACGATGGAGGCTTTTAGG	60.2	191	INTERGENIC	--	--	--
Ca4_P2_32264177	Ca4	32264177	GC/G	CCTAAAAGCCTCCATCGTCA	TGAGAAATTGGTTTCTCTTCCTC	58.9	385	INTERGENIC	--	--	--

INDEL marker IDs	Chromosomes/unanchored scaffolds	Physical positions (bp)	InDels (*Kabuli* reference genome-CDC Frontier/PII)	Forward primers (5'-3')	Reverse primers (5'-3')	Anneling temperature (0C)	Expected amplified product size (bp)	Structural annotation		Functional annotation	
								Sequence components of *kabuli* genome	*Kabuli* gene accession IDs	NCBI-KOG	TFs
Ca4_P2_32264283	Ca4	32264283	TAAAAAA/TAAAAAAA	CCTAAAAGCCTCCATCGTCA	TGCTTGTCAGCTTGTTGTGTC	60.1	682	INTERGENIC	--	--	--
Ca4_P2_32264601	Ca4	32264601	TA/TAA	GATCAAATTCTCTATGCATGTGCT	CCAATTGAAGGGTAAGTGCC	59.4	608	INTERGENIC	--	--	--
Ca4_P2_32264918	Ca4	32264918	CAA/CAAA	GCACTTACCCTTCAATTGGG	AGGAGAGGTCCATTTCTCAGG	59.7	262	INTERGENIC	--	--	--
Ca4_P2_32264980	Ca4	32264980	AAT/AATAT	GCACTTACCCTTCAATTGGG	AGGAGAGGTCCATTTCTCAGG	59.7	262	INTERGENIC	--	--	--
Ca4_P2_32291326	Ca4	32291326	T/TG	CGGACCTGCCCTACAAGTAA	CCGATCTTTGTGGTAATTGTGA	59.9	577	INTERGENIC	--	--	--
Ca4_P2_32300381	Ca4	32300381	AAAA/AAAAGAAA	AGGGAGAACCAACCAACCTT	TTCGGAGTGGTTTTAGCTTTT	58.1	395	DRR	Ca_15473	P	--
Ca4_P2_32300998	Ca4	32300998	CAAAAAAAAAAAAAA/CAAAAAAAAA	AAAAGCTAAAACCACTCCGAA	TGCACCAAATGTGTCCTTGT	60.0	581	DRR	Ca_15473	P	--
Ca4_P2_32301599	Ca4	32301599	TAAAAAAA/TAAAA	TGGATTGCAAAACTGGTTGA	CACGAGCTTACAAACCCACA	59.8	174	DRR	Ca_15472	DO	MYB_related
Ca4_P2_32301839	Ca4	32301839	ATTTTTTT/ATTTTTT	TGGATTGCAAAACTGGTTGA	AGTGGCTTCAAGCTGCAAAT	60.0	727	DRR	Ca_15473	P	--
Ca4_P2_323024	Ca4	323024	TTATATATATATATATATATATA/TTATATATATATATATATATATA	TGAGTTCCTGCACCTCACAA	TTCTTTTAAGTCCAATTCCCAG	57.4	378	INTERGENIC	--	--	--
Ca4_P2_32315268	Ca4	32315268	ATTTTTTTT/ATTTT	TTCTCCCCCAACACAAGTTC	GGCGCATCTTCACTCAAAAG	60.9	345	INTERGENIC	--	--	--
Ca4_P2_3232768	Ca4	3232768	TAAAAAAAAA/TAAAAAAAAAA	TGTAGCAAAGATGCGACCTA	AAAATGAAGTTTGGGGTCCA	59.3	867	INTERGENIC	--	--	--

INDEL marker IDs	Chromosomes/unanchored scaffolds	Physical positions (bp)	InDels (*Kabuli* reference genome-CDC Frontier/PII)	Forward primers (5'-3')	Reverse primers (5'-3')	Anneling temperature (0C)	Expected amplified product size (bp)	Structural annotation		Functional annotation	
								Sequence components of *kabuli* genome	*Kabuli* gene accession IDs	NCBI-KOG	TFs
Ca4_P2_32335179	Ca4	32335179	AAAGAAGAAGAAGAAGAAGAA/AAAGAAGAAGAAGAAGAA	AAAGTCCGTTTGTGAATCCG	TGGGCATTCAAATCAAACAA	59.9	507	INTERGENIC	--	--	--
Ca4_P2_32335777	Ca4	32335777	CA/CATTAA	ATGCCCAAGTTTTCAGTTCG	TGGGCATTCAAATCAAACAA	59.9	201	INTERGENIC	--	--	--
Ca4_P2_32353193	Ca4	32353193	GAAA/GAA	TGACATGGCTTGTTCCTTCA	CAAGTTGGGTGAACCAGGAT	59.8	736	INTERGENIC	--	--	--
Ca4_P2_32367636	Ca4	32367636	ATTTTTTTTTTTTT/ATTTTTTTTTTTT	TCCTCCTTGTGATCTTGGCT	CATTGCTCCCTCTTACTGCC	59.8	429	INTERGENIC	--	--	--
Ca4_P2_32369377	Ca4	32369377	TA/T	TTGGTTTCGTTTGCTTGTTG	AATAGTGCGCATGTGTGGTC	59.6	822	INTERGENIC	--	--	--
Ca4_P2_323717	Ca4	323717	ATTT/AT	CTGGGAATTGGACTTAAAAGAA	TTGTTGGGTTATCGTTGTCG	59.4	791	INTERGENIC	--	--	--
Ca4_P2_32373608	Ca4	32373608	ATTTTTTTT/ATTTTTTT	TCCACTTTTCAAACGAGATGG	AAGATCACGTTTCACATCGG	58.6	564	INTERGENIC	--	--	--
Ca4_P2_32390117	Ca4	32390117	GAAAAAAAAAAA/GAAAAAAAAAA	TTCAGTACAACCACGGGTGA	TCGCATGTTCTTCACAAAGG	59.8	449	INTERGENIC	--	--	--
Ca4_P2_32393229	Ca4	32393229	CATAAA/CATAAATATAAA	TGAAGGGAGACAAAGGAGGA	CGAGAGTATTCATTGTCTTGTACCAT	59.9	603	DRR	Ca_15475	--	--
Ca4_P2_32395281	Ca4	32395281	TA/TAA	GGTTAACAAATCTCCTAAAGGCA	TTTTGTTAAAGAATTGCGACCTC	59.7	523	INTERGENIC	--	--	--
Ca4_P2_32396185	Ca4	32396185	TCCC/TCC	TCGTCATTTCTTTGTTTGGTTG	TCCAATTTTCCGTGTCATGT	58.8	346	INTERGENIC	--	--	--
Ca4_P2_32399880	Ca4	32399880	CATA/CATACAAATAAAAATA	TTCTGTCCGAGTATGGGTCC	CATGATGGTTTTGTTGTGGC	59.8	835	INTERGENIC	--	--	--

INDEL marker IDs	Chromosomes/unanchored scaffolds	Physical positions (bp)	InDels (*Kabuli* reference genome-CDC Frontier/PII)	Forward primers (5'-3')	Reverse primers (5'-3')	Anneling temperature (0C)	Expected amplified product size (bp)	Structural annotation		Functional annotation	
								Sequence components of *kabuli* genome	*Kabuli* gene accession IDs	NCBI-KOG	TFs
Ca4_P2_32410630	Ca4	32410630	TA/TAA	TTATTCCACCCCACCTCAAA	CTCAGCTCATTGGTTTGGTG	59.3	262	INTERGENIC	--	--	--
Ca4_P2_32412395	Ca4	32412395	TAAAAAAA/TAAAAAA	TTCAAATCTCGCAGTTGTTTG	GAAATCCCAGTAGCAGTGGC	59.7	642	INTERGENIC	--	--	--
Ca4_P2_32413633	Ca4	32413633	TAAAAAAAAA/TAAAAAAAA	TGATCTAACAGTTGTGATTGATTGA	CAACTAACTCACACAACAGAGCG	60.0	670	INTERGENIC	--	--	--
Ca4_P2_32431643	Ca4	32431643	TTAATAATAA/TTAATAA	ACCCAACCTCATCTGCATTC	TTTCAGAAAAACTACGGAACAAA	58.0	362	DRR	Ca_15476	--	--
Ca4_P2_32431697	Ca4	32431697	A/AT	ACCCAACCTCATCTGCATTC	TTTCAGAAAAACTACGGAACAAA	58.0	362	DRR	Ca_15476	--	--
Ca4_P2_32458469	Ca4	32458469	TAAA/TAA	AATGGTCCAACCCCTTAACC	TTTGGACAATGTCGTGAAACA	60.0	526	INTERGENIC	--	--	--
Ca4_P2_32460225	Ca4	32460225	TAAAAAAAAA/TAAAAAAAAAA	CGGAGATCCATAACACGGTC	TTTGAAAGAGAAATCCACCCA	59.5	242	INTERGENIC	--	--	--
Ca4_P2_32466403	Ca4	32466403	ATT/ATTT	GCCAGTAGGAAAATCCTCCA	ACCCAAACAAGAACAAACGC	60.0	539	INTERGENIC	--	--	--
Ca4_P2_32471789	Ca4	32471789	TAAAA/TAAAAA	GCGCTATAAAAGGCTTGCAC	GAGCGCTGTAAAAGACCACC	59.9	419	INTERGENIC	--	--	--
Ca4_P2_32473583	Ca4	32473583	TTTTTTTATTTTTTATTTT/TTTTTTTATTTTTTATTTTTTTATTTT	TGATCTTAGCGATGGTTCCC	TGAGCGTTCGATTTTGTTTG	59.8	687	INTERGENIC	--	--	--
Ca4_P2_32482906	Ca4	32482906	ATTT/ATT	GTTAAAACATTTCCGCCCAA	CAAGAACGAAGAAAAACGCC	59.9	485	INTERGENIC	--	--	--
Ca4_P2_32484353	Ca4	32484353	CTTTTTT/CTTTTTTT	TTATCCTTTGCTGCCCAAAC	ATCATGTTGCCCCTAATCCA	60.2	753	INTERGENIC	--	--	--

INDEL marker IDs	Chromosomes/unanchored scaffolds	Physical positions (bp)	InDels (*Kabuli* reference genome-CDC Frontier/PII)	Forward primers (5'-3')	Reverse primers (5'-3')	Anneling temperature (0C)	Expected amplified product size (bp)	Structural annotation		Functional annotation	
								Sequence components of *kabuli* genome	*Kabuli* gene accession IDs	NCBI-KOG	TFs
Ca4_P2_32487077	Ca4	32487077	GAAA/GAAATAAAA	CGTCAATTTGAGACGACGAA	ATTTCATCAATTCGGGGTGA	60.1	618	INTERGENIC	--	--	--
Ca4_P2_32487290	Ca4	32487290	ATTTTTTTTTT/ATTTTTTTTT	TCGGAGAAACGAGATCACAA	ATTTCATCAATTCGGGGTGA	60.1	533	INTERGENIC	--	--	--
Ca4_P2_32489291	Ca4	32489291	ATT/ATTGTT	CGACAAAGAGGTGGATGGTT	CCTCCATCAATAATAATAAAACCCC	60.1	423	INTERGENIC	--	--	--
Ca4_P2_32489467	Ca4	32489467	ATTTTTTTT/ATTTTTTT	CGACAAAGAGGTGGATGGTT	TGAAGCACAAAGAGAAAATCTCA	59.1	732	INTERGENIC	--	--	--
Ca4_P2_32489750	Ca4	32489750	C/CA	GGGTTTTATTATTATTGATGGAGGG	GCAAACTCCAACTATTTTCGC	58.9	614	INTERGENIC	--	--	--
Ca4_P2_32491529	Ca4	32491529	GTT/GTTT	GGTGATGGTTTCGCCTAAAA	TCAAACACACACGAAAATAGCA	59.3	446	INTERGENIC	--	--	--
Ca4_P2_32492649	Ca4	32492649	GAAAAAA/GAAAAA	GCATTACACTGAAGCGGACA	GCCAATTTCAATTAAGCCGA	60.0	622	INTERGENIC	--	--	--
Ca4_P2_32492742	Ca4	32492742	AAGA/AA	AGTGGATTGGGTGGAAAATG	GCCAATTTCAATTAAGCCGA	60.0	296	INTERGENIC	--	--	--
Ca4_P2_32494355	Ca4	32494355	ATTTTTTTTTTT/ATTTTTTTTTTTTTTTT	ATGGGGATATGGATGGATGA	TGAGGAGCATAAATCAGAGAAAAA	59.4	388	INTERGENIC	--	--	--
Ca4_P2_32494646	Ca4	32494646	GAAAAAAA/GAAAAAAAA	CAATTCACTTGAAACGTCTCTCAT	CACAAAATCGACAAAGACATGC	60.5	386	INTERGENIC	--	--	--
Ca4_P2_32494672	Ca4	32494672	TA/T	CAATTCACTTGAAACGTCTCTCAT	CACAAAATCGACAAAGACATGC	60.5	386	INTERGENIC	--	--	--
Ca4_P2_32498847	Ca4	32498847	AG/A	TAGCACGGACCCCAATTAAG	TGAACCCCCATCAAAGGATA	60.1	897	INTERGENIC	--	--	--

INDEL marker IDs	Chromosomes/unanchored scaffolds	Physical positions (bp)	InDels (*Kabuli* reference genome-CDC Frontier/PII)	Forward primers (5'-3')	Reverse primers (5'-3')	Anneling temperature (0C)	Expected amplified product size (bp)	Structural annotation		Functional annotation	
								Sequence components of *kabuli* genome	*Kabuli* gene accession IDs	NCBI-KOG	TFs
Ca4_P2_32511427	Ca4	32511427	TTATATATATATAT/TTATATATATAT	TCCCTTTCTGACATGCATCA	TCTAGACTAGGCTGCTCCCG	59.7	468	INTERGENIC	--	--	--
Ca4_P2_32512065	Ca4	32512065	AG/A	TTTCTCCCGTTATGTGTCGG	TGAGTGCTGGGAACTAACCC	60.1	704	INTERGENIC	--	--	--
Ca4_P2_32513097	Ca4	32513097	TGGGGGG/TGGGGG	GATTTGCATTGGCCTCTTGT	ACAGTGGTTGCCATTTCCTC	60.0	479	INTERGENIC	--	--	--
Ca4_P2_32514320	Ca4	32514320	GACATTGACTTTTT/GACATTGACTTTTTTCAACATTGACTTTTT	ATTACTGGACCAGAGGCACG	AATGTCCATTCTGGGACGAG	59.9	510	INTERGENIC	--	--	--
Ca4_P2_32515383	Ca4	32515383	GAT/GATCAT	GGCTATCGACCTAGGGAAGC	ACCAACAGAACCGCCAATAG	60.0	347	INTERGENIC	--	--	--
Ca4_P2_32516312	Ca4	32516312	CAAAAAAAAA/CAAAAAAAAAA	AGCTTGTGATGTTTGGGGTC	TCCTAAGGGCTATGGGAACA	59.5	792	INTERGENIC	--	--	--
Ca4_P2_32518028	Ca4	32518028	TGGGG/TGGG	CGAGCAATGGTCCAAAGATT	AAGTCAACGCCTTTTACCCA	59.6	303	DRR	Ca_15480	--	--
Ca4_P2_32519815	Ca4	32519815	T/TA	CTTGCTGTCTGATGCCAAAA	ATTATGGCCTGATGGCTTTG	59.9	524	DRR	Ca_15480	--	--
Ca4_P2_32522290	Ca4	32522290	GAAAAAAAAA/GAAAAAAAAAA	ACTCATTCATTCCACAGCCC	TTGCTAACGGTAACACAACGA	59.3	851	INTERGENIC	--	--	--
Ca4_P2_32526682	Ca4	32526682	CAATTAATAATTAATA/CAATTAATA	GGCAAAACATTGCATGTCAG	GATCTGGGAGGAGACCATGA	60.0	677	DRR	Ca_15481	--	--
Ca4_P2_32527479	Ca4	32527479	AATAT/AATATAT	GGCGGAGTAAATTGTTGCAT	TACATGAGCAGGAGGAGGCT	60.0	333	DRR	Ca_15481	--	--
Ca4_P2_32529368	Ca4	32529368	GATATATATATAT/GATATATATATATAT	GGGTGTGATTAATGGGTTCG	TCAGGGGTTGCATGGTATTT	60.2	457	INTERGENIC	--	--	--

INDEL marker IDs	Chromosomes/unanchored scaffolds	Physical positions (bp)	InDels (*Kabuli* reference genome-CDC Frontier/PII)	Forward primers (5'-3')	Reverse primers (5'-3')	Anneling temperature (0C)	Expected amplified product size (bp)	Structural annotation		Functional annotation	
								Sequence components of *kabuli* genome	*Kabuli* gene accession IDs	NCBI-KOG	TFs
Ca4_P2_32531473	Ca4	32531473	TAA/T	TAAAATGGATGCATCGAGCC	ACAAATCAACCAGTGTCGCA	60.2	662	INTERGENIC	--	--	--
Ca4_P2_32544975	Ca4	32544975	GC/G	ACCAAAACAGAGGCAAGCAT	TGAGGCATGACGATGTTGAT	60.1	866	INTERGENIC	--	--	--
Ca4_P2_32548680	Ca4	32548680	CAAAAAAAAAA/CAAAAAAAAA	TTATTTGGCCCCACTTCTTG	TGATTGGCATGAGTGCTGAC	60.9	688	INTERGENIC	--	--	--
Ca4_P2_32548849	Ca4	32548849	GA/GAGCCA	CGAACCAAGTGAGGAAAGAGA	TGATTGGCATGAGTGCTGAC	60.9	603	INTERGENIC	--	--	--
Ca4_P2_32548891	Ca4	32548891	GT/GTTAT	CGAACCAAGTGAGGAAAGAGA	TGATTGGCATGAGTGCTGAC	60.9	603	INTERGENIC	--	--	--
Ca4_P2_32549547	Ca4	32549547	GTATA/GTA	TTGATGTCTATTGATGCAACG	ACATTGTGCCATCCCTTGAT	60.2	747	INTERGENIC	--	--	--
Ca4_P2_32550360	Ca4	32550360	ATTT/ATTTT	TGAATTAGCTTGGCCGTTTT	GAGAAAATCAACCCAACGTCA	60.0	530	INTERGENIC	--	--	--
Ca4_P2_32553993	Ca4	32553993	CAAGATGAAAGATGAAAGATGAAAGAT/CAAGATGAAAGAT	TATGATGTCGGGGTGTGAAA	ATTGCCTCCATCAAAGAAAA	57.3	756	INTERGENIC	--	--	--
Ca4_P2_32554394	Ca4	32554394	A/ATTC	AAATTAAGTGCGGAGGGGTC	ACCCATTATGGTGAAGAGCG	60.0	832	INTERGENIC	--	--	--
Ca4_P2_32555147	Ca4	32555147	TAAAAAAAA/TAAAAAAAAA	TTCACCATAATGGGTGTCCA	GGAGAATAGAGGCGGCTAGG	60.3	428	INTERGENIC	--	--	--
Ca4_P2_32555948	Ca4	32555948	AAAGAA/AAA	GTGTGTTTCCCCATACCACC	TGGGAATTTTGTTTGTGGGT	60.1	449	INTERGENIC	--	--	--
Ca4_P2_32559742	Ca4	32559742	AAAGAAAGAAGAAAGAAGA/AAAGAAAGAAGA	TTCATTTCCAGTCTGTGGCA	ACGGCCGTTATAGGTTTCCT	59.9	669	INTERGENIC	--	--	--

INDEL marker IDs	Chromosomes/unanchored scaffolds	Physical positions (bp)	InDels (*Kabuli* reference genome-CDC Frontier/PII)	Forward primers (5'-3')	Reverse primers (5'-3')	Anneling temperature (0C)	Expected amplified product size (bp)	Structural annotation		Functional annotation	
								Sequence components of *kabuli* genome	*Kabuli* gene accession IDs	NCBI-KOG	TFs
Ca4_P2_32559814	Ca4	32559814	A/AC	TTCATTTCCAGTCTGTGGCA	TGAGAAACTTTTCCGCTGCT	60.1	872	INTERGENIC	--	--	--
Ca4_P2_32562355	Ca4	32562355	CTTTTT/CTTTTTT	AAATGGAGGAGAAGGAGGGA	CGGTGAATCCTTTACGGAGA	60.1	286	INTERGENIC	--	--	--
Ca4_P2_32565478	Ca4	32565478	TC/TCCCAC	TATCAACCTATTCAGCCGCC	AGGGTTAGACGAGTTCGACG	59.4	950	DRR	Ca_15483	T	YABBY
Ca4_P2_32565748	Ca4	32565748	GATAATAATAATAATAATAATAATAATAATAA/GATAATAATAATAATAATAATAATAATAA	ATGCAACCATGGGAAGAAAT	CGTCACCACCACCACATCTA	60.4	983	DRR	Ca_15483	T	YABBY
Ca4_P2_32567747	Ca4	32567747	TAAAAA/TAAAA	GAAGGTGAGACAGCAGAGGG	TGACGTGTGTAGTTTCCCCA	60.0	589	INTERGENIC	--	--	--
Ca4_P2_32586827	Ca4	32586827	GAAAAAAAA/GAAAAAAAAA	TATTCTTTGCCGCTCCAATC	AAGGACCAAAACCAATTGTCTC	59.4	750	INTERGENIC	--	--	--
Ca4_P2_32599378	Ca4	32599378	CGG/CGGG	AGCATTGCCAATCATCTTCC	TCAAACGTCAATTGGCAAAA	60.1	757	INTERGENIC	--	--	--
Ca4_P2_32622005	Ca4	32622005	T/TTAG	CATTGTTGCGCCTTAAATGA	GAATTTGAAATGCACCCGTT	59.8	525	DRR	Ca_15485	O	--
Ca4_P2_32622348	Ca4	32622348	TATA/TATAAAAAAATCAATA	TCAATGCCATGGAAAGACAA	TGATAAAATTAAGGGATCGTGAAA	59.0	808	DRR	Ca_15485	O	--
Ca4_P2_32622405	Ca4	32622405	GTTT/GTT	TCAATGCCATGGAAAGACAA	TTCCATCTCCAACAACATCG	59.5	867	DRR	Ca_15485	O	--
Ca4_P2_32622663	Ca4	32622663	CAAAAAAA/CAAAAAAAA	TTTCACTCCATATATTTTCACTCCA	TTCCATCTCCAACAACATCG	59.5	543	DRR	Ca_15485	O	--
Ca4_P2_32623000	Ca4	32623000	TAAAAAAAAAA/TAAAAAAAAAAAA	TCGATGTTGTTGGAGATGGA	TCCATCTTTTGGAGGAATGG	59.9	566	DRR	Ca_15485	O	--

INDEL marker IDs	Chromosomes/unanchored scaffolds	Physical positions (bp)	InDels (*Kabuli* reference genome-CDC Frontier/PII)	Forward primers (5'-3')	Reverse primers (5'-3')	Anneling temperature (0C)	Expected amplified product size (bp)	Structural annotation		Functional annotation	
								Sequence components of *kabuli* genome	*Kabuli* gene accession IDs	NCBI-KOG	TFs
Ca4_P2_32626829	Ca4	32626829	TTT/TTTGAACTCTATT	CAGCTGTCCCACACTGTGAT	TGTTGCCAGTTTTGCTTGAC	59.9	144	INTRON	Ca_15485	O	--
Ca4_P2_32628615	Ca4	32628615	AA/AAGA	GGGGTGATCCGTGATATTTG	CAGTTGATTCTGGAGCCCAT	60.1	567	INTRON	Ca_15485	O	--
Ca4_P2_32631880	Ca4	32631880	CTTTCTTTTTCTTTTTCT/CTTTCTTTTTCTTTTTCTTTTTCT	TTACGTGGAGAGGGAAATGG	CACACATCACTTCAACCGTG	58.5	542	INTERGENIC	--	--	--
Ca4_P2_32633699	Ca4	32633699	CATAATAGTAATATAATAGTAAT/CATAATAGTAAT	TATACACGGGACATGAGCCA	ATCCTTCAAAGCAAAAGCCA	59.8	145	INTERGENIC	--	--	--
Ca4_P2_32634327	Ca4	32634327	TTTACTTACTTA/TTTACTTA	CAGACCCGTGTGAACACAAA	GCCTTTGGAGTTGAAGATTGA	59.3	591	INTERGENIC	--	--	--
Ca4_P2_32634672	Ca4	32634672	CTTTATCT/CTTTATCTTTTATCT	CAGACCCGTGTGAACACAAA	AGCAGATGCCTCTATCGCAT	60.0	937	INTERGENIC	--	--	--
Ca4_P2_32635524	Ca4	32635524	A/ACG	ATGCGATAGAGGCATCTGCT	TGCATTTTGCCTGCTCTATG	60.0	842	DRR	Ca_15486	R	NAC
Ca4_P2_32635587	Ca4	32635587	CATA/CA	ATGCGATAGAGGCATCTGCT	TGCATTTTGCCTGCTCTATG	60.0	842	DRR	Ca_15486	R	NAC
Ca4_P2_32646331	Ca4	32646331	CGA/C	TCAAAATCAAAGACCGAAAACA	TGCAAGTTGTGTGGTGTTGA	59.8	378	INTERGENIC	--	--	--
Ca4_P2_32646931	Ca4	32646931	TAAA/TAAAAAAA	GCGCTTTGTTCCTCACTCTC	AGTATGGGGAACAGGGGAAA	60.5	911	INTRON	Ca_15487	--	--
Ca4_P2_32647144	Ca4	32647144	TAAA/TAA	TGCATTGTTTTCAAGGGTCA	CCTAATTGCACCCCAAAATG	60.2	611	INTRON	Ca_15487	--	--
Ca4_P2_32663516	Ca4	32663516	TTTATTATTATTATTATTATTATT/TTTATTATTATTATTATT	TGCGTTTCACTCTTCCTTCC	TGTCAAGCCCAAAAACAAAA	59.2	257	INTERGENIC	--	--	--

INDEL marker IDs	Chromosomes/unanchored scaffolds	Physical positions (bp)	InDels (*Kabuli* reference genome-CDC Frontier/PII)	Forward primers (5'-3')	Reverse primers (5'-3')	Anneling temperature (0C)	Expected amplified product size (bp)	Structural annotation		Functional annotation	
								Sequence components of *kabuli* genome	*Kabuli* gene accession IDs	NCBI-KOG	TFs
Ca4_P2_32696742	Ca4	32696742	AGG/AGGG	TGTATTTGCAGGGACGTGAA	CTCATGAGAGGCGATTGTGA	59.9	467	INTERGENIC	--	--	--
Ca4_P2_32700117	Ca4	32700117	CTTTTTTT/CTTTTTTTT	TACGTGTCGCTTAGGCACTG	CGTCTTGTGCGGTGTATTTG	60.2	461	INTERGENIC	--	--	--
Ca4_P2_32747280	Ca4	32747280	GAAAA/GAAA	TCTTCCACCCAGTTTTGACC	GGAGACAGGTGTGGACGTTT	60.0	386	INTERGENIC	--	--	--
Ca4_P2_32873959	Ca4	32873959	CTTTTTTTTTTT/CTTTTTTTTTTTTT	AAAAATTATTTCCCCAAATGTCC	GGAAGCGACCACACATTTTT	60.0	219	INTERGENIC	--	--	--
Ca4_P2_32886390	Ca4	32886390	AC/ACC	AATCATCCCATGTTGCCATT	CGAGATTTCACACCAAGGGT	60.0	519	INTERGENIC	--	--	--
Ca4_P2_32888389	Ca4	32888389	TCCCC/TCCCCC	TCTGCTCTTAATTAAATCCACTCAAA	TCTTCTCCCTCCCTAGCCTC	59.9	387	DRR	Ca_15497	--	--
Ca4_P2_32909724	Ca4	32909724	TGG/TG	TTTTTAAATTGAAGCAACGGAAA	TGTCCCTCCTCCCTCTACAA	59.6	574	INTERGENIC	--	--	--
Ca4_P2_32931782	Ca4	32931782	ATTT/ATT	CCGAGCCAAAATCCAAAATA	TCCAATGAAAGCAAACCACA	60.1	867	INTERGENIC	--	--	--
Ca4_P2_33008234	Ca4	33008234	A/AC	GCGAACATGAATGTCATTGG	TTTCTTCCTGAGTTGCGCTT	60.1	832	INTERGENIC	--	--	--
Ca4_P2_33050402	Ca4	33050402	ATTTTTTTTTTT/ATTTTTTTTTT	TTCCTCACACGCAAATTGTC	AAAAGAATTTGGGGTCTTTCG	59.5	531	DRR	Ca_24017	GC	HSF
Ca4_P2_33054018	Ca4	33054018	TAA/TAAA	GGAATGGCAAACTTTCATGC	TTTGCATTACGGATTTGATGA	59.0	520	INTERGENIC	--	--	--
Ca4_P2_33054232	Ca4	33054232	TAAAAAAAA/TAAAAAAAAA	TCATCAAATCCGTAATGCAAA	GCACATGGGGTACCAGACTT	59.9	445	INTERGENIC	--	--	--

INDEL marker IDs	Chromosomes/unanchored scaffolds	Physical positions (bp)	InDels (*Kabuli* reference genome-CDC Frontier/PII)	Forward primers (5'-3')	Reverse primers (5'-3')	Anneling temperature (0C)	Expected amplified product size (bp)	Structural annotation		Functional annotation	
								Sequence components of *kabuli* genome	*Kabuli* gene accession IDs	NCBI-KOG	TFs
Ca4_P2_33055003	Ca4	33055003	CTTTTTT/CTTTTTTT	AAGTCTGGTACCCCATGTGC	TAAAGGACCAAGTCGGGAAA	59.5	645	INTERGENIC	--	--	--
Ca4_P2_33064079	Ca4	33064079	ATTGTTGTTGTTGTTGTTGTTG/ATTGTTGTTGTTGTTGTTGTTGTTG	TCGGGCCACTATCTGGATTA	GTGTGCCGTCACAAACAATC	60.0	810	INTERGENIC	--	--	--
Ca4_P2_3307079	Ca4	3307079	ANNNNNNNNNN/ANNNN	TCCCAATTGATTCGACCTTC	CAAAGACTGCATATCGGCAA	59.8	350	INTERGENIC	--	--	--
Ca4_P2_33088053	Ca4	33088053	TAAAAA/TAAAAAA	CCTGCAGTATCCTCAACTTGC	GTGCCTGGTAAATGGATGCT	60.0	790	INTERGENIC	--	--	--
Ca4_P2_33100738	Ca4	33100738	GAAAAAAAAAA/GAAAAAAAAAAAA	TTGGGTTGTGAGGAATGGAT	TCATCTGTTAGGTACCCCGC	60.0	304	INTERGENIC	--	--	--
Ca4_P2_33111076	Ca4	33111076	CTTTTTTT/CTTTTTTTT	GCATCACGCTACCATAGGCT	AAGTTTTGGGTTGCTCATGG	60.0	392	INTERGENIC	--	--	--
Ca4_P2_3313145	Ca4	3313145	AATATAT/AATAT	GCAGCCCCTCCTTTTTCTAT	TTGATGAAGGAAAAAGACATCG	59.2	698	INTERGENIC	--	--	--
Ca4_P2_33133711	Ca4	33133711	ATTTTTT/ATTTTTTT	TCAGATTTGGAATGGCTTCA	GGTACGTCCAGTCCTCTTGC	59.7	460	INTERGENIC	--	--	--
Ca4_P2_33145619	Ca4	33145619	TAAAAAAA/TAAAAAA	AAACCGTCGTTCCAGTTGTC	AGGCAACAAACATAGAATCGC	59.2	550	INTERGENIC	--	--	--
Ca4_P2_33155610	Ca4	33155610	GTTATTATTATTATTATTA/GTTATTATTATTATTA	CAAGCTCAGAAATCCAAATTCA	CCTCCAATTCAGTCTTCCCA	60.0	425	INTERGENIC	--	--	--
Ca4_P2_33170727	Ca4	33170727	TC/T	TTTTTCTTGAAAATGTTGTCGTG	TGTCATATTCGTCGGCTTTG	59.7	774	INTERGENIC	--	--	--
Ca4_P2_33184933	Ca4	33184933	TTATATATATATATAT/TTATATATATATAT	GACATTTCGAACTTGAATTTCCT	AATCAGCACTGCTCCGAACT	60.0	491	INTERGENIC	--	--	--

INDEL marker IDs	Chromosomes/unanchored scaffolds	Physical positions (bp)	InDels (*Kabuli* reference genome-CDC Frontier/PII)	Forward primers (5'-3')	Reverse primers (5'-3')	Anneling temperature (0C)	Expected amplified product size (bp)	Structural annotation		Functional annotation	
								Sequence components of *kabuli* genome	*Kabuli* gene accession IDs	NCBI-KOG	TFs
Ca4_P2_33195041	Ca4	33195041	T/TAGCATTCGGTGA	TTGTCCGTGAGAGACTGCAC	GAATCCTGGGCTGAAATCAA	60.0	595	INTERGENIC	--	--	--
Ca4_P2_33204821	Ca4	33204821	AATATATATATATATATATATAT/AATATATATATATATATAT	GCCACACTCCGAAACAAAAT	ATGGAAGTAGGTGGCAGTGG	60.0	823	INTERGENIC	--	--	--
Ca4_P2_33256637	Ca4	33256637	ATTTTTTTTT/ATTTTTTTT	CATTGATCATGGTTCTTGCC	GGCTTGTGAAAACAAATGGAA	60.0	329	INTERGENIC	--	--	--
Ca4_P2_33260832	Ca4	33260832	TGAAGAAGAAGAA/TGAAGAAGAAGAAGAA	ACATCTGGAATTTGCTTGCC	CGACTTTGGCACCATATCCT	60.0	968	INTERGENIC	--	--	--
Ca4_P2_33296074	Ca4	33296074	TAAAAAAAAAA/TAAAAAAAAAAA	TGACCTCCCACTGCATACAA	AAGAGACCGCATACGTGGAT	59.6	532	INTERGENIC	--	--	--
Ca4_P2_33308913	Ca4	33308913	GAAAA/GAAA	TCATTTTCCCCTCGTTTCTG	TTGGCCAAAACCTTTTGATG	60.8	183	DRR	Ca_24021	R	C2H2
Ca4_P2_33314599	Ca4	33314599	AC/A	ATGTCCATAACGCATGCAGA	CATCATGCCACTTTGGATTG	59.9	661	INTERGENIC	--	--	--
Ca4_P2_33315743	Ca4	33315743	CTT/CTTT	ACATGAGTCTTTGTTGCCCC	TGGCACGTGATTTGTTTGAT	60.0	477	INTERGENIC	--	--	--
Ca4_P2_33321015	Ca4	33321015	AATATA/AATATATA	TATTGTTGCACCCCCAATTT	ATGTCTGCCCCATTTTCTTG	59.9	229	INTERGENIC	--	--	--
Ca4_P2_33327822	Ca4	33327822	TAAAAAAAAAA/TAAAAAAAAAAAA	CGAAAATTCGAATGGGTAGC	GGTCAAGTGTTTGCCGAAAT	60.0	516	INTERGENIC	--	--	--
Ca4_P2_33328067	Ca4	33328067	GAAAAAAAA/GAAAAAAAAA	CGAAAATTCGAATGGGTAGC	AGTATGATGGGGTCATGGGA	60.0	631	INTERGENIC	--	--	--
Ca4_P2_33330920	Ca4	33330920	ATTTTTTT/ATTTTTTTT	CTATGGAGTTTGCCACCGAT	TTAACGGTGAGAACAAGGGG	60.0	609	INTERGENIC	--	--	--

INDEL marker IDs	Chromosomes/unanchored scaffolds	Physical positions (bp)	InDels (*Kabuli* reference genome-CDC Frontier/PII)	Forward primers (5'-3')	Reverse primers (5'-3')	Anneling temperature (0C)	Expected amplified product size (bp)	Structural annotation		Functional annotation	
								Sequence components of *kabuli* genome	*Kabuli* gene accession IDs	NCBI-KOG	TFs
Ca4_P2_33334049	Ca4	33334049	AC/ACC	ATGTTAGTTGGGGTGTGGGA	AGCAAGCACAACATAGCACG	60.1	413	INTERGENIC	--	--	--
Ca4_P2_33339958	Ca4	33339958	T/TA	TGGTGACAACAAGGTTTCCA	CCACATTTTCTCATCCACACA	59.4	437	INTERGENIC	--	--	--
Ca4_P2_33342440	Ca4	33342440	TTTTTTCTTCTT/TTTTTTCTTCTTCTTTTTCTTCTT	TGAAATTCCACCTCACCTCC	TTGGTTGAGGTTTATGCGTG	59.6	155	INTERGENIC	--	--	--
Ca4_P2_33346959	Ca4	33346959	TT/TTAT	TTTTCGGTTTGTTCAAAGGC	GGAAAACATTGGGAAACCCT	60.0	766	INTERGENIC	--	--	--
Ca4_P2_33347892	Ca4	33347892	AGG/AG	AGCCCAAGACGATCATCAAC	TCCTAAAAGACTGCCCGCTA	60.0	855	INTERGENIC	--	--	--
Ca4_P2_33349362	Ca4	33349362	GTTTTTTTTTTT/GTTTTTTTT	TTTGAAGGTGCAGACACGAG	TTTCATTGTTGCTGCCTCTG	60.0	148	INTERGENIC	--	--	--
Ca4_P2_33363121	Ca4	33363121	AAAA/AAAAGAAA	CGTTTGGTTGCACTGATGTC	AAGGTCTCAAAACGTGGTCG	60.1	610	INTERGENIC	--	--	--
Ca4_P2_33363161	Ca4	33363161	GTA/GTATA	CGTTTGGTTGCACTGATGTC	AAGGTCTCAAAACGTGGTCG	60.1	610	INTERGENIC	--	--	--
Ca4_P2_33397164	Ca4	33397164	TTATATATATATATATATA/TTATATATATATATATATATATA	ATAGGCTTTGCACTTCCCCT	CCCCAACAAAATGGAGCTAA	59.9	582	DRR	Ca_24022	--	--
Ca4_P2_33397720	Ca4	33397720	ATTTTTTTTTTT/ATTTTTTTTTT	CTCCATTTTGTTGGGGCTTA	GCCAACAAACAACCATGCTA	59.6	584	DRR	Ca_24022	--	--
Ca4_P2_33431052	Ca4	33431052	GAAAAAAAAAA/GAAAAAAAAA	TTTATGCTCCGTTTGTGCTG	CACACACCACCTCTCCCTTT	60.0	435	INTERGENIC	--	--	--
Ca4_P2_3345838	Ca4	3345838	TTATATATATATATATATATATATATATATATATA/TTATATATATATATATATATATATATATATA	TTTCATCCCCTTCACACTCC	TTTGACAAAATGACCCCAACT	59.3	456	INTERGENIC	--	--	--

INDEL marker IDs	Chromosomes/unanchored scaffolds	Physical positions (bp)	InDels (*Kabuli* reference genome-CDC Frontier/PII)	Forward primers (5'-3')	Reverse primers (5'-3')	Anneling temperature (0C)	Expected amplified product size (bp)	Structural annotation		Functional annotation	
								Sequence components of *kabuli* genome	*Kabuli* gene accession IDs	NCBI-KOG	TFs
Ca4_P2_33467450	Ca4	33467450	CAAAAAAAAAA/CAAAAAAAAAAA	TGATGTAAAAATGATGATGTGAAA	AATTTGAGTTGCAATTTGATGA	57.3	558	INTERGENIC	--	--	--
Ca4_P2_33467899	Ca4	33467899	ATTTTTT/ATTTTTTT	TCATATCATCAAATTGCAACTCA	TGATGTTAAAAGCTACAGCAACAA	59.0	455	INTERGENIC	--	--	--
Ca4_P2_33500201	Ca4	33500201	CAAAAAAAAAAA/CAAAAAAAAAA	TGAGAGAAACCTAGCTCGATGA	GGTGCTGAAAAACACAAGAGC	59.9	917	INTERGENIC	--	--	--
Ca4_P2_33517677	Ca4	33517677	TAAAAAAAA/TAAAAAAAAA	TTGGTCGTTTGAGCCTTTCT	CGAGAACACGCATTACGCTA	60.0	529	INTERGENIC	--	--	--
Ca4_P2_33557759	Ca4	33557759	GTA/G	TACCCTTTGCTAGCCCCTTT	TTTTGAGGACTTTCGGGTTG	60.1	462	INTERGENIC	--	--	--
Ca4_P2_3355845	Ca4	3355845	CGA/CGATAAGGA	GTTGGGTATTGGTGTCCCAC	GAGGGTGATCAGCTTTCTCG	60.0	589	INTERGENIC	--	--	--
Ca4_P2_33581989	Ca4	33581989	ATTTTTTTTTTTT/ATTTTTTTTTTT	TTGGGAGGAGGAGAAAGTCA	AACGGACAACCACACCTTTT	59.4	705	INTERGENIC	--	--	--
Ca4_P2_33603011	Ca4	33603011	ACC/AC	CCTCTTCGAACTTGTGAGGC	CCTTCGCACCAATAGCAAAT	60.1	552	INTRON	Ca_24027	R	--
Ca4_P2_33609998	Ca4	33609998	A/AGAT	GAAAGAACGCTGAAACCCAG	TTCAAGTCAGGTGCTCAACG	60.0	695	INTRON	Ca_24027	R	--
Ca4_P2_33615467	Ca4	33615467	ATT/ATTT	GGTCCTTGTTGAGTCCAACG	CTCAAAAACCTGAGCATGGC	60.8	646	INTERGENIC	--	--	--
Ca4_P2_33615658	Ca4	33615658	AG/A	GGTCCTTGTTGAGTCCAACG	AATGGAATAGCGGAGTGTGC	60.1	898	INTERGENIC	--	--	--
Ca4_P2_33615702	Ca4	33615702	ATTTTTTTT/ATTTTTTTTT	TGAAAATAAACTCCGCAGACG	AATGGAATAGCGGAGTGTGC	60.1	415	INTERGENIC	--	--	--

INDEL marker IDs	Chromosomes/unanchored scaffolds	Physical positions (bp)	InDels (*Kabuli* reference genome-CDC Frontier/PII)	Forward primers (5'-3')	Reverse primers (5'-3')	Anneling temperature (0C)	Expected amplified product size (bp)	Structural annotation		Functional annotation	
								Sequence components of *kabuli* genome	*Kabuli* gene accession IDs	NCBI-KOG	TFs
Ca4_P2_33619155	Ca4	33619155	CGTGT/CGTGTGT	GAACATGCTGAAAACGGGAT	TGAGGGCAATGACAAACAAA	60.1	122	INTERGENIC	--	--	--
Ca4_P2_33626327	Ca4	33626327	AA/AAAATA	TTGCACACATCTTGCACAAC	GCCTCTTTGATCGAGAACCA	60.3	463	INTERGENIC	--	--	--
Ca4_P2_33627666	Ca4	33627666	TATTTAAAATTTAAAATTTAAAATTTAAAAT/TATTTAAAATTTAAAATTTAAAAT	AGTGACATGACGGAAGTAGCA	TTGGTTCCAGTGTGCATGTT	60.0	432	INTERGENIC	--	--	--
Ca4_P2_33632691	Ca4	33632691	AAAATAAA/AAAA	AACTCACCCCTCGTTGTTTG	CGGACGATATGTTTCCTCGT	60.0	685	INTERGENIC	--	--	--
Ca4_P2_33633372	Ca4	33633372	GTTTTTTT/GTTTTTT	ACGAGGAAACATATCGTCCG	ATGTCCACACATTGGGTGTC	59.1	726	INTERGENIC	--	--	--
Ca4_P2_33633525	Ca4	33633525	ATTTTTTTT/ATTTTTTTTT	AGGATGCAAGAGAGGGATGA	ATGTCCACACATTGGGTGTC	59.1	441	INTERGENIC	--	--	--
Ca4_P2_33654066	Ca4	33654066	CAAAAAAAAAAAA/CAAAAAAAAAAA	CTTGTGTTTGTGTTGTGGGG	TTTGTTCGCACACCAAACTT	59.2	711	INTERGENIC	--	--	--
Ca4_P2_3365913	Ca4	3365913	AATATATATATATATATATATATATAT/AATATATATATATATATATATATAT	AAAGAGGGCATGACACCAAT	TCGGATCGGACACTATGACA	60.1	466	DRR	Ca_12139	--	--
Ca4_P2_33671303	Ca4	33671303	A/AC	GCCAAATGTGAAGGACCACT	GCACCAGTTCCAACGTTTTT	60.0	379	INTERGENIC	--	--	--
Ca4_P2_33714267	Ca4	33714267	T/TCTTAATC	GAACACGTTGTTAGACCGGC	TGACAAAATGGGGAGAAAGG	59.9	713	INTERGENIC	--	--	--
Ca4_P2_33714529	Ca4	33714529	ATT/ATTGTT	CGTTGTTAGACCGGCTCTTG	TGGGCTGACATAATCACCAA	59.9	940	INTERGENIC	--	--	--
Ca4_P2_33715942	Ca4	33715942	TAAAAAAAAAAAA/TAAAAAAAAAAAAAAAA	TTGGGTACGTTGGTGTAGCA	ATTGACTGGGCACCATGATT	60.2	634	INTERGENIC	--	--	--

INDEL marker IDs	Chromosomes/unanchored scaffolds	Physical positions (bp)	InDels (*Kabuli* reference genome-CDC Frontier/PII)	Forward primers (5'-3')	Reverse primers (5'-3')	Anneling temperature (0C)	Expected amplified product size (bp)	Structural annotation		Functional annotation	
								Sequence components of *kabuli* genome	*Kabuli* gene accession IDs	NCBI-KOG	TFs
Ca4_P2_33717835	Ca4	33717835	AAAAACA/AA	CCAAAAACCCCTCTTTTTCC	CCACCAAAATTTGTCCAACC	60.1	730	INTERGENIC	--	--	--
Ca4_P2_33732859	Ca4	33732859	TTCATTTTTCT/TT	ACGGTTAACGGATTACAGCG	TGTATGCGTCCGTTGAGAAG	59.9	272	INTERGENIC	--	--	--
Ca4_P2_33733792	Ca4	33733792	AATATTTGTTTATATT/AATATTTGTTTATATTTGTTTATATT	CGTTATTCCCATATGAGAGGC	TGTGATGTTGGACGTGTGTG	60.0	280	INTERGENIC	--	--	--
Ca4_P2_33736905	Ca4	33736905	ATTTTTTTT/ATTTTTTTTT	GGCTTTTCAAAGTTCATCGG	TTGAAGGATTTGAATTCCCC	58.8	789	INTERGENIC	--	--	--
Ca4_P2_33738599	Ca4	33738599	GAAAAAAAAA/GAAAAAAAAAAAAA	ATTGTTGCGCACAAATTGAA	CACCACCAAATCACAAGACG	60.0	734	INTERGENIC	--	--	--
Ca4_P2_33743516	Ca4	33743516	CAAAAAA/CAAAAA	TGTAGCTGGTGTCCACGAAG	AATGCAAGAATCCACCTTGG	59.9	435	INTERGENIC	--	--	--
Ca4_P2_33747045	Ca4	33747045	TAAAAAAAAAAAA/TAAAAAAAAAAAAA	GTAGTGGGTGAACGTGGCTT	CACCAAGGATTCATAACGGC	60.3	360	INTERGENIC	--	--	--
Ca4_P2_33780632	Ca4	33780632	AATATATATATATATATATATATATATATATATATA/AATATATATATATATATATATATATATATATATATATATATATA	GAGAGTTCGATTGCTGAGCC	ACTTTTCAGCCATGACCCAC	60.0	689	INTERGENIC	--	--	--
Ca4_P2_33783886	Ca4	33783886	TAAAAAAAAA/TAAAAAAAAAA	ATTTTGAAACAATCGGGCAA	TTGGGTGGGTACAGTTGGAT	60.1	740	INTERGENIC	--	--	--
Ca4_P2_33784625	Ca4	33784625	GTAT/GTATAT	CCGGTCTCAATTTTCCTCAA	TGTACGCTAGATTTCACTTTTGGA	60.2	440	INTERGENIC	--	--	--
Ca4_P2_33795008	Ca4	33795008	TTATATATATATATATAT/TTATATATATATAT	GTCGTGTCAATGCAACAACC	TCGACTAAGGACATTGGGCT	59.7	514	INTERGENIC	--	--	--
Ca4_P2_33807474	Ca4	33807474	TAAAAAAA/TAAAAAAAA	GTCATTTTGGCGGAACCTAA	TTGAGGTGGGATATGTTTGC	58.4	694	INTERGENIC	--	--	--

INDEL marker IDs	Chromosomes/unanchored scaffolds	Physical positions (bp)	InDels (*Kabuli* reference genome-CDC Frontier/PII)	Forward primers (5'-3')	Reverse primers (5'-3')	Anneling temperature (0C)	Expected amplified product size (bp)	Structural annotation		Functional annotation	
								Sequence components of *kabuli* genome	*Kabuli* gene accession IDs	NCBI-KOG	TFs
Ca4_P2_33813152	Ca4	33813152	TAA/TA	AGAGAAGTTGGCCGACAAAA	CTCCAATGCCCAACAAAAGT	60.0	734	INTERGENIC	--	--	--
Ca4_P2_33822409	Ca4	33822409	A/AC	TTGTCTCGGCTGCTATTGAC	AAAAACTGAGGTGGCAAACG	60.1	205	DRR	Ca_20370	--	S1Fa-like
Ca4_P2_33826708	Ca4	33826708	GTTTTTTTT/GTTTTTTT	GGACAAAGAGCATGGGTGAT	TGAAAATGAAGGGGTCAAGC	60.1	465	DRR	Ca_20369	Q	--
Ca4_P2_33833847	Ca4	33833847	TA/TAACTGCA	GCGTCATCGTCCCTACAAAT	TCCAACTCGCAAGGCTATCT	60.0	375	INTERGENIC	--	--	--
Ca4_P2_33835153	Ca4	33835153	AAGCT/A	TTTTCACCAAATCACTTGAAAGAA	TCGAGCCTTATTGTGGATGA	59.2	654	INTERGENIC	--	--	--
Ca4_P2_33835234	Ca4	33835234	TAAAAAAA/TAAAAAAAA	CCAATCAAATTGAGCATACCC	TGTGTTTGCTTTGCATGCTT	60.4	529	INTERGENIC	--	--	--
Ca4_P2_33840554	Ca4	33840554	AG/A	TATGCGCATCGTAAAATCGT	CAAAATTTTCAAACAAAACCCA	58.9	676	INTERGENIC	--	--	--
Ca4_P2_33840633	Ca4	33840633	AAAAAAATTAAAAAATTAAAAAATTAAAAA/AAAAAAAATTAAAAAATTAAAAAA	TATGCGCATCGTAAAATCGT	GATGGCTGGTAGACGGACAT	60.0	874	INTERGENIC	--	--	--
Ca4_P2_33840927	Ca4	33840927	TAAAAAAAAA/TAAAAAAAAAAA	TTTTTCCTGAGTTGGTCCTTT	GATGGCTGGTAGACGGACAT	60.0	560	INTERGENIC	--	--	--
Ca4_P2_33857426	Ca4	33857426	AAAAATTAAAAT/AAAAATTAAAATTAAAAT	GTTTTGTGATGGGGGATGAC	CAAATTTGGCGTTGTTCCTT	60.0	611	INTERGENIC	--	--	--
Ca4_P2_33857955	Ca4	33857955	TAAAAAA/TAAAAA	CAAATTTGTCTTGTGGGCCT	TTGACTTGATCTGCGTTTGC	60.0	630	INTERGENIC	--	--	--
Ca4_P2_33858628	Ca4	33858628	ATTT/ATTTT	GCAAACGCAGATCAAGTCAA	ATTATTGGGGAAATCTGGGC	60.0	841	INTERGENIC	--	--	--

INDEL marker IDs	Chromosomes/unanchored scaffolds	Physical positions (bp)	InDels (*Kabuli* reference genome-CDC Frontier/PII)	Forward primers (5'-3')	Reverse primers (5'-3')	Anneling temperature (0C)	Expected amplified product size (bp)	Structural annotation		Functional annotation	
								Sequence components of *kabuli* genome	*Kabuli* gene accession IDs	NCBI-KOG	TFs
Ca4_P2_33912297	Ca4	33912297	ATATCT/AT	AGAATCCAAATCGAAGCCCT	ACCCGCTCTAAGTCCAAAGC	60.8	606	INTERGENIC	--	--	--
Ca4_P2_33921285	Ca4	33921285	ATGCT/A	TGGGTGGTTGACACTTGAAA	CAGAGGAAAATGGGGTCAGA	60.0	560	INTRON	Ca_20365	O	GATA
Ca4_P2_33924249	Ca4	33924249	ATAGT/ATAGTAGT	CCGTCATTACATGTTGCTGG	TCGAGAGTTGCTCCTGGTTT	60.0	729	INTRON	Ca_20365	O	GATA
Ca4_P2_33983110	Ca4	33983110	T/TA	TGGAAACAAGTGGGTGTTCA	TGCATTGGTGTAGCCATAGG	59.6	716	INTERGENIC	--	--	--
Ca4_P2_33986135	Ca4	33986135	TTATATATATATATATATAT/TTATATATATATATATATATAT	CATGTTTTGCACTTGGGTTG	ATTGCAATCCCACTTGAAGC	60.1	714	INTERGENIC	--	--	--
Ca4_P2_34006503	Ca4	34006503	GA/GAGTGTTA	ACAGAGGAAGCTGCCACCTA	GAGTTCAACCAATGGCGTCT	60.1	653	INTERGENIC	--	--	--
Ca4_P2_34022046	Ca4	34022046	GAAAAAAAAAAA/GAAAAAAAAAA	TGAGCTCTGAGGGAAAGGAA	AAAAAGGAAAAGCCAGCCTC	59.8	805	INTERGENIC	--	--	--
Ca4_P2_34026279	Ca4	34026279	TAAAAAAAAAA/TAAAAAAAAAAA	AAACAACCGCCATCCAACT	TTTCATGGACAAACAAGCCA	60.1	213	DRR	Ca_20364	--	--
Ca4_P2_34039084	Ca4	34039084	TAAAAAAAAAAA/TAAAAAAAAA	TACCAAAGTGTCGCCAACAA	AAAAAGGAAAAGCCAGCCTC	59.8	742	INTERGENIC	--	--	--
Ca4_P2_34056670	Ca4	34056670	ATAAT/ATAATTAAT	AGGATGCAACAAACCCAAAG	GTCCAAATGAAAACGCCAAT	59.8	461	INTERGENIC	--	--	--
Ca4_P2_34078673	Ca4	34078673	TAAGAAGAAGAAGAAGAA/TAAGAAGAAGAAGAAGAAGAA	TTTTGTTGCCATCGTTTGAA	AAATTCCCCACCTTGGATTC	60.0	471	INTERGENIC	--	--	--
Ca4_P2_34087689	Ca4	34087689	TAAAA/TAAA	GTGTTGAGGTTGAACCAGGG	ACCAAAACAACGTGACACCA	59.9	596	INTERGENIC	--	--	--

INDEL marker IDs	Chromosomes/unanchored scaffolds	Physical positions (bp)	InDels (*Kabuli* reference genome-CDC Frontier/PII)	Forward primers (5'-3')	Reverse primers (5'-3')	Anneling temperature (0C)	Expected amplified product size (bp)	Structural annotation		Functional annotation	
								Sequence components of *kabuli* genome	*Kabuli* gene accession IDs	NCBI-KOG	TFs
Ca4_P2_34091010	Ca4	34091010	CAAAAAAAAAA/CAAAAAAAAAAAA	TGTAGGCCCACATTTTTGAA	GATGCTACATGGGAAAGGGA	59.9	124	INTERGENIC	--	--	--
Ca4_P2_34121290	Ca4	34121290	CAAAAAAAAAAAAA/CAAAAAAAAAAAAAAAA	GCATATTGGGAGAAATGAGCA	GGATGCATGGGAGTTACCAC	60.2	242	INTERGENIC	--	--	--
Ca4_P2_34127922	Ca4	34127922	ATTTTTTTTT/ATTTTTTTT	CCACCTAATCACTCATAATCGACA	GGAAAATTAAAATGTGGGAAAA	57.2	482	INTERGENIC	--	--	--
Ca4_P2_34135702	Ca4	34135702	TGG/TG	CCATCTACCTTTGTCGGTGC	GGCCTTTCCACAAAAACTCA	60.1	423	INTERGENIC	--	--	--
Ca4_P2_34169705	Ca4	34169705	CAATGA/CAATGATAATGA	TCAAAGTGGCTCACAAGTGC	TCGACTCAAGAAGGCAAGTG	59.2	781	INTERGENIC	--	--	--
Ca4_P2_34227066	Ca4	34227066	A/AG	ACGGCAAAAACCAGTGAAAT	TGAGGAAAATTGAGACCGGA	60.6	327	INTERGENIC	--	--	--
Ca4_P2_34228273	Ca4	34228273	CTTT/CT	TGGAAGTTGGGAGAAACACC	ACGATTCGTCACACAATGGA	60.0	612	INTERGENIC	--	--	--
Ca4_P2_34240189	Ca4	34240189	CC/CCTGATGC	AGCGACCAGCTCTGATTTGT	GGGAGCTGCTTGATGAGAAG	60.1	168	DRR	Ca_20356	L	--
Ca4_P2_34246830	Ca4	34246830	ATT/AT	CGTCTTCCGTACAAAGCCTC	GATTGGGGACCATTCCATTA	59.4	320	INTERGENIC	--	--	--
Ca4_P2_34247514	Ca4	34247514	TTATATATATATATATATATATA/TTATATATATATATATATATATATATATATATA	TCATTAGCTCAATCAATTACGCA	TTGATAAGATTGATCCGTTGGA	59.4	434	INTERGENIC	--	--	--
Ca4_P2_34254143	Ca4	34254143	GTTTTTTTTTTT/GTTTTTTTTTTTT	TTTTGCGACATTTGATCGTT	TGTTCTCGCACACCAAAAAG	59.9	454	INTERGENIC	--	--	--
Ca4_P2_34287834	Ca4	34287834	GT/G	GTGGAGATCACCCGAGTTGT	CAAATCCATGCTCTGCAAAA	59.8	811	INTERGENIC	--	--	--

INDEL marker IDs	Chromosomes/unanchored scaffolds	Physical positions (bp)	InDels (*Kabuli* reference genome-CDC Frontier/PII)	Forward primers (5'-3')	Reverse primers (5'-3')	Anneling temperature (0C)	Expected amplified product size (bp)	Structural annotation		Functional annotation	
								Sequence components of *kabuli* genome	*Kabuli* gene accession IDs	NCBI-KOG	TFs
Ca4_P2_34301089	Ca4	34301089	TCCC/TCC	AGTTTTGGCCGATTTGTTTG	GGATCAGGAGGAGGTGATGA	60.0	486	INTERGENIC	--	--	--
Ca4_P2_34308809	Ca4	34308809	TAAAAA/TAAAAAAA	GTTGAGCATACCGACCAACC	ATGGTCGTTTTCGGAACTTG	60.0	870	INTERGENIC	--	--	--
Ca4_P2_34309139	Ca4	34309139	CCTCTCTCTCTCTCTCTCT/CCTCTCTCTCTCTCTCT	GCTCCCAACCTTGAATTTGA	ATGGTCGTTTTCGGAACTTG	60.0	126	INTERGENIC	--	--	--
Ca4_P2_34329843	Ca4	34329843	CTTTTTTTTT/CTTTTTTTT	TGCAGACCCTTGGAAAAAGT	GCCACTATTGGCTGGGTTAC	59.5	685	INTERGENIC	--	--	--
Ca4_P2_34330740	Ca4	34330740	TAAAAAAAAAAA/TAAAAAAAAAAAA	TGCACACATCTACCCCTGAA	GGCATGGCAACTTCACTTTT	60.1	502	INTERGENIC	--	--	--
Ca4_P2_34339507	Ca4	34339507	AAAGA/AAAGAAGA	GCCTCTAGGAAACACCAGTTCA	TGTGGAAGGAAAACCAGAGG	60.1	765	INTERGENIC	--	--	--
Ca4_P2_34339778	Ca4	34339778	ATTTTTTTTTT/ATTTTTTTTTTT	CAGGATTTGGTTTAGCCATTTT	TGTGGAAGGAAAACCAGAGG	60.1	355	INTERGENIC	--	--	--
Ca4_P2_34375164	Ca4	34375164	ATTTTTTT/ATTTTTT	CAAATCTTCTTCAAATCTTGACCAT	CTATCGACCAAGCCACCATT	60.0	768	INTERGENIC	--	--	--
Ca4_P2_34382417	Ca4	34382417	GC/G	AAGGGGCTTTGAGGTAAGGA	TGGAAAATGGATGTTGACGA	59.9	572	INTERGENIC	--	--	--
Ca4_P2_34384498	Ca4	34384498	ATTTTTTTTTT/ATTTTTTTTTTT	CGATTTTTAACAGCGCGAGT	TACCCTGTGGATGGGAATGT	60.1	421	INTERGENIC	--	--	--
Ca4_P2_34388082	Ca4	34388082	CATATATATATATAT/CATATATATATATATATATAT	GTTGGTTCCCTAGCACCGTA	CTGTCAATTGAGTTGCGACG	60.4	484	INTERGENIC	--	--	--
Ca4_P2_34388181	Ca4	34388181	GAAA/GAAAA	GTTGGTTCCCTAGCACCGTA	CTGTCAATTGAGTTGCGACG	60.4	484	INTERGENIC	--	--	--

INDEL marker IDs	Chromosomes/unanchored scaffolds	Physical positions (bp)	InDels (*Kabuli* reference genome-CDC Frontier/PII)	Forward primers (5'-3')	Reverse primers (5'-3')	Anneling temperature (0C)	Expected amplified product size (bp)	Structural annotation		Functional annotation	
								Sequence components of *kabuli* genome	*Kabuli* gene accession IDs	NCBI-KOG	TFs
Ca4_P2_34390748	Ca4	34390748	TAAAAAA/TAAAAAAA	GCAAAACGTGTTCTCCCATT	AACCAATTTTCCCCATGACA	60.0	566	INTERGENIC	--	--	--
Ca4_P2_34401219	Ca4	34401219	CAAAAAAAAAAAAA/CAAAAAAAAAAAA	TGGAGAAACTCTTCCGCTGT	TCCACTCCTTCAACCGTAGG	60.1	656	INTERGENIC	--	--	--
Ca4_P2_34425896	Ca4	34425896	CTTTTTTTTTTTTTT/CTTTTTTTTTTTT	CAGCAAATTGTCGTCAAGGA	TGCAATGGCTCTGTTCTCAA	60.5	422	INTERGENIC	--	--	--
Ca4_P2_34432600	Ca4	34432600	ATTTTTTT/ATTTTTT	GGGTGAGGATTGGTCAGTCA	ACCTTTGGGTCTTGCCTTTT	60.0	407	INTERGENIC	--	--	--
Ca4_P2_34454517	Ca4	34454517	TAAAAAG/TAAAAAGAAAAAAAAG	AGGGGTGGTGATTCTCACAG	ATTTTTGGAGGACTTTCGGG	60.3	285	DRR	Ca_20352	--	B3
Ca4_P2_34458672	Ca4	34458672	T/TATC	CCAAACGTTCTCCCACAACT	AGGATGGAAGCCAGGATTTT	59.9	374	DRR	Ca_20351	--	--
Ca4_P2_34461628	Ca4	34461628	GC/G	ATTAAATGCTCCAAACGCCT	TTTCCACATTTGGTGCTTGA	60.1	781	INTERGENIC	--	--	--
Ca4_P2_34462526	Ca4	34462526	TAAAAAAAAA/TAAAAAAAA	GTGGGAAAAGATGTGGGAGA	TCGAAAGGATTTTTCCATGC	60.0	584	INTERGENIC	--	--	--
Ca4_P2_34473954	Ca4	34473954	CTTTTTTT/CTTTTTTTT	ACCCAAATCCAGTGTTCTGC	GGCCTTGTCGAATGATTGTT	59.9	839	INTERGENIC	--	--	--
Ca4_P2_34475378	Ca4	34475378	GAAAAAAA/GAAAAAAAA	CATTGTGGATGCAGTTTTGC	CGAAATAAGATGTCCACCGC	60.5	535	INTERGENIC	--	--	--
Ca4_P2_34475759	Ca4	34475759	GAAAA/GAAA	GCGGTGGACATCTTATTTCG	CGCATTGAAGCATAAACCCT	60.1	446	INTERGENIC	--	--	--
Ca4_P2_34482261	Ca4	34482261	GCCGCC/GCCGCCGTAAACCGCC	ACGCCATCTGCCATATGTTT	TTTGGGGTGATGGAAATGAT	60.0	497	INTRON	Ca_20350	R	G2-like

INDEL marker IDs	Chromosomes/unanchored scaffolds	Physical positions (bp)	InDels (*Kabuli* reference genome-CDC Frontier/PII)	Forward primers (5'-3')	Reverse primers (5'-3')	Anneling temperature (0C)	Expected amplified product size (bp)	Structural annotation		Functional annotation	
								Sequence components of *kabuli* genome	*Kabuli* gene accession IDs	NCBI-KOG	TFs
Ca4_P2_3448244	Ca4	3448244	AATATATATATATATATATATATATATAT/AATATATATATATATATATATATATATATAT	CCTTTTTGCCTCGGTGATTA	CTTCAACTCGTGCACATCGT	59.9	754	INTERGENIC	--	--	--
Ca4_P2_34518791	Ca4	34518791	ATTTTTTT/ATTTTTT	CAGCTTCCCACCAAGGTAAA	TCGATGTGAACAATAGGCCA	60.1	463	DRR	Ca_20349	--	ERF
Ca4_P2_34519402	Ca4	34519402	TAAAAAAAAA/TAAAAAAAA	GCTTCCGCAAATCCAAAATA	GGCGGTTTTTCACTTTGAGA	60.2	483	DRR	Ca_20349	--	ERF
Ca4_P2_34566354	Ca4	34566354	ATTTTTTTTT/ATTTTTTT	CGCCTAAGGACTATGCAAGC	AAAATCCAACAATCTCCCCC	60.0	761	INTERGENIC	--	--	--
Ca4_P2_34566424	Ca4	34566424	ATT/AT	CCCTGAGACTCACGAAGAGG	AAAATCCAACAATCTCCCCC	60.0	612	INTERGENIC	--	--	--
Ca4_P2_34599013	Ca4	34599013	AGGGG/AGGG	TGTGCATCGAAAAGGTGTGT	AGCCTACGGAGGACCAAAAT	60.0	568	INTERGENIC	--	--	--
Ca4_P2_34714664	Ca4	34714664	GCCC/GCC	AGGGAGGACGAAAATGACCT	CCAATAGGTGTGCTTGCGTA	59.8	454	INTERGENIC	--	--	--
Ca4_P2_34809322	Ca4	34809322	ACTCTCTCTCTCTCTCTCTCT/ACTCTCTCTCTCTCTCTCT	TCGATTTCGTTTGTTGTTGTG	TATGAATCGTGGGTTGTCCC	60.6	271	INTERGENIC	--	--	--
Ca4_P2_34938595	Ca4	34938595	CATATATATATATATATATATAT/CATATATATATATATATAT	TTTAGAAGAGGGTCCTGCGA	AGGGAGGTTTGTGTTTGACG	60.0	774	INTERGENIC	--	--	--
Ca4_P2_34989654	Ca4	34989654	A/AC	AGTCCGGATACCATGTCTCG	CGTGGCTCGTAGTGATGAGA	60.0	170	INTERGENIC	--	--	--
Ca4_P2_35132229	Ca4	35132229	GTTTTTTTTTT/GTTTTTTTTTTT	TTGCTTGATTTTGCTAGTCGT	TCTTGAGGCTGTGATGATCG	59.9	748	INTERGENIC	--	--	--
Ca4_P2_35150916	Ca4	35150916	CATATATATATATATATATATATATATAT/CATATATATATATATATATATATATATATATATATATATATAT	CGAGTTGGGTGAACAAGGAT	CTCTAAAGCGCGTGCAGATT	60.7	968	INTERGENIC	--	--	--

INDEL marker IDs	Chromosomes/unanchored scaffolds	Physical positions (bp)	InDels (*Kabuli* reference genome-CDC Frontier/PII)	Forward primers (5'-3')	Reverse primers (5'-3')	Anneling temperature (0C)	Expected amplified product size (bp)	Structural annotation		Functional annotation	
								Sequence components of *kabuli* genome	*Kabuli* gene accession IDs	NCBI-KOG	TFs
Ca4_P2_3521094	Ca4	3521094	CATATATATATATATATA/CATATATATATATATATATA	TGAAATCCAAACCCTTGCTC	GCGGTGGAGTGTCAAAATCT	60.1	248	INTRON	Ca_12150	G	--
Ca4_P2_35213666	Ca4	35213666	TAAAAAAAAA/TAAAAAAAAAA	TGGAGGAAAAGAGGGTGATG	ATTGAGGGTGATTGGTTGGA	60.2	580	INTRON	Ca_20014	U	M-type
Ca4_P2_35236009	Ca4	35236009	ATT/A	GGCCTTACCTTAAACGTGGA	GGCATTCGCAAATGTTACAA	59.6	440	INTERGENIC	--	--	--
Ca4_P2_35237197	Ca4	35237197	TTTATTATTATTATTATTATTATTATTA/TTTATTATTATTATTATTATTATTATTATTATTATTATTATTA	TCACTGCTCACACACCAACA	TTGAAATGGGGTTGAAGGAC	59.8	276	INTERGENIC	--	--	--
Ca4_P2_35246623	Ca4	35246623	TCTTCATCCTTCATCCTTCA/TCTTCATCCTTCATCCTTCATCCTTCA	TAGAAAACACGTGTGACCGC	GCGTTTGCACTGAAAGATGA	60.0	231	INTERGENIC	--	--	--
Ca4_P2_35295864	Ca4	35295864	TAA/TAAA	CACGATGAATTAAATAGCATCCA	ATTCACATGGTTGTTCGCAA	60.0	239	INTERGENIC	--	--	--
Ca4_P2_35319446	Ca4	35319446	ATTTTTTTTT/ATTTTTTTTTT	GGTCAGTTCATCCTAGGGCA	TTTTTGGTTGCCTAAGTGGG	60.0	462	INTERGENIC	--	--	--
Ca4_P2_35343662	Ca4	35343662	TCCACAACC/TCCACAACCACAACC	CCACAACATGGTGCAAACTC	AACCGGTTCAGGTTCTGTTG	60.0	679	INTRON	Ca_20018	Z	NAC
Ca4_P2_35345914	Ca4	35345914	GTTTT/GTTTTTT	TTTGTGAAGGGATTTGGCTC	ATGCCTCTCTATGCAGCGTT	60.0	763	INTRON	Ca_20018	Z	NAC
Ca4_P2_35369859	Ca4	35369859	GATTAA/GATTAATTAA	TTTAGTGCGCATGAATTTTTACA	TTTCCACAAGCTAACAAAAATGA	58.8	494	INTERGENIC	--	--	--
Ca4_P2_35431597	Ca4	35431597	CCAA/C	CCCTCACAATTTGACATTTGG	TTTTTGAATGCTTGTTTTGGA	58.3	477	INTERGENIC	--	--	--
Ca4_P2_35432013	Ca4	35432013	TTAT/TTATAT	TGTTCAACATCCACGAACAA	TGCCATACTCATGTCGGAAA	60.1	669	INTERGENIC	--	--	--

INDEL marker IDs	Chromosomes/unanchored scaffolds	Physical positions (bp)	InDels (*Kabuli* reference genome-CDC Frontier/PII)	Forward primers (5'-3')	Reverse primers (5'-3')	Anneling temperature (0C)	Expected amplified product size (bp)	Structural annotation		Functional annotation	
								Sequence components of *kabuli* genome	*Kabuli* gene accession IDs	NCBI-KOG	TFs
Ca4_P2_35432836	Ca4	35432836	TTATATATATATATATATATAT/TTATATATATATATATATATATAT	TTTCCGACATGAGTATGGCA	TTTGGAAAGTAACACTGCACG	58.9	785	INTERGENIC	--	--	--
Ca4_P2_35445772	Ca4	35445772	AATATATATATATATATATAT/AATATATATATATATAT	TGCATTCAATTAAGCTTGCG	ATGTGGGCAAGTGACAAACA	60.0	654	INTERGENIC	--	--	--
Ca4_P2_35454688	Ca4	35454688	TT/TTGACAACGCAAT	TGATGCCATGATCCTCTTCA	AGGAATGGCAGCAATGAATC	60.0	662	CDS (FRAME SHIFT)	Ca_22430	R	NAC
Ca4_P2_35457008	Ca4	35457008	TTGC/T	AATTGCAGAAAAATGCGGTC	TTGTACCAGATATTGGCCCG	60.7	776	INTERGENIC	--	--	--
Ca4_P2_35457562	Ca4	35457562	AA/AAAATTA	ATCAAATGTTCATGCGCAAA	CCCTTTCCCCCAAGTGTATT	60.0	602	INTERGENIC	--	--	--
Ca4_P2_35571122	Ca4	35571122	TAAAAAAAAAA/TAAAAAAAAA	CTTTGGAATCACGTTGCCTT	TTCATCCATTGTTGCTTCCA	60.0	498	INTERGENIC	--	--	--
Ca4_P2_35579933	Ca4	35579933	ATTTTTT/ATTTTT	TTGTGGCCCAACATTGACTA	TTGGAGAAAACAATGCGGTT	60.5	785	INTERGENIC	--	--	--
Ca4_P2_35580174	Ca4	35580174	TTTATTATTATTATTATTATTATTATTATTATTATTATTATTATTATTATT/TTTATTATTATTATTATTATTATTATTATTATTATTATTATTATTATTATT	TTGTGCCATAAAATTCCGGT	TTGGAGAAAACAATGCGGTT	60.5	494	INTERGENIC	--	--	--
Ca4_P2_35599208	Ca4	35599208	ATT/ATTTT	AGATGGCCTGGATCAAAATG	GCTCACGTTTGCCAAAAACT	60.3	689	DRR	Ca_22440	R	TCP
Ca4_P2_35617043	Ca4	35617043	CAT/CTCTTGAAT	TGCCCATGTTTACCACAGAA	AGCCAAACAGAAAGCTCCAA	60.0	841	INTRON	Ca_22440	R	TCP
Ca4_P2_35620689	Ca4	35620689	TGA/TGAGA	ACAATCCCGACACAAAGTCA	GAAGCGAGAGGTAAGCGATG	60.1	499	INTERGENIC	--	--	--
Ca4_P2_35639160	Ca4	35639160	ATT/ATTT	GGAAAAGATGGTATTTGCCG	CTTCCCTGCTGGCAACTTAG	60.0	339	INTERGENIC	--	--	--

INDEL marker IDs	Chromosomes/unanchored scaffolds	Physical positions (bp)	InDels (*Kabuli* reference genome-CDC Frontier/PII)	Forward primers (5'-3')	Reverse primers (5'-3')	Anneling temperature (0C)	Expected amplified product size (bp)	Structural annotation		Functional annotation	
								Sequence components of *kabuli* genome	*Kabuli* gene accession IDs	NCBI-KOG	TFs
Ca4_P2_3570747	Ca4	3570747	TATAC/T	GTAGTACAGCCCCGCACAAT	TGCTTCCACAATCATGAGAAA	59.3	710	DRR	Ca_12156	--	--
Ca4_P2_35779928	Ca4	35779928	AACACACACACACACACA/AACACACACACACACACACA	GGCATATATGTCAATTAGGGGA	TGCTTTTTGTGGTTGCTTTG	59.9	765	INTERGENIC	--	--	--
Ca4_P2_3578578	Ca4	3578578	GATATATATATATATATA/GATATATATATATATATA	TATCCTGATTTTGCTTGCCC	GGAAAAGTGGACCTTCAGCA	60.2	540	INTERGENIC	--	--	--
Ca4_P2_35818039	Ca4	35818039	AAATAATAATAATAATA/AAATAATAATAATA	GTTCATAACGAAGATGACAATAACA	TGCAGTCAAACTTTAAAAGGAAG	57.8	728	INTERGENIC	--	--	--
Ca4_P2_35822348	Ca4	35822348	TGG/TG	GATCAAATCGAGGACCGAAA	CGAACAAAACGATGGGAAGT	60.0	695	INTERGENIC	--	--	--
Ca4_P2_35999977	Ca4	35999977	ATTTTTTTTTT/ATTTTTTTTTTT	GCTGGCAGCACAAATAAACA	TCGAACCAATGACCTCACAA	60.1	439	INTERGENIC	--	--	--
Ca4_P2_3602818	Ca4	3602818	GTTTTTTT/GTTTTTT	GAGCATCTCTAACGGGAGGA	TCGTCTTTTATGACCAACTCCC	60.4	541	DRR	Ca_12158	--	--
Ca4_P2_36079126	Ca4	36079126	TTGTG/TTGTGTG	ATTTTGATGCTCACGGTTCC	CGGAGATGATGGTTCAGGTT	59.9	595	INTERGENIC	--	--	--
Ca4_P2_36079437	Ca4	36079437	TGTCAAGTCAA/TGTCAA	ATTTTGATGCTCACGGTTCC	GGCGTACCATGCTTTGATTT	60.0	979	CDS (FRAME SHIFT)	Ca_14855	--	--
Ca4_P2_36094744	Ca4	36094744	TAAAAAAAAAAA/TAAAAAAAAAA	CCTACCATTGCCTGACCTGT	TTCCGAGTTGTTTGAGAGGA	58.4	427	INTERGENIC	--	--	--
Ca4_P2_36175888	Ca4	36175888	AT/A	AGTTGCAGCGAGTCTGGATT	TCCGACATGCAACTCTTCAG	60.0	437	INTERGENIC	--	--	--
Ca4_P2_36245892	Ca4	36245892	TTATATATATATATATATATATATAT/TTATATATATATATATATAT	TACATGCAAGCGAAATGGAA	AGGCATGGTGTTGGAGTCAT	60.4	287	DRR	Ca_14846	O	--

INDEL marker IDs	Chromosomes/unanchored scaffolds	Physical positions (bp)	InDels (*Kabuli* reference genome-CDC Frontier/PII)	Forward primers (5'-3')	Reverse primers (5'-3')	Anneling temperature (0C)	Expected amplified product size (bp)	Structural annotation		Functional annotation	
								Sequence components of *kabuli* genome	*Kabuli* gene accession IDs	NCBI-KOG	TFs
Ca4_P2_36256375	Ca4	36256375	ATTTTTTTTTT/ATTTTTTTTTTT	GATCATCGAGTTTCCCGTGT	AGTTCTGAGGGAGGCTCACA	60.0	350	INTERGENIC	--	--	--
Ca4_P2_36335313	Ca4	36335313	ATTTTTT/ATTTTTTT	TTGCGACAAGATCAAGCAAC	TCAACACCAAACACAAGGGA	60.0	519	INTERGENIC	--	--	--
Ca4_P2_36335413	Ca4	36335413	TTTT/TTTTTTTTTGAAATTATCTTT	TTGCGACAAGATCAAGCAAC	TCAACACCAAACACAAGGGA	60.0	519	INTERGENIC	--	--	--
Ca4_P2_36336918	Ca4	36336918	TAAA/TATAAAA	ACAACAAGGGTGACCTCCAC	TGCTTGTCTATTCTGCGTCAA	59.6	736	INTERGENIC	--	--	--
Ca4_P2_36337139	Ca4	36337139	GC/G	TGCAATGTCAATATGAACCAAA	CCACAACCACTCATCACGAC	60.0	479	INTERGENIC	--	--	--
Ca4_P2_36337249	Ca4	36337249	TAAAATATTTGTTTCA/T	TGACGCAGAATAGACAAGCAA	CCACAACCACTCATCACGAC	60.0	341	INTERGENIC	--	--	--
Ca4_P2_36337338	Ca4	36337338	TTTATTATTATTATTATTATTATTATTATTATTATTATTATTATTATTATTATTATTA/TTTATTATTATTATTATTATTATTATTATTATTATTATTATTATTATTATTATTATTATTATTATTA	TGACGCAGAATAGACAAGCAA	CCACAACCACTCATCACGAC	60.0	341	INTERGENIC	--	--	--
Ca4_P2_36399797	Ca4	36399797	TG/TGG	TGTCGTTTGCAATGGAGACT	CACGTGTCATGTTCCCATGT	60.3	549	INTERGENIC	--	--	--
Ca4_P2_36472761	Ca4	36472761	TAAAAAAAAA/TAAAAAAAAAA	CGTAGGACAGAAACCGTGGTA	TATGTAGCCCGTGTCAAGCA	60.3	679	INTERGENIC	--	--	--
Ca4_P2_36529935	Ca4	36529935	TTATATATATATATATATATATATATATATA/TTATATATATATATATATATATATATATATA	TGGTGTAATTTGATTTCTTCCCT	TCTGGAAAAACTTGAACCACAA	59.6	523	INTERGENIC	--	--	--
Ca4_P2_36593028	Ca4	36593028	TAAAAAAAAAAA/TAAAAAAAAAAAA	CGCGGGGCATATTCTAGTTA	TGATTTGGCATTGTGTTTGC	60.5	527	INTERGENIC	--	--	--
Ca4_P2_36645928	Ca4	36645928	GAAAAAAAAAAA/GAAAAAAAAAA	AATTCAGTCCCATCAGCCAG	AGAGGTTTGTTTACCGCGAA	59.7	419	INTRON	Ca_14816	H	--

INDEL marker IDs	Chromosomes/unanchored scaffolds	Physical positions (bp)	InDels (*Kabuli* reference genome-CDC Frontier/PII)	Forward primers (5'-3')	Reverse primers (5'-3')	Anneling temperature (0C)	Expected amplified product size (bp)	Structural annotation		Functional annotation	
								Sequence components of *kabuli* genome	*Kabuli* gene accession IDs	NCBI-KOG	TFs
Ca4_P2_36690853	Ca4	36690853	CTTTT/CTTT	TTGACAAGCGCTGTGAAATC	CAATGTGTATTCAACGACCGA	59.5	538	INTERGENIC	--	--	--
Ca4_P2_3672023	Ca4	3672023	ATTTTTTTTTTT/ATTTTTTTTTTTT	CATTTGGACCACCTGGGTAT	GGGACAATATTAGTCACCGATAAA	58.4	614	DRR	Ca_12163	--	--
Ca4_P2_36726834	Ca4	36726834	A/ATT	GCTTGTCCGCGTAACTCTTC	GGGGTGTTGCAACCTTTAGA	60.0	283	INTERGENIC	--	--	--
Ca4_P2_36940249	Ca4	36940249	ATTTTTTTTT/ATTTTTTTT	TTTTCTGAGTTTTGCCCCAC	CAGGGTCACAAACACACACC	59.9	746	INTERGENIC	--	--	--
Ca4_P2_36952734	Ca4	36952734	ATTTTTTT/ATTTTTT	AACTCATGTGGTTTGTGGCA	ATGGGCCCTCTTTTGTCTTT	59.9	896	INTRON	Ca_14797	R	C2H2
Ca4_P2_36952818	Ca4	36952818	TT/TTGAACT	GGGTTCATGTTTGAAGGGTG	ATGGGCCCTCTTTTGTCTTT	59.9	846	INTRON	Ca_14797	R	C2H2
Ca4_P2_36953596	Ca4	36953596	AATATATATAT/AATATATATATATAT	AAAAGAGGGCCCATGAGATT	TGTGCATTGGGAACAACACT	60.0	693	INTRON	Ca_14797	R	C2H2
Ca4_P2_36954401	Ca4	36954401	TAGAGAG/TAGAG	TCTTCCACTAAAACCCACGC	TGGCACTCTCTTCTCCAGGT	60.0	751	INTRON	Ca_14797	R	C2H2
Ca4_P2_36954611	Ca4	36954611	GGTGT/GGTGTGT	GGAGGATGGATGTGTGTGTG	TGGCACTCTCTTCTCCAGGT	60.0	580	INTRON	Ca_14797	R	C2H2
Ca4_P2_36956803	Ca4	36956803	TAAAAAAA/TAAAAAA	CCTTATTTTCCCTGCCATCA	CACCAATGGCTGAAAATCCT	59.9	595	INTERGENIC	--	--	--
Ca4_P2_36966828	Ca4	36966828	ATTTTTTTTTT/ATTTTTTTTTTT	AGACGACGAATAGGGGTGTG	TTCGTGGTTTCATTGTTAAACG	59.9	710	INTERGENIC	--	--	--
Ca4_P2_36968194	Ca4	36968194	TAAAAAAAA/TAAAAAAAAA	GGCGAAAGTACCCAAACAAA	GACTTCTGCAAAGGACCTCG	60.0	426	INTERGENIC	--	--	--

INDEL marker IDs	Chromosomes/unanchored scaffolds	Physical positions (bp)	InDels (*Kabuli* reference genome-CDC Frontier/PII)	Forward primers (5'-3')	Reverse primers (5'-3')	Anneling temperature (0C)	Expected amplified product size (bp)	Structural annotation		Functional annotation	
								Sequence components of *kabuli* genome	*Kabuli* gene accession IDs	NCBI-KOG	TFs
Ca4_P2_36968275	Ca4	36968275	G/GTTAATAC	GGCGAAAGTACCCAAACAAA	GACTTCTGCAAAGGACCTCG	60.0	426	INTERGENIC	--	--	--
Ca4_P2_36969612	Ca4	36969612	CATATATATATA/CATATATATATATATATATA	CATGGGCCAAAAATAAAAAGA	GAGCTGTGGAGAAGAAAAGCA	59.7	814	INTERGENIC	--	--	--
Ca4_P2_36969837	Ca4	36969837	CTTTTTTTTTT/CTTTTTTTTTTT	AGGTCATGCCATCAAAGACA	GAGCTGTGGAGAAGAAAAGCA	59.7	145	INTERGENIC	--	--	--
Ca4_P2_36972358	Ca4	36972358	CTTT/CTT	ACAACCAACAAAATGCGACA	AGCCTTGAGCGACCACTTTA	60.0	484	INTERGENIC	--	--	--
Ca4_P2_36972412	Ca4	36972412	GTT/GTTT	ACAACCAACAAAATGCGACA	TTGAAACATTCTCAGCCTTGTT	58.9	564	INTERGENIC	--	--	--
Ca4_P2_36973113	Ca4	36973113	CATAT/CAT	GCCCCCAATTTGAATGTAGA	ATTCAATTTGCGCGAGTGTT	60.6	621	DRR	Ca_14796	B	bHLH
Ca4_P2_36973873	Ca4	36973873	TA/TAGAA	TGTGGTAAAATGCGGTCAAA	TCGGGTCATCTGCATTACAA	60.1	478	DRR	Ca_14796	B	bHLH
Ca4_P2_36977746	Ca4	36977746	C/CT	AACGGCAAAATTGTTCAAGG	CCCTCAAAAACTCAACTCCC	58.6	747	INTERGENIC	--	--	--
Ca4_P2_36977812	Ca4	36977812	AGTTTTGAAGGGTTTTGAAG/AGTTTTGAAG	AACGGCAAAATTGTTCAAGG	CCCTCAAAAACTCAACTCCC	58.6	747	INTERGENIC	--	--	--
Ca4_P2_36981556	Ca4	36981556	GCA/GCACA	TGGTTTAGGCATGTGTGGAA	CCCTTGGACCTGTTTAGCAA	60.1	532	DRR	Ca_14795	B	bHLH
Ca4_P2_36982191	Ca4	36982191	CAA/CAAAA	TACCTATCCAGCCCAAGTCG	GCAATGAAGTTCCGGGTTTA	59.9	297	DRR	Ca_14795	B	bHLH
Ca4_P2_37013372	Ca4	37013372	TA/TACTTAAATGTTAA	CACGCTTTCAACTGTCCTGA	ATTGATCATCACATTGGCGA	59.9	421	INTERGENIC	--	--	--

INDEL marker IDs	Chromosomes/unanchored scaffolds	Physical positions (bp)	InDels (*Kabuli* reference genome-CDC Frontier/PII)	Forward primers (5'-3')	Reverse primers (5'-3')	Anneling temperature (0C)	Expected amplified product size (bp)	Structural annotation		Functional annotation	
								Sequence components of *kabuli* genome	*Kabuli* gene accession IDs	NCBI-KOG	TFs
Ca4_P2_37034120	Ca4	37034120	A/AGAG	GGAGGTTATCAATGGCTGGA	TGGAGTGGAAAGGAATGGAG	60.0	536	INTERGENIC	--	--	--
Ca4_P2_37034438	Ca4	37034438	ATTTTTTT/ATTTTTT	CTCCATTCCTTTCCACTCCA	AGGGAAAACAAGGGTTTTGG	60.2	522	INTERGENIC	--	--	--
Ca4_P2_37034565	Ca4	37034565	GAAAAAAA/GAAAAAAAA	CTCCATTCCTTTCCACTCCA	AGGGAAAACAAGGGTTTTGG	60.2	522	INTERGENIC	--	--	--
Ca4_P2_37034691	Ca4	37034691	ATTTTT/ATTTT	CTCCATTCCTTTCCACTCCA	AGGGAAAACAAGGGTTTTGG	60.2	522	INTERGENIC	--	--	--
Ca4_P2_37034814	Ca4	37034814	T/TC	CTCCATTCCTTTCCACTCCA	GAGCCTTTCATTGGGTTTGA	60.1	906	INTERGENIC	--	--	--
Ca4_P2_37035544	Ca4	37035544	AC/A	TCAAACCCAATGAAAGGCTC	TCCCTTTACCTTGCCTCATTT	60.0	640	INTERGENIC	--	--	--
Ca4_P2_37035676	Ca4	37035676	GTAATAATAATAATAA/GTAATAATAATAATAA	TCAAACCCAATGAAAGGCTC	TCCCTTTACCTTGCCTCATTT	60.0	640	INTERGENIC	--	--	--
Ca4_P2_37035741	Ca4	37035741	TCC/TC	CCCAATGAAAGGCTCACAAT	TCCCTTTACCTTGCCTCATTT	60.0	635	INTERGENIC	--	--	--
Ca4_P2_37038560	Ca4	37038560	TGAGA/TGAGAGA	TGGTTTATAACACCGCTTATATTCA	AGGATTCAACCACAAAGGACA	59.4	242	INTERGENIC	--	--	--
Ca4_P2_37039540	Ca4	37039540	TAAAAAAAAAAA/TAAAAAAAA	TTTATTGACGAGCATCGTTCC	TGGCAAGTGCAACCAAAATA	60.1	386	INTERGENIC	--	--	--
Ca4_P2_37042952	Ca4	37042952	CA/CAATA	GCATGAGAATTTCCGTGTCA	TAAAACCACACAGCAAACGG	59.6	518	INTERGENIC	--	--	--
Ca4_P2_37043249	Ca4	37043249	T/TA	CCGTTTGCTGTGTGGTTTTA	TTGCAAACAAGCAACCAAAG	59.9	298	INTERGENIC	--	--	--

INDEL marker IDs	Chromosomes/unanchored scaffolds	Physical positions (bp)	InDels (*Kabuli* reference genome-CDC Frontier/PII)	Forward primers (5'-3')	Reverse primers (5'-3')	Anneling temperature (0C)	Expected amplified product size (bp)	Structural annotation		Functional annotation	
								Sequence components of *kabuli* genome	*Kabuli* gene accession IDs	NCBI-KOG	TFs
Ca4_P2_37043299	Ca4	37043299	T/TG	CCGTTTGCTGTGTGGTTTTA	TTGCAAACAAGCAACCAAAG	59.9	298	INTERGENIC	--	--	--
Ca4_P2_37043980	Ca4	37043980	CAAAA/CAAAAAAA	CTAGTCCGGTGGACCCGTAT	TCACGAAATTAGCCCTCCAA	60.6	861	INTERGENIC	--	--	--
Ca4_P2_37044142	Ca4	37044142	TAAAAAA/TAAAAAAA	TGATTGTTTTGATCCCCTCA	TCACGAAATTAGCCCTCCAA	60.6	328	INTERGENIC	--	--	--
Ca4_P2_37044759	Ca4	37044759	CGAAGAA/CGAA	TTGGAGGGCTAATTTCGTGA	TTGGGTTGGGTACAAGAGGT	59.3	557	INTERGENIC	--	--	--
Ca4_P2_37046249	Ca4	37046249	AAATTGA/AAATTGACATATTTAATTGA	CTCCCTTGTCCATATGGGGT	TGTTACGCTGGACAACTTGC	59.9	308	INTERGENIC	--	--	--
Ca4_P2_37047512	Ca4	37047512	CAAA/CA	GAAGTGGGAAATGGGTTTGA	ATGATTGTGCCTCGTGAACA	60.1	662	INTERGENIC	--	--	--
Ca4_P2_37047776	Ca4	37047776	GC/GCC	ATACTCTGCACCACACACGG	ATGATTGTGCCTCGTGAACA	60.1	241	INTERGENIC	--	--	--
Ca4_P2_37048884	Ca4	37048884	CAAAAAAAAA/CAAAAAAAA	GCATACAATAGAAACGGAGAGCA	CAGCTGAGTGCGTGAAGAAG	59.9	341	INTERGENIC	--	--	--
Ca4_P2_37049140	Ca4	37049140	TATT/TATTAATT	TTCTTCTTCACGCACTCAGC	CCCTTCTGAAGTGAGCATCTG	60.0	628	INTERGENIC	--	--	--
Ca4_P2_37055713	Ca4	37055713	A/AT	GCCGGACAGTGTATTGTGTTT	CCATATGCCACCATTTCCTT	59.6	552	INTERGENIC	--	--	--
Ca4_P2_37057959	Ca4	37057959	TA/T	TTTGTGCTCCATACACACATGA	CACCGTCTTCAAAGAATTCCA	60.1	771	INTERGENIC	--	--	--
Ca4_P2_37059578	Ca4	37059578	TCC/TC	GTCCACCTCGTTCCTGTCTT	TGGGGACTGGAAAATGGTTA	60.2	462	INTERGENIC	--	--	--

INDEL marker IDs	Chromosomes/unanchored scaffolds	Physical positions (bp)	InDels (*Kabuli* reference genome-CDC Frontier/PII)	Forward primers (5'-3')	Reverse primers (5'-3')	Anneling temperature (0C)	Expected amplified product size (bp)	Structural annotation		Functional annotation	
								Sequence components of *kabuli* genome	*Kabuli* gene accession IDs	NCBI-KOG	TFs
Ca4_P2_37069268	Ca4	37069268	GAAAAAAA/GAAAAAAAA	CCCGACATTCTGAGGTCAAT	ACTACCCTCCCCAAACAAGC	60.4	371	INTERGENIC	--	--	--
Ca4_P2_37069604	Ca4	37069604	CTGTTGTTG/CTGTTG	GCCATGCACATAGCAGAAAA	CACCGAAAAAGATGTTGTTAGTC	57.9	484	INTERGENIC	--	--	--
Ca4_P2_37070250	Ca4	37070250	TTT/TTTCATT	TCAACTCCAATTCAAGTGCG	TGTTGCGTAGCCTCTGAAAA	59.6	206	INTERGENIC	--	--	--
Ca4_P2_37070451	Ca4	37070451	T/TGACTAC	TCAACTCCAATTCAAGTGCG	GCACGAATCTGCGACAATTA	59.8	721	INTERGENIC	--	--	--
Ca4_P2_37083670	Ca4	37083670	T/TGAACACAAC	TCTCAAGACTCCAGATGGCA	CGTGCTCCATTTTTAGGCAT	60.1	558	INTERGENIC	--	--	--
Ca4_P2_37089859	Ca4	37089859	TAAAAAAAAA/TAAAAAAA	ATATCGACAATGCGCAACAA	CAACCAAGAGCGTTCTCCAT	60.3	853	INTERGENIC	--	--	--
Ca4_P2_37090867	Ca4	37090867	ATTTTTT/ATTTTTTT	CCACCCAGAAAGGAGTTCAA	TTCTCCAAGTTGTTTTGGCA	59.3	574	INTERGENIC	--	--	--
Ca4_P2_37099035	Ca4	37099035	G/GT	GGTCAACATTTGAAGAAGATTCG	CCCACTACCGGGTTTGATTA	59.7	646	INTERGENIC	--	--	--
Ca4_P2_37099831	Ca4	37099831	TAAAAAAA/TAAAAAA	TGAATAATTCGCGTACGGCT	TGTGAGGAGCTCTTTGTGGA	59.5	589	INTERGENIC	--	--	--
Ca4_P2_37101032	Ca4	37101032	GAT/GATCATAACTCCTATAT	TCAACGAGTTACCGTGCTTG	TGTGTCTCGGGTTGAGTTGA	60.3	621	INTERGENIC	--	--	--
Ca4_P2_37103905	Ca4	37103905	GTTTTTTTTTT/GTTTTTTTTTTTT	AATGGTGACGACGACAATCA	TACTGCTCCCCTTGCTCACT	60.0	665	INTERGENIC	--	--	--
Ca4_P2_37104817	Ca4	37104817	ATATTAT/ATATTATTAT	ACTACGCCATCATTTGGAGG	GGGGTGAGAGACCATTTGAA	59.9	613	INTERGENIC	--	--	--

INDEL marker IDs	Chromosomes/unanchored scaffolds	Physical positions (bp)	InDels (*Kabuli* reference genome-CDC Frontier/PII)	Forward primers (5'-3')	Reverse primers (5'-3')	Anneling temperature (0C)	Expected amplified product size (bp)	Structural annotation		Functional annotation	
								Sequence components of *kabuli* genome	*Kabuli* gene accession IDs	NCBI-KOG	TFs
Ca4_P2_37105526	Ca4	37105526	A/AAT	TTCAAATGGTCTCTCACCCC	ACGAAGGTCAAGCTCAAGGA	60.0	855	INTERGENIC	--	--	--
Ca4_P2_37106034	Ca4	37106034	GT/GTTCCGCTT	TCCTTGAGCTTGACCTTCGT	TTGCGCGAATCAAAACATTA	60.2	276	INTERGENIC	--	--	--
Ca4_P2_37107403	Ca4	37107403	TAAAAAA/TAAAAAAA	CGACGAATTTGCAGAGTCAA	TTTTTGGATTCCTCTCGTCG	60.2	533	INTERGENIC	--	--	--
Ca4_P2_37117919	Ca4	37117919	AT/A	TACGGAAATCGGCTGGATAG	GCACGATAAATGCAACCCTT	60.0	496	INTERGENIC	--	--	--
Ca4_P2_37118197	Ca4	37118197	ATTTTTTT/ATTTTTT	AAGGGTTGCATTTATCGTGC	CCAGCTCGTTTGTTTGAAGA	59.0	237	INTERGENIC	--	--	--
Ca4_P2_37120936	Ca4	37120936	GTTTTTTTTTTT/GTTTTTTTTTT	TAACTTCAGGTTGGGGACCA	TCCAAATCCCAAACAAAAGG	59.8	442	INTERGENIC	--	--	--
Ca4_P2_37121983	Ca4	37121983	ATTTTTTT/ATTTTTTTT	TCAACATGACCTCCTTATGCTTT	TCCAAACACTCCAAACACCA	60.0	407	INTERGENIC	--	--	--
Ca4_P2_37126648	Ca4	37126648	TAAAAAA/TAAAAAAA	CATCATTTCCAACAATCCCC	CACGTGCATGACCCTAAATG	60.0	242	INTERGENIC	--	--	--
Ca4_P2_37128391	Ca4	37128391	TAA/TAAA	CCTCCCCAAGTTCCATACCT	TGCATCCATCGTCAAATACAA	59.9	737	INTERGENIC	--	--	--
Ca4_P2_37128543	Ca4	37128543	TAAAAA/TAAAA	TCGCACTTTTGGAGATTGAA	TGCATCCATCGTCAAATACAA	59.9	462	INTERGENIC	--	--	--
Ca4_P2_37128775	Ca4	37128775	TAAAAA/TAAAAAA	TTCGCACTTTTGGAGATTGA	TCCCTCTCTCTTTATGCCTCTC	59.1	690	INTERGENIC	--	--	--
Ca4_P2_37128859	Ca4	37128859	G/GC	TTTGACGATGGATGCATAAGA	TTTAGGTTGGCTTTGGATGC	60.1	593	INTERGENIC	--	--	--

INDEL marker IDs	Chromosomes/unanchored scaffolds	Physical positions (bp)	InDels (*Kabuli* reference genome-CDC Frontier/PII)	Forward primers (5'-3')	Reverse primers (5'-3')	Anneling temperature (0C)	Expected amplified product size (bp)	Structural annotation		Functional annotation	
								Sequence components of *kabuli* genome	*Kabuli* gene accession IDs	NCBI-KOG	TFs
Ca4_P2_37129073	Ca4	37129073	AAGAGAGAGAGAGA/AAGAGAGAGA	TTTGACGATGGATGCATAAGA	TTTAGGTTGGCTTTGGATGC	60.1	593	INTERGENIC	--	--	--
Ca4_P2_37129106	Ca4	37129106	GAGATAGTAAAGA/GAGATAGTAAAGATAGTAAAGA	TTTGACGATGGATGCATAAGA	TTTAGGTTGGCTTTGGATGC	60.1	593	INTERGENIC	--	--	--
Ca4_P2_37130048	Ca4	37130048	ATTTTTTTTTT/ATTTTTTTTTTT	TTGCAGAAGGTTCCCAAATC	TGTTGGCCATCTTTTAGCTTT	58.9	841	INTERGENIC	--	--	--
Ca4_P2_37131217	Ca4	37131217	TC/T	TTGTGACCAAAGGTTGGAGG	AATTTGCGAGCATTACCTGC	60.2	710	INTERGENIC	--	--	--
Ca4_P2_37194951	Ca4	37194951	TATAATAATAATAATAATAATAATAATAATAATAATAA/TATAATAATAATAATAATAATAATAATAATAA	GTCTGGTCTGACCTGGAAGC	CCTGTCAGGCTCAGGCTTAT	59.5	415	INTERGENIC	--	--	--
Ca4_P2_37311820	Ca4	37311820	GATATATATATATATATATATATA/GATATATATATATATATATATATATA	CGCTGTAAAATGCATGTGCT	TTGGGTGAACCATTAGGCAT	60.2	528	INTERGENIC	--	--	--
Ca4_P2_37382618	Ca4	37382618	ATTTTTT/ATTTTTTT	TTGTGAAAAATGCACGAATCA	TTTCCAGCACTATTTCCGGT	59.6	904	INTERGENIC	--	--	--
Ca4_P2_37403650	Ca4	37403650	CTTTT/CTTTTT	CTTATCCAAAAGCGAGGCAC	TGAGTTGGACTACAAGCCATCA	60.7	597	INTERGENIC	--	--	--
Ca4_P2_37443931	Ca4	37443931	CT/CTAT	CCGCATTTTCTTCATTAGGG	ACGTTAAGGCATGAGTTGGG	60.0	364	INTERGENIC	--	--	--
Ca4_P2_37444272	Ca4	37444272	GC/GCCTTAAC	GGTTCCCAACTCATGCCTTA	TTTCACACCTGCTTCTGTGC	60.0	513	INTERGENIC	--	--	--
Ca4_P2_37462018	Ca4	37462018	ATTTTTTTTTT/ATTTTTTTTT	TCAAATCGTGGTGGAACAAA	GGAAATGGTCTTGATGGGTG	60.2	713	INTERGENIC	--	--	--
Ca4_P2_37464628	Ca4	37464628	CAAAAAAAAAAAA/CAAAAAAAAAAA	TCTCGGTCAACGGTCTCTCT	ACAACCAAACTCCATGGCTC	60.0	195	INTERGENIC	--	--	--

INDEL marker IDs	Chromosomes/unanchored scaffolds	Physical positions (bp)	InDels (*Kabuli* reference genome-CDC Frontier/PII)	Forward primers (5'-3')	Reverse primers (5'-3')	Anneling temperature (0C)	Expected amplified product size (bp)	Structural annotation		Functional annotation	
								Sequence components of *kabuli* genome	*Kabuli* gene accession IDs	NCBI-KOG	TFs
Ca4_P2_37484715	Ca4	37484715	T/TTTA	CCAAACCCTTAAACCAAGCA	GTGGCCACTGTTTCTCACCT	60.2	902	INTERGENIC	--	--	--
Ca4_P2_37490253	Ca4	37490253	TNNNNNNNNNN/TNNNNN	TCTATGCGCTGATTCAGACAA	AACATCCCCATCAATTCATCA	60.0	799	INTERGENIC	--	--	--
Ca4_P2_37519504	Ca4	37519504	CTATTTATTTATTTATTTATTT/CTATTTATTTATTTATTT	GAAAAAGGAGTTGCCACCAA	TGGGGATACGTAGGAGCAAG	60.1	306	INTERGENIC	--	--	--
Ca4_P2_37608001	Ca4	37608001	ACTCTCTCTCTCTCTC/ACTCTCTCTCTCTC	TTGGATTCCTGACACACACA	TAAAAATTTGGAGGGAGGGG	60.1	568	INTERGENIC	--	--	--
Ca4_P2_37681632	Ca4	37681632	T/TA	ATGCATGGACGCATTATTGA	AATCTGTTGAACCGACCCTG	60.0	768	INTERGENIC	--	--	--
Ca4_P2_37706092	Ca4	37706092	TC/T	GCCGGGATGTTCTTTTTGTA	GAGGGGAGGCCTTCTCATAC	60.0	818	DRR	Ca_15110	C	MYB
Ca4_P2_37706158	Ca4	37706158	G/GA	GCCGGGATGTTCTTTTTGTA	GAGGGGAGGCCTTCTCATAC	60.0	818	DRR	Ca_15110	C	MYB
Ca4_P2_37707066	Ca4	37707066	TGC/TGCTGGC	TGAGGCCTTAGCTTCGTGAT	CACAAGGACCTGACGGTTTT	60.0	555	INTERGENIC	--	--	--
Ca4_P2_37707134	Ca4	37707134	GC/GTGCCCCTCCCC	TGAGGCCTTAGCTTCGTGAT	CACAAGGACCTGACGGTTTT	60.0	555	INTERGENIC	--	--	--
Ca4_P2_37767167	Ca4	37767167	AATTA/AA	GCTGCTGCTGGTCATATTCA	CCGGAAATTTCAAAAACGAA	59.9	448	INTRON	Ca_15102	--	SAP
Ca4_P2_37789659	Ca4	37789659	AATATATATATATATATATATATATAT A/AATATATATATATATATATATATATA	GGTGGATTGGAGAAGATGGA	TCCGACAAACAGAGCTACAAGA	60.1	556	DRR	Ca_15098	--	--
Ca4_P2_3781804	Ca4	3781804	CATATTTTTTTGTCAATATATTTTTTTGTCAAT/CATATTTTTTTGTCAATATATTTTTTTGTCAATATATTTTTTTGTCAAT	TGGTTGTGCGATATTGTGCT	TCAATGCATTTTCGACATTTTT	59.5	749	INTERGENIC	--	--	--

INDEL marker IDs	Chromosomes/unanchored scaffolds	Physical positions (bp)	InDels (*Kabuli* reference genome-CDC Frontier/PII)	Forward primers (5'-3')	Reverse primers (5'-3')	Anneling temperature (0C)	Expected amplified product size (bp)	Structural annotation		Functional annotation	
								Sequence components of *kabuli* genome	*Kabuli* gene accession IDs	NCBI-KOG	TFs
Ca4_P2_37822334	Ca4	37822334	TT/TTATAGAACTCTAAT	TCAACCAAAATCCCCCAATA	CCCAATTTCGATTTACCCCT	60.0	329	INTERGENIC	--	--	--
Ca4_P2_37890823	Ca4	37890823	TTCTCTCTCTCTCTCTCTCTCTCTCTCTCTCTCTCTCTCTCTC/TTCTCTCTCTCTCTCTCTCTCTCTCTCTCTCTCTCTCTCTCTCTCTCTCTC	CACTCTTGCCGAGCACAATA	TGAAAGCCTGTGTGACATCC	59.7	740	INTERGENIC	--	--	--
Ca4_P2_37936265	Ca4	37936265	AATATATATATATATATATATATATATA/AATATATATATATATATATATATA	TCTTATTCAGCAAGCGCTCA	GTGACTGGGAAAACGTTGGT	59.9	346	INTRON	Ca_15084	--	--
Ca4_P2_38080710	Ca4	38080710	GTATATATATATATATATATATATATATATATAT/GTATATATATATATATATATATATATATATATATATATATATATATATATATAT	GCTTTTGAGGCGTAAAACCA	TTCCTCCAATAGAAATGCCG	60.0	520	INTERGENIC	--	--	--
Ca4_P2_38118271	Ca4	38118271	GTATATATATATATATATATATATATATATAT/GTATATATATATATATATATATATATATATATATATAT	GCCTTCAAAACCAAGAACCA	CGTTTGTTGCTATGAAACCG	59.2	447	INTERGENIC	--	--	--
Ca4_P2_38120609	Ca4	38120609	GAAAAAAAAAAA/GAAAAAAAAAA	CATCATCACCACCACCAAAA	CACTTCCGTCTCCAGACTCC	59.8	222	INTERGENIC	--	--	--
Ca4_P2_38231340	Ca4	38231340	TTATTATTATTATTATTATTATTATTATTATTATTATTATTATTATTATTATT/TTATTATTATTATTATTATTATTATTATTATTATTATTATTATTATTATTATT	GGCCATCAGAGAATCTTCCA	TGCACTTTTGGGAACAACAA	60.1	580	INTRON	Ca_13131	--	--
Ca4_P2_38232542	Ca4	38232542	CTATATATATATATATATATATATA/CTATATATATATATATATATATA	TTTCTGATGGATTAAATAAATTGGG	CAACGACAAATCGACAATGG	60.0	426	INTRON	Ca_13131	--	--
Ca4_P2_3825463	Ca4	3825463	CAAAAAAAAAAAAAAA/CAAAAAAAAAAAAA	TTTCATGAGTCAAGCCCAAG	CGAAATTCGGTCAAGGAAAA	60.0	412	INTERGENIC	--	--	--
Ca4_P2_3832898	Ca4	3832898	ATTT/ATTTTTT	CGGAGGGTACGAACCTGTAA	ACTGTTGTGGAAATCTGGGC	60.0	484	INTERGENIC	--	--	--
Ca4_P2_38410741	Ca4	38410741	ATT/AATAACTT	TGAAATCATACAATTTTAAGGAGGA	TTCATCCACATCAGCCAAAA	60.0	596	INTERGENIC	--	--	--
Ca4_P2_38445424	Ca4	38445424	ATATTATTATTATTATTATTATTATTATTATTATTATT/ATATTATTATTATTATTATTATTATTATTATTATTATT	ATATCGCACCGGAAAAACAA	AAAGCATCATGACCGAGACC	60.1	379	INTERGENIC	--	--	--

INDEL marker IDs	Chromosomes/unanchored scaffolds	Physical positions (bp)	InDels (*Kabuli* reference genome-CDC Frontier/PII)	Forward primers (5'-3')	Reverse primers (5'-3')	Anneling temperature (0C)	Expected amplified product size (bp)	Structural annotation		Functional annotation	
								Sequence components of *kabuli* genome	*Kabuli* gene accession IDs	NCBI-KOG	TFs
Ca4_P2_38558247	Ca4	38558247	ATTTTTT/ATTTTTTT	TGATGTGGAAATTTTTGGCA	ATTTTCGAATGAACGGTTGC	59.9	705	INTERGENIC	--	--	--
Ca4_P2_38655316	Ca4	38655316	TA/TAACTTCTCAACGGA	CGGCGATTTTTAATCCTCAA	GCGGCATTGTCGTCTTATCT	60.2	727	INTERGENIC	--	--	--
Ca4_P2_38656268	Ca4	38656268	AATATATATATATATATATATA/AATATATATATATATATATA	TTGCTGCTTTCCAGAACCTT	GGGCTAAGTGCCCCTTATTT	59.5	871	INTERGENIC	--	--	--
Ca4_P2_3868034	Ca4	3868034	GATNNNNNNNNNNAT/GAT	AGGAAGGGAACTTCAGGCAT	GCGCTTCCTGTTATCCAGAG	60.0	426	INTRON	Ca_03867	--	--
Ca4_P2_38682630	Ca4	38682630	TTATATATATATATATATA/TTATATATATATATATATATA	ACGGTATCAATTAACACAGGAAA	GTGGGGGCTTGTTGAGTTAC	59.5	707	INTERGENIC	--	--	--
Ca4_P2_38829588	Ca4	38829588	CATTATTATTATTATTATTATTATTATTATTATTATTATTATT/CATTATTATTATTATTATTATTATTATTATTATTATTATTATTATTATT	TGTGTGGTCATTGGAATCACTT	TGAGAAGAGTTGTGGCGTTG	60.0	920	INTERGENIC	--	--	--
Ca4_P2_38863836	Ca4	38863836	CATATATATATATATATATATAT/CATATATATATATATATATATATAT	AACTCAATGACATGCGACAAA	TTGAGAGGATGGTTAAAGGGA	58.6	779	INTERGENIC	--	--	--
Ca4_P2_38884678	Ca4	38884678	CAAAAAAA/CAAAAAA	GGTGGTCATGGTGTAGTTTGG	TCACACGTCCTTTCAAAATCA	59.2	575	INTERGENIC	--	--	--
Ca4_P2_38940144	Ca4	38940144	GTTTTTTTTTT/GTTTTTTTTTTT	TAAAGGACCAAGTCGGGAAA	CTCATAATCTTCCGCCCAAA	60.0	216	INTERGENIC	--	--	--
Ca4_P2_3895253	Ca4	3895253	CA/CATGGAAAA	AGGACAGCTCACGACCAAGT	AGGATGCGAATTCGAGGATA	59.6	603	INTERGENIC	--	--	--
Ca4_P2_39014915	Ca4	39014915	TTATATATATATATATATATATATATATATATATATATAT/TTATATATATATATATATATATATATAT	TGTGTTCCGACACCATCCTA	AACAGCAACAGCCATACGAA	59.3	799	INTERGENIC	--	--	--
Ca4_P2_39020626	Ca4	39020626	TAAAAAAAAA/TAAAAAAAAAA	TGGCATGACACTTAGCTCAAA	CCCCAATGTATTGGAACCAC	59.9	864	INTERGENIC	--	--	--

INDEL marker IDs	Chromosomes/unanchored scaffolds	Physical positions (bp)	InDels (*Kabuli* reference genome-CDC Frontier/PII)	Forward primers (5'-3')	Reverse primers (5'-3')	Anneling temperature (0C)	Expected amplified product size (bp)	Structural annotation		Functional annotation	
								Sequence components of *kabuli* genome	*Kabuli* gene accession IDs	NCBI-KOG	TFs
Ca4_P2_39038460	Ca4	39038460	ACTTGCTTG/ACTTG	CCGGTCACATTCAATAGCCT	ATTGCGTTGTTTCTTCCAGG	60.1	411	DRR	Ca_13078	A	--
Ca4_P2_39070213	Ca4	39070213	TAATAAATAA/TAATAA	TATTTTGGATTTTGGCTCGG	AAGGAAGCAACGAAAACGAA	59.9	446	INTERGENIC	--	--	--
Ca4_P2_39078155	Ca4	39078155	ATTTTTT/ATTTTTTT	TGTGTGCAATAGAAGGTCGC	CATCACTAGGATGCGATCACC	60.5	347	INTERGENIC	--	--	--
Ca4_P2_39147993	Ca4	39147993	AATATATATATATATATATATATAT/AATATATATATATATATATATATAT	GGAAGGAGCCCTTATCTTCG	ATGTCAATTGATTTGCGACG	59.5	422	INTERGENIC	--	--	--
Ca4_P2_39156446	Ca4	39156446	TAAAAAAAAAA/TAAAAAAAAA	GAGCATCGCAGGTTGAATTT	CCGCATGAGATATTTTGCATT	59.9	362	INTERGENIC	--	--	--
Ca4_P2_39165742	Ca4	39165742	TAAAAATAAAAAATAAAAAA/TAAAAATAAAAAATAAAAAAATAAAAAA	CGTATACCGCTGCTCATCAA	TGATGTGATTTGTGCCATGA	59.5	259	INTERGENIC	--	--	--
Ca4_P2_39249438	Ca4	39249438	CTTTTTTTT/CTTTTTTT	ATTGTTTGGCTCCACACCTC	TTTCAACAGGAGTATTTTTATGCG	59.6	245	INTERGENIC	--	--	--
Ca4_P2_39257138	Ca4	39257138	ATTTTTTTTTT/ATTTTTTTTTTT	TTTGCGTTACGAATTTGATGA	TGAATTTTCCCGACTTGGTC	59.9	181	INTERGENIC	--	--	--
Ca4_P2_39349085	Ca4	39349085	GTTAGGGTTTAGGGTTTAGGGTT/GTTAGGGTTTAGGGTTTAGGGTTTAGGGTT	AAGGGTTAGGCTTTGGGTTT	CCCTAAACCATACCTCTACCACAA	60.5	604	INTERGENIC	--	--	--
Ca4_P2_39354610	Ca4	39354610	CAAAAAAAAAAA/CAAAAAAAAAA	TTGCACTTGGTTTGACTCCA	TGGTGAGATTGTGCGAAGAG	60.0	527	INTERGENIC	--	--	--
Ca4_P2_3951584	Ca4	3951584	AT/ATATATATGT	TGGACATTTAATGGTTGCGA	TTTATCTCTCTCGTGCGTGC	59.2	563	INTERGENIC	--	--	--
Ca4_P2_39644838	Ca4	39644838	TAAAAAAA/TAAAAAA	GGAGGAAGAGATGGAGAAACCT	TGACACTCATTGCTCCTCCA	60.4	553	INTERGENIC	--	--	--

INDEL marker IDs	Chromosomes/unanchored scaffolds	Physical positions (bp)	InDels (*Kabuli* reference genome-CDC Frontier/PII)	Forward primers (5'-3')	Reverse primers (5'-3')	Anneling temperature (0C)	Expected amplified product size (bp)	Structural annotation		Functional annotation	
								Sequence components of *kabuli* genome	*Kabuli* gene accession IDs	NCBI-KOG	TFs
Ca4_P2_39647246	Ca4	39647246	CTTTT/CTTTTT	GCATGGTGCAAGATGGTTTT	TTGGATTGCATGTGTTTGAGA	60.1	272	INTERGENIC	--	--	--
Ca4_P2_39659005	Ca4	39659005	TTATATATATATATATATATATATATATATATATATATATA/TTATATATATATATATATATATATATATATATATATATATATATATATATATATATA	ACGTTGTTTCTTTTGGGACG	TGGGTGCTATTTGCAATCAA	60.1	595	DRR	Ca_14935	T	bHLH
Ca4_P2_3966806	Ca4	3966806	GAAAAAAAAAAA/GAAAAAAAAAA	CCGTCGCTAAACACACTCAA	TGCACCAACAATCCTTGCTA	60.3	814	INTERGENIC	--	--	--
Ca4_P2_39689187	Ca4	39689187	AC/ACC	ATTACCGACATTGCAGAGGG	CTCCCATTCTGCCCTTGATA	60.0	329	INTERGENIC	--	--	--
Ca4_P2_39740716	Ca4	39740716	AG/A	TCCTAGGGCTCATGAGTTGC	GAGATGTGCAGTTTAATTCGTCC	60.0	882	INTERGENIC	--	--	--
Ca4_P2_39832034	Ca4	39832034	CAAAA/CAAA	TGTGCGAATGTGGTTTTTGT	TGAACATATTGCTAGGCGCA	60.4	165	INTERGENIC	--	--	--
Ca4_P2_3989437	Ca4	3989437	AATATATATATATATATATATATAT/AATATATATATATATATATATATAT	TCCCGTTCCAAAACTTTTCA	TGATAGCACAAAATCGTGGA	57.7	594	DRR	Ca_03854	G	--
Ca4_P2_39986903	Ca4	39986903	ATTT/ATTTCGTAGGTGGTTT	CCCAGAAATCGATTAGGCAA	CCCAAGAAAGTAGCCACCAG	59.7	499	DRR	Ca_14912	--	--
Ca4_P2_39987654	Ca4	39987654	ATTTTTTTTTTT/ATTTTTTTTTT	TAAATTCCGCTGTGCACTTG	AACTTACCGATCAATTCAAATCAA	59.0	191	INTERGENIC	--	--	--
Ca4_P2_39997321	Ca4	39997321	GAAAAAAAAA/GAAAAAAAAAA	AATGCAAAGAGACGCACATT	TGGAATCCATGGCTCTAGGA	60.6	416	INTERGENIC	--	--	--
Ca4_P2_40048822	Ca4	40048822	AATATATATATATATATA/AATATATATATATATA	CCAAGTTTCAAATGGCGACT	AATTGGCCACACTCGAAAAC	60.0	203	INTERGENIC	--	--	--
Ca4_P2_40111333	Ca4	40111333	GAATAATAATAATAATAATAATAATAATAATAATAATAATAATAATA/GAATAATAATAATAATAATAATAATAATAATAATAATAATAATAATA	TTGCAAGTTGCTATCCCGTT	GATGTGACGACGGTACGAAA	59.6	723	INTERGENIC	--	--	--

INDEL marker IDs	Chromosomes/unanchored scaffolds	Physical positions (bp)	InDels (*Kabuli* reference genome-CDC Frontier/PII)	Forward primers (5'-3')	Reverse primers (5'-3')	Anneling temperature (0C)	Expected amplified product size (bp)	Structural annotation		Functional annotation	
								Sequence components of *kabuli* genome	*Kabuli* gene accession IDs	NCBI-KOG	TFs
Ca4_P2_40144715	Ca4	40144715	CT/CTCTCTAT	TTGAAGGTTCAGGCCCATAG	AATGGCGAAAACTAACACCG	60.0	507	INTRON	Ca_14905	O	--
Ca4_P2_4018431	Ca4	4018431	AATATATATATATATATATATATATATATATATA/AATATATATATATATATATATATATATATATATATATATATA	ATCGAAACACAAATGGGGAA	CTTCTGGATTGACAACGGGT	60.0	582	INTERGENIC	--	--	--
Ca4_P2_4030564	Ca4	4030564	ATTTTTTTT/ATTTTTTT	TTCACGTGACATTGCATTCAT	AGAGTGCTAAAACATCCCATCA	58.7	571	DRR	Ca_03849	--	Dof
Ca4_P2_4030815	Ca4	4030815	CAAAAA/CAAAA	TGGATCCACTAATAACATGGGA	CCTTTAAGGGGATTGAAGGA	58.1	516	DRR	Ca_03849	--	Dof
Ca4_P2_4030871	Ca4	4030871	ATTTTTTT/ATTTTTT	TGGATCCACTAATAACATGGGA	CCTTTAAGGGGATTGAAGGA	58.1	516	DRR	Ca_03849	--	Dof
Ca4_P2_4031228	Ca4	4031228	ATTTTTTT/ATTTTTT	TTGGTTGGAGGTAAATGAATGA	CCTTTAAGGGGATTGAAGGA	58.1	209	DRR	Ca_03849	--	Dof
Ca4_P2_4031381	Ca4	4031381	T/TG	AAAATTTCTCTCCCCCTCTCC	TCAAAATCAAATAAAACAAACCC	57.2	614	INTERGENIC	--	--	--
Ca4_P2_4031880	Ca4	4031880	GTTTTTTT/GTTTTTTTTT	GGGTTTGTTTTATTTGATTTTGA	GATCTGGAAAGGAGAAGGGC	60.2	238	INTERGENIC	--	--	--
Ca4_P2_4032239	Ca4	4032239	AATATATATATATA/AATATATATATA	GCGTCGTTTTAGTCGTGGTT	AGCTGGGGTTGCTTTACTGA	59.9	474	INTERGENIC	--	--	--
Ca4_P2_4033505	Ca4	4033505	AA/AAAAAGA	ATCCAACACTACCCATCCCA	ATGAGTGAAAGAGGTGGCGA	60.8	281	INTERGENIC	--	--	--
Ca4_P2_4034676	Ca4	4034676	TTGTGTGTGTGTGTGTGT/TTGTGTGTGTGTGT	CAAACGTGGACGACTTCTCA	AGGCGGCCCTTAATAGAAAA	60.1	352	INTERGENIC	--	--	--
Ca4_P2_40348298	Ca4	40348298	AATATATATATATATATATATATATATATATATATA/AATATATATATATATATATATATATATATATATATATATATATATA	CCATCCTTCGAATTTGGCTA	TGGTTAAAGGCCAGGATTTC	59.0	668	DRR	Ca_14889	PET	NAC

INDEL marker IDs	Chromosomes/unanchored scaffolds	Physical positions (bp)	InDels (*Kabuli* reference genome-CDC Frontier/PII)	Forward primers (5'-3')	Reverse primers (5'-3')	Anneling temperature (0C)	Expected amplified product size (bp)	Structural annotation		Functional annotation	
								Sequence components of *kabuli* genome	*Kabuli* gene accession IDs	NCBI-KOG	TFs
Ca4_P2_4035364	Ca4	4035364	AT/A	TTTTCTATTAAGGGCCGCCT	GTAACCTTTGCACGCTCCAT	60.1	772	INTERGENIC	--	--	--
Ca4_P2_4035909	Ca4	4035909	TTATATATA/TTATA	GCGCAAAATTGGAGAGAGAG	TTTTGTTGCTTTTGCGTTTG	59.9	192	INTERGENIC	--	--	--
Ca4_P2_4036494	Ca4	4036494	TAAAAAAA/TAAAAAAAA	TGTTCTTTGGCCCTCTTTTG	GAAAGTTGTCAGACGGAGGTTC	60.2	781	INTERGENIC	--	--	--
Ca4_P2_4039327	Ca4	4039327	TACTC/T	TTTTGGTTTGGTAATTGTGGG	TGATGGAGAAATCCTCACCC	59.9	456	INTERGENIC	--	--	--
Ca4_P2_4039648	Ca4	4039648	ACC/AC	TGCAATTACACGAGGGTTCA	AAATTCAAAAACCGGTCTGC	59.1	244	INTERGENIC	--	--	--
Ca4_P2_4040664	Ca4	4040664	TTCTC/TTC	CATCTGCGACCCCTTAACTT	TCAAAAATTTCAACCAAAACCC	60.1	392	INTERGENIC	--	--	--
Ca4_P2_4041328	Ca4	4041328	AAAAA/AAAAAAAAAATATTAAAA	GGGTTTTGGTTGAAATTTTTG	TAATGACGACCAGCGAATGA	60.2	540	INTERGENIC	--	--	--
Ca4_P2_4041384	Ca4	4041384	A/AC	GGGTTTTGGTTGAAATTTTTG	TAATGACGACCAGCGAATGA	60.2	540	INTERGENIC	--	--	--
Ca4_P2_4041545	Ca4	4041545	CTCTTCTT/CTCTT	TCATTCGCTGGTCGTCATTA	AACATGTGACCCAAGGACACT	59.3	387	INTERGENIC	--	--	--
Ca4_P2_4044991	Ca4	4044991	A/AATTGGATC	GGGTGCATTTGTTGGAACTT	TTGGTACGTGTAGAACATCGTG	58.6	676	INTERGENIC	--	--	--
Ca4_P2_4045724	Ca4	4045724	ATTTTTTTTT/ATTTTTTT	TGTTGGGTTTGTTTCCTCCT	GCTTGATAATTTAAGGGCACCA	60.3	515	INTERGENIC	--	--	--
Ca4_P2_40460426	Ca4	40460426	ATTTTTTTTTT/ATTTTTTTTTTTT	CAGCGCTTGTAAAACTCAGAA	AGCCAGGTTTGTTTCTAGCG	59.5	328	INTERGENIC	--	--	--

INDEL marker IDs	Chromosomes/unanchored scaffolds	Physical positions (bp)	InDels (*Kabuli* reference genome-CDC Frontier/PII)	Forward primers (5'-3')	Reverse primers (5'-3')	Anneling temperature (0C)	Expected amplified product size (bp)	Structural annotation		Functional annotation	
								Sequence components of *kabuli* genome	*Kabuli* gene accession IDs	NCBI-KOG	TFs
Ca4_P2_4046386	Ca4	4046386	ATTTTTTTT/ATTTTTTTTTT	TGGTGCCCTTAAATTATCAAGC	GGGGGTGTTTGGTTTTACTC	58.3	660	INTERGENIC	--	--	--
Ca4_P2_4047680	Ca4	4047680	ACCCCC/ACCCCCC	TTTGTGCTTGTGGTGATGGT	GCAACCAATTGTGTTTGTCG	60.0	465	INTRON	Ca_03848	--	bHLH
Ca4_P2_40480392	Ca4	40480392	CTTT/CTTTGCATCTTTT	CGCACCTCTATCCTCAGACC	GAGAGGGTGGCAATGGATTA	59.9	580	INTRON	Ca_14877	I	--
Ca4_P2_40485498	Ca4	40485498	GAAAAAAAA/GAAAAAAA	TTGGCTCCATCAATTGAACA	GGTCCCTTGAGCACATTAGC	59.7	625	INTERGENIC	--	--	--
Ca4_P2_40494345	Ca4	40494345	CTTTTTTTT/CTTTTTTT	CGAAATGATCTTATAAAAATGGACAA	GCAATGCTCTCCAAAGAATCA	60.3	451	INTERGENIC	--	--	--
Ca4_P2_40494662	Ca4	40494662	TAAAAAAAA/TAAAAAAA	GCAGATTGCTTGATTCTTTGG	AGAGAGGGGGAGAGAGATGG	59.8	312	INTERGENIC	--	--	--
Ca4_P2_40497979	Ca4	40497979	TATTGTGATTGTGATTGTGATT/TATTGTGATTGTGATT	CCACCAAACATCATCACAGC	TTGCATTCGAGTTGGATTTTT	59.6	403	DRR	Ca_14875	--	--
Ca4_P2_40498066	Ca4	40498066	AACATAACTAAATATTACA/AACATAACTAAATATTACATAACTAAATATTACA	CCACCAAACATCATCACAGC	TTGCATTCGAGTTGGATTTTT	59.6	403	DRR	Ca_14875	--	--
Ca4_P2_40498490	Ca4	40498490	CTTTTT/CTTTT	AAAAATCCAACTCGAATGCAA	CCATGTCCCCATTTTTCCTA	59.6	442	DRR	Ca_14875	--	--
Ca4_P2_40498650	Ca4	40498650	AAT/A	TCATTTGAAACATGGACCACA	GCTTGACAATCATGGGGTTT	59.8	262	DRR	Ca_14875	--	--
Ca4_P2_40501876	Ca4	40501876	ATTTTTTTTT/ATTTTTTTT	ACCCATGGTACTTGGTCCAC	CATGAGTTATTGATGGTGCGA	59.6	353	INTERGENIC	--	--	--
Ca4_P2_40505340	Ca4	40505340	A/AC	CGATGGTTAAGGGGATGCTA	TGAAAGCCCAAACACAATGA	60.1	591	INTERGENIC	--	--	--

INDEL marker IDs	Chromosomes/unanchored scaffolds	Physical positions (bp)	InDels (*Kabuli* reference genome-CDC Frontier/PII)	Forward primers (5'-3')	Reverse primers (5'-3')	Anneling temperature (0C)	Expected amplified product size (bp)	Structural annotation		Functional annotation	
								Sequence components of *kabuli* genome	*Kabuli* gene accession IDs	NCBI-KOG	TFs
Ca4_P2_40506424	Ca4	40506424	TTTCTT/TTT	GGATCACAACCATAAACCCG	TTCAACAAACTTTCTGGCCTC	59.3	453	INTERGENIC	--	--	--
Ca4_P2_40508840	Ca4	40508840	GAAAAAAAA/GAAAAAAAAAA	GGGGAAAAAGAAAGGAAGGA	GTGGCTGCAAAAGCTAAAGG	60.0	294	DRR	Ca_14874	--	--
Ca4_P2_40508933	Ca4	40508933	AAAATAAATAAATAAATAA/AAAATAAATAAATAAATAAATAA	GGGGAAAAAGAAAGGAAGGA	GTGGCTGCAAAAGCTAAAGG	60.0	294	DRR	Ca_14874	--	--
Ca4_P2_40510929	Ca4	40510929	T/TATTA	TTTGCTTTTGTTGTTGCTGC	GTACGAGCACGTCGACTTCA	60.1	816	INTRON	Ca_14874	--	--
Ca4_P2_40513310	Ca4	40513310	TAAA/TAAAA	TCAACCACCCAAAACAACAA	AGATTGGCTGGTTATGGACG	60.0	598	INTRON	Ca_14874	--	--
Ca4_P2_40514871	Ca4	40514871	AATA/AA	GGAGAACCAGCTTCCTCCTT	CGCTCTTGCTTCCTTTCACT	59.8	278	INTERGENIC	--	--	--
Ca4_P2_40517490	Ca4	40517490	TA/TAA	TTGCTTGTCCGTGACGTAAG	AAAAACGACGCGATATGGTT	59.5	529	INTERGENIC	--	--	--
Ca4_P2_40521264	Ca4	40521264	TTACTACTA/TTACTA	AGATGCGACACAATTCCACA	CATTAAAATTTCAAAGATTTTGCG	59.1	162	INTERGENIC	--	--	--
Ca4_P2_40521734	Ca4	40521734	ATTTTTTTTTTT/ATTTTTTTTTTTT	TCATCGGGACAACTTTGTATG	CGGCTCATCTCAATCCTTGT	60.2	655	INTERGENIC	--	--	--
Ca4_P2_40522888	Ca4	40522888	AAATATATATA/AAATATATATATTTTTAGAATATATATA	GATGGGTTGGTTTGATTTGG	CGACAATTTTGGTCTCTCCA	58.7	946	INTERGENIC	--	--	--
Ca4_P2_40526118	Ca4	40526118	A/AC	CACGGAATTGATCCCCTTATT	CCATGTCTTTAACTTCATGCAAA	59.2	520	INTERGENIC	--	--	--
Ca4_P2_4052624	Ca4	4052624	GAAAAAAA/GAAAAAAAA	CAGGCTGCATCTTTGTGTTG	GAGGGAAAGGAGAGCCAAGT	59.8	374	DRR	Ca_03848	--	bHLH

INDEL marker IDs	Chromosomes/unanchored scaffolds	Physical positions (bp)	InDels (*Kabuli* reference genome-CDC Frontier/PII)	Forward primers (5'-3')	Reverse primers (5'-3')	Anneling temperature (0C)	Expected amplified product size (bp)	Structural annotation		Functional annotation	
								Sequence components of *kabuli* genome	*Kabuli* gene accession IDs	NCBI-KOG	TFs
Ca4_P2_40527713	Ca4	40527713	GATTTAAATATATTTAAATATATTTAAATA/GATTTAAATATATTTAAATA	ACGGCAAAATTCAAACACAA	AAATATTTATATGAGTCGTACCGAGTT	57.2	308	INTERGENIC	--	--	--
Ca4_P2_40528670	Ca4	40528670	GTTTTTTTTT/GTTTTTTTT	AAATGCAACAGGAAGATGCC	TGGACTTGGAAGAGAAACGG	60.2	558	INTERGENIC	--	--	--
Ca4_P2_40529406	Ca4	40529406	CTTTTTTTTT/CTTTTTTTTTT	CCGTTTCTCTTCCAAGTCCA	CGCTAAGCACCAGTTTCCTC	60.0	608	INTERGENIC	--	--	--
Ca4_P2_40534591	Ca4	40534591	TGG/TGGG	TAGGAGACTGCTTCCGCAAT	GGTGCTCTTGACAGCACAAA	60.0	109	DRR	Ca_14873	--	--
Ca4_P2_40546225	Ca4	40546225	TAAAAA/TAAAAAA	GGCATTTTCTTTTGGTGCAT	TGTCTCTGCTGATTTGCCAC	60.0	336	INTERGENIC	--	--	--
Ca4_P2_40550374	Ca4	40550374	GTTCT/GTTCTATTCT	ACCCTGAGTGAGGGAGGATT	GTGCACTGAGGTAGGCAACA	59.9	294	INTRON	Ca_14870	--	--
Ca4_P2_40557571	Ca4	40557571	CTTTTTT/CTTTT	CTTGTGCGAACACAATGGAC	ACATTTACGGGCAAAAACGA	60.4	898	DRR	Ca_14870	--	--
Ca4_P2_40560588	Ca4	40560588	TAAAAAA/TAAAAAAA	TCATTCTCCCGATCTTCCAC	TTTCTGTGGACTCACGCATC	59.8	636	INTERGENIC	--	--	--
Ca4_P2_40561270	Ca4	40561270	TTTCAA/T	ACTGATGCGTGAGTCCACAG	GGATGTTGGCTCCCAAGTTA	59.9	785	INTERGENIC	--	--	--
Ca4_P2_40561558	Ca4	40561558	ATGCAAAATT/ATGCAAAATTGTGCAAAATT	AATTGGGTAACCCTTGGAGC	ACGCGTGGATTTATTAAGCG	60.1	768	INTERGENIC	--	--	--
Ca4_P2_4056899	Ca4	4056899	CAAAAA/CAAAA	TTCGGGAATGAGTGAAAACA	ATCTTCAGCCTTGCTTGCAT	60.0	688	INTERGENIC	--	--	--
Ca4_P2_40571626	Ca4	40571626	C/CCTA	CACTCATCATAGCCCCCTGT	AGATGCACGACAATGCTTCA	60.4	711	INTERGENIC	--	--	--

INDEL marker IDs	Chromosomes/unanchored scaffolds	Physical positions (bp)	InDels (*Kabuli* reference genome-CDC Frontier/PII)	Forward primers (5'-3')	Reverse primers (5'-3')	Anneling temperature (0C)	Expected amplified product size (bp)	Structural annotation		Functional annotation	
								Sequence components of *kabuli* genome	*Kabuli* gene accession IDs	NCBI-KOG	TFs
Ca4_P2_40571712	Ca4	40571712	T/TG	CACTCATCATAGCCCCCTGT	AGATGCACGACAATGCTTCA	60.4	711	INTERGENIC	--	--	--
Ca4_P2_40571787	Ca4	40571787	CAAAAAAAAAA/CAAAAAAAAA	CTTGGTCAAAGGCATGGAAT	GTCACTCGCTCGATCCATTT	60.2	907	INTERGENIC	--	--	--
Ca4_P2_40573928	Ca4	40573928	CAAAA/CAA	TTCGTGGAATATCACAAAAGTTC	TGGACAAAAACTCATCTGCATT	59.6	895	INTERGENIC	--	--	--
Ca4_P2_40574863	Ca4	40574863	A/AC	TGCAGATGAGTTTTTGTCCA	AAAATTAAGACAAAAGGAAGTTCAACA	59.9	658	INTERGENIC	--	--	--
Ca4_P2_40575649	Ca4	40575649	ATTTTTT/ATTTTT	TTTCTCATTTTCATGACTCCTATCA	TCACCAGTGACACGTTCGAT	60.2	375	INTERGENIC	--	--	--
Ca4_P2_40576705	Ca4	40576705	A/ATT	AAATTCTCTCCCTTTCCTTGC	TTTCATCTTCCATAGCCTCG	57.9	447	INTERGENIC	--	--	--
Ca4_P2_40578315	Ca4	40578315	GAAT/GGTCAAT	GCAGGTCTTTTTGGTGGAAA	AACCCTTCATCTTTCGCCTT	60.1	608	CDS (large-effect mutations)	Ca_14868	K	--
Ca4_P2_40593121	Ca4	40593121	CAAGT/CAAGTAAGT	CATTCCCACCATCCTTCACT	GGCCTATCGAACCACACAGT	60.0	932	INTRON	Ca_14867	R	BES1
Ca4_P2_40593400	Ca4	40593400	GTT/GTTTT	AAGGATTTGCTTGCTGATGC	GGCCTATCGAACCACACAGT	60.0	276	INTRON	Ca_14867	R	BES1
Ca4_P2_40598175	Ca4	40598175	C/CTTATGCAACGG	AGTAAGGCGGAGAATGCGTA	CGCATAATGCCTATTGCTCA	59.8	559	INTRON	Ca_14866	--	NAC
Ca4_P2_40598412	Ca4	40598412	GTATAT/GTAT	AGTGGATTGCGAAAGTGGAG	ACCATGTCCATGCACGATAA	59.8	326	INTRON	Ca_14866	--	NAC
Ca4_P2_40613144	Ca4	40613144	TATAATAATAATAATAATAA/TATAATAATAATAATAATAATAATAATAATAA	AAGTCTGGCCTACGGCCTAT	TGTCCGTTAGGGGTCTATGG	59.8	540	INTERGENIC	--	--	--

INDEL marker IDs	Chromosomes/unanchored scaffolds	Physical positions (bp)	InDels (*Kabuli* reference genome-CDC Frontier/PII)	Forward primers (5'-3')	Reverse primers (5'-3')	Anneling temperature (0C)	Expected amplified product size (bp)	Structural annotation		Functional annotation	
								Sequence components of *kabuli* genome	*Kabuli* gene accession IDs	NCBI-KOG	TFs
Ca4_P2_40614742	Ca4	40614742	CATATATATATATATA/CATATATATATATA	GAGCCCAACGATGTTCAAAT	CTGCCTTGGACCTAGTATCG	57.5	179	INTERGENIC	--	--	--
Ca4_P2_40614968	Ca4	40614968	ATTTTTTTT/ATTTTTTT	GAGCCCAACGATGTTCAAAT	TGAACCCCACCAGCATTATT	60.2	793	INTERGENIC	--	--	--
Ca4_P2_40615032	Ca4	40615032	TAA/TA	GAGCCCAACGATGTTCAAAT	TGAACCCCACCAGCATTATT	60.2	793	INTERGENIC	--	--	--
Ca4_P2_40620475	Ca4	40620475	A/AT	TATGTGAAATTGCCACCGAA	ACCTCATCGAGCCATTGATT	59.5	509	INTERGENIC	--	--	--
Ca4_P2_40621809	Ca4	40621809	AAGA/AA	TCTTTTGCACGGAGAATCAA	TTCCCGTGACGTGTGTATGT	59.9	320	INTERGENIC	--	--	--
Ca4_P2_40622124	Ca4	40622124	TAAAAAAAAAAAAA/TAAAAAAAAAAAA	ACATACACACGTCACGGGAA	TTTGCAAATGAGATTTCCTAAAGA	59.3	391	INTERGENIC	--	--	--
Ca4_P2_40624782	Ca4	40624782	TAAAAA/TAAAA	TCAAAAGAATGGAGCAAGTTGT	GGGGGCATTAAGAAGGAAAT	59.3	841	INTERGENIC	--	--	--
Ca4_P2_40626965	Ca4	40626965	TAA/TAAA	GACGTGCTCACTGAAAGGGT	CATCGAGGCCCATTATGATT	59.7	687	INTERGENIC	--	--	--
Ca4_P2_40631353	Ca4	40631353	TAAAAAA/TAAAAA	CCCAACATTAAATTGTCATTGC	GCACATCGTTTCAAAGCTCA	60.0	396	INTERGENIC	--	--	--
Ca4_P2_40631441	Ca4	40631441	TAAAAAAAA/TAAAAAAA	CCCAACATTAAATTGTCATTGC	GCACATCGTTTCAAAGCTCA	60.0	396	INTERGENIC	--	--	--
Ca4_P2_40639895	Ca4	40639895	CAAAA/CAAAAA	CAACGCTATTTACCCACCCA	TCCTATGGAACCACCAAAGC	59.9	200	INTERGENIC	--	--	--
Ca4_P2_4064097	Ca4	4064097	ATTTTTTTT/ATTTTTTTTTT	CATTTCTGCATGGCTTTGAA	TGAGGGGGAGAATCACAAAA	60.4	269	INTERGENIC	--	--	--

**INDEL marker IDs**	**Chromosomes/unanchored scaffolds**	**Physical positions (bp)**	**InDels (*Kabuli* reference genome-CDC Frontier/PII)**	**Forward primers (5'-3')**	**Reverse primers (5'-3')**	**Anneling temperature (0C)**	**Expected amplified product size (bp)**	**Structural annotation**		**Functional annotation**	
								**Sequence components of *kabuli* genome**	***Kabuli* gene accession IDs**	**NCBI-KOG**	**TFs**
Ca4_P2_40649020	Ca4	40649020	ATTTTTT/ATTTTTTTT	TGTGATTATCCGGTACCACAAC	TGTATTCAGGACCTCGCAGA	59.4	207	INTERGENIC	--	--	--
Ca4_P2_40700663	Ca4	40700663	TAA/TAAA	TGGAGCACTGCCATTGTAT	TGAAACAAAACGTTACATCAAGAGA	60.1	665	INTERGENIC	--	--	--
Ca4_P2_40700886	Ca4	40700886	AGAC/A	TCTTGATGTAACGTTTTGTTTCAGA	TGAATATTTGAGTTACGAATTTGATGA	60.1	478	INTERGENIC	--	--	--
Ca4_P2_40702737	Ca4	40702737	ACTCT/ACT	CCCCCATCATAAAATTGTGC	AAGATTAGTGGGCGACCCTT	60.0	392	INTERGENIC	--	--	--
Ca4_P2_40703571	Ca4	40703571	CA/CAAA	TGGTTGCTGAGTTTCTTCCC	GCAGTGGAGATCCAAGGGTA	60.1	437	INTERGENIC	--	--	--
Ca4_P2_40704105	Ca4	40704105	ATTTTTT/ATTTTT	GGATCTCCACTGCCAGGTTA	TTTCGTTTCCCATTCTACGC	60.1	648	INTERGENIC	--	--	--
Ca4_P2_40704362	Ca4	40704362	TA/T	GCGTAGAATGGGAAACGAAA	GAATGCAGTTAGCACGAGCA	60.2	230	INTERGENIC	--	--	--
Ca4_P2_40719941	Ca4	40719941	CTTTTTTTTTTT/CTTTTTTTTTT	TCCTCCCTTTTGTTTTGTGC	TCGTCATCACCCACACTCAT	60.0	410	INTRON	Ca_23911	BK	FAR1
Ca4_P2_40720882	Ca4	40720882	CTTTTTT/CTTTTTTT	TCATCAATGGGAACCTCACA	TGATGACTGTCAAAGAGCCG	60.0	143	DRR	Ca_23910	S	C2H2
Ca4_P2_40721323	Ca4	40721323	AT/ATT	CGGCTCTTTGACAGTCATCA	AAATGTCAACCCACTGCACA	60.0	617	DRR	Ca_23911	BK	FAR1
Ca4_P2_40726046	Ca4	40726046	GAAA/GAAAA	CAACTAATGACCGTGCCCTT	TGTACTGGGGATTGTTGCCT	60.4	454	INTRON	Ca_23910	S	C2H2
Ca4_P2_40727861	Ca4	40727861	CAAAAAAAAA/CAAAAAAAA	AGCATACATTGGCACCACAA	TTCTTTCTTGCCTTCTCCGA	60.1	375	INTERGENIC	--	--	--

INDEL marker IDs	Chromosomes/unanchored scaffolds	Physical positions (bp)	InDels (*Kabuli* reference genome-CDC Frontier/PII)	Forward primers (5'-3')	Reverse primers (5'-3')	Anneling temperature (0C)	Expected amplified product size (bp)	Structural annotation		Functional annotation	
								Sequence components of *kabuli* genome	*Kabuli* gene accession IDs	NCBI-KOG	TFs
Ca4_P2_40728044	Ca4	40728044	TATTTGTAATT/TATTTGTAATTTGTAATT	AGCATACATTGGCACCACAA	CATGCATTTCATGGTAACCG	59.8	502	INTERGENIC	--	--	--
Ca4_P2_40729597	Ca4	40729597	T/TG	TTGTGAAATTGAAAAGGTTCCA	CAACCATGTGTGAGGTCCAT	59.2	817	INTERGENIC	--	--	--
Ca4_P2_40730301	Ca4	40730301	GTTTTTT/GTTTTTTT	GGTGTTTGCGGGTTGATATT	CGTCTTCCACCTTATCCATGA	59.9	561	INTERGENIC	--	--	--
Ca4_P2_40735188	Ca4	40735188	CTTTTTAAAT/CTTTTTAAATCTTTTTTAAAT	TGCACATCTTTCTGGTGCTC	GCTTCCAATTCGATCTTCCA	60.2	802	DRR	Ca_23909	--	--
Ca4_P2_40738290	Ca4	40738290	TAA/TTTTTAATATAAACAA	TTTACCCGAGCCAATTTCAG	GGGTTTGAGAGGTGAAGTGTG	59.6	854	DRR	Ca_23908	R	NAC
Ca4_P2_40743684	Ca4	40743684	A/AC	GAATTTTGTGTGTTGCGGTG	GCGGTGTTTTTCAACACATTT	59.9	527	INTERGENIC	--	--	--
Ca4_P2_40743968	Ca4	40743968	A/AAAC	TGTTGAAAAACACCGCAAGA	GCAATGAAGTGTGTGCCAAT	59.6	575	INTERGENIC	--	--	--
Ca4_P2_40745794	Ca4	40745794	TGATAGATAGATAGATAGAT/TGATAGATAGAT	TGCTGGTGATATTGCTGCTC	CCCAACAACACAAATGCAAG	60.0	215	INTRON	Ca_23907	R	--
Ca4_P2_40753160	Ca4	40753160	TGG/TG	TAGTGAGGTGGGAAGGGATG	CCGACGGAGATGATGAATTT	59.9	804	INTERGENIC	--	--	--
Ca4_P2_40753856	Ca4	40753856	GTATATATATATATA/GTATATATA	TTACGATATTGCCGCAACTG	GCCCTTTCTTTCAGGCTTCT	60.0	444	INTRON	Ca_23905	J	--
Ca4_P2_40761334	Ca4	40761334	A/ATATAT	GAACTTTGTTATTGCATTCGTTTT	TGGATACACCGTCCAAAGTG	59.4	714	INTERGENIC	--	--	--
Ca4_P2_40765533	Ca4	40765533	TAAA/TAA	AATGAAAATGAAGGCGGTGA	CAAATGTCGTTGTCGCATCT	59.7	241	DRR	Ca_23904	Q	--

INDEL marker IDs	Chromosomes/unanchored scaffolds	Physical positions (bp)	InDels (*Kabuli* reference genome-CDC Frontier/PII)	Forward primers (5'-3')	Reverse primers (5'-3')	Anneling temperature (0C)	Expected amplified product size (bp)	Structural annotation		Functional annotation	
								Sequence components of *kabuli* genome	*Kabuli* gene accession IDs	NCBI-KOG	TFs
Ca4_P2_40765588	Ca4	40765588	T/TCA	AATGAAAATGAAGGCGGTGA	CAAATGTCGTTGTCGCATCT	59.7	241	DRR	Ca_23904	Q	--
Ca4_P2_40765765	Ca4	40765765	CGG/CGGG	AGATGCGACAACGACATTTG	TATGACGTTTGGGGTGGATT	60.1	480	DRR	Ca_23904	Q	--
Ca4_P2_40767434	Ca4	40767434	GTTTT/GTTTTT	CCAAGAAGTCCACGTTTGGT	AGGTACGTCCAGGCATCAAG	60.1	644	INTRON	Ca_23904	Q	--
Ca4_P2_40767767	Ca4	40767767	ACC/AC	CAAATTCCACTGTTTTGCGTT	TTCCAGCATGATGTGTTTTCA	60.1	227	INTRON	Ca_23904	Q	--
Ca4_P2_40767801	Ca4	40767801	ACATCAT/ACATCATCAT	TGGCATCAAAATTCTCTGTCC	AATCACCTCATTCGCAAAAA	58.2	334	INTRON	Ca_23904	Q	--
Ca4_P2_40769439	Ca4	40769439	CAAAAAA/CAAAAAAA	TGCTACGCATGCTTGAGAAC	GCCTTGTTGGGTTCATTTTG	60.3	385	INTRON	Ca_23904	Q	--
Ca4_P2_40773283	Ca4	40773283	ATTTTTTTT/ATTTTTTTTT	GTCTCTCCCCCTATCTGCCT	CAAAATATCGAGGGAGCGAA	60.2	467	INTERGENIC	--	--	--
Ca4_P2_40773929	Ca4	40773929	TTAT/TTATAT	GTGTGCACCATAGGGAAGGT	ATCCTTGCACATCCCAAAAA	60.3	405	INTERGENIC	--	--	--
Ca4_P2_40776431	Ca4	40776431	CTTTTTTTT/CTTTTTTT	AATGAAGTGTCTTAGGCGGG	GTTTGTTTCGTCCCACCAAT	59.7	579	INTERGENIC	--	--	--
Ca4_P2_40777612	Ca4	40777612	ATTTTTTTT/ATTTTTTTTT	TAAGGTCCAAAATCCAGGCA	GCTTAAGCTTTCCATTCAAAAGA	59.1	408	INTERGENIC	--	--	--
Ca4_P2_40777790	Ca4	40777790	AAAGCTTAAGCTTAAGCT/AAAGCTTAAGCT	TAAGGTCCAAAATCCAGGCA	CATAAAATGTTGCATGCGGT	59.4	791	INTERGENIC	--	--	--
Ca4_P2_40778747	Ca4	40778747	T/TA	GGGAGTTTGGAGAAGAAGGG	AATAAAGCAACAGCCCCAAT	58.6	776	INTERGENIC	--	--	--

INDEL marker IDs	Chromosomes/unanchored scaffolds	Physical positions (bp)	InDels (*Kabuli* reference genome-CDC Frontier/PII)	Forward primers (5'-3')	Reverse primers (5'-3')	Anneling temperature (0C)	Expected amplified product size (bp)	Structural annotation		Functional annotation	
								Sequence components of *kabuli* genome	*Kabuli* gene accession IDs	NCBI-KOG	TFs
Ca4_P2_4077884	Ca4	4077884	TTA/T	ATTAAAGTGGGGGCCAAATC	CCCCTTCCACAAAAAGTGAA	59.9	908	INTERGENIC	--	--	--
Ca4_P2_40779454	Ca4	40779454	A/AG	TTAATTGGGGCTGTTGCTTT	TTTGGTCCAATGCTGACAGA	60.2	585	INTERGENIC	--	--	--
Ca4_P2_4078034	Ca4	4078034	CA/CAGTA	AGCACGTGTGGAAGACATTG	CCCCTTCCACAAAAAGTGAA	59.9	654	INTERGENIC	--	--	--
Ca4_P2_40784080	Ca4	40784080	CAAAAAAAAAAAA/CAAAAAAAAAAA	AAGCACTTTTCAATTCACCCA	AATGATGCCTCCATGCTTTC	60.0	199	INTERGENIC	--	--	--
Ca4_P2_40788340	Ca4	40788340	ATTTTTTTTT/ATTTTTTTT	GCATCGGAGCATGAAATGTA	TTCGCACACGATGAAGACTAA	59.5	167	INTERGENIC	--	--	--
Ca4_P2_40790018	Ca4	40790018	A/AT	GCCCATTTTGTTCTTTGTTGA	TCATTGTCCAGTGAAGGCAA	60.2	667	INTERGENIC	--	--	--
Ca4_P2_40790090	Ca4	40790090	GAAAAAAAAAA/GAAAAAAAA	GCCCATTTTGTTCTTTGTTGA	TCATTGTCCAGTGAAGGCAA	60.2	667	INTERGENIC	--	--	--
Ca4_P2_40792698	Ca4	40792698	TAAAAA/TAAAAAA	TCAAATGCCAAAATCCATCA	GTCAAGCAAAATCCCACGAT	59.9	532	INTRON	Ca_23903	--	--
Ca4_P2_40797298	Ca4	40797298	GA/G	TTCGTTCAGTCACTCCATTTTG	TAAGGGAGGATGATTGGCAG	60.0	619	INTERGENIC	--	--	--
Ca4_P2_40797521	Ca4	40797521	ATT/AT	CAAGGTCCCCAAACATCACT	ATGGACCATCGATTCTGGAG	59.9	610	INTERGENIC	--	--	--
Ca4_P2_40799151	Ca4	40799151	TTAGCATA/TTA	CCAGCAATCTAATGAACGCA	ATGTCACCAATGGGGTTTGT	60.0	323	DRR	Ca_23902	R	--
Ca4_P2_40799742	Ca4	40799742	TCCCCC/TCCCCCC	ACAAACCCCATTGGTGACAT	TGGCTTGTAAGGCTGTGTTG	59.9	740	DRR	Ca_23902	R	--

INDEL marker IDs	Chromosomes/unanchored scaffolds	Physical positions (bp)	InDels (*Kabuli* reference genome-CDC Frontier/PII)	Forward primers (5'-3')	Reverse primers (5'-3')	Anneling temperature (0C)	Expected amplified product size (bp)	Structural annotation		Functional annotation	
								Sequence components of *kabuli* genome	*Kabuli* gene accession IDs	NCBI-KOG	TFs
Ca4_P2_40801294	Ca4	40801294	AAAA/AAAATACCAGTAAA	AGCTTGCCTCTGAGCTCTTG	TTTATGGCATGGACCAAAT	60.0	736	INTRON	Ca_23902	R	--
Ca4_P2_40802030	Ca4	40802030	A/AC	CATGAAGCCAAGAAGCAACA	TTGACACCTGTCACACATGC	59.1	518	INTRON	Ca_23902	R	--
Ca4_P2_40802414	Ca4	40802414	AATAT/AATATAT	TGACAGGTGTCAAGTTGTTGG	CGCAGTGGACTTCAAAATGA	59.8	748	INTRON	Ca_23902	R	--
Ca4_P2_40804983	Ca4	40804983	C/CAT	CCAAATCAACACCAATTCCC	ACTTGAAGCCGAAGGGTTTT	60.1	524	INTRON	Ca_23902	R	--
Ca4_P2_40805123	Ca4	40805123	AT/A	CCAAATCAACACCAATTCCC	ACTTGAAGCCGAAGGGTTTT	60.1	524	INTRON	Ca_23902	R	--
Ca4_P2_40808504	Ca4	40808504	TAAAA/TA	CGGGAAGGATCCTGACACTA	GGGCAATGGTTCTTTCTCTG	59.7	257	INTRON	Ca_23902	R	--
Ca4_P2_40809750	Ca4	40809750	A/AC	TGCAAGAACAAATCCTTCCC	GCAGCTTTTGGCAGTTCTCT	59.8	278	INTRON	Ca_23902	R	--
Ca4_P2_40810384	Ca4	40810384	G/GAAAAAA	AAAAACGGCAGTAAAAGCCA	GGGACGAGGTACATGCAGAT	60.0	298	INTRON	Ca_23902	R	--
Ca4_P2_40818336	Ca4	40818336	AA/AACA	GTTTGCGGTTATTGCTGGTT	GTTACTTGTGGGGCTGGAAA	60.0	637	INTRON	Ca_23900	R	--
Ca4_P2_4082565	Ca4	4082565	ATTT/ATT	TTGTTTCTCCCCCAGTTTTG	TTTCGTCCCGAACTTTCATC	60.1	352	INTERGENIC	--	--	--
Ca4_P2_4082985	Ca4	4082985	TTTTTT/TTTTTTTAATAAACTTTTT	TGAAAGTTCGGGACGAAATC	CAGAAAAGTGAAAAGCTGAGAAAA	59.3	519	INTERGENIC	--	--	--
Ca4_P2_40836419	Ca4	40836419	TAAAAAAAA/TAAAAAAA	GTGTCGGTCACGACATTCAG	AGATTGAAAAGTTGCACCCG	60.1	714	INTERGENIC	--	--	--

INDEL marker IDs	Chromosomes/unanchored scaffolds	Physical positions (bp)	InDels (*Kabuli* reference genome-CDC Frontier/PII)	Forward primers (5'-3')	Reverse primers (5'-3')	Anneling temperature (0C)	Expected amplified product size (bp)	Structural annotation		Functional annotation	
								Sequence components of *kabuli* genome	*Kabuli* gene accession IDs	NCBI-KOG	TFs
Ca4_P2_40837444	Ca4	40837444	TAAAAAAAAA/TAAAAAAAA	GGAAGCAGATGGAAAAGGAA	TCAACTTTGAGGACCCAACA	59.1	494	INTERGENIC	--	--	--
Ca4_P2_40842793	Ca4	40842793	TC/T	CTGGATTGAGTTGAGGCCAT	TGGACCAAACCAAGCACATA	60.0	568	INTERGENIC	--	--	--
Ca4_P2_40844159	Ca4	40844159	TAAAAAA/TAAAAAAA	AACATTAATGCAACACGCCA	GACTTCCGTCCCTTACAGAGAA	59.8	199	INTERGENIC	--	--	--
Ca4_P2_40845661	Ca4	40845661	GTATATAT/GTAT	TGTGCCTCCTTTAATTTGCC	GAAGGTTTGCTAAGCCTCCA	59.5	542	INTERGENIC	--	--	--
Ca4_P2_40850995	Ca4	40850995	GAAA/GAA	CAATCACCATTCAGCATTCG	AAGGGCATGTTCAAGGACAC	60.0	451	INTRON	Ca_23897	--	--
Ca4_P2_4086337	Ca4	4086337	TAAAAAAAAA/TAAAAAAAAAAAA	GGGGCGTGTCAAAGTTAAAA	ACCATCCGTATAAAGCACGC	60.0	453	INTERGENIC	--	--	--
Ca4_P2_40880718	Ca4	40880718	ATTTTTTTT/ATTTTTTTTT	CTTTGGGAACCAAGCACAAT	TTTCACCAAATGTGGGTCTTT	59.3	851	INTERGENIC	--	--	--
Ca4_P2_40881754	Ca4	40881754	CTTTTTTTTT/CTTTTTTTTTTTTT	ACAAATCTGAGCCATGGGAG	GGTTGATAAATGAGCAAGCCA	60.1	125	INTRON	Ca_11021	--	NAC
Ca4_P2_40887046	Ca4	40887046	GTTTTTTTT/GTTTTTTT	TGAATCCAATCCATCCAACA	AAAGAGCTAGCAATGGCGAC	59.6	426	INTERGENIC	--	--	--
Ca4_P2_40887107	Ca4	40887107	GAAAAAAAAA/GAAAAAAAA	TGAATCCAATCCATCCAACA	ACTATCGACCAACTGCCCAC	60.0	705	INTERGENIC	--	--	--
Ca4_P2_40891579	Ca4	40891579	AATATATA/AATA	GCACAAAAGCAGTGTAGCCA	CCCGAACCTCTCCACAATAA	59.9	869	INTRON	Ca_11020	R	G2-like
Ca4_P2_40891712	Ca4	40891712	TCC/TC	ATCGAATCCTCGTTGGAATG	CCCGAACCTCTCCACAATAA	59.9	725	INTRON	Ca_11020	R	G2-like

INDEL marker IDs	Chromosomes/unanchored scaffolds	Physical positions (bp)	InDels (*Kabuli* reference genome-CDC Frontier/PII)	Forward primers (5'-3')	Reverse primers (5'-3')	Anneling temperature (0C)	Expected amplified product size (bp)	Structural annotation		Functional annotation	
								Sequence components of *kabuli* genome	*Kabuli* gene accession IDs	NCBI-KOG	TFs
Ca4_P2_40893398	Ca4	40893398	TA/TAA	TGAACTCGGCTAGGAGGAAA	CGCAAGTGTTGGACAATGAT	59.6	703	DRR	Ca_11020	R	G2-like
Ca4_P2_40901024	Ca4	40901024	GAAAAAAA/GAAAAAAAA	GCGTCATACGAAACTTGTTAGTG	TTAGGAGGTCGCATCTTCGT	59.8	671	INTERGENIC	--	--	--
Ca4_P2_40901246	Ca4	40901246	AAA/AAATAA	GGGTATATTAATTTAACAACTCCCTCC	TTAGGAGGTCGCATCTTCGT	59.8	462	INTERGENIC	--	--	--
Ca4_P2_40901320	Ca4	40901320	GAA/G	GGTCCGAGATACAACCTGCT	TCAATGAAAAACATGGTGCC	59.4	480	INTERGENIC	--	--	--
Ca4_P2_40901411	Ca4	40901411	TTAT/TT	ACGAAGATGCGACCTCCTAA	AACACAAAGGAAAGCGGAAA	59.7	561	INTERGENIC	--	--	--
Ca4_P2_40901662	Ca4	40901662	TAAA/TAA	ACGAAGATGCGACCTCCTAA	AACACAAAGGAAAGCGGAAA	59.7	561	INTERGENIC	--	--	--
Ca4_P2_40904722	Ca4	40904722	ATTAAT/ATTAATCAATTTAAT	AAAGGCAACCGACTAGGGTT	GTACGCGAAGTAGGAAACGC	59.9	832	INTERGENIC	--	--	--
Ca4_P2_40904946	Ca4	40904946	CTTTTT/CTTTTTT	TCCATGCAGTTTGTCGTTGT	GTACGCGAAGTAGGAAACGC	59.9	178	INTERGENIC	--	--	--
Ca4_P2_40905620	Ca4	40905620	TAAAAAAA/TAAAAAAAA	TTTCCTACCTGTCGTTTGCC	TCCTCTTATCTTTGCCACCA	58.3	883	INTERGENIC	--	--	--
Ca4_P2_40907158	Ca4	40907158	TATACAT/TATACATAATACAT	TGTCGTTGAAATCAAAGCAAT	ATCGTTTGGGTGAAGAATCG	59.9	334	INTERGENIC	--	--	--
Ca4_P2_40909839	Ca4	40909839	GAAAAAAAAAA/GAAAAAAAAA	TGCTGATGTTCTTCCTGCAC	TGTCCATTGAGCAGAGTTGC	60.0	665	INTRON	Ca_11019	R	--
Ca4_P2_40929080	Ca4	40929080	T/TC	ATGGTGGGGGTAAGCAATCT	AAGAAGCGCGTATTGGTGTT	59.8	813	INTRON	Ca_11015	--	bZIP

INDEL marker IDs	Chromosomes/unanchored scaffolds	Physical positions (bp)	InDels (*Kabuli* reference genome-CDC Frontier/PII)	Forward primers (5'-3')	Reverse primers (5'-3')	Anneling temperature (0C)	Expected amplified product size (bp)	Structural annotation		Functional annotation	
								Sequence components of *kabuli* genome	*Kabuli* gene accession IDs	NCBI-KOG	TFs
Ca4_P2_4092954	Ca4	4092954	TTTTCTTATTAATTT/TTTT	CATTTTGAAAAAGGTTGCGG	TGCCTTAAAAGTGGATGAACG	60.1	672	INTERGENIC	--	--	--
Ca4_P2_40929606	Ca4	40929606	GAA/GA	TTTCTAACACCAATACGCGCT	TCCTCTAAATTTTTACCAACGATGA	60.2	492	INTRON	Ca_11015	--	bZIP
Ca4_P2_40931865	Ca4	40931865	TCCCC/TCCC	TGCTTGGAAAACAAGCAATTT	AACTCGTGGGAACGCACTAC	60.2	779	INTRON	Ca_11015	--	bZIP
Ca4_P2_40936303	Ca4	40936303	CATA/CA	ACGAGGTTGGATTGAACGAC	CTTTGATGGCCAAATGTGTG	60.0	689	INTERGENIC	--	--	--
Ca4_P2_40936321	Ca4	40936321	GAA/GAAA	ACGAGGTTGGATTGAACGAC	CTTTGATGGCCAAATGTGTG	60.0	689	INTERGENIC	--	--	--
Ca4_P2_40937505	Ca4	40937505	GTTTTTTTTT/GTTTTTTTTTT	TAATCCGATGGCAACATGAA	CGGTAGGAAATGATGAAACCA	59.8	615	INTERGENIC	--	--	--
Ca4_P2_40937848	Ca4	40937848	CAAA/CAA	AGTAGTTTTGGGGCATCCCT	TTTGAAAGGGTGTCGTCGTT	60.5	762	INTERGENIC	--	--	--
Ca4_P2_40937873	Ca4	40937873	TAAA/TAAAA	GACCGAAGAAACATCATGGC	TTTGAAAGGGTGTCGTCGTT	60.5	668	INTERGENIC	--	--	--
Ca4_P2_40949429	Ca4	40949429	TT/TTAT	CCATGTGCACCATAAATTGC	CAGAATGTTCTTTCGGAATGG	59.6	612	INTRON	Ca_11013	--	--
Ca4_P2_40954099	Ca4	40954099	CAAAA/CAAA	CGTCAACTGACATATGCCAACA	TTTGACGGAAACATGGTGAA	59.9	205	INTERGENIC	--	--	--
Ca4_P2_40954191	Ca4	40954191	CAAA/CAA	TTCACCATGTTTCCGTCAAA	TGTTTCTCGAGTACGAAAGAATG	58.6	368	INTERGENIC	--	--	--
Ca4_P2_4095510	Ca4	4095510	CAT/CATAT	TCGCAATCTATGTAGCACCG	AGTTGGCTCAGTAGGCTCCA	60.0	247	INTRON	Ca_03846	T	--

INDEL marker IDs	Chromosomes/unanchored scaffolds	Physical positions (bp)	InDels (*Kabuli* reference genome-CDC Frontier/PII)	Forward primers (5'-3')	Reverse primers (5'-3')	Anneling temperature (0C)	Expected amplified product size (bp)	Structural annotation		Functional annotation	
								Sequence components of *kabuli* genome	*Kabuli* gene accession IDs	NCBI-KOG	TFs
Ca4_P2_4095551	Ca4	4095551	AGTGTCTACGTGTCT/AGTGTCT	TCGCAATCTATGTAGCACCG	AGTTGGCTCAGTAGGCTCCA	60.0	247	INTRON	Ca_03846	T	--
Ca4_P2_40955675	Ca4	40955675	GAAAAAA/GAAAAA	AAACGCATTGTTTTTGTTTGA	GGAAACTTCCAAGAGGAGGG	60.0	843	INTERGENIC	--	--	--
Ca4_P2_40957631	Ca4	40957631	CTAAAAATAAAAATAAAAATAAAAATAAAAA/CTAAAAATAAAAATAAAAATAAAAA	CCGTGTTGGTTGTGTTGTTC	GTCCATTTCAATGGGTGGTC	60.0	322	INTERGENIC	--	--	--
Ca4_P2_40964102	Ca4	40964102	TT/TTCT	ATTTGGATAGGCCTGGCTTT	GCACTTTTTGATACGGGAGC	59.7	622	INTERGENIC	--	--	--
Ca4_P2_40967580	Ca4	40967580	TAAAAAAAAAA/TAAAAAAAAAAA	AATGAAAAACTTGTTGCGGC	GTTTGGAATCGGGTAACGAA	59.8	748	INTERGENIC	--	--	--
Ca4_P2_40968955	Ca4	40968955	TC/TCC	CACTGGAATGATTGCCAAAA	TTCTACCCAAGATCGGATGG	59.9	398	INTERGENIC	--	--	--
Ca4_P2_40978550	Ca4	40978550	TC/TCATGTAGGGC	TTGGGTTTCCTATTTCGTCG	GCCAATCGATTACCCTCAAA	59.9	963	INTERGENIC	--	--	--
Ca4_P2_4099530	Ca4	4099530	AT/ATT	CGTGTAAGAACACCGCAAGA	TCTGGCCACTAGCACTGATG	60.0	427	DRR	Ca_03845	R	--
Ca4_P2_40996595	Ca4	40996595	GAAAAA/GAAAA	CGGTGTTAAAAATTGGGGTG	AAACGTGGAGGTGATTTTGC	60.0	445	DRR	Ca_11010	--	--
Ca4_P2_40997097	Ca4	40997097	C/CT	GCAAAATCACCTCCACGTTT	AACGGTCGTCTCCATTCATC	59.9	762	DRR	Ca_11010	--	--
Ca4_P2_41000217	Ca4	41000217	CAA/CAATTAA	TGGTTGTGGTTTTCTCTCCA	GAACATGCAACGAGACGAGA	60.0	206	INTERGENIC	--	--	--
Ca4_P2_41006722	Ca4	41006722	CTTA/CTTAATTA	GGGATCTTGAGGCATGATGT	TTCACAATTTTGGTCCCCAT	60.0	661	INTERGENIC	--	--	--

**INDEL marker IDs**	**Chromosomes/unanchored scaffolds**	**Physical positions (bp)**	**InDels (*Kabuli* reference genome-CDC Frontier/PII)**	**Forward primers (5'-3')**	**Reverse primers (5'-3')**	**Anneling temperature (0C)**	**Expected amplified product size (bp)**	**Structural annotation**		**Functional annotation**	
								**Sequence components of *kabuli* genome**	***Kabuli* gene accession IDs**	**NCBI-KOG**	**TFs**
Ca4_P2_41009997	Ca4	41009997	CT/CTT	ACGACTTGGAGACTGTTGGG	TGATCCGCATTGACCAACTA	60.1	633	INTERGENIC	--	--	--
Ca4_P2_41012540	Ca4	41012540	TCA/T	TGCCAAAATTCTTCAATTCAAAC	CCATTTGGGAATTATGAAGCA	59.8	587	DRR	Ca_11009	--	bHLH
Ca4_P2_41012598	Ca4	41012598	T/TA	CCCAATCATGCCTTATATGC	ATTTGGGTTTCAACAAACGG	59.7	539	DRR	Ca_11009	--	bHLH
Ca4_P2_41016384	Ca4	41016384	ATTTTTTT/ATTTTTTTT	AAACACGGGGCAACATAAAG	TGTTGGTGGTGAACGAGGTA	60.0	631	INTERGENIC	--	--	--
Ca4_P2_4101732	Ca4	4101732	CTTT/CT	TCTATTGATCGACACAACTACTTTTT	TGATTTCAAAAGCCTTTCAGG	59.3	746	DRR	Ca_03844	--	--
Ca4_P2_41027713	Ca4	41027713	AATATATATATATATATATATATATATAT/AATATATATATATATATATATATATATATATATAT	TGAATCCCAAAATTTGAACCA	TTTGATCGCAAGGGTTTAGG	60.1	637	INTERGENIC	--	--	--
Ca4_P2_41029836	Ca4	41029836	AATATATATATATATATA/AATATATATATATATA	TTTCTCTCCATCGACCCATC	GAACCTTTGGGAAATCAGCA	60.1	760	INTERGENIC	--	--	--
Ca4_P2_4103051	Ca4	4103051	CA/CAAA	AACATGGCAATTGATTGAAGG	AGTCCAACTTCATTGCCTGC	60.3	496	DRR	Ca_03844	--	--
Ca4_P2_4104006	Ca4	4104006	ACCC/ACCCC	TGTGAATTTGTTGAGGTGGG	AATGTTCCAATTCGCCAGAT	59.4	223	DRR	Ca_03843	T	BBR-BPC
Ca4_P2_4104966	Ca4	4104966	AAAATAAA/AAAA	GAAGTGGGAATGGGCTGTTA	AAGTTAGGGGGACACAACCC	60.1	677	DRR	Ca_03843	T	BBR-BPC
Ca4_P2_4104993	Ca4	4104993	TTATAT/TTAT	GAAGTGGGAATGGGCTGTTA	AAGTTAGGGGGACACAACCC	60.1	677	DRR	Ca_03843	T	BBR-BPC
Ca4_P2_4107649	Ca4	4107649	CAAAAAAAAA/CAAAAAAAA	CGAGGTAATGGTGGCAACTT	TCTCACGTCGATCTCCACAG	60.0	720	INTRON	Ca_03843	T	BBR-BPC

INDEL marker IDs	Chromosomes/unanchored scaffolds	Physical positions (bp)	InDels (*Kabuli* reference genome-CDC Frontier/PII)	Forward primers (5'-3')	Reverse primers (5'-3')	Anneling temperature (0C)	Expected amplified product size (bp)	Structural annotation		Functional annotation	
								Sequence components of *kabuli* genome	*Kabuli* gene accession IDs	NCBI-KOG	TFs
Ca4_P2_4109745	Ca4	4109745	GAGT/G	CAGGCTTTCGAAAAGGACAG	TCTCTTCCCCAAGCTTCTCA	60.1	895	INTRON	Ca_03843	T	BBR-BPC
Ca4_P2_4111062	Ca4	4111062	GATAAATAAATAAATAAAT/GATAAATAAATAAAT	TTCCACATGGTCTCACCAAA	TTCCCCATGTTTGAATGGTT	60.0	608	INTRON	Ca_03843	T	BBR-BPC
Ca4_P2_4115857	Ca4	4115857	TAAAAAAA/TAAAAAA	GGTGTTGTTGGGCTTTGTCT	CCCCCTTTTTGGTCGTATCT	60.2	821	INTERGENIC	--	--	--
Ca4_P2_41159362	Ca4	41159362	ATTTTTTTTTTT/ATTTTTTTTTTTT	ATGCTTGGACCACCATTCTC	CGTGTGAAACCTTTGCTGAA	59.9	891	INTERGENIC	--	--	--
Ca4_P2_4115988	Ca4	4115988	ATTTTTT/ATTTTT	TGCAATATGAAGGTCCACCC	CCCCCTTTTTGGTCGTATCT	60.2	665	INTERGENIC	--	--	--
Ca4_P2_4116283	Ca4	4116283	TATTAAG/TATTAAGGAGATTAAG	AGATACGACCAAAAAGGGGG	GCATGTTGAGATTATGGGGC	60.3	346	INTERGENIC	--	--	--
Ca4_P2_4116323	Ca4	4116323	AAGCTAGCT/AAGCTAGCTAGCT	AGATACGACCAAAAAGGGGG	GCATGTTGAGATTATGGGGC	60.3	346	INTERGENIC	--	--	--
Ca4_P2_4116752	Ca4	4116752	TA/TACA	ACATGTTGGAAATCCGGGTA	TCGGCTACTCAAACAAAGGTG	60.3	516	INTERGENIC	--	--	--
Ca4_P2_4116866	Ca4	4116866	CCTTT/C	ACATGTTGGAAATCCGGGTA	TCGGCTACTCAAACAAAGGTG	60.3	516	INTERGENIC	--	--	--
Ca4_P2_4117353	Ca4	4117353	TAAAAAAA/TAAAAAA	CACCTTTGTTTGAGTAGCCGA	GTGAACCTCCCACACGAGTT	60.0	520	INTERGENIC	--	--	--
Ca4_P2_4118065	Ca4	4118065	CTTTTTTTTTTTTT/CTTTTTTTTTT	AAAAGGAATTTGTGGCAACG	TTTTGCAATCTATCGAGGGG	60.0	580	INTERGENIC	--	--	--
Ca4_P2_4121415	Ca4	4121415	ATT/AT	CTCATATGAACGGCCCATCT	TGTTTGTACGGACTCGACCA	60.2	403	INTERGENIC	--	--	--

INDEL marker IDs	Chromosomes/unanchored scaffolds	Physical positions (bp)	InDels (*Kabuli* reference genome-CDC Frontier/PII)	Forward primers (5'-3')	Reverse primers (5'-3')	Anneling temperature (0C)	Expected amplified product size (bp)	Structural annotation		Functional annotation	
								Sequence components of *kabuli* genome	*Kabuli* gene accession IDs	NCBI-KOG	TFs
Ca4_P2_4121591	Ca4	4121591	TA/T	CTCATATGAACGGCCCATCT	CCTGTCACTCCTTTGCGATT	60.3	733	INTERGENIC	--	--	--
Ca4_P2_4122100	Ca4	4122100	TAAAAAAAA/TAAAAAAA	AATCGCAAAGGAGTGACAGG	CACAACGACCGCATGATTAC	60.0	255	INTERGENIC	--	--	--
Ca4_P2_4126315	Ca4	4126315	TAAG/T	GCTTTGATCCATTTGCTCGT	TCAAGAAAAATATTGAGTGTCACG	58.4	883	INTERGENIC	--	--	--
Ca4_P2_41272417	Ca4	41272417	GATTATTATTATTATTATTATTATTATTATT/GATTATTATTATTATTATTATTATTATT	GGTTCACATGGAACTCCAACA	AGCCTTTTGCTTCCTTCCTC	60.0	737	INTERGENIC	--	--	--
Ca4_P2_41324242	Ca4	41324242	ATATTATTATTATTATTATTA/ATATTATTATTATTATTA	TCCAATAAAAAGTCGTCGGC	TGCGGAGAGTCAAGAAAGGT	60.0	565	INTERGENIC	--	--	--
Ca4_P2_41368775	Ca4	41368775	AT/A	TGCTTGCATGTCTTGAATTTG	CCCAATTCGCCTCATAACAT	59.8	434	DRR	Ca_10971	P	GRAS
Ca4_P2_41434988	Ca4	41434988	ATTTTTTTTTT/ATTTTTTTTT	CGGGTTTGGAGGACTTATGA	AAGCAAACATCGTTCCCCTA	59.6	586	INTERGENIC	--	--	--
Ca4_P2_41450081	Ca4	41450081	TTTCTTCTTCTTCTTCT/TTTCTTCTTCTTCT	CAACTCTTCCTGCGTCATCA	CCTGGCTTCTATGGGAAACA	60.1	538	INTRON	Ca_10962	--	--
Ca4_P2_4158092	Ca4	4158092	ATTTTTTTTTT/ATTTTTTTTT	CCTACATGTTGGAGGGGCTA	CCATTATTTCACGGGGCTTA	59.8	353	INTERGENIC	--	--	--
Ca4_P2_41581170	Ca4	41581170	GA/GATCA	ACAAAAACTCCCAATGGTCG	TAGCCAACCACACATGGAAA	60.0	737	INTERGENIC	--	--	--
Ca4_P2_4161162	Ca4	4161162	CATATATATATATATATATATATATATATATATATA/CATATATATATATATATATATATATATATATATATATATATATATA	GGGCTCGAATAAAGTCATGG	CCACCCCCACATCTCTCTAC	59.4	525	INTERGENIC	--	--	--
Ca4_P2_41623470	Ca4	41623470	TT/TTATTATTTTTAT	TTTTCTCATTGCATTTGAGGG	AATTCGCATCAGAGATTGCC	60.2	437	INTERGENIC	--	--	--

INDEL marker IDs	Chromosomes/unanchored scaffolds	Physical positions (bp)	InDels (*Kabuli* reference genome-CDC Frontier/PII)	Forward primers (5'-3')	Reverse primers (5'-3')	Anneling temperature (0C)	Expected amplified product size (bp)	Structural annotation		Functional annotation	
								Sequence components of *kabuli* genome	*Kabuli* gene accession IDs	NCBI-KOG	TFs
Ca4_P2_41678485	Ca4	41678485	TAAAAAAAAA/TAAAAAAAAAA	AGATTGAGAAAATTTTGCGTTT	TCACCAAAAAGTCACGATGC	59.7	688	DRR	Ca_10944	--	--
Ca4_P2_41713312	Ca4	41713312	TAAAAA/TAAAAAA	CCATCAAAACATGCATCCAC	CATGACAACACAACCAACCA	58.8	748	INTERGENIC	--	--	--
Ca4_P2_41775907	Ca4	41775907	GTTTTT/GTTTT	ACCTCCACAAGCCAATCAAC	TTGGAGTTCTTATGCCCCAG	60.1	352	INTRON	Ca_10937	D	M-type
Ca4_P2_41810390	Ca4	41810390	GGCA/GGCATGCA	GCCACAAGCTATGTTCACGA	GAGCTCGAAACAACAGCAAA	59.2	311	INTERGENIC	--	--	--
Ca4_P2_41815309	Ca4	41815309	GT/G	CCCCAACTTTGTCTTTTGGA	CGAAGAGGATTGCCTCAAAG	59.9	831	INTERGENIC	--	--	--
Ca4_P2_41818769	Ca4	41818769	ATTTTTTTTTTT/ATTTTTTTTTT	AGTGGAGGGTGAATCAATGG	AAGTGAAAATTCATCAGCACCA	59.6	539	INTERGENIC	--	--	--
Ca4_P2_41827147	Ca4	41827147	CTTGTTTGATTTGTTT/CTTGTTTGATTTGTTTGATTTGTTT	CGGAGAGATGCATGTTCTTTT	TCTATTGGACGAGCGTTTGA	59.4	633	INTERGENIC	--	--	--
Ca4_P2_41912274	Ca4	41912274	CATATAT/CATAT	CCATCTCATTCAAATGTTGCC	TGAGCTTTTAGGTGGCACAA	59.5	823	INTERGENIC	--	--	--
Ca4_P2_4196538	Ca4	4196538	ATTTT/ATTT	ATTGGCCGGAAATTGTACTG	TAACCCAAGGCCATATCCTG	59.8	841	DRR	Ca_03838	R	--
Ca4_P2_41995002	Ca4	41995002	ACCC/ACC	TTTCCAGGAATTGAACTCGG	CTGGAATAAATGCCCCAAGA	59.9	393	CDS (FRAME SHIFT)	Ca_10922	P	--
Ca4_P2_42078586	Ca4	42078586	AAGGCATGAAA/AGGCATGAAACACAGGCATGAAA	TCTTTTGAACACTCCCCCTG	CCAAAGGAAACCACGACAAT	59.8	810	INTERGENIC	--	--	--
Ca4_P2_42083362	Ca4	42083362	CA/CAA	TTTCAAAGGTTTGCATTGTGA	AAAATTAACCGTATGGCCGA	59.3	327	INTERGENIC	--	--	--

INDEL marker IDs	Chromosomes/unanchored scaffolds	Physical positions (bp)	InDels (*Kabuli* reference genome-CDC Frontier/PII)	Forward primers (5'-3')	Reverse primers (5'-3')	Anneling temperature (0C)	Expected amplified product size (bp)	Structural annotation		Functional annotation	
								Sequence components of *kabuli* genome	*Kabuli* gene accession IDs	NCBI-KOG	TFs
Ca4_P2_42120439	Ca4	42120439	TAAAAAAAAA/TAAAAAAAA	CACAAGCATTGGAGTCCCTT	AACTCGTGGGTTGGATTTTG	59.8	495	INTRON	Ca_10910	--	C2H2
Ca4_P2_42120489	Ca4	42120489	TAAAA/TAAA	CACAAGCATTGGAGTCCCTT	AACTCGTGGGTTGGATTTTG	59.8	495	INTRON	Ca_10910	--	C2H2
Ca4_P2_42120574	Ca4	42120574	C/CG	CACAAGCATTGGAGTCCCTT	AACTCGTGGGTTGGATTTTG	59.8	495	INTRON	Ca_10910	--	C2H2
Ca4_P2_42224113	Ca4	42224113	TAAAAAAAAA/TAAAAAAAA	CCAAATCAAACTGCATTGCT	TGTCATCCAAACCGAACAAA	59.9	679	INTERGENIC	--	--	--
Ca4_P2_42306240	Ca4	42306240	TC/T	ACGGGGTGTTACATTGTGGT	CATTCCTAGCGTTGGCTCTC	60.0	283	INTERGENIC	--	--	--
Ca4_P2_42325357	Ca4	42325357	AATTATTATTATTATTATTATTATTATTATTATTATTATTA/AATTATTATTATTATTATTATTATTATTATTATTATTATTATTATTA	AATGATCGTCTTCGGATTGG	CGTTGAATCTTGTGGGTTCA	59.5	478	INTERGENIC	--	--	--
Ca4_P2_42374619	Ca4	42374619	ATTTTTTTT/ATTTTTTT	TTTGATGTGTTCACGGCACT	CGGCATGCATGATTATTTTG	59.9	331	INTERGENIC	--	--	--
Ca4_P2_42407053	Ca4	42407053	GTTT/GTTTT	GCATAAGGATCGGGGAAACT	AATGATCGTCTTCGGATTGG	59.9	392	INTERGENIC	--	--	--
Ca4_P2_42504220	Ca4	42504220	TAAA/TAAACAAAA	TCGCTATCCCTTGGAATGAA	ACCATCATCATCCTGCACAA	59.9	401	INTRON	Ca_10898	--	WRKY
Ca4_P2_42520799	Ca4	42520799	CAAAAAAAAA/CAAAAAAAA	TATGCTCATACCGCCACAAA	CTTGGGATTTCAAGTTTTCCA	59.1	421	INTERGENIC	--	--	--
Ca4_P2_42534372	Ca4	42534372	GTTTTTTTT/GTTTTTTT	TGGGGCAGTGCAATGTATTA	CATCGGCAAGAGTTGGGTAT	60.0	574	INTERGENIC	--	--	--
Ca4_P2_42546801	Ca4	42546801	CAAAA/CAAAAA	GGTTGGTCAATATTAATTCCGC	CTAATATCATCGCCCCTTCG	59.5	686	INTERGENIC	--	--	--

INDEL marker IDs	Chromosomes/unanchored scaffolds	Physical positions (bp)	InDels (*Kabuli* reference genome-CDC Frontier/PII)	Forward primers (5'-3')	Reverse primers (5'-3')	Anneling temperature (0C)	Expected amplified product size (bp)	Structural annotation		Functional annotation	
								Sequence components of *kabuli* genome	*Kabuli* gene accession IDs	NCBI-KOG	TFs
Ca4_P2_42548370	Ca4	42548370	ATTTTTTTT/ATTTTTTTTT	CGTGCCTTTGTCGTCTCTTT	AGAGGCCCAATATCCTTGCT	60.1	528	INTERGENIC	--	--	--
Ca4_P2_42561342	Ca4	42561342	AATAGAGATAGAGATAGAGATAGAGA/AATAGAGATAGAGATAGAGA	CGGCAAAACAGAGACAATCA	TCTCACGGTTGTGAGGACTG	59.9	310	INTERGENIC	--	--	--
Ca4_P2_4263265	Ca4	4263265	CAAAAAAAAAA/CAAAAAAAAAAA	GGAGGTTGAGTCTAACGGCA	TTATGTGGGTACACAAGGTGAA	57.9	298	INTERGENIC	--	--	--
Ca4_P2_42633986	Ca4	42633986	TATAATAATAATAATAATAATAATAATAATAATA/TATAATAATAATAATAATAATAATAATAATAATAATAATA	TTGCTTTAGATGAATCAAATCCC	CGACAGGCTTAGGTTAGACCA	59.4	362	INTERGENIC	--	--	--
Ca4_P2_42693293	Ca4	42693293	TC/TCC	AAATGCTCGAACGAAAATGC	TCGAAGTTGGATGCTTTTGA	59.4	503	INTERGENIC	--	--	--
Ca4_P2_42708736	Ca4	42708736	ATTTTTTTTTTT/ATTTTTTTTTT	CATTTTCGCATCTACCTGCC	TCAGATGCAAATGGGATGAA	60.0	481	DRR	Ca_10890	R	--
Ca4_P2_42764871	Ca4	42764871	AC/ACC	ATTTCTCAAAAGCTTCGGCA	TCACAAGTGCCTCGTGTCTC	60.0	654	INTERGENIC	--	--	--
Ca4_P2_42822021	Ca4	42822021	T/TATGCAGGGAATCTGA	TTCTGCCGAGAGGAAGAGAG	GAGCACTCTTGTTGGCCTTC	60.0	707	INTERGENIC	--	--	--
Ca4_P2_42845286	Ca4	42845286	ATT/AT	TTCTAGCTGGCTTTTCTCGC	CGATTGAACCAAATTCCAGG	60.3	804	INTERGENIC	--	--	--
Ca4_P2_42945720	Ca4	42945720	CATATATATATAT/CATATATATATATAT	TGTCAATGTTACTTTATTTCAAAACTC	TTGGGTTATCTTCATTGCAAAA	59.5	739	INTERGENIC	--	--	--
Ca4_P2_43005724	Ca4	43005724	CTTTTTTTTTTT/CTTTTTTTTTT	TTGTAGTTGTATTCAATGACGAAGA	CAGAATTAATTTATTTGACAACTCCA	57.9	628	INTERGENIC	--	--	--
Ca4_P2_4302695	Ca4	4302695	ATATTATTATTATTATTATTATTATTATTATTATTATTATTATTATTAATATTATTATTATTATTA/ATATTATTATTATTATTATTATTATTATTATTATTATTATTATTATTATTAATATTATTATTATTATTA	GCATCTCCAAAATGCGATCT	TGAGTCCAAAACAAATGGTGTC	59.9	730	INTERGENIC	--	--	--

INDEL marker IDs	Chromosomes/unanchored scaffolds	Physical positions (bp)	InDels (*Kabuli* reference genome-CDC Frontier/PII)	Forward primers (5'-3')	Reverse primers (5'-3')	Anneling temperature (0C)	Expected amplified product size (bp)	Structural annotation		Functional annotation	
								Sequence components of *kabuli* genome	*Kabuli* gene accession IDs	NCBI-KOG	TFs
Ca4_P2_43222555	Ca4	43222555	TTATATATATATATATATATATATAT/TTATATATATATATATATAT	AAAGATTTAAAGGGCAAAAAGTG	CTGGCAGCTGGGAAAGATT	60.3	908	INTERGENIC	--	--	--
Ca4_P2_43278176	Ca4	43278176	GTTTTTTTT/GTTTTTTT	ACTGCGTCTCCATGGGTAAC	TTGGAATGGGCTTCTTTTTG	60.0	592	DRR	Ca_23681	--	--
Ca4_P2_43291624	Ca4	43291624	TAA/TAAAA	GAGCGATTACCGGTGTTGTT	GCCTTCAAATGTAACCCCCT	60.2	946	INTERGENIC	--	--	--
Ca4_P2_43301091	Ca4	43301091	ATAA/ATAATGTAA	ACCATAAAGGGAAACAGGGG	TCATGGTCTGCAATCTCGTC	59.8	714	INTERGENIC	--	--	--
Ca4_P2_43301725	Ca4	43301725	ATTTT/ATTT	GACGAGATTGCAGACCATGA	TGGGCCCTTAGTCTCTTTTT	57.9	753	INTERGENIC	--	--	--
Ca4_P2_43306872	Ca4	43306872	CATATATATATATATATATATATAT/CATATATATATATATATATATAT	TACCATGCAACGAAGTCCAA	AGCGCACTTGCAAAGTTTTT	60.1	440	INTERGENIC	--	--	--
Ca4_P2_43316684	Ca4	43316684	CATATATATATATATATATATATAT/CATATATATATATATATATATATATAT	GCAAATGTTTGCGACAGTTC	TTGGGTGTGTTGGAAGATGA	59.9	742	INTRON	Ca_23683	D	M-type
Ca4_P2_43330964	Ca4	43330964	TTATATATATATATATATAT/TTATATATATATATATATATAT	CATGGTGTTTGTGTGTGTGTG	CACACAATTGAACATGCGCT	60.7	135	INTERGENIC	--	--	--
Ca4_P2_43350387	Ca4	43350387	CAAAAAAAAAAA/CAAAAAAAAAA	GGACTAAATGTTTTAAAGCACGG	TTCATCATTTTGGAGAAAAGAAA	57.9	312	INTERGENIC	--	--	--
Ca4_P2_43362642	Ca4	43362642	TG/T	AGAATCACACTGCCCCAAAC	TTGCAAGGGAATTTTTGTCA	59.1	590	DRR	Ca_23687	--	AP2
Ca4_P2_4340423	Ca4	4340423	TA/T	TTGGACTAAGGGCCAATGAC	TATTGAAGTGCCAAACGACG	59.7	427	INTERGENIC	--	--	--
Ca4_P2_43483573	Ca4	43483573	ATTTTTTT/ATTTTTT	GCCTTTTTAAAATGACGACCA	GGGAATCATTTCTGTGAATTGG	60.5	383	INTERGENIC	--	--	--

INDEL marker IDs	Chromosomes/unanchored scaffolds	Physical positions (bp)	InDels (*Kabuli* reference genome-CDC Frontier/PII)	Forward primers (5'-3')	Reverse primers (5'-3')	Anneling temperature (0C)	Expected amplified product size (bp)	Structural annotation		Functional annotation	
								Sequence components of *kabuli* genome	*Kabuli* gene accession IDs	NCBI-KOG	TFs
Ca4_P2_43566104	Ca4	43566104	ATTGTGAGTTTTTTGTG/ATTGTG	AGATCCTCTCCCTTGCAAAA	TTCATTCATTGCCATTTCCA	59.9	581	INTERGENIC	--	--	--
Ca4_P2_43655718	Ca4	43655718	AATATATATATATATATATATATATATATAT/AATATATATATATATATATATATATATATATATATATAT	CCAACATACATAAGCGGTTTCA	ACGAACACGATTTCCAGGAC	60.0	162	INTERGENIC	--	--	--
Ca4_P2_43659877	Ca4	43659877	ATTTTTTTTT/ATTTTTTTT	TTTTTGCCACAGCTAGGGAG	TGGCACATCTATCCAATCACA	59.9	423	INTERGENIC	--	--	--
Ca4_P2_43680636	Ca4	43680636	ATTTTTTTTTTTTT/ATTTTTTTTTTTTTT	CACCTCTTCACCGGTCTTTC	TGGAATTCAAATCTTTCAAGCA	59.7	717	INTERGENIC	--	--	--
Ca4_P2_43892790	Ca4	43892790	AATATATATATATATATATATATATATATA/AATATATATATATATATATATA	GGAATCAATGGGGTTGAATG	GCACCACCATCGTCTACCTT	60.0	537	INTERGENIC	--	--	--
Ca4_P2_43965802	Ca4	43965802	ATTTTTTTTTTTTTTTT/ATTTTTTTTTTTTTT	CCATGGTATTAAGCTGACCCA	TGGTAGGAAGCTCTCACCGT	59.9	410	INTERGENIC	--	--	--
Ca4_P2_43975996	Ca4	43975996	TAAAAAAAAAA/TAAAAAAAAAAA	TGCCCACATGTGAGAACCTA	ACCAACATGATTCGCTCACA	60.1	381	DRR	Ca_09073	T	WRKY
Ca4_P2_43981120	Ca4	43981120	TTAGATATATA/TTA	TGTGAAGTGTGCGAAAGAGG	GGTTGGTGCATGATTTGTTG	59.8	872	INTERGENIC	--	--	--
Ca4_P2_43994137	Ca4	43994137	CTCTCTCTAT/CT	GGGAGTCACAAGAAAAGGCA	TAAAAGGGATTAGCCCGTCC	60.3	453	DRR	Ca_09076	--	ERF
Ca4_P2_44039022	Ca4	44039022	AATCTAAATATAT/AAT	GCAAAATACTAGTGCGGCGT	AACCTAGACGGCAATGATGC	60.1	567	INTERGENIC	--	--	--
Ca4_P2_44040432	Ca4	44040432	TAAAAAAA/TAAAAAAAA	GATTGCGGAATTAGCCTTCTT	TGGTTAAGTGAAGCGCAAAC	59.0	774	INTERGENIC	--	--	--
Ca4_P2_44070938	Ca4	44070938	GAAAAAAAAAAA/GAAAAAAAAAAAA	TGTGAAATTCCTGCCAAACA	AGGTTGGTGGAGCTGAAAGA	59.8	932	INTRON	Ca_09081	R	G2-like

INDEL marker IDs	Chromosomes/unanchored scaffolds	Physical positions (bp)	InDels (*Kabuli* reference genome-CDC Frontier/PII)	Forward primers (5'-3')	Reverse primers (5'-3')	Anneling temperature (0C)	Expected amplified product size (bp)	Structural annotation		Functional annotation	
								Sequence components of *kabuli* genome	*Kabuli* gene accession IDs	NCBI-KOG	TFs
Ca4_P2_44098644	Ca4	44098644	ATTTTTTT/ATTTTTTTT	TCACCTTCATCCATCGTCAA	TGTGGTGTGCAAGATGACTG	59.3	408	INTERGENIC	--	--	--
Ca4_P2_44134460	Ca4	44134460	AAC/AACACAC	TACCAGATTGCCCCACTTTC	GCCAAAACGGATGAAGTTGT	60.0	554	INTERGENIC	--	--	--
Ca4_P2_44218817	Ca4	44218817	AGG/AG	CCTTGTTGTTTTTGGCTGGT	GTGCGGGTGCATATTTTTCT	60.0	726	INTERGENIC	--	--	--
Ca4_P2_44255481	Ca4	44255481	CATATATATATATATATATATATATATAT/CATATATATATATATATATATATATATAT	GGAATTCAACTTTCCTTCTCGAT	TGCTGTTTTCTTCTTCACATCAA	59.9	528	INTERGENIC	--	--	--
Ca4_P2_44311010	Ca4	44311010	AATATATATATATATATATATATATAT/AATATATATATATATATATATATATAT	GCGTGACCAAACAAAAACAA	GTTCGGTCAATTCAATGCCT	59.9	690	INTERGENIC	--	--	--
Ca4_P2_44323140	Ca4	44323140	TCC/TCCC	TCGTGTTTGCACCTTCAGAG	ACACCATGGTCATGCGTAGA	60.0	207	INTERGENIC	--	--	--
Ca4_P2_44323786	Ca4	44323786	TATAGAATAGAATAGAA/TATAGAATAGAATAGAATAGAA	GCACCACGAACATCAATGAG	CAAGGTAGCGAGGAATAGCG	60.0	745	INTERGENIC	--	--	--
Ca4_P2_44334665	Ca4	44334665	TTCT/TT	TTGCACATGATGGAAACGAT	CTTGTGGGCCGTGAAAGTAT	60.0	837	INTERGENIC	--	--	--
Ca4_P2_44428431	Ca4	44428431	CATATATATATATATATAT/CATATATATATATATATAT	TGTTGCGGAAATGTTAGTTCA	TTCATCCATGCAATATTCAAAAA	59.3	699	INTERGENIC	--	--	--
Ca4_P2_44486854	Ca4	44486854	ATAAA/ATAAATTAAA	CTCAGGATGAACAACTGCCA	TTCGTTTATCTCTTCAATCGCA	59.9	419	INTERGENIC	--	--	--
Ca4_P2_44487366	Ca4	44487366	A/ATACATC	TGCGATTGAAGAGATAAACGAA	GAGATGCGGGGACAAGATTA	60.0	778	INTERGENIC	--	--	--
Ca4_P2_44487390	Ca4	44487390	CAAA/CAA	AAAATCCACTGGTGCAAAGC	GAGATGCGGGGACAAGATTA	60.0	405	INTERGENIC	--	--	--

INDEL marker IDs	Chromosomes/unanchored scaffolds	Physical positions (bp)	InDels (*Kabuli* reference genome-CDC Frontier/PII)	Forward primers (5'-3')	Reverse primers (5'-3')	Anneling temperature (0C)	Expected amplified product size (bp)	Structural annotation		Functional annotation	
								Sequence components of *kabuli* genome	*Kabuli* gene accession IDs	NCBI-KOG	TFs
Ca4_P2_44489599	Ca4	44489599	ATTTTTTT/ATTTTTTTT	TTCTGGACTGCTCCAAAACA	TCAATCGATCAGTTGCTTTTT	57.5	611	INTERGENIC	--	--	--
Ca4_P2_44490020	Ca4	44490020	AT/A	CAACTGATCGATTGATAGAAGCA	TGCTTCGCAAGATTAGTTAGGA	59.2	570	INTERGENIC	--	--	--
Ca4_P2_44490316	Ca4	44490316	AAAAGTGAAAGTGA/AAAAGTGA	CAACTGATCGATTGATAGAAGCA	TGCAGTCATTACTTTCGGATTG	60.1	705	INTERGENIC	--	--	--
Ca4_P2_44490357	Ca4	44490357	ATT/ATTT	TGAAAGTGAGAGGCTAAAAATCA	TGCAGTCATTACTTTCGGATTG	60.1	233	INTERGENIC	--	--	--
Ca4_P2_44492371	Ca4	44492371	ATTTTTTTT/ATTTTTTT	TAGGAAATGGGAAGGCAGTG	CATGCGAAGGAAATTGAAGA	58.8	667	INTERGENIC	--	--	--
Ca4_P2_44492961	Ca4	44492961	TAAAAAAA/TAAAAAAAA	TCTTCAATTTCCTTCGCATGT	CTCACATGATGCTCGTTGCT	60.0	810	INTERGENIC	--	--	--
Ca4_P2_44494040	Ca4	44494040	TAAA/TAA	TTGAATGCACACACAACCCT	CAAATCTTGCCTCTCTTGTTTG	59.0	675	INTERGENIC	--	--	--
Ca4_P2_44498939	Ca4	44498939	CTTT/CTT	ACCTTGGGCCTTAATTGAAA	CGGAACATCACTGTTCACAAA	59.6	410	INTERGENIC	--	--	--
Ca4_P2_44507569	Ca4	44507569	CTTTT/CTTT	AGCGGTTGTTCAAAATGACC	AAAGCAAGTCCGACCAAAAA	59.7	421	INTERGENIC	--	--	--
Ca4_P2_44507737	Ca4	44507737	TTTCTTCTT/TTTCTTCTTCTT	AGCGGTTGTTCAAAATGACC	AAAGCAAGTCCGACCAAAAA	59.7	421	INTERGENIC	--	--	--
Ca4_P2_44508190	Ca4	44508190	AT/ATT	GAAATGGATTTTTGGTCGGA	TTAAGGGAGTGCATGAGCAA	59.4	442	INTERGENIC	--	--	--
Ca4_P2_44509122	Ca4	44509122	GAAAA/GAAAAA	GCAGTGCTGAATTTTGGTGA	TGCAAAAGTTGTGTGAAACCA	60.2	494	INTERGENIC	--	--	--

INDEL marker IDs	Chromosomes/unanchored scaffolds	Physical positions (bp)	InDels (*Kabuli* reference genome-CDC Frontier/PII)	Forward primers (5'-3')	Reverse primers (5'-3')	Anneling temperature (0C)	Expected amplified product size (bp)	Structural annotation		Functional annotation	
								Sequence components of *kabuli* genome	*Kabuli* gene accession IDs	NCBI-KOG	TFs
Ca4_P2_44510362	Ca4	44510362	AACATGCACAT/AACATGCACATGCACAT	TCTTGGATTTGAGCAGCCTT	CCCGAATTTGGTTTTTGTTG	60.2	590	INTERGENIC	--	--	--
Ca4_P2_44511269	Ca4	44511269	ATTTTT/ATTTT	GATGCAGGTCATGAAACCAA	GTCCTCAACGCTTCAAGAGG	60.0	494	INTERGENIC	--	--	--
Ca4_P2_44511339	Ca4	44511339	ATTTT/ATTTTT	GATGCAGGTCATGAAACCAA	GTCCTCAACGCTTCAAGAGG	60.0	494	INTERGENIC	--	--	--
Ca4_P2_44520768	Ca4	44520768	GA/GAA	TTTGTCGTCTCTTCCTCGTG	AAGAGCACATGGGGAAATTG	59.9	513	INTERGENIC	--	--	--
Ca4_P2_44521022	Ca4	44521022	T/TA	CAATTTCCCCATGTGCTCTT	GACATTTTTGGATGATTTTGGA	58.8	290	INTERGENIC	--	--	--
Ca4_P2_44521070	Ca4	44521070	CAAAAA/CAAA	CAATTTCCCCATGTGCTCTT	TGGACTTCAAAATGATTGTTTG	57.7	418	INTERGENIC	--	--	--
Ca4_P2_44521513	Ca4	44521513	T/TCTTATA	TCCAAAATCATCCAAAAATGTC	CGGCTTTCTCTTGTGTGTCA	60.0	862	INTERGENIC	--	--	--
Ca4_P2_44522624	Ca4	44522624	GAAAAAA/GAAAAAAA	GAAGTTGCTTGCTCCGATTC	CCCTAGCAAGTGCTAACCCA	60.3	675	DRR	Ca_09124	--	ERF
Ca4_P2_44523921	Ca4	44523921	ATTTTTTTTTTT/ATTTTTTTTTTTT	GCAATGTGCTCACTTTTTGTCT	AACGAAGGAGGAGGGAAGAA	60.2	651	DRR	Ca_09124	--	ERF
Ca4_P2_44524127	Ca4	44524127	CTTTTTTTTTTTTTT/CTTTTTTTTTTTT	TGGCGAGACCTAAAACAAGG	TGAGGCTCCTCGTAATTCGT	59.8	483	DRR	Ca_09124	--	ERF
Ca4_P2_44525230	Ca4	44525230	TAAAAAAAAAAAA/TAAAAAAAA	ATGACGTTGGTGATTGGACA	GCGCTAGCAAAAATAGCCAC	60.0	884	INTERGENIC	--	--	--
Ca4_P2_44526873	Ca4	44526873	ACTCCAAA/ACTCCAAATGCTCCTCCAAA	TTCCATTCTTTTTGCCAAGG	TTGGCTCAAGTGGGATAAGC	60.2	397	INTERGENIC	--	--	--

INDEL marker IDs	Chromosomes/unanchored scaffolds	Physical positions (bp)	InDels (*Kabuli* reference genome-CDC Frontier/PII)	Forward primers (5'-3')	Reverse primers (5'-3')	Anneling temperature (0C)	Expected amplified product size (bp)	Structural annotation		Functional annotation	
								Sequence components of *kabuli* genome	*Kabuli* gene accession IDs	NCBI-KOG	TFs
Ca4_P2_44531593	Ca4	44531593	TAAAAAAAA/TAAAAAAAAA	AGATCCGCCACTGCATTTAC	TGGCAGGAGATATTTTGACTAGG	59.6	132	INTERGENIC	--	--	--
Ca4_P2_44532030	Ca4	44532030	TAAAAAAAA/TAAAAAAA	AGATCCGCCACTGCATTTAC	TCCACTCATCAGCCATGAAG	59.8	803	INTERGENIC	--	--	--
Ca4_P2_44532080	Ca4	44532080	TC/T	CCTAGTCAAAATATCTCCTGCCA	TCCACTCATCAGCCATGAAG	59.8	694	DRR	Ca_09125	L	--
Ca4_P2_44533305	Ca4	44533305	TAAAAAAAA/TAAAAAAA	CAAAATGCCGTCACAGAATG	CCTTGGTTTCGATGCCTAAA	60.1	368	DRR	Ca_09125	L	--
Ca4_P2_4454591	Ca4	4454591	CA/CAA	CCATTTTCAATGTCCCCAAC	CAGCAGCGCTTCTTTTTCTT	59.9	548	INTRON	Ca_03812	S	--
Ca4_P2_44551800	Ca4	44551800	ATT/ATTTTTT	CTATTCTGCTGTGGGGGTGT	TTTCAGACTTTGGATTCGGG	60.0	585	INTERGENIC	--	--	--
Ca4_P2_44555024	Ca4	44555024	T/TGAGATGGAGA	TTTCTCAAGGATCAAAATGGG	TGACCTGACCTATTCCCACC	59.8	629	INTERGENIC	--	--	--
Ca4_P2_44555673	Ca4	44555673	TA/T	AGGTCAGACCAGGCTTTGAA	TAAGTTAGGCCGTCCGTCAG	60.3	623	INTERGENIC	--	--	--
Ca4_P2_44558843	Ca4	44558843	AT/A	CTCTTCCACTAATCCGCAGC	TGCATGATTTTCATTTCGATG	59.5	536	INTERGENIC	--	--	--
Ca4_P2_44559914	Ca4	44559914	TTATATATATAT/TTATATATATATAT	GCGGGGCATAAACTGATAGA	CATTTTCATATCACCAAATCACG	59.2	465	INTERGENIC	--	--	--
Ca4_P2_44564446	Ca4	44564446	ATTTTTT/ATTTTTTT	TTGTGGAAAACTACAGTCCCAA	CGTCAACTTCATGTAATCTTTCG	58.8	763	INTERGENIC	--	--	--
Ca4_P2_4459373	Ca4	4459373	TAA/TA	AGCGAAGGCCTCTGTAGTCA	GGCCAAGCATTGTCGTTATT	60.0	439	DRR	Ca_03811	--	--

INDEL marker IDs	Chromosomes/unanchored scaffolds	Physical positions (bp)	InDels (*Kabuli* reference genome-CDC Frontier/PII)	Forward primers (5'-3')	Reverse primers (5'-3')	Anneling temperature (0C)	Expected amplified product size (bp)	Structural annotation		Functional annotation	
								Sequence components of *kabuli* genome	*Kabuli* gene accession IDs	NCBI-KOG	TFs
Ca4_P2_4464137	Ca4	4464137	CTTT/CTTTT	TACCACGGGTATGGATCGAG	GGGCTCACTTTGTCTCAAGC	60.0	587	INTERGENIC	--	--	--
Ca4_P2_4464383	Ca4	4464383	GAATTAAAATTA/GAATTAAAATTAAAATTA	GCTTGAGACAAAGTGAGCCC	TTTTGCCATTCCAAAGGAAG	60.0	349	INTERGENIC	--	--	--
Ca4_P2_4465005	Ca4	4465005	CATAAATAAA/CATAAA	TTACGGGACATCCTTCTTGG	CCCTCCCTCCTCGAAATCTA	60.5	820	INTERGENIC	--	--	--
Ca4_P2_4468129	Ca4	4468129	AC/A	TCCCAGTCCAGTGGAAAGAC	CTGCTCTCAGATGCTGTTGC	59.9	355	INTRON	Ca_03810	GOT	--
Ca4_P2_4468607	Ca4	4468607	TACCAAA/TA	CTGCAGGAAATTTCAGGAGC	CCGCATTGCTACTTTGTGAA	59.9	471	INTRON	Ca_03810	GOT	--
Ca4_P2_44694709	Ca4	44694709	TAGAGAG/TAGAG	GATTCGATCATCTGGGGAAA	AGGCTTCTGGTGCTGTTCTT	59.1	539	INTERGENIC	--	--	--
Ca4_P2_4470822	Ca4	4470822	ATTT/ATT	TCACATACCTGCCATCCAAA	CCATCCTGCTCCTATTCCAA	60.0	411	INTRON	Ca_03810	GOT	--
Ca4_P2_4471743	Ca4	4471743	TAAGAAAAATACAAG/TAAGAAAAATACAAGAAAAATACAAG	ACATTTGATCACGGTCCTCC	AGGCATTGATTCTTCGTTCG	60.2	615	INTRON	Ca_03810	GOT	--
Ca4_P2_44730412	Ca4	44730412	GAAA/GAA	AATGTAACAATTGCCGCACA	TGTGGTTCCACCAATTTGAA	59.8	271	INTERGENIC	--	--	--
Ca4_P2_44773439	Ca4	44773439	ATTTTTT/ATTTTTTTT	TTGTTTCACTTTTTCCATTCCA	TAGGGTTCTTGATCCATCGC	60.0	596	INTERGENIC	--	--	--
Ca4_P2_4478589	Ca4	4478589	TAAGTTAAAAGTTAAAAGTTAAAAGTTAAAA/TAAGTTAAAAGTTAAAAGTTAAAA	TGTAGACGCGCAGAGAGAGA	AATCATTTGCCTGGTGCTTT	59.6	593	INTERGENIC	--	--	--
Ca4_P2_4480054	Ca4	4480054	TT/TTATTTGAAT	TCCTCCATTTTTCCACGCTA	TTTCATCCTCCATGTGACCA	59.9	409	INTERGENIC	--	--	--

**INDEL marker IDs**	**Chromosomes/unanchored scaffolds**	**Physical positions (bp)**	**InDels (*Kabuli* reference genome-CDC Frontier/PII)**	**Forward primers (5'-3')**	**Reverse primers (5'-3')**	**Anneling temperature (0C)**	**Expected amplified product size (bp)**	**Structural annotation**		**Functional annotation**	
								**Sequence components of *kabuli* genome**	***Kabuli* gene accession IDs**	**NCBI-KOG**	**TFs**
Ca4_P2_4480136	Ca4	4480136	AATATA/AATATATA	TCCTCCATTTTTCCACGCTA	TTTCATCCTCCATGTGACCA	59.9	409	INTERGENIC	--	--	--
Ca4_P2_4480276	Ca4	4480276	AA/AAGA	TGGTCACATGGAGGATGAAA	GGTATTTTGACATGAATATCCCTT	57.5	160	DRR	Ca_03809	--	S1Fa-like
Ca4_P2_4480380	Ca4	4480380	TC/TCC	TGGTCACATGGAGGATGAAA	TTTAATACCTCGTATTTCCTATCAAGA	58.0	609	DRR	Ca_03809	--	S1Fa-like
Ca4_P2_4481135	Ca4	4481135	TAA/TAAA	TGAAATCAAATTTTCTCAAGGC	AAACGACATTACGTTATTTCTTCATT	58.6	120	DRR	Ca_03809	--	S1Fa-like
Ca4_P2_4481516	Ca4	4481516	T/TTC	TGAAATCAAATTTTCTCAAGGC	GTTCCGCCTCTTCTACCTCC	60.2	760	DRR	Ca_03809	--	S1Fa-like
Ca4_P2_4481679	Ca4	4481679	TAAAAAAAA/TAAAAAAAAA	TTTTGCTTTTGATTACTTTCTTTTT	GTTCCGCCTCTTCTACCTCC	60.2	314	DRR	Ca_03809	--	S1Fa-like
Ca4_P2_4482785	Ca4	4482785	AACA/AA	GATCACCACCACCTTGATCC	ACTTGAAGTCGCCCTAGCAA	60.0	649	INTRON	Ca_03809	--	S1Fa-like
Ca4_P2_44838736	Ca4	44838736	ATTTTTT/ATTTTTTT	TGAAGGAATGCTTCTTTGTTTG	GATGATTGCAACCAACATGG	59.8	304	INTERGENIC	--	--	--
Ca4_P2_44854776	Ca4	44854776	ATTT/ATTTT	AACTCGCCACTTAATCATTTCA	TGTTGCACTCGAACAAACTCA	60.5	400	INTERGENIC	--	--	--
Ca4_P2_4487000	Ca4	4487000	TAAA/TAAAA	GGCCAAATGAAGGACTCAAA	GAATCGTTCGGGAGAGTCTG	59.8	672	DRR	Ca_03808	--	--
Ca4_P2_4487086	Ca4	4487086	ATA/ACAGAAATATATTA	GGCCAAATGAAGGACTCAAA	CGTACGACGTCTTTCGTCAA	59.9	932	DRR	Ca_03808	--	--
Ca4_P2_4487144	Ca4	4487144	CAAAAA/CAAAAAA	GAAGTTCAACTGGCACCACA	CGTACGACGTCTTTCGTCAA	59.9	647	DRR	Ca_03808	--	--

INDEL marker IDs	Chromosomes/unanchored scaffolds	Physical positions (bp)	InDels (*Kabuli* reference genome-CDC Frontier/PII)	Forward primers (5'-3')	Reverse primers (5'-3')	Anneling temperature (0C)	Expected amplified product size (bp)	Structural annotation		Functional annotation	
								Sequence components of *kabuli* genome	*Kabuli* gene accession IDs	NCBI-KOG	TFs
Ca4_P2_4487412	Ca4	4487412	AT/ATTAATAAAGT	TCAGACTCTCCCGAACGATT	CGTACGACGTCTTTCGTCAA	59.9	281	DRR	Ca_03808	--	--
Ca4_P2_44874796	Ca4	44874796	AAATAATAATAATAATAATAAT/AAATAATAATAATAAT	GTGAGCACGCATAGCTTCAA	GCAATGGAAGATAAACCCCA	59.8	449	INTERGENIC	--	--	--
Ca4_P2_4488536	Ca4	4488536	TAAA/T	ACAGCACATTTCTGGTGGTG	TCTGCTAAACAGGGGATTGG	60.1	450	DRR	Ca_03808	--	--
Ca4_P2_44886483	Ca4	44886483	TTCT/TT	TAATGGAGGGCAATTTCGAG	AAAACACCCAACCCCCTAAT	59.4	349	INTERGENIC	--	--	--
Ca4_P2_44901004	Ca4	44901004	GT/GTGTAT	AGCTTCAAGTGGCTGAAGGA	ATGAACACGGGCGTATCTTC	60.0	527	DRR	Ca_09152	--	C2H2
Ca4_P2_44902733	Ca4	44902733	AAGAGAGAGAGAGAGAG/AAGAGAGAGAGAGAGAGAGAG	TGTGAAGCAACAAGGAACAGA	TCGATTCCCACATGACACTG	60.5	133	DRR	Ca_09152	--	C2H2
Ca4_P2_44949173	Ca4	44949173	AA/AAATTA	TGACGTGTCCCGTAACCTTT	ATAAGCCCATGTTGCGACTC	60.1	565	INTERGENIC	--	--	--
Ca4_P2_44953374	Ca4	44953374	TACACACACA/TACACA	TCCCTCCATCACCAAAACTC	ATGTGCAGTGACCAGAGCAG	60.1	349	INTRON	Ca_09159	G	--
Ca4_P2_44956370	Ca4	44956370	ATGGTG/ATG	TGTTTATCAAGGAGGGGTGTG	CAATGATCAGCAAGAAGGCA	59.9	296	INTERGENIC	--	--	--
Ca4_P2_44957130	Ca4	44957130	ATT/ATTT	TGAGCTGGTTTTAGCCGTTT	CTCACAAAGCGCTCACAAAA	60.2	788	INTERGENIC	--	--	--
Ca4_P2_44963420	Ca4	44963420	A/AG	CCACCTTGGGTGAATCAAGA	CACCGCTATTCCCAAGTGTT	60.0	750	INTERGENIC	--	--	--
Ca4_P2_44965317	Ca4	44965317	AATATATAT/AATATTATATATATAT	CCCATTCAGATTGCTTTTCC	TCACACAGACACTGCCATGA	59.9	537	INTERGENIC	--	--	--

INDEL marker IDs	Chromosomes/unanchored scaffolds	Physical positions (bp)	InDels (*Kabuli* reference genome-CDC Frontier/PII)	Forward primers (5'-3')	Reverse primers (5'-3')	Anneling temperature (0C)	Expected amplified product size (bp)	Structural annotation		Functional annotation	
								Sequence components of *kabuli* genome	*Kabuli* gene accession IDs	NCBI-KOG	TFs
Ca4_P2_44965984	Ca4	44965984	TTCAACTT/TTCAACTTAATTTCTCAACTT	ATCTTTCCCCTCCACACACA	TGGTCGCATTCGCATATAAA	60.1	601	INTERGENIC	--	--	--
Ca4_P2_44968120	Ca4	44968120	AT/ATATTATT	ATTCTCCCAATCCACCATCA	GCATGTTTAAGTGGGGCCTA	60.0	184	CDS (large-effect mutations)	Ca_09160	R	C2H2
Ca4_P2_44970459	Ca4	44970459	CAAA/CA	CATTGCACGAGCACAACTG	TGGAACGTAATTCCTCCATT	57.0	661	DRR	Ca_09160	R	C2H2
Ca4_P2_4497451	Ca4	4497451	TAGGAAG/TAG	TAAACCACATCGACACGGAA	TTATGGCATTCCATTCTCCC	59.7	631	INTRON	Ca_03808	--	--
Ca4_P2_44975345	Ca4	44975345	TCCCC/TCCCCC	CCTTCCCCTCTTTCAACACA	TTGGCTAGTAGGTGGGATGG	59.9	400	INTERGENIC	--	--	--
Ca4_P2_44978149	Ca4	44978149	AA/AATA	TTGATGGATGAGCCTCAATTT	AAGGCCAAAACCATTTTAGAGA	59.2	684	INTERGENIC	--	--	--
Ca4_P2_44983043	Ca4	44983043	TA/TAGA	GCAGAATATGCGGATCTGGT	TAGATTCCTGGCTCGGTCAT	59.7	387	DRR	Ca_09161	I	--
Ca4_P2_44986447	Ca4	44986447	AAAT/AAATAATAATAATAAT	GGGGATTTATTTTGGAGGGA	TTTGACCGACTTGTTTAGGAG	57.0	922	INTERGENIC	--	--	--
Ca4_P2_44986634	Ca4	44986634	AAATAATAATAAT/AAATAATAAT	ACGTGGACACACATTTCCCT	TTTGACCGACTTGTTTAGGAG	57.0	728	INTERGENIC	--	--	--
Ca4_P2_44987031	Ca4	44987031	TA/TAAA	CTCCTAAACAAGTCGGTCAAA	CCAAACGTTTTCATGAACTACAA	59.1	493	INTERGENIC	--	--	--
Ca4_P2_44987442	Ca4	44987442	TG/T	TCATGAAAACGTTTGGTTAATTG	AAAAGGGATACTGAATTTCATCCA	60.1	373	INTERGENIC	--	--	--
Ca4_P2_44987779	Ca4	44987779	GA/GAATA	ACTTTGGAACAAAAGGAGGAA	TGGTCAAGATGTGTTGACTTTG	58.7	570	INTERGENIC	--	--	--

INDEL marker IDs	Chromosomes/unanchored scaffolds	Physical positions (bp)	InDels (*Kabuli* reference genome-CDC Frontier/PII)	Forward primers (5'-3')	Reverse primers (5'-3')	Anneling temperature (0C)	Expected amplified product size (bp)	Structural annotation		Functional annotation	
								Sequence components of *kabuli* genome	*Kabuli* gene accession IDs	NCBI-KOG	TFs
Ca4_P2_44990917	Ca4	44990917	ATTTTTTT/ATTTTTTTT	CTTTTGCACTTTCAGGGAGG	AAATCACCAAAAGGTGAAGCA	59.6	513	DRR	Ca_09162	I	--
Ca4_P2_44993902	Ca4	44993902	ATTTTT/ATTTTTT	CACCTCTTTTGAATCGCACTC	AAAAATGGAATGCAAGTGCC	59.9	345	DRR	Ca_09163	--	--
Ca4_P2_44994360	Ca4	44994360	CAAAAA/CAAAAAA	GGCACTTGCATTCCATTTTT	ACACGTGGCATTGAACCTTT	60.4	479	DRR	Ca_09163	--	--
Ca4_P2_44994553	Ca4	44994553	GAAAAAAAAA/GAAAAAAAA	TGCATGCGAGGTATTCAAGA	ATTTGGCAACGAAGTTTTGG	60.0	827	DRR	Ca_09163	--	--
Ca4_P2_4499622	Ca4	4499622	ACTAGCTATCCGCTAGCTA/ACTAGCTA	CCCGTCCCTAAAACTCTCCT	CGTTCCACGGTTTGGTAGTT	59.9	657	INTRON	Ca_03808	--	--
Ca4_P2_44998481	Ca4	44998481	GTTA/GTTATTTA	GGTGCAAGTATTTGTGTGCG	ATTGACTCGACGAATCCACC	59.9	697	INTERGENIC	--	--	--
Ca4_P2_44999560	Ca4	44999560	GTCTCTC/GTCTC	TTCATTTTCCCACCCCTACA	TCCCTACTCAAAGGGGTCAA	59.5	630	INTERGENIC	--	--	--
Ca4_P2_45005102	Ca4	45005102	A/AAT	CTCCGTTCAAGCCTCTTGTC	GAAAGTGTCGAGCAACACGA	60.0	225	INTERGENIC	--	--	--
Ca4_P2_45010821	Ca4	45010821	AAAGAAAAAAA/AAAGAAAAAAAACAAGAAAAAAA	AGTATCGAGGAACACGTGGG	TGCACTCCAAAAATGGACAA	60.1	792	INTERGENIC	--	--	--
Ca4_P2_4502063	Ca4	4502063	C/CG	GCTGGATCCGTCGTTACAAT	CCATCTGAGAAAGCTAGCCG	60.1	441	DRR	Ca_03807	--	FAR1
Ca4_P2_4503083	Ca4	4503083	AG/A	CCTTACGACCTCCCAAATCA	AATGGACAGTCAATATGAAATCAA	57.6	464	INTRON	Ca_03807	--	FAR1
Ca4_P2_45034636	Ca4	45034636	AG/A	TGCCTACGTTTTGCATCAAG	CGCAACTGCTCAGACAGAGA	60.5	651	DRR	Ca_09166	P	--

INDEL marker IDs	Chromosomes/unanchored scaffolds	Physical positions (bp)	InDels (*Kabuli* reference genome-CDC Frontier/PII)	Forward primers (5'-3')	Reverse primers (5'-3')	Anneling temperature (0C)	Expected amplified product size (bp)	Structural annotation		Functional annotation	
								Sequence components of *kabuli* genome	*Kabuli* gene accession IDs	NCBI-KOG	TFs
Ca4_P2_45035933	Ca4	45035933	CCTTCT/CCTTCTTCT	TTGTTTGCTTTTTCTTGGGG	TTGGAGTTGTGCCAATGTGT	60.0	556	DRR	Ca_09166	P	--
Ca4_P2_45036487	Ca4	45036487	ACC/A	CACATTGGCACAACTCCAAC	GAGGCCACTTAATGCTTTGG	59.7	317	DRR	Ca_09166	P	--
Ca4_P2_4503851	Ca4	4503851	TAAAAAAAAA/TAAAAAAAA	TTCTCCCTCCGTCTCACATC	TGGAGGCAATGATTTTGTCA	60.0	421	INTRON	Ca_03807	--	FAR1
Ca4_P2_45050579	Ca4	45050579	CTTAAA/C	CGATGCCTCTGTACCAAATG	TAGCTGAGGCGGCACTATTT	60.0	662	DRR	Ca_09167	K	GATA
Ca4_P2_45051719	Ca4	45051719	TAAAAAAAA/TAAAAAAAAA	CCCCCAACTCAAATTTACGA	CATTTGGTACAGAGGCATGG	59.0	464	DRR	Ca_09167	K	GATA
Ca4_P2_45053400	Ca4	45053400	A/AAGG	TGTTTGGATAAGAAAGAGTGATCG	GAGGAACCAGCAAGAAGTGC	60.0	403	INTRON	Ca_09167	K	GATA
Ca4_P2_45054017	Ca4	45054017	CATATATATATA/CATATATATA	GCACTTCTTGCTGGTTCCTC	AACCCACAACCTCAAAATGC	59.8	731	INTERGENIC	--	--	--
Ca4_P2_4505602	Ca4	4505602	T/TA	CATTGAGAATTTGGGGACCA	TCATGTTGAAGGCTCTGTCG	60.0	891	INTERGENIC	--	--	--
Ca4_P2_45067243	Ca4	45067243	ACTCT/ACTCTCT	TCAAGATGCCAAGAGGTGAA	TCCAATTTTGGTCAAGGAGC	60.1	666	INTERGENIC	--	--	--
Ca4_P2_45068051	Ca4	45068051	CAA/CAAA	CGTGGACTTCCCGACTACAT	ACAATTTACTCCCTCCGCCT	60.0	584	DRR	Ca_09168	T	WRKY
Ca4_P2_4507391	Ca4	4507391	T/TTA	TTTGGGGACCAAAACTGAAG	GCATATACGAGGAGACCCGT	59.0	156	INTERGENIC	--	--	--
Ca4_P2_4507561	Ca4	4507561	AA/AAATA	TTTGGGGACCAAAACTGAAG	TCCGAGCAACCATACTTTCA	59.3	698	INTERGENIC	--	--	--

INDEL marker IDs	Chromosomes/unanchored scaffolds	Physical positions (bp)	InDels (*Kabuli* reference genome-CDC Frontier/PII)	Forward primers (5'-3')	Reverse primers (5'-3')	Anneling temperature (0C)	Expected amplified product size (bp)	Structural annotation		Functional annotation	
								Sequence components of *kabuli* genome	*Kabuli* gene accession IDs	NCBI-KOG	TFs
Ca4_P2_45076892	Ca4	45076892	CG/CGG	GGTCCAACATTCCTCCTTGA	TCCACAAATAACAGGTTTTCCC	60.1	606	INTERGENIC	--	--	--
Ca4_P2_4508477	Ca4	4508477	T/TA	GTCTCTTGGTTTACCGTGCC	ATGAGTGGTTTGCCACATGA	60.0	452	INTERGENIC	--	--	--
Ca4_P2_45102000	Ca4	45102000	AATATATATATATATATAT/AATATATATATATATAT	CCGGAGCTTGAACGAAAATA	TCGCGTAATACTCAAACCAAA	58.4	627	INTERGENIC	--	--	--
Ca4_P2_45113591	Ca4	45113591	GATTATTATT/GATTATTATTATT	TTCGAGGAACTTCGCAATCT	TTATGTCAAGGCTCTCCGCT	60.0	652	DRR	Ca_09171	--	MYB_related
Ca4_P2_4511887	Ca4	4511887	ATTTTT/ATTTTTT	CGTTGTTGGTGAAACTGCAT	CCGGCGAGAATTTTTCAATA	60.0	700	INTERGENIC	--	--	--
Ca4_P2_45148477	Ca4	45148477	TTGTGTGT/TTGTGT	GGGGTTGGTTTTGCTTGTTA	TTTTTCAAGTAGGGATGCGG	60.1	725	INTERGENIC	--	--	--
Ca4_P2_45154839	Ca4	45154839	TAAAA/TAAA	ATGATGTGTGCAAACGGAAA	CATCACCGTTACGGCCTATT	59.8	306	INTRON	Ca_09178	B	NAC
Ca4_P2_4517104	Ca4	4517104	GATGAATTTATTAAT/GATGAATTTATTAATGAATTTATTAAT	AAAATGCGTTTCACTCGGAC	GCACACTTGTGCTTGGAAGA	60.0	304	INTERGENIC	--	--	--
Ca4_P2_4517245	Ca4	4517245	AAGA/AA	AAAATGCGTTTCACTCGGAC	CCGTTTTTCGTCACAATTCA	59.6	550	INTERGENIC	--	--	--
Ca4_P2_4517700	Ca4	4517700	AATAT/AATATAT	TGAATTGTGACGAAAAACGG	AAGACTGGAATTGATCCACCA	59.4	717	INTERGENIC	--	--	--
Ca4_P2_4522974	Ca4	4522974	CAATTTTAACAT/C	AATGACAAATTTTCGGCCC	TACCCTCCTTCGCATCAATC	60.0	775	INTERGENIC	--	--	--
Ca4_P2_45232369	Ca4	45232369	ACTCCT/ACT	TCCTGAGGCTCACGTTTTCT	AACGATGCAACCTTACCCTG	60.0	562	DRR	Ca_09187	A	--

INDEL marker IDs	Chromosomes/unanchored scaffolds	Physical positions (bp)	InDels (*Kabuli* reference genome-CDC Frontier/PII)	Forward primers (5'-3')	Reverse primers (5'-3')	Anneling temperature (0C)	Expected amplified product size (bp)	Structural annotation		Functional annotation	
								Sequence components of *kabuli* genome	*Kabuli* gene accession IDs	NCBI-KOG	TFs
Ca4_P2_45268275	Ca4	45268275	TAAA/TAA	GCACCCTATATGCCTGGAAA	AACACGGTGCAAAATGAACA	60.0	462	INTRON	Ca_09190	--	--
Ca4_P2_4527520	Ca4	4527520	TTATATATATATATATATATATATATATATATAT/TTATATATATATATATATATATATATAT	AAGGTATGCTGTTTCAAGGGAA	AATGTGCATCTGCCACCATA	60.0	892	INTERGENIC	--	--	--
Ca4_P2_4529211	Ca4	4529211	CTTCATTTTT/CTTCATTTTTATAGTTTCATTTTT	AAGGAGATGCCAAGAGGGAT	AAGGGATGAACTACGTTGGG	58.9	346	INTRON	Ca_03806	--	--
Ca4_P2_4530457	Ca4	4530457	AT/ATT	GTGCAGCATGCGATAGCTTA	TCAAATTGGTTGGCAGCATA	60.1	923	INTRON	Ca_03806	--	--
Ca4_P2_4530702	Ca4	4530702	TAAATTGAAATTGAAATTGAAAT/TAAATTGAAATTGAAATTGAAATTGAAATTGAAAT	TATGGTGACAGCAGACCCAG	TTGTCGCCATAACTCATCCA	60.1	424	INTRON	Ca_03806	--	--
Ca4_P2_4530744	Ca4	4530744	ATT/AT	TATGGTGACAGCAGACCCAG	TTGTCGCCATAACTCATCCA	60.1	424	INTRON	Ca_03806	--	--
Ca4_P2_4532209	Ca4	4532209	TATTTGAATTTGAA/TATTTGAA	TGGGCTAGATTGACGTTGTTC	CATGGTGATTTTTGGGGTTC	60.0	144	DRR	Ca_03806	--	--
Ca4_P2_45323664	Ca4	45323664	CAA/CAATTTAAA	ATTGGTGGGTGCAATATGGT	AAACCAAACAAAAACCGCAG	60.0	837	DRR	Ca_09196	--	--
Ca4_P2_4532369	Ca4	4532369	GAAAA/GAA	TGAACCCCAAAAATCACCAT	AGGTTGAATGCAATTCACGTA	58.2	200	DRR	Ca_03806	--	--
Ca4_P2_45334533	Ca4	45334533	GTTTTTTTTTT/GTTTTTTTTTTT	TTTAATGTGGAAAGAGAGACATGAA	AAACACATTTGACCGCCATT	60.2	357	INTERGENIC	--	--	--
Ca4_P2_45348003	Ca4	45348003	TAAAAAAAAAA/TAAAAAAAAAAA	CCTTCAGATTCACCTCTCGC	AATACTCTTGCATCCGGTGG	60.0	510	INTRON	Ca_09199	F	--
Ca4_P2_45350262	Ca4	45350262	TTA/TTATA	AGAATCGTTTTGTTGGGATCA	GTCCTTGGTAGCGAGTTGGA	60.3	778	INTERGENIC	--	--	--

INDEL marker IDs	Chromosomes/unanchored scaffolds	Physical positions (bp)	InDels (*Kabuli* reference genome-CDC Frontier/PII)	Forward primers (5'-3')	Reverse primers (5'-3')	Anneling temperature (0C)	Expected amplified product size (bp)	Structural annotation		Functional annotation	
								Sequence components of *kabuli* genome	*Kabuli* gene accession IDs	NCBI-KOG	TFs
Ca4_P2_45350416	Ca4	45350416	ATTTTTTTTTT/ATTTTTTTTTTT	TGCATTTCCCTATTTAATGCAA	GTCCTTGGTAGCGAGTTGGA	60.3	684	INTERGENIC	--	--	--
Ca4_P2_45354479	Ca4	45354479	TC/T	CGTTTTGGCGTATCCATTCT	CCGGAAGAACATTCAAAGGA	60.0	760	INTERGENIC	--	--	--
Ca4_P2_45361243	Ca4	45361243	AATTATTATTATTATTATTATTATTATTATTATTATTATTATTATTATTATTATTATTATTATTAT/AATTATTATTATTATTATTATTATTATTATTATTATTATTATTATTATTATTATTATTATTAT	AAAGATTGGTTCGTGCTTGC	TCTTCATCGGACCAACAACA	60.1	526	INTERGENIC	--	--	--
Ca4_P2_45363153	Ca4	45363153	ATTTTTTT/ATTTTTTTT	GGCAAAACCAAAGAGAACCA	GATTTTTGGCTGCTTATGGC	59.7	400	INTERGENIC	--	--	--
Ca4_P2_45366346	Ca4	45366346	CAA/CA	TCATTGTCTGTTCTGCCAGG	TCAATTTTGGATTGGGGTGT	60.0	522	INTRON	Ca_09200	K	MYB
Ca4_P2_45366754	Ca4	45366754	TAAAAAAAA/TAAAAAAAAA	ACACCCCAATCCAAAATTGA	ATGCTCAAATGGGTAGCAGG	60.1	771	INTRON	Ca_09200	K	MYB
Ca4_P2_45366808	Ca4	45366808	TT/TTAT	ACACCCCAATCCAAAATTGA	ATGCTCAAATGGGTAGCAGG	60.1	771	INTRON	Ca_09200	K	MYB
Ca4_P2_45367499	Ca4	45367499	ATTTTTAATTT/ATTTTTAATTTTTTTAATTT	CCTGCTACCCATTTGAGCAT	GCAATTTCCGAGCCTGTTAT	59.2	583	INTRON	Ca_09200	K	MYB
Ca4_P2_45369151	Ca4	45369151	CTTTTTT/CTTTTT	TTGCATTCAATGTCTGCATTT	GGGCAGTCACCTCCACTAAA	60.1	575	INTERGENIC	--	--	--
Ca4_P2_45369397	Ca4	45369397	CAACAAAA/CAACAAAACAAAA	TTGCATTCAATGTCTGCATTT	GGGCAGTCACCTCCACTAAA	60.1	575	INTERGENIC	--	--	--
Ca4_P2_4537624	Ca4	4537624	TAATAAAATAAAATAAAAT/TAATAAAATAAAATAAAATAAAAT	TGCATCGCCCATATCAATAA	TATTGGACCGACCCTGAAAA	60.3	511	INTERGENIC	--	--	--
Ca4_P2_45377918	Ca4	45377918	CAAAAAAAAA/CAAAAAAAAAAA	TTTATACCATGCAAACGCTCA	CCAATGCGTTAAATTGGTCA	59.4	193	DRR	Ca_09201	--	--

INDEL marker IDs	Chromosomes/unanchored scaffolds	Physical positions (bp)	InDels (*Kabuli* reference genome-CDC Frontier/PII)	Forward primers (5'-3')	Reverse primers (5'-3')	Anneling temperature (0C)	Expected amplified product size (bp)	Structural annotation		Functional annotation	
								Sequence components of *kabuli* genome	*Kabuli* gene accession IDs	NCBI-KOG	TFs
Ca4_P2_45380400	Ca4	45380400	ACTACG/A	TTGCCGTTAATTCTTGGAGC	GTGGAGAAAACAACAACGGG	60.4	774	INTERGENIC	--	--	--
Ca4_P2_45385545	Ca4	45385545	ACT/ATTATATATCT	CACCCATAATACTTTATGAGATGGAA	CACAAATTAAGGGAAGGCGA	60.1	363	INTERGENIC	--	--	--
Ca4_P2_45389101	Ca4	45389101	T/TC	CAATTTCATTTTGTTGGGGG	ATTTGAGCATGGGGAAGATG	59.9	623	INTERGENIC	--	--	--
Ca4_P2_45390797	Ca4	45390797	AT/A	GGATTTCAACTTTGAGGGCA	CAGTAGGGTTTGGGACTCCA	60.0	252	INTERGENIC	--	--	--
Ca4_P2_45393352	Ca4	45393352	ATT/ATTT	GGCCCTTAGGCCTCATTAAC	ATCCGAAAAACCAACATTGC	59.8	155	INTERGENIC	--	--	--
Ca4_P2_4539359	Ca4	4539359	G/GC	CGTCGAGCTGAACAATGACA	GGGTGGAAATAGGTTGGGTC	60.4	604	INTERGENIC	--	--	--
Ca4_P2_45395365	Ca4	45395365	ATTTTTTTT/ATTTTTTTTT	TGATATGGGAGGTTAAGGACCA	CCCCACGAACTGTATCGTTT	59.9	604	INTERGENIC	--	--	--
Ca4_P2_4539713	Ca4	4539713	TTA/TTATA	TCGACCCAACCTATTTCCAC	GGTTAACCGTCGTAACACACTTT	59.4	340	INTERGENIC	--	--	--
Ca4_P2_45397379	Ca4	45397379	TA/TAA	CACGTGAGTGTGACATGGTG	CAATGGCAAAAATATCCCAAA	59.7	626	INTERGENIC	--	--	--
Ca4_P2_45399331	Ca4	45399331	ATTTTTTTTTTTTTTT/ATTTTTTTTTT	ACCACTTCACCACAAGAGGG	ATTCCGCTTTGCTGTCAAAC	60.3	445	INTERGENIC	--	--	--
Ca4_P2_45399782	Ca4	45399782	AG/A	GTTTGACAGCAAAGCGGAAT	ACAGCTTGCAACTGATGTGG	59.9	334	INTERGENIC	--	--	--
Ca4_P2_45405854	Ca4	45405854	TAAAAAAA/TAAAAAA	TTAATGGGAGGTCCGAAATG	TTCCACAATGCATCAAACAAA	60.0	701	INTERGENIC	--	--	--

INDEL marker IDs	Chromosomes/unanchored scaffolds	Physical positions (bp)	InDels (*Kabuli* reference genome-CDC Frontier/PII)	Forward primers (5'-3')	Reverse primers (5'-3')	Anneling temperature (0C)	Expected amplified product size (bp)	Structural annotation		Functional annotation	
								Sequence components of *kabuli* genome	*Kabuli* gene accession IDs	NCBI-KOG	TFs
Ca4_P2_45414512	Ca4	45414512	ATTTTTTTTTTT/ATTTTTTTTTT	TGCATAAATGCACGATGAAAA	GTTCAAACCGGCAATGTCTT	60.0	740	INTERGENIC	--	--	--
Ca4_P2_45414918	Ca4	45414918	GTTTTT/GTTTT	TAGTCTTGTCAACCACCCCC	ATGGTCTTGACAGGGTGGAG	60.0	126	INTERGENIC	--	--	--
Ca4_P2_45452454	Ca4	45452454	CTTTTTTTTTT/CTTTTTTTTTTT	ATTCCAAGTTTGTCCAACCC	CGGTTAAAAGGCATAACCGA	60.0	656	INTERGENIC	--	--	--
Ca4_P2_4550279	Ca4	4550279	TAAAAAA/TAAAAAAA	AACCCAGCTTATAGCGCAGA	AACAACACCGTTTACGCCTC	60.0	872	INTRON	Ca_03803	--	--
Ca4_P2_45505990	Ca4	45505990	AATTATTATTATTATTATTATTATTATTATTATTATTATTATTATTATTATTATTATTA/ATTATTATTATTATTATTATTATTATTATTATTATTATTATTATTATTATTATTATTATTATTA	GAATCACGCAGCATTCAAAA	CGGAGATTGCAAGTAAAAGAGTC	59.4	402	INTERGENIC	--	--	--
Ca4_P2_45567618	Ca4	45567618	TTATATATATATATATATAT/TTATATATATATATATAT	TCACAAATGGCAGTTTTGTCTT	CCAATCCTAGCTTCTGAAAATG	58.0	635	INTERGENIC	--	--	--
Ca4_P2_45584054	Ca4	45584054	AATA/AA	CACCTACAACCCCTGCACTT	AGCCACTATGGCATCGCTAA	60.8	504	INTERGENIC	--	--	--
Ca4_P2_45586091	Ca4	45586091	GTTTTTTTTTT/GTTTTTTTTTTT	GGAGGTCATGTGCTCCTCAT	TTTGCCCTTGTATCTGGGAC	59.9	473	INTERGENIC	--	--	--
Ca4_P2_4561239	Ca4	4561239	TTATTGAA/TTATTGAACTACTATTGAA	GTGCGATAGCTTGTTTGGGT	ACACTTCCTTGCCATGTTCC	60.0	476	INTRON	Ca_03802	UZ	--
Ca4_P2_45626888	Ca4	45626888	ATTT/ATTTT	TATCAGCAGCACCAAATGGA	GCCTGATGTTTGTCAGGGTT	60.0	286	INTRON	Ca_09219	Z	MYB_related
Ca4_P2_4565072	Ca4	4565072	TGG/TG	CTGGGGTGTCGGTTAGAAAA	CTGCTAGCTGCTCCCACTCT	59.9	161	INTRON	Ca_03802	UZ	--
Ca4_P2_4568058	Ca4	4568058	ATTTGATTTT/ATTTGATTTTTGATTTT	GTGGGGCATTACGATACCAC	GGGGAAGCCATATCACATCA	60.7	636	DRR	Ca_03802	UZ	--

INDEL marker IDs	Chromosomes/unanchored scaffolds	Physical positions (bp)	InDels (*Kabuli* reference genome-CDC Frontier/PII)	Forward primers (5'-3')	Reverse primers (5'-3')	Anneling temperature (0C)	Expected amplified product size (bp)	Structural annotation		Functional annotation	
								Sequence components of *kabuli* genome	*Kabuli* gene accession IDs	NCBI-KOG	TFs
Ca4_P2_45740248	Ca4	45740248	TTATATATATATATATATATATATAT/TTATATATATATATATATATAT	ACCAAATCATAACCGACCCA	TTGTTTTAAATCCGCATCACA	59.1	375	INTERGENIC	--	--	--
Ca4_P2_45745377	Ca4	45745377	ACC/ATTTCC	CGCAACAACGTGAGAGAAAA	AAGGCAGCTTCTTGGAGTCA	60.1	519	INTERGENIC	--	--	--
Ca4_P2_4574989	Ca4	4574989	AA/AAAGTA	TGCCACTAAATAGGGTTGGC	CGTTCTGAAAACGCGCTTAG	61.1	460	DRR	Ca_03801	--	--
Ca4_P2_4575422	Ca4	4575422	ATTTTTTTT/ATTTTTTTTTT	CGCGTTTTCAGAACGAACTA	GCATCGTTGAATGAGTTGGA	59.7	572	DRR	Ca_03801	--	--
Ca4_P2_4576739	Ca4	4576739	GAAAAA/GAAAAAA	TCCATATGATTTGAACTCGATTTTT	GGTTGGTTTAACCGTTGGAA	59.7	729	INTERGENIC	--	--	--
Ca4_P2_4577041	Ca4	4577041	GTT/GT	TTTGTTAGCATGAACAAAATGTCTT	TAAGCCAAAAATGGGAAACG	59.9	351	INTERGENIC	--	--	--
Ca4_P2_4577088	Ca4	4577088	TAAAAAAAA/TAAAAAAAAA	GAGTACACACGCGTCGACAT	TAAGCCAAAAATGGGAAACG	59.9	273	INTERGENIC	--	--	--
Ca4_P2_4582555	Ca4	4582555	ATTT/ATTTT	TTGAATTTAAATCCGGGATGA	GCTGCTTCACCTTCACCTTC	60.0	816	DRR	Ca_03800	T	--
Ca4_P2_4589864	Ca4	4589864	CTTT/CTT	TTTCGCTCAATGTGAAAAGC	AACGGAGGCAGTAATTGTCG	60.1	322	INTERGENIC	--	--	--
Ca4_P2_45902468	Ca4	45902468	TTTTTATTTTATTTTATTTTATTTTAT/TTTTTATTTTATTTTATTTTAT	ATCATGTGTAGGTCGGGGAA	TTCCGCCCCTTTCTTTCTAT	60.0	565	INTERGENIC	--	--	--
Ca4_P2_4590884	Ca4	4590884	ATAAGGTAACCT/ATAAGGTAACCTAAGGTAACCT	GCATCACATCGTTATCGTGG	AAATTCACCATTGATATGCGAA	59.3	625	INTERGENIC	--	--	--
Ca4_P2_4591345	Ca4	4591345	TAAAAAAA/TAAAAAAAA	TTCGCATATCAATGGTGAATTT	GTTGGTGTTCGACCTTCCAG	60.5	752	INTERGENIC	--	--	--

INDEL marker IDs	Chromosomes/unanchored scaffolds	Physical positions (bp)	InDels (*Kabuli* reference genome-CDC Frontier/PII)	Forward primers (5'-3')	Reverse primers (5'-3')	Anneling temperature (0C)	Expected amplified product size (bp)	Structural annotation		Functional annotation	
								Sequence components of *kabuli* genome	*Kabuli* gene accession IDs	NCBI-KOG	TFs
Ca4_P2_4593540	Ca4	4593540	CTAATTAATTAA/CTAATTAA	TTGAGGAATGTAAGGATGTCCA	TTAATGGCGGCTACGTTTTC	60.1	928	DRR	Ca_03798	--	--
Ca4_P2_45949230	Ca4	45949230	AATTATTATTATTATTATTATTATTATTATTATTATGATTATTATTATTAT/AATTATTATTATTATTATTATTATTATTATTATTATTATTATTATGATTATTATTATTAT	TCGAAAGAACCTCGACTCGT	TCAATATGAGGTCGCTTCCC	60.0	508	INTERGENIC	--	--	--
Ca4_P2_45959235	Ca4	45959235	TTTATTATTATTATTATTATTATTAT/TTTATTATTATTATTATTATTATTATTAT	GGCCTATGGCCTAGCCTACT	ACGGCCTACCATAGGCTTTT	60.0	555	INTERGENIC	--	--	--
Ca4_P2_45978843	Ca4	45978843	TATA/T	CATTTTCTTACGTTCGCCAAA	GCCATATTCGCTGGTTGATT	59.9	818	INTERGENIC	--	--	--
Ca4_P2_46001807	Ca4	46001807	GATAATAATAATAATAATAATAATAATAATAATAATAATA/GATAATAATAATAATAATAATAATAATAATAATA	TTGCTCCTACGTATCCCCAG	CTCAATCGTGGGTGACCTTT	60.0	385	INTERGENIC	--	--	--
Ca4_P2_46003281	Ca4	46003281	TTTATTATTATTATTATTATTATTATTATTATTATT/TTTATTATTATTATTATTATTATTATTATTATTATT	ATTCTCTCCCCCTGGTGTCT	TGTTTGGCCTAAAATTGGGT	59.3	724	INTERGENIC	--	--	--
Ca4_P2_46003963	Ca4	46003963	CAAAAAAAAAAAAAA/CAAAAAAAAAAAAA	TTTAGGCCAAACACCCTACG	TTGAAGAATGTCCCCACTCA	59.1	683	INTERGENIC	--	--	--
Ca4_P2_46004838	Ca4	46004838	TAAAAAA/TAAAAAAA	TGATTTCAATGAGAGGATTTTGTT	GCTCGATTGATGTTAAGGGC	59.7	425	INTERGENIC	--	--	--
Ca4_P2_46006007	Ca4	46006007	A/AT	TTTGTGTGCCGGAAAATATG	GGTGAAATGAAAGGACCCAA	59.8	793	INTERGENIC	--	--	--
Ca4_P2_46006169	Ca4	46006169	ATTTTT/ATTTT	TGCCAAAAGGCTAACAATGA	GGTGAAATGAAAGGACCCAA	59.8	692	INTERGENIC	--	--	--
Ca4_P2_46019057	Ca4	46019057	ATT/ATTT	TTTGGTCTGGATTTTCTGGG	TCATTGTTAGCCTTTTGGCA	59.3	683	INTERGENIC	--	--	--
Ca4_P2_46083062	Ca4	46083062	TTATATATATATATATATATATATA/TTATATATATATATATATATATATATA	CATACGATTTTCACACGTCACA	GGGTGCGGAGAGAGACTATT	58.4	511	INTERGENIC	--	--	--

INDEL marker IDs	Chromosomes/unanchored scaffolds	Physical positions (bp)	InDels (*Kabuli* reference genome-CDC Frontier/PII)	Forward primers (5'-3')	Reverse primers (5'-3')	Anneling temperature (0C)	Expected amplified product size (bp)	Structural annotation		Functional annotation	
								Sequence components of *kabuli* genome	*Kabuli* gene accession IDs	NCBI-KOG	TFs
Ca4_P2_46144676	Ca4	46144676	TAAAA/TAAAAA	AGTTGGAAGTCGTCGACAAAA	GTTGCGCCCATGAACTAAAT	60.0	253	INTRON	Ca_18357	--	NF-X1
Ca4_P2_46251822	Ca4	46251822	TTTATTATTATTATTATTATTATTATTATTATTATTATTATTATTATTATTATTATTATTATTATTATTATTATTATTATT/TTTATTATTATTATTATTATTATTATTATTATTATTATTATTATTATTATTATTATTATTATTAT[illegible]	TAGGCTTCTAGGTCAGGCCA	CCCATAAACATCGGGATGAG	60.1	548	INTERGENIC	--	--	--
Ca4_P2_46301292	Ca4	46301292	TAAAAAAAAAA/TAAAAAAAAA	CGCTTTTGGTCTCTAATGATTT	ATATGTCGCAGTCTCCCACC	60.0	184	INTERGENIC	--	--	--
Ca4_P2_46384693	Ca4	46384693	T/TCCCTATAAAAA	GAGGAGGGTGGAGAATGGAT	GGGTGCCTGGTACAAAACTC	59.5	708	INTERGENIC	--	--	--
Ca4_P2_46386159	Ca4	46386159	ATT/ATTT	GAGGAGGATGTCCCTAAGCC	AGAATCCAATGCTCCACAGG	60.1	779	INTRON	Ca_19274	--	Dof
Ca4_P2_46493053	Ca4	46493053	TAAA/TA	GGGAAAGTGATATTGGGGGT	CAAAGCTCCTTCAATTGTCTTG	59.0	407	INTERGENIC	--	--	--
Ca4_P2_46495714	Ca4	46495714	GATTATTATTATTATTATTATTATTATTATTATCATTATTATTAT/GATTATTATTATTATTATTATTATTATTATTATTATTATTATCATTATTATTAT	GAGGCTTCATTCCTCTCGTG	TGAATTGGTGGATCAATGAAAA	60.2	478	INTERGENIC	--	--	--
Ca4_P2_46496090	Ca4	46496090	TAAAAAAAAA/TAAAAAAAAAA	AAAGTGGGGTCAACTTTTTGAA	TGTCAAGGAGACCCACCACT	60.6	481	INTERGENIC	--	--	--
Ca4_P2_46674801	Ca4	46674801	A/AG	GGATGACTCGAAACGGTGTT	TTACCCAAAACCCCAAATCA	60.0	667	INTERGENIC	--	--	--
Ca4_P2_46677829	Ca4	46677829	AATATATATATATATATATATATATATA/AATATATATATATATATATATATATATATATA	TCCTCTCATAACGGGGTTTG	GGATGCAGATTTTCCTCGAA	60.2	330	INTERGENIC	--	--	--
Ca4_P2_46702625	Ca4	46702625	CATATATATATATATATATATATATATATATATATATA/CATATATATATATATATATATATATATATATATA	CGTCGGCAAATCCTCAAATA	CTCCTCCATTGGAATTTGGA	59.9	764	DRR	Ca_19247	I	--
Ca4_P2_4718004	Ca4	4718004	TATTTTTTGATT/TATT	GGAGTTTCACATTCACCCAAA	CATGCCAAAGCAGATGAAAA	59.8	389	INTERGENIC	--	--	--

INDEL marker IDs	Chromosomes/unanchored scaffolds	Physical positions (bp)	InDels (*Kabuli* reference genome-CDC Frontier/PII)	Forward primers (5'-3')	Reverse primers (5'-3')	Anneling temperature (0C)	Expected amplified product size (bp)	Structural annotation		Functional annotation	
								Sequence components of *kabuli* genome	*Kabuli* gene accession IDs	NCBI-KOG	TFs
Ca4_P2_47188403	Ca4	47188403	TTATATATATATATATATATATATATATAT/TTATATATATATATATATATATATATATATAT	ACGTCCACTGATTGGTCACA	ACAATAGCGCAATGCTCGTT	60.8	583	INTERGENIC	--	--	--
Ca4_P2_47193005	Ca4	47193005	TTTAT/TTTATTAT	CAATGTGGGTGAATTTGCTG	GATTCTGGCAAATTCGTCGT	60.1	287	INTRON	Ca_10862	--	--
Ca4_P2_47231412	Ca4	47231412	ATTTTTTTTTTT/ATTTTTTTTTTTT	CCCCACAAAAAGCAAATGAT	CGAGAGCTATGGAGCTTTTCA	59.7	461	INTERGENIC	--	--	--
Ca4_P2_47238699	Ca4	47238699	ATTT/ATT	TTTTGATCGAATCTGACCCA	TCGGCCATTTTTCTTCAATC	60.0	659	INTERGENIC	--	--	--
Ca4_P2_47253630	Ca4	47253630	GATATATATATATATATATATATATATATATATATATA/GATATATATATATATATATATATATATATATATATATATATATATATATATA	TTTTGCCACTTGATACAACAAAA	TTTTCGATTGTTGCTTTGCT	58.6	662	INTERGENIC	--	--	--
Ca4_P2_47277224	Ca4	47277224	TGGCTCGGCTCGGCTCGGCTC/TGGCTCGGCTCGGCTC	GGACCCATTTGGGACCTTAT	CTGCCCAGTAAGCACCAACT	60.3	657	DRR	Ca_10854	--	G2-like
Ca4_P2_47429657	Ca4	47429657	GTATATATA/GTATATATATA	GCATGTAAACCCCCAACATC	TCACTCTCACGTTTGTTGGC	59.9	931	INTRON	Ca_10845	R	ERF
Ca4_P2_47442661	Ca4	47442661	AATATATATATATATATATATATATATATAT/AATATATATATATATATATATATATATATATATATAT	TGTGGGAGCAAAATCACACT	GACGATCAAATCGGAAGAAAA	59.1	688	DRR	Ca_10843	U	--
Ca4_P2_47517234	Ca4	47517234	TAAAAAAAAAAAAA/TAAAAAAAAAAAAAAA	TGACCAAAGTGAAGCTTCTTGA	GATGGACGTTTGGCTGAGTT	60.1	579	INTERGENIC	--	--	--
Ca4_P2_47523693	Ca4	47523693	TG/T	CACCTTTCAATTGAGATCGGA	TTCTTTTGCTCCTCGTCGTT	60.0	638	INTERGENIC	--	--	--
Ca4_P2_47526364	Ca4	47526364	CAAAAAAAAA/CAAAAAAAAAA	TTGATGCATTTAAGCCCACA	CGGAAAAAGGGACCAAAACT	60.3	586	INTERGENIC	--	--	--
Ca4_P2_47542063	Ca4	47542063	TTATATATATATAT/TTATATATATATATAT	ATTTGAGTCGGGAACGAGTG	TTACATTTCGCACTCCCACA	60.1	351	INTERGENIC	--	--	--

INDEL marker IDs	Chromosomes/unanchored scaffolds	Physical positions (bp)	InDels (*Kabuli* reference genome-CDC Frontier/PII)	Forward primers (5'-3')	Reverse primers (5'-3')	Anneling temperature (0C)	Expected amplified product size (bp)	Structural annotation		Functional annotation	
								Sequence components of *kabuli* genome	*Kabuli* gene accession IDs	NCBI-KOG	TFs
Ca4_P2_47554845	Ca4	47554845	TCCCCC/TCCCCCC	AGCCGATTTTTCCTCGATTT	GGGTGTTTTGTGTTGATGGTT	59.6	365	INTERGENIC	--	--	--
Ca4_P2_4763939	Ca4	4763939	TA/TAATAA	ATGGTAATCACGTTGGGAGG	AGAATGTTGCACAATCAAGGAA	59.6	809	INTERGENIC	--	--	--
Ca4_P2_47685776	Ca4	47685776	A/AGGTTTG	AGTCAGACGCTACTCGGCAT	CCGACGAAATAAGTCCCTCA	60.1	837	INTERGENIC	--	--	--
Ca4_P2_47705093	Ca4	47705093	C/CG	TTCGCATCCGTATCAAATCA	TCACACCCATGAAGATATGAGC	60.0	269	INTERGENIC	--	--	--
Ca4_P2_47755689	Ca4	47755689	CTTTTTTTT/CTTTTTTT	TTTTCCTTTTGGTGGATTGG	TGTGCAACTTTCTCACCCAG	59.9	480	INTERGENIC	--	--	--
Ca4_P2_47793461	Ca4	47793461	TATAAATAAATAAATAAATAAA/TATAAATAAATAAATAAA	TGTGCCAATGGATTGAGAAAT	CTGCAAGCACGTATGCCTTA	60.0	396	DRR	Ca_10815	C	--
Ca4_P2_4784534	Ca4	4784534	ATTTTTTTTT/ATTTTTTTT	CCCTTCGGAAGACCACATAA	TTCATTTCTTTACCCCGTTCA	59.4	657	DRR	Ca_03778	K	G2-like
Ca4_P2_47848250	Ca4	47848250	TAAAAAAAAA/TAAAAAAAAAA	GACGGTGTTTGTAGCAGCAG	TTTCTTCCCCTTTTTGGGTT	59.8	273	INTERGENIC	--	--	--
Ca4_P2_47852567	Ca4	47852567	TAAAAAAAAA/TAAAAAAAA	AGCAGAGCAAAATTTGGTGG	TGGTATTGGTCCAACAACCTG	60.7	483	DRR	Ca_10809	O	MYB_related
Ca4_P2_47895561	Ca4	47895561	ATTT/ATTTT	AAAGGAGGCTAAATTCAAGAACC	AAAGAATGATCAAACGCAAGG	59.2	317	DRR	Ca_10803	S	--
Ca4_P2_47914059	Ca4	47914059	CAA/CA	TTGTCGTGCTTCTTCATTGC	GGTGAGAACTGTTGGGCATT	60.0	546	INTERGENIC	--	--	--
Ca4_P2_47936926	Ca4	47936926	CACATCAA/CA	AAAGGAAATTGCAGTGACGG	TGCTTGACCTGTTCGATTTG	59.8	669	CDS (large-effect mutations)	Ca_10795	--	ARF

INDEL marker IDs	Chromosomes/unanchored scaffolds	Physical positions (bp)	InDels (*Kabuli* reference genome-CDC Frontier/PII)	Forward primers (5'-3')	Reverse primers (5'-3')	Anneling temperature (0C)	Expected amplified product size (bp)	Structural annotation		Functional annotation	
								Sequence components of *kabuli* genome	*Kabuli* gene accession IDs	NCBI-KOG	TFs
Ca4_P2_47946003	Ca4	47946003	AC/A	AAGGGGAAAATTTGAACGCT	TGGTTTGGTTGTTGGTAATCA	58.8	544	INTRON	Ca_10795	--	ARF
Ca4_P2_47961030	Ca4	47961030	TG/T	CAAATTTATAACGATTCTCTCACAAA	GCAAATGCATAGAGCAGCAA	60.1	270	DRR	Ca_10794	--	ARF
Ca4_P2_47961387	Ca4	47961387	TG/T	GCTGCTCTATGCATTTGCTG	GAGCATGGACGTTTGGATTT	59.9	527	DRR	Ca_10794	--	ARF
Ca4_P2_47966378	Ca4	47966378	CTTTTTTTTTT/CTTTTTTTT	AGCAAATTGGGAACCTTGTG	CGAGAAACCCATGAGGAAAA	60.0	361	DRR	Ca_10792	--	B3
Ca4_P2_47968089	Ca4	47968089	CATAT/CATATAT	TTAAAGCAAGCGCTTTAGGC	CAGCACAAGCATGCCATAAC	60.3	651	INTERGENIC	--	--	--
Ca4_P2_47981247	Ca4	47981247	T/TC	TGCTTGACCTGTTCGATTTG	TGCAAAGATGCAAAAGATGG	59.8	676	INTRON	Ca_10790	U	ARF
Ca4_P2_47982618	Ca4	47982618	TTTTTATTTTATTTTATTTTATTTT/TTTTTATTTTATTTTATTTT	CCAAACTCCAATCAAATGGG	CAAATGAGAATTTTCTGGGCA	60.1	483	INTRON	Ca_10790	U	ARF
Ca4_P2_47982672	Ca4	47982672	TC/TCC	CCAAACTCCAATCAAATGGG	CAAATGAGAATTTTCTGGGCA	60.1	483	INTRON	Ca_10790	U	ARF
Ca4_P2_47983864	Ca4	47983864	CTATATAT/C	GCTTTGTTTCCATAAGCCCA	GGACCTCTTTCTACCCCTGG	59.9	332	INTRON	Ca_10790	U	ARF
Ca4_P2_47985512	Ca4	47985512	G/GGTCAATATTGTTAA	ATGGGAAGGGAGCTTTTTGT	TTTGGATACTATTTGCGAAGTTT	57.2	383	INTRON	Ca_10790	U	ARF
Ca4_P2_48019545	Ca4	48019545	AATATTTATTTTATATTTATTTTATATTTATTT/AATATTTATTTTATATTTATTT	AAGCAACCCCGTTATTTGTG	TTCGTACCATACCGGCAGAT	60.4	588	INTERGENIC	--	--	--
Ca4_P2_48033176	Ca4	48033176	ATTTTTTTTTT/ATTTTTTTTTTT	GGGCTCTAGGCCCATTTATT	AGAGTTGGTTTGATTGCCGT	59.6	395	INTERGENIC	--	--	--

INDEL marker IDs	Chromosomes/unanchored scaffolds	Physical positions (bp)	InDels (*Kabuli* reference genome-CDC Frontier/PII)	Forward primers (5'-3')	Reverse primers (5'-3')	Anneling temperature (0C)	Expected amplified product size (bp)	Structural annotation		Functional annotation	
								Sequence components of *kabuli* genome	*Kabuli* gene accession IDs	NCBI-KOG	TFs
Ca4_P2_48042400	Ca4	48042400	AATATATATATATATATATATATATATATATAT/AATATATATATATATATATATATATATATATATATATATAT	TTTTGGTTCACATTCACCTGAT	GGGCAAACCCCTCAGAAATA	61.2	885	DRR	Ca_10784	--	MYB_related
Ca4_P2_48116724	Ca4	48116724	AATATATATATATATA/AATATATATATATATATA	TTTGACGTTCGAAAACCAC	ATGGAACAATCGGACACCAT	60.1	433	INTERGENIC	--	--	--
Ca4_P2_48121864	Ca4	48121864	TAAAAAAAAAA/TAAAAAAAAA	AATCCATTGTTGCATCGTCA	ATTGTGGGGTGATTGGTTGT	60.0	653	DRR	Ca_10775	K	TALE
Ca4_P2_48170997	Ca4	48170997	TAAAAAAA/TAAAAAAAA	GGTGGATTGGATTGGATCAG	GGTGGGGTAGTATCCCTATCAA	59.1	670	INTERGENIC	--	--	--
Ca4_P2_48196964	Ca4	48196964	AATATATATATATATAT/AATATATATATATATATAT	GGGTTTTTACCTAGACGGTGA	TCATGACATGGCGTGGTAGT	60.0	709	INTERGENIC	--	--	--
Ca4_P2_48204434	Ca4	48204434	CT/CTT	CTCGGCATCTCAAAGTTTCTC	CCTTTGCCCTCTTCCTCTTT	59.8	391	INTERGENIC	--	--	--
Ca4_P2_48204819	Ca4	48204819	T/TAA	AAAGAGGAAGAGGGCAAAGG	GCAAACACCGATAGAGAGGG	59.7	425	INTERGENIC	--	--	--
Ca4_P2_48206793	Ca4	48206793	CAT/CATAT	TATTTGGCCAACAGGAAAGG	AGAGCTGATTTGGTTCCTCG	59.4	893	INTERGENIC	--	--	--
Ca4_P2_48207508	Ca4	48207508	AGATATAGT/A	CCGCCCCTTATCCTTATTGT	TTTCAAGCAGCTAAAACGCA	59.8	568	INTERGENIC	--	--	--
Ca4_P2_48211148	Ca4	48211148	GGAGAG/GGAG	GAGGGATGAGTATATCCCCAAA	CGTTCGTCCTTGCACCTATT	60.1	454	INTERGENIC	--	--	--
Ca4_P2_48211462	Ca4	48211462	TGGG/TGG	AATAGGTGCAAGGACGAACG	CAAGTTATCGGCTGAGAGGC	60.0	316	INTERGENIC	--	--	--
Ca4_P2_48211807	Ca4	48211807	CTATATATATA/CTATATATA	GCCTCTCAGCCGATAACTTG	ATTCCTTTACGGGGAAAACG	60.2	590	INTERGENIC	--	--	--

INDEL marker IDs	Chromosomes/unanchored scaffolds	Physical positions (bp)	InDels (*Kabuli* reference genome-CDC Frontier/PII)	Forward primers (5'-3')	Reverse primers (5'-3')	Anneling temperature (0C)	Expected amplified product size (bp)	Structural annotation		Functional annotation	
								Sequence components of *kabuli* genome	*Kabuli* gene accession IDs	NCBI-KOG	TFs
Ca4_P2_48212003	Ca4	48212003	AATATATAT/AATATATATATAT	GCCTCTCAGCCGATAACTTG	ATTCCTTTACGGGGAAAACG	60.2	590	INTERGENIC	--	--	--
Ca4_P2_48212486	Ca4	48212486	ATTTTTAATT/ATTTTTAATTTGTTTTTAATT	CGTTTTCCCCGTAAAGGAAT	TGATGCCACAATTAGCCTCTT	59.7	420	INTERGENIC	--	--	--
Ca4_P2_48213718	Ca4	48213718	A/AAAT	CAGCTCAAATGAGCAATCCA	GGTGGGTGAGGATGGTTATG	60.1	251	INTERGENIC	--	--	--
Ca4_P2_48214681	Ca4	48214681	TTTAT/TTTATTAT	GAAATGACCCCTCGAACTCA	ATGGATTTGAAAGGCGATGA	60.4	426	INTERGENIC	--	--	--
Ca4_P2_48214982	Ca4	48214982	GAAT/GAATTAAT	ATGCTTTCAAACTCATCGCC	CCATCGGATAGGTCAGAACC	59.4	525	INTERGENIC	--	--	--
Ca4_P2_48215222	Ca4	48215222	A/AAT	ATGCTTTCAAACTCATCGCC	TTGGGCTTTTGCTTTGAACT	59.9	952	INTERGENIC	--	--	--
Ca4_P2_48215838	Ca4	48215838	TTCT/TT	AGTTCAAAGCAAAAGCCCAA	TCCTCCATACCCACATCCAT	60.0	643	INTERGENIC	--	--	--
Ca4_P2_48216985	Ca4	48216985	GTATATATAT/GTATATATATAT	GGCAAGATGACATTGACGTG	TCAGCATGTTGGAGCAAATAA	59.3	469	INTERGENIC	--	--	--
Ca4_P2_48218409	Ca4	48218409	CATAT/CAT	AAATTTGAAGTCCGGCATGA	CGAGTGAGTGCAGCTGGTTA	60.2	359	INTERGENIC	--	--	--
Ca4_P2_48224503	Ca4	48224503	AGTGGTGGT/AGTGGTGGTGGTGGT	GACAGGTTTCTTCTCGCTGG	TGTTCAATCCCACCATCAAA	59.8	674	CDS (large-effect mutations)	Ca_10766	--	DBB
Ca4_P2_48224622	Ca4	48224622	CTTTTT/CTTTTTATTTTT	GACAGGTTTCTTCTCGCTGG	TGTTCAATCCCACCATCAAA	59.8	674	INTRON	Ca_10766	--	DBB
Ca4_P2_48224694	Ca4	48224694	GATTA/GATTATATTA	GACAGGTTTCTTCTCGCTGG	TGTTCAATCCCACCATCAAA	59.8	674	INTRON	Ca_10766	--	DBB

INDEL marker IDs	Chromosomes/unanchored scaffolds	Physical positions (bp)	InDels (*Kabuli* reference genome-CDC Frontier/PII)	Forward primers (5'-3')	Reverse primers (5'-3')	Anneling temperature (0C)	Expected amplified product size (bp)	Structural annotation		Functional annotation	
								Sequence components of *kabuli* genome	*Kabuli* gene accession IDs	NCBI-KOG	TFs
Ca4_P2_48227983	Ca4	48227983	TTCTCT/TTCTCTCT	AAATTCGATCTCGGCAGCTA	AGACATGGATTCGCCACTTC	60.1	594	INTERGENIC	--	--	--
Ca4_P2_48228044	Ca4	48228044	ATTGTT/ATT	AAATTCGATCTCGGCAGCTA	AGACATGGATTCGCCACTTC	60.1	594	INTERGENIC	--	--	--
Ca4_P2_48228282	Ca4	48228282	CTTTTTTTTTT/CTTTTTTTTT	TCATCTCTGTCATTTTGCTGC	AGACATGGATTCGCCACTTC	60.1	252	INTERGENIC	--	--	--
Ca4_P2_48229821	Ca4	48229821	TAAAAAAAA/TAAAAAAAAA	TTGCCCCAATAAAATACCCA	TCAAGGACCACCATTCTTCA	59.1	556	INTERGENIC	--	--	--
Ca4_P2_48230328	Ca4	48230328	A/AC	TCCTTGAGGGACCAGGATAA	ATTGTGGATTTTGTGTCGCA	60.0	431	INTERGENIC	--	--	--
Ca4_P2_48232431	Ca4	48232431	AAAA/AAAATAAA	CATGCCAAACATATGCCACT	CTGCCACCATATGTGCAAAG	60.1	729	DRR	Ca_10765	--	--
Ca4_P2_48235126	Ca4	48235126	ATTGTT/ATT	TTGGGGTACAAAAAGGTGGA	TGCTGCCTTAAGGACACTTG	59.1	519	DRR	Ca_10764	--	--
Ca4_P2_48240826	Ca4	48240826	AG/A	TCATCAAATTCGTATCGCAAA	TTCCATTCTCACCGAAATCC	59.9	322	INTRON	Ca_10764	--	--
Ca4_P2_48243356	Ca4	48243356	CCTTCT/CCT	TGCATTTGTTGGACGCTTAG	AAATCGATAGTTTTGGCCCC	60.2	601	INTERGENIC	--	--	--
Ca4_P2_48245580	Ca4	48245580	CAAA/CAA	GGTTTTGTCGATGCAAGTCA	AGGAGGATCATGCGGTACAC	60.0	580	INTERGENIC	--	--	--
Ca4_P2_48250409	Ca4	48250409	CAA/CAAA	TAGGATGCACTGTCAGCGTC	ATTACTTGTTGCAGTCCGGG	60.0	483	DRR	Ca_10763	--	--
Ca4_P2_48253836	Ca4	48253836	TAA/TA	CCAGAAACCACCACGAGATT	CCTTGCTACCAATGCTCACA	59.9	466	INTERGENIC	--	--	--

INDEL marker IDs	Chromosomes/unanchored scaffolds	Physical positions (bp)	InDels (*Kabuli* reference genome-CDC Frontier/PII)	Forward primers (5'-3')	Reverse primers (5'-3')	Anneling temperature (0C)	Expected amplified product size (bp)	Structural annotation		Functional annotation	
								Sequence components of *kabuli* genome	*Kabuli* gene accession IDs	NCBI-KOG	TFs
Ca4_P2_48254123	Ca4	48254123	A/ATTGAG	GGAGTCGACGAAACCAACTC	TTCGCATAAACCGAAGAAGTT	58.9	160	INTERGENIC	--	--	--
Ca4_P2_48254492	Ca4	48254492	TAAA/TAA	GGAGTCGACGAAACCAACTC	TTTTCTGTCCGCAAATCTGA	59.4	730	INTERGENIC	--	--	--
Ca4_P2_48254672	Ca4	48254672	AAAAATTTAAAATTTA/AAAAATTTAAAATTTAAAATTTA	TTCGAACCTTAACTTCTTCGG	TTTTCTGTCCGCAAATCTGA	59.4	601	INTERGENIC	--	--	--
Ca4_P2_48259436	Ca4	48259436	AATTTTATTTT/AATTTT	CTACTGGTAACTGGGGGCAA	GGATAAGCTGCTGAGCCAAC	60.0	652	INTRON	Ca_10761	K	B3
Ca4_P2_4826016	Ca4	4826016	AT/A	GGATTGATTGCAGTTCCAAAA	GGCAAAATGCCTTTAGACCA	60.1	445	DRR	Ca_03777	O	FAR1
Ca4_P2_48260228	Ca4	48260228	GT/GTTTTAAGTT	TGTTGCCATTTTGTTTGCAT	ACCTGCACCACAAGAAAACC	60.0	635	INTRON	Ca_10761	K	B3
Ca4_P2_48268958	Ca4	48268958	AA/AAGA	CAATCTTGCCTTCCCAAAAA	AATTGCTGCTGCTGAGGTTT	60.0	914	INTRON	Ca_10760	Q	B3
Ca4_P2_48274554	Ca4	48274554	GAAAAAAAA/GAAAAAAAAA	AGCACAAGACACCAAAACCC	GTATCTGCTGGGCTGAAAGC	60.0	240	INTRON	Ca_10760	Q	B3
Ca4_P2_48277724	Ca4	48277724	TC/T	TGATGGGTCGTGTCGTCTTA	GGCATGCCTCATATGTGTGT	59.4	427	INTERGENIC	--	--	--
Ca4_P2_48278298	Ca4	48278298	CAAAAAAAAAAA/CAAAAAAAAA	ATGTTCCGGATTTGAACTCG	CAAACCTGGGCATTGCTATT	60.0	327	INTERGENIC	--	--	--
Ca4_P2_48278382	Ca4	48278382	CGAGAGA/CGAGA	ATGTTCCGGATTTGAACTCG	ACCAACGACGAGATAGGGTG	60.0	604	INTERGENIC	--	--	--
Ca4_P2_48291800	Ca4	48291800	GACTTGATCCA/GACTTGATCCACTTGATCCA	AATAATTGGGGAGTCTGGGG	AGAGGCCTAAAAGCCCAAAG	59.9	348	INTERGENIC	--	--	--

INDEL marker IDs	Chromosomes/unanchored scaffolds	Physical positions (bp)	InDels (*Kabuli* reference genome-CDC Frontier/PII)	Forward primers (5'-3')	Reverse primers (5'-3')	Anneling temperature (0C)	Expected amplified product size (bp)	Structural annotation		Functional annotation	
								Sequence components of *kabuli* genome	*Kabuli* gene accession IDs	NCBI-KOG	TFs
Ca4_P2_48294552	Ca4	48294552	ATTTT/ATTTTT	GTTGATGGCAACCTCTTCGT	TCACATCCCCTAGAAACACCT	58.5	649	INTRON	Ca_10759	--	--
Ca4_P2_48298684	Ca4	48298684	C/CA	GCGGTGTAATCGATTGTTCA	TGAACTTCAACTTGCGGCTA	59.6	434	INTERGENIC	--	--	--
Ca4_P2_48300219	Ca4	48300219	TAAAAAAA/TAAAAAAAAAA	ACTTATTTTGGGGTCTGCCA	GCCATTGAGAAGCTCCTTTG	60.0	660	INTERGENIC	--	--	--
Ca4_P2_48300960	Ca4	48300960	TTTATTATTATTATT/TTTATTATTATTATTATT	CAAAGGAGCTTCTCAATGGC	GACAACTGTCCAAGCAAGCA	60.0	790	INTRON	Ca_10758	J	--
Ca4_P2_48308654	Ca4	48308654	C/CA	ATTTTTGTATTCGCGTCCCA	CGTAAGTCCGTTTCACCACA	59.6	194	INTERGENIC	--	--	--
Ca4_P2_48309328	Ca4	48309328	AAAAGAAA/AAAA	TTCGTAACCTGTTGACGTTGA	TAATGACGTTTGTGGAGGCA	60.1	451	INTERGENIC	--	--	--
Ca4_P2_48310611	Ca4	48310611	TG/TGCGAGAGAG	CAATGTTCAAACTGCACCGT	CAGATTCCACCCAAGGTCAA	60.9	706	INTERGENIC	--	--	--
Ca4_P2_48311635	Ca4	48311635	GTTTTTTT/GTTTTTT	GCACTTCAGCATGTTTGCAT	TTGATCTGCTTTGTGCCTTG	60.0	816	INTERGENIC	--	--	--
Ca4_P2_48314680	Ca4	48314680	ATTT/ATTTTT	GTAATGCGTTTGACCTGCAA	ATAGGCGCATCTCAATCACC	60.1	649	DRR	Ca_10756	--	BBR-BPC
Ca4_P2_48314796	Ca4	48314796	GAAAAAAAAAA/GAAAAAAAAA	GAACCTTGCTATGAAAGGAACTG	ATAGGCGCATCTCAATCACC	60.1	523	DRR	Ca_10756	--	BBR-BPC
Ca4_P2_48314877	Ca4	48314877	TA/T	TGCGCCTATCTCTCTGTGTG	TTCGATCCATTTTACCACCC	59.6	365	DRR	Ca_10756	--	BBR-BPC
Ca4_P2_48315718	Ca4	48315718	TAAAATAAAAATAAAAATAAAAAT/TAAAATAAAAATAAAAAT	TTGTCGGCATAAACATGAGAA	TAGCATTCCTCCTCTGCCAT	59.8	143	DRR	Ca_10756	--	BBR-BPC

INDEL marker IDs	Chromosomes/unanchored scaffolds	Physical positions (bp)	InDels (*Kabuli* reference genome-CDC Frontier/PII)	Forward primers (5'-3')	Reverse primers (5'-3')	Anneling temperature (0C)	Expected amplified product size (bp)	Structural annotation		Functional annotation	
								Sequence components of *kabuli* genome	*Kabuli* gene accession IDs	NCBI-KOG	TFs
Ca4_P2_48318482	Ca4	48318482	AATA/AA	CAACATATTTGCGGTTACCAA	CTCACCGTTTCACGTGATTG	60.2	920	INTERGENIC	--	--	--
Ca4_P2_48323095	Ca4	48323095	AACACACACACA/AACACACACACACA	GCAGCCAAACCAGCTTAGAC	AATTTGCAAAAATTCGGCTG	60.1	515	DRR	Ca_10754	Q	GeBP
Ca4_P2_48323556	Ca4	48323556	CTTATTATTA/CTTATTATTATTA	TTGCCACTTCCAAACACAAA	ATTTGAAATTGCTTTCCCCA	59.4	405	DRR	Ca_10754	Q	GeBP
Ca4_P2_48326584	Ca4	48326584	TAAAAA/TAAAA	ATGTCGTGCAACTGTGAACC	CGACATGATAGCCCACTTCA	59.7	289	INTERGENIC	--	--	--
Ca4_P2_48330370	Ca4	48330370	AC/A	GCAAAGGAGCTTCTCAATGG	GCATCAGGAACAACCATCCT	59.9	617	INTRON	Ca_10753	J	--
Ca4_P2_48331462	Ca4	48331462	TTATCTCCCTTAAGATAT/TTAT	CAGCTTGAAGTCCTTGCTCC	GTGGCCGTTTAATGCACTTT	60.0	834	DRR	Ca_10753	J	--
Ca4_P2_48336771	Ca4	48336771	ATT/ATTT	GGAGAGCTTAGTTGGTTAATTGG	GTGCCTCTGTTGATGCTTGA	60.0	454	INTERGENIC	--	--	--
Ca4_P2_48337515	Ca4	48337515	ATCTTTTCTTTTCTTT/ATCTTTTCTTT	CCAAAGTACACGCAAGACCA	TAATTTCCCCACCACTTGGA	60.2	840	INTERGENIC	--	--	--
Ca4_P2_48337580	Ca4	48337580	ATTTTTT/ATTTTTTT	CCAAAGTACACGCAAGACCA	TAATTTCCCCACCACTTGGA	60.2	840	INTERGENIC	--	--	--
Ca4_P2_48341983	Ca4	48341983	T/TTTATTTTTTA	CATTAGTGTGCTCCCCACCT	AATGGGTATCAAAAAGCCCC	60.0	904	INTERGENIC	--	--	--
Ca4_P2_48342297	Ca4	48342297	GC/G	TAAAGACCGTCGCCAAATTC	AATGGGTATCAAAAAGCCCC	60.0	419	INTERGENIC	--	--	--
Ca4_P2_48351799	Ca4	48351799	GTTTTTTT/GTTTTTT	TAAAAACATTTTCAGGGGCG	TCGGTAATTTTGTTCCCACA	58.9	578	INTERGENIC	--	--	--

INDEL marker IDs	Chromosomes/unanchored scaffolds	Physical positions (bp)	InDels (*Kabuli* reference genome-CDC Frontier/PII)	Forward primers (5'-3')	Reverse primers (5'-3')	Anneling temperature (0C)	Expected amplified product size (bp)	Structural annotation		Functional annotation	
								Sequence components of *kabuli* genome	*Kabuli* gene accession IDs	NCBI-KOG	TFs
Ca4_P2_48352114	Ca4	48352114	CAAAAAAA/CAAAAAA	TAAAAACATTTTCAGGGGCG	CGGTTAGGTTTGTAAGGGCA	60.0	890	INTERGENIC	--	--	--
Ca4_P2_48359177	Ca4	48359177	T/TG	CTTGGCACTGGTATGTGGTG	TTGACGAAGAAGCAACCCTT	59.9	619	DRR	Ca_10751	L	CPP
Ca4_P2_48366728	Ca4	48366728	A/AGAG	CAAGGGCTGGATTGTTTTGT	TTGGCTTTACACTTGGTCCC	60.0	673	DRR	Ca_10750	A	bHLH
Ca4_P2_48371231	Ca4	48371231	AATTAATTATTAATTATTAATTATTAAT/AATTAATTATTAATTATTAAT	CGAAATTTAGCTGCAGAGGG	TTGGGAATCAGTGAGAGCAC	58.8	543	DRR	Ca_10749	--	--
Ca4_P2_48371564	Ca4	48371564	TTAAAATAAAATAAAATA/TTAAAATAAAATA	AGTGCTCTCACTGATTCCCAA	CTATTGAAGGCCGTTGGTGT	60.0	688	DRR	Ca_10749	--	--
Ca4_P2_48386632	Ca4	48386632	TATAATAATAATAATAATA/TATAATAATAATAATAATA	TTCTCAAGGCACAATCACCA	AGCTCTGCTTTCCTCATTTTT	57.4	255	INTERGENIC	--	--	--
Ca4_P2_48389809	Ca4	48389809	ATT/ATTT	CCGTTTTCCACATTTTCGAT	CCATCAAATTCATCCACCAA	59.2	522	INTERGENIC	--	--	--
Ca4_P2_48391627	Ca4	48391627	TAAAA/TAAAAAA	TTCCATTGGGAGAGTCACCT	ACCGGTTTATGGATCCCAAT	60.3	615	INTERGENIC	--	--	--
Ca4_P2_48391760	Ca4	48391760	TCC/TCCC	TTCCATTGGGAGAGTCACCT	ACCGGTTTATGGATCCCAAT	60.3	615	INTERGENIC	--	--	--
Ca4_P2_48392399	Ca4	48392399	ATTTT/ATTTTT	TTTGTCATTGTGAATTGAATTGTTT	TCGGAAATACTCCCTCCCTT	59.9	523	INTERGENIC	--	--	--
Ca4_P2_48392635	Ca4	48392635	A/AG	TTTGTCATTGTGAATTGAATTGTTT	TCGGAAATACTCCCTCCCTT	59.9	523	INTERGENIC	--	--	--
Ca4_P2_4839789	Ca4	4839789	CTTT/CTTTTTTTT	ACGTAATACTTTGTGGCGGC	TCTCTCCTTTGTGACGGCTT	60.0	138	DRR	Ca_03775	--	--

INDEL marker IDs	Chromosomes/unanchored scaffolds	Physical positions (bp)	InDels (*Kabuli* reference genome-CDC Frontier/PII)	Forward primers (5'-3')	Reverse primers (5'-3')	Anneling temperature (0C)	Expected amplified product size (bp)	Structural annotation		Functional annotation	
								Sequence components of *kabuli* genome	*Kabuli* gene accession IDs	NCBI-KOG	TFs
Ca4_P2_48398628	Ca4	48398628	GTATA/GTATATA	ATGGCCCAATTAAGCTTTGA	CTGTTCTTGCGTGACCTTCA	60.0	565	INTERGENIC	--	--	--
Ca4_P2_48401392	Ca4	48401392	GTTT/GT	GACCAAGGCAAGCAAGAAAG	GGAAAAGGAGTGCAGTCACG	60.8	573	INTERGENIC	--	--	--
Ca4_P2_48404093	Ca4	48404093	GATTAA/GATTAATTAA	TTTGTTGCCGAGAGTCTTGA	GTGCATGGCAATTGAATGAT	59.4	410	INTERGENIC	--	--	--
Ca4_P2_48404970	Ca4	48404970	GTTTAATTT/GTTTAATTTTAATTT	TTGCATGACTGCTTTTGGAC	TTGTCATTGTTGCGGTTTGT	60.0	514	INTERGENIC	--	--	--
Ca4_P2_48405207	Ca4	48405207	TTCTCTCTC/TTCTCTC	ACAAACCGCAACAATGACAA	CTCACTCACCCGAAACCATT	60.0	485	INTERGENIC	--	--	--
Ca4_P2_4840884	Ca4	4840884	AC/A	TTGGGCCTTTGTGGAGTAAG	TACCAAAGTGGTGATGGTGG	59.3	496	DRR	Ca_03775	--	--
Ca4_P2_48410357	Ca4	48410357	T/TAAC	TTTGGATTGTTTGGTGGGTT	GAGGCATAATTTGGACGGTG	60.3	634	INTERGENIC	--	--	--
Ca4_P2_48410936	Ca4	48410936	A/AGAT	CACCGTCCAAATTATGCCTC	TGAGAACGTGCGACCTGTAG	60.0	518	INTERGENIC	--	--	--
Ca4_P2_48411282	Ca4	48411282	AATTTTTTTATTTTTTTATTTT/AATTTTTTTATTTT	CTACAGGTCGCACGTTCTCA	AAAAGATTGTTGTTCCACTTTTCA	59.1	611	INTERGENIC	--	--	--
Ca4_P2_48414216	Ca4	48414216	CATAT/CAT	ATGAGCATAGCATGTGCCAG	GCATGGTTATCGGCTGATTC	60.4	418	INTERGENIC	--	--	--
Ca4_P2_48418493	Ca4	48418493	TAAAAA/TAAAAAA	TACCCTTGCACATGGGGTAT	CCGGACAGTGTACCTTTCGT	60.0	251	INTERGENIC	--	--	--
Ca4_P2_4842046	Ca4	4842046	AAATAATAATAATAATAAT/AAATAATAATAAT	TGAGAAGAAGGGGCTTAGCA	CGAGGAGGTTATTGGCGTAA	60.1	754	INTERGENIC	--	--	--

INDEL marker IDs	Chromosomes/unanchored scaffolds	Physical positions (bp)	InDels (*Kabuli* reference genome-CDC Frontier/PII)	Forward primers (5'-3')	Reverse primers (5'-3')	Anneling temperature (0C)	Expected amplified product size (bp)	Structural annotation		Functional annotation	
								Sequence components of *kabuli* genome	*Kabuli* gene accession IDs	NCBI-KOG	TFs
Ca4_P2_48428988	Ca4	48428988	TAAAAAAAAAA/TAAAAAAAAA	CATGGAAAAGCAACAACAACA	TTTTGCTGCATTTTGCTGAC	60.0	836	INTERGENIC	--	--	--
Ca4_P2_48429106	Ca4	48429106	ATTTTTTTT/ATTTTTTT	TCCAAATCATTTTCACCAACTC	TTTTGCTGCATTTTGCTGAC	60.0	782	INTERGENIC	--	--	--
Ca4_P2_48431277	Ca4	48431277	T/TAA	TTTGTTTTGTCCCATCCCAT	TCGCCATACCTTTCGAACTT	59.7	762	INTERGENIC	--	--	--
Ca4_P2_48433198	Ca4	48433198	CAAAAAAA/CAAAAAA	AATCCTATATGGGCCGGAAC	TGATAATCTATTGGAGGCCGA	59.5	844	INTERGENIC	--	--	--
Ca4_P2_48433473	Ca4	48433473	AT/ATT	ACTTTTTCATGTCCCCCACC	AACGAAATGAACATCTGGGC	59.9	838	INTERGENIC	--	--	--
Ca4_P2_48435040	Ca4	48435040	AAAATTA/AAAATTAAATTA	AAATGAGCCAAATAGGCATGT	TTCTTTCGAAATTCAACTGCAA	59.9	399	INTERGENIC	--	--	--
Ca4_P2_48435268	Ca4	48435268	A/AG	TTGCAGTTGAATTTCGAAAGAA	TGATGTGATCCTAGGGTGAAGA	59.6	610	INTERGENIC	--	--	--
Ca4_P2_48436791	Ca4	48436791	TAA/TAAAA	TGTGACCACCACCTTGAAAA	TGGAACTCTGGTGCAAGTCA	60.4	383	INTERGENIC	--	--	--
Ca4_P2_48441518	Ca4	48441518	TTCT/TT	TTGATTTGATGGATTTATCTGGG	TTTTCTGGTGCACGAAGTTT	58.4	395	INTERGENIC	--	--	--
Ca4_P2_48443652	Ca4	48443652	TAAAAAA/TAAAAAAA	CCCAACCATCCATCCTATTG	CACCATTGGTCTCTCTATTTTGC	60.0	675	INTERGENIC	--	--	--
Ca4_P2_48443839	Ca4	48443839	AT/A	TGCCATAACTTTGAAAAATTGC	GAGTTTGAGAAGTGCAGGGC	60.0	955	INTERGENIC	--	--	--
Ca4_P2_48445750	Ca4	48445750	CTT/CT	GTAGTTGGTGGGGTGGTGTC	ATGAGGCCCAACAAACTCAC	60.0	280	INTERGENIC	--	--	--

INDEL marker IDs	Chromosomes/unanchored scaffolds	Physical positions (bp)	InDels (*Kabuli* reference genome-CDC Frontier/PII)	Forward primers (5'-3')	Reverse primers (5'-3')	Anneling temperature (0C)	Expected amplified product size (bp)	Structural annotation		Functional annotation	
								Sequence components of *kabuli* genome	*Kabuli* gene accession IDs	NCBI-KOG	TFs
Ca4_P2_48448291	Ca4	48448291	TT/TTAT	AATCCAAAACCACCCATCAA	AAAAAGCCACCTGAGCAAGA	60.0	209	INTERGENIC	--	--	--
Ca4_P2_48448806	Ca4	48448806	T/TC	TCTTGCTCAGGTGGCTTTTT	TTGATGAATTTGATATTGTTGTTGG	60.0	606	INTERGENIC	--	--	--
Ca4_P2_48448979	Ca4	48448979	TAAAAA/TAAAAAA	TTGTCGTTTGGGGGAAATTA	TGCTGGTGGGAATTTCTTTT	59.5	806	INTERGENIC	--	--	--
Ca4_P2_48449048	Ca4	48449048	GA/GAA	TTGTCGTTTGGGGGAAATTA	AGGGTCATCCAATGGTCATC	59.6	912	INTERGENIC	--	--	--
Ca4_P2_48453229	Ca4	48453229	ACTCTCTCTCTCTCTCTCTCTCT/ACTCTCTCTCTCTCTCTCTCTCT	GATGGCATTTGGAGTTTCGT	GTGTTCGGTGGTGGAAGAAT	59.8	282	INTERGENIC	--	--	--
Ca4_P2_48454259	Ca4	48454259	CTTTT/CTTT	TTGCTTTTCCTACCACCACC	CAGCAGAGTCCAATTCAGCA	60.1	376	INTERGENIC	--	--	--
Ca4_P2_48458532	Ca4	48458532	ACCCCC/ACCCC	TGCAGCATAGAGGACCTGTG	AAGAATACCATCACCCGACG	59.8	166	DRR	Ca_10747	--	--
Ca4_P2_48459552	Ca4	48459552	AT/ATT	TGGTTGGCAGTAAGATGCAA	CGTTTGTTTGACGAGCACAA	60.9	649	DRR	Ca_10747	--	--
Ca4_P2_48465591	Ca4	48465591	GAAAA/GAAA	ATAATGATCCCGGTTGGACA	GCCTTTCCTTGACAGTCTGC	60.0	699	INTRON	Ca_10746	LA	--
Ca4_P2_48465859	Ca4	48465859	GTTTTTTTTTTT/GTTTTTTTTTT	TGTGTGTCTCAGTGGTGGGT	GCCTTTCCTTGACAGTCTGC	60.0	619	INTRON	Ca_10746	LA	--
Ca4_P2_48467313	Ca4	48467313	TAG/TAGAG	CTATTTGCGTCTTTTCCCCA	ACGTGAACTTTTTGTTGCCC	60.0	202	INTRON	Ca_10746	LA	--
Ca4_P2_48471613	Ca4	48471613	ATTTT/ATTT	ATCCTCTCTCTGCGTGCATT	TATTGCACATCTGGGACCAA	59.9	921	INTRON	Ca_10746	LA	--

INDEL marker IDs	Chromosomes/unanchored scaffolds	Physical positions (bp)	InDels (*Kabuli* reference genome-CDC Frontier/PII)	Forward primers (5'-3')	Reverse primers (5'-3')	Anneling temperature (0C)	Expected amplified product size (bp)	Structural annotation		Functional annotation	
								Sequence components of *kabuli* genome	*Kabuli* gene accession IDs	NCBI-KOG	TFs
Ca4_P2_48479264	Ca4	48479264	T/TACG	AAACGAGTTTTCCATCTGCG	ACAACCTTCGCCACTCTCAC	60.3	105	INTRON	Ca_10745	L	bHLH
Ca4_P2_48481643	Ca4	48481643	ATTTTT/ATTTT	TGACCAGTCATGACAAAAATTAAA	GCTATTGGACTTCACTTGTACCAC	59.1	487	INTERGENIC	--	--	--
Ca4_P2_48483020	Ca4	48483020	GT/GTT	TTTGCAATGTTTCATGCGAT	TCTGGGTTGGATAAGCAAGG	60.1	775	INTERGENIC	--	--	--
Ca4_P2_4848593	Ca4	4848593	ATTTTTTTTT/ATTTTTTTT	TTTTGGCCCATGTCTTTTGT	TCAATTTGTTGAAAGTCACGATG	60.0	616	INTERGENIC	--	--	--
Ca4_P2_48494524	Ca4	48494524	AAGAG/AAG	GCATTCTGGCACAACAAAGA	ACTGGACCCCAAACCTCTCT	60.0	357	INTERGENIC	--	--	--
Ca4_P2_48497163	Ca4	48497163	AAATAAT/AAAT	ATGTTGCAAAACATGAGCCA	ACAAAATCGCCGATGGTAAC	59.8	768	INTRON	Ca_10742	V	GRAS
Ca4_P2_48497756	Ca4	48497756	GCTAC/GCTACTAC	AGGTGTGAGGGTTCGTCAAC	CTTCTTTGGGTCAGCCTCTG	60.0	337	CDS (large-effect mutations)	Ca_10742	V	GRAS
Ca4_P2_48506481	Ca4	48506481	C/CCGTT	ACATTTGGGCTCAGGATTTG	CGATACTTGCTGCTGGACAA	60.0	428	INTERGENIC	--	--	--
Ca4_P2_48506646	Ca4	48506646	T/TG	ACATTTGGGCTCAGGATTTG	CGATACTTGCTGCTGGACAA	60.0	428	INTERGENIC	--	--	--
Ca4_P2_48507473	Ca4	48507473	AT/ATATGTT	GGAATTCTGCACCCAACAAT	GCCCACTCCTATCTGACTCG	59.8	428	INTERGENIC	--	--	--
Ca4_P2_48514251	Ca4	48514251	ATTTTTTT/ATTTTTTTT	CGTGGAAGTGTTTTGGTTGA	TCACCTTCACCAGGGTTATTG	59.8	714	INTERGENIC	--	--	--
Ca4_P2_4851664	Ca4	4851664	CAGGAAAAGGAAAAGGAAAAGGAAAAGG/CAGGAAAAGGAAAAGGAAAAGG	TGGCATAGCAAACAGAGAAGG	GGCCAAATAAATGATGGCAC	60.2	311	INTERGENIC	--	--	--

INDEL marker IDs	Chromosomes/unanchored scaffolds	Physical positions (bp)	InDels (*Kabuli* reference genome-CDC Frontier/PII)	Forward primers (5'-3')	Reverse primers (5'-3')	Anneling temperature (0C)	Expected amplified product size (bp)	Structural annotation		Functional annotation	
								Sequence components of *kabuli* genome	*Kabuli* gene accession IDs	NCBI-KOG	TFs
Ca4_P2_48518860	Ca4	48518860	TATGTAATAAATTGAT/TAT	CATCAAGCTGTTCGTCATGC	ATCATCTTTGGCAGTAGCGG	60.2	287	INTRON	Ca_10740	--	C2H2
Ca4_P2_48520425	Ca4	48520425	TAAA/TAA	AGCATGGGTCACACACTCAA	CACCTTGTTTTGATGGAGCA	59.7	284	INTRON	Ca_10740	--	C2H2
Ca4_P2_48520759	Ca4	48520759	ATTT/ATT	AGCATGGGTCACACACTCAA	TCTCCACTATGTTCCGACCC	59.9	665	INTRON	Ca_10740	--	C2H2
Ca4_P2_48522137	Ca4	48522137	TTTATTATTATTATTATTATTATT/TTTATTATTATTATTATTATTATT	CATTGCAATCGGCCTCTATC	TCCCACTTTCACTTTGGGTC	59.9	657	DRR	Ca_10740	--	C2H2
Ca4_P2_4852416	Ca4	4852416	GAAAAAAA/GAAAAAAAA	TGGCTCCAACAACATCTTCA	ACCCCTTGGTCCAAACAAAT	60.5	549	INTERGENIC	--	--	--
Ca4_P2_48541135	Ca4	48541135	TAAAAAAAAAAAA/TAAAAAAAAA	AATTCCCACACGTACGCTTC	GATGTTCTTGGCTTGTGGCT	60.3	723	INTERGENIC	--	--	--
Ca4_P2_48575054	Ca4	48575054	TGGGG/TGGGGG	GCAACAATTGAATCAACCCC	CTTCATGAATTGCACCATGC	60.1	705	CDS (FRAME SHIFT)	Ca_23007	P	--
Ca4_P2_48575382	Ca4	48575382	TTATATATATATATATATATATATATA/TTATATATATATATATATATA	CCTGAGCCATTTGAAGAGGA	TTTCAGTTCATCGTGTCCCA	60.1	841	INTRON	Ca_23007	P	--
Ca4_P2_48576925	Ca4	48576925	AT/ATT	ATCTCACCCCAAGACAACCA	TCATTCCAGAAGGAGATGGG	60.0	211	INTRON	Ca_23007	P	--
Ca4_P2_48577228	Ca4	48577228	A/AC	AACACATCCGGGATCCATTA	TGATGACGAAGACGAAGACG	60.0	303	INTERGENIC	--	--	--
Ca4_P2_4859197	Ca4	4859197	GTCT/GT	TCCAGTTTTGGTTGCTGAGA	TAGGCATCACCGTTGTGTGT	60.0	737	INTRON	Ca_03773	--	--
Ca4_P2_4859261	Ca4	4859261	TATTAATTAATTAATTAAT/TATTAATTAATTAAT	TCCAGTTTTGGTTGCTGAGA	TAGGCATCACCGTTGTGTGT	60.0	737	INTRON	Ca_03773	--	--

INDEL marker IDs	Chromosomes/unanchored scaffolds	Physical positions (bp)	InDels (*Kabuli* reference genome-CDC Frontier/PII)	Forward primers (5'-3')	Reverse primers (5'-3')	Anneling temperature (0C)	Expected amplified product size (bp)	Structural annotation		Functional annotation	
								Sequence components of *kabuli* genome	*Kabuli* gene accession IDs	NCBI-KOG	TFs
Ca4_P2_48596162	Ca4	48596162	CATATATATATATATATATATATATATATATATA/CATATATATATATATATATATATATATATA	TGAGATTAATCGGTGGGAGC	CACTGCAAAATGTTGAAGCAA	59.9	425	INTERGENIC	--	--	--
Ca4_P2_48614082	Ca4	48614082	CAAAAAAAAAAA/CAAAAAAAAAAAA	TCACGTTTTCTCTCATCCCC	AGCCAACAAAAATGTGTCGT	58.1	599	INTERGENIC	--	--	--
Ca4_P2_48651388	Ca4	48651388	AACTTTTAAGAGTACTTTTA/AACTTTTA	AAAACCACTCCAGACATCGG	CTTGCAATACCCGCAAAGTT	60.1	682	DRR	Ca_23016	O	LBD
Ca4_P2_48651844	Ca4	48651844	CATAT/CAT	CTCCGGTGGCTAATTGTGAT	TTCAGCTTTGCGATTCATTG	60.0	442	DRR	Ca_23016	O	LBD
Ca4_P2_48662630	Ca4	48662630	ATTTTTTT/ATTTTTTTT	AACACATATGAATGCATCGAGTC	TGGCACCCGAAATGTCTAAT	60.3	399	INTERGENIC	--	--	--
Ca4_P2_48664644	Ca4	48664644	GTTTTTT/GTTTTTTTT	AAAGATCCCGTCAACCAGTTT	TCAAAGATTGGGATGAAGGC	60.0	328	INTERGENIC	--	--	--
Ca4_P2_48664816	Ca4	48664816	ATTTTTT/ATTTTT	AAAGATCCCGTCAACCAGTTT	TCAAAGATTGGGATGAAGGC	60.0	328	INTERGENIC	--	--	--
Ca4_P2_48665136	Ca4	48665136	AGG/AG	GCCTTCATCCCAATCTTTGA	CAAATGCAGTGTCCATCCAC	60.0	451	INTERGENIC	--	--	--
Ca4_P2_4866573	Ca4	4866573	GTTAAT/GTTAATATTGATTTAAT	TACCGGAAATTTCAACGAGG	CGTATTAGTAAACCCGTTGGG	58.4	430	INTERGENIC	--	--	--
Ca4_P2_4866591	Ca4	4866591	GTTT/GTT	TGGAGGGTACAAAAGCCAAC	CAAAAGTGAGGAGAGGTGAGTG	59.0	387	INTERGENIC	--	--	--
Ca4_P2_48673700	Ca4	48673700	GAAAAAAAAAA/GAAAAAAAAAAAA	TACCGAACATGCGGTGTAAG	TGACAAAAAGGAGAAGATAATGC	57.6	736	DRR	Ca_23017	--	--
Ca4_P2_48675604	Ca4	48675604	GTTTTTTTTT/GTTTTTTTT	AAAAGGAAATTGGCACGTTG	CACTTCATCATGCATCACCC	59.9	702	DRR	Ca_23018	--	LBD

INDEL marker IDs	Chromosomes/unanchored scaffolds	Physical positions (bp)	InDels (*Kabuli* reference genome-CDC Frontier/PII)	Forward primers (5'-3')	Reverse primers (5'-3')	Anneling temperature (0C)	Expected amplified product size (bp)	Structural annotation		Functional annotation	
								Sequence components of *kabuli* genome	*Kabuli* gene accession IDs	NCBI-KOG	TFs
Ca4_P2_48683487	Ca4	48683487	TAAA/TAAAA	GTGTTTGTGCGATGATTTGG	TGGCATCATAAATTTGTGTGTGTA	60.2	200	INTERGENIC	--	--	--
Ca4_P2_48687183	Ca4	48687183	TTTACTTA/TTTACTTACTTA	TTCATAATTGCTGCACCCTG	ATGGAACCCAATGATCCTGA	60.1	677	INTRON	Ca_23019	R	--
Ca4_P2_48689333	Ca4	48689333	TAT/TATAAT	AATACCTGGACAGTGGCTCG	TTTGCAGCTTGAGTGGCTTA	59.8	500	INTRON	Ca_23019	R	--
Ca4_P2_48689373	Ca4	48689373	CATAT/CAT	AATACCTGGACAGTGGCTCG	TTTGCAGCTTGAGTGGCTTA	59.8	500	INTRON	Ca_23019	R	--
Ca4_P2_48694227	Ca4	48694227	ATATTTT/AT	TGCTGCCACACCTAAACTTG	TAATCCATTGGCCCACATTT	60.0	678	INTERGENIC	--	--	--
Ca4_P2_48694298	Ca4	48694298	CTTTTTTTTT/CTTTTTTTTTT	TTACGTTGATTGAGGGGTGA	TAATCCATTGGCCCACATTT	60.0	296	INTERGENIC	--	--	--
Ca4_P2_48702506	Ca4	48702506	ATTTTTTTT/ATTTTTTT	TGTTGGAAGTGTTGGTGCAT	TTTGTCTTTCCCCTGTTTGC	60.1	493	INTRON	Ca_23020	Q	Nin-like
Ca4_P2_48708323	Ca4	48708323	TAAAAAAAA/TAAAAAAAAA	GCGCTAGAGGAACTGGATTG	TTACAAACTCAATGTCGCCG	59.7	563	DRR	Ca_23020	Q	Nin-like
Ca4_P2_48708348	Ca4	48708348	TAAAAAA/TAAAAAAA	GCGCTAGAGGAACTGGATTG	GGCGATGAATTGGTTTGTTT	59.8	944	DRR	Ca_23020	Q	Nin-like
Ca4_P2_48708820	Ca4	48708820	AC/ACTTGACCATTTC	CGGCGACATTGAGTTTGTAA	GCACGTGTTGAAATGTCTGC	60.3	630	DRR	Ca_23020	Q	Nin-like
Ca4_P2_48708876	Ca4	48708876	TTTTATTT/TTTT	CGCCACTTGTATTTCAAGCA	GCATCAGCTCTTCCAGCTTC	60.2	517	DRR	Ca_23020	Q	Nin-like
Ca4_P2_48711298	Ca4	48711298	TCACACACACA/TCACACACA	CTCTGGGCTGATCAGAAAGG	CACAAATGTGTGGCTGGAAC	60.0	732	INTRON	Ca_23021	U	--

INDEL marker IDs	Chromosomes/unanchored scaffolds	Physical positions (bp)	InDels (*Kabuli* reference genome-CDC Frontier/PII)	Forward primers (5'-3')	Reverse primers (5'-3')	Anneling temperature (0C)	Expected amplified product size (bp)	Structural annotation		Functional annotation	
								Sequence components of *kabuli* genome	*Kabuli* gene accession IDs	NCBI-KOG	TFs
Ca4_P2_48721018	Ca4	48721018	TAAAAAAAA/TAAAAAAA	CTTGGGCTTCTCCATATCCA	CTTTCCCACCTCCAGGGTAT	60.2	775	INTRON	Ca_23022	--	--
Ca4_P2_48721890	Ca4	48721890	ATTTTTT/ATTTTTTT	GACTCTCAGCATCACACGGA	GCAAGACGAATATGACCATGAA	60.0	723	INTERGENIC	--	--	--
Ca4_P2_48722275	Ca4	48722275	GTTTAATTTTATTTAATTT/GTTTAATTTTATTTAATTTTATTTAATTT	AGAACGGAAATTAGGAGGAGTT	TTCGAAAGTTGCAAGACGAA	59.6	210	INTERGENIC	--	--	--
Ca4_P2_48725595	Ca4	48725595	TAAAAAAAA/TAAAAAAA	AATAAACCGTAGGCCAGGCT	CTGGGACAACTTGTTTTGGG	60.4	798	INTERGENIC	--	--	--
Ca4_P2_48726138	Ca4	48726138	TAATG/T	CCCAAAACAAGTTGTCCCAG	TGTCGCAACTGGTTGATTGT	60.2	403	INTERGENIC	--	--	--
Ca4_P2_4873288	Ca4	4873288	AT/ATT	CACCACCCAAAGAAAGGAAA	CGGTGTGTGTGTGTGTGTGT	60.0	310	DRR	Ca_03771	Z	NAC
Ca4_P2_48733294	Ca4	48733294	ATTTTTTTTTTTT/ATTTTTTTTTTT	AGGACCAAAACGTTACCTGC	AGCGATAATGAAACGGATGC	60.1	763	INTERGENIC	--	--	--
Ca4_P2_48743012	Ca4	48743012	AAATAA/AAATAATAA	GTGCCACCTATCCTCCAGAA	TATGCGGTCAATTTCCTTCC	59.9	520	INTRON	Ca_23023	--	FAR1
Ca4_P2_4875193	Ca4	4875193	TAAAAAAAA/TAAAAAAA	TGCAAGCAAGAAACATTTGC	GCGTGAATTTTGTTGAACCTG	60.5	813	INTERGENIC	--	--	--
Ca4_P2_4875484	Ca4	4875484	GC/G	AGCCTATGGCTTGGCCTATT	GCCTAAGCTTGACTTGTGGC	60.0	687	INTERGENIC	--	--	--
Ca4_P2_48756667	Ca4	48756667	TAA/TAAA	GAAAAGGGAAATTAAATCAAAAGGA	TTTCTATTCTCGCACAGGGC	60.4	473	INTRON	Ca_23023	--	FAR1
Ca4_P2_48758212	Ca4	48758212	CTTTTTTT/CTTTTTT	ATATTGTGGGACCGATGGAA	AAAAGAATTGAGAAAGACGAAACA	58.5	895	INTRON	Ca_23023	--	FAR1

INDEL marker IDs	Chromosomes/unanchored scaffolds	Physical positions (bp)	InDels (*Kabuli* reference genome-CDC Frontier/PII)	Forward primers (5'-3')	Reverse primers (5'-3')	Anneling temperature (0C)	Expected amplified product size (bp)	Structural annotation		Functional annotation	
								Sequence components of *kabuli* genome	*Kabuli* gene accession IDs	NCBI-KOG	TFs
Ca4_P2_48758524	Ca4	48758524	TAAAAAAAA/TAAAAAAA	TGCCATATAACAAAAGACAGGTG	AAGCGACTAACATGATTTTCCA	58.8	711	INTRON	Ca_23023	--	FAR1
Ca4_P2_48758881	Ca4	48758881	TAAAAAAAA/TAAAAAAA	CATGGAAAATCATGTTAGTCGC	TTATGCAGCATGTCTCACGTC	59.9	462	INTRON	Ca_23023	--	FAR1
Ca4_P2_48759068	Ca4	48759068	TAAA/TAA	CATGGAAAATCATGTTAGTCGC	TTATGCAGCATGTCTCACGTC	59.9	462	INTRON	Ca_23023	--	FAR1
Ca4_P2_48766746	Ca4	48766746	TAAAA/TAAAAA	ATATCCAGCCGTGTTTGCAT	TTCTCCCCACCACTAACTCG	60.1	261	INTRON	Ca_23023	--	FAR1
Ca4_P2_48769675	Ca4	48769675	TAAA/TAAAAAAA	TTTCAATGAAGCCTAGTCCG	TTAAGGGTCCGCATGATTGT	60.3	605	INTRON	Ca_23023	--	FAR1
Ca4_P2_48769759	Ca4	48769759	A/AATC	TTTCAATGAAGCCTAGTCCG	TTAAGGGTCCGCATGATTGT	60.3	605	INTRON	Ca_23023	--	FAR1
Ca4_P2_48770140	Ca4	48770140	GTTTTTTT/GTTTTTT	TTTTGTTCACAATCATGCGG	TCTCCAAATGCAAACACTGC	59.8	498	INTRON	Ca_23023	--	FAR1
Ca4_P2_48772293	Ca4	48772293	GCAC/GCACAC	TGGGATTCTGCTCATCCTTC	TGTTTTTCCGTTTTCCAACC	59.8	697	DRR	Ca_23023	--	FAR1
Ca4_P2_48772701	Ca4	48772701	AT/A	GGTTGGAAAACGGAAAAACA	AAGAAAATCTAAACGCCAAGG	57.6	295	DRR	Ca_23023	--	FAR1
Ca4_P2_48772855	Ca4	48772855	T/TA	GGTTGGAAAACGGAAAAACA	CATTGGTCGGACTAACAACTCA	60.0	696	DRR	Ca_23023	--	FAR1
Ca4_P2_48775289	Ca4	48775289	TA/TAAAATTTGTAA	GATGGAGAGGGAGACGAACA	CTTTCAAATCTGTAGGGCCG	59.7	313	INTERGENIC	--	--	--
Ca4_P2_48780973	Ca4	48780973	AAATAA/AAA	TCGGCATTTTCATTGTCTCA	TGTGTACCGACCGATGTTTG	60.4	190	INTERGENIC	--	--	--

INDEL marker IDs	Chromosomes/unanchored scaffolds	Physical positions (bp)	InDels (*Kabuli* reference genome-CDC Frontier/PII)	Forward primers (5'-3')	Reverse primers (5'-3')	Anneling temperature (0C)	Expected amplified product size (bp)	Structural annotation		Functional annotation	
								Sequence components of *kabuli* genome	*Kabuli* gene accession IDs	NCBI-KOG	TFs
Ca4_P2_48781102	Ca4	48781102	TAT/TATGAT	TCGGCATTTTCATTGTCTCA	TGTGTACCGACCGATGTTTG	60.4	190	INTERGENIC	--	--	--
Ca4_P2_48781270	Ca4	48781270	AG/AGG	CAAACATCGGTCGGTACACA	GGAGGCAGGAAGTGAGATGA	60.3	596	INTERGENIC	--	--	--
Ca4_P2_48783103	Ca4	48783103	CTTTTTTTTT/CTTTTTTTTTTT	TGGTTGGAAATTTTTGAATGC	TACGCATGAGATTTCGCTCA	60.5	245	INTERGENIC	--	--	--
Ca4_P2_48783727	Ca4	48783727	AT/A	TGAGCGAAATCTCATGCGTA	TGAGATGGGAAATATAGCTGCTC	59.7	899	INTERGENIC	--	--	--
Ca4_P2_48784802	Ca4	48784802	ATT/AT	CCGGAGGAGGTTGCTAGAAT	ATCTTGGGGTAGTTGATGCG	60.0	419	INTERGENIC	--	--	--
Ca4_P2_48792291	Ca4	48792291	CTTTTTTTTTT/CTTTTTTTTT	AAACTGCGGGTATGAGGATG	TGGATTTGATGACATTTGCATT	60.2	572	INTERGENIC	--	--	--
Ca4_P2_48793498	Ca4	48793498	TA/T	ACGGAGAGTTTGGGTGTGAC	GTTATTCCACCGCGAGAAAA	60.1	451	INTERGENIC	--	--	--
Ca4_P2_48793555	Ca4	48793555	TTCTCTCTCTCTCTCTCTC/TTCTCTCTCTCTCTCTCTC	ACGGAGAGTTTGGGTGTGAC	GTTATTCCACCGCGAGAAAA	60.1	451	INTERGENIC	--	--	--
Ca4_P2_48794556	Ca4	48794556	CATT/C	TTGAAGATTGTGCTACCCGA	TTTATCCCTCCGTCTCAAAA	57.3	392	INTERGENIC	--	--	--
Ca4_P2_48797593	Ca4	48797593	ATTTTTTT/ATTTTTT	ATATTCAATGCGCCACCAAT	AAACGGTTGCATAAAAACGC	60.0	489	INTERGENIC	--	--	--
Ca4_P2_48798808	Ca4	48798808	TTATAT/TTATATAT	AACGAAAGAGAAATGGAATATGAA	ATGCTGCAAAGAACAACGTG	59.9	534	INTERGENIC	--	--	--
Ca4_P2_48798961	Ca4	48798961	ACGTC/AC	AACGAAAGAGAAATGGAATATGAA	ATGCTGCAAAGAACAACGTG	59.9	534	INTERGENIC	--	--	--

INDEL marker IDs	Chromosomes/unanchored scaffolds	Physical positions (bp)	InDels (*Kabuli* reference genome-CDC Frontier/PII)	Forward primers (5'-3')	Reverse primers (5'-3')	Anneling temperature (0C)	Expected amplified product size (bp)	Structural annotation		Functional annotation	
								Sequence components of *kabuli* genome	*Kabuli* gene accession IDs	NCBI-KOG	TFs
Ca4_P2_48799249	Ca4	48799249	TATGTTT/TATGTTTAATGTTT	TGAAATCCGCAGAAAATATGC	ATGCTGCAAAGAACAACGTG	59.9	246	INTERGENIC	--	--	--
Ca4_P2_48799420	Ca4	48799420	ATTTT/ATTT	CACGTTGTTCTTTGCAGCAT	TTGATCAGCACTTGGTTGTG	58.2	266	INTERGENIC	--	--	--
Ca4_P2_48809672	Ca4	48809672	GAAAAAAA/GAAAAAAAA	GCGAGCGAATTAAACTCACC	GGGAACATGACTTGGTTCGT	59.8	190	INTERGENIC	--	--	--
Ca4_P2_48813549	Ca4	48813549	GTTTTT/GTTTTTT	TGCCTTTGTTGATCCCATTT	CCCAAAGTCGATGGCTAAAG	59.7	324	INTERGENIC	--	--	--
Ca4_P2_48815632	Ca4	48815632	CTTTTTTTTTTT/CTTTTTTTTTTTTTT	GTGTCAATGGTCGACGCTAA	TGTGCAACGTCACCATTTTT	60.0	569	INTERGENIC	--	--	--
Ca4_P2_48818516	Ca4	48818516	TAAAAAAA/TAAAAAAAA	CCCGAAAGGCATATGAAAGA	CAACGATGTTGAAAGCCTCA	59.8	218	INTERGENIC	--	--	--
Ca4_P2_48824466	Ca4	48824466	CAAAAAAAAAAAAA/CAAAAAAAAAAA	TTTTGCTCGTTCTGTCATGG	GAATTTGGGTCCGAATCTTG	59.4	416	INTERGENIC	--	--	--
Ca4_P2_48824681	Ca4	48824681	ATTTTTTT/ATTTTTT	TTTTGCTCGTTCTGTCATGG	GAATTTGGGTCCGAATCTTG	59.4	416	INTERGENIC	--	--	--
Ca4_P2_48824745	Ca4	48824745	ATTTTTTTT/ATTTTTTT	TTTTGCTCGTTCTGTCATGG	GAATTTGGGTCCGAATCTTG	59.4	416	INTERGENIC	--	--	--
Ca4_P2_48825853	Ca4	48825853	TTAAAAATAAAAA/TTAAAAA	GATTGCGACCGGAAGATTTA	AAAATATCCCATGCCAATGC	59.6	509	INTERGENIC	--	--	--
Ca4_P2_48827857	Ca4	48827857	ATTTTTTT/ATTTTTTTT	TAGTCGGGTGTGGGGTAGAG	AGTACGACCAAAACGGTTGC	60.0	837	INTRON	Ca_23025	--	FAR1
Ca4_P2_48831208	Ca4	48831208	GTATATA/GTATATATA	TATTTCCCAAGACCTCGTGC	ATGTGGTTGTTGTTGCCTGA	60.0	255	INTERGENIC	--	--	--

INDEL marker IDs	Chromosomes/unanchored scaffolds	Physical positions (bp)	InDels (*Kabuli* reference genome-CDC Frontier/PII)	Forward primers (5'-3')	Reverse primers (5'-3')	Anneling temperature (0C)	Expected amplified product size (bp)	Structural annotation		Functional annotation	
								Sequence components of *kabuli* genome	*Kabuli* gene accession IDs	NCBI-KOG	TFs
Ca4_P2_48833902	Ca4	48833902	TAAAAAAAAAAA/TAAAAAAAAA	CGATTTTTCCGTGTCGTTTT	GAACACACCAAAAGCTCCGT	60.2	694	DRR	Ca_23026	--	FAR1
Ca4_P2_48833938	Ca4	48833938	GAA/GAAA	CGATTTTTCCGTGTCGTTTT	GAACACACCAAAAGCTCCGT	60.2	694	DRR	Ca_23026	--	FAR1
Ca4_P2_48836352	Ca4	48836352	CTTTTTT/CTTTTT	ATGGGCTTGCAAAGAGAGAG	AAGACGAACCATGAACCCAA	60.4	446	INTERGENIC	--	--	--
Ca4_P2_4884278	Ca4	4884278	CAAA/CAA	CTCCAGTTTTTCTTGCAGCC	CCCCTGATCTTTCTTTTTGG	58.6	413	INTRON	Ca_03769	Z	NAC
Ca4_P2_48846764	Ca4	48846764	ACATCATCATCATCA/ACATCATCATCA	GGTAAACGTGTGTTGCATGG	ACAGCAGTAGCCCTTTGAGC	59.6	715	INTERGENIC	--	--	--
Ca4_P2_48847771	Ca4	48847771	ATTTTTTT/ATTTTTTTT	GAAATTTGGACGAAAACGGA	GTGTATATACGCGCACCCAA	59.5	707	INTERGENIC	--	--	--
Ca4_P2_48849335	Ca4	48849335	TATAACAAT/TATAACAATAATAACAAT	TTGGGTGTATAATGATAGCGAAA	GAGGGACTAGCTACCCCGAT	59.6	441	INTERGENIC	--	--	--
Ca4_P2_48850057	Ca4	48850057	GA/GATCA	GCATCAATATGAATCACCAATCA	TGACGATTTTATTTACGCCAA	58.2	857	INTERGENIC	--	--	--
Ca4_P2_48850821	Ca4	48850821	CTTTTTTTT/CTTTTTTTTTTT	CACCACATAGTAAAACTTGGCG	TGGTTTAGAGGGGTTGATGTG	59.8	633	INTERGENIC	--	--	--
Ca4_P2_48851253	Ca4	48851253	ATTTTTTTTT/ATTTTTTTTTT	CACATCAACCCCTCTAAACCA	TGCTCCTTGTTACGGTGGAT	60.5	496	INTERGENIC	--	--	--
Ca4_P2_4888138	Ca4	4888138	CTT/CT	AAAAGCCTCACCAGGGATCT	TGCCATCTTTCTTGCTGTTG	60.0	698	INTRON	Ca_03769	Z	NAC
Ca4_P2_4890840	Ca4	4890840	TTTAGAAGTCTT/TTTAGAAGTCTTAGAAGTCTT	AACTGGATTTGACTGCACCC	TGATGGAACGATACAAGGCA	60.1	459	INTRON	Ca_03769	Z	NAC

INDEL marker IDs	Chromosomes/unanchored scaffolds	Physical positions (bp)	InDels (*Kabuli* reference genome-CDC Frontier/PII)	Forward primers (5'-3')	Reverse primers (5'-3')	Anneling temperature (0C)	Expected amplified product size (bp)	Structural annotation		Functional annotation	
								Sequence components of *kabuli* genome	*Kabuli* gene accession IDs	NCBI-KOG	TFs
Ca4_P2_4891133	Ca4	4891133	AG/A	AACTGGATTTGACTGCACCC	TGATGGAACGATACAAGGCA	60.1	459	INTRON	Ca_03769	Z	NAC
Ca4_P2_4891737	Ca4	4891737	AATTATTATTATTATTATT/AATTATTATTATTATTATT	TGCCTTGTATCGTTCCATCA	TTCCTGCTTTTTCTTTCCGA	59.9	540	INTRON	Ca_03769	Z	NAC
Ca4_P2_48921723	Ca4	48921723	TTATTA/TTATTACTATTA	AGGATGAAAATAGGCCCGAC	AGCTTACCAAGCAGGCCATA	59.9	606	INTERGENIC	--	--	--
Ca4_P2_4894281	Ca4	4894281	TATATAGATA/TATA	CTCTGTCCTTCAACTTCGGC	AATTCCCTTTCACCTTCGCT	60.1	536	DRR	Ca_03768	K	--
Ca4_P2_4894807	Ca4	4894807	TAAAAAAA/TAAAAAA	GACTTGGAGCGAAGGTGAAA	GCAATGTTGCGAGAACAAGA	60.0	705	DRR	Ca_03768	K	--
Ca4_P2_4894873	Ca4	4894873	TAAAAAAAA/TAAAAAAA	GACTTGGAGCGAAGGTGAAA	GCAATGTTGCGAGAACAAGA	60.0	705	DRR	Ca_03768	K	--
Ca4_P2_4895586	Ca4	4895586	TACACA/TACA	CCAACCGCTAACTGACCAAT	CGTGGAGGAAGCTCTAGTGG	60.0	559	DRR	Ca_03768	K	--
Ca4_P2_4897344	Ca4	4897344	ATTT/ATTTT	CTCTGCAGCCTTGATTGGTT	ATGGAGTATGGGGCTGTGAC	59.8	278	INTRON	Ca_03768	K	--
Ca4_P2_48975370	Ca4	48975370	TGG/TGGG	TGGTTGGGATTTTTGGGTTA	GACCCTCTCCTCTCAATCCC	60.0	672	INTERGENIC	--	--	--
Ca4_P2_48978055	Ca4	48978055	ATTTTTTTT/ATTTTTTT	AGGTTTGGATGAATGTGATGG	TCGAACTTTGTTTGCAACAATTA	59.7	311	DRR	Ca_20768	O	--
Ca4_P2_49008742	Ca4	49008742	CTTCTCAATTCTCAATTCTCAATTCTCAATTCTCAATTCTCAATTC/CTTCTCAATTCTCAATTCTCAATTCTCAATTCTCAATTCTCAATTC	GGGACAAAAATGAGGCGTTA	TTTTGATCACGTTCCTGCTG	59.8	639	INTERGENIC	--	--	--
Ca4_P2_49053607	Ca4	49053607	AATATATATATATATATATATATATATA/AATATATATATATATATATATATATATATA	AGAAAATTGCATTTGGCCTG	TGCTATTGTATGGCGCTCTG	60.0	261	INTRON	Ca_20775	O	--

INDEL marker IDs	Chromosomes/unanchored scaffolds	Physical positions (bp)	InDels (*Kabuli* reference genome-CDC Frontier/PII)	Forward primers (5'-3')	Reverse primers (5'-3')	Anneling temperature (0C)	Expected amplified product size (bp)	Structural annotation		Functional annotation	
								Sequence components of *kabuli* genome	*Kabuli* gene accession IDs	NCBI-KOG	TFs
Ca4_P2_49073579	Ca4	49073579	GTT/GT	GTGACTTGGAGGGAGTGGAA	ATCCGCTAACCAATCACAGC	60.1	453	INTERGENIC	--	--	--
Ca4_P2_49074099	Ca4	49074099	ATTTTTTTTTT/ATTTTTTTTT	GCTGTGATTGGTTAGCGGAT	CGACGCGGGTTACACACTA	60.7	694	INTERGENIC	--	--	--
Ca4_P2_49100863	Ca4	49100863	GAAAAAAAAA/GAAAAAAAAAA	TCATCAAATTCGTAACGCAAA	GGAGGAAGGAGAGAGGGAGA	59.9	542	INTERGENIC	--	--	--
Ca4_P2_49100951	Ca4	49100951	GA/GAAGATA	TCATCAAATTCGTAACGCAAA	GGAGGAAGGAGAGAGGGAGA	59.9	542	INTERGENIC	--	--	--
Ca4_P2_49102686	Ca4	49102686	TTATATATATATATATATATATATAT/TTATATATATATATATATATATATATATATATAT	TCGCCCTTTATTGATTTGAGA	CAATCGATGCATGCAAATTA	58.2	243	INTERGENIC	--	--	--
Ca4_P2_49121831	Ca4	49121831	TTATATATATATATATAT/TTATATATATATATATATAT	CCAAAAATCTGCCAAGAACG	TGGGGTGTAAAGTGTGTGGA	59.8	491	INTERGENIC	--	--	--
Ca4_P2_49135046	Ca4	49135046	ATATTATTATTATTATTATTATTATTATTATTATTATTATTATTATTATTATTATTATTATTATTATTATTATTAT/ATATTATTATTATTATTATTATTATTATTATTATTATTATTATTATTATTATTATTATTATTATTATTATTATTATTATTATTATTATTAT	TATAAAGCGGTTTGGTTCGG	ATCAACACAACATCCACCCA	59.7	938	INTERGENIC	--	--	--
Ca4_P2_4978107	Ca4	4978107	AAATAAATAA/AAA	GTCTAATCCCCCATTTTGGC	TCTTGTGGTCGTTGCTTGAG	60.0	557	INTRON	Ca_03758	R	--
Ca4_P2_5023916	Ca4	5023916	TTATATATATATATATATATATAT/TTATATATATATATATATAT	AAAACAGAGGGCAAGGGACT	CCATTGTGGGCTCAACTTTT	60.0	892	INTERGENIC	--	--	--
Ca4_P2_5028595	Ca4	5028595	GTTTTTTTTTT/GTTTTTTTTTTT	TGATAAAAGGGGGAAAATCC	CGCATCCCCAGATCTAACAT	59.9	163	INTERGENIC	--	--	--
Ca4_P2_5038864	Ca4	5038864	CATATATATATATATATATATATAT/CATATATATATATATATATATATATAT	CCTTAACAGCGGAGAATCTGA	AAGAGATGGCACAGTGGGAC	60.1	509	INTERGENIC	--	--	--
Ca4_P2_5081255	Ca4	5081255	GATTATTATTATTATTATTATTATTATTAT/GATTATTATTATTATTATTATTATTATTATTAT	CACCAGCAATAGCTTGCTCA	GAAAAACAGGGCTAGCAACG	59.9	711	CDS (large-effect mutations)	Ca_03748	K	Trihelix

INDEL marker IDs	Chromosomes/unanchored scaffolds	Physical positions (bp)	InDels (*Kabuli* reference genome-CDC Frontier/PII)	Forward primers (5'-3')	Reverse primers (5'-3')	Anneling temperature (0C)	Expected amplified product size (bp)	Structural annotation		Functional annotation	
								Sequence components of *kabuli* genome	*Kabuli* gene accession IDs	NCBI-KOG	TFs
Ca4_P2_5093283	Ca4	5093283	CTTTTTTTTTT/CTTTTTTTTTTT	TGCTTTTGAAAACCCTCTCC	ACAGACAACCGCATTTTTCC	60.0	687	DRR	Ca_03746	S	--
Ca4_P2_5116820	Ca4	5116820	TCAGCAGGNNNNNNNNNNNCAGCAGG/TCAGCAGG	GGATAAGGTTGCACCTTCCA	ATGCCAACATTTTGAGGAGC	60.1	143	INTRON	Ca_03742	Q	--
Ca4_P2_5190022	Ca4	5190022	TTATATATATATATATATATATATATATATATATATATATA/TTATATATATATATATATATATATATATATATATATA	ATTCAATCATGTTCCCCCAA	TTTATTTGCGCATTGACCCT	60.5	716	DRR	Ca_03734	E	--
Ca4_P2_5268963	Ca4	5268963	T/TC	GCTGCCAGATAAAGTGGGAC	GAGAGTGACGATGGATGCAA	59.8	229	INTERGENIC	--	--	--
Ca4_P2_5300098	Ca4	5300098	TAAAAAAAAAA/TAAAAAAAAA	GGCTGGAGAAAGAATATGCG	AAAAGGAGTTTGGAAGGGGA	59.9	598	INTERGENIC	--	--	--
Ca4_P2_5326448	Ca4	5326448	TTTATTATTATTATTATTATTATTATTATTATTATTATTATTATTATTATTATTATTATTAT/TTTATTATTATTATTATTATTATTATTATTATTATTATTATTATTATTATTATTATTATTATTATTAT	TTCTGGTAATTTTGTAAACATGACTG	AGCCTCAGTAGTTTGGGCCT	60.3	642	INTERGENIC	--	--	--
Ca4_P2_533246	Ca4	533246	TAAAAAAAA/TAAAAAAAAA	TGGTTTGATTTCACAAGCCAT	GAGTTAGCCACACCCCTGAA	60.1	493	INTERGENIC	--	--	--
Ca4_P2_5525661	Ca4	5525661	GAAAAAAAAAA/GAAAAAAAAA	TATTTAGCCAAAAGGGCAGC	TCCATGTGAATATGATGAACCAA	60.1	320	INTERGENIC	--	--	--
Ca4_P2_5570234	Ca4	5570234	CTTTTTTTTTT/CTTTTTTTTTTT	TGATGCATAAGTACAGGTGGG	AAGTTTTTGGCTTTAAATAGTGTCA	57.7	581	INTERGENIC	--	--	--
Ca4_P2_5574077	Ca4	5574077	TC/T	CCTGGTGAAAAACATCGCTT	CCATATGTATACCGGACGGC	60.1	585	DRR	Ca_03698	K	NF-YC
Ca4_P2_5618500	Ca4	5618500	CTTTTTTTTTT/CTTTTTTTTTTT	AAGAAATCCATTAGATGTTTTCCG	ATGGCAAAACATCAGGAACA	59.0	213	INTERGENIC	--	--	--
Ca4_P2_5622020	Ca4	5622020	TCC/TCCC	TTGATGGCGATCGTGTATGT	ATCCTGGAGGGAGGAAAAGA	60.0	150	INTRON	Ca_03688	V	--

INDEL marker IDs	Chromosomes/unanchored scaffolds	Physical positions (bp)	InDels (*Kabuli* reference genome-CDC Frontier/PII)	Forward primers (5'-3')	Reverse primers (5'-3')	Anneling temperature (0C)	Expected amplified product size (bp)	Structural annotation		Functional annotation	
								Sequence components of *kabuli* genome	*Kabuli* gene accession IDs	NCBI-KOG	TFs
Ca4_P2_5625956	Ca4	5625956	TTGT/TTGTGT	AGGTTGGCTTGTTGGTTGTC	TAGCCAGACGCATTCTTCCT	60.0	716	INTRON	Ca_03688	V	--
Ca4_P2_5642060	Ca4	5642060	TTATATATATATATATATATATATATATAT/TTATATATATATATATATATATATATATATATAT	TGGTGGTTGTTGTCTCCTCA	CACCATGATTGGGGGATAAG	60.0	463	INTERGENIC	--	--	--
Ca4_P2_5655244	Ca4	5655244	TATA/TATAAATA	TGAACCCCCACCAAAATAAA	GCACCGCTCAAAATGAAATA	58.8	928	DRR	Ca_03683	R	--
Ca4_P2_5707384	Ca4	5707384	AATATATATATATATATAT/AATATATATATATATATAT	AAGCTAAGCACCGGTAACGA	TTCACGTCTTGAAATTTTCCG	60.1	711	DRR	Ca_03679	DKL	MYB_related
Ca4_P2_5808899	Ca4	5808899	AATATATATATATATATATATATATATATATATATATA/AATATATATATATATATATATATATATATATA	CTACCTGTGCGAATTGGGAT	TGATGTGGCAATGATGACAA	59.5	765	INTERGENIC	--	--	--
Ca4_P2_5913287	Ca4	5913287	CTTTTTTTTTT/CTTTTTTTTT	TTCAAAATCTCATGACGACCC	TTTTTATAAAGTTTTCCGAAACTCG	59.2	785	INTRON	Ca_03658	--	--
Ca4_P2_5960854	Ca4	5960854	GAAAAAAAAA/GAAAAAAAAAA	AAGGTAGGTGGCATTGTCTTG	TGCTCTCATCCAAATCCCTT	59.6	997	INTERGENIC	--	--	--
Ca4_P2_5961971	Ca4	5961971	T/TGTG	CCTCTAATATCGTCGCTCCAA	ATAATCGGTGCGGATTTCCT	60.7	668	INTERGENIC	--	--	--
Ca4_P2_6145049	Ca4	6145049	ATTTTTTTTTTTT/ATTTTTTTTTTTTT	ATCCCCTCAGAATCGGCTAT	TTCGTCTATGCATTTGTTCCTG	60.1	565	INTERGENIC	--	--	--
Ca4_P2_6186297	Ca4	6186297	GTTTTTTTTT/GTTTTTTTTTT	TGTGTTTTCTCAATTTGACGATG	GCCATTTGAACCAAATTAAGAG	57.8	751	INTERGENIC	--	--	--
Ca4_P2_6200192	Ca4	6200192	GAAAAAAAAAAA/GAAAAAAAAAAAA	ATTTGGCAAACTTTGATGCC	CTTTGCAACTTTCCCCGTTA	60.1	296	INTERGENIC	--	--	--
Ca4_P2_6271908	Ca4	6271908	GTATATATATATATATATAT/GTATATATATATATATATATAT	AAAAATTACGGGATGCATAAAAA	TCCCCAAACAATGACAAAAA	58.8	775	INTERGENIC	--	--	--

INDEL marker IDs	Chromosomes/unanchored scaffolds	Physical positions (bp)	InDels (*Kabuli* reference genome-CDC Frontier/PII)	Forward primers (5'-3')	Reverse primers (5'-3')	Anneling temperature (0C)	Expected amplified product size (bp)	Structural annotation		Functional annotation	
								Sequence components of *kabuli* genome	*Kabuli* gene accession IDs	NCBI-KOG	TFs
Ca4_P2_6389441	Ca4	6389441	TT/TTCCTTATGTGGAAAT	GCCCCACCTTCAGTGTTAAA	ATGCTGGCAAATGATGTCAC	59.5	728	DRR	Ca_03602	--	ERF
Ca4_P2_6424106	Ca4	6424106	CAAAAAAAAAA/CAAAAAAAAA	GGGACCGAGATTTCATGTGT	GGCCACAAAATGTTGGATCT	59.8	762	INTERGENIC	--	--	--
Ca4_P2_6519306	Ca4	6519306	TAAAAAAAAA/TAAAAAAAAAA	TGTGGAGTGATTTTGAGGGA	CCCTCGAAGTTACTAAAGCGG	60.3	720	INTERGENIC	--	--	--
Ca4_P2_6538330	Ca4	6538330	ATTTTTTTTTTTTT/ATTTTTTTTTTTT	ATGCGACCTCCTGAAGAAGA	CGACCTAAACATACATTTTGCG	59.6	317	INTERGENIC	--	--	--
Ca4_P2_6581955	Ca4	6581955	TAAAAAAAA/TAAAAAAA	GCACGTGTGTAATGGTGCTC	TCTTGCTCAACGAAAATCCC	60.2	612	INTERGENIC	--	--	--
Ca4_P2_6596665	Ca4	6596665	TAAAAAAAAAAAAA/TAAAAAAAAAAAA	GAAGTTGCAAGTATTGGGGC	TTGGGCAAGATAAAAATCCG	59.9	405	INTERGENIC	--	--	--
Ca4_P2_664091	Ca4	664091	TTATATATATATATATATATAT/TTATATATATATATATATATATAT	AGGTGGTGGTGTTGGAAGAG	TTCACGTAAAAACCGAACAAAA	59.5	550	INTERGENIC	--	--	--
Ca4_P2_6699109	Ca4	6699109	TT/TTCTTGTCAGT	AAGCGAAAATGGTTGGATCA	TGCGTGCGTATAGGCTATTTT	59.8	172	CDS (large-effect mutations)	Ca_03576	--	WRKY
Ca4_P2_6703411	Ca4	6703411	GTTATTATTATTATTATTATTATTATTA/GTTATTATTATTATTATTATTATTATTATTA	GGGCCTAGTAGGAGGTTGCT	TGATTGGTAATGTGGCAAAA	57.5	604	INTERGENIC	--	--	--
Ca4_P2_6768891	Ca4	6768891	TTATATATATATATATATATATATAT/TTATATATATATATATATATAT	ACAACCATCCTACCAGCCAG	CAGCAACATGCCAAAAGAAA	59.8	636	INTERGENIC	--	--	--
Ca4_P2_6772676	Ca4	6772676	TTATATATATATATATATATATATAT/TTATATATATATATATATATATATATATATATAT	GGTACTTGGAGCCACATGCT	CCAAGAACCAATCCTCCAAA	59.9	895	INTERGENIC	--	--	--
Ca4_P2_6783863	Ca4	6783863	GAAAAAAAAAA/GAAAAAAAAAAA	TGGACAGAGAAGGGAGGAGA	AGCAAATTTCCAACCCAATG	59.8	435	INTERGENIC	--	--	--

INDEL marker IDs	Chromosomes/unanchored scaffolds	Physical positions (bp)	InDels (*Kabuli* reference genome-CDC Frontier/PII)	Forward primers (5'-3')	Reverse primers (5'-3')	Anneling temperature (0C)	Expected amplified product size (bp)	Structural annotation		Functional annotation	
								Sequence components of *kabuli* genome	*Kabuli* gene accession IDs	NCBI-KOG	TFs
Ca4_P2_6875620	Ca4	6875620	TGG/TG	GACAGTTTTGTGCAATCCGA	AACCTATACCCGCACAATGC	59.9	859	INTERGENIC	--	--	--
Ca4_P2_6899661	Ca4	6899661	ATTTTTTTT/ATTTTTTT	AACCTTTGCAGAAATTCCGA	AGAAATTGTCCCACCATTCG	59.8	395	INTERGENIC	--	--	--
Ca4_P2_6907181	Ca4	6907181	TTATATATATATATATAT/TTATATATATATATAT	TTTGAAATGGCGACTTCCTT	CCAAATGGCAACTTCCAACT	60.0	569	INTERGENIC	--	--	--
Ca4_P2_6907343	Ca4	6907343	TTATATATATATATATATATAT/TTATATATATATATATATAT	TTTGAAATGGCGACTTCCTT	CCAAATGGCAACTTCCAACT	60.0	569	INTERGENIC	--	--	--
Ca4_P2_6965916	Ca4	6965916	AATATATATATATATAT/AATATATATATATATATAT	TGTTTGGACTGCTTTACCCC	CCCTCCAAACTAAAATTTTCTCC	59.4	584	INTERGENIC	--	--	--
Ca4_P2_6992675	Ca4	6992675	AAGAGNNNNNNNNNNNNAGAG/AAGAG	ACTTTGCGTGTCGATAACCC	GTTCGATTCAGCCAGCAAAT	60.2	266	INTERGENIC	--	--	--
Ca4_P2_7029739	Ca4	7029739	AATATATATATATATATATATATAT/AATATATATATATATATATATATATATATATATATATAT	GCGAAAGTAGTCCCTCCATTT	AATCAAATGCAAGGTGTGGC	60.9	669	INTERGENIC	--	--	--
Ca4_P2_7089091	Ca4	7089091	GA/GAACAAAAGAGATTCACA	CTTTGCATACCCCTCTCGAC	TTGGGAGGATTGGTTTCAAG	59.9	644	INTERGENIC	--	--	--
Ca4_P2_7110091	Ca4	7110091	ACT/ACTCT	GTTGCACTGCAGCATCAAAT	AGAAGACTAGTCGGCGAACG	59.6	513	DRR	Ca_03526	--	--
Ca4_P2_7127718	Ca4	7127718	GAAAAAAAAAA/GAAAAAAAAA	AATTTGGACAATACCGCGAA	AGGGAAGGGGAAGAAGATCA	60.0	580	INTERGENIC	--	--	--
Ca4_P2_724084	Ca4	724084	TTATATATATATATATATATATATAT/TTATATATATATATATATATATATATATATATATAT	TGCAAGGGAAGTTTTTGTCTT	ACGAGCACGATGGGATAGAG	60.2	559	INTERGENIC	--	--	--
Ca4_P2_7282104	Ca4	7282104	ATATTATTATTATTATTATTATTATTATTATTA/ATATTATTATTATTATTATTATTATTATTATTATTATTA	CCCAACCCAAAAAGATGAGA	ACGGGTTATGCCTCATTTCA	60.3	764	DRR	Ca_03507	--	--

**INDEL marker IDs**	**Chromosomes/unanchored scaffolds**	**Physical positions (bp)**	**InDels (*Kabuli* reference genome-CDC Frontier/PII)**	**Forward primers (5'-3')**	**Reverse primers (5'-3')**	**Anneling temperature (0C)**	**Expected amplified product size (bp)**	**Structural annotation**		**Functional annotation**	
								**Sequence components of *kabuli* genome**	***Kabuli* gene accession IDs**	**NCBI-KOG**	**TFs**
Ca4_P2_7326848	Ca4	7326848	GAAAAAAAAAAAAAA/GAAAAAAAAAAAAAA	TTGGAATGCGTTTTTCCTTC	CGGTGGTTTTGGTTCATTCT	59.8	489	DRR	Ca_03504	R	NAC
Ca4_P2_7388463	Ca4	7388463	CAAAAAAAAAAA/CAAAAAAAAAA	TTTTCTCTAATCCCCGACTCA	TGTTGTTGTCAAACACCTCAAA	59.1	565	INTERGENIC	--	--	--
Ca4_P2_7415395	Ca4	7415395	TAA/TA	TTTCTGTCCTCACCCTCACC	TGTAGGTTTGGACGGTAGGC	60.0	535	INTRON	Ca_03496	F	YABBY
Ca4_P2_7452911	Ca4	7452911	A/ACC	TGCCAACTCGATTGAACAAG	GCACGCTAAAAGCCTGAAAC	60.0	364	INTERGENIC	--	--	--
Ca4_P2_7485052	Ca4	7485052	TC/TCGCTACGGGAAGGCAACC	CGTACACATTCCGACGATTG	ACAGGGTTAATTGAGGGGGT	59.6	643	INTERGENIC	--	--	--
Ca4_P2_7485913	Ca4	7485913	TTAATAATAATAATAATAATAATAA/TTAATAATAATAATAATAATAATAATAA	ACACGTTTTGTTTGTGGCAA	TCATTGACCAAATTTCCGGT	60.2	270	INTERGENIC	--	--	--
Ca4_P2_7503468	Ca4	7503468	AATATATATATATATATATATATATATATATATA/AATATATATATATATATATATATATATATATATATATATATATATA	TGTCCAAGTTGCAACACCAT	TAAAGACCTGGAAACCACGG	60.0	690	INTERGENIC	--	--	--
Ca4_P2_7553868	Ca4	7553868	CAAAAAAAAAAAAA/CAAAAAAAAAAAAAA	GGAGGGGTGGGAAGTAAAAA	ATGATGAGATTGAGGAGCGG	60.2	739	INTERGENIC	--	--	--
Ca4_P2_7573692	Ca4	7573692	TTATATATATATATATATATATATATA/TTATATATATATATATATATATATATA	TTGCAGACGGGACAACATAA	AAACCCAACCGGTTCCTAAC	60.1	865	INTERGENIC	--	--	--
Ca4_P2_7620418	Ca4	7620418	GATATATATATAT/GATATATATATATAT	CGATTTTTGTGAAGTGCGTC	TCTCATCGCCGACAAAGTAA	59.4	837	INTERGENIC	--	--	--
Ca4_P2_7710147	Ca4	7710147	AATATTATTATATATTA/AATATTATTATATATTATTATATATTA	TCGTTACCCCTCTCGTCATC	ACAGAATGCCTCATTGGTGG	60.9	927	DRR	Ca_03470	R	Nin-like
Ca4_P2_7735241	Ca4	7735241	TTTATTATTATTATTATTATTATTATTATTATTATTATTATTATTATT/TTTATTATTATTATTATTATTATTATTATTATTATTATTATTATTATTATTATTATT	AAGGAAACAAGAACATGCACA	CATGTCACACCGTCGTTTTT	59.5	492	INTERGENIC	--	--	--

INDEL marker IDs	Chromosomes/unanchored scaffolds	Physical positions (bp)	InDels (*Kabuli* reference genome-CDC Frontier/PII)	Forward primers (5'-3')	Reverse primers (5'-3')	Anneling temperature (0C)	Expected amplified product size (bp)	Structural annotation		Functional annotation	
								Sequence components of *kabuli* genome	*Kabuli* gene accession IDs	NCBI-KOG	TFs
Ca4_P2_7935584	Ca4	7935584	TAGTGAGTGAGTGAGTGAGTGA/TAGTGAGTGAGTGAGTGAGTGAGTGA	ACACGCATTGTTTGGTTGAA	TGCAAACAATTTGGCATGAT	59.9	592	INTERGENIC	--	--	--
Ca4_P2_7937193	Ca4	7937193	TAAAAAAAAAAA/TAAAAAAAAAA	GCTCCGGTCAAACACAATTT	GAAAATTGAAAGGCATGGGA	59.9	403	INTERGENIC	--	--	--
Ca4_P2_7938199	Ca4	7938199	TTTTTATAA/TTTTTATAATTATTTTATAA	TGCAACACACAATTGCAAAA	TGAGGGGTGGAGTTTGGTAG	60.0	532	INTERGENIC	--	--	--
Ca4_P2_7978076	Ca4	7978076	TATCTAAATCTAAAT/TATCTAAATCTAAATCTAAAT	GCACTCCATGAAACGACCTT	CCCTTACGTGGTAACATAACACA	58.9	847	INTERGENIC	--	--	--
Ca4_P2_80132	Ca4	80132	G/GA	CCGTTTGGATTGTCAGGACT	TCGTTGAAAGCTGGAGGTCT	60.0	382	INTERGENIC	--	--	--
Ca4_P2_8039801	Ca4	8039801	ATAAAATTAAAAAACTAAAATTAAAAAACTAAAA/ATAAAATTAAAAAACTAAAATTAAAAAACTAAAATTAAAAAACTAAAA	TTCGAAAATTGGATTGCCA	TTTGTGCGAGATAGGTACGAAA	59.8	267	INTERGENIC	--	--	--
Ca4_P2_8098817	Ca4	8098817	ATTTT/ATTT	CAAATGGGCCTAAAGAGCTG	GCCTCGGGGATGTTAGTGTA	60.0	463	DRR	Ca_03422	T	--
Ca4_P2_8115754	Ca4	8115754	CATATATATATATATATATATATATA/CATATATATATATATATATATATA	GCTGCTAGGGATGCTGTTTC	GAATATTGGCCAAGGCTTCA	60.0	208	INTRON	Ca_03421	--	M-type
Ca4_P2_8167140	Ca4	8167140	GCC/GC	GGGTGGGTTTAGAGGACCAT	TTTCTTGAATGGGCAAAACC	59.9	259	DRR	Ca_08305	V	GRAS
Ca4_P2_8167238	Ca4	8167238	ATCTT/ATCTTCTT	GGGTGGGTTTAGAGGACCAT	TGAAGCTTTTTGCTACAAGGAA	59.2	737	DRR	Ca_08305	V	GRAS
Ca4_P2_8167347	Ca4	8167347	ATATTATTTTTA/ATATTATTTTTATTATTTTTA	GGGTGGGTTTAGAGGACCAT	TGAAGCTTTTTGCTACAAGGAA	59.2	737	DRR	Ca_08305	V	GRAS
Ca4_P2_8169426	Ca4	8169426	GTCGATC/GTC	GACATCCGAAATGGGCTAAA	ATTTCCAAGGTCACGGTTTG	59.8	907	INTERGENIC	--	--	--

INDEL marker IDs	Chromosomes/unanchored scaffolds	Physical positions (bp)	InDels (*Kabuli* reference genome-CDC Frontier/PII)	Forward primers (5'-3')	Reverse primers (5'-3')	Anneling temperature (0C)	Expected amplified product size (bp)	Structural annotation		Functional annotation	
								Sequence components of *kabuli* genome	*Kabuli* gene accession IDs	NCBI-KOG	TFs
Ca4_P2_8169626	Ca4	8169626	TAAATAAAAA/TAAATAAAAAATAAAAA	TATGGCACCTCCAGTTGAAA	TGTTAAATCCTCCAGACGGC	60.1	858	INTERGENIC	--	--	--
Ca4_P2_8170487	Ca4	8170487	AT/A	GCCGTCTGGAGGATTTAACA	CTGCCAACTCCAAAAGATACC	58.7	914	INTERGENIC	--	--	--
Ca4_P2_8171316	Ca4	8171316	A/AT	GGTAAATGGGTTTGGGACCT	GAGCGGAAAGACCAAATTGA	60.2	337	INTERGENIC	--	--	--
Ca4_P2_8172016	Ca4	8172016	T/TC	CTAGTGTTCGGCAACAAACG	GCCTCAATTGGAGTTTGCTC	59.8	204	INTERGENIC	--	--	--
Ca4_P2_8173292	Ca4	8173292	GT/G	TCCATCACACAATTGAAGGC	TGAATGTATCGCGTTGTTGG	60.5	569	INTERGENIC	--	--	--
Ca4_P2_8178316	Ca4	8178316	TGG/TG	TGTGCTGCTCAACAGGAAAC	TGGGGGACTTAGCCCTCTAT	59.9	550	DRR	Ca_08306	R	SBP
Ca4_P2_8178926	Ca4	8178926	TTCT/TT	CGTCATTGATTGGTGTGGTC	GGCTTGAGCTTCAAGTTTGG	60.0	789	DRR	Ca_08306	R	SBP
Ca4_P2_8183444	Ca4	8183444	TAAAAAAA/TAAAAAA	CCTATTTGCTATTGCGTGGG	TCTGAGATGTAGCAGTGCGG	60.2	642	INTERGENIC	--	--	--
Ca4_P2_8185470	Ca4	8185470	TAAAAAAA/TAAAAAA	TTAGGTGTCCAGAGGTTGGG	GAAAACCACGATGCTTTTTCA	60.1	542	DRR	Ca_08308	T	--
Ca4_P2_8186328	Ca4	8186328	CTATATATATATATA/CTATATATATATA	TGCTTTTCATTTGCAGAATCC	TTTCACCTCCTGGTAGACGG	60.1	626	DRR	Ca_08308	T	--
Ca4_P2_8187730	Ca4	8187730	TCC/TC	CTAAGCCGAGCAGAAGCAAT	TCCCTTTAATGCAAAGGTGG	59.9	499	INTRON	Ca_08308	T	--
Ca4_P2_8193266	Ca4	8193266	TAAAAAAAAA/TAAAAAAAAAA	GCATCTCTCCAAGATGTCTGC	TGGGAATTGGGAGCTATCAG	60.0	620	DRR	Ca_08309	R	--

INDEL marker IDs	Chromosomes/unanchored scaffolds	Physical positions (bp)	InDels (*Kabuli* reference genome-CDC Frontier/PII)	Forward primers (5'-3')	Reverse primers (5'-3')	Anneling temperature (0C)	Expected amplified product size (bp)	Structural annotation		Functional annotation	
								Sequence components of *kabuli* genome	*Kabuli* gene accession IDs	NCBI-KOG	TFs
Ca4_P2_8204633	Ca4	8204633	CAAAAAAAAAAAA/CAAAAAAAAAA	ATTCTCTGGCAGCCATTTTG	ATTGCTCACGGAGGATTTTG	60.1	506	INTRON	Ca_08310	T	MYB_related
Ca4_P2_8205973	Ca4	8205973	ATCTT/ATCTTCTT	CGTGTCCAACATCAAAACG	TTCCAAAGAACCTACGGTGG	60.0	378	CDS (large-effect mutations)	Ca_08310	T	MYB_related
Ca4_P2_8206232	Ca4	8206232	GTATA/GTATACTATA	CAAGGGGTTGAAGTTGCATT	TTGAAGCCACCCTCTGATCT	59.8	763	INTERGENIC	--	--	--
Ca4_P2_8207569	Ca4	8207569	ATTTTTTTTT/ATTTTTTT	CTCGTGTCTTTGTGCGTTGT	TCTCTCTATCTCAGCGTCACTTT	57.5	549	INTERGENIC	--	--	--
Ca4_P2_8209784	Ca4	8209784	TAA/TA	TGAACCAGTGTTTCTCTCTGTGA	GGGCCTCTCTCTCTTGGTCT	60.0	480	INTERGENIC	--	--	--
Ca4_P2_8209835	Ca4	8209835	CTTTTTTTT/CTTTTTTT	AGACCAAGAGAGAGAGGCCC	GGGTTTGGTTTGTGCGTAGT	59.9	306	INTERGENIC	--	--	--
Ca4_P2_8210006	Ca4	8210006	AGTGTAT/A	AGACCAAGAGAGAGAGGCCC	GGGTTTGGTTTGTGCGTAGT	59.9	306	INTERGENIC	--	--	--
Ca4_P2_8210730	Ca4	8210730	TCC/TC	GGGGATACTTGCCTCAATCA	TGATGCTATTCATTTTGCGG	59.7	814	INTERGENIC	--	--	--
Ca4_P2_8216534	Ca4	8216534	GTT/GTTT	CGAATCCGCTTCGTGTTAAT	CGACCCGCTTAATATGTGGT	59.8	507	INTERGENIC	--	--	--
Ca4_P2_8216693	Ca4	8216693	AG/A	CGAATCCGCTTCGTGTTAAT	CGACCCGCTTAATATGTGGT	59.8	507	INTERGENIC	--	--	--
Ca4_P2_8216710	Ca4	8216710	T/TG	CGAATCCGCTTCGTGTTAAT	CGACCCGCTTAATATGTGGT	59.8	507	INTERGENIC	--	--	--
Ca4_P2_8220440	Ca4	8220440	AAATTATTT/AAATTATTTAATTATTT	CGGTGAAGCAACGTAGAACA	ACAGTTTTGGGTGCAAAAGG	60.0	688	DRR	Ca_08312	--	--

INDEL marker IDs	Chromosomes/unanchored scaffolds	Physical positions (bp)	InDels (*Kabuli* reference genome-CDC Frontier/PII)	Forward primers (5'-3')	Reverse primers (5'-3')	Anneling temperature (0C)	Expected amplified product size (bp)	Structural annotation		Functional annotation	
								Sequence components of *kabuli* genome	*Kabuli* gene accession IDs	NCBI-KOG	TFs
Ca4_P2_8222692	Ca4	8222692	A/AC	TTCTGTCTTTGTTGTTGCGG	TGCTAAGGATTATGCAGGGG	60.1	483	INTRON	Ca_08312	--	--
Ca4_P2_8223032	Ca4	8223032	GAAAAAAAA/GAAAAAAA	CAACCTTTGGTGTTGGCTTT	ACGCGATAATCCAATCGAAC	59.9	252	INTRON	Ca_08312	--	--
Ca4_P2_8226617	Ca4	8226617	TG/T	TATATGTGTTGCTGTGGGGG	GTACACCAAGGCGATTCGAT	60.0	969	INTERGENIC	--	--	--
Ca4_P2_8239012	Ca4	8239012	CTTTT/CTTT	TTCTCGATCCACAAAAACAAAA	CAACCTCCTCAAATTGGACA	58.5	636	INTERGENIC	--	--	--
Ca4_P2_8239816	Ca4	8239816	CTTTTTTTTTTTTT/CTTTTTTTTTTT	TGTCCAATTTGAGGAGGTTG	CTTAACTCCATCCCAACCCA	59.8	866	INTERGENIC	--	--	--
Ca4_P2_8250229	Ca4	8250229	TATTAGAAAA/TA	TTCCAGCATAACAAATGCCA	CCATGCTTCACGCCTAAAAT	60.1	354	INTRON	Ca_08315	S	--
Ca4_P2_8265225	Ca4	8265225	ACT/A	TGAGAAAAACCAGAGAAAAGGG	TTTGTAGCGTGCCTTACACG	59.9	229	INTRON	Ca_08315	S	--
Ca4_P2_8266891	Ca4	8266891	TGGGGGGGGGG/TGGGGGGGG	CCATTGAGGAAGGCATTGTT	AGCCAGTTCAGGCTACTCCA	60.0	742	INTRON	Ca_08315	S	--
Ca4_P2_8271274	Ca4	8271274	TAAAAAAAAAA/TAAAAAAAAAAA	ATTCAACGGGCCTATACGGT	TTAGTGGTGTACGTTGCGGA	60.2	794	INTRON	Ca_08315	S	--
Ca4_P2_8274882	Ca4	8274882	ATTTTTTTTTT/ATTTTTTTTTTT	CGAAGGATGGCTAATTCCAA	CCATTCATCCCCTCTCATGT	59.7	239	INTRON	Ca_08315	S	--
Ca4_P2_8280329	Ca4	8280329	GTTTT/GTTT	GATGGCGGGGTAAGGTAGTT	AACCGAGAGTTGGAGATTGG	59.1	277	INTERGENIC	--	--	--
Ca4_P2_8281328	Ca4	8281328	C/CCTA	TTGGTGTCATTCGCCTTTTT	CAATCGCAAGGATAATGGCT	60.1	711	INTERGENIC	--	--	--

INDEL marker IDs	Chromosomes/unanchored scaffolds	Physical positions (bp)	InDels (*Kabuli* reference genome-CDC Frontier/PII)	Forward primers (5'-3')	Reverse primers (5'-3')	Anneling temperature (0C)	Expected amplified product size (bp)	Structural annotation		Functional annotation	
								Sequence components of *kabuli* genome	*Kabuli* gene accession IDs	NCBI-KOG	TFs
Ca4_P2_8289104	Ca4	8289104	CAA/CA	GCGGGGTTCAAATCCTAAAT	ATAGACGCCCCAAGGTATCC	60.2	355	DRR	Ca_08316	--	NAC
Ca4_P2_8292742	Ca4	8292742	GATA/GATATATA	AGAGAAAGGGACGAGCATCA	AGGCATTTTCGTGACGAGTT	59.7	649	DRR	Ca_08317	--	--
Ca4_P2_8293335	Ca4	8293335	AT/AATT	CAATGGTTTTGCTTTTGGCT	TTCTCGAATGCGAAGAGTCA	59.7	492	INTERGENIC	--	--	--
Ca4_P2_8295749	Ca4	8295749	ATTTTTATT/ATTTTTATTATTTTTTATT	TCAACCCTCATTCAACCACA	CCTGCGACAATCTGAAGGAT	60.2	840	INTERGENIC	--	--	--
Ca4_P2_8301399	Ca4	8301399	GCAACCTAATGTTCAACCTAA/GCAACCTAA	CACAATGATGTTTGGTTCGG	AGGGGTGTTTACGCATTGAG	60.0	483	DRR	Ca_08318	S	--
Ca4_P2_8302062	Ca4	8302062	GAAAAAAAA/GAAAAAAA	TTTGAAGAATGCCTCCTGCT	TTTCGAGCCCCAGTTATTTG	60.1	490	DRR	Ca_08319	S	--
Ca4_P2_8305998	Ca4	8305998	GAAA/GAA	CATCATGCAGTCGCCTCTAA	ACCACATCTGCCATCAATCA	59.9	510	INTRON	Ca_08319	S	--
Ca4_P2_8306394	Ca4	8306394	TCA/T	TTCCGAAAAACAGAAATCCG	ATTCCCCCGAGAGTGAGATT	59.9	221	INTRON	Ca_08319	S	--
Ca4_P2_8308503	Ca4	8308503	GTTTTTT/GTTTTT	TAACAGGTGAGGGCATGTCA	TATGCAAACCCAATTGACCA	59.8	321	INTERGENIC	--	--	--
Ca4_P2_8308551	Ca4	8308551	TAA/TAAA	TAACAGGTGAGGGCATGTCA	TATGCAAACCCAATTGACCA	59.8	321	INTERGENIC	--	--	--
Ca4_P2_8309234	Ca4	8309234	TCAACACAA/TCAACACAACACAA	GAATTTAAGAGGAAGGCGATGA	TTCACCCTAATTCGTAGGCG	60.1	450	INTERGENIC	--	--	--
Ca4_P2_8310925	Ca4	8310925	CAAAAA/CAAAAAA	CATGTGTGGGACTCTTTGGA	CTCAACACGGTTCTGCTCTG	59.6	764	INTERGENIC	--	--	--

INDEL marker IDs	Chromosomes/unanchored scaffolds	Physical positions (bp)	InDels (*Kabuli* reference genome-CDC Frontier/PII)	Forward primers (5'-3')	Reverse primers (5'-3')	Anneling temperature (0C)	Expected amplified product size (bp)	Structural annotation		Functional annotation	
								Sequence components of *kabuli* genome	*Kabuli* gene accession IDs	NCBI-KOG	TFs
Ca4_P2_8313290	Ca4	8313290	ATTG/ATTGTTGTTG	GGGTCACAGGGACAAGGTAA	CACAACAACCAGTCCACCAG	60.0	154	INTERGENIC	--	--	--
Ca4_P2_8313629	Ca4	8313629	ATTTTTTTTTTTT/ATTTTTTTTTTT	CTGGTGGACTGGTTGTTGTG	GGCAGACAGGAGAGGGAGTA	59.4	563	INTERGENIC	--	--	--
Ca4_P2_8315933	Ca4	8315933	CTT/CTTATT	GGACCCGTGATGCTGTTTAT	TCTCGGTTGATGTCATTATGCT	59.6	586	INTERGENIC	--	--	--
Ca4_P2_8316304	Ca4	8316304	GTTTTTTT/GTTTTTTTT	GCATAATGACATCAACCGAGAG	AAAAGAAATGGTTGTTTCTCGTTC	59.9	235	INTERGENIC	--	--	--
Ca4_P2_8316722	Ca4	8316722	ACTCT/ACT	GAACGAGAAACAACCATTTCTTTT	CACGAGTGACACGCCATTTA	60.7	583	INTERGENIC	--	--	--
Ca4_P2_8317111	Ca4	8317111	CTTT/CTTTT	TCGTGCGCACAAGATTTTT	TGATTCGTGCAGGGATGATA	60.0	478	INTERGENIC	--	--	--
Ca4_P2_8318549	Ca4	8318549	GAAAAAAAA/GAAAAAAAAAA	AACGGAAGTTGAGTTTAACGGA	TAAAGGACCAAGTCGGGAAA	59.5	302	INTERGENIC	--	--	--
Ca4_P2_8319379	Ca4	8319379	ATTTTTT/ATTTTTTT	TTGCGTTATTTTGTTCACCG	TTATATGGATGGGGGCAAAG	59.6	782	INTERGENIC	--	--	--
Ca4_P2_8319710	Ca4	8319710	TC/T	GGGTTGTAATTGAAGGATTCCA	AGGCTGAAGACTTCGACGAG	59.7	516	INTERGENIC	--	--	--
Ca4_P2_8325707	Ca4	8325707	TA/T	ACGGGGCTGTTACTTTACCC	TTCTCAATGGGTGCGTACAA	60.1	739	INTERGENIC	--	--	--
Ca4_P2_8325805	Ca4	8325805	TAAAAAAAA/TAAAAAAAAA	TTTTTGGTGGTAGGGGAGAA	TTCTCAATGGGTGCGTACAA	60.1	357	INTERGENIC	--	--	--
Ca4_P2_8326056	Ca4	8326056	AT/ATT	TGTACGCACCCATTGAGAAA	TAATGGCTGGTTTCGTTTGG	60.9	547	INTERGENIC	--	--	--

INDEL marker IDs	Chromosomes/unanchored scaffolds	Physical positions (bp)	InDels (*Kabuli* reference genome-CDC Frontier/PII)	Forward primers (5'-3')	Reverse primers (5'-3')	Anneling temperature (0C)	Expected amplified product size (bp)	Structural annotation		Functional annotation	
								Sequence components of *kabuli* genome	*Kabuli* gene accession IDs	NCBI-KOG	TFs
Ca4_P2_8330553	Ca4	8330553	CAAA/CA	CCACCATGAATTTGCCTCTT	GGTTGAGCTGGGACACCTAA	60.1	629	DRR	Ca_08320	--	--
Ca4_P2_8333443	Ca4	8333443	GCTTCTTC/GCTTC	TCAAAGGTGATCATGGATGG	ACCCTTGCCAAAACACTACG	60.0	593	INTERGENIC	--	--	--
Ca4_P2_8334346	Ca4	8334346	TCACTATATTAACACTATATTAAC/TCACTATATTAAC	AAGTTTGCGGTGATGTGTGA	CAGCGTTATTACAAATGCCGT	60.0	438	INTERGENIC	--	--	--
Ca4_P2_8339338	Ca4	8339338	CAA/CA	AAAAACTGCCGCAAAAGCTA	GTTTGAAGATGGCGATGGTT	59.9	641	INTRON	Ca_08321	--	--
Ca4_P2_8341783	Ca4	8341783	GAAAAAA/GAAAAA	TGATCAAATCCCCAAGAAGC	TTCCTTGACTGTTGCTGGTG	59.9	235	INTRON	Ca_08321	--	--
Ca4_P2_8342083	Ca4	8342083	AATCATCAT/AATCAT	TGATCAAATCCCCAAGAAGC	CCAATCTCTTCTGCTCCCCT	60.7	951	INTRON	Ca_08321	--	--
Ca4_P2_8342865	Ca4	8342865	TAAG/TTAAGAAG	GAGCTTCAACCACAAGCACA	GCTTTTTGAGCAACTCGTCC	60.0	691	CDS (FRAME SHIFT)	Ca_08321	--	--
Ca4_P2_8344016	Ca4	8344016	CTT/CTTT	TAAGAATTTCCGCATCCCTG	CCTTTCACTCGTTCTGCTCC	60.0	336	INTRON	Ca_08321	--	--
Ca4_P2_8344606	Ca4	8344606	TAAAAAAA/TAAAAAA	GAGCAGAACGAGTGAAAGGG	CTCCTTCGCATTTCGTCTTC	60.0	545	DRR	Ca_08322	--	--
Ca4_P2_8345607	Ca4	8345607	T/TC	GTGCACGTGTAGACAGACTCC	AGGAGGTGCATCGAGGTATG	60.1	346	DRR	Ca_08322	--	--
Ca4_P2_8351185	Ca4	8351185	TAAAAAAAA/TAAAAAAA	ATTTTCGGTTTCCCTTCCAC	CGATTTCCCTTGGTCCAAAT	61.0	960	DRR	Ca_08323	--	--
Ca4_P2_8351374	Ca4	8351374	GACACACA/GACACACACA	TTTACCTCCTCAGAAGATCATTTTT	CTCCAAATCCAAATCACACG	59.0	663	DRR	Ca_08323	--	--

INDEL marker IDs	Chromosomes/unanchored scaffolds	Physical positions (bp)	InDels (*Kabuli* reference genome-CDC Frontier/PII)	Forward primers (5'-3')	Reverse primers (5'-3')	Anneling temperature (0C)	Expected amplified product size (bp)	Structural annotation		Functional annotation	
								Sequence components of *kabuli* genome	*Kabuli* gene accession IDs	NCBI-KOG	TFs
Ca4_P2_8351455	Ca4	8351455	GAAAAAAA/GAAAAAAAA	TTTACCTCCTCAGAAGATCATTTTT	GCAGATTGGCATTGATGTTG	60.1	831	DRR	Ca_08323	--	--
Ca4_P2_8353312	Ca4	8353312	AAT/AATAT	GAAAAATGGGGTCAAGGGAT	GAGAGAAAGGCTGCAACCAC	60.0	487	INTRON	Ca_08323	--	--
Ca4_P2_8353870	Ca4	8353870	CATAAAATAAAATAAAATAAAA/CATAAAATAAAATAAAA	GTGGTTGCAGCCTTTCTCTC	CAGCGCAATGAACTCAAAAA	60.0	719	INTRON	Ca_08323	--	--
Ca4_P2_8354793	Ca4	8354793	AATATAT/AATAT	CGGGGAAATTCAAAACTCAA	GGAGCAGTTGTCCCATTTGT	60.0	939	DRR	Ca_08324	T	bZIP
Ca4_P2_8357483	Ca4	8357483	CTATA/CTATATA	GCGATGCCAAATGAGAAAAT	AACTTGGGTGATCCTGAACG	60.0	559	INTRON	Ca_08324	T	bZIP
Ca4_P2_8362154	Ca4	8362154	AC/ACC	TTCGCATATGTGGCAGTCTC	CAACGATCGCTTCTCAAACA	60.0	694	INTERGENIC	--	--	--
Ca4_P2_8362831	Ca4	8362831	TT/TTAT	AGGTTCGAACGGATATGTCG	CCATGTGCAAACCTAGGAAAA	60.0	749	INTERGENIC	--	--	--
Ca4_P2_8364920	Ca4	8364920	GT/GTTGTCACTGTTTTGCT	GCTGGAACTTGGTTACGAGG	TTAGCATGCATTGGGAAACA	60.1	378	INTERGENIC	--	--	--
Ca4_P2_8368866	Ca4	8368866	CATATATATATATATATATA/CATATATATATATATATA	TCCTGCGACTCAGTTTTCCT	GCCGAAGAGAATGGTTCTTG	59.8	848	INTRON	Ca_08325	--	--
Ca4_P2_8372871	Ca4	8372871	T/TG	TCTTTTTCTGCCCCACTAGC	CAGCTGAGTTGTGAGGTCCA	60.0	150	INTERGENIC	--	--	--
Ca4_P2_8372994	Ca4	8372994	AAGATTA/AA	GGCAAAATGGACCTCACAAC	CCAATGCCACTTTATGCTCA	59.7	323	INTERGENIC	--	--	--
Ca4_P2_8373181	Ca4	8373181	TACA/TACACA	GGCAAAATGGACCTCACAAC	GGAGAGTCGGTCTTTGTGGA	60.2	706	INTERGENIC	--	--	--

INDEL marker IDs	Chromosomes/unanchored scaffolds	Physical positions (bp)	InDels (*Kabuli* reference genome-CDC Frontier/PII)	Forward primers (5'-3')	Reverse primers (5'-3')	Anneling temperature (0C)	Expected amplified product size (bp)	Structural annotation		Functional annotation	
								Sequence components of *kabuli* genome	*Kabuli* gene accession IDs	NCBI-KOG	TFs
Ca4_P2_8373461	Ca4	8373461	AAC/AACCAC	TGAGCATAAAGTGGCATTGG	GTCGGTCTTTGTGGAGAGGA	60.2	398	INTERGENIC	--	--	--
Ca4_P2_8376439	Ca4	8376439	TA/TAA	GATTCTCTCAATTTCGGCCA	TGCGATTGATTTCAGTTTTTG	58.8	373	INTERGENIC	--	--	--
Ca4_P2_8377254	Ca4	8377254	ATTTTTTTTTTT/ATTTTTTTTTTTTTT	CCACATATATCTGCCCCACC	TGACTGTGTGCACTGAAACAA	58.9	563	DRR	Ca_08327	KL	--
Ca4_P2_8377411	Ca4	8377411	AT/ATATATATATT	TAGTGTACGTGTTTGGGGCA	GGGTGAGACTGAAGCTGGTC	59.8	949	DRR	Ca_08327	KL	--
Ca4_P2_8377585	Ca4	8377585	ATTT/ATT	TTCGTCCATTTGAATCCTGA	CCTGCAAGCACTTACGACAA	60.1	891	DRR	Ca_08327	KL	--
Ca4_P2_8381452	Ca4	8381452	TAAAAAAA/TAAAAAAAA	CAATCCGTTAAGGACCTGGA	GATGATTCCCTTGCTGGAAA	60.0	388	INTRON	Ca_08327	KL	--
Ca4_P2_8384923	Ca4	8384923	AGGGGGG/AGGGGG	GCCGCAATAGTGTTTTTGGT	GCGTATCGGTTGGAATTGTT	59.8	807	INTRON	Ca_08327	KL	--
Ca4_P2_8396183	Ca4	8396183	T/TG	TTTACAACACATTGCGCACC	CGTTTATTGACGGAAGGCAT	60.0	681	DRR	Ca_08329	O	B3
Ca4_P2_8396449	Ca4	8396449	GAAAAA/GAAAAAA	CGAGCAAATGCTCAAGTGAA	CGTTTATTGACGGAAGGCAT	60.0	325	DRR	Ca_08328	R	AP2
Ca4_P2_8397091	Ca4	8397091	CTT/CT	CATCTTACCTGGCTGCATCA	AAATGCTGGAGTTGAGGGTG	60.1	547	DRR	Ca_08328	R	AP2
Ca4_P2_8400811	Ca4	8400811	GAATTACTAAT/GAATTACTAATTACTAAT	CTTAAGCATCAAAGCCCGAG	CCTCAATTGCCACCAAAACT	60.0	469	INTRON	Ca_08329	O	B3
Ca4_P2_8405694	Ca4	8405694	TAA/TAAA	GACCAGATTTCCGGTTTTCA	AACAAGCTTCGTGCTGGAAT	59.9	175	INTRON	Ca_08330	O	B3

INDEL marker IDs	Chromosomes/unanchored scaffolds	Physical positions (bp)	InDels (*Kabuli* reference genome-CDC Frontier/PII)	Forward primers (5'-3')	Reverse primers (5'-3')	Anneling temperature (0C)	Expected amplified product size (bp)	Structural annotation		Functional annotation	
								Sequence components of *kabuli* genome	*Kabuli* gene accession IDs	NCBI-KOG	TFs
Ca4_P2_8409923	Ca4	8409923	AAGAG/AAG	CGCATCAATACGACGAACAA	ACCACCACTTTGTAGCCACC	59.9	509	INTERGENIC	--	--	--
Ca4_P2_8413764	Ca4	8413764	TA/T	AATGGTGAACAAGGAGCAGG	TCAAATATTAACTCCAAGCGCA	59.8	714	INTERGENIC	--	--	--
Ca4_P2_8414127	Ca4	8414127	CAAAAAAAA/CAAAAAAAAA	TACACGTTCGGCACATCTTG	ATGCACATGGTTCTGACCAA	60.0	761	INTERGENIC	--	--	--
Ca4_P2_8415253	Ca4	8415253	GAAAAAAAAA/GAAAAAAAAAAA	CACCTGCACCGCTAAGTTTT	TTGTTGAACACCCACATGCT	60.0	274	INTERGENIC	--	--	--
Ca4_P2_8415318	Ca4	8415318	AG/AGGG	ACTTTGCCACCTCATCTGCT	TTGTTGAACACCCACATGCT	60.0	221	INTERGENIC	--	--	--
Ca4_P2_8417735	Ca4	8417735	ATTTTT/ATTTT	CACAGGGGAATAAGGTTGGA	ACAAGAGGCGGAGACAGGTA	59.9	862	DRR	Ca_08331	--	ERF
Ca4_P2_8420231	Ca4	8420231	TATATAAATGGA/T	AAAATTTGTTAAACCCCGCA	CACTTCGATTGTGTCACTCAAAA	60.2	735	DRR	Ca_08333	--	--
Ca4_P2_8422191	Ca4	8422191	GAAA/GAA	AAGACGGATGCATTTTGCTC	GACACATGCCCAACTGAATG	60.0	841	INTERGENIC	--	--	--
Ca4_P2_8423641	Ca4	8423641	TTAT/TTATAT	ACCATGTCCCCATTTTTCAA	TTCGTCATCTAAGTGTGGCG	59.9	719	INTERGENIC	--	--	--
Ca4_P2_8424598	Ca4	8424598	GA/G	GGCACCTGCTTTCAAATTGT	TCCAATTGAACCAACCAACA	59.8	463	INTERGENIC	--	--	--
Ca4_P2_8424635	Ca4	8424635	ATGT/ATGTAGTGT	GGCACCTGCTTTCAAATTGT	TCCTCTTCCTCTTACTCCTCCA	59.5	514	INTERGENIC	--	--	--
Ca4_P2_8428271	Ca4	8428271	TGATG/T	CATCTCGCCTTATCTCTCCG	GAACATTTGTGAATGGCACG	60.0	468	INTERGENIC	--	--	--

INDEL marker IDs	Chromosomes/unanchored scaffolds	Physical positions (bp)	InDels (*Kabuli* reference genome-CDC Frontier/PII)	Forward primers (5'-3')	Reverse primers (5'-3')	Anneling temperature (0C)	Expected amplified product size (bp)	Structural annotation		Functional annotation	
								Sequence components of *kabuli* genome	*Kabuli* gene accession IDs	NCBI-KOG	TFs
Ca4_P2_8430074	Ca4	8430074	AAT/AATAT	TTTTGGCATGAAAAAGGGAC	GAAATTTACCGCTTGACCGA	60.1	772	DRR	Ca_08334	--	--
Ca4_P2_8432657	Ca4	8432657	CATATA/CATA	TTTTGTGTTTGCCTTTTCCC	CATCTCATGAACCGACCCTT	59.9	479	INTRON	Ca_08335	R	--
Ca4_P2_8441953	Ca4	8441953	GTTTGTA/GTTTGTATTTTGTA	AGCAGGAGAGCTGAGATTGG	TACTGTGTATCGGGGCAACA	60.0	731	INTRON	Ca_08335	R	--
Ca4_P2_8448227	Ca4	8448227	CTTTT/CTTT	CTAGCTCTCCATCCCACAGC	TCCGAACATAATCTATCACCCA	59.3	633	DRR	Ca_08335	R	--
Ca4_P2_8449229	Ca4	8449229	ATTTTTTTTT/ATTTTTTTT	TGGAGTTTTAGCCACTTCCA	TCTCGCCAAATATCTACAATCG	59.2	337	DRR	Ca_08335	R	--
Ca4_P2_8449477	Ca4	8449477	TG/T	TGGAGTTTTAGCCACTTCCA	TCTCGCCAAATATCTACAATCG	59.2	337	DRR	Ca_08336	--	--
Ca4_P2_8451472	Ca4	8451472	GAA/GAAAA	TCCACTGATTTGCTGCTGAC	CAATGGCTGATTCACCACAC	60.0	384	CDS (FRAME SHIFT)	Ca_08336	--	--
Ca4_P2_8454111	Ca4	8454111	TC/TCC	TTCTTTGGGTCTCCCAACAC	TTATGAGCTTTGGATTGGGC	60.0	631	DRR	Ca_08337	R	MYB
Ca4_P2_8466945	Ca4	8466945	CT/C	AGGACACCAATTTGAATGGC	TGATGATGGTGTGCTTTGGT	60.0	625	INTERGENIC	--	--	--
Ca4_P2_8466970	Ca4	8466970	TTATA/TTA	AGGACACCAATTTGAATGGC	TGATGATGGTGTGCTTTGGT	60.0	625	INTERGENIC	--	--	--
Ca4_P2_8467235	Ca4	8467235	CCATCATCATCATC/CCATCATCATC	GGCAAGCATTCAATTTCCAT	GGTTGGGATGTTATGTTCGG	60.1	449	INTERGENIC	--	--	--
Ca4_P2_8475153	Ca4	8475153	ATTT/ATT	TGGACCGTACATTTTCGTGA	GTGGTAGCAGAAGGAGCAGG	60.0	829	INTRON	Ca_08339	G	--

INDEL marker IDs	Chromosomes/unanchored scaffolds	Physical positions (bp)	InDels (*Kabuli* reference genome-CDC Frontier/PII)	Forward primers (5'-3')	Reverse primers (5'-3')	Anneling temperature (0C)	Expected amplified product size (bp)	Structural annotation		Functional annotation	
								Sequence components of *kabuli* genome	*Kabuli* gene accession IDs	NCBI-KOG	TFs
Ca4_P2_8478290	Ca4	8478290	A/AT	TTTAGCTTGCCAAAACAGCA	CCTTTCTTCGTACGTCTCGC	60.0	607	INTRON	Ca_08339	G	--
Ca4_P2_8483100	Ca4	8483100	CTTTTTTTTTT/CTTTTTTTTT	ATTTCGAGGAAAAGGGAGGA	TTTCAAGAGGGGCAATTGAG	60.2	515	DRR	Ca_08340	R	--
Ca4_P2_8488611	Ca4	8488611	CG/CGG	TTCCTTATGAGTTGCACGGA	TGTTTGTGGAAATCGAACCA	59.9	252	INTERGENIC	--	--	--
Ca4_P2_8489479	Ca4	8489479	CACATACA/CACA	CCCAAATCACACACATGAGC	AGAGTTGTTTCATCTCCAAACG	58.4	415	INTERGENIC	--	--	--
Ca4_P2_8497257	Ca4	8497257	TAAACTGAAGAAACT/TAAACTGAAGAAACTGAAGAAACT	AGCGTGAGAGGAACCTGTGT	GACTGGCAACAGCAATTTGA	59.8	564	INTRON	Ca_08341	--	CPP
Ca4_P2_8503853	Ca4	8503853	AATTTTATTTT/AATTTTATTTTATTTT	CAGACATTTGAGCCGTTGAA	ACCACTTTTATCATTTGACTCCA	57.7	421	INTERGENIC	--	--	--
Ca4_P2_8504484	Ca4	8504484	ATTTTTTT/ATTTTTTTT	TTTGGAGTCAAATGATAAAAGTGG	TTTTCGAGTGCCGCTTACTT	60.0	522	INTERGENIC	--	--	--
Ca4_P2_8505276	Ca4	8505276	CATATATATATATATAT/CATATATATATATATATATATATATATATATATAT	ACAATTGTGGGGTTGCAAAT	GAAAAGTGGCCCTGTCAAAA	60.1	780	INTERGENIC	--	--	--
Ca4_P2_8509176	Ca4	8509176	CTTTT/CTT	GCCTTCTGGACAAAACGAAG	TCTGCAGGTGAAGCAACATC	60.0	578	INTRON	Ca_08343	YU	--
Ca4_P2_8514326	Ca4	8514326	CTTTT/CTTTTT	TGTCGTGCGGTAAGTTTGAC	GAAGCAAAGCTAGGGGTGTG	59.9	449	DRR	Ca_08343	YU	--
Ca4_P2_8514652	Ca4	8514652	CTTTTTTTTTTTT/CTTTTTTTT	CACACCCCTAGCTTTGCTTC	CACGAACGCCTGTGAACTTA	59.9	547	DRR	Ca_08343	YU	--
Ca4_P2_8521020	Ca4	8521020	CATA/CATATATA	AATTCAACCAAATAAATTCCCA	CACGCAAAATGAAAGGTTGA	59.7	308	INTERGENIC	--	--	--

INDEL marker IDs	Chromosomes/unanchored scaffolds	Physical positions (bp)	InDels (*Kabuli* reference genome-CDC Frontier/PII)	Forward primers (5'-3')	Reverse primers (5'-3')	Anneling temperature (0C)	Expected amplified product size (bp)	Structural annotation		Functional annotation	
								Sequence components of *kabuli* genome	*Kabuli* gene accession IDs	NCBI-KOG	TFs
Ca4_P2_8522151	Ca4	8522151	TG/T	TGTGATTGTTAGGATGATGACG	CCAAAACTGGGGAGAAACAA	59.9	288	INTERGENIC	--	--	--
Ca4_P2_8522296	Ca4	8522296	GT/GTT	GCAGTTTTGGTCCCCCTATT	GCAGTTTTGGTCCCCCTATT	60.2	328	INTERGENIC	--	--	--
Ca4_P2_8550328	Ca4	8550328	CG/C	GGGGTGTACACAAGAGGGAA	AATATAGTGGGTGCAACGGC	59.9	716	INTERGENIC	--	--	--
Ca4_P2_8572936	Ca4	8572936	CTTTTTTTTTTTT/CTTTTTTTTTTT	AATTTGAGGGCGAGTGTGTT	GTGTTTGCCGCGAGATCTAT	60.2	533	INTERGENIC	--	--	--
Ca4_P2_8575951	Ca4	8575951	TAAAAAAA/TAAAAAAAA	TCGGTTTTGATTACTCTTTCCA	CGAGAATTCATTATTTTTCATTTTCC	60.4	703	INTERGENIC	--	--	--
Ca4_P2_8609841	Ca4	8609841	CTTTTTTTTTTT/CTTTTTTTTTTTTT	GTAGGAAGTCGCCATTTGGA	AACATACGGCGAAACTCACA	59.2	612	INTERGENIC	--	--	--
Ca4_P2_8611513	Ca4	8611513	G/GATT	TTGGCCGTTGTGATGTGTAT	TGTGGGGAAAGGAATCAAAG	59.9	850	INTERGENIC	--	--	--
Ca4_P2_8611750	Ca4	8611750	AAATAATAATA/AAATAATA	TCAATTCCACTTCGGAACAA	TGTGGGGAAAGGAATCAAAG	59.9	534	INTERGENIC	--	--	--
Ca4_P2_8612629	Ca4	8612629	T/TTA	TTTCTTTCTGCTGTCGGCTT	CATGCCATCCACTATGTTGC	60.0	393	INTERGENIC	--	--	--
Ca4_P2_8613273	Ca4	8613273	TAATTAAA/TAA	CCTTTGTTTGTATTCATATGTCGG	TTTCATTTTCAACCAAATGCAC	59.9	807	INTERGENIC	--	--	--
Ca4_P2_8613689	Ca4	8613689	TTTTTCT/TT	TGCATTTGGTTGAAAATGAAA	TGGTGGGCCTACGAGTAAAG	60.1	374	INTERGENIC	--	--	--
Ca4_P2_8614008	Ca4	8614008	ATTTTTTTTT/ATTTTTTTTTT	TCATTCCCCAATAACTCCCA	AATCCAAAATTAGACGTCAACAA	57.8	384	INTERGENIC	--	--	--

**INDEL marker IDs**	**Chromosomes/unanchored scaffolds**	**Physical positions (bp)**	**InDels (*Kabuli* reference genome-CDC Frontier/PII)**	**Forward primers (5'-3')**	**Reverse primers (5'-3')**	**Anneling temperature (0C)**	**Expected amplified product size (bp)**	**Structural annotation**		**Functional annotation**	
								**Sequence components of *kabuli* genome**	***Kabuli* gene accession IDs**	**NCBI-KOG**	**TFs**
Ca4_P2_8616163	Ca4	8616163	ATTTT/ATTTTT	TTCAAAAGGCCGTAGGTTTC	TAGTTTGGAATGGTCGCACA	60.1	933	INTERGENIC	--	--	--
Ca4_P2_8616425	Ca4	8616425	TAAAAA/TAAAA	TCGACACAGCTAGCAACACA	TAGTTTGGAATGGTCGCACA	60.1	581	DRR	Ca_08355	O	--
Ca4_P2_8621480	Ca4	8621480	GAAAAAAAA/GAAAAAAAAA	GTGCATGACCCCTTCTTGTT	TGTCAGGCAACAGAAAACCA	60.3	889	INTERGENIC	--	--	--
Ca4_P2_8622368	Ca4	8622368	ATTTTCTTTT/AT	CAGTCTTTATGGGGCTTCCA	TTCCTATGTCTTTGGGTCGG	59.9	542	INTERGENIC	--	--	--
Ca4_P2_8623394	Ca4	8623394	CTAT/CT	CCTTGCATGGTGGTACTCCT	GTGGACCACACCACACAAAT	59.1	714	INTERGENIC	--	--	--
Ca4_P2_8625918	Ca4	8625918	ATTTTTTT/ATTTTTT	GGTTTGAATGTCAACACCCT	TTTTGGAACTTGAACTCCACAC	59.1	293	INTERGENIC	--	--	--
Ca4_P2_8625981	Ca4	8625981	TA/TAA	GGTTTGAATGTCAACACCCT	TTTTGGAACTTGAACTCCACAC	59.1	293	INTERGENIC	--	--	--
Ca4_P2_8626372	Ca4	8626372	ATTTT/ATTT	TGTGGAGTTCAAGTTCCAAAAG	AAGAATGAAGGGGAGGGAGA	60.0	791	INTERGENIC	--	--	--
Ca4_P2_8633429	Ca4	8633429	A/AT	GCACGAAAAATTGTGAGCAA	ATGGGCATATGGGTCAATGT	59.9	171	INTERGENIC	--	--	--
Ca4_P2_8633989	Ca4	8633989	CA/CAA	TTCCCAACTCCGTCCATTTA	TTGATCAAACGCGCAAATTA	60.2	271	INTERGENIC	--	--	--
Ca4_P2_8634285	Ca4	8634285	ATTTTTT/ATTTTTTT	TTCCCAACTCCGTCCATTTA	AAGGTGATGGTGGCTCTCAG	60.3	713	INTERGENIC	--	--	--
Ca4_P2_8635388	Ca4	8635388	TAAAAAAAA/TAAAAAAA	TTCTGAACTCGTTCCCATGA	CATGAACAATGTTTGCATTTG	57.6	658	INTERGENIC	--	--	--

INDEL marker IDs	Chromosomes/unanchored scaffolds	Physical positions (bp)	InDels (*Kabuli* reference genome-CDC Frontier/PII)	Forward primers (5'-3')	Reverse primers (5'-3')	Anneling temperature (0C)	Expected amplified product size (bp)	Structural annotation		Functional annotation	
								Sequence components of *kabuli* genome	*Kabuli* gene accession IDs	NCBI-KOG	TFs
Ca4_P2_8639881	Ca4	8639881	TA/TACAA	ACTCCCTCAATGGCAACATC	ACACCACGAACGCAACAATA	60.0	652	DRR	Ca_08357	--	--
Ca4_P2_8641169	Ca4	8641169	TAAAA/TAAA	TAGGTCAATGCAAACCCCAT	TGGAGTTCCCAATCACCAAT	60.2	197	DRR	Ca_08357	--	--
Ca4_P2_8643488	Ca4	8643488	ATTTTTTT/ATTTTTT	TGCAACGAAGCATGTGATTT	CGGTCGGTATGCTATATTCTCA	59.1	650	INTERGENIC	--	--	--
Ca4_P2_8647825	Ca4	8647825	CAA/CA	AATGGCTTCGCTATTGCCTA	AAGGACTGCGAACCTCAAAA	59.9	734	INTRON	Ca_08358	--	G2-like
Ca4_P2_8650892	Ca4	8650892	AATATA/AATATATA	TTTAAATTGAATGTGTTCGCGT	ACCACACATGATTTCTGCCA	60.0	785	INTERGENIC	--	--	--
Ca4_P2_8657818	Ca4	8657818	TAAAAAAAA/TAAAAAAA	GCACAGTGCTTAGACCCACA	CACAGCATACCCCAACACTG	60.0	448	INTERGENIC	--	--	--
Ca4_P2_8666114	Ca4	8666114	GAAAAA/GAAAAAA	AAAAGATTCCGACGAGGGAT	GGGTATCGTAGCCGTTCAAA	60.0	613	DRR	Ca_08361	T	C3H
Ca4_P2_8666608	Ca4	8666608	T/TCCTC	CCAATTTCTGATGCAAACGA	GCAAGGAGCACTTTCCTGTC	60.0	712	DRR	Ca_08361	T	C3H
Ca4_P2_8670711	Ca4	8670711	TTGATGATGATGATGA/TTGATGATGATGA	TCTTGGTGTTGTTTGTCCCA	GAGCATGGCTGACATAGCAA	60.0	862	CDS (large-effect mutations)	Ca_08361	T	C3H
Ca4_P2_8688751	Ca4	8688751	AACA/AA	AGGACCCACAACATCCACAT	ACCAATCCATGCACGCTACT	60.5	505	INTERGENIC	--	--	--
Ca4_P2_8689007	Ca4	8689007	A/AT	AGGACCCACAACATCCACAT	ACCAATCCATGCACGCTACT	60.5	505	INTERGENIC	--	--	--
Ca4_P2_8689137	Ca4	8689137	ATTTTTT/ATTTTTTT	CCCATCTTGGGTGAAGAAAG	GGTGGTTTAGACGAATAGAGACG	59.2	670	INTERGENIC	--	--	--

INDEL marker IDs	Chromosomes/unanchored scaffolds	Physical positions (bp)	InDels (*Kabuli* reference genome-CDC Frontier/PII)	Forward primers (5'-3')	Reverse primers (5'-3')	Anneling temperature (0C)	Expected amplified product size (bp)	Structural annotation		Functional annotation	
								Sequence components of *kabuli* genome	*Kabuli* gene accession IDs	NCBI-KOG	TFs
Ca4_P2_8689670	Ca4	8689670	A/ATCTC	TGCAAATTTGGTGAAAACAGA	TGTCACATTTGCAGAAGAAAAA	58.5	300	INTERGENIC	--	--	--
Ca4_P2_8690776	Ca4	8690776	T/TAA	TTTAATTGCGAGGGAGTAAAAA	GGTGTTTTGATGCGAAGTGA	59.7	335	INTERGENIC	--	--	--
Ca4_P2_8690790	Ca4	8690790	GAAAAAAA/GAAAAAAAA	TTTAATTGCGAGGGAGTAAAAA	GGTGTTTTGATGCGAAGTGA	59.7	335	INTERGENIC	--	--	--
Ca4_P2_8691223	Ca4	8691223	ACCC/ACCCC	CACGCATGCATGTTCTTTCT	CAGAGTCCAGACACACCGAA	59.9	668	INTRON	Ca_08365	G	--
Ca4_P2_8693729	Ca4	8693729	TT/TTCT	ATTTCAGTTTGCTGGGGTTG	GATGGAAGGATGGCACAAAT	59.8	414	INTRON	Ca_08365	G	--
Ca4_P2_8698891	Ca4	8698891	AAGAGTAGTAGAGTAGT/AAGAGTAGT	AAGCTGGCCACTGTAAGCAT	TCACAATCTCGACAACCCAA	60.1	246	INTRON	Ca_08365	G	--
Ca4_P2_8701352	Ca4	8701352	ATTTTTTT/ATTTTT	TTACGACTTTGAATGCCACG	TGTTTCGCAATTTAGGGGAG	60.1	503	DRR	Ca_08365	G	--
Ca4_P2_8704241	Ca4	8704241	G/GC	TTTGTACCCAACAGGGCATT	ATGCATGCGTTGACAATGTT	60.0	569	DRR	Ca_08366	J	NAC
Ca4_P2_8704735	Ca4	8704735	CTTTTTTTTT/CTTTTTTTT	CTCCAACATTGTCAACGCAT	TGAATTTGCTCCACCATTGA	60.0	249	DRR	Ca_08366	J	NAC
Ca4_P2_8705159	Ca4	8705159	GTT/GTTT	TCAATGGTGGAGCAAATTCA	GGTTCCCTTCCCCAATTTAG	59.6	399	DRR	Ca_08366	J	NAC
Ca4_P2_8705383	Ca4	8705383	CTTTTTTTTTTT/CTTTTTTTTT	CTAAATTGGGGAAGGGAACC	TTATGCCGTTACCAACAACG	59.5	695	INTERGENIC	--	--	--
Ca4_P2_8706020	Ca4	8706020	GTAATAAATAAT/GTAATAAATAATAAATAAT	TAACGTTGTTGGTAACGGCA	GACTGCACCTGCGACTATCA	60.0	451	INTERGENIC	--	--	--

INDEL marker IDs	Chromosomes/unanchored scaffolds	Physical positions (bp)	InDels (*Kabuli* reference genome-CDC Frontier/PII)	Forward primers (5'-3')	Reverse primers (5'-3')	Anneling temperature (0C)	Expected amplified product size (bp)	Structural annotation		Functional annotation	
								Sequence components of *kabuli* genome	*Kabuli* gene accession IDs	NCBI-KOG	TFs
Ca4_P2_8707105	Ca4	8707105	AAAT/A	CGAGAGGTATTTATGCTTCACTG	ATCTCTCCGACCATGTGGAA	60.5	712	INTERGENIC	--	--	--
Ca4_P2_8708587	Ca4	8708587	TAAAAAAA/TAAAAAA	GCTAAAAATTAGCGACTGGCA	CGGAATCTGCGGTGATCTAT	60.1	767	INTERGENIC	--	--	--
Ca4_P2_8709656	Ca4	8709656	CAAAAAAA/CAAAAAAAA	AATTTGTGGGTGGGCATAAA	TGCATTGAGAGAATCACCAACT	59.7	701	INTERGENIC	--	--	--
Ca4_P2_8709676	Ca4	8709676	TG/T	AATTTGTGGGTGGGCATAAA	TGCATTGAGAGAATCACCAACT	59.7	701	INTERGENIC	--	--	--
Ca4_P2_8710046	Ca4	8710046	ATTTTT/ATTTTTT	GAGTTGGTGATTCTCTCAATGC	TTGCAAAACATTGATACTTTTGGT	59.8	269	INTERGENIC	--	--	--
Ca4_P2_8713161	Ca4	8713161	ATTTTTTTTTTTTTT/ATTTTTTTTTTTT	ACCAGCTTTGGTGGATTGTC	CCCCTTTTGCCTCTCTTTCT	59.8	350	DRR	Ca_08367	R	C2H2
Ca4_P2_8719635	Ca4	8719635	ATTTTTTTT/ATTTTTTT	TCCTTGTGGATTAGGCCTTG	TTTGGAATTCTCCATCAGCC	60.0	330	INTRON	Ca_08368	Q	--
Ca4_P2_8720115	Ca4	8720115	TAAA/TA	GGCTGATGGAGAATTCCAAA	ACCAAAGACCAGATTGGCAC	60.0	860	INTRON	Ca_08368	Q	--
Ca4_P2_8720139	Ca4	8720139	AAAGT/AAAGTTTAAGT	GGCTGATGGAGAATTCCAAA	ACCAAAGACCAGATTGGCAC	60.0	860	INTRON	Ca_08368	Q	--
Ca4_P2_8723451	Ca4	8723451	CAT/CATTAT	AGAATGGTTGAGGGTCGTTG	AGCAGGTGCCCTGAGACTAA	60.0	750	INTERGENIC	--	--	--
Ca4_P2_8724841	Ca4	8724841	ATTTT/ATTTTT	AAAAACCAATACACGAAGGTTAGAA	ATGGGTTGACTTTCTCCACG	60.0	323	INTERGENIC	--	--	--
Ca4_P2_8726616	Ca4	8726616	T/TA	CAAGCATGGTCTGGCAATTA	TTGGCAAGAGCAATGAACTG	60.0	524	INTERGENIC	--	--	--

INDEL marker IDs	Chromosomes/unanchored scaffolds	Physical positions (bp)	InDels (*Kabuli* reference genome-CDC Frontier/PII)	Forward primers (5'-3')	Reverse primers (5'-3')	Anneling temperature (0C)	Expected amplified product size (bp)	Structural annotation		Functional annotation	
								Sequence components of *kabuli* genome	*Kabuli* gene accession IDs	NCBI-KOG	TFs
Ca4_P2_8727840	Ca4	8727840	ATTTTTTT/ATTTTTTTT	TGAATGCCCAAAATAAGATGA	TCACCAATCCAGCTGGTACA	60.1	425	INTERGENIC	--	--	--
Ca4_P2_8727913	Ca4	8727913	T/TCA	TGAATGCCCAAAATAAGATGA	TCACCAATCCAGCTGGTACA	60.1	425	INTERGENIC	--	--	--
Ca4_P2_8730605	Ca4	8730605	CG/C	TTCACACCAACACCCTCAAA	ATTTTGGAAGAACAATCCGC	59.0	517	INTERGENIC	--	--	--
Ca4_P2_8730686	Ca4	8730686	TAA/TA	TTCACACCAACACCCTCAAA	ATTTTGGAAGAACAATCCGC	59.0	517	INTERGENIC	--	--	--
Ca4_P2_8732648	Ca4	8732648	CTATTTTT/CTATTTTTATTTTT	CATTTTCCATGCTTGCTTTTC	TCCCACATTTTACATTGTCCC	59.5	547	INTERGENIC	--	--	--
Ca4_P2_8733054	Ca4	8733054	TGGG/TGG	AAAAATCACCCAAAATTAACATCT	CACTTTCCTTGCTCCCTCTG	60.0	867	INTERGENIC	--	--	--
Ca4_P2_8741602	Ca4	8741602	T/TACATC	TGGTGAACACGTATGCCAGT	AACCTCCTTTTGACCCACAA	59.4	771	INTERGENIC	--	--	--
Ca4_P2_8748201	Ca4	8748201	AG/A	CATTTCAATCATTCACCCCC	TCACAACATGAGAATGGGACA	60.0	218	INTERGENIC	--	--	--
Ca4_P2_8750318	Ca4	8750318	TAAAAAAA/TAAAAAAAA	CCCATGTTTTAGTGGGGATG	TGCACATTGGCTCTTTGATT	59.3	141	INTERGENIC	--	--	--
Ca4_P2_8750740	Ca4	8750740	TAAAAAAA/TAAAAAAAAA	AGAATCAAAGAGCCAATGTGC	TTGGCATACTTCCCATCCAT	60.2	863	INTERGENIC	--	--	--
Ca4_P2_8753314	Ca4	8753314	TGCTGGAACGCTGGAAC/TGCTGGAAC	TTCTTTCCCAGTCATTTCGG	CCAAATGGAGCTTCCACCTA	60.1	841	INTRON	Ca_08371	--	NAC
Ca4_P2_8753976	Ca4	8753976	GAAAAAAAA/GAAAAAAA	GGTGGAAGCTCCATTTGGTA	TAAGAAGGAACTGCGCCACT	60.0	614	INTRON	Ca_08371	--	NAC

INDEL marker IDs	Chromosomes/unanchored scaffolds	Physical positions (bp)	InDels (*Kabuli* reference genome-CDC Frontier/PII)	Forward primers (5'-3')	Reverse primers (5'-3')	Anneling temperature (0C)	Expected amplified product size (bp)	Structural annotation		Functional annotation	
								Sequence components of *kabuli* genome	*Kabuli* gene accession IDs	NCBI-KOG	TFs
Ca4_P2_8756040	Ca4	8756040	TGAAGAA/TGAA	GTTGATGAGTGCTCAAGCCA	AGCAAGCTGGTTATGGATGG	60.1	548	DRR	Ca_08371	--	NAC
Ca4_P2_8756398	Ca4	8756398	AGGG/AGG	GCTAACCTTTCCCAATTCCC	TGAATTGGCCAAAAACTCAA	59.1	458	DRR	Ca_08371	--	NAC
Ca4_P2_8758235	Ca4	8758235	TGCACGCCC/T	CATAGCAGCTTCGATGCAAA	TCACAATTTGACAACCACGG	60.4	297	INTERGENIC	--	--	--
Ca4_P2_8758495	Ca4	8758495	A/AG	TCACCATTCGTGAGTTGGAA	TGCTCATGTCTTCTAAAGATCCA	59.0	762	INTERGENIC	--	--	--
Ca4_P2_8759575	Ca4	8759575	ATTTTTT/ATTTTTTT	TGCCACTCTCAATCATTTCG	TCAGGACAAGGACATCCAAA	59.1	817	INTERGENIC	--	--	--
Ca4_P2_8764088	Ca4	8764088	CTTTTTTTTTT/CTTTTTTTTT	ATAACCAACTATGCAGGGCG	GGCAACTCGGATTTTCTTCC	60.9	528	INTERGENIC	--	--	--
Ca4_P2_8766483	Ca4	8766483	TAAG/TAAGAAG	GGTTGCGGTCGAAGTTACAT	GATGGGCCAAACTGCTTTTA	60.1	575	INTERGENIC	--	--	--
Ca4_P2_8766663	Ca4	8766663	TAAAAAAAA/TAAAAAAA	GGTTGCGGTCGAAGTTACAT	GATGGGCCAAACTGCTTTTA	60.1	575	INTERGENIC	--	--	--
Ca4_P2_8774176	Ca4	8774176	CTT/CTTT	AATGGGTTTCGTATCGTTCG	TGATTTATGTTTGTGGCGGA	59.9	574	DRR	Ca_08372	--	NAC
Ca4_P2_8775167	Ca4	8775167	ATT/ATTT	TGTTCCCTGCAATTCACATC	ACGGGTCTCGATCAATGTGT	60.4	360	DRR	Ca_08373	C	--
Ca4_P2_8779838	Ca4	8779838	GAAAAAAAAA/GAAAAAAAAAA	GGCATGCTGAGTTCTGTGAA	CAACCCACTGTCGAAAGGTT	60.0	486	INTERGENIC	--	--	--
Ca4_P2_8795294	Ca4	8795294	CT/CTAAATATGT	GAAAGACAGCACCCCAAAAA	TCAACCGTTGGAATATCACG	59.4	666	DRR	Ca_08376	--	M-type

INDEL marker IDs	Chromosomes/unanchored scaffolds	Physical positions (bp)	InDels (*Kabuli* reference genome-CDC Frontier/PII)	Forward primers (5'-3')	Reverse primers (5'-3')	Anneling temperature (0C)	Expected amplified product size (bp)	Structural annotation		Functional annotation	
								Sequence components of *kabuli* genome	*Kabuli* gene accession IDs	NCBI-KOG	TFs
Ca4_P2_8802134	Ca4	8802134	GTTT/GTT	CAGTGGCTCTGGGTATGGTT	CATTTGTCAAGTCGGGGTTT	59.8	774	DRR	Ca_08377	--	--
Ca4_P2_8805954	Ca4	8805954	T/TTACA	TTCTTCATAAGTGGGGTCGG	GCTAGGCCACTGCTTTCATC	60.0	447	INTRON	Ca_08378	AR	MYB
Ca4_P2_8808997	Ca4	8808997	TAGAGAGAGAGAGAGAGAGAGA/TAGAGAGAGAGAGAGAGA	CAAACTGTCGCTTGTGGCTA	TTCACCTCCTCCGAAATCAC	60.1	506	INTRON	Ca_08378	AR	MYB
Ca4_P2_8810050	Ca4	8810050	GAATTATAGCTTTAAAT/GAAT	ACTCCTCCCTCCTGTCTCGT	TGAATATAAATAGACCAATACCGCAA	60.0	585	INTERGENIC	--	--	--
Ca4_P2_8830201	Ca4	8830201	AATATA/AATATATA	AGAAAATCTAAACCGCGCCT	TGGACTTTCTTATTTGCCGC	60.2	303	INTERGENIC	--	--	--
Ca4_P2_8836055	Ca4	8836055	TATTTTTAATTTT/TATTTTTAATTTTTAATTTT	TGACCTGTAGGGCCGTAATC	TCACCAAATCGAAGCTCAAA	59.4	508	INTERGENIC	--	--	--
Ca4_P2_8836158	Ca4	8836158	GTT/GTTAATT	TGACCTGTAGGGCCGTAATC	TCACCAAATCGAAGCTCAAA	59.4	508	INTERGENIC	--	--	--
Ca4_P2_8836388	Ca4	8836388	CTTG/CTTGTAGTTTTG	TGACCTGTAGGGCCGTAATC	TCACCAAATCGAAGCTCAAA	59.4	508	INTERGENIC	--	--	--
Ca4_P2_8836902	Ca4	8836902	T/TG	AGCTTCGATTTGGTGAGGTG	TTGAGTTTCAATATGGTGATGTGA	59.4	854	INTERGENIC	--	--	--
Ca4_P2_8836976	Ca4	8836976	ATTTTTTTT/ATTTTTTT	AAATATTTCGGAATCATTGTGG	GGCCGATAAGCTCTCTAGAAATAA	59.5	622	INTERGENIC	--	--	--
Ca4_P2_8837184	Ca4	8837184	T/TA	AAATATTTCGGAATCATTGTGG	AATTTGGCCGATAAGCTCTC	58.4	627	INTERGENIC	--	--	--
Ca4_P2_8839277	Ca4	8839277	AAAATAAAAAAAAAATAAA/AAAA	GCCAACATCGATCGAGATAC	TTGAACGGGTTTTGTGGTCT	60.4	521	INTERGENIC	--	--	--

INDEL marker IDs	Chromosomes/unanchored scaffolds	Physical positions (bp)	InDels (*Kabuli* reference genome-CDC Frontier/PII)	Forward primers (5'-3')	Reverse primers (5'-3')	Anneling temperature (0C)	Expected amplified product size (bp)	Structural annotation		Functional annotation	
								Sequence components of *kabuli* genome	*Kabuli* gene accession IDs	NCBI-KOG	TFs
Ca4_P2_8840055	Ca4	8840055	TTAAT/TT	TCATTTAAGAATGCAAAATCACTTG	AGTCCACTTAGCCTGTCCGA	59.9	581	INTERGENIC	--	--	--
Ca4_P2_8840451	Ca4	8840451	TAAAAAAAAAAAA/TAAAAAAAAA	CCAAAATCGGACAGGCTAAG	AAAAATCTAACTTTTTGTCGGAGG	59.2	675	INTERGENIC	--	--	--
Ca4_P2_8843670	Ca4	8843670	CTATAATTTTT/CT	AGGTGAATCGCCTTCAAAAC	GGTGAAAGAGCAAAAGACGC	60.0	779	INTERGENIC	--	--	--
Ca4_P2_8858467	Ca4	8858467	TTACTAA/TTACTAATATTATAATATACTAA	TTGGTTTCCAAGTTCCCATC	TTCAACTTCGAGCAAAAGGG	60.4	602	INTRON	Ca_08382	--	MIKC
Ca4_P2_8860985	Ca4	8860985	TAAAAAAAA/TAAAAAAA	TGCAACCAACTCACATCCAT	TGCAACTTTTGAGATGCAATTT	59.8	324	DRR	Ca_08383	C	--
Ca4_P2_8861999	Ca4	8861999	CAAAAAAAA/CAAAAAAAAAAA	CGAGGGAGCATTATGCAACT	TCCTTCGCCTCTCAAGAAAA	60.1	432	DRR	Ca_08383	C	--
Ca4_P2_8864412	Ca4	8864412	TATAATAATAATAATAATAATAATAATAAT/TATAATAATAATAATAATAATAATAATAATAATAATAATAAT	TTCACCAAAGCCTCTTGCTT	GTAAAGCTCCCTTTCCCCAC	59.9	657	INTERGENIC	--	--	--
Ca4_P2_8865404	Ca4	8865404	AA/AAATATTTGTCTCTTTA	GCTGCAGCAGACTAGCAGTG	ATTCTACATTCACCCAGCGG	60.0	612	DRR	Ca_08384	O	--
Ca4_P2_8867611	Ca4	8867611	AT/A	AGCTCCCATACCAGGACCTT	GTAGCCACCATTTTCCTCCA	59.9	374	INTERGENIC	--	--	--
Ca4_P2_8868445	Ca4	8868445	A/AT	TGGTTTAGAAGTGGGTTGGC	ATTGCAGCCCAAACTTTGTC	60.1	816	INTERGENIC	--	--	--
Ca4_P2_8871044	Ca4	8871044	CATAAAAT/CATAAAATTTATAAAAT	AAAACATCGGAGTCCACGTC	AATTTCATTGCGGCAAATTC	59.9	699	INTERGENIC	--	--	--
Ca4_P2_8877564	Ca4	8877564	TTTCTTCTTCTTCTTCTT/TTTCTTCTTCTTCTTCTTCTT	CCATGCGGTAATCCCATAAC	GGTGCGAAGAAGGATCTCAG	60.0	291	INTERGENIC	--	--	--

INDEL marker IDs	Chromosomes/unanchored scaffolds	Physical positions (bp)	InDels (*Kabuli* reference genome-CDC Frontier/PII)	Forward primers (5'-3')	Reverse primers (5'-3')	Anneling temperature (0C)	Expected amplified product size (bp)	Structural annotation		Functional annotation	
								Sequence components of *kabuli* genome	*Kabuli* gene accession IDs	NCBI-KOG	TFs
Ca4_P2_8877657	Ca4	8877657	GAGAAAGAAAGAAAGAAAGAAAGAA/GAGAAAGAAAGAA	CCATGCGGTAATCCCATAAC	GGTGCGAAGAAGGATCTCAG	60.0	291	INTERGENIC	--	--	--
Ca4_P2_8879996	Ca4	8879996	AA/AAAAAAACA	AAAGGGACAGAGACGATCCA	TGTTGGTTCCATTTTCATTGG	60.6	336	INTERGENIC	--	--	--
Ca4_P2_8882798	Ca4	8882798	ACT/ACTAATGCT	CCAACTGCATTCGACGACTA	TTCCTCCATAAATTCAACATTGG	60.1	754	INTERGENIC	--	--	--
Ca4_P2_8889783	Ca4	8889783	A/AC	TTAAGAAGTATCACGCGGGC	TCCTTTCTCCGTGGTGAGTT	59.7	472	INTERGENIC	--	--	--
Ca4_P2_8890826	Ca4	8890826	CAGGTAG/CAG	TTTGAGGAGTGTGTGGTGGA	ACCCAACTCGCACTTTGAAT	59.6	360	INTERGENIC	--	--	--
Ca4_P2_8891981	Ca4	8891981	CAAA/CAA	ATACACCACAACGCGTCAAA	AAGCATGGTATTCGGGAGTG	60.0	204	DRR	Ca_08386	--	--
Ca4_P2_8893770	Ca4	8893770	ATTTTTTTTT/ATTTTTTTTTT	TGATGGTTGGGTTGTGAGAA	ACCCAAACCGGATATCAGAA	59.2	681	INTERGENIC	--	--	--
Ca4_P2_8896251	Ca4	8896251	CAAAAA/CAAAA	GTCGCTAAACTGCAAAAGGC	TTTGTCCGGATGAGTTATCAAA	59.5	477	INTERGENIC	--	--	--
Ca4_P2_8897355	Ca4	8897355	TA/T	GATCCACTCAGGGTCGTGTT	ACTTTTGCATCTGGATGGCT	59.7	461	INTERGENIC	--	--	--
Ca4_P2_8903771	Ca4	8903771	GAATTAAT/GAAT	GGATCTCATTGGTTCTATGGG	CGAGTGTGGTGGGTTTTCTT	60.0	490	INTERGENIC	--	--	--
Ca4_P2_8903859	Ca4	8903859	CATAT/CAT	GGATCTCATTGGTTCTATGGG	CGAGTGTGGTGGGTTTTCTT	60.0	490	INTERGENIC	--	--	--
Ca4_P2_8906103	Ca4	8906103	CTTTTTTTT/CTTTTTTTTTTTT	AATGAATACATTGGCCCTCG	CGTGAAAAACAACAAATCGTTC	59.5	548	INTERGENIC	--	--	--

INDEL marker IDs	Chromosomes/unanchored scaffolds	Physical positions (bp)	InDels (*Kabuli* reference genome-CDC Frontier/PII)	Forward primers (5'-3')	Reverse primers (5'-3')	Anneling temperature (0C)	Expected amplified product size (bp)	Structural annotation		Functional annotation	
								Sequence components of *kabuli* genome	*Kabuli* gene accession IDs	NCBI-KOG	TFs
Ca4_P2_8906252	Ca4	8906252	TAAA/TAAAAA	AATGAATACATTGGCCCTCG	CGTGAAAAACAACAAATCGTTC	59.5	548	INTERGENIC	--	--	--
Ca4_P2_8942163	Ca4	8942163	TAGAAAGAAAGAAAGAAAGA/TAGAAAGAAAGA	AAGGGAGAATGTTGTGGTGG	CCCAACACTGCTCAACTCAA	59.9	226	CDS (FRAME SHIFT)	Ca_08390	V	--
Ca4_P2_8977001	Ca4	8977001	ATTTTTTTTT/ATTTTTTTTTT	GGCTTATTATGGGGAGACCC	TGGATTGTTGGAGCATTTGA	60.0	806	INTERGENIC	--	--	--
Ca4_P2_9030140	Ca4	9030140	ATTTTTTTTTTT/ATTTTTTTTTTTT	TGGTGTTGGAGTCATGCTTT	AAATTTGCAATGACGGCTTC	60.1	656	INTERGENIC	--	--	--
Ca4_P2_9049533	Ca4	9049533	TTATATATATATATATATATATATATATATA/TTATATATATATATATATATATATATATATATATATATATA	AAGAATTGTCAACCAAGTTCTACC	AACATGGCGTTTTGGTTGAT	60.2	667	DRR	Ca_08398	Q	B3
Ca4_P2_9060726	Ca4	9060726	G/GAGCCTGAATTTT	GCATGGTGCAAGATGGTTTT	AAACGGCAGTTGGGTAACAC	59.9	782	INTERGENIC	--	--	--
Ca4_P2_9066423	Ca4	9066423	ACC/AC	GCAACCCGTAGGTGTAGGAA	TGTGCGTTGGTAGTCAATGTG	60.6	546	INTERGENIC	--	--	--
Ca4_P2_9066518	Ca4	9066518	ATTTTTTTT/ATTTTTTT	GCAACCCGTAGGTGTAGGAA	TGTGCGTTGGTAGTCAATGTG	60.6	546	INTERGENIC	--	--	--
Ca4_P2_9079145	Ca4	9079145	ATTTTTTTTTTT/ATTTTTTTTTT	AGTTGGAAGTCGCCAAAGAA	ATGGTGCAGTTCTTGCCATA	59.2	629	INTERGENIC	--	--	--
Ca4_P2_9176599	Ca4	9176599	ACTT/ACTTATACCTT	TGTGACCTAGACGCTTGTGC	GGAAAAGAATTGAGGAGCCC	60.0	656	INTERGENIC	--	--	--
Ca4_P2_9229798	Ca4	9229798	TGGG/TGGGG	AAGGCTCAACATGCAAAACC	TGCCAATTCGATTGGAATAA	59.0	522	INTERGENIC	--	--	--
Ca4_P2_925502	Ca4	925502	AATATATATATATATATATATATATATA/AATATATATATATATATATATATATATATATA	AATGGTGCAACACTCCATGA	CTGTCGGTTTGTTTCCCCTA	60.0	680	DRR	Ca_07727	T	--

INDEL marker IDs	Chromosomes/unanchored scaffolds	Physical positions (bp)	InDels (*Kabuli* reference genome-CDC Frontier/PII)	Forward primers (5'-3')	Reverse primers (5'-3')	Anneling temperature (0C)	Expected amplified product size (bp)	Structural annotation		Functional annotation	
								Sequence components of *kabuli* genome	*Kabuli* gene accession IDs	NCBI-KOG	TFs
Ca4_P2_9358456	Ca4	9358456	ATTTTTTTTT/ATTTTTTTTTT	CAAGAGTGGACCGACATCCT	GCGGAGAAGACAGAGACGAT	59.6	123	INTERGENIC	--	--	--
Ca4_P2_9371255	Ca4	9371255	GAAAAAAAA/GAAAAAAAAA	GGAGCATATGCCTCGTTGTT	TGGAAATTCAACTTTTTGGACA	59.5	549	INTERGENIC	--	--	--
Ca4_P2_9388140	Ca4	9388140	C/CTTCAT	TCGTGTTGCGCCTAACAAT	AGTTTTTAATCCGTGCGTCG	60.1	976	INTERGENIC	--	--	--
Ca4_P2_9388420	Ca4	9388420	TNNNNNNNNNN/TGGAAAGAAANNNNNNNNNN	CCACTTATACGGCAAAGGCT	AGTTTTTAATCCGTGCGTCG	60.1	423	INTERGENIC	--	--	--
Ca4_P2_9460620	Ca4	9460620	ATTTTTTTTT/ATTTTTTTTTT	TTGGTGGAATAAATGCCAAA	TTGCGTGTTTTAAGAATTCGG	60.1	283	INTERGENIC	--	--	--
Ca4_P2_9463038	Ca4	9463038	ATTAATTTT/ATT	CAAGAGGAAACATGGAGGGA	CCAAAAAGCAACTTTCAAGCC	61.1	632	DRR	Ca_08435	R	C2H2
Ca4_P2_9506168	Ca4	9506168	AATTATTATTATTATTATTATTATTATTATTATTATTATTATT/AATTATTATTATTATTATTATTATTATTATTATTATTATT	GGGCAAAACGCAATGTAACT	TACATCTCCAAATGCGACCA	60.1	401	INTERGENIC	--	--	--
Ca4_P2_9550177	Ca4	9550177	TAAAAAAAAAAAAAA/TAAAAAAAAAAAAAAAAAA	CCTCAGATGAGAGCCAGGAC	TTGGGCAGTACCATTTGTGT	58.9	888	INTERGENIC	--	--	--
Ca4_P2_9567689	Ca4	9567689	TTATATATATATATATATATATATATATAT/TTATATATATATATATATATATATATATATATAT	ATTGTCGTCAAATGGACCCT	CGCATGAACGTAGATATGCC	59.2	251	INTERGENIC	--	--	--
Ca4_P2_9569592	Ca4	9569592	CTT/C	CAAATGTCGAATTTAAGTATTGCG	TTGTCCTTCTCCCATTGTCC	59.9	355	INTERGENIC	--	--	--
Ca4_P2_9576810	Ca4	9576810	CGTCAGGG/CG	CCCAATTATAGCGTGGTGCT	AAAGTTTGGCCTGACTTGGA	59.7	790	INTERGENIC	--	--	--
Ca4_P2_9597551	Ca4	9597551	CTTTTTTTTTT/CTTTTTTTTTTT	TTGTTTGATACACGTGATCTGC	TGTGAAGCGAAGAGGGATTT	59.8	806	INTERGENIC	--	--	--

INDEL marker IDs	Chromosomes/unanchored scaffolds	Physical positions (bp)	InDels (*Kabuli* reference genome-CDC Frontier/PII)	Forward primers (5'-3')	Reverse primers (5'-3')	Anneling temperature (0C)	Expected amplified product size (bp)	Structural annotation		Functional annotation	
								Sequence components of *kabuli* genome	*Kabuli* gene accession IDs	NCBI-KOG	TFs
Ca4_P2_9608721	Ca4	9608721	ATTTTTTTTTT/ATTTTTTTTTTT	TTTTATGAATGAGAACTCGCAAA	TTGAATGAGAGTCGGCTCATAG	59.5	320	INTERGENIC	--	--	--
Ca4_P2_9615383	Ca4	9615383	CTCTTCTTCTTCTTCTTCTTCTTCTTCTTC/CTCTTCTTCTTCTTCTTCTTCTTCTTC	TTCTCGCCACCTCAAGTTCT	GCACAGAGCAACAGAAGCAG	59.9	661	CDS (large-effect mutations)	Ca_08443	--	MYB_related
Ca4_P2_9630289	Ca4	9630289	ATTAGTTAGTT/ATTAGTTAGTTAGTT	CCTCTTCCAAACAAATCTTCG	CCAGCTCATCAAAGCCATTT	60.2	550	INTERGENIC	--	--	--
Ca4_P2_9631458	Ca4	9631458	GTTTCCTCCTCCGTCTTT/GTTTCCTCCTCCGTCTTTCCTCCTCCGTCTTT	TTCTTCATCTTCCTCCTCCG	TTATCGGATGTATGGGAGGG	59.6	148	INTERGENIC	--	--	--
Ca4_P2_9632653	Ca4	9632653	TG/T	TGGATTGTCGTGATCTCAAA	CACACCCGTACTCAGCATTG	60.2	630	INTERGENIC	--	--	--
Ca4_P2_9633059	Ca4	9633059	TAAAAAAAAAAA/TAAAAAAAAA	CAATGCTGAGTACGGGTGTG	AACACAAGGAGGCCATGTTC	60.0	503	INTERGENIC	--	--	--
Ca4_P2_9653112	Ca4	9653112	TAAAAAAAAAA/TAAAAAAAAAAA	GGGTTGTACGATGACAGCAA	AAATCGTCGCAAATCAGTCC	60.1	208	INTERGENIC	--	--	--
Ca4_P2_9658674	Ca4	9658674	TAAAAAAA/TAAAA	TGCAATTTGATACCGAGCCT	TCCCCTTCTTTGAGGGATTT	59.9	914	INTERGENIC	--	--	--
Ca4_P2_9726284	Ca4	9726284	ATTTTTTTTTT/ATTTTTTTT	GGGCTTACCAAAAGCAGACA	GCGCCACCATGGACTACTAT	60.0	314	INTERGENIC	--	--	--
Ca4_P2_9727283	Ca4	9727283	AT/A	TGTCTGCGGTCACAAAACTC	TGGTGTCCATTTTTGCACAT	59.8	504	DRR	Ca_08452	E	--
Ca4_P2_9732872	Ca4	9732872	A/AC	CGGTGGTTGATAAAGTCGTG	GAATTCAAATGGGGAAATGC	59.2	416	INTERGENIC	--	--	--
Ca4_P2_9764739	Ca4	9764739	GTTTTTTT/GTTTTTTTT	TCAGGCGGGTTTTATAGACA	CTCTGCTCACAACCCAACAA	59.9	701	INTERGENIC	--	--	--

**INDEL marker IDs**	**Chromosomes/unanchored scaffolds**	**Physical positions (bp)**	**InDels (*Kabuli* reference genome-CDC Frontier/PII)**	**Forward primers (5'-3')**	**Reverse primers (5'-3')**	**Anneling temperature (0C)**	**Expected amplified product size (bp)**	**Structural annotation**		**Functional annotation**	
								**Sequence components of *kabuli* genome**	***Kabuli* gene accession IDs**	**NCBI-KOG**	**TFs**
Ca4_P2_978893	Ca4	978893	TAAAAAAAAA/TAAAAAAAA	TTTTGAGGTGCTTTCCCATC	CAATCCCCCAAGCTTAACAA	59.9	535	INTERGENIC	--	--	--
Ca4_P2_9797931	Ca4	9797931	CAAAAAAAAA/CAAAAAAAAAA	CTCGTAAAACACAAAGCGCA	TGAGTGTTCGACAACCAAGC	59.9	602	INTERGENIC	--	--	--
Ca4_P2_9847676	Ca4	9847676	TAAAA/TAAA	GAGTTGCAGGGGATGTAGGA	CACAAAGAAAGGCCTGGAAG	59.8	240	INTRON	Ca_08462	Z	--
Ca4_P2_9857350	Ca4	9857350	TGTGCGAGTGCGAGTGCGAGT/TGTGCGAGTGCGAGT	TGAGACGTTTTTCACACCCA	CTGCAACAATGAATCTTGAAGT	57.0	469	INTERGENIC	--	--	--
Ca4_P2_9923455	Ca4	9923455	AATATATATATATATATATA/AATATATATATATATATA	TGTGAACGTCGTCGTATGGT	CCGATAACAAAAGCTCTCGC	60.0	892	INTERGENIC	--	--	--
Ca4_P2_9946653	Ca4	9946653	TATAAATAAATAAATAAATAAATAAATA/TATAAATAAATAAATAAATAAATA	CCATATGTGTCGGAAGTCCC	TGACCAAACCCTCATTCACA	59.9	332	INTERGENIC	--	--	--
Ca4_P2_9957557	Ca4	9957557	TAAAAAAAAAAA/TAAAAAAAAAA	TGTAAGGTGCACATGCTTGC	CAGAAGGTTGAAAATGTGCG	59.3	415	INTERGENIC	--	--	--
Ca5_P2_10004188	Ca5	10004188	GTTGTTGTTATTGT/GTTGT	TCGCCATTTCCAACTACTCC	AATTTCCTCCTCCCACCAGT	59.8	703	INTERGENIC	--	--	--
Ca5_P2_10004546	Ca5	10004546	ATGT/ATGTCGGTGTTGT	GGCAGGTAGGTTAGCTGCTG	ACCTCCACCACCACTAGCAC	60.0	508	CDS (large-effect mutations)	Ca_20512	--	--
Ca5_P2_10024485	Ca5	10024485	CAAAAA/CAAAA	TGGATTGGAGTTAGCTTGGC	TCCTTGCCTTGTTTTCCATT	59.5	495	INTERGENIC	--	--	--
Ca5_P2_10024689	Ca5	10024689	TAAA/TAAAA	GGGTCTTTAGGCCCTCTTGT	ACCTCGTGTGACTGATGATGA	59.1	565	INTERGENIC	--	--	--
Ca5_P2_10025475	Ca5	10025475	GTTT/GTTTT	TGAGTGCTCGACGACATTTT	GATGTGAAGCCTCTCATTACCC	60.0	219	INTERGENIC	--	--	--

INDEL marker IDs	Chromosomes/unanchored scaffolds	Physical positions (bp)	InDels (*Kabuli* reference genome-CDC Frontier/PII)	Forward primers (5'-3')	Reverse primers (5'-3')	Anneling temperature (0C)	Expected amplified product size (bp)	Structural annotation		Functional annotation	
								Sequence components of *kabuli* genome	*Kabuli* gene accession IDs	NCBI-KOG	TFs
Ca5_P2_10025602	Ca5	10025602	TAAAAAAAA/TAAAAAAA	TGAGTGCTCGACGACATTTT	AGACCTGTTGATTGTGTTTTGG	59.0	700	INTERGENIC	--	--	--
Ca5_P2_10050680	Ca5	10050680	A/ATAT	CAATGCGAGTTCAGCCAATA	AATCGCATACCGACCAAAAC	59.8	379	INTERGENIC	--	--	--
Ca5_P2_10054215	Ca5	10054215	ACTCT/ACTCTCT	TTGCCCAACTTGATGAAACA	GCCATAGCACCTCTGTAGGC	59.9	531	DRR	Ca_20515	--	--
Ca5_P2_10079478	Ca5	10079478	G/GTGTCCGCCACGGC	CCGGAAGAAGAAGAAAACCC	TCTTCCCTTGCTCGTCATCT	60.0	776	INTERGENIC	--	--	--
Ca5_P2_10084501	Ca5	10084501	CTTGTTTTTGTTTTT/CTTGTTTTTGTTTTTGTTTTT	TTTTTGCCATTCCCATCATT	TCATGAAAATCATAAGCCACG	58.6	603	INTERGENIC	--	--	--
Ca5_P2_10087747	Ca5	10087747	TAAAAAAAAA/TAAAAAAAAAA	TTTGTTGGCTCCAAAAGTAATTC	TGTGGAACCATGCCTAACAC	59.4	770	INTERGENIC	--	--	--
Ca5_P2_10087885	Ca5	10087885	ATTTTTTT/ATTTTTTTT	TTTGTTGGCTCCAAAAGTAATTC	TAGGTATGCACGTGGTTGGA	60.0	790	INTERGENIC	--	--	--
Ca5_P2_10093890	Ca5	10093890	GT/G	AATCGGAATCAGCCAATCAG	TCAGCGTAATCCTCCAATCC	60.0	606	INTERGENIC	--	--	--
Ca5_P2_10097611	Ca5	10097611	AAAAAAATAAAAAA/AAAAAAA	TGCATTATTTCTCAGCGGGT	TCGGTTTCCTCAATACCGAT	59.4	199	INTERGENIC	--	--	--
Ca5_P2_10097809	Ca5	10097809	GTTT/GTTTT	GGGCAATGACCGATTTCTAA	CATGCCATACCAATCCATGA	60.2	554	INTERGENIC	--	--	--
Ca5_P2_10124532	Ca5	10124532	GTAACAAT/GT	TGGTTGAAACAACTCCACAAA	TGATTAAGTGGAAGCCATGTT	57.2	570	INTERGENIC	--	--	--
Ca5_P2_10128452	Ca5	10128452	CTTTTTTTTTT/CTTTTTTTTT	TTATCGCGTTAAGCCAAACC	TATGGCCAAATGGACCTCTC	59.9	616	INTRON	Ca_20519	--	--

INDEL marker IDs	Chromosomes/unanchored scaffolds	Physical positions (bp)	InDels (*Kabuli* reference genome-CDC Frontier/PII)	Forward primers (5'-3')	Reverse primers (5'-3')	Anneling temperature (0C)	Expected amplified product size (bp)	Structural annotation		Functional annotation	
								Sequence components of *kabuli* genome	*Kabuli* gene accession IDs	NCBI-KOG	TFs
Ca5_P2_10128945	Ca5	10128945	ATTTTTT/ATTTTT	GGTAGATCTTTGCTGGGTGG	TGCAACCTGAAACATTGCTC	59.8	274	INTRON	Ca_20519	--	--
Ca5_P2_10129044	Ca5	10129044	TAAAAAAA/TAAAAAA	TGGAGCAATGTTTCAGGTTG	AGTTTTCCTGGTCTGCCTCA	59.8	370	INTRON	Ca_20519	--	--
Ca5_P2_10131669	Ca5	10131669	TAAAAAAAAAAA/TAAAAAAAAAAAAAA	AATTAGACGACACGGGGTTG	CGTATCAATTTCTTTTCAAATGG	57.8	635	DRR	Ca_20519	--	--
Ca5_P2_10132207	Ca5	10132207	CTTTTTT/CTTTTT	TCCATTTGAAAAGAAATTGATACG	TCATCAACGGTCATTCAACC	59.3	737	DRR	Ca_20519	--	--
Ca5_P2_10134039	Ca5	10134039	TTATA/TTA	CTGGGGATACGTAGGAGCAA	TTCGGGAGTCGATTACGAGT	59.7	223	INTERGENIC	--	--	--
Ca5_P2_10144565	Ca5	10144565	T/TC	CTTCCCCTTGACAAACCAAA	TGTTTGCCAAAAGAAAAGGG	60.1	491	INTERGENIC	--	--	--
Ca5_P2_10150668	Ca5	10150668	CGG/CG	TAAATGGCAGGTCAATGCAC	CATTGAAAGTGTGGTGGGTG	59.8	354	INTERGENIC	--	--	--
Ca5_P2_10156657	Ca5	10156657	GTT/GT	CACCGCGAGATGTTACTTCA	GGCTTCACAAAGTTTGGGTT	59.1	281	INTRON	Ca_20521	K	--
Ca5_P2_10157748	Ca5	10157748	T/TAA	GCGGAGGATAAGGGTTTTTG	CAGCTATCGTTACACAAGCATT	57.2	692	INTERGENIC	--	--	--
Ca5_P2_10157803	Ca5	10157803	GA/GAGAAA	GCGGAGGATAAGGGTTTTTG	TTCCTTGCTTCTTTTTACGGA	59.0	917	INTERGENIC	--	--	--
Ca5_P2_10159555	Ca5	10159555	AAAAAAAA/AAAAAAAATAAAAAAA	CCAACAGGGCAAACTGAAAT	GCACAATGGGCTTATTCGAT	59.9	197	INTERGENIC	--	--	--
Ca5_P2_10159630	Ca5	10159630	TG/T	CCAACAGGGCAAACTGAAAT	GCACAATGGGCTTATTCGAT	59.9	197	INTERGENIC	--	--	--

INDEL marker IDs	Chromosomes/unanchored scaffolds	Physical positions (bp)	InDels (*Kabuli* reference genome-CDC Frontier/PII)	Forward primers (5'-3')	Reverse primers (5'-3')	Anneling temperature (0C)	Expected amplified product size (bp)	Structural annotation		Functional annotation	
								Sequence components of *kabuli* genome	*Kabuli* gene accession IDs	NCBI-KOG	TFs
Ca5_P2_10159953	Ca5	10159953	TAGAGA/TAGAGAGA	CCAACAGGGCAAACTGAAAT	GCCAAACAAGGGTTACCTGA	60.0	838	INTERGENIC	--	--	--
Ca5_P2_10164696	Ca5	10164696	AC/ACC	TTCACCCTGAAATCCTCCAG	TCCGGTTCTTACAGACCACC	60.0	636	INTRON	Ca_20522	--	C3H
Ca5_P2_10164862	Ca5	10164862	C/CTGGTT	TGTAGCCCAAACCAAATTCC	TCCGGTTCTTACAGACCACC	60.0	301	INTRON	Ca_20522	--	C3H
Ca5_P2_10167336	Ca5	10167336	ATTTTTTTT/ATTTTTTT	AGGGTTCCGGAAGAATAACC	TTCCTTCCGACAAACAAAGG	60.1	263	INTERGENIC	--	--	--
Ca5_P2_10173275	Ca5	10173275	AATATATATATATATATA/AATATATATATATATA	TGTGGTTCCATAGATGGGGT	CATTGAATCCCATTGATTGTTG	60.1	273	INTERGENIC	--	--	--
Ca5_P2_10176653	Ca5	10176653	CA/C	CTCTCAAACGGTTGCATTGA	TGTTTCAGCTGTGATGCAAA	59.0	571	INTERGENIC	--	--	--
Ca5_P2_10185527	Ca5	10185527	A/AT	AAAAAGCAGTTTCGCCAGAA	GAACATGGAGGGTGCAGAAT	59.9	109	DRR	Ca_20524	--	--
Ca5_P2_10189885	Ca5	10189885	T/TG	AGCAGCACAACTCCACACAG	TAATCTGGTCCAAGGGATGC	59.9	417	INTERGENIC	--	--	--
Ca5_P2_10191175	Ca5	10191175	CTTAAATTAT/C	TCTTGTTCGTGTGTCATAGTTCG	CATTTTTGCGTGTGTGTGCT	60.8	407	INTERGENIC	--	--	--
Ca5_P2_10223215	Ca5	10223215	CATAAAAA/C	AATGGGGGTACATGGGAAAT	TCAATTCCACTTGCAGCAATA	59.3	547	INTERGENIC	--	--	--
Ca5_P2_10223315	Ca5	10223315	ATTTTTTTTT/ATTTTTTTT	AATGGGGGTACATGGGAAAT	TCAATTCCACTTGCAGCAATA	59.3	547	INTERGENIC	--	--	--
Ca5_P2_10223381	Ca5	10223381	TA/T	AATGGGGGTACATGGGAAAT	TCAATTCCACTTGCAGCAATA	59.3	547	INTERGENIC	--	--	--

INDEL marker IDs	Chromosomes/unanchored scaffolds	Physical positions (bp)	InDels (*Kabuli* reference genome-CDC Frontier/PII)	Forward primers (5'-3')	Reverse primers (5'-3')	Anneling temperature (0C)	Expected amplified product size (bp)	Structural annotation		Functional annotation	
								Sequence components of *kabuli* genome	*Kabuli* gene accession IDs	NCBI-KOG	TFs
Ca5_P2_10226687	Ca5	10226687	ATTTTTTTTT/ATTTTTTTTTT	TTGGATTACCGGTGAATGGT	GCGGCACAACTAAACTGGAT	60.1	479	DRR	Ca_21246	I	bHLH
Ca5_P2_10226878	Ca5	10226878	ACTC/ACTCGCGGTGAAGTACCTC	TTGGATTACCGGTGAATGGT	GCTGAGGCAGCAATGTCATA	60.0	718	DRR	Ca_21246	I	bHLH
Ca5_P2_10235331	Ca5	10235331	GTTTCTTTATT/GTTTCTTTATTTTTTCTTTATT	GAAGAGAGGGATAGCAGTGGA	GTTCATGTCATGCCAGATGC	60.1	270	INTERGENIC	--	--	--
Ca5_P2_10245515	Ca5	10245515	ATTTTTTTTTT/ATTTTTTTTT	TTCGTCGAAGGAAAATATTTGAA	AAGGTTGCCTCTTCTCCCAC	60.6	838	DRR	Ca_21244	R	--
Ca5_P2_10263025	Ca5	10263025	ACAGCGGTC/AC	CATCTGAAAGGCCAGCAAAT	GGCTCCTGAGAGTTCGTGAC	60.0	179	INTERGENIC	--	--	--
Ca5_P2_10264751	Ca5	10264751	CAAAAAAA/CAAAAAAAA	TTTGAACTTTGTCCGAGGCT	ACCATCGCTGAACATGACAA	60.1	507	INTERGENIC	--	--	--
Ca5_P2_10267496	Ca5	10267496	ATTTTTT/ATTTTTTT	TCGAAACCCATAAAGGTAGCA	TTGGGATTTGTGTCTGAGCA	60.2	309	INTERGENIC	--	--	--
Ca5_P2_10267784	Ca5	10267784	TA/TAA	TGCTCAGACACAAATCCCAA	TCAAAATGCCGCTTAAAATAAAA	60.0	175	INTERGENIC	--	--	--
Ca5_P2_10268582	Ca5	10268582	A/AAAATAATG	TCGACGACAAGAGACTGAGAA	GCATAGGTGAAGGGAATCCA	59.9	623	INTERGENIC	--	--	--
Ca5_P2_10270683	Ca5	10270683	GTTT/GTTTTGCTTTT	AATTGACTCTCTGACCATTCCA	CCACCGACGAAAGGTATTGT	59.9	511	INTERGENIC	--	--	--
Ca5_P2_10276897	Ca5	10276897	GAAAAAAAAAA/GAAAAAAAAA	TGCTAACAACAATGATTGGGC	TGCTGGGAATTTCAAAAACA	59.1	442	INTERGENIC	--	--	--
Ca5_P2_10284409	Ca5	10284409	ATTTTTTTT/ATTTTTTTTT	TCACAGATCAGCGCTACACC	TTTACCCCCAACACGTAAGC	59.9	479	INTERGENIC	--	--	--

INDEL marker IDs	Chromosomes/unanchored scaffolds	Physical positions (bp)	InDels (*Kabuli* reference genome-CDC Frontier/PII)	Forward primers (5'-3')	Reverse primers (5'-3')	Anneling temperature (0C)	Expected amplified product size (bp)	Structural annotation		Functional annotation	
								Sequence components of *kabuli* genome	*Kabuli* gene accession IDs	NCBI-KOG	TFs
Ca5_P2_10287397	Ca5	10287397	AT/ATGGATTTGAGT	CCCAACGAGGAAAAACAAAA	TGTTGACTTGTGTGCGTGTC	59.3	469	INTERGENIC	--	--	--
Ca5_P2_10289518	Ca5	10289518	A/AT	TTGTGACACTTTTTACCCGTG	GTTACGGTGTGCGGTTTTCT	60.0	343	INTERGENIC	--	--	--
Ca5_P2_10290351	Ca5	10290351	CAAA/CAAAA	ATCCGAACTTCGTAGCCATC	TTTGCGCAAGGATATATGGA	59.1	842	INTERGENIC	--	--	--
Ca5_P2_10290554	Ca5	10290554	AATATATATATATATATATATAT/AATATATATATATATATATATATAT	CAATTCATAAATGTCCAATTGTTG	TTTGCGCAAGGATATATGGA	59.1	418	INTERGENIC	--	--	--
Ca5_P2_10291470	Ca5	10291470	GAAAAAAAAA/GAAAAAAAA	CACGTTGCTCTTTCCAATTT	TCAATATGCTCGTTACTTGTCTCA	59.0	569	DRR	Ca_21242	R	bZIP
Ca5_P2_10298097	Ca5	10298097	TA/TAA	CAATTTGGTGGAGAAAAATGC	TCGCATTGCCTTTAGAAAAA	58.6	668	INTERGENIC	--	--	--
Ca5_P2_10298523	Ca5	10298523	C/CCTATTT	TGTTGTTGTGACAAATCCACATT	GACACCACCGTTTCAACTCA	59.6	415	INTERGENIC	--	--	--
Ca5_P2_10299950	Ca5	10299950	CAA/CAAAA	CCTTTTCAGCATTCCAATTCA	GGTTAATTATTTTTAATGGGCGG	59.9	356	INTERGENIC	--	--	--
Ca5_P2_10300146	Ca5	10300146	GA/GATGATTAATAA	CCTTTTCAGCATTCCAATTCA	GGTTAATTATTTTTAATGGGCGG	59.9	356	INTERGENIC	--	--	--
Ca5_P2_10302322	Ca5	10302322	ATTT/A	TGATTTCATTTGAGTGACGTTG	TGGGGTCTTGTGACACCAT	59.8	645	INTERGENIC	--	--	--
Ca5_P2_10308642	Ca5	10308642	ATTTT/ATTT	TTGACATCAAACTTTCTTTGGC	TGAACATGTGTTCGATTCGTT	59.0	139	DRR	Ca_21241	--	--
Ca5_P2_10311484	Ca5	10311484	TAAAAAAA/TAAAAAAAA	AGCGCCACTGTAGCGATTAT	TCATAACCCCATTTTGGTTGA	60.0	487	INTERGENIC	--	--	--

INDEL marker IDs	Chromosomes/unanchored scaffolds	Physical positions (bp)	InDels (*Kabuli* reference genome-CDC Frontier/PII)	Forward primers (5'-3')	Reverse primers (5'-3')	Anneling temperature (0C)	Expected amplified product size (bp)	Structural annotation		Functional annotation	
								Sequence components of *kabuli* genome	*Kabuli* gene accession IDs	NCBI-KOG	TFs
Ca5_P2_10311735	Ca5	10311735	ACGAGAGAG/A	ACACGACAAACACTTTCGCA	TTCATTCGTCTTCGTCTTCTCTC	60.0	541	INTERGENIC	--	--	--
Ca5_P2_10313212	Ca5	10313212	CAAAAAAAAAAAAAA/CAAAAAAAAAAAA	GGGAGTGCATAGGACAAAACTC	TGTCCTGACGAGTCTCCAAA	59.4	225	INTERGENIC	--	--	--
Ca5_P2_10318888	Ca5	10318888	AG/A	AGTCGATGCGGGATAAATTG	GGCTGCGATTCTCCTTACAG	60.0	270	INTERGENIC	--	--	--
Ca5_P2_10322569	Ca5	10322569	ATCTCT/ATCT	TTGGCGATGCTTCTTAAGGT	TGCTGCTTTTCTTTTAGGCTC	58.9	682	DRR	Ca_21239	--	--
Ca5_P2_10331676	Ca5	10331676	ATTTTTTTT/ATTTTTTTTT	AATGCCAAAATCTCAAACCG	GCCATGTTTGTCTTTGCACT	58.8	688	DRR	Ca_21237	O	E2F/DP
Ca5_P2_10343037	Ca5	10343037	CATTTTCCCTCGTTT/CATTTTCCCTCGTTTTGATTTTCCCTCGTTT	CTGCCTATTCCCTCCATCAA	AGTAGCTCCAAGAGGCCACA	60.0	614	INTERGENIC	--	--	--
Ca5_P2_10348801	Ca5	10348801	ACC/ACCC	TGTGCCTGAAAATGATTGGA	CAGCTTCGTACACTTTGCCA	60.1	615	INTERGENIC	--	--	--
Ca5_P2_10378027	Ca5	10378027	ACC/ACCCACTCACCC	GATCGCAAGTGATGCAAAGA	TGCCACAATCCATTGTCAGT	60.0	858	INTERGENIC	--	--	--
Ca5_P2_10380821	Ca5	10380821	G/GC	TGCATTTGAGGCAGATTGAG	GGGCGCAGTCTTGATAGAAG	60.0	641	INTERGENIC	--	--	--
Ca5_P2_10387488	Ca5	10387488	TATAATAATAA/TATAATAA	TCCCATCTAAATTGATAAGTCGG	CGTCTCATTTAACTCATTCCACA	59.1	852	INTERGENIC	--	--	--
Ca5_P2_10389582	Ca5	10389582	CTACGTTA/CTA	TGGTTGCTTCATTGCTTGTG	GCGAAGGATATTCGACCAAG	59.7	216	INTERGENIC	--	--	--
Ca5_P2_10393989	Ca5	10393989	GAAAAAAAAAA/GAAAAAAAAA	CCCTACGACGTCCGTTAAAA	CCGGGAGATATGTAGGAGCA	60.1	209	INTERGENIC	--	--	--

INDEL marker IDs	Chromosomes/unanchored scaffolds	Physical positions (bp)	InDels (*Kabuli* reference genome-CDC Frontier/PII)	Forward primers (5'-3')	Reverse primers (5'-3')	Anneling temperature (0C)	Expected amplified product size (bp)	Structural annotation		Functional annotation	
								Sequence components of *kabuli* genome	*Kabuli* gene accession IDs	NCBI-KOG	TFs
Ca5_P2_10396682	Ca5	10396682	TAAAAAAAAAA/TAAAAAAAAA	TTGGAGGTCAAATCTGGGAG	AAAATTTTATGCCGCCACAC	59.8	310	INTERGENIC	--	--	--
Ca5_P2_10398127	Ca5	10398127	GAAAAAAAAAAAAA/GAAAAAAAAAAAAAAA	TCCACCAACCTTTTAACCACA	GCTGTGATGGTCATTCCTGA	59.6	897	INTERGENIC	--	--	--
Ca5_P2_10409173	Ca5	10409173	TTGTGT/TTGTGTGT	TGGCTCTTGAAAAGAAACGAA	TCAATGGTGCTTTTGTTGGA	60.1	805	DRR	Ca_21231	--	WRKY
Ca5_P2_10412522	Ca5	10412522	ATTTTTTTTTTTTT/ATTTTTTTT	TTGCATGTTATTTGTTGCGAA	TTTGCCATGTTTGGTTTATCA	58.9	614	INTRON	Ca_21231	--	WRKY
Ca5_P2_10412867	Ca5	10412867	GATA/GATACATA	TTGCATGTTATTTGTTGCGAA	GACCCTTTTGCATTGACGTT	60.0	918	INTRON	Ca_21231	--	WRKY
Ca5_P2_10413807	Ca5	10413807	GTTTTTTTTTTTTT/GTTTTTTTTTTT	TTCTCATGCTCACTGGTTGC	TCGCATTTAACCATTCACACA	60.0	569	INTERGENIC	--	--	--
Ca5_P2_10414029	Ca5	10414029	TACCCAC/TAC	TTCTCATGCTCACTGGTTGC	TCGCATTTAACCATTCACACA	60.0	569	INTERGENIC	--	--	--
Ca5_P2_10415245	Ca5	10415245	AGG/AG	GACACTCTTTAGCATTTGTCAATTT	TTTTTACCAGATCATTGGAGCA	59.6	599	INTERGENIC	--	--	--
Ca5_P2_10418988	Ca5	10418988	TTTATT/TTT	TGCTTGTGTGGAATGTGGAT	TGTGCCACGTCATTATCTTTT	58.2	408	INTERGENIC	--	--	--
Ca5_P2_10420287	Ca5	10420287	GTTTTTTTTT/GTTTTTTTT	GGTACAATTGTTGCACCACG	TTTGGGAGTAGGCCTTCCTT	60.1	702	INTERGENIC	--	--	--
Ca5_P2_10436561	Ca5	10436561	TAAAAA/TAAAAAA	CCCAATAACATACTCTATCCGAAAA	ATCCACGGATTACTCACGGA	60.3	486	INTERGENIC	--	--	--
Ca5_P2_10440868	Ca5	10440868	CTGATGATTTGATGATTT/CTGATGATTT	AAATGGTCGGACAAAATTGG	TGTTTGTTCATCCACCTCCA	59.9	566	INTERGENIC	--	--	--

INDEL marker IDs	Chromosomes/unanchored scaffolds	Physical positions (bp)	InDels (*Kabuli* reference genome-CDC Frontier/PII)	Forward primers (5'-3')	Reverse primers (5'-3')	Anneling temperature (0C)	Expected amplified product size (bp)	Structural annotation		Functional annotation	
								Sequence components of *kabuli* genome	*Kabuli* gene accession IDs	NCBI-KOG	TFs
Ca5_P2_10443343	Ca5	10443343	GTATATATATATATATATATATAT/GTATATATATATATAT	TGCCTTTTGTGTTTCCCTTC	AACGCCTTGAGCTTGAGAAA	60.1	814	INTERGENIC	--	--	--
Ca5_P2_10445932	Ca5	10445932	ACGCCCG/ACG	GTATCGCTTGGCACTTCCAT	GTGAGTGGGGCTGAGTTTGT	60.2	489	INTERGENIC	--	--	--
Ca5_P2_10446089	Ca5	10446089	TTCTCTCTCTCTC/TTCTCTCTCTC	ACAAACTCAGCCCCACTCAC	TCCCTTTTAAGGGTTGAGGG	60.3	187	INTERGENIC	--	--	--
Ca5_P2_10455362	Ca5	10455362	TTTATTATTATTATTATTATTATTA/TTTATTATTATTATTATTA	CGAAACGAAACCCAACAAGT	TGATGTGGCTTGACGTGACT	60.3	364	INTERGENIC	--	--	--
Ca5_P2_10457260	Ca5	10457260	CACAATACTC/CACAATACTCTACAATACTC	GGGGCGAGATATTCAGACAA	TGATTGTTCTAGTGGTCGCTCT	59.0	108	INTERGENIC	--	--	--
Ca5_P2_10475192	Ca5	10475192	CTTTTTTTT/CTTTTTTT	TCGTGATGTTGATTTGCGAT	GGCAAGAAGGATATGGACGA	60.0	478	INTERGENIC	--	--	--
Ca5_P2_10477577	Ca5	10477577	T/TA	AAATCTGAGCTTTGCCCTCA	GGAGGCATTGTTTCCTCAAA	60.1	832	INTERGENIC	--	--	--
Ca5_P2_10481415	Ca5	10481415	ATGTGTGTGTGTGTGTGTG/ATGTGTGTGTGTGTGTGTGTGTGTG	TGTGAGAACGTCACCCTGAG	GAAATTGCAAGGCAGAGAGG	60.0	632	INTERGENIC	--	--	--
Ca5_P2_10489248	Ca5	10489248	GTTTTTT/GTTTTTTT	TGGAAAATCCATCTGAGCAA	CTACCTCCATTTTGGGGGAT	60.0	479	INTERGENIC	--	--	--
Ca5_P2_10496217	Ca5	10496217	AAA/AAATAAATAA	TTCACTTCAACGCAATGTTGT	GGCTCTTTGTGAAGAGGGTG	59.8	776	INTERGENIC	--	--	--
Ca5_P2_10497866	Ca5	10497866	AT/ATT	GCAAACGGGTTAATTGGAGA	GTCTTATGAGCCGAAGGTCG	59.8	764	INTERGENIC	--	--	--
Ca5_P2_10498397	Ca5	10498397	ACC/AC	GGCTCATAAGACCAACGCTC	CTCTCCGTTGGTTTGTGGAT	60.0	467	INTERGENIC	--	--	--

INDEL marker IDs	Chromosomes/unanchored scaffolds	Physical positions (bp)	InDels (*Kabuli* reference genome-CDC Frontier/PII)	Forward primers (5'-3')	Reverse primers (5'-3')	Anneling temperature (0C)	Expected amplified product size (bp)	Structural annotation		Functional annotation	
								Sequence components of *kabuli* genome	*Kabuli* gene accession IDs	NCBI-KOG	TFs
Ca5_P2_10501158	Ca5	10501158	GAAAAA/GAAAAAA	GCAACAAAATAACACTTACGAAAAA	CCGACGATAAAAGTCCCTCA	60.1	596	INTERGENIC	--	--	--
Ca5_P2_10507484	Ca5	10507484	ATTTTTTTTT/ATTTTTTTTTT	TGCAAGTTAAAGAATAATTTAGAGCG	GGAAACGATTGGATTCGAGA	60.0	897	INTERGENIC	--	--	--
Ca5_P2_10507987	Ca5	10507987	GTTCTTCTTCTTCTTC/GTTCTTCTTCTTC	TCTCGAATCCAATCGTTTCC	ACCAACTTCATCAACCTCGG	60.0	143	CDS (large-effect mutations)	Ca_21230	--	--
Ca5_P2_10511429	Ca5	10511429	GTT/GTTT	AATGTGTGTGGGGATCGAAT	TAGGCCCCAACCAAGTAGTG	60.0	444	DRR	Ca_21230	--	--
Ca5_P2_10513999	Ca5	10513999	AATTATT/AATT	TTTTGTTCCAAAGAGAACACGA	AAACATCGCAGCCTTGAGTC	60.4	784	INTERGENIC	--	--	--
Ca5_P2_105147	Ca5	105147	AATATATATATATATATATATATATATATA/AATATATATATATATATATATATATATATA	CAGAACAAACACGAGAGCCA	TTTGTACCCGAGGTTATCCG	59.8	650	INTERGENIC	--	--	--
Ca5_P2_10515050	Ca5	10515050	TAATAG/T	AGAAATTGTCCCATCCTCCC	TCGTGAAACTTTGACTTTAATCG	58.5	537	INTERGENIC	--	--	--
Ca5_P2_10515370	Ca5	10515370	A/AT	CGATTAAAGTCAAAGTTTCACGA	AGCAAACCAGGTTGTCCTGT	59.6	560	INTERGENIC	--	--	--
Ca5_P2_10516286	Ca5	10516286	TTTTTATTTTATTTT/TTTTTATTTTATTTTATTTT	ATTTGGGTTGAAGTGTTGCC	ATTCAATGCAACTTCCCTGC	60.1	598	INTERGENIC	--	--	--
Ca5_P2_10516320	Ca5	10516320	A/ATGAAGCAC	ATTTGGGTTGAAGTGTTGCC	ATTCAATGCAACTTCCCTGC	60.1	598	INTERGENIC	--	--	--
Ca5_P2_10550017	Ca5	10550017	C/CATATTTTACCATTGAT	CTCCTCATATTGCAGGCGTT	CGGCTAAACAACAAGCCATT	60.1	703	DRR	Ca_21226	--	--
Ca5_P2_10550493	Ca5	10550493	TAAAAAAAA/TAAA	AATGGCTTGTTGTTTAGCCG	CAAGCAATTTCGTGTTTCCA	59.7	305	DRR	Ca_21227	C	bHLH

INDEL marker IDs	Chromosomes/unanchored scaffolds	Physical positions (bp)	InDels (*Kabuli* reference genome-CDC Frontier/PII)	Forward primers (5'-3')	Reverse primers (5'-3')	Anneling temperature (0C)	Expected amplified product size (bp)	Structural annotation		Functional annotation	
								Sequence components of *kabuli* genome	*Kabuli* gene accession IDs	NCBI-KOG	TFs
Ca5_P2_10550623	Ca5	10550623	GTTTTTTTTT/GTTTTTTTTTT	AGCCGTTGTTTGTGAAAACC	TCCACCTTGTATTATGCTTGC	57.8	788	DRR	Ca_21227	C	bHLH
Ca5_P2_10559263	Ca5	10559263	ATTTTTTTTTTTTT/ATTTTTTTTTT	CACGAATTCAGGGAGGAAAA	GCTTTTACGGAATGGCAAAA	60.1	795	INTERGENIC	--	--	--
Ca5_P2_10559602	Ca5	10559602	AT/ATTTATCATTCATATGT	TGTCATTTCAAATTAGACCCAAAA	GCTTTTACGGAATGGCAAAA	60.1	250	INTERGENIC	--	--	--
Ca5_P2_10563740	Ca5	10563740	CG/C	AAAGGCAAAATGATCCATGC	CCCTAGCTTGCTTGAACTGC	60.2	246	INTRON	Ca_21225	--	--
Ca5_P2_10564446	Ca5	10564446	TAAAAAAAA/TAAAAAAA	TCCAAATCCCAAGCTACCAA	GGGCCTCATTTCCCTTTAAC	59.8	408	INTRON	Ca_21225	--	--
Ca5_P2_10564601	Ca5	10564601	ATTTT/ATT	TCCAAATCCCAAGCTACCAA	GGGCCTCATTTCCCTTTAAC	59.8	408	INTRON	Ca_21225	--	--
Ca5_P2_10572522	Ca5	10572522	AATATATATATATATATATATATAT/AATATATATATATATAT	CGTGGACAAAGATCGTGATG	TCATTGTTAAAGAGCGGTGC	58.9	382	INTERGENIC	--	--	--
Ca5_P2_10588970	Ca5	10588970	T/TTA	TCATTCTTTTATGCGTTTGGC	GTTTTGGCGTTGGTTTCACT	60.0	792	INTERGENIC	--	--	--
Ca5_P2_10589055	Ca5	10589055	GATAATAA/GATAA	AAAGGGACCAATGAGTTTTAACC	GTTTTGGCGTTGGTTTCACT	60.0	318	INTERGENIC	--	--	--
Ca5_P2_10605600	Ca5	10605600	GT/GTT	AGTGAAACCAACGCCAAAAC	TCACTCAAAGGGCTACTGCC	60.4	272	DRR	Ca_21224	--	--
Ca5_P2_10610168	Ca5	10610168	ATTTTTTTTT/ATTTTTTTTTTT	GCTTTGCCACATGAGATTGA	GCAGTGCTGAATTTTGGTGA	59.8	519	INTERGENIC	--	--	--
Ca5_P2_10611566	Ca5	10611566	CTA/CTATA	CTAGCACCTGCAACCACTGA	GGGTTGACAGCATAAGGCAT	60.0	347	INTERGENIC	--	--	--

INDEL marker IDs	Chromosomes/unanchored scaffolds	Physical positions (bp)	InDels (*Kabuli* reference genome-CDC Frontier/PII)	Forward primers (5'-3')	Reverse primers (5'-3')	Anneling temperature (0C)	Expected amplified product size (bp)	Structural annotation		Functional annotation	
								Sequence components of *kabuli* genome	*Kabuli* gene accession IDs	NCBI-KOG	TFs
Ca5_P2_10611823	Ca5	10611823	AT/A	ATGCCTTATGCTGTCAACCC	AACTTAGAGCAAGGCGATGG	59.5	339	INTERGENIC	--	--	--
Ca5_P2_10625989	Ca5	10625989	TAAAAAAA/TAAAAAAAA	TGTAGGGGTACAGGGGAAAG	AACTTCCGTTGTTTTGCCA	60.5	165	INTERGENIC	--	--	--
Ca5_P2_1065182	Ca5	1065182	TAAAA/TAAAAA	AATGGCCACGAATCTGAAAG	ACCATTTTCAACTGCATCCC	59.8	634	INTERGENIC	--	--	--
Ca5_P2_10659656	Ca5	10659656	GTTTTTTTTTT/GTTTTTTTTT	TGCTGTCTTTTCTGCTTTGC	AGACTATGGTCCACAAGCCG	60.1	257	INTERGENIC	--	--	--
Ca5_P2_10673945	Ca5	10673945	ATTTTTTTTTT/ATTTTTTTT	CACGGCTCATTCAACAACAT	GAAGTGGGCGTTTTACCAGA	60.1	577	INTERGENIC	--	--	--
Ca5_P2_10679625	Ca5	10679625	TAAAAAAAA/TAAAAAAAAA	GGGATTGTTTTGAATATGAGGG	TCACACGATCATTTTGGCAT	59.9	596	INTERGENIC	--	--	--
Ca5_P2_10686981	Ca5	10686981	TAAAAAAAA/TAAAAAAAAAA	GCTCCTTCTGCATCAACACA	TGCCCCACATTTTAGACCTC	59.9	509	INTERGENIC	--	--	--
Ca5_P2_10687869	Ca5	10687869	TAAAAAAAA/TAAAAAAAAA	CGAGGAACGACAAGGGTAAA	TGAGTTGAGTTAAAAACCAGCAGA	60.3	673	INTERGENIC	--	--	--
Ca5_P2_10687910	Ca5	10687910	CTAT/C	CGAGGAACGACAAGGGTAAA	TGAGTTGAGTTAAAAACCAGCAGA	60.3	673	INTERGENIC	--	--	--
Ca5_P2_10689102	Ca5	10689102	TAAAA/TAA	ATGGGCCACCTATTGAGACA	TGCCAAAGAAAAAGGTTTCG	60.2	623	INTERGENIC	--	--	--
Ca5_P2_10689622	Ca5	10689622	AGAAGCATG/AG	CGAAACCTTTTTCTTTGGCA	AACGGATTGTCCCTTCATTG	59.8	418	INTERGENIC	--	--	--
Ca5_P2_10693789	Ca5	10693789	CTTGTGATTGTTTTGTTGTGATTG/CTTGTGATTG	CCAATTTGAATTGTCCGTCC	TTGCAAAAGCTGGGTCAAGT	60.8	406	INTERGENIC	--	--	--

INDEL marker IDs	Chromosomes/unanchored scaffolds	Physical positions (bp)	InDels (*Kabuli* reference genome-CDC Frontier/PII)	Forward primers (5'-3')	Reverse primers (5'-3')	Anneling temperature (0C)	Expected amplified product size (bp)	Structural annotation		Functional annotation	
								Sequence components of *kabuli* genome	*Kabuli* gene accession IDs	NCBI-KOG	TFs
Ca5_P2_10705967	Ca5	10705967	TA/TAATAATAA	AAAACAATGTCGTGCAAAAGTG	CCACCACCTTAAATGACCACA	60.7	782	INTERGENIC	--	--	--
Ca5_P2_10792462	Ca5	10792462	AATATATATATATATATAT/AATATATATATATATATAT	TCACTTTGAATTAGGGGGCA	TCATTCACTTTGTTGACCACG	59.6	634	INTERGENIC	--	--	--
Ca5_P2_10867305	Ca5	10867305	GC/G	TGTAATCATTTAGGGATGCATGA	GCACGTGCACATGTCTTACA	59.3	560	INTERGENIC	--	--	--
Ca5_P2_10995810	Ca5	10995810	AAATAATAATAATAATAATAAT/AAATAATAATAATAATAAT	ACTTCAGGTGGTCGATGTCC	GGGAGCGACCTCACACTAGA	60.4	923	INTERGENIC	--	--	--
Ca5_P2_1101216	Ca5	1101216	GATTTGAAGAATATTTGAAGAA/GATTTGAAGAATATTTGAAGAATATTTGAAGAA	TATGTGGCGATGAAGTGAGG	TCGACCCTACAAGCCAAATC	60.1	724	INTERGENIC	--	--	--
Ca5_P2_11108313	Ca5	11108313	TTATATATATATATATATATATA/TTATATATATATATATATATATATA	CCATTTCGATTTGGGAGAGA	TCACTCACATATGCACCCGT	60.0	356	INTERGENIC	--	--	--
Ca5_P2_11131180	Ca5	11131180	GT/GTT	CAAGTGCACAGCGGAATTTA	TCCGATTGGATTTTCAGGAC	59.9	148	INTERGENIC	--	--	--
Ca5_P2_11132314	Ca5	11132314	GT/G	TCGACTTGTCAATCGCTTTG	GGGGGAGATTGTTGGTTTTT	60.0	686	INTERGENIC	--	--	--
Ca5_P2_11132906	Ca5	11132906	A/AG	AAAAACCAACAATCTCCCCC	TTGGTCGACAAGCAACTGAG	60.0	716	INTERGENIC	--	--	--
Ca5_P2_11166925	Ca5	11166925	CTTTTTTTTT/CTTTTTTTTTT	GGGAGCACAGTTGGAAAATC	ACGTGGCGATATGTGAATGA	60.0	536	INTERGENIC	--	--	--
Ca5_P2_11168411	Ca5	11168411	AATA/AA	TTCAAACGGAACTGATGCTTT	TTGGTTGGGGGAGTTATTGA	60.2	954	INTERGENIC	--	--	--
Ca5_P2_11168740	Ca5	11168740	T/TA	TCGTTGAGGAAAAACAAACAAA	TTGGTTGGGGGAGTTATTGA	60.2	557	INTERGENIC	--	--	--

INDEL marker IDs	Chromosomes/unanchored scaffolds	Physical positions (bp)	InDels (*Kabuli* reference genome-CDC Frontier/PII)	Forward primers (5'-3')	Reverse primers (5'-3')	Anneling temperature (0C)	Expected amplified product size (bp)	Structural annotation		Functional annotation	
								Sequence components of *kabuli* genome	*Kabuli* gene accession IDs	NCBI-KOG	TFs
Ca5_P2_11168801	Ca5	11168801	ACTATTAGTCTATTA/ACTATTA	TCGTTGAGGAAAAACAAACAAA	TTGGTTGGGGGAGTTATTGA	60.2	557	INTERGENIC	--	--	--
Ca5_P2_11168904	Ca5	11168904	GTCTCTCT/GTCTCT	TCAATAACTCCCCCAACCAA	TCACAATTGGGTTTGGATCA	59.8	227	INTERGENIC	--	--	--
Ca5_P2_11169035	Ca5	11169035	CG/C	TCAATAACTCCCCCAACCAA	TGACGCCGTGAAAAACATTA	60.1	626	INTERGENIC	--	--	--
Ca5_P2_11233017	Ca5	11233017	TAAAAA/TAAA	ATTGGACGTATCCCGAGTTG	GTTGTGGGATGAACTGACCC	60.2	633	INTERGENIC	--	--	--
Ca5_P2_11291761	Ca5	11291761	TA/TAAGTCAACA	TTTGGTCCTTGTAACACCCC	GCGTGAAGATCATCTGGGAT	60.0	367	INTERGENIC	--	--	--
Ca5_P2_11342947	Ca5	11342947	ATATTATTATTATTATTATTATTATTATTATTATT/ATATTATTATTATTATTATTATTATTATTATTATTATTATT	TTGCAGCAAAAACAAGCAAC	CATGCTCACACAATTCCCAC	60.0	736	INTERGENIC	--	--	--
Ca5_P2_11420785	Ca5	11420785	TTATTA/TTATTACTATTA	ATTAAGTGAATGGATCGCGG	AGTGTGGAAGGACGAGCAAG	60.4	485	INTERGENIC	--	--	--
Ca5_P2_11433516	Ca5	11433516	ATT/AT	TAAGCGCACGTTCAATTGTG	AAGTTGAGCAAGTCGCAAAA	58.7	427	INTERGENIC	--	--	--
Ca5_P2_11434569	Ca5	11434569	ATATTATTATTATTATTATTATTATTATTATTATTATTATTATTATTATTATTATTA/ATATTATTATTATTATTATTATTATTATTATTATTATTATTATTATTATTATTATTATTA	TTGCAACATCCAAAATTAAACG	GGTTTTGTTTGACTCAGGAGC	58.8	430	INTERGENIC	--	--	--
Ca5_P2_114518	Ca5	114518	AATATATATATATATATATATATATATATATATATATATATATATAT/AATATATATATATATATATATATATATATATATATAT	AAGGTTATGACTATGCTTGGGG	CCATGCCATAAACACTTGTGA	59.5	930	INTERGENIC	--	--	--
Ca5_P2_11492835	Ca5	11492835	AAATAATAATAATAATAATAATAATAATAATAAT/AAATAATAATAATAATAATAATAATAATAATAAT	GGACGTTAACAACGTGTCAAAA	AATATGGGGATGCGACTACG	59.8	752	INTERGENIC	--	--	--
Ca5_P2_11496437	Ca5	11496437	TAAAATATAAAAATATAAAAATATAAAAATATAAA/TAAAATATAAAAATATAAAAATATAAAAATATAAAAATATAAA	GAAAATAAGGAAGGGACCCG	ATTCAAATAATAAAATCATCACGAAG	57.1	821	INTERGENIC	--	--	--

INDEL marker IDs	Chromosomes/unanchored scaffolds	Physical positions (bp)	InDels (*Kabuli* reference genome-CDC Frontier/PII)	Forward primers (5'-3')	Reverse primers (5'-3')	Anneling temperature (0C)	Expected amplified product size (bp)	Structural annotation		Functional annotation	
								Sequence components of *kabuli* genome	*Kabuli* gene accession IDs	NCBI-KOG	TFs
Ca5_P2_11505972	Ca5	11505972	TTTCTTC/TTTC	GGACCTAAATTTTGCAACAACA	GAACGCGAGATATTGGGTGT	60.0	199	INTERGENIC	--	--	--
Ca5_P2_11517113	Ca5	11517113	T/TA	TCGATTATGCGTAGGGAAGG	CGGGAGATACGTAGGAGCAA	60.2	248	INTERGENIC	--	--	--
Ca5_P2_115778	Ca5	115778	TTCACCGGTCA/TTCA	AAAGATTGCAATTTGCCTGC	TTTTTAGGGGGCGATTGTATT	59.7	511	INTERGENIC	--	--	--
Ca5_P2_11606213	Ca5	11606213	GATATATATATATATATATATATAT/GATATATATATATATATATATATAT	TGTGAAAAAGATGAAGTGTGAAAAA	GTTTGGCGAAAACTCTACCG	59.7	726	INTERGENIC	--	--	--
Ca5_P2_11628665	Ca5	11628665	CNNNNNNNNNN/CNN	TATCCCCGTCCATTAACCAA	GGGAGTGGTTGAGACCTTGA	60.1	248	INTERGENIC	--	--	--
Ca5_P2_11667274	Ca5	11667274	TTGCTGCTG/TTGCTG	GCTCAAAGCACATGAGGTGA	TGCCAAAGCCATGTTTCATA	60.1	377	INTERGENIC	--	--	--
Ca5_P2_1170267	Ca5	1170267	TGAGAGAGAGAGAGAGAGAG/TGAGAGAGAGAGAGAGAG	GGAGGAGAGGCAGACTTGTG	GCATGCCTCTCATTTTCCAT	60.0	587	INTERGENIC	--	--	--
Ca5_P2_11729570	Ca5	11729570	AAATAATAATAA/AAATAATAA	CACGGTCCCTAGAGCCAATA	CGGGAGATACGTAGGAGCAA	60.2	672	INTERGENIC	--	--	--
Ca5_P2_11878820	Ca5	11878820	CATATATATATATATATATATATATAT/CATATATATATATATATATATAT	TATTTTGGATTTTGGCTCGG	AGAGGGGTTTGTGTTTGACG	60.0	599	INTERGENIC	--	--	--
Ca5_P2_11879108	Ca5	11879108	TAA/TAAA	AAGATTTCCTCCCTCCTCCA	CTTTAACATGCGTTTCCGGT	60.0	327	INTERGENIC	--	--	--
Ca5_P2_11886997	Ca5	11886997	GATAATAATAATAATAATAATAA/GATAATAATAATAATAATAATAA	TGCCTTCGGTTTTCCTATTG	AATGTTTGGAATTCCCCCTC	60.0	888	INTERGENIC	--	--	--
Ca5_P2_11983861	Ca5	11983861	GTCT/GATCTTCT	CTGCTGTCAACTCCACTCCA	TCAAAATCCCCCAATAACCA	60.0	713	DRR	Ca_17106	--	--

INDEL marker IDs	Chromosomes/unanchored scaffolds	Physical positions (bp)	InDels (*Kabuli* reference genome-CDC Frontier/PII)	Forward primers (5'-3')	Reverse primers (5'-3')	Anneling temperature (0C)	Expected amplified product size (bp)	Structural annotation		Functional annotation	
								Sequence components of *kabuli* genome	*Kabuli* gene accession IDs	NCBI-KOG	TFs
Ca5_P2_12040897	Ca5	12040897	T/TTTTAAATGAAG	TCGATGATGTTAACTCGCAA	GCGGTGTCATTTTGTTTCAA	59.6	636	INTERGENIC	--	--	--
Ca5_P2_12129667	Ca5	12129667	CT/C	CGTATTATTTTGGGGGTTTGC	GCTCACAACTCGGTCTTTCC	59.9	449	INTERGENIC	--	--	--
Ca5_P2_1230643	Ca5	1230643	TT/TTCCGT	ACGAAATCGAAGAGAAGCGA	TCCAGGACACAAACAATCCA	59.9	455	INTERGENIC	--	--	--
Ca5_P2_12366498	Ca5	12366498	CTTTTT/CTTTT	ATAAGGCTGCTGCTGTTGGT	GCAATTGTTTGTTTTCGGCT	60.1	351	INTERGENIC	--	--	--
Ca5_P2_1239625	Ca5	1239625	AATATATATATATATATATATA/AATATATATATATATATATATATA	GGTTGATTTTGAATAGGTGTCG	ATTAAATTAAGGGGCCGCAG	60.3	107	INTERGENIC	--	--	--
Ca5_P2_12441998	Ca5	12441998	AAATAATAATAATAATAATAATAATAATAATAATAATAATAATAATAATAATAATAAT/AAATAATAATAATAATAATAATAATAATAATAATAATAATAATAATAATAATAAT	CATCAGGCCTATTCAAGGCT	TTTTGTTGCCGCAGTAGTTT	58.5	469	INTERGENIC	--	--	--
Ca5_P2_12448859	Ca5	12448859	ATTTT/ATTTTT	TTCTACCCGAAATTTGTGCC	GTCAGTCAGCCTCTCTTGCC	60.1	361	INTERGENIC	--	--	--
Ca5_P2_1245935	Ca5	1245935	ATTTTTTTTTTTT/ATTTTTTTTTTTTTT	TTGACTGGGCAAAATCAACA	TTTTATGGCTTAGAGGTGTTCAAA	59.3	206	INTERGENIC	--	--	--
Ca5_P2_12475737	Ca5	12475737	GATATATATATATATATATATATATATA/GATATATATATATATATATATATATATATATA	GAGAACGCGACCTCCTATTG	CTGGCAAATGCTAGCAGAAA	59.2	529	INTERGENIC	--	--	--
Ca5_P2_12578471	Ca5	12578471	TATAAAATAAAATAA/TATAAAATAAAATAAAATAA	CTGCATAGGCTATTGGCTGA	GCCACCCTCGTACTCCTCTT	60.6	445	INTERGENIC	--	--	--
Ca5_P2_12606519	Ca5	12606519	AAGAGAG/AAGAG	TGTGTTTGGTTGAAAGCAATG	GAAGAAGCAGTCAAGGTGGC	60.0	540	INTRON	Ca_17071	PT	--
Ca5_P2_12606549	Ca5	12606549	CGG/C	TGTGTTTGGTTGAAAGCAATG	GAAGAAGCAGTCAAGGTGGC	60.0	540	INTRON	Ca_17071	PT	--

INDEL marker IDs	Chromosomes/unanchored scaffolds	Physical positions (bp)	InDels (*Kabuli* reference genome-CDC Frontier/PII)	Forward primers (5'-3')	Reverse primers (5'-3')	Anneling temperature (0C)	Expected amplified product size (bp)	Structural annotation		Functional annotation	
								Sequence components of *kabuli* genome	*Kabuli* gene accession IDs	NCBI-KOG	TFs
Ca5_P2_12619050	Ca5	12619050	AATATATATATATATATATATATA/AATATATATATATATATATATATATATATA	TCCTAACCTTTTGACTAATTTTTCTTC	TCAATCCTCTTAAGATATTTCCCTG	59.1	685	INTERGENIC	--	--	--
Ca5_P2_12631984	Ca5	12631984	GCGTC/GC	GGTTATCACCGTGGTGCTCT	TGGCTTCTGGTTGGATTTTC	60.1	648	INTERGENIC	--	--	--
Ca5_P2_1286848	Ca5	1286848	TAAAAAAAAAAAA/TAAAAAAAAAAA	CTCATTGAAATTCTCATTCGGA	CGTTCACTTTCCACAAAAACAA	60.0	494	INTERGENIC	--	--	--
Ca5_P2_1298884	Ca5	1298884	CAAAAAAAAAAA/CAAAAAAAAAA	TTGAGCACAAGAACCACTTCA	GGTCGTTGTGGATTTTAGCG	60.5	674	DRR	Ca_26278	R	NAC
Ca5_P2_132937	Ca5	132937	CAA/CAAA	CCAAAGACACAGCAAAGCAA	CACTTCCACCAGTTGCAAAA	59.7	569	INTERGENIC	--	--	--
Ca5_P2_133085	Ca5	133085	ACC/AC	GCCATGGAGTGCTGGTTATT	GCGTTGTGAAAACAGGACAA	59.7	709	INTERGENIC	--	--	--
Ca5_P2_133283	Ca5	133283	AATATAT/AATAT	ACCTTTACATGGTGCCAAGC	GCGTTGTGAAAACAGGACAA	59.7	433	INTERGENIC	--	--	--
Ca5_P2_133428	Ca5	133428	GAACA/GAACAATTTTTATAATAACA	ACCTTTACATGGTGCCAAGC	GCGTTGTGAAAACAGGACAA	59.7	433	INTERGENIC	--	--	--
Ca5_P2_13624288	Ca5	13624288	ATTTT/ATTTTTT	TGAAAATGTTGTTGGTAGTGGC	CGACGGTGCCAATTTTCTAT	60.0	948	INTERGENIC	--	--	--
Ca5_P2_13628890	Ca5	13628890	TTGT/TT	CAAAGCATAGATCGGACCGT	CAACAATGACGCAACCATTT	59.4	799	INTERGENIC	--	--	--
Ca5_P2_136326	Ca5	136326	A/AT	TCGAATGTCAACAAACAAGCA	ATTCCTGCACACCACTTTCC	60.0	433	INTERGENIC	--	--	--
Ca5_P2_13636401	Ca5	13636401	A/AC	ATCGACAAACCAACATGCAA	GGGAAACTCACTTGCGGTTA	60.1	735	INTERGENIC	--	--	--

INDEL marker IDs	Chromosomes/unanchored scaffolds	Physical positions (bp)	InDels (*Kabuli* reference genome-CDC Frontier/PII)	Forward primers (5'-3')	Reverse primers (5'-3')	Anneling temperature (0C)	Expected amplified product size (bp)	Structural annotation		Functional annotation	
								Sequence components of *kabuli* genome	*Kabuli* gene accession IDs	NCBI-KOG	TFs
Ca5_P2_13636726	Ca5	13636726	T/TCCA	TGAAATGCAGTACCATAAGTCGAT	GGGAAACTCACTTGCGGTTA	60.1	629	INTERGENIC	--	--	--
Ca5_P2_13636921	Ca5	13636921	CTGT/CT	TAACCGCAAGTGAGTTTCCC	GCGGTGGCTGAGTTTCTTAC	59.9	387	INTERGENIC	--	--	--
Ca5_P2_13640097	Ca5	13640097	AATATATATATATATATATATATATATA/AATATATATATATATATATATATA	CCCAACAATCACATTCAACTCA	CTGCGATCCGATAACCTCAT	60.1	557	INTERGENIC	--	--	--
Ca5_P2_136504	Ca5	136504	AAAAAAGAAAAAGAAAA/AAAAAAGAAAAAGAAAAAAGAAAAAGAAAA	TTTCTAACCCGGAACACGTC	ATTCCTGCACACCACTTTCC	60.0	189	INTERGENIC	--	--	--
Ca5_P2_13702174	Ca5	13702174	ATTTTTTTTTTT/ATTTTTTTTTTTT	ACAGCCCAGGTGTATTTTGC	ATGGATGCAATGAACATGGA	59.7	341	INTERGENIC	--	--	--
Ca5_P2_13714998	Ca5	13714998	TA/T	TGATGTGAGGTGACAGGCAT	GTAGCGTGGAGAGGAGCAAC	60.0	374	INTERGENIC	--	--	--
Ca5_P2_1377883	Ca5	1377883	TCC/TC	GACTCTTTCCATAGCGTGCC	CACCTTGTAGCCAACGGTTT	60.0	720	INTERGENIC	--	--	--
Ca5_P2_13806811	Ca5	13806811	CATCATATC/CATC	GCCAGAAAACTTTGCAGACC	TCTTTATCCGCTGCGATTTT	59.8	308	INTERGENIC	--	--	--
Ca5_P2_1381338	Ca5	1381338	CTTT/CTT	TCCTGGGCTTAATTCAATCG	CCCGTGATAGGCAGTACGTT	60.0	646	INTERGENIC	--	--	--
Ca5_P2_13825062	Ca5	13825062	ATATTATTATTATTATTATTATTATCATTATTATTATTAATATGATTATTATTATTATTATTATTATTATTATTATTATTATTATTATTATTATTATT/ATATTATTATTATTATTATTATTATTATTATCATTATTATTATTAATATGATT[illegible]	TGGTTTCATAACGTTTGTCCA	TGATTTGGTCATTTGGTATTCA	57.9	823	INTERGENIC	--	--	--
Ca5_P2_13854486	Ca5	13854486	ATA/ATACTCGATGGTA	GCTTGTCCGCGTAACTCTTC	CCAATGTAGTGGTTGATGCG	60.0	911	INTERGENIC	--	--	--
Ca5_P2_13855690	Ca5	13855690	TA/TAA	CCAGGTTTGCACTCAACTCA	GGGTGATGAGGACTCAGGAA	60.0	573	INTERGENIC	--	--	--

INDEL marker IDs	Chromosomes/unanchored scaffolds	Physical positions (bp)	InDels (*Kabuli* reference genome-CDC Frontier/PII)	Forward primers (5'-3')	Reverse primers (5'-3')	Anneling temperature (0C)	Expected amplified product size (bp)	Structural annotation		Functional annotation	
								Sequence components of *kabuli* genome	*Kabuli* gene accession IDs	NCBI-KOG	TFs
Ca5_P2_13858932	Ca5	13858932	A/AC	GATTCCCCATTCTTAACGCC	TCGCGAAGAGATAGAAGGGA	60.1	554	INTERGENIC	--	--	--
Ca5_P2_138852	Ca5	138852	ATTTTTTTTTT/ATTTTTTTTT	ATCTCCTGCTGCTGTTGCTT	ACTCCAAACAAAGGCTCCCT	60.1	609	INTRON	Ca_18170	--	--
Ca5_P2_13923143	Ca5	13923143	GTATATATATATATATATATATATATATATATATATAT/GTATATATATATATATATATATATATATATATATATATAT	GATCTGCCCTTGCTTCTTTG	CTACCGCGATCAAATACGGT	60.0	749	INTERGENIC	--	--	--
Ca5_P2_1392326	Ca5	1392326	TTATATATATATATATATATATATATATA/TTATATATATATATATATATATATATATATATATATATA	TGCGCCGAGAGTAGATTTTT	AGCACCGTAGCATCAAGGTT	59.8	386	INTERGENIC	--	--	--
Ca5_P2_14022502	Ca5	14022502	ATTT/ATTTT	GGAAATGGATGTCTGCCAAT	TTGCAATCTCATCAACTCGG	59.8	318	INTERGENIC	--	--	--
Ca5_P2_14067521	Ca5	14067521	TAAAAAAA/TAAAAAAAA	ACCACATGATGCTCTCCACA	TCAGTAGCACATGGGTAGCG	59.9	840	INTERGENIC	--	--	--
Ca5_P2_14084083	Ca5	14084083	A/ACAAGGATG	TGTTGTTGTGATTGTGCCCT	TTAGCATGCACCTTGAAAGC	59.1	385	DRR	Ca_20825	K	--
Ca5_P2_14249901	Ca5	14249901	CAATAATAATAATAATAATAATAATAATA/CAATAATAATAATAATAATAATAATA	AGTTTTCTCCCGAAGACACAA	GTGGCATGCAACCCAATTAT	60.6	329	INTERGENIC	--	--	--
Ca5_P2_14273213	Ca5	14273213	TATT/TATTGGATT	AATGGCCACGAATCTGAAAG	ATTTTCAACGGCATCCCATA	60.2	500	INTERGENIC	--	--	--
Ca5_P2_14307259	Ca5	14307259	TTTATTATTATTATTATTATTATTATTATTATTATTATTATTATTATTATTATTAT/TTTATTATTATTATTATTATTATTATTATTATTATTATTATTATTATTATTATTATTATTATTAT	GCTCTCCAACAACAAATCAACA	CACTTGAAATTTTAGGCCGC	59.7	798	INTERGENIC	--	--	--
Ca5_P2_14316093	Ca5	14316093	TTATATATATA/TTATATATATATATATATATATATATA	TTTACGGAGGCCCCTAATTC	TGAAGAGGGATTGGTATGGC	59.9	627	INTERGENIC	--	--	--
Ca5_P2_14330970	Ca5	14330970	ATTTTTTTTTT/ATTTTTTTTTTT	TGATTAAGAAGTTTTGATTTGCG	GGTTTGGACAATCTGCCTTG	60.5	795	INTERGENIC	--	--	--

INDEL marker IDs	Chromosomes/unanchored scaffolds	Physical positions (bp)	InDels (*Kabuli* reference genome-CDC Frontier/PII)	Forward primers (5'-3')	Reverse primers (5'-3')	Anneling temperature (0C)	Expected amplified product size (bp)	Structural annotation		Functional annotation	
								Sequence components of *kabuli* genome	*Kabuli* gene accession IDs	NCBI-KOG	TFs
Ca5_P2_14394771	Ca5	14394771	AG/A	GGCTCGTCGAACTTTTTCAG	GTGGGAGACAGGTGTGGAGT	60.0	831	INTERGENIC	--	--	--
Ca5_P2_14394852	Ca5	14394852	CTTTTT/CTTTT	TCTTCCACCCAGTTTTCACC	TTGAGGGCTATGTTTTTCGG	60.1	337	INTERGENIC	--	--	--
Ca5_P2_146635	Ca5	146635	GAAAAAAAA/GAAAAAAAAA	TGCATTTATTACTTCTTTACCAACA	CGAGGAAGGTAATACCATTTTGA	59.4	712	INTERGENIC	--	--	--
Ca5_P2_1475597	Ca5	1475597	GAAAAAA/GAAAAA	ATGCTAAGGGTGGTGCTTTG	TTGAGCCTTCACGACATTTG	59.8	213	INTERGENIC	--	--	--
Ca5_P2_1485293	Ca5	1485293	ATTTT/ATTT	CAAATGCCCAACATTTAACG	AGTGAAACCAACGCCAAAAC	60.0	506	INTERGENIC	--	--	--
Ca5_P2_149074	Ca5	149074	TT/TTAT	TTCGGTTGGTTTTGCAATTT	TGCGGAGTTGGATCTCTCTT	60.0	200	INTERGENIC	--	--	--
Ca5_P2_1511121	Ca5	1511121	CTTT/CTT	GGGACTGATTCGTCCTTTCA	TGAACGTAGAGTTTGCACCG	59.9	473	INTERGENIC	--	--	--
Ca5_P2_15126551	Ca5	15126551	CTTTTTTTTTTTT/CTTTTTTTTTTTTTTT	CCTGTGGTGAGTGTTGATGG	AGACAGAAAGGGCGAATCAA	59.8	596	INTERGENIC	--	--	--
Ca5_P2_15176756	Ca5	15176756	AATATATATATATATATATATATATATAT/AATATATATATATATATATATATATATATAT	ACATTTAGGGCCACTTGGTG	GTTGGTTCCCTAGCACCGTA	60.0	725	INTERGENIC	--	--	--
Ca5_P2_15184248	Ca5	15184248	AGGGGGGG/AGGGGGGGG	TCTAACCCGGAATCCTTTGA	TGAGGATTGAACGTAACCCA	59.0	300	DRR	Ca_21631	--	--
Ca5_P2_152255	Ca5	152255	CATATATATATATATAT/CATATATATATATAT	TTTTGGCCCCTAAGACACAC	TTTAATTGCACGCTCACGTC	59.9	331	INTERGENIC	--	--	--
Ca5_P2_15237764	Ca5	15237764	GATATTCATTTAT/GAT	GTGCGGTCCTTAATTTGCAT	GTTGTGGGATGAACTGACCC	60.2	697	INTERGENIC	--	--	--

INDEL marker IDs	Chromosomes/unanchored scaffolds	Physical positions (bp)	InDels (*Kabuli* reference genome-CDC Frontier/PII)	Forward primers (5'-3')	Reverse primers (5'-3')	Anneling temperature (0C)	Expected amplified product size (bp)	Structural annotation		Functional annotation	
								Sequence components of *kabuli* genome	*Kabuli* gene accession IDs	NCBI-KOG	TFs
Ca5_P2_1525359	Ca5	1525359	AATATATATATATATATATATATATATATATATATATATATATATATAT/AATATATATATATATATATATATATATATATATATATATAT	TTAGCTTGGTCCATTGCCTC	CCCATAACCTCTCCAAACCA	59.8	693	INTERGENIC	--	--	--
Ca5_P2_152864	Ca5	152864	TAAAAAAA/TAAAAAA	GACGTGAGCGTGCAATTAAA	TCCACCCAACATATTTTCATCA	60.1	575	INTERGENIC	--	--	--
Ca5_P2_153302	Ca5	153302	AAGTTA/AA	TGATGAAAATATGTTGGGTGGA	AGGAATTGCGAGGCCTAAAA	61.0	420	INTERGENIC	--	--	--
Ca5_P2_15338517	Ca5	15338517	GTTATTATTATTATTATTATTATTATTATTATTATTATTATT/GTTATTATTATTATTATTATTATTATTATTATTATTATTATTATT	TCAAGGCCACCACATTTACA	TTCCAAAGGTCGTATTTCCG	59.9	828	INTERGENIC	--	--	--
Ca5_P2_15339602	Ca5	15339602	GAAAAAAAAAA/GAAAAAAAAA	AGGAGAGAAAAAGGGGGAAA	GGGAAACCCACCTCTTGATT	60.2	211	INTERGENIC	--	--	--
Ca5_P2_15544446	Ca5	15544446	AG/A	TGGGCTTCAATTTATTCCTTG	AGAATGTTGGATGGTCTGCC	59.9	812	INTERGENIC	--	--	--
Ca5_P2_155468	Ca5	155468	C/CGTTTTCT	GAGTGGTCACTCACGGGTTT	TTGGAAAATTGAGGGTGTGG	60.7	689	INTERGENIC	--	--	--
Ca5_P2_15559013	Ca5	15559013	CAAAAAAAAAAA/CAAAAAAAAAAAA	CACCAAAGCCCCCTTAATTT	TTTTGTGGGCTTAGTGGACC	60.0	533	INTERGENIC	--	--	--
Ca5_P2_155645	Ca5	155645	TAAAAAA/TAAAAAAA	GAGTGGTCACTCACGGGTTT	CCAATTTTCCCACTTGGTTG	60.2	773	INTERGENIC	--	--	--
Ca5_P2_15618875	Ca5	15618875	GAAAAAAAAAA/GAAAAAAAAAAA	TGAACAAAGGAGGCACAATG	TTCAAGGGGCATTGAGGTAG	60.1	498	INTERGENIC	--	--	--
Ca5_P2_15716555	Ca5	15716555	ATTTTTTTTTTTT/ATTTTTTTTTTTTT	TTTGTGCAGACTGCGTTTGT	TTGCAATGTCAACAACAGAGG	59.7	859	INTERGENIC	--	--	--
Ca5_P2_15756736	Ca5	15756736	GACAC/GAC	GCGCGATTTACCAAAAATGT	TTTGAGTGGTGGGTTGTGAA	60.0	431	INTERGENIC	--	--	--

INDEL marker IDs	Chromosomes/unanchored scaffolds	Physical positions (bp)	InDels (*Kabuli* reference genome-CDC Frontier/PII)	Forward primers (5'-3')	Reverse primers (5'-3')	Anneling temperature (0C)	Expected amplified product size (bp)	Structural annotation		Functional annotation	
								Sequence components of *kabuli* genome	*Kabuli* gene accession IDs	NCBI-KOG	TFs
Ca5_P2_15765125	Ca5	15765125	AATATATATATATATATATAT/AATATATATATATATATATATATAT	TCAATCCTACATTCTGGGGC	TGGTTCCAACAATCTCTCCC	59.9	510	INTERGENIC	--	--	--
Ca5_P2_15773178	Ca5	15773178	GATTATTATTATTATTATTATTATTATTATTATTATTATTATTATT/GATTATTATTATTATTATTATTATTATTATTATTATTATTATTATTATTATT	GCCTTAAAATTTGGCAATGG	TCTTCGCAAATCAAAGAATGA	59.8	807	INTERGENIC	--	--	--
Ca5_P2_15794387	Ca5	15794387	GTATTATTATTATTATTATTATTATTATTATTATTATTATTATTATTATTATTATTATTATTATTATGATTATTATTAT/GTATTATTATTATTATTATTATTATTATTATTATTATTATTATTATTATTATTATTATTATTATTA [illegible]	CAAGGGTCAAACTTGCCAAT	ATTATCTGTCCCCAGCAACG	60.0	417	INTERGENIC	--	--	--
Ca5_P2_15794643	Ca5	15794643	TAAAAAAA/TAAAAAAAA	AAGGGTCAAACTTGCCAATG	TGGGATTTTGACCATCGTTT	60.2	542	INTERGENIC	--	--	--
Ca5_P2_15835478	Ca5	15835478	CAAAA/CAAA	TGCATGATTCCAGAATTCCA	AGCGGTGAGGAGAACTCTGA	60.1	690	INTERGENIC	--	--	--
Ca5_P2_15840606	Ca5	15840606	T/TA	TCACTGCTTCTGCCAAAATG	GTGACAACTGGGGAGGAAGA	60.1	829	INTERGENIC	--	--	--
Ca5_P2_15877436	Ca5	15877436	GC/G	GGGCTACATCCAGTCCTCAC	TTTGGCTGTAGAGGTTCCAAA	59.7	474	INTERGENIC	--	--	--
Ca5_P2_158794	Ca5	158794	TAAAAAA/TAAAAA	TGATGCGTCATGGACATAAA	TTTTAAAAATGTCCATTTACACCG	59.3	498	INTERGENIC	--	--	--
Ca5_P2_15918467	Ca5	15918467	CG/C	TTGCTCCTACGTATCCCCAG	CCAGCGCGGTAAACACTTAT	60.2	591	INTERGENIC	--	--	--
Ca5_P2_15918981	Ca5	15918981	TTTATTATTATTATTATTATTATTATTATTATTATTATTATTATTATTATT/TTTATTATTATTATTATTATTATTATTATTATTATTATTATTATTATTATTATTATT	CCTCCCCCATTTGTCTTCTT	GCCTGATGTTCGAATTTTTCA	60.1	462	INTERGENIC	--	--	--
Ca5_P2_15929348	Ca5	15929348	ACCC/ACC	TGATGACGAAGACGAAGACG	AACACATCCGGGATCCATTA	60.0	416	INTERGENIC	--	--	--
Ca5_P2_15931643	Ca5	15931643	AATTTATATT/AATTTATATTTATATT	GATGGTGTGAGTGGAGCTGA	GCTGAAGCTGAATACAGGGC	60.0	931	INTERGENIC	--	--	--

INDEL marker IDs	Chromosomes/unanchored scaffolds	Physical positions (bp)	InDels (*Kabuli* reference genome-CDC Frontier/PII)	Forward primers (5'-3')	Reverse primers (5'-3')	Anneling temperature (0C)	Expected amplified product size (bp)	Structural annotation		Functional annotation	
								Sequence components of *kabuli* genome	*Kabuli* gene accession IDs	NCBI-KOG	TFs
Ca5_P2_16109117	Ca5	16109117	ATTTTTTTTTTTT/ATTTTTTTTTT	CACCACTGAAACCAAACCCT	CAAGTGGAACCCACCAATTT	59.7	188	INTERGENIC	--	--	--
Ca5_P2_161153	Ca5	161153	CTT/CTTAAATT	CTGATACTCCACTTTGTCGTGTG	GCATAATCCGCTCACCCTAA	60.1	819	INTERGENIC	--	--	--
Ca5_P2_16126632	Ca5	16126632	CTATATATATATATATA/CTATATATATATATATA	GCCACGATTTATACCGCTTT	ATGGAAGAAAAGATGCCACG	60.1	388	INTRON	Ca_17911	C	MYB
Ca5_P2_16238083	Ca5	16238083	AATATATATATATATATATATATATATATA/AATATATATATATATATATATATATATATATA	CTGATGCGGCCATTAAAACT	TGTTCCCCCAGAAACATCAT	60.2	409	DRR	Ca_17916	K	MYB
Ca5_P2_162548	Ca5	162548	TTTATT/TTT	TGTCCTGTGCAAGAGAGGC	TCCATGACAGTTCAAGGATCTC	59.1	456	INTERGENIC	--	--	--
Ca5_P2_16319965	Ca5	16319965	TGGG/TGGGG	TTGTGTGCAGGATCCACATT	TTGACCATTGCACGAAACAT	60.0	502	INTERGENIC	--	--	--
Ca5_P2_1633237	Ca5	1633237	CAAAAAAAAAAAA/CAAAAAAAAAAA	TGCATTGTGCTAGATGGTCTT	TGTTCCGATTGTATTTTGAGGA	59.5	699	INTERGENIC	--	--	--
Ca5_P2_1634715	Ca5	1634715	ATTTTTTTTTTTTTTT/ATTTTTTTTTTTTTTT	TGTTACATGGTTGCTCCCAA	CGATACTCACCCCTCGTTGT	60.0	829	INTERGENIC	--	--	--
Ca5_P2_16363340	Ca5	16363340	GTT/GTTT	AATCCTGGTGCACGATTTTC	ACATCGCCATTACCGAACTC	60.0	445	DRR	Ca_17921	R	MYB_related
Ca5_P2_16382084	Ca5	16382084	TAAAAAAAAAA/TAAAAAAAAA	CTCCCTCTGCAACACACAGA	TGCAGATAAATGGCCAAACA	60.1	469	INTERGENIC	--	--	--
Ca5_P2_16393546	Ca5	16393546	CTTTTTTTTTT/CTTTTTTTTT	CACAACTCCGCCATTACCTT	GTTGCAAGTTGCCGTGTCTA	59.9	713	INTERGENIC	--	--	--
Ca5_P2_164138	Ca5	164138	T/TGTAAATG	GGTAAAAGAGACTCGCGTGG	CTGATGTGCTCGTTGTTGCT	60.1	695	INTERGENIC	--	--	--

INDEL marker IDs	Chromosomes/unanchored scaffolds	Physical positions (bp)	InDels (*Kabuli* reference genome-CDC Frontier/PII)	Forward primers (5'-3')	Reverse primers (5'-3')	Anneling temperature (0C)	Expected amplified product size (bp)	Structural annotation		Functional annotation	
								Sequence components of *kabuli* genome	*Kabuli* gene accession IDs	NCBI-KOG	TFs
Ca5_P2_16470095	Ca5	16470095	AAATAATAATAATAATAATAATAATAATAATAATAATAATAATAATAATAATAATAA/AAATAATAATAATAATAATAATAATAATAATAATAATAATAATAATAATAATAATAATAATAA	CATCCATTTGGCTTTTGACA	TCGGAAAGTTGGCCAATAAA	60.4	789	INTERGENIC	--	--	--
Ca5_P2_165547	Ca5	165547	TTATA/TTATATATA	ATTAATTGGATTTGGCAGCG	ATTTTGGTTTGCCCACACTC	59.8	572	INTRON	Ca_18171	--	NAC
Ca5_P2_16633256	Ca5	16633256	GTTTTTTTTTTTT/GTTTTTTTTTTTTTT	GGAATCCATTCGTGATTTGC	GTATGCCAAGTCCAGGAAGC	59.7	628	INTERGENIC	--	--	--
Ca5_P2_166515	Ca5	166515	TTAATAATAATAAT/TTAATAATAATAATAAT	ATTCTGCAGGAGGCAACAGT	TTGGATTCCAGTCCCATAGC	59.9	285	CDS (large-effect mutations)	Ca_18171	--	NAC
Ca5_P2_166546	Ca5	166546	CAATCCAAATCCAAATCCAAATCCAAATC/CAATCCAAATCCAAATCCAAATCCAAATCCAAATC	ATTCTGCAGGAGGCAACAGT	TTGGATTCCAGTCCCATAGC	59.9	285	CDS (large-effect mutations)	Ca_18171	--	NAC
Ca5_P2_16715406	Ca5	16715406	GAAAAAAAAA/GAAAAAAAAAA	TTGCGTTACGGATTTGATGA	GCTAAATTACATTCGTGGTCCC	59.7	240	INTERGENIC	--	--	--
Ca5_P2_16765808	Ca5	16765808	TAGA/TA	CGATCGTGCTACGTCTTCAA	TGGTGGGAATGTTGTGATTG	60.2	677	DRR	Ca_17941	R	HSF
Ca5_P2_1677289	Ca5	1677289	CTATATATATATATATATATATATATATAT/CTATATATATATATATATATATATATATATATAT	AAGCGCTGTAAAAGGTGCAT	ATGTGAATCCCACCAGGAAA	60.2	794	INTERGENIC	--	--	--
Ca5_P2_168125	Ca5	168125	T/TG	AGAGAGGGCTTCGTTCAACA	CGAGACCATTGAGTCCAAAAA	60.1	577	DRR	Ca_18171	--	NAC
Ca5_P2_168371	Ca5	168371	TAAAAAAAA/TAAAAAAAAA	TTTTTGGACTCAATGGTCTCG	ACCACTGAAACCAACCACAT	57.7	564	DRR	Ca_18171	--	NAC
Ca5_P2_16881029	Ca5	16881029	GGTTGTTGTTGTTGTT/GGTTGTTGTTGTTGTTGTT	TGCATGCTTAATTTGACAGTGA	TGATTTAACGGTTGTGATTGATT	58.4	779	INTERGENIC	--	--	--
Ca5_P2_16889364	Ca5	16889364	AATATATATATATATATATAT/AATATATATATATATATATATATAT	GGAGAAAATTCATTTGGCATT	CCTTTGCATGGGACAGTTTT	60.0	985	INTERGENIC	--	--	--

INDEL marker IDs	Chromosomes/unanchored scaffolds	Physical positions (bp)	InDels (*Kabuli* reference genome-CDC Frontier/PII)	Forward primers (5'-3')	Reverse primers (5'-3')	Anneling temperature (0C)	Expected amplified product size (bp)	Structural annotation		Functional annotation	
								Sequence components of *kabuli* genome	*Kabuli* gene accession IDs	NCBI-KOG	TFs
Ca5_P2_1695092	Ca5	1695092	TTTCNNNNNNNNNNTTC/TTTC	TCCACTTCACAAAGCAGCAC	GCTGCAAGTGCAGAGCATAG	59.9	282	INTERGENIC	--	--	--
Ca5_P2_16958159	Ca5	16958159	GA/G	GGGAAGGATGGAATTTTGGT	TATTTTGGATTTTGGCTCGG	59.9	417	INTERGENIC	--	--	--
Ca5_P2_16958294	Ca5	16958294	AT/A	GGGAAGGATGGAATTTTGGT	GGTGGAGAGTGAGGTGGTGT	60.0	684	INTERGENIC	--	--	--
Ca5_P2_16961163	Ca5	16961163	TTTATTATTATTATTATTATTATTATTATTATTATTATTATTATTATTATTAT/TTTATTATTATTATTATTATTATTATTATTATTATTATTATTAT	TCAATCCGTTTCTTTTCGGA	TTGAAGCCGAGAAAACGACT	60.0	672	INTERGENIC	--	--	--
Ca5_P2_16964758	Ca5	16964758	CA/CAA	TTGTCGTCTCGATGTAAGCG	TCTCAATTCAAATAGGCCCG	60.0	296	INTERGENIC	--	--	--
Ca5_P2_16970696	Ca5	16970696	GATAATAATAATAATAATAATAATAATAATAATAATAATAATAATAATAATAATA/GATAATAATAATAATAATAATAATAATAATAATAATAATAATAATAATAATAATAATAATAATA	TTGCTCCTACGTATCCCCAG	CCTCCCCGTCCATTTTTATT	60.0	594	INTERGENIC	--	--	--
Ca5_P2_17007480	Ca5	17007480	AATATATATATATATATA/AATATATATATATATATA	TGCAATCAATTTTTGAGCCA	TGGTGGTTGTTGTCTCCTCA	60.1	626	INTERGENIC	--	--	--
Ca5_P2_17020837	Ca5	17020837	TAGAGAGA/TAGAGA	CCTACGGATGACCTCTCTCG	GGTTTAACATCCCCACAAGC	59.3	773	INTERGENIC	--	--	--
Ca5_P2_170263	Ca5	170263	ATT/ATTTT	TCCAAGACCAGGCTAAATGC	CATGGACAAAATGCACAAGG	60.0	504	INTERGENIC	--	--	--
Ca5_P2_1709441	Ca5	1709441	T/TC	AAGCTTGATAGCCCACATGC	ACAAAATGTTTCCCCCATCA	60.0	453	INTERGENIC	--	--	--
Ca5_P2_1709760	Ca5	1709760	A/ATT	TGATGGGGGAAACATTTTGT	AAAAAGGGCATGTGAATGATCT	59.8	247	INTERGENIC	--	--	--
Ca5_P2_171078	Ca5	171078	TAAA/TA	AATGGTTTTCAGAGCGGAGA	GCAAATTTTGTGTGTGCTTTG	59.3	723	INTERGENIC	--	--	--

INDEL marker IDs	Chromosomes/unanchored scaffolds	Physical positions (bp)	InDels (*Kabuli* reference genome-CDC Frontier/PII)	Forward primers (5'-3')	Reverse primers (5'-3')	Anneling temperature (0C)	Expected amplified product size (bp)	Structural annotation		Functional annotation	
								Sequence components of *kabuli* genome	*Kabuli* gene accession IDs	NCBI-KOG	TFs
Ca5_P2_171559	Ca5	171559	A/AT	CAAAGCACACACAAAATTTGCTA	GCCATCCATCTACAAGCCAT	59.9	277	INTERGENIC	--	--	--
Ca5_P2_17169621	Ca5	17169621	CAAAA/CAAAAA	CCTCGAAGGACCGTAAATCA	GTGGCACCTCGGTAAACAGT	60.0	427	INTERGENIC	--	--	--
Ca5_P2_17244450	Ca5	17244450	AG/A	GTACCCTCCTCCTCGGTCTC	TTGTGTTTCAACAACTCCCAA	59.1	729	INTERGENIC	--	--	--
Ca5_P2_17258502	Ca5	17258502	TTAATAATAATAATAATAATAATAATAA/TTAATAATAATAATAATAATAATAATAATAA	ATGTGGGAGACATGTGTGGA	TTAAGCCACAAGTGGGGAAC	60.0	712	INTERGENIC	--	--	--
Ca5_P2_17263854	Ca5	17263854	TTATATATATATATATATATATATATATATATATATATATATATAT/TTATATATATATATATATATATATATATATATATATAT	GCTTTTATCTTGGCCCTCCT	AATTGTGGATGCAGAGTGTCC	60.0	288	INTERGENIC	--	--	--
Ca5_P2_17314539	Ca5	17314539	TAAAAAAAAAA/TAAAAAAAAAAA	TCGATTATGCGTAGGGAAGG	TTGCGACAACTTGGTGATTC	59.7	824	INTERGENIC	--	--	--
Ca5_P2_173324	Ca5	173324	ATTTTTTTTTTTT/ATTTTTTTTTTT	TGTTTATCCACAAGCTTTCCG	ACCGTGAGATGCCACATTTT	60.4	270	DRR	Ca_18172	J	--
Ca5_P2_17448710	Ca5	17448710	CACTATACAA/CACTATACAACTATACAA	TGTGGATGTTCAAGGGACAA	ACAAATGGCGTATGCCTCTC	60.1	864	INTERGENIC	--	--	--
Ca5_P2_17494887	Ca5	17494887	CTGA/CTGACCTCCTATATGTGA	CTACGCCAAGAAACTGCTCC	GTACGCGTCCAGACATAGCA	59.9	245	INTERGENIC	--	--	--
Ca5_P2_1749550	Ca5	1749550	ANNNNNNNNNN/ANNNNN	TCCACTTCACAAAGCAGCAC	TGGTGTGTCGTCAAGAATGA	58.6	398	INTERGENIC	--	--	--
Ca5_P2_17503626	Ca5	17503626	TATCAT/TAT	ATGGGGATAGCTTTGAGGGT	CTCCTTTCCCTGCTGCTATG	60.0	601	INTERGENIC	--	--	--
Ca5_P2_17526738	Ca5	17526738	T/TG	GATTGGGGGAAGATGAGGAT	TTTGCGTTACGAATTTGATGA	59.2	465	INTERGENIC	--	--	--

INDEL marker IDs	Chromosomes/unanchored scaffolds	Physical positions (bp)	InDels (*Kabuli* reference genome-CDC Frontier/PII)	Forward primers (5'-3')	Reverse primers (5'-3')	Anneling temperature (0C)	Expected amplified product size (bp)	Structural annotation		Functional annotation	
								Sequence components of *kabuli* genome	*Kabuli* gene accession IDs	NCBI-KOG	TFs
Ca5_P2_17575418	Ca5	17575418	GTT/GT	TCTCCATTCCTGTCCGTAGG	TTAAGTCAGGGGCTCAGGAA	59.8	523	INTERGENIC	--	--	--
Ca5_P2_17612584	Ca5	17612584	CATTCATAAAAATTCA/CATTCATAAAAATTCATAAAAATTCA	GTCTCTTGCTCTCCCACCAA	TTTATGTGGTGAATTGGCGA	59.9	769	INTERGENIC	--	--	--
Ca5_P2_17613064	Ca5	17613064	GAAAAAAAAAAA/GAAAAAAAAAA	CGCCAATTCACCACATAAAA	TGCTTTCCAAGATGGATGTG	59.6	293	INTERGENIC	--	--	--
Ca5_P2_17672056	Ca5	17672056	GTTTT/GTTTTT	CTGTTCATCGCACGCACTAT	AGAATACGTGGTAGGGCACG	60.0	446	INTERGENIC	--	--	--
Ca5_P2_17726441	Ca5	17726441	ATTTTTTTTTT/ATTTTTTTTT	GGTCCAATTCCCACATTGTC	TCTGATTTGGGCTGCCTAGT	59.8	294	INTERGENIC	--	--	--
Ca5_P2_17733319	Ca5	17733319	A/ATCGGTTCAGC	CGCGATGTCGTCTAAAGTGA	GAGGGGAGGGTTTTTGCTAC	59.9	583	INTERGENIC	--	--	--
Ca5_P2_17757862	Ca5	17757862	TC/TCC	AGAGTCTTTCCAAACGGCCT	TGTTGGAATCAAGTTGGTTTT	57.1	815	DRR	Ca_22474	--	--
Ca5_P2_17760753	Ca5	17760753	TAATAAAAATAAAAATAAAAATAAAA/TAATAAAAATAAAAATAAAAATAAAAATAAAA	TGAAACCGCGTTTGATTGTA	GGTTGGGACCACTTCTCAAA	59.9	168	INTERGENIC	--	--	--
Ca5_P2_17763580	Ca5	17763580	CATATATATATATATAT/CATATATATATATAT	TCAGGGGCCTATGATTTGAC	TGTGCAACTTTGCAACCTAGA	59.5	462	INTERGENIC	--	--	--
Ca5_P2_17763617	Ca5	17763617	TTTATTATTATTATTATTATTATTATTATTATTATTATTATTATTATT/TTTATTATTATTATTATTATTATTATTATTATTATTATTATTATTATT	TCAGGGGCCTATGATTTGAC	TGTGCAACTTTGCAACCTAGA	59.5	462	INTERGENIC	--	--	--
Ca5_P2_17852569	Ca5	17852569	ATTTTTTTTTT/ATTTTTTTTTTT	AGCCAGTCGAAGCACATTCT	TCCCCTTGAATTTTGGAGTG	59.9	791	DRR	Ca_22847	--	--
Ca5_P2_17876530	Ca5	17876530	CAA/CAAA	TTTCATCCATCACCGACAAA	CTCAACGAATGCCTGAAACA	59.8	573	INTERGENIC	--	--	--

**INDEL marker IDs**	**Chromosomes/unanchored scaffolds**	**Physical positions (bp)**	**InDels (*Kabuli* reference genome-CDC Frontier/PII)**	**Forward primers (5'-3')**	**Reverse primers (5'-3')**	**Anneling temperature (0C)**	**Expected amplified product size (bp)**	**Structural annotation**		**Functional annotation**	
								**Sequence components of *kabuli* genome**	***Kabuli* gene accession IDs**	**NCBI-KOG**	**TFs**
Ca5_P2_17876693	Ca5	17876693	CACAAA/CAA	TGTTTCAGGCATTCGTTGAG	TCAATACCATTTTCACCGCA	59.9	471	INTERGENIC	--	--	--
Ca5_P2_179179	Ca5	179179	CAAAAAAA/CAAAAAAAA	TTAGCAGCAGACGCAAGAGA	GACTCTGCTGGTTTGGAAGC	60.0	340	INTRON	Ca_18172	J	--
Ca5_P2_180097	Ca5	180097	ATTTTTTTTT/ATTTTTTTT	ATGACCAAGGAGGCACAGAC	GAATCAGAATGACGATTGTTTGA	59.1	782	INTERGENIC	--	--	--
Ca5_P2_18015209	Ca5	18015209	TAAA/TAA	GCTTATCCCGGAATTTGACA	ATCGCATTCAGACGACAAAA	59.3	759	DRR	Ca_22851	--	--
Ca5_P2_180472	Ca5	180472	A/AC	CCCTCAAAACAAGGTTGTTAGTTT	TGACAAATTTGATTGGAGAGTCA	59.6	913	INTERGENIC	--	--	--
Ca5_P2_18049660	Ca5	18049660	CATATATATATAT/CATATATATAT	GCACGTTGTAAAGAGTGGACTG	AAAAGGCCCACAAGAGTTGA	59.7	694	INTERGENIC	--	--	--
Ca5_P2_18072367	Ca5	18072367	TAAAAAAAAAA/TAAAAAAAAA	CGAAAAGAAAATGGTGCAGTT	GTTCAACTGACGGGGAGAAA	60.1	613	INTERGENIC	--	--	--
Ca5_P2_18136111	Ca5	18136111	CTTTTTTTTTTT/CTTTTTTTTTT	CGGGTCATTTTCTTTCTCCA	TTGTGGTCCTTTAAGATGGTTG	59.0	327	INTERGENIC	--	--	--
Ca5_P2_18176797	Ca5	18176797	TAAAAAAAAAA/TAAAAAAAAAAAA	TGGGTTGAAAGTTGGATGTG	ATCCTGGTTGGGGAAGGTAG	60.2	473	INTERGENIC	--	--	--
Ca5_P2_18181708	Ca5	18181708	GTTTTTTTTTT/GTTTTTTTTTTT	ACGGTTAACGGATGACGAAG	TGTAACAAACCTGCAATGGG	59.4	595	INTERGENIC	--	--	--
Ca5_P2_18191704	Ca5	18191704	ATTTTTTTT/ATTTTTTTTT	TGAAATCCGCCTAAATCCTG	TAATTGGGGAACCAAAAAGC	58.9	715	INTERGENIC	--	--	--
Ca5_P2_1819638	Ca5	1819638	GAAAAAAAA/GAAAAAAAAAA	TCTGGAGTTTTCGAGCGATT	GATGAAATCGAGGACCAGGA	60.0	384	INTERGENIC	--	--	--

INDEL marker IDs	Chromosomes/unanchored scaffolds	Physical positions (bp)	InDels (*Kabuli* reference genome-CDC Frontier/PII)	Forward primers (5'-3')	Reverse primers (5'-3')	Anneling temperature (0C)	Expected amplified product size (bp)	Structural annotation		Functional annotation	
								Sequence components of *kabuli* genome	*Kabuli* gene accession IDs	NCBI-KOG	TFs
Ca5_P2_1824949	Ca5	1824949	TCC/TCCC	GCCCAAAAAGTCCAAATGAA	GCTAGGGTGGAAGCCTCTCT	60.0	810	DRR	Ca_26714	--	--
Ca5_P2_18261167	Ca5	18261167	CAAAAAAAAAAAA/CAAAAAAAAAAA	ACCATGCAAAGACATCCACA	AACCGGGTACCAAATTCACA	60.1	697	INTERGENIC	--	--	--
Ca5_P2_18299239	Ca5	18299239	GTTTTTTTTT/GTTTTTTTTTT	ATCACAAATTTTCGCGACCT	CGGGATTGTGAAGAAGTGGT	60.0	880	INTERGENIC	--	--	--
Ca5_P2_18300997	Ca5	18300997	TA/TAA	TAGAAGCTGAGGGTGGCAGT	AAATTGAACATGCCTCGACC	59.9	657	INTERGENIC	--	--	--
Ca5_P2_18310904	Ca5	18310904	GTTTTTTTTTTTT/GTTTTTTTTTTTTT	ATGGGTAAAAACGCATTGCT	CCCATCACCCTCCCTATTTT	60.0	314	INTERGENIC	--	--	--
Ca5_P2_18311599	Ca5	18311599	GAATAATAATAATAATAATAATAATAATAATAATAATAATAA/GAATAATAATAATAATAATAATAATAATAATAATAATAA	AAAAGCCATGCAAACCAAAG	GAAGAAGATGGGTTGGCTGA	60.2	589	INTERGENIC	--	--	--
Ca5_P2_18404984	Ca5	18404984	CATATATATATATATATATATATA/CATATATATATATATATATATATATATATA	ATTTTGGACAACAGGTTCGC	GCAAGAACATGTGAGGAGCA	60.0	397	INTERGENIC	--	--	--
Ca5_P2_18428828	Ca5	18428828	CGG/CG	CGATTGACGAATCGATTGAA	GTTTAGGCACGTAGGGACCA	60.0	348	INTERGENIC	--	--	--
Ca5_P2_18589511	Ca5	18589511	TTTATTATTATTATTATTATTATTATTATTATTATTATTATTATTATTATTATTATTATTATTAT/TTTATTATTATTATTATTATTATTATTATTATTATTATTATTATTATTATTATTATTATTATTATTATTATTAT	CACGTTCCCACACTCTCTCA	TCCATTTACGGCAAATTCAA	59.0	829	INTERGENIC	--	--	--
Ca5_P2_18593873	Ca5	18593873	TTA/T	GGTCTGTCCAATCAGACCGT	TCCATTAAGCCCCTCAACAA	60.4	600	INTERGENIC	--	--	--
Ca5_P2_18612826	Ca5	18612826	GTATATATATATATATATATATATATATAT/GTATATATATATATATATATATATATATATATAT	GCAATTAAAGTTGCCTTGGAA	TGCATAGGTCCCATAAAAACG	59.8	374	DRR	Ca_24762	QR	B3
Ca5_P2_18645920	Ca5	18645920	ATTTTTTTTTTTTT/ATTTTTTTTTTTT	GGGTGGGAATAGGTCAGGTT	GGTTTGCGAGGATTGTGACT	60.1	779	INTERGENIC	--	--	--

INDEL marker IDs	Chromosomes/unanchored scaffolds	Physical positions (bp)	InDels (*Kabuli* reference genome-CDC Frontier/PII)	Forward primers (5'-3')	Reverse primers (5'-3')	Anneling temperature (0C)	Expected amplified product size (bp)	Structural annotation		Functional annotation	
								Sequence components of *kabuli* genome	*Kabuli* gene accession IDs	NCBI-KOG	TFs
Ca5_P2_18683897	Ca5	18683897	AGCG/AGCGCGAGTCATCAGCTCGCG	ATGGCCGTTCTTAGTTGGTG	GTACAAAGGGCAGGGACGTA	60.0	395	INTRAGENIC	--	--	--
Ca5_P2_18761912	Ca5	18761912	TAAAATAAAAAATAAAAAATAAAAAATAAA/TAAAATAAAAAATAAAAAATAAA	CCGCATAAACAAAATCTTCCA	AGAAAAACCTCCCTGCGAAT	60.1	504	INTERGENIC	--	--	--
Ca5_P2_18774822	Ca5	18774822	CA/CAA	TAGACGTTGGGGACACATGA	GGATCTCTTCTCATGGCTGG	59.8	562	INTERGENIC	--	--	--
Ca5_P2_18813953	Ca5	18813953	AAATAATAATAATAATAATAATAATAATAATAACAATAATAATAACAATAATAATAATAATAATAATAATAATAAT/AAATAATAATAATAATAATAATAATAACAATAATAATAACAATAATAATAATAATAATAATAATAATAAT	AAGAGACTTTTGGGGCAACA	ATGCATTTGCAACTCATCTTT	57.4	505	INTRON	Ca_24488	--	--
Ca5_P2_18821116	Ca5	18821116	AGAAACTTTCGAAACTTTCGAAACTTTCGAA/AGAAACTTTCGAAACTTTCGAA	TGAGTTTTGTACCACGCACC	GCCAAACGTTCGAAATGAAA	61.0	789	INTERGENIC	--	--	--
Ca5_P2_188946	Ca5	188946	CTT/CTTT	CCCCATTTTTACAATCCACG	CATGTATGAGCAGCCACACC	60.1	366	INTERGENIC	--	--	--
Ca5_P2_18944656	Ca5	18944656	CAAAA/CAA	TCCTGTCATGTGCTTTGAGC	CTAACCTCCCAGGACCCAAT	60.2	131	INTERGENIC	--	--	--
Ca5_P2_190126	Ca5	190126	AAA/AAATTAA	CCCAAAAACTAGTGAGGCGA	AAATCCTAAAAGCACGGGTT	57.7	828	INTERGENIC	--	--	--
Ca5_P2_1903216	Ca5	1903216	GA/GAA	TGCATAGGCCTCTTGTCCTT	AGTTCCGCGCTAGATTTCAA	60.0	553	INTERGENIC	--	--	--
Ca5_P2_191179	Ca5	191179	CCAA/CCAACAA	GCAAGTGATGATGTAGCGGA	GCTCCAAAGTGCAAGTGTGA	60.0	605	INTERGENIC	--	--	--
Ca5_P2_191704	Ca5	191704	CGGA/C	TCACACTTGCACTTTGGAGC	TTGTTGGGATCCCTGAAGAG	60.0	484	INTERGENIC	--	--	--
Ca5_P2_191792	Ca5	191792	ACTATCTATCTATCTA/ACTATCTATCTATCTATCTA	TCACACTTGCACTTTGGAGC	CTTGGAATCGAAACCGTTGT	60.0	680	INTERGENIC	--	--	--

INDEL marker IDs	Chromosomes/unanchored scaffolds	Physical positions (bp)	InDels (*Kabuli* reference genome-CDC Frontier/PII)	Forward primers (5'-3')	Reverse primers (5'-3')	Anneling temperature (0C)	Expected amplified product size (bp)	Structural annotation		Functional annotation	
								Sequence components of *kabuli* genome	*Kabuli* gene accession IDs	NCBI-KOG	TFs
Ca5_P2_1925917	Ca5	1925917	T/TG	TCCTGGTCCTCGATTTCATC	TCGAGGATAGGGAGACCCTT	60.0	790	INTERGENIC	--	--	--
Ca5_P2_19338107	Ca5	19338107	TAAAAAAAAAA/TAAAAAAAAAAA	TCAACTGTGGGGTTTCAATTC	GGTCAGACCACCTTTACCCA	59.8	873	DRR	Ca_23510	R	MYB
Ca5_P2_19351420	Ca5	19351420	ATTTTTTTT/ATTTTTTT	CAAGACCCGCACAAATACAA	AGGCAACACTTCTTGGTTGTC	59.2	618	INTERGENIC	--	--	--
Ca5_P2_19386415	Ca5	19386415	GTTTTT/GTTTT	GAGATTCCCCATTCTCAACG	TTCGCAACTCCACTCTTCCT	60.0	596	INTERGENIC	--	--	--
Ca5_P2_19391673	Ca5	19391673	ATTTTTT/ATTTTTTT	CATGTTCGGCTGCACTTCTA	GATTTTGGGCAATTTTACGTC	58.5	663	INTERGENIC	--	--	--
Ca5_P2_19542912	Ca5	19542912	GATGTATATGGAT/GATGTATATGGATATGTATATGGAT	GGCCACCTTACTGAGGATGA	CTCAACCCTTCCATATCCCA	59.7	627	INTRON	Ca_23525	--	--
Ca5_P2_19579350	Ca5	19579350	A/AC	ACGAACGTTGAAAATCCAGC	GTATCAAGTGCACAGCGGAA	59.9	489	INTERGENIC	--	--	--
Ca5_P2_19591188	Ca5	19591188	CATATATATATATATATATATATATATATATAT/CATATATATATATATATATATATATATATATATATATATATATAT	CCACAGCTTCAACAAAAGCA	TGGATGATTTGATGTCACATAGG	59.7	525	INTERGENIC	--	--	--
Ca5_P2_19592020	Ca5	19592020	AA/AATAATA	TGGATCTCAGTCACACTCACG	GGTAAAATCCACTCTTCCCCA	60.2	548	INTERGENIC	--	--	--
Ca5_P2_19593344	Ca5	19593344	GTATATATATATATATATATAT/GTATATATATATATATATAT	CATTCATTCACGCTCATTGG	ACGAATTGGTTTGGTTCAGG	59.8	521	INTERGENIC	--	--	--
Ca5_P2_195971	Ca5	195971	GATAT/GATATTTTGTTAAGTGTTATAT	ATCACGGAAAGCTTGTGTCC	TTTCACCTTCATCCTCTGGC	60.2	866	INTRON	Ca_18174	L	--
Ca5_P2_19614516	Ca5	19614516	CAATAATAATAATAATAATAATAATAATAATAA/CAATAATAATAATAATAATAATAATAATAATAATAATAA	AAGATTCATTGACAACTGACCA	CATATCTTTACTTTCGGCCACA	59.1	425	INTERGENIC	--	--	--

INDEL marker IDs	Chromosomes/unanchored scaffolds	Physical positions (bp)	InDels (*Kabuli* reference genome-CDC Frontier/PII)	Forward primers (5'-3')	Reverse primers (5'-3')	Anneling temperature (0C)	Expected amplified product size (bp)	Structural annotation		Functional annotation	
								Sequence components of *kabuli* genome	*Kabuli* gene accession IDs	NCBI-KOG	TFs
Ca5_P2_19640668	Ca5	19640668	GTATATATATATATATATAT/GTATATATATATATAT	TGCACTCGAGATTGTGCTTC	AAAACAAAAAGCATGGTCGG	60.0	558	INTERGENIC	--	--	--
Ca5_P2_19658678	Ca5	19658678	CAAAAAAAAA/CAAAAAAAA	TTCGGGAGTCGATTACGAGT	CGCTGCACCATGTTATTCAC	60.1	546	INTRON	Ca_22824	O	--
Ca5_P2_1970073	Ca5	1970073	ATTTTTTTT/ATTTTTTTTT	GATGAAATCGAGGACTGGGA	TTCGGACTAGACGAGGGCTA	60.0	596	INTERGENIC	--	--	--
Ca5_P2_19705315	Ca5	19705315	TTATATATATATATATATATATATATATAT/TTATATATATATATATATATATATATATATATATATATATATATATAT	TTGGAAGGAGCCCTTACCTT	CTGCCACGTCACCCTTTTAT	60.0	742	INTERGENIC	--	--	--
Ca5_P2_19725885	Ca5	19725885	CG/C	GCCCAGGAAAAAGAAAGGAC	CCCAATCTCAGGCTCTTCAG	59.9	112	INTERGENIC	--	--	--
Ca5_P2_19725939	Ca5	19725939	CTGA/CTGATGA	GCCCAGGAAAAAGAAAGGAC	TCGGTATTTGCCATGAGACA	60.1	405	INTERGENIC	--	--	--
Ca5_P2_19746235	Ca5	19746235	TAAAAAAA/TAAAAAAAA	ACCATATCGGCATAGTGCAT	CCGAGCCAAAATCCAAAATA	59.9	259	INTERGENIC	--	--	--
Ca5_P2_19815809	Ca5	19815809	CA/CATACAGTGA	TTTTGGGTTGGGATGACATT	ACCAGGAAAAGAAGAAGGGC	59.7	378	INTERGENIC	--	--	--
Ca5_P2_19824929	Ca5	19824929	TAAAAA/TTAAAAAAAAA	TTCGCAACTCCACTCTTCCT	ATCACGCAACATAACACCCA	59.8	387	INTERGENIC	--	--	--
Ca5_P2_19830708	Ca5	19830708	T/TCG	TACCGCAATCAGAAGAACCC	TGCGCCTCTTCTAAAATCGT	60.0	537	INTERGENIC	--	--	--
Ca5_P2_198698	Ca5	198698	AA/AAGA	CCGGAGCACGTAAAGCTTAG	GTGGCATCCACAATGACAAA	60.4	488	DRR	Ca_18174	L	--
Ca5_P2_19899300	Ca5	19899300	GATATATATATATATATATATATATATATATA/GATATATATATATATATATATATATATA	AACCCTTGTAAGCGCTGTCT	TGCTTGGACGAATTAAAATAAAGA	59.2	518	INTERGENIC	--	--	--

INDEL marker IDs	Chromosomes/unanchored scaffolds	Physical positions (bp)	InDels (*Kabuli* reference genome-CDC Frontier/PII)	Forward primers (5'-3')	Reverse primers (5'-3')	Anneling temperature (0C)	Expected amplified product size (bp)	Structural annotation		Functional annotation	
								Sequence components of *kabuli* genome	*Kabuli* gene accession IDs	NCBI-KOG	TFs
Ca5_P2_19899928	Ca5	19899928	AATATATATATATATATATATATATATATATATATATATAT/AATATATATATATATATATATATATATATATATATATATATATATATATATATAT	ATGACATGTCGTGCGATAAA	GGTTGCGTGATTGCCTTAGT	60.1	483	INTERGENIC	--	--	--
Ca5_P2_19922331	Ca5	19922331	A/AG	ATGTTGACTACGCGGGAGAC	CCCCTTTTTGCACATGATCT	59.9	334	INTERGENIC	--	--	--
Ca5_P2_19994870	Ca5	19994870	GTTTTTTTTTTTTT/GTTTTTTTTTTTT	CAAACTTGTGTAACCCATCCTG	GCAAACACGTGAAAAGGGTT	60.0	508	INTERGENIC	--	--	--
Ca5_P2_2000745	Ca5	2000745	T/TGC	TATGAAAATCGCCCGAAAAG	TTCTGGGTCCTCGATTTCAC	60.1	380	INTERGENIC	--	--	--
Ca5_P2_200719	Ca5	200719	GTTTT/GTTT	CGATCACCTCCAACCAAAAT	AAGTGTTTCCGCATGGTAGG	60.0	665	DRR	Ca_18175	--	--
Ca5_P2_2007457	Ca5	2007457	GCCC/GCC	TCCAGATGAAATCGAGGACC	TCCAGATGAAATCGAGGACC	60.0	712	INTERGENIC	--	--	--
Ca5_P2_20087836	Ca5	20087836	GAAGCCTATTTAAAA/GAAGCCTATTTAAAAGCCTATTTAAAA	AAACGAATTTTGAGGCCCTT	GGCTAGGCTTAGGCCATTTT	59.7	697	INTERGENIC	--	--	--
Ca5_P2_20126776	Ca5	20126776	A/AACACCGCCGCCGC	ACCTCTGTCCACTGACCACC	TGTTCACCGAAACCAAATCA	59.9	254	INTERGENIC	--	--	--
Ca5_P2_202427	Ca5	202427	T/TG	TGGTGCCACGTTCTTCTTCT	ACGAGTTGGATTGGGTTGAC	59.8	612	INTRON	Ca_18175	--	--
Ca5_P2_202535	Ca5	202535	TAAAAAAA/TAAAAAA	TTGGACTTGAAGTTGACATTGC	ACGAGTTGGATTGGGTTGAC	59.8	269	INTRON	Ca_18175	--	--
Ca5_P2_20430611	Ca5	20430611	AAAAATAAAAAAATAAAAAAATAAAAAAATAAAAAATAAAA/AAAAATAAAAAAATAAAAAATAAAAAAATAAAAAAATAAAAAAATAAAA	AGACAAAAGGGCCAACAAAA	AACACATATTAAATTGGGGCTCA	59.6	193	INTERGENIC	--	--	--
Ca5_P2_20438947	Ca5	20438947	AT/A	AGTTTGGAACCAAGCCACTG	TAAGCCGTGGGAACGTTAAG	60.1	343	INTERGENIC	--	--	--

INDEL marker IDs	Chromosomes/unanchored scaffolds	Physical positions (bp)	InDels (*Kabuli* reference genome-CDC Frontier/PII)	Forward primers (5'-3')	Reverse primers (5'-3')	Anneling temperature (0C)	Expected amplified product size (bp)	Structural annotation		Functional annotation	
								Sequence components of *kabuli* genome	*Kabuli* gene accession IDs	NCBI-KOG	TFs
Ca5_P2_20455904	Ca5	20455904	TTTATTATTATTATTATTATTATTATTATT ATTATTATTATTGTTATTATTGTTATTAT TATTATTATTATTATTATTATTATTATTA TT/TTTATTATTATTATTATTATTATTATT ATTATTATTATTGTTATTATTGTTATTAT [illegible]	TCACTTTTCCAGCCTCC ACT	CCCGGTCAAACTCCAA ATTA	59.8	729	DRR	Ca_18207	--	--
Ca5_P2_20515946	Ca5	20515946	GAAAAAAAAAAA/GAAAAAAAAAA	TGTCAACAGCAGAGGC TGAG	TGGTTCCAACAATCTC CTCC	59.9	577	INTERGENIC	--	--	--
Ca5_P2_20518047	Ca5	20518047	ATATTATTATTATTATTATTATTATTATT ATTATTAT/ATATTATTATTATTATTATTA TTATTATTATTATTATTAT	TGGCCTACTTATGACCT GGC	TGAGTCCGGCCTACGA TTTA	60.6	592	INTERGENIC	--	--	--
Ca5_P2_20547334	Ca5	20547334	AACCAACACCAACACCAACA/AACCAAC ACCAACACCAACACCAACA	AGGAGATGGAGCGTTG TTTG	TTGGTTAGCGTGCATC TGAG	60.0	462	INTERGENIC	--	--	--
Ca5_P2_206513	Ca5	206513	ATT/AT	ATCACGCAGATGATGG CATA	AAAGGCAAAAGGATGG GAGT	59.9	426	INTRON	Ca_18175	--	--
Ca5_P2_20743462	Ca5	20743462	AA/AATA	AAAATAAAGCATGGCGT TGG	AGTTTGTAAACCGTGC GTCG	61.1	258	INTERGENIC	--	--	--
Ca5_P2_20759180	Ca5	20759180	CAATAATAATAATAATAATAATAATAATA ATAATAATAATAAT/CAATAATAATAATA ATAATAATAATAATAATAAT	AACAGGAGGTCGTTGT GACC	ATCAGGTTCCGTTGCA AGAC	60.1	757	INTERGENIC	--	--	--
Ca5_P2_20781552	Ca5	20781552	GATATATATATATATATATATATATATAT/ GATATATATATATATATATATATATATAT AT	GCTGAGGTGGGAAACA TTTG	CCAAATCAGAACCAAA GGCA	61.0	598	DRR	Ca_18223	--	ARF
Ca5_P2_208088	Ca5	208088	TA/T	TGTTTGGGGAACTTGC TTTC	GCCAACATGCGACTCT GTTA	59.9	578	INTERGENIC	--	--	--
Ca5_P2_208132	Ca5	208132	TAATGAATGAATGAA/TAATGAATGAAT GAATGAA	TGTTTGGGGAACTTGC TTTC	GCCAACATGCGACTCT GTTA	59.9	578	INTERGENIC	--	--	--
Ca5_P2_2082706	Ca5	2082706	TA/TAA	CAAGCATGATCCAGGG AGAT	CCATAAACGGAATTAAT GGACC	59.5	475	INTERGENIC	--	--	--
Ca5_P2_2083274	Ca5	2083274	ATATTATTATTATTATTATTATTATTATT ATTATTATTATTATTATTATTA/ATATTAT TATTATTATTATTATTATTATTATTATTA TTATTATTATTA	CCATTAATTCCGTTTAT GGTGAA	TTTCCTAAATTTCGAGG ACAAA	57.9	501	INTERGENIC	--	--	--

INDEL marker IDs	Chromosomes/unanchored scaffolds	Physical positions (bp)	InDels (*Kabuli* reference genome-CDC Frontier/PII)	Forward primers (5'-3')	Reverse primers (5'-3')	Anneling temperature (0C)	Expected amplified product size (bp)	Structural annotation		Functional annotation	
								Sequence components of *kabuli* genome	*Kabuli* gene accession IDs	NCBI-KOG	TFs
Ca5_P2_208514	Ca5	208514	ATTTTTTTTTTTT/ATTTTTTTTTT	ATTCCAATTCGGATGTGCTC	AACAAGTGATGGTGATGCCA	60.0	407	INTERGENIC	--	--	--
Ca5_P2_20895157	Ca5	20895157	TTTATTATTATTATTATTATTATTATTATTATTATTATT/TTTATTATTATTATTATTATTATTATTATTATTATTATTATT	ATGAAGTGATGCCCTTACCG	TTGGATGGAGGTTGTGTTGA	59.9	303	INTERGENIC	--	--	--
Ca5_P2_209767	Ca5	209767	GAAAAAAAA/GAAAAAAAAAA	AAAACATTCAACCAACCACCA	TTAGACCATCCACAACACGG	59.4	320	DRR	Ca_18176	--	C2H2
Ca5_P2_210018	Ca5	210018	TTATACTTATCTAT/TTAT	TTAAACACCCGTGTTGTGGA	AAATGGCTTAGGGAGGGAGA	60.0	607	DRR	Ca_18176	--	C2H2
Ca5_P2_2100697	Ca5	2100697	ATTTTTTT/ATTTTTT	TGGACACGACGGTGAAATAA	TTCGGTTCTTGTTTGCCTCT	59.9	642	INTERGENIC	--	--	--
Ca5_P2_21009024	Ca5	21009024	CTATATATATATATATATATATATATAT/CTATATATATATATATATATATATATATATATAT	AACAGATTTTAACCCGTGCG	CGCAATGCGAGAGTTATCGT	61.3	275	INTERGENIC	--	--	--
Ca5_P2_210200	Ca5	210200	ATTTTTTTTT/ATTTTTTTTTT	TTAAACACCCGTGTTGTGGA	AAATGGCTTAGGGAGGGAGA	60.0	607	DRR	Ca_18176	--	C2H2
Ca5_P2_210510	Ca5	210510	ATT/ATTT	TCTCCCTCCCTAAGCCATTT	GAATTATCCGCTGCTTGCAT	60.2	201	DRR	Ca_18176	--	C2H2
Ca5_P2_210754	Ca5	210754	TAAAAAAAAAAAA/TAAAAAAAAAAAAA	TCTCCCTCCCTAAGCCATTT	TGGCTCAGAGCTGATTCTGAT	60.1	746	DRR	Ca_18176	--	C2H2
Ca5_P2_21085449	Ca5	21085449	A/AT	TTAGGCGACCACTGTATCCC	TGAGCCACTTTGATCAGCAC	60.0	758	INTERGENIC	--	--	--
Ca5_P2_21106062	Ca5	21106062	CATATATATATATATATATATATATATATAT/CATATATATATATATATATATATATATATATATATAT	GATTTTGACGATGCCTTGGT	TTCTTTCCTTGCTCACCTTCA	60.0	443	INTERGENIC	--	--	--
Ca5_P2_21106566	Ca5	21106566	TAGAA/TAGAAGAA	TGAAGGTGAGCAAGGAAAGAA	AAAAACCACAAGTGTGCTTAGG	58.4	236	INTERGENIC	--	--	--

INDEL marker IDs	Chromosomes/unanchored scaffolds	Physical positions (bp)	InDels (*Kabuli* reference genome-CDC Frontier/PII)	Forward primers (5'-3')	Reverse primers (5'-3')	Anneling temperature (0C)	Expected amplified product size (bp)	Structural annotation		Functional annotation	
								Sequence components of *kabuli* genome	*Kabuli* gene accession IDs	NCBI-KOG	TFs
Ca5_P2_21121401	Ca5	21121401	CACATATA/CATA	TGTCACCATTCAAGGAAGCA	TGCTACTAGGCCCTCAGCAT	60.0	721	INTERGENIC	--	--	--
Ca5_P2_21144099	Ca5	21144099	ATATTATTATTATTATTATTATTATTATTATTATTATTATTATT/ATATTATTATTATTATTATTATTATTATTATTATTATTATTATTATTATTATT	AATCGTTGGTGGCCTTAGTG	AGGGGAAAACCTTGCTCCTA	60.1	484	INTERGENIC	--	--	--
Ca5_P2_21152020	Ca5	21152020	ATTTTTT/ATTTTTTT	AAATTGTGGGTTTCGCGTAG	GAGGTAGGTGGCAAAGGACA	60.1	459	INTERGENIC	--	--	--
Ca5_P2_2140832	Ca5	2140832	G/GC	GCCCGGATTGCAAGTATAAA	TTTGTGAGAGGGTGGGAGTC	60.1	549	INTRON	Ca_26505	B	--
Ca5_P2_21411216	Ca5	21411216	TAAAAAAAA/TAAAAAAAAAAA	CCAAAGTGGGTCAATAACGC	CTTCGACAGCCAGTTCATCA	60.0	883	INTERGENIC	--	--	--
Ca5_P2_21459937	Ca5	21459937	GATATATATATATATATAT/GATATATATATATATAT	AAATTGAAGCTCGAATCCCA	GCCCTGTTTGTTTGCATTGT	60.9	671	INTERGENIC	--	--	--
Ca5_P2_21498233	Ca5	21498233	AT/ATTGGTCCAAACGTT	CCAGCTCCTTTGGTTCTCAC	GGGCATTTGAGAAGGTACGA	60.1	826	INTERGENIC	--	--	--
Ca5_P2_21501337	Ca5	21501337	AAT/AATAT	GCGTCGTACGGAGACAAATTA	TGACGAAGGAAAACCATGTG	59.5	340	INTERGENIC	--	--	--
Ca5_P2_21623515	Ca5	21623515	AAATTAATT/AAATT	AGGTCATTTTCATCCACCCA	ACACACCTATGGGATGCACA	59.8	678	INTERGENIC	--	--	--
Ca5_P2_21754940	Ca5	21754940	AAATAATAATAATAATAATAATAATAATAATAATAATAATAATA/AAATAATAATAATAATAATAATAATAATAATAATAATAATAATAATAATAATAATAATA	TAAACATGTGTTGCATCCGC	TTGCAGCAAAAACAAGCAAC	60.0	396	INTERGENIC	--	--	--
Ca5_P2_21755455	Ca5	21755455	CATAG/CATAGTATAG	TGGCTTTGTTGATCTCATGC	ACCAATGCAAGCTGGAAGAA	60.8	363	INTERGENIC	--	--	--
Ca5_P2_21756986	Ca5	21756986	GAAAAAAAA/GAAAAAAAAA	AAACCTGGATGGGAATTCAA	TCGACTTGCTTTGGCTTTTT	60.0	439	INTERGENIC	--	--	--

INDEL marker IDs	Chromosomes/unanchored scaffolds	Physical positions (bp)	InDels (*Kabuli* reference genome-CDC Frontier/PII)	Forward primers (5'-3')	Reverse primers (5'-3')	Anneling temperature (0C)	Expected amplified product size (bp)	Structural annotation		Functional annotation	
								Sequence components of *kabuli* genome	*Kabuli* gene accession IDs	NCBI-KOG	TFs
Ca5_P2_21762261	Ca5	21762261	TG/T	TACCTTAACGCCTCCACCAC	CAAGGTGAAGAGGCTTTTGC	60.0	708	INTERGENIC	--	--	--
Ca5_P2_21779978	Ca5	21779978	TAAAAAAAAAA/TAAAAAAAAAAA	TGGTTGTTCAATTCATGTTCAA	CACCCAATGGTGATTAGCAA	59.4	696	INTERGENIC	--	--	--
Ca5_P2_217922	Ca5	217922	CTT/CTTAGTTT	AAGGACATCCACAATGGAAA	ATCACAACACCACCACGCTA	60.0	406	INTERGENIC	--	--	--
Ca5_P2_218483	Ca5	218483	ATCTCTC/ATCTCTCTC	TAGCGTGGTGGTGTTGTGAT	CCTTTCAAGGTCAGCCACAT	60.1	739	INTERGENIC	--	--	--
Ca5_P2_218592	Ca5	218592	TATAATAATAATAATAATA/TATAATAATAATAATAATAATA	TAGCGTGGTGGTGTTGTGAT	CCTTTCAAGGTCAGCCACAT	60.1	739	INTERGENIC	--	--	--
Ca5_P2_21866401	Ca5	21866401	CT/CTTT	GGTTGAGAGGGCTCAACAAG	GCTTCGTTCTTCGTTCTTCG	60.1	615	INTERGENIC	--	--	--
Ca5_P2_21866481	Ca5	21866481	GA/GAA	TCCGATTGGATTTTCAGGAC	GCTTCGTTCTTCGTTCTTCG	60.1	437	INTERGENIC	--	--	--
Ca5_P2_21874875	Ca5	21874875	GAAAAAAAAAAAA/GAAAAAAAAAAAAAA	TAAACACTTAACCGGGGCTG	ACTTTCAGCTGGGCACACTT	59.9	424	INTERGENIC	--	--	--
Ca5_P2_21883868	Ca5	21883868	AATATATATATATATATATA/AATATATATATATATATATATA	GCTCCATTTGTGGTTCCAAT	CTATTTTGCTATGCCGCTCC	59.8	405	INTRON	Ca_24467	--	C2H2
Ca5_P2_21899576	Ca5	21899576	TAAAAAAAAAAAA/TAAAAAAAAAAA	TGACCTGACCTGGCCTATTC	CCCAAACAAAGCCTTATGGA	59.9	519	INTERGENIC	--	--	--
Ca5_P2_21899696	Ca5	21899696	TAAAAAAA/TAAAAAA	TGACCTGACCTGGCCTATTC	CCCAAACAAAGCCTTATGGA	59.9	519	INTERGENIC	--	--	--
Ca5_P2_219030	Ca5	219030	TAAAAAAA/TAAAAAAAAA	TTGAGATGTGGCTGACCTTG	CTGGAGCTGTCTCCAACACA	60.0	613	INTERGENIC	--	--	--

INDEL marker IDs	Chromosomes/unanchored scaffolds	Physical positions (bp)	InDels (*Kabuli* reference genome-CDC Frontier/PII)	Forward primers (5'-3')	Reverse primers (5'-3')	Anneling temperature (0C)	Expected amplified product size (bp)	Structural annotation		Functional annotation	
								Sequence components of *kabuli* genome	*Kabuli* gene accession IDs	NCBI-KOG	TFs
Ca5_P2_21965060	Ca5	21965060	AG/AGG	ACGCTCAGAGTTCACTGGGT	GGCAAATCGATTGGACAAGT	59.9	365	INTERGENIC	--	--	--
Ca5_P2_220686	Ca5	220686	TAAAAAAAA/TAAAAAAA	GCAATCTCCAAACAAGCCAT	TCTTCTTCCGTTCCCATTTG	60.0	188	INTERGENIC	--	--	--
Ca5_P2_2207983	Ca5	2207983	AATATATATATATATATATATATATATATATATATATATATA/AATATATATATATATATATATATATATATATATATATATATATA	TATTTTGGATTTTGGCTCGG	AGGAAGAGTGGAGTTGCGAA	60.0	528	INTERGENIC	--	--	--
Ca5_P2_22145277	Ca5	22145277	ATTTTTTT/ATTTTTTTT	GGCTTTTCAACTTTCACCCA	TGGTCGCCAATTATGTCTCA	60.1	693	INTERGENIC	--	--	--
Ca5_P2_22173040	Ca5	22173040	AATATATATATATATATATATATATATATA/AATATATATATATATATATATATATATA	ATCTCATGCGTGTGGATTGA	GCGTGTAGGCGAAGAGTTGT	60.5	798	INTERGENIC	--	--	--
Ca5_P2_22218019	Ca5	22218019	TAAATTAAAATTAAAATTAAA/TAAATTAAAATTAAAATTAAAATTAAAATTAAA	AAATCCTGCATTTGTGAGGG	TGCTTATTGTGGCTGCTTTG	60.0	676	INTERGENIC	--	--	--
Ca5_P2_222796	Ca5	222796	CAAGCAAAGCAAAGCAAAGCAAAGCAAAG/CAAGCAAAGCAAAGCAAAG	AGCCATGTTTTGTCCTCACC	CGGCACAAGAAAGGTGTTTT	60.1	190	INTERGENIC	--	--	--
Ca5_P2_2231251	Ca5	2231251	GTATATATATATATATATATATAT/GTATATATATATATATATATATATAT	AAACTGGTTGTGTGGAAGCC	GGTGTGCAAGAGGACTGGAT	60.1	270	INTERGENIC	--	--	--
Ca5_P2_22350080	Ca5	22350080	CAAAAAAAAAAAAA/CAAAAAAAAAAAA	TGCCTCTGGAACACCTAACC	ATTTGTTCTGGTGAGGGCAC	60.0	665	INTERGENIC	--	--	--
Ca5_P2_22362048	Ca5	22362048	GGAAAG/GGAAAGAGAAGAAAG	CATGCGCAACTTCTTGGTAA	CACAAAAGCTTGCGCTTACA	60.2	701	INTERGENIC	--	--	--
Ca5_P2_223736	Ca5	223736	CCTACT/CCTACTACTGCTACT	CCATCACCACTCCTCCAACT	TTTTTCAGCAAAGCTCAGCA	59.9	331	CDS (large-effect mutations)	Ca_18177	--	ZF-HD
Ca5_P2_224582	Ca5	224582	GTTTTTTT/GTTTTTT	GGACTCTGACCTGAGTTGCC	TTCACATATCGCTCCATCCA	60.0	444	DRR	Ca_18177	--	ZF-HD

INDEL marker IDs	Chromosomes/unanchored scaffolds	Physical positions (bp)	InDels (*Kabuli* reference genome-CDC Frontier/PII)	Forward primers (5'-3')	Reverse primers (5'-3')	Anneling temperature (0C)	Expected amplified product size (bp)	Structural annotation		Functional annotation	
								Sequence components of *kabuli* genome	*Kabuli* gene accession IDs	NCBI-KOG	TFs
Ca5_P2_22485566	Ca5	22485566	TAATAAATAAATAAATAAAT/TAATAAATAAATAAATAAATAAAT	TTTTCCTGGGTTTGAACTCG	CCCACAAAACAAAGGAGGAA	59.9	624	INTERGENIC	--	--	--
Ca5_P2_225405	Ca5	225405	TTCTCTC/TTCTC	CGGATATGCCATCACAGTTG	GGCCCCATTGATGGTTACTA	59.6	834	DRR	Ca_18177	--	ZF-HD
Ca5_P2_225505	Ca5	225505	ATTTTTTTTT/ATTTTTTTT	CGGATATGCCATCACAGTTG	GGCCCCATTGATGGTTACTA	59.6	834	DRR	Ca_18177	--	ZF-HD
Ca5_P2_22631833	Ca5	22631833	TGAGAGAGAGAGAGAGAGAGAGAGAGAGAGAGAGA/TGAGAGAGAGAGAGAGAGAGAGAGAGAGAGAGAGAGA	TGGAGAATTGGCTGGAGTTC	GCTTAAACTCATGGCGAAGC	60.0	749	INTRON	Ca_20079	--	--
Ca5_P2_22639111	Ca5	22639111	TAAAAAAAAAAA/TAAAAAAAAAAAA	CCGATTTCCATTCACTCCAT	TGCGAACTTGTTAATCTGGC	58.9	281	INTERGENIC	--	--	--
Ca5_P2_22659274	Ca5	22659274	AATATATATATATATATATATATATATATA/AATATATATATATATA	CGGCGACTTTTCATTTGATT	AATCGGCATTTCAAACTTGG	59.9	501	INTERGENIC	--	--	--
Ca5_P2_22659444	Ca5	22659444	AATATATATATATATATATATATATATATA/AATATATATATATATATATATATATATATATATATA	CCAAGTTTGAAATGCCGATT	GCTGCATGTTCTTATGCTCG	59.6	485	INTERGENIC	--	--	--
Ca5_P2_227141	Ca5	227141	AA/AAATA	GAGTATGGTTGGCCGAAAAA	TGGATGCTGTTGGATTTTGA	60.0	721	INTERGENIC	--	--	--
Ca5_P2_2274980	Ca5	2274980	GAAAAAAAAAAAA/GAAAAAAAAAAAAAA	TGACACTGAAGTCTGAACCGA	CTCATTTGGAACGGAGGAAA	60.0	593	INTERGENIC	--	--	--
Ca5_P2_22865562	Ca5	22865562	GTTTTTTT/GTTTTTT	TTGGATTTGTCAGGCATCAA	TCAACGAATCATAGAGCTGAAA	57.7	762	INTERGENIC	--	--	--
Ca5_P2_22917650	Ca5	22917650	CTTTTTTTTT/CTTTTTTTTTT	TCACACGCACACATTAAGCC	GCAGTGGATAAGGCCGATAA	60.1	916	INTERGENIC	--	--	--
Ca5_P2_22918447	Ca5	22918447	T/TG	TTATCGGCCTTATCCACTGC	GGATTCCACCAAATTCAGGA	59.7	721	INTERGENIC	--	--	--

INDEL marker IDs	Chromosomes/unanchored scaffolds	Physical positions (bp)	InDels (*Kabuli* reference genome-CDC Frontier/PII)	Forward primers (5'-3')	Reverse primers (5'-3')	Anneling temperature (0C)	Expected amplified product size (bp)	Structural annotation		Functional annotation	
								Sequence components of *kabuli* genome	*Kabuli* gene accession IDs	NCBI-KOG	TFs
Ca5_P2_22918526	Ca5	22918526	AT/ATT	TTATCGGCCTTATCCACTGC	TGAAAATCCATGAACCGTGA	59.9	915	INTERGENIC	--	--	--
Ca5_P2_22919284	Ca5	22919284	ATTTT/A	TCACGGTTCATGGATTTTCA	TTCGGAGATTCCACCAAATC	59.9	639	DRR	Ca_21962	--	--
Ca5_P2_22919631	Ca5	22919631	AG/A	GATTTGGTGGAATCTCCGAA	GCCACCGACTACTTCTCCCT	60.6	336	DRR	Ca_21962	--	--
Ca5_P2_22919722	Ca5	22919722	GTT/GT	GATTTGGTGGAATCTCCGAA	GCCACCGACTACTTCTCCCT	60.6	336	DRR	Ca_21962	--	--
Ca5_P2_2295220	Ca5	2295220	GAA/GA	GCACCAGCATAAACTGCACA	GCAGGAGTTTTCCTTGCATT	59.3	739	INTERGENIC	--	--	--
Ca5_P2_22955537	Ca5	22955537	ATATAGAACCTATAGAACCTA/ATATAGAACCTA	GCTCAAAGCACATGACAGGA	GATTGACGCATCAAAGGGTT	59.9	777	INTERGENIC	--	--	--
Ca5_P2_22981606	Ca5	22981606	CT/CTT	TCAATTTCGCTTCTCGTGTG	TTGTTGGAGATTTTGGAGGG	59.9	349	INTERGENIC	--	--	--
Ca5_P2_23051252	Ca5	23051252	ATTTTTTTTT/ATTTTTTTTTT	TGGGACTAAAACATAGCGCC	ACGGATGAGTGACGTGACAA	60.2	118	DRR	Ca_21972	U	--
Ca5_P2_23096429	Ca5	23096429	TGTG/TGTGAGTG	GAATGTGCCGCCTATTGTTT	TGTCCTCCCTTAATTGCACA	59.1	662	INTERGENIC	--	--	--
Ca5_P2_23111759	Ca5	23111759	ACCCCC/ACCCCCC	TCTCACGGTTGTGAGGACTG	TAGAGAAGTGTGCACCGTGG	59.9	600	INTERGENIC	--	--	--
Ca5_P2_23184362	Ca5	23184362	AATTATTATTATTATTATTATTA/AATTATTATTATTATTATTATTATTA	GAAAAAGCGCTGTAAATGGC	ATCCCACCAACCAAGACAAA	60.2	725	INTERGENIC	--	--	--
Ca5_P2_2323461	Ca5	2323461	GAAAA/GAAAAA	GCGCCAGATTTCAAACACTA	ACCAGCAGACCAAAATGGAC	60.0	262	INTERGENIC	--	--	--

INDEL marker IDs	Chromosomes/unanchored scaffolds	Physical positions (bp)	InDels (*Kabuli* reference genome-CDC Frontier/PII)	Forward primers (5'-3')	Reverse primers (5'-3')	Anneling temperature (0C)	Expected amplified product size (bp)	Structural annotation		Functional annotation	
								Sequence components of *kabuli* genome	*Kabuli* gene accession IDs	NCBI-KOG	TFs
Ca5_P2_2323758	Ca5	2323758	TGA/TGGAGGA	GTCCATTTTGGTCTGCTGGT	TCCCTGTCCTCGATTTCATC	60.0	425	INTERGENIC	--	--	--
Ca5_P2_2327252	Ca5	2327252	TAA/TAAA	TCGAAATGAAATCGAGGACC	ACAGACGAGGGCTAGGGATT	60.1	721	INTERGENIC	--	--	--
Ca5_P2_23306940	Ca5	23306940	CTTTTTTTTTT/CTTTTTTTTTTT	GTATTGAGGGGGTTGGAGGT	CCCTTTCAACCTTTTGCATC	59.5	352	INTERGENIC	--	--	--
Ca5_P2_23426466	Ca5	23426466	TTCTCTCTCTCTCTCTCTCTCTCTCTCTC/TTCTCTCTCTCTCTCTCTCTCTCTC	GCTCGAATAAAATCGCCAAA	GTTGAGGAGGTGCCATAGGA	60.1	783	INTERGENIC	--	--	--
Ca5_P2_23476079	Ca5	23476079	CAAAAAAAAAAAAA/CAAAAAAAAAAAA	GCACATGAGATCTTGAGGCA	TTTTGGTCCCCCAAACATAA	60.0	102	INTERGENIC	--	--	--
Ca5_P2_23547474	Ca5	23547474	TAAAAAAAAAA/TAAAAAAAAAAA	AGCCCATGAAAATAACCCAA	CCAAATTATGCGAAAAACGG	60.3	484	INTERGENIC	--	--	--
Ca5_P2_23585549	Ca5	23585549	TTT/TTTAAACATACTT	TTGCCACTGACTTGTGCAAT	CAGGGATCTCCCAAAATGTG	60.3	622	INTERGENIC	--	--	--
Ca5_P2_236073	Ca5	236073	CGAAGAAAAT/CGAAGAAAATGAAGAAAATGAAGAAAAT	TTGAATTGACCGGAGGAATC	GGGATAGGGAGGGTTGTGTT	60.1	680	INTERGENIC	--	--	--
Ca5_P2_23608112	Ca5	23608112	GCCTCACCCTCATCCTCACC/GCCTCACCCTCATCCTCACCCTCATCCTCACC	CAACTTCGAGGGGAATTTGA	CTGGTATGGGTGCTTCTGGT	60.0	529	CDS (large-effect mutations)	Ca_17689	--	--
Ca5_P2_23645517	Ca5	23645517	T/TCAGA	CCCCATGTTGGATAAACCAG	TCTTGGTTCTTCAATTGCCC	60.1	839	INTERGENIC	--	--	--
Ca5_P2_23663097	Ca5	23663097	TAATGAATGAATGAA/TAATGAATGAA	GAGGGTTTTGGGTCAGTGAA	AGTTTTCAAGGGTAGCGGGT	60.0	789	INTERGENIC	--	--	--
Ca5_P2_23663124	Ca5	23663124	C/CGA	GAGGGTTTTGGGTCAGTGAA	AGTTTTCAAGGGTAGCGGGT	60.0	789	INTERGENIC	--	--	--

INDEL marker IDs	Chromosomes/unanchored scaffolds	Physical positions (bp)	InDels (*Kabuli* reference genome-CDC Frontier/PII)	Forward primers (5'-3')	Reverse primers (5'-3')	Anneling temperature (0C)	Expected amplified product size (bp)	Structural annotation		Functional annotation	
								Sequence components of *kabuli* genome	*Kabuli* gene accession IDs	NCBI-KOG	TFs
Ca5_P2_23664574	Ca5	23664574	TT/TTCCAACT	CAAGAAGGAACGAGGTGCTC	TAGCCTAGAGCAGGGGTGAA	60.0	626	INTERGENIC	--	--	--
Ca5_P2_23672060	Ca5	23672060	TC/TCGCCC	TGAGTAAGGGGGCTGAAATG	TGAGAGACGGTGGTTTACCC	60.0	822	CDS (FRAME SHIFT)	Ca_17683	--	--
Ca5_P2_236749	Ca5	236749	AT/ATT	AACACAACCCTCCCTATCCC	CCAACAAATGCATGTCCATC	59.8	819	INTERGENIC	--	--	--
Ca5_P2_23675051	Ca5	23675051	GT/G	TCCATGCACATTGCTCTCTC	TGGGTTATTTTCATGGGCTT	59.3	514	INTERGENIC	--	--	--
Ca5_P2_236894	Ca5	236894	CCACA/CCA	CCCTCACAAATTACAATCCCA	CCAACAAATGCATGTCCATC	59.8	763	INTERGENIC	--	--	--
Ca5_P2_237297	Ca5	237297	CTGTCAT/CTGTCATGTCAT	TCATTGCAATACCGGGAAAT	TGAATTTGGATAGGTGCAAAAA	59.5	233	INTERGENIC	--	--	--
Ca5_P2_237378	Ca5	237378	GA/GACAGAATGCAAA	TCATTGCAATACCGGGAAAT	TGCAACTCATGTGTGAATCG	59.2	614	INTERGENIC	--	--	--
Ca5_P2_23750817	Ca5	23750817	CTTTTTTTTT/CTTTTTTTTTT	CGATTTGGGTCTTTTGCATT	GGTCACATAGCGCTCTCACA	60.0	710	INTERGENIC	--	--	--
Ca5_P2_2375435	Ca5	2375435	TTTA/T	ATGTTTTGGATTTTGGCTCG	AGGAAGAGTGGAGTTGCGAA	60.0	571	INTRON	Ca_18664	--	B3
Ca5_P2_23770283	Ca5	23770283	ATTTTT/ATTTTTT	TTGGCCTTCCTTTTACATGC	CCATCAAGCGTCTGTTGAGA	60.0	882	INTERGENIC	--	--	--
Ca5_P2_23771514	Ca5	23771514	G/GAAATAATAATAA	ACCGATTTTGCCCTACCTTT	AAGTTTTTGGCATTGCTTGC	60.3	326	INTERGENIC	--	--	--
Ca5_P2_237891	Ca5	237891	GT/GTT	CATGCACGATTCACACATGA	GTGTCGTCCAACCTCTCCAT	60.0	486	INTERGENIC	--	--	--

INDEL marker IDs	Chromosomes/unanchored scaffolds	Physical positions (bp)	InDels (*Kabuli* reference genome-CDC Frontier/PII)	Forward primers (5'-3')	Reverse primers (5'-3')	Anneling temperature (0C)	Expected amplified product size (bp)	Structural annotation		Functional annotation	
								Sequence components of *kabuli* genome	*Kabuli* gene accession IDs	NCBI-KOG	TFs
Ca5_P2_23812349	Ca5	23812349	TAAAAAAAAAAA/TAAAAAAAAAAAAAAAAA	ATTGCTAGCCACATTTTGGG	CGCTATGATAGGATGGTCCAA	59.9	664	INTERGENIC	--	--	--
Ca5_P2_239370	Ca5	239370	CAA/CAAA	AACTCGCATGTATTCCCCAC	TGGATTCCCAAACACTAGCC	59.9	597	INTERGENIC	--	--	--
Ca5_P2_23976521	Ca5	23976521	GT/GTT	TTGGTGAAAGATTTTGTGCG	TTCAATGACAAAGGGGGAGA	60.4	759	INTERGENIC	--	--	--
Ca5_P2_239777	Ca5	239777	CTTT/CTTTT	CTCAACGCTGCCACAATAGA	TGGATTCCCAAACACTAGCC	59.9	473	INTERGENIC	--	--	--
Ca5_P2_23990846	Ca5	23990846	GCCC/GCC	TTCAGCTGGTTGCACCATAG	AGTTGGAAGTCGCCATTTTG	60.1	261	INTERGENIC	--	--	--
Ca5_P2_24097297	Ca5	24097297	AAGCTTTGGATAC/A	CAGAGCCAACACTACAGGCA	ATTTGAAGGGCCATGTGAAG	59.9	329	CDS (large-effect mutations)	Ca_17656	S	--
Ca5_P2_2410636	Ca5	2410636	GATAATAATAATAATAATAATAATAATA/GATAATAATAATAATAATAATAATA	AAATTGAACATGCTCGGACC	CCGTCGAAAGTTTCTGCTTC	60.0	465	INTRON	Ca_18664	--	B3
Ca5_P2_241183	Ca5	241183	ATTTTTTT/ATTTTTTTT	AAAACACAACCCTTCAAGAGACA	GGGACGTCTTCGCATACATT	60.0	129	INTERGENIC	--	--	--
Ca5_P2_2424723	Ca5	2424723	CAAAAAAAAA/CAAAAAAAA	GCAAGGAAAACGAAAATGGA	TCCAGATCGAATTAATGGCA	59.1	509	INTRON	Ca_18664	--	B3
Ca5_P2_24302587	Ca5	24302587	ATTTTTTTTTTTT/ATTTTTTTTTTT	TGCCCATTTAAAACTTGGGT	CCAATGACGACTTCCCACTT	60.0	158	INTERGENIC	--	--	--
Ca5_P2_243413	Ca5	243413	CTAAT/CT	CAGAAAGTAAACGGGCCAAG	TGAGGTCGATAAACATAAAAATACG	58.6	491	INTERGENIC	--	--	--
Ca5_P2_243436	Ca5	243436	CAA/CA	CAGAAAGTAAACGGGCCAAG	TCGCGCAAAATAACATTTGA	60.2	951	INTERGENIC	--	--	--

INDEL marker IDs	Chromosomes/unanchored scaffolds	Physical positions (bp)	InDels (*Kabuli* reference genome-CDC Frontier/PII)	Forward primers (5'-3')	Reverse primers (5'-3')	Anneling temperature (0C)	Expected amplified product size (bp)	Structural annotation		Functional annotation	
								Sequence components of *kabuli* genome	*Kabuli* gene accession IDs	NCBI-KOG	TFs
Ca5_P2_244132	Ca5	244132	TAAAAAAAAAA/TAAAAAAAAA	TTTTGCGCGAGAATTGATAA	TTTCAGAGGTTTGTTGCATTTTT	60.0	360	INTERGENIC	--	--	--
Ca5_P2_244348	Ca5	244348	TA/TAATACAAA	TTTTGCGCGAGAATTGATAA	GCACTTACCACCGCTCTCTT	59.5	918	INTERGENIC	--	--	--
Ca5_P2_24470830	Ca5	24470830	GATATATATATATATATATATATATATATATATATATA/GATATATATATATATATATATATATATA	TGCAACTGCATGACAAATCA	CCCATTTTGTTCCTCCTCAA	59.9	792	INTERGENIC	--	--	--
Ca5_P2_24602434	Ca5	24602434	GTAAATAAATAAATAAATAAATAAATAA/GTAAATAAATAAATAAATAAATAAATAAATAAATAA	TCACATCAGTTAGGCCACCA	GTTTCGAACACCGGACCTTA	60.0	235	INTRON	Ca_15595	G	bHLH
Ca5_P2_24698571	Ca5	24698571	CTTTTTTTTTTTT/CTTTTTTTTTTT	TACAACCTTATTCGCTCCCG	TCCTGTCCATCAAACTGATCATA	59.4	382	INTERGENIC	--	--	--
Ca5_P2_24701450	Ca5	24701450	CCATACATACATACATACATACATACATACATACATACATACATACATACATACA/CCATACATACATACATACATACATACATACATACATACATACATACATACATACATACATACA	CGTGTCCGAGTTAACAGCAA	TGTGTGTGGGTTATCACGCT	60.0	621	INTERGENIC	--	--	--
Ca5_P2_24764505	Ca5	24764505	TT/TTTCTTTGATTTTATCT	ATTTCTTGCCCTTTGTCCCT	CGGAACGTCATTTTTCACCT	60.0	505	INTERGENIC	--	--	--
Ca5_P2_24881793	Ca5	24881793	TAAAAAAAAAAAA/TAAAAAAAAAAA	CCGAAAGAATAGCTGGTGGA	TTCAAAAATCTGACGGAGGG	60.0	681	INTERGENIC	--	--	--
Ca5_P2_24897143	Ca5	24897143	AATATATATATATATATATATATAT/AATATATATATATATATATATATATATAT	TGTTTAAGTGGTGGGGCTTC	CTTGTCGCTGATACATGCGT	59.9	845	DRR	Ca_15612	--	C2H2
Ca5_P2_24932738	Ca5	24932738	ATTTTTTTT/ATTTTTTT	TGAAGAAACTTTTCCGCTGC	TGGAAATTTTACGGTGCAGA	59.2	710	INTERGENIC	--	--	--
Ca5_P2_24961934	Ca5	24961934	GAAAAATAAAAAAAAAATAAAAAAAAAATAAAAA/GAAAAATAAAAAAAAAAATAAAAA	TACCCTTTGCTAGCCCCTTT	TTTGAAGGACTTTCGGGTTG	60.1	449	INTERGENIC	--	--	--
Ca5_P2_24982766	Ca5	24982766	GTATATATATATATATATATAT/GTATATATATATATATATATATATAT	CTTGGCCAGAAAATTGGAAA	CGTTGAATACGCCTAACGGT	60.0	676	INTERGENIC	--	--	--

INDEL marker IDs	Chromosomes/unanchored scaffolds	Physical positions (bp)	InDels (*Kabuli* reference genome-CDC Frontier/PII)	Forward primers (5'-3')	Reverse primers (5'-3')	Anneling temperature (0C)	Expected amplified product size (bp)	Structural annotation		Functional annotation	
								Sequence components of *kabuli* genome	*Kabuli* gene accession IDs	NCBI-KOG	TFs
Ca5_P2_25027142	Ca5	25027142	TAAAAAAAAAAAAAA/TAAAAAAAAA	AAATGGAATAACTTTGAAAATGGAA	CTCCATCGCACACTTTCTCA	60.0	602	INTERGENIC	--	--	--
Ca5_P2_25050486	Ca5	25050486	AATATATATATATATATATATATATATATATATATAT/AATATATATATATATATATATATATATATATATATATATATATAT	AGTGGCGGAGCTTGATAGAA	GGGTCCAAGTTGAACTAAACCA	60.3	677	INTERGENIC	--	--	--
Ca5_P2_25109370	Ca5	25109370	T/TATTA	ACCACCAAAAAGGGTTGGTT	CGCATTCCAGATATGCAACA	60.6	500	INTERGENIC	--	--	--
Ca5_P2_25129964	Ca5	25129964	T/TA	ATCTCCAAGCTCGGCTGTAA	TTCTTTGGCCTAGTGTTGGG	60.1	211	INTERGENIC	--	--	--
Ca5_P2_25186556	Ca5	25186556	GAAAAAAAA/GAAAAAAAAA	TCAGAGGTACATTCGCCAGA	GTCAATTTGATAGGCCCCCT	60.2	686	DRR	Ca_15627	L	--
Ca5_P2_25202952	Ca5	25202952	ATTTTTTTTTTTTT/ATTTTTTTTTTTT	AACCAGGGTCTATTCCATGC	TTGGTAATGCGTTGATGGTG	60.4	790	DRR	Ca_15629	O	--
Ca5_P2_2521445	Ca5	2521445	ATTACAGAGTTACA/ATTACAGAGTTACAGAGTTACA	CTGGGCAAGGGATCTGAATA	GCACATTATACCGCGCTTTT	60.1	603	DRR	Ca_18666	--	Trihelix
Ca5_P2_25230946	Ca5	25230946	C/CT	TGTGGAACAATCGATTTGGA	AGTGTTTGAGGTGGTGAGGG	60.0	420	INTERGENIC	--	--	--
Ca5_P2_25232479	Ca5	25232479	GTTTT/GTTTTT	CCACCCTGATTTTTGGAGAA	AAAATCCAACAATCTCCCCC	60.0	790	INTERGENIC	--	--	--
Ca5_P2_25240045	Ca5	25240045	CAAAAAAAAAAA/CAAAAAAAAAAAA	CAGACTTTCCAGCACTTCAATG	TTTGGCCAGACCACCTTTAC	60.0	924	INTERGENIC	--	--	--
Ca5_P2_25242251	Ca5	25242251	CTGT/CTGTTATAAAAATTGT	CCTTGCTGGTTGAATCTGGT	TGCATAATCGATTGGCACAT	59.9	593	INTERGENIC	--	--	--
Ca5_P2_25250090	Ca5	25250090	ATCTT/AT	TGCTCTGACTCATTCAACCG	TCTTTAGCTAGACGCCCTGC	59.8	638	INTERGENIC	--	--	--

INDEL marker IDs	Chromosomes/unanchored scaffolds	Physical positions (bp)	InDels (*Kabuli* reference genome-CDC Frontier/PII)	Forward primers (5'-3')	Reverse primers (5'-3')	Anneling temperature (0C)	Expected amplified product size (bp)	Structural annotation		Functional annotation	
								Sequence components of *kabuli* genome	*Kabuli* gene accession IDs	NCBI-KOG	TFs
Ca5_P2_25287994	Ca5	25287994	CAAAAA/CAAAA	TTGCTTGAAGACATGAACGC	TGGATGTCAAGCAGTCTTGC	60.0	493	INTERGENIC	--	--	--
Ca5_P2_25288223	Ca5	25288223	AGAAACTTGTGATTGAGGAAAC/AGAAAC	TTGCTTGAAGACATGAACGC	TGGATGTCAAGCAGTCTTGC	60.0	493	INTERGENIC	--	--	--
Ca5_P2_25345595	Ca5	25345595	GAAAAAAA/GAAAAAA	TCCAATGAAAAGAGAAATGTTGAA	CACTCCACATTCGAAAACGA	59.7	122	INTERGENIC	--	--	--
Ca5_P2_25364005	Ca5	25364005	GTATATATATATATATATAT/GTATATATATATATATAT	TTTTTCTCTTATAAAGGGACCAGAAA	TGGACCAACTTGCACACATT	60.0	552	INTERGENIC	--	--	--
Ca5_P2_25484902	Ca5	25484902	TAAG/T	TGAAATGATACGGTGTCGGA	TGATTGGTTATTGCAGTTATGTGTT	59.7	683	INTERGENIC	--	--	--
Ca5_P2_2552337	Ca5	2552337	AATATATATATATATATATATATATATATAT/AATATATATATATATATATATATATATATATATATATATATATAT	TTCGAACGTGAATCGTGAAA	ATTGGCAATAGCAACAACCC	59.8	236	INTERGENIC	--	--	--
Ca5_P2_25555823	Ca5	25555823	ATATG/A	GGATCGGTCGGTGGATAGTA	TTCGAGTTATTACGAAAATGAAAAA	58.4	317	INTERGENIC	--	--	--
Ca5_P2_25565924	Ca5	25565924	TTATATATATATATATAT/TTATATATATATATATAT	AGCCCATTTCCAACACTTTG	AAACCTTGGAAGGCCAACTT	60.0	632	INTRON	Ca_09033	QR	B3
Ca5_P2_25600693	Ca5	25600693	A/AT	CGTGCACTTTCACGACAAAT	TTGCTGTACTTGGTTGCTCG	60.1	801	INTRON	Ca_09030	E	--
Ca5_P2_25623890	Ca5	25623890	GTTTTTTTTTTTTTT/GTTTTTTTTTTTTTTTT	TGACGAAGCAATCTTACCGA	TGTTGTCCAACCCTCTTCTCT	58.8	459	INTERGENIC	--	--	--
Ca5_P2_25636144	Ca5	25636144	AAAAAAATAAAAA/AAAAAAATAAAAAATAAAAA	TACCCTCCAACAAGCCAATC	GGGAACACTCAAATCCTCCTT	59.4	512	INTERGENIC	--	--	--
Ca5_P2_25653033	Ca5	25653033	AAATTAA/AAA	GTGATCGTCTTCGGATTGGT	CATAAGGATCGGGGAATCAA	59.7	628	INTERGENIC	--	--	--

INDEL marker IDs	Chromosomes/unanchored scaffolds	Physical positions (bp)	InDels (*Kabuli* reference genome-CDC Frontier/PII)	Forward primers (5'-3')	Reverse primers (5'-3')	Anneling temperature (0C)	Expected amplified product size (bp)	Structural annotation		Functional annotation	
								Sequence components of *kabuli* genome	*Kabuli* gene accession IDs	NCBI-KOG	TFs
Ca5_P2_25657788	Ca5	25657788	CAAA/CAAAA	GACTCGCAATCGTCTTCACA	AGATTCCTCGAGTTTCCCGT	60.1	343	INTERGENIC	--	--	--
Ca5_P2_25661861	Ca5	25661861	TAAAAAAAAA/TAAAAAAAAAA	GGAGTGACAATGCCCTCAAT	TCCGCTTGGGACAATTTTAG	60.1	432	INTERGENIC	--	--	--
Ca5_P2_25662749	Ca5	25662749	AATATATATATATATATATATATATATATATATA/AATATATATATATATATATATATATATATATATATA	CCTACGTATCTCCTGGTGCG	TCAAAAAGGAGGATCGGAGA	59.7	772	INTERGENIC	--	--	--
Ca5_P2_25690803	Ca5	25690803	CATATATATATATATATATATATATATATATATATATA/CATATATATATATATATATATATATATATATATATATATATA	AGCGATTTTGACACCAATCC	TGAACTTATGAATGCCAATCCA	60.3	936	DRR	Ca_09027	U	bHLH
Ca5_P2_25726766	Ca5	25726766	AG/A	TCAAAGCGCGAGGAAATAGT	ATTGTTGCCCAATGGAGAAG	59.9	315	INTERGENIC	--	--	--
Ca5_P2_2577647	Ca5	2577647	TTATATATATATATATATATATATATATATA/TTATATATATATATATATATATATATATATATATATATA	GATGAAGAGGGTGTGAGGGA	TGGAGGGCGCTATTTCTATG	60.2	597	INTERGENIC	--	--	--
Ca5_P2_25785107	Ca5	25785107	CATA/CA	TGCCCGTGCTAATAATTTTG	AAGCACGGAAACAACGATTT	59.6	580	INTERGENIC	--	--	--
Ca5_P2_25803856	Ca5	25803856	TAAAAAAAAAAAAA/TAAAAAAAAAAA	ATCCGATAAATCGCATTGTG	CCCACAAGGTCCGAGAATTA	59.9	203	DRR	Ca_09020	--	--
Ca5_P2_25803970	Ca5	25803970	TGGGGG/TGGGG	GGGGCGTTACATGTGGTATC	TGTAGGGCACATTGGGTTTT	60.2	510	DRR	Ca_09020	--	--
Ca5_P2_25836482	Ca5	25836482	AG/A	AAGGCAACAACAAACAAGCC	ATGCCATTCAGCACATTTGA	60.1	344	INTERGENIC	--	--	--
Ca5_P2_25876394	Ca5	25876394	ATTTTTTTTTT/ATTTTTTTTTTT	TTGCCTCATATCGAGACTTTCA	GGGTAATCGTGCTACATGGG	60.2	899	INTERGENIC	--	--	--
Ca5_P2_25890397	Ca5	25890397	ACCCCCC/ACCCCC	GATTGCTTTCTGGGTGGAAA	TGCAGGGTTTAACGATAGCC	60.1	812	INTERGENIC	--	--	--

INDEL marker IDs	Chromosomes/unanchored scaffolds	Physical positions (bp)	InDels (*Kabuli* reference genome-CDC Frontier/PII)	Forward primers (5'-3')	Reverse primers (5'-3')	Anneling temperature (0C)	Expected amplified product size (bp)	Structural annotation		Functional annotation	
								Sequence components of *kabuli* genome	*Kabuli* gene accession IDs	NCBI-KOG	TFs
Ca5_P2_25915705	Ca5	25915705	ATTTTTTTTTT/ATTTTTTTTTTT	TAGGGCATTTTGGTCCGTAG	CCAAACAGAGCTAGAATTGGA	57.1	943	INTERGENIC	--	--	--
Ca5_P2_25922321	Ca5	25922321	TTTATTATTATTATTATTATTATTATTATTATTATTATTATTATTATTA/TTTATTATTATTATTATTATTATTATTATTATTATTATTATTA	AGGCGTTTGTGTATGGAAGG	ATTGGACAAAAGGAGGACCC	60.2	580	INTERGENIC	--	--	--
Ca5_P2_25988402	Ca5	25988402	TAAAAAAAA/TAAAAAAAAA	TGAGTTTGACATGGCAATTTTT	CCTCCATGGAATCATCGTCT	59.9	377	INTERGENIC	--	--	--
Ca5_P2_26078186	Ca5	26078186	AAGA/AA	TCATGAAAATGCAAGTTCGAC	GCATTGCCATGATTTGAAGA	59.6	793	INTERGENIC	--	--	--
Ca5_P2_260923	Ca5	260923	CATAT/CATATAT	CTTCGGCCTAAAGATTTTGC	ATTGCGGTTGTTCGGTTATC	59.8	358	DRR	Ca_18180	A	--
Ca5_P2_261589	Ca5	261589	CAA/CAAAA	CTTGTTGCACCACGAGACTT	CATTTTACTTGTGCGAGCGA	60.0	612	DRR	Ca_18180	A	--
Ca5_P2_26213471	Ca5	26213471	ATATTATTATTATTATTATTATTATTATTATTATTATTATTATTATTAT/ATATTATTATTATTATTATTATTATTATTATTATTATTAT	TGAGTTAAAACTAACAAACGAAGATAA	TTTTGTAATTGTATTTCATAACGAAGA	57.6	792	INTERGENIC	--	--	--
Ca5_P2_262311	Ca5	262311	CTTTT/CTTT	TCGCTCGCACAAGTAAAATG	GGTCGATGCGTAGTTTCCAT	60.0	859	INTRON	Ca_18180	A	--
Ca5_P2_26280416	Ca5	26280416	CTTTTTTTTTT/CTTTTTTTTTTT	GGGGAAAAAGGTACAAGCCT	AGCGACCTCCTACTTGGTGA	59.9	251	INTERGENIC	--	--	--
Ca5_P2_2628560	Ca5	2628560	AT/ATATATT	TTGTTTCTCCCCAGTTTTGG	TTTTAGCATTGACCGCTCCT	59.8	357	INTERGENIC	--	--	--
Ca5_P2_26329315	Ca5	26329315	CATATATATATATATATAT/CATATATATATATATATATAT	AAGCTACGACATGATGGCAA	CACGGTGGTCATCGACATAG	60.0	525	INTERGENIC	--	--	--
Ca5_P2_26379323	Ca5	26379323	TAAAAAAAAAA/TAAAAAAAAAAA	CCAAGTGCAAAATAAACACGAA	TGACAAAAACTCACACATCCAA	59.1	513	INTERGENIC	--	--	--

INDEL marker IDs	Chromosomes/unanchored scaffolds	Physical positions (bp)	InDels (*Kabuli* reference genome-CDC Frontier/PII)	Forward primers (5'-3')	Reverse primers (5'-3')	Anneling temperature (0C)	Expected amplified product size (bp)	Structural annotation		Functional annotation	
								Sequence components of *kabuli* genome	*Kabuli* gene accession IDs	NCBI-KOG	TFs
Ca5_P2_26430612	Ca5	26430612	AATATATATATATATATATATATATATATATATATATATA/AATATATATATATATATATATATATATATA	GCGGCTCACACACACTTAGA	CTGGAGGTCACCTTCGACAT	60.1	693	INTERGENIC	--	--	--
Ca5_P2_26457056	Ca5	26457056	AG/AGTAACATGGG	GCACTAGGCTTCAAACAGGC	CACATTCTTGCACGGTTCAT	59.6	450	INTERGENIC	--	--	--
Ca5_P2_26498909	Ca5	26498909	CTTTTTTTT/CTTTTTTTTT	TTTTGGCACAATTGATCCTG	TGCCATGTAAGATTTTTGCG	59.7	569	INTERGENIC	--	--	--
Ca5_P2_26543232	Ca5	26543232	TTATATATATATATATATATATATATATAT/TTATATATATATATATATATATATATATATAT	TAGCGTGTAACCAGTGCGTC	CGGTCTTGGAGTCGTGTTTT	60.1	233	INTERGENIC	--	--	--
Ca5_P2_26571749	Ca5	26571749	TTTCATGACTTCAT/TTTCAT	TCCTAAAACGTCGAAACCAAA	TCACAATCTTAAAATGCGAAAAA	58.8	371	DRR	Ca_08965	--	--
Ca5_P2_26739811	Ca5	26739811	AG/A	GATTGAGGGGCAAATCTTGA	GATGTTGCTTCCACGTCCTT	60.1	532	INTERGENIC	--	--	--
Ca5_P2_26816643	Ca5	26816643	AATATATATATATATATATA/AATATATATATATATATATATA	CGCACAAAAATCATGTGGAC	GGACACCCCAAATTTCTCAA	59.8	896	INTERGENIC	--	--	--
Ca5_P2_26848995	Ca5	26848995	TAAAAAAAAAAA/TAAAAAAAAAA	AGGCCATTGTGTTGAGGTGT	TGGCATTCAAGATTTGACCA	60.0	490	INTERGENIC	--	--	--
Ca5_P2_26852877	Ca5	26852877	TGAA/TGAACGTCGTCGATGTTAGGAA	CCATTGCTTGCTCGCTAAAT	CCTTCAAAGAATTTGGTTGGG	60.7	218	INTERGENIC	--	--	--
Ca5_P2_26889418	Ca5	26889418	TAAAAAAAAAA/TAAAAAAAAA	TCATCGCAGGTGTACCATGT	TCCCTTTCGCAGACAAGAAT	59.8	712	INTERGENIC	--	--	--
Ca5_P2_26904550	Ca5	26904550	GTTTTTTTTTT/GTTTTTTTTT	CGTGGTAGACCTTCGGTGAT	TCCTTCACGAGTAAAATGTCTCA	58.9	786	INTERGENIC	--	--	--
Ca5_P2_26962541	Ca5	26962541	CAGAGAGAGAGAGAGAGAGAGAGAGAGAGAGA/CAGAGAGAGAGAGAGAGAGAGAGAGAGAGAGA	GATTGACGCATGCGAGTAGA	ACCACTCAAAGTGTTTGGGC	60.0	774	INTERGENIC	--	--	--

INDEL marker IDs	Chromosomes/unanchored scaffolds	Physical positions (bp)	InDels (*Kabuli* reference genome-CDC Frontier/PII)	Forward primers (5'-3')	Reverse primers (5'-3')	Anneling temperature (0C)	Expected amplified product size (bp)	Structural annotation		Functional annotation	
								Sequence components of *kabuli* genome	*Kabuli* gene accession IDs	NCBI-KOG	TFs
Ca5_P2_26994998	Ca5	26994998	TC/TCC	GGAGCGGGAATATGACTGAA	GGGAGAGGTTGGGAGCTTAC	60.1	492	DRR	Ca_08931	R	MYB
Ca5_P2_26995171	Ca5	26995171	TAG/TAGCACAAGCACTAAG	GGAGCGGGAATATGACTGAA	GCGGCAGAAGAACCTATTTG	59.8	576	DRR	Ca_08931	R	MYB
Ca5_P2_26996169	Ca5	26996169	TG/TGATCG	ATGTTGGGAACTTGCACTCC	AACTATGTCCTGTGGACCGC	60.0	458	DRR	Ca_08931	R	MYB
Ca5_P2_26996806	Ca5	26996806	A/AAAAGCAAAATTA	TCGTTTTGGGTGAATTGTGA	TGCCTCCTCGTTCCTTTTTA	59.8	410	INTERGENIC	--	--	--
Ca5_P2_27000467	Ca5	27000467	AA/AAGA	TTGGCACTGAATAGTGCTCG	TCGAAGCTGTGCTAATGGTG	60.0	361	INTERGENIC	--	--	--
Ca5_P2_27000752	Ca5	27000752	CTTTTTTTTTTTTTTT/CTTTTTTTTTTTTT	TTGGCACTGAATAGTGCTCG	CGTCCCGAATAGACGAACAT	60.0	698	INTERGENIC	--	--	--
Ca5_P2_27000834	Ca5	27000834	ATCTCT/ATCT	TTGGCACTGAATAGTGCTCG	CTCAGCGGGTAGAGTATCGC	60.0	940	INTERGENIC	--	--	--
Ca5_P2_27072859	Ca5	27072859	GAAAAAAAAAAAA/GAAAAAAAAAAA	CATGCCCCTCTTGCAATACT	TGGCTCTTGTGACTTTGGTG	59.9	671	INTERGENIC	--	--	--
Ca5_P2_27081316	Ca5	27081316	TAAAAAAAAA/TAAAAAAAA	CCACTCCAAAGTCCCCAATA	GGCAGAGCTTGAACGAAAAC	60.0	643	INTERGENIC	--	--	--
Ca5_P2_27099226	Ca5	27099226	GAA/GAAA	TGGCAGACAAACCAAACAAA	TGGTTTTGCCATGAGACAGA	60.2	406	INTERGENIC	--	--	--
Ca5_P2_27193838	Ca5	27193838	GTTTTTTTTT/GTTTTTTTTTT	TTCAAGATATAAAGAGGCCGGA	TGACACGGGACGTCGTAGTA	60.2	350	INTERGENIC	--	--	--
Ca5_P2_27198500	Ca5	27198500	AC/ATGAATTC	TGTCTGCCCTCTCTGATCCT	AGGGTTGAAAAAGTAAAGATTAAAAA	57.4	592	DRR	Ca_08920	O	--

INDEL marker IDs	Chromosomes/unanchored scaffolds	Physical positions (bp)	InDels (*Kabuli* reference genome-CDC Frontier/PII)	Forward primers (5'-3')	Reverse primers (5'-3')	Anneling temperature (0C)	Expected amplified product size (bp)	Structural annotation		Functional annotation	
								Sequence components of *kabuli* genome	*Kabuli* gene accession IDs	NCBI-KOG	TFs
Ca5_P2_27204653	Ca5	27204653	GAAAAAA/GAAAAA	GCAAAGCGATCACAAACTCA	GAAATGCTTCTTTCGGCTTG	60.0	329	INTERGENIC	--	--	--
Ca5_P2_27249998	Ca5	27249998	AATATATATATATATATATATATATATATA/AATATATATATATATATATATATATATATATA	TTGCATTTGTTTTCCCATGA	TGTATCGGGATGCGACATAG	59.5	234	INTERGENIC	--	--	--
Ca5_P2_27252852	Ca5	27252852	ATTTTTTTTTT/ATTTTTTTTTTT	TAATTGGCGTCACTCAACCA	TTGAAAATTTGAGTTATGCATTTGA	59.9	659	INTERGENIC	--	--	--
Ca5_P2_27278537	Ca5	27278537	GTTTTTTTTTT/GTTTTTTTTTTT	TCAACGTTTTTGGAAGGTCA	TGACGCAAAAGAGGAGACAA	59.6	464	INTERGENIC	--	--	--
Ca5_P2_27332965	Ca5	27332965	TAAAAAAAA/TAAAAAAA	TTGAAGGCTAGATGGCATGA	ATTGGTTTGGCTCAGTTTGG	60.0	315	DRR	Ca_08909	L	--
Ca5_P2_27339973	Ca5	27339973	ATTTTTTTTT/ATTTTTTTT	CGGCTGAGATTAATTATTGTGTGT	TTTCATTTCACCAAAGACTAAAACA	59.2	697	INTERGENIC	--	--	--
Ca5_P2_27364017	Ca5	27364017	AAAAAAAAATAAAA/AAAAAAAAATAAAAAAAATAAAA	CAAGAAGAGTCCATTGGGGA	GGTAAGACCACTCGATCAATCA	59.1	748	INTERGENIC	--	--	--
Ca5_P2_27369515	Ca5	27369515	TAAAAAAAA/TAAAAAAAAA	GCCCACATTCACTCCAACTT	GCTGCAAGAAAGTTAGCAAGG	59.3	360	INTERGENIC	--	--	--
Ca5_P2_27389155	Ca5	27389155	A/AAAAAAT	CACGAGCTTCAAGAGAGCCT	ATGAGATCGTTGTTGGGAGC	60.1	507	INTERGENIC	--	--	--
Ca5_P2_27389757	Ca5	27389757	TTATATATATATATATATATATA/TTATATATATATATATATA	GCTCCCAACAACGATCTCAT	GCACGATTGTCATGTCGAGT	59.7	898	INTERGENIC	--	--	--
Ca5_P2_27393057	Ca5	27393057	CAGTCATA/CAGTCATAGAGTAGTCATA	TGGTGCTGATGAATTTTCACTT	TGCAATTTCCCTTACGCTTT	59.7	639	INTERGENIC	--	--	--
Ca5_P2_27445899	Ca5	27445899	CATTAATATTAATATTAATATTAATATTAATATTAATAT/CATTAATATTAATATTAATATTAATATTAATAT	TGCCAGAGCAACTAGGCTTT	ATTGTTGTCCAGAAGCCTGC	60.3	474	INTRON	Ca_08906	--	bHLH

INDEL marker IDs	Chromosomes/unanchored scaffolds	Physical positions (bp)	InDels (*Kabuli* reference genome-CDC Frontier/PII)	Forward primers (5'-3')	Reverse primers (5'-3')	Anneling temperature (0C)	Expected amplified product size (bp)	Structural annotation		Functional annotation	
								Sequence components of *kabuli* genome	*Kabuli* gene accession IDs	NCBI-KOG	TFs
Ca5_P2_27458409	Ca5	27458409	ATTATATATAAAATTTTATATATAAAATTTTAT/ATTATATATAAAATTTTATATATAAAATTTTATATATAAAATTTTAT	CAATTGGTCGGCAGAGTTTT	CCCACAACCATGCAATAGAA	59.4	531	INTERGENIC	--	--	--
Ca5_P2_27460399	Ca5	27460399	GAAAAAAAAAA/GAAAAAAAAA	GCACATGTGTTGGCAACTTT	AAAGCTCACCAAATGCCACT	59.7	456	INTERGENIC	--	--	--
Ca5_P2_27472549	Ca5	27472549	AG/A	GCATTATGCCGTCATCATTTT	TTCGCATTCAAAATTGTTGTT	58.2	491	INTERGENIC	--	--	--
Ca5_P2_27477754	Ca5	27477754	ATTTTTTTATTTTTTTTTTTATTTTTTTT/ATTTTTTTATTTTTTTTTTTATTTTTTTTTTATTTTTTTT	CCGCCCTCCTTTTCTAGTCT	AAAAACCCAAAATATGCCCC	59.9	678	INTRON	Ca_08905	--	bHLH
Ca5_P2_2754705	Ca5	2754705	AG/A	GAGCTTTTGAGCCGAAACAC	GGGTTGTAATGGGGCTTAGG	60.6	597	INTERGENIC	--	--	--
Ca5_P2_27603143	Ca5	27603143	GATATATATATATATAT/GATATATATATATAT	CTTGCCTTCATGCGAATTTT	GATTTCAAAGATGCAGGGGA	60.0	555	DRR	Ca_08896	--	--
Ca5_P2_27675113	Ca5	27675113	GATATATATATATATATATATATA/GATATATATATATATATATATATATA	TTGCCACTTGATACAGCAAAA	ATCGGTGGGAATAAGAGCCT	59.9	891	INTERGENIC	--	--	--
Ca5_P2_276846	Ca5	276846	T/TGCATCGGTTCA	TTGTTGCTTTTTCTTCGGCT	ACAGGCACTCCAGGATCAAC	60.1	330	INTRON	Ca_18181	D	--
Ca5_P2_27718024	Ca5	27718024	TAAAAAAA/TAAAAAAAA	TGGCAACAGAAACCAAACAA	TTCTTTTAGCCCGTTGCTGT	59.9	573	INTERGENIC	--	--	--
Ca5_P2_27724317	Ca5	27724317	GAAAAAA/GAAAAAAA	CCAACTCTTCCTTTGCTTGC	TGCAAAACTACAACGATTCTCC	59.3	820	INTERGENIC	--	--	--
Ca5_P2_27724401	Ca5	27724401	GATATATATATATATATA/GATATATATATATATATA	ACATCATTGGCATTGGGTTT	TGCAAAACTACAACGATTCTCC	59.3	678	INTERGENIC	--	--	--
Ca5_P2_2774211	Ca5	2774211	CTCT/CTCTTTCCCTTAATCT	ATCCGACATTTCTTTGGCAG	CAAAGCAAGGTGAAGTGCAA	60.0	842	INTERGENIC	--	--	--

INDEL marker IDs	Chromosomes/unanchored scaffolds	Physical positions (bp)	InDels (*Kabuli* reference genome-CDC Frontier/PII)	Forward primers (5'-3')	Reverse primers (5'-3')	Anneling temperature (0C)	Expected amplified product size (bp)	Structural annotation		Functional annotation	
								Sequence components of *kabuli* genome	*Kabuli* gene accession IDs	NCBI-KOG	TFs
Ca5_P2_27800920	Ca5	27800920	AC/A	TCTCAACAAGCACAATTGCC	TTTATTTGAGCCAAGGGGTG	59.9	112	INTERGENIC	--	--	--
Ca5_P2_27880873	Ca5	27880873	GAAAAAAAAAAA/GAAAAAAAAAA	GGCAGCTTCTGTTTTGTCAAC	CATTTCTCCTCCCAAAGGGT	60.3	786	INTERGENIC	--	--	--
Ca5_P2_27906217	Ca5	27906217	AATATATATATATATATATATATATAT/AATATATATATATATATATATATAT	TCCAACAACATCAAATTCATCA	TGTCAACTCTTCCAGCACCA	60.4	630	INTERGENIC	--	--	--
Ca5_P2_27945125	Ca5	27945125	TAATAATAA/TAATAATAAAAATAATAA	TGGCTACATCCAGTCCCTTC	TTGGGCAAATTTAGTTCTTGTTT	59.1	579	INTERGENIC	--	--	--
Ca5_P2_27953689	Ca5	27953689	TA/T	AACAACAGCCCCAATGTAGC	ACGCTTTTCAAACAAACGCT	59.9	794	INTERGENIC	--	--	--
Ca5_P2_27998972	Ca5	27998972	GTGCGTA/G	GTGTGTGTGTGTGTGTGCG	TGCATGGACTAGCCTTGAAC	58.9	365	INTERGENIC	--	--	--
Ca5_P2_28012407	Ca5	28012407	AATATATATATATATAT/AATATATATATATAT	TTGAAGGAAGATGAATCGTTGA	ATGCGGATACTCTCTGCCAC	60.2	622	DRR	Ca_13417	S	--
Ca5_P2_280542	Ca5	280542	AATTATTATTATT/AATTATTATTATTATT	CAAACAAGCTTTTTCACTTTCTCA	AGGACCTCCCTTTTTCTCCA	60.0	636	DRR	Ca_18183	--	ARF
Ca5_P2_28055720	Ca5	28055720	AATATATATATATATATATATATATATATATA/AATATATATATATATATATATATATATATATATATATATATATA	CAAATGGCGACTTCCAACTT	CATGCCATAGCTGCATGTTC	60.3	713	INTERGENIC	--	--	--
Ca5_P2_28068856	Ca5	28068856	CTATATATATATATATATATATA/CTATATATATATATATATATATATA	GATTCGTCGGTTCACTCGTT	TGATGATGAATGTTGCACGA	59.6	139	INTERGENIC	--	--	--
Ca5_P2_28087394	Ca5	28087394	TATAA/T	TTTGCTTTTTGCAAGCACAC	GACATTTCGAACTTGAATTTCCT	58.7	509	INTERGENIC	--	--	--
Ca5_P2_28090344	Ca5	28090344	AATATATATATATATATATATATATATATA/AATATATATATATATATATATATATATATATATATATATA	CACCATTTGGAACTTCGGAT	CAATTGCCAACTGAGAAAATGT	59.1	725	INTERGENIC	--	--	--

INDEL marker IDs	Chromosomes/unanchored scaffolds	Physical positions (bp)	InDels (*Kabuli* reference genome-CDC Frontier/PII)	Forward primers (5'-3')	Reverse primers (5'-3')	Anneling temperature (0C)	Expected amplified product size (bp)	Structural annotation		Functional annotation	
								Sequence components of *kabuli* genome	*Kabuli* gene accession IDs	NCBI-KOG	TFs
Ca5_P2_28103137	Ca5	28103137	ATTTTTTTTTT/ATTTTTTTTTTT	CGTCTTTAAACGGTCGCTTC	AAAAAGAAAATTTGGGCGGT	59.8	700	INTERGENIC	--	--	--
Ca5_P2_28131912	Ca5	28131912	ATTT/ATT	TGCCATATTCAGGAGTGTGC	ATGTACATTGCATGAGGCCA	60.0	850	INTERGENIC	--	--	--
Ca5_P2_28136431	Ca5	28136431	AATATATATATATATATA/AATATATATATATATATA	CTCAAAACTCAACTTCGCCA	TTTGGTTGCAAAATGTGTGG	60.4	135	INTERGENIC	--	--	--
Ca5_P2_28155675	Ca5	28155675	TAT/TATCAT	TGTTTTTGTTTTCTTTCACACGA	CGGACAAACGTGGATGATAA	59.4	543	INTERGENIC	--	--	--
Ca5_P2_28187994	Ca5	28187994	AATATATATATATAT/AATATATATATATATATAT	CAAGAAAAGTAACCATCGTAATGA	TGTGGATTCATGATCCTTGTG	59.4	494	INTERGENIC	--	--	--
Ca5_P2_28188039	Ca5	28188039	T/TC	CAAGAAAAGTAACCATCGTAATGA	TGTGGATTCATGATCCTTGTG	59.4	494	INTERGENIC	--	--	--
Ca5_P2_28192036	Ca5	28192036	CATATATATATATATATATATAT/CATATATATATATATATATATATAT	CCACATGCTCATTGTGTGACT	CCATTCTCCTTCACCCAGAC	59.5	653	INTERGENIC	--	--	--
Ca5_P2_28224948	Ca5	28224948	TTATATATATATATATATAT/TTATATATATATATATATAT	GACCCACGTATGAAGGTGAAA	TATTGAGGGCAAATCCCAGA	60.4	486	INTERGENIC	--	--	--
Ca5_P2_28234704	Ca5	28234704	AATATATATATATATATATATAT/AATATATATATATATATATATAT	TTTCAAGCTTTTGATGCACG	TTTCTTTTCCAGTTCCACCG	60.1	751	INTERGENIC	--	--	--
Ca5_P2_28238690	Ca5	28238690	GTTTTTTTTT/GTTTTTTTTTT	CAGGTACGAGAGTTGCGTCA	TAAGATTCTCCGGGCATCAC	60.0	827	INTERGENIC	--	--	--
Ca5_P2_28241348	Ca5	28241348	TAAAAAAA/TAAAAAAAA	GGGCTAAAGTCATTGGGTGA	GCCATATGGATTTGTCTGCAT	59.8	649	DRR	Ca_13400	--	ARF
Ca5_P2_28246811	Ca5	28246811	AATATATATATATATATA/AATATATATATATATATATATATATATATATA	TGCCCTCGTCAAACTAACAA	GTCTTTTGTGAGGGCAAAGG	59.7	791	INTERGENIC	--	--	--

INDEL marker IDs	Chromosomes/unanchored scaffolds	Physical positions (bp)	InDels (*Kabuli* reference genome-CDC Frontier/PII)	Forward primers (5'-3')	Reverse primers (5'-3')	Anneling temperature (0C)	Expected amplified product size (bp)	Structural annotation		Functional annotation	
								Sequence components of *kabuli* genome	*Kabuli* gene accession IDs	NCBI-KOG	TFs
Ca5_P2_28247768	Ca5	28247768	TAAAAAAAAAA/TAAAAAAAAAAA	ACCACCTTATTTGTGGCTGC	TCAGGCTTAGGCCTCAAAAA	60.0	796	INTERGENIC	--	--	--
Ca5_P2_28291153	Ca5	28291153	CAAAAAAAAAAA/CAAAAAAAAAA	CACGTCCCTAACATTTTGCC	TGTTCCTGATATTTTGCCGA	59.1	400	INTERGENIC	--	--	--
Ca5_P2_28310357	Ca5	28310357	AAATAATAATAATAATAATAATAATAATAATAATAATAATAATAATAATAATAATAAT/AAATAATAATAATAATAATAATAATAATAATAATAATAATAATAATAATAATAATAATAATAAT	CCAACATGTGGGATAACTTGTG	TGGCCTGACCTATTCTCACC	60.1	635	INTERGENIC	--	--	--
Ca5_P2_28342481	Ca5	28342481	TTCAAAATCAAAATCAAAATCAAAATCA/TTCAAAATCAAAATCAAAATCA	CTCCTTAAGTGTACGGCCCA	GGACTATTTTCGCGAATGAGTAA	59.7	329	INTERGENIC	--	--	--
Ca5_P2_28366916	Ca5	28366916	TTATATATATATATATATATATATATATATATAT/TTATATATATATATATATATATATATATATATATATAT	TGAATGCACTCAAAAGCACA	TACAAAGACGTTGTCGTCGC	59.9	747	INTERGENIC	--	--	--
Ca5_P2_28370910	Ca5	28370910	TTATATATATATATATATATATATATATAT/TTATATATATATATATATATATATATATAT	CTAGTTCGTAACCCGTGCGT	AGGCATGGTGTTGGAGTCAT	60.4	260	INTERGENIC	--	--	--
Ca5_P2_28411984	Ca5	28411984	ATTTTTTTTTTTTTT/ATTTTTTTTTTTTT	TTTCCTTTGTACCCCCACAC	ATCTGTTTTACGGCTGGGTG	60.0	153	DRR	Ca_13382	--	SAP
Ca5_P2_28413401	Ca5	28413401	A/AT	TTCTCCTTGAAGCTGTCGGT	ATTGCCAACATTTCCGGTAG	59.8	252	INTERGENIC	--	--	--
Ca5_P2_28588926	Ca5	28588926	TGGG/TGG	CAACTGCTCCTGTTCTCCAA	CTAGGAGCATGTCGCTCTCC	60.1	412	INTERGENIC	--	--	--
Ca5_P2_286315	Ca5	286315	ACC/ACCC	ATGATTTGAATTTGCGAGGC	TCCAACGGGGTTTAAGAGTG	60.0	821	INTERGENIC	--	--	--
Ca5_P2_286412	Ca5	286412	GTTTTTTT/GTTTTTTTT	TGCTGCTACTACTGCCATCG	TCCAACGGGGTTTAAGAGTG	60.0	760	INTERGENIC	--	--	--
Ca5_P2_28702762	Ca5	28702762	TG/T	TCATTTTATCACAAGGGTAGTTTTG	TGAATTATACCGTTTGATGCAC	57.6	621	INTERGENIC	--	--	--

INDEL marker IDs	Chromosomes/unanchored scaffolds	Physical positions (bp)	InDels (*Kabuli* reference genome-CDC Frontier/PII)	Forward primers (5'-3')	Reverse primers (5'-3')	Anneling temperature (0C)	Expected amplified product size (bp)	Structural annotation		Functional annotation	
								Sequence components of *kabuli* genome	*Kabuli* gene accession IDs	NCBI-KOG	TFs
Ca5_P2_287058	Ca5	287058	ATTTTTTT/ATTTTTTTT	CACTCTTAAACCCCGTTGGA	CACTGTTCCTTTGTGGGAGG	60.5	700	DRR	Ca_18184	R	B3
Ca5_P2_28763604	Ca5	28763604	CATTATTATTATTATTATTATTATTATTATTATTATTAT/CATTATTATTATTATTATTATTATTATTATTATTATTAT	CCCAAATGCCCTACTCTGAA	TTTCATTTCATTTTCAAGTTATCAA	57.1	729	DRR	Ca_13353	--	--
Ca5_P2_288048	Ca5	288048	CTT/CTTTT	GGACCAGCAAGAAGCAACTC	TGTGAGAAATGGGAATTGGA	58.9	933	DRR	Ca_18185	R	B3
Ca5_P2_28839815	Ca5	28839815	ATTTTTTTTTT/ATTTTTTTTTTTT	CGGCATAATTTGGTGAATTG	CCACGTCAACGCAAAGTAGA	59.9	845	INTERGENIC	--	--	--
Ca5_P2_28864108	Ca5	28864108	AATATATATATATATATATATATAT/AATATATATATATATATATATAT	AAAAGGGACGATACTTGCCA	TCCTCTCAAAAGCCCTCAAA	59.9	787	DRR	Ca_13346	--	NF-YC
Ca5_P2_28865362	Ca5	28865362	TTCT/TT	TTTAGTGGTGCAAGTGCATGT	CGAAAGCTAGCCATGAGGTT	59.5	104	INTERGENIC	--	--	--
Ca5_P2_288684	Ca5	288684	ATACTTACTT/ATACTT	CTTGGACTCCAGTGCCTCAT	GTGCATTTTGCAGGCAATTA	59.7	474	DRR	Ca_18185	R	B3
Ca5_P2_28921728	Ca5	28921728	CAAAAAAAAAA/CAAAAAAAAAAAA	TTTTCTCCCTCACCAACGTC	TGGTACCCGTCCAAAGGATA	60.2	596	INTERGENIC	--	--	--
Ca5_P2_289542	Ca5	289542	CATATATATATATATA/CATATATATATATATATA	GCAAGAACAGGTGTCATGGA	AAAATGCGAGAAAATGTGGC	60.1	611	INTRON	Ca_18185	R	B3
Ca5_P2_28974386	Ca5	28974386	TAAAAAAAAAA/TAAAAAAAAAAAA	GCCATAATCGAGAGGAATGG	ATTAATCCCCGTCAAGACCC	60.0	527	INTERGENIC	--	--	--
Ca5_P2_28978028	Ca5	28978028	A/ATAATG	AGCAGCACATCAAAATGCAG	TTGTGCTTGTCGTAAGTGGG	59.8	354	INTERGENIC	--	--	--
Ca5_P2_28978125	Ca5	28978125	C/CCG	TGCAGAACCAAAACAAGCAG	GGACTAGGTCAGCATTTCGC	59.8	845	INTERGENIC	--	--	--

INDEL marker IDs	Chromosomes/unanchored scaffolds	Physical positions (bp)	InDels (*Kabuli* reference genome-CDC Frontier/PII)	Forward primers (5'-3')	Reverse primers (5'-3')	Anneling temperature (0C)	Expected amplified product size (bp)	Structural annotation		Functional annotation	
								Sequence components of *kabuli* genome	*Kabuli* gene accession IDs	NCBI-KOG	TFs
Ca5_P2_29097154	Ca5	29097154	ATTTTTTTTT/ATTTTTTTTTT	GCTGCATGCGTTACGAGATA	AAATTCACGGTGTCATCTTCG	60.0	271	INTERGENIC	--	--	--
Ca5_P2_29098754	Ca5	29098754	ATTTTTTTTTTT/ATTTTTTTTTT	CATCATGTTCAATGAAAGCCA	CCATCCATTATTCCAACCCA	60.4	822	INTERGENIC	--	--	--
Ca5_P2_291054	Ca5	291054	TAAAAAAAAA/TAAAAAAAAAAA	TGCTTCATATGCTTGACCACA	TGGTGCTTCTTTGGCTCTTT	60.0	476	INTRON	Ca_18185	R	B3
Ca5_P2_29111048	Ca5	29111048	AGNNNNNNNNNNG/AG	TGCGAGTGAATCTGAGGATG	TGCGCAGCATTATCTGAAAC	60.0	548	INTRON	Ca_23826	I	--
Ca5_P2_291688	Ca5	291688	ATTTTTT/ATTTTTTTT	AAAGAGCCAAAGAAGCACCA	AGCAAATGCGACTGTGATTG	59.9	722	DRR	Ca_18186	--	--
Ca5_P2_291943	Ca5	291943	GCGCAACCGCAA/GCGCAACCGCAACCGCAA	TTCGTGGCTTAATCCCACTC	GTCTGGTGCTCTTCCCAATC	59.7	517	DRR	Ca_18186	--	--
Ca5_P2_292149	Ca5	292149	A/AC	AAAGTGCCGAAGTTTGCAGT	TGCTTTCCCTTCAACAGTCA	59.4	485	DRR	Ca_18186	--	--
Ca5_P2_29247345	Ca5	29247345	ATTTTTTTTTT/ATTTTTTTTTTT	CGTGGAAAACTTCCCTCAAA	TGACGAGCGTAAGAACATGC	60.0	853	INTERGENIC	--	--	--
Ca5_P2_29351977	Ca5	29351977	CTATTATTATTATTATTATTATTATTATTATTATTATT/CTATTATTATTATTATTATTATTATTATTATTATT	GAGCCGAAAACGTATCCTAA	TTAGGCGCGTTTCAAGAACT	60.0	701	INTERGENIC	--	--	--
Ca5_P2_29413662	Ca5	29413662	CATATATATATATATATATATATATATA/CATATATATATATATATATATATATA	GCTGCTCCTTGGGAAGTAAA	ACCAAGTTGGCTGCTCTGTT	59.9	575	INTERGENIC	--	--	--
Ca5_P2_29418887	Ca5	29418887	TTATATATATATATATA/TTATATATATATATATATA	CCCAAGCTAGAAGTGTCACGA	TTGCATGGGCATAGTGAGAG	59.8	671	DRR	Ca_16633	G	--
Ca5_P2_29568691	Ca5	29568691	ATTTTTTTTT/ATTTTTTTTTT	AATTGTGCACATTGTTGCGT	CACCGGTTCAATTTTCGATT	59.8	489	INTERGENIC	--	--	--

INDEL marker IDs	Chromosomes/unanchored scaffolds	Physical positions (bp)	InDels (*Kabuli* reference genome-CDC Frontier/PII)	Forward primers (5'-3')	Reverse primers (5'-3')	Anneling temperature (0C)	Expected amplified product size (bp)	Structural annotation		Functional annotation	
								Sequence components of *kabuli* genome	*Kabuli* gene accession IDs	NCBI-KOG	TFs
Ca5_P2_29572290	Ca5	29572290	ATTTTTTTTTTTTT/ATTTTTTTTTTTTTT	CGGTCCATCTCGTAGGTCAT	ATGGCTGATAATTTACGCGG	60.0	560	INTERGENIC	--	--	--
Ca5_P2_29592860	Ca5	29592860	AATATATATATATATATATATATATATATA/AATATATATATATATATATATATATATATA	TTTGATTCTTTTGAGCGATTCAT	GTCGTACCCTTTGGGAGACA	60.0	744	INTRON	Ca_16645	T	MYB
Ca5_P2_29601698	Ca5	29601698	ATTATTTATTTATTTAT/ATTATTTATTTATTTATTTATTTAT	ATCTTTGGTTGGTTGGTTGC	TGCATATCCATTGAGTTTCACA	59.0	607	INTERGENIC	--	--	--
Ca5_P2_29614403	Ca5	29614403	ATTTTTTTT/ATTTTTTT	TGGAAGTTACAATCACGTGTAAAAA	CGTGTTCCAGAAATTTTTGTTT	58.2	641	INTERGENIC	--	--	--
Ca5_P2_29665140	Ca5	29665140	ATCACATTTCACATTTCACATTT/ATCACATTTCACATTTCACATTTCACATTT	TGGAAAGCGATTTATCCTGG	AGAGATCTTCTTGCGGTGGA	60.0	579	INTERGENIC	--	--	--
Ca5_P2_29689908	Ca5	29689908	CTTTTTTTTTTT/CTTTTTTTTTT	AATCAAAATTTTCAAACAAAACTCA	CATCAAAATTTTCAACCAAAACC	59.6	488	INTERGENIC	--	--	--
Ca5_P2_29720043	Ca5	29720043	TTATATATATATATATATATA/TTATATATATATATATATATATA	GCAGTTTGAAAAGCTTACACGA	CCAATAGAGGTGCGACCAAT	60.0	726	DRR	Ca_16654	--	MYB
Ca5_P2_29778022	Ca5	29778022	CAAAAAAAAAAAAA/CAAAAAAAAAAAAAAAA	GCTGCTGGCACATCTACTCA	TAACTGCAACAGATGGTCGC	59.9	547	INTERGENIC	--	--	--
Ca5_P2_29778782	Ca5	29778782	TAAAAAAAAAA/TAAAAAAAAAAA	TTCGATTCACCCGAAGAAAT	GTGGCGTGAAACAACAAATG	60.0	329	INTERGENIC	--	--	--
Ca5_P2_29827143	Ca5	29827143	TAAAAAAAAAAA/TAAAAAAAAAA	ATCTGGTTAACGGATGCGAG	TTGGGACTGACAGAAAGAAAAA	58.9	669	INTERGENIC	--	--	--
Ca5_P2_29881935	Ca5	29881935	CTATATATATATATATATATATATATATATATATATA/CTATATATATATATATATATATATATATATATATATATATA	ACATGCTTGGAGAGTTGCCT	TCCATGCAATTCGTTTTTGA	60.1	425	INTRON	Ca_16670	K	--
Ca5_P2_29904685	Ca5	29904685	GAAAAAAAAA/GAAAAAAAA	GCTTCCACGTTTGATTTGGT	TGGGAAGATTACGCACTTCA	59.3	631	INTERGENIC	--	--	--

INDEL marker IDs	Chromosomes/unanchored scaffolds	Physical positions (bp)	InDels (*Kabuli* reference genome-CDC Frontier/PII)	Forward primers (5'-3')	Reverse primers (5'-3')	Anneling temperature (0C)	Expected amplified product size (bp)	Structural annotation		Functional annotation	
								Sequence components of *kabuli* genome	*Kabuli* gene accession IDs	NCBI-KOG	TFs
Ca5_P2_29925024	Ca5	29925024	CATATATATATATATATATATATATATATATATATATATA/CATATATATATATATATATATATATATATA	ATTCCCAAAGCACCCATGTA	ACTCCCACCCCCAATTAAAA	60.4	652	DRR	Ca_16672	--	--
Ca5_P2_29933300	Ca5	29933300	TAAAAAAAAAAA/TAAAAAAAAAA	TGTTTCAAGCATGCATTTAATTTT	TCAGCGTACACTTTTCGACG	60.1	758	INTERGENIC	--	--	--
Ca5_P2_29933558	Ca5	29933558	AATATATATATATATATATATATATAT/AATATATATATATATATATATATATATATATATATATAT	GACCTTTTCAAAGGATGCATTA	TCAGCGTACACTTTTCGACG	60.1	369	INTERGENIC	--	--	--
Ca5_P2_29933647	Ca5	29933647	GATATATATATATA/GATATATATATATATA	GACCTTTTCAAAGGATGCATTA	CGTGATGACAGACCAACACC	60.0	713	INTERGENIC	--	--	--
Ca5_P2_29938246	Ca5	29938246	ATT/ATTT	GCAAAAACAGCGACACTGAA	TTGTGTGGGAAAGGTGACAA	60.0	764	DRR	Ca_16674	--	--
Ca5_P2_29957696	Ca5	29957696	TAAAAAAAAAAA/TAAAAAAAAAAAAA	TTGGAGTTCCGGAAAAGATG	CCCCTAGAGCCCCTCTCTTA	59.8	206	INTERGENIC	--	--	--
Ca5_P2_29971249	Ca5	29971249	AATA/AATATGGGATTCCATA	CGTAAGTTGCCTGGAATGGT	CGTAGCAATCTGTCTGGCAA	60.0	399	DRR	Ca_04674	E	--
Ca5_P2_29978017	Ca5	29978017	CTATTATTATTATTATTATTATTATTATTATTATTA/CTATTATTATTATTATTATTATTATTATTA	CATCCATTCTCCAAACACCT	TGTGATATGGATGAGATGAAGCA	60.5	806	INTERGENIC	--	--	--
Ca5_P2_30012116	Ca5	30012116	CTATATATATATATATAT/CTATATATATATATAT	TGGGTATTAGTCCGACCAGG	AAAGTATCCAACTGTGCCAGC	59.3	286	INTERGENIC	--	--	--
Ca5_P2_30078059	Ca5	30078059	CTTTTTTTTTT/CTTTTTTTTTTT	GCACAAGTGAAATAGCAAAGCA	TTTGCGTTACGAATTTGATGA	59.2	591	INTERGENIC	--	--	--
Ca5_P2_30078290	Ca5	30078290	GAAAAAAAAAA/GAAAAAAAAA	TCATCAAATTCGTAACGCAAA	TCACACCCGAGATAAGTTCTCA	59.7	190	INTERGENIC	--	--	--
Ca5_P2_30080567	Ca5	30080567	CAAAAAAAAAA/CAAAAAAAAA	AGATTTGGTGATTGGGCAAG	CATGCTAAATTGATTTTTCAGTCTTC	59.6	326	INTERGENIC	--	--	--

INDEL marker IDs	Chromosomes/unanchored scaffolds	Physical positions (bp)	InDels (*Kabuli* reference genome-CDC Frontier/PII)	Forward primers (5'-3')	Reverse primers (5'-3')	Anneling temperature (0C)	Expected amplified product size (bp)	Structural annotation		Functional annotation	
								Sequence components of *kabuli* genome	*Kabuli* gene accession IDs	NCBI-KOG	TFs
Ca5_P2_30106797	Ca5	30106797	ATAGGTCA/ATAGGTCAGGTCAAGCTAGGTCA	GCCAGCAACCTCCAATTAAG	TGCACATTGACCCAAATTGT	59.8	779	INTERGENIC	--	--	--
Ca5_P2_30106907	Ca5	30106907	TAAAAAAAA/TAAAAAAAAAA	GCCAGCAACCTCCAATTAAG	TGCACATTGACCCAAATTGT	59.8	779	INTERGENIC	--	--	--
Ca5_P2_30107423	Ca5	30107423	TG/TGG	CAATTTGGGTCAATGTGCAA	GCCAACCTAGTCTCAGAAGCA	59.6	475	INTERGENIC	--	--	--
Ca5_P2_30107954	Ca5	30107954	AAACA/AA	TCATTGCATTCATTCACCAAA	TCCAACAGGATTCGTGACAA	60.1	709	INTERGENIC	--	--	--
Ca5_P2_30114886	Ca5	30114886	TTATATATATATATATATATATATATATA/TTATATATATATATATATATATATA	CGGGTGGCTTAGTGAGAAGT	AAACTCTTTCCGCTCACCTTT	59.4	325	INTERGENIC	--	--	--
Ca5_P2_30115828	Ca5	30115828	CATATATATATATATATATATATATA/CATATATATATATATATATA	TCCGGGTTTTGTTTTTCAGT	GACTGATTAACCGACATAATTCGT	58.5	378	INTERGENIC	--	--	--
Ca5_P2_30121861	Ca5	30121861	ATAT/ATATATATTTAT	CATGCGTGAATTTGATCGTC	AAACGCGTCACTTTACCTTCTC	59.8	224	INTERGENIC	--	--	--
Ca5_P2_30126893	Ca5	30126893	ATTTTTTTTT/ATTTTTTTTTT	GTGGCTTCCAAAGAGAAACG	TTCGGTACACAATTTTCCCC	59.7	612	INTERGENIC	--	--	--
Ca5_P2_30128968	Ca5	30128968	GATTATAATTAAAAATA/GATTATAATTAAAAATAATTATAATTAAAAATA	TGTTTCTCTCTCCGCTCACA	CCGCCGACTATACCTGTTTT	59.1	681	INTERGENIC	--	--	--
Ca5_P2_30131050	Ca5	30131050	AATATATATATATATATATATATA/AATATATATATATATATATA	CAAATTTCGAGCAACAATGC	AACACAAATGTTAAATTGCGAAGA	59.9	571	INTERGENIC	--	--	--
Ca5_P2_30135601	Ca5	30135601	CAT/CATAT	CTGGTCCTCCTTCTGTCGAG	ATCCCATCAAAATAGCTGCG	60.1	426	DRR	Ca_04690	J	E2F/DP
Ca5_P2_30284983	Ca5	30284983	ATATTATTATTATTATTATTATTATTATTATTATTATTGTTATTGTTATTATTATTATTAT/ATATTATTATTATTATTATTATTATTATTATTATTATTATTATTATTGTTATTGTTATTATTATTATTAT	TGTTGTCACATGCTTTGGGT	TTGTGCACATTTTATTCTTCAAA	57.5	651	INTERGENIC	--	--	--

INDEL marker IDs	Chromosomes/unanchored scaffolds	Physical positions (bp)	InDels (*Kabuli* reference genome-CDC Frontier/PII)	Forward primers (5'-3')	Reverse primers (5'-3')	Anneling temperature (0C)	Expected amplified product size (bp)	Structural annotation		Functional annotation	
								Sequence components of *kabuli* genome	*Kabuli* gene accession IDs	NCBI-KOG	TFs
Ca5_P2_30325134	Ca5	30325134	ATTTTTTTTTTT/ATTTTTTTTTT	CGACTATTGGACTGAGCAAGC	GTGATTTCAGAATACGCCCG	60.5	497	INTERGENIC	--	--	--
Ca5_P2_30338194	Ca5	30338194	TTATATATATATATATA/TTATATATATATATA	TGAAATCTCAATAAATGGCGG	CATCGATGGTTGATAAGAAAATG	58.5	154	INTERGENIC	--	--	--
Ca5_P2_30355494	Ca5	30355494	CATATATATATATATATATATA/CATATATATATATATATATATA	CCTTTGGTTCAAGTAATGGCA	TGCACACACCAATTCCAAGT	60.0	531	INTERGENIC	--	--	--
Ca5_P2_30468145	Ca5	30468145	GTATATATATATATATATATA/GTATATATATATATATATATA	TGCGTAAAAAGTCAAATAAGTGAA	GCAGTTTTATTCAACCAAATCAA	58.2	241	DRR	Ca_04729	--	--
Ca5_P2_30533386	Ca5	30533386	GTATATATATATATATATATATATATATATATATA/GTATATATATATATATATATATATATATATATATATATATATATATA	AAAATTCGCACTCATTTCCG	AAACTAAAACGGAGGGGCAT	59.8	357	DRR	Ca_04741	--	bHLH
Ca5_P2_30551642	Ca5	30551642	AT/ATT	CAAGAGCCATCGTCATCAGA	AGCTGATGCCAAGCTCAAAT	60.0	838	INTRON	Ca_04744	R	C3H
Ca5_P2_30587292	Ca5	30587292	TCTTCTCCTTCTCCTTCTCCTTCTCCTTCTCTTTCTCCTTCTCCTTCTCCATCTCCTTCTCCTT/TCTTCTCCTTCTCCTTCTCCTTCTCTTTCTCCTTCTCCTTCTCCATCTCCTTCTCCTT	GGGGCAACCCCTAATAAAAA	CATCAAGGAGACGCTGAACA	60.0	590	CDS (large-effect mutations)	Ca_04750	K	C3H
Ca5_P2_30610017	Ca5	30610017	AAATAATAATAATAATAATAATAATAATAATA/AAATAATAATAATAATAATAATAATAATA	TTGCAATCAAACTTCTTGAAAA	GCCATTCAATGAACAACGTG	60.0	604	INTERGENIC	--	--	--
Ca5_P2_30610925	Ca5	30610925	GTTTTTTTT/GTTTTTTT	ATAATCATCCACCCGATCCA	TGTCAACCCTAAAGACGCAA	59.3	976	INTERGENIC	--	--	--
Ca5_P2_3061873	Ca5	3061873	GATAATAATAATAATAATAATAATAATAATAATAATAATAATAATAATAATA/GATAATAATAATAATAATAATAATAATAATAATAATAATAATAATAATA	TTGCTCCTACGTATCCCCAG	TTATTTCACCGTCGTGTCCA	60.0	332	INTERGENIC	--	--	--
Ca5_P2_30712358	Ca5	30712358	G/GGATAGAAA	TATTGGATTGGGCCAAACAT	TTCGTTGGTTTTGGGTTCTC	59.9	534	INTERGENIC	--	--	--
Ca5_P2_30819736	Ca5	30819736	A/AT	GGAAAGGAAGGGAAAAGGTG	CATCATTGTTGGATATGCGG	59.8	428	INTERGENIC	--	--	--

INDEL marker IDs	Chromosomes/unanchored scaffolds	Physical positions (bp)	InDels (*Kabuli* reference genome-CDC Frontier/PII)	Forward primers (5'-3')	Reverse primers (5'-3')	Anneling temperature (0C)	Expected amplified product size (bp)	Structural annotation		Functional annotation	
								Sequence components of *kabuli* genome	*Kabuli* gene accession IDs	NCBI-KOG	TFs
Ca5_P2_30826086	Ca5	30826086	TAAAAAAA/TAAAAAAAAA	AATTTTCCGGGCTCAAGAAT	TGTCGTGAGCGAAGGTGTAA	60.4	668	INTERGENIC	--	--	--
Ca5_P2_30828016	Ca5	30828016	CAGTTAGTT/CAGTT	TGGAAAAGAGAGTGGGTGGT	TTGGTTTTTCGGATCTTTGC	60.1	598	INTERGENIC	--	--	--
Ca5_P2_30828251	Ca5	30828251	CACAA/CA	TGGAAAAGAGAGTGGGTGGT	TTGGTTTTTCGGATCTTTGC	60.1	598	INTERGENIC	--	--	--
Ca5_P2_30832548	Ca5	30832548	AGGG/A	TCCAAAAACCCAGAATCCAA	TTCGTCGTTGATGCTTTTTG	59.8	633	DRR	Ca_04780	R	G2-like
Ca5_P2_30832859	Ca5	30832859	CAAAAAAAAAAA/CAAAAAAAAA	GACGATGTCTGAAATGCCAA	TTCGTCGTTGATGCTTTTTG	59.8	482	DRR	Ca_04780	R	G2-like
Ca5_P2_30834614	Ca5	30834614	AAATAATAATAATAATAATA/AAATAATAATAATAATAATAATAATA	CGACGATTTCTCCGAATTGT	TTTATCGGATTTTTAAAGGGAAA	58.2	420	INTERGENIC	--	--	--
Ca5_P2_30835479	Ca5	30835479	TTATATATAT/TTATATATATAT	TCGGCGATGTAAAATTCTCG	TGATAGACAAGCCACATGCC	59.7	495	INTERGENIC	--	--	--
Ca5_P2_30840881	Ca5	30840881	AT/ATAATT	CCAACTTCGTGAATTGGTAAAA	CACTCATAAGTTACTTGAGCTATGCTT	58.8	472	INTERGENIC	--	--	--
Ca5_P2_30841076	Ca5	30841076	T/TGTATAC	CCAACTTCGTGAATTGGTAAAA	CACTCATAAGTTACTTGAGCTATGCTT	58.8	472	INTERGENIC	--	--	--
Ca5_P2_30844172	Ca5	30844172	C/CTCTT	CCAAACTCATCCACCTCCTC	AAACGATGCAATGGTTGAGAG	60.1	248	INTERGENIC	--	--	--
Ca5_P2_30844785	Ca5	30844785	C/CAAGACT	TGATTTGTTGACGAAGTAGAAGAGA	TGCGAACACAACAACCATTT	60.0	231	INTERGENIC	--	--	--
Ca5_P2_30844820	Ca5	30844820	TTGTTGTGTT/TTGTT	TATGTTTTCGAGCGGTTGCT	AAGTTGAAGGAAGGTCACGA	57.4	465	INTERGENIC	--	--	--

INDEL marker IDs	Chromosomes/unanchored scaffolds	Physical positions (bp)	InDels (*Kabuli* reference genome-CDC Frontier/PII)	Forward primers (5'-3')	Reverse primers (5'-3')	Anneling temperature (0C)	Expected amplified product size (bp)	Structural annotation		Functional annotation	
								Sequence components of *kabuli* genome	*Kabuli* gene accession IDs	NCBI-KOG	TFs
Ca5_P2_30848351	Ca5	30848351	GAAAAA/GAAAA	CCTTGTAAAGAAAGCGGCAG	TTTTTCCAACCGAGCATTTC	60.1	717	INTERGENIC	--	--	--
Ca5_P2_30848614	Ca5	30848614	GTTTTTTTTT/GTTTTTTTT	TCCTTGCACGTGTTGTCACT	TTTTTCCAACCGAGCATTTC	60.1	214	INTERGENIC	--	--	--
Ca5_P2_30849042	Ca5	30849042	CAAAA/CAAA	GAAATGCTCGGTTGGAAAAA	GCATGCCTTTATTAGAGCGG	59.8	526	INTERGENIC	--	--	--
Ca5_P2_30852049	Ca5	30852049	TAAA/TAAAA	TCCGTACGGTTACGGTTTTC	TTGGGTGCTGGTTCTTCTTC	60.2	509	INTERGENIC	--	--	--
Ca5_P2_30858994	Ca5	30858994	TTT/TTTATT	ATGAAGGCTGCGTTCTGACT	GGCCCTTGGAAGTCACATAA	59.9	214	DRR	Ca_04781	--	NF-YB
Ca5_P2_30859236	Ca5	30859236	ATTTTTTT/ATTTTTT	ATGAAGGCTGCGTTCTGACT	AACCATCAACCACTGGAACC	59.7	740	INTERGENIC	--	--	--
Ca5_P2_30860226	Ca5	30860226	TT/TTCT	TATTTGAGCGTGTGAACCGA	TTAGCAACTGATCGCCACTG	60.0	687	INTERGENIC	--	--	--
Ca5_P2_30860398	Ca5	30860398	GAAAAAAA/GAAAAAA	TATTTGAGCGTGTGAACCGA	TTAGCAACTGATCGCCACTG	60.0	687	INTERGENIC	--	--	--
Ca5_P2_30860511	Ca5	30860511	CAA/CAAA	ATAGCAACACTCCATCCGCT	TTAGCAACTGATCGCCACTG	60.0	580	INTERGENIC	--	--	--
Ca5_P2_30861561	Ca5	30861561	TA/T	TTACCTACTCCGAATGCCCA	TTCACAGTTCAGTTTAGTTTGAATGA	59.3	572	INTERGENIC	--	--	--
Ca5_P2_30861801	Ca5	30861801	TTAAT/TTAATGTAAT	TTACCTACTCCGAATGCCCA	TGATGGTGGTGAGGATCAAA	59.9	658	INTERGENIC	--	--	--
Ca5_P2_30864475	Ca5	30864475	TAA/TAAA	TTCTGCAAGACGGTTCTGTG	CGGATGTCTACCCTCGTGTT	60.0	260	INTRON	Ca_04782	T	bHLH

INDEL marker IDs	Chromosomes/unanchored scaffolds	Physical positions (bp)	InDels (*Kabuli* reference genome-CDC Frontier/PII)	Forward primers (5'-3')	Reverse primers (5'-3')	Anneling temperature (0C)	Expected amplified product size (bp)	Structural annotation		Functional annotation	
								Sequence components of *kabuli* genome	*Kabuli* gene accession IDs	NCBI-KOG	TFs
Ca5_P2_30868972	Ca5	30868972	AT/A	ACCATTCATTTTCTGCAGCC	ATTTCCATGTCCAACAAGGC	59.8	526	DRR	Ca_04782	T	bHLH
Ca5_P2_30873220	Ca5	30873220	AA/AAGA	GGGACCGTCGGAATTTTTAT	GCACGGCATCAACCTAAAAT	60.0	674	INTRON	Ca_04783	--	BES1
Ca5_P2_30875873	Ca5	30875873	ATT/ATTT	GTCGTCCAGCGGTTAGGATA	AAGAGAATTAGAGAAGAGTGCGG	58.4	318	DRR	Ca_04783	--	BES1
Ca5_P2_30876347	Ca5	30876347	AAACAA/AAA	AATTGTTTACCGCACTCTTCTC	CCGGCGAAATTAATATGGTG	60.2	471	INTERGENIC	--	--	--
Ca5_P2_30876698	Ca5	30876698	GTTTTTTTTTTTT/GTTTTTTTTT	CGCCGGAGAAAAATGTTAAA	ATGCAACTGACCAATTTAATGA	57.2	539	INTERGENIC	--	--	--
Ca5_P2_30877095	Ca5	30877095	ATTT/ATT	TTCACGAAGTATGAGTTTTTGATGA	ACCCACTGGACTGCTGCTAT	59.8	409	INTERGENIC	--	--	--
Ca5_P2_30877283	Ca5	30877283	TAA/TA	TTCACGAAGTATGAGTTTTTGATGA	ATTGTTAACGGTCTCCGTCG	60.0	735	INTERGENIC	--	--	--
Ca5_P2_30877568	Ca5	30877568	CATTATTATTATTATTA/CATTATTATTATTATTATTATTATTA	TGCAAATAGCAGCAGTCCAG	ATTGTTAACGGTCTCCGTCG	60.0	351	INTERGENIC	--	--	--
Ca5_P2_30880041	Ca5	30880041	TA/TACACAA	CCTGTATTCTGCCTCAGCGT	CAACCTAAGATTGGCCGTGT	60.0	494	DRR	Ca_04784	R	--
Ca5_P2_30882274	Ca5	30882274	TTTAGACAAATTAGACA/TTTAGACA	AGGTTTGGGTGGTTTCAATG	AAACTCGGTCTCAAGGGGTT	60.0	780	INTERGENIC	--	--	--
Ca5_P2_30886397	Ca5	30886397	CTT/CTTT	GTGACATATTGAGGGTGGGG	CTCCCCAACGTAGAACCAAA	60.0	791	INTRON	Ca_04785	R	--
Ca5_P2_30898589	Ca5	30898589	ATTTTTTT/ATTTTTT	TATGGTGTGGTGAACGATGG	CGAACATGAACTTGCTCGAA	60.0	914	INTERGENIC	--	--	--

INDEL marker IDs	Chromosomes/unanchored scaffolds	Physical positions (bp)	InDels (*Kabuli* reference genome-CDC Frontier/PII)	Forward primers (5'-3')	Reverse primers (5'-3')	Anneling temperature (0C)	Expected amplified product size (bp)	Structural annotation		Functional annotation	
								Sequence components of *kabuli* genome	*Kabuli* gene accession IDs	NCBI-KOG	TFs
Ca5_P2_30898776	Ca5	30898776	AG/A	CCCGGATGGAGATGGTAGAT	CGAACATGAACTTGCTCGAA	60.0	642	INTERGENIC	--	--	--
Ca5_P2_30900578	Ca5	30900578	AAAA/AAAAATAAA	AGTGTCACGTGAGAGTGCCA	TCAAAGGAGGCAAATGGTAAA	59.6	570	DRR	Ca_04787	T	MYB_related
Ca5_P2_30900953	Ca5	30900953	TA/TAACA	CGGTCCGACTTGTCTTTATCA	GGAATTGGAAGTCGGATGAA	59.9	367	DRR	Ca_04787	T	MYB_related
Ca5_P2_30909427	Ca5	30909427	ACT/A	TTCGTGAAAAATGTTTGTTTGG	GACGTTACAAAAGCATGAATCG	59.7	674	INTERGENIC	--	--	--
Ca5_P2_30909997	Ca5	30909997	TATTAAT/TATTAATTAAT	CGTTTGGTTCATGTTTTCCTT	ATCGAGGAAGAGAATGGGGT	59.9	243	INTERGENIC	--	--	--
Ca5_P2_30910017	Ca5	30910017	T/TC	CGTTTGGTTCATGTTTTCCTT	ATCGAGGAAGAGAATGGGGT	59.9	243	INTERGENIC	--	--	--
Ca5_P2_30910282	Ca5	30910282	ATTTTTTTTT/ATTTTTTTT	ACCCCATTCTCTTCCTCGAT	ACGGTCACATCTTCCACTCC	60.0	526	INTERGENIC	--	--	--
Ca5_P2_30917795	Ca5	30917795	G/GA	GAACCAAAGTTGTGGAACAAAA	GTGCGTAAGTGCAGTTGGAG	59.5	526	INTERGENIC	--	--	--
Ca5_P2_30919604	Ca5	30919604	TTATA/TTATATA	TGGATACATCTCAATGGGCA	CTCCCACAACGCGATAAACT	60.1	737	DRR	Ca_04789	--	--
Ca5_P2_30923030	Ca5	30923030	TTATAATTTA/TTATAATTTATATAATTTA	GACTTCAACCATCACCCACC	ACCAACCAACCACTCCACTC	59.9	411	INTERGENIC	--	--	--
Ca5_P2_30924630	Ca5	30924630	CGG/CGGG	ATGCTTTGTCTCAAGGTCGG	TTTGAAATTAAACGACGGGG	59.8	549	INTERGENIC	--	--	--
Ca5_P2_30927465	Ca5	30927465	TAAAAAAAA/TAAAAAAAAA	TTCGATGCAAGACACTACCAA	CGATTTATGCGGAGACTCGT	60.2	723	INTERGENIC	--	--	--

INDEL marker IDs	Chromosomes/unanchored scaffolds	Physical positions (bp)	InDels (*Kabuli* reference genome-CDC Frontier/PII)	Forward primers (5'-3')	Reverse primers (5'-3')	Anneling temperature (0C)	Expected amplified product size (bp)	Structural annotation		Functional annotation	
								Sequence components of *kabuli* genome	*Kabuli* gene accession IDs	NCBI-KOG	TFs
Ca5_P2_30927659	Ca5	30927659	TACA/TA	TTGAAGAAGGGTAATGTCGATTT	CGATTTATGCGGAGACTCGT	60.2	116	INTERGENIC	--	--	--
Ca5_P2_30927704	Ca5	30927704	GTATATA/GTATATATATA	TTGAAGAAGGGTAATGTCGATTT	CGATTTATGCGGAGACTCGT	60.2	116	INTERGENIC	--	--	--
Ca5_P2_30928660	Ca5	30928660	CAAAAGTCAACA/CAAAAGTCAACACACGTAAAAAGTCAACA	AAGAAGCACGTCATTTGCTG	GTTTGAACCAGTGCGATCCT	60.1	668	INTERGENIC	--	--	--
Ca5_P2_30929556	Ca5	30929556	T/TAAGTAGACTTAAC	AACAAGGATCGCACTGGTTC	GGTTCACCACCAACCTGACT	59.9	590	INTERGENIC	--	--	--
Ca5_P2_30932039	Ca5	30932039	AAAGATAAGATAAGA/AAAGATAAGA	TGAGTAACTTCGAACGTGCAG	AGTAGTGTTGGCCTGTGCCT	59.8	553	INTERGENIC	--	--	--
Ca5_P2_30934463	Ca5	30934463	ATTT/ATT	GCCAAATTAAAATACGGCACA	GCAAGGGGTGAGAAAATGAA	60.1	927	DRR	Ca_04790	--	--
Ca5_P2_30936563	Ca5	30936563	T/TC	GCAGTTGTGCACGTTGATTT	TGAGTGACGGTTTGACTGGA	60.3	456	INTERGENIC	--	--	--
Ca5_P2_30937344	Ca5	30937344	CAA/CAAA	GGCTGGCTAAAAGATACCCC	TGCACCTGATGTTGTGGAAT	60.0	590	DRR	Ca_04792	--	--
Ca5_P2_30941228	Ca5	30941228	TAAAAAAAAA/TAAAAAAAA	TGCTTTGACAAATGGAGTGG	TCTCGAGATCGAATTTGAGACTT	59.5	233	INTERGENIC	--	--	--
Ca5_P2_30941710	Ca5	30941710	CAAAAAAAAAA/CAAAAAAAAA	TCTCAAATTCGATCTCGAGAAAA	TCGATAGGGTTTAATGGAACAA	58.5	574	INTERGENIC	--	--	--
Ca5_P2_30942392	Ca5	30942392	CTCTT/CTCTTCTT	TTATAAATTTGGGCACCCGT	GCCATTTTGACCGACCTAGA	60.1	155	INTERGENIC	--	--	--
Ca5_P2_30948697	Ca5	30948697	CAA/CAAA	ATATTGTTGGTAGCGTCGGC	TGTGCGGAGACATCTTATGC	59.8	601	INTERGENIC	--	--	--

INDEL marker IDs	Chromosomes/unanchored scaffolds	Physical positions (bp)	InDels (*Kabuli* reference genome-CDC Frontier/PII)	Forward primers (5'-3')	Reverse primers (5'-3')	Anneling temperature (0C)	Expected amplified product size (bp)	Structural annotation		Functional annotation	
								Sequence components of *kabuli* genome	*Kabuli* gene accession IDs	NCBI-KOG	TFs
Ca5_P2_30948949	Ca5	30948949	TAGAAGAAG/TAGAAG	ATATTGTTGGTAGCGTCGGC	TGTGCGGAGACATCTTATGC	59.8	601	INTERGENIC	--	--	--
Ca5_P2_30949176	Ca5	30949176	CAAT/CAATTAAT	ATGGCATAAGATGTCTCCGC	TGCATGAGGGTAGAGACTATCAA	58.9	338	INTERGENIC	--	--	--
Ca5_P2_30950079	Ca5	30950079	ATCTC/ATC	TGTTAATGTTTATGGCCGCA	CAGCGTCTTGTCAAATTGTGT	58.8	508	INTERGENIC	--	--	--
Ca5_P2_30951992	Ca5	30951992	TAAAAAAAAA/TAAAAAAAAAAA	TGTTGGTAGGGGTGGGAATA	TTGAATGTGTTTTGTTGGATGA	58.9	398	DRR	Ca_04794	--	--
Ca5_P2_30957314	Ca5	30957314	AATTTTG/A	AGCCTCCTACAACCTTTCCC	TGATCCAAGTTTAGTTTGCAATTC	59.6	467	INTERGENIC	--	--	--
Ca5_P2_30957781	Ca5	30957781	A/ATTT	TTTGAATTGCAAACTAAACTTGGA	CTAACAAGAAATCCGCGCTC	60.0	641	INTERGENIC	--	--	--
Ca5_P2_30959016	Ca5	30959016	CTATAT/CTAT	GGGGCATACTGCTACGATGT	TTCGCTACACCTTGACCACA	60.3	639	INTERGENIC	--	--	--
Ca5_P2_30959641	Ca5	30959641	AT/ATGATACT	TGTGGTCAAGGTGTAGCGAA	TGCCATGTATTCTGAGCGTC	59.8	425	INTERGENIC	--	--	--
Ca5_P2_30959956	Ca5	30959956	GTTTTTTTTTTT/GTTTTTTTTTT	CTTTGAAGCTCACCCTCCAC	ACCTTTGACACGAAACGGAA	60.5	748	INTERGENIC	--	--	--
Ca5_P2_30961401	Ca5	30961401	CATAT/CAT	GGACGATCGATGGAGACAGT	GAAGCCAAGATAGCACAGGC	60.0	383	INTERGENIC	--	--	--
Ca5_P2_30961731	Ca5	30961731	A/AC	GCCTGTGCTATCTTGGCTTC	AGGATTGCTCCAGCAAAGAA	60.0	493	INTRON	Ca_04795	R	--
Ca5_P2_30961814	Ca5	30961814	CTAA/CTAATTAA	GCCTGTGCTATCTTGGCTTC	AGGATTGCTCCAGCAAAGAA	60.0	493	INTRON	Ca_04795	R	--

INDEL marker IDs	Chromosomes/unanchored scaffolds	Physical positions (bp)	InDels (*Kabuli* reference genome-CDC Frontier/PII)	Forward primers (5'-3')	Reverse primers (5'-3')	Anneling temperature (0C)	Expected amplified product size (bp)	Structural annotation		Functional annotation	
								Sequence components of *kabuli* genome	*Kabuli* gene accession IDs	NCBI-KOG	TFs
Ca5_P2_30971169	Ca5	30971169	TAAAAAAA/TAAAAAA	TTCCACCAATCTCTGCAAAA	TTGAAAAGCCCATTTTGACC	59.9	450	INTRON	Ca_04796	T	--
Ca5_P2_30977866	Ca5	30977866	TAAAAAAAA/TAAAAAAA	GCATATCTTGGCCCTTCAAA	CTGAGGACCGGGACTCAATA	60.1	522	INTRON	Ca_04796	T	--
Ca5_P2_30986745	Ca5	30986745	GATAAAT/GATAAATTTGTCATTAATAAAT	AAAAAGACTGCAACAAGAAAAACT	TCTCATAACTGCATCCGCTG	60.0	957	INTERGENIC	--	--	--
Ca5_P2_30987073	Ca5	30987073	TAAAAAA/TAAAAAAA	TGTGGAATCACCGACAAAAA	TCTCATAACTGCATCCGCTG	60.0	268	INTERGENIC	--	--	--
Ca5_P2_30987368	Ca5	30987368	AT/ATT	TCAGCGGATGCAGTTATGAG	GCGACTACTGTGCGAGTCAC	59.7	501	INTERGENIC	--	--	--
Ca5_P2_30988109	Ca5	30988109	TCCTTCTCGA/T	TAGTCGCACTATTGCACGCT	ATTCTCCGTTCAGGGTTGTG	60.0	851	INTERGENIC	--	--	--
Ca5_P2_30994608	Ca5	30994608	TAAA/TAAAA	TTTGCCTCCTCGTGAGTTCT	GTTTCCCGCACATTGATCTT	59.9	540	INTRON	Ca_04797	K	--
Ca5_P2_30997360	Ca5	30997360	GA/GAA	TTGGTGCCAAATTGGTGTAA	CTCTCGACTCTCAAATTCCGA	59.6	419	DRR	Ca_04797	K	--
Ca5_P2_30998862	Ca5	30998862	TTAT/TT	ATAGATGCTGCGGCCATTAG	AATATAGAAACCAAATAACCCGTTT	57.7	166	INTERGENIC	--	--	--
Ca5_P2_30999647	Ca5	30999647	A/AC	CATTTTCAATAAATCTGAAATGATTGT	GCAATGTTATGCAAGCAGCA	61.0	206	DRR	Ca_04798	--	--
Ca5_P2_31000378	Ca5	31000378	CAAAAA/CAAAAAAA	CAAGCGTATCCATAGTCCACC	ACTCTCCCCTCCCAACCTAA	59.9	487	DRR	Ca_04798	--	--
Ca5_P2_31001905	Ca5	31001905	AAT/AATATAT	CGACTCACGACAGAGGATGA	CGAAGAGGAAATTCTTGTTTGTTT	60.0	836	INTERGENIC	--	--	--

**INDEL marker IDs**	**Chromosomes/unanchored scaffolds**	**Physical positions (bp)**	**InDels (*Kabuli* reference genome-CDC Frontier/PII)**	**Forward primers (5'-3')**	**Reverse primers (5'-3')**	**Anneling temperature (0C)**	**Expected amplified product size (bp)**	**Structural annotation**		**Functional annotation**	
								**Sequence components of *kabuli* genome**	***Kabuli* gene accession IDs**	**NCBI-KOG**	**TFs**
Ca5_P2_31002201	Ca5	31002201	TAAAAAAAAA/TAAAAAAAA	TCAACCACCCACAAGATTCA	CGAAGAGGAAATTCTTGTTTGTTT	60.0	653	INTERGENIC	--	--	--
Ca5_P2_31004244	Ca5	31004244	GTTT/GTTTTT	TGGCTTAATATTGTGGCAGATG	GAAAAAGCACCCCAGAAAAA	59.2	175	INTERGENIC	--	--	--
Ca5_P2_31004299	Ca5	31004299	CTTGTAATT/CT	TGATTTCTGCTTGACAAGTGC	ATGGCCAAGAATTTCACAGG	59.9	546	INTERGENIC	--	--	--
Ca5_P2_31009263	Ca5	31009263	CTTATTAAT/CTTATTAATTATTAAT	TCATTGGCATGAAACCAGAG	TGAATGTTGGAAACAAGCCA	60.1	318	INTERGENIC	--	--	--
Ca5_P2_31009609	Ca5	31009609	CAA/CAAAA	TGGCTTGTTTCCAACATTCA	CTTCCCTTCCATTTCCTTCC	59.9	693	INTERGENIC	--	--	--
Ca5_P2_31012665	Ca5	31012665	TA/T	GTTCCGCCTCTTAAACCTCC	CCCTCACACTCAACACACAA	58.1	745	INTERGENIC	--	--	--
Ca5_P2_31013035	Ca5	31013035	GTTTTT/GTTTTTT	GCCCAACCTTAAGAGGCTTT	CACTTGCTAATTGCAACCGA	59.9	616	INTERGENIC	--	--	--
Ca5_P2_31014350	Ca5	31014350	CTTTTTTTTT/CTTTTTTTT	ACTTTTGACCCTGTTGGGTG	ATGTGAAAAACTTCACGGGG	59.8	636	INTERGENIC	--	--	--
Ca5_P2_31015449	Ca5	31015449	AA/AATTCTTTTA	TTCATCCAATCCATCCACCT	TTGCCCGCTTTTTATTATGG	59.9	747	INTERGENIC	--	--	--
Ca5_P2_31029239	Ca5	31029239	AAACAACAACAACAACAACAACAA/AAACAACAACAACAA	TTTCATTCGGTTTTTGGGAG	GATCGGGTGATTCAATTAGTTTG	59.7	872	INTERGENIC	--	--	--
Ca5_P2_31029772	Ca5	31029772	ATTTTTTTTT/ATTTTTTTTTT	TCAAACTAATTGAATCACCCGA	TTTTGCATTTAATTTCAAGAACCTT	59.5	509	INTERGENIC	--	--	--
Ca5_P2_31031627	Ca5	31031627	TA/TAA	ATGCGAAATCCGTCTCATTC	CAAAACACCTCGCACAGAGA	60.0	853	INTRON	Ca_04801	B	MYB_related

INDEL marker IDs	Chromosomes/unanchored scaffolds	Physical positions (bp)	InDels (*Kabuli* reference genome-CDC Frontier/PII)	Forward primers (5'-3')	Reverse primers (5'-3')	Anneling temperature (0C)	Expected amplified product size (bp)	Structural annotation		Functional annotation	
								Sequence components of *kabuli* genome	*Kabuli* gene accession IDs	NCBI-KOG	TFs
Ca5_P2_31031889	Ca5	31031889	AATAATATAATAT/AATAATAT	AGCTCTCTGTGCCATTGCTT	ATAGTTGCCGCGAACAAAAC	60.1	531	INTRON	Ca_04801	B	MYB_related
Ca5_P2_31032598	Ca5	31032598	AA/AATCAAATTA	AGCCTTCTGCTGATGCTGAT	ACCAAACTCAGGCGACAAGT	59.8	329	DRR	Ca_04802	--	--
Ca5_P2_31038384	Ca5	31038384	ATACTACT/ATACT	TCAAAACACAATCCTTCCTTCA	TGGAATAATGACGGTGACGA	59.9	625	INTERGENIC	--	--	--
Ca5_P2_31041985	Ca5	31041985	ACT/ACTCT	TCATCAACTTTCCGTGTCCA	CCATTTGCTGTTACGATGCT	58.8	477	INTERGENIC	--	--	--
Ca5_P2_31044270	Ca5	31044270	GTTTAGACTTTTA/GTTTA	GGCCCAAAAGAGGTTCCTAC	TTCGAAATGGACAAATTCTTACAA	59.9	216	DRR	Ca_04803	O	--
Ca5_P2_31044407	Ca5	31044407	ATA/ATAATGTTA	GGCCCAAAAGAGGTTCCTAC	TGACACCTTTAAATAATTCCATGA	57.7	574	DRR	Ca_04803	O	--
Ca5_P2_31045566	Ca5	31045566	AT/ATT	AACTCACTTCCACAATGCCC	CACTTGCAAATCAACCCCTT	60.0	231	INTRON	Ca_04803	O	--
Ca5_P2_31046318	Ca5	31046318	TTGTTTATTAGT/TT	TGGTTTATTCCTTCAACCGC	CACGTGATTTCTCCATCGTG	60.1	730	INTRON	Ca_04803	O	--
Ca5_P2_31050359	Ca5	31050359	CATTCATATTCAT/CATTCATATTCATATTCAT	AAGAAGGAGGAAGAGCAGGG	TGATCCGCATAAAGCATTCA	60.2	189	INTERGENIC	--	--	--
Ca5_P2_31051735	Ca5	31051735	AA/AAGAACAAAATA	TGAAATTGACTGAGAAATTGCAT	AAGAAATGCAAGGGGCATTA	59.5	223	INTERGENIC	--	--	--
Ca5_P2_31051998	Ca5	31051998	TAAAAAAAAA/TAAAAAAAA	CATAATGCCCCTTGCATTTC	TATCCACATGGCAAGATCCA	59.9	382	INTERGENIC	--	--	--
Ca5_P2_31052451	Ca5	31052451	TAAAAAAAAAA/TAAAAAAAAA	TGGATCTTGCCATGTGGATA	TCTGAAACCTGGTGGGAGAC	60.1	581	INTERGENIC	--	--	--

INDEL marker IDs	Chromosomes/unanchored scaffolds	Physical positions (bp)	InDels (*Kabuli* reference genome-CDC Frontier/PII)	Forward primers (5'-3')	Reverse primers (5'-3')	Anneling temperature (0C)	Expected amplified product size (bp)	Structural annotation		Functional annotation	
								Sequence components of *kabuli* genome	*Kabuli* gene accession IDs	NCBI-KOG	TFs
Ca5_P2_31054226	Ca5	31054226	TAAAAAAA/TAAAAAAAA	TTGCTTCTTTAATGGCCACC	AAACGGAGGAGTGTTTCCCT	60.0	291	INTRON	Ca_04804	--	NAC
Ca5_P2_31062689	Ca5	31062689	TCC/TC	CTATTGTGGCTTCCATCGGT	CAGCAAGGCCAATACCAAAT	60.0	599	INTRON	Ca_04805	R	--
Ca5_P2_31063645	Ca5	31063645	CCTAGCTTG/CTTAAGACAACACTAGCTTG	CCCAGTTCAGTGGTTCTGGT	CTGTTCCTGTCTCAGGGAGC	60.0	824	INTRON	Ca_04805	R	--
Ca5_P2_3106419	Ca5	3106419	CACA/CACATAACTACA	CAATTTTCGTGATGTGGCAG	TGAGTGATGCGAGTTGTTCG	61.0	749	INTERGENIC	--	--	--
Ca5_P2_31065540	Ca5	31065540	TATAA/TATAAATAA	GTGCATCCTTGTTTTCGGTT	GTGATGACTTCAAGGGGCAT	59.9	938	INTRON	Ca_04805	R	--
Ca5_P2_31068913	Ca5	31068913	A/AT	GAAAACTCGACCTCCTATTGAA	TAAAAGCCGCCCATTTAGAA	59.7	595	DRR	Ca_04806	--	--
Ca5_P2_31072626	Ca5	31072626	CAA/CA	CCTTCACAGCAGCAACAAAA	AGGGAGCAATGCATGAAAAC	60.1	566	INTERGENIC	--	--	--
Ca5_P2_31072835	Ca5	31072835	A/AC	GTTTTCATGCATTGCTCCCT	TTCAACCCAATGCACTTCAA	60.1	622	INTERGENIC	--	--	--
Ca5_P2_31073126	Ca5	31073126	CTTTT/CTTTTT	GTTTTCATGCATTGCTCCCT	TTCAACCCAATGCACTTCAA	60.1	622	INTERGENIC	--	--	--
Ca5_P2_31073172	Ca5	31073172	GTTTTTTTTT/GTTTTTTTTTT	GTTTTCATGCATTGCTCCCT	TTCAACCCAATGCACTTCAA	60.1	622	INTERGENIC	--	--	--
Ca5_P2_31076855	Ca5	31076855	T/TG	AATAGGCACATGTCCATCCC	CCATTGGTTCATTTTTGGTG	58.7	637	INTERGENIC	--	--	--
Ca5_P2_31078017	Ca5	31078017	TTATA/TTA	GGGACATGCGTTTATCAAAGT	TCCATAAACCCCATAATCAAAA	58.2	473	INTERGENIC	--	--	--

INDEL marker IDs	Chromosomes/unanchored scaffolds	Physical positions (bp)	InDels (*Kabuli* reference genome-CDC Frontier/PII)	Forward primers (5'-3')	Reverse primers (5'-3')	Anneling temperature (0C)	Expected amplified product size (bp)	Structural annotation		Functional annotation	
								Sequence components of *kabuli* genome	*Kabuli* gene accession IDs	NCBI-KOG	TFs
Ca5_P2_31078519	Ca5	31078519	AT/ATTT	TTTTGATTATGGGGTTTATGGA	ACCAACACCAACTATTGGCA	58.9	473	INTERGENIC	--	--	--
Ca5_P2_31083532	Ca5	31083532	AAGTAGTTG/AAGTAGTTGTAGTAGTTG	TCTTATCCCCTCTCCCCTGT	TTACTTGTTTGCCCCGAATC	59.9	777	DRR	Ca_04808	--	--
Ca5_P2_31084271	Ca5	31084271	TAAC/TAACTCAAC	GATTCGGGGCAAACAAGTAA	ATTTCACCCCAACCCTTAGC	60.2	524	DRR	Ca_04808	--	--
Ca5_P2_31087296	Ca5	31087296	GAAAAAAAAAAAA/GAAAAAAAAAAA	TGCACTTTCCGATCAATCAA	TCCAACTAAAATTTTGGGGTTTT	60.0	140	DRR	Ca_04809	O	--
Ca5_P2_31092038	Ca5	31092038	TAAAAA/TAAAA	ATTTAGGGGGAACGCCTTTA	AACTGTTGTGAGAGCCGCTT	60.1	317	INTRON	Ca_04809	O	--
Ca5_P2_31094152	Ca5	31094152	AATATATA/AATATA	TTTCATGCAATTCAAAAATCG	CTGCCCCAATGTCTAATGCT	60.1	438	DRR	Ca_04810	O	--
Ca5_P2_31098359	Ca5	31098359	TC/T	GAGGCCATTCAAACCAAAAA	CCACGGTTTGTCTTTGACCT	60.0	662	INTRON	Ca_04811	--	NF-YC
Ca5_P2_31102459	Ca5	31102459	AAAA/AAAACAAA	CATTTGGATCGGCAATTTTC	CATCCCAGATTTTTGTGGGT	59.6	728	INTERGENIC	--	--	--
Ca5_P2_31108640	Ca5	31108640	CAAAAAAA/CAAAAAAAAA	TTGAGCCCTATCTTGTTGGG	AAACCATCTGGGGTTTTTCC	60.0	442	INTRON	Ca_04812	R	--
Ca5_P2_31109120	Ca5	31109120	AATAAAATA/AA	AGTCAAGCCTCACTCCCAGA	TCCCGATTTTTGGTCAACAT	60.2	434	INTRON	Ca_04812	R	--
Ca5_P2_31110488	Ca5	31110488	AGGATAATG/AG	GGTGAGTCAACGGCATTTTT	CCGGGTGCAAGTTCAGTAAA	61.0	279	INTERGENIC	--	--	--
Ca5_P2_31111351	Ca5	31111351	C/CGTATAAT	TTTGTTTACAAGGGTAGTTTGTCA	CATTTAGGATTCGTTTGATATGGA	59.3	485	INTERGENIC	--	--	--

INDEL marker IDs	Chromosomes/unanchored scaffolds	Physical positions (bp)	InDels (*Kabuli* reference genome-CDC Frontier/PII)	Forward primers (5'-3')	Reverse primers (5'-3')	Anneling temperature (0C)	Expected amplified product size (bp)	Structural annotation		Functional annotation	
								Sequence components of *kabuli* genome	*Kabuli* gene accession IDs	NCBI-KOG	TFs
Ca5_P2_31111411	Ca5	31111411	ATTTTTTTTTTT/ATTTTTTTTTT	TTTGTTTACAAGGGTAGTTTGTCA	TGAAAACCCAAAACACGGAT	60.2	560	INTERGENIC	--	--	--
Ca5_P2_31111925	Ca5	31111925	TAAAAAA/TAAAAA	ATCCGTGTTTTGGGTTTTCA	TGGGTCATTGAAGTGCTAATTG	60.0	457	INTERGENIC	--	--	--
Ca5_P2_31111985	Ca5	31111985	AT/A	ATCCGTGTTTTGGGTTTTCA	TGGGTCATTGAAGTGCTAATTG	60.0	457	INTERGENIC	--	--	--
Ca5_P2_31112728	Ca5	31112728	TAAAAAA/TAAAAA	CAATTAGCACTTCAATGACCCA	CGTGTCATCATTTCACCGAC	60.0	784	INTERGENIC	--	--	--
Ca5_P2_31113202	Ca5	31113202	ATTTTTTTT/ATTTTTTT	GTCGGTGAAATGATGACACG	GAGGACGTCTTTTTCGTCCA	60.2	317	INTERGENIC	--	--	--
Ca5_P2_31113335	Ca5	31113335	AGACG/AG	GTCGGTGAAATGATGACACG	GAGGACGTCTTTTTCGTCCA	60.2	317	INTERGENIC	--	--	--
Ca5_P2_31115282	Ca5	31115282	AA/AATA	CCATCTCCAACAATATCAAATTCA	CCCGCAATTGCAAGAGTAAT	60.1	428	INTERGENIC	--	--	--
Ca5_P2_31118727	Ca5	31118727	GTTTTTTT/GTTTTTT	ATCCAACGGTTAACGAATGC	CTCTCATTTCAATGCACCCA	59.6	606	DRR	Ca_04814	--	NF-YC
Ca5_P2_31121983	Ca5	31121983	TCAACTCCAACTCCAA/TCAACTCCAACTCCAACTCCAA	CTGCTGCCAAACTTCTCCTC	CCGTTTGTGCAGTGTTCATC	60.2	614	INTRON	Ca_04814	--	NF-YC
Ca5_P2_31123518	Ca5	31123518	GAAAAAAAAAA/GAAAAAAAAA	TTGGAGTTTTTGTTTCACAATGTT	AGAACACACTCTTGCCGCTT	60.1	505	INTERGENIC	--	--	--
Ca5_P2_31129813	Ca5	31129813	GTAA/G	GAGAGGGTGAAAAATGGCAA	TACCATTCCCAACCACACCT	60.1	430	INTERGENIC	--	--	--
Ca5_P2_31130050	Ca5	31130050	TG/T	GAGAGGGTGAAAAATGGCAA	TACCATTCCCAACCACACCT	60.1	430	INTERGENIC	--	--	--

INDEL marker IDs	Chromosomes/unanchored scaffolds	Physical positions (bp)	InDels (*Kabuli* reference genome-CDC Frontier/PII)	Forward primers (5'-3')	Reverse primers (5'-3')	Anneling temperature (0C)	Expected amplified product size (bp)	Structural annotation		Functional annotation	
								Sequence components of *kabuli* genome	*Kabuli* gene accession IDs	NCBI-KOG	TFs
Ca5_P2_31132248	Ca5	31132248	GTTT/GTT	TAATGTGCTGCAAACCAGGA	CAGGGAATTGATTCGAGCAT	60.0	409	INTRON	Ca_04815	--	--
Ca5_P2_31149820	Ca5	31149820	AATGAGATGAGATGAGATGAGATGA/AATGAGATGAGATGAGATGA	CGCCGCCTATACAACAAAAT	AAGCTTGTGGAAAGGGAAT	59.9	400	INTRON	Ca_04818	--	GRAS
Ca5_P2_31150042	Ca5	31150042	T/TAA	CGCCGCCTATACAACAAAAT	GCGTCAAGGAAAGCTAATCG	60.0	939	INTRON	Ca_04818	--	GRAS
Ca5_P2_31153699	Ca5	31153699	TAAAAAAAAA/TAAAAAAAA	TTTAAAGGGGCTCACTTGCT	TTTTGGCATAAAATTGAAGTATGG	59.3	552	INTERGENIC	--	--	--
Ca5_P2_31153866	Ca5	31153866	ATTT/ATT	TGAACAATATTTCTCTTCCAGCA	TTGTGTGGAGGTAAAAGCCC	60.0	885	INTERGENIC	--	--	--
Ca5_P2_31153903	Ca5	31153903	CAAAAAA/CAAAAA	TGAACAATATTTCTCTTCCAGCA	TTGTGTGGAGGTAAAAGCCC	60.0	885	INTERGENIC	--	--	--
Ca5_P2_31153994	Ca5	31153994	TAAAAAA/TAAAAA	GACCATACTTCAATTTTATGCCAA	TTGTGTGGAGGTAAAAGCCC	60.0	432	INTERGENIC	--	--	--
Ca5_P2_31154231	Ca5	31154231	TG/TGAG	GACCATACTTCAATTTTATGCCAA	TTGTGTGGAGGTAAAAGCCC	60.0	432	INTERGENIC	--	--	--
Ca5_P2_31155852	Ca5	31155852	CTAATAATAATAATAATAATAATAAT/CTAATAATAATAATAATAATAATAATAAT	GGTGGGTCGCTTTTTAATGA	AAGGGGAAAGGAAAAGGAAA	59.0	253	INTERGENIC	--	--	--
Ca5_P2_31170171	Ca5	31170171	GCC/GC	CAGCCCGGATAACACATTCT	GGCCCCATTTACAGGAATTT	60.0	612	DRR	Ca_04822	TU	C3H
Ca5_P2_31175434	Ca5	31175434	GTT/GT	CCTTCAAGCCCCTAAGATCC	AGTTTGCTCCATGGGAATTG	59.9	252	INTRON	Ca_04822	TU	C3H
Ca5_P2_31177378	Ca5	31177378	ATCTA/ATCTATTCTA	CAAGAGCCATCTCATCCCAT	GAGCTGTGTGCAGCCTACAA	60.2	452	INTRON	Ca_04822	TU	C3H

INDEL marker IDs	Chromosomes/unanchored scaffolds	Physical positions (bp)	InDels (*Kabuli* reference genome-CDC Frontier/PII)	Forward primers (5'-3')	Reverse primers (5'-3')	Anneling temperature (0C)	Expected amplified product size (bp)	Structural annotation		Functional annotation	
								Sequence components of *kabuli* genome	*Kabuli* gene accession IDs	NCBI-KOG	TFs
Ca5_P2_31182029	Ca5	31182029	GAAGAA/GAAGAAGAAGAAGAA	ACAGCCTCAGCTCCACTGAT	ATGAAATTGCTCCGTTGAGG	60.1	382	INTERGENIC	--	--	--
Ca5_P2_31183009	Ca5	31183009	G/GATGC	CCAAACACTCAAACCGACTC	AAAGACCAAATTGGACACTTCA	58.6	817	DRR	Ca_04823	--	WOX
Ca5_P2_31183454	Ca5	31183454	AGTTAAAAATG/AGTTAAAAATGTTTGTTAAAAATG	GGAGCTGATTGATAGATTTGGAA	TTTCTTGTCCCCAAATGTCTT	58.5	567	DRR	Ca_04823	--	WOX
Ca5_P2_31183515	Ca5	31183515	TTGT/TT	TTCACATACACCCAAGTTTGAGA	TTTCTTGTCCCCAAATGTCTT	58.5	272	DRR	Ca_04823	--	WOX
Ca5_P2_31183794	Ca5	31183794	CAAAAAAA/CAAAAAA	AAGACATTTGGGGACAAGAAA	ATGCCTACTGTGAACCAACG	58.7	628	DRR	Ca_04823	--	WOX
Ca5_P2_31184967	Ca5	31184967	TC/T	CAACCATTGAAGGGAGCCTA	AATGATGGGTTTGCGTTAGC	60.0	653	DRR	Ca_04823	--	WOX
Ca5_P2_31185925	Ca5	31185925	CTTTTTTTTTT/CTTTTTTT	ATTTGAAGCTGGAGAGGGGT	TCCATCACCTCTCAACACGA	60.2	529	INTRON	Ca_04823	--	WOX
Ca5_P2_31186832	Ca5	31186832	TCC/TCCC	GTGTCTTTCGGTGGAGGAAA	TGACGTTGATAGAAACGAAGACA	59.8	478	INTERGENIC	--	--	--
Ca5_P2_31187813	Ca5	31187813	CAAAAAAA/CAAAAAA	GAGGAATGCTAATGTGTCCCA	AAAGTGTTTCTCGCCTCGAA	60.0	603	INTERGENIC	--	--	--
Ca5_P2_31188289	Ca5	31188289	TAAAA/TAAA	AAAGTGTTCGAGGCGAGAAA	GCATGAGTTCATTGGTGGTG	60.0	479	INTERGENIC	--	--	--
Ca5_P2_31189292	Ca5	31189292	ATCTT/ATCTTCTT	TTTCGTCGTAAAATTTCTTTATTTGA	GTCGGAATTCGAAGTGTGGT	60.0	540	INTERGENIC	--	--	--
Ca5_P2_31191176	Ca5	31191176	AATATATATATATATA/AATATATATATATA	AACCGTGTACGAGCGGTATT	TGTGTTTGCAAGGAAAATCC	58.6	619	INTRON	Ca_04824	CR	NAC

INDEL marker IDs	Chromosomes/unanchored scaffolds	Physical positions (bp)	InDels (*Kabuli* reference genome-CDC Frontier/PII)	Forward primers (5'-3')	Reverse primers (5'-3')	Anneling temperature (0C)	Expected amplified product size (bp)	Structural annotation		Functional annotation	
								Sequence components of *kabuli* genome	*Kabuli* gene accession IDs	NCBI-KOG	TFs
Ca5_P2_31191876	Ca5	31191876	G/GT	GGATTTTCCTTGCAAACACAC	GTTTCCATGCTTCCACCTGT	60.0	665	INTRON	Ca_04824	CR	NAC
Ca5_P2_31192988	Ca5	31192988	TAAAAAAAA/TAAAAAAA	TGGCAGGGTGGTAAAAAGAA	AAGTATCCATTGGCTCCGTG	60.0	557	DRR	Ca_04825	R	WOX
Ca5_P2_31193802	Ca5	31193802	T/TTC	TTAATTCCAATGCGGAAAGC	CCTTTCCAATGCACCAAACT	60.0	367	DRR	Ca_04825	R	WOX
Ca5_P2_31196205	Ca5	31196205	GT/GTT	GGGTTTGAAAGGGGAGAGAG	TACCGTGACAAACAAGCGAG	59.9	217	INTERGENIC	--	--	--
Ca5_P2_31197193	Ca5	31197193	TA/T	TTGGCTAAATACCTCTCGTTCC	GTCGGTTGAGCTAGGGCTTA	59.5	488	INTERGENIC	--	--	--
Ca5_P2_31229749	Ca5	31229749	GATATATA/GGATATATATA	TCGGCTCCTTTTCATTTACG	CTACCGATTCGCAATCCAAT	59.9	272	INTERGENIC	--	--	--
Ca5_P2_31229830	Ca5	31229830	TAAAAAAAA/TAAAAAAAAA	TCGGCTCCTTTTCATTTACG	CACACTTTCGTTGCGTCACT	59.9	622	INTERGENIC	--	--	--
Ca5_P2_31230495	Ca5	31230495	AC/A	AGTGACGCAACGAAAGTGTG	AAAGAGGTTTGACTGATTTTGAGA	58.5	511	INTERGENIC	--	--	--
Ca5_P2_31231114	Ca5	31231114	GTTTTTTTTTT/GTGTGTTTTTTTTTTT	TCTCAAATGGTACTTGGAACAA	AATTCACGTTGAGTGACCCAT	59.3	185	DRR	Ca_04829	--	--
Ca5_P2_31231138	Ca5	31231138	TAAAATAAATAATAAA/TAAAATAAATAATAAAAATAAATAATAAA	TCTCAAATGGTACTTGGAACAA	AATTCACGTTGAGTGACCCAT	59.3	185	DRR	Ca_04829	--	--
Ca5_P2_31241531	Ca5	31241531	CTTTTTTTT/CTTTTTTTTTT	CGATGAACGCAAACTCAAGA	TAATCAACCAAAACCCCCAA	60.0	689	INTRON	Ca_04830	R	--
Ca5_P2_31254755	Ca5	31254755	GTTT/GTT	CTCACTTTTCCATTTCCAAACA	CCGACATGACAATGATCAAGA	59.5	313	INTERGENIC	--	--	--

INDEL marker IDs	Chromosomes/unanchored scaffolds	Physical positions (bp)	InDels (*Kabuli* reference genome-CDC Frontier/PII)	Forward primers (5'-3')	Reverse primers (5'-3')	Anneling temperature (0C)	Expected amplified product size (bp)	Structural annotation		Functional annotation	
								Sequence components of *kabuli* genome	*Kabuli* gene accession IDs	NCBI-KOG	TFs
Ca5_P2_31255133	Ca5	31255133	TAAAAAAAAAAAA/TAAAAAAAAAA	TCTTGATCATTGTCATGTCGG	CACCTGCACCTGTCAGAGAA	60.0	573	INTERGENIC	--	--	--
Ca5_P2_31255359	Ca5	31255359	GAATAATAACAATAATA/GAATAATA	TCTTGATCATTGTCATGTCGG	CACCTGCACCTGTCAGAGAA	60.0	573	INTERGENIC	--	--	--
Ca5_P2_31255829	Ca5	31255829	TG/T	AGACGGCAACAAATACTGGC	TGTCTATTTTGGTGTGCGTAAAA	59.6	673	INTERGENIC	--	--	--
Ca5_P2_31256076	Ca5	31256076	TA/TAA	TTTTTGTTGTAACGATGGCTAA	CGCTCAAACACACGTTCATT	59.8	672	INTERGENIC	--	--	--
Ca5_P2_31257262	Ca5	31257262	TATAATAATAATAA/TATAATAATAATAATAATAATAATAA	AAACAGTCACCAAACCAGGC	CACCAAAATGTTGCCTTCAA	59.6	218	INTERGENIC	--	--	--
Ca5_P2_31264749	Ca5	31264749	TTATATATATATAT/TTATATATATATATAT	CTGGAGCTGGTGTGTCATTG	GGCCATTTGCAAAAACTTGT	60.0	374	DRR	Ca_04832	T	WRKY
Ca5_P2_31268811	Ca5	31268811	TAAAAA/TAAAAAA	TCTCCAACGGTTAACTCAACA	TGTGTCAAGTCATCACGGAAA	60.1	523	INTERGENIC	--	--	--
Ca5_P2_31271824	Ca5	31271824	TA/T	CTGCAAGAGGTTTGGCTTTC	TTGATCCCTAGCATGACAAGAA	59.7	719	INTRON	Ca_04833	T	WRKY
Ca5_P2_31272097	Ca5	31272097	AG/A	ACCTATGGTTACGCAGCACC	TGGGATTTTCTGCTTCTTTTATG	59.6	693	INTRON	Ca_04833	T	WRKY
Ca5_P2_31272560	Ca5	31272560	ATTTT/ATTTTT	TTCTTGTCATGCTAGGGATCAAT	TGAAACGGACCTAATCCCTG	59.9	861	INTRON	Ca_04833	T	WRKY
Ca5_P2_31287214	Ca5	31287214	TAAAAAAA/TAAAAAAAAA	TTCTGACGTGACGTATTCTCATC	TATCGCTCAAACAAGCAACG	60.0	470	INTERGENIC	--	--	--
Ca5_P2_31292148	Ca5	31292148	TC/TCC	TCCATCAGCTACCTCCATCC	GTTTGATTTTTGGTGGTGGG	60.1	725	INTERGENIC	--	--	--

INDEL marker IDs	Chromosomes/unanchored scaffolds	Physical positions (bp)	InDels (*Kabuli* reference genome-CDC Frontier/PII)	Forward primers (5'-3')	Reverse primers (5'-3')	Anneling temperature (0C)	Expected amplified product size (bp)	Structural annotation		Functional annotation	
								Sequence components of *kabuli* genome	*Kabuli* gene accession IDs	NCBI-KOG	TFs
Ca5_P2_31292993	Ca5	31292993	GTTG/GTTGAATTCTTTG	AAATGCGGGTTTTCACTGTC	TCTGCTTCCCGTTGTAGCTT	60.0	527	INTERGENIC	--	--	--
Ca5_P2_31302356	Ca5	31302356	CAA/CA	CTTCCTGCAGGGGTATTTCA	ATGCATCCCTGCGAATTAAC	59.9	179	INTERGENIC	--	--	--
Ca5_P2_31305290	Ca5	31305290	CTT/CTTT	ATCCCTCCTCCCTCTTTTGA	CACGGCAATTTCTCCCTAAA	60.1	633	INTERGENIC	--	--	--
Ca5_P2_31311316	Ca5	31311316	CTTTT/CTTTTT	TTGGCACATATGGGGTTTTT	CCAACGCATAAGTCATGTGG	60.0	730	DRR	Ca_04834	--	Nin-like
Ca5_P2_31311984	Ca5	31311984	AACACAC/AACACACAC	CCACATGACTTATGCGTTGG	CATTCACACACATTGCACCA	60.0	312	DRR	Ca_04834	--	Nin-like
Ca5_P2_31317675	Ca5	31317675	TTAAAGTAAA/TTAAA	TGTTTAGGGAATCTGACGCC	TTGCACACAAAACTAAGTGGC	58.9	602	INTERGENIC	--	--	--
Ca5_P2_31319933	Ca5	31319933	ATTTTTTTTTT/ATTTTTTTT	CCTTTACGCCTTGACTCTGC	GATCGATTTCGTGAATTGAAAA	59.1	387	INTERGENIC	--	--	--
Ca5_P2_31321508	Ca5	31321508	TAAAAAAA/TAAAAAAAA	GGGCTTCGTACCAAAAACAA	AAAGAAAACCATGTGCCCAA	60.3	565	INTERGENIC	--	--	--
Ca5_P2_31325751	Ca5	31325751	TAATAAATAAATAAAT/TAATAAATAAATAAATAAATAAAT	TCATACACATTACCCACGCTTT	ACTCGCATAATGGTATGGGC	59.8	598	INTERGENIC	--	--	--
Ca5_P2_31327026	Ca5	31327026	T/TA	AAGGCCTTGTACGGTCATTG	TTGGCATAGTTCTGAAAGACCA	59.7	602	INTERGENIC	--	--	--
Ca5_P2_31333124	Ca5	31333124	CAAAAAAAAAAA/CAAAAAAAAAAAA	CACAGCAGTTTCCATGCAGT	ATGTAAGTCACCCCAAACGC	59.9	236	INTRON	Ca_04835	P	--
Ca5_P2_31333502	Ca5	31333502	TAAAAAAAAAAAAA/TAAAAAAAAAAA	CACAGCAGTTTCCATGCAGT	CCATTCTGAAGAGTTTGCGG	60.8	523	INTRON	Ca_04835	P	--

INDEL marker IDs	Chromosomes/unanchored scaffolds	Physical positions (bp)	InDels (*Kabuli* reference genome-CDC Frontier/PII)	Forward primers (5'-3')	Reverse primers (5'-3')	Anneling temperature (0C)	Expected amplified product size (bp)	Structural annotation		Functional annotation	
								Sequence components of *kabuli* genome	*Kabuli* gene accession IDs	NCBI-KOG	TFs
Ca5_P2_31333742	Ca5	31333742	CAA/CA	GCGTTTGGGGTGACTTACAT	TGGTTCGTTCGTGTGTTGAT	60.0	780	INTRON	Ca_04835	P	--
Ca5_P2_31334829	Ca5	31334829	TATTATAAAAATT/TATT	AATGTCCCCAACCAATTTGA	ATGGGTGATAAATACCTGTTTTG	57.0	774	INTERGENIC	--	--	--
Ca5_P2_31335103	Ca5	31335103	TAA/TAAA	CATTAACGATGGGTTTTCTTTTT	TGAATGTATTGCAATTGTTTTGA	58.2	745	INTERGENIC	--	--	--
Ca5_P2_31337412	Ca5	31337412	AATA/AATATATA	TGATAAAGGAAAGAGCGCGT	TTTTGCAAATTTTCAGAGATCAA	58.9	722	INTERGENIC	--	--	--
Ca5_P2_31338733	Ca5	31338733	GA/GAA	TGAAGAGCTTTGAGAAGAGGAAA	TGCGTTCAGACCAAAACAAG	59.9	562	INTERGENIC	--	--	--
Ca5_P2_31340520	Ca5	31340520	CAACAAACAAA/CAACAAA	AGGCCCAAACCATACAATCA	CACCAGACATCACCACCTTG	60.0	536	INTERGENIC	--	--	--
Ca5_P2_31342686	Ca5	31342686	TAAAAAAA/TAAAAAAAA	ACCCCTTTCAATTGTGATCG	CCGTCCACAACCGTTCTATT	59.9	858	INTERGENIC	--	--	--
Ca5_P2_31355229	Ca5	31355229	TGGAAGGAAGGAAG/TGGAAGGAAG	AATGCCAACAACAACAACCA	AGCTCTTGGGATTTGCTTCA	60.0	699	INTERGENIC	--	--	--
Ca5_P2_31361479	Ca5	31361479	CAAAA/CAAA	GACGGGCTTTTTGTATTCCA	GACATGGGCCTATTGCCTAGT	60.4	461	INTERGENIC	--	--	--
Ca5_P2_31361550	Ca5	31361550	ATGTTT/ATGTTTGTTT	GACGGGCTTTTTGTATTCCA	GACATGGGCCTATTGCCTAGT	60.4	461	INTERGENIC	--	--	--
Ca5_P2_31364177	Ca5	31364177	TTTTC/TTTTCTTTC	TCAACAACCAACATCAAAGTGA	TTTCCCGATTTGGTCCTTTA	59.4	809	INTERGENIC	--	--	--
Ca5_P2_31365376	Ca5	31365376	A/AT	TAACCTGATTCAGAAGCGGC	GTGTCGATAGGACCGCAGTT	60.1	638	INTERGENIC	--	--	--

INDEL marker IDs	Chromosomes/unanchored scaffolds	Physical positions (bp)	InDels (*Kabuli* reference genome-CDC Frontier/PII)	Forward primers (5'-3')	Reverse primers (5'-3')	Anneling temperature (0C)	Expected amplified product size (bp)	Structural annotation		Functional annotation	
								Sequence components of *kabuli* genome	*Kabuli* gene accession IDs	NCBI-KOG	TFs
Ca5_P2_31365681	Ca5	31365681	AAATA/AA	TAACCTGATTCAGAAGCGGC	GTGTCGATAGGACCGCAGTT	60.1	638	INTERGENIC	--	--	--
Ca5_P2_31371469	Ca5	31371469	ATTTTTTTTT/ATTTTTTTT	CAAACGGCTGGAGCATAAAT	GTCCGTTCTTTTGCCATGTT	60.0	717	DRR	Ca_04838	S	--
Ca5_P2_31375160	Ca5	31375160	ATTT/ATT	TTGTCCGTTTGTTACGTGGA	TCCAAAGCCTGAAGCTCAAT	60.0	429	INTERGENIC	--	--	--
Ca5_P2_31375255	Ca5	31375255	GT/GTCATGAATGAAATT	TTGTCCGTTTGTTACGTGGA	TCCAAAGCCTGAAGCTCAAT	60.0	429	INTERGENIC	--	--	--
Ca5_P2_31375720	Ca5	31375720	A/AT	ATTGAGCTTCAGGCTTTGGA	TCGGGTTGAAATTGTTAGGC	59.9	295	INTERGENIC	--	--	--
Ca5_P2_31375927	Ca5	31375927	GTTT/GTTTTT	ATTGAGCTTCAGGCTTTGGA	CAATTCAAATGTTTCAAAACAACA	59.0	767	INTERGENIC	--	--	--
Ca5_P2_31386154	Ca5	31386154	ATA/ATATTTAGTA	ATGTCGACTGCGTGACTACG	TTGCTCTTACAGGCGAAAGG	60.5	759	DRR	Ca_04840	S	--
Ca5_P2_31392948	Ca5	31392948	T/TGTAATG	TTGGTGGAGAAACTGATAAACG	TAGGGTCGTGTCAGTGGTGA	60.2	259	INTERGENIC	--	--	--
Ca5_P2_31393346	Ca5	31393346	GTATATATATA/GTATATATATATA	TCACCACTGACACGACCCTA	TGCCCGTAGTTTTTCTTGTG	58.8	522	INTERGENIC	--	--	--
Ca5_P2_31396550	Ca5	31396550	GAGACCAGACCAG/GAGACCAG	GAGCATTAGTGCTTGGCACA	CTGTGATCTGCCAGTTGCAT	59.9	335	INTRON	Ca_04842	--	MIKC
Ca5_P2_31401482	Ca5	31401482	GTGTTGACT/GTGTTGACTACCTGTTGACT	TCATGGGAGAACCAAAGGAG	GCTGTCCTCCCATTCCACTA	60.1	626	DRR	Ca_04843	--	--
Ca5_P2_31408248	Ca5	31408248	ATTTCATTT/ATTTCATTTTCATTT	ATTTGTCTTCCGTTTGTCCG	AACGAGTGGACCTGGTAACG	60.0	555	INTRON	Ca_04843	--	--

INDEL marker IDs	Chromosomes/unanchored scaffolds	Physical positions (bp)	InDels (*Kabuli* reference genome-CDC Frontier/PII)	Forward primers (5'-3')	Reverse primers (5'-3')	Anneling temperature (0C)	Expected amplified product size (bp)	Structural annotation		Functional annotation	
								Sequence components of *kabuli* genome	*Kabuli* gene accession IDs	NCBI-KOG	TFs
Ca5_P2_31408843	Ca5	31408843	ATGAAATTGAAATTG/ATGAAATTG	CCGGCAAGTGATTGTTAGGT	TCACACTCACACTCTTCCGC	60.0	291	INTERGENIC	--	--	--
Ca5_P2_31409770	Ca5	31409770	CA/CAAAAAAATTAA	AGGGCGGATTAGTTTTCGAG	TTGAAGAATTTGATGTTGTTGGA	59.5	557	INTERGENIC	--	--	--
Ca5_P2_31412254	Ca5	31412254	AATTA/AA	CACGTGTCCATCAAAATTGC	TGCAAGCGAATCAAACAAAA	60.4	721	INTERGENIC	--	--	--
Ca5_P2_31423555	Ca5	31423555	GATTTAATTTA/GATTTA	TGGATTTGTTGTGCTACCCA	TCGAGACGGAAGAGAACGAT	60.0	301	INTERGENIC	--	--	--
Ca5_P2_31429862	Ca5	31429862	ATTTTTTTTTTT/ATTTTTTTTT	CAAAGGAAAATCGCCACAGT	AGGGCCTACAGCCTCTGTTT	60.3	809	INTERGENIC	--	--	--
Ca5_P2_31434842	Ca5	31434842	TA/TCATATAATAAA	AGGCGTCTTCCAACTTTTCA	TGTCACCAATCGGATTTCAA	59.9	615	INTERGENIC	--	--	--
Ca5_P2_31435124	Ca5	31435124	CTTTTT/CTTTTTT	TTGAAATCCGATTGGTGACA	ATTTGGTGTCCCTTGTCTCG	60.0	538	INTERGENIC	--	--	--
Ca5_P2_31435328	Ca5	31435328	ATTTTTT/ATTTTT	TTGAAATCCGATTGGTGACA	ATTTGGTGTCCCTTGTCTCG	60.0	538	INTERGENIC	--	--	--
Ca5_P2_31435353	Ca5	31435353	AAA/AAAAATAA	TTGAAATCCGATTGGTGACA	ATTTGGTGTCCCTTGTCTCG	60.0	538	INTERGENIC	--	--	--
Ca5_P2_31441846	Ca5	31441846	TAAAAAAAA/TAAAAAAA	AATGATCGTTGAAGCATCCC	GCCTCAATGACCCTTGACAT	59.9	742	INTERGENIC	--	--	--
Ca5_P2_31442562	Ca5	31442562	CT/C	GTCAAGGGTCATTGAGGCAT	GCCTAGTAGAACCAAGGGGG	60.0	557	INTERGENIC	--	--	--
Ca5_P2_31444478	Ca5	31444478	AC/ACC	AACAGCGACAACTTTTTGCC	TCCGAAATGTGCTTATTGGG	60.8	550	INTERGENIC	--	--	--

INDEL marker IDs	Chromosomes/unanchored scaffolds	Physical positions (bp)	InDels (*Kabuli* reference genome-CDC Frontier/PII)	Forward primers (5'-3')	Reverse primers (5'-3')	Anneling temperature (0C)	Expected amplified product size (bp)	Structural annotation		Functional annotation	
								Sequence components of *kabuli* genome	*Kabuli* gene accession IDs	NCBI-KOG	TFs
Ca5_P2_31444498	Ca5	31444498	CTTTTTTTTTT/CTTTTTTTT	AACAGCGACAACTTTTTGCC	TCCGAAATGTGCTTATTGGG	60.8	550	INTERGENIC	--	--	--
Ca5_P2_31455329	Ca5	31455329	CT/CTT	TTGCTTTTAGGGCGTCATCT	TATGCACGTTGTTGGGCTAA	60.1	458	INTERGENIC	--	--	--
Ca5_P2_31456132	Ca5	31456132	TAAAAAA/TAAAAA	GAAAAAGAATCCACGACCGA	GGCGTGGAAGATATCGAAAA	60.0	443	INTERGENIC	--	--	--
Ca5_P2_31456646	Ca5	31456646	CTTTTTTTTTTTTT/CTTTTTTTTTTTT	TTTTCGATATCTTCCACGCC	TATGGCAAAAAGTTGTCGCA	60.3	727	INTERGENIC	--	--	--
Ca5_P2_31457540	Ca5	31457540	ATT/ATTT	TGCGACAACTTTTTGCCATA	TGGGGCTGCTTGTTTAAGAT	59.7	813	INTERGENIC	--	--	--
Ca5_P2_31460612	Ca5	31460612	AC/A	TTTGATCAATTCACGCGTTC	TGGCACGTGAACATTTCTTT	59.2	874	DRR	Ca_04847	--	--
Ca5_P2_31463048	Ca5	31463048	GTTTTTT/GTTTTTTT	GAGGTGATCCAAAGCCATGT	ATAAACAGGGACCATGCCAC	59.7	771	INTERGENIC	--	--	--
Ca5_P2_31465989	Ca5	31465989	TTATC/TTATCTATC	TTGCACAGCTGCAAATTCA	TAGGGGTATGGCCACTTTTG	59.8	555	INTERGENIC	--	--	--
Ca5_P2_31474537	Ca5	31474537	C/CA	TTTTTGTTGTAGTTTTCTTTTTGGG	TTGGAGAAATCCAAATAACCTC	57.2	640	INTRON	Ca_04848	--	--
Ca5_P2_31474883	Ca5	31474883	ATATAATTGTTATAATTGT/ATATAATTGT	CGATAGAATCATGCTACAAAATGTG	AGTGCTCTCCCCAAGCTACA	60.0	705	INTRON	Ca_04848	--	--
Ca5_P2_31481808	Ca5	31481808	TG/T	GATGGTTTGGTTGTTTTGGG	ACTACAGTCCACCGGCCTTA	59.6	507	INTERGENIC	--	--	--
Ca5_P2_31489071	Ca5	31489071	TA/TAA	GATTCAGGGCCACGTCATAG	TGCACCATGCAGTATCAACA	59.7	359	INTRON	Ca_04850	E	--

INDEL marker IDs	Chromosomes/unanchored scaffolds	Physical positions (bp)	InDels (*Kabuli* reference genome-CDC Frontier/PII)	Forward primers (5'-3')	Reverse primers (5'-3')	Anneling temperature (0C)	Expected amplified product size (bp)	Structural annotation		Functional annotation	
								Sequence components of *kabuli* genome	*Kabuli* gene accession IDs	NCBI-KOG	TFs
Ca5_P2_31504924	Ca5	31504924	GGTGTGTGTGT/GGTGTGTGTGTGT	GCATGTGGAACACACCTCAC	AAATGGCACATTGAAAAGGC	59.9	805	INTRON	Ca_04853	Q	--
Ca5_P2_31505255	Ca5	31505255	ATTTTTTTT/ATTTTTTTTT	ATTTCGCGGGAGGAGTAAGT	TGGGGGAGAGTTGTCTTTTG	60.1	691	INTRON	Ca_04853	Q	--
Ca5_P2_31505281	Ca5	31505281	TAAAAAAAAAAA/TAAAAAAAAAA	TTTACCCACTTGGAGTTCGC	TGGGGGAGAGTTGTCTTTTG	60.1	568	INTRON	Ca_04853	Q	--
Ca5_P2_31506180	Ca5	31506180	TAAA/TAAAA	GGCCCATCCATTTACCTTCT	CCAGCTGCTTTGTGAAATGA	60.0	748	INTRON	Ca_04853	Q	--
Ca5_P2_31507991	Ca5	31507991	TTATA/TTA	GTCTTCGCCTATGTTTGGGA	GAATTCGGCTTTGCAAGACT	59.5	341	INTRON	Ca_04853	Q	--
Ca5_P2_31509118	Ca5	31509118	ACCCCC/ACCCC	TGGCCAATACCCCAAAGTAA	CTGAATGCGGAAGTTAAGGG	59.7	596	INTRON	Ca_04853	Q	--
Ca5_P2_31509396	Ca5	31509396	GTTTTTTTTTTT/GTTTTTTTTTTTTTTTT	TGGCCAATACCCCAAAGTAA	CTGAATGCGGAAGTTAAGGG	59.7	596	INTRON	Ca_04853	Q	--
Ca5_P2_31526667	Ca5	31526667	CTTTTTT/CTTTTTTT	GGATGTTTATTGGTCATTTTTCG	TTGTGAAATGCACGAGAGAA	58.0	254	INTERGENIC	--	--	--
Ca5_P2_31528715	Ca5	31528715	CTTTGCTTTT/CTTTGCTTTTTGCTTTT	TCCATAAACCAAATCGAGCA	CACCGTGAAAGAAAAAGATGAG	58.9	806	INTERGENIC	--	--	--
Ca5_P2_31528942	Ca5	31528942	GA/G	CAATTTTGATCATTCAATCTTTGC	CACCGTGAAAGAAAAAGATGAG	58.9	454	INTERGENIC	--	--	--
Ca5_P2_31532842	Ca5	31532842	CTT/CTTT	AAGCAAAAGGGAGGGATGAT	CCTGAAAACTCAACCCAAGG	59.6	577	INTERGENIC	--	--	--
Ca5_P2_31535070	Ca5	31535070	T/TGTTATTTA	CACTCTTCTTTCCATCCCCA	GAAGTCATTCACTGAAGCCCA	60.2	687	INTERGENIC	--	--	--

INDEL marker IDs	Chromosomes/unanchored scaffolds	Physical positions (bp)	InDels (*Kabuli* reference genome-CDC Frontier/PII)	Forward primers (5'-3')	Reverse primers (5'-3')	Anneling temperature (0C)	Expected amplified product size (bp)	Structural annotation		Functional annotation	
								Sequence components of *kabuli* genome	*Kabuli* gene accession IDs	NCBI-KOG	TFs
Ca5_P2_31535350	Ca5	31535350	A/AGTTGGTT	CTCCCTCGATTATGATGTCCA	AATTGACGGGTTCAAGCAGA	60.6	771	INTERGENIC	--	--	--
Ca5_P2_31539135	Ca5	31539135	GTTGAA/GTTGAATTGAA	GCACATATGAAGTAATCGAGCG	AACAGACCATTATCCTGGTCAAC	59.3	209	INTERGENIC	--	--	--
Ca5_P2_31539485	Ca5	31539485	TAAAAA/TAAAA	CGCACATATGAAGTAATCGAGC	AAACGCCTTTTGTGAACACC	60.0	863	INTERGENIC	--	--	--
Ca5_P2_31539844	Ca5	31539844	GTTTT/GTT	TGTTTGAAGGACCCAATGAA	ACCGGAGAGGGCATAAAAGT	60.0	146	INTERGENIC	--	--	--
Ca5_P2_31545245	Ca5	31545245	AT/ATT	GTAATTTTGCGCCGATTGTT	TTTTGCACACGCAAATTCAT	60.1	588	INTERGENIC	--	--	--
Ca5_P2_31550362	Ca5	31550362	TA/TAA	CAGATACCATCAGCGCTTCA	CAAGGAAACGAAAAGCATCA	58.9	848	INTERGENIC	--	--	--
Ca5_P2_31551541	Ca5	31551541	GAA/GA	TTACAGATGCACGGCTGAAG	GGAGTGAAATCCCAGCAAAA	60.1	213	INTERGENIC	--	--	--
Ca5_P2_31551885	Ca5	31551885	GTTTTTTTTT/GTTTTTTTT	TTACAGATGCACGGCTGAAG	TGTGAGATTCATCTAAATTGAACATT	58.2	747	INTERGENIC	--	--	--
Ca5_P2_31552568	Ca5	31552568	ATTTTTTT/ATTTTTT	AATCAAATGTGGGTACGGGA	CGAGCTTTTCCACTCTAGCAA	59.8	190	INTERGENIC	--	--	--
Ca5_P2_31553334	Ca5	31553334	GT/GTGTAT	TTCAGGCATTGAAGAGCAGA	AACGGTTACGCAGCAGAGAT	59.9	286	INTERGENIC	--	--	--
Ca5_P2_31553471	Ca5	31553471	CAA/CAAA	TTCAGGCATTGAAGAGCAGA	AACGGTTACGCAGCAGAGAT	59.9	286	INTERGENIC	--	--	--
Ca5_P2_31555586	Ca5	31555586	GTTTT/GTTT	AAGGTGGGACCCCAATTATC	CTGAAAGGATCTGACTGGCG	60.9	620	DRR	Ca_04857	K	--

INDEL marker IDs	Chromosomes/unanchored scaffolds	Physical positions (bp)	InDels (*Kabuli* reference genome-CDC Frontier/PII)	Forward primers (5'-3')	Reverse primers (5'-3')	Anneling temperature (0C)	Expected amplified product size (bp)	Structural annotation		Functional annotation	
								Sequence components of *kabuli* genome	*Kabuli* gene accession IDs	NCBI-KOG	TFs
Ca5_P2_31563195	Ca5	31563195	AATAA/AATAAGTGATAA	CACTCCCTGTCTCGTTTTCC	CGAACGAGACTGTCAGCAAG	59.8	313	INTERGENIC	--	--	--
Ca5_P2_31563660	Ca5	31563660	TA/T	CTCCATGATTCTTTTCCCCA	GTGAAGCACGAGAGTAGGGG	59.9	564	INTERGENIC	--	--	--
Ca5_P2_31564314	Ca5	31564314	TAAAAAAAA/TAAAAAA	CCCCTACTCTCGTGCTTCAC	CTCATATGTGAAATCCCGCC	60.3	917	INTERGENIC	--	--	--
Ca5_P2_31566906	Ca5	31566906	CACA/CCAACA	TTTGGGACAATTTTGTTTTGC	TGGGTCAAGTTTAGGTCGCT	59.7	716	DRR	Ca_04858	E	--
Ca5_P2_31567967	Ca5	31567967	CTTATTA/CTTA	CACATTTGCAATGTTTCTTCG	CCGGTCAATATTTTGCGAGT	60.0	536	DRR	Ca_04858	E	--
Ca5_P2_31568304	Ca5	31568304	AATATATATAT/AATATATAT	TCAACCCACGTGCTTTTGTA	CCGGTCAATATTTTGCGAGT	60.0	196	INTERGENIC	--	--	--
Ca5_P2_31568829	Ca5	31568829	CTTTTTTTT/CTTTTTTT	TGCGACGGAATGTCTCATTA	GCATTGGCATGTGAAAAATG	59.9	721	INTERGENIC	--	--	--
Ca5_P2_31569100	Ca5	31569100	CAAT/C	GCCGACAAACAAATACAGTGA	GCATTGGCATGTGAAAAATG	59.9	204	INTERGENIC	--	--	--
Ca5_P2_31584538	Ca5	31584538	TAAAAAA/TAAAAAAA	TTTGAGGCACCGATGATAGA	GAGGAGGATGTGTCCACGTT	60.0	530	INTERGENIC	--	--	--
Ca5_P2_31585122	Ca5	31585122	GTTTT/GTTTTT	AACGTGGACACATCCTCCTC	CTAGCTGAAGTTCATGCGCC	61.1	181	DRR	Ca_04861	R	--
Ca5_P2_31585436	Ca5	31585436	TAAAAAAAAA/TAAAAAAAAAAAAA	AACGTGGACACATCCTCCTC	ACACGGAGGGAGTGTTGGTA	60.4	954	DRR	Ca_04861	R	--
Ca5_P2_31585638	Ca5	31585638	TTTAAATATTAAATATTAAAT/TTTAAATATTAAATATTAAATATTAAAT	CACAGAATTTGGTGGCATTG	TGATCATGCGTGGATTTTGT	59.9	745	DRR	Ca_04861	R	--

INDEL marker IDs	Chromosomes/unanchored scaffolds	Physical positions (bp)	InDels (*Kabuli* reference genome-CDC Frontier/PII)	Forward primers (5'-3')	Reverse primers (5'-3')	Anneling temperature (0C)	Expected amplified product size (bp)	Structural annotation		Functional annotation	
								Sequence components of *kabuli* genome	*Kabuli* gene accession IDs	NCBI-KOG	TFs
Ca5_P2_31586253	Ca5	31586253	TTATCAAAAAGAAAATATCAA/TTATCAA	TCCACGCATGATCAAATTGT	TATTGCTCAAAGTGGAGCCC	60.2	640	DRR	Ca_04861	R	--
Ca5_P2_31587183	Ca5	31587183	CAA/C	TGGAACCAAAACATGCTTCA	TAACGGGTGAGTGAAAAGCC	60.1	682	INTRON	Ca_04861	R	--
Ca5_P2_31593650	Ca5	31593650	AATAT/AAT	CCCGATACAAGTCGGACACT	AATTTAATCCTCCATCCCCG	60.0	576	INTRON	Ca_04861	R	--
Ca5_P2_31596027	Ca5	31596027	A/AGAGATATATTG	CAATATCAAACATCGCCACTCA	TATTGAGGGTGGTGGCTTTC	59.9	616	INTRON	Ca_04861	R	--
Ca5_P2_31596044	Ca5	31596044	GATATAT/GATAT	CAATATCAAACATCGCCACTCA	TATTGAGGGTGGTGGCTTTC	59.9	616	INTRON	Ca_04861	R	--
Ca5_P2_31597751	Ca5	31597751	CTT/CT	AACCCACAAATCCAACACAG	CCGCATTGAGTCTCCATTTT	60.1	473	INTERGENIC	--	--	--
Ca5_P2_31599963	Ca5	31599963	AC/ACC	GGTATGTGGAGAGCATCACG	TGGTTGCAAAGTGTGTGTGA	59.8	231	INTERGENIC	--	--	--
Ca5_P2_31602427	Ca5	31602427	AACC/A	ACTGAGCCTTCGTTGAATGG	TGGACCAATTTCGTGAATGA	59.9	573	INTERGENIC	--	--	--
Ca5_P2_31602535	Ca5	31602535	CAAAAAAAAAA/CAAAAAAAA	ACTGAGCCTTCGTTGAATGG	TGGACCAATTTCGTGAATGA	59.9	573	INTERGENIC	--	--	--
Ca5_P2_31606958	Ca5	31606958	CA/C	GGGGGTGATTCTGGAATTTT	CAAAAGATTCCTCCGATCCA	60.0	485	DRR	Ca_04862	IR	--
Ca5_P2_31609157	Ca5	31609157	AATAATAATAATAATAA/AATAATAATAATAATAATAA	TGATCCCAAGTGGCCTAAAG	GCAGCCTGCTTTTAATGGAC	59.9	290	DRR	Ca_04863	--	--
Ca5_P2_31609524	Ca5	31609524	T/TGTATATGAA	GCAGGCTGCCTATTTGAGTC	ATGTAGCAAACTTGCGGGTC	60.1	627	DRR	Ca_04863	--	--

INDEL marker IDs	Chromosomes/unanchored scaffolds	Physical positions (bp)	InDels (*Kabuli* reference genome-CDC Frontier/PII)	Forward primers (5'-3')	Reverse primers (5'-3')	Anneling temperature (0C)	Expected amplified product size (bp)	Structural annotation		Functional annotation	
								Sequence components of *kabuli* genome	*Kabuli* gene accession IDs	NCBI-KOG	TFs
Ca5_P2_31610317	Ca5	31610317	AA/AAAGAAAAATA	TCCCAATCAAATCGGGAATA	ATGGACCAAACCTTCATTGG	59.6	180	DRR	Ca_04863	--	--
Ca5_P2_31610741	Ca5	31610741	CAAAAA/CAAAAAA	ACAACCCAATCCCAATCAAA	GGGGAGGAGAAGGAAATCAG	60.0	939	DRR	Ca_04863	--	--
Ca5_P2_31610778	Ca5	31610778	TAAAAAAAA/TAAAAAAA	ACAACCCAATCCCAATCAAA	GGGGAGGAGAAGGAAATCAG	60.0	939	DRR	Ca_04863	--	--
Ca5_P2_31611802	Ca5	31611802	TGGG/TGG	TTCTTACCTGGGCTGAATGG	GCAGGCTGTTTCACAACTGA	60.0	689	INTERGENIC	--	--	--
Ca5_P2_31613486	Ca5	31613486	GATATA/GATATATCATATA	TCAACGAGTCGTAAAAGGGG	GTCCCAACTCGGAATCTGAA	60.1	418	DRR	Ca_04864	R	B3
Ca5_P2_31621509	Ca5	31621509	CT/CTGTATATGTACTTT	ATCCGAGTTTGCGTATGTCC	GGGACGTGGAGATTGTTGAT	59.8	635	INTERGENIC	--	--	--
Ca5_P2_31622785	Ca5	31622785	C/CT	GGCGTTTTTGTTTTGGAGAA	CAAGGCAACAAACGATAGCA	59.9	328	INTRON	Ca_04865	J	GRAS
Ca5_P2_31623290	Ca5	31623290	TGG/TGGG	GCAAGTCCTTGCAGCCTAAC	TTGAAGGATCAAAGGGTTCG	60.0	356	INTRON	Ca_04865	J	GRAS
Ca5_P2_316293	Ca5	316293	AATATATATATATATATATATAT/AATATATATATATATATAT	TTCTACCTTTCTCGCCGCTA	TTAATTGAAAGTGGGGGTGAA	59.3	562	INTERGENIC	--	--	--
Ca5_P2_31630101	Ca5	31630101	T/TG	TCAATCACCATTAATCACAATCAA	CTGGGGATTGATTCATTTGG	60.1	341	INTERGENIC	--	--	--
Ca5_P2_31630256	Ca5	31630256	AATCACATCACATCACATCA/AATCACATCACATCA	CCAAATGAATCAATCCCCAG	GATGATCCCTGAGGCAGAAA	60.2	438	INTERGENIC	--	--	--
Ca5_P2_31634691	Ca5	31634691	TA/T	GAAGCACCAGCAAAGTAGCC	TTGACATTGACCGATATATTTAGTCTT	58.6	373	INTERGENIC	--	--	--

INDEL marker IDs	Chromosomes/unanchored scaffolds	Physical positions (bp)	InDels (*Kabuli* reference genome-CDC Frontier/PII)	Forward primers (5'-3')	Reverse primers (5'-3')	Anneling temperature (0C)	Expected amplified product size (bp)	Structural annotation		Functional annotation	
								Sequence components of *kabuli* genome	*Kabuli* gene accession IDs	NCBI-KOG	TFs
Ca5_P2_31639443	Ca5	31639443	TAAAAAAAAAAAAAA/TAAAAAAAAAAAA	CGGTCAACTACGATGTTGCTT	TTTCACAATTTTGGTCCATCT	57.0	541	INTERGENIC	--	--	--
Ca5_P2_31639526	Ca5	31639526	TAAAA/TAAAAA	CGGTCAACTACGATGTTGCTT	AAATTGATTCGCACTTTTGGTT	59.9	634	INTERGENIC	--	--	--
Ca5_P2_31642001	Ca5	31642001	ATTTT/ATTTTT	TGGCAACTAAGGCATCTCTG	TTGGGGATGAAAATGGAAAA	60.1	779	INTERGENIC	--	--	--
Ca5_P2_31648841	Ca5	31648841	CTTGATT/CT	TGACTGGTTTGGCACTTCAG	GAGATTTTGAGCCAGCTTCG	60.1	811	DRR	Ca_04866	A	GATA
Ca5_P2_31648877	Ca5	31648877	TTTAAATCT/TTTAAATCTTTAAATCT	TGACTGGTTTGGCACTTCAG	GAGATTTTGAGCCAGCTTCG	60.1	811	DRR	Ca_04866	A	GATA
Ca5_P2_31652822	Ca5	31652822	TA/T	AGGTATGTTTTCCGGCACAG	CTCCAACCGTTATGGCAACT	60.0	514	INTRON	Ca_04866	A	GATA
Ca5_P2_31656236	Ca5	31656236	CTAGTTGTA/CTA	AACTTTCACCTGCACCAACC	ATACCGAGCTCCCTTCGATT	60.1	695	INTERGENIC	--	--	--
Ca5_P2_31656516	Ca5	31656516	AA/AATGTTTAATAGA	GACAAAAGCATGGTGGTCTG	ATACCGAGCTCCCTTCGATT	60.1	221	INTERGENIC	--	--	--
Ca5_P2_31658056	Ca5	31658056	ATTT/ATT	CAGCATTGCACAACCAAACT	CAGATTTCCTCACCTTCACACA	60.1	618	INTERGENIC	--	--	--
Ca5_P2_31658090	Ca5	31658090	GTTTTTTTT/GTTTTTTT	CAGCATTGCACAACCAAACT	CAGATTTCCTCACCTTCACACA	60.1	618	INTERGENIC	--	--	--
Ca5_P2_31659792	Ca5	31659792	TTT/TTTAATT	TAACCTACACCGCATCCACA	TTTTATCACATCCTTCATTTGGG	60.1	667	INTERGENIC	--	--	--
Ca5_P2_31662583	Ca5	31662583	TAAAAAAA/TAAAAAAAAA	CAATGGCTCTTGATGGTTCC	AAGTGGTGGCCTCTGTTTTT	58.7	854	INTERGENIC	--	--	--

INDEL marker IDs	Chromosomes/unanchored scaffolds	Physical positions (bp)	InDels (*Kabuli* reference genome-CDC Frontier/PII)	Forward primers (5'-3')	Reverse primers (5'-3')	Anneling temperature (0C)	Expected amplified product size (bp)	Structural annotation		Functional annotation	
								Sequence components of *kabuli* genome	*Kabuli* gene accession IDs	NCBI-KOG	TFs
Ca5_P2_31667322	Ca5	31667322	TAAA/TAAAA	GTGGCGAAGAAGGAGGTAGA	CAAGTCCTGCAGCTGATTTTT	59.5	941	INTERGENIC	--	--	--
Ca5_P2_31669569	Ca5	31669569	GTTTT/GTTTTT	CAACATCGGTTGGGTCTTTC	CAGAGCAGTTTGCAGTTTGG	59.6	319	INTERGENIC	--	--	--
Ca5_P2_31678718	Ca5	31678718	AAAGAAGA/AAAGA	GCCATGAGATTTTTCGTGCT	CTCTGCATGCACCTAGACCA	60.0	214	INTERGENIC	--	--	--
Ca5_P2_31681318	Ca5	31681318	AT/ATTATACTTT	ACTACTCACCCACACTCCCG	AGCAGCAGCAGCAGTAATCA	59.9	442	DRR	Ca_04870	--	--
Ca5_P2_31685020	Ca5	31685020	CTTTTTTT/CTTTTTTTT	AGGCTGTGACCCTAGTTCCA	TTCATTTTGTGGCGTGTCAT	60.0	725	INTERGENIC	--	--	--
Ca5_P2_31685319	Ca5	31685319	ATTTT/ATTTTT	AACCGAAATGTTTCCCACAC	TTCATTTTGTGGCGTGTCAT	60.0	600	INTERGENIC	--	--	--
Ca5_P2_31687603	Ca5	31687603	ATTTCTTTCTTTCTTTCTT/ATTTCTTTCTTTCTT	TCACTCCTGATTTTGAGGGC	TTGCCAGAATCAATGGATGA	60.0	505	INTERGENIC	--	--	--
Ca5_P2_31691708	Ca5	31691708	TA/TAA	CCGTTCAACCTCTGCATCTT	TTGAGTGATGTGGCAAAGAGA	59.4	746	DRR	Ca_04871	OJ	bZIP
Ca5_P2_31693187	Ca5	31693187	AATA/AA	GGCTAAGCCCCAAGAATCTC	TGTGAAAATAACACAGGTAGAACCA	59.9	662	INTERGENIC	--	--	--
Ca5_P2_31694006	Ca5	31694006	GGAATGAATGAATGAATGA/GGAATGAATGAATGAATGAATGA	CCAAGCATGCTCATCAGAAA	TGAGAATGGGATCAGGGAAG	60.0	484	INTRON	Ca_04872	--	G2-like
Ca5_P2_31699777	Ca5	31699777	GTTTTTTTTTT/GTTTTTTTT	CCACAAGAACAATTATGCCAAA	ATGCATTCGCATAAGTCACG	59.7	613	INTERGENIC	--	--	--
Ca5_P2_31699872	Ca5	31699872	CTT/CTTT	CCACAAGAACAATTATGCCAAA	ATGCATTCGCATAAGTCACG	59.7	613	INTERGENIC	--	--	--

INDEL marker IDs	Chromosomes/unanchored scaffolds	Physical positions (bp)	InDels (*Kabuli* reference genome-CDC Frontier/PII)	Forward primers (5'-3')	Reverse primers (5'-3')	Anneling temperature (0C)	Expected amplified product size (bp)	Structural annotation		Functional annotation	
								Sequence components of *kabuli* genome	*Kabuli* gene accession IDs	NCBI-KOG	TFs
Ca5_P2_31701207	Ca5	31701207	TTAT/TTATAT	ATACGCATGCATCAACTCCA	GAAGCAACTTCCATCGGAGA	60.3	614	INTERGENIC	--	--	--
Ca5_P2_31701551	Ca5	31701551	TTTA/TTTAATTA	CATCTCCGATGGAAGTTGCT	CCCAAAATCAACCAAAGCTC	59.5	559	INTERGENIC	--	--	--
Ca5_P2_31705393	Ca5	31705393	AAC/AACGTAC	ATTTGTGTTGTGGGGTCCAT	CCCACCTTGTGCTGTACCTT	60.0	231	INTRON	Ca_04873	--	G2-like
Ca5_P2_31706402	Ca5	31706402	TAAAAAAAAAA/TAAAAAAAA	AACGGTGTAGCATTCGGTGT	CGTGGGTCAGTTGAGTTTGA	59.7	367	INTRON	Ca_04873	--	G2-like
Ca5_P2_31707305	Ca5	31707305	ATTTTTTTT/ATTTTTTTTTT	CGCCAGTTTAGATGATGCAA	ATTTGGGTGACACTTCCTGC	60.0	505	INTRON	Ca_04873	--	G2-like
Ca5_P2_31710056	Ca5	31710056	CA/CAGA	GCATTGAGTCAAACAGCCAA	TTTTGCTTGAAGCCAGGTCT	60.0	353	DRR	Ca_04874	--	--
Ca5_P2_31714891	Ca5	31714891	T/TG	GCAGCATCAAACACCCCTAT	GTGCACTTTGCCTCCACTTT	60.3	171	INTRON	Ca_04875	--	FAR1
Ca5_P2_31716024	Ca5	31716024	AAA/AAATAA	CAATGAAACCCTGCAACCTT	CACGAGAATCTCCCCTACCA	60.1	717	INTRON	Ca_04875	--	FAR1
Ca5_P2_31716104	Ca5	31716104	TC/T	CAATGAAACCCTGCAACCTT	CACGAGAATCTCCCCTACCA	60.1	717	INTRON	Ca_04875	--	FAR1
Ca5_P2_31719625	Ca5	31719625	CA/CATA	AGTTTTTCCATTGGCGTTCA	TTGGTTTGTGGAAGAAGGGT	59.4	895	INTERGENIC	--	--	--
Ca5_P2_31720853	Ca5	31720853	CCTTCTTC/CCTTC	TCCTATACACGTCGGTGATGTT	TCCATTTTTCAGATGGACCC	59.7	521	INTERGENIC	--	--	--
Ca5_P2_31720937	Ca5	31720937	TTA/T	CGTCGGTGATGTTTTTCCTT	TCCATTTTTCAGATGGACCC	59.7	512	INTERGENIC	--	--	--

INDEL marker IDs	Chromosomes/unanchored scaffolds	Physical positions (bp)	InDels (*Kabuli* reference genome-CDC Frontier/PII)	Forward primers (5'-3')	Reverse primers (5'-3')	Anneling temperature (0C)	Expected amplified product size (bp)	Structural annotation		Functional annotation	
								Sequence components of *kabuli* genome	*Kabuli* gene accession IDs	NCBI-KOG	TFs
Ca5_P2_31722791	Ca5	31722791	ATAGCTA/ATAGCTAGCTA	TAGGTGAAAGCATGCAAACG	GCTTGTGCCTACCAAAGTCC	59.7	486	DRR	Ca_04876	R	bHLH
Ca5_P2_31726199	Ca5	31726199	T/TC	AGCAACCTCTGTCCACGACT	CATCATTGTCAACCCCAAAA	59.2	556	INTRON	Ca_04876	R	bHLH
Ca5_P2_31726458	Ca5	31726458	ATTTTTTTT/ATTTTTTTTTT	GCAACCTCTGTCCACGACTT	TTGGAGCACGTAGAATTGGA	59.3	772	INTRON	Ca_04876	R	bHLH
Ca5_P2_31726817	Ca5	31726817	ATTTTTT/ATTTTTTT	TCCAATTCTACGTGCTCCAA	CTCAAACCAAGCGACCTTGT	60.3	565	INTRON	Ca_04876	R	bHLH
Ca5_P2_31728220	Ca5	31728220	GATATATATATATATATAT/GATATATATATATATAT	ATGGTTGCTCATAGACATCGT	GCATCACCTCCACCTTGAAT	59.9	830	INTERGENIC	--	--	--
Ca5_P2_31728341	Ca5	31728341	GATATA/GATATATATA	TTTTTCTTTGGAAATTTTGACC	GCATCACCTCCACCTTGAAT	59.9	501	INTERGENIC	--	--	--
Ca5_P2_31730522	Ca5	31730522	CTTT/CTT	CCATAGATGTGACTCGTATGATTG	GCTTCCAACTCCAAGCAAGA	60.5	710	INTERGENIC	--	--	--
Ca5_P2_31732127	Ca5	31732127	CTGT/CT	TGCTAGCTTCTTCCCACGTT	AAAACGAACAAAAATAACATTCG	57.4	306	INTERGENIC	--	--	--
Ca5_P2_31747598	Ca5	31747598	T/TC	TTTAGTTGGTGAGTCGAGCG	ACATGGAAAACATGAGGGAA	57.8	553	INTERGENIC	--	--	--
Ca5_P2_31754975	Ca5	31754975	TTAT/TT	TTTGGTGCGAGTACACTTCC	TGAGGATTTGGTTGAGAGGTG	60.1	261	INTERGENIC	--	--	--
Ca5_P2_31755077	Ca5	31755077	CTTT/CT	TACACTTCCGTGCACCGTTA	TGAGGATTTGGTTGAGAGGTG	60.1	250	INTERGENIC	--	--	--
Ca5_P2_31759396	Ca5	31759396	G/GC	CCACAAGCAGCACAAAGTGT	GGATTAGCGCGTCTGACTTC	60.0	490	DRR	Ca_04880	O	MYB_related

INDEL marker IDs	Chromosomes/unanchored scaffolds	Physical positions (bp)	InDels (*Kabuli* reference genome-CDC Frontier/PII)	Forward primers (5'-3')	Reverse primers (5'-3')	Anneling temperature (0C)	Expected amplified product size (bp)	Structural annotation		Functional annotation	
								Sequence components of *kabuli* genome	*Kabuli* gene accession IDs	NCBI-KOG	TFs
Ca5_P2_31760008	Ca5	31760008	GTTT/GTT	GAAGTCAGACGCGCTAATCC	AATGCTTATGCAGGAGTGGG	60.1	660	DRR	Ca_04880	O	MYB_related
Ca5_P2_31760331	Ca5	31760331	TCCC/TCC	CTCCAATGTAGAGGGGTCCA	TTGTTCATCGTAACCCCACA	59.8	316	DRR	Ca_04880	O	MYB_related
Ca5_P2_31760524	Ca5	31760524	ATT/ATGAGGGATTT	CTCCAATGTAGAGGGGTCCA	TTGTGATCGGAAAATTGGTTT	59.3	766	INTERGENIC	--	--	--
Ca5_P2_31762104	Ca5	31762104	TAA/TA	TAATGTGAACGCGCATGATT	AAGAGCAACCTAGTCCGCAA	60.0	229	DRR	Ca_04881	--	--
Ca5_P2_31769395	Ca5	31769395	ATTTATT/A	CTCCAACGGTGAACTCAACA	TTGGAGTTGTGCCAATGTGT	60.0	563	INTERGENIC	--	--	--
Ca5_P2_31772153	Ca5	31772153	AATTAATATTAATAT/AATTAATAT	CTTGTGCCCACACACATTTT	TCCATTGTTGTTGTCGTCGT	60.0	498	INTERGENIC	--	--	--
Ca5_P2_31773021	Ca5	31773021	GTTTTTTTTTTT/GTTTTTTTTTT	CGACGACAACAACAATGGAC	AACACGGTGTGAGTCTTCCC	60.0	647	INTRON	Ca_04882	Z	MYB_related
Ca5_P2_31777053	Ca5	31777053	ATTTTTTT/ATTTTTT	AAACACATCCCACAGGCTTC	GGCAAGAGCAAGACATGTGA	60.0	931	INTRON	Ca_04882	Z	MYB_related
Ca5_P2_31787653	Ca5	31787653	CA/C	GAACCCCAAACACGCTTCTA	GCTTTAACGGCGCATATGTT	60.1	626	DRR	Ca_04883	--	--
Ca5_P2_31792803	Ca5	31792803	CTTTTTT/CTTTT	TATTGGTTGCAGGTCGATGA	TTTAAAAGATGGGAGCGGTG	60.1	308	DRR	Ca_04884	--	--
Ca5_P2_31793044	Ca5	31793044	TGGG/TGG	TATTGGTTGCAGGTCGATGA	TGCCTTAGCATTCTCAGTGG	59.0	797	DRR	Ca_04884	--	--
Ca5_P2_31793281	Ca5	31793281	ATTTTTTTT/ATTTTTTT	TAGCCTTTGAAGATGCGGTT	TTTTGAAGATCATCCCTCCG	60.0	891	DRR	Ca_04884	--	--

INDEL marker IDs	Chromosomes/unanchored scaffolds	Physical positions (bp)	InDels (*Kabuli* reference genome-CDC Frontier/PII)	Forward primers (5'-3')	Reverse primers (5'-3')	Anneling temperature (0C)	Expected amplified product size (bp)	Structural annotation		Functional annotation	
								Sequence components of *kabuli* genome	*Kabuli* gene accession IDs	NCBI-KOG	TFs
Ca5_P2_31797813	Ca5	31797813	TAAA/TAAAA	GCACAACAAGCATTCGTCAT	GATAATTGCGGGGTTGCTTA	59.9	171	DRR	Ca_04885	--	--
Ca5_P2_31797845	Ca5	31797845	ACCCC/ACCCCCCC	GCACAACAAGCATTCGTCAT	TAAAGTTGGAAAACCCTCGG	59.1	557	DRR	Ca_04885	--	--
Ca5_P2_31805131	Ca5	31805131	GAAAAAA/GAAAAAAA	TGCAGTGGATAGATAACGGG	TCTTTTGGCAGTGTCAGCAC	60.0	919	INTERGENIC	--	--	--
Ca5_P2_31805298	Ca5	31805298	A/AGT	ATCCCCGAAACTTACAATGC	TCTTTTGGCAGTGTCAGCAC	60.0	355	INTERGENIC	--	--	--
Ca5_P2_31806703	Ca5	31806703	GTTTTTTTTTTTTTTTTTT/GTTTTTTTTTTT	TTCGTACTTTACCAAATCTTAAACACA	AGATGGAAGGCGTACATTGG	60.0	390	INTERGENIC	--	--	--
Ca5_P2_31807113	Ca5	31807113	CG/C	CCAATGTACGCCTTCCATCT	CCCACACGCGTCTACAATTT	60.9	596	INTERGENIC	--	--	--
Ca5_P2_31807148	Ca5	31807148	ATTTTTTTTT/ATTTTTTTT	CCAATGTACGCCTTCCATCT	CCCACACGCGTCTACAATTT	60.9	596	INTERGENIC	--	--	--
Ca5_P2_31807328	Ca5	31807328	TAAAAAAAAAAAA/TAAAAAAAAA	CCAATGTACGCCTTCCATCT	ACGACCCACCGTGTATGATT	60.1	837	INTERGENIC	--	--	--
Ca5_P2_31807637	Ca5	31807637	GCAC/G	AAATTGTAGACGCGTGTGGG	TTGCATTGTTCACATGCTCA	59.8	465	INTERGENIC	--	--	--
Ca5_P2_31807859	Ca5	31807859	TCC/TC	AATCATACACGGTGGGTCGT	TTGCATTGTTCACATGCTCA	59.8	224	INTERGENIC	--	--	--
Ca5_P2_31807893	Ca5	31807893	CAAA/CAAAA	AATCATACACGGTGGGTCGT	ACGTTATCTATGAGGCCCCC	60.2	713	INTERGENIC	--	--	--
Ca5_P2_31809508	Ca5	31809508	TAAAAAAAAA/TAAAAAAAA	TCTGTTCTCGACGTTGTTCG	CAAAAGAACATGGACCACGA	59.5	637	INTRON	Ca_04886	Z	--

INDEL marker IDs	Chromosomes/unanchored scaffolds	Physical positions (bp)	InDels (*Kabuli* reference genome-CDC Frontier/PII)	Forward primers (5'-3')	Reverse primers (5'-3')	Anneling temperature (0C)	Expected amplified product size (bp)	Structural annotation		Functional annotation	
								Sequence components of *kabuli* genome	*Kabuli* gene accession IDs	NCBI-KOG	TFs
Ca5_P2_31812979	Ca5	31812979	AAAA/AAAATTTGGAAA	CATATCCCGATTCCCTCAAA	TTGGAACGAAAAGGTTGAAAA	59.6	518	DRR	Ca_04886	Z	--
Ca5_P2_31835187	Ca5	31835187	AG/A	AGAGTGCATCCTATGCGACC	CCAACAAAAGAGGGGGAGTT	60.3	211	INTERGENIC	--	--	--
Ca5_P2_31835201	Ca5	31835201	TAAAAAAAAAA/TAAAAAAA	AGAGTGCATCCTATGCGACC	CCAACAAAAGAGGGGGAGTT	60.3	211	INTERGENIC	--	--	--
Ca5_P2_31836426	Ca5	31836426	ATTTTAAATT/ATTTTAAATTATATGCTTTTTAAATT	TGGGCTACAAAAATGCATCA	ACTGACCATCCTCATTTGGC	59.9	808	INTERGENIC	--	--	--
Ca5_P2_31837580	Ca5	31837580	TAAAAAAA/TAAAAAA	TTTCACACCGTTCACGAAAA	CATCATCAACCAACCCTGTG	59.8	564	INTERGENIC	--	--	--
Ca5_P2_31837773	Ca5	31837773	GGTAATATATAT/G	TTTCACACCGTTCACGAAAA	CATCATCAACCAACCCTGTG	59.8	564	INTERGENIC	--	--	--
Ca5_P2_31843831	Ca5	31843831	T/TA	TACCAACGGCTAAACCGAAG	TTAACGGCCCTGTTCTTGAC	60.1	684	INTERGENIC	--	--	--
Ca5_P2_31844171	Ca5	31844171	CGG/CG	CAAGAACAGGGCCGTTAAGA	CGGTAGCCTAATGAACTCGC	59.9	371	INTERGENIC	--	--	--
Ca5_P2_31847356	Ca5	31847356	T/TAATC	GGACTATTCAACCATGCGCT	CGCCATTTGTTTTGTTTCAA	59.6	701	INTERGENIC	--	--	--
Ca5_P2_31853293	Ca5	31853293	ATTATAA/ATTATAACTTTATAA	TTCAAAGTGACAAACATCTCCA	CTTTCTTTGACCATCACGCA	59.8	237	INTERGENIC	--	--	--
Ca5_P2_31859212	Ca5	31859212	AAATTAA/AAA	TGTTTTGTCTCCAAGCAGAAGA	TGAAGAACCCTAAACCAAGGAA	60.0	499	INTERGENIC	--	--	--
Ca5_P2_31859391	Ca5	31859391	CATTTTTTATTTTTTATTTTTTATTTT/CATTTTTTATTTTTTATTTT	TGTTTTGTCTCCAAGCAGAAGA	CTCTCCCTCGCCGTACAATA	60.2	874	INTERGENIC	--	--	--

INDEL marker IDs	Chromosomes/unanchored scaffolds	Physical positions (bp)	InDels (*Kabuli* reference genome-CDC Frontier/PII)	Forward primers (5'-3')	Reverse primers (5'-3')	Anneling temperature (0C)	Expected amplified product size (bp)	Structural annotation		Functional annotation	
								Sequence components of *kabuli* genome	*Kabuli* gene accession IDs	NCBI-KOG	TFs
Ca5_P2_31859850	Ca5	31859850	AAATACTAATACTAATA/AAATACTAATACTAATACTAATACTAATA	GTATTGTACGGCGAGGGAGA	TTTGACATCACATTTCATGGATTA	59.2	602	INTERGENIC	--	--	--
Ca5_P2_31861858	Ca5	31861858	CT/CTCTCTAT	TCAATCTCCATCTTCCCCTG	CACCTTTTTGTTAGCCCCAA	60.0	634	INTRON	Ca_04892	--	--
Ca5_P2_31862121	Ca5	31862121	TA/TAA	TCAATCTCCATCTTCCCCTG	TGTCTCCGCAACCTCTTCTT	60.0	901	INTRON	Ca_04892	--	--
Ca5_P2_31869313	Ca5	31869313	TAA/TA	TCTTTTCACGGAAGAAGGGA	TTTGGTGCCTTTTATAATGAATGA	59.8	645	DRR	Ca_04893	--	--
Ca5_P2_31869532	Ca5	31869532	TAA/TA	GCTGGAGGCACTTCAAGAGT	TTGGTCATTGAAATAATTGGGTT	59.5	788	DRR	Ca_04893	--	--
Ca5_P2_31871185	Ca5	31871185	TAGAGAGAGAGAGAG/TAGAGAGAGAGAG	GAATGAGGTTCGGGAAAGAA	TCGAATAAACACGCTACCCC	60.0	143	INTERGENIC	--	--	--
Ca5_P2_31882128	Ca5	31882128	TAAAAAAAA/TAAAAAAAAA	AGCTCTTATGCAATGGGTCG	TATGCTACGACGCAACGAGT	59.5	457	INTERGENIC	--	--	--
Ca5_P2_31882431	Ca5	31882431	TAGAGAGAGAGAGAGAGAGAGAG/TAGAGAGAGAGAGAGAG	AGCTCTTATGCAATGGGTCG	CCTCCAATTTCCAAGTTTTGTT	59.4	611	INTERGENIC	--	--	--
Ca5_P2_31883506	Ca5	31883506	ATTTTTTTTTTT/ATTTTTTTTTT	CATTTTCCGAGCAATCCACT	GCAGTTAAAATCCATACTAATTCACG	59.4	828	INTERGENIC	--	--	--
Ca5_P2_31883810	Ca5	31883810	AAAATTA/AAAATTATAAATTA	TGTGGTGTTGTTAAATCAGGATG	TGATTGCGGGATTTTATGGT	60.2	780	INTERGENIC	--	--	--
Ca5_P2_31886554	Ca5	31886554	AATA/AA	AGGCTTCAAGGTCAGACCAG	AATGGCTGGGCAGTAAAACA	60.5	235	INTERGENIC	--	--	--
Ca5_P2_31887650	Ca5	31887650	G/GGAAT	TGAATTGATACGCCATTTCG	ATGCACCAGTCACACCTCAC	59.6	484	INTERGENIC	--	--	--

INDEL marker IDs	Chromosomes/unanchored scaffolds	Physical positions (bp)	InDels (*Kabuli* reference genome-CDC Frontier/PII)	Forward primers (5'-3')	Reverse primers (5'-3')	Anneling temperature (0C)	Expected amplified product size (bp)	Structural annotation		Functional annotation	
								Sequence components of *kabuli* genome	*Kabuli* gene accession IDs	NCBI-KOG	TFs
Ca5_P2_31887987	Ca5	31887987	ATTTTTTTTT/ATTTTTTTTTTTT	GTGAGGTGTGACTGGTGCAT	TTCAAGGACATGAATTTTGCC	59.9	722	INTERGENIC	--	--	--
Ca5_P2_31889225	Ca5	31889225	GTTTATTTTATTT/GTTTATTTTATTTATTTTATTT	AGATTGTGCAAAGTGGGGAC	GTAAATGACCCTGGGGGAGT	60.1	618	INTERGENIC	--	--	--
Ca5_P2_31892271	Ca5	31892271	GTTTTTTT/GTTTTTT	TCCTCAAGCATGCAAAACAG	GTATTTTCCAAACCGAGCCA	59.9	596	INTRON	Ca_04896	--	WRKY
Ca5_P2_31894832	Ca5	31894832	GTATGTTTGGTTATGTTTGGTT/GTATGTTTGGTT	TGATCAGCCATTGAATTGGA	GCTTTTCATTGCCTTCAAGC	60.0	877	DRR	Ca_04896	--	WRKY
Ca5_P2_31897937	Ca5	31897937	AAGCTA/AAGCTAGCTA	TTCTTCCAGGGTGCCATATC	CTGCTCTGGATAATCCCCAA	60.0	428	INTRON	Ca_04897	--	--
Ca5_P2_31897997	Ca5	31897997	A/AT	TTCTTCCAGGGTGCCATATC	CTGCTCTGGATAATCCCCAA	60.0	428	INTRON	Ca_04897	--	--
Ca5_P2_31899382	Ca5	31899382	G/GT	CAGGACTGCTGCCACAGTAA	AAAAACAACGCACCATCCAT	60.2	878	INTERGENIC	--	--	--
Ca5_P2_31900131	Ca5	31900131	ATTTTTTTT/ATTTTTTTTT	CTGCAAAGGGTAGTTCAGCC	AAGTGTTGCAGTTCACAGCG	60.1	360	INTERGENIC	--	--	--
Ca5_P2_31900463	Ca5	31900463	T/TC	CGCTGTGAACTGCAACACTT	ATTTCGTGGGTGCCAATAAC	59.7	250	INTERGENIC	--	--	--
Ca5_P2_31903196	Ca5	31903196	TTTTATAATATTATT/TTT	CAAGTTTCTTGGAAGCCTGC	CATCCAATCCATCCCAAAAG	60.1	563	INTERGENIC	--	--	--
Ca5_P2_31905197	Ca5	31905197	CTTTTTTT/CTTTTTTTT	TGTGTGAGAAAACTTGTAGAGATGC	TGTTTCCCAATAGACCCCAG	59.8	653	INTERGENIC	--	--	--
Ca5_P2_31905904	Ca5	31905904	GTTT/GTT	ACACGTCTGGGGTCTATTGG	TGATTCAAACCTCAAAAATCAAAA	59.9	872	INTERGENIC	--	--	--

INDEL marker IDs	Chromosomes/unanchored scaffolds	Physical positions (bp)	InDels (*Kabuli* reference genome-CDC Frontier/PII)	Forward primers (5'-3')	Reverse primers (5'-3')	Anneling temperature (0C)	Expected amplified product size (bp)	Structural annotation		Functional annotation	
								Sequence components of *kabuli* genome	*Kabuli* gene accession IDs	NCBI-KOG	TFs
Ca5_P2_31906133	Ca5	31906133	TAAAAAA/TAAAAAAA	CGTAGGTGTACAAATCCCGA	CTCCCTTTCATTGCATGGTT	59.9	887	INTERGENIC	--	--	--
Ca5_P2_31906416	Ca5	31906416	GTT/GTTT	CAATTTTGATTTTTGAGGTTTGAA	CTCCCTTTCATTGCATGGTT	59.9	202	INTERGENIC	--	--	--
Ca5_P2_31915574	Ca5	31915574	TAAAA/TAAA	GCGGCTTTTAGCAATTCAAG	ACGCGTCAATTTGGAGAAAC	60.1	583	INTERGENIC	--	--	--
Ca5_P2_31919399	Ca5	31919399	ATT/ATTTATTT	CCCTCTCTCCCTCAGTTCCT	ACTCCTCCTAGCTCCCCTTG	59.8	485	INTERGENIC	--	--	--
Ca5_P2_31921838	Ca5	31921838	GAAAAAAAA/GA	TTGTCTTGGTCGTGACTCCA	TTGCAAAAAGGAAGTACCCG	60.1	606	INTERGENIC	--	--	--
Ca5_P2_31922417	Ca5	31922417	TTAT/TT	CACGCACACCTCAACTCAAT	ATGCCCACTTTTTCTCATGC	60.1	718	INTERGENIC	--	--	--
Ca5_P2_31932559	Ca5	31932559	AAACAACA/AAACAACAACA	CAAATGGGAGGAAAAACCAA	TCATCAAAAGGGAAACCTGC	60.1	284	DRR	Ca_04898	--	--
Ca5_P2_31938363	Ca5	31938363	TTTTTCTCTCTCTTTT/TTTTT	GTGGTGCCACTCCCACTATT	ATTGCTGGTGGTCCAAACTC	60.0	174	INTERGENIC	--	--	--
Ca5_P2_31938862	Ca5	31938862	AT/ATCTTAATTGT	GTTTGGACCACCAGCAATCT	TTTTCCATGGTTTTCGCACT	60.5	760	INTERGENIC	--	--	--
Ca5_P2_31939101	Ca5	31939101	AATA/AA	TGCAAACATTGGTTAATGGAGA	TTTTCCATGGTTTTCGCACT	60.5	550	INTERGENIC	--	--	--
Ca5_P2_31941021	Ca5	31941021	AATATATATATATATATATATATATATA/AATATATATATATA	CGTAAACAAGGCCCAAACAC	GCCCCCTTCAATAAATCCAT	60.0	568	INTERGENIC	--	--	--
Ca5_P2_31941334	Ca5	31941334	ATTTTT/ATTTTTT	ATGGATTTATTGAAGGGGGC	GCGATTTATATGGTGCCGTC	60.3	417	INTERGENIC	--	--	--

INDEL marker IDs	Chromosomes/unanchored scaffolds	Physical positions (bp)	InDels (*Kabuli* reference genome-CDC Frontier/PII)	Forward primers (5'-3')	Reverse primers (5'-3')	Anneling temperature (0C)	Expected amplified product size (bp)	Structural annotation		Functional annotation	
								Sequence components of *kabuli* genome	*Kabuli* gene accession IDs	NCBI-KOG	TFs
Ca5_P2_31941372	Ca5	31941372	ATTTTT/ATTTTTT	ATGGATTTATTGAAGGGGGC	GCGATTTATATGGTGCCGTC	60.3	417	INTERGENIC	--	--	--
Ca5_P2_31943086	Ca5	31943086	TAAAAAAA/TAAAAAA	TGTGGCACTTAGATGTCATTTTG	TTGCCAACAATTAAAGCGAC	58.8	445	INTERGENIC	--	--	--
Ca5_P2_31943734	Ca5	31943734	TAAAAAAAAA/TAAAAAAA	TTCTTTGTCCTTGCTTGCCT	TGTTGTTGGACCATTATGCAC	59.3	630	INTERGENIC	--	--	--
Ca5_P2_31944532	Ca5	31944532	ATTTTTTT/ATTTTTTTT	CTGCAAAATGCAAATCATGC	AAGGTGAATGCAATCCCAAG	59.9	413	INTERGENIC	--	--	--
Ca5_P2_31944876	Ca5	31944876	AC/A	CTTGGGATTGCATTCACCTT	TGTACGTTGTTGTGTGTTTGACA	60.0	418	INTERGENIC	--	--	--
Ca5_P2_31945685	Ca5	31945685	ATTTTTTT/ATTTTTTTT	GGAAGCTCCTTCAACTCGTG	GGCGAGGTTGGATTGTAAAG	59.6	149	INTERGENIC	--	--	--
Ca5_P2_31945710	Ca5	31945710	TTTT/TTTTAGCTACGTTT	GGAAGCTCCTTCAACTCGTG	GGCGAGGTTGGATTGTAAAG	59.6	149	INTERGENIC	--	--	--
Ca5_P2_31946772	Ca5	31946772	TAAAAAAA/TAAAAAAAA	GAAAGACCAGCAAGGGAGAG	GCCATTTTCGAGTACGGTGT	60.0	266	INTERGENIC	--	--	--
Ca5_P2_31953492	Ca5	31953492	CAAA/CAAAA	GGCAAACCCATCCAGAAGTA	TGGCTACCAAAGTCCCAAAC	60.0	486	INTRON	Ca_04899	S	ARF
Ca5_P2_31958667	Ca5	31958667	ATTTTTTT/ATTTTTTTT	CCATTGTAAAACGATTTTCTCCA	CAAGACTACGGGCAAGTTTGA	60.3	542	INTERGENIC	--	--	--
Ca5_P2_31959175	Ca5	31959175	ATTT/ATTTT	CAAACTTGCCCGTAGTCTTGA	CAGTGGACAAAGGTGTGTGG	60.0	338	INTERGENIC	--	--	--
Ca5_P2_31959447	Ca5	31959447	CTTTTTTTTT/CTTTTTTTTTT	CCACACACCTTTGTCCACTG	GCTTAGGTTAGGTACCGTTTGG	59.1	454	INTERGENIC	--	--	--

INDEL marker IDs	Chromosomes/unanchored scaffolds	Physical positions (bp)	InDels (*Kabuli* reference genome-CDC Frontier/PII)	Forward primers (5'-3')	Reverse primers (5'-3')	Anneling temperature (0C)	Expected amplified product size (bp)	Structural annotation		Functional annotation	
								Sequence components of *kabuli* genome	*Kabuli* gene accession IDs	NCBI-KOG	TFs
Ca5_P2_31959719	Ca5	31959719	TA/T	CCACACACCTTTGTCCACTG	TTGGGTTGAGTTTCATGGGT	60.2	960	INTERGENIC	--	--	--
Ca5_P2_31960083	Ca5	31960083	TTCTCT/TTCT	TCAAGTTGGAGATTGAACCC	CTTGGGTTGAGTTTCATGGG	60.3	202	INTERGENIC	--	--	--
Ca5_P2_31960544	Ca5	31960544	TAAAAAA/TAAAAA	ACCCATGAAACTCAACCCAA	TTTAATTTCATTGCGGGCAT	60.3	585	INTERGENIC	--	--	--
Ca5_P2_31964037	Ca5	31964037	T/TG	CCAACAAGACTGACCCCACT	TCATTGTGATGCTCTCACCG	60.8	877	INTERGENIC	--	--	--
Ca5_P2_31964201	Ca5	31964201	GTTTTTTTTTTT/GTTTTTTTTTT	CCAACTTGCCTGGTCGATTA	TCATTGTGATGCTCTCACCG	60.8	778	INTERGENIC	--	--	--
Ca5_P2_31967141	Ca5	31967141	AATATATATATAT/AATATATATATATAT	ACGGGTCATTTTAAACACGC	TTCGCCACCAAGTCATGATA	60.1	550	DRR	Ca_04900	--	--
Ca5_P2_31969297	Ca5	31969297	GAATAATAA/GAATAATAATAA	GATGGAAGAGTGACGGTGGT	CCGCAATCCTCACCTGTATT	60.0	174	INTERGENIC	--	--	--
Ca5_P2_31969588	Ca5	31969588	CTGCATTTGCATTT/CTGCATTTGCATTTGCATTT	GATGGAAGAGTGACGGTGGT	CATTCCCTAGTGACAGCGAG	58.5	740	INTERGENIC	--	--	--
Ca5_P2_31970233	Ca5	31970233	CATATATATA/CATATATATATA	CGCTGTCACTAGGGAATGTGT	CTGTTTTTACCACCCAAGCG	60.5	435	INTERGENIC	--	--	--
Ca5_P2_31971491	Ca5	31971491	ATTTTT/ATTTT	CGTCGTTTCAGATCAGGGTT	GTATGGGTTGTATGGGACCG	59.9	876	INTERGENIC	--	--	--
Ca5_P2_31972825	Ca5	31972825	A/AG	CTTTGAAAAACGAACCCACC	CAACTAATCGAGGAAGTCCCA	59.2	759	INTERGENIC	--	--	--
Ca5_P2_31973164	Ca5	31973164	CTTT/CTTTT	TGGGACTTCCTCGATTAGTTG	CGACGTTGTTTTGTTCGTCA	60.7	492	INTERGENIC	--	--	--

INDEL marker IDs	Chromosomes/unanchored scaffolds	Physical positions (bp)	InDels (*Kabuli* reference genome-CDC Frontier/PII)	Forward primers (5'-3')	Reverse primers (5'-3')	Anneling temperature (0C)	Expected amplified product size (bp)	Structural annotation		Functional annotation	
								Sequence components of *kabuli* genome	*Kabuli* gene accession IDs	NCBI-KOG	TFs
Ca5_P2_31974245	Ca5	31974245	GTTTTTTTTTTT/GTTTTTTTT	GGCTAAGTTTTCTTTGGGGG	GCACTCCATGAATCAAAGCA	59.8	696	INTERGENIC	--	--	--
Ca5_P2_31976038	Ca5	31976038	CTTATTTATTTATTTATT/CTTATTTATTTATTTATTTATT	AGCAACGGTGTAAAGGGAAA	CGGTAGCCAGGCTGATATGT	60.1	397	INTERGENIC	--	--	--
Ca5_P2_31977940	Ca5	31977940	ATGT/AT	AAGGGGACGGAGTTAGAAGG	TTTGAGTGTTGGAGGAACCC	59.9	655	INTERGENIC	--	--	--
Ca5_P2_31978151	Ca5	31978151	T/TG	CCCTCCTGCTTCCATTAACA	AAATCAACATGCTTGGGGAG	59.9	301	INTERGENIC	--	--	--
Ca5_P2_31978727	Ca5	31978727	GTATATATATATATATAT/GTATATATATAT	GGGTTCCTCCAACACTCAAA	GGGCAACAATTTTTCTCCAA	59.9	710	INTERGENIC	--	--	--
Ca5_P2_31980468	Ca5	31980468	AAAC/AAACAAC	ATGAACTACCCGACGACCAA	TTCACGCATAGAAAGCATCG	60.0	425	DRR	Ca_04901	R	--
Ca5_P2_31981912	Ca5	31981912	TA/TATGAAATATTCTAA	AATCTGGGAGCCTTGTGTTG	GGTTTCAATCTGCTGCCAAT	60.1	703	INTRON	Ca_04901	R	--
Ca5_P2_31988086	Ca5	31988086	GAAAAAAAAA/GAAAAAAAA	CCATCAGCAGAACTGCAAAA	ATGAGCAAAAGACCACCACC	60.0	195	INTRON	Ca_04901	R	--
Ca5_P2_31989784	Ca5	31989784	ATT/AT	ATTCGTTTCCCCTGTTTCCT	CGGAAGTTCTCAAGAGACGG	60.0	258	INTERGENIC	--	--	--
Ca5_P2_31991616	Ca5	31991616	CTATTATTATTATT/CTATTATTATTATTATTATT	CCCGCGTGTAATTTAATGCT	TCCATTCCAAGCTCCAAATC	60.0	455	INTRON	Ca_04902	R	--
Ca5_P2_31991736	Ca5	31991736	CAA/CA	GATTTGGAGCTTGGAATGGA	CTGGATGAACGGAACCATCT	59.9	406	INTRON	Ca_04902	R	--
Ca5_P2_31999075	Ca5	31999075	TGGGGG/TGGGGGG	AACGGTTATTTTCCGGCTTT	TCACACCTCTCCCCCTATGA	60.5	165	DRR	Ca_04903	--	S1Fa-like

INDEL marker IDs	Chromosomes/unanchored scaffolds	Physical positions (bp)	InDels (*Kabuli* reference genome-CDC Frontier/PII)	Forward primers (5'-3')	Reverse primers (5'-3')	Anneling temperature (0C)	Expected amplified product size (bp)	Structural annotation		Functional annotation	
								Sequence components of *kabuli* genome	*Kabuli* gene accession IDs	NCBI-KOG	TFs
Ca5_P2_31999312	Ca5	31999312	GA/G	AACGGTTATTTTCCGGCTTT	TTACAACAGCACGTGCAACC	60.8	433	DRR	Ca_04903	--	S1Fa-like
Ca5_P2_32000614	Ca5	32000614	AC/A	ATCACTGTGGGGAAGGAGTG	TTGAAGGTTGTCGTGTTGGA	60.1	745	DRR	Ca_04903	--	S1Fa-like
Ca5_P2_32005179	Ca5	32005179	ATTTTT/ATTTTTT	AAACCCGATAAAAAGCGTGA	ACAGAAAAAGCGGATAGGCA	59.8	526	DRR	Ca_04904	Z	MYB_related
Ca5_P2_32005545	Ca5	32005545	GTTTTTT/GTTTTTTT	TGCCTATCCGCTTTTTCTGT	CCGCACTTGATTTGTGACAT	59.6	550	DRR	Ca_04904	Z	MYB_related
Ca5_P2_32007977	Ca5	32007977	CAATAAAAAA/CAATAAAAAATAAATAAAAAA	GATTGATTCCATGCGTCCTC	AACGGTGTCACCTCCTCAAC	60.0	842	INTRON	Ca_04904	Z	MYB_related
Ca5_P2_32015160	Ca5	32015160	CA/CACTATATA	AGGAAGTGGACAATGGTTGG	TAATGCAAACTTTGGGGGAG	59.9	784	INTERGENIC	--	--	--
Ca5_P2_32017949	Ca5	32017949	ATTT/ATT	TTTCAAGTGTGAGAGGCAAA	CGATTGCAAATAAAGCAGCA	60.0	816	DRR	Ca_04905	--	--
Ca5_P2_32018567	Ca5	32018567	CTAATAATAATAATAATAAT/CTAATAATAATAATAAT	CAATCTTGTTGCCTGCTCAA	TCACGGTGAGTCGTTGTCAT	60.2	451	INTRON	Ca_04906	S	--
Ca5_P2_32020735	Ca5	32020735	GAAAAAAAAAAAA/GAAAAAAAAAAAAA	GAGTAAAGCAGGTGCGAAGG	TCCCAGTAAGGCTAGCTCCA	60.0	765	INTRON	Ca_04906	S	--
Ca5_P2_32021738	Ca5	32021738	CAAA/CA	CTTGGGAAGGTCACAACGAT	TACTGTCAGCATGGCACCTC	59.9	659	INTRON	Ca_04906	S	--
Ca5_P2_32024431	Ca5	32024431	ATTTTTT/ATTTTTTT	AACCCCATTTCAACCATCAA	TCCATATTCGGTAGCCGTTC	59.9	627	DRR	Ca_04906	S	--
Ca5_P2_32037572	Ca5	32037572	CG/C	GCAAGTGACTCCAAGGAAGC	ATGTGGGTGGGTGAAATGAT	59.9	296	INTRON	Ca_04908	KT	DBB

INDEL marker IDs	Chromosomes/unanchored scaffolds	Physical positions (bp)	InDels (*Kabuli* reference genome-CDC Frontier/PII)	Forward primers (5'-3')	Reverse primers (5'-3')	Anneling temperature (0C)	Expected amplified product size (bp)	Structural annotation		Functional annotation	
								Sequence components of *kabuli* genome	*Kabuli* gene accession IDs	NCBI-KOG	TFs
Ca5_P2_32040453	Ca5	32040453	T/TC	ATAGTATCGTTTGTGGCGGC	TTTGCACATACATGACCAATCA	59.9	482	DRR	Ca_04909	K	MIKC
Ca5_P2_32041461	Ca5	32041461	CTT/CT	GAAGTAGCGGTCATGCCAAT	ATAGTGGAGGAGCTGGAGCA	60.0	338	DRR	Ca_04909	K	MIKC
Ca5_P2_32044892	Ca5	32044892	T/TC	CTGTTTTTCACCGCCAAAAT	TCTTCCCTAATCACGCATCC	60.0	763	INTERGENIC	--	--	--
Ca5_P2_32062626	Ca5	32062626	ATTTTTTTTTTT/ATTTTTTTTTT	GAGTGGGGGATGTCAATGAG	TAGGCTGCCGTACAATACGA	59.3	340	INTERGENIC	--	--	--
Ca5_P2_32062989	Ca5	32062989	ATATTATTATTATTATT/ATATTATTATTATT	GAGGGAGAGCTCATGTGGAA	TTCGTGCATGCAAATTGAGT	60.3	376	INTERGENIC	--	--	--
Ca5_P2_32065425	Ca5	32065425	C/CG	TATTTTGTCAGCAGCACCCC	ACCTGAGATGCAAAATTCGG	60.1	124	INTERGENIC	--	--	--
Ca5_P2_32068203	Ca5	32068203	TT/TTGATAAATGAAT	TGAAAAATGGAATCCCACTAGC	GAGCATTTAGTGGAGCGGAG	60.0	683	INTERGENIC	--	--	--
Ca5_P2_32080664	Ca5	32080664	GAAAAAAAAA/GAAAAAAAAAA	GGTTGGTGACTTGGTGGTGT	TCCATGATAAATGTGTGGAAAA	57.9	745	DRR	Ca_04910	C	--
Ca5_P2_32084497	Ca5	32084497	T/TA	TTTGGCCGTTTTAAATGAGG	CAATGTTTTCATGACGTGGC	60.0	358	INTRON	Ca_04911	K	LBD
Ca5_P2_32086717	Ca5	32086717	GAATTAA/GAATTAATTAA	TTGCATGAGGTTTTGCCATA	GGTCCCAAAATGTGACATCC	60.0	336	DRR	Ca_04911	K	LBD
Ca5_P2_32086876	Ca5	32086876	ATTTTTT/ATTTTT	TTAAACCTTGCGGTTCCATC	GGTCCCAAAATGTGACATCC	60.0	287	DRR	Ca_04911	K	LBD
Ca5_P2_32087284	Ca5	32087284	TAA/TAAA	GGATGTCACATTTTGGGACC	TTGAGTTATACCATGCTTCTCAACA	60.1	553	DRR	Ca_04911	K	LBD

INDEL marker IDs	Chromosomes/unanchored scaffolds	Physical positions (bp)	InDels (*Kabuli* reference genome-CDC Frontier/PII)	Forward primers (5'-3')	Reverse primers (5'-3')	Anneling temperature (0C)	Expected amplified product size (bp)	Structural annotation		Functional annotation	
								Sequence components of *kabuli* genome	*Kabuli* gene accession IDs	NCBI-KOG	TFs
Ca5_P2_32087684	Ca5	32087684	ACTTCTT/ACTT	AAAACCATGTTGCCACAAACT	TGTGTGTGGTGGGTCAATCT	59.8	930	DRR	Ca_04911	K	LBD
Ca5_P2_32087748	Ca5	32087748	TAAAAAA/TAAAAAAAAAAA	TTCTAAATGTTGAGAAGCATGG	TGTGTGTGGTGGGTCAATCT	59.8	630	DRR	Ca_04911	K	LBD
Ca5_P2_32090869	Ca5	32090869	GGGTATTAT/G	TCAGGGATGACAATTGGTAGAG	TCGTCCGTAATAAGTGACCCA	60.4	231	INTERGENIC	--	--	--
Ca5_P2_32096296	Ca5	32096296	AGAT/AGATGAT	TTGTGGATACCAAAAGAATCTCAA	CACACCACCTCCTCCTGTTT	60.0	648	INTERGENIC	--	--	--
Ca5_P2_32097444	Ca5	32097444	CTTAATTA/CTTA	GCTCACTGCCAACTTTCTCC	GCAATAGATAGCGCACACGA	60.0	761	INTERGENIC	--	--	--
Ca5_P2_32098338	Ca5	32098338	CTTTTTTTTTT/CTTTTTTTTT	TTGTGTTGTCAGCGAAGGAG	CGACAAGGGCAAGAAGAAAG	60.0	573	INTRON	Ca_04912	--	ERF
Ca5_P2_32100080	Ca5	32100080	CATAT/CATATATAT	GTGGCATCAGGAATAGCGTT	CACCTAAAGGTCCCAACCAA	59.8	480	INTRON	Ca_04912	--	ERF
Ca5_P2_32100682	Ca5	32100682	ATTTTTTTTTT/ATTTTTTTTT	TAAGGCGGCTGACTCTCATT	TGCTCCCTTGCAAATTTCTT	59.8	772	INTRON	Ca_04912	--	ERF
Ca5_P2_32104880	Ca5	32104880	TAAAAAAAAAA/TAAAAAAAAA	TTTCAAACCCCGATTAACCA	TGTTTCTCGTTGCCCGTAGT	60.7	835	INTERGENIC	--	--	--
Ca5_P2_32111441	Ca5	32111441	CATT/CATTAATT	TCTCCCACATTAGCTAACCAAAA	TGTGCAACAATGTCATACCG	59.0	367	INTERGENIC	--	--	--
Ca5_P2_32113178	Ca5	32113178	ATTTTTTT/ATTTTTTTT	ACGGCCACATGCATTTTACT	TCCGATTGATTTATTTGAGCA	58.2	304	INTERGENIC	--	--	--
Ca5_P2_32115801	Ca5	32115801	TGTACGTAC/TGTAC	CTCGATTCATCGTCATTGGTT	GAATGGCTCAAGGGAGATGA	60.2	582	INTERGENIC	--	--	--

INDEL marker IDs	Chromosomes/unanchored scaffolds	Physical positions (bp)	InDels (*Kabuli* reference genome-CDC Frontier/PII)	Forward primers (5'-3')	Reverse primers (5'-3')	Anneling temperature (0C)	Expected amplified product size (bp)	Structural annotation		Functional annotation	
								Sequence components of *kabuli* genome	*Kabuli* gene accession IDs	NCBI-KOG	TFs
Ca5_P2_32119098	Ca5	32119098	GAT/GATAT	TTGTCGGTGAAGTTGTTTGG	TCAATTGGAAGCACATCGAC	59.7	451	INTERGENIC	--	--	--
Ca5_P2_32119133	Ca5	32119133	CAGA/C	GTCGGTGAAGTTGTTTGGGT	TCAATTGGAAGCACATCGAC	59.7	449	INTERGENIC	--	--	--
Ca5_P2_32138752	Ca5	32138752	TAAGAAGAA/TAAGAA	GCCGTTTGTAATGGTGGACT	CGAAGTCGCAAAACACGTTA	59.9	575	INTERGENIC	--	--	--
Ca5_P2_32144176	Ca5	32144176	ATTTTTTTTT/ATTTTTTTTTTTT	AATGCACTGTGATTTGCGTC	TTGGGGTCACTAATTGGAGC	59.9	438	DRR	Ca_04915	O	--
Ca5_P2_32147774	Ca5	32147774	A/AATTTAATCGATC	GTGCAGAAGAGGAGAATGGC	ACAAATCAGCCCGACGTTAC	60.0	574	DRR	Ca_04917	G	C2H2
Ca5_P2_32149202	Ca5	32149202	TCAC/TCACAC	AAATTCCATTTTCGGGAACC	GGTGGGAATCCTTGGAAAAT	60.0	466	DRR	Ca_04917	G	C2H2
Ca5_P2_32155171	Ca5	32155171	CAAAA/CAAAAA	CGGACATGACGTTCAACCTA	GTCAAATTGGCCCACTCACT	60.0	514	INTRON	Ca_04917	G	C2H2
Ca5_P2_32159188	Ca5	32159188	TAAA/TAAAA	CCTCAACCTGATGGGACACT	CAAATGTGCTCCACCTTGAA	59.7	618	INTRON	Ca_04917	G	C2H2
Ca5_P2_32160479	Ca5	32160479	ATTTTTTT/ATTTTTTTT	CGCCCTACTCTTGGTCTCTG	AAAATTTTGGTGTTCGCCAC	59.8	380	INTRON	Ca_04917	G	C2H2
Ca5_P2_32161908	Ca5	32161908	CT/CTCTATAT	ATTTTCGACTCCTCCTTCCA	GGAGGGGATTTGAACTTGGT	60.2	442	INTRON	Ca_04917	G	C2H2
Ca5_P2_32162925	Ca5	32162925	TGAGAGAGAGAGAGAGAGAGAGAG/TGAGAGAGAGAGAGAGAGAGAGAGAGAG	CACGTGCCTTTTCTCACTCA	ATCTTGTTGGAGCAGCGTTT	59.9	408	INTRON	Ca_04917	G	C2H2
Ca5_P2_32163070	Ca5	32163070	CCTTCTT/CCTTCTTCTT	CACGTGCCTTTTCTCACTCA	GTTGATCTTTACGGCCTCCA	60.1	552	INTRON	Ca_04917	G	C2H2

INDEL marker IDs	Chromosomes/unanchored scaffolds	Physical positions (bp)	InDels (*Kabuli* reference genome-CDC Frontier/PII)	Forward primers (5'-3')	Reverse primers (5'-3')	Anneling temperature (0C)	Expected amplified product size (bp)	Structural annotation		Functional annotation	
								Sequence components of *kabuli* genome	*Kabuli* gene accession IDs	NCBI-KOG	TFs
Ca5_P2_32164917	Ca5	32164917	ATTTTTTT/ATTTTTTTT	GAAGTCGGTTCAACGTGGTT	GCGACACGACACGTAAGAGA	60.1	153	INTRON	Ca_04917	G	C2H2
Ca5_P2_32165510	Ca5	32165510	CATATATATATATA/CATATATATATATATA	TCCCTTAATGCTGATGGAGG	TCCAACAACCAATCATTCAAA	58.9	296	INTRON	Ca_04917	G	C2H2
Ca5_P2_32182918	Ca5	32182918	AT/A	ATTGGTCCCAATCATGGAAA	ACGGTTCAAGCGGTAGTTTG	60.2	240	INTERGENIC	--	--	--
Ca5_P2_32186163	Ca5	32186163	T/TC	TCTTCCCTGTTTGAGCCATC	TACAGTTTTTGGAGGTGGGG	59.8	353	INTERGENIC	--	--	--
Ca5_P2_32190466	Ca5	32190466	TAAAAAAAAAA/TAAAAAAAAAAA	TGAATGCAAAAGCTTCAAACA	TAAATTTCCGGGTCATGCTT	59.4	550	DRR	Ca_04918	K	TALE
Ca5_P2_32192146	Ca5	32192146	AT/ATGTGT	TATAAACGTGCTGTGGCTGC	TGCAATGGCTAGTAAAGCGA	59.6	618	INTERGENIC	--	--	--
Ca5_P2_32196357	Ca5	32196357	TC/TCC	AGCATTTCCAAAGGGGACTC	CCAAAACGGTGGACTGGTTA	60.8	779	INTRON	Ca_04919	K	TALE
Ca5_P2_32201181	Ca5	32201181	T/TGTGTGTG	ATTCGATCCTCCTCTTCCGT	TTCTCCTGCCTCTCTCCAAA	60.1	892	INTRON	Ca_04920	M	--
Ca5_P2_32201348	Ca5	32201348	A/AC	ATGGAGTTGGCGTTTCTGTT	TTCTCCTGCCTCTCTCCAAA	60.1	772	INTRON	Ca_04920	M	--
Ca5_P2_32207299	Ca5	32207299	TAA/TA	GGTCCCTGTCGGTCAGTTTA	GATTGGTTTGTTTTCGTCGG	60.3	796	INTERGENIC	--	--	--
Ca5_P2_32210939	Ca5	32210939	C/CG	GCTATTGGTGGTCGTCGTTT	TCCAATCTACCGGGACGTAG	59.9	686	INTRON	Ca_04921	O	FAR1
Ca5_P2_32213085	Ca5	32213085	TAAA/TAAAAAAA	TGCAGAAACATTTCCGATGA	TGGAACTAACTTTTGGAATGGG	60.2	700	DRR	Ca_04922	--	--

INDEL marker IDs	Chromosomes/unanchored scaffolds	Physical positions (bp)	InDels (*Kabuli* reference genome-CDC Frontier/PII)	Forward primers (5'-3')	Reverse primers (5'-3')	Anneling temperature (0C)	Expected amplified product size (bp)	Structural annotation		Functional annotation	
								Sequence components of *kabuli* genome	*Kabuli* gene accession IDs	NCBI-KOG	TFs
Ca5_P2_32217559	Ca5	32217559	TGC/TGCGC	TCTTCTTCGTGTTGTCGCTG	TTAGCAGCATTCCCCCATAC	59.9	152	INTERGENIC	--	--	--
Ca5_P2_32225711	Ca5	32225711	CTAAAATAAAATATAAAATAAA/CTAAAATAAA	TTACCTCTCACGTGCAATCG	AACAAATATGGACAAGCCGC	60.0	639	INTERGENIC	--	--	--
Ca5_P2_32225894	Ca5	32225894	CAAAAAAAAAAAA/CAAAAAAAAAA	GCGGCTTGTCCATATTTGTT	TTCATCATTATCGGAGCCGT	60.4	245	INTERGENIC	--	--	--
Ca5_P2_32226752	Ca5	32226752	ACC/ACCC	TTGGGCGACGTTACTTTTAGA	GGAGTGGGTTATTCTTTTGGC	59.8	189	DRR	Ca_04923	T	WRKY
Ca5_P2_32226879	Ca5	32226879	TT/TTATAT	TGGCCAAAAGAATAACCCAC	AAAATTTGAATGATTTCCTCGTG	59.4	142	DRR	Ca_04923	T	WRKY
Ca5_P2_32227379	Ca5	32227379	GCC/GC	GCTCAAATTTAAGGGTATGTTTGG	TGTGCATCCCTTGCAAATTA	60.1	679	DRR	Ca_04923	T	WRKY
Ca5_P2_32232650	Ca5	32232650	ATTT/ATT	AAAACATCCACCAAGACGAAA	TGGTGGGAAGAATGAAATGA	58.9	355	INTERGENIC	--	--	--
Ca5_P2_32240947	Ca5	32240947	ATTTTTTTT/ATTTTTTTTT	TTCAGCCTCCCTTTCCTACA	CAACCTCTTTTGGCCATGAT	59.9	622	INTERGENIC	--	--	--
Ca5_P2_32241464	Ca5	32241464	GTAATAATAATAATA/GTAATAATAATAATAATAATA	ATCATGGCCAAAAGAGGTTG	TCGGTTTGGTTTGGGTTAAA	60.2	724	INTERGENIC	--	--	--
Ca5_P2_32253033	Ca5	32253033	ATTTGTGTTTGTG/ATTTGTG	TTGCAACCAATTCGAGTGAA	CACGAATGGTGAAAGGGTCT	60.0	387	DRR	Ca_04924	--	ARF
Ca5_P2_32261714	Ca5	32261714	TGG/TG	TATTTGACCCGATACCCGAA	GCATCTCCTTCAGCGTCTTC	60.1	554	INTERGENIC	--	--	--
Ca5_P2_32267524	Ca5	32267524	T/TCCTCCATTTTG	TAACGAATGGCCAAAAGGAG	GATGAATGTGCTTCTCGCAA	60.0	663	DRR	Ca_04925	U	--

INDEL marker IDs	Chromosomes/unanchored scaffolds	Physical positions (bp)	InDels (*Kabuli* reference genome-CDC Frontier/PII)	Forward primers (5'-3')	Reverse primers (5'-3')	Anneling temperature (0C)	Expected amplified product size (bp)	Structural annotation		Functional annotation	
								Sequence components of *kabuli* genome	*Kabuli* gene accession IDs	NCBI-KOG	TFs
Ca5_P2_32268040	Ca5	32268040	CGTGTG/CGTGTGTG	TTGCGAGAAGCACATTCATC	TTCCGAAGTTCGCACCTTAT	59.7	356	DRR	Ca_04925	U	--
Ca5_P2_32274951	Ca5	32274951	CTTTT/CTTT	GCACCGAGTGTCAAACTCAA	TAAAAGCCGCCAAAATGAAG	60.2	548	INTERGENIC	--	--	--
Ca5_P2_32279253	Ca5	32279253	AATTATTATTATTATTATTATTATTATTATTATTA/AATTATTATTATTATTATTATTATTATTATTATTATTATTA	TTCGAAATGAAATCATAGGTAGCA	AACCAGCTTTTTAGTTCGTCCTT	59.7	458	INTERGENIC	--	--	--
Ca5_P2_32279611	Ca5	32279611	ATTTTTT/ATTTTTTTT	AAGGACGAACTAAAAAGCTGG	TTTCTTCTTGAGTTGGGAGGA	58.9	519	INTERGENIC	--	--	--
Ca5_P2_32286131	Ca5	32286131	CAAAAAAAAAA/CAAAAAAAAA	ATCAACGAAGGACGGTGAAG	TCGCAAAAGTAGTCCCCTCA	60.8	527	INTERGENIC	--	--	--
Ca5_P2_32300873	Ca5	32300873	CGGG/CGG	TAAAGGTGGTGGTAGGGTGC	TCATGATGAATCGGAACCAA	59.9	621	INTERGENIC	--	--	--
Ca5_P2_32301086	Ca5	32301086	TTTTATTTATTTATTTATTTATTT/TTTTATTTATTTATTTATTT	CCTGAAAACACGGGATCTGT	TCATGATGAATCGGAACCAA	59.9	377	INTERGENIC	--	--	--
Ca5_P2_32301196	Ca5	32301196	AATATATATATATATA/AATATATATATATATATATA	CCTGAAAACACGGGATCTGT	GCCCATGAGTCATCCAGTTT	59.9	820	INTERGENIC	--	--	--
Ca5_P2_323043	Ca5	323043	CTTTATTTATTTAT/CTTTATTTATTTATTTAT	ATGGTCTCAAAGATGCAGGG	TGTGGCGAAAAACAAATCAA	60.1	795	INTERGENIC	--	--	--
Ca5_P2_32307961	Ca5	32307961	ATACATTACATTACATTACATTA/ATACATTACATTACATTA	TTGGGATTTTCATTCATCCC	GCATTTCTGTGAATTTGGGG	60.3	524	INTERGENIC	--	--	--
Ca5_P2_32312463	Ca5	32312463	A/ATCACTCATAT	TTCTAACCCAAACCCCTGTG	GCTTCGATCCCCTATTCTCC	60.0	777	DRR	Ca_04928	--	bHLH
Ca5_P2_32314942	Ca5	32314942	GAAAAAAA/GAAAAAA	AATTGATGATGATGCTTGCG	ATTATCTGCCATGCTCCCAC	59.9	626	INTERGENIC	--	--	--

INDEL marker IDs	Chromosomes/unanchored scaffolds	Physical positions (bp)	InDels (*Kabuli* reference genome-CDC Frontier/PII)	Forward primers (5'-3')	Reverse primers (5'-3')	Anneling temperature (0C)	Expected amplified product size (bp)	Structural annotation		Functional annotation	
								Sequence components of *kabuli* genome	*Kabuli* gene accession IDs	NCBI-KOG	TFs
Ca5_P2_32319718	Ca5	32319718	TTA/TTAGTA	TGATTCATTGCATGTTCATGTT	GGTGTCGGCAAAAGACAAAT	60.0	796	INTERGENIC	--	--	--
Ca5_P2_32320368	Ca5	32320368	TCC/TC	ATTTGTCTTTTGCCGACACC	ATCGACTGCATGAAGCTCCT	60.0	606	INTERGENIC	--	--	--
Ca5_P2_32324347	Ca5	32324347	GCTCTCTCTCTCTCTCTC/GCTCTCTCTCTCTCTC	TGATGCCACGTGAGAAAGAG	CTGTTCAAGATGCAGGCAAA	60.0	899	DRR	Ca_04930	I	bHLH
Ca5_P2_32325553	Ca5	32325553	TAAAAAAAA/TAAAAAAAAA	TGCCCAATATCTGTTCACCA	ATGCATCCTGTGCCATACAA	60.0	609	DRR	Ca_04930	I	bHLH
Ca5_P2_32329993	Ca5	32329993	AGG/AGGG	GGAGGCATCGATGTTAGGAA	CGCATTGTTTTTGAAGCTGA	60.0	808	INTRON	Ca_04931	T	--
Ca5_P2_3234176	Ca5	3234176	ATTTTTTTTTTT/ATTTTTTTTTTTT	GATGTCGTTGCGAGAGTGAA	TGAACAAAGCAAAGCGTCAT	59.5	169	INTERGENIC	--	--	--
Ca5_P2_32348939	Ca5	32348939	TTT/TTTTGGAAGTT	GCAAGTTGGTGGAGATTGAAG	GCTGAAGCAGAACCTTGTCC	60.0	476	DRR	Ca_04932	QI	GeBP
Ca5_P2_32349699	Ca5	32349699	TAAAAA/TAAAAAA	AGGGGCCATGTGTAAGTACG	CATTAACACCGATGAGGGCT	60.0	566	DRR	Ca_04933	--	--
Ca5_P2_32349746	Ca5	32349746	CTTTT/CTTT	AGGGGCCATGTGTAAGTACG	CATTAACACCGATGAGGGCT	60.0	566	DRR	Ca_04933	--	--
Ca5_P2_32350759	Ca5	32350759	TAAAAAA/TAAAAAAA	ACACTTACGGAACATTGTGCT	TGCACAGCAGAATTAGTGGG	59.9	357	DRR	Ca_04933	--	--
Ca5_P2_32352329	Ca5	32352329	ATTTTT/ATTTTTT	TTTGGTTTTCACAGGAAGCC	AAGGATGCGTTTGGATTGAC	59.9	420	INTERGENIC	--	--	--
Ca5_P2_32352849	Ca5	32352849	ATTTTT/ATTTTTTT	AAGTCAATCCAAACGCATCC	CCCTGGTGGAGAACAGAAAA	60.1	908	INTERGENIC	--	--	--

INDEL marker IDs	Chromosomes/unanchored scaffolds	Physical positions (bp)	InDels (*Kabuli* reference genome-CDC Frontier/PII)	Forward primers (5'-3')	Reverse primers (5'-3')	Anneling temperature (0C)	Expected amplified product size (bp)	Structural annotation		Functional annotation	
								Sequence components of *kabuli* genome	*Kabuli* gene accession IDs	NCBI-KOG	TFs
Ca5_P2_3235916	Ca5	3235916	ATTTTTTTTTTTTTTT/ATATATTTTTTTTTTTTTTTT	TGGATGTAGCTATCGCGTTG	CAAGAACGAGGAACACAAGAAA	59.4	189	INTERGENIC	--	--	--
Ca5_P2_32362062	Ca5	32362062	TTAT/TTATAT	ACGGGAAAGAATGGGATTTC	GGGAACAGGAACACAAGGAA	59.9	394	INTERGENIC	--	--	--
Ca5_P2_32391991	Ca5	32391991	AAATAAT/AAAT	AATGGTTGGGGGAAGAAATC	GGATGAGGGACCTCAATGAA	59.9	731	INTRON	Ca_04938	--	bZIP
Ca5_P2_32396570	Ca5	32396570	TATAATAATAATAATAAT/TATAATAATAATAAT	CAGCTGTTCAGATCCGGTTT	TGGATCGAGAAGAAAGTGGC	60.3	810	INTERGENIC	--	--	--
Ca5_P2_32435142	Ca5	32435142	TC/T	CCATCGCAAGGCTGTAAAAT	ACATGTCAAGTGGGGGTGTT	60.1	873	INTERGENIC	--	--	--
Ca5_P2_32522095	Ca5	32522095	TTATATATATATATA/TTATATATATATA	GCTGTTATGTTTGGTTCGCA	GGGTTTTGCCTTCTCCACTC	61.0	614	INTERGENIC	--	--	--
Ca5_P2_32540371	Ca5	32540371	TA/T	TGGTTGCTTGTTGGTTTCTG	GTGAAAGAGCGTTAGAGCGG	60.2	706	INTERGENIC	--	--	--
Ca5_P2_32649599	Ca5	32649599	ATTTTTTTTTTT/ATTTTTTTTTT	TCGGACAAGTTAGCAAAAACAA	CCGTATTTTTGAATATCAGTGGC	59.8	561	INTERGENIC	--	--	--
Ca5_P2_32694142	Ca5	32694142	AAATAATAATAATAATAATAATAATAA/AAATAATAATAATAATAATAATAATAATAA	GCCATGATATTTTGCCGATAA	GATCGCAACCTGAGGATGTT	60.1	553	DRR	Ca_04975	--	SBP
Ca5_P2_32704938	Ca5	32704938	CATATATATATATATATATATATATATAT/CATATATATATATATATATATATATATATATATATATAT	ATAAGCAACGGCTCACTGGT	GGAGACCAAAATTGCGAATC	59.5	648	DRR	Ca_04976	--	--
Ca5_P2_32736061	Ca5	32736061	TTT/TTTCCTT	AAAAGGGTGTCACAAGTGGG	TGAAAAGGATGAAAGTCGCA	59.4	250	INTRON	Ca_04978	T	--
Ca5_P2_32745764	Ca5	32745764	TAAA/TAAAA	AGGCATCACATTGAGGGACT	CCTCGGTGACCCTGAATAAA	59.9	148	INTERGENIC	--	--	--

INDEL marker IDs	Chromosomes/unanchored scaffolds	Physical positions (bp)	InDels (*Kabuli* reference genome-CDC Frontier/PII)	Forward primers (5'-3')	Reverse primers (5'-3')	Anneling temperature (0C)	Expected amplified product size (bp)	Structural annotation		Functional annotation	
								Sequence components of *kabuli* genome	*Kabuli* gene accession IDs	NCBI-KOG	TFs
Ca5_P2_32769672	Ca5	32769672	GATATATATATATATATATATATATATATATATA/GATATATATATATATATATATATATATATA	CATGGCGTGAGTGAGTGAGT	TCACGGTTCCATGAAGAACA	60.1	760	INTERGENIC	--	--	--
Ca5_P2_32848829	Ca5	32848829	GAAAAAAAAAA/GAAAAAAAAAAA	AACATTTACCAGTCCAGCGG	TCACCGTATGCAAAGACGAG	59.9	848	INTERGENIC	--	--	--
Ca5_P2_32849733	Ca5	32849733	GATATATATATATATATATATATATAT/GATATATATATATATATATATATATATAT	GATGTACCAAGACATCGCCA	CCAGTTAACCGACATAGTCTGC	58.8	187	INTERGENIC	--	--	--
Ca5_P2_32912336	Ca5	32912336	AATATATATATATATATATATATATAT/AATATATATATATATATATATATATATATATAT	ACCCCCTCACCTCCTTATTG	TTTTCTTGCGTCACTTGTTGA	59.5	499	INTERGENIC	--	--	--
Ca5_P2_32950457	Ca5	32950457	AAATAATAATAATAATAATAATAATAATAATAATAATAATAATAATAAT/AAATAATAATAATAATAATAATAATAATAATAATAATAATAATAATAAT	ATTGCCACATTTCATGCTCA	GAATTCCCTACAATCGGCAA	59.9	345	INTERGENIC	--	--	--
Ca5_P2_33056739	Ca5	33056739	AATATATATATATATATATATATATATAT/AATATATATATATATATATATATATATATATATATATATATATAT	TTGAGTTTGCTTGTTCACAATTT	TTTCTTTCGAACAATTTCAGCA	59.9	483	INTERGENIC	--	--	--
Ca5_P2_33146690	Ca5	33146690	TAAAAAAAAA/TAAAAAAAAAA	TACCCGAACAACTTTGACCC	TGCTTTCGTGATTCCAACAA	60.2	731	DRR	Ca_05009	D	YABBY
Ca5_P2_33288448	Ca5	33288448	AAATAATTTGTAATAATTTGTAATAATTTGT/AAATAATTTGTAATAATTTGT	TGTCGCGAACTCAAATTTTC	GTCTTGCAAGTTGAAGCCAA	59.1	134	INTERGENIC	--	--	--
Ca5_P2_33353284	Ca5	33353284	AATATATATATATATA/AATATATATATATATA	AATGAAGGGAAATCGGTGTG	CAGGCGGTTTGATTAACGAT	60.0	536	INTERGENIC	--	--	--
Ca5_P2_3337230	Ca5	3337230	CATATATATATAT/CATATATATATATAT	TCATCCCGAATCAGTTCACA	GGCATGATGCAAGGGATAGT	59.9	291	DRR	Ca_18697	--	--
Ca5_P2_33395030	Ca5	33395030	TAAAAAAAAA/TAAAAAAAAAA	GGGATGCCACGATGTAAAAT	AAAATGTTAGGGACGCGTTG	60.0	504	INTERGENIC	--	--	--
Ca5_P2_334333	Ca5	334333	ATT/AT	CGTTCCACACAGCTTCTCAA	ACATGTTTCCCAAGAGGTGC	60.0	882	INTRON	Ca_18190	H	--

INDEL marker IDs	Chromosomes/unanchored scaffolds	Physical positions (bp)	InDels (*Kabuli* reference genome-CDC Frontier/PII)	Forward primers (5'-3')	Reverse primers (5'-3')	Anneling temperature (0C)	Expected amplified product size (bp)	Structural annotation		Functional annotation	
								Sequence components of *kabuli* genome	*Kabuli* gene accession IDs	NCBI-KOG	TFs
Ca5_P2_33441420	Ca5	33441420	ATTT/ATTTT	GTGTGTGAGAGGTTGTTGGG	TAACTCGAACCAACCGATCC	59.9	204	INTERGENIC	--	--	--
Ca5_P2_33444959	Ca5	33444959	GATATA/GATATATA	TTATGGAGGCCACATTCACA	GAAATTAAGGCCTCTTGATCTTTTT	59.6	961	DRR	Ca_01912	O	MYB_related
Ca5_P2_33456475	Ca5	33456475	TTATGTTAAATATGTTAA/TTATGTTAAATATGTTAAATATGTTAA	GATGAAGTGCTGATCAGAGGC	ACCAGGAAACATTGCCAGAC	60.0	789	DRR	Ca_01911	O	MYB_related
Ca5_P2_33457018	Ca5	33457018	TTTTATTTATTTATTTA/TTTTATTTATTTATTTATTTA	GTCTGGCAATGTTTCCTGGT	GGTGGTGTATGTATATCTTTACGGG	59.8	430	DRR	Ca_01911	O	MYB_related
Ca5_P2_33457459	Ca5	33457459	ATT/ATTT	TCTTCAAATTAAAATTCCACCCA	GTTGAAATTTTGGGTGTCGG	60.2	750	DRR	Ca_01911	O	MYB_related
Ca5_P2_33457924	Ca5	33457924	ATTTTTTT/ATTTTTTTT	CCGACACCCAAAATTTCAAC	GCCCTCAGATGAGAGTCAGG	59.9	680	DRR	Ca_01911	O	MYB_related
Ca5_P2_33506470	Ca5	33506470	TTATATATATATATAT/TTATATATATATATATAT	CCATAAGCCTAAATCTCTTGCC	AGATATAGCGTGTGAGGCCC	59.2	503	INTERGENIC	--	--	--
Ca5_P2_3353130	Ca5	3353130	A/AC	GCGGTCACCTCCACATAGTT	TGTGTGGAGCCCATATGAAA	59.9	659	INTERGENIC	--	--	--
Ca5_P2_33542100	Ca5	33542100	GTTTATTTATTTATTTAT/GTTTATTTATTTATTTATTTATTTAT	AATTCGGTTTTTGGCGTGTA	GTTAAGGGACCACGAATGCT	59.1	569	INTERGENIC	--	--	--
Ca5_P2_33600042	Ca5	33600042	CT/C	TAACCATTCCACAGCCACAA	CTTAGAGGTCGCTTCAACGG	60.0	680	DRR	Ca_01896	--	--
Ca5_P2_33632878	Ca5	33632878	ATTTTTTTTT/ATTTTTTTT	ACAATCGTCCAGCTTTGGAG	CATGCCACAAAAATTCAACG	60.0	723	INTERGENIC	--	--	--
Ca5_P2_33687861	Ca5	33687861	TTATATATATATATAT/TTATATATATATAT	GACTTCCTAAGGAGGGTGGC	ATTTCCACGGCAAATTCGTA	60.3	381	DRR	Ca_01887	--	--

INDEL marker IDs	Chromosomes/unanchored scaffolds	Physical positions (bp)	InDels (*Kabuli* reference genome-CDC Frontier/PII)	Forward primers (5'-3')	Reverse primers (5'-3')	Anneling temperature (0C)	Expected amplified product size (bp)	Structural annotation		Functional annotation	
								Sequence components of *kabuli* genome	*Kabuli* gene accession IDs	NCBI-KOG	TFs
Ca5_P2_337016	Ca5	337016	ATTT/ATTTT	CCAAAACTGGGGAGAAACAA	TGATATTGGAGGGGGCATAA	60.1	306	DRR	Ca_18190	H	--
Ca5_P2_33701975	Ca5	33701975	AGGGGG/AGGGG	GGGAGTAGGGTGGAGTAGCC	GAGCCACCAGAGATCCTCAC	59.8	279	DRR	Ca_01884	K	MYB
Ca5_P2_33794381	Ca5	33794381	AATATATATATATAT/AATATATATATATATAT	GCAAGGGTACCCACGTCTAA	TAATTTCCACCACACCCCAT	59.9	439	INTRON	Ca_01877	Q	--
Ca5_P2_33798172	Ca5	33798172	TAAAAAAA/TAAAAAAAA	GCGGAATGACCTACCGAATA	CATCACATCACTATGCTCCCTC	59.6	714	INTERGENIC	--	--	--
Ca5_P2_3379986	Ca5	3379986	GAAA/GAA	TCCCTTTACTGAATTAATGTGGC	GCAGCACAGGTCGATTACAA	59.9	199	INTERGENIC	--	--	--
Ca5_P2_33804970	Ca5	33804970	GTTTTTTTTTTT/GTTTTTTTTTT	CGAAAATTTGAAAATCGGGA	CCACAATGTTTTTCGCTCCT	60.1	178	INTERGENIC	--	--	--
Ca5_P2_33822575	Ca5	33822575	G/GC	GGCGTGTTGAATACCGTTCT	GTCGACTCCCAGTTTCTCCA	60.2	949	DRR	Ca_01875	--	--
Ca5_P2_33876092	Ca5	33876092	TTATTATTATTATTATTATT/TTATTATTATTATTATTATT	TTGAAAATGAAGCCACCACA	TTTCCCACTCGAAACCTGTC	60.1	379	INTRON	Ca_01865	--	bZIP
Ca5_P2_33883232	Ca5	33883232	AATTTAACTATATTT/AATTT	TGACATAAAGCACCACTGTTCA	TCCTACGGTCTTGTTTTGGG	60.0	559	INTERGENIC	--	--	--
Ca5_P2_33888841	Ca5	33888841	ATTTTTTTTTT/ATTTTTTTTTTT	AACTTCCAAGTGACGTGTTGG	CTGGACAAGTCTTTGTCATTGAT	58.2	702	INTERGENIC	--	--	--
Ca5_P2_34016644	Ca5	34016644	TTTATTATTATTATTATTATTATTAT/TTTATTATTATTATTATTATTATTATTATTAT	TAAGTGTGAATGTGGGACGG	AAAACACGCACTTTCTGCAA	59.5	621	INTERGENIC	--	--	--
Ca5_P2_34072036	Ca5	34072036	CCCAAAGCAGAT/C	CATTGGTGTTGTCTGTTCGG	ACCGTTCCGTTTGTTCTGAC	60.0	262	INTERGENIC	--	--	--

INDEL marker IDs	Chromosomes/unanchored scaffolds	Physical positions (bp)	InDels (*Kabuli* reference genome-CDC Frontier/PII)	Forward primers (5'-3')	Reverse primers (5'-3')	Anneling temperature (0C)	Expected amplified product size (bp)	Structural annotation		Functional annotation	
								Sequence components of *kabuli* genome	*Kabuli* gene accession IDs	NCBI-KOG	TFs
Ca5_P2_34164848	Ca5	34164848	C/CA	CTTCCAGGGGATGCAAGATA	CGTAGGCAATAAGACACGCA	59.9	377	DRR	Ca_01832	--	NF-YB
Ca5_P2_34219298	Ca5	34219298	CATATATATATATATATATATATATATATATATATATATATA/CATATATATATATATATATATATATATATATATA	TTGGGTTTTAGAAGGTGGGA	TGCGTTGATTTAAGGTGCTC	58.9	499	INTERGENIC	--	--	--
Ca5_P2_34355845	Ca5	34355845	AATATATATATATATATATATATATATATA/AATATATATATATATATATA	TGGGTTTTTCAATTGTTGGG	AGCCTAAATCCATGGTTATTTTT	57.3	736	DRR	Ca_01810	--	--
Ca5_P2_34408923	Ca5	34408923	ATTTTTTTTTT/ATTTTTTTTT	CAAAACAAATGATTTTCCACCA	CGAAACGATGGAAATCAACA	59.5	720	INTERGENIC	--	--	--
Ca5_P2_3451613	Ca5	3451613	CATATATATATATAT/CATATATATATATATAT	GTTGGTTCCCTAGCACCGTA	ATGAAAACGCCAATGAGGAC	59.9	859	INTERGENIC	--	--	--
Ca5_P2_34545559	Ca5	34545559	TTTA/TTTAATTA	CCAAGCACATACCATTGTGG	TTTTTGCATCCATAAAAACGC	60.0	355	INTERGENIC	--	--	--
Ca5_P2_34548859	Ca5	34548859	GAAAAAAAA/GAAAAAAA	ATTGATTTCGCAATAAGCGG	CCTAGCCGTCTCATTTGGTT	59.2	629	INTERGENIC	--	--	--
Ca5_P2_34581854	Ca5	34581854	TAAAAAAAAAAAAA/TAAAAAAAAAAAAAA	AAAAATTGAGCCATCCATGC	TCCCTCGTTTCATTTCCCTA	59.5	202	INTERGENIC	--	--	--
Ca5_P2_34585257	Ca5	34585257	GTA/GTAGGGTGTTAATA	CGGGAGATACGTAGGAGCAA	ATAAATGTCGCGGCCTAAAA	59.6	386	INTERGENIC	--	--	--
Ca5_P2_34629428	Ca5	34629428	A/ACTTAAAAAGTTG	TTAGGTTGAGGCAGTTCGCT	TTTGGGTTTCGACCATAAAAA	59.3	587	INTERGENIC	--	--	--
Ca5_P2_34698063	Ca5	34698063	GATAATAATAATAATAATAATAATAATAATAATAA/GATAATAATAATAATAATAATAATAATAATAATAA	ATGGCTTTTCCTACCTTCGG	ACCAAAACAAAAACCAACGG	59.7	560	INTERGENIC	--	--	--
Ca5_P2_34714609	Ca5	34714609	TTTATTATTATTATTATT/TTTATTATTATTATT	GTTGTAGCATTGCAGCCGTA	TCCTGCATTCACAACATCAAA	60.1	140	INTERGENIC	--	--	--

INDEL marker IDs	Chromosomes/unanchored scaffolds	Physical positions (bp)	InDels (*Kabuli* reference genome-CDC Frontier/PII)	Forward primers (5'-3')	Reverse primers (5'-3')	Anneling temperature (0C)	Expected amplified product size (bp)	Structural annotation		Functional annotation	
								Sequence components of *kabuli* genome	*Kabuli* gene accession IDs	NCBI-KOG	TFs
Ca5_P2_34834106	Ca5	34834106	GA/GAGGAAATAAGATTA	ATTGTGTGCAGTCATGCGTC	TCCTCATCCAGCAAACACAA	60.2	552	INTERGENIC	--	--	--
Ca5_P2_34860685	Ca5	34860685	CTTTTTTTTTTTTT/CTTTTTTTTTTTT	TGGCTTACCTGTTTTGGAGC	AGTGGGTGACACACATCCAA	59.8	477	INTERGENIC	--	--	--
Ca5_P2_34871969	Ca5	34871969	GA/GAAATGAA	TGACGTTGCATTCACCAAAT	AGGGAGGACGAAAATGACCT	59.9	609	INTERGENIC	--	--	--
Ca5_P2_34981999	Ca5	34981999	ATTTTTTTTT/ATTTTTTTT	ATGTACCCGCACCAGAACTC	TTCAGGTTTCTCCACCCAAC	59.9	249	INTERGENIC	--	--	--
Ca5_P2_34991564	Ca5	34991564	AATATATATATATATATATATATATATATATATATATAT/AATATATATATATATATATATATATATATATATATATATATATATAT	GATTCCCAAATGGCTTGAAA	TATTTTTGGTCGGCCATGTT	60.2	876	INTERGENIC	--	--	--
Ca5_P2_35072594	Ca5	35072594	GATATATATATATATATATATAT/GATATATATATATATATATATATATATATATAT	GGTAGAGGCACATGGGAAAA	CTTGAACTCTCACATGCATGAAC	59.8	760	INTERGENIC	--	--	--
Ca5_P2_35104483	Ca5	35104483	TTATATATATATATATATAT/TTATATATATATATATATATATAT	GCTTCACCCACCTTCCACTA	TCTTGTTTTAGAGTGTTGCAAATGA	60.2	833	INTERGENIC	--	--	--
Ca5_P2_35190711	Ca5	35190711	GAAAA/GAAAAA	TGTCACTTTTTGTTTCGTCCT	TGAAATGTTGCCCACTGTGT	60.0	454	DRR	Ca_01716	O	--
Ca5_P2_35231327	Ca5	35231327	ATTTTTTTT/ATTTTTTTTT	CACGAACCATAGCATGGGA	ATTGGATCGGGCCTAGTGTA	59.4	834	INTERGENIC	--	--	--
Ca5_P2_35247530	Ca5	35247530	TAAAAAAAAA/TAAAAAAAAAA	GCTCACACGAGTGTTAATGGAA	GAAAAGTAGCAAGTACGGGTCG	60.2	584	INTERGENIC	--	--	--
Ca5_P2_35328160	Ca5	35328160	ATTTTTTTTT/ATTTTTTTTTT	ACCACGTGACCCCTTAACTT	TGATGAAATTACGTTATATTGAGGATG	59.6	204	INTERGENIC	--	--	--
Ca5_P2_35485623	Ca5	35485623	TAAAAAAAAA/TAAAAAAAAAA	CCCAACTTCTTCATTGCCTC	GGACCCAGCAGAGTTGGTAA	60.1	639	INTERGENIC	--	--	--

INDEL marker IDs	Chromosomes/unanchored scaffolds	Physical positions (bp)	InDels (*Kabuli* reference genome-CDC Frontier/PII)	Forward primers (5'-3')	Reverse primers (5'-3')	Anneling temperature (0C)	Expected amplified product size (bp)	Structural annotation		Functional annotation	
								Sequence components of *kabuli* genome	*Kabuli* gene accession IDs	NCBI-KOG	TFs
Ca5_P2_35527896	Ca5	35527896	ATTTTTTTT/ATTTTTTTTT	AAATAAAAGGGGAAGGTGGC	CGTTTAACTCTGCGTGGGAT	60.1	717	INTERGENIC	--	--	--
Ca5_P2_35572820	Ca5	35572820	AC/A	ACATAAGCCAAACAACCCCA	AGAGCGATCTAGTCCGCAAA	60.1	892	INTERGENIC	--	--	--
Ca5_P2_35599782	Ca5	35599782	TTATATATATATATATATATATA/TTATATATATATATATATATA	ATTTCCAACTGTTTGGGCAG	TACTAGATCGCGTCCCACG	59.8	665	INTERGENIC	--	--	--
Ca5_P2_35607633	Ca5	35607633	CAATAATAATAATAATAATAATAATAATAAAAATAATAATAATAATAA/CAATAATAATAATAATAATAATAATAATAATAATAAAAATAATAATAATAATAA	TCGTTTACAGCATCGACCAA	GAACAATCTTTGCACGTCCC	60.5	531	INTERGENIC	--	--	--
Ca5_P2_35608840	Ca5	35608840	ATTTT/ATTT	TCTTAACGCACGCATTTCTG	GCAGTCTTGGTGTTGGGTTT	60.0	528	INTERGENIC	--	--	--
Ca5_P2_35734976	Ca5	35734976	CNNNNNNNNNN/CNNN	TTTTGGGCAACTTGGTTAGG	TCAATATAAACGGCGAAGGG	59.9	570	INTERGENIC	--	--	--
Ca5_P2_3575663	Ca5	3575663	A/AC	TTTGACGAAGAGCCATGTTG	CGGGAATCAGTTTTGAGGAA	60.0	254	INTERGENIC	--	--	--
Ca5_P2_357876	Ca5	357876	A/AC	GTGAACAAGCCCATTGGAGT	GGATTGGCAATTTCATTTGG	60.1	161	INTERGENIC	--	--	--
Ca5_P2_35983003	Ca5	35983003	GTTATTATTATTATTATT/GTTATTATTATTATTATTATT	ATCACGGGATACGACCACAT	AGTGGAGAGAGAGCGGTTGA	60.1	730	DRR	Ca_01625	--	bHLH
Ca5_P2_36021839	Ca5	36021839	ACC/AC	CACCGTTCCTTCCTGACAGT	GTGAAAATGAGTTCGTCCGC	60.6	112	INTERGENIC	--	--	--
Ca5_P2_360986	Ca5	360986	ATTTTTTTTTTT/ATTTTTTTTTT	TGTGAGTTTAAGGGAGGGGA	GCATCCAGGATCAGAGGTGT	60.1	390	DRR	Ca_18192	P	--
Ca5_P2_36120649	Ca5	36120649	GA/GATAA	GCTTTCAGGCCAGACTATGC	AGGGGTAGGAATAGGCCAGA	59.9	118	INTERGENIC	--	--	--

INDEL marker IDs	Chromosomes/unanchored scaffolds	Physical positions (bp)	InDels (*Kabuli* reference genome-CDC Frontier/PII)	Forward primers (5'-3')	Reverse primers (5'-3')	Anneling temperature (0C)	Expected amplified product size (bp)	Structural annotation		Functional annotation	
								Sequence components of *kabuli* genome	*Kabuli* gene accession IDs	NCBI-KOG	TFs
Ca5_P2_3615116	Ca5	3615116	TAGAAGAAGAAGAAGAAGAAGAAGAAGAAGAAGAAGAAGAA/TAGAAGAAGAAGAAGAAGAAGAAGAAGAAGAAGAAGAAGAA	TTGAGGGTGACATTGCAAAA	TAAATTGGTCAACGGCACAA	60.0	415	INTERGENIC	--	--	--
Ca5_P2_36157082	Ca5	36157082	ATTT/ATT	TGAGTTTTTCTTCTACATACCACCA	TTGTTCGCAACCATCAAGAG	59.8	358	INTERGENIC	--	--	--
Ca5_P2_36176137	Ca5	36176137	AATATATATATAT/AATATATATATATAT	TCATTGACATTTTGTCCCCA	TTGGTTGGAAAGGTTTGTCA	59.0	617	DRR	Ca_01608	--	--
Ca5_P2_36212540	Ca5	36212540	GTT/GTTAGATT	TTTCATTGTTTCCGTCACCA	TTGAGTCAATGCAAGAGTCCTT	59.0	874	DRR	Ca_01603	I	B3
Ca5_P2_36213208	Ca5	36213208	CAT/CATTGAT	GGAATCGCCACAATGGTAAG	AAGGATCTCGAGAGCACGAA	60.1	408	DRR	Ca_01603	I	B3
Ca5_P2_36214320	Ca5	36214320	G/GC	TACCAACCCAACAATGCAGA	TGGTGATGGTGATGGAACTG	60.4	475	DRR	Ca_01603	I	B3
Ca5_P2_36285568	Ca5	36285568	TAAAAAAAA/TAAAAAAAAA	GGCTTAACGCTCTGTCGTCT	AGCAGCTGCCATTTGAGAAT	60.0	630	INTERGENIC	--	--	--
Ca5_P2_36308359	Ca5	36308359	TTTATT/TTTATTGGTATTATTATTATT	GTTTGTGGCGGATTTTGTTT	CCGATACAACGGAATTGTCC	60.2	423	DRR	Ca_01593	Z	--
Ca5_P2_36375832	Ca5	36375832	AATATATATATATATATATATATATATATATAT/AATATATATATATATATATATATATATAT	GTGCGCGAGTGAAATAGGAT	AGCACGATGCAAGAGTTGTG	60.1	580	INTERGENIC	--	--	--
Ca5_P2_36414199	Ca5	36414199	ATTTTTTT/ATTTTTT	AACTCGCAATGCATCCAATC	TGTGACCATGATTTTGTGGG	60.2	240	INTERGENIC	--	--	--
Ca5_P2_36445067	Ca5	36445067	ATTTTT/ATTTT	ACCAGCTTCCACCCCTCTAT	GAAACGAAGCTTTGGAGCAC	60.0	687	INTRON	Ca_01575	--	--
Ca5_P2_36491208	Ca5	36491208	AG/AGG	TTCGCAACTCCACTCTTCCT	AACACAACGTGACACCCAAA	59.9	476	DRR	Ca_01569	D	M-type

INDEL marker IDs	Chromosomes/unanchored scaffolds	Physical positions (bp)	InDels (*Kabuli* reference genome-CDC Frontier/PII)	Forward primers (5'-3')	Reverse primers (5'-3')	Anneling temperature (0C)	Expected amplified product size (bp)	Structural annotation		Functional annotation	
								Sequence components of *kabuli* genome	*Kabuli* gene accession IDs	NCBI-KOG	TFs
Ca5_P2_36551187	Ca5	36551187	TAAAAAAAAAAA/TAAAAAAAAAA	CATACACGGAAGCACCAATG	TTACAGCCGGCCATCTTATC	60.1	323	INTERGENIC	--	--	--
Ca5_P2_36583877	Ca5	36583877	CATATATATATATATATAT/CATATATATATATATATATATAT	TCACTTTTAGGATCCGTTTGA	CACAATGGACCATGCACTTC	60.0	809	INTERGENIC	--	--	--
Ca5_P2_36612442	Ca5	36612442	TAAAAA/TAAAA	AAGTGGTTGAGGACGATGGA	ACGTGACAAAAGGACTTGGG	60.0	336	INTERGENIC	--	--	--
Ca5_P2_36634446	Ca5	36634446	GTTTTTTTTTTTTTT/GTTTTTTTTTTTTT	AAATGGGAAGAGGAGGAGGA	ATTTAATGGGCTGTTGTCCG	59.8	671	INTERGENIC	--	--	--
Ca5_P2_36640481	Ca5	36640481	GAAAAAAAAAA/GAAAAAAAAAAA	TGCACGAGAGGATTTGTTTG	TGGCAATAGGGGTGGAAATA	60.1	375	INTERGENIC	--	--	--
Ca5_P2_36675050	Ca5	36675050	GAAAAAAAAAA/GAAAAAAAAA	AGGTGTGTTTTGACGGCTCT	GACCTTCATTTATTCAAATGGATTCT	60.0	815	DRR	Ca_01554	--	NAC
Ca5_P2_36688846	Ca5	36688846	CATTATTATTATTATTAT/CATTATTATTAT	AGCAGGATTGGGCCTTTAAT	GGTCATTTTTAAGCTTCGATGG	60.0	466	INTERGENIC	--	--	--
Ca5_P2_36740490	Ca5	36740490	ATAT/ATATTTAT	TATTGTCTAACGCGCCTCAA	TCACTTGCTTCGGTCTGTCA	60.6	606	INTERGENIC	--	--	--
Ca5_P2_36742914	Ca5	36742914	CTTTTTTTTTTT/CTTTTTTTTTT	CAAATGTTGGGCTTGGAGTC	ATTTCTTTGGGGATTTTGGG	60.0	351	INTERGENIC	--	--	--
Ca5_P2_36897567	Ca5	36897567	TTATATATATATATATATATATATATAT/TTATATATATATATATATATATATATATAT	AGTCCCCATCTCAACTCGTG	TGATTTGGACGGAGAAAACC	59.9	383	INTERGENIC	--	--	--
Ca5_P2_37010323	Ca5	37010323	TAT/TAGTATATTGAT	TGCGTGAAAGGTTGAAAATG	TTGGTTTCATTGTGGGGAAT	60.0	540	INTERGENIC	--	--	--
Ca5_P2_37015456	Ca5	37015456	TTATATATATATATA/TTATATATATATATATA	TTGCATTTTGGTCTCTCTCTCA	TGTCAGCATCATGCACTTCA	60.0	534	INTERGENIC	--	--	--

INDEL marker IDs	Chromosomes/unanchored scaffolds	Physical positions (bp)	InDels (*Kabuli* reference genome-CDC Frontier/PII)	Forward primers (5'-3')	Reverse primers (5'-3')	Anneling temperature (0C)	Expected amplified product size (bp)	Structural annotation		Functional annotation	
								Sequence components of *kabuli* genome	*Kabuli* gene accession IDs	NCBI-KOG	TFs
Ca5_P2_37027315	Ca5	37027315	ATTTTTTTTT/ATTTTTTTT	GTTCGAAATGCTGCTTCCTC	GGACATCCTCCTCCATCTGA	60.0	433	INTERGENIC	--	--	--
Ca5_P2_37056417	Ca5	37056417	CTATATATATATATATATATA/CTATATATATATATATA	CCGATTAGAGATCAATGCAGAG	TATGCATTGGGCGAGTGTTA	60.1	334	INTERGENIC	--	--	--
Ca5_P2_37070230	Ca5	37070230	CTTTTT/CTTTTTT	CCAACCAATTCAACGGAAAC	GGGCCAATTATATTGGACAGA	58.8	704	DRR	Ca_01509	J	GRAS
Ca5_P2_37218586	Ca5	37218586	TATACATACATACATACATACATA/TATACATACATACATACATA	CACTCGTATCATGCTTGTCG	CTTTTTCGCCACCATTTGTT	60.0	680	INTERGENIC	--	--	--
Ca5_P2_37228639	Ca5	37228639	AG/AGG	CAAGTTCGTGCCCATTCTTT	AGCAGCTCGAATCCAACACT	60.0	480	INTERGENIC	--	--	--
Ca5_P2_37229002	Ca5	37229002	CCACACACACACACACACACA/CCACACACACACACACACA	GAGCTGCTGAGCTGGACTTT	GCAACTGACTTGTTGTGCGT	60.0	281	INTERGENIC	--	--	--
Ca5_P2_37235701	Ca5	37235701	AATATATATATATATATATATATATA/AATATATATATATATATATATATA	GTTGGAGCCAAAAACTAGCG	ACATTCATTGGCCCTTTTGA	60.3	577	INTERGENIC	--	--	--
Ca5_P2_37244251	Ca5	37244251	CATATATATATATATATATATATATATATATAT/CATATATATATATATATATATATAT	TTTGGACAAACCCAATCTCA	GTACCGTGCGATCCAATTTA	58.5	690	INTERGENIC	--	--	--
Ca5_P2_37288205	Ca5	37288205	TAAAAAAA/TAAAAAAAA	TCACGCGCATTTATGAATTT	ACGCAAACTGAGTCTGTCCA	59.5	596	INTERGENIC	--	--	--
Ca5_P2_37303563	Ca5	37303563	GTGTGTGTGT/GTGTGTGTGTATGTGTGTGT	GATTTATTGTGTGCGTGCGT	AAAGGATCAACTCGGGAAAAA	59.9	183	INTERGENIC	--	--	--
Ca5_P2_37347650	Ca5	37347650	ATTTTTTTTTTTT/ATTTTTTTTTTT	CTAGGTCTCGGCCAAAACAA	TTTGCGTTACGAATTTGATGA	59.2	448	INTERGENIC	--	--	--
Ca5_P2_37401293	Ca5	37401293	T/TCCGGATTCA	TCGTTGCACTTGATAAACGC	TATATTCGAATCCGAAGCGG	60.0	132	INTERGENIC	--	--	--

INDEL marker IDs	Chromosomes/unanchored scaffolds	Physical positions (bp)	InDels (*Kabuli* reference genome-CDC Frontier/PII)	Forward primers (5'-3')	Reverse primers (5'-3')	Anneling temperature (0C)	Expected amplified product size (bp)	Structural annotation		Functional annotation	
								Sequence components of *kabuli* genome	*Kabuli* gene accession IDs	NCBI-KOG	TFs
Ca5_P2_3742109	Ca5	3742109	TT/TTACATAT	ATTTACGAGCAGCGGAAAGA	TGGTCATGCAGAAACTCAGC	60.0	605	INTERGENIC	--	--	--
Ca5_P2_3742639	Ca5	3742639	TA/TAA	TGCTGAGTTTCTGCATGACC	CCCGTGATAGGCAGTACGTT	60.0	597	INTERGENIC	--	--	--
Ca5_P2_3755353	Ca5	3755353	ATTTTTTTT/ATTTTTTTTT	AGCATTGTGAACAAGGTCCC	CCTGCTTGTTGTGGTGAGTG	60.3	487	INTERGENIC	--	--	--
Ca5_P2_37666217	Ca5	37666217	TAAAAAAAAAAA/TAAAAAAAAAA	CTGTTGATGCCTTGTCGGTA	GAAAGCAAGGGGAATCATCA	60.0	448	INTRON	Ca_01456	R	ERF
Ca5_P2_37673409	Ca5	37673409	G/GT	GGGAAACGCAAGAAATGAGA	ACGGTTGAGAATTGAGTGGC	60.1	396	DRR	Ca_01455	TU	--
Ca5_P2_37676399	Ca5	37676399	GTTTTTTTTTT/GTTTTTTTTTTT	TAGCCTCAATCTCACTCGCA	AAATGGTAAACACCGCGAAG	60.0	797	INTERGENIC	--	--	--
Ca5_P2_37713383	Ca5	37713383	TAAACAAAACAAAACAAAACAAAACAAA/TAAACAAAACAAAACAAAACAAA	CATCGTGTGTCCATCCTTTG	TCTCGTATTTTCCGCCAGTT	59.7	658	INTERGENIC	--	--	--
Ca5_P2_37757226	Ca5	37757226	CAAAAAAA/CAAAAAAAA	AGCATTATAGGGAGCGCAGA	ATTCTAGGACGAAGCCGTCA	59.8	379	INTERGENIC	--	--	--
Ca5_P2_37799581	Ca5	37799581	CTTTTT/CTTTTTT	GCACCGTGATTTTGATGATG	ATCAAGTCAGTGCTGCATCG	60.0	594	DRR	Ca_01443	--	--
Ca5_P2_378415	Ca5	378415	T/TG	AGCTTTAGCTTGAGGGGGAG	TCGATTTAATCCGCAGAAGG	60.2	510	DRR	Ca_18193	E	--
Ca5_P2_37868766	Ca5	37868766	CATTATATTATATTATATTATATTATAT/CATTATATTATATTATATTATATTATATTATAT	GCGAAAACTTTGGTGGTGTT	ACAAGATCCACACCGCTACC	60.0	541	INTRON	Ca_01437	H	--
Ca5_P2_3787032	Ca5	3787032	TATAATAATAATAATAATAATAATAATAATAATAATAATATTAATAGTAATAACAATATTAATAATAATAATAATAATAACAATATTAATAATAATAATAATAATAATAATATTAATAATAATAATAATAATAATAATAATAATAA/TATAATAATAATAATAATAATAAT[illegible]	CATACAATAAGATCCATCGCTGA	GATCCTTGCACACATCTAACG	58.2	680	INTERGENIC	--	--	--

INDEL marker IDs	Chromosomes/unanchored scaffolds	Physical positions (bp)	InDels (*Kabuli* reference genome-CDC Frontier/PII)	Forward primers (5'-3')	Reverse primers (5'-3')	Anneling temperature (0C)	Expected amplified product size (bp)	Structural annotation		Functional annotation	
								Sequence components of *kabuli* genome	*Kabuli* gene accession IDs	NCBI-KOG	TFs
Ca5_P2_37903499	Ca5	37903499	T/TATA	TCACGTGACACACGAACAAA	GCACCTATTTATCGGGATGC	59.4	279	INTERGENIC	--	--	--
Ca5_P2_37930695	Ca5	37930695	TT/TTTTCATAGGT	TCCTTGCTCCACTTTTCCAT	TGGTTGATTGGCTGATTAATTG	59.8	644	INTERGENIC	--	--	--
Ca5_P2_37951256	Ca5	37951256	CAGAGA/CAGAGAGA	GAGCTTTAAATCAGGATGCGA	AAAATCTCTCGCGCTGTCAT	60.0	716	INTERGENIC	--	--	--
Ca5_P2_37955644	Ca5	37955644	AATATATATATATATATATATATATAT/AATATATATATATATATATATATATATAT	TTGGAGAGGTGCATGGTAAA	TCGTTGTCTTTTTGTCTTGCC	60.3	603	INTERGENIC	--	--	--
Ca5_P2_37980921	Ca5	37980921	TGGG/TGG	TGATTTATGATTGTGGGCGA	GTTTGGATGCGTGTTTCCTT	60.0	704	DRR	Ca_01423	U	bHLH
Ca5_P2_37981277	Ca5	37981277	ATT/AT	AAGGAAACACGCATCCAAAC	TGCGCAAAATCGTAGACAAG	60.0	311	DRR	Ca_01422	B	MYB_related
Ca5_P2_37982128	Ca5	37982128	CGCCAATA/C	AGCTCCAAGAGCAGTTCCAA	ATCACGTCCCCTACTTCACG	60.0	599	DRR	Ca_01423	U	bHLH
Ca5_P2_37994601	Ca5	37994601	AT/ATTCTCCT	TTGTTGATGGCTGATTGCAT	GTTTTAGCACCAGCTTTGGC	59.9	943	INTRON	Ca_01421	B	--
Ca5_P2_37997822	Ca5	37997822	TAAAAAA/TAAAAAAA	GCTATTGCAAACTCCAATCAA	TGGTGAACACTCCGGATTTA	59.0	494	DRR	Ca_01421	B	--
Ca5_P2_38000622	Ca5	38000622	ATTTTTTTTTTTT/ATTTTTTTTTTT	GCCATGTGCGTGATTTTTCT	TGGAATAAAATGCCAAAACAAA	59.4	643	INTERGENIC	--	--	--
Ca5_P2_38003361	Ca5	38003361	GAAAAAAAAAA/GAAAAAAAAAAA	CTTGTTGCCATTTTGCTTGA	TGGCTTCGACATAACAATCG	59.7	713	INTERGENIC	--	--	--
Ca5_P2_38005397	Ca5	38005397	CTCT/C	TGAGAGACCTTGATTGATACAAATG	TCAATTTGGTTCCAACGTCAT	60.2	540	INTERGENIC	--	--	--

INDEL marker IDs	Chromosomes/unanchored scaffolds	Physical positions (bp)	InDels (*Kabuli* reference genome-CDC Frontier/PII)	Forward primers (5'-3')	Reverse primers (5'-3')	Anneling temperature (0C)	Expected amplified product size (bp)	Structural annotation		Functional annotation	
								Sequence components of *kabuli* genome	*Kabuli* gene accession IDs	NCBI-KOG	TFs
Ca5_P2_38007676	Ca5	38007676	CTTTTTTTTTT/CTTTTTTTTT	TTTGACGGGTCTCATTCTGA	TGACACATTTATTCGACCGC	59.5	462	INTERGENIC	--	--	--
Ca5_P2_38008849	Ca5	38008849	AAGA/AA	TGATGATGAAATGGACCCAA	GGGGATTTTCGTTTTGGTTT	60.0	365	INTERGENIC	--	--	--
Ca5_P2_38016624	Ca5	38016624	TAAGAAA/TAA	ATCCTCGAGAAGGAAGGAGC	CCTTTTCCCTCCAATGTCAA	59.9	276	INTRON	Ca_01419	A	--
Ca5_P2_38017775	Ca5	38017775	CTTTTTT/CTTTTT	TGATCCCATGTGCACAAACT	CTATCCACACAACGTCGAGC	59.3	405	INTRON	Ca_01419	A	--
Ca5_P2_38019468	Ca5	38019468	ATTTTTTTT/ATTTTTTT	TTTTTCCAGCTTTGGACCAG	CAAACGTAACCATTGTGCCA	60.4	644	DRR	Ca_01419	A	--
Ca5_P2_38021365	Ca5	38021365	AAAGCA/AAAGCAAGCA	TCTTGCATTTTCCTCAGCCT	ATTTTGGCAGCTGTCTTTGG	60.2	507	INTERGENIC	--	--	--
Ca5_P2_38023980	Ca5	38023980	AACACAC/AACAC	CCGCAGTAAGAGTTTGACCA	TTCGGAGGAATGGTTGTTTC	59.9	548	INTERGENIC	--	--	--
Ca5_P2_38026164	Ca5	38026164	ATTTTTTTTTT/AATTTTTTTTTTTT	ATGCCAAAAATAAACGCCAG	ATTGCAATGAAAAGGCAACC	59.9	738	INTERGENIC	--	--	--
Ca5_P2_38027300	Ca5	38027300	ACT/A	TTGTTCTCCACCACCATTTG	TGCTTACAAGGTATTCTTGCCA	59.8	916	INTERGENIC	--	--	--
Ca5_P2_38027475	Ca5	38027475	T/TA	TGGAGTGATACACACTTTTGTGG	TGCTTACAAGGTATTCTTGCCA	59.8	446	INTERGENIC	--	--	--
Ca5_P2_38028893	Ca5	38028893	CTATTTATTTATTTATTT/CTATTTATTTATTT	TCAATCACCTTAGAATCTCACCC	CGAAGTGGTTAGATCGGCTC	59.8	603	DRR	Ca_01418	R	DBB
Ca5_P2_38031714	Ca5	38031714	ATTTTTT/ATTTTT	GACTGGAAACCTTCCGTTGA	TCTGTTTTCTCGCACCACTG	60.0	612	INTRON	Ca_01418	R	DBB

INDEL marker IDs	Chromosomes/unanchored scaffolds	Physical positions (bp)	InDels (*Kabuli* reference genome-CDC Frontier/PII)	Forward primers (5'-3')	Reverse primers (5'-3')	Anneling temperature (0C)	Expected amplified product size (bp)	Structural annotation		Functional annotation	
								Sequence components of *kabuli* genome	*Kabuli* gene accession IDs	NCBI-KOG	TFs
Ca5_P2_38032027	Ca5	38032027	ATCTCTCTCTC/ATCTCTCTCTCTC	CAGTGGTGCGAGAAAACAGA	TTCCATAATGTCCACGCAAA	59.9	429	INTERGENIC	--	--	--
Ca5_P2_38034605	Ca5	38034605	CG/CGG	CTGCTATTGCGATCCCTCTC	TTTTGAACGCAAGAAAACAGAA	59.9	800	INTERGENIC	--	--	--
Ca5_P2_38034644	Ca5	38034644	GTAT/GTATATAT	CTGCTATTGCGATCCCTCTC	TTTTGAACGCAAGAAAACAGAA	59.9	800	INTERGENIC	--	--	--
Ca5_P2_38034841	Ca5	38034841	TTTA/TTTAATTA	TCGTCCGATTTATACCGACA	CGACGAGGTATATGCCAACA	59.6	856	INTERGENIC	--	--	--
Ca5_P2_38042134	Ca5	38042134	T/TA	TTGTCATAGGTTATACGGTAGGTCTC	GGGGACCAAAACCACAATTA	59.5	265	INTERGENIC	--	--	--
Ca5_P2_38043572	Ca5	38043572	TAAAA/TAAA	AGGAGGCTAATTGAGGGAGC	TGTGGGTGCATGGTAGAAAA	60.0	519	INTERGENIC	--	--	--
Ca5_P2_38045991	Ca5	38045991	ATT/ATTTTCTT	TGACATTTTCAAATGCCCAG	TGAAATGCGAAAGAGGTGAA	59.4	652	INTERGENIC	--	--	--
Ca5_P2_38048496	Ca5	38048496	CGAGTT/C	GTTGCATTTGGGCAATTTCT	CTCCTTCAGCAAAGCATACG	58.7	527	INTERGENIC	--	--	--
Ca5_P2_38049343	Ca5	38049343	TAAAAAAAAAA/TAAAAAAAAA	AAACTCGCAAACATCTATCTCG	TCCTTTCATGATTATGCATTTGA	59.4	732	INTERGENIC	--	--	--
Ca5_P2_38051854	Ca5	38051854	TTTTTCTTT/TTTT	AAAAGTTGGCCTTTCCTAACAA	TCAATTGTGTGCATCAGTTTCA	60.2	534	INTERGENIC	--	--	--
Ca5_P2_38052063	Ca5	38052063	CATATGAATATATGAAT/CATATGAAT	CAACAAAATTTCCCAAAAATTGA	TTTTTGGCAGTTATCGACATT	57.3	822	INTERGENIC	--	--	--
Ca5_P2_38052150	Ca5	38052150	TAAAAAAAAAA/TAAAAAAAAA	CAACAAAATTTCCCAAAAATTGA	TTTTTGGCAGTTATCGACATT	57.3	822	INTERGENIC	--	--	--

INDEL marker IDs	Chromosomes/unanchored scaffolds	Physical positions (bp)	InDels (*Kabuli* reference genome-CDC Frontier/PII)	Forward primers (5'-3')	Reverse primers (5'-3')	Anneling temperature (0C)	Expected amplified product size (bp)	Structural annotation		Functional annotation	
								Sequence components of *kabuli* genome	*Kabuli* gene accession IDs	NCBI-KOG	TFs
Ca5_P2_38058498	Ca5	38058498	AG/A	TTGTTGACAACTTGCCGAAG	GCGGTACTATTTAGGCCGAT	58.3	492	DRR	Ca_01417	R	--
Ca5_P2_38058853	Ca5	38058853	CTTT/CTTTTT	CGGCCTAAATAGTACCGCAA	GGGTCCATGAAAAACATTCG	60.2	557	DRR	Ca_01417	R	--
Ca5_P2_38066285	Ca5	38066285	CAAGATGAAGACGAAGATGAAGA/CAAGATGAAGATGAAGACGAAGATGAAGA	CCGTCCTCTTCTTCGACAAC	CGGCGATCTTGGACCTATTA	60.1	307	CDS (large-effect mutations)	Ca_01413	--	--
Ca5_P2_38067303	Ca5	38067303	TTATAT/TT	TGAAATCAAACACCCACATCA	AACTGTCTTGTTTGGATTGTGC	59.1	600	DRR	Ca_01413	--	--
Ca5_P2_38067587	Ca5	38067587	TTAAAAA/TTAAAAAAATTACATATAAAAA	TGAAATCAAACACCCACATCA	GCGACAGATTCAACTCACAGA	59.0	676	DRR	Ca_01413	--	--
Ca5_P2_38067693	Ca5	38067693	CTATATA/CTATATATA	GAGCACAATAATTTTCGTCAACA	GCGACAGATTCAACTCACAGA	59.0	593	DRR	Ca_01413	--	--
Ca5_P2_38067755	Ca5	38067755	AGACAG/A	AGCAATGTGCACAATCCAAA	GCGACAGATTCAACTCACAGA	59.0	106	DRR	Ca_01413	--	--
Ca5_P2_38068190	Ca5	38068190	GAAAAGAAAAAGA/GAAAAGAAAAAGAAAAAGA	GCAATGTGCACAATCCAAAC	CCCTTACCTGCTAAGCGAAA	59.5	580	DRR	Ca_01413	--	--
Ca5_P2_38070976	Ca5	38070976	TAA/TAAA	CGAATAATGTCGCGATCAAC	TCAAACACGCCTCATTTTGT	59.2	975	INTERGENIC	--	--	--
Ca5_P2_38071203	Ca5	38071203	AG/A	TTTTGGGAAAAAGATTTCGATT	CTCAAACACGCCTCATTTTG	59.3	435	INTERGENIC	--	--	--
Ca5_P2_38071355	Ca5	38071355	AG/A	TTTTGGGAAAAAGATTTCGATT	TACTCGTGAGTGGCGAGTGT	59.5	744	INTERGENIC	--	--	--
Ca5_P2_38071525	Ca5	38071525	TATTGAT/TAT	CAAAATGAGGCGTGTTTGAG	GGGTTGAGAAATGTGAGGGA	59.9	484	INTERGENIC	--	--	--

INDEL marker IDs	Chromosomes/unanchored scaffolds	Physical positions (bp)	InDels (*Kabuli* reference genome-CDC Frontier/PII)	Forward primers (5'-3')	Reverse primers (5'-3')	Anneling temperature (0C)	Expected amplified product size (bp)	Structural annotation		Functional annotation	
[truncated: 570,085 more chars]
