# Supplementary material for: A High-Resolution InDel (Insertion–Deletion) Markers-Anchored Consensus Genetic Map Identifies Major QTLs Governing Pod Number and Seed Yield in Chickpea
Source: Front Plant Sci. 2016 Sep 16;7:1362. doi: 10.3389/fpls.2016.01362 (PMC5025440; doi:10.3389/fpls.2016.01362)
Supplement: Supplementary file 4 [file Table4.PDF]

Table S4. InDel markers (15640) differentiating high pod number-containing parental accession (Pusa 256) and homozygous bulk (HPNB) of a mapping population (Pusa 256 x ILWC 46) from reference *kabuli* accession (CDC Frontier)

INDEL marker IDs	Chromosomes/ unanchored scaffolds	Physical positions (bp)	InDels (*Kabuli* reference genome-CDC Frontier/PII)	Forward primers (5'-3')	Reverse primers (5'-3')	Anneling temperature (0C)	Expected amplified product size (bp)	Structural annotation		Functional annotation	
								Sequence components of *kabuli* genome	*Kabuli* gene accession IDs	NCBI-KOG	TFs
C11122410_P3_2331	C11122410	2331	G/GCGT	GAAAAACACATTCCCGTTGC	TTTTGTGTGTCAGTTGCGCT	60.4	133	INTERGENIC	--	--	--
C11154862_P3_3633	C11154862	3633	CTT/CT	GCCAAGTTCCCACTTAGATCC	GATAGAAGAGGCCGAAACCC	60.0	583	INTERGENIC	--	--	--
C11169592_P3_2162	C11169592	2162	TAAAAAAAAA/TAAAAAAAAAA	TTATTTCAACCGTTTCTCTTTCTT	TGTGCACATCAAACATATGACA	57.7	911	INTERGENIC	--	--	--
C11172910_P3_8105	C11172910	8105	AATATATATATATAT/AATATATATATATATAT	TTCCATCACCAACAAGACCA	GTATTTGCAGGTCCCGTGTC	59.9	287	INTRON	Ca_27557	P	--
C11177592_P3_13168	C11177592	13168	CAAA/CAA	TTGTTGTTGTACATTAACGGGTC	TTTAATTGGAGCATCAACGAAT	58.8	229	URR	Ca_27747	--	--
C11177592_P3_7683	C11177592	7683	CAAA/CAA	CAGGCACACTGCACAAAGTT	TCCCTCGGATTATGTGAAGC	59.9	533	INTRON	Ca_27746	O	bHLH
C11178116_P3_12920	C11178116	12920	CTTATTATTATTATTATTATTATTATTATTATTATTATTATTATTATTATTATT/CTTATTATTATTATTATTATTATTATTATTATTATTATTATTATTATTATT	TGCACATTGGGTCAGACATT	AAACCAACATCCCATAAAGGAG	60.0	786	INTERGENIC	--	--	--
C11181782_P3_14863	C11181782	14863	A/AC	TGAACCCTAGGTTCGTCCAC	TGGGCTTTGATACGTGGTTT	60.0	539	INTERGENIC	--	--	--
C11181782_P3_18097	C11181782	18097	AATATAT/AATATATAT	TTGCACAAAATGACTGGACC	GTTGGCTTGTGGTTGGACTT	59.5	754	INTERGENIC	--	--	--
C11181782_P3_18610	C11181782	18610	TG/T	AAGTCCAACCACAAGCCAAC	GGAGTGTTCGAGAGCCTTTG	60.0	510	INTERGENIC		--	--

INDEL marker IDs	Chromosomes/ unanchored scaffolds	Physical positions (bp)	InDels (*Kabuli* reference genome-CDC Frontier/PII)	Forward primers (5'-3')	Reverse primers (5'-3')	Anneling temperature (0C)	Expected amplified product size (bp)	Structural annotation		Functional annotation	
								Sequence components of *kabuli* genome	*Kabuli* gene accession IDs	NCBI-KOG	TFs
Ca1_P3_10057638	Ca1	10057638	CATATATATATATATATAT/CATATATAT ATATAT	TGATGGGAGCAATGAA TGAA	TCAAATTCCGACTTGA GGGA	60.0	542	INTERGENIC	--	--	--
Ca1_P3_10183238	Ca1	10183238	ACCC/ACC	CCAATAAGCAAACCGG TCAC	ACCATTGCATTTGTTG CTCA	60.4	177	INTERGENIC	--	--	--
Ca1_P3_10428835	Ca1	10428835	GTTTTT/GTTTTTT	TCAAACAAAGCCTAAAA CATCA	TCATGCCTTCTTTCACT TATTCA	57.5	361	INTERGENIC	--	--	--
Ca1_P3_10513925	Ca1	10513925	CTTATTTATTTATTTATTTATTTATTTAT TTATTTATTTATT/CTTATTTATTTATTTA TTTATTTATTTATTTATTTATTTATTTAT T	CCAGATTTCCTTTTCCC CTC	GGGTAGGCCACAGACT GTCTTA	59.9	634	INTERGENIC	--	--	--
Ca1_P3_10577010	Ca1	10577010	TA/TATATACA	AAGGAAGGCCTACTCC CAAA	GGTGGTCCAAATAGTA CCCG	60.1	919	INTERGENIC	--	--	--
Ca1_P3_10705135	Ca1	10705135	CA/CACGGA	TTGCCCTTGGTATTCTT TGC	ACTGGATGTACGGAGG AACG	60.1	365	INTRON	Ca_02693	R	--
Ca1_P3_10843023	Ca1	10843023	TT/TTATTAT	CCGATATCTTCCTGCG ATGT	CACGGTTCAAAGTGAC AACTTC	60.1	192	INTRON	Ca_02674	G	NAC
Ca1_P3_10858035	Ca1	10858035	TTTCTTCTTCTTCTTCTTCTTCTTC TT/TTTCTTCTTCTTCTTCTTCTTCTTCT TCTTCTT	TCATTTGGAAAAGCACA CCA	CCACGAAGTCCCCAAG ATAA	60.1	237	INTERGENIC	--	--	--
Ca1_P3_10893701	Ca1	10893701	CCTCTCTCTCTCTCTCTCT/CCTCTCTC TCTCTCTCT	TTGACTGATTGAGGGC TAAGG	ATTTTGCGACCCAAAT TGTC	59.3	186	INTERGENIC	--	--	--
Ca1_P3_10927883	Ca1	10927883	GC/G	GTTGGAGCCCAAAGTT GTGT	TTGATCGTGACGTTAT TCGC	60.0	596	DRR	Ca_02668	D	M-type

**INDEL marker IDs**	**Chromosomes/ unanchored scaffolds**	**Physical positions (bp)**	**InDels (*Kabuli* reference genome-CDC Frontier/PII)**	**Forward primers (5'-3')**	**Reverse primers (5'-3')**	**Anneling temperature (0C)**	**Expected amplified product size (bp)**	**Structural annotation**		**Functional annotation**	
								**Sequence components of *kabuli* genome**	***Kabuli* gene accession IDs**	**NCBI-KOG**	**TFs**
Ca1_P3_10950952	Ca1	10950952	CATTATTATTATTATTATTATTATTATTATTATTATTATTATTATTATTATTATTATTATTATT/CATTATTATTATTATTATTATTATTATTATTATTATTATTATTATTATTATTATTATTATTATTATTATT	AATGAAGTGGCACATGGTGA	CATCCGTGTTTCGTGGTATG	60.0	396	INTERGENIC	--	--	--
Ca1_P3_11093084	Ca1	11093084	TTATATATATATATATATATA/TTATATATATATATATATATATA	TCACTTCACCACTTTTGGACC	ACCGTCGACAATCAATCATC	60.0	412	INTERGENIC	--	--	--
Ca1_P3_11148737	Ca1	11148737	CAATAATAATAAT/CAATAATAAT	CGTGTAATTGGTAATGTGGCA	GGCTAACTTGAAGTCGTGGC	59.4	693	INTERGENIC	--	--	--
Ca1_P3_11169889	Ca1	11169889	GAAAAA/GAAAA	TGGTCAGCTGCTTGGTACTG	CCACAAGAGTTTTGCCATTG	60.0	878	DRR	Ca_02641	--	--
Ca1_P3_11170701	Ca1	11170701	GGAGTTGTGTGTCGTGAGTTGTGTG/GGAGTTGTGTGTCGTGAGTTGTGTGTCGTGAGTTGTGTG	GCAATGGCAAAACTCTTGTG	ATCTCGAAAAGGGGTTCCAT	59.3	901	DRR	Ca_02642	U	--
Ca1_P3_11190216	Ca1	11190216	ATTACT/ATTACTTACT	TATGGCTAGCTGGTGAAGCA	TAGAATCCCACGCAGCTCTT	59.6	304	INTRON	Ca_02639	--	--
Ca1_P3_11195317	Ca1	11195317	TTCTCTCTCTCTCTCTCTCTCTCTC/TTCTCTCTCTCTCTCTCTCTC	AGGTGGTAAAGACACGTGGG	TGAACTCGTCACGAACCTTG	59.9	338	DRR	Ca_02638	--	--
Ca1_P3_11290997	Ca1	11290997	A/AG	CTGACAATTTTTGTCCGCCT	TCTTTCGCACAAGGTAGGCT	60.1	884	INTRON	Ca_02624	--	MYB_related
Ca1_P3_11331696	Ca1	11331696	AATTTTATTTTATTT/AATTTTATTTTATTTTATTT	CCTCTGCTCACACCCTTTTC	CACCATTTATCTGTTTTGCGTT	59.8	860	INTERGENIC	--	--	--
Ca1_P3_11418092	Ca1	11418092	ATTTTTTTTTTTT/ATTTTTTTTTTT	AAACTCACGATCTCCGAACG	TCCTCCCTCCTTCTTGTTGA	60.3	519	INTERGENIC	--	--	--

INDEL marker IDs	Chromosomes/ unanchored scaffolds	Physical positions (bp)	InDels (*Kabuli* reference genome-CDC Frontier/PII)	Forward primers (5'-3')	Reverse primers (5'-3')	Anneling temperature (0C)	Expected amplified product size (bp)	Structural annotation		Functional annotation	
								Sequence components of *kabuli* genome	*Kabuli* gene accession IDs	NCBI-KOG	TFs
Ca1_P3_11457087	Ca1	11457087	ATTTTTTTTTTT/ATTTTTTTTTT	TGTCCCAAAATGCAAAACAA	GGAGATGACACATCCTATAACTTTTT	59.9	667	INTERGENIC	--	--	--
Ca1_P3_11540175	Ca1	11540175	AATATATATATATATATATATATATATATATATATATATAT/AATATATATATATATATATATATATATATATATATATATATATAT	GCGAAGGAAAGAAGTTCACG	CATTTAAAAGTTCAAAGGCCTGA	60.0	728	DRR	Ca_02595	KB	--
Ca1_P3_11558313	Ca1	11558313	TTATATATATATATATATATATATATATATATATATATATAT/TTATATATATATATATATATATATATATATATATATAT	TGGGTCACTAAAACCTTGCC	GCTTCAACATATTTGGAATGTCA	60.0	679	INTERGENIC	--	--	--
Ca1_P3_11668592	Ca1	11668592	TG/T	AGCAAAAAGGGGTTGAGGTT	CTTCTGAATTTCTGGCCTGC	60.0	327	INTERGENIC	--	--	--
Ca1_P3_11735185	Ca1	11735185	GTATTATTATTATTATT/GTATTATTATTATTATTATTATTATT	TGACAGTCAGAGTGCGATCC	TCTCTGGCAACCGTTCTTCT	60.0	441	INTERGENIC	--	--	--
Ca1_P3_11788825	Ca1	11788825	AATATATATATATATA/AATATATATATATATATA	TGGGAATAGGTCAGACCAGG	CCAAGTTGTTTGAGAGAATTTGTTT	59.9	696	INTERGENIC	--	--	--
Ca1_P3_1186147	Ca1	1186147	CTTTTT/CTTTTTTT	TATCGCAGAATTTTCGTCCC	AAGAGTTGCACCAATGGACC	60.0	320	INTRON	Ca_00147	EH	bHLH
Ca1_P3_12079599	Ca1	12079599	TC/T	CGGATGAATCAATCCTCACC	ACCGTTGACCGTTTTGAAAT	60.3	512	INTERGENIC	--	--	--
Ca1_P3_12092743	Ca1	12092743	AATATATATATATATATATAT/AATATATATATATATATATATATATATAT	CGGCAATTTTGAGTTGATTTG	CATCGGATGGTCCAGAAAGT	60.5	825	INTERGENIC	--	--	--
Ca1_P3_12102708	Ca1	12102708	AT/A	AATGTGCTTGACACTCGTGG	CACAACCCAAGTCAAACCAA	59.8	245	INTRON	Ca_02545	--	--

INDEL marker IDs	Chromosomes/ unanchored scaffolds	Physical positions (bp)	InDels (*Kabuli* reference genome-CDC Frontier/PII)	Forward primers (5'-3')	Reverse primers (5'-3')	Anneling temperature (0C)	Expected amplified product size (bp)	Structural annotation		Functional annotation	
								Sequence components of *kabuli* genome	*Kabuli* gene accession IDs	NCBI-KOG	TFs
Ca1_P3_12110141	Ca1	12110141	ATTTTTTTTTTTTTT/ATTTTTTTTTT	TGAATTGGGCCAAGACATTT	TGGCAGACCACAGATAACCA	60.3	943	INTERGENIC	--	--	--
Ca1_P3_12112319	Ca1	12112319	C/CTTGG	GCGAAATTATTTGACGGAGTTT	CATTTCAGAAGTGAAGAGCAACA	59.5	874	INTERGENIC	--	--	--
Ca1_P3_12232952	Ca1	12232952	TAAAA/TAAAAA	GACCGTGGGCTATAAGTGGA	AAGCTGAAATCATGGGTGGA	60.0	542	DRR	Ca_02532	--	--
Ca1_P3_12253965	Ca1	12253965	CAAA/CA	AATCGCGTTTCCTGAAGCTA	TGAGGATTCAACGCACAAAA	60.0	546	INTERGENIC	--	--	--
Ca1_P3_12376905	Ca1	12376905	GGTTGTT/GGTT	GAAGGTTTGCTACGCAGGAG	TCCACATTGCTTCTCCAAGA	60.0	143	INTERGENIC	--	--	--
Ca1_P3_12420239	Ca1	12420239	CAAAAAAAAA/CAAAAAAAAAA	AAATGCAATCTTGGTTTCCA	TTTTGGTAATGTGCCCATGATA	58.1	595	INTERGENIC	--	--	--
Ca1_P3_12425557	Ca1	12425557	CTTTTTTTTT/CTTTTTTTTTT	GCTAAGGCGACGTGTTGAAT	AGCAATCATTTGAACTGGGC	60.3	892	INTERGENIC	--	--	--
Ca1_P3_12452777	Ca1	12452777	AATATATATATATATATATATATATATA/AATATATATATATATATATATATATA	TTTTTGAAGGATATTGATGCGA	CTCGAGCTAGCCATCCTCAG	59.6	734	INTERGENIC	--	--	--
Ca1_P3_12526252	Ca1	12526252	CTATATATATATATATATATATATATATATAT/CTATATATATATATATATATATATATATATATATATAT	TTTGGTGCACTGGTTCTGAG	AAATGGCACCTCACCAAAAC	59.9	449	INTERGENIC	--	--	--
Ca1_P3_12536004	Ca1	12536004	AAAAAATAAAAATAAAAATAAAAA/AAAAAATAAAAATAAAAA	CGCTTAATACCGACATGGCT	ACGCAGGCAGAAAACTTACC	60.1	952	INTERGENIC	--	--	--

INDEL marker IDs	Chromosomes/ unanchored scaffolds	Physical positions (bp)	InDels (*Kabuli* reference genome-CDC Frontier/PII)	Forward primers (5'-3')	Reverse primers (5'-3')	Anneling temperature (0C)	Expected amplified product size (bp)	Structural annotation		Functional annotation	
								Sequence components of *kabuli* genome	*Kabuli* gene accession IDs	NCBI-KOG	TFs
Ca1_P3_12544202	Ca1	12544202	TTATATATATATATAT/TTATATATATATAT	TCGGTGTCTCCCTTCATTTC	TCATTCAAGGATGCAACTGG	60.1	656	INTERGENIC	--	--	--
Ca1_P3_12553366	Ca1	12553366	ACACAATCAATTCACAA/ACACAA	CCTATTACAGCGCTTTTTCCA	CGCTATAAAAGGGTGCTTGC	59.4	544	INTERGENIC	--	--	--
Ca1_P3_12616198	Ca1	12616198	ATTTTTTTTT/ATTTTTTT	ATGGCCACAATCACAACAAA	TGGGTGGTAGAAGAAAACGTG	59.8	409	INTERGENIC	--	--	--
Ca1_P3_12644693	Ca1	12644693	TA/T	TTGATTTTCAATTGGGAGCC	TGCCAGGATGTAACCCTTTC	59.9	807	INTERGENIC	--	--	--
Ca1_P3_12644727	Ca1	12644727	TGTA/TGTACGTA	TTGATTTTCAATTGGGAGCC	TGCCAGGATGTAACCCTTTC	59.9	807	INTERGENIC	--	--	--
Ca1_P3_12644844	Ca1	12644844	CGG/CG	ACCCAACCGCAAAAACAATA	TGCCAGGATGTAACCCTTTC	60.2	440	INTERGENIC	--	--	--
Ca1_P3_12644922	Ca1	12644922	AC/A	ACCCAACCGCAAAAACAATA	TGCCAGGATGTAACCCTTTC	60.2	440	INTERGENIC	--	--	--
Ca1_P3_12645367	Ca1	12645367	TAAAAAAAA/TAAAAAAAAA	GAAAGGGTTACATCCTGGCA	TTGGATAAAATTCAAGTGTTTGACA	59.9	511	INTERGENIC	--	--	--
Ca1_P3_12647652	Ca1	12647652	CTTTTTTTTTTTT/CTTTTTTTTTT	TGCTTCCAATTTCAAACACAA	TTTTTCCCTCGATTTAATCCTTT	59.2	751	INTERGENIC	--	--	--
Ca1_P3_12653983	Ca1	12653983	TAA/TA	AAAAATGTTCACGCTCCCTC	TTGCTTCAAGATGATGTGGC	59.2	123	DRR	Ca_02490	--	--

INDEL marker IDs	Chromosomes/ unanchored scaffolds	Physical positions (bp)	InDels (*Kabuli* reference genome-CDC Frontier/PII)	Forward primers (5'-3')	Reverse primers (5'-3')	Anneling temperature (0C)	Expected amplified product size (bp)	Structural annotation		Functional annotation	
								Sequence components of *kabuli* genome	*Kabuli* gene accession IDs	NCBI-KOG	TFs
Ca1_P3_12690899	Ca1	12690899	TTAAATAAATAAATAAATAA/TTAAATAAATAAATAAATAAATAA	TGCTTCATACCAACGTTCCA	CAGAGTTTTAAACGTATGACGGTG	60.1	516	INTERGENIC	--	--	--
Ca1_P3_12721902	Ca1	12721902	CATATATATATATATATATATATAT/CATATATATATATATATATATAT	CGCATGATTCAAACAAATGG	CCAACTCAGGTTCAGCAAGA	59.9	746	DRR	Ca_02480	--	--
Ca1_P3_12727627	Ca1	12727627	TTATATATATATATATATATATAT/TTATATATATATATATAT	CCTTACGGACAACCAAGCAT	GCAAAAGAATTTGCAACAAAGA	60.0	419	INTERGENIC	--	--	--
Ca1_P3_12774149	Ca1	12774149	TTAAT/TTAATCGTTATAAT	GAAGCAGCCTCAACCTCAAC	TCCGCCACCTCTAAATATGAA	60.0	782	INTERGENIC	--	--	--
Ca1_P3_12776177	Ca1	12776177	T/TC	ATCTCCCCGTCAGATATCCC	GACAATAACCACCCAGTGCC	60.1	225	INTERGENIC	--	--	--
Ca1_P3_12776605	Ca1	12776605	GAAAAAA/GAAA	GGCACTGGGTGGTTATTGTC	CAATCTTGTTACACAGACGGC	60.2	800	INTERGENIC	--	--	--
Ca1_P3_12783563	Ca1	12783563	CATATA/CATA	GCCACACAAAAAGGAAGGAA	ACAATGTGGCCTCCTTGAAC	60.1	287	INTERGENIC	--	--	--
Ca1_P3_12905675	Ca1	12905675	CAAA/CAA	TTGGGAGGATGCCAAATTAC	TTGAATAACCTCCACGCACA	59.8	941	INTERGENIC	--	--	--
Ca1_P3_12927388	Ca1	12927388	GAAAAAAA/GAAAAAA	ACCTACGACATCGGGTTTTG	TGGAAGAAACCAGAGTTGGG	59.9	919	INTERGENIC	--	--	--
Ca1_P3_12947634	Ca1	12947634	ATAAATTTAAA/ATAAATTTAAAATTTAAATTTAAA	AACTTTGTGGTCGTTTTGGC	TGCTTATTGTGGCTGCTTTG	60.0	647	INTERGENIC	--	--	--

INDEL marker IDs	Chromosomes/ unanchored scaffolds	Physical positions (bp)	InDels (*Kabuli* reference genome-CDC Frontier/PII)	Forward primers (5'-3')	Reverse primers (5'-3')	Anneling temperature (0C)	Expected amplified product size (bp)	Structural annotation		Functional annotation	
								Sequence components of *kabuli* genome	*Kabuli* gene accession IDs	NCBI-KOG	TFs
Ca1_P3_12956423	Ca1	12956423	CAAAAAAA/CAAAAA	TTGTCGGATACCTGCTCAGT	TTGCAAATTCGGACAAACAA	58.3	455	INTERGENIC	--	--	--
Ca1_P3_1297317	Ca1	1297317	ATTTTTTTTTT/ATTTTTTTTT	TGCATGAACAAAGGGACAAG	TAACATCCCGCGTAACATGA	59.7	625	INTERGENIC	--	--	--
Ca1_P3_12985940	Ca1	12985940	ATT/ATTT	TTGATGAGGATCTTTCTATGGGA	TCAACAAGTTATTCGAAAGGCA	59.9	364	INTERGENIC	--	--	--
Ca1_P3_12988934	Ca1	12988934	C/CGGG	ATCGGCATCCTTCCTTCTTT	GACGCTAAGCCCCTTTCTCT	60.0	354	INTERGENIC	--	--	--
Ca1_P3_13007507	Ca1	13007507	TTAT/TTATATAT	CACGGGTCCAGATGGTTAGT	CCAAGCATCTTGGTGCATAA	59.8	322	INTERGENIC	--	--	--
Ca1_P3_13014725	Ca1	13014725	TTCT/TT	AAAATGAAGTCAGTTTTTCAAAAGG	AGCTTGATTTCCACCACGAC	59.2	766	INTERGENIC	--	--	--
Ca1_P3_13060064	Ca1	13060064	AAATAATAATAATAATAATAATAATAATAATAATAATAATAATAATAA/AAATAATAATAATAATAATAATAATAATAATAATAATAATAATAATAATAATAATAA	TCCTGAAACAAATGCACCAA	GGTCTGAAATAAGGCCGTGA	60.1	463	DRR	Ca_14070	O	--
Ca1_P3_13084713	Ca1	13084713	TAAAAAAA/TAAAAAAAA	TCACTGTGCAGATAACAACAACA	GGACACCGTCTTATCACCCA	59.3	570	INTERGENIC	--	--	--
Ca1_P3_13117590	Ca1	13117590	TAATAAAAATAAAAATAAAA/TAATAAAAATAAAA	TGTTGTTTACGGAATTTGGTTTT	TTTCGTTGTATATTAAAGCACGG	59.7	547	INTERGENIC	--	--	--
Ca1_P3_13487363	Ca1	13487363	TAAAAAAAAAAAA/TAAAAAAAAAAAAA	GATGATTGCAGCAAGTTGGA	TCGTGTTGGCTAATTCAACG	59.8	558	INTERGENIC	--	--	--

INDEL marker IDs	Chromosomes/ unanchored scaffolds	Physical positions (bp)	InDels (*Kabuli* reference genome-CDC Frontier/PII)	Forward primers (5'-3')	Reverse primers (5'-3')	Anneling temperature (0C)	Expected amplified product size (bp)	Structural annotation		Functional annotation	
								Sequence components of *kabuli* genome	*Kabuli* gene accession IDs	NCBI-KOG	TFs
Ca1_P3_13570743	Ca1	13570743	AT/ATCTCCTCCT	CCGGAAGAACATTCAAAGGA	TTCAAACCCAAACCTCTTCG	60.0	460	INTERGENIC	--	--	--
Ca1_P3_13671513	Ca1	13671513	AACA/AA	CGAGGAAGAGGTGGATTCAA	TGGTTTATGATAGGTTCAATTTGG	60.2	348	INTERGENIC	--	--	--
Ca1_P3_13671962	Ca1	13671962	ATTT/ATT	CCAAATTGAACCTATCATAAACCA	AAAAGACCAAATCGGGAAAAA	59.2	748	INTERGENIC	--	--	--
Ca1_P3_13672615	Ca1	13672615	AC/A	TTTTTCCCGATTTGGTCTTTT	TTTCCCGATTTGCTCCTTTA	59.8	448	INTERGENIC	--	--	--
Ca1_P3_13679372	Ca1	13679372	CTTTT/CTTT	AGGTGCTCTCCTCTCCACAG	TTTGGCATGGATAGGGGTTA	59.6	429	DRR	Ca_14133	--	ERF
Ca1_P3_13683980	Ca1	13683980	TAAA/TAAAA	ATCGAAATGGGTGCAAAGTC	GGTTGCCTTGGTATGGAGAA	59.9	621	DRR	Ca_14134	--	MYB_related
Ca1_P3_13692764	Ca1	13692764	CTTTT/CTTTTT	CGCCAGTGGTTATGCTCTTA	AAGGAACCACACTTGAACCG	58.9	875	INTERGENIC	--	--	--
Ca1_P3_13697453	Ca1	13697453	TAAAAAAA/TAAAAAAAA	TCTGAAAACAATTTCCCCTTG	CTAGACCATGGCCGTTCAAT	59.1	865	DRR	Ca_14135	--	bHLH
Ca1_P3_13698221	Ca1	13698221	TG/T	TTGAACGGCCATGGTCTAGT	ATGGAATTATGGTGGCGTGT	60.5	611	DRR	Ca_14135	--	bHLH
Ca1_P3_13701840	Ca1	13701840	AGAATGTATAT/A	TGCAAAACCAAGTCTGTCCA	ACCTTGCCCCTGAAGTAGGT	60.3	632	INTRON	Ca_14135	--	bHLH

INDEL marker IDs	Chromosomes/ unanchored scaffolds	Physical positions (bp)	InDels (*Kabuli* reference genome-CDC Frontier/PII)	Forward primers (5'-3')	Reverse primers (5'-3')	Anneling temperature (0C)	Expected amplified product size (bp)	Structural annotation		Functional annotation	
								Sequence components of *kabuli* genome	*Kabuli* gene accession IDs	NCBI-KOG	TFs
Ca1_P3_13703551	Ca1	13703551	GTTTTTTTTTT/GTTTTTTTTTTTT	TTTCCTCCATCTTGTAC GGC	AACCTCAAAAGGATGA CCCC	60.1	533	INTRON	Ca_14135	--	bHLH
Ca1_P3_13704468	Ca1	13704468	ATTTTTTT/ATTTTTT	TCATTAACGGTGCTCTA TGCC	GGATATGCAATTGGGA TGCT	60.1	981	INTRON	Ca_14135	--	bHLH
Ca1_P3_13710997	Ca1	13710997	TAAAAAAAAAA/TAAAAAAAAA	CCACCGAAAAACTTCAT CCA	CCGGAAAACTTCATTC CAAA	60.9	411	INTERGENIC	--	--	--
Ca1_P3_13841782	Ca1	13841782	GTTTTTTTTTT/GTTTTTTTTTTT	CATCTCCTATTTGCGG CCTA	TGAATCAATGCAACTTT TTATGC	60.2	465	INTERGENIC	--	--	--
Ca1_P3_13915867	Ca1	13915867	CAAAAAAAA/CAAAAAAAAA	TACAATTGTGTGCCGT CGAT	ATAGTCAAGAGGCGCT TCCA	60.0	528	INTERGENIC	--	--	--
Ca1_P3_13939746	Ca1	13939746	TA/TACTTTATTAAAAAA	TCAGGTGTCTCATCCA CTGC	TGGATTTTCAATGCCA GACA	59.8	755	DRR	Ca_07137	--	--
Ca1_P3_13950121	Ca1	13950121	AATATATATATATATATATATAT/AATAT ATATATATATATATAT	CAAATTTTGGACGGAG AGGA	ACGGGGATAATGGTTT TGTG	60.0	463	INTERGENIC	--	--	--
Ca1_P3_1397435	Ca1	1397435	CA/C	TGGAAACAGCAACGTG GTAG	CAAAGGGTTGGATTTT CCAC	59.8	555	INTERGENIC	--	--	--
Ca1_P3_14094110	Ca1	14094110	TA/TAA	GAATTTCTTTTTGGGAG GAGAA	CCAACTACATTGTTTGT GCCA	58.7	442	INTERGENIC	--	--	--
Ca1_P3_14194799	Ca1	14194799	ATTTTTTTTT/ATTTTTTTT	CGGTGATATTTGGGGA AATG	AAAGACTTTTTCGATG GCATGT	60.0	762	INTRON	Ca_07116	V	--

INDEL marker IDs	Chromosomes/ unanchored scaffolds	Physical positions (bp)	InDels (*Kabuli* reference genome-CDC Frontier/PII)	Forward primers (5'-3')	Reverse primers (5'-3')	Anneling temperature (0C)	Expected amplified product size (bp)	Structural annotation		Functional annotation	
								Sequence components of *kabuli* genome	*Kabuli* gene accession IDs	NCBI-KOG	TFs
Ca1_P3_14309917	Ca1	14309917	G/GAA	AACCATTGGAAGGCAAAATG	TCTTCCACCTCTTTCGCATC	59.8	106	INTERGENIC	--	--	--
Ca1_P3_14399975	Ca1	14399975	TAAAAAAAA/TAAAAAAA	AGACGTGATCCGACAAAAAGA	GCACCAAAACGGGATAAAAA	59.7	585	INTERGENIC	--	--	--
Ca1_P3_14400629	Ca1	14400629	TCAAAGCAA/TCAA	TGATGCGGCATTGAAGTTAG	TGGGAAACACGGATACAAGA	59.8	290	INTERGENIC	--	--	--
Ca1_P3_14426009	Ca1	14426009	CATATATATATATATATATATATATAT/CATATATATATATATATATATATATATATAT	CAGCCAAGACCCAATTTCAT	ACTCTCCCAAAAAGGCGATT	59.9	432	INTERGENIC	--	--	--
Ca1_P3_14575406	Ca1	14575406	CATATATATATATATATATATATATATA/CATATATATATATATATATATATATATATATATA	TGGTTAGAAATGGAGGAGCC	TGGTGGTGAAAGATTTGGGT	59.1	790	DRR	Ca_07077	--	NAC
Ca1_P3_14660648	Ca1	14660648	T/TA	GGAAATTGAATCAGCAGGGA	AGATGATGCGACCTCTTGCT	60.0	170	INTERGENIC	--	--	--
Ca1_P3_14682464	Ca1	14682464	CTTTT/CTTT	TCCATTGTTGAGCGAAAACA	TGGATGAAGGATCAACCACA	60.2	659	INTERGENIC	--	--	--
Ca1_P3_14695327	Ca1	14695327	CTTTTTTT/CTTTTTT	ATTACAGATGTGGGCCGTTC	CGAGAATCATGTCATGTGGG	59.8	572	DRR	Ca_07061	--	ERF
Ca1_P3_1470537	Ca1	1470537	T/TC	AAGTGGCGATAGGGTTTGTG	CAAAATGTGGCATATGAGCTG	60.0	834	INTERGENIC	--	--	--
Ca1_P3_14727063	Ca1	14727063	CT/C	ATAGCTGGCGGGTCACATAC	TGCGTCCATTGAAAATGATG	60.0	452	INTERGENIC	--	--	--

**INDEL marker IDs**	**Chromosomes/ unanchored scaffolds**	**Physical positions (bp)**	**InDels (*Kabuli* reference genome-CDC Frontier/PII)**	**Forward primers (5'-3')**	**Reverse primers (5'-3')**	**Anneling temperature (0C)**	**Expected amplified product size (bp)**	**Structural annotation**		**Functional annotation**	
								**Sequence components of *kabuli* genome**	***Kabuli* gene accession IDs**	**NCBI-KOG**	**TFs**
Ca1_P3_14843454	Ca1	14843454	GTTT/GTTTT	ATCGATTGAACCAAATCCCA	TTCTAGCTGGCTTTTCTCGC	60.1	802	INTERGENIC	--	--	--
Ca1_P3_14870030	Ca1	14870030	CAATAATAATAATAATAATAATAATAATAATAAT/CAATAATAATAATAATAATAATAATAATAATAAT	TCCTTTTCCATATGCTGCAA	TTTCAAACCAAAACAAACCAAA	59.3	684	INTERGENIC	--	--	--
Ca1_P3_14891282	Ca1	14891282	AATATATATATATATATATATATATATAT/AATATATATATATATATATAT	TCACAAGATAGTTTTGCCTTTTT	CAAAATCATCGGAACGATTACA	57.2	360	INTERGENIC	--	--	--
Ca1_P3_14995819	Ca1	14995819	TTGTTATGTTAT/TTGTTATGTTATGTTAT	CAGCGCTTTTCCCAAATAAG	CCAAAAAGGGTGGAGATTGA	59.8	359	INTERGENIC	--	--	--
Ca1_P3_14995847	Ca1	14995847	ACATGCATGCA/ACATGCA	CAGCGCTTTTCCCAAATAAG	CCAAAAAGGGTGGAGATTGA	59.8	359	INTERGENIC	--	--	--
Ca1_P3_15037301	Ca1	15037301	CATATATATATATATA/CATATATATATATATATA	GTCACACGAGCATGGAAAGT	TAACCTGGCCAGTCACCATT	58.7	578	INTERGENIC	--	--	--
Ca1_P3_15380342	Ca1	15380342	ATTCTTCTTCTTCTTCTTCTT/ATTCTTCTTCTTCTTCTTCTTCTTCTT	GGCTAAGCCATAAGTTCCCA	TGAACAGGATTTGGACGACA	59.2	238	INTERGENIC	--	--	--
Ca1_P3_15667505	Ca1	15667505	AC/A	TCGGGCAAAAATTCTGACAT	CAATTCCCACAGGTACGCTT	60.4	447	INTERGENIC	--	--	--
Ca1_P3_15773203	Ca1	15773203	A/ATATATT	CCTAAAGAAAGATGGGCCTG	TTTGTTCTTTGGGCCTTCTG	58.8	288	INTERGENIC	--	--	--
Ca1_P3_15835234	Ca1	15835234	TTTATTATTATTATTATTATTATTATTATTATT/TTTATTATTATTATTATTATTATTATTATT	TCGTTTGCCGTTAACTAGGG	TTGGCCTAACCTGAAAGCAT	60.1	768	INTERGENIC	--	--	--

INDEL marker IDs	Chromosomes/ unanchored scaffolds	Physical positions (bp)	InDels (*Kabuli* reference genome-CDC Frontier/PII)	Forward primers (5'-3')	Reverse primers (5'-3')	Anneling temperature (0C)	Expected amplified product size (bp)	Structural annotation		Functional annotation	
								Sequence components of *kabuli* genome	*Kabuli* gene accession IDs	NCBI-KOG	TFs
Ca1_P3_15890229	Ca1	15890229	ATTTTT/ATTTTTT	AATTCAAATGCGCTGCTTCT	TTGTCAATTACTTGCATCTCCC	60.0	707	DRR	Ca_06963	--	--
Ca1_P3_15928922	Ca1	15928922	TTTATTATTATTATTATTATTATTATTATTATTATTATTATTATTATTATTA/TTTATTATTATTATTATTATTATTATTATTATTATTATTATTATTA	GGGATAGATTTCTTTGAATGTGC	TTTTTCTGATGGGGTTGCAT	59.0	553	INTERGENIC	--	--	--
Ca1_P3_15952106	Ca1	15952106	TTCTCTCTCTCTCTCTCTCTCTCTCTCTCTCTCTCTCTCTCTCTC/TTCTCTCTCTCTCTCTCTCTCTCTCTCTCTCTCTCTCTCTC	AGCGGCTCTTTCAATTTTCA	TGAGGTAGCCAAATCCAAGG	60.0	334	INTERGENIC	--	--	--
Ca1_P3_16106451	Ca1	16106451	ACCC/ACCCC	AGCCTTTGGCTGTCACTCAT	CCATAATACAAACTGTAGCCGC	59.9	341	INTERGENIC	--	--	--
Ca1_P3_16106562	Ca1	16106562	ATTTTTT/ATTTT	AGCCTTTGGCTGTCACTCAT	TGGGATAATTTCTAAACGAAAAA	59.9	795	INTERGENIC	--	--	--
Ca1_P3_16146114	Ca1	16146114	ATTTTT/ATTTTTT	TACCTTCCCTGCGCATAATC	AGAAGGCTACCACTACGCGA	60.1	529	INTERGENIC	--	--	--
Ca1_P3_16158710	Ca1	16158710	AG/A	AGTCCGCAACCCATTTAGTG	TTTGGGGAATGCTTAAAACC	60.0	619	INTERGENIC	--	--	--
Ca1_P3_16159268	Ca1	16159268	TAAAAAAAAAAAAAAAAA/TAAAAAAAAAAAAAAAAAAAA	ATGGTTTTAAGCATTCCCCA	TTAAGTTGCGAGGTTGCTCA	59.3	457	INTERGENIC	--	--	--
Ca1_P3_16166780	Ca1	16166780	TAAA/TAA	TGTCCAAAAACGGACAAACA	AAAACGTGCCATGCTAAAAA	60.0	551	DRR	Ca_06938	R	--
Ca1_P3_16166882	Ca1	16166882	AC/A	CCAGAGGTGTCCACTGATTACA	TTTTTAAAACGTGCCATGCT	60.0	495	DRR	Ca_06938	R	--

INDEL marker IDs	Chromosomes/ unanchored scaffolds	Physical positions (bp)	InDels (*Kabuli* reference genome-CDC Frontier/PII)	Forward primers (5'-3')	Reverse primers (5'-3')	Anneling temperature (0C)	Expected amplified product size (bp)	Structural annotation		Functional annotation	
								Sequence components of *kabuli* genome	*Kabuli* gene accession IDs	NCBI-KOG	TFs
Ca1_P3_16197666	Ca1	16197666	TAAAA/TAAAAA	TTGGTAAGTGGAAGAAGATGAGA	TTGGAGGCAACCAACAAAAT	58.0	789	INTERGENIC	--	--	--
Ca1_P3_16206173	Ca1	16206173	TAAAA/TAAAAA	TTTTCCCTAAAATGTCACCAAA	GGGGCTTACGAGTACAGACTCA	58.5	644	INTERGENIC	--	--	--
Ca1_P3_16206852	Ca1	16206852	ATATTAT/ATAT	GAGTCTGTACTCGTAAGCCCCT	ATTTTGACAATGGCCACACA	58.9	697	INTERGENIC	--	--	--
Ca1_P3_16213026	Ca1	16213026	CGAGTAGAGTA/CGAGTA	TTTGGAATGCTTCTGAGGCT	TTTCGTTACGGGTCCTTTTG	60.0	682	INTRON	Ca_06936	--	bHLH
Ca1_P3_16360387	Ca1	16360387	AAAAAAAACTATAAAAAAAC/AAAAAAAACTATAAAAAAACTATAAAAAAAC	TGAGCATATAACAGCCCCAA	CAATTGCCTCAGTCCTCCAT	59.1	565	INTERGENIC	--	--	--
Ca1_P3_16421756	Ca1	16421756	TAAAAAAAAA/TAAAAAAAAAA	CTTTGCTGCCCTTTGAAGTC	TTAGAGTTGTGCCATCACGG	60.0	580	INTRON	Ca_06921	PT	FAR1
Ca1_P3_16505055	Ca1	16505055	CAAAAAAAAAAA/CAAAAAAAAAA	GCGAATACAAGTTGGGCAAT	TGACGTGCCAATGCATATTT	60.0	713	INTERGENIC	--	--	--
Ca1_P3_16509431	Ca1	16509431	CA/C	AAATACGGTGCTTTTGGTGC	TCCGACGTGTAACTTCCCAT	60.0	714	INTRON	Ca_06913	--	--
Ca1_P3_16580518	Ca1	16580518	GTTTTTTTTTTT/GTTTTTTTTTTTTTTTTT	CATCTGCATGGAAATCGTGT	AAGAGCCTTGGGAAGAGGAG	59.5	496	INTERGENIC	--	--	--
Ca1_P3_16629521	Ca1	16629521	TTTAATTAATTAATTAATT/TTTAATTAATTAATTAATTAATTAATT	AACGTTTGAGGATTCCGTTG	GAAACTCCAGCTCCAAAACG	60.0	370	INTERGENIC	--	--	--

INDEL marker IDs	Chromosomes/ unanchored scaffolds	Physical positions (bp)	InDels (*Kabuli* reference genome-CDC Frontier/PII)	Forward primers (5'-3')	Reverse primers (5'-3')	Anneling temperature (0C)	Expected amplified product size (bp)	Structural annotation		Functional annotation	
								Sequence components of *kabuli* genome	*Kabuli* gene accession IDs	NCBI-KOG	TFs
Ca1_P3_16661578	Ca1	16661578	TAAAAAAAAAAA/TAAAAAAAAAAAA	TGCATCTCATATGGGTAGTGTG	CAGCGACAATATTATTTATCCCG	58.5	320	INTERGENIC	--	--	--
Ca1_P3_16738344	Ca1	16738344	AAATAATAATAATAATAATAATAATAATAATAATAATAATAATAATAATAAT/AAATAATAATAATAATAATAATAATAATAATAATAATAATAATAATAATAATAAT	TCTCCCAAATTTTGATCCCA	ATGCATGCACGTTGATTTTT	60.2	815	INTERGENIC	--	--	--
Ca1_P3_16739207	Ca1	16739207	TTAATAATAATAATAATAATAATAATAATAATA/TTAATAATAATAATAATAATAATAATAATA	CCACTCATCGTGTAAGCTTTTC	TTCACAATTTGGGAGGGAAG	58.9	630	INTERGENIC	--	--	--
Ca1_P3_16748809	Ca1	16748809	GAAAAAAA/GAAAAAAAA	TTGGGTCACCAAAGGATTTC	GGATGGGAGGAGAAACTTCC	59.8	353	INTERGENIC	--	--	--
Ca1_P3_16753431	Ca1	16753431	ATTT/ATTTT	GTCAATGCGGGTTTTCAAGT	TGTTGCATTTGGGTTCGATA	60.0	724	INTERGENIC	--	--	--
Ca1_P3_16757631	Ca1	16757631	AATATATATATATATATATATATATATA/AATATATATATATATATATATATATATATATATATATATA	GATTCAAGACGAGACGCAAA	AAAAATGTTTTTCAATAAGCAAATC	59.0	562	INTERGENIC	--	--	--
Ca1_P3_1688068	Ca1	1688068	ATTTTTT/ATTTTT	TTGTGCATAGTACCGCCAAG	ACGACATGGGTTTACGGTTC	59.8	575	INTERGENIC	--	--	--
Ca1_P3_1705040	Ca1	1705040	GT/GTATAT	CTGAACCTTTAATTTCAGTCACAA	ATTAATCTTCGCCTTGGTGC	57.6	760	INTERGENIC	--	--	--
Ca1_P3_17188029	Ca1	17188029	TTAT/TT	TAGACTTCCGATGCCACACC	TTTCAATTGCGAGCATGAGA	61.1	102	INTERGENIC	--	--	--
Ca1_P3_1720459	Ca1	1720459	TA/T	CCATGACGCCAACTTCACTA	GGCGAAATCAGCTTTTTGAA	59.7	546	DRR	Ca_00215	R	--

INDEL marker IDs	Chromosomes/ unanchored scaffolds	Physical positions (bp)	InDels (*Kabuli* reference genome-CDC Frontier/PII)	Forward primers (5'-3')	Reverse primers (5'-3')	Anneling temperature (0C)	Expected amplified product size (bp)	Structural annotation		Functional annotation	
								Sequence components of *kabuli* genome	*Kabuli* gene accession IDs	NCBI-KOG	TFs
Ca1_P3_17261345	Ca1	17261345	GTGGTGTATAATG/GTG	TGAACGACGTGTTGAA GTGC	TCCGTTCATTGCATGG TAGA	60.9	331	DRR	Ca_22776	--	--
Ca1_P3_1733198	Ca1	1733198	AAAACAA/AAA	GACCAAATCCACCTTCT CCA	GAGGCAGAATGCTCCA AAAA	59.9	693	INTRON	Ca_00217	--	TALE
Ca1_P3_17339203	Ca1	17339203	TTTATTATTATTATTATTATTATTA/TTTA TTATTATTATTATTATTA	TGGCTTCAAGGTTGAC CACT	CTAGCCCGGAGAGCTA CCTT	60.7	236	INTERGENIC	--	--	--
Ca1_P3_1736894	Ca1	1736894	ATT/AT	CGTCAATGAGTGCTGG AAAA	GGACCCAAAACTTCAC CTCA	59.8	528	INTERGENIC	--	--	--
Ca1_P3_1738013	Ca1	1738013	TAAAAAAA/TAAAAAA	CTAGGATGGAATCCTC TGCC	AGTGCTGCCTCCGTAA GAAA	58.7	638	INTERGENIC	--	--	--
Ca1_P3_1740906	Ca1	1740906	GTTTTTTT/GTTTTTT	GGACAGGATCTTCTGG TGGA	CTTTCTCAATGCAGCA ACCA	60.0	660	INTRON	Ca_00218	--	TALE
Ca1_P3_17436816	Ca1	17436816	A/AC	GACGAAAAGATCACCC TCACA	CATGTGCCACAGGATC ATTC	60.1	512	INTERGENIC	--	--	--
Ca1_P3_1744972	Ca1	1744972	CA/CACAGTTCCACTTTA	ACCCTGTGGCACAATA CCAT	AGCACCGATCAAATCA CACA	60.1	820	INTRON	Ca_00219	--	TALE
Ca1_P3_1746242	Ca1	1746242	ACAAAT/A	AATGCAAAAAGCCGAC ATCT	GTGGGAGTCGTGAAAC TCGT	59.7	281	INTRON	Ca_00219	--	TALE
Ca1_P3_1746561	Ca1	1746561	AT/ATTCTGTCATAATT	ACACGAGTTTCACGAC TCCC	TTGGGTTGAAACTTGA AGGTG	60.2	423	INTRON	Ca_00219	--	TALE

INDEL marker IDs	Chromosomes/ unanchored scaffolds	Physical positions (bp)	InDels (*Kabuli* reference genome-CDC Frontier/PII)	Forward primers (5'-3')	Reverse primers (5'-3')	Anneling temperature (0C)	Expected amplified product size (bp)	Structural annotation		Functional annotation	
								Sequence components of *kabuli* genome	*Kabuli* gene accession IDs	NCBI-KOG	TFs
Ca1_P3_1746940	Ca1	1746940	CT/CTAGT	ACGAGTTTCACGACTCCCAC	CATGATGGAGAAATGGAGATGA	60.2	733	INTRON	Ca_00219	--	TALE
Ca1_P3_17476559	Ca1	17476559	ATTTTTTTT/ATTTTTTTTT	ACCTTCTCACTCAAACCCCC	CAACATCATTGGCAGCAAAA	60.3	457	INTERGENIC	--	--	--
Ca1_P3_17498653	Ca1	17498653	TTATATATATATATATATATATATAT/TTATATATATATATATATATATATATAT	TCATGCCCTAACACCCTCTC	GATGCGACCTCCTATGGTGT	60.1	268	INTERGENIC	--	--	--
Ca1_P3_17532277	Ca1	17532277	CTTTTTT/CTTTTTTT	AAAATTGGCAAATGTTTGTGTG	TCAATATTGGGGCAAAGCAT	59.8	700	INTERGENIC	--	--	--
Ca1_P3_17598565	Ca1	17598565	CTTTTTTTTTT/CTTTTTTTTTTT	TCATACGAGGTTTCTTGGGT	TCTCCAAATTTTTCAACAAACTCA	57.1	482	INTERGENIC	--	--	--
Ca1_P3_17634013	Ca1	17634013	TTATATATATATATATAT/TTATATATATATATATATAT	TGATTGGATGTGGGTGTGAG	TTTCAAAGGCAACAACCCTC	60.4	529	INTERGENIC	--	--	--
Ca1_P3_1777780	Ca1	1777780	TAAAAAAA/TAAAAAA	CGTCACAAAGCATCACAGGT	CTACGGTGCAAACAGAACCA	59.8	563	INTERGENIC	--	--	--
Ca1_P3_1777834	Ca1	1777834	CTCT/CTCTATCT	CGTCACAAAGCATCACAGGT	ATTGCACAACGGCTACAACA	59.8	906	INTERGENIC	--	--	--
Ca1_P3_1779893	Ca1	1779893	A/AAGT	GGTGAATGTTTGCTTCCTCAA	TGCATCGTCACTTTATACGAAA	60.1	222	INTERGENIC	--	--	--
Ca1_P3_1779944	Ca1	1779944	TAAAA/T	GGTGAATGTTTGCTTCCTCAA	AATAAATCACTTTTATTTTCCCGTT	60.1	637	INTERGENIC	--	--	--

INDEL marker IDs	Chromosomes/ unanchored scaffolds	Physical positions (bp)	InDels (*Kabuli* reference genome-CDC Frontier/PII)	Forward primers (5'-3')	Reverse primers (5'-3')	Anneling temperature (0C)	Expected amplified product size (bp)	Structural annotation		Functional annotation	
								Sequence components of *kabuli* genome	*Kabuli* gene accession IDs	NCBI-KOG	TFs
Ca1_P3_1780151	Ca1	1780151	TAAAAAAA/TAAAAAAAA	GGTGAATGTTTGCTTCCTCAA	TGGTTGAGATGCATCAAAATAAAT	60.1	843	INTERGENIC	--	--	--
Ca1_P3_1781854	Ca1	1781854	GAAAAA/GAAAAAA	AATTTCCATGCACAAGGTCC	CCAAGCCCAAAGTATGGAAA	59.8	723	INTRON	Ca_00224	S	--
Ca1_P3_1781920	Ca1	1781920	CAAAA/CAAAAAA	AATTTCCATGCACAAGGTCC	CCAAGCCCAAAGTATGGAAA	59.8	723	INTRON	Ca_00224	S	--
Ca1_P3_17843943	Ca1	17843943	ATTTTTTT/ATTTTTTTT	TCATGGCAATTTGTCAGCTC	TTGTTTCATCACTCCCTCCC	59.8	552	INTERGENIC	--	--	--
Ca1_P3_1785166	Ca1	1785166	AG/A	TCTACTTGCGGCAACTTGTG	CATCATTCCAATCAAGCACG	60.1	319	DRR	Ca_00224	S	--
Ca1_P3_1785215	Ca1	1785215	A/AAATG	TCTACTTGCGGCAACTTGTG	CATCATTCCAATCAAGCACG	60.1	319	DRR	Ca_00224	S	--
Ca1_P3_1787307	Ca1	1787307	GTTTTTTTTTTT/GTTTTTTTTT	TAACCTACGCCACACCAACA	CAACACCACCACCTTCTCCT	60.0	272	INTERGENIC	--	--	--
Ca1_P3_1793129	Ca1	1793129	TG/T	TGGTTTCCCCTGCTACAAAC	ATATGCCCTCACCAAACTGC	60.0	497	INTRON	Ca_00226	D	MYB_related
Ca1_P3_179359	Ca1	179359	AATATATATATATATATATATATATATATATATATATAT/AATATATATATATATATATATATATATATATAT	CCTCAAACAATCGCAACCAT	ATTTTGGGGAAGTTGGATTC	60.9	726	INTERGENIC	--	--	--
Ca1_P3_17936377	Ca1	17936377	CTATATATATATATATATATATATATATATAT/CTATATATATATATATATATATATATATATATAT	CGGACAATGTCGGTTAATTG	CCTTTGCAAGCGACTTTCTT	58.9	219	INTERGENIC	--	--	--

INDEL marker IDs	Chromosomes/ unanchored scaffolds	Physical positions (bp)	InDels (*Kabuli* reference genome-CDC Frontier/PII)	Forward primers (5'-3')	Reverse primers (5'-3')	Anneling temperature (0C)	Expected amplified product size (bp)	Structural annotation		Functional annotation	
								Sequence components of *kabuli* genome	*Kabuli* gene accession IDs	NCBI-KOG	TFs
Ca1_P3_1794406	Ca1	1794406	GTTT/GTT	TCTTGAACGAGAAAAC CGCT	ACGGTTTCTTGCTTTTT CCC	60.0	365	INTERGENIC	--	--	--
Ca1_P3_1800007	Ca1	1800007	AGTT/A	TGTTGCACAAGGTCCA GAAG	TTTTGGCCAAGAGATG GATG	59.9	601	DRR	Ca_00227	P	--
Ca1_P3_1801730	Ca1	1801730	ACACACAACGAGTC/ACACACAACGAG TCACACAACGAGTC	CAGGGAAAATTCCAAG CTGA	TGGACAAGAGCATCTG CAAC	60.2	565	CDS (large-effect mutations)	Ca_00228	F	MYB_related
Ca1_P3_1801900	Ca1	1801900	CTTTT/CTTTTT	GACACACAACGAGTCG AGGA	TGGACAAGAGCATCTG CAAC	59.9	310	DRR	Ca_00227	P	--
Ca1_P3_18088	Ca1	18088	TA/TAA	CCGCTTGTTTTTCTTTG CAG	TTGATTGCTTGTTGCT CCAG	60.9	543	INTERGENIC	--	--	--
Ca1_P3_1811462	Ca1	1811462	AA/AATCTTCTTA	AACGGCAAGAACAGAG CAAT	CGCCTCAAAGTACGTT CACA	59.9	611	INTERGENIC	--	--	--
Ca1_P3_1811791	Ca1	1811791	A/ACT	TGTGAACGTACTTTGA GGCG	TGGTCAACATGCTGTT CCAT	59.9	653	INTERGENIC	--	--	--
Ca1_P3_1817917	Ca1	1817917	G/GGAA	CTTCTCTGGCCATTTTT CCA	ATTGATTCGGCATAGC CAAG	60.2	752	INTERGENIC	--	--	--
Ca1_P3_18181716	Ca1	18181716	AATATATATATATATATATATATATATA/ AATATATATATATATATATATATATATAT ATATA	CGAATACTCATGGTTTT TGATCTG	ACCCAACGTAGCACGT TACC	59.9	626	INTERGENIC	--	--	--
Ca1_P3_1819582	Ca1	1819582	TAAAAAAA/TAAAAAAAA	AAAATTGATTCGTCACT CCATACA	CAGCACCAGCAACTCC TACA	59.8	434	INTERGENIC	--	--	--

INDEL marker IDs	Chromosomes/ unanchored scaffolds	Physical positions (bp)	InDels (*Kabuli* reference genome-CDC Frontier/PII)	Forward primers (5'-3')	Reverse primers (5'-3')	Anneling temperature (0C)	Expected amplified product size (bp)	Structural annotation		Functional annotation	
								Sequence components of *kabuli* genome	*Kabuli* gene accession IDs	NCBI-KOG	TFs
Ca1_P3_1822026	Ca1	1822026	TC/T	ATGTTTGGGGCTATCG ACTG	GAGGAATTTTGCTCTC GTGC	60.0	449	INTERGENIC	--	--	--
Ca1_P3_1823990	Ca1	1823990	TGAAGAAGAAGAAGAAGA/TGAAGAAG AAGAAGAAGAAGA	CTCTGCGTCTTCCCTTT CAC	GAGCAATTTCCATAAC CCCA	60.0	554	CDS (large-effect mutations)	Ca_00230	K	--
Ca1_P3_18298335	Ca1	18298335	TCC/TC	GGTAATGCCTCGTACA AAACAA	CATTCCTTATGCCTCC TACACC	59.1	751	INTERGENIC	--	--	--
Ca1_P3_18299880	Ca1	18299880	TTCCTATCCTATCC/TTCCTATCC	TGGATCACTCACCTCTC TTCC	TGGAAGTGGTGGTTTT GTTG	59.2	185	INTERGENIC	--	--	--
Ca1_P3_18300620	Ca1	18300620	GTTTTTTTTT/GTTTTTTT	TTTCCATGAGTTATATT TGTCACGA	TGGCCTTCAATTTTCTT GCT	59.8	134	INTERGENIC	--	--	--
Ca1_P3_18300800	Ca1	18300800	ATTT/ATT	AGCAAGAAAATTGAAG GCCA	GGAAGCTTTAAAGGGA GCAAA	59.8	161	INTERGENIC	--	--	--
Ca1_P3_18325523	Ca1	18325523	AGG/AG	CGGATCATTACACACTT GCG	GCCAAAGCCATGTTTT CAAT	60.1	324	INTERGENIC	--	--	--
Ca1_P3_18328170	Ca1	18328170	GTTTTTT/GTTTTTTT	TTAAAACCACAAAATGG GGC	GAGGCAAAGCTTCCAA ATCA	59.7	475	INTERGENIC	--	--	--
Ca1_P3_18328227	Ca1	18328227	GTCTATCT/GTCT	TTAAAACCACAAAATGG GGC	GAGGCAAAGCTTCCAA ATCA	59.7	475	INTERGENIC	--	--	--
Ca1_P3_1835748	Ca1	1835748	TTAT/TT	TTCTTGGAATCTCCACC ACC	TTTCTTGGGCCTTAGG GTTT	59.9	680	INTERGENIC	--	--	--

INDEL marker IDs	Chromosomes/ unanchored scaffolds	Physical positions (bp)	InDels (*Kabuli* reference genome-CDC Frontier/PII)	Forward primers (5'-3')	Reverse primers (5'-3')	Anneling temperature (0C)	Expected amplified product size (bp)	Structural annotation		Functional annotation	
								Sequence components of *kabuli* genome	*Kabuli* gene accession IDs	NCBI-KOG	TFs
Ca1_P3_18364053	Ca1	18364053	GAAA/GAAAA	CCCGTAGCAAGACTTGAAGC	TTGGAGAAACTTTTCCGCTG	60.0	627	INTERGENIC	--	--	--
Ca1_P3_1836845	Ca1	1836845	TATTTTAATT/TATTTTAATTTTAATT	CATTTGCATACCGTTTCACG	AGCACCACTTCTTCATTCAAAA	60.0	704	INTERGENIC	--	--	--
Ca1_P3_1837155	Ca1	1837155	CAAAAAAA/CAAAAAA	GGGAGCTACACATGCGTCTT	AAGCACCACTTCTTCATTCAAA	60.3	686	INTERGENIC	--	--	--
Ca1_P3_1837913	Ca1	1837913	TA/TAA	TCGTACTGATTAGTGAAGTGTGAGG	CAGTCAAATCGAAGCCATCA	59.8	617	INTERGENIC	--	--	--
Ca1_P3_1838743	Ca1	1838743	TAAAAAAA/TAAAAAA	AAGATGGGTCATAATTAAGGTGGA	ATGAGCCCAAAAGTACACCG	60.0	630	INTERGENIC	--	--	--
Ca1_P3_1839822	Ca1	1839822	TAAAAAA/TAAAAAAA	TTCACTCGATTATTGGTGGG	TTTGAAAACGATGCCCATAA	58.4	162	INTERGENIC	--	--	--
Ca1_P3_1841034	Ca1	1841034	TTTCTTCT/TTTCT	TCAAGGTGTTGATTCGGTAGG	TTTTGCAACCCGGTCTATTC	60.0	595	INTERGENIC	--	--	--
Ca1_P3_1841061	Ca1	1841061	TAAAA/TAAAAAAAA	TCAAGGTGTTGATTCGGTAGG	TTTTGCAACCCGGTCTATTC	60.0	595	INTERGENIC	--	--	--
Ca1_P3_1841424	Ca1	1841424	A/AT	GAATAGACCGGGTTGCAAAA	CCTCTTTCCGGACAACACAT	59.9	302	INTERGENIC	--	--	--
Ca1_P3_1841544	Ca1	1841544	A/AATGATTATTCGT	ATGTGTTGTCCGGAAAGAGG	TGAATCCGGAATAGAACCGA	60.0	147	INTERGENIC	--	--	--

INDEL marker IDs	Chromosomes/ unanchored scaffolds	Physical positions (bp)	InDels (*Kabuli* reference genome-CDC Frontier/PII)	Forward primers (5'-3')	Reverse primers (5'-3')	Anneling temperature (0C)	Expected amplified product size (bp)	Structural annotation		Functional annotation	
								Sequence components of *kabuli* genome	*Kabuli* gene accession IDs	NCBI-KOG	TFs
Ca1_P3_18441535	Ca1	18441535	CCGGACGG/CCGG	TTGACTGAGCAAGGCACAAG	TGGCATTGATGATGGCTTTA	60.2	382	INTERGENIC	--	--	--
Ca1_P3_1844205	Ca1	1844205	TTATG/T	TTGGGCGGAGAAAGTAAGAA	TCGACAACCATCTCCATTCC	59.8	548	INTERGENIC	--	--	--
Ca1_P3_1847538	Ca1	1847538	TAA/TA	TGCCCCCTTCTTCTCCTATT	CCCGACAGTGAGTCCAAGAT	60.0	188	DRR	Ca_00232	K	MYB
Ca1_P3_1847825	Ca1	1847825	TAGAA/TAGAAGAA	TGCCCCCTTCTTCTCCTATT	GCTGCATGCTTTAGAGAAAGG	60.0	461	DRR	Ca_00232	K	MYB
Ca1_P3_1850030	Ca1	1850030	CAATTAATTA/CAATTA	GTTGTCCGTCCTTCCTGGTA	GGACAACCGAAGAGGATCAA	60.0	563	INTRON	Ca_00232	K	MYB
Ca1_P3_18514111	Ca1	18514111	TTATATATATATATATATATATATATAT/TTATATATATATATATATATATATAT	CACATCAAAACACACAATTGGA	GCACTTGGAGTGGCCAATAA	59.3	663	INTERGENIC	--	--	--
Ca1_P3_1852604	Ca1	1852604	TA/TAA	ACACGGCGTTCATTTTCTTC	CAAACCATGGGAGAGAGAGC	60.1	843	INTERGENIC	--	--	--
Ca1_P3_1858938	Ca1	1858938	GA/GACA	CTCCTTCAACCACAAGAGCC	TGATCCAATTGATGGCACAC	59.8	677	INTRON	Ca_00233	FP	bHLH
Ca1_P3_18609095	Ca1	18609095	ATATTATTATTAT/ATATTATTATTATTAT	CCCTCTACGAATTCCAAAGAA	CGATTTGCTTTCAATTTTGG	59.6	540	INTERGENIC	--	--	--
Ca1_P3_1862254	Ca1	1862254	TTAATAA/TTAATAATAA	TGCGTTGAAGAGGATGACTG	TCATAATTTTGAGCCGGTCC	60.0	466	INTERGENIC	--	--	--

INDEL marker IDs	Chromosomes/ unanchored scaffolds	Physical positions (bp)	InDels (*Kabuli* reference genome-CDC Frontier/PII)	Forward primers (5'-3')	Reverse primers (5'-3')	Anneling temperature (0C)	Expected amplified product size (bp)	Structural annotation		Functional annotation	
								Sequence components of *kabuli* genome	*Kabuli* gene accession IDs	NCBI-KOG	TFs
Ca1_P3_18625845	Ca1	18625845	CACTAC/CAC	GGGGAGCTTCTAAAAGCCAT	TCCATCGATGATATGCCTCA	59.7	851	INTERGENIC	--	--	--
Ca1_P3_1865337	Ca1	1865337	TAGGAGG/TAGGAGGAGG	ATTGGGTATTGCAGACGAGC	TGGTCACAGCAGTCAATGGT	60.1	517	DRR	Ca_00234	--	--
Ca1_P3_18683661	Ca1	18683661	CTTATTATTATTATTATTATTATTATTATTATTATT/CTTATTATTATTATTATTATTATTATTATTATTATTATTATTATT	ACCTCCCGCTAATGTCAATG	ATCGTGGAACACCAACCAAT	60.0	764	INTERGENIC	--	--	--
Ca1_P3_186939	Ca1	186939	TAA/TAAA	CTCTTGGGGGATCTTCATCA	GCATGCCATGTAGTGCATTT	60.0	329	DRR	Ca_00014	--	--
Ca1_P3_1871868	Ca1	1871868	CAAAAAAAAA/CAAAAAAAAAA	GGGCATAACAGTTCCATGCT	TTCAAGAACATTGTGACGCC	60.0	638	INTERGENIC	--	--	--
Ca1_P3_1875806	Ca1	1875806	T/TATA	TCCAAGGTGGGTCAAAAGAC	ACGTTGTTTTTAATTCGCCG	59.9	574	INTERGENIC	--	--	--
Ca1_P3_1876420	Ca1	1876420	AT/ATACGTAGTTCGGTTGAGT	CGGGTCTTCAAAATGGAATG	CAAAGGAAACGTGCTTCCAG	60.3	861	INTERGENIC	--	--	--
Ca1_P3_1882088	Ca1	1882088	CCA/CCACA	TTTTCAAGAAGTCGCATCCC	TGTATCGGGGTCTGTCCAAT	60.2	269	INTERGENIC	--	--	--
Ca1_P3_1882286	Ca1	1882286	CTAAT/CT	CGCATCCCCATAATATGACC	GGTGCAAATGCAAGAGTCAA	60.0	653	INTERGENIC	--	--	--
Ca1_P3_18823381	Ca1	18823381	TTAATAATAATAATAATAA/TTAATAATAATAATAA	TCATCACAATGGTGGAAATCA	TAACCCAAAAATCCCAACCA	59.8	388	INTERGENIC	--	--	--

INDEL marker IDs	Chromosomes/ unanchored scaffolds	Physical positions (bp)	InDels (*Kabuli* reference genome-CDC Frontier/PII)	Forward primers (5'-3')	Reverse primers (5'-3')	Anneling temperature (0C)	Expected amplified product size (bp)	Structural annotation		Functional annotation	
								Sequence components of *kabuli* genome	*Kabuli* gene accession IDs	NCBI-KOG	TFs
Ca1_P3_18824350	Ca1	18824350	GT/GTAGGAGGAGAT	GCGTTTTGAAAAATCGGTGT	TGAGGAGACAACAACCACCA	60.0	296	INTERGENIC	--	--	--
Ca1_P3_1883449	Ca1	1883449	CTATA/CTA	GCACGTGGCCCACTTATACT	ATGGAGTGAGTCGGCCATAC	60.0	770	DRR	Ca_00237	A	--
Ca1_P3_18843377	Ca1	18843377	ATTTTTTTTT/ATTTTTTTT	TAGTGCAAGCCTGCTCAGTG	GGACAGCAACTCAGCAATCA	60.3	392	INTERGENIC	--	--	--
Ca1_P3_18868658	Ca1	18868658	CGG/CGGGAATGG	AATCGATTGATTCAGCCCAG	CGTTTTTCAAAGCGAGGGTA	60.0	707	INTERGENIC	--	--	--
Ca1_P3_1890401	Ca1	1890401	AATATATATATATATA/AATATATATATATATATATA	TTTCCTGAAGGAGGATGGTG	TATCACTTTGATCTCCGCCC	60.0	710	INTERGENIC	--	--	--
Ca1_P3_1894075	Ca1	1894075	GAAAAAAAAAAA/GAAAAAAAAAA	TGCATTTCATGCTTCAAACAA	TGCACCATTGAAGAAACCAA	60.2	324	INTERGENIC	--	--	--
Ca1_P3_1894477	Ca1	1894477	TATAATAATAATAATAATAATAATAATAATAATAATA/TATAATAATAATAATAATAATAATAATAATAATA	TGGTTTCTTCAATGGTGCAT	GCAGCTTTTTCATGTGTCCA	59.0	298	INTERGENIC	--	--	--
Ca1_P3_19025184	Ca1	19025184	A/ATTG	CGGGATCCTTGAACTTGGTA	CTTGTGCAAAGGTTCCATCA	59.9	533	INTERGENIC	--	--	--
Ca1_P3_19048181	Ca1	19048181	TAAAAAAAAAA/TAAAAAAAAAAA	CTCCTCCCTGGTTGTGTTGT	TGGTGAATAAGAGGGGACAAA	60.0	102	INTERGENIC	--	--	--
Ca1_P3_1906299	Ca1	1906299	AAG/A	GCCATGTCTTCAGATGGTGA	TTGATTTGATCAGGTAACGCC	59.6	566	DRR	Ca_00238	R	--

INDEL marker IDs	Chromosomes/ unanchored scaffolds	Physical positions (bp)	InDels (*Kabuli* reference genome-CDC Frontier/PII)	Forward primers (5'-3')	Reverse primers (5'-3')	Anneling temperature (0C)	Expected amplified product size (bp)	Structural annotation		Functional annotation	
								Sequence components of *kabuli* genome	*Kabuli* gene accession IDs	NCBI-KOG	TFs
Ca1_P3_19152828	Ca1	19152828	ATTTTTTTTTT/ATTTTTTTTTTT	TCAGCTGAGACTGAAACTCAAAA	AGTGGCATAGGAGTGCCATC	59.3	406	INTERGENIC	--	--	--
Ca1_P3_19168	Ca1	19168	GCTCTCT/GCTCT	CAGAACCGAGGAATTAAGCG	AGGCTTGAAGCGTGGTAATG	59.8	637	INTERGENIC	--	--	--
Ca1_P3_1927607	Ca1	1927607	G/GA	TCCGAAGGGAAAGTGAAGAA	TTCAATGGTTGATGCCATGT	59.8	626	INTERGENIC	--	--	--
Ca1_P3_1930262	Ca1	1930262	ATTTTTTTT/ATTTTTTTTTT	GGCAACATTTTACAAAAGTTAAGG	AGTTTGATTAATGTCGGGCG	58.3	318	INTERGENIC	--	--	--
Ca1_P3_19422873	Ca1	19422873	CTTATTTATTT/CTTATTT	GCCCATAACCTAGCCCATTT	TCGTGTCCCAAAACTCAATG	60.2	713	INTERGENIC	--	--	--
Ca1_P3_19423044	Ca1	19423044	TAAAAAAA/TAAAAAA	TCTTTGAGTTGTTAGGATACAAGTGG	TCGATTATGCGTAGGGAAGG	60.0	728	INTERGENIC	--	--	--
Ca1_P3_19423865	Ca1	19423865	ATTATTTATTTATTTATTTATTTA/ATTATTTATTTATTTATTTATTTATTTA	ACCGTTTTTAACGGATGTCG	TGTTCAAACCCGGGAGTAAG	59.9	525	INTERGENIC	--	--	--
Ca1_P3_19431535	Ca1	19431535	TCA/T	TCTGATGCAGTGTTTGGCTC	GTGATAAAGGCGTCAACGGT	60.0	688	INTERGENIC	--	--	--
Ca1_P3_19498678	Ca1	19498678	TTTTGATCCTTTGAT/TTTTGAT	GCCCGAGTTAACAACAAACG	ATCGATTTCCTCAGCACTGG	60.5	374	INTERGENIC	--	--	--
Ca1_P3_19567652	Ca1	19567652	T/TCCCATGCC	CGAAGACCGAAATGCTGATT	GGTCAAACCGTCAAAAAGGA	60.2	321	INTRON	Ca_08866	J	G2-like

INDEL marker IDs	Chromosomes/ unanchored scaffolds	Physical positions (bp)	InDels (*Kabuli* reference genome-CDC Frontier/PII)	Forward primers (5'-3')	Reverse primers (5'-3')	Anneling temperature (0C)	Expected amplified product size (bp)	Structural annotation		Functional annotation	
								Sequence components of *kabuli* genome	*Kabuli* gene accession IDs	NCBI-KOG	TFs
Ca1_P3_1965007	Ca1	1965007	TG/T	CCACCCCAAGAAGAAAAACA	TTTCCCAAGCCACTAACCAC	59.9	704	DRR	Ca_00244	--	B3
Ca1_P3_19661093	Ca1	19661093	TTATATATATATATATATATATATATATATATATA/TTATATATATATATATATATATATATATA	GGCCATCACAAATGGGTAAG	GGGAGTGGAGTTAATGGTCG	60.2	581	INTERGENIC	--	--	--
Ca1_P3_19673403	Ca1	19673403	AATATATATATATATATATATATATATATA/AATATATATATATATATATATATATATA	TGTAGGTGATCCTTCCACCG	AGGGACTTCGTCCCCTTAAA	60.9	508	INTERGENIC	--	--	--
Ca1_P3_1969241	Ca1	1969241	AAATTAA/AAA	AGAAGAGAACGGTTGCGTGT	GAAAACTACCACGGCCAAAA	59.9	604	INTERGENIC	--	--	--
Ca1_P3_1969508	Ca1	1969508	ATT/ATTT	TTTTGGCCGTGGTAGTTTTC	ATATTAGTTGTGGGCGCGAG	60.0	519	INTERGENIC	--	--	--
Ca1_P3_1969676	Ca1	1969676	TGG/TGGGG	TTTTGGCCGTGGTAGTTTTC	TTCGAATTTTCCCGTACCTG	60.0	724	INTERGENIC	--	--	--
Ca1_P3_1971360	Ca1	1971360	GTTAAATTTTA/GTTAAATTTTAAATTTTA	CTGCATTTTGAGCCACTTGA	TTATTCCACAAGCAATGCGA	60.0	724	INTERGENIC	--	--	--
Ca1_P3_1971572	Ca1	1971572	AA/AAGAAATGCA	ATGTGCATCACCTCTGACGA	GCTGCCATGGAGGTAGTCAT	60.3	807	INTERGENIC	--	--	--
Ca1_P3_1972044	Ca1	1972044	ACTTCTTCT/ACTTCTTCTTCT	GCCCTCAGCCTCACTTTATG	AGGGAGGCTACGAACGATTT	59.8	356	INTERGENIC	--	--	--
Ca1_P3_1973631	Ca1	1973631	TG/T	TTTTTGACAAGGCAGTGCAG	CAGCCACTGAAAGTAACGCA	60.0	290	INTRON	Ca_00246	A	--

INDEL marker IDs	Chromosomes/ unanchored scaffolds	Physical positions (bp)	InDels (*Kabuli* reference genome-CDC Frontier/PII)	Forward primers (5'-3')	Reverse primers (5'-3')	Anneling temperature (0C)	Expected amplified product size (bp)	Structural annotation		Functional annotation	
								Sequence components of *kabuli* genome	*Kabuli* gene accession IDs	NCBI-KOG	TFs
Ca1_P3_19752414	Ca1	19752414	TAAAAAAAAAAAAAA/TAAAAAAAAAAAAA	TTTTTCGATATCTCAATTTCTCTTCA	TACATTAACTCGCGGGAACC	59.7	277	INTERGENIC	--	--	--
Ca1_P3_1979080	Ca1	1979080	CTTCTTA/CTTCTTAGTTCTTA	CGTAGGGAGGAGGTTGTTGA	TAGTGACGACTTGGGCACAG	60.1	481	INTERGENIC	--	--	--
Ca1_P3_1981299	Ca1	1981299	AATATATATA/AATATATATATA	GGCAACGAACAACAAGGTTT	TGTTGAAATTTGGAATTCATGT	60.0	565	INTERGENIC	--	--	--
Ca1_P3_1981984	Ca1	1981984	ATG/ATGTTAGAGATTAAATTG	TGTCATTTTGACAGTATCATTTTGATT	GGATAGCTTTGGCTGTCTCG	60.1	316	INTERGENIC	--	--	--
Ca1_P3_1987558	Ca1	1987558	ATAGTAG/ATAG	GTGGCATAGGCTCCAATCAT	GGAATGGTGCTGGACATTTT	59.9	922	DRR	Ca_00248	T	--
Ca1_P3_1989750	Ca1	1989750	TC/T	AGCCCTTTGTTCTTTTGGGT	TACCATCGCCATTGAAACAA	60.0	474	INTERGENIC	--	--	--
Ca1_P3_1989852	Ca1	1989852	AAA/AAATAA	TTGGGCTTATTGGGTCACTC	TATCCAATCCCAACCCAAAA	59.9	115	INTERGENIC	--	--	--
Ca1_P3_19903627	Ca1	19903627	TA/TAATTGGCAGCA	TGGGCTGAAAAGCAACTTCT	ACCAGTGCTGTTCCCAAATC	60.0	233	INTRON	Ca_08847	K	--
Ca1_P3_1992977	Ca1	1992977	TAC/TACAC	AGCCTGCACCTGCACTTAAT	ATTATCGGCATTCAAGGTGG	59.9	185	INTRON	Ca_00249	CR	HB-other
Ca1_P3_1998783	Ca1	1998783	GAAAAAAAAA/GAAAAAAAAAA	CTAGCACCTTTAGGGGGAGG	CGACCGATATTCGTCCACTC	60.1	686	INTERGENIC	--	--	--

INDEL marker IDs	Chromosomes/ unanchored scaffolds	Physical positions (bp)	InDels (*Kabuli* reference genome-CDC Frontier/PII)	Forward primers (5'-3')	Reverse primers (5'-3')	Anneling temperature (0C)	Expected amplified product size (bp)	Structural annotation		Functional annotation	
								Sequence components of *kabuli* genome	*Kabuli* gene accession IDs	NCBI-KOG	TFs
Ca1_P3_2003340	Ca1	2003340	ACT/A	GTGTTTGGGTTTCGCAGTTT	CAATTGCAAGCAACACGTTTA	60.0	604	INTERGENIC	--	--	--
Ca1_P3_2004325	Ca1	2004325	AC/ACCATC	TACCTGGTGTCATTGCTTGG	CATATCCTCATGGCAGTCCC	59.6	625	INTRON	Ca_00252	--	TCP
Ca1_P3_2004592	Ca1	2004592	T/TA	TCAGCAGTTGCTACCTCCCT	CGCCAAGAACAATAATGCCT	60.0	447	INTRON	Ca_00252	--	TCP
Ca1_P3_2007237	Ca1	2007237	GGTG/GG	TCTCTCACACCCAAACCACA	TGTGAATTTTTGCCACTCAAA	60.1	741	DRR	Ca_00252	--	TCP
Ca1_P3_2008759	Ca1	2008759	GCCCC/GCC	ATCTTGTCACCGCGAGTCTT	TGGTATGTATGAAGAAATCCGAA	59.9	181	INTERGENIC	--	--	--
Ca1_P3_20097334	Ca1	20097334	CNNNNNNNNNNNNNN/CNNN	TTTCGTTTGCGATAGTGATCC	TCATTTCAAGATCCTGGTTCC	60.1	250	INTERGENIC	--	--	--
Ca1_P3_2010658	Ca1	2010658	CTATAT/CTATATAT	TTGGAGACAACACAATGGGA	TTGCCCTGATGGTGTCAGTA	59.9	656	INTERGENIC	--	--	--
Ca1_P3_2015582	Ca1	2015582	TTGT/TT	CTCTAGAACCCCCAACTCCC	CTCCAATGTTGGGAGACTACG	59.9	499	DRR	Ca_00253	E	--
Ca1_P3_2016428	Ca1	2016428	GAAAA/GAAA	TCTCTTTGACCCTTTGGCAC	GCAACACATGCATTTTGGTC	60.2	319	DRR	Ca_00253	E	--
Ca1_P3_2017039	Ca1	2017039	GAAAGNNNNNNNNNNNAAAG/GAAAG	ATCATCATTGCTCTGCTCCC	TTGTTCTGAAACGTGGGTGA	60.2	289	DRR	Ca_00253	E	--

INDEL marker IDs	Chromosomes/ unanchored scaffolds	Physical positions (bp)	InDels (*Kabuli* reference genome-CDC Frontier/PII)	Forward primers (5'-3')	Reverse primers (5'-3')	Anneling temperature (0C)	Expected amplified product size (bp)	Structural annotation		Functional annotation	
								Sequence components of *kabuli* genome	*Kabuli* gene accession IDs	NCBI-KOG	TFs
Ca1_P3_2019732	Ca1	2019732	ATTTTTT/ATTTTTTT	AACTCAACCGGAGGTCCTTT	GCACATTTAGGTGTGGAGCA	60.0	745	INTRON	Ca_00254	FP	bHLH
Ca1_P3_2055085	Ca1	2055085	ATTTTT/ATTTTTT	CCTGTTATTTGCGTGCTTCA	TCACATTCATGCCAGTTGGT	59.9	495	DRR	Ca_00260	T	M-type
Ca1_P3_2056660	Ca1	2056660	CATTGAAATTGAAATT/CATTGAAATT	TGAAATGTTACGGCTTGCTTT	ATTGACAACGTCCTTCCACA	59.8	465	INTERGENIC	--	--	--
Ca1_P3_2057962	Ca1	2057962	GAAAAGTTA/GA	TGTGCATGAAAGCAAAATTGA	GGCCGTGAAAGATATTTGGA	60.2	712	DRR	Ca_00261	--	--
Ca1_P3_2058843	Ca1	2058843	CAAAA/CAA	GAGAAAGGGGAGGAGGATTG	AGACTCTGCAGCCACCTCAT	60.0	357	DRR	Ca_00261	--	--
Ca1_P3_2073961	Ca1	2073961	CTTTT/CTTT	AGAGGCACGAGATTTCCAGA	AAATATCCCCGCAATAAGGG	60.0	336	CDS (FRAME SHIFT)	Ca_00263	T	M-type
Ca1_P3_2084021	Ca1	2084021	GAAAAAAAAAAA/GAAAAAAAAAAAAA	ATGCACACACACGCTTTCTC	GGCAACTACTTGGGCATTGT	59.9	476	INTERGENIC	--	--	--
Ca1_P3_2085412	Ca1	2085412	AAGTTTTAT/AAGTTTTATAGAGTTTTAT	TTTTTGGGCTCACACAATTT	CAATTGTCCAAAACTCTTATCCAA	58.1	723	INTERGENIC	--	--	--
Ca1_P3_2085895	Ca1	2085895	GAAAAATAAAAA/GAAAAATAAAAATAAAAA	TGTTTTTGGATAAGAGTTTTGGA	TCCTCAATCGCGTTCTTTCT	57.9	626	INTERGENIC	--	--	--
Ca1_P3_2088365	Ca1	2088365	AAAACA/AA	ACAGCAACCAAGAGTTGTGC	CAGAAAAAGCTATTCAGGCCA	58.9	529	INTERGENIC	--	--	--

INDEL marker IDs	Chromosomes/ unanchored scaffolds	Physical positions (bp)	InDels (*Kabuli* reference genome-CDC Frontier/PII)	Forward primers (5'-3')	Reverse primers (5'-3')	Anneling temperature (0C)	Expected amplified product size (bp)	Structural annotation		Functional annotation	
								Sequence components of *kabuli* genome	*Kabuli* gene accession IDs	NCBI-KOG	TFs
Ca1_P3_2088423	Ca1	2088423	ATT/AT	ACAGCAACCAAGAGTTGTGC	GCAGGTGGCATCAATTTACC	58.9	548	INTERGENIC	--	--	--
Ca1_P3_2088688	Ca1	2088688	TT/TTGAATATTTAAATTAT	ACAGCAACCAAGAGTTGTGC	GCATCCGGCCAAAATAAATA	58.9	654	INTERGENIC	--	--	--
Ca1_P3_2099355	Ca1	2099355	ATTTTTTTTTTTTT/ATTTTTTTTTT	GGAAAGGCTGAGGCCTAATAA	TTGGTAATTTGAAGTTCTTTTGGA	59.7	738	INTERGENIC	--	--	--
Ca1_P3_2100521	Ca1	2100521	ATTT/ATT	TGATGTCAAATGATCTTGTCCTG	CAATGCGTGCCGTGTAATAA	60.0	563	DRR	Ca_00267	D	NAC
Ca1_P3_2102738	Ca1	2102738	ATTTTTTTTT/ATTTTTTTTTT	GAGCCAAGTTGCTCCTTCAC	CACAGGCTTGTTGGATAGCA	60.0	729	INTRON	Ca_00267	D	NAC
Ca1_P3_2113146	Ca1	2113146	TAAAAAAAAAA/TAAAAAAAA	TGTGGACACGTTAAGTTGTGG	TGATCTCGCCCTATTATTCAAAA	59.5	409	INTERGENIC	--	--	--
Ca1_P3_2115049	Ca1	2115049	TAAAAAAAAA/TAAAAAAAAAAA	TGGACTAAAATTGCTTTTTGGAA	TTAGAAAGAGCCACACGCAA	60.0	897	INTERGENIC	--	--	--
Ca1_P3_2116628	Ca1	2116628	A/AC	TTGCGTTCATTTACGTTTGA	TCAATTCACTTCATGCCCAA	57.8	636	INTERGENIC	--	--	--
Ca1_P3_2116994	Ca1	2116994	AGTGTGTGTGTGTGT/AGTGTGTGTGTGT	CATGGATGGCCACATATCAA	TTGCAATTGTGTCTTTTGCC	60.2	148	INTERGENIC	--	--	--
Ca1_P3_2118056	Ca1	2118056	CAGAG/CAGAGAG	CCATCAAATTCGTTGATTTCC	GAAGCTTCTGCTCATTTGCC	59.3	311	INTERGENIC	--	--	--

INDEL marker IDs	Chromosomes/ unanchored scaffolds	Physical positions (bp)	InDels (*Kabuli* reference genome-CDC Frontier/PII)	Forward primers (5'-3')	Reverse primers (5'-3')	Anneling temperature (0C)	Expected amplified product size (bp)	Structural annotation		Functional annotation	
								Sequence components of *kabuli* genome	*Kabuli* gene accession IDs	NCBI-KOG	TFs
Ca1_P3_2119695	Ca1	2119695	AATTTCTATTTCTAT/AATTTCTATTTCTATTTCTAT	TCCGACGTTTTTCAATTTCA	AATGGTGTCTGGGGACATTT	59.1	176	INTERGENIC	--	--	--
Ca1_P3_2120405	Ca1	2120405	TTTAATTTAATTAA/TTTAATTTAATTAATTTAATTAA	TGTATGGAAAGAGGTAAAATGGAA	AACGACAAATGTTGCCATGA	59.0	532	INTERGENIC	--	--	--
Ca1_P3_2121780	Ca1	2121780	TA/TAAAAAAAGAAAGA	AACGCAAAATCAAACACCAA	TTGGCCAAATCACTGGTACA	59.1	752	INTERGENIC	--	--	--
Ca1_P3_2130717	Ca1	2130717	G/GAA	TCATTCTCTAGAACGCGGCT	TGCTTGTGGGATGAATAGCA	60.1	350	INTERGENIC	--	--	--
Ca1_P3_2131678	Ca1	2131678	TAT/TATTGAT	AGGAATGCATCGAATCCAAG	CCACAAGTGCCATTCAGCTA	60.0	516	INTERGENIC	--	--	--
Ca1_P3_2132014	Ca1	2132014	G/GTGC	CATGGATGGTTAGCCCAAAG	AATGACCAACCCACAAGTGC	60.3	107	INTERGENIC	--	--	--
Ca1_P3_213789	Ca1	213789	TATAATAATAATAATAATAATAATAATAATAAT/TATAATAATAATAATAATAATAAT	TACAGCATAGCCCAGCACAG	GTTTTCGGCGACGTATTCAT	60.0	504	INTERGENIC	--	--	--
Ca1_P3_2140468	Ca1	2140468	TTTTA/T	GGATCAAATTTGTTGACCTGG	AACCGTCCCATGTGGTAACT	59.3	513	INTERGENIC	--	--	--
Ca1_P3_21595611	Ca1	21595611	TTATATATATATATATATATATATATATATATATATATATATATATA/TTATATATATATATATATATATATATATATATATATATATATATATATATATA	GGCATTATGGTCACCCACTT	TGTCAAGTCCGTAGGACGAA	59.7	512	INTERGENIC	--	--	--
Ca1_P3_21607967	Ca1	21607967	CTTTTTTTTTTTT/CTTTTTTTTTT	GTGTTGGTGTTTTGACGCAC	TCATCAAATTCGTAACGCAAA	60.1	593	INTERGENIC	--	--	--

**INDEL marker IDs**	**Chromosomes/ unanchored scaffolds**	**Physical positions (bp)**	**InDels (*Kabuli* reference genome-CDC Frontier/PII)**	**Forward primers (5'-3')**	**Reverse primers (5'-3')**	**Anneling temperature (0C)**	**Expected amplified product size (bp)**	**Structural annotation**		**Functional annotation**	
								**Sequence components of *kabuli* genome**	***Kabuli* gene accession IDs**	**NCBI-KOG**	**TFs**
Ca1_P3_21673033	Ca1	21673033	AAATAATAATAATAATAATAATAATAATAATAATAATAATAATAATAATAATAATAATAATAA/AAATAATAATAATAATAATAATAATAATAATAATAATAATAATAATAATAATAATAATAATAA	TGGAAAGAACACTACAAAGCAAA	TTTTGCAACCCACTCTTGAA	59.0	311	INTERGENIC	--	--	--
Ca1_P3_2169418	Ca1	2169418	CTATTATTATTATTATTATT/CTATTATTATTATTATTATTATT	TCGCTGAAAAAGAAAGGTGA	ATGTAGGTGATGGGATGGGA	58.6	509	DRR	Ca_00273	Z	--
Ca1_P3_21746056	Ca1	21746056	AC/ACACATATCAGCTTTC	CTATTGCCCCCTCCTCCTAC	TACCGATACCTCCAAGTGCC	59.9	585	INTRON	Ca_22116	S	--
Ca1_P3_21772275	Ca1	21772275	CATATATATATATAT/CATATATATATATATAT	CTTTTGTCTCCCCATCCTCA	AAAACTGAGACGAAAAAGTTGTCC	60.0	342	DRR	Ca_22115	--	MYB
Ca1_P3_21862084	Ca1	21862084	ATT/ATTT	CGTATGTTCTCCACGACCTG	TGTTTTTGAATTGCATCGAGA	59.2	941	INTERGENIC	--	--	--
Ca1_P3_21862720	Ca1	21862720	TTATATATATATATATATATATATATATA/TTATATATATATATATATATATATATATATATATA	TCTCGATGCAATTCAAAAACA	GGATGATCATGTCCACCCTC	59.3	257	INTERGENIC	--	--	--
Ca1_P3_21938049	Ca1	21938049	CACTGAGTCCAGA/CACTGAGTCCAGACTGAGTCCAGA	TGTCAAAGGCCAAGATTCCT	CCTCACATCAAGAGCAGCAA	59.7	526	INTRON	Ca_22106	--	MIKC
Ca1_P3_21970751	Ca1	21970751	AAATAATAATAATAATA/AAATAATAATAATAATAATA	TGAATAATGAATGCCACCCA	TCCTCAATTTTTGCCCGTTA	59.7	529	INTERGENIC	--	--	--
Ca1_P3_21999732	Ca1	21999732	AGGGGG/AGGG	CGCCTTTGGCTATTCATCAT	TCCCTTATTGCCCAAACATC	60.1	492	DRR	Ca_22101	R	MYB
Ca1_P3_22029011	Ca1	22029011	TTATATATATATATATATATATATATA/TTATATATATATATATATA	CGAATCTTCACCCTCTTTGG	GCCGGATCCAAAATCAAAAT	59.7	839	INTERGENIC	--	--	--

INDEL marker IDs	Chromosomes/ unanchored scaffolds	Physical positions (bp)	InDels (*Kabuli* reference genome-CDC Frontier/PII)	Forward primers (5'-3')	Reverse primers (5'-3')	Anneling temperature (0C)	Expected amplified product size (bp)	Structural annotation		Functional annotation	
								Sequence components of *kabuli* genome	*Kabuli* gene accession IDs	NCBI-KOG	TFs
Ca1_P3_22131328	Ca1	22131328	AATATATAT/AATATATATAT	GTGTGGACATGCCTAAAACG	GTTGCAATTGAGGTGCTGAA	59.1	403	INTERGENIC	--	--	--
Ca1_P3_22131513	Ca1	22131513	GAAAAA/GAAAA	GTGTGGACATGCCTAAAACG	GTTGCAATTGAGGTGCTGAA	59.1	403	INTERGENIC	--	--	--
Ca1_P3_22201000	Ca1	22201000	TAAAATATACAAAATATA/TAAAATATA	GCTTTACCCCCAACACGTAA	TCACAGATCAGCGCTACACC	59.9	531	INTERGENIC	--	--	--
Ca1_P3_22204858	Ca1	22204858	TTATATATATATATATATAT/TTATATATATATATATAT	TGACATTTCGAACTTGAGTTTCC	CACAAGTCGCCATTTGGAAT	60.5	532	DRR	Ca_20635	--	TALE
Ca1_P3_2222887	Ca1	2222887	AATATATATATA/AATATATATATATATA	TTGCGTAGTATGGGAAAGTCAA	CTCCCGGTAACTCAATCCAA	59.6	774	INTERGENIC	--	--	--
Ca1_P3_22298046	Ca1	22298046	CTAA/CTAATAA	TGAAATTTAGATGATTGCCGC	GATTTGCATATGGGTGGTCC	60.1	821	DRR	Ca_20631	--	SAP
Ca1_P3_2236260	Ca1	2236260	TAGGAG/TAG	TTCAAGCTGCAAACAAGTGG	CTCAAGGGACGGAATTCAAA	60.0	182	INTRON	Ca_00279	F	--
Ca1_P3_22415512	Ca1	22415512	AATATATATATATA/AATATATATATATATA	TTTTTGATGTACAAGTTAGCACCA	CCACAGTTGCAACAAAGAATG	58.8	519	INTERGENIC	--	--	--
Ca1_P3_2249259	Ca1	2249259	AGT/A	AACACATGGATGTGGGGATT	AACATTTTACGCCTTCGGTG	59.9	284	DRR	Ca_00281	O	--
Ca1_P3_2250936	Ca1	2250936	GA/GAA	TGAATATCCAGAGATCGCCC	CATTGCCCTTTCACCCTTTA	60.0	858	INTRON	Ca_00281	O	--

INDEL marker IDs	Chromosomes/ unanchored scaffolds	Physical positions (bp)	InDels (*Kabuli* reference genome-CDC Frontier/PII)	Forward primers (5'-3')	Reverse primers (5'-3')	Anneling temperature (0C)	Expected amplified product size (bp)	Structural annotation		Functional annotation	
								Sequence components of *kabuli* genome	*Kabuli* gene accession IDs	NCBI-KOG	TFs
Ca1_P3_22513646	Ca1	22513646	ATGT/ATGTGT	ACCAGCATGATGACCAGACA	GACGCCTGACGGTTATTGTT	60.1	676	INTERGENIC	--	--	--
Ca1_P3_2257348	Ca1	2257348	TTAATTACTAATT/TTAATT	AGCTCCCATGATTTTGTTGG	TCACAAAAAGAGATTCGCTTGA	59.9	718	INTERGENIC	--	--	--
Ca1_P3_22621219	Ca1	22621219	TAAAAAAAAAA/TAAAAAAAAA	CATTTTTCCTCTGCACAACG	GCCGTCTTTTAGAAACATCGT	59.3	409	INTERGENIC	--	--	--
Ca1_P3_2263763	Ca1	2263763	T/TTAA	CATACACACCCCACAACCAA	CGGATGTGGTGACTCATTTG	60.1	391	INTERGENIC	--	--	--
Ca1_P3_22663917	Ca1	22663917	CAA/CAAAA	CAAATCAGCAACAGCAGGAA	CGATCCCACCTCTAACCAAC	60.0	666	INTRON	Ca_20616	--	bHLH
Ca1_P3_2273946	Ca1	2273946	CTTT/CTTTT	TTCCATTGTCCTTGTGGGAT	AACCACACACAACAGCCAAA	60.2	738	INTRON	Ca_00285	A	MYB_related
Ca1_P3_2280712	Ca1	2280712	A/AG	CCTTCGTTTTCATGCCTGTT	AACTGCTTTGAAGCACCGTT	60.1	779	DRR	Ca_00285	A	MYB_related
Ca1_P3_22877317	Ca1	22877317	GTTTTTTTTTT/GTTTTTTTTT	AGTGATACTGTGTTTGATTTCCTTT	TTCTTCTTCTCCAAGTGCGG	57.5	606	INTERGENIC	--	--	--
Ca1_P3_22885854	Ca1	22885854	TTA/TTATA	TGATTTGGGGTTTTGGGTAA	GGGTGAGTTCTTTGAGGGTG	60.0	531	INTERGENIC	--	--	--
Ca1_P3_22886112	Ca1	22886112	GAA/GAAA	TGATTTGGGGTTTTGGGTAA	GGGTGAGTTCTTTGAGGGTG	60.0	531	INTERGENIC	--	--	--

INDEL marker IDs	Chromosomes/ unanchored scaffolds	Physical positions (bp)	InDels (*Kabuli* reference genome-CDC Frontier/PII)	Forward primers (5'-3')	Reverse primers (5'-3')	Anneling temperature (0C)	Expected amplified product size (bp)	Structural annotation		Functional annotation	
								Sequence components of *kabuli* genome	*Kabuli* gene accession IDs	NCBI-KOG	TFs
Ca1_P3_22896361	Ca1	22896361	CAAAAAAAA/CAAAAAA	TGTTGTGTGAACTGATTTTGGA	GCGATGTAGAATCGGGTGTT	59.1	481	INTERGENIC	--	--	--
Ca1_P3_2293523	Ca1	2293523	CATATATATATATATAT/CATATATATATAT	TTCCCAAACATAACCCCAGA	CCTTTGATCCGACTTGTGGT	60.2	618	INTRON	Ca_00287	--	--
Ca1_P3_23006705	Ca1	23006705	GC/GCC	TTAATGAGGGGCTGAATTGG	GTTTCGAAGCGAAAAACTGC	59.9	426	INTERGENIC	--	--	--
Ca1_P3_23011755	Ca1	23011755	AC/ACCAGGGCATTTGGC	GCCAAGCAAGGTCTTACTGC	CCTTCAACTATTTGCAGCCC	60.0	106	INTERGENIC	--	--	--
Ca1_P3_23012426	Ca1	23012426	TTATATATATATATATATATATATAT/ TTATATATATATATATATAT	ACCATTAAGTCGACCTTCACA	ATATCGAGGCTGGTCCAACA	57.2	729	INTERGENIC	--	--	--
Ca1_P3_2313379	Ca1	2313379	GTTTT/GTT	GCCAAAGGCATTCATTGTTT	TGTCACTAAAAGGGTTCCCG	59.9	528	INTRON	Ca_00290	--	--
Ca1_P3_2315915	Ca1	2315915	AACGTTACG/AACG	ACCGTCGGAGACAAAAACAC	AGCAAACAAGCAAAGCCAAC	60.0	412	INTERGENIC	--	--	--
Ca1_P3_2317093	Ca1	2317093	TG/T	TTGTGGTTGGTGGTGATGTC	CCTGGGTACATCATAACATTTTTC	60.3	664	INTERGENIC	--	--	--
Ca1_P3_2324181	Ca1	2324181	TAAAAA/TAAAA	TGGGCCTCAACAAAGCTTAC	TCTTGGAAGTGCTCAGCTTAAT	60.2	668	DRR	Ca_00291	V	NAC
Ca1_P3_2324632	Ca1	2324632	ATTTTTTTT/ATTTTTTTTTTT	TTAAGCTGAGCACTTCCAAGA	GAATCCGAAACCACACGAAT	57.9	523	DRR	Ca_00291	V	NAC

INDEL marker IDs	Chromosomes/ unanchored scaffolds	Physical positions (bp)	InDels (*Kabuli* reference genome-CDC Frontier/PII)	Forward primers (5'-3')	Reverse primers (5'-3')	Anneling temperature (0C)	Expected amplified product size (bp)	Structural annotation		Functional annotation	
								Sequence components of *kabuli* genome	*Kabuli* gene accession IDs	NCBI-KOG	TFs
Ca1_P3_2332780	Ca1	2332780	CTT/CT	CAAAAATGGCACATGCAAAG	TTCACCAATTCCGTGTTTCA	60.1	399	INTERGENIC	--	--	--
Ca1_P3_2332911	Ca1	2332911	GAAAA/GAAA	CAAAAATGGCACATGCAAAG	TTCACCAATTCCGTGTTTCA	60.1	399	INTERGENIC	--	--	--
Ca1_P3_23386171	Ca1	23386171	GTTATTATTATTATTATTATTATTATTATTATTATTATTATTAT/GTTATTATTATTATTATTATTATTATTATTATTATTATTAT	ACAAGAATGGCAAATGGAGC	TTTGCCCCACTAACTCTTGG	60.1	537	INTERGENIC	--	--	--
Ca1_P3_23426237	Ca1	23426237	TTATATATATATATATATATATATATATATATAT/TTATATATATATATATATATATATATATATAT	AAGTTAAGGAACCACGAGTGC	AAACATGTTGTTGATGCGTACA	58.4	256	DRR	Ca_19317	--	--
Ca1_P3_2344725	Ca1	2344725	GTTG/GTTGTAATTG	TCACTCCATCACAACCATTCA	GGGGCCATAGATTCTCAACA	60.0	552	INTERGENIC	--	--	--
Ca1_P3_2348339	Ca1	2348339	CACCCTA/CA	ATCTTTTGTTTGCCCCACAG	GCCTGTAGTGGAGTTTTGGG	60.0	661	DRR	Ca_00295	--	Dof
Ca1_P3_2348529	Ca1	2348529	TAA/TAAA	ATCTTTTGTTTGCCCCACAG	GCCTGTAGTGGAGTTTTGGG	60.0	661	DRR	Ca_00295	--	Dof
Ca1_P3_2351581	Ca1	2351581	GAAAAAA/GAAAAA	TCTTGCATTCTCGTTTTACACAA	GCTTTGATTTTGCTTCGTCG	59.8	279	INTERGENIC	--	--	--
Ca1_P3_23516838	Ca1	23516838	CAAAAAA/CAAAAA	GGAATCCAAACCAAAACCCT	TTGGTGTGGTTTTTGTGGAA	60.0	634	INTERGENIC	--	--	--
Ca1_P3_2352395	Ca1	2352395	TTGT/TT	CCTTTGGAAAAGAAAATGTAGAATG	AAACAAGCAGCTTCTCTGCC	59.5	336	INTERGENIC	--	--	--

**INDEL marker IDs**	**Chromosomes/ unanchored scaffolds**	**Physical positions (bp)**	**InDels (*Kabuli* reference genome-CDC Frontier/PII)**	**Forward primers (5'-3')**	**Reverse primers (5'-3')**	**Anneling temperature (0C)**	**Expected amplified product size (bp)**	**Structural annotation**		**Functional annotation**	
								**Sequence components of *kabuli* genome**	***Kabuli* gene accession IDs**	**NCBI-KOG**	**TFs**
Ca1_P3_23565928	Ca1	23565928	AA/AACATCATATTA	GCGGTTAAGCCAATTTTGAG	TGGTGTAACGAGCATCAACAA	59.7	450	INTERGENIC	--	--	--
Ca1_P3_23569704	Ca1	23569704	ATATTATTATTATTATTATTATTATTATTATTATTATTATTATTATTATTATTATTATTAT/ATATTATTATTATTATTATTATTATTATTATTATTATTATTATTATTATTATTATTATTAT	GCTATTGAATGCGTCTTTCG	TTCAAATTGTGGCGACTTTG	58.5	876	INTERGENIC	--	--	--
Ca1_P3_2358909	Ca1	2358909	ATTT/ATT	TTCATCAAGAGACAAGTGAACCA	AATAGGCGCGAATTTAGGGT	59.8	516	DRR	Ca_00296	L	--
Ca1_P3_2363035	Ca1	2363035	AGGG/AGG	TTGGCTTCTTGGTTCTGCTT	CTTTGTGTCATCGCATCACC	60.0	584	INTERGENIC	--	--	--
Ca1_P3_2363092	Ca1	2363092	CTTATAT/CTTATATTATAT	TTGGCTTCTTGGTTCTGCTT	CTTTGTGTCATCGCATCACC	60.0	584	INTERGENIC	--	--	--
Ca1_P3_2364127	Ca1	2364127	TAAAAAA/TAAAAA	CCCAACAAGCCATAAAGCAT	ATCTCGATACAACGATCGCC	60.0	595	DRR	Ca_00300	--	--
Ca1_P3_23643612	Ca1	23643612	TAGAG/TAG	TCGGAATGGTCTGACATCAA	CAATAGGTGGAGGTGCGATT	60.0	830	INTERGENIC	--	--	--
Ca1_P3_23651952	Ca1	23651952	TTATATATATATATATATATATATAT/TTATATATATATATATATATAT	GCGTAGTGGTAGCCTTTTGG	AGTTGGATGTTGGGGAATCA	59.8	582	INTERGENIC	--	--	--
Ca1_P3_23683980	Ca1	23683980	A/AAAGGAAGGAGCGTAGCAC	AAAATGAGGAAAGAGGTCATAAAAA	CGTTGACCAATGCAAAAGAG	58.7	101	DRR	Ca_19303	R	MYB
Ca1_P3_2370923	Ca1	2370923	CA/CAA	CTATGGAAAATGCTCCCGAA	CCGAAACTCACAATGAACCA	60.0	507	INTRON	Ca_00301	DR	--

**INDEL marker IDs**	**Chromosomes/ unanchored scaffolds**	**Physical positions (bp)**	**InDels (*Kabuli* reference genome-CDC Frontier/PII)**	**Forward primers (5'-3')**	**Reverse primers (5'-3')**	**Anneling temperature (0C)**	**Expected amplified product size (bp)**	**Structural annotation**		**Functional annotation**	
								**Sequence components of *kabuli* genome**	***Kabuli* gene accession IDs**	**NCBI-KOG**	**TFs**
Ca1_P3_23722429	Ca1	23722429	CAAAAAAAA/CAAAAAAA	CTTTTACAGCGCTTTTTGGG	AAGAACAGAGGGAACGCAGA	59.9	395	INTERGENIC	--	--	--
Ca1_P3_23736065	Ca1	23736065	TACAAAAATAA/TA	GAAGGCCAAGAAATGACCAA	CATGCTGAGAAATAAAGTTGCG	60.1	712	INTRON	Ca_19300	--	SAP
Ca1_P3_2376563	Ca1	2376563	CG/C	GCAATTTCTGAAAGGTGGGA	ATGAATTGTGCTGCTTGCTG	60.1	583	DRR	Ca_00302	R	NAC
Ca1_P3_23776181	Ca1	23776181	C/CTTATA	CGACAAATGGTTGTGGAGTG	CTTGTTAACCGGACGGATCT	60.0	785	INTERGENIC	--	--	--
Ca1_P3_23784964	Ca1	23784964	ATTTTTTTT/ATTTTTT	GTCAACCAATTGACCCCAAC	TGCCAAGAAAACACCATGAA	60.1	663	INTERGENIC	--	--	--
Ca1_P3_23824031	Ca1	23824031	AAG/AAGCCTAG	CCAAGGAAGGACGAAAATGA	TTTTCGGTGATTCAAAAGCC	60.0	390	INTERGENIC	--	--	--
Ca1_P3_23838777	Ca1	23838777	ATGTGTGTGTGT/ATGTGTGT	AATGCCCACCCATAATTTCA	TGTACGAGAAGGTCGTGTGC	60.0	593	INTERGENIC	--	--	--
Ca1_P3_2386580	Ca1	2386580	GTATATATATATATATATATATATATATATATATAT/GTATATATATATATATATATATATATATAT	TCCCGTGTTTGTTTATCTATCA	TGAGACACGTGTTTTTAACCG	57.2	207	INTERGENIC	--	--	--
Ca1_P3_23881803	Ca1	23881803	CA/CAA	CCATTTTCATTCGTCGCTTT	TACCGCGACCGACTCTACTT	60.1	747	INTERGENIC	--	--	--
Ca1_P3_23966814	Ca1	23966814	GA/GAA	AACTGTGGAGGAATCCATGC	GCTTCAAGTCTTGCTACGGG	59.9	490	INTERGENIC	--	--	--

INDEL marker IDs	Chromosomes/ unanchored scaffolds	Physical positions (bp)	InDels (*Kabuli* reference genome-CDC Frontier/PII)	Forward primers (5'-3')	Reverse primers (5'-3')	Anneling temperature (0C)	Expected amplified product size (bp)	Structural annotation		Functional annotation	
								Sequence components of *kabuli* genome	*Kabuli* gene accession IDs	NCBI-KOG	TFs
Ca1_P3_24030518	Ca1	24030518	CG/CGTGTTG	CCAATTCAAAAGCTCCCAAA	CAGATCCTCCTTTCCCAACA	60.0	424	DRR	Ca_19291	OK	--
Ca1_P3_2406732	Ca1	2406732	GAGAAGAAGAA/GAGAAGAA	CACGCAAACACTGAATTTGG	ACAAAAATGGGTCCCTCCTT	60.1	777	INTERGENIC	--	--	--
Ca1_P3_24098971	Ca1	24098971	GATATATATATATATATATATATATATATATA/GATATATATATATATATATATATATATATATATATATATATATATA	AGGCATGGTGTTGGAGTCAT	TGCATTTTTCCACGTTCAAG	60.4	526	INTERGENIC	--	--	--
Ca1_P3_2411747	Ca1	2411747	T/TA	TGTGTCTTGTTGGGGAAACA	TGACGCTTTTGAAGCAGTTG	60.0	547	INTERGENIC	--	--	--
Ca1_P3_2412541	Ca1	2412541	TTTATTA/TTTA	AGGACAATAGGACGAGGCAA	AGTCACAGAGCATGCACCAC	59.7	436	INTERGENIC	--	--	--
Ca1_P3_24154735	Ca1	24154735	ATTTTT/ATTTTTT	GGGTGCCATCATAAGCAAGT	CAGATACTGCAGCCTCCACA	60.0	516	INTERGENIC	--	--	--
Ca1_P3_2416502	Ca1	2416502	GA/G	CAAAATCAGTCGCATGTTGG	TAGCCTTTTTGTTGGTTGGC	60.1	248	INTERGENIC	--	--	--
Ca1_P3_24180187	Ca1	24180187	TAAAAAAAA/TAAAAAAA	TTGCATCGATCATTGTTGAC	GAGTTGTTGTTCTTCCAACCAA	58.0	642	INTERGENIC	--	--	--
Ca1_P3_24242300	Ca1	24242300	TAAAAAAAAAAA/TAAAAAAAAAA	ACCAACTCATCCCTTTCCCT	ATGATTGCAGCATTTCACGA	59.8	638	INTERGENIC	--	--	--
Ca1_P3_24392807	Ca1	24392807	CAAATT/C	CCGAGCATCGATTTGGTAAT	TGATGGTGAAAGTGGGTTGA	59.9	376	INTERGENIC	--	--	--

INDEL marker IDs	Chromosomes/ unanchored scaffolds	Physical positions (bp)	InDels (*Kabuli* reference genome-CDC Frontier/PII)	Forward primers (5'-3')	Reverse primers (5'-3')	Anneling temperature (0C)	Expected amplified product size (bp)	Structural annotation		Functional annotation	
								Sequence components of *kabuli* genome	*Kabuli* gene accession IDs	NCBI-KOG	TFs
Ca1_P3_2446992	Ca1	2446992	CAAA/CAAAA	TGGACTCATCATGGATCGAA	GTAGGTGGGTCCACCATGTC	60.0	470	INTERGENIC	--	--	--
Ca1_P3_2447611	Ca1	2447611	ATTTAGTTTTA/ATTTA	CTGTGGTCCCCATCCATTAC	AGAAATTTGAGATTACCCTTTTCA	60.1	387	INTERGENIC	--	--	--
Ca1_P3_2447832	Ca1	2447832	CCTTC/CCTTCTTC	CTGTGGTCCCCATCCATTAC	AACTGGATAGGGAGAATTTTGTT	60.1	751	INTERGENIC	--	--	--
Ca1_P3_2452489	Ca1	2452489	CTTTT/CTTTTT	TTGCAAAGCTGTTGAGGTTG	TTACCGTGAGACGAAGGGAC	60.0	347	DRR	Ca_00310	--	--
Ca1_P3_2452798	Ca1	2452798	TAT/TATCAT	GTCCCTTCGTCTCACGGTAA	TCAAACACGCACATAAAAAGG	60.1	729	DRR	Ca_00310	--	--
Ca1_P3_24719200	Ca1	24719200	AATATATATATATATATATATATATATATATATA/AATATATATATATATATATATATATATATATATA	TCTTTGTTTCAACGGTCAATG	ATTATTTCCTGTTCGGGGGA	58.7	661	DRR	Ca_18334	--	--
Ca1_P3_24801961	Ca1	24801961	CTTTTTTTTT/CTTTTTTTTTT	TTTCAATGATGTTGGGTTGC	CAGCATATCCCCAACAAAAGA	59.4	998	INTERGENIC	--	--	--
Ca1_P3_24856702	Ca1	24856702	TCATA/T	TTGGTTGGGTTTGTGATGAG	TTGGGCAGGTAAAAACAACTTT	59.4	183	INTERGENIC	--	--	--
Ca1_P3_25004655	Ca1	25004655	TC/TCCTGGAGCGC	ACTTTCATGAAGACACCGGG	CTTCGAATCCAACCTTGGTG	60.0	586	INTERGENIC	--	--	--
Ca1_P3_2529049	Ca1	2529049	AATATATATATATATATA/AATATATATATATATATA	GTCCACTATGGTCGAAGCCT	AACGCAAGACGAAGCTGTTT	59.2	657	DRR	Ca_00315	R	WRKY

INDEL marker IDs	Chromosomes/ unanchored scaffolds	Physical positions (bp)	InDels (*Kabuli* reference genome-CDC Frontier/PII)	Forward primers (5'-3')	Reverse primers (5'-3')	Anneling temperature (0C)	Expected amplified product size (bp)	Structural annotation		Functional annotation	
								Sequence components of *kabuli* genome	*Kabuli* gene accession IDs	NCBI-KOG	TFs
Ca1_P3_25296360	Ca1	25296360	GAAAAAAAA/GAAAAAAAAAAA	GGCGATTCTATGTCCATTGC	TCAACAAAATCACAGGCCAC	60.4	697	INTERGENIC	--	--	--
Ca1_P3_25296432	Ca1	25296432	CTTT/CTTTT	GTTGGTGGCTGGACTCAGAT	TTCCCTAGTTGTTTGGGTGC	60.1	817	INTERGENIC	--	--	--
Ca1_P3_25376447	Ca1	25376447	ATTTTTTTTTTTT/ATTTTTTTTTT	AGCGACGACAAGTCACTGAG	TCATTGTTTGTGTTTGGCGT	59.2	719	INTERGENIC	--	--	--
Ca1_P3_25381451	Ca1	25381451	AC/A	TTTAAGCCCTTATTCCCGGT	ATGGCGGCATAGTATCAAGC	59.8	455	INTERGENIC	--	--	--
Ca1_P3_25382154	Ca1	25382154	GAA/GAAA	GAATCACTAGGCTTGCCAGG	GGGGTGAGAGAACGTTTGAA	59.8	714	INTERGENIC	--	--	--
Ca1_P3_25382333	Ca1	25382333	AAC/ATGGCTAC	CAAATGCCACACATTTAACGA	GGGGTGAGAGAACGTTTGAA	59.5	255	INTERGENIC	--	--	--
Ca1_P3_25437512	Ca1	25437512	AATATATATATATATATATATATATATATATATATATATATATA/AATATATATATATATATATATATATATATATATATATATATATATATATATA	GACTTTGATGCAAGTCACCG	CTCCCATCAGTTTAATCGGC	59.3	875	INTERGENIC	--	--	--
Ca1_P3_25477131	Ca1	25477131	AATTATT/AATTATTAAAATTATATTATT	CCCATGCATGCTTCATAGAG	TTCTCGAAGATCCGACAGGT	59.3	782	INTERGENIC	--	--	--
Ca1_P3_25509778	Ca1	25509778	AATATATATATATATATATATATATATATATATATATATCTATATATATAT/AATATATATATATATATATATATATATATATATATATATATATATATATATATATCTATATATATAT	ATTTTGGCTCGGAGGTTTTT	GTTTTCGAATCAACGAGGGA	59.9	550	INTERGENIC	--	--	--
Ca1_P3_25519824	Ca1	25519824	CAGACCCA/CAGACCCAAACATAAGACCCA	TCAGGGACAAAATTGCATGA	CGTTCCTCTGAAGGTTACGAA	60.0	382	INTERGENIC	--	--	--

INDEL marker IDs	Chromosomes/ unanchored scaffolds	Physical positions (bp)	InDels (*Kabuli* reference genome-CDC Frontier/PII)	Forward primers (5'-3')	Reverse primers (5'-3')	Anneling temperature (0C)	Expected amplified product size (bp)	Structural annotation		Functional annotation	
								Sequence components of *kabuli* genome	*Kabuli* gene accession IDs	NCBI-KOG	TFs
Ca1_P3_25629959	Ca1	25629959	AATATATATATATATATAT/AATATATATATATATATATAT	CAAAAATCCTTCGGCGAATA	ATTTCACTCATGCCTCCACC	60.0	895	INTERGENIC	--	--	--
Ca1_P3_25786546	Ca1	25786546	TACTC/T	ATCGATTTCGCGAGTCTTGT	GGAGAGTAATCGATTGGGCA	59.8	531	INTERGENIC	--	--	--
Ca1_P3_25796612	Ca1	25796612	GAAAAAAAAAAA/GAAAAAAAAA	GTGCCCCCAAACTACTTTCA	AAGTCCCGGTAAGGTTGAGC	60.0	638	INTERGENIC	--	--	--
Ca1_P3_25875193	Ca1	25875193	CAAA/CAAAA	GAGTGTTAGTAGGCGGCAGG	CTAGGGGTCCTTCCTTTTGG	59.9	576	INTERGENIC	--	--	--
Ca1_P3_26034127	Ca1	26034127	TAAAAAAAAA/TAAAAAAAAAA	TAACCAAAATGCCACAACGA	TATGATCCACCACCGACTCA	60.0	835	DRR	Ca_18591	--	SBP
Ca1_P3_26133146	Ca1	26133146	CTT/CTTGGCTTT	GCCGAACCCTTTTCTCCTAC	CCACAACAAAACGAAAGATCAA	60.1	241	INTERGENIC	--	--	--
Ca1_P3_26246371	Ca1	26246371	C/CG	GCGCCTTAAAAGGATTTTCC	CCCGTGATAGGCAGTACGTT	60.0	873	INTERGENIC	--	--	--
Ca1_P3_26279948	Ca1	26279948	ATTTTTTTT/ATTTTTTT	GTTTTGAAAGCAGCCTGGAG	TGCTTGATGGCAAAAACAGA	60.0	653	INTERGENIC	--	--	--
Ca1_P3_2633537	Ca1	2633537	CTTTTTTT/CTTTTTT	GGACAATTGTTTTAAATGTGATGG	TCACGCTTCGACTAAGCAAA	59.5	709	INTERGENIC	--	--	--
Ca1_P3_26377123	Ca1	26377123	ATTTTTTT/ATTTTTTTT	TGTTCTAGATGGGGTTTGGC	GCCACTATCCAACAAATGGC	59.9	655	INTERGENIC	--	--	--

INDEL marker IDs	Chromosomes/ unanchored scaffolds	Physical positions (bp)	InDels (*Kabuli* reference genome-CDC Frontier/PII)	Forward primers (5'-3')	Reverse primers (5'-3')	Anneling temperature (0C)	Expected amplified product size (bp)	Structural annotation		Functional annotation	
								Sequence components of *kabuli* genome	*Kabuli* gene accession IDs	NCBI-KOG	TFs
Ca1_P3_26447765	Ca1	26447765	ATATTATTATTATTATTATTATTATTATT/ATATTATTATTATTATTATTATTATTATTATTATT	AAATAGGGCCACACCAAAAA	TTAAATCGGAGGTATGGGGA	59.3	320	INTERGENIC	--	--	--
Ca1_P3_26447923	Ca1	26447923	CACATATCTG/C	AAATAGGGCCACACCAAAAA	GTAATGGGTATGGGTACGGG	59.3	420	INTERGENIC	--	--	--
Ca1_P3_26463148	Ca1	26463148	CTTTTTTTT/CTTTTTTTTT	GGCTAAATTACATTTGTGGTCCC	AAACGCGAAAATTAGAAACAAGA	60.8	551	INTERGENIC	--	--	--
Ca1_P3_26473008	Ca1	26473008	CTAAATT/CTAAATTCTGTAAATT	CCATTGACCCTTGCAAACTT	CTGCAGGAAGCTAAAATCCG	60.0	368	DRR	Ca_18608	R	NAC
Ca1_P3_2653521	Ca1	2653521	T/TC	GGTGGGTGACCCTCACTTTA	CATTCCAGTTAGTTGAACGGGT	59.8	525	INTERGENIC	--	--	--
Ca1_P3_2655382	Ca1	2655382	CTTT/CTT	TCCGTGGAAATGAACCTAGC	CGCGTGATGATTGCTAAAAA	60.1	228	INTERGENIC	--	--	--
Ca1_P3_2656323	Ca1	2656323	TTATTATTATTATTAATATTATTATTA/TTATTATTATTA	TTGCACAATTTCTGATTGGC	GATCATTGCATCCCCATTCT	59.7	569	INTERGENIC	--	--	--
Ca1_P3_2656475	Ca1	2656475	A/AG	TTGCACAATTTCTGATTGGC	GATCATTGCATCCCCATTCT	59.7	569	INTERGENIC	--	--	--
Ca1_P3_2661346	Ca1	2661346	TAAAA/TAAAAA	CGCCGTTTTTCTTCAAACTC	TGGTGTTCATTTTGATTCAGGA	59.9	840	INTERGENIC	--	--	--
Ca1_P3_2661645	Ca1	2661645	TA/TAA	AACTTTCATACAAACTCACACCATTT	TAGCGTGTTCGAGTGCAGAG	59.3	449	INTERGENIC	--	--	--

**INDEL marker IDs**	**Chromosomes/ unanchored scaffolds**	**Physical positions (bp)**	**InDels (*Kabuli* reference genome-CDC Frontier/PII)**	**Forward primers (5'-3')**	**Reverse primers (5'-3')**	**Anneling temperature (0C)**	**Expected amplified product size (bp)**	**Structural annotation**		**Functional annotation**	
								**Sequence components of *kabuli* genome**	***Kabuli* gene accession IDs**	**NCBI-KOG**	**TFs**
Ca1_P3_2662211	Ca1	2662211	AATATA/AATA	CTCTGCACTCGAACACGCTA	TTTGTCTCTCCCACTCCCAC	60.3	345	INTERGENIC	--	--	--
Ca1_P3_2663715	Ca1	2663715	TGAAAAGAAAA/TGAAAA	GTTGCCATGCCTATAACGGT	TGAAGACCATGGACACCAAC	59.9	547	INTERGENIC	--	--	--
Ca1_P3_2668175	Ca1	2668175	AC/A	AATGCATCACGTGGGATTTT	CGATTGCACCCGTAGTTGTA	60.2	206	INTERGENIC	--	--	--
Ca1_P3_26681887	Ca1	26681887	AAAAAAACAAAAAACAAAAAACAAAA/AAAAAAACAAAAAACAAAA	CCAGACACAACTCAACCCCT	CTTTTGGCTCCCCCTAAGTC	60.0	476	INTERGENIC	--	--	--
Ca1_P3_2668242	Ca1	2668242	GTTTTTTTT/GTTTTTT	AATGCATCACGTGGGATTTT	CGATTGCACCCGTAGTTGTA	60.2	206	INTERGENIC	--	--	--
Ca1_P3_26683215	Ca1	26683215	T/TAACTAGAAG	AATCCTTAACCCTTCCCCCT	GATCTCGAAGACCCACCAAA	60.0	473	INTERGENIC	--	--	--
Ca1_P3_26683611	Ca1	26683611	TG/T	CAATTGTGTAAAACGTGCGG	TTGCATTTGGTTTGACTCCA	60.0	538	INTERGENIC	--	--	--
Ca1_P3_2669284	Ca1	2669284	TAAAAAAAAAAAAA/TAAAAAAAAAA	CCCCAAACTTTGAAGCAACT	TTTGTGCTGCTATGTGCCTC	59.2	344	DRR	Ca_00327	--	ERF
Ca1_P3_2669686	Ca1	2669686	TAAAAAAAA/TAAAAAAA	GAGGCACATAGCAGCACAAA	TTTTTAAAACACCAACGGGC	60.0	379	DRR	Ca_00327	--	ERF
Ca1_P3_2669957	Ca1	2669957	CATAAA/CA	GAGGCACATAGCAGCACAAA	AGCCAGATATCAGACCGCAC	60.0	818	DRR	Ca_00327	--	ERF

INDEL marker IDs	Chromosomes/ unanchored scaffolds	Physical positions (bp)	InDels (*Kabuli* reference genome-CDC Frontier/PII)	Forward primers (5'-3')	Reverse primers (5'-3')	Anneling temperature (0C)	Expected amplified product size (bp)	Structural annotation		Functional annotation	
								Sequence components of *kabuli* genome	*Kabuli* gene accession IDs	NCBI-KOG	TFs
Ca1_P3_2670991	Ca1	2670991	CT/CTT	TTCGCCCACTATCAACACAA	AACTTCCCTTACCCTTCCGA	60.1	851	DRR	Ca_00327	--	ERF
Ca1_P3_26725325	Ca1	26725325	CTATATATATATATATATAT/CTATATATATATATATAT	AATTAGCAGCGGAAGGAAAG	GCATCACAGTTAAATCATCGC	58.6	352	INTERGENIC	--	--	--
Ca1_P3_26729744	Ca1	26729744	TA/T	ATCCGCTTACATTTGGTTCG	GTTGGGGTGAGTTGACGAGT	60.0	557	INTERGENIC	--	--	--
Ca1_P3_2674516	Ca1	2674516	CTAGATTGGAT/CTAGATTGGATTAGATTGGAT	TGTCGGAATATGGGAAGCTC	GCCGTTGTTTTCAGATCCAT	60.0	934	INTERGENIC	--	--	--
Ca1_P3_2678904	Ca1	2678904	ATTTTTT/ATTTTTTT	TCGGGTTATAAGCCACACAA	CAAGGAATGAGGGATAAGGATG	59.0	434	INTERGENIC	--	--	--
Ca1_P3_2679246	Ca1	2679246	CTTTTTTTT/CTTTTTTTTTT	CATCCTTATCCCTCATTCCTTG	ATGGTGGCCGGTAGACCTAT	59.8	723	INTERGENIC	--	--	--
Ca1_P3_2680668	Ca1	2680668	TA/TATTATTTAAA	AAACTTAACCGGGAAACAAAAA	TGGTAAATACCTCCGCACTTT	59.0	506	INTERGENIC	--	--	--
Ca1_P3_26815473	Ca1	26815473	GTTTTTTTTTTTTT/GTTTTTTTTTTT	TGGATGTTTTTGGCTCATCA	CCTGAAAAAGCTTGGAGTGG	60.0	336	INTERGENIC	--	--	--
Ca1_P3_26826581	Ca1	26826581	TAA/TAAA	CACAGAACAAATATTGTTGGTGATAA	AGGCACAACAAGCAAGAAATC	59.3	435	INTERGENIC	--	--	--
Ca1_P3_26845597	Ca1	26845597	ATTTTTTTTT/ATTTTTTTTTT	TCCCTCCAAACTTCTTGAGTCT	TGGATGCAATATTACTTTCTTCG	59.4	387	INTERGENIC	--	--	--

INDEL marker IDs	Chromosomes/ unanchored scaffolds	Physical positions (bp)	InDels (*Kabuli* reference genome-CDC Frontier/PII)	Forward primers (5'-3')	Reverse primers (5'-3')	Anneling temperature (0C)	Expected amplified product size (bp)	Structural annotation		Functional annotation	
								Sequence components of *kabuli* genome	*Kabuli* gene accession IDs	NCBI-KOG	TFs
Ca1_P3_2686786	Ca1	2686786	AACA/AA	TGGTTTCACGTTTGCCATTA	CAAATTTCACGCAACAATCG	60.0	957	DRR	Ca_00328	--	MIKC
Ca1_P3_26868947	Ca1	26868947	ATTTTTTTTTT/ATTTTTTTTT	AGTATGAAAGCTCCGAGGCA	TTTTTGCCCATTCAAACACA	60.0	454	INTERGENIC	--	--	--
Ca1_P3_26894031	Ca1	26894031	GAATTTGTAATTATTG/GAATTTGTAATTATTGTAATTTGTAATTATTG	ATGCACACACTTTTGATGCC	TCATACAAATTCGAGGTTCCAA	59.6	670	INTERGENIC	--	--	--
Ca1_P3_26894935	Ca1	26894935	GAAAAAAAAA/GAAAAAAAA	CGAAGAAGAAAATGCGACAA	AAAAACAAAATCACAGGTAACCAAA	59.0	512	INTERGENIC	--	--	--
Ca1_P3_2690867	Ca1	2690867	ATTTTTTTTT/ATTTTTTTT	GAGCTTTGCTTTGGGATCTG	CAAACGGAACCTCAACCATC	60.0	714	INTERGENIC	--	--	--
Ca1_P3_2691872	Ca1	2691872	ATTAAGT/ATTAAGTTTAAGT	TTAGCTCAGCTTGCACAGGA	GCATTGTACATTCTCGTGCG	59.9	541	DRR	Ca_00330	--	--
Ca1_P3_2693080	Ca1	2693080	TAAAAAAAA/TAAAAAAAAA	CAACCAGTAATAATGCGCCA	CGTGACAGAGGACGCTTGTA	59.6	468	DRR	Ca_00330	--	--
Ca1_P3_26958642	Ca1	26958642	TT/TTTGAT	TGCTCCATTTGATTGCTGAC	CCGACCAATAGACTTTTGTTGA	59.8	663	INTERGENIC	--	--	--
Ca1_P3_2698195	Ca1	2698195	A/ACTT	TTCGCGCATATTTTAGTGAAAG	TTTGTGCCCCATCAACTACA	59.4	609	INTERGENIC	--	--	--
Ca1_P3_26989616	Ca1	26989616	AAATAATAATAATAATAATAATAATAATAATAATAATAATAA/AAATAATAATAATAATAATAATAATAATAATAATAA	GAGGTCGTTGACCGGATATG	CGTGTGATTGGTAATGTGGC	60.3	639	INTERGENIC	--	--	--

INDEL marker IDs	Chromosomes/ unanchored scaffolds	Physical positions (bp)	InDels (*Kabuli* reference genome-CDC Frontier/PII)	Forward primers (5'-3')	Reverse primers (5'-3')	Anneling temperature (0C)	Expected amplified product size (bp)	Structural annotation		Functional annotation	
								Sequence components of *kabuli* genome	*Kabuli* gene accession IDs	NCBI-KOG	TFs
Ca1_P3_2699190	Ca1	2699190	AGTTGTGTGTTGTGTGTTGTGTGT/AGTTGTGTGT	TCAATGTCAAACAAAAGCCAA	CCTATTCCGCCAAAAACAAA	59.2	538	INTERGENIC	--	--	--
Ca1_P3_2699403	Ca1	2699403	T/TG	TCAATGTCAAACAAAAGCCAA	CCTATTCCGCCAAAAACAAA	59.2	538	INTERGENIC	--	--	--
Ca1_P3_2699414	Ca1	2699414	TAAAAAA/TAAAAAAA	TCAATGTCAAACAAAAGCCAA	CCTATTCCGCCAAAAACAAA	59.2	538	INTERGENIC	--	--	--
Ca1_P3_27034910	Ca1	27034910	T/TA	GACTGGACGTAACCCACGTT	GGCAACTAAAGATCATGCCG	59.9	601	INTERGENIC	--	--	--
Ca1_P3_27049303	Ca1	27049303	TAAAAAAAAAAAA/TAAAAAAAAAAA	CTCCAGGTGCAATAGGGAAA	AAAGAACATTTCCAGTGCGG	60.1	581	INTERGENIC	--	--	--
Ca1_P3_27104159	Ca1	27104159	AT/A	AGTGCCATTTGTGTTGTAGGG	AGGGGTGTGGGAAGAAAAAT	59.9	399	INTERGENIC	--	--	--
Ca1_P3_2711863	Ca1	2711863	CATAATAATAATAATAATA/CATAATAATAATAATAATAATA	TTCGGTGCTACATATTCCTCAA	CCCGTTTCAGCTCAATCAAT	59.6	448	DRR	Ca_00334	G	--
Ca1_P3_2712445	Ca1	2712445	GTT/GTTT	TTGATCTGCGAGGTGAAGTG	TCCAACCTTTCCAATTGAGG	60.0	637	DRR	Ca_00334	G	--
Ca1_P3_2712542	Ca1	2712542	CATA/CA	TTGATCTGCGAGGTGAAGTG	TCCAACCTTTCCAATTGAGG	60.0	637	DRR	Ca_00334	G	--
Ca1_P3_27137277	Ca1	27137277	TAAAA/TAAAAACAGAAAAA	TCACACAAGAATGGAGCGAA	TGGGCTATGTTTGTTTTCCC	60.4	463	DRR	Ca_20311	KR	--

INDEL marker IDs	Chromosomes/ unanchored scaffolds	Physical positions (bp)	InDels (*Kabuli* reference genome-CDC Frontier/PII)	Forward primers (5'-3')	Reverse primers (5'-3')	Anneling temperature (0C)	Expected amplified product size (bp)	Structural annotation		Functional annotation	
								Sequence components of *kabuli* genome	*Kabuli* gene accession IDs	NCBI-KOG	TFs
Ca1_P3_27143610	Ca1	27143610	AATATATATATATATATATATATATATA/ AATATATATATATATATATATATATATATAT ATATATA	GTTCAATCAATACTCGG CGG	GGCATCTTTGATCAAT GGTCTT	60.5	681	INTERGENIC	--	--	--
Ca1_P3_27204677	Ca1	27204677	C/CT	GCCGAAGTACCAATTA CCGA	TACGGTGCTAGGGAAC CAAC	60.0	705	INTERGENIC	--	--	--
Ca1_P3_27219176	Ca1	27219176	CTATTATTATTATTATTATTATTATTATT ATTATTATTATTATTGTTATTATTATTAT TATTATTATTATTATTATTATTA/CTATT ATTATTATTATTATTATTATTATTATTAT TATTATTATTATTGTTATTATTATTATTA [illegible]	TGAGGGAGATATTTGG TGGG	TACGGCGCTGTTCTTT TCTT	59.7	277	INTERGENIC	--	--	--
Ca1_P3_27232314	Ca1	27232314	AATATATATATATATATATATATATATAT/A ATATATATATATATATATATATATATATATA TATAT	CTTGGGAACTGGAGAG CAAG	AGGGAGGTTTGTGTTT GACG	60.0	682	INTERGENIC	--	--	--
Ca1_P3_2725497	Ca1	2725497	TAA/TA	GTGTAACCTCACGGGA AGGA	GGGTCAATTTGAAACA ACGG	60.0	660	INTERGENIC	--	--	--
Ca1_P3_2727935	Ca1	2727935	AT/A	CAAACGCAAATTGAGC GATA	CATTTTGAAAGATTCAC ATTCATTT	59.8	717	INTERGENIC	--	--	--
Ca1_P3_2728007	Ca1	2728007	CTA/CTACTTA	CAAACGCAAATTGAGC GATA	CATTTTGAAAGATTCAC ATTCATTT	59.8	717	INTERGENIC	--	--	--
Ca1_P3_27282795	Ca1	27282795	CAGA/CA	AAAATCCAACAATCTCC CCC	CCCTGAGACTCACGAA GAGG	60.0	561	INTERGENIC	--	--	--
Ca1_P3_27282873	Ca1	27282873	GTATA/GTATATA	AAAATCCAACAATCTCC CCC	CCCTGAGACTCACGAA GAGG	60.0	561	INTERGENIC	--	--	--
Ca1_P3_2729433	Ca1	2729433	ATTTTTTT/ATTTTTT	ACAGAATCAAAAGCATG GGG	TTCATTCCACTTCCAC CTCC	59.9	740	INTRON	Ca_00335	O	MYB

INDEL marker IDs	Chromosomes/ unanchored scaffolds	Physical positions (bp)	InDels (*Kabuli* reference genome-CDC Frontier/PII)	Forward primers (5'-3')	Reverse primers (5'-3')	Anneling temperature (0C)	Expected amplified product size (bp)	Structural annotation		Functional annotation	
								Sequence components of *kabuli* genome	*Kabuli* gene accession IDs	NCBI-KOG	TFs
Ca1_P3_27335479	Ca1	27335479	CATATATATATATATATATATATATAT/CATATATATATATATATATATATAT	TGAAGAAGCTGAAGGGGAAA	TGAGTGATGTGATGTGCGAA	59.9	138	INTERGENIC	--	--	--
Ca1_P3_27346637	Ca1	27346637	GTATATATATATATATATATATAT/GTATATATATATATATATAT	CTTCCGTTCACTGGTGACAA	TGTTGTTGGTGGAGCACAAT	59.7	704	INTERGENIC	--	--	--
Ca1_P3_2745007	Ca1	2745007	GTTTTTTTTTT/GTTTTTTTTT	GAACGAACGGTGTGATGATG	GGGCCCACAATAACACAAAC	60.0	513	INTERGENIC	--	--	--
Ca1_P3_2747689	Ca1	2747689	AT/ATT	TTGCTGAGCTGAAGCAGAAA	CTTGGGGATCAGCTTATGGA	60.0	961	DRR	Ca_00338	R	NAC
Ca1_P3_27597440	Ca1	27597440	ATT/AT	AAAATCCAACAATCTCCCCC	CCCTGAGACTCACGAAGAGG	60.0	559	DRR	Ca_25069	--	--
Ca1_P3_2762193	Ca1	2762193	ATTTTTTT/ATTTTTT	ACCTTTGGGTTGCCTCCTAC	TGGAAGGATGGATGCTTAGG	60.4	625	INTERGENIC	--	--	--
Ca1_P3_2762478	Ca1	2762478	ATCCATCCTTCCATCCT/ATCCATCCT	TATTTGGACAAACGAAGGGC	GCATAAATCGTGCTCGACAA	59.9	530	INTERGENIC	--	--	--
Ca1_P3_27637609	Ca1	27637609	GAA/GA	CCTGAAATCTACCAGGCGAA	GGTTTGGATTTCTTTGGGGT	60.2	740	INTERGENIC	--	--	--
Ca1_P3_2765671	Ca1	2765671	GTTTTTTTTT/GTTTTTTT	TAAAAAGGGCCGATATGCAC	CATGCACGATTGTGGTAATTG	59.9	564	INTERGENIC	--	--	--
Ca1_P3_2766077	Ca1	2766077	CTTATGTT/CTTATGTTATGTT	AAAACAATTGATTGGGCTCC	CCAATCATACGCAGGTGAAA	58.9	561	INTERGENIC	--	--	--

INDEL marker IDs	Chromosomes/ unanchored scaffolds	Physical positions (bp)	InDels (*Kabuli* reference genome-CDC Frontier/PII)	Forward primers (5'-3')	Reverse primers (5'-3')	Anneling temperature (0C)	Expected amplified product size (bp)	Structural annotation		Functional annotation	
								Sequence components of *kabuli* genome	*Kabuli* gene accession IDs	NCBI-KOG	TFs
Ca1_P3_2766176	Ca1	2766176	A/AG	AAAACAATTGATTGGGCTCC	CCAATCATACGCAGGTGAAA	58.9	561	INTERGENIC	--	--	--
Ca1_P3_2766346	Ca1	2766346	AATATA/AATATATA	TTTCACCTGCGTATGATTGG	TGACCTCTTATGCTTCTCGAAAT	59.5	501	INTERGENIC	--	--	--
Ca1_P3_27665616	Ca1	27665616	CGTTTCA/C	TCAGGGATAGGTTTCACTTCG	TTCAAACCAAATGCCAATGA	59.2	717	INTRON	Ca_25073	--	--
Ca1_P3_2766600	Ca1	2766600	CTTTTTTTT/CTTTTTTT	TTTCACCTGCGTATGATTGG	TTGAAAGTTGAAACGTGGGA	59.5	779	INTERGENIC	--	--	--
Ca1_P3_27668412	Ca1	27668412	GAAAAAAAAAAA/GAAAAAAAAAA	CCTTGATTGAGAACACCCCA	GGACTAGGTCCCGTGGTTTT	60.9	593	INTRON	Ca_25073	--	--
Ca1_P3_2766944	Ca1	2766944	ATTTTTTTTT/ATTTTTTTT	TTTCGAGAAGCATAAGAGGTCA	AAACCATGCACCTCATCACA	59.1	359	INTERGENIC	--	--	--
Ca1_P3_2769230	Ca1	2769230	TT/TGAATGCAGT	AGCGTGAAGACTCCGATGTT	TGCATCACTTTATTTGCCCA	59.9	374	INTERGENIC	--	--	--
Ca1_P3_2774451	Ca1	2774451	AATATATATA/AATATATATATATATATATA	CTTTTGGGGCACCACTGTAT	TTGGATTTTTCGCTGCAAGT	59.9	667	INTERGENIC	--	--	--
Ca1_P3_2774917	Ca1	2774917	TA/T	AACTTGCAGCGAAAAATCCA	TTTCCACTAATTGTGCCTTCC	60.8	517	INTERGENIC	--	--	--
Ca1_P3_2779177	Ca1	2779177	CATATA/CATA	ATGATGAAGGGGAAAAGGGT	TGACTGCATGTAGTTGTGGG	59.6	512	INTERGENIC	--	--	--

INDEL marker IDs	Chromosomes/ unanchored scaffolds	Physical positions (bp)	InDels (*Kabuli* reference genome-CDC Frontier/PII)	Forward primers (5'-3')	Reverse primers (5'-3')	Anneling temperature (0C)	Expected amplified product size (bp)	Structural annotation		Functional annotation	
								Sequence components of *kabuli* genome	*Kabuli* gene accession IDs	NCBI-KOG	TFs
Ca1_P3_2779559	Ca1	2779559	AATATATATATATATAT/AATATATATAT AT	AAGGGTCCTCTCAATC GTCA	GTGAGGTTGGTCAACT CCGT	59.7	715	INTERGENIC	--	--	--
Ca1_P3_27902762	Ca1	27902762	AAA/AAATTAA	TTTTGCAAGAGCGTCAA ATG	GGCTTCATAAAAGGTG GCAA	60.0	592	INTERGENIC	--	--	--
Ca1_P3_2793456	Ca1	2793456	GAAAAAAAAAAA/GAAAAAAAAA	TGTAGGCCTTGTGTGG ATCA	ATCCCTCCCTCGTCAA AATC	60.1	487	DRR	Ca_00343	T	NAC
Ca1_P3_2794449	Ca1	2794449	TA/TAAAACCACA	TTTTTGGTTCAAATGAT TGGTC	TGTCAAAACGAAAACG TTGAA	58.8	560	DRR	Ca_00343	T	NAC
Ca1_P3_2797230	Ca1	2797230	A/AC	TGTCTTAAAATCTGTGC ATTCAA	AAATAGTTGACAAACC GCCG	57.5	581	INTERGENIC	--	--	--
Ca1_P3_2797821	Ca1	2797821	CTTATTTT/CTTATTTTTTATTTT	ACGGCGGTTTGTCAAC TATT	GGGTGTTTTTGTCATT TGGG	59.5	743	INTERGENIC	--	--	--
Ca1_P3_2800036	Ca1	2800036	A/AAT	AAAATAAATCGTTGTGG CCG	ACTTGCTCGCTCTTGC TCTC	59.8	683	INTERGENIC	--	--	--
Ca1_P3_2802455	Ca1	2802455	ATTTTT/ATTTT	GGGTTACAGACCGACC TTGA	GCGGATTTGTCCATCT TTGT	60.0	765	DRR	Ca_00344	--	NAC
Ca1_P3_2803396	Ca1	2803396	GTGATGATG/GTGATG	ACAACGGAATTGGTCA TGGT	TTCCTAATTCCAATCCC ATGT	60.1	360	INTERGENIC	--	--	--
Ca1_P3_28170186	Ca1	28170186	CTTTTTTTTTT/CTTTTTTTTTTT	TCCAACGCCTTCTTTTC AAT	GTGCGATCCGTTGAAG TTCT	59.7	811	INTERGENIC	--	--	--

INDEL marker IDs	Chromosomes/ unanchored scaffolds	Physical positions (bp)	InDels (*Kabuli* reference genome-CDC Frontier/PII)	Forward primers (5'-3')	Reverse primers (5'-3')	Anneling temperature (0C)	Expected amplified product size (bp)	Structural annotation		Functional annotation	
								Sequence components of *kabuli* genome	*Kabuli* gene accession IDs	NCBI-KOG	TFs
Ca1_P3_2825323	Ca1	2825323	GTTTTATTTTTTATTTTTT/GTTTTATTTTTTATTTTTTATTTTTT	ACTTGGTTCTGTTGTTGGGC	TCCACCTCTCACTTTGGCTT	60.0	870	INTERGENIC	--	--	--
Ca1_P3_2826980	Ca1	2826980	CATA/C	CGCCCTGCCTCAAATTATTA	TTTAATGTTGTTGTTGAAGATGAAAA	60.1	962	INTERGENIC	--	--	--
Ca1_P3_2828086	Ca1	2828086	ATAATTA/ATAATTAATTA	TGGAACTCCAACATCCAACA	TTTCCCCAAAATTGCAAAAG	59.9	597	INTERGENIC	--	--	--
Ca1_P3_2828117	Ca1	2828117	TAG/TAGAG	TGGAACTCCAACATCCAACA	TTTCCCCAAAATTGCAAAAG	59.9	597	INTERGENIC	--	--	--
Ca1_P3_2830013	Ca1	2830013	AAAATATTATTTTAAATATT/AAAATATTATTTTAAATATTATTTTAAATATT	ATCCGGGTCCAAAGTTTCTT	AAAAGCATGCAAAAGCATCA	59.8	774	INTERGENIC	--	--	--
Ca1_P3_2831499	Ca1	2831499	GTTTTTTTTTTTTTT/GTTTTTTTTTTTT	CCTCTGACCACATGGGACTT	TCAAAAACAAAAGCGCACTG	60.0	625	DRR	Ca_00347	--	ARF
Ca1_P3_28322869	Ca1	28322869	GAAAAAAAAAA/GAAAAAAAAA	GAGAACAACAACTTGACCCAAA	GTTACCAGCCCATCAATGCT	59.1	376	INTERGENIC	--	--	--
Ca1_P3_28339292	Ca1	28339292	GCTCTCTCTCTCTCTCTCTCTCTCTCTCTCTCTCTCTCTCTCT/GCTCTCTCTCTCTCTCTCTCTCTCTCTCTCTCTCTCTCTCTCTCT	CGCCTTTCTGTTTTCCACAT	TGGTGTGCTTGGTGATGAAT	60.1	305	INTERGENIC	--	--	--
Ca1_P3_2838327	Ca1	2838327	CTTTTTTTTTT/CTTTTTTTTTTTT	TGCTCCCTCCATCTATCCAC	TTGCATTGTAATTGTGTTCGAC	60.0	437	INTERGENIC	--	--	--
Ca1_P3_2840718	Ca1	2840718	GTTTTTTTTTT/GTTTTTTTTT	GCTACATGTAACGCAGCGAA	ATTTAAGATGGCGATGTGCC	60.0	512	INTERGENIC	--	--	--

INDEL marker IDs	Chromosomes/ unanchored scaffolds	Physical positions (bp)	InDels (*Kabuli* reference genome-CDC Frontier/PII)	Forward primers (5'-3')	Reverse primers (5'-3')	Anneling temperature (0C)	Expected amplified product size (bp)	Structural annotation		Functional annotation	
								Sequence components of *kabuli* genome	*Kabuli* gene accession IDs	NCBI-KOG	TFs
Ca1_P3_2841066	Ca1	2841066	CTGTG/CTGTGTG	GCTACATGTAACGCAGCGAA	ATTTAAGATGGCGATGTGCC	60.0	512	INTERGENIC	--	--	--
Ca1_P3_2842423	Ca1	2842423	TTTTCTTTC/TTTTC	TTTCACAAATTAACATTTGCATGA	ATTCGAATTCTGCCACTGAA	59.4	357	INTERGENIC	--	--	--
Ca1_P3_2842728	Ca1	2842728	ATTTT/ATTTTT	TCAGTGGCAGAATTCGAATAAA	TTGCAACAGCTTGTTGAATGT	59.7	403	INTERGENIC	--	--	--
Ca1_P3_2844156	Ca1	2844156	TT/TTAT	AAAACGGGTTACCACTCACG	CAACATATCAATGGAACATGGC	59.9	216	INTERGENIC	--	--	--
Ca1_P3_2844599	Ca1	2844599	TAAAAAAA/TAAAAAA	AGGAGCGCGTGTTTCTTTTA	TGTTTCGTCTTTGAAAGGTGG	60.0	705	INTERGENIC	--	--	--
Ca1_P3_2845108	Ca1	2845108	TAAAAA/TAAA	CCACCTTTCAAAGACGAAACA	GCTTGATTTCAAAATCTCCTCC	60.1	686	INTERGENIC	--	--	--
Ca1_P3_2845159	Ca1	2845159	TAAAAAAAA/TAAAAAAA	CCACCTTTCAAAGACGAAACA	GCTTGATTTCAAAATCTCCTCC	60.1	686	INTERGENIC	--	--	--
Ca1_P3_28458887	Ca1	28458887	AAATAATAATAATAATAATAATAATA/AAATAATAATAATAATAATAATAATAATAATA	CGTCGTTTGTTTTGACACATC	CGTTGTTTGTTTCGACACATCT	59.1	612	INTERGENIC	--	--	--
Ca1_P3_2846472	Ca1	2846472	CATAT/CAT	GTTTGAACACGGGTGCTTTT	TTTGGTTAAGGGTTTGGCTG	60.0	756	INTERGENIC	--	--	--
Ca1_P3_2850106	Ca1	2850106	ATTTT/ATT	TAGGTGGGGCTCTCATTGTC	GTTTCTCCATGCGCAAAAAT	60.1	902	INTERGENIC	--	--	--

INDEL marker IDs	Chromosomes/ unanchored scaffolds	Physical positions (bp)	InDels (*Kabuli* reference genome-CDC Frontier/PII)	Forward primers (5'-3')	Reverse primers (5'-3')	Anneling temperature (0C)	Expected amplified product size (bp)	Structural annotation		Functional annotation	
								Sequence components of *kabuli* genome	*Kabuli* gene accession IDs	NCBI-KOG	TFs
Ca1_P3_2850297	Ca1	2850297	TTATATATATATATA/TTATATATATATA	ATCGCGTGATTAGATG CACA	GTTTCTCCATGCGCAA AAAT	60.3	301	INTERGENIC	--	--	--
Ca1_P3_28550132	Ca1	28550132	ATTTTTTTTTT/ATTTTTTT	TGGAAGCACGCTTAAG GACT	TTTCCCAATCGATTTG CTTC	60.0	664	INTERGENIC	--	--	--
Ca1_P3_28550236	Ca1	28550236	T/TG	CTGACACAGGGGGAGA AAAA	TTTCCCAATCGATTTG CTTC	60.1	159	INTERGENIC	--	--	--
Ca1_P3_2861840	Ca1	2861840	CAAA/CAAAA	CGCCTTGAAAACCCTC AGTA	CTAATCCCTTGCCCTC AACA	60.2	666	INTRON	Ca_00348	R	--
Ca1_P3_2861879	Ca1	2861879	A/AG	CGCCTTGAAAACCCTC AGTA	CTAATCCCTTGCCCTC AACA	60.2	666	INTRON	Ca_00348	R	--
Ca1_P3_2862459	Ca1	2862459	GAA/GA	TGTTGAGGGCAAGGGA TTAG	CGGAGAGAATTTGGCC ATTA	60.1	326	INTRON	Ca_00348	R	--
Ca1_P3_2867984	Ca1	2867984	AAT/AATGAT	ATGAGAAAGGGGAAGA GGGA	AAGGGGTGAACAGTGA GGTG	60.0	646	INTERGENIC	--	--	--
Ca1_P3_2868551	Ca1	2868551	TTTGTT/TTTGTTGTT	CACCTCACTGTTCACC CCTT	CATCCACTGTCGACAA CACC	60.0	697	INTRON	Ca_00349	--	--
Ca1_P3_28711868	Ca1	28711868	TAGAGAGAGAGAGAGAGAGAG/TAGA GAGAGAGAGAGAGAG	TGTTGGGTTGACAAAAA CCA	TTAATGGGGAAGCACA AAGC	59.8	532	INTERGENIC	--	--	--
Ca1_P3_28713213	Ca1	28713213	TTA/TTAGAAAGAGTGTA	TTCATTTCTCTGATTCC CCG	TCCGCATTATAACCTC TGCC	60.0	425	INTERGENIC	--	--	--

INDEL marker IDs	Chromosomes/ unanchored scaffolds	Physical positions (bp)	InDels (*Kabuli* reference genome-CDC Frontier/PII)	Forward primers (5'-3')	Reverse primers (5'-3')	Anneling temperature (0C)	Expected amplified product size (bp)	Structural annotation		Functional annotation	
								Sequence components of *kabuli* genome	*Kabuli* gene accession IDs	NCBI-KOG	TFs
Ca1_P3_28716048	Ca1	28716048	CATATATATATATATATAT/CATATATATATATAT	CGCAAAGATTTGGGTTTGAT	TGGACCCTGAATTACGAAGG	59.9	776	INTERGENIC	--	--	--
Ca1_P3_2875002	Ca1	2875002	CTTTTTTTTTT/CTTTTTTTTT	TGCCAAAGGTAATTTTTCCG	GCCCGCAAAAGTTAAAAGAA	59.9	149	DRR	Ca_00349	--	--
Ca1_P3_2875348	Ca1	2875348	CAAAAA/CAAAA	TGCCAAAGGTAATTTTTCCG	GATGGCCTAATGCAAACCAT	59.9	879	DRR	Ca_00349	--	--
Ca1_P3_2875382	Ca1	2875382	CATAAATAAATAAATAAATA/CATAAATAAATAAATAAATAAATAAATA	TGCCAAAGGTAATTTTTCCG	GATGGCCTAATGCAAACCAT	59.9	879	DRR	Ca_00349	--	--
Ca1_P3_2884045	Ca1	2884045	TAAA/TAA	GAGCTTCTCCACCTCACGTC	GTGGCATTCAGGTGAGGAAT	60.0	412	DRR	Ca_00351	T	M-type
Ca1_P3_2884405	Ca1	2884405	AGAGACATT/A	ATTCCTCACCTGAATGCCAC	TGAAAGCATCATGCACACAA	59.9	511	DRR	Ca_00351	T	M-type
Ca1_P3_2884853	Ca1	2884853	GTTTT/GTTT	TCTCCGTCGTGTTGTGTTGT	ATTGCGGAGTTAACAATGGC	60.2	743	DRR	Ca_00351	T	M-type
Ca1_P3_2885377	Ca1	2885377	ATTTTTTT/ATTTTTTTT	GCCATTGTTAACTCCGCAAT	CATTTGGAGAGGGCATAGGA	60.0	360	DRR	Ca_00351	T	M-type
Ca1_P3_2885733	Ca1	2885733	CTGT/CT	GCCATTGTTAACTCCGCAAT	TTTGAAAGCACAGCACTTGG	60.0	670	INTERGENIC	--	--	--
Ca1_P3_2886588	Ca1	2886588	CTT/CTTT	GGGCGTTCTCTCTTCATCAG	TCCACCGACTGTGTCATTGT	60.0	671	DRR	Ca_00352	--	--

INDEL marker IDs	Chromosomes/ unanchored scaffolds	Physical positions (bp)	InDels (*Kabuli* reference genome-CDC Frontier/PII)	Forward primers (5'-3')	Reverse primers (5'-3')	Anneling temperature (0C)	Expected amplified product size (bp)	Structural annotation		Functional annotation	
								Sequence components of *kabuli* genome	*Kabuli* gene accession IDs	NCBI-KOG	TFs
Ca1_P3_28906702	Ca1	28906702	TAAAAAAA/TAAAAAA	TATGGAGAGCAGCGACTCAA	TGTTGGGTTGCACAACATTC	59.7	184	INTERGENIC	--	--	--
Ca1_P3_28910299	Ca1	28910299	CATT/CATTAATT	GAAAATGATCGGTGGAGGAA	CTTGCGTGTTGCACGAGTAT	59.9	620	DRR	Ca_18486	R	--
Ca1_P3_28910417	Ca1	28910417	CTTTTTTT/CTTTTTTTTTTT	GAGTCATTGGTGCCTTGGTT	CTTGCGTGTTGCACGAGTAT	60.0	385	DRR	Ca_18486	R	--
Ca1_P3_28910492	Ca1	28910492	GATTAA/GATTAATTAA	GAGTCATTGGTGCCTTGGTT	CTTGCGTGTTGCACGAGTAT	60.0	385	DRR	Ca_18486	R	--
Ca1_P3_28930406	Ca1	28930406	T/TA	TCAAAGACATGGTGCCTTCA	TTGGTGCCAATTTATGTATTTCA	60.2	605	INTERGENIC	--	--	--
Ca1_P3_28936099	Ca1	28936099	ATTTTTTTT/ATTTTTTTTTTT	GAAATTTGACCAGAGCTGCC	TCGGTCACCGTTTTTAGTTT	59.8	465	INTERGENIC	--	--	--
Ca1_P3_28939130	Ca1	28939130	TTATATATATAT/TTATATATATATAT	TGGATATACGCACACCCAAT	AGTTACCGATCAATGGTGCC	58.7	634	INTERGENIC	--	--	--
Ca1_P3_28941503	Ca1	28941503	CAA/CAAAA	TGGGGTTTAGGGTGTCACAT	CTTTTGTAAAGGCAGCTCCG	60.1	207	INTERGENIC	--	--	--
Ca1_P3_28941872	Ca1	28941872	GAAAAAAA/GAAAAAAAAAA	TGGGGTTTAGGGTGTCACAT	TCAATTTGGATTTCTTGGTGG	60.1	847	INTERGENIC	--	--	--
Ca1_P3_28943560	Ca1	28943560	GG/GGTG	ACTTGGGACAAATCACATGG	TGATCACCAGCTTTCCAATG	58.3	252	INTERGENIC	--	--	--

INDEL marker IDs	Chromosomes/ unanchored scaffolds	Physical positions (bp)	InDels (*Kabuli* reference genome-CDC Frontier/PII)	Forward primers (5'-3')	Reverse primers (5'-3')	Anneling temperature (0C)	Expected amplified product size (bp)	Structural annotation		Functional annotation	
								Sequence components of *kabuli* genome	*Kabuli* gene accession IDs	NCBI-KOG	TFs
Ca1_P3_28944045	Ca1	28944045	TAAAAAA/TAAAAA	TTGCCTTTAATGGACCAAGG	CAATATGAAGTCTGGTGCCAA	59.9	345	INTERGENIC	--	--	--
Ca1_P3_28944496	Ca1	28944496	TTATATATAT/TTATATAT	TTGGCACCAGACTTCATATTG	AAATGTACTTTGCGGCACAT	58.6	775	INTERGENIC	--	--	--
Ca1_P3_28944803	Ca1	28944803	AACTA/AA	TTTCAATTGCATTAATCCGTTC	CAATAAAAATTGCGGTCCGA	59.0	963	INTERGENIC	--	--	--
Ca1_P3_28945159	Ca1	28945159	GTTT/GTTTT	TGCCGCAAAGTACATTTATCA	CAATAAAAATTGCGGTCCGA	59.2	369	INTERGENIC	--	--	--
Ca1_P3_2898163	Ca1	2898163	CAAAAAAAAAAA/CAAAAAAAAA	TGGTGTTTACCACTCCGTCA	TTAAATCACAGTGGCGGTCA	60.0	368	INTRON	Ca_00354	--	bHLH
Ca1_P3_2900742	Ca1	2900742	AAC/A	CTGCAGGGATCTGAGTGACA	GGAATGGTTTGGGGAATTTT	60.0	637	INTRON	Ca_00354	--	bHLH
Ca1_P3_2905447	Ca1	2905447	GGAT/GGATCACTGAGTGAT	CTGGAACTGCTTTCTTTGCC	GGGGGATAGGATGGAAGTGT	60.0	810	DRR	Ca_00354	--	bHLH
Ca1_P3_2910422	Ca1	2910422	TAAGAAGAAGAAGAA/TAAGAAGAAGAA	TCCAACACCAGAATCACCAA	CCATTCCCCAAAACAAAACA	59.9	382	INTERGENIC	--	--	--
Ca1_P3_2910652	Ca1	2910652	CAATAATAATAATAATAATA/CAATAATAATAATAATAATA	TCCAACACCAGAATCACCAA	GGGATTTGTCAGTGTAGCCAA	59.9	562	INTERGENIC	--	--	--
Ca1_P3_2910946	Ca1	2910946	CAAAAAAAAAA/CAAAAAAAA	TGTTTTGTTTTGGGGAATGG	TTTCTTCACCGCTTAATTTGAA	60.6	929	INTERGENIC	--	--	--

INDEL marker IDs	Chromosomes/ unanchored scaffolds	Physical positions (bp)	InDels (*Kabuli* reference genome-CDC Frontier/PII)	Forward primers (5'-3')	Reverse primers (5'-3')	Anneling temperature (0C)	Expected amplified product size (bp)	Structural annotation		Functional annotation	
								Sequence components of *kabuli* genome	*Kabuli* gene accession IDs	NCBI-KOG	TFs
Ca1_P3_2912085	Ca1	2912085	GTTTTTTTT/GTTTTTTTTT	GAACCAAAGTAAAACCGGCA	TGGTCGATGGAATGAGTCAA	60.0	763	INTERGENIC	--	--	--
Ca1_P3_29124803	Ca1	29124803	ATTT/ATTTT	AGTGGCATTGGGCAAATTAC	ACCAAATATACGCGTCTGCC	59.8	350	INTERGENIC	--	--	--
Ca1_P3_2912876	Ca1	2912876	GGTAC/GGTACGTAC	TGTCATCCCTAGCAACACGA	TGTAATGCATTTCCCCTTGTC	60.3	192	INTERGENIC	--	--	--
Ca1_P3_2914650	Ca1	2914650	CTTTTTTTTTTT/CTTTTTTTT	TGTGATGGTTCATGCAGAAAG	CAGAAAGGTCTGACCCGAAA	59.7	722	DRR	Ca_00357	--	WOX
Ca1_P3_2914725	Ca1	2914725	ATT/AT	TGTGATGGTTCATGCAGAAAG	CAGAAAGGTCTGACCCGAAA	59.7	722	DRR	Ca_00357	--	WOX
Ca1_P3_2915161	Ca1	2915161	TAAAAAAAAAAA/TAAAAAAAA	CGTTGGTACCATTCCGTTTC	CATATGGTGAAAAGCGGTGA	60.2	715	DRR	Ca_00357	--	WOX
Ca1_P3_2919131	Ca1	2919131	CAAAAAAAAAAA/CAAAAAAAAAA	GTATCCCATCCCCTGCAATA	TTCCATGTTCCCTTTGCTTC	59.6	202	INTERGENIC	--	--	--
Ca1_P3_2920371	Ca1	2920371	TTGATAT/TTGATATATATGGAGGTGATAT	TCACGTGAGCTATAGCCATAAA	TGACAGCTGAAAGCACAACC	57.7	245	INTERGENIC	--	--	--
Ca1_P3_2920887	Ca1	2920887	TAAT/TAATAAAT	ATTGCCATGGATTTGACGTT	AAGGCTTACGATGTTTGTGC	60.2	770	INTERGENIC	--	--	--
Ca1_P3_2921722	Ca1	2921722	CAATA/CA	CAACTGGGCCAAATGCTTAT	TGATGATCACACGAGGGAGA	60.0	570	INTERGENIC	--	--	--

INDEL marker IDs	Chromosomes/ unanchored scaffolds	Physical positions (bp)	InDels (*Kabuli* reference genome-CDC Frontier/PII)	Forward primers (5'-3')	Reverse primers (5'-3')	Anneling temperature (0C)	Expected amplified product size (bp)	Structural annotation		Functional annotation	
								Sequence components of *kabuli* genome	*Kabuli* gene accession IDs	NCBI-KOG	TFs
Ca1_P3_2922769	Ca1	2922769	CAAAA/CAAAAAA	TTTGTGGATCGTATTGCGAA	AAATGCATTCCTTCATTCTTTCA	60.1	604	INTERGENIC	--	--	--
Ca1_P3_2922949	Ca1	2922949	CTTTTTTTTTTTT/CTTTTTTTTTTT	TTTGTGGATCGTATTGCGAA	AGCATATGGTCCCCTTTTCA	60.1	947	INTERGENIC	--	--	--
Ca1_P3_2923053	Ca1	2923053	TATGAA/TATGAATGAATGAA	CGCGAAATCTTATTTTCATGG	TGATGCGATTGTAGTCCCAA	59.6	606	INTERGENIC	--	--	--
Ca1_P3_2923274	Ca1	2923274	A/ATATTAAAATAAT	CGCGAAATCTTATTTTCATGG	TGATGCGATTGTAGTCCCAA	59.6	606	INTERGENIC	--	--	--
Ca1_P3_2923437	Ca1	2923437	GTCTCTCTCT/GTCTCTCTCTCTCTCTCTCT	TGACATATGAAAAGGGGACCA	TGATGCGATTGTAGTCCCAA	60.2	134	INTERGENIC	--	--	--
Ca1_P3_2923894	Ca1	2923894	TAAAAAAAAA/TAAAAAAAA	TTGGGACTACAATCGCATCA	ATTGACCCGATTTGCTGAAC	60.1	718	INTERGENIC	--	--	--
Ca1_P3_2923967	Ca1	2923967	TAAA/TAAAA	TTGGGACTACAATCGCATCA	ATTGACCCGATTTGCTGAAC	60.1	718	INTERGENIC	--	--	--
Ca1_P3_2923992	Ca1	2923992	TTA/T	TTGGGACTACAATCGCATCA	ATTGACCCGATTTGCTGAAC	60.1	718	INTERGENIC	--	--	--
Ca1_P3_2924514	Ca1	2924514	GAAAAAAAAAAAA/GAAAAAAAAAA	GTTCAGCAAATCGGGTCAAT	TGAGTTAATGCGGCCTAACC	59.9	595	INTERGENIC	--	--	--
Ca1_P3_2931986	Ca1	2931986	ATTTTTT/ATTTTT	CTTTAGCTGGGCCTCCTTCT	TTTGTATGAGTGCGGAACCA	60.0	570	DRR	Ca_00358	E	--

INDEL marker IDs	Chromosomes/ unanchored scaffolds	Physical positions (bp)	InDels (*Kabuli* reference genome-CDC Frontier/PII)	Forward primers (5'-3')	Reverse primers (5'-3')	Anneling temperature (0C)	Expected amplified product size (bp)	Structural annotation		Functional annotation	
								Sequence components of *kabuli* genome	*Kabuli* gene accession IDs	NCBI-KOG	TFs
Ca1_P3_2932356	Ca1	2932356	TAA/TA	GAATCTGGTTCCGCACTCAT	AGTTCGATTGTGACCGTGTG	60.1	400	DRR	Ca_00358	E	--
Ca1_P3_2933661	Ca1	2933661	AATG/A	TTACGGTAACACCACCAGCA	AAGCATTCACCGGTTGGTAG	60.0	336	INTRON	Ca_00358	E	--
Ca1_P3_2933961	Ca1	2933961	AAGAGA/AAGA	CTACCAACCGGTGAATGCTT	CCCCCACTTTCATTCAGTAAA	60.0	641	INTRON	Ca_00358	E	--
Ca1_P3_2934264	Ca1	2934264	CA/CAAA	AACCCTTCATGATTGCAGGA	CACGGTGTTGATGTTGAAGG	60.5	797	INTRON	Ca_00358	E	--
Ca1_P3_29342752	Ca1	29342752	ATAGTA/ATA	AGGTTCGCGTTTGATTGTTC	TTTACGCATGCAAAAAGCAG	60.1	342	INTERGENIC	--	--	--
Ca1_P3_2934879	Ca1	2934879	T/TACTC	TCATCAGCAAAGTTGGCAAG	ATCTGGTTCGGATTGTTGGA	60.0	432	INTRON	Ca_00358	E	--
Ca1_P3_2937429	Ca1	2937429	ACAAGTG/A	AAAACGGTGATGATTTGGGA	TCTCGTACCGGACCAGAGTC	60.2	199	CDS (large-effect mutations)	Ca_00358	E	--
Ca1_P3_29386755	Ca1	29386755	T/TATATATA	TCGTTTCCCAGAGAAGCAAC	AGAAAATGCGGGTTATGTCG	60.4	743	DRR	Ca_22561	R	HB-other
Ca1_P3_29386975	Ca1	29386975	AATATATATATATATATATATATATATAT/AATATATATATATATATATATATATATATATATATAT	TCGTTTCCCAGAGAAGCAAC	AGAAAATGCGGGTTATGTCG	60.4	743	DRR	Ca_22561	R	HB-other
Ca1_P3_2938785	Ca1	2938785	T/TTG	GGCGGATAGACAAAATCGAA	GTCGCGAAAAGATTTGCACT	60.0	640	INTERGENIC	--	--	--

INDEL marker IDs	Chromosomes/ unanchored scaffolds	Physical positions (bp)	InDels (*Kabuli* reference genome-CDC Frontier/PII)	Forward primers (5'-3')	Reverse primers (5'-3')	Anneling temperature (0C)	Expected amplified product size (bp)	Structural annotation		Functional annotation	
								Sequence components of *kabuli* genome	*Kabuli* gene accession IDs	NCBI-KOG	TFs
Ca1_P3_2938872	Ca1	2938872	ATTTTTT/ATTTTT	GGCGGATAGACAAAATCGAA	GCAACCGTGTGGATATGTTG	60.0	703	INTERGENIC	--	--	--
Ca1_P3_29402165	Ca1	29402165	TG/TGTAG	GGCCGTAAACCAGACTCAAA	GGGGTAAGTTTCCTTGACCA	60.1	627	INTERGENIC	--	--	--
Ca1_P3_29406026	Ca1	29406026	CTTTTTTTTTT/CTTTTTTTTTTT	ATAAAGGGACCAACCGTGAA	TGGGACCAAATTGAACATAAAA	59.3	482	INTERGENIC	--	--	--
Ca1_P3_29422590	Ca1	29422590	TT/TTATGAT	AGTGGATCGGGTGAAACAAG	GTCGCGTCGAAAACTAGCTC	60.0	783	DRR	Ca_22560	--	--
Ca1_P3_2945732	Ca1	2945732	AA/AATTA	ATCAAATTGCGTTGGTAGCC	TGGCGATGGATACACGATAA	60.0	885	INTERGENIC	--	--	--
Ca1_P3_2947472	Ca1	2947472	TA/TAATAGA	TTTCAGTTCGATACGTCAAAACA	ACCACGTGTGGTTGTTGAAT	59.7	403	INTERGENIC	--	--	--
Ca1_P3_29524255	Ca1	29524255	ATTTTTTTTTTT/ATTTTTTTTTTTTT	GGCTTCTTTCCAAAATGCAG	CACCATATTCCCCCAAAACA	59.8	669	INTERGENIC	--	--	--
Ca1_P3_2954111	Ca1	2954111	ATTTTTT/ATTTTTTT	TTTGTGGGTTGAATTGGGAT	GGTCCAAAAGTAAGGGGAAAA	60.0	888	INTERGENIC	--	--	--
Ca1_P3_2956674	Ca1	2956674	TTATATATAT/TTATAT	CCCATCTCAAAGGAAGTTGC	CCACTGCTGCGCTATTATGA	59.7	641	INTERGENIC	--	--	--
Ca1_P3_2959903	Ca1	2959903	TTTTTTT/TTTTTTATTTTTT	TGTTATGATCCGTAAACGATGG	TTGCCGCCAATAAATATTAAAAA	59.7	554	INTERGENIC	--	--	--

INDEL marker IDs	Chromosomes/ unanchored scaffolds	Physical positions (bp)	InDels (*Kabuli* reference genome-CDC Frontier/PII)	Forward primers (5'-3')	Reverse primers (5'-3')	Anneling temperature (0C)	Expected amplified product size (bp)	Structural annotation		Functional annotation	
								Sequence components of *kabuli* genome	*Kabuli* gene accession IDs	NCBI-KOG	TFs
Ca1_P3_2960148	Ca1	2960148	ATTTTTTTT/ATTTTTTTTT	TGTTATGATCCGTAAACGATGG	CGGGATAGACCCTTATCACAGA	59.7	758	INTERGENIC	--	--	--
Ca1_P3_29618796	Ca1	29618796	GACTT/GACTTTACTT	TGCCTACATCCCAGACACAA	AGCGGCCTTCTCATTAACAA	60.1	433	INTERGENIC	--	--	--
Ca1_P3_29636835	Ca1	29636835	AA/AAGA	TCACAAATCTCAACCCTCCC	ATCAGTTGGAGCCTGGTTGT	59.9	497	INTRON	Ca_24382	AR	--
Ca1_P3_29637072	Ca1	29637072	GAAAAAAAAA/GAAAAAAAAAAA	ACAACCAGGCTCCAACTGAT	ATGCTGCCAGCCTACAAAAT	59.6	503	INTRON	Ca_24382	AR	--
Ca1_P3_2966305	Ca1	2966305	T/TTC	CCACGTCTGTAAATTCGCTTT	GCTCGCCACTCTCTTTCTCT	59.3	785	INTERGENIC	--	--	--
Ca1_P3_29709028	Ca1	29709028	ACC/AC	AAAGATTTGTTGAGGCGTGG	GACGAGAATGGAGATGGGAA	60.1	580	INTERGENIC	--	--	--
Ca1_P3_2976356	Ca1	2976356	TCACA/TCA	GGAAGTCTTTGCAGGGCATA	TTTGGCCCAATAAGTTGGAG	60.2	638	INTRON	Ca_00361	--	--
Ca1_P3_29791416	Ca1	29791416	GAAAA/GAAAAA	TCCGATAAAATCTCCGTTCG	AAAAACACGTTCCCCTCCTT	60.0	323	INTERGENIC	--	--	--
Ca1_P3_29805099	Ca1	29805099	GATATATATATATATA/GATATATATATATA	TACGAACCCTCACAACCACA	CGTTCTTTGAATGAGGGCTG	60.0	712	INTERGENIC	--	--	--
Ca1_P3_29818737	Ca1	29818737	ACTA/ACTAAATTACCGAGGTGCTA	CTCGGAATTCCAAACCAAAA	ATCGATGGCTCAATTGGTTC	59.9	359	INTERGENIC	--	--	--

INDEL marker IDs	Chromosomes/ unanchored scaffolds	Physical positions (bp)	InDels (*Kabuli* reference genome-CDC Frontier/PII)	Forward primers (5'-3')	Reverse primers (5'-3')	Anneling temperature (0C)	Expected amplified product size (bp)	Structural annotation		Functional annotation	
								Sequence components of *kabuli* genome	*Kabuli* gene accession IDs	NCBI-KOG	TFs
Ca1_P3_29821471	Ca1	29821471	ATT/AT	GCCTCCGAGTTCAATACCAA	TGTCGTGTTTGACGTGTTCA	60.1	473	INTERGENIC	--	--	--
Ca1_P3_29825526	Ca1	29825526	ATTTTT/ATTT	CAAGACTTAAGGCTCCCCCT	CCCATTTGCCTAACCAAGAA	59.7	500	INTERGENIC	--	--	--
Ca1_P3_29842423	Ca1	29842423	AC/A	TCATCAAAGGCATGCAAATC	GACCAAAACACAACGCAATG	59.6	359	INTERGENIC	--	--	--
Ca1_P3_29843804	Ca1	29843804	G/GA	ATGGATCAAGAGAAGGGGCT	CCTATGCGAAGTGGTGGTTT	60.0	535	INTERGENIC	--	--	--
Ca1_P3_29844165	Ca1	29844165	TTTATT/TTT	ATGGATCAAGAGAAGGGGCT	CCTATGCGAAGTGGTGGTTT	60.0	535	INTERGENIC	--	--	--
Ca1_P3_29846530	Ca1	29846530	CTTTT/CTTTTT	TGGATTGGGGATCATTCATT	TCAACATTTGGAAGCACAAGA	60.0	897	DRR	Ca_24388	--	--
Ca1_P3_29846735	Ca1	29846735	AC/AATATATATATATC	AGAACGGTTTTCTGTCCGTG	TCAACATTTGGAAGCACAAGA	60.1	689	DRR	Ca_24388	--	--
Ca1_P3_29846941	Ca1	29846941	TAAAAAAAAAA/TAAAAAAAA	TGTTGCCAACTGAAAATCGT	ATACAGACACCGAATGGGGA	59.2	828	DRR	Ca_24388	--	--
Ca1_P3_29847994	Ca1	29847994	TAAAAAA/TAAAAAAAA	TCTTTTCATCAGGCCCAATC	GGAAAGTGAGGGAACCAATG	60.0	228	INTRON	Ca_24388	--	--
Ca1_P3_29848026	Ca1	29848026	ATGT/AT	TCTTTTCATCAGGCCCAATC	GGAAAGTGAGGGAACCAATG	60.0	228	INTRON	Ca_24388	--	--

INDEL marker IDs	Chromosomes/ unanchored scaffolds	Physical positions (bp)	InDels (*Kabuli* reference genome-CDC Frontier/PII)	Forward primers (5'-3')	Reverse primers (5'-3')	Anneling temperature (0C)	Expected amplified product size (bp)	Structural annotation		Functional annotation	
								Sequence components of *kabuli* genome	*Kabuli* gene accession IDs	NCBI-KOG	TFs
Ca1_P3_29853119	Ca1	29853119	TAA/TA	TCCACGAGATTTTCCTTTGG	TGTCATCATTCTCTGCTGGC	60.0	357	INTERGENIC	--	--	--
Ca1_P3_29853730	Ca1	29853730	GTGGAAGTCT/GTGGAAGTCTGGAAGTCT	TTGCATTCCTAGGTTCAGCC	TATCGCTCGACCTCTGCTTT	60.2	602	INTERGENIC	--	--	--
Ca1_P3_29856647	Ca1	29856647	CTTT/CTTTT	GCATTCCCTGCTCAAGACTC	AGACAAGGGAGAGTCCCACA	60.0	812	INTERGENIC	--	--	--
Ca1_P3_2989173	Ca1	2989173	TC/T	CATTGGATCCCCTTTTTGAA	AGAGCCCTCCGTTTTACCAT	59.7	233	INTRON	Ca_00363	PET	NAC
Ca1_P3_2993633	Ca1	2993633	TACACA/TACACACA	GCTGTTCTCTGTTGCTTCCC	TGACCATTCCCTACCCCATA	60.0	278	INTERGENIC	--	--	--
Ca1_P3_29945246	Ca1	29945246	AAAATCGATTAGTAAATCGATTAGT/AAAATCGATTAGT	GAGAAATTAGCCCATTGCCA	CGATTTCCCAATCGGTTTTA	60.0	281	INTERGENIC	--	--	--
Ca1_P3_29994387	Ca1	29994387	GTTTT/GTTTTT	TCCAGCTTCCTCGAAACCTA	AACAAGACCTGCGAAAATCG	59.9	480	INTERGENIC	--	--	--
Ca1_P3_3002623	Ca1	3002623	TCA/TCACA	TCTTCATTGATTTCCCTCGG	CCAAGCAAGGGAACGTAGAA	60.0	523	DRR	Ca_00364	A	bHLH
Ca1_P3_3003040	Ca1	3003040	ATTTTTT/ATTTTTTT	TCTTCATTGATTTCCCTCGG	ACCCATATTGCATGCCTTTC	60.0	955	DRR	Ca_00364	A	bHLH
Ca1_P3_30073453	Ca1	30073453	ACT/ACTCT	AGAAGAAATGGTGGTGACCG	CACTTCCAAGGACTGCATCA	60.0	472	DRR	Ca_22471	I	--

INDEL marker IDs	Chromosomes/ unanchored scaffolds	Physical positions (bp)	InDels (*Kabuli* reference genome-CDC Frontier/PII)	Forward primers (5'-3')	Reverse primers (5'-3')	Anneling temperature (0C)	Expected amplified product size (bp)	Structural annotation		Functional annotation	
								Sequence components of *kabuli* genome	*Kabuli* gene accession IDs	NCBI-KOG	TFs
Ca1_P3_30097998	Ca1	30097998	ATTTTTTTTTT/ATTTTTTTTTTTT	TCACCGGTCGTGATACAAAA	CGTCTCTGTAGGTTCTCGCC	60.0	520	INTERGENIC	--	--	--
Ca1_P3_30100111	Ca1	30100111	GAAAAAAAAA/GAAAAAAAAAA	GGAAAAGGTGTCCAACCAAG	TTGCCCACCCCTACTAAGTG	59.4	571	INTERGENIC	--	--	--
Ca1_P3_3013603	Ca1	3013603	ATTTTTTTTT/ATTTTTTTT	GCGTTAAAAGTGGTGGTGGT	TCACCAATGTAATAGCCGCA	59.9	620	INTERGENIC	--	--	--
Ca1_P3_3013867	Ca1	3013867	GAAAAA/GAAAA	CAAGTTCAGCAACACCAGGA	AACCGACAAGGCAAGAAAGA	59.9	614	INTERGENIC	--	--	--
Ca1_P3_30153108	Ca1	30153108	AAGCTAG/AAG	CGAATCATGCGCAAGAACTA	GCAAATTTTTCATCATCGCA	60.0	821	INTERGENIC	--	--	--
Ca1_P3_30154835	Ca1	30154835	TTATATATATATATATATATATATATATATATATATATATAT/TTATATATATATATATATATATATATATATAT	TTTGGTGTTCCAACGATTCA	CAAACCCTTTACCACCAAGC	59.9	525	INTERGENIC	--	--	--
Ca1_P3_3015571	Ca1	3015571	CAAAAA/CAAAA	AGGCAACCAATCTATGCCAC	TGGGATGTGAGAAATGCAAA	60.0	565	INTERGENIC	--	--	--
Ca1_P3_3018574	Ca1	3018574	CAATTAATTAATT/CAATTAATT	AGCCAACAGCCCAACATAAA	AAGGTTCATGTCCAAATCGC	60.5	242	INTERGENIC	--	--	--
Ca1_P3_3020286	Ca1	3020286	A/AATAAATAAT	CAAGCAACATGCTGAAGGAA	ATGTCCTTAGTGTCACGGGG	60.0	808	INTERGENIC	--	--	--
Ca1_P3_3020354	Ca1	3020354	AGG/AGGG	CAAGCAACATGCTGAAGGAA	ATGTCCTTAGTGTCACGGGG	60.0	808	INTERGENIC	--	--	--

INDEL marker IDs	Chromosomes/ unanchored scaffolds	Physical positions (bp)	InDels (*Kabuli* reference genome-CDC Frontier/PII)	Forward primers (5'-3')	Reverse primers (5'-3')	Anneling temperature (0C)	Expected amplified product size (bp)	Structural annotation		Functional annotation	
								Sequence components of *kabuli* genome	*Kabuli* gene accession IDs	NCBI-KOG	TFs
Ca1_P3_3020776	Ca1	3020776	CAATTTAATTTA/CAATTTAATTTAATTTA	CCCCGTGACACTAAGGACAT	GAAGGTCGCATCTCCATGAT	59.8	513	INTERGENIC	--	--	--
Ca1_P3_3020844	Ca1	3020844	GAAAAAA/GAAAAAAA	CCCCGTGACACTAAGGACAT	GAAGGTCGCATCTCCATGAT	59.8	513	INTERGENIC	--	--	--
Ca1_P3_30243449	Ca1	30243449	AGG/AG	CCAAACCAAATAAAACATTCCAA	GAACTCTGCCTTCCAGCAAC	60.0	463	INTERGENIC	--	--	--
Ca1_P3_30264384	Ca1	30264384	TAAAAA/TAAAA	TGTGCAAATAACGCAGATTC	GGGTTTTATTGCAATCTCATGG	57.4	511	INTERGENIC	--	--	--
Ca1_P3_30273424	Ca1	30273424	ACTCTTTCNNNNNNNNNNCTCTTTC/ACTCTTTC	GCCCCAACACCATACTCACT	TTTGGATCAAAAATTTGGGG	59.9	450	INTERGENIC	--	--	--
Ca1_P3_30287511	Ca1	30287511	GTTTT/GTTT	CCATCAAACATCGTCATGGA	ACTCAGATTGGTCTGTGGGG	60.3	305	INTERGENIC	--	--	--
Ca1_P3_3030441	Ca1	3030441	AATATATAT/AATATAT	AATAGTGAGGGGTGGAGGCT	TGATGGATGAGACAAAATGCC	60.0	415	DRR	Ca_00367	--	--
Ca1_P3_30316576	Ca1	30316576	ATTTTTTTT/ATTTTTTT	TTCAAAGGTTGTGTTTCCCC	GGCTACACCAACATCGACCT	59.8	598	INTERGENIC	--	--	--
Ca1_P3_3033831	Ca1	3033831	ATATCTATCTATC/ATATCTATC	GTGTTTGGCTTTGCAACTGA	AGGAGAAGCTGACGGAACAA	59.9	675	DRR	Ca_00368	L	--
Ca1_P3_30352443	Ca1	30352443	CACA/CACAACA	TCATCTTGGGAGGTCTCGAT	TTGTGGTCTAGGCGTCAAAA	59.6	673	INTERGENIC	--	--	--

INDEL marker IDs	Chromosomes/ unanchored scaffolds	Physical positions (bp)	InDels (*Kabuli* reference genome-CDC Frontier/PII)	Forward primers (5'-3')	Reverse primers (5'-3')	Anneling temperature (0C)	Expected amplified product size (bp)	Structural annotation		Functional annotation	
								Sequence components of *kabuli* genome	*Kabuli* gene accession IDs	NCBI-KOG	TFs
Ca1_P3_3035970	Ca1	3035970	TTCT/TT	AAGGAAGAAGCGCAATTGAA	ATCCATCCCCCTCATTCTCT	60.0	463	DRR	Ca_00370	--	ARF
Ca1_P3_30366063	Ca1	30366063	GA/GAA	GGCAATGTGTTGGTCATAGC	AAACAAAAGCGAGAACGCAT	59.0	560	DRR	Ca_22460	--	--
Ca1_P3_3037452	Ca1	3037452	AT/ATTTAAAGTATTTGTT	ACACCAGCCGTTGAATAAGG	ATAACGGTGGAGCCTGTTTG	60.0	916	INTRON	Ca_00370	--	ARF
Ca1_P3_3039653	Ca1	3039653	GTTTTT/GTTTT	CGGAATTTAGCTTTGGTTCTTG	GGCGTTTTGTTGATTCTGTG	60.1	902	INTERGENIC	--	--	--
Ca1_P3_3039698	Ca1	3039698	ATT/ATTT	CGGAATTTAGCTTTGGTTCTTG	TAACGACGCGTGACAATGTT	60.1	937	INTERGENIC	--	--	--
Ca1_P3_30398618	Ca1	30398618	GTTTTTTTTTT/GTTTTTTTTTTTT	TTAGGCCCAGTACAGTTCGG	GGGGTGCTCAACTTGTTTGT	60.1	901	INTERGENIC	--	--	--
Ca1_P3_30399037	Ca1	30399037	CATATATATATATATATATATAT/CATATATATATATATATATATATAT	TAACGTGGCAAAGGTAAGGG	GGGGTGCTCAACTTGTTTGT	60.0	232	INTERGENIC	--	--	--
Ca1_P3_3039989	Ca1	3039989	T/TG	TTCTCCGCTAATTTCTCAACC	ATCCCCCATCTGATTTGTCA	58.5	683	INTERGENIC	--	--	--
Ca1_P3_30406481	Ca1	30406481	ATGTGTGTGTGTG/ATGTGTGTGTGTGTG	GTTGCGGGATTGAGAGATGT	TGGTTGGAAGGAAGCAGACT	60.1	459	INTERGENIC	--	--	--
Ca1_P3_30440874	Ca1	30440874	AAATAATAATAATAATAATAATAATAATAATAATAATAATAATAATAATAATAATAATAATAAT/AAATAATAATAATAATAATAATAATAATAATAATAATAATAATAATAATAATAATAATAATAAT	GAAATGCGGATGTGAGGTTT	ATGCTTTTGCCAATGAGGTC	59.9	612	INTERGENIC	--	--	--

INDEL marker IDs	Chromosomes/ unanchored scaffolds	Physical positions (bp)	InDels (*Kabuli* reference genome-CDC Frontier/PII)	Forward primers (5'-3')	Reverse primers (5'-3')	Anneling temperature (0C)	Expected amplified product size (bp)	Structural annotation		Functional annotation	
								Sequence components of *kabuli* genome	*Kabuli* gene accession IDs	NCBI-KOG	TFs
Ca1_P3_3045928	Ca1	3045928	TGC/TGCGGC	ACCGTGCAATGCAGATGTAA	TGATGCAGCCTAAGTGGATG	60.1	398	DRR	Ca_00371	I	--
Ca1_P3_30475415	Ca1	30475415	A/AC	ACGGCAAGACCCTAAAACCT	AAACTTCTACTCGGGCCCAT	60.0	906	INTERGENIC	--	--	--
Ca1_P3_30475519	Ca1	30475519	ATTT/ATTTT	AGGATGCATGGGATGAAAAG	AAACTTCTACTCGGGCCCAT	59.9	766	INTERGENIC	--	--	--
Ca1_P3_3049357	Ca1	3049357	ATT/AT	ATCCTTAAAGAGAGGGGCCA	AAACCCAGGAAAGGGTCACT	60.0	888	INTRON	Ca_00371	I	--
Ca1_P3_3049531	Ca1	3049531	GTATAT/GTATATAT	TGATCCTACTCAATCCCCCA	CCAATTGCTTTGACCGTTTT	60.3	919	INTRON	Ca_00371	I	--
Ca1_P3_30508648	Ca1	30508648	CAAAATAAAAT/CAAAAT	AGCCCCCTTGACTGAATCTT	AGGACACATTTGTTCCGAGC	60.1	144	INTERGENIC	--	--	--
Ca1_P3_3051042	Ca1	3051042	AAAAATAGGA/AAAAATAGGAAAATAGGA	CCGATAGATGGTCACACCAA	ACAAGCAAGAAAGAATCGGG	59.4	571	INTERGENIC	--	--	--
Ca1_P3_3051782	Ca1	3051782	CGG/CG	TGCAGGTACAACCTCCATTTC	TCTCCACGAGCTGAAACCTT	60.0	854	INTERGENIC	--	--	--
Ca1_P3_3055342	Ca1	3055342	AC/ACC	CAATGGCGGTTTCAATTTTT	AGAAATGGTTGATGTGGGGA	59.8	913	DRR	Ca_00372	S	--
Ca1_P3_30682856	Ca1	30682856	CATATATATATATATATATATAT/CATATATATATATATATAT	GTTGGTTCCCTAGCACCGTA	TCCGCCTAGGAGATAGCAGA	60.0	672	INTERGENIC	--	--	--

INDEL marker IDs	Chromosomes/ unanchored scaffolds	Physical positions (bp)	InDels (*Kabuli* reference genome-CDC Frontier/PII)	Forward primers (5'-3')	Reverse primers (5'-3')	Anneling temperature (0C)	Expected amplified product size (bp)	Structural annotation		Functional annotation	
								Sequence components of *kabuli* genome	*Kabuli* gene accession IDs	NCBI-KOG	TFs
Ca1_P3_3069205	Ca1	3069205	CTTTTTTTTTTT/CTTTTTTTTT	ATAATGGGGAAGGGAAATGC	TATTTGATATGTGGCCCGGT	60.0	607	INTRON	Ca_00374	EH	bHLH
Ca1_P3_3071270	Ca1	3071270	AGAAAAAATTTAATT/AGAAAAAATTTAATTGAAAAAATTTAATT	AGGCACAAATGGAGCAAATC	TCCATTCGACCATTTTCACA	60.1	623	DRR	Ca_00374	EH	bHLH
Ca1_P3_30726711	Ca1	30726711	GTATTATTATTATTATTATTATTATT/GTATTATTATTATTATTATTATT	CTTGCCTGTGTCGAAGATGA	CCGGAACCAAACCCTTATTT	60.0	301	INTERGENIC	--	--	--
Ca1_P3_3072719	Ca1	3072719	A/ATAAG	GCGAATGGTTGAAACTGAAA	CGTTGGAACTCCCAATGACT	58.7	563	INTERGENIC	--	--	--
Ca1_P3_3073454	Ca1	3073454	CAA/CA	CCTCCTTACGGCGAAACATA	TTTTGGAAAGGTGGGTGTGT	60.1	508	INTRON	Ca_00375	--	bHLH
Ca1_P3_30739956	Ca1	30739956	TAAAAA/TAAAAAA	TTCGGAGCACAACCTACACA	GAATGGTGGCCATTATTAAGGT	60.3	382	DRR	Ca_22454	OE	WRKY
Ca1_P3_30740000	Ca1	30740000	TTATATATATATATATATATATATATA/TTATATATATATATATATATATATATA	TTCGGAGCACAACCTACACA	GAATGGTGGCCATTATTAAGGT	60.3	382	DRR	Ca_22454	OE	WRKY
Ca1_P3_30745555	Ca1	30745555	G/GA	TTTTTGGGTTTCCTTTTCCC	TGCAATATGTATCGTGGATGG	60.1	817	INTERGENIC	--	--	--
Ca1_P3_3074826	Ca1	3074826	A/AT	CACGAGGTTTGACCTCTTCC	CGTGTGAACATTCATACACCTCA	59.7	578	INTRON	Ca_00375	--	bHLH
Ca1_P3_3075028	Ca1	3075028	A/AT	CACGAGGTTTGACCTCTTCC	CGATCGTCGCATTTTTCATA	59.7	841	INTRON	Ca_00375	--	bHLH

INDEL marker IDs	Chromosomes/ unanchored scaffolds	Physical positions (bp)	InDels (*Kabuli* reference genome-CDC Frontier/PII)	Forward primers (5'-3')	Reverse primers (5'-3')	Anneling temperature (0C)	Expected amplified product size (bp)	Structural annotation		Functional annotation	
								Sequence components of *kabuli* genome	*Kabuli* gene accession IDs	NCBI-KOG	TFs
Ca1_P3_3075180	Ca1	3075180	CT/CTTT	GAGGTGTATGAATGTTCACACGTA	CGATCGTCGCATTTTTCATA	59.0	285	INTRON	Ca_00375	--	bHLH
Ca1_P3_3075382	Ca1	3075382	ATTTTTT/ATTTTTTT	TTGTTGATGCATGTGAGATAAATG	TCATGGCAGTCTGCAAAAAG	59.9	336	INTRON	Ca_00375	--	bHLH
Ca1_P3_30755262	Ca1	30755262	CAAAAAAAAAAA/CAAAAAAAAAA	AAGAGAGGTCACTTTGGCGA	TGACTGAGACAGCTCGATGG	60.0	489	INTERGENIC	--	--	--
Ca1_P3_30770184	Ca1	30770184	TAAA/TAAAA	AGTGGCTTGGAGGAGTTGGT	CGTTCGGCTTTCTTCTTCTG	61.1	535	INTERGENIC	--	--	--
Ca1_P3_3084291	Ca1	3084291	AT/ATT	TCAAATCCAACTCCTCGTCC	AGCCACCGCAAAATTTACAC	60.1	576	INTRON	Ca_00376	--	bHLH
Ca1_P3_3089236	Ca1	3089236	ATTT/ATT	GCAAAACGTCTTATCGGGAA	TGCAAACTATTTGACATTGACG	60.1	591	INTERGENIC	--	--	--
Ca1_P3_3089287	Ca1	3089287	TTGTAAT/TT	GCAAAACGTCTTATCGGGAA	TGCAAACTATTTGACATTGACG	60.1	591	DRR	Ca_00378	I	--
Ca1_P3_3091233	Ca1	3091233	AATAAATTCTCAATAT/AAT	TCAGACTTATGGTCGGCACA	AACCAGATGGTTCGTGGAAG	60.3	233	DRR	Ca_00378	I	--
Ca1_P3_3092960	Ca1	3092960	ATGTTGTTGTT/ATGTTGTTGTTGTTGTT	TTTTCGCCGCTTAGTTGTCT	TCTGGTTCCAAAAGCAATCA	60.0	120	INTERGENIC	--	--	--
Ca1_P3_3093078	Ca1	3093078	AT/A	TTTTCGCCGCTTAGTTGTCT	GGCCTATATACAATGGCGGA	60.0	623	INTERGENIC	--	--	--

INDEL marker IDs	Chromosomes/ unanchored scaffolds	Physical positions (bp)	InDels (*Kabuli* reference genome-CDC Frontier/PII)	Forward primers (5'-3')	Reverse primers (5'-3')	Anneling temperature (0C)	Expected amplified product size (bp)	Structural annotation		Functional annotation	
								Sequence components of *kabuli* genome	*Kabuli* gene accession IDs	NCBI-KOG	TFs
Ca1_P3_30936047	Ca1	30936047	ATTTTTTT/ATTTTTTTT	ACGGTAGGGTTACGATGCAG	AGGTCACACAGCACTTGCAC	60.0	564	INTERGENIC	--	--	--
Ca1_P3_30943497	Ca1	30943497	G/GGATAATAATAATAAT	TCATCTCGCTCTCCACCTTT	GGAAGCGACCTGCTATTGAG	60.0	965	INTERGENIC	--	--	--
Ca1_P3_30969530	Ca1	30969530	ATACTTGT/AT	GTTATCCGGGTTAATTGGCA	AAAACGCTCGCTTTGTTAGC	59.7	631	INTERGENIC	--	--	--
Ca1_P3_31005556	Ca1	31005556	TATTAATTAATTAATTAATTAAT/TATTAATTAATTAATTAAT	CAAAAGCTTTGAGGAGCGAT	GTTTCGGTTTTGACCACCAT	59.6	598	INTERGENIC	--	--	--
Ca1_P3_3102430	Ca1	3102430	GTTTTTTTTTTT/GTTTTTTTTTT	ATTCAAGTGGGACGGTTCAC	TATCGTTGCCACCAATTTCA	59.8	216	INTRON	Ca_00379	--	bHLH
Ca1_P3_3104925	Ca1	3104925	CTTTTTTT/CTTTTTTTT	TGTGTCGTGTTGACAATCACTT	ACCCGAATTTAGTGCACGTC	59.1	795	DRR	Ca_00379	--	bHLH
Ca1_P3_31072943	Ca1	31072943	TAAAAAAA/TAAAAAA	CGCTCAGCTCGGGTATAGAG	CAAACCGGATGTGACAGTTG	60.1	615	INTERGENIC	--	--	--
Ca1_P3_3107522	Ca1	3107522	ATTTTTT/ATTTTTTT	CACGCTCGCAATGATAAAGA	AAACGTGATCGAACGAATCC	60.0	695	INTERGENIC	--	--	--
Ca1_P3_31090559	Ca1	31090559	CAA/CA	CGCTCGCTCATTGAATAACA	CTCCACGACCATCCTGAAAT	60.0	417	INTRON	Ca_25826	S	--
Ca1_P3_3110575	Ca1	3110575	TATTATAATTATAATTATAATTATAATTA/TATTATAATTATAATTATAATTA	TTCCATTTCTCACTTGCTCG	TGGAAGCATGATTATGGTGTG	59.0	285	INTERGENIC	--	--	--

INDEL marker IDs	Chromosomes/ unanchored scaffolds	Physical positions (bp)	InDels (*Kabuli* reference genome-CDC Frontier/PII)	Forward primers (5'-3')	Reverse primers (5'-3')	Anneling temperature (0C)	Expected amplified product size (bp)	Structural annotation		Functional annotation	
								Sequence components of *kabuli* genome	*Kabuli* gene accession IDs	NCBI-KOG	TFs
Ca1_P3_3111052	Ca1	3111052	GTTTTTTTTT/GTTTTTTTTTT	CACACCATAATCATGCTTCCA	ATACATTCGGCAAAGCCAAC	59.4	517	INTERGENIC	--	--	--
Ca1_P3_3111391	Ca1	3111391	AGG/AGGG	GTTGGCTTTGCCGAATGTAT	CGTTGCATTTTCAAGTCTCG	60.0	254	INTERGENIC	--	--	--
Ca1_P3_3111483	Ca1	3111483	TAAAA/TAAA	GTTGGCTTTGCCGAATGTAT	CGTTGCATTTTCAAGTCTCG	60.0	254	INTERGENIC	--	--	--
Ca1_P3_31117965	Ca1	31117965	AATATA/AATA	GGGACATCAGAATGCTCACA	TGCGACACACGAATTACACA	59.6	536	INTERGENIC	--	--	--
Ca1_P3_31118295	Ca1	31118295	T/TG	TCGTGTGTCGCACATAAACA	CATGCAAATTCAAAGCTAAAACA	59.7	566	INTERGENIC	--	--	--
Ca1_P3_31125934	Ca1	31125934	T/TTA	TTTGTTGTGTTGCCAAGTCAG	TTCCTGCAGGTATCACATTTTT	59.8	368	INTERGENIC	--	--	--
Ca1_P3_31147497	Ca1	31147497	TAAAAA/TAAAA	AGCACCCCTCAAAGCATAAA	ATTACAAGGAAGGGGGTTGG	59.7	862	INTERGENIC	--	--	--
Ca1_P3_31163466	Ca1	31163466	T/TAGA	ACTTTTGCGGAGAAGCACAT	CGTTCGTCTTTTCCTCTTGC	59.9	360	INTERGENIC	--	--	--
Ca1_P3_31188680	Ca1	31188680	CTTTTTTTTTT/CTTTTTTTTTTT	TATCAGAGTTCTCCCCACCG	GGTGTGAGTTGGCGGTTATT	60.1	536	INTERGENIC	--	--	--
Ca1_P3_31194406	Ca1	31194406	ATTTGAAGCTATTGTTTGAA/ATTTGAAGCTATTGTTTGAAGCTATTGTTTGAA	AACCACAACCACATCAATGC	CAACAACCATGCACTCTTGG	59.3	677	INTERGENIC	--	--	--

INDEL marker IDs	Chromosomes/ unanchored scaffolds	Physical positions (bp)	InDels (*Kabuli* reference genome-CDC Frontier/PII)	Forward primers (5'-3')	Reverse primers (5'-3')	Anneling temperature (0C)	Expected amplified product size (bp)	Structural annotation		Functional annotation	
								Sequence components of *kabuli* genome	*Kabuli* gene accession IDs	NCBI-KOG	TFs
Ca1_P3_3124668	Ca1	3124668	CAT/CATATAT	TTTACGGTGCATCAATTTGC	CATTCGTTGGTTTGTGCATC	59.6	133	INTRON	Ca_00382	--	--
Ca1_P3_3124734	Ca1	3124734	AATATATATAT/AATATATATATATAT	GATGCACAAACCAACGAATG	TGTTGCCCTTTCATTTGTCA	60.0	551	INTRON	Ca_00382	--	--
Ca1_P3_3125340	Ca1	3125340	GATAAATAAATAAATAAATAAATAAA/GATAAATAAATAAATAAATAAATAAATAAATAAA	ACCACTGAATCATGCACCAA	CAGTCTGCAGTGACCTTCCA	60.0	103	INTRON	Ca_00382	--	--
Ca1_P3_3125906	Ca1	3125906	GAAAAA/GAAAAAA	GAAATCCACACCATCGAACC	TGATTCATGTGCCTTACCCA	60.2	595	INTRON	Ca_00382	--	--
Ca1_P3_3125978	Ca1	3125978	CAA/CAAA	GAAATCCACACCATCGAACC	TGATTCATGTGCCTTACCCA	60.2	595	INTRON	Ca_00382	--	--
Ca1_P3_31305353	Ca1	31305353	A/AC	TGCTCATCAGTAATTGCACAAA	GAAGGCTTGTCCCATTTTCA	59.4	327	INTERGENIC	--	--	--
Ca1_P3_31329661	Ca1	31329661	ATTTTTTTTTTT/ATTTTTTTTTTTTT	GATTGAGGGGCAAATCTTGA	TTGTTTCCATGTCCGTCTCA	60.0	531	INTERGENIC	--	--	--
Ca1_P3_3133490	Ca1	3133490	AATTATATATTATA/AATTATA	CCACATTTTGCCACTTTTCA	TCGCCTTTTAAACCAACCAG	59.6	853	INTERGENIC	--	--	--
Ca1_P3_31341315	Ca1	31341315	CTT/CTTT	TGTCCATTTGAAACTTCGGTT	AGAGCAAAATCGCTCCAAAA	59.5	772	INTERGENIC	--	--	--
Ca1_P3_31354820	Ca1	31354820	AAAG/A	CGGTCTTCTCCACAAAGAGC	TTAAAGGCATCTACCCGTGC	60.0	718	INTERGENIC	--	--	--

INDEL marker IDs	Chromosomes/ unanchored scaffolds	Physical positions (bp)	InDels (*Kabuli* reference genome-CDC Frontier/PII)	Forward primers (5'-3')	Reverse primers (5'-3')	Anneling temperature (0C)	Expected amplified product size (bp)	Structural annotation		Functional annotation	
								Sequence components of *kabuli* genome	*Kabuli* gene accession IDs	NCBI-KOG	TFs
Ca1_P3_31368685	Ca1	31368685	CATATATATATATATATAT/CATATATATATATATATATAT	GAAACAAGTACCCTGCCCAA	CCGCAGAAAGTTTTCCACAT	60.0	807	INTRON	Ca_21855	O	--
Ca1_P3_3137798	Ca1	3137798	TAAAAAAAAAAA/TAAAAAAAAAAAA	ATCGGGGTGGACAAAACATA	ATCGATCAGTGGGGAATTTG	60.1	217	INTERGENIC	--	--	--
Ca1_P3_3138107	Ca1	3138107	A/ATT	ATCGGGGTGGACAAAACATA	GGGCTTTAAATTGAGTGAGGC	60.1	683	INTERGENIC	--	--	--
Ca1_P3_3138261	Ca1	3138261	AATATATATATATATA/AATATATATATATATATA	ATCGATCAATTGCTTGGACC	GGGCTTTAAATTGAGTGAGGC	59.9	472	INTERGENIC	--	--	--
Ca1_P3_31384103	Ca1	31384103	GAAAAAAAAA/GAAAAAAAAAA	TCCAATTGGCTACAGAACCC	TGAATTCTGGTGACTCGTGG	59.9	563	DRR	Ca_21856	--	--
Ca1_P3_31442895	Ca1	31442895	T/TA	AATTTCCGAGCACGCATAAC	TCCACGAGTATGATTTGTGGTT	60.1	299	INTERGENIC	--	--	--
Ca1_P3_31442996	Ca1	31442996	ACACCAC/ACAC	AATTTCCGAGCACGCATAAC	CTCGGGTAGGATCCAACAAA	60.1	743	INTERGENIC	--	--	--
Ca1_P3_31448865	Ca1	31448865	GTTTTTTTTTT/GTTTTTTTTT	TAAGGGTCCCACCCATCTTT	TAATTGCTCCTGCGGTCTTT	60.5	737	INTERGENIC	--	--	--
Ca1_P3_31477691	Ca1	31477691	CAAAA/CAAAAA	AAGGGAAACGGGTACCAAAC	ACCCGTAAACCCCAAATCTC	60.1	629	INTERGENIC	--	--	--
Ca1_P3_31478810	Ca1	31478810	TAAAAAAA/TAAAAAAAA	AATTTGGAGCTGTTTTGGGA	TGGTTCCCCACTTAACCTCA	59.5	338	INTERGENIC	--	--	--

INDEL marker IDs	Chromosomes/ unanchored scaffolds	Physical positions (bp)	InDels (*Kabuli* reference genome-CDC Frontier/PII)	Forward primers (5'-3')	Reverse primers (5'-3')	Anneling temperature (0C)	Expected amplified product size (bp)	Structural annotation		Functional annotation	
								Sequence components of *kabuli* genome	*Kabuli* gene accession IDs	NCBI-KOG	TFs
Ca1_P3_3148374	Ca1	3148374	TAAAAAA/TAAAAAAA	AATACCCCCATGACCCATCT	GTGGACACCAGATACGCCTT	60.3	579	INTRON	Ca_00385	--	--
Ca1_P3_31490463	Ca1	31490463	GATATATATATA/GATATATATATATA	CATTCATTTGCAAAACGCC	GGCGGTCTAAGGTAACCACA	60.1	818	INTERGENIC	--	--	--
Ca1_P3_31490990	Ca1	31490990	ATTTTTTTTTT/ATTTTTTTTT	TGTGGTTACCTTAGACCGCC	TGTCATTTTTGGCGTAGTGG	60.0	703	DRR	Ca_21862	--	Trihelix
Ca1_P3_31491889	Ca1	31491889	CGCTTTTTAGCTTTTTA/CGCTTTTTA	CCACTACGCCAAAAATGACA	GCTGGGCAAGGGATCTAAA	59.6	865	DRR	Ca_21862	--	Trihelix
Ca1_P3_31498685	Ca1	31498685	AGG/AG	TTGGAGCTTCAGCATTTGTG	CCTCTTTTCTTCGCATTTCG	60.0	747	DRR	Ca_21863	--	M-type
Ca1_P3_3155018	Ca1	3155018	GAAAAAAAA/GAAAAAAAAA	ATAGATCAACGCCGTCAACC	CATGTGCTCTGTTCTTCCCA	60.0	591	INTERGENIC	--	--	--
Ca1_P3_3155301	Ca1	3155301	T/TTA	GGGTTGCTTTGGATTGAAGA	ATGCATTTGCAGTTTGCACT	60.1	772	INTERGENIC	--	--	--
Ca1_P3_31592114	Ca1	31592114	CT/CTTT	TCAGCTGCAGTTCCAACATC	GCACAAGTCCTTGAGGAAGC	60.0	375	INTRON	Ca_21868	F	--
Ca1_P3_31592347	Ca1	31592347	GAA/GAAAA	CCTGTGTAGCCAGCTTCCTC	TAGCCTCATTCACTCTCGCA	60.0	618	INTRON	Ca_21868	F	--
Ca1_P3_31600651	Ca1	31600651	AC/A	GGATGGAAGAGAAATTGGGA	AGGATCATGTTTGTGGTTGC	58.9	899	INTERGENIC	--	--	--

INDEL marker IDs	Chromosomes/ unanchored scaffolds	Physical positions (bp)	InDels (*Kabuli* reference genome-CDC Frontier/PII)	Forward primers (5'-3')	Reverse primers (5'-3')	Anneling temperature (0C)	Expected amplified product size (bp)	Structural annotation		Functional annotation	
								Sequence components of *kabuli* genome	*Kabuli* gene accession IDs	NCBI-KOG	TFs
Ca1_P3_31602245	Ca1	31602245	TTATATATATATATATATATATA/TTATATATATATATATATA	TTTCGTGTGCGTCAAATCAT	AGAGGCACACGGGTCTAATG	60.1	756	INTERGENIC	--	--	--
Ca1_P3_31647394	Ca1	31647394	GTTTTTTTTTTT/GTTTTTTTTTTTTTT	TCTCTTATTCTCTTCGCCGC	TGTTCGGATGAATGTTCCTTT	59.7	454	INTERGENIC	--	--	--
Ca1_P3_31664962	Ca1	31664962	AA/AAATA	TTCACTCGCGTTTGTCACTC	TAGGGGGCCTTTAGTGTGTG	60.0	603	INTERGENIC	--	--	--
Ca1_P3_31768486	Ca1	31768486	TT/TTTTTTAT	ACACATTATATTAAATGCGCGA	TGCCAAACGATAGGTTGTTG	57.4	646	INTERGENIC	--	--	--
Ca1_P3_31783386	Ca1	31783386	AT/ATT	TGGATTGGGCTGGAATTAAA	TATAACCGACGAGGGACGAG	60.3	466	INTERGENIC	--	--	--
Ca1_P3_31783811	Ca1	31783811	TTAATAATAATAATAATAATAATAATAATAATAAT/TTAATAATAATAATAATAATAATAATAAT	ATACTGCCTTCGATCCGTTG	CGGGAAAAAGGGTGGTAAAT	60.1	477	INTERGENIC	--	--	--
Ca1_P3_31894698	Ca1	31894698	AATATATATATATATATATATATATA/AATATATATATATATATATA	CTGGCCTGTAAGCCCATTTA	TTCCGAGTTGTTTGAGAGGA	60.1	602	INTERGENIC	--	--	--
Ca1_P3_31995500	Ca1	31995500	CTTT/CTTTT	TGCTTTGAAGACCATGTGGA	AAAAGGATTCATGCGTGTCC	60.2	354	INTERGENIC	--	--	--
Ca1_P3_32019371	Ca1	32019371	AATATATATATATATATATATATATATATATATATATATATA/AATATATATATATATATATATATATATATATATATATATATATATATATATATA	GGTGCACGTGTAGCACAAAC	TTGCCACAGTTAACCACCAA	60.2	422	INTERGENIC	--	--	--
Ca1_P3_32030892	Ca1	32030892	AATATATATATATATATATATATATATATATATATAT/AATATATATATATATATATATATATATATATAT	CATGGATGGGTTTTGTACCC	TTGGAAGGAGCCCTTACCTT	59.9	771	INTERGENIC	--	--	--

INDEL marker IDs	Chromosomes/ unanchored scaffolds	Physical positions (bp)	InDels (*Kabuli* reference genome-CDC Frontier/PII)	Forward primers (5'-3')	Reverse primers (5'-3')	Anneling temperature (0C)	Expected amplified product size (bp)	Structural annotation		Functional annotation	
								Sequence components of *kabuli* genome	*Kabuli* gene accession IDs	NCBI-KOG	TFs
Ca1_P3_32037255	Ca1	32037255	CA/CAA	GGGAAAACTTTTGTGTGGGA	TTGGCAAATCGATTAGACTGG	59.8	802	INTERGENIC	--	--	--
Ca1_P3_32037334	Ca1	32037334	CTTTTT/CTTTT	TCCAGTCATGTGGCTTCTCA	CTGAGCTTGTGACCAAATCG	60.4	559	INTERGENIC	--	--	--
Ca1_P3_32038703	Ca1	32038703	GAAAAAAAA/GAAAAAA	AAAATCCAACAATCTCCCCC	TGGAAGCACGCTTAAGGACT	60.0	683	INTERGENIC	--	--	--
Ca1_P3_32078246	Ca1	32078246	TA/TAAAAA	CTCGGTTCGAACAGGAGAAG	TATGTCAACAACCCCCTGGT	60.0	702	INTRON	Ca_26405	--	--
Ca1_P3_32079499	Ca1	32079499	A/AG	ACCCCAGAATAAAAGGGGTG	GAGGGCTCTCAGCCACATAG	60.0	341	INTRON	Ca_26405	--	--
Ca1_P3_3209604	Ca1	3209604	ATTTTTTTT/ATTTTTTT	TCAATAGGGTCGTTTCTCGG	TTACATTTTGGAAGTGGGGC	60.1	392	INTERGENIC	--	--	--
Ca1_P3_32147050	Ca1	32147050	T/TA	CGGCCTCCAAATCTTTAACA	TTGTCATGAAAGTCTCGCCT	60.1	670	INTERGENIC	--	--	--
Ca1_P3_32153452	Ca1	32153452	TTTATTTTAT/TTTATTTTATATTATTTTAT	GTTTTGGCGTTGGTTTCACT	TGGGTTTGACCCTATGTGTG	60.0	353	INTERGENIC	--	--	--
Ca1_P3_32153737	Ca1	32153737	TTATATATATATAT/TTATATATATATATAT	CACACATAGGGTCAAACCCA	ATACATGGATTGGTTGGGGA	59.3	585	INTERGENIC	--	--	--
Ca1_P3_32154060	Ca1	32154060	TGTATTGTAAGTATTGTAAG/TGTATTGTAAG	ATGGAATACTCCCCAACCAA	ACTTGCAAAATCCAAATGCC	59.1	446	INTERGENIC	--	--	--

INDEL marker IDs	Chromosomes/ unanchored scaffolds	Physical positions (bp)	InDels (*Kabuli* reference genome-CDC Frontier/PII)	Forward primers (5'-3')	Reverse primers (5'-3')	Anneling temperature (0C)	Expected amplified product size (bp)	Structural annotation		Functional annotation	
								Sequence components of *kabuli* genome	*Kabuli* gene accession IDs	NCBI-KOG	TFs
Ca1_P3_32189060	Ca1	32189060	ATTTT/ATTTTT	ATTAACGCCATTGCCTTGTC	GCTTTTTCCTCGCTTCCTTT	60.0	632	DRR	Ca_26126	QI	bHLH
Ca1_P3_32191667	Ca1	32191667	ATTTTTTTTT/ATTTTTTT	CATCATGATCAAGGGACCAA	AAGTTAGCGGTCGGGAGAAT	59.3	413	INTERGENIC	--	--	--
Ca1_P3_32244195	Ca1	32244195	A/AG	TGAATAGAGGGAAACGGGTG	CAGTGACCTTGTGGAGAGCA	59.9	640	INTERGENIC	--	--	--
Ca1_P3_32259713	Ca1	32259713	TAACAA/TAACAACAA	CCAAAAATTCGTCGCAAACT	CCGGATTCACACTTCCGATA	60.1	616	INTERGENIC	--	--	--
Ca1_P3_32312793	Ca1	32312793	GTT/GT	GATTGGCTCAGCAGAAGTCC	TTTCAAGCAGCGGAAAAAGT	60.0	561	INTERGENIC	--	--	--
Ca1_P3_32312813	Ca1	32312813	ATTT/ATTTT	GATTGGCTCAGCAGAAGTCC	TTTCAAGCAGCGGAAAAAGT	60.0	561	INTERGENIC	--	--	--
Ca1_P3_32430396	Ca1	32430396	TG/T	TCGTTTGAGCCTTTCGAACT	GCAATCACAATGCAGCAAAC	60.0	473	INTERGENIC	--	--	--
Ca1_P3_3244914	Ca1	3244914	CATATATATATATATAT/CATATATATATATATAT	CTTGATCACCACCACTCCCT	TCAATTAAAAACCGGGTCAA	60.0	797	INTERGENIC	--	--	--
Ca1_P3_32485193	Ca1	32485193	CAAAAAAAAAA/CAAAAAAAAAAA	ACAATGTAGCAAAGACGGGG	CTTGTGCGATGCACAGATAA	60.0	692	DRR	Ca_26796	Z	--
Ca1_P3_32801754	Ca1	32801754	ATTTTTTTTTT/ATTTTTTTTTTT	CTGATTGTAGCCTTGTGCGA	CAGCAGAAACAGAAGTGGCA	60.0	369	INTERGENIC	--	--	--

INDEL marker IDs	Chromosomes/ unanchored scaffolds	Physical positions (bp)	InDels (*Kabuli* reference genome-CDC Frontier/PII)	Forward primers (5'-3')	Reverse primers (5'-3')	Anneling temperature (0C)	Expected amplified product size (bp)	Structural annotation		Functional annotation	
								Sequence components of *kabuli* genome	*Kabuli* gene accession IDs	NCBI-KOG	TFs
Ca1_P3_32852360	Ca1	32852360	CATATATATATATATAT/CATATATATAT	GTATTTCGGATCTTGGCTCG	AATGGAGGAAGGAGGGAGAA	59.7	415	INTERGENIC	--	--	--
Ca1_P3_3287281	Ca1	3287281	TAAAAAAA/TAAAAAAAA	TGCTGAAAGAGTTGTCGGTG	AGAGGGACCACAAAGTCAAA	60.0	516	INTRON	Ca_00398	--	LBD
Ca1_P3_32876010	Ca1	32876010	AAACAACAACAACAACAACAACAA/AAACAACAACAACAACAACAACAACAACAA	CCAACCAAAACAAAATTCCC	ACACTACGGTGCTTGTGAGC	59.2	572	INTERGENIC	--	--	--
Ca1_P3_32906329	Ca1	32906329	TTATATATATATATATAT/TTATATATATATAT	TCATTAAATCAAAGGCACACAAA	TCGTATGTCATAGCGGTGCT	59.5	488	INTERGENIC	--	--	--
Ca1_P3_32943900	Ca1	32943900	CACTTAC/CAC	CGAGAAGACCCTTTTTGAAGC	CTTTGACTTCCCTTCCCCTC	60.4	801	INTERGENIC	--	--	--
Ca1_P3_329558	Ca1	329558	TAAAAAAA/TAAAAAA	TCGGTTTGATTCGATTTTGG	ATTCAGTCTGTCGTGGGACC	60.8	490	INTERGENIC	--	--	--
Ca1_P3_32960646	Ca1	32960646	CA/CACATAAAAATGTA	CCGAGCCAAAATCCAAAATA	ACTTCATTGTCCTCCCATGC	59.9	215	INTERGENIC	--	--	--
Ca1_P3_33018948	Ca1	33018948	CTT/CT	TTCGCAACTCCACTCTTCCT	CACATCATCACAACATGCCA	60.0	481	INTERGENIC	--	--	--
Ca1_P3_33034774	Ca1	33034774	AATATATATATATATA/AATATATATATA	ACCCCAACTTGTAAAGGCAA	TCACGTTGATTGAGGGATGA	59.5	618	INTERGENIC	--	--	--
Ca1_P3_33102523	Ca1	33102523	TTT/TTTATT	AAGGCTAAATCACATTCGTGG	TCACTTCCGTCCATGTCAAA	59.1	835	INTERGENIC	--	--	--

INDEL marker IDs	Chromosomes/ unanchored scaffolds	Physical positions (bp)	InDels (*Kabuli* reference genome-CDC Frontier/PII)	Forward primers (5'-3')	Reverse primers (5'-3')	Anneling temperature (0C)	Expected amplified product size (bp)	Structural annotation		Functional annotation	
								Sequence components of *kabuli* genome	*Kabuli* gene accession IDs	NCBI-KOG	TFs
Ca1_P3_33131597	Ca1	33131597	TC/TCAAC	TCCACCCACGATACTG ATGA	ACAGATTCTCCACAAT GGGC	59.9	529	INTERGENIC	--	--	--
Ca1_P3_33147438	Ca1	33147438	CATATATATATATATATATAT/CATATAT ATATATATATATATATATATAT	TTAATGGTGGCTGCAA CAAA	CCTCCATGGTTGTTGT TTGA	60.1	617	INTERGENIC	--	--	--
Ca1_P3_33154204	Ca1	33154204	AGCGCTGTAAAATGGGCGCTGTAAAA TGGGCGCTGT/AGCGCTGTAAAATGG GCGCTGTAAAATGGGCGCTGTAAAAT GGGCGCTGT	TTCAATGAAAGCGCAT GTGT	GGGATTGACCAAAACT GTCG	60.3	344	INTERGENIC	--	--	--
Ca1_P3_33160136	Ca1	33160136	T/TA	CGCGAGTAATGAAAAT GGGT	GGACTTCCAATCAGTT CCCA	60.0	533	INTERGENIC	--	--	--
Ca1_P3_33182977	Ca1	33182977	TCCC/TCC	ATGCCCGTGAATATCT GTCC	GAGGTGTCTCAGGTG GGGTA	59.8	597	INTRON	Ca_19440	A	--
Ca1_P3_33187249	Ca1	33187249	GTAT/GTATAT	TAAAGGTTCCCTCGCAT CTG	CACCACAGAAGAACTT GGCA	60.2	530	INTRON	Ca_19440	A	--
Ca1_P3_33228816	Ca1	33228816	AATATATATATATATATATATATATATAT ATAT/AATATATATATATATATATATATA TATATATATAT	TACCTTTGTGCTTCCCC ATT	ACCAAACACCCCCAAT ATGA	59.4	842	INTERGENIC	--	--	--
Ca1_P3_33247147	Ca1	33247147	TA/T	GTGTTGGACCGGTTCT TGAT	GTCTGATGACAAAAGG GGGA	59.8	643	INTERGENIC	--	--	--
Ca1_P3_33286471	Ca1	33286471	ATTT/ATTTT	ATTATCAGAGAGTCCC CGGC	CAGATTGGCACCTATG AGCA	60.4	879	INTERGENIC	--	--	--
Ca1_P3_33291292	Ca1	33291292	TCC/TC	AGACATCGCTCATACC CCTG	CCGTTGGAGTTGTGTC AATG	60.1	594	INTERGENIC	--	--	--

INDEL marker IDs	Chromosomes/ unanchored scaffolds	Physical positions (bp)	InDels (*Kabuli* reference genome-CDC Frontier/PII)	Forward primers (5'-3')	Reverse primers (5'-3')	Anneling temperature (0C)	Expected amplified product size (bp)	Structural annotation		Functional annotation	
								Sequence components of *kabuli* genome	*Kabuli* gene accession IDs	NCBI-KOG	TFs
Ca1_P3_33304561	Ca1	33304561	CA/CAA	AACGTACTGCCTATCACGGG	TTTTGTAAATCGTGCAGACCC	60.0	340	INTERGENIC	--	--	--
Ca1_P3_33322247	Ca1	33322247	CTTTTTT/CTTTT	TGGACAACGTTCCAAAAACA	GCCAATGCAACACAAACAAC	60.0	470	DRR	Ca_19441	--	MIKC
Ca1_P3_33336930	Ca1	33336930	GAAAAA/GAAAA	CAATGGCAACAAGGTCAATG	TCACATCTGTTGGTAACGGG	60.0	856	INTRON	Ca_19441	--	MIKC
Ca1_P3_33347713	Ca1	33347713	CTTTTTTT/CTTTTTT	TGCAAAGCGATCTTCCCTAT	AGCTCGGGTTATGGTTGATG	59.8	425	INTRON	Ca_19441	--	MIKC
Ca1_P3_33365935	Ca1	33365935	CTTTTTTTTTT/CTTTTTTTTTTTTT	CCACGTGTGGTCCCTTAACT	TTTTTCTCCCGATTTGGTCA	59.9	485	INTRON	Ca_19441	--	MIKC
Ca1_P3_33366605	Ca1	33366605	TAAAAAAAA/TAAAAAAA	TGACCAAATCGGGAGAAAAA	ACCCTTCTTCCACCTTGGAT	60.4	741	INTRON	Ca_19441	--	MIKC
Ca1_P3_33407768	Ca1	33407768	TA/TAA	TGGCCATCAATTAATCCACTC	GATCTGCAACTGGGGGTAAA	59.8	230	INTERGENIC	--	--	--
Ca1_P3_33426830	Ca1	33426830	GATATATATATATATATATA/GATATATATATATATATATATA	AATCTGGCTTGGAGAATGGA	AACCATATGGGTTGTTGTCCTT	59.6	768	DRR	Ca_19444	--	--
Ca1_P3_33452743	Ca1	33452743	CCTTCTTCTTCTTCTTCTTCTTCTTCTTCTTCTTCTTCTT/CCTTCTTCTTCTTCTTCTTCTTCTTCTTCTTCTTCTT	TTGGATCCAGAGGCAGAGTT	TGAAGAAGAGGAGCCCAAGA	59.8	477	INTERGENIC	--	--	--
Ca1_P3_33476619	Ca1	33476619	CTTT/CTT	ACCAGCAACCATAAAAACGC	AATTGGTTTCATGACCTGCAC	60.0	450	INTERGENIC	--	--	--

INDEL marker IDs	Chromosomes/ unanchored scaffolds	Physical positions (bp)	InDels (*Kabuli* reference genome-CDC Frontier/PII)	Forward primers (5'-3')	Reverse primers (5'-3')	Anneling temperature (0C)	Expected amplified product size (bp)	Structural annotation		Functional annotation	
								Sequence components of *kabuli* genome	*Kabuli* gene accession IDs	NCBI-KOG	TFs
Ca1_P3_33502148	Ca1	33502148	TTT/TTTATT	TCCCTTTTAAATAAACATGTGCAA	CACCCCTTACCTCGACTGAA	59.8	725	INTERGENIC	--	--	--
Ca1_P3_33514102	Ca1	33514102	ATTTTTTTTTTT/ATTTTTTTTTT	CAATGTCGAGAAACTTCAAGCA	AATTCAAGTCGGAAAGCGAA	60.4	784	INTERGENIC	--	--	--
Ca1_P3_3352776	Ca1	3352776	C/CT	ACCCTGAAAATCAGGACGTG	CTCCGAATTTCAAGGGAAAA	60.0	239	INTERGENIC	--	--	--
Ca1_P3_33564950	Ca1	33564950	TAACATCCATTTGAAA/TAACATCCATTTGAAAACATCCATTTGAAA	AGATGGGTGCATAGGAGTGG	ACGGCTGATGGAATCAGAAC	60.0	611	INTERGENIC	--	--	--
Ca1_P3_33567505	Ca1	33567505	CATATATATATATATATATAT/CATATATATATATATAT	GCATTCCCTCATTGTAACTCG	GGGTTTGAGTTTGATGACAGG	59.6	711	INTERGENIC	--	--	--
Ca1_P3_33603009	Ca1	33603009	C/CCCTACAAGAAG	CAAATCTTCTTCAAATCTTGACCAT	AGGCACATGTTGGCTTCTTC	59.9	285	INTERGENIC	--	--	--
Ca1_P3_33604424	Ca1	33604424	GAAAAAAAAAAA/GAAAAAAAAAA	TGATCATGACGTTGATGGCT	TGTGGTTAGGTGAGCTGGAA	60.1	551	INTERGENIC	--	--	--
Ca1_P3_33638489	Ca1	33638489	ATT/ATTT	CGCAGATTGATCTTGAAGCA	TGATCTTTTGCTTGGTGCTG	60.1	581	INTERGENIC	--	--	--
Ca1_P3_33653494	Ca1	33653494	TTTATTATTATTATTATTATTA/TTTATTATTATTATTATTATTA	TGGGCAAAATGTTAAGAACACA	AGAACAAATGCATGATTCTTGG	60.4	573	INTERGENIC	--	--	--
Ca1_P3_33658726	Ca1	33658726	ATTT/ATT	ATGTGAATCCCACCAGGAAA	TTGCACAGCCAAGTTCGTAG	60.2	778	INTERGENIC	--	--	--

INDEL marker IDs	Chromosomes/ unanchored scaffolds	Physical positions (bp)	InDels (*Kabuli* reference genome-CDC Frontier/PII)	Forward primers (5'-3')	Reverse primers (5'-3')	Anneling temperature (0C)	Expected amplified product size (bp)	Structural annotation		Functional annotation	
								Sequence components of *kabuli* genome	*Kabuli* gene accession IDs	NCBI-KOG	TFs
Ca1_P3_33665321	Ca1	33665321	ATTTTTTT/ATTTTTT	AGTGCAAACTGCAAATCACG	CTGTACGGGTTGGATCGAGT	59.9	420	INTERGENIC	--	--	--
Ca1_P3_33681689	Ca1	33681689	TA/TAA	AACGTTGTGGAGGAAATTGG	GCCTCAAGCAATTTGTGGAT	59.8	801	INTERGENIC	--	--	--
Ca1_P3_33746473	Ca1	33746473	GTATATATATATATATAT/GTATATATATAT	CCAATCGATTTCCTCCAAAA	TTTCATCAAAAACCCCCTTG	59.9	779	INTERGENIC	--	--	--
Ca1_P3_33777775	Ca1	33777775	ATTT/ATT	CATGCTCAATACACGGATGG	AACGTTCTTGAGTGGCTTGG	59.9	870	INTERGENIC	--	--	--
Ca1_P3_33781062	Ca1	33781062	CTTTTTTT/CTTTTTTTTT	AATTTCCCAATCACCCACAA	TCCACAATGTCTCCCAACAA	60.0	517	CDS (FRAME SHIFT)	Ca_19460	--	--
Ca1_P3_33795058	Ca1	33795058	ATTTTTTTTTT/ATTTTTTTT	TTGGGGTTTGGCAGTTTTAG	CTTGTTCGGGTACCTCGTGT	60.0	502	INTERGENIC	--	--	--
Ca1_P3_33803595	Ca1	33803595	AC/ACC	TATTCAGATCCCTTGCCCAG	TGTACGAGAAGGTCGTGTGC	60.0	758	INTRON	Ca_19463	--	Trihelix
Ca1_P3_33812037	Ca1	33812037	GTTTTTTTTTT/GTTTTTTTTT	GAAAGCGCATGCATTTTACA	TAGCCGCATCTCATCAATCA	59.8	706	INTERGENIC	--	--	--
Ca1_P3_33905908	Ca1	33905908	GAATAATAATAATAATAATAATAATAATAATAATAATAATAATAATAATAATTGTAATAATAATAATAATAATAATAATAATAATAATAATAATAATAATAATAATAATAATAATAATAATAATAATAATA/GAATAA[illegible]	TGTGGAGGTGAGGTTCCTTC	TCCACAATGTAACGCTCCAG	60.1	454	INTERGENIC	--	--	--
Ca1_P3_34137124	Ca1	34137124	GTTTTTTTT/GTTTTTTT	GCAAGCATTGTCCTCGACTT	CATTGGTGGAATGTGTGCAT	60.4	587	DRR	Ca_25472	QI	bHLH

INDEL marker IDs	Chromosomes/ unanchored scaffolds	Physical positions (bp)	InDels (*Kabuli* reference genome-CDC Frontier/PII)	Forward primers (5'-3')	Reverse primers (5'-3')	Anneling temperature (0C)	Expected amplified product size (bp)	Structural annotation		Functional annotation	
								Sequence components of *kabuli* genome	*Kabuli* gene accession IDs	NCBI-KOG	TFs
Ca1_P3_34140254	Ca1	34140254	TAAAA/TAAA	AATCCAATTTGCATGCTTCA	GACCAAAACCGTCTTTTCCA	59.1	210	INTRON	Ca_25472	QI	bHLH
Ca1_P3_34145521	Ca1	34145521	TAAAA/TAAA	CGCACACATTAAGCCTTAGC	TTCTTTTATTCAAATCGGACATCA	58.6	530	INTERGENIC	--	--	--
Ca1_P3_34160007	Ca1	34160007	C/CAT	TGGAGACACTTGCCCTCAAT	TTTTATGTCAGGGCGAAACC	60.7	501	INTERGENIC	--	--	--
Ca1_P3_34160476	Ca1	34160476	TG/TGG	GGTTTCGCCCTGACATAAAA	ACTCTGGACACTTGGGTTGC	59.9	773	DRR	Ca_25473	O	--
Ca1_P3_341635	Ca1	341635	TGATTGGGATT/TGATTGGGATTTGGATTGGGATT	GAGGAAGTGGTGTCGTTGGT	CGGTTTGGTGGAAAAAGAGA	60.0	652	CDS (large-effect mutations)	Ca_00034	S	B3
Ca1_P3_34165386	Ca1	34165386	GAAAAAAA/GAAAAAAAAA	AACCCAATAATCTCTCTGGGC	ACATTGAAAGGAGAACCGCT	59.4	443	INTERGENIC	--	--	--
Ca1_P3_34169923	Ca1	34169923	GAA/GAAA	CCATTTCCTTTGTCCTCGTG	AAGCCTTGGAAGGAGAAAGC	60.5	538	INTERGENIC	--	--	--
Ca1_P3_34177476	Ca1	34177476	TAAAAAAAAA/TAAAAAAAA	AAGAACATGGATGGGGATGA	TTCATAAACGCAACGTGCTC	60.1	412	INTERGENIC	--	--	--
Ca1_P3_34179876	Ca1	34179876	ATCTTTCTTT/ATCTTT	CAACAGAATGGTGGCTAGGG	TTTGGATAATGGGCAAAAGG	60.5	160	INTERGENIC	--	--	--
Ca1_P3_34215575	Ca1	34215575	G/GTA	GTTGAGCCAAAAACCGACAT	CGACTCCAGCCTCTTCATTC	60.0	435	INTERGENIC	--	--	--

INDEL marker IDs	Chromosomes/ unanchored scaffolds	Physical positions (bp)	InDels (*Kabuli* reference genome-CDC Frontier/PII)	Forward primers (5'-3')	Reverse primers (5'-3')	Anneling temperature (0C)	Expected amplified product size (bp)	Structural annotation		Functional annotation	
								Sequence components of *kabuli* genome	*Kabuli* gene accession IDs	NCBI-KOG	TFs
Ca1_P3_34231675	Ca1	34231675	GAAAAAAA/GAAAAAAAA	CAACAGAGCACTTTCCACGA	AATATCCTGGTGCACCCAAC	60.0	545	INTERGENIC	--	--	--
Ca1_P3_34250780	Ca1	34250780	CTT/CTTCATTTTTATTTT	TTGGAGTCGAAGTGAAACCC	ACATTCAGGTGGTGGTGGAT	60.1	561	INTERGENIC	--	--	--
Ca1_P3_34263760	Ca1	34263760	TA/TTGCACA	ATCCAAAATGGGATGGATGA	GTTCGGAACATGCTGTCCTT	60.0	703	INTRON	Ca_25475	M	MYB_related
Ca1_P3_34277520	Ca1	34277520	TCTTTCTCTCTCTACTTTCTCTCTCTACTTTCTCTCTC/TCTCTACTTTCTCTCTCTACTTTCTCTCTCTACTTTCTCTCTC	GAGCAACAGCGAAAAGGAAC	CATAATGCATTGCGTCAACC	60.0	452	INTERGENIC	--	--	--
Ca1_P3_34278298	Ca1	34278298	ATTTTT/ATTTTTT	TGGAGCGACTTGTAGTGTTTG	CCAAAATGAAGGGTCTCAATTC	59.0	588	INTERGENIC	--	--	--
Ca1_P3_34279803	Ca1	34279803	TAATAAGCA/T	AGGTTGGTCTGGCTCAAAAA	AAGTTGGGTCGTCGTCAGAG	59.7	418	INTERGENIC	--	--	--
Ca1_P3_34281146	Ca1	34281146	AAATAATAATAATAATAAT/AAATAAAAATAATAATAATAATAATAAT	TTCGTACCCTCATCCCTCAC	AGAGTAGCAGCCCTATGCCA	59.9	566	INTERGENIC	--	--	--
Ca1_P3_34301461	Ca1	34301461	GTATATATATATAT/GTATATATATATATATAT	TCGGTTGGAGAAGATGAAAGA	CAATATTGTGTTTGGGCCGT	59.8	368	INTERGENIC	--	--	--
Ca1_P3_34313358	Ca1	34313358	GTG/GTGATG	GGGGGTAATAGGATGTCACG	TTATCGCAGATTGTCCCACA	59.1	590	INTRON	Ca_25478	R	ARR-B
Ca1_P3_34331823	Ca1	34331823	CTCA/CTCAAGTCA	TGATGAATTTGATGTTGTTGGA	AAAACCCCTGCTTCCAATTT	58.9	407	INTERGENIC	--	--	--

INDEL marker IDs	Chromosomes/ unanchored scaffolds	Physical positions (bp)	InDels (*Kabuli* reference genome-CDC Frontier/PII)	Forward primers (5'-3')	Reverse primers (5'-3')	Anneling temperature (0C)	Expected amplified product size (bp)	Structural annotation		Functional annotation	
								Sequence components of *kabuli* genome	*Kabuli* gene accession IDs	NCBI-KOG	TFs
Ca1_P3_34332058	Ca1	34332058	CATATAT/CATATATAT	AATTGGAAGCAGGGGTTTTT	TGAACCCGTTATAAATATTTTTCCT	59.8	356	INTERGENIC	--	--	--
Ca1_P3_343407	Ca1	343407	CATA/CA	ATCAACTTCAGGTTGGGTGG	CCAAAGATGAGCCCAAGAAA	59.8	704	INTERGENIC	--	--	--
Ca1_P3_34348577	Ca1	34348577	TAAA/TAA	TTTGTGGACACGACTACCGA	TGGGCAAAAGGAATTTCAAG	60.2	252	INTERGENIC	--	--	--
Ca1_P3_34352352	Ca1	34352352	ACTCT/ACT	TTCTTCCACACTTGCGTCTG	TTTGACTAGTTTGTCCCCGC	60.0	463	INTERGENIC	--	--	--
Ca1_P3_34362589	Ca1	34362589	GATAAATA/GA	GGAGGAAACAAGCGTACGAA	TAAGGAGGCCTTTTACAGCG	60.2	859	INTERGENIC	--	--	--
Ca1_P3_34364559	Ca1	34364559	T/TCTC	TGGGAAAACCTTGACCAATC	TCATGAGTGGGAGTGAACCA	59.8	865	INTERGENIC	--	--	--
Ca1_P3_34386272	Ca1	34386272	GTTT/GTTTT	AATCTGGAGGGCGGTAAGTT	ATATCGGTCAAACGCACACA	60.0	413	INTERGENIC	--	--	--
Ca1_P3_343924	Ca1	343924	AACACACA/AACACACACA	TTTCTTGGGCTCATCTTTGG	CCTTGATTTTGCCACAACCT	60.2	404	INTERGENIC	--	--	--
Ca1_P3_34434177	Ca1	34434177	AC/A	TGGCCAATGCTAATGTGGTA	TGAAGCGCTGTAAAATGTGC	60.0	386	INTERGENIC	--	--	--
Ca1_P3_344347	Ca1	344347	A/AT	AGGTTGTGGCAAAATCAAGG	CAACGGCTTCTTTTACGGAG	60.0	404	INTERGENIC	--	--	--

INDEL marker IDs	Chromosomes/ unanchored scaffolds	Physical positions (bp)	InDels (*Kabuli* reference genome-CDC Frontier/PII)	Forward primers (5'-3')	Reverse primers (5'-3')	Anneling temperature (0C)	Expected amplified product size (bp)	Structural annotation		Functional annotation	
								Sequence components of *kabuli* genome	*Kabuli* gene accession IDs	NCBI-KOG	TFs
Ca1_P3_34469736	Ca1	34469736	AT/ATT	CACATAAAGGCATCATCCCC	ATTAAAGGGGCAACCGATTC	60.2	553	INTERGENIC	--	--	--
Ca1_P3_34487555	Ca1	34487555	TAACAACAACAACAACAACAACAACAACAA/TAACAACAACAACAACAACAACAACAA	GGCAACAACAGCAGTAACGA	ATTCAGGCAAAAGGGGAGTT	59.9	304	INTERGENIC	--	--	--
Ca1_P3_34505064	Ca1	34505064	TTCTCTCTCTCTCTCTCTCTCTCTCTCTCTCTCTCTCTC/TTCTCTCTCTCTCTCTCTCTCTCTCTCTCTCTCTCTCTCTC	ACAAGTCCATGTGGCAATGA	CGGGTGGTGAAATAAAATGG	60.0	124	INTERGENIC	--	--	--
Ca1_P3_345335	Ca1	345335	TTTATT/TTT	CGACCATCTTTTTGGAAGGA	CCTCTGCTTCCTTCGTTTTG	60.0	742	INTRON	Ca_00035	O	--
Ca1_P3_345766	Ca1	345766	GAA/GA	TCAACTTTGAGGTCTCCGGT	TCACTTCCGTTTCTGCCTCT	59.7	182	INTRON	Ca_00035	O	--
Ca1_P3_346007	Ca1	346007	CCTTCTTCTTC/CCTTCTTCTTCTTC	AGAGGCAGAAACGGAAGTGA	AGCGAGAAATTCGAAAGCAA	60.0	238	INTRON	Ca_00035	O	--
Ca1_P3_34642112	Ca1	34642112	TGG/TG	GAGGCGTCTTGCTATGCTTT	GAGAGATGTGGCTTTGGAGG	59.6	882	INTERGENIC	--	--	--
Ca1_P3_34647162	Ca1	34647162	TGG/TG	TGGATGAATGCTTGGATGAA	TAGTCCAACAATGGCCAACA	60.0	978	INTERGENIC	--	--	--
Ca1_P3_34666828	Ca1	34666828	TAAAAAAAAA/TAAAAAAAA	TGTTTCAGGATGTCGACCAA	GTTACCGCAGAGGGACGTAA	60.1	640	INTERGENIC	--	--	--
Ca1_P3_34682200	Ca1	34682200	ATTTTTT/ATTTTTTT	CATTGCTTGACCTTGGTCTG	TCACCAAACCTTGGGTTTCT	59.3	646	INTERGENIC	--	--	--

INDEL marker IDs	Chromosomes/ unanchored scaffolds	Physical positions (bp)	InDels (*Kabuli* reference genome-CDC Frontier/PII)	Forward primers (5'-3')	Reverse primers (5'-3')	Anneling temperature (0C)	Expected amplified product size (bp)	Structural annotation		Functional annotation	
								Sequence components of *kabuli* genome	*Kabuli* gene accession IDs	NCBI-KOG	TFs
Ca1_P3_34682417	Ca1	34682417	AATATATATATATATATATATATATATATATAT/AATATATATATATATATATATATATATATATATATATATATATAT	TATTTTGGATTTTGGCTCGG	CCAACACCGTTTTTCTTCGT	59.9	500	INTERGENIC	--	--	--
Ca1_P3_34768259	Ca1	34768259	AAAATAAATA/AAAATAAATAAATA	ATCGCCAGTGTCAATTTTCC	TGCGATTTTACGTGTATATGTGAG	59.9	677	INTERGENIC	--	--	--
Ca1_P3_347792	Ca1	347792	CTTCT/CTTCTTTCT	CAGCCGTTCAGCTGTTATGA	AAGAACTGCCACCAGGTTTG	60.0	511	DRR	Ca_00036	J	--
Ca1_P3_34810237	Ca1	34810237	GTTTTT/GTTTT	ATTGCCTCGCTTGAAGAATG	TTTGGATGAGCAACAAGCAG	60.4	598	INTERGENIC	--	--	--
Ca1_P3_34814277	Ca1	34814277	T/TG	GCGTGAACAAAAATCAAGCA	TCGAAGACGTCTCGTTGTTG	59.9	452	INTERGENIC	--	--	--
Ca1_P3_34853862	Ca1	34853862	CAAAAAAAAAAA/CAAAAAAAAA	AGTTTGGCTAGATGGGCCTT	TAATTCAAACCGCACTGCAA	60.1	256	INTERGENIC	--	--	--
Ca1_P3_34854959	Ca1	34854959	TTATATA/TTATA	AGAACTTAGCCAACGAGGCA	TGTGGTGCCCTAAATGTGAA	60.0	485	INTERGENIC	--	--	--
Ca1_P3_34865996	Ca1	34865996	TAATAAATAAATAAA/TAATAAATAAATAAATAAA	TTGTTCGAGAATTTGGAGGG	TGAGGTGGTGACCAATTGAA	60.0	462	DRR	Ca_21411	--	MYB_related
Ca1_P3_34895156	Ca1	34895156	ATCTCTCTCTCTCTC/ATCTCTCTCTCTCTCTCTCTCTCTCTCTCTC	TTTCAAGTTTTCCGGAGTGC	GTGGGAATCATATGGGGATG	60.2	883	INTERGENIC	--	--	--
Ca1_P3_34913480	Ca1	34913480	AAT/AATAT	TTGTGTTCACCGAGGTTGTC	CAACAACCAACTTCGTGCAT	59.6	572	INTERGENIC	--	--	--

INDEL marker IDs	Chromosomes/ unanchored scaffolds	Physical positions (bp)	InDels (*Kabuli* reference genome-CDC Frontier/PII)	Forward primers (5'-3')	Reverse primers (5'-3')	Anneling temperature (0C)	Expected amplified product size (bp)	Structural annotation		Functional annotation	
								Sequence components of *kabuli* genome	*Kabuli* gene accession IDs	NCBI-KOG	TFs
Ca1_P3_34952469	Ca1	34952469	AAATAATAATAATAATAA/AAATAATAATAATAA	CTTCAACCAATGCCTCCACT	AAAAAGGAGGTGCGACAAAA	60.1	603	INTERGENIC	--	--	--
Ca1_P3_349576	Ca1	349576	ATTTTT/ATTTT	CAATTGGGGAACAAAATTGG	AAATCAGTTTTGGCGATTGG	60.0	550	DRR	Ca_00035	O	--
Ca1_P3_34971020	Ca1	34971020	CAAAAAAA/CAAAAA	AAATACCCCTTTTTCCCACG	TGAAAACGCTCAATGCCATA	60.0	689	INTERGENIC	--	--	--
Ca1_P3_34983014	Ca1	34983014	TGAAGAAG/TGAAG	ACGCAAAGCTTCCTTTTTGA	TTCAACGCATCCCTTAAACC	60.0	617	INTERGENIC	--	--	--
Ca1_P3_34989112	Ca1	34989112	CAT/CATAT	CAGGATAGTCGACGTGCAAA	TCTTGGCATAGCAAGAAGCA	59.9	406	INTERGENIC	--	--	--
Ca1_P3_35026117	Ca1	35026117	CAAAAAAA/CAAAAAAAA	AAACCAAAACCTCAACACGC	AATGTTCTGGAGAGGCGAGA	60.0	624	INTERGENIC	--	--	--
Ca1_P3_35044743	Ca1	35044743	AG/A	GTACCGAATAGCCCGCATAA	CCAGTCCATATCGGGTCAGT	60.0	654	INTERGENIC	--	--	--
Ca1_P3_35056040	Ca1	35056040	TAAAAA/TAAAAAAA	TTTCCATAATGTAATGGGCTGA	GGGAATTGGATTGAAGCAGA	59.3	401	INTERGENIC	--	--	--
Ca1_P3_35056113	Ca1	35056113	TAAAAAAA/TAAAAAAAA	ACAGAAGAGGGCGCACATAC	GGGAATTGGATTGAAGCAGA	60.3	297	INTERGENIC	--	--	--
Ca1_P3_35056786	Ca1	35056786	AACATCTAGCTTTACA/AACA	TCTGCTTCAATCCAATTCCC	TGAATAGAACCAGCCCAAAA	60.0	667	INTERGENIC	--	--	--

INDEL marker IDs	Chromosomes/ unanchored scaffolds	Physical positions (bp)	InDels (*Kabuli* reference genome-CDC Frontier/PII)	Forward primers (5'-3')	Reverse primers (5'-3')	Anneling temperature (0C)	Expected amplified product size (bp)	Structural annotation		Functional annotation	
								Sequence components of *kabuli* genome	*Kabuli* gene accession IDs	NCBI-KOG	TFs
Ca1_P3_35065402	Ca1	35065402	CAA/CA	CCAACCAAAACCCTTCTCAA	GTACATGGTAACCGGATGGG	59.9	309	INTERGENIC	--	--	--
Ca1_P3_35072166	Ca1	35072166	CTA/CTATATA	AGTTGGCTCACTAGCACCGT	CACAAGTGGATGAACAACCG	59.9	696	INTERGENIC	--	--	--
Ca1_P3_35086819	Ca1	35086819	AATATATATATATATATATATATA/AATATATATATATATA	CTGCTGAAGAAGCCAAATCC	CTTGATTGAAAGGGGAACCA	60.0	718	INTERGENIC	--	--	--
Ca1_P3_35143888	Ca1	35143888	TT/TTAT	CGCTTGTTTTGGTTTAATTCG	GAGCATGATGGAGCAACTCA	59.6	618	INTERGENIC	--	--	--
Ca1_P3_35147370	Ca1	35147370	CTTT/CTT	TCTATCGTGGTTCATTGCCA	TCTGAATGTCAATCCTCCGA	60.1	631	INTERGENIC	--	--	--
Ca1_P3_35150218	Ca1	35150218	CA/C	TGCAACATTCCTCCAAGGAT	CATGGACACGATCGTCACTT	60.5	302	INTERGENIC	--	--	--
Ca1_P3_35150770	Ca1	35150770	TC/TCC	GTGACGATCGTGTCCATGTC	CAAGCCTCTCCAAGAAATCG	60.0	715	INTERGENIC	--	--	--
Ca1_P3_35159500	Ca1	35159500	CTTTTT/CTTTTTT	CTAGCGCACCCTAAGACTGG	TTGGGTTTTGGTGTCCTTGT	60.0	427	INTERGENIC	--	--	--
Ca1_P3_35165919	Ca1	35165919	GTTTTTT/GTTTTTTTTTTTT	TCATCGCAGGTGTACCATGT	CACCTAAGGGAATCGGTCAA	60.0	377	INTERGENIC	--	--	--
Ca1_P3_35166209	Ca1	35166209	TAAAAAAAAAA/TAAAAAAAA	AAGAGAGGTCACTTTGGCGA	CAATTCCCACAGGTACGCTT	60.0	852	INTERGENIC	--	--	--

INDEL marker IDs	Chromosomes/ unanchored scaffolds	Physical positions (bp)	InDels (*Kabuli* reference genome-CDC Frontier/PII)	Forward primers (5'-3')	Reverse primers (5'-3')	Anneling temperature (0C)	Expected amplified product size (bp)	Structural annotation		Functional annotation	
								Sequence components of *kabuli* genome	*Kabuli* gene accession IDs	NCBI-KOG	TFs
Ca1_P3_35168994	Ca1	35168994	AAT/AATAT	GTGATAATTCCCCATGTGCC	TCAACGCCTCTTCTCGATTT	60.0	350	INTERGENIC	--	--	--
Ca1_P3_35169414	Ca1	35169414	TATTTAATTT/TATTT	GAAATCGAGAAGAGGCGTTG	TGTCTCGAAAAAGCGAGATG	60.0	636	INTERGENIC	--	--	--
Ca1_P3_35169496	Ca1	35169496	T/TC	GAAATCGAGAAGAGGCGTTG	TGTCTCGAAAAAGCGAGATG	60.0	636	INTERGENIC	--	--	--
Ca1_P3_35178858	Ca1	35178858	A/AC	CAAAAATGCACGTGGAAGAG	TCCTAGAATTGAAAAACGGCA	59.3	849	INTERGENIC	--	--	--
Ca1_P3_35179269	Ca1	35179269	GGA/GGAGA	GGCAGTTATTCCCTTCCATTC	CCTTGCATGCCTTCAAAAAT	59.8	842	INTERGENIC	--	--	--
Ca1_P3_35186704	Ca1	35186704	GAAATG/GAAATGATTAAAATG	GGTCAGTGAGCCAAGGGATA	CCGTTTAAACCCACCTGCTA	60.1	729	INTERGENIC	--	--	--
Ca1_P3_3518856	Ca1	3518856	AATGATTTTAGGGTTCATGATTTTAGGGTTCATGA/AATGATTTTAGGGTTCATGATTTTAGGGTTCATGATTTTAGGGTTCATGA	AAAGAACCTTGCAGAAGGCA	TGTTAGGGAGTTCGGGACAC	60.0	758	INTERGENIC	--	--	--
Ca1_P3_35232976	Ca1	35232976	TTTTT/TTTTTATTAGATTTT	TGCATAATTTCCTACTCTTCCACA	CACAAAACAAAGCCAACCCT	60.0	306	INTERGENIC	--	--	--
Ca1_P3_35236411	Ca1	35236411	ATTATT/ATTATTGTTATT	ATGTGCCAAAAGAGGAGGAA	GGGATGCGACCTTGTACTGT	59.7	704	INTERGENIC	--	--	--
Ca1_P3_35238941	Ca1	35238941	CTTTTT/CTTTTTT	AAAATGCATTGCCTCTGGTC	TTTATTTACCCAAACGAAAACAA	60.1	824	INTERGENIC	--	--	--

INDEL marker IDs	Chromosomes/ unanchored scaffolds	Physical positions (bp)	InDels (*Kabuli* reference genome-CDC Frontier/PII)	Forward primers (5'-3')	Reverse primers (5'-3')	Anneling temperature (0C)	Expected amplified product size (bp)	Structural annotation		Functional annotation	
								Sequence components of *kabuli* genome	*Kabuli* gene accession IDs	NCBI-KOG	TFs
Ca1_P3_35240205	Ca1	35240205	ATAT/ATATTAT	GTAGCATTTTTGGCCTCCCT	GTCGAGAAGAAGGCTTGCAT	60.5	782	DRR	Ca_26097	QI	bHLH
Ca1_P3_35244173	Ca1	35244173	TAAAA/TAAA	ATTTGGATCGGTGTGGTTTT	TCGGAAATGTTGCTGAGATG	59.2	553	INTERGENIC	--	--	--
Ca1_P3_35245591	Ca1	35245591	GGGA/GGGAGGA	TTTCCCGACTTGGTCCTTTA	CTCTTCCCCAAATCCCTCTT	59.5	697	INTERGENIC	--	--	--
Ca1_P3_35258289	Ca1	35258289	A/AC	TGTCATTGCATTTGTTCATCA	CCCACCCTAGGATCTCTGCT	58.5	256	INTERGENIC	--	--	--
Ca1_P3_35260458	Ca1	35260458	ATT/ATTT	ATGGCTCATACTGGGATTGC	AAGACTGGGTTTGCCTGCTA	59.9	592	INTERGENIC	--	--	--
Ca1_P3_35261821	Ca1	35261821	ATT/AT	TGGATTGTCATGGTCTGGAA	AATGGTCTTGAATTTTGCCG	59.9	640	INTERGENIC	--	--	--
Ca1_P3_35265473	Ca1	35265473	TAAAAAAA/TAAAAAA	TTCCCTGAATCGAGTGAACC	TTTTCCAATAACTCTACCGCGT	60.1	674	INTERGENIC	--	--	--
Ca1_P3_35281444	Ca1	35281444	ATTTTTTTT/ATTTTTTT	ACAAAGGCCTGCCTAGATCA	TTTTTGATGTCTCTCTGTTCAAAT	59.8	591	INTRON	Ca_26099	QI	bHLH
Ca1_P3_35281570	Ca1	35281570	TC/T	ATGACACGTCATTCGTTTCG	GGTTCGAGACATCCATAATTCA	59.6	733	INTRON	Ca_26099	QI	bHLH
Ca1_P3_35281940	Ca1	35281940	TA/TAA	AAACGTGATCTATACAGTTTCCAAAA	TTCACATCTGGAAACAACGC	59.4	529	INTRON	Ca_26099	QI	bHLH

INDEL marker IDs	Chromosomes/ unanchored scaffolds	Physical positions (bp)	InDels (*Kabuli* reference genome-CDC Frontier/PII)	Forward primers (5'-3')	Reverse primers (5'-3')	Anneling temperature (0C)	Expected amplified product size (bp)	Structural annotation		Functional annotation	
								Sequence components of *kabuli* genome	*Kabuli* gene accession IDs	NCBI-KOG	TFs
Ca1_P3_35283249	Ca1	35283249	TT/TTTAAATGT	ATGCATTTGTTTTGGCATGT	TGAAGTGTTCTGCAAAATTAGTGA	58.9	612	INTERGENIC	--	--	--
Ca1_P3_35283797	Ca1	35283797	TGGGGG/TGGGG	GGGTGTGTTTGATAGGAGGG	CTCCCCTTCCATCTCCTCTT	59.3	139	INTERGENIC	--	--	--
Ca1_P3_35284017	Ca1	35284017	ATTT/ATT	GAGATGGAAGGGGAGGAAAG	GCACAAAAGAAAAATACAAGTCCA	60.0	273	INTERGENIC	--	--	--
Ca1_P3_35284253	Ca1	35284253	TT/TTGT	GAGATGGAAGGGGAGGAAAG	TCTGATCAAATATGTGGCAGAAA	60.0	657	INTERGENIC	--	--	--
Ca1_P3_35284860	Ca1	35284860	CAAAAA/CAA	TGAAGGGAGTGCTATTTGGT	TCACCTTTTGACTTCCTCATGTT	57.3	108	INTERGENIC	--	--	--
Ca1_P3_35285638	Ca1	35285638	TATT/TATTAATT	GACCTTATGCTCGTGCAATGT	GCCAGAGTGCAATAGGAAGG	60.2	419	INTERGENIC	--	--	--
Ca1_P3_35290921	Ca1	35290921	ATTT/ATTTT	CACACCTAATGCCTGTCACG	TGATGGACCAAACAAGTCCA	60.2	760	INTERGENIC	--	--	--
Ca1_P3_35291933	Ca1	35291933	TTCGATGAGAAAGTCGATGA/TTCGATGA	CTGCACCAAGAACACCTCAA	TCTTGCATGGCAAGTATCCA	59.9	724	INTERGENIC	--	--	--
Ca1_P3_35301437	Ca1	35301437	ATTTTTTTTTTT/ATTTTTTTTT	GCTTTCATGGTGTGCTCTGA	TTAGGCCCAGTGAAGTTTGG	60.0	560	INTERGENIC	--	--	--
Ca1_P3_35310736	Ca1	35310736	ATTTTTTTTT/ATTTTTTTT	TTGATGCAACCTGTGACTCC	TTGGATCCATCCCCTAGATG	59.7	572	INTERGENIC	--	--	--

**INDEL marker IDs**	**Chromosomes/ unanchored scaffolds**	**Physical positions (bp)**	**InDels (*Kabuli* reference genome-CDC Frontier/PII)**	**Forward primers (5'-3')**	**Reverse primers (5'-3')**	**Anneling temperature (0C)**	**Expected amplified product size (bp)**	**Structural annotation**		**Functional annotation**	
								**Sequence components of *kabuli* genome**	***Kabuli* gene accession IDs**	**NCBI-KOG**	**TFs**
Ca1_P3_35310757	Ca1	35310757	ATTTTT/ATTTT	TTGATGCAACCTGTGACTCC	TTGGATCCATCCCCTAGATG	59.7	572	INTERGENIC	--	--	--
Ca1_P3_35337257	Ca1	35337257	CTTTTTT/CTTTTTTT	TACATGCACACACTCCACCA	TGGGAGCCTCAATTGCTTAG	59.6	290	INTERGENIC	--	--	--
Ca1_P3_35353052	Ca1	35353052	G/GTTGA	TCAGCAGCAAATAGGCTTCA	AGCCCATTCAGATTTTGTGG	59.7	352	INTERGENIC	--	--	--
Ca1_P3_35353239	Ca1	35353239	GAA/GAAA	CCAACAACTTCCTTTCGAGC	AGCCCATTCAGATTTTGTGG	59.9	147	INTERGENIC	--	--	--
Ca1_P3_35372027	Ca1	35372027	CA/CAA	TGTGAGGATGGTGAGTTGTGA	AAGCGTAAAAGAGGGGGTGT	60.1	309	INTERGENIC	--	--	--
Ca1_P3_35405146	Ca1	35405146	ATCCTG/A	TCCTCAAGCTTTTTATTCACTTTTT	CACAAGTGGTAGTTTTGAAGGAAA	59.0	752	INTERGENIC	--	--	--
Ca1_P3_35430888	Ca1	35430888	CG/C	ATAATGTTCTTGGTTCGGCG	GATGCAGGTCATGAAACCAA	60.0	844	INTERGENIC	--	--	--
Ca1_P3_35460079	Ca1	35460079	AAAAGTGTTATGA/AA	AACCCATAACTGCGAGACCTT	ATGCAGCAATTTCCATCCTC	60.0	601	INTERGENIC	--	--	--
Ca1_P3_35537314	Ca1	35537314	T/TG	ACATGGAACCCATCCAAAGA	AATCGATTGATTCAGCCCAG	60.2	475	INTERGENIC	--	--	--
Ca1_P3_35551205	Ca1	35551205	CATCGTTT/C	CTAAGGCATATGGCCTCCAA	TGAAGACAAGGCGTTATCCA	60.1	644	INTERGENIC	--	--	--

INDEL marker IDs	Chromosomes/ unanchored scaffolds	Physical positions (bp)	InDels (*Kabuli* reference genome-CDC Frontier/PII)	Forward primers (5'-3')	Reverse primers (5'-3')	Anneling temperature (0C)	Expected amplified product size (bp)	Structural annotation		Functional annotation	
								Sequence components of *kabuli* genome	*Kabuli* gene accession IDs	NCBI-KOG	TFs
Ca1_P3_35553773	Ca1	35553773	GTATCCTA/GTA	GTCGAGGAGATTGCAAAAGC	TTTGAATCGTCTTCACTGCG	60.0	469	INTERGENIC	--	--	--
Ca1_P3_35569645	Ca1	35569645	TAAAAAA/TAAAAA	TGAGTGCACCTTTTACAGCG	TTGCACGCTTAGTATGCACC	60.1	256	DRR	Ca_24107	--	--
Ca1_P3_35572190	Ca1	35572190	TTG/T	CTCTTTCGCCCTCTTGATTG	CGAAACATGCACAAGGATGA	59.9	370	INTERGENIC	--	--	--
Ca1_P3_35610504	Ca1	35610504	AT/ATATATATGT	TATTTTGGATTTTGGCTCGG	TTTTCCTTTCTTTTTCCCCC	59.9	261	INTERGENIC	--	--	--
Ca1_P3_35646771	Ca1	35646771	C/CGT	TGAAACAGTGGGGAAATATGC	ACAAGCTCTCAAACGCACCT	59.8	674	INTERGENIC	--	--	--
Ca1_P3_35679606	Ca1	35679606	CAAAAAAAAAAA/CAAAAAAAAAA	GTGCACAACCTTGTGGTGTC	TAGCCAACATGCCATACGTC	60.1	604	INTERGENIC	--	--	--
Ca1_P3_35711382	Ca1	35711382	GCTTACTTA/GCTTA	CAAATGGAGACGTGAGGGTT	CGCACCAAATAAACCGAAGA	60.0	222	DRR	Ca_24110	K	--
Ca1_P3_35913252	Ca1	35913252	TA/TAAA	CATCATTGTTTGGGCTTGTG	TTTATTGAGGATGGCGAAGG	60.0	798	INTERGENIC	--	--	--
Ca1_P3_35977319	Ca1	35977319	TA/TAA	AACTCGTTGGAACTTCCCTCT	GCAAGCAGCCTTGTTGACTA	59.2	540	INTERGENIC	--	--	--
Ca1_P3_36044248	Ca1	36044248	CGG/CG	CGATTTCCCTGCATGTTTTT	GCCTGTGATAGGCGGTACAT	59.9	540	INTERGENIC	--	--	--

INDEL marker IDs	Chromosomes/ unanchored scaffolds	Physical positions (bp)	InDels (*Kabuli* reference genome-CDC Frontier/PII)	Forward primers (5'-3')	Reverse primers (5'-3')	Anneling temperature (0C)	Expected amplified product size (bp)	Structural annotation		Functional annotation	
								Sequence components of *kabuli* genome	*Kabuli* gene accession IDs	NCBI-KOG	TFs
Ca1_P3_360763	Ca1	360763	CTACG/CTACGTACG	TCATCCGTGGTCATTTTGAA	ACCGCCTTCTCACCCTATCT	59.9	352	INTRON	Ca_00037	E	--
Ca1_P3_36171485	Ca1	36171485	TA/T	TTCGGTTGTTGTTCACTCCA	TAAAACCCATTGGTCCCTTT	60.1	749	INTERGENIC	--	--	--
Ca1_P3_36220499	Ca1	36220499	TAAAAAAAAAAAA/TAAAAAAAAAAA	TGTTTTACCTGCTTGCTCCA	CCAAAGAATATGTTCCATTGGTT	59.5	589	INTERGENIC	--	--	--
Ca1_P3_36283336	Ca1	36283336	ATT/AT	GAGCATAGTGAGTGCGCTTG	TGATGGCTGAAATGGAATGA	59.8	531	INTERGENIC	--	--	--
Ca1_P3_36473784	Ca1	36473784	AA/AATA	TTTTGGATTTCGGTTTTGAA	TTGACCAAAATTCAAGGAGACA	58.1	284	DRR	Ca_21691	--	--
Ca1_P3_36489661	Ca1	36489661	TTATATATATATATATATATATATATATATATATATATATA/TTATATATATATATATATATATATATATATATATA	TTTGCCATGGGAATGTTTTT	TGCACAGTGACAGACCAACA	60.2	871	DRR	Ca_21692	R	--
Ca1_P3_36724754	Ca1	36724754	CTTTTTTTT/CTTTTTTTTTT	TTGCACCACACTTCTTCACC	CTTATTTCGACGCTTTTCGC	59.7	279	INTERGENIC	--	--	--
Ca1_P3_36727739	Ca1	36727739	TCC/TC	AGGCTGCATTTTAGTCCACG	ACGACCACTGCAAACACAAA	60.3	218	INTERGENIC	--	--	--
Ca1_P3_36860487	Ca1	36860487	AAGAGAGAGAGAGAGAGAGAGAGA/AAGAGAGAGAGAGAGAGAGAGAGA	AGAAGGAGTGTGTGCAGGCT	CATCATCATCACGCCTCATC	60.1	645	INTERGENIC	--	--	--
Ca1_P3_36869128	Ca1	36869128	CA/CAAA	TCCACAACAACCTACGTCCA	CCCAGAAATCGATTAGGCAA	60.0	555	INTERGENIC	--	--	--

INDEL marker IDs	Chromosomes/ unanchored scaffolds	Physical positions (bp)	InDels (*Kabuli* reference genome-CDC Frontier/PII)	Forward primers (5'-3')	Reverse primers (5'-3')	Anneling temperature (0C)	Expected amplified product size (bp)	Structural annotation		Functional annotation	
								Sequence components of *kabuli* genome	*Kabuli* gene accession IDs	NCBI-KOG	TFs
Ca1_P3_36897436	Ca1	36897436	TTATATATATATATATATATATATA/TTATATATATATATATATATATATA	CACTAGCATCGTAGCGTGGA	TGTGGAAATTTGTGGGTGAA	60.0	415	INTERGENIC	--	--	--
Ca1_P3_37031419	Ca1	37031419	TNNNNNNNNNN/TNNNNNNNNN	GGGTGAATGGATTTGTCCAC	TTGAAATGTCATGGATGGGA	60.0	537	INTERGENIC	--	--	--
Ca1_P3_37078918	Ca1	37078918	A/AAATTAATG	TGTGACACCCTAAACCCTAAGAA	TTGATTGCTTGCGTGTTCTC	59.9	499	INTERGENIC	--	--	--
Ca1_P3_37081733	Ca1	37081733	CAAAA/CAAA	CCCACAACAATCGTTAAGCA	TGTTCTTCGTATTTGGCGTG	59.6	518	INTERGENIC	--	--	--
Ca1_P3_37122104	Ca1	37122104	CAAAAAAAAAA/CAAAAAAAAAAAAA	CGTATCTCGTATCACCGACG	AGTTGCGCTCCTCCTAATCA	59.2	873	INTERGENIC	--	--	--
Ca1_P3_37122520	Ca1	37122520	AGG/AG	TGCAATCATTGGTGCTTAGG	AAAAAGCGCCGTAAAATGAG	59.7	710	INTERGENIC	--	--	--
Ca1_P3_37122699	Ca1	37122699	GTTT/GTT	GGAGGAGCGCAACTTAATCA	AAAAAGCGCCGTAAAATGAG	60.4	372	INTERGENIC	--	--	--
Ca1_P3_37122810	Ca1	37122810	TACAAC/TACAACAAC	GGAGGAGCGCAACTTAATCA	AAAAAGCGCCGTAAAATGAG	60.4	372	INTERGENIC	--	--	--
Ca1_P3_37128515	Ca1	37128515	ATTTTTTT/ATTTT	CAATTCCCACAGGTACGCTT	TTGGTCTCATCAATGATTGTAAAAA	60.0	568	INTERGENIC	--	--	--
Ca1_P3_37197766	Ca1	37197766	GTTT/GTT	TGAGAGGAAAGAAGACAACCATC	TTTTCTTTTTAAAGCGGCGA	59.7	605	DRR	Ca_21831	L	C3H

INDEL marker IDs	Chromosomes/ unanchored scaffolds	Physical positions (bp)	InDels (*Kabuli* reference genome-CDC Frontier/PII)	Forward primers (5'-3')	Reverse primers (5'-3')	Anneling temperature (0C)	Expected amplified product size (bp)	Structural annotation		Functional annotation	
								Sequence components of *kabuli* genome	*Kabuli* gene accession IDs	NCBI-KOG	TFs
Ca1_P3_37211046	Ca1	37211046	AGTAGTGTAGTGT/AGTAGTGT	GTTGCGGCAGTTCTAC ATTG	ATGGATTCAACTCTTC CCCC	59.3	524	INTERGENIC	--	--	--
Ca1_P3_37243078	Ca1	37243078	AAATTAT/AAATTATAGAATTAT	TCATCCACATCAGCCAA AAA	TTTGTGCATTTTAATAA TTGGAAG	60.0	490	INTERGENIC	--	--	--
Ca1_P3_37412282	Ca1	37412282	CCATG/CCATGCATG	CGTGGATGATGCTTTT CTCA	TGCTTCTCATCTGCTT GGTG	59.8	580	DRR	Ca_21842	R	GRAS
Ca1_P3_37420494	Ca1	37420494	ATTTTTTTTT/ATTTTTTT	GGGAAAAGGAAACCCA AAAA	TAGTGGAGTCCCCGTC AAGT	60.1	630	INTERGENIC	--	--	--
Ca1_P3_37420598	Ca1	37420598	ATTTT/ATTT	GGGAAAAGGAAACCCA AAAA	TGAGTGAGGGAGACAA AGGG	60.1	950	INTERGENIC	--	--	--
Ca1_P3_37421082	Ca1	37421082	CCTCACTCACTCACTCACTCAC/CCTC ACTCACTCACTCAC	ACTTGACGGGGACTCC ACTA	ACATGTTCCTTGTGCT CGTG	59.6	637	INTERGENIC	--	--	--
Ca1_P3_37424329	Ca1	37424329	CATATATATATATATATATATAT/CATAT ATATATATATATAT	AAAAAGTATTGAACAAA CACGCT	TGAAATCCAAATTAAAT TGCTGAA	57.2	549	INTERGENIC	--	--	--
Ca1_P3_37424731	Ca1	37424731	ATTTTTT/ATTTTTTT	GCAATTTAATTTGGATT TCATGG	AGTTTGGTTCAATTGTT TCGAT	59.6	104	INTERGENIC	--	--	--
Ca1_P3_37424983	Ca1	37424983	AC/A	GCAATTTAATTTGGATT TCATGG	TCACATCCATCCACGG TACA	59.6	761	INTERGENIC	--	--	--
Ca1_P3_37425070	Ca1	37425070	ATTTTTTTTTT/ATTTTTTTT	GCAATTTAATTTGGATT TCATGG	TCACATCCATCCACGG TACA	59.6	761	INTERGENIC	--	--	--

INDEL marker IDs	Chromosomes/ unanchored scaffolds	Physical positions (bp)	InDels (*Kabuli* reference genome-CDC Frontier/PII)	Forward primers (5'-3')	Reverse primers (5'-3')	Anneling temperature (0C)	Expected amplified product size (bp)	Structural annotation		Functional annotation	
								Sequence components of *kabuli* genome	*Kabuli* gene accession IDs	NCBI-KOG	TFs
Ca1_P3_37425133	Ca1	37425133	TTATATATA/TTATATA	GCAATTTAATTTGGATTTCATGG	TCACATCCATCCACGGTACA	59.6	761	INTERGENIC	--	--	--
Ca1_P3_37425153	Ca1	37425153	G/GAT	GCAATTTAATTTGGATTTCATGG	TCACATCCATCCACGGTACA	59.6	761	INTERGENIC	--	--	--
Ca1_P3_37425439	Ca1	37425439	TG/T	TGTACCGTGGATGGATGTGA	TGACACGTATCAATTTAGGGAGAA	60.8	222	INTERGENIC	--	--	--
Ca1_P3_37425620	Ca1	37425620	ACG/A	TGTACCGTGGATGGATGTGA	TATGCATCCTCACGGTTTTG	60.8	675	INTERGENIC	--	--	--
Ca1_P3_37425673	Ca1	37425673	ATTTTTTTTTT/ATTTTTTTTTTTT	TGTACCGTGGATGGATGTGA	TATGCATCCTCACGGTTTTG	60.8	675	INTERGENIC	--	--	--
Ca1_P3_37426537	Ca1	37426537	ATT/AT	CTTCAAAACCGTGAGGATGC	TCTGAGCTTCTGATGCTTGC	60.6	916	INTERGENIC	--	--	--
Ca1_P3_37433380	Ca1	37433380	CAA/CAACGTAAA	AATAAATTAGTGCAGCAAACCAA	GGCAAAGAGAGGAAAGTGGA	58.0	756	INTERGENIC	--	--	--
Ca1_P3_374423	Ca1	374423	GTT/GT	TGGCTTGCAGATGGATAATG	TAGCAAACAATGGACGGACA	59.6	718	INTRON	Ca_00038	S	--
Ca1_P3_374767	Ca1	374767	TCA/TCACA	AACACGGCAGTGGTATCTCC	CATCATCAAGGCCATCCTCT	60.0	722	INTRON	Ca_00038	S	--
Ca1_P3_37489932	Ca1	37489932	CAAAAA/CAAAA	ATTGTCCAGTTTCCTCACCG	CGATTGGTAAATCGATTGGG	60.0	594	INTERGENIC	--	--	--

INDEL marker IDs	Chromosomes/ unanchored scaffolds	Physical positions (bp)	InDels (*Kabuli* reference genome-CDC Frontier/PII)	Forward primers (5'-3')	Reverse primers (5'-3')	Anneling temperature (0C)	Expected amplified product size (bp)	Structural annotation		Functional annotation	
								Sequence components of *kabuli* genome	*Kabuli* gene accession IDs	NCBI-KOG	TFs
Ca1_P3_37489959	Ca1	37489959	AT/ATT	ATTGTCCAGTTTCCTCACCG	CGATTGGTAAATCGATTGGG	60.0	594	INTERGENIC	--	--	--
Ca1_P3_37490066	Ca1	37490066	A/AATGTAT	ATTGTCCAGTTTCCTCACCG	TGAACTCTGAGCGTGTGACC	60.0	775	INTERGENIC	--	--	--
Ca1_P3_3764450	Ca1	3764450	GATATATATATATATATATATATA/GATATATATATATATATATATATATA	TTTTGGAGGTGTGATTAATGTATTTT	TGGTTGATGTGTCGAGTGTCT	59.2	515	INTERGENIC	--	--	--
Ca1_P3_37693180	Ca1	37693180	AATTAT/AAT	TGTGGAACATTCTCTGCGTC	TTCCCGAGAGATTTGTCTTCA	59.8	593	DRR	Ca_23533	--	G2-like
Ca1_P3_37697426	Ca1	37697426	GTTTTTT/GTTTTTTTT	TATTGGGTAAGTCCACGGCT	TGGGGTGTACTGGAAAACTTG	59.5	644	INTERGENIC	--	--	--
Ca1_P3_37701615	Ca1	37701615	ATTTTTTTT/ATTTTTTTTTT	TCCGAAAAACTCGCAATAGG	TCACGTGATGCGACCATAAT	60.2	807	INTERGENIC	--	--	--
Ca1_P3_37702469	Ca1	37702469	ATTACCTTACCTTACCTTA/ATTACCTTACCTTACCTTACCTTA	TTTTGTCCCCACAAGTGACA	ATTGGTGTGTCTGGAGAGGC	60.0	703	INTERGENIC	--	--	--
Ca1_P3_37713033	Ca1	37713033	TG/T	GGTGTTGGAGGTGTAATGGG	CATGTCCCGTATTCGCTTTT	60.1	639	INTERGENIC	--	--	--
Ca1_P3_37714757	Ca1	37714757	ATTT/ATTTT	TCGCTCACATATAAGGTTGCAT	TTCGCCCATATTTCCTTTTG	59.6	640	INTERGENIC	--	--	--
Ca1_P3_37714818	Ca1	37714818	CAAAAAAA/CAAAAAA	TCGCTCACATATAAGGTTGCAT	TTCGCCCATATTTCCTTTTG	59.6	640	INTERGENIC	--	--	--

INDEL marker IDs	Chromosomes/ unanchored scaffolds	Physical positions (bp)	InDels (*Kabuli* reference genome-CDC Frontier/PII)	Forward primers (5'-3')	Reverse primers (5'-3')	Anneling temperature (0C)	Expected amplified product size (bp)	Structural annotation		Functional annotation	
								Sequence components of *kabuli* genome	*Kabuli* gene accession IDs	NCBI-KOG	TFs
Ca1_P3_37715518	Ca1	37715518	ATTTTTTTT/ATTTTTTT	TCCCTGAAAATCATTTGTTTGA	TGAAATGGCCATCACTGTTC	59.4	891	INTERGENIC	--	--	--
Ca1_P3_37721826	Ca1	37721826	AAAA/AAAATAAA	TCCATAGCACCTCTTGATTTTG	CGTTGATGGCATCTTGATTG	59.2	420	DRR	Ca_23534	--	--
Ca1_P3_37724987	Ca1	37724987	A/ATT	ACCCTGTTGCTGCCAAATAC	GCTCCAATTCCCTCAGATCA	60.0	664	INTRON	Ca_23534	--	--
Ca1_P3_37737691	Ca1	37737691	AAAATAAATAAATAAATAAATAAA/AAAATAAATAAATAAATAAA	AGGGACCACTATGACCGTTT	TCCTGCAATCACCCTTTTTC	58.4	481	INTERGENIC	--	--	--
Ca1_P3_37739006	Ca1	37739006	T/TA	GCATTCTTGATTCAGAAGATGG	TGGAATACTGGACGTAGCCC	58.8	500	INTERGENIC	--	--	--
Ca1_P3_37747193	Ca1	37747193	GTTTTT/GTTTTTT	TCCATTAAAAATTATGTTGAAAGTCG	TTCTAATCGCCATCAAACCC	59.7	443	INTERGENIC	--	--	--
Ca1_P3_37747588	Ca1	37747588	GAA/GAAA	AATTGAATGGGTTTGATGGC	GCTCATTGCTTCACACCGTA	59.6	780	INTERGENIC	--	--	--
Ca1_P3_37751911	Ca1	37751911	GAAAAAAAAAA/GAAAAAAAAAAA	GGACAAAATGCATCTTCTTTCAA	GTTGGCCTTTGTGGACTGTT	60.5	684	INTERGENIC	--	--	--
Ca1_P3_37758416	Ca1	37758416	AATATATATAT/AATATATAT	AATGCAAATTTTTCCCACCA	AGCGAATGATTTCAACCACC	60.2	791	INTERGENIC	--	--	--
Ca1_P3_37758491	Ca1	37758491	TA/TAA	TGACGAGGTTCGTGATGATG	AGCGAATGATTTCAACCACC	60.7	489	INTERGENIC	--	--	--

INDEL marker IDs	Chromosomes/ unanchored scaffolds	Physical positions (bp)	InDels (*Kabuli* reference genome-CDC Frontier/PII)	Forward primers (5'-3')	Reverse primers (5'-3')	Anneling temperature (0C)	Expected amplified product size (bp)	Structural annotation		Functional annotation	
								Sequence components of *kabuli* genome	*Kabuli* gene accession IDs	NCBI-KOG	TFs
Ca1_P3_37759391	Ca1	37759391	GT/G	CCATCTTGATCCTTTGCCTC	TCAAACGGTCAAATTGGATG	59.6	268	INTERGENIC	--	--	--
Ca1_P3_37780594	Ca1	37780594	TAAAAAAAAA/TAAAAAAAAAAAAA	CCGGAAGTCCTACTGGTTCA	CCACTAGAAACCTGGTTGGA	60.1	501	INTERGENIC	--	--	--
Ca1_P3_37794492	Ca1	37794492	ATTTTTTTTTT/ATTTTTTTTT	TTTGATTTGCATTCAGCAGG	TTTGGAAAAACCGTACTGGC	59.8	445	DRR	Ca_23537	--	NAC
Ca1_P3_37794748	Ca1	37794748	CT/CTT	TTTGATTTGCATTCAGCAGG	TTTGGAAAAACCGTACTGGC	59.8	445	DRR	Ca_23537	--	NAC
Ca1_P3_37795802	Ca1	37795802	CAAAAAAAAAAA/CAAAAAAAAAAAAAAA	CACTTTGAAGACATGCAAGAGG	ATGCATTTGATTGGGGACTT	59.9	491	INTRON	Ca_23537	--	NAC
Ca1_P3_37795956	Ca1	37795956	GTTTTTTT/GTTTTTTTT	CACTTTGAAGACATGCAAGAGG	TACTCCCCATTTTCCTCACG	59.9	696	INTRON	Ca_23537	--	NAC
Ca1_P3_37798578	Ca1	37798578	CAAAAAAA/CAAAAAA	GGCATGTGCATCCTTGTAGA	GGAATCACATCCTGGTGCTT	59.7	644	INTRON	Ca_23537	--	NAC
Ca1_P3_37798793	Ca1	37798793	TATTA/TATTATTAATATCTGATTA	TGTGGAACTTGATTTTGGTGA	TATCGCCTCATGGAATCACA	59.0	583	INTRON	Ca_23537	--	NAC
Ca1_P3_37800107	Ca1	37800107	TA/TATTTAAA	TGGGTCCTAAACCGCAATAC	CAAAAATGATGCAACCATCG	59.8	371	INTERGENIC	--	--	--
Ca1_P3_37809250	Ca1	37809250	ACTTTTCCTTTTCCT/ACTTTTCCT	TGCCTTTGGGGATTTTATCA	TTTTGCATTAAAAATGGATGAA	60.3	579	INTERGENIC	--	--	--

INDEL marker IDs	Chromosomes/ unanchored scaffolds	Physical positions (bp)	InDels (*Kabuli* reference genome-CDC Frontier/PII)	Forward primers (5'-3')	Reverse primers (5'-3')	Anneling temperature (0C)	Expected amplified product size (bp)	Structural annotation		Functional annotation	
								Sequence components of *kabuli* genome	*Kabuli* gene accession IDs	NCBI-KOG	TFs
Ca1_P3_37810493	Ca1	37810493	ATTTTTTTTT/ATTTTTTTT	TTTTCCCTTCCATCCAACTTT	CGGCAAAAGACTCAAGAACC	59.8	620	INTERGENIC	--	--	--
Ca1_P3_3783626	Ca1	3783626	TATTTGCATTTG/TATTTG	TTCACAATTACCACAAAGATCG	GCGACTATCCAGGAAATCCA	57.7	433	INTERGENIC	--	--	--
Ca1_P3_37836904	Ca1	37836904	CAGTTAGTTAGTTAGTTAGTTAGTTA/CAGTTAGTTAGTTAGTTAGTTAGTTAGTTAGTTA	ATGGATGTAGCGATTACGCC	CCAATACTCCACCTGCGATT	60.0	442	INTERGENIC	--	--	--
Ca1_P3_37837915	Ca1	37837915	GTTTTTTTTT/GTTTTTTTT	TGGAGAAAATAAAAAGAAGAGTTCA	CTCAAATTCTGTTTGGGGGA	57.8	918	INTERGENIC	--	--	--
Ca1_P3_37852569	Ca1	37852569	TAAAAAAA/TAAAAAA	TGTGGTATGTGCGGTTCCTA	TTTGATGGGTTCATGTTCCA	60.0	814	INTERGENIC	--	--	--
Ca1_P3_37852817	Ca1	37852817	AAGCA/AAGCAGCA	CACGTAGCAAAAATAATTGGCA	TCATCCTGCCGGTTAGTACC	60.0	765	INTERGENIC	--	--	--
Ca1_P3_37861907	Ca1	37861907	TGG/TGGG	TTAGGGATTTGACCGTCGAT	GATCGAAGGCGAAGTAATGC	59.4	953	INTERGENIC	--	--	--
Ca1_P3_37869799	Ca1	37869799	AC/ACC	TTCCTGATTCCAGATTTGCC	AAAGATGGGGTCAGTGCATC	60.0	371	INTRON	Ca_23539	KT	--
Ca1_P3_37875883	Ca1	37875883	TTAT/TT	GCTGGGACTTATTTCCGTGA	TGCCAGGTGACAAAATCTCA	60.1	201	INTRON	Ca_23539	KT	--
Ca1_P3_37887479	Ca1	37887479	GTTTTTTTTTT/GTTTTTTTTTTTT	GATTGCAAAATCCGGAAAAA	TGGGCACTATTGTTTGGACA	59.9	530	INTRON	Ca_23539	KT	--

INDEL marker IDs	Chromosomes/ unanchored scaffolds	Physical positions (bp)	InDels (*Kabuli* reference genome-CDC Frontier/PII)	Forward primers (5'-3')	Reverse primers (5'-3')	Anneling temperature (0C)	Expected amplified product size (bp)	Structural annotation		Functional annotation	
								Sequence components of *kabuli* genome	*Kabuli* gene accession IDs	NCBI-KOG	TFs
Ca1_P3_37913622	Ca1	37913622	AATATATATATATA/AATATATATATATATATA	ACGTTCGCCACCTATCTCAT	CCTTATGCGGTAATCCCTGA	59.6	563	INTERGENIC	--	--	--
Ca1_P3_37913861	Ca1	37913861	GTATATATATATAT/GTATATATATATATATAT	GGAGTGAATTTGTGTTGCTCC	TGAGGGTAACAGGGAAGGTG	59.6	611	INTERGENIC	--	--	--
Ca1_P3_37913991	Ca1	37913991	CCAATT/CCAATTCCAATTTCAATT	TCAGGGATTACCGCATAAGG	TGAGGGTAACAGGGAAGGTG	59.9	365	INTERGENIC	--	--	--
Ca1_P3_37917967	Ca1	37917967	ATATGACAGTTA/ATA	TCCACAAGTTATTCGACGAAAC	TTCCATTGATCTACGTTAATGTTTG	59.1	558	INTERGENIC	--	--	--
Ca1_P3_37927331	Ca1	37927331	TAAA/TAAATTGTATCAAA	ATTTTGATCGCGGAAGTTTG	TCAATCAACCAGATTTTGCATT	60.1	553	INTERGENIC	--	--	--
Ca1_P3_37927484	Ca1	37927484	A/AT	TCAAGGTACCGATAATCGCA	CTCAATCAACCAGATTTTGCAT	59.1	387	INTERGENIC	--	--	--
Ca1_P3_37927862	Ca1	37927862	G/GTA	TGCAAAATCTGGTTGATTGAG	TCCCAGATCAAAATTGGGTT	58.8	745	INTERGENIC	--	--	--
Ca1_P3_37928765	Ca1	37928765	TAAA/TTTTGATGAAA	CCGTGCAACAATGTCAATTC	TGGTTGCATGTTTGTAACTTGA	60.0	176	INTERGENIC	--	--	--
Ca1_P3_37929646	Ca1	37929646	TAAAAAAAA/TAAAAAAAAAA	TGTGACGGTTAATATGGTGGTC	TTGACTTGTTTTGCGGATTG	59.6	817	INTERGENIC	--	--	--
Ca1_P3_37929753	Ca1	37929753	ATTTTTTTTT/ATTTTTTTTTTT	TGTGACGGTTAATATGGTGGTC	TTGACTTGTTTTGCGGATTG	59.6	817	INTERGENIC	--	--	--

INDEL marker IDs	Chromosomes/ unanchored scaffolds	Physical positions (bp)	InDels (*Kabuli* reference genome-CDC Frontier/PII)	Forward primers (5'-3')	Reverse primers (5'-3')	Anneling temperature (0C)	Expected amplified product size (bp)	Structural annotation		Functional annotation	
								Sequence components of *kabuli* genome	*Kabuli* gene accession IDs	NCBI-KOG	TFs
Ca1_P3_37954014	Ca1	37954014	AATATATATATATATATA/AATATATATATATATATATA	TCTTTTCTCCGATCCCATTG	TCGCGTAGTGGTAGCCTTCT	60.0	415	INTERGENIC	--	--	--
Ca1_P3_37954337	Ca1	37954337	GTTTTT/GTTT	TCTTTTCTCCGATCCCATTG	CGACTCCCGAACTCAAAATC	60.0	868	INTERGENIC	--	--	--
Ca1_P3_37993308	Ca1	37993308	CAAAAAAAAAAAAAAA/CAAAAAAAAA	TCATCGCAGGTGTACCATGT	GGATTGGTAAAGGGCTCACA	60.0	482	INTERGENIC	--	--	--
Ca1_P3_37993665	Ca1	37993665	CAAAAAA/CAAAAA	GTGAGCCCTTTACCAATCCA	CAATTCCCACAGGTACGCTT	59.9	654	INTERGENIC	--	--	--
Ca1_P3_38024874	Ca1	38024874	TATGAATGAAT/TATGAATGAATGAAT	GCAAGCTCAGTTCCAGTTCC	TGCATCAATTATGGCTCCAA	60.0	655	INTRON	Ca_27443	S	--
Ca1_P3_38070666	Ca1	38070666	T/TA	AACCATTGGGGGAGAATAGAA	TTGTTAGAACCATTGGGGGA	59.7	362	INTERGENIC	--	--	--
Ca1_P3_38070895	Ca1	38070895	ATGTGTGT/ATGTGT	CTCCCCCAATGGTTCTAACA	CACTGGAATCCGTTTCTTTTG	59.8	587	INTERGENIC	--	--	--
Ca1_P3_38071534	Ca1	38071534	TACACACAC/TACACAC	CTCCTCCTCATTCACCCAAC	ACACACCCACTCTCCTTTGC	59.5	189	INTERGENIC	--	--	--
Ca1_P3_38105826	Ca1	38105826	AAGACGAAATTTCT/A	GCTCAAATGAGTAGTGAATGCC	GGGTGTTGCAATTTTCTCCT	58.9	143	INTERGENIC	--	--	--
Ca1_P3_38109587	Ca1	38109587	ATTT/ATTTT	ATTATGAAATTCCCTTGGCG	TCTCTCACCCCCTACCTCAA	58.9	597	INTERGENIC	--	--	--

INDEL marker IDs	Chromosomes/ unanchored scaffolds	Physical positions (bp)	InDels (*Kabuli* reference genome-CDC Frontier/PII)	Forward primers (5'-3')	Reverse primers (5'-3')	Anneling temperature (0C)	Expected amplified product size (bp)	Structural annotation		Functional annotation	
								Sequence components of *kabuli* genome	*Kabuli* gene accession IDs	NCBI-KOG	TFs
Ca1_P3_38130303	Ca1	38130303	C/CG	AAAGGGGGAGAACCATCTGT	TGCATTTGCTTGACTTGGAG	59.8	680	INTERGENIC	--	--	--
Ca1_P3_38131605	Ca1	38131605	C/CT	CGTAACTCGTGTTGGGTGAA	GAAGCAAGAGGCAACTCCAC	59.6	379	INTERGENIC	--	--	--
Ca1_P3_38142065	Ca1	38142065	A/AGT	GCTCTAACAGCGTAGCAAAAA	TATGAGGTGGCAGCAAGAGA	57.6	321	INTERGENIC	--	--	--
Ca1_P3_38147657	Ca1	38147657	TC/TCC	TGTTGACACATCACATCCTTAACA	TTTCATGTCTCATTTCCCCA	60.3	858	INTERGENIC	--	--	--
Ca1_P3_38148096	Ca1	38148096	CAAAAAAAAAA/CAAAAAAAA	TGGGGAAATGAGACATGAAA	ACGTTGCGCAGGTGTAATTT	58.9	567	INTERGENIC	--	--	--
Ca1_P3_38169087	Ca1	38169087	TTATATATAT/TTATATATATATATAT	AAAATCTTTGTGAAATCGGCA	TGCTGCAACTTCACGCTACT	59.6	583	INTERGENIC	--	--	--
Ca1_P3_38185371	Ca1	38185371	TAAA/TAA	GTTCCATGAACGCCTCAGTT	GAGCATGGAAGGCATCTAGC	60.1	407	INTERGENIC	--	--	--
Ca1_P3_38187356	Ca1	38187356	AT/ATT	ATCACTCCACCGACTTCCAC	GAGTAGGGCTGTGCATGGTT	60.0	602	INTERGENIC	--	--	--
Ca1_P3_38197697	Ca1	38197697	TAAAAAAA/TAAAAAA	TTAGCGACCAATATACCGGC	AACCAAATGTTTCGGGATCA	60.0	289	DRR	Ca_13974	L	C2H2
Ca1_P3_38197951	Ca1	38197951	GATATATAT/GATATATATAT	TTAGCGACCAATATACCGGC	ATTATGCTGCAATCCCTTCG	60.0	857	DRR	Ca_13974	L	C2H2

INDEL marker IDs	Chromosomes/ unanchored scaffolds	Physical positions (bp)	InDels (*Kabuli* reference genome-CDC Frontier/PII)	Forward primers (5'-3')	Reverse primers (5'-3')	Anneling temperature (0C)	Expected amplified product size (bp)	Structural annotation		Functional annotation	
								Sequence components of *kabuli* genome	*Kabuli* gene accession IDs	NCBI-KOG	TFs
Ca1_P3_38203782	Ca1	38203782	CTTT/CTT	CTACGTTGAATGGCATGTGG	GTCTTGAGACGTCGGGTGAT	60.0	528	INTERGENIC	--	--	--
Ca1_P3_38241608	Ca1	38241608	TGTCACTA/T	GTTGGGAGACTTGTGCCCTA	ACCGAGTGTCGAACTCAAGG	60.1	763	INTERGENIC	--	--	--
Ca1_P3_38244609	Ca1	38244609	GAAAAAAAAT/GAAAAAAAATTTGAAAAAAAAAT	GCGGAAATATTTTTGCAGGA	AACCAATTGCATCACCCTTC	60.0	523	INTERGENIC	--	--	--
Ca1_P3_38244809	Ca1	38244809	TAAAGAA/TAA	GCTACACAGGACTTCTGGGC	GATCTTGGTCTTGGAAACGC	59.9	492	INTERGENIC	--	--	--
Ca1_P3_38245861	Ca1	38245861	AAATAATAATAATAATAA/AAATAATAATAATAATAATAA	GAATAGCTGGAACTGGCATCA	TGCCACATCACAACCCTAAA	60.2	816	INTERGENIC	--	--	--
Ca1_P3_38278656	Ca1	38278656	C/CG	TCCAGCATTGCCTTTTCTTT	GCATGGGTGAGAATGGAGTT	59.8	687	DRR	Ca_13968	O	--
Ca1_P3_38282680	Ca1	38282680	G/GATAAAATCAATT	CTACATTAGCGTCCCGATCC	GCTCAATGCAAACCAAAACA	59.6	312	INTERGENIC	--	--	--
Ca1_P3_38283096	Ca1	38283096	ATTTTTTTTT/ATTTTTTTT	ATTCGTAGCAACGCATCCTT	GTGGGCCAAAAGCTACACAT	59.7	482	INTERGENIC	--	--	--
Ca1_P3_38284193	Ca1	38284193	TC/T	GCCAACACATGGTGAAAGTG	AACAAAGTTTGCATTTGGGC	60.0	939	INTERGENIC	--	--	--
Ca1_P3_38286027	Ca1	38286027	TAAAAAAAAA/TAAAAAAAAAAAAAA	AACCAAATTGCTCCCATGAA	TTGAGCATCACCAAATTCCA	60.3	490	INTERGENIC	--	--	--

INDEL marker IDs	Chromosomes/ unanchored scaffolds	Physical positions (bp)	InDels (*Kabuli* reference genome-CDC Frontier/PII)	Forward primers (5'-3')	Reverse primers (5'-3')	Anneling temperature (0C)	Expected amplified product size (bp)	Structural annotation		Functional annotation	
								Sequence components of *kabuli* genome	*Kabuli* gene accession IDs	NCBI-KOG	TFs
Ca1_P3_38303577	Ca1	38303577	TT/TTGT	GGTCGTGCTGCAAGGATTAT	GTGTGGTACTCATGCGATGC	60.1	873	INTERGENIC	--	--	--
Ca1_P3_38335041	Ca1	38335041	GC/G	TCCACACCAATGGATGCTAA	TCGCGAACTACGTGACAATC	59.9	459	INTERGENIC	--	--	--
Ca1_P3_38338783	Ca1	38338783	A/AG	GGTTTTTCGAATTGGGGATT	TGGTTCATGTTGGTCACATTC	60.0	756	DRR	Ca_13967	--	--
Ca1_P3_38339024	Ca1	38339024	TTTTC/TTTTCTTTC	TTGTATCATGTCCAATGGTCG	CCGAACGCTATCCAACTCTC	59.3	957	DRR	Ca_13967	--	--
Ca1_P3_38340566	Ca1	38340566	AT/A	ATTTTAATGAATGCCGACGC	TTCAAATCCTCGGCCATAAG	59.9	913	INTERGENIC	--	--	--
Ca1_P3_38353011	Ca1	38353011	ATT/ATTT	GTGGTGATTGGAGTGTGTCG	CAAATTCCCCTCCATGTTTG	60.0	710	INTERGENIC	--	--	--
Ca1_P3_38393649	Ca1	38393649	GTTTTTTTTT/GTTTTTTTT	GTTGAGGCAGTAGCCTTTGC	CGTTCTTCCTTGCCTTTGTC	60.0	280	INTERGENIC	--	--	--
Ca1_P3_38472540	Ca1	38472540	TAAAAAAAA/TAAAAAAAAA	TTCATGCTGTTTCTTGGCAT	TGATGAATTCAACGCCGATA	59.3	570	INTERGENIC	--	--	--
Ca1_P3_38476810	Ca1	38476810	TTAT/TTATAT	TTGCAAGTACGTTCTCCAAAA	GTGTTACACGCCCTTCATCC	58.5	280	INTERGENIC	--	--	--
Ca1_P3_38494341	Ca1	38494341	TAAGAAGAAGAAGAAGAAGAAGAA/TAAGAAGAAGAAGAAGAAGAA	ATCCGTCTTCAAGTTGTGGC	ACGCCCCTTGAGTGAGATTA	60.1	462	INTERGENIC	--	--	--

INDEL marker IDs	Chromosomes/ unanchored scaffolds	Physical positions (bp)	InDels (*Kabuli* reference genome-CDC Frontier/PII)	Forward primers (5'-3')	Reverse primers (5'-3')	Anneling temperature (0C)	Expected amplified product size (bp)	Structural annotation		Functional annotation	
								Sequence components of *kabuli* genome	*Kabuli* gene accession IDs	NCBI-KOG	TFs
Ca1_P3_38495552	Ca1	38495552	AAT/AATAATAATTAT	AACACCTTCCTGATCCTCCC	TGGCGAACTTTTAAGAATGGA	60.3	659	INTERGENIC	--	--	--
Ca1_P3_385001	Ca1	385001	G/GT	TAGTATGCTTCGCTGCCCTT	GTGCGCTTCACAAGAAATCA	60.0	471	INTERGENIC	--	--	--
Ca1_P3_38510213	Ca1	38510213	TTTTATTTAT/TTTTATTTATTTAT	TCAGGCTTGAATGATTTCCC	GCATGGGCTGGTTTTACAAT	60.0	205	DRR	Ca_13961	--	bHLH
Ca1_P3_38528019	Ca1	38528019	ATTTTTTTTTT/ATTTTTTTTTTTT	TGGGGATCCTCACCTACAAG	ATTTGACTCGTGTGCGACTG	59.9	796	INTERGENIC	--	--	--
Ca1_P3_38553789	Ca1	38553789	TAAAAAAAAAA/TAAAAAAAAA	GAAACCACCGAAAAACTCCA	TGCATGTTAGTAGGCGTTGG	59.9	720	DRR	Ca_13958	--	--
Ca1_P3_38555062	Ca1	38555062	ATTTCTTTT/ATTTT	AACCAATTTGTAAAGTGCGGA	TGTTGCGCTCATTCTTTTTG	59.5	246	DRR	Ca_13958	--	--
Ca1_P3_38559279	Ca1	38559279	CGTT/CGTTGTT	CCTAACCTGTGTCCTTCCCA	GCATCGCTGGGTAATTTGTT	60.0	826	DRR	Ca_13957	--	--
Ca1_P3_38559526	Ca1	38559526	ATTT/ATTTT	GTGCAAAACAGACGTTCAGC	GCATCGCTGGGTAATTTGTT	59.5	363	DRR	Ca_13957	--	--
Ca1_P3_38565326	Ca1	38565326	A/AT	TCAATGCCAAATGTTTTTATCG	AAGTGCATGCGTGTCTTTTG	59.8	735	INTERGENIC	--	--	--
Ca1_P3_38571843	Ca1	38571843	CAAA/CAAAA	CACCTGACACTGCAAACCAG	TGGTCAAAGATTTTCATCAATCA	60.3	617	INTERGENIC	--	--	--

INDEL marker IDs	Chromosomes/ unanchored scaffolds	Physical positions (bp)	InDels (*Kabuli* reference genome-CDC Frontier/PII)	Forward primers (5'-3')	Reverse primers (5'-3')	Anneling temperature (0C)	Expected amplified product size (bp)	Structural annotation		Functional annotation	
								Sequence components of *kabuli* genome	*Kabuli* gene accession IDs	NCBI-KOG	TFs
Ca1_P3_38573120	Ca1	38573120	A/AG	TTTCATTTGGTGGTGCTTCA	ACCACTTTTGTGACTTGGGC	60.1	839	INTERGENIC	--	--	--
Ca1_P3_38596761	Ca1	38596761	CTGTGTG/CTGTG	AACGAGATAGCTGCACGGAT	TGCTCATGACCTTTTTGTGG	59.9	729	INTERGENIC	--	--	--
Ca1_P3_38597715	Ca1	38597715	ATAATTTTAATTTTAATTT/ATAATTTTAATTT	CATTTGCCTCCCTTGTTGTT	TGTAGTTGCTGCAAACGAGG	60.0	551	DRR	Ca_13956	--	--
Ca1_P3_38597934	Ca1	38597934	CAAAAA/CAAAAAA	CATTTGCCTCCCTTGTTGTT	TGTAGTTGCTGCAAACGAGG	60.0	551	DRR	Ca_13956	--	--
Ca1_P3_38613531	Ca1	38613531	GAAAAAA/GAAAAA	CCTTCCTTCATGGTGATTTGA	TCCCTGATCACACTTCCTCTG	59.9	610	INTERGENIC	--	--	--
Ca1_P3_38614804	Ca1	38614804	TTAAGATATAGCAGTAA/TTAA	TGATGGATTTGGGTTCCATT	GGATGATGCATTTGATGCTTT	60.0	756	INTERGENIC	--	--	--
Ca1_P3_38632783	Ca1	38632783	T/TG	TAAATTCGAAATTCGGCCAG	CCACTCCTTGGAGGAATTGA	60.0	250	INTERGENIC	--	--	--
Ca1_P3_38636457	Ca1	38636457	AAT/AATAT	AATATCCCAACAGCCTCACG	TTGATTGAAACATTGATTTCCG	60.0	710	INTERGENIC	--	--	--
Ca1_P3_38656733	Ca1	38656733	AT/AAATCATTATT	AATTGGGTGTTGGTGGTTTC	TCACTGATGGGATTGAACTGTC	59.6	708	INTERGENIC	--	--	--
Ca1_P3_38657360	Ca1	38657360	TAAAAAAA/TAAAAAA	CCATCAGTGATAACTTGTCCCA	CACAGGACAAGTGCAATCTCA	59.9	451	INTERGENIC	--	--	--

INDEL marker IDs	Chromosomes/ unanchored scaffolds	Physical positions (bp)	InDels (*Kabuli* reference genome-CDC Frontier/PII)	Forward primers (5'-3')	Reverse primers (5'-3')	Anneling temperature (0C)	Expected amplified product size (bp)	Structural annotation		Functional annotation	
								Sequence components of *kabuli* genome	*Kabuli* gene accession IDs	NCBI-KOG	TFs
Ca1_P3_38671088	Ca1	38671088	CTTGTGATT/CTTGTGATTATTTTGTTGTGATT	TGTTTGGGTCCTTGATGGTT	TTAGAGCGGAGCTTCTTCCA	60.2	825	INTERGENIC	--	--	--
Ca1_P3_38672855	Ca1	38672855	CTG/C	AACCCTCACTCTCGCTCAAA	GCCTTAGTTTGTTGGGGACA	60.0	570	INTERGENIC	--	--	--
Ca1_P3_38707461	Ca1	38707461	CAAAAAAAAAAA/CAAAAAAAAAAAAAAA	AGTCGGATCTAGTCCCCCAT	TTTTCATGGCAACCGTTGTA	59.8	508	INTERGENIC	--	--	--
Ca1_P3_38735998	Ca1	38735998	TC/T	TTCTCCCCTCTCAATCATGC	TGGATTGACATAAATTGAAAGAAA	60.2	573	INTERGENIC	--	--	--
Ca1_P3_38744020	Ca1	38744020	ATTTTTTTTT/ATTTTTTTT	CGGTTGTCATGAAAATGGTTC	TGTGCTTGTCCCACACACTT	60.2	658	INTERGENIC	--	--	--
Ca1_P3_38751694	Ca1	38751694	CCTTCTTCTT/CCTTCTT	TCAAATCATGAGCCCATTTG	ATACCATTTGTGTGGCCGTT	59.5	437	INTERGENIC	--	--	--
Ca1_P3_38759350	Ca1	38759350	CAAAAAAAAAAA/CAAAAAAAAAA	TGTACTCCCCCAGACGTAGG	TCAAATTTCGGGTTTATGGC	60.0	575	INTERGENIC	--	--	--
Ca1_P3_38759673	Ca1	38759673	TTTTATTT/TTTT	CAATTGCCATGAAAATGGTTT	TGTGCTTGTCCCACACACTT	59.7	397	INTERGENIC	--	--	--
Ca1_P3_38771127	Ca1	38771127	GTTATTATTATTAT/GTTATTATTATTATTATTAT	CCTTGTGGGAAGAAGCTTGA	ATATTGAGCGTCGTAACCCG	60.4	657	INTERGENIC	--	--	--
Ca1_P3_38796233	Ca1	38796233	GAAA/GAAAA	AACCATTGTGGGGTCGATTA	CGGTGATCAAACTTATGGGG	60.1	628	INTERGENIC	--	--	--

INDEL marker IDs	Chromosomes/ unanchored scaffolds	Physical positions (bp)	InDels (*Kabuli* reference genome-CDC Frontier/PII)	Forward primers (5'-3')	Reverse primers (5'-3')	Anneling temperature (0C)	Expected amplified product size (bp)	Structural annotation		Functional annotation	
								Sequence components of *kabuli* genome	*Kabuli* gene accession IDs	NCBI-KOG	TFs
Ca1_P3_38798126	Ca1	38798126	CAAAA/CAAAAA	AGGACTGGACGTAGCCTTCA	GTGTCCCAGAGCTCGTTAGC	59.9	626	INTERGENIC	--	--	--
Ca1_P3_388495	Ca1	388495	ACTCTCTCTCTCTCTCTCTCT/ACTCTCTCTCTCTCTCTCT	GCCTTGCTGTTCTTGACCTC	TAGCTGTGCATGCTCAAAGG	60.0	618	DRR	Ca_00040	--	TCP
Ca1_P3_38855200	Ca1	38855200	TGG/TG	TTAGATGGGGCACAGGAATC	TTCCCTTGTCATTGGTCCTC	59.9	526	INTERGENIC	--	--	--
Ca1_P3_38875335	Ca1	38875335	GAAAA/GAAAAA	ACCGAATCCAGTTGAACGAC	GCGGTTCTCTTCGCTTATGT	60.0	412	INTERGENIC	--	--	--
Ca1_P3_38875592	Ca1	38875592	TCA/T	ACCGAATCCAGTTGAACGAC	GGTCAATCCATCCATAGCCA	60.0	516	INTERGENIC	--	--	--
Ca1_P3_38882967	Ca1	38882967	GTTAGGA/GTTAGGACTTAGGA	ATTAGGAACCCTGGCATGTG	GGCAGCATTTGGTGAACTTT	59.8	464	DRR	Ca_13949	G	--
Ca1_P3_38885860	Ca1	38885860	AC/A	CCAGAAACATTTGGGCAGTT	GACCGACCGTCTGTTGTTCT	60.0	790	INTERGENIC	--	--	--
Ca1_P3_38886569	Ca1	38886569	TAAAAAAAA/TAAAAAAAAAAA	GCAACACTAGAACAACAGACGG	ATTTATCCATCCATGTCGCC	59.8	786	INTERGENIC	--	--	--
Ca1_P3_38914248	Ca1	38914248	CCTTCAACTTCA/CCTTCA	ATGGATGGCGTGGAGTAAAT	TCGTAGGGAAATTGGATTGC	59.3	430	INTERGENIC	--	--	--
Ca1_P3_38922345	Ca1	38922345	CACA/CACAACA	CAAAATGGCTCCAACTCCAT	AACACAAGAAATGCCGGTTC	59.9	754	INTERGENIC	--	--	--

INDEL marker IDs	Chromosomes/ unanchored scaffolds	Physical positions (bp)	InDels (*Kabuli* reference genome-CDC Frontier/PII)	Forward primers (5'-3')	Reverse primers (5'-3')	Anneling temperature (0C)	Expected amplified product size (bp)	Structural annotation		Functional annotation	
								Sequence components of *kabuli* genome	*Kabuli* gene accession IDs	NCBI-KOG	TFs
Ca1_P3_38923411	Ca1	38923411	TAT/TATCTTGAT	GCACATTCGAACCTTTCCAT	TTAGGGCACATGCATTTCAA	59.9	642	INTERGENIC	--	--	--
Ca1_P3_38930606	Ca1	38930606	GAAA/GAAAA	TAAAGGACCAAGTCGGGAAG	TTGTCGAGCACGATTTATGC	59.2	491	INTERGENIC	--	--	--
Ca1_P3_38931461	Ca1	38931461	CTTTTTTTT/CTTTTTTTTTT	TGAAGATTGGGAGGATGGAG	TTCATGGATTGGTGAGGTGA	60.0	423	INTERGENIC	--	--	--
Ca1_P3_38933493	Ca1	38933493	ACCC/ACCCC	TGTCGCAACCTAACATTTCG	ATACGCATAATCGACTCCCG	59.7	167	INTERGENIC	--	--	--
Ca1_P3_38933903	Ca1	38933903	CCACA/CCACACA	TGTCGCAACCTAACATTTCG	TCTCGTTTTTAAATAAACCCAAA	59.7	657	INTERGENIC	--	--	--
Ca1_P3_38943612	Ca1	38943612	ATTTAAA/ATTTAAATGTTCTCTTTAAA	GCTCTTGTGAGTTGGTGTTGC	TGGCCTCTTCGGTACTGATT	60.9	571	INTERGENIC	--	--	--
Ca1_P3_38943899	Ca1	38943899	TAAAAAAA/TAAAAAA	AATCAGTACCGAAGAGGCCA	TGATGGATCCCCTTCATAAAT	59.7	451	INTERGENIC	--	--	--
Ca1_P3_38951140	Ca1	38951140	CAA/CAAAA	AACTTTCCCAAGGCTCCAGT	GTTGTTGCTTCTGCTCCTCC	60.1	680	INTERGENIC	--	--	--
Ca1_P3_39019566	Ca1	39019566	ATA/ATAAATTA	CTCCTCCTCCCTCCATTGAT	ACGAACACCCTTCGTCATTC	60.4	684	INTRON	Ca_13946	--	--
Ca1_P3_39019605	Ca1	39019605	TA/T	CTCCTCCTCCCTCCATTGAT	ACGAACACCCTTCGTCATTC	60.4	684	INTRON	Ca_13946	--	--

INDEL marker IDs	Chromosomes/ unanchored scaffolds	Physical positions (bp)	InDels (*Kabuli* reference genome-CDC Frontier/PII)	Forward primers (5'-3')	Reverse primers (5'-3')	Anneling temperature (0C)	Expected amplified product size (bp)	Structural annotation		Functional annotation	
								Sequence components of *kabuli* genome	*Kabuli* gene accession IDs	NCBI-KOG	TFs
Ca1_P3_39025647	Ca1	39025647	AACTTACTTA/AACTTA	TGTGCACACTTAACATCAGTCAA	GCAAGCTTTTTCAGGTGTCC	59.3	627	INTRON	Ca_13946	--	--
Ca1_P3_39028216	Ca1	39028216	TAAAAAAAAAA/TAAAAAAAAAAAA	TCATGGAAGGAGCAATCACA	TAAGGTTTAGGATGCCCCAA	60.2	764	INTRON	Ca_13946	--	--
Ca1_P3_39041564	Ca1	39041564	TAAA/TAA	GAAAAAGCGCTGTTGTAGGG	TGGCCGCACAATATGTAGAG	59.9	435	INTRON	Ca_13946	--	--
Ca1_P3_39053033	Ca1	39053033	ATTTTTTTTT/ATTTTTTTTTT	TGAGTGCAGAAGCTCCAAGA	TTTACCCAGAATGTCCCTGC	59.9	644	INTRON	Ca_13946	--	--
Ca1_P3_39056415	Ca1	39056415	T/TG	CCTTTTACCGCGCTTATTTG	TCCCAAGGAACGACCTCTTA	59.7	781	INTRON	Ca_13946	--	--
Ca1_P3_39067582	Ca1	39067582	GAAAAAAA/GAAAAAA	ACCAAAGGATGGCAATGAAG	TAGCTTGGAAAGCTGCCATT	59.9	422	INTRON	Ca_13946	--	--
Ca1_P3_39070514	Ca1	39070514	GAAAAAAAAAAAA/GAAAAAAAAA	TCACTGACAGGGCTGGATAA	GAGCCTACAAAATCAAACTCCG	59.2	884	INTRON	Ca_13946	--	--
Ca1_P3_39070800	Ca1	39070800	T/TC	TGGAAAATGCAATCCTCAGA	AATTGATAACCCACCGCAAG	59.2	609	INTRON	Ca_13946	--	--
Ca1_P3_39073326	Ca1	39073326	GTT/GTTT	GAAAACATGCCCATGGATCT	GCATTAAGGTGCGATGAATG	59.8	578	DRR	Ca_13946	--	--
Ca1_P3_39073753	Ca1	39073753	TCAGAAA/TA	CATTCATCGCACCTTAATGC	TTGAAGAAGCATCCCTCTGA	59.1	309	DRR	Ca_13946	--	--

INDEL marker IDs	Chromosomes/ unanchored scaffolds	Physical positions (bp)	InDels (*Kabuli* reference genome-CDC Frontier/PII)	Forward primers (5'-3')	Reverse primers (5'-3')	Anneling temperature (0C)	Expected amplified product size (bp)	Structural annotation		Functional annotation	
								Sequence components of *kabuli* genome	*Kabuli* gene accession IDs	NCBI-KOG	TFs
Ca1_P3_39075214	Ca1	39075214	T/TA	TGCTCCTCTTTCCTGGATTG	TTCTGCACACGGGACACTTA	60.3	489	INTERGENIC	--	--	--
Ca1_P3_39077300	Ca1	39077300	AA/AACA	CCATCTCTTGGATTCTTGACG	AGGTTTTGGTTGTTTTTGCAT	59.7	532	INTERGENIC	--	--	--
Ca1_P3_390880	Ca1	390880	TGAGA/TGAGAGAGAGAATGAGAGAGA	TTTGTGGGGAAGCCTACTTG	CCCACGAGTGGTCTTCTGTT	60.1	475	INTERGENIC	--	--	--
Ca1_P3_390995	Ca1	390995	ACTCTCTCTCTCTCTCTCT/ACTCTCTCT	TGCTTGCTCGGTTATGTGAG	GAGTGGGCAAGGAAATTTGA	60.0	522	INTERGENIC	--	--	--
Ca1_P3_39108521	Ca1	39108521	CAAAA/CAAA	GCGTTCGGACCAGATAAAAG	GAAAACAAAAAGCGAGGCAC	59.7	685	INTERGENIC	--	--	--
Ca1_P3_39112897	Ca1	39112897	ACCC/ACCCC	CATCGAAAAGCCACGAAAAG	CTTTCGGGTCAAGTAGCAGC	60.7	711	DRR	Ca_13944	O	--
Ca1_P3_39135722	Ca1	39135722	ACAAAATAAATTTTTTCAAAATAAATTT/ ACAAAATAAATTTTTTCAAAATAAATTTTTTCAAAATAAATTT	ACGCGTTCTTTCGACAAGTT	GCATATTGTGCATTGTGGGA	59.9	611	INTERGENIC	--	--	--
Ca1_P3_39141870	Ca1	39141870	AATATAATATATAATATATAATAT/AATATAATATATAATAT	CAGCAAAACAACGCACATTC	AACAAGTGGGTGACCTGCAT	60.3	644	INTERGENIC	--	--	--
Ca1_P3_39147028	Ca1	39147028	AT/ATT	AAAATGATCAGGGAAAATCATGT	ACAACTTCTCATGCTCGCAA	58.7	339	DRR	Ca_13942	--	ARF
Ca1_P3_39166873	Ca1	39166873	GTTTTATTTTATTTTAT/GTTTTATTTTAT	ATAATTGCGTTAGCGATGCC	GGTGTCATCGACCACACATC	60.1	428	INTRON	Ca_13941	--	Trihelix

INDEL marker IDs	Chromosomes/ unanchored scaffolds	Physical positions (bp)	InDels (*Kabuli* reference genome-CDC Frontier/PII)	Forward primers (5'-3')	Reverse primers (5'-3')	Anneling temperature (0C)	Expected amplified product size (bp)	Structural annotation		Functional annotation	
								Sequence components of *kabuli* genome	*Kabuli* gene accession IDs	NCBI-KOG	TFs
Ca1_P3_39180438	Ca1	39180438	AGTGTTATGTGTTA/AGTGTTA	GTGGTAATCGCGTTGAAGGT	GAGTCCCTGACTTAAGGCCC	60.0	319	INTERGENIC	--	--	--
Ca1_P3_39251292	Ca1	39251292	CAAA/CAAAA	GATCACCTTCACATCCCACC	TTGTCGGCAGGGGTAGTAAC	60.2	651	INTERGENIC	--	--	--
Ca1_P3_39261977	Ca1	39261977	A/AT	GGTTTGCTGTCGAGCCTTAG	AAACACAAGTCCAATCAAAAGG	60.0	421	INTERGENIC	--	--	--
Ca1_P3_3929921	Ca1	3929921	AT/ATTATTATTATTATTTT	TTGTCTTTTGTGGTAGCCCC	GGATCATGTCCGAGCATTTT	60.0	412	DRR	Ca_00475	LK	Nin-like
Ca1_P3_39319575	Ca1	39319575	TTTATTTATTTGCTTATTTATTT/TTTATTTATTTGCTTATTTATTTGCTTATTTATTT	TTTGCCTCTTCTCCTCCAGA	CCACCAAATACAAATAAATTACTTACG	60.1	970	INTERGENIC	--	--	--
Ca1_P3_39391530	Ca1	39391530	AATA/AATATA	GCATGACATGGAATCGTCTC	ATGGGGTAGCTTTGGCTTCT	59.0	413	INTERGENIC	--	--	--
Ca1_P3_39402730	Ca1	39402730	TAAAAAAAAAAA/TAAAAAAAAAAAAAA	TCCAAACATTATTTCTTGTTTTCG	TTTTGCAACCATTGATTAGAGA	59.5	325	INTERGENIC	--	--	--
Ca1_P3_39423396	Ca1	39423396	TAA/TA	GGAAAGTTGAGTTGGAAGATGTTT	CCCCTCTCTCCCAAATTCTC	59.9	632	INTERGENIC	--	--	--
Ca1_P3_39423621	Ca1	39423621	TAAAAAA/TAAAAAAA	CTCTGGTAAGCGGATCTCGT	CCCCTCTCTCCCAAATTCTC	59.5	311	INTERGENIC	--	--	--
Ca1_P3_39430506	Ca1	39430506	ATT/ATTTT	TGCATCTCCCTCAAGCTTTT	TGGAAAATTTTGGGGTTCAA	60.0	976	INTERGENIC	--	--	--

INDEL marker IDs	Chromosomes/ unanchored scaffolds	Physical positions (bp)	InDels (*Kabuli* reference genome-CDC Frontier/PII)	Forward primers (5'-3')	Reverse primers (5'-3')	Anneling temperature (0C)	Expected amplified product size (bp)	Structural annotation		Functional annotation	
								Sequence components of *kabuli* genome	*Kabuli* gene accession IDs	NCBI-KOG	TFs
Ca1_P3_39431958	Ca1	39431958	CAAA/CAA	TCAAGTGGAAGGGGGTAATG	ACCCCCAATCTGCATAAAAA	59.8	902	INTERGENIC	--	--	--
Ca1_P3_39432122	Ca1	39432122	CTTTTTTTT/CTTTTTTTTTTTTT	CAATTGCACAAATCATTGTTGA	CAGGTGCATTATTCGGTGTG	59.5	870	INTERGENIC	--	--	--
Ca1_P3_39433392	Ca1	39433392	CTTTTTTT/CTTTTTT	TATCCATACCACCCTGCCAT	CTCAGGATCCTCAACCCAAA	60.0	378	INTRON	Ca_13931	Z	NAC
Ca1_P3_39442792	Ca1	39442792	A/ACT	TGAATGGCATGCAAGAAAAG	GCCAGTGTTCGTGCTTCATA	59.8	631	INTRON	Ca_13931	Z	NAC
Ca1_P3_39444043	Ca1	39444043	TAGTTAA/TA	AATGATGGAAAAGCCGAATG	GGAGGGACCTGAAGTCACAA	59.9	838	INTRON	Ca_13931	Z	NAC
Ca1_P3_39446823	Ca1	39446823	GATT/G	GTTGTAAGCTTGCAGGAGGG	TTTTCGCCCCAATAAAGTTG	59.9	713	DRR	Ca_13931	Z	NAC
Ca1_P3_39447104	Ca1	39447104	TTAATG/TTAATGTTGATCTAATG	CAACTTTATTGGGGCGAAAA	CCCTCGATTAATTCTCTTGAGGT	59.9	253	DRR	Ca_13931	Z	NAC
Ca1_P3_39450720	Ca1	39450720	TAAAAAA/TAAAAAAA	CCTGTGCCTTTGCTTTATTTG	TTGCATAAATGGGAGGGAAG	59.8	705	INTERGENIC	--	--	--
Ca1_P3_39462763	Ca1	39462763	TA/TAA	AGCGGCGCTTTATACCTTTT	GGGCCGGGAATTAATTAGAA	60.2	642	INTERGENIC	--	--	--
Ca1_P3_39463854	Ca1	39463854	AAATTTAAT/AAAT	ATATTGAGAGGTTGCGTGGG	ACCATCTCGATGCAACACAA	60.0	556	INTERGENIC	--	--	--

INDEL marker IDs	Chromosomes/ unanchored scaffolds	Physical positions (bp)	InDels (*Kabuli* reference genome-CDC Frontier/PII)	Forward primers (5'-3')	Reverse primers (5'-3')	Anneling temperature (0C)	Expected amplified product size (bp)	Structural annotation		Functional annotation	
								Sequence components of *kabuli* genome	*Kabuli* gene accession IDs	NCBI-KOG	TFs
Ca1_P3_39464239	Ca1	39464239	T/TA	TTGTGTTGCATCGAGATGGT	CAAAGCCGGAAAAGTTTCAA	60.1	560	INTERGENIC	--	--	--
Ca1_P3_39468902	Ca1	39468902	CATATATATATATATAT/CATATATATATATATATATAT	GAAGGAAACATGCATGGAAAA	CTGAACCCTGGTGGAACTGT	59.9	696	INTERGENIC	--	--	--
Ca1_P3_39492770	Ca1	39492770	A/AC	ACCGAGTGTCGAACTCAAGG	GCAGTGCTGAATTTTGGTGA	60.3	902	INTERGENIC	--	--	--
Ca1_P3_39508256	Ca1	39508256	GAAAAAAAAA/GAAAAAAAAAAA	ACCTTTAGACTTGGGGCACA	CGGTTACCATTGTAAAAACAATCA	59.6	254	INTERGENIC	--	--	--
Ca1_P3_39511343	Ca1	39511343	CA/CAA	CGACGAAAGTGATGTGCATT	TGGATTTCACAATCATGGGTT	59.7	136	INTERGENIC	--	--	--
Ca1_P3_39541335	Ca1	39541335	AATATATATATATATATATATA/AATATATATATATATATATATATA	TTCCTTTTCCTCCTGACCCT	CAAAAGATTCGGGCATTTGT	60.0	627	INTERGENIC	--	--	--
Ca1_P3_39550969	Ca1	39550969	C/CG	CGTTTTTCAAAGCGAGGGTA	AATCGATTGATTCAGCCCAG	60.2	782	INTERGENIC	--	--	--
Ca1_P3_39594388	Ca1	39594388	CTTTTTTTTTTT/CTTTTTTTTTTTTT	TGAAATGTGGGCTTTTCACA	AGCTCCAGCCTTTGAAATTG	60.1	543	INTERGENIC	--	--	--
Ca1_P3_39598978	Ca1	39598978	TC/TCC	TCAAATTTGGCAACCTCACA	GCGAAGATGGTGCCAATAAT	60.1	162	INTERGENIC	--	--	--
Ca1_P3_39608275	Ca1	39608275	AGGG/AGGGG	GGTCGACACATGGAGAGTTG	TTATTACCAAACTGCCCCCA	59.1	637	INTERGENIC	--	--	--

INDEL marker IDs	Chromosomes/ unanchored scaffolds	Physical positions (bp)	InDels (*Kabuli* reference genome-CDC Frontier/PII)	Forward primers (5'-3')	Reverse primers (5'-3')	Anneling temperature (0C)	Expected amplified product size (bp)	Structural annotation		Functional annotation	
								Sequence components of *kabuli* genome	*Kabuli* gene accession IDs	NCBI-KOG	TFs
Ca1_P3_39652228	Ca1	39652228	ACCC/ACCCC	GGAGTGACAATGCCCTCAAT	CGGGAGATACGTAGGAGCAA	59.9	774	INTERGENIC	--	--	--
Ca1_P3_39659397	Ca1	39659397	CAAAAAAAAA/CAAAAAAAAAAA	TCATGACAATGCAAGTTCGAC	TTCATTCGTGTCTGCTTTGG	59.7	483	DRR	Ca_13922	IR	--
Ca1_P3_39673797	Ca1	39673797	ATTTTT/ATTTTTT	GCCTCAAAAGCGAAATTTGT	TTGGAACACATAAAAATTAATAATCCA	59.3	759	DRR	Ca_13921	--	--
Ca1_P3_39675342	Ca1	39675342	CT/CTCTGAT	CACCAAAGACAGGACCACCT	CCTAGGGTTTTTGGTTGGGT	60.0	784	DRR	Ca_13920	--	--
Ca1_P3_39682708	Ca1	39682708	GCT/G	TCAATTGGCATTTTGCACAT	AAATTCAGCACTGCACCAAA	59.9	802	INTERGENIC	--	--	--
Ca1_P3_39702404	Ca1	39702404	TAAAA/TAAA	TTGTATAACCTCCATGATTTTGC	ACCAGGAAAGAGAGGTGGGT	58.1	615	INTERGENIC	--	--	--
Ca1_P3_39727320	Ca1	39727320	GATATGATATA/GATATGATATATGATATA	GAGGGTAGCCCAATTGTTCA	GGGCCAAAAGTTACTTGGGT	59.9	452	INTRON	Ca_13917	C	--
Ca1_P3_39743942	Ca1	39743942	TAAAA/TAAAAA	TTCCATTTCCTCCACAAAGC	TGCTACAACCGCATTGTCAT	60.1	563	DRR	Ca_13915	--	--
Ca1_P3_39744308	Ca1	39744308	CTATATATATATATATATATATATATATATATATATATATATA/CTATATATATATATATATATATATATATATATATATATATATATATA	ATGACAATGCGGTTGTAGCA	TCCATTTGCACTGACCCATA	60.1	667	DRR	Ca_13915	--	--
Ca1_P3_39766448	Ca1	39766448	AT/ATCTT	AAAGCCACTATCAGGGTGGA	TCCAAATCCATTTCCAATCC	59.6	701	INTERGENIC	--	--	--

INDEL marker IDs	Chromosomes/ unanchored scaffolds	Physical positions (bp)	InDels (*Kabuli* reference genome-CDC Frontier/PII)	Forward primers (5'-3')	Reverse primers (5'-3')	Anneling temperature (0C)	Expected amplified product size (bp)	Structural annotation		Functional annotation	
								Sequence components of *kabuli* genome	*Kabuli* gene accession IDs	NCBI-KOG	TFs
Ca1_P3_39782012	Ca1	39782012	TA/T	TTTTCCTAGACCATCTG CGG	CGATCAAATATTTTTCA CCGAA	60.2	678	INTERGENIC	--	--	--
Ca1_P3_39816033	Ca1	39816033	CTTTTTT/CTTTTT	ATGCTAACTCACGCGTT GTA	AATATCGGCATTTGTC GGTT	57.1	688	INTERGENIC	--	--	--
Ca1_P3_39844169	Ca1	39844169	ATT/ATCACTTT	AATTAATATTCGCGCAA CGC	AATGGATGCATCAAAT GGGT	60.1	879	INTRON	Ca_13909	--	--
Ca1_P3_39847076	Ca1	39847076	TTATATATATATATATATATATATATAT/ TTATATATATATATATAT	ACGAATTGGAGACGAA CACC	GCGGGAATAATTTCCT CCAA	60.0	491	INTERGENIC	--	--	--
Ca1_P3_39847433	Ca1	39847433	AAATA/AAATAATA	GAGGAAATTATTCCCG CAAA	TTTGGACTTCGTCGTT TTGA	59.0	735	INTERGENIC	--	--	--
Ca1_P3_39850133	Ca1	39850133	GTTAGTAATTCAAAAATTA/GTTA	CACAGCACCAGGTTAT CCCT	CTCCTTGTGGAGGGAC CATA	60.0	563	INTERGENIC	--	--	--
Ca1_P3_39913212	Ca1	39913212	GTTTTTTTTTT/GTTTTTTTTT	TTGCTCCGAAAAAGATA GACC	GAGTTGAGGTATGACC ATTGGA	58.5	596	INTERGENIC	--	--	--
Ca1_P3_39913516	Ca1	39913516	TTTATTATTATTATTATTATTATTATT/TT TATTATTATTATTATT	TTGCTCCGAAAAAGATA GACC	CCCGGAGAGATAACAA CGAA	58.5	979	INTERGENIC	--	--	--
Ca1_P3_39949927	Ca1	39949927	AACACA/AACA	TTCATTCTCGTGTTTGC ACC	GGAAAGCCGTCACAAA TGTT	59.7	421	INTERGENIC	--	--	--
Ca1_P3_39955215	Ca1	39955215	GTA/GTATA	CAAATGGATGAAGAGG AAAAGG	AACGACTTGCAATTGT CCCT	59.9	435	INTERGENIC	--	--	--

INDEL marker IDs	Chromosomes/ unanchored scaffolds	Physical positions (bp)	InDels (*Kabuli* reference genome-CDC Frontier/PII)	Forward primers (5'-3')	Reverse primers (5'-3')	Anneling temperature (0C)	Expected amplified product size (bp)	Structural annotation		Functional annotation	
								Sequence components of *kabuli* genome	*Kabuli* gene accession IDs	NCBI-KOG	TFs
Ca1_P3_39990882	Ca1	39990882	ATATTATTATTATTATTATT/ATATTATT ATTATTATTATTATT	CGGATCAATGGAGAAG AAGG	AGCTGACTCCCATTGA AAGC	59.6	778	INTERGENIC	--	--	--
Ca1_P3_40015677	Ca1	40015677	CTTTT/CTTTTT	CAACTTGGTCAAAGATT TTCTTCA	CCAATAGTGGTTGCCT TCGT	59.7	573	INTERGENIC	--	--	--
Ca1_P3_40026256	Ca1	40026256	GAA/GA	CATTGAGGCAAGGGGT AAGA	CGAATTGTTCGTGAGT AAAATGA	60.1	658	INTERGENIC	--	--	--
Ca1_P3_40032224	Ca1	40032224	GCTCT/GCT	TGAGTAACCGTGCATT CAGC	TAATGCTGTGCTTCTC GGTG	59.9	278	INTRON	Ca_13904	OE	WRKY
Ca1_P3_40033057	Ca1	40033057	CAGATAGATAGATAGATAGATA/CAGAT AGATAGATAGATAGATAGATA	ATCCCGGTGACATCTA CTCG	CGTGAGTCCGGCATAT TCTT	60.0	248	INTRON	Ca_13904	OE	WRKY
Ca1_P3_400621	Ca1	400621	ATT/AT	TGCCCAAAACACCACA CTAA	GCGTTGAACGGTTTTT GAAT	60.0	442	DRR	Ca_00042	J	GRAS
Ca1_P3_40073823	Ca1	40073823	ATTTTTTTTTTTTTTT/ATTTTTTTTTTTT	CAAGCTCGGACTTGAA CCTC	ACTCCCCCTCAATATG TGCT	60.0	323	INTERGENIC	--	--	--
Ca1_P3_40081687	Ca1	40081687	AATTTATATAT/AAT	TTTCCACCATTGGATGA TGA	TTGCCTGAAAAAGAGA TGTCAA	59.7	475	INTERGENIC	--	--	--
Ca1_P3_40087661	Ca1	40087661	C/CTATAACTATT	CGGGGAATGAATGTTA TTGG	TACAACCCCCTTCATC TTCG	60.0	249	DRR	Ca_13902	J	NAC
Ca1_P3_4009762	Ca1	4009762	AT/ATT	CGCACACACTTTCTAC GCAT	TTGATCTGGTCAAATA AGCCA	59.9	841	INTERGENIC	--	--	--

INDEL marker IDs	Chromosomes/ unanchored scaffolds	Physical positions (bp)	InDels (*Kabuli* reference genome-CDC Frontier/PII)	Forward primers (5'-3')	Reverse primers (5'-3')	Anneling temperature (0C)	Expected amplified product size (bp)	Structural annotation		Functional annotation	
								Sequence components of *kabuli* genome	*Kabuli* gene accession IDs	NCBI-KOG	TFs
Ca1_P3_40147055	Ca1	40147055	CGTTTCAAATTAAGT/CGT	ACGAAACAATCATTTGAACGTA	AGCGTTGCCAAAAATAATGC	57.3	532	INTERGENIC	--	--	--
Ca1_P3_40169550	Ca1	40169550	AT/A	TTCCAGTGGCTAACAATCCC	AAATGGGACTCACACAACCA	59.9	490	INTERGENIC	--	--	--
Ca1_P3_401873	Ca1	401873	TAAA/TAAAA	CAAAATTACTGCAGGCCCAT	TGTCCACCACTGTCACCACT	60.0	708	INTERGENIC	--	--	--
Ca1_P3_40196351	Ca1	40196351	CAAA/CAA	ACATGCCAGGTGACAAAACA	TGTTAACCGATTCGTCCAGA	60.0	912	INTERGENIC	--	--	--
Ca1_P3_40209764	Ca1	40209764	GATATATATATATA/GATATATATATA	ACGGCAAGACCCTAAAACCT	CTTGTTTTTCACCGACCGTT	60.0	518	INTERGENIC	--	--	--
Ca1_P3_40224367	Ca1	40224367	AGG/AG	CAACGCAAAATTGAACTCACC	TCTTTGCCTCATAGGAGCGT	60.5	201	INTERGENIC	--	--	--
Ca1_P3_40297702	Ca1	40297702	TAAAAAAAAA/TAAAAAAAAAAAA	TCCTCAGGTTTAGTTTCTTCAAA	ATTGCCACGTTTTGGACTTC	57.3	396	INTERGENIC	--	--	--
Ca1_P3_40298582	Ca1	40298582	TAAAAAAAAAAA/TAAAAAAAAA	TTTTAAAGATGCTGAAACAAACAAA	AGCCGTGTTGATTTTCGCTA	59.3	251	INTERGENIC	--	--	--
Ca1_P3_40298972	Ca1	40298972	AC/A	TAGCGAAAATCAACACGGCT	ATCGAAACATCGCTCTGGAT	60.8	706	INTERGENIC	--	--	--
Ca1_P3_40299346	Ca1	40299346	AACACACAC/AACACAC	CAAATTTGGCATATCCCTCC	GAATGAAGATGGCCTTGGAA	59.2	532	INTERGENIC	--	--	--

INDEL marker IDs	Chromosomes/ unanchored scaffolds	Physical positions (bp)	InDels (*Kabuli* reference genome-CDC Frontier/PII)	Forward primers (5'-3')	Reverse primers (5'-3')	Anneling temperature (0C)	Expected amplified product size (bp)	Structural annotation		Functional annotation	
								Sequence components of *kabuli* genome	*Kabuli* gene accession IDs	NCBI-KOG	TFs
Ca1_P3_40303709	Ca1	40303709	TGG/TG	TGACCAATGCCTTGAGAACA	CAAGGAGAGGCGAGCTTAAA	60.2	602	INTERGENIC	--	--	--
Ca1_P3_40319622	Ca1	40319622	CTATTATTATT/CTATTATT	TCTCCGTAATTGGTGTGCAA	AAACCAAAATCCATGCCAAA	60.1	771	DRR	Ca_13894	R	C3H
Ca1_P3_40320081	Ca1	40320081	CTCTTCTT/CTCTT	TTGGCATGGATTTTGGTTTT	GCTGGTGTCCACAGACAATG	60.2	380	INTERGENIC	--	--	--
Ca1_P3_40326561	Ca1	40326561	GTT/GTTT	CCGGCAAATATGACGAACTT	AAACCAAGAACAAAATGGCG	60.0	540	INTERGENIC	--	--	--
Ca1_P3_40329164	Ca1	40329164	AT/A	GCCGATTGTCTCAGGAAGAG	TACGCAAATCCCACACAAAA	60.0	419	INTERGENIC	--	--	--
Ca1_P3_40334577	Ca1	40334577	TGGGGGGG/TGGGGGG	TTTTCAGGCATTGCATTCAT	AATTTTCCCCAACAAAAGGG	59.1	762	DRR	Ca_13893	S	--
Ca1_P3_40355583	Ca1	40355583	TAAAA/TAAAAA	GGAATTCTCAGGTTTTCGCA	TTCATGGTTGCTTTAAGGGG	60.2	616	INTRON	Ca_13893	S	--
Ca1_P3_40370849	Ca1	40370849	GATAGACTTAAT/GAT	AAACTCAAATCCATCGGTCG	TTGTGTGAGAAGCTGCCCTA	59.9	724	INTERGENIC	--	--	--
Ca1_P3_40392920	Ca1	40392920	AT/A	AATTGCTTCTTGCTTGCCTT	TTGACCAACTATCACCAAGCC	59.1	594	INTERGENIC	--	--	--
Ca1_P3_40398387	Ca1	40398387	CT/C	TGAAAAATACTTTTGGGCCG	AAAACGCCATGGATTGTCAT	59.9	858	INTERGENIC	--	--	--

INDEL marker IDs	Chromosomes/ unanchored scaffolds	Physical positions (bp)	InDels (*Kabuli* reference genome-CDC Frontier/PII)	Forward primers (5'-3')	Reverse primers (5'-3')	Anneling temperature (0C)	Expected amplified product size (bp)	Structural annotation		Functional annotation	
								Sequence components of *kabuli* genome	*Kabuli* gene accession IDs	NCBI-KOG	TFs
Ca1_P3_403989	Ca1	403989	CT/CTCGTATTGACAGT	TTGCACCTTATGCTTCACCA	CAATGGGAGCGCAGTTTTAT	60.3	228	INTERGENIC	--	--	--
Ca1_P3_40408351	Ca1	40408351	CAACGTATCAAA/CAA	TCACAAACAATTGCGAAAACA	CCACCTCCATGCCTCTAAAA	60.1	657	INTERGENIC	--	--	--
Ca1_P3_40408466	Ca1	40408466	CTTATTTATTTATTTA/CTTATTTATTTATTTATTTA	TCACAAACAATTGCGAAAACA	CCACCTCCATGCCTCTAAAA	60.1	657	INTERGENIC	--	--	--
Ca1_P3_40408801	Ca1	40408801	ATT/ATTGAGCCAACTT	TTTTAGAGGCATGGAGGTGG	TTTTGCAAATGTTGGGTTCA	60.1	343	INTERGENIC	--	--	--
Ca1_P3_40411055	Ca1	40411055	CTATTTTTATATATTTT/CTATTTTTATATATTTTTATATATTTT	TGGTTGTCTTTGTGGCATGT	AACGCCACAAAATTTGATCC	60.0	460	INTERGENIC	--	--	--
Ca1_P3_40411589	Ca1	40411589	TAAAAAAAAAAA/TAAAAAAAAAAAAA	TTTGGGGCATTAGAGCAATC	GGGTGTTACCCGTACTTCCT	60.0	363	INTERGENIC	--	--	--
Ca1_P3_40421450	Ca1	40421450	ATTTTTT/ATTTTTTT	GGTGGTCTTGAGCGAGTAGC	CGGTCTTTGGCTTGGAAATA	60.0	583	DRR	Ca_13890	A	--
Ca1_P3_40421787	Ca1	40421787	GT/GTT	TATTTCCAAGCCAAAGACCG	TCCTTCATCCGAACATACCA	60.1	217	DRR	Ca_13890	A	--
Ca1_P3_40424994	Ca1	40424994	C/CCAAAATATTATAA	TGCAAAACTTTGTTCATCGAG	GCATCATTCTCCTTCGAGACA	59.0	748	INTERGENIC	--	--	--
Ca1_P3_40425232	Ca1	40425232	AAA/AAATAA	CATTTTTAAGCTTTTGTTGCCA	GCATCATTCTCCTTCGAGACA	59.3	513	INTERGENIC	--	--	--

INDEL marker IDs	Chromosomes/ unanchored scaffolds	Physical positions (bp)	InDels (*Kabuli* reference genome-CDC Frontier/PII)	Forward primers (5'-3')	Reverse primers (5'-3')	Anneling temperature (0C)	Expected amplified product size (bp)	Structural annotation		Functional annotation	
								Sequence components of *kabuli* genome	*Kabuli* gene accession IDs	NCBI-KOG	TFs
Ca1_P3_40425317	Ca1	40425317	GTCTC/GTCTCTC	CATTTTTAAGCTTTTGTTGCCA	TTTCATTAAGGTGAATCGTTCAGA	59.3	608	INTERGENIC	--	--	--
Ca1_P3_40425540	Ca1	40425540	ATTTTTTTT/ATTTTTTTTT	TGTCTCGAAGGAGAATGATGC	AAACCGGTTCAAAAATGCAC	60.4	600	INTERGENIC	--	--	--
Ca1_P3_4049247	Ca1	4049247	TAA/TA	CAAGATGAGCAGCATGGAAA	TTGATGTCGATGGCTACGAA	59.9	677	DRR	Ca_00486	K	--
Ca1_P3_40535882	Ca1	40535882	AATATATATATATATATATATAT/AATATATATATATATATATATATAT	GATGATGATGATGTCGTGCC	ATCACAGTTTGGCCAAGTCC	59.9	311	INTERGENIC	--	--	--
Ca1_P3_406876	Ca1	406876	C/CCCTCTTT	TGACACAAAATCAGGCCAAA	ACACATGGCAAAATTTCACG	60.1	629	INTRON	Ca_00043	--	--
Ca1_P3_40735911	Ca1	40735911	AATATATATATATATATATATATATATATATATATATATAT/AATATATATATATATATATATATATATATATAT	AAATGCAAAATTTTAAGCGTGA	CCATTGGAAAGTGTGTAGTGC	58.8	533	INTERGENIC	--	--	--
Ca1_P3_4078386	Ca1	4078386	GCACA/GCA	AATTTTAGGCCGTGACATCG	TTCACTAGGACGACGAGGCT	60.0	575	INTERGENIC	--	--	--
Ca1_P3_40889769	Ca1	40889769	TTATATA/TTA	GCATCTCTTGATGCCCTTTT	AACATACGCCATACCCAAGC	59.3	289	INTERGENIC	--	--	--
Ca1_P3_40890013	Ca1	40890013	ATT/ATTT	GCTTGGGTATGGCGTATGTT	TACGAACACTTGCAAATCCG	59.9	243	INTERGENIC	--	--	--
Ca1_P3_40890559	Ca1	40890559	ATTTTT/ATTTTTT	CGGATTTGCAAGTGTTCGTA	TGGGGTTTAGGATATCACATCA	59.7	707	INTERGENIC	--	--	--

INDEL marker IDs	Chromosomes/ unanchored scaffolds	Physical positions (bp)	InDels (*Kabuli* reference genome-CDC Frontier/PII)	Forward primers (5'-3')	Reverse primers (5'-3')	Anneling temperature (0C)	Expected amplified product size (bp)	Structural annotation		Functional annotation	
								Sequence components of *kabuli* genome	*Kabuli* gene accession IDs	NCBI-KOG	TFs
Ca1_P3_40890662	Ca1	40890662	CAAA/CA	CAAGAAACAAATTGCAATGGA	TCAAGTTTGGGAGGAACCAC	58.7	704	INTERGENIC	--	--	--
Ca1_P3_4105789	Ca1	4105789	A/AGCACT	AAAAGTTTGCTGAGGAGCCA	AAAGGGCAAGGATGTGTTTG	60.0	163	INTRON	Ca_00493	D	TCP
Ca1_P3_41069860	Ca1	41069860	TG/T	GACCAGCTCCAAACCATCAT	ATTTATGGGTTGCTTGAGGG	59.9	179	INTERGENIC	--	--	--
Ca1_P3_41076452	Ca1	41076452	T/TAAC	AAATGGAACAGAAGCAACCG	TGTGCACATGTCTCGATTGAC	60.1	650	INTERGENIC	--	--	--
Ca1_P3_41247330	Ca1	41247330	AATATATATATATATATATATATA/AATATATATATATATATATATATATA	AAAGACCAAGCTCTTTCATTGC	GAATGTTGTCCTTCGCACCT	59.9	688	INTERGENIC	--	--	--
Ca1_P3_41500233	Ca1	41500233	CTTTTTTTTT/CTTTTTTTTTT	TCCAGGTAACGTTTTGGTCC	TGGAAAGAAAACGGATGAGG	59.8	136	INTERGENIC	--	--	--
Ca1_P3_41535010	Ca1	41535010	TCCCC/TCCC	GGAACTCCCGGATTAAATTG	GCGACCATCTGTTGCAGTTA	58.4	635	INTERGENIC	--	--	--
Ca1_P3_4164104	Ca1	4164104	TCAGC/TC	CGATAGCCAAATGAGTTCGTC	GTAACTGCTGTGAAGCACCG	59.7	131	INTERGENIC	--	--	--
Ca1_P3_4164590	Ca1	4164590	GTT/GT	ATGAGTTCGTCCGTCAGCTT	CGTGTTCATAGAAATTGCCG	59.9	617	INTERGENIC	--	--	--
Ca1_P3_41659303	Ca1	41659303	GATAATAATAATAATAATAATAATAATAATAATA/GATAATAATAATAATAATAATAATAATAATAATA	ATGCATAAGCAGCAGACACG	CAGAAGATTTTCTTCCTGCCA	60.0	773	INTERGENIC	--	--	--

INDEL marker IDs	Chromosomes/ unanchored scaffolds	Physical positions (bp)	InDels (*Kabuli* reference genome-CDC Frontier/PII)	Forward primers (5'-3')	Reverse primers (5'-3')	Anneling temperature (0C)	Expected amplified product size (bp)	Structural annotation		Functional annotation	
								Sequence components of *kabuli* genome	*Kabuli* gene accession IDs	NCBI-KOG	TFs
Ca1_P3_41695402	Ca1	41695402	CTA/CTATA	TACTTCCATGCAAACCAGCA	CCAACTCTGAAATACAAAAGCCA	60.3	486	INTERGENIC	--	--	--
Ca1_P3_41704419	Ca1	41704419	CATAATAAT/CATAAT	GGGGAATTTTTCATTTTGTGG	TGTTTTGGTTTCAAGGGACA	60.4	311	INTERGENIC	--	--	--
Ca1_P3_41704503	Ca1	41704503	CTTTTTTTT/CTTTTTT	GGGGAATTTTTCATTTTGTGG	TGTTTTGGTTTCAAGGGACA	60.4	311	INTERGENIC	--	--	--
Ca1_P3_4171296	Ca1	4171296	T/TCATTTTTTAGCTTA	CAACAAAACGCGCTAATGAC	CGAGATTTTGAGGCATGTTG	59.4	522	INTERGENIC	--	--	--
Ca1_P3_41718814	Ca1	41718814	T/TG	GGGTCGAATGTCGATTCTGT	TCATCTCTCAGCCCCACTCT	59.9	156	INTERGENIC	--	--	--
Ca1_P3_417847	Ca1	417847	CCAAAGCAA/CCAA	TAAGGGTAAGGCCACCAAAG	AACCGTTATACGGCAACGAC	59.1	594	INTERGENIC	--	--	--
Ca1_P3_41797428	Ca1	41797428	CCTTCTT/CCTT	CTCACAGCCATCACCACAAC	GGAAAAGATGGGTGGGTTTT	60.2	383	CDS (large-effect mutations)	Ca_22519	--	--
Ca1_P3_41858276	Ca1	41858276	ANNNNNNNNNN/A	CTCATGTGTCCGAATTGTGG	CGTGGCCTACTACCGTTCAT	60.0	681	CDS (FRAME SHIFT)	Ca_22522	--	NF-YB
Ca1_P3_41912357	Ca1	41912357	AATATATATATATATATATATAT/AATATATATATATATATAT	TATGGTTCAAATTGCGACGA	TGTTATATTTTGTCATGTCTTCGG	60.1	689	INTERGENIC	--	--	--
Ca1_P3_41998443	Ca1	41998443	CG/CGG	CCATTTTTCTCACGTGCTTTC	ATCCCTCTATTGGTGGGTCC	59.7	339	INTERGENIC	--	--	--

INDEL marker IDs	Chromosomes/ unanchored scaffolds	Physical positions (bp)	InDels (*Kabuli* reference genome-CDC Frontier/PII)	Forward primers (5'-3')	Reverse primers (5'-3')	Anneling temperature (0C)	Expected amplified product size (bp)	Structural annotation		Functional annotation	
								Sequence components of *kabuli* genome	*Kabuli* gene accession IDs	NCBI-KOG	TFs
Ca1_P3_42021612	Ca1	42021612	TAAA/TAAAA	CTCCTTCAGTTTTGAGACACGA	TCGATTATGCGTAGGGAAGG	59.5	442	INTERGENIC	--	--	--
Ca1_P3_42026909	Ca1	42026909	AACAACAACGAC/AACAACAACGACAACAACGAC	GACGACGACAACATGACGAC	TGTTGTTGCCGTTGTTGTTT	60.2	374	INTERGENIC	--	--	--
Ca1_P3_42053807	Ca1	42053807	CGTTGTTGTTGTTGTTGTTGTTGTTGT/CGTTGTTGTTGTTGTTGTTGTTGT	GGGATCTTTTTGGCTGATGA	CTCATCCTCGCCATTGTTTT	60.0	452	INTERGENIC	--	--	--
Ca1_P3_42079737	Ca1	42079737	AATATATATATATATATATATATATA/AATATATATATATATATATATATATATA	GTTGCAACGAACCAACCTTT	GCTACTTTGCAAGTGGATGAAG	60.0	730	INTERGENIC	--	--	--
Ca1_P3_42098363	Ca1	42098363	CTATATATATATATATATAT/CTATATATATATATATATATAT	ACGTTCTTTTTGGGTTCGTG	ATCCCATTAGCCACATTCCA	60.0	266	INTERGENIC	--	--	--
Ca1_P3_422421	Ca1	422421	CTTTTT/CTTTTTT	TGCAGGAACATAAATTGCCA	CCCTGCTGCTCTCTGATTTC	60.1	564	INTRON	Ca_00046	--	NF-YC
Ca1_P3_4228091	Ca1	4228091	TAA/TA	TACAAATGTGTTGGAGCGGA	TCTTCTGCGGTGATTGTGAG	60.1	396	INTERGENIC	--	--	--
Ca1_P3_422976	Ca1	422976	ATATATATGCCTATATATG/ATATATATG	GAAATCAGAGAGCAGCAGGG	CTAGCGCCTTCTTACATCGC	60.1	768	DRR	Ca_00047	--	--
Ca1_P3_4231040	Ca1	4231040	CTA/CTATATA	CCTTCGAATCTTTCCCATCA	TCGAGCTAATTCCATCACCC	60.0	778	INTERGENIC	--	--	--
Ca1_P3_4233360	Ca1	4233360	TTTAAAATTAAAATTAAAATT/TTTAAAATTAAAATT	TAATGGGCTTCGTGGGTTAG	GTGCAGCACATTCGAGAGAA	60.0	683	DRR	Ca_00504	--	--

INDEL marker IDs	Chromosomes/ unanchored scaffolds	Physical positions (bp)	InDels (*Kabuli* reference genome-CDC Frontier/PII)	Forward primers (5'-3')	Reverse primers (5'-3')	Anneling temperature (0C)	Expected amplified product size (bp)	Structural annotation		Functional annotation	
								Sequence components of *kabuli* genome	*Kabuli* gene accession IDs	NCBI-KOG	TFs
Ca1_P3_4234905	Ca1	4234905	A/AC	TGGAGACAAAAGGAGAATCCA	TTCGTTTACCGGATGTCCTC	59.7	759	DRR	Ca_00504	--	--
Ca1_P3_4239941	Ca1	4239941	CGGG/CGGGG	CGACATGGACGCTAATTCCT	TAGGGCCACCTACTGCTGTC	60.1	327	INTERGENIC	--	--	--
Ca1_P3_4240341	Ca1	4240341	CGTGT/CGTGTATATAGCTTTTGTGT	TTGGATGAGACCCAAGTGGT	AATCGTGTTTGAAAAGGCGT	60.4	606	INTERGENIC	--	--	--
Ca1_P3_424349	Ca1	424349	TC/T	TGATAACGAAGCCACCATCA	TCATGCAGAGCTGGTTCTTG	60.1	475	DRR	Ca_00047	--	--
Ca1_P3_4244246	Ca1	4244246	A/ACAAC	TGCTGTCCAAACGAGACATT	GTGGAGGTTTGGCAAGTGTT	59.3	675	DRR	Ca_00505	--	--
Ca1_P3_425468	Ca1	425468	T/TAACCGAA	TACAAAGCTGGTGTGCTTGC	AGCTTGGAGGTTTGCGTAGA	60.1	886	DRR	Ca_00048	U	--
Ca1_P3_4255726	Ca1	4255726	TAAAAAA/TAAAAA	CTTTTGCGCTTCACAAATCA	TCAAAGCAAAGTACCCCCAC	60.0	584	INTRON	Ca_00507	--	--
Ca1_P3_42615631	Ca1	42615631	ATTTTTTTT/ATTTTTTTTT	GCAGCAAATTCCGAAGGTAG	GGTCAATCAAATTCGACGGT	59.8	529	INTERGENIC	--	--	--
Ca1_P3_4279698	Ca1	4279698	TAA/TAAA	CTCAAAGGGTTCCTTCCACA	CAAAGTTTGGACAAACGTGAA	60.1	706	INTERGENIC	--	--	--
Ca1_P3_42810991	Ca1	42810991	AATATATATATATATATATATATA/AATATATATATATATATATA	GTCCACATCATTCAAACCCC	AATGGGTCTTTCACGAATCAA	60.0	837	INTERGENIC	--	--	--

INDEL marker IDs	Chromosomes/ unanchored scaffolds	Physical positions (bp)	InDels (*Kabuli* reference genome-CDC Frontier/PII)	Forward primers (5'-3')	Reverse primers (5'-3')	Anneling temperature (0C)	Expected amplified product size (bp)	Structural annotation		Functional annotation	
								Sequence components of *kabuli* genome	*Kabuli* gene accession IDs	NCBI-KOG	TFs
Ca1_P3_4286155	Ca1	4286155	TTTATTATTATTATTATTATTATTATTATTATTATTATTATTATTATTATTA/TTTATTATTATTATTATTATTATTATTATTATTATTATTATTATTATTATTATTA	ACAAGGGTGCTGCTATTTGC	TTTTGATGAGTCACCAGACAAGTT	60.3	839	INTERGENIC	--	--	--
Ca1_P3_43049776	Ca1	43049776	CTATATATATATATATATATATATATA/CTATATATATATATATATATATATATATA	TGATCCTTCCGATCTTCCTG	AATGACGACGACGATGATGA	60.2	102	INTERGENIC	--	--	--
Ca1_P3_43081583	Ca1	43081583	A/AGTACGTACTGTAACGTAC	TGTTCTGAGACCCGAAATCC	TGGGCGTTATCTTTCCATTT	60.1	328	INTERGENIC	--	--	--
Ca1_P3_43119967	Ca1	43119967	AATATATATATATATATATATA/AATATATATATATATA	TTTCCGGGAATAAATCCCTC	GAAGGAGAGTTCGCGTTGAC	60.1	361	INTERGENIC	--	--	--
Ca1_P3_43188274	Ca1	43188274	AATATATATATATATAT/AATATATATATATAT	CACCTTTTAACAAATTGACCATTG	AGCAAGAACAGCCGCATAGT	59.7	916	INTERGENIC	--	--	--
Ca1_P3_43262365	Ca1	43262365	GAA/GA	CGTATCTCCTAGTGCGATGGA	AAAAGGGGTTGATTTCCTTCA	60.2	792	INTERGENIC	--	--	--
Ca1_P3_43262446	Ca1	43262446	GAATAAAATAAAATAAAATAAAATAAAATAAA/GAATAAAATAAAATAAAATAAAATAAAATAAAATAAA	TCTCCTAGTGCGATGGAGAAA	TTAAGTTGGTTTGGGCTTGG	60.0	883	INTERGENIC	--	--	--
Ca1_P3_43375332	Ca1	43375332	GAAAAAAAAAAA/GAAAAAAAAAA	TTTTCGAAATGGAACCTTCG	TACCATGCACCATCACACCT	60.0	511	INTERGENIC	--	--	--
Ca1_P3_43389439	Ca1	43389439	TAAAAAAAAA/TAAAAAAAAAA	GTGTAATGGGGTCAAATGGC	CAGTTGAAAAAGGGCTTTCG	60.1	758	DRR	Ca_13577	D	--
Ca1_P3_43438412	Ca1	43438412	CGTCGTGTCGTGT/CGTCGTGT	CATGAACGACGTTTGATTGG	TGGCCACCAAATTAATGACA	60.0	575	INTERGENIC	--	--	--

INDEL marker IDs	Chromosomes/ unanchored scaffolds	Physical positions (bp)	InDels (*Kabuli* reference genome-CDC Frontier/PII)	Forward primers (5'-3')	Reverse primers (5'-3')	Anneling temperature (0C)	Expected amplified product size (bp)	Structural annotation		Functional annotation	
								Sequence components of *kabuli* genome	*Kabuli* gene accession IDs	NCBI-KOG	TFs
Ca1_P3_43438547	Ca1	43438547	T/TAC	GGGGCGTTACATGTGGTATC	TTCATAGAGGTTACACAATCACACA	60.1	300	INTERGENIC	--	--	--
Ca1_P3_43459831	Ca1	43459831	TTATATATATATAT/TTATATATATAT	TTGCATTGCACTTTGTTTGA	ACGCAATGCTGTTTCTCGTA	58.9	404	INTERGENIC	--	--	--
Ca1_P3_43550360	Ca1	43550360	ATTT/ATT	TGCAGAGTAATTCGGTTTGTTTT	GGCATTAAGCCTCAAATCCA	60.1	458	INTERGENIC	--	--	--
Ca1_P3_43595794	Ca1	43595794	AAAGAAGAAGAAGAAGAAGAAGAAGAAGAA/AAAGAAGAAGAAGAAGAAGAAGAAGAAGAA	GGAAGCCTGGAATGACACAT	TTCAATTCGGATTTTGACCC	59.9	555	INTERGENIC	--	--	--
Ca1_P3_43638401	Ca1	43638401	A/ATATGTCTTT	CGCTTCATGTGTGTTGCTTT	ACCCCTCCTAAGTGGGTTGA	59.9	857	INTERGENIC	--	--	--
Ca1_P3_43687147	Ca1	43687147	TAA/TA	TGTGGCTAAGATTAGAATCCCAT	TTGATGCAAACGTAATTCATAAAGA	59.0	586	INTERGENIC	--	--	--
Ca1_P3_43687166	Ca1	43687166	GAAAAAAAAAAA/GAAAAAAAAAA	TGTGGCTAAGATTAGAATCCCAT	TTGATGCAAACGTAATTCATAAAGA	59.0	586	INTERGENIC	--	--	--
Ca1_P3_43696370	Ca1	43696370	TAAA/TAA	TGATCTTCAGCTTCACCGAA	AGCCGTCATGACTTTTGTCC	59.5	200	INTERGENIC	--	--	--
Ca1_P3_43729030	Ca1	43729030	TA/TAGAGAGAGAGA	CAGAACCCCATAGACCCAAA	TGTGTAGACACCCCAGGACA	59.8	351	INTERGENIC	--	--	--
Ca1_P3_43734484	Ca1	43734484	GTATATATATATATATATATATATATATATATATATATATATATATATATATATATATAT/GTATATATATATATATATATATATATATATATATATATATATAT	TGCATCAAACTTGTCGGAAA	TGTGCAGGCAAGAACTCAAC	60.2	869	INTERGENIC	--	--	--

INDEL marker IDs	Chromosomes/ unanchored scaffolds	Physical positions (bp)	InDels (*Kabuli* reference genome-CDC Frontier/PII)	Forward primers (5'-3')	Reverse primers (5'-3')	Anneling temperature (0C)	Expected amplified product size (bp)	Structural annotation		Functional annotation	
								Sequence components of *kabuli* genome	*Kabuli* gene accession IDs	NCBI-KOG	TFs
Ca1_P3_43781549	Ca1	43781549	CG/CGTGGTGGTTAG	ATCGGTGAACGAGGATCAAG	CTTTCCTTTCCGGTGAACAA	60.1	747	INTRON	Ca_13549	--	--
Ca1_P3_43865009	Ca1	43865009	TAAAAAAAAA/TAAAAAAAA	AAACATCAAATTCACCCCCA	TTTGATTTGTGGTATGGAGCA	60.0	747	INTERGENIC	--	--	--
Ca1_P3_43867975	Ca1	43867975	AATATATATATATATATATATATATATATA/AATATATATATATATATATATATATATA	TTGCTGGCATAAGTGAATCG	CCGTGAATTTTCATGGAATAAA	59.8	711	INTERGENIC	--	--	--
Ca1_P3_43963718	Ca1	43963718	AAATAATAATAATAATAATAATAATAATAATAATAATA/AAATAATAATAATAATAATAATAATAATA	ACTTGGTGTTGGTGTGTCCA	TAGTCGTGGTGTATGCGGAA	59.9	529	DRR	Ca_13536	--	--
Ca1_P3_44080228	Ca1	44080228	AATATATATATATATATATATATATATAT/AATATATATATATATATATATATATATATATATAT	TGCAAATTTCATCAAATCCA	CCACATCATTTAAAACTCGAATG	57.5	761	INTERGENIC	--	--	--
Ca1_P3_44093089	Ca1	44093089	G/GAA	AAAGAGCCAAGAGCCAAACA	TATTTTGGAGCCCAGTGGTG	60.0	522	DRR	Ca_13529	S	--
Ca1_P3_44245473	Ca1	44245473	GT/GTT	GAGAAACAAGGTGTGGAGCC	TGTTTCAACCCATAGAGGGC	59.7	380	INTERGENIC	--	--	--
Ca1_P3_44247311	Ca1	44247311	GTTTTTTTTTT/GTTTTTTTTT	TTCTCCAGACTCTAGCCCGA	AGCTTCTCAGTAGGGGGCTC	60.1	592	INTERGENIC	--	--	--
Ca1_P3_44351850	Ca1	44351850	TTATATATATATATATA/TTATATATATATATATATA	TGAAATTCGGAAGAACCAGG	CGCATTCCCTTCACCTCTAA	60.0	111	INTERGENIC	--	--	--
Ca1_P3_44368738	Ca1	44368738	GATAATAATAATAATAATAATAATAATAATAATAATAATAATAATAA/GATAATAATAATAATAATAATAATAATAATAATAATAATAA	TTGTAACTCCCCGAATTGTTG	AGAGGCAAACAAGAACCGAA	59.8	534	INTERGENIC	--	--	--

INDEL marker IDs	Chromosomes/ unanchored scaffolds	Physical positions (bp)	InDels (*Kabuli* reference genome-CDC Frontier/PII)	Forward primers (5'-3')	Reverse primers (5'-3')	Anneling temperature (0C)	Expected amplified product size (bp)	Structural annotation		Functional annotation	
								Sequence components of *kabuli* genome	*Kabuli* gene accession IDs	NCBI-KOG	TFs
Ca1_P3_4444544	Ca1	4444544	AATATATATATATATATATATATA/AATATATATATATATATATA	AATCGATTGGCTGAAATCCA	GACATTTAACAGCGCCCATT	60.4	663	INTERGENIC	--	--	--
Ca1_P3_44467119	Ca1	44467119	GTT/GTTT	ATGCAGCTCAAAAACAGCCT	GGTTCACCCAAAGATGGCTA	60.0	537	INTERGENIC	--	--	--
Ca1_P3_44467151	Ca1	44467151	GTTT/GTTTT	ATGCAGCTCAAAAACAGCCT	GGTTCACCCAAAGATGGCTA	60.0	537	INTERGENIC	--	--	--
Ca1_P3_44543117	Ca1	44543117	GGAGAGAGAGA/GGAGAGAGAGAGA	CCGACAGGGTCTTAGCTGAC	AGAAGCCATTCGTGCAAAGT	59.9	454	INTERGENIC	--	--	--
Ca1_P3_44650256	Ca1	44650256	T/TC	TCGTTTGCGTCATTGGATTA	AGGGAGGACGAAAATGACCT	60.1	444	INTERGENIC	--	--	--
Ca1_P3_44715452	Ca1	44715452	AATATATATATATATATATATATATATATATAT/AATATATATATATATATATATATATATATATATATATATAT	AGTTCGAGCTTGGGACTTCA	TTTCGGGAAAGAAACAATAGGA	60.0	704	INTERGENIC	--	--	--
Ca1_P3_44734189	Ca1	44734189	TC/T	TTTCACCATTGGTCTCCACA	GTGGCGAATCATCACAAATG	59.9	429	INTERGENIC	--	--	--
Ca1_P3_44745225	Ca1	44745225	A/AC	TCCGGATATAGTCCTGCCAC	TCCTTGTGAACGACTTGGTG	59.9	632	INTERGENIC	--	--	--
Ca1_P3_44776081	Ca1	44776081	AATATATATATATATATATATATATATATATATATATATATATA/AATATATATATATATATATATATATATATATATATATATATATATATATATA	TGGAATACCATATATTTTACGTTTTCA	TGACGGATCCTTTCTTGACA	59.1	795	INTERGENIC	--	--	--
Ca1_P3_44847079	Ca1	44847079	ATTTTTTTTTTT/ATATTTTTTTTTTTTTTT	GACATGCTCAGTAATTGCCC	AAAATCCAACAATCTCCCCC	58.2	373	INTERGENIC	--	--	--

INDEL marker IDs	Chromosomes/ unanchored scaffolds	Physical positions (bp)	InDels (*Kabuli* reference genome-CDC Frontier/PII)	Forward primers (5'-3')	Reverse primers (5'-3')	Anneling temperature (0C)	Expected amplified product size (bp)	Structural annotation		Functional annotation	
								Sequence components of *kabuli* genome	*Kabuli* gene accession IDs	NCBI-KOG	TFs
Ca1_P3_44847313	Ca1	44847313	TATAATAATAATAATAATAATA/TATAATAATAATAATAATA	TGTTTTCTGCCTTTTTGCCT	AAAATCCAACAATCTCCCCC	59.9	274	INTERGENIC	--	--	--
Ca1_P3_44982741	Ca1	44982741	TAAAAAAAAA/TAAAAAAAAAA	CCTATCTATCCCGAAACGCA	ACCTTCCGCCAATAAATGTT	60.1	197	INTRON	Ca_25713	--	WRKY
Ca1_P3_45057162	Ca1	45057162	CATAAA/CATAAATAAA	TTGCTTGAATAGGCAATGGA	ACGTCGACGACATCAAAGTG	59.3	645	INTERGENIC	--	--	--
Ca1_P3_45057964	Ca1	45057964	CAAA/CAA	AAACTAGATTATGGCCTGCG	TGCTCGCAATTAAAACATGC	57.0	426	INTERGENIC	--	--	--
Ca1_P3_45104438	Ca1	45104438	TTAATAATAATAATAATAATAATAATAATAATAATAATAATAATAATAATAATAATAATAATAATAATAA/TTAATAATAATAATAATAATAATAATAATAATAATAATAATAATAATAATAATAATAATAATAATAATAATAATAA	TTTCAGGCGATTTTCGTACC	AAGTGGGGAACGTTGACTTG	60.1	744	INTERGENIC	--	--	--
Ca1_P3_45124635	Ca1	45124635	GTATATATATATATATATATATATA/GTATATATATATATATATATATATATA	TCCGAGACACAAGACCAACA	TTACGAACACAATTGTTGCCA	60.3	836	INTERGENIC	--	--	--
Ca1_P3_45186673	Ca1	45186673	AATATATATATA/AATATATATATATATA	AAGTGGGGGTTTGTGTTTGA	ATTGAGATGGGAGTGAAGCG	60.2	378	INTERGENIC	--	--	--
Ca1_P3_45206253	Ca1	45206253	AATATATATATATATATATATATATATATATATA/AATATATATATATATATATATATATATATATATATATATATA	CCTCTTTCTCATGCCCTTCA	AAGATTTTACGCGATTTTACGTG	60.3	651	INTERGENIC	--	--	--
Ca1_P3_45231841	Ca1	45231841	TT/TATTGTGGCAGT	TGCTATCAATTGTGCTCCCA	TTCATTGATCGGGGCTTAAC	60.2	619	INTERGENIC	--	--	--
Ca1_P3_45232359	Ca1	45232359	G/GGCAATAATGGA	GTTAAGCCCCGATCAATGAA	CATCCCCCTCTTTAACCCAT	59.9	515	INTERGENIC	--	--	--

INDEL marker IDs	Chromosomes/ unanchored scaffolds	Physical positions (bp)	InDels (*Kabuli* reference genome-CDC Frontier/PII)	Forward primers (5'-3')	Reverse primers (5'-3')	Anneling temperature (0C)	Expected amplified product size (bp)	Structural annotation		Functional annotation	
								Sequence components of *kabuli* genome	*Kabuli* gene accession IDs	NCBI-KOG	TFs
Ca1_P3_45238172	Ca1	45238172	T/TGACA	ATTCTTTTTGGAGCGCAGAA	CCAATTAGGTTTACGGCGAA	60.0	401	INTERGENIC	--	--	--
Ca1_P3_45243775	Ca1	45243775	T/TATGCACTTCTTTTCTC	TGTGCCAAGGTTTCAGAACA	CTTTTATTGCAAGACCCCGA	60.3	605	DRR	Ca_26020	--	--
Ca1_P3_45244574	Ca1	45244574	TC/TCATAAAGCTCAC	TCGGGGTCTTGCAATAAAAG	GACAATGGGAATTCGCCTAA	60.1	532	CDS (FRAME SHIFT)	Ca_26020	--	--
Ca1_P3_45246034	Ca1	45246034	C/CGTCTTGTT	CGATTTCGCTTTTAGCATCC	CAATTGCGATATTATGGGGG	59.8	253	INTERGENIC	--	--	--
Ca1_P3_45251376	Ca1	45251376	C/CCTATGA	CTAGCTCTGGGTTCGAGTGG	ATCCGACTAGTTCCGGGTTC	60.0	247	INTERGENIC	--	--	--
Ca1_P3_45253870	Ca1	45253870	TA/T	TGCCAATAAGGCAATGTGAA	TGGATTAGGAGCGTGAAAACA	60.1	478	INTERGENIC	--	--	--
Ca1_P3_45392704	Ca1	45392704	ATTT/ATTTT	GGGGGCTCTTCATTTTTCTC	CACAGTTTCATCGCTCCTCA	60.0	476	INTERGENIC	--	--	--
Ca1_P3_45398138	Ca1	45398138	CATATATATATATATATATATATATATATAT/CATATATATATATATATATATAT	GTTGGTTCCCTAGCACCGTA	TTAATGCGTTGTGGGTGAAA	60.0	524	INTERGENIC	--	--	--
Ca1_P3_45443277	Ca1	45443277	GTAATAATAATAATAATAATAATAATAATAATAATAATAATAATAATAATAATAATAATAGTAATAGTAATAATAATAATAATAATAATAACAATAATAATAATAATAATAATAATAATAGTAATAGTAATAATAATAAT[illegible]	CCCACATTCATGACGTGTTC	CTTAGAGGTGGGGAACGTGA	59.8	741	INTERGENIC	--	--	--
Ca1_P3_45497100	Ca1	45497100	CTTTTTTTTTTT/CTTTTTTTTTTTTT	AGTATAAGTGGGGCCGTGTG	TGGATTGTGCCAACTTTTCA	59.9	237	INTERGENIC	--	--	--

**INDEL marker IDs**	**Chromosomes/ unanchored scaffolds**	**Physical positions (bp)**	**InDels (*Kabuli* reference genome-CDC Frontier/PII)**	**Forward primers (5'-3')**	**Reverse primers (5'-3')**	**Anneling temperature (0C)**	**Expected amplified product size (bp)**	**Structural annotation**		**Functional annotation**	
								**Sequence components of *kabuli* genome**	***Kabuli* gene accession IDs**	**NCBI-KOG**	**TFs**
Ca1_P3_45566205	Ca1	45566205	GT/GTT	ATTTACCGACGGACAAGCTG	AAGAGGATGAGCAACGCACT	60.1	635	DRR	Ca_24438	--	--
Ca1_P3_45570477	Ca1	45570477	TTATATATATATATATATATATATATATATATATATATATATATATATAT/TTATATATATATATATATATATATATATATATATATATAT	TCCCGTAACAAACCAATGCT	TTCACGCAGCTAGGGAAGAT	60.4	943	INTERGENIC	--	--	--
Ca1_P3_45609121	Ca1	45609121	C/CT	TCGCGTGAGAAAATAATCCC	CGAACCCTCGGTAAACAAAA	60.0	295	INTERGENIC	--	--	--
Ca1_P3_45609373	Ca1	45609373	AAA/AAATTACAA	TTTTGTTTACCGAGGGTTCG	CAATGATCTTGAAGGCACCA	60.0	142	INTERGENIC	--	--	--
Ca1_P3_45778776	Ca1	45778776	AT/ATTTGT	TGATGATTTTGGGCACTGAA	CTTGGAAAATCGGGGTGTTA	60.0	815	INTERGENIC	--	--	--
Ca1_P3_45840868	Ca1	45840868	ATTTTTTTTTTTTT/ATTTTTTTTTTTT	TGTGAACAACTTGGTGGGAA	ATGGGGAAAATAAACCTGCC	60.0	610	INTERGENIC	--	--	--
Ca1_P3_46056038	Ca1	46056038	ATTTTTTTTT/ATTTTTTTTTT	ACCAGAGTTTGAGCATTGGG	TGCCATCACGGAAGACATAA	60.1	670	INTERGENIC	--	--	--
Ca1_P3_46088799	Ca1	46088799	CTATATATATATATATATATATATATATATAT/CTATATATATATATATATATATATATATATATAT	TAAGTCCGTAAGTGGTGCCC	GGGCTTTCCAAAGTGTTATCC	60.0	586	INTERGENIC	--	--	--
Ca1_P3_46102663	Ca1	46102663	AT/A	TGACACGAGGAAAACGATGA	GGATGGGCAAGGAATTGTTA	60.2	671	INTERGENIC	--	--	--
Ca1_P3_46148657	Ca1	46148657	AATATATATATATATATATATATATATATATATATATATATATATATAT/AATATATATATATATATATATATATATATATATATATATATATATATATATATATATATAT	CCCAATCTTGGGGGATAAAT	TTGCTCACAACACACCCATT	59.8	162	INTERGENIC	--	--	--

INDEL marker IDs	Chromosomes/ unanchored scaffolds	Physical positions (bp)	InDels (*Kabuli* reference genome-CDC Frontier/PII)	Forward primers (5'-3')	Reverse primers (5'-3')	Anneling temperature (0C)	Expected amplified product size (bp)	Structural annotation		Functional annotation	
								Sequence components of *kabuli* genome	*Kabuli* gene accession IDs	NCBI-KOG	TFs
Ca1_P3_46178255	Ca1	46178255	AATTTATAATTTAT/AAT	TCAAACATGCTCTGAATGCC	AACCAATACATAACATCACAGTGC	59.8	570	INTERGENIC	--	--	--
Ca1_P3_4618143	Ca1	4618143	ATTTTTTTTT/ATTTTTTTTTT	ATTGCTGCAAACAACACGAG	TTATCCGGTTTTGGTTGAAAA	59.9	574	INTRON	Ca_00546	A	MYB
Ca1_P3_46268212	Ca1	46268212	TA/T	AAACGCAATCCTTGAGTTCG	CATGACTTGGATTGGTCGTG	60.2	226	INTERGENIC	--	--	--
Ca1_P3_46270342	Ca1	46270342	CA/CATATA	GAAGGTCAGTCTGCTATTCTGC	ATGTGTTGTACCTGCCGTGA	58.2	928	INTERGENIC	--	--	--
Ca1_P3_46278771	Ca1	46278771	ATGGCCTGGCCTGGCCTGGCCTG/ATGGCCTGGCCTG	ACCTTCAGTCAGGGGCCTAT	AAGTCTGGTCTGGCCTGAAA	60.0	538	INTERGENIC	--	--	--
Ca1_P3_46283206	Ca1	46283206	C/CTAATTATAA	AGCTGCCATGCCTAATGAAT	CAATGGGCTTCTTACCTGGA	59.7	425	INTERGENIC	--	--	--
Ca1_P3_46286614	Ca1	46286614	CTTTT/CTTT	CCAAGTTATGCTTGATCGCA	CGAGAACCATCCTGCATTTT	59.8	820	INTERGENIC	--	--	--
Ca1_P3_46286661	Ca1	46286661	GTAGGGATA/GTA	CCAAGTTATGCTTGATCGCA	CGAGAACCATCCTGCATTTT	59.8	820	INTERGENIC	--	--	--
Ca1_P3_46286821	Ca1	46286821	AAACAA/AAA	TCCCAAAAATGGCTTAGGAA	CGAGAACCATCCTGCATTTT	59.5	502	INTERGENIC	--	--	--
Ca1_P3_46347636	Ca1	46347636	CATATATATAT/CATATATATATAT	GGATTTCTTACAATCTGCCCA	TTCTCCAGGATCAGGACTGG	59.0	862	INTERGENIC	--	--	--

INDEL marker IDs	Chromosomes/ unanchored scaffolds	Physical positions (bp)	InDels (*Kabuli* reference genome-CDC Frontier/PII)	Forward primers (5'-3')	Reverse primers (5'-3')	Anneling temperature (0C)	Expected amplified product size (bp)	Structural annotation		Functional annotation	
								Sequence components of *kabuli* genome	*Kabuli* gene accession IDs	NCBI-KOG	TFs
Ca1_P3_46352138	Ca1	46352138	CACAAC/CACAACAAC	AGCGATGCATGTCTCAAGTG	TGCCCTTCTTCTTTTCCTAGTG	60.0	396	INTERGENIC	--	--	--
Ca1_P3_46417421	Ca1	46417421	TGAGAGAGAGAGAGAGAGA/TGAGAGAGAGAGAGAGA	TCGAATGGTGTTTTAACTCCTACA	CTGTTTTGGCCTCCACAAAT	59.9	541	INTERGENIC	--	--	--
Ca1_P3_46491737	Ca1	46491737	CAAG/CAAGAAG	GATGCATCTCACCAAGGGTT	CGGTGCAAACTCTACGTTCA	59.9	420	INTERGENIC	--	--	--
Ca1_P3_4649534	Ca1	4649534	CTT/C	CTTGGACCCAACGCATAGTT	CACTCATTCATTGGTGCGTT	60.0	423	INTERGENIC	--	--	--
Ca1_P3_46515935	Ca1	46515935	TTTATTTATT/TTT	TTGGTGCATTATGTCCGTGT	TCATGGATGTTTGGATGTTTG	59.8	569	INTERGENIC	--	--	--
Ca1_P3_46523548	Ca1	46523548	CAAAAAAAAAAAAAAAA/CAAAAAAAAAAAAA	GTGGACAAACCTGCATTCCT	ACCATGCACAGTTGCTGAGA	60.0	675	INTERGENIC	--	--	--
Ca1_P3_46524349	Ca1	46524349	TAAAAAAAAA/TAAAAAAAAAAA	TGAAGCATGCACAAATCCTC	TTGATGTAGCAACGGCTGAG	59.8	843	INTERGENIC	--	--	--
Ca1_P3_46526481	Ca1	46526481	CAAAAAAA/CAAAAAAAA	TTAAAATTCCGAGGCGAAAA	ACATGGTTGTGGTGGTGATG	59.7	698	INTERGENIC	--	--	--
Ca1_P3_46526676	Ca1	46526676	TAAAA/TAAA	TTAAAATTCCGAGGCGAAAA	ACATGGTTGTGGTGGTGATG	59.7	698	INTERGENIC	--	--	--
Ca1_P3_46528558	Ca1	46528558	CT/CTT	TTAAAATTCCCTCCTCGGCT	AACTCTGGGAACCCTCCTGT	60.0	398	INTERGENIC	--	--	--

INDEL marker IDs	Chromosomes/ unanchored scaffolds	Physical positions (bp)	InDels (*Kabuli* reference genome-CDC Frontier/PII)	Forward primers (5'-3')	Reverse primers (5'-3')	Anneling temperature (0C)	Expected amplified product size (bp)	Structural annotation		Functional annotation	
								Sequence components of *kabuli* genome	*Kabuli* gene accession IDs	NCBI-KOG	TFs
Ca1_P3_46528735	Ca1	46528735	GTGACAGCCTGACAG/GTGACAG	TTAAAATTCCCTCCTCGGCT	GGGGAAAACATCCCGTTATT	60.0	865	INTERGENIC	--	--	--
Ca1_P3_46528763	Ca1	46528763	G/GC	TTAAAATTCCCTCCTCGGCT	GGGGAAAACATCCCGTTATT	60.0	865	INTERGENIC	--	--	--
Ca1_P3_46530950	Ca1	46530950	CAAATTGAAAATTGAAAATT/CAAATTGAAAATT	ATTTGAGAATGTTCGGGCAA	GTCTGCGGATAATTTCGCTT	60.4	665	DRR	Ca_21551	M	--
Ca1_P3_46531041	Ca1	46531041	T/TG	GCGAAAATAATGTTGATGCAGA	GTCTGCGGATAATTTCGCTT	60.1	139	DRR	Ca_21551	M	--
Ca1_P3_46539303	Ca1	46539303	TTCAAATCTTCATCAAATCTTC/TTCAAATCTTC	TCAAGCCAATCTTCCCAAAC	TCTGATGACAAAAGGGGGAG	60.1	757	INTERGENIC	--	--	--
Ca1_P3_46539360	Ca1	46539360	CAAA/CAAAA	TCCCAAACAGCCTGATAACC	CTGATGACAAAAGGGGGAGA	59.9	744	INTERGENIC	--	--	--
Ca1_P3_46633354	Ca1	46633354	GTATATATATATATATATATAT/GTATATATATATATATAT	CGACATGGCTCTGATACCAA	TCAGACCGCCTTTACCAATC	59.7	615	INTERGENIC	--	--	--
Ca1_P3_46662012	Ca1	46662012	AAT/AATAT	GCATTTACGTCGATATTTATGAGTG	TCAGCCAAAAAGCTGATGTG	59.1	617	INTERGENIC	--	--	--
Ca1_P3_46831943	Ca1	46831943	CTTTT/CTTT	GTGGTTGGTTTGCAAGAGGT	AACTGGGTGAGAGCCTGAGA	60.0	725	INTRON	Ca_25116	U	--
Ca1_P3_46867363	Ca1	46867363	CTTTT/CTTT	GCCCTCAGATGAGAGTCAGG	CAAACCTTTCATGCGGGTAT	59.9	836	DRR	Ca_25118	--	--

INDEL marker IDs	Chromosomes/ unanchored scaffolds	Physical positions (bp)	InDels (*Kabuli* reference genome-CDC Frontier/PII)	Forward primers (5'-3')	Reverse primers (5'-3')	Anneling temperature (0C)	Expected amplified product size (bp)	Structural annotation		Functional annotation	
								Sequence components of *kabuli* genome	*Kabuli* gene accession IDs	NCBI-KOG	TFs
Ca1_P3_4687490	Ca1	4687490	TAAAAAAAAAA/TAAAAAAAAAAA	ACAACCAACTATTTTGCGGG	TGTATGTTTATGCGCACACG	59.9	766	INTERGENIC	--	--	--
Ca1_P3_46896717	Ca1	46896717	AATATATATATATATATATA/AATATATATATATATATATATATA	CACGATGCATTGGTGTAGGT	TCGTTCAATAACCTTTTTCTTGG	59.4	555	INTERGENIC	--	--	--
Ca1_P3_47059710	Ca1	47059710	TTTATTATTATTATTATTATTATTATTATTATTATTATTA/TTTATTATTATTATTATTATTATTATTATTATTATTATTATTATTATTA	GGAGAACGTTAAGGCAGCAG	GGCAATAAGAACCAAAGGCA	60.0	642	INTERGENIC	--	--	--
Ca1_P3_47080923	Ca1	47080923	ATTTTTTTTTTT/ATTTTTTTTTT	CCTTTGAGTTGTTAGGACGCA	ACGTATCTCCTGGTGGGATG	60.3	417	INTERGENIC	--	--	--
Ca1_P3_47084853	Ca1	47084853	AATATATATATATATATATATATATATA/AATATATATATATATATATATATA	TTTGTGTTTTCACAAGAGGACAA	GACCGTTTCAGTCAAAGTAAATGA	59.7	673	INTERGENIC	--	--	--
Ca1_P3_47161229	Ca1	47161229	TAGAG/TAG	TCTGCAATGAAAGCAAACACA	AGTTTTGGCTCCCTATCGGT	60.4	829	INTERGENIC	--	--	--
Ca1_P3_4718653	Ca1	4718653	CATAT/CAT	TTTGACACGACCAACCAAAA	TGGACACAGCAAGTTAAGCG	60.0	781	INTRON	Ca_00555	T	MYB_related
Ca1_P3_4720443	Ca1	4720443	AT/A	TCAGAGTTCATTGCAAACGG	GGGATTCCCATTTTCCATCT	59.8	691	INTRON	Ca_00555	T	MYB_related
Ca1_P3_4720541	Ca1	4720541	ATTTTTTTTTT/ATTTTTTTT	GCTTTACAATGCACCTGTTGA	GGGATTCCCATTTTCCATCT	58.8	359	INTRON	Ca_00555	T	MYB_related
Ca1_P3_4722248	Ca1	4722248	GAAGAAA/GAA	TAACAAAGGTTTTGCCTGCC	AAAGTGGAAGGCACATTTGG	60.1	533	INTRON	Ca_00555	T	MYB_related

INDEL marker IDs	Chromosomes/ unanchored scaffolds	Physical positions (bp)	InDels (*Kabuli* reference genome-CDC Frontier/PII)	Forward primers (5'-3')	Reverse primers (5'-3')	Anneling temperature (0C)	Expected amplified product size (bp)	Structural annotation		Functional annotation	
								Sequence components of *kabuli* genome	*Kabuli* gene accession IDs	NCBI-KOG	TFs
Ca1_P3_47266775	Ca1	47266775	CATACTTCGTAATAAAA/CA	GAATGGACCTATTCGTTGTGC	CAATTCGAAACGCAATCCTT	59.4	409	INTERGENIC	--	--	--
Ca1_P3_47321461	Ca1	47321461	AACA/AACATGCGACAAAAACACA	GGTTGGTAGAGGAATAGCGG	ATTGGGATTTTGTTCGTTGC	58.7	827	DRR	Ca_12921	--	--
Ca1_P3_47331266	Ca1	47331266	ATTTTTTT/ATTTTTTTT	TCGTTCCATATTCGGTAGCC	TCCTCCTAATGTGATTGCCC	59.9	503	DRR	Ca_12920	--	--
Ca1_P3_47337298	Ca1	47337298	C/CAAATATATACT	ATTGGGCATAGGAAGCACTG	AGTTGACCACGGACCCATAG	60.1	533	DRR	Ca_12919	--	--
Ca1_P3_47371134	Ca1	47371134	CA/CATTTTA	ATTGTTTTGGACCATGCCTT	TCCAAGCACAACAACAAAGC	59.3	356	INTERGENIC	--	--	--
Ca1_P3_4742142	Ca1	4742142	TAAA/TAA	GACCGCGTAGATGTGGAACT	GGATGAGACCTCCTACTTGCC	60.1	677	INTERGENIC	--	--	--
Ca1_P3_47572869	Ca1	47572869	TAAAAAAAAA/TAAAAAAAAAA	TGTATGCATGAAGCAATAGCAG	GCAAAAAGAGGTTTGATCGG	59.0	407	INTERGENIC	--	--	--
Ca1_P3_47599399	Ca1	47599399	AGGGGGG/AGGGGG	TCCTACGTATCTTCCGGTGC	ATCATCAAATCGACCTTCGC	60.1	791	INTERGENIC	--	--	--
Ca1_P3_47630703	Ca1	47630703	TAAAAAGAAAAAGAAAAAGAAA/TAAAAAGAAAAAGAAAAAGAAAAAGAAA	CGTGTCCGGCAAAGTAAAAT	GTGATGCACGCAGTAATTGG	60.0	517	INTERGENIC	--	--	--
Ca1_P3_47710001	Ca1	47710001	AATTATTATTATTATTATT/AATTATTATTATTATT	CATCACATCACCTACACGCC	TTGACGGTGGTTTTATTTTCG	60.0	916	INTERGENIC	--	--	--

INDEL marker IDs	Chromosomes/ unanchored scaffolds	Physical positions (bp)	InDels (*Kabuli* reference genome-CDC Frontier/PII)	Forward primers (5'-3')	Reverse primers (5'-3')	Anneling temperature (0C)	Expected amplified product size (bp)	Structural annotation		Functional annotation	
								Sequence components of *kabuli* genome	*Kabuli* gene accession IDs	NCBI-KOG	TFs
Ca1_P3_47712006	Ca1	47712006	AT/ATT	CCCTCTATCCACCCAGTGAA	GATTTTCAACAATTTGGGGG	59.9	501	INTERGENIC	--	--	--
Ca1_P3_47730180	Ca1	47730180	GATATATATATATATATATA/GATATATATATATATATATATA	TGCGTATCTTAGGAATGCGA	GGCTAGACGAGTTCGACGAC	59.4	321	INTERGENIC	--	--	--
Ca1_P3_47771262	Ca1	47771262	TT/TTTAT	GGGTTACAAGCTGGACCAAA	GACACGCGTCATTTCTTCAA	60.0	755	INTERGENIC	--	--	--
Ca1_P3_47842080	Ca1	47842080	GTTATTATTATTATTATTATTATTATTATTATTATTATTATTATTAT/GTTATTATTATTATTATTATTATTATTATTATTATTATTATTATTAT	TGAACAGAAACAATGGGCAA	CGATGATTTTAGAACCGATTTCA	60.1	509	INTERGENIC	--	--	--
Ca1_P3_47875198	Ca1	47875198	AT/ATT	GAAAAACAAGACCCGCGATA	TTCCTATTGCTCACCCCTTG	60.1	466	INTERGENIC	--	--	--
Ca1_P3_47913934	Ca1	47913934	CTTTA/CTTTATTTA	ATACCCAGGGACGGTTTTTC	ATGTACGTGGCTTTGCATGA	60.1	383	DRR	Ca_12886	O	E2F/DP
Ca1_P3_47916750	Ca1	47916750	ATT/ATTTCAGGTTT	GTTGGTGATGCCACTGAATG	GAGAGAAAGGCAAGTGTGGG	60.0	979	DRR	Ca_12885	O	M-type
Ca1_P3_48052485	Ca1	48052485	TCTC/TCTCCTC	AATTGGAGGGGCGATATTTT	TGGCATGGGATCCATAATTT	59.6	336	DRR	Ca_12870	--	--
Ca1_P3_48055075	Ca1	48055075	GTATATATATATATATATATATATATATATATA/GTATATATATATATATATATATATATATATATATATA	TGACACTCGCATCTTTGTGA	GGCCATATCCGAAAACATTG	59.0	432	INTERGENIC	--	--	--
Ca1_P3_48071541	Ca1	48071541	TATAAAT/TAT	TGGTGGTGAATACTACGGCA	GGCTCTTGAGGCACTTGTTC	60.0	651	INTERGENIC	--	--	--

INDEL marker IDs	Chromosomes/ unanchored scaffolds	Physical positions (bp)	InDels (*Kabuli* reference genome-CDC Frontier/PII)	Forward primers (5'-3')	Reverse primers (5'-3')	Anneling temperature (0C)	Expected amplified product size (bp)	Structural annotation		Functional annotation	
								Sequence components of *kabuli* genome	*Kabuli* gene accession IDs	NCBI-KOG	TFs
Ca1_P3_48126788	Ca1	48126788	GAATAATAATAATAATAATAATAATAATAATAATAATAATAATAATAATAAT/GAATAATAATAATAATAATAATAATAATAAT	TCATTGAACTCTTTCTTCTTTGAA	AAAAATTGAAGGCGAATGGA	57.4	708	INTERGENIC	--	--	--
Ca1_P3_48172069	Ca1	48172069	AAATAATAATAATAATAATAATAATAATAATAATAATA/AAATAATAATAATAATAATAATAATAATAATAATAATAATAATAATAATAATAATA	TAAATGGCAGGTAAAGGCGA	AGACTTTGCTGCCACCTCAT	60.6	315	DRR	Ca_12862	R	WRKY
Ca1_P3_48185936	Ca1	48185936	GA/GAA	CCCGGCAATTGTGTTACTCT	TAGGTCCGTGGGAGAACAAG	60.0	735	INTRON	Ca_12861	--	--
Ca1_P3_48200764	Ca1	48200764	AT/A	TTTATTTGGTCAACAAAGTAGTATCAA	TCATTTTCTCAAGTCGGGCT	57.6	322	INTERGENIC	--	--	--
Ca1_P3_48217476	Ca1	48217476	TTCTCTCTCTCTCTCTCT/TTCTCTCTCTCTCTCT	CAACCAAAATTCAACGCAAA	ACATGTTAGGCTCTGGCAGG	59.6	295	DRR	Ca_12859	K	HB-other
Ca1_P3_48239496	Ca1	48239496	GGGAG/GG	TCATGTTCAATGCCGTTCTC	CAGGAAGGGCACCAAGAATA	59.7	314	CDS (large-effect mutations)	Ca_12858	--	--
Ca1_P3_48331419	Ca1	48331419	AATTATTATTATTATTATTATTATTATTATTATT/AATTATTATTATTATTATTATTATTATTATTATTATT	CATTGCGTTGTCACAAAATCA	AAGTAACTGGCACCACCAGG	60.6	395	INTERGENIC	--	--	--
Ca1_P3_4900100	Ca1	4900100	TATTCAAT/TATTCAATTCAAT	TCTTTGCAGCATGCTTTTTG	TTTCTGAAGCGACCAATTCC	60.1	561	DRR	Ca_00575	--	--
Ca1_P3_4952260	Ca1	4952260	GATATATATATATATATATATATATATATAT/GATATATATATATATATATATATATATATATATAT	TGCACTAGACCCAGTGCTAGTTA	AGCCACAACGACGAGTTTCT	59.1	939	INTERGENIC	--	--	--
Ca1_P3_4957879	Ca1	4957879	CAAAAAAAAAAAA/CAAAAAAAAAAA	TCATGACAATGCAAATTCGAC	TTATTCGTGTGTGCCTTGGA	59.5	459	DRR	Ca_00586	T	WRKY

INDEL marker IDs	Chromosomes/ unanchored scaffolds	Physical positions (bp)	InDels (*Kabuli* reference genome-CDC Frontier/PII)	Forward primers (5'-3')	Reverse primers (5'-3')	Anneling temperature (0C)	Expected amplified product size (bp)	Structural annotation		Functional annotation	
								Sequence components of *kabuli* genome	*Kabuli* gene accession IDs	NCBI-KOG	TFs
Ca1_P3_5040464	Ca1	5040464	ACC/AC	CAAAATGCTCGCAATGAGAA	GAACTCCCCATGTGGTTGAT	60.0	635	INTERGENIC	--	--	--
Ca1_P3_5106336	Ca1	5106336	CT/CTCTAT	CGAGTGATTTGCTTCATTGG	TGTTACGCATTGTTTGGGAA	59.3	651	INTERGENIC	--	--	--
Ca1_P3_5139845	Ca1	5139845	TTTCTTCTTCTTCTTCTTCTTCTTCTTCTTCTTCTT/TTTCTTCTTCTTCTTCTTCTTCTTCTTCTT	ACGTCTGAATCGGATTTTGC	CCCAGAAGAAGATGGAGCTG	60.1	306	INTERGENIC	--	--	--
Ca1_P3_5139903	Ca1	5139903	TTTCTTCTTCTTCTTCTTCTTCTTCTTCTTCTTCTTCTTCTTCTTCTTCTTCTTC/TTTCTTCTTCTTCTTCTTCTTCTTCTTCTTCTTCTTCTTCTTCTTCTTCTTC	ACGTCTGAATCGGATTTTGC	CCCAGAAGAAGATGGAGCTG	60.1	306	INTERGENIC	--	--	--
Ca1_P3_5180264	Ca1	5180264	CTAATAATAATAATAATAATAATAATAATAATAAT/CTAATAATAATAATAATAATAATAATAATAATAATAAT	ATGCATGGAGCTATAAGCCG	TTCAAAGTTGGAATAACCCTTCTC	60.2	684	INTERGENIC	--	--	--
Ca1_P3_524043	Ca1	524043	ATTTTTTTTTT/ATTTTTTTTT	ATTTTAGGGGATGGTGGAGG	CGAGTGTCCAATGAAAAACCA	60.0	456	INTERGENIC	--	--	--
Ca1_P3_5242725	Ca1	5242725	CATATATATATATATA/CATATATATATATATATA	TATTTGGCATTGGTGGTTGA	ATTTTCTCGAGGCCATGTCA	59.8	805	INTERGENIC	--	--	--
Ca1_P3_5273611	Ca1	5273611	TTATATATATATATATATATATATAT/TTATATATATATATATATATATATATATATATAT	TGCATTAATTTTGAAAACGTCAA	TGTTTGCCACGGTTGTTAAA	59.5	593	INTERGENIC	--	--	--
Ca1_P3_5275130	Ca1	5275130	AATATATATATATATATA/AATATATATATATATATA	CCATTCTCACCCGCAATAGT	CCAGCCTCAAATCATCCCTA	60.0	445	INTERGENIC	--	--	--
Ca1_P3_5290057	Ca1	5290057	ACC/AC	AGAAACGGTCACCTTCCCTT	CTCATTGCTTCCCAAACCAT	60.0	811	INTRON	Ca_00616	P	--

INDEL marker IDs	Chromosomes/ unanchored scaffolds	Physical positions (bp)	InDels (*Kabuli* reference genome-CDC Frontier/PII)	Forward primers (5'-3')	Reverse primers (5'-3')	Anneling temperature (0C)	Expected amplified product size (bp)	Structural annotation		Functional annotation	
								Sequence components of *kabuli* genome	*Kabuli* gene accession IDs	NCBI-KOG	TFs
Ca1_P3_5347843	Ca1	5347843	TT/TTAT	ATGAGACAGCAACGAC AACG	CGCTTCTTACTTGTAC GGTCG	59.9	652	INTERGENIC	--	--	--
Ca1_P3_5349356	Ca1	5349356	ACC/AC	TGAACTAGATCATCTGC GTAAAAT	GGGAAAGGGAGAGAG TACGG	57.2	660	INTERGENIC	--	--	--
Ca1_P3_5351086	Ca1	5351086	CAAA/CAA	TGTGGCAGACAGCCAA CTAA	TTTGGCAATCGAAAAT TGTG	60.4	607	INTERGENIC	--	--	--
Ca1_P3_5371220	Ca1	5371220	CAAAAAAAAAAA/CAAAAAAAAA	TGAAATATCACACAGCC CAAA	TTTGCGACAAAAATCA AAAAGA	59.0	474	DRR	Ca_00623	S	--
Ca1_P3_5372427	Ca1	5372427	A/ATTATT	CCAACCCTGTCCCTCTT ACA	TGATGGATCATGTGTT TGTGG	60.0	381	INTERGENIC	--	--	--
Ca1_P3_5375317	Ca1	5375317	A/AAAAGTTT	TATGATGTGCTTGTGG AGCC	GCCCTAATAAGCCAAC CCAT	59.7	515	INTERGENIC	--	--	--
Ca1_P3_5390962	Ca1	5390962	CTT/CT	TGTTCAGCAGAAGCAT GTCC	GGGACCAAATCAAGTG CCTA	60.0	808	INTERGENIC	--	--	--
Ca1_P3_5391770	Ca1	5391770	GAAAAAAA/GAAAAAAAA	TAGGCACTTGATTTGGT CCC	TCAACCAAACAAATCC ACCA	59.9	726	INTERGENIC	--	--	--
Ca1_P3_5393298	Ca1	5393298	ATTTTTTTT/ATTTTTTTTTT	AACTTCCTTGACATGGC CTC	TGATGTTGTTGGAGAT GGAAA	59.1	505	INTERGENIC	--	--	--
Ca1_P3_5393351	Ca1	5393351	ATTTTTTT/ATTTTTTTT	AACTTCCTTGACATGGC CTC	TGATGTTGTTGGAGAT GGAAA	59.1	505	INTERGENIC	--	--	--

**INDEL marker IDs**	**Chromosomes/ unanchored scaffolds**	**Physical positions (bp)**	**InDels (*Kabuli* reference genome-CDC Frontier/PII)**	**Forward primers (5'-3')**	**Reverse primers (5'-3')**	**Anneling temperature (0C)**	**Expected amplified product size (bp)**	**Structural annotation**		**Functional annotation**	
								**Sequence components of *kabuli* genome**	***Kabuli* gene accession IDs**	**NCBI-KOG**	**TFs**
Ca1_P3_5412267	Ca1	5412267	TAAAAAAA/TAAAAAA	ACCGAGTTGTCCTACCTGGA	CTTAGCCGGAGTCCAACATC	59.6	896	INTERGENIC	--	--	--
Ca1_P3_5418395	Ca1	5418395	GTTTTTTTTTTTT/GTTTTTTTTTT	GGGGCCATAGTGAGTTGAAA	TCAGCCAAAAGATGACCAAA	59.9	215	INTERGENIC	--	--	--
Ca1_P3_5421386	Ca1	5421386	AA/AAATA	GCTGGGTTTCAGGTTTGAGA	CAGGGTGGCTCTTGCTAAAC	60.2	579	INTERGENIC	--	--	--
Ca1_P3_5422480	Ca1	5422480	TCA/T	TCCAACGAATAATTTTAGTCCCTC	TGGTTTTTACTCATCGCACA	59.8	455	INTERGENIC	--	--	--
Ca1_P3_5422837	Ca1	5422837	GTATAATGTT/GTATAATGTTATAATGTT	TGTGCGATGAGTAAAAACCA	GGGAATACAAAAACTACCCCG	57.8	497	INTERGENIC	--	--	--
Ca1_P3_5423457	Ca1	5423457	CAGAGA/CAGA	CGGGGTAGTTTTTGTATTCCC	GGTGACGCCTTTTAATTTGC	59.6	640	INTERGENIC	--	--	--
Ca1_P3_5425431	Ca1	5425431	TAA/TAAGAA	TGCCTTGCTTATTGGGATTT	TCCCGAACCTGTGAGGTTAT	59.5	457	INTERGENIC	--	--	--
Ca1_P3_5427164	Ca1	5427164	ACTC/ACTCTC	CAAAACACGAGACAAAGCCA	GCGATGGGATGGAGTTAGAA	59.9	535	INTERGENIC	--	--	--
Ca1_P3_5428791	Ca1	5428791	TTATTATAT/TTAT	TTAATGGGTAGGTGGGTGGA	TTTTCAGCGGTGTCACTGAG	60.0	839	INTERGENIC	--	--	--
Ca1_P3_5429701	Ca1	5429701	TT/TTTAAAAAT	CTCAGTGACACCGCTGAAAA	AGGTATGTCCACGCACAACA	60.0	653	INTERGENIC	--	--	--

INDEL marker IDs	Chromosomes/ unanchored scaffolds	Physical positions (bp)	InDels (*Kabuli* reference genome-CDC Frontier/PII)	Forward primers (5'-3')	Reverse primers (5'-3')	Anneling temperature (0C)	Expected amplified product size (bp)	Structural annotation		Functional annotation	
								Sequence components of *kabuli* genome	*Kabuli* gene accession IDs	NCBI-KOG	TFs
Ca1_P3_5433441	Ca1	5433441	TC/T	TCGGGATTTGTTTGTTTACCA	GGGCCCTATGCCTTTTTATG	60.2	511	INTERGENIC	--	--	--
Ca1_P3_5436359	Ca1	5436359	CTTATTATTATTATTA/CTTATTATTATTA	AGATGGGATCGAATGAATGG	ACCCCCGCACACTCATTAT	59.7	375	INTERGENIC	--	--	--
Ca1_P3_5446706	Ca1	5446706	GTTTTT/GTTTTTTT	TGACGCAGTCTCATAGTCGG	GCAATTGTCAAGCACCATGT	60.0	336	DRR	Ca_00630	--	--
Ca1_P3_5455204	Ca1	5455204	TTGTT/TTGTTAATGTT	TGGTTGAACTGGGACCATTA	GGCATCAACAACAGCAAGAA	58.8	300	INTERGENIC	--	--	--
Ca1_P3_5460761	Ca1	5460761	ATTTTTTTTT/ATTTTTTTT	TTTTTGTACGAGTTGCCATTT	CCATGTGAATTGCACACCAT	57.4	456	INTERGENIC	--	--	--
Ca1_P3_5470320	Ca1	5470320	AAGGAGGAG/AAGGAG	TTGCTGACGAGTTTCATTCG	TGATAATATCCTCGGGGCTG	60.0	588	INTRON	Ca_00632	O	--
Ca1_P3_5482656	Ca1	5482656	A/AATTTATT	TCTCATCCCAACACAAACACA	GATTTGCTGCCACTCCTCTT	60.0	650	DRR	Ca_00633	--	S1Fa-like
Ca1_P3_5484846	Ca1	5484846	CTTT/CTTTT	CGCGCGTTGTGTTTATAAGT	TTACCAAGATTGCCCCTCTG	58.9	444	INTERGENIC	--	--	--
Ca1_P3_5484865	Ca1	5484865	ATTTTTTT/ATTTTTT	CGCGCGTTGTGTTTATAAGT	TTACCAAGATTGCCCCTCTG	58.9	444	INTERGENIC	--	--	--
Ca1_P3_5500587	Ca1	5500587	TAAAAAA/TAAAAAAA	CACTCACTCACACACACATGC	TTTCTCATCGATTCCCTTCC	58.7	677	INTERGENIC	--	--	--

INDEL marker IDs	Chromosomes/ unanchored scaffolds	Physical positions (bp)	InDels (*Kabuli* reference genome-CDC Frontier/PII)	Forward primers (5'-3')	Reverse primers (5'-3')	Anneling temperature (0C)	Expected amplified product size (bp)	Structural annotation		Functional annotation	
								Sequence components of *kabuli* genome	*Kabuli* gene accession IDs	NCBI-KOG	TFs
Ca1_P3_5501358	Ca1	5501358	CAA/CA	TTTCCCCCAACCAATGAATA	CTTGCTTTGTGCCATGAAGA	60.0	523	INTERGENIC	--	--	--
Ca1_P3_5510732	Ca1	5510732	ACTTTCTTTCTTTCT/ACTTTCTTTCT	TTTTTGGAGCCTCAGAATGG	ACACAGCCCACCCAAAATAA	60.2	659	INTRON	Ca_00636	--	FAR1
Ca1_P3_5513383	Ca1	5513383	GAAA/GAA	TTTCCAACATGCGTCTCTCA	CATTGTGTGCCTTGCAGTTT	60.4	610	INTRON	Ca_00636	--	FAR1
Ca1_P3_5640191	Ca1	5640191	CTCTGAGTCTGAGTCTGAGTCTGAGTCTG/CTCTGAGTCTGAGTCTGAGTCTGAGTCTGAGTCTG	TCTCGAAGCAACACTCATGG	ATGCCGGAGGTCTAAGGTTT	60.0	661	CDS (large-effect mutations)	Ca_00652	V	--
Ca1_P3_5664340	Ca1	5664340	ATTTTTTT/ATTTTTTTT	TGGAAACTAATCTTGTTCGAGG	GTGCTTGCATGTGAAGGAGA	58.4	507	DRR	Ca_00656	--	WRKY
Ca1_P3_5670926	Ca1	5670926	GTATATATATATATATATATA/GTATATATATATATATATATATA	CGCAACGACAAGAAATTCAA	TGCATGTTCTTACGCTCGTC	59.8	716	INTERGENIC	--	--	--
Ca1_P3_5754591	Ca1	5754591	CATA/CA	AACCTCCAGATCCTCGAACA	CTCCACAAAGAAAAGGGCAA	59.7	344	DRR	Ca_00667	J	TALE
Ca1_P3_5803481	Ca1	5803481	TTAAGATAAGAT/TTAAGAT	GCTCATGAGTTCCCTACCCA	GTCAGGGGCTCGTGTATGAT	60.1	560	INTRON	Ca_00671	O	FAR1
Ca1_P3_5804081	Ca1	5804081	AT/ATT	AATAACACGGGCACAAGAGG	AGGTCCACCAGGTTTGTCAG	60.0	457	INTRON	Ca_00671	O	FAR1
Ca1_P3_5804105	Ca1	5804105	AG/A	AATAACACGGGCACAAGAGG	AGGTCCACCAGGTTTGTCAG	60.0	457	INTRON	Ca_00671	O	FAR1

INDEL marker IDs	Chromosomes/ unanchored scaffolds	Physical positions (bp)	InDels (*Kabuli* reference genome-CDC Frontier/PII)	Forward primers (5'-3')	Reverse primers (5'-3')	Anneling temperature (0C)	Expected amplified product size (bp)	Structural annotation		Functional annotation	
								Sequence components of *kabuli* genome	*Kabuli* gene accession IDs	NCBI-KOG	TFs
Ca1_P3_582587	Ca1	582587	TAAAA/TAAA	TGCGCTAGTTTGTGGATGTC	GACAAAACAGGAAGGGCAAA	59.9	614	DRR	Ca_00065	A	--
Ca1_P3_5952722	Ca1	5952722	TTATATATATATATATATATATATAT/TTATATATATATATATATATATAT	TTCCCACACACACAAAATCC	GTGGTGGCGCCTTATGTAAC	59.2	756	INTERGENIC	--	--	--
Ca1_P3_5998961	Ca1	5998961	TATAATAATAATAATAATAATAATAATAATAATAATAATAATAATAATAATAATAATA/TATAATAATAATAATAATAATAATAATAATAATAATAATAATAATAATAATAATAATAATAATAATAATAATA	ATAGCGCACCGTCTCTCTGT	ATTTTGTGGCACATGAAGTCT	60.0	872	DRR	Ca_00687	--	--
Ca1_P3_6129000	Ca1	6129000	CTTTTT/CTTT	CGTCATGTATCTTGGTCGGA	TGTTTAACACATTAACATGACCCA	59.5	556	INTERGENIC	--	--	--
Ca1_P3_6130817	Ca1	6130817	TAAA/TAA	GAACATCACAGACCCACACG	TCCTCTTCCACAAACCATCC	60.0	670	DRR	Ca_00698	--	--
Ca1_P3_6135478	Ca1	6135478	TAAAA/TAAA	TAGCACATCCTCCCCTCAAC	TGGATAACCAAAGGTGGCTC	60.1	313	INTERGENIC	--	--	--
Ca1_P3_6135753	Ca1	6135753	CTTTTT/CTTTT	AACACTTGAGCCACCTTTGG	CCCACTTTGAGTAAACCACGA	60.1	370	INTERGENIC	--	--	--
Ca1_P3_6138299	Ca1	6138299	AT/ATT	GGCACGATGTTCTTGATTTTT	CCGATTGTTGATCGAGGTTT	59.1	846	INTERGENIC	--	--	--
Ca1_P3_6141472	Ca1	6141472	CTTT/CTT	AGCAATTCTTCCATCCCACA	ACATTGTGTGGAACAAGGCA	60.5	157	INTERGENIC	--	--	--
Ca1_P3_6148078	Ca1	6148078	CAA/CA	TTGCTATTCCCTTTCCCCTT	CTCTCATCTCTGGCCTTTGG	59.9	558	DRR	Ca_00699	S	--

INDEL marker IDs	Chromosomes/ unanchored scaffolds	Physical positions (bp)	InDels (*Kabuli* reference genome-CDC Frontier/PII)	Forward primers (5'-3')	Reverse primers (5'-3')	Anneling temperature (0C)	Expected amplified product size (bp)	Structural annotation		Functional annotation	
								Sequence components of *kabuli* genome	*Kabuli* gene accession IDs	NCBI-KOG	TFs
Ca1_P3_6150658	Ca1	6150658	GAAAAAAAAAAAAAAA/GAAAAAAAAAAAAAAAAAA	CGCTCATAGACCTGATTGACC	TGAAGTTCGCAACGAATCAG	59.7	419	INTERGENIC	--	--	--
Ca1_P3_6156071	Ca1	6156071	AACACAC/AACAC	TTCGTGAAAGCTTCCGTCTT	TTTAACCGCTGGTTTTACCG	60.0	315	DRR	Ca_00700	G	--
Ca1_P3_6156406	Ca1	6156406	TTA/TTATA	TTGGCAAAAATGCGATAACA	TCCATCAAACCAAATGAACA	60.1	847	DRR	Ca_00700	G	--
Ca1_P3_6158170	Ca1	6158170	ATTTT/ATTT	TGGTTTGTCATTCCGGTCAT	ATGTCTCATTCATGCTCCCC	61.2	359	INTERGENIC	--	--	--
Ca1_P3_6158400	Ca1	6158400	TAAAA/TAAAAA	GGGGAGCATGAATGAGACAT	AAACCGCGACAATTTAATCTC	59.9	387	INTERGENIC	--	--	--
Ca1_P3_6166149	Ca1	6166149	TCACAC/TCAC	AACGTGACACATTCGCACTC	CTTTGGTTCCAACGTATAGTTTTT	59.8	862	INTERGENIC	--	--	--
Ca1_P3_6175294	Ca1	6175294	GTTTT/GTTT	CCTTAACTGGGTCACGTGCT	CGATCTCATGCCACAACTTT	60.2	521	INTERGENIC	--	--	--
Ca1_P3_6177278	Ca1	6177278	TAAAAAA/TAAAAA	CCAAACTTTGTTTTAAAATACATCAGA	CTTTGGCATTCCTCATTTTCT	59.1	866	INTERGENIC	--	--	--
Ca1_P3_6202765	Ca1	6202765	T/TATA	CGCTTCCTTCTTCACACACA	CTCGTGTCTCAAAACTCAAGGA	60.0	877	INTERGENIC	--	--	--
Ca1_P3_6203281	Ca1	6203281	GTTTTTTTTT/GTTTTTTTTTT	CTCCTTGAGTTTTGAGACACGA	TCGATTATGCGTAGGGAAGG	59.5	453	INTERGENIC	--	--	--

INDEL marker IDs	Chromosomes/ unanchored scaffolds	Physical positions (bp)	InDels (*Kabuli* reference genome-CDC Frontier/PII)	Forward primers (5'-3')	Reverse primers (5'-3')	Anneling temperature (0C)	Expected amplified product size (bp)	Structural annotation		Functional annotation	
								Sequence components of *kabuli* genome	*Kabuli* gene accession IDs	NCBI-KOG	TFs
Ca1_P3_6204822	Ca1	6204822	AAGA/AA	TGGTTTGGTTGCATCACTTT	TCGAAATTTGTGTTCCAAAAGA	59.0	543	DRR	Ca_07880	T	--
Ca1_P3_6205233	Ca1	6205233	CAAAAAAAAAAA/CAAAAAAAAAAAA	GGGTATTGGATAAAAATTTCAAAAA	TGAAAATTAAGGGACGGGTTT	58.7	604	DRR	Ca_07880	T	--
Ca1_P3_6206056	Ca1	6206056	ACCCC/ACCCCC	TGTGCTCCAATCATGGATGT	CGCACTGGTTGAAAGCATAA	59.9	323	CDS (FRAME SHIFT)	Ca_07880	T	--
Ca1_P3_6206345	Ca1	6206345	CATATATATATA/CATATATATATATA	TGTGCTCCAATCATGGATGT	CGAACAAAAGGAGAATCTGACA	59.9	380	INTERGENIC	--	--	--
Ca1_P3_6206568	Ca1	6206568	CTTTTTT/CTTTTTTTT	TTATGCTTTCAACCAGTGCG	TGTCGGATAAGTGTGATATATTCG	59.9	747	INTERGENIC	--	--	--
Ca1_P3_6212978	Ca1	6212978	AATATATATATAT/AATATATATATATAT	GTGCGCAGTACAATTCTCCA	CAACCGCTCCCAAATAGAAA	59.9	573	INTERGENIC	--	--	--
Ca1_P3_6214084	Ca1	6214084	TAAACTAATTTTTTAATTGAAACTA/TAAACTAATTTTTTAATTGAAACTAATTTTTTAATTGAAACTA	TGTCACCGTTATACGAGTTTTAGA	TTGGCGTAGTGAAACGAACA	58.0	618	INTERGENIC	--	--	--
Ca1_P3_6259182	Ca1	6259182	AATGGTCATGGTCATGGTCA/AATGGTCATGGTCA	TGATGATTTTGCTCTTCCCC	GTCCACTCCACCGATCAACT	60.0	575	INTRON	Ca_07885	R	bHLH
Ca1_P3_6267078	Ca1	6267078	GAAA/GA	TTTCTTGCACTGGTTTGTGC	TGGGTCCCTCATTGTACTCC	59.9	560	INTERGENIC	--	--	--
Ca1_P3_6268966	Ca1	6268966	GAAAAAA/GAAAAA	ATGACTGCGTGCTGAGTTGA	TTCTTTTCCAAACCACCAAA	60.6	634	INTERGENIC	--	--	--

**INDEL marker IDs**	**Chromosomes/ unanchored scaffolds**	**Physical positions (bp)**	**InDels (*Kabuli* reference genome-CDC Frontier/PII)**	**Forward primers (5'-3')**	**Reverse primers (5'-3')**	**Anneling temperature (0C)**	**Expected amplified product size (bp)**	**Structural annotation**		**Functional annotation**	
								**Sequence components of *kabuli* genome**	***Kabuli* gene accession IDs**	**NCBI-KOG**	**TFs**
Ca1_P3_6277414	Ca1	6277414	ATT/ATTT	TACTCCCATCCATGCAATCA	CCAATATGGAGTCCACGTCA	59.9	491	DRR	Ca_07888	--	--
Ca1_P3_6279828	Ca1	6279828	CTTTTTT/CTTTTT	ATCCGACGGTAACAGAAACG	CTGACCGACAACACTCCAAA	60.0	620	INTERGENIC	--	--	--
Ca1_P3_6284717	Ca1	6284717	ATTTTTTTTTTT/ATTTTTTTTTT	TACCTGCCGATTCTCATTCC	CTTGCAAGGTTTGTGCATGT	60.0	543	DRR	Ca_07889	--	--
Ca1_P3_6291176	Ca1	6291176	A/AG	CAGTGGCTTCTCATTTGGGT	TCCTTTCCTATTTCTCTCCAACA	60.1	553	INTERGENIC	--	--	--
Ca1_P3_6291858	Ca1	6291858	G/GGA	GTGCTATCAAATTTTGCGGA	ACTTATTGGGGAGTCTGGGG	58.8	429	INTERGENIC	--	--	--
Ca1_P3_6292164	Ca1	6292164	TT/TTGGATCCT	CAAGTGCATTTGGTCCATTG	TTAGTGTGTTGGGGAGGAGC	60.0	368	INTERGENIC	--	--	--
Ca1_P3_6293340	Ca1	6293340	TGATCGAT/TGAT	CATGCATTCCAATCTCACCA	GCTCTGCTTTTGTTATTTCCCA	60.5	471	INTERGENIC	--	--	--
Ca1_P3_6293614	Ca1	6293614	GATTAT/GAT	CATGCATTCCAATCTCACCA	TATCTTTTTGGTGGGCCAAG	60.5	786	INTERGENIC	--	--	--
Ca1_P3_6293780	Ca1	6293780	TAAA/TAAAA	ACACACTGATCGATGATCCC	TATCTTTTTGGTGGGCCAAG	58.3	716	INTERGENIC	--	--	--
Ca1_P3_6294872	Ca1	6294872	T/TC	AGTTTCCACGTTCACATCCC	ATGGTGGTTTTTAGAGCCCC	59.8	904	INTERGENIC	--	--	--

**INDEL marker IDs**	**Chromosomes/ unanchored scaffolds**	**Physical positions (bp)**	**InDels (*Kabuli* reference genome-CDC Frontier/PII)**	**Forward primers (5'-3')**	**Reverse primers (5'-3')**	**Anneling temperature (0C)**	**Expected amplified product size (bp)**	**Structural annotation**		**Functional annotation**	
								**Sequence components of *kabuli* genome**	***Kabuli* gene accession IDs**	**NCBI-KOG**	**TFs**
Ca1_P3_6298179	Ca1	6298179	ATTTT/ATTTTT	AACCAAGTTGGGGAAGGTTT	ATCGACAGAAGGCAGACTGG	59.7	421	DRR	Ca_07890	--	--
Ca1_P3_6298845	Ca1	6298845	T/TG	GAGGCAAAATTGGCTCTTGT	TAATTGACGACGACGACGAG	59.3	124	DRR	Ca_07890	--	--
Ca1_P3_6298941	Ca1	6298941	GTT/GTATTT	CTCGTCGTCGTCGTCAATTA	TTCAATGACATGATTGGAGACTTT	59.9	110	DRR	Ca_07890	--	--
Ca1_P3_6299112	Ca1	6299112	TGTGCATCTCATAAG/TG	CTCGTCGTCGTCGTCAATTA	TGAAAGCTCATTTTTCGTATGTTG	59.9	439	DRR	Ca_07890	--	--
Ca1_P3_6299712	Ca1	6299712	T/TGTTG	CAACATACGAAAAATGAGCTTTCA	GTGACATCAAGCCGGATTCT	60.5	895	DRR	Ca_07890	--	--
Ca1_P3_6299741	Ca1	6299741	GTT/GT	CAACATACGAAAAATGAGCTTTCA	GTGACATCAAGCCGGATTCT	60.5	895	DRR	Ca_07890	--	--
Ca1_P3_6300049	Ca1	6300049	TAAAA/TAAAAA	GTCCGAAAATTCATTCATTAAAA	GTGACATCAAGCCGGATTCT	57.3	544	INTERGENIC	--	--	--
Ca1_P3_6307314	Ca1	6307314	GTATAT/GTATATATAT	TGTGAAGTCATCCATGGTCAA	TTGCAGCATCTTTGTTCTCC	60.0	593	INTERGENIC	--	--	--
Ca1_P3_6310776	Ca1	6310776	TAA/TAAA	CATTGTGGCACTGAACGACT	CCGGGTAGAATACCGTGTTG	59.8	780	INTERGENIC	--	--	--
Ca1_P3_6311738	Ca1	6311738	ATAGT/AT	TTTTTGTGACTGTTGACTGCAT	TTTTCCCCTCATCGTTTGTC	58.3	275	INTERGENIC	--	--	--

INDEL marker IDs	Chromosomes/ unanchored scaffolds	Physical positions (bp)	InDels (*Kabuli* reference genome-CDC Frontier/PII)	Forward primers (5'-3')	Reverse primers (5'-3')	Anneling temperature (0C)	Expected amplified product size (bp)	Structural annotation		Functional annotation	
								Sequence components of *kabuli* genome	*Kabuli* gene accession IDs	NCBI-KOG	TFs
Ca1_P3_6312511	Ca1	6312511	ATTTTTTTTT/ATTTTTTTT	TGTGATTTCGGGATCAAATCT	TCCCTTAATTTCATTTGCGG	59.4	291	INTERGENIC	--	--	--
Ca1_P3_6317540	Ca1	6317540	TTG/T	ACAGGGCGATGACAAGAAAG	AAGATCCCCCATGGAAACTC	60.3	364	INTRON	Ca_07892	B	MYB_related
Ca1_P3_6328156	Ca1	6328156	TCAATGTTAATAC/T	TGGATCCACCATTTCCATTT	ACTGGTGGTGGGATTGATGT	60.0	763	DRR	Ca_07893	--	--
Ca1_P3_6332779	Ca1	6332779	CT/CTT	TTTTTCTGCCTGTTCTGCCT	TCATGATCGCGAAGTTGAAA	60.0	746	INTRON	Ca_07893	--	--
Ca1_P3_6336949	Ca1	6336949	TAA/TA	TGAGAAACGCGAAAACAATTT	TGGGCCGAATTATTTATCCA	59.8	345	INTERGENIC	--	--	--
Ca1_P3_6337132	Ca1	6337132	AAA/AAATAA	TCGGCCCACATTATTGTTTT	ATAAATCCCACAAGTGCCCA	60.2	258	INTERGENIC	--	--	--
Ca1_P3_6354181	Ca1	6354181	TAA/TAATGGGAAA	TGGTGGAAGCAAATAAAGGC	TAAGTGGCAGATGGGGAAAG	60.1	330	INTERGENIC	--	--	--
Ca1_P3_6354364	Ca1	6354364	ACAACAAATCAA/ACAACAAATCAACAAATCAA	CTTTCCCCATCTGCCACTTA	TTCCCCTCTCTTGCACATTC	60.1	373	INTERGENIC	--	--	--
Ca1_P3_6362169	Ca1	6362169	ACTAGCTA/ACTA	CTGCCACTGCAGACACTGTT	CCAAGTTCCATACCACCACC	60.1	232	INTRON	Ca_07898	--	--
Ca1_P3_6362466	Ca1	6362466	TAAAAA/TAAAAAA	GGTATGGAACTTGGGGTGTG	AGAAACATGCGCTCCAAACT	60.1	370	INTRON	Ca_07898	--	--

INDEL marker IDs	Chromosomes/ unanchored scaffolds	Physical positions (bp)	InDels (*Kabuli* reference genome-CDC Frontier/PII)	Forward primers (5'-3')	Reverse primers (5'-3')	Anneling temperature (0C)	Expected amplified product size (bp)	Structural annotation		Functional annotation	
								Sequence components of *kabuli* genome	*Kabuli* gene accession IDs	NCBI-KOG	TFs
Ca1_P3_6363555	Ca1	6363555	CAA/CAAA	GTTTCGGTTTGACGGAAGTC	CGTGCCATATGACCCTCTTT	59.6	806	INTERGENIC	--	--	--
Ca1_P3_6369470	Ca1	6369470	G/GA	TCAAAATTTTCGAACAAAACCC	ATTGCATATGCGGTCCCTTA	60.2	392	INTERGENIC	--	--	--
Ca1_P3_6370146	Ca1	6370146	CTT/C	GGAGAGATTGGGGGCATATT	AGTTTGTTGTTGCTTGCTGG	60.1	323	INTERGENIC	--	--	--
Ca1_P3_6370396	Ca1	6370396	TAATAA/TA	GGAGAGATTGGGGGCATATT	CCTTTAGCTTGGGCCATTTT	60.1	691	INTERGENIC	--	--	--
Ca1_P3_6374117	Ca1	6374117	CAA/CA	TGCCATTCAAAAATAAGGCA	TCCATCTTGGACCAATCTCA	59.1	792	INTERGENIC	--	--	--
Ca1_P3_6374330	Ca1	6374330	AAAGAAGAAGAAGAA/AAAGAAGAAGAA	CAAAATGATGGACACCCTTG	TCCATCTTGGACCAATCTCA	58.8	291	INTERGENIC	--	--	--
Ca1_P3_6374648	Ca1	6374648	ACTTCTT/ACTT	CAAAATGATGGACACCCTTG	CCAAACAAACCCCTTAAGTGAA	58.8	658	INTERGENIC	--	--	--
Ca1_P3_6376706	Ca1	6376706	TTGGATAAATTGTGGATAAATTGTG/TTGGATAAATTGTG	GCTCGTCCAAATATTTACGCA	CCCAGCAGTGAAGCAAACTT	60.1	379	INTERGENIC	--	--	--
Ca1_P3_6383346	Ca1	6383346	TAAGATAAAAGATAA/TAAGATAA	TACCGACATCGTTGTTTCCA	TTGACAACGAATGAAGCAGC	60.0	600	DRR	Ca_07899	--	--
Ca1_P3_6384180	Ca1	6384180	TTATAT/TTAT	TAGGCTCAAATCCACAACCC	TTGGAATAAAACATGTAAAAACTTCG	59.9	380	INTERGENIC	--	--	--

INDEL marker IDs	Chromosomes/ unanchored scaffolds	Physical positions (bp)	InDels (*Kabuli* reference genome-CDC Frontier/PII)	Forward primers (5'-3')	Reverse primers (5'-3')	Anneling temperature (0C)	Expected amplified product size (bp)	Structural annotation		Functional annotation	
								Sequence components of *kabuli* genome	*Kabuli* gene accession IDs	NCBI-KOG	TFs
Ca1_P3_6389745	Ca1	6389745	GTTT/GTT	TTTGTTGGAACTGGAC CACA	TCCATTACCTCCAAGC CAAG	60.0	233	DRR	Ca_07902	R	AP2
Ca1_P3_6403212	Ca1	6403212	AA/AATAATA	TCACGCACGTTAAACTT CCA	TGCACCGACCTACACA CATT	60.3	501	INTERGENIC	--	--	--
Ca1_P3_6406443	Ca1	6406443	TAAAAA/TAAAAAA	TCATTTTGTCACCTGCT CCA	TGCAAACCGTTGTGAT CAAT	60.2	158	DRR	Ca_07904	--	--
Ca1_P3_6413936	Ca1	6413936	ATTTTTTT/ATTTTTTTT	GGTGGCTTATGACATG CTGA	GCTTTTGCAACTTTCCT TGC	59.7	567	INTRON	Ca_07905	P	MIKC
Ca1_P3_6415075	Ca1	6415075	GTAATAATAATAATAATAAT/GTAATAAT AATAATAATAATAATAAT	TCCCTCCTTTTCTCCCT TGT	CCCTCGTTACATGTGC CTTT	60.0	971	INTRON	Ca_07905	P	MIKC
Ca1_P3_6425270	Ca1	6425270	GTT/GTTT	TCTTCTCTGGCAACCG TTCT	AGCCACCGTTGATGAA AATC	60.0	479	INTERGENIC	--	--	--
Ca1_P3_6425437	Ca1	6425437	G/GTTT	GATTTTCATCAACGGTG GCT	CGGTTACATGTTGGAT GGTG	59.9	113	INTERGENIC	--	--	--
Ca1_P3_6425687	Ca1	6425687	GAATGAAT/GAATGAATTTAATGAAT	GATTTTCATCAACGGTG GCT	TGGCTTGGATTTGGGA ATTA	59.9	795	INTERGENIC	--	--	--
Ca1_P3_6426698	Ca1	6426698	T/TA	TTTCTTTCTCACCATCC GCT	GGGTTGTCATAGCCCA AAGA	59.8	699	INTERGENIC	--	--	--
Ca1_P3_6436385	Ca1	6436385	GTACTTGTTTTACTTGT/GTACTTGT	ATCCAAACTCCCTTCTT GCC	TCGAAAGGAGGCAAAA TCAA	60.4	409	INTRON	Ca_07907	T	C3H

INDEL marker IDs	Chromosomes/ unanchored scaffolds	Physical positions (bp)	InDels (*Kabuli* reference genome-CDC Frontier/PII)	Forward primers (5'-3')	Reverse primers (5'-3')	Anneling temperature (0C)	Expected amplified product size (bp)	Structural annotation		Functional annotation	
								Sequence components of *kabuli* genome	*Kabuli* gene accession IDs	NCBI-KOG	TFs
Ca1_P3_6436872	Ca1	6436872	GAAAAAA/GAAAAAAA	AAAATCAATTTTCACCGCCA	AAAATTGCAGGGCTTTTTCC	60.3	295	INTRON	Ca_07907	T	C3H
Ca1_P3_6446871	Ca1	6446871	ATTTTTTT/ATTTTTT	CTAAACAACATGGTGGCACG	TTGGTCCTTACACCCTCACA	60.0	771	INTERGENIC	--	--	--
Ca1_P3_6452850	Ca1	6452850	ATT/ATTT	GGAGCCCAAACCAAGTCATA	GACTGGGACTTCTGGGACAA	59.9	714	INTERGENIC	--	--	--
Ca1_P3_6453816	Ca1	6453816	AT/ATT	GGTCTTGCTGACCTTGAAGC	CACGTTAAAATCTCTAACGATGAA	60.0	644	DRR	Ca_07909	--	Trihelix
Ca1_P3_6462386	Ca1	6462386	TG/TGG	GATCCGCGCGAATACTAAGA	ATAAGCTTTGTTACGCATCACTT	60.3	362	INTERGENIC	--	--	--
Ca1_P3_6463116	Ca1	6463116	TAAAAAAAAAA/TAAAAAAA	AACCATGAGGTCATAACGCC	TCATAACAAAATGCTTTCTTCAACA	59.8	332	INTERGENIC	--	--	--
Ca1_P3_6464875	Ca1	6464875	AT/A	CACATCCAATTGTGAATCCG	CAGAACATTGGCAACAGTGC	59.8	631	INTERGENIC	--	--	--
Ca1_P3_6468779	Ca1	6468779	TAAA/TAA	GAAGGGGTCTCCTCAACACA	TTTGGGTCAATCGATTTGGT	60.1	690	INTRON	Ca_07910	S	--
Ca1_P3_6470423	Ca1	6470423	AAA/AAAGAA	CGCAATGATTTCGAGAAAGA	ATCGAATCCCGAATTAGCCT	59.0	217	INTRON	Ca_07910	S	--
Ca1_P3_6471650	Ca1	6471650	ACGT/A	GAGTTGAAGGTTGGGGATGA	CGATGGTTCACTTGAACGTTAT	59.9	735	INTERGENIC	--	--	--

**INDEL marker IDs**	**Chromosomes/ unanchored scaffolds**	**Physical positions (bp)**	**InDels (*Kabuli* reference genome-CDC Frontier/PII)**	**Forward primers (5'-3')**	**Reverse primers (5'-3')**	**Anneling temperature (0C)**	**Expected amplified product size (bp)**	**Structural annotation**		**Functional annotation**	
								**Sequence components of *kabuli* genome**	***Kabuli* gene accession IDs**	**NCBI-KOG**	**TFs**
Ca1_P3_6475844	Ca1	6475844	C/CA	TGAGGACTGAACGTAACCCA	ATCAGGGAACCCCTAAAAGC	59.1	603	INTERGENIC	--	--	--
Ca1_P3_6476072	Ca1	6476072	GAAT/GAATTAAT	CAATTCAAACCCCCTTCCTT	AAATGATGCTCAAGCGACAA	60.2	623	INTERGENIC	--	--	--
Ca1_P3_6480157	Ca1	6480157	ATTTTT/ATTTT	AAGCGGCAGCATGTCTTACT	AACCAAGGAAGCAAAAAGCA	60.0	643	INTRON	Ca_07911	D	B3
Ca1_P3_6481070	Ca1	6481070	TA/T	TTTCACACCCACTTAAGCCC	AATGGGATTGTGGGATCGTA	60.0	433	INTRON	Ca_07911	D	B3
Ca1_P3_6488951	Ca1	6488951	ATTTTTTT/ATTTTTT	AATGCATCTTGGGCTGAAAC	TGGCATGAGAATTCTGGACA	60.1	363	INTRON	Ca_07911	D	B3
Ca1_P3_6493108	Ca1	6493108	ATTT/ATT	TGCAGAATTGCAGCACCTAC	GAAGACCGCCATGTTGAGTT	60.0	695	DRR	Ca_07911	D	B3
Ca1_P3_6512969	Ca1	6512969	TAAAAAAAAAA/TAAAAAAAA	GTTGGTGATGGCTGATTCCT	TGTCTGTGTCTTCTATGGTGGG	59.9	762	DRR	Ca_07914	R	G2-like
Ca1_P3_6514272	Ca1	6514272	CTGTGT/CTGT	GAATTGTACGCAGGACGGTT	AAGTTGGATCAACCCAATTTAGA	60.0	533	DRR	Ca_07914	R	G2-like
Ca1_P3_6515179	Ca1	6515179	CAT/CATAT	ACCGAAATGTGGTGCACTTA	CAACCGTCTCAGCCTTCTTC	59.1	460	INTERGENIC	--	--	--
Ca1_P3_6516147	Ca1	6516147	GTTTTTTTTTTTTTT/GTTTTTTTTTTT	CTATTAAGCGCGCACACAAA	CAGCATGCCAACCAACATAG	60.0	530	INTRON	Ca_07915	--	--

INDEL marker IDs	Chromosomes/ unanchored scaffolds	Physical positions (bp)	InDels (*Kabuli* reference genome-CDC Frontier/PII)	Forward primers (5'-3')	Reverse primers (5'-3')	Anneling temperature (0C)	Expected amplified product size (bp)	Structural annotation		Functional annotation	
								Sequence components of *kabuli* genome	*Kabuli* gene accession IDs	NCBI-KOG	TFs
Ca1_P3_6518494	Ca1	6518494	TAAAAAAAA/TAAAAAAAAAAA	TGGTTCGACCAACACAAATG	GAGCTTGTGCTTTCCCAATC	60.4	709	DRR	Ca_07915	--	--
Ca1_P3_6518769	Ca1	6518769	CA/CATA	TGACTCGTTTCAACTCACTCAA	TTTAGATAAACAACGGCGGG	58.6	579	DRR	Ca_07915	--	--
Ca1_P3_6527452	Ca1	6527452	AA/AAATAGA	CAAGATTCAATTCTCCCCCA	TTGAATCAAACGAATACCACAA	59.9	683	INTERGENIC	--	--	--
Ca1_P3_6527529	Ca1	6527529	TTAT/TTATAT	CAAGATTCAATTCTCCCCCA	TTGAATCAAACGAATACCACAA	59.9	683	INTERGENIC	--	--	--
Ca1_P3_6532709	Ca1	6532709	T/TG	GGGGTGTCAAGATGTTGGTT	TTTTTGGCTCCTACCACCAC	59.7	894	INTERGENIC	--	--	--
Ca1_P3_6532843	Ca1	6532843	CAAA/CAAAA	CAGCACCGATATTGATAAGCA	TTTTTGGCTCCTACCACCAC	58.8	396	INTERGENIC	--	--	--
Ca1_P3_6534234	Ca1	6534234	TATAAATAAA/TATAAA	AAATTTGAGGGGCACAACTG	TACAACTTTTCTGGGCCGAC	60.0	669	INTERGENIC	--	--	--
Ca1_P3_6534439	Ca1	6534439	CA/CAA	AAATTTGAGGGGCACAACTG	TACAACTTTTCTGGGCCGAC	60.0	669	INTERGENIC	--	--	--
Ca1_P3_6534633	Ca1	6534633	A/AC	TTACTGGGCCACGTTTACAA	TGGTTCCAAAGGAAAAGCAT	59.1	328	INTERGENIC	--	--	--
Ca1_P3_6537729	Ca1	6537729	A/ACTACTAAATTTGAAG	TTTGCCTCAAAGGACCAATC	TCACTTGGTCTTTCGGAACC	60.1	457	INTRON	Ca_07918	--	--

INDEL marker IDs	Chromosomes/ unanchored scaffolds	Physical positions (bp)	InDels (*Kabuli* reference genome-CDC Frontier/PII)	Forward primers (5'-3')	Reverse primers (5'-3')	Anneling temperature (0C)	Expected amplified product size (bp)	Structural annotation		Functional annotation	
								Sequence components of *kabuli* genome	*Kabuli* gene accession IDs	NCBI-KOG	TFs
Ca1_P3_6538753	Ca1	6538753	AT/ATT	CAAGGAAAGGGAATGGCATA	AATTGAAGGCTGAAACGTGG	59.9	787	DRR	Ca_07919	R	bZIP
Ca1_P3_6546764	Ca1	6546764	TCCCC/TCCC	TGGGACATGGCATCTCATAA	ATTCCAAACCACCGCTATTG	59.9	265	INTERGENIC	--	--	--
Ca1_P3_6546870	Ca1	6546870	TAAAAAA/TAAAA	TGGGACATGGCATCTCATAA	TTGGAGGGGACATGTTTGAT	59.9	753	INTERGENIC	--	--	--
Ca1_P3_6550647	Ca1	6550647	ATTTTTT/ATTTTTTT	ATGTGAGTCTTGGGCCACTC	CTTGTAGCCATGCCAAAACA	60.1	392	DRR	Ca_07920	P	MYB_related
Ca1_P3_6739259	Ca1	6739259	TTTAATGTTTGTTAATGTTTGTTAA/TTTAATGTTTGTTAA	TTGGTGTGTGAAGTGGGAAA	GATCACGTGTGAATTGGGAA	60.0	704	INTRON	Ca_07938	--	G2-like
Ca1_P3_6806099	Ca1	6806099	CA/C	TCATGCCTTCAAATTCCACA	ATGCGTGCTTCAAAACTCAA	60.0	970	DRR	Ca_07947	S	--
Ca1_P3_6972063	Ca1	6972063	CTATTATTATTATTATTATTATTATTATTATTATTATTATTATTATTATTATTATTATTATTATTATTATTATTAT/CTATTATTATTATTATTATTATTATTATTATTATTATTATTATTATTATTATTATTATTATTATTATTATTATT[illegible]	GTTTTCGTTCTCTCCTCCCC	CCGGTGCATTTTAACCTTGT	60.1	578	INTERGENIC	--	--	--
Ca1_P3_6999458	Ca1	6999458	TTATATATATATATATATATATATATAT/TTATATATATATATATATATATATATATAT	AACAACGGCTTTCATTGACTTT	TTTTTCCTGAAACAGGCAGT	60.0	795	INTERGENIC	--	--	--
Ca1_P3_7215414	Ca1	7215414	AA/AACA	AACCCCCAACTACAAGGTCC	CAATTTGACAAGCCCCCTAA	60.1	639	INTERGENIC	--	--	--
Ca1_P3_7274145	Ca1	7274145	AATATATATATATATATATATATATATA/AATATATATATATATATATATATATA	TGAATGAACTTTTATCCTGCCA	CAGCGTGAACGACTTCAAAA	59.6	539	INTERGENIC	--	--	--

INDEL marker IDs	Chromosomes/ unanchored scaffolds	Physical positions (bp)	InDels (*Kabuli* reference genome-CDC Frontier/PII)	Forward primers (5'-3')	Reverse primers (5'-3')	Anneling temperature (0C)	Expected amplified product size (bp)	Structural annotation		Functional annotation	
								Sequence components of *kabuli* genome	*Kabuli* gene accession IDs	NCBI-KOG	TFs
Ca1_P3_7382853	Ca1	7382853	GAAAAA/GAAAAAA	TCTCATTTGTACGGAAGGGAA	AACGAGGGCTCCTTATGTGA	59.6	646	INTERGENIC	--	--	--
Ca1_P3_7404769	Ca1	7404769	TGG/TG	CCCCGACTCAACATCTTCAT	GTTTTCTCCAGCTCGTGAGG	59.9	688	DRR	Ca_07993	--	--
Ca1_P3_7590751	Ca1	7590751	AG/A	TTCGCTTCCTTCGCTATTGT	GACCCATCTTTCTGCTCCAA	60.0	344	CDS (FRAME SHIFT)	Ca_08013	--	HD-ZIP
Ca1_P3_7630412	Ca1	7630412	G/GACAAAA	TCGTTGCGAAGATAGTGTGG	AGCATGAGTTGCACAAGTGG	59.9	768	CDS (large-effect mutations)	Ca_08016	--	HD-ZIP
Ca1_P3_7634633	Ca1	7634633	ATTTTTTTT/ATTTTTTTTTT	TCCAGCACAAATACCTTTTTACC	TGAATTCGCGTTGTTTGTGT	59.4	666	INTERGENIC	--	--	--
Ca1_P3_7636063	Ca1	7636063	GATTTATTTATTT/GATTTATTT	CCGGGAAACTTGGTTTAACA	CTGCCCATTTACCTCCTTGA	59.8	370	INTERGENIC	--	--	--
Ca1_P3_7642434	Ca1	7642434	TA/TAA	TGCCACACAAAAATCTTGGA	TAACCTACGCTTGTGGGGTC	60.1	219	INTERGENIC	--	--	--
Ca1_P3_7648137	Ca1	7648137	GAAAA/GAAAAA	TGTTGCCTGCTATGTGAAGC	AGATGCCTTAATGCAATGCC	60.0	778	INTRON	Ca_08018	--	bZIP
Ca1_P3_7649138	Ca1	7649138	GTGA/GTGAAAATTTTTTTGATGA	ACCCAGCACTTCCATTTGTC	GCCTATTCCCACCCCTACAT	60.0	642	DRR	Ca_08019	Z	--
Ca1_P3_7653498	Ca1	7653498	TAAAAAAAAAAAA/TAAAAAAAAAA	AGTGTTGAGGGCATTTTTGG	TGAGTCCAAAAGCATAAATGAGA	60.0	573	INTERGENIC	--	--	--

INDEL marker IDs	Chromosomes/ unanchored scaffolds	Physical positions (bp)	InDels (*Kabuli* reference genome-CDC Frontier/PII)	Forward primers (5'-3')	Reverse primers (5'-3')	Anneling temperature (0C)	Expected amplified product size (bp)	Structural annotation		Functional annotation	
								Sequence components of *kabuli* genome	*Kabuli* gene accession IDs	NCBI-KOG	TFs
Ca1_P3_7659682	Ca1	7659682	CGGGGGGGGGGGGG/CGGGGGGGG	TTAGCCTCTCGCCCTTGTAA	TCGAAATAGCAAAACCGTCC	60.0	593	INTERGENIC	--	--	--
Ca1_P3_7660044	Ca1	7660044	CTAA/CTAATTAA	TTAGCCTCTCGCCCTTGTAA	TCGAAATAGCAAAACCGTCC	60.0	593	INTERGENIC	--	--	--
Ca1_P3_7665875	Ca1	7665875	TA/T	GAATGGAATCAATTGGGTGG	AGGAAGAGACCCAACAAGCA	60.0	256	DRR	Ca_08020	--	--
Ca1_P3_7670112	Ca1	7670112	ATTTT/ATTTTT	TTTCAATCAAAACCCTTGCC	TCGCATAAAGTTGACTCCCC	59.9	868	INTERGENIC	--	--	--
Ca1_P3_7670138	Ca1	7670138	AAATAA/AAA	TTTCAATCAAAACCCTTGCC	TCGCATAAAGTTGACTCCCC	59.9	868	INTERGENIC	--	--	--
Ca1_P3_7671456	Ca1	7671456	TC/T	GGCCTTGAACGTTTAACCAA	AAGCGCTTGTTTGAAGTGGT	60.0	514	INTERGENIC	--	--	--
Ca1_P3_7671768	Ca1	7671768	ACCCC/ACCCCC	GGCCTTGAACGTTTAACCAA	TGTCGTCATAAGGTGGTGGA	60.0	699	INTERGENIC	--	--	--
Ca1_P3_7675303	Ca1	7675303	AA/AATA	CATGCGATTTCTTAGCTTAATTTT	TCGGATTTAACTTGGGTCTCA	58.2	858	INTERGENIC	--	--	--
Ca1_P3_7675315	Ca1	7675315	AA/AAGA	TGGTATTGAATTTTATGGATGTTTG	TCGGATTTAACTTGGGTCTCA	59.1	505	INTERGENIC	--	--	--
Ca1_P3_7678036	Ca1	7678036	TAAAAAAAA/TAAAAAAA	ATCGGAATCAAACCACTTCG	ACTTTGTCATCTGTGTGCGG	59.9	852	INTERGENIC	--	--	--

INDEL marker IDs	Chromosomes/ unanchored scaffolds	Physical positions (bp)	InDels (*Kabuli* reference genome-CDC Frontier/PII)	Forward primers (5'-3')	Reverse primers (5'-3')	Anneling temperature (0C)	Expected amplified product size (bp)	Structural annotation		Functional annotation	
								Sequence components of *kabuli* genome	*Kabuli* gene accession IDs	NCBI-KOG	TFs
Ca1_P3_7685475	Ca1	7685475	AA/AACAATAGGA	TGTCGGGCCTTCTAAT GAAC	CGCTCCAACAGCTAAC ATCA	60.1	165	INTERGENIC	--	--	--
Ca1_P3_7687191	Ca1	7687191	AC/A	CACGACACAAAATTTCA CTTATGA	GGATGGACTTCATGCT TCCT	59.1	369	INTERGENIC	--	--	--
Ca1_P3_7700414	Ca1	7700414	TG/T	TGGATGTTGATTTTTCC CAAA	TCAAGGTTGGCCAAGA AAAG	60.2	708	INTERGENIC	--	--	--
Ca1_P3_7706630	Ca1	7706630	TAAAAAAA/TAAAAAAAAA	TTTGGTGTCGGTTTGA CAAT	AAATTTCCAGACACAT GCCC	58.9	685	INTERGENIC	--	--	--
Ca1_P3_7707553	Ca1	7707553	A/ATTTT	GCAACCATGTGAGAAG CAAA	TAGCAGGTGGTTGTGG ATCA	59.8	431	INTERGENIC	--	--	--
Ca1_P3_7711941	Ca1	7711941	AGTT/A	CGCATCAGCCAACTAC TCAA	GCCTATGACGAAGCTT CCAA	60.0	314	DRR	Ca_08023	K	bHLH
Ca1_P3_7712937	Ca1	7712937	CAAA/CAAAA	CACGACCTAAAACCGA ATGC	CCCCTTCATGTTGTTC GTTT	60.5	428	INTERGENIC	--	--	--
Ca1_P3_7726229	Ca1	7726229	CAAAAAAAA/CAAAAAAAAA	TTTAGTGCCCGAGAGG AAGA	TTTGAGTCCCAACCAA AGGA	59.9	394	INTERGENIC	--	--	--
Ca1_P3_7726542	Ca1	7726542	TGCGCG/TATATGCGCGCG	GCGTGATCCATCATTT GACA	GGAAATTGCTCAAAAC TCACG	60.5	566	INTERGENIC	--	--	--
Ca1_P3_7727805	Ca1	7727805	CAAT/CAATAAT	TGAGAGATCAACCGTC TTGCT	CCATTGGAGAAAGCTT GGAA	60.0	420	INTERGENIC	--	--	--

INDEL marker IDs	Chromosomes/ unanchored scaffolds	Physical positions (bp)	InDels (*Kabuli* reference genome-CDC Frontier/PII)	Forward primers (5'-3')	Reverse primers (5'-3')	Anneling temperature (0C)	Expected amplified product size (bp)	Structural annotation		Functional annotation	
								Sequence components of *kabuli* genome	*Kabuli* gene accession IDs	NCBI-KOG	TFs
Ca1_P3_7728817	Ca1	7728817	ATTTTTTTT/ATTTTTTTTT	CATCTGATCCAAAATTGAGCC	AAAACCCATTTTGCAAATCAA	59.5	390	INTERGENIC	--	--	--
Ca1_P3_7748872	Ca1	7748872	CTATATATATATATATATATATATATATATATATATATATAT/CTATATATATATATATATATATATATATATATATATATATAT	TTCCAACTTAATGCACAAAAAGAA	TCTCCATCACTCTCCTTTACGA	60.0	234	INTERGENIC	--	--	--
Ca1_P3_7750166	Ca1	7750166	ATTT/ATTTT	TTGCAATAGGCGTTGAAGTTT	AGGTCAAGGGTTGGGCTACT	59.8	649	INTERGENIC	--	--	--
Ca1_P3_7750284	Ca1	7750284	AAAGAA/AAA	TTGACAATAGAATCGATCAAAAACA	AGGTCAAGGGTTGGGCTACT	59.9	312	INTERGENIC	--	--	--
Ca1_P3_7752633	Ca1	7752633	A/AC	CCTCGTAAGCCTTAGGGTCC	GGCATGGCTCATGGTAAATC	60.1	783	DRR	Ca_08027	--	GRAS
Ca1_P3_7753341	Ca1	7753341	TAAAAAAAAAA/TAAAAAAAAA	TAAATAGGGTCCACCATGCC	CGGTACGTAAATGGGTTCGT	59.6	263	DRR	Ca_08027	--	GRAS
Ca1_P3_7756723	Ca1	7756723	TTCTCT/TTCT	GAAAGCAAATCTTCCATCCAA	CTCCGATTGTCGGTGCTAAT	59.1	793	INTERGENIC	--	--	--
Ca1_P3_7765877	Ca1	7765877	ATTTTTTTTTTTT/ATTTTTTTTTT	GCGAATCTGGATCTGAAGAAA	CCCTCTGCAATGTCGGTAAT	59.4	458	INTERGENIC	--	--	--
Ca1_P3_7774878	Ca1	7774878	T/TC	ATGTTTGCTTTAGGAGCCCA	ATCAGGCTTGGTTCAACGAT	59.7	422	INTERGENIC	--	--	--
Ca1_P3_7777758	Ca1	7777758	GATATATATATATATATATATATATATATATATAT/GATATATATATATATATATATATATATAT	TTCCATGACGATTTGACGAG	CGTCGCACGGAAATAATGTA	59.6	552	INTERGENIC	--	--	--

INDEL marker IDs	Chromosomes/ unanchored scaffolds	Physical positions (bp)	InDels (*Kabuli* reference genome-CDC Frontier/PII)	Forward primers (5'-3')	Reverse primers (5'-3')	Anneling temperature (0C)	Expected amplified product size (bp)	Structural annotation		Functional annotation	
								Sequence components of *kabuli* genome	*Kabuli* gene accession IDs	NCBI-KOG	TFs
Ca1_P3_7778630	Ca1	7778630	CTTT/C	CCACACACATCAACCACCAC	GGGCGATTGACTTGAGACAT	60.8	444	INTERGENIC	--	--	--
Ca1_P3_7782186	Ca1	7782186	C/CGTAATATAATTT	TTGTCGAAAAGAGGAAGTGCT	CAAGACACACCCTTGGGATT	59.1	448	INTERGENIC	--	--	--
Ca1_P3_7784640	Ca1	7784640	T/TG	ATTGGGCATGTTTCGTCTGT	TGGGTAGACCTCTTTGACCG	60.4	411	INTERGENIC	--	--	--
Ca1_P3_7784813	Ca1	7784813	CA/C	ATTGGGCATGTTTCGTCTGT	TGGGTAGACCTCTTTGACCG	60.4	411	INTERGENIC	--	--	--
Ca1_P3_7786049	Ca1	7786049	GTTAATT/GTT	ATAGGGGAGGGGTTGTCTTG	ACGACCAGCTGGGTATGAAC	60.2	553	INTERGENIC	--	--	--
Ca1_P3_7789078	Ca1	7789078	TAAAAAAAAAAAAA/TAAAAAAAAAA	AGGGAAGAAGCACGATCTTT	AGATGTTAACCCGCGTTTTG	58.0	728	INTERGENIC	--	--	--
Ca1_P3_7791444	Ca1	7791444	CTTTTTTTT/CTTTTTTTTT	TGGCTCACTCTTGTTGCATT	GGATGGATCTTTCACCTCCA	59.4	493	INTERGENIC	--	--	--
Ca1_P3_7810052	Ca1	7810052	TCTAA/TCTAACTAA	CTTTGTGGGCAAACCAAACT	GGGTGGTTGAAACTTCAGGA	60.0	521	DRR	Ca_08031	--	FAR1
Ca1_P3_7841172	Ca1	7841172	A/AC	TTTTGGCGTTGTTGATGTGT	GTACCACACGGGAGCTTCAT	60.0	839	INTERGENIC	--	--	--
Ca1_P3_7841312	Ca1	7841312	TT/TTGT	CACGACCGTCCGATTAAGAT	GTTGTACCACACGGGAGCTT	60.0	473	INTERGENIC	--	--	--

INDEL marker IDs	Chromosomes/ unanchored scaffolds	Physical positions (bp)	InDels (*Kabuli* reference genome-CDC Frontier/PII)	Forward primers (5'-3')	Reverse primers (5'-3')	Anneling temperature (0C)	Expected amplified product size (bp)	Structural annotation		Functional annotation	
								Sequence components of *kabuli* genome	*Kabuli* gene accession IDs	NCBI-KOG	TFs
Ca1_P3_7844969	Ca1	7844969	TAA/TA	TGAATGGACCTCCAAC GAAT	TTCAAAAATGAATTTTA GGAAGAGC	60.3	434	DRR	Ca_08034	--	GRAS
Ca1_P3_7893707	Ca1	7893707	CAAAAAAAAA/CAAAAAAAA	AGAACGGCAAGTTAAT GCAA	TCTGTGGTTGCGAAGT TTTG	58.4	703	INTERGENIC	--	--	--
Ca1_P3_7899699	Ca1	7899699	ATTTTTTTTT/ATTTTTTTT	AACGTTGGGTGCAAAA AGTC	TTCCTGGAAAGTTGTT GGAGA	60.0	565	INTERGENIC	--	--	--
Ca1_P3_7914166	Ca1	7914166	T/TG	TGCATAGGTTGTTGTG GGAA	GACGAGAGGTTGGAG ACTCG	60.0	939	INTERGENIC	--	--	--
Ca1_P3_7915897	Ca1	7915897	GAAAA/GAAAATAAAA	CTAACCTCTGGAGCAC CGAC	GCACAATACAAGGCTG AGCA	59.9	459	INTERGENIC	--	--	--
Ca1_P3_7918757	Ca1	7918757	A/AT	TGGAAGTTGTTAAATGT GTCCTAAA	CATCCATTGGAAAGGG TGAC	59.1	700	DRR	Ca_08041	J	--
Ca1_P3_7929093	Ca1	7929093	GA/G	AATGCCTAAAAGCAATG CAAA	AACACGATGAGATGGA AGGG	59.7	527	INTERGENIC	--	--	--
Ca1_P3_7929432	Ca1	7929432	T/TTA	ATATCCGCAAGCTGAAT CGT	GGTTTGAACCCTCCAC AAGA	59.7	677	INTERGENIC	--	--	--
Ca1_P3_7930160	Ca1	7930160	GAAAAAAAAA/GAAAAAAAAAA	GGGTTCAAACCCCACT TTTT	AAACGGGCCTCTGAAA TCTT	60.1	701	INTERGENIC	--	--	--
Ca1_P3_7933130	Ca1	7933130	TG/TGG	TCCTGCATAACACCATT GGA	CCTTCACAAACTTGAA GGACC	59.9	883	INTERGENIC	--	--	--

INDEL marker IDs	Chromosomes/ unanchored scaffolds	Physical positions (bp)	InDels (*Kabuli* reference genome-CDC Frontier/PII)	Forward primers (5'-3')	Reverse primers (5'-3')	Anneling temperature (0C)	Expected amplified product size (bp)	Structural annotation		Functional annotation	
								Sequence components of *kabuli* genome	*Kabuli* gene accession IDs	NCBI-KOG	TFs
Ca1_P3_7933454	Ca1	7933454	A/ATG	CGCAACTAATTTCCTTCAACAA	CCTTCACAAACTTGAAGGACC	59.3	591	INTERGENIC	--	--	--
Ca1_P3_7941806	Ca1	7941806	TG/TGG	CCAAAACAGGCCAACATTCT	GGGCAAGGATCAAGAAACAA	60.0	197	DRR	Ca_08044	QI	B3
Ca1_P3_7942966	Ca1	7942966	TAAA/TAAAA	GCACTTTCCATCCTTTTGGA	GAAAAGGCCTCCAACACAAA	60.1	378	INTRON	Ca_08044	QI	B3
Ca1_P3_7947029	Ca1	7947029	CAAAAA/CAAA	AACGATTTTTCACGCTTGCT	CAATGATTGGAATTTGGAGGA	59.9	693	INTERGENIC	--	--	--
Ca1_P3_7947965	Ca1	7947965	TAAAAAAAAA/TAAAAAAA	TCATGGTATTACCCGTACACTTACA	TTTTTATTCTCGTGGGAGTGAG	59.6	540	DRR	Ca_08045	QI	B3
Ca1_P3_7952570	Ca1	7952570	ATT/ATTTTTTT	GCCAACCAGGTGTGAATCTT	AAATCCATCAACGAGCCACT	60.0	577	INTRON	Ca_08045	QI	B3
Ca1_P3_7952716	Ca1	7952716	CTTTTTTTTT/CTTTTTTTT	GCCAACCAGGTGTGAATCTT	AAATCCATCAACGAGCCACT	60.0	577	INTRON	Ca_08045	QI	B3
Ca1_P3_7954789	Ca1	7954789	TTAT/TT	CCCCTCGGAACTTTCATTTT	TCGTAATTGCTTTCTGGCCT	60.3	558	INTRON	Ca_08045	QI	B3
Ca1_P3_7956095	Ca1	7956095	TGATAGATAGA/TGATAGA	GGACACGCAGAGAAGCAGTT	ACACGCTAATTTTTGCCACC	60.6	757	INTRON	Ca_08045	QI	B3
Ca1_P3_7957918	Ca1	7957918	C/CT	CAACACACACACACACACAAAA	CGGTAGATCCCAACCAAAAA	59.0	633	INTERGENIC	--	--	--

**INDEL marker IDs**	**Chromosomes/ unanchored scaffolds**	**Physical positions (bp)**	**InDels (*Kabuli* reference genome-CDC Frontier/PII)**	**Forward primers (5'-3')**	**Reverse primers (5'-3')**	**Anneling temperature (0C)**	**Expected amplified product size (bp)**	**Structural annotation**		**Functional annotation**	
								**Sequence components of *kabuli* genome**	***Kabuli* gene accession IDs**	**NCBI-KOG**	**TFs**
Ca1_P3_7964474	Ca1	7964474	GTTTTTTTTTT/GTTTTTTTT	TGCTTTGCAATAAAAAC GTTGA	AGCAAATTTCCGGATC TCCT	60.6	476	INTERGENIC	--	--	--
Ca1_P3_7975712	Ca1	7975712	TAAAAAA/TAAAAA	GCTCCTTGCAAATGGC TATC	GCCTCAAGTGATGAGA ATTGG	59.8	967	INTERGENIC	--	--	--
Ca1_P3_7987454	Ca1	7987454	TAAAAAAAAA/TAAAAAAAAAA	GGGGGTTACTTAGGGT CAGC	TCGAATATTTAAAAAGA TTGGTGAAA	59.8	371	INTERGENIC	--	--	--
Ca1_P3_7987594	Ca1	7987594	A/AC	GGGGGTTACTTAGGGT CAGC	CAAAGTGTAGAATGGT TACGGATG	59.8	794	INTERGENIC	--	--	--
Ca1_P3_7988388	Ca1	7988388	ATT/ATTTT	TCAATCCAAACAAGTCC AGAA	AATGCATGCTTTGGTT CCTC	58.2	761	DRR	Ca_08049	--	WRKY
Ca1_P3_7990610	Ca1	7990610	AATATATATATA/AATATATA	AGTTGGCCATGGATGA TTGT	GCAAATGTGTTGTCAA TGGG	60.2	349	INTRON	Ca_08049	--	WRKY
Ca1_P3_7991381	Ca1	7991381	ACACACACCTCTTCA/ACA	TGGTGCAGGAATACAA ACCA	CACGTGGCACCGTTAA TATG	60.0	382	INTRON	Ca_08049	--	WRKY
Ca1_P3_7992031	Ca1	7992031	GTAA/GTAATTAA	TGATTCTCTCCCATGCC ATT	TTCTTCAGCTGGTGTT GCAC	60.4	450	INTRON	Ca_08049	--	WRKY
Ca1_P3_7992083	Ca1	7992083	CTC/CTCTTC	TGATTCTCTCCCATGCC ATT	TTCTTCAGCTGGTGTT GCAC	60.4	450	CDS (large-effect mutations)	Ca_08049	--	WRKY
Ca1_P3_7992471	Ca1	7992471	TGTAGTA/TGTAGTAGTA	GTGCAACACCAGCTGA AGAA	CAACATCATGATCAAT GGCA	60.0	310	CDS (large-effect mutations)	Ca_08049	--	WRKY

INDEL marker IDs	Chromosomes/ unanchored scaffolds	Physical positions (bp)	InDels (*Kabuli* reference genome-CDC Frontier/PII)	Forward primers (5'-3')	Reverse primers (5'-3')	Anneling temperature (0C)	Expected amplified product size (bp)	Structural annotation		Functional annotation	
								Sequence components of *kabuli* genome	*Kabuli* gene accession IDs	NCBI-KOG	TFs
Ca1_P3_7994035	Ca1	7994035	TAAAAAAAA/TAAAAAAAAA	CACCTTGCATGTGTATGAATGA	TACTTGCCGCCTCTTTTGTT	59.5	250	INTERGENIC	--	--	--
Ca1_P3_7994334	Ca1	7994334	AATTAT/AATTATTAT	AACAAAAGAGGCGGCAAGTA	GGGCATGGGTTAGCTAGTGA	59.9	705	INTERGENIC	--	--	--
Ca1_P3_7995324	Ca1	7995324	ATAGTAGT/ATAGTAGTAGT	CGGATTGTTTTCGATATGGG	AAGGGATTTTTATTCACAAAACAA	60.1	527	INTERGENIC	--	--	--
Ca1_P3_7995361	Ca1	7995361	TAAAAAA/TAAAAAAA	CGGATTGTTTTCGATATGGG	TCCTTGATTATAGGGCACTCAA	60.1	663	INTERGENIC	--	--	--
Ca1_P3_7995599	Ca1	7995599	CTTTTTTT/CTTTTTTTTT	CGGATTGTTTTCGATATGGG	TCATTGAGGATATTTGCGAGG	60.1	867	INTERGENIC	--	--	--
Ca1_P3_7995701	Ca1	7995701	ATTAAATGAGTGTTAAAT/ATTAAAT	GCGTAAGTTATGCATGCCAAT	GGTGCAAGGGAAGAAAATGA	60.0	902	INTERGENIC	--	--	--
Ca1_P3_7995766	Ca1	7995766	CAAAAAAA/CAAAAAAAA	GCCAATTGCGTGTAGCTGT	GGTGCAAGGGAAGAAAATGA	59.9	887	INTERGENIC	--	--	--
Ca1_P3_7996929	Ca1	7996929	ATT/ATTAATAAATTT	TCCCCGGTCACACAATAATC	TTCTCAAGCACCAAGCACAC	60.6	185	INTERGENIC	--	--	--
Ca1_P3_8000792	Ca1	8000792	G/GC	CCAACCCTTCACACTCGTTT	TCCAACCCAAATTTCAAACA	60.0	878	INTERGENIC	--	--	--
Ca1_P3_8003548	Ca1	8003548	T/TAA	CATTTGGCATCGATATGCAC	GGACACAAAATATAATTGAGCCA	59.9	867	INTERGENIC	--	--	--

INDEL marker IDs	Chromosomes/ unanchored scaffolds	Physical positions (bp)	InDels (*Kabuli* reference genome-CDC Frontier/PII)	Forward primers (5'-3')	Reverse primers (5'-3')	Anneling temperature (0C)	Expected amplified product size (bp)	Structural annotation		Functional annotation	
								Sequence components of *kabuli* genome	*Kabuli* gene accession IDs	NCBI-KOG	TFs
Ca1_P3_8003639	Ca1	8003639	AC/A	TTGGTCATCTCCAAAG GTCC	GGACACAAAATATAATT GAGCCA	59.9	631	INTERGENIC	--	--	--
Ca1_P3_8004049	Ca1	8004049	AAT/AATATATAT	GGCTCAATTATATTTTG TGTCCC	TCATGATATTATGCAC CGGG	58.8	551	INTERGENIC	--	--	--
Ca1_P3_8017505	Ca1	8017505	TAAA/TAAAA	GGCGAAACTCGTGAGG ATAC	TCAAACCATCGAACCA AACA	59.7	754	INTERGENIC	--	--	--
Ca1_P3_8017527	Ca1	8017527	CATAATAATAATAATAATA/CATAATAAT AATAATAATAATAATA	GGCGAAACTCGTGAGG ATAC	TCAAACCATCGAACCA AACA	59.7	754	INTERGENIC	--	--	--
Ca1_P3_8018022	Ca1	8018022	G/GAAACAT	TGTTTGGTTCGATGGTT TGA	TGTATTGGGCCAGTTT CACA	59.9	544	INTERGENIC	--	--	--
Ca1_P3_8018283	Ca1	8018283	GAAA/GAA	TGTTTGGTTCGATGGTT TGA	TGTATTGGGCCAGTTT CACA	59.9	544	INTERGENIC	--	--	--
Ca1_P3_8019062	Ca1	8019062	AT/A	GACCGTGACTGTGAGC AAGA	AGTTTGTGGACTTCGG GTTG	60.0	463	INTERGENIC	--	--	--
Ca1_P3_8020277	Ca1	8020277	ATTT/ATTTGTTT	TCGCGGTGAATATCAC AAGA	GTCGATTCAAGTTGGC GAAT	60.2	756	INTERGENIC	--	--	--
Ca1_P3_8023347	Ca1	8023347	T/TG	TAGGCCAGCTTCGAAA CATT	TGTTGGGATCACCACT AGGC	59.8	761	INTERGENIC	--	--	--
Ca1_P3_8031692	Ca1	8031692	TTA/TTATA	ATCTTCTTTTTGTGGGG CCT	CTTGGCAGATCTCGAG TATGAA	59.9	397	INTERGENIC	--	--	--

INDEL marker IDs	Chromosomes/ unanchored scaffolds	Physical positions (bp)	InDels (*Kabuli* reference genome-CDC Frontier/PII)	Forward primers (5'-3')	Reverse primers (5'-3')	Anneling temperature (0C)	Expected amplified product size (bp)	Structural annotation		Functional annotation	
								Sequence components of *kabuli* genome	*Kabuli* gene accession IDs	NCBI-KOG	TFs
Ca1_P3_8031727	Ca1	8031727	CG/C	ATCTTCTTTTTGTGGGGCCT	CTTGGCAGATCTCGAGTATGAA	59.9	397	INTERGENIC	--	--	--
Ca1_P3_8032266	Ca1	8032266	T/TTTA	TCGTAAAGTTTGCAGAGAGAAAA	TGAGGGTTTGACTCTTTCCATT	58.4	511	INTERGENIC	--	--	--
Ca1_P3_8033455	Ca1	8033455	CTTTTTTTTT/CTTTTTTTTTTTT	TCACACCTTCATTCCCTTCC	CCCAGGAGAAGAAAATCGTG	59.9	766	INTERGENIC	--	--	--
Ca1_P3_8037389	Ca1	8037389	TG/T	CCATGATATCCTGCTGCTGA	CCTGTAACCTGCAAGGTGGT	59.8	535	INTRON	Ca_08051	M	FAR1
Ca1_P3_8038627	Ca1	8038627	TGGGGGG/TGGGGG	GCACCTACTCGGGATTGAAG	TCAGCAAAATCAATTCGTGG	59.7	676	DRR	Ca_08051	M	FAR1
Ca1_P3_8038814	Ca1	8038814	GTTTTTTT/GTTTTTTTT	GCACCTACTCGGGATTGAAG	TGGTTGAGCATTTGCCATTA	59.7	707	DRR	Ca_08051	M	FAR1
Ca1_P3_8041307	Ca1	8041307	T/TG	CGGCGGCAATTGTTATAAGT	CAATTTCAAGCAAAATCGCA	60.0	890	INTERGENIC	--	--	--
Ca1_P3_8041385	Ca1	8041385	CTTT/CTT	TCGAAGATGGTGGTTGATGA	CAATTTCAAGCAAAATCGCA	60.0	780	INTERGENIC	--	--	--
Ca1_P3_8041662	Ca1	8041662	TAAC/TAACATTAAAC	CCCTTCATTGGAGATGTTCC	TTGGGGACACTAAGAGGTGG	59.3	866	INTERGENIC	--	--	--
Ca1_P3_8050233	Ca1	8050233	ATTTTTTT/ATTTTTT	TCGACACATCATCACCTTTTG	TGGTTGTGTTGAGTTTGGGA	59.6	288	INTERGENIC	--	--	--

INDEL marker IDs	Chromosomes/ unanchored scaffolds	Physical positions (bp)	InDels (*Kabuli* reference genome-CDC Frontier/PII)	Forward primers (5'-3')	Reverse primers (5'-3')	Anneling temperature (0C)	Expected amplified product size (bp)	Structural annotation		Functional annotation	
								Sequence components of *kabuli* genome	*Kabuli* gene accession IDs	NCBI-KOG	TFs
Ca1_P3_8061072	Ca1	8061072	C/CTAAA	CAACGTTGGATGGTGG TGTA	GTTTAGGACCCGTGCA TTTG	60.3	571	INTERGENIC	--	--	--
Ca1_P3_8062163	Ca1	8062163	TCATCCACATCCACAT/TCATCCACAT	TTATTCGCCCAACAACA CAA	TGAAGATGAAGATGAG GGAGG	60.0	459	INTERGENIC	--	--	--
Ca1_P3_8062288	Ca1	8062288	TG/T	TCATCCACATCCACATT CTCA	TCATTGGTAGTACGTG AACCGA	59.9	366	INTERGENIC	--	--	--
Ca1_P3_8063349	Ca1	8063349	CTGGTTGGT/CTGGT	GCAGTATGGCAAGAGC AACA	TCATCACATTAAGGCC AGCA	60.0	486	INTRON	Ca_08053	O	MYB_related
Ca1_P3_8090682	Ca1	8090682	ATTTTTT/ATTTTT	GAGGGGCTACCACCAA ATTC	CCCACACAAACAACTG CATC	60.7	622	INTERGENIC	--	--	--
Ca1_P3_8092031	Ca1	8092031	CTTTT/CTTT	TCATCTTCGCCAGAGTT TCA	AAGGCATTGGGAAATG GTAA	59.5	480	INTERGENIC	--	--	--
Ca1_P3_8101729	Ca1	8101729	CAGAGA/CAGA	CTTGACTCTTTGTCTCC GCC	GCCATTCGTGTCCTGA GAAT	60.0	346	DRR	Ca_08056	C	--
Ca1_P3_8123087	Ca1	8123087	AC/ACTTTTCAAGC	CAAGGCCCTTCCTATT GTCA	TGACTGAACCATCCAA CGAA	60.1	138	DRR	Ca_08058	R	--
Ca1_P3_8125888	Ca1	8125888	GTT/GACATATTAAAATT	TGTTGGGTTACGAGGG AGTC	TTGCGGGTTTTTAACC TGAG	60.0	526	INTERGENIC	--	--	--
Ca1_P3_8126149	Ca1	8126149	TAAAAAAAA/TAAAA	TCAAAATTTGTCCACGT CCA	AATTTACAAACACGGC GACC	59.9	478	INTERGENIC	--	--	--

INDEL marker IDs	Chromosomes/ unanchored scaffolds	Physical positions (bp)	InDels (*Kabuli* reference genome-CDC Frontier/PII)	Forward primers (5'-3')	Reverse primers (5'-3')	Anneling temperature (0C)	Expected amplified product size (bp)	Structural annotation		Functional annotation	
								Sequence components of *kabuli* genome	*Kabuli* gene accession IDs	NCBI-KOG	TFs
Ca1_P3_8127150	Ca1	8127150	T/TG	CCCTCCTTCCAGAAAACCTT	AAAGGGCAAGAAAGAAACGTC	59.5	157	INTERGENIC	--	--	--
Ca1_P3_8127206	Ca1	8127206	AG/A	CCCTCCTTCCAGAAAACCTT	AAAGGGCAAGAAAGAAACGTC	59.5	157	INTERGENIC	--	--	--
Ca1_P3_8130349	Ca1	8130349	ATTTT/ATTT	TTACTCACCAGGAACCGTCC	GTTGCCGGGTAAGTTTTCAA	60.0	557	INTRON	Ca_08059	P	--
Ca1_P3_8130436	Ca1	8130436	ATTTTT/ATTTT	TTACTCACCAGGAACCGTCC	GTTGCCGGGTAAGTTTTCAA	60.0	557	INTRON	Ca_08059	P	--
Ca1_P3_8139974	Ca1	8139974	AAGTTT/A	TTGGAGCACAGAAATTGGGT	GGAAACACAAGGGATGTTGG	60.5	409	INTRON	Ca_08061	--	C2H2
Ca1_P3_8282795	Ca1	8282795	TTCTCTCTCTCTCTCTCTCTCTCTCTCT/TTCTCTCTCTCTCTCTCTCTCTCT	ACGCCCGCATAGAACATAAG	CTGAACGAGCAAGGGAGTTC	60.1	661	INTERGENIC	--	--	--
Ca1_P3_831015	Ca1	831015	TG/TCTCTTATAAATGAG	GCCGGATTAAATATAAATCCACA	TGACCTAATAAGCCTGTTTGTTT	59.2	595	DRR	Ca_00101	--	--
Ca1_P3_839232	Ca1	839232	AGTGT/AGTGTGT	AAGGATTGTGAAATCGCCAC	TAGATGCCCGTCATTGTTGA	59.9	680	INTERGENIC	--	--	--
Ca1_P3_8428302	Ca1	8428302	TAAAA/TAAA	TGCACGTAGAATCGCCTAAA	TGTTAACACGCCCATTTTTAT	59.5	263	INTERGENIC	--	--	--
Ca1_P3_8430467	Ca1	8430467	TT/TTATTATTAT	AGGTCATGGGCTGGATGTAG	ACCCTTTCACTGCGCTCTTA	60.0	612	INTERGENIC	--	--	--

**INDEL marker IDs**	**Chromosomes/ unanchored scaffolds**	**Physical positions (bp)**	**InDels (*Kabuli* reference genome-CDC Frontier/PII)**	**Forward primers (5'-3')**	**Reverse primers (5'-3')**	**Anneling temperature (0C)**	**Expected amplified product size (bp)**	**Structural annotation**		**Functional annotation**	
								**Sequence components of *kabuli* genome**	***Kabuli* gene accession IDs**	**NCBI-KOG**	**TFs**
Ca1_P3_8457640	Ca1	8457640	ATTATTTATTTATTTATTTATTTATTTATTTATTTATTTATTTATTTATTTATTTA/ATTATTTATTTATTTATTTATTTATTTATTTATTTATTTATTTATTTATTTATTTATTTATTTA	ATTACTGTCGGCACGCTTCT	CGTTGACACCTGGCACTAGA	59.9	237	DRR	Ca_08081	--	--
Ca1_P3_8669425	Ca1	8669425	TA/T	AGCCAGGCCATGTAACAAAC	GATGGAGAGGGTCATCCAGA	60.0	234	INTRON	Ca_02944	P	GRAS
Ca1_P3_89142	Ca1	89142	AATATATATATATATATATATATATATATATATATATATATATATAT/AATATATATATATATATATATATATATATATATAT	CACCCCTACCTTCACCTTCA	CCGTGAAATGTCAACTCGTG	60.0	571	INTERGENIC	--	--	--
Ca1_P3_8968122	Ca1	8968122	AA/AACA	CAAGCTCCCGAATCAAACAC	CACAAAACCGAAAAGGGAGA	60.6	305	INTERGENIC	--	--	--
Ca1_P3_9108079	Ca1	9108079	CTATATATATATATATAT/CTATATATATATATAT	GAGGCCATTGAATCTCCAAA	CGCCCTTAAAAATTAGGTGCT	60.0	672	INTERGENIC	--	--	--
Ca1_P3_9130103	Ca1	9130103	TTATATATATATATATATATAT/TTATATATATATATATATAT	GGGTCAAAATTCGTTGACCAT	TACGGGTCAAGACAGGTTCG	61.0	550	INTERGENIC	--	--	--
Ca1_P3_915168	Ca1	915168	CTTTTTTTTTT/CTTTTTTTTT	CGTGCAAGGTGGTTTCTTTT	AAATGACACCCCCAAAATCA	60.1	446	INTERGENIC	--	--	--
Ca1_P3_9223996	Ca1	9223996	TAAAAAAAAAA/TAAAAAAAAAAA	AATGGATTCTTCCGCTCAAA	ACAGAACGGTGCGTTGTAAA	59.6	489	INTERGENIC	--	--	--
Ca1_P3_9236712	Ca1	9236712	AT/A	TTTTTGCTTAAATCCGCAGG	CAAGCTCACCAAGTCACGAA	60.2	729	INTERGENIC	--	--	--
Ca1_P3_9394926	Ca1	9394926	CG/C	CAAGTCCCCACTTGGAAAAA	CGGGCTCTAAATATCACCTACG	59.9	441	INTERGENIC	--	--	--

INDEL marker IDs	Chromosomes/ unanchored scaffolds	Physical positions (bp)	InDels (*Kabuli* reference genome-CDC Frontier/PII)	Forward primers (5'-3')	Reverse primers (5'-3')	Anneling temperature (0C)	Expected amplified product size (bp)	Structural annotation		Functional annotation	
								Sequence components of *kabuli* genome	*Kabuli* gene accession IDs	NCBI-KOG	TFs
Ca1_P3_9396	Ca1	9396	CAAAAA/CAAAA	TTGGCAAATCGATTGAAACA	TTGATCCTTGCAGTCACTCG	60.1	906	DRR	Ca_00001	--	--
Ca1_P3_9418383	Ca1	9418383	ACACATTT/A	AGTGGCTGAACACACTGACA	TCAGCTGAGAAAGTCACCGA	57.8	916	INTERGENIC	--	--	--
Ca1_P3_9449325	Ca1	9449325	GTTTTTTTTTTTT/GTTTTTTTTTTTTT	CGAAGCATCACAGTCAAGGA	CGATTCGCTGAATGACAAAA	60.0	435	DRR	Ca_02851	R	--
Ca1_P3_9652123	Ca1	9652123	GAAATTTTTTTGCCAAATTTTTTT/GAAATTTTTTT	ATTTTGACCAAATCCAGCCC	CATTTTCAAGCATGTAAGTAATCAAA	61.1	881	INTERGENIC	--	--	--
Ca1_P3_9871519	Ca1	9871519	ATT/A	CTTAGTCCACGTGCAAACGA	AAAGTGCGGGCCAATATCTA	59.9	600	INTERGENIC	--	--	--
Ca1_P3_9926230	Ca1	9926230	CAAAAAAAAAAA/CAAAAAAAAAAAA	GGAACCTGAAAGTGCGTGTT	TTTTGTTTTTGCACCGATCA	60.2	284	INTERGENIC	--	--	--
Ca2_P3_10009732	Ca2	10009732	GATATATATATATATATATATATATATATATATATATAT/GATATATATATATATATATATATATATATATATAT	TGTCACATTTACTCAACCGCA	AAAATTGATTCGTGTGATTGGA	60.2	287	INTERGENIC	--	--	--
Ca2_P3_10081600	Ca2	10081600	CAAAAAAAAAAAAAAA/CAAAAAAAAAAAAAA	TTTTGATTGATTGATGATGGG	TGCAAATTCAACCTCACGAA	58.3	552	INTERGENIC	--	--	--
Ca2_P3_10200721	Ca2	10200721	ATTTTTTTTTT/ATTTTTTTTT	GGATGAGCATTCAATTTCAAAGA	GGTCTGTACATCTTCAATGGCA	60.4	510	INTERGENIC	--	--	--
Ca2_P3_1022421	Ca2	1022421	TAAA/TAA	GCCATTCTTTCTGCCATTTC	TTCCATCAGTCAGTGGGTTG	59.7	729	INTRON	Ca_17008	E	C3H

INDEL marker IDs	Chromosomes/ unanchored scaffolds	Physical positions (bp)	InDels (*Kabuli* reference genome-CDC Frontier/PII)	Forward primers (5'-3')	Reverse primers (5'-3')	Anneling temperature (0C)	Expected amplified product size (bp)	Structural annotation		Functional annotation	
								Sequence components of *kabuli* genome	*Kabuli* gene accession IDs	NCBI-KOG	TFs
Ca2_P3_1033751	Ca2	1033751	CATTTATTA/C	TGTGTGATTTTCAATAGTCACCG	AAGTTAAGGGACCACGGGAT	59.9	845	INTERGENIC	--	--	--
Ca2_P3_10373779	Ca2	10373779	TTATATATATATATATATATATA/TTATATATATATATATATA	AAATGCATCGAAACATCAAAAA	GGTCCCAGAAATAAGTGCCA	59.5	382	INTERGENIC	--	--	--
Ca2_P3_10498057	Ca2	10498057	CTTTTTTTTTTT/CTTTTTTTTTTTT	TGACCAAATCGGAAAGAAAAA	AGGGAATGAGAGCAATGACG	59.6	454	INTERGENIC	--	--	--
Ca2_P3_10563482	Ca2	10563482	GATATATATATATA/GATATATATATATATA	TTGCATCTTCGTTGATCGTC	GACAATTCTGAGTGCCATGC	59.8	510	INTERGENIC	--	--	--
Ca2_P3_10702214	Ca2	10702214	AAATAATAATAATAATAATAATAATAATAATAATAAT/AAATAATAATAATAATAATAATAATAATAATAATAATAAT	TGGGTGATGTGAGTTTGTTCA	ATGAGCCTCCTATGTCCCCT	60.0	415	INTERGENIC	--	--	--
Ca2_P3_10720766	Ca2	10720766	ATTTTTTTTTTT/ATTTTTTTTTTTT	GTTTGTCCCCAAGCAACAAT	TTGGTCGTTTGAGCCTTTCT	59.8	796	INTERGENIC	--	--	--
Ca2_P3_10779124	Ca2	10779124	TTA/TTACATA	CACTTTTCTTATGCACCGCA	GCAGCGAGTAGGAGAAATGC	59.9	663	INTERGENIC	--	--	--
Ca2_P3_10789942	Ca2	10789942	ATTTTTTT/ATTTTTTTT	TTGAATCTGCACCCAAATGA	GTGGCTTCATAAGGCAGAGG	60.0	888	INTERGENIC	--	--	--
Ca2_P3_10833947	Ca2	10833947	CATATATATATATATATAT/CATATATATATATATATAT	GTTGGTTCCCTAGCACCGTA	ACCCAATTACTGGAGATGCG	60.0	650	INTERGENIC	--	--	--
Ca2_P3_10879782	Ca2	10879782	CCAGC/CCAGCAGC	GTTGGTTCCCTAGCACCGTA	TGCCTTATGGGTGAAATGGT	60.0	594	INTERGENIC	--	--	--

INDEL marker IDs	Chromosomes/ unanchored scaffolds	Physical positions (bp)	InDels (*Kabuli* reference genome-CDC Frontier/PII)	Forward primers (5'-3')	Reverse primers (5'-3')	Anneling temperature (0C)	Expected amplified product size (bp)	Structural annotation		Functional annotation	
								Sequence components of *kabuli* genome	*Kabuli* gene accession IDs	NCBI-KOG	TFs
Ca2_P3_10904528	Ca2	10904528	ACCCC/ACCC	CACCAACAATTGGCATCAAG	CCCACATTTCTTTGGCAGTT	60.0	374	INTERGENIC	--	--	--
Ca2_P3_10916127	Ca2	10916127	TAAAAAAAAAA/TAAAAAAAAAAA	GCAAGAACGATGCAGTGAAA	GTTTGGGTCCAAAGAAGCAA	60.0	671	DRR	Ca_17556	--	--
Ca2_P3_10927606	Ca2	10927606	T/TAATAATAATAATAA	TCACGTGAGTTCCCAATGAA	ATGGAGGCTCTTGTTGAGGA	60.1	624	INTERGENIC	--	--	--
Ca2_P3_10929028	Ca2	10929028	GATATATATATATATATATATATA/GATATATATATATATATATA	TTTTCCCCGACTTAGTCCTTT	TGAAGCCATGGATCGTAACA	59.1	663	INTERGENIC	--	--	--
Ca2_P3_10948001	Ca2	10948001	ATTA/ATTACACTGACGTTA	TGATTCTTGATCATGTTGTTTTCA	CCATCCTCACTCCAAAGACAA	59.5	702	INTERGENIC	--	--	--
Ca2_P3_11063661	Ca2	11063661	TATT/TATTGTGAATT	TTGCATGCTTTTATTGACATTG	AGTCGTTCTTTGGAGGGGTT	58.7	543	INTERGENIC	--	--	--
Ca2_P3_11085781	Ca2	11085781	AATATATATATATATATATATATATATATATATATTTATATATATATATAT/AATATATATATATATATATATATATATATTTATATATATATAT	TTTGGTTTTGAACTCGGAGG	CACCGTAGTGTGGTGGTGAG	60.1	660	INTERGENIC	--	--	--
Ca2_P3_11097797	Ca2	11097797	T/TTA	GCCCTCAGATGAGAGTCAGG	CCCAAAGAAAACCAAACCAA	59.9	480	INTERGENIC	--	--	--
Ca2_P3_11136560	Ca2	11136560	GAAAAAAAAA/GAAAAAAAAAA	TGACCAATGGAAAAAGGGAG	TGAAAATTTTGGGGTGTTACAA	59.9	300	INTERGENIC	--	--	--
Ca2_P3_11144082	Ca2	11144082	CTTTTT/CTTTT	TGCGAGCTTTCCTTTTTCAT	AAGGAGCTCTGGGACACTGA	60.0	214	INTERGENIC	--	--	--

INDEL marker IDs	Chromosomes/ unanchored scaffolds	Physical positions (bp)	InDels (*Kabuli* reference genome-CDC Frontier/PII)	Forward primers (5'-3')	Reverse primers (5'-3')	Anneling temperature (0C)	Expected amplified product size (bp)	Structural annotation		Functional annotation	
								Sequence components of *kabuli* genome	*Kabuli* gene accession IDs	NCBI-KOG	TFs
Ca2_P3_11144239	Ca2	11144239	T/TACTC	TCAGTGTCCCAGAGCTCCTT	TCAATTGGCATCAGAGCAAG	60.0	402	INTERGENIC	--	--	--
Ca2_P3_11156323	Ca2	11156323	ACC/ACCC	AATCCTAAGCGCCATCTGAA	ATGCCCATGTGTGAAGACAA	59.8	525	INTERGENIC	--	--	--
Ca2_P3_11215907	Ca2	11215907	TAAAAAAAAAA/TAAAAAAAAA	GCGAGTTTTAGAACCATCAAGC	TTGGATCCGTTTGTTCAAGA	60.3	967	INTERGENIC	--	--	--
Ca2_P3_11220118	Ca2	11220118	ANNNNNNNNNN/ANNNNNNNN	TTGCACTTGGTTTGACTCCA	CTCTCAACCCTCTCGTTTGC	60.3	939	INTERGENIC	--	--	--
Ca2_P3_11240445	Ca2	11240445	ATTTTTT/ATTTTTTT	TCGACCGAATCAAACAATGA	ACGTTTCCTCACGTTTCCAC	60.0	622	INTERGENIC	--	--	--
Ca2_P3_11256709	Ca2	11256709	AC/A	AAGAAAGACAAGGGGCCATC	CCATGCTTTTGGTTTGTTGA	60.4	215	INTERGENIC	--	--	--
Ca2_P3_11321715	Ca2	11321715	TAAAAAAAAAA/TAAAAAAAAAAA	GCCTTAGTTGCGCGTAAAAC	CAACTCCACAAGGCCTCACT	59.9	398	INTERGENIC	--	--	--
Ca2_P3_11625278	Ca2	11625278	AATATATATATATATATATATATATAT A/AATATATATATATATATATATATATAT ATATATA	GTTTAGCGCTCCTCCATCAT	CTGCCCGTAAAAATCTTCCA	59.3	839	INTERGENIC	--	--	--
Ca2_P3_11869889	Ca2	11869889	TTAACTATAACTAT/TTAACTAT	TGCAAGGAAGCAGATCGAAT	AGCAAACATCCAAACAAGCC	60.9	537	INTERGENIC	--	--	--
Ca2_P3_12048675	Ca2	12048675	AATAATTTATAATTTATA/AATAATTTATA	CAAACATGCTGATGTGGACC	CCATTGGTAAATGGCTAACAA	60.0	278	INTERGENIC	--	--	--

INDEL marker IDs	Chromosomes/ unanchored scaffolds	Physical positions (bp)	InDels (*Kabuli* reference genome-CDC Frontier/PII)	Forward primers (5'-3')	Reverse primers (5'-3')	Anneling temperature (0C)	Expected amplified product size (bp)	Structural annotation		Functional annotation	
								Sequence components of *kabuli* genome	*Kabuli* gene accession IDs	NCBI-KOG	TFs
Ca2_P3_12181425	Ca2	12181425	TTATATATATATATATATATATA/TTATATATATATATATATATA	CGGCGAAATAAATTCACCAT	GGAAGTCGTAGGGTTGGGAT	59.8	578	INTERGENIC	--	--	--
Ca2_P3_1221818	Ca2	1221818	ATTTTTTTTTT/ATTTTTTTTTTT	TAGGTGCCCAACATAGCACA	ACAAAATGGTTCTCGAAATGA	60.1	978	INTERGENIC	--	--	--
Ca2_P3_12322193	Ca2	12322193	TTCACTCACTC/TTCACTC	ACTGCAACCATCTTTCCTGG	AGAGACCCAAGTGAGGCTGA	60.1	576	INTRON	Ca_18064	E	--
Ca2_P3_12360564	Ca2	12360564	TAAAAAAAAAAAA/TAAAAAAAAAAA	TCGCACTCACACAACAAATC	CCTCTTCACTTTTTGGAAGGG	59.8	155	INTERGENIC	--	--	--
Ca2_P3_12384713	Ca2	12384713	AAAATAAATAAATAAATAAATAAATAAATAAATAAATAAA/AAAATAAATAAATAAATAAATAAATAAATAAATAAATAAA	CCTACGTATCTCCTGGTGCG	TTAATCGTTGCGTTGCTGAG	60.7	649	INTERGENIC	--	--	--
Ca2_P3_12475607	Ca2	12475607	TTGTATTTGATGTAT/TTGTAT	TCAAAGAGAAATGACTTTGTCCA	TCACTTTGCTTTGTGTGTCTCA	58.9	852	INTERGENIC	--	--	--
Ca2_P3_12567970	Ca2	12567970	ATTTTTTTTTTT/ATTTTTTTTTTTTT	GGAAGTTGCCTTTCCCTTTC	TCGCAAGGATCTACACACTGA	60.1	743	INTERGENIC	--	--	--
Ca2_P3_12589348	Ca2	12589348	AATATATATATATATATATATATATATATATAT/AATATATATATATATATATATATATATATATAT	CCAGCTCGGTTCAGTTTTTC	TTTCGATCAGACGGTTAAATTG	59.9	637	INTERGENIC	--	--	--
Ca2_P3_12634541	Ca2	12634541	CTTTTTTTT/CTTTTTTT	GTTCAAAGCAGCTGCAACAA	CCACAACTACCCTTGGCATT	60.2	236	INTERGENIC	--	--	--
Ca2_P3_12805309	Ca2	12805309	ATTTTTTT/ATTTTTTTT	TTTTGAACTACGGCACACACA	AGCACTAAATGGGTGGAACC	60.2	836	INTERGENIC	--	--	--

INDEL marker IDs	Chromosomes/ unanchored scaffolds	Physical positions (bp)	InDels (*Kabuli* reference genome-CDC Frontier/PII)	Forward primers (5'-3')	Reverse primers (5'-3')	Anneling temperature (0C)	Expected amplified product size (bp)	Structural annotation		Functional annotation	
								Sequence components of *kabuli* genome	*Kabuli* gene accession IDs	NCBI-KOG	TFs
Ca2_P3_12931745	Ca2	12931745	A/AGC	GTTGATGCTCCAACAAGAGC	TTTGACCAAGTCGAAAACAAGA	58.4	470	INTERGENIC	--	--	--
Ca2_P3_12933106	Ca2	12933106	A/AT	AGGTTGAAAGAAGCCCCACT	ATCGGTTGGTTGGTGAAATG	60.1	834	INTERGENIC	--	--	--
Ca2_P3_12933331	Ca2	12933331	TTATATATATATATATATATATAT/TTATATATATATATATATATATATATATATATATAT	TGGAGTTTTGGTCTCCATATTT	AGGGACATGCAATGGGTTAG	57.6	618	INTERGENIC	--	--	--
Ca2_P3_12933573	Ca2	12933573	CACCAACCAACC/CACCAACC	TCATTGACAAGGAAAGAACATGA	CGAATTGGTTTTGGGATTTG	59.6	413	INTERGENIC	--	--	--
Ca2_P3_12934202	Ca2	12934202	AAA/AAACAA	AGAAAGCAAGCAAATCCCAA	TTCCAACACCTCACGATTCA	59.8	488	INTERGENIC	--	--	--
Ca2_P3_12934976	Ca2	12934976	TATCAATC/TATC	TGCCTCAAAAATCTGGTGTG	CTGCTCCAAACTGATCACGA	59.7	516	INTERGENIC	--	--	--
Ca2_P3_12937651	Ca2	12937651	ATTTT/ATTTTT	GATTGCTTTCCTCTATGCCG	GTTTTGGCGTTGGTTTCACT	59.8	353	INTERGENIC	--	--	--
Ca2_P3_12939688	Ca2	12939688	AATATATA/AATATA	TTTTGGTGTTGAGGGTGTCA	TTAATCAAAGCGGCAAAAGAA	60.0	564	INTERGENIC	--	--	--
Ca2_P3_12941200	Ca2	12941200	CAAAA/CAAA	TGTGCTTCTTCCTGATTTTGG	GGCCAAAGACGGATATGGTA	60.2	888	INTERGENIC	--	--	--
Ca2_P3_12941800	Ca2	12941800	T/TA	CATATCCGTCTTTGGCCATT	CCTTGAACAATTTGGGCAGT	59.8	643	INTERGENIC	--	--	--

INDEL marker IDs	Chromosomes/ unanchored scaffolds	Physical positions (bp)	InDels (*Kabuli* reference genome-CDC Frontier/PII)	Forward primers (5'-3')	Reverse primers (5'-3')	Anneling temperature (0C)	Expected amplified product size (bp)	Structural annotation		Functional annotation	
								Sequence components of *kabuli* genome	*Kabuli* gene accession IDs	NCBI-KOG	TFs
Ca2_P3_12947970	Ca2	12947970	G/GT	TGCTTTTATGAGCATTACGGG	CGACTTCAAATCCCTTCGAG	60.1	777	INTERGENIC	--	--	--
Ca2_P3_12952557	Ca2	12952557	ACATATCATATCA/ACATATCA	AAAGCATTTTATCCCGACCA	TTTTTCATCTCACTCTCTCCCA	59.4	787	INTERGENIC	--	--	--
Ca2_P3_12952944	Ca2	12952944	TGTCATGATTAT/TGTCATGATTATAGTCATGATTAT	TGGGAGAGAGTGAGATGAAAAA	ACGGATGTCTAATCCGTTGG	58.9	308	INTERGENIC	--	--	--
Ca2_P3_12960292	Ca2	12960292	GT/GTTAT	TTCATACATCCAATCGTGGTTT	CCACAAGAAGCAACACAAGC	59.2	527	INTERGENIC	--	--	--
Ca2_P3_13079939	Ca2	13079939	ATTTTTTT/ATTTTTTTT	GCAGCAAAATGAAAATTGGG	CGCAACAAAATGGAAGATCA	60.4	541	INTERGENIC	--	--	--
Ca2_P3_13141012	Ca2	13141012	CT/C	TTGGGGTCCCCTACCACTAT	TAGAGGGCTCAAAACTGCAA	60.4	771	INTERGENIC	--	--	--
Ca2_P3_13187716	Ca2	13187716	ATTTATTTTATTTTATTTTA/ATTTATTTTATTTTA	CCAAATTCGGCTGTTTCATT	CGATTTAATTTGGTTGGTAATGG	59.9	457	INTRON	Ca_11682	K	Trihelix
Ca2_P3_13191440	Ca2	13191440	AT/ATTTTATT	CGAGGGTAATGAATGTCGAA	TCCCACCCCTACCAATTACA	58.6	532	INTRON	Ca_11682	K	Trihelix
Ca2_P3_13191472	Ca2	13191472	ATTATT/ATTATTCTTATT	CGAGGGTAATGAATGTCGAA	TCCCACCCCTACCAATTACA	58.6	532	INTRON	Ca_11682	K	Trihelix
Ca2_P3_13247575	Ca2	13247575	ATTTTTTTT/ATTTTTTT	TGAAAAGCTAAATGCATGGAA	TCATGACCATGGACCCACTA	58.4	937	INTERGENIC	--	--	--

INDEL marker IDs	Chromosomes/ unanchored scaffolds	Physical positions (bp)	InDels (*Kabuli* reference genome-CDC Frontier/PII)	Forward primers (5'-3')	Reverse primers (5'-3')	Anneling temperature (0C)	Expected amplified product size (bp)	Structural annotation		Functional annotation	
								Sequence components of *kabuli* genome	*Kabuli* gene accession IDs	NCBI-KOG	TFs
Ca2_P3_1350612	Ca2	1350612	TAAAAA/TAAAA	GCGTTGTAACTGATTTGCGA	ATCAAACGGGTTTAAGTGCG	59.9	272	INTERGENIC	--	--	--
Ca2_P3_13622321	Ca2	13622321	TTATATATATATATATATATATATATAT/TTATATATATATATATATATATATATATATAT	TTGTCTTTTGTTTAGCACATCTCA	GAAGCTCACTAAGCACTTGCC	59.0	491	INTERGENIC	--	--	--
Ca2_P3_13639596	Ca2	13639596	TAATTAAATAAAAT/TAAT	AAGCCAGCCGCATGTAAATA	TTCACTTCCTCTGCATCTTTCA	60.6	788	INTERGENIC	--	--	--
Ca2_P3_13662185	Ca2	13662185	CATATATATATATATATATATA/CATATATATATATATATATATA	GGGGGACCAATTCCATAAAT	AAGTGAATCAATTGTGTGGTCTTT	59.7	724	DRR	Ca_11645	R	C2H2
Ca2_P3_13713037	Ca2	13713037	ATTTTTTTTTTTT/ATTTTTTTTTTT	TCTCCAACAACAACAACATGAA	TCCTGAGTGTTTGTCCATCAA	59.1	515	INTERGENIC	--	--	--
Ca2_P3_13728931	Ca2	13728931	TTTATTATTATTATTATTATTATTATTATTATTATTATTATTATT/TTTATTATTATTATTATTATTATTATTATTATTATTATTATT	AGAGGCAAACAAGAACCGAA	GAGGACCAAAGACCTCCACA	59.9	417	INTERGENIC	--	--	--
Ca2_P3_13794561	Ca2	13794561	CTTTTTTT/CTTTTTT	GATTCGGTGGAGAATCCAAA	CACCGGTGGTAACGAGAACT	59.9	834	INTRON	Ca_11639	--	SAP
Ca2_P3_13956600	Ca2	13956600	AATTATTATT/AATTATTATTATT	TGAAATTTGTGATTTAGTTGCTTAAAA	AAACAAGTTGGTGGTGGAGC	59.6	686	INTERGENIC	--	--	--
Ca2_P3_13957974	Ca2	13957974	A/AG	TTAGCCATTGTGCGTAGCTG	GTGGGGATAGTAGTGGGGGT	60.0	218	INTERGENIC	--	--	--
Ca2_P3_13983591	Ca2	13983591	ATTTTTTTTTT/ATTTTTTTTTTT	ACACGAGGCGGAGAAATAAA	TGCAATTGTGGTCCATTTGT	59.7	893	INTERGENIC	--	--	--

INDEL marker IDs	Chromosomes/ unanchored scaffolds	Physical positions (bp)	InDels (*Kabuli* reference genome-CDC Frontier/PII)	Forward primers (5'-3')	Reverse primers (5'-3')	Anneling temperature (0C)	Expected amplified product size (bp)	Structural annotation		Functional annotation	
								Sequence components of *kabuli* genome	*Kabuli* gene accession IDs	NCBI-KOG	TFs
Ca2_P3_14017645	Ca2	14017645	ATATTATTATTATTATTAT/ATATTATTATTATTAT	ACAAAAAGGTGCTGCCAAAC	TCGACCCCCTTTTATACCAA	60.2	329	INTERGENIC	--	--	--
Ca2_P3_14049093	Ca2	14049093	GAAAAAAAAAAA/GAAAAAAAAAA	GTAGGAAGTCGCCATTTGGA	TGCGCTCCTATGAGGTTTTT	60.1	180	INTERGENIC	--	--	--
Ca2_P3_14086143	Ca2	14086143	C/CT	GCTTCTGATGGTGCACTTGA	TGGCCTAAAGCAAGCTCCTA	60.0	371	INTERGENIC	--	--	--
Ca2_P3_14117374	Ca2	14117374	AATATATATATATATATATATATATATAT/AATATATATATATATATATATATATATATATAT	ACCTACGGATGTTAGCCGTG	GGTCCATGCAAAATCATTCA	60.0	768	INTERGENIC	--	--	--
Ca2_P3_14196319	Ca2	14196319	T/TGGA	TTTGCAAACCCTATCATCCC	TTGGAGCTTCAGCATTTGTG	59.8	729	CDS (large-effect mutations)	Ca_11619	--	Trihelix
Ca2_P3_14200236	Ca2	14200236	A/AT	GCGCTGTAAAATGTGCAAAA	TCGGCTATATGATTGTCCCA	59.9	258	DRR	Ca_11619	--	Trihelix
Ca2_P3_15280624	Ca2	15280624	AATATATATATATATATATATATATATATATATATA/AATATATATATATATATATATATATATATATA	AGAGGCAGAAATGGTGGTGT	AAGATTTTAGGCGATTCTACGTG	59.6	659	INTERGENIC	--	--	--
Ca2_P3_15296907	Ca2	15296907	TTATATATATATATATATATATATATA/TTATATATATATATATATATATATATATATATA	ATCAGTCCTCGCCTCCAATA	TTGAGGCATGGTGTGGTTTA	59.7	619	DRR	Ca_18563	--	--
Ca2_P3_15302288	Ca2	15302288	AATATATATATATATATATATATA/AATATATATATATATATATATATATATATATA	TTCTCCTCCCAACCCTTTTT	TGAATGTGCTTTTTCTCCGA	59.9	262	INTERGENIC	--	--	--
Ca2_P3_15313880	Ca2	15313880	ATATTATTATTATTATTATTATTATTATTATTATTATTATTATTATTATTATTATTATTATTATT/ATATTATTATTATTATTATTATTATTATTATTATTATTATTATTATTATT	GCAAAAAGCAGCGCAATAAT	ACGAATCTAACGGTGGTTGC	60.4	360	INTERGENIC	--	--	--

INDEL marker IDs	Chromosomes/ unanchored scaffolds	Physical positions (bp)	InDels (*Kabuli* reference genome-CDC Frontier/PII)	Forward primers (5'-3')	Reverse primers (5'-3')	Anneling temperature (0C)	Expected amplified product size (bp)	Structural annotation		Functional annotation	
								Sequence components of *kabuli* genome	*Kabuli* gene accession IDs	NCBI-KOG	TFs
Ca2_P3_15458001	Ca2	15458001	AATATATATATATATATATATATATAT/AATATATATATATATATATATATATATAT	TGTGATGTCACCATGACAAAAA	TAGTCGAAACGTTTGCTTGC	59.9	571	INTERGENIC	--	--	--
Ca2_P3_15589036	Ca2	15589036	ATTT/ATTTT	ATACGCCTCAACAAAATGGC	CTGCTTTGCTTGATTCCGTT	60.0	376	INTERGENIC	--	--	--
Ca2_P3_15593194	Ca2	15593194	G/GT	TGCTTCCAATCCGGTATTTC	CAACGCTTGATGTTAGATTGGA	59.9	789	INTERGENIC	--	--	--
Ca2_P3_15598813	Ca2	15598813	TA/TAA	GGAGGCGCATTAGATATGGA	GCCTCGTGAGTTGCATCATA	60.0	518	INTERGENIC	--	--	--
Ca2_P3_15812866	Ca2	15812866	TAAAAAAAAA/TAAAAAAAAAA	CATTGGCGGAGCTTTACAAT	CCATGGCCACTTCAAGATTT	60.1	298	INTERGENIC	--	--	--
Ca2_P3_15904424	Ca2	15904424	TTAAAATTAAAATAAAATTAAAAT/TTAAAATTAAAATAAAATTAAAATAAAATTAAAAT	TTAGGCCGCCACAAAGTATC	GAGGGTAAAGGCATCCACAA	60.1	524	INTERGENIC	--	--	--
Ca2_P3_15918431	Ca2	15918431	GTATATATATATATATAT/GTATATATATATATATATAT	CTTGGCCAGAAAATCGGTAA	AGGATGCATGGGATGAAAAG	60.1	691	INTERGENIC	--	--	--
Ca2_P3_15920758	Ca2	15920758	GTATATATATATATATAT/GTATATATATATATAT	TTCGGAAGTCCATCGGTATC	GCAAATGCTCCCCTAATTGA	59.9	546	INTERGENIC	--	--	--
Ca2_P3_1640062	Ca2	1640062	ATTTTTTTTT/ATTTTTT	AGATCATGTGCAAAAAGGGG	AGTAACCGTGAGGGAACGTG	59.9	509	INTERGENIC	--	--	--
Ca2_P3_16496636	Ca2	16496636	GTCAAAC/GTCAAACTAAAAGGACTATTCAAAC	TATGGACTTTGTGGTGGCAA	TTGGCACGTACTCCCATACA	60.0	441	INTERGENIC	--	--	--

INDEL marker IDs	Chromosomes/ unanchored scaffolds	Physical positions (bp)	InDels (*Kabuli* reference genome-CDC Frontier/PII)	Forward primers (5'-3')	Reverse primers (5'-3')	Anneling temperature (0C)	Expected amplified product size (bp)	Structural annotation		Functional annotation	
								Sequence components of *kabuli* genome	*Kabuli* gene accession IDs	NCBI-KOG	TFs
Ca2_P3_16514436	Ca2	16514436	AC/A	TGTGCTGGAAAGGCACATAG	GTCTCCCCCACTACAGTCCA	59.9	812	INTERGENIC	--	--	--
Ca2_P3_16516440	Ca2	16516440	AAGGGA/AA	CACATGGCTGAACGAGAAGA	TCGCCACCAAACATCTTGTA	60.0	315	INTERGENIC	--	--	--
Ca2_P3_16604975	Ca2	16604975	TAACAACA/TAACA	CCGGTGAACAAAAACGAACT	ACCCCCTCAATTAACCCTGT	60.0	851	INTERGENIC	--	--	--
Ca2_P3_16623329	Ca2	16623329	GTAATAATAATAATAATAATAATAATAA/GTAATAATAATAATAATAATAATAATAATAATAATAA	TATGGGTCAAACCCAAAACC	TTCATGCAAAGTCGAAGCAC	59.5	337	INTERGENIC	--	--	--
Ca2_P3_16665400	Ca2	16665400	T/TC	TAATTTACACACGCTGCCGA	TCGTTACTGCTGTTGTTGCC	60.3	440	INTERGENIC	--	--	--
Ca2_P3_16695716	Ca2	16695716	CTAATAATAATAATAATAATAATAATAATAATAATA/CTAATAATAATAATAATAATAATAATAATAATA	ATTTTGCCAAATTCACCGTC	CCAGCAGTAACGTTGTTGGA	59.8	449	INTERGENIC	--	--	--
Ca2_P3_16712405	Ca2	16712405	CATATATATATATATATATAT/CATATATATATATATATATATAT	AAATTCGCACACCACAAAAA	TGTTTAATTCGGCAAGCACA	59.1	607	INTERGENIC	--	--	--
Ca2_P3_16961941	Ca2	16961941	AATATATATATATATATATATATATATATATATATATATATA/AATATATATATATATATATATATATATATATATATATATATA	AAATGGTTCATTCATCGGTGT	TGAATAATTCTTGACCATCCATGT	59.2	499	INTERGENIC	--	--	--
Ca2_P3_16998327	Ca2	16998327	ACTCT/ACT	TCATGCCGAACAAAAATTCA	CAAGGGTGTGCAAGAGGTTT	60.1	345	INTERGENIC	--	--	--
Ca2_P3_17061321	Ca2	17061321	TAATATATA/TAATATATAAATATATA	TTTGGCTCGGAAGCTTTTTA	AGGAAGAGTGGAGTTGCGAA	60.0	498	INTERGENIC	--	--	--

INDEL marker IDs	Chromosomes/ unanchored scaffolds	Physical positions (bp)	InDels (*Kabuli* reference genome-CDC Frontier/PII)	Forward primers (5'-3')	Reverse primers (5'-3')	Anneling temperature (0C)	Expected amplified product size (bp)	Structural annotation		Functional annotation	
								Sequence components of *kabuli* genome	*Kabuli* gene accession IDs	NCBI-KOG	TFs
Ca2_P3_17061679	Ca2	17061679	T/TCCA	TTCGCAACTCCACTCTTCCT	CGGTAAAAATCTCTGCTCGC	60.0	190	INTERGENIC	--	--	--
Ca2_P3_17196393	Ca2	17196393	CTTTTTTTT/CTTTTTTT	CCGAGTGGAGCTCCTTATTG	ACTTGCAATCGTCTCAAGGC	59.8	382	INTERGENIC	--	--	--
Ca2_P3_1728113	Ca2	1728113	AATTATTATTATTATTATTATTATTATTATTATTATT/AATTATTATTATTATTATTATTATTATTATTATT	GAAAAGACACACATATTTGGCA	GTTTGTTGTGGTTGCAGTGG	57.7	491	INTERGENIC	--	--	--
Ca2_P3_17360962	Ca2	17360962	AAG/AAAAGGTTCAGCAG	TTGTGGTTTGGACTGTGGAA	AGCTAAAGGCCCAAAGAAGC	60.0	275	INTERGENIC	--	--	--
Ca2_P3_17377695	Ca2	17377695	CAAAAAAAAAA/CAAAAAAAAAAA	ACTCGAAATTTATGCCGCAC	AACGCACATTTTTATTTGGACTT	60.1	741	INTERGENIC	--	--	--
Ca2_P3_17551687	Ca2	17551687	G/GC	ATGTGGTTGATAAGGCCTCG	AGCAAATGCAATGCTCTGAA	60.0	398	CDS (FRAME SHIFT)	Ca_16000	OE	--
Ca2_P3_17552188	Ca2	17552188	T/TTTCCTTAGTTC	TGGCAAATTTGAAGAGGACA	GGCAACATTTTTCTCGCATT	59.2	583	DRR	Ca_16000	OE	--
Ca2_P3_17799541	Ca2	17799541	TTGT/TTGTGTGT	CAAAGGACATGATTGGGTGA	AAATCCCTGAACTCGTGGTG	59.3	547	INTERGENIC	--	--	--
Ca2_P3_17930071	Ca2	17930071	CAAAAAAAAAAAA/CAAAAAAAAAAA	CATAAGAAGCGGGAAGGTTG	GGGTGTTACAATTGGCCATC	59.7	463	INTERGENIC	--	--	--
Ca2_P3_17994871	Ca2	17994871	TTATATATATATATATATATATATATATATATATATATATA/TTATATATATATATATATATATATATATATATATATATATATATA	TGTGGCCCTATGTACTGCTG	TTGGAAGGAGCCCTTACCTT	59.7	799	INTERGENIC	--	--	--

INDEL marker IDs	Chromosomes/ unanchored scaffolds	Physical positions (bp)	InDels (*Kabuli* reference genome-CDC Frontier/PII)	Forward primers (5'-3')	Reverse primers (5'-3')	Anneling temperature (0C)	Expected amplified product size (bp)	Structural annotation		Functional annotation	
								Sequence components of *kabuli* genome	*Kabuli* gene accession IDs	NCBI-KOG	TFs
Ca2_P3_18101343	Ca2	18101343	A/AT	ACTGCGGTGAATCCTTTACG	AATTCCTTCCCACACAACCA	60.1	469	INTERGENIC	--	--	--
Ca2_P3_18159995	Ca2	18159995	TTTATTATTATTATTATTATTATTATTATTATTATTA/TTTATTATTATTATTATTATTATTATTATTATTA	AGGGACACCTCATTTGTTCG	GAAGCGACCACCTGAAGAAC	60.0	510	INTERGENIC	--	--	--
Ca2_P3_18170028	Ca2	18170028	C/CTA	AGCCAAGTCCCTGTTACCCT	TTGTCCGTGAAAGACTGCAC	60.0	395	INTERGENIC	--	--	--
Ca2_P3_18243615	Ca2	18243615	CATATATATATATATATATATATATATAT/CATATATATATATATATATATATATATAT	AATGGCTTGGCTTATGTTGG	TGAGCTTGCAATGAGTTGTTC	60.0	479	INTERGENIC	--	--	--
Ca2_P3_1835747	Ca2	1835747	TTATATATATATATATATATATATAT/TTATATATATATATATAT	CACAATGCACCATGTGACAA	TGGAACTGAAGCCATATCCC	60.0	831	INTRON	Ca_12601	--	--
Ca2_P3_1842322	Ca2	1842322	AG/AGGCAGG	CGGCTTCTGACACACAAAGA	CTAAATGCAGAAAAAGCCGC	60.0	395	INTRON	Ca_12600	--	SBP
Ca2_P3_1853896	Ca2	1853896	AATATATATATATATATATATATAT/AATATATATATATATATATATATATAT	TGAGTCCGCTTCCCAATTAC	TTGAGCACGACTTTTTGCAC	60.1	536	INTERGENIC	--	--	--
Ca2_P3_18610007	Ca2	18610007	TAA/TAACAA	TTGATCCCTTATTTCCTGCG	TTATTTCACCGTCGTGTCCA	60.0	492	INTERGENIC	--	--	--
Ca2_P3_18752437	Ca2	18752437	GAATAATAATAATAATAATAATAATAATAATA/GAATAATAATAATAATAATAATAATAATA	TTTGGCCAATGAATGTTTGA	GCCTGGCTGTACTTTGGATT	59.9	558	INTERGENIC	--	--	--
Ca2_P3_18764616	Ca2	18764616	GAATAATAATAATAATAATAATA/GAATAATAATAATAATA	ATTTGCCTCCCTCAACCTTT	TGTCTTCATGGGTGCATTGT	59.9	841	INTERGENIC	--	--	--

INDEL marker IDs	Chromosomes/ unanchored scaffolds	Physical positions (bp)	InDels (*Kabuli* reference genome-CDC Frontier/PII)	Forward primers (5'-3')	Reverse primers (5'-3')	Anneling temperature (0C)	Expected amplified product size (bp)	Structural annotation		Functional annotation	
								Sequence components of *kabuli* genome	*Kabuli* gene accession IDs	NCBI-KOG	TFs
Ca2_P3_18833380	Ca2	18833380	TAAAAAAAAAAA/TAAAAAAAAAAAA	TCGAAAAATCCCTTGAAAAAGA	GGACCTATCAAGTGAGCTTGTG	60.0	551	INTERGENIC	--	--	--
Ca2_P3_18905246	Ca2	18905246	GTTTTTTTTTTT/GTTTTTTTTTTTT	TGTGAGCTCAATTATTGGAACAG	TACTCAACCACCACCGTCAA	59.3	595	INTERGENIC	--	--	--
Ca2_P3_19219016	Ca2	19219016	ATTTT/ATTT	CCCGTGAGATCCTCCTAACA	TGAATCTACGGCTTTCTGGG	60.1	498	INTERGENIC	--	--	--
Ca2_P3_1935888	Ca2	1935888	CAAAAAAAAAAAA/CAAAAAAAAAAA	CGAATTTTAAGACCACCCAAA	ACATGCAATTGTGTGCCTTC	59.0	398	INTERGENIC	--	--	--
Ca2_P3_19387244	Ca2	19387244	TTATATATATATATATAT/TTATATATATATATATATAT	CGACATTGTAGCACCCCTTT	TTTAGATCCCTTGCACGACC	60.0	191	INTERGENIC	--	--	--
Ca2_P3_19513567	Ca2	19513567	TG/T	GCCCCTGAGTCAATACCAAA	ATCGCCAAACATTCAACAAA	59.9	615	INTERGENIC	--	--	--
Ca2_P3_19574229	Ca2	19574229	GGAGA/GGA	ACCGGAGAAGAGAAATGGGT	TTGGCTAGTCCACCATCTCC	59.9	761	INTERGENIC	--	--	--
Ca2_P3_19772797	Ca2	19772797	CT/CTT	GTTTAGGCACGTAGGGACCA	CGTTTAATTTCGGGACTGGA	60.0	417	INTERGENIC	--	--	--
Ca2_P3_19791685	Ca2	19791685	C/CG	TAGGCCATTTTCGTCCTCAC	AATCCAATGACACAAACGCA	60.1	392	INTERGENIC	--	--	--
Ca2_P3_20175480	Ca2	20175480	GATATATATATATATATATA/GATATATATATATATATATATA	TTTCAGAGTGTCTGTGGTTAGCA	GATCTGAGGTGTCATGGGCT	60.0	669	INTERGENIC	--	--	--

INDEL marker IDs	Chromosomes/ unanchored scaffolds	Physical positions (bp)	InDels (*Kabuli* reference genome-CDC Frontier/PII)	Forward primers (5'-3')	Reverse primers (5'-3')	Anneling temperature (0C)	Expected amplified product size (bp)	Structural annotation		Functional annotation	
								Sequence components of *kabuli* genome	*Kabuli* gene accession IDs	NCBI-KOG	TFs
Ca2_P3_20233917	Ca2	20233917	ACATAATCATAATCATAAT/ACATAATCATAATCATAATCATAAT	TTTTGCAGCTTTGCATTTTG	GCCATGACAAAGAGGGAGAA	60.0	930	INTERGENIC	--	--	--
Ca2_P3_20348649	Ca2	20348649	AATATATATATATATATATAT/AATATATATATATATATATATAT	TTCGTGGTTTACAAAATTCAATACA	GGTCGAAATTGGTGTTGGTC	59.7	664	INTERGENIC	--	--	--
Ca2_P3_20474785	Ca2	20474785	GAAAA/GAAAAA	ATACTGGCTCGGAGGCTTTT	CGTAGCGTGGTAGTGAGCAA	60.2	614	INTERGENIC	--	--	--
Ca2_P3_20544865	Ca2	20544865	CATATATATATATATATATATATATATATATAT/CATATATATATATATATATATATATATATATATATATAT	AGGAAGAGTGGAGTTGCGAA	CCAAGCATCATTCTCCGTTT	60.0	720	INTERGENIC	--	--	--
Ca2_P3_20647114	Ca2	20647114	AATATATATATATATATATAT/AATATATATATATATATATATAT	ATCACTCGCACAACAAACCA	TTGGAAGGAGCCCTTACCTT	60.2	920	INTERGENIC	--	--	--
Ca2_P3_20649381	Ca2	20649381	GAAAAA/GAAAA	ATATTGGCTCGGAGGCTTTT	AAATGGGAGAAGGAGGGAGA	60.1	385	INTERGENIC	--	--	--
Ca2_P3_20715795	Ca2	20715795	T/TCATCA	GACATTGCATTTTGGACACG	AAATGGATTTCAGCCAATCG	60.0	539	INTERGENIC	--	--	--
Ca2_P3_2080611	Ca2	2080611	TAAA/TAAAA	AAGCGATGACTGTGTGCTTG	AACCTCTCCTCGATCACCCT	60.1	376	INTRON	Ca_12572	O	B3
Ca2_P3_21000306	Ca2	21000306	TTCCAATC/TTC	GCCATGTCAAAGTATCGCCT	GCCCCGTATTTACCAATGTG	60.1	431	INTERGENIC	--	--	--
Ca2_P3_21163296	Ca2	21163296	CAAAAAA/CAAAAA	GGCCACAAACTGGGAAGTTA	CCCTGTGACAAACACAATCG	60.0	288	INTERGENIC	--	--	--

INDEL marker IDs	Chromosomes/ unanchored scaffolds	Physical positions (bp)	InDels (*Kabuli* reference genome-CDC Frontier/PII)	Forward primers (5'-3')	Reverse primers (5'-3')	Anneling temperature (0C)	Expected amplified product size (bp)	Structural annotation		Functional annotation	
								Sequence components of *kabuli* genome	*Kabuli* gene accession IDs	NCBI-KOG	TFs
Ca2_P3_21308575	Ca2	21308575	TTTTCCTT/TTTTCCTTTGTTTCTATTTCCTT	TTTCGAAGCAACGAGGAAGT	TTGGTATTGAACTCGGAGGC	60.0	474	INTERGENIC	--	--	--
Ca2_P3_21308629	Ca2	21308629	CAAA/CAA	TTTCGAAGCAACGAGGAAGT	TTGGTATTGAACTCGGAGGC	60.0	474	INTERGENIC	--	--	--
Ca2_P3_21308673	Ca2	21308673	TAA/TCAA	GTTGGTTCCCTAGCACCGTA	AAATTCGACTTCCGTTCCCT	60.0	905	INTERGENIC	--	--	--
Ca2_P3_21309961	Ca2	21309961	A/AT	AGCCTTGGGTTTCCTTTGTT	CAATTGAGTTGCGACGAGAG	60.0	294	INTERGENIC	--	--	--
Ca2_P3_21314611	Ca2	21314611	AG/AGG	CAAACCCTTTCAAGGTGCAT	GCACTGCCCCAAGAATTTTA	60.0	443	INTERGENIC	--	--	--
Ca2_P3_2140867	Ca2	2140867	TCACACACACACAC/TCACACACACACACACAC	TGAAATCAAAATCCATCGCA	TCGATTTAAATGAATGAATGTCG	60.0	642	INTERGENIC	--	--	--
Ca2_P3_2169056	Ca2	2169056	C/CG	AACCTCTCCAAGAGGATCGG	GGAGAGTGAAACGGATGGAA	60.6	648	INTERGENIC	--	--	--
Ca2_P3_2169184	Ca2	2169184	GTTTT/GTTT	TCTCACGGTTTCAACTTCCC	GTCTGATGGAGGAGGCAGAG	60.1	447	INTERGENIC	--	--	--
Ca2_P3_21695844	Ca2	21695844	GTTATTATTATTATTATTATTATTATTATTAATATTATTATTATTATTATTA/GTTATTATTATTATTATTATTATTATTATTATTAATATTATTATTATTATTA	GATGAAATTCAGGCTCGCTC	TTGACAAGGGAAAACCTTGC	59.9	444	INTERGENIC	--	--	--
Ca2_P3_21699071	Ca2	21699071	AAATAATAATAATAATAATAATAA/AAATAATAATAATAATAATAATAATAATAATAA	GGCCTAGGCAAAAATTAGGG	AGGTTGCCCTTTCAGGTTTT	59.9	210	INTERGENIC	--	--	--

INDEL marker IDs	Chromosomes/ unanchored scaffolds	Physical positions (bp)	InDels (*Kabuli* reference genome-CDC Frontier/PII)	Forward primers (5'-3')	Reverse primers (5'-3')	Anneling temperature (0C)	Expected amplified product size (bp)	Structural annotation		Functional annotation	
								Sequence components of *kabuli* genome	*Kabuli* gene accession IDs	NCBI-KOG	TFs
Ca2_P3_21717164	Ca2	21717164	TA/T	GGTAGAGGCAGGCCATATCA	GATTTGTGAGGGATTTTTCAGC	60.1	841	INTERGENIC	--	--	--
Ca2_P3_2174282	Ca2	2174282	ATTTTTTTTTTTTT/ATTTTTTTTTTT	TGACATCCATCTTATGTGGGTC	GGTCTACTCATGTTGAAAGACGC	59.7	674	INTERGENIC	--	--	--
Ca2_P3_21798248	Ca2	21798248	TAAAAAAAAAAAA/TAAAAAAAAAAAAA	AGCTTTGATTTCTCCATCGC	TCAATCGAGTCACCACCAAA	59.4	370	INTERGENIC	--	--	--
Ca2_P3_21865327	Ca2	21865327	TTATATATATATATATATATATATATATATAT/TTATATATATATATATATATATATAT	AACACCAGTGTACTTCCCCG	AACGAAGTGGTATCTTCCTATTGAA	59.9	884	INTERGENIC	--	--	--
Ca2_P3_21889862	Ca2	21889862	G/GGT	CGATGTTAGTGGTGACCGTG	CTGCTTGAGATGGCGTTGTA	60.0	606	INTERGENIC	--	--	--
Ca2_P3_22111543	Ca2	22111543	T/TA	ATTCCCCCTTCATCTTGGAC	AAATTAAAGTTTCAAACCACGG	60.1	522	INTERGENIC	--	--	--
Ca2_P3_22286038	Ca2	22286038	GAAGCGC/GAAGCGCACCCAAGCGC	GGGAGGCAACCCAATTTTAT	GCTTAGATCATCCGCTCCTG	60.0	368	INTERGENIC	--	--	--
Ca2_P3_22455964	Ca2	22455964	GTATATATATA/GTATATATATATA	AGCGCTGTAAAATGTCCACC	CTGGGCAAGGGATCTGAATA	60.1	470	DRR	Ca_14999	--	Trihelix
Ca2_P3_224634	Ca2	224634	TCATTTATAGCATTTATAG/TCATTTATAG	GTTGGGTCACCGTTTCACTT	GCTCAACATTGGTTGCCTTT	59.9	657	INTRON	Ca_16974	--	--
Ca2_P3_22468520	Ca2	22468520	CAAAAAAAAAA/CAAAAAAAAAAA	TGGCCATGTGCATTGTTTAT	AGGCATCAACGCCTAAATTG	59.8	674	INTERGENIC	--	--	--

INDEL marker IDs	Chromosomes/ unanchored scaffolds	Physical positions (bp)	InDels (*Kabuli* reference genome-CDC Frontier/PII)	Forward primers (5'-3')	Reverse primers (5'-3')	Anneling temperature (0C)	Expected amplified product size (bp)	Structural annotation		Functional annotation	
								Sequence components of *kabuli* genome	*Kabuli* gene accession IDs	NCBI-KOG	TFs
Ca2_P3_22500042	Ca2	22500042	ANNNNNNNNNN/ANN	CGTTCTGCATTTCTGGTTCC	ACTTTTCACCATGTCCCTCG	60.6	180	INTERGENIC	--	--	--
Ca2_P3_22517081	Ca2	22517081	ATTTTTTT/ATTTTTTTT	TGTGCAATTTCTCCACCAAA	CAGTTAACAGCCACCTTCCA	60.1	149	INTRON	Ca_14997	G	--
Ca2_P3_22527944	Ca2	22527944	TAAAAAAA/TAAAAAAAA	TGCTCCCTCATTCTTCCATT	ATCTTGGAGCCCCTGATCTT	59.6	713	INTERGENIC	--	--	--
Ca2_P3_22579550	Ca2	22579550	CT/CTTT	CTAAGCCCTCACAAAGGCAG	AGTTGAAGCTTCTGGGACGA	60.0	292	DRR	Ca_14994	--	--
Ca2_P3_22650634	Ca2	22650634	TG/T	GAGAGGAGGGTTCCCAGAGT	GTGCATCCTCCATCTTTGGT	59.7	550	DRR	Ca_14991	--	--
Ca2_P3_22667401	Ca2	22667401	TAAAA/TAAA	ACCAAAGATGGAGGATGCAC	CCCAGAACTCAGGTTTCACC	59.9	258	INTERGENIC	--	--	--
Ca2_P3_22682677	Ca2	22682677	A/AATAATAATT	CCCGTCAATTTTTGAGTGGT	CAATTCCCACAGGTACGCTT	59.8	762	INTERGENIC	--	--	--
Ca2_P3_22779378	Ca2	22779378	T/TAAA	TGAACCAGTCACCAAAACCA	TGCCTATGTCACCAATTCCA	60.0	722	INTERGENIC	--	--	--
Ca2_P3_23050911	Ca2	23050911	AATG/AATGGTCTATTGGCTTATG	TATGGGGATCGGAAATCAAA	ACAATCGAAATTTTAGGCCG	60.1	907	INTERGENIC	--	--	--
Ca2_P3_23058191	Ca2	23058191	GTTTTTTTTTTT/GTTTTTTTTTTTT	GAAACTGTGTCACGAGGCAA	AGAGCCGTCCAGAAAAATCA	59.9	121	INTERGENIC	--	--	--

INDEL marker IDs	Chromosomes/ unanchored scaffolds	Physical positions (bp)	InDels (*Kabuli* reference genome-CDC Frontier/PII)	Forward primers (5'-3')	Reverse primers (5'-3')	Anneling temperature (0C)	Expected amplified product size (bp)	Structural annotation		Functional annotation	
								Sequence components of *kabuli* genome	*Kabuli* gene accession IDs	NCBI-KOG	TFs
Ca2_P3_23183113	Ca2	23183113	TAAAAAA/TAAAAAAA	TGGATACATATGCCGCTCAA	ACCGTGGACCACCTTTGATA	60.1	565	DRR	Ca_14971	--	--
Ca2_P3_23193466	Ca2	23193466	TA/T	CCACAAATGCTCATCGTGAC	TTTGCAGCCACAAAAGACAG	60.1	561	INTERGENIC	--	--	--
Ca2_P3_2345426	Ca2	2345426	GG/GGTTCAG	GGACAAAGCTGCAATGATGA	ACAGAAACATGCCAACTCCC	59.8	381	INTRON	Ca_12544	S	BBR-BPC
Ca2_P3_23472039	Ca2	23472039	CTTTATTTATTTATTTATTTATTTATTTATTTATTTAT/CTTTATTTATTTATTTATTTATTTATTTATTTATTTATTTATTTATTTATTTATTTAT	TTTTGCTAACCACACCCCTC	TGACCACGTGTTTTTGGGTA	60.0	434	INTRON	Ca_14958	--	--
Ca2_P3_23495114	Ca2	23495114	ATTTTTTT/ATTTTTT	GCTCCCCATGAGCACTAGAA	AGGCTCTCAATGCCAACCTA	60.4	710	INTERGENIC	--	--	--
Ca2_P3_23509535	Ca2	23509535	AAAAACTTAAAACTTAAAACTTA/AAAAACTTAAAACTTA	CCCTCTTGAATTTTTGTTTTCC	CCCACACCCTCGACTAAAGA	59.0	542	INTERGENIC	--	--	--
Ca2_P3_23819744	Ca2	23819744	ATTT/ATT	TTGTTGTAGGGCACATTGGA	TGTTCGTTTGACGAGAGCAC	60.0	391	DRR	Ca_14945	--	--
Ca2_P3_2388005	Ca2	2388005	GTTTTTTTTT/GTTTTTTTTTT	GCCAACAACGTTACGAGCTA	CTTGCTAAATGCCCCACTTC	59.0	581	INTERGENIC	--	--	--
Ca2_P3_23926547	Ca2	23926547	CAAAAAAAAAAAAAA/CAAAAAAAAAAAAAAAA	GGCCATTATTTGGATGGATG	CAAAATTGCTCGCATCTTCA	60.0	875	INTERGENIC	--	--	--
Ca2_P3_23932608	Ca2	23932608	TAAATTAATGAATAAAAATTAAT/TAAATTAAT	AAGGCATCCAATCAAAGCAC	CTCAAGATTTTAGCGCCGTC	60.1	460	INTERGENIC	--	--	--

INDEL marker IDs	Chromosomes/ unanchored scaffolds	Physical positions (bp)	InDels (*Kabuli* reference genome-CDC Frontier/PII)	Forward primers (5'-3')	Reverse primers (5'-3')	Anneling temperature (0C)	Expected amplified product size (bp)	Structural annotation		Functional annotation	
								Sequence components of *kabuli* genome	*Kabuli* gene accession IDs	NCBI-KOG	TFs
Ca2_P3_23989802	Ca2	23989802	AAAAACAAAACAAAA/AAAAACAAAACAAAACAAAA	AATTCGTTGAGCCGGTCTAGT	AGCACGGGGAGTATGAGAAA	60.1	421	INTERGENIC	--	--	--
Ca2_P3_23990182	Ca2	23990182	AAAAACAAAACAAAA/AAAAACAAAACAAAACAAAA	TCTCATACTCCCCGTGCTTT	ATCCCCTACACCAGATGCAC	59.7	565	INTERGENIC	--	--	--
Ca2_P3_24019689	Ca2	24019689	TAAAAAAAAAAAAAA/TAAAAAAAAAAAAA	TGCCTTGAACAACCACTGTC	CCTTTCAGAGAATCGTGGGA	59.7	584	INTERGENIC	--	--	--
Ca2_P3_24102909	Ca2	24102909	GT/GTT	TGGGGGATCCAAGTTAAAAA	GAAAACTCTCCGGCATTCAA	59.2	428	CDS (FRAME SHIFT)	Ca_21670	S	--
Ca2_P3_24251584	Ca2	24251584	ACCCCC/ACCCCCC	GATTGCTTTCTGGGTGGAAA	TGATCAACCCTTGGTGTGAA	60.1	652	INTERGENIC	--	--	--
Ca2_P3_24252139	Ca2	24252139	TAA/TAAGAAA	GATCCTCAAATCGACCTCCA	TGTCACCATTGGTCTCCAAA	60.0	465	INTERGENIC	--	--	--
Ca2_P3_24393200	Ca2	24393200	G/GAAGATA	TCCTAGAACATGCATCACGC	TACCCTTTGCTAGCCCCTTT	59.8	505	INTERGENIC	--	--	--
Ca2_P3_24685078	Ca2	24685078	TAAAAAAAA/TAAAAAAAAA	TTCCACACTCCTTCAGCCAT	CCAACAAATGGAGAAGCCAT	60.7	665	INTRON	Ca_22893	--	--
Ca2_P3_24687137	Ca2	24687137	ATTTTTTTTTTT/ATTTTTTTTTTTT	TGCTCAAATTATCCGGTGAAA	TGAAATTGAGATAACTGACGTGC	60.4	664	INTERGENIC	--	--	--
Ca2_P3_24943252	Ca2	24943252	CTTT/CTT	AACCCCTCCAAAGAACGACT	AGGGCCCCACTAACTCCTTA	60.0	680	INTERGENIC	--	--	--

INDEL marker IDs	Chromosomes/ unanchored scaffolds	Physical positions (bp)	InDels (*Kabuli* reference genome-CDC Frontier/PII)	Forward primers (5'-3')	Reverse primers (5'-3')	Anneling temperature (0C)	Expected amplified product size (bp)	Structural annotation		Functional annotation	
								Sequence components of *kabuli* genome	*Kabuli* gene accession IDs	NCBI-KOG	TFs
Ca2_P3_24948166	Ca2	24948166	CTTTTT/CTTTT	GGTGAGACTTTCGAGGGCTA	GGCATTGAACGTTTTCACAA	59.4	295	INTERGENIC	--	--	--
Ca2_P3_24948276	Ca2	24948276	GAAAA/GAAA	AAACGTCCAAGGTAAGGGCT	GGCATTGAACGTTTTCACAA	60.0	255	INTERGENIC	--	--	--
Ca2_P3_25025231	Ca2	25025231	TAA/TA	CAGCCACCCACCTACAAAGT	ATAGCACCAACAAACCCAGC	60.0	829	INTRON	Ca_14236	--	CO-like
Ca2_P3_2506639	Ca2	2506639	T/TAATAATAA	GCGACTTTATCGCCTAGCAC	GGGTGTGTATGGCTTCCAAT	60.0	912	INTERGENIC	--	--	--
Ca2_P3_25078728	Ca2	25078728	GATATATATATATATATATAT/GATATATATATATATATAT	TCACAACAAAGGAAATGATTCAA	TTGCACACATAAAGTGTTCGG	59.5	503	INTERGENIC	--	--	--
Ca2_P3_25084292	Ca2	25084292	CTTTTTT/CTTTTTTT	GAAACAGCGAGAATGTGCAA	GCGCGTCTTTTATAGCGTTT	60.0	768	INTERGENIC	--	--	--
Ca2_P3_25135786	Ca2	25135786	GTT/GTTT	GGAAGCTGACTTTTTGGCAT	TGGCTTGATTCCTTTTGCTT	59.3	160	INTERGENIC	--	--	--
Ca2_P3_25290391	Ca2	25290391	A/AGAAGG	AGGGTTGAAGCATTTGATGG	CCCTCCAGAGAAAAGCAGAA	59.9	356	DRR	Ca_14263	R	C3H
Ca2_P3_25444353	Ca2	25444353	CAAA/CAA	AAAATACCGTGCCGTTTCTG	TGAGAAGTTGGTGTGCTTGC	60.0	475	DRR	Ca_14272	Q	B3
Ca2_P3_2546989	Ca2	2546989	AT/ATCATAGTTT	CCCGTCGATCAAAAGAAAAA	TTGAATTGCTTTCCAGTGTCC	60.0	705	INTRON	Ca_10592	R	--

INDEL marker IDs	Chromosomes/ unanchored scaffolds	Physical positions (bp)	InDels (*Kabuli* reference genome-CDC Frontier/PII)	Forward primers (5'-3')	Reverse primers (5'-3')	Anneling temperature (0C)	Expected amplified product size (bp)	Structural annotation		Functional annotation	
								Sequence components of *kabuli* genome	*Kabuli* gene accession IDs	NCBI-KOG	TFs
Ca2_P3_25527401	Ca2	25527401	GATAATAATAATAATAATAATAATAATAATAA/GATAATAATAATAATAATAATAATAATAATAATAA	ACCCTTCAATTTCGACATGC	CCAATTGGAATTTGCAACAA	59.9	598	INTERGENIC	--	--	--
Ca2_P3_25529864	Ca2	25529864	GTTTTTT/GTTTTTTTTT	AGCAGCCCAAGCAAGACTTA	TCATGCCGATTGATAGAGGTT	60.2	553	INTERGENIC	--	--	--
Ca2_P3_25530031	Ca2	25530031	A/ATACCCTTG	AGCAGCCCAAGCAAGACTTA	ATCCGACCGTTGAAGATTTG	60.2	724	INTERGENIC	--	--	--
Ca2_P3_25535050	Ca2	25535050	CTT/CTTCTTTTTTTT	CCAGATTTGAACTGGGGAAA	AATCAAGTGGGTTCGAGTGG	59.9	551	DRR	Ca_14283	R	MYB
Ca2_P3_25554310	Ca2	25554310	TTGTGTGTGTGTGT/TTGTGTGTGTGT	ATGCGGCAGGTATGAGAACA	ATATCAACGCCCTTTTGCAT	61.6	473	INTERGENIC	--	--	--
Ca2_P3_25567949	Ca2	25567949	TTGTGTGT/TTGTGT	CATATGCAACACTGGCTTGG	TCAACCTTTTGAAATTATTTGCC	60.1	363	DRR	Ca_14285	R	bZIP
Ca2_P3_25733071	Ca2	25733071	GGCCGC/GGCCGCCGC	CTCAGAAAGCGGATCAAAGG	CTCAACCAACCACCCACTCT	59.9	477	INTERGENIC	--	--	--
Ca2_P3_25791084	Ca2	25791084	ATTTTTTTTTTTTTTT/ATTTTTTTTTTTTT	GCAAGTTGAGCCTCATGGAT	GCCTCGGTTGAAAATTGAAA	60.2	452	INTERGENIC	--	--	--
Ca2_P3_25845983	Ca2	25845983	TGG/TG	AGTGTTTTGAGGGTCATCCG	ATTCCATGCTTGCTATTCGG	60.0	647	INTERGENIC	--	--	--
Ca2_P3_26043745	Ca2	26043745	AGGG/AGG	AAATTGTCACCGCCTCATTT	ACCCAATGATTCGTCCAAAA	59.4	343	INTERGENIC	--	--	--

INDEL marker IDs	Chromosomes/ unanchored scaffolds	Physical positions (bp)	InDels (*Kabuli* reference genome-CDC Frontier/PII)	Forward primers (5'-3')	Reverse primers (5'-3')	Anneling temperature (0C)	Expected amplified product size (bp)	Structural annotation		Functional annotation	
								Sequence components of *kabuli* genome	*Kabuli* gene accession IDs	NCBI-KOG	TFs
Ca2_P3_26109346	Ca2	26109346	AATATATATATATATATATATA/AATATATATATATATATATATATA	GGGTGAACCAAAAGAGAGCA	TCCCACAACAATAGGCACAA	60.2	962	INTERGENIC	--	--	--
Ca2_P3_26113486	Ca2	26113486	CTTTTTTTTTTTT/CTTTTTTTTTTTTTT	AGGAGTGTGTAGGGGTCACG	GATGCGAGTTTCTCCAAACC	60.0	137	INTERGENIC	--	--	--
Ca2_P3_2624920	Ca2	2624920	CGGCTGGCT/CGGCT	GGCCAAGTTGCTGAAGGATA	CGCTAAGAGGCCGTTATCAA	60.2	610	INTRON	Ca_10586	--	--
Ca2_P3_263972	Ca2	263972	TAAAAAAAAAAAAA/TAAAAAAAAAAAA	TTGGGATTAAAAACCCATTCC	TGCACGTTCTTCTCCTCCTT	59.9	467	INTERGENIC	--	--	--
Ca2_P3_26480524	Ca2	26480524	GAAAA/GAAA	ATGGGGAAAATTAACCCAGC	GTACGTGGAAGGAAAGCGTC	60.0	612	INTERGENIC	--	--	--
Ca2_P3_26488356	Ca2	26488356	C/CAT	TAACCAGCCATTAAGGCGTC	GCCACTCAAACAACCATCCT	60.1	565	INTERGENIC	--	--	--
Ca2_P3_26504194	Ca2	26504194	AGG/AG	CCGACGTCCAGTCAGAATTT	GGATTGGAGTTGGTTGGATG	60.1	319	INTERGENIC	--	--	--
Ca2_P3_26516638	Ca2	26516638	ATTTTT/ATTTTTT	AGGGTTTCTCACCCATTTCC	GCAGCTTCTTCCCGTTGTAG	60.2	552	INTERGENIC	--	--	--
Ca2_P3_26570798	Ca2	26570798	GAAAAAAAAAAA/GAAAAAAAAAA	GGTGGGCATGAGATGAGAGT	TTTACGGGGAAGAGAAGTGC	60.1	782	INTERGENIC	--	--	--
Ca2_P3_26811414	Ca2	26811414	TAAAAAAA/TAAAAAAAA	GGGCTTGTTTGATTGTGTTTT	GGCTCCATTCTTTGCTCTTG	59.0	584	INTERGENIC	--	--	--

INDEL marker IDs	Chromosomes/ unanchored scaffolds	Physical positions (bp)	InDels (*Kabuli* reference genome-CDC Frontier/PII)	Forward primers (5'-3')	Reverse primers (5'-3')	Anneling temperature (0C)	Expected amplified product size (bp)	Structural annotation		Functional annotation	
								Sequence components of *kabuli* genome	*Kabuli* gene accession IDs	NCBI-KOG	TFs
Ca2_P3_2692293	Ca2	2692293	ATTTTTTTT/ATTTTTTT	AAAGGTGATGAAGCAGATTTTGA	TGGTGTCCTCTCTTCTCTAATCG	60.1	478	INTERGENIC	--	--	--
Ca2_P3_27240694	Ca2	27240694	GTATATATATATATATATATATATATAT/GTATATATATATATATATATAT	CCAAAGTTGGATTCAAAGTGAAG	CTGGAAGGGGAGATTGTTGA	60.0	921	INTERGENIC	--	--	--
Ca2_P3_2726175	Ca2	2726175	TTATATATATATATATATATATATAT/TTATATATATATATATATATAT	CGTTTCGGGTTTCGTAAAAA	TTGTTGACAATTCGATCCAAAG	60.0	302	DRR	Ca_10571	Q	B3
Ca2_P3_27420609	Ca2	27420609	CATATATATATATATATATATATATATATATAT/CATATATATATATATATATATATATATATATATAT	TTTAGATCCCTTGCACGACC	CTTCGACACTGTAGCACCCA	60.1	204	DRR	Ca_15661	--	--
Ca2_P3_27526456	Ca2	27526456	TTATATATATATATATATATATATA/TTATATATATATATATATATATATA	CGAGCCCAAAATCCAAAATA	CTCAATGCGGGTTTACGATT	59.9	485	INTERGENIC	--	--	--
Ca2_P3_27628273	Ca2	27628273	ATTTTTTTTT/ATTTTTTTTTT	ATGTGCAACTGCCACCAATA	TTGTGGGTGATTGAATGTGC	60.0	486	INTERGENIC	--	--	--
Ca2_P3_27647945	Ca2	27647945	T/TC	ATGATTTGGAGCCATTCCAG	CAAGTCAGTGGCCAAAATCC	59.9	244	INTERGENIC	--	--	--
Ca2_P3_27715399	Ca2	27715399	AATATATATATATATATATATATATATATATAT/AATATATATATATATATATATATATATATATATAT	AAGTCGCATCACCGATATTGT	CAAGGCGATTTGAATTGGTT	59.5	372	INTERGENIC	--	--	--
Ca2_P3_27729659	Ca2	27729659	AATATATATATATATATATATATATATATATATAT/AATATATATATATATATATATATATATATATATAT	TTTGGGGCTACTTTCCACTG	GGGATTGAATATATGGTCCCG	60.1	712	INTERGENIC	--	--	--
Ca2_P3_27776478	Ca2	27776478	GTTTTTTTTTT/GTTTTTTTTTTT	CAATGAACTTGGTCAACCCC	GCCCATGTCTGCATAGTAACAA	60.2	407	INTERGENIC	--	--	--

INDEL marker IDs	Chromosomes/ unanchored scaffolds	Physical positions (bp)	InDels (*Kabuli* reference genome-CDC Frontier/PII)	Forward primers (5'-3')	Reverse primers (5'-3')	Anneling temperature (0C)	Expected amplified product size (bp)	Structural annotation		Functional annotation	
								Sequence components of *kabuli* genome	*Kabuli* gene accession IDs	NCBI-KOG	TFs
Ca2_P3_27776538	Ca2	27776538	TAAAAAAA/TAAAAAA	CAATGAACTTGGTCAACCCC	GCCCATGTCTGCATAGTAACAA	60.2	407	INTERGENIC	--	--	--
Ca2_P3_27826409	Ca2	27826409	TG/TGG	TATTGGGGGATTTTGGTTGA	CCCACTTATAAGGGCTGCAA	60.0	310	INTRON	Ca_15681	U	--
Ca2_P3_28037628	Ca2	28037628	AATATATATATATATATATATATATATATAT/AATATATATATATATATATATATATATAT	CTAGTGTTGCACGGGTCAAA	CATAACGAATTCTCCCACCG	59.8	768	INTERGENIC	--	--	--
Ca2_P3_28064133	Ca2	28064133	GTATATATATATATATAT/GTATATATATATATATATATATAT	TCAAAATGCCACAATTCCAA	ACATATACCATTGGGGCGAT	59.9	612	INTERGENIC	--	--	--
Ca2_P3_2807369	Ca2	2807369	ATTTT/AT	TTGGCTTCTTTGGCAAGTCT	CACCATCATCATCGCTCATC	60.0	756	CDS (large-effect mutations)	Ca_10561	Q	Nin-like
Ca2_P3_28104611	Ca2	28104611	GGAGAGAGAGAGAGAGAGAGAGAGAGAG/GGAGAGAGAGAGAGAGAGAGAGAGAGAGAGAG	TCGTTTCTCCTCAATTTGTGTG	CTTCGGAAGTCTCCACTCCA	60.1	499	INTERGENIC	--	--	--
Ca2_P3_28202546	Ca2	28202546	TATAATAATAATAATAATAATAATAATAATAATAATAATAAT/TATAATAATAATAATAATAATAATAATAATAATAAT	TGAACCACATCCCTTATGCTT	CCATGAATGAGAGTGCAAAGG	59.4	632	INTERGENIC	--	--	--
Ca2_P3_28203929	Ca2	28203929	CTCTTCATCT/CTCTTCATCTTCATCT	GACCACGGCACTTCAAACTT	AACGAACCGTCCATCTCAAC	60.2	153	INTERGENIC	--	--	--
Ca2_P3_28262473	Ca2	28262473	GAA/GAAAA	CTGAGACACGGCGACAATAA	ATCGGAGCAATTCTGCATTC	59.9	305	INTERGENIC	--	--	--
Ca2_P3_28365177	Ca2	28365177	AATATATATATATATATATATAT/AATATATATATATATATATAT	TCGGTCTCCTCATTTGATCC	GGGCCACATGTAATGCTTCT	60.0	768	INTERGENIC	--	--	--

INDEL marker IDs	Chromosomes/ unanchored scaffolds	Physical positions (bp)	InDels (*Kabuli* reference genome-CDC Frontier/PII)	Forward primers (5'-3')	Reverse primers (5'-3')	Anneling temperature (0C)	Expected amplified product size (bp)	Structural annotation		Functional annotation	
								Sequence components of *kabuli* genome	*Kabuli* gene accession IDs	NCBI-KOG	TFs
Ca2_P3_28421823	Ca2	28421823	GTTTTTT/GTTTTTTT	CTACACCCCAAAAACCTCCA	TGTATTGTGGACCAGCCTCA	59.8	500	INTERGENIC	--	--	--
Ca2_P3_28464988	Ca2	28464988	TAAAAAAAAA/TAAAAATAAAAAAAAAA	TCATCGCAGGTGTACCATGT	AACATTGCACCGGTTTGATT	60.0	414	INTERGENIC	--	--	--
Ca2_P3_28465098	Ca2	28465098	CAAAAAAAAAAAA/CAAAAAAAAAAAAAA	TCATCGCAGGTGTACCATGT	AACATTGCACCGGTTTGATT	60.0	414	INTERGENIC	--	--	--
Ca2_P3_28465231	Ca2	28465231	CAAAAA/CAAAA	TCATCGCAGGTGTACCATGT	GGTATCAGAGCCCACCATGT	60.0	908	INTERGENIC	--	--	--
Ca2_P3_28465620	Ca2	28465620	A/AT	AGCTCCATTGCAAAAACACC	GCAATTCCCACAGGTATGCT	60.1	553	INTERGENIC	--	--	--
Ca2_P3_28585157	Ca2	28585157	GTATTATTATTATTATTATTA/GTATTATTATTATTATTATTATTA	CCTCAACTCGTTCAAAGGGA	AAAACTTCACCAAAATGCCG	60.2	650	DRR	Ca_14363	--	--
Ca2_P3_28617666	Ca2	28617666	TTTTATTTATTTATTTATT/TTTTATTTATTTATTTATTTATTTATT	ATCGACTCCCGAACTCAAAA	CCACGGTTCTTAGAGCCATTA	59.7	621	INTERGENIC	--	--	--
Ca2_P3_2862408	Ca2	2862408	CAGT/CAGTAAGT	TCCAGAATGTTCATGGTTTGAG	TTGGAATCATGGAAAGACACC	60.0	522	DRR	Ca_10558	--	ERF
Ca2_P3_28723744	Ca2	28723744	AAAAACACACA/AACA	AAAAAGCGCTGTTCATTTCAA	AAAAAGCCTCCACCAAAGGT	59.9	511	DRR	Ca_14359	R	--
Ca2_P3_2887257	Ca2	2887257	ATTTTTTTTTT/ATTTTTTTTTTT	AATCGAGACAAATTCGGGTG	GAGTAGGGCACTCTAGCACCTT	59.9	322	INTERGENIC	--	--	--

INDEL marker IDs	Chromosomes/ unanchored scaffolds	Physical positions (bp)	InDels (*Kabuli* reference genome-CDC Frontier/PII)	Forward primers (5'-3')	Reverse primers (5'-3')	Anneling temperature (0C)	Expected amplified product size (bp)	Structural annotation		Functional annotation	
								Sequence components of *kabuli* genome	*Kabuli* gene accession IDs	NCBI-KOG	TFs
Ca2_P3_28874011	Ca2	28874011	CTTT/CTT	TGATGGAATGCAATGA AATGA	TCCGTGCATGCCATAA TCTA	59.9	386	INTERGENIC	--	--	--
Ca2_P3_28937196	Ca2	28937196	GTTTTTTTTTTTTTT/GTTTTTTTTTTTTT	TTTGAAAGTACGTGGC CAGA	TGTTATTGGGTCAGGG GAAA	59.3	585	INTERGENIC	--	--	--
Ca2_P3_28984959	Ca2	28984959	CT/CTT	TTGACATTGCTCGTTCC TTG	TGGATGTCATTGATGA TCCTTT	59.8	848	INTERGENIC	--	--	--
Ca2_P3_28989281	Ca2	28989281	AAGAGAGAGAGAGAGAGAGAGAGAGA/A AGAGAGAGAGAGAGAGAGAGAGAGA	TCGACCCGGTAAATGA GAAG	GGAACAGGTTCTTCCA CCAA	60.1	914	DRR	Ca_14339	K	MYB
Ca2_P3_2907015	Ca2	2907015	AATATATATATATATATATATATATA/AATA TATATATATATATATATATATA	TTTCTCTGTGTTTATTG TGCGTT	TTTTTGACTGACGCCT CAAAT	58.9	577	INTERGENIC	--	--	--
Ca2_P3_29072040	Ca2	29072040	ATCTGCAGACTTT/ATCTGCAGACTTTC TGCAGACTTT	TTGCTGGCAGAACAAT CAAG	TGTGGGAAGCACCATG TTTA	60.0	546	DRR	Ca_14332	--	--
Ca2_P3_29072081	Ca2	29072081	TTATATATATATATATATATATATATATATA TA/TTATATATATATATATATATATATATATAT ATA	TTGCTGGCAGAACAAT CAAG	TGTGGGAAGCACCATG TTTA	60.0	546	DRR	Ca_14332	--	--
Ca2_P3_29172910	Ca2	29172910	CTTTTTTTTTTT/CTTTTTTTTTT	CATGTCATAGGCAATGT GGC	GTTTTGACGACCGCGT AGAT	60.0	807	INTERGENIC	--	--	--
Ca2_P3_29184413	Ca2	29184413	GAAAAAAAAA/GAAAAAAAAAA	TGGTGGGTGATTGCTG ATTA	AAGATTGGAACTTGGG CCTT	59.9	435	INTERGENIC	--	--	--
Ca2_P3_29195958	Ca2	29195958	CATATATATATATATATATA/CATATATA TATATATATATATA	TTGTTTCGGAGGGTTG ACTC	CCACCGATTGAAACAT ACCA	60.1	612	DRR	Ca_14321	PT	C3H

INDEL marker IDs	Chromosomes/ unanchored scaffolds	Physical positions (bp)	InDels (*Kabuli* reference genome-CDC Frontier/PII)	Forward primers (5'-3')	Reverse primers (5'-3')	Anneling temperature (0C)	Expected amplified product size (bp)	Structural annotation		Functional annotation	
								Sequence components of *kabuli* genome	*Kabuli* gene accession IDs	NCBI-KOG	TFs
Ca2_P3_29215461	Ca2	29215461	TTAT/TTATAT	TACTTTAGCAACCACCCGCT	ATGACTCCAACACCATGCCT	59.8	873	INTERGENIC	--	--	--
Ca2_P3_29220253	Ca2	29220253	GATATATATATATATATATATATA/GATATATATATATATATATATATA	AGGCATGGTGTTGGAGTCAT	CTAGTTCGTAACCCGTGCGT	60.4	253	INTERGENIC	--	--	--
Ca2_P3_29239996	Ca2	29239996	TAAAA/TAA	GGCTGCTGATCCTGTTCTTC	CTGACGGACGGACTCATTTT	60.0	482	INTERGENIC	--	--	--
Ca2_P3_29240150	Ca2	29240150	TC/TCACC	CTTAGAGCATCTCCAACGGC	GAAGCATCGTTCCTTCCTGA	60.0	134	INTERGENIC	--	--	--
Ca2_P3_29240382	Ca2	29240382	TCCC/TCC	CTTAGAGCATCTCCAACGGC	CCTTGCACTATCATTTTCGGA	60.0	803	INTERGENIC	--	--	--
Ca2_P3_29252004	Ca2	29252004	GTT/GT	TCAAAGGTGCACTCTTGCTATT	TGGCTTCCGGAAAGACTAAA	59.0	502	INTERGENIC	--	--	--
Ca2_P3_29356033	Ca2	29356033	CTTTTTTTTTTT/CTTTTTTTTTT	TGAAGTCAACCCGTCAACTG	AGGTGCAATTCCATCTACCG	59.7	638	INTERGENIC	--	--	--
Ca2_P3_2941315	Ca2	2941315	GGG/GGGCCGG	CAAACCCATTGGTCCCTTTA	AAGTTTGAATCTTGAGGGTGG	59.7	695	INTERGENIC	--	--	--
Ca2_P3_29417303	Ca2	29417303	TA/TAA	CCCAGAAATCGATTAGGCAA	TCAACGTTCGTTTTGGTTCA	60.0	522	INTERGENIC	--	--	--
Ca2_P3_29427514	Ca2	29427514	GAAAA/GAAAAA	TTCAAACCCCCTTTCTTGTG	TGGAAATCGTTTTGCTTTGA	59.9	735	INTERGENIC	--	--	--

INDEL marker IDs	Chromosomes/ unanchored scaffolds	Physical positions (bp)	InDels (*Kabuli* reference genome-CDC Frontier/PII)	Forward primers (5'-3')	Reverse primers (5'-3')	Anneling temperature (0C)	Expected amplified product size (bp)	Structural annotation		Functional annotation	
								Sequence components of *kabuli* genome	*Kabuli* gene accession IDs	NCBI-KOG	TFs
Ca2_P3_29444244	Ca2	29444244	ATTTT/ATTTTT	AAATCCGACTGTCTAACCCG	TTTTCTCCTCCAATTTAGTCCAA	59.1	826	INTERGENIC	--	--	--
Ca2_P3_29445834	Ca2	29445834	CG/CGACTG	TCACCCACACTCTCAATCCA	CCCTACCTAGGGGTGGACAT	60.1	419	INTERGENIC	--	--	--
Ca2_P3_29445927	Ca2	29445927	AACCCGAACCC/AACCCGAACCCACCCGAACCC	TGTCTAACCCGTCTAACCCG	CCCTACCTAGGGGTGGACAT	60.0	138	INTERGENIC	--	--	--
Ca2_P3_29458705	Ca2	29458705	GATATATATATATATATATATATATATATATATA/GATATATATATATATATATATATATATATATATATATATATATATATATA	CTGGTGTTGGAGTCATGGTTT	TAGTGTGTAACCCGTGCGTG	59.9	240	INTERGENIC	--	--	--
Ca2_P3_2950890	Ca2	2950890	TAAAA/TAAAAA	GAAGGGACTGCGGCATAATA	TCCCCTTTCTTTCTGTCCCT	60.1	125	INTERGENIC	--	--	--
Ca2_P3_2960458	Ca2	2960458	GAAA/GAA	TATGGGTGGAAGTCAGGGAG	CAAGCACCTTGAAAGCATGA	59.9	832	INTRON	Ca_10547	O	--
Ca2_P3_29608982	Ca2	29608982	TAAAAAAA/TAAAAAA	TTTGCCACAAACAAAACGTG	TGAAATTTCCCTACGATGCC	60.6	504	INTERGENIC	--	--	--
Ca2_P3_2962089	Ca2	2962089	ATATTATT/ATTATTATT	ATGTCACCCAGACATGCAAA	TGGTGCTCTCCACATACCAA	60.0	719	DRR	Ca_10547	O	--
Ca2_P3_29646744	Ca2	29646744	T/TC	TCACGGGTCGACACAATTTA	GGTAAGGGCTCCTTCCAAAC	60.0	845	INTERGENIC	--	--	--
Ca2_P3_29659079	Ca2	29659079	CAAAAAAAAAAA/CAAAAAAAAAA	CGACGATCTCATTTAGCAATCA	TTTGCATTACGGATTTGATGA	60.2	384	INTERGENIC	--	--	--

INDEL marker IDs	Chromosomes/ unanchored scaffolds	Physical positions (bp)	InDels (*Kabuli* reference genome-CDC Frontier/PII)	Forward primers (5'-3')	Reverse primers (5'-3')	Anneling temperature (0C)	Expected amplified product size (bp)	Structural annotation		Functional annotation	
								Sequence components of *kabuli* genome	*Kabuli* gene accession IDs	NCBI-KOG	TFs
Ca2_P3_2969250	Ca2	2969250	TAAATTAATGTAAC/TAAATTAATGTAACAAATTAATGTAAC	TTCCAAAATCCCTCACATCC	CGGAACATCGTGTTCTCCTT	59.7	408	DRR	Ca_10546	--	--
Ca2_P3_2975084	Ca2	2975084	GTATTATT/GTATT	ATCTACGAAGACGTGGTGGG	TGAGGATTAATTGGCTGCTCT	60.0	852	INTERGENIC	--	--	--
Ca2_P3_2975267	Ca2	2975267	GAAAAAAAA/GAAAAAAA	TTCACGACTTGGAAAGGACC	TGATCGCCATTGATTGAGAA	60.1	750	INTERGENIC	--	--	--
Ca2_P3_2977761	Ca2	2977761	A/ACTT	CAACTTGCCAAACAGCTTCA	TGGTGCAAGGATAGTACGACA	60.0	620	INTERGENIC	--	--	--
Ca2_P3_29829880	Ca2	29829880	T/TA	CTTTTCGTCGGAGTTTGTCC	TCGTCCTCTGGTTTTCTCGT	59.7	476	INTERGENIC	--	--	--
Ca2_P3_29829940	Ca2	29829940	TT/TTTCGAAAAAAT	CTTTTCGTCGGAGTTTGTCC	TCGTCCTCTGGTTTTCTCGT	59.7	476	INTERGENIC	--	--	--
Ca2_P3_2984403	Ca2	2984403	GAT/GATATAT	GCTCACAACAGCAAAAACCA	TCTCTTTCTTAGACCGGAGCA	59.9	443	INTERGENIC	--	--	--
Ca2_P3_29847360	Ca2	29847360	CTTTTTTTTTTTT/CTTTTTTTTTT	AACGTTCTCCGTTTTTCAGC	GTGCGTCATGCAAATATGGT	59.4	838	INTERGENIC	--	--	--
Ca2_P3_2987706	Ca2	2987706	TAAAAAAA/TAAAAAAAAAA	TGTTTCAAAGCTCCATTTGC	GCCGACCATGGAGTAATTGT	58.9	458	INTERGENIC	--	--	--
Ca2_P3_29892926	Ca2	29892926	CAAAAAAAAA/CAAAAAAAA	CAAAAAGGTCATTTCAGCGA	TTGAATTCGAGAATTTGGGC	58.9	689	INTERGENIC	--	--	--

INDEL marker IDs	Chromosomes/ unanchored scaffolds	Physical positions (bp)	InDels (*Kabuli* reference genome-CDC Frontier/PII)	Forward primers (5'-3')	Reverse primers (5'-3')	Anneling temperature (0C)	Expected amplified product size (bp)	Structural annotation		Functional annotation	
								Sequence components of *kabuli* genome	*Kabuli* gene accession IDs	NCBI-KOG	TFs
Ca2_P3_29956158	Ca2	29956158	TTGTGTGTGTGTGTGTGTG/TTGTGTGTGTGTGTGTGTG	CCGCATAATATTCAATGCACTC	TTAAGGTCAACCCCGTTTTG	59.5	597	INTERGENIC	--	--	--
Ca2_P3_2996172	Ca2	2996172	ATTT/ATT	TTCTAAGGACCAACGCAACA	GTATGATCGATTATGCGCGA	59.3	434	INTERGENIC	--	--	--
Ca2_P3_29962401	Ca2	29962401	T/TTATGC	ACATCTGAAGGGCCAACATC	CCTCCTAATCATGGCTTCCA	59.9	544	INTRON	Ca_16094	A	--
Ca2_P3_2996631	Ca2	2996631	CAAAAAAAAA/CAAAAAAAA	CTCGAGTCGGTGTTGTGATG	TTCTTCACGATCATCCATGC	60.3	633	INTERGENIC	--	--	--
Ca2_P3_29977480	Ca2	29977480	AT/A	TCAAACTAGCACATCATAGAAAACAA	CCAAATTCAGTGTTTGATGAACT	59.3	648	INTERGENIC	--	--	--
Ca2_P3_29996298	Ca2	29996298	CAAAAAA/CAAAAAAA	CCACGAATCTCTTGTGGATG	GCTTTAGTGAGGAGGGGAGA	59.1	681	DRR	Ca_16090	--	--
Ca2_P3_3004473	Ca2	3004473	TC/TCC	AAACACATGGGCGAATAAGC	CCATGCATTTGTCACCCTAA	60.0	374	INTERGENIC	--	--	--
Ca2_P3_30050486	Ca2	30050486	CAGAAGAAGAAGAAGAAGAAGAAGAA/CAGAAGAAGAAGAAGAAGAAGAAGAAGAA	AATCGGAGAAATGAAGCCCT	TTGAAAATGAAGGTGGGGTC	60.0	157	INTERGENIC	--	--	--
Ca2_P3_3006310	Ca2	3006310	C/CAGTGGTAGT	TGCATGGAATGAAGGACAAA	TCTATCTTTTCCTCCGCCAC	60.0	402	DRR	Ca_10544	G	--
Ca2_P3_3009059	Ca2	3009059	ACCCCC/ACCCC	ATTCGCTTGCCTCTTTGGTA	CCCGAACTTTCTGTGCATT	59.8	658	INTRON	Ca_10544	G	--

INDEL marker IDs	Chromosomes/ unanchored scaffolds	Physical positions (bp)	InDels (*Kabuli* reference genome-CDC Frontier/PII)	Forward primers (5'-3')	Reverse primers (5'-3')	Anneling temperature (0C)	Expected amplified product size (bp)	Structural annotation		Functional annotation	
								Sequence components of *kabuli* genome	*Kabuli* gene accession IDs	NCBI-KOG	TFs
Ca2_P3_30095996	Ca2	30095996	TAAAAAAA/TAAAAAAAAA	CATTCCCGCTACATTTTGGT	GGAGAGAGGCCAATTTTTCA	59.8	226	INTERGENIC	--	--	--
Ca2_P3_30096215	Ca2	30096215	TTAAAAATAA/TTAAAAATAAAAATAA	CATTCCCGCTACATTTTGGT	CTCTAGATGGAGGGTGACGG	59.8	780	INTERGENIC	--	--	--
Ca2_P3_30096276	Ca2	30096276	TA/TAAA	CATTCCCGCTACATTTTGGT	GAAGCTGCTTGTGTGCCATA	59.8	876	INTERGENIC	--	--	--
Ca2_P3_30096869	Ca2	30096869	CATATATATATATATATATATATATATATATATATATATAT/CATATATATATATATATATATATATATATATATATATATATATATATAT	CATTGCAGGTGCTATGGTTG	TTCTCTTATGAAGCGGAGGA	60.1	759	INTERGENIC	--	--	--
Ca2_P3_3010652	Ca2	3010652	TAAAAAAAAAAA/TAAAAAAAAA	TCGCTCGCTGATGACTAATG	GTGGTGATTGGGATTGGTTC	60.1	514	INTRON	Ca_10544	G	--
Ca2_P3_30187523	Ca2	30187523	GATATATATATATATATATATATATATA/GATATATATATATATATATATA	GGACCATAGGTCAGGGGTTT	GAGGTCTGGTAACTCAAATAGGAA	60.1	870	INTERGENIC	--	--	--
Ca2_P3_3019233	Ca2	3019233	ATTTT/ATTTTT	GGGATTACTTTCGCGATTCA	TGCTTTGTTTGCAAAATGGT	60.0	783	INTERGENIC	--	--	--
Ca2_P3_3019419	Ca2	3019419	CAAAAAAA/CAAAAAA	TTTCAACCATCAACATTTGACC	TGCTTTGTTTGCAAAATGGT	59.7	541	INTERGENIC	--	--	--
Ca2_P3_3020087	Ca2	3020087	GTTTTT/GTTTT	ACCATTTTGCAAACAAAGCA	CACGTCTTGCAAATCCTCAC	59.2	725	INTERGENIC	--	--	--
Ca2_P3_3020857	Ca2	3020857	AT/ATT	GCATAATCTATATCGGCTTCTTCA	ATTTCGGAAACGTTTTGCAG	58.9	628	INTERGENIC	--	--	--

INDEL marker IDs	Chromosomes/ unanchored scaffolds	Physical positions (bp)	InDels (*Kabuli* reference genome-CDC Frontier/PII)	Forward primers (5'-3')	Reverse primers (5'-3')	Anneling temperature (0C)	Expected amplified product size (bp)	Structural annotation		Functional annotation	
								Sequence components of *kabuli* genome	*Kabuli* gene accession IDs	NCBI-KOG	TFs
Ca2_P3_3022026	Ca2	3022026	A/AT	CATAAGTCTGGCCTACGGCT	TTGGTGCATTTGTTTCAGGA	59.4	283	INTERGENIC	--	--	--
Ca2_P3_3022835	Ca2	3022835	TTAA/TTAATAA	CCCGACTCGAAAGTTTCTCA	AAAATTGACACGGCTTCGTC	60.4	544	INTERGENIC	--	--	--
Ca2_P3_3026756	Ca2	3026756	TAAAAAAAA/TAAAAAAAAA	TTTGAGGTCACGTTTAGCGA	TCATTGTGGGTGCATTTTGT	59.5	231	INTERGENIC	--	--	--
Ca2_P3_3027521	Ca2	3027521	GAAAAAAAAAA/GAAAAAAAAA	TTCTCGTGGACGTTTCTCAA	AAAGTTGTGGGCATGGAGAC	59.4	664	INTERGENIC	--	--	--
Ca2_P3_30291445	Ca2	30291445	TTATATATATATATATATATATATAT/TTATATATATATATATATATATATATATATATATAT	CCCTTTGTGCTCCACTCAAT	CCATTTCTCCCACAAAGGAA	60.1	791	INTERGENIC	--	--	--
Ca2_P3_3031259	Ca2	3031259	ATTTTT/ATTTTTT	TCAATTGCGATGGATAGAGAAA	AATTGGTGTACCGATTGCGT	59.7	647	INTERGENIC	--	--	--
Ca2_P3_3031554	Ca2	3031554	C/CCAAAATATCT	CATCTTGTCCATCAAACGGA	GGTGTACCGATTGCGTATCC	59.5	279	INTERGENIC	--	--	--
Ca2_P3_3033690	Ca2	3033690	TTCT/TTCTGTCTCTCTCT	TCAAATGATGGTGCAAAGGA	TTGATCCTTAAGGCATTCGG	60.0	446	INTERGENIC	--	--	--
Ca2_P3_30340682	Ca2	30340682	TAAAAAAAAAAAA/TAAAAAAAAAAA	TGTCTTATCCTGAACTCAAACTCG	ATTTGGTCAACCACCCAAAA	59.8	537	INTERGENIC	--	--	--
Ca2_P3_30341034	Ca2	30341034	TAA/TAAA	TGTCTTATCCTGAACTCAAACTCG	ATTTGGTCAACCACCCAAAA	59.8	537	INTERGENIC	--	--	--

INDEL marker IDs	Chromosomes/ unanchored scaffolds	Physical positions (bp)	InDels (*Kabuli* reference genome-CDC Frontier/PII)	Forward primers (5'-3')	Reverse primers (5'-3')	Anneling temperature (0C)	Expected amplified product size (bp)	Structural annotation		Functional annotation	
								Sequence components of *kabuli* genome	*Kabuli* gene accession IDs	NCBI-KOG	TFs
Ca2_P3_30342173	Ca2	30342173	CA/CATA	TGAATCATTGACAAAAGACTCAATC	CTAGCCCAAGATCCAAACCA	59.5	572	INTERGENIC	--	--	--
Ca2_P3_30344799	Ca2	30344799	GTTTATTTATTTAT/GTTTATTTATTTATTTATTTAT	TTCATACTTGGTTGGGGACC	GGATGATTTCGCCCAACTTA	59.6	408	INTERGENIC	--	--	--
Ca2_P3_30344848	Ca2	30344848	TAAAAAAAAAAAAA/TAAAAAAAAAA	TTCATACTTGGTTGGGGACC	GGATGATTTCGCCCAACTTA	59.6	408	INTERGENIC	--	--	--
Ca2_P3_30347371	Ca2	30347371	TGG/TGGG	CACTCCATTTTTGATTGAAACG	AGTTAACAACCGCGATTTGG	59.5	454	INTRON	Ca_12518	GE	bZIP
Ca2_P3_30352364	Ca2	30352364	ATTTTTTTT/ATTTTTT	TAAAAAGTGGTTCGGTTCGG	TTATTTTTCCCTTTTGGCCC	60.0	826	INTERGENIC	--	--	--
Ca2_P3_30352714	Ca2	30352714	GAAAA/GAAAAA	GGGCCAAAAGGGAAAAATAA	CCTGTCACTCTCCACTTCACC	60.1	369	INTERGENIC	--	--	--
Ca2_P3_30355281	Ca2	30355281	ATATTTTATTTTATTT/ATATTTTATTTTATTTTATTT	TGTAATTGGGTTCCTTTCGC	CAAGGTCAACTCTTGCACAAA	59.9	965	DRR	Ca_12517	--	ERF
Ca2_P3_30367278	Ca2	30367278	AG/AGG	ACCCATTTCATTTGCATGGT	AGGTCATTTGGTGGTGGAAG	60.1	463	INTRON	Ca_12515	Q	--
Ca2_P3_30367772	Ca2	30367772	TA/TATTTGTTAAA	CTTCCACCACCAAATGACCT	TACCTGGCGCGTTTTATTTT	59.8	300	DRR	Ca_12515	Q	--
Ca2_P3_30369374	Ca2	30369374	TAAAA/TAAAAA	TGTACTTCCTCCGATTCCAAA	TGAATGACGACGACGGTAAA	59.6	632	DRR	Ca_12515	Q	--

INDEL marker IDs	Chromosomes/ unanchored scaffolds	Physical positions (bp)	InDels (*Kabuli* reference genome-CDC Frontier/PII)	Forward primers (5'-3')	Reverse primers (5'-3')	Anneling temperature (0C)	Expected amplified product size (bp)	Structural annotation		Functional annotation	
								Sequence components of *kabuli* genome	*Kabuli* gene accession IDs	NCBI-KOG	TFs
Ca2_P3_30376142	Ca2	30376142	AA/AATA	TAGCCACCCCAAAAATCAAA	CCAGGAATCATCTCAGCCAC	60.3	857	INTERGENIC	--	--	--
Ca2_P3_3038190	Ca2	3038190	AAC/AACATAC	ACTTGCACTTTGGAGTGCCT	TGTGAACTCTTGGCATCATTG	59.9	682	INTERGENIC	--	--	--
Ca2_P3_3038248	Ca2	3038248	GAAAAAAA/GAAAAAAAA	ACTTGCACTTTGGAGTGCCT	CCCTTGTGCCTTATGGTTTG	59.9	784	INTERGENIC	--	--	--
Ca2_P3_30385637	Ca2	30385637	TTAATA/TTAATAATA	TTGACGGTCCTTTGTATTTGC	ACAGAAAATGTCGTCCCTGC	60.0	384	INTERGENIC	--	--	--
Ca2_P3_30394771	Ca2	30394771	TAAAAATAATTATAAAAAA/TAAAAA	GGAAAGAAGTGATTTGGATGG	AGGCTTTTGTTGTTGTTGGG	58.5	814	INTERGENIC	--	--	--
Ca2_P3_30395203	Ca2	30395203	CATATATATA/CATATATATATA	CCCAACAACAACAAAAGCCT	AAACCAGCATGACAAAAGCA	60.0	289	INTERGENIC	--	--	--
Ca2_P3_30395506	Ca2	30395506	TTCGAT/TT	CCCAACAACAACAAAAGCCT	CCAAATGAAACAATCGAGGAA	60.0	780	INTERGENIC	--	--	--
Ca2_P3_30395542	Ca2	30395542	CAAAAA/CAAAAAA	CCCAACAACAACAAAAGCCT	CCAAATGAAACAATCGAGGAA	60.0	780	INTERGENIC	--	--	--
Ca2_P3_30399984	Ca2	30399984	CATATATATATATATAT/CATATATATATATAT	AGGTGGGCCCATTACTTGAT	AGTGGAGGACCACTTCCTCA	60.6	836	INTERGENIC	--	--	--
Ca2_P3_30401507	Ca2	30401507	TT/TTGT	GCATCACAAAGGGTTTTTGG	TCGATGCGAATACAACTAAATCA	60.3	610	INTERGENIC	--	--	--

INDEL marker IDs	Chromosomes/ unanchored scaffolds	Physical positions (bp)	InDels (*Kabuli* reference genome-CDC Frontier/PII)	Forward primers (5'-3')	Reverse primers (5'-3')	Anneling temperature (0C)	Expected amplified product size (bp)	Structural annotation		Functional annotation	
								Sequence components of *kabuli* genome	*Kabuli* gene accession IDs	NCBI-KOG	TFs
Ca2_P3_30402988	Ca2	30402988	ATT/AT	TACCGGATTGGTAACGTGGT	GCGTTTGATGCATATTTTCG	60.1	419	DRR	Ca_12511	--	--
Ca2_P3_30403193	Ca2	30403193	ATTGAGTT/ATTGAGTTGAGTT	AAAGTTGCACCAAGAGGTGG	TCTCTGGCCTTCGTTAGTCAA	60.1	989	DRR	Ca_12511	--	--
Ca2_P3_30410480	Ca2	30410480	TAGGAG/TAGGAGGAG	CGCAAAACATTTGTTTATCCTA	TAAGGGGACGTCGAAAGTTG	57.0	884	INTERGENIC	--	--	--
Ca2_P3_30414684	Ca2	30414684	T/TTAA	AACATATTCTGGCACAGCCAC	ACTTGTGTGTTAAACGGGGC	60.0	611	INTRON	Ca_12510	--	--
Ca2_P3_30415849	Ca2	30415849	CTTTTTT/CTTTTT	GCAACAACCCGTTGAAGATT	GATGGGATATGGACACCGTC	60.0	472	INTRON	Ca_12510	--	--
Ca2_P3_30419465	Ca2	30419465	ATAATTT/ATAATTTATTATTCGTAATTT	AGTGGTTGCAATCATCACCA	CGGTGGAGAACAGAATTTCAA	60.0	654	DRR	Ca_12509	C	M-type
Ca2_P3_3042033	Ca2	3042033	TTTTGTTTGCTT/TTT	TGCCAAATCATGCGTTTTTA	CTCTGATGCGAAGGTTGACA	60.1	100	INTRON	Ca_10543	--	--
Ca2_P3_30423136	Ca2	30423136	CTATATATATATATATATATATATA/CTATATATATATATATATATATATATATATATA	CACCATGGCCTAGCATTTTC	TCCCCATTGAATTCCCATAA	60.5	303	INTRON	Ca_12509	C	M-type
Ca2_P3_30432943	Ca2	30432943	GTTTTTTTTTT/GTTTTTTTT	ATGAATCAAACGAACACCGA	TTACAGTTAAGGGCCAACCG	59.0	778	INTERGENIC	--	--	--
Ca2_P3_30433806	Ca2	30433806	CTTTT/CTTTTT	CGGTTGGCCCTTAACTGTAA	TCCTCAAACCCAAAATGGTC	60.0	674	INTERGENIC	--	--	--

INDEL marker IDs	Chromosomes/ unanchored scaffolds	Physical positions (bp)	InDels (*Kabuli* reference genome-CDC Frontier/PII)	Forward primers (5'-3')	Reverse primers (5'-3')	Anneling temperature (0C)	Expected amplified product size (bp)	Structural annotation		Functional annotation	
								Sequence components of *kabuli* genome	*Kabuli* gene accession IDs	NCBI-KOG	TFs
Ca2_P3_30445916	Ca2	30445916	TAAAAAA/TAAAAAAA	CAAAAATATATCGATCGCAACAA	AATTGAAAGCTGCATTGGCT	59.0	406	INTERGENIC	--	--	--
Ca2_P3_30447122	Ca2	30447122	CATATATATATATAT/CATATATATATATATATATATATAT	TGGTGCACACAATACAATCAAA	CAAGGCCGAGAAGACAAAAG	59.9	225	INTERGENIC	--	--	--
Ca2_P3_30447321	Ca2	30447321	TAAAAAA/TAAAAAAA	CTTTTGTCTTCTCGGCCTTG	AGCTTTCCAACCCCTTTTGT	60.0	155	INTERGENIC	--	--	--
Ca2_P3_30447611	Ca2	30447611	A/AT	CTTTTGTCTTCTCGGCCTTG	CGTGCTCATCTCCATTGTTG	60.0	891	INTERGENIC	--	--	--
Ca2_P3_30447653	Ca2	30447653	AC/A	CTTTTGTCTTCTCGGCCTTG	ATCTCCTCAGCATTGTCGCT	60.0	943	INTERGENIC	--	--	--
Ca2_P3_30449164	Ca2	30449164	TAAAAAAA/TAAAAAAAA	TGTTGCAAAGAGAAAGAACTTCA	TGACGGTGGCAATCGATATT	59.2	557	DRR	Ca_12507	--	--
Ca2_P3_3045119	Ca2	3045119	AA/AAGTGAGTCTA	CCTGATGTGCATGTGGAAAG	CGTTCCAACACATAGCTCCA	60.1	553	INTRON	Ca_10543	--	--
Ca2_P3_3045472	Ca2	3045472	TAG/TAGAG	TGGAGCTATGTGTTGGAACG	CTGACGGATCTTGGGTAGGA	59.7	356	INTRON	Ca_10543	--	--
Ca2_P3_30456719	Ca2	30456719	TAAAAAAAA/TAAAAAAA	TTCCTGATTCCGATCTCACC	CATACCTTGGGGATGCAAAG	60.0	601	DRR	Ca_12504	--	WOX
Ca2_P3_30457207	Ca2	30457207	CAAAAAAAAA/CAAAAAAAA	TGCATCCCCAAGGTATGACT	CCACCATTAGATTTGCGGTT	60.3	649	DRR	Ca_12504	--	WOX

INDEL marker IDs	Chromosomes/ unanchored scaffolds	Physical positions (bp)	InDels (*Kabuli* reference genome-CDC Frontier/PII)	Forward primers (5'-3')	Reverse primers (5'-3')	Anneling temperature (0C)	Expected amplified product size (bp)	Structural annotation		Functional annotation	
								Sequence components of *kabuli* genome	*Kabuli* gene accession IDs	NCBI-KOG	TFs
Ca2_P3_30457303	Ca2	30457303	A/AT	TGCATCCCCAAGGTATGACT	CCACCATTAGATTTGCGGTT	60.3	649	DRR	Ca_12504	--	WOX
Ca2_P3_30461229	Ca2	30461229	AG/A	TCCCATTCAATCAAGACGTG	TTTTTGTCTCACATTGATTGTCTTT	59.5	666	INTERGENIC	--	--	--
Ca2_P3_30463432	Ca2	30463432	TAAAAAAAAAA/TAAAAAAAA	ATTCTCAACCCTTGGTGTGC	AGTGTTTGTTTGGGAGGTGG	60.0	670	INTERGENIC	--	--	--
Ca2_P3_30463925	Ca2	30463925	AT/A	CCACCTCCCAAACAAACACT	TGTGGTCTCCGATAAATCAAAA	59.9	430	INTERGENIC	--	--	--
Ca2_P3_30464092	Ca2	30464092	G/GAAATAAGTTGAT	CCACCTCCCAAACAAACACT	GGCAAGTTGCTTTTACCAAAAC	59.9	602	INTERGENIC	--	--	--
Ca2_P3_30464150	Ca2	30464150	CTT/CTTT	CCACCTCCCAAACAAACACT	GGCAAGTTGCTTTTACCAAAAC	59.9	602	INTERGENIC	--	--	--
Ca2_P3_30473533	Ca2	30473533	TAAA/TAAAA	TGAATCGTGTGAGAGAACGAA	TGCTGATTTTGCTCCTCATCT	59.4	313	INTERGENIC	--	--	--
Ca2_P3_30473938	Ca2	30473938	TTTTTTA/T	AGATGAGGAGCAAAATCAGCA	TGTGTTCCTGGAGCATTTGA	60.0	624	INTERGENIC	--	--	--
Ca2_P3_30477581	Ca2	30477581	G/GC	TCACTGAATGCAAGCCATTT	TTCACGCACGAACTCATCTC	59.3	712	DRR	Ca_12502	G	--
Ca2_P3_30478050	Ca2	30478050	CTTTTTTTTTTT/CTTTTTTTTT	GTGCGTGAAGGGATGAAAAT	CCCTCACTTCTTCAAGGCAC	59.9	388	DRR	Ca_12502	G	--

INDEL marker IDs	Chromosomes/ unanchored scaffolds	Physical positions (bp)	InDels (*Kabuli* reference genome-CDC Frontier/PII)	Forward primers (5'-3')	Reverse primers (5'-3')	Anneling temperature (0C)	Expected amplified product size (bp)	Structural annotation		Functional annotation	
								Sequence components of *kabuli* genome	*Kabuli* gene accession IDs	NCBI-KOG	TFs
Ca2_P3_3047861	Ca2	3047861	ATTTTTTTT/ATTTTTTTTT	TGAGGGTCCAGTTTTTCCAC	TGAGCTTAAATTAACGAGCCAA	59.9	446	DRR	Ca_10543	--	--
Ca2_P3_30481892	Ca2	30481892	AACATA/AA	TCAATGCATGGAAAATAATCTCT	TGGTCCTCATCCATCAAACA	57.8	401	INTERGENIC	--	--	--
Ca2_P3_30481983	Ca2	30481983	ATATTATTT/ATATTATTTAAATTATTATTT	TCAATGCATGGAAAATAATCTCT	TGGTCCTCATCCATCAAACA	57.8	401	INTERGENIC	--	--	--
Ca2_P3_30484577	Ca2	30484577	ATT/A	ACATGAGACCGTCAATGCAA	CCACAGGACTGAAGAAGTTGTG	60.1	135	INTERGENIC	--	--	--
Ca2_P3_30484802	Ca2	30484802	A/ATT	ACATGAGACCGTCAATGCAA	CGAAAAATCAAAATAACATCGC	60.1	386	INTERGENIC	--	--	--
Ca2_P3_3048873	Ca2	3048873	CTT/CTTT	TAGGCGGCGGAGTGTATAAT	CCCGTTCATTGAGTTTAAGAAA	59.6	699	DRR	Ca_10543	--	--
Ca2_P3_30489467	Ca2	30489467	ATTTTTTTTT/ATTTTTTTT	TGCAAAGTTTGATGGAGTGC	TAGAACCCCAATTTTGTCCG	59.8	363	INTERGENIC	--	--	--
Ca2_P3_30490221	Ca2	30490221	T/TA	GGAAAAATTGCATTTGGGAA	GTTGGCTTACACATTGGGCT	59.8	578	INTERGENIC	--	--	--
Ca2_P3_30490973	Ca2	30490973	TTCAACACTTCAC/T	AGCCCAATGTGTAAGCCAAC	AAGCTTTTGCAAATGGGAGA	60.0	410	INTERGENIC	--	--	--
Ca2_P3_30494831	Ca2	30494831	GAAAAAAAAA/GAAAAAAAAAA	TGAGAACGCCCATGTCAATA	TCTCTGGCAACCGTTCTTCT	60.1	404	INTERGENIC	--	--	--

INDEL marker IDs	Chromosomes/ unanchored scaffolds	Physical positions (bp)	InDels (*Kabuli* reference genome-CDC Frontier/PII)	Forward primers (5'-3')	Reverse primers (5'-3')	Anneling temperature (0C)	Expected amplified product size (bp)	Structural annotation		Functional annotation	
								Sequence components of *kabuli* genome	*Kabuli* gene accession IDs	NCBI-KOG	TFs
Ca2_P3_3049734	Ca2	3049734	AGAT/A	CGATCTCATATGTGGGGACC	TGCAATCGAAAGAAGTGTGG	60.2	536	INTERGENIC	--	--	--
Ca2_P3_30503807	Ca2	30503807	TAA/TAAA	GTTCTCACTATGCCCCCAAA	AGTAATTAGCCCGAATCGCA	59.9	404	INTERGENIC	--	--	--
Ca2_P3_30505914	Ca2	30505914	ATTTTTTTTTT/ATTTTTTTTTTT	TTTAACGCTTCTCGCGTTTT	CGTGCTCCATAGTTCCCAAC	60.0	730	INTRON	Ca_12501	GMW	--
Ca2_P3_30506609	Ca2	30506609	GTT/GT	GTGTTTTGGTGAGCGTTTGA	GGTACAAATCCCACCATTGC	59.7	522	INTRON	Ca_12501	GMW	--
Ca2_P3_30507919	Ca2	30507919	CT/CTT	TCATCTTTGATGCATTTCGC	TGCATTTGCGACAAGAAAAG	59.8	503	INTRON	Ca_12501	GMW	--
Ca2_P3_30509747	Ca2	30509747	CAAAA/CAA	GGCCACATAATCCGTACCAC	AACACATTTGTGCAGCGAAG	60.1	586	DRR	Ca_12500	--	--
Ca2_P3_30510130	Ca2	30510130	ATTTTTTTTTTTTTTT/ATTTTTTTTTTTTT	AGACCCCACGGTTAACATGA	AGCACCTTACAAGGGGGAGT	60.2	700	DRR	Ca_12500	--	--
Ca2_P3_30513681	Ca2	30513681	TGAGA/TGA	GTGGGGTCTAACAAACGTGG	TCGCATTACAATTCGGATCA	60.3	185	INTERGENIC	--	--	--
Ca2_P3_30520425	Ca2	30520425	GAAAAAAAAA/GAAAAAAAAAAA	TTGACGTAGGGTCTTGGGTC	ATGTCCTGCTTCCATTCCAC	60.0	745	INTERGENIC	--	--	--
Ca2_P3_30522102	Ca2	30522102	TAAAAAAAA/TAAAAAAAAA	TCGTTCAACTAGTGCGGCTA	TTTTGGTGCCAAATTCTTCTC	59.6	822	INTERGENIC	--	--	--

INDEL marker IDs	Chromosomes/ unanchored scaffolds	Physical positions (bp)	InDels (*Kabuli* reference genome-CDC Frontier/PII)	Forward primers (5'-3')	Reverse primers (5'-3')	Anneling temperature (0C)	Expected amplified product size (bp)	Structural annotation		Functional annotation	
								Sequence components of *kabuli* genome	*Kabuli* gene accession IDs	NCBI-KOG	TFs
Ca2_P3_30522129	Ca2	30522129	A/AG	TCGTTCAACTAGTGCGGCTA	TTTTGGTGCCAAATTCTTCTC	59.6	822	INTERGENIC	--	--	--
Ca2_P3_30526988	Ca2	30526988	GAAAA/GAAA	CTCGTGAAGGATTAGGTCGG	TTGCAACGCATCTCAATCTC	59.7	353	INTERGENIC	--	--	--
Ca2_P3_30530043	Ca2	30530043	TAA/TAAA	AAGGTTCAACGACAGATGCC	AGGAGAAGAAGGATGAGCCC	60.1	451	DRR	Ca_12498	J	--
Ca2_P3_30537433	Ca2	30537433	AAAGAA/AAA	ACATGGTAACTGCGATGCAA	GTAGCTGCAGTTGCTACCCC	60.1	329	DRR	Ca_12497	J	--
Ca2_P3_30540749	Ca2	30540749	CTTTTTT/CTTTTTTT	TCGCAAACAGAGAAGAAACAAA	TGTCCTTGTTGTTAGCGCCT	60.0	204	INTERGENIC	--	--	--
Ca2_P3_30550219	Ca2	30550219	TCC/TC	TTTCTTTATTCTCGGTGGCG	TGCATTCGATCAAGCTCAAC	60.2	640	INTERGENIC	--	--	--
Ca2_P3_30553379	Ca2	30553379	GAAAA/GAAAAA	TTTTTATGAGGCGTGGAAGG	CCATGAACACGCCAAACATA	60.1	874	INTERGENIC	--	--	--
Ca2_P3_30553679	Ca2	30553679	TAAAAAAA/TAAAAAA	TGCACTTTATCACTTGGTAATCG	TGTTTGCGAGCTTCAATGAC	59.2	561	INTERGENIC	--	--	--
Ca2_P3_30555764	Ca2	30555764	AAG/AAGACACGTGTAG	AAAACATAACCTTTTGCCCG	TCACTCCAAGCTTCCCAAAT	59.0	257	INTERGENIC	--	--	--
Ca2_P3_30556664	Ca2	30556664	TAAAAA/TAAAAAAAAA	TGGGCATGTGATCCTGAATA	AGTTTTGGTGGACAAGGCAC	59.9	631	INTERGENIC	--	--	--

INDEL marker IDs	Chromosomes/ unanchored scaffolds	Physical positions (bp)	InDels (*Kabuli* reference genome-CDC Frontier/PII)	Forward primers (5'-3')	Reverse primers (5'-3')	Anneling temperature (0C)	Expected amplified product size (bp)	Structural annotation		Functional annotation	
								Sequence components of *kabuli* genome	*Kabuli* gene accession IDs	NCBI-KOG	TFs
Ca2_P3_30558124	Ca2	30558124	TAAA/TAAAA	TGTTTAAGGGTGATGC GACA	AGGCTTCATAGATGGG GTGA	60.1	948	INTERGENIC	--	--	--
Ca2_P3_30558600	Ca2	30558600	CTAT/CTATATAT	GATGCATACAAAAGTCA CCCC	GGATTGATGAAGTGGT TGGG	59.3	254	INTERGENIC	--	--	--
Ca2_P3_30559073	Ca2	30559073	AG/A	TGCATACAAAAGTCACC CCA	AAAAATTATGGGCTTT GGGC	60.0	982	INTERGENIC	--	--	--
Ca2_P3_30565181	Ca2	30565181	CTTTTT/CTTTTTT	TGGTCCCCTCAACCAC TAAG	TGTCTCTGTTTGGTTG CAAAA	60.0	708	INTERGENIC	--	--	--
Ca2_P3_30566132	Ca2	30566132	AT/ATT	CAGAGACAAAAGAGGG AACCA	TTGTGTTGTTGTTGTG TGCG	59.3	884	INTERGENIC	--	--	--
Ca2_P3_30567133	Ca2	30567133	TAAAAAAAA/TAAAAAAA	AAATTACCGCAACAATT GGC	GAAGCGCAACTAAACC AAGC	59.8	539	INTERGENIC	--	--	--
Ca2_P3_30569147	Ca2	30569147	TCCCCC/TCCCC	GAAAATGTGGTTCACTC GGG	CCGTCCAATTTTCTCTT GGA	60.4	875	INTRON	Ca_12495	E	--
Ca2_P3_30569240	Ca2	30569240	AT/ATT	AGTTTTGCAGCTTTTGC CTC	CCGTCCAATTTTCTCTT GGA	59.6	609	INTRON	Ca_12495	E	--
Ca2_P3_30571103	Ca2	30571103	TAAAAAA/TAAAAA	TTTGGAGTTTGACCATG TGA	GAAATGGCACCAACCT CATC	57.5	706	INTRON	Ca_12495	E	--
Ca2_P3_30574200	Ca2	30574200	ATTTTTT/ATTTTTTT	AAATGAAAAGGGGCCA AAGT	AATTTCCCGTTGCAAT GTCT	59.8	859	INTERGENIC	--	--	--

INDEL marker IDs	Chromosomes/ unanchored scaffolds	Physical positions (bp)	InDels (*Kabuli* reference genome-CDC Frontier/PII)	Forward primers (5'-3')	Reverse primers (5'-3')	Anneling temperature (0C)	Expected amplified product size (bp)	Structural annotation		Functional annotation	
								Sequence components of *kabuli* genome	*Kabuli* gene accession IDs	NCBI-KOG	TFs
Ca2_P3_30577110	Ca2	30577110	CTTT/CTT	GTTCTCGTGGCTCAGGACTC	CCATTGGCTTTTGCTCATTT	60.0	388	DRR	Ca_12493	--	--
Ca2_P3_30578105	Ca2	30578105	AC/A	GCCCCTGCACTAGTTTGAAG	GGCTTGTTAGTCTGCTTGCC	59.9	583	DRR	Ca_12493	--	--
Ca2_P3_30585791	Ca2	30585791	CAT/CATAAAT	GAAACCCTAACGCCATGAAA	TGTTTAACAAATGTCTGAAAGACG	59.9	631	INTERGENIC	--	--	--
Ca2_P3_30585996	Ca2	30585996	TA/TAA	ACAAATGGAGAGTGGCGAAG	TGTTTAACAAATGTCTGAAAGACG	60.3	465	INTERGENIC	--	--	--
Ca2_P3_30615674	Ca2	30615674	ACTAA/ACTAAATCTAA	TACTAAAACGGCAAAACCCG	TCCTGTGCGTAAGACTGGTG	60.0	533	INTRON	Ca_12491	--	--
Ca2_P3_30622722	Ca2	30622722	A/ACCGTAATCC	CTTGGTCAGAATCACGGAGC	CACGTTGACCCTTTTTGTCA	60.8	380	INTRON	Ca_12490	R	--
Ca2_P3_30622746	Ca2	30622746	AC/A	CTTGGTCAGAATCACGGAGC	CACGTTGACCCTTTTTGTCA	60.8	380	INTRON	Ca_12490	R	--
Ca2_P3_30623593	Ca2	30623593	TTATGTTTTAT/TTATGTTTTATATGTTTTAT	AGTAGAGCCCGACAAGATGC	CCGCAATCAATTTAGCGAAT	59.5	483	INTERGENIC	--	--	--
Ca2_P3_30630371	Ca2	30630371	ATTTA/ATTTATTTTA	TGCGTGTATCATTTTCAACTCA	GGAGATCCAATAGATGGCGA	59.2	841	INTERGENIC	--	--	--
Ca2_P3_30633064	Ca2	30633064	CTTTTTTT/CTTTTTTTT	TCAAATTGGAAGAGATATCAAGCA	CGAGAAGTGGAATGGTTTCAA	60.1	448	INTERGENIC	--	--	--

**INDEL marker IDs**	**Chromosomes/ unanchored scaffolds**	**Physical positions (bp)**	**InDels (*Kabuli* reference genome-CDC Frontier/PII)**	**Forward primers (5'-3')**	**Reverse primers (5'-3')**	**Anneling temperature (0C)**	**Expected amplified product size (bp)**	**Structural annotation**		**Functional annotation**	
								**Sequence components of *kabuli* genome**	***Kabuli* gene accession IDs**	**NCBI-KOG**	**TFs**
Ca2_P3_30636206	Ca2	30636206	TAAAAAAA/TAAAAAAAA	TGAATTGGTGTTATAAA AAGACGG	GGTCACGGTGTCAGTG AGTG	59.4	249	INTERGENIC	--	--	--
Ca2_P3_30637681	Ca2	30637681	ATTTTT/ATTTT	GGAAAGGGAGCCTGAA GAGA	GGGTCTTTTTGCAAAT TTGATT	60.8	455	INTERGENIC	--	--	--
Ca2_P3_30639797	Ca2	30639797	GAAAA/GAA	CACCAATTCCATGCATT TCA	GGACAGAGTTCGGTGT GGAT	60.3	460	INTERGENIC	--	--	--
Ca2_P3_30642821	Ca2	30642821	ATT/AT	TGTAATTTCATTCCGTG GCA	AAAGTTTCGTAATTTTT GCATGG	59.9	467	INTERGENIC	--	--	--
Ca2_P3_30652935	Ca2	30652935	TAA/TA	TTTCTTCAATCTTAATG GTTTTTGG	CATTCGACCAAGGAAA GTTCA	59.8	432	INTERGENIC	--	--	--
Ca2_P3_3065499	Ca2	3065499	TGGG/TGG	ACTTTACCACCCCGTTG TTG	GGTACTGTTCACTGCG CAAA	59.8	174	INTRON	Ca_10542	Z	--
Ca2_P3_30665443	Ca2	30665443	ATTTTTTTTTT/ATTTTTTTT	TCATGGTGGACCGTAA TCTG	CTACTGCCTTGGATTC CTTTT	59.4	403	INTRON	Ca_12489	QI	bHLH
Ca2_P3_30667413	Ca2	30667413	ATTT/ATT	TTTCTTGTTTTCTCCAC CGC	TGAAGCAGACTGCAGA ATCG	60.2	461	INTRON	Ca_12489	QI	bHLH
Ca2_P3_30670732	Ca2	30670732	AAATAATAATAA/AAATAATAATAATAA	CCGTGTTTCTTCACCGT TTT	TGGTAGGTGAAGGTGC AACA	60.0	660	DRR	Ca_12489	QI	bHLH
Ca2_P3_30671540	Ca2	30671540	TAAAAAA/TAAAAA	TTCGATTGATGGTTAAG AGACA	ATAACGTGTTGGATCA GCCC	57.4	697	DRR	Ca_12489	QI	bHLH

INDEL marker IDs	Chromosomes/ unanchored scaffolds	Physical positions (bp)	InDels (*Kabuli* reference genome-CDC Frontier/PII)	Forward primers (5'-3')	Reverse primers (5'-3')	Anneling temperature (0C)	Expected amplified product size (bp)	Structural annotation		Functional annotation	
								Sequence components of *kabuli* genome	*Kabuli* gene accession IDs	NCBI-KOG	TFs
Ca2_P3_30672058	Ca2	30672058	ATTTT/ATT	GGGCTGATCCAACACG TTAT	CCATGGGAAACGTTGG TAAC	59.8	396	DRR	Ca_12489	QI	bHLH
Ca2_P3_30676605	Ca2	30676605	GAAAAA/GAAAA	GCTCCAGCAAAAATAG AAATGG	TCATCAATCAAACTACT TACGCAGA	60.1	504	INTERGENIC	--	--	--
Ca2_P3_30676782	Ca2	30676782	ACTAGC/ACTAGCTAGC	GCTCCAGCAAAAATAG AAATGG	TCATCAATCAAACTACT TACGCAGA	60.1	504	INTERGENIC	--	--	--
Ca2_P3_30678822	Ca2	30678822	ATT/ATTTTT	CAAACCCAAAACCTTTT CCA	CTCACGCATCTCCAAA CTCA	59.8	369	INTERGENIC	--	--	--
Ca2_P3_30687952	Ca2	30687952	CA/C	ATCAATGGTCATGCAG CAAA	GACTCTCCTGGCAGCA AAAG	60.1	563	INTRON	Ca_12488	F	--
Ca2_P3_30690585	Ca2	30690585	AAATAATAATAATAATAA/AAATAATAAT AATAATAATAATAATAA	TATTCGCATCTGTGTGC TCC	CGTGGTTGAGTAGATG TGCC	59.8	670	INTERGENIC	--	--	--
Ca2_P3_30695617	Ca2	30695617	CTTTTTTTTTT/CTTTTTTTTTTT	CCCATGCATATCACGTT TTG	ACGCAGGGCCATGATA CTAC	59.8	361	DRR	Ca_12487	KLB	YABBY
Ca2_P3_30695734	Ca2	30695734	AA/AAAATTAACA	CCCATGCATATCACGTT TTG	ACGCAGGGCCATGATA CTAC	59.8	361	DRR	Ca_12487	KLB	YABBY
Ca2_P3_30700575	Ca2	30700575	CAAGTAAG/CAAG	CGCTAAAGGCACCTCA AAAG	GCAGCTTTCTGTTAGT GGGC	60.0	583	INTRON	Ca_12487	KLB	YABBY
Ca2_P3_30700832	Ca2	30700832	ATCCAAGTCCAAGTC/ATCCAAGTC	GCCCACTAACAGAAAG CTGC	GCAGAATCCAGGGCA GATAA	60.0	460	INTRON	Ca_12487	KLB	YABBY

INDEL marker IDs	Chromosomes/ unanchored scaffolds	Physical positions (bp)	InDels (*Kabuli* reference genome-CDC Frontier/PII)	Forward primers (5'-3')	Reverse primers (5'-3')	Anneling temperature (0C)	Expected amplified product size (bp)	Structural annotation		Functional annotation	
								Sequence components of *kabuli* genome	*Kabuli* gene accession IDs	NCBI-KOG	TFs
Ca2_P3_30702625	Ca2	30702625	GAAAAA/GAAAAAA	CTTCCGCCATCAATGACAC	AATCTCTTCAATCCCCCGTT	60.1	566	INTRON	Ca_12487	KLB	YABBY
Ca2_P3_30704006	Ca2	30704006	TAAAA/TAAA	CCGTATGTATGCGTGACACC	GGAAGGTGAATGATCTTAAAATAACG	59.9	902	INTERGENIC	--	--	--
Ca2_P3_30704638	Ca2	30704638	TA/TAA	AAATTCAGTGCAACGCACAT	CCCACTTTGGATAACAATTCC	59.2	445	INTERGENIC	--	--	--
Ca2_P3_30710239	Ca2	30710239	C/CT	TGCCAGCTGATATTGCAAAG	CTTCAGAGGCAGAACAAGCA	60.0	563	DRR	Ca_12486	T	--
Ca2_P3_30719499	Ca2	30719499	AT/A	CACGGTTGTGGGGACTAAAC	AAGAACCATCCAGCGGAAC	60.3	988	INTERGENIC	--	--	--
Ca2_P3_30719829	Ca2	30719829	A/AT	CAACGGACAAGAAAATCTCACA	TCACAAATTAACGGCCACAA	60.1	804	INTERGENIC	--	--	--
Ca2_P3_30720601	Ca2	30720601	TAAAAA/TAAAAAA	TTGTGGCCGTTAATTTGTGA	TTGTTGTGGATGCGGTTAAA	60.0	917	INTERGENIC	--	--	--
Ca2_P3_30735781	Ca2	30735781	ATTTTTTTTTT/ATTTTTTTTT	TTTAGAGCCAATGGAGGGTG	AGGCCCGTATCATTTATCCC	60.1	573	INTRON	Ca_12484	T	RAV
Ca2_P3_30737308	Ca2	30737308	TAAAAAAAAA/TAAAAAAAAAAA	TTTTGCCATGGTCTTGGATT	TCAAGAAAATTGCCAACCCT	60.3	618	DRR	Ca_12484	T	RAV
Ca2_P3_30737463	Ca2	30737463	TAAAAAA/TAAAAAAA	TTTTGCCATGGTCTTGGATT	TGGGCCCTCATGATATTGAT	60.3	720	DRR	Ca_12484	T	RAV

INDEL marker IDs	Chromosomes/ unanchored scaffolds	Physical positions (bp)	InDels (*Kabuli* reference genome-CDC Frontier/PII)	Forward primers (5'-3')	Reverse primers (5'-3')	Anneling temperature (0C)	Expected amplified product size (bp)	Structural annotation		Functional annotation	
								Sequence components of *kabuli* genome	*Kabuli* gene accession IDs	NCBI-KOG	TFs
Ca2_P3_30737660	Ca2	30737660	CAAAAAAA/CAAAAAA	TTTTGCCATGGTCTTG GATT	TGGGCCCTCATGATAT TGAT	60.3	720	DRR	Ca_12484	T	RAV
Ca2_P3_30738426	Ca2	30738426	AATATATATATATAT/AATATATATATAT	TGTGCACAATTTTCATG GGT	GAAGTTGAGCCCACAC ACAA	59.8	968	DRR	Ca_12483	T	FAR1
Ca2_P3_30742309	Ca2	30742309	CAGAGAGAG/CAGAGAGAGAGAGAG	CGTTGCGGTAACAAAC AATG	TTGGAGAAAAGGCAGG CTTA	60.0	349	INTERGENIC	--	--	--
Ca2_P3_30742679	Ca2	30742679	TAAAAAA/TAAAAAAAA	TGTTTGCTTCATTGGCA GAG	AAATTTGATGTAGAATA TAACCCAAAA	60.0	674	INTERGENIC	--	--	--
Ca2_P3_30766209	Ca2	30766209	CAAAAAAAA/CAAAAAAAAAA	TGTCGTGCGAGAAAAA TGAG	GTTGCTTTCGGATAGC TTGC	60.0	770	DRR	Ca_12479	K	ERF
Ca2_P3_30771512	Ca2	30771512	TAAAAA/TAAAA	GACCACCTTTGTGGAC CAGT	AATTTGATGGTTATGG GCGA	59.9	713	INTRON	Ca_12479	K	ERF
Ca2_P3_30790808	Ca2	30790808	GAAAAAAAA/GAAAAAAAAA	CACTCAGGGGATGAAT GTACG	CGAAGTTTGGACATGA GCTG	60.4	480	INTERGENIC	--	--	--
Ca2_P3_30792322	Ca2	30792322	TAAAAAAAAAA/TAAAAAAAAA	TCCCCAAAACAAATCAA ACA	TCAGCATCATAATTGG CACG	58.8	771	INTERGENIC	--	--	--
Ca2_P3_30794400	Ca2	30794400	CTTTTTTT/CTTTTTT	GAGTTGAATGCAGCTG ACCA	TGCATTTCACTGGAGA CGTT	60.0	741	INTERGENIC	--	--	--
Ca2_P3_30796973	Ca2	30796973	CAAA/CAAAA	CGTGTCCCTGATATTTT GCC	GGGACAGTTTTGTTTG TGGC	60.3	108	INTERGENIC	--	--	--

INDEL marker IDs	Chromosomes/ unanchored scaffolds	Physical positions (bp)	InDels (*Kabuli* reference genome-CDC Frontier/PII)	Forward primers (5'-3')	Reverse primers (5'-3')	Anneling temperature (0C)	Expected amplified product size (bp)	Structural annotation		Functional annotation	
								Sequence components of *kabuli* genome	*Kabuli* gene accession IDs	NCBI-KOG	TFs
Ca2_P3_30805003	Ca2	30805003	T/TA	TGCTGAATTGGATAGA GGGG	TTTTTGGTTTGTGTACA TTTGTTTT	60.0	838	INTERGENIC	--	--	--
Ca2_P3_30806790	Ca2	30806790	T/TA	GAAGTCAACATGTGCA TGGG	TTTAAAATTATGCGAC GGCA	60.0	606	INTERGENIC	--	--	--
Ca2_P3_30809205	Ca2	30809205	AG/AGG	ACATGAGGCTCTCCTA CCCA	TGTTCACCAACAAAATT GACAAA	59.7	592	INTERGENIC	--	--	--
Ca2_P3_30839646	Ca2	30839646	G/GA	TTGATTTCTCCATCGCA CTG	GGAGTCGATTATGCGT AGGG	59.8	268	DRR	Ca_12475	--	--
Ca2_P3_30840473	Ca2	30840473	AG/A	GCATGCGAACATGAAT TTTG	CCGGTGGATTGGATGT TAAA	60.1	589	DRR	Ca_12475	--	--
Ca2_P3_30840723	Ca2	30840723	TC/T	GCATGCGAACATGAAT TTTG	GGCCACTCATGTTGTC TCCT	60.1	788	DRR	Ca_12475	--	--
Ca2_P3_30842777	Ca2	30842777	AA/AATAGGAGCTAA	ACGTTAAGGGGGCAAT TACA	CGTGACTTCAGGGGCA ATAG	59.3	728	INTERGENIC	--	--	--
Ca2_P3_30843796	Ca2	30843796	ACTAGCTAG/ACTAG	AGCAATCCCCTATTCAT CCA	TTCATCTAACGCAGTC GTGG	59.3	746	INTERGENIC	--	--	--
Ca2_P3_30844156	Ca2	30844156	TTTAA/TTTAATTAA	TCCACACTCTTGTAGGT TGCAT	CAGAATCAGCACGCAA ACAT	59.7	854	INTERGENIC	--	--	--
Ca2_P3_30847372	Ca2	30847372	ATTTTTT/ATTTTTTT	GGTATGGGATGGGTGT GAGA	GACACCCTAAGCTCCA CACAA	60.6	192	INTERGENIC	--	--	--

INDEL marker IDs	Chromosomes/ unanchored scaffolds	Physical positions (bp)	InDels (*Kabuli* reference genome-CDC Frontier/PII)	Forward primers (5'-3')	Reverse primers (5'-3')	Anneling temperature (0C)	Expected amplified product size (bp)	Structural annotation		Functional annotation	
								Sequence components of *kabuli* genome	*Kabuli* gene accession IDs	NCBI-KOG	TFs
Ca2_P3_30849743	Ca2	30849743	ATT/ATTT	TCAACTTAGATATGCCG TCCG	TGGGTTGGTTTGAATC ATCA	60.1	410	INTERGENIC	--	--	--
Ca2_P3_30861518	Ca2	30861518	GAAA/GAAAA	CCAAAGGTGGGTGAAG AAGA	CAGGCCCAATTCACAA AGTT	60.1	554	INTERGENIC	--	--	--
Ca2_P3_30861798	Ca2	30861798	A/AAT	AACTTTGTGAATTGGG CCTG	TTTGCATGCCGATAAT TTGA	60.0	365	INTERGENIC	--	--	--
Ca2_P3_30861870	Ca2	30861870	A/AT	AACTTTGTGAATTGGG CCTG	TTTGCATGCCGATAAT TTGA	60.0	365	INTERGENIC	--	--	--
Ca2_P3_30862016	Ca2	30862016	GAAAAAAAAAA/GAAAAAAA	AACTTTGTGAATTGGG CCTG	CCACATTTTGACCTAC TTTCACA	60.0	867	INTERGENIC	--	--	--
Ca2_P3_30862283	Ca2	30862283	TAAAAAAAAA/TAAAAAAAAAAA	TTATCGGCATGCAAAAC AAA	GTTGGGTGACAGACAG CACA	60.1	794	INTERGENIC	--	--	--
Ca2_P3_30864595	Ca2	30864595	ATTTTTTTTT/ATTTTTTTTTTT	TCAAAGAGCGGATAAT AGGTTTT	CTTTCGAAATCCCTCA GCTT	58.4	769	INTERGENIC	--	--	--
Ca2_P3_30865173	Ca2	30865173	GAAAAAAAA/GAAAAAAA	GCTGAGGGATTTCGAA AGTAA	ATAATACCCCTCAGGG TGGC	58.5	576	INTERGENIC	--	--	--
Ca2_P3_30876227	Ca2	30876227	AT/ATTT	GCAGCCAAATTTTCTTG AGC	CAAAGCAGCATTCAGG ACAA	60.0	678	INTRON	Ca_12473	U	--
Ca2_P3_30877201	Ca2	30877201	GGAGAG/GGAG	TCATCTGCGGTTCAAG AGTG	AGGAAATCAGCCTGAA GCAA	60.0	928	INTRON	Ca_12473	U	--

INDEL marker IDs	Chromosomes/ unanchored scaffolds	Physical positions (bp)	InDels (*Kabuli* reference genome-CDC Frontier/PII)	Forward primers (5'-3')	Reverse primers (5'-3')	Anneling temperature (0C)	Expected amplified product size (bp)	Structural annotation		Functional annotation	
								Sequence components of *kabuli* genome	*Kabuli* gene accession IDs	NCBI-KOG	TFs
Ca2_P3_30880412	Ca2	30880412	CTTT/CTTTT	AAAACCACCAGAGACCGTTG	GAATTGCATCTCCCCAAAGA	60.0	167	INTRON	Ca_12473	U	--
Ca2_P3_30886527	Ca2	30886527	CTTTTT/CTTTTTT	AGGATGCAACCACCTACACC	GAGTTTTGGAAGAAAAACGAACA	59.9	755	INTERGENIC	--	--	--
Ca2_P3_30889956	Ca2	30889956	TGAA/T	CTTGGCAGACTGAGTTCACG	CATTTGTTGAATGTTGCCCA	59.6	875	INTERGENIC	--	--	--
Ca2_P3_30890128	Ca2	30890128	AAAATAAATAAATAAATAAA/AAAATAAATAAATAAA	GATGATGTAAATGGGAGCCAA	CATTTGTTGAATGTTGCCCA	59.8	697	INTERGENIC	--	--	--
Ca2_P3_30890182	Ca2	30890182	AAA/AAAGAA	GATGATGTAAATGGGAGCCAA	GGAATTGATCCACAACTCGG	59.8	958	INTERGENIC	--	--	--
Ca2_P3_30892153	Ca2	30892153	AA/AATGTACA	AGGATGGGTTTCTGTGTTGC	CTAAGGGGCAAAAGCGTACA	60.0	617	INTRON	Ca_12472	--	--
Ca2_P3_30895897	Ca2	30895897	TTATATATATATATATATATATATATATA/TTATATATATATATATATATA	TGCCAAAAAGAAAAAGGAATG	GCCAAGTTGTATAGGACAGCTC	59.2	566	DRR	Ca_12472	--	--
Ca2_P3_30896118	Ca2	30896118	ATT/AT	TGCCAAAAAGAAAAAGGAATG	TGTTAAAGCCAAAAACCCCA	59.2	742	DRR	Ca_12472	--	--
Ca2_P3_30896877	Ca2	30896877	ATTTTTT/ATTTTT	TGGGGTTTTTGGCTTTAACA	AGAGAAGCCCACTCCAACTG	60.3	919	INTERGENIC	--	--	--
Ca2_P3_30897629	Ca2	30897629	TAAAAAAAA/TAAAAAAA	CCAACAGATCAAGGGGAATG	ATCGAGATCTCACCGTGGAA	60.3	548	INTERGENIC	--	--	--

INDEL marker IDs	Chromosomes/ unanchored scaffolds	Physical positions (bp)	InDels (*Kabuli* reference genome-CDC Frontier/PII)	Forward primers (5'-3')	Reverse primers (5'-3')	Anneling temperature (0C)	Expected amplified product size (bp)	Structural annotation		Functional annotation	
								Sequence components of *kabuli* genome	*Kabuli* gene accession IDs	NCBI-KOG	TFs
Ca2_P3_30900169	Ca2	30900169	TGAGTAG/T	GTCTATGCAGGAGCCAGAGC	TATTCCGTGGACCCTTCATC	60.1	510	INTRON	Ca_12471	--	--
Ca2_P3_30900645	Ca2	30900645	T/TA	GATGAAGGGTCCACGGAATA	TTCAGAACCGGAGTGGTTTC	59.8	668	INTRON	Ca_12471	--	--
Ca2_P3_30900923	Ca2	30900923	TAAAAAAAAAA/TAAAAAAAA	CATGGTCAAATTGGGTGGAT	CTTCCCTTGACGAATGGTGT	60.4	767	DRR	Ca_12470	DO	--
Ca2_P3_30901376	Ca2	30901376	CTTT/CTTTTTTCTTTTTT	TCATTGGTGTTTACCGGGAT	TGCAGTGCTTCCATTCTCAG	60.1	323	DRR	Ca_12470	DO	--
Ca2_P3_30901717	Ca2	30901717	TG/T	TCATTGGTGTTTACCGGGAT	TAGCCTTTTGCAATATGGGG	60.1	886	DRR	Ca_12470	DO	--
Ca2_P3_30902626	Ca2	30902626	T/TC	CCCCATATTGCAAAAGGCTA	TGGGGAATCGCAAGATAAAC	59.9	839	DRR	Ca_12471	--	--
Ca2_P3_3090420	Ca2	3090420	CCTCTCTCTCTCTCTCTCTCTCTCTC/CCTCTCTCTCTCTCTCTCTCTCTC	TACAAGCACTCTCTCCCGCT	TTTCCATGCCCAAAAGTCTC	60.2	257	INTERGENIC	--	--	--
Ca2_P3_30915268	Ca2	30915268	A/ACAATCACTAAG	GAAGTGAAGTGGTTCTTTGCG	CAAGACCTTGTGGCTCATCA	59.9	783	INTRON	Ca_12470	DO	--
Ca2_P3_30916172	Ca2	30916172	TCC/TCCC	TGAATTGCCACAGGTCTTGA	TAAAGAGTGGCACCCCTCAC	60.2	411	INTRON	Ca_12470	DO	--
Ca2_P3_30925833	Ca2	30925833	ACC/ACCC	ATGAAGGATGGGTGAAGCAG	CAGGATGACTGCTATTGCGA	60.1	649	INTRON	Ca_12470	DO	--

INDEL marker IDs	Chromosomes/ unanchored scaffolds	Physical positions (bp)	InDels (*Kabuli* reference genome-CDC Frontier/PII)	Forward primers (5'-3')	Reverse primers (5'-3')	Anneling temperature (0C)	Expected amplified product size (bp)	Structural annotation		Functional annotation	
								Sequence components of *kabuli* genome	*Kabuli* gene accession IDs	NCBI-KOG	TFs
Ca2_P3_30927686	Ca2	30927686	TAAAAAAAAA/TAAAAAAAAAA	TTTGGGGCAGCAGATAAAAC	GCCAGATGAGCACACAAGAA	60.1	506	INTRON	Ca_12470	DO	--
Ca2_P3_30931955	Ca2	30931955	ACC/AC	GCATCTTCCTTCGTCCTACG	AAGAAGTTGGATGCCAAGGA	59.8	116	INTRON	Ca_12470	DO	--
Ca2_P3_30933751	Ca2	30933751	TC/T	TCGAAACGTGTGGTGATGAT	ACAATTCCACGCAATGTGAG	60.0	895	INTERGENIC	--	--	--
Ca2_P3_30935935	Ca2	30935935	GAACAAC/GAAC	ATCGATTAGTTCGGGTGACG	CGAGAAAGAGAGAGGCCAAG	60.0	578	INTERGENIC	--	--	--
Ca2_P3_30936877	Ca2	30936877	TAAAAAAA/TAAAAAAAA	TTGCACACAAGCGTTAGAGC	ATCAACCGCACACAATTCAA	60.2	393	INTERGENIC	--	--	--
Ca2_P3_30940918	Ca2	30940918	TAAAAAAAAAAA/TAAAAAAAAAA	CAATTTTGCTTGCAAATGTCA	AGGGTACGGTCGGGTTAGTT	59.7	506	INTERGENIC	--	--	--
Ca2_P3_30943416	Ca2	30943416	TAAAAA/TAAA	CGAACTCACCTGATGCTCAA	GCCAATATGTTCATTGACGG	60.0	607	INTERGENIC	--	--	--
Ca2_P3_30951832	Ca2	30951832	T/TACTC	TTCCTCCAGACAAAGGTTGG	ATGTCGGGATCCTCACTGTC	60.1	904	INTRON	Ca_12469	U	bHLH
Ca2_P3_30957370	Ca2	30957370	T/TA	TGCTCAATTGTTGCCTCTTG	CGACTAGCTCGGCTACAACC	60.0	361	DRR	Ca_12469	U	bHLH
Ca2_P3_30959076	Ca2	30959076	TAAAAAA/TAAAAAAA	TTGGTAAACCGATTAATCCCC	AACGGAACGAGCAAAGAAAG	59.9	386	DRR	Ca_12469	U	bHLH

INDEL marker IDs	Chromosomes/ unanchored scaffolds	Physical positions (bp)	InDels (*Kabuli* reference genome-CDC Frontier/PII)	Forward primers (5'-3')	Reverse primers (5'-3')	Anneling temperature (0C)	Expected amplified product size (bp)	Structural annotation		Functional annotation	
								Sequence components of *kabuli* genome	*Kabuli* gene accession IDs	NCBI-KOG	TFs
Ca2_P3_30962317	Ca2	30962317	CTTTTT/CTTTTTT	TTGTAAGTGTTATGTGCTGCCA	GGAGCAGTTCAATGGGCTTA	59.3	545	DRR	Ca_12468	K	MYB
Ca2_P3_30963140	Ca2	30963140	GT/G	TAAGCCCATTGAACTGCTCC	CACAATTGCCTACACCATCG	60.2	780	INTERGENIC	--	--	--
Ca2_P3_30968038	Ca2	30968038	CAAA/CAA	GTGTGCGAAAGGTTGGTTTT	CGAACCTACCCGATCAAGAA	60.0	168	INTERGENIC	--	--	--
Ca2_P3_30968401	Ca2	30968401	CAAAAAAAA/CAAAAAAAAA	TTCTTGATCGGGTAGGTTCG	ATTTTGCTCCCGTCTCCTTT	60.1	507	INTERGENIC	--	--	--
Ca2_P3_30969700	Ca2	30969700	AACACA/AACA	AAGGCACATCACGTTCAATTT	AACTTCTTTAGAGTGCGCCG	59.5	764	INTERGENIC	--	--	--
Ca2_P3_30982651	Ca2	30982651	AT/ATACT	ACAAAATGGGATGGATGGAA	TAATCCCAAGGCGATGCTAC	60.0	517	INTERGENIC	--	--	--
Ca2_P3_30997225	Ca2	30997225	A/AT	TCACCCTCGTATCCACAACA	ATGTGGCCATTGACAAGTGA	60.0	245	DRR	Ca_12465	--	ERF
Ca2_P3_31004156	Ca2	31004156	TAAA/TAAAA	AACGGTCCTCTTAGGACATCA	ACATGGGAATCCAAAGACCA	58.7	562	DRR	Ca_12464	--	LBD
Ca2_P3_31006068	Ca2	31006068	CTTTTTTTT/CTTTTTTTTT	TTGCAAATTTCTTGCACTCG	ACCATCCACTGTCCACATGA	60.0	477	INTERGENIC	--	--	--
Ca2_P3_31066601	Ca2	31066601	TTGTGTGTGTGTGTGTGTGTGTGT/TTGTGTGTGTGTGTGTGTGT	GATTCGTGAGACCGTTGGAT	AAGACATGTCCATAACGAACCA	59.9	640	INTRON	Ca_12458	M	--

INDEL marker IDs	Chromosomes/ unanchored scaffolds	Physical positions (bp)	InDels (*Kabuli* reference genome-CDC Frontier/PII)	Forward primers (5'-3')	Reverse primers (5'-3')	Anneling temperature (0C)	Expected amplified product size (bp)	Structural annotation		Functional annotation	
								Sequence components of *kabuli* genome	*Kabuli* gene accession IDs	NCBI-KOG	TFs
Ca2_P3_31104284	Ca2	31104284	TTGTGTG/TTGTG	TCACAAATGGCAACCTTTCA	CGGAAGAGGTTTGGTGTGTT	60.1	862	INTRON	Ca_12452	F	--
Ca2_P3_31111896	Ca2	31111896	TAAAAAAA/TAAAAAAAA	TCGGTAATTTGTGTCCCTCA	AGAAGTGATTGCTTGGTCGG	59.0	680	INTRON	Ca_12451	F	--
Ca2_P3_31131055	Ca2	31131055	ATTTTTTTTTT/ATTTTTTTTT	TAATGGAGGATTCGGGTTTG	CCCTACACATTTTAGTCAAATCACC	59.8	493	INTERGENIC	--	--	--
Ca2_P3_31240228	Ca2	31240228	TCAA/T	CTGCCCCCACTATTTCTCAA	GAAACACTTGGCATGTTAAGGA	60.1	479	INTERGENIC	--	--	--
Ca2_P3_31280508	Ca2	31280508	GC/GCC	CCACTGTCGCACACAAAATC	TGCTTCCTTTTCCATCATCC	60.2	834	INTERGENIC	--	--	--
Ca2_P3_3128367	Ca2	3128367	AATATATATATATATATATATATATATAT/AATATATATATATATATATATATATATATATAT	TTGTTTGACATTTTGCTTCCA	TGTAAGCCTGCAGAGTGGTG	59.2	396	INTERGENIC	--	--	--
Ca2_P3_31294019	Ca2	31294019	AG/AGAGG	AGACCGTCGGTTTCAATGAC	AAAAATCTTTAACAAGCGGGT	60.0	494	INTERGENIC	--	--	--
Ca2_P3_31323113	Ca2	31323113	GATATATATATATATATATA/GATATATATATATATATATATA	TGTTCACCACCAAACTGGAA	TTCATTTTAAAGGCATCGTGA	60.0	901	INTERGENIC	--	--	--
Ca2_P3_31390103	Ca2	31390103	AATATATATATATATATATATATATATATATATATATAT/AATATATATATATATATATATATATATATATATATATATATATATATATATATATATAT	TGCCTGTGCACTTTTTCTTG	CAAATTTGCAACTCAAATCTTCA	60.0	568	INTERGENIC	--	--	--
Ca2_P3_31534378	Ca2	31534378	CG/CGG	AGGAAAGCAACGTGAACAGC	CCAAGACAGGCAAAGAAAGC	60.4	519	INTERGENIC	--	--	--

INDEL marker IDs	Chromosomes/ unanchored scaffolds	Physical positions (bp)	InDels (*Kabuli* reference genome-CDC Frontier/PII)	Forward primers (5'-3')	Reverse primers (5'-3')	Anneling temperature (0C)	Expected amplified product size (bp)	Structural annotation		Functional annotation	
								Sequence components of *kabuli* genome	*Kabuli* gene accession IDs	NCBI-KOG	TFs
Ca2_P3_31542752	Ca2	31542752	TAAAAAA/TAAAAAAA	TCTGCATCCATGAATCAATGT	TACCGTTTTATCGTCAGCCC	58.9	511	INTERGENIC	--	--	--
Ca2_P3_31551913	Ca2	31551913	TTGTG/TTG	TTGAAGTTGAAGTCGGACCC	CTTGGCACTCAATATCGCAC	60.1	521	INTERGENIC	--	--	--
Ca2_P3_31577288	Ca2	31577288	CTAATAATAATAATAATAATAATAATAATAATAATAATAATAATAATAATA/CTAATAATAATAATAATAATAATAATAATAATAATAATAATAATAATAATAATAATAATAATA	TTGCATTTGCCTCACTTGAA	TGGTGAGAAACGTTGGATGA	60.4	805	INTERGENIC	--	--	--
Ca2_P3_31647740	Ca2	31647740	CT/CTCTTGTGAT	CGAGTTGCAGCCACTGTTTA	ATGGCGACTTGCTTCAGAGT	60.1	552	INTERGENIC	--	--	--
Ca2_P3_31727649	Ca2	31727649	AATATATATATATATATATAT/AATATATATATATATATAT	TCAAAATTGTCGGCAAAATAAA	TCCCAACCACCAAGTAAAGC	59.5	763	INTERGENIC	--	--	--
Ca2_P3_31825776	Ca2	31825776	TAATATAATAATAA/TA	CTTTGAGCTTTGGCCACTTC	AAGATGCCTCTGGGGCTAAT	60.0	945	INTRON	Ca_17837	J	--
Ca2_P3_31846061	Ca2	31846061	TTTATTATTATTATTATTATTATTATTATTATTATT/TTTATTATTATTATTATTATTATTATTATTATTATT	GATTGGAGCACCATCCTCTC	TTCGAGACAAAACGTTCAACC	59.6	805	INTERGENIC	--	--	--
Ca2_P3_31857388	Ca2	31857388	ATTT/ATT	GCCTAGACTTAAGCCGGTGG	GCACTCTCAGTGTAACAAGTTTGA	61.1	532	INTERGENIC	--	--	--
Ca2_P3_31857743	Ca2	31857743	ATTTTTTTT/ATTTTTTT	CAAACTTGTTACACTGAGAGTGCAT	CATGCTTTCTCTACCACCTCAA	59.8	630	INTERGENIC	--	--	--
Ca2_P3_31858178	Ca2	31858178	TTTAATATT/TTT	TGTGTTTCAGAGCTGCTAGTTGA	ATGAAAGGCATCCCAAAACA	60.2	368	INTERGENIC	--	--	--

INDEL marker IDs	Chromosomes/ unanchored scaffolds	Physical positions (bp)	InDels (*Kabuli* reference genome-CDC Frontier/PII)	Forward primers (5'-3')	Reverse primers (5'-3')	Anneling temperature (0C)	Expected amplified product size (bp)	Structural annotation		Functional annotation	
								Sequence components of *kabuli* genome	*Kabuli* gene accession IDs	NCBI-KOG	TFs
Ca2_P3_31882952	Ca2	31882952	CTTTATTTATTTAT/CTTTATTTATTTAT TTAT	TTTCTGGTGGGATCAG CTCT	GACATCCTGCGCCAGT TATT	59.8	591	INTRON	Ca_17830	D	LBD
Ca2_P3_31883010	Ca2	31883010	AAGAG/AAG	TTTCTGGTGGGATCAG CTCT	GACATCCTGCGCCAGT TATT	59.8	591	INTRON	Ca_17830	D	LBD
Ca2_P3_31883642	Ca2	31883642	TAA/TAAA	AATAACTGGCGCAGGA TGTC	TAAAGATGGCACCAGG AAGG	60.1	632	INTRON	Ca_17830	D	LBD
Ca2_P3_31898002	Ca2	31898002	TAAAAA/TAAAAAA	TAAATTGGCCGACCAA AAAG	CCAAGGGGTAGACATG GAAA	59.9	838	INTRON	Ca_17828	I	C3H
Ca2_P3_31905190	Ca2	31905190	ATTTT/ATTTTT	AATAAGGTGGTTTGGG GGAG	GGTATTCGTTGCGTCA TGTG	60.0	450	INTRON	Ca_17827	--	C3H
Ca2_P3_31922902	Ca2	31922902	CATA/CATATATA	AAAGGTGTTGGGATTG TCATT	GCTTGTTATGGCTTGA TCACC	58.3	394	INTERGENIC	--	--	--
Ca2_P3_31926664	Ca2	31926664	GTTTTTTT/GTTTTTTTT	GGTAACACACCATTTTC CGC	TCTTTGGACATCGTCA TGGA	60.2	649	DRR	Ca_17824	--	ERF
Ca2_P3_31933147	Ca2	31933147	AAGTTTACTA/AA	ACGGAGCCAAACAAGA AAAA	ACGAATTGGCATGGCT AAAG	59.7	706	INTERGENIC	--	--	--
Ca2_P3_31933297	Ca2	31933297	ATTTTTTTT/ATTTTTTTTT	TTCAGTGTACTCCCTCC GTCT	ACGAATTGGCATGGCT AAAG	58.8	315	INTERGENIC	--	--	--
Ca2_P3_31939481	Ca2	31939481	TTTGATTGATTGATTG/TTTGATTGATT G	TCTTAAAACCCAAACCG TGAA	GAGCTTTTGGGGGATA GAGG	59.5	490	DRR	Ca_17823	R	C3H

INDEL marker IDs	Chromosomes/ unanchored scaffolds	Physical positions (bp)	InDels (*Kabuli* reference genome-CDC Frontier/PII)	Forward primers (5'-3')	Reverse primers (5'-3')	Anneling temperature (0C)	Expected amplified product size (bp)	Structural annotation		Functional annotation	
								Sequence components of *kabuli* genome	*Kabuli* gene accession IDs	NCBI-KOG	TFs
Ca2_P3_31939517	Ca2	31939517	ACT/ACTCT	TCTTAAAACCCAAACCG TGAA	GAGCTTTTGGGGGATA GAGG	59.5	490	DRR	Ca_17823	R	C3H
Ca2_P3_31943749	Ca2	31943749	TAAA/TAAAAAAAAA	ACCATGTGCGATCAAG CATA	CCACGTGGAGGGTAAT ATGG	60.1	894	INTERGENIC	--	--	--
Ca2_P3_31944652	Ca2	31944652	T/TC	TCCACGTGGCAACTCA AATA	TGAAATTTGGAGTCGT ACCCT	60.1	834	INTERGENIC	--	--	--
Ca2_P3_31958606	Ca2	31958606	AT/ATGTT	ACGTTGTCGGACATTC ATCA	TTGCAACTGACTTGAA TCCG	60.0	606	INTERGENIC	--	--	--
Ca2_P3_31963928	Ca2	31963928	GTACTT/GTACTTACTT	CCTCGAGAAACCCAGC TAGA	GCATTCATGAGTTTGC CCTT	59.6	187	INTERGENIC	--	--	--
Ca2_P3_31964191	Ca2	31964191	AT/ATT	AAGGGCAAACTCATGA ATGC	CAAGGAAGGAGGAAAA GGAGA	60.1	609	INTERGENIC	--	--	--
Ca2_P3_31964265	Ca2	31964265	TG/T	AAGGGCAAACTCATGA ATGC	GGTGTGACAAAGGAAA CGTG	60.1	674	INTERGENIC	--	--	--
Ca2_P3_31964493	Ca2	31964493	AC/ACC	AAGGGCAAACTCATGA ATGC	TTGATGGTCACCTTTG TTGC	60.1	792	INTERGENIC	--	--	--
Ca2_P3_31964883	Ca2	31964883	GTTTTTTTTTTTTTT/GTTTTTTTTTT	GCAACAAAGGTGACCA TCAA	TTGTGCTTTGTGAGGG TGAG	59.5	195	INTERGENIC	--	--	--
Ca2_P3_31977188	Ca2	31977188	TTAGTAG/TTAG	CTCCTTTTCCCTGTCTT CCC	GTCCCATGTTAAATGC TCCG	60.0	826	INTRON	Ca_17820	--	--

INDEL marker IDs	Chromosomes/ unanchored scaffolds	Physical positions (bp)	InDels (*Kabuli* reference genome-CDC Frontier/PII)	Forward primers (5'-3')	Reverse primers (5'-3')	Anneling temperature (0C)	Expected amplified product size (bp)	Structural annotation		Functional annotation	
								Sequence components of *kabuli* genome	*Kabuli* gene accession IDs	NCBI-KOG	TFs
Ca2_P3_31981326	Ca2	31981326	AT/ATTT	ACGTTGTGTGGACAATGAGG	TCCGGAGGGTTTTTCTACCT	59.4	353	INTERGENIC	--	--	--
Ca2_P3_31981661	Ca2	31981661	TA/TAAA	CCCTCCGGACTCAATAAACA	GTGCAGATCGCTAGTGTCCA	59.9	663	INTERGENIC	--	--	--
Ca2_P3_31983759	Ca2	31983759	ATTTTTTTTT/ATTTTTTTTTTT	GACACTTGTTGCGTATGGGA	CAATGGAGATTCCAACCTGT	59.6	555	INTERGENIC	--	--	--
Ca2_P3_31983903	Ca2	31983903	CTA/CTATA	GACACTTGTTGCGTATGGGA	AGCCTTGCAGTGATGTGTGA	59.6	858	INTERGENIC	--	--	--
Ca2_P3_31990534	Ca2	31990534	GTTTTTTTTTTTTT/GTTTTTTTTTTTT	GCTAAGTGCAATACTCGCCA	TTGCACCATTTGTTATGATGG	59.1	136	INTERGENIC	--	--	--
Ca2_P3_32003428	Ca2	32003428	GTTT/GTTTT	AAGATGTTGTTTGCACGCAT	CCATCTATCACTTGCAGCAGTC	59.2	719	INTERGENIC	--	--	--
Ca2_P3_32004747	Ca2	32004747	ATTT/ATT	CGAATGAGAAATGGTGGACA	CTTCACCCCGCTTCATTTT	59.5	741	INTERGENIC	--	--	--
Ca2_P3_32015355	Ca2	32015355	TAAAAAA/TAAAAA	ATTTGCACAAAAGGAGGGAA	GCTATGCACATACGGCTGAA	59.5	654	INTRON	Ca_17819	V	GeBP
Ca2_P3_32032083	Ca2	32032083	ATATTTTTATTTTT/ATATTTTTATTTTTATTTTT	TGACAACGCATACGTGGATT	AAATAACACGAAATTCTAGCCCA	60.0	312	INTERGENIC	--	--	--
Ca2_P3_32034535	Ca2	32034535	AATATATA/AATATATATATATATA	CGGTGTTGAAGATGGGAAAT	GCACTTTTGAAGGCTGAAGG	59.8	874	INTRON	Ca_17818	R	--

INDEL marker IDs	Chromosomes/ unanchored scaffolds	Physical positions (bp)	InDels (*Kabuli* reference genome-CDC Frontier/PII)	Forward primers (5'-3')	Reverse primers (5'-3')	Anneling temperature (0C)	Expected amplified product size (bp)	Structural annotation		Functional annotation	
								Sequence components of *kabuli* genome	*Kabuli* gene accession IDs	NCBI-KOG	TFs
Ca2_P3_32038853	Ca2	32038853	TGCC/TGCCGGCC	TCCAAAGACAACTGCTCAAGG	TTCGGGGTATTACAACGTCTTT	60.4	728	DRR	Ca_17818	R	--
Ca2_P3_32048288	Ca2	32048288	AC/A	CCAAATCGAACTCTCTCCTCA	CGTGATGCACCGATAAATTG	59.4	634	DRR	Ca_17817	R	--
Ca2_P3_32048344	Ca2	32048344	CTTTTTT/CTTTTTTT	CCAAATCGAACTCTCTCCTCA	CGTGATGCACCGATAAATTG	59.4	634	DRR	Ca_17817	R	--
Ca2_P3_32057057	Ca2	32057057	ATTTTTT/ATTTTTTT	AACCATCAATGTTTCGTGAGC	TTTCTTTGGCAGAAATCATGG	60.0	575	INTERGENIC	--	--	--
Ca2_P3_32069395	Ca2	32069395	TTATATATATATATAT/TTATATATATATATATATAT	ACCCCGTTGATCATAAGTCG	AAAATCCTGGCAGTCATCGT	59.8	922	DRR	Ca_17815	U	--
Ca2_P3_32069472	Ca2	32069472	CTTTTTT/CTTTTTTT	GGACCTTGTTAAATGTTGGCA	AAAATCCTGGCAGTCATCGT	59.9	836	DRR	Ca_17815	U	--
Ca2_P3_32070956	Ca2	32070956	TAAAAAAAAAAAA/TAAAAAAAAAAAAAA	TTTTTCCGACTTGGTCCTTT	GGAGCTGAAACTAACGCAGC	58.7	814	INTERGENIC	--	--	--
Ca2_P3_32078280	Ca2	32078280	ACTCT/ACTCTCT	CAAATTTGGGACTTCCTTTTT	GAGCCTCTATGTGGTTGCAC	57.3	624	INTERGENIC	--	--	--
Ca2_P3_32081128	Ca2	32081128	GTTT/GTT	TCAGCTGGTGCAAAACAAAG	CCCTTCATCTCTACTCACCTGG	60.0	226	INTERGENIC	--	--	--
Ca2_P3_32094277	Ca2	32094277	GTTTTTTTTTTT/GTTTTTTTTT	CCCTTTGAGAGACATAATTGGG	GCTTCAGAGTCGGATTCAGG	59.8	479	INTERGENIC	--	--	--

INDEL marker IDs	Chromosomes/ unanchored scaffolds	Physical positions (bp)	InDels (*Kabuli* reference genome-CDC Frontier/PII)	Forward primers (5'-3')	Reverse primers (5'-3')	Anneling temperature (0C)	Expected amplified product size (bp)	Structural annotation		Functional annotation	
								Sequence components of *kabuli* genome	*Kabuli* gene accession IDs	NCBI-KOG	TFs
Ca2_P3_32095584	Ca2	32095584	CTATATATATATATA/CTATATATATATATATA	CGATTTTTCTTAATTCACCGC	CCATGTTGGTTTCTTTTCCAA	58.8	761	INTERGENIC	--	--	--
Ca2_P3_32095820	Ca2	32095820	CAT/CATAT	CAAGGATAATCTTAAATGTGGTTGG	GACATGATCTTGCAGCCAAA	60.0	596	INTERGENIC	--	--	--
Ca2_P3_32104385	Ca2	32104385	TAAAAAAA/TAAAAAAAAA	ATATTGCACACTGCATCACG	TGCAAAAATGGAGGGATTCT	58.2	760	INTERGENIC	--	--	--
Ca2_P3_32110176	Ca2	32110176	AAAACAAA/AAAA	GCACCCTTCAAGAAGCAAAC	TTGGTCCATCGAATCACTTG	59.9	221	INTERGENIC	--	--	--
Ca2_P3_32126579	Ca2	32126579	C/CCT	AGACAAGAAACCCAACGACG	CAAATACCCCCAGTCCCTTT	60.1	897	INTRON	Ca_17812	O	--
Ca2_P3_32127033	Ca2	32127033	TGC/TGCGC	TGCAAGTTGTGATGGGTTGT	TGTCTGTTCTGCACGGAGTC	60.0	650	INTRON	Ca_17812	O	--
Ca2_P3_32130215	Ca2	32130215	ATTGAA/ATTGAATTTGAATTTGAA	CCGGGTCAGTTTCCTTCTTT	ACTCCACCAACTTCCAATGC	60.5	388	INTRON	Ca_17811	J	--
Ca2_P3_32143712	Ca2	32143712	ATTTTTTTT/ATTTTTTTTT	CCGTGAGGTTTAAGGGATCA	GGATAACAAGCCACGAACCT	59.9	974	INTRON	Ca_17808	--	G2-like
Ca2_P3_32145004	Ca2	32145004	TA/TACA	GGCCTTCAAAATTCAATGAGA	TGGATACCTAGCACCTTAGCAA	59.1	598	INTRON	Ca_17808	--	G2-like
Ca2_P3_32147211	Ca2	32147211	TAAA/TA	TCATGCATCTAAGAAATGAACCA	ATGAGAAGGCAAAGGAAGCA	59.6	517	INTRON	Ca_17808	--	G2-like

INDEL marker IDs	Chromosomes/ unanchored scaffolds	Physical positions (bp)	InDels (*Kabuli* reference genome-CDC Frontier/PII)	Forward primers (5'-3')	Reverse primers (5'-3')	Anneling temperature (0C)	Expected amplified product size (bp)	Structural annotation		Functional annotation	
								Sequence components of *kabuli* genome	*Kabuli* gene accession IDs	NCBI-KOG	TFs
Ca2_P3_32152633	Ca2	32152633	T/TA	GAGTTGGGTAGGGTAGGGGA	TTTCCCCTTTTGCTTGTCAT	60.2	393	INTERGENIC	--	--	--
Ca2_P3_32161753	Ca2	32161753	TCCCC/TCCC	AGGCCATTAAGCATGACACC	TTCTTGGAACCCTGCATACC	60.0	867	INTERGENIC	--	--	--
Ca2_P3_32161954	Ca2	32161954	ATTTT/ATT	GTCCCCAGACTTGGCTGTAA	AAACATTTGCGGTTTCTTGG	60.1	638	INTERGENIC	--	--	--
Ca2_P3_32162834	Ca2	32162834	GTTTTTTTTTT/GTTTTTTTTTTTT	GGCCTTCACAATAAGCCAAA	TGGACAAAATCATGTCTCTTTGA	60.1	803	INTERGENIC	--	--	--
Ca2_P3_32173774	Ca2	32173774	TC/T	ATGTTTTGATTGGGGCTTTG	TAAATCAGCCCTTTTGGTGG	59.8	631	DRR	Ca_17806	--	WRKY
Ca2_P3_32176027	Ca2	32176027	ATTTT/ATTT	ACCAAAGGAAGATGACGTGG	ACAACTTCTGATGCTCGCAA	60.0	514	INTERGENIC	--	--	--
Ca2_P3_32203753	Ca2	32203753	TTTTTTATATAT/TT	GGGCAATTCCTGCAATTTTA	GATGCACCGAATAAGGAAGC	59.9	390	INTRON	Ca_10295	--	G2-like
Ca2_P3_32209131	Ca2	32209131	T/TG	GCAAAGATAGACACCACCCC	AACCATTCCCATGGAACAAA	59.4	577	INTERGENIC	--	--	--
Ca2_P3_32209334	Ca2	32209334	CTATATATA/CTATA	TCCATGGGAATGGTTTTGAT	TTGAAGAGGAAGTTTTGGCA	60.0	342	INTERGENIC	--	--	--
Ca2_P3_32209671	Ca2	32209671	TC/TCC	TCCATGGGAATGGTTTTGAT	TTTTAGTGTGCACCAATGTCAA	60.0	899	INTERGENIC	--	--	--

INDEL marker IDs	Chromosomes/ unanchored scaffolds	Physical positions (bp)	InDels (*Kabuli* reference genome-CDC Frontier/PII)	Forward primers (5'-3')	Reverse primers (5'-3')	Anneling temperature (0C)	Expected amplified product size (bp)	Structural annotation		Functional annotation	
								Sequence components of *kabuli* genome	*Kabuli* gene accession IDs	NCBI-KOG	TFs
Ca2_P3_32210099	Ca2	32210099	CA/CAAATA	TCAGCTTCAATTATCTC CAACAA	TCGGCGACAAAAATCA CATA	58.9	644	INTERGENIC	--	--	--
Ca2_P3_32210241	Ca2	32210241	TA/T	TGACATTGGTGCACAC TAAAAA	CCACGTCATCTTCCTT TGGT	59.1	505	INTERGENIC	--	--	--
Ca2_P3_32211817	Ca2	32211817	ATATTATTATTATTATTATTATTATTATT ATTATTATTATTAT/ATATTATTATTATT ATTATTATTATTATTATTATTAT	ATGGAATTCTGAATGGT GGG	CATGTGGATCGAAATG ATTTTT	59.6	365	INTERGENIC	--	--	--
Ca2_P3_32213171	Ca2	32213171	GTGAT/GTGATGAT	CAACATGACAAGGACT TTCCAA	TTACCGGTATCGCAAG GAAC	60.0	529	INTERGENIC	--	--	--
Ca2_P3_32213424	Ca2	32213424	CAAA/CAAAA	AGGGATAGATAACGGC ACGA	AAGGCCCTGCTAGAAT GATG	59.6	504	INTERGENIC	--	--	--
Ca2_P3_32218161	Ca2	32218161	GTTTTTTTTTTT/GTTTTTTTTTT	ATTGTGCCGGAGCAAC TACT	TGTTACGGCTGTGAAT CTGG	59.8	138	INTERGENIC	--	--	--
Ca2_P3_32218604	Ca2	32218604	TATCTAAATCT/TATCTAAATCTAAATCT	ATTGTGCCGGAGCAAC TACT	TTGGAAATTCAAAAAG GGTAAGA	59.8	966	INTERGENIC	--	--	--
Ca2_P3_32221226	Ca2	32221226	ATTTTTTTTTTTTTTT/ATTTTTTTTTTTT T	GCTTCAGTTGTTCATCG CAA	CATTGTCCAAACTGGC ACAT	60.0	507	INTERGENIC	--	--	--
Ca2_P3_32221530	Ca2	32221530	ATAATAATA/ATAATAATATTAATAATA	GCTTCAGTTGTTCATCG CAA	CGTGCAAAAATGGAGG AAGT	60.0	836	INTERGENIC	--	--	--
Ca2_P3_32232018	Ca2	32232018	ATTTTTT/ATTTTT	CCCTCCTTCCAGCAAC ATTA	CGAGGAACTTTTGCAG ATGA	60.1	754	INTERGENIC	--	--	--

INDEL marker IDs	Chromosomes/ unanchored scaffolds	Physical positions (bp)	InDels (*Kabuli* reference genome-CDC Frontier/PII)	Forward primers (5'-3')	Reverse primers (5'-3')	Anneling temperature (0C)	Expected amplified product size (bp)	Structural annotation		Functional annotation	
								Sequence components of *kabuli* genome	*Kabuli* gene accession IDs	NCBI-KOG	TFs
Ca2_P3_32234513	Ca2	32234513	AAAATTTGA/AAAATTTGAATTAAATTTGA	TCGCATCCGTATTCGTTTTT	TAACCCGGTTTATCCAATCG	60.5	778	INTERGENIC	--	--	--
Ca2_P3_32245950	Ca2	32245950	TA/TAGA	AACCGACAAAAGATTGCATGT	GCAGTTGCACAAGGTGGTAA	59.5	586	INTERGENIC	--	--	--
Ca2_P3_32255605	Ca2	32255605	A/AG	GGTAGAGGAGGTGTTGGTGG	TACGGGGCCAAAATAATCAA	59.4	435	DRR	Ca_10289	K	TALE
Ca2_P3_32259695	Ca2	32259695	TCAC/TCACAC	AAATGCAACTCCTTCCTCCA	TCAGCTTAAGGCTCTTGCTGT	59.7	833	INTRON	Ca_10289	K	TALE
Ca2_P3_32260990	Ca2	32260990	GAGAA/GA	TGTTGTTTCCTCCAAACCCT	TTTTACCTTCTTTTCCGGGG	59.4	157	INTERGENIC	--	--	--
Ca2_P3_32261025	Ca2	32261025	AAAGAGAAGAGA/AAAGAGAAGAGAAGAGA	CCCCGGAAAAGAAGGTAAAA	CCTCTCTGTCTCCACCTGCT	60.3	126	INTERGENIC	--	--	--
Ca2_P3_32261783	Ca2	32261783	ATT/ATTGTTT	CAAAAGGTGCGTTGATAATAACC	TTGTTGATTGAATTGACCAAACT	59.8	290	INTERGENIC	--	--	--
Ca2_P3_32262011	Ca2	32262011	TAAAAAA/TAAAAAAA	CAAAAGGTGCGTTGATAATAACC	CCTTCACAAATCCCAACCTG	59.8	714	INTERGENIC	--	--	--
Ca2_P3_32262374	Ca2	32262374	GATATATA/GATATA	CAGGTTGGGATTTGTGAAGG	GCCCGAATTGGTTGAAATAA	60.3	292	INTERGENIC	--	--	--
Ca2_P3_32262643	Ca2	32262643	CAT/CATAT	TTATTTCAACCAATTCGGGC	GCCGGTGACATTTTGTTTTC	59.8	485	INTERGENIC	--	--	--

INDEL marker IDs	Chromosomes/ unanchored scaffolds	Physical positions (bp)	InDels (*Kabuli* reference genome-CDC Frontier/PII)	Forward primers (5'-3')	Reverse primers (5'-3')	Anneling temperature (0C)	Expected amplified product size (bp)	Structural annotation		Functional annotation	
								Sequence components of *kabuli* genome	*Kabuli* gene accession IDs	NCBI-KOG	TFs
Ca2_P3_32262689	Ca2	32262689	T/TCTA	TTATTTCAACCAATTCGGGC	GCCGGTGACATTTTGTTTTC	59.8	485	INTERGENIC	--	--	--
Ca2_P3_32262880	Ca2	32262880	G/GGAA	TTATTTCAACCAATTCGGGC	ATTGCCAACAACACACCAAA	59.8	747	INTERGENIC	--	--	--
Ca2_P3_32282292	Ca2	32282292	CATTAGTGATT/CATTAGTGATTAGTGATT	ACCCTCCAGGGTGCTAAGAT	GCCAACATTTTCTGGTTGCT	60.0	726	INTRON	Ca_10287	S	--
Ca2_P3_32290065	Ca2	32290065	GTTAATTA/GTTA	ACTGATCGATGACCATGCCT	TCGTGCTCACATTTCCAAAA	60.5	941	INTERGENIC	--	--	--
Ca2_P3_32290375	Ca2	32290375	TAAAA/TAAA	TGATGCATTTGCCTCATTTC	TCGTGCTCACATTTCCAAAA	59.6	344	INTERGENIC	--	--	--
Ca2_P3_32290432	Ca2	32290432	TG/T	TGATGCATTTGCCTCATTTC	TCGTGCTCACATTTCCAAAA	59.6	344	INTERGENIC	--	--	--
Ca2_P3_32293814	Ca2	32293814	CT/CTTTGT	TGTATTTGGACGGGTGACAA	TACACCCACGAAACGTGAAA	59.8	571	INTERGENIC	--	--	--
Ca2_P3_32326709	Ca2	32326709	TG/T	AGAGTTTGAACCCGGACCTT	AGTGATACTAACCGGTGGCG	60.0	571	INTRON	Ca_10285	R	--
Ca2_P3_32328208	Ca2	32328208	CAACGTCACTAACGTCACTA/CAACGTCACTA	TCTGAAGGTTCAATCCCACC	ATGGACAGGCGGTCTATTTG	59.9	520	DRR	Ca_10285	R	--
Ca2_P3_32339187	Ca2	32339187	CCTCT/CCTCTCT	AGCAACCAAAATTTCCCATT	TGGGACAATGACATCAAACAA	58.4	754	DRR	Ca_10284	R	C3H

**INDEL marker IDs**	**Chromosomes/ unanchored scaffolds**	**Physical positions (bp)**	**InDels (*Kabuli* reference genome-CDC Frontier/PII)**	**Forward primers (5'-3')**	**Reverse primers (5'-3')**	**Anneling temperature (0C)**	**Expected amplified product size (bp)**	**Structural annotation**		**Functional annotation**	
								**Sequence components of *kabuli* genome**	***Kabuli* gene accession IDs**	**NCBI-KOG**	**TFs**
Ca2_P3_32341023	Ca2	32341023	GAAT/GAATGTTAAAAT	CAACCACCTTAAATGACCACAA	AAAAACAATGTCATGCAGAGG	59.8	682	INTERGENIC	--	--	--
Ca2_P3_32347455	Ca2	32347455	ATTTTTTT/ATTTT	ACAAGCTGTTCCCTCCAGAA	TGAGGTTCTTGCACAACTGC	59.8	678	INTRON	Ca_10283	--	--
Ca2_P3_32348598	Ca2	32348598	T/TAA	GGAGATATTTGGGTCAGCGA	AGACTCCACCGAGCGAGATA	60.0	265	INTRON	Ca_10283	--	--
Ca2_P3_32349885	Ca2	32349885	ATGT/ATGTGT	AAGTTGGAGGCTCAAAAGCA	AAAATTGCAAATGCACACCA	60.0	661	INTRON	Ca_10283	--	--
Ca2_P3_32349934	Ca2	32349934	CAAAA/CAA	ATTCTGACGCCTCTTGCACT	AAAATTGCAAATGCACACCA	60.0	390	INTRON	Ca_10283	--	--
Ca2_P3_32353407	Ca2	32353407	TGAAC/T	GAACGACAACAATTTCGCCT	CAATGGATTTCTTTGGCCTT	60.1	513	DRR	Ca_10283	--	--
Ca2_P3_32354453	Ca2	32354453	ATTT/ATT	TTCCAGCCAACTCCTGATTC	TTTAGTGGTCCCTTGCCAAC	60.2	548	DRR	Ca_10283	--	--
Ca2_P3_32356323	Ca2	32356323	CAAA/CAAAA	GCTAGTTGCTGTGAATGGCA	ACGTGTGGATGATTGGATGA	60.0	711	INTERGENIC	--	--	--
Ca2_P3_32359801	Ca2	32359801	AGAG/A	TTGATGAGTTCGGTCATGGA	AGCCGCTTAATTGATCGTTG	60.0	195	INTERGENIC	--	--	--
Ca2_P3_32361145	Ca2	32361145	ATT/ATTT	TGGGATTACTGCATTTTCTGG	GCCGTTTCATATGTCGGTTT	59.9	610	INTERGENIC	--	--	--

INDEL marker IDs	Chromosomes/ unanchored scaffolds	Physical positions (bp)	InDels (*Kabuli* reference genome-CDC Frontier/PII)	Forward primers (5'-3')	Reverse primers (5'-3')	Anneling temperature (0C)	Expected amplified product size (bp)	Structural annotation		Functional annotation	
								Sequence components of *kabuli* genome	*Kabuli* gene accession IDs	NCBI-KOG	TFs
Ca2_P3_32366421	Ca2	32366421	ATCAAAATTCAAAATT/ATCAAAATT	TCGTGACTTTGTCCATCATTTC	GCTTGGCAACATTCTTCACA	60.0	739	INTERGENIC	--	--	--
Ca2_P3_32370223	Ca2	32370223	AAAAAATTAATGAAAAATTAATGAAAAATTAA/AAAAAATTAATGAAAAATTAA	TCAAGCAAGTCACAACATTTCA	TGCTATTTTAATTGTGAGCATGAGA	59.4	637	INTERGENIC	--	--	--
Ca2_P3_32375875	Ca2	32375875	TAAAGAAAAAGAAAAAGAAA/TAAAGAAAAAGAAAAAGAAAAAGAAA	CATCTGCATACCCAAACACG	GAGTGTTCCAGCGGTCTCTC	60.0	168	DRR	Ca_10281	J	MYB
Ca2_P3_3237800	Ca2	3237800	CAAAAA/CAAAA	TAACAATCGTCGATGGAAGC	CAATTGGGACCTTGATCTCC	58.7	469	INTERGENIC	--	--	--
Ca2_P3_32378634	Ca2	32378634	GTTTTT/GTTTT	GTGGAAGGTCGGTGGTCTTA	ACCACACCCAATCCTTTCAA	60.0	144	INTERGENIC	--	--	--
Ca2_P3_32379585	Ca2	32379585	AATTAATTTT/AATTAATTTTGATTAATTTT	TGTGCGAGTTTATTGCGGTA	CATTTGGTTCAAAATGTCATGG	60.3	624	INTERGENIC	--	--	--
Ca2_P3_32393042	Ca2	32393042	ATTTTTTTT/ATTTTTTTTT	TGCCCATCCTTATGCTCTTC	ATCACGTGCAACCACAAGTC	60.2	626	INTERGENIC	--	--	--
Ca2_P3_32393090	Ca2	32393090	ATT/ATTTT	TGCCCATCCTTATGCTCTTC	ATCACGTGCAACCACAAGTC	60.2	626	INTERGENIC	--	--	--
Ca2_P3_32393117	Ca2	32393117	T/TC	TGCCCATCCTTATGCTCTTC	ATCACGTGCAACCACAAGTC	60.2	626	INTERGENIC	--	--	--
Ca2_P3_32393187	Ca2	32393187	AATAT/A	TGCCCATCCTTATGCTCTTC	ATCACGTGCAACCACAAGTC	60.2	626	INTERGENIC	--	--	--

INDEL marker IDs	Chromosomes/ unanchored scaffolds	Physical positions (bp)	InDels (*Kabuli* reference genome-CDC Frontier/PII)	Forward primers (5'-3')	Reverse primers (5'-3')	Anneling temperature (0C)	Expected amplified product size (bp)	Structural annotation		Functional annotation	
								Sequence components of *kabuli* genome	*Kabuli* gene accession IDs	NCBI-KOG	TFs
Ca2_P3_32393441	Ca2	32393441	TGG/TGGG	TCGCACTTTTTCGTGTAGGA	GGGCTTGTTTGACGCATTAT	59.5	588	INTERGENIC	--	--	--
Ca2_P3_32393633	Ca2	32393633	TAACATAATAA/TA	GACTTGTGGTTGCACGTGAT	GGGCTTGTTTGACGCATTAT	59.6	445	INTERGENIC	--	--	--
Ca2_P3_32395073	Ca2	32395073	TCTCTCA/T	AATGCAGGTTTTCACAATGC	GTGGGGTTTGTGGTCCATAG	58.6	418	INTERGENIC	--	--	--
Ca2_P3_32398215	Ca2	32398215	TTTAATTTATTAATTTATTAATTTATTAATT/TTTAATTTATTAATTTATTAATT	TTGTGTTGTGTGCCCCTTTA	TTGGATCTTGATCTCTCGCA	60.0	524	INTERGENIC	--	--	--
Ca2_P3_32408047	Ca2	32408047	ATTTTTT/ATTTTTTT	TGTCAACTGTTCCTGTGGGA	TGAACGCAACTGGTAGCAAG	60.1	613	INTRON	Ca_10279	--	bHLH
Ca2_P3_32409946	Ca2	32409946	ATTTTTT/ATTTT	TGGAGAGTTGAGAAGCCAGG	TACTCGAGGCCTCTCGCTAC	60.5	850	INTRON	Ca_10279	--	bHLH
Ca2_P3_32415535	Ca2	32415535	T/TCC	GGGATTGTGTTCATTCCAGG	ATCGATATACCAACGGCTGC	60.2	398	INTRON	Ca_10278	--	bHLH
Ca2_P3_32417829	Ca2	32417829	TTCT/TT	TTGGATACATGCCACCAGAG	GCCTAGGATCCACGAGTTTG	59.5	601	INTRON	Ca_10278	--	bHLH
Ca2_P3_32422110	Ca2	32422110	ATTTTTTTTTT/ATTTTTTTTT	TATCAAAACCCAAGCCCAAG	TTTGTAATTGTCGCAACCCC	59.9	327	DRR	Ca_10277	--	--
Ca2_P3_32436418	Ca2	32436418	CG/C	TCACATGGTGGCATCCTAGA	CGAGTCATCCATGCTCTGTT	60.1	302	DRR	Ca_10275	O	C2H2

INDEL marker IDs	Chromosomes/ unanchored scaffolds	Physical positions (bp)	InDels (*Kabuli* reference genome-CDC Frontier/PII)	Forward primers (5'-3')	Reverse primers (5'-3')	Anneling temperature (0C)	Expected amplified product size (bp)	Structural annotation		Functional annotation	
								Sequence components of *kabuli* genome	*Kabuli* gene accession IDs	NCBI-KOG	TFs
Ca2_P3_32436855	Ca2	32436855	TAAAA/TAAA	CAGAGCATGGATGACTCGTAA	TGAAAATCACTCGTTTTATCTTAATC	58.9	658	INTERGENIC	--	--	--
Ca2_P3_32437527	Ca2	32437527	ATTTTTT/ATTTTT	TTTTATGTTTCATGTCTCATTTTGA	TGCACAAACAATCCAATGAA	57.8	537	INTERGENIC	--	--	--
Ca2_P3_32437597	Ca2	32437597	ATTTTTT/ATTTTTTT	TTTTATGTTTCATGTCTCATTTTGA	TGCACAAACAATCCAATGAA	57.8	537	INTERGENIC	--	--	--
Ca2_P3_32453109	Ca2	32453109	ATTTTTTT/ATTTTTTTTT	TCTTCACACAATCCCACCAA	CTCAAGTCGACAAGCCTTCC	59.9	659	INTERGENIC	--	--	--
Ca2_P3_32477117	Ca2	32477117	TTTGTT/TTTGTTGTT	CTGCAATGATAATTGGGGCT	CAACTGCAATGTTCCATATTCG	59.9	555	DRR	Ca_10273	R	C3H
Ca2_P3_32480547	Ca2	32480547	CTACTTA/CTA	TCCACATGCTTCCAATCAAA	TGTTGTGTGTCAGAAACGTTCA	60.0	559	INTERGENIC	--	--	--
Ca2_P3_32510389	Ca2	32510389	TGGGGGGGGGG/TGGGGGGGGG	TACTGGCTGATTTGCGACAG	CATATTTGACAAGGACCGGG	60.0	880	INTRON	Ca_10271	O	GATA
Ca2_P3_32520939	Ca2	32520939	ATTTTTTT/ATTTTTT	AACATGGAAGATCGGTTGGA	GATCAATGCGAGTTTGTTGG	60.3	318	INTERGENIC	--	--	--
Ca2_P3_3262653	Ca2	3262653	TTATATATATATATATATATATATA/TTATATATATATATATATATATATA	TGAGAAAATGAAAGAATTAATGGTC	AAGTGACAAAAACCGCAACC	57.9	740	DRR	Ca_10525	Q	Nin-like
Ca2_P3_32672427	Ca2	32672427	TC/T	ATTCAACGTTTCTGGATGGC	ATAAGGCAGTGGCGTAGAGC	59.9	452	DRR	Ca_10254	DR	--

INDEL marker IDs	Chromosomes/ unanchored scaffolds	Physical positions (bp)	InDels (*Kabuli* reference genome-CDC Frontier/PII)	Forward primers (5'-3')	Reverse primers (5'-3')	Anneling temperature (0C)	Expected amplified product size (bp)	Structural annotation		Functional annotation	
								Sequence components of *kabuli* genome	*Kabuli* gene accession IDs	NCBI-KOG	TFs
Ca2_P3_32713700	Ca2	32713700	TTTTTTTTT/TTTTTTTTTTT	AACGCATCGGTAAACACTCC	GAGGTACTACAGCTTGCCCC	60.0	234	INTERGENIC	--	--	--
Ca2_P3_32713890	Ca2	32713890	AAAAACAAAA/AAAAACAAAACAAAA	AACGCATCGGTAAACACTCC	TGTTTTGCAGCAAGATGTCC	60.0	695	INTERGENIC	--	--	--
Ca2_P3_32861621	Ca2	32861621	AATATATATATATATAT/AATATATATATATATATAT	TTTTTCTGTTTCATTTTCACTTCAA	TTGAAAACACCAAAAAGGGG	59.3	584	INTERGENIC	--	--	--
Ca2_P3_32934778	Ca2	32934778	TTAAATAAATAAATAAATAAA/TTAAATAAATAAATAAATAAATAAA	ACAGCGACACGAAGGACTCT	GGAACAAGTTTCCGTGAAGG	60.1	396	INTRON	Ca_10230	OJ	MYB_related
Ca2_P3_32938114	Ca2	32938114	AATATATATATATATATATATAT/AATATATATATATATATATATATAT	ATTTGATGTGGGTGTGCGTA	ATTAAAGGGCAACCCCAAAT	59.8	303	INTERGENIC	--	--	--
Ca2_P3_32970157	Ca2	32970157	TAATCAAAGTGGTATAAATCAA/TAATCAA	GGTCAATTTCGCGAATTAGTTA	TCAGATTCAGCTGGACAAAGA	58.3	609	INTERGENIC	--	--	--
Ca2_P3_33030318	Ca2	33030318	AATATATATATATATATATATATATAT/AATATATATATATATATATATATATAT	CAGCATGGGTTCATGCACTA	GCAAATTGGAATGAGTTGGTC	60.7	601	INTERGENIC	--	--	--
Ca2_P3_33068395	Ca2	33068395	TTATATATATATATATATATATAT/TTATATATATATATATATATATATATATAT	CTCTTTTAGATCGCAACCGC	TAGGTTGGAGTTGGGACCAG	60.0	881	DRR	Ca_10217	--	--
Ca2_P3_3312282	Ca2	3312282	TGAAGAAGAAGAAGAAGAAGAA/TGAAGAAGAAGAAGAAGAAGAAGAA	AATTGAGCCTTTTGCCATTG	TCCCTCCCTTCACTCTCCTT	60.1	215	INTERGENIC	--	--	--
Ca2_P3_3321788	Ca2	3321788	TT/TTAGCCCAAAAT	GAGGGTGGGGATCAAACTCT	ACCACAACGGACAAAAGAGG	60.3	152	CDS (FRAME SHIFT)	Ca_10520	O	--

INDEL marker IDs	Chromosomes/ unanchored scaffolds	Physical positions (bp)	InDels (*Kabuli* reference genome-CDC Frontier/PII)	Forward primers (5'-3')	Reverse primers (5'-3')	Anneling temperature (0C)	Expected amplified product size (bp)	Structural annotation		Functional annotation	
								Sequence components of *kabuli* genome	*Kabuli* gene accession IDs	NCBI-KOG	TFs
Ca2_P3_33257195	Ca2	33257195	ATTTTTTTTT/ATTTTTTTTTT	ATCGCATTTTGCCTTTGTTT	GCTGTCGTTTGCAGCATAAA	59.6	801	INTERGENIC	--	--	--
Ca2_P3_33278516	Ca2	33278516	ATGTTGTTGTTGTTGTT/ATGTTGTTGTTGTTGTTGTT	AGGGGTCACATGGTTTTGAA	AGGTGGTTGCTGATAGGTGG	60.2	335	INTERGENIC	--	--	--
Ca2_P3_33306373	Ca2	33306373	TTATATATATATATATATA/TTATATATATATATA	GAATCCGTTTCCGAGTTTGA	TCAATGGAAATCAAAGTTGAAGG	60.1	644	INTERGENIC	--	--	--
Ca2_P3_33398330	Ca2	33398330	TCACA/TCA	AGTCAATGGCATGAGAAGGC	AAATGCAGGGTCACAACACA	60.2	513	DRR	Ca_10186	T	M-type
Ca2_P3_33405227	Ca2	33405227	AATATATATATA/AATATATATA	AAAGAGACATGACTTTGTCCATCA	TTTCGCTACCCATCATACCC	60.0	586	INTERGENIC	--	--	--
Ca2_P3_33447451	Ca2	33447451	AATATATATATATATATATATATATATATATATATAT/AATATATATATATATATATATATATAT	CCTGAAACAAACGCATCCAT	TTCTTGCCACTTGAGTGTGG	60.9	487	INTERGENIC	--	--	--
Ca2_P3_33523362	Ca2	33523362	GTTTTTTTTTT/GTTTTTTTTT	TCAGCTGGAGTGGATGAGTG	TGCAAGCATTGGTTGAAATG	60.0	447	DRR	Ca_10181	--	--
Ca2_P3_33565335	Ca2	33565335	TAAAAAAAAAA/TAAAAAAAAAAA	CGTCGTTTGAGTACGATGTTG	TAACTTTTGGCTGGCATGTG	59.3	754	INTERGENIC	--	--	--
Ca2_P3_33565533	Ca2	33565533	CTTTT/CTTTTT	AATGAAGATGCGTGCAATTTA	TAACTTTTGGCTGGCATGTG	58.3	159	INTERGENIC	--	--	--
Ca2_P3_33639211	Ca2	33639211	GAA/GAAA	TGGCAAACATGTTGTCCTAAA	AGAGAGCAAACTATTCCGTCG	59.1	230	INTERGENIC	--	--	--

INDEL marker IDs	Chromosomes/ unanchored scaffolds	Physical positions (bp)	InDels (*Kabuli* reference genome-CDC Frontier/PII)	Forward primers (5'-3')	Reverse primers (5'-3')	Anneling temperature (0C)	Expected amplified product size (bp)	Structural annotation		Functional annotation	
								Sequence components of *kabuli* genome	*Kabuli* gene accession IDs	NCBI-KOG	TFs
Ca2_P3_33809112	Ca2	33809112	GATATACATATACATATACATATACATATACATAT/GATATACATATACATATACATATACATAT	TTAATCAAATGCACCGACCA	GCGATCGTGAGTGAGTTCAA	59.9	462	DRR	Ca_10155	--	--
Ca2_P3_33887480	Ca2	33887480	TTATATATATATATATATATATAT/TTATATATATATATATATATATAT	GTCCATCAAAATGGACCACC	ATTCATGCCTTTTGGCACTT	60.0	703	DRR	Ca_10149	--	--
Ca2_P3_33947237	Ca2	33947237	TTATATATATATATATATATATATA/TTATATATATATATATATATATATA	TCCTTCGCCAATATTTTGTTTT	TTTGAGTCGGAGTTGTCGGT	59.9	426	INTERGENIC	--	--	--
Ca2_P3_33959397	Ca2	33959397	ATATTTATTTATTT/ATATTTATTT	GGCTAATTTTCGTTATATGGATGC	ACTAGTGATGGCCGAAATGG	60.1	764	INTERGENIC	--	--	--
Ca2_P3_33978678	Ca2	33978678	CATATATATATATATAT/CATATATATATATATAT	AGGAAGAGTGGAGTTGCGAA	TGCGTTACGGTGAAATGGTA	60.0	364	INTERGENIC	--	--	--
Ca2_P3_33984310	Ca2	33984310	CTATATATATATATATATATATATA/CCATATATATATATATATATATATATATA	TGGCTATGATCACAATTGGC	CCCTTTGCAAGAGACTCCAA	59.5	869	INTERGENIC	--	--	--
Ca2_P3_33984629	Ca2	33984629	CAAAA/CAAA	AACAAAGCAGAAATATCCTGTCG	AGCATAGCCAAAACCCCTTT	59.7	389	INTERGENIC	--	--	--
Ca2_P3_33986265	Ca2	33986265	TT/TTATAT	AACTTTGGATCTACACGCGA	ACTCCAAGATCATCATCGGC	58.4	623	INTERGENIC	--	--	--
Ca2_P3_34028835	Ca2	34028835	AC/A	AAAGGGGTTTTGGCTATGCT	CATGATCATAATTGCATGGAAAA	60.0	229	INTERGENIC	--	--	--
Ca2_P3_34087701	Ca2	34087701	CATATATATATATATAT/CATATATATATATATATATAT	ATGGTGGTGAGCAACTTTCC	AACAAACAGCCATTCCAAGG	60.0	707	INTERGENIC	--	--	--

INDEL marker IDs	Chromosomes/ unanchored scaffolds	Physical positions (bp)	InDels (*Kabuli* reference genome-CDC Frontier/PII)	Forward primers (5'-3')	Reverse primers (5'-3')	Anneling temperature (0C)	Expected amplified product size (bp)	Structural annotation		Functional annotation	
								Sequence components of *kabuli* genome	*Kabuli* gene accession IDs	NCBI-KOG	TFs
Ca2_P3_34093725	Ca2	34093725	TTATATATATATATATATATAT/TTATATATATATATATATATATATAT	CAATCCTTTGGCTTTTGGAA	TGCAGCACATGACTTTTCCT	60.0	509	INTERGENIC	--	--	--
Ca2_P3_34213186	Ca2	34213186	T/TAAG	GACACCATTATCGAAGGCGT	AGTATCGGACACCAAAAGCG	60.0	249	INTERGENIC	--	--	--
Ca2_P3_34224429	Ca2	34224429	TAAAAA/TAAAA	AGGGGTGGAAGGAGAGAGAA	AACGGAAAACGTCAATCTGG	60.2	698	INTERGENIC	--	--	--
Ca2_P3_34224923	Ca2	34224923	ATTTTTTTTT/ATTTTTTTTTT	CCAGATTGACGTTTTCCGTT	TGGGATGAGTTTGTTTGAGTTT	60.0	366	INTERGENIC	--	--	--
Ca2_P3_34272124	Ca2	34272124	AATATATATATATATATATATATATA/AATATATATATATATATATATA	GGCACCTCATATGCAACCTT	TGGCACCAAACTTACACCAA	60.0	652	DRR	Ca_15251	--	WRKY
Ca2_P3_3428568	Ca2	3428568	AAATAATAATAATAATAATAATAA/AAATAATAATAATAATAATAA	TTGATTGACACGTTTACTTGCAT	GGAGCAAACACATAAGGGTG	59.6	688	INTERGENIC	--	--	--
Ca2_P3_34331090	Ca2	34331090	CTTTT/CTTT	TTTTGCAGTTGAAGTATCACAAA	TTCACGCTCCACAACAAGAG	57.6	698	INTERGENIC	--	--	--
Ca2_P3_34344410	Ca2	34344410	TATTTATTCATTTATT/TATTTATT	GTCGTTCAATTTCTGTGCCA	GCACCAAACACATCAACCAA	59.7	231	DRR	Ca_15262	--	bZIP
Ca2_P3_34353539	Ca2	34353539	CATATATATATATATATATATATATATATAT/CATATATATATATATATATATATATATAT	CATGAGCTTGAGTCGCAAAA	TAGGAGGCATATGTGAGGGG	60.1	760	INTERGENIC	--	--	--
Ca2_P3_34353805	Ca2	34353805	GTTTTTTTT/GTTTTTTTTT	AGTTGAAGGAGTGCATGAGGA	TAGGAGGCATATGTGAGGGG	59.9	633	INTERGENIC	--	--	--

INDEL marker IDs	Chromosomes/ unanchored scaffolds	Physical positions (bp)	InDels (*Kabuli* reference genome-CDC Frontier/PII)	Forward primers (5'-3')	Reverse primers (5'-3')	Anneling temperature (0C)	Expected amplified product size (bp)	Structural annotation		Functional annotation	
								Sequence components of *kabuli* genome	*Kabuli* gene accession IDs	NCBI-KOG	TFs
Ca2_P3_34355206	Ca2	34355206	CTTGGGCT/CTTGGGCTTTGTTGGGCT	TCTTTGATGCAATGCCAGAG	ATCATTCGAGGTGCCATCAT	59.9	975	INTERGENIC	--	--	--
Ca2_P3_34355457	Ca2	34355457	TAAAAAA/TAAAAAAA	ATTCTTGGGCTGTGTTTTGG	GGGTGATGAGGACTGGTTGT	60.0	725	INTERGENIC	--	--	--
Ca2_P3_34357732	Ca2	34357732	CATATATATATATATATATATATATATATAT/CATATATATATATATATATATATATATATATATATATAT	CATGAGCTTGAGTCGCAAAA	GCAACAAGATGGGGACAAGT	60.1	654	INTERGENIC	--	--	--
Ca2_P3_34450150	Ca2	34450150	TTATATATATATATATATATATATATATAT/TTATATATATATATATATATATAT	AGCTAGCTCAGAATGCAGCC	CCCACAAAACTTGCTTCGTT	59.9	324	INTERGENIC	--	--	--
Ca2_P3_34499214	Ca2	34499214	A/AAAATT	ATGGGATTCTTGAGCCTGTG	CCTTGGGAGCAGTCTCTGTC	60.1	680	INTERGENIC	--	--	--
Ca2_P3_34540051	Ca2	34540051	TAAAAAAAAAAAA/TAAAAAAAAAAA	GAATTTGCAGAAGCACACGA	TTCCATAGCTAACACGTGCG	60.0	456	INTERGENIC	--	--	--
Ca2_P3_34562775	Ca2	34562775	TCTTTA/TCTTTACTTTA	ATAGCGTGCAACGATCCTCT	CATAGCGCCCATAAAAGGAA	59.9	515	INTERGENIC	--	--	--
Ca2_P3_34683422	Ca2	34683422	TA/T	GTTTTTCGCAACCACCTTTC	TCGAGTTCAAATTTTGGTTGG	59.6	115	INTERGENIC	--	--	--
Ca2_P3_34683764	Ca2	34683764	TC/TCC	GTTTTTCGCAACCACCTTTC	CGCTTTTTGCACTAACCTCC	59.6	682	INTERGENIC	--	--	--
Ca2_P3_34684386	Ca2	34684386	CAACGTTAAC/CAAC	ACAATACTGCGGCCTGTTTC	GATTCCGATCGTTTTTCACG	60.1	830	INTERGENIC	--	--	--

INDEL marker IDs	Chromosomes/ unanchored scaffolds	Physical positions (bp)	InDels (*Kabuli* reference genome-CDC Frontier/PII)	Forward primers (5'-3')	Reverse primers (5'-3')	Anneling temperature (0C)	Expected amplified product size (bp)	Structural annotation		Functional annotation	
								Sequence components of *kabuli* genome	*Kabuli* gene accession IDs	NCBI-KOG	TFs
Ca2_P3_34684591	Ca2	34684591	TTATATATATATATATATATATATATATA/TTATATATATATATATATATATATATA	TGGAGTTTTGTGTGATGGACA	GATTCCGATCGTTTTTCACG	60.0	758	INTERGENIC	--	--	--
Ca2_P3_34684688	Ca2	34684688	CATCTT/C	CGTACGTAGCAAATGCAACAA	ATTCCGATCGTTTTTCACGA	59.8	229	INTERGENIC	--	--	--
Ca2_P3_34684923	Ca2	34684923	ATTTTTTT/ATTTTTTTT	TCGTGAAAAACGATCGGAAT	TGCTTGATTTTAATGGATGCC	60.4	550	INTERGENIC	--	--	--
Ca2_P3_34684966	Ca2	34684966	TAAAAA/TAAAA	CGTGAAAAACGATCGGAATC	TTGCCCCTTAAATGACATGC	60.4	593	INTERGENIC	--	--	--
Ca2_P3_34685695	Ca2	34685695	ATTTTTT/ATTTTTTT	CCCCATTTTGTTTCTCTCCA	TTGTTTCTCCCCAGTTTTGG	59.9	234	INTERGENIC	--	--	--
Ca2_P3_34685921	Ca2	34685921	TAAAAAAA/TAAAAA	CCAAAACTGGGGAGAAACAA	TACGATAGGGGGATCTCACG	59.9	503	INTERGENIC	--	--	--
Ca2_P3_34706742	Ca2	34706742	GAAAAA/GAAAA	CTCTCTCTCCATCTCGGCAC	AAAGTAGCCGGCAAGGTCTC	60.1	459	INTERGENIC	--	--	--
Ca2_P3_34883646	Ca2	34883646	GATATATATATATATATATATATATATATATATATAT/GATATATATATATATATATATATATATATATAT	AGTCCCTTCGAAAAATGGCT	TTTGAAGGGGGAGAAGAGGT	60.1	650	INTRON	Ca_16880	S	--
Ca2_P3_34983521	Ca2	34983521	AATATATATATATATATATATATATATATATATATATA/AATATATATATATATATATATATATATATATA	CAAATGCATGCTCCCCTAAT	CTAAAAGCCCAAAGCTGCAC	59.9	450	INTERGENIC	--	--	--
Ca2_P3_35031076	Ca2	35031076	TTATATATATATATATATATATATATATATAT/TTATATATATATATATATATATATATATATAT	CGTAACTCTCAATTGGCAATGTT	TGTCACCATGCATATCCCAC	60.4	788	INTERGENIC	--	--	--

INDEL marker IDs	Chromosomes/ unanchored scaffolds	Physical positions (bp)	InDels (*Kabuli* reference genome-CDC Frontier/PII)	Forward primers (5'-3')	Reverse primers (5'-3')	Anneling temperature (0C)	Expected amplified product size (bp)	Structural annotation		Functional annotation	
								Sequence components of *kabuli* genome	*Kabuli* gene accession IDs	NCBI-KOG	TFs
Ca2_P3_35180722	Ca2	35180722	GTATATATATATATATATAT/GTATATATATATATATATATATATAT	TTTAACCGCAATTTAATTCATCA	GGTCTCCCTATATCACGCCA	58.6	301	INTERGENIC	--	--	--
Ca2_P3_35258590	Ca2	35258590	A/ATT	AAAAAGTTTTAACACGGAGGTTTTT	TGTTGTTGGATTGGACATGG	59.8	487	INTERGENIC	--	--	--
Ca2_P3_35273988	Ca2	35273988	CTAGATATTT/CTAGATATTTAGATATTT	GATTAAGCGATGGAGGACCA	CCTTTCCTTTTCCCTCCAAA	60.0	555	INTERGENIC	--	--	--
Ca2_P3_35288109	Ca2	35288109	ATTTTTT/ATTTTTTT	TGCTTTATAGTGCTATTATGTGCCA	CGTCATTAAAAGGGAAACCAA	60.1	757	INTERGENIC	--	--	--
Ca2_P3_35288459	Ca2	35288459	TT/TTAT	TTCGATGGTATGCAGTAATTGG	TTCCCATGAAAGAGAGGTGG	59.9	433	INTERGENIC	--	--	--
Ca2_P3_35297767	Ca2	35297767	CTGAACTG/CTGAACTGAATTGAACTG	ACCAGGAAAAGAAGAAGGGC	TTTTGGGTTGGGATGACATT	59.7	535	INTERGENIC	--	--	--
Ca2_P3_35298056	Ca2	35298056	TA/TAA	AATGTCATCCCAACCCAAAA	TCGCAATAAAAACATATAAAACCA	60.0	190	INTERGENIC	--	--	--
Ca2_P3_35303488	Ca2	35303488	ATTTTTT/ATTTTTTT	TTCCGAATAAGGATTTCTTGG	ATGGTTTGGGGAAAAGAACC	58.2	137	INTERGENIC	--	--	--
Ca2_P3_35304698	Ca2	35304698	C/CG	GCTTGGAAACAGCCTCAGAC	CAGGGTGGTACTGCAATGTG	60.0	536	INTERGENIC	--	--	--
Ca2_P3_35329169	Ca2	35329169	AATATATATATATATATATATATATATAT/AATATATATATATATATATATATAT	CAATTTTGGAAGACGATTTTGA	TGTCCCGAAAAAGTTCATCC	59.1	330	INTERGENIC	--	--	--

INDEL marker IDs	Chromosomes/ unanchored scaffolds	Physical positions (bp)	InDels (*Kabuli* reference genome-CDC Frontier/PII)	Forward primers (5'-3')	Reverse primers (5'-3')	Anneling temperature (0C)	Expected amplified product size (bp)	Structural annotation		Functional annotation	
								Sequence components of *kabuli* genome	*Kabuli* gene accession IDs	NCBI-KOG	TFs
Ca2_P3_35332736	Ca2	35332736	AATATATATATATATATATATATATATATAT ATATATATATATATA/AATATATATATAT ATATATATATATATATATATA	TCGAAAGTTTCCATCAA TACGA	TGGCGGTTGTAAAATG ATGA	59.6	604	INTERGENIC	--	--	--
Ca2_P3_35425064	Ca2	35425064	GAAAAAAAAAA/GAAAAAAAAAAA	TGTCGAAATGATTAGCA AAAGAA	ACATTTTCCCTCCCTT GGTC	58.9	257	INTERGENIC	--	--	--
Ca2_P3_35426514	Ca2	35426514	TAAAAAAAAAAA/TAAAAAAAAAAAA	TTCATCTATTCCATCGA TTCCA	TTGTTTGATTTATGCCC TTGC	59.4	346	INTERGENIC	--	--	--
Ca2_P3_35481606	Ca2	35481606	T/TTCTGGTTGA	TGAAAATGTGCATGCTT GGT	GTCAGAGGAAGGAGG CACTG	60.1	410	CDS (large-effect mutations)	Ca_09709	L	--
Ca2_P3_35508606	Ca2	35508606	GAAAAAAAAA/GAAAAAAAA	CAATTTTCCTCTTCCCT AACAAA	ATTTCGAGTGGAGTGG GTTG	58.7	266	INTERGENIC	--	--	--
Ca2_P3_35556151	Ca2	35556151	AATATATATATATAT/AATATATATATAT	ATCAAAGGACCCATGC TGAG	AGTGTTTCTTTGGGTG GACG	60.1	852	DRR	Ca_09720	B	--
Ca2_P3_35581740	Ca2	35581740	AATATATATATATATATATATATATATA/ AATATATATATATATATATATATATA	TTGCCACTTGATACAGC AAAA	TTGAAAGGATAACATG GCCG	59.4	690	INTERGENIC	--	--	--
Ca2_P3_35648295	Ca2	35648295	CTTTTTTTTTTTTT/CTTTTTTTTTTTT	TGATACACGGTCCACA CTTCA	TCGCTTTCCACCAAAT TTTC	60.0	463	INTERGENIC	--	--	--
Ca2_P3_35713957	Ca2	35713957	T/TCTTCTTCAAATTCAGCAG	CAACGGTTAACTTCCCT CCA	GTAGCGGCCATAAACC AGAA	60.0	813	INTERGENIC	--	--	--
Ca2_P3_35726243	Ca2	35726243	CTTTTTTTTTTTTT/CTTTTTTTTTTTTTT	AACTTCACAGGCCTCG TTGT	CAATGAACAAGGTTTG GATGAA	59.8	902	INTERGENIC	--	--	--

INDEL marker IDs	Chromosomes/ unanchored scaffolds	Physical positions (bp)	InDels (*Kabuli* reference genome-CDC Frontier/PII)	Forward primers (5'-3')	Reverse primers (5'-3')	Anneling temperature (0C)	Expected amplified product size (bp)	Structural annotation		Functional annotation	
								Sequence components of *kabuli* genome	*Kabuli* gene accession IDs	NCBI-KOG	TFs
Ca2_P3_3575424	Ca2	3575424	TAAAAAAAAAAAA/TAAAAAAAAAAAAA	AATTTAGCGTGGTGTTTCCG	AAGGACAATGGGGACATCAA	60.0	815	INTERGENIC	--	--	--
Ca2_P3_35789864	Ca2	35789864	AATTTGATTT/AATTTGATTTGATTT	TGCCTGCGAGATCTTAATCA	CCAAATCGAACCCAAATCAC	59.5	365	INTERGENIC	--	--	--
Ca2_P3_35815027	Ca2	35815027	ATTTTTTTT/ATTTTTTT	TAAGACCATGCATCTCACGC	CGTGCATGGATCAAACTAGG	59.8	650	INTERGENIC	--	--	--
Ca2_P3_35834317	Ca2	35834317	TTATATATATATATATATATATATAT/TTATATATATATATATATATAT	CAAACCGTATTAGCCTCACCA	GGTTGAAGATGTTCGTCGGT	60.0	675	INTRON	Ca_09758	O	MYB
Ca2_P3_35846531	Ca2	35846531	ATT/ATTT	TTATTTGTTCGGTGGCAACA	TGATCGGAATGCGAATAACA	60.0	455	DRR	Ca_09760	J	--
Ca2_P3_35848640	Ca2	35848640	TAAAAAAAA/TAAAAAAAAA	CAGGGGTGATAGGATCAGGA	CCCCAACCTTCACTTTTTCC	59.9	323	INTRON	Ca_09761	E	MYB
Ca2_P3_35848756	Ca2	35848756	TGG/TG	CAGGGGTGATAGGATCAGGA	ACAAGCAGGCCACAAAGTCT	59.9	804	INTRON	Ca_09761	E	MYB
Ca2_P3_35853362	Ca2	35853362	A/AT	TCTACGCTTGTACGTGCTGA	GAAAGAACAAGCTGGGCAAC	58.2	615	INTERGENIC	--	--	--
Ca2_P3_35853696	Ca2	35853696	AAATAA/AAA	GTTGCCCAGCTTGTTCTTTC	GGGAGAGTGGGTGTTTATGC	59.9	404	INTERGENIC	--	--	--
Ca2_P3_35853969	Ca2	35853969	TATTGCATTG/TATTG	GTTGCCCAGCTTGTTCTTTC	GCATGCATGTGTCTTTGGAT	59.9	644	INTERGENIC	--	--	--

INDEL marker IDs	Chromosomes/ unanchored scaffolds	Physical positions (bp)	InDels (*Kabuli* reference genome-CDC Frontier/PII)	Forward primers (5'-3')	Reverse primers (5'-3')	Anneling temperature (0C)	Expected amplified product size (bp)	Structural annotation		Functional annotation	
								Sequence components of *kabuli* genome	*Kabuli* gene accession IDs	NCBI-KOG	TFs
Ca2_P3_35855468	Ca2	35855468	CTTTATGTT/CT	TGATTGCGAAAATGGAAAAG	AGCCTCTGAAGCAAAAACCA	58.7	429	INTERGENIC	--	--	--
Ca2_P3_35855865	Ca2	35855865	CAATGAATGA/CAATGA	TGGTTTTTGCTTCAGAGGCT	CTCAACATGTCCACCAGCAC	60.0	592	INTERGENIC	--	--	--
Ca2_P3_35859193	Ca2	35859193	GAAAAAAAA/GAAAAAAAAAA	AACAATGCCAACATTACGCA	TACTGGAGGCTGTTGGAAGG	60.0	720	INTRON	Ca_09762	R	--
Ca2_P3_35860824	Ca2	35860824	TAA/TA	GGCCTTTGTGATACCCTCAA	GGACCAAACTGTTGGACGAT	59.9	221	INTRON	Ca_09762	R	--
Ca2_P3_35866879	Ca2	35866879	TAAAAAAAAAA/TAAAAAAA	AAGGGCATGCATGAAAGTCT	TCGAGATGTCCCCTTAATGC	59.7	578	INTERGENIC	--	--	--
Ca2_P3_35892945	Ca2	35892945	CAAA/CAA	CTCCAAATGGCAACCAAAAT	TTTAGGGGTGGACAAAATTCA	59.8	510	INTERGENIC	--	--	--
Ca2_P3_35893338	Ca2	35893338	TAAAAAAAA/TAAAAAAAAA	CTCCAAATGGCAACCAAAAT	ACTTTTGGGCCAAGCTAACC	59.8	611	INTERGENIC	--	--	--
Ca2_P3_35893619	Ca2	35893619	TAAAAA/TAAAAAA	GGTTAGCTTGGCCCAAAAGT	GGGTAGAGTGGAGGAAAGGG	60.5	628	INTERGENIC	--	--	--
Ca2_P3_35893811	Ca2	35893811	ATTTTTTT/ATTTTTTTT	GGTTAGCTTGGCCCAAAAGT	GGGTAGAGTGGAGGAAAGGG	60.5	628	INTERGENIC	--	--	--
Ca2_P3_35895369	Ca2	35895369	GT/GTT	CCAAAGGGTAAACGTGTCGT	TGCATGGAATCCAATTCTGA	59.9	510	INTRON	Ca_09764	T	--

INDEL marker IDs	Chromosomes/ unanchored scaffolds	Physical positions (bp)	InDels (*Kabuli* reference genome-CDC Frontier/PII)	Forward primers (5'-3')	Reverse primers (5'-3')	Anneling temperature (0C)	Expected amplified product size (bp)	Structural annotation		Functional annotation	
								Sequence components of *kabuli* genome	*Kabuli* gene accession IDs	NCBI-KOG	TFs
Ca2_P3_35896805	Ca2	35896805	TG/T	GTTGATGATGAATGGGGTCC	CCAGGAGGAGATTGGAATGA	60.0	371	INTRON	Ca_09764	T	--
Ca2_P3_35897304	Ca2	35897304	AAGTTGTAGTTGTA/AAGTTGTA	TCATTCCAATCTCCTCCTGG	TTTACCTCGAGCTGGGAGAA	60.0	776	INTRON	Ca_09764	T	--
Ca2_P3_35899592	Ca2	35899592	TAA/TAAA	GGTGGAACATCTCCAAGGAA	CTCAGACTACAACCTCGCCC	59.9	768	DRR	Ca_09764	T	--
Ca2_P3_35899927	Ca2	35899927	C/CTTTTT	AAGGAAAGGGAGACACATCG	TCCTCTCTTCCACTGCGATT	59.1	910	DRR	Ca_09764	T	--
Ca2_P3_35905548	Ca2	35905548	CA/CAA	AATCTCCGCAAAAGTCAGGA	GAACATCAACCTTTGGCTGC	59.8	635	INTRON	Ca_09765	G	--
Ca2_P3_35914177	Ca2	35914177	A/AT	ACAAATGCATGGGAATGACA	GGGGGAGACTCATCACTCAA	59.8	182	DRR	Ca_09766	--	ERF
Ca2_P3_35915430	Ca2	35915430	TTCTTCTTCATC/TTC	TGGCTTCTTCTATGGCTGCT	CCTCTTCCTGCACCTGAAAG	60.1	169	CDS (large-effect mutations)	Ca_09767	M	--
Ca2_P3_35918565	Ca2	35918565	TAAAAAAA/TAAAAAAAA	TGCATGTCCATATTATTGCCC	TTTTCAACGCTACCTTCATCA	60.6	462	DRR	Ca_09767	M	--
Ca2_P3_35924105	Ca2	35924105	GACTTATAAATTTCA/GACTTATAAATTTCACTTATAAATTTCA	TCCAACCTCAATCACCAATG	GGATGTGAAAGGGTCTCCAA	59.3	543	INTERGENIC	--	--	--
Ca2_P3_35925644	Ca2	35925644	TG/T	TTGTTCAGTGACCTGCAAGC	TCACGTGTTGGTGGGTTATC	60.0	758	INTERGENIC	--	--	--

INDEL marker IDs	Chromosomes/ unanchored scaffolds	Physical positions (bp)	InDels (*Kabuli* reference genome-CDC Frontier/PII)	Forward primers (5'-3')	Reverse primers (5'-3')	Anneling temperature (0C)	Expected amplified product size (bp)	Structural annotation		Functional annotation	
								Sequence components of *kabuli* genome	*Kabuli* gene accession IDs	NCBI-KOG	TFs
Ca2_P3_35928233	Ca2	35928233	GTT/GTTT	TGTTTTTGATTTTATTG TTTGGTGA	ACAAAAATGACCAGCA ACCG	59.7	820	INTERGENIC	--	--	--
Ca2_P3_35936801	Ca2	35936801	ATAAAAGC/A	TTCTTGACCAATGAATG CCA	GGGAGTGCCTCCACTT TTCT	60.0	711	INTRON	Ca_09768	--	SAP
Ca2_P3_35938206	Ca2	35938206	AAGA/AA	TGCATATATTGCTGCAG GTTG	ATCAATGAAGTTTGGC CTGC	59.7	453	INTRON	Ca_09768	--	SAP
Ca2_P3_35939216	Ca2	35939216	G/GT	TGTAAAAGTTGAGTGG TGTGTTTC	TTTGCCTTAGAAACCG GAGA	58.3	320	INTERGENIC	--	--	--
Ca2_P3_35939475	Ca2	35939475	CAAAAAAAA/CAAAAAAAAAA	TCTCCGGTTTCTAAGG CAAA	GGGCTCCACAAAAATC TCAA	59.8	413	INTERGENIC	--	--	--
Ca2_P3_35939549	Ca2	35939549	TATAATA/TATA	TCTCCGGTTTCTAAGG CAAA	GGGCTCCACAAAAATC TCAA	59.8	413	INTERGENIC	--	--	--
Ca2_P3_35940064	Ca2	35940064	CAAA/CA	TTGAGATTTTTGTGGAG CCC	AAGGAGGATTAGGGAT GCGT	60.1	826	INTERGENIC	--	--	--
Ca2_P3_35941960	Ca2	35941960	TGAATAC/TGAATACGAATAC	GGATGGATTGAAATTG CAGG	TTTCGCCATCCAAGAT TTTC	60.3	423	INTERGENIC	--	--	--
Ca2_P3_35942197	Ca2	35942197	T/TA	GGATGGATTGAAATTG CAGG	TTTCGCCATCCAAGAT TTTC	60.3	423	INTERGENIC	--	--	--
Ca2_P3_35942518	Ca2	35942518	ATT/ATTT	GAAAATCTTGGATGGC GAAA	TTTTTGCCGTTATGCG TGTA	60.0	264	DRR	Ca_09769	--	--

INDEL marker IDs	Chromosomes/ unanchored scaffolds	Physical positions (bp)	InDels (*Kabuli* reference genome-CDC Frontier/PII)	Forward primers (5'-3')	Reverse primers (5'-3')	Anneling temperature (0C)	Expected amplified product size (bp)	Structural annotation		Functional annotation	
								Sequence components of *kabuli* genome	*Kabuli* gene accession IDs	NCBI-KOG	TFs
Ca2_P3_35943158	Ca2	35943158	TCCCC/TCCCCCC	TGAATCCACGCTCCAACATA	GCAATTGTTATTGTGTGCGG	60.1	434	DRR	Ca_09769	--	--
Ca2_P3_35944288	Ca2	35944288	TAAAAAAA/TAAAAAAAA	GCATATGCAAAATGCCACTG	CTGTTTTGCGCCATAGGATT	60.1	352	CDS (FRAME SHIFT)	Ca_09769	--	--
Ca2_P3_35944964	Ca2	35944964	CAAAA/CA	TCCAACCATACCTGAAAGGC	CTCGTGCGAACTTGTGTGTT	59.9	313	CDS (large-effect mutations)	Ca_09769	--	--
Ca2_P3_35945318	Ca2	35945318	CCAA/C	AACATCATACTTTTGCGGGC	GCCTGAAATATGCCGAGGTA	60.0	417	INTRON	Ca_09769	--	--
Ca2_P3_35945786	Ca2	35945786	CTATA/CTATATATA	TACCTCGGCATATTTCAGGC	ATGCAGGTTTTGGATTTTGC	60.1	776	INTRON	Ca_09769	--	--
Ca2_P3_35955676	Ca2	35955676	GTTTTTTT/GTTTTTTTT	TGAATTTGCAGTGTGGGGTA	TGAACAAGATTGCACCGTTT	60.0	667	INTERGENIC	--	--	--
Ca2_P3_35955931	Ca2	35955931	TCACACA/TCACA	AAATGTCAATTTCAAACCCCC	GTGATCCCTTTGTCCCAAAA	59.9	792	INTERGENIC	--	--	--
Ca2_P3_35960202	Ca2	35960202	CAAAAAA/CAAAAAAA	AGAGGACTTGTGTTGCATCC	TCCATTATAAGAGAATGTCCCCA	57.7	707	DRR	Ca_09770	--	--
Ca2_P3_35960498	Ca2	35960498	TAA/TA	TGTGGCAAGTTTTAAACAAATGA	TGAGTGGAGTGTGAATGAAAATG	59.6	663	DRR	Ca_09770	--	--
Ca2_P3_35961760	Ca2	35961760	C/CTTATCT	TCATCAAAGACATTCGTGCC	TTCGACAATATGGCCAATGA	59.7	573	INTERGENIC	--	--	--

INDEL marker IDs	Chromosomes/ unanchored scaffolds	Physical positions (bp)	InDels (*Kabuli* reference genome-CDC Frontier/PII)	Forward primers (5'-3')	Reverse primers (5'-3')	Anneling temperature (0C)	Expected amplified product size (bp)	Structural annotation		Functional annotation	
								Sequence components of *kabuli* genome	*Kabuli* gene accession IDs	NCBI-KOG	TFs
Ca2_P3_35961921	Ca2	35961921	TAAAA/TAAA	AGACGCTCATCACTCAATTTCA	TTCGACAATATGGCCAATGA	59.9	251	INTERGENIC	--	--	--
Ca2_P3_35962691	Ca2	35962691	ATTTTTTT/ATTTTTTTT	TGAGTCATGAGATCGACGAAA	TTCAAATTTAACGGTTGGATCA	59.4	367	INTERGENIC	--	--	--
Ca2_P3_35962726	Ca2	35962726	GAAAA/GAAAAA	TGAGTCATGAGATCGACGAAA	TTCAAATTTAACGGTTGGATCA	59.4	367	INTERGENIC	--	--	--
Ca2_P3_35963890	Ca2	35963890	AATATAT/AATAT	GTCAAGGAAACTCGATCCCA	TACCAAAGCGGCAGAGAGAT	60.1	683	INTERGENIC	--	--	--
Ca2_P3_35965669	Ca2	35965669	GTTTTAATTTTT/GTTTT	TGGAAGAATGCTTTCAAGGG	ACATTCCATGGGCTGCTTAG	60.2	636	DRR	Ca_09771	J	NAC
Ca2_P3_35965829	Ca2	35965829	CAAAAA/CA	ATTTTTCTGCTAGCGGGACC	ACATTCCATGGGCTGCTTAG	60.6	260	DRR	Ca_09771	J	NAC
Ca2_P3_35966120	Ca2	35966120	TT/TTAT	AGCCCATGGAATGTGAAAAG	CCAAAACCTTTGCCTTGAAA	59.9	506	DRR	Ca_09771	J	NAC
Ca2_P3_35967176	Ca2	35967176	T/TG	TTGAGGGGAGGTAAGCCTTT	GGTGTCTTTGGATGGAGTGG	60.1	253	INTRON	Ca_09771	J	NAC
Ca2_P3_35967204	Ca2	35967204	TTTCTCTTTACTT/TTT	TTGAGGGGAGGTAAGCCTTT	TGATAGGAGGAAGCGTGACC	60.1	583	INTRON	Ca_09771	J	NAC
Ca2_P3_35971845	Ca2	35971845	TAT/TATATCACAT	GCGGAGCAGAACCCTTATTA	TGACTTTTTATTTTTCCGCACTC	59.3	955	INTERGENIC	--	--	--

INDEL marker IDs	Chromosomes/ unanchored scaffolds	Physical positions (bp)	InDels (*Kabuli* reference genome-CDC Frontier/PII)	Forward primers (5'-3')	Reverse primers (5'-3')	Anneling temperature (0C)	Expected amplified product size (bp)	Structural annotation		Functional annotation	
								Sequence components of *kabuli* genome	*Kabuli* gene accession IDs	NCBI-KOG	TFs
Ca2_P3_35987159	Ca2	35987159	GCAGTTGAAAACAG/GCAG	ATCTCATAATGGCGAGGCTG	AAATTATGGAGGCCAATCC	60.2	416	DRR	Ca_09772	T	LBD
Ca2_P3_35987956	Ca2	35987956	ATGTTGT/ATGT	GGATTGGCCTCCCATAATTT	GCGTTTTCAAGGTTGGATGT	60.0	851	DRR	Ca_09772	T	LBD
Ca2_P3_35995293	Ca2	35995293	ATGATCCTTTTT/AT	CGTGTGAGTTTCGTCGCTAA	TGGTTTGGGTGGTTCATTTT	60.1	499	INTERGENIC	--	--	--
Ca2_P3_35995557	Ca2	35995557	TAAAAAAAA/TAAAAAAAAAAAA	CGTGTGAGTTTCGTCGCTAA	TGGTTTGGGTGGTTCATTTT	60.1	499	INTERGENIC	--	--	--
Ca2_P3_35996261	Ca2	35996261	TTCTCT/TTCT	CCATTCGTCAACGGCATAAT	AAAATCAACGACGATGAATAAGAA	60.7	330	INTERGENIC	--	--	--
Ca2_P3_35999918	Ca2	35999918	CAAAAA/CAAAAAA	GGAATCAAAGCGGAATTCAA	AAGGAGCATGGTCCAATCAC	60.0	221	INTRON	Ca_09774	--	FAR1
Ca2_P3_36000699	Ca2	36000699	TTTATATTTTTTAATA/TTTATATTTTTTAATAATTATATTTTTTAATA	CACGCAATATGTTAAGGGGG	TGATATGCACCACCATCACC	60.2	424	INTERGENIC	--	--	--
Ca2_P3_36028104	Ca2	36028104	TAACAAACAAA/TAACAAA	AGGGAGAGAGAGGAAGGCTG	CTTGAAATCGTGACCTGCAA	60.1	282	DRR	Ca_09778	P	--
Ca2_P3_36028825	Ca2	36028825	CAAA/CAA	TGGCACAGTCAGAGTGGTTT	CGACGACTTCCACTTCATTG	59.3	576	DRR	Ca_09778	P	--
Ca2_P3_36029029	Ca2	36029029	AC/ACGC	TGGCACAGTCAGAGTGGTTT	CGACGACTTCCACTTCATTG	59.3	576	DRR	Ca_09778	P	--

INDEL marker IDs	Chromosomes/ unanchored scaffolds	Physical positions (bp)	InDels (*Kabuli* reference genome-CDC Frontier/PII)	Forward primers (5'-3')	Reverse primers (5'-3')	Anneling temperature (0C)	Expected amplified product size (bp)	Structural annotation		Functional annotation	
								Sequence components of *kabuli* genome	*Kabuli* gene accession IDs	NCBI-KOG	TFs
Ca2_P3_36029534	Ca2	36029534	A/AAGTT	CCAGGCACGTGGTAGCTAAT	AAAGCCCTAGCGGGTTACAT	60.2	824	DRR	Ca_09778	P	--
Ca2_P3_36029693	Ca2	36029693	CAAGTAAGTA/CAAGTA	TGGAATGAAATTATGTGGCAA	TTTGCCAGCTGTTCAATCTG	58.9	503	DRR	Ca_09778	P	--
Ca2_P3_36036557	Ca2	36036557	ATTTTTTT/ATTTTTT	CCCACTTGAGCCAATTTTGT	GCAACATCTCATTGGTGGTG	60.0	580	INTERGENIC	--	--	--
Ca2_P3_36045387	Ca2	36045387	TAAA/TA	AAAGTTGTGACGGTTTCTTGTG	GGCCAAATACAACTTAGCCG	59.2	315	DRR	Ca_09781	--	--
Ca2_P3_36051944	Ca2	36051944	ACCCC/ACCC	CCTTTTTGGGCGACATTTTA	TGAAAACAAGCTTTGAAAAGGA	59.9	248	INTERGENIC	--	--	--
Ca2_P3_36053697	Ca2	36053697	TAAA/TAAAA	CATGTCTCAAGGCACGAAGA	CGTAGACTCGTAGGGTGGGA	60.0	635	INTERGENIC	--	--	--
Ca2_P3_36056071	Ca2	36056071	TAC/T	CATGCAGGGCAATATTGATG	TCTGCATTTTCCAGGCTCTT	59.9	504	DRR	Ca_09783	--	--
Ca2_P3_36059042	Ca2	36059042	GA/GATA	TTGAAATCTTGAAGCCGGAC	GAAAAGACCAACTCTTTGCGA	60.2	510	INTERGENIC	--	--	--
Ca2_P3_36060625	Ca2	36060625	TAA/TAAA	TTTTGAAGTTGAAGAAGGTGGTT	AAACCACGTCAAACCTCACC	59.2	616	INTERGENIC	--	--	--
Ca2_P3_36064276	Ca2	36064276	GGAGAGAGAGAGAGAGA/GGAGAGAGAGAGAGA	GTGCATTGGCAATTTTACCC	GGGGGAAGCTTAACCAAAAG	60.2	423	INTERGENIC	--	--	--

INDEL marker IDs	Chromosomes/ unanchored scaffolds	Physical positions (bp)	InDels (*Kabuli* reference genome-CDC Frontier/PII)	Forward primers (5'-3')	Reverse primers (5'-3')	Anneling temperature (0C)	Expected amplified product size (bp)	Structural annotation		Functional annotation	
								Sequence components of *kabuli* genome	*Kabuli* gene accession IDs	NCBI-KOG	TFs
Ca2_P3_36064305	Ca2	36064305	AAGAGAGAGAGAGAGAG/AAGAGAGAGAGAGAG	GTGCATTGGCAATTTTACCC	GGGGGAAGCTTAACCAAAAG	60.2	423	INTERGENIC	--	--	--
Ca2_P3_36065466	Ca2	36065466	TTCTCTCTCTCTCTCTCT/TTCTCTCTCTCTCTCTCTCT	TGGGCACTCTCTTCCACTCT	GAACCACCAAACCAAACACC	60.0	500	INTERGENIC	--	--	--
Ca2_P3_36089547	Ca2	36089547	GAAAA/GAAAAA	TTGGTTGCTTCTTGTGCTTG	TTGAATGCTTTGCAGTTTGC	60.0	351	DRR	Ca_09786	--	GRAS
Ca2_P3_36090973	Ca2	36090973	CAGAGAGAGAGAGA/CAGAGAGAGAGAGAGA	GGCATCCAAGATACGAGCAT	GCGGCTGTACGTTATTGACA	60.1	556	DRR	Ca_09786	--	GRAS
Ca2_P3_36104944	Ca2	36104944	TGAG/TGAGAG	GAGATCGCGTTTGTGAGGTA	GCATGACTACGCCCCACTAT	58.9	464	INTRON	Ca_09789	R	MIKC
Ca2_P3_36105866	Ca2	36105866	GAAAA/GAAAAA	TGACAAGGTGAGACCCACAA	GACAGAATTGGAGTGGGGAA	60.1	633	DRR	Ca_09789	R	MIKC
Ca2_P3_36111217	Ca2	36111217	AA/AATA	CAAATTCAAAATCCAAAACGG	AAGGGAAGCGACTCAAACAA	59.3	845	INTERGENIC	--	--	--
Ca2_P3_36113875	Ca2	36113875	ATACTTTTACTTTT/ATACTTTT	CCACACTTGGCCTACATGTTT	TCGACTCACATGGAAAATTGA	59.9	341	INTERGENIC	--	--	--
Ca2_P3_36114736	Ca2	36114736	AG/A	TTTGAAAGGAAACAAACTTCCA	CTCGATTCCGTAAAAACCCA	58.8	729	INTERGENIC	--	--	--
Ca2_P3_36130204	Ca2	36130204	GAAAAAAAAA/GAAAAAAAAAAAAAA	ACTTACCTGGAGAGCGCAAA	AATTATCAGCCGCCACAATC	60.0	729	INTRON	Ca_09792	J	B3

INDEL marker IDs	Chromosomes/ unanchored scaffolds	Physical positions (bp)	InDels (*Kabuli* reference genome-CDC Frontier/PII)	Forward primers (5'-3')	Reverse primers (5'-3')	Anneling temperature (0C)	Expected amplified product size (bp)	Structural annotation		Functional annotation	
								Sequence components of *kabuli* genome	*Kabuli* gene accession IDs	NCBI-KOG	TFs
Ca2_P3_36146623	Ca2	36146623	GAAAAAA/GAAAAA	AAACAGCAACAAAAATCCCAA	TTTGGAAATCAAGAGGTGGG	59.5	201	INTERGENIC	--	--	--
Ca2_P3_36146925	Ca2	36146925	TTTATTATTATTAT/TTTATTATTATTATTAT	AGGGAAACCCACCTCTTGAT	AAATCCAAAGTGCTTGCGTT	59.8	623	INTERGENIC	--	--	--
Ca2_P3_36152551	Ca2	36152551	TAAAAAAAAAA/TAAAAAAAAAAAA	CGAGGTTCCTCTTAGTTCTCTTG	GCACATTCAAATCGCTTCAA	58.7	804	DRR	Ca_09793	O	--
Ca2_P3_36177227	Ca2	36177227	TGGGTTTAGGGTTTAGGGTTTAGGGTTTAGG/TGGGTTTAGGGTTTAGGGTTTAGGGTTTAGGGTTTAGGGTTTAGG	TTGATGTTTGCATGATGGGT	TCTTGCATGGCCTTCAACTA	59.8	427	INTERGENIC	--	--	--
Ca2_P3_36237151	Ca2	36237151	A/AG	AACTTAGCTGGCAGTCTCGC	AGCAAGCTGTGGTTGGTCTT	59.8	534	DRR	Ca_09807	I	--
Ca2_P3_36274282	Ca2	36274282	AACAAATACAAATACAAAT/AACAAATACAAAT	TTATTCATCTCCTGGCGTCC	GCTTCATCAGGCATTCCATT	60.0	728	DRR	Ca_09811	G	--
Ca2_P3_36372982	Ca2	36372982	GATTATTATTATTATTAATATTATTATTA/GATTATTATTATTATTATTATTATTAATATTATTATTA	TGAGCTGAAATTGTTCGTCG	CCAATCCTCGTTGTCAAGGT	60.0	274	CDS (large-effect mutations)	Ca_09826	--	HD-ZIP
Ca2_P3_36401773	Ca2	36401773	TATAATAATAATAATA/TATAATAATAATA	GGATAAGGATCTGCATCGGA	ATCCATTTTTGTGGAACCAA	60.0	434	DRR	Ca_09829	R	--
Ca2_P3_36423377	Ca2	36423377	TAAAAAA/TAAAAAAA	TAAGGGTTTGGGCCAATTTT	CCACTTCGAAAGCGAAAGAG	60.5	565	INTERGENIC	--	--	--
Ca2_P3_36444443	Ca2	36444443	TAATAAATAAATAAATAAATAAATAAA/TAATAAATAAATAAATAAATAAA	AAATCATCAAGCCATGAGGC	AGAGTTGCACACAGCTGCAC	60.0	242	INTRON	Ca_09833	I	--

INDEL marker IDs	Chromosomes/ unanchored scaffolds	Physical positions (bp)	InDels (*Kabuli* reference genome-CDC Frontier/PII)	Forward primers (5'-3')	Reverse primers (5'-3')	Anneling temperature (0C)	Expected amplified product size (bp)	Structural annotation		Functional annotation	
								Sequence components of *kabuli* genome	*Kabuli* gene accession IDs	NCBI-KOG	TFs
Ca2_P3_3644473	Ca2	3644473	CATATATATATATATATATATATATATATATA/CATATATATATATATATATATATATATATATATATATATATATA	GTCCCAACCTGCTTTTGTTT	AGCAAAGTTTTTGCACCCAT	59.1	759	INTERGENIC	--	--	--
Ca2_P3_36491896	Ca2	36491896	AG/A	CTCATGCATGGCAATTATGG	CATCCATGTGTGATTCTGCC	59.9	584	DRR	Ca_09837	--	--
Ca2_P3_36551423	Ca2	36551423	TTATATATATATATATATATATATATATATATATAT/TTATATATATATATATATATATATATATATATATATAT	CAGACACACACACATATGGGATA	TCATCCACCTCCAACCAAAT	58.3	661	DRR	Ca_09842	--	--
Ca2_P3_36574982	Ca2	36574982	GAAACCCAAAACCCAAAACCCAAAACC/GAAACCCAAAACCCAAAACC	AAATTTGAATCCCGAATCCC	TCGAGCTGAGATTTTTGGGT	60.0	552	INTERGENIC	--	--	--
Ca2_P3_36600441	Ca2	36600441	AATTATTATTATTATTATTATTATTA/AATTATTATTATTATTATTATTATTATTA	GGAGAACGTTAAGGCAGCAG	AGGTCAAGTTTCGAGGACGA	60.0	704	INTERGENIC	--	--	--
Ca2_P3_36631768	Ca2	36631768	TTATATATATATAT/TTATATATATATATATAT	TCTCCTACCTCTGGCCAACA	CTCCAACATATGCATGGCAC	60.8	100	INTERGENIC	--	--	--
Ca2_P3_37373	Ca2	37373	TTATATATATATATATATATAT/TTATATATATATATATAT	TCATGGTCTGGGTTTGAACT	TGAATCGCGAAATTAGTCCC	58.0	708	INTERGENIC	--	--	--
Ca2_P3_3771259	Ca2	3771259	T/TC	GCTCGACGGAATAGCATTTT	TGGCCAAGCATGATCTAACA	59.3	612	DRR	Ca_10483	E	--
Ca2_P3_377449	Ca2	377449	ATTTTTTTTTTTTT/ATTTTTTTTTTTT	TTTGCCGTTTGAACTGTGAG	CTCTCGCATCCTATTCCAGC	59.9	146	INTERGENIC	--	--	--
Ca2_P3_3857450	Ca2	3857450	TTTTATTTATTTATTTATTTATTTATTT/TTTTATTTATTTATTTATTTATTTATTTATTTATTTATTTATTT	GGGAAATTGTGGTGTGAGGT	TTCGTGCAACGCTATAATTCC	59.7	513	INTERGENIC	--	--	--

INDEL marker IDs	Chromosomes/ unanchored scaffolds	Physical positions (bp)	InDels (*Kabuli* reference genome-CDC Frontier/PII)	Forward primers (5'-3')	Reverse primers (5'-3')	Anneling temperature (0C)	Expected amplified product size (bp)	Structural annotation		Functional annotation	
								Sequence components of *kabuli* genome	*Kabuli* gene accession IDs	NCBI-KOG	TFs
Ca2_P3_3874478	Ca2	3874478	CCTTCTTC/CCTTCTTCTTC	TTTGCATCCTAAGGACCACAC	AGAGGTGGAGAAGCTGGTCA	60.0	515	CDS (large-effect mutations)	Ca_10470	GC	HSF
Ca2_P3_3901844	Ca2	3901844	AC/ACTTCACGAGC	GTCGCTATCTCCCCAGTGAA	CATTCTGTGCCGCTATCTGA	60.2	248	DRR	Ca_10466	GT	--
Ca2_P3_3949908	Ca2	3949908	TAAAAAAAAAAAA/TAAAAAAAAAAAAA	TCAAAGAATAGAGGTATTGTCTCCG	CTAAAGCCTGCCCATTTCTG	60.0	795	INTERGENIC	--	--	--
Ca2_P3_3955516	Ca2	3955516	TATAATAATAATAATAATAATAA/TATAATAATAATAATAATAATAATAA	TTTCCGATTTCGAGGTGTTC	TGAATAGTGGAAAACACAACAATG	60.1	385	INTERGENIC	--	--	--
Ca2_P3_4026182	Ca2	4026182	TTTATTATTATTATTATTATTATTATTATTATTATTATTATTATTATTATTATTATTATTATT/TTTATTATTATTATTATTATTATTATTATTATTATTATTATTATTATTATTATTATTATTATTATTATT	TCAAATATGATTTTTCACTGACCA	GGGACACATCAAACCTGACC	58.5	510	INTERGENIC	--	--	--
Ca2_P3_404702	Ca2	404702	TTTTTAATATTTTAATATTTTAAT/TTTTTAATATTTTAATATTTTAATATTTTAAT	ATGGATCAGCTCAACCCAAT	AACCTAAACGCAAGGTCTCG	59.4	225	INTERGENIC	--	--	--
Ca2_P3_412601	Ca2	412601	CAAAAAAAAAA/CAAAAAAAAAAA	GGTAGTGACAAAATCGGCTCA	TCGTCTCCAAACCACAACAA	60.1	587	INTERGENIC	--	--	--
Ca2_P3_416248	Ca2	416248	TTATATATATATATATATATATATATATAT/TTATATATATATATATATATATAT	ATAAATGAAGGGGTGGGAGG	GTTTCCCATAAACAGCACCC	60.0	882	INTERGENIC	--	--	--
Ca2_P3_42172	Ca2	42172	TTATATATATATATATATATATATA/TTATATATATATATATATATATATATATA	TGGCAGTCTGTTTCACGTTT	CAAGGGACCAGATTCTGCAT	59.3	553	INTERGENIC	--	--	--
Ca2_P3_4266265	Ca2	4266265	A/ATTAATCCGAAAG	TGGACGTTTAACGGTTGACA	CCCAGTAGAAAAGAAGGGGC	60.0	705	INTERGENIC	--	--	--

INDEL marker IDs	Chromosomes/ unanchored scaffolds	Physical positions (bp)	InDels (*Kabuli* reference genome-CDC Frontier/PII)	Forward primers (5'-3')	Reverse primers (5'-3')	Anneling temperature (0C)	Expected amplified product size (bp)	Structural annotation		Functional annotation	
								Sequence components of *kabuli* genome	*Kabuli* gene accession IDs	NCBI-KOG	TFs
Ca2_P3_4275332	Ca2	4275332	GAAAAAAAAA/GAAAAAAAAAAA	TACCCTTTGCTAGCCCCTTT	TCCTAGAACATGCATCACGC	60.1	523	INTERGENIC	--	--	--
Ca2_P3_4285697	Ca2	4285697	TAAAA/TAAA	CCAGTCCCGAAATTAAACGA	ACTTGCGATAATCGATTGGG	59.9	613	INTERGENIC	--	--	--
Ca2_P3_4353642	Ca2	4353642	CAAAAAAAAAA/CAAAAAAAAAAA	AGAATGTTCTCCACAACGCC	ATGGGGTTCCTTTTATTGGC	60.1	500	INTRON	Ca_21108	GC	HSF
Ca2_P3_4370645	Ca2	4370645	CAAAAAAAAAAA/CAAAAAAAAAA	TTGGAACAGAACCATCACCA	TTCGAATTCAAACACGTGACA	59.9	480	DRR	Ca_21107	GC	HSF
Ca2_P3_4412680	Ca2	4412680	CAAAAAAAA/CAAAAAAA	TTGAGGTAGCCCAATCCAAG	TGCACCCACTAACGATCAAA	60.1	539	INTERGENIC	--	--	--
Ca2_P3_4466454	Ca2	4466454	CAAAAAAAAAAAAAA/CAAAAAAAAAAAA	AAAGCAGGGCATCAGAAAAA	CCACTCCATTTTCCACAACC	59.8	827	INTERGENIC	--	--	--
Ca2_P3_4502043	Ca2	4502043	ANNNNNNNNNN/ANNNNNN	GCTTTGGGTGAACAAATGCT	AGGCACCCTTCAACAAAATG	60.1	258	INTERGENIC	--	--	--
Ca2_P3_4513905	Ca2	4513905	GAAAAAAAAAAA/GAAAAAAAAAAAA	ACGCGTTGAAGTGTTGTTTG	GTGGTTGCGGAGATAGTGGT	59.8	944	INTERGENIC	--	--	--
Ca2_P3_453297	Ca2	453297	TTATATATATATATATATAT/TTATATATATATATATATATAT	TTCAAACCAAATGATCCAATGA	ATAAATGTTGGGTGGCCAAA	60.2	685	DRR	Ca_16985	L	--
Ca2_P3_4539194	Ca2	4539194	AATATATATATATATATATATAT/AATATATATATATATATATATAT	TTTGCCTTCCACTTTTGTCC	ACCAACCTGCATACCCTGTC	60.1	890	INTERGENIC	--	--	--

INDEL marker IDs	Chromosomes/ unanchored scaffolds	Physical positions (bp)	InDels (*Kabuli* reference genome-CDC Frontier/PII)	Forward primers (5'-3')	Reverse primers (5'-3')	Anneling temperature (0C)	Expected amplified product size (bp)	Structural annotation		Functional annotation	
								Sequence components of *kabuli* genome	*Kabuli* gene accession IDs	NCBI-KOG	TFs
Ca2_P3_4549506	Ca2	4549506	AATATATATATATATATATATATATATATATATATATATAT/AATATATATATATATATATATATATATATATATATATATATAT	TTTGATGCCCCGATTAAAAA	CATCGCTTCCAAAGTTGGTT	60.3	665	INTERGENIC	--	--	--
Ca2_P3_4595333	Ca2	4595333	CAATAATAATAATAATAATAATAATAATAATAATAATAATA/CAATAATAATAATAATAATAATAATAATAATAATA	GTGGACCAAGCCCATTAAAA	TCACTCCTCAAGTGGAACCC	59.8	124	INTERGENIC	--	--	--
Ca2_P3_4628652	Ca2	4628652	AG/A	TGAGGTGTCTTGAGATGGACC	TCCCCCTCACTCAACTTTTG	60.1	754	INTERGENIC	--	--	--
Ca2_P3_4639205	Ca2	4639205	TAAAAAAAAAAA/TAAAAAAAAAA	AACACTCGTACCTTTATTTCTGGG	ATGCAGCAATTTCCATCCTC	59.8	950	INTERGENIC	--	--	--
Ca2_P3_4728500	Ca2	4728500	CAAAAAAAAAAAAAAA/CAAAAAAAAAAAAA	CTCCGAAAATGGCAGAATTG	TGAATTCAAGTGATAATTGCTCG	60.6	826	INTERGENIC	--	--	--
Ca2_P3_4812881	Ca2	4812881	ATTTTTTT/ATTTTTTTTT	AACCAGTTCCGTTCCAAGTG	TCCCCCAAACAGAATTTGAG	60.0	731	DRR	Ca_21090	--	MYB
Ca2_P3_4832915	Ca2	4832915	ACC/AC	ATACATCACGACGCCACTCA	AGAGAAGGGCACGAGATCAA	60.1	581	INTERGENIC	--	--	--
Ca2_P3_4891048	Ca2	4891048	TTTTATTTATTTATTTATTTATTTATTT/TTTTATTTATTTATTTATTTATTTATTTATTT	AACGGTGTGAAAGGGAAATG	TGAATTGAATTTGGAAGGGG	59.8	515	INTERGENIC	--	--	--
Ca2_P3_4891335	Ca2	4891335	AAATTGAAG/A	AACGGTGTGAAAGGGAAATG	TGAATTGAATTTGGAAGGGG	59.8	515	INTERGENIC	--	--	--
Ca2_P3_4892794	Ca2	4892794	CATATATATATATATATATATATATATATATAT/CATATATATATATATATATATATATATATATATATATATAT	GCGGACATAAGGAAGATGTGA	AGGTCTGCGAAGATGGAAAA	60.1	606	INTERGENIC	--	--	--

INDEL marker IDs	Chromosomes/ unanchored scaffolds	Physical positions (bp)	InDels (*Kabuli* reference genome-CDC Frontier/PII)	Forward primers (5'-3')	Reverse primers (5'-3')	Anneling temperature (0C)	Expected amplified product size (bp)	Structural annotation		Functional annotation	
								Sequence components of *kabuli* genome	*Kabuli* gene accession IDs	NCBI-KOG	TFs
Ca2_P3_4894086	Ca2	4894086	TA/TAGACAACTCAA	TTGAACTACAACCAGCGCAC	TTTTGTTCAATTTCTTGCTCCA	59.9	493	INTERGENIC	--	--	--
Ca2_P3_4894751	Ca2	4894751	CTATATA/CTATATATA	AAAGGGAAGAAATTTCGGGA	AACCGGACGATCATTTGAAG	59.9	498	INTERGENIC	--	--	--
Ca2_P3_4894836	Ca2	4894836	ATGATT/AT	AAAGGGAAGAAATTTCGGGA	AACCGGACGATCATTTGAAG	59.9	498	INTERGENIC	--	--	--
Ca2_P3_4915954	Ca2	4915954	AAATAATAATAATAATAATAATAATAATAATAATAATAATAATAATAA/AAATCATAATAATAATAATAATAATAATAATAATAATAATAATAATAATAATAATAA	TTATCGGACATTTTCCCGAG	CTAGGTCGCCCTTTCAGGTT	59.9	504	INTERGENIC	--	--	--
Ca2_P3_5096452	Ca2	5096452	AATATATATATATATATATATATATAT/AATATATATATATATATATATATATAT	GTTTTGTGGTGCAGTTTGGA	TTTGAAAGAGTCCGAATTCAAATA	59.6	570	INTERGENIC	--	--	--
Ca2_P3_5108884	Ca2	5108884	TAAAAAAAAAAAA/TAAAAAAAAAAA	CGTTCTCCGTTTTCTGTTTTC	TACCGTGTGCGAAATACCAA	58.9	925	INTERGENIC	--	--	--
Ca2_P3_5139260	Ca2	5139260	AATATATATATATATATATATATATATATATATATATAT/AATATATATATATATATATATATATATATATATATATATATAT	GTAATTGACGCGTGTGATGC	GATAAAATGCAGAGGCGGAG	60.2	537	INTERGENIC	--	--	--
Ca2_P3_5559488	Ca2	5559488	TAAAAAAAAAAA/TAAAAAAAAAAAAA	CCCTTTCCAAACCTCACAAA	AAATAAACCGAAGCTGCACG	59.9	474	DRR	Ca_14657	K	--
Ca2_P3_5595728	Ca2	5595728	AATATATATATATATATATATATA/AATATATATATATATATATATATA	TTGCCACTTGATACAGCAAAA	CGCGTATTTATCGTGTGGAA	59.4	652	INTERGENIC	--	--	--
Ca2_P3_5620818	Ca2	5620818	ATTTTTT/ATTTTTTT	TTCGATTTGACATAAAAGACACAAA	TGTTTTAATTGTAAGCATGAGAAGTTG	59.9	526	INTERGENIC	--	--	--

INDEL marker IDs	Chromosomes/ unanchored scaffolds	Physical positions (bp)	InDels (*Kabuli* reference genome-CDC Frontier/PII)	Forward primers (5'-3')	Reverse primers (5'-3')	Anneling temperature (0C)	Expected amplified product size (bp)	Structural annotation		Functional annotation	
								Sequence components of *kabuli* genome	*Kabuli* gene accession IDs	NCBI-KOG	TFs
Ca2_P3_5674299	Ca2	5674299	TCTTTACTTTACTT/TCTTTACTTTACTTTACTT	ATTAGTCCACTGTGCTGGGG	GCTTCCAAATTGCTTGTTCC	60.0	815	INTRON	Ca_14646	O	FAR1
Ca2_P3_5818662	Ca2	5818662	TTCAT/TT	CGGTTGGAAACGAAGAATGT	CTGGGTAGGCACGTTTGATT	60.0	524	INTRON	Ca_19687	--	--
Ca2_P3_5994105	Ca2	5994105	AATATATATATATATA/AATATATATATATATATA	GCGGTGTTGAATTTCGTTCT	ACACTCCACACCCCAACAAT	60.1	233	INTERGENIC	--	--	--
Ca2_P3_6047361	Ca2	6047361	AATTATTATTATTATTATTATTATTATTATTATTAT/AATTATTATTATTATTATTATTATTATTATTATTATTATTATTATTATTAT	ACCTTCCACACAAGCCAAAC	AGGCCTTTATGTGGCCTTTT	60.0	456	INTRON	Ca_19707	K	MYB
Ca2_P3_6056245	Ca2	6056245	TATAAATAAATAAATAAATAAATAAATAAATAAATAAATAAATAAATAAATAAA/TATAAATAAATAAATAAATAAATAAATAAATAAATAAATAAATAAATAAATAAA	ATCCCAGTTTTGGTTGTTGG	TCGTGGTCCAGAAAACATGA	59.7	489	INTRON	Ca_19707	K	MYB
Ca2_P3_6075442	Ca2	6075442	CAAAA/CAAA	CGGAGTCTAATGTGGACGGT	TGCGTATGCACTCTTATCGC	60.0	711	INTRON	Ca_19709	--	--
Ca2_P3_6329097	Ca2	6329097	ATAGCTAG/ATAG	TGCCTAATTCAGATCAAAAGGAA	GTGCGGTAATAGCGGGTTTA	60.1	142	INTERGENIC	--	--	--
Ca2_P3_6338672	Ca2	6338672	TTATATATATATATATATATATATATATATATA/TTATATATATATATATATATATATATATATATATATATA	CTTTATGCGGCGTTCAAAGT	TGCATGGTTGGAAGAAATTG	60.3	505	DRR	Ca_20930	S	--
Ca2_P3_6505178	Ca2	6505178	TA/TAA	CGTAATAACGGCGAGGAAAA	TGAGCAGGGGGAAGAGTAGA	60.1	548	INTERGENIC	--	--	--
Ca2_P3_6513260	Ca2	6513260	AAATAATAATAATAATAATAATAATAATAATAAT/AAATAATAATAATAATAATAATAATAATAAT	ATGGTCCAAGGAAATTGTGC	AGTCGTCGAGGCCAAATCTA	59.8	265	INTERGENIC	--	--	--

INDEL marker IDs	Chromosomes/ unanchored scaffolds	Physical positions (bp)	InDels (*Kabuli* reference genome-CDC Frontier/PII)	Forward primers (5'-3')	Reverse primers (5'-3')	Anneling temperature (0C)	Expected amplified product size (bp)	Structural annotation		Functional annotation	
								Sequence components of *kabuli* genome	*Kabuli* gene accession IDs	NCBI-KOG	TFs
Ca2_P3_6640316	Ca2	6640316	TAAAAAA/TAAAAAAA	ATGGCCACGAATCTGAAGAG	ACGGTTGTTTTGGCATTGAT	60.2	890	INTERGENIC	--	--	--
Ca2_P3_6640457	Ca2	6640457	T/TA	AAAAAGGTCCTAGGGTGGGA	ACGGTTGTTTTGGCATTGAT	59.8	473	INTERGENIC	--	--	--
Ca2_P3_6641737	Ca2	6641737	CATATAT/CATATATATAT	GTATTCAGGGGCGAAGGAAT	AATGCAAACGACAAAGGAGG	60.3	500	INTERGENIC	--	--	--
Ca2_P3_6641916	Ca2	6641916	TG/T	GTTCTATGGGGACAGGGTCA	TATCAACTACCAGAGCGGGG	59.8	476	INTERGENIC	--	--	--
Ca2_P3_6642487	Ca2	6642487	ATTTT/ATTTTTTTTT	CCCCGCTCTGGTAGTTGATA	ACCCACTTTGAGAAACGTGC	60.1	479	INTERGENIC	--	--	--
Ca2_P3_6643843	Ca2	6643843	CTTTTTTTTTTT/CTTTTTTTT	ATGGAACAAGCAGCGAAGTT	GTTGATACGAGGGGGTTGAA	59.9	537	INTERGENIC	--	--	--
Ca2_P3_6648530	Ca2	6648530	TAAAAAA/TAAAAAAAA	TTATCGATCGTCGGTGTCAA	TTCCATATGCACCTCCCATT	60.1	612	INTERGENIC	--	--	--
Ca2_P3_6656104	Ca2	6656104	CG/CGGG	GAGGACAAGGCCAATTTGAA	CCACCCACTTCGAGAAACAT	60.1	955	INTERGENIC	--	--	--
Ca2_P3_6657720	Ca2	6657720	AATG/AATGATG	AAGAATTGTTGTCCCCCTCC	GGAACCAAAACAAAAGGCAA	60.2	635	INTERGENIC	--	--	--
Ca2_P3_6680911	Ca2	6680911	AAATAATAATAAT/AAATAATAATAATAATAAT	GAACGAAAATGTGTTTTATGGC	GGCGTTGCCTACACTCAAAT	58.5	381	INTERGENIC	--	--	--

INDEL marker IDs	Chromosomes/ unanchored scaffolds	Physical positions (bp)	InDels (*Kabuli* reference genome-CDC Frontier/PII)	Forward primers (5'-3')	Reverse primers (5'-3')	Anneling temperature (0C)	Expected amplified product size (bp)	Structural annotation		Functional annotation	
								Sequence components of *kabuli* genome	*Kabuli* gene accession IDs	NCBI-KOG	TFs
Ca2_P3_6769002	Ca2	6769002	ACCCC/ACCC	TGACCCGTCGTGACTACAAA	AGCGGTGATGCTTTCTCAAT	60.2	265	INTERGENIC	--	--	--
Ca2_P3_6811440	Ca2	6811440	ATTTTTT/ATTTTT	TATGTGGGTTTGCTTGGTGA	AGGAAGAGTGGAGTTGCGAA	60.0	283	INTERGENIC	--	--	--
Ca2_P3_6831436	Ca2	6831436	AC/A	CCTGTGACATGTTGCCTTTG	AAATGGGCTGAGTTTGAACA	60.2	360	INTERGENIC	--	--	--
Ca2_P3_7043443	Ca2	7043443	AATATATATATATATATATATATAT/AATATATATATATATATATATATATATAT	ATGGGTCGTGTTCGATTGTT	AACCAACTATTGACCCCAAAAA	60.2	267	INTERGENIC	--	--	--
Ca2_P3_7144237	Ca2	7144237	A/AG	GGACCGGAATCATAATGCAA	TTTTTCCGTAAAAGTCCCCC	60.7	743	INTERGENIC	--	--	--
Ca2_P3_7171869	Ca2	7171869	TTAT/TT	CGGCCATTAAATTTCTGACA	TTGCATTATGTTTCTGGCAAA	58.6	320	INTERGENIC	--	--	--
Ca2_P3_7171912	Ca2	7171912	TATTTAATTTAATTTAATT/TATTTAATTTAATT	CGGCCATTAAATTTCTGACA	TCATTTCATAACGAGTGACCCA	58.6	584	INTERGENIC	--	--	--
Ca2_P3_7172121	Ca2	7172121	CTATATATATATAT/CTATATATATAT	TTTGCCAGAAACATAATGCAA	TCATTTCATAACGAGTGACCCA	59.2	285	INTERGENIC	--	--	--
Ca2_P3_7172172	Ca2	7172172	ATTTTTTTTT/ATTTTTTTTTT	TTTGCCAGAAACATAATGCAA	TTTTGGAAAAATGAAGGACCA	59.2	774	INTERGENIC	--	--	--
Ca2_P3_7206191	Ca2	7206191	CGG/CG	TATTGTACATGGCGACCGAA	TCACGGGTCAACACGATTTA	60.0	165	INTERGENIC	--	--	--

INDEL marker IDs	Chromosomes/ unanchored scaffolds	Physical positions (bp)	InDels (*Kabuli* reference genome-CDC Frontier/PII)	Forward primers (5'-3')	Reverse primers (5'-3')	Anneling temperature (0C)	Expected amplified product size (bp)	Structural annotation		Functional annotation	
								Sequence components of *kabuli* genome	*Kabuli* gene accession IDs	NCBI-KOG	TFs
Ca2_P3_7276294	Ca2	7276294	CAAAA/CAAAAA	TCCACCACAATCGTTAAGCA	AGCGGAGATTTTACCGGATT	60.1	676	INTERGENIC	--	--	--
Ca2_P3_7302005	Ca2	7302005	TTATATATATATATATATATA/TTATATATATATATATATATATA	TGTAGCAAAACGTGCAGACC	GGATATTTTGCTCTCTCGCAA	59.9	159	INTERGENIC	--	--	--
Ca2_P3_7312990	Ca2	7312990	GTATATATATATATATATATATATATAT/GTATATATATATATATATATATATATATATATAT	TATTTTGGATTTTGGCTCGG	CGCGAGTAAATCCGGTAAAA	59.9	682	INTERGENIC	--	--	--
Ca2_P3_7528105	Ca2	7528105	CAAAAAAAA/CAAAAAAA	AGAGGCCAACCTCTGTCTCA	TCGTCAGGAGGTTGATGATG	60.0	313	INTERGENIC	--	--	--
Ca2_P3_753916	Ca2	753916	CTTTTTTTTTT/CTTTTTTTTT	TTGCCCATTAACTTTACCCG	ACTTGTGCATTTGCATTAGTTC	59.8	555	DRR	Ca_17000	--	ARF
Ca2_P3_7546466	Ca2	7546466	TATAAATAAATAAATAAA/TATAAATAAATAAATAAATAAA	TGCTCTTCGCAACACATTTC	TCCCAATTCATTCGCTTTTC	60.0	432	INTRON	Ca_18113	--	--
Ca2_P3_7557453	Ca2	7557453	TTATATATATATATATAT/TTATATATATATATATATAT	CGCACACACGAATATTCACC	ACGACAACATGGTGGAACAC	60.0	465	INTERGENIC	--	--	--
Ca2_P3_7617731	Ca2	7617731	CTAAAATAAAATAAAA/CTAAAATAAAATAAAATAAAATAAAA	CCAAGATCTCGTAGTGCAACA	TGGGTTTAGTCCGTTTTTCTTC	58.9	468	INTERGENIC	--	--	--
Ca2_P3_7657049	Ca2	7657049	ATTTTT/ATTTTTT	TTATGTGGGGGATTTGGATG	TTCCACGTCCTTTTCATTCC	60.4	705	INTERGENIC	--	--	--
Ca2_P3_7657147	Ca2	7657147	CTTTTT/CTTTTTT	TTATGTGGGGGATTTGGATG	TTCCACGTCCTTTTCATTCC	60.4	705	INTERGENIC	--	--	--

INDEL marker IDs	Chromosomes/ unanchored scaffolds	Physical positions (bp)	InDels (*Kabuli* reference genome-CDC Frontier/PII)	Forward primers (5'-3')	Reverse primers (5'-3')	Anneling temperature (0C)	Expected amplified product size (bp)	Structural annotation		Functional annotation	
								Sequence components of *kabuli* genome	*Kabuli* gene accession IDs	NCBI-KOG	TFs
Ca2_P3_7657698	Ca2	7657698	CTTTTTTTT/CTTTTTTT	CCTCTTAGGATGTGCCCAAA	CGTTTGAATTGATGGAAAGTGA	60.1	573	DRR	Ca_18120	--	--
Ca2_P3_7668490	Ca2	7668490	CCTTC/CCTTCTTC	CCAACACAAGCACCAACATC	TGTCCGTCCATCAATGAAAA	60.0	435	INTERGENIC	--	--	--
Ca2_P3_7668820	Ca2	7668820	GTTATTATTATTATTATTAT/GTTATTATTATTAT	CCAACACAAGCACCAACATC	CACTGATGAAGAGGCGAACA	60.0	725	INTERGENIC	--	--	--
Ca2_P3_7669241	Ca2	7669241	TAA/TAAA	TGTTCGCCTCTTCATCAGTG	TGTGTTTTGGGTTCGACTAGG	60.0	475	INTERGENIC	--	--	--
Ca2_P3_7669996	Ca2	7669996	TTTAT/TTTATATTAT	AGTCATTTCCTTCGTGAGCAA	AAGGTTTAAATCTCGATGAATGAGT	59.9	553	INTERGENIC	--	--	--
Ca2_P3_7676116	Ca2	7676116	TTTTTTATTTTTATTTTTAT/TTTTTTATTTTTATTTTTATTTTTAT	TGTACCGTGAATGTAAACTCATTT	TGGCATTTGCACCTTCATTA	57.7	263	INTERGENIC	--	--	--
Ca2_P3_7676173	Ca2	7676173	TAAAAAAAAAA/TAAAAAAAAA	TGTACCGTGAATGTAAACTCATTT	TGGCATTTGCACCTTCATTA	57.7	263	INTERGENIC	--	--	--
Ca2_P3_7684165	Ca2	7684165	CAAAAAAAAAAAA/CAAAAAAAAAAA	TGTGAAGAGCACTGGGAAGA	GGTGCGCCTTTTACAATGTT	59.5	755	DRR	Ca_18121	R	ERF
Ca2_P3_7687260	Ca2	7687260	ATAAGTAA/ATAAGTAAGTAA	GATGGCAACAAGATGTGTGG	TCCCTGAGTATTCTGGTCGG	60.0	582	INTRON	Ca_18122	--	--
Ca2_P3_7695044	Ca2	7695044	CTTTTTTTTTTT/CTTTTTTTTTT	AGGATTGGATGTAGCTGCCA	TTTCCCGACTTGGTCCTTTA	60.6	751	INTERGENIC	--	--	--

INDEL marker IDs	Chromosomes/ unanchored scaffolds	Physical positions (bp)	InDels (*Kabuli* reference genome-CDC Frontier/PII)	Forward primers (5'-3')	Reverse primers (5'-3')	Anneling temperature (0C)	Expected amplified product size (bp)	Structural annotation		Functional annotation	
								Sequence components of *kabuli* genome	*Kabuli* gene accession IDs	NCBI-KOG	TFs
Ca2_P3_7698335	Ca2	7698335	ATTTTTTT/ATTTTTTTT	ACCCTTCGCACTCTTTTTGA	CACTTTCATCCAAATCTCCACA	59.9	610	INTERGENIC	--	--	--
Ca2_P3_7699980	Ca2	7699980	TAAACAAACAA/TAAACAA	AAAATGGAGGCATCATCAGC	GCAAAACCGACTTGTAAGGG	60.0	632	INTRON	Ca_18123	R	ERF
Ca2_P3_7705481	Ca2	7705481	AATTGTGA/AA	ATTCCTGCGGTACAACCATC	GGTGTTTGTGGAAATGGGTC	59.8	897	INTERGENIC	--	--	--
Ca2_P3_7707114	Ca2	7707114	AT/A	ACAGGCAAGAAAGTGGATGA	TTATCGATTAACCCCGCAAC	58.3	604	INTERGENIC	--	--	--
Ca2_P3_7714112	Ca2	7714112	TG/TTGG	CTCACTCGATCGATGGTTCA	TGACAATGCTGGCTGTTAGC	59.8	588	INTERGENIC	--	--	--
Ca2_P3_7717172	Ca2	7717172	ATTGGTTGG/ATTGG	TCATTCTTGTGGGTGTTGGA	TGAGACATGCAATGACGGAT	59.9	410	DRR	Ca_18124	--	--
Ca2_P3_7721082	Ca2	7721082	TAAAAAAAA/TAAAAAAAAA	TTCACCCCTTCATCCTATGG	CATACCATTTGGTGGGTTTG	59.7	671	INTERGENIC	--	--	--
Ca2_P3_7722069	Ca2	7722069	TAAGCGA/TA	TATGCACGCGAATCTTTGAC	AAACGGAAGTTTCTGCAAGC	59.8	784	INTERGENIC	--	--	--
Ca2_P3_7723559	Ca2	7723559	TAAAAAAAAA/TAAAAAAAA	CGTGTCTTATAAAAATTGCGGTT	TTTGCAAGCCAAGCACATAC	59.5	558	INTERGENIC	--	--	--
Ca2_P3_7724864	Ca2	7724864	TA/TAAAGAGATTGAGA	ACCAGAGACACTGCAGCAAA	AACAAGATTCTCGAATGCTCC	59.6	464	INTERGENIC	--	--	--

INDEL marker IDs	Chromosomes/ unanchored scaffolds	Physical positions (bp)	InDels (*Kabuli* reference genome-CDC Frontier/PII)	Forward primers (5'-3')	Reverse primers (5'-3')	Anneling temperature (0C)	Expected amplified product size (bp)	Structural annotation		Functional annotation	
								Sequence components of *kabuli* genome	*Kabuli* gene accession IDs	NCBI-KOG	TFs
Ca2_P3_7725184	Ca2	7725184	ATTTTTTT/ATTTTTTTT	GGTGCGGTTGTTGTGTTTTT	GTGTCACACCCTTCAGGACC	60.8	862	INTERGENIC	--	--	--
Ca2_P3_7729483	Ca2	7729483	TAAA/TAAAA	AAAGGAAGAAGGGGTGCATT	ATGTTCTAAAGCCCATGCCA	59.9	843	INTERGENIC	--	--	--
Ca2_P3_7744794	Ca2	7744794	CATGACATGAATGA/CATGA	CATTTTGATTGGGAATGTGC	CAACAACAGTCCAAAGTAGGCA	58.8	585	INTERGENIC	--	--	--
Ca2_P3_7745257	Ca2	7745257	CAAAA/CAAAAAA	TGGCATGACATGAATGAAGAA	GAGCAATTTGTGTGGACCCT	60.1	760	INTERGENIC	--	--	--
Ca2_P3_7749605	Ca2	7749605	AAAATAAATAAATAAATAAATAAA/AAAATAAATAAATAAATAAATAAATAAATAAA	CAACCCTTATGGATTCCGTC	GGAGAAGATGGTTGCAGCTC	59.2	613	INTERGENIC	--	--	--
Ca2_P3_7749962	Ca2	7749962	TT/TTAT	GGAGCTGCAACCATCTTCTC	TGGATTTGGGCTGAATTAGG	60.0	321	INTERGENIC	--	--	--
Ca2_P3_7750643	Ca2	7750643	CAGCTTAG/CAGCTTAGCTTAG	CACACTCACACACCCTATGGA	TTTGCAAGCTCAAGTGGACA	59.5	398	INTERGENIC	--	--	--
Ca2_P3_7753009	Ca2	7753009	A/AC	AAGTGGCGAAGCTTGTTGAT	AGCTTGCCATTGCAGCTACT	59.9	797	INTRON	Ca_18125	--	MYB_related
Ca2_P3_7753358	Ca2	7753358	C/CAAT	CGGAGGTTTGGTGTAAACTGA	AGCTTGCCATTGCAGCTACT	60.0	447	INTRON	Ca_18125	--	MYB_related
Ca2_P3_7756359	Ca2	7756359	CAAA/CAAAA	AACCAGCATGTGTTGTTGGA	TGTGGTGGGGGAATTAAAAA	60.0	399	INTERGENIC	--	--	--

INDEL marker IDs	Chromosomes/ unanchored scaffolds	Physical positions (bp)	InDels (*Kabuli* reference genome-CDC Frontier/PII)	Forward primers (5'-3')	Reverse primers (5'-3')	Anneling temperature (0C)	Expected amplified product size (bp)	Structural annotation		Functional annotation	
								Sequence components of *kabuli* genome	*Kabuli* gene accession IDs	NCBI-KOG	TFs
Ca2_P3_7760357	Ca2	7760357	ATGTGTGTGTGTGTGT/ATGTGTGTGTGT	AATGCCCACCCATAATTTCA	TGCCATAAAAAGCTAAAAAGCA	60.0	282	INTERGENIC	--	--	--
Ca2_P3_7771633	Ca2	7771633	TTAT/TT	CATCCATGGTACCTTAATTTAATTTTT	TAAATAACACCCGTGGTCCC	59.1	512	INTERGENIC	--	--	--
Ca2_P3_7771725	Ca2	7771725	CAAAAAA/CAAAAAAA	TGTTTTAAAATGTCAACAATGTTATCC	TAAATAACACCCGTGGTCCC	59.5	423	INTERGENIC	--	--	--
Ca2_P3_7772281	Ca2	7772281	TAAAAA/TAAAAAAAAA	TTAAGGGACCACGGGTGTTA	CAATTGCACAAAGGCTGAAA	60.2	328	INTERGENIC	--	--	--
Ca2_P3_7772344	Ca2	7772344	CAAAAAAA/CAAAAAAAA	TTAAGGGACCACGGGTGTTA	CAATTGCACAAAGGCTGAAA	60.2	328	INTERGENIC	--	--	--
Ca2_P3_7772575	Ca2	7772575	AGG/AGGG	TTAAGGGACCACGGGTGTTA	TCCCACCTCTCAATTTTTCAA	60.2	898	INTERGENIC	--	--	--
Ca2_P3_7773617	Ca2	7773617	TTAAATAAAAT/TTAAATAAAATAAATAAAAT	GCTGAGCCCAAACAAGCTAA	CCACTGTGGAAAGGTAAATGC	60.5	845	INTERGENIC	--	--	--
Ca2_P3_7773852	Ca2	7773852	ATTAAATATTTTAAATATT/ATTAAATATT	CGTCAAAAACTTCAATGAATATGG	GGATGGATGTATGTGATGGGA	59.8	410	INTERGENIC	--	--	--
Ca2_P3_7773914	Ca2	7773914	TAAA/TAAAA	CGTCAAAAACTTCAATGAATATGG	GGATGGATGTATGTGATGGGA	59.8	410	INTERGENIC	--	--	--
Ca2_P3_7780730	Ca2	7780730	ATTTTTTTTTTT/ATTTTTTTTTT	TTGTCTCCATCTCCAGCCTT	TGTTGTTTGAGTTGGGACACA	59.8	995	INTERGENIC	--	--	--

INDEL marker IDs	Chromosomes/ unanchored scaffolds	Physical positions (bp)	InDels (*Kabuli* reference genome-CDC Frontier/PII)	Forward primers (5'-3')	Reverse primers (5'-3')	Anneling temperature (0C)	Expected amplified product size (bp)	Structural annotation		Functional annotation	
								Sequence components of *kabuli* genome	*Kabuli* gene accession IDs	NCBI-KOG	TFs
Ca2_P3_7789494	Ca2	7789494	TAAAAAA/TAAAAAAA	ACATGAACATGTTAAATTTTCTGAAC	ACCAATTGGCCAACTCTCAC	57.7	580	INTERGENIC	--	--	--
Ca2_P3_7793328	Ca2	7793328	GAAAAA/GAAAA	ATATCCCCTCCAACGAGGAA	TTTATTGCGGTTTCCCTTCA	60.7	877	INTERGENIC	--	--	--
Ca2_P3_7797097	Ca2	7797097	TAAAAAAAA/TAAAAAAA	GGGGACTCCAAGACTTCACA	GCCCGTACTCAGTTCTTGCT	60.1	699	INTERGENIC	--	--	--
Ca2_P3_7798797	Ca2	7798797	TTTTCTTT/TTTT	GGAACAACGGATTCTGTCCA	TGGTCTCGGTCACTATTTAAGTTTT	60.9	603	INTERGENIC	--	--	--
Ca2_P3_7801882	Ca2	7801882	CATATAT/CATAT	TTCGGAGTTGCAGATGATTG	TCATTTCTTCACCTTGAACCA	59.8	203	DRR	Ca_18127	--	ERF
Ca2_P3_7811963	Ca2	7811963	CAAAAAAA/CAAAAAAAA	ACTAACTCGGTGGGGAACAA	TCCCTTGTTCTTGCTGTGAA	59.4	330	INTERGENIC	--	--	--
Ca2_P3_7813181	Ca2	7813181	GAAAAAA/GAAAAAAA	TAGAAGGTCGCTTCTTCGGA	CACCTCACCTACACGTCCCT	60.1	771	INTERGENIC	--	--	--
Ca2_P3_7818010	Ca2	7818010	GATA/GATATTCTAATA	TGGACATAGAAAAACCAAATGGA	ATGTGGAAGTTGAACCCCAA	60.6	598	DRR	Ca_18128	QR	B3
Ca2_P3_7818292	Ca2	7818292	TGGGG/TGGG	TTGGGGTTCAACTTCCACAT	TGTGAATGGGGATGATTTTG	60.2	426	DRR	Ca_18128	QR	B3
Ca2_P3_7823490	Ca2	7823490	ATTTTTTTTTTTT/ATTTTTTTTTT	TCAACCGTTCAATTCACCAA	TGCTTTTGAAAACGACACCA	59.9	734	INTERGENIC	--	--	--

INDEL marker IDs	Chromosomes/ unanchored scaffolds	Physical positions (bp)	InDels (*Kabuli* reference genome-CDC Frontier/PII)	Forward primers (5'-3')	Reverse primers (5'-3')	Anneling temperature (0C)	Expected amplified product size (bp)	Structural annotation		Functional annotation	
								Sequence components of *kabuli* genome	*Kabuli* gene accession IDs	NCBI-KOG	TFs
Ca2_P3_7824127	Ca2	7824127	A/AGGTTATT	GAAAGTTTTGCCACCAATTCA	GCATACCCATACCACCAAAGA	60.0	960	INTERGENIC	--	--	--
Ca2_P3_7844408	Ca2	7844408	CAAAAAAAAAA/CAAAAAAAAA	GTCCACTGTGCCACATCATC	AACTGCTTTGAAGCACCGTT	60.0	611	INTERGENIC	--	--	--
Ca2_P3_7844534	Ca2	7844534	CTTTTTTTT/CTTTTTTT	AACGGTGCTTCAAAGCAGTT	TGCCACATCACCCTAAATCA	59.9	451	INTERGENIC	--	--	--
Ca2_P3_7875411	Ca2	7875411	CTTTTTTT/CTTTTTT	TGTTGCGAAAAGTGGAAAAA	TCGAAGCTTGTTTCGGAAGT	59.3	805	INTERGENIC	--	--	--
Ca2_P3_7893121	Ca2	7893121	GCC/GC	AAACAAATTCGCGGTGCTAC	CGAACGGTGTCATGATTTGA	60.1	525	INTERGENIC	--	--	--
Ca2_P3_7893479	Ca2	7893479	AGCAAAA/AGCAAAAATGCAAAA	AAACAAATTCGCGGTGCTAC	TGATGAAACCACCCAGTTGA	60.1	690	INTERGENIC	--	--	--
Ca2_P3_7894741	Ca2	7894741	TA/TAA	GTCATGGTTGTAGCATCCCC	GATGGGGATGGAAGATGAAA	60.2	639	INTERGENIC	--	--	--
Ca2_P3_7913902	Ca2	7913902	GAAAAAAAAAA/GAAAAAAAAA	TCAAATCTACCTCTAACGATTCACA	AAGGACAGTTGTCCCAATGC	59.2	326	DRR	Ca_18139	FE	--
Ca2_P3_7914260	Ca2	7914260	TG/T	CAAGAGCACCAGCCACAATA	TGTACCCCAAGACACCCATT	59.9	298	INTERGENIC	--	--	--
Ca2_P3_7917938	Ca2	7917938	TTTGAAATTGAAATTGAAATT/TTTGAAATTGAAATTGAAATTGAAATT	GTCGGAGATCCCACAAAAGA	TGGAACCAAATCTTCGTTGA	60.1	884	INTERGENIC	--	--	--

INDEL marker IDs	Chromosomes/ unanchored scaffolds	Physical positions (bp)	InDels (*Kabuli* reference genome-CDC Frontier/PII)	Forward primers (5'-3')	Reverse primers (5'-3')	Anneling temperature (0C)	Expected amplified product size (bp)	Structural annotation		Functional annotation	
								Sequence components of *kabuli* genome	*Kabuli* gene accession IDs	NCBI-KOG	TFs
Ca2_P3_7926426	Ca2	7926426	CTTT/CTT	CTTGGCGAAGTGAAGGGATA	GAGGCTTGGTTCCCTAATCC	60.2	471	INTRON	Ca_18142	U	--
Ca2_P3_7929173	Ca2	7929173	CTGT/CTGTGT	GTGGGTATCAGGCTCTTCCA	CCAAGCCCTCATTCACTTGT	60.1	144	DRR	Ca_18143	--	--
Ca2_P3_7929427	Ca2	7929427	CATTT/CATTTTTATTTTGATCCAATTT	GTGGGTATCAGGCTCTTCCA	GCATTGGTTTCAGCATTGTG	60.1	782	DRR	Ca_18142	U	--
Ca2_P3_7929592	Ca2	7929592	AT/A	ACAAGTGAATGAGGGCTTGG	CTTGAATATTGTCGCCGGTT	60.1	778	DRR	Ca_18142	U	--
Ca2_P3_7933051	Ca2	7933051	AC/A	CCCCAAAAAGTGCTTCAAAA	TGACAAGCACGCTTAAGGAA	60.1	694	INTERGENIC	--	--	--
Ca2_P3_7933550	Ca2	7933550	TAAAAAAAA/TAAAAAAAAA	TTCCTTAAGCGTGCTTGTCA	CCAAAATAACCCACAATCGG	59.6	539	INTERGENIC	--	--	--
Ca2_P3_7938788	Ca2	7938788	AA/AACA	TGATCAAATAATTGAAACAAGCG	GCCGTATTGGGAGAAGATGA	59.2	589	INTRON	Ca_18144	--	--
Ca2_P3_7940111	Ca2	7940111	ATTTTTTT/ATTTTTT	TCTTGACCGTTAGATTGAACCA	CAAAATGGACAAAATTGGAGC	59.6	714	INTERGENIC	--	--	--
Ca2_P3_7940136	Ca2	7940136	ATTTTTTT/ATTTTTTTT	TCTTGACCGTTAGATTGAACCA	CAAAATGGACAAAATTGGAGC	59.6	714	INTERGENIC	--	--	--
Ca2_P3_7941058	Ca2	7941058	TAAAAAAAA/TAAAAAAA	CAGTGTCATGGTTGAGGCAC	GAAGTTGGAACGACGGTGAT	60.2	437	INTERGENIC	--	--	--

INDEL marker IDs	Chromosomes/ unanchored scaffolds	Physical positions (bp)	InDels (*Kabuli* reference genome-CDC Frontier/PII)	Forward primers (5'-3')	Reverse primers (5'-3')	Anneling temperature (0C)	Expected amplified product size (bp)	Structural annotation		Functional annotation	
								Sequence components of *kabuli* genome	*Kabuli* gene accession IDs	NCBI-KOG	TFs
Ca2_P3_7941503	Ca2	7941503	GTTTTT/GTTTT	ATCACCGTCGTTCCAACTTC	TGGGAATATGAGTTATTGGCA	60.0	402	INTERGENIC	--	--	--
Ca2_P3_7982128	Ca2	7982128	CTTTTTTTTTTT/CTTTTTTTTT	GCCCGAATCAAGTAGTGGAA	ATCAATGGAGGATAGCGACG	60.1	644	INTERGENIC	--	--	--
Ca2_P3_8016754	Ca2	8016754	ATTTTTTTTTTT/ATTTTTTTTTTTT	TTTCCCCTGTACCTCCACAG	CCCCCTTTGTCACAACCTAA	60.0	671	INTERGENIC	--	--	--
Ca2_P3_8063038	Ca2	8063038	TTATATATATATATATATATATATATAT/TTATATATATATATATATATATATATATAT	ATAAAACAAAAACGGGTGCG	AAATGGCCTCCCAATTCTCT	59.9	602	INTERGENIC	--	--	--
Ca2_P3_8159118	Ca2	8159118	TTATATATATATATATATATATA/TTATATATATATATATATATATA	TAGCCCTTTAATGCATTGGG	CATGATTTGTGTGCGGTTCT	59.9	513	INTERGENIC	--	--	--
Ca2_P3_8159863	Ca2	8159863	AATATATATATATATATATATATATATAT/AATATATATATATATATATATATATATATATATATATATATAT	TATGCCGTGAAACGAGAGTG	ATTATGCTGATTTGCTCGCC	59.9	725	INTERGENIC	--	--	--
Ca2_P3_8174591	Ca2	8174591	TTTATTATTATTATTATTATTATTATTATTATTATTAT/TTTATTATTATTATTATTATTATTATTATTATTATTATTATTATTAT	CAAATGGGGGTATTTTGGTG	TATTTGGTTGTCCATGTGGC	59.9	963	DRR	Ca_18154	U	WOX
Ca2_P3_8308316	Ca2	8308316	TAAAAAAAA/TAAAAAAA	AATTGGGACCATTTGATAGGC	GCCTCTACCATCTTTGACAGC	60.0	171	INTERGENIC	--	--	--
Ca2_P3_8328118	Ca2	8328118	TATTTATTTATGCATTT/TATTTATTTATGCATTTATTTATGCATTT	AGGTTCAATTTGGTGGCAAG	TTGAATTGTTATGCCCGTGA	60.0	570	INTRON	Ca_21469	--	C2H2
Ca2_P3_8343398	Ca2	8343398	AATATATATATATAT/AATATATATATATATATAT	CCAGTGTTTGACCTCCAAGC	TTTGCAACAACGCGAAATAG	60.7	743	INTERGENIC	--	--	--

INDEL marker IDs	Chromosomes/ unanchored scaffolds	Physical positions (bp)	InDels (*Kabuli* reference genome-CDC Frontier/PII)	Forward primers (5'-3')	Reverse primers (5'-3')	Anneling temperature (0C)	Expected amplified product size (bp)	Structural annotation		Functional annotation	
								Sequence components of *kabuli* genome	*Kabuli* gene accession IDs	NCBI-KOG	TFs
Ca2_P3_8374636	Ca2	8374636	AATATATATATATATATATATATAT/AATATATATATATATATATAT	AAAGGGACAATGAAGGCAAG	TTGAAAATGTGATGTTTCTTCCA	59.2	636	INTERGENIC	--	--	--
Ca2_P3_8375527	Ca2	8375527	TCAAATCCAAATCCAAAT/TCAAATCCAAAT	GGGCCGTGAGAGTGTTATGT	GCTGCAAAGCCTACCAAGAC	60.0	573	DRR	Ca_21461	T	bHLH
Ca2_P3_8401872	Ca2	8401872	CTTTT/CTTTTT	AAGATCCCCTTTCCATGTCA	GAACTTCCACCAGACCATCC	59.3	470	DRR	Ca_21459	--	WRKY
Ca2_P3_8420270	Ca2	8420270	AACA/AACACA	CCAAGTTTGAAATGCCGATT	GCTGCATGATCTTATGCTCG	59.9	451	INTERGENIC	--	--	--
Ca2_P3_8478800	Ca2	8478800	AATTT/A	CCCGTATGAATGAACGGAAC	CTACCCCCACATTTCAATCG	60.2	519	INTERGENIC	--	--	--
Ca2_P3_8478910	Ca2	8478910	ACT/A	CCCGTATGAATGAACGGAAC	CTACCCCCACATTTCAATCG	60.2	519	INTERGENIC	--	--	--
Ca2_P3_8688633	Ca2	8688633	GAAAAAAAAAAAA/GAAAAAAAAAAA	ATCATAGCAGGCCCAATGTC	ATTTCCTAAACCCAACCCCA	59.9	665	DRR	Ca_22311	--	NF-X1
Ca2_P3_8718137	Ca2	8718137	TTTCATCA/T	GGGACTTGCACTTTGTGCTT	GGGAGCCTTATACCTCTGGG	60.3	590	INTRON	Ca_22308	U	--
Ca2_P3_8839936	Ca2	8839936	TAATAGGAAATAGGAAAT/TAATAGGAAATAGGAAATAGGAAAT	ATGGGCCCCTCATTTATACC	CGTGGATTGAACCACATCAC	59.9	257	INTRON	Ca_22301	--	GRAS
Ca2_P3_88585	Ca2	88585	TG/T	TGAATTTGACGAAATTTGCATT	GGCTATGAGTGCTCCATTTGA	59.5	752	INTERGENIC	--	--	--

INDEL marker IDs	Chromosomes/ unanchored scaffolds	Physical positions (bp)	InDels (*Kabuli* reference genome-CDC Frontier/PII)	Forward primers (5'-3')	Reverse primers (5'-3')	Anneling temperature (0C)	Expected amplified product size (bp)	Structural annotation		Functional annotation	
								Sequence components of *kabuli* genome	*Kabuli* gene accession IDs	NCBI-KOG	TFs
Ca2_P3_903067	Ca2	903067	TTATATTAAAATATATTAAAATATA/TTATATTAAAATATA	GCACATCACTTATGATTGCGAG	TAGGAGGATAGACACCGCCA	60.7	516	INTERGENIC	--	--	--
Ca2_P3_9379100	Ca2	9379100	ATTTTTTTTTTT/ATTTTTTTTTT	GTGCAACCACTTGAAGCAAA	GCCCTCAGATGAGAGACAGG	59.9	620	DRR	Ca_20030	F	--
Ca2_P3_9428131	Ca2	9428131	GTATATATATATATAT/GTATATATATATAT	GAAAATGCTAATGGTTGCGG	TTCCAAAACACTTACCCCCA	60.5	657	INTERGENIC	--	--	--
Ca2_P3_9494071	Ca2	9494071	T/TA	GAACTGCAACAACAACGACAA	GGTCTCGAAATCCCTGATGA	59.8	609	INTERGENIC	--	--	--
Ca2_P3_9500229	Ca2	9500229	CGTATGCTAGACCTGG/CGTATGCTAGACCTGGGTATGCTAGACCTGG	AGAGGTTGGGGGAGAAGAAA	GCAACAACAGCAGAGACAATG	60.0	213	INTERGENIC	--	--	--
Ca2_P3_9689510	Ca2	9689510	TTATATATATATATATATATATATA/TTATATATATATATATATATATATATA	GTGAGTTGAGCATCGCAAAA	GATTTGCGGTAGATATTTGCATC	60.0	928	INTERGENIC	--	--	--
Ca2_P3_9690407	Ca2	9690407	TAAAAAAAAAAAAA/TAAAAAAAAAAAA	TGATGCAAATATCTACCGCAA	TTCAAGGGACGTAAATGCAA	59.2	558	INTERGENIC	--	--	--
Ca2_P3_9694074	Ca2	9694074	ATTTTTTTTTTT/ATTTTTTTTTTTT	TGAGTTGTGGTGAAGTGGGA	GCACTCCTTTACAACGCACA	60.1	256	INTERGENIC	--	--	--
Ca2_P3_9694226	Ca2	9694226	TTATATATATATATATATATATA/TTATATATATATATATATATA	TGTGCGTTGTAAAGGAGTGC	CGTCGGAATCCAAAAGTCAT	59.9	504	INTERGENIC	--	--	--
Ca2_P3_976095	Ca2	976095	ATTTTTTTT/ATTTTTTTTT	TTTGATGAGCAAACGAAAAA	TGATGATTGGAAGGGATGAA	57.0	575	INTERGENIC	--	--	--

INDEL marker IDs	Chromosomes/ unanchored scaffolds	Physical positions (bp)	InDels (*Kabuli* reference genome-CDC Frontier/PII)	Forward primers (5'-3')	Reverse primers (5'-3')	Anneling temperature (0C)	Expected amplified product size (bp)	Structural annotation		Functional annotation	
								Sequence components of *kabuli* genome	*Kabuli* gene accession IDs	NCBI-KOG	TFs
Ca2_P3_981417	Ca2	981417	ACTCTCTCTCTCTCTCTCTCTCTCTCTCTCTCT/ACTCTCTCTCTCTCTCTCTCTCTCTCTCTCTCTCTCTCTCTCTCTCTCT	ACATAATTCCCAACCGACCA	CCAAAGGGGTGAGTTTTTGA	60.1	328	INTERGENIC	--	--	--
Ca2_P3_9970688	Ca2	9970688	AT/ATTTTTCT	TTGATAGTTGATCGAATGTTTGATATT	TCCCCACAAACTTATGAGCC	59.3	888	INTERGENIC	--	--	--
Ca3_P3_10159468	Ca3	10159468	T/TA	TGGGGCAAACAAAGCTATTC	CTAAGGGCGCTACAACAAGC	60.1	107	INTERGENIC	--	--	--
Ca3_P3_10159672	Ca3	10159672	AGGGGGGG/AGGGGGGGG	CACACCAACCCTTTCGTTCT	TGCTCCCACACACACAAACT	60.0	277	INTERGENIC	--	--	--
Ca3_P3_10350276	Ca3	10350276	CAAGAAG/CAAGAAGAAG	ATTGGCAAATCGGTTCAGTC	GCCAAACCCTTACGAGATCA	59.9	459	DRR	Ca_18818	--	--
Ca3_P3_1038019	Ca3	1038019	AAT/AATAATCAT	GGAGCAAAATGTGTTTTATGGA	GGAAACGGTCTTCCCTTTTT	59.0	173	INTERGENIC	--	--	--
Ca3_P3_10398321	Ca3	10398321	G/GATTGATCTTTCCC	CGGATTGCCAAGTACAAGGT	CAAACCACAACCAGCACAAC	60.0	770	DRR	Ca_18819	J	--
Ca3_P3_10581730	Ca3	10581730	AATATATATATATATATATAT/AATATATATATATATATAT	TCGAATATTCAAGGTTTGTTGA	TTCCCCTTGTGTGGCTAATC	57.3	253	INTERGENIC	--	--	--
Ca3_P3_10581834	Ca3	10581834	GTTTTTTTTTTTT/GTTTTTTTTTTT	GATTAGCCACACAAGGGGAA	GCAAAATTTTCAAACGCTCC	59.9	162	INTERGENIC	--	--	--
Ca3_P3_10846962	Ca3	10846962	AATATATATATATATATATATATATATATA/AATATATATATATATATATATATATATATATATA	CCAAATGACGACTTCCCACT	CAAGACGTCGTCAAAATCCC	60.0	683	INTERGENIC	--	--	--

INDEL marker IDs	Chromosomes/ unanchored scaffolds	Physical positions (bp)	InDels (*Kabuli* reference genome-CDC Frontier/PII)	Forward primers (5'-3')	Reverse primers (5'-3')	Anneling temperature (0C)	Expected amplified product size (bp)	Structural annotation		Functional annotation	
								Sequence components of *kabuli* genome	*Kabuli* gene accession IDs	NCBI-KOG	TFs
Ca3_P3_10911530	Ca3	10911530	TTT/TTTATT	CCTGACATGGCCTGACCTAT	AAGCCTGGCCTACGGTCTAT	60.0	528	INTERGENIC	--	--	--
Ca3_P3_10923237	Ca3	10923237	T/TCC	GCTGATTGAAATCGAGCACA	TTAAAACAAGCACCAAGGGC	60.0	347	INTERGENIC	--	--	--
Ca3_P3_10937275	Ca3	10937275	TATAATAATAATAATAATAATAATAATAATAATAATAATAATAATAATAATAAT/TATAATAATAATAATAATAATAATAATAATAATAATAATAAT	CCAATGACCTTGAAATCCAAA	ACCGAATGCATCACGTACAA	59.8	561	INTERGENIC	--	--	--
Ca3_P3_1103988	Ca3	1103988	CATATATATATATATATATATATAT/CATATATATATATATATATATATAT	ATCAGTTGCCCTTCCTTCCT	GCGTTACGGGTAAAAAGGTG	60.1	229	INTERGENIC	--	--	--
Ca3_P3_11182328	Ca3	11182328	GAAAAAAAAAA/GAAAAAAAAAAA	AGGTGCCCTTCTTTTTGGTT	CCATCGTCGACCTTGAGAAT	60.0	527	INTERGENIC	--	--	--
Ca3_P3_11279419	Ca3	11279419	CTATTATTATTATTATTATTA/CTATTATTATTATTATTATTATTA	TTCCAGGGCAGACTTCTTGT	GGCCCATTATGACCAAAACTT	59.8	641	INTERGENIC	--	--	--
Ca3_P3_11306806	Ca3	11306806	TTATATATATATATATATATATATATATATATAT/TTATATATATATATATATATATATATATAT	CGTGTGAGAAAACCTCGACA	CCGATTTCCCAAAGGTTTTT	59.9	275	INTERGENIC	--	--	--
Ca3_P3_11326575	Ca3	11326575	G/GTGC	TCCATGGTCTGCCATCAATA	ACTTGGCCACCTGATTTCAC	59.9	496	INTERGENIC	--	--	--
Ca3_P3_11462251	Ca3	11462251	ATTTTTTTTT/ATTTTTTTTTT	ATGATTCCCCAACATCCAAA	CACGAGTCCGATAAGGGTGT	60.0	149	INTERGENIC	--	--	--
Ca3_P3_1149320	Ca3	1149320	ATTTTTTTTT/ATTTTTTTTTT	TGTGCAATCGTTTTAACCCA	CCCTTACCACAAAATCAACTCA	60.0	719	INTERGENIC	--	--	--

INDEL marker IDs	Chromosomes/ unanchored scaffolds	Physical positions (bp)	InDels (*Kabuli* reference genome-CDC Frontier/PII)	Forward primers (5'-3')	Reverse primers (5'-3')	Anneling temperature (0C)	Expected amplified product size (bp)	Structural annotation		Functional annotation	
								Sequence components of *kabuli* genome	*Kabuli* gene accession IDs	NCBI-KOG	TFs
Ca3_P3_11497478	Ca3	11497478	TTTATTATT/TTTATT	GGGTTTGCACTTGAAG GTGT	CCTCCGAGCCAAAATA CAAA	60.0	617	INTERGENIC	--	--	--
Ca3_P3_11519916	Ca3	11519916	A/ACAGACTTATACTGTTT	CCGAGCCAAAATCCAA AATA	AATTTCAAACGCAAAC CAGG	59.9	198	INTERGENIC	--	--	--
Ca3_P3_11531328	Ca3	11531328	CAAAAAAAA/CAAAAAAAAA	TTTCCTTTCAACCCAGC ATC	CGATGCAGTCGTCTCT TTGA	60.1	482	INTERGENIC	--	--	--
Ca3_P3_11537812	Ca3	11537812	CTTTTTTT/CTTTTTTTT	GATGCAACATCTTTTTA ATCTTTGA	CCTACGTATCTCCTGG TGCG	58.8	688	INTERGENIC	--	--	--
Ca3_P3_11720789	Ca3	11720789	ATTATTTATTTATTTATT/ATTATTTATTT ATTTATTTATTTATT	CCTTCTCGTGTTTGCAC CTT	TGCCATGTTTTAATGG CTGT	60.3	620	INTERGENIC	--	--	--
Ca3_P3_11753481	Ca3	11753481	T/TTA	TTGTTCAAAAATTCAGC AAAAA	GTTGGTGGGTATAAGG GGCT	57.6	517	INTERGENIC	--	--	--
Ca3_P3_11765428	Ca3	11765428	ATTT/ATTTT	GATCAGATCTGGCAGC AACA	TGGCATACATCTAGGG GGAG	60.0	574	DRR	Ca_19390	--	--
Ca3_P3_11902496	Ca3	11902496	TTATATATATATATATATATATAT/TTAT ATATATATATATATATATATATATAT	CCAACGCATTTAACAAC ACG	TTTACAGCTTACTCAG GCGGA	60.0	740	INTERGENIC	--	--	--
Ca3_P3_11919676	Ca3	11919676	CNNNNNNNNNNNNNNNNNNNN/CNNN NNNNNN	CGGAAATTGAAATCGG AAAA	ATCGCGAAAGTAGTCT CCCA	59.9	633	INTERGENIC	--	--	--
Ca3_P3_1199360	Ca3	1199360	TAAATCTTGATCAAATCTT/TAAATCTT GATCAAATCTTGATCAAATCTT	CGATGCTTTTCTCCTTC AGC	TGTCCCTCAAATTCTT GGTTG	60.1	654	INTERGENIC	--	--	--

INDEL marker IDs	Chromosomes/ unanchored scaffolds	Physical positions (bp)	InDels (*Kabuli* reference genome-CDC Frontier/PII)	Forward primers (5'-3')	Reverse primers (5'-3')	Anneling temperature (0C)	Expected amplified product size (bp)	Structural annotation		Functional annotation	
								Sequence components of *kabuli* genome	*Kabuli* gene accession IDs	NCBI-KOG	TFs
Ca3_P3_11996299	Ca3	11996299	GAAAAAAAAAA/GAAAAAAAAAAA	TGACAATTTCGTCTCCGTACC	TGCCATTGAAGAGTTCTGGA	60.0	351	INTERGENIC	--	--	--
Ca3_P3_1206042	Ca3	1206042	AT/ATATTCT	GGCCGCCACAAAATATTAAC	TTTGAATTGGGGAGAGATGG	59.3	683	INTERGENIC	--	--	--
Ca3_P3_12231461	Ca3	12231461	ACAAACAAGCAAACAAGCAAA/ACAAACAAGCAAA	TCACGGTTGTGAAGTCTGGA	CCTCAAAAGATGACGCGATT	60.3	398	INTERGENIC	--	--	--
Ca3_P3_12263072	Ca3	12263072	A/AGAATCACAT	CAAAGAAGGGGGTTTGAATTG	TCCACTTTGGGTTAACCAGG	60.7	230	INTERGENIC	--	--	--
Ca3_P3_12390760	Ca3	12390760	AATATATATATATATATATATATATATAT/AATATATATATATATATATATATAT	TTGGGTTTGCATAATGTCCA	CTAGGTGTCGGTCCGAGCTA	59.8	885	INTERGENIC	--	--	--
Ca3_P3_12597173	Ca3	12597173	GATATATATATATATATATATATATAT/GATATATATATATATATATATATATATATATAT	TATGCGGTGAAATGATGGAA	TGGCAAACCAAAGACACTGA	59.9	338	DRR	Ca_21021	--	Dof
Ca3_P3_12643711	Ca3	12643711	TTATATATATATATATAT/TTATATATATATATATATATAT	GACGCGTAGTTTCGAGGATG	GAAGAAAGAAGTTCCGCGTG	60.8	501	INTERGENIC	--	--	--
Ca3_P3_1267466	Ca3	1267466	ATGTT/ATGTTGTT	CAAGTCACATCGCAAACACC	AGGGGACTACTTTCGCGATT	60.2	599	DRR	Ca_19422	J	--
Ca3_P3_12715466	Ca3	12715466	T/TACACATTAAAG	TATTCCCGATTAATGCAGCC	GTCCCGAATGCAATGTCTCT	59.9	667	INTERGENIC	--	--	--
Ca3_P3_12819743	Ca3	12819743	GAAAAAAAAA/GAAAAAAAAAA	TTGGAAGAAGATTGGACAAAGA	CTCCGCATCAATTTATCCCT	58.8	509	INTERGENIC	--	--	--

INDEL marker IDs	Chromosomes/ unanchored scaffolds	Physical positions (bp)	InDels (*Kabuli* reference genome-CDC Frontier/PII)	Forward primers (5'-3')	Reverse primers (5'-3')	Anneling temperature (0C)	Expected amplified product size (bp)	Structural annotation		Functional annotation	
								Sequence components of *kabuli* genome	*Kabuli* gene accession IDs	NCBI-KOG	TFs
Ca3_P3_12922330	Ca3	12922330	TCC/TC	TTACCAAATCGATGCTCGTG	ATTTGGAAATCGACGTGCTC	59.7	356	INTERGENIC	--	--	--
Ca3_P3_12969818	Ca3	12969818	TAAAAAAA/TAAAAAAAA	TTACACCAATTCCCCCAAGA	CATGCAAGGTTGGGCTAGAT	60.2	400	INTERGENIC	--	--	--
Ca3_P3_12997438	Ca3	12997438	GTATATATATATATATATATATATATATATAT/GTATATATATATATATATATATATAT	GAAGGAAGGAGGGAGAATGG	TGCGCTTTTATTCCAACACA	60.0	648	INTERGENIC	--	--	--
Ca3_P3_13062124	Ca3	13062124	TAAAAAAAAAAAAA/TAAAAAAAAAAAA	TTGACGAGGCTTGGAAAAAT	GCCTCTCACTTGCCTCTCAC	59.7	619	INTERGENIC	--	--	--
Ca3_P3_13072537	Ca3	13072537	CATAG/CTGTGATAGATAG	TCAGATCGTTCCCTGTTTCC	TGAAGCATATTCTGAGGGGG	60.1	376	INTERGENIC	--	--	--
Ca3_P3_13096897	Ca3	13096897	TCT/TCTGGAACT	TCTGGTCGACTTCTGCCTTT	TCATCAGTATCGTGGGTGGA	60.0	775	INTERGENIC	--	--	--
Ca3_P3_13097015	Ca3	13097015	ATTTTTTT/ATTTTTT	CCTTCTTTACGGGACCCAAT	TCATCAGTATCGTGGGTGGA	60.2	364	INTERGENIC	--	--	--
Ca3_P3_13097036	Ca3	13097036	A/AT	CCTTCTTTACGGGACCCAAT	TCATCAGTATCGTGGGTGGA	60.2	364	INTERGENIC	--	--	--
Ca3_P3_13105438	Ca3	13105438	CAC/CACTCTTTCACCCCTTAC	ATCCAACATCCCGATAACGA	AAAGGCTATTTGCTCGTGGA	60.2	288	INTERGENIC	--	--	--
Ca3_P3_13163471	Ca3	13163471	AT/ATATTT	TGCAAAGTGAGGATTGACCA	TTTTGGGTCTTGGATTTTGG	60.2	420	INTERGENIC	--	--	--

INDEL marker IDs	Chromosomes/ unanchored scaffolds	Physical positions (bp)	InDels (*Kabuli* reference genome-CDC Frontier/PII)	Forward primers (5'-3')	Reverse primers (5'-3')	Anneling temperature (0C)	Expected amplified product size (bp)	Structural annotation		Functional annotation	
								Sequence components of *kabuli* genome	*Kabuli* gene accession IDs	NCBI-KOG	TFs
Ca3_P3_13169603	Ca3	13169603	ATT/ATTTTTTTT	TAGCTGGCAGGGTCTCAGTT	CGAAAGAAAGTTTCCCCTCC	60.0	419	INTERGENIC	--	--	--
Ca3_P3_13171985	Ca3	13171985	GATATATATATATATATATATATA/GATATATATATATATATATATATATA	TCCCAACTACTGGGAATCCA	TAGGGGAATGACTCCACCAC	60.3	414	INTERGENIC	--	--	--
Ca3_P3_13185454	Ca3	13185454	AT/ATTT	GCACACGACCTTCTCGTACA	AATCTGGATCGGGGAAAATC	59.9	302	CDS (FRAME SHIFT)	Ca_22687	--	Trihelix
Ca3_P3_1320883	Ca3	1320883	CAAAAAAAAA/CAAAAAAAA	CATGAATTGATTGGATTGCG	AAAAATGAAAGGTTGGCACG	59.9	750	INTERGENIC	--	--	--
Ca3_P3_13319796	Ca3	13319796	TAATAAATAAATAAATAAATAAATAAATAAATAAATAAATAAATAAAT/TAATAAATAAATAAATAAATAAATAAATAAATAAATAAATAAATAAATAAAT	GGGAAGTGCTCGTAAATCCA	GTGCAACATGAGAGGAAGCA	60.1	431	INTERGENIC	--	--	--
Ca3_P3_13363177	Ca3	13363177	CTTTTTTTTTTT/CTTTTTTTTTTTTTT	CACCGATCAGAGGATGAACA	AGAAACGCATTTGGTCTTGG	59.6	169	DRR	Ca_22692	--	--
Ca3_P3_13462560	Ca3	13462560	GATATATATATATATATATAT/GATATATATATATATATATAT	TGGAAAAAGGGCAATTGAAG	GCATGCAATGGATGATGAAG	60.0	316	INTERGENIC	--	--	--
Ca3_P3_13647076	Ca3	13647076	AATATATATATATATATATAT/AATATATATATATATATATAT	TTGCACCTTATTTGATGCGT	TCTCTCTCTCATGCCACCCT	59.2	183	INTERGENIC	--	--	--
Ca3_P3_1386021	Ca3	1386021	T/TTTA	AAAGAACATTTCCAATGCGG	ACGTATCCCCAGGTGCAATA	59.9	704	INTERGENIC	--	--	--
Ca3_P3_13935709	Ca3	13935709	AGAT/AGATGAT	CAGTACCCCTTTGGAGCTTG	ATTTTCGGGCAATTTCACTG	59.7	127	INTERGENIC	--	--	--

INDEL marker IDs	Chromosomes/ unanchored scaffolds	Physical positions (bp)	InDels (*Kabuli* reference genome-CDC Frontier/PII)	Forward primers (5'-3')	Reverse primers (5'-3')	Anneling temperature (0C)	Expected amplified product size (bp)	Structural annotation		Functional annotation	
								Sequence components of *kabuli* genome	*Kabuli* gene accession IDs	NCBI-KOG	TFs
Ca3_P3_14226734	Ca3	14226734	CAAATAAATAAATAAATAAATAAATAAATAAATAAATAAATAAATAAATAAATAA/CAAATAAATAAATAAATAAATAAATAAATAAATAAATAAATAAATAAATAAATAAATAA	TTTTGGGGTTTAGGGTGTCA	CGACCTCTCTTGCAGATCAA	60.2	865	INTERGENIC	--	--	--
Ca3_P3_14454312	Ca3	14454312	GTTTTTTTTTTT/GTTTTTTTTTTTT	ACCAAGCACAGCGACTTTCT	CGTTGCAATTGAACAAACGA	60.1	634	INTRON	Ca_22513	K	G2-like
Ca3_P3_14492105	Ca3	14492105	AT/ATCACT	TGTGTTCCATTTCCTCCACA	TAATGGAACCCGGATGTGTT	59.9	577	INTRON	Ca_22511	--	--
Ca3_P3_14493863	Ca3	14493863	GTATATATATATATATATATATATATAT/GTATATATATATATATATATATATATATATATATAT	ATCGGAAAATCCCTACCAGC	TTGGTGGAACAAAACGACAA	60.3	762	INTERGENIC	--	--	--
Ca3_P3_14496111	Ca3	14496111	CATATATAT/CATATATATAT	TTTATTCGGAAGTCCATCGG	GCAAATGCTCCCCTAATTGA	59.9	629	INTERGENIC	--	--	--
Ca3_P3_14496130	Ca3	14496130	GTATATATATATATATATATATATAT/GTATATATATATATATATATATATAT	TTTATTCGGAAGTCCATCGG	GCAAATGCTCCCCTAATTGA	59.9	629	INTERGENIC	--	--	--
Ca3_P3_14503491	Ca3	14503491	AATATATATATATATATATATATATATAT/AATATATATATATATATATATAT	TGCGTTACGGTGAAATGGTA	AGGAAGAGTGGAGTTGCGAA	60.0	374	INTERGENIC	--	--	--
Ca3_P3_14520599	Ca3	14520599	GTA/GTATA	GACGAAATGTGCTTTTGGGT	CCACCAGTTTTGTGATGCAA	60.0	222	DRR	Ca_22510	--	--
Ca3_P3_14615218	Ca3	14615218	ATTTTTTTTTT/ATTTTTTTTT	CCCATCCAAAACCTCATTTG	AGGCATGGTTGTCTTTCCAG	60.2	557	INTERGENIC	--	--	--
Ca3_P3_14653135	Ca3	14653135	T/TACCGGGGTCC	AGGAGCTTCTGCCATTGAAA	TAAAAGGAGCAAAGGCCAGA	60.0	408	INTRON	Ca_22505	O	--

INDEL marker IDs	Chromosomes/ unanchored scaffolds	Physical positions (bp)	InDels (*Kabuli* reference genome-CDC Frontier/PII)	Forward primers (5'-3')	Reverse primers (5'-3')	Anneling temperature (0C)	Expected amplified product size (bp)	Structural annotation		Functional annotation	
								Sequence components of *kabuli* genome	*Kabuli* gene accession IDs	NCBI-KOG	TFs
Ca3_P3_14653349	Ca3	14653349	A/AG	AGGAGCTTCTGCCATTGAAA	TAAAAGGAGCAAAGGCCAGA	60.0	408	INTRON	Ca_22505	O	--
Ca3_P3_14801451	Ca3	14801451	GATATATATATATATATATATAT/GATATATATATATATATATATATAT	ATGAGCCACAAGCTCCAAGT	TGCCATTTGGTGAACGATTA	59.9	357	INTERGENIC	--	--	--
Ca3_P3_1481439	Ca3	1481439	TATAATAATAATAATAATAATAATAATAATAATAATAATAATAATAATAATAATAATAATAATAATAATAATAATAATAATA/TATAATAATAATAATAATAATAATAATAATAATAATAATAATAATAATAATAATA[illegible]	TTTGATGTTTCTGCGACCAG	TGGCATGTGAAGTGTCTTTTG	59.8	918	INTERGENIC	--	--	--
Ca3_P3_14843003	Ca3	14843003	AATATATATATATATATATATATATATATA/AATATATATATATATATATATATATATATATA	GAGAAAGAAGATATCGGATTCTCAA	AAAAATCAACGCAGTGGGAG	59.3	356	INTERGENIC	--	--	--
Ca3_P3_1486345	Ca3	1486345	ATCTCTCTCTCTCTCTCTCTCTCTCTC/ATCTCTCTCTCTCTCTCTCTCTCTCTCTC	TTGAAACCTTTGTTAGGAAGCA	TCCTCCCACCTCACAATAGAA	58.9	371	DRR	Ca_19415	K	MYB
Ca3_P3_14874833	Ca3	14874833	GCC/GCCC	AGGTCATTTTCGTCCTCCCT	CAAACACACCTGTTGGATGC	59.9	679	INTERGENIC	--	--	--
Ca3_P3_14876392	Ca3	14876392	AT/ATT	GCATCCCACAAGTGTGTTTG	AGGGAGGACGAAAATGACCT	60.0	656	INTERGENIC	--	--	--
Ca3_P3_14880871	Ca3	14880871	TG/TGTCG	TTTCGGTCATTTTCGTCCTC	ACACACCTATCGGATGCACA	60.1	616	INTERGENIC	--	--	--
Ca3_P3_1488532	Ca3	1488532	TTATATATATATATATATATATA/TTATATATATATATATATATA	TCCAAACACCTTCACCATGA	CTGATCAGTGCTGTGGCATT	59.9	507	INTERGENIC	--	--	--
Ca3_P3_14902851	Ca3	14902851	GAA/GAAA	CGGTGGGGAGAACTCTGATA	TTCACATGACATCATCCCGT	60.1	474	INTERGENIC	--	--	--

INDEL marker IDs	Chromosomes/ unanchored scaffolds	Physical positions (bp)	InDels (*Kabuli* reference genome-CDC Frontier/PII)	Forward primers (5'-3')	Reverse primers (5'-3')	Anneling temperature (0C)	Expected amplified product size (bp)	Structural annotation		Functional annotation	
								Sequence components of *kabuli* genome	*Kabuli* gene accession IDs	NCBI-KOG	TFs
Ca3_P3_14906453	Ca3	14906453	TGGGGG/TGGGGGG	TTGGTCTTGGGAAGTCAAGC	AACTCTGTTGATTGGCGACC	60.2	449	INTERGENIC	--	--	--
Ca3_P3_14919264	Ca3	14919264	GTTT/GTTTT	CGATTGTGTTTGTCACAGGG	GAAGCAAATCGATTGGGAAA	60.0	303	INTERGENIC	--	--	--
Ca3_P3_14944530	Ca3	14944530	A/AT	CTCTCACCCAAGAAGATCGC	TGGCAAATCGATTGAAAGAA	60.0	590	INTERGENIC	--	--	--
Ca3_P3_15039270	Ca3	15039270	TC/T	CAACACGATCATGACAAGCA	GCATTTTGCATTTGTCAACG	59.2	677	INTERGENIC	--	--	--
Ca3_P3_15039370	Ca3	15039370	ATTTTTTTTT/ATTTTTTTT	TGGTATTGGTAGGGATCTTTGG	GCATTTTGCATTTGTCAACG	60.1	568	INTERGENIC	--	--	--
Ca3_P3_15072147	Ca3	15072147	AATATATATATATATATATATATATA/AATATATATATATATATATATATA	GAGTGGTGTTGGAGTCATGTTT	AGACATGTGTTTCCCTTCCG	59.0	424	DRR	Ca_17011	--	--
Ca3_P3_15101056	Ca3	15101056	TAAAAAAAAA/TAAAAAAAAAA	AAGTGAATCCAAACATGCCC	AAAAATTGATGGGTGTGGGA	59.8	825	INTRON	Ca_17014	--	GRAS
Ca3_P3_15231571	Ca3	15231571	ATGAACTGAACTGAACTGAA/ATGAACTGAACTGAA	ACCAGGAAAAGAAGAAGGGC	TTGGTTTATTTGGGTTGGGA	59.7	462	INTERGENIC	--	--	--
Ca3_P3_15320734	Ca3	15320734	GGAGAGAGAGAGAGAGAGAGAGAG/GGAGAGAGAGAGAGAGAGAG	TGTGCCTTCCTTACTGTGATG	GCATTGTGCATTTCATTTGC	58.8	675	INTERGENIC	--	--	--
Ca3_P3_1533357	Ca3	1533357	TCAAAA/TCAAAACTCCAAAA	ACCCAAAAGCTAACTTGCCA	GCATCAACAAAGCAGCAGAA	59.7	533	INTERGENIC	--	--	--

INDEL marker IDs	Chromosomes/ unanchored scaffolds	Physical positions (bp)	InDels (*Kabuli* reference genome-CDC Frontier/PII)	Forward primers (5'-3')	Reverse primers (5'-3')	Anneling temperature (0C)	Expected amplified product size (bp)	Structural annotation		Functional annotation	
								Sequence components of *kabuli* genome	*Kabuli* gene accession IDs	NCBI-KOG	TFs
Ca3_P3_1534003	Ca3	1534003	AAACTGCAACTAACT/AAACTGCAACTAACTGCAACTAACT	TTCTGCTGCTTTGTTGATGC	GTGGATGGGTGTTTTCCATC	60.1	774	INTERGENIC	--	--	--
Ca3_P3_15396841	Ca3	15396841	CATATATATATATATATATATATATATATATATATATATATA/CATATATATATATATATATATATATATATATATATATATATATATATATATATA	TCAATGCGGTCTCTGAAGTG	CACATGCTAATCGACACCGT	60.0	412	DRR	Ca_17029	--	Trihelix
Ca3_P3_15424721	Ca3	15424721	ATTTTTTTTTTT/ATTTTTTTTTT	TTCGTGCCTATATGTAAGATCCA	GTCGCATTTTCAGACAACCA	58.7	295	INTRON	Ca_17030	OE	WRKY
Ca3_P3_15463471	Ca3	15463471	CATATATATATATATATATA/CATATATATATATATATATA	TTTGTGGATTTGCATTGATGA	TGAATTCACCGTAAACGGAA	59.9	783	INTERGENIC	--	--	--
Ca3_P3_15519811	Ca3	15519811	TAT/TATGCAAT	CCAATCCCATGACCAATTTC	TAACGCACCGTGCCTAATTT	60.0	502	INTERGENIC	--	--	--
Ca3_P3_15618505	Ca3	15618505	AG/AGG	CAGGATCTTGCAAAGGCTTC	TCTTTTGCAGATCAAATACACCA	60.0	813	INTERGENIC	--	--	--
Ca3_P3_15658492	Ca3	15658492	T/TTCCAC	AAAGCATTCCCACTACGGTG	TTGTCACAGCCTAAAACCCC	60.0	343	DRR	Ca_17043	--	--
Ca3_P3_15658525	Ca3	15658525	TC/TCATTCC	AAAGCATTCCCACTACGGTG	TTGTCACAGCCTAAAACCCC	60.0	343	DRR	Ca_17043	--	--
Ca3_P3_15660475	Ca3	15660475	TA/TAA	AAGTGAGCTAACGGGGGTTT	TGGACAGAGCAAGTGAGGTG	60.0	140	INTERGENIC	--	--	--
Ca3_P3_15694473	Ca3	15694473	TAAAAAAAAA/TAAAAAAAAAA	TTATCACCAACGAATGTGCC	GCATCCAACTTGCTCACTCA	59.4	340	INTERGENIC	--	--	--

INDEL marker IDs	Chromosomes/ unanchored scaffolds	Physical positions (bp)	InDels (*Kabuli* reference genome-CDC Frontier/PII)	Forward primers (5'-3')	Reverse primers (5'-3')	Anneling temperature (0C)	Expected amplified product size (bp)	Structural annotation		Functional annotation	
								Sequence components of *kabuli* genome	*Kabuli* gene accession IDs	NCBI-KOG	TFs
Ca3_P3_15806766	Ca3	15806766	GAAAAAAAAAAAAAAAAAA/GAAAAAAAAAAAAAAA	AGATTGTTGGGGTTGACTGC	CGTTGCCTTGTTTGTTGCTA	60.0	926	INTERGENIC	--	--	--
Ca3_P3_15824653	Ca3	15824653	C/CCTGCTT	CCCTCAAGACTCCCACGATA	CATTGGCTTGAAGTGGGTCT	60.1	268	INTERGENIC	--	--	--
Ca3_P3_15844609	Ca3	15844609	C/CTAGG	ATGGGTAGTAAGCCCCGTCT	TCTTTCCAAAGGATGGATGG	59.8	831	INTERGENIC	--	--	--
Ca3_P3_15868884	Ca3	15868884	AT/ATTATTATT	AATCGTGCGTTTTTGCTTTT	AAATTTCGATGATCCAACGG	59.8	833	INTERGENIC	--	--	--
Ca3_P3_16094863	Ca3	16094863	CTTTTTT/CTTTTTTT	TCTCCGCATAAGCTTTCGAT	TGATTGCAAATAGCACCCAA	59.9	248	DRR	Ca_17065	--	--
Ca3_P3_16107009	Ca3	16107009	TAAAA/TAAAAA	CCAAAATCTTGATGTGGCAA	CAGATGGAAATCCTCCTCCA	59.5	654	INTERGENIC	--	--	--
Ca3_P3_16108101	Ca3	16108101	TAAAAAAAAAAA/TAAAAAAAAA	AAAACAGGGCAAGAAAGCAA	AACAGCAAAAGAGAAGGCGA	59.9	292	INTERGENIC	--	--	--
Ca3_P3_16133157	Ca3	16133157	GTTTTATTTTATTTTATTT/GTTTTATTTTATTTTATTTTATTT	CGTCTCTTGAAGCGATGAAA	CATTGTTGGTCACTAGCAAATCA	59.1	698	INTERGENIC	--	--	--
Ca3_P3_16133384	Ca3	16133384	CAATAATAATAATAATAATAATAATAATAATAATAATA/CAATAATAATAATAATAATAATAATAATAATAATAATAATA	CGTCTCTTGAAGCGATGAAA	ATAAGCCGTCAATCTGCCAC	59.1	807	INTERGENIC	--	--	--
Ca3_P3_16133499	Ca3	16133499	TA/TACACAAAGAA	TTTGCCCCATTATAAACAAAA	ATAAGCCGTCAATCTGCCAC	57.2	625	INTERGENIC	--	--	--

INDEL marker IDs	Chromosomes/ unanchored scaffolds	Physical positions (bp)	InDels (*Kabuli* reference genome-CDC Frontier/PII)	Forward primers (5'-3')	Reverse primers (5'-3')	Anneling temperature (0C)	Expected amplified product size (bp)	Structural annotation		Functional annotation	
								Sequence components of *kabuli* genome	*Kabuli* gene accession IDs	NCBI-KOG	TFs
Ca3_P3_16137267	Ca3	16137267	CATATATATATATATATATA/CATATATATATATATATATATA	TGGCACATCTAAAAATAGGAGGA	AGAAAGTCGCATCCCAGAGA	60.0	544	INTERGENIC	--	--	--
Ca3_P3_16163642	Ca3	16163642	TAAAAAAAAAAA/TAAAAAAAAAA	TTCAAAATCGCAAACACTGC	ACCCACAAAACGCAAAGATT	59.9	795	INTERGENIC	--	--	--
Ca3_P3_16166714	Ca3	16166714	TTTTG/TTTTGCTTTG	TCAACCATCTGGGTTGGAAT	CCCCTAGCAAAGGAAACAAA	60.2	674	INTERGENIC	--	--	--
Ca3_P3_16171083	Ca3	16171083	T/TG	TACAGCGCTTCATTCCACAG	CGTGCATGAGCAAAAGAAAA	60.0	468	INTERGENIC	--	--	--
Ca3_P3_16182804	Ca3	16182804	ATTTTTTTT/ATTTTTTTTTT	CCGGCATCACCTATCAGAGT	TCCGACCTCCTTTGAGAAGA	60.1	583	INTERGENIC	--	--	--
Ca3_P3_16182900	Ca3	16182900	A/ATAATAATAAT	CCGGCATCACCTATCAGAGT	TCCGACCTCCTTTGAGAAGA	60.1	583	INTERGENIC	--	--	--
Ca3_P3_16190775	Ca3	16190775	CA/C	TCGCGGTTCTTGATGATGTA	AGCTCGACTTATCCTTCGCA	60.2	423	INTERGENIC	--	--	--
Ca3_P3_16195131	Ca3	16195131	AT/ATT	TGATAAAAGACTAACGATGCAAACA	TAAACCATCAATCTGCCACG	60.1	655	INTERGENIC	--	--	--
Ca3_P3_16195368	Ca3	16195368	CATATAT/CATATATAT	ACGTGGCAGATTGATGGTTT	TTTGATGTTGGCGTAGAGGA	60.4	350	INTERGENIC	--	--	--
Ca3_P3_16195709	Ca3	16195709	AC/A	ACGTGGCAGATTGATGGTTT	GAACGTACGGAAGAGAAGCG	60.4	754	INTERGENIC	--	--	--

INDEL marker IDs	Chromosomes/ unanchored scaffolds	Physical positions (bp)	InDels (*Kabuli* reference genome-CDC Frontier/PII)	Forward primers (5'-3')	Reverse primers (5'-3')	Anneling temperature (0C)	Expected amplified product size (bp)	Structural annotation		Functional annotation	
								Sequence components of *kabuli* genome	*Kabuli* gene accession IDs	NCBI-KOG	TFs
Ca3_P3_16197261	Ca3	16197261	TCTTAA/T	CTGCATCGAATTAATGCAACA	AAGGGGGATCATGGAAAACT	59.7	316	DRR	Ca_18251	--	--
Ca3_P3_16212096	Ca3	16212096	TAA/TAAA	AATGCACTTTGCCACTGTTG	CCAAGATTTTGCCAGATCAA	59.8	550	INTERGENIC	--	--	--
Ca3_P3_16212378	Ca3	16212378	A/AACATTC	ATGCACTTTGCCACTGTTGT	GAGGGGATGATGCTTCAAAA	59.2	879	INTERGENIC	--	--	--
Ca3_P3_16230171	Ca3	16230171	ATTTTTTTTTTT/ATTTTTTTTTT	TAGAGTTCTCCCCACCGCTA	TCTAAGACACGGAGGAGGGA	59.8	568	INTERGENIC	--	--	--
Ca3_P3_16331794	Ca3	16331794	TTC/TTCTC	AGGAGACTTGTGCCCTAGCA	TCAAGATTTGCCCCTCAATC	60.0	953	INTERGENIC	--	--	--
Ca3_P3_16331942	Ca3	16331942	TAA/TA	AAACGCAATCCTTGAGTTCG	TCAAGATTTGCCCCTCAATC	60.2	373	INTERGENIC	--	--	--
Ca3_P3_16347875	Ca3	16347875	CAAAAAA/CAAAAAAA	GCGTTTTTATGGTTGCTGGT	TATGGAAGGTGAGACAGGGG	60.0	690	INTERGENIC	--	--	--
Ca3_P3_16372476	Ca3	16372476	GATATATATATATATATAT/GATATATATATATATATATATAT	ATTTTGCAAATGGCGACTTC	CATTTCATTCATGAGTTGCACA	60.1	414	INTERGENIC	--	--	--
Ca3_P3_16471674	Ca3	16471674	CA/CAA	GTGAGGACTGGACGTAACCC	TCCTTGCGGAGTTGTTCTTT	59.4	505	INTERGENIC	--	--	--
Ca3_P3_16476513	Ca3	16476513	TNNNNNNNNNN/TNNN	GGCTCACATGTGGAGGCTAT	CCAATGCCATACCATAGAGGA	60.1	344	INTERGENIC	--	--	--

INDEL marker IDs	Chromosomes/ unanchored scaffolds	Physical positions (bp)	InDels (*Kabuli* reference genome-CDC Frontier/PII)	Forward primers (5'-3')	Reverse primers (5'-3')	Anneling temperature (0C)	Expected amplified product size (bp)	Structural annotation		Functional annotation	
								Sequence components of *kabuli* genome	*Kabuli* gene accession IDs	NCBI-KOG	TFs
Ca3_P3_16544552	Ca3	16544552	GTTATTATTATTATTATTATTATTATTATTATTATTATTATTA/GTTATTATTATTATTATTATTATTATTATTATTA	TGCTCGACATGTTTCTCCTT	CCACACAAACTGACAACTCCA	58.4	605	INTERGENIC	--	--	--
Ca3_P3_16793468	Ca3	16793468	TTATA/TTATATA	TAATCAAATGCCCCCAATGT	TTGAGTTCAAGCGTGAGTGG	60.0	851	INTERGENIC	--	--	--
Ca3_P3_168691	Ca3	168691	TTGTGTG/TTGTGTGTG	ATGGTGGCATTATTGTGGGT	CCAAGATAAGCTAGGCGTGC	59.9	267	DRR	Ca_22938	V	NAC
Ca3_P3_16885390	Ca3	16885390	CTATATATATATATATATA/CTATATATATATATATA	CATTTTCATCAAAGGTCGCA	CAAATTTGCCACTGTCTTGC	59.7	244	INTERGENIC	--	--	--
Ca3_P3_16954725	Ca3	16954725	CATATATATATATATATATATATA/CATATATATATATATATATATATATA	TAAATGACCAAGCCGGAAAA	CGTTTGATCGATTGCAGTTC	60.4	573	INTERGENIC	--	--	--
Ca3_P3_17039245	Ca3	17039245	CAAAAAA/CAAAAAAAA	TTCTAACTTTGCTGTCCCATTTT	GGGGTAAAACCAGGAGGAAG	59.2	750	INTERGENIC	--	--	--
Ca3_P3_17039468	Ca3	17039468	C/CA	TGTTTTGAGGACAAATGTTTTGA	GGGGTAAAACCAGGAGGAAG	59.5	301	INTERGENIC	--	--	--
Ca3_P3_17135834	Ca3	17135834	TCTC/TCTCTTTCCCTC	CAACGCGCTTTTAGATGAGA	AAAGCTTTGAAGCAGGGTCA	59.2	655	INTERGENIC	--	--	--
Ca3_P3_17187813	Ca3	17187813	CT/CTTT	AAACAAATAAGCCTGGCGTG	GGAGAGACCAAGCTCTCGAA	60.1	462	INTRON	Ca_24618	S	B3
Ca3_P3_1719355	Ca3	1719355	ATATATCTATAT/ATATAT	TTTCCCTCCAAACACACACA	CTTTTGATGCCCCGATTAAA	60.0	351	INTERGENIC	--	--	--

INDEL marker IDs	Chromosomes/ unanchored scaffolds	Physical positions (bp)	InDels (*Kabuli* reference genome-CDC Frontier/PII)	Forward primers (5'-3')	Reverse primers (5'-3')	Anneling temperature (0C)	Expected amplified product size (bp)	Structural annotation		Functional annotation	
								Sequence components of *kabuli* genome	*Kabuli* gene accession IDs	NCBI-KOG	TFs
Ca3_P3_17201946	Ca3	17201946	TTATATATATATATATATATATATATATATATATAT/TTATATATATATATATATATATATATATATATAT	AAAATCAAACATGGTGGGGA	AAGGCATGATGTTGGAGTCA	60.0	398	DRR	Ca_24619	S	--
Ca3_P3_17244270	Ca3	17244270	AATATATATATATATATATATATA/AATATATATATATATATATATA	AAGAGCGGTGTTGGAGTCAT	ATCTAGCCGCCTCCATTTCT	59.7	624	INTERGENIC	--	--	--
Ca3_P3_17300298	Ca3	17300298	A/AAAAAAAC	TTTAGGGTCTTGTTGTGGGC	TCGAATTTGTGCCTTAGCAA	60.0	396	INTRON	Ca_23343	T	WRKY
Ca3_P3_173424	Ca3	173424	TAAAAAAAAAAAAA/TAAAAAAAAAAAA	GAGAGCGACAACACTGTCAAA	GTGGGAGGCGTGTTGTTATT	59.1	646	INTERGENIC	--	--	--
Ca3_P3_17474697	Ca3	17474697	AATATATATATATATATATATATATATAT/AATATATATATATATATATATATATATATATATAT	CCAACATGCCGACAAACTTA	TACCATTCACATGCAATGCC	59.6	765	INTERGENIC	--	--	--
Ca3_P3_17491839	Ca3	17491839	AATATATATATATATA/AATATATATATATA	TGAATTCATGGCTAAAAAGCCT	GCAAAGATGATAATTCGGCAA	60.1	654	INTERGENIC	--	--	--
Ca3_P3_17491912	Ca3	17491912	ATTT/ATTTTTTT	TGAATTCATGGCTAAAAAGCCT	GCAAAGATGATAATTCGGCAA	60.1	654	INTERGENIC	--	--	--
Ca3_P3_17556672	Ca3	17556672	CTTTTTTT/CTTTTTT	ACATCCCTATTCACCTCCCC	GCAAACCGAAGGAAGACGTA	60.0	405	INTERGENIC	--	--	--
Ca3_P3_17579559	Ca3	17579559	TAAAA/TAAATAAAAA	TGCATTTTCACCAACATCGT	AATCACCGAGCTGAAATCAAA	60.0	912	INTERGENIC	--	--	--
Ca3_P3_17708984	Ca3	17708984	CATATATATATATATATA/CATATATATATATATATATATA	TCACTCAATTTCCACAACAACC	CCCAAAAAGGATATCCCAGAA	59.9	174	INTERGENIC	--	--	--

INDEL marker IDs	Chromosomes/ unanchored scaffolds	Physical positions (bp)	InDels (*Kabuli* reference genome-CDC Frontier/PII)	Forward primers (5'-3')	Reverse primers (5'-3')	Anneling temperature (0C)	Expected amplified product size (bp)	Structural annotation		Functional annotation	
								Sequence components of *kabuli* genome	*Kabuli* gene accession IDs	NCBI-KOG	TFs
Ca3_P3_17725458	Ca3	17725458	CATATATATATATATATATAT/CATATATATATATATATATATAT	AGAAGAAGGAGGATTCGCGT	GCGATTTGGGAGAGAAACAG	60.3	572	INTERGENIC	--	--	--
Ca3_P3_18191123	Ca3	18191123	A/AC	GGGGAGGAACAATCTTGGAT	CCTTCCTCAATCCTCCATCA	60.1	292	DRR	Ca_21141	--	--
Ca3_P3_18241779	Ca3	18241779	CTATATATATATATA/CTATATATATATATATA	CTCTACCACGCGATTACGGT	GGAAGTGATGGGTGAAATGG	60.2	390	INTERGENIC	--	--	--
Ca3_P3_18272727	Ca3	18272727	A/AC	TTTGCCAATCGATTATCGTG	GGGCAATCGACTGACTGAAT	59.5	683	INTERGENIC	--	--	--
Ca3_P3_18487937	Ca3	18487937	AAAGA/AAAGAGATGAAGA	AAAGGAATGCGGTTTGATTG	CAGAAGAGGCGTCTCCAAAC	59.9	706	INTERGENIC	--	--	--
Ca3_P3_18707832	Ca3	18707832	AAATAATAATAATAATAATAATAATAATAATAATAATAATAATAATAATAATAATAATAATA/AAATAATAATAATAATAATAATAATAATAATAATAATAATAATAATAATAATAATAATAATAATAATAATAATAATA	CCATTTTAATGCGAAAAGGGT	GACCGTGGGATTGACAGAAT	60.2	597	INTERGENIC	--	--	--
Ca3_P3_18725170	Ca3	18725170	TTTATTATTATTATTATTATTATTATTATTATT/TTTATTATTATTATTATTATTATTATTATTATT	TTGGAGTGAATTGTGCCATC	TTATACTTTGGCCGATGTGG	59.5	602	INTERGENIC	--	--	--
Ca3_P3_18943181	Ca3	18943181	AAT/A	TGGAGTGTTGATGTCGTTGTC	AAATCGAAGATCGCAACAGT	59.6	428	INTERGENIC	--	--	--
Ca3_P3_19089322	Ca3	19089322	T/TATC	CACCGTTGACCAGTTTGATG	CACGGGAAACTCGATGATCT	60.0	713	INTERGENIC	--	--	--
Ca3_P3_19124579	Ca3	19124579	TAAAAAAAAAAA/TAAAAAAAAAA	CACTTGTTGTTGGGTGGTGA	GGGCAAACAAGCAAGACAAT	60.5	302	DRR	Ca_19329	--	MYB_related

INDEL marker IDs	Chromosomes/ unanchored scaffolds	Physical positions (bp)	InDels (*Kabuli* reference genome-CDC Frontier/PII)	Forward primers (5'-3')	Reverse primers (5'-3')	Anneling temperature (0C)	Expected amplified product size (bp)	Structural annotation		Functional annotation	
								Sequence components of *kabuli* genome	*Kabuli* gene accession IDs	NCBI-KOG	TFs
Ca3_P3_19170970	Ca3	19170970	GAAAAAAAAAA/GAAAAAAAAAAA	TGCAGTTCTGAATTGGTTTGA	TTTTGGAAATTTGTTTTTCTCG	59.3	391	INTERGENIC	--	--	--
Ca3_P3_19196479	Ca3	19196479	CTATATAT/CTATATATATAT	GGTTCATTGCCATGTTGTGA	AAATGTGAATCCCACCAGGA	60.4	633	INTERGENIC	--	--	--
Ca3_P3_19358625	Ca3	19358625	GTTTTTTTTTTTTT/GTTTTTTTT	AAGATTTGGTCCGTTATGCG	GTGGGAATGAACAAAGGACG	60.0	517	INTERGENIC	--	--	--
Ca3_P3_19359038	Ca3	19359038	TAAAA/TAAA	CGTCCTTTGTTCATTCCCAC	TGAGGGCCTTTAGAATCGTG	60.4	670	INTERGENIC	--	--	--
Ca3_P3_19359334	Ca3	19359334	CCA/C	AGGGAAATCAACGACAGAGC	TGAGGGCCTTTAGAATCGTG	59.3	365	INTERGENIC	--	--	--
Ca3_P3_19362553	Ca3	19362553	TAAAAA/TAAAA	CCAAAATGATGTTGCAGGGT	TCATCATCATTGAAAATCCGTT	60.8	361	INTERGENIC	--	--	--
Ca3_P3_19366983	Ca3	19366983	ATTTTTTTTT/ATTTTTTTTTT	AGCTACTCGTCATCCTCCGA	TTAAAAGTGAACACACGCCG	60.0	807	DRR	Ca_19336	--	--
Ca3_P3_19367869	Ca3	19367869	TGCTAGCT/TGCT	ATTGATTCAAGGATGAGCGG	GCTGCAACATTTTCAAAGCA	60.0	665	DRR	Ca_19336	--	--
Ca3_P3_19368565	Ca3	19368565	GTTTTT/GTTTTTT	GGTGATGCATGATGATTTGC	TGGGTTAGCTTTTGGGATTG	59.9	630	DRR	Ca_19336	--	--
Ca3_P3_19369038	Ca3	19369038	CCAACAAC/CCAAC	CAATCCCAAAAGCTAACCCA	AAGAATCTGCGCCAACATCT	59.9	173	INTERGENIC	--	--	--

INDEL marker IDs	Chromosomes/ unanchored scaffolds	Physical positions (bp)	InDels (*Kabuli* reference genome-CDC Frontier/PII)	Forward primers (5'-3')	Reverse primers (5'-3')	Anneling temperature (0C)	Expected amplified product size (bp)	Structural annotation		Functional annotation	
								Sequence components of *kabuli* genome	*Kabuli* gene accession IDs	NCBI-KOG	TFs
Ca3_P3_19371062	Ca3	19371062	TAAAAAAAA/TAAAAAAAAA	TTCATTGGATTGTTTGTGGG	GAATTCTGCGTTTTTCCTGC	59.2	845	INTERGENIC	--	--	--
Ca3_P3_19373696	Ca3	19373696	TAA/TA	GCTGAATGCGTGAAGAACAA	GGGTAGAAAACACGCGGAAT	60.0	339	INTERGENIC	--	--	--
Ca3_P3_19375049	Ca3	19375049	AAAAGAAA/AAAAGAAAGAAA	TCTCCGTTCCATTTACACGA	AACTCCAAATCCCTGCTCAA	59.1	365	INTERGENIC	--	--	--
Ca3_P3_19375109	Ca3	19375109	GAGAAGAAGAAGAAGAAGA/GAGAGAGAGAGAGAAGAAGAAGAAGAAGAAGA	TCTCCGTTCCATTTACACGA	AACTCCAAATCCCTGCTCAA	59.1	365	INTERGENIC	--	--	--
Ca3_P3_19376077	Ca3	19376077	A/ATTT	TGACCTTGTGCATTGTTTTGA	GCAAACTCAACCTTGCCAAT	60.1	333	INTERGENIC	--	--	--
Ca3_P3_19381558	Ca3	19381558	TTATTATTAT/TTATTATTATTAT	GGAGAAAAGCATCGAAAAGC	ACCTAACCCTCCAAGCCAGT	59.0	338	INTERGENIC	--	--	--
Ca3_P3_19382845	Ca3	19382845	TTT/TTTATT	TAGGGGCATATGGTCTCGTC	ACCACATGATGCTCTCCACA	59.9	782	INTERGENIC	--	--	--
Ca3_P3_19383912	Ca3	19383912	CAAA/CAA	GTCAGGTCAGGCTGGAAAAA	TCGTCTTAATCCACTTGGTGC	60.2	436	DRR	Ca_19337	--	--
Ca3_P3_19386426	Ca3	19386426	AG/A	GTCAAGCGACTGCATTGAAA	GAAGCTCCCAAGAAGTGTCG	60.0	694	INTRON	Ca_19337	--	--
Ca3_P3_19388879	Ca3	19388879	TAAAAA/TAAAA	GAGGTGCTAAGTTCAAACACGA	GTTTTGCACACACAACCGAC	59.4	335	DRR	Ca_19338	--	--

INDEL marker IDs	Chromosomes/ unanchored scaffolds	Physical positions (bp)	InDels (*Kabuli* reference genome-CDC Frontier/PII)	Forward primers (5'-3')	Reverse primers (5'-3')	Anneling temperature (0C)	Expected amplified product size (bp)	Structural annotation		Functional annotation	
								Sequence components of *kabuli* genome	*Kabuli* gene accession IDs	NCBI-KOG	TFs
Ca3_P3_19402891	Ca3	19402891	AGA/AAGATGA	ACCCAATCCAAGTTTGTCCA	GAAGGCGACCTCAAAGACTG	60.2	632	INTERGENIC	--	--	--
Ca3_P3_19403411	Ca3	19403411	T/TTCG	AGGGTAATCCGCCAGTCTTT	GCAACATTCGTCCTCTCCAT	60.0	674	INTERGENIC	--	--	--
Ca3_P3_1955332	Ca3	1955332	CAAA/CAA	TCATGGTTCTTTTCGAAGTGG	CACCCACTCATCCTTCACAA	60.1	930	INTERGENIC	--	--	--
Ca3_P3_1956429	Ca3	1956429	GATATATATATATATAT/GATATATATATATATAT	TGAAAGCGTTTCTGCAAGTG	GAAAGCAACATGGGAAGGAA	60.2	766	INTERGENIC	--	--	--
Ca3_P3_1964709	Ca3	1964709	A/AT	TCTCTTTTCTCGAACCGCAT	AAACGCAATCCTTGAGTTCG	60.0	317	INTERGENIC	--	--	--
Ca3_P3_19753066	Ca3	19753066	ATTTTTTTT/ATTTTTTTTT	TTCTCACCAGTTTTGGTCCC	GCACATGAGATCTTGAGGCA	59.9	107	INTERGENIC	--	--	--
Ca3_P3_19893481	Ca3	19893481	AATATATATATATATATATATATATATATAT/AATATATATATATATATATATAT	TTGAGGATCACCGATATTGAAA	TTCAAACAAGGGTTGCCTTT	59.4	474	INTERGENIC	--	--	--
Ca3_P3_19960460	Ca3	19960460	A/AT	CCTAGTTGGGTGAGGTTTGC	TTACGTATCTCTCGGTGCGA	59.6	792	INTERGENIC	--	--	--
Ca3_P3_19960902	Ca3	19960902	TAAAAAAAAAAA/TAAAAAAAAAA	AGCTTTGATTTCTCCATCGC	GGTATTAGCACCCTACGGCA	59.4	243	INTERGENIC	--	--	--
Ca3_P3_19967887	Ca3	19967887	T/TCCATTG	TGGTCATCCATCAAACGAAA	ATCCATTCCCCACATTGAAA	59.9	645	INTERGENIC	--	--	--

INDEL marker IDs	Chromosomes/ unanchored scaffolds	Physical positions (bp)	InDels (*Kabuli* reference genome-CDC Frontier/PII)	Forward primers (5'-3')	Reverse primers (5'-3')	Anneling temperature (0C)	Expected amplified product size (bp)	Structural annotation		Functional annotation	
								Sequence components of *kabuli* genome	*Kabuli* gene accession IDs	NCBI-KOG	TFs
Ca3_P3_19989742	Ca3	19989742	CAAAAAAAAAA/CAAAAAAAAA	TGGGCAATGCATGAAAAGTA	CCCATGCATGCAATACAAAG	60.1	582	INTERGENIC	--	--	--
Ca3_P3_20004221	Ca3	20004221	TAA/TA	GGTTGTGTGTGGCTTTGATG	ACGGAGAAGTGGCTCAGAAA	60.0	441	INTERGENIC	--	--	--
Ca3_P3_20011561	Ca3	20011561	TTGACACTT/TTGACACTTGACACTT	GCAATTGCAAGAGCGTGATA	TTTGGCATGCAATCAAACAT	60.0	277	DRR	Ca_09404	--	--
Ca3_P3_20052639	Ca3	20052639	AATTATTATTATTATTATTATTATTATTATTATTATTATTAT/AATTATTATTATTATTATTATTATTATTATTAT	ATCGTGTGCAAAGGAAGGAT	TGTGCTCAAATAAATAACGTGTCTC	59.6	856	INTERGENIC	--	--	--
Ca3_P3_20128194	Ca3	20128194	A/AATT	GGGGCCAATGTAAAAAGGTT	CCCAATTAATGGGAAGGGTAA	60.1	111	INTERGENIC	--	--	--
Ca3_P3_20221448	Ca3	20221448	TATAATAATAATAATAATAATAATAATAATAATAATAATAATAATAATAATATTAATAATAATAATAA/TATAATAATAATAATAATAATAATAATAATAATAATAATAATAATAATAATAATAATAATATTAATAATAATAATAA	CCTGAGTGTTTTCGAGAGGC	GGGAAACCCACCTCTTGATT	60.0	772	INTERGENIC	--	--	--
Ca3_P3_20315844	Ca3	20315844	ATTTTTT/ATTTTTTT	CGCAAACACACTTGAACTGC	AAAGCATGTCACCAAATAAGCA	60.5	184	INTERGENIC	--	--	--
Ca3_P3_20322285	Ca3	20322285	CTTTTTTT/CTTTTTTTT	AGAGCTCTCGGTGGTTTTCA	AAGTTTTCTTTTGCTGCGGA	60.0	532	INTERGENIC	--	--	--
Ca3_P3_20339565	Ca3	20339565	TAA/TAAA	GGCAAAATGAATTTCCCTCA	AAACGCAATCCTTGAGTTCG	59.9	417	INTERGENIC	--	--	--
Ca3_P3_20424638	Ca3	20424638	CAAAAAAAAA/CAAAAAAAA	TACATTGGTTTGTGCGATGC	TGACTATGGGAGGTCAGGAAA	60.5	636	INTERGENIC	--	--	--

INDEL marker IDs	Chromosomes/ unanchored scaffolds	Physical positions (bp)	InDels (*Kabuli* reference genome-CDC Frontier/PII)	Forward primers (5'-3')	Reverse primers (5'-3')	Anneling temperature (0C)	Expected amplified product size (bp)	Structural annotation		Functional annotation	
								Sequence components of *kabuli* genome	*Kabuli* gene accession IDs	NCBI-KOG	TFs
Ca3_P3_20498750	Ca3	20498750	GAAAAATTAAAAAATTAAAAATTAAAAA/GAAAAATTAAAAA	CGTCTGTAATTTTGGCACATTC	GGTCCGAGGCATAATCCATA	59.5	496	DRR	Ca_09440	GC	HSF
Ca3_P3_20617847	Ca3	20617847	AATATATATATATATATATATATA/AATATATATATATATATATATATA	AATCGATTGGCTGAAATCCA	TCGAGTTTGAGCATGTGAGG	60.4	809	INTERGENIC	--	--	--
Ca3_P3_20750487	Ca3	20750487	CTTTATTTATTTTATTTATTTTATTTATTTTATTTATTTTATTTATTTTATTT/CTTTATTTATTTTATTTATTTTATTTATTTTATTTATTTTATTTATTTTATTTATTTTATTT	TGTTATGTTGGGTTTGGGCT	TGCAGATGGGTTGAGTGTGT	60.2	875	INTERGENIC	--	--	--
Ca3_P3_20811683	Ca3	20811683	CATAATAATAATAATAATAATAATAATAATAATAATAATA/CATAATAATAATAATAATAATAATAATAATAATAATAATAATAATAATA	ATTCCACATAAGCGTTTGCC	CGATTGTGGACAATGGAAGA	60.0	361	INTERGENIC	--	--	--
Ca3_P3_20949286	Ca3	20949286	CTATATATATATATATATATA/CTATATATATATATATATATATA	GCCGTTACACACACTTCCCT	TCAACCGTCAAATTAAAAGTTCTG	60.0	661	INTERGENIC	--	--	--
Ca3_P3_20956913	Ca3	20956913	ATTTTTTTTTT/ATTTTTTTTT	TTGCAAGGCCCAATAAAAAC	TTTTCCGGAAGCCACTTATG	59.9	445	DRR	Ca_09466	--	--
Ca3_P3_20988635	Ca3	20988635	AATATATATATATATATATATATATATATATATATATATAT/AATATATATATATATATATATATATATATATATATATATATATAT	CAAGCTGAAGGACTTCCACA	ATTATGTGGCGAGTGACGGT	59.0	477	INTERGENIC	--	--	--
Ca3_P3_21068795	Ca3	21068795	TA/T	ACAGGAGGTCCTGCTCTTCC	ATCCACCAGGGTCAATTCAA	60.8	483	INTRON	Ca_09474	J	--
Ca3_P3_21097843	Ca3	21097843	ATTTTTTTTT/ATTTTTTTTTT	GGCATTTTGATTTGATTCGC	CAAATTTTGTGTTTCAAAAACCATT	60.4	653	INTERGENIC	--	--	--
Ca3_P3_21192862	Ca3	21192862	AATATATATATATATATATATATATATATATATAT/AATATATATATATATATATATATATATATATAT	TGTCTTTCCTCGTTTTTCAATG	TTTGTAGTTCACCAGCAGGG	59.2	414	INTERGENIC	--	--	--

INDEL marker IDs	Chromosomes/ unanchored scaffolds	Physical positions (bp)	InDels (*Kabuli* reference genome-CDC Frontier/PII)	Forward primers (5'-3')	Reverse primers (5'-3')	Anneling temperature (0C)	Expected amplified product size (bp)	Structural annotation		Functional annotation	
								Sequence components of *kabuli* genome	*Kabuli* gene accession IDs	NCBI-KOG	TFs
Ca3_P3_21207756	Ca3	21207756	TAAAAAAAAAA/TAAAAAAAAA	TAACCATTGAACCGGC TTTT	GAGACTTCCTCCCCAT CTCC	59.5	501	INTERGENIC	--	--	--
Ca3_P3_21208119	Ca3	21208119	CTATATATATATATATATATATATAT/CT ATATATATATATATATATATATAT	GGAGATGGGGAGGAA GTCTC	TCTTGAAGCTAACGGA TTTCG	60.0	300	INTERGENIC	--	--	--
Ca3_P3_21252842	Ca3	21252842	CTTTTTGTAG/CTTTTTGTAGGATTTTT TGTAG	CTCCTTTTGCTTCTTCC ACG	TCCCTCCAACACACAA CAAA	60.0	331	INTERGENIC	--	--	--
Ca3_P3_21283219	Ca3	21283219	AATATATATATATATATATATATAT/AAT ATATATATATATATATATATAT	AAAAACTCCGCCACAAA ATG	ACAACTCCAACGGAAA AACG	60.0	506	INTERGENIC	--	--	--
Ca3_P3_21311568	Ca3	21311568	GAAAAAAAAA/GAAAAAAAAAA	CACGTCACATCGCAATT TTT	CATGTCATTTCAACCC ACGA	59.6	542	INTERGENIC	--	--	--
Ca3_P3_21419450	Ca3	21419450	CA/CAA	CTTCCGTCGAAAAGGT CAAA	GGGATCATTCTTCAGC CTCA	60.2	447	INTERGENIC	--	--	--
Ca3_P3_21583634	Ca3	21583634	CTTTTTTTTTTTTTT/CTTTTTTTTT	CAATTCCCACAGGTAC GCTT	AAGAGAGGTCACTTTG GCGA	60.0	812	INTERGENIC	--	--	--
Ca3_P3_21602656	Ca3	21602656	TAAAAAAAAAAA/TAAAAAAAAAA	TTGTGACTCAATTGGC CTTG	TTCGTTTTTAGTGATGT TGAATTTG	59.7	958	INTERGENIC	--	--	--
Ca3_P3_21627195	Ca3	21627195	ATCTCTCTCTCTCTCTCTCTCTCT/ATC TCTCTCTCTCTCTCTCTCTCTCTCTCT CTCT	GAGCAAGCAAGTCAAC CACA	CCACCACCAGAGGAGT CTGT	60.0	644	INTERGENIC	--	--	--
Ca3_P3_21635343	Ca3	21635343	TTCATATCATATCATATCATATCA/TTCA TATCATATCATATCA	TGGCGAGAAGGAGACT TTGT	TCCTCCTCCACAACAT GACA	60.0	608	INTRON	Ca_09516	--	--

INDEL marker IDs	Chromosomes/ unanchored scaffolds	Physical positions (bp)	InDels (*Kabuli* reference genome-CDC Frontier/PII)	Forward primers (5'-3')	Reverse primers (5'-3')	Anneling temperature (0C)	Expected amplified product size (bp)	Structural annotation		Functional annotation	
								Sequence components of *kabuli* genome	*Kabuli* gene accession IDs	NCBI-KOG	TFs
Ca3_P3_21635589	Ca3	21635589	GCATCATCA/GCATCA	AAGCATGCCAAACCAAAAAC	TGAACATCAAAGATCTGCGG	60.0	521	INTRON	Ca_09516	--	--
Ca3_P3_21696134	Ca3	21696134	CTATTATTATTATTATTATTATTATTATTATTATTATTATTATTATT/CTATTATTATTATTATTATTATTATTATTATTATTATTATTATTATTATT	TTTTTCAACAACTCTCTCCGA	GCAAATGCAGACCAGAATCA	57.6	532	INTERGENIC	--	--	--
Ca3_P3_21884477	Ca3	21884477	CATATATATATATATATAT/CATATATATATATATAT	CCGCGAGTAATTCCGATAAA	AGGGGCAACTCCTTTCTCAT	60.1	948	INTERGENIC	--	--	--
Ca3_P3_21897869	Ca3	21897869	ATATTATTATTATTATTATTATTATTA/ATATTATTATTATTATTATTATTATTA	GAGTTGAGTGTTGCCAGGGT	AGCTACCAAGCTGATGGTGAA	60.2	296	INTERGENIC	--	--	--
Ca3_P3_21989057	Ca3	21989057	CTATATATATATATATATATATATA/CTATATATATATATATATATATATATATATA	GGGACAACTGATGTAATTTAGCC	ACAAAGATTCGCGGATTGAC	58.9	515	INTERGENIC	--	--	--
Ca3_P3_22026351	Ca3	22026351	GTTTTT/GTTTTTT	ACCGCTATAGTTTGGGGCTT	CGGAGCTGGTTTCTCATTGT	60.0	635	INTRON	Ca_20423	Z	B3
Ca3_P3_22114232	Ca3	22114232	ATTTTTTTTT/ATTTTTTTT	AAAAGTTGACGTGGCATTGA	ACTCGTATTTTACCCGTGCG	59.2	579	INTERGENIC	--	--	--
Ca3_P3_22165515	Ca3	22165515	TAAACAAAAAAACAAAAAAACAAAAAAA/TAAACAAAAAAACAAAAAAA	TCCAATGTGTGACCAAACAGA	CACCGGCTTCAAGATGTTTT	60.0	211	DRR	Ca_20412	U	--
Ca3_P3_22377503	Ca3	22377503	TGTT/TGTTAGTT	TTCATTAACGCTTATTCCCTCA	TGCAACTCTAACAGCGACAA	58.8	694	INTERGENIC	--	--	--
Ca3_P3_22378443	Ca3	22378443	AAATAATAATAATAATAATAATAATAATAATAATAATAA/AAATAATAATAATAATAATAATAATAATAATAA	TTCGAGATAGCCTTGCGAAT	TTGTTTGATGAGGCATGACA	59.9	721	INTERGENIC	--	--	--

INDEL marker IDs	Chromosomes/ unanchored scaffolds	Physical positions (bp)	InDels (*Kabuli* reference genome-CDC Frontier/PII)	Forward primers (5'-3')	Reverse primers (5'-3')	Anneling temperature (0C)	Expected amplified product size (bp)	Structural annotation		Functional annotation	
								Sequence components of *kabuli* genome	*Kabuli* gene accession IDs	NCBI-KOG	TFs
Ca3_P3_22438235	Ca3	22438235	GAAAAAAAAAA/GAAAAAAAAAAA	TTCCAAACAAAGCCTTTTGC	TAGCAACGGAACAAACCACA	60.2	690	INTRON	Ca_06267	--	HD-ZIP
Ca3_P3_22481840	Ca3	22481840	ATTTTTTTTT/ATTTTTTTT	AAAAACAGAGGTGGCTCCTG	TGGAGATTGATCTCAAGCGTT	59.3	595	INTERGENIC	--	--	--
Ca3_P3_22487747	Ca3	22487747	TACAGTGA/TA	GTAATGCCCCGTGAGTAGGA	CCCAATTGATTCCAAAAGAAA	60.0	778	INTERGENIC	--	--	--
Ca3_P3_22498984	Ca3	22498984	CAGA/CA	GCCCCTTATGACCAAAGTGA	AGAGGTGAAAGAAGTGGCGA	59.9	518	INTERGENIC	--	--	--
Ca3_P3_22922150	Ca3	22922150	ATTTTTTTTT/ATTTTTTTTTT	AAAATACATTGCCGGTGGAA	TGTCTCAAAAATGAGTTATCAATTAAG	60.2	477	INTERGENIC	--	--	--
Ca3_P3_22951325	Ca3	22951325	GCC/GCCC	CAAACTGTGCAATTACGGATT	AGTGGGAGACGAGGTGTCTG	57.7	795	INTERGENIC	--	--	--
Ca3_P3_23014028	Ca3	23014028	CAAAAAAAAAAAAAAA/CAAAAAAAAAAAAA	GGATGTTGCACAATCAAGGA	GGCAGTGGTAGTGAAGCACA	59.5	370	INTERGENIC	--	--	--
Ca3_P3_23016776	Ca3	23016776	TCCTACCT/TCCT	GCACACCTATGTGGTCTTGC	CCATTTTGTCGAAGTTTGGTT	59.2	615	DRR	Ca_06208	QR	B3
Ca3_P3_23063614	Ca3	23063614	TTATATATATATATATATATATATA/TTATATATATATATATATATATATATA	GTAACTTGCACCGACCCAAT	ATGACGGTGTTTGGAGACAA	59.9	599	INTERGENIC	--	--	--
Ca3_P3_23253882	Ca3	23253882	GAAAAAAAAAAA/GAAAAAAAAAA	TGGGTTGCGATCGTGTATTA	TCAGTCAGTTGCAACATAGGAGA	60.0	327	INTERGENIC	--	--	--

INDEL marker IDs	Chromosomes/ unanchored scaffolds	Physical positions (bp)	InDels (*Kabuli* reference genome-CDC Frontier/PII)	Forward primers (5'-3')	Reverse primers (5'-3')	Anneling temperature (0C)	Expected amplified product size (bp)	Structural annotation		Functional annotation	
								Sequence components of *kabuli* genome	*Kabuli* gene accession IDs	NCBI-KOG	TFs
Ca3_P3_23551096	Ca3	23551096	AACAATTTACAATTTACGATTTACAATTT/AACAATTTACAATTTACAATTTACGATTTACAATTT	TCCCATTTTGATTCCCTTCA	TCCCTAAATCGCTGAATCTGA	60.2	363	INTERGENIC	--	--	--
Ca3_P3_23651728	Ca3	23651728	TTTATTATTATTATTATTATTATTATTATTATT/TTTATTATTATTATTATTATTATTATTATTATTATTATT	GGTACGTTTGGTCTTGCAGC	TGAGCGCTTATGATCCATTTT	60.7	529	DRR	Ca_06162	--	NAC
Ca3_P3_236571	Ca3	236571	CGAGAGAGAGAGAGA/CGAGAGAGAGAGA	CAATTCACAGCAAAAGCGAA	CCTAGGGTTTCTTCCCCTCA	60.0	191	INTERGENIC	--	--	--
Ca3_P3_23691313	Ca3	23691313	CTTTTTTTT/CTTTTTTTTT	TGTCAGCAACCCTTGTCTTG	TTGACGTCACCAATTCAACC	59.9	590	INTERGENIC	--	--	--
Ca3_P3_23759284	Ca3	23759284	GA/GAA	CCATCTCAAACGTGCTCTCA	TTTTGGGTGATGTGGGAAAT	60.0	181	DRR	Ca_06153	GC	--
Ca3_P3_23762724	Ca3	23762724	ATTTTTTTTTTT/ATTTTTTTTTTTT	TTGTGCCACCTAAAAGCTCA	GCTGAATGTGGACAATCTTCTTT	59.5	336	DRR	Ca_06152	QI	bHLH
Ca3_P3_23855473	Ca3	23855473	AAT/AATTAT	CGCCGTTAATTAGTGTTTGGA	TTTTTCTTCCAACGGTGAGG	60.0	674	INTERGENIC	--	--	--
Ca3_P3_23864075	Ca3	23864075	ATTTTTTTTT/ATTTTTTTT	TTCCAATTCTGGTGAGTAATGA	TCCTGGAAGATGAAAACTCAGA	57.2	843	INTERGENIC	--	--	--
Ca3_P3_23876448	Ca3	23876448	AATATATATATATATAT/AATATATATATATATATATATAT	CATAATCCCATCGCTCTCGT	TCGGTTAACTGGCCAAACTC	60.1	568	DRR	Ca_06137	--	--
Ca3_P3_23881356	Ca3	23881356	TTATATATATATATATAT/TTATATATATATATATATATATAT	ACAGCCAACAACCACATCAA	CGTAAGTGTGACCGACGCTA	60.0	864	INTERGENIC	--	--	--

INDEL marker IDs	Chromosomes/ unanchored scaffolds	Physical positions (bp)	InDels (*Kabuli* reference genome-CDC Frontier/PII)	Forward primers (5'-3')	Reverse primers (5'-3')	Anneling temperature (0C)	Expected amplified product size (bp)	Structural annotation		Functional annotation	
								Sequence components of *kabuli* genome	*Kabuli* gene accession IDs	NCBI-KOG	TFs
Ca3_P3_23881409	Ca3	23881409	ATTTTTTTT/ATTTTTTTTT	TGGTACTGGAGGCCACCTAC	CGTAAGTGTGACCGACGCTA	60.0	522	INTERGENIC	--	--	--
Ca3_P3_24011215	Ca3	24011215	ATT/ATTT	TCATGAAGATTTGGAAGAGAAAGA	TGTTGCTACGCTGAAACTGG	59.4	272	INTERGENIC	--	--	--
Ca3_P3_24232615	Ca3	24232615	CAAAAAAAAAA/CAAAAAAAAA	AGCAATTTTTCGGCTAAGCA	TCTACGGCACGAATGATGAA	60.0	706	INTERGENIC	--	--	--
Ca3_P3_24272959	Ca3	24272959	GAATAATAA/GAATAATAATAA	GCATGTCCGTTACCTCCACT	GCGATTTATTATTCAGAGTTATTAGCG	60.0	646	INTERGENIC	--	--	--
Ca3_P3_24410514	Ca3	24410514	GAAAAAAAAAAA/GAAAAAAAAAAAA	CAAAAGCACTGGGAGGAGAG	TCCCTGCCTTAACCTTGACA	60.0	519	INTERGENIC	--	--	--
Ca3_P3_24414416	Ca3	24414416	AATATATATATATATATAT/AATATATATATATATATAT	TGGAGGCAAATACACTCCCT	AAATGCGGACTATGTCGGTT	59.6	434	INTERGENIC	--	--	--
Ca3_P3_24439636	Ca3	24439636	ATTTTTTTTTT/ATTTTTTTTT	ACTTTGCAAGCTTTTGCGAT	AACAATGTATGCACAGGCAAG	60.0	503	INTERGENIC	--	--	--
Ca3_P3_24470665	Ca3	24470665	GATATATATATATATATAT/GATATATATATATATATAT	CAATGTGTGGCATTTTGGTC	CCCTATTATTGCATCCAGTTCC	59.8	325	INTERGENIC	--	--	--
Ca3_P3_24550785	Ca3	24550785	AATATATATATATATATATAT/AATATATATATATATATATATATAT	TCGTGCATATCAATTAAACTTAGGA	CCGGTTCAATGATGTGACCT	59.2	664	INTERGENIC	--	--	--
Ca3_P3_24554701	Ca3	24554701	ATGTG/ATG	GGAGTATTGTGAACTCCACACG	AGATTTTGATTGATTGGCGG	59.5	764	INTERGENIC	--	--	--

INDEL marker IDs	Chromosomes/ unanchored scaffolds	Physical positions (bp)	InDels (*Kabuli* reference genome-CDC Frontier/PII)	Forward primers (5'-3')	Reverse primers (5'-3')	Anneling temperature (0C)	Expected amplified product size (bp)	Structural annotation		Functional annotation	
								Sequence components of *kabuli* genome	*Kabuli* gene accession IDs	NCBI-KOG	TFs
Ca3_P3_24731209	Ca3	24731209	ATTTTTTTTTTTT/ATTTTTTTTTTTTTT	GGGGTTTCACTGGTTGAAGA	GCTTTGACCCCAGAATTCAA	59.9	467	INTERGENIC	--	--	--
Ca3_P3_24791040	Ca3	24791040	GAAAAAA/GAAAAA	GGGACCACGAATGTAATTTAGC	TGCGAGCATAAGAAGTTGTTG	59.7	740	INTERGENIC	--	--	--
Ca3_P3_24796858	Ca3	24796858	AA/AACATTATCACAAACTA	AAAACCAAATGCTTCACATGC	GGCAATGACTTGTCCCTGTT	60.0	784	INTERGENIC	--	--	--
Ca3_P3_24837729	Ca3	24837729	TAAAAAAAA/TAAAAAAA	ATGCCAAATTGCCAAATACC	GATTGGTTGGACAAAGGGAA	59.7	434	INTERGENIC	--	--	--
Ca3_P3_24912627	Ca3	24912627	AAATAATAATAATAATAATAATAATAATAATA/AAATAATAATAATAATAATAATAATAATAATAATA	CGTAGAGGCGAAAATGTGAA	TGAATCAAATTCAACGCACA	58.9	490	INTERGENIC	--	--	--
Ca3_P3_24956986	Ca3	24956986	TAAAAAAAAAA/TAAAAAAAAAAA	TAAAGGACCAAGTCGGGAAA	CGAGAAAAATGATTATCCCTCAA	59.5	315	DRR	Ca_06033	--	Trihelix
Ca3_P3_24957469	Ca3	24957469	TAA/TA	TTGAGGGATAATCATTTTTCTCG	GAAAAAGCGCTGTAAATGGC	59.5	820	DRR	Ca_06033	--	Trihelix
Ca3_P3_24969495	Ca3	24969495	CATATATATATATATATATATATATATATATATA/CATATATATATATATATATATATATATATATATATATATATA	TTGAAAATGTGATTCGATTGC	TTGAGGATTATTGCCTTGGG	58.6	469	DRR	Ca_06032	--	ERF
Ca3_P3_24984768	Ca3	24984768	CTTTTTTTTT/CTTTTTTTT	TATATGGGCTGCTCTCCCTG	TTTTGCTTTTGAAGCCAACC	60.2	663	INTRON	Ca_06031	--	SAP
Ca3_P3_25112902	Ca3	25112902	ATTTTTTTTTT/ATTTTTTTTTTT	ATCATGCCCCGTAGTGTAGC	ATGCAGGAATTTGTGAAGGC	60.0	780	INTRON	Ca_06017	--	--

INDEL marker IDs	Chromosomes/ unanchored scaffolds	Physical positions (bp)	InDels (*Kabuli* reference genome-CDC Frontier/PII)	Forward primers (5'-3')	Reverse primers (5'-3')	Anneling temperature (0C)	Expected amplified product size (bp)	Structural annotation		Functional annotation	
								Sequence components of *kabuli* genome	*Kabuli* gene accession IDs	NCBI-KOG	TFs
Ca3_P3_25195854	Ca3	25195854	AAGAATACTCTATGTT/AAGAATACTCTATGTTAGAATACTCTATGTT	TGCCCTAATTAAGGGAATAAATG	GTGGTTTGGGCTACTTTCCA	58.5	267	INTERGENIC	--	--	--
Ca3_P3_25252671	Ca3	25252671	GATATATATATA/GATATATATATATA	CCAACCATGTTGACATTTGC	GGTGCATTTTATGGCTGCTT	59.8	361	INTRON	Ca_06003	--	--
Ca3_P3_25254854	Ca3	25254854	AATATATATATATATATATATATATATATATATATATAT/AATATATATATATATATATATATAT	CCTTGTGGTTCACCATGGAC	GACCAATGTGGTCGATTCAA	61.2	334	INTERGENIC	--	--	--
Ca3_P3_25314505	Ca3	25314505	GAAAAAAAAAAA/GAAAAAAAAAAAA	CCCTCGAATTTGTTTTGGAA	TGGAAAATGATTGTTTTTGGG	59.9	711	INTERGENIC	--	--	--
Ca3_P3_25413762	Ca3	25413762	GATATAT/GATAT	TCTAAAAGGTACCGGTGCTCA	TCTGCAACCAAAAACATCCA	59.8	316	INTERGENIC	--	--	--
Ca3_P3_25425291	Ca3	25425291	AATATATATATATATATATATATATATA/AATATATATATATATATATATATATATATATATATATA	GGCATCGACATGAGAGATGA	ACGGTTTGATCCATTTGCTC	59.8	635	INTERGENIC	--	--	--
Ca3_P3_25427001	Ca3	25427001	GTATATATATATATATATAT/GTATATATATATATATATAT	ACCAGCATGATGACCAGACA	TAGGCTTGGTGGACCAGTTC	60.1	318	INTERGENIC	--	--	--
Ca3_P3_25464472	Ca3	25464472	AAGACAGAGAGA/AAGA	CCGTTTCCTCTGTGTCCAAT	GCTGTTAAAGGTTGACCCCA	60.0	656	INTERGENIC	--	--	--
Ca3_P3_25508320	Ca3	25508320	GT/GTT	TTGCAATCGAATGACCTCAC	CCTTATCGGTTCCGAAATCA	59.7	490	INTRON	Ca_05985	--	CO-like
Ca3_P3_25519863	Ca3	25519863	AATATATATATATATATATATATATATATAT/AATATATATATATATATATATATATAT	TCAGTCAACCGCACATAAAAG	TCATCTATTCAAAAAGTCTCACCG	58.8	541	INTERGENIC	--	--	--

INDEL marker IDs	Chromosomes/ unanchored scaffolds	Physical positions (bp)	InDels (*Kabuli* reference genome-CDC Frontier/PII)	Forward primers (5'-3')	Reverse primers (5'-3')	Anneling temperature (0C)	Expected amplified product size (bp)	Structural annotation		Functional annotation	
								Sequence components of *kabuli* genome	*Kabuli* gene accession IDs	NCBI-KOG	TFs
Ca3_P3_25585812	Ca3	25585812	TAAAAAAAA/TAAAAAAA	TGTTTCGTTTTTGTTCCCTCT	TCCATTGCAATCGTACTTAATGA	58.7	735	DRR	Ca_05976	--	B3
Ca3_P3_25600130	Ca3	25600130	T/TTTGT	CAATCACGTGAATAAAAGCAGC	CTCAAATGCATGAAATGAGCA	59.8	811	INTERGENIC	--	--	--
Ca3_P3_25684585	Ca3	25684585	GTATATATATATATATATATATATATATATATATATATA/GTATATATATATATATATATATATATATATATATATATA	GTGTGCTTGTTGATGTTGCC	AACAATTCCATGCGCATTTC	60.2	452	DRR	Ca_23891	E	bHLH
Ca3_P3_25758872	Ca3	25758872	TAAAAAAAAA/TAAAAAAAA	GGATCCGGTGCTACTCCATA	ATTTGAGGTTGTGATTATGCAA	59.9	713	INTERGENIC	--	--	--
Ca3_P3_2580419	Ca3	2580419	GGAGAGAGAGAGAGAGAGAGAGAGAGAGAGAGAGAGAGA/GGAGAGAGAGAGAGAGAGAGAGAGAGAGAGAGAGA	GTGGAAAAAGGCAAGAGTGC	CGCATTTCATCCTCACCATA	59.9	266	DRR	Ca_22145	--	--
Ca3_P3_25866798	Ca3	25866798	AATATATATATATATATATATATATATATATATATATA/AATATATATATATATATATATATATATATATATATATATATATATA	AGCCAATTAGGCAACCACAA	TTCCCCATTCAGAGTTAGGC	60.5	871	INTERGENIC	--	--	--
Ca3_P3_25872845	Ca3	25872845	ACTAAATTGGTCGCTAAAT/ACTAAAT	TGTGTACAAGATTGTGAAGGCA	CACGTAAGATCGCCAAAATC	59.2	680	INTERGENIC	--	--	--
Ca3_P3_25888533	Ca3	25888533	GAAA/G	CAGGTCATGCCAGACTTTGA	TCTGACCCGACCTGGTCTAC	59.8	578	INTERGENIC	--	--	--
Ca3_P3_25888907	Ca3	25888907	GTTTTTTT/GTTTTTTTT	CAGGTCATGCCAGACTTTGA	TCTGACCCGACCTGGTCTAC	59.8	578	INTERGENIC	--	--	--
Ca3_P3_25889145	Ca3	25889145	ATTTTTTTT/ATTTTTTTTT	GTAGACCAGGTCGGGTCAGA	TGATATGTCACATTGTCGCAC	60.1	403	INTERGENIC	--	--	--

INDEL marker IDs	Chromosomes/ unanchored scaffolds	Physical positions (bp)	InDels (*Kabuli* reference genome-CDC Frontier/PII)	Forward primers (5'-3')	Reverse primers (5'-3')	Anneling temperature (0C)	Expected amplified product size (bp)	Structural annotation		Functional annotation	
								Sequence components of *kabuli* genome	*Kabuli* gene accession IDs	NCBI-KOG	TFs
Ca3_P3_25891751	Ca3	25891751	TAAA/TAAAA	CGGTTCCTAATTGAAAAGGC	CAACGCTTTGTTCTCTCAACA	58.7	341	INTERGENIC	--	--	--
Ca3_P3_25916961	Ca3	25916961	AATATATATATATATATATATATATA/AATATATATATATATATATATATATATATA	CAAAAATCCGCACTCACCTT	TCGTCTTAATTTATGCGGGG	60.1	427	INTERGENIC	--	--	--
Ca3_P3_25950181	Ca3	25950181	AGATGGGGATGGGGATG/AGATGGGGATG	ACTTGCACCCCATCAGTTTC	TCCCCATCTTCATCTTCGTC	60.0	410	INTERGENIC	--	--	--
Ca3_P3_26014278	Ca3	26014278	TATAATAATAATAATAATAATAATAATAATAATAATAATAATAATAATAATATTAATAATAATAATAA/TATAATAATAATAATAATAATAATAATAATAATAATAATAATATTAATAATAATAATAA	GCCCTCATACCCAATGAAGA	GAAAAAGGGAAACCCACCTC	59.9	781	INTERGENIC	--	--	--
Ca3_P3_26071358	Ca3	26071358	TAAA/TAAAA	TGTGTTTGTTTGGTTTCATCTTG	TGTCAACGTGATTGGAGCAT	59.9	563	INTRON	Ca_08292	--	MYB_related
Ca3_P3_26071877	Ca3	26071877	TAAA/TAAAA	TGCTCCAATCACGTTGACAT	AGACGCTGCAACATCAAAGC	60.1	397	INTRON	Ca_08292	--	MYB_related
Ca3_P3_26072078	Ca3	26072078	T/TAATGGT	TGCTCCAATCACGTTGACAT	GACACGCGAAATAAAAGGAA	60.1	777	INTRON	Ca_08292	--	MYB_related
Ca3_P3_26114065	Ca3	26114065	AG/A	TTTATCCTGGGGACTTCGTG	AGGAAAATACACGGGCAAGA	59.9	364	INTRON	Ca_08292	--	MYB_related
Ca3_P3_26114282	Ca3	26114282	ATTATAGTTTTTATT/AT	TTTATCCTGGGGACTTCGTG	ATACACCGATGGAGGGCTTT	59.9	838	INTRON	Ca_08292	--	MYB_related
Ca3_P3_26114943	Ca3	26114943	GTTTT/GTTT	TGGCATGGTACTCAACCAGA	TTCACATGAATGATTGTTGGGT	60.1	545	INTRON	Ca_08292	--	MYB_related

INDEL marker IDs	Chromosomes/ unanchored scaffolds	Physical positions (bp)	InDels (*Kabuli* reference genome-CDC Frontier/PII)	Forward primers (5'-3')	Reverse primers (5'-3')	Anneling temperature (0C)	Expected amplified product size (bp)	Structural annotation		Functional annotation	
								Sequence components of *kabuli* genome	*Kabuli* gene accession IDs	NCBI-KOG	TFs
Ca3_P3_26115029	Ca3	26115029	AACG/A	TGGCATGGTACTCAACCAGA	TTCACATGAATGATTGTTGGGT	60.1	545	INTRON	Ca_08292	--	MYB_related
Ca3_P3_26159882	Ca3	26159882	CATATATAT/CATATAT	TTGACCAGACCGAGATCACA	GGGGCATTATGGACTTGAAA	60.2	779	INTERGENIC	--	--	--
Ca3_P3_26215963	Ca3	26215963	T/TATATATATATATA	TTTATGGGAGCTCTTGCCAC	TTGACCCCTCAAAGAGATGG	60.2	742	INTERGENIC	--	--	--
Ca3_P3_26328372	Ca3	26328372	AAGAGAGAGAGAGAGAGAGAGAGA/AAGAGAGAGAGAGAGAGAGAGAGA	TTGTTGAATTTGATGTCGTTGA	TCCCTGATTTTGTAAGACCAA	59.1	327	INTERGENIC	--	--	--
Ca3_P3_26336721	Ca3	26336721	GATATATATATATATATATATATATATA/GATATATATATATATATATATATATATATATA	AGAAAGGCCGTTTTCTGGAT	ACCACCGACCACCTACTTCA	60.1	574	INTRON	Ca_08268	YU	bHLH
Ca3_P3_26508807	Ca3	26508807	AATATATATATATATAT/AATATATATATATATATAT	AACCACCGCAAAACTACCAC	AAAACTTTTGCATGCGTGTG	59.9	514	DRR	Ca_08257	--	NAC
Ca3_P3_26584162	Ca3	26584162	TTTTATTT/TTTTATTTATTT	CAGAACAAATGACGTGTCCC	TCCTGGAGAAGCGACCTCTA	59.0	533	INTERGENIC	--	--	--
Ca3_P3_26628740	Ca3	26628740	AATATATATATATATATATATATATATATATAT/AATATATATATATATATATATATATATAT	TGTGATTTTGAGTTTCCTCTCG	CTTGCACGTATGCAAGAACA	59.4	342	INTERGENIC	--	--	--
Ca3_P3_26682986	Ca3	26682986	GTTATTATTATTATTATTATTATTATTATTATTATTATTATTATTATTA/GTTATTATTATTATTATTATTATTATTATTATTATTATTA	GTTGGCTTTTGTACCCCTCA	GCTGTCATATCATGGGCAAG	60.0	734	INTERGENIC	--	--	--
Ca3_P3_26724618	Ca3	26724618	TCC/T	AGGAGTGTTTTTGGGGTTTG	GCCTCTGATGGTGTCAACTG	58.9	569	DRR	Ca_08235	J	--

INDEL marker IDs	Chromosomes/ unanchored scaffolds	Physical positions (bp)	InDels (*Kabuli* reference genome-CDC Frontier/PII)	Forward primers (5'-3')	Reverse primers (5'-3')	Anneling temperature (0C)	Expected amplified product size (bp)	Structural annotation		Functional annotation	
								Sequence components of *kabuli* genome	*Kabuli* gene accession IDs	NCBI-KOG	TFs
Ca3_P3_26872317	Ca3	26872317	CAGAGAGAGAGAGAGAGAGAGAGAG/ CAGAGAGAGAGAGAGAGAGAGAGAGAG	CAATGTGCTCGATTTCTCCA	TTACATTCCTTGCCTCGTCA	59.8	389	INTERGENIC	--	--	--
Ca3_P3_26922565	Ca3	26922565	TAAAAAAAAAA/TAAAAAAAAAAA	GGCCATTTTTCCCCCTAATA	ATTGAGGGATCTGGAGGCTT	60.0	494	INTERGENIC	--	--	--
Ca3_P3_27073479	Ca3	27073479	TTTT/TTTTTTATTT	ATCATTTTCTTCAATGCGGA	ATTGGCGTCAACCTTGTTTC	58.2	727	DRR	Ca_08197	E	--
Ca3_P3_27142432	Ca3	27142432	CATATATATATATATATA/CATATATATATATATATA	GCTTCTTTCACACCCACGTT	GCCGTAACGTAACCGTCAGT	60.2	547	INTERGENIC	--	--	--
Ca3_P3_27263608	Ca3	27263608	TC/TCC	TTGTTTTCTTCGTTTTGGGG	TGGATGGTTATCATTCATTGG	59.9	544	INTERGENIC	--	--	--
Ca3_P3_27273898	Ca3	27273898	GCTCTCTCTCTCTCTCTCTCT/GCTCTCTCTCTCTCTCTCTCTCTCT	TCCTTCATCCACCAGACTCC	TGCCACATCCAACACTTTGT	60.0	500	INTERGENIC	--	--	--
Ca3_P3_27278816	Ca3	27278816	AAATAATAATAATAATAATAATAATAATAATAATAATAATAATAATAA/AAATAATAATAATAATAATAATAATAATAATAATAATAATAATAATAA	ACACGTCCGTATTCTTTGCC	TAGAATGTGGCCATGTGGAA	60.0	484	INTERGENIC	--	--	--
Ca3_P3_27299542	Ca3	27299542	ATTTTTTTTTTT/ATTTTTTTTTT	TGCTTTTTCAAAGGGAATGG	TGTCGAGTTCGGTCATTTTG	60.0	531	INTERGENIC	--	--	--
Ca3_P3_27300846	Ca3	27300846	CTTTTTTTTTTTT/CTTTTTTTTTTT	TGATCCCTTTCCTCATTTCAA	AAATTTAGTTTATGCGCTTTGTCA	59.5	359	INTERGENIC	--	--	--
Ca3_P3_27310680	Ca3	27310680	CTTTTTTTTTTTT/CTTTTTTTTTTT	CATGTGACCAAGGACCAACTT	AAACCAACCTTGCATTGACA	59.9	606	INTERGENIC	--	--	--

INDEL marker IDs	Chromosomes/ unanchored scaffolds	Physical positions (bp)	InDels (*Kabuli* reference genome-CDC Frontier/PII)	Forward primers (5'-3')	Reverse primers (5'-3')	Anneling temperature (0C)	Expected amplified product size (bp)	Structural annotation		Functional annotation	
								Sequence components of *kabuli* genome	*Kabuli* gene accession IDs	NCBI-KOG	TFs
Ca3_P3_27340222	Ca3	27340222	AA/AAGGA	TGATGAAATTTGCATTAAATCG	CGGGGTTATACGCTGACACT	57.7	402	INTERGENIC	--	--	--
Ca3_P3_27365754	Ca3	27365754	AAATAATAATAATAATAATAATAAT/AAATAATAATAATAATAATAATAATAATAAT	TGATACAGGAAAATCAATTGCG	CAACTTTGATGGCAGAAGCA	60.0	752	INTERGENIC	--	--	--
Ca3_P3_27378702	Ca3	27378702	CAA/CAAA	GTCGGGGACTAGTGGACTCA	CGGGGAAGTTCTAAGGTGCT	60.1	388	INTERGENIC	--	--	--
Ca3_P3_27382854	Ca3	27382854	GTTTTTTTTTTTT/GTTTTTTTTTTT	GCGGTAAAAGAAGGCAAGTG	TGTGGGTACCTTAAACCCTAAAAA	59.9	591	INTERGENIC	--	--	--
Ca3_P3_27490383	Ca3	27490383	CATATAT/CATAT	TTCCCTTGCACTCAAGAACA	GGGCAAATCATTGTTTCGTT	59.4	390	DRR	Ca_08157	Q	B3
Ca3_P3_27687227	Ca3	27687227	A/ATAGTATAG	TGGCGATTATGTGTCCAATG	CGAAAGTGGATCCTGGAAAA	60.3	347	CDS (FRAME SHIFT)	Ca_08137	S	--
Ca3_P3_27854154	Ca3	27854154	TGG/TG	TCGATAATGAGTGATTAGGTGGG	ACCTCACCCTCGAATGTCAC	60.2	459	INTERGENIC	--	--	--
Ca3_P3_27854804	Ca3	27854804	T/TA	GGATTGAGAGGGGAGAAAGG	TTTGAAAGCAAGAGCGTCAA	60.0	516	INTERGENIC	--	--	--
Ca3_P3_27856755	Ca3	27856755	TAAAAAAAAA/TAAAAAAAA	CGAAGATGAAGAGGCTTGAAA	CAACAACTTCTGATGCTCGC	59.6	593	INTERGENIC	--	--	--
Ca3_P3_27892125	Ca3	27892125	CAAAAAAA/CAAAAAAAAA	AGGGGCACCAGTACTCTCCT	GAAATGGCCGACTTCATTGT	60.1	508	INTRON	Ca_08120	A	--

INDEL marker IDs	Chromosomes/ unanchored scaffolds	Physical positions (bp)	InDels (*Kabuli* reference genome-CDC Frontier/PII)	Forward primers (5'-3')	Reverse primers (5'-3')	Anneling temperature (0C)	Expected amplified product size (bp)	Structural annotation		Functional annotation	
								Sequence components of *kabuli* genome	*Kabuli* gene accession IDs	NCBI-KOG	TFs
Ca3_P3_27937082	Ca3	27937082	TAAAAAAAAA/TAAAAAAAA	CCCTTTCCCATTAGTGCTCA	GCCATTTGCAAAGACACTTG	60.1	821	INTERGENIC	--	--	--
Ca3_P3_2795118	Ca3	2795118	CAAAAAAAAAA/CAAAAAAAAAAA	AAGTTTGGGGTTGCTCTTGA	TTTGGAGGACTTTTGGGTTG	59.7	307	INTERGENIC	--	--	--
Ca3_P3_28008657	Ca3	28008657	CTAATAATAATAATAATAATAATAATAATAATAATAATAATAATAATAATAA/CTAATAATAATAATAATAATAATAATAATAATAATAATAATAATAATAATAATAA	GTCGGCCTTGAGTAAATGGA	TCACCCCAAACATCACTTCA	60.1	645	INTERGENIC	--	--	--
Ca3_P3_28014908	Ca3	28014908	TAAAAAAAAAAA/TAAAAAAAAAAAA	TTAGCTTGCTAGCCCAGGAC	AATCCACGCACTTATTTGCC	59.6	360	INTERGENIC	--	--	--
Ca3_P3_28052758	Ca3	28052758	CACAGAC/CAC	ACTTCCGTTCACACGTTTCC	CGATGCAGGTGAAGATCAAA	60.0	481	INTERGENIC	--	--	--
Ca3_P3_28052899	Ca3	28052899	TTGTTCTATATAAATGTT/TTGTT	TTTGATCTTCACCTGCATCG	GCCAAACTAGCGCCTAAACA	59.8	269	INTERGENIC	--	--	--
Ca3_P3_28113252	Ca3	28113252	ATTTTTTTT/ATTTTTTT	TCCTTCTCCTCTTCCTTCCA	TGTTTGTGCAATTGAATTATAGTTATG	58.9	849	INTERGENIC	--	--	--
Ca3_P3_28188438	Ca3	28188438	CAAAA/CAAAAA	ACCCATAACGCACTCGTTTC	TCCTCCATTTTTCGTTTTCG	60.0	434	INTERGENIC	--	--	--
Ca3_P3_28191077	Ca3	28191077	CTTTTTTTTT/CTTTTTTTTTT	TAAGATGCGTGGGTTTAGGG	GCTTGTGTTTAAAGCGCTCC	60.0	659	INTERGENIC	--	--	--
Ca3_P3_2820552	Ca3	2820552	CAAAAAAAAAAAA/CAAAAAAAAAAAAA	AAAAGGCAAGAAATGGGCTT	TCATATATGGGGGAGTGGGA	60.1	369	INTERGENIC	--	--	--

INDEL marker IDs	Chromosomes/ unanchored scaffolds	Physical positions (bp)	InDels (*Kabuli* reference genome-CDC Frontier/PII)	Forward primers (5'-3')	Reverse primers (5'-3')	Anneling temperature (0C)	Expected amplified product size (bp)	Structural annotation		Functional annotation	
								Sequence components of *kabuli* genome	*Kabuli* gene accession IDs	NCBI-KOG	TFs
Ca3_P3_28262509	Ca3	28262509	GTTTTTTTT/GTTTTTTTTT	TACTTTTACCCCCATTGCCA	AAACACCCATCCCACGTATG	60.2	414	INTERGENIC	--	--	--
Ca3_P3_28357344	Ca3	28357344	TTTATTATTATTATTATTATTATTATTATTATTATTAT/TTTATTATTATTATTATTATTATTATTATTAT	CGTGTCTCCAATATTTTGCCT	TCCTCATTGAAATTTCCCCA	59.1	440	INTERGENIC	--	--	--
Ca3_P3_28461083	Ca3	28461083	ATTTTTTTTTT/ATTTTTTTTTTT	AATTGGATCCCCAACCTTTC	CCCCCTACCATTAAATTCCC	60.0	158	DRR	Ca_07158	--	--
Ca3_P3_28511565	Ca3	28511565	AATATATATATATATAT/AATATATATATATAT	TGGGATAGAGTTTGCTTGCC	CCCTCCCATGTTTTCCACTA	60.2	642	INTERGENIC	--	--	--
Ca3_P3_28533888	Ca3	28533888	ATTTTTTTT/ATTTTTTTTT	ATCCAATAATGGGTGTGGGA	TGGAGATAGCTGGGGAAGAA	59.9	477	INTERGENIC	--	--	--
Ca3_P3_2855993	Ca3	2855993	TAAA/TAA	CTTGGTTGGAATTGCATGTG	CGTTTTGGAAGGTAGGGTGT	60.0	514	INTERGENIC	--	--	--
Ca3_P3_28684295	Ca3	28684295	TTTTATTTATTTATTTATTTATTTATTTAT/TTTTATTTATTTATTTATTTATTTATTTAT	GCCCCTACTTGTAGCACTGG	CAAACAATAGATGGCACCGA	59.8	253	INTERGENIC	--	--	--
Ca3_P3_28743912	Ca3	28743912	ATGTGTGTGTGTGTGTGTGTGTGTG/ATGTGTGTGTGTGTGTGTGTGTGTG	ATCCCATAATCCCCTTTGGA	GTAAGGATGCCTGGTTTGGA	60.3	548	INTERGENIC	--	--	--
Ca3_P3_28797825	Ca3	28797825	A/AG	CATAGTCTGCGAAACCAGCA	GGTGGAATTGATCGGAGAGA	60.0	454	INTERGENIC	--	--	--
Ca3_P3_28799402	Ca3	28799402	AATATA/AATA	TCTAGCAAGTTCGCGCAATA	TTTGCTGCTGCAATTTAACAA	59.7	795	INTERGENIC	--	--	--

INDEL marker IDs	Chromosomes/ unanchored scaffolds	Physical positions (bp)	InDels (*Kabuli* reference genome-CDC Frontier/PII)	Forward primers (5'-3')	Reverse primers (5'-3')	Anneling temperature (0C)	Expected amplified product size (bp)	Structural annotation		Functional annotation	
								Sequence components of *kabuli* genome	*Kabuli* gene accession IDs	NCBI-KOG	TFs
Ca3_P3_28799571	Ca3	28799571	AG/A	TCTTGGACCTCACTGGGTTT	TGTCCTTGATCTTGTTTCCG	59.5	836	INTERGENIC	--	--	--
Ca3_P3_28799969	Ca3	28799969	GTA/GTATA	TGCAGCAGCAAAACTTGTGT	CGAATTAAAAACTCAAGGGTTCA	60.6	302	INTERGENIC	--	--	--
Ca3_P3_28800295	Ca3	28800295	AT/A	CGGAAACAAGATCAAGGACA	TGTACCGGCGTGTATGAAAA	58.7	654	INTERGENIC	--	--	--
Ca3_P3_2886351	Ca3	2886351	AATATATATATATATATATAT/AATATATATATATATATAT	TGTTTTTGTAAAGTAAAAGCCCC	CATGTGTTTGACGATGTGAATTT	58.7	548	INTERGENIC	--	--	--
Ca3_P3_28976687	Ca3	28976687	AATTATTATTATTATTATTATTATTATTATTATTATTATTATT/AATTATTATTATTATTATTATTATTATTATTATTATTATT	ATTCGCAGCCCATAATTTCA	TGGATTAATCATGAAATACCAACAA	60.4	445	INTERGENIC	--	--	--
Ca3_P3_28984656	Ca3	28984656	GTCTCTCT/GTCTCT	GAGAACGCAAGCTAACGGAC	GGATCAGAGAAAACGTGGGA	60.0	326	INTERGENIC	--	--	--
Ca3_P3_28986892	Ca3	28986892	GTTTTTTTTTTT/GTTTTTTTTTTTT	ATTTCTTTCCCGAACCGACT	TGATTCACGGATTATGCGAA	59.9	546	INTERGENIC	--	--	--
Ca3_P3_28994933	Ca3	28994933	AC/A	CAAATTAAGCAGAGCCCGTC	TTTTGGACAATGTCAACCCA	59.8	165	INTRON	Ca_07207	KTDL	--
Ca3_P3_28995118	Ca3	28995118	CAA/CAAA	CAAATTAAGCAGAGCCCGTC	CGGAATTTGAACAAAACGCT	59.8	769	INTRON	Ca_07207	KTDL	--
Ca3_P3_28996658	Ca3	28996658	ATT/ATTT	AGGGCTTTGGTCGTGTATTG	AACAATTGCGGGTGTAGAGG	60.0	861	INTRON	Ca_07207	KTDL	--

INDEL marker IDs	Chromosomes/ unanchored scaffolds	Physical positions (bp)	InDels (*Kabuli* reference genome-CDC Frontier/PII)	Forward primers (5'-3')	Reverse primers (5'-3')	Anneling temperature (0C)	Expected amplified product size (bp)	Structural annotation		Functional annotation	
								Sequence components of *kabuli* genome	*Kabuli* gene accession IDs	NCBI-KOG	TFs
Ca3_P3_28999636	Ca3	28999636	CT/CTT	TCTGAATTTCTGTACCG CCC	GGGATAGGGCATGTGT GAGT	60.1	536	INTRON	Ca_07207	KTDL	--
Ca3_P3_29095257	Ca3	29095257	TAAAAAAAAAA/TAAAAAAAAAAA	GTGGGTCGAATCAACA TTCA	GAAATGGCTTTTTAATC CACG	59.3	370	INTERGENIC	--	--	--
Ca3_P3_29097133	Ca3	29097133	CTACTTATTTA/C	AAAATGCTGCACTCAAA CGA	CACGGGTTGCGTATAT CCAT	59.5	233	INTERGENIC	--	--	--
Ca3_P3_29174780	Ca3	29174780	TTATATATATATATAT/TTATATATATAT ATATATATAT	ATCCACCCCACATTCAA ATC	GGAGAGAAATACGCGT GGAG	59.5	439	INTERGENIC	--	--	--
Ca3_P3_29263411	Ca3	29263411	TAAAAAAAA/TAAAAAAA	TTCACAAATGGACCGT GAAA	TTGCAACTTTACGAAA CCCC	59.9	537	DRR	Ca_07235	IR	Nin-like
Ca3_P3_29263688	Ca3	29263688	A/AAAGGT	TTGAAGATCTCTGTGG CGAA	CTTCTCTCCCCAGCTT GTTG	59.5	762	DRR	Ca_07235	IR	Nin-like
Ca3_P3_29446926	Ca3	29446926	GCA/GCACA	CAGCAGGGGCATACGT TAAT	GATTCCCCTCCCTGAT TCTC	60.0	306	INTERGENIC	--	--	--
Ca3_P3_2944898	Ca3	2944898	GAAAAAAAAAAAAA/GAAAAAAAAAAAA AAA	TCGGCAGATTGTGCAT AAAA	GACGAGTGGGCCAAA GAATA	60.2	838	INTERGENIC	--	--	--
Ca3_P3_29486404	Ca3	29486404	TAAAAAAAAA/TAAAAAAAAAA	TCACCCCAAACCAAAA GAAG	GAATTAAGACGTGGCC GGTA	59.9	592	INTRON	Ca_07254	--	C2H2
Ca3_P3_29512683	Ca3	29512683	AATATATATATATATATATAT/AATATAT ATATATATATAT	TGCAATGAATGGAATCT TGG	TCCAAAACAACAAGGA CACG	59.5	380	INTERGENIC	--	--	--

**INDEL marker IDs**	**Chromosomes/ unanchored scaffolds**	**Physical positions (bp)**	**InDels (*Kabuli* reference genome-CDC Frontier/PII)**	**Forward primers (5'-3')**	**Reverse primers (5'-3')**	**Anneling temperature (0C)**	**Expected amplified product size (bp)**	**Structural annotation**		**Functional annotation**	
								**Sequence components of *kabuli* genome**	***Kabuli* gene accession IDs**	**NCBI-KOG**	**TFs**
Ca3_P3_29613965	Ca3	29613965	AAATAATAATAATAATAATAA/AAATAATAATAATAATAATAATAATAATAATAATAA	CGGCAAAATATAACGAACACG	CATGAGGATGCGACCATCTA	60.4	500	DRR	Ca_07267	--	--
Ca3_P3_29658789	Ca3	29658789	ATTTTTTT/ATTTTTTTT	TGGCCTACTTATGGCCTGAC	AAGTCTGGCCTACGGCCTAT	60.1	619	INTERGENIC	--	--	--
Ca3_P3_29699118	Ca3	29699118	ATATTT/AT	AATACACCAGGAGCAATCGG	CAACTTTGGATTTGCTACCCC	60.0	732	INTERGENIC	--	--	--
Ca3_P3_29740808	Ca3	29740808	A/ATATATAT	TTTGGCCCGTTGTTATTCTC	GAAGCACCAGTCCTTACCACA	59.9	906	INTERGENIC	--	--	--
Ca3_P3_29746184	Ca3	29746184	TTATATATATATATATATATATATAT/TTATATATATATATATATATATATATATATATATATATATAT	CGTGATCAGAAGCGTGATTG	TCCTTGACTGAAACCCCAAC	60.4	649	INTERGENIC	--	--	--
Ca3_P3_29767581	Ca3	29767581	AATATATATATATATATATATATATAT/AATATATATATATATAT	AGCAAGGTCGACTTTCTCCA	TGGCACAACTCTAACAACCAA	60.0	694	DRR	Ca_07284	--	bHLH
Ca3_P3_29768034	Ca3	29768034	CTTTTTTTTTTT/CTTTTTTTTTTTT	TTGGTTGTTAGAGTTGTGCCA	GCGACCACACAATATCAACG	59.2	550	DRR	Ca_07284	--	bHLH
Ca3_P3_29809142	Ca3	29809142	ATAT/AATTAT	GCTCATGCCTCCTTCAAAAA	AAGCAGCCATAATGACAGGG	60.3	856	INTERGENIC	--	--	--
Ca3_P3_29829081	Ca3	29829081	CAAAAA/CAAAA	TTCATTCATCGCATTGAGGA	CTTTGAATCCTTGCCCAAAA	60.2	337	INTERGENIC	--	--	--
Ca3_P3_29848442	Ca3	29848442	AATATATATATATATATATATATATATATATATATATATATATAT/AATATATATATATATATATATATATATATATATATATATATATATATATATATAT	GCTGGTTCCAACACATGAGA	GATGCTAGCGCTTCACAAGA	59.7	258	INTERGENIC	--	--	--

**INDEL marker IDs**	**Chromosomes/ unanchored scaffolds**	**Physical positions (bp)**	**InDels (*Kabuli* reference genome-CDC Frontier/PII)**	**Forward primers (5'-3')**	**Reverse primers (5'-3')**	**Anneling temperature (0C)**	**Expected amplified product size (bp)**	**Structural annotation**		**Functional annotation**	
								**Sequence components of *kabuli* genome**	***Kabuli* gene accession IDs**	**NCBI-KOG**	**TFs**
Ca3_P3_29849306	Ca3	29849306	AATTATTATTATTATTATTATTATTATTATTATTATTATTATTATTATTATTATTATT/AATTATTATTATTATTATTATTATTATTATTATTATTATTATTATTATTATTATTATTATT	TGGCAATCATTTGCTTTTTG	AGAGGCATTGACTTTGGTGG	59.7	228	INTERGENIC	--	--	--
Ca3_P3_29950436	Ca3	29950436	ATATTATTATTATTATTATTATTATTATTATTATTATTATTATTATT/ATATTATTATTATTATTATTATTATTATTATTATTATTATTATTATTATTATTATT	TTCCGGAGAACTTTTTCCAA	TGACGTCTCTTCCTCGTGTG	59.7	333	INTERGENIC	--	--	--
Ca3_P3_30036759	Ca3	30036759	TTCTCTCTCTCTCTCTCTCTCTCTCTCTCTC/TTCTCTCTCTCTCTCTCTCTCTCTCTCTCTCTCTCTC	CTTATGCGGAGCTTACCCTG	TCAATTCGTTTGTTGCTGGA	59.9	756	INTRON	Ca_07319	P	--
Ca3_P3_30049696	Ca3	30049696	CATATATATATATATATATATATATATAT/CATATATATATATATATATATATATATATAT	TCCCCTTTTCCTCATCCTCT	TGCACTTTGTTCCCTCAACA	60.0	546	INTRON	Ca_07320	--	--
Ca3_P3_30072883	Ca3	30072883	GGTGAGGTG/GGTGAGGTGTGAGGTG	CACCTCACGCACCACATTAC	CACCAATTATGCCCCTTGTC	60.0	267	INTERGENIC	--	--	--
Ca3_P3_30100381	Ca3	30100381	ATATTATTATTATTATTATTATT/ATATTATTATTATTATTATT	TTGCACATTTACTCAACCGC	ATTCCAGAAATGCGACCTTG	59.7	319	INTERGENIC	--	--	--
Ca3_P3_30231295	Ca3	30231295	CACATA/CA	AGGCTCACTAAGCCCACAAA	CGAGTTTGCAAATGTGGCT	59.9	474	INTERGENIC	--	--	--
Ca3_P3_30415056	Ca3	30415056	AGT/AGAAAGAGTGT	TGGATACAAATGTCAACAAACTATCA	CGCATATTGTTTTATGCACCA	59.7	505	INTERGENIC	--	--	--
Ca3_P3_30417410	Ca3	30417410	ATTTTTTT/ATTTTTTTT	CCCCATAACGCATAATCACG	TCATTCTTTTTCTGAAATTCATGC	61.1	714	INTERGENIC	--	--	--
Ca3_P3_30484112	Ca3	30484112	TATCTTCTTCT/TATCTTCTTCTTCATCTTCATCTTCTTCT	TTGGATTCTTTTTCGCTGCT	TGTCATCCCAAACCCAATTT	60.0	189	INTRON	Ca_07369	--	--

INDEL marker IDs	Chromosomes/ unanchored scaffolds	Physical positions (bp)	InDels (*Kabuli* reference genome-CDC Frontier/PII)	Forward primers (5'-3')	Reverse primers (5'-3')	Anneling temperature (0C)	Expected amplified product size (bp)	Structural annotation		Functional annotation	
								Sequence components of *kabuli* genome	*Kabuli* gene accession IDs	NCBI-KOG	TFs
Ca3_P3_30602335	Ca3	30602335	TTG/T	GCCCTCAGATGAGAGTCAGG	AAATTAAACCCTCATCCCCG	59.9	842	INTERGENIC	--	--	--
Ca3_P3_30707652	Ca3	30707652	CTTTTTTTTTTTTTTTT/CTTTTTTTTTTTTTT	TGGTTCCAATCTCTACCGTCTT	TGAAAAAGAATAGCCACATATTCC	60.0	575	INTERGENIC	--	--	--
Ca3_P3_30894316	Ca3	30894316	A/AGAACTGGAGG	AATTTTCCTACTTGGGCGGT	AGTGAAATGCTCCACAGCCT	59.8	230	INTERGENIC	--	--	--
Ca3_P3_30899185	Ca3	30899185	G/GACA	CTTTAGTGGGGAGCCATGAA	GAAATTCGCCATTTTCTGGA	60.1	520	CDS (large-effect mutations)	Ca_12186	O	--
Ca3_P3_30927472	Ca3	30927472	ATTTT/ATTT	GCAATTTTGACCGCGATTTA	TGGTAGGAATGCCTCCTGAA	60.9	708	DRR	Ca_12190	--	--
Ca3_P3_30930504	Ca3	30930504	GTTTTTATTTTTATTTTTA/GTTTTTATTTTTA	GCATGGACCGTTTAAAATTG	TTTAGGTGGAAAATTCATTAGAAAA	58.0	818	INTERGENIC	--	--	--
Ca3_P3_30941967	Ca3	30941967	TTATATATATATATATATATATATATATATATATATA/TTATATATATATATATATATATATATATATATATA	GGGAAAACAATTATCCGAATC	ATGGATGCCACCTCTTGAGT	57.4	565	INTERGENIC	--	--	--
Ca3_P3_3094451	Ca3	3094451	CTTTTTTTTTTTT/CTTTTTTTTTTT	TTTGGAATCTTGTGAGGAGAA	TCAACAAGCCCCCATGTAAT	57.4	167	INTERGENIC	--	--	--
Ca3_P3_30964476	Ca3	30964476	TTATATATATATATATATA/TTATATATATATATATATATA	TGGACAATTTTCTTTGTCTTTTGA	GCAAGAACACATTTACGCGA	60.0	800	INTERGENIC	--	--	--
Ca3_P3_31029281	Ca3	31029281	ATTTTTTT/ATTTTTTTTT	TGGTCACAAAGGTGAAACTCA	ATTGGTTCGATTCTTCCGTG	59.2	952	INTERGENIC	--	--	--

INDEL marker IDs	Chromosomes/ unanchored scaffolds	Physical positions (bp)	InDels (*Kabuli* reference genome-CDC Frontier/PII)	Forward primers (5'-3')	Reverse primers (5'-3')	Anneling temperature (0C)	Expected amplified product size (bp)	Structural annotation		Functional annotation	
								Sequence components of *kabuli* genome	*Kabuli* gene accession IDs	NCBI-KOG	TFs
Ca3_P3_31087418	Ca3	31087418	TTATATATATATATATATATATATATATATAT/TTATATATATATATATATATATATATATATATATATATAT	AACACATTTATTCGACCGCA	CACGGTAAGGGACAACTGTG	59.0	533	INTERGENIC	--	--	--
Ca3_P3_31183232	Ca3	31183232	TATATA/TATATAAATATA	CACCGTATTACACCAAAACGA	TTTCGACGACAACAACAAGG	58.5	599	INTERGENIC	--	--	--
Ca3_P3_3122065	Ca3	3122065	TAAAAAAAAAA/TAAAAAAAAAAA	TGCCTAAGGCGTATGATTCC	CGGGGAATCAGCTTTATTGA	60.1	156	INTERGENIC	--	--	--
Ca3_P3_31266098	Ca3	31266098	TC/T	TGCGTGCGTCTTTGATTAAC	TAGCCCAAGTCAACCGAATC	59.9	446	INTERGENIC	--	--	--
Ca3_P3_31266903	Ca3	31266903	ATTTTTTTT/ATTTTTTTTT	CACGCATCATTCCTTCATTG	TTTGTTTCCCACTTTTTGCC	60.1	600	INTERGENIC	--	--	--
Ca3_P3_31283918	Ca3	31283918	GTTTTTT/GTTTTTTT	AGCCTTCGCAGTCTTTTCAA	ACTGCGCTTTCTGCTTCAAT	60.1	667	DRR	Ca_12224	S	--
Ca3_P3_31285016	Ca3	31285016	A/AT	CGTCGTTTTCAACTGGGAAT	GGATACGAAGTCGGTTGCAT	60.0	328	INTERGENIC	--	--	--
Ca3_P3_31285044	Ca3	31285044	TATAATAATA/TATAATA	CGTCGTTTTCAACTGGGAAT	GGATACGAAGTCGGTTGCAT	60.0	328	INTERGENIC	--	--	--
Ca3_P3_31286034	Ca3	31286034	GTTTTTTT/GTTTTTTTTTTT	TGGTAAATTGTTCGTGTTTGAA	AACTTTGACATTGGGTCGGT	58.1	391	INTERGENIC	--	--	--
Ca3_P3_31289874	Ca3	31289874	ATTTTT/ATT	GGTTCAAACGTGTTCCCTTG	TTAAAGAGCGACCTCCCTTC	60.4	460	DRR	Ca_12225	P	ERF

INDEL marker IDs	Chromosomes/ unanchored scaffolds	Physical positions (bp)	InDels (*Kabuli* reference genome-CDC Frontier/PII)	Forward primers (5'-3')	Reverse primers (5'-3')	Anneling temperature (0C)	Expected amplified product size (bp)	Structural annotation		Functional annotation	
								Sequence components of *kabuli* genome	*Kabuli* gene accession IDs	NCBI-KOG	TFs
Ca3_P3_31290388	Ca3	31290388	AG/A	TTTAAAGAAGGGAGGT CGCTC	TTGAAGCAGTGTTGTG GGAA	59.8	632	DRR	Ca_12225	P	ERF
Ca3_P3_31292004	Ca3	31292004	TTCT/TT	CCAAACACTTTAATTAG CTTAACATTT	AATTGGCTTGATGTCC CAAC	57.8	419	INTERGENIC	--	--	--
Ca3_P3_31296714	Ca3	31296714	ATTTTTTT/ATTTTTT	TCGGTTTTAAGGGAAG CAAA	ACACACGCGTCAAACT CAAA	59.7	365	INTRON	Ca_12226	K	MYB
Ca3_P3_31303690	Ca3	31303690	GAAAA/GAAA	TCCCTCAATTGCATCAA ACA	ACAACAGATGGCTGCA CAAG	60.0	482	DRR	Ca_12228	--	--
Ca3_P3_31307321	Ca3	31307321	GACTTATA/GA	TCTTGGGACATGTGAA TGTGA	GTGAGCCCACATTTTG TCCT	60.0	886	INTRON	Ca_12229	--	Dof
Ca3_P3_31307610	Ca3	31307610	CAAACGCACATAA/CA	TCGTTTCCAAATCCAAC ACA	GTGAGCCCACATTTTG TCCT	59.9	214	INTRON	Ca_12229	--	Dof
Ca3_P3_31310435	Ca3	31310435	CTAGT/CT	CTCTCCCACTGTGGCT CTTC	AAAGCCAAATCCCCAT ATCC	60.0	573	INTRON	Ca_12229	--	Dof
Ca3_P3_31311879	Ca3	31311879	TTATA/TTATATA	TTTCACGTTCTTCAAAC CCA	TTATTCATTCGCTCGT GCAG	59.1	352	DRR	Ca_12230	--	Dof
Ca3_P3_31312021	Ca3	31312021	TAAA/TAAATAAAA	TTTCACGTTCTTCAAAC CCA	TTATTCATTCGCTCGT GCAG	59.1	352	DRR	Ca_12230	--	Dof
Ca3_P3_31320036	Ca3	31320036	AATATAT/AATAT	GGTATGGAAGCCTTGT GCAT	AAACATGGCATCACCC AAAT	60.0	494	DRR	Ca_12232	U	bHLH

INDEL marker IDs	Chromosomes/ unanchored scaffolds	Physical positions (bp)	InDels (*Kabuli* reference genome-CDC Frontier/PII)	Forward primers (5'-3')	Reverse primers (5'-3')	Anneling temperature (0C)	Expected amplified product size (bp)	Structural annotation		Functional annotation	
								Sequence components of *kabuli* genome	*Kabuli* gene accession IDs	NCBI-KOG	TFs
Ca3_P3_31320091	Ca3	31320091	TGAG/TGAGAG	GGTATGGAAGCCTTGTGCAT	AAACATGGCATCACCCAAAT	60.0	494	DRR	Ca_12232	U	bHLH
Ca3_P3_31322105	Ca3	31322105	TAAAAAAAAAA/TAAAAAAAAA	GTTTCAACATGACCATCCCC	CCCCCTGAAAGATACCCAAT	60.0	868	INTRON	Ca_12232	U	bHLH
Ca3_P3_31322668	Ca3	31322668	TAAAAA/TAAAA	ATTGGGTATCTTTCAGGGGG	ACTCTTTCGAATGTGGTGCC	60.0	229	INTRON	Ca_12232	U	bHLH
Ca3_P3_31324543	Ca3	31324543	TAG/TAGTAATGTAATAAAG	CCTGGGTCCAATCCTTACCT	TCAGCTTATTAGGGCATGGG	60.2	660	INTRON	Ca_12232	U	bHLH
Ca3_P3_31325736	Ca3	31325736	ATTT/ATTTT	ATTAATTGGGCTGACGTGGA	GTCCACATTAGGGACACGCT	60.3	426	INTERGENIC	--	--	--
Ca3_P3_31327189	Ca3	31327189	TAAAAAAAAA/TAAAAAAAAAA	CAAATGCATGGACAAACTGG	GGATCATTCGCGACCATAAT	60.0	426	INTERGENIC	--	--	--
Ca3_P3_31328179	Ca3	31328179	GTTTTTTTTT/GTTTTTTT	TCCAACTAACCGCAATTCAA	TCATGGCAAATTTAGGATGTTG	59.2	447	INTERGENIC	--	--	--
Ca3_P3_31335663	Ca3	31335663	TTATATATATAT/TTATATATAT	GTTTGAGACCCTGCTTTTGC	AAGGATACACCGCACGTTTC	59.9	767	DRR	Ca_12233	S	bZIP
Ca3_P3_31338630	Ca3	31338630	ATTT/ATT	TATCGTTCGTTGCTCTCGTG	GGCCGAAAGTTATTACGTGG	60.0	870	INTERGENIC	--	--	--
Ca3_P3_31339236	Ca3	31339236	T/TC	CCAACCCATGTACCGACATT	AGAGACCGTTTCATGAGATTTG	60.5	525	INTERGENIC	--	--	--

INDEL marker IDs	Chromosomes/ unanchored scaffolds	Physical positions (bp)	InDels (*Kabuli* reference genome-CDC Frontier/PII)	Forward primers (5'-3')	Reverse primers (5'-3')	Anneling temperature (0C)	Expected amplified product size (bp)	Structural annotation		Functional annotation	
								Sequence components of *kabuli* genome	*Kabuli* gene accession IDs	NCBI-KOG	TFs
Ca3_P3_31343206	Ca3	31343206	AG/A	TGGTTTGAAATACTGGTCATGC	GATCACGGGAGGATCGACTA	59.9	257	DRR	Ca_12234	--	--
Ca3_P3_31343763	Ca3	31343763	TAAA/TAAAA	AATAGTCGATCCTCCCGTGAT	CCATGCTCAAGTCTAGGTTCG	59.8	510	DRR	Ca_12234	--	--
Ca3_P3_31345376	Ca3	31345376	AAATGGAAT/AAAT	ATGGAAAGCCAGCTGAAGAA	CTATGTTGTGGGAAGGTGGG	60.0	592	INTERGENIC	--	--	--
Ca3_P3_31349079	Ca3	31349079	CAT/CATGAT	TAAAGGCATTTGCGTGTTGA	TTTTGGTGAAAGCCAATAACG	60.3	512	INTERGENIC	--	--	--
Ca3_P3_31350671	Ca3	31350671	ATCGAGT/ATCGAGTCGAGT	TCTTAGCGTAGATTCGCCGT	AACATCTGGGCTAATCGGTG	60.0	280	INTERGENIC	--	--	--
Ca3_P3_31350700	Ca3	31350700	TCATG/TCATGCATG	TCTTAGCGTAGATTCGCCGT	AACATCTGGGCTAATCGGTG	60.0	280	INTERGENIC	--	--	--
Ca3_P3_31350961	Ca3	31350961	GTTTAATTTAATTT/GTTTAATTT	TCTTAGCGTAGATTCGCCGT	TGTCATTGAGGTGGGTGTTG	60.0	781	INTERGENIC	--	--	--
Ca3_P3_31351028	Ca3	31351028	A/ATAT	CACCGATTAGCCCAGATGTT	TGTCATTGAGGTGGGTGTTG	60.0	521	INTERGENIC	--	--	--
Ca3_P3_31370335	Ca3	31370335	ATATTAT/ATATTATTAT	GGAGATGATGACGCCAAAGT	TGGTCTGACCCTTAAATTGGA	60.1	679	INTERGENIC	--	--	--
Ca3_P3_31375853	Ca3	31375853	TAATAAATAAATAA/TAATAAATAAATAAATAA	TGGTTGTCAGAGCAGGTTCA	CATATGTGAACCATGGCAGC	60.4	334	INTERGENIC	--	--	--

INDEL marker IDs	Chromosomes/ unanchored scaffolds	Physical positions (bp)	InDels (*Kabuli* reference genome-CDC Frontier/PII)	Forward primers (5'-3')	Reverse primers (5'-3')	Anneling temperature (0C)	Expected amplified product size (bp)	Structural annotation		Functional annotation	
								Sequence components of *kabuli* genome	*Kabuli* gene accession IDs	NCBI-KOG	TFs
Ca3_P3_31381469	Ca3	31381469	TC/T	TAAACCTTGGCTGGGTCAAC	TTGTTTATTTTGTTCGTCGTGTT	60.0	403	INTERGENIC	--	--	--
Ca3_P3_31383053	Ca3	31383053	CTTTTT/CTTTT	CCCTAAATCAACGGGAGTCA	AGCCGTCACTCTTTCTCCAA	59.9	285	INTERGENIC	--	--	--
Ca3_P3_31383299	Ca3	31383299	GATAAAAA/GATAAAAATAAAAA	TTGGAGAAAGAGTGACGGCT	GAGGTGTTTGTTTCGCGTCT	60.0	671	INTERGENIC	--	--	--
Ca3_P3_31385464	Ca3	31385464	T/TA	TTTCGATTGATTTTGCATTTTG	TTGTGACCCCCTCTATTTTGA	59.9	736	INTERGENIC	--	--	--
Ca3_P3_31396349	Ca3	31396349	CT/CTTT	TTCCTCTTGTGCGTTGTTGA	GGGAGTGACCAAAACTGTCG	60.4	251	INTERGENIC	--	--	--
Ca3_P3_31408939	Ca3	31408939	TAAAAAAAAA/TAAAAAAAA	TTAAAATTCATCCCCTCCCC	AGATTTCCTTTGCATGGGTG	60.0	828	INTRON	Ca_12237	O	--
Ca3_P3_31415275	Ca3	31415275	T/TG	GTAGCTAACCCAACCGGTGA	GGGCCCGTTATATATTGGCT	60.0	565	INTERGENIC	--	--	--
Ca3_P3_31440549	Ca3	31440549	ATTT/ATTTT	GCGGTTGTCATTTCTCGTTT	TTGTTAACGAATTGCACCGA	60.1	583	DRR	Ca_12242	--	--
Ca3_P3_31441706	Ca3	31441706	AT/ATGAAATT	AACAATTTTTGGAACCCCTAA	TTTGCAAGTGCAACTTCTGC	57.5	669	DRR	Ca_12242	--	--
Ca3_P3_31442973	Ca3	31442973	CTTTTT/CTTTTTT	TAGATGATTTTGGCAAGGGC	CTGGACTTTCGTCATCAGCA	60.0	457	INTRON	Ca_12242	--	--

INDEL marker IDs	Chromosomes/ unanchored scaffolds	Physical positions (bp)	InDels (*Kabuli* reference genome-CDC Frontier/PII)	Forward primers (5'-3')	Reverse primers (5'-3')	Anneling temperature (0C)	Expected amplified product size (bp)	Structural annotation		Functional annotation	
								Sequence components of *kabuli* genome	*Kabuli* gene accession IDs	NCBI-KOG	TFs
Ca3_P3_31443022	Ca3	31443022	CGT/C	TAGATGATTTTGGCAAGGGC	CTGGACTTTCGTCATCAGCA	60.0	457	INTRON	Ca_12242	--	--
Ca3_P3_31443328	Ca3	31443328	ATTTTT/ATTTTTT	TGCTGATGACGAAAGTCCAG	CATATTGGATGGGATGGTCC	60.0	302	INTRON	Ca_12242	--	--
Ca3_P3_31445239	Ca3	31445239	ATA/ATATGTA	GGCCGGATTAGTTGAGTTTG	AATCGTTGGTAGGCGAGAAA	59.6	536	DRR	Ca_12243	G	--
Ca3_P3_31445406	Ca3	31445406	ATT/AT	GGCACAATGCAAAAAGAATTG	AGCGCAAACTAAACCGGTAA	60.5	297	DRR	Ca_12243	G	--
Ca3_P3_31445620	Ca3	31445620	ATCATT/AT	TTACCGGTTTAGTTTGCGCT	TGCAAATTGGGATGACAAAA	59.8	416	DRR	Ca_12243	G	--
Ca3_P3_31448937	Ca3	31448937	ATTT/ATTTAGAAAGTTT	TGGCCACCACTAACACAAAA	ACAGGTGATCCTCTTTCCCC	60.0	757	INTERGENIC	--	--	--
Ca3_P3_31451366	Ca3	31451366	CTTTT/CTTT	TGGATTTTACAAACGCCCTC	TCTGGCCTGACCTGAGCTAT	59.9	947	INTERGENIC	--	--	--
Ca3_P3_31465126	Ca3	31465126	GCAAT/GCAATCTACAAT	TTCCTGATTCACAACTCCCC	TCATGAGTGCACAAAGCACA	59.9	205	DRR	Ca_12245	P	--
Ca3_P3_31466998	Ca3	31466998	CTTTTTTTT/CTTTTTTTTT	GAAACCACGAGCAAGAAAGC	GCTCTCGCAAGAACAAAACC	60.0	759	INTERGENIC	--	--	--
Ca3_P3_31475626	Ca3	31475626	TAT/TATTCATATAGAT	ATCTTGTCGTGCCGTCATTT	TCACCAAACAAATCTGAACCA	60.5	540	INTERGENIC	--	--	--

INDEL marker IDs	Chromosomes/ unanchored scaffolds	Physical positions (bp)	InDels (*Kabuli* reference genome-CDC Frontier/PII)	Forward primers (5'-3')	Reverse primers (5'-3')	Anneling temperature (0C)	Expected amplified product size (bp)	Structural annotation		Functional annotation	
								Sequence components of *kabuli* genome	*Kabuli* gene accession IDs	NCBI-KOG	TFs
Ca3_P3_31475946	Ca3	31475946	CT/CTGGTATTT	CGTGCCGTCATTTTCAAGTT	TCACCAAACAAATCTGAACCA	61.1	533	INTERGENIC	--	--	--
Ca3_P3_31476572	Ca3	31476572	AAATA/AA	TGGTTGTTTCACTCACGTTTG	GGATGGCAATCCTCCATTAC	59.6	519	INTERGENIC	--	--	--
Ca3_P3_31476761	Ca3	31476761	CTTATTAT/CTTATTATTAT	TGGTTGTTTCACTCACGTTTG	AGGGTGTCACAAATGGAAGC	59.6	612	INTERGENIC	--	--	--
Ca3_P3_31477247	Ca3	31477247	TTTTATTTATTTATTTATTTATT/TTTTATTTATTTATTTATTTATTTATTTATT	GCTTCCATTTGTGACACCCT	ACAAAAGGCCACTTCCAATG	60.0	596	INTERGENIC	--	--	--
Ca3_P3_31482869	Ca3	31482869	CGTTATAAAGG/CG	CTTCCTTGACACGCTAAGGG	AAGTTCGTTTTGTTTTGCGG	59.9	618	INTERGENIC	--	--	--
Ca3_P3_31488477	Ca3	31488477	CCTCT/CCT	AGGCCCTTATGACGTTGATG	ACACATTCATTCGCCAAACA	60.0	496	INTRON	Ca_12247	T	--
Ca3_P3_31489433	Ca3	31489433	ATTT/ATTTTT	GGTGCCAAGAGACCAGAGAG	AGTGGAGCTAGCAGGAACCA	60.0	383	INTRON	Ca_12247	T	--
Ca3_P3_31491087	Ca3	31491087	CTTTTT/CTTTT	CCCCCAAACCTTCAAAAAGT	CGTTTGATAATTTGAGGGGC	60.2	421	DRR	Ca_12247	T	--
Ca3_P3_31499028	Ca3	31499028	ATTTTTTTTT/ATTTTTTTTTT	GGCCAAGTTTTTGTATTGGC	GTCCTTCCTGCCTCGAAAAT	59.5	348	DRR	Ca_12249	I	--
Ca3_P3_31499806	Ca3	31499806	CATATCAATATCAATATCAATATCAATAT/CATATCAATATCAATATCAATAT	GCGGCAAATAACATGTGAAA	TCCTAGACTTGTTGGGGCAC	59.6	565	DRR	Ca_12248	T	--

INDEL marker IDs	Chromosomes/ unanchored scaffolds	Physical positions (bp)	InDels (*Kabuli* reference genome-CDC Frontier/PII)	Forward primers (5'-3')	Reverse primers (5'-3')	Anneling temperature (0C)	Expected amplified product size (bp)	Structural annotation		Functional annotation	
								Sequence components of *kabuli* genome	*Kabuli* gene accession IDs	NCBI-KOG	TFs
Ca3_P3_31508746	Ca3	31508746	TAAA/TAA	GGGCAAAAACTAAAGAGGGG	TTGGATCCCACCAAGAAAAG	59.9	650	INTRON	Ca_12249	I	--
Ca3_P3_31519164	Ca3	31519164	C/CT	TACACGTGGCCTCAAACAAA	TCAAGCATGGTTCCAATCAG	60.1	364	INTERGENIC	--	--	--
Ca3_P3_31519218	Ca3	31519218	AA/AACAGGA	TACACGTGGCCTCAAACAAA	AAGCCTGCAATGGATGAAAG	60.1	800	INTERGENIC	--	--	--
Ca3_P3_31520896	Ca3	31520896	ATTTTTTTTTT/ATTTTTTTTTTTT	GGCAACAACACTTGCAGCTA	GCAGCAGTCAGCACTTGTTC	60.1	428	INTRON	Ca_12251	--	--
Ca3_P3_31530376	Ca3	31530376	AAT/AATGCAT	CCAATCGTAGGAAGGCCATA	TGGGAAATAGATCGGTCTCG	59.9	730	INTRON	Ca_12252	E	TALE
Ca3_P3_31530486	Ca3	31530486	TA/TACTTGA	AACTCCTTCTGCACATTGGG	TGGGAAATAGATCGGTCTCG	60.1	449	INTRON	Ca_12252	E	TALE
Ca3_P3_31530560	Ca3	31530560	TTGGGT/TTGGGTGGGT	AACTCCTTCTGCACATTGGG	TGGGAAATAGATCGGTCTCG	60.1	449	INTRON	Ca_12252	E	TALE
Ca3_P3_31531338	Ca3	31531338	CATGTAATGTAAT/CATGTAAT	CCGTACCAATCATCCCAATC	GCCGTTATCGGACCTTTGTA	60.0	427	INTRON	Ca_12252	E	TALE
Ca3_P3_31560705	Ca3	31560705	A/AAG	TGTTCACTGCCAAACTCTGC	GCAGTGCACAACTGATGGTT	60.0	585	INTERGENIC	--	--	--
Ca3_P3_31563003	Ca3	31563003	CATA/CATATA	GAATGGTGTTCCTTTGTCCC	AATTTCACCCAAAGACACGG	59.2	274	INTERGENIC	--	--	--

INDEL marker IDs	Chromosomes/ unanchored scaffolds	Physical positions (bp)	InDels (*Kabuli* reference genome-CDC Frontier/PII)	Forward primers (5'-3')	Reverse primers (5'-3')	Anneling temperature (0C)	Expected amplified product size (bp)	Structural annotation		Functional annotation	
								Sequence components of *kabuli* genome	*Kabuli* gene accession IDs	NCBI-KOG	TFs
Ca3_P3_31563949	Ca3	31563949	CAAAA/CAAA	TGCTTCCAACTCCTTATCCAA	GATGATGGCTTACTTCGGGA	59.7	357	INTERGENIC	--	--	--
Ca3_P3_31563986	Ca3	31563986	TATTAATTA/TATTA	TGCTTCCAACTCCTTATCCAA	GATGATGGCTTACTTCGGGA	59.7	357	INTERGENIC	--	--	--
Ca3_P3_31565099	Ca3	31565099	TAAAAAAAAA/TAAAAAAAA	GTAGCAATCTCAAGGCCGAA	CCACCCCAACCATGTTAATC	60.4	501	DRR	Ca_12255	--	G2-like
Ca3_P3_31565430	Ca3	31565430	GACGCCA/GA	GATTAACATGGTTGGGGTGG	TGCAAGCATGTGGTTCATTT	59.9	258	DRR	Ca_12255	--	G2-like
Ca3_P3_31566099	Ca3	31566099	CG/C	AAAGGGCATGGGGAAAATAG	TTTTCATGCATTTGAGACGC	60.1	338	DRR	Ca_12255	--	G2-like
Ca3_P3_31566407	Ca3	31566407	T/TCAGTACCATATTAAAACC	GCGTCTCAAATGCATGAAAA	GCAGATAATGGCTACGCCTC	59.8	441	DRR	Ca_12255	--	G2-like
Ca3_P3_31567532	Ca3	31567532	TC/TCTCTGC	TGGAGGTACCTGCCTTGTTC	ATCCCCTTCGCTAGTACGGT	60.1	754	INTRON	Ca_12255	--	G2-like
Ca3_P3_31569678	Ca3	31569678	TAAAA/TAAAAA	TGTCAGTGCTGTGTGCGTTA	TTTGTTGAATACCGTTGGCA	60.1	635	INTRON	Ca_12255	--	G2-like
Ca3_P3_31574191	Ca3	31574191	ATTT/ATTTT	TAGCGCATTTATATTGCCCC	CAATCGCAGCGACGTAGATA	59.9	767	INTERGENIC	--	--	--
Ca3_P3_31576727	Ca3	31576727	AATATATATATATATATATATATATA/AATATATATATATATATA	GGGGTTGTGTTTGGATGAAG	AACCTCCATGGACATAGTTGAAA	60.2	946	INTERGENIC	--	--	--

INDEL marker IDs	Chromosomes/ unanchored scaffolds	Physical positions (bp)	InDels (*Kabuli* reference genome-CDC Frontier/PII)	Forward primers (5'-3')	Reverse primers (5'-3')	Anneling temperature (0C)	Expected amplified product size (bp)	Structural annotation		Functional annotation	
								Sequence components of *kabuli* genome	*Kabuli* gene accession IDs	NCBI-KOG	TFs
Ca3_P3_31578728	Ca3	31578728	CATAT/CAT	TCCATGAAATTGAAATAAAACGC	GCCCGTTAGGATGTGGAGTA	60.2	715	DRR	Ca_12256	--	--
Ca3_P3_31578868	Ca3	31578868	TAAAAA/TAAAAAA	GCAGGACATGAAGATTTGGG	GCCCGTTAGGATGTGGAGTA	60.5	273	DRR	Ca_12256	--	--
Ca3_P3_31583199	Ca3	31583199	C/CG	AACTGGAGAAGGGGGAAAAA	TTTGCCTGGATTTTGGTTTC	59.9	430	DRR	Ca_12257	J	WRKY
Ca3_P3_31583283	Ca3	31583283	ATTGT/ATTGTTGT	AACTGGAGAAGGGGGAAAAA	TTTGCCTGGATTTTGGTTTC	59.9	430	DRR	Ca_12257	J	WRKY
Ca3_P3_31588307	Ca3	31588307	T/TC	CTTCCAAATACCTCCCCCAT	CCGTGGCCTTCTCTACTCTG	60.0	346	DRR	Ca_12258	BK	G2-like
Ca3_P3_31589916	Ca3	31589916	CCT/CCTCT	TGCTTGTTCAAAATTGTCGC	TTTCACCCATCACTGCTTCA	59.9	399	INTRON	Ca_12258	BK	G2-like
Ca3_P3_31593525	Ca3	31593525	GCTCCA/G	GAAGCTCCTGGTTCAACTGC	GGCACCGAATCCAAAAGTTA	60.0	770	CDS (FRAME SHIFT)	Ca_12258	BK	G2-like
Ca3_P3_31602650	Ca3	31602650	AC/A	GCACGAGCACAACTTCAAAA	AAAGAACCATCATCGCAGGT	60.0	618	INTERGENIC	--	--	--
Ca3_P3_31602911	Ca3	31602911	ATGTT/ATGTTGTT	ACCGAATATTCCATGCTCCA	CCAAGTGATGTGTCATGGCT	60.3	565	INTERGENIC	--	--	--
Ca3_P3_31603278	Ca3	31603278	CA/C	ACCGAATATTCCATGCTCCA	CCAAGTGATGTGTCATGGCT	60.3	565	INTERGENIC	--	--	--

INDEL marker IDs	Chromosomes/ unanchored scaffolds	Physical positions (bp)	InDels (*Kabuli* reference genome-CDC Frontier/PII)	Forward primers (5'-3')	Reverse primers (5'-3')	Anneling temperature (0C)	Expected amplified product size (bp)	Structural annotation		Functional annotation	
								Sequence components of *kabuli* genome	*Kabuli* gene accession IDs	NCBI-KOG	TFs
Ca3_P3_31604363	Ca3	31604363	AATATATA/AATATATATA	GATGAGCAAAGGGCATGATT	CAAATCAGCAGATTCCAGCA	60.0	393	INTERGENIC	--	--	--
Ca3_P3_31604401	Ca3	31604401	TCTTACTTA/TCTTA	GATGAGCAAAGGGCATGATT	CAAATCAGCAGATTCCAGCA	60.0	393	INTERGENIC	--	--	--
Ca3_P3_31605810	Ca3	31605810	CAAAAAAATATCATTAAAAAAATAT/CAAAAAAATAT	ACGGGAATAGGCCACTCTTT	GAGGATACCCATTTGCGAGA	60.0	855	INTERGENIC	--	--	--
Ca3_P3_31606081	Ca3	31606081	T/TTTTTC	GCCACTGCATTAATTGACGA	GAGGATACCCATTTGCGAGA	59.7	254	INTERGENIC	--	--	--
Ca3_P3_31606915	Ca3	31606915	ATCTTCTTCTTCTTC/ATCTTCTTCTTCTTCTTC	GTGGCAGCTGCAAGAATACA	ATGCCATTTGGCCCAATAAT	60.0	894	CDS (large-effect mutations)	Ca_12260	--	--
Ca3_P3_31607443	Ca3	31607443	G/GT	TTTGACATTTGAGAAATGGGG	CCAATTGCTGCTACTCCTCC	59.8	209	INTRON	Ca_12260	--	--
Ca3_P3_31610292	Ca3	31610292	TAAAAAAAAAA/TAAAAAAAA	GCATTCATTGGTGTGAGTGG	TTTGATGGTGTGGTTTCACG	60.0	695	INTERGENIC	--	--	--
Ca3_P3_31631967	Ca3	31631967	AATAT/AAT	ATTTCAGCAGTCAACCCCAG	TCGAATTCCACAAATGCGTA	60.1	738	INTRON	Ca_12262	T	--
Ca3_P3_31632588	Ca3	31632588	ATTTT/ATTT	TGTTCAAAATGCGCTACAGG	AGCCAATTGTATGGGAGACG	59.9	392	INTRON	Ca_12262	T	--
Ca3_P3_31633512	Ca3	31633512	GTT/GTTT	AAATGCCTGGTTTTTGTTGC	CCTCCAGTTTCTTGGCATGT	60.0	288	INTRON	Ca_12262	T	--

INDEL marker IDs	Chromosomes/ unanchored scaffolds	Physical positions (bp)	InDels (*Kabuli* reference genome-CDC Frontier/PII)	Forward primers (5'-3')	Reverse primers (5'-3')	Anneling temperature (0C)	Expected amplified product size (bp)	Structural annotation		Functional annotation	
								Sequence components of *kabuli* genome	*Kabuli* gene accession IDs	NCBI-KOG	TFs
Ca3_P3_31637386	Ca3	31637386	GAAAAAAAA/GAAAAAAAAAA	TGATACCACCCAGTTTCACTTC	GCACAAGCAGAGCAAAATGA	59.0	254	DRR	Ca_12264	--	NF-YB
Ca3_P3_31641658	Ca3	31641658	TAATTAAAATTAAAAT/TAATTAAAATTAAAATTAAAAT	GCTCACCAGGAATTTTTCCA	CAGCCATGTACAAGCTGCAT	60.1	903	DRR	Ca_12265	--	--
Ca3_P3_31820476	Ca3	31820476	GAAAAAAAAAAAAA/GAAAAAAAAAAAA	CAGTCACCATCACCAACAGC	TCACGTGTTCCAGCATTTTG	60.2	360	INTERGENIC	--	--	--
Ca3_P3_31846181	Ca3	31846181	CCTCTCTCTCTCTCTCTCTCTCTCTCT/CCTCTCTCTCTCTCTCTCTCTCTCTCT	GCCACTACCAGCCATTGTTT	CGCATCCAACCTGTACACAC	60.0	365	DRR	Ca_12288	G	--
Ca3_P3_3185857	Ca3	3185857	TAAAAAAAAAA/TAAAAAAAA	CGCCTCTCTTTTTCTATGCG	TGTTGCTGTTGTTGCCTCTC	60.1	468	INTERGENIC	--	--	--
Ca3_P3_31895120	Ca3	31895120	CTAATATCTTAA/CTAATATCTTAATATCTTAA	GCCCATCAAACTCTGTGGAT	ATCGGGTGTCAATTGTCGAT	59.9	771	INTERGENIC	--	--	--
Ca3_P3_31918341	Ca3	31918341	TAATCTGTGAAAT/TAATCTGTGAAATCTGTGAAAT	TGGTCCAAAAACCACTCACA	TGAATCACCATTTGCCAGAA	60.0	761	INTERGENIC	--	--	--
Ca3_P3_31939882	Ca3	31939882	TAAAAA/TAAAA	GCACGGAAACAGCGATTTTA	GCGGTTGTCCGTCCTAATAA	61.1	896	INTERGENIC	--	--	--
Ca3_P3_3194968	Ca3	3194968	CA/CAA	CATCCAGGAAGGCAGAAAAC	GGAGGCAAACCAAGTGAAAA	59.7	201	INTERGENIC	--	--	--
Ca3_P3_3198316	Ca3	3198316	TTTATTATTATTATTATTATTATTATTATTATTATTATTATTATTATTATTAT/TTTATTATTATTATTATTATTATTATTATTATTATTATTATTATTATTATTAT	GTTGGACAACATCCGAACAA	GCAAATTTGGGAAGCTTGAA	59.4	575	INTERGENIC	--	--	--

INDEL marker IDs	Chromosomes/ unanchored scaffolds	Physical positions (bp)	InDels (*Kabuli* reference genome-CDC Frontier/PII)	Forward primers (5'-3')	Reverse primers (5'-3')	Anneling temperature (0C)	Expected amplified product size (bp)	Structural annotation		Functional annotation	
								Sequence components of *kabuli* genome	*Kabuli* gene accession IDs	NCBI-KOG	TFs
Ca3_P3_3235341	Ca3	3235341	AGCTTCAGTCGCT/AGCT	TGGAGTGAGCTGTGGTTGAG	TGGTCACCACTTGGTGTTTG	60.0	840	INTERGENIC	--	--	--
Ca3_P3_32354219	Ca3	32354219	ATTTTTTTTTT/ATTTTTTTTTTT	TGGTTGTTAGCATGCAAGTG	TTCACAACCCACATTTGGAC	58.3	508	DRR	Ca_11959	O	MYB_related
Ca3_P3_3235471	Ca3	3235471	TAAA/TAA	ATAGGCCAAGCAACCAAAGA	GACGATGGTGAAGATGACGA	59.7	663	INTERGENIC	--	--	--
Ca3_P3_32379633	Ca3	32379633	TTAATAATAATAATAA/TTAATAATAATAATAA	TGCATTTTGCATTTTGTTGTT	TGCGGATTGTATTTTGGGTT	59.1	347	INTRON	Ca_11961	--	FAR1
Ca3_P3_32399895	Ca3	32399895	CATATATATATATATATATATATATATATATATAT/CATATATATATATATATATATATATATATATATATATATATATATATAT	TTTCTTTCCGAAAATGGAGA	TGTGAATCCCGCTTTAACAA	57.4	523	INTERGENIC	--	--	--
Ca3_P3_32458289	Ca3	32458289	CAAAAA/CAAA	CCTCCTCACGCAATTTTTGT	TCAACAGCGTCTTGTGGAAG	60.1	692	INTRON	Ca_11969	J	--
Ca3_P3_32486522	Ca3	32486522	AAATAATAATAATAATAATAATAATAATAATAAT/AAATAATAATAATAATAATAATAATAATAAT	AACCACTTTTAAATCGATGCG	TGCAGTAGTTTGCTGCCATC	59.1	498	DRR	Ca_11974	--	--
Ca3_P3_32551513	Ca3	32551513	TAAAAAAAAAAAA/TAAAAAAAAAAA	AATGCATTGGACCTACCGAC	TTTGGCTGACCTTGATCCTC	59.8	303	DRR	Ca_11981	R	ZF-HD
Ca3_P3_32563568	Ca3	32563568	TTATATATATATATATATAT/TTATATATATATATATATATAT	CGAAAGAAAAAGAAAACATCGC	GAAGGTGGCTCGGCATATAA	60.2	574	INTERGENIC	--	--	--
Ca3_P3_32574160	Ca3	32574160	TCC/TCCC	TCCTACCATCTAGCAACGGC	TGCTTACCGGGATTTTCAAC	60.2	555	INTERGENIC	--	--	--

INDEL marker IDs	Chromosomes/ unanchored scaffolds	Physical positions (bp)	InDels (*Kabuli* reference genome-CDC Frontier/PII)	Forward primers (5'-3')	Reverse primers (5'-3')	Anneling temperature (0C)	Expected amplified product size (bp)	Structural annotation		Functional annotation	
								Sequence components of *kabuli* genome	*Kabuli* gene accession IDs	NCBI-KOG	TFs
Ca3_P3_32616318	Ca3	32616318	GTTTTTTTTTTTTT/GTTTTTTTTTTT	TGTGGTTGTTGTGGGCTTAG	CGAATGTGAGGTTTGGGACT	59.6	512	INTERGENIC	--	--	--
Ca3_P3_32671018	Ca3	32671018	CATATATATATATATATATATATATATA/CATATATATATATATATATATATATATATATATATA	TGGTTGGAGAGCAAAGAAGG	TTAGTGGATTGCTTCCCTGG	60.4	655	DRR	Ca_11996	--	--
Ca3_P3_32795871	Ca3	32795871	ATTTTTT/ATTTTTTT	TTGATGGGTCTGGATGTTTT	TTGAAATATCCAAAAACTGTTACAAA	57.8	297	INTERGENIC	--	--	--
Ca3_P3_32803256	Ca3	32803256	AATATATATATATATATATATATATAT/AATATATATATATATATATATATAT	GCTACGACGCTGCGATTATT	ACCCAAGACCTTGTTGATGC	60.4	458	INTRON	Ca_12014	L	--
Ca3_P3_32843639	Ca3	32843639	TGG/TG	CATTCCACACCTCACTCCGT	TGCATTCACGTGGATCTCTT	61.0	817	DRR	Ca_12020	--	--
Ca3_P3_32853814	Ca3	32853814	CTTAATATGA/CTTAATATGATTAATATGA	GAAGGAATCATCATGTGGCA	TTGCTCAAAACCAAGCTTCA	59.5	891	INTRON	Ca_12021	O	--
Ca3_P3_32881199	Ca3	32881199	ATTTTTTTT/ATTTTTTTTT	CAGTGGAAAAAGGAGAGGACA	TAAACACGGGGGAGAAGAGA	59.3	440	INTERGENIC	--	--	--
Ca3_P3_32886904	Ca3	32886904	CTTTTTTTTTT/CTTTTTTTTTTT	TCTCCGTTGGTTTGTTTTCC	ATTGCTGATGAAGGTGGAGG	59.9	541	INTERGENIC	--	--	--
Ca3_P3_32896213	Ca3	32896213	ATTT/ATT	TGAGAGTTGTGAGCCAATCG	GAAAGACTTGGTTCGCGTTC	60.0	277	DRR	Ca_12026	--	--
Ca3_P3_32961119	Ca3	32961119	ATTTTT/ATTTT	TGAGAGGGGTTCATAAGCCT	TCCTACGCTTTCGCTTTCAT	58.7	409	INTERGENIC	--	--	--

INDEL marker IDs	Chromosomes/ unanchored scaffolds	Physical positions (bp)	InDels (*Kabuli* reference genome-CDC Frontier/PII)	Forward primers (5'-3')	Reverse primers (5'-3')	Anneling temperature (0C)	Expected amplified product size (bp)	Structural annotation		Functional annotation	
								Sequence components of *kabuli* genome	*Kabuli* gene accession IDs	NCBI-KOG	TFs
Ca3_P3_32996406	Ca3	32996406	GATATATATATATAT/GATATATATATAT	ATGTTTGCAGGAGGAG CTGT	ACCAAATGGAGAAAGG ACCC	59.9	782	INTERGENIC	--	--	--
Ca3_P3_33046145	Ca3	33046145	TTTATTATTATTATTATTAT/TTTATTATT ATTATTATTATTAT	TATTCACCACCCTACCC CAA	AAAAGGTTGCACAATG ACAAA	60.0	738	INTERGENIC	--	--	--
Ca3_P3_33099785	Ca3	33099785	TAAAAAAA/TAAAAAAAA	GCTTTTTCACTCTCGGT TGC	ATATGAGTTACGGGAT GCGG	60.0	683	INTERGENIC	--	--	--
Ca3_P3_33145027	Ca3	33145027	AATATATATATATATATATAT/AATATAT ATATATATATATATATATAT	CTTCCTAAAGTCGTGCT GCC	GCGGCAATTTTTGTTG ATAA	60.0	295	INTERGENIC	--	--	--
Ca3_P3_33204887	Ca3	33204887	TA/TAA	GCACATGCCAAACTCG TAAA	AAATGCTGCGTTCCAT CTTT	59.7	295	INTERGENIC	--	--	--
Ca3_P3_33229820	Ca3	33229820	AATATATATATATATATA/AATATATATA TATATATATATATATATATA	AGCGTTGGTTTAACATT GCC	AGGCAAATGGGGAAAG AGAT	60.0	609	INTRON	Ca_12058	J	--
Ca3_P3_33346296	Ca3	33346296	GATATATATATATATATATATATA/GATA TATATATATATATATATATA	ATTTTGCCACATGATAC GCA	GCTTCACACGGAAACA ACAA	60.0	531	DRR	Ca_23675	--	--
Ca3_P3_33387153	Ca3	33387153	AATATATATATATATATATATATATATAT /AATATATATATATATATATATATATATA TATATATATATATAT	ATACCTACGGATTTGTG CCG	TTTGTTGTTGAAGATG GCAA	59.8	553	INTERGENIC	--	--	--
Ca3_P3_33419630	Ca3	33419630	TTTATTATTATTATTATTATTATTATTAT TATTATTATTATTATTATTATTATT/TTTA TTATTATTATTATTATTATTATTATTATT ATTATTATTATTATTATT	TGTGCTCCCCTTTGTCT TCT	TTTTTACGGGTTGATG AGGC	59.8	691	INTERGENIC	--	--	--
Ca3_P3_33545135	Ca3	33545135	C/CTATCAGTAAACATT	ACGTCATCAGTCGAAG TCCC	AGTTTGCGTAGGTTGT TGGG	60.1	329	INTERGENIC	--	--	--

INDEL marker IDs	Chromosomes/ unanchored scaffolds	Physical positions (bp)	InDels (*Kabuli* reference genome-CDC Frontier/PII)	Forward primers (5'-3')	Reverse primers (5'-3')	Anneling temperature (0C)	Expected amplified product size (bp)	Structural annotation		Functional annotation	
								Sequence components of *kabuli* genome	*Kabuli* gene accession IDs	NCBI-KOG	TFs
Ca3_P3_33579150	Ca3	33579150	AAAGNNNNNNNNNNNAAG/AAAG	TGTGGGAGCTGAAAGTGTTG	TTCCTTGCCATGTCACCATA	59.9	175	INTERGENIC	--	--	--
Ca3_P3_33581359	Ca3	33581359	TAAAAAAAAAAA/TAAAAAAAAAA	TGGGTCAAGATTAGAGCCGT	CAATTTGACAAGCCCCCTAA	59.7	610	INTERGENIC	--	--	--
Ca3_P3_33630409	Ca3	33630409	CTTTTTT/CTTTTTTTTTT	ATAGTGACATCCAGGCCCAG	TCATCGCAGGTGTACCATGT	60.0	651	INTERGENIC	--	--	--
Ca3_P3_33693334	Ca3	33693334	AATATATATATATATATATATAT/AATATATATATATATATAT	CTCACCATAAACCACCCACG	CGTCCCGTCCAAATCTTCTA	61.2	464	INTERGENIC	--	--	--
Ca3_P3_33828272	Ca3	33828272	TTTATTATTATTATTATTATTATTATTATTATTATTATTATTATTATTATTATT/TTTATTATTATTATTATTATTATTATTATTATTATTATTATTATTATT	AGAGGCAAACAAGAACCGAA	TGCATATAAGGAATTGACGCC	59.9	318	INTERGENIC	--	--	--
Ca3_P3_33862493	Ca3	33862493	ATATTATTATTATTATTATTATTATTATT/ATATTATTATTATTATTATTATTATT	GGGGATATAGTTCCGCCAAT	GCCCAGTAGTTTTGGGGATT	60.0	606	DRR	Ca_20881	R	--
Ca3_P3_33877257	Ca3	33877257	AATATATATATATATAT/AATATATATATATATATAT	CTCACTTCCGCTCATGTCAA	ATATGGCAAGGATGGGAAAA	60.0	287	INTERGENIC	--	--	--
Ca3_P3_33945517	Ca3	33945517	ATAATGTTA/ATA	CGGTTGAAACTGACACGAAA	CCAAGTTGCAAAACCCTGAT	59.7	783	INTERGENIC	--	--	--
Ca3_P3_3394940	Ca3	3394940	GAAAAA/GAAAA	TTTCAATTGCATTAAGTGGGA	CCCGGGAGTAGGAATTTGAT	58.1	498	INTERGENIC	--	--	--
Ca3_P3_3395133	Ca3	3395133	AAACAAC/AAAC	TTTCAATTGCATTAAGTGGGA	ATGGACTGTTTGGCAACCTC	58.1	836	INTERGENIC	--	--	--

INDEL marker IDs	Chromosomes/ unanchored scaffolds	Physical positions (bp)	InDels (*Kabuli* reference genome-CDC Frontier/PII)	Forward primers (5'-3')	Reverse primers (5'-3')	Anneling temperature (0C)	Expected amplified product size (bp)	Structural annotation		Functional annotation	
								Sequence components of *kabuli* genome	*Kabuli* gene accession IDs	NCBI-KOG	TFs
Ca3_P3_33954378	Ca3	33954378	TTATATATATATATATATATAT/TTATATATATATATATATATAT	GGCTTCAAAGCCAACATCAT	TTTGTGCTGCCAACATTTTC	60.1	440	INTERGENIC	--	--	--
Ca3_P3_33954528	Ca3	33954528	ATATTATTATTATTATTATTATTATTATTATTATTATTATTATTATTATTATTATT/ATATTATTATTATTATTATTATTATTATTATTATTATTATTATTATTATTATTATTATT	GGCTTCAAAGCCAACATCAT	TCATACAAAAACCAAACGGG	60.1	622	INTERGENIC	--	--	--
Ca3_P3_33983043	Ca3	33983043	TA/TAGACACTGA	GGTGTTTTACGAATGGGGTG	GGGACTCATTTATGCCGATG	60.1	353	INTERGENIC	--	--	--
Ca3_P3_33989572	Ca3	33989572	AC/A	TGCAAACCTATGGCTACACG	TCCCTAGGGTCAGCTTGAAA	59.8	190	INTERGENIC	--	--	--
Ca3_P3_34128563	Ca3	34128563	TTATATATATATATA/TTATATATATATA	TCAAGTTTGGGAGGAACACC	CGGGTCAGGACTACATTTGC	59.9	590	INTERGENIC	--	--	--
Ca3_P3_34264150	Ca3	34264150	TAAAAAAAAA/TAAAAAAAA	AATGGAAAGAGCATTGGGTG	GCAAACAAACAAAAACACATTGA	59.9	709	INTERGENIC	--	--	--
Ca3_P3_34297920	Ca3	34297920	GAATAAATAAATAAATAAATAAATAAATAAA/GAATAAATAAATAAATAAATAAATAAATAAATAAATAAA	CCTCATGGGGAGCAAAATAA	TTCAGTTCACTGCGCATTTC	59.9	607	INTERGENIC	--	--	--
Ca3_P3_34307680	Ca3	34307680	ATTTTTTTTTT/ATTTTTTTTTTT	TGAATTTGCAATTGGTCGAA	TGTCTTTGGTCTTTGCAATTATTA	60.1	607	INTERGENIC	--	--	--
Ca3_P3_34448761	Ca3	34448761	GATATATATATATATATATATATATA/GATATATATATATATATATATATATATA	TTTCCACACATGATCCAAAAA	GTGGAGAAAGAGTGGCCTTG	58.9	543	INTERGENIC	--	--	--
Ca3_P3_34474422	Ca3	34474422	TAAAAAAAA/TAAAAAAA	AAAAATCAATTTATGCCGTTGT	TTATGCCGGATTGACACTCTT	57.7	478	INTERGENIC	--	--	--

INDEL marker IDs	Chromosomes/ unanchored scaffolds	Physical positions (bp)	InDels (*Kabuli* reference genome-CDC Frontier/PII)	Forward primers (5'-3')	Reverse primers (5'-3')	Anneling temperature (0C)	Expected amplified product size (bp)	Structural annotation		Functional annotation	
								Sequence components of *kabuli* genome	*Kabuli* gene accession IDs	NCBI-KOG	TFs
Ca3_P3_34557168	Ca3	34557168	TGAGAGAGAGAGAGAGAGAGAGAGAGAGAGAG/TGAGAGAGAGAGAGAGAGAGAGAGAGAGAG	TGTTCAATTATTTTGGGATGGA	TCCTCCAATTTTCTTTCCCC	59.2	582	DRR	Ca_00726	S	--
Ca3_P3_34578013	Ca3	34578013	AGAGG/AGAGGAGG	CCAAACCGCAAGATTGATTT	TTTCCCAATATTCTGCCTCG	59.9	480	INTERGENIC	--	--	--
Ca3_P3_34646219	Ca3	34646219	ATTTTTTTTTTT/ATTTTTTTTTT	TTTTGTTTGCGGTGCATAGA	TGGGCCAAGACTCCTTTTTA	60.3	602	INTERGENIC	--	--	--
Ca3_P3_34790945	Ca3	34790945	TTATATATATATATATATATATATATATATATATATATAT/TTATATATATATATATATATATATATATATATATAT	TTATCATGCCCTGTGTGTTTT	GGTAAGAACAGTGAAGCAACCA	58.0	589	INTERGENIC	--	--	--
Ca3_P3_34813314	Ca3	34813314	GTTTTTTTTTTT/GTTTTTTTTTTTTT	AAAAATGCAAAAACACCATGAA	AAGTCGCCATTTGAAACTCG	59.4	340	INTERGENIC	--	--	--
Ca3_P3_34879718	Ca3	34879718	ATTTTTTT/ATTTTTT	GGTAAAAGGTGTAACCGAAGAAA	GCAATTAGATGGGTCACGCT	58.7	447	INTERGENIC	--	--	--
Ca3_P3_34966563	Ca3	34966563	GATATATATATATATATATATATAT/GATATATATATATATATATATATAT	TTTTCCCGTCAAACCAAGTC	AAAATTTCGAGCGTTTCTCG	59.9	437	INTERGENIC	--	--	--
Ca3_P3_35016413	Ca3	35016413	ATTTTTTTTTT/ATTTTTTTTT	AGGCTTAAGAGAGGGGTCCA	CCCTAAAATTTTGTGGGCTT	60.2	342	INTERGENIC	--	--	--
Ca3_P3_35017508	Ca3	35017508	GATATATATATATATATATATATAT/GATATATATATATATATATATATAT	TCAATGCCATGTTAAGGCAA	GGTAAAAACATATGCGCCTG	60.1	460	INTERGENIC	--	--	--
Ca3_P3_35033877	Ca3	35033877	GTTTTTTTTTTT/GTTTTTTTTTTTT	TGCTTCGGCTGTATTTTTCA	CACACACACACACACGAAGG	59.4	410	DRR	Ca_00777	R	--

INDEL marker IDs	Chromosomes/ unanchored scaffolds	Physical positions (bp)	InDels (*Kabuli* reference genome-CDC Frontier/PII)	Forward primers (5'-3')	Reverse primers (5'-3')	Anneling temperature (0C)	Expected amplified product size (bp)	Structural annotation		Functional annotation	
								Sequence components of *kabuli* genome	*Kabuli* gene accession IDs	NCBI-KOG	TFs
Ca3_P3_35130213	Ca3	35130213	ATTTTTTTTTT/ATTTTTTTTTTT	CCGGAAAACTTCATTCCAAA	TCTGGAAACCACCGAAAAAC	59.9	382	INTERGENIC	--	--	--
Ca3_P3_35213222	Ca3	35213222	TC/T	AACTTCATTCACGTGCAAAAT	TGATATTGCAAGAAACCCCA	57.3	302	INTERGENIC	--	--	--
Ca3_P3_3523859	Ca3	3523859	CTATATATATATATATATA/CTATATATATATATATATA	GTTTGCAGGAAGAGGCAAAG	GCCAGCAGCCTTCAAGATAG	60.0	712	DRR	Ca_21350	J	--
Ca3_P3_3524015	Ca3	3524015	AATATATATATATAT/AATATATATATATATATATAT	GTTTGCAGGAAGAGGCAAAG	GCCAGCAGCCTTCAAGATAG	60.0	712	DRR	Ca_21350	J	--
Ca3_P3_35251407	Ca3	35251407	CTTTTT/CTTTT	TTCGTCCCTCACCATACACA	CGGGTTGTTTGTCCATTTTC	60.0	588	INTERGENIC	--	--	--
Ca3_P3_35258173	Ca3	35258173	TAAAAAAAAAAA/TAAAAAAAAAA	TCTGCCATGTTCAGTTTGGA	TCAAATCCGAACCCATGAAT	60.2	606	INTERGENIC	--	--	--
Ca3_P3_35264037	Ca3	35264037	GAAAAAAAA/GAAAAAAA	GAATCCAAGGTTGGTTGGTG	ATGCTTAGGCATGCTGGATT	60.2	566	INTERGENIC	--	--	--
Ca3_P3_35267953	Ca3	35267953	CATATATATATATATATATATATATATATATAT/CATATATATATATATATATATATATATATATAT	ACCGATATTTGAGCACTGCC	CCTATTCCACGAGAAAATCTGC	60.1	284	DRR	Ca_00804	--	--
Ca3_P3_35271726	Ca3	35271726	TCC/TC	TTATCCCCACAACACAAGCA	TGTCTGGAACAAACCCATCA	60.0	681	INTERGENIC	--	--	--
Ca3_P3_35272345	Ca3	35272345	ATATTAATTATTAATTATTAA/ATATTAATTATTAA	CTTTCCTTCTTCCTTGTGCG	GTACGTGACTGGTGCCCTTT	60.0	502	INTERGENIC	--	--	--

INDEL marker IDs	Chromosomes/ unanchored scaffolds	Physical positions (bp)	InDels (*Kabuli* reference genome-CDC Frontier/PII)	Forward primers (5'-3')	Reverse primers (5'-3')	Anneling temperature (0C)	Expected amplified product size (bp)	Structural annotation		Functional annotation	
								Sequence components of *kabuli* genome	*Kabuli* gene accession IDs	NCBI-KOG	TFs
Ca3_P3_35433457	Ca3	35433457	AAT/AATAATTATTATTATTAT	TTATCGTTTTTCTCCCACCG	CCCACCTTGAATGATCTGCT	59.9	277	DRR	Ca_00826	--	B3
Ca3_P3_3554927	Ca3	3554927	TTATATATATA/TTATATATATATA	TTGGAGAAACTTTTCCGCTG	GCACCCTAAACCCTAAACCC	60.4	440	INTERGENIC	--	--	--
Ca3_P3_35679181	Ca3	35679181	ATTTTTTTT/ATTTTTTT	TTCATTCTTTGGCTTGGGAC	CGTCATGGTCGTGGCTTTAT	60.1	959	DRR	Ca_00856	R	SBP
Ca3_P3_35687478	Ca3	35687478	TATAATAATAATAATAATAATAATAATAATAATAATAATAA/TATAATAATAATAATAATAATAATAATAATAATAATAA	GGAAAATGAAAATGGCCAAC	TGTTTGACTTGTCCGAAAACTT	59.3	604	INTERGENIC	--	--	--
Ca3_P3_35706538	Ca3	35706538	TAAAAAAAAAAA/TAAAAAAAAAAAAA	CGTTCACTCGGCCACTATTT	TTTGGGAAGTGAGAGAGAAAGC	60.1	666	INTERGENIC	--	--	--
Ca3_P3_35712646	Ca3	35712646	ATTTTTTTTTTT/ATTTTTTTTTT	GGATTCCAACCGGATTCTTT	AATCACTGGATATGGCCGAG	60.1	794	INTERGENIC	--	--	--
Ca3_P3_35772940	Ca3	35772940	TAAAAAAA/TAAAAAAAA	ATGTGGCTCGAGGTCAAAAT	CTATCATCCGAAGAGCTGCC	59.6	782	INTERGENIC	--	--	--
Ca3_P3_35798935	Ca3	35798935	CATAGAATAGAATAGAATAGAAT/CATAGAATAGAATAGAAT	AGCTCAGTGCAACGATTGTG	AGTGCATCATTTGTCCACCA	60.1	355	INTERGENIC	--	--	--
Ca3_P3_35805111	Ca3	35805111	CATATATATAT/CATATAT	TTTAGCATCGTGGTTTCCTTT	TGCTAAAATAAACTCCGCCAA	58.8	521	INTERGENIC	--	--	--
Ca3_P3_35817728	Ca3	35817728	ATTTTTTTTT/ATTTTTTTTTT	AAAATGATGGCGTGAGATGA	TCTTGGGTCAAAAGACACCC	59.1	565	INTERGENIC	--	--	--

INDEL marker IDs	Chromosomes/ unanchored scaffolds	Physical positions (bp)	InDels (*Kabuli* reference genome-CDC Frontier/PII)	Forward primers (5'-3')	Reverse primers (5'-3')	Anneling temperature (0C)	Expected amplified product size (bp)	Structural annotation		Functional annotation	
								Sequence components of *kabuli* genome	*Kabuli* gene accession IDs	NCBI-KOG	TFs
Ca3_P3_35834316	Ca3	35834316	GATATATATATATATATAT/GATATATATATATATATAT	GCTCCCCCTTCATGTGACTA	CCTTTTCGCCATCTTTGTGT	60.1	595	INTERGENIC	--	--	--
Ca3_P3_35848866	Ca3	35848866	ATTTTTTTTTTT/ATTTTTTTTTT	TTGGTTTCCAACACTCTCCC	AATGAACACTTCACCGAGGG	59.9	573	DRR	Ca_00873	S	--
Ca3_P3_35863496	Ca3	35863496	A/AG	CGTGAAGATATTGTTGCTTTGG	CCTTTCTACGACCAATTCCG	59.6	383	INTERGENIC	--	--	--
Ca3_P3_35878677	Ca3	35878677	TAAAAAAAAAA/TAAAAAAAAA	TCCCTTCCCTTTGTTGTGTC	TTTTATGTTTCGCGGGGATA	59.9	753	INTERGENIC	--	--	--
Ca3_P3_35886280	Ca3	35886280	GAAAAAAAA/GAAAAAAA	CATGATGTGGTCGAAAAGGA	TGCAAGCAATGATGAAAACTC	59.5	209	INTERGENIC	--	--	--
Ca3_P3_35913625	Ca3	35913625	TTATATATATATATATA/TTATATATATATATA	TCGTTACCCCTCTCGTCAAC	TGTCTCGTGTGGTAAGGGTG	60.1	686	DRR	Ca_00883	R	ERF
Ca3_P3_35947619	Ca3	35947619	CAA/CA	GATGGGGTGCATTTCTGAAT	TTGAAATTTTGACCGCATCA	59.8	830	DRR	Ca_00889	J	--
Ca3_P3_35950711	Ca3	35950711	ATTTA/ATTTAAAATTACATGCAATTTTA	CATGCGATGGAACTGAATTG	GTAAAGTTGGCACAGGGGAA	60.1	479	INTERGENIC	--	--	--
Ca3_P3_35951796	Ca3	35951796	ACACATGCA/ACA	AACGTTTGAATGCACATGGA	ATAAGCGGTTCTTTCACGGA	60.0	593	INTERGENIC	--	--	--
Ca3_P3_35953275	Ca3	35953275	ATTTTTTTT/ATTTTTTTTT	TTCATTAAGGGCAGACCCAC	TGGAGCCAGAGTTAAATGGG	59.9	781	INTERGENIC	--	--	--

INDEL marker IDs	Chromosomes/ unanchored scaffolds	Physical positions (bp)	InDels (*Kabuli* reference genome-CDC Frontier/PII)	Forward primers (5'-3')	Reverse primers (5'-3')	Anneling temperature (0C)	Expected amplified product size (bp)	Structural annotation		Functional annotation	
								Sequence components of *kabuli* genome	*Kabuli* gene accession IDs	NCBI-KOG	TFs
Ca3_P3_35953332	Ca3	35953332	ATTTTTT/ATTTTTTT	TTCATTAAGGGCAGACCCAC	TGGAGCCAGAGTTAAATGGG	59.9	781	INTERGENIC	--	--	--
Ca3_P3_35953562	Ca3	35953562	TA/T	TGATGGCAGCTACCAATTTT	AAGAAGGGTGGAGCCAGAGT	58.2	209	INTERGENIC	--	--	--
Ca3_P3_35953819	Ca3	35953819	TA/T	ACTCTGGCTCCACCCTTCTT	ATTGCGGGTATAGAGACGGA	60.3	293	INTERGENIC	--	--	--
Ca3_P3_35959327	Ca3	35959327	GAAAAAAA/GAAAAAAAA	TGTTGACCAATAAGCAAAATTGA	TGACACACAACATACAAGCCA	59.5	360	DRR	Ca_00891	V	GRAS
Ca3_P3_35959933	Ca3	35959933	GTTTTTTT/GTTTTTTTT	TCTGAACAAATGATCGGGTG	ACATGCAATTTCCATCCACA	59.5	164	DRR	Ca_00891	V	GRAS
Ca3_P3_35959985	Ca3	35959985	CAAAAAAAAAAAAAAA/CAAAAAAAAAAAA	TCTGAACAAATGATCGGGTG	ACATGCAATTTCCATCCACA	59.5	164	DRR	Ca_00891	V	GRAS
Ca3_P3_35960562	Ca3	35960562	AGTTTGATT/AGTTTGATTGTTTGATT	TCCGACTTGGTAAAACAGCA	TTTGGACGAAATAAATTTGAAAAA	59.3	398	DRR	Ca_00891	V	GRAS
Ca3_P3_35960756	Ca3	35960756	TT/TTATATAT	GGATGCTACATTTCTTGGTGG	GGTGATTCTTCTGGTGGGAA	59.4	776	DRR	Ca_00891	V	GRAS
Ca3_P3_35963251	Ca3	35963251	AG/A	ATAGCCCGACTTTGACCACA	GGAAATTCGGTGCTTGAGAC	60.5	148	INTERGENIC	--	--	--
Ca3_P3_35963324	Ca3	35963324	ATTT/ATTTTT	AGCTGTCTCAAGCACCGAAT	ACAATCCATAATTTTTCCCTCA	60.0	104	INTERGENIC	--	--	--

INDEL marker IDs	Chromosomes/ unanchored scaffolds	Physical positions (bp)	InDels (*Kabuli* reference genome-CDC Frontier/PII)	Forward primers (5'-3')	Reverse primers (5'-3')	Anneling temperature (0C)	Expected amplified product size (bp)	Structural annotation		Functional annotation	
								Sequence components of *kabuli* genome	*Kabuli* gene accession IDs	NCBI-KOG	TFs
Ca3_P3_35980134	Ca3	35980134	T/TGTGA	TAGTGGCTGGGTAACG GAAC	CAGCAAAAGCGAAATC AAGA	60.0	515	INTERGENIC	--	--	--
Ca3_P3_35986617	Ca3	35986617	CCA/CCACA	GGTGGATTCTTTGCTT CCAA	AGGTGCAAGTGCAAGT TCCT	60.1	479	INTRON	Ca_00893	--	--
Ca3_P3_35992940	Ca3	35992940	TAAAAAAA/TAAAAAA	AGAAGCACAAGTCCCC TGAA	GCTCATGTGTAAAGCC GTGA	59.8	710	DRR	Ca_00894	--	--
Ca3_P3_35993475	Ca3	35993475	CATATA/CATA	TCACGGCTTTACACATG AGC	TTGATTCGAACCCAAA ATCT	59.9	515	DRR	Ca_00894	--	--
Ca3_P3_35994174	Ca3	35994174	CAAAA/CAAA	TTGGGTTCGAATCAATA TCTTACA	GAATTCGGAACAAAAC GTTCA	59.7	625	INTERGENIC	--	--	--
Ca3_P3_35994489	Ca3	35994489	CT/CTAAATGTT	TGAATGATGTTTCATTC CATCA	GAATTCGGAACAAAAC GTTCA	58.8	311	INTERGENIC	--	--	--
Ca3_P3_35994561	Ca3	35994561	TAAAAAAAA/TAAAAAAA	AACGTTTTGTTCCGAAT TCAA	GGGAAGTTGGAAGGT GAAAA	59.5	517	INTERGENIC	--	--	--
Ca3_P3_35994788	Ca3	35994788	TCAC/T	AAATTTGGTGCTCCATG GTC	GTGCTCCATTTTCTTCA CGG	59.8	464	INTERGENIC	--	--	--
Ca3_P3_36000193	Ca3	36000193	AATATATATA/AATATATATATA	TCCAAATCATCCGCACA TAA	TCGGTAAATAAGTTTTT GGACACA	59.9	662	INTERGENIC	--	--	--
Ca3_P3_36013137	Ca3	36013137	CTTTTTTT/CTTTTTT	TTATACCATGGATAGCC GCC	ACCTTAGGGTTGCTTG GGTT	59.8	617	DRR	Ca_00899	O	CAMTA

INDEL marker IDs	Chromosomes/ unanchored scaffolds	Physical positions (bp)	InDels (*Kabuli* reference genome-CDC Frontier/PII)	Forward primers (5'-3')	Reverse primers (5'-3')	Anneling temperature (0C)	Expected amplified product size (bp)	Structural annotation		Functional annotation	
								Sequence components of *kabuli* genome	*Kabuli* gene accession IDs	NCBI-KOG	TFs
Ca3_P3_36016763	Ca3	36016763	TAA/TA	CGCGGAACAAGAATCAAAAT	ATCTCGCGAAGGTGAGAGAA	60.1	384	DRR	Ca_00900	O	CAMTA
Ca3_P3_36016803	Ca3	36016803	GTTTTTTTTTTTT/GTTTTTTTTTT	CGCGGAACAAGAATCAAAAT	ATCTCGCGAAGGTGAGAGAA	60.1	384	DRR	Ca_00900	O	CAMTA
Ca3_P3_36017650	Ca3	36017650	G/GAT	GGAAAACACATATTACGGAGGAA	GAAGGAGAATGTGGCAGCTC	59.3	771	DRR	Ca_00900	O	CAMTA
Ca3_P3_36017693	Ca3	36017693	TAAAAA/TAAAAAAA	GGAAAACACATATTACGGAGGAA	GAAGGAGAATGTGGCAGCTC	59.3	771	DRR	Ca_00900	O	CAMTA
Ca3_P3_36019725	Ca3	36019725	GAAA/GAA	ACCTTTGGATTCATGGTTGG	CCGATAGCCATTTGGACACT	59.6	614	INTERGENIC	--	--	--
Ca3_P3_36020115	Ca3	36020115	ATTTTT/ATTTT	AGTGTCCAAATGGCTATCGG	TCACTAACGCAACCTTCACCT	60.0	487	INTERGENIC	--	--	--
Ca3_P3_36020456	Ca3	36020456	CAAA/CAAAA	ACCACAACCGTCCAATGTTT	TTTCAAGATGCTTGACGAAGAA	60.1	736	INTERGENIC	--	--	--
Ca3_P3_36022347	Ca3	36022347	AC/ACC	AACATTTACAAACATAAACGACGAA	TGGAGATGGAAATGGAGAGG	59.0	524	DRR	Ca_00901	O	CAMTA
Ca3_P3_36022379	Ca3	36022379	TCC/TC	AACATTTACAAACATAAACGACGAA	TGGAGATGGAAATGGAGAGG	59.0	524	DRR	Ca_00901	O	CAMTA
Ca3_P3_36024202	Ca3	36024202	TAGGACACTGA/TA	ATACCATGGATAGCCGCAGA	TTCACATTTCGAATCCATGC	60.5	523	INTERGENIC	--	--	--

INDEL marker IDs	Chromosomes/ unanchored scaffolds	Physical positions (bp)	InDels (*Kabuli* reference genome-CDC Frontier/PII)	Forward primers (5'-3')	Reverse primers (5'-3')	Anneling temperature (0C)	Expected amplified product size (bp)	Structural annotation		Functional annotation	
								Sequence components of *kabuli* genome	*Kabuli* gene accession IDs	NCBI-KOG	TFs
Ca3_P3_36025628	Ca3	36025628	A/AT	ACGCCAGTCACACAGACAGA	GAAAGACGGTTTAGTTCCGTTG	60.5	506	INTERGENIC	--	--	--
Ca3_P3_36025951	Ca3	36025951	GTCATTT/GT	CAACGGAACTAAACCGTCTTTC	CATTTAGGGGCGTAGCAAAT	60.0	391	INTERGENIC	--	--	--
Ca3_P3_36032000	Ca3	36032000	TAATTTGAAATTTGAAATT/TAATTTGAAATT	TGGTAAGGACAAAAAGAGTTCCA	AACGCTGAACCAAAATGACC	60.0	801	INTERGENIC	--	--	--
Ca3_P3_36032624	Ca3	36032624	CAAA/CAAAA	GGTCATTTTGGTTCAGCGTT	CCATGCATGAATCCTTGAAA	60.0	614	DRR	Ca_00902	S	bZIP
Ca3_P3_36033414	Ca3	36033414	ATTTTTTTTTTT/ATTTTTTTTTT	CTTCTCACCCCCTCAAAACA	CACCAAATGTCATTACAGCCA	60.1	530	DRR	Ca_00902	S	bZIP
Ca3_P3_36035545	Ca3	36035545	GTTTTTTTTT/GTTTTTTTT	GTTCCCAACTTCCCATTTCC	TTACCTGTTTGGCATGGACA	60.5	559	INTRON	Ca_00902	S	bZIP
Ca3_P3_3603826	Ca3	3603826	C/CT	AAGCAGCTAGACCACCTCCA	ATCCTTGGAATTATTGGCCC	60.0	679	DRR	Ca_21347	J	--
Ca3_P3_36039531	Ca3	36039531	ACAATAGCA/ACAATAGCAATAGCA	CCTCTCTAGCTCAGAGCCCA	TACGCCGATAGTGCAACAAA	59.8	423	INTRON	Ca_00902	S	bZIP
Ca3_P3_36040095	Ca3	36040095	TAAAAAAAAAA/TAAAAAAAAA	TGTTGCACTATCGGCGTAAA	TTTAAAACAGGCAGGGTTGC	60.3	782	INTRON	Ca_00902	S	bZIP
Ca3_P3_36041204	Ca3	36041204	ATT/AT	TTCACACTCTCCAGTTCGGA	AAGTCGACGTTTGTGAAGAGTTC	59.4	699	INTERGENIC	--	--	--

INDEL marker IDs	Chromosomes/ unanchored scaffolds	Physical positions (bp)	InDels (*Kabuli* reference genome-CDC Frontier/PII)	Forward primers (5'-3')	Reverse primers (5'-3')	Anneling temperature (0C)	Expected amplified product size (bp)	Structural annotation		Functional annotation	
								Sequence components of *kabuli* genome	*Kabuli* gene accession IDs	NCBI-KOG	TFs
Ca3_P3_36041740	Ca3	36041740	T/TAAAGAAC	AAAGAACTCTTCACAAACGTCG	TTGAGGGTCCAAAACTGGAG	58.6	627	INTERGENIC	--	--	--
Ca3_P3_36042558	Ca3	36042558	ATTTTTT/ATTTTTTT	CTCCAGTTTTGGACCCTCAA	TTTCCAAAATAAACTCGACGC	60.1	430	INTERGENIC	--	--	--
Ca3_P3_36043107	Ca3	36043107	TAA/TAAA	ACGCGTCGAGTTTATTTTGG	GTCCGTTGTGATGGCTTTTT	60.1	746	INTERGENIC	--	--	--
Ca3_P3_36045627	Ca3	36045627	C/CA	TGTGATTGAAAAGATGTGTCAAAA	GGGGTACGGAGTGGAGGTAT	59.5	622	INTERGENIC	--	--	--
Ca3_P3_36048215	Ca3	36048215	CTTTTTTTTTTT/CTTTTTTTTTT	AACCGCCCATCTATCCCTAC	GGACGTTGAAAAGCAGAAGC	60.2	468	INTRON	Ca_00903	A	--
Ca3_P3_36052612	Ca3	36052612	GTCACTTTCACTTT/GTCACTTT	CATCAATGCACGACCCATAC	TAAGTGAGCGCATCCATTTG	59.8	639	INTERGENIC	--	--	--
Ca3_P3_36068600	Ca3	36068600	TC/TCC	TAGGGGGCTCTTTAGCCATT	TCCAGCTTTTGCAGGTCTTT	60.1	414	INTRON	Ca_00904	A	--
Ca3_P3_36076512	Ca3	36076512	AGAATT/A	GCATTTGTTTCAACAAGGTAAGG	TTTGGAGGACAAAAACTGGG	59.9	395	INTERGENIC	--	--	--
Ca3_P3_36086627	Ca3	36086627	AC/ACC	ATTCCAACGGAAAACATCCA	CTCCCAAACAAAGCCTACGA	60.2	605	INTRON	Ca_00907	I	--
Ca3_P3_36087073	Ca3	36087073	AGGGGG/AGGGGGG	GGTCCATGGAAGCTTTTCAA	GTTGTTGATGGCGTTTTGTG	60.1	757	INTRON	Ca_00907	I	--

INDEL marker IDs	Chromosomes/ unanchored scaffolds	Physical positions (bp)	InDels (*Kabuli* reference genome-CDC Frontier/PII)	Forward primers (5'-3')	Reverse primers (5'-3')	Anneling temperature (0C)	Expected amplified product size (bp)	Structural annotation		Functional annotation	
								Sequence components of *kabuli* genome	*Kabuli* gene accession IDs	NCBI-KOG	TFs
Ca3_P3_36088323	Ca3	36088323	TTATATAT/TTATAT	AACCGAACCTTGCCAT AGTG	AGACAGATTACCCACG GCTG	60.0	355	INTERGENIC	--	--	--
Ca3_P3_36088514	Ca3	36088514	CAAAA/CAAA	AACCGAACCTTGCCAT AGTG	TCAGCACGCTTTTTAG TAGGTG	60.0	968	INTERGENIC	--	--	--
Ca3_P3_36088538	Ca3	36088538	AATATATATATATA/AATATATATATA	GTGCTGAACCCTCCAT TGTT	TCAGCACGCTTTTTAG TAGGTG	60.0	951	INTERGENIC	--	--	--
Ca3_P3_36089824	Ca3	36089824	AAT/AATTCCATCTAT	GGGATCAACAAGTAAA AGCTCC	AAACTGGTTTGAGTGA CGGC	59.1	709	INTERGENIC	--	--	--
Ca3_P3_36089940	Ca3	36089940	GTAAA/G	AATACGAATCATTGGAG CTTTTT	AAACTGGTTTGAGTGA CGGC	58.3	167	INTERGENIC	--	--	--
Ca3_P3_36090593	Ca3	36090593	G/GT	CCCCGTAACGGAGGAT ACTT	TTGATGTAATGATTATG TGATTTGTCA	60.2	441	INTERGENIC	--	--	--
Ca3_P3_36092177	Ca3	36092177	CAAAAAAAAA/CAAAAAAAAAA	ACATTCGTGACACGGT GAAA	GGTCTAGCCTATTCCA CCCC	60.0	756	INTERGENIC	--	--	--
Ca3_P3_36097089	Ca3	36097089	TATAA/T	GCCATTTCTTAGAAGCC CCT	TTTTGCCTGGATATTC CCTG	59.7	485	INTERGENIC	--	--	--
Ca3_P3_36099581	Ca3	36099581	TAAA/TAAAAAA	CATTTCGGAGGTAGGT GGAA	TGCCTTACTGATTTGC CTCC	59.9	421	DRR	Ca_00908	K	--
Ca3_P3_36102327	Ca3	36102327	GTT/GT	CCTGCAGGCAAGCAAA TAAT	GGTATTGTGGTGGGAT GGTC	60.2	486	INTRON	Ca_00908	K	--

INDEL marker IDs	Chromosomes/ unanchored scaffolds	Physical positions (bp)	InDels (*Kabuli* reference genome-CDC Frontier/PII)	Forward primers (5'-3')	Reverse primers (5'-3')	Anneling temperature (0C)	Expected amplified product size (bp)	Structural annotation		Functional annotation	
								Sequence components of *kabuli* genome	*Kabuli* gene accession IDs	NCBI-KOG	TFs
Ca3_P3_36105355	Ca3	36105355	CTTTTTT/CTTTTT	GAGTGTGGGAAGGATCGAAA	CCTGGAGAAGGCTGTCAAAG	60.1	372	INTERGENIC	--	--	--
Ca3_P3_36105726	Ca3	36105726	TC/T	CTTTGACAGCCTTCTCCAGG	AGGCTCATAAAACGCCTCAA	60.0	440	INTERGENIC	--	--	--
Ca3_P3_36111673	Ca3	36111673	CTT/CTTT	CTAGGGTTTTTCTTTCGCCC	GATGCATTGCAATTTGTTCA	60.1	462	INTERGENIC	--	--	--
Ca3_P3_36113136	Ca3	36113136	GAAAAAAAA/GAAAAAAA	TCAAGCTCTTGAAGCATTGG	GCCATATCCATTTCGTTTGC	59.1	608	DRR	Ca_00910	--	--
Ca3_P3_36113210	Ca3	36113210	CTTTTTTTTTT/CTTTTTTTTTTT	TCAAGCTCTTGAAGCATTGG	GCCATATCCATTTCGTTTGC	59.1	608	DRR	Ca_00910	--	--
Ca3_P3_36116816	Ca3	36116816	CT/CTCATTAT	CGAAAAACCGCCATAAACAT	TGGCTCAATCATTCATCTGC	59.8	337	DRR	Ca_00911	D	--
Ca3_P3_36116832	Ca3	36116832	TT/TTATTACT	CGAAAAACCGCCATAAACAT	TGGCTCAATCATTCATCTGC	59.8	337	DRR	Ca_00911	D	--
Ca3_P3_36119727	Ca3	36119727	G/GA	GCATATTCATCACCCCCATC	TCCTTCTTGCAGGTGCTTTT	60.0	376	INTRON	Ca_00911	D	--
Ca3_P3_36119763	Ca3	36119763	ACTT/ACTTCTT	GCATATTCATCACCCCCATC	TCCTTCTTGCAGGTGCTTTT	60.0	376	CDS (large-effect mutations)	Ca_00911	D	--
Ca3_P3_36123210	Ca3	36123210	G/GA	TTGTTGTAGGGGAGACTGGG	TACTAGCCGATTTTGTGGGG	60.0	549	INTERGENIC	--	--	--

INDEL marker IDs	Chromosomes/ unanchored scaffolds	Physical positions (bp)	InDels (*Kabuli* reference genome-CDC Frontier/PII)	Forward primers (5'-3')	Reverse primers (5'-3')	Anneling temperature (0C)	Expected amplified product size (bp)	Structural annotation		Functional annotation	
								Sequence components of *kabuli* genome	*Kabuli* gene accession IDs	NCBI-KOG	TFs
Ca3_P3_36123248	Ca3	36123248	ACAG/ACAGGAGTAGCAG	TTGTTGTAGGGGAGACTGGG	TACTAGCCGATTTTGTGGGG	60.0	549	INTERGENIC	--	--	--
Ca3_P3_36128528	Ca3	36128528	GATATAT/GATAT	ACTAACGGAACGACGAATGG	AAAGTCGAGGTGCTTTTTGC	60.0	657	INTERGENIC	--	--	--
Ca3_P3_36129194	Ca3	36129194	GTTAAATTA/GTTAAATTAAATTA	GCAAAAAGCACCTCGACTTT	CACACCAATCAAACGAGCAA	59.5	696	DRR	Ca_00912	YU	--
Ca3_P3_36129411	Ca3	36129411	ATT/ATTT	CGTCACGTTAGTGTAAAGGCG	CACACCAATCAAACGAGCAA	60.7	443	DRR	Ca_00912	YU	--
Ca3_P3_36138844	Ca3	36138844	AG/A	TGGCTTGAGTGTGATGATGAA	GGGTGTTGGCAATATTGGAT	60.3	806	INTERGENIC	--	--	--
Ca3_P3_36139586	Ca3	36139586	CT/CTGT	ATGCAAATTACCCAAGACGC	TTATACCGGCTTCGTTGACC	60.0	580	INTERGENIC	--	--	--
Ca3_P3_36140094	Ca3	36140094	CAA/CAAA	GGTCAACGAAGCCGGTATAA	CGAAAAATAACACCAATGCAAA	60.0	551	INTERGENIC	--	--	--
Ca3_P3_36153591	Ca3	36153591	ATTTTTT/ATTTTTTT	TCAAAGTTCATTTCATGTTCCG	ATTTGACAAAAGCCCGAAAA	60.0	526	DRR	Ca_00916	A	GeBP
Ca3_P3_36155823	Ca3	36155823	TAAAAAAAAA/TAAAAAAAA	GTGCTTTCTGAGCTCTTCGG	CCCGTGATTTTCCGTTCTTA	60.3	659	INTRON	Ca_00916	A	GeBP
Ca3_P3_36156758	Ca3	36156758	TAAAAAAAA/TAAAAAAAAAAA	TGAATCAATCATCGCCAAAA	CATGACTTAATGGCGTGGTG	60.0	435	INTRON	Ca_00916	A	GeBP

INDEL marker IDs	Chromosomes/ unanchored scaffolds	Physical positions (bp)	InDels (*Kabuli* reference genome-CDC Frontier/PII)	Forward primers (5'-3')	Reverse primers (5'-3')	Anneling temperature (0C)	Expected amplified product size (bp)	Structural annotation		Functional annotation	
								Sequence components of *kabuli* genome	*Kabuli* gene accession IDs	NCBI-KOG	TFs
Ca3_P3_36157270	Ca3	36157270	ACCC/ACC	AAAAATGTCACCACGC CATT	GTCTTTCCCCATCCCT TTGT	60.2	209	INTRON	Ca_00916	A	GeBP
Ca3_P3_36163828	Ca3	36163828	GTTTTTTTTTTT/GTTTTTTTTTT	AATCGTTTGAAAGACGT CGAA	TCAAACGGACCTCTCC ATAAA	59.7	573	INTERGENIC	--	--	--
Ca3_P3_36180666	Ca3	36180666	CATA/CATATATA	ACTCGGCTTGGGAATA CCAT	AGATCGACGCATAAGT GGCT	60.7	736	INTRON	Ca_00921	Q	B3
Ca3_P3_36187931	Ca3	36187931	TAA/TAACTGTTTAAAA	ACAATTGAAAAACAAGG GGG	GGGCCTAGTCCTATGC TTCA	58.8	514	INTERGENIC	--	--	--
Ca3_P3_36188427	Ca3	36188427	TAA/TAAA	GCATAGGACTAGGCCC AAAA	GGGAAGCGTCACACTA AAGC	59.2	521	DRR	Ca_00922	Q	B3
Ca3_P3_36192498	Ca3	36192498	AA/AAGA	TGACATCTCCTCCAACC ACA	TGGACCAATTTTCGTA TTCACTC	60.1	332	INTRON	Ca_00922	Q	B3
Ca3_P3_36197056	Ca3	36197056	TTGTGT/TTGT	GTGGCGCAAAAAGAAA AGAG	TGCAGTAACTTCAACG GGTCT	60.0	756	INTRON	Ca_00923	--	bHLH
Ca3_P3_36197389	Ca3	36197389	CTTTT/CTTTTT	TCAACGTTCCATCTTCT CTCA	TTTGGATTTAGCCGTT GGAC	58.4	635	INTRON	Ca_00923	--	bHLH
Ca3_P3_36197502	Ca3	36197502	AACACACA/AACACA	TCAACGTTCCATCTTCT CTCA	TTTGGATTTAGCCGTT GGAC	58.4	635	INTRON	Ca_00923	--	bHLH
Ca3_P3_36203687	Ca3	36203687	GGTG/GGTGTG	CACCGTGAAGATTGTG GATG	TGTGAGGGATTTGTGC ATGT	60.0	292	INTERGENIC	--	--	--

INDEL marker IDs	Chromosomes/ unanchored scaffolds	Physical positions (bp)	InDels (*Kabuli* reference genome-CDC Frontier/PII)	Forward primers (5'-3')	Reverse primers (5'-3')	Anneling temperature (0C)	Expected amplified product size (bp)	Structural annotation		Functional annotation	
								Sequence components of *kabuli* genome	*Kabuli* gene accession IDs	NCBI-KOG	TFs
Ca3_P3_36204191	Ca3	36204191	TG/T	ACATGCACAAATCCCTCACA	GGTAGCAAAGCAATTTCGGA	60.0	435	DRR	Ca_00925	--	--
Ca3_P3_36204205	Ca3	36204205	ATTT/ATTTT	ACATGCACAAATCCCTCACA	GGTAGCAAAGCAATTTCGGA	60.0	435	DRR	Ca_00925	--	--
Ca3_P3_36204911	Ca3	36204911	ATATTAAATTT/ATATTAAATTTATTAAATTT	GTCGTTGGAGTCTTTGCGAT	TTCACCAAATGTTGACCGAA	60.3	452	DRR	Ca_00925	--	--
Ca3_P3_36205967	Ca3	36205967	GT/GTCT	GCATGGCATCAACAAGAATG	CATCAGAAGTCTGTTGCCGA	60.1	467	DRR	Ca_00925	--	--
Ca3_P3_36207784	Ca3	36207784	GA/GAA	TGGGTTCATTAGCCACATCA	GTTCAAACTTCGTTGCCTCC	59.9	237	INTRON	Ca_00925	--	--
Ca3_P3_36211079	Ca3	36211079	GAAAAAAAAAA/GAAAAAAAAAAA	GTGAGTGAGCCGTGTACGAA	TGAGTAAGGTGGGGAGGTTG	59.9	301	INTERGENIC	--	--	--
Ca3_P3_36212061	Ca3	36212061	ATTTTTTTTTT/ATTTTTTTTT	TGGAGATGGAAAGTGGGAAG	CCAGAAATTTTAACGAACGAGG	60.0	326	INTRON	Ca_00926	--	LSD
Ca3_P3_36212647	Ca3	36212647	ATTT/ATT	TCCGTGCAAATATAGCACATTC	TTGGCTGAGAACAACAATGC	60.0	504	INTRON	Ca_00926	--	LSD
Ca3_P3_36213542	Ca3	36213542	GTGATGATGAT/GTGATGAT	AAAATTATTGTGGCCGAGCA	CCGCACCAAGTATGAAGAGC	60.5	414	DRR	Ca_00926	--	LSD
Ca3_P3_36215526	Ca3	36215526	TA/TAA	TTGATTGCCGACAGTCTTTG	CGCAATTTTGAATGTCATCATC	59.8	622	DRR	Ca_00927	--	CO-like

**INDEL marker IDs**	**Chromosomes/ unanchored scaffolds**	**Physical positions (bp)**	**InDels (*Kabuli* reference genome-CDC Frontier/PII)**	**Forward primers (5'-3')**	**Reverse primers (5'-3')**	**Anneling temperature (0C)**	**Expected amplified product size (bp)**	**Structural annotation**		**Functional annotation**	
								**Sequence components of *kabuli* genome**	***Kabuli* gene accession IDs**	**NCBI-KOG**	**TFs**
Ca3_P3_36215688	Ca3	36215688	ATTTTT/ATTTT	TGCTGTAACGATGATG CCAC	CAATGTGGATGCCTTT GTTG	60.7	981	DRR	Ca_00927	--	CO-like
Ca3_P3_36215864	Ca3	36215864	CTTTT/CTT	TGATGACATTCAAAATT GCG	CCTGTTTGTTTGGCTT GGAT	58.1	582	DRR	Ca_00927	--	CO-like
Ca3_P3_36219676	Ca3	36219676	ATTTTTTT/ATTTTTT	TTCACGCCCAAAGGAA ATAC	CCCACCAATAAGAGAG CCAA	59.9	512	INTERGENIC	--	--	--
Ca3_P3_36220254	Ca3	36220254	T/TG	TTGGCTCTCTTATTGGT GGG	ACCGGTGATGACTCCA GTTC	60.1	265	DRR	Ca_00928	--	--
Ca3_P3_36220304	Ca3	36220304	GAAAAAAAAAAAAA/GAAAAAAAAAAA	TTGGCTCTCTTATTGGT GGG	AATTCGGCGCTTTAGG ATTT	60.1	446	DRR	Ca_00928	--	--
Ca3_P3_36224870	Ca3	36224870	AAGAGAGAGAGAGAGAGAGAG/AAGA GAGAGAGAGAGAGAGAGAGAGAG	ACCTGTGTGACTCTGA GGGG	CCCAGTAACCGCTCTC TCTG	60.2	721	INTERGENIC	--	--	--
Ca3_P3_36233651	Ca3	36233651	AGTTGTTGTT/AGTTGTT	GCAATGCCAACAGATG AAAA	AATCACACCTTTAAATC GGCA	59.7	536	DRR	Ca_00930	O	--
Ca3_P3_36233714	Ca3	36233714	CTTTTTTTT/CTTTTTTT	GCAATGCCAACAGATG AAAA	AATCACACCTTTAAATC GGCA	59.7	536	DRR	Ca_00930	O	--
Ca3_P3_36237362	Ca3	36237362	ATTT/ATTTT	TCGCAAGATGAGTCTT GGTG	AATGGGCTTTGAAGGG TCTT	60.0	596	DRR	Ca_00931	O	--
Ca3_P3_36240433	Ca3	36240433	TAAAAAAAAA/TAAAAAAAA	TTGTTTTGAATTTGGGC CAG	TTCCAAAATCTCACCAT TCATT	60.8	859	INTERGENIC	--	--	--

INDEL marker IDs	Chromosomes/ unanchored scaffolds	Physical positions (bp)	InDels (*Kabuli* reference genome-CDC Frontier/PII)	Forward primers (5'-3')	Reverse primers (5'-3')	Anneling temperature (0C)	Expected amplified product size (bp)	Structural annotation		Functional annotation	
								Sequence components of *kabuli* genome	*Kabuli* gene accession IDs	NCBI-KOG	TFs
Ca3_P3_36242951	Ca3	36242951	GTTTTTTT/GTTTTTTTT	TGCTGCAAAAGACAATTTCG	AAGTGGTGGTGCTGTTTTCC	60.0	907	INTERGENIC	--	--	--
Ca3_P3_36251365	Ca3	36251365	TAA/TA	GGTAAATTCGGTGGAACCAA	TCTGCCTGTTGTTTTTGACG	59.7	336	INTERGENIC	--	--	--
Ca3_P3_36254231	Ca3	36254231	GTTTTTTTTT/GTTTTTTTT	TGCCGATGTAATCTACCGAA	CGCCATTAGAAGTGGGAAAA	59.1	652	DRR	Ca_00934	O	--
Ca3_P3_36256005	Ca3	36256005	CTAAAGATA/CTAAAGATATAAAGATA	CCCTCCCTTGTTGACGAATA	ATTGGCGAAGAAATGCAATC	59.9	705	DRR	Ca_00935	--	--
Ca3_P3_36256160	Ca3	36256160	CAAA/CAAAA	CCCTCCCTTGTTGACGAATA	ATTGGCGAAGAAATGCAATC	59.9	705	DRR	Ca_00935	--	--
Ca3_P3_36258385	Ca3	36258385	G/GA	CAATTGTTTTTCCCTCCCCT	CGGTTTCCTTTTCATAAGCG	60.2	469	DRR	Ca_00936	--	MYB_related
Ca3_P3_36261656	Ca3	36261656	CAAAAA/CAAAA	TGAAGTGCAAAGGTTGATGC	AGGAGCTCACCGACAATGAT	59.8	493	INTRON	Ca_00936	--	MYB_related
Ca3_P3_36263206	Ca3	36263206	CTAT/C	GCATGAGTTGGATTGCATTG	TGCCCTAAATAGCATTGGATG	60.1	301	INTERGENIC	--	--	--
Ca3_P3_36263271	Ca3	36263271	TAAAAAAAA/TAAAAAAA	GCATGAGTTGGATTGCATTG	TGCCCTAAATAGCATTGGATG	60.1	301	INTERGENIC	--	--	--
Ca3_P3_36264829	Ca3	36264829	GAACGGAAA/GAA	ATCACCTGCAACATGGATCA	TTAATTAGGCACACCACGCA	59.9	706	INTERGENIC	--	--	--

INDEL marker IDs	Chromosomes/ unanchored scaffolds	Physical positions (bp)	InDels (*Kabuli* reference genome-CDC Frontier/PII)	Forward primers (5'-3')	Reverse primers (5'-3')	Anneling temperature (0C)	Expected amplified product size (bp)	Structural annotation		Functional annotation	
								Sequence components of *kabuli* genome	*Kabuli* gene accession IDs	NCBI-KOG	TFs
Ca3_P3_36268678	Ca3	36268678	ATTTTTTTTTT/ATTTTTTTTTTTT	ATCGGTATCGGAGCTTGATG	CATGGTTTTGAAAAATGGGG	60.1	967	INTRON	Ca_00937	J	Dof
Ca3_P3_36269424	Ca3	36269424	TGTTGTTAGTTGTTA/TGTTGTTA	CAAAACCATGCCAGGAATCT	CCGGTACCTGCTCTTACTGC	59.9	752	INTRON	Ca_00937	J	Dof
Ca3_P3_36270571	Ca3	36270571	TTTTTA/T	TGATGTTGAATCAGGTCCGA	GTGCCGTCGTCAGTTCAATA	60.0	811	INTRON	Ca_00937	J	Dof
Ca3_P3_36274695	Ca3	36274695	ATAAAATATA/ATAAAATATATAATTAAAATATA	TGAGTTGCTTTAAAAATTCGCA	GGAGACAAATATAATTCACCACCA	59.9	541	DRR	Ca_00937	J	Dof
Ca3_P3_36275195	Ca3	36275195	AAT/AATAT	TTTTGGTGGTGAATTATATTTGTCTC	TGCATGACAAGTGGCTCTTC	59.6	392	DRR	Ca_00937	J	Dof
Ca3_P3_36277496	Ca3	36277496	A/AG	GGCTTGGGAAGATAAGAGGG	CAGATCCAAAACCACCGAAT	60.0	602	INTRON	Ca_00938	R	--
Ca3_P3_36278881	Ca3	36278881	GA/GAA	GGTCTTCAAGTTGCTGGCAT	CAATGGACGGCTTACCTGTT	60.3	164	INTRON	Ca_00938	R	--
Ca3_P3_36284489	Ca3	36284489	ATTTTTTTT/ATTTTTTT	ACCCCTCCTGTCGTGTATTG	AGCCACATTCACAAACCACA	59.8	449	INTERGENIC	--	--	--
Ca3_P3_36291066	Ca3	36291066	ATTTTTTT/ATTTTTTTT	TCCCTATCTCATTTTACCGTGA	GGTTTGGTTCCTTCGGTTTT	58.6	745	INTERGENIC	--	--	--
Ca3_P3_36302805	Ca3	36302805	C/CA	ATCGTGGCAACATTTGAGGT	CACGTGTCAAACTTGCCAAT	60.4	995	INTERGENIC	--	--	--

INDEL marker IDs	Chromosomes/ unanchored scaffolds	Physical positions (bp)	InDels (*Kabuli* reference genome-CDC Frontier/PII)	Forward primers (5'-3')	Reverse primers (5'-3')	Anneling temperature (0C)	Expected amplified product size (bp)	Structural annotation		Functional annotation	
								Sequence components of *kabuli* genome	*Kabuli* gene accession IDs	NCBI-KOG	TFs
Ca3_P3_36307639	Ca3	36307639	GTCTTATCTTAT/GTCTTATCTTATCTTAT	GTGTTCCGCGGTTAACAACT	TGGCTAATAAGCCTACGCAAA	60.0	409	INTERGENIC	--	--	--
Ca3_P3_36308411	Ca3	36308411	GAAAAAAAAA/GAAAAAAAAAAA	TTTGCCACTTGATACAGCAGA	GCCCAAGTTTGAGTCCAAAG	59.5	733	INTERGENIC	--	--	--
Ca3_P3_36319172	Ca3	36319172	A/AT	AAAGGAATTGCAAGGACCAA	CGCGGTGATTTTATTGCTCT	59.5	616	INTRON	Ca_00941	D	TCP
Ca3_P3_36324828	Ca3	36324828	T/TG	TCCATGAAACATTCCCCATT	CGCACTAAAACGACGAACAA	60.0	454	INTERGENIC	--	--	--
Ca3_P3_36326987	Ca3	36326987	AT/ATATTT	CATTTTTGCATCACACTGCC	TGGTTGCAAGGAGAAAGTGA	60.1	565	INTERGENIC	--	--	--
Ca3_P3_36327195	Ca3	36327195	ATTTTTTTTT/ATTTTTTTTTT	CACTTTCTCCTTGCAACCAA	TGGAGAAACAATGCAAATGG	58.9	512	INTERGENIC	--	--	--
Ca3_P3_36331408	Ca3	36331408	TGG/TG	TGTAGGTTTTTGACCGGAGG	TCACCAAGACCAAACAGGCT	60.0	494	INTRON	Ca_00942	--	HB-other
Ca3_P3_36334629	Ca3	36334629	TAAAAAAAAA/TAAAAAAAA	AACGGCGAATACTAACACGG	GCCCAAACGAAACCTATCAA	60.0	372	INTRON	Ca_00942	--	HB-other
Ca3_P3_36334754	Ca3	36334754	ATTTTTT/ATTTTTTT	AACGGCGAATACTAACACGG	GCGGAATACCACGAGAAAAA	60.0	697	INTRON	Ca_00942	--	HB-other
Ca3_P3_36336902	Ca3	36336902	TAAA/TAA	CCCATCTCCAACTCCTTCAA	GGTGCTAAGTTTTGCCGTGT	60.0	818	INTRON	Ca_00942	--	HB-other

INDEL marker IDs	Chromosomes/ unanchored scaffolds	Physical positions (bp)	InDels (*Kabuli* reference genome-CDC Frontier/PII)	Forward primers (5'-3')	Reverse primers (5'-3')	Anneling temperature (0C)	Expected amplified product size (bp)	Structural annotation		Functional annotation	
								Sequence components of *kabuli* genome	*Kabuli* gene accession IDs	NCBI-KOG	TFs
Ca3_P3_36340133	Ca3	36340133	A/AGTAGAGATATAATTG	TAGCCCGTCACAATTTAGGG	CTCTTTCTTTCCCTCCGCTC	60.0	910	DRR	Ca_00942	--	HB-other
Ca3_P3_36342047	Ca3	36342047	CAA/CAAAA	ACAAATGCCTCGATCTGTCC	GGCGGTGATGCTTCAATACT	60.1	367	INTERGENIC	--	--	--
Ca3_P3_36342426	Ca3	36342426	GAAAAAA/GAAAAAAA	AGTATTGAAGCATCACCGCC	TCGAGGCATTTGTCTGAGAG	60.1	195	INTERGENIC	--	--	--
Ca3_P3_36344245	Ca3	36344245	CATAT/CAT	GGCCTTTTGATCCTCTAGGC	TGCAATTTTTATCACATCCCC	60.2	777	DRR	Ca_00943	R	C2H2
Ca3_P3_36349322	Ca3	36349322	TTCTC/TTCTCTC	TGACATTTTCCAAGGTCGAA	GTTGACGGTCGATTGAAACA	59.1	594	INTERGENIC	--	--	--
Ca3_P3_36353533	Ca3	36353533	CTTTT/CTTT	TCAAGTTGCAGGCTCAAAAA	TGACTTGGTTTTGTATTCGGC	59.6	524	INTERGENIC	--	--	--
Ca3_P3_36361587	Ca3	36361587	CAA/CA	ACTCATCTCCAACACGAGGG	CTGTCAACAAATCGCAGTCG	60.1	465	INTERGENIC	--	--	--
Ca3_P3_36361613	Ca3	36361613	GACA/GA	ACTCATCTCCAACACGAGGG	TTGCTGCTGATTTTCTCGTC	60.1	769	INTERGENIC	--	--	--
Ca3_P3_36363297	Ca3	36363297	CAAATAAA/CAAATAAATAAA	CAGTGTGAGTTAGGAATGGCAA	TTCCGTCCATATGCATTGATT	60.2	145	DRR	Ca_00945	--	NF-X1
Ca3_P3_36371582	Ca3	36371582	ATTTTTTT/ATTTTTT	CAAAACATGCACCTAAAAACCA	GGGGGACTAGAAAACCCAAG	59.9	500	INTERGENIC	--	--	--

INDEL marker IDs	Chromosomes/ unanchored scaffolds	Physical positions (bp)	InDels (*Kabuli* reference genome-CDC Frontier/PII)	Forward primers (5'-3')	Reverse primers (5'-3')	Anneling temperature (0C)	Expected amplified product size (bp)	Structural annotation		Functional annotation	
								Sequence components of *kabuli* genome	*Kabuli* gene accession IDs	NCBI-KOG	TFs
Ca3_P3_36371828	Ca3	36371828	GT/GTTAT	GGGTTTTCTAGTCCCCCAAG	TGTTTTTCATTATGTGCCCG	59.8	483	INTERGENIC	--	--	--
Ca3_P3_36376514	Ca3	36376514	CTAT/CT	CACACTGGATCTAAACCTCGG	TCCACCACGATTGTTTTTCA	59.6	353	DRR	Ca_00946	D	WRKY
Ca3_P3_36381118	Ca3	36381118	T/TTTTTGAAAGGGCA	AAAAAGCGCGGGTTAATTTT	CCATGCTTCCCACGTCTAAT	60.0	544	INTERGENIC	--	--	--
Ca3_P3_36383650	Ca3	36383650	TAA/TAAA	CTGACACGTATGATGGGTGC	CAAAACAGTGCCAAACATGC	60.0	332	INTERGENIC	--	--	--
Ca3_P3_36384085	Ca3	36384085	TAAAAAAAAAAAAAAA/TAAAAAAAAAAAA	TGGCACTGTTTTGGTCTTTG	TGGACCCAAAATTTCCCATA	59.7	605	INTERGENIC	--	--	--
Ca3_P3_36389750	Ca3	36389750	GATTTTTATTTTTATTTTT/GATTTTTATTTTT	TGCCACCATTATGCACCTTA	GGACTATTTGGGCTGCGATA	60.0	524	DRR	Ca_00948	R	NAC
Ca3_P3_36397763	Ca3	36397763	TACCACCACCACCACCACCA/TACCACCACCACCACCA	CCCTCCAATTCAGCATGTTT	GCCTTTGTCCGTGCATATTT	59.9	546	CDS (large-effect mutations)	Ca_00950	P	--
Ca3_P3_36402333	Ca3	36402333	TA/T	GGCCTCACACTCCATCAAAT	GCTCAATGTTGCGTTTAGCA	59.9	316	INTRON	Ca_00951	H	--
Ca3_P3_36403180	Ca3	36403180	TA/T	AACCGGAAGGATCTGTGTTG	TTTTCACATTTCAACCGCAC	60.0	953	DRR	Ca_00951	H	--
Ca3_P3_36403564	Ca3	36403564	TCA/T	TGAGGTGCTAACACCGAAGA	AGTGACAACCCCTCAATTCG	59.4	223	DRR	Ca_00951	H	--

INDEL marker IDs	Chromosomes/ unanchored scaffolds	Physical positions (bp)	InDels (*Kabuli* reference genome-CDC Frontier/PII)	Forward primers (5'-3')	Reverse primers (5'-3')	Anneling temperature (0C)	Expected amplified product size (bp)	Structural annotation		Functional annotation	
								Sequence components of *kabuli* genome	*Kabuli* gene accession IDs	NCBI-KOG	TFs
Ca3_P3_36413392	Ca3	36413392	T/TAATAAAAAA	ATGCCAAATCCAAAAGCACT	TTGAAAACGATGCCAAACTC	59.6	460	INTRON	Ca_00954	G	--
Ca3_P3_36414069	Ca3	36414069	TTT/TTTATT	GATGCGCTATCCAACCTCAC	GCCGCCCACATATCATTTAT	60.6	659	INTRON	Ca_00954	G	--
Ca3_P3_36415214	Ca3	36415214	AAAGAAG/AAAG	GAGTCAACCTACCGGACCAA	TGGGAATGACCTTTGCTTTC	60.0	466	INTRON	Ca_00954	G	--
Ca3_P3_36420971	Ca3	36420971	AATATATATATATAT/AATATATATAT	TGGCATCGTGATTTCTACCA	GGCCATGGTGATTGTAAAGG	60.1	746	INTERGENIC	--	--	--
Ca3_P3_36426774	Ca3	36426774	TAGATGAAGATGAAGATGAAGATGA/TAGATGAAGATGAAGATGAAGATGAAGATGA	TCAAAGGAAGCTTAGCCCAA	GCAAACGCTTCTCTGGTTTC	60.0	861	CDS (large-effect mutations)	Ca_00956	GM	WRKY
Ca3_P3_36429496	Ca3	36429496	TAAAAAAA/TAAA	CCATCTTGCGAAATGTGAAG	GTTACAGCTGGTGCATCCCT	59.3	590	INTERGENIC	--	--	--
Ca3_P3_36431342	Ca3	36431342	T/TGAAC	CCCATTTGCTGTATTTGCCT	TTGCATCAGGCTTGTACCTG	60.0	694	INTRON	Ca_00957	H	--
Ca3_P3_36432148	Ca3	36432148	TA/TAA	CTTCTTGCAGGGAACGAAAG	AGTGCTCCAGCAACGAGAAT	60.0	378	INTRON	Ca_00957	H	--
Ca3_P3_36434602	Ca3	36434602	CGT/CGTGT	TGCAAGGACAAACATAAGCG	AAAAAGGATGGATCGGGAAT	59.9	511	DRR	Ca_00957	H	--
Ca3_P3_36443050	Ca3	36443050	AT/A	TTGCAATTGAAGTTCCCACA	GGCAAGCTCATTTCTGCTTC	60.1	427	INTRON	Ca_00959	R	--

INDEL marker IDs	Chromosomes/ unanchored scaffolds	Physical positions (bp)	InDels (*Kabuli* reference genome-CDC Frontier/PII)	Forward primers (5'-3')	Reverse primers (5'-3')	Anneling temperature (0C)	Expected amplified product size (bp)	Structural annotation		Functional annotation	
								Sequence components of *kabuli* genome	*Kabuli* gene accession IDs	NCBI-KOG	TFs
Ca3_P3_36447139	Ca3	36447139	CAT/CATAT	GGATGAGTGCAATTTGGGAT	AATGAATGGCTGCGCTAGTT	59.8	473	DRR	Ca_00960	O	--
Ca3_P3_36447416	Ca3	36447416	AGCTGC/AGCTGCTGC	GGACACCAACGTCTCCACTT	GAGCAAGAAGTGTCGAAGCC	60.0	210	CDS (large-effect mutations)	Ca_00960	O	--
Ca3_P3_36447887	Ca3	36447887	TCCC/TCC	GGCTTCGACACTTCTTGCTC	TTGCAGTTGATGTCTGGCTC	60.1	785	INTRON	Ca_00960	O	--
Ca3_P3_36448772	Ca3	36448772	CAAAAAAAA/CAAAAAAA	TTCCTCCTAAGCCTCCCATT	CAATGTAGGAGTGCGAAGCA	60.0	489	INTRON	Ca_00960	O	--
Ca3_P3_36476148	Ca3	36476148	A/AATACAT	TGGCAAATCGATTGACAAAA	ACATCCAGTCCTGCAAATCC	60.1	844	INTERGENIC	--	--	--
Ca3_P3_36481853	Ca3	36481853	TA/TAA	TCCTCGAGATCCAAAAGCAT	GTCCAATTCCCCCTTTCATT	59.8	272	INTERGENIC	--	--	--
Ca3_P3_36534011	Ca3	36534011	ATTTTTTTTT/ATTTTTTTT	TTACTCATCCATCGTTCGCA	TCAGCGTCGTCAAATATTGC	60.2	415	INTERGENIC	--	--	--
Ca3_P3_36551603	Ca3	36551603	AATATATATATATATATATATATATATATATA/AATATATATATATATATATATATATATATATATATATATATATA	TCACACACCTCGTTGAAAGC	TCAACCGGACGTACTCAACA	59.9	613	INTERGENIC	--	--	--
Ca3_P3_36560978	Ca3	36560978	T/TA	ATCTGATTTGAGCAATGGGC	GGTAAGGCCATTTTGGGTAA	60.0	452	INTERGENIC	--	--	--
Ca3_P3_36582832	Ca3	36582832	TAAAAAAAAAA/TAAAAAAAAAAA	ACCCTCCGTCTGTTTCACAC	GGGAATGCGGTGGATAAATA	60.0	682	INTERGENIC	--	--	--

INDEL marker IDs	Chromosomes/ unanchored scaffolds	Physical positions (bp)	InDels (*Kabuli* reference genome-CDC Frontier/PII)	Forward primers (5'-3')	Reverse primers (5'-3')	Anneling temperature (0C)	Expected amplified product size (bp)	Structural annotation		Functional annotation	
								Sequence components of *kabuli* genome	*Kabuli* gene accession IDs	NCBI-KOG	TFs
Ca3_P3_36668334	Ca3	36668334	GTTTT/GTTTTT	TGGGTAAAAGCTCGAGTTCC	CAGTGGTGGAAAAAGGCAAT	59.3	499	INTERGENIC	--	--	--
Ca3_P3_36810363	Ca3	36810363	TTCTCTCTCTCTCTCTCTCTCTCTCTCTCTCTCTCTCTCT/TTCTCTCTCTCTCTCTCTCTCTCTCTCTCTCTCTCTCT	GGCAGATTCCTCACCCTACA	TGGATTCGTTGCATTGGTAA	60.1	474	DRR	Ca_01004	--	bZIP
Ca3_P3_36829016	Ca3	36829016	TAAAAAAAA/TAAAAAAA	CGTTCTTTGAATGAGTGACCAA	AGTGCAAAGAGGTTGCAAGG	60.1	321	INTERGENIC	--	--	--
Ca3_P3_36829634	Ca3	36829634	CTATATATATATATA/CTATATATATATA	ACCTTGCAACCTCTTTGCAC	TTTTAAAATGTTGTCGGGTGG	60.3	545	INTERGENIC	--	--	--
Ca3_P3_36834575	Ca3	36834575	TAATGC/T	GTACCTTCCACCCCCATTTT	TTTCGGAAGATTGGTCTTCAA	59.9	674	DRR	Ca_01008	K	GRAS
Ca3_P3_3688489	Ca3	3688489	AAATCGATTTCCCAATCGATTT/AAATCGATTT	GTGCCAGTCGATTGCCTAAT	CAACCACGAGGACAACAATG	60.1	292	INTERGENIC	--	--	--
Ca3_P3_36984584	Ca3	36984584	ATTTTTTT/ATTTTTTTT	CAGCGTTCATCACTGCCTAA	CTTCGGATGGAAAGTGGAAA	60.0	516	INTERGENIC	--	--	--
Ca3_P3_37000758	Ca3	37000758	TTAACTAACTAACTAACTAAC/TTAACTAACTAACTAAC	TGTGTGCTAACCAAATGCAAA	TGAATTGGATGCATCATCGT	60.2	664	INTRON	Ca_01028	--	--
Ca3_P3_37017817	Ca3	37017817	AATATATATATATATATATATATATATATATATA/AATATATATATATATATATATATATATA	CAGAAGGCCAGGAGAAGAAA	TGTGCGTGAAGTTTCATGGT	59.5	707	INTERGENIC	--	--	--
Ca3_P3_37030241	Ca3	37030241	ATAT/ATATTTAT	GAAAAGCCCATTTTGTCTCG	AAAGGTTTGAAAATGGATGGTC	59.7	593	DRR	Ca_01031	G	C3H

INDEL marker IDs	Chromosomes/ unanchored scaffolds	Physical positions (bp)	InDels (*Kabuli* reference genome-CDC Frontier/PII)	Forward primers (5'-3')	Reverse primers (5'-3')	Anneling temperature (0C)	Expected amplified product size (bp)	Structural annotation		Functional annotation	
								Sequence components of *kabuli* genome	*Kabuli* gene accession IDs	NCBI-KOG	TFs
Ca3_P3_37047269	Ca3	37047269	ATTTTTTTTT/ATTTTTTTT	CAGTTTTCAGAGTTGCATTTTCA	ATGAATGAAGGGGCTGAATG	59.4	474	INTERGENIC	--	--	--
Ca3_P3_37048753	Ca3	37048753	AATATATATATATATATATATATATAT/AATATATATATATATATATATATATATATATATAT	TTGATTCTATTCGTCTTTGGTTCA	CAACTTGCCGCAAAGGTATT	60.0	633	INTERGENIC	--	--	--
Ca3_P3_37078968	Ca3	37078968	CT/C	GGAAAGTAGCTGCTGTCGCT	AATGGAGTGCCAGACAAACC	59.8	556	INTRON	Ca_01034	R	--
Ca3_P3_37088296	Ca3	37088296	ATTTTTT/ATTTTT	CAAGAGGCAAACCGTTCAAT	TGCAACAAATTGTCAATCTCAA	60.1	478	INTERGENIC	--	--	--
Ca3_P3_3712841	Ca3	3712841	CATATATATATATATATATATATAT/CATATATATATATATATATATAT	TGACACACCCACCCACTAAA	GACTGAGATGATGATTCACTTTGC	59.8	520	INTERGENIC	--	--	--
Ca3_P3_37134779	Ca3	37134779	ACCCCC/ACCCCCC	TGCATGTCCGATTGAAATGT	ACGCCACTGTCCTTGTTACC	59.9	917	INTERGENIC	--	--	--
Ca3_P3_37150967	Ca3	37150967	ATTTTTTTT/ATTTTTTTTT	TTTTGCTTGCGTGAAAATTG	CCAAATGATAATCCATGGGC	59.9	604	INTERGENIC	--	--	--
Ca3_P3_37163693	Ca3	37163693	AAGTCCGTTAACCACTTCAGTCC/AAGTCCGTTAACCACTTCAGTCCGTTAACCACTTCAGTCC	CCAGTTAGGGGTCGAACTCA	CGTTCCAAAAACCAAATGCT	60.1	227	INTRON	Ca_01047	U	--
Ca3_P3_37192959	Ca3	37192959	T/TCA	TCAAATTTCATGTGCGCTGT	TAATAGTCCGGCCCACTTTG	60.3	478	INTERGENIC	--	--	--
Ca3_P3_37211475	Ca3	37211475	GAACAACAACA/GAACAACAACAACAACAACA	TGGTCAAATTCTTGGAGCAA	TGATCACACCCCAAGTTTTG	59.2	336	CDS (large-effect mutations)	Ca_01053	--	bHLH

INDEL marker IDs	Chromosomes/ unanchored scaffolds	Physical positions (bp)	InDels (*Kabuli* reference genome-CDC Frontier/PII)	Forward primers (5'-3')	Reverse primers (5'-3')	Anneling temperature (0C)	Expected amplified product size (bp)	Structural annotation		Functional annotation	
								Sequence components of *kabuli* genome	*Kabuli* gene accession IDs	NCBI-KOG	TFs
Ca3_P3_37247726	Ca3	37247726	GTATATATATATATATATATATATATAT A/GTATATATATATATATATATATA	GCCTGTTTGCTTTGGG ATTA	GGTGACACGCATTTTT GTGA	60.1	856	INTERGENIC	--	--	--
Ca3_P3_37348088	Ca3	37348088	TAAAAAAAAA/TAAAAAAAAAA	TCGGCGGACTTACCAA ATAA	GGGAACTTTTTAAGTG GGGC	60.4	438	INTERGENIC	--	--	--
Ca3_P3_37396352	Ca3	37396352	CAATTGGGAATTGG/CAATTGGGAATT GGGAATTGG	CCTTTGCCTTTCTGAGA TGG	GCGGTGAATATCCAAA GGAC	59.8	481	INTERGENIC	--	--	--
Ca3_P3_37464289	Ca3	37464289	TAAAAAAAAA/TAAAAAAAAAA	ATGTGCAGCCCTGCAT ACTA	GCTGCTGCCTGCATGT ATAA	59.3	824	DRR	Ca_01080	--	--
Ca3_P3_37719535	Ca3	37719535	TGG/TGGG	TTGGGTCAAGAATTCAT CTCG	CCCAAAATGTGGCTTG AAAT	60.1	581	INTERGENIC	--	--	--
Ca3_P3_37756750	Ca3	37756750	ATTTTTTTTT/ATTTTTTTTTT	AATAGGCCAGGTCAGG CTTT	GGATTCGTTGATGATG GCTT	60.1	656	INTERGENIC	--	--	--
Ca3_P3_37828643	Ca3	37828643	CCAGAC/CC	GGGAATAGGCTAGACC GTCC	GCTTGGCCTACGACCT ATCA	59.9	512	INTERGENIC	--	--	--
Ca3_P3_37841677	Ca3	37841677	CAAA/CAAAA	CATGTTGTTGGCTATG GTGC	TGCAGATCGCATCTGA AAAC	60.0	293	INTRON	Ca_01123	U	GATA
Ca3_P3_37875029	Ca3	37875029	GT/GTT	ATGGCGTGACTGAACA TGAA	AAGGGCAAGATCCCAA AAAC	60.1	672	INTERGENIC	--	--	--
Ca3_P3_37978022	Ca3	37978022	TTTATTATTATTATTATTATTATTATTAT TATTAT/TTTATTATTATTATTATTATTAT TATTATTAT	TTTGGTTTCTGCTTGCC TCT	CCTTGCTACTCGTATC CCCA	60.0	273	INTERGENIC	--	--	--

INDEL marker IDs	Chromosomes/ unanchored scaffolds	Physical positions (bp)	InDels (*Kabuli* reference genome-CDC Frontier/PII)	Forward primers (5'-3')	Reverse primers (5'-3')	Anneling temperature (0C)	Expected amplified product size (bp)	Structural annotation		Functional annotation	
								Sequence components of *kabuli* genome	*Kabuli* gene accession IDs	NCBI-KOG	TFs
Ca3_P3_37989883	Ca3	37989883	GTATATATATATATATATATA/GTATATATATATATATATATATATATATA	TGTCACGGGTGGACAAATTA	TAGGCGATTGTAGGGCTTTG	59.8	749	INTERGENIC	--	--	--
Ca3_P3_37991623	Ca3	37991623	TAAAAAAAAA/TAAAAAAAAAA	TAGATTCCCCACCAGTCCAG	TGGTACGCGACAATTTTTGTT	59.9	793	DRR	Ca_01140	--	--
Ca3_P3_38024661	Ca3	38024661	CAAAAAAAAAA/CAAAAAAAAAAA	CATGTATATCATTGCAATTCGTGTT	TTGTGATTTTATTAAGAGGGTAGTTTG	60.0	322	DRR	Ca_01143	S	--
Ca3_P3_3804615	Ca3	3804615	TA/T	CGCCATAAAGGATCAAGCAT	AACTTGATGCCGAGTAGCGT	60.1	317	INTERGENIC	--	--	--
Ca3_P3_38064707	Ca3	38064707	TTATATATATATATATATATATATATATATAT/TTATATATATATATATATATATATATATATATATAT	ATTTGCACCCACCACATTT	CGCCATATTCAAACAAAATTCA	60.1	944	INTERGENIC	--	--	--
Ca3_P3_38065578	Ca3	38065578	CATATATATATATATATATATATATATATAT/CATATATATATATATATATATATATAT	ATGTTTAATAGGCCGACGCC	TATCAATTGGTTGGTCCGCT	61.2	521	INTERGENIC	--	--	--
Ca3_P3_38073514	Ca3	38073514	CAAAAAAAAAAAAA/CAAAAAAAAAAAAA	CTTCTGAATGGGTTTGGTGG	ATGGACTCAATTGGGAGTGG	60.3	439	DRR	Ca_01151	DL	--
Ca3_P3_38096030	Ca3	38096030	TTATATATATATATATATATATATATAT/TTATATATATATATATATATATATATATATATAT	TCACGCGATCTAATCTCGACT	AGGGGTGTGAATATGTCCGA	60.0	543	INTERGENIC	--	--	--
Ca3_P3_38137580	Ca3	38137580	GATATATATA/GATATA	GCTTGCAAAAGGAATGAAGC	CAGAGCATGACGGTGAAAGA	60.0	205	INTERGENIC	--	--	--
Ca3_P3_38143286	Ca3	38143286	CATTATTATTATTATTA/CATTATTATTATTATTATTATTATTATTA	TCGCCCTATAATGCCAATTC	CCTCCATAAGGCCAAACAAA	59.9	450	INTERGENIC	--	--	--

INDEL marker IDs	Chromosomes/ unanchored scaffolds	Physical positions (bp)	InDels (*Kabuli* reference genome-CDC Frontier/PII)	Forward primers (5'-3')	Reverse primers (5'-3')	Anneling temperature (0C)	Expected amplified product size (bp)	Structural annotation		Functional annotation	
								Sequence components of *kabuli* genome	*Kabuli* gene accession IDs	NCBI-KOG	TFs
Ca3_P3_38146206	Ca3	38146206	ATC/ATCGCTC	CAACCCTTCTGTGCCAATTT	ATTTCGCAATGCTCGTCTTT	60.0	545	INTRON	Ca_01158	C	--
Ca3_P3_38149213	Ca3	38149213	TTTTATTTATTTATTTAT/TTTTATTTATTTATTTATTTAT	CACGAATGCCTTTGGCTAGT	CGATTTCAAACTCAAGACAAAATG	60.3	586	DRR	Ca_01159	R	MYB
Ca3_P3_38156087	Ca3	38156087	GTATATCTTGTATAT/GTATATCTTGTATATATCTTGTATAT	TGCACGGAATGTACACCCTA	TGGTTTTACCCCTCTTGCAG	60.0	753	DRR	Ca_01160	L	MYB_related
Ca3_P3_38156627	Ca3	38156627	ACTCT/ACT	CTGCAAGAGGGGTAAAACCA	ATGTTCTGAAAATGAGGCGG	60.1	560	INTRON	Ca_01160	L	MYB_related
Ca3_P3_38170265	Ca3	38170265	TAA/TA	CCCAGATCTGCCTAATGGAA	GATGGGTTTGGAACATCCAG	60.0	594	INTRON	Ca_01160	L	MYB_related
Ca3_P3_38170925	Ca3	38170925	CAAT/CAATCAACGCCATAAT	GAACAAATGCAGTGGCTGAA	TGGCGAGGAAGCTTGTAGAT	59.8	327	INTRON	Ca_01160	L	MYB_related
Ca3_P3_38174096	Ca3	38174096	AGG/AG	CGAAACACGTTACCATCATCC	CCGATGAGTGTGGATGTGAA	60.2	410	DRR	Ca_01161	--	NF-YA
Ca3_P3_38174447	Ca3	38174447	A/AT	TTCACATCCACACTCATCGG	CTGGCACTGGCATTCCTAAT	60.5	628	DRR	Ca_01161	--	NF-YA
Ca3_P3_3818047	Ca3	3818047	G/GGA	ATGCTCTGCACTTGCAGCTA	TTCATCGCCTCAAAGTGTTG	59.9	707	INTERGENIC	--	--	--
Ca3_P3_38181335	Ca3	38181335	TAAAAAA/TAAAAAAA	AAAAGCCAAAACCATGCAAC	TGGGGTTCTAACTCTCGCAC	60.0	699	INTERGENIC	--	--	--

INDEL marker IDs	Chromosomes/ unanchored scaffolds	Physical positions (bp)	InDels (*Kabuli* reference genome-CDC Frontier/PII)	Forward primers (5'-3')	Reverse primers (5'-3')	Anneling temperature (0C)	Expected amplified product size (bp)	Structural annotation		Functional annotation	
								Sequence components of *kabuli* genome	*Kabuli* gene accession IDs	NCBI-KOG	TFs
Ca3_P3_38182839	Ca3	38182839	AGTGTGTGTGTGTGTGT/AGTGTGTGTGTGT	AAAGCGTCTTTCATTAATAGTGTGTG	GGTTTTGCCAACATCTGCTT	60.0	319	INTERGENIC	--	--	--
Ca3_P3_38183694	Ca3	38183694	TAAA/TAAAA	CGACACGCACAAACCTTTTA	AACCTTCTTCGTTTCGTGGA	59.8	856	INTERGENIC	--	--	--
Ca3_P3_38203857	Ca3	38203857	CT/CTT	AGGAACTGGGCATGGTGTAG	TATTGGATTCCTCCAAAGCG	60.0	870	INTRON	Ca_01163	E	--
Ca3_P3_38206767	Ca3	38206767	AAGAG/AAG	CTATCCTGGAGAAGGGGGAG	GCTATTGCATTGGAGTTGCTC	60.0	706	DRR	Ca_01163	E	--
Ca3_P3_38208512	Ca3	38208512	TCCC/TCC	TTTGCGTTACGAATTTGATGA	TGACCCTTCATTCTTCCCAG	59.2	624	INTERGENIC	--	--	--
Ca3_P3_38208620	Ca3	38208620	CATAT/CAT	AAATCCTCATTCCCAATCAAA	TGACCCTTCATTCTTCCCAG	58.4	222	INTERGENIC	--	--	--
Ca3_P3_38208680	Ca3	38208680	ATTTTTTTTT/ATTTTTTTTTT	AAATCCTCATTCCCAATCAAA	TGACCCTTCATTCTTCCCAG	58.4	222	INTERGENIC	--	--	--
Ca3_P3_38212764	Ca3	38212764	CTTGTTT/CTT	ATGCAGACTTTGCTGCTCAA	TCAGAGGGATCGGACATAGG	59.7	267	INTRON	Ca_01164	T	WRKY
Ca3_P3_38215460	Ca3	38215460	CT/CTATAGT	ACTCTTCTTTGGTCGCGATG	CAACTCATTTCCAGGCAACA	60.4	452	DRR	Ca_01164	T	WRKY
Ca3_P3_38215897	Ca3	38215897	CTT/CTTCTCTTCATT	TGTTGCCTGGAAATGAGTTG	TGCTGCATTGAAATCGAGTC	59.7	688	DRR	Ca_01165	R	--

INDEL marker IDs	Chromosomes/ unanchored scaffolds	Physical positions (bp)	InDels (*Kabuli* reference genome-CDC Frontier/PII)	Forward primers (5'-3')	Reverse primers (5'-3')	Anneling temperature (0C)	Expected amplified product size (bp)	Structural annotation		Functional annotation	
								Sequence components of *kabuli* genome	*Kabuli* gene accession IDs	NCBI-KOG	TFs
Ca3_P3_38219046	Ca3	38219046	TCC/TCCC	GGTGAGGTTCTTGCAA CCAT	TATGCATGGTGAAAAA GCGA	60.0	655	DRR	Ca_01166	Z	B3
Ca3_P3_38219080	Ca3	38219080	TAAAAA/TAAAAAA	GGTGAGGTTCTTGCAA CCAT	TATGCATGGTGAAAAA GCGA	60.0	655	DRR	Ca_01166	Z	B3
Ca3_P3_38219214	Ca3	38219214	TTCT/TT	CTACCGGCTACTTTCTT GCG	TATGCATGGTGAAAAA GCGA	60.0	463	DRR	Ca_01166	Z	B3
Ca3_P3_38219924	Ca3	38219924	AC/ACC	CATTCCAATTTCATCTG CACC	CGAGGGAACGAAATCT GTGT	60.3	469	DRR	Ca_01166	Z	B3
Ca3_P3_38221107	Ca3	38221107	TCTCCTC/TCTC	CCACTGGTGGATTCAG AGGT	GAGAACTCATGTGCCG GAAT	60.0	566	CDS (large-effect mutations)	Ca_01166	Z	B3
Ca3_P3_38224413	Ca3	38224413	TAAACAAACAAACAAACAAACAAA/TAA ACAAACAAACAAACAAA	GCTGAGGAGAAATTTC GTGG	CGACTTTTCTTCTGCC ATCTG	59.8	154	INTRON	Ca_01166	Z	B3
Ca3_P3_38225211	Ca3	38225211	ATGTTGTTG/ATGTTG	TTAGAGAACGGAAACC TGGC	ACAGGTTGGTTTCTTG TCCG	59.3	363	INTERGENIC	--	--	--
Ca3_P3_38225828	Ca3	38225828	TAA/TA	CCACCATCCTTGTGCTT TTT	CCCCTCTCCCACGTTT AGTT	60.0	444	DRR	Ca_01167	R	--
Ca3_P3_38225921	Ca3	38225921	TA/T	CCACCATCCTTGTGCTT TTT	TTTGAGAGGTTGGATG AGCC	60.0	989	DRR	Ca_01167	R	--
Ca3_P3_38232333	Ca3	38232333	TC/T	GAAATCCAGCCATGAA GGAA	CCATCTGTTTTACGAT GAAAGTCA	60.0	335	DRR	Ca_01169	G	--

INDEL marker IDs	Chromosomes/ unanchored scaffolds	Physical positions (bp)	InDels (*Kabuli* reference genome-CDC Frontier/PII)	Forward primers (5'-3')	Reverse primers (5'-3')	Anneling temperature (0C)	Expected amplified product size (bp)	Structural annotation		Functional annotation	
								Sequence components of *kabuli* genome	*Kabuli* gene accession IDs	NCBI-KOG	TFs
Ca3_P3_38232966	Ca3	38232966	ATTT/ATT	TGTGTATGATGAAGAC GCATGT	ATTGGCAATCGCGAAA AA	59.1	251	DRR	Ca_01169	G	--
Ca3_P3_3825225	Ca3	3825225	TA/T	ATTCGACTAGTGGTGG TGCC	TACCGCCACAAAATCT CTCC	60.0	851	DRR	Ca_21335	--	--
Ca3_P3_38289059	Ca3	38289059	T/TA	CTTTCCTTCACAACCCT CCA	GAAAAATGAAGGAGGT GGCA	60.1	547	INTERGENIC	--	--	--
Ca3_P3_38333156	Ca3	38333156	TTATATATATATATATATATATAT/TTAT ATATATATATATATATAT	CCTGCCTTGCACCTAT CAGT	TAGGTGTGATTGCCCT TTTT	60.3	683	DRR	Ca_01182	R	--
Ca3_P3_3847338	Ca3	3847338	CATATATATATAT/CATATATATATATAT AT	AGGAAGAGTGGAGTTG CGAA	ATTTTGGTTCGGAGGC TTTT	60.0	496	INTERGENIC	--	--	--
Ca3_P3_38618211	Ca3	38618211	TAAAAAAAAA/TAAAAAAAAAA	ATGAGGAACGAAACCG ACAG	TTTTCAGCACATTCTTT TTGG	60.1	632	INTERGENIC	--	--	--
Ca3_P3_38624777	Ca3	38624777	TTTCTTCTTCTTCTTCTTCTTCTTC/TTT CTTCTTCTTCTTCTTCTTCTTCTTC TTC	GAAGCAATTCCCTTTCC CTC	CAAAGTAAACCCCACC GCTA	60.0	431	CDS (large-effect mutations)	Ca_01218	--	--
Ca3_P3_38640396	Ca3	38640396	T/TTTAG	GCTGCACAAAGACGAT GAAA	TTCAAGGGTTTCGGAT TGAA	60.0	519	INTERGENIC	--	--	--
Ca3_P3_38665334	Ca3	38665334	CTT/CTTCATTT	CCCCTTCATCAGTGGT GAGT	TCTTCTCCTCCCAAAA GCAA	60.0	257	INTERGENIC	--	--	--
Ca3_P3_38677144	Ca3	38677144	TAT/TATAAT	TGGAAATATGCTTGGC ATCA	TCTTTTGTACCCGCAA ATCC	60.0	741	INTRON	Ca_01223	S	--

INDEL marker IDs	Chromosomes/ unanchored scaffolds	Physical positions (bp)	InDels (*Kabuli* reference genome-CDC Frontier/PII)	Forward primers (5'-3')	Reverse primers (5'-3')	Anneling temperature (0C)	Expected amplified product size (bp)	Structural annotation		Functional annotation	
								Sequence components of *kabuli* genome	*Kabuli* gene accession IDs	NCBI-KOG	TFs
Ca3_P3_38690878	Ca3	38690878	GAAAAATAAAAATAAAAA/GAAAAATAAAAA	TTGGCATAATCCTCATTGACA	CCAAAACAAAGACCTCCGAA	59.0	469	DRR	Ca_01225	--	NAC
Ca3_P3_38729060	Ca3	38729060	GATATATATATATA/GATATATATATATATA	CAGATCATGTCGGAGCTTCA	CCTTTTCCTTCTTGCCCTTC	59.9	535	INTERGENIC	--	--	--
Ca3_P3_38735210	Ca3	38735210	GTGTTG/GTG	CACCTGCACAAACTTCCAAA	TGGTGTTACTCCCTTCCTTCA	59.7	220	INTERGENIC	--	--	--
Ca3_P3_3873650	Ca3	3873650	GAAAAAAAAAA/GAAAAAAAAAAA	GCTGTTGGTTAGTCGGGGTA	GCAGTAATGCATCCGTTGAA	60.0	631	INTERGENIC	--	--	--
Ca3_P3_3876327	Ca3	3876327	AAATAATAATAATAATAATAATAGTAATAATAATAATAATAATAAT/AAATAATAATAATAATAATAATAATAGTAATAATAATAATAATAATAAT	TCAAACCCGGGTGTAAGGT	CAAAATTTTAGGCCGCGATA	60.2	371	INTERGENIC	--	--	--
Ca3_P3_38900161	Ca3	38900161	GACAACA/GACA	GCCATTCCATTAAAGGGTCA	TCTGCCTAATCAAACATGCC	59.8	891	INTERGENIC	--	--	--
Ca3_P3_38901208	Ca3	38901208	TAA/TAAA	CTTGCACATGCAACCAGGTA	ATTGTTCATGTTGTGTGGCG	60.7	625	INTERGENIC	--	--	--
Ca3_P3_38975771	Ca3	38975771	GTATTATTATTATTATTATTATTATT/GTATTATTATTATTATTATTATTATTATT	ATGGCTAGACGAGTTCGACG	TAATAGGAGGTCGCTGGACG	60.4	494	INTERGENIC	--	--	--
Ca3_P3_3898691	Ca3	3898691	CAAAAAAAAAAAA/CAAAAAAAAAAAAA	TTGGTTACATTTCGAAAACGG	CCTGGAAAATCAAAAGCCAA	59.9	839	INTRON	Ca_21332	E	--
Ca3_P3_3900290	Ca3	3900290	TTTTATTTATTTATTTATTTATTT/TTTTATTTATTTATTTATTT	CTGCAAGTGGTGTGTTTGCT	AATTACTCACGGCCCAAAAA	59.9	621	INTRON	Ca_21332	E	--

**INDEL marker IDs**	**Chromosomes/ unanchored scaffolds**	**Physical positions (bp)**	**InDels (*Kabuli* reference genome-CDC Frontier/PII)**	**Forward primers (5'-3')**	**Reverse primers (5'-3')**	**Anneling temperature (0C)**	**Expected amplified product size (bp)**	**Structural annotation**		**Functional annotation**	
								**Sequence components of *kabuli* genome**	***Kabuli* gene accession IDs**	**NCBI-KOG**	**TFs**
Ca3_P3_39027474	Ca3	39027474	CGGGG/CGGGGG	CATATGCCACATACGCAAGG	CAAATGTTAATGGCGGTGTG	60.0	586	INTRON	Ca_01265	YU	--
Ca3_P3_39155160	Ca3	39155160	AATATATATATATATATATAT/AATATATATATATATATATAT	ATTAACGCATCACCAACCAA	TAAATTTTCGCAAGCTCGAC	58.9	369	INTERGENIC	--	--	--
Ca3_P3_3920259	Ca3	3920259	AG/A	TTTGTGATGGCGACTCTGAT	ATCAAGAAATGCGGGAGTTG	59.2	425	DRR	Ca_21330	O	WRKY
Ca3_P3_39298050	Ca3	39298050	AAGAGAGAGAGAGAGAGAG/AAGAGAGAGAGAGAGAG	CCGAAATCACATGACAGTGG	TTTTGAGAAGCCGAGGAAGA	60.0	181	INTERGENIC	--	--	--
Ca3_P3_39317786	Ca3	39317786	CATATATATATATATATATAT/CATATATATATATATATAT	AGGACCATTCCATCCTGTCA	TGCATCCAAACGAGCACTAA	60.3	664	INTERGENIC	--	--	--
Ca3_P3_39355383	Ca3	39355383	CATATATATATATATATATATATAT/CATATATATATATATATATATATAT	TCGTAGGACCTCAAGTTGTATGAG	TTGGCCAAATCTTCGATCTC	59.7	338	INTERGENIC	--	--	--
Ca3_P3_39413210	Ca3	39413210	CATATATATATATATATATATATATATATATAT/CATATATATATATATATATATATATAT	GGCATTCAATGGATATTGGG	ACGTTATCCGATCAAAACCG	60.0	357	INTERGENIC	--	--	--
Ca3_P3_39518552	Ca3	39518552	AAATAATAATAATAATAATAATAATAATAATAATA/AAATAATAATAATAATAATAATAATAATAATAATAATA	ATTCCATGCAAACCACCATT	ACCACTGTAGCATTTTGGGG	60.1	423	INTRON	Ca_01323	--	NAC
Ca3_P3_39571556	Ca3	39571556	GAAAAAAAAA/GAAAAAAAA	AATTTGTTTCGGCAATTCCA	GCAAAAGTGGCTGATGACAA	60.3	553	INTERGENIC	--	--	--
Ca3_P3_39573787	Ca3	39573787	GGTAATTGTAATTGTAATTGTAATTGTAA/GGTAATTGTAATTGTAATTGTAA	TGTGCAAAACTTGCAAAAGG	GTATGTTTGGCAATTTGGGG	59.9	934	DRR	Ca_01331	--	Dof

INDEL marker IDs	Chromosomes/ unanchored scaffolds	Physical positions (bp)	InDels (*Kabuli* reference genome-CDC Frontier/PII)	Forward primers (5'-3')	Reverse primers (5'-3')	Anneling temperature (0C)	Expected amplified product size (bp)	Structural annotation		Functional annotation	
								Sequence components of *kabuli* genome	*Kabuli* gene accession IDs	NCBI-KOG	TFs
Ca3_P3_39611324	Ca3	39611324	TA/T	TGTTTGCCTTTGTTGAAGCA	CCCAATTTCGAAGCTCTCTG	60.4	198	INTRON	Ca_01338	--	--
Ca3_P3_39617624	Ca3	39617624	CTTTTTTTTTTTTTT/CTTTTTTTTTTTTT	GCGTTCTCCCAAAACCATAA	TTACAAACATCAAAGATTTTGGC	59.9	793	INTERGENIC	--	--	--
Ca3_P3_39627249	Ca3	39627249	TTATATATATATATATATATATATATATATATATATATATATATATATAT/TTATATATATATATATATATATATATATATATATATATATAT	CCTTCTCAACAAGCACATCA	TGAAAGCTTAAAAAGTCATCTTACCA	57.4	590	INTERGENIC	--	--	--
Ca3_P3_39632172	Ca3	39632172	CTTTTTTTT/CTTTTTTTTT	CCATCATTCCGTCCTCACTT	TGATTATTCGGTTGGGCTTC	59.9	434	INTERGENIC	--	--	--
Ca3_P3_3963300	Ca3	3963300	CAAAAAAAAA/CAAAAAAAAAAAAA	TCCATTGTGTGCCACAGTCT	TCTAGTCCGCGACTCAACCT	60.2	505	INTERGENIC	--	--	--
Ca3_P3_39706199	Ca3	39706199	TTATATATATATATATATA/TTATATATATATATATA	TGCTCAAACCAAATATGGCA	TTGGGCAAAACTCCATCTTC	60.1	770	INTERGENIC	--	--	--
Ca3_P3_39713077	Ca3	39713077	GAAA/GAAAA	CACAAAGTTTCATCAATCCAAGA	TCGACTCCTGTTCAACCAAG	59.1	442	DRR	Ca_01353	J	--
Ca3_P3_39836659	Ca3	39836659	ATTTTTTTTTT/ATTTTTTTTTTT	GCATGTGATTATGGAATCGG	ACCCGCGTCACATTAGTTTC	58.8	554	INTERGENIC	--	--	--
Ca3_P3_39866735	Ca3	39866735	C/CT	ACAGTCTTTGGTTCGATGGG	CTGCCCCAAAAGACACAAAT	60.0	288	INTRON	Ca_01372	--	WRKY
Ca3_P3_39866810	Ca3	39866810	ATGG/ATGGTGG	ACAGTCTTTGGTTCGATGGG	CTGCCCCAAAAGACACAAAT	60.0	288	INTRON	Ca_01372	--	WRKY

INDEL marker IDs	Chromosomes/ unanchored scaffolds	Physical positions (bp)	InDels (*Kabuli* reference genome-CDC Frontier/PII)	Forward primers (5'-3')	Reverse primers (5'-3')	Anneling temperature (0C)	Expected amplified product size (bp)	Structural annotation		Functional annotation	
								Sequence components of *kabuli* genome	*Kabuli* gene accession IDs	NCBI-KOG	TFs
Ca3_P3_39926798	Ca3	39926798	CT/CTGTAT	CATCTGCAGCCAAAAACTCA	GTGTGGGCTTTTCCTTTGAA	60.0	418	INTERGENIC	--	--	--
Ca3_P3_39963962	Ca3	39963962	AGATCAGGATC/AGATC	TTGCAGCCAACACTGAACTC	CGTTCTTCGCCTTTCTCAAC	60.0	578	CDS (large-effect mutations)	Ca_01380	--	--
Ca3_P3_4006367	Ca3	4006367	A/AATGGTGTACT	TGTTCCAAGAGGGCATTACA	GACGTGATGCTCACTCCAGA	59.1	590	INTERGENIC	--	--	--
Ca3_P3_4017573	Ca3	4017573	TAA/TAAA	ACCACAGATCACCAGTCGCT	ACGTCCAAGGTAAGGGCTTT	60.7	683	INTERGENIC	--	--	--
Ca3_P3_4042829	Ca3	4042829	GATATATATATATATATATATATAT/GATATATATATATATATATATATAT	ACACGAAGAGCAGGATCGTT	AAACAAAAATAAAAACTCGGAGACA	59.9	743	DRR	Ca_21325	QR	--
Ca3_P3_4073796	Ca3	4073796	CT/CTT	CTCAACATCCACCATGCAAT	TGCAAATTTCATCAAATGCATAA	59.4	746	INTERGENIC	--	--	--
Ca3_P3_4132295	Ca3	4132295	TAAAAAAAAAAAAAA/TAAAAAAAAAAAAAAAAAA	CGTGTGAGAAAACCTCGACA	CAAACCCAAGTTCGAAATGC	59.9	261	INTERGENIC	--	--	--
Ca3_P3_4132392	Ca3	4132392	TTATATATATATATATATAT/TTATATATATATATATATATATATAT	CGTGTGAGAAAACCTCGACA	GAAGGCCAACTTCCAAATGA	59.9	738	INTERGENIC	--	--	--
Ca3_P3_4137886	Ca3	4137886	TTATATATATATATATATATATATATATATATATA/TTATATATATATATATATATATATATATATATATATATATATATATA	ATGTTCTCCTCCCCTTTTCA	GTTCCGACGAAAACGACATT	58.6	783	INTERGENIC	--	--	--
Ca3_P3_414861	Ca3	414861	T/TG	CTATGCAACCACAGCTTCCA	GCAGATTCCGGTTCAAAAGA	59.9	438	INTRON	Ca_22928	O	--

INDEL marker IDs	Chromosomes/ unanchored scaffolds	Physical positions (bp)	InDels (*Kabuli* reference genome-CDC Frontier/PII)	Forward primers (5'-3')	Reverse primers (5'-3')	Anneling temperature (0C)	Expected amplified product size (bp)	Structural annotation		Functional annotation	
								Sequence components of *kabuli* genome	*Kabuli* gene accession IDs	NCBI-KOG	TFs
Ca3_P3_4152638	Ca3	4152638	CTTTTTTTTTT/CTTTTTTTTT	TGATGCAAAGTGTGCTGTGA	TTTTTGGTTGGGTGGGTAAA	60.0	675	INTERGENIC	--	--	--
Ca3_P3_4378055	Ca3	4378055	AATATATATATATATATATATATATA/AATATATATATATATATATATA	TATTTTGGATTTTGGCTCGG	GTTTTCGATTCAACGAGGGA	59.9	502	INTERGENIC	--	--	--
Ca3_P3_4527615	Ca3	4527615	AATATATATATATATATATA/AATATATATATATATATA	TTTGGTATTGACTCAGGGGC	TTGGAAGGAGCCCTTACCTT	59.9	711	INTERGENIC	--	--	--
Ca3_P3_454494	Ca3	454494	AGGG/AGG	GGATTGGAGTGTTCAGCCAT	TTGTCATTAGTTTCAATCTCACCAA	59.9	460	INTERGENIC	--	--	--
Ca3_P3_454633	Ca3	454633	GATATATATATATAT/GATATATATATAT	GGATTGGAGTGTTCAGCCAT	TTGTCATTAGTTTCAATCTCACCAA	59.9	460	INTERGENIC	--	--	--
Ca3_P3_4596733	Ca3	4596733	A/AAGT	GTGCACACGGGTTACACAGT	GTGCGGTGGTTGAAGGTAGT	59.5	159	INTERGENIC	--	--	--
Ca3_P3_4689138	Ca3	4689138	TAAAAAA/TAAAAAAA	CAGCTTGCATTTGCTTTTGA	TTGCTCCTACGTATCTCCCG	60.1	888	INTERGENIC	--	--	--
Ca3_P3_4761643	Ca3	4761643	TAAAAAAAAAAAAAAAAAAAAA/TAAAAAAAAAAAAAAAAAAAAAA	TGCGCAAGAGATGATTGAGT	CCGTTGATTTGATGTGTTCG	59.6	556	INTERGENIC	--	--	--
Ca3_P3_4787200	Ca3	4787200	CA/CATA	GTGTCGGATGTCTGACGCTA	GACGACTTGTCACTGGACGA	59.9	450	INTERGENIC	--	--	--
Ca3_P3_4842537	Ca3	4842537	TAAAAAAAAAAA/TAAAAAAAAAA	ACAAAGCTGGTCGAAGAGGA	TCTTGGACTCGCTAAGCCAT	60.0	453	INTERGENIC	--	--	--

INDEL marker IDs	Chromosomes/ unanchored scaffolds	Physical positions (bp)	InDels (*Kabuli* reference genome-CDC Frontier/PII)	Forward primers (5'-3')	Reverse primers (5'-3')	Anneling temperature (0C)	Expected amplified product size (bp)	Structural annotation		Functional annotation	
								Sequence components of *kabuli* genome	*Kabuli* gene accession IDs	NCBI-KOG	TFs
Ca3_P3_4938478	Ca3	4938478	ATTT/ATTTT	TCAACTCAACCCGAAACACA	TGTTTGAATGTGTGGCATTTG	60.1	813	INTERGENIC	--	--	--
Ca3_P3_4943052	Ca3	4943052	GTTTTTTTTTTT/GTTTTTTTTTT	GCCACAAAATTGGTCCAAAA	TATGTTGGTTTAGGGGGCTG	60.7	582	INTERGENIC	--	--	--
Ca3_P3_4965548	Ca3	4965548	ATTTT/ATTTTT	CGGGGAATGTGATTGATAGG	GTTGGAGAGATCGGTGGAAA	60.1	948	INTERGENIC	--	--	--
Ca3_P3_4965627	Ca3	4965627	T/TGAG	TGCAAATCTGGAGCAGAGAA	GTTGGAGAGATCGGTGGAAA	59.7	510	INTERGENIC	--	--	--
Ca3_P3_4966098	Ca3	4966098	CTTTTTTTT/CTTTTTTT	TTTCCACCGATCTCTCCAAC	AAACCCGCTCCTTTCAAAAT	60.1	514	INTERGENIC	--	--	--
Ca3_P3_4975195	Ca3	4975195	AATATATATATATATA/AACATATATATATATATATA	CAAAGTTCAATACGCCAGCA	AGGTTTTGGCGTAACTCCCT	59.9	455	INTERGENIC	--	--	--
Ca3_P3_5089285	Ca3	5089285	CAAAAAAAAAA/CAAAAAAAAAAA	TGCCAAGAAAACACCATGAA	GCCAACCAACATTGTGTCAG	60.1	700	INTERGENIC	--	--	--
Ca3_P3_5175484	Ca3	5175484	TGAGAGAGAGAGAGAGAGAGAGAAAGAGAGAGAGAGAGAGA/TGAGAGAGAGAGAGAGAGAGAGAGAAAGAGAGAGAGAGAGAGA	CGCTGAATCACCACATCATC	ACCGATAACATTTTGGCTGC	60.1	664	INTRON	Ca_23872	--	--
Ca3_P3_5269970	Ca3	5269970	CAAACAATAA/CAA	GATTGGCACGTCGATTAACA	TTCTCGCTTTCGTTTCGTTT	59.5	563	INTERGENIC	--	--	--
Ca3_P3_5270123	Ca3	5270123	GAA/GA	ACGAAACGAAAGCGAGAAAA	CGACCTCGAAATAATCGGAA	60.0	521	INTERGENIC	--	--	--

**INDEL marker IDs**	**Chromosomes/ unanchored scaffolds**	**Physical positions (bp)**	**InDels (*Kabuli* reference genome-CDC Frontier/PII)**	**Forward primers (5'-3')**	**Reverse primers (5'-3')**	**Anneling temperature (0C)**	**Expected amplified product size (bp)**	**Structural annotation**		**Functional annotation**	
								**Sequence components of *kabuli* genome**	***Kabuli* gene accession IDs**	**NCBI-KOG**	**TFs**
Ca3_P3_5270478	Ca3	5270478	TATCAATC/TATC	ACGAAACGAAAGCGAGAAAA	CGACCTCGAAATAATCGGAA	60.0	521	INTERGENIC	--	--	--
Ca3_P3_5278342	Ca3	5278342	ATT/AT	CACCCCAGTTTTGAGGGTAA	TTTGCCAATCGATTATCGTG	59.8	549	DRR	Ca_20669	--	--
Ca3_P3_5286221	Ca3	5286221	ATTT/ATT	CACCCCTCCCAAAACATCT	TTGTGAGTGCATTTGATTTTGTT	59.8	876	INTERGENIC	--	--	--
Ca3_P3_5288032	Ca3	5288032	AATATATATATATATATATATATAT/AATATATATATATATATATAT	AGAAGGTCGCATCTCCAAGA	TGGCACTTGTATTCACTCGC	60.0	456	INTERGENIC	--	--	--
Ca3_P3_5347423	Ca3	5347423	CAATAATAATAATAATAATAATAATAATAATAATAA/CAATAATAATAATAATAATAATAATAATAA	TTGCTCCTACGTATCTCCCG	TTGTGACAATTTGATGGTTCTAAA	60.2	563	INTERGENIC	--	--	--
Ca3_P3_5403691	Ca3	5403691	ACTCTCTCTCTCTCTCTCT/ACTCTCTCTCTCTCTCT	GAGAGGAGCCAACTGGAACA	TGAACCTGTTGAAGTGAAGCA	60.4	326	INTERGENIC	--	--	--
Ca3_P3_5447948	Ca3	5447948	TAAAAAAAAAAA/TAAAAAAAAAA	GCATGAAGACGAATGAGCAA	CATCCCTCTAACCCCACAAA	60.0	667	DRR	Ca_20658	--	--
Ca3_P3_5486984	Ca3	5486984	ATTTTTTTTTTT/ATTTTTTTTTT	AGGCCTGATCTACGGCCTAT	CAACAGGGGCCTATGGTATG	60.1	637	INTERGENIC	--	--	--
Ca3_P3_5502243	Ca3	5502243	TG/T	TGCGTTTTTCTGCATTTCTG	ATTCTGCGCGACTATCAACC	60.0	661	INTERGENIC	--	--	--
Ca3_P3_5502450	Ca3	5502450	GTATATATATATATATA/GTATATATATATA	TGCGTTTTTCTGCATTTCTG	ATTCTGCGCGACTATCAACC	60.0	661	INTERGENIC	--	--	--

INDEL marker IDs	Chromosomes/ unanchored scaffolds	Physical positions (bp)	InDels (*Kabuli* reference genome-CDC Frontier/PII)	Forward primers (5'-3')	Reverse primers (5'-3')	Anneling temperature (0C)	Expected amplified product size (bp)	Structural annotation		Functional annotation	
								Sequence components of *kabuli* genome	*Kabuli* gene accession IDs	NCBI-KOG	TFs
Ca3_P3_5507601	Ca3	5507601	ATTT/ATTTT	TATTTTGGATTTTGGGCTCG	GGAGGGATAAGGTTTCTCGC	59.9	427	INTERGENIC	--	--	--
Ca3_P3_5507746	Ca3	5507746	AATATATATATATATATATATATATATATATATAT/AATATATATATATATATATATATATATATATATATATATATATATATATAT	TATTTTGGATTTTGGGCTCG	GGAGGGATAAGGTTTCTCGC	59.9	427	INTERGENIC	--	--	--
Ca3_P3_5520403	Ca3	5520403	GTATATATATATATATATATATATATATA/GTATATATATATATATATATATATA	CAAATGACGACGACGATGAC	GAATGATGACAATGCGGTTG	60.1	503	INTERGENIC	--	--	--
Ca3_P3_5587239	Ca3	5587239	CTTTTTTTTTTT/CTTTTTTTTTTTT	GTGATAAAGGCGTCAACGGT	TCTGATGCAGTGTTTGGCTC	60.0	691	INTERGENIC	--	--	--
Ca3_P3_5659123	Ca3	5659123	C/CTCGACG	TGTTGATGTAATACTCTTCAATGATGT	AGAGCTAGCTGGTTCTCCCC	58.2	593	INTERGENIC	--	--	--
Ca3_P3_5659736	Ca3	5659736	T/TCGG	CTAGCTCTGGGTTCGAGTGG	GGAGGGATTACGGGAGAGAG	60.0	742	INTERGENIC	--	--	--
Ca3_P3_5689325	Ca3	5689325	ATTT/ATT	TTGGTCAATGTGGCTTCTCA	TGAACTCTGAGCGTGTGACC	60.2	439	INTERGENIC	--	--	--
Ca3_P3_5698499	Ca3	5698499	TTATAT/TTATATAT	GAGGATTTGGGGGAGAAGAG	AGAAGGGAGGAGATGGAAGC	60.0	397	INTERGENIC	--	--	--
Ca3_P3_5723134	Ca3	5723134	TTATATATATATATATATATATATATATATATATATAT/TTATATATATATATATATATATATATATATATATATATATATATATATAT	GGATGGAGTCTTGGGTGAAA	TGATGTTTGTATTGGGGAAAAA	59.9	424	INTERGENIC	--	--	--
Ca3_P3_5805263	Ca3	5805263	GT/GTT	CAATTGGTCGAAAGACGGAT	TTCGAATGAACATAATTGAACTTT	59.9	164	INTERGENIC	--	--	--

INDEL marker IDs	Chromosomes/ unanchored scaffolds	Physical positions (bp)	InDels (*Kabuli* reference genome-CDC Frontier/PII)	Forward primers (5'-3')	Reverse primers (5'-3')	Anneling temperature (0C)	Expected amplified product size (bp)	Structural annotation		Functional annotation	
								Sequence components of *kabuli* genome	*Kabuli* gene accession IDs	NCBI-KOG	TFs
Ca3_P3_5822155	Ca3	5822155	TATA/TATACTTAAACTAATAATA	TGCCTGTTTCCTTGCTTTCT	TTGAAACCCAAGAGGGTACG	60.0	378	INTERGENIC	--	--	--
Ca3_P3_5994220	Ca3	5994220	TGAGTAAAG/TGAGTAAAGAGTAAAG	AAAGCAGTCTGCAAGGGAAA	GTGCAACCATCATGCAAAAG	60.0	382	INTRON	Ca_27241	--	--
Ca3_P3_6072190	Ca3	6072190	ATTTTTTTTTT/ATTTTTTTTTTT	GTGTGACCAGCTCGTTTGTG	CCAACGAGGACCCAATTCTA	60.4	498	INTERGENIC	--	--	--
Ca3_P3_6088066	Ca3	6088066	CATC/CATCGTCAGAATC	ATGAGGCGCATTCAATTTTC	TTGCTCTTTGGTAGGTCGCT	60.0	812	INTERGENIC	--	--	--
Ca3_P3_613347	Ca3	613347	ATTTTTTTTTTT/ATTTTTTTTTT	TGGAGCTTTGTTTTGGACCT	AGTAAGTGTCGTCGTTGGGC	59.7	409	INTERGENIC	--	--	--
Ca3_P3_6243942	Ca3	6243942	CTTATT/CTT	TGATGGACTTCGTGCTCTTG	CCTCAGGATAAAACAGCCCA	60.0	298	INTERGENIC	--	--	--
Ca3_P3_6301895	Ca3	6301895	TCCCCC/TCCCC	CATTGGAAGCTTTAGGCTCG	GGGTTTTGAGGAGGTTGTCA	60.0	372	INTERGENIC	--	--	--
Ca3_P3_6335739	Ca3	6335739	AATATATATATATATATATATATATATATAT/AATATATATATATATATATATATATATATATATATATAT	CTTGGGAACTGGAGAGCAAG	GTTGGTTCCCTAGCACCGTA	60.0	762	INTERGENIC	--	--	--
Ca3_P3_6345015	Ca3	6345015	CAAAAAAAAAAA/CAAAAAAAAAA	CAGCTGTTCCGATGTCTTCA	GCTCATGGGATGTCAACCTT	60.0	979	DRR	Ca_24832	--	B3
Ca3_P3_6352791	Ca3	6352791	AATATATATATATATATATAT/AATATATATATATATATATAT	TTTTTATGTTGGGCCCATGT	ATGCGCTTGACAAGAATGTG	60.1	655	INTERGENIC	--	--	--

INDEL marker IDs	Chromosomes/ unanchored scaffolds	Physical positions (bp)	InDels (*Kabuli* reference genome-CDC Frontier/PII)	Forward primers (5'-3')	Reverse primers (5'-3')	Anneling temperature (0C)	Expected amplified product size (bp)	Structural annotation		Functional annotation	
								Sequence components of *kabuli* genome	*Kabuli* gene accession IDs	NCBI-KOG	TFs
Ca3_P3_6353414	Ca3	6353414	TTTTTTTATTTT/TTTTTTTATTTTTTATTTT	ATTTTAGGCCGCGACAACTA	TGGACATCCAGAAGGCTACC	59.7	427	INTERGENIC	--	--	--
Ca3_P3_6386934	Ca3	6386934	TGG/TGGG	TGTACGAGAAGGTCGTGTGC	GCCCAACAGCAAGCTATTTC	59.9	574	INTRON	Ca_24834	--	Trihelix
Ca3_P3_6548012	Ca3	6548012	CT/CTT	TTATGCCGTTTGGAGTTTCC	TTCAATTGCCTTGTGGCATA	59.9	615	INTERGENIC	--	--	--
Ca3_P3_656777	Ca3	656777	ATTCAATATTTCAA/ATTCAATATTTCAATATTTCAA	CAATGAACAATGCAACCCAG	GAGGGAAATGGATGGTTTGA	60.0	384	INTERGENIC	--	--	--
Ca3_P3_6587330	Ca3	6587330	T/TA	TTGAAGGGTTGCATGAATGA	GGAGGTCGAAAATGACCAAA	60.0	741	INTERGENIC	--	--	--
Ca3_P3_6621889	Ca3	6621889	AG/AGG	ATGATGGCCCCCTAAAATTC	AATCCAATGACACAAACGCA	60.0	573	INTERGENIC	--	--	--
Ca3_P3_6636800	Ca3	6636800	TG/T	ATTCCAAATGAGTCATCGGC	GTTGATCGGGCCACAATATC	59.9	632	INTRON	Ca_25775	G	MYB_related
Ca3_P3_6663794	Ca3	6663794	TAAAAAAAAAAAA/TAAAAAAAAAA	AAGGCTCAACATGCAAAACC	AATGCCCACCCATAATTTCA	60.1	324	INTERGENIC	--	--	--
Ca3_P3_6687388	Ca3	6687388	ATTTTTTTT/ATTTTTTT	GAACAATGTGGGCAGGAAGT	GTGGTTGCCAGGCTTCTTTA	60.0	563	DRR	Ca_25772	A	--
Ca3_P3_6694741	Ca3	6694741	TTCTCTCTCTCTCTCTC/TTCTCTCTCTCTCTCTCTC	TTTGTCACGTCAGACAAGGC	AATCGGTGTAGAGGCAATGG	59.9	660	INTERGENIC	--	--	--

INDEL marker IDs	Chromosomes/ unanchored scaffolds	Physical positions (bp)	InDels (*Kabuli* reference genome-CDC Frontier/PII)	Forward primers (5'-3')	Reverse primers (5'-3')	Anneling temperature (0C)	Expected amplified product size (bp)	Structural annotation		Functional annotation	
								Sequence components of *kabuli* genome	*Kabuli* gene accession IDs	NCBI-KOG	TFs
Ca3_P3_6721949	Ca3	6721949	CTTTTTTTTTTTT/CTTTTTTTTTT	TGGTGAGGAAGGGACAACTC	TAAAGGACCAAGTCGGGAAA	60.1	637	INTERGENIC	--	--	--
Ca3_P3_6772905	Ca3	6772905	GTTT/GTT	TCCCTTTGTCTTTTCGCACT	CACTGCCGTTCTTCACTTCA	59.9	383	DRR	Ca_16559	R	NAC
Ca3_P3_6774064	Ca3	6774064	T/TTAA	TTTGCTGGACTTTGAAGCCT	CCCGGTCCTAGAGAGAAAGA	60.0	640	DRR	Ca_16559	R	NAC
Ca3_P3_6780457	Ca3	6780457	A/ACAAT	GGATGCTTCGTTTGGTGTTT	TTTGAACCAAGATGCAGCAG	60.0	832	INTRON	Ca_16557	R	NAC
Ca3_P3_6790470	Ca3	6790470	T/TA	GGCTGGCTACTTAGGGATCA	GACCTCGTCCAGCTTTTCAG	59.3	582	INTERGENIC	--	--	--
Ca3_P3_6820763	Ca3	6820763	CTTTTTTTT/CTTTTTTTTT	GTGCAGTGCAATCCGATTTA	TTACGATGGTTGTGGTGAGG	59.7	415	INTERGENIC	--	--	--
Ca3_P3_6821985	Ca3	6821985	TTATATATATATATATATATATATATATA/TTATATATATATATATATATATATATATATATATATATATA	GCGTGATCCCAAGCTACAAT	CATCTTTTAAGGTGCGGGTG	60.1	139	INTERGENIC	--	--	--
Ca3_P3_6838667	Ca3	6838667	AGCA/AGCAGGATCCAATGCAATGGCA	GGAACCCCACAATCATCATC	GCAGCCAATTTTGTTGCATA	60.0	547	CDS (large-effect mutations)	Ca_16550	R	bHLH
Ca3_P3_6959714	Ca3	6959714	TCCCCC/TCCCC	GCACTTTTGGTCCCCCTATT	GATTCTAGTCTCTTGCGCCG	60.2	420	DRR	Ca_16543	--	G2-like
Ca3_P3_7076805	Ca3	7076805	C/CCCGAATGT	TCTTCGATTCCATTCCAACC	TTGGGGGAGACGATATTCTG	59.9	701	INTERGENIC	--	--	--

INDEL marker IDs	Chromosomes/ unanchored scaffolds	Physical positions (bp)	InDels (*Kabuli* reference genome-CDC Frontier/PII)	Forward primers (5'-3')	Reverse primers (5'-3')	Anneling temperature (0C)	Expected amplified product size (bp)	Structural annotation		Functional annotation	
								Sequence components of *kabuli* genome	*Kabuli* gene accession IDs	NCBI-KOG	TFs
Ca3_P3_7106684	Ca3	7106684	ANNNNNNNNNN/ANNN	CATTTGTGGGATTTGGCTCT	CTATAGCGGCGGTATGGTGT	59.9	379	INTRON	Ca_16539	--	bHLH
Ca3_P3_7141941	Ca3	7141941	GATATATATATATATATATA/GATATATATATATATATATA	ATGTGAATCCCACCAGGAAA	GCGCTAGAGCGCTGTAAAAG	60.2	474	INTERGENIC	--	--	--
Ca3_P3_7223058	Ca3	7223058	TA/TATACA	AGGTTATGTCGTCCGTCAGG	GCGTAGTGGGAGCTTGTAGG	60.0	767	INTERGENIC	--	--	--
Ca3_P3_7312652	Ca3	7312652	TAAAAAAAAA/TAAAAAAAAAA	CGCAAGATTTTACCTACCTGTG	TGATCAAGCTTCCAGTCCAA	58.8	720	DRR	Ca_16529	--	LFY
Ca3_P3_7377309	Ca3	7377309	TAAAAAA/TAAAAAAA	CATATTCGCATTCCCAAACC	ACCTGCAAACCCAACTCAAC	60.2	306	INTERGENIC	--	--	--
Ca3_P3_7474683	Ca3	7474683	CATAGAAAAATGTATAGAAAAATGTAT/CATAGAAAAATGTAT	TACCAACAAAGCTCCCAACC	GCAAGGTCCACCACTGAAAT	60.0	191	INTERGENIC	--	--	--
Ca3_P3_7580880	Ca3	7580880	TAAAAAAAAA/TAAAAAAAAAA	GTGGTTCCGGAAAAGATGAA	CCCCATCCATTTCCCTATCT	59.9	373	INTERGENIC	--	--	--
Ca3_P3_7773838	Ca3	7773838	AATATATATATATATAT/AATATATATATATAT	CATGCGAGTTGTAACACCCT	CGTGCATACTTAATATCAGTCAAGTG	58.7	365	INTERGENIC	--	--	--
Ca3_P3_7922530	Ca3	7922530	TTATATATATATATATATAT/TTATATATATATATAT	AGGAAGAGTGGAGTTGCGAA	TATTTTGGATTTTGGCTCGG	60.0	504	INTERGENIC	--	--	--
Ca3_P3_7959750	Ca3	7959750	TTAATTTGATTATGTTAAGTAATTTGATTATGTTAA/TTAATTTGATTATGTTAAGTAATTTGATTATGTTAAGTAATTTGATTATGTTAA	TTGGCATAGGTCATGGAAGTT	TTTGCCAATGTTGCTAGCTG	59.4	848	INTERGENIC	--	--	--

INDEL marker IDs	Chromosomes/ unanchored scaffolds	Physical positions (bp)	InDels (*Kabuli* reference genome-CDC Frontier/PII)	Forward primers (5'-3')	Reverse primers (5'-3')	Anneling temperature (0C)	Expected amplified product size (bp)	Structural annotation		Functional annotation	
								Sequence components of *kabuli* genome	*Kabuli* gene accession IDs	NCBI-KOG	TFs
Ca3_P3_799026	Ca3	799026	AACACACA/AACACA	TTGCAGCCACATTTGTTTGT	TCCTTTGTGCTACCATTCCA	60.2	592	INTERGENIC	--	--	--
Ca3_P3_8245494	Ca3	8245494	TCT/TCTTCTGCT	TGCGCCTCTTCTAAAATCGT	TATCGCAATCAGAGGAACCC	60.0	670	INTERGENIC	--	--	--
Ca3_P3_8393314	Ca3	8393314	GTATATATATATATATATAT/GTATATATATATATATATATATAT	GTTGGTTCCCTAGCACCGTA	TATTTTGGATTTTGGCTCGG	60.0	602	INTERGENIC	--	--	--
Ca3_P3_8443852	Ca3	8443852	AATATATATATATATATATATATATAT/AATATATATATATATATATATATATATATAT	ACCGTAGCATGGAAAGTTGG	ATGCGACTTGGGAATTTGAG	60.0	699	INTERGENIC	--	--	--
Ca3_P3_8523296	Ca3	8523296	ATTTTTTTTTT/ATTTTTTTT	CACATGACAAAATTGGTGGAA	CGTTAAGGCACATCTCACTCC	59.3	508	INTERGENIC	--	--	--
Ca3_P3_8535141	Ca3	8535141	ATTTTTTTTTTT/ATTTTTTTTTT	GGACAAAGCCTAATGGTGGA	AATGGATTTGGCACAAAAGG	59.9	589	INTERGENIC	--	--	--
Ca3_P3_8535668	Ca3	8535668	TA/TAAAA	AACCTGCACTTCCTTTGACG	TTGGTCTTCTGTTCTTGGCA	60.3	586	INTERGENIC	--	--	--
Ca3_P3_8563440	Ca3	8563440	CATATATATATATATATATATATATA/CATATATATATATATATATATA	ATCTTGGCAACGGCTAAAAA	AGAGCAAAATGGAGGCAAGA	59.7	527	INTERGENIC	--	--	--
Ca3_P3_8602266	Ca3	8602266	AATATATATATATA/AATATATATATATATA	GCTCAACAGAGACCACCACA	CGTATTCGTGTGTGAAAGCG	59.9	835	INTERGENIC	--	--	--
Ca3_P3_8716119	Ca3	8716119	AATATATATATATATATATATATATATATATATATATATATATATAT/AATATATATATATATATATATATATATATATATATATATATATATATATATATATAT	ACGGCAATCATTGAAGTTGA	AAAAACCACAAACAACGAAAAA	59.1	316	INTERGENIC	--	--	--

INDEL marker IDs	Chromosomes/ unanchored scaffolds	Physical positions (bp)	InDels (*Kabuli* reference genome-CDC Frontier/PII)	Forward primers (5'-3')	Reverse primers (5'-3')	Anneling temperature (0C)	Expected amplified product size (bp)	Structural annotation		Functional annotation	
								Sequence components of *kabuli* genome	*Kabuli* gene accession IDs	NCBI-KOG	TFs
Ca3_P3_8756604	Ca3	8756604	CT/CTGTTTTAAAAT	TGGGTTGGAACACTTCATCA	ATTCAAGCATTCGATGGAGG	59.9	249	INTERGENIC	--	--	--
Ca3_P3_8775439	Ca3	8775439	GTATATATATATATATATATATATATATATATATAT/GTATATATATATATATATATATATATATATATATATATATAT	AGTCGCATCTTCGTCGTTTC	GGCATCGGGAGTCTGTTTTA	60.4	537	INTERGENIC	--	--	--
Ca3_P3_8826227	Ca3	8826227	CAAAAAAAAAAA/CAAAAAAAAAA	CTTGCGGTTGGAATGAAGTT	TATACACGGAACGAAAGCCC	60.1	589	INTERGENIC	--	--	--
Ca3_P3_8942193	Ca3	8942193	AT/ATT	TGGGGAAAAACCCTTACCTT	CACATGATTTCAATTGGCTTTG	59.7	326	INTERGENIC	--	--	--
Ca3_P3_9092559	Ca3	9092559	AA/AACA	CATGTCCCAAAAATATGCCC	TCTTTCAAGTGGGTTGGGTC	60.0	304	INTERGENIC	--	--	--
Ca3_P3_9186815	Ca3	9186815	CTATATATATATATATATATATATA/CTATATATATATATATATATATATATATA	TCCACAAACACGATAACATTGA	TGTAACAAGCATTTGTCGGG	58.9	693	INTERGENIC	--	--	--
Ca3_P3_9289555	Ca3	9289555	TTATATATATATATATATATATAT/TTATATATATATATATATATATATATATATAT	GAACCAAGTTGCATGGGAAT	CCTAGGGACTTCGTGGTTGA	59.8	651	INTERGENIC	--	--	--
Ca3_P3_9291365	Ca3	9291365	CATATATATATATATATATAT/CATATATATATATATATATATAT	TGTGTGGTGGTCAATTTGCT	CCCGATCTCAACTTGTGGTT	60.0	654	INTERGENIC	--	--	--
Ca3_P3_939073	Ca3	939073	GTT/GTTTT	ATTGGTTCTGCTCTGGGTTG	GGGACCACAGCACCTATGTT	60.1	296	INTERGENIC	--	--	--
Ca3_P3_9396146	Ca3	9396146	CATATATATATAT/CATATATATATATATAT	TCAAAGCAAATCATGTCCCA	TTGTAGGGGGAGAGATCGTG	60.0	757	DRR	Ca_20968	--	ERF

INDEL marker IDs	Chromosomes/ unanchored scaffolds	Physical positions (bp)	InDels (*Kabuli* reference genome-CDC Frontier/PII)	Forward primers (5'-3')	Reverse primers (5'-3')	Anneling temperature (0C)	Expected amplified product size (bp)	Structural annotation		Functional annotation	
								Sequence components of *kabuli* genome	*Kabuli* gene accession IDs	NCBI-KOG	TFs
Ca3_P3_9399660	Ca3	9399660	ATTTT/ATTTTT	CAACGGATAGAAACGAAAAGAAA	CGTGAAATGTGACCATAAAAACA	59.7	683	INTERGENIC	--	--	--
Ca3_P3_9414969	Ca3	9414969	CATAT/CAT	TGTCTTGAGCATGCCTTGTC	CGTTCTCCATGCTGTTCTCA	60.0	372	INTERGENIC	--	--	--
Ca3_P3_9427257	Ca3	9427257	TTTA/T	CATGCGTTCCTTGTGGTAGA	GGCCTAAGATCATAGCAATTCAA	59.7	165	INTERGENIC	--	--	--
Ca3_P3_9500843	Ca3	9500843	CAATCGATTTCC/CAATCGATTTCCAAATCGATTTCC	CCCGTGAGATCCTCCTAACA	CATGCAGGAATTCAGCAAAA	60.1	498	INTERGENIC	--	--	--
Ca3_P3_9551898	Ca3	9551898	CAAAAAAAA/CAAAAAAA	AGACTTGGCTGATGCGTTTT	GGTTGTGACTTCGACGGAAT	59.9	696	INTERGENIC	--	--	--
Ca3_P3_9577851	Ca3	9577851	TCCC/TAGGGAACCC	TTGCGTTCAAAGACTCGATG	TGAATTGCAGAATCCCATGA	60.0	575	INTRON	Ca_20977	--	--
Ca3_P3_9651257	Ca3	9651257	CTTTTTTT/CTTTTTT	CTCCAAACTGGGGTGTCACT	CGGTCTCAACCTCTCCCATA	60.0	882	DRR	Ca_20979	--	--
Ca3_P3_9812322	Ca3	9812322	CT/C	CTCGTGTCTAGGGATGGGAA	CGAAAAGCGACAAAGAAAGG	60.1	528	INTERGENIC	--	--	--
Ca3_P3_9940820	Ca3	9940820	TCAATTGCAATT/TCAATT	TCAGGGATTACCGCATAAGG	CATTCTTCCACTAGGCACGC	59.9	293	INTERGENIC	--	--	--
Ca3_P3_9953160	Ca3	9953160	GAAAAAA/GAAAAAAA	TGCCACGTTTCTATTTCCCT	ATCACGCCATAAGCACCTTC	59.6	511	INTERGENIC	--	--	--

INDEL marker IDs	Chromosomes/ unanchored scaffolds	Physical positions (bp)	InDels (*Kabuli* reference genome-CDC Frontier/PII)	Forward primers (5'-3')	Reverse primers (5'-3')	Anneling temperature (0C)	Expected amplified product size (bp)	Structural annotation		Functional annotation	
								Sequence components of *kabuli* genome	*Kabuli* gene accession IDs	NCBI-KOG	TFs
Ca4_P3_10033685	Ca4	10033685	TTATATATATATATATATAT/TTATATATATATATATATATATAT	CTAGTTCGTAACCCGTGCGT	AGGCATGGTGTTGGAGTCAT	60.2	248	INTERGENIC	--	--	--
Ca4_P3_10108026	Ca4	10108026	GTAATAATAATAATAATAATAATAATAAT/GTAATAATAATAATAATAATAATAATAATAAT	AACCGAATGAAATGAAACGG	AAGCGCACAGGAAAACCTTA	59.8	551	INTERGENIC	--	--	--
Ca4_P3_10173137	Ca4	10173137	ATTTTTTTT/ATTTTTTTTT	CCCGTTATTTTTGACGAAACA	TGTGTTGAGTGGTGGCATTT	59.9	791	INTERGENIC	--	--	--
Ca4_P3_10215060	Ca4	10215060	AATATATATATATATATATATATATAT/AATATATATATATATATATAT	CTTGCACACAATTGCAAAGTC	CAAGGCCAAAGAGGTCAGAG	59.4	321	INTERGENIC	--	--	--
Ca4_P3_10233239	Ca4	10233239	GACAATTATTTGGTACAATTAT/GACAATTATTTGGTACAATTATTTGGTACAATTAT	TGTCGGTTGTTGCTTGAAGA	ATACAATCATTTTCCCGCCA	60.4	403	INTERGENIC	--	--	--
Ca4_P3_10241994	Ca4	10241994	TAAAAAAAAAAA/TAAAAAAAAAAAA	CCTGCACCATTTCTCTTGGT	TTTTCTGGTAACTCGGACCC	60.1	649	INTERGENIC	--	--	--
Ca4_P3_10242563	Ca4	10242563	TTATATATATATATATATATATATAT/TTATATATATATATATATATATATATATATATATAT	GGGTCCGAGTTACCAGAAAA	AAACCTGAAAACAACGGACG	59.0	802	INTERGENIC	--	--	--
Ca4_P3_10247608	Ca4	10247608	CAATATTGTAAAATA/CAATATTGTAAAATAATATTGTAAAATA	GAGTTAGGGTTGGTGAGGCA	TTTTTCACGTCGCATAAATCT	60.1	236	INTERGENIC	--	--	--
Ca4_P3_10253630	Ca4	10253630	GAAAAAAAA/GAAAAAAA	TGTGTTTGAATGGGCAAAAA	CTTCATTTAAACGCCCCAAA	59.9	312	INTERGENIC	--	--	--
Ca4_P3_10261671	Ca4	10261671	ATTTTTT/ATTTTT	TGGATTTTGGACTTGGTCTTG	AACAACACGTGATTTGAATTGC	60.0	654	INTERGENIC	--	--	--

INDEL marker IDs	Chromosomes/ unanchored scaffolds	Physical positions (bp)	InDels (*Kabuli* reference genome-CDC Frontier/PII)	Forward primers (5'-3')	Reverse primers (5'-3')	Anneling temperature (0C)	Expected amplified product size (bp)	Structural annotation		Functional annotation	
								Sequence components of *kabuli* genome	*Kabuli* gene accession IDs	NCBI-KOG	TFs
Ca4_P3_10270991	Ca4	10270991	ATATTATTATTATTATTATTATTATTATT/ATATTATTATTATTATTATTATTATTATT	GTCACTGCCGGAGTACCAAT	CCCTCCTGCTGTTGTGAAAT	60.0	223	INTRON	Ca_08494	I	NF-YB
Ca4_P3_10330675	Ca4	10330675	GTTTTTTTTT/GTTTTTTTTTT	TGATTTGTGATGCACATTTGG	TTGCATGAAAAATTGTGAATGAT	60.4	747	INTERGENIC	--	--	--
Ca4_P3_10439513	Ca4	10439513	GAAAAAAAAAA/GAAAAAAAAA	CCTCTCTGCCTGAAGTGACC	GCAAGGATGCATTTGAAACA	60.0	516	INTRON	Ca_04281	Z	--
Ca4_P3_10456572	Ca4	10456572	ATTTTTTTT/ATTTTTTT	ATCGATCAAAACCCGTGAAG	CGTTGTTGGTAAGGGAATCG	59.9	454	INTERGENIC	--	--	--
Ca4_P3_10466054	Ca4	10466054	CTTTTTTTTTTT/CTTTTTTTTTTTT	TGGCCTATGCATCAACACAT	TGCTGATTTTGGGTTCTTCC	60.0	367	INTRON	Ca_04283	--	--
Ca4_P3_10612304	Ca4	10612304	TTTATTATTATTATTATTATTATTATTATTATTATTATTATTATT/TTTATTATTATTATTATTATTATTATTATTATTATTATT	AATCACGCAAACAAGTGTCG	AGCGACATAAATGGCTGCTA	59.8	408	INTERGENIC	--	--	--
Ca4_P3_10657750	Ca4	10657750	T/TCTAACACTCTTTCC	ATACAAAAACATTTGGGCCG	TTCACATGAAGTGGCGAGAC	59.7	417	DRR	Ca_04301	--	--
Ca4_P3_10666722	Ca4	10666722	CTT/CTGTTGTTT	AACTCTGCCATTGAATTGGG	CTCGATGATCTGCCGTAACA	59.9	481	DRR	Ca_04303	--	STAT
Ca4_P3_10784287	Ca4	10784287	TAAAAAAAAAAA/TAAAAAAAAAA	TGGGCCGAATTTTGTGATAG	CGCACAATTGATACATCAACAC	60.8	486	DRR	Ca_04314	U	--
Ca4_P3_10820773	Ca4	10820773	T/TAATA	GTTTACCACGCCGACAGTTT	TGGACAATCTTCTTTGTGAGC	60.0	770	INTERGENIC	--	--	--

INDEL marker IDs	Chromosomes/ unanchored scaffolds	Physical positions (bp)	InDels (*Kabuli* reference genome-CDC Frontier/PII)	Forward primers (5'-3')	Reverse primers (5'-3')	Anneling temperature (0C)	Expected amplified product size (bp)	Structural annotation		Functional annotation	
								Sequence components of *kabuli* genome	*Kabuli* gene accession IDs	NCBI-KOG	TFs
Ca4_P3_10828911	Ca4	10828911	AAAAAATTGATATTTAAAAATTGATATTTAAAAATTGATA/AAAAAATTGATATTTAAAAATTGATATTTAAAAATTGATATTTAAAAATTGATA	TGCTAGGAGGAAAGTTTTTGG	AGAATCATGTTTATGATTGGAAATTA	58.5	629	INTERGENIC	--	--	--
Ca4_P3_10855427	Ca4	10855427	GTATATATATATATATATATATATATATAT/GTATATATATATATATATATATATATATAT	AATTGTTGGCACCAGTCACA	GCAAACACAAAAGCAACCAA	60.0	773	INTERGENIC	--	--	--
Ca4_P3_11046179	Ca4	11046179	TAAAATAAAATAAAA/TAAAATAAAATAAAAATAAAATAAAA	CTCTCCGTTCCGATCTTCAG	TTTTTACGTACCACATCCATTATGA	59.9	454	INTERGENIC	--	--	--
Ca4_P3_11154234	Ca4	11154234	AATATATATATATATATATATATATATATATATAT/AATATATATATATATATATATATATATATAT	TGCAAACTCGGGATTCCTTA	TTGGTATTCTTTTGAGGCATTG	60.6	823	INTERGENIC	--	--	--
Ca4_P3_11198337	Ca4	11198337	A/ATCTT	CTTTCAAACGACCCTTCCAA	ATTTCGTCAAGCATTCCCTG	60.1	570	INTERGENIC	--	--	--
Ca4_P3_11209323	Ca4	11209323	TAAAAAAA/TAAAAAA	TTAGTGTTGAATTGACGGCG	CAATGCCTAGAATCACGGGT	59.7	960	INTERGENIC	--	--	--
Ca4_P3_11209656	Ca4	11209656	GTATATA/GTATATATA	TTGGCTTAGGCTGACATGAA	CAATGCCTAGAATCACGGGT	59.4	217	INTERGENIC	--	--	--
Ca4_P3_11211716	Ca4	11211716	AC/ACTCCC	GGAACATGGAATGTTGGAGG	GCAAAGTGCATGAAAAGCAA	60.2	500	INTRON	Ca_04353	U	M-type
Ca4_P3_11216142	Ca4	11216142	CGAGA/CGA	GCATAACCAATGCTCTTGGAA	ATTAAGACACTGGCGCGAAG	60.1	523	DRR	Ca_04353	U	M-type
Ca4_P3_11216218	Ca4	11216218	TAAAAAAAAA/TAAAAAAA	GCATAACCAATGCTCTTGGAA	TCAAACCAAGGATGATGCAA	60.1	853	DRR	Ca_04353	U	M-type

INDEL marker IDs	Chromosomes/ unanchored scaffolds	Physical positions (bp)	InDels (*Kabuli* reference genome-CDC Frontier/PII)	Forward primers (5'-3')	Reverse primers (5'-3')	Anneling temperature (0C)	Expected amplified product size (bp)	Structural annotation		Functional annotation	
								Sequence components of *kabuli* genome	*Kabuli* gene accession IDs	NCBI-KOG	TFs
Ca4_P3_11216741	Ca4	11216741	AC/A	TTGCATCATCCTTGGTTTGA	ACTGCATTCCGTCCAAAATC	60.0	229	INTERGENIC	--	--	--
Ca4_P3_11229115	Ca4	11229115	ATATTATTA/ATATTA	CCATGTAAGTGAGTGAAATCGG	GTGCACTTAGGCTTGCATGA	59.5	702	INTRON	Ca_04355	G	--
Ca4_P3_11238142	Ca4	11238142	ATATTATTATTATTATTATT/ATATTATTATTATTATT	CCCTTTGTCCTACAGTGAACG	TTCTTCCGGAACCATTATAAAAA	59.6	327	DRR	Ca_04357	O	NAC
Ca4_P3_11240162	Ca4	11240162	G/GA	TTCAACATGGTCCATTCCTTC	GGCAGCCGATATTTTGTCAT	59.8	623	INTRON	Ca_04357	O	NAC
Ca4_P3_11240985	Ca4	11240985	CCTTACTT/CCTT	ACAAAATATCGGCTGCCAAG	CCTCTCTCCCTCTCGCTTTT	60.1	622	INTRON	Ca_04357	O	NAC
Ca4_P3_11255585	Ca4	11255585	T/TA	TCAGGTGGATCCGACAAAAT	ATACTCTTGGCTGCGCTGTT	60.3	385	INTRON	Ca_04358	R	--
Ca4_P3_11258051	Ca4	11258051	TAAAA/TAAAAAA	GCAAATGCCTTGTACGGTTT	CCCTTCCTTCAGGTCATCAA	60.0	346	INTRON	Ca_04358	R	--
Ca4_P3_11263655	Ca4	11263655	CAAAA/CAAA	GCACGGGTAGGAGTTTGTGT	TGCACTCTCCAATCTCGTTG	60.0	414	INTERGENIC	--	--	--
Ca4_P3_11266524	Ca4	11266524	TTTCTT/TTT	ATGGCAATGAGGATGGTTGT	ACTCACAATGCTTAACCGCC	60.2	440	INTERGENIC	--	--	--
Ca4_P3_11268024	Ca4	11268024	AAAGAA/AAA	TAGTGCCCTCCAAAAGGATG	GCGTTGAGGTGTCAACTGTG	60.1	733	INTERGENIC	--	--	--

INDEL marker IDs	Chromosomes/ unanchored scaffolds	Physical positions (bp)	InDels (*Kabuli* reference genome-CDC Frontier/PII)	Forward primers (5'-3')	Reverse primers (5'-3')	Anneling temperature (0C)	Expected amplified product size (bp)	Structural annotation		Functional annotation	
								Sequence components of *kabuli* genome	*Kabuli* gene accession IDs	NCBI-KOG	TFs
Ca4_P3_11270494	Ca4	11270494	CATATAT/CATAT	TGTTGACAAACACAAAGACCG	ATCCACCATCAATGCCATCT	59.6	645	INTERGENIC	--	--	--
Ca4_P3_11273043	Ca4	11273043	TCAAATTCTCCA/TCA	TGATCTTCACCGAACTGCAC	ACCTCAGCAATATCATCGGC	59.8	544	INTRON	Ca_04359	--	--
Ca4_P3_11273251	Ca4	11273251	A/ATATAT	CCCCTCATTCAAATTCTCCA	GTAACGGTTGCGGATCATCT	59.9	638	INTRON	Ca_04359	--	--
Ca4_P3_11281622	Ca4	11281622	TG/T	TTTAAGGCATGTGAGTTCAGC	TGCAAAATCAATACATGGTTCG	58.0	641	DRR	Ca_04359	--	--
Ca4_P3_11291621	Ca4	11291621	ATTT/ATTTT	GATGAAAGTTTTTGGTGTGGC	AGCGCAGTGTTAACGTTCCT	59.5	946	INTERGENIC	--	--	--
Ca4_P3_11297413	Ca4	11297413	TGAA/T	AATTGCTTGTGCTGTTGTGC	AATGAAGCCAACCCTGTGAC	59.9	662	INTERGENIC	--	--	--
Ca4_P3_11298187	Ca4	11298187	CTTTTTTTTTTTT/CTTTTTTTTT	AAATGCTCCAAAAACCAACG	ATTAAAATGCCAGCAGTGGG	60.0	471	INTERGENIC	--	--	--
Ca4_P3_11300403	Ca4	11300403	ATATTATT/ATATT	GTGTTACTTCCGCCGTTGAT	TGTCAAGTTGTTTCATGCTGC	60.0	185	INTERGENIC	--	--	--
Ca4_P3_11306565	Ca4	11306565	TG/TGG	ACCATAAGGTGACGAGTGCC	GTCAACCATTGGATTTTGGC	60.0	453	DRR	Ca_04362	--	--
Ca4_P3_11311023	Ca4	11311023	CATTATTATTATTATTATTAT/CATTATTATTATTATTATTATTAT	ATGTTTATGTGCCGTCCCTC	GGCCAACCATCAACCATATC	59.8	434	CDS (large-effect mutations)	Ca_04364	D	WRKY

INDEL marker IDs	Chromosomes/ unanchored scaffolds	Physical positions (bp)	InDels (*Kabuli* reference genome-CDC Frontier/PII)	Forward primers (5'-3')	Reverse primers (5'-3')	Anneling temperature (0C)	Expected amplified product size (bp)	Structural annotation		Functional annotation	
								Sequence components of *kabuli* genome	*Kabuli* gene accession IDs	NCBI-KOG	TFs
Ca4_P3_11311504	Ca4	11311504	A/ACAC	TGATGGTTGGCCTAAATGGT	TTGCTGAAGACTGATGCCAC	60.2	463	DRR	Ca_04363	--	--
Ca4_P3_11317243	Ca4	11317243	ATCTCTCTCTCTCTCTC/ATCTCTCTCTCTCTC	CCTTCCACTGCCAAAACATT	CAATTGGTCGGTTTCTGGTT	60.0	430	DRR	Ca_04364	D	WRKY
Ca4_P3_11318155	Ca4	11318155	ATTTTTTTTTT/ATTTTTTT	ATTCTCATGGCAAGGGATTG	TGCGTAATGGACCAATTCAA	59.9	909	INTERGENIC	--	--	--
Ca4_P3_11322920	Ca4	11322920	AAGCTCCTCTTC/A	TCGATGGCATGATGATTGTT	AAGAGGATTGGAGATAGGAGAGC	59.9	371	INTERGENIC	--	--	--
Ca4_P3_11323452	Ca4	11323452	CTTT/CT	AAGCTCTCCTATCTCCAATCCTC	GAAATCCATCATTTCAACCAAAA	59.4	559	DRR	Ca_04365	--	M-type
Ca4_P3_11323964	Ca4	11323964	GAA/GAAA	TTGGTTGAAATGATGGATTTCTT	GGCCGTTGATTTGTTCTGTT	59.7	579	DRR	Ca_04365	--	M-type
Ca4_P3_11326193	Ca4	11326193	CATATATA/CATA	CACCTGGATTGTTTGTGTGC	ATATTGGTCAAGCTGCTGGC	60.0	272	INTRON	Ca_04365	--	M-type
Ca4_P3_11330842	Ca4	11330842	G/GAA	CTTTGTAGACGAGGCCGAAG	CTTCCCCGAAAAACCACTCT	60.0	402	DRR	Ca_04367	U	FAR1
Ca4_P3_11338339	Ca4	11338339	AAGA/AAGAGA	ACGGATAAAATGCCCATTGA	ATTGGTGTGAGTGCTGTGGA	60.2	821	INTERGENIC	--	--	--
Ca4_P3_11344375	Ca4	11344375	TG/TGG	TGGAGGGAGGTTGGTATTTG	AAGACAGAGGAGCACCAGGA	59.8	369	INTRON	Ca_04368	R	--

INDEL marker IDs	Chromosomes/ unanchored scaffolds	Physical positions (bp)	InDels (*Kabuli* reference genome-CDC Frontier/PII)	Forward primers (5'-3')	Reverse primers (5'-3')	Anneling temperature (0C)	Expected amplified product size (bp)	Structural annotation		Functional annotation	
								Sequence components of *kabuli* genome	*Kabuli* gene accession IDs	NCBI-KOG	TFs
Ca4_P3_11359837	Ca4	11359837	ACCC/ACC	TGTTGCGGTTGTATGG TGTC	TGACATTGGGGGATTC ATTT	60.4	455	INTERGENIC	--	--	--
Ca4_P3_11360175	Ca4	11360175	CTGATTTTG/CTGATTTTGATTTTG	AAATGAATCCCCCAATG TCA	TTATCAATCGAAAAAGA AACGC	60.0	212	INTERGENIC	--	--	--
Ca4_P3_11368276	Ca4	11368276	CG/C	TGATGAATTTTCATTCA CCAATC	TCCCAAGGTCAATTAG GCAG	58.8	116	INTERGENIC	--	--	--
Ca4_P3_11370815	Ca4	11370815	TAAA/TAAAAA	GAGATGGTGAAATCCA ACGC	AGGGAGGGGATGAGA GAAAA	60.5	641	INTERGENIC	--	--	--
Ca4_P3_11372269	Ca4	11372269	TGGGG/TGGGGG	CTTATGCCGCACAATG AAAA	CTCAATCACCCCATTC TCGT	59.7	670	INTERGENIC	--	--	--
Ca4_P3_11376038	Ca4	11376038	CAATTTTTTAATTTTT/CAATTTTTTAAT TTTTTAATTTTT	GCCACCACCCAACACT AAAT	GCCCCTCACTTCAACA CAGT	59.7	844	INTERGENIC	--	--	--
Ca4_P3_11399144	Ca4	11399144	TTA/T	CGGAATTGATGGTTGA AAGG	ATGCAACCGCTTCAAT TTTT	60.3	743	INTERGENIC	--	--	--
Ca4_P3_11411081	Ca4	11411081	AGG/AGGG	GGGTGAGACCAGGACG ACTA	TGGCAAGTACGATTTT CAACTG	60.1	543	INTRON	Ca_04375	--	bZIP
Ca4_P3_11415027	Ca4	11415027	A/AG	CACGTGGCACATTGTC ACTA	AACAACGACCTCCGAA CATC	59.2	545	INTERGENIC	--	--	--
Ca4_P3_11416261	Ca4	11416261	TAAA/TAA	TGTGTCCGGAATAACG AAAA	GCCATTGTTAACGGTG GTTT	59.0	711	INTERGENIC	--	--	--

INDEL marker IDs	Chromosomes/ unanchored scaffolds	Physical positions (bp)	InDels (*Kabuli* reference genome-CDC Frontier/PII)	Forward primers (5'-3')	Reverse primers (5'-3')	Anneling temperature (0C)	Expected amplified product size (bp)	Structural annotation		Functional annotation	
								Sequence components of *kabuli* genome	*Kabuli* gene accession IDs	NCBI-KOG	TFs
Ca4_P3_11424676	Ca4	11424676	CTGTT/CTGTTGTT	TCTGCCGGAAGAATTACCAC	CAGCACAGCACCTAGCAGAG	60.1	422	DRR	Ca_04377	D	--
Ca4_P3_11434874	Ca4	11434874	AATTTTAGATTTT/AATTTTAGATTTTAGATTTT	TTTTTCCGTCCACTCCTTTG	CCGGTCTTCCAATGTCAACT	60.1	368	INTRON	Ca_04378	S	--
Ca4_P3_11450457	Ca4	11450457	AA/AACACTTA	GGGGAGGCCTTAAGTAGTGG	CATGGTTGCTTGACCTTGAA	60.0	363	INTERGENIC	--	--	--
Ca4_P3_11451772	Ca4	11451772	TGTAA/T	TGACGGATACTCCAACACCA	CAAACCCACCAGATCCAACT	60.0	845	INTRON	Ca_04381	--	--
Ca4_P3_11452051	Ca4	11452051	AG/AGG	TGGTGGTTTTGCTTTGGTAA	GCAACACGAAGCTGAAAACA	59.1	913	INTRON	Ca_04381	--	--
Ca4_P3_11452126	Ca4	11452126	AATATATATATA/AATATATATATATA	GGAATCAAACTCTCCGGATCT	GCAACACGAAGCTGAAAACA	59.5	398	INTRON	Ca_04381	--	--
Ca4_P3_11452533	Ca4	11452533	TGAGA/TGAGAGA	TGTTTTCAGCTTCGTGTTGC	CCATACTACCTCTGCCCACC	60.0	324	DRR	Ca_04381	--	--
Ca4_P3_11453272	Ca4	11453272	ACC/AC	AAAGTTTAGTCAGCTGAAATCGC	TGTCTTCGTGCAAAATGGAG	59.1	273	DRR	Ca_04381	--	--
Ca4_P3_11454920	Ca4	11454920	TCC/TCCC	AGAAAAACATAACGCGGTGG	TGGAACCTCGTCTTGGAAAG	60.0	417	INTERGENIC	--	--	--
Ca4_P3_11471153	Ca4	11471153	CAAA/CAAAA	TTCGATTGTTCAAACTCATTCAA	TTGGCCATCGTTTGAATTTT	59.6	787	INTERGENIC	--	--	--

INDEL marker IDs	Chromosomes/ unanchored scaffolds	Physical positions (bp)	InDels (*Kabuli* reference genome-CDC Frontier/PII)	Forward primers (5'-3')	Reverse primers (5'-3')	Anneling temperature (0C)	Expected amplified product size (bp)	Structural annotation		Functional annotation	
								Sequence components of *kabuli* genome	*Kabuli* gene accession IDs	NCBI-KOG	TFs
Ca4_P3_11472631	Ca4	11472631	AC/A	TTTCTTGTCTCCTCGGTTGG	TTTGTCATGTTCACGCACTG	60.2	632	INTERGENIC	--	--	--
Ca4_P3_11476079	Ca4	11476079	GAA/GAATAAATAA	AACCTCATGCAATGACACCA	GATCGGCTTCATGGTTTTGT	60.0	329	INTERGENIC	--	--	--
Ca4_P3_11494600	Ca4	11494600	ATAAATTTAAATTTAAATTTA/ATAAATTTAAATTTA	CAGGTTTTCTCTCTCGCACC	CAACACAACCCGTGAACATC	60.0	398	INTERGENIC	--	--	--
Ca4_P3_11507043	Ca4	11507043	ATTT/ATTTT	TTATCCCATGCATAGATCCG	TTATGGCAAGAATAGTTTTATCATTTT	58.4	796	INTERGENIC	--	--	--
Ca4_P3_11512594	Ca4	11512594	TA/TAA	AATCCAAAGTGGCGTGTTTC	TCATTCGTGTGTTGCAAAAGT	60.0	581	INTERGENIC	--	--	--
Ca4_P3_11513722	Ca4	11513722	CTGATATTT/CT	AAAGGAATGTCGAAGCTCCA	CCAGCCCATTACATAAATCCA	59.8	489	INTERGENIC	--	--	--
Ca4_P3_11514646	Ca4	11514646	AT/ATT	AAGAGTTGAAACTCGGCGAA	AAGCCACATTTTGGGTCAAG	60.0	700	INTERGENIC	--	--	--
Ca4_P3_11519668	Ca4	11519668	CTATTAT/CTAT	TCAGGCAAACAAGCAGACAC	ATGGTTGGTTCGGAGACTTG	60.0	381	INTERGENIC	--	--	--
Ca4_P3_11534681	Ca4	11534681	ATTTTTT/ATTTTTTT	TTTTTGGGGCTTTAATACCG	TAAGGCCCAAGAGAAAAGGG	59.0	685	INTERGENIC	--	--	--
Ca4_P3_11537100	Ca4	11537100	CTATA/CTATATATA	TCAATCTCCCGTTTAATGGTTT	CACGCTTATGCAGACGAAAA	59.7	206	INTERGENIC	--	--	--

INDEL marker IDs	Chromosomes/ unanchored scaffolds	Physical positions (bp)	InDels (*Kabuli* reference genome-CDC Frontier/PII)	Forward primers (5'-3')	Reverse primers (5'-3')	Anneling temperature (0C)	Expected amplified product size (bp)	Structural annotation		Functional annotation	
								Sequence components of *kabuli* genome	*Kabuli* gene accession IDs	NCBI-KOG	TFs
Ca4_P3_11539392	Ca4	11539392	TA/TATGA	TGCGGTTTTCACAAAATGAC	CAACGGTTCATGATCCAATCT	59.6	192	INTERGENIC	--	--	--
Ca4_P3_11543619	Ca4	11543619	AAATAACTG/AAATAACTGTATAATAACTG	CACAGTTCGAGTTGGTGCAT	TGGTGATTATGGTTCTTTTGGA	59.8	224	INTERGENIC	--	--	--
Ca4_P3_11544381	Ca4	11544381	C/CGA	AAGGGAAAGATGGAGACCAA	AATCGATGGCAAGGAATCTG	58.6	534	INTERGENIC	--	--	--
Ca4_P3_11547482	Ca4	11547482	ATTTTTTTTT/ATTTTT	TCCCCAAGCCTACAAACAAC	TGTTCATCGCAGATCCTTTCT	60.0	552	INTERGENIC	--	--	--
Ca4_P3_11547604	Ca4	11547604	TCC/TC	TCCCCAAGCCTACAAACAAC	TGTTCATCGCAGATCCTTTCT	60.0	552	INTERGENIC	--	--	--
Ca4_P3_11549653	Ca4	11549653	TAA/TAAAA	AAATTCGTAAAGGTCCACCG	CCAACGGTGTGCATAATGAG	58.9	729	INTERGENIC	--	--	--
Ca4_P3_11550574	Ca4	11550574	AT/ATTTAATTT	CTCATTATGCACACCGTTGG	CCCCAAAATGTTATGCCCTA	60.0	809	INTERGENIC	--	--	--
Ca4_P3_11551467	Ca4	11551467	TTTTTTTTAATTTTTTTA/TTTTTTTTA	ATCCAAAAGCCCCCAATAAC	TCACTGTTGCATCTCCAAGC	60.0	844	INTERGENIC	--	--	--
Ca4_P3_11552635	Ca4	11552635	AG/A	ATGGGGTGCCATACTCCATA	TGCGTTTCGATGATAACCAA	60.0	563	INTRON	Ca_04385	K	Trihelix
Ca4_P3_11555971	Ca4	11555971	CATATATATAT/CATATAT	CATTATTGGATTCGTTGGGG	GGGTGATAGATTCTTGATGCAAT	60.0	362	DRR	Ca_04385	K	Trihelix

INDEL marker IDs	Chromosomes/ unanchored scaffolds	Physical positions (bp)	InDels (*Kabuli* reference genome-CDC Frontier/PII)	Forward primers (5'-3')	Reverse primers (5'-3')	Anneling temperature (0C)	Expected amplified product size (bp)	Structural annotation		Functional annotation	
								Sequence components of *kabuli* genome	*Kabuli* gene accession IDs	NCBI-KOG	TFs
Ca4_P3_11576043	Ca4	11576043	CAAAAAAAA/CAAAAAAA	TCCTCAGATCTCCACATCCC	CTCAGGTGGTGGAGTTGGAT	60.0	327	INTRON	Ca_04386	K	Trihelix
Ca4_P3_11635720	Ca4	11635720	CTTCCCCATTCCCCA/CTTCCCCA	TCTGCATTTGTTTGCTGAGG	AAAGCCGCATTTTAGAAGCA	60.0	500	INTERGENIC	--	--	--
Ca4_P3_1169667	Ca4	1169667	TTTATTATTATTATTATTATTATTATTATTATTATTATTATTATTAT/TTTATTATTATTATTTTTATTATTATTATTATTATTATTATTATTATTATTATTATTATTATTAT	TTTTAGCCCCGTTTTGTTCA	TAAGGTGTCCGACATCAGATT	60.5	701	DRR	Ca_07748	OU	--
Ca4_P3_11713997	Ca4	11713997	AATATATATATATATATATATATATATATAT/AATATATATATATATATATATATATATATATAT	AATTTATCCCAAAGGCACCA	TGGCGTTCTAAATTGCCAGT	59.3	603	INTERGENIC	--	--	--
Ca4_P3_11769762	Ca4	11769762	TA/T	TCCATAGTGGAGATGCGACA	GGAACATGCGAATCACAAAA	60.2	666	INTERGENIC	--	--	--
Ca4_P3_11785607	Ca4	11785607	TAAAAAAAAAAAA/TAAAAAAAAAAA	GGATATGAGTGGGCACGAAC	GAGCGTGAGAACAACGATCA	60.3	588	INTERGENIC	--	--	--
Ca4_P3_11797493	Ca4	11797493	TTTATTATTATTATTATTATTATTATTATTATTATTATTA/TTTATTATTATTATTATTATTATTATTATTATTA	TCTGAACGTCATTTCACCGA	TGTTCACCAACTTGTTTGTTGTT	60.2	935	INTERGENIC	--	--	--
Ca4_P3_11818956	Ca4	11818956	CAAA/CAAAA	CCAAAACATGAAAGCAACAAAA	TAACATTGATGGCAGAGGCA	60.0	170	INTERGENIC	--	--	--
Ca4_P3_11913229	Ca4	11913229	TTATATATATATATATATATATATATATATATATAT/TTATATATATATATATATATATATATATATATATATATATAT	CGAAAGAGGTTCAATTGTTGC	TTTCATGTTGTCGCGATACG	59.7	358	INTERGENIC	--	--	--
Ca4_P3_11931582	Ca4	11931582	TGAAAAACAAGAAGAAAAACAA/TGAAAAACAAGAAGAAAAACAAGAAGAAAAACAA	CAAAGAACCTGTGACGAGCA	TTTAGTTAACGCAGGCACCC	60.0	480	INTRON	Ca_04415	O	Trihelix

INDEL marker IDs	Chromosomes/ unanchored scaffolds	Physical positions (bp)	InDels (*Kabuli* reference genome-CDC Frontier/PII)	Forward primers (5'-3')	Reverse primers (5'-3')	Anneling temperature (0C)	Expected amplified product size (bp)	Structural annotation		Functional annotation	
								Sequence components of *kabuli* genome	*Kabuli* gene accession IDs	NCBI-KOG	TFs
Ca4_P3_12050020	Ca4	12050020	T/TG	CCGAACTAATTTGCCAGAAA	GACCAAGTCACAAAGGGAAGA	57.9	793	INTERGENIC	--	--	--
Ca4_P3_12090377	Ca4	12090377	TTATATATATATATATATATATATAT/TTATATATATATATATATATATATATATAT	ATTTGGTTCTGCGTTTGACC	ACTAGGTTGGAGCATGAGGC	60.0	567	INTERGENIC	--	--	--
Ca4_P3_12127716	Ca4	12127716	AAATAATAATAATAATAATAATAATAAT/AAATAATAATAATAATAATAATAAT	CATTCGGTGGACCTTTTACG	TGAAGAGACGAAGCTGCAAA	60.4	343	INTERGENIC	--	--	--
Ca4_P3_12136812	Ca4	12136812	AATATATATATATATATATATATATATATATAT/AATATATATATATATATATATATATATAT	ACGCGGAGCTAAAATCTTGA	GGGGGAAGACCCATTACAGT	60.0	880	INTERGENIC	--	--	--
Ca4_P3_12222188	Ca4	12222188	G/GT	TCATTCATCACAAAGGTGGC	TGTGCAGTCGGTGTTGATTT	59.5	521	INTERGENIC	--	--	--
Ca4_P3_12225286	Ca4	12225286	G/GAGTCTC	TCATGGAGGCAAAGAAGGAG	TGACATGTGGATCGTGGTTT	60.3	724	DRR	Ca_04444	--	--
Ca4_P3_12265944	Ca4	12265944	ACTTCTTCTT/ACTTCTTCTTCTT	AAAAACATGGGGTGAAGCTG	TGCTGTTTTCAATGCCTCTG	60.0	874	CDS (large-effect mutations)	Ca_04450	Q	C2H2
Ca4_P3_12270457	Ca4	12270457	TCAGAC/TCAGACCAGAC	GGTCGTCGGTAGGGGTTTAT	GTGGAGAGTATGGGTGGGAA	60.1	552	INTRON	Ca_04451	--	C2H2
Ca4_P3_1237884	Ca4	1237884	AATATATATATATATATATATATATA/AATATATATATATATATATA	GTGGTCTGGAACATCGTCCT	TTGCAGTTGCAATATCGTTG	60.0	863	INTERGENIC	--	--	--
Ca4_P3_12393377	Ca4	12393377	TAAAAAAAAAAAAAAAA/TAAAAAAAAAAAAAA	TGTGCTGTTGTGATTAGGGG	AGCGTTCAAGGTGCAAAAGT	59.6	659	DRR	Ca_04463	QI	bHLH

INDEL marker IDs	Chromosomes/ unanchored scaffolds	Physical positions (bp)	InDels (*Kabuli* reference genome-CDC Frontier/PII)	Forward primers (5'-3')	Reverse primers (5'-3')	Anneling temperature (0C)	Expected amplified product size (bp)	Structural annotation		Functional annotation	
								Sequence components of *kabuli* genome	*Kabuli* gene accession IDs	NCBI-KOG	TFs
Ca4_P3_12478630	Ca4	12478630	ATTTTTT/ATTTTTTT	TCTTTCACACCAACCACCAA	CACTTATGATGCCCAAGGGT	60.0	789	INTERGENIC	--	--	--
Ca4_P3_12479687	Ca4	12479687	GA/GAA	GATGCCCCACAACCACTACT	CGAGACCAAGAAACGGAAAA	59.9	164	INTERGENIC	--	--	--
Ca4_P3_12482146	Ca4	12482146	GTTT/GTT	GGGCACTAAAAACACGCATT	TGCACATTCGTATCTTCCCA	60.0	234	INTERGENIC	--	--	--
Ca4_P3_12490666	Ca4	12490666	TGTAGTAGTAGTAGTAGTAGTAGTAGTA/TGTAGTAGTAGTAGTAGTAGTAGTAGTAGTAGTAGTAGTAGTAGTA	GGAATATGGCAGCAGACGAT	GCCTGCATATGGCTAAGAGC	60.1	734	DRR	Ca_04472	R	NAC
Ca4_P3_12493651	Ca4	12493651	ATTTTTT/ATTTTTTTT	ATTCCTCGTCCGTTTTCAGA	TTTTTCTTCCTGATTTGGTCC	59.7	293	DRR	Ca_04473	--	--
Ca4_P3_12506839	Ca4	12506839	CGG/CG	ATGTGCTTTCTGGGCTGTCT	CCGCTTGTTGTGTGTAGGAG	59.9	270	INTERGENIC	--	--	--
Ca4_P3_12510823	Ca4	12510823	TTATATATATATATA/TTATATATATATA	TTAGCGGTAAAATTCACGCA	CATCATCGCAACAAAGATGG	59.4	467	INTERGENIC	--	--	--
Ca4_P3_12512980	Ca4	12512980	CACAATT/C	CATGCGCCTTCTTGCATTAT	AAGGGTGTGAGGAAGTCACG	61.1	261	INTERGENIC	--	--	--
Ca4_P3_12520618	Ca4	12520618	AATATATATATATAT/AATATATATATATATAT	TCAAAGCTGCATTTTGTTGC	TTCGTATGCTATGCACGCTC	60.0	441	INTERGENIC	--	--	--
Ca4_P3_12528995	Ca4	12528995	CTTTT/CTTT	TAACGCTAGGGTCCCATGTC	TCGTTTTGATCATTTATCGTTTTT	60.0	720	INTERGENIC	--	--	--

INDEL marker IDs	Chromosomes/ unanchored scaffolds	Physical positions (bp)	InDels (*Kabuli* reference genome-CDC Frontier/PII)	Forward primers (5'-3')	Reverse primers (5'-3')	Anneling temperature (0C)	Expected amplified product size (bp)	Structural annotation		Functional annotation	
								Sequence components of *kabuli* genome	*Kabuli* gene accession IDs	NCBI-KOG	TFs
Ca4_P3_12529201	Ca4	12529201	TAAAAAAAAAA/TAAAAAAAA	CATGGCTCCAAGAAGCTTAAA	TTCGTTTTGATCATTTATCGTTTT	59.5	527	INTERGENIC	--	--	--
Ca4_P3_12531241	Ca4	12531241	CTTTTT/CTTTTTTT	CCTCAACCTGACCCAGTTGT	AATTTCAAGGCCATGTGAGG	60.0	944	INTRON	Ca_04476	--	--
Ca4_P3_12531515	Ca4	12531515	ATT/ATTT	TTTGCTTCCAATTGGGTGAT	AATTTCAAGGCCATGTGAGG	60.3	712	INTRON	Ca_04476	--	--
Ca4_P3_12532996	Ca4	12532996	C/CA	ATGAATTTGATGAGCAGGGC	GTTCCAGTTGCCCTGTGTTT	60.0	349	DRR	Ca_04477	K	MYB
Ca4_P3_12535193	Ca4	12535193	GAAAAAAAA/GAAAAAA	CGAAGCCTGCAACTTTTACC	CCAAACATGCAGGTTCCTCT	59.9	231	DRR	Ca_04476	--	--
Ca4_P3_12545942	Ca4	12545942	GTTTTTTTTTTT/GTTTTTTTTT	GTCATTTCCTTCCCCTGACA	CATTTTTGACAACCGATTGTTTT	59.9	444	INTERGENIC	--	--	--
Ca4_P3_12548954	Ca4	12548954	TAAAA/TAAAAA	AAGCATGCGAATGTATTACTGC	CATGAGGGCTTAAACAAGGAA	59.3	487	INTERGENIC	--	--	--
Ca4_P3_12551844	Ca4	12551844	TGA/TGATGAAGA	CCATTCCTTTGCAAACCTGT	CCAATCAAAGCAGGTTGGTT	60.0	472	INTERGENIC	--	--	--
Ca4_P3_12570942	Ca4	12570942	T/TC	GGCTCACATCAGGAATGGAT	CACCAGATCCCAATTTGCTT	59.9	654	DRR	Ca_04479	E	--
Ca4_P3_12571013	Ca4	12571013	GTATATA/GTATA	GGCTCACATCAGGAATGGAT	GGCTCTTGGTCATTGGATGT	59.9	968	DRR	Ca_04479	E	--

INDEL marker IDs	Chromosomes/ unanchored scaffolds	Physical positions (bp)	InDels (*Kabuli* reference genome-CDC Frontier/PII)	Forward primers (5'-3')	Reverse primers (5'-3')	Anneling temperature (0C)	Expected amplified product size (bp)	Structural annotation		Functional annotation	
								Sequence components of *kabuli* genome	*Kabuli* gene accession IDs	NCBI-KOG	TFs
Ca4_P3_12571781	Ca4	12571781	ACCCC/ACCCCC	ACATCCAATGACCAAGAGCC	CCACTTTCGGAACAATTTTGA	59.9	394	INTERGENIC	--	--	--
Ca4_P3_12577562	Ca4	12577562	T/TC	TCTGTCAGCCAATGCACTTC	CAGGAATGCCAAAACCAAAT	60.0	409	INTRON	Ca_04480	L	--
Ca4_P3_12582939	Ca4	12582939	TTAATATAA/TTAA	CAAGCTTGTACCTGGGGTGT	CCGAAAAAGCATCACTGGTT	60.0	503	INTRON	Ca_04480	L	--
Ca4_P3_12584518	Ca4	12584518	CTATG/CTATGTATG	GCCTGAATGCCCATGTATCT	CAAGTGGATTTGCACCAATG	59.9	536	DRR	Ca_04480	L	--
Ca4_P3_12588239	Ca4	12588239	ATTTTTTTTTTT/ATTTTTTTTT	ACAATTCCGAAGATACCCCC	GATGTGGTCGTTGTCGAGTG	60.0	506	INTERGENIC	--	--	--
Ca4_P3_12593930	Ca4	12593930	AATATATAT/AATATATATAT	TCAACTTGTTTTGACAAGGGC	TCTCCCATAGCTGCACACTG	60.1	733	INTERGENIC	--	--	--
Ca4_P3_12604256	Ca4	12604256	G/GTCAATTTAGTCT	CCTTCTTAATGAAGCCGTGG	TTGAGAGAGAAAGAGGGACGA	59.7	149	INTERGENIC	--	--	--
Ca4_P3_12605516	Ca4	12605516	CAA/CAAAAAAAA	GCTACGCAGTTCGTTTTATTGA	CAACGGAGGTGAGAAGGGTA	59.5	827	INTERGENIC	--	--	--
Ca4_P3_12637168	Ca4	12637168	TAAAAAAAAA/TAAAAAAAAAAA	GTGGCTGCATTGATATGGTG	CAAAATCACTTCAGGTGCCA	60.0	697	DRR	Ca_04486	--	--
Ca4_P3_12674571	Ca4	12674571	TAAAATAAAAA/TAAAATAAAAAAATAAAAA	CCAACGTCCAGAAAAATACCA	GCAAAAGTTAAAACGGAAGGG	59.8	620	INTERGENIC	--	--	--

INDEL marker IDs	Chromosomes/ unanchored scaffolds	Physical positions (bp)	InDels (*Kabuli* reference genome-CDC Frontier/PII)	Forward primers (5'-3')	Reverse primers (5'-3')	Anneling temperature (0C)	Expected amplified product size (bp)	Structural annotation		Functional annotation	
								Sequence components of *kabuli* genome	*Kabuli* gene accession IDs	NCBI-KOG	TFs
Ca4_P3_12832103	Ca4	12832103	GTT/GT	GCGTTTCCAATGGATGAAGT	ATGCCGAAAAAGACTGGTCA	59.9	203	DRR	Ca_04501	T	ARR-B
Ca4_P3_13159056	Ca4	13159056	TTATATATATATATATATATATATA/TTATATATATATATATATATATATATA	GATCCAATGTGGTTTGACCC	AGGCTAGATTGGGGGTTTTG	60.0	454	INTERGENIC	--	--	--
Ca4_P3_13320887	Ca4	13320887	AATATATATATATATATATATA/AATATATATATATATATATATATA	TATGCGCCTCAGCTTATCCT	CCGAGGAAACAATTGGATGT	60.0	600	INTERGENIC	--	--	--
Ca4_P3_13411051	Ca4	13411051	TGTTAGTTTTATATTAGTTAGTTTTATATTAGTTAGTTTTAT/TGTTAGTTTTATATTAGTTAGTTTTATATTAGTTAGTTTTATATTAGTTAGTTTTAT	ACATTGCGTCAAATCCAACA	AAATCTCGGTGTTCCTCGAA	60.0	720	DRR	Ca_04562	R	HD-ZIP
Ca4_P3_13433311	Ca4	13433311	TA/T	CGAGCAAACACAATGAATGG	TTGGTTGGTTCATTGGTTGA	60.1	569	INTERGENIC	--	--	--
Ca4_P3_13479788	Ca4	13479788	AT/ATTT	TCACACCCAAAATTCAAAGTACA	TGTGGCATAAACATTGGGAA	59.4	390	INTERGENIC	--	--	--
Ca4_P3_13480535	Ca4	13480535	TAAAAAAAAAAA/TAAAAAAAAAAAAA	TGAAAACATTTCAACAACCAAAA	TTAGGTGTGAGGGTGCATGA	59.4	472	INTERGENIC	--	--	--
Ca4_P3_13509527	Ca4	13509527	ATTTT/ATTTTATTAAAAAATTTATTTTT	CCTCTTCACGGTGCTCATTT	CCTCGTTTTAAGCCCAATCA	60.3	158	INTERGENIC	--	--	--
Ca4_P3_13546142	Ca4	13546142	TAAA/TAA	GAGATTCACGAAGACCGAGG	GCCAATGCTAATCTGCAACA	59.8	665	DRR	Ca_04571	T	--
Ca4_P3_13555112	Ca4	13555112	CTAGCTA/CTAGCTAATAGCTA	TCGTTTTGTGACATGGGAAC	CAGCCACATGCCAGTAGAAG	59.4	229	DRR	Ca_04572	--	--

INDEL marker IDs	Chromosomes/ unanchored scaffolds	Physical positions (bp)	InDels (*Kabuli* reference genome-CDC Frontier/PII)	Forward primers (5'-3')	Reverse primers (5'-3')	Anneling temperature (0C)	Expected amplified product size (bp)	Structural annotation		Functional annotation	
								Sequence components of *kabuli* genome	*Kabuli* gene accession IDs	NCBI-KOG	TFs
Ca4_P3_13579808	Ca4	13579808	CAA/CAAA	TTCCCAATTTTTCTTTCAATTTT	GCACTCACGACTCACGAGAA	58.2	579	INTERGENIC	--	--	--
Ca4_P3_13579909	Ca4	13579909	ATTT/AT	TTCCCAATTTTTCTTTCAATTTT	GCACTCACCAAGCAATAGCA	58.2	666	INTERGENIC	--	--	--
Ca4_P3_13587853	Ca4	13587853	T/TA	CAATCCTTCATGCAACCAGT	AGCCAACCTTGCAAGTCCTA	58.6	808	INTRON	Ca_04577	--	HB-other
Ca4_P3_13589679	Ca4	13589679	TAAAAAAAA/TAAAAAAA	GGGAGATGAGGCATGTGAAT	GAACCCCAATGAAATGTTGG	59.9	796	INTRON	Ca_04577	--	HB-other
Ca4_P3_13603773	Ca4	13603773	TAAAAAAA/TAAAAAAAA	AGCAATAGCCACATTCGTCC	TGAACCAATAGCTTCGTGGA	60.1	368	INTERGENIC	--	--	--
Ca4_P3_13608282	Ca4	13608282	ATTTTTTTT/ATTTTTTTTTT	TCTTTGTCCAAAGGCCAATC	CACATTTTGCAAGGTTCGTG	60.1	795	INTERGENIC	--	--	--
Ca4_P3_13614309	Ca4	13614309	C/CTCACCTG	CATTTGATTCGAGTGGCACA	GTGGTCGGTTTAAGTGCGTT	60.7	373	INTERGENIC	--	--	--
Ca4_P3_13615727	Ca4	13615727	ATTTTTTTTT/ATTTTTTTT	TGCTAGACTACCATGCCACG	GGGTAAGCTGATAGGGACCG	59.9	665	INTERGENIC	--	--	--
Ca4_P3_13616063	Ca4	13616063	ATTTT/ATTTTT	TCACATTTTAAGGTAAAGTGGAAAGA	CTTGGGATTGATGGCTTTGT	59.5	743	INTERGENIC	--	--	--
Ca4_P3_13617085	Ca4	13617085	CTTTTTTTTTTTT/CTTTTTTTTTT	TGGGTTGTGTTCAGAAGCAG	AAAAGCTGAAGAAAAGGCCC	59.9	436	DRR	Ca_04579	--	--

INDEL marker IDs	Chromosomes/ unanchored scaffolds	Physical positions (bp)	InDels (*Kabuli* reference genome-CDC Frontier/PII)	Forward primers (5'-3')	Reverse primers (5'-3')	Anneling temperature (0C)	Expected amplified product size (bp)	Structural annotation		Functional annotation	
								Sequence components of *kabuli* genome	*Kabuli* gene accession IDs	NCBI-KOG	TFs
Ca4_P3_13617448	Ca4	13617448	TG/TGG	GGGCCTTTTCTTCAGCTTTT	TGCATGTTATCCGCAATCTC	59.8	713	DRR	Ca_04579	--	--
Ca4_P3_13626293	Ca4	13626293	TTAATA/TTA	TTTCAACTCGAGCATTGTGC	AGCACCTTAACTTCCACCCC	60.0	374	INTERGENIC	--	--	--
Ca4_P3_13627308	Ca4	13627308	A/AT	TTGGCGTGAAAGACAACATT	TGTTGGGTGAGTTTTCCACA	59.2	938	INTERGENIC	--	--	--
Ca4_P3_13629536	Ca4	13629536	TA/TAA	TGTGTGGATGAATGGTTCAAA	TTTTATTTCAGGCCGTTTGC	59.8	531	INTERGENIC	--	--	--
Ca4_P3_13630632	Ca4	13630632	AA/AAGAGTCTA	TATGCAGGCCCAAACTCTCT	TTTTCACAATCAATGCCCAA	59.8	370	INTERGENIC	--	--	--
Ca4_P3_13631062	Ca4	13631062	GCC/GC	TTGGGCATTGATTGTGAAAA	TCGCAACTCGTATAGGACCA	59.9	271	INTERGENIC	--	--	--
Ca4_P3_13631101	Ca4	13631101	GTTTTTTTT/GTTTTTTTTT	TTGGGCATTGATTGTGAAAA	TCGCAACTCGTATAGGACCA	59.9	271	INTERGENIC	--	--	--
Ca4_P3_13636270	Ca4	13636270	TAAA/TAA	CTTGGCAATTGGACACCTTT	ATTGACCCATTTCATTCCCA	60.0	570	DRR	Ca_04581	--	--
Ca4_P3_13637017	Ca4	13637017	GAAAA/GAAA	CATTTGAACAATGGCACAGG	GATCTGGAGACAGAGGCGAC	60.0	482	DRR	Ca_04581	--	--
Ca4_P3_13638865	Ca4	13638865	AC/ACC	GTCAGGCCAGACCGATAAAA	GGTGAAAGGTGACGGGTAGA	60.1	231	DRR	Ca_04582	--	--

INDEL marker IDs	Chromosomes/ unanchored scaffolds	Physical positions (bp)	InDels (*Kabuli* reference genome-CDC Frontier/PII)	Forward primers (5'-3')	Reverse primers (5'-3')	Anneling temperature (0C)	Expected amplified product size (bp)	Structural annotation		Functional annotation	
								Sequence components of *kabuli* genome	*Kabuli* gene accession IDs	NCBI-KOG	TFs
Ca4_P3_13643579	Ca4	13643579	GTTTT/GTTT	ATAGTTGTTGAAGGGG CACG	TGAGCAAATTTCCATC AACTT	60.0	657	INTERGENIC	--	--	--
Ca4_P3_13643715	Ca4	13643715	GTT/GTTT	ATAGTTGTTGAAGGGG CACG	GGGTGAAGAATGTATG CCAAA	60.0	798	INTERGENIC	--	--	--
Ca4_P3_13643836	Ca4	13643836	ATTTTTT/ATTTTTTT	ATAGTTGTTGAAGGGG CACG	GGGTGAAGAATGTATG CCAAA	60.0	798	INTERGENIC	--	--	--
Ca4_P3_13655282	Ca4	13655282	TTATA/TTATAGGATATA	AATTCACAAGGTCCAGT GGC	GGGCATGATTTTTCCC CTAT	60.0	790	INTERGENIC	--	--	--
Ca4_P3_13657394	Ca4	13657394	CAAAAAAA/CAAAAAAAA	TCGTTTTTCAACCATCA GTCA	TCTCCCAATGTGATTG CTCA	59.2	823	INTERGENIC	--	--	--
Ca4_P3_13657824	Ca4	13657824	ATATT/AT	TGCATGCATGAATAGT GTTTGA	TCTCCCAATGTGATTG CTCA	60.1	395	INTERGENIC	--	--	--
Ca4_P3_13661362	Ca4	13661362	T/TATTTA	TTCCTTCGTAAATGGCC TTG	TTCATCTCCCGTCTGA GCTT	60.1	335	INTRON	Ca_04585	R	bHLH
Ca4_P3_13666161	Ca4	13666161	ACTG/ACTGCTG	CCCATTTCACCTCTTTG GAA	TTCCCGCAACAAATAG ATCC	59.9	840	CDS (large-effect mutations)	Ca_04586	Q	B3
Ca4_P3_13668027	Ca4	13668027	CAAATTAATTA/CAAATTAATTAAATTAA TTA	TCCAAGTTCCAAGGAT GAGC	TGTTGATGCGACTGGT TTTT	60.2	289	DRR	Ca_04586	Q	B3
Ca4_P3_13671186	Ca4	13671186	TCAA/T	CGTCCATTGTGGTTAT GCTC	TTCCTAAAAAGTTAAAC CGGCA	59.0	685	INTERGENIC	--	--	--

INDEL marker IDs	Chromosomes/ unanchored scaffolds	Physical positions (bp)	InDels (*Kabuli* reference genome-CDC Frontier/PII)	Forward primers (5'-3')	Reverse primers (5'-3')	Anneling temperature (0C)	Expected amplified product size (bp)	Structural annotation		Functional annotation	
								Sequence components of *kabuli* genome	*Kabuli* gene accession IDs	NCBI-KOG	TFs
Ca4_P3_13674173	Ca4	13674173	TAAAAAAAAAAAAAAA/TAAAAAAAAAAAA	AACCCCTTCGAGTTGTTCCT	TTGCTGACAGACACGAGACC	60.0	479	DRR	Ca_04587	Q	B3
Ca4_P3_13675043	Ca4	13675043	CAAA/CAAAAA	CGACGAATTCCCTTCAACAT	GTGGGGGATTGTGAGAGAAA	59.9	459	DRR	Ca_04587	Q	B3
Ca4_P3_13676728	Ca4	13676728	AATATAT/AATAT	CCACTTGTGGAAGCGTGAT	TGACAAAATGTTCATGGAAGAAA	59.7	775	DRR	Ca_04588	E	--
Ca4_P3_13678246	Ca4	13678246	C/CT	CCGACGAATTTCCTTCAAAA	TGTTCGAGTCGGTGAGACAG	60.0	583	DRR	Ca_04588	E	--
Ca4_P3_13682163	Ca4	13682163	TAAAAAAAAA/TAAAAAAAA	GGCGTCATAAGTAGGGTGGA	CACCTTCTTATTTTCTCTCCGA	60.0	479	INTERGENIC	--	--	--
Ca4_P3_13687679	Ca4	13687679	CAGTTTAGTTTAGTTTAGTTTA/CAGTTTAGTTTAGTTTA	TGGACTCGGTATCAACCACA	GACGAGCTGCGGTATTCTTC	60.0	248	INTRON	Ca_04589	--	--
Ca4_P3_13692284	Ca4	13692284	ATTTTTTTTT/ATTTTTTTT	TATTGGATGTGTTGGAGCGA	CGGAATATTATGTCCCGAGTTC	60.1	175	INTERGENIC	--	--	--
Ca4_P3_13693484	Ca4	13693484	ATTTTTTTTT/ATTTTTTTTTT	TCGAAGCTAGATCGGGAAGA	TGAAGGACAGTGTCGGTGAA	60.1	974	DRR	Ca_04590	--	--
Ca4_P3_13700242	Ca4	13700242	GCC/GC	ATTTGTTTCCCAAACCCTCC	CCTGTTGGTGCCCTTTAGAA	60.0	863	INTRON	Ca_04591	--	--
Ca4_P3_13700589	Ca4	13700589	GATATATATATATA/GATATATATATATATA	CCAAATCCCAAATCAGCATC	GCCCATACGCCTCTTAACAA	60.3	825	INTRON	Ca_04591	--	--

INDEL marker IDs	Chromosomes/ unanchored scaffolds	Physical positions (bp)	InDels (*Kabuli* reference genome-CDC Frontier/PII)	Forward primers (5'-3')	Reverse primers (5'-3')	Anneling temperature (0C)	Expected amplified product size (bp)	Structural annotation		Functional annotation	
								Sequence components of *kabuli* genome	*Kabuli* gene accession IDs	NCBI-KOG	TFs
Ca4_P3_13700936	Ca4	13700936	AT/ATT	TCCGATAGAAATGGGACAGC	TTTTGCCTTGTAACTTGGGG	60.0	463	INTRON	Ca_04591	--	--
Ca4_P3_13706359	Ca4	13706359	TAAAAAAA/TAAAAAA	GATGGCTTAGTGGTGGAGGA	TTGAAGGCTATAAGCCCCCT	60.1	479	INTRON	Ca_04592	R	--
Ca4_P3_13708395	Ca4	13708395	CAAA/CAA	TTGGAGGACCAAAACTGGAG	GGCTTGGACAATTTTGTGTG	60.1	795	INTRON	Ca_04592	R	--
Ca4_P3_13710634	Ca4	13710634	CAAAAAAAA/CAAAAAAA	GCTCCAAATGTTGGACCCTA	GGGTTAAATGCTTGCGTGTT	59.9	855	DRR	Ca_04593	R	--
Ca4_P3_13712060	Ca4	13712060	AAAA/AAAAGAATTTCAAA	TAGCAAGAGATGTTGCGGTG	AGGAAATGAAGGAAACGGGT	60.0	898	INTERGENIC	--	--	--
Ca4_P3_13712227	Ca4	13712227	GCC/GC	ATTTGGCCCCCTATCAAAAT	AGGAAATGAAGGAAACGGGT	59.5	668	INTERGENIC	--	--	--
Ca4_P3_13721306	Ca4	13721306	CATATATATATAT/CATATATATAT	TCCTTGAAAAGGAAGCCAAA	TGGTTCAAGTTCCATGTCCA	59.8	190	DRR	Ca_04596	--	B3
Ca4_P3_13743876	Ca4	13743876	GAA/GAAGCATATGCAA	CTGGTTCATGCCCCTACAAT	TCAAAGCACTAGCCACATGC	59.8	740	DRR	Ca_04598	R	MYB_related
Ca4_P3_13749552	Ca4	13749552	AA/AAGA	TTGACTTCTCCTTGGTTGCC	TTTTTGTTGACATGAGGCCA	60.2	758	INTRON	Ca_04599	R	MIKC
Ca4_P3_13750930	Ca4	13750930	TTATA/TTATATATA	TGCCTTTTATAATTGATTGTAGGTTG	TCGCTCACTCTCTTACCTCCA	59.7	908	INTERGENIC	--	--	--

INDEL marker IDs	Chromosomes/ unanchored scaffolds	Physical positions (bp)	InDels (*Kabuli* reference genome-CDC Frontier/PII)	Forward primers (5'-3')	Reverse primers (5'-3')	Anneling temperature (0C)	Expected amplified product size (bp)	Structural annotation		Functional annotation	
								Sequence components of *kabuli* genome	*Kabuli* gene accession IDs	NCBI-KOG	TFs
Ca4_P3_13751977	Ca4	13751977	TAAAAAAAA/TAAAAAAAAAA	TTTTGCATCTCATCACG AGC	CCTGATTGCAAGTTCA TCAAA	60.0	890	INTERGENIC	--	--	--
Ca4_P3_13759058	Ca4	13759058	GAAAAAAAA/GAAAAAAAAA	TTCTGCATACCCTGCAC AAG	AGGCATCATGGTTTCG TTTC	59.9	695	INTRON	Ca_04600	--	NF-YB
Ca4_P3_13770606	Ca4	13770606	TCACA/T	AAGCACCCATCCTGAA ATTG	TTTCACCGCGATAAAA ATCA	59.9	586	INTERGENIC	--	--	--
Ca4_P3_13770963	Ca4	13770963	TC/T	CCAACAATTAGGAGCC CTTTT	GGCCACTGAGCCACTG TATT	59.5	681	INTERGENIC	--	--	--
Ca4_P3_13771017	Ca4	13771017	TTAA/T	CCAACAATTAGGAGCC CTTTT	GGCCACTGAGCCACTG TATT	59.5	681	INTERGENIC	--	--	--
Ca4_P3_13775947	Ca4	13775947	TAA/TAAATTAAAAA	CCACGTGGGATGAAGA GATT	TACAGTGCATTTTGTG GGGA	59.9	616	INTERGENIC	--	--	--
Ca4_P3_13776143	Ca4	13776143	A/AG	AACTGTGCGGCCTAAA TAGC	GTATGGAGCCCAATTT GGAA	59.4	201	INTERGENIC	--	--	--
Ca4_P3_13776936	Ca4	13776936	GTATATATATATA/GTATATATATA	TGTGTCTGCCCATGAG TGTT	ATATGCGGTTTCATCC TTGC	60.2	301	INTERGENIC	--	--	--
Ca4_P3_13777793	Ca4	13777793	CAGA/CAGAGA	ATTCCCTTTAACCCCCA ATG	GAAAGGCTGCAGTGAT GGAT	59.9	741	INTERGENIC	--	--	--
Ca4_P3_13779676	Ca4	13779676	CTTTTTTTTTT/CTTTTTTTTTTTTTTTT	CATTTTTCTCGAAATGG GGA	ACCAGCCACATAAGCC AAAC	59.9	685	INTRON	Ca_04602	I	--

INDEL marker IDs	Chromosomes/ unanchored scaffolds	Physical positions (bp)	InDels (*Kabuli* reference genome-CDC Frontier/PII)	Forward primers (5'-3')	Reverse primers (5'-3')	Anneling temperature (0C)	Expected amplified product size (bp)	Structural annotation		Functional annotation	
								Sequence components of *kabuli* genome	*Kabuli* gene accession IDs	NCBI-KOG	TFs
Ca4_P3_13789143	Ca4	13789143	CAA/CAAA	ATCCACCACTCAAAGTTGGC	TTGGTACGAAACATCCATCAA	60.0	517	DRR	Ca_04603	Q	--
Ca4_P3_13792182	Ca4	13792182	T/TA	GGCAACATAGATCCTCTCACC	GCTCACTCCGTCAGGTTAGG	58.6	719	INTRON	Ca_04604	Q	--
Ca4_P3_13792300	Ca4	13792300	T/TTA	GGCAACATAGATCCTCTCACC	GCTCACTCCGTCAGGTTAGG	58.6	719	INTRON	Ca_04604	Q	--
Ca4_P3_13792402	Ca4	13792402	ATTAAAATTTTAAAATTTT/ATTAAAATTTT	GGCAACATAGATCCTCTCACC	GCTCACTCCGTCAGGTTAGG	58.6	719	INTRON	Ca_04604	Q	--
Ca4_P3_13793997	Ca4	13793997	GTA/G	CCAGCACCCGTTTTTAATGT	AAATGTTGGGCTAGACATGGA	59.9	360	INTERGENIC	--	--	--
Ca4_P3_13796561	Ca4	13796561	TAAAAA/TAAAAAA	CCGAAACAGGGTGGAAAGTA	ATCGACGATTATGCCCTGTC	60.0	913	INTERGENIC	--	--	--
Ca4_P3_13798999	Ca4	13798999	CTAATTAAT/CTAAT	AATTTCGGAAACCTCGTGTG	AACTCCATTGTGGATGACCC	60.0	846	DRR	Ca_04605	R	--
Ca4_P3_13807289	Ca4	13807289	TAAAAAA/TAAAAA	ACAAGAAAAGCACAGGCACC	TGATCAAAGCCGTCATGTCT	60.3	576	INTERGENIC	--	--	--
Ca4_P3_13807671	Ca4	13807671	CAAAAAAA/CAAAAAA	GCCACAACTGGGTAGGTTTT	GGTTTCAACTCGAGTCGTCG	59.0	885	INTERGENIC	--	--	--
Ca4_P3_13808256	Ca4	13808256	TT/TATATTCT	TCGAGTTGAAACCCGAGAAA	CGGAGAAGTGAACGTGGAAT	60.7	525	INTERGENIC	--	--	--

INDEL marker IDs	Chromosomes/ unanchored scaffolds	Physical positions (bp)	InDels (*Kabuli* reference genome-CDC Frontier/PII)	Forward primers (5'-3')	Reverse primers (5'-3')	Anneling temperature (0C)	Expected amplified product size (bp)	Structural annotation		Functional annotation	
								Sequence components of *kabuli* genome	*Kabuli* gene accession IDs	NCBI-KOG	TFs
Ca4_P3_13808549	Ca4	13808549	ATTTTTTTTT/ATTTTTTTT	TCGAGTTGAAACCCGAGAAA	CGGAGAAGTGAACGTGGAAT	60.7	525	INTERGENIC	--	--	--
Ca4_P3_13810887	Ca4	13810887	TTGA/TTGAATGA	GATTAGGTGTTGCCTGACGC	GCTGAGATGAAGGCAAAAATG	60.7	281	INTERGENIC	--	--	--
Ca4_P3_13825682	Ca4	13825682	ATTTTTTT/ATTTTTTTT	AACTCAGATTTCAACTTCGGAAA	TCAAAGTGCAACAACCACCT	59.3	595	INTERGENIC	--	--	--
Ca4_P3_13826117	Ca4	13826117	ATTTTT/ATTTTTT	AGGTGGTTGTTGCACTTTGA	CAAATTTGTTCCGCTTCACA	59.2	474	INTERGENIC	--	--	--
Ca4_P3_13826688	Ca4	13826688	ATT/AT	TGGAACTATGTGAAGCGGAA	GTTGAGGAAAACGCAGGAAG	59.3	898	INTERGENIC	--	--	--
Ca4_P3_13827539	Ca4	13827539	GGTTCTGTT/GGTTCTGTTCTGTT	CTTCCTGCGTTTTCCTCAAC	GGAGTTTCGGTATGCTTTGC	59.9	635	INTERGENIC	--	--	--
Ca4_P3_13827753	Ca4	13827753	GTATATATATATA/GTATATATATATATA	CATGTCAGCAAAGCATACCG	CAATAGTGTTTGCACGTGGG	60.3	542	INTERGENIC	--	--	--
Ca4_P3_13828480	Ca4	13828480	G/GT	GTGGGGTCAAGGCTTAACAA	GCCTAGGCCATGTCCAATAA	60.0	148	INTERGENIC	--	--	--
Ca4_P3_13828607	Ca4	13828607	TG/T	GTGGGGTCAAGGCTTAACAA	ACACGTCACTTGCACACACA	60.0	498	INTERGENIC	--	--	--
Ca4_P3_13829467	Ca4	13829467	AT/ATT	TGAGAATTTGCATGCCTCAC	AGCGAAGTGCTAGCAACACA	59.8	671	INTERGENIC	--	--	--

INDEL marker IDs	Chromosomes/ unanchored scaffolds	Physical positions (bp)	InDels (*Kabuli* reference genome-CDC Frontier/PII)	Forward primers (5'-3')	Reverse primers (5'-3')	Anneling temperature (0C)	Expected amplified product size (bp)	Structural annotation		Functional annotation	
								Sequence components of *kabuli* genome	*Kabuli* gene accession IDs	NCBI-KOG	TFs
Ca4_P3_13830386	Ca4	13830386	ACGATGAGTCGATGA/ACGATGA	CGCCTAAGTTAAGCCAATCC	AGAACGGTGGTCATACGAGG	58.9	661	INTERGENIC	--	--	--
Ca4_P3_13836175	Ca4	13836175	GAAAAAAAAAA/GAAAAAAAAA	ATGCTTTATCTTTGGCCCCT	TTTGGTTTCCTTGCTTGTCC	59.9	719	DRR	Ca_04608	--	--
Ca4_P3_13836254	Ca4	13836254	TGAAAGAA/TGAAAGAAGAAAGAA	CTGGTCCAACACACCAACAC	TTTGGTTTCCTTGCTTGTCC	59.9	459	DRR	Ca_04608	--	--
Ca4_P3_13839771	Ca4	13839771	CTGCTGCTG/CTGCTGCTGTTGCTGCTG	CGCTGGAGAAGTTTCACCTC	TTCACAGATTCAAACGCAGC	60.0	526	CDS (large-effect mutations)	Ca_04608	--	--
Ca4_P3_13853886	Ca4	13853886	CAAAAAA/CAAAAAAA	TGGATTCATGCACGTTTTTC	CCTTCTTTCCCACAATGTTGA	59.5	770	INTERGENIC	--	--	--
Ca4_P3_13854087	Ca4	13854087	TAAAAAAAAA/TAAAAAAA	CGATCACTGACCATTATATGCAC	CCTTCTTTCCCACAATGTTGA	59.4	611	INTERGENIC	--	--	--
Ca4_P3_13856631	Ca4	13856631	TAAA/TAA	AACACACCAATTTCGAACCC	CCAACCCAATAACTCCTATCG	59.7	388	INTERGENIC	--	--	--
Ca4_P3_13856659	Ca4	13856659	TAAAAAAAAAAA/TAAAAAAAAAA	AACACACCAATTTCGAACCC	CCAACCCAATAACTCCTATCG	59.7	388	INTERGENIC	--	--	--
Ca4_P3_13856903	Ca4	13856903	TCAATTGAA/T	AACACACCAATTTCGAACCC	CCAACCCAATAACTCCTATCG	59.7	388	INTERGENIC	--	--	--
Ca4_P3_13857193	Ca4	13857193	ATTTTTTTTT/ATTTTTTTT	CGATAGGAGTTATTGGGTTGG	GAAAACAGCCGAGGAACAGA	58.5	717	INTERGENIC	--	--	--

INDEL marker IDs	Chromosomes/ unanchored scaffolds	Physical positions (bp)	InDels (*Kabuli* reference genome-CDC Frontier/PII)	Forward primers (5'-3')	Reverse primers (5'-3')	Anneling temperature (0C)	Expected amplified product size (bp)	Structural annotation		Functional annotation	
								Sequence components of *kabuli* genome	*Kabuli* gene accession IDs	NCBI-KOG	TFs
Ca4_P3_13857943	Ca4	13857943	T/TG	TGTTTTCACTGTCCGTGCAT	GCAATGGGAGCAACGTAAAT	60.2	696	INTERGENIC	--	--	--
Ca4_P3_13861025	Ca4	13861025	GAAAAA/GAAA	CAATCGGACATGTATTCCCC	AATTTGCCCTCACAATGGTC	60.0	421	DRR	Ca_04609	R	--
Ca4_P3_13865867	Ca4	13865867	ACAC/A	TGACAAACGGATCCTCAAAG	GTCTCCAGCAAGTGTCGTGA	58.7	548	INTRON	Ca_04610	R	--
Ca4_P3_13904337	Ca4	13904337	TAAAAAAAA/TAAAAAAA	GCCACTTTTCATCGATGGTT	TGAAAATCGGGACTTTGGTC	59.9	218	INTERGENIC	--	--	--
Ca4_P3_13905983	Ca4	13905983	GACACACACA/GACACACA	TGTATGCGGCTGTTGAGAAG	CGGGTTGAGGATTGATCTGT	60.0	449	INTERGENIC	--	--	--
Ca4_P3_13908536	Ca4	13908536	TC/T	CTCCTCCGATGTCTGGAAAA	CGCGGTTGCTTCAATTCTAT	60.2	232	INTERGENIC	--	--	--
Ca4_P3_13909254	Ca4	13909254	CA/CAA	CATCACGATTTGACGAGTTGA	AAAAACATGACTCCAAACACCA	59.7	636	INTERGENIC	--	--	--
Ca4_P3_1405750	Ca4	1405750	CATATATATATATATATATATA/CATATATATATATATATATA	GGCCACGTGGAAGTAACTGT	ATTTGGTGCCAAAGGAATTG	60.0	432	INTERGENIC	--	--	--
Ca4_P3_14238993	Ca4	14238993	TTATATATATATA/TTATATATATA	TGCTTTACACAAGCAAGCCA	CGACAGAGACGAGGGAAGTC	60.6	170	DRR	Ca_04659	K	M-type
Ca4_P3_14280610	Ca4	14280610	AATATATATATATATATATATATATAT/AATATATATATATATATATATATATATAT	GTCTAAGGCTTGCTACCCCC	AGAGGCTTCTCGTTGGGTTT	60.1	596	INTERGENIC	--	--	--

INDEL marker IDs	Chromosomes/ unanchored scaffolds	Physical positions (bp)	InDels (*Kabuli* reference genome-CDC Frontier/PII)	Forward primers (5'-3')	Reverse primers (5'-3')	Anneling temperature (0C)	Expected amplified product size (bp)	Structural annotation		Functional annotation	
								Sequence components of *kabuli* genome	*Kabuli* gene accession IDs	NCBI-KOG	TFs
Ca4_P3_1437324	Ca4	1437324	ATTTTTTTTTT/ATTTTTTTTT	TTCGAAATTTGTGGTGCAAG	GATGGCAAGAGTCAATTGAAGA	59.7	827	DRR	Ca_07772	D	bHLH
Ca4_P3_14397300	Ca4	14397300	GATATATATATATATATATATGTATATATATATATA/GATATATATATATATATATATATATATGTATATATATATATA	TAAATGCATGCTCCCCTGAT	AACACATCCGGGATCCATTA	60.4	273	INTERGENIC	--	--	--
Ca4_P3_14398861	Ca4	14398861	TCAACAACAA/TCAACAACAACAA	GGCAAGTACAAAGGGCATGT	GTTGCTGGAAGGCAGAGTTC	60.0	426	CDS (large-effect mutations)	Ca_05664	--	--
Ca4_P3_14405624	Ca4	14405624	ATGTTGTT/ATGTTGTTGTT	CGGTGGTTGCTCTCTCTTTC	GGGGTACAATGTGGAACCAA	60.0	440	DRR	Ca_05663	E	MYB
Ca4_P3_14405755	Ca4	14405755	ATCTCTCTCT/ATCTCTCT	ATGTGCATGCCAAGTTGTGT	ACCTATAAGCCCTTTGCGCT	60.0	963	DRR	Ca_05663	E	MYB
Ca4_P3_14406133	Ca4	14406133	CAAA/CAAAA	TTGGTTCCACATTGTACCCC	CCATTGGTTGGGATATGGAG	60.5	892	DRR	Ca_05663	E	MYB
Ca4_P3_14539491	Ca4	14539491	TTATATATATATATATATATATATATATAT/TTATATATATATATATATATATATATAT	TTCTTTACAGGGACATGATTGA	CTTGTGCTCATGCAAGCAAT	57.2	605	DRR	Ca_05648	--	--
Ca4_P3_14558930	Ca4	14558930	TATAATAATAATAATAATAATAAT/TATAATAATAATAATAATAATAAT	AAACTGTGTTGCCCGGATAG	GCCGGAATCAAAATTCAAGA	60.0	384	INTERGENIC	--	--	--
Ca4_P3_14625891	Ca4	14625891	TTATATATATATATATAT/TTATATATATATATATAT	TGCTTGCTTGTGCTCAGACT	AAAAAGCATCATTTTTCACTGGA	59.9	415	INTERGENIC	--	--	--
Ca4_P3_14665183	Ca4	14665183	TTATATATATATATATAT/TTATATATATATAT	CGTGCCCTTTTATTCTTAAGGT	TGTTTATGCGTGTGGCATTT	58.8	735	INTERGENIC	--	--	--

INDEL marker IDs	Chromosomes/ unanchored scaffolds	Physical positions (bp)	InDels (*Kabuli* reference genome-CDC Frontier/PII)	Forward primers (5'-3')	Reverse primers (5'-3')	Anneling temperature (0C)	Expected amplified product size (bp)	Structural annotation		Functional annotation	
								Sequence components of *kabuli* genome	*Kabuli* gene accession IDs	NCBI-KOG	TFs
Ca4_P3_14677950	Ca4	14677950	CAAAAAAA/CAAAAAAAA	TGTTGGGCAAAATAAATTGAGA	CAAACCTTACCACCGTCTTG	59.5	424	INTERGENIC	--	--	--
Ca4_P3_14836473	Ca4	14836473	GAAAAAAAAAAA/GAAAAAAAAAAAA	TGAGACACGCTACAGCCATC	ATTCCCATTCCACCCTTTTC	60.0	241	INTRON	Ca_05618	--	--
Ca4_P3_1491046	Ca4	1491046	AATATATATATATATATATAT/AATATATATATATATATATATAT	GGCTGAATTGATTAAATTTGTCG	GAGTTGCAAGGGGACCAATA	59.9	584	DRR	Ca_07779	--	WRKY
Ca4_P3_14928511	Ca4	14928511	CATAT/CATATAT	CGGGACATGCTATTAGGTGC	AAATTTGCCAATCCTTGTCG	60.5	246	INTERGENIC	--	--	--
Ca4_P3_14967549	Ca4	14967549	GTAATAATAATAATAATAATAATAATAATAA/GTAATAATAATAATAATAATAATAATAATAATAATAA	CAAAGTGCTTTTGTACCCCC	CCGTTTGTTTTGGGACATCT	59.5	899	INTERGENIC	--	--	--
Ca4_P3_14997131	Ca4	14997131	A/AC	CTAACGGGTACCGGTCAAAA	AGCCATTCAGTTGTCGTGTG	59.9	311	INTERGENIC	--	--	--
Ca4_P3_15012755	Ca4	15012755	AATATATATATATATA/AATATATATATATATATATA	TACTCCCAAATGGCGACTTC	GACATTTCGAACTTGAATTTCCT	60.1	322	INTERGENIC	--	--	--
Ca4_P3_15014116	Ca4	15014116	TGGGGG/TGGGGGG	CTGCGTTGATGGACTCAGAA	ATCCGACACATTGCACAAAA	60.0	325	INTERGENIC	--	--	--
Ca4_P3_15048181	Ca4	15048181	TA/TAA	CAAAAGCTGTAACACCCCGT	AATCGATTGATTCAGCCCAG	60.0	528	INTERGENIC	--	--	--
Ca4_P3_15095206	Ca4	15095206	TTATATATATATATATATATAT/TTATATATATATATATATATATAT	TGACCGCTAAGTTGTTGTGC	TTTTACCACTGCACACGCAT	59.9	359	DRR	Ca_05591	D	LBD

INDEL marker IDs	Chromosomes/ unanchored scaffolds	Physical positions (bp)	InDels (*Kabuli* reference genome-CDC Frontier/PII)	Forward primers (5'-3')	Reverse primers (5'-3')	Anneling temperature (0C)	Expected amplified product size (bp)	Structural annotation		Functional annotation	
								Sequence components of *kabuli* genome	*Kabuli* gene accession IDs	NCBI-KOG	TFs
Ca4_P3_15392307	Ca4	15392307	TA/TACACTAA	GGTCTCACCAACACTG TGGA	CGGTTTTGGCGTCTTT ATGT	59.6	428	DRR	Ca_05557	R	B3
Ca4_P3_15438623	Ca4	15438623	ATTTTTTTT/ATTTTTTTTT	CCGTGTTACAAAACTTC ACCG	GACCATCAATTTCACA GGCA	60.4	682	INTERGENIC	--	--	--
Ca4_P3_15455600	Ca4	15455600	GTTTTTTTTTT/GTTTTTTTTTTT	TTTCCCTCGATTCCCTT TTT	CATCTTGCCAAGTTTG GGTT	59.9	592	INTRON	Ca_05551	A	bHLH
Ca4_P3_15465052	Ca4	15465052	T/TG	GCAACTCATTCGTCCC ATTT	GGAAACGAAAAATTTG CAGC	59.9	712	INTERGENIC	--	--	--
Ca4_P3_15509140	Ca4	15509140	TAAAAAAAAA/TAAAAAAAAAA	GGTCCGTAATCAAAGG CAAA	CCACGAAGAATACTTC CCCA	59.9	525	INTERGENIC	--	--	--
Ca4_P3_1559727	Ca4	1559727	G/GT	CACCATGGCATTTGAA CTTG	GGAGATTGCACTGTTG CAGA	60.0	741	INTRON	Ca_07784	--	--
Ca4_P3_15607878	Ca4	15607878	GTTTTTTTTTTT/GTTTTTTTTTTTTT	AGAGATGAAACCTGAG AAATTCA	TGTTTTTCCACTAACCT AAAACG	57.1	681	INTERGENIC	--	--	--
Ca4_P3_15643039	Ca4	15643039	T/TTACTGTAGGGA	TGCACCGACAAACTGA AGTC	ACGCGTGAGTTTGCTC ACTA	59.9	592	INTERGENIC	--	--	--
Ca4_P3_15780348	Ca4	15780348	TACACACACA/TACACACA	CAACTTTAAAAAGGACC AAAATGAA	TTGTGCCACGACCAAT TAAA	59.8	537	INTERGENIC	--	--	--
Ca4_P3_15786146	Ca4	15786146	TA/T	AAAGAAGGAAGGGAAA TCGG	CTGGTGATGGCGTTAA TTTG	59.5	533	INTERGENIC	--	--	--

INDEL marker IDs	Chromosomes/ unanchored scaffolds	Physical positions (bp)	InDels (*Kabuli* reference genome-CDC Frontier/PII)	Forward primers (5'-3')	Reverse primers (5'-3')	Anneling temperature (0C)	Expected amplified product size (bp)	Structural annotation		Functional annotation	
								Sequence components of *kabuli* genome	*Kabuli* gene accession IDs	NCBI-KOG	TFs
Ca4_P3_15884633	Ca4	15884633	GAAAAAAAA/GAAAAAAAAA	TTGTGTGGCTTAGAAGGGCT	CCAGGCCGAGTAGGTGTCTA	59.9	449	INTERGENIC	--	--	--
Ca4_P3_15936441	Ca4	15936441	TCC/TC	AATTTTTCCCCACAACCCAT	CACCAAAAATCTCCCTCCAA	60.3	596	INTERGENIC	--	--	--
Ca4_P3_15988705	Ca4	15988705	CTAT/CT	GGTGCCAACCTCATTGAAAC	TGAAACACTTGTGCGCTGTT	60.4	830	INTERGENIC	--	--	--
Ca4_P3_15989879	Ca4	15989879	TTAT/TT	TCATACACTTGTTTGATACACACTTTC	AGCTCGTTTGTTGGACAATCT	58.7	772	INTERGENIC	--	--	--
Ca4_P3_15998419	Ca4	15998419	G/GCTTAC	TTTTTCCAAACCAGGAAACG	CCAATTACCACAAGCTTCCAA	59.9	300	INTERGENIC	--	--	--
Ca4_P3_16051344	Ca4	16051344	CTG/CTGTG	GAGGCATGACTTCAACAGCA	TGGAAAAGGCACCTGTCAGT	60.0	600	INTRON	Ca_05504	J	bHLH
Ca4_P3_16065224	Ca4	16065224	CCTCTC/CCTC	TCAATCACCTCACCTCACCT	GGCGACTTCCATTGGACTAA	58.1	310	INTERGENIC	--	--	--
Ca4_P3_16065708	Ca4	16065708	ACATCAT/ACAT	TTAGTCCAATGGAAGTCGCC	ATGCAAAACCCTGCTTCATC	60.1	706	INTERGENIC	--	--	--
Ca4_P3_16067505	Ca4	16067505	CAAAAAAAA/CAAAAAAA	GGCTAAATTAAATCACTGGTCCC	TACATCTGCGGCCCTTTAAC	60.1	572	INTERGENIC	--	--	--
Ca4_P3_16072660	Ca4	16072660	ATATTAT/ATATTATTAT	GTCACAGCTCTGATGCCAAA	GCGATACGGAGTGGACATCT	60.0	787	INTERGENIC	--	--	--

INDEL marker IDs	Chromosomes/ unanchored scaffolds	Physical positions (bp)	InDels (*Kabuli* reference genome-CDC Frontier/PII)	Forward primers (5'-3')	Reverse primers (5'-3')	Anneling temperature (0C)	Expected amplified product size (bp)	Structural annotation		Functional annotation	
								Sequence components of *kabuli* genome	*Kabuli* gene accession IDs	NCBI-KOG	TFs
Ca4_P3_16079766	Ca4	16079766	TAAAAAAAAAA/TAAAAAAAAA	TGGCTACTTAAAGGGCCAAA	GAAAGGGATCACGTGAGGAA	59.7	916	INTERGENIC	--	--	--
Ca4_P3_16080327	Ca4	16080327	CTTTTTTTTTTTTT/CTTTTTTTTTTT	TTCCTCACGTGATCCCTTTC	ACAATTGCTCCAACGGTCAT	60.1	567	INTERGENIC	--	--	--
Ca4_P3_16099117	Ca4	16099117	TAAAAAAA/TAAAAAA	TTCAATTCAAAATATAAACGGCA	AGGTTGACGATTGGTGTTCC	58.6	398	DRR	Ca_05500	--	--
Ca4_P3_16146480	Ca4	16146480	CT/C	AAAGAAGCAGAGGAAGCGTG	GCAAACGTATGATGGGGACT	59.8	337	DRR	Ca_05497	I	bHLH
Ca4_P3_1615805	Ca4	1615805	AATATATATATATATATATATATATATATATATATATATATA/AATATATATATATATATATATATATATATATATATATATATATA	CGGTTTGCATAATGGACAGA	AAGACATCCGATCAGCTTGC	59.5	731	INTERGENIC	--	--	--
Ca4_P3_16225256	Ca4	16225256	TTTATTATTATTATTATTAT/TTTATTATTATTATTAT	GGTGAGGTTGATGCTTTGGT	AGAAAACGATGGTACGGTGG	60.0	908	DRR	Ca_05490	--	--
Ca4_P3_16238878	Ca4	16238878	TTATATATATATATATATATATATA/TTATATATATATATATATATATA	TGTTGTTGCGTTATTCCAGC	CACCCATCGTTTCCCTCTTA	59.7	555	INTERGENIC	--	--	--
Ca4_P3_16278961	Ca4	16278961	CACCACTACCACTACCACTACCA/CACCACTACCACTACCACTACCACTACCA	TCCCGTGAAAGTTTTCCATC	ACACTCACACTCCCACTCCC	59.9	778	CDS (large-effect mutations)	Ca_05487	K	CO-like
Ca4_P3_16298566	Ca4	16298566	AATATATATATATATATATATATATATATATATATATA/AATATATATATATATATATATATATATATATATATATATATATATATA	AAAAACTGAGCACACAATCATAAA	TCCCATTAAGACCCATCAGC	57.9	562	INTERGENIC	--	--	--
Ca4_P3_16305794	Ca4	16305794	ATTTTTTTTTTTT/ATTTTTTTTTTT	ACATGGTGTGAAAATCGGGT	CGGGGTATTACGTATTTGCG	60.1	544	INTERGENIC	--	--	--

INDEL marker IDs	Chromosomes/ unanchored scaffolds	Physical positions (bp)	InDels (*Kabuli* reference genome-CDC Frontier/PII)	Forward primers (5'-3')	Reverse primers (5'-3')	Anneling temperature (0C)	Expected amplified product size (bp)	Structural annotation		Functional annotation	
								Sequence components of *kabuli* genome	*Kabuli* gene accession IDs	NCBI-KOG	TFs
Ca4_P3_16481759	Ca4	16481759	CA/CAAA	TCGAGACACCAACACCGTAA	AATTCGTCAAACCCACAACC	60.2	732	INTERGENIC	--	--	--
Ca4_P3_16545258	Ca4	16545258	AATATATATATATATATATAT/AATATATATATATATATAT	TTTGGGCTTACTTTTGTGAGAA	CAAAGAGTTACAACGATGCGA	58.9	313	INTERGENIC	--	--	--
Ca4_P3_16605966	Ca4	16605966	TTATA/TTA	GGAATCAAATTCGAAGCCAA	CCTCTGACTCCGACGCTAAC	60.0	364	INTERGENIC	--	--	--
Ca4_P3_16711599	Ca4	16711599	TTATATATATATATATATATATATATAT/TTATATATATATATATATATATATATATATATATATAT	GCCCTCAATACGGTACCTCAC	TATCGTTGCTTGCCAACTCA	60.8	726	INTERGENIC	--	--	--
Ca4_P3_16728462	Ca4	16728462	TAAA/TAAAA	CATGGGGATGCTGCTATTTT	CAACTTCCTCTTTTGGCACA	59.9	388	DRR	Ca_05454	O	MYB
Ca4_P3_16729722	Ca4	16729722	CAAAAAAAA/CAAAAAAAAA	AATTATCATCGTCGCGTTCC	TGATTTTTCACCTTCCTGCC	59.9	467	DRR	Ca_05453	--	--
Ca4_P3_1677374	Ca4	1677374	TAAAAAAA/TAAAAA	TCCAAACTAGTCCGACGTGA	AGCTGCTGGAGGAGTTGGTA	59.3	450	INTRON	Ca_07795	--	--
Ca4_P3_16776651	Ca4	16776651	TAAAAAAAAAAAA/TAAAAAAAAAAA	CGCTTTTTGGTGAGGACAGT	AGAACACAAAGGGCACCAAC	60.3	553	DRR	Ca_05447	U	MIKC
Ca4_P3_16793556	Ca4	16793556	AG/AGG	ACACTTGCTATGAAACGGGG	ACGCTTTGCAAGGTCTGAAT	60.0	590	INTERGENIC	--	--	--
Ca4_P3_16826129	Ca4	16826129	A/AAATT	TTTTATCCACCAAAAATTCAAAAA	TGATTTTTCAGGAGGCCACT	58.9	521	DRR	Ca_05439	--	--

INDEL marker IDs	Chromosomes/ unanchored scaffolds	Physical positions (bp)	InDels (*Kabuli* reference genome-CDC Frontier/PII)	Forward primers (5'-3')	Reverse primers (5'-3')	Anneling temperature (0C)	Expected amplified product size (bp)	Structural annotation		Functional annotation	
								Sequence components of *kabuli* genome	*Kabuli* gene accession IDs	NCBI-KOG	TFs
Ca4_P3_16827901	Ca4	16827901	AATATTGATTATA/AATATTGATTATATTGATTATA	ACGCCAACATTTGTGAGTGA	TCGGATTCACTTAGTTGCACA	60.2	853	INTERGENIC	--	--	--
Ca4_P3_16846815	Ca4	16846815	CACCTTCTTTT/CACCTTCTTTTACCTTCTTTT	ACATGTGCCTTGGGACTAGC	GCGAAAAGTCGAAGATCCTG	60.1	571	CDS (FRAME SHIFT)	Ca_05437	--	--
Ca4_P3_16864661	Ca4	16864661	ATTTTTT/ATTTT	ACACTTTACGTCGGTGGGAG	CGCATGCAAGAATAACTCCA	60.0	424	INTERGENIC	--	--	--
Ca4_P3_16933042	Ca4	16933042	TAAAAAAAAAA/TAAAAAAAAA	CCAAATAAACTACTTTTGTGAGACGA	CACACTCTGCCTCCACTGAA	60.0	388	INTERGENIC	--	--	--
Ca4_P3_16943222	Ca4	16943222	TATGAATGAAT/TATGAATGAATGAAT	CTCAAGCTCAGTCCCAGTCC	TGCGTTTTGTTTGGTGTAGC	60.0	668	INTERGENIC	--	--	--
Ca4_P3_16960625	Ca4	16960625	GTT/GTTT	GCGAATTGCAGACTGAAACA	CTGGAAAATCCTCCTCCTCC	60.0	925	DRR	Ca_05428	--	--
Ca4_P3_16981152	Ca4	16981152	AATATATATATATATATATATATATATATA/AATATATATATATATATATATATATATATATATA	TTCGAGTAAAAGTCGGATGC	TTTAATTCAAAGGGTGACAAAAA	58.0	469	INTERGENIC	--	--	--
Ca4_P3_16990316	Ca4	16990316	ATTTTTTTTTT/ATTTTTTTTTTT	TTGCCCAAGATTTTCCATTT	TGAGGGCTTTGGGATGTAAC	59.4	501	INTRON	Ca_05426	U	--
Ca4_P3_17003120	Ca4	17003120	ATT/ATTTT	CCATGAGGTGTTAGCCCAAT	TCTCAACATGCTCTTGGCTG	59.8	207	INTERGENIC	--	--	--
Ca4_P3_17006862	Ca4	17006862	TTAT/TT	TCCTTCACAACTTCCTCGCT	CCGTCCAAGTTTGTCCTGTT	60.0	741	INTRON	Ca_05423	--	--

INDEL marker IDs	Chromosomes/ unanchored scaffolds	Physical positions (bp)	InDels (*Kabuli* reference genome-CDC Frontier/PII)	Forward primers (5'-3')	Reverse primers (5'-3')	Anneling temperature (0C)	Expected amplified product size (bp)	Structural annotation		Functional annotation	
								Sequence components of *kabuli* genome	*Kabuli* gene accession IDs	NCBI-KOG	TFs
Ca4_P3_17010005	Ca4	17010005	AAAAAATAATATC/AAAAAATAATATCATTTAAAAATAATATC	ACGGCCTAGCTTATTCCCAT	GTATGCAAATGGGTGCTGTG	60.0	690	DRR	Ca_05423	--	--
Ca4_P3_17010261	Ca4	17010261	GAGACA/GAGACAGACA	GTTAGCCTCACAGCACCCAT	GCTTCTAACTCGGCAAGTGG	60.1	342	DRR	Ca_05422	--	G2-like
Ca4_P3_17011310	Ca4	17011310	TTA/TTATA	TTTTGGAAGGGTGAGGACTG	TTTGGGCAAAATCTACGGTC	60.1	723	INTRON	Ca_05422	--	G2-like
Ca4_P3_17014729	Ca4	17014729	ATTCAAAA/ATTCAAAATTTTCAAAA	TTTTGTGGAATTGCCAATCA	AAAGCGAGGCAGTTGAGAAA	59.9	570	INTERGENIC	--	--	--
Ca4_P3_17024740	Ca4	17024740	T/TC	GGACATATTTGTCACAATCCCA	ACCAATTCCATTCCATTCCA	59.6	723	INTERGENIC	--	--	--
Ca4_P3_17030182	Ca4	17030182	TAA/TAAAA	AAGATTTTGTGTTTGAACCCTCA	TCTCGCCTTTTGTATCGTCA	59.9	426	DRR	Ca_05420	R	bHLH
Ca4_P3_17044153	Ca4	17044153	A/AT	AAACAATTTACGCGAATGCC	TAGTTCCAAGAGAAGGGCGA	60.0	665	INTERGENIC	--	--	--
Ca4_P3_17050258	Ca4	17050258	AGG/AGGG	GCGAAATGTTCTTCTCCTGC	TTTGGATAACCACCCCTACG	60.0	887	INTRON	Ca_05418	DA	--
Ca4_P3_17063499	Ca4	17063499	GTTTTTTTTTATAATTTTTTTTT/GTTTTTTTT	AAACGGATTGATTTGTTATTGAGA	TCACCGTATAAAGCTTTTCTTTTG	59.0	595	INTERGENIC	--	--	--
Ca4_P3_17064843	Ca4	17064843	TAAA/TAAAA	TAACAAAATGGCATGTCGGA	TGCCTTATGATGTTTGGATGA	59.9	774	INTERGENIC	--	--	--

INDEL marker IDs	Chromosomes/ unanchored scaffolds	Physical positions (bp)	InDels (*Kabuli* reference genome-CDC Frontier/PII)	Forward primers (5'-3')	Reverse primers (5'-3')	Anneling temperature (0C)	Expected amplified product size (bp)	Structural annotation		Functional annotation	
								Sequence components of *kabuli* genome	*Kabuli* gene accession IDs	NCBI-KOG	TFs
Ca4_P3_17065936	Ca4	17065936	CTTTTTTTTTTT/CTTTTTTTTT	TCAAATACAATTGTGGTTCATTCA	CCTCACCTTCTTCCATCACC	59.3	542	INTERGENIC	--	--	--
Ca4_P3_17067831	Ca4	17067831	TAAAAAAAA/TAAAAAAAAA	TTCACCAAACAGCCAGTATCC	GTGAGTCATGGGACGGTTCT	60.0	402	INTERGENIC	--	--	--
Ca4_P3_17073988	Ca4	17073988	A/AGTTAC	AATGCTAAACACACGAGGCA	TTTCACTCCCACCACAACAA	59.3	882	INTERGENIC	--	--	--
Ca4_P3_17081756	Ca4	17081756	TTATA/TTATATA	TACCGGAAGATTCGGAAATG	CTGCAGGTAAGCATCCCAAT	59.9	694	INTRON	Ca_05415	--	--
Ca4_P3_17082004	Ca4	17082004	TGAT/TGATCGAT	TTTTGACAATGGGCACTCAA	CTGCAGGTAAGCATCCCAAT	60.1	295	DRR	Ca_05415	--	--
Ca4_P3_17089902	Ca4	17089902	ATTTTTTT/ATTTTTTTT	CCAACATTAAAACGGCCCTA	TGTGCTAGGATTGCACGGTA	59.8	574	INTERGENIC	--	--	--
Ca4_P3_17090015	Ca4	17090015	ATATTTATTTATTTATTTATTT/ATATTTATTTATTTATTT	CCAACATTAAAACGGCCCTA	GCATTTTCACAATGTGGTGC	59.8	698	INTERGENIC	--	--	--
Ca4_P3_17094592	Ca4	17094592	TA/T	TGCTTCAAAGAAAAATCGGA	TGGGATTACCTGACCCCATA	58.5	190	INTERGENIC	--	--	--
Ca4_P3_17096036	Ca4	17096036	CTTTCAA/CTTTCAAGTTTCAA	CTGCAATTGTCAAGCTCCAA	TCCATATGTGCCCATTGCTA	60.0	276	INTERGENIC	--	--	--
Ca4_P3_17097898	Ca4	17097898	CATAT/CAT	GTGGCTTTTTGTCCGACATT	CAGCTGCCCTCACAACAATA	60.0	821	INTERGENIC	--	--	--

INDEL marker IDs	Chromosomes/ unanchored scaffolds	Physical positions (bp)	InDels (*Kabuli* reference genome-CDC Frontier/PII)	Forward primers (5'-3')	Reverse primers (5'-3')	Anneling temperature (0C)	Expected amplified product size (bp)	Structural annotation		Functional annotation	
								Sequence components of *kabuli* genome	*Kabuli* gene accession IDs	NCBI-KOG	TFs
Ca4_P3_17099062	Ca4	17099062	A/AC	TTTTCGGTTCGAAAGACACC	AGGAAGCTGAACAAGCTCCA	60.1	267	INTERGENIC	--	--	--
Ca4_P3_17101199	Ca4	17101199	A/AC	TGGTTTGATTTATTGTGGTGC	GTTCTTTGAAATTGCACGAATA	58.4	812	DRR	Ca_05414	--	--
Ca4_P3_17102148	Ca4	17102148	AA/AAGA	ATGACCCAACTCTCGACACC	TCAGGTGATTGTTGACCCAA	60.0	733	DRR	Ca_05414	--	--
Ca4_P3_17114726	Ca4	17114726	GAAAA/GAAAAA	CAAAAACAAGGGCACCAACT	TATTTGAAACGAACGGTCCC	60.0	736	DRR	Ca_05412	CR	Nin-like
Ca4_P3_17129875	Ca4	17129875	CAT/CATTAT	CCCATCCTCTAAAGCACAGC	GCTAGGGATGAAGGAAGCCT	59.8	530	INTRON	Ca_05411	CR	Nin-like
Ca4_P3_17131617	Ca4	17131617	TAAAAAAAA/TAAAAAAAAA	CATGCCTTCTAACACAGCGA	TTGTGAAGGTGATTTGCTCG	60.0	517	INTRON	Ca_05411	CR	Nin-like
Ca4_P3_17134644	Ca4	17134644	TAAAAAAA/TAAAAAAAAAA	AGATCTACCATTTGCGTCCG	TTGGAAACAATTCAAGGCAA	60.1	731	INTRON	Ca_05411	CR	Nin-like
Ca4_P3_17140665	Ca4	17140665	GGGTGAAAAAAGAA/G	ACGGCTACGAGTTGTTGCTT	GCAAAAACTTGATACGATTGAAA	59.9	522	DRR	Ca_05410	CR	Nin-like
Ca4_P3_17141537	Ca4	17141537	ATT/AT	TGCAAAATCAAACATAAAGGTTGT	AAACTGAAAGGCCTAATTCCAA	59.8	233	DRR	Ca_05410	CR	Nin-like
Ca4_P3_17141571	Ca4	17141571	TAAAAAA/TAAAAAAA	TGCAAAATCAAACATAAAGGTTGT	AAACTGAAAGGCCTAATTCCAA	59.8	233	DRR	Ca_05410	CR	Nin-like

INDEL marker IDs	Chromosomes/ unanchored scaffolds	Physical positions (bp)	InDels (*Kabuli* reference genome-CDC Frontier/PII)	Forward primers (5'-3')	Reverse primers (5'-3')	Anneling temperature (0C)	Expected amplified product size (bp)	Structural annotation		Functional annotation	
								Sequence components of *kabuli* genome	*Kabuli* gene accession IDs	NCBI-KOG	TFs
Ca4_P3_17143567	Ca4	17143567	GGCA/G	TTCGACCAAGCATGATTTTG	TTGAGGATCGAAATGACCAA	59.7	237	INTRON	Ca_05410	CR	Nin-like
Ca4_P3_17146270	Ca4	17146270	AGGGG/AGGG	ATTCCAGCTCCCACAATCAC	GGCTCTTGATGGGTTGTGTT	59.9	442	DRR	Ca_05409	K	--
Ca4_P3_17147517	Ca4	17147517	G/GACGCTCATCA	ATTTCCCAACAAGATTCCCC	GGCGTTCCAAGTGAATGTTT	60.0	627	DRR	Ca_05409	K	--
Ca4_P3_17157766	Ca4	17157766	TTAAT/TTAATAAT	AGAACCTTGCTATGCGTCGT	CATGGTGTGGACAAGATTGG	59.9	501	DRR	Ca_05407	--	--
Ca4_P3_17168225	Ca4	17168225	GAAAAAAA/GAAAAAAAAAA	CTCTACCCTTGAGAGTGTGTGG	TGGACGGAGTATGCATTATTGA	58.9	581	INTERGENIC	--	--	--
Ca4_P3_17168501	Ca4	17168501	TAAAAAA/TAAAAAAAA	CTCTACCCTTGAGAGTGTGTGG	TGGACGGAGTATGCATTATTGA	58.9	581	INTERGENIC	--	--	--
Ca4_P3_17172307	Ca4	17172307	ATT/AT	TCTCCAGCATGTAATTACGGTTA	AAACGAAACATCCTCGTTGC	58.7	368	INTERGENIC	--	--	--
Ca4_P3_17195362	Ca4	17195362	TAA/TAAA	TGTTGAACAAAATTCAAAATTCAAA	AAAAACTATTCATCGAAATATCACCC	59.8	194	INTERGENIC	--	--	--
Ca4_P3_17195413	Ca4	17195413	A/AG	TGTTGAACAAAATTCAAAATTCAAA	AAAAACTATTCATCGAAATATCACCC	59.8	194	INTERGENIC	--	--	--
Ca4_P3_17196085	Ca4	17196085	TTCTCTCTCTCTCTCTCTCTCTCTCTCT/TTCTCTCTCTCTCTCTCTCTCTCTCTCTCT	CCTCAACCATGGCCTGTACT	AGTTGCATTGGAGACAAGGG	60.0	118	INTERGENIC	--	--	--

INDEL marker IDs	Chromosomes/ unanchored scaffolds	Physical positions (bp)	InDels (*Kabuli* reference genome-CDC Frontier/PII)	Forward primers (5'-3')	Reverse primers (5'-3')	Anneling temperature (0C)	Expected amplified product size (bp)	Structural annotation		Functional annotation	
								Sequence components of *kabuli* genome	*Kabuli* gene accession IDs	NCBI-KOG	TFs
Ca4_P3_17206969	Ca4	17206969	TAAAAAAAA/TAAAAAAA	CCTGGCAGATGGCAATAGAT	GAAAATGGATCCGACGAAGA	60.1	593	INTERGENIC	--	--	--
Ca4_P3_17207193	Ca4	17207193	TA/T	CCTGGCAGATGGCAATAGAT	GAAAATGGATCCGACGAAGA	60.1	593	INTERGENIC	--	--	--
Ca4_P3_17208586	Ca4	17208586	TACAC/TACACAC	TGTAAGATGGGTGTGGCTCA	GGTCCCTAATGCAGTTGACC	60.1	487	INTRON	Ca_05402	C	--
Ca4_P3_17210802	Ca4	17210802	ATG/ATGGTG	AGGGGGAATTCAGAAGGCTA	CCTCTTCTCCACCCTGCATA	60.0	581	INTRON	Ca_05402	C	--
Ca4_P3_17214261	Ca4	17214261	GCTATC/G	GCTTTTTAGGCTGGGGAATC	AATAATTTGGTCAGGCGTGC	60.0	194	DRR	Ca_05402	C	--
Ca4_P3_17214540	Ca4	17214540	A/ATATAG	GCTTTTTAGGCTGGGGAATC	TTGGTCAATGCGGAAGTGTA	60.0	627	DRR	Ca_05402	C	--
Ca4_P3_17214599	Ca4	17214599	AC/ACTTC	GCTTTTTAGGCTGGGGAATC	TTGGTCAATGCGGAAGTGTA	60.0	627	DRR	Ca_05402	C	--
Ca4_P3_1721745	Ca4	1721745	GATAATAATAATAATAA/GATAATAATAATAA	AAGCGGAAAGGAGCTAAAGG	AACAAAATTACGAGTGGCCG	60.0	200	INTERGENIC	--	--	--
Ca4_P3_17219514	Ca4	17219514	CTTATTTATTTATTTAT/CTTATTTATTTATTTATTTATTTATTTAT	GGAATGAGAGAGAGCGTTGG	TTATGCGATGCCAATTCAAA	60.0	563	INTRON	Ca_05401	O	--
Ca4_P3_17223748	Ca4	17223748	GAAAAAA/GAAAAAAAA	TCACCAACATGGTATGACACAA	GGCCTGGTTCTCAAAATTCA	59.7	368	DRR	Ca_05401	O	--

INDEL marker IDs	Chromosomes/ unanchored scaffolds	Physical positions (bp)	InDels (*Kabuli* reference genome-CDC Frontier/PII)	Forward primers (5'-3')	Reverse primers (5'-3')	Anneling temperature (0C)	Expected amplified product size (bp)	Structural annotation		Functional annotation	
								Sequence components of *kabuli* genome	*Kabuli* gene accession IDs	NCBI-KOG	TFs
Ca4_P3_17223825	Ca4	17223825	GAAAAGAAAAA/GAAAA	TTTTCTGCATATCACTGCGA	GGCCTGGTTCTCAAAATTCA	58.0	246	DRR	Ca_05401	O	--
Ca4_P3_17227245	Ca4	17227245	ATTTT/ATTT	CATTGACGACCAGGTTTGTG	TCAGACACCGTTGTGGGATA	60.0	732	INTRON	Ca_05400	YU	--
Ca4_P3_17238317	Ca4	17238317	CTTTATTT/CTTTATTTATTT	AAAAATGGGGTGGATGAACA	TTCCAATGTTGCAGAAGCAG	60.0	439	DRR	Ca_05400	YU	--
Ca4_P3_17240545	Ca4	17240545	GAAAAAAAA/GAAAAAAAAA	TGGTCTAAAAAGATTGGCCG	TACGTGGCAAACATCCAAAA	60.1	537	INTERGENIC	--	--	--
Ca4_P3_17242500	Ca4	17242500	T/TAA	CTTGATGGCTTCAACTTGGG	AACTAATTTTTGGTTCCACTATTTTT	60.6	766	INTERGENIC	--	--	--
Ca4_P3_17243538	Ca4	17243538	AT/A	TTCACACATGCATTGTTTACCA	TGCAAAACTGCTCTACCCAA	59.9	422	INTERGENIC	--	--	--
Ca4_P3_17243952	Ca4	17243952	A/AT	AGCTTTGCGATTCTATTGGG	CAACCGTTTGAAAATGACGA	59.3	501	INTERGENIC	--	--	--
Ca4_P3_17247653	Ca4	17247653	GAAAAAAAA/GAAAAAAAAAA	TTTATGTTTGGCCAACTCCC	TTTTTAGGGACGCAATTCAAA	59.8	539	INTERGENIC	--	--	--
Ca4_P3_17249235	Ca4	17249235	AATATAGAT/AAT	TTTCCAACTCAAAAGAGGGC	CAATACGGGCTCAACTTGGT	59.3	601	INTERGENIC	--	--	--
Ca4_P3_17254453	Ca4	17254453	ATTTTTTTTT/ATTTTTTTTTT	CCAAGCGACTCACTTCATCA	TTGAAAGGGCAAAATTACACA	60.0	413	INTERGENIC	--	--	--

INDEL marker IDs	Chromosomes/ unanchored scaffolds	Physical positions (bp)	InDels (*Kabuli* reference genome-CDC Frontier/PII)	Forward primers (5'-3')	Reverse primers (5'-3')	Anneling temperature (0C)	Expected amplified product size (bp)	Structural annotation		Functional annotation	
								Sequence components of *kabuli* genome	*Kabuli* gene accession IDs	NCBI-KOG	TFs
Ca4_P3_17257056	Ca4	17257056	ATTTT/ATTT	CCAACAAGCATTACATTGCG	ACGTTGACATTCCAGCATGA	60.1	423	INTERGENIC	--	--	--
Ca4_P3_17257163	Ca4	17257163	ATTTTTTT/ATTTTTT	TGTACGCCAAAGTAATGCCA	TCCAATGAAATTAAACGCAAA	60.1	897	INTERGENIC	--	--	--
Ca4_P3_17257804	Ca4	17257804	TG/T	TTTGCGTTTAATTTCATTGGA	ATCCAACCTGCCATGTCCTA	58.2	283	INTERGENIC	--	--	--
Ca4_P3_17259358	Ca4	17259358	TAAA/TAAAA	ATGGGTGCATTTGTTTCAGA	GCCTGAGTTTGGCATACGAT	59.0	338	INTERGENIC	--	--	--
Ca4_P3_17259694	Ca4	17259694	TG/T	ATCGTATGCCAAACTCAGGC	CGTGTGTGCATTGGAATTGT	60.1	422	INTERGENIC	--	--	--
Ca4_P3_17259744	Ca4	17259744	GTTTTTTTTTT/GTTTTTTT	ATCGTATGCCAAACTCAGGC	CGTGTGTGCATTGGAATTGT	60.1	422	INTERGENIC	--	--	--
Ca4_P3_17262149	Ca4	17262149	ATT/AT	ATTTGTCTGATCGGTACGGC	CAAATCCGAATCCTCGTTGT	60.0	677	INTRON	Ca_05398	R	--
Ca4_P3_17263445	Ca4	17263445	CT/CTT	CGGTTCAATTTTCAAACGGT	GGTTCAAAATGGCGTTGAGT	59.8	658	INTRON	Ca_05398	R	--
Ca4_P3_17267236	Ca4	17267236	GT/GTT	GCTTAATGTGCCTTGGGCTA	ATAGGAGGAGGAGCACGGAT	60.2	274	DRR	Ca_05397	S	--
Ca4_P3_17267576	Ca4	17267576	AGGG/AGG	ATCCGTGCTCCTCCTCCTAT	CCCGGAAAAATGAAGAACAA	60.1	507	DRR	Ca_05398	R	--

INDEL marker IDs	Chromosomes/ unanchored scaffolds	Physical positions (bp)	InDels (*Kabuli* reference genome-CDC Frontier/PII)	Forward primers (5'-3')	Reverse primers (5'-3')	Anneling temperature (0C)	Expected amplified product size (bp)	Structural annotation		Functional annotation	
								Sequence components of *kabuli* genome	*Kabuli* gene accession IDs	NCBI-KOG	TFs
Ca4_P3_17268685	Ca4	17268685	TGG/TGGG	CATCCCATCCAGGACCTTTA	CTGGTTCCCGGTTGAACTTA	59.7	308	DRR	Ca_05398	R	--
Ca4_P3_17271595	Ca4	17271595	TTTATATATTATATATT/TTTATATATT	CGCTTGGAAGAAGGTAGACG	CATTCGCATTTAGAATCAACTCA	60.0	593	INTERGENIC	--	--	--
Ca4_P3_17280110	Ca4	17280110	ATT/AT	CATTGGCTGCTTTGTTGCTA	GCAATATGGAGCCACGGTAT	60.0	596	DRR	Ca_05395	R	--
Ca4_P3_17284874	Ca4	17284874	GATA/GA	TTGAATATGAGCCTCCAAGAGA	CTTGTTGAGGCTTTGGGTGT	58.9	794	DRR	Ca_05393	R	--
Ca4_P3_17285160	Ca4	17285160	ACT/A	CATTTCCTTTGAGTCATGAGAGG	CTTGTTGAGGCTTTGGGTGT	60.1	310	DRR	Ca_05393	R	--
Ca4_P3_17285484	Ca4	17285484	GTTTTTTTTTTTTT/GTTTTTTTTTTT	ACACCCAAAGCCTCAACAAG	GCTTTCTATTTTTGCGGGTG	60.1	624	DRR	Ca_05393	R	--
Ca4_P3_17286530	Ca4	17286530	TA/T	GGCCCCTGTTATCCCTAAAG	CTAGGGACCATCGCAAGTTC	59.8	551	INTRON	Ca_05393	R	--
Ca4_P3_17286686	Ca4	17286686	CATTGGATTGGATTG/CATTGGATTG	TGCATTTCATGCCTTTGCTA	TGATTAGCCCGTATTTTGGG	60.4	648	INTRON	Ca_05393	R	--
Ca4_P3_17286899	Ca4	17286899	ATCA/ATCATGTCA	GGTGATCATGCACTCCATTG	TGATTAGCCCGTATTTTGGG	59.9	389	INTRON	Ca_05393	R	--
Ca4_P3_17290871	Ca4	17290871	GTTTAATT/GTTTAATTTTAATT	CCAAGTATGGCAGGTGGAAT	TCATGAGCTAGAATGGTTGTAAAAA	59.8	615	INTERGENIC	--	--	--

INDEL marker IDs	Chromosomes/ unanchored scaffolds	Physical positions (bp)	InDels (*Kabuli* reference genome-CDC Frontier/PII)	Forward primers (5'-3')	Reverse primers (5'-3')	Anneling temperature (0C)	Expected amplified product size (bp)	Structural annotation		Functional annotation	
								Sequence components of *kabuli* genome	*Kabuli* gene accession IDs	NCBI-KOG	TFs
Ca4_P3_17295286	Ca4	17295286	CTTT/CTTTTTT	TTCATTGGGGTTTCACCTTT	TGATGCAGCAAAATCAGGAA	59.3	854	INTRON	Ca_05392	R	--
Ca4_P3_17295338	Ca4	17295338	A/AG	TTCATTGGGGTTTCACCTTT	TCTCACAAGCACACTCCTGG	59.3	964	INTRON	Ca_05392	R	--
Ca4_P3_17297039	Ca4	17297039	TAAAAAA/TAAAAAAA	TTTGCCTCAGCTGTGTTGAC	TCATCAGCTGGACAACTTCG	60.0	607	INTRON	Ca_05392	R	--
Ca4_P3_17313148	Ca4	17313148	TC/T	AGAGGATGCAGCTATGCGTT	CAACATTTTGGGAGGGAAGA	60.0	170	INTERGENIC	--	--	--
Ca4_P3_17313409	Ca4	17313409	GAAAAAA/GAAAAAAA	AGAGGATGCAGCTATGCGTT	CAGCCCAAGTTTTGAAAAAGA	60.0	573	INTERGENIC	--	--	--
Ca4_P3_17313521	Ca4	17313521	TAAA/TAA	GCTAGACAGGTTTGCCTTCG	AAGGCTCCTGCAAGCATTTA	60.0	966	INTERGENIC	--	--	--
Ca4_P3_17314228	Ca4	17314228	CAAAAAAA/CAAAAAA	TAAATGCTTGCAGGAGCCTT	CCTTTTGTTCTCAATCGCGT	60.0	366	INTERGENIC	--	--	--
Ca4_P3_17314494	Ca4	17314494	TA/T	TAAATGCTTGCAGGAGCCTT	CTTCCCCACTCACAGTCCAT	60.0	950	DRR	Ca_05390	M	--
Ca4_P3_17314621	Ca4	17314621	ATTTTTT/ATTTTT	ACGCGATTGAGAACAAAAGG	CTTCCCCACTCACAGTCCAT	60.2	604	DRR	Ca_05390	M	--
Ca4_P3_17314848	Ca4	17314848	CAA/CAAACCGAACTAGAA	TTGTAAAAACTGCATCAATCGAA	CTTCCCCACTCACAGTCCAT	59.7	488	DRR	Ca_05390	M	--

INDEL marker IDs	Chromosomes/ unanchored scaffolds	Physical positions (bp)	InDels (*Kabuli* reference genome-CDC Frontier/PII)	Forward primers (5'-3')	Reverse primers (5'-3')	Anneling temperature (0C)	Expected amplified product size (bp)	Structural annotation		Functional annotation	
								Sequence components of *kabuli* genome	*Kabuli* gene accession IDs	NCBI-KOG	TFs
Ca4_P3_17314963	Ca4	17314963	TGGGG/TGGGGG	ATGGACTGTGAGTGGGGAAG	CAATGGAGAGGTTCGTGACA	60.0	156	DRR	Ca_05390	M	--
Ca4_P3_17317797	Ca4	17317797	A/AT	CATCAGGCACCAAGGAAAAT	TCCCACTTTTGAGGTTGGTC	59.9	459	INTRON	Ca_05390	M	--
Ca4_P3_17317872	Ca4	17317872	TGGG/TGG	CATCAGGCACCAAGGAAAAT	ACAGCGTGAAACAAGATCCC	59.9	875	INTRON	Ca_05390	M	--
Ca4_P3_17317909	Ca4	17317909	ATTTTT/ATTTTTT	CTCAAAAGTGGGATAGGGCA	ACAGCGTGAAACAAGATCCC	60.1	429	INTRON	Ca_05390	M	--
Ca4_P3_17318086	Ca4	17318086	A/AG	CTCAAAAGTGGGATAGGGCA	ACAGCGTGAAACAAGATCCC	60.1	429	INTRON	Ca_05390	M	--
Ca4_P3_17318109	Ca4	17318109	AA/AATATTA	CTCAAAAGTGGGATAGGGCA	ACAGCGTGAAACAAGATCCC	60.1	429	INTRON	Ca_05390	M	--
Ca4_P3_17318183	Ca4	17318183	T/TCTA	CTCAAAAGTGGGATAGGGCA	ACAGCGTGAAACAAGATCCC	60.1	429	INTRON	Ca_05390	M	--
Ca4_P3_17321954	Ca4	17321954	TA/TATATATATATAAA	CTGCCGACCTGAATACCAAT	TGCCTTTAATGAAACACACCA	60.0	541	INTERGENIC	--	--	--
Ca4_P3_17322303	Ca4	17322303	CATA/CA	TGAGGATAAAATGGTGTGTTTCA	TGCGGTTAGATTTGCTCACTT	59.4	733	INTERGENIC	--	--	--
Ca4_P3_17327734	Ca4	17327734	ATTTT/ATTT	CGAACACAACGATAAGATCTCG	TGCACTTAATTTGCCAACGA	59.8	876	INTERGENIC	--	--	--

INDEL marker IDs	Chromosomes/ unanchored scaffolds	Physical positions (bp)	InDels (*Kabuli* reference genome-CDC Frontier/PII)	Forward primers (5'-3')	Reverse primers (5'-3')	Anneling temperature (0C)	Expected amplified product size (bp)	Structural annotation		Functional annotation	
								Sequence components of *kabuli* genome	*Kabuli* gene accession IDs	NCBI-KOG	TFs
Ca4_P3_17328665	Ca4	17328665	GAA/GAAAA	ACACGTGCAATATCGGTGAA	TCCTCCCTCTTTATCATGCC	60.0	750	INTERGENIC	--	--	--
Ca4_P3_17329245	Ca4	17329245	TCACA/TCA	GGCATGATAAAGAGGGAGGA	TAACCTCTCACGTGCAATCG	59.1	386	INTERGENIC	--	--	--
Ca4_P3_17337403	Ca4	17337403	CAA/CAAA	AATGGCTGCCGTTATTTTTG	AACAACATTTGCCTTCCCTG	60.0	198	INTERGENIC	--	--	--
Ca4_P3_17339892	Ca4	17339892	ATTT/ATT	TTTTGGGGAACACAACCTTT	GACATTTCTCATAGGGGCGA	59.3	297	INTERGENIC	--	--	--
Ca4_P3_17345629	Ca4	17345629	GA/G	AAGAACAGCAGCAGCCAACT	TGAGCCTCATAGACACGACG	60.2	436	INTRON	Ca_05389	A	--
Ca4_P3_17360539	Ca4	17360539	TAA/TAAA	GTGTGCGATTGAGTTGAACG	CTTGCTGGGCTTTTCAAGAC	60.3	579	INTRON	Ca_05388	T	NAC
Ca4_P3_17364116	Ca4	17364116	GAAA/GAAAA	AAATTTTCAGATCCACCCCC	CTTTGTGCAGGTTCATCCCT	60.0	550	INTRON	Ca_05388	T	NAC
Ca4_P3_17367149	Ca4	17367149	AATAT/AAT	GCAGCTAAAGGAGAGTGCCA	AGACAGTTCGCTCGCAAATC	60.7	506	INTERGENIC	--	--	--
Ca4_P3_17375846	Ca4	17375846	CTTTTTTTT/CTTTTTTT	TTGTTGCACACTTCAAGGAGA	TCGTAGTTGGTCACCGATCA	59.5	435	INTERGENIC	--	--	--
Ca4_P3_17379518	Ca4	17379518	TG/T	AACAAATGGTGCATCACGAA	TCTTCATTGGTCTCGTGCTT	60.0	740	INTRON	Ca_05387	R	--

INDEL marker IDs	Chromosomes/ unanchored scaffolds	Physical positions (bp)	InDels (*Kabuli* reference genome-CDC Frontier/PII)	Forward primers (5'-3')	Reverse primers (5'-3')	Anneling temperature (0C)	Expected amplified product size (bp)	Structural annotation		Functional annotation	
								Sequence components of *kabuli* genome	*Kabuli* gene accession IDs	NCBI-KOG	TFs
Ca4_P3_17380447	Ca4	17380447	CG/C	TAGGGCTTTGCATTGATTCC	CAACCTCATCCACTTTCCGT	60.0	352	INTRON	Ca_05387	R	--
Ca4_P3_17381165	Ca4	17381165	TAAAAAAA/TAAAAA	GGGTCCTTTTGTTGTGGTGT	GGGATGGGAATAGGCTAAACTT	59.7	627	INTERGENIC	--	--	--
Ca4_P3_17386248	Ca4	17386248	AT/ATT	CCAAAAGGCGACCATAAAAA	TCAAGTAGCAGCGCAACATC	59.9	536	INTERGENIC	--	--	--
Ca4_P3_17395103	Ca4	17395103	TAAAAAA/TAAAAAAA	GCGTGGGTAGCAACTTTCAT	TCGATGGTCATTTTTGTTGC	60.1	378	INTRON	Ca_05386	K	--
Ca4_P3_17396948	Ca4	17396948	ATTTTTTTTTTT/ATTTTTTTTT	CTTGCAAATTGTTGAAGCCA	CAACTTGTCCTTAGCGGCTC	59.8	229	INTRON	Ca_05386	K	--
Ca4_P3_17397039	Ca4	17397039	C/CGCAAG	TGAGTGCTTCCGTTTGACTG	GCAGCCATCAGGAAAAAGAG	60.0	500	CDS (FRAME SHIFT)	Ca_05386	K	--
Ca4_P3_17408958	Ca4	17408958	ATGTTGT/ATGT	CCAAAGAATTTTCCGACCAA	CTCCTCCCCTCAAAATCTCC	59.9	567	INTERGENIC	--	--	--
Ca4_P3_17423076	Ca4	17423076	TAA/TAAA	GTGTGGGTTGGCTTTCATCT	TCCGCAAACAATGCAAAATA	60.0	252	INTERGENIC	--	--	--
Ca4_P3_17423293	Ca4	17423293	TTATATA/TTA	GTGTGGGTTGGCTTTCATCT	GAAACGCTATGATGCCGTAAA	60.0	492	INTERGENIC	--	--	--
Ca4_P3_17426153	Ca4	17426153	GTTTTTTTT/GTTTTTTT	TTTATGCGACATCGAAATTCA	AAAGTGGAAATGGATCAACAAA	59.2	278	INTERGENIC	--	--	--

INDEL marker IDs	Chromosomes/ unanchored scaffolds	Physical positions (bp)	InDels (*Kabuli* reference genome-CDC Frontier/PII)	Forward primers (5'-3')	Reverse primers (5'-3')	Anneling temperature (0C)	Expected amplified product size (bp)	Structural annotation		Functional annotation	
								Sequence components of *kabuli* genome	*Kabuli* gene accession IDs	NCBI-KOG	TFs
Ca4_P3_17430156	Ca4	17430156	GT/GTAGAT	TGCGGACGATACACCAAATA	CAGAGGATGCAAAAGCAACA	60.0	761	INTRON	Ca_05384	K	--
Ca4_P3_17430506	Ca4	17430506	GTTTTTTT/GTTTTTTTT	TGTTGCTTTTGCATCCTCTG	CAAGGTTAAGAGCGCCTTTG	60.0	485	INTRON	Ca_05384	K	--
Ca4_P3_17431572	Ca4	17431572	GC/G	TCTGTCATTCCTGCCATTTTT	TCGCAATGTAGCTCCCTCTT	59.6	858	INTRON	Ca_05384	K	--
Ca4_P3_17435255	Ca4	17435255	GTACTACTA/GTACTA	TCAAGCTGCTTTCAATGGTG	GAGGCCAATGGAGTGGTTTA	60.0	241	INTRON	Ca_05384	K	--
Ca4_P3_17435927	Ca4	17435927	TG/T	TAAACCACTCCATTGGCCTC	TCCATATCATCTTGAGGGGC	59.9	718	INTRON	Ca_05384	K	--
Ca4_P3_17436584	Ca4	17436584	CAG/CAGAG	AAGATCCTGCCCCTCAAGAT	GCAAGGTCTCCAAAAGCAAG	60.0	945	INTRON	Ca_05384	K	--
Ca4_P3_17437453	Ca4	17437453	T/TG	CTTGCTTTTGGAGACCTTGC	CATCCAAGGCAAGGAACAAT	60.0	463	INTRON	Ca_05384	K	--
Ca4_P3_17443455	Ca4	17443455	TAA/TA	GTTGGTGTTGGCTTTTGCTT	ACATAGAGCTGTGCGTGGTG	60.2	246	DRR	Ca_05384	K	--
Ca4_P3_17443644	Ca4	17443644	GAAAAAAAAA/GAAAAAAAA	GTTGGTGTTGGCTTTTGCTT	CGTTGGTTTACAGAGGGCAT	60.2	768	DRR	Ca_05384	K	--
Ca4_P3_17445199	Ca4	17445199	A/AC	CCCCTTTCAACATTCCTCAA	GAGGCACGCAAATCTTTGTT	59.9	292	INTERGENIC	--	--	--

INDEL marker IDs	Chromosomes/ unanchored scaffolds	Physical positions (bp)	InDels (*Kabuli* reference genome-CDC Frontier/PII)	Forward primers (5'-3')	Reverse primers (5'-3')	Anneling temperature (0C)	Expected amplified product size (bp)	Structural annotation		Functional annotation	
								Sequence components of *kabuli* genome	*Kabuli* gene accession IDs	NCBI-KOG	TFs
Ca4_P3_17447166	Ca4	17447166	ATTTTTT/ATTTTTTTT	TACTCGTTTCGTTGGG CTTC	AACATCGTCTCCCAAT TTCA	60.2	452	INTERGENIC	--	--	--
Ca4_P3_17458594	Ca4	17458594	ATTT/ATTTCTATAATTTTGTTT	TGCCTCTACCTTTTCTT TGATTG	TCATTCAACATTTGGC CATT	59.8	213	INTERGENIC	--	--	--
Ca4_P3_17464214	Ca4	17464214	CTATATAT/CTATATATATAT	ACCACTCACCAACCTTC CAG	AACCCGGAGACCCAAT TTAG	60.0	635	INTRON	Ca_05381	O	GRAS
Ca4_P3_17464247	Ca4	17464247	CA/CGAGGA	ACCACTCACCAACCTTC CAG	AACCCGGAGACCCAAT TTAG	60.0	635	INTRON	Ca_05381	O	GRAS
Ca4_P3_17465788	Ca4	17465788	TC/T	GCAAGAGCATTACAGG AGGC	CAACCCTAGTGACCCA TGCT	60.0	794	INTRON	Ca_05381	O	GRAS
Ca4_P3_17475440	Ca4	17475440	GT/GTTGTTATT	CGGGCAGTAGCATTAG ATGG	GGATGAATGGGAGGT GGTTA	60.6	210	INTERGENIC	--	--	--
Ca4_P3_17480298	Ca4	17480298	ATATTATTATTATTATTAT/ATATTATTA TTAT	TTGTTGGCCGTCATAT GCTA	GACCAATTACAAGCGA GGGA	60.1	617	INTRON	Ca_05379	O	--
Ca4_P3_17487664	Ca4	17487664	GTTTTT/GTTTTTT	TGGGAAATTGCAAGAC AACA	TCTTTCCACGGATCCT GTTC	60.1	291	INTRON	Ca_05379	O	--
Ca4_P3_17489508	Ca4	17489508	ATTTTTT/ATTTTTTTTT	GTTTTCTCCACCCAAAA GCA	CCAGCTTGTGACAAAA AGCA	60.1	488	DRR	Ca_05379	O	--
Ca4_P3_17489726	Ca4	17489726	AC/ACC	ATAAAATGTAGATGCCG CCG	CCAGCTTGTGACAAAA AGCA	60.0	349	DRR	Ca_05379	O	--

INDEL marker IDs	Chromosomes/ unanchored scaffolds	Physical positions (bp)	InDels (*Kabuli* reference genome-CDC Frontier/PII)	Forward primers (5'-3')	Reverse primers (5'-3')	Anneling temperature (0C)	Expected amplified product size (bp)	Structural annotation		Functional annotation	
								Sequence components of *kabuli* genome	*Kabuli* gene accession IDs	NCBI-KOG	TFs
Ca4_P3_17494020	Ca4	17494020	GAAA/GAAAA	GGGTGTGGAAAAATGAAAGC	AGAATTGCTGCTGCTCAGGT	59.4	734	INTRON	Ca_05378	--	--
Ca4_P3_17527235	Ca4	17527235	CGG/CG	ATTCCCCGTTCAGTCATCAG	CGCCTGCCTTCTTAACTTTG	59.9	468	INTRON	Ca_05374	R	NAC
Ca4_P3_17527426	Ca4	17527426	GTGATT/GTGATTGATT	CAAAGTTAAGAAGGCAGGCG	CAAAATTGGTGGGATTTTGG	60.0	578	INTRON	Ca_05374	R	NAC
Ca4_P3_17527467	Ca4	17527467	AGT/AGTGT	CAAAGTTAAGAAGGCAGGCG	CAAAATTGGTGGGATTTTGG	60.0	578	INTRON	Ca_05374	R	NAC
Ca4_P3_17528276	Ca4	17528276	TGG/TGGG	AAGCTTTCCATAAGCCGTGA	GACAACGCAGAAGCAGAACA	59.8	911	INTRON	Ca_05374	R	NAC
Ca4_P3_17530366	Ca4	17530366	GTTTTTTTTTT/GTTTTTTTTTTTT	GGTCTATGGCGGAAAGATGA	TCAAAATCTCGATGCAGTTCA	60.0	577	DRR	Ca_05373	O	--
Ca4_P3_17533077	Ca4	17533077	CAGTG/CAGTGCATAAGTG	TGGTGAGTTGTACCGTGCTC	AAAATGGCACCAGCAAAAAG	59.8	358	INTERGENIC	--	--	--
Ca4_P3_17534710	Ca4	17534710	ATATTT/ATT	CTGGATCCCCTCGGAAGTAT	TAGAAACCGCACACACACAA	60.3	317	INTERGENIC	--	--	--
Ca4_P3_17545627	Ca4	17545627	TAAAAAAAA/TAAAAAAA	AGTTTAGGGGCAAGCACTGA	TGCATCGGAGATCACATCTT	59.9	363	INTERGENIC	--	--	--
Ca4_P3_17547225	Ca4	17547225	TTTATT/TTT	CAACATCAGATGCACATGGA	TTCGTGATCGTTTGGTGAAA	59.0	845	INTERGENIC	--	--	--

INDEL marker IDs	Chromosomes/ unanchored scaffolds	Physical positions (bp)	InDels (*Kabuli* reference genome-CDC Frontier/PII)	Forward primers (5'-3')	Reverse primers (5'-3')	Anneling temperature (0C)	Expected amplified product size (bp)	Structural annotation		Functional annotation	
								Sequence components of *kabuli* genome	*Kabuli* gene accession IDs	NCBI-KOG	TFs
Ca4_P3_17599940	Ca4	17599940	CTTTTT/CTTTTTT	ATCAACGAAATGAAGTCGCC	CGGTCAAATAGGCCTACCAA	60.1	417	INTERGENIC	--	--	--
Ca4_P3_17600797	Ca4	17600797	TC/T	AAGGGTGATGGACGAAAAAC	TTAAATGGTCGCATAGAGCAA	58.9	682	INTERGENIC	--	--	--
Ca4_P3_17600811	Ca4	17600811	TAAAAAA/TAAAAAAA	AAGGGTGATGGACGAAAAAC	TCCAACAACATCAAATTCATCC	58.9	951	INTERGENIC	--	--	--
Ca4_P3_17601510	Ca4	17601510	TC/T	GGATGAATTTGATGTTGTTGGA	CCGTGTCTATCCCCATTTTG	59.7	515	INTERGENIC	--	--	--
Ca4_P3_17601888	Ca4	17601888	TAAA/TAAAA	CAAAATGGGGATAGACACGG	TTTCAAATGAACGTTTGCTACA	60.2	440	INTERGENIC	--	--	--
Ca4_P3_17602358	Ca4	17602358	GT/GTT	TTTGAATGTGTATTGTAGCAAACG	CCTATTCTCGAGAGCAACCG	59.2	322	INTERGENIC	--	--	--
Ca4_P3_17604832	Ca4	17604832	ATTTTTTTT/ATTTTTTTTTTTTTTT	GCCCAAGGCCAAAGTTAGTA	TTCACACCTCACAGCAGGAC	59.2	336	INTRON	Ca_05371	S	--
Ca4_P3_17617099	Ca4	17617099	T/TATG	GAGAGTGGGTCGTTGATGGT	TTTGATCACATTCCCCAACA	60.0	633	INTERGENIC	--	--	--
Ca4_P3_17618987	Ca4	17618987	TATCATCAT/TATCAT	GCCGATCTTCAACTTCAACC	TCGAGCTAGAAGCACCAACA	59.7	470	INTERGENIC	--	--	--
Ca4_P3_17620504	Ca4	17620504	T/TG	CTCTGTGTGACACCCCATTG	CTAATCCAAACCAGGCCAAA	60.0	719	INTRON	Ca_05368	F	--

INDEL marker IDs	Chromosomes/ unanchored scaffolds	Physical positions (bp)	InDels (*Kabuli* reference genome-CDC Frontier/PII)	Forward primers (5'-3')	Reverse primers (5'-3')	Anneling temperature (0C)	Expected amplified product size (bp)	Structural annotation		Functional annotation	
								Sequence components of *kabuli* genome	*Kabuli* gene accession IDs	NCBI-KOG	TFs
Ca4_P3_17622678	Ca4	17622678	ATTTTTT/ATTTTT	CCTGAAAGGCATGGCTGTAT	TGCTTCCTTCCTTGAAGCAT	60.1	289	INTRON	Ca_05368	F	--
Ca4_P3_17626407	Ca4	17626407	AGGT/AGGTGGT	TGTCCCTTTCTCGTGCTCTT	GTCCAACTCCGGTTTAATGC	60.0	285	DRR	Ca_05368	F	--
Ca4_P3_17627644	Ca4	17627644	TCAC/TC	TTCGATCAAATTCCTCCACC	TGACATGGACTGAGACAATGAA	59.9	741	INTERGENIC	--	--	--
Ca4_P3_1763030	Ca4	1763030	ATTT/ATTTT	AAAGGCATCATTCACGTTCA	ACATTTTGGAAGGGCATCTG	59.1	757	INTERGENIC	--	--	--
Ca4_P3_17632165	Ca4	17632165	TTTTTGCTT/TTTTTGCTTCAATTTTGCTT	ACACAGATCCCATCCCATGT	ATTTACGGCCACCAAAACTG	60.1	575	INTRON	Ca_05367	Z	WRKY
Ca4_P3_17637226	Ca4	17637226	CTATT/C	ACACAAGTTAGGTGGGGCAG	CTGCATCTGTAGTGAGGCCA	60.0	346	INTRON	Ca_05367	Z	WRKY
Ca4_P3_17640054	Ca4	17640054	TTTATTATTATTATTATTATT/TTTATTATTATTATTATT	TATGAGTGCTGGTTTGAGCG	GAACCCTTTCCATCGAGTCA	60.0	779	DRR	Ca_05367	Z	WRKY
Ca4_P3_17640878	Ca4	17640878	T/TC	GACTCGATGGAAAGGGTTCA	AGCCAACAAAACCACCAAAG	60.1	634	DRR	Ca_05367	Z	WRKY
Ca4_P3_1764308	Ca4	1764308	AT/ATGT	AGAGGCATTGCTCATCAACC	TCGAAAAGATGCAAGACCAA	60.2	754	INTERGENIC	--	--	--
Ca4_P3_17645379	Ca4	17645379	ATTA/ATTATCTTTA	TCAAAAGCTCAAAGCAACCA	TGATGTGATATGCAATTCTACGA	59.6	600	DRR	Ca_05366	--	--

INDEL marker IDs	Chromosomes/ unanchored scaffolds	Physical positions (bp)	InDels (*Kabuli* reference genome-CDC Frontier/PII)	Forward primers (5'-3')	Reverse primers (5'-3')	Anneling temperature (0C)	Expected amplified product size (bp)	Structural annotation		Functional annotation	
								Sequence components of *kabuli* genome	*Kabuli* gene accession IDs	NCBI-KOG	TFs
Ca4_P3_1764559	Ca4	1764559	TAAAAAAAAAA/TAAAAAAAAAAA	GCCAACTCATGTCTTTGTCG	ATTCTCGTGAGTGGGGTTTG	59.3	758	INTERGENIC	--	--	--
Ca4_P3_17650086	Ca4	17650086	T/TGTA	TGAGAGGTTGGTGCTTATTTTG	TGCGGTTCCTATAAAATGGG	59.3	322	INTERGENIC	--	--	--
Ca4_P3_1765263	Ca4	1765263	CTTTTA/C	GGTCATGCGTGGGCTACTAT	TTTTCTGAAATTTTGGATGAATG	60.0	497	INTERGENIC	--	--	--
Ca4_P3_1765326	Ca4	1765326	GA/G	GGTCATGCGTGGGCTACTAT	TTTTCTGAAATTTTGGATGAATG	60.0	497	INTERGENIC	--	--	--
Ca4_P3_17654597	Ca4	17654597	TCACACAC/TCACACACAC	TCCAATTGACACCACTGCTC	TTTTCAACATTCTAAAAATGGGC	59.7	542	DRR	Ca_05364	S	--
Ca4_P3_17654627	Ca4	17654627	AT/ATAAAACTTT	TCCAATTGACACCACTGCTC	TTTTCAACATTCTAAAAATGGGC	59.7	542	DRR	Ca_05364	S	--
Ca4_P3_17658409	Ca4	17658409	GT/GTT	TCTTGTTGCTCCCCTTCATC	ACACACGTGCATTCGGTAAA	60.2	702	INTERGENIC	--	--	--
Ca4_P3_17670952	Ca4	17670952	T/TG	TGTTGACTCTTGGCTGTTGC	CGACATCATGGATTGTTTGG	60.0	659	INTERGENIC	--	--	--
Ca4_P3_17672684	Ca4	17672684	GATA/GA	TGTAGTCCCTCTGTCCCAAAA	CGGAAGATACCAAGTCGCAT	59.6	433	DRR	Ca_05360	--	--
Ca4_P3_1767777	Ca4	1767777	AATTAAAATTATT/AATT	GTACTGATGCACGCTTCCAA	TGAGAATCCTTGTGGTGGTG	59.9	494	INTERGENIC	--	--	--

INDEL marker IDs	Chromosomes/ unanchored scaffolds	Physical positions (bp)	InDels (*Kabuli* reference genome-CDC Frontier/PII)	Forward primers (5'-3')	Reverse primers (5'-3')	Anneling temperature (0C)	Expected amplified product size (bp)	Structural annotation		Functional annotation	
								Sequence components of *kabuli* genome	*Kabuli* gene accession IDs	NCBI-KOG	TFs
Ca4_P3_1767872	Ca4	1767872	ATTTTTTTT/ATTTTTTT	GTACTGATGCACGCTTCCAA	TGAGAATCCTTGTGGTGGTG	59.9	494	INTERGENIC	--	--	--
Ca4_P3_17678728	Ca4	17678728	ATTTTTTTTTTT/ATTTTTTTTTTTTTTT	CACCATGAAACCTTGCTCCT	GTTGCAGAGATGAATGGGGT	60.1	499	INTERGENIC	--	--	--
Ca4_P3_17681279	Ca4	17681279	CTTTT/CTTT	GTGTTTTAACCCAGCCCAAA	GACACGTACCACGAGCTTCA	59.8	389	DRR	Ca_05359	--	--
Ca4_P3_17690587	Ca4	17690587	TGGG/TGGGG	CGTTCTCAGAGGGAAATGGA	TCCCAAAGTGAAATGCATCA	60.2	611	INTRON	Ca_05356	TU	--
Ca4_P3_17693931	Ca4	17693931	CAAA/CAAAA	ATCCCACGCTCAGTCATTTC	TGAAGAATTGCCCTCAGACC	60.1	390	INTRON	Ca_05356	TU	--
Ca4_P3_17698774	Ca4	17698774	CAAAAAAAA/CAAAAAAAAA	TGCTTGCAGTCTGAAATTCG	ATCACGGATTGACCTTGGAG	60.1	532	INTERGENIC	--	--	--
Ca4_P3_17706860	Ca4	17706860	TATATTCGATATT/TATATT	TTGCATGCTTGGATTCAAAA	CCATCGAAAAGGAATGGCTA	60.2	413	DRR	Ca_05355	R	SBP
Ca4_P3_17715218	Ca4	17715218	GAAAAAAAAAA/GAAAAAAAA	CTGTTCCTCACATCACGCAT	GGAGTCACCCTCCAAATTGA	59.7	143	INTERGENIC	--	--	--
Ca4_P3_17715291	Ca4	17715291	CTT/CT	TCAATTTGGAGGGTGACTCC	TACCAGCTTTGGTTTCTGCC	59.9	250	INTERGENIC	--	--	--
Ca4_P3_17715589	Ca4	17715589	TAAAAAA/TAAAAAAAA	TCAATTTGGAGGGTGACTCC	AAAAATTGTTGCACCATCCC	59.9	372	INTERGENIC	--	--	--

INDEL marker IDs	Chromosomes/ unanchored scaffolds	Physical positions (bp)	InDels (*Kabuli* reference genome-CDC Frontier/PII)	Forward primers (5'-3')	Reverse primers (5'-3')	Anneling temperature (0C)	Expected amplified product size (bp)	Structural annotation		Functional annotation	
								Sequence components of *kabuli* genome	*Kabuli* gene accession IDs	NCBI-KOG	TFs
Ca4_P3_17721817	Ca4	17721817	AGCCAACTTG/AG	CACAATTTGGCAGGGATTTT	TAATTTGCGGGTATGCTTCC	59.8	874	DRR	Ca_05352	KL	bHLH
Ca4_P3_17721863	Ca4	17721863	TA/TAA	CAGCCAACTTGAGCAACAAA	TAATTTGCGGGTATGCTTCC	60.0	426	DRR	Ca_05352	KL	bHLH
Ca4_P3_17736173	Ca4	17736173	TTTAAGAAAATTAA/TTTAA	CAGTGAAGGATGATGAAGAAAAA	AAACGTGGCCCTATAAGCCT	58.4	403	INTERGENIC	--	--	--
Ca4_P3_17738806	Ca4	17738806	TAAAAAAAAAA/TAAAAAAAAAAA	GGAGTCATGGAATTTGGTCG	TTGCTGTATCAAGTGGCAAAA	60.3	810	INTERGENIC	--	--	--
Ca4_P3_17738882	Ca4	17738882	AC/A	TTCTTTCTCTTGGTGTTGCG	TTGCTGTATCAAGTGGCAAAA	59.0	745	INTERGENIC	--	--	--
Ca4_P3_17746874	Ca4	17746874	G/GA	CGATGGCAATTTTGGTTTCT	ACTCGTCGCTGTCTTGGACT	59.9	543	INTERGENIC	--	--	--
Ca4_P3_17747209	Ca4	17747209	TAAAAAAAAAA/TAAAAAAAA	AGTCCAAGACAGCGACGAGT	CCCAAGAGAGTTGTGGGCTA	60.1	313	INTERGENIC	--	--	--
Ca4_P3_17748069	Ca4	17748069	AG/A	CCCATTTACGAAGAATCCGA	CGTACCTTTCGTTGCCATTT	59.9	287	INTERGENIC	--	--	--
Ca4_P3_17748105	Ca4	17748105	GTTT/GTTTT	CCCATTTACGAAGAATCCGA	CGTACCTTTCGTTGCCATTT	59.9	287	INTERGENIC	--	--	--
Ca4_P3_17748605	Ca4	17748605	ATTTTTTTTT/ATTTTTTTTTT	AAATGGCAACGAAAGGTACG	TGCTCAACAACCTGAGCATC	60.0	423	DRR	Ca_05350	R	NF-YB

INDEL marker IDs	Chromosomes/ unanchored scaffolds	Physical positions (bp)	InDels (*Kabuli* reference genome-CDC Frontier/PII)	Forward primers (5'-3')	Reverse primers (5'-3')	Anneling temperature (0C)	Expected amplified product size (bp)	Structural annotation		Functional annotation	
								Sequence components of *kabuli* genome	*Kabuli* gene accession IDs	NCBI-KOG	TFs
Ca4_P3_17749950	Ca4	17749950	T/TC	AGTGGCAATTTGGTGCTCTT	GTGCTTATGTGGCAAACCCT	59.7	762	DRR	Ca_05351	R	WOX
Ca4_P3_1775612	Ca4	1775612	TT/TTTCAATTTTTGCT	GAGAAATGGAGAGGGAAGGG	TCGTCTGACACCACCAACAT	60.0	718	DRR	Ca_07800	K	HSF
Ca4_P3_17757375	Ca4	17757375	T/TC	TAAACCAACCCCACACCATT	ACAATGCCTTTTTGCCACTC	59.9	252	DRR	Ca_05349	O	--
Ca4_P3_17765368	Ca4	17765368	AA/AAGA	TAAACCGTGGTAAAGGCTGG	TCCGATCCCGATGTACTCTC	60.0	602	INTRON	Ca_05349	O	--
Ca4_P3_17766884	Ca4	17766884	A/AAAT	TGCACCAGACATATTTTCGC	AAAAGCCACACAGGATGGAC	59.7	873	DRR	Ca_05348	--	--
Ca4_P3_17774621	Ca4	17774621	A/AC	ACATTTGTTTTCACTCCGGC	TGCAGCCACGTGTTTATTTC	60.0	736	DRR	Ca_05347	R	--
Ca4_P3_17774656	Ca4	17774656	TATG/TATGAATG	ACATTTGTTTTCACTCCGGC	TGCAGCCACGTGTTTATTTC	60.0	736	DRR	Ca_05347	R	--
Ca4_P3_17775890	Ca4	17775890	TAAA/TA	TGCATTCAGCAAAAAGTACCA	GGCACTGCCGTCAAAAGTAT	59.4	525	DRR	Ca_05347	R	--
Ca4_P3_17776534	Ca4	17776534	GTAA/GTAATTAA	GCAGTGCCATTGAACAGCTA	GTACTTGTTTCCGGCGTGAT	60.0	694	INTERGENIC	--	--	--
Ca4_P3_17777647	Ca4	17777647	CATCTGAATCTGAA/CATCTGAA	CCTTAGGGGAGATTTGCATTC	TGCAGATAAATGGCCAAACA	59.9	852	INTERGENIC	--	--	--

INDEL marker IDs	Chromosomes/ unanchored scaffolds	Physical positions (bp)	InDels (*Kabuli* reference genome-CDC Frontier/PII)	Forward primers (5'-3')	Reverse primers (5'-3')	Anneling temperature (0C)	Expected amplified product size (bp)	Structural annotation		Functional annotation	
								Sequence components of *kabuli* genome	*Kabuli* gene accession IDs	NCBI-KOG	TFs
Ca4_P3_1777960	Ca4	1777960	CATAAAAAT/CATAAAAATAAAAAT	AGGGGACGTCTCCCATTTAT	AGATAGGACCCCCACCATTC	59.7	705	INTRON	Ca_07800	K	HSF
Ca4_P3_17786473	Ca4	17786473	TTTTTGT/TT	CGCGCCAACTTTACTTCTTC	TCCAAAATTTAGTGGCAGCA	60.0	621	INTERGENIC	--	--	--
Ca4_P3_1779497	Ca4	1779497	GTT/GTTT	CACCCCAAAGCAACAAGAAT	TTGGCTTAACATGCCTTGAA	60.0	575	INTERGENIC	--	--	--
Ca4_P3_17823791	Ca4	17823791	TAAAAAAAAAAAA/TAAAAAAAAAA	TCTCATGTCTTGCAAAATTCTTCT	TTTGTGATTGATATTTGCCGTT	59.4	354	INTERGENIC	--	--	--
Ca4_P3_17825306	Ca4	17825306	TAAAA/TAAA	AATCCATCAAACTCGGTTGC	TGCTATTTGGTTGGCCTTTT	59.9	301	INTERGENIC	--	--	--
Ca4_P3_17825598	Ca4	17825598	ACC/ACCC	AATCCATCAAACTCGGTTGC	GGGCCCCGTAAGTATTCAGT	59.9	679	INTERGENIC	--	--	--
Ca4_P3_17825906	Ca4	17825906	ATTTTTTT/ATTTTTT	AAAAGGCCAACCAAATAGCA	CCTCAATTCTCGATTTCCCA	59.6	555	INTERGENIC	--	--	--
Ca4_P3_17825981	Ca4	17825981	ATTTTTTTT/ATTTTTTT	GGGGCCCCATAATCATATTT	CCTCAATTCTCGATTTCCCA	59.7	164	INTERGENIC	--	--	--
Ca4_P3_17827610	Ca4	17827610	A/AT	TCAACTTCGGTGCTTGACAG	TTGGGGTACACGAACAATGA	60.0	141	INTERGENIC	--	--	--
Ca4_P3_17828981	Ca4	17828981	ATT/ATTTT	ATGGTTTGTCTCCACTTGGC	AAAACCAAGAACGGTGATGC	60.0	414	INTERGENIC	--	--	--

INDEL marker IDs	Chromosomes/ unanchored scaffolds	Physical positions (bp)	InDels (*Kabuli* reference genome-CDC Frontier/PII)	Forward primers (5'-3')	Reverse primers (5'-3')	Anneling temperature (0C)	Expected amplified product size (bp)	Structural annotation		Functional annotation	
								Sequence components of *kabuli* genome	*Kabuli* gene accession IDs	NCBI-KOG	TFs
Ca4_P3_17830486	Ca4	17830486	ATTTTTTTTT/ATTTTTTTTTT	TGGCTTGCATTCTCATTCAG	TTTGGATGCATAGGCCATTT	59.9	296	INTERGENIC	--	--	--
Ca4_P3_17831112	Ca4	17831112	TAA/TA	CACTCCAAGCCTCTTATGGG	GGAAAGATGGACACACGAAAA	59.7	605	INTERGENIC	--	--	--
Ca4_P3_17831943	Ca4	17831943	ATT/AT	TTTCTTCGCAGCATACCACA	ACCCTTATTTCTTCGCTCCG	60.4	491	INTERGENIC	--	--	--
Ca4_P3_17843666	Ca4	17843666	GAA/GAACAA	ACCCGTTTCTTTCACACCAG	CCAATCGAGTGTCAGCAAGA	60.0	398	INTERGENIC	--	--	--
Ca4_P3_17844265	Ca4	17844265	AGG/AGGG	AGGAGCACCACATTTATGCC	GAGAATGCCGATTCCGTTTA	60.0	362	DRR	Ca_18621	J	--
Ca4_P3_17845307	Ca4	17845307	TAAGAGTAATGAA/TAA	TACCATTCACCCGTTCACCT	CTGGGGTCATTTTGGAGAAA	60.2	535	DRR	Ca_18621	J	--
Ca4_P3_17846018	Ca4	17846018	AAATA/AA	TTCACCAAATTCATGTCAAAGC	ACAACATGGGTCGGTCTCTC	60.0	737	DRR	Ca_18621	J	--
Ca4_P3_17848856	Ca4	17848856	AATAAATTATTATAA/AATAA	TCTGCTGAGACCCGTCTTTT	AAGCCAGGGCCAGAAATTAT	60.0	602	INTRON	Ca_18621	J	--
Ca4_P3_17849398	Ca4	17849398	AGGG/AGGGG	AGTGTAAGTGCCCAAGGTGG	CCGTTCGTTAAGTGCGAAAT	60.0	156	INTRON	Ca_18621	J	--
Ca4_P3_1786291	Ca4	1786291	GTTAGTTAGTT/GTTAGTTAGTTTGTTATTAGTTAGTT	AAATCGTGTCAACCGCTACC	ATCGTCGTCTCTCACCATCC	60.0	870	INTRON	Ca_07801	T	--

INDEL marker IDs	Chromosomes/ unanchored scaffolds	Physical positions (bp)	InDels (*Kabuli* reference genome-CDC Frontier/PII)	Forward primers (5'-3')	Reverse primers (5'-3')	Anneling temperature (0C)	Expected amplified product size (bp)	Structural annotation		Functional annotation	
								Sequence components of *kabuli* genome	*Kabuli* gene accession IDs	NCBI-KOG	TFs
Ca4_P3_17865279	Ca4	17865279	CTTTTTTTTTTTT/CTTTTTTTTTT	ATATGGAAAGTCGCCG TTTG	CCGAGGCAATAAAACA CCTC	60.0	494	INTERGENIC	--	--	--
Ca4_P3_17865546	Ca4	17865546	TAAAAAA/TAAAAAAA	GAGGTGTTTTATTGCCT CGG	TGTTAATCCCAATTCAA TATCCC	59.6	133	INTERGENIC	--	--	--
Ca4_P3_17871181	Ca4	17871181	TAAAAAAAAAA/TAAAAAAAAA	GGGGTATTTGGACGAA TGTG	CGAAAGCGATTGTTTA CGGT	60.1	651	DRR	Ca_18624	TU	WOX
Ca4_P3_17873043	Ca4	17873043	TAAA/TA	CATTGAAATGCATGGA CCAA	TTTTCTCCATGCACCC TACC	60.3	885	INTERGENIC	--	--	--
Ca4_P3_17873058	Ca4	17873058	T/TA	CATTGAAATGCATGGA CCAA	TTTTCTCCATGCACCC TACC	60.3	885	INTERGENIC	--	--	--
Ca4_P3_17873426	Ca4	17873426	AAGGA/AAGGAGGA	CGAATGGAAAAAGCGA GTTC	CAATAATCCCCGCTCT TGAA	59.8	303	INTERGENIC	--	--	--
Ca4_P3_1789478	Ca4	1789478	GC/GCC	TCAAATCAGGATGAGG GAGG	CTGGTTGGCCTTGAGT GAAT	60.0	270	INTRON	Ca_07801	T	--
Ca4_P3_1791824	Ca4	1791824	GA/G	CGATATTGTCAAATGGC GTG	TTTTGGATTCCTCCCT CCTT	60.0	644	INTRON	Ca_07801	T	--
Ca4_P3_1794143	Ca4	1794143	TAA/TAAA	TTGGTAGCAAAGGACC CAAC	CAGCTTCTTTCCACTG AGGG	60.0	385	INTRON	Ca_07801	T	--
Ca4_P3_17942733	Ca4	17942733	CTATATATATATATAT/CTATATATATAT ATATAT	CATCGTCAGCACATCA CAAA	CTTAGGAGGTCTTGCC CCTC	59.2	447	INTERGENIC	--	--	--

INDEL marker IDs	Chromosomes/ unanchored scaffolds	Physical positions (bp)	InDels (*Kabuli* reference genome-CDC Frontier/PII)	Forward primers (5'-3')	Reverse primers (5'-3')	Anneling temperature (0C)	Expected amplified product size (bp)	Structural annotation		Functional annotation	
								Sequence components of *kabuli* genome	*Kabuli* gene accession IDs	NCBI-KOG	TFs
Ca4_P3_1795184	Ca4	1795184	AATAT/AATATAT	GGTTTGAAAGATCCACCCAA	CAACCCATCATCCCAGTTTC	59.8	317	INTRON	Ca_07801	T	--
Ca4_P3_17959700	Ca4	17959700	C/CATTCCCTT	AGTGGCATGTGGGGATTAAA	TTTGGTGGAAATGGCATGTA	60.2	168	INTERGENIC	--	--	--
Ca4_P3_17962638	Ca4	17962638	GATATATATATATATATATA/GATATATATATATATATATATA	CTTCAGCGTGCAAAATTCAA	CACGTGCATGCTTGTGTTTA	60.0	700	INTRON	Ca_18636	--	--
Ca4_P3_1801951	Ca4	1801951	TAAAAAAAAAA/TAAAAAAAAAAA	CGTAGCAAGTGCCACGTAGA	ATTTTACGACCTCGCACCAC	60.1	813	INTRON	Ca_07801	T	--
Ca4_P3_18033913	Ca4	18033913	CT/CTT	CTGAGTGACCTCCCTTCCAA	TCATTCCAGAAGGAGATGGG	60.2	433	INTRON	Ca_18645	--	--
Ca4_P3_18041622	Ca4	18041622	CTTTTTTT/CTTTTTTTT	CCTGTTTTCCTCCACCCTTT	ATGGGGACTTGGGGAGTAAC	60.3	660	INTERGENIC	--	--	--
Ca4_P3_18061059	Ca4	18061059	TATAATAATAATAATAATAATAATAA/TATAATAATAATAATAATAATAATAATAATAA	GAAGCGACCACCTGAAGAAC	CAGAACAAATGACGTGTCCC	59.9	536	INTERGENIC	--	--	--
Ca4_P3_18091373	Ca4	18091373	TCATCAC/T	CCACGGATGTGATTTAGCCT	GAAGCACCGTTCCTTCTTGA	60.0	401	INTERGENIC	--	--	--
Ca4_P3_18111455	Ca4	18111455	ATTTTTTTTTT/ATTTTTTTTTTT	TCACCATCATTTTCATCGAGTC	TGTGGTTGTCGTTTACTCACG	59.9	472	INTERGENIC	--	--	--
Ca4_P3_18121565	Ca4	18121565	TAAGAAG/TAAG	GCTGCACCATGAGATCTTGA	ATGGTGCTGGAAGTTGCTTT	60.0	440	INTRON	Ca_18652	--	--

INDEL marker IDs	Chromosomes/ unanchored scaffolds	Physical positions (bp)	InDels (*Kabuli* reference genome-CDC Frontier/PII)	Forward primers (5'-3')	Reverse primers (5'-3')	Anneling temperature (0C)	Expected amplified product size (bp)	Structural annotation		Functional annotation	
								Sequence components of *kabuli* genome	*Kabuli* gene accession IDs	NCBI-KOG	TFs
Ca4_P3_1818718	Ca4	1818718	AATATATATATA/AATATATATA	TTGCACTGTTTGGTGAAAGG	CAACCGATCCAACTTCAACA	59.7	218	INTERGENIC	--	--	--
Ca4_P3_18196212	Ca4	18196212	ATTT/ATTTAATCTTTTT	GTGGTCCAGATTCTCCCAAA	TGTCGCTTCAGCAAAAAGAA	59.9	549	INTERGENIC	--	--	--
Ca4_P3_1822667	Ca4	1822667	TAAAAAAA/TAAAAAA	TTGATTTGTGTTGTTCTGGGTC	TTGAAAAGGGGTTCCTCAAA	59.9	604	INTERGENIC	--	--	--
Ca4_P3_1823869	Ca4	1823869	CTC/CTCTAATC	GCATCAACATGCTTACACACG	GTGAAAAGCGTTTTGACCCT	60.2	444	INTERGENIC	--	--	--
Ca4_P3_1824490	Ca4	1824490	ATTTTTTTT/ATTTTTTTTT	TTGAATTTTCATTGGTCCAAAA	CGAGCCAAAACCGTACAAAT	59.3	788	INTERGENIC	--	--	--
Ca4_P3_1830211	Ca4	1830211	CTATATATATATATATA/CTATATATATATATATATA	ATGATCTACGGATGCTTGGC	CAATCAACCCCAAGCAGTTT	60.1	554	DRR	Ca_07803	T	--
Ca4_P3_18314259	Ca4	18314259	CAAAAAAAA/CAAAAAAAAA	TCACTGAAGGCTCAAGCTCA	TTCATCATTGTCGTTCCAGG	59.9	348	INTERGENIC	--	--	--
Ca4_P3_18388650	Ca4	18388650	ATATTA/ATA	AGTTATCGATCACGGATGCC	CGTGTATATTCGCGCACTCA	59.9	514	INTERGENIC	--	--	--
Ca4_P3_1841038	Ca4	1841038	CA/CAAA	AGCCAGCAGATGGAAGAGAA	GCCAAAGCATTTGACATTGA	60.1	638	DRR	Ca_07805	--	--
Ca4_P3_1841092	Ca4	1841092	TTC/T	AGCCAGCAGATGGAAGAGAA	GCCAAAGCATTTGACATTGA	60.1	638	DRR	Ca_07805	--	--

INDEL marker IDs	Chromosomes/ unanchored scaffolds	Physical positions (bp)	InDels (*Kabuli* reference genome-CDC Frontier/PII)	Forward primers (5'-3')	Reverse primers (5'-3')	Anneling temperature (0C)	Expected amplified product size (bp)	Structural annotation		Functional annotation	
								Sequence components of *kabuli* genome	*Kabuli* gene accession IDs	NCBI-KOG	TFs
Ca4_P3_18446227	Ca4	18446227	GTTTTTT/GTTTTTTT	TCTCACGGTTGTGAGGACTG	GGTGGAGGTGAAGGTAAACAA	59.9	304	INTERGENIC	--	--	--
Ca4_P3_1849410	Ca4	1849410	TAAAAAAA/TAAAAAA	CCTCTTTGTTCTCACGCGAT	ATCAACTGAAACGACCTGGC	60.4	879	INTERGENIC	--	--	--
Ca4_P3_1849451	Ca4	1849451	CTTTTTTTT/CTTTTTTTTT	GGATTTTCCATGAGAAAGGCTA	ATCAACTGAAACGACCTGGC	59.6	689	INTERGENIC	--	--	--
Ca4_P3_1849594	Ca4	1849594	CT/CTAAT	GGATTTTCCATGAGAAAGGCTA	ATCAACTGAAACGACCTGGC	59.6	689	INTERGENIC	--	--	--
Ca4_P3_1849730	Ca4	1849730	ATTTT/ATTTTT	TTTGGGAGCAATAAATTTTCAA	ATCAACTGAAACGACCTGGC	58.7	228	INTERGENIC	--	--	--
Ca4_P3_1855105	Ca4	1855105	TTTAT/TTTATTAT	CACGACCCTTTCGATTGTTT	AAACCAGCTGCCAATTCTGT	60.0	430	INTERGENIC	--	--	--
Ca4_P3_18554841	Ca4	18554841	TTATATATATATATATATATATATATATATAT/TTATATATATATATATATATATATATATAT	TGGACCATATGTGTTGGGTC	CCCGGATAATTGTTCACTTTG	59.1	809	INTERGENIC	--	--	--
Ca4_P3_18582757	Ca4	18582757	GTATATATATATATATAT/GTATATATATAT	CGAAGATTTTATGACACGGGA	CGGTTAATTTCCACACCGAC	59.9	806	INTERGENIC	--	--	--
Ca4_P3_1860443	Ca4	1860443	CTTTTTT/CTTTTTTT	TGCCTTCAAAGTTCTGCATC	TGGGAATGGATTTATCGGAA	59.0	455	INTERGENIC	--	--	--
Ca4_P3_1861881	Ca4	1861881	GTTTATTTATTTATTTATTTATT/GTTTATTTATTTATTTATTTATTTATT	TGAGGACAAAAGATCCCCTG	AGGGTCCACAGAAAATCGTG	60.0	870	INTERGENIC	--	--	--

**INDEL marker IDs**	**Chromosomes/ unanchored scaffolds**	**Physical positions (bp)**	**InDels (*Kabuli* reference genome-CDC Frontier/PII)**	**Forward primers (5'-3')**	**Reverse primers (5'-3')**	**Anneling temperature (0C)**	**Expected amplified product size (bp)**	**Structural annotation**		**Functional annotation**	
								**Sequence components of *kabuli* genome**	***Kabuli* gene accession IDs**	**NCBI-KOG**	**TFs**
Ca4_P3_1864781	Ca4	1864781	CTATTATTATTATTATT/CTATTATTATTATTATTATT	CTTCTGGGTGTCAGCAATGA	AATCAAATCAACGTGTGCCA	59.8	432	INTRON	Ca_07807	P	MIKC
Ca4_P3_18674763	Ca4	18674763	AATATATATATATATATATAT/AATATATATATATATATATATAT	CATGAGGGTTAGGTTTCGCT	ACTTGCATCCACGTACTCCC	59.2	649	INTERGENIC	--	--	--
Ca4_P3_18676206	Ca4	18676206	A/AGGTTGATGACACC	TCGTAACGCAAAGCTTCAAA	GCAGGACGTTTCCATTTACC	59.6	551	INTERGENIC	--	--	--
Ca4_P3_18703975	Ca4	18703975	GACACA/GACA	CCACATTGACATAAACATCTTCTCA	TCAACAACGTTGCTCTTTTTG	60.3	173	INTERGENIC	--	--	--
Ca4_P3_1875003	Ca4	1875003	GAAAAAA/GAAAAAAA	ATTTCTGCTCTCCTGGCAAA	GGAGGTTGAGTTGCACCATT	60.0	833	INTRON	Ca_07808	--	MYB_related
Ca4_P3_1883350	Ca4	1883350	ATTTTTTTT/ATTTTTTTTT	ATTATCATGCGTGCGTCAAC	TACTTGAAACAGGCCGGAAG	59.6	421	INTERGENIC	--	--	--
Ca4_P3_18836993	Ca4	18836993	TGG/TGGG	AGTGGAAAAAGCAGAAGGCA	CAACTGCTAGACGTTGGGC	60.0	137	INTERGENIC	--	--	--
Ca4_P3_18883677	Ca4	18883677	AATATATATATATATATATATATATATATATA/AATATATATATATATATATATATATATATATATATATATA	AAAATGTAGGGTGGACGGTG	TATTCCCCCAATGAAGGACA	59.7	750	DRR	Ca_13985	E	WRKY
Ca4_P3_19045820	Ca4	19045820	A/AT	GGGCTTGCTTGGTTTGAATA	TCAACTGCACGTGTTAAATGC	60.1	825	INTERGENIC	--	--	--
Ca4_P3_19046060	Ca4	19046060	CAAAAAAA/CAAAAAAAA	TGAAAATGAGATTAGAACCACCAA	TCTTCACCTGCATCGAAATG	59.9	677	INTERGENIC	--	--	--

INDEL marker IDs	Chromosomes/ unanchored scaffolds	Physical positions (bp)	InDels (*Kabuli* reference genome-CDC Frontier/PII)	Forward primers (5'-3')	Reverse primers (5'-3')	Anneling temperature (0C)	Expected amplified product size (bp)	Structural annotation		Functional annotation	
								Sequence components of *kabuli* genome	*Kabuli* gene accession IDs	NCBI-KOG	TFs
Ca4_P3_1907150	Ca4	1907150	TATAAAAAAATAAAA/TATAAAAAATAAAAAATAAAAA	AAAGAGTTTAAAGGCGGGGA	GCGGTCATATTCCGAATGAA	60.1	437	INTERGENIC	--	--	--
Ca4_P3_19081618	Ca4	19081618	CAATAATAATAATAATAATAATAA/CAATAATAATAATAATAATAATAA	CGACACTTCAACACAAAATTCA	TGTTGAAAACAACATGTTCCC	58.7	893	INTERGENIC	--	--	--
Ca4_P3_19228202	Ca4	19228202	TATG/TATGATCGTGATG	GCAATCTGGAAAGATGGGAA	AAATTCGCCCTGTGTTTTTG	60.0	288	CDS (large-effect mutations)	Ca_14021	T	WRKY
Ca4_P3_19294563	Ca4	19294563	T/TATATAC	GCATACTCTGTCCTTTCGTCC	GGCTGCCATCACTTCATCTC	58.8	586	INTERGENIC	--	--	--
Ca4_P3_19342121	Ca4	19342121	TAAAAAAAA/TAAAAAAAAA	TTCCGAAAATTCACATGCAC	GAATTTGGATTATTCGATTCTCTGA	59.5	388	INTERGENIC	--	--	--
Ca4_P3_1935392	Ca4	1935392	TAAAAAAAAA/TAAAAAAAAAA	GCGTTGCACGGGTATATTTT	TCAACCAAAAAGCTGACGTG	59.9	371	INTERGENIC	--	--	--
Ca4_P3_19369951	Ca4	19369951	CAAAAA/CAAAAAA	CCCATGTTTAAGGGTGGTTG	AATTTTTCACGACCTTCTTCAA	60.1	561	INTERGENIC	--	--	--
Ca4_P3_19370452	Ca4	19370452	CAAAAAAAAAAA/CAAAAAAAAAAAA	GGATTGAGATATTCTTCGTCCA	TCGCTCATGTGAGAGACCAC	58.1	265	INTERGENIC	--	--	--
Ca4_P3_19387051	Ca4	19387051	CAAAA/CAA	GGAGTACTCTGACAACGTCCAA	ACGGTCTTCCAATCGAGATG	59.3	256	DRR	Ca_14033	O	--
Ca4_P3_19397426	Ca4	19397426	GAAAA/GAAA	GCCAACATATGCTTGATGGA	TTGAACTTTCTCCGTTTGGG	59.5	667	INTERGENIC	--	--	--

INDEL marker IDs	Chromosomes/ unanchored scaffolds	Physical positions (bp)	InDels (*Kabuli* reference genome-CDC Frontier/PII)	Forward primers (5'-3')	Reverse primers (5'-3')	Anneling temperature (0C)	Expected amplified product size (bp)	Structural annotation		Functional annotation	
								Sequence components of *kabuli* genome	*Kabuli* gene accession IDs	NCBI-KOG	TFs
Ca4_P3_19406743	Ca4	19406743	AAAACCAAATACACACTACAA/AAAACCAAATACACACTACAAACCAAATACACACTACAA	ACCATCCGTCACAAAAACAA	CAACTCCACCAAACCACCTT	58.9	576	INTERGENIC	--	--	--
Ca4_P3_19425608	Ca4	19425608	GTTTTTTTTTTTTT/GTTTTTTTTTTTT	AGAACCCGACGACTGCTCTA	TATTTGGGCTTTAACCGACG	60.0	324	INTERGENIC	--	--	--
Ca4_P3_19576423	Ca4	19576423	CATATATATATATATATATATATATAT/CATATATATATATATATATATATATATAT	CCGATCGGAAAACAAAAGTAA	TCCATATTCGGTAGCCGTTC	59.1	939	INTERGENIC	--	--	--
Ca4_P3_1958352	Ca4	1958352	TAAAAAA/TAAAAA	AACAAGCCCAATTTTGCAGT	TGGAGATTGTTCACCCATCA	59.6	463	INTERGENIC	--	--	--
Ca4_P3_19643688	Ca4	19643688	CTTTTT/CTTTTTT	GGTGCAGTTGTTTGATGCAC	ATGCGATTTACATGGTTGGC	60.2	255	INTERGENIC	--	--	--
Ca4_P3_19678061	Ca4	19678061	C/CGGCTGGTACAA	ATTTTGGCAACGGCTGATAC	CCACAATCCTCCGTTGTCTT	60.0	492	INTERGENIC	--	--	--
Ca4_P3_19780699	Ca4	19780699	CTTGTAATTTGTAATTTGT/CTTGTAATTTGTAATTTGTAATTTGT	AGCAAACCACACAACCACAA	TTTGTGACCACCACTTCCAA	60.0	484	INTERGENIC	--	--	--
Ca4_P3_19789343	Ca4	19789343	A/AG	CTAGGGGATTGCATTCCAAA	TTTTCCTGCCAAACACCTTC	59.9	177	INTERGENIC	--	--	--
Ca4_P3_19883616	Ca4	19883616	ATTTTTTT/ATTTTTTTT	CATGTGCAATTTCTTGTGGG	TTTTTGTGTGCAGAGATGGG	60.0	572	INTERGENIC	--	--	--
Ca4_P3_19886492	Ca4	19886492	CATTTCTTATTTCTT/CATTTCTT	GACCCCCTGACAAAGACAGA	AGAGGGTTGTGCACTTGAGC	60.1	415	INTERGENIC	--	--	--

INDEL marker IDs	Chromosomes/ unanchored scaffolds	Physical positions (bp)	InDels (*Kabuli* reference genome-CDC Frontier/PII)	Forward primers (5'-3')	Reverse primers (5'-3')	Anneling temperature (0C)	Expected amplified product size (bp)	Structural annotation		Functional annotation	
								Sequence components of *kabuli* genome	*Kabuli* gene accession IDs	NCBI-KOG	TFs
Ca4_P3_2002357	Ca4	2002357	ATTCTTCTTCTTCTTCTTCTTCTTCTTCTT/ATTCTTCTTCTTCTTCTTCTTCTTCTTCTTCTTCTT	ATGGCTGCTTTTGCTGATCT	TAGAGGCTCCCCACTCTCAA	60.0	377	DRR	Ca_07818	--	--
Ca4_P3_20026462	Ca4	20026462	TTTTTTT/TTTTTTTATTTTTT	GACGGATCTTCTCTGGAACG	TGGAGAGGGAAGAGGTGAAA	59.8	118	INTERGENIC	--	--	--
Ca4_P3_20051846	Ca4	20051846	GTAT/GTATGTGATTAGTGACTAT	CACGAAGCGTATTGACTCCA	AAATTTGAAGGCGCTTTTTG	59.9	432	INTERGENIC	--	--	--
Ca4_P3_20180492	Ca4	20180492	CAAAAAAAAAAAAAAA/CAAAAAAAAAAAAAA	TGGTAAGCTAGATTGAGGGCA	ATCATCACCCCAGGATTTCA	59.9	224	DRR	Ca_17139	R	MYB
Ca4_P3_20233525	Ca4	20233525	A/AG	ATATTCAGATCCCTTGCCCA	CTTTGCCAATTCCAGTCCAT	59.3	136	DRR	Ca_17143	--	Trihelix
Ca4_P3_20266418	Ca4	20266418	T/TGC	CTGCAGAATGTTTGCAGAATG	ACCATCAAGGACCCAAACAA	59.5	508	INTERGENIC	--	--	--
Ca4_P3_2030157	Ca4	2030157	GAAAAAAA/GAAAAAAAA	TGAAAGTGCCAGAAAACCCT	CGAATGTTTATGGTTGGGGT	59.7	354	INTERGENIC	--	--	--
Ca4_P3_2032207	Ca4	2032207	TC/T	TGCTGAGTTCGAGGAAAGGT	CACATCTCTTGTTCGCTCCA	60.0	517	CDS (FRAME SHIFT)	Ca_07822	D	--
Ca4_P3_2032547	Ca4	2032547	CTGTGTGTGT/CTGTGTGT	TGCTGAGTTCGAGGAAAGGT	CACATCTCTTGTTCGCTCCA	60.0	517	INTRON	Ca_07822	D	--
Ca4_P3_2032733	Ca4	2032733	A/AT	TGGAGCGAACAAGAGATGTG	AGCAGCTGCACCACTAATCA	60.0	234	INTRON	Ca_07822	D	--

INDEL marker IDs	Chromosomes/ unanchored scaffolds	Physical positions (bp)	InDels (*Kabuli* reference genome-CDC Frontier/PII)	Forward primers (5'-3')	Reverse primers (5'-3')	Anneling temperature (0C)	Expected amplified product size (bp)	Structural annotation		Functional annotation	
								Sequence components of *kabuli* genome	*Kabuli* gene accession IDs	NCBI-KOG	TFs
Ca4_P3_2034429	Ca4	2034429	C/CA	ACCAGATACCGCACCAAAAG	TTCTGCATACTTGCTCCACG	60.0	352	INTRON	Ca_07822	D	--
Ca4_P3_2034663	Ca4	2034663	TACA/TACACA	ACCAGATACCGCACCAAAAG	AGTTGGCAAGTTTGTCAGGC	60.0	570	INTRON	Ca_07822	D	--
Ca4_P3_2035282	Ca4	2035282	GTTTTTT/GTTTTTTT	CGCGCCAGAAGTATGTGATA	TGGGCAGCTTAGACTGGACT	59.9	275	INTRON	Ca_07822	D	--
Ca4_P3_2035697	Ca4	2035697	ATTT/AT	AGTCCAGTCTAAGCTGCCCA	TGGAATCATGCCATGCTAGA	60.0	436	DRR	Ca_07823	R	MYB
Ca4_P3_2035798	Ca4	2035798	A/AGTGT	AGTCCAGTCTAAGCTGCCCA	TGGAATCATGCCATGCTAGA	60.0	436	DRR	Ca_07823	R	MYB
Ca4_P3_20615648	Ca4	20615648	ATTTTTTTTTTTTTT/ATTTTTTTTTTTTT	CCATTGTCGTTGTGTTTTGC	TGAATAATCTGCACGTCCCA	60.0	460	INTERGENIC	--	--	--
Ca4_P3_20650435	Ca4	20650435	TTATATATATATATATATATATATAT/TTATATATATATATATATATAT	CGTGTGAGAAAACCTCGACA	CACCCTTACAAGTCGCCATT	59.9	733	INTERGENIC	--	--	--
Ca4_P3_20709240	Ca4	20709240	ATTTTTTT/ATTTTTTTT	ACTTTGCCCATCAGTTTTGC	TCCGTTGGTGAAAGTTTGAA	60.1	251	INTERGENIC	--	--	--
Ca4_P3_20773849	Ca4	20773849	CTTATTATTATTAT/CTTATTATTATTATTAT	TCTCGGCTGAAGGAACAGAT	AGCGGAACAACGACAAGATT	60.0	524	INTRON	Ca_17170	--	E2F/DP
Ca4_P3_20798832	Ca4	20798832	TTTTATTTATTTATTTATT/TTTTATTTATTTATT	TAGTGCTTTTGCATGCCTCA	TTTGTCCTGGGAAAATGCTC	60.5	316	INTERGENIC	--	--	--

INDEL marker IDs	Chromosomes/ unanchored scaffolds	Physical positions (bp)	InDels (*Kabuli* reference genome-CDC Frontier/PII)	Forward primers (5'-3')	Reverse primers (5'-3')	Anneling temperature (0C)	Expected amplified product size (bp)	Structural annotation		Functional annotation	
								Sequence components of *kabuli* genome	*Kabuli* gene accession IDs	NCBI-KOG	TFs
Ca4_P3_20884613	Ca4	20884613	AATATATATATATATATATATAT/AATATATATATATATATATATATAT	TCGGTTATAGAGATTAAACTCGGG	CAGAAAATAGAAACGGAAAATTCG	59.9	391	INTERGENIC	--	--	--
Ca4_P3_20962664	Ca4	20962664	AATATATATATATATATATATATATATATAT/AATATATATATATATATATATATATATATATATATAT	TGGTCAAAATCTTTCTTTGGAA	GATTTAACCATTTCATCCTCATTG	58.7	743	INTERGENIC	--	--	--
Ca4_P3_20962865	Ca4	20962865	TTATATATATATATATATATATAT/TTATATATATATATATATATATAT	AAGAAAGGAGAATGATAAGATCTCAAA	GCCAACTGCTTCAGCTTTTC	59.3	827	INTERGENIC	--	--	--
Ca4_P3_20977808	Ca4	20977808	TA/TAA	ACATTGCCTTGTGCATACCA	CTCAAGCAAACACCTCGACA	60.0	493	INTERGENIC	--	--	--
Ca4_P3_21047816	Ca4	21047816	TATAATAATAATAATAATAATAA/TATAATAATAATAATAATAATAATAA	CCTACCGGACCGTTTGTCTA	GCAGATCAATTTAACCGCATT	60.0	711	INTERGENIC	--	--	--
Ca4_P3_21071879	Ca4	21071879	T/TA	AATTGAGTGATCGAGTCGGC	ATGACGCGTGTCCAATTACA	60.2	837	INTERGENIC	--	--	--
Ca4_P3_21072127	Ca4	21072127	AG/A	TCTGTTTGGCAAGAAGAAGAAA	ATGACGCGTGTCCAATTACA	59.1	716	INTERGENIC	--	--	--
Ca4_P3_21082826	Ca4	21082826	AAATAATAATAATAATAATAATAA/AAATAATAATAATAATAA	GTGCGTGCTTTGGGATTTTA	CGAACTGAAGAACGATTAGGTG	61.0	332	INTERGENIC	--	--	--
Ca4_P3_21134274	Ca4	21134274	TTATATATATATATATATATAT/TTATATATATATATATATATATAT	ACAAGTCGCCTTTTGGAATG	TCACTCCCAAATCTCGAACA	60.1	521	INTERGENIC	--	--	--
Ca4_P3_2121744	Ca4	2121744	TCCC/TCCCC	TGCCATAAGCAAATCACCAA	ACTGGAAAACCGTGCATAGC	60.1	708	DRR	Ca_07832	O	--

INDEL marker IDs	Chromosomes/ unanchored scaffolds	Physical positions (bp)	InDels (*Kabuli* reference genome-CDC Frontier/PII)	Forward primers (5'-3')	Reverse primers (5'-3')	Anneling temperature (0C)	Expected amplified product size (bp)	Structural annotation		Functional annotation	
								Sequence components of *kabuli* genome	*Kabuli* gene accession IDs	NCBI-KOG	TFs
Ca4_P3_21226048	Ca4	21226048	AAA/AAAAAGGAA	CACGGCTCTACGAAGGAAAG	TGAACACCCCCAAGGTTAAG	60.0	287	INTERGENIC	--	--	--
Ca4_P3_21230858	Ca4	21230858	CATATATATATATATATATA/CATATATATATATATA	GGTCTGCATTTTACACCGCT	AGTGCCCTCGTCAAACAAAT	60.1	920	DRR	Ca_19058	--	Trihelix
Ca4_P3_21261688	Ca4	21261688	AATATATATATATATATATATATATATATATATATA/AATATATATATATATATATATATATATATATATATATATATATATA	GAAAAATTTGCTGAGCTGGTG	TGCGACAGAATCTTAATGTGC	59.9	485	INTERGENIC	--	--	--
Ca4_P3_21308758	Ca4	21308758	CAAAAAAA/CAAAAAA	TTGCTCCTCTGGTCCTCACT	CGTTCAACGTTCTCCATTTTT	60.0	854	INTERGENIC	--	--	--
Ca4_P3_21426612	Ca4	21426612	ATTTTT/ATTTTTT	TGCAGATAAATGGCCAAACA	AGAACGGTTGCCAGAGAAGA	60.1	370	INTERGENIC	--	--	--
Ca4_P3_21529168	Ca4	21529168	CAAAA/CAA	TGGGGGATTGTATTCCAAAA	TATGGAATGGACCTCCTGCT	60.0	499	INTERGENIC	--	--	--
Ca4_P3_21529241	Ca4	21529241	TATGAATGAA/TATGAA	TGGGGGATTGTATTCCAAAA	TATGGAATGGACCTCCTGCT	60.0	499	INTERGENIC	--	--	--
Ca4_P3_21622605	Ca4	21622605	TAATGTTAAAA/TAATGTTAAAAAAATGTTAAAA	CGTGTATATACGCACACCCAA	GTTTTGGCGTTGGTTTCACT	59.4	805	INTERGENIC	--	--	--
Ca4_P3_21622678	Ca4	21622678	ATGTG/ATGTGTG	CGTGTATATACGCACACCCAA	GTTTTGGCGTTGGTTTCACT	59.4	805	INTERGENIC	--	--	--
Ca4_P3_21703341	Ca4	21703341	AATATATATATATATATATATA/AATATATATATATATATATATATATA	TAAAGGACCAAGTCGGGAAA	TGCACGTGTATACGAGACGG	59.5	373	INTERGENIC	--	--	--

INDEL marker IDs	Chromosomes/ unanchored scaffolds	Physical positions (bp)	InDels (*Kabuli* reference genome-CDC Frontier/PII)	Forward primers (5'-3')	Reverse primers (5'-3')	Anneling temperature (0C)	Expected amplified product size (bp)	Structural annotation		Functional annotation	
								Sequence components of *kabuli* genome	*Kabuli* gene accession IDs	NCBI-KOG	TFs
Ca4_P3_21728459	Ca4	21728459	TAA/TAATAATAATAAAAA	CCATTTCGTTGCTCCATCTT	TGGTTTTTGTTGTTTGGTTTCT	60.1	267	INTERGENIC	--	--	--
Ca4_P3_21755010	Ca4	21755010	GAAAAAAA/GAAAAAA	CGATCAAGTGTCGAAAAACTACC	TGATGGAACCAAAGTCCAAA	60.0	448	INTERGENIC	--	--	--
Ca4_P3_21767863	Ca4	21767863	ATATTATTATTATTATTATTATTATTATTATTAT/ATATTATTATTATTATTATTATTATTATTAT	AATCACCCGAAACGAGATTG	TCGTTTTTCACATAAGCGTTTT	59.9	632	INTERGENIC	--	--	--
Ca4_P3_21790580	Ca4	21790580	CAAAAAAAAA/CAAAAAAAAAAAA	GGGGAATTTAGAAAATCGTGC	CACCATCCTGCATGTGAATC	59.8	921	INTERGENIC	--	--	--
Ca4_P3_21804779	Ca4	21804779	CTGT/CTGTTATGT	CCGCATCATAAACAGCCTTT	CTAAAGCTGGTGCTTTTGGC	60.1	159	INTERGENIC	--	--	--
Ca4_P3_21933188	Ca4	21933188	GAAAAAAAAAAA/GAAAAAAAAAA	GTTGGGCAATGGATCTGAAT	TGCTCACCGTTTGATCTTTG	59.8	862	DRR	Ca_20146	--	--
Ca4_P3_21936546	Ca4	21936546	TTTATTATTATTATTATTATTATTATTATTATTATTATTATTATTATTATTATTATTATTATTATTAT/TTTATTATTATTATTATTATTATTATTATTATTATTATTATTATTATTATTATTATTATTATTATTAT	TGAACACCCCCAAGGTTAAG	TGGTTGCAGGTAAGGGTAGG	59.8	532	INTERGENIC	--	--	--
Ca4_P3_21986042	Ca4	21986042	AATATATATATATATATATATATATATATATAT/AATATATATATATATATATATATATAT	TAATGAGCTTGGAACGACCC	GGCGGGAGATCAGAACATT	60.1	344	INTERGENIC	--	--	--
Ca4_P3_22052693	Ca4	22052693	TTTATTATTATTATTATTATTATTATTATTATT/TTTATTATTATTATTATTATTATTATTATTATT	AACATGTCCAAACGTTGCTG	GGATGCGACCTCCTACTACG	59.6	505	INTERGENIC	--	--	--
Ca4_P3_22073219	Ca4	22073219	AATATATATATATATATATATATA/AATATATATATATATATATATATATA	TGCAATTGACCTGTAAATCCTG	CCTGACAATTTTCCTCCTCG	60.0	820	INTERGENIC	--	--	--

INDEL marker IDs	Chromosomes/ unanchored scaffolds	Physical positions (bp)	InDels (*Kabuli* reference genome-CDC Frontier/PII)	Forward primers (5'-3')	Reverse primers (5'-3')	Anneling temperature (0C)	Expected amplified product size (bp)	Structural annotation		Functional annotation	
								Sequence components of *kabuli* genome	*Kabuli* gene accession IDs	NCBI-KOG	TFs
Ca4_P3_22134752	Ca4	22134752	TT/TTGTTGT	ATTTTTGGTGCATGGTTTCC	TTTGATGCCCACATTGAAGA	59.7	900	DRR	Ca_20134	J	--
Ca4_P3_22134995	Ca4	22134995	CATAT/CAT	GCACCTATGGTGGCAAAAAC	TTTGATGCCCACATTGAAGA	60.4	506	DRR	Ca_20134	J	--
Ca4_P3_22233819	Ca4	22233819	TTATATATATATATATATATATATAT/TTATATATATATATATATATAT	GTGAAATTGAGGCATGAGCA	CGAAGCTCCCTACTTCGTTG	59.8	843	INTERGENIC	--	--	--
Ca4_P3_2233798	Ca4	2233798	CT/C	TTCATTCTCGGGGAACTAGC	TTGTTATTTAGTCTTGCTTGCCA	59.3	264	INTERGENIC	--	--	--
Ca4_P3_22366283	Ca4	22366283	CTTTTTT/CTTTTTTT	TGGGTGAAATCCATTGAGGT	GTTCTGGAACATTTCCCCCT	60.2	494	INTERGENIC	--	--	--
Ca4_P3_22441886	Ca4	22441886	CTA/CTATA	TCCAATGGTGGAAAATAAACG	TCAATCACAAATTTCAAGGAGG	59.7	848	INTERGENIC	--	--	--
Ca4_P3_22546535	Ca4	22546535	AATATATATATATA/AATATATATATATATA	CATTTTGGTTTCAAAGCCGT	CCAGGGGATGAGATCATAGG	60.0	714	INTERGENIC	--	--	--
Ca4_P3_22649924	Ca4	22649924	A/AT	CCGTCCAAATCAAATGAAAAC	CATTTACCATGTGCTTTTTATGC	59.3	491	INTERGENIC	--	--	--
Ca4_P3_22663018	Ca4	22663018	ATTTTTTTT/ATTTTTTTTT	ACCCCCTCTTCTCGTGTTTT	AGGCTCAGGCAACTAGGGAT	60.0	536	INTERGENIC	--	--	--
Ca4_P3_22666443	Ca4	22666443	ATTTCTACTTT/ATTT	CATCCAGTGGTTGCGAGTTA	GTGTACAAACCCATGTTGCG	59.7	632	INTERGENIC	--	--	--

INDEL marker IDs	Chromosomes/ unanchored scaffolds	Physical positions (bp)	InDels (*Kabuli* reference genome-CDC Frontier/PII)	Forward primers (5'-3')	Reverse primers (5'-3')	Anneling temperature (0C)	Expected amplified product size (bp)	Structural annotation		Functional annotation	
								Sequence components of *kabuli* genome	*Kabuli* gene accession IDs	NCBI-KOG	TFs
Ca4_P3_22667999	Ca4	22667999	ATTTTTTTT/ATTTTTTTTT	GTGAAGCTAGCAAGCCGAAG	TGAATGAAAGGACGGGAAAC	60.3	656	INTERGENIC	--	--	--
Ca4_P3_22674188	Ca4	22674188	CAAAAAAAAAA/CAAAAAAAAA	AATGCATGTTGCACCACAGT	GCTGAAAAGGAAGGGAAAGG	60.0	292	INTERGENIC	--	--	--
Ca4_P3_22702413	Ca4	22702413	CTTGAAA/CTTGAAATTTTTTTGAAA	GTCATTTGAAACGTGGGCTT	CATGTTGGAGGGGCTAAAAA	60.0	717	INTERGENIC	--	--	--
Ca4_P3_22702531	Ca4	22702531	TT/TTTTTTTTCT	GGCTTAAGAGAGGGGGTTCA	CATGTTGGAGGGGCTAAAAA	60.6	314	INTERGENIC	--	--	--
Ca4_P3_22703292	Ca4	22703292	AT/ATGT	GTGCAAACAAGGAGCAACAA	AATATCGGACCAAGACGACG	59.9	717	INTERGENIC	--	--	--
Ca4_P3_22719475	Ca4	22719475	A/AT	ACACGTCATTTGTTTTGGCA	TGTTGCAATAGAAGGTCGCA	60.0	390	INTERGENIC	--	--	--
Ca4_P3_22723588	Ca4	22723588	AC/A	CATGACGACTTTACCCAGCA	AAGAGAGGAGAGGTAGCGGG	59.7	784	DRR	Ca_14476	--	--
Ca4_P3_22723629	Ca4	22723629	CTTTTT/CTTTT	TCTAACACCGGCAACATCAA	AGGCCACGAAAGTGAAGAGA	60.1	527	DRR	Ca_14476	--	--
Ca4_P3_22727990	Ca4	22727990	TAAAAAAAAA/TAAAAAAAA	AACCTATTTATCCAACTAACAAATGTC	GGGACAAACGTTTTTGCTATG	57.4	257	INTERGENIC	--	--	--
Ca4_P3_22728327	Ca4	22728327	ATTTTTTTTT/ATTTTTTTT	GCAAAAACGTTTGTCCCTTG	CCATCGTAGTTGACAAATGGC	60.5	315	INTERGENIC	--	--	--

INDEL marker IDs	Chromosomes/ unanchored scaffolds	Physical positions (bp)	InDels (*Kabuli* reference genome-CDC Frontier/PII)	Forward primers (5'-3')	Reverse primers (5'-3')	Anneling temperature (0C)	Expected amplified product size (bp)	Structural annotation		Functional annotation	
								Sequence components of *kabuli* genome	*Kabuli* gene accession IDs	NCBI-KOG	TFs
Ca4_P3_22728505	Ca4	22728505	GTTG/GTTGTTTTTG	GCAAAAACGTTTGTCCCTTG	TCAGTGTTGTTCAGCACGTT	60.5	459	INTERGENIC	--	--	--
Ca4_P3_22750911	Ca4	22750911	CTAATTAAT/CTAAT	CAAGTGTCTCATTGCAAATTCA	CCGAGGCCTCTTATTCTCAA	58.8	955	INTERGENIC	--	--	--
Ca4_P3_22752532	Ca4	22752532	CTAA/CTAATAA	AGGACAAATCCGCAACAAAC	GCCGAGGAATATGTTTCATTC	60.0	523	INTERGENIC	--	--	--
Ca4_P3_22752758	Ca4	22752758	ATTTTTT/ATTTTTTT	AGTGGTGGAGGGTATCAACG	CAAAGCCGACTCAGATCACA	59.8	853	INTERGENIC	--	--	--
Ca4_P3_22762970	Ca4	22762970	ATTTTTTTTTT/ATTTTTTTT	TGGTGTATTTTTGCCGATGA	ATTGACCTTGGGTTGTGCAT	59.9	699	INTERGENIC	--	--	--
Ca4_P3_22763617	Ca4	22763617	CAAAAAAAAAA/CAAAAAAAAA	ATGCACAACCCAAGGTCAAT	AAAGTGCTTGTTTGGTTCGG	60.2	434	INTERGENIC	--	--	--
Ca4_P3_22766863	Ca4	22766863	T/TGG	GCATAATCAAAAGCATTGGC	ATCGATTCTCAGTTGGACCG	58.3	756	INTERGENIC	--	--	--
Ca4_P3_22768044	Ca4	22768044	CAA/CA	AGGATGACCTCGTGAGAGGA	GGTCTGCATTTAGCCTTTGC	59.8	257	INTERGENIC	--	--	--
Ca4_P3_22780788	Ca4	22780788	TAAAAA/TAAA	GTCCCACCCATTGTTCGTAG	TCCACACCGTTTTTGATATTG	60.2	668	INTERGENIC	--	--	--
Ca4_P3_22782656	Ca4	22782656	TTATAAATATACAT/TTATAAATATACATATAAATATACAT	AAGAGCAGGACAAATTAATGAGA	TTTGGCATTGATGAATTGGA	57.2	733	INTERGENIC	--	--	--

INDEL marker IDs	Chromosomes/ unanchored scaffolds	Physical positions (bp)	InDels (*Kabuli* reference genome-CDC Frontier/PII)	Forward primers (5'-3')	Reverse primers (5'-3')	Anneling temperature (0C)	Expected amplified product size (bp)	Structural annotation		Functional annotation	
								Sequence components of *kabuli* genome	*Kabuli* gene accession IDs	NCBI-KOG	TFs
Ca4_P3_22791089	Ca4	22791089	CAAAAAAAAAAAAA/CAAAAAAAAAAA	AAATGCGCAAATGCATACAA	GTGACTTTAGAGCCGCCATC	60.1	691	INTERGENIC	--	--	--
Ca4_P3_22792153	Ca4	22792153	GAAAAAAAAA/GAAAAAAAAAA	CTCATTTTCATTCACCGGCT	ACGAAGCATTAGGCATGGAT	60.1	931	DRR	Ca_14472	--	--
Ca4_P3_22793825	Ca4	22793825	GTTTTTTTTTTT/GTTTTTTTTT	AGGAGGCAAGACGAAAACAG	TGAAGTTCTGCCACTCGTCA	59.5	572	DRR	Ca_14472	--	--
Ca4_P3_22797432	Ca4	22797432	CTTTTTT/CTTTTTTT	TGCGCTGGGGATTTTTATAC	TTATTTCGGAGCGCAATACC	59.9	455	INTERGENIC	--	--	--
Ca4_P3_22799504	Ca4	22799504	AAATAA/AAA	CCAACACACCAAGACAGTGG	TCACACACAAATCACACCGA	60.0	213	INTERGENIC	--	--	--
Ca4_P3_22799819	Ca4	22799819	TAAAAAAA/TAAAAAAAA	CCAACACACCAAGACAGTGG	TTCGATCAATCGGTCACAAA	60.0	807	INTERGENIC	--	--	--
Ca4_P3_22803782	Ca4	22803782	TA/T	CCAAGTTCCTCAAAAGCAGG	ATCCTGCCGTCATATGCTTC	59.8	682	INTRON	Ca_14471	Q	Nin-like
Ca4_P3_22806695	Ca4	22806695	TA/T	AAAAGAGCCGTCACATCCAC	CTGCCTGCAAATCACAAATG	60.1	591	INTRON	Ca_14471	Q	Nin-like
Ca4_P3_22808161	Ca4	22808161	A/AC	CATGGTTTTCGCTTACCGTT	TTGGGTGATGTGGCAAAATA	60.0	559	DRR	Ca_14470	H	--
Ca4_P3_22808420	Ca4	22808420	GAAAA/GAAA	CATGGTTTTCGCTTACCGTT	TTGGGTGATGTGGCAAAATA	60.0	559	DRR	Ca_14471	Q	Nin-like

INDEL marker IDs	Chromosomes/ unanchored scaffolds	Physical positions (bp)	InDels (*Kabuli* reference genome-CDC Frontier/PII)	Forward primers (5'-3')	Reverse primers (5'-3')	Anneling temperature (0C)	Expected amplified product size (bp)	Structural annotation		Functional annotation	
								Sequence components of *kabuli* genome	*Kabuli* gene accession IDs	NCBI-KOG	TFs
Ca4_P3_22816167	Ca4	22816167	A/AGGGAATAG	TCCCAAATGAGTTGGTGGAT	AAGAGGAGACGGAGACTGCC	60.2	107	INTERGENIC	--	--	--
Ca4_P3_22816244	Ca4	22816244	AGGGG/AGG	TCCCAAATGAGTTGGTGGAT	CAATTCCAAATCAAAGCAAAC	60.2	358	INTERGENIC	--	--	--
Ca4_P3_22818473	Ca4	22818473	GTATATATATAT/GTATATATATATAT	CAATTGCATGTTGAAATGGTG	TGGCCATATTGTTTTTGCAT	59.8	821	INTERGENIC	--	--	--
Ca4_P3_22824416	Ca4	22824416	CCGT/CCGTACGT	TCTATTCCCGGTGTTGAAGC	TGCACTCTAATGGGATTATTTTGA	60.1	147	INTERGENIC	--	--	--
Ca4_P3_22825336	Ca4	22825336	TTT/TTTGTT	TGGCTTCCATAGTAAGAGAGTGAA	AAATAACGTGTCACGGGGAT	59.5	546	INTERGENIC	--	--	--
Ca4_P3_22867161	Ca4	22867161	ATTTT/ATTTTT	TCACCACTGACCAACCCTTT	CACCACCACCACAAAACACT	60.4	485	INTERGENIC	--	--	--
Ca4_P3_22867230	Ca4	22867230	TTCTATTCAT/TTCTATTCATCTATTCAT	TCACCACTGACCAACCCTTT	CACCACCACCACAAAACACT	60.4	485	INTERGENIC	--	--	--
Ca4_P3_22867454	Ca4	22867454	GAAAAAAAAAA/GAAAAAAAAA	GATCTTCATCCCCTGCAGAC	CACCACCACCACAAAACACT	59.6	292	INTERGENIC	--	--	--
Ca4_P3_22870716	Ca4	22870716	A/AC	GGAATCGGTGAAAACCAAAA	AGGTAACGCGATTTTATGCG	59.8	786	INTERGENIC	--	--	--
Ca4_P3_22874517	Ca4	22874517	TAAAAA/TAAAAAA	GAACTCCAGATATGGCGGAA	ACATTGGCCTCTCCATCAAC	60.0	336	INTERGENIC	--	--	--

INDEL marker IDs	Chromosomes/ unanchored scaffolds	Physical positions (bp)	InDels (*Kabuli* reference genome-CDC Frontier/PII)	Forward primers (5'-3')	Reverse primers (5'-3')	Anneling temperature (0C)	Expected amplified product size (bp)	Structural annotation		Functional annotation	
								Sequence components of *kabuli* genome	*Kabuli* gene accession IDs	NCBI-KOG	TFs
Ca4_P3_22877356	Ca4	22877356	CAAAAAAAAAAAA/CAAAAAAAAAA	AATTCAATGAAGTCGCCACC	GGAGATACGCAGGAGCAAGA	59.9	362	INTERGENIC	--	--	--
Ca4_P3_22880077	Ca4	22880077	GTTTTTT/GTTTTTTT	GTGACCGTCTTCGGATTGTT	TTTTGCCTCGAAAAAGCAGT	60.0	360	INTERGENIC	--	--	--
Ca4_P3_22880803	Ca4	22880803	TCCCCC/TCCCC	AGTTACAACGTGGGGACGAG	CCTCGAATTACGAAGGACCA	60.0	359	INTERGENIC	--	--	--
Ca4_P3_2288480	Ca4	2288480	AA/AATTCATGAATTTGTA	CACCCCTGTGCTTAATGCTT	ACCGAGTGTCGAACTCAAGG	60.1	523	INTERGENIC	--	--	--
Ca4_P3_22889580	Ca4	22889580	AC/A	GGGTCAGGATACTTACAACCCA	TCCACCCCCAAATTTTATCA	60.1	717	INTERGENIC	--	--	--
Ca4_P3_22890995	Ca4	22890995	AGCAAGATTTT/AGCAAGATTTTATTTTTGGCAAGATTTT	TTGCGATTTTGCTCCTACCT	AGGCGCAACTTGCATTTACT	59.8	526	INTERGENIC	--	--	--
Ca4_P3_22897920	Ca4	22897920	ATTTTTTTTT/ATTTTTTTTTT	GGTTGGCTTGTGATCTCCAT	TGCTTGTGGTCTGAAACCTG	59.9	463	INTERGENIC	--	--	--
Ca4_P3_22898104	Ca4	22898104	ATTTTTTT/ATTTTTTTT	GGTTGGCTTGTGATCTCCAT	CCCTTTAAGTGGGGTTCCAT	59.9	797	INTERGENIC	--	--	--
Ca4_P3_22911497	Ca4	22911497	AC/A	TTTGTTGCATTTGTTTTCTTCG	GAAGACTCTCCACCGTCAGG	60.1	441	DRR	Ca_14462	J	--
Ca4_P3_22915979	Ca4	22915979	GTTTTT/GTTTTTT	GGTGGAAGGCTTACGAAACA	ACTCGGTGGATGGAAGACAC	60.1	333	INTERGENIC	--	--	--

INDEL marker IDs	Chromosomes/ unanchored scaffolds	Physical positions (bp)	InDels (*Kabuli* reference genome-CDC Frontier/PII)	Forward primers (5'-3')	Reverse primers (5'-3')	Anneling temperature (0C)	Expected amplified product size (bp)	Structural annotation		Functional annotation	
								Sequence components of *kabuli* genome	*Kabuli* gene accession IDs	NCBI-KOG	TFs
Ca4_P3_22921547	Ca4	22921547	TAA/TAAAATAAATAAAAAA	TCGTCCGAACACAATCACAT	TGATCTCGTGCTTCTACGTGTT	60.0	311	INTERGENIC	--	--	--
Ca4_P3_22936991	Ca4	22936991	CAAAAAAAAAAAAA/CAAAAAAAAAAA	TGTGAGCAAATGGGAAAAGA	GTTTTGGGTCCTTGGGAGTT	59.2	828	INTRON	Ca_14461	--	--
Ca4_P3_22938166	Ca4	22938166	ATTGTTG/ATTG	GATCTTTTTGGGCCAGTCAA	CCAAGATGGTTCTTGCCATT	60.1	343	INTRON	Ca_14461	--	--
Ca4_P3_22947175	Ca4	22947175	CAAAA/CAAA	AACCACCACGGATCAAAGAG	TTAAATGGGGGATTGTTGGA	60.0	966	INTRON	Ca_14461	--	--
Ca4_P3_22947623	Ca4	22947623	TTGTTCCAACCCTGTTCCAACCC/TTGTTCCAACCC	ACGCCCCATAGTTACCTTCC	TTAAATGGGGGATTGTTGGA	60.2	531	INTRON	Ca_14461	--	--
Ca4_P3_22965696	Ca4	22965696	TAAAAAAAAAAA/TAAAAAAAAAA	CGGCTTTGTCACATTTGTTG	AATTCAAACCCCACTTCCTT	60.1	369	INTERGENIC	--	--	--
Ca4_P3_22981123	Ca4	22981123	ATTTTTTTT/ATTTTTTTTTTT	CGACGAAACGCCTTAAAAAG	TTTTGTCATGTTTGCGAGGT	59.9	797	INTERGENIC	--	--	--
Ca4_P3_22981343	Ca4	22981343	ATTTTTTTT/ATTTTTTT	TCCGCCGTTCTAAATTCTTG	CAAGCCCTCTCCCTCTCTTC	60.2	874	INTERGENIC	--	--	--
Ca4_P3_22984986	Ca4	22984986	CTTTTTTT/CTTTTTTTT	CCTCATTTCTTCGTTTACGTGG	AATGATCGTTGGCTAGTGGG	60.9	563	INTERGENIC	--	--	--
Ca4_P3_22986496	Ca4	22986496	TAAAAAAAAAA/TAAAAAAAAA	ACTTTAGCGCGCCTTTCAT	GCCAACAAAAGAGGAAGAAAA	60.0	984	INTERGENIC	--	--	--

**INDEL marker IDs**	**Chromosomes/ unanchored scaffolds**	**Physical positions (bp)**	**InDels (*Kabuli* reference genome-CDC Frontier/PII)**	**Forward primers (5'-3')**	**Reverse primers (5'-3')**	**Anneling temperature (0C)**	**Expected amplified product size (bp)**	**Structural annotation**		**Functional annotation**	
								**Sequence components of *kabuli* genome**	***Kabuli* gene accession IDs**	**NCBI-KOG**	**TFs**
Ca4_P3_23002623	Ca4	23002623	TTTACTATTACTATT/TTTACTATTACTATTACTATT	TTTATGGCGGCTGGTTTTAC	ACGATGCATTTGATTGATGTT	60.0	546	INTERGENIC	--	--	--
Ca4_P3_23014862	Ca4	23014862	CATATATA/CATATATATA	GAACATGTGCGTGCTACGTT	GCTGTTCAAAGGGCTAGACG	59.8	756	INTERGENIC	--	--	--
Ca4_P3_23027613	Ca4	23027613	AGG/AGGG	GATGTTGGGAAGGGTGGTTA	AAGGTTGTGGATCGATTCGT	59.6	622	INTERGENIC	--	--	--
Ca4_P3_23030651	Ca4	23030651	ATTGACACATGACTTTGACACA/ATTGACACA	TGGACGTGAGTGGTTCACAT	TTGAACACATCATTTAGATACACAAAA	60.0	304	INTERGENIC	--	--	--
Ca4_P3_23032839	Ca4	23032839	ATAAAAAT/A	TGGTTGTTGTGTTATCGTTGC	TTCATGTCGGGATCATCAGA	59.5	895	INTERGENIC	--	--	--
Ca4_P3_23049056	Ca4	23049056	TGGG/TGGGG	ATAGGCGAGAAGAGCAACCA	TATTTTGAACGATCGGGAGG	60.0	783	INTERGENIC	--	--	--
Ca4_P3_23056430	Ca4	23056430	TAAAAAAAA/TAAAAAAAAA	ATGAATAGCATGCCACGACA	TCATGTGGGCTCATTTGGTA	60.1	771	INTERGENIC	--	--	--
Ca4_P3_23056577	Ca4	23056577	TAAAAAAAAAA/TAAAAAAAAA	ATGAATAGCATGCCACGACA	TCATGTGGGCTCATTTGGTA	60.1	771	INTERGENIC	--	--	--
Ca4_P3_23059712	Ca4	23059712	CTTTTT/CTTT	TCATTCGTTTGTGCAGTTTTG	TTTAATTTCCCTGCACCCCT	59.8	612	INTERGENIC	--	--	--
Ca4_P3_23067580	Ca4	23067580	TTGAATGAATGAATGAATGAATGAAT/TTGAATGAATGAATGAATGAAT	CGGAAATAACTGACCGCAAT	GGAATCGAAAGTCGTGGAAA	60.0	373	INTERGENIC	--	--	--

INDEL marker IDs	Chromosomes/ unanchored scaffolds	Physical positions (bp)	InDels (*Kabuli* reference genome-CDC Frontier/PII)	Forward primers (5'-3')	Reverse primers (5'-3')	Anneling temperature (0C)	Expected amplified product size (bp)	Structural annotation		Functional annotation	
								Sequence components of *kabuli* genome	*Kabuli* gene accession IDs	NCBI-KOG	TFs
Ca4_P3_23067690	Ca4	23067690	GTTTTTTTTT/GTTTTTTTTTT	CGGAAATAACTGACCG CAAT	GGAATCGAAAGTCGTG GAAA	60.0	373	INTERGENIC	--	--	--
Ca4_P3_23067844	Ca4	23067844	GTAATTAAATAATTAAATAA/GTAATTAA ATAA	CGGAAATAACTGACCG CAAT	TTCATTCGCAAATTTTA CCG	60.0	608	INTERGENIC	--	--	--
Ca4_P3_23076109	Ca4	23076109	CTTT/CTTTT	TGGCATTGCAAGAAGA AACA	CCTCTTTCAACACCTTT GGC	60.4	760	INTERGENIC	--	--	--
Ca4_P3_23078922	Ca4	23078922	CAAAAAAAAAAAA/CAAAAAAAAAAAAA AAA	CCTGTTTTGGTTCAAGC CAT	ATGTTAAGGGACCGCA AATG	60.0	446	INTERGENIC	--	--	--
Ca4_P3_23099904	Ca4	23099904	TTAT/TT	ACGATGCACGAATGTG ATGT	GTCTGATCTGGCCTGA AAGC	60.0	658	INTERGENIC	--	--	--
Ca4_P3_23102309	Ca4	23102309	GATA/GA	TGAGTCCCAAACTTTTT GTCG	TCCTAGGACCCTCCCA ACTT	60.1	197	INTERGENIC	--	--	--
Ca4_P3_23107392	Ca4	23107392	T/TTTTTTA	GGCCAAATTGCGTAAA ACAT	AGTGACAGCCGAAGCA AAGT	59.8	445	DRR	Ca_14456	--	--
Ca4_P3_23117240	Ca4	23117240	TC/TCAAC	GCACATTCGAACCTTTC CAT	GATCATCGAGTTTCCC GTGT	59.9	739	INTERGENIC	--	--	--
Ca4_P3_23118669	Ca4	23118669	ACC/AC	TCCATCAAATGCTTTCA CCA	AGCACCAAACCCTTTT CCTT	60.0	578	INTERGENIC	--	--	--
Ca4_P3_23134474	Ca4	23134474	ATTGTTGTTGT/ATTGTTGT	ATGCTGCGAAATTGTT GAAA	AAAAGCGTTGTAAATT GGATTGA	59.3	342	INTERGENIC	--	--	--

INDEL marker IDs	Chromosomes/ unanchored scaffolds	Physical positions (bp)	InDels (*Kabuli* reference genome-CDC Frontier/PII)	Forward primers (5'-3')	Reverse primers (5'-3')	Anneling temperature (0C)	Expected amplified product size (bp)	Structural annotation		Functional annotation	
								Sequence components of *kabuli* genome	*Kabuli* gene accession IDs	NCBI-KOG	TFs
Ca4_P3_23135135	Ca4	23135135	ATTTTTTTT/ATTTTTTT	ATTCAGCAATGAGAGG TCCG	CCCTTCTGTGTTCATC CCAC	60.2	266	INTERGENIC	--	--	--
Ca4_P3_23135482	Ca4	23135482	GAAAAAA/GAAAAA	ATTCAGCAATGAGAGG TCCG	GATGATGCATGCCATT TGAG	60.2	752	INTERGENIC	--	--	--
Ca4_P3_23135678	Ca4	23135678	CGG/CG	GGGATGAACACAGAAG GGTG	GATGATGCATGCCATT TGAG	60.4	504	INTERGENIC	--	--	--
Ca4_P3_23145296	Ca4	23145296	TAAAAAAA/TAAAAAAAA	TGCATGTTTCATGGTGA TCC	CACCAATTACAGTGGC ATCAA	60.3	680	INTERGENIC	--	--	--
Ca4_P3_23145659	Ca4	23145659	ATTTTTTT/ATTTTTT	TGGGAACTTAGGATTC CGTTC	CAAATTTGAATCCGCC TACTG	60.3	587	INTERGENIC	--	--	--
Ca4_P3_23145983	Ca4	23145983	GATATATA/GATA	GCTTGAGGATTCAGTA GGCG	GAGTGGAGGACCACAT GTCA	60.0	408	INTERGENIC	--	--	--
Ca4_P3_23272983	Ca4	23272983	ATTTTTTTTT/ATTTTTTTT	ACCACATCGGAAGAAA CGAC	GGACGAAGTAGAACG GACCA	60.0	596	INTERGENIC	--	--	--
Ca4_P3_23307347	Ca4	23307347	CATATATATATATATATA/CATATATA TATATATATA	TACTGTCCTTGCTCCA GGCT	TGACACGTCAACGGCA TAAT	60.0	781	INTERGENIC	--	--	--
Ca4_P3_23361352	Ca4	23361352	TCAGTC/TCAGTCCCAGTC	CAAGCCCAATCAAAAC CTGT	GAGCTTGCTGGAACTG GAAC	60.0	355	CDS (large-effect mutations)	Ca_14445	--	--
Ca4_P3_23421612	Ca4	23421612	TT/TTGT	GATGCCAGATGGGTAT GCTT	CGTTGCAATTGATGAG GATG	59.9	703	INTERGENIC	--	--	--

INDEL marker IDs	Chromosomes/ unanchored scaffolds	Physical positions (bp)	InDels (*Kabuli* reference genome-CDC Frontier/PII)	Forward primers (5'-3')	Reverse primers (5'-3')	Anneling temperature (0C)	Expected amplified product size (bp)	Structural annotation		Functional annotation	
								Sequence components of *kabuli* genome	*Kabuli* gene accession IDs	NCBI-KOG	TFs
Ca4_P3_23480946	Ca4	23480946	AATATATATATATATATATATATATATAT A/AATATATATATATATATATATATA	GGGGTGGATTTTTGTC TTCA	AACTTGCACCCGCAGA TACT	59.8	514	INTERGENIC	--	--	--
Ca4_P3_23525491	Ca4	23525491	GTTTTTTTTTT/GTTTTTTTTT	TGAGATTATATGCACGC GGA	TGAATCGCGAAAGTAG TCCC	60.2	574	INTERGENIC	--	--	--
Ca4_P3_23721197	Ca4	23721197	TAAAACA/TAAAACAAAACA	ATTTGTGTGCATTTGCC TGA	TGGGCAATCGACTGAC TAAA	60.1	353	INTERGENIC	--	--	--
Ca4_P3_23828073	Ca4	23828073	G/GAACAGCAGA	TGTTGAAAACGGTCCG TACA	CTTCATCCGAAGAGGC AGAG	60.0	744	INTERGENIC	--	--	--
Ca4_P3_23850671	Ca4	23850671	CTTTTTTTTTT/CTTTTTTTTTTT	TGACGCTTTCACACAC GAAT	AAAATGTGTTTTATGG GTTAAGCTC	60.3	566	INTERGENIC	--	--	--
Ca4_P3_23942700	Ca4	23942700	TAAAAAAAAAAAAA/TAAAAAAAAAAAA	TTGTCTCAACAATCTCC CCC	AAGGACCAAAATGGAA AGGG	59.9	581	INTERGENIC	--	--	--
Ca4_P3_23965497	Ca4	23965497	CTTTTTTTTTT/CTTTTTTTTT	TTCATGCAAAATGTTCA AGACC	AATGGGGTTTAGGGTT TTCG	60.0	607	INTERGENIC	--	--	--
Ca4_P3_24115264	Ca4	24115264	AAATAATAATAATAATAATA/AAATAATA ATAATAATAATAATAATA	CACACGTGCATGGATA GAGG	TTCACCGTGGATGAAT TTGA	60.1	341	INTERGENIC	--	--	--
Ca4_P3_24147447	Ca4	24147447	TAA/TAAAAA	GGAAAACGACTTACAC CCGA	TCCCCTTTGTCTTCTT GTGG	60.0	813	INTERGENIC	--	--	--
Ca4_P3_24259985	Ca4	24259985	TAAAAAAA/TAAAAAAAA	TAGTGGAAAATTGAGG GCCA	AAGGAGAGATAAGCTC CGGC	60.4	626	INTERGENIC	--	--	--

INDEL marker IDs	Chromosomes/ unanchored scaffolds	Physical positions (bp)	InDels (*Kabuli* reference genome-CDC Frontier/PII)	Forward primers (5'-3')	Reverse primers (5'-3')	Anneling temperature (0C)	Expected amplified product size (bp)	Structural annotation		Functional annotation	
								Sequence components of *kabuli* genome	*Kabuli* gene accession IDs	NCBI-KOG	TFs
Ca4_P3_24326964	Ca4	24326964	TAAAAAAAAAAA/TAAAAAAAAAA	CTGGGCAAGGGATCTG AATA	AATGCCCACCCATAAT TTCA	60.0	646	INTERGENIC	--	--	--
Ca4_P3_24355412	Ca4	24355412	AATATATATATAT/AATATATATATATAT	TGCCATTTTCAAATTTG TGG	AGGCACACCTTGTAGC CAAC	59.4	376	INTERGENIC	--	--	--
Ca4_P3_24362931	Ca4	24362931	TAA/TA	GGTTTAGATGGGGGAT CGTT	CCCCGAGTTGGTCCTT TATT	60.0	947	INTERGENIC	--	--	--
Ca4_P3_24373144	Ca4	24373144	GAAAA/GAAAAA	GGCGTAAAACCCAAAA CGTA	TTGTTTGCGAGAGATT GCAC	59.9	270	INTERGENIC	--	--	--
Ca4_P3_24425317	Ca4	24425317	GTTT/GTTTT	TGGGGTGTTTCATAGC ATCA	ACCGTTTAATTGGCGA ACAG	59.9	267	INTERGENIC	--	--	--
Ca4_P3_24427573	Ca4	24427573	GTT/GTTT	CTGGAAAATCCTCCTC CTCC	TTCGTCTCAACAATCT CCCC	60.0	346	INTERGENIC	--	--	--
Ca4_P3_24438141	Ca4	24438141	CAAAAAA/CAAAAA	CCGCTCTGGAAATCATT CAT	AAGACTCGGGATAATG GCCT	60.0	551	DRR	Ca_20867	--	--
Ca4_P3_24445284	Ca4	24445284	GC/GCC	CTCGCCAAATCCATACA CCT	CACAGCACCAGGTTAT CCCT	60.0	383	INTERGENIC	--	--	--
Ca4_P3_24446747	Ca4	24446747	GATAATAATAATAATAATAATAATAATA ATAATAATA/GATAATAATAATAATAATA ATAATA	TGAGGTTAGGAAGTGT GGGG	TCCATCAAGATTCGAC CACA	60.0	300	INTERGENIC	--	--	--
Ca4_P3_2445623	Ca4	2445623	AATATATATATATATATATATATATATAT ATATATATA/AATATATATATATATATAT ATATATATATATATATATATATATATATA TA	GGAGGTCACAACCACT CCAA	CGGACTTTCGACTTTC TTGC	61.0	654	INTERGENIC	--	--	--

INDEL marker IDs	Chromosomes/ unanchored scaffolds	Physical positions (bp)	InDels (*Kabuli* reference genome-CDC Frontier/PII)	Forward primers (5'-3')	Reverse primers (5'-3')	Anneling temperature (0C)	Expected amplified product size (bp)	Structural annotation		Functional annotation	
								Sequence components of *kabuli* genome	*Kabuli* gene accession IDs	NCBI-KOG	TFs
Ca4_P3_24473624	Ca4	24473624	T/TA	GAGCTTAGACCGCCACAAAG	TTGTAGTTGAGTTCGCACAAAAA	60.0	743	INTERGENIC	--	--	--
Ca4_P3_24478956	Ca4	24478956	CAA/CAAA	CGGTTCTACACAAGTTGCGA	TCGATTATGCGTAGGGAAGG	59.9	746	INTERGENIC	--	--	--
Ca4_P3_24479035	Ca4	24479035	ATTTTTTT/ATTTTTTTTTT	CGGTTCTACACAAGTTGCGA	TCGATTATGCGTAGGGAAGG	59.9	746	INTERGENIC	--	--	--
Ca4_P3_24488859	Ca4	24488859	TAAA/TAAAA	TTGGAACTCATGCAAATGGA	CGATACTCACCCCTCGTTGT	60.0	557	INTERGENIC	--	--	--
Ca4_P3_24502640	Ca4	24502640	ATTTTTTTTT/ATTTTTTTTTT	TTGCCTGTTACCCTATTCGC	TCTTGCCACGAACGTATCAC	60.1	938	INTERGENIC	--	--	--
Ca4_P3_24510502	Ca4	24510502	CTGGAACATTGGAACATTG/CTGGAACATTG	CTGCAAAAGCCCTCACAAAT	TCCTCTTGCACACCACAAGA	60.2	969	INTERGENIC	--	--	--
Ca4_P3_24518915	Ca4	24518915	GTTTTTTTTTT/GTTTTTTTTT	TCATGTTCGGACAAGTGAGC	TGGTTCCAACAACATTTCCA	59.8	437	INTERGENIC	--	--	--
Ca4_P3_24553809	Ca4	24553809	ATTTTTTTT/ATTTTTTTTT	TGGTGCGATTGTGGAAAATA	TTCTTTGGTGGTTGAATTTTGTT	59.9	695	INTERGENIC	--	--	--
Ca4_P3_24597173	Ca4	24597173	TAAAAAAAAAAA/TAAAAAAAAAA	TGTCACGACAATATTGGATGATT	CCTTTAGAGGACCACCAATGTC	59.2	482	INTERGENIC	--	--	--
Ca4_P3_24607917	Ca4	24607917	GATGAATGAATGAATGAATGAATGAATGAATGA/GATGAATGAATGAATGAATGAATGAATGA	CTTCAGGGCCGAAAATAACA	CGAAAACATCCCTCCTTTCA	60.1	404	INTRON	Ca_20857	--	--

INDEL marker IDs	Chromosomes/ unanchored scaffolds	Physical positions (bp)	InDels (*Kabuli* reference genome-CDC Frontier/PII)	Forward primers (5'-3')	Reverse primers (5'-3')	Anneling temperature (0C)	Expected amplified product size (bp)	Structural annotation		Functional annotation	
								Sequence components of *kabuli* genome	*Kabuli* gene accession IDs	NCBI-KOG	TFs
Ca4_P3_24624388	Ca4	24624388	AA/AATA	TTCACTTGGGCTAAATGCCT	TTGAACAATTCCAAGGCACA	59.7	693	INTERGENIC	--	--	--
Ca4_P3_24624996	Ca4	24624996	CTTTTTTTTTT/CTTTTTTTTTTT	GTGTGCCTTGGAATTGTTCA	CATTTGCCAACTCACCAAAA	59.5	461	INTERGENIC	--	--	--
Ca4_P3_24651558	Ca4	24651558	TAAA/TAA	CAAGTGCCTTATTGCAAATTCA	TTTTCATCCGGTAGAAGTCGTT	60.1	464	INTERGENIC	--	--	--
Ca4_P3_24677817	Ca4	24677817	ATTTTTTTTT/ATTTTTTTTTT	CACGAAAACCTTAAACCCCA	TCTTAAAAGCGATGTAAAGGGC	59.8	526	INTERGENIC	--	--	--
Ca4_P3_24693623	Ca4	24693623	TC/TCC	AGGATGTGCTTCCAGGATTG	CTTGCCGAACCATACCTTGT	60.1	594	INTERGENIC	--	--	--
Ca4_P3_24748886	Ca4	24748886	GAAAAAAAAA/GAAAAAAAA	CACGACCACATCTGTGAGAAA	TTCCAAAATTTCTTCAACACTCAA	59.7	629	INTERGENIC	--	--	--
Ca4_P3_24810166	Ca4	24810166	GAAAAAAAAAA/GAAAAAAAAAAA	GCTTTTCCTCCTGCCTTTTT	TTCTTGGATATTGGCCTTCC	59.8	815	DRR	Ca_20849	--	--
Ca4_P3_24828297	Ca4	24828297	ATTTTTTTTTT/ATTTTTTTTTTT	TGGAATTTCAGACTGCCTTG	GGGCACGAAGAAGCACTATC	58.8	716	INTERGENIC	--	--	--
Ca4_P3_24874840	Ca4	24874840	ATACTCATTGTAACCCTATTA/ATA	CACTGCTCGCAGAATGGTAA	AGGGATAACCTGGTGCTGTG	60.0	539	INTERGENIC	--	--	--
Ca4_P3_24897433	Ca4	24897433	GAAAAAAAAA/GAAAAAAAAAA	TGCACAAACCTTTCAATGCT	TTTTTGAATTTTGAACGAAACA	59.3	775	INTERGENIC	--	--	--

INDEL marker IDs	Chromosomes/ unanchored scaffolds	Physical positions (bp)	InDels (*Kabuli* reference genome-CDC Frontier/PII)	Forward primers (5'-3')	Reverse primers (5'-3')	Anneling temperature (0C)	Expected amplified product size (bp)	Structural annotation		Functional annotation	
								Sequence components of *kabuli* genome	*Kabuli* gene accession IDs	NCBI-KOG	TFs
Ca4_P3_24997166	Ca4	24997166	T/TG	TGTTGAATGCTGAAACAGCC	CATCGGGGCTATGAGACCTA	59.8	626	INTERGENIC	--	--	--
Ca4_P3_25036917	Ca4	25036917	T/TA	ATCCAAGTTGAGCAAGCATT	CATTGAGACTTTGCCACGAA	57.4	605	INTERGENIC	--	--	--
Ca4_P3_25046968	Ca4	25046968	TGCTGAGCTGA/TGCTGA	ATGTTTCGTGCAATGGTCAA	AAGGTCAAGGGCCAAAAACT	60.0	601	INTERGENIC	--	--	--
Ca4_P3_25071336	Ca4	25071336	CA/C	TGAGACTGCTTGGCTACCCT	ATCTGAATCATCCCAAGCCA	60.0	488	INTERGENIC	--	--	--
Ca4_P3_25077409	Ca4	25077409	ATTTTTTTT/ATTTTTTT	TGCAAAAACCTCAAGCAATG	TTGTTATGGCTGGGGCTAAG	59.8	777	INTERGENIC	--	--	--
Ca4_P3_25088043	Ca4	25088043	CCT/CCTCCTTCTTCT	AACCTTCGCAAGAACCCTTC	ATCAATCTCGTTTTCCTGGG	60.6	219	INTERGENIC	--	--	--
Ca4_P3_25104814	Ca4	25104814	ACT/ACTCT	GATCGCAACTTGGATGTTCA	GGAAGGAAAGCTACCAAGGC	59.7	667	DRR	Ca_16560	--	--
Ca4_P3_25135426	Ca4	25135426	GTTTTTTT/GTTTTTTTT	ATCAGTCCTTGGCAGCATCT	GCAAAATATGGCTGATGCAA	59.8	289	INTERGENIC	--	--	--
Ca4_P3_25145575	Ca4	25145575	TAAAAAAAA/TAAAAAAAAA	TCACATGATCGAACGAAGACA	CCCATGTGCATGAGACTTTG	60.3	845	INTERGENIC	--	--	--
Ca4_P3_25146347	Ca4	25146347	GAAAAAAAA/GAAAAAAA	TCATCCTTGCCTCGATTAGG	GGAAATGCAAATCAAAACCAA	60.2	566	INTERGENIC	--	--	--

INDEL marker IDs	Chromosomes/ unanchored scaffolds	Physical positions (bp)	InDels (*Kabuli* reference genome-CDC Frontier/PII)	Forward primers (5'-3')	Reverse primers (5'-3')	Anneling temperature (0C)	Expected amplified product size (bp)	Structural annotation		Functional annotation	
								Sequence components of *kabuli* genome	*Kabuli* gene accession IDs	NCBI-KOG	TFs
Ca4_P3_25152424	Ca4	25152424	ATTTTTTTTTTTT/ATTTTTTTTTTT	GGTTTGGATGATTTTG GTGG	TGGCAACACATGAACC TGAT	60.0	361	INTRON	Ca_16561	Q	B3
Ca4_P3_25168771	Ca4	25168771	AA/AATA	GACCAAGGCAAGCAAG AAAG	GTGGAAGTTGAAACGA CGGT	60.0	178	INTERGENIC	--	--	--
Ca4_P3_25177814	Ca4	25177814	CAGAGAGA/CAGAGA	TCCTTGATCGGTTAAGT CGG	TAAAGGACCAAGTCGG GAAA	60.1	373	INTERGENIC	--	--	--
Ca4_P3_25178138	Ca4	25178138	GAAAAAAAAA/GAAAAAAAAAAAA	TTTCCCGACTTGGTCCT TTA	TGACTAACGCATTGCC AAAA	59.5	619	INTERGENIC	--	--	--
Ca4_P3_25182596	Ca4	25182596	TATATATATATA/TATATATATATAAATA TATATATA	TGGTAACTTGCATTCAC CCA	CTTTTGGTCCCCGACA TAGA	60.0	252	INTRON	Ca_16562	--	--
Ca4_P3_25187640	Ca4	25187640	ATTTTTTTTTT/ATTTTTTTTT	GGTAGGGAGGAGAGTT TGGC	TCGAGCTCAACCTTAA ATCCA	60.1	614	INTRON	Ca_16562	--	--
Ca4_P3_2521098	Ca4	2521098	AAAAAAATTTAATAAACAAAAAATTTAA TAAACAAAAAATT/AAAAAAATTTAATAA ACAAAAAATT	GCACCACCACTACCGT CTTT	TAAGTTCACGGCCACA TTCA	60.0	883	INTERGENIC	--	--	--
Ca4_P3_25214452	Ca4	25214452	CC/CCGAACTC	TCTCCATTTGCATAAAG CCC	GGTGTGACCCTTCCTT TTCA	60.0	347	INTERGENIC	--	--	--
Ca4_P3_25218739	Ca4	25218739	GATGAT/GATGATTATGAT	GCAGCAAATTCCAGAT GCTT	GAACTGCTCTCAATGT CCCC	60.4	611	INTERGENIC	--	--	--
Ca4_P3_25249365	Ca4	25249365	C/CA	GTTAAGCCGTAAACGC CAAC	TGCGTCAAATCTCTGG TGAG	59.7	128	INTERGENIC	--	--	--

INDEL marker IDs	Chromosomes/ unanchored scaffolds	Physical positions (bp)	InDels (*Kabuli* reference genome-CDC Frontier/PII)	Forward primers (5'-3')	Reverse primers (5'-3')	Anneling temperature (0C)	Expected amplified product size (bp)	Structural annotation		Functional annotation	
								Sequence components of *kabuli* genome	*Kabuli* gene accession IDs	NCBI-KOG	TFs
Ca4_P3_25251614	Ca4	25251614	TCC/TCCC	TGAGAAGGGCGAATAC CAAC	TAAATTTGGGGGTTGG ATGA	60.1	450	DRR	Ca_16566	O	WRKY
Ca4_P3_25252210	Ca4	25252210	GTT/GT	AAGGGGGCCCTAATAA GGTT	GATGTGCCTGGTTGGA AAGT	60.0	855	DRR	Ca_16567	CD	--
Ca4_P3_25258427	Ca4	25258427	GAAAAAAAAA/GAAAAAAAAAA	TTTGTGCGCAAACAACA GA	TGTGGGGAGCAAAAGT GTTT	60.0	626	INTERGENIC	--	--	--
Ca4_P3_25280052	Ca4	25280052	CTTT/CTT	CTCACTTTTGTTCCCCC AAA	TGGTGCAAGGTCTAAA GGAGA	59.9	292	INTERGENIC	--	--	--
Ca4_P3_25287465	Ca4	25287465	CAAAAAAAAA/CAAAAAAA	CTAGCGCCACTCCTGT AACC	TCCGATGGACACAAGT CTCA	59.9	330	INTERGENIC	--	--	--
Ca4_P3_25292705	Ca4	25292705	GTTAATTAA/GTTAA	GGCAGAAATTTGAACG TGGT	GGACAAAAGAAGAGCA AAAAGG	60.0	227	INTERGENIC	--	--	--
Ca4_P3_25295191	Ca4	25295191	CTTT/CTTTT	TTTGCGAACAAATTCAT ATCCTT	GTTGCACCAATTGCAC AGTC	59.9	705	INTERGENIC	--	--	--
Ca4_P3_25347808	Ca4	25347808	TAAAAAAAA/TAAAAAAA	CAAAGGGCGACCTCCT TAAA	GAGCTCCAAAGTAGGG TTTTCTT	61.4	278	INTERGENIC	--	--	--
Ca4_P3_25349410	Ca4	25349410	TA/TAA	TCGTTTGTTTTGGCACA TCT	TCAAACCAAAAGAGCA GGAAG	59.2	349	INTERGENIC	--	--	--
Ca4_P3_25352610	Ca4	25352610	T/TA	GGAAATGGTTCTTTGAA AGCC	AGCCTTACGCATGTAT TGCC	59.9	719	INTERGENIC	--	--	--

INDEL marker IDs	Chromosomes/ unanchored scaffolds	Physical positions (bp)	InDels (*Kabuli* reference genome-CDC Frontier/PII)	Forward primers (5'-3')	Reverse primers (5'-3')	Anneling temperature (0C)	Expected amplified product size (bp)	Structural annotation		Functional annotation	
								Sequence components of *kabuli* genome	*Kabuli* gene accession IDs	NCBI-KOG	TFs
Ca4_P3_25358083	Ca4	25358083	TAAAAAAAA/TAAAAAAAAA	AAAATTCTGTTTGGGGGACC	ATTACACGCTTGCAGACGAA	60.0	886	INTERGENIC	--	--	--
Ca4_P3_25367189	Ca4	25367189	A/AT	TTTATTGTAGGTGGAGGCGG	GGCCAAATTGATGCAAAGTT	60.0	271	INTERGENIC	--	--	--
Ca4_P3_25371828	Ca4	25371828	ATTT/ATTTT	TGAAGCTCCGAAACATAGCC	TAAGGCGCCAGACTCAAGTT	60.4	283	INTERGENIC	--	--	--
Ca4_P3_25393862	Ca4	25393862	TATTAAAATT/TATTAAAATTAAAATT	CAATTGGATTGTGGCTTCCT	CAGGCGTCGAACAATTCATA	59.9	909	INTERGENIC	--	--	--
Ca4_P3_25434376	Ca4	25434376	TAAAAAAAAAAA/TAAAAAAAA	TTTGTCCACCAAGAAAAGGC	TCCGCTCCTTACCATCACTC	60.1	732	DRR	Ca_16576	O	MIKC
Ca4_P3_25457334	Ca4	25457334	ATTTTTTT/ATTTTTTTT	CATTTTACTCATCGATCTTTGTGG	TGGACAAAATCATGTCTCTTTGA	59.9	336	DRR	Ca_16577	--	--
Ca4_P3_25457701	Ca4	25457701	AATATATATATATATATATATATATATATA/AATATATATATATATATATATATATATATATATA	TCAAAGAGACATGATTTTGTCCA	TCACTTTGCTTTGTGTGTCTCA	59.6	524	DRR	Ca_16577	--	--
Ca4_P3_25470351	Ca4	25470351	CTTTTTTTTT/CTTTTTTTTTT	TGCAATTATCCAAAGATGTATGAAA	TCCTTTTGTCCGTCGTCTCT	59.8	479	INTERGENIC	--	--	--
Ca4_P3_25495365	Ca4	25495365	TAACATAAC/T	GGGAAGCTGGTTTTGTTGTT	AATGGTCTTTCAATGGGACG	59.1	783	INTERGENIC	--	--	--
Ca4_P3_25501013	Ca4	25501013	TAAAAAAAA/TAAAAAAAAA	GGGCATTGGTAACTCCAAAA	CCAATTGTGCTCTCCTCTCC	59.8	909	INTERGENIC	--	--	--

INDEL marker IDs	Chromosomes/ unanchored scaffolds	Physical positions (bp)	InDels (*Kabuli* reference genome-CDC Frontier/PII)	Forward primers (5'-3')	Reverse primers (5'-3')	Anneling temperature (0C)	Expected amplified product size (bp)	Structural annotation		Functional annotation	
								Sequence components of *kabuli* genome	*Kabuli* gene accession IDs	NCBI-KOG	TFs
Ca4_P3_2550484	Ca4	2550484	CT/CTGGAATTAGAATGTGTGTT	ATTGTTTGTCCAGCGATTCC	TCTGCAATGACCCTCTTGTG	59.9	433	INTERGENIC	--	--	--
Ca4_P3_25543812	Ca4	25543812	TTTTCTTCTTTCTTCTTTC/TTTTCTTCTTTC	AGGACTTTCGACCACCACAC	TACCCTTTGCTAGCCCCTTT	60.0	415	INTERGENIC	--	--	--
Ca4_P3_25580326	Ca4	25580326	ATTTTTTTTTTTTT/ATTTTTTTTTTTTTTT	TCGTGGATAAAATAAATCAAATCAA	GCAAATCCATTGGCAATTCT	58.9	860	INTERGENIC	--	--	--
Ca4_P3_25590282	Ca4	25590282	TAAAAAAAA/TAAAAAAAAA	GCATGGATGCAACCTCCTAT	TGACCCCACACTGCAAATTA	59.9	400	INTERGENIC	--	--	--
Ca4_P3_25596032	Ca4	25596032	TTAT/TTATAT	AGTGATGTGGGCAACAATGA	TGCATTTTACAGCGCTTTTG	60.0	515	INTERGENIC	--	--	--
Ca4_P3_25596215	Ca4	25596215	ATGTGTGTGTGTGT/ATGTGTGTGTGT	TTCTCACGAAAGCGCTGTAA	GGGCAAGGGGATCTGAATA	59.8	585	DRR	Ca_16586	--	Trihelix
Ca4_P3_25630012	Ca4	25630012	TG/T	AGTAGACTTGCCTCCCGGTT	CCTTGTTGTGGAGTGTGGTG	60.1	788	INTERGENIC	--	--	--
Ca4_P3_25641770	Ca4	25641770	CT/CTT	TGGTTCCAACAGTACATGCAA	TCCCAGAACACCACAACAAA	60.0	454	INTERGENIC	--	--	--
Ca4_P3_25671775	Ca4	25671775	TTTTCTTTCTTTC/TTTTCTTTCTTTCTTTC	CCAGGATGCTTCCCTGATAA	AGAGCGCGAACTGTCAAAAT	60.0	809	INTERGENIC	--	--	--
Ca4_P3_25694964	Ca4	25694964	CAAAAAAAAA/CAAAAAAAAAAAA	CCACTTCGCTTAGGGATCAA	TATGGGCATTCTTCTTTGGG	60.2	675	INTERGENIC	--	--	--

INDEL marker IDs	Chromosomes/ unanchored scaffolds	Physical positions (bp)	InDels (*Kabuli* reference genome-CDC Frontier/PII)	Forward primers (5'-3')	Reverse primers (5'-3')	Anneling temperature (0C)	Expected amplified product size (bp)	Structural annotation		Functional annotation	
								Sequence components of *kabuli* genome	*Kabuli* gene accession IDs	NCBI-KOG	TFs
Ca4_P3_2570743	Ca4	2570743	GATATATATATATATATATATATAT/GATATATATATATATATATATATAT	AACTCGAGGAGAGAGGAGGG	GGGAATGAAAACAACAATGGA	59.9	866	INTERGENIC	--	--	--
Ca4_P3_25718304	Ca4	25718304	GTATA/GTA	AACACAAAGGGTTGATTCGC	AACCTTGGCAACAAGTGGAC	60.0	568	DRR	Ca_16594	--	--
Ca4_P3_25725070	Ca4	25725070	CAAAAAAAAA/CAAAAAAAAAA	TCTTCGAAATGCAACTTGGT	TTTTAATTTTTGGAGGGGGT	58.4	130	INTERGENIC	--	--	--
Ca4_P3_25727547	Ca4	25727547	ATTTTTTTTT/ATTTTTTTTTT	TGGGTGGATGGTAAGAAAATG	TCAACTCATGGCTTTCCACA	59.7	709	INTERGENIC	--	--	--
Ca4_P3_25744614	Ca4	25744614	TAAAAAAA/TAAAAAA	TTTGCAAAAAGGCTTCACAA	GCATATCGAAGGATGCCATT	59.5	405	INTERGENIC	--	--	--
Ca4_P3_25754372	Ca4	25754372	AAAAATAAAATAAAATAAA/AAAAATAAAATAAA	AATGGGCGCTGTTAAATGTC	TCATTTAAACCACTGCGTCAA	60.0	750	INTERGENIC	--	--	--
Ca4_P3_25758636	Ca4	25758636	CTTTT/CTTTTTT	TGCACTTCAGTTAAATTGGGC	AGAACTTGCGTGGTGATTCC	60.1	530	INTERGENIC	--	--	--
Ca4_P3_25760041	Ca4	25760041	ATTTTTTT/ATTTTTT	CACAACGGAATTTGGGTTTC	TTGAGCTTGCAACTTGATCACT	60.2	565	INTERGENIC	--	--	--
Ca4_P3_25768326	Ca4	25768326	TAAAAAA/TAAAAAAA	ATGTTTCGTGCAATGGTCAA	CACATGGCTGAACGAGAAGA	60.0	382	INTERGENIC	--	--	--
Ca4_P3_25774052	Ca4	25774052	TAAAAAAAAAAA/TAAAAAAAAAA	TGTCAAATCCAACGCAAAGA	TCGCAACCAAACAAAGCATA	60.2	661	INTERGENIC	--	--	--

INDEL marker IDs	Chromosomes/ unanchored scaffolds	Physical positions (bp)	InDels (*Kabuli* reference genome-CDC Frontier/PII)	Forward primers (5'-3')	Reverse primers (5'-3')	Anneling temperature (0C)	Expected amplified product size (bp)	Structural annotation		Functional annotation	
								Sequence components of *kabuli* genome	*Kabuli* gene accession IDs	NCBI-KOG	TFs
Ca4_P3_25779217	Ca4	25779217	CTTAATTAATTAATTAATTAATTA/CTTAATTAATTAATTAATTA	ATACCGAGATGGACACCACC	TAACCCTTCAACATCTGGGC	59.7	367	DRR	Ca_16597	R	NAC
Ca4_P3_25783388	Ca4	25783388	CTTTT/CTTTTT	GCGTGATGCATGATCTAGGA	TCTTTTGATTCCGAGTTGGG	59.8	658	INTERGENIC	--	--	--
Ca4_P3_25803073	Ca4	25803073	ATTTTTTTTTT/ATTTTTTTTT	TGAGCCTCAACAGCAAAATG	CTCCAAAGCTGCAATGTTGA	60.0	711	INTERGENIC	--	--	--
Ca4_P3_25806778	Ca4	25806778	CTTTTTTTTT/CTTTTTTTT	CGTTACACTTGCCTATGGAACA	GGTCCGAACTGTGACAAGGT	60.1	423	INTERGENIC	--	--	--
Ca4_P3_25822369	Ca4	25822369	CAAAAAAA/CAAAAAA	TGTTTGAACCCGAGACTTCAC	CGTGGCATTTGAAAAAGCAT	60.1	611	INTERGENIC	--	--	--
Ca4_P3_25823927	Ca4	25823927	T/TG	CACAGGTGGTGCGTTGTTTA	TCACAACCACAACCACATCA	60.6	170	INTERGENIC	--	--	--
Ca4_P3_25830554	Ca4	25830554	CAAAAAAAAAA/CAAAAAAAAA	ATCGTGCACATTCCCATTTT	TTCAAATTGCTCCTTCTGGG	60.2	621	INTERGENIC	--	--	--
Ca4_P3_25831604	Ca4	25831604	TCCCC/TCCCCC	GGTGAACCGACACTGGTTTT	TCCAACATTTATGCAAGGCA	59.9	301	INTERGENIC	--	--	--
Ca4_P3_25834950	Ca4	25834950	AAGACGTTCCGTA/AA	TCGCTGCTTATTCATGTTCA	ACCACCAGTGAGGATTTTGC	58.0	336	INTERGENIC	--	--	--
Ca4_P3_25843650	Ca4	25843650	T/TCAG	AACCTGTTGGCTAAAGCCCT	TCAATGGCCCCTGAAGTTAC	60.1	862	INTRON	Ca_16601	R	--

INDEL marker IDs	Chromosomes/ unanchored scaffolds	Physical positions (bp)	InDels (*Kabuli* reference genome-CDC Frontier/PII)	Forward primers (5'-3')	Reverse primers (5'-3')	Anneling temperature (0C)	Expected amplified product size (bp)	Structural annotation		Functional annotation	
								Sequence components of *kabuli* genome	*Kabuli* gene accession IDs	NCBI-KOG	TFs
Ca4_P3_25847698	Ca4	25847698	T/TG	CATGCTACCAAAGCGATTGA	ATCGATTTCGCGAGTCTTGT	59.8	370	INTERGENIC	--	--	--
Ca4_P3_25869082	Ca4	25869082	ATTTTT/ATTTT	TATTTGTCGATTTGCGTGGA	TTATAAGTGCGGTTAGCGGG	60.1	693	INTERGENIC	--	--	--
Ca4_P3_25884525	Ca4	25884525	TA/T	AGCAACCAAATCGAAACACC	GGGAAATTTTGGAACCAATG	60.0	307	INTERGENIC	--	--	--
Ca4_P3_2589226	Ca4	2589226	GTTGCAAGTTGCAAG/GTTGCAAGTTGCAAGTTGCAAG	AAGATCGATCAGTTCCACCG	ATTGGGAACCAACTGACGAG	60.1	396	INTERGENIC	--	--	--
Ca4_P3_25897872	Ca4	25897872	GGTGTACCGTGTA/GGTGTA	AGTGCCCAACTTTCACCTTC	TCACGATGCACCCAAGAATA	59.2	493	INTERGENIC	--	--	--
Ca4_P3_25906423	Ca4	25906423	TC/T	TAATGGCATTGGTACAGGCA	AAAAATCCTGAATGTCCCCC	60.0	472	INTERGENIC	--	--	--
Ca4_P3_25909415	Ca4	25909415	ATTTTTTTTTTTTT/ATTTTTTTTTTT	CTTGTGCTTCGACTGGATCA	GCTCCGGCTGAGAACAATAC	60.0	817	DRR	Ca_16605	R	--
Ca4_P3_25916811	Ca4	25916811	CTTTTTTTTTTT/CTTTTTTTTTTTTT	ATGGTAACAGATATCGCCGC	ATTGTAGCATTGCCCAAAGG	60.0	627	DRR	Ca_16606	I	--
Ca4_P3_25919215	Ca4	25919215	GTTTTTTTTTTTTTT/GTTTTTTTTTTTTTTTT	AAATCGACAGTCCTTGCCAC	TGCAGGTCTTGAGCTTGATG	60.1	629	INTRON	Ca_16606	I	--
Ca4_P3_25927650	Ca4	25927650	AGG/AGGG	ATCACAACTGGGAACCCAAA	AAGCTCCAAGACCAGATGGA	60.2	470	INTERGENIC	--	--	--

INDEL marker IDs	Chromosomes/ unanchored scaffolds	Physical positions (bp)	InDels (*Kabuli* reference genome-CDC Frontier/PII)	Forward primers (5'-3')	Reverse primers (5'-3')	Anneling temperature (0C)	Expected amplified product size (bp)	Structural annotation		Functional annotation	
								Sequence components of *kabuli* genome	*Kabuli* gene accession IDs	NCBI-KOG	TFs
Ca4_P3_25927721	Ca4	25927721	CTTTTTT/CTTTTTTT	GTTTCACGGAGAAAAT CCCA	AAGCTCCAAGACCAGA TGGA	59.9	441	INTERGENIC	--	--	--
Ca4_P3_25941584	Ca4	25941584	A/AG	CGATCGTTGTTGGTAT GTCG	AGCTGACCAAATGCGT AAGG	60.0	244	INTERGENIC	--	--	--
Ca4_P3_25947026	Ca4	25947026	GAAAACACAAAA/GAAAA	CACTAGAAGCGCGTGT CAGA	TTGTGAACGCCAATGG TAAA	60.3	924	INTERGENIC	--	--	--
Ca4_P3_25956176	Ca4	25956176	ATTTTTTTTTTTTT/ATTTTTTTTTT	ATATTGGGACCGGTTA AGGG	TTTGGGTGTGTTGACT GCAT	59.9	784	INTERGENIC	--	--	--
Ca4_P3_25958010	Ca4	25958010	TTATATATATAT/TTATATATATATAT	TCGGGGAAATCAATTC GTTA	TGGAAGTCATCGACCC ATTT	60.3	673	INTERGENIC	--	--	--
Ca4_P3_25959074	Ca4	25959074	GCC/GC	TGAAGATGAAGAAGAG GGCAA	TGTGGCATAAGGAGGA GTTTC	59.9	376	INTERGENIC	--	--	--
Ca4_P3_25962811	Ca4	25962811	GTATATATAT/GTATATATATAT	TGGTTGGAAGCTCCAG AAGT	GAAAAGGTGAGGAATC GCTG	59.8	275	DRR	Ca_16607	--	B3
Ca4_P3_25967972	Ca4	25967972	ATTTTTTTT/ATTTTTTTTT	TTGTACCCTTGTGCAAC CAA	TGGTTGGATACTTGCA ATGG	60.0	180	INTERGENIC	--	--	--
Ca4_P3_25968820	Ca4	25968820	GTTT/GTTTT	TTCTTCTGCCCAAAAAT GCT	CCTCAAGCAGACCTTT GGAG	59.8	412	INTERGENIC	--	--	--
Ca4_P3_25969511	Ca4	25969511	ACC/AC	TCCCTTGCCTCTAGCC AGTA	GCAGATGTTCTCAACA GCCA	60.0	350	INTERGENIC	--	--	--

INDEL marker IDs	Chromosomes/ unanchored scaffolds	Physical positions (bp)	InDels (*Kabuli* reference genome-CDC Frontier/PII)	Forward primers (5'-3')	Reverse primers (5'-3')	Anneling temperature (0C)	Expected amplified product size (bp)	Structural annotation		Functional annotation	
								Sequence components of *kabuli* genome	*Kabuli* gene accession IDs	NCBI-KOG	TFs
Ca4_P3_25970159	Ca4	25970159	AC/A	TGGTTGGGTCTTGGTTTGTT	CACCTGCTATGGATGCAATG	60.2	709	INTERGENIC	--	--	--
Ca4_P3_25971278	Ca4	25971278	GATTGAAGAA/GA	GCTTCGCCAAGCTTACAATC	CCAACGATCATCAAAGGGAT	60.0	268	INTERGENIC	--	--	--
Ca4_P3_25972652	Ca4	25972652	TGGG/TGG	TTGTCTCAACAATCTCCCCC	CTCAGTTCCTAGCACGAGGG	59.9	676	INTERGENIC	--	--	--
Ca4_P3_25975114	Ca4	25975114	TGGGG/TGGGGG	ACACATGTTGGCCAATGAGA	GAGTCCGATTTCCATGAGGA	60.0	621	CDS (FRAME SHIFT)	Ca_16608	--	--
Ca4_P3_25978445	Ca4	25978445	CT/CTTT	TGCCTCAAAGGCTTCATCTT	AGCACTCATCCTCGGGTAGA	60.0	435	INTERGENIC	--	--	--
Ca4_P3_25979689	Ca4	25979689	TG/TGG	GTCCTCGTCAGGAGGTTGAG	CCACTGTTGCTGGCAGATAA	59.8	707	INTERGENIC	--	--	--
Ca4_P3_25982514	Ca4	25982514	CAATAATA/CAATAATAATA	GCGCAAAAACAAGAAGGAAG	GGTCCAGGTTGTTTGGAGAA	60.0	290	INTERGENIC	--	--	--
Ca4_P3_25984075	Ca4	25984075	TA/TAA	CGATTTTTAACAGCGTGGGT	TGGGTCCATGTTCCAATTTT	60.0	276	INTERGENIC	--	--	--
Ca4_P3_25984442	Ca4	25984442	TGAGAG/TGAGAGAG	AGATCCTGTTGACACGGCTT	CGGTTGTTGTTGTGATTTGC	59.7	524	INTERGENIC	--	--	--
Ca4_P3_25987244	Ca4	25987244	AATATATATAT/AATATATATATATATATAT	TCTACTCTCGTCGCAGCTCA	TGCTTAGGCATTTACCCCAG	60.0	250	INTERGENIC	--	--	--

INDEL marker IDs	Chromosomes/ unanchored scaffolds	Physical positions (bp)	InDels (*Kabuli* reference genome-CDC Frontier/PII)	Forward primers (5'-3')	Reverse primers (5'-3')	Anneling temperature (0C)	Expected amplified product size (bp)	Structural annotation		Functional annotation	
								Sequence components of *kabuli* genome	*Kabuli* gene accession IDs	NCBI-KOG	TFs
Ca4_P3_25987622	Ca4	25987622	TCC/TC	TTCAACTGGGGTAAATGCCT	TGTTCTAGTCCAGATCAAAACGA	59.4	323	INTERGENIC	--	--	--
Ca4_P3_25988978	Ca4	25988978	AAAGAAGAAGAAG/AAAGAAGAAG	AAGCTGGATGTGGACTTTGC	AGTTCCAATTGGCCTCCTTT	60.3	404	INTERGENIC	--	--	--
Ca4_P3_25989281	Ca4	25989281	TCCCC/TCCC	AAGCTGGATGTGGACTTTGC	AGTTCCAATTGGCCTCCTTT	60.3	404	INTERGENIC	--	--	--
Ca4_P3_25989322	Ca4	25989322	GTTTTT/GTTTTTT	GTACGCGATCTAGTCCCCAA	CCGGGGAGAAAGTTCTAGTG	60.1	168	INTERGENIC	--	--	--
Ca4_P3_25994448	Ca4	25994448	GTTTTTTTTTTT/GTTTTTTTTTT	ATCCACAACCGAGTTAGCGT	TCGAGCCTTTTTGTCTGATG	59.6	623	INTERGENIC	--	--	--
Ca4_P3_25994543	Ca4	25994543	GTTTTTT/GTTTTTTT	TTAAGATTGAGGGGTGTGGG	CACCACTTCATTCGGGACTT	59.8	516	INTERGENIC	--	--	--
Ca4_P3_25994721	Ca4	25994721	AC/A	TTAAGATTGAGGGGTGTGGG	CACCACTTCATTCGGGACTT	59.8	516	INTERGENIC	--	--	--
Ca4_P3_25995802	Ca4	25995802	ATTTT/ATTT	CCCTAAATCAAAGCAGCGTC	TGCACTGTTGTTGTTGCTGA	59.8	470	INTERGENIC	--	--	--
Ca4_P3_25995987	Ca4	25995987	A/AT	CCCTAAATCAAAGCAGCGTC	TGCACTGTTGTTGTTGCTGA	59.8	470	INTERGENIC	--	--	--
Ca4_P3_26000260	Ca4	26000260	GTTTTT/GTTT	GGGCATTGCTTTGTTTTTGT	CAATGGAAGTGGTTGTGTCG	60.0	414	INTRON	Ca_16610	C	--

INDEL marker IDs	Chromosomes/ unanchored scaffolds	Physical positions (bp)	InDels (*Kabuli* reference genome-CDC Frontier/PII)	Forward primers (5'-3')	Reverse primers (5'-3')	Anneling temperature (0C)	Expected amplified product size (bp)	Structural annotation		Functional annotation	
								Sequence components of *kabuli* genome	*Kabuli* gene accession IDs	NCBI-KOG	TFs
Ca4_P3_26009631	Ca4	26009631	A/AC	GGAACTTCTCTTGTGGCAGC	TATTCCTCTGATTGGGTCGC	60.0	148	INTERGENIC	--	--	--
Ca4_P3_26015572	Ca4	26015572	ATTTTTTTTTT/ATTTTTTTTT	TGCGAGGGCTAGCTTAAAAA	TGAGACAAAAGTTTGCGGTG	60.1	635	INTERGENIC	--	--	--
Ca4_P3_26018351	Ca4	26018351	TAAAAAAAA/TAAAAAAAAA	AGAACATGGTGGATGCACAG	TCCAAGAGCTTGGTCTGACA	59.6	373	INTERGENIC	--	--	--
Ca4_P3_26033150	Ca4	26033150	TA/TAA	CCTTTGAACCAAGGAAGCCT	TGGCGTCCCAATTATTTTTC	60.6	528	INTERGENIC	--	--	--
Ca4_P3_26051597	Ca4	26051597	AATAT/AAT	GTCTTCCCCTTGTCAAACCA	TCAAAAATTTGGATTCGGGA	59.9	225	INTERGENIC	--	--	--
Ca4_P3_26051855	Ca4	26051855	CTTTTTTTTTTTTT/CTTTTTTTTTT	TTTAACGGACGTTGTAGGGC	CTTCCACGGGATGTGTTTCT	60.0	448	INTERGENIC	--	--	--
Ca4_P3_26069953	Ca4	26069953	ATTTAAATTAATAATTTTAAATTA/ATTTAAATTA	CATCCACCTAAAGTTTTTACCCA	AACCGTGGGGGTATAAGGAC	59.3	895	INTERGENIC	--	--	--
Ca4_P3_26078815	Ca4	26078815	GTTTTTTTTT/GTTTTTTTTTTTT	TTATTGTGAGTCCGTGCAGC	GCTTTCAGACAAACCCTTGG	59.9	348	INTERGENIC	--	--	--
Ca4_P3_26091108	Ca4	26091108	ATTTTTTTTT/ATTTTTTTTTTT	AGGTGCATTTCTGAAGGTGG	TGTTCAAGATCATTCAAATGCC	60.1	564	INTERGENIC	--	--	--
Ca4_P3_26091418	Ca4	26091418	CATATATATATAT/CATATATATATATAT	AGGTGCATTTCTGAAGGTGG	GGGGCATTTTGTTGCATATT	60.1	729	INTERGENIC	--	--	--

INDEL marker IDs	Chromosomes/ unanchored scaffolds	Physical positions (bp)	InDels (*Kabuli* reference genome-CDC Frontier/PII)	Forward primers (5'-3')	Reverse primers (5'-3')	Anneling temperature (0C)	Expected amplified product size (bp)	Structural annotation		Functional annotation	
								Sequence components of *kabuli* genome	*Kabuli* gene accession IDs	NCBI-KOG	TFs
Ca4_P3_2609181	Ca4	2609181	T/TGTGCTATA	GCTTCTGCTAGGCATCCATC	ATGCATGTGCTGATGCTTGT	59.9	907	DRR	Ca_12062	--	--
Ca4_P3_26104167	Ca4	26104167	TTTTTTT/TTTTTTTTATTTTTT	TTGAGATGATTTGGCAACCA	CGTGAACGGACCCAGTTACTA	60.0	589	INTERGENIC	--	--	--
Ca4_P3_26173966	Ca4	26173966	CATAT/CAT	AGAGTGCATGTGGGATAGGG	TTCCATGATGCTCTTTTGGG	60.0	563	DRR	Ca_16612	--	HD-ZIP
Ca4_P3_26176783	Ca4	26176783	TAAAAAAAAA/TAAAAAAAA	CACAATCAATCACGCCCTTA	TGTCATTTTTGGCGTAGTGC	59.5	500	INTERGENIC	--	--	--
Ca4_P3_26179133	Ca4	26179133	GTTTTTTTTT/GTTTTTTTT	AGGAACTTTGTGTGGATGGG	TGGAAAATGCAATTCAACCA	59.8	743	INTERGENIC	--	--	--
Ca4_P3_26179923	Ca4	26179923	ATTTTTTTT/ATTTTTTT	CAAAACAACTCATGTTGGTCG	CCCATCATGAAATCCAAACC	59.1	976	INTERGENIC	--	--	--
Ca4_P3_26226774	Ca4	26226774	GGNNNNNNNNNNG/GG	GCCAGCAGTTCTTCAGGTTC	AACATGGCCCAAGAACAGAC	60.0	357	INTERGENIC	--	--	--
Ca4_P3_26259040	Ca4	26259040	ATTTTTTTTTT/ATTTTTTTTT	TTCTCGAATCAATTCCGTCC	CTCAGAGGCCGACCTATACA	60.0	888	INTERGENIC	--	--	--
Ca4_P3_26263716	Ca4	26263716	CAAAAAAAAA/CAAAAAAAAAA	CCTTGAGTTCGACACTCGGT	AGACAAACCATCCACCTTCG	60.3	670	INTERGENIC	--	--	--
Ca4_P3_26287529	Ca4	26287529	GTTTTTTTTTTTTTT/GTTTTTTTTTTTTT	AAGCCTGACCTACGGCCTAT	TGGCCTACTTATGGCCTGAC	60.1	537	DRR	Ca_25308	--	--

INDEL marker IDs	Chromosomes/ unanchored scaffolds	Physical positions (bp)	InDels (*Kabuli* reference genome-CDC Frontier/PII)	Forward primers (5'-3')	Reverse primers (5'-3')	Anneling temperature (0C)	Expected amplified product size (bp)	Structural annotation		Functional annotation	
								Sequence components of *kabuli* genome	*Kabuli* gene accession IDs	NCBI-KOG	TFs
Ca4_P3_26301785	Ca4	26301785	GAAAAAAAAA/GAAAAAAAAAA	ACCACTACGCCACACATCAA	GTGACAGTACTGGGGGAAGC	60.0	428	INTERGENIC	--	--	--
Ca4_P3_26304368	Ca4	26304368	ATTTTTTTTT/ATTTTTTTTTT	CCGATTAACCTTAAACGCCA	TTTAAAGTCCCGACCCATGA	60.0	508	INTERGENIC	--	--	--
Ca4_P3_26306719	Ca4	26306719	C/CA	CAACCCTTTGCATTCCTCAT	AATGCCCATGTGAAACCTTC	59.9	662	DRR	Ca_25307	S	--
Ca4_P3_26313656	Ca4	26313656	ATTTTTTTTTTT/ATTTTTTTTTT	TGAGGTATGCCCAAAGAAGG	TTAGGCCGTAGTCAGGCTGT	60.1	837	INTERGENIC	--	--	--
Ca4_P3_26339385	Ca4	26339385	AA/AAGAAGTA	TCAAGACGTTGATCTCGTGC	CATCTTCATTGCCTCCCATT	60.0	397	INTERGENIC	--	--	--
Ca4_P3_26350658	Ca4	26350658	TGGGG/TGGG	CTCTGACATGCGACAATGCT	GTTTGGTCGGGTTAAGGGAT	60.0	555	INTRON	Ca_25303	I	--
Ca4_P3_26354768	Ca4	26354768	AGGG/AGGGG	TGCTGGCAGGTTTATGTTTG	TCACTGCCTTCCTTACGAGG	59.7	534	DRR	Ca_25303	I	--
Ca4_P3_26364469	Ca4	26364469	CA/CAAA	TATTTGCCATCTGCACCTCA	ATTGAAAAGGCCAGGAAACA	60.2	815	INTERGENIC	--	--	--
Ca4_P3_26369131	Ca4	26369131	ATTTTTTTTTT/ATTTTTTTTT	TTCTGTGCATTTGAATGGGA	CCCCAAACCCTACGAACATA	60.0	718	INTERGENIC	--	--	--
Ca4_P3_26398945	Ca4	26398945	AATTTAATTT/AATTTAATTTATCTATTTAATTT	GTGGCGAATTGATTTGTCCT	CGACGTAATCTGGGTTGGTT	59.9	413	INTERGENIC	--	--	--

INDEL marker IDs	Chromosomes/ unanchored scaffolds	Physical positions (bp)	InDels (*Kabuli* reference genome-CDC Frontier/PII)	Forward primers (5'-3')	Reverse primers (5'-3')	Anneling temperature (0C)	Expected amplified product size (bp)	Structural annotation		Functional annotation	
								Sequence components of *kabuli* genome	*Kabuli* gene accession IDs	NCBI-KOG	TFs
Ca4_P3_26404295	Ca4	26404295	ATTTTTTTTTT/ATTTTTTTTT	TTTGGGTCCTTGGTGGTTAG	CCCACTTGGTAAAGACCACTG	59.8	430	INTERGENIC	--	--	--
Ca4_P3_26410577	Ca4	26410577	TGGAGGAG/TGGAG	ATGGCATGTGATTGAGTGGA	GATGACTCTTCGTGGGGGTA	59.9	425	INTERGENIC	--	--	--
Ca4_P3_26411287	Ca4	26411287	GTTT/GTTTT	TGTTCTGCTTAAGGGGCCTA	GATCAAGAGGTCACCGGTTC	59.8	841	INTERGENIC	--	--	--
Ca4_P3_26411531	Ca4	26411531	GAAAAAAAA/GAAAAAAAAAA	TGTAACCAACATTTGCTTTCTTT	CGCGCGGAAACAACTATTAT	57.9	681	INTERGENIC	--	--	--
Ca4_P3_26418036	Ca4	26418036	TCACACACACACACACA/TCACACACACACACACACA	CCAAAATTCAAACACCACCC	AACCCTGATTGACGGTGAAG	60.1	542	DRR	Ca_25299	--	--
Ca4_P3_26456492	Ca4	26456492	T/TG	GCGAGCATGAGAAGTTGTTG	AATTCAATGGGAAAAACCACA	59.6	713	INTERGENIC	--	--	--
Ca4_P3_26460247	Ca4	26460247	TCCC/TCC	GTGGCTGAAACAACTGATGG	TTTTTGGATAATGTTGCTTGTGA	59.1	423	INTERGENIC	--	--	--
Ca4_P3_26461127	Ca4	26461127	CAAAAA/CAAAA	TTTGCCCCATGTTTTCTCTC	GGGCGTAAATTCCAACACAT	60.1	486	INTERGENIC	--	--	--
Ca4_P3_26483173	Ca4	26483173	TAAAAAAA/TAAAAAA	TTTTGAAAACCAATGCACCA	CGCTATATCACAGCTTGGCA	59.9	413	INTRON	Ca_25006	O	C2H2
Ca4_P3_26489826	Ca4	26489826	AC/A	TTGGTTGATCGGCTAATTGA	TCCGAGTAACCCGTTTTGAC	59.1	435	INTERGENIC	--	--	--

INDEL marker IDs	Chromosomes/ unanchored scaffolds	Physical positions (bp)	InDels (*Kabuli* reference genome-CDC Frontier/PII)	Forward primers (5'-3')	Reverse primers (5'-3')	Anneling temperature (0C)	Expected amplified product size (bp)	Structural annotation		Functional annotation	
								Sequence components of *kabuli* genome	*Kabuli* gene accession IDs	NCBI-KOG	TFs
Ca4_P3_26492251	Ca4	26492251	ACTC/AC	CAAATGCACATGAGAACCGT	AATGATCCGGAACCATTTCA	59.6	519	INTERGENIC	--	--	--
Ca4_P3_26495554	Ca4	26495554	GAAAGTTGTTC/GAAAGTTGTTCAAAGTTGTTC	AACGATTTATCCTGGTCCCC	CCCGAATACAAACCCAAGTC	60.0	844	INTERGENIC	--	--	--
Ca4_P3_26498981	Ca4	26498981	GC/G	GGTTTATTTCCGCAGTTTTCC	TCAACGGACAACACGCTAAG	59.8	321	INTERGENIC	--	--	--
Ca4_P3_26518861	Ca4	26518861	AT/A	GGGTTCAGTTCACTCAAGGG	TGAAGCATATGTATTTTCACCACA	59.5	633	DRR	Ca_25005	R	NAC
Ca4_P3_26519944	Ca4	26519944	CATTATTATTATTATT/CATTATTATTATT	GGCAGCGGGTACGTAAAATA	CATTTGTGCAACAATGAGGG	60.0	414	INTERGENIC	--	--	--
Ca4_P3_26523067	Ca4	26523067	ATTTTTTTT/ATTTTTTTTT	TATCTGCCAGGGAGAAAACG	TGCCATGAAACGAAAGAAAA	60.2	362	DRR	Ca_25004	P	--
Ca4_P3_26525402	Ca4	26525402	GTTTTTTTTTT/GTTTTTTTTTTTT	TGTAGCTACCGCATTGATGG	GCTGCAAGGGATGATTTGAT	59.7	436	INTERGENIC	--	--	--
Ca4_P3_26547528	Ca4	26547528	AT/A	TCCAAAACTTGTATATCCTCAAGC	TTTGTGCTTCTGCAACAAGG	58.8	815	INTERGENIC	--	--	--
Ca4_P3_26552272	Ca4	26552272	TAAAAAAA/TAAAAAA	TCCTGGTTCACTCAATGTGG	CTCAAGTTGAAGGCCCAAAG	59.5	818	INTERGENIC	--	--	--
Ca4_P3_26581837	Ca4	26581837	TTTCCTTCCTTCCTT/TTTCCTTCCTTCCTTCCTT	GGAGAAGGAGGGAGAAATGG	ATGCATTACGGGTGAAATGG	60.0	263	DRR	Ca_25001	--	--

INDEL marker IDs	Chromosomes/ unanchored scaffolds	Physical positions (bp)	InDels (*Kabuli* reference genome-CDC Frontier/PII)	Forward primers (5'-3')	Reverse primers (5'-3')	Anneling temperature (0C)	Expected amplified product size (bp)	Structural annotation		Functional annotation	
								Sequence components of *kabuli* genome	*Kabuli* gene accession IDs	NCBI-KOG	TFs
Ca4_P3_26593036	Ca4	26593036	CAT/CATAT	AAAATCATGATCAAGGAGGCA	TTTGGTGGTGGTGGTATGTG	59.5	254	INTERGENIC	--	--	--
Ca4_P3_26616894	Ca4	26616894	AACATTTTATACATTTTATACATTTTATACATTTTA/AACATTTTATACATTTTATACATTTTATACATTTTATACATTTTA	TTGCTGGATTGAAAACCCTC	CCAAAACCCATCATAATCCG	60.1	881	INTRON	Ca_25000	E	bHLH
Ca4_P3_26620833	Ca4	26620833	GTT/GT	AAAGAAGGAAGGGTCTTGCC	TCAAAATCGAACCAAATCACA	59.7	593	INTERGENIC	--	--	--
Ca4_P3_26621141	Ca4	26621141	TAAAAA/TAAA	CGGTGCCGTTTTAAGAGAAA	GCGATTCAAAAGACATGCAA	60.2	685	INTERGENIC	--	--	--
Ca4_P3_26622087	Ca4	26622087	ATTTTTTTTTT/ATTTTTTTTTTT	TACCCCAATCAAAATGACCC	TGCACCAACAGTATCGAACAA	59.5	363	INTERGENIC	--	--	--
Ca4_P3_26649182	Ca4	26649182	TA/TAA	TGAGTGAGATGCCAGTGGAG	TGAGTGGCTTCAAGATTGACC	60.0	631	DRR	Ca_24999	L	--
Ca4_P3_26662668	Ca4	26662668	ATTTTTTTTTT/ATTTTTTTTT	GTGACGTGGGGGAGTTAAGA	CAACGACCTTAACATTTGGTG	60.0	799	INTERGENIC	--	--	--
Ca4_P3_26664091	Ca4	26664091	TAAAAAAAAA/TAAAAAAAAAA	TGATACAATTGATTCGGCCA	TTTTTGTCACGCGAGCTCTA	59.9	371	INTERGENIC	--	--	--
Ca4_P3_26696179	Ca4	26696179	AAAATGCAAATGCAAAT/AAAATGCAAATGCAAATGCAAAT	ATCACCCCAGACAGAATCCA	ATTTGACACCTCGTCGTTCC	60.3	439	DRR	Ca_28173	R	MYB
Ca4_P3_26749373	Ca4	26749373	TAA/TAAA	TGGCTTTAGGAGCATCACAA	CTATTGAGCCCTCGTCTTCG	59.4	140	INTERGENIC	--	--	--

INDEL marker IDs	Chromosomes/ unanchored scaffolds	Physical positions (bp)	InDels (*Kabuli* reference genome-CDC Frontier/PII)	Forward primers (5'-3')	Reverse primers (5'-3')	Anneling temperature (0C)	Expected amplified product size (bp)	Structural annotation		Functional annotation	
								Sequence components of *kabuli* genome	*Kabuli* gene accession IDs	NCBI-KOG	TFs
Ca4_P3_26749648	Ca4	26749648	ATTTTT/ATTTT	CGAAGACGAGGGCTCAATAG	GGATGGGATCATTTGCTTTTT	60.0	599	INTERGENIC	--	--	--
Ca4_P3_26752542	Ca4	26752542	CATATATA/CATATATATA	TGAAGACTTTGGAGTGACCG	CCGAAGATTTTGCGAGTGTT	58.9	267	INTERGENIC	--	--	--
Ca4_P3_26753796	Ca4	26753796	GT/GATTTAT	AGAGCGGCAACTCAAACAAT	TGCAGGGGACATCTCTTACC	59.9	613	INTERGENIC	--	--	--
Ca4_P3_26763494	Ca4	26763494	TAAAAAAA/TAAAAAAAA	AAAAATACCGCGTCATTTCATT	TCGCTCAAAAACACAACGAG	59.8	572	INTERGENIC	--	--	--
Ca4_P3_26770441	Ca4	26770441	CAAAAAAAAAAAAAAAA/CAAAAAAAAAAAAAA	TGCTGCAACAATATGTCCCT	AATCCACATGCTAAGCTCGAA	59.2	731	INTERGENIC	--	--	--
Ca4_P3_26775300	Ca4	26775300	TAAAAAAAAAAA/TAAAAAAAAAAAA	ACGGCGCTAAACCAACATAG	TTGTGTGCTTAACCTTTGCG	60.2	320	INTERGENIC	--	--	--
Ca4_P3_26785592	Ca4	26785592	ATT/AT	TGCACATGAATTTTTGCTCC	TGCCACATACAATGTCAAGTCA	59.7	494	INTERGENIC	--	--	--
Ca4_P3_26847495	Ca4	26847495	A/AC	AGCTTCAAGTCGGATCGAAA	AAAAATCTTGTGGGAATAATTCAA	60.0	216	INTERGENIC	--	--	--
Ca4_P3_26853441	Ca4	26853441	ATTTTTTTT/ATTTTTTTTT	TGAGATTGTCAATGTTGGCG	TGGAAATGAAATCTTGAGGTATGA	60.7	393	INTERGENIC	--	--	--
Ca4_P3_26903304	Ca4	26903304	T/TG	ACTCGATTGGTCCATGCTTC	ATGTTTTGGACGCACACAGA	60.1	532	INTERGENIC	--	--	--

INDEL marker IDs	Chromosomes/ unanchored scaffolds	Physical positions (bp)	InDels (*Kabuli* reference genome-CDC Frontier/PII)	Forward primers (5'-3')	Reverse primers (5'-3')	Anneling temperature (0C)	Expected amplified product size (bp)	Structural annotation		Functional annotation	
								Sequence components of *kabuli* genome	*Kabuli* gene accession IDs	NCBI-KOG	TFs
Ca4_P3_26905366	Ca4	26905366	TAAAAAAAAAA/TAAAAAAAAA	TGAAAGCCCAAAACTAATCAAGA	ATTGCTCGGTCGGTTTAGTC	60.1	471	DRR	Ca_20443	R	G2-like
Ca4_P3_26953409	Ca4	26953409	CAAA/CAAAA	CAATGCATTTCTTTTTGCCA	TACACCAAACTGCCAACCAA	59.7	533	INTERGENIC	--	--	--
Ca4_P3_26955373	Ca4	26955373	CTTTTTTTTTT/CTTTTTTTTTTTT	TGAAGCTGATTGTTTGACCG	CGTGTTTTGAAGCACCGATA	59.8	604	INTERGENIC	--	--	--
Ca4_P3_26970810	Ca4	26970810	TAAAAAAAA/TAAAAAAA	GAAAAGCTGAAACTGTATTTTGGAA	AACAGTGCCGATGAATAGTTTT	60.1	306	DRR	Ca_20452	A	--
Ca4_P3_27007761	Ca4	27007761	TAAAAAAAA/TAAAAAAAAA	TGTGATTCCTTGCTCGAGTG	TGCGCACATTTATCATTTAGTCA	60.0	445	INTERGENIC	--	--	--
Ca4_P3_27017251	Ca4	27017251	GTTTTTTTTT/GTTTTTTTTTT	CCTTTGCATCTCAGGGACAT	TGAGACCGGGTTTGAGTACC	60.1	453	INTERGENIC	--	--	--
Ca4_P3_27149358	Ca4	27149358	ATTTTTTTT/ATTTTTTT	CGATGATGCTACCAGAGGGT	CGGGGATTCCCATATCACTA	60.1	611	INTERGENIC	--	--	--
Ca4_P3_27161752	Ca4	27161752	ATTTTTT/ATTTTTTT	TTTCAATGCAGTTCGATTTTTG	TTTTCAAAATCCCGACGTTA	60.1	504	INTERGENIC	--	--	--
Ca4_P3_27171856	Ca4	27171856	CAAAAA/CAAAA	CGGCTATAAGCCAGGACAAG	AAATCGTTTTGGTGGCTTTG	59.9	379	INTERGENIC	--	--	--
Ca4_P3_27174775	Ca4	27174775	TTGTTTCAAA/TTGTTTCAAAAGGATGTTTCAAA	ACCAAAAAGCTGGAGAGCAA	CCAAGCTCTTGGTGCTTGTT	60.0	512	INTERGENIC	--	--	--

INDEL marker IDs	Chromosomes/ unanchored scaffolds	Physical positions (bp)	InDels (*Kabuli* reference genome-CDC Frontier/PII)	Forward primers (5'-3')	Reverse primers (5'-3')	Anneling temperature (0C)	Expected amplified product size (bp)	Structural annotation		Functional annotation	
								Sequence components of *kabuli* genome	*Kabuli* gene accession IDs	NCBI-KOG	TFs
Ca4_P3_27193093	Ca4	27193093	GTTATTTTTTATTTTTTATTTTTTT/GTTATTTTTTATTTTTTATTTTTTTATTTTTT	GTAGAATGCCCCAACAAAGC	TTTCATCCTGCAATGTGTGA	59.6	463	INTERGENIC	--	--	--
Ca4_P3_27196099	Ca4	27196099	GAT/GATAT	CTGAAAACTCACAGGTGCCA	TCTTCCTCTAACTCGCGGTC	59.9	591	INTERGENIC	--	--	--
Ca4_P3_27198549	Ca4	27198549	ACCCCCC/ACCCCCCC	TGCTTCCAAAAATCATCGTG	AAGGCTTCGAAGGTTTGACA	59.7	520	DRR	Ca_20458	--	NF-YB
Ca4_P3_27233281	Ca4	27233281	ATAATTCACAAGGTAATTAATTCACAAGGTA/ATAATTCACAAGGTAATTAATTCACAAGGTAATTAATTCACAAGGTA	CCAACGCTATTGGGTTTCAG	GCAGGAGAGAGAAAACGTGG	60.5	891	INTRON	Ca_20460	--	--
Ca4_P3_27260077	Ca4	27260077	GAAA/GAAAA	GCGTCCAAGTCTTTGAGGAA	TTTGGGGTTGCATAATTTGA	60.4	498	INTERGENIC	--	--	--
Ca4_P3_27267123	Ca4	27267123	TAAAAA/TAAAA	CCCTCAACTCAACTGAGATCC	TGCATTGGTTTGAATTTGGA	58.7	568	DRR	Ca_20461	R	--
Ca4_P3_27274392	Ca4	27274392	GAA/GA	AGTCCCACCCTTCCTCTGAT	TTGCCAACCGATTGTGTTTA	59.9	805	INTERGENIC	--	--	--
Ca4_P3_27283830	Ca4	27283830	GTTT/GTTTT	TATTGGTCGTCAAGCAGCAG	TTTCCCAATCGATTTGCTTC	60.0	681	DRR	Ca_20463	--	--
Ca4_P3_27312714	Ca4	27312714	ATA/ATAATTA	AGTGGATTTTGTGGCTGCTT	TGATGGCGACTCATTTTTCA	59.7	546	INTERGENIC	--	--	--
Ca4_P3_27331583	Ca4	27331583	CAAAAA/CAAAA	TGAAGCATTGATTCCATTTAGG	TTGTTGGTTTGGGATTGTTG	59.1	375	INTERGENIC	--	--	--

INDEL marker IDs	Chromosomes/ unanchored scaffolds	Physical positions (bp)	InDels (*Kabuli* reference genome-CDC Frontier/PII)	Forward primers (5'-3')	Reverse primers (5'-3')	Anneling temperature (0C)	Expected amplified product size (bp)	Structural annotation		Functional annotation	
								Sequence components of *kabuli* genome	*Kabuli* gene accession IDs	NCBI-KOG	TFs
Ca4_P3_27334621	Ca4	27334621	TGG/TGGG	CACGCTTACATGACATTCCA	TGTGGACTCCCAATTGAAAA	58.1	101	INTERGENIC	--	--	--
Ca4_P3_27349697	Ca4	27349697	AAA/AAAAAGAA	TGGGGCACAAAATATGTGAA	CCCCAGCATTCTTTAGACCA	59.8	584	INTERGENIC	--	--	--
Ca4_P3_27365593	Ca4	27365593	GATTTGA/GATTTGAAGAAAATTTGA	GCCGAATGGTTAGATGGCTA	CAACCTGACAACCCGAGATT	60.1	905	INTERGENIC	--	--	--
Ca4_P3_27368144	Ca4	27368144	GAAAAAAAA/GAAAAAAA	AGGCATTCAGCTCGACAACT	ATTTTGCACATGGTCTTCCC	60.0	800	INTERGENIC	--	--	--
Ca4_P3_27368649	Ca4	27368649	ATTTTTTTT/ATTTTTTT	GGGAAGACCATGTGCAAAAT	TTGTTCCAAGCCCTAAATGC	59.8	543	INTERGENIC	--	--	--
Ca4_P3_27375475	Ca4	27375475	T/TG	TGGCTGATGTGTGTTTGGAT	TGCAGCACAGGAAAAGAATG	60.0	548	INTRON	Ca_21323	--	--
Ca4_P3_27376069	Ca4	27376069	TAA/TAAA	ATCCTCTCCATTCCTTGCCT	TTGCTTTCACAGTCCAATGC	60.0	304	INTRON	Ca_21323	--	--
Ca4_P3_27378491	Ca4	27378491	TAAAAAAAAAA/TAAAAAAAAA	GGATCCGTCTCCTGCATAAA	TTTGCACAGGTTGTTTGTTG	60.0	585	DRR	Ca_21323	--	--
Ca4_P3_27379933	Ca4	27379933	CAAAAAAA/CAAAAAAAA	ATGGTACAAAGTGGCAAGGC	GAGAAAAGCAGGGTGAATGC	60.0	748	DRR	Ca_21323	--	--
Ca4_P3_27381951	Ca4	27381951	AG/AGAAAGTGGTAGCG	GAAAAGATCGAATCGGGTGA	GTGGAGACACCGGAAGATGT	60.0	525	INTERGENIC	--	--	--

INDEL marker IDs	Chromosomes/ unanchored scaffolds	Physical positions (bp)	InDels (*Kabuli* reference genome-CDC Frontier/PII)	Forward primers (5'-3')	Reverse primers (5'-3')	Anneling temperature (0C)	Expected amplified product size (bp)	Structural annotation		Functional annotation	
								Sequence components of *kabuli* genome	*Kabuli* gene accession IDs	NCBI-KOG	TFs
Ca4_P3_27383955	Ca4	27383955	GTTTTTTT/GTTTTTT	CGTCGATGTTGATTCC CTCT	TTTCGCATCTTGCTTCA CTG	60.1	927	INTERGENIC	--	--	--
Ca4_P3_27395535	Ca4	27395535	TAAAAAAA/TAAAAAAAAA	ATTTTGTTTGGCCGTTT GAG	GCATCACGGTACCATA GGCT	60.0	542	INTERGENIC	--	--	--
Ca4_P3_27403057	Ca4	27403057	ATTTTTT/ATTTTTTT	CGCAACAACGAAGCAA AATA	GCACGGTTGAGTGAAA AAGG	59.9	579	INTERGENIC	--	--	--
Ca4_P3_27436244	Ca4	27436244	T/TAG	ACCGCTTGTGAGACCA AATC	CCATTTTGGGACATTG TGTG	60.1	580	INTERGENIC	--	--	--
Ca4_P3_27491121	Ca4	27491121	ATTTTTTTT/ATTTTTTT	ATGCAGCACCTAGTTTT GGC	CATGTTCGTCGCTCTC ATGT	60.3	531	INTERGENIC	--	--	--
Ca4_P3_27491848	Ca4	27491848	ATTTTTTT/ATTTTTT	TACGCGCTTTCTTTCAT TCA	CGTCCCCTTCCTCAAA CATA	59.6	503	INTERGENIC	--	--	--
Ca4_P3_27499638	Ca4	27499638	TA/T	ATATGATCTAGCGCCC ACCA	ATTTTGGCAGACCAAA GGTG	60.5	763	INTERGENIC	--	--	--
Ca4_P3_27508639	Ca4	27508639	T/TC	TCCAATGGAGATCAAT GCAA	AACATAATGCCATGCC CAAT	60.0	233	INTERGENIC	--	--	--
Ca4_P3_27513599	Ca4	27513599	ATTTTTTT/ATTTTTTTT	TGATGGGGTTTTGTGT GAGA	ATGCATTACACGACCG CATA	59.9	612	INTERGENIC	--	--	--
Ca4_P3_27516071	Ca4	27516071	CAAAGGTTTTTGAACTTGAAAGGTTTT TGAACTTGAAAGGTTTTTGAACTTGA/C AAAGGTTTTTGAACTTGAAAGGTTTTT GAACTTGA	TTGATCGAAGACGTTT GCAG	TATCACCCAAAGGAAG ACGG	60.0	201	INTERGENIC	--	--	--

INDEL marker IDs	Chromosomes/ unanchored scaffolds	Physical positions (bp)	InDels (*Kabuli* reference genome-CDC Frontier/PII)	Forward primers (5'-3')	Reverse primers (5'-3')	Anneling temperature (0C)	Expected amplified product size (bp)	Structural annotation		Functional annotation	
								Sequence components of *kabuli* genome	*Kabuli* gene accession IDs	NCBI-KOG	TFs
Ca4_P3_27535888	Ca4	27535888	CTTTTT/CTTTT	TCCTTATTGTATGGCCCCAG	ATGCCGCTTGTGTTCTTCTT	59.8	496	INTERGENIC	--	--	--
Ca4_P3_27537181	Ca4	27537181	AGTGTGTGTGTGT/AGTGTGTGTGTGTGT	TTTCATCCTCTCTCACGGCT	TCTTCATGGCAACACTTCCA	60.0	478	INTERGENIC	--	--	--
Ca4_P3_27537614	Ca4	27537614	GAAA/GAAAA	TGGAAGTGTTGCCATGAAGA	CGACTTCAAGCTTCATCCGT	60.2	195	INTERGENIC	--	--	--
Ca4_P3_27539521	Ca4	27539521	TAA/TAACATAGTGAA	CTTGTGAAAGCCTCAAAGCC	TTGATTTCGTGAGTTCATTTCAA	60.0	776	INTERGENIC	--	--	--
Ca4_P3_27539986	Ca4	27539986	ATA/ATAGATTA	TTGAAATGAACTCACGAAATCAA	CACCCTCGCTTAGAAAAACG	59.6	542	INTERGENIC	--	--	--
Ca4_P3_27548804	Ca4	27548804	TAAAA/TAAA	TTCCCCATTCTTAACACCCA	TTCGCAACTCCACTCTTCCT	60.2	672	INTERGENIC	--	--	--
Ca4_P3_27563516	Ca4	27563516	CTTTTT/CTTTTTT	TTTGGTCAACTTTTTGGCCT	GGTTTGGGGTTGTCAAGCTA	59.6	599	INTRON	Ca_21314	--	--
Ca4_P3_27573953	Ca4	27573953	GAAAAAAAAAA/GAAAAAAAAA	CGTGTGGGGATGCTTAACTT	AACCACTCAAGTGTTTGGGC	60.0	453	INTRON	Ca_21314	--	--
Ca4_P3_27576276	Ca4	27576276	GAA/GAAA	TCGCACAAAAGGTTAGGTCA	GCAACCAATTCGCTGAAAGT	59.3	698	INTRON	Ca_21314	--	--
Ca4_P3_27594072	Ca4	27594072	CTTTTTTT/CTTTTTTTTT	ATCAGAGGTTGCGCAAAAAT	TTTCCTTCCAAATTTACGCC	59.7	668	INTRON	Ca_21314	--	--

INDEL marker IDs	Chromosomes/ unanchored scaffolds	Physical positions (bp)	InDels (*Kabuli* reference genome-CDC Frontier/PII)	Forward primers (5'-3')	Reverse primers (5'-3')	Anneling temperature (0C)	Expected amplified product size (bp)	Structural annotation		Functional annotation	
								Sequence components of *kabuli* genome	*Kabuli* gene accession IDs	NCBI-KOG	TFs
Ca4_P3_27614005	Ca4	27614005	ATTTTTTTTTTTTT/ATTTTTTTTTTTT	TAAAAAGGAACATGGG CAGC	CCCACAGTCGATGTTG TTTG	60.1	821	INTRON	Ca_21314	--	--
Ca4_P3_27615030	Ca4	27615030	TAAAAAAAAAAA/TAAAAAAAAAA	GCTGAGCAAGGATCT GAAT	TTTAATGCTGCCAATG ACCA	59.4	833	DRR	Ca_21313	--	--
Ca4_P3_27631111	Ca4	27631111	GTTTTTTT/GTTTTTT	ATCAACGCGATAATTG GAGC	TGCATGCAACAATCAA GAAA	60.1	813	INTERGENIC	--	--	--
Ca4_P3_27692193	Ca4	27692193	TAAAAAAAA/TAAAAAAA	TTGGTCGCAAATAAAAC TAACAAA	TGACAATTACATGGGG ACGA	59.9	247	INTERGENIC	--	--	--
Ca4_P3_27692676	Ca4	27692676	GACA/GA	TCGTCCCCATGTAATTG TCA	TTGCATATTAGTATTGC AGTGTCAAA	59.8	639	INTERGENIC	--	--	--
Ca4_P3_27698175	Ca4	27698175	CAAAAAAAAAAA/CAAAAAAAAAAAA	CTAACCTTGCATGGGG AAAA	ATTCATGGTGCTTTCTT GGC	59.9	274	DRR	Ca_21307	--	--
Ca4_P3_27744353	Ca4	27744353	ATTCAACCCCCTCTTC/ATTCAACCCCC TCTTCAACCCCCTCTTC	CCATCTTTGGGTGAAC CAGT	CCTCACCGCAACCACT ATTT	59.8	481	DRR	Ca_21304	--	--
Ca4_P3_27746278	Ca4	27746278	CTTTTTTTTTTT/CTTTTTTTT	AGTTTTCTGTGGGTTCA CCG	TGCCACATCACCCTAA ATCA	60.0	546	INTERGENIC	--	--	--
Ca4_P3_27761523	Ca4	27761523	CTTTTTT/CTTTTTTT	TTTTGCGACACGTTTTA TGC	GACACGACGATGATGA CCAC	59.7	120	INTERGENIC	--	--	--
Ca4_P3_27796696	Ca4	27796696	AATATATATATATATATATATATATAT/AAT ATATATATATATATATAT	GGCAGAAGTGAAACAA TGATGA	TAATAAAGGTGGTGGT CGGC	60.1	731	INTERGENIC	--	--	--

INDEL marker IDs	Chromosomes/ unanchored scaffolds	Physical positions (bp)	InDels (*Kabuli* reference genome-CDC Frontier/PII)	Forward primers (5'-3')	Reverse primers (5'-3')	Anneling temperature (0C)	Expected amplified product size (bp)	Structural annotation		Functional annotation	
								Sequence components of *kabuli* genome	*Kabuli* gene accession IDs	NCBI-KOG	TFs
Ca4_P3_27872586	Ca4	27872586	CTTTTTTTTTT/CTTTTTTTTTTTTT	GGGTTGTAACGTGGTCAAGG	CGCCAAAACTTGACTTCACTC	60.3	466	INTERGENIC	--	--	--
Ca4_P3_27878476	Ca4	27878476	ATTTTTTTTTTTTT/ATTTTTTTTTTT	ATCCTCACCTGCAAGACCAT	TGTCTCGAATTACCCCGAAG	59.5	640	INTERGENIC	--	--	--
Ca4_P3_27902426	Ca4	27902426	T/TC	CTGATTTGGCTCTGATGGCT	ATCATAGATCGCGAGCATCC	60.4	686	INTERGENIC	--	--	--
Ca4_P3_27919988	Ca4	27919988	ATTTTTTTTTTTT/ATTTTTTTTTTT	ATCAACGGATTGTGTTGGGT	GTATGTGGGTTTTGATGGGC	60.1	210	INTERGENIC	--	--	--
Ca4_P3_27923862	Ca4	27923862	CTATATATATATATATAT/CTATATATATATATATAT	CATTAATTTTGCGAGGAGGC	AATGGGTCTAAGTGCATGGC	59.7	783	DRR	Ca_21301	--	C2H2
Ca4_P3_27928820	Ca4	27928820	CACAAC/CACAACAAC	AGCGATGCATGTCTCAAGTG	AGCGCTTTGTGACCTCCTTA	60.0	754	INTERGENIC	--	--	--
Ca4_P3_27954238	Ca4	27954238	A/AT	ACTTCTTGACCATGCCAACC	CCATTAGTGGTTGTTGCCCT	60.0	466	INTERGENIC	--	--	--
Ca4_P3_27966402	Ca4	27966402	AA/AAGA	CCATTAACCAAGTTGTGCAAGA	ACACATTGGTCCGTGTTTGA	60.0	498	INTERGENIC	--	--	--
Ca4_P3_27971871	Ca4	27971871	GCTTCAC/G	TGTGTTTCGAGTTTTCGCTG	TGCCATGACAGAACCAATGT	60.0	412	INTRON	Ca_21299	T	--
Ca4_P3_27973163	Ca4	27973163	GTTTTTTTTTTTT/GTTTTTTTTTT	GTGCTCAAAATTTGGTGGCT	TTTGGGTGATTTTAACCTGTCA	60.1	610	DRR	Ca_21299	T	--

INDEL marker IDs	Chromosomes/ unanchored scaffolds	Physical positions (bp)	InDels (*Kabuli* reference genome-CDC Frontier/PII)	Forward primers (5'-3')	Reverse primers (5'-3')	Anneling temperature (0C)	Expected amplified product size (bp)	Structural annotation		Functional annotation	
								Sequence components of *kabuli* genome	*Kabuli* gene accession IDs	NCBI-KOG	TFs
Ca4_P3_28020575	Ca4	28020575	TG/T	GGGAGCAAAAGCTTTG AATG	AATGAGACCTACGTCA CGGG	59.8	427	INTERGENIC	--	--	--
Ca4_P3_28020842	Ca4	28020842	GTTT/GTTTT	CCCGTGACGTAGGTCT CATT	CCAACCATTGTTGCTT CCTT	60.0	335	INTERGENIC	--	--	--
Ca4_P3_28026871	Ca4	28026871	TAAGAA/TAAGAAGAA	GACATCACACCAATTC GACG	TTTTGAAGGAGATGGG GTTG	60.0	885	INTERGENIC	--	--	--
Ca4_P3_28046427	Ca4	28046427	ANNNNNNNNNNNN/ANNNNNNNNNNN	TGTGTAACCATTGTGG GAGG	TGAGGATGGCCAAGTC TCTT	59.3	782	INTERGENIC	--	--	--
Ca4_P3_28052191	Ca4	28052191	CATAT/CATATAT	TTTGGACACGGATGAA GAAA	TTAGGCTAGCACTGCA TCCAT	59.1	860	INTERGENIC	--	--	--
Ca4_P3_28071934	Ca4	28071934	CTTTTTTTTTT/CTTTTTTTTTTTT	CCAGGAATCGACCATC AAGT	CTATTTGACTTGGCTC GGGA	59.9	681	INTERGENIC	--	--	--
Ca4_P3_28109349	Ca4	28109349	ATTTTTT/ATTTTTTT	CGGGGGTAGGGTTTTA GAGA	GCTGCTTGGTTGTGCT ATGA	60.3	491	INTERGENIC	--	--	--
Ca4_P3_28131693	Ca4	28131693	TT/TTCGGTGGCT	TAGCAAGAACACCATC GCAA	TTAACCCTCAATTGCC GTTC	60.4	463	INTERGENIC	--	--	--
Ca4_P3_28148545	Ca4	28148545	ATACTTACT/ATACT	TGGTCATAATCGACGTT GGA	CTGCAAGCAGCAGAAG AACA	59.9	956	INTERGENIC	--	--	--
Ca4_P3_28153242	Ca4	28153242	CAA/C	TTGGCAGTGGTTCATTT TCA	AAAAGCTTGCGAACAT CACC	60.1	365	INTERGENIC	--	--	--

INDEL marker IDs	Chromosomes/ unanchored scaffolds	Physical positions (bp)	InDels (*Kabuli* reference genome-CDC Frontier/PII)	Forward primers (5'-3')	Reverse primers (5'-3')	Anneling temperature (0C)	Expected amplified product size (bp)	Structural annotation		Functional annotation	
								Sequence components of *kabuli* genome	*Kabuli* gene accession IDs	NCBI-KOG	TFs
Ca4_P3_28159950	Ca4	28159950	ATTTTTT/ATTTTTTT	AGTCAATGGTTGTGGG CTCT	TGCAACCTCATACTGG GAAA	59.6	584	INTERGENIC	--	--	--
Ca4_P3_28160876	Ca4	28160876	TAAAAAAAAAAAAAA/TAAAAAAAAAA	TGTCAAAACAAACAACG TGTCA	GTCCGTTTGTGATGGC TTTT	60.1	755	INTERGENIC	--	--	--
Ca4_P3_28160912	Ca4	28160912	TGG/TG	TGTCAAAACAAACAACG TGTCA	GTCCGTTTGTGATGGC TTTT	60.1	755	INTERGENIC	--	--	--
Ca4_P3_28167031	Ca4	28167031	CAAAAAA/CAAAAAAA	TTGAGATGGTTGCTTT GCAG	GCCCATTGGAGATTGA AAAA	60.0	661	DRR	Ca_23172	--	Dof
Ca4_P3_28169092	Ca4	28169092	GC/G	TTTTTGCCCATTCAAAC ACA	AAATGCGTCAGCCCAG TAAG	59.9	829	INTERGENIC	--	--	--
Ca4_P3_28169919	Ca4	28169919	TGGG/TGG	CTTACTGGGCTGACGC ATTT	CCGGAAATTTAAGGCA CAAA	60.3	696	INTERGENIC	--	--	--
Ca4_P3_28170171	Ca4	28170171	TG/TGG	GCCTTAAATTTCCGGT GGTT	AACCTCCATTGCCCAT GATA	60.2	235	INTERGENIC	--	--	--
Ca4_P3_28177214	Ca4	28177214	ATTTTTTTTTT/ATTTTTTTTT	CCAATTCATGAAATCGG TCC	TTCTATGGGACAGCCC AATC	60.1	615	INTERGENIC	--	--	--
Ca4_P3_28179172	Ca4	28179172	TAAAAAAAAA/TAAAAAAAAAA	CGGCTCACGTTTATTTG TTG	TTCCACATTTTTCCCCT CAA	59.2	485	INTERGENIC	--	--	--
Ca4_P3_28180097	Ca4	28180097	ATTTTTTTTT/ATTTTTTT	TCGAGCATGCTATAGT CCGA	ACCAATGTCCAACGGG AATA	59.5	869	INTERGENIC	--	--	--

INDEL marker IDs	Chromosomes/ unanchored scaffolds	Physical positions (bp)	InDels (*Kabuli* reference genome-CDC Frontier/PII)	Forward primers (5'-3')	Reverse primers (5'-3')	Anneling temperature (0C)	Expected amplified product size (bp)	Structural annotation		Functional annotation	
								Sequence components of *kabuli* genome	*Kabuli* gene accession IDs	NCBI-KOG	TFs
Ca4_P3_28180264	Ca4	28180264	TAAAA/TAAA	GGAAGAGCGAGAAGGGAGAT	ACCAATGTCCAACGGGAATA	59.9	325	INTERGENIC	--	--	--
Ca4_P3_28184807	Ca4	28184807	ATTT/ATTTT	GACCTTTGTTGACCTTGCGT	TGTTGAGTTGGAGATGAAGCA	60.2	595	INTERGENIC	--	--	--
Ca4_P3_28184868	Ca4	28184868	AA/AATA	GACCTTTGTTGACCTTGCGT	TGTTGAGTTGGAGATGAAGCA	60.2	595	INTERGENIC	--	--	--
Ca4_P3_28205709	Ca4	28205709	TTATATATATA/TTATATATA	GGTGGAATTCAACGTGAAGG	AACATTCTGGAGGCATTTGG	60.4	673	INTERGENIC	--	--	--
Ca4_P3_28206465	Ca4	28206465	AACA/AACACA	CCAAATGCCTCCAGAATGTT	TCCTGTCCAGAAGCTATGCC	59.9	643	INTERGENIC	--	--	--
Ca4_P3_28209971	Ca4	28209971	T/TA	ATTGATGGAGCGAATCGAAC	AATTCCTCTTTTGGTGGGCT	60.0	891	INTERGENIC	--	--	--
Ca4_P3_28211715	Ca4	28211715	TCC/TC	AAATTTTGGGCCAATACGTG	TCGTTGGAAAATGTCTGCAT	59.7	683	INTERGENIC	--	--	--
Ca4_P3_28213499	Ca4	28213499	CTTTTTTTT/CTTTTTTTTT	ACTGACCTGACCTAGGCGTG	GTTGCAAAATTGGTGTCTGC	60.3	646	INTERGENIC	--	--	--
Ca4_P3_28254483	Ca4	28254483	CAACAA/CAACAAGACAAACAA	GGGGAACTCTCCAAGTCACA	CGTGGAAACCAACCTTGTCT	60.1	703	INTERGENIC	--	--	--
Ca4_P3_28268353	Ca4	28268353	ATGTGTGTGTGTGTGT/ATGTGTGTGTGTGTGTGT	TCATGCATTCTCGAACCAAA	CTCGCTCCACACAATCTTCA	60.2	888	INTERGENIC	--	--	--

INDEL marker IDs	Chromosomes/ unanchored scaffolds	Physical positions (bp)	InDels (*Kabuli* reference genome-CDC Frontier/PII)	Forward primers (5'-3')	Reverse primers (5'-3')	Anneling temperature (0C)	Expected amplified product size (bp)	Structural annotation		Functional annotation	
								Sequence components of *kabuli* genome	*Kabuli* gene accession IDs	NCBI-KOG	TFs
Ca4_P3_28274294	Ca4	28274294	GAAAAAAAAAA/GAAAAAAAAA	CCAGAAGCAGAAAAACCAGG	TTTGAAACTGTCGCGACTCA	59.8	631	INTERGENIC	--	--	--
Ca4_P3_28274340	Ca4	28274340	CAAAAAAAAAA/CAAAAAAAAA	CCAGAAGCAGAAAAACCAGG	TTTGAAACTGTCGCGACTCA	59.8	631	INTERGENIC	--	--	--
Ca4_P3_28281450	Ca4	28281450	ATTTTTTTTTTT/ATTTTTTTTTTTTT	AATCGAACGACTGAGAACGG	GTTCTTTTGGCCACTTGCAT	60.3	261	INTRON	Ca_23166	TR	FAR1
Ca4_P3_28281644	Ca4	28281644	CTAT/CTATATAT	ATGCAAGTGGCCAAAAGAAC	ACCAAGAAATGCCTTCTGGA	60.1	405	INTRON	Ca_23166	TR	FAR1
Ca4_P3_28281945	Ca4	28281945	CTTTTTTT/CTTTTTT	ATGCAAGTGGCCAAAAGAAC	GCTGCCACATGAATACAACC	60.1	673	INTRON	Ca_23166	TR	FAR1
Ca4_P3_28282579	Ca4	28282579	CA/C	TCATGTGGCAGCATTAAGGA	TTCCCCGAGTCTTTTGTTTTT	60.2	620	DRR	Ca_23166	TR	FAR1
Ca4_P3_28284800	Ca4	28284800	AAAGAAGAAGAAGAAGAAGAAG/AAAGAAGAAGAAGAAG	TTGAATCCTCATTTCTGCCC	CCTTCCACCGAGTCAACATT	60.0	813	INTERGENIC	--	--	--
Ca4_P3_28289460	Ca4	28289460	TAAAAAAAAAAAA/TAAAAAAAAA	TTGCATTGCACTTCTTTTGC	CGGTGATGCCTCTGTTTCTT	60.0	585	INTERGENIC	--	--	--
Ca4_P3_28295619	Ca4	28295619	ATT/AT	CAACCATCCCCTTTCTTTGA	CCACTTGACCCAATTAAGCA	59.9	545	INTERGENIC	--	--	--
Ca4_P3_28306538	Ca4	28306538	CTTTTTTTT/CTTTTTTTTT	GGGTTTGTCTTTTGCGAGAA	AATTGTCCCATCCCTTACCC	60.2	772	INTERGENIC	--	--	--

INDEL marker IDs	Chromosomes/ unanchored scaffolds	Physical positions (bp)	InDels (*Kabuli* reference genome-CDC Frontier/PII)	Forward primers (5'-3')	Reverse primers (5'-3')	Anneling temperature (0C)	Expected amplified product size (bp)	Structural annotation		Functional annotation	
								Sequence components of *kabuli* genome	*Kabuli* gene accession IDs	NCBI-KOG	TFs
Ca4_P3_28322339	Ca4	28322339	A/ACCGT	TGCGTACACGAAAAAGCAAC	TGTTGTTGCCGACTGAGAAG	59.9	333	INTERGENIC	--	--	--
Ca4_P3_28335781	Ca4	28335781	A/AT	CCCAGTTCTTGAGCATGACA	TGGACAAAACTTTGGTGCTTC	59.8	783	INTERGENIC	--	--	--
Ca4_P3_28337463	Ca4	28337463	AGTCTAT/A	ACAGCCGAGGACTTAAACCA	GACCATCTGGTCAACGCTTC	59.7	768	INTERGENIC	--	--	--
Ca4_P3_28338890	Ca4	28338890	CAAAAAAAAA/CAAAAAAAA	TATTTCATGGGGCTCAAAGC	TAACCGCCTATTTTCTTGCG	60.0	555	INTERGENIC	--	--	--
Ca4_P3_28342637	Ca4	28342637	GCCC/GCCCC	TCACAAATCCTGGTGGTTGA	GGAAGAAAGCAATGGTGGAA	59.9	359	INTERGENIC	--	--	--
Ca4_P3_28346696	Ca4	28346696	A/AT	ACCACAGGTGAAAGAAACCG	TTTTCAACTCTATCCGTAGCCC	60.0	340	INTERGENIC	--	--	--
Ca4_P3_28349257	Ca4	28349257	TT/TTATGAGTAATACTAT	CTTTGCGATCAAATGGTCAA	TCAAGCTCAAAACGTGATGC	59.7	592	INTERGENIC	--	--	--
Ca4_P3_28351973	Ca4	28351973	A/AT	CCCTCTTTTTAAAACGGAGACA	TGCATTGCAAACTCAGAGG	59.6	326	INTERGENIC	--	--	--
Ca4_P3_28360547	Ca4	28360547	ATTTTT/ATTTT	ATGGACATAGCAATTGGCGT	GTCATTTTGGTGAACCGGAG	60.4	520	INTRON	Ca_23163	AYT	MYB
Ca4_P3_28362288	Ca4	28362288	TA/T	CAGCTGTTAGAGGGTGAGGG	TTCCATAATTCCTGCTTCTTCC	59.9	742	INTRON	Ca_23163	AYT	MYB

INDEL marker IDs	Chromosomes/ unanchored scaffolds	Physical positions (bp)	InDels (*Kabuli* reference genome-CDC Frontier/PII)	Forward primers (5'-3')	Reverse primers (5'-3')	Anneling temperature (0C)	Expected amplified product size (bp)	Structural annotation		Functional annotation	
								Sequence components of *kabuli* genome	*Kabuli* gene accession IDs	NCBI-KOG	TFs
Ca4_P3_28367927	Ca4	28367927	GGA/GGAGA	GAAAAGACGGCGATGAAGAA	ACTATGGAGCTGGATGGCTG	60.3	803	DRR	Ca_23163	AYT	MYB
Ca4_P3_28379686	Ca4	28379686	TAAA/TAAAAAAA	TCGGATCTGAACCCATTAGC	TTTTCTTTCTTGTAATGTAAACTGCC	60.0	505	INTERGENIC	--	--	--
Ca4_P3_28390462	Ca4	28390462	TAA/TAAA	GTCATCAAAAGCCGACGAAT	GGCCCACAATTTTGAAGAGA	60.1	786	INTERGENIC	--	--	--
Ca4_P3_28390811	Ca4	28390811	CTTG/CTTGTTTGTATTTG	TGATGGGCACATGAATCAAT	GGCCCACAATTTTGAAGAGA	59.7	283	INTERGENIC	--	--	--
Ca4_P3_28402964	Ca4	28402964	CTTTTTTTT/CTTTTTTT	CATTCTTCTTCATTAAGTGGGCA	TTGACGTGTCAACCTTGCTC	60.5	650	INTERGENIC	--	--	--
Ca4_P3_28403960	Ca4	28403960	TCTCCT/TCTCCTCCT	CGTTTAAGTCGACGTGTCCAT	TGCACTTGTTCACGTGAGTG	60.0	721	INTERGENIC	--	--	--
Ca4_P3_28404551	Ca4	28404551	AAG/AAGAG	GGAATTCAATTGATTGGGGA	CGTGTTGATGAACCATCATTG	59.6	460	INTERGENIC	--	--	--
Ca4_P3_28407155	Ca4	28407155	ATTTTTTT/ATTTTTTTT	TTGGAACAATGGCCACCTAT	CCTGGTTCCCTTTATGACCA	60.2	456	INTERGENIC	--	--	--
Ca4_P3_28407567	Ca4	28407567	CAAAAAAAA/CAAAAAAA	TGGTCATAAAGGGAACCAGG	GGAGGGGGTGAATTAGGTGT	59.8	146	INTERGENIC	--	--	--
Ca4_P3_28408105	Ca4	28408105	AAAAGAAA/AAAA	TCAAAGTGGTTGGAAAAGGG	CCCAACAATTTGAAGTGGCT	59.9	179	INTERGENIC	--	--	--

INDEL marker IDs	Chromosomes/ unanchored scaffolds	Physical positions (bp)	InDels (*Kabuli* reference genome-CDC Frontier/PII)	Forward primers (5'-3')	Reverse primers (5'-3')	Anneling temperature (0C)	Expected amplified product size (bp)	Structural annotation		Functional annotation	
								Sequence components of *kabuli* genome	*Kabuli* gene accession IDs	NCBI-KOG	TFs
Ca4_P3_28408255	Ca4	28408255	GGGAAAAGGAAAAGGAAAAGGAAAAG/ GGGAAAAGGAAAAGGAAAAG	CAAAGTGGTTGGAAAA GGGA	AGCCTCGACAGGCTTA CAAA	59.9	666	INTERGENIC	--	--	--
Ca4_P3_28419575	Ca4	28419575	TCA/TCAACACAACTTCCA	TCTTGCTTTTCAAATTG CCA	GCTGGCTTGATGATAG GAGC	59.4	582	INTERGENIC	--	--	--
Ca4_P3_28422388	Ca4	28422388	TA/TAA	TGAAGGAAACTTTGATG CCA	TTGGGTGATGGTAGTT TCAATG	59.2	534	INTERGENIC	--	--	--
Ca4_P3_28423269	Ca4	28423269	T/TA	TAGGTTTCCCCATGTG GTGT	AGCACTTGCCCCTTAG GAGT	60.1	697	INTERGENIC	--	--	--
Ca4_P3_28443084	Ca4	28443084	AAT/AATAT	CGATGAACTTGCGGTT TCTA	CCACATTGTTTCCAAC TCCA	58.9	658	INTERGENIC	--	--	--
Ca4_P3_28444987	Ca4	28444987	GTTTTTT/GTTTTTTT	TGCACTGGCTCAGATG ATTC	TTTCATCCAAAGGTTTT GACC	60.0	649	INTERGENIC	--	--	--
Ca4_P3_28454722	Ca4	28454722	TTAT/TTATTAT	GCCATTTGCCTAACCAA AAA	CTTGGTTCCAACAATC TCCC	59.9	493	INTERGENIC	--	--	--
Ca4_P3_28483864	Ca4	28483864	CTGAAT/C	GAATGCCACCAGGAAA AGAA	TTGGTTTATTTGGGTT GGGA	60.1	392	INTERGENIC	--	--	--
Ca4_P3_28501292	Ca4	28501292	AAATATTTTATTATT/AAATATTTTATTA TTAATATTTTATTATT	GCACGCACACACATAT ACCC	TTTGCAGGCACAAAAG ACAG	59.9	747	INTERGENIC	--	--	--
Ca4_P3_28508068	Ca4	28508068	AT/A	TGAAGGCCTAATAAACA CACCA	AGGTGGAGGCGTATGA TGTT	59.5	441	INTERGENIC	--	--	--

INDEL marker IDs	Chromosomes/ unanchored scaffolds	Physical positions (bp)	InDels (*Kabuli* reference genome-CDC Frontier/PII)	Forward primers (5'-3')	Reverse primers (5'-3')	Anneling temperature (0C)	Expected amplified product size (bp)	Structural annotation		Functional annotation	
								Sequence components of *kabuli* genome	*Kabuli* gene accession IDs	NCBI-KOG	TFs
Ca4_P3_28510451	Ca4	28510451	TAAAAAAAA/TAAAAAAAAAAA	AGGAAATTTCGCCCAAGAAC	GCACATTTTCCAAGGGCTTA	60.4	427	INTERGENIC	--	--	--
Ca4_P3_28510638	Ca4	28510638	GTCAAGATTTGATCAAGATTTG/GTCAAGATTTG	TAAGCCCTTGGAAAATGTGC	TTCGATGATCTTCTTCTTCAACC	60.1	424	INTERGENIC	--	--	--
Ca4_P3_28524237	Ca4	28524237	ATTTTTTTT/ATTTTTTTTT	TTGGAAATGGTTTCTCCTGC	GCAAATGGTGTTGTCATTGC	60.1	579	INTERGENIC	--	--	--
Ca4_P3_28536428	Ca4	28536428	ATTTTTTTTTTT/ATTTTTTTTTTTTT	TTGTAACACCCCGTTTTTCG	CCTCAGATGAGAGCCAGGAC	60.8	433	INTERGENIC	--	--	--
Ca4_P3_28552997	Ca4	28552997	CTTTTTTTTTTT/CTTTTTTTTTT	TTTGCATGGAGATGGTTTGA	TCATGCGGTTAAAATGTGGA	60.0	437	INTERGENIC	--	--	--
Ca4_P3_28556813	Ca4	28556813	TAAAAAAAAA/TAAAAAAAAAA	GCAAAAATTCATTCAAAAATTCG	TGGTGGTTGGGTGAAGAAGT	59.9	295	INTERGENIC	--	--	--
Ca4_P3_28590176	Ca4	28590176	CTTTTT/CTTTTTT	CCACCTCCACCATTCCTCTA	CAGCACCACAAACATCCAAG	59.9	476	INTERGENIC	--	--	--
Ca4_P3_28591975	Ca4	28591975	TTATATATATATATATA/TTATATATATATATATATATATA	ATCGATGCGACCACATGTAA	TGGGGTTCAAAGAAAGGTCA	60.0	245	INTERGENIC	--	--	--
Ca4_P3_28601578	Ca4	28601578	AAAAGGATCAAAGG/AAAAGGATCAAAGGATCAAAGG	TGCGATCGACAAGAACATTT	AGATGTTTGCTGCGGAAGAT	59.3	377	INTERGENIC	--	--	--
Ca4_P3_28616334	Ca4	28616334	GTTTTTTTTTT/GTTTTTTTTTTT	ATTGCATGTTGGAGGAAAGC	GGTGTACATGTGACAAGGCG	60.1	752	INTERGENIC	--	--	--

INDEL marker IDs	Chromosomes/ unanchored scaffolds	Physical positions (bp)	InDels (*Kabuli* reference genome-CDC Frontier/PII)	Forward primers (5'-3')	Reverse primers (5'-3')	Anneling temperature (0C)	Expected amplified product size (bp)	Structural annotation		Functional annotation	
								Sequence components of *kabuli* genome	*Kabuli* gene accession IDs	NCBI-KOG	TFs
Ca4_P3_28622827	Ca4	28622827	ATGTGT/ATGTGTGT	ATATGGTTGCGCACAA ATGA	CGGCACACATTCAACA TTTC	60.0	475	INTERGENIC	--	--	--
Ca4_P3_28625916	Ca4	28625916	GAAAAAAAAAA/GAAAAAAAAAAAAA	GACACTCTCGGACCAC CACT	AGAGTCTTCTGTTGCG CTCC	60.2	350	INTERGENIC	--	--	--
Ca4_P3_28662523	Ca4	28662523	ACC/ACCC	AGGGAGGACGAAAATG ACCT	ATTTCTGGACTCCCTT GGCT	59.9	214	INTERGENIC	--	--	--
Ca4_P3_28712353	Ca4	28712353	GAAA/GAA	AGGGAGGACAAAAACG ACCT	CGAAAATGAAAACGGG AGAA	60.0	553	INTERGENIC	--	--	--
Ca4_P3_28714992	Ca4	28714992	GATAT/GAT	AGAGCCAACGCTCATA GGAA	TTAAAATTCCCTCCTC GGCT	60.0	531	INTERGENIC	--	--	--
Ca4_P3_28792571	Ca4	28792571	GTT/G	GGGATCTCGGAAGTGT TGAA	TGCAACGTGGCTAAAC AAAG	60.1	146	INTERGENIC	--	--	--
Ca4_P3_28793395	Ca4	28793395	TAA/TAAAAACAA	CCAAAATTACACCAACG GTTC	TTGTTCGCATGCTCAT TCTC	59.2	275	INTERGENIC	--	--	--
Ca4_P3_28806476	Ca4	28806476	T/TA	TGTTTGTTTTTGCAAGC AGG	GCAAGAAGCCATCCAC AAGT	59.9	351	INTERGENIC	--	--	--
Ca4_P3_28806715	Ca4	28806715	CAAA/CAAAA	TGTTTGTTTTTGCAAGC AGG	CATCAACGTCTTTTGG AGCC	59.9	624	INTERGENIC	--	--	--
Ca4_P3_28817710	Ca4	28817710	CTTTTTTT/CTTTTTTTT	CCTCCACGCCCTCATT ATTA	GCAATTCGGGCTTCTA GTTG	59.9	315	INTERGENIC	--	--	--

INDEL marker IDs	Chromosomes/ unanchored scaffolds	Physical positions (bp)	InDels (*Kabuli* reference genome-CDC Frontier/PII)	Forward primers (5'-3')	Reverse primers (5'-3')	Anneling temperature (0C)	Expected amplified product size (bp)	Structural annotation		Functional annotation	
								Sequence components of *kabuli* genome	*Kabuli* gene accession IDs	NCBI-KOG	TFs
Ca4_P3_28821762	Ca4	28821762	CAA/CAAA	ATTCAGAATGGCGGATCTTG	TAACATGCACTTTTGCGCTT	60.0	305	INTERGENIC	--	--	--
Ca4_P3_28822073	Ca4	28822073	TA/TAA	ATTCAGAATGGCGGATCTTG	AACACCATTTGCGGAAGAAG	60.0	646	INTERGENIC	--	--	--
Ca4_P3_28822607	Ca4	28822607	TAAAA/TAAA	CTTCTTCCGCAAATGGTGTT	TGTATTACCATGATTTTGTTGACTGA	60.1	448	INTERGENIC	--	--	--
Ca4_P3_28828821	Ca4	28828821	AAT/A	GGATTTATTGGGTTGATCGC	GGCTTAGGCCACCACAAATA	59.2	507	INTERGENIC	--	--	--
Ca4_P3_28828876	Ca4	28828876	GTTTTTTTTTTT/GTTTTTTTTTTTTTT	GTGGCCTAAGCCCTCACAA	TCTTTATTTGTGGCGGTCAA	61.2	108	INTERGENIC	--	--	--
Ca4_P3_28832888	Ca4	28832888	TGGTGTATA/T	CAGTTGTGCACTTTGAATGTTG	AAAATCACATGGAGGAACAAAAA	59.3	665	INTERGENIC	--	--	--
Ca4_P3_28833469	Ca4	28833469	TTATATATATATATATATATATAT/TTATATATATATATATATATAT	TTTTGTTCCTCCATGTGATTTTT	CAACGTTTTATTTCAATTTCATGTG	59.7	426	INTERGENIC	--	--	--
Ca4_P3_28833682	Ca4	28833682	ATTGAAATAAAACGTTG/ATTG	TTTTTGTTCCTCCATGTGATTTT	GAAAGAGCATTGTGTTGCCA	59.7	503	INTERGENIC	--	--	--
Ca4_P3_28853222	Ca4	28853222	CAAAAAAAAA/CAAAAAAAAAA	TCCATTTTGGTCCATGAGAA	TTTTTCTTCTTCGTCCTCGC	58.9	435	INTERGENIC	--	--	--
Ca4_P3_28855738	Ca4	28855738	TT/TTAAAAT	TCGGATTCTTTCAATCCCTC	GTTTAATCACCCTGGGCCTT	59.1	386	INTERGENIC	--	--	--

INDEL marker IDs	Chromosomes/ unanchored scaffolds	Physical positions (bp)	InDels (*Kabuli* reference genome-CDC Frontier/PII)	Forward primers (5'-3')	Reverse primers (5'-3')	Anneling temperature (0C)	Expected amplified product size (bp)	Structural annotation		Functional annotation	
								Sequence components of *kabuli* genome	*Kabuli* gene accession IDs	NCBI-KOG	TFs
Ca4_P3_28871485	Ca4	28871485	AA/AATTCATGAAAATA	CTCCTACTTGCGCACACAAA	ACAGTGCCTTCCATAAGCCT	60.1	233	DRR	Ca_23703	R	YABBY
Ca4_P3_28893508	Ca4	28893508	CTTTTTTTTTTTTTTTTT/CTTTTTTTTTTT	CCAAGCTCTTGGAGTTCGTT	TCTCAACGTGAGCCACAAAG	59.5	706	INTERGENIC	--	--	--
Ca4_P3_28915111	Ca4	28915111	TAAAAAAAA/TTAAAAAAAAAA	AAATGTTCCACATGATCGCA	TCCAAAAATAACGTTTTGCCA	59.9	400	INTERGENIC	--	--	--
Ca4_P3_28915187	Ca4	28915187	GTTTTTTTTT/GTTTTTTTT	AAATGTTCCACATGATCGCA	TCCAAAAATAACGTTTTGCCA	59.9	400	INTERGENIC	--	--	--
Ca4_P3_28936822	Ca4	28936822	CTT/CT	TAAAGACATTGGGTGGAGGC	TGGACTTTGATGACTGCTCG	59.9	415	INTERGENIC	--	--	--
Ca4_P3_28949557	Ca4	28949557	CAAAAAAAA/CAAAAAAA	TTTCATCATTTCTGGAGGCA	GTGAGGATGAGCTGACACGA	59.2	528	DRR	Ca_23705	J	--
Ca4_P3_28955741	Ca4	28955741	CAT/CATAT	TTTTAATGGGCTTGACCCAC	TTTCAAAATGGGTAGAGGCG	59.8	858	INTERGENIC	--	--	--
Ca4_P3_28965039	Ca4	28965039	ATTTTTTTT/ATTTTTTTTT	CTTTGGTCATACGGCATGTG	TTGAGTTGGGGGAGAAAAGA	60.0	168	INTERGENIC	--	--	--
Ca4_P3_28981567	Ca4	28981567	GAAAAAAAA/GAAAAAAAAA	GCCCTCAGATGAGAGTCAGG	AACTTTACCGCGATTGTTGG	59.9	778	INTERGENIC	--	--	--
Ca4_P3_28992327	Ca4	28992327	TC/T	CAAGTCCATGCAGTAAGCGA	GGAACCTGGAACACCGTAGA	60.0	836	INTRON	Ca_23706	--	NF-YB

INDEL marker IDs	Chromosomes/ unanchored scaffolds	Physical positions (bp)	InDels (*Kabuli* reference genome-CDC Frontier/PII)	Forward primers (5'-3')	Reverse primers (5'-3')	Anneling temperature (0C)	Expected amplified product size (bp)	Structural annotation		Functional annotation	
								Sequence components of *kabuli* genome	*Kabuli* gene accession IDs	NCBI-KOG	TFs
Ca4_P3_28992492	Ca4	28992492	CA/CAAA	GGCGGTACAATTGCTTGATT	GGAACCTGGAACACCGTAGA	60.0	592	INTRON	Ca_23706	--	NF-YB
Ca4_P3_28992636	Ca4	28992636	AAT/AATAT	GGCGGTACAATTGCTTGATT	GGAACCTGGAACACCGTAGA	60.0	592	INTRON	Ca_23706	--	NF-YB
Ca4_P3_28993058	Ca4	28993058	CTTT/CT	TCTACGGTGTTCCAGGTTCC	AATGGATGGTTAACGAGTTGC	60.0	500	INTRON	Ca_23706	--	NF-YB
Ca4_P3_28996068	Ca4	28996068	CTTTTTTTTTTTT/CTTTTTTTTTT	TAACAGCATTGCATTGGGAA	TTCCAACACTAAAGGGGTGC	60.1	243	DRR	Ca_23706	--	NF-YB
Ca4_P3_28997297	Ca4	28997297	GTT/GTTT	AGACGGAGGGAGTATCCGTT	CTGGGAGCTTCCGAAAAAGT	60.0	784	DRR	Ca_23706	--	NF-YB
Ca4_P3_29008246	Ca4	29008246	GTTTT/GTTT	CCTACAATCCCAAGACAAGGA	ATTAATGCCCATGAAGGCAC	59.0	252	INTERGENIC	--	--	--
Ca4_P3_29036852	Ca4	29036852	CAAA/CA	TTGAGCCATGGATTTGATGA	TCCATTCATTCCTTGCCATT	60.0	806	INTERGENIC	--	--	--
Ca4_P3_29037043	Ca4	29037043	GAAAA/GAAAAAAA	CCATTGAGCTACACTGTGCG	TTGGACAACGCTTCAAACTG	60.5	434	INTERGENIC	--	--	--
Ca4_P3_29046225	Ca4	29046225	TAAAAAAA/TAAAAAA	TTTCAACGCGCTTTAGACAAT	TGACCAGGCTTGTTACTCAAA	59.9	740	INTERGENIC	--	--	--
Ca4_P3_29049004	Ca4	29049004	ATT/ATTT	GTTGAATGCCACCATGACTG	TGGGGCATTCCATATTTTGT	60.0	425	INTERGENIC	--	--	--

INDEL marker IDs	Chromosomes/ unanchored scaffolds	Physical positions (bp)	InDels (*Kabuli* reference genome-CDC Frontier/PII)	Forward primers (5'-3')	Reverse primers (5'-3')	Anneling temperature (0C)	Expected amplified product size (bp)	Structural annotation		Functional annotation	
								Sequence components of *kabuli* genome	*Kabuli* gene accession IDs	NCBI-KOG	TFs
Ca4_P3_29052231	Ca4	29052231	GTAATAA/GTTAATAA	TGTTTGCTTTGAGTTTGGCA	AGAGGACACGTGTTGGAACC	60.4	584	INTERGENIC	--	--	--
Ca4_P3_29053469	Ca4	29053469	ACCCC/ACCC	CCTGTTGCAATGTTGATTGG	ACATCAGAGAAATCACCGCC	60.0	855	INTERGENIC	--	--	--
Ca4_P3_29053917	Ca4	29053917	ATTTTTTT/ATTTTTTTT	GGCGGTGATTTCTCTGATGT	TGAAAGGTTCTCATGTGCATTT	60.1	548	INTERGENIC	--	--	--
Ca4_P3_29061010	Ca4	29061010	ATTTTTTT/ATTTTTTTT	AACGCGATGTGGAGGTTTAG	TTATATTGGCAACCGGCCTA	60.1	287	INTERGENIC	--	--	--
Ca4_P3_29065949	Ca4	29065949	AATATATATATAT/AATATATATAT	GGATCAAGATGCTCTAGTTCCAA	CATAACATTTGCGCCACATC	59.7	547	INTERGENIC	--	--	--
Ca4_P3_29068157	Ca4	29068157	C/CTCAAATAG	CTTGGCTACCACCACGAAAT	ATCCAAGGGAGAAAGAAGGG	60.0	767	INTERGENIC	--	--	--
Ca4_P3_29070680	Ca4	29070680	T/TATTAA	ATACCAGACGCGATAGGTGG	GAACCCTTACTATCGGTGGC	60.0	461	INTERGENIC	--	--	--
Ca4_P3_29085344	Ca4	29085344	AATCGATC/AATC	TCGCGAATCTGCTCTTGAAT	CGCCGCCAATTTTATTTTTA	61.0	556	INTERGENIC	--	--	--
Ca4_P3_29085408	Ca4	29085408	CTGG/CTGGAGATGG	TCGCGAATCTGCTCTTGAAT	CGCCGCCAATTTTATTTTTA	61.0	556	INTERGENIC	--	--	--
Ca4_P3_29085455	Ca4	29085455	TAAAAAAAAAAA/TAAAAAAAAAAAAA	GCAAGGTCTTTCCCTTGTCA	CGCCGCCAATTTTATTTTTA	60.2	278	INTERGENIC	--	--	--

INDEL marker IDs	Chromosomes/ unanchored scaffolds	Physical positions (bp)	InDels (*Kabuli* reference genome-CDC Frontier/PII)	Forward primers (5'-3')	Reverse primers (5'-3')	Anneling temperature (0C)	Expected amplified product size (bp)	Structural annotation		Functional annotation	
								Sequence components of *kabuli* genome	*Kabuli* gene accession IDs	NCBI-KOG	TFs
Ca4_P3_29085860	Ca4	29085860	TAAAAAA/TAAAAAAA	TAAAAATAAAATTGGCGGCG	TCCAATTCAATTCATGGGGT	59.9	348	INTERGENIC	--	--	--
Ca4_P3_29088324	Ca4	29088324	CAAA/CAAAA	CCCCTGTCTTTCAGATTAGCC	GGCTCAAGACTGCATTCACA	60.1	468	INTERGENIC	--	--	--
Ca4_P3_29094195	Ca4	29094195	TCTA/T	TCGGTAGCTGGCTGAAATCT	TGCGTTGTCGCGTAAGTAAC	60.0	470	INTRON	Ca_23708	C	HD-ZIP
Ca4_P3_29094516	Ca4	29094516	ATTTTTTT/ATTTTTTTT	TCGGTAGCTGGCTGAAATCT	TGCGTTGTCGCGTAAGTAAC	60.0	470	INTRON	Ca_23708	C	HD-ZIP
Ca4_P3_29100516	Ca4	29100516	GAA/GAAA	CCAAGGGGCACTGTTGTACT	ACAGATCCATCGGGTTTCAG	60.0	578	INTERGENIC	--	--	--
Ca4_P3_29100812	Ca4	29100812	GGTAGTA/GGTA	TCAAGAAATAGTGGGGGCAG	TGGAGAAACTCTTCCGCTGT	60.1	840	INTERGENIC	--	--	--
Ca4_P3_29109089	Ca4	29109089	GAA/GA	TTGTTGGTTGGTCTCGTAAAA	AAGGCATACACATGGCAACA	58.2	379	INTERGENIC	--	--	--
Ca4_P3_29109343	Ca4	29109343	CAAA/CAAAA	CGAAGTCACCGTTGTGTCTC	CGCAATTGGAATACTCCCTC	59.3	398	INTERGENIC	--	--	--
Ca4_P3_29119825	Ca4	29119825	AT/ATAAATTGT	TTCACACCTGTTTTTATGGGC	TTGTTTTCTTCCCCTTTGTCA	59.9	431	INTERGENIC	--	--	--
Ca4_P3_29121532	Ca4	29121532	CAAAAAAA/CAAAAAAAA	TTTGTGTCTTCTCTCGTCGG	TGTGTGAGATGCAAAAGCAA	59.0	571	INTERGENIC	--	--	--

INDEL marker IDs	Chromosomes/ unanchored scaffolds	Physical positions (bp)	InDels (*Kabuli* reference genome-CDC Frontier/PII)	Forward primers (5'-3')	Reverse primers (5'-3')	Anneling temperature (0C)	Expected amplified product size (bp)	Structural annotation		Functional annotation	
								Sequence components of *kabuli* genome	*Kabuli* gene accession IDs	NCBI-KOG	TFs
Ca4_P3_29124667	Ca4	29124667	ATTTTTTTT/ATTTTTTT	GAACTTTGGGACTTGGTGGA	GAGCTCAAGTTATGGCCTGC	59.9	303	INTERGENIC	--	--	--
Ca4_P3_29155695	Ca4	29155695	ATTTTTT/ATTTTTTT	CAAATCGGTTGGTTTGATCC	AAAAGATCTCGACCCTTTTGG	60.2	505	DRR	Ca_23711	--	--
Ca4_P3_29156658	Ca4	29156658	CATGCAAATGCAAA/CATGCAAA	AGTTGGTTTGGTGGTGAAGG	GCATGGTAAAGGCTGTTGGT	59.9	841	DRR	Ca_23711	--	--
Ca4_P3_29157805	Ca4	29157805	GTTTTTTTTTTTTTTTTT/GTTTTTTTTTTTTT	TGCTGTTGTTGGTGGTTGTT	TCTCCACTCCTCTCTCCTCA	60.0	418	INTERGENIC	--	--	--
Ca4_P3_29158154	Ca4	29158154	T/TG	TTTGGGGAGACTGAAGATGG	GCTCACGTTTTTGTAATGTCCA	60.0	713	INTERGENIC	--	--	--
Ca4_P3_29160022	Ca4	29160022	GAAAAAAA/GAAAAAAAA	AACAAATGACATCGCAGGAA	GGAAATGCATGAATTGAAACTG	59.1	461	INTERGENIC	--	--	--
Ca4_P3_29191237	Ca4	29191237	CTTTTTTTT/CTTTTTTTTT	CCAACTTGGTTCCAACAATCT	GGAGAAAAGTTAGGGGGTGC	58.9	119	INTERGENIC	--	--	--
Ca4_P3_29195942	Ca4	29195942	T/TA	TGTCATCTTCAGATGCAAACG	ACTGCACAATCCTGCAACAG	59.9	718	INTERGENIC	--	--	--
Ca4_P3_29196512	Ca4	29196512	CTATAT/CTATATAT	AATCAACGGCAAATCGTTTC	ATGGTTTGATCCATCGGGTA	59.9	466	INTERGENIC	--	--	--
Ca4_P3_29211121	Ca4	29211121	AT/A	GATGTTTAAGGTCTGTTTGGCA	AACTTCAGCCGTCATTGGTT	59.1	641	DRR	Ca_14151	--	--

INDEL marker IDs	Chromosomes/ unanchored scaffolds	Physical positions (bp)	InDels (*Kabuli* reference genome-CDC Frontier/PII)	Forward primers (5'-3')	Reverse primers (5'-3')	Anneling temperature (0C)	Expected amplified product size (bp)	Structural annotation		Functional annotation	
								Sequence components of *kabuli* genome	*Kabuli* gene accession IDs	NCBI-KOG	TFs
Ca4_P3_29221169	Ca4	29221169	A/AG	TAACCATGGTCATCCCTCGT	ATGTTAACGTTCGGAGTGCC	60.2	374	INTERGENIC	--	--	--
Ca4_P3_29222326	Ca4	29222326	ATTTTTTT/ATTTTTTTT	TGCACCTCTCTATGTGCGTG	TTCGTTCCGGACAAATCTTC	61.0	342	INTERGENIC	--	--	--
Ca4_P3_29233350	Ca4	29233350	TT/TTCT	TTGTCGTGAGAGGGATAGGG	CTCCAGCCTTGCATCATTTT	60.1	381	INTERGENIC	--	--	--
Ca4_P3_29240532	Ca4	29240532	AA/AAGA	AGGGGGAGATTGTTGGAATC	TTCCTAGCTTGGTCAACAACAA	60.1	611	INTERGENIC	--	--	--
Ca4_P3_29241687	Ca4	29241687	TG/T	TCCTCAAAAGTTTGGGGTTG	ATCTCAAATTGAACCCGCAC	59.9	450	INTERGENIC	--	--	--
Ca4_P3_29243330	Ca4	29243330	CAAA/CAAAA	TGTGGCAGCTACGTTCAGTC	GTTGACCTCGCACAAGGAAT	60.1	553	INTERGENIC	--	--	--
Ca4_P3_29245084	Ca4	29245084	ATA/ATACTTA	GCCCTTTGCAAGCTATGAAG	TGGTGCAATTTGGTTGGTAA	60.0	418	INTERGENIC	--	--	--
Ca4_P3_29245578	Ca4	29245578	T/TA	TTACCAACCAAATTGCACCA	CCCTAATGGGCCAAATTACA	59.8	583	INTERGENIC	--	--	--
Ca4_P3_29262137	Ca4	29262137	AAT/AATTAT	TTCAAAGGTGCATTGTCGAT	CACTGCGACGAAACTTACCA	59.1	619	INTERGENIC	--	--	--
Ca4_P3_29280793	Ca4	29280793	TAAAAAAAAAA/TAAAAAAAAAAAAAAA	TGGTCCATCAAAACAAGCTG	TCCTCGCCCAATTTGTAGAC	59.7	662	INTERGENIC	--	--	--

INDEL marker IDs	Chromosomes/ unanchored scaffolds	Physical positions (bp)	InDels (*Kabuli* reference genome-CDC Frontier/PII)	Forward primers (5'-3')	Reverse primers (5'-3')	Anneling temperature (0C)	Expected amplified product size (bp)	Structural annotation		Functional annotation	
								Sequence components of *kabuli* genome	*Kabuli* gene accession IDs	NCBI-KOG	TFs
Ca4_P3_29280898	Ca4	29280898	AAATAGATATTTTTCTATAATAGATATTTTTCTATAATAGATATTTTTC/AAATAGATATTTTTCTATAATAGATATTTTTC	GGGTCCTTGATGTGATGTAGG	TCCTCGCCCAATTTGTAGAC	59.3	514	INTERGENIC	--	--	--
Ca4_P3_29314266	Ca4	29314266	TAAAA/TAAA	AGTCTTGCTTGCTTTGGCTT	CGAAACCAATTCTCTCCCAA	59.3	569	INTERGENIC	--	--	--
Ca4_P3_29314421	Ca4	29314421	GAAAAAAAAAAA/GAAAAAAAAAA	AGTCTTGCTTGCTTTGGCTT	CGAAACCAATTCTCTCCCAA	59.3	569	INTERGENIC	--	--	--
Ca4_P3_29315886	Ca4	29315886	CTTTTTTTTT/CTTTTTTTTTT	CGTGGCAACCCAAGACTTAT	TCAATGTTCAAGAATCGGTCC	60.0	733	INTERGENIC	--	--	--
Ca4_P3_29319511	Ca4	29319511	A/AC	GAATCTTCGATGCCTTGGTC	TTGGGAAATGATTGCTTTTTG	59.6	531	INTERGENIC	--	--	--
Ca4_P3_29333372	Ca4	29333372	TTG/TTGAAAAACATATG	GATTCGGAGAACCAGGCATA	TCATTGCAGGGTATGCAAAA	60.0	717	INTERGENIC	--	--	--
Ca4_P3_29338120	Ca4	29338120	GAAAAAAAAAA/GAAAAAAAAA	TTAGAAGAAAGGGAAGGGGC	TGAGGTTCTGCAAAGAGGTATT	59.7	637	INTERGENIC	--	--	--
Ca4_P3_29360599	Ca4	29360599	TAA/TAAA	CAAAGTAAGAGCGCAACACG	TGCAAGTATCCACCAAAATGC	59.7	441	INTERGENIC	--	--	--
Ca4_P3_29360677	Ca4	29360677	TAAAAAA/TAAAAAAA	CAAAGTAAGAGCGCAACACG	TGCAAGTATCCACCAAAATGC	59.7	441	INTERGENIC	--	--	--
Ca4_P3_29361271	Ca4	29361271	TT/TTTTATATATATATATAT	TTGGTGGATACTTGCACAAAA	TGCAGTACCGTCCATAAATCC	59.1	693	INTERGENIC	--	--	--

INDEL marker IDs	Chromosomes/ unanchored scaffolds	Physical positions (bp)	InDels (*Kabuli* reference genome-CDC Frontier/PII)	Forward primers (5'-3')	Reverse primers (5'-3')	Anneling temperature (0C)	Expected amplified product size (bp)	Structural annotation		Functional annotation	
								Sequence components of *kabuli* genome	*Kabuli* gene accession IDs	NCBI-KOG	TFs
Ca4_P3_29369624	Ca4	29369624	TAAAAAAAAAAA/TAAAAAAAAAA	TCTCTTTGTGATTTGGGGATG	AAATGTACGGCGGTTAGTGG	59.9	557	INTERGENIC	--	--	--
Ca4_P3_29369690	Ca4	29369690	TAAAAAAAA/TAAAAAAA	TCTCTTTGTGATTTGGGGATG	AAATGTACGGCGGTTAGTGG	59.9	557	INTERGENIC	--	--	--
Ca4_P3_29376833	Ca4	29376833	T/TA	ATTTTTCCACCCCTAAGGCA	GCCGCGAGTTGTTCAATTAT	60.7	507	INTERGENIC	--	--	--
Ca4_P3_29377748	Ca4	29377748	GAAAAAAAAAA/GAAAAAAAAAAA	CATCCTTACGCGATCTCCAT	AATGGATACGACCCATTCCA	60.1	491	INTERGENIC	--	--	--
Ca4_P3_29379238	Ca4	29379238	CAAAAAAA/CAAAAAA	ATGCAAGTGGGCCTAGACAG	CACTTCGTTTTGGAATCGGT	60.3	630	INTERGENIC	--	--	--
Ca4_P3_29386753	Ca4	29386753	T/TG	AGGAGAAATGCGAACCATTG	CGACTCCAGTGAATTCGTGA	60.1	391	INTERGENIC	--	--	--
Ca4_P3_29415806	Ca4	29415806	GACAC/GAC	TCTAGAATCAGGAGTGCCGC	GATGACCATTTTCCATTGGG	60.5	463	INTERGENIC	--	--	--
Ca4_P3_29418053	Ca4	29418053	GAATAAATAAATAAA/GAATAAATAAATAAATAAA	TTGCTCCTACGTATCTCCCG	AACTTCCACCGTACACCACC	60.2	836	INTERGENIC	--	--	--
Ca4_P3_29418531	Ca4	29418531	TT/TTAT	GGTGGTGTACGGTGGAAGTT	GTCAAGGCAGACCAGAAAGC	59.7	383	INTERGENIC	--	--	--
Ca4_P3_29419619	Ca4	29419619	ATTTTT/ATTTT	GCCCCCAAAAAGAAAGTCTC	GGCATAAGCTTGCATTGTGA	60.1	682	INTERGENIC	--	--	--

INDEL marker IDs	Chromosomes/ unanchored scaffolds	Physical positions (bp)	InDels (*Kabuli* reference genome-CDC Frontier/PII)	Forward primers (5'-3')	Reverse primers (5'-3')	Anneling temperature (0C)	Expected amplified product size (bp)	Structural annotation		Functional annotation	
								Sequence components of *kabuli* genome	*Kabuli* gene accession IDs	NCBI-KOG	TFs
Ca4_P3_29444899	Ca4	29444899	GTTTTTTTTTT/GTTTTTTTTT	TCGAAATTTTAGGCCACGAC	CCAGAAGGCTACCACTACGC	60.1	415	INTERGENIC	--	--	--
Ca4_P3_29463257	Ca4	29463257	GAAAAAAA/GAAAAAA	TGCTTGGTTCTTGAGCCTCT	ATGTTCGTGTTTTTCCCCAA	60.1	297	INTERGENIC	--	--	--
Ca4_P3_29475056	Ca4	29475056	G/GA	GGTTCACCCAAAGATGGCTA	AGAGCAACACCCATCCTCAC	59.9	272	DRR	Ca_14157	R	--
Ca4_P3_29501722	Ca4	29501722	TGG/TGGG	ATGGAGGTGCAGGGAAGAAT	TGAGTGGAAAATGGCATCAA	60.9	790	INTERGENIC	--	--	--
Ca4_P3_29509804	Ca4	29509804	GTTTTTTTTTTT/GTTTTTTTTTT	GGCCGATAAGACAATCATTCA	ATCGGAAAAACCCTATGGGA	59.9	420	INTERGENIC	--	--	--
Ca4_P3_29520331	Ca4	29520331	TATATATATAAAG/T	AAAAATCGAAACACATGGGG	TTTCCGAAACTCAAAAAGGG	59.7	320	INTERGENIC	--	--	--
Ca4_P3_29530159	Ca4	29530159	TAAAAAAAAA/TAAAAAAAA	ACTGCCAGATCCAACCAAAC	CACACCACACCCCTTTCTCT	60.0	518	INTRON	Ca_14161	--	--
Ca4_P3_29582409	Ca4	29582409	TAAAA/TAAA	AGCTGTATTGGGCCTCCTTT	GCCGAATAAAACCCTCACAA	60.1	212	INTERGENIC	--	--	--
Ca4_P3_29595669	Ca4	29595669	T/TA	TGCACGTACTTCGCATTCTT	TAGCAACAGGCCAGAGAGGT	59.5	458	INTERGENIC	--	--	--
Ca4_P3_29603133	Ca4	29603133	ATTTTT/ATTTTTT	CCATCAAATTTGTGATTGGTTTT	GGTCCACTCAAACGACAACA	60.0	732	INTERGENIC	--	--	--

INDEL marker IDs	Chromosomes/ unanchored scaffolds	Physical positions (bp)	InDels (*Kabuli* reference genome-CDC Frontier/PII)	Forward primers (5'-3')	Reverse primers (5'-3')	Anneling temperature (0C)	Expected amplified product size (bp)	Structural annotation		Functional annotation	
								Sequence components of *kabuli* genome	*Kabuli* gene accession IDs	NCBI-KOG	TFs
Ca4_P3_29619182	Ca4	29619182	AG/A	TTGTCGTGGTGATTAATGAAAAA	GCCGACACCTTCAATCAAAT	59.4	751	INTERGENIC	--	--	--
Ca4_P3_29620388	Ca4	29620388	ATTTTTTT/ATTTTTTTT	TGGGAGGATAGCATTCTTGG	TATCTTCCACCAATGTCGCA	60.0	543	INTERGENIC	--	--	--
Ca4_P3_29624503	Ca4	29624503	CCACA/CCA	TGCACAAGACCATGCTAACC	CCTTGATTGTGGCACCTTTT	59.7	619	INTERGENIC	--	--	--
Ca4_P3_29630388	Ca4	29630388	TAAAAAAAA/TAAAAAAA	TACGGATTTCAGAGCGGAGT	AGCTAGGCTTACCAACGCAA	59.8	524	INTERGENIC	--	--	--
Ca4_P3_29642172	Ca4	29642172	ATTT/ATT	GAGTGCGCATCATTGAAAAA	GTGACCAAAAGGGAGTTTCG	59.8	624	INTERGENIC	--	--	--
Ca4_P3_29646991	Ca4	29646991	GGATCG/GG	AGGATTCGGTCTGTGTTTGG	TCAGGACTGCAAATAACCCA	60.0	555	DRR	Ca_14165	S	--
Ca4_P3_29648827	Ca4	29648827	CTTTT/CTTT	GCCTTCGGTTTATTGTTGGA	TGGAAATAGGCCATACCGAG	59.9	714	INTERGENIC	--	--	--
Ca4_P3_29650782	Ca4	29650782	A/AT	ATTTTCCAGGTGCATTTTCG	CCTAGAGCTTGGTTTGGCAG	59.9	492	INTERGENIC	--	--	--
Ca4_P3_29651625	Ca4	29651625	CTC/CTCAAAATC	GAGCAACAAACCCTTGTGGT	GGCCAAAAACATTGCTTTGA	60.0	833	INTERGENIC	--	--	--
Ca4_P3_29671539	Ca4	29671539	GTTATGG/GTTATGGACTTATGG	CACTTGTGTTGGAACGTTGG	TGCATGTCAGCCATACCAAT	60.0	158	DRR	Ca_14166	Z	MYB_related

INDEL marker IDs	Chromosomes/ unanchored scaffolds	Physical positions (bp)	InDels (*Kabuli* reference genome-CDC Frontier/PII)	Forward primers (5'-3')	Reverse primers (5'-3')	Anneling temperature (0C)	Expected amplified product size (bp)	Structural annotation		Functional annotation	
								Sequence components of *kabuli* genome	*Kabuli* gene accession IDs	NCBI-KOG	TFs
Ca4_P3_29672778	Ca4	29672778	ATATGATAGCCAAC/ATATGATAGCCAACTATGATAGCCAAC	CCCAACATGGCCTTAAAAGA	GTTAGCTGCTGCCAAAGGAC	59.9	346	DRR	Ca_14166	Z	MYB_related
Ca4_P3_29684929	Ca4	29684929	ATTTTTTTTT/ATTTTTTTTTT	GCCCTGGCCTAAGAATAACC	CAAGCCAACAGAACCTGGAT	59.9	562	INTRON	Ca_14166	Z	MYB_related
Ca4_P3_29689261	Ca4	29689261	CTATA/CTATATA	GAAACGGCTGCATACAAGGT	GGAAGCAACGAGGATGTGAT	60.1	212	INTRON	Ca_14166	Z	MYB_related
Ca4_P3_29690022	Ca4	29690022	GAAAAAAAA/GAAAAAAAAAA	GAGGATAAGCCATGGATGTCA	AGCAAGCCAAGAGAATCGAA	59.9	524	INTRON	Ca_14166	Z	MYB_related
Ca4_P3_29700063	Ca4	29700063	ATTTT/ATTT	TGGCCTTATTTGCTTTTTGG	CCTACACCCATACGTACCCG	60.1	716	INTERGENIC	--	--	--
Ca4_P3_29703578	Ca4	29703578	A/AG	TTTGTATGTACGCGTCATGTTT	TTGGTTGTGTTGGTCGAGAA	58.2	488	INTERGENIC	--	--	--
Ca4_P3_29712197	Ca4	29712197	ATTTTTTTT/ATTTTTTT	CAGCCTTCGAAAATCCACAT	GGGGGTTTGAGTAAGCAAAAA	60.1	567	INTERGENIC	--	--	--
Ca4_P3_29712689	Ca4	29712689	CAAA/CAA	TTTGCTTACTCAAACCCCCA	AGTTCGGTAGTGGCTTCCCT	60.5	837	INTERGENIC	--	--	--
Ca4_P3_29768507	Ca4	29768507	ATTTTT/ATTTT	CACACAATTGCGAAGTCTCG	TTGCTTTTTCTTCTTCATCGG	60.4	529	INTERGENIC	--	--	--
Ca4_P3_29774862	Ca4	29774862	T/TC	TGTGCTTTTCAAATCATGCC	CATTTGAGGAGCAGCAACAA	59.7	826	DRR	Ca_14170	K	HD-ZIP

INDEL marker IDs	Chromosomes/ unanchored scaffolds	Physical positions (bp)	InDels (*Kabuli* reference genome-CDC Frontier/PII)	Forward primers (5'-3')	Reverse primers (5'-3')	Anneling temperature (0C)	Expected amplified product size (bp)	Structural annotation		Functional annotation	
								Sequence components of *kabuli* genome	*Kabuli* gene accession IDs	NCBI-KOG	TFs
Ca4_P3_29776445	Ca4	29776445	TAAAAAAAAA/TAAAAAAAAAA	TATTTTGGGCCTGGTGTTGT	TGGGCCACCCTATACCAATA	60.2	729	INTRON	Ca_14170	K	HD-ZIP
Ca4_P3_29811970	Ca4	29811970	AGGCGCGC/AGGCGCGCAGGGCGCGC	GCGTTTTTATGGTTGCTGGT	ACTTTCATGAAGACACCGGG	60.0	152	INTERGENIC	--	--	--
Ca4_P3_29813940	Ca4	29813940	CTATATATATATATATATATATATATATATATAT/CTATATATATATATATATATATATATATATATATATATAT	CCACGTAATTATCACGCCAA	CCCTGAAGTCATTCCAGCAT	59.4	483	INTERGENIC	--	--	--
Ca4_P3_29826112	Ca4	29826112	ATTTTTTTTTTT/ATTTTTTTTTT	TTTGCACAAGCGCTGTAAAC	AATCTCATCAACTCGGTCGG	60.1	833	INTERGENIC	--	--	--
Ca4_P3_29826826	Ca4	29826826	TGTG/T	CGAACCTACGAGGCTAAACG	GGGTGTCGAAACATTCCATC	59.9	290	INTERGENIC	--	--	--
Ca4_P3_29864644	Ca4	29864644	ATTT/ATT	TTTCTCCCGCTTTTGTCAAG	TATACAAGGCCTCTTGCGCT	60.4	725	INTERGENIC	--	--	--
Ca4_P3_29901176	Ca4	29901176	TAAAAAAAAAA/TAAAAAAAAA	CGAATTCAATGGAGTTGCCT	TCGATCCCACCGAGAGATAC	60.1	363	INTERGENIC	--	--	--
Ca4_P3_29916991	Ca4	29916991	TC/T	GAGCAACGTCTGGTTTGGTT	GGAGGAGCAGAAGCAACAAC	60.2	363	INTERGENIC	--	--	--
Ca4_P3_29924754	Ca4	29924754	TTATATATATATA/TTATATATATATATA	GGGAATCCCCTGAAGGTAAA	AGAGGGTCAACTCTGTCCGA	60.1	915	INTERGENIC	--	--	--
Ca4_P3_29925363	Ca4	29925363	CAAAAAAA/CAAAAAAAA	ATCGGACAGAGTTGACCCTC	GAATGGGCTGGCTTGACTAA	59.1	394	INTERGENIC	--	--	--

INDEL marker IDs	Chromosomes/ unanchored scaffolds	Physical positions (bp)	InDels (*Kabuli* reference genome-CDC Frontier/PII)	Forward primers (5'-3')	Reverse primers (5'-3')	Anneling temperature (0C)	Expected amplified product size (bp)	Structural annotation		Functional annotation	
								Sequence components of *kabuli* genome	*Kabuli* gene accession IDs	NCBI-KOG	TFs
Ca4_P3_29930734	Ca4	29930734	TAA/TCAAA	GCTCATAATTGCGGGTCATT	AGGTATGGACAAAATTGCGG	59.9	547	INTERGENIC	--	--	--
Ca4_P3_29931074	Ca4	29931074	AGATGA/AGA	TGTGCACCAATATCTGCCAT	GAAGAAAACCCGGGATCAGT	60.0	670	INTERGENIC	--	--	--
Ca4_P3_29931459	Ca4	29931459	TTTTTATTTT/TTTTT	CCGCAATTTTGTCCATACCT	CCAAAAAGTAGGGGGAGAGAA	59.8	545	INTERGENIC	--	--	--
Ca4_P3_29933754	Ca4	29933754	ATTTTTTT/ATTTTTTTT	AGCCACACGATGTTGATTGA	TGATTAGGGAATCACCCGTT	60.1	845	INTERGENIC	--	--	--
Ca4_P3_29939743	Ca4	29939743	CAA/CA	TCCTTTTCGAGACTCAGGGA	GCACCACCTGTTTGATTTCC	59.9	718	INTERGENIC	--	--	--
Ca4_P3_29944759	Ca4	29944759	TAAAAAAAA/TAAAAAAAAA	AAATGCAATAGCAGGTTGGC	ACCGAAAATTTTATTGGCGA	60.1	309	INTERGENIC	--	--	--
Ca4_P3_29944817	Ca4	29944817	TCCC/TCCCC	AAATGCAATAGCAGGTTGGC	ACCGAAAATTTTATTGGCGA	60.1	309	INTERGENIC	--	--	--
Ca4_P3_29974650	Ca4	29974650	ACT/AACTCTTAATATTGTCT	GGGTTTGTAACCACACCACA	GTTGAGTTGGCCGCATTATT	59.2	416	INTERGENIC	--	--	--
Ca4_P3_29975043	Ca4	29975043	CTTTTTTT/CTTTTTT	AATAATGCGGCCAACTCAAC	CCAAGAACAATCTTGGAATGC	60.0	491	INTERGENIC	--	--	--
Ca4_P3_29986497	Ca4	29986497	GTGCGAATACATGCG/GTGCG	AAAAAGGATATAAATACCCGCAA	AAAAATCTTCTCCACAAGATGAA	58.1	449	INTERGENIC	--	--	--

INDEL marker IDs	Chromosomes/ unanchored scaffolds	Physical positions (bp)	InDels (*Kabuli* reference genome-CDC Frontier/PII)	Forward primers (5'-3')	Reverse primers (5'-3')	Anneling temperature (0C)	Expected amplified product size (bp)	Structural annotation		Functional annotation	
								Sequence components of *kabuli* genome	*Kabuli* gene accession IDs	NCBI-KOG	TFs
Ca4_P3_29987350	Ca4	29987350	TTTCAA/TTTCAATTCAA	TCACCAAGCAACCAAGAAAA	CATAGGAAATGGTGGTGCCT	59.3	161	INTERGENIC	--	--	--
Ca4_P3_29987923	Ca4	29987923	GATGGTAT/GATGGTATGGTAT	GAGCAGAGCTGCCTAATGCT	GGCGTTGTTGTAGAGTGCAA	59.9	419	INTERGENIC	--	--	--
Ca4_P3_30025836	Ca4	30025836	TAA/TAAA	GTGAACAATGACCAAGCCAA	CCTGTCTCACGACATCCAAA	59.5	383	INTERGENIC	--	--	--
Ca4_P3_30040584	Ca4	30040584	GAAAA/GAAAAAA	TAGCCGCGGTTAAGTGAAGT	TGGAACAAGAGAGGGATTGG	59.9	551	INTERGENIC	--	--	--
Ca4_P3_30044755	Ca4	30044755	TAGAGA/TAGA	GTGTGGAAAAAGACCAGGGA	CATCAACCACTTATGCACGC	59.9	858	DRR	Ca_14181	--	S1Fa-like
Ca4_P3_30049126	Ca4	30049126	G/GAA	TGAGGGCAAACAACTATTCAA	TTTGACCAATGTGCAATTCAA	58.3	771	INTERGENIC	--	--	--
Ca4_P3_30049460	Ca4	30049460	C/CTAGT	GAGTGGGAGAGAAATGTTTAGTGAA	TGACAAATCCCCTTTTCCAA	60.1	233	INTERGENIC	--	--	--
Ca4_P3_30053805	Ca4	30053805	ATTTTTTTTT/ATTTTTTTTTT	TTTGCGGTGTTTCTGTCTTG	CAACTAAGACGATGGCGGAT	59.9	557	INTERGENIC	--	--	--
Ca4_P3_30076786	Ca4	30076786	GTTTTTTTT/GTTTTTTTTTT	GCACTTCCGCTAAAAGCATC	AAGCGCTCCCAAATATGCTA	60.0	432	INTERGENIC	--	--	--
Ca4_P3_30077264	Ca4	30077264	ATTTTTT/ATTTTTTT	GCATATTTGGGAGCGCTTTA	TGGATGATTGTGACAAAATTGC	60.2	508	INTERGENIC	--	--	--

INDEL marker IDs	Chromosomes/ unanchored scaffolds	Physical positions (bp)	InDels (*Kabuli* reference genome-CDC Frontier/PII)	Forward primers (5'-3')	Reverse primers (5'-3')	Anneling temperature (0C)	Expected amplified product size (bp)	Structural annotation		Functional annotation	
								Sequence components of *kabuli* genome	*Kabuli* gene accession IDs	NCBI-KOG	TFs
Ca4_P3_30077287	Ca4	30077287	CTTTTTTTT/CTTTTTTTTTT	GCATATTTGGGAGCGCTTTA	TGGATGATTGTGACAAAATTGC	60.2	508	INTERGENIC	--	--	--
Ca4_P3_30081112	Ca4	30081112	CCCTT/CCCTTCCTT	CACGGGACAAATCTTTGACAT	TCGTCCAAGTTCATCATCACA	59.8	941	INTERGENIC	--	--	--
Ca4_P3_30084699	Ca4	30084699	GTTTTTTTTT/GTTTTTTTT	CGAAATTTTGTAGCCGGAAA	AAGTTGCATTGTGGTGGTCA	60.1	277	INTERGENIC	--	--	--
Ca4_P3_30086898	Ca4	30086898	ACTTTCT/ACT	ACGCCGAAAAACACTTTCAC	TCCATTCGACTGTCCTTCAA	60.2	630	INTERGENIC	--	--	--
Ca4_P3_30091332	Ca4	30091332	GA/GAA	ACACAGTGGGGCATTTTAGC	AAAAAGGAAGAGGCATTTTGAA	60.0	380	INTERGENIC	--	--	--
Ca4_P3_30092872	Ca4	30092872	A/AT	GATGCGACCATCTACTGCAA	TGTCAAAACAAACGATGTGCT	59.8	347	INTERGENIC	--	--	--
Ca4_P3_30092938	Ca4	30092938	CT/CTTT	GATGCGACCATCTACTGCAA	TGATGTGTCCCTAATATTTTCCG	59.8	529	INTERGENIC	--	--	--
Ca4_P3_30093195	Ca4	30093195	AAATAATAATAATAATAATAATAATAATAATAATAATAATAATAATAATAA/AAATAATAATAATAATAATAATAATAATAATAATAATAATAATAA	AGCACATCGTTTGTTTTGACA	AACTGCATTCCTTTTCTCCAA	59.2	656	INTERGENIC	--	--	--
Ca4_P3_30102122	Ca4	30102122	CTCTTCCAAT/CTCTTCCAATTCTTCCAAT	TGATTCTTCAATTGCCACCA	ACGAGAGACAAGTGAGGGGA	60.0	580	INTERGENIC	--	--	--
Ca4_P3_30102438	Ca4	30102438	CTT/CT	TCCCCTCACTTGTCTCTCGT	GTGTTTGGGATTGGGAAATG	59.8	609	INTERGENIC	--	--	--

INDEL marker IDs	Chromosomes/ unanchored scaffolds	Physical positions (bp)	InDels (*Kabuli* reference genome-CDC Frontier/PII)	Forward primers (5'-3')	Reverse primers (5'-3')	Anneling temperature (0C)	Expected amplified product size (bp)	Structural annotation		Functional annotation	
								Sequence components of *kabuli* genome	*Kabuli* gene accession IDs	NCBI-KOG	TFs
Ca4_P3_30104588	Ca4	30104588	AATATA/AATA	TAGTATGTGGTGCCACGGAA	TCCCTTTCCATTACACAGCC	60.0	464	INTRON	Ca_14183	T	--
Ca4_P3_30111928	Ca4	30111928	CAA/C	CGAATTTGGATGGATTGTTT	TTGTAAATCCCCATTTGGTCA	57.4	897	INTERGENIC	--	--	--
Ca4_P3_30116632	Ca4	30116632	ATTTTTTTTTT/ATTTTTTTT	GATCACGCCAGTTGTTGGTA	TGTCGATTGCGAAGAGTTGT	59.6	896	INTERGENIC	--	--	--
Ca4_P3_30122125	Ca4	30122125	AA/AACATGTTA	ATGTCAATGGGCCACTTAGC	TCAAATCACCTCCCACAACA	60.0	441	INTERGENIC	--	--	--
Ca4_P3_30122538	Ca4	30122538	ATT/AT	ATGTCAATGGGCCACTTAGC	TTGTTTGTTGTGCTTTGACCA	60.0	764	INTERGENIC	--	--	--
Ca4_P3_30122790	Ca4	30122790	A/AG	TGTTGTGGGAGGTGATTTGA	ATCTTTTTGGGAGGTAGCGG	59.9	741	INTERGENIC	--	--	--
Ca4_P3_30122891	Ca4	30122891	CAA/CAAA	TGTTGTGGGAGGTGATTTGA	GGGTCAAACCTCAAAGACGA	59.9	862	INTERGENIC	--	--	--
Ca4_P3_30130046	Ca4	30130046	GTTTTTTTTTT/GTTTTTTTTT	ATGGTTTGTTGGTGTGGGAT	GCTTGACTGCAAAAATCGGT	60.0	463	INTERGENIC	--	--	--
Ca4_P3_30130092	Ca4	30130092	GAAAAAAA/GAAAAAA	ATGGTTTGTTGGTGTGGGAT	GCTTGACTGCAAAAATCGGT	60.0	463	INTERGENIC	--	--	--
Ca4_P3_30133481	Ca4	30133481	ATGTGT/ATGT	CGTCAATTGATGTCGTTCACTT	TCCAGTGCCCTTCATTTCTC	60.0	559	INTERGENIC	--	--	--

INDEL marker IDs	Chromosomes/ unanchored scaffolds	Physical positions (bp)	InDels (*Kabuli* reference genome-CDC Frontier/PII)	Forward primers (5'-3')	Reverse primers (5'-3')	Anneling temperature (0C)	Expected amplified product size (bp)	Structural annotation		Functional annotation	
								Sequence components of *kabuli* genome	*Kabuli* gene accession IDs	NCBI-KOG	TFs
Ca4_P3_30135919	Ca4	30135919	TTTGTTG/TTTG	CTGATCATCGTTCCCTTGGT	TTGAAGGAGGCACGTCTTTT	59.9	211	INTERGENIC	--	--	--
Ca4_P3_30138209	Ca4	30138209	CAAAAAAAA/CAAAAAAA	GCATGCAAACATGGCTCTAA	CAAATTTGGTTGGGTCTTGG	59.8	553	INTERGENIC	--	--	--
Ca4_P3_30139366	Ca4	30139366	TGAAAA/T	GAACAAGTCAGAGGGCTTGC	TGTGTGCCATTTCCTGAAGA	60.0	321	INTERGENIC	--	--	--
Ca4_P3_30146206	Ca4	30146206	CTTT/CTT	TTTTTACCGCCAATGTCCTC	TGTTGTCCAACACCAAAATGA	59.9	596	INTERGENIC	--	--	--
Ca4_P3_30147920	Ca4	30147920	ATTTTTTTTTT/ATTTTTTTTTTTT	ATCACCACTTCTTGCATCCC	CATCTGCACGATTTTCCTTTT	59.9	536	INTRON	Ca_14185	--	--
Ca4_P3_30169936	Ca4	30169936	CAAAAAAAAAAAA/CAAAAAAAAA	CTAACGATTTGCTTCGCCTC	TCCATGGAATACAAGTGGCA	60.0	405	INTERGENIC	--	--	--
Ca4_P3_30172265	Ca4	30172265	TC/T	CTCAACAGCCTTATGTGCGA	TGCGATGGAATAATGTGTCG	60.0	752	INTERGENIC	--	--	--
Ca4_P3_3017713	Ca4	3017713	ATTATTTTAAATTTATTTTAAATTTATTTTAAATTTATTTTAAATTTATTTTAAATT/ATTATTTTAAATTTATTTTAAATTTATTTTAAATTTATTTTAAATTTATTTTAAATTTATTTTAAATT	GACAAATTAGTGGGGCAACC	TTGTATTGTTGGTGAGGCCA	59.3	902	INTERGENIC	--	--	--
Ca4_P3_30184517	Ca4	30184517	TA/T	CGAGGGGCGTAAAAATCATA	GCGAGATTTGTTTCCGTTGT	59.9	579	INTERGENIC	--	--	--
Ca4_P3_30184775	Ca4	30184775	TG/T	ACAACGGAAACAAATCTCGC	GGGGGTTAGGAGTAAGAAAGGA	60.1	116	INTERGENIC	--	--	--

INDEL marker IDs	Chromosomes/ unanchored scaffolds	Physical positions (bp)	InDels (*Kabuli* reference genome-CDC Frontier/PII)	Forward primers (5'-3')	Reverse primers (5'-3')	Anneling temperature (0C)	Expected amplified product size (bp)	Structural annotation		Functional annotation	
								Sequence components of *kabuli* genome	*Kabuli* gene accession IDs	NCBI-KOG	TFs
Ca4_P3_30185012	Ca4	30185012	TAAAAAAAA/TAAAAAAAAA	ACAACGGAAACAAATCTCGC	TTGGCACTGAGTTAGCGATG	60.1	583	INTERGENIC	--	--	--
Ca4_P3_30188259	Ca4	30188259	TAA/TA	CTCCAAAACATGGTATGCCC	CACCATCAGCAGCCTAAACA	60.2	690	INTRON	Ca_14186	--	SAP
Ca4_P3_30189123	Ca4	30189123	AAGAG/AAG	TTCGTTCCTGAATTTTTCGG	TGTTCCGAATGGAAGACACA	60.0	391	INTERGENIC	--	--	--
Ca4_P3_30197839	Ca4	30197839	ATT/ATTTTTTT	GCCCATGTTTAATTAGGCCC	GCAAATGACAGAGACAAAGGC	60.5	236	INTERGENIC	--	--	--
Ca4_P3_30200799	Ca4	30200799	AGGGG/AGGG	GGGTCATTGTTCCTCAAACC	ATGGGGTCCTTCCAAAATTC	59.2	908	INTERGENIC	--	--	--
Ca4_P3_30205980	Ca4	30205980	T/TGTGAG	TCCCACTGCATTGTAGGACA	AGCTCGAGGGATCAAATCAA	60.1	537	DRR	Ca_14187	--	--
Ca4_P3_30206114	Ca4	30206114	ATTTTTTT/ATTTTTT	GTGGCTCAGAAGTGCATTGA	TTGCAAGCATCCATATCCAA	60.0	832	DRR	Ca_14187	--	--
Ca4_P3_30206308	Ca4	30206308	ATTT/ATTTT	TTTGAAGGCAACGAAAATGC	TTGCAAGCATCCATATCCAA	61.1	517	DRR	Ca_14187	--	--
Ca4_P3_30206461	Ca4	30206461	TAAAAAAAAAAA/TAAAAAAAAA	TCCCTCGAGCTATAGGATGC	ATGCAATGGTTTATTCCCCA	59.4	643	DRR	Ca_14187	--	--
Ca4_P3_30206666	Ca4	30206666	TA/TAA	TCCCTCGAGCTATAGGATGC	ATGCAATGGTTTATTCCCCA	59.4	643	DRR	Ca_14187	--	--

INDEL marker IDs	Chromosomes/ unanchored scaffolds	Physical positions (bp)	InDels (*Kabuli* reference genome-CDC Frontier/PII)	Forward primers (5'-3')	Reverse primers (5'-3')	Anneling temperature (0C)	Expected amplified product size (bp)	Structural annotation		Functional annotation	
								Sequence components of *kabuli* genome	*Kabuli* gene accession IDs	NCBI-KOG	TFs
Ca4_P3_30207330	Ca4	30207330	GAA/GA	CCAATCACCAAACTACCGGA	GACGAAATTGTGTGCCATTG	60.7	503	DRR	Ca_14187	--	--
Ca4_P3_30211443	Ca4	30211443	T/TA	ACATCAAACAAACAGCGCAC	CCGCTAAAATTGCCCAATAA	59.8	714	INTERGENIC	--	--	--
Ca4_P3_30226696	Ca4	30226696	TTCTC/TTCTCTC	GCTTATGCAATGCACGAAAA	CATGGGTTGAAATGGAGACA	59.8	528	INTERGENIC	--	--	--
Ca4_P3_30226757	Ca4	30226757	GAAAAAAA/GAAAAAA	GCTTATGCAATGCACGAAAA	CATGGGTTGAAATGGAGACA	59.8	528	INTERGENIC	--	--	--
Ca4_P3_30241127	Ca4	30241127	AT/ATT	ATCAGGCTCAATCGGTTGTC	GGAAAGTGAGGGAACCAATG	60.1	262	INTERGENIC	--	--	--
Ca4_P3_30247800	Ca4	30247800	CAAAAAAAAAAA/CAAAAAAAAAAAA	TTGAAGGCTTTGTGCAAGTG	TTCTCTTGGTTTTGCCTGCT	60.0	388	INTERGENIC	--	--	--
Ca4_P3_3027721	Ca4	3027721	GAAAAAAAAAAAA/GAAAAAAAAAAA	TGGTAGCAAACATCAGCAGC	GTTGGTTCTTTTGGCAAGGA	60.0	308	DRR	Ca_12100	R	NAC
Ca4_P3_30327391	Ca4	30327391	ATTTTTTT/ATTTTTTTT	CTCACGACTAAGGGTGGGAA	TTTTTCCTTCCTTGGGCTCT	60.1	497	INTERGENIC	--	--	--
Ca4_P3_30351190	Ca4	30351190	ATTTTTTT/ATTTTTT	CCGTGCACTCTCACCATCTA	CGAGAGGGAAATGCCTACAA	59.9	781	INTERGENIC	--	--	--
Ca4_P3_30351459	Ca4	30351459	CTTTTTTT/CTTTTTTTT	TCTTTGAAACACATCACTTTCG	AAAATTTGATCGGTGGTGGA	57.9	314	INTERGENIC	--	--	--

INDEL marker IDs	Chromosomes/ unanchored scaffolds	Physical positions (bp)	InDels (*Kabuli* reference genome-CDC Frontier/PII)	Forward primers (5'-3')	Reverse primers (5'-3')	Anneling temperature (0C)	Expected amplified product size (bp)	Structural annotation		Functional annotation	
								Sequence components of *kabuli* genome	*Kabuli* gene accession IDs	NCBI-KOG	TFs
Ca4_P3_30355803	Ca4	30355803	ACATACATAACACAATCATACATAACACAATCATACATAACACAATCA/ACATACATAACACAATCATACATAACACAATCA	TGCATGACGTACGAAGCATT	GACCGTATAGCAGCTGAGCC	60.3	451	INTERGENIC	--	--	--
Ca4_P3_30371345	Ca4	30371345	GAAAAAAAAA/GAAAAAAAAAA	TCACGTGCTTGCATTTCTTC	TCACCGGAAATTTCATTCGT	60.0	224	INTERGENIC	--	--	--
Ca4_P3_30382163	Ca4	30382163	CAAAAAAAA/CAAAAAAAAA	TCTCCGATGATTGGTGAAAA	TTGGTATTGAAAGATTGGCCT	59.0	696	INTERGENIC	--	--	--
Ca4_P3_30404870	Ca4	30404870	AATAACAT/AAT	TTAACTGTTGGTGCGCAGTC	CGTCACATTTAACACGCCAT	59.9	798	INTERGENIC	--	--	--
Ca4_P3_30408624	Ca4	30408624	TC/T	TGCCTACGTCGTGGGATTAT	TCATCAAATCCGTAACGCAA	60.4	361	INTERGENIC	--	--	--
Ca4_P3_30408872	Ca4	30408872	AA/AAAAGA	TTGCGTTACGGATTTGATGA	AGACTTTCGACCGACGCTAA	60.1	485	INTERGENIC	--	--	--
Ca4_P3_30409376	Ca4	30409376	AAAGAA/AAA	TTAGCGTCGGTCGAAAGTCT	ATGACTTTAGCGTCGGATGG	60.0	441	INTERGENIC	--	--	--
Ca4_P3_30419460	Ca4	30419460	GGAAAGGG/GGAAAGGGAGGAGATGAAAGGG	TGAGGTCAGCACATTCTTGC	TTTTTAGCCCCCTCACACAT	60.0	272	DRR	Ca_14197	--	--
Ca4_P3_30421939	Ca4	30421939	AC/ACACC	TTTAACCCTTTGACTTCAAGGAA	TGGAATGTCACTTGGCAGAA	59.2	883	INTERGENIC	--	--	--
Ca4_P3_30462755	Ca4	30462755	GGAA/G	GAATTTTATTGCGATTTCATGG	TCGGCTAAATTACATTCGTGG	58.5	513	DRR	Ca_14198	--	G2-like

INDEL marker IDs	Chromosomes/ unanchored scaffolds	Physical positions (bp)	InDels (*Kabuli* reference genome-CDC Frontier/PII)	Forward primers (5'-3')	Reverse primers (5'-3')	Anneling temperature (0C)	Expected amplified product size (bp)	Structural annotation		Functional annotation	
								Sequence components of *kabuli* genome	*Kabuli* gene accession IDs	NCBI-KOG	TFs
Ca4_P3_30465071	Ca4	30465071	TAAA/TAA	CAAGCAAATTTGTGAG CACC	TCAAGCGACTCAAATC GTGA	59.3	399	INTERGENIC	--	--	--
Ca4_P3_30482085	Ca4	30482085	TGAA/TGAAGTTTAGAA	TTTGGAACAGAGGTTTT GCC	GGGTATGGGGACCAAT TTTT	60.1	720	DRR	Ca_14201	--	SBP
Ca4_P3_30502836	Ca4	30502836	ATTTTTTTT/ATTTTTTT	AATGTAGCATGTGCGT AGCG	CAATTCATTTGATTGTG TTGCAT	59.9	303	INTERGENIC	--	--	--
Ca4_P3_30504576	Ca4	30504576	CAAAAAAAAAAAAA/CAAAAAAAAAAAA	TGACACATCAACAATCC AACAA	GTTGCCTGACAACATC ATGC	59.9	604	INTERGENIC	--	--	--
Ca4_P3_30523182	Ca4	30523182	GAAAAAAAAAAAAA/GAAAAAAAAAAA	TCCTCTCTACACCCACC CTG	AGCCGATTGTCTTCTG GATG	60.1	535	INTERGENIC	--	--	--
Ca4_P3_30528133	Ca4	30528133	ATTTTTTTTT/ATTTTTTTT	TTTTCCTCCGAATTGTG TGA	ACGCATCGTACTCTTT GCAC	59.1	429	INTERGENIC	--	--	--
Ca4_P3_30535730	Ca4	30535730	GACCAAAGATA/G	CCATTCAGAATTAGAAA ATCACCA	CCAAATTTCCCAAGGG TTCT	59.4	582	INTERGENIC	--	--	--
Ca4_P3_30535832	Ca4	30535832	TATAAATAAATAAATAAA/TATAAATAAA TAAATAAATAAATAAATAAA	CCATTCAGAATTAGAAA ATCACCA	CCAAATTTCCCAAGGG TTCT	59.4	582	INTERGENIC	--	--	--
Ca4_P3_30536932	Ca4	30536932	GTTTTTTTTT/GTTTTTTTTTT	ATCATTGAACGACCGG AAAC	CGGATACAATGGCAAT CAAA	59.8	431	INTRON	Ca_14204	--	NAC
Ca4_P3_30537523	Ca4	30537523	CA/CATA	TTTGATTGCCATTGTAT CCG	CAAAATGGAAATGCAA GAACAA	59.4	803	INTRON	Ca_14204	--	NAC

INDEL marker IDs	Chromosomes/ unanchored scaffolds	Physical positions (bp)	InDels (*Kabuli* reference genome-CDC Frontier/PII)	Forward primers (5'-3')	Reverse primers (5'-3')	Anneling temperature (0C)	Expected amplified product size (bp)	Structural annotation		Functional annotation	
								Sequence components of *kabuli* genome	*Kabuli* gene accession IDs	NCBI-KOG	TFs
Ca4_P3_30538166	Ca4	30538166	AAAGAAG/AAAGAAGAAG	TTGTTCTTGCATTTCCA TTTTG	CGCCATCTTCCTCTTC TTTG	60.0	380	CDS (large-effect mutations)	Ca_14204	--	NAC
Ca4_P3_30539573	Ca4	30539573	A/AATTG	CCGTGTAGCCAATTTCA CCT	TACTCCAGGTTCTGCC CTTG	60.0	517	DRR	Ca_14204	--	NAC
Ca4_P3_30540068	Ca4	30540068	ATTTTTTT/ATTTTTTTT	GATTTATCGTCGCTCTT GCC	CAACTGGATCTGCCAC GTTA	59.8	268	DRR	Ca_14204	--	NAC
Ca4_P3_30542137	Ca4	30542137	TAAAAAA/TAAAAAAA	TAAGGCATGATAAGGC CGAC	GTCATGTCACCCGTGA TGAG	60.1	299	DRR	Ca_14205	--	--
Ca4_P3_30542725	Ca4	30542725	AT/A	ACTCATCACGGGTGAC ATGA	CCGAGTATGATGGTTG GCTT	60.0	767	DRR	Ca_14205	--	--
Ca4_P3_30542760	Ca4	30542760	AT/A	TGCCCAAGCTCAATTAA TCC	CCGAGTATGATGGTTG GCTT	60.0	362	DRR	Ca_14205	--	--
Ca4_P3_3055139	Ca4	3055139	CGG/CG	CAACGGCTACCACATG TCAG	CTAGCTTGCTTGCATG GTGA	60.2	481	INTERGENIC	--	--	--
Ca4_P3_30551565	Ca4	30551565	TAAAAAAAAAAAA/TAAAAAAAAA	CCACCGTTTCTAACACC ATTG	TGATATGGCAATCCCT GACA	60.3	813	INTERGENIC	--	--	--
Ca4_P3_30551689	Ca4	30551689	ATTT/ATTTT	CGGAAATGGACTTTAC GAATG	TGATATGGCAATCCCT GACA	59.5	548	INTERGENIC	--	--	--
Ca4_P3_30551948	Ca4	30551948	ATTTTT/ATTTT	TGTCAGGGATTGCCAT ATCA	CAACCAAAACATTCGT GGTG	59.9	480	INTERGENIC	--	--	--

INDEL marker IDs	Chromosomes/ unanchored scaffolds	Physical positions (bp)	InDels (*Kabuli* reference genome-CDC Frontier/PII)	Forward primers (5'-3')	Reverse primers (5'-3')	Anneling temperature (0C)	Expected amplified product size (bp)	Structural annotation		Functional annotation	
								Sequence components of *kabuli* genome	*Kabuli* gene accession IDs	NCBI-KOG	TFs
Ca4_P3_30551971	Ca4	30551971	GAAAAAAAAA/GAAAAAAAAAA	TGTCAGGGATTGCCATATCA	CAACCAAAACATTCGTGGTG	59.9	480	INTERGENIC	--	--	--
Ca4_P3_30558234	Ca4	30558234	GTTTTTTT/GTTTTTTTT	GCGAAATCTAGGGGCAACTT	TTGATAGATGCCAAAAGGGG	60.6	725	INTERGENIC	--	--	--
Ca4_P3_30561689	Ca4	30561689	TAAAAA/TAAAA	GGAGGCACCATACTTTTCCA	AGAATGGCCACTTCAAGTCAA	59.9	198	INTERGENIC	--	--	--
Ca4_P3_30573766	Ca4	30573766	T/TC	CATTATGATCACAGACGATGGTG	TCCTCTTCGATCAAGCATCC	60.3	259	DRR	Ca_14206	A	--
Ca4_P3_30583372	Ca4	30583372	AT/ATTATTGATAAATATTTGT	AAAATGGGAGGCATGTAACG	ACGCTCGTCAATTTCAAAAA	59.8	541	INTERGENIC	--	--	--
Ca4_P3_30583911	Ca4	30583911	GAAAAAA/GAAAAAAA	CCATTTTTGAAATTGACGAGC	GCAAATTGAGGTCACTCAACC	59.6	300	INTERGENIC	--	--	--
Ca4_P3_30595068	Ca4	30595068	GAAAAAAAAA/GAAAAAAAAAA	ATCTCACAGTGTGCGTCTGC	CCGTCAAGTGGATCAGTGAA	60.1	305	INTERGENIC	--	--	--
Ca4_P3_30595283	Ca4	30595283	CTGTGTGT/CTGTGT	ACGTGAATCTCGTCTTCGCT	CAAAATTGTGGTTGCACTCG	60.0	520	INTRON	Ca_14207	--	bHLH
Ca4_P3_30601684	Ca4	30601684	ACCTCCC/ACC	GGCAATAGAGAAAAAGGGGG	TTTCTACTTGTGTGTGGCCG	59.9	702	DRR	Ca_14207	--	bHLH
Ca4_P3_30604376	Ca4	30604376	CT/CTT	AATGCCCCACTTTTAATCCC	CGGAGGCTACCGATACCATA	60.0	185	INTRON	Ca_14208	T	--

INDEL marker IDs	Chromosomes/ unanchored scaffolds	Physical positions (bp)	InDels (*Kabuli* reference genome-CDC Frontier/PII)	Forward primers (5'-3')	Reverse primers (5'-3')	Anneling temperature (0C)	Expected amplified product size (bp)	Structural annotation		Functional annotation	
								Sequence components of *kabuli* genome	*Kabuli* gene accession IDs	NCBI-KOG	TFs
Ca4_P3_30606887	Ca4	30606887	TAAAAAAAAA/TAAAAAAAAAA	GCAATTTCAGCACGACTCAA	ATGTGCAGTTTTTGCCACTG	60.0	294	INTERGENIC	--	--	--
Ca4_P3_30608454	Ca4	30608454	AC/A	TTGCCTCACGTGAAGAATGA	CATTATGATGTTTGGCACGC	60.4	631	INTERGENIC	--	--	--
Ca4_P3_30621604	Ca4	30621604	ATTTTTTTTTTT/ATTTTTTTTTTTT	TGGGCTTCAATTTAGTCCTTG	CTGATGACAAAAGGGGGAGA	59.2	516	INTERGENIC	--	--	--
Ca4_P3_30646682	Ca4	30646682	AT/ATTCGT	GTGGCGAACCAGGATACATT	CTTGGCGCTCCTTTAAGTTG	59.8	260	INTERGENIC	--	--	--
Ca4_P3_30653740	Ca4	30653740	AC/ACC	TGCCCATTGATGCTAAACAA	GAATGATGTCGCGGTAGGAT	60.1	513	INTERGENIC	--	--	--
Ca4_P3_30655579	Ca4	30655579	TAAAAAAAAAA/TAAAAAAAAA	TTTTCTGGCCTAGCTCCTTG	TGCTCAAAGCATGTTGTTTCT	59.6	488	INTERGENIC	--	--	--
Ca4_P3_30686877	Ca4	30686877	CTTTTTTTTTTTT/CTTTTTTTTTTT	TTTGATCTTGAGTCCTCGGG	TGGGAAATTCAGAAAACAACG	60.2	714	INTERGENIC	--	--	--
Ca4_P3_30687261	Ca4	30687261	TA/TATCA	TCATCATTCATAGCTTGGGCT	AAATTTCCCAAGCAAAAGGG	59.7	393	INTERGENIC	--	--	--
Ca4_P3_30691980	Ca4	30691980	AAACTTAACTTAA/AAACTTAA	CACAATGCAGTCCATTACGG	AACTCAACGGGGGTTAGGAG	60.0	577	INTERGENIC	--	--	--
Ca4_P3_30702309	Ca4	30702309	CAAAAAAAAAA/CAAAAAAAAAAA	GATAACATCGTGCACTTGCG	GGGATAAATGGGCACACATC	60.3	189	INTERGENIC	--	--	--

INDEL marker IDs	Chromosomes/ unanchored scaffolds	Physical positions (bp)	InDels (*Kabuli* reference genome-CDC Frontier/PII)	Forward primers (5'-3')	Reverse primers (5'-3')	Anneling temperature (0C)	Expected amplified product size (bp)	Structural annotation		Functional annotation	
								Sequence components of *kabuli* genome	*Kabuli* gene accession IDs	NCBI-KOG	TFs
Ca4_P3_30752597	Ca4	30752597	TAAAAAAA/TAAAAAAAA	CGAGACACACATTTTTCACCA	GAGACACCCTTGTGCGTTTT	59.6	874	INTERGENIC	--	--	--
Ca4_P3_30753677	Ca4	30753677	ATTTTTTTT/ATTTTTTT	TTGAATGACAAACATTGAACTACAGA	TGGGAATTTATTTCGCGAGT	60.0	347	INTERGENIC	--	--	--
Ca4_P3_30764018	Ca4	30764018	TTAATAATAATAATAATAATAATA/TTAATAATAATAATAATAATAATAATA	TTGAGATCAGAGGGTGCAAA	TTGCAGGGATGTTGTTCAAA	59.4	179	INTERGENIC	--	--	--
Ca4_P3_30772711	Ca4	30772711	CTTTTTT/CTTTTTTT	TCAACGACGATCTTGTTCAAA	GGCTTCAATTGGGACTCAAA	59.3	732	INTERGENIC	--	--	--
Ca4_P3_30772757	Ca4	30772757	ATTTTTTTTTT/ATTTTTTTTTTT	TCGTACTTTGTTTTGATTGCG	GCACCTATGAACCGTCCAAT	58.9	907	INTERGENIC	--	--	--
Ca4_P3_30772971	Ca4	30772971	CAAAAAAAAA/CAAAAAAAAAA	TTTGAGTCCCAATTGAAGCC	GCACCTATGAACCGTCCAAT	60.1	321	INTERGENIC	--	--	--
Ca4_P3_30773714	Ca4	30773714	CTTTTTTTTT/CTTTTTTTTTT	ATCCGATTGGACGGTTCATA	GCAAAATCAATTTATGCTGGAA	60.2	855	INTERGENIC	--	--	--
Ca4_P3_30774640	Ca4	30774640	TAAAAAAAAAAA/TAAAAAAAAAAAA	CGAATCTGTAGCCGAAAAGC	TTGGGCTTATGTTACGAGTTCA	60.0	249	INTERGENIC	--	--	--
Ca4_P3_30774863	Ca4	30774863	TA/T	CGAATCTGTAGCCGAAAAGC	GGAAAGACCCCGAATAATGA	60.0	770	INTERGENIC	--	--	--
Ca4_P3_30775212	Ca4	30775212	CTTTTTTTTTT/CTTTTTTTTT	TGAACTCGTAACATAAGCCCAA	TGGGAATGCTTTCTTATGGG	59.6	931	INTERGENIC	--	--	--

INDEL marker IDs	Chromosomes/ unanchored scaffolds	Physical positions (bp)	InDels (*Kabuli* reference genome-CDC Frontier/PII)	Forward primers (5'-3')	Reverse primers (5'-3')	Anneling temperature (0C)	Expected amplified product size (bp)	Structural annotation		Functional annotation	
								Sequence components of *kabuli* genome	*Kabuli* gene accession IDs	NCBI-KOG	TFs
Ca4_P3_30776003	Ca4	30776003	GTATATATATATATATATAT/GTATATAT ATATATATATATATATATATATAT	AGAAAGCATTCCCAAG AGGC	AATGTTTTGGGGTCTC CTCC	60.7	747	INTERGENIC	--	--	--
Ca4_P3_30801791	Ca4	30801791	ACTC/ACTCTC	GGAATCCCTACATGCC TTTG	ATTACATGCCATAGTG GGGG	59.4	404	INTERGENIC	--	--	--
Ca4_P3_30824879	Ca4	30824879	AT/A	ACCGAGTGTCGAACTC AAGG	ACCAGCAACCATAAAA ACGC	60.3	823	INTERGENIC	--	--	--
Ca4_P3_30868287	Ca4	30868287	TAAAAAA/TAAAAAAA	CAAAGAATCGTGCAAG AGCA	CGATAGTGCCATGTTT GCAT	60.1	730	INTERGENIC	--	--	--
Ca4_P3_30883366	Ca4	30883366	ACTCT/ACT	TTGAAGCATGCATTTGA AGC	TGTGCTTTCTTCTCTG GCCT	60.0	897	INTERGENIC	--	--	--
Ca4_P3_30883608	Ca4	30883608	GAAA/GAA	TGAAATTCATTCACGCT CCA	TGTGCTTTCTTCTCTG GCCT	60.2	495	INTERGENIC	--	--	--
Ca4_P3_30928717	Ca4	30928717	TAAAAAAA/TAAAAAAAA	CAATGTCCATGTCGAC CAAA	TCTGTCGCACCATCTC AGTC	60.4	949	INTERGENIC	--	--	--
Ca4_P3_30946923	Ca4	30946923	AG/A	CAACCAGACAGTCAGC CTCA	TTGCAACACAAGGTGA GAGG	60.0	963	DRR	Ca_14215	--	ERF
Ca4_P3_30952582	Ca4	30952582	CTTT/CTTTT	GGGTCCAACAATCTCC CTCT	GCCATTTGCCTAACCA AAAA	60.3	499	INTERGENIC	--	--	--
Ca4_P3_30953391	Ca4	30953391	GCACATACACATACACATACAC/GCAC ATACACATACAC	TTTTTGGTTAGGCAAAT GGC	TGACTACACCAATGCA CCCT	59.9	538	INTERGENIC	--	--	--

INDEL marker IDs	Chromosomes/ unanchored scaffolds	Physical positions (bp)	InDels (*Kabuli* reference genome-CDC Frontier/PII)	Forward primers (5'-3')	Reverse primers (5'-3')	Anneling temperature (0C)	Expected amplified product size (bp)	Structural annotation		Functional annotation	
								Sequence components of *kabuli* genome	*Kabuli* gene accession IDs	NCBI-KOG	TFs
Ca4_P3_30955630	Ca4	30955630	TAAAAAAAAA/TAAAAAAAA	CAAAGCCATGTTTCTTTCCA	TGAGCAAATGAGGCAAAATG	58.7	579	INTERGENIC	--	--	--
Ca4_P3_30959148	Ca4	30959148	TAAAATAAAAAAATAAAAAA/TAAAATAAAAAA	TGTTTTGAAAGATTTATGATGGGA	CACCTCAGGCACAATAAATCG	59.7	464	INTERGENIC	--	--	--
Ca4_P3_30959450	Ca4	30959450	CAAAAAAAAA/CAAAAAAAAAA	TGTTTTGAAAGATTTATGATGGGA	TTGAACCGGTTGGTGTTTTT	59.7	848	INTERGENIC	--	--	--
Ca4_P3_30968151	Ca4	30968151	CTTTTTTTT/CTTTTTTT	CACATCCCTTGCCATTTCTT	CCCGCATTTGTAACCATCTT	59.9	827	INTERGENIC	--	--	--
Ca4_P3_31002155	Ca4	31002155	GAGACA/GAGACAGACA	GCTGTTGAAAGATGCAACCA	TGACAAGCAAGTGATCGTCTG	59.8	607	INTERGENIC	--	--	--
Ca4_P3_31016630	Ca4	31016630	AAAAGTTTCTAAA/AAAAGTTTCTAAAGTTTCTAAA	CTTGCGTGAGTTTTGGTTCA	TGAAAAGAGGAAGAAAGAGGAAGA	59.9	978	INTERGENIC	--	--	--
Ca4_P3_31024618	Ca4	31024618	TAAAAAAAAA/TAAAAAAA	CGTCCTTGAAATCGTTTGGT	GCTCCCTTAACCAAAATCCC	60.0	724	INTERGENIC	--	--	--
Ca4_P3_31051791	Ca4	31051791	TG/T	AAACAAAATGCACCTTTGGAA	TTGATTTCGTTTCCTCGGAC	59.5	872	INTERGENIC	--	--	--
Ca4_P3_31101573	Ca4	31101573	TGTCATTCCC/T	AATCGGACAAGAAACCGATCT	AGTTGTGACCCTCCGATACG	60.0	260	INTERGENIC	--	--	--
Ca4_P3_31104781	Ca4	31104781	TG/T	ACTGACGCTTTGTCATGTCG	GTGCTTGGGGTTTTGTCTGT	59.9	610	INTRON	Ca_14221	U	bHLH

INDEL marker IDs	Chromosomes/ unanchored scaffolds	Physical positions (bp)	InDels (*Kabuli* reference genome-CDC Frontier/PII)	Forward primers (5'-3')	Reverse primers (5'-3')	Anneling temperature (0C)	Expected amplified product size (bp)	Structural annotation		Functional annotation	
								Sequence components of *kabuli* genome	*Kabuli* gene accession IDs	NCBI-KOG	TFs
Ca4_P3_31107506	Ca4	31107506	CAGTTAGTTAGTT/CAGTTAGTT	GTGCACCATAGAATTG GCCT	GGGACTGAATCCGACT TGAA	60.0	668	INTERGENIC	--	--	--
Ca4_P3_31118703	Ca4	31118703	GA/G	CAGGCAATTTGTTTGAC CCT	TTGGATGCTACTAGTG GGGG	60.0	634	INTERGENIC	--	--	--
Ca4_P3_31140324	Ca4	31140324	TAAAA/TAAAAA	ACTTCCATTCGCTTCAG CAT	AAATGATTCTGGTGGG GACA	59.8	536	INTRON	Ca_14223	--	bHLH
Ca4_P3_31151009	Ca4	31151009	TAAAAAAAAAA/TAAAAAAAAA	GTATTGTTTGTTTTCGG CCC	CCAATGGTTCAATACG ACCC	59.3	565	INTERGENIC	--	--	--
Ca4_P3_31172943	Ca4	31172943	TA/T	TCGCCTAAGGTAAGGG CTTT	TTACACCAATTCCCCC AAGA	60.2	421	INTERGENIC	--	--	--
Ca4_P3_31192815	Ca4	31192815	TGG/TG	TTGAAGGAGAAGCCAG AGTCA	TGAGGCATGCAGAAAC GTAG	60.1	238	INTERGENIC	--	--	--
Ca4_P3_31200114	Ca4	31200114	TA/TAGACCA	TTGTCTTAGGCAGCAC TGGA	TGGTGAACGAAAGTGA CGTG	59.6	903	INTERGENIC	--	--	--
Ca4_P3_31222344	Ca4	31222344	ATTTTTTTTTT/ATTTTTTTTTTTT	TTGAACGCTACATTCAC GTTG	TTTGAAAATCCCAAATT GCC	59.8	748	INTERGENIC	--	--	--
Ca4_P3_31222775	Ca4	31222775	CAAAAAAA/CAAAAAAAAA	GAGGGCAATTTGGGAT TTTC	TGACAAGTTTATGTGT GGGGG	60.6	523	INTERGENIC	--	--	--
Ca4_P3_31226486	Ca4	31226486	GTTGGTGTTTCATATCCAT/GT	TTCCCAATGGTGGACT GTCT	CTGTAGGGCCACCTCC AATA	60.4	458	INTRON	Ca_14227	C	--

INDEL marker IDs	Chromosomes/ unanchored scaffolds	Physical positions (bp)	InDels (*Kabuli* reference genome-CDC Frontier/PII)	Forward primers (5'-3')	Reverse primers (5'-3')	Anneling temperature (0C)	Expected amplified product size (bp)	Structural annotation		Functional annotation	
								Sequence components of *kabuli* genome	*Kabuli* gene accession IDs	NCBI-KOG	TFs
Ca4_P3_31228892	Ca4	31228892	TAAAAAA/TAAAAAAA	GAAGCCAAGTGATTGA AGGTG	CAGTGCAACGGAAGGA CATA	59.7	870	DRR	Ca_14227	C	--
Ca4_P3_31230420	Ca4	31230420	TTCTCTCTCTCT/TTCTCTCTCT	CTTTCCATCTCACTCCC TCG	TAGCCGACAGGGTCTC AGTT	59.8	435	INTERGENIC	--	--	--
Ca4_P3_31248634	Ca4	31248634	ACC/AC	TGGGTCATTTCTCATTA TGTCG	TGCTATGCCTTTTGAT CGC	59.8	645	DRR	Ca_14228	--	Trihelix
Ca4_P3_31254902	Ca4	31254902	TAAAAAAAAAAA/TAAAAAAAAAAAAA	CCCTCTTCTCTTGTTTG GTTTG	GGAAACTGCTCTGGTA AGCG	60.1	338	DRR	Ca_14229	R	bHLH
Ca4_P3_31255343	Ca4	31255343	GAAAA/GAAAAA	CGCTTACCAGAGCAGT TTCC	GAGAGAAGTGTCTTTG CGGG	60.0	681	DRR	Ca_14229	R	bHLH
Ca4_P3_31258911	Ca4	31258911	GTT/GT	CAGGTCGTGCCAATTC TTTT	GGCTAGGATTTGCAAC AGGT	60.1	264	INTERGENIC	--	--	--
Ca4_P3_31261697	Ca4	31261697	AGAAAC/AGAAACTATATGAAAC	GAGCGAGCGCTAGTAA ATGC	TCGTCTCCTCTCATTT GTGAAA	60.3	617	DRR	Ca_14230	R	MYB_related
Ca4_P3_31261725	Ca4	31261725	T/TC	TTGAAATTGGAAGATGC GTG	TCGTCTCCTCTCATTT GTGAAA	59.7	560	DRR	Ca_14230	R	MYB_related
Ca4_P3_3126592	Ca4	3126592	TAAAAAAAAAA/TAAAAAAAAAAA	AGGCAACATTTAAAACG CCT	TCTCAACCGCCTCTCT CTTG	58.8	402	INTERGENIC	--	--	--
Ca4_P3_31268906	Ca4	31268906	TAA/TA	TCCAATCTATCTCGCAC TCG	GAAAACCCAAAACACG GAAA	59.0	491	INTERGENIC	--	--	--

INDEL marker IDs	Chromosomes/ unanchored scaffolds	Physical positions (bp)	InDels (*Kabuli* reference genome-CDC Frontier/PII)	Forward primers (5'-3')	Reverse primers (5'-3')	Anneling temperature (0C)	Expected amplified product size (bp)	Structural annotation		Functional annotation	
								Sequence components of *kabuli* genome	*Kabuli* gene accession IDs	NCBI-KOG	TFs
Ca4_P3_31275708	Ca4	31275708	ATT/ATTT	AACCACAAAACGTGCAACTTC	TTGTGCGTTGTTGGTATTTTG	60.1	533	INTERGENIC	--	--	--
Ca4_P3_31276483	Ca4	31276483	AAATA/AA	CACGTCGAATATCAACAAAACTT	TTCTCACAACCATACAACCATGT	58.2	415	INTERGENIC	--	--	--
Ca4_P3_31278515	Ca4	31278515	T/TA	CTCTCGCCATTCACAAACAC	TAGGAAGTTGGACAGGTGGC	59.3	773	INTERGENIC	--	--	--
Ca4_P3_31281553	Ca4	31281553	CAA/CA	TAGTACCTGTACCCGCACCC	ACAAAACGCCAATGACACAA	59.9	471	INTERGENIC	--	--	--
Ca4_P3_31290902	Ca4	31290902	GAAAAAAAAAA/GAAAAAAAAAAA	TCTGCAGGTCATGAAACCAA	TTCATCCCAAGCAGGAAAAC	60.2	847	INTERGENIC	--	--	--
Ca4_P3_31291385	Ca4	31291385	A/ATTT	GTTTTCCTGCTTGGGATGAA	ACTCCGACAATGGAACAAGG	60.1	339	INTERGENIC	--	--	--
Ca4_P3_31303190	Ca4	31303190	CTTTTTTTTTTT/CTTTTTTTTTT	GAGGTCCCGTCCAACCTAAC	TCAGCACCGAGAGTGTCAAC	60.7	488	INTRON	Ca_14231	--	bHLH
Ca4_P3_31307742	Ca4	31307742	CAAAAA/CAAAA	GGTCGCGTAGACCTTCAGAG	AAGCATTTGATCGTCTGGCT	60.0	166	INTERGENIC	--	--	--
Ca4_P3_31316566	Ca4	31316566	GAAA/GAA	CATTGTTTTGCTGTTGTGGG	CCTCACGAGCTGCTTTAACC	60.0	664	INTERGENIC	--	--	--
Ca4_P3_31319642	Ca4	31319642	GAA/GAAAA	CTTTGAGCACAAACAAGGCA	TCATCCGTGCAAAGTATCCA	60.0	422	INTERGENIC	--	--	--

INDEL marker IDs	Chromosomes/ unanchored scaffolds	Physical positions (bp)	InDels (*Kabuli* reference genome-CDC Frontier/PII)	Forward primers (5'-3')	Reverse primers (5'-3')	Anneling temperature (0C)	Expected amplified product size (bp)	Structural annotation		Functional annotation	
								Sequence components of *kabuli* genome	*Kabuli* gene accession IDs	NCBI-KOG	TFs
Ca4_P3_31348460	Ca4	31348460	TAAAAAAAAAA/TAAAAAAAAAAA	GTCACCTTTTGGCGATGTCT	AAACGCAACCTTTGATAATCG	60.1	700	INTERGENIC	--	--	--
Ca4_P3_31349174	Ca4	31349174	TAAAAAAAAAA/TAAAAAAAAAAA	TCGATTATCAAAGGTTGCGTT	TCCAACTACAATCCTTCCGC	59.6	779	INTERGENIC	--	--	--
Ca4_P3_31387262	Ca4	31387262	CTTTTTT/CTTTTTTT	CGAAACCGTAACCCTACGAA	GCCACTTACAGCGCTTTTTC	60.0	580	INTERGENIC	--	--	--
Ca4_P3_31445256	Ca4	31445256	ATT/ATTT	AGATCTATGCATCGGCAACC	AGGGTACTACAGCTGCCCCT	60.1	338	INTERGENIC	--	--	--
Ca4_P3_31463046	Ca4	31463046	AAGTAGTAGT/AAGTAGT	TAAAACGTGCGGAACGAAAT	GCTAACTGATGCAAGGAGCC	60.5	716	INTERGENIC	--	--	--
Ca4_P3_31509721	Ca4	31509721	CATATATATATATATATATATAT/CATATATATATATATATATAT	TGTTCACTAGCACCGTAGCG	TTATGGAGCCCCGATTAAAA	60.1	570	INTERGENIC	--	--	--
Ca4_P3_31523716	Ca4	31523716	AAATAATAATAATAATAATAATAATAATAATAATAATAATAATAATAATAA/AAATAATAATAATAATAATAATAATAATAATAATAATAATAATAATAATAATAATAATAA	GAAACTCTCACAAGGGTGGC	TGGCTTTAGGAACCGTGTTT	59.7	832	INTERGENIC	--	--	--
Ca4_P3_31527261	Ca4	31527261	GTTTTTTTTTTTT/GTTTTTTTTTTT	TGCACATCATCTGCGTTTTT	TCAGCCTTTGCACACATGAT	60.3	189	INTERGENIC	--	--	--
Ca4_P3_31530944	Ca4	31530944	ATCTCTCTCTCTCTCTCTC/ATCTCTCTCTCTCTCTC	AAAGGAGGCTGGAAGGTCAT	TGCAGCTGTGATGTTGTTCA	60.1	207	INTERGENIC	--	--	--
Ca4_P3_31534512	Ca4	31534512	TCCCCC/TCCCC	CAAGCCAACGTGTGTTTGTT	GCAGGGATGTGAACCAAATAA	59.7	746	INTERGENIC	--	--	--

INDEL marker IDs	Chromosomes/ unanchored scaffolds	Physical positions (bp)	InDels (*Kabuli* reference genome-CDC Frontier/PII)	Forward primers (5'-3')	Reverse primers (5'-3')	Anneling temperature (0C)	Expected amplified product size (bp)	Structural annotation		Functional annotation	
								Sequence components of *kabuli* genome	*Kabuli* gene accession IDs	NCBI-KOG	TFs
Ca4_P3_31560244	Ca4	31560244	TAT/TATGACTTATTAATAGTAAT	CGCTTGTGGGAATACAGGTT	TTCCAAAATCATGCGTTGAA	60.0	315	INTERGENIC	--	--	--
Ca4_P3_31562617	Ca4	31562617	CAA/C	ACTTTTGCAAGCACAAATCG	CGTTGTTAACAGAGCACGGA	59.0	646	INTERGENIC	--	--	--
Ca4_P3_31565109	Ca4	31565109	CAGAGA/CAGA	TTGAATAGGGTTGCCTGGAG	GATGGACTGCATGGAAGGTT	60.1	844	INTRON	Ca_15438	--	--
Ca4_P3_31566588	Ca4	31566588	GGAAAGAGA/GGAAAGAGAAAGAGA	GCAAAGAAAACATGCGATGA	TGATGTTTTATGTCGCCGAA	59.8	317	DRR	Ca_15438	--	--
Ca4_P3_31570022	Ca4	31570022	TGGGGGG/TGGGGGGG	TATTTTGGATTTTGGCTCGG	AGGAAGAGTGGAGTTGCGAA	59.9	505	INTERGENIC	--	--	--
Ca4_P3_31611840	Ca4	31611840	GTTTTTTTTTT/GTTTTTTTTTTTT	CGTTTTCAGCTGAGCAAATTC	TTGATCCATTGGGATTGTGA	60.0	392	INTERGENIC	--	--	--
Ca4_P3_31611867	Ca4	31611867	AAT/AATAT	CGTTTTCAGCTGAGCAAATTC	TTGATCCATTGGGATTGTGA	60.0	392	INTERGENIC	--	--	--
Ca4_P3_31617249	Ca4	31617249	TAAAAAAAAAA/TAAAAAAAAA	CACGTTTATCGTGAGGCAAA	CTATGCAACATGTCACACGC	59.7	597	INTERGENIC	--	--	--
Ca4_P3_31619550	Ca4	31619550	CTTATATTA/CTTA	TTGATTTGCCCTCCTCTCAT	TAATCTTGATTCCCCACCCA	59.6	576	INTERGENIC	--	--	--
Ca4_P3_31619831	Ca4	31619831	CA/CAA	TATGAATTGGGTGGGGAATC	TCACGAGCCACAAAGTAAAAA	59.4	285	INTERGENIC	--	--	--

INDEL marker IDs	Chromosomes/ unanchored scaffolds	Physical positions (bp)	InDels (*Kabuli* reference genome-CDC Frontier/PII)	Forward primers (5'-3')	Reverse primers (5'-3')	Anneling temperature (0C)	Expected amplified product size (bp)	Structural annotation		Functional annotation	
								Sequence components of *kabuli* genome	*Kabuli* gene accession IDs	NCBI-KOG	TFs
Ca4_P3_31620795	Ca4	31620795	TT/TTAAT	TGAACGTGGCTTATTTG TCG	GATCGCATACAACAAC CGTG	59.7	352	INTERGENIC	--	--	--
Ca4_P3_31620834	Ca4	31620834	AAAAAGAAAAGAAAAGAAA/AAAAAGAA AAGAAA	TGAACGTGGCTTATTTG TCG	GATCGCATACAACAAC CGTG	59.7	352	INTERGENIC	--	--	--
Ca4_P3_31625976	Ca4	31625976	ATTTTTTTTT/ATTTTTTTTTT	TGATTCCAACAATCATC CCC	CTACGACACGACTTCT GCCA	60.5	546	INTERGENIC	--	--	--
Ca4_P3_31626384	Ca4	31626384	ATG/ATGAAGTG	TGATTCCAACAATCATC CCC	TGGCTGCAGTTCTCAA CATC	60.5	624	INTERGENIC	--	--	--
Ca4_P3_31626899	Ca4	31626899	GTTTTTTTTT/GTTTTTTTTTT	GATGTTGAGAACTGCA GCCA	AACCCCCAGGTTTTGA AAGT	60.0	862	INTERGENIC	--	--	--
Ca4_P3_31626932	Ca4	31626932	ATTTTTT/ATTTTT	GATGTTGAGAACTGCA GCCA	AACCCCCAGGTTTTGA AAGT	60.0	862	INTERGENIC	--	--	--
Ca4_P3_31630531	Ca4	31630531	AGG/AG	AGTTGAAGGTGCAAAC ACGA	AATTTACTTGGAGGGT CGGG	59.3	288	INTERGENIC	--	--	--
Ca4_P3_31636819	Ca4	31636819	CAA/CAAA	TCTCAACATCTCGTAGC GCA	TTCATGATTCATTGGG TCATCT	60.7	771	INTERGENIC	--	--	--
Ca4_P3_31636858	Ca4	31636858	ATTTTT/ATTTT	TGGTGATTAGATTTCG GCAA	TTCATGATTCATTGGG TCATCT	59.1	467	INTERGENIC	--	--	--
Ca4_P3_31641182	Ca4	31641182	CAAAAAA/CAAAAAAA	TTGTGATCGATCGAATC CTCT	GCTTCCTTCCATTCGG GTAT	59.6	423	INTERGENIC	--	--	--

INDEL marker IDs	Chromosomes/ unanchored scaffolds	Physical positions (bp)	InDels (*Kabuli* reference genome-CDC Frontier/PII)	Forward primers (5'-3')	Reverse primers (5'-3')	Anneling temperature (0C)	Expected amplified product size (bp)	Structural annotation		Functional annotation	
								Sequence components of *kabuli* genome	*Kabuli* gene accession IDs	NCBI-KOG	TFs
Ca4_P3_31644451	Ca4	31644451	TA/T	AATGTCGGGAGTTTTC ATCG	GCCATCTTAGGCTTTC CCTT	59.9	953	INTRON	Ca_15442	U	EIL
Ca4_P3_31649942	Ca4	31649942	CT/CTT	CGTGCCCTGCAGGTAT TTAT	ATTGACCGGTGGAAGA CATT	60.0	454	INTRON	Ca_15442	U	EIL
Ca4_P3_31649993	Ca4	31649993	GAAAAA/GAAAA	CGTGCCCTGCAGGTAT TTAT	ATTGACCGGTGGAAGA CATT	60.0	454	INTRON	Ca_15442	U	EIL
Ca4_P3_31652021	Ca4	31652021	CTGTTGTT/CTGTT	CCATTACCCTTTCGTGA TGC	TGTCGCTTATGTTTGA GAACAA	60.3	442	DRR	Ca_15442	U	EIL
Ca4_P3_31652107	Ca4	31652107	TAAAA/TAAA	CCATTACCCTTTCGTGA TGC	TGAAAGCAAAGCCACG TATG	60.3	796	DRR	Ca_15442	U	EIL
Ca4_P3_31667051	Ca4	31667051	GAAAA/GAAA	TGAGCTTGATTCCCCAA TTC	CAAGGGAAACCATTGG AAGA	60.0	646	INTERGENIC	--	--	--
Ca4_P3_31667470	Ca4	31667470	CTTT/CTT	TGATCTTCCAATGGTTT CCC	TTGGACAAAGCTCACA GCAC	59.7	197	INTERGENIC	--	--	--
Ca4_P3_31669067	Ca4	31669067	ATTTTTTT/ATTTTTTTT	ACTGAGCCTCCACCAA AGAA	CCTTTGACTCACCCGT TAGC	59.8	641	INTERGENIC	--	--	--
Ca4_P3_31679449	Ca4	31679449	TA/T	AATTTTCCACAACAACC GGA	TGTCTCCATTTCTCTTC CGTG	60.2	154	INTERGENIC	--	--	--
Ca4_P3_31712621	Ca4	31712621	CTATATATATATATATATATATA/CTATA TATATATATATATATATATATATATA	TTTTCACAAAAATCCAA AGGG	CAAAATGGACAAAATT GGGG	59.0	393	INTERGENIC	--	--	--

INDEL marker IDs	Chromosomes/ unanchored scaffolds	Physical positions (bp)	InDels (*Kabuli* reference genome-CDC Frontier/PII)	Forward primers (5'-3')	Reverse primers (5'-3')	Anneling temperature (0C)	Expected amplified product size (bp)	Structural annotation		Functional annotation	
								Sequence components of *kabuli* genome	*Kabuli* gene accession IDs	NCBI-KOG	TFs
Ca4_P3_3174653	Ca4	3174653	A/ATATT	AATCAACGGTGGGGATACAA	CGGACCTAAAAGCTTATTTCACA	60.1	568	INTERGENIC	--	--	--
Ca4_P3_31759099	Ca4	31759099	CTTTTTTTTTTT/CTTTTTTTTTT	TCTCCCCACCATCCACTTAG	CTCCACCTCCCCTATTCAT	59.9	847	INTRON	Ca_15447	R	bZIP
Ca4_P3_31772554	Ca4	31772554	AC/A	TTATCCTCGCCAATGACCTC	ATTCCACCAACAATATGGGC	60.0	561	INTERGENIC	--	--	--
Ca4_P3_31806633	Ca4	31806633	ATGTGACTAATTTT/ATGTGACTAATTTTGTGACTAATTTT	ACAAAAGCCTGGATAGCGAA	TCACATATACCCTTAAAATCGCA	59.8	410	INTERGENIC	--	--	--
Ca4_P3_31810307	Ca4	31810307	ATAAAGTAAAGTA/ATAAAGTA	CAGATCCAGTGGTGCAGAAA	CCTCAAAGAAGCTTGGGCTA	59.8	916	INTERGENIC	--	--	--
Ca4_P3_31822803	Ca4	31822803	ATTTTTTT/ATTTTTTTT	CACAGATTTCTCGAGGCACA	TCTAAAGCTCATGTCCCGCT	60.0	841	INTERGENIC	--	--	--
Ca4_P3_31859170	Ca4	31859170	GATAATA/GATA	CCTCGATGGATAAGGGTGAA	AAAATTGTCGACCAATCACTTG	59.9	418	INTERGENIC	--	--	--
Ca4_P3_31859597	Ca4	31859597	TAAAAAAAAA/TAAAAAAAAAAA	GTTGGAGCCCCCACTTTATT	TGCAAGCAGCATCAGAGAAC	60.2	793	INTERGENIC	--	--	--
Ca4_P3_31868533	Ca4	31868533	AGTGAGGTGAGG/AGTGAGG	CTAAAGCCATTGGGCATTGT	TGTCATCTATAAAAGCCCCACA	60.0	155	INTERGENIC	--	--	--
Ca4_P3_31871158	Ca4	31871158	AACACACA/AACACACACA	AAGGTGTGTTGCTAATCGGT	AAAAGGAATTTCTCAAATTGATGG	57.2	394	INTERGENIC	--	--	--

INDEL marker IDs	Chromosomes/ unanchored scaffolds	Physical positions (bp)	InDels (*Kabuli* reference genome-CDC Frontier/PII)	Forward primers (5'-3')	Reverse primers (5'-3')	Anneling temperature (0C)	Expected amplified product size (bp)	Structural annotation		Functional annotation	
								Sequence components of *kabuli* genome	*Kabuli* gene accession IDs	NCBI-KOG	TFs
Ca4_P3_31885190	Ca4	31885190	CTATTTATTTATTTAT/CTATTTATTTAT TTATTTAT	GGGTCCCTTATGTGAT GCAG	CCCAATCCCAAATCAA TCAC	60.3	352	INTERGENIC	--	--	--
Ca4_P3_31894163	Ca4	31894163	AAGAGAGA/AAGAGAGAGA	CCCCACCAAACCAATC ATAG	AAACCCCGCATATCTT TTCC	60.0	928	INTERGENIC	--	--	--
Ca4_P3_31894568	Ca4	31894568	ATTTTTTTTTTT/ATTTTTTTTT	TTTGCTCGCACAACACT AGG	GAAGGCCCTCAAAATC ATCA	60.1	645	INTERGENIC	--	--	--
Ca4_P3_31910459	Ca4	31910459	ATTTTTTTTT/ATTTTTTTTTTTT	TCCATGACTTATTTGTT GTGGC	CTCAAATTCTGTTTGG GGGA	59.9	315	INTERGENIC	--	--	--
Ca4_P3_31929869	Ca4	31929869	TAATAAATAAATAAATAAA/TAATAAATA AATAAA	CCATGCTTTCAAAAATG CCT	TTTGGAAGACTTTTGG GCTC	60.1	961	INTERGENIC	--	--	--
Ca4_P3_31934155	Ca4	31934155	ATTTTTTTTT/ATTTTTTTT	ACAGTTTTACCTCCCAC CCC	GTCTTTTCTTCCCCTTT CCG	60.1	835	INTERGENIC	--	--	--
Ca4_P3_31934186	Ca4	31934186	TATTATTATTATTATT/TATTATTATTATT ATTATT	ACACTCTTTTCTCACCC CCA	GTCTTTTCTTCCCCTTT CCG	59.5	750	INTERGENIC	--	--	--
Ca4_P3_31938648	Ca4	31938648	TTA/T	AATGCTCTACAGGAGC TGGC	GAGGAGGTCCAGGAT GATGA	59.6	782	INTRON	Ca_15455	--	Dof
Ca4_P3_31950817	Ca4	31950817	ATTTTTTTTTTT/ATTTTTTTTTT	GAGCAACAGTCAGTGG CGTA	CAACGCGAGCAAAATA TCAA	60.1	742	INTERGENIC	--	--	--
Ca4_P3_31962932	Ca4	31962932	T/TA	TGTGGAAAAGGAACAT GCAA	GATGTCACCACATCAC TGCC	60.1	444	INTERGENIC	--	--	--

INDEL marker IDs	Chromosomes/ unanchored scaffolds	Physical positions (bp)	InDels (*Kabuli* reference genome-CDC Frontier/PII)	Forward primers (5'-3')	Reverse primers (5'-3')	Anneling temperature (0C)	Expected amplified product size (bp)	Structural annotation		Functional annotation	
								Sequence components of *kabuli* genome	*Kabuli* gene accession IDs	NCBI-KOG	TFs
Ca4_P3_31965959	Ca4	31965959	TAAAAAAA/TAAAAAAAA	TTCATTGCAAGTTCCGTGAG	TTCAAGCCCTTCCTTTTCCT	59.8	627	INTERGENIC	--	--	--
Ca4_P3_31968115	Ca4	31968115	AAT/AATAT	CCAATGATAATGGGGGTTTG	CCATTGCAGGTTTCATTGTG	59.9	794	INTRON	Ca_15456	--	--
Ca4_P3_31969931	Ca4	31969931	ATTTT/ATTT	ATTCACGCAGTCAGCACATC	TTCGAAGGGAGTATGCAAGG	59.9	574	CDS (FRAME SHIFT)	Ca_15456	--	--
Ca4_P3_31978850	Ca4	31978850	CAA/CAAA	ACATTCTTATGTCGGGGCAG	ACAGCCTTTTCTCCATCCCT	60.0	605	DRR	Ca_15457	S	--
Ca4_P3_31988631	Ca4	31988631	GAA/GAAA	ATAAGAAGCAACAAGGCCCA	TGCAGTGGGTGCACATTTAT	59.7	631	INTERGENIC	--	--	--
Ca4_P3_31989159	Ca4	31989159	ATTTTTTTTT/ATTTTTTTT	AGAGGTGGCAAAATTGATGG	GGGAGTGGGAGGAATCTTTC	59.9	557	INTERGENIC	--	--	--
Ca4_P3_31992893	Ca4	31992893	CTGAGTTAA/CTGAGTTAATGAGTTAA	TGACACAATTGCTTATGCGG	TTCCAACGGTCATAAAAGGC	60.7	869	DRR	Ca_15458	--	--
Ca4_P3_32003422	Ca4	32003422	CAAAAAA/CAAAA	ATCAGCGAGGATGCAGAACT	AACTTGGGGATGAAAAATGC	60.0	611	INTERGENIC	--	--	--
Ca4_P3_32005104	Ca4	32005104	GACTACTACT/GACTACT	CACTTGACAGGGGTGTTCCT	CATGTGACAACAAGCCTCCA	60.0	596	INTERGENIC	--	--	--
Ca4_P3_32005564	Ca4	32005564	TAAC/TAACTCAAC	CATTGGAGGCTTGTTGTCAC	AGGTGCACGCTTCAGCTATT	59.1	674	INTERGENIC	--	--	--

INDEL marker IDs	Chromosomes/ unanchored scaffolds	Physical positions (bp)	InDels (*Kabuli* reference genome-CDC Frontier/PII)	Forward primers (5'-3')	Reverse primers (5'-3')	Anneling temperature (0C)	Expected amplified product size (bp)	Structural annotation		Functional annotation	
								Sequence components of *kabuli* genome	*Kabuli* gene accession IDs	NCBI-KOG	TFs
Ca4_P3_32007324	Ca4	32007324	AT/ACCAATT	CCATTAGTCTTGCCCTTTGC	CAAATAAAATCATGGCTGCAC	59.7	450	INTERGENIC	--	--	--
Ca4_P3_32008196	Ca4	32008196	TC/T	TCTGAGCACACTTGTGGGAC	TTGCGAAGTCAAGCAAACTG	59.9	418	INTERGENIC	--	--	--
Ca4_P3_32012018	Ca4	32012018	AT/ATT	GGACAAGAGAGAAGAGGGAAAA	ATCGGCTTGCTATGTTTGCT	59.0	380	INTERGENIC	--	--	--
Ca4_P3_32016200	Ca4	32016200	TGAGATACC/TGAGATACCGAGATACC	TCTAAATGTGGGTCGTGCAA	GAAGGATAAACATTGGGGCA	60.1	279	INTERGENIC	--	--	--
Ca4_P3_32018236	Ca4	32018236	A/AGG	GAAAGACAAATCGTTCGGGA	CCACTTCTTCCCAAATCCAA	60.1	431	INTERGENIC	--	--	--
Ca4_P3_32018461	Ca4	32018461	CTT/CTTT	GAAAGACAAATCGTTCGGGA	CCACTTCTTCCCAAATCCAA	60.1	431	INTERGENIC	--	--	--
Ca4_P3_32019814	Ca4	32019814	ATTTTTT/ATTTTTTT	TGAAGCGGTAATAAGAAGAGTGG	CGCTGAATCTCATCAAAAGTTG	59.8	255	INTERGENIC	--	--	--
Ca4_P3_32034763	Ca4	32034763	ATT/ATTT	CGTCCACTTCCACTACCGAT	ATTAAAATTTGGAGCGGGGA	60.0	553	INTERGENIC	--	--	--
Ca4_P3_32034803	Ca4	32034803	TAAAAAAAAAAA/TAAAAAAAA	CGTCCACTTCCACTACCGAT	ATTAAAATTTGGAGCGGGGA	60.0	553	INTERGENIC	--	--	--
Ca4_P3_32038234	Ca4	32038234	G/GA	ACAATGCCCGACTTTACTGG	ACTATGAGGTTTTGGTGGCG	60.0	812	INTERGENIC	--	--	--

INDEL marker IDs	Chromosomes/ unanchored scaffolds	Physical positions (bp)	InDels (*Kabuli* reference genome-CDC Frontier/PII)	Forward primers (5'-3')	Reverse primers (5'-3')	Anneling temperature (0C)	Expected amplified product size (bp)	Structural annotation		Functional annotation	
								Sequence components of *kabuli* genome	*Kabuli* gene accession IDs	NCBI-KOG	TFs
Ca4_P3_32040799	Ca4	32040799	CCTCTCTCTCT/CCTCTCTCT	GCAGTGGTCCTCTTAA TGGC	TGTTCGTGCTTTGTAC CCAA	59.7	241	INTRON	Ca_15459	--	--
Ca4_P3_32046867	Ca4	32046867	TAA/T	ACTAGTGCCTTTGAAG CCGA	GTTCATGCAAAGAGCA GCAA	60.0	712	DRR	Ca_15459	--	--
Ca4_P3_32047003	Ca4	32047003	TTAATA/TTA	GCTGGTTTTCATGGGT CATT	GTTCATGCAAAGAGCA GCAA	59.8	474	DRR	Ca_15459	--	--
Ca4_P3_32051935	Ca4	32051935	ATTTTTTTTTTT/ATTTTTTTTT	TTGAAGATGGTGAATG TGGAA	TATGACCCGTTGGATT CGAT	59.0	797	INTERGENIC	--	--	--
Ca4_P3_32053962	Ca4	32053962	ATTTTTTTTT/ATTTTTTTT	TGCATGATTAGGGTTA GCTTTTC	ATGCTCCCCTGACATT CTTG	59.7	258	INTERGENIC	--	--	--
Ca4_P3_32056772	Ca4	32056772	ATT/ATTT	CCAGATCCACCACCAA AATC	ATAAAAGCGAAGCGAA GCAA	60.2	683	INTERGENIC	--	--	--
Ca4_P3_32056846	Ca4	32056846	TAAAA/TAAAAA	CCAGATCCACCACCAA AATC	ATAAAAGCGAAGCGAA GCAA	60.2	683	INTERGENIC	--	--	--
Ca4_P3_32057189	Ca4	32057189	AATATAT/AATAT	CGCTGCTCTTCCTTGTT CTC	AAAGCGAAGCGAAGCA ATAA	60.3	294	INTERGENIC	--	--	--
Ca4_P3_32057442	Ca4	32057442	TAAAAAAA/TAAAAAAAA	TATTGCTTCGCTTCGCT TTT	GGGAAACTGCCCCTAA TTCT	60.1	451	INTERGENIC	--	--	--
Ca4_P3_32061418	Ca4	32061418	T/TGTTTTTTTA	ATCTGTGGCGAAGAGT CCAG	TGTTCAAGGGATCAAA AGGG	60.4	359	INTRON	Ca_15460	--	--

INDEL marker IDs	Chromosomes/ unanchored scaffolds	Physical positions (bp)	InDels (*Kabuli* reference genome-CDC Frontier/PII)	Forward primers (5'-3')	Reverse primers (5'-3')	Anneling temperature (0C)	Expected amplified product size (bp)	Structural annotation		Functional annotation	
								Sequence components of *kabuli* genome	*Kabuli* gene accession IDs	NCBI-KOG	TFs
Ca4_P3_32067701	Ca4	32067701	GAAAAA/GAAAAAA	TGTTGCATTTCTGTCGGAAG	CGAAGATTCGGTTGATGGTT	59.8	701	INTERGENIC	--	--	--
Ca4_P3_32103137	Ca4	32103137	TAAA/TAAAA	TAGATCATGCGGCTGTTTCA	ACCACCATTCATCCGAGAAG	60.4	683	INTRON	Ca_15462	--	--
Ca4_P3_32105124	Ca4	32105124	TAAAAA/TAAAAAA	TTTGGGGGTACAGAAGCAAC	GGCCATTATTGATCCCTTCC	60.0	477	INTRON	Ca_15462	--	--
Ca4_P3_32105685	Ca4	32105685	T/TC	GAGGAAGGGATCAATAATGGC	TTGGATGATGGTGATTGATGA	59.8	650	INTRON	Ca_15462	--	--
Ca4_P3_32107042	Ca4	32107042	AAGAT/A	TTGCAACAAGCGGTAATTGA	CACCACCATGCTCTTCAAAA	60.3	647	INTRON	Ca_15462	--	--
Ca4_P3_32117339	Ca4	32117339	CAAAAA/CAAAA	CCCCAACAAAATGACGTCTC	TCCCAACTCTTCGTTCATCC	60.4	410	INTERGENIC	--	--	--
Ca4_P3_32118643	Ca4	32118643	AAAT/A	GCCAAAGGTAACAAAGGCAA	TGATTTCTTCATTGCATCGG	60.1	569	INTERGENIC	--	--	--
Ca4_P3_32121497	Ca4	32121497	TAAAAAAAA/TAAAAAAAAAA	TGGCATACGACCAACTCAAA	TTTGCTTTACAAGCTTGGGG	60.1	440	INTERGENIC	--	--	--
Ca4_P3_32136390	Ca4	32136390	TC/TCC	AAGCTTCTTAGGCCTGTCCC	TCCTTTTCTCACTTCCCCTTT	59.8	637	INTERGENIC	--	--	--
Ca4_P3_32137832	Ca4	32137832	CAAAAAAAA/CAAAAAAAAAA	CGACAGCACATTAGATAAACCG	ATGGATGATGGGCAGTAAGC	59.7	806	INTERGENIC	--	--	--

INDEL marker IDs	Chromosomes/ unanchored scaffolds	Physical positions (bp)	InDels (*Kabuli* reference genome-CDC Frontier/PII)	Forward primers (5'-3')	Reverse primers (5'-3')	Anneling temperature (0C)	Expected amplified product size (bp)	Structural annotation		Functional annotation	
								Sequence components of *kabuli* genome	*Kabuli* gene accession IDs	NCBI-KOG	TFs
Ca4_P3_32137903	Ca4	32137903	GAAAAA/GAAAAAA	CGACAGCACATTAGAT AAACCG	ATGGATGATGGGCAGT AAGC	59.7	806	INTERGENIC	--	--	--
Ca4_P3_32139759	Ca4	32139759	AAAAGCTTT/AAAAGCTTTAAAGCTTT	TGTGGTTTGGATTGGG TTTT	ACTTCAAAAATTGAGG GGCA	60.1	736	INTERGENIC	--	--	--
Ca4_P3_32143162	Ca4	32143162	GAAAAAAA/GAAAAAAAA	AATTCCCTTGTGTGTGC CTT	CCGTTCAAGCAATGAT AACG	59.5	424	INTERGENIC	--	--	--
Ca4_P3_32143723	Ca4	32143723	G/GGA	CGTTATCATTGCTTGAA CGG	CGTACAAGAACAACAG GCAAA	59.2	581	INTERGENIC	--	--	--
Ca4_P3_32143997	Ca4	32143997	TC/T	TTTGCCTGTTGTTCTTG TACG	TTGAGCACTTACTGGT GGGA	58.9	381	INTERGENIC	--	--	--
Ca4_P3_32144702	Ca4	32144702	ATGAGTTTGAG/ATGAGTTTGAGTTTG AG	CCACACAGACCAACAA CGAC	GATGCAAGCTCCATCT GACA	60.0	461	INTERGENIC	--	--	--
Ca4_P3_32148957	Ca4	32148957	GAAAAAAA/GAAAAAA	AAGGTGGAAGAAAAGG CAAAA	TGCCCATAACTGCACA ACAT	60.1	663	INTERGENIC	--	--	--
Ca4_P3_32149191	Ca4	32149191	CTTTTTTT/CTTTTTT	CGGTGAAGGGACCACA CTAT	TGCCCATAACTGCACA ACAT	59.8	404	INTERGENIC	--	--	--
Ca4_P3_32155616	Ca4	32155616	TAAA/TAA	CCGTACAACCAAGTCC CATC	GAAATGGAAACGATCC CAAC	60.2	151	INTERGENIC	--	--	--
Ca4_P3_32167743	Ca4	32167743	A/AG	GCAGCATATGATCGCA AAAA	AAAGTCCCACTTGATG GTGG	59.8	565	INTERGENIC	--	--	--

INDEL marker IDs	Chromosomes/ unanchored scaffolds	Physical positions (bp)	InDels (*Kabuli* reference genome-CDC Frontier/PII)	Forward primers (5'-3')	Reverse primers (5'-3')	Anneling temperature (0C)	Expected amplified product size (bp)	Structural annotation		Functional annotation	
								Sequence components of *kabuli* genome	*Kabuli* gene accession IDs	NCBI-KOG	TFs
Ca4_P3_32169313	Ca4	32169313	ATTTTTTTT/ATTTTTTTTT	TACGTTAACGGTGACCACGA	GCACTTTGTAAACGTCCCGT	60.0	762	INTERGENIC	--	--	--
Ca4_P3_32179289	Ca4	32179289	CTTTTTT/CTTTTT	GTTGGCATTCCTCTTTTGGA	TGGAAGTGAACTAAACCCCTTT	60.1	826	INTERGENIC	--	--	--
Ca4_P3_32179682	Ca4	32179682	CAATAATCAAACAAA/CAATAATCAAACAAATAATCAAACAAA	TTGCATACCTCATACCATCCC	TTGATGCATTACCTCCCACA	59.7	731	INTERGENIC	--	--	--
Ca4_P3_32181885	Ca4	32181885	GTTT/GTTTT	AGATTTAAGGGAGGGGGCTC	CGAACAATTCGTCCTTGGTT	60.8	401	INTERGENIC	--	--	--
Ca4_P3_32185519	Ca4	32185519	ACCAAA/ACCAAATGGTCAAGGGAGCCAAA	GTGGTTCGGAACACGCTATT	ACATGGTTTGGCACAAGTGA	60.0	480	DRR	Ca_15464	R	ERF
Ca4_P3_32187077	Ca4	32187077	CTGTG/CTGTGTG	TTAAACATTCATTTCCGCCG	GCTTGGTTTGTGGCATGTTA	60.8	385	INTERGENIC	--	--	--
Ca4_P3_32202833	Ca4	32202833	TGA/TGAAGA	GATGAAGCCAATGATGCTGA	ACCCTGCAGCTACTTCCTGA	59.8	499	CDS (large-effect mutations)	Ca_15465	--	--
Ca4_P3_32206950	Ca4	32206950	CA/C	ATCTGGCAAACCCTTCAAGA	GAGGAGGTGGAACTTGTGGA	59.7	852	INTRON	Ca_15466	E	--
Ca4_P3_32216199	Ca4	32216199	ATTTTTTTT/ATTTTTTT	ATAAAGGTCAACGCACGCTT	GAAATCAACGGATCTCCATCA	59.8	384	INTERGENIC	--	--	--
Ca4_P3_32216251	Ca4	32216251	GTTTTTT/GTTTTTTTT	ATAAAGGTCAACGCACGCTT	GAAATCAACGGATCTCCATCA	59.8	384	INTERGENIC	--	--	--

INDEL marker IDs	Chromosomes/ unanchored scaffolds	Physical positions (bp)	InDels (*Kabuli* reference genome-CDC Frontier/PII)	Forward primers (5'-3')	Reverse primers (5'-3')	Anneling temperature (0C)	Expected amplified product size (bp)	Structural annotation		Functional annotation	
								Sequence components of *kabuli* genome	*Kabuli* gene accession IDs	NCBI-KOG	TFs
Ca4_P3_32216472	Ca4	32216472	ACT/A	ATAAAGGTCAACGCAC GCTT	GAAATCAACGGATCTC CATCA	59.8	384	INTERGENIC	--	--	--
Ca4_P3_32218753	Ca4	32218753	AT/ATT	GCATTGAAGGGGTCAA CACT	GAGGGAGCAAATCGGT ATCA	60.0	390	INTERGENIC	--	--	--
Ca4_P3_32225930	Ca4	32225930	GAAAAAAA/GAAAAAAAA	TCGTCGCATTGGTGTA ATGT	TCCGTGAGGACACAAG TGAG	60.0	506	INTERGENIC	--	--	--
Ca4_P3_32227293	Ca4	32227293	AAAAA/AAAAATTAAAA	TCTTCACAACCCCTTGA ACC	TTCTTCCAAACGAGCC TCTC	59.9	868	INTERGENIC	--	--	--
Ca4_P3_32227770	Ca4	32227770	GT/GTGATATATGTT	GAAGAGAGGCTCGTTT GGAA	CTAATGGCTCCCGAAT TCAA	59.6	474	INTERGENIC	--	--	--
Ca4_P3_32241433	Ca4	32241433	ATT/A	ACAATTGTTGCACCAC GAGA	ATCGCAAAAGTAGTCC CCCT	60.2	148	INTERGENIC	--	--	--
Ca4_P3_32242240	Ca4	32242240	TATTAAATTAAA/TATTAAA	TGCTAATGCTTTTCAAG GGC	GCGATGCACAGGTAAA CAAA	60.3	482	INTERGENIC	--	--	--
Ca4_P3_32243226	Ca4	32243226	TAAAAAAAA/TAAAAAAAAA	TTGAATCAATGGGGAC GAAT	GTCTTGCAATGCTCCA GTGA	60.1	405	INTERGENIC	--	--	--
Ca4_P3_32256317	Ca4	32256317	A/AC	AAACAAAACTCATCCTT CCCAA	CGTGAGTCTCGTTGAA GCAA	59.9	336	INTERGENIC	--	--	--
Ca4_P3_32263797	Ca4	32263797	CAAAAAAA/CAAAAAA	ACACATGTCCCAGTTG ACGA	TGACGATGGAGGCTTT TAGG	60.0	682	INTERGENIC	--	--	--

INDEL marker IDs	Chromosomes/ unanchored scaffolds	Physical positions (bp)	InDels (*Kabuli* reference genome-CDC Frontier/PII)	Forward primers (5'-3')	Reverse primers (5'-3')	Anneling temperature (0C)	Expected amplified product size (bp)	Structural annotation		Functional annotation	
								Sequence components of *kabuli* genome	*Kabuli* gene accession IDs	NCBI-KOG	TFs
Ca4_P3_32263961	Ca4	32263961	AAA/AAATAA	GATCAAATCCCCTATGTATGTGC	TGACGATGGAGGCTTTTAGG	59.6	191	INTERGENIC	--	--	--
Ca4_P3_32264113	Ca4	32264113	TAAAAAAA/TAAAAAA	CCTAAAAGCCTCCATCGTCA	TGAGAAATTGGTTTCTCTTCCTC	60.2	385	INTERGENIC	--	--	--
Ca4_P3_32264177	Ca4	32264177	GC/G	CCTAAAAGCCTCCATCGTCA	TGAGAAATTGGTTTCTCTTCCTC	60.2	385	INTERGENIC	--	--	--
Ca4_P3_32264980	Ca4	32264980	AAT/AATAT	GCACTTACCCTTCAATTGGG	AGGAGAGGTCCATTTCTCAGG	59.4	262	INTERGENIC	--	--	--
Ca4_P3_32266919	Ca4	32266919	GAAT/GAATCAAT	GTCCTCACTTGGCTTCTTGC	CAAATCTGCCAAAACCACCT	60.0	567	INTERGENIC	--	--	--
Ca4_P3_32297893	Ca4	32297893	CTGAAT/CTGAATGAAT	GCGGTGCGATATTCTTTGTT	TGGAAAATGTTGGAGCATGA	60.1	471	INTERGENIC	--	--	--
Ca4_P3_32298277	Ca4	32298277	T/TC	TCATGCTCCAACATTTTCCA	CCAAAACCAAACCTAAGGCA	60.0	587	INTERGENIC	--	--	--
Ca4_P3_32301466	Ca4	32301466	CAAAAAAAA/CAAAAAAAAA	AACCATCCCACTTGATGGAA	TCAACCAGTTTTGCAATCCA	60.2	186	DRR	Ca_15472	DO	MYB_related
Ca4_P3_32301599	Ca4	32301599	TAAAAAAA/TAAAA	TGGATTGCAAAACTGGTTGA	CACGAGCTTACAAACCCACA	60.1	174	DRR	Ca_15472	DO	MYB_related
Ca4_P3_323024	Ca4	323024	TTATATATATATATATATATATA/TTATATATATATATATATATATATATA	TGAGTTCCTGCACCTCACAA	TTCTTTTAAGTCCAATTCCCAG	60.4	378	INTERGENIC	--	--	--

INDEL marker IDs	Chromosomes/ unanchored scaffolds	Physical positions (bp)	InDels (*Kabuli* reference genome-CDC Frontier/PII)	Forward primers (5'-3')	Reverse primers (5'-3')	Anneling temperature (0C)	Expected amplified product size (bp)	Structural annotation		Functional annotation	
								Sequence components of *kabuli* genome	*Kabuli* gene accession IDs	NCBI-KOG	TFs
Ca4_P3_32322091	Ca4	32322091	AA/AAGA	CGAACTTGGAGACTGAACGA	GATGGCCATGAATTGCTTTT	59.0	276	INTERGENIC	--	--	--
Ca4_P3_32335179	Ca4	32335179	AAAGAAGAAGAAGAAGAAGAA/AAAGAAGAAGAAGAAGAA	AAAGTCCGTTTGTGAATCCG	TGGGCATTCAAATCAAACAA	60.0	507	INTERGENIC	--	--	--
Ca4_P3_32370221	Ca4	32370221	GTTTTTTTT/GTTTTTTTTT	GACCACACATGCGCACTATT	TCATGCCCTTACGATCAACA	59.6	648	INTERGENIC	--	--	--
Ca4_P3_32384144	Ca4	32384144	AAAAAAATAAAA/AAAAAAATAAAAAATAAAA	GCTTGTGGTTGTCGGATCTT	ATGAGTTGCTTTTGGTTCCG	60.1	667	INTERGENIC	--	--	--
Ca4_P3_32384172	Ca4	32384172	TAAAAAA/TAAAAAAA	GCTTGTGGTTGTCGGATCTT	ATGAGTTGCTTTTGGTTCCG	60.1	667	INTERGENIC	--	--	--
Ca4_P3_32384505	Ca4	32384505	ATTTATTTTTATTTTTATTTTTATT/ATTTATTTTTATTTTTATT	CGGAACCAAAAGCAACTCAT	CGCCTAATTTTGTCCGCTAC	60.1	159	INTERGENIC	--	--	--
Ca4_P3_32388402	Ca4	32388402	ATTTTTTTT/ATTTTTTTTT	TCAATTGCTTGTTGCCTCTG	AGAGACATAACAAAGGGCCG	60.0	606	INTERGENIC	--	--	--
Ca4_P3_32388731	Ca4	32388731	GTTTAATTATTTA/GTTTAATTATTTAATTATTTA	CGGCCCTTTGTTATGTCTCT	TTTGGGCGAATTCCATTTTA	59.2	692	INTERGENIC	--	--	--
Ca4_P3_32390488	Ca4	32390488	TAGAGAGAGAGAGAGAGAGAGAGA/TAGAGAGAGAGAGAGA	TGGCACAAAAATCCAAATCA	CCTCGTCGGAGCTATTAACG	59.9	836	INTERGENIC	--	--	--
Ca4_P3_32396185	Ca4	32396185	TCCC/TCC	TCGTCATTTCTTTGTTTGGTTG	TCCAATTTTCCGTGTCATGT	60.0	346	INTERGENIC	--	--	--

INDEL marker IDs	Chromosomes/ unanchored scaffolds	Physical positions (bp)	InDels (*Kabuli* reference genome-CDC Frontier/PII)	Forward primers (5'-3')	Reverse primers (5'-3')	Anneling temperature (0C)	Expected amplified product size (bp)	Structural annotation		Functional annotation	
								Sequence components of *kabuli* genome	*Kabuli* gene accession IDs	NCBI-KOG	TFs
Ca4_P3_32399880	Ca4	32399880	CATA/CATACAAATAAAAATA	TTCTGTCCGAGTATGGGTCC	CATGATGGTTTTGTTGTGGC	59.9	835	INTERGENIC	--	--	--
Ca4_P3_32409390	Ca4	32409390	TAAAATAAAAAAATAAAAAA/TAAAATAAAAAA	GACCCCATACGAAGGTGAAA	CGATGTATTTTGGCGGATTC	59.8	569	INTERGENIC	--	--	--
Ca4_P3_32412395	Ca4	32412395	TAAAAAAA/TAAAAAA	TTCAAATCTCGCAGTTGTTTG	GAAATCCCAGTAGCAGTGGC	59.0	642	INTERGENIC	--	--	--
Ca4_P3_32414230	Ca4	32414230	ATATTTTTATTTT/ATATTTT	CCACCAATAGGGAGAGAAGAAA	CTTTAAGCTCCGAAACGACG	59.6	136	INTERGENIC	--	--	--
Ca4_P3_32415458	Ca4	32415458	CTTTT/CTTTTT	AACATGGAGGGTCATGGAAA	AACTTTGTCGATTAAGCACGG	60.2	435	INTERGENIC	--	--	--
Ca4_P3_32416231	Ca4	32416231	ATT/AT	CCCACCATTGAAATTTGGAG	GAAGGAGGGGAAATTTTGACA	60.2	680	INTERGENIC	--	--	--
Ca4_P3_32416840	Ca4	32416840	ATTTTTTT/ATTTTTT	TTGCAGCCTTAATTAGCCAAA	GCATGACTTTTGGTCGTGTG	59.9	436	INTERGENIC	--	--	--
Ca4_P3_32416873	Ca4	32416873	AG/A	TTGCAGCCTTAATTAGCCAAA	TCACCGATCAAAAGGAATGA	59.9	645	INTERGENIC	--	--	--
Ca4_P3_32432090	Ca4	32432090	T/TA	TGATAAATTTGGGTCATGTATTTTT	TTGGATCATAGATTTCTGAATGATAA	57.9	564	INTERGENIC	--	--	--
Ca4_P3_32454180	Ca4	32454180	TAA/TAAA	GCGGATACATTTCTTTTGGG	AAACAAGCGGAAGAGCTTGA	59.4	310	INTERGENIC	--	--	--

INDEL marker IDs	Chromosomes/ unanchored scaffolds	Physical positions (bp)	InDels (*Kabuli* reference genome-CDC Frontier/PII)	Forward primers (5'-3')	Reverse primers (5'-3')	Anneling temperature (0C)	Expected amplified product size (bp)	Structural annotation		Functional annotation	
								Sequence components of *kabuli* genome	*Kabuli* gene accession IDs	NCBI-KOG	TFs
Ca4_P3_32458469	Ca4	32458469	TAAA/TAA	AATGGTCCAACCCCTTAACC	TTTGGACAATGTCGTGAAACA	59.9	526	INTERGENIC	--	--	--
Ca4_P3_32460179	Ca4	32460179	TTATATATATATATATATATATATAT/TTATATATATAT	CGGAGATCCATAACACGGTC	TTTGAAAGAGAAATCCACCCA	60.3	242	INTERGENIC	--	--	--
Ca4_P3_32482372	Ca4	32482372	AGGGG/AGGGGG	TACCAAACCCCTTGGTCATC	TTTTGGGCGGAAATGTTTTA	59.6	583	INTERGENIC	--	--	--
Ca4_P3_32494139	Ca4	32494139	GATAT/GAT	CGTCATTACCCGTTGAAACC	TGAGGAGCATAAATCAGAGAAAAA	60.2	786	INTERGENIC	--	--	--
Ca4_P3_32496585	Ca4	32496585	TATTTATTTTTA/TATTTATTTTTAAAATTTATTTTTA	CCACAATCCACCGTCTACCT	CCCAGACCCCTCATAATCAA	59.8	565	INTERGENIC	--	--	--
Ca4_P3_32498847	Ca4	32498847	AG/A	TAGCACGGACCCCAATTAAG	TGAACCCCCATCAAAGGATA	60.0	897	INTERGENIC	--	--	--
Ca4_P3_32511208	Ca4	32511208	GAAAAAAAAA/GAAAAAAAAAA	ACTGTTTTGCCCAGAAATGC	TGATGCATGTCAGAAAGGGA	60.1	475	INTERGENIC	--	--	--
Ca4_P3_32513097	Ca4	32513097	TGGGGGG/TGGGGG	GATTTGCATTGGCCTCTTGT	ACAGTGGTTGCCATTTCCTC	60.1	479	INTERGENIC	--	--	--
Ca4_P3_32515308	Ca4	32515308	ATGTG/ATG	GGCTATCGACCTAGGGAAGC	ACCAACAGAACCGCCAATAG	60.2	347	INTERGENIC	--	--	--
Ca4_P3_32523443	Ca4	32523443	TAAAAAAA/TAAAAAA	GAAGGAGGAGGAGAGTGCAG	ATTGAACACGACGCAAATCA	59.1	391	INTERGENIC	--	--	--

INDEL marker IDs	Chromosomes/ unanchored scaffolds	Physical positions (bp)	InDels (*Kabuli* reference genome-CDC Frontier/PII)	Forward primers (5'-3')	Reverse primers (5'-3')	Anneling temperature (0C)	Expected amplified product size (bp)	Structural annotation		Functional annotation	
								Sequence components of *kabuli* genome	*Kabuli* gene accession IDs	NCBI-KOG	TFs
Ca4_P3_32525507	Ca4	32525507	CAAAA/CAAAAA	GTCATGCATATGTTGG ACGC	AAGTTTTCTTCCCGCT CACA	60.0	460	INTERGENIC	--	--	--
Ca4_P3_32530130	Ca4	32530130	TC/T	AAATACCATGCAACCCC TGA	CTTTGTGTGCAATTCA AGCC	60.2	928	INTERGENIC	--	--	--
Ca4_P3_32531473	Ca4	32531473	TAA/T	TAAAATGGATGCATCGA GCC	ACAAATCAACCAGTGT CGCA	60.9	662	INTERGENIC	--	--	--
Ca4_P3_32547296	Ca4	32547296	AA/AACATTTATCAATTA	TGCGAGATTTTGAGTTC AACC	TGCTTATCTTGAACGG GACC	60.2	325	INTERGENIC	--	--	--
Ca4_P3_32557354	Ca4	32557354	TAAATAAAAT/TAAATAAAATAAAAT	AGGCAAAATACATGCAA TCAT	TGCGTTTTGAGATTCG AAGA	57.1	497	INTERGENIC	--	--	--
Ca4_P3_32562355	Ca4	32562355	CTTTTT/CTTTTTT	AAATGGAGGAGAAGGA GGGA	CGGTGAATCCTTTACG GAGA	60.0	286	INTERGENIC	--	--	--
Ca4_P3_32593055	Ca4	32593055	TAAAAAAAAA/TAAAAAAAAAA	ATGCAACCCTTTTACAA CGC	CAAGGAAGAATGGTGT CGCT	60.0	658	INTERGENIC	--	--	--
Ca4_P3_32622503	Ca4	32622503	ACC/AC	TCAATGCCATGGAAAG ACAA	TTCCATCTCCAACAAC ATCG	60.0	867	DRR	Ca_15485	O	--
Ca4_P3_32623000	Ca4	32623000	TAAAAAAAAAA/TAAAAAAAAAAAA	TCGATGTTGTTGGAGA TGGA	TCCATCTTTTGGAGGA ATGG	60.0	566	DRR	Ca_15485	O	--
Ca4_P3_32630240	Ca4	32630240	TAA/TAAA	GTTTCTCCCCAGTCCT CCTC	CTGAAATTGGAACGTG GGTT	60.1	533	INTRON	Ca_15485	O	--

INDEL marker IDs	Chromosomes/ unanchored scaffolds	Physical positions (bp)	InDels (*Kabuli* reference genome-CDC Frontier/PII)	Forward primers (5'-3')	Reverse primers (5'-3')	Anneling temperature (0C)	Expected amplified product size (bp)	Structural annotation		Functional annotation	
								Sequence components of *kabuli* genome	*Kabuli* gene accession IDs	NCBI-KOG	TFs
Ca4_P3_32639909	Ca4	32639909	AC/A	GCGTTACGGTCTTCTG CTTC	TTTGTTAATGCATCTGC CCA	60.0	453	INTERGENIC	--	--	--
Ca4_P3_32647144	Ca4	32647144	TAAA/TAA	TGCATTGTTTTCAAGGG TCA	CCTAATTGCACCCCAA AATG	60.1	611	INTRON	Ca_15487	--	--
Ca4_P3_32647560	Ca4	32647560	CTTT/CTT	TTCCCCTGTTCCCCATA CTT	AGAATACCCCAGCAGC AATG	60.5	524	INTRON	Ca_15487	--	--
Ca4_P3_32647717	Ca4	32647717	TAAAAAAAAAAA/TAAAAAAAAAA	GGGTGCAATTAGGGAT GAGA	AGAATACCCCAGCAGC AATG	59.9	313	INTRON	Ca_15487	--	--
Ca4_P3_32650495	Ca4	32650495	AAGAG/AAGAGAG	TTTCAAACAAAACCCAT GAAA	CACAACAACATTGAGG GTGC	58.0	603	DRR	Ca_15487	--	--
Ca4_P3_3265055	Ca4	3265055	GAAAAA/GAAAAAA	GTTGCTGTTGCTGTTG CTGT	TTCCTGTGGGACAAGA AAGG	60.1	735	INTERGENIC	--	--	--
Ca4_P3_32663516	Ca4	32663516	TTTATTATTATTATTATTATTATT/TTTAT TATTATTATTATT	TGCGTTTCACTCTTCCT TCC	TGTCAAGCCCAAAAAC AAAA	60.4	257	INTERGENIC	--	--	--
Ca4_P3_32700117	Ca4	32700117	CTTTTTTT/CTTTTTTTT	TACGTGTCGCTTAGGC ACTG	CGTCTTGTGCGGTGTA TTTG	60.1	461	INTERGENIC	--	--	--
Ca4_P3_32886390	Ca4	32886390	AC/ACC	AATCATCCCATGTTGCC ATT	CGAGATTTCACACCAA GGGT	60.0	519	INTERGENIC	--	--	--
Ca4_P3_32908126	Ca4	32908126	ATTTTTTTTTTTTT/ATTTTTTTTTTTTTT TT	TTGGGTAAAAAGGACG CAAC	GAGGGACCAAAATTGC AAAA	60.0	678	INTERGENIC	--	--	--

INDEL marker IDs	Chromosomes/ unanchored scaffolds	Physical positions (bp)	InDels (*Kabuli* reference genome-CDC Frontier/PII)	Forward primers (5'-3')	Reverse primers (5'-3')	Anneling temperature (0C)	Expected amplified product size (bp)	Structural annotation		Functional annotation	
								Sequence components of *kabuli* genome	*Kabuli* gene accession IDs	NCBI-KOG	TFs
Ca4_P3_3304220	Ca4	3304220	AAATAATAATAATAATAATAATAATAATAATAAT/AAATAATAATAATAATAATAATAATAAT	AATATCGTCGGGAAATGCTG	GAAAGGTCGGTCAAAAAGCA	59.9	465	INTERGENIC	--	--	--
Ca4_P3_33113244	Ca4	33113244	GAAA/GAA	TGGGATTCCTCTCTCCCACT	GGCAAACTTGTCCACTTGGT	61.0	274	INTERGENIC	--	--	--
Ca4_P3_33176272	Ca4	33176272	ATTTTTTTTT/ATTTTTTTTTT	ATCCACGGACAATCCATTTG	TGGATGTCTTGTTCTGGGTTC	60.6	478	INTERGENIC	--	--	--
Ca4_P3_33195041	Ca4	33195041	T/TAGAATTCGGTGA	TTGTCCGTGAGAGACTGCAC	GAATCCTGGGCTGAAATCAA	60.0	595	INTERGENIC	--	--	--
Ca4_P3_33207850	Ca4	33207850	TAAATAAAATAAAATAAAATAAAA/TAAATAAAATAAAATAAAATAAAATAAAA	TTGTTTCTCCCCAGTTTTGG	GTGAAATCGTTCCCTGAGGA	59.9	745	INTERGENIC	--	--	--
Ca4_P3_33251796	Ca4	33251796	GTTTTTTTTTT/GTTTTTTTTTTTT	ATCAAAGACATGCAAGGGCT	AGCACCTAGGGCATGGTATG	59.7	816	INTERGENIC	--	--	--
Ca4_P3_33256637	Ca4	33256637	ATTTTTTTTT/ATTTTTTTT	CATTGATCATGGTTCTTGCC	GGCTTGTGAAAACAAATGGAA	58.9	329	INTERGENIC	--	--	--
Ca4_P3_33270889	Ca4	33270889	AAGCTGGAAATTCCA/AA	CAGGTTCAGCACGATCAAGA	CTGGATGAGGAACTTGGGAA	60.0	127	CDS (FRAME SHIFT)	Ca_24019	--	--
Ca4_P3_33295958	Ca4	33295958	TAAAAAAAAAAA/TAAAAAAAAAA	TGACCTCCCACTGCATACAA	AAGAGACCGCATACGTGGAT	60.1	532	INTERGENIC	--	--	--
Ca4_P3_33298619	Ca4	33298619	T/TTAG	AGCTCCTAGACCACCTGCAA	TTTGGGTGGCATTCTTTTTC	60.0	448	INTERGENIC	--	--	--

INDEL marker IDs	Chromosomes/ unanchored scaffolds	Physical positions (bp)	InDels (*Kabuli* reference genome-CDC Frontier/PII)	Forward primers (5'-3')	Reverse primers (5'-3')	Anneling temperature (0C)	Expected amplified product size (bp)	Structural annotation		Functional annotation	
								Sequence components of *kabuli* genome	*Kabuli* gene accession IDs	NCBI-KOG	TFs
Ca4_P3_33304176	Ca4	33304176	GAAAAAAAAAAAA/GAAAAAAAAAAA	TCCATCTCCAACAACATCAAA	AAATTTTGGATGGAGCCCTT	59.0	600	INTERGENIC	--	--	--
Ca4_P3_33308913	Ca4	33308913	GAAAA/GAAA	TCATTTTCCCCTCGTTTCTG	TTGGCCAAAACCTTTTGATG	60.0	183	DRR	Ca_24021	R	C2H2
Ca4_P3_33310326	Ca4	33310326	GAAAAAAAA/GAAAAAAA	TGCCTTGCTTTCTGGTTTTC	CGGTATATGTCACACTTCCGA	60.4	597	DRR	Ca_24021	R	C2H2
Ca4_P3_33312811	Ca4	33312811	CCGATCGA/CCGA	TTCACAGGCAAGAAATGTGC	CTGAATGGAAGTGGGAAGGA	59.8	131	INTERGENIC	--	--	--
Ca4_P3_33327000	Ca4	33327000	CA/C	AATATTCCCGTCGAATGTGC	TTTATTGCAACATCGCCTTTT	59.8	662	INTERGENIC	--	--	--
Ca4_P3_33327822	Ca4	33327822	TAAAAAAAAAA/TAAAAAAAAAAAA	CGAAAATTCGAATGGGTAGC	GGTCAAGTGTTTGCCGAAAT	59.5	516	INTERGENIC	--	--	--
Ca4_P3_33328067	Ca4	33328067	GAAAAAAAA/GAAAAAAAAA	CGAAAATTCGAATGGGTAGC	AGTATGATGGGGTCATGGGA	59.5	631	INTERGENIC	--	--	--
Ca4_P3_33329397	Ca4	33329397	TAAAAAAAA/TAAAAAAAAA	ACGTGGTCTCACGATAAGGC	CTCCATGTGAAAGTGCCAAA	60.1	552	INTERGENIC	--	--	--
Ca4_P3_33344478	Ca4	33344478	CATTTATTTATTTATTTATTT/CATTTATTTATTTATTT	AAGTTGATCTCAGAGCCCGA	ACAATCGGCTCACCAATTTC	60.0	824	INTERGENIC	--	--	--
Ca4_P3_33346959	Ca4	33346959	TT/TTAT	TTTTCGGTTTGTTCAAAGGC	GGAAAACATTGGGAAACCCT	60.1	766	INTERGENIC	--	--	--

INDEL marker IDs	Chromosomes/ unanchored scaffolds	Physical positions (bp)	InDels (*Kabuli* reference genome-CDC Frontier/PII)	Forward primers (5'-3')	Reverse primers (5'-3')	Anneling temperature (0C)	Expected amplified product size (bp)	Structural annotation		Functional annotation	
								Sequence components of *kabuli* genome	*Kabuli* gene accession IDs	NCBI-KOG	TFs
Ca4_P3_33347892	Ca4	33347892	AGG/AG	AGCCCAAGACGATCAT CAAC	TCCTAAAAGACTGCCC GCTA	60.1	855	INTERGENIC	--	--	--
Ca4_P3_33348179	Ca4	33348179	ATT/AT	AGGTTGAGCGATTGGA GAAA	TCCTAAAAGACTGCCC GCTA	59.8	378	INTERGENIC	--	--	--
Ca4_P3_33348851	Ca4	33348851	T/TCTTATA	TTTGGCATCTTCTCAGG CTT	CTCGTGTCTGCACCTT CAAA	60.0	799	INTERGENIC	--	--	--
Ca4_P3_33353051	Ca4	33353051	TTG/TTGTG	ACCAATGCCGCTTAATT GAC	AGACGATCAATTGGTT TGCC	60.0	396	INTERGENIC	--	--	--
Ca4_P3_33413394	Ca4	33413394	ATTTTTTTTTT/ATTTTTTTTT	TAGAGCGGGGCTCACT TAAA	GCGGAGATTGCCATAT CACT	60.0	447	INTERGENIC	--	--	--
Ca4_P3_33460204	Ca4	33460204	AAACTTAACTTA/AAACTTA	CGAATGCACTTGAAGA CGAA	GACAAAAGCTACGGTG GAGC	60.0	309	DRR	Ca_24024	C	M-type
Ca4_P3_33533176	Ca4	33533176	CAAAAAAAAAAA/CAAAAAAAAAA	GAACCAAGAGGCGATT GAAG	TCCATTTTGGGTGAAC CATT	59.8	216	INTERGENIC	--	--	--
Ca4_P3_33557759	Ca4	33557759	GTA/G	TACCCTTTGCTAGCCC CTTT	TTTTGAGGACTTTCGG GTTG	60.1	462	INTERGENIC	--	--	--
Ca4_P3_3355845	Ca4	3355845	CGA/CGATAAGGA	GTTGGGTATTGGTGTC CCAC	GAGGGTGATCAGCTTT CTCG	60.0	589	INTERGENIC	--	--	--
Ca4_P3_3356082	Ca4	3356082	A/AGCCAAGC	TCACCGGAAAGTAACT CGCT	ATCGATATTCTCCGCA CCTG	59.9	622	INTERGENIC	--	--	--

INDEL marker IDs	Chromosomes/ unanchored scaffolds	Physical positions (bp)	InDels (*Kabuli* reference genome-CDC Frontier/PII)	Forward primers (5'-3')	Reverse primers (5'-3')	Anneling temperature (0C)	Expected amplified product size (bp)	Structural annotation		Functional annotation	
								Sequence components of *kabuli* genome	*Kabuli* gene accession IDs	NCBI-KOG	TFs
Ca4_P3_33603011	Ca4	33603011	ACC/AC	CCTCTTCGAACTTGTGAGGC	CCTTCGCACCAATAGCAAAT	60.0	552	INTRON	Ca_24027	R	--
Ca4_P3_33615467	Ca4	33615467	ATT/ATTT	GGTCCTTGTTGAGTCCAACG	CTCAAAAACCTGAGCATGGC	60.5	646	INTERGENIC	--	--	--
Ca4_P3_33615658	Ca4	33615658	AG/A	GGTCCTTGTTGAGTCCAACG	AATGGAATAGCGGAGTGTGC	60.5	898	INTERGENIC	--	--	--
Ca4_P3_33617796	Ca4	33617796	AAAAGAA/AAA	CCCCGATTTGGTCCTTTATT	TGCTAGTTGTTGCGACCAAA	60.0	765	INTERGENIC	--	--	--
Ca4_P3_33618635	Ca4	33618635	T/TGTAGTGAA	GTTCGGCCAGATGCTGTAAT	CAGTTTGCTGCAACCATCAT	60.1	277	INTERGENIC	--	--	--
Ca4_P3_33619158	Ca4	33619158	GTATATATATATATATATATATATAT/GTATATATATATATAT	GAACATGCTGAAAACGGGAT	TGAGGGCAATGACAAACAAA	59.9	122	INTERGENIC	--	--	--
Ca4_P3_33619321	Ca4	33619321	TAAAAAAAA/TAAAAA	GAACATGCTGAAAACGGGAT	CCAATTCCAAGGTCATCTGG	59.9	448	INTERGENIC	--	--	--
Ca4_P3_33620200	Ca4	33620200	TACACA/TACA	GCCATGCTACCATTTGGATT	TTACCGACGAACAAGCAGTG	59.8	603	INTERGENIC	--	--	--
Ca4_P3_33626156	Ca4	33626156	TG/T	AGGGAGTAGGAGGGGAGTGA	GCCTCTTTGATCGAGAACCA	60.1	809	INTERGENIC	--	--	--
Ca4_P3_33633372	Ca4	33633372	GTTTTTTT/GTTTTTT	ACGAGGAAACATATCGTCCG	ATGTCCACACATTGGGTGTC	60.0	726	INTERGENIC	--	--	--

INDEL marker IDs	Chromosomes/ unanchored scaffolds	Physical positions (bp)	InDels (*Kabuli* reference genome-CDC Frontier/PII)	Forward primers (5'-3')	Reverse primers (5'-3')	Anneling temperature (0C)	Expected amplified product size (bp)	Structural annotation		Functional annotation	
								Sequence components of *kabuli* genome	*Kabuli* gene accession IDs	NCBI-KOG	TFs
Ca4_P3_33646948	Ca4	33646948	AAGAGAGAGAGAGAGAGAGAGAGAG/ AAGAGAGAGAGAGAGAGAGAGAGAGAG	TTTTGGATTGCTCCAGAACC	GCTGATTCACTTGTGCAGGA	60.1	359	INTRON	Ca_24030	L	bHLH
Ca4_P3_33654066	Ca4	33654066	CAAAAAAAAAAAA/CAAAAAAAAAAA	CTTGTGTTTGTGTTGTGGGG	TTTGTTCGCACACCAAACTT	59.9	711	INTERGENIC	--	--	--
Ca4_P3_33685602	Ca4	33685602	TT/TTAT	GAAGAGTTACGCGGACAAGC	AGGAAGAGTGGAGTTGCGAA	60.0	768	INTERGENIC	--	--	--
Ca4_P3_33705709	Ca4	33705709	A/AT	ACAAACGCGATGTGAACAAA	TCCCCCTTATTCAGAAGGCT	60.2	564	INTERGENIC	--	--	--
Ca4_P3_33713040	Ca4	33713040	C/CAAAAAGTT	GAGATGGTGCCATGAGTTGA	AGTGAAGGGATTGGATGCAG	59.6	393	INTERGENIC	--	--	--
Ca4_P3_33714267	Ca4	33714267	T/TCTTAATC	GAACACGTTGTTAGACCGGC	TGACAAAATGGGGAGAAAGG	60.6	713	INTERGENIC	--	--	--
Ca4_P3_33714529	Ca4	33714529	ATT/ATTGTT	CGTTGTTAGACCGGCTCTTG	TGGGCTGACATAATCACCAA	60.8	940	INTERGENIC	--	--	--
Ca4_P3_33715954	Ca4	33715954	A/AAAAAT	TTGGGTACGTTGGTGTAGCA	ATTGACTGGGCACCATGATT	60.0	634	INTERGENIC	--	--	--
Ca4_P3_33717821	Ca4	33717821	TAAAAAAAAAAAAAAAAAA/TAAAAAAAAAAA	CCAAAAACCCCTCTTTTTCC	CCACCAAAATTTGTCCAACC	59.8	730	INTERGENIC	--	--	--
Ca4_P3_33732916	Ca4	33732916	CAAAAAAAAAAAA/CAAAAAAAAAA	ACGGTTAACGGATTACAGCG	TGCACGTCTGTCGAGAAGTT	60.0	482	INTERGENIC	--	--	--

INDEL marker IDs	Chromosomes/ unanchored scaffolds	Physical positions (bp)	InDels (*Kabuli* reference genome-CDC Frontier/PII)	Forward primers (5'-3')	Reverse primers (5'-3')	Anneling temperature (0C)	Expected amplified product size (bp)	Structural annotation		Functional annotation	
								Sequence components of *kabuli* genome	*Kabuli* gene accession IDs	NCBI-KOG	TFs
Ca4_P3_33733792	Ca4	33733792	AATATTTGTTTATATT/AATATTTGTTTATATTTGTTTATATT	CGTTATTCCCATATGAGAGGC	TGTGATGTTGGACGTGTGTG	58.5	280	INTERGENIC	--	--	--
Ca4_P3_33743516	Ca4	33743516	CAAAAAA/CAAAAA	TGTAGCTGGTGTCCACGAAG	AATGCAAGAATCCACCTTGG	59.9	435	INTERGENIC	--	--	--
Ca4_P3_33763932	Ca4	33763932	GTTTTTTTTTTT/GTTTTTTTTTTTTT	TTGAACACCTTGGGAAGAGG	CCCAAACCGCAAACTAAAAA	60.1	787	INTRON	Ca_20373	--	--
Ca4_P3_33788165	Ca4	33788165	GAAAAAAAAAAA/GAAAAAAAAA	CGTGGGATGAGTGGAACTCT	TGAAGAAAAGGGCATGAACC	60.1	499	INTERGENIC	--	--	--
Ca4_P3_33828542	Ca4	33828542	AATA/AATATA	TACATTGGTGCAACCTGCAT	TTGCCATGACACACCTGATT	60.0	703	INTRON	Ca_20369	Q	--
Ca4_P3_33835105	Ca4	33835105	CAAAAAAA/CAAAAAAAAA	TTTTCACCAAATCACTTGAAAGAA	TCGAGCCTTATTGTGGATGA	60.0	654	INTERGENIC	--	--	--
Ca4_P3_33835153	Ca4	33835153	AAGCT/A	TTTTCACCAAATCACTTGAAAGAA	TCGAGCCTTATTGTGGATGA	60.0	654	INTERGENIC	--	--	--
Ca4_P3_33836766	Ca4	33836766	GAAAAAAAAA/GAAAAAAAAAAA	GTTTTGTGATGGGGGATGAC	CCATTGAAAAACGAACGACC	60.0	205	INTERGENIC	--	--	--
Ca4_P3_33843176	Ca4	33843176	TAAAAAAAAA/TAAAAAAAAAA	CGCTTCTTCTGTCCAACACA	TGGTGAAACCCACAAGACAA	60.0	291	INTERGENIC	--	--	--
Ca4_P3_33845460	Ca4	33845460	ATTTT/ATTT	TTCAAAACGTAAAATTAAGTGCGA	TGTAGGGATGCTCAACTTGG	60.1	592	INTERGENIC	--	--	--

INDEL marker IDs	Chromosomes/ unanchored scaffolds	Physical positions (bp)	InDels (*Kabuli* reference genome-CDC Frontier/PII)	Forward primers (5'-3')	Reverse primers (5'-3')	Anneling temperature (0C)	Expected amplified product size (bp)	Structural annotation		Functional annotation	
								Sequence components of *kabuli* genome	*Kabuli* gene accession IDs	NCBI-KOG	TFs
Ca4_P3_33857426	Ca4	33857426	AAAAATTAAAAT/AAAAATTAAAATTAAAAT	GTTTTGTGATGGGGGATGAC	CAAATTTGGCGTTGTTCCTT	60.0	611	INTERGENIC	--	--	--
Ca4_P3_33884406	Ca4	33884406	GTTTTTTTTT/GTTTTTTTTTT	AGGCTAGTGCATCATCCAGG	GTACCCCAATTTTTCCCGTT	60.2	303	INTERGENIC	--	--	--
Ca4_P3_33912297	Ca4	33912297	ATATCT/AT	AGAATCCAAATCGAAGCCCT	ACCCGCTCTAAGTCCAAAGC	60.0	606	INTERGENIC	--	--	--
Ca4_P3_33921285	Ca4	33921285	ATGCT/A	TGGGTGGTTGACACTTGAAA	CAGAGGAAAATGGGGTCAGA	60.0	560	INTRON	Ca_20365	O	GATA
Ca4_P3_33922360	Ca4	33922360	CAAA/CAAAA	AGTCAGCCTGCGCTACAAGT	GCCAGACCATAAGTAGGCCA	60.2	365	INTRON	Ca_20365	O	GATA
Ca4_P3_33924249	Ca4	33924249	ATAGT/ATAGTAGT	CCGTCATTACATGTTGCTGG	TCGAGAGTTGCTCCTGGTTT	60.0	729	INTRON	Ca_20365	O	GATA
Ca4_P3_33953368	Ca4	33953368	CAT/CATATCATAT	TATTTTGGGATTGTGGGCTC	AGATTAGGGCCTCAGGAACC	59.8	523	INTERGENIC	--	--	--
Ca4_P3_33972054	Ca4	33972054	AAACAACA/AAACAACAACA	TGAGTTCCAAAATCGGGAAA	AATTGGCTAAGCAAGCTCCA	60.4	500	INTERGENIC	--	--	--
Ca4_P3_34006503	Ca4	34006503	GA/GAGTGTTA	ACAGAGGAAGCTGCCACCTA	GAGTTCAACCAATGGCGTCT	60.0	653	INTERGENIC	--	--	--
Ca4_P3_34016739	Ca4	34016739	CTT/CT	TTGCTTTCTGAATGGCAGTG	TGGAAGGATGGGTTGAGAAG	60.0	492	INTERGENIC	--	--	--

INDEL marker IDs	Chromosomes/ unanchored scaffolds	Physical positions (bp)	InDels (*Kabuli* reference genome-CDC Frontier/PII)	Forward primers (5'-3')	Reverse primers (5'-3')	Anneling temperature (0C)	Expected amplified product size (bp)	Structural annotation		Functional annotation	
								Sequence components of *kabuli* genome	*Kabuli* gene accession IDs	NCBI-KOG	TFs
Ca4_P3_34055460	Ca4	34055460	GAAAATTAATGAAAAAATTAATGAAAAAATTAAT/GAAAATTAATGAAAAAATTAAT	CGGTTGAATAAATGTGGCAA	ACCAGCTGAAGGCAAAAATG	59.4	524	INTERGENIC	--	--	--
Ca4_P3_34070757	Ca4	34070757	ATTTTTTTTTT/ATTTTTTTTTTT	TTCCACAAAAATGAGCTCAAAA	GGAGATCAAAATTGCCGACT	59.7	901	INTERGENIC	--	--	--
Ca4_P3_34083271	Ca4	34083271	AGGGG/AGGG	AAAGGGACCAATGGATTTGA	AATCGGACTTACGGTGAACG	59.2	330	INTERGENIC	--	--	--
Ca4_P3_34086894	Ca4	34086894	ATTTTTTTTT/ATTTTTTTTTT	TGAAAGTTTGAAGGGGTTGG	AATTTCTTCCCGAAGCATCC	59.9	431	INTERGENIC	--	--	--
Ca4_P3_34121290	Ca4	34121290	CAAAAAAAAAAAAA/CAAAAAAAAAAAAAAAA	GCATATTGGGAGAAATGAGCA	GGATGCATGGGAGTTACCAC	60.1	242	INTERGENIC	--	--	--
Ca4_P3_34169598	Ca4	34169598	TTATATATATATATATATATATATATATAT/TTATATATATATATAT	TCAAAGTGGCTCACAAGTGC	TCGACTCAAGAAGGCAAGTG	60.0	781	INTERGENIC	--	--	--
Ca4_P3_34169705	Ca4	34169705	CAATGA/CAATGATAATGA	TCAAAGTGGCTCACAAGTGC	TCGACTCAAGAAGGCAAGTG	60.0	781	INTERGENIC	--	--	--
Ca4_P3_34188062	Ca4	34188062	GCCC/GCC	TTGTCGTGAGAATCGCGTAG	TGAGCCCTTGACTTAATGCC	60.0	427	INTERGENIC	--	--	--
Ca4_P3_34227066	Ca4	34227066	A/AG	ACGGCAAAAACCAGTGAAAT	TGAGGAAAATTGAGACCGGA	59.5	327	INTERGENIC	--	--	--
Ca4_P3_34228273	Ca4	34228273	CTTT/CT	TGGAAGTTGGGAGAAACACC	ACGATTCGTCACACAATGGA	59.9	612	INTERGENIC	--	--	--

INDEL marker IDs	Chromosomes/ unanchored scaffolds	Physical positions (bp)	InDels (*Kabuli* reference genome-CDC Frontier/PII)	Forward primers (5'-3')	Reverse primers (5'-3')	Anneling temperature (0C)	Expected amplified product size (bp)	Structural annotation		Functional annotation	
								Sequence components of *kabuli* genome	*Kabuli* gene accession IDs	NCBI-KOG	TFs
Ca4_P3_34239824	Ca4	34239824	GTTTTTTTT/GTTTTTTT	GGTTTTACGGCAACATCAGG	ACAAATCAGAGCTGGTCGCT	60.4	711	DRR	Ca_20356	L	--
Ca4_P3_34245869	Ca4	34245869	ATTTTTTTT/ATTTTTTTTTT	AAAGAGAATGGGTGTGGGTG	ACTTGGGAACCCGAAGAAGT	59.8	212	INTERGENIC	--	--	--
Ca4_P3_34257490	Ca4	34257490	CTTT/CT	TCCATTTCCGAGAAATCCAT	CACAAAGACCAAAGCCCAAT	59.3	490	INTERGENIC	--	--	--
Ca4_P3_34262481	Ca4	34262481	TAAAAAAAAA/TAAAAAAAAAA	GACCACCTACGCCACACTTT	TGTTCCAATTCCTCGTGTCA	60.0	807	INTERGENIC	--	--	--
Ca4_P3_34262693	Ca4	34262693	AATATATATATATATATATATATATATATATATA/AATATATATATATATATATATATA	TCTTGCCACCAACAAAACAA	TGTTCCAATTCCTCGTGTCA	60.1	223	INTERGENIC	--	--	--
Ca4_P3_34329843	Ca4	34329843	CTTTTTTTTT/CTTTTTTTT	TGCAGACCCTTGGAAAAAGT	GCCACTATTGGCTGGGTTAC	59.7	685	INTERGENIC	--	--	--
Ca4_P3_34339778	Ca4	34339778	ATTTTTTTTTT/ATTTTTTTTTTT	CAGGATTTGGTTTAGCCATTTT	TGTGGAAGGAAAACCAGAGG	59.4	355	INTERGENIC	--	--	--
Ca4_P3_34375164	Ca4	34375164	ATTTTTTT/ATTTTTT	CAAATCTTCTTCAAATCTTGACCAT	CTATCGACCAAGCCACCATT	59.9	768	INTERGENIC	--	--	--
Ca4_P3_34388181	Ca4	34388181	GAAA/GAAAA	GTTGGTTCCCTAGCACCGTA	CTGTCAATTGAGTTGCGACG	60.0	484	INTERGENIC	--	--	--
Ca4_P3_34405663	Ca4	34405663	GAAAAAAAAAA/GAAAAAAAAAAA	GTTCGAAAACTGCAGCAACA	ATGATTCATCAGAGTCCGCC	60.0	280	INTERGENIC	--	--	--

INDEL marker IDs	Chromosomes/ unanchored scaffolds	Physical positions (bp)	InDels (*Kabuli* reference genome-CDC Frontier/PII)	Forward primers (5'-3')	Reverse primers (5'-3')	Anneling temperature (0C)	Expected amplified product size (bp)	Structural annotation		Functional annotation	
								Sequence components of *kabuli* genome	*Kabuli* gene accession IDs	NCBI-KOG	TFs
Ca4_P3_34454517	Ca4	34454517	TAAAAAG/TAAAAAGAAAAAAAAG	AGGGGTGGTGATTCTCACAG	ATTTTTGGAGGACTTTCGGG	60.0	285	DRR	Ca_20352	--	B3
Ca4_P3_34458862	Ca4	34458862	CAAAA/CAAAAA	CCAAACGTTCTCCCACAACT	GAGGATGCCAAAATGCCTAA	60.0	744	DRR	Ca_20351	--	--
Ca4_P3_34468802	Ca4	34468802	GTTTTTTT/GTTTTTTTT	GGGAAGCTTGTTTGCTTCTG	TAAGAGTGAGTGGGTTGGGC	60.0	353	INTERGENIC	--	--	--
Ca4_P3_34471336	Ca4	34471336	ATTATTTATTTATTTATTTAT/ATTATTTATTTATTTATTTATTTAT	AACGTGACATTTTCGGAAGC	TCAAGTTTTCCGATATAGCCGT	60.1	577	INTERGENIC	--	--	--
Ca4_P3_34473954	Ca4	34473954	CTTTTTTT/CTTTTTTTT	ACCCAAATCCAGTGTTCTGC	GGCCTTGTCGAATGATTGTT	60.0	839	INTERGENIC	--	--	--
Ca4_P3_34482261	Ca4	34482261	GCCGCC/GCCGCCGTAAACCGCC	ACGCCATCTGCCATATGTTT	TTTGGGGTGATGGAAATGAT	60.4	497	INTRON	Ca_20350	R	G2-like
Ca4_P3_34492879	Ca4	34492879	TAAAAAAAAA/TAAAAAAAAAA	CTGATCAGTCGTTGGGAGGT	TGAGTTGATCGTTTCCACCA	60.1	538	DRR	Ca_20350	R	G2-like
Ca4_P3_34525292	Ca4	34525292	TAAAA/TAAAAA	GGACAACCTGGGAAAAGTGA	CTTCGATCCGTGTAAGCCAT	59.9	212	INTERGENIC	--	--	--
Ca4_P3_34566361	Ca4	34566361	TTTCT/TT	CGCCTAAGGACTATGCAAGC	AAAATCCAACAATCTCCCCC	60.0	761	INTERGENIC	--	--	--
Ca4_P3_34566424	Ca4	34566424	ATT/AT	CCCTGAGACTCACGAAGAGG	AAAATCCAACAATCTCCCCC	60.0	612	INTERGENIC	--	--	--

INDEL marker IDs	Chromosomes/ unanchored scaffolds	Physical positions (bp)	InDels (*Kabuli* reference genome-CDC Frontier/PII)	Forward primers (5'-3')	Reverse primers (5'-3')	Anneling temperature (0C)	Expected amplified product size (bp)	Structural annotation		Functional annotation	
								Sequence components of *kabuli* genome	*Kabuli* gene accession IDs	NCBI-KOG	TFs
Ca4_P3_34756	Ca4	34756	AATATATATATATATATATATA/AATATATATATATATATATA	ACACATGACTTCCTTTGCCC	TTGCAACAACAAAAACTGCC	60.0	673	INTERGENIC	--	--	--
Ca4_P3_34885596	Ca4	34885596	A/ACGAT	GAATGTGGGGGCTGTTCTTA	TTAATCTCGGGCGAAATACG	59.9	631	INTERGENIC	--	--	--
Ca4_P3_3493500	Ca4	3493500	TGAGAGAGAGAGAGAGAGA/TGAGAGAGAGAGAGAGA	TTCACGAAATTGGTCCCTTT	TGTCCCCACTTGACAAAAAGA	59.4	529	INTERGENIC	--	--	--
Ca4_P3_34938595	Ca4	34938595	CATATATATATATATATATATAT/CATATATATATATATATATAT	TTTAGAAGAGGGTCCTGCGA	AGGGAGGTTTGTGTTTGACG	59.9	774	INTERGENIC	--	--	--
Ca4_P3_35033996	Ca4	35033996	TTTCCG/T	TTCAATGTCTCCAACCACCA	TGCAACTGAGAAATTGCGAC	59.9	520	INTERGENIC	--	--	--
Ca4_P3_35213666	Ca4	35213666	TAAAAAAAAA/TAAAAAAAAAA	TGGAGGAAAAGAGGGTGATG	ATTGAGGGTGATTGGTTGGA	60.0	580	INTRON	Ca_20014	U	M-type
Ca4_P3_3529103	Ca4	3529103	GATATATATATATATATATATATA/GATATATATATATATATATATATA	ATGCACTTGGGCCAGATTAG	AACTCGTTTTGATATGGGCG	60.1	442	INTERGENIC	--	--	--
Ca4_P3_35319446	Ca4	35319446	ATTTTTTTTT/ATTTTTTTTTT	GGTCAGTTCATCCTAGGGCA	TTTTTGGTTGCCTAAGTGGG	60.1	462	INTERGENIC	--	--	--
Ca4_P3_35345914	Ca4	35345914	GTTTT/GTTTTTT	TTTGTGAAGGGATTTGGCTC	ATGCCTCTCTATGCAGCGTT	60.1	763	INTRON	Ca_20018	Z	NAC
Ca4_P3_35352556	Ca4	35352556	CTTTT/CTTTTT	CCATCAAAGTCCACGCCTAT	TCTCAGAGAACGACCTCCTATT	60.0	166	INTERGENIC	--	--	--

INDEL marker IDs	Chromosomes/ unanchored scaffolds	Physical positions (bp)	InDels (*Kabuli* reference genome-CDC Frontier/PII)	Forward primers (5'-3')	Reverse primers (5'-3')	Anneling temperature (0C)	Expected amplified product size (bp)	Structural annotation		Functional annotation	
								Sequence components of *kabuli* genome	*Kabuli* gene accession IDs	NCBI-KOG	TFs
Ca4_P3_35366554	Ca4	35366554	TTAGCTGATAG/TTAGCTGATAGCTGATAG	TCGAAAAACAAAAATAACCCG	GATTTACCATATACTCCTTCGCTT	59.0	498	INTERGENIC	--	--	--
Ca4_P3_35432013	Ca4	35432013	TTAT/TTATAT	TGTTCAACATCCACGAACAA	TGCCATACTCATGTCGGAAA	58.5	669	INTERGENIC	--	--	--
Ca4_P3_35432836	Ca4	35432836	TTATATATATATATATATATAT/TTATATATATATATATATATAT	TTTCCGACATGAGTATGGCA	TTTGGAAAGTAACACTGCACG	60.1	785	INTERGENIC	--	--	--
Ca4_P3_35433246	Ca4	35433246	TAAAAA/TAAAAAA	CACGTGCAGTGTTACTTTCCA	TGACGAATTTCAAAAACCTGC	59.8	491	INTERGENIC	--	--	--
Ca4_P3_35433291	Ca4	35433291	TTATATATATATATATATATATAT/TTATATATATATATATATATATATATATATATAT	CACGTGCAGTGTTACTTTCCA	TGACGAATTTCAAAAACCTGC	59.8	491	INTERGENIC	--	--	--
Ca4_P3_35457008	Ca4	35457008	TTGC/T	AATTGCAGAAAAATGCGGTC	TTGTACCAGATATTGGCCCG	60.1	776	INTERGENIC	--	--	--
Ca4_P3_3548502	Ca4	3548502	TC/T	CACCTGCAGTGAAAAGACCA	AGGCAGAAATGTGGATGGAG	59.9	380	INTERGENIC	--	--	--
Ca4_P3_35523540	Ca4	35523540	AAT/AATAT	ACATAGCATCTGTCCTGGGC	TCATGGGTTCGTAACAGCAA	60.1	340	INTRON	Ca_22434	--	--
Ca4_P3_35708239	Ca4	35708239	GTTTTTT/GTTTTT	AAAGGACCAAGTCGGAAAAA	TGCTTCAAGATCAATCTGCG	58.7	578	INTERGENIC	--	--	--
Ca4_P3_35720299	Ca4	35720299	TTTATTATTATTATTATTATTATTATTATTATTATT/TTTATTATTATTATTATTATTATTATTATT	GCTATAGCTGGCGACTCCAC	ACTCTTCCAAAGTAGGCGCA	60.0	688	INTERGENIC	--	--	--

INDEL marker IDs	Chromosomes/ unanchored scaffolds	Physical positions (bp)	InDels (*Kabuli* reference genome-CDC Frontier/PII)	Forward primers (5'-3')	Reverse primers (5'-3')	Anneling temperature (0C)	Expected amplified product size (bp)	Structural annotation		Functional annotation	
								Sequence components of *kabuli* genome	*Kabuli* gene accession IDs	NCBI-KOG	TFs
Ca4_P3_35779928	Ca4	35779928	AACACACACACACACACA/AACACACACACACACACACACA	GGCATATATGTCAATTAGGGGA	TGCTTTTTGTGGTTGCTTTG	57.9	765	INTERGENIC	--	--	--
Ca4_P3_35822348	Ca4	35822348	TGG/TG	GATCAAATCGAGGACCGAAA	CGAACAAAACGATGGGAAGT	60.0	695	INTERGENIC	--	--	--
Ca4_P3_3582998	Ca4	3582998	TATAGATAGATAGATAG/TATAGATAGATAGATAGATAG	CCACAAATGATGTGCCAAAG	TGGGTTTTAATTTTGCGGAG	60.0	326	INTERGENIC	--	--	--
Ca4_P3_35975112	Ca4	35975112	TTTATTATTATTATTATTATTATTATTATTATTATTATTATTAT/TTTATTATTATTATTATTATTATTATTATTATTATTATTATTATTATTATTAT	AGCCAAAAGTGGGTAACGTG	TTTTGTGTAACGCCCAGTCA	60.0	193	INTERGENIC	--	--	--
Ca4_P3_3602818	Ca4	3602818	GTTTTTTT/GTTTTTT	GAGCATCTCTAACGGGAGGA	TCGTCTTTTATGACCAACTCCC	59.4	541	DRR	Ca_12158	--	--
Ca4_P3_36079126	Ca4	36079126	TTGTG/TTGTGTG	ATTTTGATGCTCACGGTTCC	CGGAGATGATGGTTCAGGTT	59.9	595	INTERGENIC	--	--	--
Ca4_P3_36079437	Ca4	36079437	TGTCAAGTCAA/TGTCAA	ATTTTGATGCTCACGGTTCC	GGCGTACCATGCTTTGATTT	59.9	979	CDS (FRAME SHIFT)	Ca_14855	--	--
Ca4_P3_36138277	Ca4	36138277	ATT/AT	ACCATGAAAGACATGGGAGC	CGGAGTACCCGACCAAACTA	59.9	436	INTERGENIC	--	--	--
Ca4_P3_36175888	Ca4	36175888	AT/A	AGTTGCAGCGAGTCTGGATT	TCCGACATGCAACTCTTCAG	60.0	437	INTERGENIC	--	--	--
Ca4_P3_36299251	Ca4	36299251	ATTTT/ATT	AACCCAAGAGCGCACATATT	GAGCCAAACACTGCATCAGA	59.6	444	INTERGENIC	--	--	--

INDEL marker IDs	Chromosomes/ unanchored scaffolds	Physical positions (bp)	InDels (*Kabuli* reference genome-CDC Frontier/PII)	Forward primers (5'-3')	Reverse primers (5'-3')	Anneling temperature (0C)	Expected amplified product size (bp)	Structural annotation		Functional annotation	
								Sequence components of *kabuli* genome	*Kabuli* gene accession IDs	NCBI-KOG	TFs
Ca4_P3_36300197	Ca4	36300197	GGTCGGCGTCGGCGT/GGTCGGCGTCGGCGTCGGCGT	ACATGTTGCCGTTGTCAAAA	AACCCAGGCCCAAACTAACT	60.0	312	INTERGENIC	--	--	--
Ca4_P3_36310356	Ca4	36310356	ATTTT/ATTTTT	TTGATTCTTTTTAAAATTTTGCTCA	TTGGTGCGTTTCTCTAAACA	58.5	765	INTERGENIC	--	--	--
Ca4_P3_36335652	Ca4	36335652	TT/TTAT	TCCCTTGTGTTTGGTGTTGA	ACCCTAGGGGTATGAGCGAT	60.0	470	INTERGENIC	--	--	--
Ca4_P3_36336918	Ca4	36336918	TAAA/TATAAAA	ACAACAAGGGTGACCTCCAC	TGCTTGTCTATTCTGCGTCAA	59.9	736	INTERGENIC	--	--	--
Ca4_P3_36337139	Ca4	36337139	GC/G	TGCAATGTCAATATGAACCAAA	CCACAACCACTCATCACGAC	58.9	479	INTERGENIC	--	--	--
Ca4_P3_36337249	Ca4	36337249	TAAAATATTTGTTTCA/T	TGACGCAGAATAGACAAGCAA	CCACAACCACTCATCACGAC	59.6	341	INTERGENIC	--	--	--
Ca4_P3_36337433	Ca4	36337433	AT/ATTTTTTAACTAAATT	TTGACGCAGAATAGACAAGCA	TGAGTTTGTGATCGCTTTGC	59.6	709	INTERGENIC	--	--	--
Ca4_P3_36390176	Ca4	36390176	TATAATAATAA/TATAATAATAATAA	TATGCACGCTCTCTCTCCCT	AGGAGGAACCAAAACCGTCT	60.1	611	INTERGENIC	--	--	--
Ca4_P3_36394814	Ca4	36394814	AGAAGAT/AGAAGATAAAGAAGATGAAGAT	CCAACTGTTCTTCTCCCCAA	CTTCAATTCCTCAGAAGCGG	60.1	605	CDS (large-effect mutations)	Ca_14837	--	--
Ca4_P3_36410224	Ca4	36410224	A/AT	GGTGGGGTAGACACATGGAA	TCAATGTAAGACTCATTTTATTTCCC	60.6	693	INTERGENIC	--	--	--

INDEL marker IDs	Chromosomes/ unanchored scaffolds	Physical positions (bp)	InDels (*Kabuli* reference genome-CDC Frontier/PII)	Forward primers (5'-3')	Reverse primers (5'-3')	Anneling temperature (0C)	Expected amplified product size (bp)	Structural annotation		Functional annotation	
								Sequence components of *kabuli* genome	*Kabuli* gene accession IDs	NCBI-KOG	TFs
Ca4_P3_36424107	Ca4	36424107	TAAAAAAAAA/TAAAAAAA	GCAGTCGCACTGCACATAAT	ACAACAGCGCTTGTCAAAAA	59.9	788	DRR	Ca_14835	K	M-type
Ca4_P3_36427743	Ca4	36427743	TTT/TTTTCTT	ATGCATCTATTGAATGCCCA	TAGCATCCAGCGCTGTAAAA	58.9	148	INTERGENIC	--	--	--
Ca4_P3_36448542	Ca4	36448542	CTTT/CTTTT	CCAAAATATGGACATTCACGG	CTGCATGGCTTGAATAGCAA	60.1	928	INTRON	Ca_14833	S	--
Ca4_P3_36450396	Ca4	36450396	GTCTT/GTCTTCTT	GACACCAAATTGGGAAAGGA	GCGGTGACATGTACGATTTG	59.8	710	DRR	Ca_14833	S	--
Ca4_P3_36450797	Ca4	36450797	TTGTGT/TTGT	ATCACAAGCGATGTCAGTGC	GCGGTGACATGTACGATTTG	59.9	192	DRR	Ca_14833	S	--
Ca4_P3_36455905	Ca4	36455905	A/AC	GGTGGAGAGCTATTTGAGCG	AAGCAAAACTTCAGGAGCGA	60.0	668	INTRON	Ca_14832	T	YABBY
Ca4_P3_36457501	Ca4	36457501	TT/TTGTTGAT	TGGGGGTCAATTTGAATGTT	ACCAATCTGTGGACTGCTCA	60.0	516	DRR	Ca_14832	T	YABBY
Ca4_P3_36463027	Ca4	36463027	CAAAAA/CAAA	TCAGCCATATGACCAATCCA	AAACCCCCATTTGTTGTTGA	59.9	692	INTRON	Ca_14831	--	--
Ca4_P3_36465756	Ca4	36465756	TC/T	CAGGAATTCGATCAGGGAAA	GTTTGAAGGCCTTGTCTTGC	60.0	256	INTRON	Ca_14830	E	--
Ca4_P3_36474350	Ca4	36474350	ACATAATTCA/ACATAATTCATAATTCA	CAAACCACATCACCCTCCTT	GCTACACCGACCACCTGAAT	59.8	585	INTERGENIC	--	--	--

INDEL marker IDs	Chromosomes/ unanchored scaffolds	Physical positions (bp)	InDels (*Kabuli* reference genome-CDC Frontier/PII)	Forward primers (5'-3')	Reverse primers (5'-3')	Anneling temperature (0C)	Expected amplified product size (bp)	Structural annotation		Functional annotation	
								Sequence components of *kabuli* genome	*Kabuli* gene accession IDs	NCBI-KOG	TFs
Ca4_P3_36478044	Ca4	36478044	AATATA/AATA	AAAGTGATAACCGATGAGTGACAA	AAGGCTCCACATGAACAAGG	59.9	706	INTERGENIC	--	--	--
Ca4_P3_36496727	Ca4	36496727	ATTT/ATTTT	TGGCTTTCACATTGTTTCCA	CAGTCCTGCTTTATGGCTGA	60.1	500	INTERGENIC	--	--	--
Ca4_P3_36502639	Ca4	36502639	TCCCC/TC	GTGGCAAAATGGATCGAACT	GCCGTCTAGTGTTTCTTTAATGACT	59.9	551	INTERGENIC	--	--	--
Ca4_P3_36530142	Ca4	36530142	TAAAAAAAAA/TAAAAAAAA	TTGGTGTAATTTGATTTCTTCCC	TGAACTGTGTAGCCAAAAACG	59.3	778	INTERGENIC	--	--	--
Ca4_P3_36537541	Ca4	36537541	TTT/TTTCTT	TCGAAACGCTTATCTTGGCT	GGGACCGCCTATGTATGAGA	60.0	382	DRR	Ca_14825	--	B3
Ca4_P3_36545458	Ca4	36545458	GACACACACA/GACACACA	CCTCCGCAAAAAGGATATGA	GTCACATGGAAAGGGCATCT	60.0	915	INTERGENIC	--	--	--
Ca4_P3_36565480	Ca4	36565480	T/TAAACCCC	CACGCTTTCTCCCAAAACAT	GGCACTGAGATCAACATGAGG	60.1	603	DRR	Ca_14822	--	--
Ca4_P3_36569199	Ca4	36569199	TTGTC/TTGTCCGTGTC	ATGATAGCATGTGCACGGAA	TGTATGCTGAGGGGATGTCA	60.1	656	INTRON	Ca_14821	DT	--
Ca4_P3_36588362	Ca4	36588362	TTTAAATATTAAATATT/TTTAAATATTAAATATTAAATATT	CCCCCTTCCAAAATTGAGAT	GAAGTGGCACATGGTGATTG	60.1	332	INTERGENIC	--	--	--
Ca4_P3_36589789	Ca4	36589789	TAAA/TAAAAA	TTTTAATCCTAAACAAATGAGTCCAA	TGGAGGAACAATGAATGTCG	59.4	496	INTERGENIC	--	--	--

INDEL marker IDs	Chromosomes/ unanchored scaffolds	Physical positions (bp)	InDels (*Kabuli* reference genome-CDC Frontier/PII)	Forward primers (5'-3')	Reverse primers (5'-3')	Anneling temperature (0C)	Expected amplified product size (bp)	Structural annotation		Functional annotation	
								Sequence components of *kabuli* genome	*Kabuli* gene accession IDs	NCBI-KOG	TFs
Ca4_P3_36593400	Ca4	36593400	TAA/TAAGTAAGAAGAA	TTGCAAACACAATGCCAAAT	TCTCAGGTTGAGTTGAGGGG	60.0	430	INTERGENIC	--	--	--
Ca4_P3_36607300	Ca4	36607300	TTT/TTTTTTAGTT	ACGACATCCACACTCAACCA	TGTGGGACGGATGGAGTATT	60.0	360	INTERGENIC	--	--	--
Ca4_P3_36608717	Ca4	36608717	TCC/TC	GCTCTGACCAAATCTGACCC	TTCAGCAGCGTGAATCAAGT	59.7	361	INTERGENIC	--	--	--
Ca4_P3_36608761	Ca4	36608761	CGCTGCTG/CGCTG	GCTCTGACCAAATCTGACCC	CTTTCGCGACCATGGTAATC	59.7	601	INTERGENIC	--	--	--
Ca4_P3_36609336	Ca4	36609336	AC/A	CATGGTCGCGAAAGTCAATA	CAACACGACAACACAAAGGG	59.7	829	INTERGENIC	--	--	--
Ca4_P3_36616620	Ca4	36616620	AGTGTGT/AGTGTGTGT	TGAAGTGAGTGCAATCCAGC	TCTGGAACCAATTCCCTTTG	60.0	858	DRR	Ca_14818	I	WRKY
Ca4_P3_36620937	Ca4	36620937	CCATTCA/CCA	ACTATGCTCTTGTTTGGCCC	TCAAAATCATGTTGGAGGCA	59.2	622	CDS (FRAME SHIFT)	Ca_14818	I	WRKY
Ca4_P3_36622112	Ca4	36622112	AGTGTTACGTGTTACGTGTT/AGTGTTACGTGTT	ACAAGTCAAAACCGCCTGAC	ATGCTGCCATCAGTAGGTCC	60.2	427	INTRON	Ca_14818	I	WRKY
Ca4_P3_36638657	Ca4	36638657	GAAAA/GAAAAA	CTGGGATTATGGGGTCATTG	ATTCAGCTTAGTGGCAAGGC	60.0	446	DRR	Ca_14816	H	--
Ca4_P3_36638983	Ca4	36638983	TA/T	CTGGGATTATGGGGTCATTG	ATTCAGCTTAGTGGCAAGGC	60.0	446	DRR	Ca_14816	H	--

INDEL marker IDs	Chromosomes/ unanchored scaffolds	Physical positions (bp)	InDels (*Kabuli* reference genome-CDC Frontier/PII)	Forward primers (5'-3')	Reverse primers (5'-3')	Anneling temperature (0C)	Expected amplified product size (bp)	Structural annotation		Functional annotation	
								Sequence components of *kabuli* genome	*Kabuli* gene accession IDs	NCBI-KOG	TFs
Ca4_P3_36643235	Ca4	36643235	T/TG	ACGCAATACATCTCAAGCCC	AATGGTAGCTGGGACAATGC	60.1	811	INTRON	Ca_14816	H	--
Ca4_P3_36644531	Ca4	36644531	ACTCTC/ACTC	CAACACCAAATTGCCAAGTG	AGCTGCCTCAGTTCCACTGT	60.0	870	INTRON	Ca_14816	H	--
Ca4_P3_36646334	Ca4	36646334	ACACTT/A	GGAACAAAGGTGGGACAAAA	GGGGGATATGTGGTTCCAGT	59.8	823	INTRON	Ca_14816	H	--
Ca4_P3_36651037	Ca4	36651037	ACAAAATTGAGCTTCAAACCAAAATTG/ACAAAATTGAGCTTCAAACCAAAATTGAGCTTCAAACCAAAATTG	GCACATGGCTGGCTCTACTA	ATGGGACCAGTGATGTTGGT	59.1	675	INTERGENIC	--	--	--
Ca4_P3_36726687	Ca4	36726687	ATTTTT/ATTTT	GCTTGTCCGCGTAACTCTTC	GGGGTGTTGCAACCTTTAGA	60.0	283	INTERGENIC	--	--	--
Ca4_P3_36779245	Ca4	36779245	TATAATAATAATAATAATAATAATAATAATAATAATAATAA/TATAATAATAATAATAATAATAATAATAATAATAATAATAA	AGCTTAGTTCAACATGGTCCG	CGGTCGACATATGGGTCAG	59.3	621	INTERGENIC	--	--	--
Ca4_P3_3680781	Ca4	3680781	GTTTTTTTTTTT/GTTTTTTTTTT	CCCAAAGACACATTTTGGGT	TTGCAAATGCCATTAAGCTG	59.7	452	INTERGENIC	--	--	--
Ca4_P3_3683925	Ca4	3683925	ATTTTTTTTT/ATTTTTTTTTT	CGACTGACCACATGGATTGT	CGAGTCATCTTATTGTTTGCCA	59.4	734	INTERGENIC	--	--	--
Ca4_P3_36911328	Ca4	36911328	GATATATATATATATATATA/GATATATATATATATATATATATATA	ATGTGAATCCCACCAGGAAA	TTACAACGCTTTGGCAACAA	60.2	523	INTERGENIC	--	--	--
Ca4_P3_36952734	Ca4	36952734	ATTTTTTT/ATTTTTT	AACTCATGTGGTTTGTGGCA	ATGGGCCCTCTTTTGTCTTT	60.0	896	INTRON	Ca_14797	R	C2H2

INDEL marker IDs	Chromosomes/ unanchored scaffolds	Physical positions (bp)	InDels (*Kabuli* reference genome-CDC Frontier/PII)	Forward primers (5'-3')	Reverse primers (5'-3')	Anneling temperature (0C)	Expected amplified product size (bp)	Structural annotation		Functional annotation	
								Sequence components of *kabuli* genome	*Kabuli* gene accession IDs	NCBI-KOG	TFs
Ca4_P3_36953596	Ca4	36953596	AATATATATAT/AATATATATATATAT	AAAAGAGGGCCCATGAGATT	TGTGCATTGGGAACAACACT	59.9	693	INTRON	Ca_14797	R	C2H2
Ca4_P3_36954611	Ca4	36954611	GGTGT/GGTGTGT	GGAGGATGGATGTGTGTGTG	TGGCACTCTCTTCTCCAGGT	59.8	580	INTRON	Ca_14797	R	C2H2
Ca4_P3_36956074	Ca4	36956074	TAAAAAAAA/TAAAAAAAAAAAA	TGTTCCAGGTTGATTCCTCC	TGATGGCAGGGAAAATAAGG	59.9	963	INTERGENIC	--	--	--
Ca4_P3_36956803	Ca4	36956803	TAAAAAAA/TAAAAAA	CCTTATTTTCCCTGCCATCA	CACCAATGGCTGAAAATCCT	59.9	595	INTERGENIC	--	--	--
Ca4_P3_36960688	Ca4	36960688	ATATCTTTGTTATTAT/ATATCTTTGTTATTATCTTTGTTATTAT	TTGATTTTGATGCAGGTGGA	CAAATACCTGTTGCCGAGGT	60.0	818	INTERGENIC	--	--	--
Ca4_P3_36968132	Ca4	36968132	ATTTTTTTT/ATTTTTTTTT	GGCGAAAGTACCCAAACAAA	GACTTCTGCAAAGGACCTCG	60.0	426	INTERGENIC	--	--	--
Ca4_P3_36968275	Ca4	36968275	G/GTTAATAC	GGCGAAAGTACCCAAACAAA	GACTTCTGCAAAGGACCTCG	60.0	426	INTERGENIC	--	--	--
Ca4_P3_36969612	Ca4	36969612	CATATATATATA/CATATATATATATATATATA	CATGGGCCAAAAATAAAAAGA	GAGCTGTGGAGAAGAAAAGCA	59.0	814	INTERGENIC	--	--	--
Ca4_P3_36969837	Ca4	36969837	CTTTTTTTTTT/CTTTTTTTTTTT	AGGTCATGCCATCAAAGACA	GAGCTGTGGAGAAGAAAAGCA	59.1	145	INTERGENIC	--	--	--
Ca4_P3_36972358	Ca4	36972358	CTTT/CTT	ACAACCAACAAAATGCGACA	AGCCTTGAGCGACCACTTTA	60.0	484	INTERGENIC	--	--	--

INDEL marker IDs	Chromosomes/ unanchored scaffolds	Physical positions (bp)	InDels (*Kabuli* reference genome-CDC Frontier/PII)	Forward primers (5'-3')	Reverse primers (5'-3')	Anneling temperature (0C)	Expected amplified product size (bp)	Structural annotation		Functional annotation	
								Sequence components of *kabuli* genome	*Kabuli* gene accession IDs	NCBI-KOG	TFs
Ca4_P3_36972540	Ca4	36972540	AATATATATATATA/AATATATATATATATATATATA	TAAAGTGGTCGCTCAAGGCT	TCTACATTCAAATTGGGGGC	60.0	608	INTERGENIC	--	--	--
Ca4_P3_36973113	Ca4	36973113	CATAT/CAT	GCCCCCAATTTGAATGTAGA	ATTCAATTTGCGCGAGTGTT	59.8	621	DRR	Ca_14796	B	bHLH
Ca4_P3_36973873	Ca4	36973873	TA/TAGAA	TGTGGTAAAATGCGGTCAAA	TCGGGTCATCTGCATTACAA	60.0	478	DRR	Ca_14796	B	bHLH
Ca4_P3_37013372	Ca4	37013372	TA/TACTTAAATGTTAA	CACGCTTTCAACTGTCCTGA	ATTGATCATCACATTGGCGA	60.0	421	INTERGENIC	--	--	--
Ca4_P3_37034054	Ca4	37034054	CTTT/CTTTT	GGAGGTTATCAATGGCTGGA	TGGAGTGGAAAGGAATGGAG	59.9	536	INTERGENIC	--	--	--
Ca4_P3_37035741	Ca4	37035741	TCC/TC	CCCAATGAAAGGCTCACAAT	TCCCTTTACCTTGCCTCATTT	59.9	635	INTERGENIC	--	--	--
Ca4_P3_37035891	Ca4	37035891	AT/ATT	AAATGAGGCAAGGTAAAGGGA	AGAATGCGCTCATGCTTCAC	60.0	504	INTERGENIC	--	--	--
Ca4_P3_37040426	Ca4	37040426	TAAAAAAA/TAAAAAAAA	GCGCTACAGAGAGCCTTCAC	GGTCATAACAAAGGGCCAGA	60.3	648	INTERGENIC	--	--	--
Ca4_P3_37042884	Ca4	37042884	A/AC	GCATGAGAATTTCCGTGTCA	TAAAACCACACAGCAAACGG	59.7	518	INTERGENIC	--	--	--
Ca4_P3_37043299	Ca4	37043299	T/TG	CCGTTTGCTGTGTGGTTTTA	TTGCAAACAAGCAACCAAAG	59.6	298	INTERGENIC	--	--	--

INDEL marker IDs	Chromosomes/ unanchored scaffolds	Physical positions (bp)	InDels (*Kabuli* reference genome-CDC Frontier/PII)	Forward primers (5'-3')	Reverse primers (5'-3')	Anneling temperature (0C)	Expected amplified product size (bp)	Structural annotation		Functional annotation	
								Sequence components of *kabuli* genome	*Kabuli* gene accession IDs	NCBI-KOG	TFs
Ca4_P3_37046683	Ca4	37046683	A/AT	GCAAGTTGTCCAGCGTAACA	CCCATTTCCCACTTCAATTT	59.9	870	INTERGENIC	--	--	--
Ca4_P3_37059204	Ca4	37059204	ATCTTCATTTTTTCTTCATT/ATCTTCATT	CAGTTCAACAATTTGACTGAGAAA	ATGGGACGATGTGGATCAAT	58.5	784	INTERGENIC	--	--	--
Ca4_P3_37076395	Ca4	37076395	A/AGT	TTCCAAATTGCAAAACATGC	TGGTATTGTGGTGGCAAAGA	59.5	370	INTERGENIC	--	--	--
Ca4_P3_37082858	Ca4	37082858	GTTTTTTTTTTT/GTTTTTTTT	GAAGTTGTTTGGGTTGGCAT	ATTGCAAATTGAGAGCCCAG	59.8	391	INTERGENIC	--	--	--
Ca4_P3_37083111	Ca4	37083111	ATTTT/ATTT	GAAGTTGTTTGGGTTGGCAT	TTTGCCATCTGGAGTCTTGA	59.8	798	INTERGENIC	--	--	--
Ca4_P3_37083670	Ca4	37083670	T/TGAACACAAC	TCTCAAGACTCCAGATGGCA	CGTGCTCCATTTTTAGGCAT	59.5	558	INTERGENIC	--	--	--
Ca4_P3_37089859	Ca4	37089859	TAAAAAAAAA/TAAAAAAA	ATATCGACAATGCGCAACAA	CAACCAAGAGCGTTCTCCAT	60.1	853	INTERGENIC	--	--	--
Ca4_P3_37098592	Ca4	37098592	AAATAAT/AAAT	GTGCTATTGAGTGGTGCCAA	CGAATCTTCTTCAAATGTTGACC	59.7	369	INTERGENIC	--	--	--
Ca4_P3_37098679	Ca4	37098679	GAAGATTCGATTAAGATT/GAAGATT	GTGCTATTGAGTGGTGCCAA	TCTTCTTCTTCTTCAGCCTTGA	59.7	734	INTERGENIC	--	--	--
Ca4_P3_37104335	Ca4	37104335	TTTTATTT/TTTT	GTGAGCAAGGGGAGCAGTAG	CCTCCAAATGATGGCGTAGT	60.0	401	INTERGENIC	--	--	--

INDEL marker IDs	Chromosomes/ unanchored scaffolds	Physical positions (bp)	InDels (*Kabuli* reference genome-CDC Frontier/PII)	Forward primers (5'-3')	Reverse primers (5'-3')	Anneling temperature (0C)	Expected amplified product size (bp)	Structural annotation		Functional annotation	
								Sequence components of *kabuli* genome	*Kabuli* gene accession IDs	NCBI-KOG	TFs
Ca4_P3_37105524	Ca4	37105524	AA/AACA	TTCAAATGGTCTCTCACCCC	ACGAAGGTCAAGCTCAAGGA	59.9	855	INTERGENIC	--	--	--
Ca4_P3_37117919	Ca4	37117919	AT/A	TACGGAAATCGGCTGGATAG	GCACGATAAATGCAACCCTT	60.1	496	INTERGENIC	--	--	--
Ca4_P3_37119347	Ca4	37119347	TAAAAAAA/TAAAAAAAA	AAAACATGTGCGACCCTAGA	CCCAACCATTTTGAAACCAC	58.2	555	INTERGENIC	--	--	--
Ca4_P3_37121983	Ca4	37121983	ATTTTTTT/ATTTTTTTT	TCAACATGACCTCCTTATGCTTT	TCCAAACACTCCAAACACCA	60.0	407	INTERGENIC	--	--	--
Ca4_P3_37128543	Ca4	37128543	TAAAAA/TAAAA	TCGCACTTTTGGAGATTGAA	TGCATCCATCGTCAAATACAA	59.4	462	INTERGENIC	--	--	--
Ca4_P3_37128859	Ca4	37128859	G/GC	TTTGACGATGGATGCATAAGA	TTTAGGTTGGCTTTGGATGC	59.1	593	INTERGENIC	--	--	--
Ca4_P3_37131327	Ca4	37131327	TAA/TAATAGTTTAAAAA	CAACTCAAACCACCTTCATTCA	AATTTGCGAGCATTACCTGC	60.0	633	INTERGENIC	--	--	--
Ca4_P3_37173541	Ca4	37173541	ATTTTTT/ATTTT	TTTGGTGTAGGAGGAGGTCG	AAGGAGTTGTCGTGGATTGG	60.1	665	DRR	Ca_15153	A	bHLH
Ca4_P3_37199729	Ca4	37199729	GG/GGTCG	GGCGACACTTCAATCCCTAA	AGGCCTTTTTCATTTGGTCA	60.1	492	INTERGENIC	--	--	--
Ca4_P3_37199973	Ca4	37199973	GCC/GCCC	GGCGACACTTCAATCCCTAA	AATGCAAATTTTATCAAATGCAAA	60.1	940	INTERGENIC	--	--	--

INDEL marker IDs	Chromosomes/ unanchored scaffolds	Physical positions (bp)	InDels (*Kabuli* reference genome-CDC Frontier/PII)	Forward primers (5'-3')	Reverse primers (5'-3')	Anneling temperature (0C)	Expected amplified product size (bp)	Structural annotation		Functional annotation	
								Sequence components of *kabuli* genome	*Kabuli* gene accession IDs	NCBI-KOG	TFs
Ca4_P3_37218174	Ca4	37218174	TTC/T	AATTCAAACAGCGCAATGAA	TTATTTTTCCCAACATCGGC	59.3	893	INTERGENIC	--	--	--
Ca4_P3_37221016	Ca4	37221016	A/AG	TGGTTGTCGACTTGTCTTGC	ATGCGGTAGCTTTTGATTCG	59.9	468	INTERGENIC	--	--	--
Ca4_P3_37223150	Ca4	37223150	TT/TTAT	ACCTAACATGGGATTGAGCG	ATTTTTAAGCACGCCCATTG	60.0	757	INTERGENIC	--	--	--
Ca4_P3_37225875	Ca4	37225875	T/TG	TCAAATTGGAGTGGGATGAA	TTTGAAGAATGTCACGGTGAAG	58.9	685	INTERGENIC	--	--	--
Ca4_P3_37233320	Ca4	37233320	ATTTTTTTTT/ATTTTTTTT	CAACCAAACTCTCCGACAAGA	TATGAAATGACGCAGGTGGA	60.3	436	INTERGENIC	--	--	--
Ca4_P3_37247959	Ca4	37247959	TAAA/TAAAA	CGAATTTTGTGTACCGAATTTTT	AGGAGAAATGAACATCCACG	59.3	553	INTERGENIC	--	--	--
Ca4_P3_37249911	Ca4	37249911	GAAAAA/GAAAAAA	TCGTCGATGAGATCAAATATGC	GGCACAAAAGAGCTGGTCAT	60.1	251	INTERGENIC	--	--	--
Ca4_P3_37283555	Ca4	37283555	GT/GTT	TGAATGAAATATTGGATACGTTTATGA	GCAGGGCATTTGTCTCTAGC	59.9	530	INTERGENIC	--	--	--
Ca4_P3_37283825	Ca4	37283825	T/TTTTAACA	TGCAACCTTTTTGATCTAGCA	GCAGGGCATTTGTCTCTAGC	58.6	196	INTERGENIC	--	--	--
Ca4_P3_37302615	Ca4	37302615	TAAAA/TAAA	CATTCAGCCTGTTTTCACCA	TCCTTGCTTTAAAAGGGGGT	59.7	667	DRR	Ca_15145	J	--

INDEL marker IDs	Chromosomes/ unanchored scaffolds	Physical positions (bp)	InDels (*Kabuli* reference genome-CDC Frontier/PII)	Forward primers (5'-3')	Reverse primers (5'-3')	Anneling temperature (0C)	Expected amplified product size (bp)	Structural annotation		Functional annotation	
								Sequence components of *kabuli* genome	*Kabuli* gene accession IDs	NCBI-KOG	TFs
Ca4_P3_37303497	Ca4	37303497	ATTTTTT/ATTTT	CAGTCGAAATACCAGCCCAT	TTTCATTGCGGGACATACAA	60.0	113	INTRON	Ca_15145	J	--
Ca4_P3_37306375	Ca4	37306375	C/CCT	TCAATGAAGTCCCACGTGAA	TGCAGTCGAAACCAAAAGTG	60.1	381	INTERGENIC	--	--	--
Ca4_P3_37312922	Ca4	37312922	GAAAAAA/GAAAAAAA	AATGCATGCAGTTCAATTCG	TTATAAAAACCTCCTCCACAAAA	59.7	436	DRR	Ca_15144	O	--
Ca4_P3_37313817	Ca4	37313817	CTTTT/CTTTTT	CCTTCTCAAGATCGCCTTTG	CCTTGCAACCATCATTTTCA	59.9	445	DRR	Ca_15144	O	--
Ca4_P3_37331887	Ca4	37331887	CAA/CAAAA	TGACCGACGACTACAATTTCA	AATGGATCACAAAAATCATGTGT	59.2	782	INTERGENIC	--	--	--
Ca4_P3_37333057	Ca4	37333057	CTTATT/CTT	TCTGTAATCGGTTTGGGCTC	CAATTCGTTCAAGTTCGGGT	60.1	905	INTERGENIC	--	--	--
Ca4_P3_37339630	Ca4	37339630	AC/ACC	TTGGGTCTTGCAAAGGAATC	TGCCATTGTGTGCAGGTATT	60.1	317	INTRON	Ca_15142	--	--
Ca4_P3_37345139	Ca4	37345139	TAA/TAAA	TTTGCTTTTAGGACTTGCACC	TGGACTCTGCAGAAAACACG	59.4	754	INTERGENIC	--	--	--
Ca4_P3_37346206	Ca4	37346206	TTTTATTTATTTATTT/TTTTATTTATTTATTTATTT	GCCCCAACGTGAACATTATT	TCAATAATGCTGCCATCCAA	59.7	208	INTERGENIC	--	--	--
Ca4_P3_37362171	Ca4	37362171	GTTGT/GTTGTTTGT	CCATTGGTTTTTGCTTCGTT	CCACGCCTGGAGTTTATTGT	60.0	947	INTRON	Ca_15140	S	--

INDEL marker IDs	Chromosomes/ unanchored scaffolds	Physical positions (bp)	InDels (*Kabuli* reference genome-CDC Frontier/PII)	Forward primers (5'-3')	Reverse primers (5'-3')	Anneling temperature (0C)	Expected amplified product size (bp)	Structural annotation		Functional annotation	
								Sequence components of *kabuli* genome	*Kabuli* gene accession IDs	NCBI-KOG	TFs
Ca4_P3_37366698	Ca4	37366698	AAA/AAACCAA	TTACCCTCTGTTGCTTTGCC	ACCATGGACACCGTTTGTTT	60.2	430	DRR	Ca_15139	R	NF-YA
Ca4_P3_37377123	Ca4	37377123	TTA/TTATA	CCTTTTAGCTAATTGATCGGAGA	ATATCCGGGGGAGAAATGAG	59.0	682	INTERGENIC	--	--	--
Ca4_P3_37385575	Ca4	37385575	TTT/TTTAATT	AAGCCTAGGACAACCTGCAA	CATGTGACCAAATCCAATCG	59.9	448	DRR	Ca_15138	T	HSF
Ca4_P3_37391739	Ca4	37391739	T/TC	TTGCTAGTTGCTGATGTGGC	TGGCAAATGTCACAAGGTGT	60.0	269	INTERGENIC	--	--	--
Ca4_P3_37397649	Ca4	37397649	AC/ACAATC	AATGAGGAACCAATGGTGGA	GCTTATGCATGCCCTTCATT	60.2	233	DRR	Ca_15137	R	MYB
Ca4_P3_37403650	Ca4	37403650	CTTTT/CTTTTT	CTTATCCAAAAGCGAGGCAC	TGAGTTGGACTACAAGCCATCA	59.8	597	INTERGENIC	--	--	--
Ca4_P3_37404660	Ca4	37404660	TAAAAAA/TAAAAAAAA	CGTTAAGGGTGTAATTGGCTG	TGATGGACCCAAGCATTTTT	59.5	702	INTERGENIC	--	--	--
Ca4_P3_37409343	Ca4	37409343	ATTTTTT/ATTTTTTT	TGAGGCAGTTTGAACAGTCG	ACCCGATTATCATGGAGCAA	60.0	347	DRR	Ca_15136	--	--
Ca4_P3_37417887	Ca4	37417887	TTT/TTTACCTT	AACCCGACCCGAAAATAAAC	ATTCTCCGGTACGGACTCCT	60.1	338	INTRON	Ca_15135	--	--
Ca4_P3_37423475	Ca4	37423475	T/TGATTG	CATTGACCCAATTCATGCTG	TTATTCAGGGTGGATGGGAA	59.9	483	INTRON	Ca_15134	T	--

INDEL marker IDs	Chromosomes/ unanchored scaffolds	Physical positions (bp)	InDels (*Kabuli* reference genome-CDC Frontier/PII)	Forward primers (5'-3')	Reverse primers (5'-3')	Anneling temperature (0C)	Expected amplified product size (bp)	Structural annotation		Functional annotation	
								Sequence components of *kabuli* genome	*Kabuli* gene accession IDs	NCBI-KOG	TFs
Ca4_P3_37443931	Ca4	37443931	CT/CTAT	CCGCATTTTCTTCATTAGGG	ACGTTAAGGCATGAGTTGGG	59.5	364	INTERGENIC	--	--	--
Ca4_P3_37455037	Ca4	37455037	AC/A	TTTTGATAGAGCCCAATGGC	CAGCCTGATGAACAAACCAA	60.0	282	INTRON	Ca_15130	S	--
Ca4_P3_37475806	Ca4	37475806	ATTTT/ATTT	TTCTATGGCTGCCGTTCTCT	GTCAAATTTCCCGTCCAAAA	60.0	557	INTERGENIC	--	--	--
Ca4_P3_37483206	Ca4	37483206	TAAAAAAAAAAAAAA/TAAAAAAAAAAAAAAAA	AAATTGGGTGATGTGGTGGT	CCTAGAAGATGAGGGTGCCA	60.0	424	INTERGENIC	--	--	--
Ca4_P3_37483889	Ca4	37483889	CA/CAA	TGGCACCCTCATCTTCTAGG	TGCTTGGTTTAAGGGTTTGG	60.2	734	INTERGENIC	--	--	--
Ca4_P3_37490253	Ca4	37490253	TNNNNNNNNNN/TNNNNN	TCTATGCGCTGATTCAGACAA	AACATCCCCATCAATTCATCA	59.6	799	INTERGENIC	--	--	--
Ca4_P3_37502468	Ca4	37502468	ATTTT/ATTTTT	GCAAGTTATTGGTCCAGCGT	AAAAGGGGTCATTTGGTTCC	60.1	577	DRR	Ca_15128	--	--
Ca4_P3_37508480	Ca4	37508480	ATTTTTTT/ATTTTTTTT	CCACCATTATAAGGATGCGA	GCCAAAACGGAGAAGAAACA	58.5	513	INTERGENIC	--	--	--
Ca4_P3_37519504	Ca4	37519504	CTATTTATTTATTTATTTATTT/CTATTTATTTATTTATTT	GAAAAAGGAGTTGCCACCAA	TGGGGATACGTAGGAGCAAG	60.1	306	INTERGENIC	--	--	--
Ca4_P3_37542772	Ca4	37542772	TGGGGG/TG	AATTGAAGTACCCGCACCTG	CAAATCCAACTGTTGCCCAT	60.0	693	INTRON	Ca_15126	--	--

INDEL marker IDs	Chromosomes/ unanchored scaffolds	Physical positions (bp)	InDels (*Kabuli* reference genome-CDC Frontier/PII)	Forward primers (5'-3')	Reverse primers (5'-3')	Anneling temperature (0C)	Expected amplified product size (bp)	Structural annotation		Functional annotation	
								Sequence components of *kabuli* genome	*Kabuli* gene accession IDs	NCBI-KOG	TFs
Ca4_P3_37554271	Ca4	37554271	TA/T	AGTGGCTCAAACAAGG GATG	CTTTCTTGTGCAAGGT GCAA	60.1	450	INTRON	Ca_15125	K	MYB_related
Ca4_P3_37607126	Ca4	37607126	T/TATTA	TTTCCCTACAGCAAAAG CATC	GCTGATTGGTGTGACA GGAC	59.4	454	INTERGENIC	--	--	--
Ca4_P3_37608001	Ca4	37608001	ACTCTCTCTCTCTCTC/ACTCTCTCTCT CTCTCTC	TTGGATTCCTGACACAC ACA	TAAAAATTTGGAGGGA GGGG	58.4	568	INTERGENIC	--	--	--
Ca4_P3_37609204	Ca4	37609204	GTGCT/GT	CGTGTCCTGATGTCCA ACAC	AGCGCTGTAGCCGTTA TTTG	60.0	510	INTRON	Ca_15121	--	ERF
Ca4_P3_37619516	Ca4	37619516	A/AAG	TCTGGATGGTGACGAA ATGA	ACTCTACTCCGTCCGT CTCA	60.0	613	INTERGENIC	--	--	--
Ca4_P3_37633640	Ca4	37633640	AAAGAA/AAA	TATCCGTTTGTCCGCTT TTC	GAGCACTTGGGCTTTT CAAG	60.1	844	DRR	Ca_15118	--	--
Ca4_P3_37646806	Ca4	37646806	C/CAA	GCTCTGGTGGGTGATT TGTT	ATGTTCCGTTGGAAAG CATC	60.0	641	INTRON	Ca_15117	T	LBD
Ca4_P3_37652154	Ca4	37652154	TTAGTA/TTA	ACCACGCCACAACAAG TGTA	GAGGGAAACGTCCAAT GCTA	60.1	620	DRR	Ca_15115	T	TALE
Ca4_P3_37671559	Ca4	37671559	CAA/CA	TTTCCACCCAGAAAGC AATC	GCAAAACAGAGGCAAC ATCA	60.1	250	INTERGENIC	--	--	--
Ca4_P3_37678492	Ca4	37678492	TAAGAAGAA/TAAGAAGAAGAA	TGGAACATTTGAAGCA CCCT	ATTCTGGAGCCACAGA CCAC	60.5	624	INTERGENIC	--	--	--

INDEL marker IDs	Chromosomes/ unanchored scaffolds	Physical positions (bp)	InDels (*Kabuli* reference genome-CDC Frontier/PII)	Forward primers (5'-3')	Reverse primers (5'-3')	Anneling temperature (0C)	Expected amplified product size (bp)	Structural annotation		Functional annotation	
								Sequence components of *kabuli* genome	*Kabuli* gene accession IDs	NCBI-KOG	TFs
Ca4_P3_37683070	Ca4	37683070	ATTT/ATTTTT	CATTGATTCTTGCCATG CAG	AATGCGAAGGGTTGAG ATTG	60.2	630	INTERGENIC	--	--	--
Ca4_P3_37683443	Ca4	37683443	CGTCAGTGTCAGT/CGTCAGT	CAATCTCAACCCTTCGC ATT	ACAAGGCCACATTGTC ATCA	60.1	652	INTRON	Ca_15113	S	--
Ca4_P3_37683526	Ca4	37683526	CTTTTTTTTT/CTTTT	CAATCTCAACCCTTCGC ATT	ACAAGGCCACATTGTC ATCA	60.1	652	INTRON	Ca_15113	S	--
Ca4_P3_37688244	Ca4	37688244	GTATTAA/GTATTAAATTATTAA	TTTAAAGTCCCCATCAC CCA	CCGAGCCTTACAAGGA CAAC	60.2	684	INTRON	Ca_15113	S	--
Ca4_P3_37688621	Ca4	37688621	ATCTTCTTC/ATCTTC	GGATTTCCTGTGAGCT GCAT	AGAATTGGAATGCCTG AACG	60.2	492	INTRON	Ca_15113	S	--
Ca4_P3_37696707	Ca4	37696707	TA/TATAGACATAA	AATGTTTTTGGTAATCC CGGT	AACATTGGTGGTGCAA GACA	59.5	607	INTERGENIC	--	--	--
Ca4_P3_37701270	Ca4	37701270	TG/TGG	GTCCCAATGACTCCAA CTCC	TCCAATTAATGCGGTG TTCA	59.4	246	DRR	Ca_15111	F	--
Ca4_P3_37701400	Ca4	37701400	ATTTTTTTTTT/ATTTTTTTTTTT	TGAACACCGCATTAATT GGA	GGGTTTTTGTTCGAAG ATTTTG	59.9	262	DRR	Ca_15111	F	--
Ca4_P3_37703548	Ca4	37703548	TT/TTAT	AACCACTCAGCACCGT TTTC	CCAACAGACCTTCCCC ACTA	60.2	239	INTERGENIC	--	--	--
Ca4_P3_37703661	Ca4	37703661	T/TA	AACCACTCAGCACCGT TTTC	CCAACAGACCTTCCCC ACTA	60.2	239	INTERGENIC	--	--	--

INDEL marker IDs	Chromosomes/ unanchored scaffolds	Physical positions (bp)	InDels (*Kabuli* reference genome-CDC Frontier/PII)	Forward primers (5'-3')	Reverse primers (5'-3')	Anneling temperature (0C)	Expected amplified product size (bp)	Structural annotation		Functional annotation	
								Sequence components of *kabuli* genome	*Kabuli* gene accession IDs	NCBI-KOG	TFs
Ca4_P3_37703958	Ca4	37703958	C/CAAG	TAGTGGGGAAGGTCTGTTGG	TGTTGCTGCGTCTTGAAATC	60.0	733	INTERGENIC	--	--	--
Ca4_P3_37704228	Ca4	37704228	T/TG	AGGAGGGATCAAACCGAACT	TGTTGCTGCGTCTTGAAATC	59.9	472	INTERGENIC	--	--	--
Ca4_P3_37705943	Ca4	37705943	GA/GATAA	GCCGGGATGTTCTTTTTGTA	TTTACTGAATGCCATGCTGC	59.9	679	DRR	Ca_15110	C	MYB
Ca4_P3_37706000	Ca4	37706000	GCC/GCCTTAACC	GCCGGGATGTTCTTTTTGTA	TTTACTGAATGCCATGCTGC	59.9	679	DRR	Ca_15110	C	MYB
Ca4_P3_37706092	Ca4	37706092	TC/T	GCCGGGATGTTCTTTTTGTA	GAGGGGAGGCCTTCTCATAC	59.9	818	DRR	Ca_15110	C	MYB
Ca4_P3_37706158	Ca4	37706158	G/GA	GCCGGGATGTTCTTTTTGTA	GAGGGGAGGCCTTCTCATAC	59.9	818	DRR	Ca_15110	C	MYB
Ca4_P3_37707068	Ca4	37707068	C/CTGGCAAGGAGCTACT	TGAGGCCTTAGCTTCGTGAT	CACAAGGACCTGACGGTTTT	60.0	555	INTERGENIC	--	--	--
Ca4_P3_37707688	Ca4	37707688	T/TTC	AAAACCGTCAGGTCCTTGTG	TGAGCGTTTTCTCGGTCTTT	60.0	386	INTERGENIC	--	--	--
Ca4_P3_37709272	Ca4	37709272	CCT/CCTTTCT	GTCTATCGGAGAGCCAGTCG	CCCTTCACCTCTTTCACCAA	60.0	109	INTERGENIC	--	--	--
Ca4_P3_37710905	Ca4	37710905	TGAGAGAG/TGAGAG	ACGTGTTGAGCTAACCCTGC	TGTGAGTTTGTTTGCAAGAGC	60.3	854	INTRON	Ca_15109	--	--

**INDEL marker IDs**	**Chromosomes/ unanchored scaffolds**	**Physical positions (bp)**	**InDels (*Kabuli* reference genome-CDC Frontier/PII)**	**Forward primers (5'-3')**	**Reverse primers (5'-3')**	**Anneling temperature (0C)**	**Expected amplified product size (bp)**	**Structural annotation**		**Functional annotation**	
								**Sequence components of *kabuli* genome**	***Kabuli* gene accession IDs**	**NCBI-KOG**	**TFs**
Ca4_P3_37712217	Ca4	37712217	GATATATATATATATATATATATATATATA/GATATATATATATATATATATATATATA	ACCGTTTGCTGCTTAATGCT	TTAAATTTGGCCCTCCAATG	59.9	600	INTRON	Ca_15109	--	--
Ca4_P3_37716531	Ca4	37716531	ATG/A	TGCATAGATTCCCATTCCCT	TTTACCTCACATTGGCCTCC	59.3	344	DRR	Ca_15109	--	--
Ca4_P3_37721837	Ca4	37721837	CT/CTGT	TACACGTCAAAGGCAAGTGG	GGGGAAGAGTGGCATATTCA	59.8	978	DRR	Ca_15108	R	NAC
Ca4_P3_37729255	Ca4	37729255	T/TG	TCAATGCACAAAACGTGGTT	GAGGCGCGATCTAGCTTATG	60.0	794	INTERGENIC	--	--	--
Ca4_P3_37746382	Ca4	37746382	GTTTTT/GTTTT	TTGCAATAGGCATAGGACCC	TTGTTTCTCCCCAGTTTTGG	59.9	448	INTERGENIC	--	--	--
Ca4_P3_37769216	Ca4	37769216	TAA/TA	GGAAGTACGTGGAGGTGGAA	GAGACGAGGTGAACGGAGAG	60.0	441	INTERGENIC	--	--	--
Ca4_P3_37784787	Ca4	37784787	TAA/TAAA	CAAACGTGGTGTTCAGTAGCA	TCTCCACGTTTCCATCTTCC	59.8	836	INTERGENIC	--	--	--
Ca4_P3_3781804	Ca4	3781804	CATATTTTTTTGTCAATATATTTTTTTGTCAAT/CATATTTTTTTGTCAATATATTTTTTTGTCAATATATTTTTTTGTCAAT	TGGTTGTGCGATATTGTGCT	TCAATGCATTTTCGACATTTTT	60.1	749	INTERGENIC	--	--	--
Ca4_P3_37824259	Ca4	37824259	CTAATCTTAGGAGATAATCT/CTAATCT	TTGCCAATTTTGTTCAGCAG	AAAGCTTAGGGCCCTTTTGA	59.8	696	INTERGENIC	--	--	--
Ca4_P3_37855733	Ca4	37855733	CTATAATTATATATATAATTATATA/CTATAATTATATA	CGGTTCGAAGCTTTGATGTT	TTTGAATTGTATTGTGTTTAATCGAA	60.2	605	INTERGENIC	--	--	--

INDEL marker IDs	Chromosomes/ unanchored scaffolds	Physical positions (bp)	InDels (*Kabuli* reference genome-CDC Frontier/PII)	Forward primers (5'-3')	Reverse primers (5'-3')	Anneling temperature (0C)	Expected amplified product size (bp)	Structural annotation		Functional annotation	
								Sequence components of *kabuli* genome	*Kabuli* gene accession IDs	NCBI-KOG	TFs
Ca4_P3_37859268	Ca4	37859268	AATT/AATTAGATT	CAAACTGTGCCAGCTT CGTA	TGCCCTAGCTATTGCC AAAC	60.1	693	INTRON	Ca_15089	--	--
Ca4_P3_37860226	Ca4	37860226	TAAAAAA/TAAAAA	TTTCATGGAGTGAAGG GAGG	CCAAGACCAGCATCAG ACAA	60.0	502	INTRON	Ca_15089	--	--
Ca4_P3_37860521	Ca4	37860521	CTTA/CTTAATTA	TGGTCCCTTGTTGCCT TTAG	AATGGACGGCTATGAG TTGC	60.1	739	INTRON	Ca_15089	--	--
Ca4_P3_37861985	Ca4	37861985	C/CAA	CTCCGTGGTACCCTCC TACA	ACAATCCCTTGTGGAA GTGC	60.0	663	INTRON	Ca_15089	--	--
Ca4_P3_37866708	Ca4	37866708	TATAATAATAATAATAATAATAA/TATAA TAATAATAATAATAATAATAA	TCTTGAATTTGGGTGCA TTTC	GGTTGCTTTGAGACGG ATGT	59.9	687	INTERGENIC	--	--	--
Ca4_P3_37866908	Ca4	37866908	ATTTTT/ATTT	CAAAGCCATGGTTTCC CTTA	TTTCATCATAATGGAGT ACGGA	59.9	219	INTERGENIC	--	--	--
Ca4_P3_37871651	Ca4	37871651	ATTTTTT/ATTTTT	ACCCTACCCATTGTCAA CCA	TGGATGGTTATGATTT GCTGA	60.1	668	INTERGENIC	--	--	--
Ca4_P3_37877856	Ca4	37877856	ATTT/ATT	CGGGCCACATTCAAAC TACT	GCATTGGAATGCACTC ATCTT	60.0	476	INTERGENIC	--	--	--
Ca4_P3_37896451	Ca4	37896451	TG/T	TCATTGTGCTTGGAGTT TGC	CACGGAAGAAGCAAGG AAAA	59.8	123	INTERGENIC	--	--	--
Ca4_P3_37909050	Ca4	37909050	GTTTTTTT/GTTTTTTTTTT	TGCATTTTAGGTCCATG ATGA	ATTTGACCACCGAAAG ATGC	59.0	190	INTERGENIC	--	--	--

INDEL marker IDs	Chromosomes/ unanchored scaffolds	Physical positions (bp)	InDels (*Kabuli* reference genome-CDC Frontier/PII)	Forward primers (5'-3')	Reverse primers (5'-3')	Anneling temperature (0C)	Expected amplified product size (bp)	Structural annotation		Functional annotation	
								Sequence components of *kabuli* genome	*Kabuli* gene accession IDs	NCBI-KOG	TFs
Ca4_P3_37919621	Ca4	37919621	TTTTTTT/TCAAAATTAATTTTTT	CACACTTTCAACTTAAGGGGTTG	ACAGGGGTGATGAGGACAAG	59.9	573	INTERGENIC	--	--	--
Ca4_P3_37933855	Ca4	37933855	TATAATAATAAT/TATAATAATAATAATAATAAT	CAATTGGCCGACATACACAC	GGGCAAACAGTCAAAGGAAA	59.8	494	DRR	Ca_15084	--	--
Ca4_P3_37935122	Ca4	37935122	CTTATTATTA/CTTATTA	TTTTGAAAGAAACAATCATGCAG	GGTGGGATTGTGAAATGGAC	59.3	731	DRR	Ca_15084	--	--
Ca4_P3_37936676	Ca4	37936676	CATATAT/CAT	CAACGTTTTCCCAGTCACCT	TTGGTCCAGCATCATTTTCA	60.0	613	INTRON	Ca_15084	--	--
Ca4_P3_37937708	Ca4	37937708	GTTT/GTTTT	CAAAAGAGGTTGAAACAAGGAGA	TGCCCGCTCTGAAAATTAAC	59.8	416	INTRON	Ca_15084	--	--
Ca4_P3_37939769	Ca4	37939769	TT/TTATTATTAT	ATGGGGGCTTATCGAAGAGT	GGGCTTGTGAAATGGACCTA	59.9	729	INTRON	Ca_15084	--	--
Ca4_P3_37977728	Ca4	37977728	CGTGGTGGT/CGTGGT	GTTCCCAACTTGTGTGCCTT	TGCATTTCATCACAGACATTGA	60.0	543	INTERGENIC	--	--	--
Ca4_P3_37978815	Ca4	37978815	ATTC/ATTCTTC	CAAACACGCCCTTACCACTT	TGGTGATCGGAAACTGATGA	60.0	112	CDS (large-effect mutations)	Ca_13148	--	--
Ca4_P3_37980637	Ca4	37980637	A/AAT	GCTATGTCGCCACAATCCTC	GGCAAAGCAATAATGGAAGC	60.6	652	DRR	Ca_13148	--	--
Ca4_P3_37989769	Ca4	37989769	C/CA	AAACAAACCGATCCTTAATCTCC	TCAATTACCAAAAGGGCCAG	59.7	454	INTERGENIC	--	--	--

INDEL marker IDs	Chromosomes/ unanchored scaffolds	Physical positions (bp)	InDels (*Kabuli* reference genome-CDC Frontier/PII)	Forward primers (5'-3')	Reverse primers (5'-3')	Anneling temperature (0C)	Expected amplified product size (bp)	Structural annotation		Functional annotation	
								Sequence components of *kabuli* genome	*Kabuli* gene accession IDs	NCBI-KOG	TFs
Ca4_P3_37990861	Ca4	37990861	ATTTTTTTT/ATTTTTTTTTT	TCCATCGAGGAAAACG AATC	AAACGAGAAAAACGCA TTGG	60.0	666	INTERGENIC	--	--	--
Ca4_P3_38017794	Ca4	38017794	TTATAT/TTATATAT	ACAACTTCTCATGCTCG CAA	GCGAAGGGAAACATGT CATT	59.6	503	DRR	Ca_13143	E	--
Ca4_P3_38021208	Ca4	38021208	TA/TAAAAA	AAGATTGATTTTCATGG ATTGACA	TTGGTGGATGTGAGGA AACA	59.7	584	INTRON	Ca_13143	E	--
Ca4_P3_38028818	Ca4	38028818	T/TG	ACTCAACCCCACAAAAC GAG	GTTGAAATCCTGCCAC CACT	60.0	584	INTRON	Ca_13142	O	ERF
Ca4_P3_38046129	Ca4	38046129	TCA/TCACA	GGGAGGAAGGTGTCTT TGGT	TGGGTCCAAAAATTTT CAATG	60.3	358	INTERGENIC	--	--	--
Ca4_P3_38049243	Ca4	38049243	CAAA/CAAAA	GAGAGGACCACCCTCT TTCC	TCACTCCGCTGCATGT CTAC	60.1	573	INTERGENIC	--	--	--
Ca4_P3_38064052	Ca4	38064052	T/TA	AAAGCTTCATCCTTCCA ACG	TGTGCGATTAGAAACA CTACCAA	59.3	351	INTERGENIC	--	--	--
Ca4_P3_38076613	Ca4	38076613	GTTTTTTT/GTTTTTTTTT	CGAAATTGCTGGTGGC TACT	GGGCATACAACTGTGC TTCA	60.3	720	INTERGENIC	--	--	--
Ca4_P3_38077899	Ca4	38077899	ATAATTAATTAAT/ATAATTAAT	AATGCTTTTGGTGGTCT TGG	CATAAGAGCGAGGTGC AACA	60.0	331	INTERGENIC	--	--	--
Ca4_P3_38085487	Ca4	38085487	AT/A	TCCCCTTTCTTACGAAT CCC	CACTTGGAAGAGGGCA ACAT	60.3	476	INTERGENIC	--	--	--

INDEL marker IDs	Chromosomes/ unanchored scaffolds	Physical positions (bp)	InDels (*Kabuli* reference genome-CDC Frontier/PII)	Forward primers (5'-3')	Reverse primers (5'-3')	Anneling temperature (0C)	Expected amplified product size (bp)	Structural annotation		Functional annotation	
								Sequence components of *kabuli* genome	*Kabuli* gene accession IDs	NCBI-KOG	TFs
Ca4_P3_38118268	Ca4	38118268	CAT/CATAT	GCCTTCAAAACCAAGAACCA	CGTTTGTTGCTATGAAACCG	60.1	447	INTERGENIC	--	--	--
Ca4_P3_38120410	Ca4	38120410	AG/A	AAACCTGAACCACTACCCCA	CACTTCCGTCTCCAGACTCC	59.3	460	INTERGENIC	--	--	--
Ca4_P3_38121246	Ca4	38121246	ATTTTTTT/ATTTTTT	TGAGCCATGGGACAATTATG	AGATCCTTTTACAACGGCCC	59.4	583	INTERGENIC	--	--	--
Ca4_P3_38129704	Ca4	38129704	AATTATATATATTA/AATTA	AGTTATGGGTCCCAATGCAC	TTGCCTTCTTATAGGCCCAA	59.7	433	INTERGENIC	--	--	--
Ca4_P3_38129851	Ca4	38129851	GAAAAAAAA/GAAAAAAAAAAA	AGTTATGGGTCCCAATGCAC	GCCCCTTGCTCATTTTATCA	59.7	595	INTERGENIC	--	--	--
Ca4_P3_38132858	Ca4	38132858	GTTTT/GTTTTT	CGTTAGACAAGTTGGCTGCTT	TTGTCTTGTCGTCGCATTTC	59.6	173	INTRON	Ca_13139	--	ERF
Ca4_P3_38140746	Ca4	38140746	AGAGAT/A	GGTGGTGTTTTTCCCAGTTG	TGAAGCACAACGCCTATCTG	60.2	775	INTERGENIC	--	--	--
Ca4_P3_38170210	Ca4	38170210	TAAAAA/TAAA	AGCTTTGCAGGACAAGATCG	ATTGCAACCAAACCACATCA	60.5	710	INTERGENIC	--	--	--
Ca4_P3_38170494	Ca4	38170494	TTG/T	TCTTTTGACCCTCTTTGTTGAGA	AAATACTGATTGCAACCAAACC	60.3	434	INTERGENIC	--	--	--
Ca4_P3_38197317	Ca4	38197317	GAATAATAATAATAATAATAA/GAATAATAATAATAATAA	ATATGTCCAGGTGCACGACA	TTCTTCAATGGTGGGCTTTC	60.0	559	INTRON	Ca_13134	GE	HB-other

INDEL marker IDs	Chromosomes/ unanchored scaffolds	Physical positions (bp)	InDels (*Kabuli* reference genome-CDC Frontier/PII)	Forward primers (5'-3')	Reverse primers (5'-3')	Anneling temperature (0C)	Expected amplified product size (bp)	Structural annotation		Functional annotation	
								Sequence components of *kabuli* genome	*Kabuli* gene accession IDs	NCBI-KOG	TFs
Ca4_P3_38200388	Ca4	38200388	T/TA	ACATGGAGCGTTTGTGTGAA	TCTTGGGATCACCAACCAAT	60.2	590	INTERGENIC	--	--	--
Ca4_P3_38200490	Ca4	38200490	GTTTTTTTT/GTTTTTTTTTTTTTT	ACATGGAGCGTTTGTGTGAA	TCTTGGGATCACCAACCAAT	60.2	590	INTERGENIC	--	--	--
Ca4_P3_38231337	Ca4	38231337	AC/A	GGCCATCAGAGAATCTTCCA	TGCACTTTTGGGAACAACAA	60.2	580	INTRON	Ca_13131	--	--
Ca4_P3_38237743	Ca4	38237743	CTTTTTTTTT/CTTTTTTTTTTTTTT	CTCCATAGCCACAACAAGCA	TCCACAGGTTGCAATCACAT	59.9	604	INTRON	Ca_13131	--	--
Ca4_P3_38239017	Ca4	38239017	TAAAAAA/TAAAAAAA	CGGCTCGGGTATCAGATTTA	TATTTGCAACCTTGATGCCA	60.1	693	INTRON	Ca_13131	--	--
Ca4_P3_38240819	Ca4	38240819	ACCC/ACC	CTGGTGAAACTCCGTATCCAA	TTGGATGTGCACGATGTTTT	60.0	258	INTRON	Ca_13131	--	--
Ca4_P3_38248300	Ca4	38248300	T/TTG	CGGAACCGAAATCGAAAATA	TTGAAACCAAAGCCATCCTC	59.9	515	INTERGENIC	--	--	--
Ca4_P3_38294135	Ca4	38294135	A/AAT	AGATTTTGTTGCTGAATGATGG	CAGGTCGCTGGATTCAAAGT	59.1	946	DRR	Ca_13127	--	--
Ca4_P3_38311578	Ca4	38311578	C/CTAGTAT	CGAGCAATAGGAGGCTGTTC	GGCACTTCAAAAAGAGGTGG	60.0	550	INTERGENIC	--	--	--
Ca4_P3_38318785	Ca4	38318785	ATTTTTT/ATTTTTTT	TGAATTAGGGTGGTTTGTTTAAGA	TCATTCTTTCACATTGAATTTGCT	59.0	832	INTERGENIC	--	--	--

INDEL marker IDs	Chromosomes/ unanchored scaffolds	Physical positions (bp)	InDels (*Kabuli* reference genome-CDC Frontier/PII)	Forward primers (5'-3')	Reverse primers (5'-3')	Anneling temperature (0C)	Expected amplified product size (bp)	Structural annotation		Functional annotation	
								Sequence components of *kabuli* genome	*Kabuli* gene accession IDs	NCBI-KOG	TFs
Ca4_P3_38321094	Ca4	38321094	GAAAAA/GAAAA	TCCTATGGTCGTAGGTGGCT	AGGAAAATGCAGGACCACAC	59.6	935	INTRON	Ca_13122	C	bHLH
Ca4_P3_3833618	Ca4	3833618	T/TGACAGGATAG	GCCCAGATTTCCACAACAGT	CAACCCATTCCTTCATTGCT	60.0	919	INTERGENIC	--	--	--
Ca4_P3_38367094	Ca4	38367094	T/TAGATTGGAA	TCGTTTAGCGGTGAAACTCA	CAAGCTCTTGCCATGTTTGA	59.5	158	DRR	Ca_13120	S	--
Ca4_P3_38368647	Ca4	38368647	A/ATTCTACTTTCAATT	TATGGTTGGATTTTCCGCTC	TTGTCTTCCCCGTTTAGTGG	59.9	751	INTERGENIC	--	--	--
Ca4_P3_38377327	Ca4	38377327	TTT/TTTGAATT	TGATTGTGATGCACCGTAAAA	AGCGAAAGTTCTTGGAGGGT	60.0	470	INTERGENIC	--	--	--
Ca4_P3_38435446	Ca4	38435446	ATT/ATTT	AGCGACATTAACCAACGACC	CAATTTCCCCATCCAATACG	60.0	479	INTERGENIC	--	--	--
Ca4_P3_38438657	Ca4	38438657	TAATAAAAA/TAATAAAAAATAAAAA	TGGCACAAAAACGGTTACAA	TGTCCTTTTGCAACTTCATCA	60.0	516	INTERGENIC	--	--	--
Ca4_P3_38450725	Ca4	38450725	AGT/AGTGT	GCCATGTCATTTCTGACCCT	TTTCTGTTGATGCCTGCTTG	59.9	644	INTRON	Ca_13113	O	--
Ca4_P3_38457252	Ca4	38457252	CTGTGT/CTGT	CGATCACCAAGTCCCCTAAA	CCACTCCCTCCAATCTTCAA	59.9	344	INTERGENIC	--	--	--
Ca4_P3_38466655	Ca4	38466655	CT/CTT	CACCAATCGATGCAAATGAG	TCACAATGGTTCCTCATCCA	60.1	627	INTERGENIC	--	--	--

INDEL marker IDs	Chromosomes/ unanchored scaffolds	Physical positions (bp)	InDels (*Kabuli* reference genome-CDC Frontier/PII)	Forward primers (5'-3')	Reverse primers (5'-3')	Anneling temperature (0C)	Expected amplified product size (bp)	Structural annotation		Functional annotation	
								Sequence components of *kabuli* genome	*Kabuli* gene accession IDs	NCBI-KOG	TFs
Ca4_P3_38479454	Ca4	38479454	CAAAA/CAAA	GGTGGGATCCATTTGAGTTG	CCGAAGGCTTTCTTCAACAG	60.2	676	INTERGENIC	--	--	--
Ca4_P3_38480745	Ca4	38480745	TGG/TG	GTGGAGCTTCTTTGGGTGAC	ATCACCACCGAAAAACTTGG	59.7	844	INTERGENIC	--	--	--
Ca4_P3_38482887	Ca4	38482887	CAAAAA/CAAAA	CAAATGAGAGCGTTAAGGCAG	TGTCCTTCAATTTCATTTCCAA	60.0	501	INTERGENIC	--	--	--
Ca4_P3_38482961	Ca4	38482961	A/AC	TTGGAAATGAAATTGAAGGACA	TTGTTGATTTCTAACCTTGCTCA	59.4	193	INTERGENIC	--	--	--
Ca4_P3_38483484	Ca4	38483484	AATA/AATATATA	TGAGCAAGGTTAGAAATCAACAA	ACCATTGGTTTCCTTTGCAC	58.9	470	INTERGENIC	--	--	--
Ca4_P3_38483773	Ca4	38483773	TAGAGAGAGAAA/TAGAGAGAGAAAGAGAGAGAGAAA	CAGTGTGCAAAGGAAACCAA	TCCAGAAGCAGCAAAGATGA	59.7	405	INTERGENIC	--	--	--
Ca4_P3_38484063	Ca4	38484063	TAGA/TA	TCATCTTTGCTGCTTCTGGA	CTACCTCATGCTTGGCATCC	59.7	299	INTRON	Ca_13111	K	MIKC
Ca4_P3_38484127	Ca4	38484127	TTTATT/TTT	TCATCTTTGCTGCTTCTGGA	CTACCTCATGCTTGGCATCC	59.7	299	INTRON	Ca_13111	K	MIKC
Ca4_P3_38484269	Ca4	38484269	ATTG/ATTGTTG	TCATCTTTGCTGCTTCTGGA	TGCCAGATCTGTTCATGGAT	59.7	523	INTRON	Ca_13111	K	MIKC
Ca4_P3_38484662	Ca4	38484662	TA/TAA	GGATCCGATCTGCTCTCTTG	TCAACTTGCATGCCATCATT	59.9	239	INTRON	Ca_13111	K	MIKC

INDEL marker IDs	Chromosomes/ unanchored scaffolds	Physical positions (bp)	InDels (*Kabuli* reference genome-CDC Frontier/PII)	Forward primers (5'-3')	Reverse primers (5'-3')	Anneling temperature (0C)	Expected amplified product size (bp)	Structural annotation		Functional annotation	
								Sequence components of *kabuli* genome	*Kabuli* gene accession IDs	NCBI-KOG	TFs
Ca4_P3_38484870	Ca4	38484870	CATATA/CATA	AATGATGGCATGCAAGTTGA	GCCTGTTTCTCCCGGATAGT	60.1	268	INTRON	Ca_13111	K	MIKC
Ca4_P3_384893	Ca4	384893	AATATATATATATATATATATATATATATATATA/AATATATATATATATATATATATATATA	CACACACAACTGCGACATCTT	TCATGCTTGTTCTGGATTCG	59.8	680	INTERGENIC	--	--	--
Ca4_P3_38495273	Ca4	38495273	ATTTTTTGTTTTTT/ATTTTTT	GCACATGTGTGGTCCTCATC	TGTTGGTGTGGGTGATGTCT	60.0	680	INTERGENIC	--	--	--
Ca4_P3_38502864	Ca4	38502864	T/TA	AAGCGCAAAGAGATGGAAAA	TGAACTTCAATTTCGCCAGC	60.0	889	INTRON	Ca_13110	G	--
Ca4_P3_38503644	Ca4	38503644	TTATATATATATAT/TTATATATATATATATATATATATATATATAT	CGGCTTTGATGCATTGATAC	CCCAACGTTTAGCCACCTTA	59.1	506	INTRON	Ca_13110	G	--
Ca4_P3_38506244	Ca4	38506244	GCACC/G	AAAGGAGCAGTGACTGGCAT	AAAAGGGACGGAGGGAGTAA	59.9	915	INTRON	Ca_13110	G	--
Ca4_P3_38508113	Ca4	38508113	TGGG/TGGGG	TGGGGTAGCTTCCGTCATAG	AAATTGGGGGAAATCAAAGG	60.1	549	INTRON	Ca_13110	G	--
Ca4_P3_38514954	Ca4	38514954	CAAA/CA	TGCCTACGTTTTTAGGCCAC	GGCCTGATGTTGCTTGGTAT	60.1	743	INTRON	Ca_13110	G	--
Ca4_P3_38523744	Ca4	38523744	ATTTTTT/ATTTTTTT	ACTTTGCGTTGGGAGAAAAA	CGTTCATTTTGAAACAGATTGG	59.7	601	INTRON	Ca_13110	G	--
Ca4_P3_38524170	Ca4	38524170	TG/T	CCAATCTGTTTCAAAATGAACG	GGTGGTTAAGAGGAGTTGAAGTG	59.5	238	INTRON	Ca_13110	G	--

INDEL marker IDs	Chromosomes/ unanchored scaffolds	Physical positions (bp)	InDels (*Kabuli* reference genome-CDC Frontier/PII)	Forward primers (5'-3')	Reverse primers (5'-3')	Anneling temperature (0C)	Expected amplified product size (bp)	Structural annotation		Functional annotation	
								Sequence components of *kabuli* genome	*Kabuli* gene accession IDs	NCBI-KOG	TFs
Ca4_P3_38536489	Ca4	38536489	GCAACAACA/GCAACA	GCATCCCCTTCCAACTGTTA	TTCCAATCCATCCTCACACA	59.9	818	DRR	Ca_13110	G	--
Ca4_P3_38545997	Ca4	38545997	ATTTTTTTTTT/ATTTTTTTTT	CTCACGCGAGAAGTCAAGAA	GAGATTGGATAAAAACATCGCA	59.3	287	INTERGENIC	--	--	--
Ca4_P3_38558247	Ca4	38558247	ATTTTTT/ATTTTTTT	TGATGTGGAAATTTTTGGCA	ATTTTCGAATGAACGGTTGC	59.9	705	INTERGENIC	--	--	--
Ca4_P3_38558416	Ca4	38558416	CAAAAAAAA/CAAAAAAAAAA	ACGCCGGAGTCATAATGGTA	ATTTTCGAATGAACGGTTGC	60.4	583	INTERGENIC	--	--	--
Ca4_P3_38561994	Ca4	38561994	ATT/AT	GGACTGGACGTAACCCACTG	GTTGTGGGCCACTCTCTGAT	60.4	603	INTERGENIC	--	--	--
Ca4_P3_38563052	Ca4	38563052	T/TCA	ATTTCCCGCAGGAACGTAAT	GCAATCCATGCCTCCTAGTC	60.7	679	INTERGENIC	--	--	--
Ca4_P3_38564518	Ca4	38564518	T/TC	CGCTCAAATCCATTTTCACC	TGTAGCATTTGATGGGTTCAA	60.4	447	INTERGENIC	--	--	--
Ca4_P3_38567221	Ca4	38567221	GAATAAAA/GAA	GACAAAAATGAGAAAGACCTGGA	AAATGCTGCCGAAGAAGCTA	59.6	824	INTERGENIC	--	--	--
Ca4_P3_38567924	Ca4	38567924	A/ATTAAGAG	TAGCTTCTTCGGCAGCATTT	TGGCTTGCTTAAAAGTCGAGA	60.1	316	INTERGENIC	--	--	--
Ca4_P3_38568495	Ca4	38568495	ATATT/ATATTATT	CGAAATTGACAAAATCACAATCA	TGTCCATGAAGAAAGAGCCC	59.9	340	INTERGENIC	--	--	--

INDEL marker IDs	Chromosomes/ unanchored scaffolds	Physical positions (bp)	InDels (*Kabuli* reference genome-CDC Frontier/PII)	Forward primers (5'-3')	Reverse primers (5'-3')	Anneling temperature (0C)	Expected amplified product size (bp)	Structural annotation		Functional annotation	
								Sequence components of *kabuli* genome	*Kabuli* gene accession IDs	NCBI-KOG	TFs
Ca4_P3_38573615	Ca4	38573615	GAATTGGAAATTGGAAATTG/GAATTGGAAATTGGAAATTGGAAATTG	CCGTTTGGTGTTGAAAAGGT	CCACGTGGCACTCATCTCTA	59.9	179	INTERGENIC	--	--	--
Ca4_P3_38573732	Ca4	38573732	ATTTTTTT/ATTTTTT	TGGGAATTGGAAATTGGAAA	ATCAACACGGTTGGTTACCG	60.1	219	INTERGENIC	--	--	--
Ca4_P3_38575831	Ca4	38575831	ATT/AT	TAACCCATCAGTCAGCCTCC	CATGCCACTTTTCCCAAATC	60.1	790	INTERGENIC	--	--	--
Ca4_P3_38578752	Ca4	38578752	ATTTTATATTTTTTATATTTTTT/ATTTTATATTTTTT	AATCTCATTGCCTCGTTGCT	TGGAAGCACTCAAATCCACA	59.8	759	INTERGENIC	--	--	--
Ca4_P3_38588079	Ca4	38588079	GAAAAAAAAA/GAAAAAAA	GCTCACTCACATTAACTGCAACA	TCGATAATTTTGATCTCTCCATCA	60.4	576	INTERGENIC	--	--	--
Ca4_P3_38618852	Ca4	38618852	CATAA/CA	GCCGACAGAGAAAATCCAAA	ATTGGGGAAGGCCTTGATAA	60.2	532	INTERGENIC	--	--	--
Ca4_P3_38626236	Ca4	38626236	TCC/TCCCC	GGCTAAATTACATCTGCGGTC	GTTTTCCCGAATTGGTCCTT	58.7	511	INTERGENIC	--	--	--
Ca4_P3_38626867	Ca4	38626867	TAATAAGAGTAAA/TAATAAGAGTAAATAAGAGTAAA	AAGGACCAATTCGGGAAAAC	TCAAGAGATCGTGGAAGCAA	60.2	707	INTERGENIC	--	--	--
Ca4_P3_38649950	Ca4	38649950	TAAAAA/TAAAAAAA	ACGCGGTAGCAGAAAAGATG	TGGAAGATCCTTGTACTTTTTGA	60.4	144	INTERGENIC	--	--	--
Ca4_P3_38656268	Ca4	38656268	AATATATATATATATATATATA/AATATATATATATATATATA	TTGCTGCTTTCCAGAACCTT	GGGCTAAGTGCCCCTTATTT	60.0	871	INTERGENIC	--	--	--

INDEL marker IDs	Chromosomes/ unanchored scaffolds	Physical positions (bp)	InDels (*Kabuli* reference genome-CDC Frontier/PII)	Forward primers (5'-3')	Reverse primers (5'-3')	Anneling temperature (0C)	Expected amplified product size (bp)	Structural annotation		Functional annotation	
								Sequence components of *kabuli* genome	*Kabuli* gene accession IDs	NCBI-KOG	TFs
Ca4_P3_38667296	Ca4	38667296	ATTTTCTAACAACCTTTTCTAA/ATTTTCTAA	TGCTTGCATGTTAGGAGTGC	GCTTTGCCAGGCTTTATCTG	60.0	576	INTRON	Ca_13100	L	CPP
Ca4_P3_38684912	Ca4	38684912	TTGTGTAAACTCTG/TTG	AACCATGGGGCTATAGGCTT	GCTCAACACTTCGTCATAGATTCA	59.8	487	INTERGENIC	--	--	--
Ca4_P3_38686412	Ca4	38686412	AA/AAGGTGGAAGA	AGGTCAAGCACCCACTTCAG	TTCACAATCTTCGGGTCCTC	60.3	323	INTERGENIC	--	--	--
Ca4_P3_38705713	Ca4	38705713	ATTTTTTT/ATTTTTTTTT	TTGGGTACAACTCCGTCACA	TCAAAATCTTCCTTCAATGCAA	60.0	715	INTERGENIC	--	--	--
Ca4_P3_38707255	Ca4	38707255	TG/TGCCGGTCCAAG	TGAACACCCCAATTTCCATT	TCGGTTTGAGTTAAGGTGGC	60.0	632	INTERGENIC	--	--	--
Ca4_P3_38708333	Ca4	38708333	GTTTT/GTTTTT	AATGCATGAAGCCACAACAC	TTTCCAGCAGAGCCCATAGT	59.6	404	INTERGENIC	--	--	--
Ca4_P3_38719493	Ca4	38719493	GAAA/GAAAA	TGTGCAATTTGCCAAAAGAA	TTTCCACTTTCTCCCCCTTT	60.2	577	DRR	Ca_13098	GMW	NAC
Ca4_P3_38727283	Ca4	38727283	GAAAAAAAAAAA/GAAAAAAAA	GCTGATGGTGCATTGTTGTT	GCCTCACCGTGTCACCTAAT	59.6	818	INTERGENIC	--	--	--
Ca4_P3_38728191	Ca4	38728191	A/AC	TCCATCTTCGTGTTCTTCCTC	TCGATTCACACCACTCGTGA	59.3	681	INTERGENIC	--	--	--
Ca4_P3_38729171	Ca4	38729171	TAC/T	AAAGGAGATGCTCCCCCTAA	CGTCACAATGACCTTTCCCT	60.0	480	INTERGENIC	--	--	--

INDEL marker IDs	Chromosomes/ unanchored scaffolds	Physical positions (bp)	InDels (*Kabuli* reference genome-CDC Frontier/PII)	Forward primers (5'-3')	Reverse primers (5'-3')	Anneling temperature (0C)	Expected amplified product size (bp)	Structural annotation		Functional annotation	
								Sequence components of *kabuli* genome	*Kabuli* gene accession IDs	NCBI-KOG	TFs
Ca4_P3_38732253	Ca4	38732253	TATAAATAAATAAATAAA/TATAAATAAATAAA	AATCTGGTTTTGGTGCAAGG	GTTTGGGATTGTCAGGCTGT	60.0	458	INTERGENIC	--	--	--
Ca4_P3_38734850	Ca4	38734850	TAAAA/TAAAAA	GAGACCAAATCTCATCCCCA	GCGAAATCGTGTGAACCTCT	59.9	800	INTERGENIC	--	--	--
Ca4_P3_38734922	Ca4	38734922	CACACATAATATATACA/CACACATAATATATACACATAATATATACA	GAGACCAAATCTCATCCCCA	GCGAAATCGTGTGAACCTCT	59.9	800	INTERGENIC	--	--	--
Ca4_P3_38738477	Ca4	38738477	GTATAT/GTATATAT	TGGGGGAGTTCATAGGATCA	TGGTGCGTTAAGATGTGGAA	60.3	216	INTERGENIC	--	--	--
Ca4_P3_38740713	Ca4	38740713	CAAAAA/CAAAA	ACAATTATTTCACCTGCGGC	TTGAGGAAAAGAACCGTTGG	60.0	422	INTERGENIC	--	--	--
Ca4_P3_38741986	Ca4	38741986	TAATT/TAATTAAAATAAAATT	GCCATAAACACAACCCTTTCT	AAATACGAGCAAACAAAAACAAA	58.1	680	INTERGENIC	--	--	--
Ca4_P3_38744817	Ca4	38744817	CAAAAA/CAAAA	GAAGCACCATCGACCACTTT	TGGTGGCACTAGGTGTATTGA	60.1	194	INTERGENIC	--	--	--
Ca4_P3_38758073	Ca4	38758073	GTTTTT/GTTT	TAGACAGGTTGGTCCCTCCC	ACCGTACAATCAAACCGCTC	61.3	840	INTRON	Ca_13097	--	--
Ca4_P3_38758645	Ca4	38758645	AAA/AAACAA	GAGCGGTTTGATTGTACGGT	AACATTGCCATTCCAAGACC	60.0	514	INTRON	Ca_13097	--	--
Ca4_P3_38766114	Ca4	38766114	AA/AAAAAAAATATATATATA	ACCGACAGCATCCTCAATCT	CGCCAATTTAAAACCACGAT	59.7	677	INTERGENIC	--	--	--

INDEL marker IDs	Chromosomes/ unanchored scaffolds	Physical positions (bp)	InDels (*Kabuli* reference genome-CDC Frontier/PII)	Forward primers (5'-3')	Reverse primers (5'-3')	Anneling temperature (0C)	Expected amplified product size (bp)	Structural annotation		Functional annotation	
								Sequence components of *kabuli* genome	*Kabuli* gene accession IDs	NCBI-KOG	TFs
Ca4_P3_38773383	Ca4	38773383	TAAAAAA/TAAAAAAA	CGGTTGAGTCATATTG GTTCG	ATTTGGGAAACCGACA CTTG	60.4	591	INTERGENIC	--	--	--
Ca4_P3_38813013	Ca4	38813013	AC/A	TGGAATTCGAGAGGAT GACC	TGCCATCCAGTTTTCA TTTG	60.0	519	INTERGENIC	--	--	--
Ca4_P3_38816584	Ca4	38816584	AATTTATATTTA/AATTTATATTTATATT TA	ATGCCTTTACATGCACA AAAT	GCAAATATTGTCTGCC AAAAGTG	57.2	724	DRR	Ca_13093	K	NF-YB
Ca4_P3_38823148	Ca4	38823148	CTTTTTTTT/CTTTTTTTTTTTT	TTGGATATTCATTGCAT CATCTAGT	GCATTGAGAATTGTCT TCCACA	59.0	888	INTERGENIC	--	--	--
Ca4_P3_38826268	Ca4	38826268	AT/ATTTT	CCCTTGTCATCTTTGTT GGC	TTGGCGTTGGACTGAT GTTA	60.5	156	INTERGENIC	--	--	--
Ca4_P3_38829588	Ca4	38829588	CATTATTATTATTATTATTATTATTATTA TTATTATTATTATTATT/CATTATTATTA TTATTATTATTATTATTATTATTATTATT ATTATTATTATT	TGTGTGGTCATTGGAA TCACTT	TGAGAAGAGTTGTGGC GTTG	60.3	920	INTERGENIC	--	--	--
Ca4_P3_38829902	Ca4	38829902	GTT/GTTT	ACTAAGGAGAATTGGG CCGT	TGAGAAGAGTTGTGGC GTTG	60.0	245	INTERGENIC	--	--	--
Ca4_P3_38832483	Ca4	38832483	AAAGA/AAAGAAGA	CCCTTGACTCCGGTTT ATCA	TCTTCCCAGTGTTTCC CATC	59.9	263	DRR	Ca_13091	S	--
Ca4_P3_38832626	Ca4	38832626	CAAA/CAA	CCCTTGACTCCGGTTT ATCA	CATCGGTCGATTCCAT ATCC	59.9	795	DRR	Ca_13092	J	--
Ca4_P3_38883985	Ca4	38883985	ATAATT/ATAATTAATT	CCTCAAATTAATCATGT GTCGATT	AAACTACACCATGACC ACCCA	59.3	592	INTERGENIC	--	--	--

INDEL marker IDs	Chromosomes/ unanchored scaffolds	Physical positions (bp)	InDels (*Kabuli* reference genome-CDC Frontier/PII)	Forward primers (5'-3')	Reverse primers (5'-3')	Anneling temperature (0C)	Expected amplified product size (bp)	Structural annotation		Functional annotation	
								Sequence components of *kabuli* genome	*Kabuli* gene accession IDs	NCBI-KOG	TFs
Ca4_P3_38916952	Ca4	38916952	TAAAAA/TAAAAAA	ATCCGTCGAGTCAAAACACC	GCTATTAGAGAGGTCGGCCC	60.0	572	INTERGENIC	--	--	--
Ca4_P3_38917638	Ca4	38917638	CAAA/CAA	GGGCCGACCTCTCTAATAGC	TGATGTTGCAATGGACGTTT	60.2	535	INTERGENIC	--	--	--
Ca4_P3_38925649	Ca4	38925649	ATTA/ATTAATAATATACAATTTA	GAGTATGTGCCCCACTCCAT	TGCACGTTGAGACGACAACT	59.8	496	INTERGENIC	--	--	--
Ca4_P3_38927088	Ca4	38927088	TAAAA/TAAA	TTTGAGGAAAAGGAAAGCCA	GGACCATTTTGCCAGTCAGT	59.8	200	INTRON	Ca_13085	--	--
Ca4_P3_38932264	Ca4	38932264	TT/TTACAAAAATAT	AAGCCGAACTTCAAGAACCA	TGCCGTTTTCAACAAAACAA	59.9	367	INTERGENIC	--	--	--
Ca4_P3_38936372	Ca4	38936372	ACTAATGCT/ACT	TACGCATTTCACGTGCTCTC	TTCAAACTCATCGATTCAGGG	60.0	597	INTERGENIC	--	--	--
Ca4_P3_38949892	Ca4	38949892	GT/G	TCAAATCAGTATGCGAAGCG	ACCCACACAACACACATGCT	60.0	360	INTERGENIC	--	--	--
Ca4_P3_38954815	Ca4	38954815	CAGTTAGT/CAGTTAGTTAGT	CACTCTCAAATCGCCATCAA	AGCATGTCACCCCAAAACTC	59.8	839	INTERGENIC	--	--	--
Ca4_P3_38955357	Ca4	38955357	GATAAAATA/GATA	GAGTTTTGGGGTGACATGCT	CATCCTGCACAATGCTGTTC	60.0	251	INTERGENIC	--	--	--
Ca4_P3_38955492	Ca4	38955492	TACACA/TACACACA	GAGTTTTGGGGTGACATGCT	GCTCCCTTTAGATTCTCGTCA	60.0	498	INTERGENIC	--	--	--

INDEL marker IDs	Chromosomes/ unanchored scaffolds	Physical positions (bp)	InDels (*Kabuli* reference genome-CDC Frontier/PII)	Forward primers (5'-3')	Reverse primers (5'-3')	Anneling temperature (0C)	Expected amplified product size (bp)	Structural annotation		Functional annotation	
								Sequence components of *kabuli* genome	*Kabuli* gene accession IDs	NCBI-KOG	TFs
Ca4_P3_38955534	Ca4	38955534	GTGGGT/G	GAGTTTTGGGGTGACATGCT	GCTCCCTTTAGATTCTCGTCA	60.0	498	INTERGENIC	--	--	--
Ca4_P3_38965155	Ca4	38965155	TATAATAA/TATAA	AGCCATGCCAGATTATTTGC	CAGTGCGATCCATCTCTGAA	60.1	221	INTRON	Ca_13082	O	MYB_related
Ca4_P3_38965739	Ca4	38965739	TC/T	TTCAGAGATGGATCGCACTG	TTTAAGCCACCCAAGGTTTG	59.9	536	INTRON	Ca_13082	O	MYB_related
Ca4_P3_38970599	Ca4	38970599	CCTT/C	AGCGTACGAGAAGGAGTCCA	ACCAAAATGGCAATGAGGAC	60.0	510	INTERGENIC	--	--	--
Ca4_P3_38984036	Ca4	38984036	TC/TCC	AAACCACGACACACCAACAA	TCAAATGTGGTTAAATGATGACTG	59.9	872	DRR	Ca_13081	H	--
Ca4_P3_38984191	Ca4	38984191	TCC/TCCC	TGTGAAATGGTTGCACATGA	TTGTAACTTGATTCGGAAAAATGA	59.5	843	DRR	Ca_13081	H	--
Ca4_P3_38994620	Ca4	38994620	ATGTATT/ATGTATTGTATT	GCATACCCTTTTCCGGTCTT	GCATGCTCTAATCCATTCCAA	60.3	769	INTERGENIC	--	--	--
Ca4_P3_38997590	Ca4	38997590	TCAA/T	ACCCACCACTGATTCCATGT	TGTCTTCAACTAGCACTCGGG	60.1	480	INTERGENIC	--	--	--
Ca4_P3_39013061	Ca4	39013061	TAAAAAA/TAAAAAAA	ACCAACGGAGAGTTTGCCTA	TTCACTTCGACAGCAAAACG	59.7	613	INTERGENIC	--	--	--
Ca4_P3_39015473	Ca4	39015473	TTATATA/TTATA	AAGCCTTCGTATGGCTGTTG	CCTTTCCTTTGGGTGAAGAA	60.3	551	INTRON	Ca_13079	R	DBB

INDEL marker IDs	Chromosomes/ unanchored scaffolds	Physical positions (bp)	InDels (*Kabuli* reference genome-CDC Frontier/PII)	Forward primers (5'-3')	Reverse primers (5'-3')	Anneling temperature (0C)	Expected amplified product size (bp)	Structural annotation		Functional annotation	
								Sequence components of *kabuli* genome	*Kabuli* gene accession IDs	NCBI-KOG	TFs
Ca4_P3_39017863	Ca4	39017863	GTTTTTTTTT/GTTTTTTTTTTT	TCGTTGCGGTATGTGTCATT	TCCAAACATGGCTCTTCAAA	60.0	768	DRR	Ca_13079	R	DBB
Ca4_P3_39018704	Ca4	39018704	T/TGG	TGCTCTTAGCCACATCATTG	TGCGAAACGCAATTATTTCA	57.4	443	DRR	Ca_13079	R	DBB
Ca4_P3_39024713	Ca4	39024713	ATTTTTTT/ATTTTTT	AGAAGGGGATGCCAAAAAGT	CCCAGAGTCCCAATTTCTCA	59.9	594	INTERGENIC	--	--	--
Ca4_P3_39033106	Ca4	39033106	GCC/GCCC	GGATTTCCCAAACAGCAAAA	TGCCTTACCAAAGGAATTGG	59.9	523	INTRON	Ca_13078	A	--
Ca4_P3_39038460	Ca4	39038460	ACTTGCTTG/ACTTG	CCGGTCACATTCAATAGCCT	ATTGCGTTGTTTCTTCCAGG	60.0	411	DRR	Ca_13078	A	--
Ca4_P3_39044673	Ca4	39044673	AGTGTGT/AGTGT	AGGGGATTCTTTTGACAGGG	GGAGAAGACGAAACTGTCGC	60.3	794	INTRON	Ca_13077	O	WRKY
Ca4_P3_39063852	Ca4	39063852	TCACAC/TCACACAC	TTGCCACTTGATACAATGAAGC	CGTGTGGTTGTCAATTTTGC	60.1	453	INTERGENIC	--	--	--
Ca4_P3_39077079	Ca4	39077079	TGAAAAAACACACGAAAAAA/TGAAAAAA	TGGTCAAATAAAACGTGGCA	TGAGTCGTGGACTATGTCGC	60.0	461	INTERGENIC	--	--	--
Ca4_P3_39081038	Ca4	39081038	CCACA/CCACACA	TGTTCAGGTTTCGGGATTCT	CTCGAGAAGAGGGAGCAATG	59.5	469	INTRON	Ca_13074	PT	FAR1
Ca4_P3_3912089	Ca4	3912089	CTATATATATATATATAT/CTATATATATATATATATATATATATATATAT	TCCCTTAATTTTGTTGTTGTTGAA	GTTTGCAAGTACGTGGTGGA	59.8	457	INTRON	Ca_03861	--	--

INDEL marker IDs	Chromosomes/ unanchored scaffolds	Physical positions (bp)	InDels (*Kabuli* reference genome-CDC Frontier/PII)	Forward primers (5'-3')	Reverse primers (5'-3')	Anneling temperature (0C)	Expected amplified product size (bp)	Structural annotation		Functional annotation	
								Sequence components of *kabuli* genome	*Kabuli* gene accession IDs	NCBI-KOG	TFs
Ca4_P3_39165742	Ca4	39165742	TAAAAATAAAAAATAAAAAA/TAAAAATAAAAAATAAAAAATAAAAAA	CGTATACCGCTGCTCATCAA	TGATGTGATTTGTGCCATGA	59.9	259	INTERGENIC	--	--	--
Ca4_P3_39186722	Ca4	39186722	C/CTCT	AAAATTCTGTTTGGGGACAAAA	CTTTTGTTGACTTGGCCCAT	59.7	833	INTERGENIC	--	--	--
Ca4_P3_39257138	Ca4	39257138	ATTTTTTTTTT/ATTTTTTTTTTT	TTTGCGTTACGAATTTGATGA	TGAATTTTCCCGACTTGGTC	59.2	181	INTERGENIC	--	--	--
Ca4_P3_39348944	Ca4	39348944	GTTT/GTT	AAGGGTTAGGCTTTGGGTTT	CCCTAAACCATACCTCTACCACAA	59.0	604	INTERGENIC	--	--	--
Ca4_P3_3948347	Ca4	3948347	TAAAAAAAAAA/TAAAAAAAAAAA	TTTGCATTGTCAATCAGTCAC	CAAGGGTATTTTTACCACACACA	57.2	514	INTERGENIC	--	--	--
Ca4_P3_39689187	Ca4	39689187	AC/ACC	ATTACCGACATTGCAGAGGG	CTCCCATTCTGCCCTTGATA	60.0	329	INTERGENIC	--	--	--
Ca4_P3_39775285	Ca4	39775285	TTATATATATATATATATATATATATATATATATATAT/TTATATATATATATATATATATATATATATAT	TTGAGAAGTCGCAACAAACG	TGCACGAAGTTTAGGAAGGTT	60.0	831	INTERGENIC	--	--	--
Ca4_P3_39834326	Ca4	39834326	ACCCCC/ACCCCCC	TCAGTATTATGCGGATGCCA	CCGACATCTGAGGGGACTAA	60.1	231	INTRON	Ca_14923	E	--
Ca4_P3_39838128	Ca4	39838128	GT/GCAAT	TCACAATTCCTGGCAATCAA	TGAAGATGGTGGATGGATGA	60.0	502	INTERGENIC	--	--	--
Ca4_P3_3989437	Ca4	3989437	AATATATATATATATATATATATAT/AATATATATATATATATATATATATATATATAT	TCCCGTTCCAAAACTTTTCA	TGATAGCACAAAATCGTGGA	60.5	594	DRR	Ca_03854	G	--

INDEL marker IDs	Chromosomes/ unanchored scaffolds	Physical positions (bp)	InDels (*Kabuli* reference genome-CDC Frontier/PII)	Forward primers (5'-3')	Reverse primers (5'-3')	Anneling temperature (0C)	Expected amplified product size (bp)	Structural annotation		Functional annotation	
								Sequence components of *kabuli* genome	*Kabuli* gene accession IDs	NCBI-KOG	TFs
Ca4_P3_3992081	Ca4	3992081	TAAAAAA/TAAAAAAA	TCGGACGTCTATGACACAGC	CGATGGTTCTGGTTCTGGTT	59.9	645	INTERGENIC	--	--	--
Ca4_P3_3993001	Ca4	3993001	CCC/CCCATTATATTAGGACC	TTAGCATCGTGCTGTTCCAG	TCTCCCGATTTTCCAACAAG	60.0	480	INTRON	Ca_03853	--	--
Ca4_P3_3997061	Ca4	3997061	AG/A	GGAAAAGAAACGGTGCTCAG	GGTGCAAGCTTTCCTTCTTG	59.9	468	DRR	Ca_03852	--	--
Ca4_P3_40004864	Ca4	40004864	TTATATATATATATATATATATATATATATATATATAT/TTATATATATATATATATATATAT	TTTTGACAGGCCAAACTCAA	ACTCTTATGGGTCCCCTGCT	59.3	713	INTERGENIC	--	--	--
Ca4_P3_40048822	Ca4	40048822	AATATATATATATATATA/AATATATATATATATA	CCAAGTTTCAAATGGCGACT	AATTGGCCACACTCGAAAAC	60.1	203	INTERGENIC	--	--	--
Ca4_P3_4008216	Ca4	4008216	TTATATA/TTATA	GCTTCCTAAGAAACCTCATTCAA	TGGTTTTAATTTAGAGTTGATCCAAG	58.9	796	INTERGENIC	--	--	--
Ca4_P3_4008273	Ca4	4008273	TTTTAAAATATATTTAAAATA/TTTTAAAATATATTTAAAATATATTTAAAATA	GCTTCCTAAGAAACCTCATTCAA	TGGTTTTAATTTAGAGTTGATCCAAG	58.9	796	INTERGENIC	--	--	--
Ca4_P3_40111947	Ca4	40111947	GAATAATAATAATAATAATAATAATAATA/GAATAATAATAATAATAATAATAATA	TGCAAGTTGCTATCCCATTG	TTGCAATACGGATCACAAAAA	59.7	753	INTERGENIC	--	--	--
Ca4_P3_4011601	Ca4	4011601	AGT/A	TTTTGGGCTCCAAATTTACG	TGAATGGTTAATGGATGACAGTG	59.9	348	INTERGENIC	--	--	--
Ca4_P3_4013283	Ca4	4013283	GTA/GTAAAGTGTGATGCTATA	TTGCCAACAAATTAGTGTTTGTG	CTTTGCCAAGCAAATCAACA	60.0	508	INTERGENIC	--	--	--

INDEL marker IDs	Chromosomes/ unanchored scaffolds	Physical positions (bp)	InDels (*Kabuli* reference genome-CDC Frontier/PII)	Forward primers (5'-3')	Reverse primers (5'-3')	Anneling temperature (0C)	Expected amplified product size (bp)	Structural annotation		Functional annotation	
								Sequence components of *kabuli* genome	*Kabuli* gene accession IDs	NCBI-KOG	TFs
Ca4_P3_40143450	Ca4	40143450	AAATAATAATAATAATAA/AAATAATAATAATAATAATAA	TCGACGCATGTTTTATCCAA	TTACCACTTCAAGTTTGCCG	60.1	150	INTERGENIC	--	--	--
Ca4_P3_4024785	Ca4	4024785	TAATTTTAAAATTTTAAAAT/TAATTTTAAAATTTTAAAATTTTAAAAT	GAATGACCAATTTCCAGAATCA	ACCGAGCCATAGTGACCAAC	58.9	625	INTERGENIC	--	--	--
Ca4_P3_40279996	Ca4	40279996	ATTTTTTTTT/ATTTTTTTTTT	ACTGGGCTCACTCTTGTGCT	TTTTCTCAAAGCCAAAACCG	60.1	600	DRR	Ca_14893	S	--
Ca4_P3_4028300	Ca4	4028300	GTATATATATA/GTATATATATATA	CATGGACACAACTCAGTGCC	TTCTTGAACCACCACCAACA	60.2	596	INTERGENIC	--	--	--
Ca4_P3_4029055	Ca4	4029055	GATCATCATCATCAT/GATCATCATCATCATCATCAT	ACATTCCTGTTGGTGGTGGT	ACATTCCAGTCCAAAACCCA	60.1	612	CDS (large-effect mutations)	Ca_03849	--	Dof
Ca4_P3_40320788	Ca4	40320788	GATATATATATATATATATATATATATATATA/GATATATATATATATATATATATATATA	CCTAGCCAATTCACAAACCA	CGACCTTGTGTTGAGGATGA	58.6	756	INTERGENIC	--	--	--
Ca4_P3_4032239	Ca4	4032239	AATATATATATATA/AATATATATATA	GCGTCGTTTTAGTCGTGGTT	AGCTGGGGTTGCTTTACTGA	60.2	474	INTERGENIC	--	--	--
Ca4_P3_4032914	Ca4	4032914	A/AACATTCCGCTTG	ATCCAAACTCCACCGTTGAC	CAGCACTTTGTTAAGCTGCAA	59.8	357	INTERGENIC	--	--	--
Ca4_P3_40348298	Ca4	40348298	AATATATATATATATATATATATATATATATATATA/AATATATATATATATATATATATATATATATATATATA	CCATCCTTCGAATTTGGCTA	TGGTTAAAGGCCAGGATTTC	60.0	668	DRR	Ca_14889	PET	NAC
Ca4_P3_4036494	Ca4	4036494	TAAAAAAA/TAAAAAAAA	TGTTCTTTGGCCCTCTTTTG	GAAAGTTGTCAGACGGAGGTTC	60.2	781	INTERGENIC	--	--	--

INDEL marker IDs	Chromosomes/ unanchored scaffolds	Physical positions (bp)	InDels (*Kabuli* reference genome-CDC Frontier/PII)	Forward primers (5'-3')	Reverse primers (5'-3')	Anneling temperature (0C)	Expected amplified product size (bp)	Structural annotation		Functional annotation	
								Sequence components of *kabuli* genome	*Kabuli* gene accession IDs	NCBI-KOG	TFs
Ca4_P3_4037177	Ca4	4037177	GTTTTTTTTTTTT/GTTTTTTTTT	AAAGTGAAAGCCACTCCCAA	AATGTTCGCCACCACTTTTC	59.7	369	INTERGENIC	--	--	--
Ca4_P3_4041384	Ca4	4041384	A/AC	GGGTTTTGGTTGAAATTTTTG	TAATGACGACCAGCGAATGA	58.4	540	INTERGENIC	--	--	--
Ca4_P3_40423953	Ca4	40423953	TTATATATATATATATATATAT/TTATATATATATATATATAT	TTGACACTTTTTCTCTGATGTTCA	TGAAATTCGTGAACGGATGA	58.9	594	INTERGENIC	--	--	--
Ca4_P3_4044064	Ca4	4044064	TAAA/TAAAA	AAGATTGACTTGTGCATCGC	AAGTTCCAACAAATGCACCC	58.9	660	INTERGENIC	--	--	--
Ca4_P3_4044468	Ca4	4044468	GAAAAAAAA/GAAAAAAAAA	CTCGAGCCTAATGACTTCGC	AAGTTCCAACAAATGCACCC	60.1	440	INTERGENIC	--	--	--
Ca4_P3_40460426	Ca4	40460426	ATTTTTTTTTT/ATTTTTTTTTTTT	CAGCGCTTGTAAAACTCAGAA	AGCCAGGTTTGTTTCTAGCG	58.4	328	INTERGENIC	--	--	--
Ca4_P3_4046043	Ca4	4046043	TAAAA/TAAAAA	TGTTGGGTTTGTTTCCTCCT	GCTTGATAATTTAAGGGCACCA	59.4	515	INTERGENIC	--	--	--
Ca4_P3_40461135	Ca4	40461135	TNNNNNNNNNNNN/TNNNNNNNNNN	CGCTAGAAACAAACCTGGCT	AAATGCCTGCCAAAGAACAT	59.5	485	INTERGENIC	--	--	--
Ca4_P3_4046386	Ca4	4046386	ATTTTTTTT/ATTTTTTTTTT	TGGTGCCCTTAAATTATCAAGC	GGGGGTGTTTGGTTTTACTC	60.3	660	INTERGENIC	--	--	--
Ca4_P3_40479668	Ca4	40479668	TCCC/TCC	CTCCCTAACCCTTCTCCCTG	CGGATATTGGCTGGCTTGTA	60.1	622	INTRON	Ca_14877	I	--

INDEL marker IDs	Chromosomes/ unanchored scaffolds	Physical positions (bp)	InDels (*Kabuli* reference genome-CDC Frontier/PII)	Forward primers (5'-3')	Reverse primers (5'-3')	Anneling temperature (0C)	Expected amplified product size (bp)	Structural annotation		Functional annotation	
								Sequence components of *kabuli* genome	*Kabuli* gene accession IDs	NCBI-KOG	TFs
Ca4_P3_40480392	Ca4	40480392	CTTTTTTTTT/CTTTGCATCTTTTTTTTTT	CGCACCTCTATCCTCAGACC	GAGAGGGTGGCAATGGATTA	59.8	580	INTRON	Ca_14877	I	--
Ca4_P3_40484412	Ca4	40484412	TATAATAA/TATAA	GAGGGAACCAGGATTTCTCC	ACCACCACCTTCACCACAAT	59.9	764	DRR	Ca_14877	I	--
Ca4_P3_40491458	Ca4	40491458	AAG/A	TCCGTTAGATTGCTGAATGC	CATCTCGAATGGCTTCCAAT	58.9	628	INTRON	Ca_14876	--	HD-ZIP
Ca4_P3_40494265	Ca4	40494265	GGAAG/GG	CGAAATGATCTTATAAAAATGGACAA	GCAATGCTCTCCAAAGAATCA	59.7	451	INTERGENIC	--	--	--
Ca4_P3_40499362	Ca4	40499362	CA/CAA	TGAAACAAACAATTAAGTCCGTG	AAAGGGAAGTTTCTCGAGCC	59.1	802	DRR	Ca_14875	--	--
Ca4_P3_40499622	Ca4	40499622	ATTTTT/ATTTT	GGCCAAATTCATCCAACAAA	GGGGTGGGTTGAAGAAAAAT	60.7	501	INTRON	Ca_14875	--	--
Ca4_P3_40505340	Ca4	40505340	A/AC	CGATGGTTAAGGGGATGCTA	TGAAAGCCCAAACACAATGA	59.9	591	INTERGENIC	--	--	--
Ca4_P3_40506274	Ca4	40506274	CAGTATAATAGTA/CAGTATAATAGTATAATAGTA	GTGGCTGTTTATGGATTGGG	TGATCCCCTTCATTCCTTCA	60.2	239	INTERGENIC	--	--	--
Ca4_P3_4050866	Ca4	4050866	TTA/TTATA	ATCATCCCTCATGGTGGAAA	CCTACTTCAAACGCCCATGT	60.1	519	INTRON	Ca_03848	--	bHLH
Ca4_P3_40508933	Ca4	40508933	AAAATAAATAAATAAATAA/AAAATAAATAAATAAATAAATAA	GGGGAAAAAGAAAGGAAGGA	GTGGCTGCAAAAGCTAAAGG	59.5	294	DRR	Ca_14874	--	--

INDEL marker IDs	Chromosomes/ unanchored scaffolds	Physical positions (bp)	InDels (*Kabuli* reference genome-CDC Frontier/PII)	Forward primers (5'-3')	Reverse primers (5'-3')	Anneling temperature (0C)	Expected amplified product size (bp)	Structural annotation		Functional annotation	
								Sequence components of *kabuli* genome	*Kabuli* gene accession IDs	NCBI-KOG	TFs
Ca4_P3_40519165	Ca4	40519165	TAAA/TAAAA	TTCTTACAATTACCAAACACGCA	TGTCGACCATGTGAATTAGAGAA	59.6	488	INTERGENIC	--	--	--
Ca4_P3_40519544	Ca4	40519544	CTTTATTTA/CTTTATTTATTTA	ACAATTACCAAACACGCACA	TGTCGACCATGTGAATTAGAGAA	57.5	483	INTERGENIC	--	--	--
Ca4_P3_40522888	Ca4	40522888	AAATATATATA/AAATATATATATTTTTAGAATATATATA	GATGGGTTGGTTTGATTTGG	CGACAATTTTGGTCTCTCCA	60.0	946	INTERGENIC	--	--	--
Ca4_P3_40526118	Ca4	40526118	A/AC	CACGGAATTGATCCCCTTATT	CCATGTCTTTAACTTCATGCAAA	60.0	520	INTERGENIC	--	--	--
Ca4_P3_40534846	Ca4	40534846	GTTT/GTT	TAGGAGACTGCTTCCGCAAT	TTCTCTTGCACGTTGAATCG	60.0	639	DRR	Ca_14873	--	--
Ca4_P3_4055008	Ca4	4055008	CCACAC/CCAC	TTCGTTGTCATGCTCAGGTA	TCATCTAAATCGGTGCCAAA	57.8	855	INTERGENIC	--	--	--
Ca4_P3_40550485	Ca4	40550485	GGAG/GGAGAG	ACCCTGAGTGAGGGAGGATT	CACCCAACAAGAGCTTCCAT	59.9	533	INTRON	Ca_14870	--	--
Ca4_P3_40571626	Ca4	40571626	C/CTA	CACTCATCATAGCCCCCTGT	AGATGCACGACAATGCTTCA	60.0	711	INTERGENIC	--	--	--
Ca4_P3_40576705	Ca4	40576705	A/ATT	AAATTCTCTCCCTTTCCTTGC	TTTCATCTTCCATAGCCTCG	58.8	447	INTERGENIC	--	--	--
Ca4_P3_40584112	Ca4	40584112	ATATCGT/AT	TGATATGCGGTAGCCATCAA	GACCCAAATCTGGCTTCAAA	60.1	547	INTRON	Ca_14868	K	--

INDEL marker IDs	Chromosomes/ unanchored scaffolds	Physical positions (bp)	InDels (*Kabuli* reference genome-CDC Frontier/PII)	Forward primers (5'-3')	Reverse primers (5'-3')	Anneling temperature (0C)	Expected amplified product size (bp)	Structural annotation		Functional annotation	
								Sequence components of *kabuli* genome	*Kabuli* gene accession IDs	NCBI-KOG	TFs
Ca4_P3_40587958	Ca4	40587958	CAA/CAAA	CTTGCACACAATGCGATCAT	GGGGTTTGTCATTCCATAAAAA	60.7	464	DRR	Ca_14868	K	--
Ca4_P3_4060243	Ca4	4060243	TAAAAAAAA/TAAAAAAA	TGTGACCACGAAGTTTTGGA	CATTTGTTGAAGGCACCAGA	60.1	158	INTERGENIC	--	--	--
Ca4_P3_40622737	Ca4	40622737	CAAAA/CAAA	TTTGCAAATGATGACTTCCTACT	TGTTCAAAGCTCAACCCCTC	58.0	578	INTERGENIC	--	--	--
Ca4_P3_40639757	Ca4	40639757	AAAATATTATAAATATTA/AAAATATTA	CCCCTGCCAAAATACTTTCA	TCCTATGGAACCACCAAAGC	59.9	752	INTERGENIC	--	--	--
Ca4_P3_40640309	Ca4	40640309	T/TAA	TGCCATAATTGGTTGCAAAA	AAATGATCAAGTCAGGAAGAAAAA	59.9	605	INTRON	Ca_14865	--	--
Ca4_P3_40640542	Ca4	40640542	CA/CAAGTGA	TGCCATAATTGGTTGCAAAA	TGAATTTGATGTTGTTGAAGATGA	59.9	807	INTRON	Ca_14865	--	--
Ca4_P3_40647639	Ca4	40647639	TAA/TA	TATTGCTCTGGCTTGTGGTG	TCATGTTGGGTCAGATGGAA	59.9	663	DRR	Ca_14865	--	--
Ca4_P3_40648444	Ca4	40648444	CTAATAATAATAATAATAA/CTAATAATAATAA	ATTTGGTGAAGGCTGATTGG	TGTGGTACCGGATAATCACAAA	59.9	838	DRR	Ca_14865	--	--
Ca4_P3_40648481	Ca4	40648481	AG/AGG	ATTTGGTGAAGGCTGATTGG	TGTGGTACCGGATAATCACAAA	59.9	838	DRR	Ca_14865	--	--
Ca4_P3_40649020	Ca4	40649020	ATTTTTT/ATTTTTTTT	TGTGATTATCCGGTACCACAAC	TGTATTCAGGACCTCGCAGA	59.6	207	INTERGENIC	--	--	--

INDEL marker IDs	Chromosomes/ unanchored scaffolds	Physical positions (bp)	InDels (*Kabuli* reference genome-CDC Frontier/PII)	Forward primers (5'-3')	Reverse primers (5'-3')	Anneling temperature (0C)	Expected amplified product size (bp)	Structural annotation		Functional annotation	
								Sequence components of *kabuli* genome	*Kabuli* gene accession IDs	NCBI-KOG	TFs
Ca4_P3_40650076	Ca4	40650076	TG/TGTTGCCG	GTTCGACTTCGTCGCTAAGG	CCAGCTAAGCCACTTTCACC	60.0	258	CDS (large-effect mutations)	Ca_14864	T	--
Ca4_P3_40653220	Ca4	40653220	TAA/TAAA	TTCAGTTGGTGGGTTGACCT	CAATGAGTGTGTTTATTGCGA	60.4	769	DRR	Ca_14864	T	--
Ca4_P3_4068418	Ca4	4068418	ATTTTTTTTTTT/ATTTTTTTTT	ATAGATGGTCGCATTCTGGG	TCATCAAATCCGTAACGCAA	59.9	698	INTERGENIC	--	--	--
Ca4_P3_4068666	Ca4	4068666	ATTTTTTTTTTTT/ATTTTTTTTTTTTTT	TTGCGTTACGGATTTGATGA	CAGAATTGACCAAGCGATCA	60.1	569	INTERGENIC	--	--	--
Ca4_P3_40704105	Ca4	40704105	ATTTTTT/ATTTTT	GGATCTCCACTGCCAGGTTA	TTTCGTTTCCCATTCTACGC	60.1	648	INTERGENIC	--	--	--
Ca4_P3_40724110	Ca4	40724110	TAATGGAGAA/TAA	ATGGAGGGAGAAGGAAATGC	CCATTTTCGATGCTTGGACT	60.4	398	INTRON	Ca_23910	S	C2H2
Ca4_P3_40730301	Ca4	40730301	GTTTTTT/GTTTTTTT	GGTGTTTGCGGGTTGATATT	CGTCTTCCACCTTATCCATGA	59.7	561	INTERGENIC	--	--	--
Ca4_P3_40734535	Ca4	40734535	GTTTT/GTTT	ATTAATGGTCACGACCCTGC	CACTCGCAAAGACCAGATCA	59.8	293	INTRON	Ca_23909	--	--
Ca4_P3_40738290	Ca4	40738290	T/TTTTTAATATAAAC	TTTACCCGAGCCAATTTCAG	GGGTTTGAGAGGTGAAGTGTG	60.1	854	DRR	Ca_23908	R	NAC
Ca4_P3_40743126	Ca4	40743126	AATATATATATATAT/AATATATATATATATATATATATATATATATAT	CACAAACACAACATGCGACA	ACCGCAACACACAAAATTCA	60.2	373	INTERGENIC	--	--	--

INDEL marker IDs	Chromosomes/ unanchored scaffolds	Physical positions (bp)	InDels (*Kabuli* reference genome-CDC Frontier/PII)	Forward primers (5'-3')	Reverse primers (5'-3')	Anneling temperature (0C)	Expected amplified product size (bp)	Structural annotation		Functional annotation	
								Sequence components of *kabuli* genome	*Kabuli* gene accession IDs	NCBI-KOG	TFs
Ca4_P3_40746698	Ca4	40746698	CAA/CA	CAGTGTTGTGTCCGATGTCC	GTACCAGCAGCATTTGCTCA	60.0	410	INTRON	Ca_23907	R	--
Ca4_P3_40763607	Ca4	40763607	CAAAAA/CAAAA	GCTCTGCTTGATTGAAAATGC	CCAATGCATGAAATTTGTTCTT	60.0	696	INTERGENIC	--	--	--
Ca4_P3_40765533	Ca4	40765533	TAAA/TAA	AATGAAAATGAAGGCGGTGA	CAAATGTCGTTGTCGCATCT	60.4	241	DRR	Ca_23904	Q	--
Ca4_P3_40769867	Ca4	40769867	AACTTACTT/AACTT	TGCTACGCATGCTTGAGAAC	TAAAAGTTTGCATGCCCCTC	60.2	628	INTRON	Ca_23904	Q	--
Ca4_P3_40777635	Ca4	40777635	ATTTTTT/ATTTTTTT	TAAGGTCCAAAATCCAGGCA	GCTTAAGCTTTCCATTCAAAAGA	60.4	408	INTERGENIC	--	--	--
Ca4_P3_40783879	Ca4	40783879	TCTAATTG/T	TCTTTTTCTATCACATGGTCAACA	AATGATGCCTCCATGCTTTC	58.7	732	INTERGENIC	--	--	--
Ca4_P3_40789596	Ca4	40789596	A/AATATAAT	ATGGCCATGTCCCAATATCT	TCAACAAAGAACAAAATGGGC	59.1	724	INTERGENIC	--	--	--
Ca4_P3_40790090	Ca4	40790090	GAAAAAAAAAA/GAAAAAAAA	GCCCATTTTGTTCTTTGTTGA	TCATTGTCCAGTGAAGGCAA	60.0	667	INTERGENIC	--	--	--
Ca4_P3_40790403	Ca4	40790403	AT/ATCT	TGTTGATTTTGTTTGTCACGA	TCATTGTCCAGTGAAGGCAA	57.7	472	INTERGENIC	--	--	--
Ca4_P3_40792698	Ca4	40792698	TAAAAA/TAAAAAA	TCAAATGCCAAAATCCATCA	GTCAAGCAAAATCCCACGAT	59.9	532	INTRON	Ca_23903	--	--

INDEL marker IDs	Chromosomes/ unanchored scaffolds	Physical positions (bp)	InDels (*Kabuli* reference genome-CDC Frontier/PII)	Forward primers (5'-3')	Reverse primers (5'-3')	Anneling temperature (0C)	Expected amplified product size (bp)	Structural annotation		Functional annotation	
								Sequence components of *kabuli* genome	*Kabuli* gene accession IDs	NCBI-KOG	TFs
Ca4_P3_40797470	Ca4	40797470	CAAAAAAA/CAAAAAAAAAA	CAAGGTCCCCAAACATCACT	ATGGACCATCGATTCTGGAG	59.8	610	INTERGENIC	--	--	--
Ca4_P3_40803911	Ca4	40803911	AA/AATAGTA	AGAGATAGCGAGGCACCAAA	TGAATTGGTCATTTCCCCTC	60.0	597	INTRON	Ca_23902	R	--
Ca4_P3_40805123	Ca4	40805123	AT/A	CCAAATCAACACCAATTCCC	ACTTGAAGCCGAAGGGTTTT	60.0	524	INTRON	Ca_23902	R	--
Ca4_P3_40807547	Ca4	40807547	TCAACAACAA/TCAACAACAACAA	CACCTGAAAGCTCCCTCAAG	CGACCTTGAAGGCTTAGTCG	60.0	505	INTRON	Ca_23902	R	--
Ca4_P3_40808926	Ca4	40808926	TAAAAAAAAAA/TAAAAAAAAAAAA	CGGGAAGGATCCTGACACTA	GGTTGCATAACAAACAGCGA	60.1	669	INTRON	Ca_23902	R	--
Ca4_P3_40809750	Ca4	40809750	A/AC	TGCAAGAACAAATCCTTCCC	GCAGCTTTTGGCAGTTCTCT	60.1	278	INTRON	Ca_23902	R	--
Ca4_P3_40810756	Ca4	40810756	GA/GAA	AGGCCTTTCCGGTAACAAGT	GTTGCGAATCAACTGCAAGA	60.0	394	INTRON	Ca_23902	R	--
Ca4_P3_40818336	Ca4	40818336	AA/AACA	GTTTGCGGTTATTGCTGGTT	GTTACTTGTGGGGCTGGAAA	60.0	637	INTRON	Ca_23900	R	--
Ca4_P3_40819629	Ca4	40819629	TCAACAACAACAA/TCAACAACAACAACAA	GGCCTGGAACACTTTCCATA	CAAGGGGGTGAAAAATCAGA	59.9	407	INTRON	Ca_23900	R	--
Ca4_P3_4083518	Ca4	4083518	ATATTAT/ATATTATTAT	TCTTATGAATGAGACAGAGGCA	CCAAGACCCCGAGTTAGAGA	57.6	491	INTERGENIC	--	--	--

INDEL marker IDs	Chromosomes/ unanchored scaffolds	Physical positions (bp)	InDels (*Kabuli* reference genome-CDC Frontier/PII)	Forward primers (5'-3')	Reverse primers (5'-3')	Anneling temperature (0C)	Expected amplified product size (bp)	Structural annotation		Functional annotation	
								Sequence components of *kabuli* genome	*Kabuli* gene accession IDs	NCBI-KOG	TFs
Ca4_P3_40837782	Ca4	40837782	A/AAT	TGAATTGTTGGGTCCTCAAAG	CAAATAACTGTTGAAAAATCATCTGAA	60.0	191	INTERGENIC	--	--	--
Ca4_P3_40842793	Ca4	40842793	TC/T	CTGGATTGAGTTGAGGCCAT	TGGACCAAACCAAGCACATA	60.1	568	INTERGENIC	--	--	--
Ca4_P3_40845528	Ca4	40845528	GCC/GC	TTTTTCAAGCAAATTGTTCCC	TGCATAAACGTCAAAGGCAA	59.1	476	INTERGENIC	--	--	--
Ca4_P3_40847080	Ca4	40847080	AATATATAT/AATAT	CCCACCCCCATAAAACACTA	GGTGATTGTTTGAGCCACCT	59.5	147	INTERGENIC	--	--	--
Ca4_P3_40847227	Ca4	40847227	GT/GTCAT	GGTGGCTCAAACAATCACCT	TGTCCAAAAATCGGAAGAAAA	60.0	355	INTERGENIC	--	--	--
Ca4_P3_40851940	Ca4	40851940	AG/A	AAGCTATGTGGCAATTTGGG	AATCATGCCTTTGAAGTGGG	60.0	471	INTRON	Ca_23897	--	--
Ca4_P3_4086522	Ca4	4086522	GATATA/GATATATA	GCGTGCTTTATACGGATGGT	TGATGGAGGCCTAAACAAGG	60.0	499	INTERGENIC	--	--	--
Ca4_P3_40880303	Ca4	40880303	GA/GAA	GGAAGTTTCCAAAGCACGAG	AAGTTCCATTGCTTGCTGGT	59.9	411	INTERGENIC	--	--	--
Ca4_P3_40893226	Ca4	40893226	CTCTATCTAT/CTCTAT	TGAACTCGGCTAGGAGGAAA	TATATGGGGGTCCATTCCTG	59.9	294	DRR	Ca_11020	R	G2-like
Ca4_P3_40893508	Ca4	40893508	AG/A	TGAACTCGGCTAGGAGGAAA	CGCAAGTGTTGGACAATGAT	59.9	703	DRR	Ca_11020	R	G2-like

INDEL marker IDs	Chromosomes/ unanchored scaffolds	Physical positions (bp)	InDels (*Kabuli* reference genome-CDC Frontier/PII)	Forward primers (5'-3')	Reverse primers (5'-3')	Anneling temperature (0C)	Expected amplified product size (bp)	Structural annotation		Functional annotation	
								Sequence components of *kabuli* genome	*Kabuli* gene accession IDs	NCBI-KOG	TFs
Ca4_P3_40899332	Ca4	40899332	AT/ATATATTTAATTT	AGGACCTTGGACACAACAGG	TTTGGCTATTTCAGCCCATC	60.0	509	INTERGENIC	--	--	--
Ca4_P3_40901246	Ca4	40901246	AAA/AAATAA	GGGTATATTAATTTAACAACTCCCTCC	TTAGGAGGTCGCATCTTCGT	59.7	462	INTERGENIC	--	--	--
Ca4_P3_40901320	Ca4	40901320	GAA/G	GGTCCGAGATACAACCTGCT	TCAATGAAAAACATGGTGCC	59.2	480	INTERGENIC	--	--	--
Ca4_P3_40907158	Ca4	40907158	TATACAT/TATACATAATACAT	TGTCGTTGAAATCAAAGCAAT	ATCGTTTGGGTGAAGAATCG	58.3	334	INTERGENIC	--	--	--
Ca4_P3_40922455	Ca4	40922455	CTTTTTTTTTT/CTTTTTTTTTTTT	CAGCATGCATCTGGAAAGAA	AATGAGGCGGTGGTATCTTG	59.9	534	INTRON	Ca_11016	R	--
Ca4_P3_4092489	Ca4	4092489	TAA/TAAAACGAA	TGAGGTGAGATGCAAGAGCA	AATTCACATGCGAAGCTCTG	60.7	142	INTERGENIC	--	--	--
Ca4_P3_40925099	Ca4	40925099	CTATATA/CTATA	TCATGAAACTTGAAAGGCCC	CATGAAGTAGGTTGACCAGGC	60.1	317	DRR	Ca_11016	R	--
Ca4_P3_40929080	Ca4	40929080	T/TC	ATGGTGGGGGTAAGCAATCT	AAGAAGCGCGTATTGGTGTT	60.6	813	INTRON	Ca_11015	--	bZIP
Ca4_P3_40929966	Ca4	40929966	TAAAAAAAAA/TAAAAAAA	TCATCGTTGGTAAAAATTTAGAGGA	TTAAGATGGTTGAGGCCTTATT	60.2	418	INTRON	Ca_11015	--	bZIP
Ca4_P3_4093453	Ca4	4093453	CTAATTAAGTAATAATTAAGTAA/CTAATTAAGTAA	CGTTCATCCACTTTTAAGGCA	AAAAATCTAGGCGTTTTGGTC	60.1	746	INTERGENIC	--	--	--

INDEL marker IDs	Chromosomes/ unanchored scaffolds	Physical positions (bp)	InDels (*Kabuli* reference genome-CDC Frontier/PII)	Forward primers (5'-3')	Reverse primers (5'-3')	Anneling temperature (0C)	Expected amplified product size (bp)	Structural annotation		Functional annotation	
								Sequence components of *kabuli* genome	*Kabuli* gene accession IDs	NCBI-KOG	TFs
Ca4_P3_40934743	Ca4	40934743	A/AGC	GCTCGTCAACAACGTGAGAA	TGCAGATTGCAAAGACAAGG	60.0	689	INTERGENIC	--	--	--
Ca4_P3_40935397	Ca4	40935397	GAATATA/G	GATGGTGGAGGTAGGTGGTG	TGGTGGATCATTCGTGAAAA	60.2	789	INTERGENIC	--	--	--
Ca4_P3_40943811	Ca4	40943811	GTTTT/GTTTTT	AGGGTTTGGTGTTGAGATGC	CGCACGAGATAATAATGCCA	60.0	575	INTERGENIC	--	--	--
Ca4_P3_40944823	Ca4	40944823	CAAAGAAA/CAAAGAAAGAAA	GCACATTGGATGTTTGATGG	TGGGTTTTGTTTGAAATTTTTG	59.8	698	INTERGENIC	--	--	--
Ca4_P3_40950208	Ca4	40950208	AA/AATTA	CACGATTGGGGTGAAAAATC	TTGTTGGCATCAATGCAAGT	60.2	718	INTRON	Ca_11013	--	--
Ca4_P3_40954191	Ca4	40954191	CAAA/CAA	TTCACCATGTTTCCGTCAAA	TGTTTCTCGAGTACGAAAGAATG	59.9	368	INTERGENIC	--	--	--
Ca4_P3_40955257	Ca4	40955257	GAAA/GAA	TTGTTTTGCGGTTACAAGCA	TCCGTTTTTATTTTGGTCGC	60.3	820	INTERGENIC	--	--	--
Ca4_P3_40958047	Ca4	40958047	GTTT/GTTTT	CCGTGTTGGTTGTGTTGTTC	TTTGTGGGTGTTTGAGACCA	59.9	558	INTERGENIC	--	--	--
Ca4_P3_40978550	Ca4	40978550	TC/TCATGTAGGGC	TTGGGTTTCCTATTTCGTCG	GCCAATCGATTACCCTCAAA	59.9	963	INTERGENIC	--	--	--
Ca4_P3_41015733	Ca4	41015733	TAAAAAA/TAAAAAAA	CAAATTTTGATGATCTCACACGA	GCCCCATAAAAAGATGCTCA	60.0	504	INTERGENIC	--	--	--

INDEL marker IDs	Chromosomes/ unanchored scaffolds	Physical positions (bp)	InDels (*Kabuli* reference genome-CDC Frontier/PII)	Forward primers (5'-3')	Reverse primers (5'-3')	Anneling temperature (0C)	Expected amplified product size (bp)	Structural annotation		Functional annotation	
								Sequence components of *kabuli* genome	*Kabuli* gene accession IDs	NCBI-KOG	TFs
Ca4_P3_41027713	Ca4	41027713	AATATATATATATATATATATATATATATAT/AATATATATATATATATATATATATATATATATAT	TGAATCCCAAAATTTGAACCA	TTTGATCGCAAGGGTTTAGG	60.2	637	INTERGENIC	--	--	--
Ca4_P3_41029578	Ca4	41029578	A/AC	TTTCTCTCCATCGACCCATC	CCACGTGGGAAGTAGTCAGA	60.0	266	INTERGENIC	--	--	--
Ca4_P3_41029836	Ca4	41029836	AATATATATATATATATA/AATATATATATATATA	TTTCTCTCCATCGACCCATC	GAACCTTTGGGAAATCAGCA	60.0	760	INTERGENIC	--	--	--
Ca4_P3_4103235	Ca4	4103235	ACTC/ACTCCTC	AACATGGCAATTGATTGAAGG	AGTCCAACTTCATTGCCTGC	59.8	496	DRR	Ca_03844	--	--
Ca4_P3_4104310	Ca4	4104310	ATTTT/ATTTTT	AGGACCCAGCAAGGAAAACT	TAACAGCCCATTCCCACTTC	60.1	722	DRR	Ca_03843	T	BBR-BPC
Ca4_P3_41061116	Ca4	41061116	ATT/AT	TTCTTCAGACCCATCTCTTGC	TCGTCGATGACTACGATGGA	59.4	392	INTERGENIC	--	--	--
Ca4_P3_41116006	Ca4	41116006	CAATAATAATAATAATAATAATAATAATAATAATAATAATAATAATAATAATAATA/CAATAATAATAATAATAATAATAATAATAATAATAATAATAATAATAATAATAATAATAATAA	TGTGAACCACATTGAATCCTG	TGGATACCGGGCAGTTTTTA	59.4	642	INTRON	Ca_10997	L	NF-YB
Ca4_P3_4115753	Ca4	4115753	TAA/TA	GGTGTTGTTGGGCTTTGTCT	CCCCCTTTTTGGTCGTATCT	60.0	821	INTERGENIC	--	--	--
Ca4_P3_41162022	Ca4	41162022	CATATATATATATATATATATA/CATATATATATATATATATATATA	TTTGATTGTCCTGCACCAAA	TGGTAGCCTTGAACTTGTGC	60.1	261	INTERGENIC	--	--	--
Ca4_P3_41172721	Ca4	41172721	AC/A	TCAAGTTAAAGGTGGAGCGG	TTCCTCCCTTTCTTTGACAAC	60.2	761	INTERGENIC	--	--	--

INDEL marker IDs	Chromosomes/ unanchored scaffolds	Physical positions (bp)	InDels (*Kabuli* reference genome-CDC Frontier/PII)	Forward primers (5'-3')	Reverse primers (5'-3')	Anneling temperature (0C)	Expected amplified product size (bp)	Structural annotation		Functional annotation	
								Sequence components of *kabuli* genome	*Kabuli* gene accession IDs	NCBI-KOG	TFs
Ca4_P3_4117947	Ca4	4117947	ATTTTT/ATTTTTT	AACTCGTGTGGGAGGTTCAC	TTTTGCAATCTATCGAGGGG	60.0	955	INTERGENIC	--	--	--
Ca4_P3_4118588	Ca4	4118588	AATGCATTATG/AATG	CCCCTCGATAGATTGCAAAA	TAGGGTATAGCATGGGCAGG	60.0	301	INTERGENIC	--	--	--
Ca4_P3_4120892	Ca4	4120892	G/GA	ATGGTCGAAACCAATTGAGC	TTTTGGAGGATTGAGATGGG	59.9	660	INTERGENIC	--	--	--
Ca4_P3_4126019	Ca4	4126019	AGCTTAAAC/A	TGGTTAGGGAGCAAAGGCTA	ATAAGGAAAGTGGGGGCATT	59.8	477	INTERGENIC	--	--	--
Ca4_P3_41268887	Ca4	41268887	AATATATATATATATATATA/AATATATATATATATATA	AAACCTCGTCAATTGGCTCA	CAATTGGTGGAACAATCGTG	60.6	299	INTERGENIC	--	--	--
Ca4_P3_4129605	Ca4	4129605	TAAAAAAAA/TAAAAAAA	TTGACGAACCAACTCATCCA	CCACGCACTACTTCAACGAC	60.1	851	INTERGENIC	--	--	--
Ca4_P3_41306366	Ca4	41306366	CTTTTTTTTTT/CTTTTTTTTTTTT	GGTAGCATCCAACCATCTAGACA	GGGGATGTGAACACTTAAGCA	60.4	157	INTERGENIC	--	--	--
Ca4_P3_4132036	Ca4	4132036	ATTTTT/ATTTT	GAGAGAGAAATACCACAAATTGGAA	GATGAGAATGAAAATAGGAGGGA	59.9	376	INTERGENIC	--	--	--
Ca4_P3_41320371	Ca4	41320371	TGGGG/TGGG	TGGAGCATATGCGTAGCAAG	GCTTGCTTTTGCCCATACAT	60.0	761	DRR	Ca_10977	K	LBD
Ca4_P3_4134423	Ca4	4134423	CAAATAATTAAAAAAATAAT/CAAATAAT	GGATTAGGTCGTTGTTTCCC	GGCGATAGTGATCCAGCCTA	58.4	856	INTERGENIC	--	--	--

INDEL marker IDs	Chromosomes/ unanchored scaffolds	Physical positions (bp)	InDels (*Kabuli* reference genome-CDC Frontier/PII)	Forward primers (5'-3')	Reverse primers (5'-3')	Anneling temperature (0C)	Expected amplified product size (bp)	Structural annotation		Functional annotation	
								Sequence components of *kabuli* genome	*Kabuli* gene accession IDs	NCBI-KOG	TFs
Ca4_P3_4136593	Ca4	4136593	TAGTAAG/TAGTAAGTAAG	GTGGATGAACCAATGG ACAA	CCGATCTAAATAAAAAT TTGGGG	59.2	103	INTERGENIC	--	--	--
Ca4_P3_41376499	Ca4	41376499	TAAA/TAAAA	TATTCGCGAAATGGGT TTCT	CCTGCAGGTTCTTCGT CTTC	59.5	447	DRR	Ca_10970	--	NF-YB
Ca4_P3_4142760	Ca4	4142760	CAAAAAA/CAAAAA	ATCCATCCAATGATTCC CAA	TGATGGCCTGTTCTCT GTTG	60.0	422	DRR	Ca_03842	--	--
Ca4_P3_4150033	Ca4	4150033	GTTTTTTTTT/GTTTTTTT	TCTGCCGTATGAGTTC GATG	CTCCAACCCCCTCCAT ATTT	59.8	133	INTERGENIC	--	--	--
Ca4_P3_4154282	Ca4	4154282	TCAAG/TCAAGGAAGTTGTCCAAG	CAATCCATTGAAAACCC CAC	ACTCTCAAGGCTGCCA AAAA	60.0	653	CDS (FRAME SHIFT)	Ca_03840	R	--
Ca4_P3_4155952	Ca4	4155952	AT/ATT	TTGGGCAATAGGAGAT GAGG	TTGCAAAGGAAAATGG AAGG	60.0	542	INTRON	Ca_03840	R	--
Ca4_P3_4156202	Ca4	4156202	ACTT/ACTTCTT	TTGTGGTCCTTCCATTT TCC	AGCTGCAAGGAGCTAT CCAA	59.8	316	DRR	Ca_03840	R	--
Ca4_P3_41581170	Ca4	41581170	GA/GATCA	ACAAAAACTCCCAATGG TCG	TAGCCAACCACACATG GAAA	59.8	737	INTERGENIC	--	--	--
Ca4_P3_4162107	Ca4	4162107	AATTAAATT/AATTAAATTTATTAAATT	AGGGAGAGAGGCATGA GACA	ATGTGCGACATGAAAA CCAA	59.9	735	INTERGENIC	--	--	--
Ca4_P3_4162204	Ca4	4162204	GTTTTTTTT/GTTTTTTTTTT	AGGGAGAGAGGCATGA GACA	ATGTGCGACATGAAAA CCAA	59.9	735	INTERGENIC	--	--	--

INDEL marker IDs	Chromosomes/ unanchored scaffolds	Physical positions (bp)	InDels (*Kabuli* reference genome-CDC Frontier/PII)	Forward primers (5'-3')	Reverse primers (5'-3')	Anneling temperature (0C)	Expected amplified product size (bp)	Structural annotation		Functional annotation	
								Sequence components of *kabuli* genome	*Kabuli* gene accession IDs	NCBI-KOG	TFs
Ca4_P3_4163076	Ca4	4163076	GTTTTTTT/GTTTTTT	TATTGCAGCAACACATGCAA	TGCAAATCCATAGACTCCAAGA	59.9	854	INTERGENIC	--	--	--
Ca4_P3_4171930	Ca4	4171930	CCT/CCTCTGTCTTATCTCTCT	GAGGCATGAGACGGAGGTAG	GATTGACATGTTCACGCCAC	59.8	608	INTERGENIC	--	--	--
Ca4_P3_4173017	Ca4	4173017	ATTTGTGTTTTTTTTTTT/ATTT	GTCTTGGGGCTGATGTTGTT	TCCTTGTTATTGGGGTGTCC	60.0	796	INTERGENIC	--	--	--
Ca4_P3_4173103	Ca4	4173103	TAAAAAAAAAAA/TAAAAAAAAA	GTCTTGGGGCTGATGTTGTT	TCCTTGTTATTGGGGTGTCC	60.0	796	INTERGENIC	--	--	--
Ca4_P3_4173930	Ca4	4173930	GAAAAAA/GAAAAAAA	GGACACCCCAATAACAAGGA	TCTTGACATAAAACTATTTTTCAGCG	59.6	699	INTERGENIC	--	--	--
Ca4_P3_4174299	Ca4	4174299	TGA/TGAGA	CAGAAATGTCTGAAAGTGTAGGGTT	TCGTCACTCATCGGTTGAAA	60.0	376	INTERGENIC	--	--	--
Ca4_P3_4174846	Ca4	4174846	TAAA/TAA	TTCAACCGATGAGTGACGAA	CGATGTCCAACCCTCGTTAT	60.2	460	INTERGENIC	--	--	--
Ca4_P3_4176207	Ca4	4176207	TAAGTCAATGCATAA/TA	AGAGTCAGCCATCATCGACC	GGGAATGGGGATAGGTTCAT	60.2	709	INTERGENIC	--	--	--
Ca4_P3_4179576	Ca4	4179576	CTTTTTT/CTTTTTTT	CCTATTTGCTTGGTTGGCAT	TGGTTGGTGATTCGTTTGAA	60.0	522	INTERGENIC	--	--	--
Ca4_P3_41806206	Ca4	41806206	CTA/CTAATA	TGCCTTGAATCTCTTCTTGGA	TGGAAAACCATAATTGAGCG	59.9	905	INTRON	Ca_10934	QR	B3

INDEL marker IDs	Chromosomes/ unanchored scaffolds	Physical positions (bp)	InDels (*Kabuli* reference genome-CDC Frontier/PII)	Forward primers (5'-3')	Reverse primers (5'-3')	Anneling temperature (0C)	Expected amplified product size (bp)	Structural annotation		Functional annotation	
								Sequence components of *kabuli* genome	*Kabuli* gene accession IDs	NCBI-KOG	TFs
Ca4_P3_41806539	Ca4	41806539	ATTTT/ATT	TGGTGCTTGTAGTTGATGAGG	TTTGGGTCAGGACAAGAAGG	58.8	949	INTRON	Ca_10934	QR	B3
Ca4_P3_41806627	Ca4	41806627	ATT/AT	AACACATCCAAATCACATGCT	ATTTGGAGCCCACTTGTTTG	57.9	964	INTRON	Ca_10934	QR	B3
Ca4_P3_41808242	Ca4	41808242	TA/TAGA	TGCAAAACTAGCCGAGTGAA	ATGCGCCGAAAACACTTATT	59.6	498	DRR	Ca_10934	QR	B3
Ca4_P3_418090	Ca4	418090	CTTT/CTTTTTTT	TGTGCGCGTACACTTTCTTT	CCAAGTTCCTTTTGATGGGA	59.5	565	INTERGENIC	--	--	--
Ca4_P3_41823400	Ca4	41823400	A/AAT	GGAAATTTACTTGAATTCATGGCT	TGGAACAATTGAACACCGTT	59.8	420	INTERGENIC	--	--	--
Ca4_P3_41823714	Ca4	41823714	TATT/TATTAATT	GGAAATTTACTTGAATTCATGGCT	CAATTGTTCGTGTTGCGTCT	59.8	689	INTERGENIC	--	--	--
Ca4_P3_41823980	Ca4	41823980	CATCAGA/CA	CATCAAGACCATTTCCAGCA	CAATTGTTCGTGTTGCGTCT	59.6	114	INTERGENIC	--	--	--
Ca4_P3_41826984	Ca4	41826984	TA/T	TGCACCTGAAAATTCAGACC	AAACAAGCCCATGGAAAATG	58.7	508	INTERGENIC	--	--	--
Ca4_P3_41827335	Ca4	41827335	CATGA/CATGATGA	ATTTTCCATGGGCTTGTTTG	TACGATGAATCTCGGGCTCT	59.8	619	INTERGENIC	--	--	--
Ca4_P3_41832968	Ca4	41832968	AATATATATATATA/AATATATATATA	TTTCCATTGCACTCACTCCA	CCTATCATGGCCTGACCTGT	60.2	758	INTERGENIC	--	--	--

INDEL marker IDs	Chromosomes/ unanchored scaffolds	Physical positions (bp)	InDels (*Kabuli* reference genome-CDC Frontier/PII)	Forward primers (5'-3')	Reverse primers (5'-3')	Anneling temperature (0C)	Expected amplified product size (bp)	Structural annotation		Functional annotation	
								Sequence components of *kabuli* genome	*Kabuli* gene accession IDs	NCBI-KOG	TFs
Ca4_P3_4194540	Ca4	4194540	TAATG/T	ATCTTCGGCAAAAGAAGCAA	AGAGAAGACTCACTGGGGCA	60.0	840	INTRON	Ca_03838	R	--
Ca4_P3_4196255	Ca4	4196255	CTATAAATT/CTATAAATTAATATAAATT	ATTGGCCGGAAATTGTACTG	TCAGGGGACCATAAAAGAAGG	59.8	378	DRR	Ca_03838	R	--
Ca4_P3_4196538	Ca4	4196538	ATTTT/ATTT	ATTGGCCGGAAATTGTACTG	TAACCCAAGGCCATATCCTG	59.8	841	DRR	Ca_03838	R	--
Ca4_P3_42005183	Ca4	42005183	GTTATTATTATTATTATTATTATTA/GTTATTATTATTATTATTATTATTATTA	GCAAGTGCACCCTTTGATTT	TCACATCATCTCCCCAAACA	60.1	447	INTRON	Ca_10921	PT	C3H
Ca4_P3_42078586	Ca4	42078586	AAGGCATGAAA/AAGGCATGAAACACAGGCATGAAA	TCTTTTGAACACTCCCCCTG	CCAAAGGAAACCACGACAAT	60.1	810	INTERGENIC	--	--	--
Ca4_P3_42120439	Ca4	42120439	TAAAAAAAAA/TAAAAAAAA	CACAAGCATTGGAGTCCCTT	AACTCGTGGGTTGGATTTTG	60.1	495	INTRON	Ca_10910	--	C2H2
Ca4_P3_42120489	Ca4	42120489	TAAAA/TAAA	CACAAGCATTGGAGTCCCTT	AACTCGTGGGTTGGATTTTG	60.1	495	INTRON	Ca_10910	--	C2H2
Ca4_P3_42120574	Ca4	42120574	C/CG	CACAAGCATTGGAGTCCCTT	AACTCGTGGGTTGGATTTTG	60.1	495	INTRON	Ca_10910	--	C2H2
Ca4_P3_4214660	Ca4	4214660	GAAAAAAAAAAAA/GAAAAAAAAAAAAA	TACATAGCACGCTGAGGACG	CCAAGTTTTTGGAGTAGAGCG	60.0	509	INTERGENIC	--	--	--
Ca4_P3_42213086	Ca4	42213086	TTATATATATATATATATATATATATATATATATATA/TTATATATATATATATATATATATATATATATATATATATATA	CTCTGCATTTGTTTCCCCAT	CCCTTATCGTGTTTGCACCT	59.9	752	INTERGENIC	--	--	--

INDEL marker IDs	Chromosomes/ unanchored scaffolds	Physical positions (bp)	InDels (*Kabuli* reference genome-CDC Frontier/PII)	Forward primers (5'-3')	Reverse primers (5'-3')	Anneling temperature (0C)	Expected amplified product size (bp)	Structural annotation		Functional annotation	
								Sequence components of *kabuli* genome	*Kabuli* gene accession IDs	NCBI-KOG	TFs
Ca4_P3_42325397	Ca4	42325397	AATATATATATATATATATA/AATATATATATATATATATATA	AATGATCGTCTTCGGATTGG	CGTTGAATCTTGTGGGTTCA	59.9	478	INTERGENIC	--	--	--
Ca4_P3_4242385	Ca4	4242385	TTATATATATATATATATATATATATAT/TTATATATATATATATATATATATATATATATATAT	GAACCATTGAGAGGCCGTT	TCGGTTTCCGAGTAGTGGAC	60.1	359	INTERGENIC	--	--	--
Ca4_P3_42516686	Ca4	42516686	CAAAA/CAAAAAA	GTCCTTTGCCATCTACCTCG	ATCTGCCAGAGCGATGTCTT	59.7	695	DRR	Ca_10897	--	Dof
Ca4_P3_42517427	Ca4	42517427	AATATATATATATATATATATATATATATATATATA/AATATATATATATATATATATATATA	ATAATGTTCAGCACCCGAGG	GAGCTGTCCACGACACTCAA	60.0	466	INTRON	Ca_10897	--	Dof
Ca4_P3_42546801	Ca4	42546801	CAAAA/CAAAAA	GGTTGGTCAATATTAATTCCGC	CTAATATCATCGCCCCTTCG	59.6	686	INTERGENIC	--	--	--
Ca4_P3_42546916	Ca4	42546916	TTAAAAAAAATAAAAAA/TTAAAAAA	AAAGTGAGGAGTTTTGCCCC	CTAATATCATCGCCCCTTCG	60.5	401	INTERGENIC	--	--	--
Ca4_P3_42548370	Ca4	42548370	ATTTTTTTT/ATTTTTTTTT	CGTGCCTTTGTCGTCTCTTT	AGAGGCCCAATATCCTTGCT	60.4	528	INTERGENIC	--	--	--
Ca4_P3_42574723	Ca4	42574723	AGGG/AGGGG	TCGTCATCACACAAGGTGGT	CAGGTTGTAAGCAAAAGCCC	60.0	227	INTERGENIC	--	--	--
Ca4_P3_4261669	Ca4	4261669	TAAAAAAAAAAA/TAAAAAAAAAA	TATGTTCAAATGGATGGCGA	AAGGGCATTTCATATTTCGG	59.9	308	INTERGENIC	--	--	--
Ca4_P3_42644681	Ca4	42644681	GTATATATATATATATATATATATATATATAT/GTATATATATATATATATATATATATATAT	ACAGTTTTGGTCCCTCCCTT	CTTTACAACACGGACGCTCA	59.8	528	INTERGENIC	--	--	--

INDEL marker IDs	Chromosomes/ unanchored scaffolds	Physical positions (bp)	InDels (*Kabuli* reference genome-CDC Frontier/PII)	Forward primers (5'-3')	Reverse primers (5'-3')	Anneling temperature (0C)	Expected amplified product size (bp)	Structural annotation		Functional annotation	
								Sequence components of *kabuli* genome	*Kabuli* gene accession IDs	NCBI-KOG	TFs
Ca4_P3_42672856	Ca4	42672856	CATTT/CATTTGGAGTATATTT	TGTTGTTGTTGTTGTTG TTGTTG	GGGCATAAAGACATGC CAAT	59.5	551	INTERGENIC	--	--	--
Ca4_P3_4269269	Ca4	4269269	TTATATATATATATATATATATAT/TTAT ATATATATATATATATATATATAT	ACACGGTGATCGTCAG TGAA	ACACGTGACACTTACC TAGCAA	60.2	596	INTERGENIC	--	--	--
Ca4_P3_4273619	Ca4	4273619	ATTTTTTTTTT/ATTTTTTTT	GCGCCCGTTGAAAATA ATAA	ACACGTGTGGACGTGA TTTG	59.9	408	INTERGENIC	--	--	--
Ca4_P3_42776316	Ca4	42776316	TTTTTATTTTATTTTATTTTATTTTATT/T TTTTATTTTATTTTATTTTATT	AGGAAATGCTAGGTTG TGCTG	AAAACACCAAATCGGT GAGC	59.4	533	INTERGENIC	--	--	--
Ca4_P3_42845286	Ca4	42845286	ATT/AT	TTCTAGCTGGCTTTTCT CGC	CGATTGAACCAAATTC CAGG	59.9	804	INTERGENIC	--	--	--
Ca4_P3_42993153	Ca4	42993153	GCCC/GCC	ACAGTGTTTCGGATTCT GGG	GTTTCGTACCCCTCGA CAAA	60.0	757	INTERGENIC	--	--	--
Ca4_P3_43005724	Ca4	43005724	CTTTTTTTTTTT/CTTTTTTTTTT	TTGTAGTTGTATTCAAT GACGAAGA	CAGAATTAATTTATTTG ACAACTCCA	58.0	628	INTERGENIC	--	--	--
Ca4_P3_43177763	Ca4	43177763	TCCCCC/TCCCCCC	GTGCCAGTCGATTGCC TAAT	TGCCTTAACTGACAAA GGGG	60.1	562	INTERGENIC	--	--	--
Ca4_P3_43252814	Ca4	43252814	TA/TAA	TGTTTTGAAAATTAATT GCTATTTGG	CCACAAACAGAGAAGT GGCA	59.7	393	INTERGENIC	--	--	--
Ca4_P3_43304756	Ca4	43304756	A/ATATTG	TGAGCAAGCTGAAAGA AGCA	AACACTCCTGCACGAC ACAC	60.0	264	DRR	Ca_23682	K	TALE

INDEL marker IDs	Chromosomes/ unanchored scaffolds	Physical positions (bp)	InDels (*Kabuli* reference genome-CDC Frontier/PII)	Forward primers (5'-3')	Reverse primers (5'-3')	Anneling temperature (0C)	Expected amplified product size (bp)	Structural annotation		Functional annotation	
								Sequence components of *kabuli* genome	*Kabuli* gene accession IDs	NCBI-KOG	TFs
Ca4_P3_43308710	Ca4	43308710	TT/TTAAT	ATCCTTGAGCAGCACCAAAT	TGGACTACCAATGAAGGAATTG	59.7	611	INTERGENIC	--	--	--
Ca4_P3_43317546	Ca4	43317546	TAGAAGAAGAAGAAGAAGAA/TAGAAGAAGAAGAA	CATGTGGAGTGATATGGCCT	TGCTTGGTAAAAAGAATGACAAA	58.4	757	INTRON	Ca_23683	D	M-type
Ca4_P3_43318250	Ca4	43318250	T/TTA	AGTTCAGACGGCGAGAGAAG	CCCATGTATGGTTAGTGGGG	59.7	540	INTRON	Ca_23683	D	M-type
Ca4_P3_43339918	Ca4	43339918	ATT/ATTT	TCATTCTCTTCATCGAAGATTTCA	TGGAGAATAATTAACAGAGCAGCA	60.2	565	INTERGENIC	--	--	--
Ca4_P3_43346773	Ca4	43346773	TAA/TA	TTTCCAATGGAGGTGATTCC	CTACATTTGCGCCTCTGTCA	59.7	324	DRR	Ca_23685	--	S1Fa-like
Ca4_P3_43349693	Ca4	43349693	TA/T	TATGGGGAAGAAACCAGCAG	TGTGGTTTCCCATACCAATTC	60.1	665	INTERGENIC	--	--	--
Ca4_P3_43369208	Ca4	43369208	CAGAG/CAG	TGTGAATGTTGCTGCCATTT	ACAGCAATGCTTGTGATCCT	60.1	464	INTERGENIC	--	--	--
Ca4_P3_43389738	Ca4	43389738	TAA/TA	TCACATTGTGGAATGTTGAGAA	TCCATGTATAATTACGCCTTTAAGAA	59.0	232	INTERGENIC	--	--	--
Ca4_P3_43399815	Ca4	43399815	TC/T	AACAATTTTCCAAGTCAACCG	CCACAAAGAGCAACAAACATTG	59.0	597	INTERGENIC	--	--	--
Ca4_P3_43425153	Ca4	43425153	CGCATGCATG/CGCATG	TTAATCCGAAACGATCCGAC	AACTTTTTGAAGTCACGCCG	59.9	370	INTERGENIC	--	--	--

INDEL marker IDs	Chromosomes/ unanchored scaffolds	Physical positions (bp)	InDels (*Kabuli* reference genome-CDC Frontier/PII)	Forward primers (5'-3')	Reverse primers (5'-3')	Anneling temperature (0C)	Expected amplified product size (bp)	Structural annotation		Functional annotation	
								Sequence components of *kabuli* genome	*Kabuli* gene accession IDs	NCBI-KOG	TFs
Ca4_P3_43426311	Ca4	43426311	CAA/CAAAA	ATTCACACATTTGGCGTTGA	TCCGATGCATCTATTTTGGA	60.0	629	INTERGENIC	--	--	--
Ca4_P3_43432356	Ca4	43432356	GTCACACTCA/GTCACACTCACACTCA	TGAGAAGTCACATGGCTTGG	GAGGGACCAAGGGAATCAAT	59.8	793	INTERGENIC	--	--	--
Ca4_P3_43440070	Ca4	43440070	TAAA/TAAAA	GACAATGGGCCAATTTGATT	GATTTTGCTTCCTGCTTTGC	59.6	699	INTRON	Ca_23689	--	--
Ca4_P3_43443823	Ca4	43443823	CA/CACTTA	GCAAGAGTCTCCCTCGTTTG	CGCCTCGTAAACATAATTGGA	60.0	391	DRR	Ca_23689	--	--
Ca4_P3_43445155	Ca4	43445155	TAAAAAAAAAAAA/TAAAAAAAAAAA	CAAAGGCTTGGTTGTCCATT	GTCAATGCCAAATGGTTGAA	60.0	458	INTERGENIC	--	--	--
Ca4_P3_43450204	Ca4	43450204	A/AT	TTCGTGTCAGCAAGACCAAG	AGGGAGATTCTTGGAGAGAGC	60.0	194	DRR	Ca_23690	--	--
Ca4_P3_43485060	Ca4	43485060	ATT/ATTT	TTTTCCATATGATCACGCCA	TGGCCCAACATATAAAGGGA	59.9	388	INTERGENIC	--	--	--
Ca4_P3_43518348	Ca4	43518348	AATTTTTTTTATTTTTTTTATTTTTT/AATTTTTTTATTTTT	GTGAGTCATGGGACGGTTCT	CTAGTTGGCCCATGGAATGT	60.0	749	INTRON	Ca_23694	--	bHLH
Ca4_P3_43535197	Ca4	43535197	A/ATTTTTTGATGG	TCGGGATTTTGATGTTGGTT	AGGAAAATGAATCATGGGCA	60.2	473	INTERGENIC	--	--	--
Ca4_P3_43535291	Ca4	43535291	CATTATTAT/CATTAT	TCGGGATTTTGATGTTGGTT	AGGAAAATGAATCATGGGCA	60.2	473	INTERGENIC	--	--	--

INDEL marker IDs	Chromosomes/ unanchored scaffolds	Physical positions (bp)	InDels (*Kabuli* reference genome-CDC Frontier/PII)	Forward primers (5'-3')	Reverse primers (5'-3')	Anneling temperature (0C)	Expected amplified product size (bp)	Structural annotation		Functional annotation	
								Sequence components of *kabuli* genome	*Kabuli* gene accession IDs	NCBI-KOG	TFs
Ca4_P3_43549693	Ca4	43549693	ATTTTT/ATTTTTT	TGGATTATTCGCGCTTCTTT	GCTTTGAAAAACGGGGTGT	59.8	545	INTERGENIC	--	--	--
Ca4_P3_43564232	Ca4	43564232	CTTTTTTT/CTTTTTT	TCAAACAAAATTGGGTTACAACA	TTTGGTAATCACAAAAATCCAAAA	59.3	664	INTERGENIC	--	--	--
Ca4_P3_43564696	Ca4	43564696	AATATTTTATATTTT/AATATTTT	CCTACATAATTTTCTCATGTTGGA	GGGAAGGGTTGTAAGTCTGGA	57.3	367	INTERGENIC	--	--	--
Ca4_P3_4363529	Ca4	4363529	ATTTTTTT/ATTTTTTTT	CACCCATCCCATCCACTTAC	CAACCCAAACCCAAACAATC	60.1	684	DRR	Ca_03825	--	WRKY
Ca4_P3_4371615	Ca4	4371615	ATTTTTTTTTTTTT/ATTTTTTTTTTT	TGGCACAATAAATCAAACCA	GCAAAAATCGAACTGCATTG	57.5	512	INTERGENIC	--	--	--
Ca4_P3_4400297	Ca4	4400297	CCTCATGCTCATGCT/CCTCATGCTCATGCTCATGCT	CGCATGTTTTTCTGGGTTCT	TTTGGACCGACTTACCCAAG	60.1	227	INTRON	Ca_03821	R	--
Ca4_P3_44006518	Ca4	44006518	CACCCCTAATAAACCCCTAATAA/CACCCCTAATAA	CGTGCCATCTCAATTACCCT	CGAGGACCAAAATCCAAAAA	60.0	655	INTERGENIC	--	--	--
Ca4_P3_44057615	Ca4	44057615	TTTATTATTATTATTATTATTATTATT/TTTATTATTATTATTATTATTATT	AATGCGTGTTCTTGCTGTTG	CTTGGCCAAAGGAATCAAAA	59.9	198	INTRON	Ca_09080	K	ARR-B
Ca4_P3_4406517	Ca4	4406517	TA/T	CAAGGTAATGCATTGTCATCAAA	CGATTGCTTGCGTGTATGAG	59.9	734	INTERGENIC	--	--	--
Ca4_P3_44065193	Ca4	44065193	AAGAGAGAGAGAGAGAGAGAGAGAGAGAGAGAGAGAGAGAGAGAGAGAGA/AAGAGAGAGAGAGAGAGAGAGAGAGAGAGAGAGAGAGAGA	TAGCTTGCCACCTCCCATAC	CATCAGAAAACCCTGGCACT	60.1	295	INTRON	Ca_09081	R	G2-like

INDEL marker IDs	Chromosomes/ unanchored scaffolds	Physical positions (bp)	InDels (*Kabuli* reference genome-CDC Frontier/PII)	Forward primers (5'-3')	Reverse primers (5'-3')	Anneling temperature (0C)	Expected amplified product size (bp)	Structural annotation		Functional annotation	
								Sequence components of *kabuli* genome	*Kabuli* gene accession IDs	NCBI-KOG	TFs
Ca4_P3_44083427	Ca4	44083427	TTATATATATATATATATATA/TTATATATATATATATATA	TTGGCAGAGGTGAAGGTAGG	AAACCAACCATGCATAACCC	60.2	565	INTERGENIC	--	--	--
Ca4_P3_44134460	Ca4	44134460	AAC/AACACAC	TACCAGATTGCCCCACTTTC	GCCAAAACGGATGAAGTTGT	59.9	554	INTERGENIC	--	--	--
Ca4_P3_44295072	Ca4	44295072	T/TGTAGTA	ATGATGAATCGGTTTTTGCC	AGTTTTTCCAACCTCAGCCA	59.8	305	CDS (large-effect mutations)	Ca_09103	--	--
Ca4_P3_44323786	Ca4	44323786	TATAGAATAGAATAGAA/TATAGAATAGAATAGAATAGAA	GCACCACGAACATCAATGAG	CAAGGTAGCGAGGAATAGCG	60.1	745	INTERGENIC	--	--	--
Ca4_P3_44344897	Ca4	44344897	ATTTTTTTTTTT/ATTTTTTTTTT	TTGATTATTGATTACCCACGGA	TACCCATTGATCCGATTCGT	59.2	510	INTERGENIC	--	--	--
Ca4_P3_44428431	Ca4	44428431	CATATATATATATATATATAT/CATATATATATATATATATATAT	TGTTGCGGAAATGTTAGTTCA	TTCATCCATGCAATATTCAAAAA	59.2	699	INTERGENIC	--	--	--
Ca4_P3_44479699	Ca4	44479699	TTATATATATATATATATATATATATATATAT/TTATATATATATATATATATATATATATATATATATATATATATAT	CAATGATCCCAAAAATTTAACCA	ATATTGATGGGGCCGATTTT	59.9	701	INTERGENIC	--	--	--
Ca4_P3_44487366	Ca4	44487366	A/ATACATC	TGCGATTGAAGAGATAAACGAA	GAGATGCGGGGACAAGATTA	59.9	778	INTERGENIC	--	--	--
Ca4_P3_44488143	Ca4	44488143	AAATAATAATAAT/AAATAATAATAATAAT	AAACCCAATTGTTGATGGGA	GCGACCATCTGCTTTCGTAT	60.0	336	INTERGENIC	--	--	--
Ca4_P3_44491772	Ca4	44491772	ATAGCTAG/ATAGCTAGCTAG	GGCTGAATAATTGTGTCGGG	CACTGCCTTCCCATTTCCTA	60.3	314	INTERGENIC	--	--	--

INDEL marker IDs	Chromosomes/ unanchored scaffolds	Physical positions (bp)	InDels (*Kabuli* reference genome-CDC Frontier/PII)	Forward primers (5'-3')	Reverse primers (5'-3')	Anneling temperature (0C)	Expected amplified product size (bp)	Structural annotation		Functional annotation	
								Sequence components of *kabuli* genome	*Kabuli* gene accession IDs	NCBI-KOG	TFs
Ca4_P3_4450094	Ca4	4450094	ATTTTTTT/ATTTTTT	AAAACCAAGGAGGAGGAGGA	GTCGATCGAGTTGCTTCACA	60.0	312	INTRON	Ca_03813	T	WRKY
Ca4_P3_44507737	Ca4	44507737	TTTCTTCTT/TTTCTTCTTCTT	AGCGGTTGTTCAAAATGACC	AAAGCAAGTCCGACCAAAAA	60.0	421	INTERGENIC	--	--	--
Ca4_P3_44508264	Ca4	44508264	TTCCCTGAT/TAT	GAAATGGATTTTTGGTCGGA	TGTGCATCCGTGATACATGA	59.7	961	INTERGENIC	--	--	--
Ca4_P3_44509122	Ca4	44509122	GAAAA/GAAAAA	GCAGTGCTGAATTTTGGTGA	TGCAAAAGTTGTGTGAAACCA	59.8	494	INTERGENIC	--	--	--
Ca4_P3_44520768	Ca4	44520768	GA/GAA	TTTGTCGTCTCTTCCTCGTG	AAGAGCACATGGGGAAATTG	59.0	513	INTERGENIC	--	--	--
Ca4_P3_44521022	Ca4	44521022	T/TA	CAATTTCCCCATGTGCTCTT	GACATTTTTGGATGATTTTGGA	59.9	290	INTERGENIC	--	--	--
Ca4_P3_44521070	Ca4	44521070	CAAAAA/CAAA	CAATTTCCCCATGTGCTCTT	TGGACTTCAAAATGATTGTTTG	59.9	418	INTERGENIC	--	--	--
Ca4_P3_44522624	Ca4	44522624	GAAAAAA/GAAAAAAA	GAAGTTGCTTGCTCCGATTC	CCCTAGCAAGTGCTAACCCA	60.0	675	DRR	Ca_09124	--	ERF
Ca4_P3_44522868	Ca4	44522868	A/AG	ACAATCCCTTCGTTCCCTCT	CCCTAGCAAGTGCTAACCCA	59.9	516	DRR	Ca_09124	--	ERF
Ca4_P3_44525079	Ca4	44525079	AGTATTTTTCTTA/AGTATTTTTCTTATGTATTTTTCTTA	ATGACGTTGGTGATTGGACA	TGGAGTAATCCCTAATCATTTCA	59.8	357	INTERGENIC	--	--	--

INDEL marker IDs	Chromosomes/ unanchored scaffolds	Physical positions (bp)	InDels (*Kabuli* reference genome-CDC Frontier/PII)	Forward primers (5'-3')	Reverse primers (5'-3')	Anneling temperature (0C)	Expected amplified product size (bp)	Structural annotation		Functional annotation	
								Sequence components of *kabuli* genome	*Kabuli* gene accession IDs	NCBI-KOG	TFs
Ca4_P3_44525239	Ca4	44525239	AAAAGAA/AAA	ATGACGTTGGTGATTGGACA	GCGCTAGCAAAAATAGCCAC	59.8	884	INTERGENIC	--	--	--
Ca4_P3_44530462	Ca4	44530462	TAAAAAAAAA/TAAAAAAAAAA	CTCAAGGGTTTTTCCCACAA	GCGATGCGTGTTCCTAAGAT	59.9	567	INTERGENIC	--	--	--
Ca4_P3_44532030	Ca4	44532030	TAAAAAAAA/TAAAAAAA	AGATCCGCCACTGCATTTAC	TCCACTCATCAGCCATGAAG	60.1	803	INTERGENIC	--	--	--
Ca4_P3_44532080	Ca4	44532080	TC/T	CCTAGTCAAAATATCTCCTGCCA	TCCACTCATCAGCCATGAAG	59.6	694	DRR	Ca_09125	L	--
Ca4_P3_44533123	Ca4	44533123	TAAAAAAA/TAAAAAAAA	CAAAATGCCGTCACAGAATG	CCTTGGTTTCGATGCCTAAA	60.1	368	DRR	Ca_09125	L	--
Ca4_P3_44540317	Ca4	44540317	TAAAAAAAAA/TAAAAAAAAAA	CACTCGATTTGACCCCTCTC	CCTACGATCTTAGACACGACCA	59.7	822	INTERGENIC	--	--	--
Ca4_P3_44540601	Ca4	44540601	TAAAA/TAAAAA	AACAATGGGGTCTATCGTGTG	GGACCCATGGTCAAGAAAGA	59.7	678	INTERGENIC	--	--	--
Ca4_P3_44540632	Ca4	44540632	TAAA/TAAAA	AACAATGGGGTCTATCGTGTG	GATTGGAACCATCGAGGAGA	59.7	896	INTERGENIC	--	--	--
Ca4_P3_44540915	Ca4	44540915	CAT/CATTGTGTTAT	TTTCTTGACCATGGGTCCTT	TTGGCCAAAAACGGTAAAAA	59.4	272	INTERGENIC	--	--	--
Ca4_P3_44543641	Ca4	44543641	TAAAA/TAAAAA	CAAATATGTTGGGCCAATCC	GATCAAGTCCAGGGTTGTGG	60.0	473	INTERGENIC	--	--	--

INDEL marker IDs	Chromosomes/ unanchored scaffolds	Physical positions (bp)	InDels (*Kabuli* reference genome-CDC Frontier/PII)	Forward primers (5'-3')	Reverse primers (5'-3')	Anneling temperature (0C)	Expected amplified product size (bp)	Structural annotation		Functional annotation	
								Sequence components of *kabuli* genome	*Kabuli* gene accession IDs	NCBI-KOG	TFs
Ca4_P3_44545143	Ca4	44545143	AATAT/AAT	TTTTCAGCCCATTCCTTTTG	TAATATGCAAGTGGAGGCCC	60.0	910	INTERGENIC	--	--	--
Ca4_P3_44551806	Ca4	44551806	ATTTTTTT/ATTTTTTTTTTTT	CTATTCTGCTGTGGGGGTGT	CCATGGATAATTGATTCGGG	60.0	854	INTERGENIC	--	--	--
Ca4_P3_44555024	Ca4	44555024	T/TGAGATGGAGA	TTTCTCAAGGATCAAAATGGG	TGACCTGACCTATTCCCACC	59.0	629	INTERGENIC	--	--	--
Ca4_P3_44558843	Ca4	44558843	AT/A	CTCTTCCACTAATCCGCAGC	TGCATGATTTTCATTTCGATG	60.0	536	INTERGENIC	--	--	--
Ca4_P3_44559914	Ca4	44559914	TTATATATATAT/TTATATATATATAT	GCGGGGCATAAACTGATAGA	CATTTTCATATCACCAAATCACG	60.1	465	INTERGENIC	--	--	--
Ca4_P3_44561281	Ca4	44561281	ATTTTTTT/ATTTTTTTT	TCAAATCAAAGAATCAACAACAAAA	TTCGGTTAATTTTGCCAGGA	59.9	561	INTERGENIC	--	--	--
Ca4_P3_44563494	Ca4	44563494	CTTCTTTT/CTT	GGCAAATCACATGCTAGGCT	TCGTCCAATCTTGTGGTTCA	60.2	513	INTERGENIC	--	--	--
Ca4_P3_4456690	Ca4	4456690	TAAAAAAAAAAAAA/TAAAAAAAAAA	GTTTTGCAAATGTGCCATATAA	TCCATTTGCATATGAGGCAT	57.7	764	INTERGENIC	--	--	--
Ca4_P3_44567598	Ca4	44567598	AGG/AGGG	AAAATTTTACGAGGATGAAGAAGTT	CTCGTCCATGAATCGGTTTT	58.0	732	INTERGENIC	--	--	--
Ca4_P3_44569146	Ca4	44569146	AGT/A	CGGAATTGGGATGAGAAAGA	GGACCTGTTTGTGTGGTGTG	60.0	986	DRR	Ca_09128	--	--

INDEL marker IDs	Chromosomes/ unanchored scaffolds	Physical positions (bp)	InDels (*Kabuli* reference genome-CDC Frontier/PII)	Forward primers (5'-3')	Reverse primers (5'-3')	Anneling temperature (0C)	Expected amplified product size (bp)	Structural annotation		Functional annotation	
								Sequence components of *kabuli* genome	*Kabuli* gene accession IDs	NCBI-KOG	TFs
Ca4_P3_44578743	Ca4	44578743	GAAAA/GAAAAA	TCATGAAGGAAAGCCAAACC	ATGGCTTGCAAGGTCATAGG	60.1	845	INTRON	Ca_09129	T	WRKY
Ca4_P3_44578984	Ca4	44578984	TAAGA/TA	TCCAAGCTACCTCTTGGCAT	ATGGCTTGCAAGGTCATAGG	59.8	394	INTRON	Ca_09129	T	WRKY
Ca4_P3_44581219	Ca4	44581219	GAAAAAAAA/GAAAAAAAAA	ATGATCGCAAAATGGAGAGG	CCTTTTGGCTGTACTGAGGC	60.0	597	INTERGENIC	--	--	--
Ca4_P3_44581553	Ca4	44581553	AATA/AA	GCCTCAGTACAGCCAAAAGG	GATGATGAGGTGCTTGCTCA	59.9	282	INTERGENIC	--	--	--
Ca4_P3_44584688	Ca4	44584688	ATTTTTTTTTTT/AATTTTTTTTTTTTT	TTCCTGGACCAAATTTGACA	TGCGAAACTTCCTAAATGCAA	58.9	837	DRR	Ca_09130	--	--
Ca4_P3_44585709	Ca4	44585709	GAATTAAATATAAATTAAATATA/GAATTAAATATAAATTAAATATAAATTAAATATA	TTTCTATTTGGGGCCTAGCA	TCTCATTGTCACCGTTGCAT	59.7	587	INTERGENIC	--	--	--
Ca4_P3_44588070	Ca4	44588070	ATTTTT/ATTTTTT	GACTTTGATGAGATACCATGCAG	TGCTTTAAAAGGAATCATGCAA	58.7	700	INTERGENIC	--	--	--
Ca4_P3_44601700	Ca4	44601700	TAAA/TAA	TGCAGGGATGAAATTCAAAAA	AGGACACGGATTCATTTGGA	60.4	380	INTERGENIC	--	--	--
Ca4_P3_44606783	Ca4	44606783	ATTTT/ATTTTT	TTTCTTTCCTACATAGCCAACCA	TGCGTGAGAAATGATTCTTGA	60.0	926	INTERGENIC	--	--	--
Ca4_P3_44607654	Ca4	44607654	TC/T	CACGCTTTAGGTTTGGCATT	AACAAAATTGTGCCGGAGAC	60.1	750	INTERGENIC	--	--	--

INDEL marker IDs	Chromosomes/ unanchored scaffolds	Physical positions (bp)	InDels (*Kabuli* reference genome-CDC Frontier/PII)	Forward primers (5'-3')	Reverse primers (5'-3')	Anneling temperature (0C)	Expected amplified product size (bp)	Structural annotation		Functional annotation	
								Sequence components of *kabuli* genome	*Kabuli* gene accession IDs	NCBI-KOG	TFs
Ca4_P3_44611189	Ca4	44611189	TTCTC/TTC	TTTTTGCCCCTATCACCAAG	CGGTACAAAGTGGGATCTGG	59.9	621	INTERGENIC	--	--	--
Ca4_P3_44613140	Ca4	44613140	T/TA	CCATCGTTGAAACGTTGTGAT	TGCGAGTTTCTTTATCATTTCC	60.8	428	INTERGENIC	--	--	--
Ca4_P3_44617698	Ca4	44617698	TAAAAAA/TAAAAAAA	GCGTGAACACTGCACACTTT	CAAAAGGATTCAAACGCCTC	60.0	492	INTERGENIC	--	--	--
Ca4_P3_44621340	Ca4	44621340	GATTTTAAATTTTAAATTTTA/GATTTTAAATTTTA	TACGTGCCTCCTCCAATTTC	TTACAGGGAAGCATGGAAGC	60.1	489	INTERGENIC	--	--	--
Ca4_P3_44622646	Ca4	44622646	CT/CTGTCAGTTT	TGTTGAGTCGCCGTTAAATG	ACGCAAAGCACACACAAGAG	59.7	701	INTERGENIC	--	--	--
Ca4_P3_44633134	Ca4	44633134	ATTTTT/ATTTT	TCCAAAACCTCAGCAAGTGA	ACAAATAATGGAGGCAACGC	59.4	805	INTERGENIC	--	--	--
Ca4_P3_44640556	Ca4	44640556	TG/T	GTTCTCATGGCCGGTAGTGT	ACAATCAGGATCGGTCGAAG	60.0	698	INTERGENIC	--	--	--
Ca4_P3_44640891	Ca4	44640891	TA/T	TTGCACCAGATTTTATTGGG	CAAAATTTGCGACTTGCTCA	58.5	748	INTERGENIC	--	--	--
Ca4_P3_44654593	Ca4	44654593	GAA/GAATCTAA	CCCATTCTTTTGGGTCATTTT	GAGCCACCGTGATATTGTCC	60.0	462	DRR	Ca_09137	R	NF-YA
Ca4_P3_44662720	Ca4	44662720	TAA/TAAA	TTGGGTTGAAACATGTCAGG	TTGATGTTGGTGGGATGATG	59.4	469	INTERGENIC	--	--	--

INDEL marker IDs	Chromosomes/ unanchored scaffolds	Physical positions (bp)	InDels (*Kabuli* reference genome-CDC Frontier/PII)	Forward primers (5'-3')	Reverse primers (5'-3')	Anneling temperature (0C)	Expected amplified product size (bp)	Structural annotation		Functional annotation	
								Sequence components of *kabuli* genome	*Kabuli* gene accession IDs	NCBI-KOG	TFs
Ca4_P3_44670060	Ca4	44670060	AC/ACC	TGCCTAAGACCCCAAATCAA	GCCACTTCTGGAATCACCAT	60.4	682	DRR	Ca_09140	E	--
Ca4_P3_44672472	Ca4	44672472	G/GTT	TTTGATCGAACAATCACCGA	CACATTCACATTGAATTTTGGAA	60.0	407	DRR	Ca_09139	--	--
Ca4_P3_44675409	Ca4	44675409	CGATTGATTGATTG/CGATTGATTG	TGCTTCAGCACTCTGCATCT	GTTTTGGAAAGGTTTGCAGC	59.9	519	INTRON	Ca_09140	E	--
Ca4_P3_44676740	Ca4	44676740	T/TG	TAAGGGCCCCATCATGACTA	GAACAACGGAGGAGGTGGTA	60.3	462	INTERGENIC	--	--	--
Ca4_P3_44678267	Ca4	44678267	CTAATTAATA/CTA	CATTTTGCCGATCAGAGTCA	GCAGGAACATGTGGGCTAGT	59.8	694	INTERGENIC	--	--	--
Ca4_P3_44679413	Ca4	44679413	CATA/CATATATA	AATCCTCTATGGATGCCGTG	TTGTGGGATTAAAAGTCGAAAAA	59.9	707	INTERGENIC	--	--	--
Ca4_P3_44679485	Ca4	44679485	TCATG/TCATGCATG	AATCCTCTATGGATGCCGTG	TTGTGGGATTAAAAGTCGAAAAA	59.9	707	INTERGENIC	--	--	--
Ca4_P3_44680416	Ca4	44680416	TTTATTATTATT/TTTATTATTATTATTATT	TGCCATCTTAAGTGACACGC	AGGAACACATTGTGGGTGGT	59.9	206	INTERGENIC	--	--	--
Ca4_P3_44685070	Ca4	44685070	TAAAAAAA/TAAAAAAAA	TGTTTAGAGTCAGTGGGCTTCA	CCCCTTAATTACTTGTGTTGGG	59.9	129	INTERGENIC	--	--	--
Ca4_P3_44691108	Ca4	44691108	TTAATAATAATAATAATAAT/TTAATAATAATAATAAT	CGAAAATGGAGCAATGAGGT	TGGGTTGAAAAATAAATGGAGG	60.1	539	INTERGENIC	--	--	--

INDEL marker IDs	Chromosomes/ unanchored scaffolds	Physical positions (bp)	InDels (*Kabuli* reference genome-CDC Frontier/PII)	Forward primers (5'-3')	Reverse primers (5'-3')	Anneling temperature (0C)	Expected amplified product size (bp)	Structural annotation		Functional annotation	
								Sequence components of *kabuli* genome	*Kabuli* gene accession IDs	NCBI-KOG	TFs
Ca4_P3_44691175	Ca4	44691175	TCCC/TCC	ACGAAAATGGAGCAATGAGG	TGTGTTGGGGTGAGTGAAAA	60.1	912	INTERGENIC	--	--	--
Ca4_P3_44694055	Ca4	44694055	TTAT/TT	GAACGAGCTTGCATTGATGA	AATCTTGAATGCCCGATTTG	60.0	245	INTERGENIC	--	--	--
Ca4_P3_44694278	Ca4	44694278	ATTTTTT/ATTTTTTT	GAACGAGCTTGCATTGATGA	TGGAGAATTAAGCAAAGGGG	60.0	602	INTERGENIC	--	--	--
Ca4_P3_44694688	Ca4	44694688	TAGA/TAGAGA	GATTCGATCATCTGGGGAAA	AGGCTTCTGGTGCTGTTCTT	59.8	539	INTERGENIC	--	--	--
Ca4_P3_44694709	Ca4	44694709	TAGAGAG/TAGAG	GATTCGATCATCTGGGGAAA	AGGCTTCTGGTGCTGTTCTT	59.8	539	INTERGENIC	--	--	--
Ca4_P3_44706448	Ca4	44706448	CAAAAAAAAA/CAAAAAAAA	ACTACTCCTCCCCTGGGAAA	GGTACTAGAGTTGCGGTGGC	59.9	811	INTRON	Ca_09141	O	--
Ca4_P3_44706474	Ca4	44706474	CAAAAAAAAA/CAAAAAAAA	ACTACTCCTCCCCTGGGAAA	GGTACTAGAGTTGCGGTGGC	59.9	811	INTRON	Ca_09141	O	--
Ca4_P3_44706655	Ca4	44706655	GAAA/GAA	AATAAACAAAGCACGCCTGG	GGTACTAGAGTTGCGGTGGC	60.1	576	INTRON	Ca_09141	O	--
Ca4_P3_44708231	Ca4	44708231	CAAAAAAAAAA/CAAAAAAAAAAAA	AGTCCAGTGGGCTGAAACAG	CAAGTTCATTGCACCGTTTTT	60.3	759	INTERGENIC	--	--	--
Ca4_P3_44710328	Ca4	44710328	TTCTCTCT/TTCTCT	AGGAAGTGTCGCGGTTAAGA	TTTTTGTTGAATGGTGGAAAT	59.9	517	INTERGENIC	--	--	--

INDEL marker IDs	Chromosomes/ unanchored scaffolds	Physical positions (bp)	InDels (*Kabuli* reference genome-CDC Frontier/PII)	Forward primers (5'-3')	Reverse primers (5'-3')	Anneling temperature (0C)	Expected amplified product size (bp)	Structural annotation		Functional annotation	
								Sequence components of *kabuli* genome	*Kabuli* gene accession IDs	NCBI-KOG	TFs
Ca4_P3_44710559	Ca4	44710559	GAAAAAA/GAAAAAAA	AGGAGTGGATGAGCCAAAAA	GGTGCTAAGTTCAAACACACAAA	59.7	822	INTERGENIC	--	--	--
Ca4_P3_4471270	Ca4	4471270	TTA/T	TTGGAATAGGAGCAGGATGG	GGAGGACCGTGATCAAATGT	60.0	424	INTRON	Ca_03810	GOT	--
Ca4_P3_44714173	Ca4	44714173	T/TC	AAGCTGAAATCGCTGAGAGC	TGGTACCCCATCCAAGGTAA	59.9	414	INTERGENIC	--	--	--
Ca4_P3_44714845	Ca4	44714845	A/ACACCC	GAAATGAGGAAGAGCACCCA	GGCGTAGTGCGTGTTGTAAA	60.2	457	INTERGENIC	--	--	--
Ca4_P3_44718399	Ca4	44718399	GGAGTGGTT/GGAGTGGTTGAGTGGTT	CAAAGATCGCGTCAATGAGA	TGCAGTCGATAAATTCACGC	59.9	617	INTERGENIC	--	--	--
Ca4_P3_44725079	Ca4	44725079	TTGT/TT	TTCAACCCCCTTTTAGTGAA	TGTCTTTAGTGTGGGGGTGG	57.2	838	INTERGENIC	--	--	--
Ca4_P3_44726033	Ca4	44726033	GTTTT/GTTT	TTCATGGCATCACAACCAAT	TATCCTCTCTCACGGTCCCA	59.8	155	INTERGENIC	--	--	--
Ca4_P3_44726266	Ca4	44726266	TAAA/TAAAA	TTCATGGCATCACAACCAAT	CAATTGCGGGTGGATTATTT	59.8	751	INTERGENIC	--	--	--
Ca4_P3_44727882	Ca4	44727882	AATTTT/AATTTTAAAATATTTT	CTCATTTTGCCAGTCCATTT	TTGATCTCGTGGTGAATACTCG	57.7	379	INTERGENIC	--	--	--
Ca4_P3_44730412	Ca4	44730412	GAAA/GAA	AATGTAACAATTGCCGCACA	TGTGGTTCCACCAATTTGAA	60.0	271	INTERGENIC	--	--	--

INDEL marker IDs	Chromosomes/ unanchored scaffolds	Physical positions (bp)	InDels (*Kabuli* reference genome-CDC Frontier/PII)	Forward primers (5'-3')	Reverse primers (5'-3')	Anneling temperature (0C)	Expected amplified product size (bp)	Structural annotation		Functional annotation	
								Sequence components of *kabuli* genome	*Kabuli* gene accession IDs	NCBI-KOG	TFs
Ca4_P3_44738779	Ca4	44738779	TT/TTAT	GGTTGCCTCTTTTACATGGG	TCATGCATGGTTGGAAAAGA	59.4	649	INTERGENIC	--	--	--
Ca4_P3_44747851	Ca4	44747851	ATTTTTTT/ATTTTTTTTT	TGGACTTGTGAAGCAAGCCT	CTTTCCCCGTGTGCTTACAT	61.0	580	INTERGENIC	--	--	--
Ca4_P3_44748836	Ca4	44748836	GATTAATTAATTAATTA/GATTAATTAATTAATTAATTA	CGCGTGTGATCATGAAAAAT	TTGCCTTTGTTTTTCCTTCA	59.5	732	INTERGENIC	--	--	--
Ca4_P3_44750728	Ca4	44750728	CAAAATTAAAATTAAA/CAAAATTAAA	CGGTCTAGGAAAAACACCACA	AAAACTTAATGTTTGACGAGTTGG	60.0	845	INTERGENIC	--	--	--
Ca4_P3_44750767	Ca4	44750767	TTAATAATAATAATAAT/TTAATAATAATAATAATAAT	CGGTCTAGGAAAAACACCACA	AAAACTTAATGTTTGACGAGTTGG	60.0	845	INTERGENIC	--	--	--
Ca4_P3_4475741	Ca4	4475741	CTATATA/CTATA	ACCATCATCTGAGTCCAGGC	TCAAGGGTTGGTCCAAGAAG	60.1	673	INTRON	Ca_03810	GOT	--
Ca4_P3_44766738	Ca4	44766738	CTTTTTTTT/CTTTTTT	CTTTGCATGTGATTGTTGGG	GAGCTAGGGCATTGTTCAGC	60.0	727	INTERGENIC	--	--	--
Ca4_P3_44771286	Ca4	44771286	CT/C	ATCGGATGTAAAATTCCCCC	TTGCTTCAGTTGGTGCATTC	59.9	411	INTERGENIC	--	--	--
Ca4_P3_44776874	Ca4	44776874	TTAT/TT	GCCTCACTTTTATCCCCCTC	AACCTTACGATCCAAAGCCA	59.9	644	INTERGENIC	--	--	--
Ca4_P3_4478589	Ca4	4478589	TAAGTTAAAAGTTAAAAGTTAAAAGTTAAAA/TAAGTTAAAAGTTAAAAGTTAAAA	TGTAGACGCGCAGAGAGAGA	AATCATTTGCCTGGTGCTTT	60.0	593	INTERGENIC	--	--	--

INDEL marker IDs	Chromosomes/ unanchored scaffolds	Physical positions (bp)	InDels (*Kabuli* reference genome-CDC Frontier/PII)	Forward primers (5'-3')	Reverse primers (5'-3')	Anneling temperature (0C)	Expected amplified product size (bp)	Structural annotation		Functional annotation	
								Sequence components of *kabuli* genome	*Kabuli* gene accession IDs	NCBI-KOG	TFs
Ca4_P3_44787142	Ca4	44787142	CTGT/CTGTGT	TGGCTTCATTTTCCATG TCA	ATTCCAAGAATAGCCG CCTT	60.0	246	INTRON	Ca_09144	--	S1Fa-like
Ca4_P3_4480276	Ca4	4480276	AA/AAGA	TGGTCACATGGAGGAT GAAA	GGTATTTTGACATGAA TATCCCTT	59.9	160	DRR	Ca_03809	--	S1Fa-like
Ca4_P3_4481679	Ca4	4481679	TAAAAAAAA/TAAAAAAAAA	TTTTGCTTTTGATTACT TTCTTTTT	GTTCCGCCTCTTCTAC CTCC	57.2	314	DRR	Ca_03809	--	S1Fa-like
Ca4_P3_4482906	Ca4	4482906	C/CG	GATCACCACCACCTTG ATCC	ACTTGAAGTCGCCCTA GCAA	60.2	649	INTRON	Ca_03809	--	S1Fa-like
Ca4_P3_44833451	Ca4	44833451	CAAAA/CAAAAAA	AATACCCCACAAAAATG CCA	CGATCGGGAGAAACAG TGAT	60.1	417	INTRON	Ca_09148	K	--
Ca4_P3_44838837	Ca4	44838837	TC/T	TGAAGGAATGCTTCTTT GTTTG	GATGATTGCAACCAAC ATGG	59.4	304	INTERGENIC	--	--	--
Ca4_P3_44845646	Ca4	44845646	TCAAACAAAC/TCAAAC	AGTCCACACAAGCCTT CACC	GGAGCTTTTTCGTATG TGCC	60.2	797	INTERGENIC	--	--	--
Ca4_P3_44850456	Ca4	44850456	ATCTTTTT/ATCTTTTTCTTTTT	GTTCCAACCAGGACCT GAAG	AGCTGGAGAGCAGAAT TCCA	59.5	746	INTRON	Ca_09149	--	--
Ca4_P3_44863007	Ca4	44863007	GAAAAAAA/GAAAAAA	TGAGCATGCAAAGTCC AATC	CAACCCTCCCAGTTTC ACAT	59.8	285	INTERGENIC	--	--	--
Ca4_P3_44865492	Ca4	44865492	TA/TAGA	TGACCATTGTGTGAAAA TGAGA	TGGTCGTAAAGTGCAT TGGA	59.0	685	INTERGENIC	--	--	--

INDEL marker IDs	Chromosomes/ unanchored scaffolds	Physical positions (bp)	InDels (*Kabuli* reference genome-CDC Frontier/PII)	Forward primers (5'-3')	Reverse primers (5'-3')	Anneling temperature (0C)	Expected amplified product size (bp)	Structural annotation		Functional annotation	
								Sequence components of *kabuli* genome	*Kabuli* gene accession IDs	NCBI-KOG	TFs
Ca4_P3_44870852	Ca4	44870852	TCCC/TC	CGCGGGGATACGTTAG TTTA	GGCATGTCTAGCAGCA ATCA	60.0	572	INTRON	Ca_09151	R	--
Ca4_P3_4487086	Ca4	4487086	ATA/ACAGAAATATATTA	GGCCAAATGAAGGACT CAAA	CGTACGACGTCTTTCG TCAA	60.1	932	DRR	Ca_03808	--	--
Ca4_P3_44871777	Ca4	44871777	TTCACCACAAATAT/TT	TATTGGCGATTGGTTT GTGA	TTGCCAACCTTTGAAC AGTG	59.9	627	DRR	Ca_09151	R	--
Ca4_P3_44873586	Ca4	44873586	A/ATATAGCCCTATG	TGAAGGAGTTTGAAGC AGGAA	AGAAGCTTCAATGGTG TGGG	60.0	190	INTERGENIC	--	--	--
Ca4_P3_4487412	Ca4	4487412	AT/ATTAATAAAGT	TCAGACTCTCCCGAAC GATT	CGTACGACGTCTTTCG TCAA	59.8	281	DRR	Ca_03808	--	--
Ca4_P3_44887114	Ca4	44887114	GAAACAAA/GAAACAAAAACAAA	ATTAGGGGGTTGGGTG TTTT	AAAGGGCCACTCATGT TTTG	59.4	519	INTERGENIC	--	--	--
Ca4_P3_44887376	Ca4	44887376	TA/T	CAAAACATGAGTGGCC CTTT	AGTGTGGGTAACGGGT ATGG	60.0	487	INTERGENIC	--	--	--
Ca4_P3_4489505	Ca4	4489505	TTATA/TTA	AGTGGTCTGCAACATA GGGG	GAAATGCAAAATGCCT TGGT	60.0	698	INTRON	Ca_03808	--	--
Ca4_P3_44897706	Ca4	44897706	ATTTTTTTT/ATTTTTTTTTTT	ATTTGTTGGAGGATGG GTCA	CACTCTTGTTCACGGC AAAA	60.2	623	INTERGENIC	--	--	--
Ca4_P3_44898118	Ca4	44898118	ACTAATATCTAA/ACTAATATCTAATATC TAA	TTTTGCCGTGAACAAGA GTG	CGATTGCACACGAGGA TATG	59.9	753	INTERGENIC	--	--	--

INDEL marker IDs	Chromosomes/ unanchored scaffolds	Physical positions (bp)	InDels (*Kabuli* reference genome-CDC Frontier/PII)	Forward primers (5'-3')	Reverse primers (5'-3')	Anneling temperature (0C)	Expected amplified product size (bp)	Structural annotation		Functional annotation	
								Sequence components of *kabuli* genome	*Kabuli* gene accession IDs	NCBI-KOG	TFs
Ca4_P3_44901004	Ca4	44901004	GT/GTGTAT	AGCTTCAAGTGGCTGAAGGA	ATGAACACGGGCGTATCTTC	60.1	527	DRR	Ca_09152	--	C2H2
Ca4_P3_44905023	Ca4	44905023	CAAAA/CAAAAA	TACAGTGTTGTGGTGCCGTT	GACCAGTAAATGCCTTCCCA	60.1	516	INTRON	Ca_09153	--	--
Ca4_P3_44905077	Ca4	44905077	TTCTCT/TTCT	TACAGTGTTGTGGTGCCGTT	GACCAGTAAATGCCTTCCCA	60.1	516	INTRON	Ca_09153	--	--
Ca4_P3_44917486	Ca4	44917486	AATATATATATA/AATATATATATATA	TTCCACCTTCAATGCAACAA	TTTGGAATTGGTCCAAGAGC	60.1	899	DRR	Ca_09155	Q	--
Ca4_P3_44922267	Ca4	44922267	CTTTTTT/CTTTTTTT	TGATTTGAGGGAGTTGGAGTG	CCCATAATGTCCCGATTCTG	60.1	676	INTERGENIC	--	--	--
Ca4_P3_44927225	Ca4	44927225	TTT/TTTATT	CGGGAGATACGTAGGAGCAA	GCTTGTGCATATCCCAACCT	60.2	438	INTERGENIC	--	--	--
Ca4_P3_44933948	Ca4	44933948	TATCCAATTAAT/TAT	TGCGGCTCTTTTGAGACTTT	CCTCACTCTCGTTCACCCAT	60.1	875	INTERGENIC	--	--	--
Ca4_P3_44940291	Ca4	44940291	TAGAGAGAGA/TAGAGAGAGAGA	CAATTATTTGGTGCAAAAGCA	TGCTCTGATATGAGGCGTTG	58.7	538	INTERGENIC	--	--	--
Ca4_P3_44948033	Ca4	44948033	TAAGCT/TAAGCTATTTTCACAAGCT	ATAGGGGCTGCAAAGAGGTC	TCCTTTCATGCTTGTGCATC	60.6	975	INTRON	Ca_09157	G	--
Ca4_P3_44949173	Ca4	44949173	AA/AAATTA	TGACGTGTCCCGTAACCTTT	ATAAGCCCATGTTGCGACTC	60.4	565	INTERGENIC	--	--	--

INDEL marker IDs	Chromosomes/ unanchored scaffolds	Physical positions (bp)	InDels (*Kabuli* reference genome-CDC Frontier/PII)	Forward primers (5'-3')	Reverse primers (5'-3')	Anneling temperature (0C)	Expected amplified product size (bp)	Structural annotation		Functional annotation	
								Sequence components of *kabuli* genome	*Kabuli* gene accession IDs	NCBI-KOG	TFs
Ca4_P3_4499622	Ca4	4499622	ACTAGCTATCCGCTAGCTA/ACTAGCTA	CCCGTCCCTAAAACTCTCCT	CGTTCCACGGTTTGGTAGTT	59.6	657	INTRON	Ca_03808	--	--
Ca4_P3_45053104	Ca4	45053104	ATTTTTT/ATTTTTTTT	ATTGCCCTTCTTGCTTTCCT	TACCCCACTTTGGAGGAGTG	60.2	785	INTRON	Ca_09167	K	GATA
Ca4_P3_45059546	Ca4	45059546	AAAACTACTT/AAAACTACTTAAACTACTT	CCCATTTCTTGTTGTGTTTCTG	ATGATGTTGTTGCATGTGGC	59.5	117	INTERGENIC	--	--	--
Ca4_P3_45059808	Ca4	45059808	TTA/TTATA	GCCACATGCAACAACATCAT	AAATCCCCGAGCTTTGATTC	60.4	595	INTERGENIC	--	--	--
Ca4_P3_45066858	Ca4	45066858	GATATATATATATA/GATATATATATA	GGAGGGACCATGTTTTTGTG	TTCACCTCTTGGCATCTTGA	60.2	544	INTERGENIC	--	--	--
Ca4_P3_4507040	Ca4	4507040	C/CTATTTTAGCTG	GCGAGACGTGGAATTTTGTT	CTCTTCAGTTTTGGTCCCCA	60.1	319	INTERGENIC	--	--	--
Ca4_P3_4507391	Ca4	4507391	T/TTA	TTTGGGGACCAAAACTGAAG	GCATATACGAGGAGACCCGT	59.9	156	INTERGENIC	--	--	--
Ca4_P3_4507561	Ca4	4507561	AAT/AAATAT	TTTGGGGACCAAAACTGAAG	TCCGAGCAACCATACTTTCA	59.9	698	INTERGENIC	--	--	--
Ca4_P3_45081374	Ca4	45081374	ATTTT/ATTTTT	TCGTTTTCCTTCAAAGGTGG	TGCTGAGTTATGGCGTCTTG	60.1	917	INTERGENIC	--	--	--
Ca4_P3_45097980	Ca4	45097980	T/TTTTGA	CCCAATTTCAATCCTCCAGA	GAGGCGGGGTGTATCACTAA	59.9	454	INTERGENIC	--	--	--

INDEL marker IDs	Chromosomes/ unanchored scaffolds	Physical positions (bp)	InDels (*Kabuli* reference genome-CDC Frontier/PII)	Forward primers (5'-3')	Reverse primers (5'-3')	Anneling temperature (0C)	Expected amplified product size (bp)	Structural annotation		Functional annotation	
								Sequence components of *kabuli* genome	*Kabuli* gene accession IDs	NCBI-KOG	TFs
Ca4_P3_45102000	Ca4	45102000	AATATATATATATATATAT/AATATATATATATAT	CCGGAGCTTGAACGAAAATA	TCGCGTAATACTCAAACCAAA	60.2	627	INTERGENIC	--	--	--
Ca4_P3_45102037	Ca4	45102037	CAATAATAATAATAATAATAAT/CAATAATAATAATAATAATAATAAT	CAGCACATCACAATCATCCA	TCGCGTAATACTCAAACCAAA	59.0	335	INTERGENIC	--	--	--
Ca4_P3_45108280	Ca4	45108280	A/ATT	CAGAAACAGTGAGCCAAGCA	AAAAACATTCCCGCCTTTTA	60.2	188	DRR	Ca_09170	--	--
Ca4_P3_45113591	Ca4	45113591	GATTATTATT/GATTATTATTATT	TTCGAGGAACTTCGCAATCT	TTATGTCAAGGCTCTCCGCT	60.0	652	DRR	Ca_09171	--	MYB_related
Ca4_P3_45115919	Ca4	45115919	CTT/CTTATT	TGCAGTGTTTGATTACGGGA	GGTGAAGATGATTCAATTTCCTTT	60.1	774	INTERGENIC	--	--	--
Ca4_P3_45116369	Ca4	45116369	TA/T	AAAGGAAATTGAATCATCTTCACC	GCAATTTCCGCTTTCTGTTT	59.7	385	INTERGENIC	--	--	--
Ca4_P3_45122277	Ca4	45122277	TAA/TAAA	GCGAGCGTATTGGTATTGGT	ATGTGACAAATTGGGGGAAA	60.0	551	DRR	Ca_09172	T	--
Ca4_P3_45126116	Ca4	45126116	CGTTGTTGTT/CGTTGTT	AACATTGGGACTGCACACAA	GGCGGATGACGAGAATATGT	60.0	913	DRR	Ca_09173	T	NAC
Ca4_P3_45132405	Ca4	45132405	CT/CTTT	AGCTCGATCGGAGACAGGTA	GCAAAGTTGTAGGTTTCGGC	60.0	347	INTRON	Ca_09175	S	--
Ca4_P3_45145450	Ca4	45145450	GAAAAAAAAAA/GAAAAAAAA	GCCTTGTCTGGATTGCATTT	GAAAAACAATTTGGCTATTCACC	60.1	926	INTERGENIC	--	--	--

INDEL marker IDs	Chromosomes/ unanchored scaffolds	Physical positions (bp)	InDels (*Kabuli* reference genome-CDC Frontier/PII)	Forward primers (5'-3')	Reverse primers (5'-3')	Anneling temperature (0C)	Expected amplified product size (bp)	Structural annotation		Functional annotation	
								Sequence components of *kabuli* genome	*Kabuli* gene accession IDs	NCBI-KOG	TFs
Ca4_P3_45153271	Ca4	45153271	CT/CTT	GACTGAGCCTTGCATCAACA	CATTGCGGTTTAGAAGGCAT	60.0	511	INTRON	Ca_09178	B	NAC
Ca4_P3_45154998	Ca4	45154998	CTGTG/CTG	ATGATGTGTGCAAACGGAAA	CTCACAACGTGGAAGTGCAG	60.0	544	INTRON	Ca_09178	B	NAC
Ca4_P3_45159393	Ca4	45159393	GTTT/GTTTTT	TGGAGGCTTGCTTCAGATTT	GACTTGTCCATGAAGGGCAT	60.0	793	INTRON	Ca_09179	P	--
Ca4_P3_45161056	Ca4	45161056	T/TG	GGATTGTGGAATTGGATTGG	CAAGACACGTGCACACACAG	60.0	496	INTRON	Ca_09179	P	--
Ca4_P3_45165137	Ca4	45165137	A/AC	TTGTTCTTCCTCCCCCTTCT	TTCAGCCCGCTAGCTATGAT	60.0	669	DRR	Ca_09179	P	--
Ca4_P3_45166587	Ca4	45166587	AATAT/AAT	TTGTGTGCTGCAAGGTTCTT	ACACCAAATTCGGCAAACTC	59.5	518	INTERGENIC	--	--	--
Ca4_P3_4516907	Ca4	4516907	AC/A	AAAATGCGTTTCACTCGGAC	GCACACTTGTGCTTGGAAGA	60.1	304	INTERGENIC	--	--	--
Ca4_P3_4516975	Ca4	4516975	ATT/A	AAAATGCGTTTCACTCGGAC	GCACACTTGTGCTTGGAAGA	60.1	304	INTERGENIC	--	--	--
Ca4_P3_4517051	Ca4	4517051	ATTT/ATTTT	AAAATGCGTTTCACTCGGAC	GCACACTTGTGCTTGGAAGA	60.1	304	INTERGENIC	--	--	--
Ca4_P3_4517104	Ca4	4517104	GATGAATTTATTAAT/GATGAATTTATTAATGAATTTATTAAT	AAAATGCGTTTCACTCGGAC	GCACACTTGTGCTTGGAAGA	60.1	304	INTERGENIC	--	--	--

INDEL marker IDs	Chromosomes/ unanchored scaffolds	Physical positions (bp)	InDels (*Kabuli* reference genome-CDC Frontier/PII)	Forward primers (5'-3')	Reverse primers (5'-3')	Anneling temperature (0C)	Expected amplified product size (bp)	Structural annotation		Functional annotation	
								Sequence components of *kabuli* genome	*Kabuli* gene accession IDs	NCBI-KOG	TFs
Ca4_P3_4517245	Ca4	4517245	AAGA/AA	AAAATGCGTTTCACTCGGAC	CCGTTTTTCGTCACAATTCA	60.1	550	INTERGENIC	--	--	--
Ca4_P3_45182355	Ca4	45182355	AATATATATATATATATATATATAT/AATATATATATATATATAT	TCATTGTCTTGGTCTTCCCC	GTGGTATTTAGTAAAATGAATGTGTCA	59.9	428	INTERGENIC	--	--	--
Ca4_P3_45185940	Ca4	45185940	T/TG	TACTCGAGCACATGCAAACC	CTTTGAGGCCTCCATAGCTG	59.9	510	INTRON	Ca_09180	K	TALE
Ca4_P3_45194041	Ca4	45194041	CAATA/CA	GAGCGAGTCAAAACCGAGTC	GCAAGCAATCAAGTAAAAATGG	60.0	597	INTERGENIC	--	--	--
Ca4_P3_45198346	Ca4	45198346	CGG/CG	TTGACTGAATGGAAAGCCAA	ACAATCAAGCGTCGGAAAAC	59.2	569	INTERGENIC	--	--	--
Ca4_P3_45199389	Ca4	45199389	TA/TAA	GCCAAACTCGTTGATTGGTT	TGTGGGTCTCATAAGGACGA	60.0	573	DRR	Ca_09182	--	--
Ca4_P3_45201732	Ca4	45201732	TAA/TAAA	GTGAAACTCCGGTTTGAGGA	GCACCAAGCACAGAACAAAA	60.1	699	INTRON	Ca_09182	--	--
Ca4_P3_45205185	Ca4	45205185	TC/T	GGAAATGGGAGGAGGGAATA	TCGTGTTTTGAATAAACAGGAAAA	60.1	300	INTERGENIC	--	--	--
Ca4_P3_45205721	Ca4	45205721	ACC/AC	CGTATTTTGGCTTAAATATTTCCAA	CCTCCAACCCACTGATCCTA	59.7	451	INTERGENIC	--	--	--
Ca4_P3_4521076	Ca4	4521076	AATATATATATATATATA/AATATATATATATATA	CCACAGAAAATGTTGGGCTT	TCGTAAAATAGTTGGCCGGA	60.0	568	INTERGENIC	--	--	--

INDEL marker IDs	Chromosomes/ unanchored scaffolds	Physical positions (bp)	InDels (*Kabuli* reference genome-CDC Frontier/PII)	Forward primers (5'-3')	Reverse primers (5'-3')	Anneling temperature (0C)	Expected amplified product size (bp)	Structural annotation		Functional annotation	
								Sequence components of *kabuli* genome	*Kabuli* gene accession IDs	NCBI-KOG	TFs
Ca4_P3_45210841	Ca4	45210841	CAAA/CA	TGGATCTTTTGTTGTCCCCT	ATGCTAAGCCACCAACCAAC	59.4	680	DRR	Ca_09183	O	--
Ca4_P3_45221189	Ca4	45221189	GTTTTT/GTTTT	GCACTCAAAGAGCACCATCA	CTGGCTCCGAAATCTATTGG	60.0	505	DRR	Ca_09184	A	C2H2
Ca4_P3_45222131	Ca4	45222131	TA/TAA	CCACCTTGTTCCCACTCCTA	TCCAATGAATTTGGAGAGCC	60.0	334	DRR	Ca_09184	A	C2H2
Ca4_P3_45222380	Ca4	45222380	TA/T	TTAGCCAATCACCCCAAAAC	ATACGAAGCAACAAGCACCC	59.8	353	DRR	Ca_09184	A	C2H2
Ca4_P3_45223304	Ca4	45223304	ATTACT/ATGAATTACTTACT	TGAAGAGGGGAAATATGCAA	GAAAACGCTTTTATCATCATCCA	58.2	442	INTERGENIC	--	--	--
Ca4_P3_4522341	Ca4	4522341	ATTTTTTTTTT/ATTTTTTTTT	TTTGTCCTGTGTTGTTTTATGTTT	GGGCCGAAAATTTGTCATT	57.7	276	INTERGENIC	--	--	--
Ca4_P3_45223941	Ca4	45223941	AGG/AGGG	CAGTTGATTGCATGCCTTATTT	TAGTAACATGCTGCACCGCT	59.1	493	INTERGENIC	--	--	--
Ca4_P3_45226065	Ca4	45226065	CTTTTTTTTT/CTTTTTTTTTTT	GAAAGACGGCTTGTTGCTTC	GAGACCGGCCAGGAACTAAT	60.0	688	INTRON	Ca_09185	S	--
Ca4_P3_45226893	Ca4	45226893	TTC/TTCGTC	ATTAGTTCCTGGCCGGTCTC	GGAGAGAGGACCCACAAACA	60.5	868	DRR	Ca_09185	S	--
Ca4_P3_45228406	Ca4	45228406	ATTATT/ATTATTATTTTTATT	GAGAGGGTGGCTAGTTGCTG	TTGACCCATTTCGTATCACTG	60.0	672	DRR	Ca_09185	S	--

INDEL marker IDs	Chromosomes/ unanchored scaffolds	Physical positions (bp)	InDels (*Kabuli* reference genome-CDC Frontier/PII)	Forward primers (5'-3')	Reverse primers (5'-3')	Anneling temperature (0C)	Expected amplified product size (bp)	Structural annotation		Functional annotation	
								Sequence components of *kabuli* genome	*Kabuli* gene accession IDs	NCBI-KOG	TFs
Ca4_P3_45228759	Ca4	45228759	CAATATATCTAATATATC/CAATATATC	TCAGTGATACGAAATGGGTCA	TTTGGTGATTGGCACAACAT	59.0	484	INTERGENIC	--	--	--
Ca4_P3_45232759	Ca4	45232759	TAAAAAA/TAAAAA	TGGCAAAATCCTTCACAACA	AACGATGCAACCTTACCCTG	60.1	181	DRR	Ca_09187	A	--
Ca4_P3_4523289	Ca4	4523289	TT/TTATAT	TGATAGAAAAATGATGCAAGAAGG	AATTTTCCATGCCAACTGGA	59.6	454	INTERGENIC	--	--	--
Ca4_P3_4523557	Ca4	4523557	GAAAA/GAAAAA	GATTGATGCGAAGGAGGGTA	AACACACCAACCACCACTCA	60.0	490	INTERGENIC	--	--	--
Ca4_P3_45236013	Ca4	45236013	CATGTGCGAACAATG/CATGTGCGAACAATGTGCGAACAATG	CAAACCTGAAAAGGGCACAT	TAACGATGGGATTGGTCCAT	60.0	461	INTRON	Ca_09187	A	--
Ca4_P3_45245377	Ca4	45245377	TC/TCC	AGCATTGGAACCCAAATGAG	AAAAACGCAGTATCCATACAAAA	59.9	797	DRR	Ca_09188	--	B3
Ca4_P3_45247621	Ca4	45247621	TA/TAA	GTTGGAAGCCATGGTGAACT	TTTTGGTTTTCAAGTATTGGCA	60.0	430	INTERGENIC	--	--	--
Ca4_P3_45250720	Ca4	45250720	GTTTTTTTTT/GTTTTTTTTTTT	GGGACCCCACAAAGAAACTT	TCCGGAGGTCAACACAAAAT	60.2	706	INTERGENIC	--	--	--
Ca4_P3_45261057	Ca4	45261057	ATT/A	TGTATGTGGGAAATGGCAGA	CATAAAGCGGGTGAAGGGTA	59.9	816	DRR	Ca_09189	O	--
Ca4_P3_45265560	Ca4	45265560	TC/T	GGAAAGTCTCGATGGTTGGA	GCCCAAATGAAGCAAAACAT	60.1	664	INTRON	Ca_09190	--	--

INDEL marker IDs	Chromosomes/ unanchored scaffolds	Physical positions (bp)	InDels (*Kabuli* reference genome-CDC Frontier/PII)	Forward primers (5'-3')	Reverse primers (5'-3')	Anneling temperature (0C)	Expected amplified product size (bp)	Structural annotation		Functional annotation	
								Sequence components of *kabuli* genome	*Kabuli* gene accession IDs	NCBI-KOG	TFs
Ca4_P3_45267760	Ca4	45267760	GT/GTAATCGTAATT	ATTGATGGCAAGAAAG GTGG	TTTCCAGGCATATAGG GTGC	59.9	758	INTRON	Ca_09190	--	--
Ca4_P3_45269812	Ca4	45269812	TTATAT/TTAT	GAAGCATGGATGCTGG ATTT	TGACAGGCAACATTAT CCGA	60.0	742	DRR	Ca_09190	--	--
Ca4_P3_45274571	Ca4	45274571	T/TG	TCGAACGACAATCCAC ACAT	CGTGGGGGAAATTTTT CTTT	60.0	518	DRR	Ca_09191	O	--
Ca4_P3_4527820	Ca4	4527820	GAAAAAAAA/GAAAAAAA	TTTTCACTCGATACGGC TTTT	AATGTGCATCTGCCAC CATA	58.9	163	INTERGENIC	--	--	--
Ca4_P3_4527882	Ca4	4527882	AGG/AG	AAAAAGTTGGAGAAGG CGAA	AATGTGCATCTGCCAC CATA	58.9	118	INTERGENIC	--	--	--
Ca4_P3_45289876	Ca4	45289876	GAAAAAAAAAA/GAAAAAAAA	AAGCGAGCATCAGAGA AAGC	CTTGATGGCAAAACCC AAGT	59.9	269	INTERGENIC	--	--	--
Ca4_P3_45308407	Ca4	45308407	ACCAT/ACCATCCAT	TGTTAATCCAAACATGG CCC	GTTAGCAACATGTGCA TCGG	60.6	267	INTERGENIC	--	--	--
Ca4_P3_4532626	Ca4	4532626	GTATTATTA/GTATTA	TGAACCCCAAAAATCAC CAT	CAAACTCATAGCGGGC ATTT	60.0	684	DRR	Ca_03806	--	--
Ca4_P3_4532666	Ca4	4532666	TAAAA/TAAAAA	TGAACCCCAAAAATCAC CAT	CAAACTCATAGCGGGC ATTT	60.0	684	DRR	Ca_03806	--	--
Ca4_P3_45331096	Ca4	45331096	TAT/TATGAT	CACATGTCCATTCGGA GTGT	TCGACCAAGTATCAGC AAACC	59.4	748	INTERGENIC	--	--	--

INDEL marker IDs	Chromosomes/ unanchored scaffolds	Physical positions (bp)	InDels (*Kabuli* reference genome-CDC Frontier/PII)	Forward primers (5'-3')	Reverse primers (5'-3')	Anneling temperature (0C)	Expected amplified product size (bp)	Structural annotation		Functional annotation	
								Sequence components of *kabuli* genome	*Kabuli* gene accession IDs	NCBI-KOG	TFs
Ca4_P3_45335482	Ca4	45335482	ATTTT/ATTT	GTTCCGATCACATGGCTTCT	TGAGACGCTTTCGTCACAAC	60.1	406	INTRON	Ca_09198	L	--
Ca4_P3_45341608	Ca4	45341608	AAGAGAATCGGA/AAGAGAATCGGAGAGAATCGGA	GCTGGACAAGTCTGCCAAG	CTAGTGTGCAGTGGTGCCAT	59.6	874	DRR	Ca_09198	L	--
Ca4_P3_45343872	Ca4	45343872	TCC/TCCCC	ATTAAGCGGAACAGAAGCGA	ATGCTGGAATTGTCTGGGAG	60.0	662	INTRON	Ca_09199	F	--
Ca4_P3_45350416	Ca4	45350416	ATTTTTTTTTT/ATTTTTTTT	TGCATTTCCCTATTTAATGCAA	GTCCTTGGTAGCGAGTTGGA	59.5	684	INTERGENIC	--	--	--
Ca4_P3_45351213	Ca4	45351213	A/AGT	TCAATCGTCTGCAAATCCAC	TCATTTATTCGACTGTCGGC	59.7	446	INTERGENIC	--	--	--
Ca4_P3_45363153	Ca4	45363153	ATTTTTTT/ATTTTTTTT	GGCAAAACCAAAGAGAACCA	GATTTTTGGCTGCTTATGGC	60.1	400	INTERGENIC	--	--	--
Ca4_P3_45368976	Ca4	45368976	ATTTTTTTT/ATTTTTTTTT	TGAATTGGTTGAGTGAAAGGAA	CAATGAAACTTTATGAAAGATAAATGA	59.6	618	INTERGENIC	--	--	--
Ca4_P3_4537550	Ca4	4537550	AATAT/AATATATAGATAT	TGCATCGCCCATATCAATAA	TATTGGACCGACCCTGAAAA	59.9	511	INTERGENIC	--	--	--
Ca4_P3_45380400	Ca4	45380400	ACTACG/A	TTGCCGTTAATTCTTGGAGC	GTGGAGAAAACAACAACGGG	60.2	774	INTERGENIC	--	--	--
Ca4_P3_45382285	Ca4	45382285	AATAT/AAT	CCTCAATGGAGGAACAAAGG	GCTGCGCACATCATCTCTAA	59.5	448	INTERGENIC	--	--	--

INDEL marker IDs	Chromosomes/ unanchored scaffolds	Physical positions (bp)	InDels (*Kabuli* reference genome-CDC Frontier/PII)	Forward primers (5'-3')	Reverse primers (5'-3')	Anneling temperature (0C)	Expected amplified product size (bp)	Structural annotation		Functional annotation	
								Sequence components of *kabuli* genome	*Kabuli* gene accession IDs	NCBI-KOG	TFs
Ca4_P3_4538536	Ca4	4538536	T/TC	CCGTACCAAAACAATC GTCC	TGCATCTATTCCAACC GTCA	60.2	597	INTERGENIC	--	--	--
Ca4_P3_45385545	Ca4	45385545	ACT/ATTATATATCT	CACCCATAATACTTTAT GAGATGGAA	CACAAATTAAGGGAAG GCGA	59.6	363	INTERGENIC	--	--	--
Ca4_P3_45388943	Ca4	45388943	TGGGGG/TGGGG	TGCTCATGGCTCAAGT TTGT	GAAAAATCGGAAGCTG GACA	59.4	465	INTERGENIC	--	--	--
Ca4_P3_45399782	Ca4	45399782	AG/A	GTTTGACAGCAAAGCG GAAT	ACAGCTTGCAACTGAT GTGG	60.3	334	INTERGENIC	--	--	--
Ca4_P3_45401996	Ca4	45401996	TAAAAAAAAA/TAAAAAAAA	TTGATAGTGGCTGGCA CAAA	GGGGTTTTGGTTTTGG TTTT	60.3	480	INTERGENIC	--	--	--
Ca4_P3_45404851	Ca4	45404851	GTTTCTTTTTTCT/GTTTCTTTTTTCTTT TTTCT	TTTCTTTTGTGTTTGCA GCG	GGGAGGAAAGTTGAAG TTGAAA	60.0	400	INTERGENIC	--	--	--
Ca4_P3_45407639	Ca4	45407639	TAA/TA	TCCCTTTGAAATTGTGG AGG	TAGCAACGCCAGTTGC ATAG	59.9	536	INTERGENIC	--	--	--
Ca4_P3_45411021	Ca4	45411021	AG/A	TGGTCCAACTCTCATG GTCA	TTTAAAAGAACCCACC GCAC	60.1	760	INTERGENIC	--	--	--
Ca4_P3_45414589	Ca4	45414589	CTT/CTTT	TGCATAAATGCACGAT GAAAA	GTTCAAACCGGCAATG TCTT	60.1	740	INTERGENIC	--	--	--
Ca4_P3_45416085	Ca4	45416085	AAAAGAA/AAA	CCAATTCATGGTACAAG GGG	CCTGTTAAACACACCC CCAC	60.0	404	INTRON	Ca_09202	K	TALE

INDEL marker IDs	Chromosomes/ unanchored scaffolds	Physical positions (bp)	InDels (*Kabuli* reference genome-CDC Frontier/PII)	Forward primers (5'-3')	Reverse primers (5'-3')	Anneling temperature (0C)	Expected amplified product size (bp)	Structural annotation		Functional annotation	
								Sequence components of *kabuli* genome	*Kabuli* gene accession IDs	NCBI-KOG	TFs
Ca4_P3_45416148	Ca4	45416148	CAAAA/CAAA	CCAATTCATGGTACAAGGGG	CCTGTTAAACACACCCCCAC	60.0	404	INTRON	Ca_09202	K	TALE
Ca4_P3_45416537	Ca4	45416537	CTATAGT/CT	GTGGGGGTGTGTTTAACAGG	TCCCATTCCATTTTGAAAGC	60.1	358	INTRON	Ca_09202	K	TALE
Ca4_P3_45421314	Ca4	45421314	CTTTTTT/CTTTTTTTTTTTT	TTCAACGGAGTTACAGCAAAAA	CAAATGTCACCCTTAGTTGACG	59.8	257	DRR	Ca_09202	K	TALE
Ca4_P3_45431503	Ca4	45431503	TGAGAGA/TGAGA	GAGGGCAAGACCAAGATCAA	TCACATGAATGCACACAACAA	60.2	742	INTERGENIC	--	--	--
Ca4_P3_45431951	Ca4	45431951	AATATATATATATATATATATA/AATATATATATATATA	TTTGTTGTGTGCATTCATGTG	ATCACTTTTGTGCGTGGAAA	59.0	598	INTERGENIC	--	--	--
Ca4_P3_45441612	Ca4	45441612	CTATATATATATATATATATATATATATATAT/CTATATATATATATATATATATATAT	TCTTGTCGCCTTTCCAAACT	ATGGGTTCATGTTTGACCGT	59.9	225	INTERGENIC	--	--	--
Ca4_P3_45441733	Ca4	45441733	T/TTTAA	ACGGTCAAACATGAACCCAT	ATTGTCGTGAGAATCGAGGC	60.1	525	INTERGENIC	--	--	--
Ca4_P3_45476868	Ca4	45476868	GAGAAAGAAAGAAAGAAAGAAAGAAAGAAAGAAAGA/GAGAAAGAAAGAAAGAAAGAAAGAAAGAAAGAAAGAAAGA	AGCCCAATTGACAAAACTGG	ACTCCATATGACGTGGCACA	60.0	571	INTERGENIC	--	--	--
Ca4_P3_4547758	Ca4	4547758	CAAA/CAAAAA	TTGCTAATTTTCGGATGCCT	TCTCGAGTTCAATGCGTCAC	59.7	617	INTRON	Ca_03803	--	--
Ca4_P3_4549797	Ca4	4549797	TTTC/TTTCTTC	GGGCAATTTGGAAGTTACGA	TCTGCGCTATAAGCTGGGTT	59.9	383	INTRON	Ca_03803	--	--

INDEL marker IDs	Chromosomes/ unanchored scaffolds	Physical positions (bp)	InDels (*Kabuli* reference genome-CDC Frontier/PII)	Forward primers (5'-3')	Reverse primers (5'-3')	Anneling temperature (0C)	Expected amplified product size (bp)	Structural annotation		Functional annotation	
								Sequence components of *kabuli* genome	*Kabuli* gene accession IDs	NCBI-KOG	TFs
Ca4_P3_45505990	Ca4	45505990	AATTATTATTATTATTATTATTATTATTATTATTATTATTATTATTATTATTATTA/AATTATTATTATTATTATTATTATTATTATTATTATTATTATTATTATTATTATTATTATTA	GAATCACGCAGCATTCAAAA	CGGAGATTGCAAGTAAAAGAGTC	59.8	402	INTERGENIC	--	--	--
Ca4_P3_45532146	Ca4	45532146	TTTATTATTATTATTATTATTATTATTATTATTATTATTATTATTATTATTATT/TTTATTATTATTATTATTATTATTATTATTATTATTATTATTATT	GAACCCAGGAACCAAAGACA	ATAGCGGAGACTTTGGGGTT	59.9	632	INTERGENIC	--	--	--
Ca4_P3_45582823	Ca4	45582823	GTTTTTTTTTTTTT/GTTTTTTTTTTTT	TGGGATGTGACTAAATGCGA	GGGACCAACAATAAAAACGAAA	60.1	366	INTERGENIC	--	--	--
Ca4_P3_45586091	Ca4	45586091	GTTTTTTTTTT/GTTTTTTTTTTT	GGAGGTCATGTGCTCCTCAT	TTTGCCCTTGTATCTGGGAC	60.1	473	INTERGENIC	--	--	--
Ca4_P3_4561606	Ca4	4561606	AG/A	GGAACATGGCAAGGAAGTGT	TCGGGCCATTTAGCTCATAC	60.0	609	INTRON	Ca_03802	UZ	--
Ca4_P3_45645932	Ca4	45645932	GT/G	AACTTGATGCCGAGTAGCGT	GCCGAATAAAACCCTCACAA	59.9	713	INTERGENIC	--	--	--
Ca4_P3_45692476	Ca4	45692476	TAAAAAAA/TAAAAAAAA	TGTTGTTGTCGCAATGGAAT	CACAAAGGCGTCAAAACTCA	60.0	804	INTERGENIC	--	--	--
Ca4_P3_4570498	Ca4	4570498	GAAA/GAAAA	ACTGTTGCTCCTGGTGAGGT	TCATGGGACTGGACAAAACA	59.8	559	INTRON	Ca_03801	--	--
Ca4_P3_45740248	Ca4	45740248	TTATATATATATATATATATATATATAT/TTATATATATATATATATATAT	ACCAAATCATAACCGACCCA	TTGTTTTAAATCCGCATCACA	60.1	375	INTERGENIC	--	--	--
Ca4_P3_45756329	Ca4	45756329	TC/TCAC	TATTTTGGATTTTGGCTCGG	AACAGAGGGGTGTGGAGTTG	59.9	512	DRR	Ca_18385	--	--

INDEL marker IDs	Chromosomes/ unanchored scaffolds	Physical positions (bp)	InDels (*Kabuli* reference genome-CDC Frontier/PII)	Forward primers (5'-3')	Reverse primers (5'-3')	Anneling temperature (0C)	Expected amplified product size (bp)	Structural annotation		Functional annotation	
								Sequence components of *kabuli* genome	*Kabuli* gene accession IDs	NCBI-KOG	TFs
Ca4_P3_4582555	Ca4	4582555	ATTT/ATTTT	TTGAATTTAAATCCGGGATGA	GCTGCTTCACCTTCACCTTC	59.2	816	DRR	Ca_03800	T	--
Ca4_P3_4588591	Ca4	4588591	TAAAAAAAAA/TAAAAAAAA	TTTTCCTCCATCGTTGATCC	GACCACGTGTACCGTTGAAA	59.9	785	INTERGENIC	--	--	--
Ca4_P3_4589409	Ca4	4589409	AATTATTA/AATTATTAGATTATTA	TCATTTTCCGGCTGAGTACA	TCGAAAGTTGGCTTTTCACA	59.3	832	INTERGENIC	--	--	--
Ca4_P3_4589677	Ca4	4589677	GACA/GA	TGGATTATCAATTGGGTAGGAA	AACGGAGGCAGTAATTGTCG	58.3	817	INTERGENIC	--	--	--
Ca4_P3_4590029	Ca4	4590029	C/CAT	TTTCGCTCAATGTGAAAAGC	CCACGATAACGATGTGATGC	59.0	749	INTERGENIC	--	--	--
Ca4_P3_4593293	Ca4	4593293	A/ACTAAT	TGGATCAACACGTGGAAAAA	ACAATTGTTGCCAGGGGTAA	59.9	667	DRR	Ca_03798	--	--
Ca4_P3_4593540	Ca4	4593540	CTAATTAATTAA/CTAATTAA	TTGAGGAATGTAAGGATGTCCA	TTAATGGCGGCTACGTTTTC	59.4	928	DRR	Ca_03798	--	--
Ca4_P3_4594832	Ca4	4594832	GAAA/GAAAAATAAA	TATGAGTGGGGATGATGGGT	CCTTGTTTCCCGCACATAAT	60.0	403	INTERGENIC	--	--	--
Ca4_P3_46001807	Ca4	46001807	GATAATAATAATAATAATAATAATAATAATAATAATAATAATA/GATAATAATAATAATAATAATAATAATAATAATAATAATAATA	TTGCTCCTACGTATCCCCAG	CTCAATCGTGGGTGACCTTT	60.1	385	INTERGENIC	--	--	--
Ca4_P3_46004838	Ca4	46004838	TAAAAAA/TAAAAAAA	TGATTTCAATGAGAGGATTTTGTT	GCTCGATTGATGTTAAGGGC	59.0	425	INTERGENIC	--	--	--

INDEL marker IDs	Chromosomes/ unanchored scaffolds	Physical positions (bp)	InDels (*Kabuli* reference genome-CDC Frontier/PII)	Forward primers (5'-3')	Reverse primers (5'-3')	Anneling temperature (0C)	Expected amplified product size (bp)	Structural annotation		Functional annotation	
								Sequence components of *kabuli* genome	*Kabuli* gene accession IDs	NCBI-KOG	TFs
Ca4_P3_46144676	Ca4	46144676	TAAAA/TAAAAA	AGTTGGAAGTCGTCGACAAAA	GTTGCGCCCATGAACTAAAT	59.8	253	INTRON	Ca_18357	--	NF-X1
Ca4_P3_46377599	Ca4	46377599	CATATATATATATATATATATATATATATATATATAT/CATATATATATATATATATATATATATAT	ATGGGAACCCACAAACAATC	CCACATGCTATTTTAATCTTTGGA	59.5	732	INTERGENIC	--	--	--
Ca4_P3_46378685	Ca4	46378685	GTTATTATTATTAT/GTTATTATTATTATTAT	TCAACCTCTCACTCCAACCA	AGGATAACATGCTTTGGTGTTG	59.2	551	INTERGENIC	--	--	--
Ca4_P3_46493029	Ca4	46493029	AATATATATATATATATATATATATA/AATATATATATATATATATATA	GGGAAAGTGATATTGGGGGT	CAAAGCTCCTTCAATTGTCTTG	59.9	407	INTERGENIC	--	--	--
Ca4_P3_46494989	Ca4	46494989	GATGATATAT/GAT	TGAATTCGACTTGATTTCCAA	CTTGTTTGGAACCCATTGCT	58.2	622	INTERGENIC	--	--	--
Ca4_P3_46496090	Ca4	46496090	TAAAAAAAAA/TAAAAAAAAAA	AAAGTGGGGTCAACTTTTTGAA	TGTCAAGGAGACCCACCACT	59.9	481	INTERGENIC	--	--	--
Ca4_P3_46508599	Ca4	46508599	AATATATATATATATATATATATA/AATATATATATATATATATA	AAAGGGGGAGTATTGTTGGG	CAAAGCTCTTTCATAATTGTCTTGA	60.0	492	INTERGENIC	--	--	--
Ca4_P3_46537753	Ca4	46537753	TATAATAATAATAATAATAATAATAATAATAATAATAATAATAATAATA/TATAATAATAATAATAATAATAATAATAATAATAATAATAATAATAATAATAATAATA	CAGCGAAATGTTAAGTGCTTTT	CGTTCTCTCCTCCCTTCCTC	58.6	610	INTERGENIC	--	--	--
Ca4_P3_46555948	Ca4	46555948	TATTTTAATTTTAATT/TATTTTAATT	GCTATGGGTTCAAGTTTCGG	CACTTCCCCTAATGTCCTGG	59.6	785	INTERGENIC	--	--	--
Ca4_P3_46638195	Ca4	46638195	TTATATATATATATATA/TTATATATATATATA	GCGTGGAAATGAGCAATTTT	GATTGAATTGCGGTGAAGGT	60.1	328	INTERGENIC	--	--	--

INDEL marker IDs	Chromosomes/ unanchored scaffolds	Physical positions (bp)	InDels (*Kabuli* reference genome-CDC Frontier/PII)	Forward primers (5'-3')	Reverse primers (5'-3')	Anneling temperature (0C)	Expected amplified product size (bp)	Structural annotation		Functional annotation	
								Sequence components of *kabuli* genome	*Kabuli* gene accession IDs	NCBI-KOG	TFs
Ca4_P3_46674801	Ca4	46674801	A/AG	GGATGACTCGAAACGG TGTT	TTACCCAAAACCCCAA ATCA	60.0	667	INTERGENIC	--	--	--
Ca4_P3_46677829	Ca4	46677829	AATATATATATATATATATATATATA/ AATATATATATATATATATATATATAT ATATATATA	TCCTCTCATAACGGGG TTTG	GGATGCAGATTTTCCT CGAA	59.9	330	INTERGENIC	--	--	--
Ca4_P3_46707184	Ca4	46707184	AATAT/AATATATAT	AGGGATGGATTAAAAA CGGG	AGATTTAGAGGGGGCA CACA	60.0	302	INTERGENIC	--	--	--
Ca4_P3_46711100	Ca4	46711100	TAAAAA/TAAAAAA	CCTTTTTCTTGGTGGG GAAT	TGTATGAGACGATGTC ACTTTGAA	60.2	629	INTERGENIC	--	--	--
Ca4_P3_46711273	Ca4	46711273	AATA/AA	TTGACCAAAATCGCTG CTAA	AGACTAATGACCTCGC CACG	59.4	880	INTERGENIC	--	--	--
Ca4_P3_46711486	Ca4	46711486	ATTTTTTTTT/ATTTTTTTTTT	AAAGGTGATTTCGCTAT CGG	ATGCTTTTTCACCATCC TCG	59.2	915	INTERGENIC	--	--	--
Ca4_P3_46711628	Ca4	46711628	GTTATAG/GTTATAGACTTATAG	TCATTTACTCGTGCATG AAAGTT	ATGCTTTTTCACCATCC TCG	58.8	685	INTERGENIC	--	--	--
Ca4_P3_46716313	Ca4	46716313	GTA/G	AGATTGACGAGGAAGC TGGA	GCATGGGGATAAAAGC AAGA	60.0	592	INTRON	Ca_19246	S	--
Ca4_P3_46716677	Ca4	46716677	TAAA/TAA	AGATTGACGAGGAAGC TGGA	CTCTGCTTGCAGGGTT AAGG	60.0	947	INTRON	Ca_19246	S	--
Ca4_P3_46730464	Ca4	46730464	CTTTTT/CTTTTTT	CATTAGTCCACGTTGCT TCG	TAACAGACCCGTGCAA TGAG	59.3	271	INTRON	Ca_19245	P	--

INDEL marker IDs	Chromosomes/ unanchored scaffolds	Physical positions (bp)	InDels (*Kabuli* reference genome-CDC Frontier/PII)	Forward primers (5'-3')	Reverse primers (5'-3')	Anneling temperature (0C)	Expected amplified product size (bp)	Structural annotation		Functional annotation	
								Sequence components of *kabuli* genome	*Kabuli* gene accession IDs	NCBI-KOG	TFs
Ca4_P3_46732510	Ca4	46732510	G/GCA	CGGTATAGCCTGGGAAATGA	GATGGAAGGAATGCCAAAGA	59.9	766	DRR	Ca_19245	P	--
Ca4_P3_46734440	Ca4	46734440	CACAT/CACATTCATCTACAT	TGATTCCCTTTCGAACAAGC	CCACTCCCAGATCTTTGCAT	60.2	748	INTRON	Ca_19244	--	bZIP
Ca4_P3_46759531	Ca4	46759531	CG/C	TGGATTTTGTTTGAAGATGATGA	TCTTTTGCGGTGCCTTAACT	59.4	173	INTERGENIC	--	--	--
Ca4_P3_46770853	Ca4	46770853	AT/A	AAGTCAAGGAACCACGGATG	AAAGCAAAGCCACCAAAATG	60.0	575	INTRON	Ca_19241	OE	WRKY
Ca4_P3_4693642	Ca4	4693642	GAAAAAAAAAAA/GAAAAAAAAAAAAA	CTCGCTACATTCCACCCAGT	CTTAGGAGTGGTGCTCCTCG	60.1	643	INTERGENIC	--	--	--
Ca4_P3_47193005	Ca4	47193005	TTTAT/TTTATTAT	CAATGTGGGTGAATTTGCTG	GATTCTGGCAAATTCGTCGT	60.0	287	INTRON	Ca_10862	--	--
Ca4_P3_47193746	Ca4	47193746	CTACT/CTACTCCTCCCCTTTATACT	TGAAATCCCTCCTCTTCCCT	AATCCTCCAAGTCCAACTGC	60.0	162	INTRON	Ca_10862	--	--
Ca4_P3_47197291	Ca4	47197291	CATACCGATACCGA/CATACCGA	CCATGACAAAATAGGAGGCAC	TTCACCAAGTGTTGCTTCCA	59.4	467	DRR	Ca_10862	--	--
Ca4_P3_47206262	Ca4	47206262	A/ATAT	TCACCATTCAATCATTTTTGG	CCCGAGAAAGATTTTACGCA	58.3	550	INTERGENIC	--	--	--
Ca4_P3_47206655	Ca4	47206655	CTTTTTTT/CTTTTT	TGCGTAAAATCTTTCTCGGG	TGATGAAGTCGTTGAAGTTAGGA	60.2	454	INTERGENIC	--	--	--

INDEL marker IDs	Chromosomes/ unanchored scaffolds	Physical positions (bp)	InDels (*Kabuli* reference genome-CDC Frontier/PII)	Forward primers (5'-3')	Reverse primers (5'-3')	Anneling temperature (0C)	Expected amplified product size (bp)	Structural annotation		Functional annotation	
								Sequence components of *kabuli* genome	*Kabuli* gene accession IDs	NCBI-KOG	TFs
Ca4_P3_47211846	Ca4	47211846	TG/TGG	TTGAAATGCAACCTTGTCCA	AAGCCCCTGCTTTGATTTCT	60.1	774	DRR	Ca_10861	--	LBD
Ca4_P3_47211909	Ca4	47211909	GCTTGACTCTTGACTCTTGACT/GCTTGACTCTTGACT	CAGCGGGGGAATATTATCAA	AAGCCCCTGCTTTGATTTCT	59.8	542	DRR	Ca_10861	--	LBD
Ca4_P3_47226196	Ca4	47226196	GAAAAAAAAA/GAAAAAAAAAAA	TAGCCTTGACCCAAACCAAC	CCATTCAAATCCTTCATGGC	60.0	684	INTRON	Ca_10859	F	--
Ca4_P3_47228872	Ca4	47228872	T/TA	CCCTCATCCACACCTGTTTT	TGATTTTGATGAAAAGTTTCGC	59.8	489	INTERGENIC	--	--	--
Ca4_P3_47238699	Ca4	47238699	ATTT/ATT	TTTTGATCGAATCTGACCCA	TCGGCCATTTTTCTTCAATC	59.0	659	INTERGENIC	--	--	--
Ca4_P3_47239694	Ca4	47239694	A/AT	TCAATTTGCATTTGGTCGAA	TGAAACGTTTTTAATGATTTGATTG	60.1	491	INTERGENIC	--	--	--
Ca4_P3_47239747	Ca4	47239747	A/AT	TCAATTTGCATTTGGTCGAA	AATGATGCACGACAATTAAGC	60.1	655	INTERGENIC	--	--	--
Ca4_P3_47239785	Ca4	47239785	T/TA	TCAATTTGCATTTGGTCGAA	CGTTGATTAATGATGCACGA	60.1	663	INTERGENIC	--	--	--
Ca4_P3_47242175	Ca4	47242175	AAATAATAATAATAATAATAATAA/AAATAATAATAATAATAA	CGCTCCTAATTCCCACACAT	TTTCTCGCATGCATTCTCAC	60.0	835	INTERGENIC	--	--	--
Ca4_P3_47249263	Ca4	47249263	GAAA/GAA	TTCTCTTCCTTCTACGGCCA	TCAAAATTGATTTCCAACCTCA	59.9	663	DRR	Ca_10856	--	B3

INDEL marker IDs	Chromosomes/ unanchored scaffolds	Physical positions (bp)	InDels (*Kabuli* reference genome-CDC Frontier/PII)	Forward primers (5'-3')	Reverse primers (5'-3')	Anneling temperature (0C)	Expected amplified product size (bp)	Structural annotation		Functional annotation	
								Sequence components of *kabuli* genome	*Kabuli* gene accession IDs	NCBI-KOG	TFs
Ca4_P3_47249646	Ca4	47249646	TTAATTCTATAATTAAAACTAATTCTAT/ TTAATTCTATAATTAAAACTAATTCTAT AATTAAAACTAATTCTAT	TTGAACAATTTGAGGTT GGAAA	GATTTGATTTGATGGG ACCG	59.5	258	DRR	Ca_10856	--	B3
Ca4_P3_47306163	Ca4	47306163	AATATATATATATATATATATAT/AATAT ATATATATATATATATATATATAT	GCAAATATCGGTCAATC ATCA	GCATGGAGAAATTGGA AGGA	58.4	545	INTERGENIC	--	--	--
Ca4_P3_47410517	Ca4	47410517	CAAAA/CAAA	TTCTAACGCAAAGGCA GGTT	GAAGCTATGGTGGAGA CCCA	59.9	304	INTERGENIC	--	--	--
Ca4_P3_47457546	Ca4	47457546	CATTATTATTATTATTATTA/CATTATTA TTATTATTATTATTATTA	GGTTGGGATGAAAACT GGAA	CCTGGAAAACATTAATT CCCC	59.8	543	INTERGENIC	--	--	--
Ca4_P3_4763939	Ca4	4763939	TATAATAATAATAATAATAATAATAATA ATAATAATAATAATAATAATAATAA/TAT AATAATAATAATAATAATAATAATAATA ATAATAATAATAATAATAATAATAATAA TAA	ATGGTAATCACGTTGG GAGG	AGAATGTTGCACAATC AAGGAA	59.7	809	INTERGENIC	--	--	--
Ca4_P3_47669226	Ca4	47669226	AG/A	GATTCACCCAAAGTGG GCTA	AGCTGCTAAATCCTCC GTCA	59.9	611	INTERGENIC	--	--	--
Ca4_P3_47685878	Ca4	47685878	AATATATATATATATA/AATATATATATA TATATATATATATA	AAATTAAAGTTTCAAAC CACGG	CCGACGAAATAAGTCC CTCA	57.3	667	INTERGENIC	--	--	--
Ca4_P3_4769266	Ca4	4769266	TTATATATATATATATATAT/TTATATAT ATATATATATATAT	ATACCGATGCAGTACC CGAG	TGTTTGCCAACCATAT GTGAA	60.0	375	INTERGENIC	--	--	--
Ca4_P3_47757234	Ca4	47757234	CTATATATATATATATATATATATATAT/ CTATATATATATATATATATATATATATA TATATAT	TCTCTGTCCGTTTCCTT GCT	CCTTGGGTTGCTGAAA ATTG	60.0	203	INTERGENIC	--	--	--
Ca4_P3_47762380	Ca4	47762380	CT/CTTT	TGCTGGTTTTAATTTTG GGG	ATTGTGGGGTCTGAAA GTGC	59.8	727	INTERGENIC	--	--	--

INDEL marker IDs	Chromosomes/ unanchored scaffolds	Physical positions (bp)	InDels (*Kabuli* reference genome-CDC Frontier/PII)	Forward primers (5'-3')	Reverse primers (5'-3')	Anneling temperature (0C)	Expected amplified product size (bp)	Structural annotation		Functional annotation	
								Sequence components of *kabuli* genome	*Kabuli* gene accession IDs	NCBI-KOG	TFs
Ca4_P3_47961387	Ca4	47961387	TG/T	GCTGCTCTATGCATTTGCTG	GAGCATGGACGTTTGGATTT	59.7	527	DRR	Ca_10794	--	ARF
Ca4_P3_47982672	Ca4	47982672	TC/TCC	CCAAACTCCAATCAAATGGG	CAAATGAGAATTTTCTGGGCA	60.2	483	INTRON	Ca_10790	U	ARF
Ca4_P3_47991117	Ca4	47991117	GTTTTTT/GTTTTTTTT	ATTAGCCACGACGATGGTTC	TGGTGTACATGTAGGCGTGC	60.0	564	DRR	Ca_10789	--	B3
Ca4_P3_48074355	Ca4	48074355	TG/T	GGTCGACATGACATCACTAAATG	AGTGTCAAAGGGGGAGGAAG	59.4	601	INTERGENIC	--	--	--
Ca4_P3_48202910	Ca4	48202910	AAATAA/AAA	TCGTCCTTACACCGGAAAAC	CAAGTTTCGGTTGACTATACACG	60.0	638	INTERGENIC	--	--	--
Ca4_P3_48206793	Ca4	48206793	CAT/CATAT	TATTTGGCCAACAGGAAAGG	AGAGCTGATTTGGTTCCTCG	59.9	893	INTERGENIC	--	--	--
Ca4_P3_48208929	Ca4	48208929	TC/T	AAGTTTTTGAATACCGCAAAAT	AAGCTGCGATGGGACAATAC	57.0	490	INTERGENIC	--	--	--
Ca4_P3_48218216	Ca4	48218216	TATTA/TATTAATTA	CAACTTGAATTTGGTTTGGCT	TCTGTCGGCAGATAACCCTT	59.1	885	INTERGENIC	--	--	--
Ca4_P3_48218409	Ca4	48218409	CATAT/CAT	AAATTTGAAGTCCGGCATGA	CGAGTGAGTGCAGCTGGTTA	60.4	359	INTERGENIC	--	--	--
Ca4_P3_48243356	Ca4	48243356	CCTTCT/CCT	TGCATTTGTTGGACGCTTAG	AAATCGATAGTTTTGGCCCC	59.9	601	INTERGENIC	--	--	--

INDEL marker IDs	Chromosomes/ unanchored scaffolds	Physical positions (bp)	InDels (*Kabuli* reference genome-CDC Frontier/PII)	Forward primers (5'-3')	Reverse primers (5'-3')	Anneling temperature (0C)	Expected amplified product size (bp)	Structural annotation		Functional annotation	
								Sequence components of *kabuli* genome	*Kabuli* gene accession IDs	NCBI-KOG	TFs
Ca4_P3_48247972	Ca4	48247972	TA/TACAA	AAAATTGCCCTGTTTCCTTG	TCCAGTAGCAAAATGGCACA	59.1	195	INTRON	Ca_10763	--	--
Ca4_P3_48254564	Ca4	48254564	ATAAAA/ATAAAAAGTAAAA	GGAGTCGACGAAACCAACTC	TTTTCTGTCCGCAAATCTGA	59.7	730	INTERGENIC	--	--	--
Ca4_P3_48254866	Ca4	48254866	AT/A	AATCAGATTTGCGGACAGAAA	CGTATTTGCAAAAATTCATTCG	59.7	269	INTERGENIC	--	--	--
Ca4_P3_48268958	Ca4	48268958	AA/AAGA	CAATCTTGCCTTCCCAAAAA	AATTGCTGCTGCTGAGGTTT	60.0	914	INTRON	Ca_10760	Q	B3
Ca4_P3_48279856	Ca4	48279856	A/AC	TATCCTCCAACACCCGAGAC	CGATTTTAACTTTACCGTGACCA	59.9	673	INTERGENIC	--	--	--
Ca4_P3_48301621	Ca4	48301621	CCT/C	GGCAAAGGCAGAGAAGATTG	GGGAACAAAACAGCTTCCAA	60.0	187	CDS (FRAME SHIFT)	Ca_10758	J	--
Ca4_P3_4830250	Ca4	4830250	A/ATTATTATAGTTAT	CAACATGGGCAACAAGACAC	CCCTTTCCCCTTCATCATTT	60.0	273	INTERGENIC	--	--	--
Ca4_P3_48309823	Ca4	48309823	CAAATTTAAATTT/CAAATTT	TGCCTCCACAAACGTCATTA	TGAAAACCCTAATCTCCGCA	60.1	832	INTERGENIC	--	--	--
Ca4_P3_48310392	Ca4	48310392	CACCGTTAGTTTT/C	TTTCTGCTGCGGAGATTAGG	TCGAAGCAAAAACACATTGC	60.5	419	INTERGENIC	--	--	--
Ca4_P3_48314680	Ca4	48314680	ATTT/ATTTTT	GTAATGCGTTTGACCTGCAA	ATAGGCGCATCTCAATCACC	59.7	649	DRR	Ca_10756	--	BBR-BPC

**INDEL marker IDs**	**Chromosomes/ unanchored scaffolds**	**Physical positions (bp)**	**InDels (*Kabuli* reference genome-CDC Frontier/PII)**	**Forward primers (5'-3')**	**Reverse primers (5'-3')**	**Anneling temperature (0C)**	**Expected amplified product size (bp)**	**Structural annotation**		**Functional annotation**	
								**Sequence components of *kabuli* genome**	***Kabuli* gene accession IDs**	**NCBI-KOG**	**TFs**
Ca4_P3_48314994	Ca4	48314994	CGAGTAGAGT/CGAGT	TGCGCCTATCTCTCTGTGTG	TTCGATCCATTTTACCACCC	60.2	365	DRR	Ca_10756	--	BBR-BPC
Ca4_P3_48317503	Ca4	48317503	CATATATATATATATATATATATAT/CATATATATATATATATATATAT	CAAATCGGAAAATCATGGGA	TGGACTTCTTCACCTTTCCA	60.6	731	INTERGENIC	--	--	--
Ca4_P3_48318546	Ca4	48318546	ATTTTTATTTTTT/ATTTTT	TGTCTTTTGTCGATATACTTATTTGC	CTCACCGTTTCACGTGATTG	58.0	867	INTERGENIC	--	--	--
Ca4_P3_48323352	Ca4	48323352	CCAAACACAAACACAAACAC/CCAAACACAAACAC	TTTTCTGCAGCCGAATTTTT	CATTTGAAATTGCTTTCCCC	59.8	625	DRR	Ca_10755	--	bZIP
Ca4_P3_48332119	Ca4	48332119	ATTTTT/ATT	AAAGTGCATTAAACGGCCAC	TGTTCCTCGAAGGATTGGTC	60.0	455	DRR	Ca_10753	J	--
Ca4_P3_48339026	Ca4	48339026	ATT/ATTT	TGCCAACATGCATCTATCGT	TGCATGACACATCTATCAAATACG	60.1	419	INTERGENIC	--	--	--
Ca4_P3_48340135	Ca4	48340135	TAAA/T	TCCAAACAAAGTGGAGATGC	TTGGTCCCTGGTAGCTTACG	58.7	369	INTERGENIC	--	--	--
Ca4_P3_48351278	Ca4	48351278	TAATTCCAATTCCAATT/TAATTCCAATTCCAATTCCAATT	GACACGTGATGTCTCTGTAATGG	AATGTATGTATCGAGAGCGCC	59.5	651	INTERGENIC	--	--	--
Ca4_P3_48391760	Ca4	48391760	TCC/TCCC	TTCCATTGGGAGAGTCACCT	ACCGGTTTATGGATCCCAAT	59.5	615	INTERGENIC	--	--	--
Ca4_P3_4839788	Ca4	4839788	A/ATTTTT	ACGTAATACTTTGTGGCGGC	TCTCTCCTTTGTGACGGCTT	60.0	138	DRR	Ca_03775	--	--

INDEL marker IDs	Chromosomes/ unanchored scaffolds	Physical positions (bp)	InDels (*Kabuli* reference genome-CDC Frontier/PII)	Forward primers (5'-3')	Reverse primers (5'-3')	Anneling temperature (0C)	Expected amplified product size (bp)	Structural annotation		Functional annotation	
								Sequence components of *kabuli* genome	*Kabuli* gene accession IDs	NCBI-KOG	TFs
Ca4_P3_48404970	Ca4	48404970	GTTTAATTT/GTTTAATTTTAATTT	TTGCATGACTGCTTTTG GAC	TTGTCATTGTTGCGGT TTGT	59.8	514	INTERGENIC	--	--	--
Ca4_P3_4840884	Ca4	4840884	AC/A	TTGGGCCTTTGTGGAG TAAG	TACCAAAGTGGTGATG GTGG	60.1	496	DRR	Ca_03775	--	--
Ca4_P3_48411520	Ca4	48411520	ATTTTTTT/ATTTTTTTTT	CTACAGGTCGCACGTT CTCA	TCGAAACAGACTTTTT GGGC	60.0	795	INTERGENIC	--	--	--
Ca4_P3_48414498	Ca4	48414498	ATCAATTTCA/ATCA	ATGAGCATAGCATGTG CCAG	ATGCACCCATTCACAT TCAA	59.9	664	INTERGENIC	--	--	--
Ca4_P3_48441010	Ca4	48441010	TAA/TAAA	TCCGAGTCACAGATCC ATCA	TCATGCATTAATGTCAA CCTCA	60.2	338	INTERGENIC	--	--	--
Ca4_P3_48443075	Ca4	48443075	ATTT/ATTTT	CCGGTTGTTAGCTTGT TTGC	CAATAGGATGGATGGT TGGG	60.7	609	INTERGENIC	--	--	--
Ca4_P3_48453417	Ca4	48453417	ATATTTATTTATTTATTTATT/ATATTTAT TTATTTATTTATTTATT	ACCACCACCACCACTA CCAT	CACCTCCCCCTGTTTA TTCA	60.0	561	INTERGENIC	--	--	--
Ca4_P3_48453751	Ca4	48453751	A/AT	GTTCCACCACCGCAAA TATC	GGTGGTGGTAGGAAAA GCAA	60.2	742	INTERGENIC	--	--	--
Ca4_P3_48454259	Ca4	48454259	CTTTT/CTTT	TTGCTTTTCCTACCACC ACC	CAGCAGAGTCCAATTC AGCA	60.0	376	INTERGENIC	--	--	--
Ca4_P3_48454771	Ca4	48454771	GTGCTGTTGCTGTTGCTGTTGCT/GTG CTGTTGCTGTTGCT	TGCTGAATTGGACTCT GCTG	TCACTTGATCCTCCAA ACCC	60.1	548	INTRON	Ca_10747	--	--

INDEL marker IDs	Chromosomes/ unanchored scaffolds	Physical positions (bp)	InDels (*Kabuli* reference genome-CDC Frontier/PII)	Forward primers (5'-3')	Reverse primers (5'-3')	Anneling temperature (0C)	Expected amplified product size (bp)	Structural annotation		Functional annotation	
								Sequence components of *kabuli* genome	*Kabuli* gene accession IDs	NCBI-KOG	TFs
Ca4_P3_48461232	Ca4	48461232	TAGAG/TAG	TGCAAAGTTCGATCGG AATAA	AATTTCTCACCACCAC CCAC	60.6	483	INTERGENIC	--	--	--
Ca4_P3_48465591	Ca4	48465591	GAAAA/GAAA	ATAATGATCCCGGTTG GACA	GCCTTTCCTTGACAGT CTGC	60.0	699	INTRON	Ca_10746	LA	--
Ca4_P3_48465859	Ca4	48465859	GTTTTTTTTTTT/GTTTTTTTTTT	TGTGTGTCTCAGTGGT GGGT	GCCTTTCCTTGACAGT CTGC	60.0	619	INTRON	Ca_10746	LA	--
Ca4_P3_48473482	Ca4	48473482	TG/TGG	CTCCACGGGGTAACCC TAAT	TAATGGAACCATGACC ACCC	60.1	652	DRR	Ca_10745	L	bHLH
Ca4_P3_48493001	Ca4	48493001	CTTTTTT/CTTTTTTT	TGGATTCTTTTTGGCTA GAACC	TTGTTAGCCTGTCGCT CTCA	59.6	456	INTERGENIC	--	--	--
Ca4_P3_48495755	Ca4	48495755	T/TTTTG	CGATCTTCCACCGTCA GATT	CAATCCAAGCAACCGA AACT	60.1	684	INTERGENIC	--	--	--
Ca4_P3_48497163	Ca4	48497163	AAATAAT/AAAT	ATGTTGCAAAACATGAG CCA	ACAAAATCGCCGATGG TAAC	60.1	768	INTRON	Ca_10742	V	GRAS
Ca4_P3_48507473	Ca4	48507473	AT/ATATGTT	GGAATTCTGCACCCAA CAAT	GCCCACTCCTATCTGA CTCG	59.8	428	INTERGENIC	--	--	--
Ca4_P3_4851248	Ca4	4851248	TAA/TA	GGAATGCATGGTATCC AACC	CAGCACTTCTCCCTTT TCCTT	60.0	678	INTERGENIC	--	--	--
Ca4_P3_4851664	Ca4	4851664	CAGGAAAAGGAAAAGGAAAAGGAAAA GG/CAGGAAAAGGAAAAGGAAAAGG	TGGCATAGCAAACAGA GAAGG	GGCCAAATAAATGATG GCAC	60.4	311	INTERGENIC	--	--	--

INDEL marker IDs	Chromosomes/ unanchored scaffolds	Physical positions (bp)	InDels (*Kabuli* reference genome-CDC Frontier/PII)	Forward primers (5'-3')	Reverse primers (5'-3')	Anneling temperature (0C)	Expected amplified product size (bp)	Structural annotation		Functional annotation	
								Sequence components of *kabuli* genome	*Kabuli* gene accession IDs	NCBI-KOG	TFs
Ca4_P3_48518535	Ca4	48518535	AATAT/AATATAT	TGGTGGTTGACCACAAAAGA	ATCATCTTTGGCAGTAGCGG	60.0	927	INTRON	Ca_10740	--	C2H2
Ca4_P3_48518860	Ca4	48518860	TATGTAATAAATTGAT/TAT	CATCAAGCTGTTCGTCATGC	ATCATCTTTGGCAGTAGCGG	60.4	287	INTRON	Ca_10740	--	C2H2
Ca4_P3_4852153	Ca4	4852153	TATTTAATTT/TATTT	TGGCTCCAACAACATCTTCA	ACCCCTTGGTCCAAACAAAT	60.2	549	INTERGENIC	--	--	--
Ca4_P3_48574983	Ca4	48574983	AAAATTAAATTAAATTAAA/AAAATTAAATTAAA	GCAACAATTGAATCAACCCC	CTTCATGAATTGCACCATGC	60.2	705	INTRON	Ca_23007	P	--
Ca4_P3_48576925	Ca4	48576925	AT/ATT	ATCTCACCCCAAGACAACCA	TCATTCCAGAAGGAGATGGG	60.4	211	INTRON	Ca_23007	P	--
Ca4_P3_48577228	Ca4	48577228	A/AC	AACACATCCGGGATCCATTA	TGATGACGAAGACGAAGACG	60.0	303	INTERGENIC	--	--	--
Ca4_P3_4858835	Ca4	4858835	G/GGT	TTCAGGCCAATTTTACGAGG	ATGTTCATTTTCTTCGCGCT	60.1	753	INTRON	Ca_03773	--	--
Ca4_P3_4859197	Ca4	4859197	GTCT/GT	TCCAGTTTTGGTTGCTGAGA	TAGGCATCACCGTTGTGTGT	59.4	737	INTRON	Ca_03773	--	--
Ca4_P3_4859261	Ca4	4859261	TATTAATTAATTAATTAAT/TATTAATTAATTAAT	TCCAGTTTTGGTTGCTGAGA	TAGGCATCACCGTTGTGTGT	59.4	737	INTRON	Ca_03773	--	--
Ca4_P3_48596162	Ca4	48596162	CATATATATATATATATATATATATATATATA/CATATATATATATATATATATATATATATA	TGAGATTAATCGGTGGGAGC	CACTGCAAAATGTTGAAGCAA	60.0	425	INTERGENIC	--	--	--

INDEL marker IDs	Chromosomes/ unanchored scaffolds	Physical positions (bp)	InDels (*Kabuli* reference genome-CDC Frontier/PII)	Forward primers (5'-3')	Reverse primers (5'-3')	Anneling temperature (0C)	Expected amplified product size (bp)	Structural annotation		Functional annotation	
								Sequence components of *kabuli* genome	*Kabuli* gene accession IDs	NCBI-KOG	TFs
Ca4_P3_48632628	Ca4	48632628	CTATATATATATATATATA/CTATATATATATATATATATA	CCATGCGAGAAATTGAGGAT	TTTTCCCCTTTTTCACTCCA	60.0	438	INTERGENIC	--	--	--
Ca4_P3_48662498	Ca4	48662498	A/ATATATCAAACT	CGTTATAATATCGCGTTTTGATTAGA	AAATAAATAGCGCTGGCACC	59.8	609	INTERGENIC	--	--	--
Ca4_P3_48664333	Ca4	48664333	G/GGGTTGGAGGTAT	GTGCCGCATTCATCCTTAGT	AAACTGGTTGACGGGATCTTT	60.1	504	INTERGENIC	--	--	--
Ca4_P3_48670171	Ca4	48670171	AATTTA/AATTTATTTA	TATCTCCATGGCGTCACAAA	CTGCGCCGACTAACAATACA	60.1	589	INTERGENIC	--	--	--
Ca4_P3_48670468	Ca4	48670468	GATA/GATAAAATCTTATA	CTGTATTGTTAGTCGGCGCA	CCGACCCAACACGTTATGA	59.9	620	INTERGENIC	--	--	--
Ca4_P3_48683487	Ca4	48683487	TAAA/TAAAA	GTGTTTGTGCGATGATTTGG	TGGCATCATAAATTTGTGTGTGTA	60.0	200	INTERGENIC	--	--	--
Ca4_P3_48684109	Ca4	48684109	TAAA/TAAAA	CGACAAAATTTAGTTGAATCTCTATGA	GTGCAAATATCAACGCCCTT	59.2	649	INTERGENIC	--	--	--
Ca4_P3_48684917	Ca4	48684917	ATTTTTTTT/ATTTTTT	ATCGCAACAACGCATTAAAA	ATCCAACACCCAAACCCATA	59.2	838	DRR	Ca_23019	R	--
Ca4_P3_48685664	Ca4	48685664	AAAGTTAAG/AAAGTTAAGTTAAG	ATGGGGTGGAGGAAAATAGG	GGGTGATTGATCGTTCAAAAG	60.0	757	DRR	Ca_23019	R	--
Ca4_P3_48685955	Ca4	48685955	TGAA/T	GGCAAAGCCTATCACTGCTC	CTCCAAAAATGGACGGATTC	60.0	810	DRR	Ca_23019	R	--

INDEL marker IDs	Chromosomes/ unanchored scaffolds	Physical positions (bp)	InDels (*Kabuli* reference genome-CDC Frontier/PII)	Forward primers (5'-3')	Reverse primers (5'-3')	Anneling temperature (0C)	Expected amplified product size (bp)	Structural annotation		Functional annotation	
								Sequence components of *kabuli* genome	*Kabuli* gene accession IDs	NCBI-KOG	TFs
Ca4_P3_48692254	Ca4	48692254	GTGATAT/GTGATATGATAT	TCCCGACATTGTGGGTTAAT	TTGTGAAAATGTTGATGGTCTTG	60.1	749	INTERGENIC	--	--	--
Ca4_P3_48694696	Ca4	48694696	ACG/A	AAATGTGGGCCAATGGATTA	GGGCTGTTGTTCGTCAAAAT	60.0	620	INTERGENIC	--	--	--
Ca4_P3_48708820	Ca4	48708820	AC/ACTTGACCATTTC	CGGCGACATTGAGTTTGTAA	GCACGTGTTGAAATGTCTGC	59.7	630	DRR	Ca_23020	Q	Nin-like
Ca4_P3_48708876	Ca4	48708876	TTTTATTT/TTTT	CGCCACTTGTATTTCAAGCA	GCATCAGCTCTTCCAGCTTC	59.9	517	DRR	Ca_23020	Q	Nin-like
Ca4_P3_48716729	Ca4	48716729	AATA/AATACATGGTTACATA	GACCAGAAAGCCCAATTGAA	CCGATTCGTGGTCTGTTTTT	60.1	576	INTRON	Ca_23021	U	--
Ca4_P3_48724001	Ca4	48724001	GATATATATATATATA/GATATATATATATATATATATA	TTCTTCAGGTGGTTGCTTCC	TTTTGGTTCGTGGCTTTTTC	60.2	154	INTERGENIC	--	--	--
Ca4_P3_4873540	Ca4	4873540	TACACACACACACACACAC/TACACACACACACACAC	CACCACCCAAAGAAAGGAAA	ATGATTGCTCCCTAGCAGGA	59.9	466	DRR	Ca_03771	Z	NAC
Ca4_P3_48758329	Ca4	48758329	C/CAAGT	TGCTCAATTCGAACACAATCA	AAGCGACTAACATGATTTTCCA	60.3	904	INTRON	Ca_23023	--	FAR1
Ca4_P3_48769759	Ca4	48769759	A/AATC	TTTCAATGAAGCCTAGTCCG	TTAAGGGTCCGCATGATTGT	58.0	605	INTRON	Ca_23023	--	FAR1
Ca4_P3_48772855	Ca4	48772855	T/TA	GGTTGGAAAACGGAAAAACA	CATTGGTCGGACTAACAACTCA	59.8	696	DRR	Ca_23023	--	FAR1

INDEL marker IDs	Chromosomes/ unanchored scaffolds	Physical positions (bp)	InDels (*Kabuli* reference genome-CDC Frontier/PII)	Forward primers (5'-3')	Reverse primers (5'-3')	Anneling temperature (0C)	Expected amplified product size (bp)	Structural annotation		Functional annotation	
								Sequence components of *kabuli* genome	*Kabuli* gene accession IDs	NCBI-KOG	TFs
Ca4_P3_48789337	Ca4	48789337	GTATA/GTATATA	TTTCCTTGTTGTGGAATTTGC	ACCGTCAAATTTTTCGATGC	60.0	555	DRR	Ca_23024	--	FAR1
Ca4_P3_48793977	Ca4	48793977	TAAAAA/TAAAAAAA	TTACGGTCGAAAATGAAGGG	TCGGGTAGCACAATCTTCAA	59.9	640	INTERGENIC	--	--	--
Ca4_P3_48794099	Ca4	48794099	TAA/TA	TTACGGTCGAAAATGAAGGG	TCGGGTAGCACAATCTTCAA	59.9	640	INTERGENIC	--	--	--
Ca4_P3_48798808	Ca4	48798808	TTATAT/TTATATAT	AACGAAAGAGAAATGGAATATGAA	ATGCTGCAAAGAACAACGTG	58.3	534	INTERGENIC	--	--	--
Ca4_P3_48813159	Ca4	48813159	TAAAA/TAAAAA	TGGCTTCACCAATGATTTGA	AATGGGATCAACAAAGGCAA	60.0	322	INTERGENIC	--	--	--
Ca4_P3_48837715	Ca4	48837715	ATT/ATTT	TGTGTAACGGGTAAGAAATACGG	CAAAATACATCATCACGCGG	60.2	630	INTERGENIC	--	--	--
Ca4_P3_48849335	Ca4	48849335	TATAACAAT/TATAACAATAATAACAAT	TTGGGTGTATAATGATAGCGAAA	GAGGGACTAGCTACCCCGAT	58.6	441	INTERGENIC	--	--	--
Ca4_P3_48850057	Ca4	48850057	GA/GATCA	GCATCAATATGAATCACCAATCA	TGACGATTTTATTTACGCCAA	59.7	857	INTERGENIC	--	--	--
Ca4_P3_4888138	Ca4	4888138	CTT/CT	AAAAGCCTCACCAGGGATCT	TGCCATCTTTCTTGCTGTTG	60.1	698	INTRON	Ca_03769	Z	NAC
Ca4_P3_4891737	Ca4	4891737	AATTATTATTATTATTATT/AATTATTATTATTATTATTATT	TGCCTTGTATCGTTCCATCA	TTCCTGCTTTTTCTTTCCGA	60.1	540	INTRON	Ca_03769	Z	NAC

INDEL marker IDs	Chromosomes/ unanchored scaffolds	Physical positions (bp)	InDels (*Kabuli* reference genome-CDC Frontier/PII)	Forward primers (5'-3')	Reverse primers (5'-3')	Anneling temperature (0C)	Expected amplified product size (bp)	Structural annotation		Functional annotation	
								Sequence components of *kabuli* genome	*Kabuli* gene accession IDs	NCBI-KOG	TFs
Ca4_P3_4891996	Ca4	4891996	GAAAAAAAA/GAAAAAAAAA	TCGGAAAGAAAAAGCAGGAA	GGCAAATTGCTACTGAAGGG	59.9	265	INTRON	Ca_03769	Z	NAC
Ca4_P3_4892454	Ca4	4892454	C/CT	CAGTAGCAATTTGCCCTTCC	GCTTGGATTGATGGACAGGT	59.7	552	INTRON	Ca_03769	Z	NAC
Ca4_P3_4893509	Ca4	4893509	CTTT/CTTTT	CAGCGTTTTGTCGCAAGTAA	GCCGAAGTTGAAGGACAGAG	60.1	480	INTERGENIC	--	--	--
Ca4_P3_48939492	Ca4	48939492	ATCT/ATCTCT	TGTCTCATGATTGTCCAGGG	CCCATTGTGCAGGAGAATTT	59.5	249	DRR	Ca_20762	R	NAC
Ca4_P3_4897344	Ca4	4897344	ATTT/ATTTT	CTCTGCAGCCTTGATTGGTT	ATGGAGTATGGGGCTGTGAC	60.4	278	INTRON	Ca_03768	K	--
Ca4_P3_48975362	Ca4	48975362	GAAAA/GAAAAA	TGGTTGGGATTTTTGGGTTA	GACCCTCTCCTCTCAATCCC	60.0	672	INTERGENIC	--	--	--
Ca4_P3_48975458	Ca4	48975458	AC/A	TGGTTGGGATTTTTGGGTTA	GACCCTCTCCTCTCAATCCC	60.0	672	INTERGENIC	--	--	--
Ca4_P3_4900827	Ca4	4900827	CT/CTT	TCCAAATTTTGACCATTGTCTG	TGAACCACCAATCATATGCC	59.8	536	INTERGENIC	--	--	--
Ca4_P3_4902894	Ca4	4902894	TAATAAAATAAAATAAAA/TAATAAAATAAAA	CCCCGTGAGTTGAATTTTGT	AAATCAGGGCAAAATGGTGA	59.8	748	INTERGENIC	--	--	--
Ca4_P3_49073579	Ca4	49073579	GTT/GT	GTGACTTGGAGGGAGTGGAA	ATCCGCTAACCAATCACAGC	60.1	453	INTERGENIC	--	--	--

INDEL marker IDs	Chromosomes/ unanchored scaffolds	Physical positions (bp)	InDels (*Kabuli* reference genome-CDC Frontier/PII)	Forward primers (5'-3')	Reverse primers (5'-3')	Anneling temperature (0C)	Expected amplified product size (bp)	Structural annotation		Functional annotation	
								Sequence components of *kabuli* genome	*Kabuli* gene accession IDs	NCBI-KOG	TFs
Ca4_P3_4908883	Ca4	4908883	T/TA	CAAACTTTTTCCCACGCAAT	TCATCATTGTGCCGTCATTT	60.0	318	DRR	Ca_03767	F	--
Ca4_P3_49160495	Ca4	49160495	TGGGTTTAGG/TGGGTTTAGGGTTTAGG	CCGGGGAGAATAGGAAGAAC	CCCCAAACCCTAAACCCTAA	59.9	459	INTERGENIC	--	--	--
Ca4_P3_4917872	Ca4	4917872	ATTTTTT/ATTTTTT	TCAATGTCATCACAACATGTCC	AAGTTGATGAAAATTGGCGG	59.3	522	INTERGENIC	--	--	--
Ca4_P3_4923832	Ca4	4923832	CAAAA/CAAA	AGGTGAGGCAGGCAGTAAAA	TTTTTCGGACTTGGTCCTTT	59.9	783	INTERGENIC	--	--	--
Ca4_P3_4925562	Ca4	4925562	TT/TTCT	GAAAGATGCCCTAAGGAGCC	ATGCCAATTGCTTTATTGGG	60.2	893	INTERGENIC	--	--	--
Ca4_P3_4934099	Ca4	4934099	ATTTTTTTTT/ATTTTTTTT	TGTGCAACTTTTTAATTCAATTCG	CAACAAACGAGGGACCAGTT	60.4	657	INTERGENIC	--	--	--
Ca4_P3_5028595	Ca4	5028595	GTTTTTTTTTT/GTTTTTTTTTTT	TGATAAAAGGGGGAAAATCC	CGCATCCCCAGATCTAACAT	57.8	163	INTERGENIC	--	--	--
Ca4_P3_5190499	Ca4	5190499	GTTTTTTT/GTTTTTTTT	CGCGTGCAAAAAGTTGTTTA	GATCCTTGAGCAGTTGAGCC	59.9	674	DRR	Ca_03734	E	--
Ca4_P3_526483	Ca4	526483	TGAGAGAGAGAGAGAGAGAGAGAGAGAGAG/TGAGAGAGAGAGAGAGAGAGAGAGAGAG	GGGATTTTGAAAAACGCTCA	AAACGGTGTGGTCCAAAAAG	60.1	524	INTERGENIC	--	--	--
Ca4_P3_5306497	Ca4	5306497	CGGGG/CGGG	ATGGGAGAACGCTGCATTAC	TAGATGATCGCACGAGATGC	60.1	257	DRR	Ca_03727	QR	B3

INDEL marker IDs	Chromosomes/ unanchored scaffolds	Physical positions (bp)	InDels (*Kabuli* reference genome-CDC Frontier/PII)	Forward primers (5'-3')	Reverse primers (5'-3')	Anneling temperature (0C)	Expected amplified product size (bp)	Structural annotation		Functional annotation	
								Sequence components of *kabuli* genome	*Kabuli* gene accession IDs	NCBI-KOG	TFs
Ca4_P3_5472447	Ca4	5472447	TGAGATAGAGATAGAGATAGAGATAGAGA/TGAGATAGAGATAGAGATAGAGATAGAGATAGAGA	GATACGAATTCGGTGCTTCC	TTGAAATTTTTGTCATTCTCCAAA	59.5	677	INTERGENIC	--	--	--
Ca4_P3_5618841	Ca4	5618841	TTTATTATTATTATTATTATTATTATTA/TTTATTATTATTATTATTATTATTATTATTA	GTGTTCCTGATGTTTTGCCA	GCAACTTCGAGATGCGACTT	59.5	752	INTERGENIC	--	--	--
Ca4_P3_5629484	Ca4	5629484	CATATATATATATATATATATATATATATAT/CATATATATATATATATATATATATATATATAT	TATCGCAAACCGGTCAATTT	GGGTCACGTGGATTTTGTCT	60.3	906	INTERGENIC	--	--	--
Ca4_P3_5642060	Ca4	5642060	TTATATATATATATATATATATATATATATAT/TTATATATATATATATATATATATATATATATATAT	TGGTGGTTGTTGTCTCCTCA	CACCATGATTGGGGGATAAG	60.1	463	INTERGENIC	--	--	--
Ca4_P3_5670743	Ca4	5670743	CGGGG/CGGG	CACCCAAACCACCTAACTCG	GTGCATGATCAGATCGCTGT	60.4	647	INTRON	Ca_03681	BDT	MYB_related
Ca4_P3_5712745	Ca4	5712745	CTTTTTTTT/CTTTTTTT	TGTTCCCTCAAAAGGAATGC	AACAATGTGAGAGCTTCCCC	60.1	714	INTERGENIC	--	--	--
Ca4_P3_571676	Ca4	571676	CTATATATATATATATATATATATAT/CTATATATATATATATATATATATAT	GTGGTGGCCATAATGCTTCT	TGCGCTTAGCTTTGATTCCT	60.0	731	INTERGENIC	--	--	--
Ca4_P3_5793201	Ca4	5793201	TAAAAA/TAAA	GACTGCCACCGACCACTTAT	GGTCACACCGTTCAGCTTTT	60.0	469	INTERGENIC	--	--	--
Ca4_P3_5931137	Ca4	5931137	ATATTATTATTATTATTATTATTATTATTATTATTATTATTATTATTATTATTATTATTATTATTATT/ATATTATTATTATTATTATTATTATTATTATTATTATTATTATTATTATTATTATTATTATTATTATTATTATTATTATTATT	CCAGCCCAATTTACATGCTT	CCGCCCATTTCAACTTTTTA	60.0	527	INTERGENIC	--	--	--
Ca4_P3_6117419	Ca4	6117419	AG/A	TCGGACAATTTTATGCCTCC	ATCAAAGGACGACCCAGTTG	59.9	743	DRR	Ca_03633	--	--

INDEL marker IDs	Chromosomes/ unanchored scaffolds	Physical positions (bp)	InDels (*Kabuli* reference genome-CDC Frontier/PII)	Forward primers (5'-3')	Reverse primers (5'-3')	Anneling temperature (0C)	Expected amplified product size (bp)	Structural annotation		Functional annotation	
								Sequence components of *kabuli* genome	*Kabuli* gene accession IDs	NCBI-KOG	TFs
Ca4_P3_6182357	Ca4	6182357	TTATA/TTATATA	ACAACCAAATGATCGAAGCC	TGGCCCTCTAATTCTGCACT	59.9	743	INTRON	Ca_03625	K	TALE
Ca4_P3_6209314	Ca4	6209314	TTATATATATATATATATATA/TTATATATATATATATATA	AAACTGTTAGACTTTGCAGGATGA	TCGCCCCTTAAATTTGACAC	59.4	205	INTERGENIC	--	--	--
Ca4_P3_6280527	Ca4	6280527	TTTATTATTATTATTATTATTATTATTATTATTATTATTATTATTATTATTATTATTATTAT/TTTATTATTATTATTATTATTATTATTATTATTATTATTATTATTATTAT	ATTACAGGCTGCCAACAACC	TCAACAAACACAGCAGCAGA	60.0	336	INTRON	Ca_03615	E	--
Ca4_P3_6324755	Ca4	6324755	ATTAATAATTTAATAATTTAATAATT/ATTAATAATTTAATAATT	TGGGTGGAACATGACAGAGA	ATTTTGCCATTTGGGATTGA	60.1	588	INTERGENIC	--	--	--
Ca4_P3_6526377	Ca4	6526377	AATATATATATATATATAT/AATATATATATATATAT	AGCACGGCCGACTTTATTTT	ATGCCAAACTTTCAGCCATC	61.0	706	INTERGENIC	--	--	--
Ca4_P3_6530860	Ca4	6530860	CATATATATATATATATAT/CATATATATATATATAT	AATTGTGGGTCTGACGTAAAAA	ATCCACAAACCCAATGAAGC	58.5	728	INTERGENIC	--	--	--
Ca4_P3_6542618	Ca4	6542618	TAAAAAAAAAA/TAAAAAAAAA	CCACTTTGGTCTGCCATTCT	GCTCACGCGGTTAAAGACTC	60.1	706	INTERGENIC	--	--	--
Ca4_P3_6549650	Ca4	6549650	TAAAAAA/TAAAAA	TTTCGAAAATGCCAAACCTC	AGCCCACAGACCTCTCAAAA	60.1	370	INTERGENIC	--	--	--
Ca4_P3_6596665	Ca4	6596665	TAAAAAAAAAAAAA/TAAAAAAAAAAAA	GAAGTTGCAAGTATTGGGGC	TTGGGCAAGATAAAAATCCG	59.6	405	INTERGENIC	--	--	--
Ca4_P3_6603972	Ca4	6603972	TAAAAA/TAAAA	ACATCCCAACCCCTTTCACT	TTGTCGGACCAAGTCAACTG	60.6	140	INTERGENIC	--	--	--

INDEL marker IDs	Chromosomes/ unanchored scaffolds	Physical positions (bp)	InDels (*Kabuli* reference genome-CDC Frontier/PII)	Forward primers (5'-3')	Reverse primers (5'-3')	Anneling temperature (0C)	Expected amplified product size (bp)	Structural annotation		Functional annotation	
								Sequence components of *kabuli* genome	*Kabuli* gene accession IDs	NCBI-KOG	TFs
Ca4_P3_6699109	Ca4	6699109	TT/TTCTTGTCAGT	AAGCGAAAATGGTTGGATCA	TGCGTGCGTATAGGCTATTTT	60.4	172	CDS (large-effect mutations)	Ca_03576	--	WRKY
Ca4_P3_6732926	Ca4	6732926	TTTTTTTT/TTTTTTTTT	AAAAACACTCGCCGTGAGAT	TCCAACCGTGAACTCATCAA	59.7	435	INTERGENIC	--	--	--
Ca4_P3_6907181	Ca4	6907181	TTATATATATATATATATAT/TTATATATATATATAT	TTTGAAATGGCGACTTCCTT	CCAAATGGCAACTTCCAACT	59.7	569	INTERGENIC	--	--	--
Ca4_P3_6923630	Ca4	6923630	AATATATATATATATATATATA/AATATATATATATATATATATATA	CGTTTGGTCTATCGTCGGTT	AGTGGGGGACTGTGTTGAAG	60.0	277	DRR	Ca_03547	Z	--
Ca4_P3_6965934	Ca4	6965934	AATATATATATATATATATATA/AATATATATATATATATA	TGTTTGGACTGCTTTACCCC	CCCTCCAAACTAAAATTTTCTCC	60.0	584	INTERGENIC	--	--	--
Ca4_P3_6966183	Ca4	6966183	TAAAAAAAA/TAAAAAAAAA	TTTTGAGCCAAAAATCACCTG	TAGATGGGAGGAGTTCCGCT	60.1	964	INTERGENIC	--	--	--
Ca4_P3_6998635	Ca4	6998635	CGGGGG/CGGGG	ACTTTGCGTGTCGATAACCC	GGCAGCGAAAAGATCAAATC	60.0	345	INTERGENIC	--	--	--
Ca4_P3_7089091	Ca4	7089091	GA/GAACAAAAGAGATTCACA	CTTTGCATACCCCTCTCGAC	TTGGGAGGATTGGTTTCAAG	59.7	644	INTERGENIC	--	--	--
Ca4_P3_7106451	Ca4	7106451	AATATATATATATATATATATATATATATATATAT/AATATATATATATATATATATATATATATATATATATATATAT	GGAGTCGCCACCAAAGTTTA	ACAACGACTCAAATGCGACA	60.1	512	DRR	Ca_03527	--	--
Ca4_P3_7110091	Ca4	7110091	ACT/ACTCT	GTTGCACTGCAGCATCAAAT	AGAAGACTAGTCGGCGAACG	59.9	513	DRR	Ca_03526	--	--

INDEL marker IDs	Chromosomes/ unanchored scaffolds	Physical positions (bp)	InDels (*Kabuli* reference genome-CDC Frontier/PII)	Forward primers (5'-3')	Reverse primers (5'-3')	Anneling temperature (0C)	Expected amplified product size (bp)	Structural annotation		Functional annotation	
								Sequence components of *kabuli* genome	*Kabuli* gene accession IDs	NCBI-KOG	TFs
Ca4_P3_7132134	Ca4	7132134	TAAAAAAAAAAAAA/TAAAAAAAAAAAA	CACACCAGGGGAGACTCACT	GTCTCCCTACCCCCACATTT	60.2	595	INTERGENIC	--	--	--
Ca4_P3_7180004	Ca4	7180004	ATTTTTTTT/ATTTTTTT	ATGGTTACCAGCTTTGGCCT	TATAGGTGGTGTTTGGCACG	60.9	427	DRR	Ca_03517	--	GRAS
Ca4_P3_719535	Ca4	719535	TTTTTTTAAAATAATTTTTTAAAAT/TTTTTTTAAAAT	GCAACAAGTCAGCAACGGTA	CATGTTTGATTCCATTTTCCA	59.9	675	INTERGENIC	--	--	--
Ca4_P3_7213997	Ca4	7213997	TAAAAAAAAAAA/TAAAAAAAAAA	GCATGCAAAATTTTATATTGTTATTGT	TGCTCATGTTCTTCTGGTCAA	58.9	849	INTERGENIC	--	--	--
Ca4_P3_7221356	Ca4	7221356	ATTTTTTTTT/ATTTTTTTT	ACAAAACTCGTCTCCGCCTA	AAGCCAATTCGATTGTGTCG	59.9	836	INTERGENIC	--	--	--
Ca4_P3_7278135	Ca4	7278135	TTATATATATATATATATATATATATATATATATAT/TTATATATATATATATATATATATAT	GGGACAATCCGCTTTGATTA	TGGATTTTGCCTCCTTCATC	59.9	468	INTERGENIC	--	--	--
Ca4_P3_730137	Ca4	730137	TTATATATATATATATATATAT/TTATATATATATATATATAT	GTCAACCTTGGTGCTTGGTT	AAGTATTTCCGCCTGTCACG	60.0	775	DRR	Ca_07702	G	--
Ca4_P3_7359661	Ca4	7359661	ATTTT/ATTT	ATGTGGGAACAATTGAAGGC	CGCAGCAACATTCAAGAAAA	59.8	578	INTERGENIC	--	--	--
Ca4_P3_7360660	Ca4	7360660	TTTTATTTATTTATTTATTTATTTATTTATTTA/TTTTATTTATTTATTTATTTATTTATTTA	ACTACGAAAACGGTCATCCG	CAAGCATGCTCTATCTCTTTACG	60.0	380	INTERGENIC	--	--	--
Ca4_P3_7388463	Ca4	7388463	CAAAAAAAAAAA/CAAAAAAAAAA	TTTTCTCTAATCCCCGACTCA	TGTTGTTGTCAAACACCTCAAA	58.8	565	INTERGENIC	--	--	--

INDEL marker IDs	Chromosomes/ unanchored scaffolds	Physical positions (bp)	InDels (*Kabuli* reference genome-CDC Frontier/PII)	Forward primers (5'-3')	Reverse primers (5'-3')	Anneling temperature (0C)	Expected amplified product size (bp)	Structural annotation		Functional annotation	
								Sequence components of *kabuli* genome	*Kabuli* gene accession IDs	NCBI-KOG	TFs
Ca4_P3_7591008	Ca4	7591008	CATATATATATATATATATATATATATA/CATATATATATATATATATATATATATATA	CCATCCACAGTTTTCTCCGT	GACACGGTTGTTTAATTATAATCGC	60.0	817	INTRON	Ca_03476	--	MYB
Ca4_P3_7710147	Ca4	7710147	AATATTATTATATATTA/AATATTATTATATATTATTATATATTA	TCGTTACCCCTCTCGTCATC	ACAGAATGCCTCATTGGTGG	60.1	927	DRR	Ca_03470	R	Nin-like
Ca4_P3_7773804	Ca4	7773804	CTTTTTTTTTT/CTTTTTTTTTTTTT	CTTTGCCACGGTTCAATTCT	GGACAAAAAGTTGGCAAAGG	60.1	226	DRR	Ca_03463	--	--
Ca4_P3_7783679	Ca4	7783679	GATATATATATATATATATATATATATATATATA/GATATATATATATATATATATATATATATATATATA	GGAATATTCATTTGCATGTTGG	GACAAAACGTCGCACAAAAA	59.2	639	INTERGENIC	--	--	--
Ca4_P3_7798738	Ca4	7798738	CA/C	CATTAAAAGTGCCGTGTTTGAA	TCTGTGCACTCCACCTGTTC	60.0	758	INTRON	Ca_03459	--	--
Ca4_P3_7825515	Ca4	7825515	TAAAAAAAAAAA/TAAAAAAAAAAAA	TGCACAAAAATAAAAACATTTCAA	AAAGGATGGAAGCCCATTTT	58.7	959	DRR	Ca_03457	G	NAC
Ca4_P3_7835255	Ca4	7835255	AAATATAATATAATATAATATAATATAA/AAATATAATATAATATAATATAATATAATATAA	GCATATTCATGGCATACCCC	CAACCAGTGATGTTGGTTGC	60.0	865	INTRON	Ca_03455	--	--
Ca4_P3_7937193	Ca4	7937193	TAAAAAAAAAAA/TAAAAAAAAAA	GCTCCGGTCAAACACAATTT	GAAAATTGAAAGGCATGGGA	60.0	403	INTERGENIC	--	--	--
Ca4_P3_7978076	Ca4	7978076	TATCTAAATCTAAAT/TATCTAAATCTAAATCTAAAT	GCACTCCATGAAACGACCTT	CCCTTACGTGGTAACATAACACA	60.1	847	INTERGENIC	--	--	--
Ca4_P3_8003681	Ca4	8003681	GTATATATATATATATATATATATAT/GTATATATATATATATATATATATATATAT	AATGTGTCCTCGCCGTTAAA	TAGGGGGATGATGTGGAAAA	60.5	565	INTERGENIC	--	--	--

INDEL marker IDs	Chromosomes/ unanchored scaffolds	Physical positions (bp)	InDels (*Kabuli* reference genome-CDC Frontier/PII)	Forward primers (5'-3')	Reverse primers (5'-3')	Anneling temperature (0C)	Expected amplified product size (bp)	Structural annotation		Functional annotation	
								Sequence components of *kabuli* genome	*Kabuli* gene accession IDs	NCBI-KOG	TFs
Ca4_P3_80132	Ca4	80132	G/GA	CCGTTTGGATTGTCAGGACT	TCGTTGAAAGCTGGAGGTCT	60.0	382	INTERGENIC	--	--	--
Ca4_P3_8098817	Ca4	8098817	ATTTT/ATTT	CAAATGGGCCTAAAGAGCTG	GCCTCGGGGATGTTAGTGTA	59.8	463	DRR	Ca_03422	T	--
Ca4_P3_8109119	Ca4	8109119	TAAAAAAA/TAAAAAAAA	TAATCCACGTGTTGAGGGGT	AAGGAACCACTGGTGCTATTT	60.2	449	INTERGENIC	--	--	--
Ca4_P3_8109188	Ca4	8109188	CTTTTTTTTTT/CTTTTTTTTTTT	TAATCCACGTGTTGAGGGGT	CAAAATTTTTAAACAAAACCGACA	60.2	804	INTERGENIC	--	--	--
Ca4_P3_813651	Ca4	813651	ATTATTATTATTATTATTATTATTATTATTATT/ATTATTATTATTATTATTATTATTATTATTATTATT	TGTCACATTTATTCAACCGCA	TGACTCAAAGTGCAACCAGC	60.0	862	INTERGENIC	--	--	--
Ca4_P3_8167017	Ca4	8167017	CAAAA/CAAA	GGGTGGGTTTAGAGGACCAT	TTTCTTGAATGGGCAAAACC	60.1	259	DRR	Ca_08305	V	GRAS
Ca4_P3_8167238	Ca4	8167238	ATCTT/ATCTTCTT	GGGTGGGTTTAGAGGACCAT	TGAAGCTTTTTGCTACAAGGAA	60.1	737	DRR	Ca_08305	V	GRAS
Ca4_P3_8167347	Ca4	8167347	ATATTATTTTTA/ATATTATTTTTATTATTTTTA	GGGTGGGTTTAGAGGACCAT	TGAAGCTTTTTGCTACAAGGAA	60.1	737	DRR	Ca_08305	V	GRAS
Ca4_P3_8169626	Ca4	8169626	TAAATAAAAA/TAAATAAAAAATAAAAA	TATGGCACCTCCAGTTGAAA	TGTTAAATCCTCCAGACGGC	59.1	858	INTERGENIC	--	--	--
Ca4_P3_8170540	Ca4	8170540	TAAAAAA/TAAAAA	TCAACCCCTTTGTAGTTTCACA	CTGCCAACTCCAAAAGATACC	59.5	713	INTERGENIC	--	--	--

INDEL marker IDs	Chromosomes/ unanchored scaffolds	Physical positions (bp)	InDels (*Kabuli* reference genome-CDC Frontier/PII)	Forward primers (5'-3')	Reverse primers (5'-3')	Anneling temperature (0C)	Expected amplified product size (bp)	Structural annotation		Functional annotation	
								Sequence components of *kabuli* genome	*Kabuli* gene accession IDs	NCBI-KOG	TFs
Ca4_P3_8171433	Ca4	8171433	ATATTCAGAATTAT/ATAT	TCAATTTGGTCTTTCCG CTC	AAAGTTTTGCTCCAGT CCCA	60.2	451	INTERGENIC	--	--	--
Ca4_P3_8172016	Ca4	8172016	T/TC	CTAGTGTTCGGCAACA AACG	GCCTCAATTGGAGTTT GCTC	59.4	204	INTERGENIC	--	--	--
Ca4_P3_8173142	Ca4	8173142	TAAAAAAAAA/TAAAAAAAA	TCCATCACACAATTGAA GGC	TGAATGTATCGCGTTG TTGG	59.5	569	INTERGENIC	--	--	--
Ca4_P3_8173292	Ca4	8173292	GT/G	TCCATCACACAATTGAA GGC	TGAATGTATCGCGTTG TTGG	59.5	569	INTERGENIC	--	--	--
Ca4_P3_8173963	Ca4	8173963	TTAT/TTATAT	GAAGCTCGCTTTATCCA ACG	TCCACGGAATTTTCTG CTTC	60.0	147	INTERGENIC	--	--	--
Ca4_P3_8174018	Ca4	8174018	TAA/TAAA	GAAGCTCGCTTTATCCA ACG	TCCACGGAATTTTCTG CTTC	60.0	147	INTERGENIC	--	--	--
Ca4_P3_8178683	Ca4	8178683	TCCC/TCCCC	CGTCATTGATTGGTGT GGTC	GCCTTGTCAGATTGGA TGCT	59.8	574	DRR	Ca_08307	R	B3
Ca4_P3_8183910	Ca4	8183910	TAAA/TAA	AACTCAATCCAACCAAG CCA	TCACCACACCAACCTC AAAA	60.5	548	INTERGENIC	--	--	--
Ca4_P3_8183976	Ca4	8183976	TAAAAAAA/TAAAAAAAA	AACTCAATCCAACCAAG CCA	TCACCACACCAACCTC AAAA	60.5	548	INTERGENIC	--	--	--
Ca4_P3_8185194	Ca4	8185194	AGTGTGT/AGTGTGTGT	TGGTCTGGTTGGCCAA TACT	CCCAACCTCTGGACAC CTAA	60.4	212	DRR	Ca_08308	T	--

INDEL marker IDs	Chromosomes/ unanchored scaffolds	Physical positions (bp)	InDels (*Kabuli* reference genome-CDC Frontier/PII)	Forward primers (5'-3')	Reverse primers (5'-3')	Anneling temperature (0C)	Expected amplified product size (bp)	Structural annotation		Functional annotation	
								Sequence components of *kabuli* genome	*Kabuli* gene accession IDs	NCBI-KOG	TFs
Ca4_P3_8186328	Ca4	8186328	CTATATATATATATA/CTATATATATATA	TGCTTTTCATTTGCAGAATCC	TTTCACCTCCTGGTAGACGG	60.2	626	DRR	Ca_08308	T	--
Ca4_P3_8192071	Ca4	8192071	GAAAAAAAAAAAAA/GAAAAAAAAAA	GAATGCGTATTCAAGGGTCTAA	TTTTTGACGTTGCGTTTTCC	58.3	240	DRR	Ca_08309	R	--
Ca4_P3_8193266	Ca4	8193266	TAAAAAAAAA/TAAAAAAAAAA	GCATCTCTCCAAGATGTCTGC	TGGGAATTGGGAGCTATCAG	60.0	620	DRR	Ca_08309	R	--
Ca4_P3_8203863	Ca4	8203863	TAAA/TAA	GCTGCACAACCTACACCAAA	ACAAAGCCTTGTTTTGTGGG	59.8	471	INTRON	Ca_08310	T	MYB_related
Ca4_P3_8204473	Ca4	8204473	CAA/C	GAGTCCAATTCTCTGGCAGC	TTGTAATTCGGAAGGTTGGC	60.0	213	INTRON	Ca_08310	T	MYB_related
Ca4_P3_8205973	Ca4	8205973	ATCTT/ATCTTCTT	CGTGTCCAACATCAAAAACG	TTCCAAAGAACCTACGGTGG	60.0	378	CDS (large-effect mutations)	Ca_08310	T	MYB_related
Ca4_P3_8206232	Ca4	8206232	GTATA/GTATACTATA	CAAGGGGTTGAAGTTGCATT	TTGAAGCCACCCTCTGATCT	60.0	763	INTERGENIC	--	--	--
Ca4_P3_8207569	Ca4	8207569	ATTTTTTTTT/ATTTTTTT	CTCGTGTCTTTGTGCGTTGT	TCTCTCTATCTCAGCGTCACTTT	59.9	549	INTERGENIC	--	--	--
Ca4_P3_8208104	Ca4	8208104	ATTTTTTTTT/ATTTTTTTTTTT	TGTTGCAACCATTTTTACATAGTG	GTGGAGATCGATGCTTGGTT	59.0	532	INTERGENIC	--	--	--
Ca4_P3_8210483	Ca4	8210483	GATAT/GATATAT	GGCCAGGAAATCAACTTCAA	ATGAATTGCATGCATGGTGA	60.1	439	INTERGENIC	--	--	--

INDEL marker IDs	Chromosomes/ unanchored scaffolds	Physical positions (bp)	InDels (*Kabuli* reference genome-CDC Frontier/PII)	Forward primers (5'-3')	Reverse primers (5'-3')	Anneling temperature (0C)	Expected amplified product size (bp)	Structural annotation		Functional annotation	
								Sequence components of *kabuli* genome	*Kabuli* gene accession IDs	NCBI-KOG	TFs
Ca4_P3_8210730	Ca4	8210730	TCC/TC	GGGGATACTTGCCTCA ATCA	TGATGCTATTCATTTTG CGG	59.9	814	INTERGENIC	--	--	--
Ca4_P3_8211793	Ca4	8211793	AG/A	CCAATATGTGATGGTT GTTTGG	CTACAATGGAATGAGG CGCT	60.0	473	INTERGENIC	--	--	--
Ca4_P3_8216116	Ca4	8216116	ATTTTTTT/ATTTTTT	TCAAAATGCACGAGATT AGCA	CGAAGCGGATTCGAAC TAAG	59.5	745	INTERGENIC	--	--	--
Ca4_P3_8216693	Ca4	8216693	AG/A	CGAATCCGCTTCGTGT TAAT	CGACCCGCTTAATATG TGGT	60.1	507	INTERGENIC	--	--	--
Ca4_P3_8217219	Ca4	8217219	AG/A	ACCACATATTAAGCGG GTCG	ATGTTGGTGTTGGTGG AGGT	59.8	781	INTERGENIC	--	--	--
Ca4_P3_8220440	Ca4	8220440	AAATTATTT/AAATTATTTAATTATTT	CGGTGAAGCAACGTAG AACA	ACAGTTTTGGGTGCAA AAGG	59.9	688	DRR	Ca_08312	--	--
Ca4_P3_8221295	Ca4	8221295	TACACAC/TACAC	AACACAAGGAGGGAAA CACG	TTGTGGTGGAAAAGTC ATGG	60.0	136	DRR	Ca_08312	--	--
Ca4_P3_8225755	Ca4	8225755	AAAT/A	GTAGAAGAGGGGAAGG TGGC	GTGTACGGCGGATTTC TTGT	60.1	611	INTERGENIC	--	--	--
Ca4_P3_8226133	Ca4	8226133	CATATATAT/CATATAT	ACAAGAAATCCGCCGT ACAC	CCCCCTAATTCCACTT TACCA	60.0	613	INTERGENIC	--	--	--
Ca4_P3_8227216	Ca4	8227216	ATT/ATTTT	AATCGAATCGCCTTGG TGTA	GCGACATCATTTAAAAT AAATCACA	60.5	223	INTERGENIC	--	--	--

INDEL marker IDs	Chromosomes/ unanchored scaffolds	Physical positions (bp)	InDels (*Kabuli* reference genome-CDC Frontier/PII)	Forward primers (5'-3')	Reverse primers (5'-3')	Anneling temperature (0C)	Expected amplified product size (bp)	Structural annotation		Functional annotation	
								Sequence components of *kabuli* genome	*Kabuli* gene accession IDs	NCBI-KOG	TFs
Ca4_P3_8232418	Ca4	8232418	TAT/TATAAT	CAGTATGGACGAATGG GAGG	GGTCTACTCTCGAACC ACCG	60.3	563	INTERGENIC	--	--	--
Ca4_P3_8237892	Ca4	8237892	TAAAAAAAAAA/TAAAAAAAAA	TCCAATCCTCAACCTCG TTC	CTTGCGTGTTCCCAAT TTTT	60.1	364	INTERGENIC	--	--	--
Ca4_P3_8239012	Ca4	8239012	CTTTT/CTTT	TTCTCGATCCACAAAAA CAAAA	CAACCTCCTCAAATTG GACA	59.6	636	INTERGENIC	--	--	--
Ca4_P3_8281442	Ca4	8281442	TATA/T	TTTGCTATCTCTTTCCC CACA	CAATCGCAAGGATAAT GGCT	59.7	328	INTERGENIC	--	--	--
Ca4_P3_8282938	Ca4	8282938	ATTTTTTTT/ATTTTTTT	TTGCATGCATTGAGATT TAAAGA	TTTTGTGTTCGTTTATT GTAACCAA	59.7	607	INTERGENIC	--	--	--
Ca4_P3_8293335	Ca4	8293335	A/AAT	CAATGGTTTTGCTTTTG GCT	TTCTCGAATGCGAAGA GTCA	60.1	492	INTERGENIC	--	--	--
Ca4_P3_8294555	Ca4	8294555	TATACATA/TATA	TCGTGTGACCCCAACA ACTA	GTGATCGATGGCTTTT TGGT	60.0	484	INTERGENIC	--	--	--
Ca4_P3_8295749	Ca4	8295749	ATTTTTATT/ATTTTTATTATTTTTTATT	TCAACCCTCATTCAACC ACA	CCTGCGACAATCTGAA GGAT	59.9	840	INTERGENIC	--	--	--
Ca4_P3_8295853	Ca4	8295853	TAA/TA	GCAGGTGGGAATGGTT CTAA	TGTCTGGTGGATGAAA CCAA	59.9	805	INTERGENIC	--	--	--
Ca4_P3_8297213	Ca4	8297213	TAAAAAAA/TAAAAAAAA	GGGTTTTGTTTGAAAAA TTTGAT	TGGTGCTAACATGTGG CTAA	58.4	676	INTERGENIC	--	--	--

INDEL marker IDs	Chromosomes/ unanchored scaffolds	Physical positions (bp)	InDels (*Kabuli* reference genome-CDC Frontier/PII)	Forward primers (5'-3')	Reverse primers (5'-3')	Anneling temperature (0C)	Expected amplified product size (bp)	Structural annotation		Functional annotation	
								Sequence components of *kabuli* genome	*Kabuli* gene accession IDs	NCBI-KOG	TFs
Ca4_P3_8300267	Ca4	8300267	TATAAAA/TATAAAATAAAA	GCTTCTTGGGCATTGATTGT	ACAAATGAAACGCAGCTTCC	60.1	605	DRR	Ca_08319	S	--
Ca4_P3_8307784	Ca4	8307784	ATTT/ATT	TACACCCATTGACGTGGCTA	TCAATCATGTCTGCATACCG	60.0	694	INTERGENIC	--	--	--
Ca4_P3_8309234	Ca4	8309234	TCAACACAA/TCAACACAACACAA	GAATTTAAGAGGAAGGCGATGA	TTCACCCTAATTCGTAGGCG	59.7	450	INTERGENIC	--	--	--
Ca4_P3_8309328	Ca4	8309328	TTGGTGGT/TTGGT	CATCCCAGGATCAACACTCA	TTCACCCTAATTCGTAGGCG	59.5	272	INTERGENIC	--	--	--
Ca4_P3_8309498	Ca4	8309498	A/ACC	CATCCCAGGATCAACACTCA	TTCACCCTAATTCGTAGGCG	59.5	272	INTERGENIC	--	--	--
Ca4_P3_8311844	Ca4	8311844	TG/T	TTACTCCCTCCTTCCCATGA	ATGGATGGGGGATAAAAAGG	59.5	433	INTERGENIC	--	--	--
Ca4_P3_8312228	Ca4	8312228	GTTAT/GT	CCTTTTTATCCCCCATCCAT	TAACCGCATCTCCAAATTCC	59.8	705	INTERGENIC	--	--	--
Ca4_P3_8313629	Ca4	8313629	ATTTTTTTTTTTT/ATTTTTTTTTTT	CTGGTGGACTGGTTGTTGTG	GGCAGACAGGAGAGGGAGTA	60.0	563	INTERGENIC	--	--	--
Ca4_P3_8315749	Ca4	8315749	TAGA/TA	CCTTTACAAAAAGTGCCCCA	TCTCGGTTGATGTCATTATGCT	60.0	853	INTERGENIC	--	--	--
Ca4_P3_8315933	Ca4	8315933	CTT/CTTATT	GGACCCGTGATGCTGTTTAT	TCTCGGTTGATGTCATTATGCT	59.8	586	INTERGENIC	--	--	--

INDEL marker IDs	Chromosomes/ unanchored scaffolds	Physical positions (bp)	InDels (*Kabuli* reference genome-CDC Frontier/PII)	Forward primers (5'-3')	Reverse primers (5'-3')	Anneling temperature (0C)	Expected amplified product size (bp)	Structural annotation		Functional annotation	
								Sequence components of *kabuli* genome	*Kabuli* gene accession IDs	NCBI-KOG	TFs
Ca4_P3_8316304	Ca4	8316304	GTTTTTTT/GTTTTTTTT	GCATAATGACATCAACCGAGAG	AAAAGAAATGGTTGTTTCTCGTTC	59.6	235	INTERGENIC	--	--	--
Ca4_P3_8316423	Ca4	8316423	TAAAAAAA/TAAAAAA	GCATAATGACATCAACCGAGAG	CACGAGTGACACGCCATTTA	59.6	794	INTERGENIC	--	--	--
Ca4_P3_8316722	Ca4	8316722	ACTCT/ACT	GAACGAGAAACAACCATTTCTTTT	CACGAGTGACACGCCATTTA	59.9	583	INTERGENIC	--	--	--
Ca4_P3_8320140	Ca4	8320140	C/CG	CTTTGCCCCCATCCATATAA	AGGCTGAAGACTTCGACGAG	59.6	358	INTERGENIC	--	--	--
Ca4_P3_8320529	Ca4	8320529	CAAATATTA/CAAATATTAAATATTA	AAAATCTTCCGGTGTCTCCA	TTTTCCCAAGTTGGAAGTGG	59.5	668	INTERGENIC	--	--	--
Ca4_P3_8321641	Ca4	8321641	A/AGAACCGCT	GGACCATTCAAAAATCGACG	CCTGCTTGATGCATGTCACT	60.3	108	INTERGENIC	--	--	--
Ca4_P3_8325629	Ca4	8325629	CTTTTTTTTT/CTTTTTTTTTT	ACGGGGCTGTTACTTTACCC	TTCTCAATGGGTGCGTACAA	60.2	739	INTERGENIC	--	--	--
Ca4_P3_8331477	Ca4	8331477	TTA/TTATA	AAGGGTATGCTGCCATGAAC	TGCTGCTACTGGCAAAAATG	60.0	666	DRR	Ca_08320	--	--
Ca4_P3_8333720	Ca4	8333720	GTA/GTATA	CCACACGTAGTGTTTTGGCA	TCACACATCACCGCAAACTT	60.6	346	INTERGENIC	--	--	--
Ca4_P3_8333830	Ca4	8333830	ATTTTTTTTTT/ATTTTTTTTTTT	ACGTAGTGTTTTGGCAAGGG	TCACACATCACCGCAAACTT	60.0	342	INTERGENIC	--	--	--

INDEL marker IDs	Chromosomes/ unanchored scaffolds	Physical positions (bp)	InDels (*Kabuli* reference genome-CDC Frontier/PII)	Forward primers (5'-3')	Reverse primers (5'-3')	Anneling temperature (0C)	Expected amplified product size (bp)	Structural annotation		Functional annotation	
								Sequence components of *kabuli* genome	*Kabuli* gene accession IDs	NCBI-KOG	TFs
Ca4_P3_8334346	Ca4	8334346	TCACTATATTAACACTATATTAAC/TCACTATATTAAC	AAGTTTGCGGTGATGTGTGA	CAGCGTTATTACAAATGCCGT	60.2	438	INTERGENIC	--	--	--
Ca4_P3_8342083	Ca4	8342083	AATCATCAT/AATCAT	TGATCAAATCCCCAAGAAGC	CCAATCTCTTCTGCTCCCCT	60.0	951	INTRON	Ca_08321	--	--
Ca4_P3_8345936	Ca4	8345936	AAAATCAC/AAAATCACTAAATCAC	CATACCTCGATGCACCTCCT	TGTGCGACCAAAGGTTACAA	60.1	213	DRR	Ca_08322	--	--
Ca4_P3_8348600	Ca4	8348600	TAAAAA/TAAAAAA	TCAAGCAAAGGCTTCGAGAT	AAACGGCTTGTGGTTGTTTC	60.1	551	INTRON	Ca_08322	--	--
Ca4_P3_8372871	Ca4	8372871	T/TG	TCTTTTTCTGCCCCACTAGC	CAGCTGAGTTGTGAGGTCCA	59.5	150	INTERGENIC	--	--	--
Ca4_P3_8373461	Ca4	8373461	AAC/AACCAC	TGAGCATAAAGTGGCATTGG	GTCGGTCTTTGTGGAGAGGA	59.7	398	INTERGENIC	--	--	--
Ca4_P3_8373669	Ca4	8373669	TAGAAA/TAGAAAGAAA	TGAATTGGATCCTCTCCACC	AAGCCGAGTCTGGCCTATTT	59.9	680	INTERGENIC	--	--	--
Ca4_P3_8377059	Ca4	8377059	CAA/CAAA	TAGTGTACGTGTTTGGGGCA	TCAGGATTCAAATGGACGAA	60.0	249	DRR	Ca_08327	KL	--
Ca4_P3_8381452	Ca4	8381452	TAAAAAAA/TAAAAAAAA	CAATCCGTTAAGGACCTGGA	GATGATTCCCTTGCTGGAAA	59.9	388	INTRON	Ca_08327	KL	--
Ca4_P3_8382072	Ca4	8382072	AT/ATTT	TTTCCAGCAAGGGAATCATC	GCATTCAAATCTGGGTTGGT	60.0	534	INTRON	Ca_08327	KL	--

INDEL marker IDs	Chromosomes/ unanchored scaffolds	Physical positions (bp)	InDels (*Kabuli* reference genome-CDC Frontier/PII)	Forward primers (5'-3')	Reverse primers (5'-3')	Anneling temperature (0C)	Expected amplified product size (bp)	Structural annotation		Functional annotation	
								Sequence components of *kabuli* genome	*Kabuli* gene accession IDs	NCBI-KOG	TFs
Ca4_P3_8382639	Ca4	8382639	ATTTTTTTTT/ATTTTTTTTTT	TGGTGCAATAAATGAGTCGG	GGGATGGTAGCAAGGAAACA	59.5	378	INTRON	Ca_08327	KL	--
Ca4_P3_8389681	Ca4	8389681	CATA/CA	CGGCAAACATGAAGATCTGA	CGTGGCTTAACCTTGGACAT	59.8	312	INTRON	Ca_08327	KL	--
Ca4_P3_8390760	Ca4	8390760	CG/CGG	TCCCTAATCAGAACCATCGC	CGTCGCTATTCTGACTCCCT	60.0	238	INTERGENIC	--	--	--
Ca4_P3_8400811	Ca4	8400811	GAATTACTAAT/GAATTACTAATTACTAAT	CTTAAGCATCAAAGCCCGAG	CCTCAATTGCCACCAAAACT	60.0	469	INTRON	Ca_08329	O	B3
Ca4_P3_8400838	Ca4	8400838	CAAAAAAAA/CAAAAAAAAAA	CTTAAGCATCAAAGCCCGAG	CCTCAATTGCCACCAAAACT	60.0	469	INTRON	Ca_08329	O	B3
Ca4_P3_8401589	Ca4	8401589	TG/T	AGTTTTGGTGGCAATTGAGG	TGAAAATTGATCAGGGTCAAAA	60.0	769	INTERGENIC	--	--	--
Ca4_P3_8407015	Ca4	8407015	CTATATAT/CTATAT	TGCTAATCATCCGTGCTCTG	GTGGATGAGCAACCACCTTT	60.0	521	INTRON	Ca_08330	O	B3
Ca4_P3_8408638	Ca4	8408638	TC/T	CGCTGAAACAATTGCAGAAA	GGGAGGTCCACTCAAAGTGA	60.0	450	INTERGENIC	--	--	--
Ca4_P3_8408879	Ca4	8408879	TAAAA/TAAAAA	TCACTTTGAGTGGACCTCCC	TCCTACAAAATCACGGACACC	60.1	469	INTERGENIC	--	--	--
Ca4_P3_8408977	Ca4	8408977	AT/ATT	TCACTTTGAGTGGACCTCCC	TCCTACAAAATCACGGACACC	60.1	469	INTERGENIC	--	--	--

INDEL marker IDs	Chromosomes/ unanchored scaffolds	Physical positions (bp)	InDels (*Kabuli* reference genome-CDC Frontier/PII)	Forward primers (5'-3')	Reverse primers (5'-3')	Anneling temperature (0C)	Expected amplified product size (bp)	Structural annotation		Functional annotation	
								Sequence components of *kabuli* genome	*Kabuli* gene accession IDs	NCBI-KOG	TFs
Ca4_P3_8409923	Ca4	8409923	AAGAG/AAG	CGCATCAATACGACGAACAA	ACCACCACTTTGTAGCCACC	60.7	509	INTERGENIC	--	--	--
Ca4_P3_8414127	Ca4	8414127	CAAAAAAAA/CAAAAAAAAA	TACACGTTCGGCACATCTTG	ATGCACATGGTTCTGACCAA	60.7	761	INTERGENIC	--	--	--
Ca4_P3_8415318	Ca4	8415318	AG/AGGG	ACTTTGCCACCTCATCTGCT	TTGTTGAACACCCACATGCT	59.9	221	INTERGENIC	--	--	--
Ca4_P3_8424082	Ca4	8424082	GAAA/GAAAA	CGCCACACTTAGATGACGAA	AACGATTAGCGAATGTTGGG	59.9	433	INTERGENIC	--	--	--
Ca4_P3_8425720	Ca4	8425720	TAAAAAAA/TAAAAAAAA	GGGATTTGTTCGATGCACTT	GGCACAAGGGTTTTTCAAGA	59.9	340	INTERGENIC	--	--	--
Ca4_P3_8441953	Ca4	8441953	GTTTGTA/GTTTGTATTTTGTA	AGCAGGAGAGCTGAGATTGG	TACTGTGTATCGGGGCAACA	59.7	731	INTRON	Ca_08335	R	--
Ca4_P3_8447485	Ca4	8447485	GATGTCAATTTAA/G	CAGGCATTGGACACTTTTCA	AAACCTCATGGCACAACTCC	59.7	294	INTRON	Ca_08335	R	--
Ca4_P3_8454109	Ca4	8454109	C/CATTTT	TTCTTTGGGTCTCCCAACAC	TTATGAGCTTTGGATTGGGC	59.9	631	DRR	Ca_08337	R	MYB
Ca4_P3_8458330	Ca4	8458330	A/ATC	GAGTTCTTTGAAGGGCTCACA	TCCCCTACCCAAAATTGGAA	59.5	569	INTERGENIC	--	--	--
Ca4_P3_8459318	Ca4	8459318	ATTTTTTTT/ATTTTTTT	TCAGAAATTGAAAATGAAATGGC	GGTTGTTGGTAGTAGACCTGTGG	60.3	836	INTERGENIC	--	--	--

INDEL marker IDs	Chromosomes/ unanchored scaffolds	Physical positions (bp)	InDels (*Kabuli* reference genome-CDC Frontier/PII)	Forward primers (5'-3')	Reverse primers (5'-3')	Anneling temperature (0C)	Expected amplified product size (bp)	Structural annotation		Functional annotation	
								Sequence components of *kabuli* genome	*Kabuli* gene accession IDs	NCBI-KOG	TFs
Ca4_P3_8459775	Ca4	8459775	G/GA	TGGACCACAGGTCTAC TACCAA	TGTACCACTTTTGGTG ACCG	59.5	260	INTERGENIC	--	--	--
Ca4_P3_8459845	Ca4	8459845	ATTTT/ATTTTT	TGGACCACAGGTCTAC TACCAA	TGTACCACTTTTGGTG ACCG	59.5	260	INTERGENIC	--	--	--
Ca4_P3_8462210	Ca4	8462210	T/TTGTTTAGA	TCCGTGCACTTATTTTT CCC	ATTTCCAGCCAAGTGA GGTG	59.9	471	INTERGENIC	--	--	--
Ca4_P3_8466945	Ca4	8466945	CT/C	AGGACACCAATTTGAAT GGC	TGATGATGGTGTGCTT TGGT	59.8	625	INTERGENIC	--	--	--
Ca4_P3_8468674	Ca4	8468674	AGATTC/A	CCGAACATAACATCCCA ACC	AGAACCCCTCAAATCG GAGT	60.1	851	INTERGENIC	--	--	--
Ca4_P3_8472031	Ca4	8472031	ATTT/ATTTT	CTTCAACTTGCGCCAAT ACA	GTGGCAACGTATGACA TGGT	59.9	682	DRR	Ca_08339	G	--
Ca4_P3_8475153	Ca4	8475153	ATTT/ATT	TGGACCGTACATTTTC GTGA	GTGGTAGCAGAAGGA GCAGG	60.0	829	INTRON	Ca_08339	G	--
Ca4_P3_8476352	Ca4	8476352	AC/A	AGTGGGACTGTGGGAC AAAG	TGCATGTTTGCCTCTA GGTG	60.0	761	INTRON	Ca_08339	G	--
Ca4_P3_8476596	Ca4	8476596	GAAAAAA/GAAAAAAA	ACCTGCAGATGGAAGC TTGT	TGCAGTGTGTGCGTGA TAGA	59.9	459	INTRON	Ca_08339	G	--
Ca4_P3_8478290	Ca4	8478290	A/AT	TTTAGCTTGCCAAAACA GCA	CCTTTCTTCGTACGTC TCGC	59.6	607	INTRON	Ca_08339	G	--

INDEL marker IDs	Chromosomes/ unanchored scaffolds	Physical positions (bp)	InDels (*Kabuli* reference genome-CDC Frontier/PII)	Forward primers (5'-3')	Reverse primers (5'-3')	Anneling temperature (0C)	Expected amplified product size (bp)	Structural annotation		Functional annotation	
								Sequence components of *kabuli* genome	*Kabuli* gene accession IDs	NCBI-KOG	TFs
Ca4_P3_8481188	Ca4	8481188	ATCTCTCTCTCTCTCT/ATCTCTCTCTCTCT	AGGGAAAAGTACCGGCAAAT	ATAGATCCAAACGGCACAGC	59.8	463	INTERGENIC	--	--	--
Ca4_P3_8482101	Ca4	8482101	C/CTCATGTTGAAG	GGTTTTGTGCGCTTTTCAAT	TGACTCTCCTCCTCTCCTCAA	60.1	403	DRR	Ca_08340	R	--
Ca4_P3_8483100	Ca4	8483100	CTTTTTTTTTT/CTTTTTTTTT	ATTTCGAGGAAAAGGGAGGA	TTTCAAGAGGGGCAATTGAG	60.0	515	DRR	Ca_08340	R	--
Ca4_P3_8486769	Ca4	8486769	CAGAAAGA/CAGAAAGAAAGA	CAACTGCACATGCACACAAG	ACAGCAGGGCTGAATATTGG	59.9	733	INTRON	Ca_08340	R	--
Ca4_P3_8486971	Ca4	8486971	AT/ATT	CAACTGCACATGCACACAAG	ACAGCAGGGCTGAATATTGG	59.9	733	INTRON	Ca_08340	R	--
Ca4_P3_8490497	Ca4	8490497	TAAAAAAAA/TAAAAAAAAAA	TATGCCTTAGCGGTTTTTGG	GGTCCTTTGATCCCACGTTA	60.1	498	INTERGENIC	--	--	--
Ca4_P3_8490694	Ca4	8490694	TTATATATATATATA/TTATATATATATATATATATA	TATGCCTTAGCGGTTTTTGG	GGTCCTTTGATCCCACGTTA	60.1	498	INTERGENIC	--	--	--
Ca4_P3_8491035	Ca4	8491035	AAGA/AA	TAACGTGGGATCAAAGGACC	ATATGAAACCACCAACCCCA	59.8	385	INTERGENIC	--	--	--
Ca4_P3_8497257	Ca4	8497257	TAAACTGAAGAAACT/TAAACTGAAGAAACTGAAGAAACT	AGCGTGAGAGGAACCTGTGT	GACTGGCAACAGCAATTTGA	59.9	564	INTRON	Ca_08341	--	CPP
Ca4_P3_8503853	Ca4	8503853	AATTTTATTTT/AATTTTATTTTATTTT	CAGACATTTGAGCCGTTGAA	ACCACTTTTATCATTTGACTCCA	59.8	421	INTERGENIC	--	--	--

INDEL marker IDs	Chromosomes/ unanchored scaffolds	Physical positions (bp)	InDels (*Kabuli* reference genome-CDC Frontier/PII)	Forward primers (5'-3')	Reverse primers (5'-3')	Anneling temperature (0C)	Expected amplified product size (bp)	Structural annotation		Functional annotation	
								Sequence components of *kabuli* genome	*Kabuli* gene accession IDs	NCBI-KOG	TFs
Ca4_P3_8504070	Ca4	8504070	GGTAGA/G	CAGACATTTGAGCCGTTGAA	TTCGAGTGCCGCTTACTTTT	59.8	916	INTERGENIC	--	--	--
Ca4_P3_8513082	Ca4	8513082	GTTCTTC/GTTC	CTTCCTCTTCGGCAGTCAAC	AACACTGTCCTCGGCCATAC	60.0	356	CDS (large-effect mutations)	Ca_08343	YU	--
Ca4_P3_8514326	Ca4	8514326	CTTTT/CTTTTT	TGTCGTGCGGTAAGTTTGAC	GAAGCAAAGCTAGGGGTGTG	59.8	449	DRR	Ca_08343	YU	--
Ca4_P3_8514546	Ca4	8514546	A/AG	TGTCGTGCGGTAAGTTTGAC	GAAGCAAAGCTAGGGGTGTG	59.8	449	DRR	Ca_08343	YU	--
Ca4_P3_8514652	Ca4	8514652	CTTTTTTTTTTTT/CTTTTTTTT	CACACCCCTAGCTTTGCTTC	CACGAACGCCTGTGAACTTA	59.9	547	DRR	Ca_08343	YU	--
Ca4_P3_8514977	Ca4	8514977	G/GT	CACACCCCTAGCTTTGCTTC	CACGAACGCCTGTGAACTTA	59.9	547	DRR	Ca_08343	YU	--
Ca4_P3_8516971	Ca4	8516971	AT/ATGTCAAATTAAGCTT	AAAAATGAGGGGCGTGAGTA	AGGTTGAGCCCCTTGAAAAT	59.6	392	DRR	Ca_08344	--	--
Ca4_P3_8521823	Ca4	8521823	AACA/AA	AATTTGGCAAAAGGAGATGC	AATAGGGGGACCAAAACTGC	59.2	816	INTERGENIC	--	--	--
Ca4_P3_8522151	Ca4	8522151	TG/T	TGTGATTGTTAGGATGATGACG	CCAAAACTGGGGAGAAACAA	58.5	288	INTERGENIC	--	--	--
Ca4_P3_8614404	Ca4	8614404	TAAAAAAAA/TAAAAAAAAA	GTAGGCCCACCATGATTTTG	TCAAACATAAAAGATTGCTTGCAT	60.2	787	INTERGENIC	--	--	--

INDEL marker IDs	Chromosomes/ unanchored scaffolds	Physical positions (bp)	InDels (*Kabuli* reference genome-CDC Frontier/PII)	Forward primers (5'-3')	Reverse primers (5'-3')	Anneling temperature (0C)	Expected amplified product size (bp)	Structural annotation		Functional annotation	
								Sequence components of *kabuli* genome	*Kabuli* gene accession IDs	NCBI-KOG	TFs
Ca4_P3_8616583	Ca4	8616583	GATGT/GATGTATGT	GCGAGAATACAATTTCC AGCA	ACTCGATCGTGGAAGC AAGT	60.2	324	DRR	Ca_08355	O	--
Ca4_P3_8617685	Ca4	8617685	GTTTTTTTT/GTTTTTTTTTTT	CCAGCCAACCATTTCT GTTT	ATTGCGTGAGATTTGG TTCC	60.0	504	DRR	Ca_08355	O	--
Ca4_P3_8623305	Ca4	8623305	CAAAA/CAA	CCTTGCATGGTGGTAC TCCT	GCCACAACCTTATAAC CGCT	60.0	511	INTERGENIC	--	--	--
Ca4_P3_8623394	Ca4	8623394	CTAT/CT	CCTTGCATGGTGGTAC TCCT	GTGGACCACACCACAC AAAT	60.0	714	INTERGENIC	--	--	--
Ca4_P3_8625981	Ca4	8625981	TA/TAA	GGTTTGAATGTCAACAC CCT	TTTTGGAACTTGAACT CCACAC	57.3	293	INTERGENIC	--	--	--
Ca4_P3_8626276	Ca4	8626276	CAAA/CAAAA	TGTGGAGTTCAAGTTC CAAAAG	ATTCTGCAACTAGTGG CGCT	59.3	602	INTERGENIC	--	--	--
Ca4_P3_8626895	Ca4	8626895	G/GT	CTCCCTCCCCTTCATTC TTC	CATCAGTAGCAGGTGG AGCA	60.0	378	INTERGENIC	--	--	--
Ca4_P3_8636225	Ca4	8636225	GTT/GTTT	GGGAAACCAAGGAGTG TGAA	GCATGCGATCGTAATT CCTT	59.9	771	INTERGENIC	--	--	--
Ca4_P3_8637240	Ca4	8637240	GACA/GACACA	TTTGATAGTTTTGGTGC CTCG	GTCCCCCATTCACATT TTTG	60.1	442	INTERGENIC	--	--	--
Ca4_P3_8641591	Ca4	8641591	ATTTTTT/ATTTTT	TGGTGATTGGGAACTC CATT	TCACGAAATCTAAATCA CGCA	60.2	305	DRR	Ca_08357	--	--

INDEL marker IDs	Chromosomes/ unanchored scaffolds	Physical positions (bp)	InDels (*Kabuli* reference genome-CDC Frontier/PII)	Forward primers (5'-3')	Reverse primers (5'-3')	Anneling temperature (0C)	Expected amplified product size (bp)	Structural annotation		Functional annotation	
								Sequence components of *kabuli* genome	*Kabuli* gene accession IDs	NCBI-KOG	TFs
Ca4_P3_8647825	Ca4	8647825	CAA/CA	AATGGCTTCGCTATTGCCTA	AAGGACTGCGAACCTCAAAA	59.8	734	INTRON	Ca_08358	--	G2-like
Ca4_P3_8650948	Ca4	8650948	TTAAAAAATAAAAAATAAAAAA/TTAAAAAATAAAAAA	TTTAAATTGAATGTGTTCGCGT	ACCACACATGATTTCTGCCA	59.5	785	INTERGENIC	--	--	--
Ca4_P3_8651348	Ca4	8651348	CAT/CATAT	GCATTATTCCCTTGCCTACG	TGGGGGCACTTTAGAACTTG	59.6	185	INTERGENIC	--	--	--
Ca4_P3_8689670	Ca4	8689670	A/ATCTC	TGCAAATTTGGTGAAAACAGA	TGTCACATTTGCAGAAGAAAAA	59.2	300	INTERGENIC	--	--	--
Ca4_P3_8690461	Ca4	8690461	TAAAAA/TAAAA	CACACTTTAAGATTTGTTCCCCA	GCATGCGTGTCGTGAAAATA	60.3	674	INTERGENIC	--	--	--
Ca4_P3_8690768	Ca4	8690768	TAAAAAAA/TAAAAAAAAA	GTTTTAATGCAAGCCATGTAGA	GGTGTTTTGATGCGAAGTGA	57.1	397	INTERGENIC	--	--	--
Ca4_P3_8690790	Ca4	8690790	GAAAAAAA/GAAAAAAAA	TTTAATTGCGAGGGAGTAAAAA	GGTGTTTTGATGCGAAGTGA	58.0	335	INTERGENIC	--	--	--
Ca4_P3_8691223	Ca4	8691223	ACCC/ACCCC	CACGCATGCATGTTCTTTCT	CAGAGTCCAGACACACCGAA	59.9	668	INTRON	Ca_08365	G	--
Ca4_P3_8691733	Ca4	8691733	ATATT/AT	TTCGGTGTGTCTGGACTCTG	TCACAAATAACAACCGCCAA	59.9	460	INTRON	Ca_08365	G	--
Ca4_P3_8694140	Ca4	8694140	TC/T	ATTTGTGCCATCCTTCCATC	GAAGCTTTCCAGTACCGACG	59.8	360	INTRON	Ca_08365	G	--

INDEL marker IDs	Chromosomes/ unanchored scaffolds	Physical positions (bp)	InDels (*Kabuli* reference genome-CDC Frontier/PII)	Forward primers (5'-3')	Reverse primers (5'-3')	Anneling temperature (0C)	Expected amplified product size (bp)	Structural annotation		Functional annotation	
								Sequence components of *kabuli* genome	*Kabuli* gene accession IDs	NCBI-KOG	TFs
Ca4_P3_8698891	Ca4	8698891	AAGAGTAGTAGAGTAGT/AAGAGTAGT	AAGCTGGCCACTGTAA GCAT	TCACAATCTCGACAAC CCAA	59.9	246	INTRON	Ca_08365	G	--
Ca4_P3_8703614	Ca4	8703614	CTCTTCTT/CTCTT	TTGGGTGTGGGGATTC TTAC	AATGCCCTGTTGGGTA CAAA	59.6	568	DRR	Ca_08366	J	NAC
Ca4_P3_8706854	Ca4	8706854	TAAAAA/TAAAAAA	CGAATTACGAAAAAGG CAGG	ATCTCTCCGACCATGT GGAA	59.7	935	INTERGENIC	--	--	--
Ca4_P3_8710207	Ca4	8710207	CAAAA/CAAA	GAGTTGGTGATTCTCT CAATGC	CGATGAATGTTCTAAT CAACGC	58.8	725	INTERGENIC	--	--	--
Ca4_P3_8711163	Ca4	8711163	CTTTTTTTTTTT/CTTTTTTTTT	CGCACCACTCTCATCA CCTA	CTTTGCCAACCAAACC TTGT	59.9	826	INTERGENIC	--	--	--
Ca4_P3_8711880	Ca4	8711880	ACTC/ACTCTC	CAAGGTTTGGTTGGCA AAGT	GGGGGTGTTTCCATGT TATG	60.0	429	INTERGENIC	--	--	--
Ca4_P3_8712182	Ca4	8712182	CAACAACAACAACAACAACAACAA/CAA CAACAACAACAACAACAACAACAA	ACTGCAGAGGGAGATG GAGA	CCCTTGATTGTCCTTG TGCT	59.9	190	CDS (large-effect mutations)	Ca_08367	R	C2H2
Ca4_P3_8727347	Ca4	8727347	TAAAAAAAAAAAA/TAAAAAAAAA	CGGGTCTCTTTGACTC TTCG	TCATCTTATTTTGGGCA TTCA	60.0	563	INTERGENIC	--	--	--
Ca4_P3_8728353	Ca4	8728353	TTTATT/TTTATTATT	AATCCAACGCTCTTTCA ATCA	GGAACCAAACCAGCAT GAAT	59.7	343	INTERGENIC	--	--	--
Ca4_P3_8730794	Ca4	8730794	ATTTTTTTTT/ATTTTTTTT	TTCACACCAACACCCTC AAA	ATTTTGGAAGAACAAT CCGC	60.0	517	INTERGENIC	--	--	--

INDEL marker IDs	Chromosomes/ unanchored scaffolds	Physical positions (bp)	InDels (*Kabuli* reference genome-CDC Frontier/PII)	Forward primers (5'-3')	Reverse primers (5'-3')	Anneling temperature (0C)	Expected amplified product size (bp)	Structural annotation		Functional annotation	
								Sequence components of *kabuli* genome	*Kabuli* gene accession IDs	NCBI-KOG	TFs
Ca4_P3_8731216	Ca4	8731216	TAAAAAAA/TAAAAAAAA	ATTCTGCGGATTGTTCTTCC	ACAATTGTGTGGGTGTGCAT	59.1	508	INTERGENIC	--	--	--
Ca4_P3_8733054	Ca4	8733054	TGGG/TGG	AAAAATCACCCAAAATTAACATCT	CACTTTCCTTGCTCCCTCTG	57.2	867	INTERGENIC	--	--	--
Ca4_P3_8741602	Ca4	8741602	T/TACATC	TGGTGAACACGTATGCCAGT	AACCTCCTTTTGACCCACAA	60.0	771	INTERGENIC	--	--	--
Ca4_P3_8741921	Ca4	8741921	AATA/AA	ATTGTTGCGGAAAAGTTTGA	AACCTCCTTTTGACCCACAA	58.3	408	INTERGENIC	--	--	--
Ca4_P3_8742911	Ca4	8742911	AAT/A	AGGTGCTCCACCAAAATCAG	CCACATCTTAATGTTTTGTTTGGT	60.1	547	INTERGENIC	--	--	--
Ca4_P3_8750740	Ca4	8750740	TAAAAAAA/TAAAAAAAAA	AGAATCAAAGAGCCAATGTGC	TTGGCATACTTCCCATCCAT	59.3	863	INTERGENIC	--	--	--
Ca4_P3_8753314	Ca4	8753314	TGCTGGAACGCTGGAAC/TGCTGGAAC	TTCTTTCCCAGTCATTTCGG	CCAAATGGAGCTTCCACCTA	60.0	841	INTRON	Ca_08371	--	NAC
Ca4_P3_8754248	Ca4	8754248	T/TCGTAGATGATG	CATGGAAAATTTGCGATCCT	TAAGAAGGAACTGCGCCACT	59.9	139	INTRON	Ca_08371	--	NAC
Ca4_P3_8757082	Ca4	8757082	GATGGATATGGATA/GATGGATA	TGAGTTTTTGGCCAATTCAA	GCAGGTGCAGAAAGATTACCA	59.1	605	DRR	Ca_08371	--	NAC
Ca4_P3_8757256	Ca4	8757256	CATATA/CATATATA	TGGATATGGTGCAGATACGAA	AGGTGCATGAGTATCAATGTCAA	59.0	595	DRR	Ca_08371	--	NAC

INDEL marker IDs	Chromosomes/ unanchored scaffolds	Physical positions (bp)	InDels (*Kabuli* reference genome-CDC Frontier/PII)	Forward primers (5'-3')	Reverse primers (5'-3')	Anneling temperature (0C)	Expected amplified product size (bp)	Structural annotation		Functional annotation	
								Sequence components of *kabuli* genome	*Kabuli* gene accession IDs	NCBI-KOG	TFs
Ca4_P3_8757388	Ca4	8757388	CTTTTTTTTTTTT/CTTTTTTTTT	AAAGATGACAATTGAGGCCA	GGTTGTTCAAGGTGCATGAG	58.1	804	DRR	Ca_08371	--	NAC
Ca4_P3_8758399	Ca4	8758399	TAA/TA	CATAGCAGCTTCGATGCAAA	TCACAATTTGACAACCACGG	60.1	297	INTERGENIC	--	--	--
Ca4_P3_8758495	Ca4	8758495	A/AG	TCACCATTCGTGAGTTGGAA	TGCTCATGTCTTCTAAAGATCCA	60.1	762	INTERGENIC	--	--	--
Ca4_P3_8762153	Ca4	8762153	ATTTTT/ATTTT	TCATAAAGCTGTTGCCAAAAA	ATAGAGTGGGGTGTTGCCAG	58.5	546	INTERGENIC	--	--	--
Ca4_P3_8762736	Ca4	8762736	ATTTTTT/ATTTTTTT	CTGGCAACACCCCACTCTAT	TTTCAAGCTAAATGGACCCG	60.0	617	INTERGENIC	--	--	--
Ca4_P3_8768314	Ca4	8768314	GTTTTTTTTT/GTTTTTTTTTTT	CTCACCCTTTCTCCTCCTCC	AACGTCGTCGTCATTCACTG	60.2	399	INTERGENIC	--	--	--
Ca4_P3_8769706	Ca4	8769706	AGTCT/A	TTAGCACCGGAACTTTCCAT	ATCGGAATCCAGGTGAGTTG	59.6	452	INTERGENIC	--	--	--
Ca4_P3_8774335	Ca4	8774335	CT/C	GACGATTGCGAATTTGAGGT	TGATTTATGTTTGTGGCGGA	60.1	295	DRR	Ca_08373	C	--
Ca4_P3_8774388	Ca4	8774388	TAAAAAA/TAAA	GACGATTGCGAATTTGAGGT	TGATTTATGTTTGTGGCGGA	60.1	295	DRR	Ca_08373	C	--
Ca4_P3_8775167	Ca4	8775167	ATT/ATTT	TGTTCCCTGCAATTCACATC	ACGGGTCTCGATCAATGTGT	59.5	360	DRR	Ca_08373	C	--

INDEL marker IDs	Chromosomes/ unanchored scaffolds	Physical positions (bp)	InDels (*Kabuli* reference genome-CDC Frontier/PII)	Forward primers (5'-3')	Reverse primers (5'-3')	Anneling temperature (0C)	Expected amplified product size (bp)	Structural annotation		Functional annotation	
								Sequence components of *kabuli* genome	*Kabuli* gene accession IDs	NCBI-KOG	TFs
Ca4_P3_8785179	Ca4	8785179	AGGTTTTATT/A	ACAATGACAGGCAAGGAAGG	GGAACTTTCCACACTCCCAA	60.1	274	DRR	Ca_08374	I	G2-like
Ca4_P3_8789366	Ca4	8789366	CAAAA/CAAA	TTCTTGGAAGATCCACAGCC	TCATTGATTGGGCTTTCCTC	60.2	719	INTRON	Ca_08375	R	NF-YA
Ca4_P3_8795294	Ca4	8795294	CT/CTAAATATGT	GAAAGACAGCACCCCAAAAA	TCAACCGTTGGAATATCACG	60.1	666	DRR	Ca_08376	--	M-type
Ca4_P3_8795841	Ca4	8795841	A/AC	GTGGTCATGTGACCCTCGAT	GTAGCCCATGCCCTCATAAA	60.8	873	DRR	Ca_08376	--	M-type
Ca4_P3_8816656	Ca4	8816656	AT/A	ATCGTTGTCGTAGATTGGGC	TTGGATCAAAATCTGAAGCG	60.0	789	INTERGENIC	--	--	--
Ca4_P3_8836388	Ca4	8836388	CTTG/CTTGTAGTTTTG	TGACCTGTAGGGCCGTAATC	TCACCAAATCGAAGCTCAAA	60.0	508	INTERGENIC	--	--	--
Ca4_P3_8840055	Ca4	8840055	TTAAT/TT	TCATTTAAGAATGCAAAATCACTTG	AGTCCACTTAGCCTGTCCGA	59.6	581	INTERGENIC	--	--	--
Ca4_P3_8841401	Ca4	8841401	ATTTTTTTT/ATTTTTTT	TTTGGTGACATTTTAGCAAAGAC	TCTTCTTCATTCACTTGTTTGTCA	58.4	229	INTERGENIC	--	--	--
Ca4_P3_8858467	Ca4	8858467	TTACTAA/TTACTAATATTATAATATACTAA	TTGGTTTCCAAGTTCCCATC	TTCAACTTCGAGCAAAAGGG	59.8	602	INTRON	Ca_08382	--	MIKC
Ca4_P3_8859654	Ca4	8859654	CAA/CAAA	TTCACCAAAGCCTCTTGCTT	TATGCGCTCGTTTTGAAGTG	60.0	540	INTERGENIC	--	--	--

INDEL marker IDs	Chromosomes/ unanchored scaffolds	Physical positions (bp)	InDels (*Kabuli* reference genome-CDC Frontier/PII)	Forward primers (5'-3')	Reverse primers (5'-3')	Anneling temperature (0C)	Expected amplified product size (bp)	Structural annotation		Functional annotation	
								Sequence components of *kabuli* genome	*Kabuli* gene accession IDs	NCBI-KOG	TFs
Ca4_P3_8860985	Ca4	8860985	TAAAAAAAA/TAAAAAAA	TGCAACCAACTCACATCCAT	TGCAACTTTTGAGATGCAATTT	60.0	324	DRR	Ca_08383	C	--
Ca4_P3_8864397	Ca4	8864397	A/ATT	TTCACCAAAGCCTCTTGCTT	GTAAAGCTCCCTTTCCCCAC	60.0	657	INTERGENIC	--	--	--
Ca4_P3_8864736	Ca4	8864736	TGGGG/TGG	TTTTTGGCTTATGGGGTGAG	TCAGCGTATTGCGAAACAAC	59.9	912	INTERGENIC	--	--	--
Ca4_P3_8867611	Ca4	8867611	AT/A	AGCTCCCATACCAGGACCTT	GTAGCCACCATTTTCCTCCA	60.0	374	INTERGENIC	--	--	--
Ca4_P3_8869508	Ca4	8869508	GC/G	CCCTCCCCTCAAAAGAAAAG	TGGAGGAGAAGTTTTGGGTG	60.0	653	INTERGENIC	--	--	--
Ca4_P3_8870614	Ca4	8870614	TAAAA/TAAA	AAAACATCGGAGTCCACGTC	TTTTGCCCTGTTTATTTTTCG	60.0	522	INTERGENIC	--	--	--
Ca4_P3_8871044	Ca4	8871044	CATAAAAT/CATAAAATTTATAAAAT	AAAACATCGGAGTCCACGTC	AATTTCATTGCGGCAAATTC	60.0	699	INTERGENIC	--	--	--
Ca4_P3_8876702	Ca4	8876702	A/AAGGAAT	ACTTCCCATGCCTTTGTGTC	TTGGTGGTGTTGATGCTGTT	60.0	393	INTRON	Ca_08385	U	B3
Ca4_P3_8880111	Ca4	8880111	AC/A	AAGGGACAGAGACGATCCAA	CATAATCGGCCTGATTGTTGT	59.7	830	INTERGENIC	--	--	--
Ca4_P3_8880284	Ca4	8880284	ATTTT/ATTTTT	CCAATGAAAATGGAACCAACA	TTTTTGTCATAATCGGCCTG	60.6	523	INTERGENIC	--	--	--

INDEL marker IDs	Chromosomes/ unanchored scaffolds	Physical positions (bp)	InDels (*Kabuli* reference genome-CDC Frontier/PII)	Forward primers (5'-3')	Reverse primers (5'-3')	Anneling temperature (0C)	Expected amplified product size (bp)	Structural annotation		Functional annotation	
								Sequence components of *kabuli* genome	*Kabuli* gene accession IDs	NCBI-KOG	TFs
Ca4_P3_8882798	Ca4	8882798	ACT/ACTAATGCT	CCAACTGCATTCGACGACTA	TTCCTCCATAAATTCAACATTGG	59.9	754	INTERGENIC	--	--	--
Ca4_P3_8884654	Ca4	8884654	ATTTTTTTT/ATTTTTTT	ACCTGACCTATTCCCATCCC	GTCGATCTCGCTCACTCCAC	60.0	866	INTERGENIC	--	--	--
Ca4_P3_8891264	Ca4	8891264	AAATTAATTAA/AAATTAA	AATTCAAAGTGCGAGTTGGG	CCCGATGTGTACCCTCTGAT	60.1	753	DRR	Ca_08386	--	--
Ca4_P3_8891981	Ca4	8891981	CAAA/CAA	ATACACCACAACGCGTCAAA	AAGCATGGTATTCGGGAGTG	60.0	204	DRR	Ca_08386	--	--
Ca4_P3_8892700	Ca4	8892700	AAGA/AA	CCTCAGCCGCTACTCAGCTA	CATGTCGTCAACCGTAATCG	60.8	958	DRR	Ca_08386	--	--
Ca4_P3_8893972	Ca4	8893972	GTTTTTT/GTTTTTTT	AGGTGTTTTGGTTGGAGGTG	GGAAAGCCAGCCATACTTCA	59.9	967	INTERGENIC	--	--	--
Ca4_P3_8894416	Ca4	8894416	CTATATATATATAT/CTATATATATATATATATAT	TGATATCCGGTTTGGGTGTT	CTCCCGTTATCCGAAACAGA	60.1	569	INTERGENIC	--	--	--
Ca4_P3_8895815	Ca4	8895815	G/GA	CCGCGTATTATGTCATGTTTTG	GCCTTTTGCAGTTTAGCGAC	60.3	691	INTERGENIC	--	--	--
Ca4_P3_8897355	Ca4	8897355	TA/T	GATCCACTCAGGGTCGTGTT	ACTTTTGCATCTGGATGGCT	60.0	461	INTERGENIC	--	--	--
Ca4_P3_8901196	Ca4	8901196	GT/GTTT	CCCTCCATTACAGGGGATTT	AAGCTCATCACCGATCATCC	60.0	813	INTERGENIC	--	--	--

INDEL marker IDs	Chromosomes/ unanchored scaffolds	Physical positions (bp)	InDels (*Kabuli* reference genome-CDC Frontier/PII)	Forward primers (5'-3')	Reverse primers (5'-3')	Anneling temperature (0C)	Expected amplified product size (bp)	Structural annotation		Functional annotation	
								Sequence components of *kabuli* genome	*Kabuli* gene accession IDs	NCBI-KOG	TFs
Ca4_P3_8901492	Ca4	8901492	TTTATATGTTATA/T	GCAACAAATTATCTTGCCGTC	AAGCTCATCACCGATCATCC	59.6	334	INTERGENIC	--	--	--
Ca4_P3_8904444	Ca4	8904444	CA/CTCTTAA	AAGAAAACCCACCACACTCG	ATGATTTCTGCATTCTCGGC	60.0	865	INTERGENIC	--	--	--
Ca4_P3_8907266	Ca4	8907266	ATATTATTATTATTATTATTATTATTATTATTATTATTATTATTATTATTATTATTATTATTATTATTATTATT/ATATTATTATTATTATTATTATTATTATTATTATTATTATTATTATTATTATTATT	TGCCAAGTAGCTGAACCACA	TTCATCCATCCCTGTCCATT	60.4	741	INTERGENIC	--	--	--
Ca4_P3_8946430	Ca4	8946430	TGAGAGAGAGAGAGA/TGAGAGAGAGAGAGAGA	ACAGCAACCGAAACTGCTTT	GGGGAGCATTGGTCACTAAA	59.9	432	INTERGENIC	--	--	--
Ca4_P3_89549	Ca4	89549	AAACAA/AAA	TGGATTGTCAGGACTGGATG	TCTGCCAAGTCACGAATCAC	59.5	979	INTERGENIC	--	--	--
Ca4_P3_9028821	Ca4	9028821	TTATATATATATATATATAT/TTATATATATATATATATATAT	ATGGAACAATCGGACACCAT	CTGTCAACTCACTCATCAAATCG	60.1	422	INTERGENIC	--	--	--
Ca4_P3_9030140	Ca4	9030140	ATTTTTTTTTTT/ATTTTTTTTTTTT	TGGTGTTGGAGTCATGCTTT	AAATTTGCAATGACGGCTTC	59.1	656	INTERGENIC	--	--	--
Ca4_P3_9041838	Ca4	9041838	AGTG/A	GAAGCAGACACCAAAATCAAAA	CCAAAGAGGTGAAACTGGGA	59.2	500	INTERGENIC	--	--	--
Ca4_P3_9066423	Ca4	9066423	ACC/AC	GCAACCCGTAGGTGTAGGAA	TGTGCGTTGGTAGTCAATGTG	60.0	546	INTERGENIC	--	--	--
Ca4_P3_9079145	Ca4	9079145	ATTTTTTTTTTT/ATTTTTTTTTT	AGTTGGAAGTCGCCAAAGAA	ATGGTGCAGTTCTTGCCATA	59.9	629	INTERGENIC	--	--	--

INDEL marker IDs	Chromosomes/ unanchored scaffolds	Physical positions (bp)	InDels (*Kabuli* reference genome-CDC Frontier/PII)	Forward primers (5'-3')	Reverse primers (5'-3')	Anneling temperature (0C)	Expected amplified product size (bp)	Structural annotation		Functional annotation	
								Sequence components of *kabuli* genome	*Kabuli* gene accession IDs	NCBI-KOG	TFs
Ca4_P3_9130579	Ca4	9130579	CATATATATATATAT/CATATATATATAT	AATTCTGACATGCGAATCCC	TGTAAAGCGCTTCTAAGTCTCG	59.9	589	INTERGENIC	--	--	--
Ca4_P3_9229798	Ca4	9229798	TGGG/TGGGG	AAGGCTCAACATGCAAAACC	TGCCAATTCGATTGGAATAA	60.1	522	INTERGENIC	--	--	--
Ca4_P3_9264863	Ca4	9264863	AT/A	CAACTTGAAAAGAACGGGAGA	TTTTAAGCCACACCACACCA	59.3	615	INTERGENIC	--	--	--
Ca4_P3_932693	Ca4	932693	ATATTATTATTATTATTATTATTATTATTATTATTATTATTATTATTATTATT/ATATTATTATTATTATTATTATTATTATTATTATTATTATTATTATTATTATT	GCAAAACATCCAAAACCCTG	CCTTGTGCTTCTCTTCTGCC	60.3	217	INTERGENIC	--	--	--
Ca4_P3_9339969	Ca4	9339969	TAAAAAAAAAAAA/TAAAAAAAAAAA	TGAGGCCCAGTTTCTTTCTG	TACACCCACATAGTGGGCAA	60.4	856	INTRON	Ca_08423	Q	E2F/DP
Ca4_P3_9351475	Ca4	9351475	TAAAAAAAAAAAAA/TAAAAAAAAAAAAAA	GGTGTTATCCCGCTCATGTT	CTATTAGCCTTGCTGAGCCC	59.8	683	DRR	Ca_08424	K	--
Ca4_P3_9358456	Ca4	9358456	ATTTTTTTTT/ATTTTTTTTTT	CAAGAGTGGACCGACATCCT	GCGGAGAAGACAGAGACGAT	60.1	123	INTERGENIC	--	--	--
Ca4_P3_9371255	Ca4	9371255	GAAAAAAAA/GAAAAAAAAA	GGAGCATATGCCTCGTTGTT	TGGAAATTCAACTTTTTGGACA	60.1	549	INTERGENIC	--	--	--
Ca4_P3_9407946	Ca4	9407946	ACTTTGTCTTTGTCT/ACTTTGTCTTTGTCTTTGTCT	GTTGTCAGCTTCCCGAAGAG	CTCGGATCCTTCATGAGTTTG	60.0	607	DRR	Ca_08430	A	--
Ca4_P3_9540231	Ca4	9540231	TTA/TTATTTCTTCTTAATA	ACCACATGGAAAAGCCAAAG	ACACCCTTCTCTCCCCATTT	60.0	363	INTRON	Ca_08439	--	bHLH

INDEL marker IDs	Chromosomes/ unanchored scaffolds	Physical positions (bp)	InDels (*Kabuli* reference genome-CDC Frontier/PII)	Forward primers (5'-3')	Reverse primers (5'-3')	Anneling temperature (0C)	Expected amplified product size (bp)	Structural annotation		Functional annotation	
								Sequence components of *kabuli* genome	*Kabuli* gene accession IDs	NCBI-KOG	TFs
Ca4_P3_9562230	Ca4	9562230	A/ATATATAT	TTGGTGAATGGTGAACCGTA	ATTAGTGGGTTGCTTGTGGC	59.8	773	DRR	Ca_08440	--	bHLH
Ca4_P3_9576810	Ca4	9576810	CGTCAGGG/CG	CCCAATTATAGCGTGGTGCT	AAAGTTTGGCCTGACTTGGA	60.0	790	INTERGENIC	--	--	--
Ca4_P3_9625757	Ca4	9625757	ATATTATTATTATTATTATTATTATTATTATT/ATATTATTATTATTATTATTATTATTATTATTATTATT	ATGTGCGAGAAAGGGAGAGA	CACTTCGGTCATTCTCTTCAAA	60.0	543	INTERGENIC	--	--	--
Ca4_P3_9630152	Ca4	9630152	GAA/GA	CCTCTTCCAAACAAATCTTCG	TCAAAATATGGCATGTGGGA	58.8	455	INTERGENIC	--	--	--
Ca4_P3_9630289	Ca4	9630289	ATTAGTTAGTT/ATTAGTTAGTTAGTT	CCTCTTCCAAACAAATCTTCG	CCAGCTCATCAAAGCCATTT	58.8	550	INTERGENIC	--	--	--
Ca4_P3_9632653	Ca4	9632653	TG/T	TGGATTGTCGTGATCTCAAA	CACACCCGTACTCAGCATTG	57.6	630	INTERGENIC	--	--	--
Ca4_P3_9633059	Ca4	9633059	TAAAAAAAAAAA/TAAAAAAAAA	CAATGCTGAGTACGGGTGTG	AACACAAGGAGGCCATGTTC	60.2	503	INTERGENIC	--	--	--
Ca4_P3_9704009	Ca4	9704009	AAGTTAGGAGTTAGGAGTTAGGAGTTAGGAGTTAGG/AAGTTAGGAGTTAGGAGTTAGGAGTTAGGAGTTAGGAGTTAGG	TTCCAAAAGCAAACACACCA	CTCTTTCTTGACCTGCCCTG	60.1	745	DRR	Ca_08447	Q	Nin-like
Ca4_P3_9722503	Ca4	9722503	AATATATATATATATATATATATATATATATAT/AATATATATATATATATATATATATATATATATATATATAT	AGTGTTTAACCCATGCGACC	TGTTTTGCCATTAGATACACCAA	59.9	549	INTERGENIC	--	--	--
Ca4_P3_9726284	Ca4	9726284	ATTTTTTTTTT/ATTTTTTTT	GGGCTTACCAAAAGCAGACA	GCGCCACCATGGACTACTAT	60.2	314	INTERGENIC	--	--	--

INDEL marker IDs	Chromosomes/ unanchored scaffolds	Physical positions (bp)	InDels (*Kabuli* reference genome-CDC Frontier/PII)	Forward primers (5'-3')	Reverse primers (5'-3')	Anneling temperature (0C)	Expected amplified product size (bp)	Structural annotation		Functional annotation	
								Sequence components of *kabuli* genome	*Kabuli* gene accession IDs	NCBI-KOG	TFs
Ca4_P3_9754081	Ca4	9754081	ATGT/ATGTTGT	TCACCGTGAAAACGATGAGA	CAAAAGCAAATCCTTTGGGA	60.2	362	INTERGENIC	--	--	--
Ca4_P3_978893	Ca4	978893	TAAAAAAAAA/TAAAAAAAA	TTTTGAGGTGCTTTCCCATC	CAATCCCCCAAGCTTAACAA	60.1	535	INTERGENIC	--	--	--
Ca4_P3_9797931	Ca4	9797931	CAAAAAAAAA/CAAAAAAAAAA	CTCGTAAAACACAAAGCGCA	TGAGTGTTCGACAACCAAGC	60.1	602	INTERGENIC	--	--	--
Ca4_P3_9894681	Ca4	9894681	CA/CAA	AAACTGAAAGCATTGGGTGG	TCAGACCGGAGGAATTATGC	60.0	738	DRR	Ca_08466	--	--
Ca4_P3_9967472	Ca4	9967472	ATTTTT/ATTTTTT	CCCCTTTTATGAAATGGTTGAA	TGAGACTGAAATCGGGTCTTTT	60.0	945	INTERGENIC	--	--	--
Ca4_P3_9992046	Ca4	9992046	AAGAGAGAGAGAGAGAGAGAGAGAGAGAGAGAG/AAGAGAGAGAGAGAGAGAGAGAGAGAGAGAGAG	CAGACCTCTGTGTCTCCGGT	GGTATGAGCCTACGTGGGAA	60.3	857	INTRON	Ca_08473	TO	--
Ca5_P3_10120706	Ca5	10120706	ATTTTTTTTT/ATTTTTTTTTT	ATTGCAAGAGAGGGGGAGAT	CAGTTGCAAGCATCTCCAAA	60.0	549	INTERGENIC	--	--	--
Ca5_P3_10335145	Ca5	10335145	GTTTTTTTTTTT/GTTTTTTTTTTTT	CTCAACCCCTACAAAACCGA	ATACGGGAAAAGGGGAATTG	60.0	396	INTRON	Ca_21237	O	E2F/DP
Ca5_P3_10419875	Ca5	10419875	AC/A	GGTACAATTGTTGCACCACG	TGTACATTGTGGCAGCTTGA	59.9	536	INTERGENIC	--	--	--
Ca5_P3_10420287	Ca5	10420287	GTTTTTTTTT/GTTTTTTTT	GGTACAATTGTTGCACCACG	TTTGGGAGTAGGCCTTCCTT	59.9	702	INTERGENIC	--	--	--

INDEL marker IDs	Chromosomes/ unanchored scaffolds	Physical positions (bp)	InDels (*Kabuli* reference genome-CDC Frontier/PII)	Forward primers (5'-3')	Reverse primers (5'-3')	Anneling temperature (0C)	Expected amplified product size (bp)	Structural annotation		Functional annotation	
								Sequence components of *kabuli* genome	*Kabuli* gene accession IDs	NCBI-KOG	TFs
Ca5_P3_10420664	Ca5	10420664	AATATATATATATATATATATA/AATATATATATATATATATATATA	AAGGAAGGCCTACTCCCAAA	TACCAAGGCGTCGTCAAAAT	60.1	597	INTERGENIC	--	--	--
Ca5_P3_10572522	Ca5	10572522	AATATATATATATATATATATATAT/AATATATATATATATATAT	CGTGGACAAAGATCGTGATG	TCATTGTTAAAGAGCGGTGC	60.1	382	INTERGENIC	--	--	--
Ca5_P3_10585651	Ca5	10585651	AG/A	GGGGGAGATTGTTGGATTTT	TCGACTTGTCAATCGCTTTG	60.0	634	INTERGENIC	--	--	--
Ca5_P3_10634338	Ca5	10634338	AC/ACC	AATTTTTCAGTCCCGTGTCG	ATCCCTCAAAGGGCTACTGC	60.0	229	INTERGENIC	--	--	--
Ca5_P3_10641003	Ca5	10641003	AGGGGG/AGGGGGG	CGCCACTGTCATAAGGGATT	CAAGTTGGGTGAACCAGGAT	60.0	520	INTERGENIC	--	--	--
Ca5_P3_1065182	Ca5	1065182	TAAAA/TAAAAA	AATGGCCACGAATCTGAAAG	ACCATTTTCAACTGCATCCC	60.1	634	INTERGENIC	--	--	--
Ca5_P3_10686981	Ca5	10686981	TAAAAAAAA/TAAAAAAAAAAA	GCTCCTTCTGCATCAACACA	TGCCCCACATTTTAGACCTC	60.0	509	INTERGENIC	--	--	--
Ca5_P3_10687869	Ca5	10687869	TAAAAAAAA/TAAAAAAAAA	CGAGGAACGACAAGGGTAAA	TGAGTTGAGTTAAAAACCAGCAGA	60.1	673	INTERGENIC	--	--	--
Ca5_P3_10688557	Ca5	10688557	AGTTGTT/AGTT	TCCATCAAACTGATCCTAAGGG	TGGGGTTTAGATGATGGCTC	60.3	179	INTERGENIC	--	--	--
Ca5_P3_10688722	Ca5	10688722	C/CCACAT	GAGCCATCATCTAAACCCCA	TTCAACTTGGAATTCGGAAAA	59.9	462	INTERGENIC	--	--	--

INDEL marker IDs	Chromosomes/ unanchored scaffolds	Physical positions (bp)	InDels (*Kabuli* reference genome-CDC Frontier/PII)	Forward primers (5'-3')	Reverse primers (5'-3')	Anneling temperature (0C)	Expected amplified product size (bp)	Structural annotation		Functional annotation	
								Sequence components of *kabuli* genome	*Kabuli* gene accession IDs	NCBI-KOG	TFs
Ca5_P3_10689102	Ca5	10689102	TAAAA/TAA	ATGGGCCACCTATTGAGACA	TGCCAAAGAAAAAGGTTTCG	60.3	623	INTERGENIC	--	--	--
Ca5_P3_10689356	Ca5	10689356	CT/C	CGAAACCTTTTTCTTTGGCA	GGATTGTCCCTTCATTGGTG	60.2	415	INTERGENIC	--	--	--
Ca5_P3_10689622	Ca5	10689622	AGAAGCATG/AG	CGAAACCTTTTTCTTTGGCA	AACGGATTGTCCCTTCATTG	60.2	418	INTERGENIC	--	--	--
Ca5_P3_10693789	Ca5	10693789	CTTGTGATTGTTTTGTTGTGATTG/CTTGTGATTG	CCAATTTGAATTGTCCGTCC	TTGCAAAAGCTGGGTCAAGT	60.2	406	INTERGENIC	--	--	--
Ca5_P3_10867305	Ca5	10867305	GC/G	TGTAATCATTTAGGGATGCATGA	GCACGTGCACATGTCTTACA	59.3	560	INTERGENIC	--	--	--
Ca5_P3_10955133	Ca5	10955133	CAAA/CAAAA	CCGGTGGATGAGAAGGAGTA	TTGGAGGAGGAGATTGGTTG	60.1	485	INTERGENIC	--	--	--
Ca5_P3_10980425	Ca5	10980425	TATAATAATAATAATAATAATAATAATAATAATAATAATAATAATAATAATAATAATAATAATAATAATAA/TATAATAATAATAATAATAATAATAATAATAATAATAATAATAATAATAATAATAATAATAATAATAATAATAATAAT[illegible]	AAAACCAATAATGGCAACGC	GGATTGGTTACGCTTGTTTGA	59.8	591	INTERGENIC	--	--	--
Ca5_P3_1101216	Ca5	1101216	GATTTGAAGAATATTTGAAGAA/GATTTGAAGAATATTTGAAGAATATTTGAAGAA	TATGTGGCGATGAAGTGAGG	TCGACCCTACAAGCCAAATC	59.7	724	INTERGENIC	--	--	--
Ca5_P3_11131260	Ca5	11131260	CA/CAAA	CAAGTGCACAGCGGAATTTA	GGTTGAGAGGGCTCAACAAG	59.9	325	INTERGENIC	--	--	--
Ca5_P3_11138840	Ca5	11138840	GT/G	TCGACTTGTCAATCGCTTTG	TCGCATTAACTACGAACGCA	60.0	676	INTERGENIC	--	--	--

INDEL marker IDs	Chromosomes/ unanchored scaffolds	Physical positions (bp)	InDels (*Kabuli* reference genome-CDC Frontier/PII)	Forward primers (5'-3')	Reverse primers (5'-3')	Anneling temperature (0C)	Expected amplified product size (bp)	Structural annotation		Functional annotation	
								Sequence components of *kabuli* genome	*Kabuli* gene accession IDs	NCBI-KOG	TFs
Ca5_P3_11168411	Ca5	11168411	AATA/AA	TTCAAACGGAACTGATGCTTT	TTGGTTGGGGGAGTTATTGA	59.7	954	INTERGENIC	--	--	--
Ca5_P3_11168707	Ca5	11168707	CTTTTTTTT/CTTTTTTTTTT	TCGTTGAGGAAAAACAAACAAA	TTGGTTGGGGGAGTTATTGA	59.6	557	INTERGENIC	--	--	--
Ca5_P3_11168737	Ca5	11168737	ATGT/A	TCGTTGAGGAAAAACAAACAAA	TTGGTTGGGGGAGTTATTGA	59.6	557	INTERGENIC	--	--	--
Ca5_P3_11168801	Ca5	11168801	ACTATTAGTCTATTA/ACTATTA	TCGTTGAGGAAAAACAAACAAA	TTGGTTGGGGGAGTTATTGA	59.6	557	INTERGENIC	--	--	--
Ca5_P3_11168904	Ca5	11168904	GTCTCTCT/GTCTCT	TCAATAACTCCCCCAACCAA	TCACAATTGGGTTTGGATCA	60.2	227	INTERGENIC	--	--	--
Ca5_P3_11169035	Ca5	11169035	CG/C	TCAATAACTCCCCCAACCAA	TGACGCCGTGAAAAACATTA	60.2	626	INTERGENIC	--	--	--
Ca5_P3_11169613	Ca5	11169613	C/CG	TGAAGCAGTTGTTTGGGTGA	GAGCCCAAATCCAAAATTCA	60.3	225	CDS (FRAME SHIFT)	Ca_23744	A	--
Ca5_P3_11291761	Ca5	11291761	TA/TAAGTCAACA	TTTGGTCCTTGTAACACCCC	GCGTGAAGATCATCTGGGAT	59.7	367	INTERGENIC	--	--	--
Ca5_P3_11414411	Ca5	11414411	A/AACCT	TCTGAATCGGAGTACCCCAC	AATGGCTTTCCTTCGTGATG	59.9	770	INTERGENIC	--	--	--
Ca5_P3_11420782	Ca5	11420782	TTA/TTACTA	ATTAAGTGAATGGATCGCGG	AGTGTGGAAGGACGAGCAAG	59.9	485	INTERGENIC	--	--	--

INDEL marker IDs	Chromosomes/ unanchored scaffolds	Physical positions (bp)	InDels (*Kabuli* reference genome-CDC Frontier/PII)	Forward primers (5'-3')	Reverse primers (5'-3')	Anneling temperature (0C)	Expected amplified product size (bp)	Structural annotation		Functional annotation	
								Sequence components of *kabuli* genome	*Kabuli* gene accession IDs	NCBI-KOG	TFs
Ca5_P3_11494502	Ca5	11494502	AATATATATATATATATATATATATATATATATATAT/AATATATATATATATATATATATATATATATATATATATATAT	AAACCTACCAATTGGCATCC	CTTCGGGTTGTTTGAAAAGG	58.8	594	INTERGENIC	--	--	--
Ca5_P3_11517113	Ca5	11517113	T/TA	TCGATTATGCGTAGGGAAGG	CGGGAGATACGTAGGAGCAA	60.1	248	INTERGENIC	--	--	--
Ca5_P3_115778	Ca5	115778	TTCACCGGTCA/TTCA	AAAGATTGCAATTTGCCTGC	TTTTTAGGGGGCGATTGTATT	60.2	511	INTERGENIC	--	--	--
Ca5_P3_11587595	Ca5	11587595	TTATATATATATATATATAT/TTATATATATATATATAT	TCGAACTACCTTCCCCCTTT	TTGTTGGAGTGGTTTTGACG	59.9	756	DRR	Ca_23350	--	Trihelix
Ca5_P3_11666989	Ca5	11666989	T/TGTTTGAC	GCCAATAGAGGATGGAAGCA	TATGCCAAAGCCATGTTTCA	60.2	533	INTERGENIC	--	--	--
Ca5_P3_11729570	Ca5	11729570	AAATAATAATAA/AAATAATAA	CACGGTCCCTAGAGCCAATA	CGGGAGATACGTAGGAGCAA	60.1	672	INTERGENIC	--	--	--
Ca5_P3_11807512	Ca5	11807512	ATTTTTTTTTTTT/ATTTTTTTTTTT	CCTAGTGCTGCTCTGCCTTT	ACCTACCAATTTTTCGCACG	59.8	428	INTERGENIC	--	--	--
Ca5_P3_11878820	Ca5	11878820	CATATATATATATATATATATATATATAT/CATATATATATATATATATATATATAT	TATTTTGGATTTTGGCTCGG	AGAGGGGTTTGTGTTTGACG	59.9	599	INTERGENIC	--	--	--
Ca5_P3_11879038	Ca5	11879038	CG/C	AAGATTTCCTCCCTCCTCCA	CTTTAACATGCGTTTCCGGT	60.0	327	INTERGENIC	--	--	--
Ca5_P3_11879108	Ca5	11879108	TAA/TAAA	AAGATTTCCTCCCTCCTCCA	CTTTAACATGCGTTTCCGGT	60.0	327	INTERGENIC	--	--	--

INDEL marker IDs	Chromosomes/ unanchored scaffolds	Physical positions (bp)	InDels (*Kabuli* reference genome-CDC Frontier/PII)	Forward primers (5'-3')	Reverse primers (5'-3')	Anneling temperature (0C)	Expected amplified product size (bp)	Structural annotation		Functional annotation	
								Sequence components of *kabuli* genome	*Kabuli* gene accession IDs	NCBI-KOG	TFs
Ca5_P3_11947363	Ca5	11947363	TAAAAAA/TAAAAAAA	TCCACTGTGCCACATCATTT	TGTGTTTGTGGTGCGATTTT	60.0	729	INTERGENIC	--	--	--
Ca5_P3_11983861	Ca5	11983861	G/GATCTTCGATCCT	CTGCTGTCAACTCCACTCCA	TCAAAATCCCCCAATAACCA	60.0	713	DRR	Ca_17106	--	--
Ca5_P3_12095777	Ca5	12095777	CAAAAAAAAA/CAAAAAA	CTCTTTAAGACGTTTCGCGG	TGCTCGCCTAATTTTCGAGT	60.0	461	INTERGENIC	--	--	--
Ca5_P3_1230643	Ca5	1230643	TT/TTCCGT	ACGAAATCGAAGAGAAGCGA	TCCAGGACACAAACAATCCA	60.1	455	INTERGENIC	--	--	--
Ca5_P3_12448859	Ca5	12448859	ATTTT/ATTTTT	TTCTACCCGAAATTTGTGCC	GTCAGTCAGCCTCTCTTGCC	59.9	361	INTERGENIC	--	--	--
Ca5_P3_12635364	Ca5	12635364	CAAAAAAAAA/CAAAAAAAAAAA	TGGAACCAAACACACACCAT	AGCGAAAAGAAGATTGGTCG	59.7	642	INTERGENIC	--	--	--
Ca5_P3_12650184	Ca5	12650184	TGG/TG	CTTGCCCAGGCAATAACATC	AGGTTAAATGCGGGTGTGAG	60.5	392	INTRON	Ca_17067	T	Dof
Ca5_P3_1301357	Ca5	1301357	AGGG/AGG	AGGGATAACCTGGTGCTGTG	GCTGTCGAGTTCCCAACAAT	60.0	597	INTERGENIC	--	--	--
Ca5_P3_131995	Ca5	131995	TAAAAAAA/TAAAAAAAA	ATCCAAGTATCCAACGGCTG	CACCTCTGCCCACTTCATTT	60.0	493	INTERGENIC	--	--	--
Ca5_P3_132937	Ca5	132937	CAA/CAAA	CCAAAGACACAGCAAAGCAA	CACTTCCACCAGTTGCAAAA	60.0	569	INTERGENIC	--	--	--

INDEL marker IDs	Chromosomes/ unanchored scaffolds	Physical positions (bp)	InDels (*Kabuli* reference genome-CDC Frontier/PII)	Forward primers (5'-3')	Reverse primers (5'-3')	Anneling temperature (0C)	Expected amplified product size (bp)	Structural annotation		Functional annotation	
								Sequence components of *kabuli* genome	*Kabuli* gene accession IDs	NCBI-KOG	TFs
Ca5_P3_133428	Ca5	133428	GAACA/GAACAATTTTTATAATAACA	ACCTTTACATGGTGCCAAGC	GCGTTGTGAAAACAGGACAA	60.0	433	INTERGENIC	--	--	--
Ca5_P3_133648	Ca5	133648	GAAAAA/GAAAA	TTGTCCTGTTTTCACAACGC	TCAACAATTTGTGGGCTTCA	59.7	574	INTERGENIC	--	--	--
Ca5_P3_133927	Ca5	133927	ATTT/ATTTT	TTGTCCTGTTTTCACAACGC	TCAACAATTTGTGGGCTTCA	59.7	574	INTERGENIC	--	--	--
Ca5_P3_133972	Ca5	133972	CAAAAAA/CAAAAA	TTGTCCTGTTTTCACAACGC	TCAACAATTTGTGGGCTTCA	59.7	574	INTERGENIC	--	--	--
Ca5_P3_134014	Ca5	134014	TA/T	CAGGACCGGTTTCTTACTGA	TCAACAATTTGTGGGCTTCA	57.8	265	INTERGENIC	--	--	--
Ca5_P3_13615970	Ca5	13615970	CT/CTT	CGAGTGACTGCAAGGATCAA	ATCCAACTGAGGCCATGAAC	60.0	547	INTERGENIC	--	--	--
Ca5_P3_13624288	Ca5	13624288	ATTTT/ATTTTTT	TGAAAATGTTGTTGGTAGTGGC	CGACGGTGCCAATTTTCTAT	59.9	948	INTERGENIC	--	--	--
Ca5_P3_13628890	Ca5	13628890	TTGT/TT	CAAAGCATAGATCGGACCGT	CAACAATGACGCAACCATTT	60.1	799	INTERGENIC	--	--	--
Ca5_P3_13636401	Ca5	13636401	A/AC	ATCGACAAACCAACATGCAA	GGGAAACTCACTTGCGGTTA	60.0	735	INTERGENIC	--	--	--
Ca5_P3_13636921	Ca5	13636921	CTGT/CT	TAACCGCAAGTGAGTTTCCC	GCGGTGGCTGAGTTTCTTAC	60.1	387	INTERGENIC	--	--	--

INDEL marker IDs	Chromosomes/ unanchored scaffolds	Physical positions (bp)	InDels (*Kabuli* reference genome-CDC Frontier/PII)	Forward primers (5'-3')	Reverse primers (5'-3')	Anneling temperature (0C)	Expected amplified product size (bp)	Structural annotation		Functional annotation	
								Sequence components of *kabuli* genome	*Kabuli* gene accession IDs	NCBI-KOG	TFs
Ca5_P3_136504	Ca5	136504	AAAAAAGAAAAAGAAAA/AAAAAAGAAAAAGAAAAAGAAAAAGAAAA	TTTCTAACCCGGAACACGTC	ATTCCTGCACACCACTTTCC	60.0	189	INTERGENIC	--	--	--
Ca5_P3_13702174	Ca5	13702174	ATTTTTTTTTTT/ATTTTTTTTTTTT	ACAGCCCAGGTGTATTTTGC	ATGGATGCAATGAACATGGA	60.0	341	INTERGENIC	--	--	--
Ca5_P3_1377883	Ca5	1377883	TCC/TC	GACTCTTTCCATAGCGTGCC	CACCTTGTAGCCAACGGTTT	59.8	720	INTERGENIC	--	--	--
Ca5_P3_13858932	Ca5	13858932	A/AC	GATTCCCCATTCTTAACGCC	TCGCGAAGAGATAGAAGGGA	60.6	554	INTERGENIC	--	--	--
Ca5_P3_13970765	Ca5	13970765	T/TG	TATTTGGACAAACGAAGGGC	CCCCTTTTGCTTGTTGGTTA	59.9	282	INTERGENIC	--	--	--
Ca5_P3_13971864	Ca5	13971864	AATATATATATATATATATATATATATA/AATATATATATATATATATATATATATATA	ATGGAGACTTTCTGCAGCGT	TCCTCCGGCTGCTAAATAAA	60.0	691	INTERGENIC	--	--	--
Ca5_P3_14015904	Ca5	14015904	AAGAGAGAGAGAGAGAGAGAGAGAGAGAGAGAGAGAGAGAGAGAGA/AAGAGAGAGAGAGAGAGAGAGAGAGAGAGAGAGAGAGAGAGAGA	ATAGCTGTGAGGGGGTGATG	TGTGCCTACAACCAACCAAA	60.0	490	INTERGENIC	--	--	--
Ca5_P3_14022502	Ca5	14022502	ATTT/ATTTT	GGAAATGGATGTCTGCCAAT	TTGCAATCTCATCAACTCGG	59.8	318	INTERGENIC	--	--	--
Ca5_P3_14022676	Ca5	14022676	T/TC	CGGACCGAGTTGATGAGATT	TGCTTTGAAGACCATGTGGA	60.1	618	INTERGENIC	--	--	--
Ca5_P3_14153085	Ca5	14153085	TAAAA/TAAA	TGAAAATTCCAAGGCACACA	GCAAGAAAAGCTTGTGAATGTTT	60.1	484	INTERGENIC	--	--	--

INDEL marker IDs	Chromosomes/ unanchored scaffolds	Physical positions (bp)	InDels (*Kabuli* reference genome-CDC Frontier/PII)	Forward primers (5'-3')	Reverse primers (5'-3')	Anneling temperature (0C)	Expected amplified product size (bp)	Structural annotation		Functional annotation	
								Sequence components of *kabuli* genome	*Kabuli* gene accession IDs	NCBI-KOG	TFs
Ca5_P3_14223524	Ca5	14223524	TAAAAAAA/TAAAAAA	TTGGATTTTGGGTAGTGGGA	TTTGAATGTGAAAAGGAACAGTG	60.2	805	INTERGENIC	--	--	--
Ca5_P3_14300751	Ca5	14300751	G/GA	TTTGCGTTACGAATTTGATGA	AAATGAACGAATTTGCACTCG	59.2	595	INTERGENIC	--	--	--
Ca5_P3_14394852	Ca5	14394852	CTTTTT/CTTTT	TCTTCCACCCAGTTTTCACC	TTGAGGGCTATGTTTTTCGG	59.9	337	INTERGENIC	--	--	--
Ca5_P3_1457283	Ca5	1457283	GA/GAA	TTTTCCACAAAATCCAACCA	TACGGTGCTAGGGAACCAAC	58.8	274	INTERGENIC	--	--	--
Ca5_P3_149074	Ca5	149074	TT/TTAT	TTCGGTTGGTTTTGCAATTT	TGCGGAGTTGGATCTCTCTT	60.3	200	INTERGENIC	--	--	--
Ca5_P3_151448	Ca5	151448	TAT/TTTATATTGAT	ATGACTGGGTGGCAAAACTC	CATTTTGGCGGTACTCGTTT	60.0	669	INTERGENIC	--	--	--
Ca5_P3_15219291	Ca5	15219291	AATCAAGTGATCAAGTGATCAA/AATCAAGTGATCAAGTGATCAAGTGATCAA	TGTGTTAGTCGTGTTTGGCA	CCTTTAGATTTTGCATCGCC	58.8	378	DRR	Ca_21632	--	Trihelix
Ca5_P3_15358764	Ca5	15358764	CTTTTTTTTTTT/CTTTTTTTTTT	AAAGGCAAGGCTCGTTACAA	TTGTGCTATGGTGCTTTTGC	59.9	392	INTERGENIC	--	--	--
Ca5_P3_15444111	Ca5	15444111	TTCTCTCTCTCTCTCTCTCTC/TTCTCTCTCTCTCTCTC	CATGGTTCTTGGTCTGGCTT	CAACCACTAGACAGGCAGCA	60.1	367	INTERGENIC	--	--	--
Ca5_P3_154620	Ca5	154620	CATATATATATATATAT/CATATATATATATAT	CTCGACACCTTGAGACGTGA	CAAGCGTGAGATGAGCAAGA	60.0	492	INTERGENIC	--	--	--

INDEL marker IDs	Chromosomes/ unanchored scaffolds	Physical positions (bp)	InDels (*Kabuli* reference genome-CDC Frontier/PII)	Forward primers (5'-3')	Reverse primers (5'-3')	Anneling temperature (0C)	Expected amplified product size (bp)	Structural annotation		Functional annotation	
								Sequence components of *kabuli* genome	*Kabuli* gene accession IDs	NCBI-KOG	TFs
Ca5_P3_15699246	Ca5	15699246	T/TAC	GCAGCTGCGAAGCTAACTTAAT	CTAATCCACAGGACGAAGCC	60.2	900	INTERGENIC	--	--	--
Ca5_P3_15756736	Ca5	15756736	GACAC/GAC	GCGCGATTTACCAAAAATGT	TTTGAGTGGTGGGTTGTGAA	60.0	431	INTERGENIC	--	--	--
Ca5_P3_15773222	Ca5	15773222	TT/TTAT	GCCTTAAAATTTGGCAATGG	TCTTCGCAAATCAAAAGAATGA	59.4	807	INTERGENIC	--	--	--
Ca5_P3_1586054	Ca5	1586054	TAAAA/TAAA	CTGTTTTCTACCCCCACCCT	AGCGTTGTAAAAGATGCGCT	60.2	727	INTERGENIC	--	--	--
Ca5_P3_15871940	Ca5	15871940	ATTTTTT/ATTTTT	TGAGGATGAATGCATGGAAG	TTGAAATGAGCATGATGGGA	59.6	163	INTERGENIC	--	--	--
Ca5_P3_158794	Ca5	158794	TAAAAAA/TAAAAA	TGATGCGTCATGGACATAAA	TTTTAAAAATGTCCATTTACACCG	58.5	498	INTERGENIC	--	--	--
Ca5_P3_1591818	Ca5	1591818	ATTTTTTTTTT/ATTTTTTTTT	CTGACATCGCATTTCGCTTA	GCCCTCAGATGAGAGTCAGG	60.0	788	INTERGENIC	--	--	--
Ca5_P3_15929124	Ca5	15929124	ATAT/ATATGTAT	TGATGACGAAGACGAAGACG	AACACATCCGGGATCCATTA	60.0	416	INTERGENIC	--	--	--
Ca5_P3_15929247	Ca5	15929247	AACCAC/AAC	TGATGACGAAGACGAAGACG	AACACATCCGGGATCCATTA	60.0	416	INTERGENIC	--	--	--
Ca5_P3_16152519	Ca5	16152519	AG/AGTG	CTGCCCACCGTTGTTAATCT	CTGGGAATCGTTCAAAAGGA	60.0	369	INTERGENIC	--	--	--

INDEL marker IDs	Chromosomes/ unanchored scaffolds	Physical positions (bp)	InDels (*Kabuli* reference genome-CDC Frontier/PII)	Forward primers (5'-3')	Reverse primers (5'-3')	Anneling temperature (0C)	Expected amplified product size (bp)	Structural annotation		Functional annotation	
								Sequence components of *kabuli* genome	*Kabuli* gene accession IDs	NCBI-KOG	TFs
Ca5_P3_16251480	Ca5	16251480	T/TAGTGTAGAAAGAGTG	TCAGAATCAACCGAGTTCCC	TAAACACACTCGTCCCCACA	60.1	249	INTERGENIC	--	--	--
Ca5_P3_162548	Ca5	162548	TTTATT/TTT	TGTCCTGTGCAAGAGAGGC	TCCATGACAGTTCAAGGATCTC	60.1	456	INTERGENIC	--	--	--
Ca5_P3_16319965	Ca5	16319965	TGGG/TGGGG	TTGTGTGCAGGATCCACATT	TTGACCATTGCACGAAACAT	60.0	502	INTERGENIC	--	--	--
Ca5_P3_1633237	Ca5	1633237	CAAAAAAAAAAAA/CAAAAAAAAAAA	TGCATTGTGCTAGATGGTCTT	TGTTCCGATTGTATTTTGAGGA	58.4	699	INTERGENIC	--	--	--
Ca5_P3_16340358	Ca5	16340358	AATATATATATATATATATATAT/AATATATATATATATATAT	ACTCGGATGCTTTTTAGCCA	GTTGGTTCCCTAGCACCGTA	59.8	626	INTERGENIC	--	--	--
Ca5_P3_16363340	Ca5	16363340	GTT/GTTT	AATCCTGGTGCACGATTTTC	ACATCGCCATTACCGAACTC	59.9	445	DRR	Ca_17921	R	MYB_related
Ca5_P3_166014	Ca5	166014	AAA/AAATTAA	GAGTGTGGGCAAACCAAAAT	CGCTGCACGGTAATTAGGAT	59.8	559	INTRON	Ca_18171	--	NAC
Ca5_P3_166515	Ca5	166515	TTAATAATAATAAT/TTAATAATAATAATAAT	ATTCTGCAGGAGGCAACAGT	TTGGATTCCAGTCCCATAGC	59.9	285	CDS (large-effect mutations)	Ca_18171	--	NAC
Ca5_P3_16715406	Ca5	16715406	GAAAAAAAAA/GAAAAAAAAAA	TTGCGTTACGGATTTGATGA	GCTAAATTACATTCGTGGTCCC	60.1	240	INTERGENIC	--	--	--
Ca5_P3_16765808	Ca5	16765808	TAGA/TA	CGATCGTGCTACGTCTTCAA	TGGTGGGAATGTTGTGATTG	60.0	677	DRR	Ca_17941	R	HSF

INDEL marker IDs	Chromosomes/ unanchored scaffolds	Physical positions (bp)	InDels (*Kabuli* reference genome-CDC Frontier/PII)	Forward primers (5'-3')	Reverse primers (5'-3')	Anneling temperature (0C)	Expected amplified product size (bp)	Structural annotation		Functional annotation	
								Sequence components of *kabuli* genome	*Kabuli* gene accession IDs	NCBI-KOG	TFs
Ca5_P3_1678181	Ca5	1678181	TTATATATATAT/TTATATATATATATAT	CGGTGAATCCTTTACGGAGA	AGGAAGAGTGGAGTTGCGAA	60.1	384	INTERGENIC	--	--	--
Ca5_P3_16881790	Ca5	16881790	GATATATATATATATATATATAT/GATATATATATATATATATATATAT	AATCAATCACAACCGTTAAATCA	CACGTTGCTCTTGGTCTTGA	58.4	902	INTERGENIC	--	--	--
Ca5_P3_168831	Ca5	168831	GTGGTTGGTT/GTGGTT	CGAGATACGCGTCATTAAAAA	TCGTCGAGAACAAGTGCATC	58.0	358	INTERGENIC	--	--	--
Ca5_P3_16948578	Ca5	16948578	CATATATATATATATATAT/CATATATATATATATATATAT	GTTTTCGAATCAACGAGGGA	GCTCAGAGGCTTTTTAGCCA	60.1	509	INTERGENIC	--	--	--
Ca5_P3_16961163	Ca5	16961163	TTTATTATTATTATTATTATTATTATTATTATTATTATTATTATTAT/TTTATTATTATTATTATTATTATTATTATTATTATTATTAT	TCAATCCGTTTCTTTTCGGA	TTGAAGCCGAGAAAACGACT	60.6	672	INTERGENIC	--	--	--
Ca5_P3_17015113	Ca5	17015113	TTTATTATTATTATTATTATTATTATTATTATTATTATTATT/TTTATTATTATTATTATTATTATTATTATTATTATTATTATT	TGAAAATGGAGAAGGATGGG	TCCTCCAAGTTCCAACAACA	59.9	457	INTERGENIC	--	--	--
Ca5_P3_17048541	Ca5	17048541	AATATATATATATATATATATATAT/AATATATATATATATATATATATAT	GGTCAGCATTTACCCCATGA	TTTCTCCACCTTCCTTGGTG	60.7	534	INTERGENIC	--	--	--
Ca5_P3_171078	Ca5	171078	TAAA/TA	AATGGTTTTCAGAGCGGAGA	GCAAATTTTGTGTGTGCTTTG	59.8	723	INTERGENIC	--	--	--
Ca5_P3_17244450	Ca5	17244450	AG/A	GTACCCTCCTCCTCGGTCTC	TTGTGTTTCAACAACTCCCAA	60.1	729	INTERGENIC	--	--	--
Ca5_P3_17314539	Ca5	17314539	TAAAAAAAAAA/TAAAAAAAAAAA	TCGATTATGCGTAGGGAAGG	TTGCGACAACTTGGTGATTC	60.1	824	INTERGENIC	--	--	--

INDEL marker IDs	Chromosomes/ unanchored scaffolds	Physical positions (bp)	InDels (*Kabuli* reference genome-CDC Frontier/PII)	Forward primers (5'-3')	Reverse primers (5'-3')	Anneling temperature (0C)	Expected amplified product size (bp)	Structural annotation		Functional annotation	
								Sequence components of *kabuli* genome	*Kabuli* gene accession IDs	NCBI-KOG	TFs
Ca5_P3_17448710	Ca5	17448710	CACTATACAA/CACTATACAACTATACAA	TGTGGATGTTCAAGGGACAA	ACAAATGGCGTATGCCTCTC	59.9	864	INTERGENIC	--	--	--
Ca5_P3_17494887	Ca5	17494887	CTGA/CTGACCTCCTATATGTGA	CTACGCCAAGAAACTGCTCC	GTACGCGTCCAGACATAGCA	60.0	245	INTERGENIC	--	--	--
Ca5_P3_17526738	Ca5	17526738	T/TG	GATTGGGGGAAGATGAGGAT	TTTGCGTTACGAATTTGATGA	60.1	465	INTERGENIC	--	--	--
Ca5_P3_17612584	Ca5	17612584	CATTCATAAAAATTCA/CATTCATAAAAATTCATAAAAATTCA	GTCTCTTGCTCTCCCACCAA	TTTATGTGGTGAATTGGCGA	60.4	769	INTERGENIC	--	--	--
Ca5_P3_17733319	Ca5	17733319	A/ATCGGTTCAGC	CGCGATGTCGTCTAAAGTGA	GAGGGGAGGGTTTTTGCTAC	60.0	583	INTERGENIC	--	--	--
Ca5_P3_17763617	Ca5	17763617	TTTATTATTATTATTATTATTATTATTATTATTATTATTATTATTATT/TTTATTATTATTATTATTATTATTATTATTATTATTATTATTATT	TCAGGGGCCTATGATTTGAC	TGTGCAACTTTGCAACCTAGA	59.9	462	INTERGENIC	--	--	--
Ca5_P3_17837198	Ca5	17837198	GAAA/GAAAAA	TACGTGTTAACGTGGTCGGA	CCCGCCTGACAGTTAATCAT	60.0	372	INTERGENIC	--	--	--
Ca5_P3_178618	Ca5	178618	TAAAAAAAAAAA/TAAAAAAAAAA	TGCTTTCGTCAACCTCAGTG	TCTCTTGCGTCTGCTGCTAA	60.0	717	INTRON	Ca_18172	J	--
Ca5_P3_178743	Ca5	178743	CCTAA/CCTAACTACTCCACTAA	TGCTTTCGTCAACCTCAGTG	TCTCTTGCGTCTGCTGCTAA	60.0	717	INTRON	Ca_18172	J	--
Ca5_P3_17875475	Ca5	17875475	GAAAAAAAA/GAAAAAAAAA	TGCAGAACCTTTCGCTTCTT	CACATTTGGAGGCAAAATCA	60.1	385	INTERGENIC	--	--	--

INDEL marker IDs	Chromosomes/ unanchored scaffolds	Physical positions (bp)	InDels (*Kabuli* reference genome-CDC Frontier/PII)	Forward primers (5'-3')	Reverse primers (5'-3')	Anneling temperature (0C)	Expected amplified product size (bp)	Structural annotation		Functional annotation	
								Sequence components of *kabuli* genome	*Kabuli* gene accession IDs	NCBI-KOG	TFs
Ca5_P3_17876530	Ca5	17876530	CAA/CAAA	TTTCATCCATCACCGACAAA	CTCAACGAATGCCTGAAACA	59.9	573	INTERGENIC	--	--	--
Ca5_P3_17876693	Ca5	17876693	CACAAA/CAA	TGTTTCAGGCATTCGTTGAG	TCAATACCATTTTCACCGCA	59.8	471	INTERGENIC	--	--	--
Ca5_P3_17876974	Ca5	17876974	ATTTTT/ATTTTTTT	TGTTTCAGGCATTCGTTGAG	TCAATACCATTTTCACCGCA	59.8	471	INTERGENIC	--	--	--
Ca5_P3_1794712	Ca5	1794712	AATTATTATTATTATTATTATTATTATTATTATTATTATTATTATTATT/AATTATTATTATTATTATTATTATTATTATTATTATTATTATT	GAGAAGGCGAGAAGGAAGGT	GCAAGGTTTAGGTGGGTCAA	60.0	605	INTERGENIC	--	--	--
Ca5_P3_179816	Ca5	179816	ACC/ACCC	GCTTCCAAACCAGCAGAGTC	AAGTGTGTGCAGGCCGTAG	60.0	542	INTERGENIC	--	--	--
Ca5_P3_18015209	Ca5	18015209	TAAA/TAA	GCTTATCCCGGAATTTGACA	ATCGCATTCAGACGACAAAA	59.9	759	DRR	Ca_22851	--	--
Ca5_P3_18049660	Ca5	18049660	CATATATATATAT/CATATATATAT	GCACGTTGTAAAGAGTGGACTG	AAAAGGCCCACAAGAGTTGA	59.8	694	INTERGENIC	--	--	--
Ca5_P3_180539	Ca5	180539	CTTTTTTTTTT/CTTTTTTTTT	TGGATACATGTTTAATGGTGCTT	TGACAAATTTGATTGGAGAGTCA	58.4	673	INTERGENIC	--	--	--
Ca5_P3_18179865	Ca5	18179865	CAAAAAAAAAAAA/CAAAAAAAAAAA	GGCAAAATATCTTTCATTCCG	TCTGAACAGTGTTGCCACAGA	58.6	352	INTERGENIC	--	--	--
Ca5_P3_18191704	Ca5	18191704	ATTTTTTTT/ATTTTTTTTT	TGAAATCCGCCTAAATCCTG	TAATTGGGGAACCAAAAAGC	60.0	715	INTERGENIC	--	--	--

INDEL marker IDs	Chromosomes/ unanchored scaffolds	Physical positions (bp)	InDels (*Kabuli* reference genome-CDC Frontier/PII)	Forward primers (5'-3')	Reverse primers (5'-3')	Anneling temperature (0C)	Expected amplified product size (bp)	Structural annotation		Functional annotation	
								Sequence components of *kabuli* genome	*Kabuli* gene accession IDs	NCBI-KOG	TFs
Ca5_P3_18299239	Ca5	18299239	GTTTTTTTTT/GTTTTTTTTTT	ATCACAAATTTTCGCGACCT	CGGGATTGTGAAGAAGTGGT	59.6	880	INTERGENIC	--	--	--
Ca5_P3_18539073	Ca5	18539073	ATTTTT/ATTTT	ACACTGAGACGGCGAAAATC	GAACCGGTGACCTTCAGTGT	60.3	739	INTERGENIC	--	--	--
Ca5_P3_18593864	Ca5	18593864	GTTTTTTTTTT/GTTTTTTTT	GGTCTGTCCAATCAGACCGT	TCCATTAAGCCCCTCAACAA	60.0	600	INTERGENIC	--	--	--
Ca5_P3_18612826	Ca5	18612826	GTATATATATATATATATATATATATATAT/GTATATATATATATATATATATATATATATATATATATAT	GCAATTAAAGTTGCCTTGGAA	TGCATAGGTCCCATAAAAACG	59.2	374	DRR	Ca_24762	QR	B3
Ca5_P3_18679735	Ca5	18679735	AC/ACCAGTCCC	TCCCTTTCCAGAAAATTCCA	TAAGGGGCACATTTTCGAGT	59.5	625	INTERGENIC	--	--	--
Ca5_P3_18683897	Ca5	18683897	AGCG/AGCGCGAGTCATCAGCTCGCG	ATGGCCGTTCTTAGTTGGTG	GTACAAAGGGCAGGGACGTA	60.0	395	INTRAGENIC	--	--	--
Ca5_P3_1876426	Ca5	1876426	AGGGGG/AGGGGGG	TCTCCAAATGATTGGAAGGC	TCAAGGAACGTTCTCCGTTT	60.0	631	INTERGENIC	--	--	--
Ca5_P3_18814300	Ca5	18814300	ATT/ATTTTATTT	CCGCCCAAACCATATTTATT	GATTTGGGCAGGAAACTGAA	58.7	805	INTRON	Ca_24488	--	--
Ca5_P3_188946	Ca5	188946	CTT/CTTT	CCCCATTTTTACAATCCACG	CATGTATGAGCAGCCACACC	60.0	366	INTERGENIC	--	--	--
Ca5_P3_18944656	Ca5	18944656	CAAAA/CAA	TCCTGTCATGTGCTTTGAGC	CTAACCTCCCAGGACCCAAT	60.0	131	INTERGENIC	--	--	--

INDEL marker IDs	Chromosomes/ unanchored scaffolds	Physical positions (bp)	InDels (*Kabuli* reference genome-CDC Frontier/PII)	Forward primers (5'-3')	Reverse primers (5'-3')	Anneling temperature (0C)	Expected amplified product size (bp)	Structural annotation		Functional annotation	
								Sequence components of *kabuli* genome	*Kabuli* gene accession IDs	NCBI-KOG	TFs
Ca5_P3_18962064	Ca5	18962064	AAATAATAATAATAATAATAATAATAATAATAATAATAATAA/AAATAATAATAATAATAATAATAATAATAATAATAATAATAATAATAA	GCCCTCTCCTCGTGTTACAA	CCGAAAATAGCGATGAGAGC	60.3	394	INTERGENIC	--	--	--
Ca5_P3_1903216	Ca5	1903216	GA/GAA	TGCATAGGCCTCTTGTCCTT	AGTTCCGCGCTAGATTTCAA	59.8	553	INTERGENIC	--	--	--
Ca5_P3_191620	Ca5	191620	CTTT/CTT	GGTTTGCAGTGGAGTGGAGT	TTGTTGGGATCCCTGAAGAG	60.2	719	INTERGENIC	--	--	--
Ca5_P3_191704	Ca5	191704	CGGA/C	TCACACTTGCACTTTGGAGC	TTGTTGGGATCCCTGAAGAG	60.0	484	INTERGENIC	--	--	--
Ca5_P3_191792	Ca5	191792	ACTATCTATCTATCTA/ACTATCTATCTATCTATCTA	TCACACTTGCACTTTGGAGC	CTTGGAATCGAAACCGTTGT	60.0	680	INTERGENIC	--	--	--
Ca5_P3_192919	Ca5	192919	CGTGGAGGTGGAGGTGGAGGTG/CGTGGAGGTGGAGGTG	CGGTGATGACGAGATTGATG	TTCTTCATCCTCCTCCCCTT	60.1	463	CDS (large-effect mutations)	Ca_18174	L	--
Ca5_P3_19366220	Ca5	19366220	AAT/AATGGTTTAGGGAAAGATAT	GGCACCGTATTTTTGCAAGT	TGACCATAGAGCCGTGAGTG	60.0	365	INTERGENIC	--	--	--
Ca5_P3_19542771	Ca5	19542771	CA/C	GGCCACCTTACTGAGGATGA	AGCATGCTTCCACAAAGGAT	60.1	196	INTRON	Ca_23525	--	--
Ca5_P3_19542868	Ca5	19542868	TGAAGAAGAAGA/TGAAGAAGAAGAAGA	GGCCACCTTACTGAGGATGA	CTCAACCCTTCCATATCCCA	60.1	627	INTRON	Ca_23525	--	--
Ca5_P3_19579350	Ca5	19579350	A/AC	ACGAACGTTGAAAATCCAGC	GTATCAAGTGCACAGCGGAA	60.1	489	INTERGENIC	--	--	--

INDEL marker IDs	Chromosomes/ unanchored scaffolds	Physical positions (bp)	InDels (*Kabuli* reference genome-CDC Frontier/PII)	Forward primers (5'-3')	Reverse primers (5'-3')	Anneling temperature (0C)	Expected amplified product size (bp)	Structural annotation		Functional annotation	
								Sequence components of *kabuli* genome	*Kabuli* gene accession IDs	NCBI-KOG	TFs
Ca5_P3_195971	Ca5	195971	GATAT/GATATTTTGTTAAGTGTTATAT	ATCACGGAAAGCTTGTGTCC	TTTCACCTTCATCCTCTGGC	60.1	866	INTRON	Ca_18174	L	--
Ca5_P3_19658678	Ca5	19658678	CAAAAAAAAA/CAAAAAAAA	TTCGGGAGTCGATTACGAGT	CGCTGCACCATGTTATTCAC	59.7	546	INTRON	Ca_22824	O	--
Ca5_P3_19689124	Ca5	19689124	ATTTTTTTTT/ATTTTTTTT	CATTGCAAACGAAAGGGAAT	ATGCGGCCTCCTTATTCTTT	59.9	678	INTERGENIC	--	--	--
Ca5_P3_19725940	Ca5	19725940	TGA/TGACGA	GCCCAGGAAAAAGAAAGGAC	TCGGTATTTGCCATGAGACA	60.1	405	INTERGENIC	--	--	--
Ca5_P3_19746235	Ca5	19746235	TAAAAAAA/TAAAAAAAA	ACCATATCGGCATAGTGCAT	CCGAGCCAAAATCCAAAATA	57.9	259	INTERGENIC	--	--	--
Ca5_P3_197674	Ca5	197674	CA/CATA	TGAATTTTTCTGTTTGGGGC	GAACCCGATTCATAACCCCT	59.9	309	INTRON	Ca_18174	L	--
Ca5_P3_19770978	Ca5	19770978	TTATATATATATATATATATATATATATATATATATAT/TTATATATATATATATATATATATATATAT	TGCAGGTGAAAGAAATTGAAGA	CTGGCCTATTGCCATCCTTA	59.9	790	INTERGENIC	--	--	--
Ca5_P3_19815809	Ca5	19815809	CA/CATACAGTGA	TTTTGGGTTGGGATGACATT	ACCAGGAAAAGAAGAAGGGC	60.0	378	INTERGENIC	--	--	--
Ca5_P3_19905604	Ca5	19905604	CAAAAAA/CAAAAAAA	CTCCTTTCTATCACCGTCGC	TATGGCAAATGAGAGGGAGG	59.8	155	INTERGENIC	--	--	--
Ca5_P3_19922331	Ca5	19922331	A/AG	ATGTTGACTACGCGGGAGAC	CCCCTTTTTGCACATGATCT	60.1	334	INTERGENIC	--	--	--

INDEL marker IDs	Chromosomes/ unanchored scaffolds	Physical positions (bp)	InDels (*Kabuli* reference genome-CDC Frontier/PII)	Forward primers (5'-3')	Reverse primers (5'-3')	Anneling temperature (0C)	Expected amplified product size (bp)	Structural annotation		Functional annotation	
								Sequence components of *kabuli* genome	*Kabuli* gene accession IDs	NCBI-KOG	TFs
Ca5_P3_19929068	Ca5	19929068	ATTT/ATT	CTCGTGGGTAAAGTTGCTCC	GGATTCGAACTGAAATGTCG	59.7	429	INTERGENIC	--	--	--
Ca5_P3_1995521	Ca5	1995521	ATGTT/ATGTTGTT	TCTTCGTTTTGCAGTCAAACTT	TCTTCGTTTTATGGTTTTATTTCA	59.1	279	INTERGENIC	--	--	--
Ca5_P3_19962026	Ca5	19962026	TTATCAATAT/TTATCAATATCAATAT	CTTAGACCGGCACTCGTAGG	GGGCTCAAAGAGTCTGCAAG	59.9	314	INTERGENIC	--	--	--
Ca5_P3_19973066	Ca5	19973066	ATTTTTTTTTTTTT/ATTTTTTTTTTTTTT	GCTCGAGCACACATTGTCAT	ACATGTCCCTTCTCCTCCCT	59.9	545	INTERGENIC	--	--	--
Ca5_P3_200719	Ca5	200719	GTTTT/GTTT	CGATCACCTCCAACCAAAAT	AAGTGTTTCCGCATGGTAGG	59.8	665	DRR	Ca_18175	--	--
Ca5_P3_20087836	Ca5	20087836	GAAGCCTATTTAAAA/GAAGCCTATTTAAAAGCCTATTTAAAA	AAACGAATTTTGAGGCCCTT	GGCTAGGCTTAGGCCATTTT	59.9	697	INTERGENIC	--	--	--
Ca5_P3_20107894	Ca5	20107894	ATTTTTTTTTT/ATTTTTTTTTTT	TTTGAAGCCATTGAGTCGTG	CTCACCCACCTTCACCATTT	59.8	207	INTERGENIC	--	--	--
Ca5_P3_20118512	Ca5	20118512	TAAAAAAAAAAAAA/TAAAAAAAAAAAA	AAAAATTTGCCACTGTTCCG	AATTGAGTTGCGGCGATAAT	60.0	184	INTERGENIC	--	--	--
Ca5_P3_20118559	Ca5	20118559	AATATATATATATATATATATATAT/AATATATATATATATATATATAT	GTAAAACTTTCGCTCGCGTC	TGCATCTGTCAATTGAGTTGC	60.0	400	INTERGENIC	--	--	--
Ca5_P3_202535	Ca5	202535	TAAAAAAA/TAAAAAA	TTGGACTTGAAGTTGACATTGC	ACGAGTTGGATTGGGTTGAC	60.2	269	INTRON	Ca_18175	--	--

INDEL marker IDs	Chromosomes/ unanchored scaffolds	Physical positions (bp)	InDels (*Kabuli* reference genome-CDC Frontier/PII)	Forward primers (5'-3')	Reverse primers (5'-3')	Anneling temperature (0C)	Expected amplified product size (bp)	Structural annotation		Functional annotation	
								Sequence components of *kabuli* genome	*Kabuli* gene accession IDs	NCBI-KOG	TFs
Ca5_P3_20306122	Ca5	20306122	CAAAAAAAA/CAAAAAAAAA	TGCAAAAACCACTTTCAAGG	TTGACCAATTTTGTGGCTTG	58.8	615	INTERGENIC	--	--	--
Ca5_P3_20432870	Ca5	20432870	GATAATAATAATAATAATAATAATAATAATAATAATAATAATAATAA/GATAATAATAATAATAATAATAATAATAATAATAATAATAATAATAATAA	TTAAATGTGCATGTGTGGCA	TTTTCTTTCACGAAGTGGGG	59.6	503	INTERGENIC	--	--	--
Ca5_P3_20438947	Ca5	20438947	AT/A	AGTTTGGAACCAAGCCACTG	TAAGCCGTGGGAACGTTAAG	60.1	343	INTERGENIC	--	--	--
Ca5_P3_204596	Ca5	204596	GTT/GTTT	TTTGTTGTGCTTTGACCCAA	TGCTGTTCTTGGTGTTCCTG	60.1	373	INTRON	Ca_18175	--	--
Ca5_P3_20520449	Ca5	20520449	CTTT/CTT	TCTCCCTCCTTCTCCCATTT	TTGGAAGGAGCCCTTACCTT	60.0	278	INTERGENIC	--	--	--
Ca5_P3_20547334	Ca5	20547334	AACCAACACCAACACCAACA/AACCAACACCAACACCAACACCAACA	AGGAGATGGAGCGTTGTTTG	TTGGTTAGCGTGCATCTGAG	60.3	462	INTERGENIC	--	--	--
Ca5_P3_20614227	Ca5	20614227	TAAAAAAAAAAAAA/TAAAAAAAAAAA	ATGCTCATGCAACCAATCTC	GAAAAGAGGAATGCGGAATG	58.7	608	INTERGENIC	--	--	--
Ca5_P3_20740644	Ca5	20740644	CATATATATATATATATATA/CATATATATATATATATATATA	CAAAGTTTGCCACATCAGGA	CAAACGTGGCAAAGAAACAC	59.7	936	INTERGENIC	--	--	--
Ca5_P3_20743462	Ca5	20743462	AA/AATA	AAAATAAAGCATGGCGTTGG	AGTTTGTAAACCGTGCGTCG	60.0	258	INTERGENIC	--	--	--
Ca5_P3_20781552	Ca5	20781552	GATATATATATATATATATATATATATAT/GATATATATATATATATATATATATATATATAT	GCTGAGGTGGGAAACATTTG	CCAAATCAGAACCAAAGGCA	60.5	598	DRR	Ca_18223	--	ARF

INDEL marker IDs	Chromosomes/ unanchored scaffolds	Physical positions (bp)	InDels (*Kabuli* reference genome-CDC Frontier/PII)	Forward primers (5'-3')	Reverse primers (5'-3')	Anneling temperature (0C)	Expected amplified product size (bp)	Structural annotation		Functional annotation	
								Sequence components of *kabuli* genome	*Kabuli* gene accession IDs	NCBI-KOG	TFs
Ca5_P3_208514	Ca5	208514	ATTTTTTTTTTTT/ATTTTTTTTTT	ATTCCAATTCGGATGTGCTC	AACAAGTGATGGTGATGCCA	59.9	407	INTERGENIC	--	--	--
Ca5_P3_20895157	Ca5	20895157	TTTATTATTATTATTATTATTATTATTATTATTATTATT/TTTATTATTATTATTATTATTATTATTATTATTATTATTATTATT	ATGAAGTGATGCCCTTACCG	TTGGATGGAGGTTGTGTTGA	60.0	303	INTERGENIC	--	--	--
Ca5_P3_209466	Ca5	209466	AAAAAACTTAAAAACTTAAAA/AAAAAACTTAAAAACTTAAAAACTTAAAA	AATTGAAGCATCCCATAAACG	TTAGACCATCCACAACACGG	59.0	698	DRR	Ca_18176	--	C2H2
Ca5_P3_209690	Ca5	209690	ATTTTTTT/ATTTTTTTT	AAAACATTCAACCAACCACCA	TTAGACCATCCACAACACGG	60.1	320	DRR	Ca_18176	--	C2H2
Ca5_P3_209767	Ca5	209767	GAAAAAAAA/GAAAAAAAAAA	AAAACATTCAACCAACCACCA	TTAGACCATCCACAACACGG	60.1	320	DRR	Ca_18176	--	C2H2
Ca5_P3_209793	Ca5	209793	AAATTAAT/AAATTAATTAAT	AAAACATTCAACCAACCACCA	TTAGACCATCCACAACACGG	60.1	320	DRR	Ca_18176	--	C2H2
Ca5_P3_2100697	Ca5	2100697	ATTTTTTT/ATTTTTT	TGGACACGACGGTGAAATAA	TTCGGTTCTTGTTTGCCTCT	60.0	642	INTERGENIC	--	--	--
Ca5_P3_2101143	Ca5	2101143	TTTATTATTATTATTATTAT/TTTATTATTATTATTATTATTAT	AGAGGCAAACAAGAACCGAA	ACCGTGTGAATAATTGTCGG	59.9	565	INTERGENIC	--	--	--
Ca5_P3_210200	Ca5	210200	ATTTTTTTTT/ATTTTTTTTTT	TTAAACACCCGTGTTGTGGA	AAATGGCTTAGGGAGGGAGA	59.9	607	DRR	Ca_18176	--	C2H2
Ca5_P3_210510	Ca5	210510	ATT/ATTT	TCTCCCTCCCTAAGCCATTT	GAATTATCCGCTGCTTGCAT	60.0	201	DRR	Ca_18176	--	C2H2

INDEL marker IDs	Chromosomes/ unanchored scaffolds	Physical positions (bp)	InDels (*Kabuli* reference genome-CDC Frontier/PII)	Forward primers (5'-3')	Reverse primers (5'-3')	Anneling temperature (0C)	Expected amplified product size (bp)	Structural annotation		Functional annotation	
								Sequence components of *kabuli* genome	*Kabuli* gene accession IDs	NCBI-KOG	TFs
Ca5_P3_21085449	Ca5	21085449	A/AT	TTAGGCGACCACTGTATCCC	TGAGCCACTTTGATCAGCAC	60.0	758	INTERGENIC	--	--	--
Ca5_P3_21238726	Ca5	21238726	CAAAA/CAAA	CGCAAGATCCTTCTGACACA	TGATGTGATTGTGCCGAAAT	60.0	603	INTERGENIC	--	--	--
Ca5_P3_213050	Ca5	213050	GTTTTTTTTTTT/GTTTTTTTTTT	CAACAAAACTTGGTTGGGTAGA	TTTGCATTGTGTTGAAGATGA	59.0	322	INTERGENIC	--	--	--
Ca5_P3_213294	Ca5	213294	AAAAA/AAAAAGAAAA	CAACAAAACTTGGTTGGGTAGA	TTTTCCATGTGACATACGATGA	59.0	874	INTERGENIC	--	--	--
Ca5_P3_21405763	Ca5	21405763	TTATATATATATATATATATAT/TTATATATATATATATATATAT	AACCACTTATGGCGTAACGG	GAGTTGTCCTCGCCATCAAT	59.9	360	INTERGENIC	--	--	--
Ca5_P3_2140832	Ca5	2140832	G/GC	GCCCGGATTGCAAGTATAAA	TTTGTGAGAGGGTGGGAGTC	59.9	549	INTRON	Ca_26505	B	--
Ca5_P3_21462460	Ca5	21462460	TTTATTATTATTATTATTATTATTATTATTATTAT/TTTATTATTATTATTATTATTATTATTATTAT	AATTGGAGATGAGCGGTTTG	ACGGACCAAATTCCACTACG	60.1	933	INTERGENIC	--	--	--
Ca5_P3_21596936	Ca5	21596936	AATATATATATATATATATATATATATA/AATATATATATATATATATATATATATATATA	CCTCCTCCCAAGGCTTATTC	TGGTGAGCAAATTTCCCTTC	60.0	876	INTERGENIC	--	--	--
Ca5_P3_21598205	Ca5	21598205	TA/TAA	TTGGTGTGGATTGTCGATTG	GGGGAAATCGATTGAATGAA	60.4	953	INTERGENIC	--	--	--
Ca5_P3_21623515	Ca5	21623515	AAATTAATT/AAATT	AGGTCATTTTCATCCACCCA	ACACACCTATGGGATGCACA	60.2	678	INTERGENIC	--	--	--

INDEL marker IDs	Chromosomes/ unanchored scaffolds	Physical positions (bp)	InDels (*Kabuli* reference genome-CDC Frontier/PII)	Forward primers (5'-3')	Reverse primers (5'-3')	Anneling temperature (0C)	Expected amplified product size (bp)	Structural annotation		Functional annotation	
								Sequence components of *kabuli* genome	*Kabuli* gene accession IDs	NCBI-KOG	TFs
Ca5_P3_2169747	Ca5	2169747	CTATTTATTTATTTATTTATTT/CTATTTATTTATTTATTTATTTATTT	GTTGTGGGTGGTGGACTTCT	TGGGGATACGTAGGAGCAAG	59.9	471	INTRON	Ca_26506	B	--
Ca5_P3_218592	Ca5	218592	TATAATAATAATAATAATA/TATAATAATAATAATAATAATA	TAGCGTGGTGGTGTTGTGAT	CCTTTCAAGGTCAGCCACAT	60.0	739	INTERGENIC	--	--	--
Ca5_P3_21883868	Ca5	21883868	AATATATATATATATATATA/AATATATATATATATATATATA	GCTCCATTTGTGGTTCCAAT	CTATTTTGCTATGCCGCTCC	59.8	405	INTRON	Ca_24467	--	C2H2
Ca5_P3_21899576	Ca5	21899576	TAAAAAAAAAAAA/TAAAAAAAAAAA	TGACCTGACCTGGCCTATTC	CCCAAACAAAGCCTTATGGA	60.1	519	INTERGENIC	--	--	--
Ca5_P3_219030	Ca5	219030	TAAAAAAA/TAAAAAAAAA	TTGAGATGTGGCTGACCTTG	CTGGAGCTGTCTCCAACACA	59.8	613	INTERGENIC	--	--	--
Ca5_P3_21910420	Ca5	21910420	AC/A	CTCCAACCTCGTTCCATTGT	AAAGTTTGCAGCAGGAGGAA	60.0	751	INTERGENIC	--	--	--
Ca5_P3_2192617	Ca5	2192617	A/ACAC	ACAAACCCATTTTTCGCAAC	CAGCGCTTTTCCCAAATAAG	59.8	454	INTERGENIC	--	--	--
Ca5_P3_21965060	Ca5	21965060	AG/AGG	ACGCTCAGAGTTCACTGGGT	GGCAAATCGATTGGACAAGT	59.9	365	INTERGENIC	--	--	--
Ca5_P3_22173724	Ca5	22173724	TAAAAAAA/TAAAAAA	ACAACTCTTCGCCTACACGC	AGGACTTCGGGAGTTTGGTT	60.5	753	INTERGENIC	--	--	--
Ca5_P3_222234	Ca5	222234	TT/TTAAT	ACCGCTTTACAGAACAACCG	GGTGAGGACAAAACATGGCT	60.2	538	INTERGENIC	--	--	--

INDEL marker IDs	Chromosomes/ unanchored scaffolds	Physical positions (bp)	InDels (*Kabuli* reference genome-CDC Frontier/PII)	Forward primers (5'-3')	Reverse primers (5'-3')	Anneling temperature (0C)	Expected amplified product size (bp)	Structural annotation		Functional annotation	
								Sequence components of *kabuli* genome	*Kabuli* gene accession IDs	NCBI-KOG	TFs
Ca5_P3_222733	Ca5	222733	CTCTTTCT/CTCTTTCTTTCT	AGCCATGTTTTGTCCTCACC	TGCTTGCACTATCAGCCATC	60.0	110	INTERGENIC	--	--	--
Ca5_P3_223736	Ca5	223736	CCTACT/CCTACTACTGCTACT	CCATCACCACTCCTCCAACT	TTTTTCAGCAAAGCTCAGCA	60.0	331	CDS (large-effect mutations)	Ca_18177	--	ZF-HD
Ca5_P3_22389657	Ca5	22389657	AT/ATT	TGCCTTTGCTAAGTCCCACT	TAAATAGCACCTGCGGTTCC	59.9	432	INTERGENIC	--	--	--
Ca5_P3_22391502	Ca5	22391502	GAAAAAAAAAAAAAAAA/GAAAAAAAAAAAAA	CCATTGAGGAATGTTGGCTT	CTGATTGGGGTTTGGCTAGA	59.9	613	INTERGENIC	--	--	--
Ca5_P3_224526	Ca5	224526	TTA/TTATAAGTACAGATA	GGACTCTGACCTGAGTTGCC	TTCACATATCGCTCCATCCA	59.8	444	DRR	Ca_18177	--	ZF-HD
Ca5_P3_224582	Ca5	224582	GTTTTTTT/GTTTTTT	GGACTCTGACCTGAGTTGCC	TTCACATATCGCTCCATCCA	59.8	444	DRR	Ca_18177	--	ZF-HD
Ca5_P3_22613210	Ca5	22613210	GAAAAAAAAAA/GAAAAAAAAAAA	ATGACAAGTCCACCCAGACC	TGCATTGAAAACGATCCAAA	59.8	387	INTERGENIC	--	--	--
Ca5_P3_22615880	Ca5	22615880	AT/ATATGTGGGTGTTCTCT	TGAGTGGGTGGGTACTGACA	TGATTGAGAGGGTTGGGAAC	60.0	448	DRR	Ca_20080	--	SAP
Ca5_P3_226338	Ca5	226338	CAAAAAAAAA/CAAAAAAAA	TTGCAGTTTGAATCACTGCC	TTTCAAGTGAGTGCCGAGTG	59.8	577	INTERGENIC	--	--	--
Ca5_P3_22659444	Ca5	22659444	AATATATATATATATATATATATATATATA/AATATATATATATATATATATATATATATATATA	CCAAGTTTGAAATGCCGATT	GCTGCATGTTCTTATGCTCG	59.9	485	INTERGENIC	--	--	--

INDEL marker IDs	Chromosomes/ unanchored scaffolds	Physical positions (bp)	InDels (*Kabuli* reference genome-CDC Frontier/PII)	Forward primers (5'-3')	Reverse primers (5'-3')	Anneling temperature (0C)	Expected amplified product size (bp)	Structural annotation		Functional annotation	
								Sequence components of *kabuli* genome	*Kabuli* gene accession IDs	NCBI-KOG	TFs
Ca5_P3_2276073	Ca5	2276073	AAATAATAATAATAATAATAATAATAATAATAATAATAA/AAATAATAATAATAATAATAATAATAATAATAATAATAATAA	CCAAGAAATTGGATTCGCAC	AAGTGCGGTTTGTCATTCAA	60.4	784	INTERGENIC	--	--	--
Ca5_P3_22785999	Ca5	22785999	TAAAAAAAAAAAAA/TAAAAAAAAAAAA	CACACAACCGCATTATCCTG	TGACTCGAACCCAATCCTTT	60.0	758	INTERGENIC	--	--	--
Ca5_P3_22918101	Ca5	22918101	AC/ACC	TTATCGGCCTTATCCACTGC	ACCGAGATTGTTTCAGCCAA	60.1	214	INTERGENIC	--	--	--
Ca5_P3_22918444	Ca5	22918444	TATT/TATTGATT	TTATCGGCCTTATCCACTGC	GGATTCCACCAAATTCAGGA	60.1	721	INTERGENIC	--	--	--
Ca5_P3_22918526	Ca5	22918526	AT/ATT	TTATCGGCCTTATCCACTGC	TGAAAATCCATGAACCGTGA	60.1	915	INTERGENIC	--	--	--
Ca5_P3_22919631	Ca5	22919631	AG/A	GATTTGGTGGAATCTCCGAA	GCCACCGACTACTTCTCCCT	59.9	336	DRR	Ca_21962	--	--
Ca5_P3_22919722	Ca5	22919722	GTT/GT	GATTTGGTGGAATCTCCGAA	GCCACCGACTACTTCTCCCT	59.9	336	DRR	Ca_21962	--	--
Ca5_P3_2294066	Ca5	2294066	GTTTTTTTTT/GTTTTTTTTTT	CTCAGATGACAGCCAGGACA	TGGTACATGTAACCCACCGA	60.0	277	INTERGENIC	--	--	--
Ca5_P3_22955537	Ca5	22955537	ATATAGAACCTATAGAACCTA/ATATAGAACCTA	GCTCAAAGCACATGACAGGA	GATTGACGCATCAAAGGGTT	60.0	777	INTERGENIC	--	--	--
Ca5_P3_22973493	Ca5	22973493	TTC/T	TTGAATTCGACAAGCACTCG	CAGATGCACCAGAATCGAGA	60.0	664	INTERGENIC	--	--	--

INDEL marker IDs	Chromosomes/ unanchored scaffolds	Physical positions (bp)	InDels (*Kabuli* reference genome-CDC Frontier/PII)	Forward primers (5'-3')	Reverse primers (5'-3')	Anneling temperature (0C)	Expected amplified product size (bp)	Structural annotation		Functional annotation	
								Sequence components of *kabuli* genome	*Kabuli* gene accession IDs	NCBI-KOG	TFs
Ca5_P3_22981606	Ca5	22981606	CT/CTT	TCAATTTCGCTTCTCGTGTG	TTGTTGGAGATTTTGGAGGG	60.0	349	INTERGENIC	--	--	--
Ca5_P3_23184336	Ca5	23184336	TTAATAATAATAATAATAATAATAATAAT/TTAATAATAATAATAATAATAATAATAATAAT	AGCAGCGCGTTTTTAGAAGA	ACCCAACCTCCCCATTAAAA	60.3	770	INTERGENIC	--	--	--
Ca5_P3_2323461	Ca5	2323461	GAAAA/GAAAAA	GCGCCAGATTTCAAACACTA	ACCAGCAGACCAAAATGGAC	58.9	262	INTERGENIC	--	--	--
Ca5_P3_2327252	Ca5	2327252	TAA/TAAA	TCGAAATGAAATCGAGGACC	ACAGACGAGGGCTAGGGATT	60.0	721	INTERGENIC	--	--	--
Ca5_P3_23275268	Ca5	23275268	TAAAAAAAAA/TAAAAAAAAAA	AAGCCTAATTTCAGCCAGCA	TGATAGCCTCCAACGACAAA	60.0	482	INTERGENIC	--	--	--
Ca5_P3_23533132	Ca5	23533132	TTTATTATTATTATTATTATTATTATTATTATTATTATTATTATCATTATTATTATTATTATT/TTTATTATTATTATTATTATTATTATTATTATTATTATTATTATTATCATTATTATTATTATTATT	CCGCAAGATACGCAATATCA	TTCTTGCAATGATTGACCCA	59.7	613	INTERGENIC	--	--	--
Ca5_P3_23552592	Ca5	23552592	CATAAATAAATAAATAAATAAATAAATAAATAAATAAATA/CATAAATAAATAAATAAATAAATAAATAAATAAATAAATAAATAAATA	AAGCCGACAACAAATCCAAC	TGAATGGAACAGCATTGGAA	60.0	298	INTERGENIC	--	--	--
Ca5_P3_23557622	Ca5	23557622	C/CAGCT	GTGACTGGGGACTGGTGACT	CTGTGACTAGGGAAGGCTCG	60.0	122	INTERGENIC	--	--	--
Ca5_P3_236073	Ca5	236073	CGAAGAAAAT/CGAAGAAAATGAAGAAAATGAAGAAAAT	TTGAATTGACCGGAGGAATC	GGGATAGGGAGGGTTGTGTT	59.9	680	INTERGENIC	--	--	--
Ca5_P3_23643411	Ca5	23643411	ATTTTTTT/ATTTTTTTT	AAGCAATCAACGCATTCACA	TTGACTCTCGAAATGTCCCC	60.3	462	INTERGENIC	--	--	--

INDEL marker IDs	Chromosomes/ unanchored scaffolds	Physical positions (bp)	InDels (*Kabuli* reference genome-CDC Frontier/PII)	Forward primers (5'-3')	Reverse primers (5'-3')	Anneling temperature (0C)	Expected amplified product size (bp)	Structural annotation		Functional annotation	
								Sequence components of *kabuli* genome	*Kabuli* gene accession IDs	NCBI-KOG	TFs
Ca5_P3_23663097	Ca5	23663097	TAATGAATGAATGAA/TAATGAATGAA	GAGGGTTTTGGGTCAGTGAA	AGTTTTCAAGGGTAGCGGGT	59.9	789	INTERGENIC	--	--	--
Ca5_P3_23663124	Ca5	23663124	C/CGA	GAGGGTTTTGGGTCAGTGAA	AGTTTTCAAGGGTAGCGGGT	59.9	789	INTERGENIC	--	--	--
Ca5_P3_23664574	Ca5	23664574	TT/TTCCAACT	CAAGAAGGAACGAGGTGCTC	TAGCCTAGAGCAGGGGTGAA	60.0	626	INTERGENIC	--	--	--
Ca5_P3_236674	Ca5	236674	TG/T	AACACAACCCTCCCTATCCC	CCAACAAATGCATGTCCATC	60.1	819	INTERGENIC	--	--	--
Ca5_P3_23672060	Ca5	23672060	TC/TCGCCC	TGAGTAAGGGGGCTGAAATG	TGAGAGACGGTGGTTTACCC	60.1	822	CDS (FRAME SHIFT)	Ca_17683	--	--
Ca5_P3_23673793	Ca5	23673793	T/TACGAACGT	AAATCATCGCACCTACGGTC	GACAATCGTTCGGACTTGGT	60.0	497	INTERGENIC	--	--	--
Ca5_P3_23675051	Ca5	23675051	GT/G	TCCATGCACATTGCTCTCTC	TGGGTTATTTTCATGGGCTT	60.0	514	INTERGENIC	--	--	--
Ca5_P3_236893	Ca5	236893	ACC/A	CCCTCACAAATTACAATCCCA	CCAACAAATGCATGTCCATC	59.7	763	INTERGENIC	--	--	--
Ca5_P3_23691134	Ca5	23691134	ATTTTTTTTTT/ATTTTTTTTT	AACAACACAACATGCGGGTA	AGTTTGGTTTTCACATCCGC	59.9	360	INTERGENIC	--	--	--
Ca5_P3_237297	Ca5	237297	CTGTCAT/CTGTCATGTCAT	TCATTGCAATACCGGGAAAT	TGAATTTGGATAGGTGCAAAAA	60.2	233	INTERGENIC	--	--	--

INDEL marker IDs	Chromosomes/ unanchored scaffolds	Physical positions (bp)	InDels (*Kabuli* reference genome-CDC Frontier/PII)	Forward primers (5'-3')	Reverse primers (5'-3')	Anneling temperature (0C)	Expected amplified product size (bp)	Structural annotation		Functional annotation	
								Sequence components of *kabuli* genome	*Kabuli* gene accession IDs	NCBI-KOG	TFs
Ca5_P3_238011	Ca5	238011	GTTTTTT/GTTTTT	CATGCACGATTCACACATGA	GTGTCGTCCAACCTCTCCAT	60.1	486	INTERGENIC	--	--	--
Ca5_P3_23812349	Ca5	23812349	TAAAAAAAAAAA/TAAAAAAAAAAAAAAAAAA	ATTGCTAGCCACATTTTGGG	CGCTATGATAGGATGGTCCAA	60.0	664	INTERGENIC	--	--	--
Ca5_P3_239546	Ca5	239546	AATA/AATATA	CTCAACGCTGCCACAATAGA	TGGATTCCCAAACACTAGCC	60.0	473	INTERGENIC	--	--	--
Ca5_P3_239777	Ca5	239777	CTTT/CTTTT	CTCAACGCTGCCACAATAGA	TGGATTCCCAAACACTAGCC	60.0	473	INTERGENIC	--	--	--
Ca5_P3_23995501	Ca5	23995501	CATATATATATATATATATATA/CATATATATATATATATATA	TTGCGAGCATGAGAAGTTGT	AAAAACCACCTAATCGCTCA	59.6	714	INTERGENIC	--	--	--
Ca5_P3_240184	Ca5	240184	ATTT/ATTTT	GGCTAGTGTTTGGGAATCCA	ACGGTGTGTTGGGTTAAAGG	59.9	820	INTERGENIC	--	--	--
Ca5_P3_24054306	Ca5	24054306	CTATTATTATTATTATTATTATTATTATTATTATTAT/CTATTATTATTATTATTATTATTATTATTATTATTAT	TCAATAGAAGGTCGCATCCC	TCGCACATCTACCTAAACGC	60.0	759	INTERGENIC	--	--	--
Ca5_P3_240662	Ca5	240662	T/TCAATCCA	CCTTTAACCCAACACACCGT	TTGGTGCCTACAACATATTTTCC	59.8	268	INTERGENIC	--	--	--
Ca5_P3_24097297	Ca5	24097297	AAGCTTTGGATAC/A	CAGAGCCAACACTACAGGCA	ATTTGAAGGGCCATGTGAAG	60.0	329	CDS (large-effect mutations)	Ca_17656	S	--
Ca5_P3_241028	Ca5	241028	GAAAAAAAA/GAAAAAAA	CCTTTAACCCAACACACCGT	GGGACGTCTTCGCATACATT	59.8	624	INTERGENIC	--	--	--

INDEL marker IDs	Chromosomes/ unanchored scaffolds	Physical positions (bp)	InDels (*Kabuli* reference genome-CDC Frontier/PII)	Forward primers (5'-3')	Reverse primers (5'-3')	Anneling temperature (0C)	Expected amplified product size (bp)	Structural annotation		Functional annotation	
								Sequence components of *kabuli* genome	*Kabuli* gene accession IDs	NCBI-KOG	TFs
Ca5_P3_241392	Ca5	241392	TAAA/TAAAA	AATGTATGCGAAGACGTCCC	GGAGCTCAACAACCTCCTTG	60.0	511	INTERGENIC	--	--	--
Ca5_P3_2418306	Ca5	2418306	ATTTTTTTTTTTTTT/ATTTTTTTTTTTTT	GCTCTGCATTTTCCTTGAGC	TGAGAAATTTTTCCGCTGCT	60.1	328	INTRON	Ca_18664	--	B3
Ca5_P3_24246490	Ca5	24246490	TG/TGCGAAAGAG	TTGAATAGACGGATTCGGGA	TCTATAATGCATGGTTGCCG	60.4	118	CDS (FRAME SHIFT)	Ca_15567	S	--
Ca5_P3_24297832	Ca5	24297832	AATATATATATATATA/AATATATATATATATATA	CCCAAGTTTTAAATGGGCAA	GGAGGCCACCTTCAACATTA	59.8	556	INTERGENIC	--	--	--
Ca5_P3_245247	Ca5	245247	CTTTTT/CTTTT	CTTCACAGCACAGAAGACGC	GGAATGGCAGGAATCTGAAA	59.8	337	INTERGENIC	--	--	--
Ca5_P3_245296	Ca5	245296	TTAATAATAATAATAATAAT/TTAATAATAATAATAATAATAAT	CTTCACAGCACAGAAGACGC	GGAATGGCAGGAATCTGAAA	59.8	337	INTERGENIC	--	--	--
Ca5_P3_24602434	Ca5	24602434	GTAAATAAATAAATAAATAAATAAATAA/GTAAATAAATAAATAAATAAATAAATAAATAAATAA	TCACATCAGTTAGGCCACCA	GTTTCGAACACCGGACCTTA	60.1	235	INTRON	Ca_15595	G	bHLH
Ca5_P3_24625027	Ca5	24625027	GT/G	GGTCCAAACAATGGCAAATC	ACCCACAACTCCGTATGTCA	60.2	503	INTERGENIC	--	--	--
Ca5_P3_24631329	Ca5	24631329	AGTAT/A	TGGATGACATTTCTACGGCA	ATTTATCCCCCTCCCAAGTG	60.1	500	INTERGENIC	--	--	--
Ca5_P3_24701450	Ca5	24701450	CCATACATACATACATACATACATACATACATACATACATACATACATACATACA/CCATACATACATACATACATACATACATACATACATACATACATACATACATACATACATACA	CGTGTCCGAGTTAACAGCAA	TGTGTGTGGGTTATCACGCT	59.9	621	INTERGENIC	--	--	--

INDEL marker IDs	Chromosomes/ unanchored scaffolds	Physical positions (bp)	InDels (*Kabuli* reference genome-CDC Frontier/PII)	Forward primers (5'-3')	Reverse primers (5'-3')	Anneling temperature (0C)	Expected amplified product size (bp)	Structural annotation		Functional annotation	
								Sequence components of *kabuli* genome	*Kabuli* gene accession IDs	NCBI-KOG	TFs
Ca5_P3_247324	Ca5	247324	TG/T	TTTGGGAATCTAATGGCAGC	CACCCACTTGGAGAGTGTCA	60.0	635	INTRON	Ca_18179	R	--
Ca5_P3_24865224	Ca5	24865224	TAAAAAAAA/TAAAAAAA	TGGAATGTAAATTGGGGGAA	GTTGGCCACCGACATTAGTT	60.0	381	INTERGENIC	--	--	--
Ca5_P3_2486684	Ca5	2486684	AATATATATATATATATATATATATATA/AATATATATATATATATATATATATATATATATATATATATA	CACCTTGTAGAGGGGAGCAA	CTGTCAATTGAGTTGCGATGA	60.2	280	INTERGENIC	--	--	--
Ca5_P3_24886870	Ca5	24886870	TAATAAATAAATAAATAAA/TAATAAATAAATAAATAAATAAATAAATAAA	TGTTGACGTGAGACCCAAAG	GGCCCAACAACAAAGCTAAG	59.7	550	INTERGENIC	--	--	--
Ca5_P3_24915736	Ca5	24915736	CTTTTTTTTTTT/CTTTTTTTTTTTT	TTCATTCATGTGTGTTTTGGAA	TCATGACAATGCAAGTTCGAC	58.9	493	INTERGENIC	--	--	--
Ca5_P3_25027142	Ca5	25027142	TAAAAAAAAAAAAAA/TAAAAAAAAA	AAATGGAATAACTTTGAAAATGGAA	CTCCATCGCACACTTTCTCA	59.2	602	INTERGENIC	--	--	--
Ca5_P3_25080544	Ca5	25080544	TTATATATATATATATATATATATATATATAT/TTATATATATATATATATATATATATATATATATATATAT	AGAATGTCCCCAGCTGTCAC	AAGCAACCAACACATCCACA	60.1	498	INTERGENIC	--	--	--
Ca5_P3_25109370	Ca5	25109370	TTTATTATTATTATTATTATTATTATTATTATTATTATTATTATTATTATTATT/TTTATTATTATTATTATTATTATTATTATTATTATTATTATTATTATTATTATTATTATTATTATTATT	ACCACCAAAAAGGGTTGGTT	CGCATTCCAGATATGCAACA	60.5	500	INTERGENIC	--	--	--
Ca5_P3_25171173	Ca5	25171173	ATT/AT	TCATGGTGTAAGGATGACCG	GGTTTAGCCCATATGGTTTGC	59.4	190	INTERGENIC	--	--	--
Ca5_P3_2521445	Ca5	2521445	ATTACAGAGTTACA/ATTACAGAGTTACAGAGTTACA	CTGGGCAAGGGATCTGAATA	GCACATTATACCGCGCTTTT	60.0	603	DRR	Ca_18666	--	Trihelix

INDEL marker IDs	Chromosomes/ unanchored scaffolds	Physical positions (bp)	InDels (*Kabuli* reference genome-CDC Frontier/PII)	Forward primers (5'-3')	Reverse primers (5'-3')	Anneling temperature (0C)	Expected amplified product size (bp)	Structural annotation		Functional annotation	
								Sequence components of *kabuli* genome	*Kabuli* gene accession IDs	NCBI-KOG	TFs
Ca5_P3_25230946	Ca5	25230946	C/CT	TGTGGAACAATCGATTTGGA	AGTGTTTGAGGTGGTGAGGG	59.9	420	INTERGENIC	--	--	--
Ca5_P3_25232481	Ca5	25232481	T/TC	CCACCCTGATTTTTGGAGAA	AAAATCCAACAATCTCCCCC	59.9	790	INTERGENIC	--	--	--
Ca5_P3_25287994	Ca5	25287994	CAAAAA/CAAAA	TTGCTTGAAGACATGAACGC	TGGATGTCAAGCAGTCTTGC	60.0	493	INTERGENIC	--	--	--
Ca5_P3_25303659	Ca5	25303659	C/CCTCTCAA	TTGATCTTGCCTCTTTGCCT	TGTTTATTGCATCAGCCCAA	60.0	623	INTERGENIC	--	--	--
Ca5_P3_2551220	Ca5	2551220	ATTTTTTTTTT/ATTTTTTTTT	GGCTAGACGAGTTCGACGAC	GGTTGTTGAATTTTGTTATTGGG	60.0	365	INTERGENIC	--	--	--
Ca5_P3_25531858	Ca5	25531858	TAAAAA/TAAAAAAAA	ATTGGGCAATCGATTTGGTA	AGGGAGGTGGAGGTGAAAGT	60.2	299	INTERGENIC	--	--	--
Ca5_P3_25544088	Ca5	25544088	TAAAAAAAAAA/TAAAAAAAAAAA	TTCACAACCATTCAATTCCAA	TGGTTATGCATGTTCGTCGT	58.9	175	INTERGENIC	--	--	--
Ca5_P3_25555104	Ca5	25555104	CTTT/CT	ACACCAGAAATGCCACACAA	TGAGATAGCAGTGTAGTGAAAGGG	60.0	419	INTERGENIC	--	--	--
Ca5_P3_25574427	Ca5	25574427	CATATATATATATATATATATATATATAT/CATATATATATATATATATATATATATATAT	TTTTTGCAATCCACTCTTGAA	GATTGTTACACAATTCTTCACGC	58.4	301	INTERGENIC	--	--	--
Ca5_P3_25600693	Ca5	25600693	A/AT	CGTGCACTTTCACGACAAAT	TTGCTGTACTTGGTTGCTCG	59.8	801	INTRON	Ca_09030	E	--

INDEL marker IDs	Chromosomes/ unanchored scaffolds	Physical positions (bp)	InDels (*Kabuli* reference genome-CDC Frontier/PII)	Forward primers (5'-3')	Reverse primers (5'-3')	Anneling temperature (0C)	Expected amplified product size (bp)	Structural annotation		Functional annotation	
								Sequence components of *kabuli* genome	*Kabuli* gene accession IDs	NCBI-KOG	TFs
Ca5_P3_25662749	Ca5	25662749	AATATATATATATATATATATATATATATATAT ATATA/AATATATATATATATATATATATAT ATATATATATATATA	CCTACGTATCTCCTGG TGCG	TCAAAAAGGAGGATCG GAGA	60.7	772	INTERGENIC	--	--	--
Ca5_P3_25692452	Ca5	25692452	TTATATATATATATATATATATATATATAT/ TTATATATATATATATATATATATATATATATA TATATAT	GAGGTCGGGCGATAGT TGTA	GAGGGGAGAAGGGAA ATGAG	60.1	924	INTERGENIC	--	--	--
Ca5_P3_25726766	Ca5	25726766	AG/A	TCAAAGCGCGAGGAAA TAGT	ATTGTTGCCCAATGGA GAAG	60.0	315	INTERGENIC	--	--	--
Ca5_P3_2577647	Ca5	2577647	TTATATATATATATATATATATATATATATA /TTATATATATATATATATATATATATATAT ATA	GATGAAGAGGGTGTGA GGGA	TGGAGGGCGCTATTTC TATG	60.0	597	INTERGENIC	--	--	--
Ca5_P3_25802252	Ca5	25802252	ATTTTT/ATTTTTTT	TGATGGACTGGAATTG TCAAAG	TGCCAAGAAAACACCA TGAA	60.0	846	INTERGENIC	--	--	--
Ca5_P3_25803856	Ca5	25803856	TAAAAAAAAAAAA/TAAAAAAAAAAA	ATCCGATAAATCGCATT GTG	CCCACAAGGTCCGAGA ATTA	58.5	203	DRR	Ca_09020	--	--
Ca5_P3_25803970	Ca5	25803970	TGGGGG/TGGGG	GGGGCGTTACATGTGG TATC	TGTAGGGCACATTGGG TTTT	60.1	510	DRR	Ca_09020	--	--
Ca5_P3_25832709	Ca5	25832709	TTTT/TTTTAATTT	AGCTTGCCAACTTCTG GGTA	GAAAACGTGTGGGAGA AGGA	59.9	835	INTERGENIC	--	--	--
Ca5_P3_25836761	Ca5	25836761	GC/GCTTTC	AAGGCAACAACAAACAA GCC	CCCAAAGATGGCTACT TCCA	60.2	718	INTERGENIC	--	--	--
Ca5_P3_25961156	Ca5	25961156	TAAAAAAAA/TAAAAAAA	CTCCTTTATTGGGCCAT GTG	GGCGACGACATCTCCT ACAT	60.3	539	INTERGENIC	--	--	--

INDEL marker IDs	Chromosomes/ unanchored scaffolds	Physical positions (bp)	InDels (*Kabuli* reference genome-CDC Frontier/PII)	Forward primers (5'-3')	Reverse primers (5'-3')	Anneling temperature (0C)	Expected amplified product size (bp)	Structural annotation		Functional annotation	
								Sequence components of *kabuli* genome	*Kabuli* gene accession IDs	NCBI-KOG	TFs
Ca5_P3_260923	Ca5	260923	CATAT/CATATAT	CTTCGGCCTAAAGATTTTGC	ATTGCGGTTGTTCGGTTATC	59.0	358	DRR	Ca_18180	A	--
Ca5_P3_261320	Ca5	261320	TCAC/TC	GATAACCGAACAACCGCAAT	CGGAGCTTCGGTAGGACATA	59.8	754	DRR	Ca_18179	R	--
Ca5_P3_26153652	Ca5	26153652	GGT/GGTAACTTTAGT	GATTCTGAAACCATTGGGG	GGTACGAATTTTTCGGATTGA	59.2	625	INTERGENIC	--	--	--
Ca5_P3_26207850	Ca5	26207850	TTCTCTCTCTCTCTCTCTCTCTCTC/TTCTCTCTCTCTCTCTCTCTCTC	TTCGCTACCTCATCCAAACC	GATCGACGATTTCACGTCCT	60.1	168	INTERGENIC	--	--	--
Ca5_P3_262311	Ca5	262311	CTTTT/CTTT	TCGCTCGCACAAGTAAAATG	GGTCGATGCGTAGTTTCCAT	60.0	859	INTRON	Ca_18180	A	--
Ca5_P3_26260310	Ca5	26260310	TTTATTATTATTATTATTATTATTATTATTATTATTATTATTATTATTATTATTATTATTATTATTATTAT/TTTATTATTATTATTATTATTATTATTATTATTATTATTATTATTATTATTATTATTATTATTATTATTAT	TCAAACCCTTAAACCCCAAA	TGGTGGTGGATGAGATGAGA	59.3	626	INTERGENIC	--	--	--
Ca5_P3_26280416	Ca5	26280416	CTTTTTTTTTT/CTTTTTTTTTTT	GGGGAAAAAGGTACAAGCCT	AGCGACCTCCTACTTGGTGA	59.5	251	INTERGENIC	--	--	--
Ca5_P3_2628560	Ca5	2628560	AT/ATATATT	TTGTTTCTCCCCAGTTTTGG	TTTTAGCATTGACCGCTCCT	59.9	357	INTERGENIC	--	--	--
Ca5_P3_26430487	Ca5	26430487	CTTTTTTTTTTTTT/CTTTTTTTTTTTTTTT	GCGGCTCACACACACTTAGA	CTGGAGGTCACCTTCGACAT	60.1	693	INTERGENIC	--	--	--
Ca5_P3_26430640	Ca5	26430640	TATATATATAA/T	GCGGCTCACACACACTTAGA	CTGGAGGTCACCTTCGACAT	60.1	693	INTERGENIC	--	--	--

INDEL marker IDs	Chromosomes/ unanchored scaffolds	Physical positions (bp)	InDels (*Kabuli* reference genome-CDC Frontier/PII)	Forward primers (5'-3')	Reverse primers (5'-3')	Anneling temperature (0C)	Expected amplified product size (bp)	Structural annotation		Functional annotation	
								Sequence components of *kabuli* genome	*Kabuli* gene accession IDs	NCBI-KOG	TFs
Ca5_P3_26446975	Ca5	26446975	AATATATATATATATATATATA/AATATATATATATATATATATATA	TGTTGATTGATGGTGGGAGA	TCGACTCTAATAAATCTCATTCATTTC	59.9	818	DRR	Ca_08973	J	--
Ca5_P3_265081	Ca5	265081	TGAAAGTAGAAAGTAGAAAG/TGAAAGTAGAAAG	CTGGCACCGGATACTTCATT	TCGACATGGAAAGGGAACTC	60.0	696	INTRON	Ca_18180	A	--
Ca5_P3_26550928	Ca5	26550928	ATTTTTTTTTTT/ATTTTTTTTTTTT	ATTGTGCTGATTGCGAAGAA	TCTGCTAACGCTTCGAATGAT	59.4	573	INTERGENIC	--	--	--
Ca5_P3_26559939	Ca5	26559939	AATATATATATATATAT/AATATATATATATATATAT	TTTTTGGGTTGAGGACAAGG	GCACTATCCTATCAAATGGTCTCTC	59.9	145	DRR	Ca_08966	--	bHLH
Ca5_P3_26605667	Ca5	26605667	TT/TTTTTTGAT	CTGCTCCTTAACGTTCTCCG	TTTCTCTTTTGGTGGTTGCC	60.0	187	DRR	Ca_08963	--	M-type
Ca5_P3_26656175	Ca5	26656175	TATCATTATCATCATTATCATCATTATCATCAT/TATCATTATCATCATTATCATCAT	ACACCGCAAAAGTTTCCATC	GGTTGACCCATTTTTCCCTT	60.0	542	DRR	Ca_08956	--	--
Ca5_P3_26904550	Ca5	26904550	GTTTTTTTTTT/GTTTTTTTTT	CGTGGTAGACCTTCGGTGAT	TCCTTCACGAGTAAAATGTCTCA	60.0	786	INTERGENIC	--	--	--
Ca5_P3_26906467	Ca5	26906467	GTAATAATAATAATAATAATAATAATAATAATAATAATAATA/GTAATAATAATAATAATAATAATAATAATAATAATAATAATAATAATAATAATA	GGCAAGAGAGGTTGACCAAT	TCGAAAATGTTTTCAAAAGCC	59.1	911	INTERGENIC	--	--	--
Ca5_P3_26989148	Ca5	26989148	AG/A	CCAAAGCAAGCCAGAAAGAC	GCGAGGTGGAACCACACTAT	60.0	609	INTERGENIC	--	--	--
Ca5_P3_26994530	Ca5	26994530	G/GGT	TAGATTAGCGCCCAGCCTTA	TTCAGTCATATTCCCGCTCC	60.0	280	DRR	Ca_08932	R	MYB

INDEL marker IDs	Chromosomes/ unanchored scaffolds	Physical positions (bp)	InDels (*Kabuli* reference genome-CDC Frontier/PII)	Forward primers (5'-3')	Reverse primers (5'-3')	Anneling temperature (0C)	Expected amplified product size (bp)	Structural annotation		Functional annotation	
								Sequence components of *kabuli* genome	*Kabuli* gene accession IDs	NCBI-KOG	TFs
Ca5_P3_26994998	Ca5	26994998	TC/TCC	GGAGCGGGAATATGACTGAA	GGGAGAGGTTGGGAGCTTAC	60.0	492	DRR	Ca_08931	R	MYB
Ca5_P3_26995171	Ca5	26995171	TAG/TAGCACAAGCACTAAG	GGAGCGGGAATATGACTGAA	GCGGCAGAAGAACCTATTTG	60.0	576	DRR	Ca_08931	R	MYB
Ca5_P3_26996169	Ca5	26996169	TG/TGATCG	ATGTTGGGAACTTGCACTCC	AACTATGTCCTGTGGACCGC	60.0	458	DRR	Ca_08931	R	MYB
Ca5_P3_26996186	Ca5	26996186	GGGCGG/GGCGGCGG	CTCCTTGTCCTTTCAGGCAG	AGTACTGACCACACCGAGGG	60.0	172	DRR	Ca_08931	R	MYB
Ca5_P3_26996806	Ca5	26996806	A/AAAAGCAAAATTA	TCGTTTTGGGTGAATTGTGA	TGCCTCCTCGTTCCTTTTTA	59.9	410	INTERGENIC	--	--	--
Ca5_P3_27000468	Ca5	27000468	A/AGAT	TTGGCACTGAATAGTGCTCG	TCGAAGCTGTGCTAATGGTG	60.0	361	INTERGENIC	--	--	--
Ca5_P3_27000514	Ca5	27000514	A/ATCGA	TTGGCACTGAATAGTGCTCG	TCGAAGCTGTGCTAATGGTG	60.0	361	INTERGENIC	--	--	--
Ca5_P3_27000752	Ca5	27000752	CTTTTTTTTTTTTTTT/CTTTTTTTTTTTTT	TTGGCACTGAATAGTGCTCG	CGTCCCGAATAGACGAACAT	60.0	698	INTERGENIC	--	--	--
Ca5_P3_27000834	Ca5	27000834	ATCTCT/ATCT	TTGGCACTGAATAGTGCTCG	CTCAGCGGGTAGAGTATCGC	60.0	940	INTERGENIC	--	--	--
Ca5_P3_27046097	Ca5	27046097	TTATATATATATATATATATATATATATATA/TTATATATATATATATATATATATA	TGGAGAAGGAAACTGGAGGA	TGTGCCCAAAAATGTTTCAA	59.8	776	INTRON	Ca_08930	--	MYB_related

INDEL marker IDs	Chromosomes/ unanchored scaffolds	Physical positions (bp)	InDels (*Kabuli* reference genome-CDC Frontier/PII)	Forward primers (5'-3')	Reverse primers (5'-3')	Anneling temperature (0C)	Expected amplified product size (bp)	Structural annotation		Functional annotation	
								Sequence components of *kabuli* genome	*Kabuli* gene accession IDs	NCBI-KOG	TFs
Ca5_P3_27099226	Ca5	27099226	GAA/GAAA	TGGCAGACAAACCAAACAAA	TGGTTTTGCCATGAGACAGA	60.1	406	INTERGENIC	--	--	--
Ca5_P3_27119906	Ca5	27119906	AATATATATATATATATA/AATATATATATATATATATA	TTAACGATTGGAATTTAGTGCAT	TGGTCTCATCCGAAAGCTTATT	57.8	874	INTERGENIC	--	--	--
Ca5_P3_27204653	Ca5	27204653	GAAAAAA/GAAAAA	GCAAAGCGATCACAAACTCA	GAAATGCTTCTTTCGGCTTG	60.0	329	INTERGENIC	--	--	--
Ca5_P3_27249906	Ca5	27249906	CATATATATATATATATATATATATATATA/CATATATATATATATATATATATATATA	TTGCATTTGTTTTCCCATGA	TGTATCGGGATGCGACATAG	59.9	234	INTERGENIC	--	--	--
Ca5_P3_27249998	Ca5	27249998	AATATATATATATATATATATATATATA/AATATATATATATATATATATATATATATATA	TTGCATTTGTTTTCCCATGA	TGTATCGGGATGCGACATAG	59.9	234	INTERGENIC	--	--	--
Ca5_P3_27472549	Ca5	27472549	AG/A	GCATTATGCCGTCATCATTTT	TTCGCATTCAAAATTGTTGTT	59.8	491	INTERGENIC	--	--	--
Ca5_P3_27532068	Ca5	27532068	CAAAAAAAAAAAA/CAAAAAAAAAAA	GGATTTAGCGGAAGGGGATA	TACACCCAAGGGAGGGTACA	60.2	544	DRR	Ca_08901	L	--
Ca5_P3_27603143	Ca5	27603143	GATATATATATATATAT/GATATATATATATAT	CTTGCCTTCATGCGAATTTT	GATTTCAAAGATGCAGGGGA	60.2	555	DRR	Ca_08896	--	--
Ca5_P3_27718024	Ca5	27718024	TAAAAAAA/TAAAAAAAA	TGGCAACAGAAACCAAACAA	TTCTTTTAGCCCGTTGCTGT	60.1	573	INTERGENIC	--	--	--
Ca5_P3_27724401	Ca5	27724401	GATATATATATATATATATA/GATATATATATATATATA	ACATCATTGGCATTGGGTTT	TGCAAAACTACAACGATTCTCC	60.1	678	INTERGENIC	--	--	--

INDEL marker IDs	Chromosomes/ unanchored scaffolds	Physical positions (bp)	InDels (*Kabuli* reference genome-CDC Frontier/PII)	Forward primers (5'-3')	Reverse primers (5'-3')	Anneling temperature (0C)	Expected amplified product size (bp)	Structural annotation		Functional annotation	
								Sequence components of *kabuli* genome	*Kabuli* gene accession IDs	NCBI-KOG	TFs
Ca5_P3_2774211	Ca5	2774211	CTCT/CTCTTTCCCTTAATCT	ATCCGACATTTCTTTGGCAG	CAAAGCAAGGTGAAGTGCAA	60.1	842	INTERGENIC	--	--	--
Ca5_P3_27776620	Ca5	27776620	ATTTTTTTTTT/ATTTTTTTTTTT	TCCAAACTTGGGTTGGTTTC	CATGAGCATGCAAGATGGAT	59.8	603	INTERGENIC	--	--	--
Ca5_P3_27848020	Ca5	27848020	CATATATATATATATATATAT/CATATATATATATATATATATAT	CAAGCACCCCTTTGCATTAT	TGACAAAATTGTAATTTCTCATTTCA	60.0	309	INTERGENIC	--	--	--
Ca5_P3_27855551	Ca5	27855551	AATATATATATATATATATATATATATATATAT/AATATATATATATATATATATATATATATATATATATATATATAT	TTATGGCATGTGATTCTTTCAA	GCAAGCATATGCACACAAAAA	58.2	477	INTERGENIC	--	--	--
Ca5_P3_27953406	Ca5	27953406	A/ATG	AACAACAGCCCCAATGTAGC	ATCATCCCGTCCATTACGAA	60.0	599	CDS (FRAME SHIFT)	Ca_13422	--	Trihelix
Ca5_P3_27953689	Ca5	27953689	TA/T	AACAACAGCCCCAATGTAGC	ACGCTTTTCAAACAAACGCT	60.0	794	INTERGENIC	--	--	--
Ca5_P3_27962429	Ca5	27962429	TTATATATATATATATATATATATATATATATAT/TTATATATATATATATATATATATATATATATATATATAT	TCCAAGAGTCGGTTGTGTGA	TGGACCAACCAAATTTTAGTGA	60.3	555	INTERGENIC	--	--	--
Ca5_P3_28131912	Ca5	28131912	ATTT/ATT	TGCCATATTCAGGAGTGTGC	ATGTACATTGCATGAGGCCA	59.7	850	INTERGENIC	--	--	--
Ca5_P3_28188267	Ca5	28188267	TAAAAAAAAA/TAAAAAAAAAAA	CAAGAAAAGTAACCATCGTAATGA	GATCCATGTGAATTGGTTTGG	57.5	670	INTERGENIC	--	--	--
Ca5_P3_28192036	Ca5	28192036	CATATATATATATATATATAT/CATATATATATATATATATATATAT	CCACATGCTCATTGTGTGACT	CCATTCTCCTTCACCCAGAC	59.6	653	INTERGENIC	--	--	--

INDEL marker IDs	Chromosomes/ unanchored scaffolds	Physical positions (bp)	InDels (*Kabuli* reference genome-CDC Frontier/PII)	Forward primers (5'-3')	Reverse primers (5'-3')	Anneling temperature (0C)	Expected amplified product size (bp)	Structural annotation		Functional annotation	
								Sequence components of *kabuli* genome	*Kabuli* gene accession IDs	NCBI-KOG	TFs
Ca5_P3_28305464	Ca5	28305464	GTGTATATATG/GTGTATATATGTATATATG	AATATGTGGCGATTAATCTATTGTT	TGGTTGATCATTTCGTTAGTACG	57.3	960	INTERGENIC	--	--	--
Ca5_P3_28366916	Ca5	28366916	TTATATATATATATATATATATATATATATAT/TTATATATATATATATATATATATATATATATATAT	TGAATGCACTCAAAAGCACA	TACAAAGACGTTGTCGTCGC	59.0	747	INTERGENIC	--	--	--
Ca5_P3_28370910	Ca5	28370910	TTATATATATATATATATATATATATATAT/TTATATATATATATATATATATATATATAT	CTAGTTCGTAACCCGTGCGT	AGGCATGGTGTTGGAGTCAT	60.2	260	INTERGENIC	--	--	--
Ca5_P3_28399764	Ca5	28399764	GT/GTACTAGCATCCT	AGAGTCGCCAGCTGAACAAT	TGATTTCTCCATAGCACCGA	60.0	502	INTERGENIC	--	--	--
Ca5_P3_28413401	Ca5	28413401	AATATATA/ATATATATA	TTCTCCTTGAAGCTGTCGGT	ATTGCCAACATTTCCGGTAG	60.0	252	INTERGENIC	--	--	--
Ca5_P3_28463884	Ca5	28463884	TAAAAAAAAA/TAAAAAAAAAA	TGTTTCCTTTCGACACATTGA	TGTTGAGCCCAAAATACAAGG	59.2	590	INTERGENIC	--	--	--
Ca5_P3_28463958	Ca5	28463958	ATTTTTTTT/ATTTTTTTTT	TGTTTCCTTTCGACACATTGA	TGTTGAGCCCAAAATACAAGG	59.2	590	INTERGENIC	--	--	--
Ca5_P3_28519997	Ca5	28519997	TATAATAATAAT/TATAATAATAATAAT	TTTCGAATGATGAACTTTTTATGT	CCATTTCTCCCATAACGCAT	57.0	822	INTERGENIC	--	--	--
Ca5_P3_28588926	Ca5	28588926	TGGG/TGG	CAACTGCTCCTGTTCTCCAA	CTAGGAGCATGTCGCTCTCC	59.0	412	INTERGENIC	--	--	--
Ca5_P3_286315	Ca5	286315	ACC/ACCC	ATGATTTGAATTTGCGAGGC	TCCAACGGGGTTTAAGAGTG	60.0	821	INTERGENIC	--	--	--

INDEL marker IDs	Chromosomes/ unanchored scaffolds	Physical positions (bp)	InDels (*Kabuli* reference genome-CDC Frontier/PII)	Forward primers (5'-3')	Reverse primers (5'-3')	Anneling temperature (0C)	Expected amplified product size (bp)	Structural annotation		Functional annotation	
								Sequence components of *kabuli* genome	*Kabuli* gene accession IDs	NCBI-KOG	TFs
Ca5_P3_28668837	Ca5	28668837	TTATATATAT/TTATATATATAT	AGCTAAAATAGGGGGACCGA	TGAGAGGTTGTTGTGGCAAA	59.9	242	INTERGENIC	--	--	--
Ca5_P3_28766272	Ca5	28766272	ACC/AC	ATGAAAACGTTTGCTCCAGG	GCCAAGTCCCATCAACTTGT	60.1	160	INTERGENIC	--	--	--
Ca5_P3_28870694	Ca5	28870694	TTATATATATATATATA/TTATATATATATATA	CGGTCGACCACTTGTCCTAT	CCATCATCATGCGTCTTACG	60.0	669	INTERGENIC	--	--	--
Ca5_P3_28918279	Ca5	28918279	GC/GCCACACATC	CTGGTTTGCATAGCACCAAC	AAAGGCCCACAAATAAGCCT	59.2	320	INTRON	Ca_13343	--	ERF
Ca5_P3_28943369	Ca5	28943369	CTTTTTTTTTT/CTTTTTTTTTTT	AGGACAAAAGGGCAATAGCA	CACACTTCGATTGTGCCACT	59.7	425	INTERGENIC	--	--	--
Ca5_P3_28977874	Ca5	28977874	CAATGCAGAA/CAATGCAGAATAATGCAGAA	TGCAGAACCAGAACAAGCAC	TCTGGATTTTGATGTGCTGC	60.0	137	INTERGENIC	--	--	--
Ca5_P3_289898	Ca5	289898	ATT/ATTT	AAAGTGCCGAAGTTTGCAGT	TTTTCCCTTCAACAGTCATGG	59.9	351	INTRON	Ca_18185	R	B3
Ca5_P3_291054	Ca5	291054	TAAAAAAAAA/TAAAAAAAAAAA	TGCTTCATATGCTTGACCACA	TGGTGCTTCTTTGGCTCTTT	60.3	476	INTRON	Ca_18185	R	B3
Ca5_P3_291688	Ca5	291688	ATTTTTT/ATTTTTTTT	AAAGAGCCAAAGAAGCACCA	AGCAAATGCGACTGTGATTG	60.0	722	DRR	Ca_18186	--	--
Ca5_P3_291943	Ca5	291943	GCGCAACCGCAA/GCGCAACCGCAACCGCAA	TTCGTGGCTTAATCCCACTC	GTCTGGTGCTCTTCCCAATC	60.1	517	DRR	Ca_18186	--	--

INDEL marker IDs	Chromosomes/ unanchored scaffolds	Physical positions (bp)	InDels (*Kabuli* reference genome-CDC Frontier/PII)	Forward primers (5'-3')	Reverse primers (5'-3')	Anneling temperature (0C)	Expected amplified product size (bp)	Structural annotation		Functional annotation	
								Sequence components of *kabuli* genome	*Kabuli* gene accession IDs	NCBI-KOG	TFs
Ca5_P3_292149	Ca5	292149	A/AC	AAAGTGCCGAAGTTTG CAGT	TGCTTTCCCTTCAACA GTCA	59.9	485	DRR	Ca_18186	--	--
Ca5_P3_292231	Ca5	292231	C/CA	AAAGTGCCGAAGTTTG CAGT	TGCTTTCCCTTCAACA GTCA	59.9	485	DRR	Ca_18186	--	--
Ca5_P3_29247345	Ca5	29247345	ATTTTTTTTTT/ATTTTTTTTTTT	CGTGGAAAACTTCCCT CAAA	TGACGAGCGTAAGAAC ATGC	60.1	853	INTERGENIC	--	--	--
Ca5_P3_29302461	Ca5	29302461	AC/A	CCCAAATCCATCACAAT CCT	GGAGTGTCGTCAACAG CAGA	59.6	720	DRR	Ca_16624	--	--
Ca5_P3_29384323	Ca5	29384323	CTTTTTTTTTTT/CTTTTTTTTT	CTTGGACAATTGAGGC ATTTC	ATAGACAAACGTCCGC CTTC	59.6	545	INTERGENIC	--	--	--
Ca5_P3_29413662	Ca5	29413662	CATATATATATATATATATATATATA/ CATATATATATATATATATATATA	GCTGCTCCTTGGGAAG TAAA	ACCAAGTTGGCTGCTC TGTT	59.5	575	INTERGENIC	--	--	--
Ca5_P3_29418196	Ca5	29418196	CAAAAAAAAA/CAAAAAAAA	TGAAAAACACGGAGTC AAACA	CGTGACACTTCTAGCT TGGGA	59.2	512	INTERGENIC	--	--	--
Ca5_P3_29418887	Ca5	29418887	TTATATATATATATATA/TTATATATATA TATATATA	CCCAAGCTAGAAGTGT CACGA	TTGCATGGGCATAGTG AGAG	60.4	671	DRR	Ca_16633	G	--
Ca5_P3_29601698	Ca5	29601698	ATTATTTATTTATTTAT/ATTATTTATTTA TTTATTTATTTAT	ATCTTTGGTTGGTTGGT TGC	TGCATATCCATTGAGT TTCACA	59.8	607	INTERGENIC	--	--	--
Ca5_P3_29627877	Ca5	29627877	TTAATAATAATAATAATAATAATAATAA TAATAATAATAATAATAATAAT/TTAATA ATAATAATAATAATAATAATAATAATAA TAATAATAATAATAATAAT	TGGTGATATTTTTGTGT GCG	GTGTTGGGCCTAAATG AGGA	58.0	563	INTERGENIC	--	--	--

INDEL marker IDs	Chromosomes/ unanchored scaffolds	Physical positions (bp)	InDels (*Kabuli* reference genome-CDC Frontier/PII)	Forward primers (5'-3')	Reverse primers (5'-3')	Anneling temperature (0C)	Expected amplified product size (bp)	Structural annotation		Functional annotation	
								Sequence components of *kabuli* genome	*Kabuli* gene accession IDs	NCBI-KOG	TFs
Ca5_P3_29638227	Ca5	29638227	CAA/CAACAATAA	CAATCATCCAGAGTTGTAGGAGG	AATGTCCGTGAGGTCACTCC	60.0	566	INTERGENIC	--	--	--
Ca5_P3_29663443	Ca5	29663443	AATATATATATATATATA/AATATATATATATATATATA	CTCCTCCACTAAAAATAATTCCAA	GGGGAGGAGCTTCCTATATCC	57.9	756	INTERGENIC	--	--	--
Ca5_P3_29664249	Ca5	29664249	GAAAAAAAAAAA/GAAAAAAAAAA	ATTCATAAACACATGGCCCC	TAGGGTTAGGTGAGGGGCTT	59.5	255	INTERGENIC	--	--	--
Ca5_P3_29778035	Ca5	29778035	A/AAAT	GCTGCTGGCACATCTACTCA	TAACTGCAACAGATGGTCGC	60.2	547	INTERGENIC	--	--	--
Ca5_P3_29827143	Ca5	29827143	TAAAAAAAAAAA/TAAAAAAAAAA	ATCTGGTTAACGGATGCGAG	TTGGGACTGACAGAAAGAAAAA	60.1	669	INTERGENIC	--	--	--
Ca5_P3_29856414	Ca5	29856414	AATATATATATATATATATATATATATATAT/AATATATATATATATATATATATATATATATATATATATATAT	CACCTTCGTTCGGCCTAATA	CGTGGACAAAGATCGTGATG	60.1	947	DRR	Ca_16668	E	--
Ca5_P3_29901145	Ca5	29901145	GTATATATATATATATATATAT/GTATATATATATATATATATATATATATATAT	GCAGTTTGCGGAGGATTAAG	ATGACGCGGGTGATTTAGTC	59.8	646	INTERGENIC	--	--	--
Ca5_P3_29911055	Ca5	29911055	AATATATATATATATATATATATATATATATATAT/AATATATATATATATATATATAT	TGGGATAAGGCAGAAGATGG	AATTGAGATGTCTGGCCCAC	60.0	545	INTERGENIC	--	--	--
Ca5_P3_29917084	Ca5	29917084	ATTTTTTTT/ATTTTTTTTT	CAACAACAAAGTGCATTGGG	TGGCTTGTTGGATTTAGGACA	60.0	449	INTERGENIC	--	--	--
Ca5_P3_29932346	Ca5	29932346	CATA/CATATA	AAGAGGCGAGCAATTTTTCA	AAATTGGATCCTCGTGATGC	60.0	242	INTERGENIC	--	--	--

INDEL marker IDs	Chromosomes/ unanchored scaffolds	Physical positions (bp)	InDels (*Kabuli* reference genome-CDC Frontier/PII)	Forward primers (5'-3')	Reverse primers (5'-3')	Anneling temperature (0C)	Expected amplified product size (bp)	Structural annotation		Functional annotation	
								Sequence components of *kabuli* genome	*Kabuli* gene accession IDs	NCBI-KOG	TFs
Ca5_P3_29933558	Ca5	29933558	AATATATATATATATATATATATATATATAT/AATATATATATATATATATATATATATATATATATAT	GACCTTTTCAAAGGATGCATTA	TCAGCGTACACTTTTCGACG	58.3	369	INTERGENIC	--	--	--
Ca5_P3_29933647	Ca5	29933647	GATATATATATATA/GATATATATATATATA	GACCTTTTCAAAGGATGCATTA	CGTGATGACAGACCAACACC	58.3	713	INTERGENIC	--	--	--
Ca5_P3_29938246	Ca5	29938246	ATT/ATTT	GCAAAAACAGCGACACTGAA	TTGTGTGGGAAAGGTGACAA	60.0	764	DRR	Ca_16674	--	--
Ca5_P3_29964353	Ca5	29964353	ATAT/A	ATTCGAATTCGGGACATCAC	AATACGTGAACGCAACCACA	59.8	383	INTERGENIC	--	--	--
Ca5_P3_300955	Ca5	300955	GATTTTTTTTTT/GATTTTTTTTTTATATATTTTTTTTT	GGAGCGAAAGAGAAGAGGGT	GGTCTCCCAGACCTACCTCC	60.0	210	INTERGENIC	--	--	--
Ca5_P3_30108030	Ca5	30108030	A/ATAAT	TCATTGCATTCATTCACCAAA	TGGACGGGAGATTGTCTTTC	59.9	945	INTERGENIC	--	--	--
Ca5_P3_30109678	Ca5	30109678	CATAAATAAATAAATAAATA/CATAAATAAATAAATAAATAAATA	CGACCAATTGTCTTTTCCGT	GAATTTCAGCCCAAGTTCCA	60.0	749	INTERGENIC	--	--	--
Ca5_P3_30114886	Ca5	30114886	TTATATATATATATATATATATATATATATATA/TTATATATATATATATATATATATATATA	CGGGTGGCTTAGTGAGAAGT	AAACTCTTTCCGCTCACCTTT	59.4	325	INTERGENIC	--	--	--
Ca5_P3_30119778	Ca5	30119778	CTTTTTTTTTT/CTTTTTTTTTTT	AGGGAACGAAGTGCACAACT	TGCAAATTTCATCAAATGCATAA	59.8	771	INTERGENIC	--	--	--
Ca5_P3_30121835	Ca5	30121835	TTATATATATATATATATATATATATATATAT/TTATATATATATATATATATATATATATATATATATATATAT	CATGCGTGAATTTGATCGTC	AAACGCGTCACTTTACCTTCTC	60.1	224	INTERGENIC	--	--	--

INDEL marker IDs	Chromosomes/ unanchored scaffolds	Physical positions (bp)	InDels (*Kabuli* reference genome-CDC Frontier/PII)	Forward primers (5'-3')	Reverse primers (5'-3')	Anneling temperature (0C)	Expected amplified product size (bp)	Structural annotation		Functional annotation	
								Sequence components of *kabuli* genome	*Kabuli* gene accession IDs	NCBI-KOG	TFs
Ca5_P3_30126893	Ca5	30126893	ATTTTTTTTT/ATTTTTTTTTT	GTGGCTTCCAAAGAGAAACG	TTCGGTACACAATTTTCCCC	59.9	612	INTERGENIC	--	--	--
Ca5_P3_30128968	Ca5	30128968	GATTATAATTAAAAATA/GATTATAATTAAAAATAATTATAATTAAAAATA	TGTTTCTCTCTCCGCTCACA	CCGCCGACTATACCTGTTTT	59.7	681	INTERGENIC	--	--	--
Ca5_P3_30131050	Ca5	30131050	AATATATATATATATATATATATA/AATATATATATATATATATATATATATATA	CAAATTTCGAGCAACAATGC	AACACAAATGTTAAATTGCGAAGA	59.3	571	INTERGENIC	--	--	--
Ca5_P3_30141422	Ca5	30141422	TATTTTCCTTCATT/TATT	TACTGACGTGAGTCCCACCA	TGTTAGATACGTGTCGCGGA	60.2	637	INTRON	Ca_04690	J	E2F/DP
Ca5_P3_30253731	Ca5	30253731	AATATATATATATATATATATATATATAT/AATATATATATATATATATATATATATAT	CAAATTTGATCTTGCTTCTTTCAA	TTGCAGGTCATAACCTTTTGT	59.8	873	INTERGENIC	--	--	--
Ca5_P3_30284983	Ca5	30284983	ATATTATTATTATTATTATTATTATTATTATTATTATTGTTATTGTTATTATTATTATTAT/ATATTATTATTATTATTATTATTATTATTATTATTATTATTATTATTATTGTTATTGTTATTATTATTATTAT	TGTTGTCACATGCTTTGGGT	TTGTGCACATTTTATTCTTCAAA	60.0	651	INTERGENIC	--	--	--
Ca5_P3_30478691	Ca5	30478691	GTTTTTTTTTTT/GTTTTTTTTTTTT	TTCGTCAATTCTTTTGACAACG	AAAACAGACGGGTCCACAAA	60.1	423	INTERGENIC	--	--	--
Ca5_P3_30478963	Ca5	30478963	TGA/T	TTGTGGACCCGTCTGTTTTT	CAACACATGCCCTGAATCTG	60.4	436	INTERGENIC	--	--	--
Ca5_P3_30481129	Ca5	30481129	GTTTTTTTT/GTTTTTTTTT	CAAACACGCCCTCAATCTTT	AGTCCCATCCAAATTCCCTC	60.1	203	INTERGENIC	--	--	--
Ca5_P3_30540589	Ca5	30540589	TATC/TATCATC	CATTAATAGCTGCACGCCAA	AAGTTTTGGGACAAAATTGAACA	59.9	643	DRR	Ca_04742	--	--

**INDEL marker IDs**	**Chromosomes/ unanchored scaffolds**	**Physical positions (bp)**	**InDels (*Kabuli* reference genome-CDC Frontier/PII)**	**Forward primers (5'-3')**	**Reverse primers (5'-3')**	**Anneling temperature (0C)**	**Expected amplified product size (bp)**	**Structural annotation**		**Functional annotation**	
								**Sequence components of *kabuli* genome**	***Kabuli* gene accession IDs**	**NCBI-KOG**	**TFs**
Ca5_P3_30610017	Ca5	30610017	AAATAATAATAATAATAATAATAATAATAATA/AAATAATAATAATAATAATAATAATAATA	TTGCAATCAAACTTCTTGAAAA	GCCATTCAATGAACAACGTG	57.6	604	INTERGENIC	--	--	--
Ca5_P3_30612967	Ca5	30612967	TAAAAAAAAA/TAAAAAAAAAA	CGGCATATCATCACCCTTTT	ACCCCCGAAAAAGTGCTAGT	59.8	378	INTERGENIC	--	--	--
Ca5_P3_30731838	Ca5	30731838	TTATATATATATATATATATAT/TTATATATATATATATATATATATATATAT	TTCTGAAACAAACGCACCAA	TCAGAATAGGCTAGGCCGTC	60.3	406	INTERGENIC	--	--	--
Ca5_P3_30777454	Ca5	30777454	TTATATATATATATATATATATATATATATAT/TTATATATATATATATATATATATATAT	AAACCTTTGAGAAATCGGCA	AGGAAGCCCAAGTTCCAAAT	59.7	382	INTERGENIC	--	--	--
Ca5_P3_30793756	Ca5	30793756	GTTTTATTTTTTT/GTTTTATTTTTTTATTTTTTT	AGTTATGGTGGGGCAGTGAG	TTTGAACCAAACGACTTCCC	60.0	623	INTERGENIC	--	--	--
Ca5_P3_30880201	Ca5	30880201	CA/CAA	CCTGTATTCTGCCTCAGCGT	CAACCTAAGATTGGCCGTGT	60.4	494	DRR	Ca_04784	R	--
Ca5_P3_30920900	Ca5	30920900	ATTTTTTTTTTTT/ATTTTTTTTTTT	CCATATTCTGGCTTGGGCTA	CGAACACCAAAACAAAACGA	60.1	824	DRR	Ca_04789	--	--
Ca5_P3_30972147	Ca5	30972147	TTATATATATATATATATA/TTATATATATATATATATATATA	GCCTTTGGGGTGATGAAGTA	GCTTCTTGGCAATCTTCCTG	59.9	559	INTRON	Ca_04796	T	--
Ca5_P3_30990200	Ca5	30990200	ATTT/ATTTTTTT	GAATCCCCAAACAAAACCCT	CACACCACCAGCATTTTGAC	60.0	540	INTERGENIC	--	--	--
Ca5_P3_31002418	Ca5	31002418	TAAAAAAAAAA/TAAAAAAAAAAA	AAGCAAACAAACAAGAATTTCCTC	CGAACTTAGGCTCAAATTCCC	60.0	497	INTERGENIC	--	--	--

INDEL marker IDs	Chromosomes/ unanchored scaffolds	Physical positions (bp)	InDels (*Kabuli* reference genome-CDC Frontier/PII)	Forward primers (5'-3')	Reverse primers (5'-3')	Anneling temperature (0C)	Expected amplified product size (bp)	Structural annotation		Functional annotation	
								Sequence components of *kabuli* genome	*Kabuli* gene accession IDs	NCBI-KOG	TFs
Ca5_P3_31075075	Ca5	31075075	TAAAAAAAAAA/TAAAAAAAAA	ATACGAGGAGGCGTGAGCTA	GCAGTGAATCTTGCGTCGTA	60.0	712	INTERGENIC	--	--	--
Ca5_P3_31129774	Ca5	31129774	TAGAGAG/TAGAG	TCGAGTTCACACATTCCATCA	TACCATTCCCAACCACACCT	60.1	795	INTERGENIC	--	--	--
Ca5_P3_31131906	Ca5	31131906	TC/T	GGTGTTCAACAGGATGCCTT	CAGGGAATTGATTCGAGCAT	60.0	608	INTRON	Ca_04815	--	--
Ca5_P3_31133268	Ca5	31133268	TG/T	ACACACCATTAGGCTCACCC	GGTCTCCGCGTTCTGTGTAT	59.9	139	DRR	Ca_04815	--	--
Ca5_P3_31133950	Ca5	31133950	A/AC	GAGGAGATTGGGGAGCAACT	CTCATTGGCCATGAAAGGAT	60.6	539	DRR	Ca_04815	--	--
Ca5_P3_31136023	Ca5	31136023	AAAAT/A	TCACAATTTCCCAACCCAAT	AATTTCTACCAATGCGTCCG	60.0	796	INTRON	Ca_04816	G	Trihelix
Ca5_P3_31143475	Ca5	31143475	GTAACTAACTAACTAACTAACT/GTAACTAACTAACTAACT	CAAAGCTGCAACAAATGGAA	GTTGGTGTTGGGAGAAGGAA	59.8	663	INTRON	Ca_04817	PET	NAC
Ca5_P3_3115746	Ca5	3115746	CAAAAAAAA/CAAAAAAA	GCAACATGGGATGATTCAGA	TCCAAGTCGTTCAGCTTGTG	59.5	628	INTERGENIC	--	--	--
Ca5_P3_31158732	Ca5	31158732	AATTTGGAATATTTGGAAT/AATTTGGAAT	TTCATCGTGTGCAACCAAAT	CCATTTGCTCTACCTCTCGC	60.0	840	DRR	Ca_04819	--	--
Ca5_P3_31167819	Ca5	31167819	A/AC	TGTGAACGATGAAGCAGAGG	AGCTGTGGAGCAAACCAACT	60.0	623	INTRON	Ca_04821	--	--

INDEL marker IDs	Chromosomes/ unanchored scaffolds	Physical positions (bp)	InDels (*Kabuli* reference genome-CDC Frontier/PII)	Forward primers (5'-3')	Reverse primers (5'-3')	Anneling temperature (0C)	Expected amplified product size (bp)	Structural annotation		Functional annotation	
								Sequence components of *kabuli* genome	*Kabuli* gene accession IDs	NCBI-KOG	TFs
Ca5_P3_31181722	Ca5	31181722	AATGAATTATGAATTATGAATTATGAATT/AATGAATTATGAATTATGAATT	TCAGGCGTTAAATCTCGCTT	ATCAGTGGAGCTGAGGCTGT	60.0	439	INTRON	Ca_04822	TU	C3H
Ca5_P3_31183021	Ca5	31183021	ATTT/ATTTTTT	CCAAACACTCAAACCGACTC	AAAGACCAAATTGGACACTTCA	58.2	817	DRR	Ca_04823	--	WOX
Ca5_P3_31237915	Ca5	31237915	TAAAAAAA/TAAAAAAAA	ACACAAATTCGCCAAAATCC	CATCATGCCTCAAGATCACG	59.8	768	INTERGENIC	--	--	--
Ca5_P3_31382813	Ca5	31382813	GTT/GTTT	GAATTGGAGAGGATGCCGTA	GAACAAGGACTGCGTTGTCA	60.0	415	INTRON	Ca_04840	S	--
Ca5_P3_31594106	Ca5	31594106	TAAAAAAAAA/TAAAAAAAA	GTATGGGCGGAGAAACTCAA	GCAGACAATAGTGGGCTAAAAA	60.1	813	INTRON	Ca_04861	R	--
Ca5_P3_31621509	Ca5	31621509	CT/CTGTATATCTACTTT	ATCCGAGTTTGCGTATGTCC	GGGACGTGGAGATTGTTGAT	60.0	635	INTERGENIC	--	--	--
Ca5_P3_316293	Ca5	316293	AATATATATATATATATATATAT/AATATATATATATATATAT	TTCTACCTTTCTCGCCGCTA	TTAATTGAAAGTGGGGGTGAA	60.1	562	INTERGENIC	--	--	--
Ca5_P3_317066	Ca5	317066	TAAA/TAAAA	GAGCATTGATCCGGATTGTAA	TTTGACGATGCAACGGTTTA	59.9	544	INTERGENIC	--	--	--
Ca5_P3_31809508	Ca5	31809508	TAAAAAAAAA/TAAAAAAAA	TCTGTTCTCGACGTTGTTCG	CAAAAGAACATGGACCACGA	60.0	637	INTRON	Ca_04886	Z	--
Ca5_P3_31821908	Ca5	31821908	ATTTTTTTTTTT/ATTTTTTTTTT	TGGGTGGTATCCAAGAAAGC	TGCTCTTTGCGACCCTTACT	59.9	631	INTERGENIC	--	--	--

INDEL marker IDs	Chromosomes/ unanchored scaffolds	Physical positions (bp)	InDels (*Kabuli* reference genome-CDC Frontier/PII)	Forward primers (5'-3')	Reverse primers (5'-3')	Anneling temperature (0C)	Expected amplified product size (bp)	Structural annotation		Functional annotation	
								Sequence components of *kabuli* genome	*Kabuli* gene accession IDs	NCBI-KOG	TFs
Ca5_P3_31842102	Ca5	31842102	AATTATTATTATTATTATTATTATTATTATTATTATTATTATTATTAT/AATTATTATTATTATTATTATTATTATTATTATTATTATTATTATTAT	GCCTCGTTGTCATGCCTAGT	ATTTCAAAGGGCCACCTCTT	60.3	793	INTERGENIC	--	--	--
Ca5_P3_31847315	Ca5	31847315	ATTTTTTTTTTTT/ATTTTTTTTTTT	GGACTATTCAACCATGCGCT	CGCCATTTGTTTTGTTTCAA	60.1	701	INTERGENIC	--	--	--
Ca5_P3_31930749	Ca5	31930749	GATATATATATATATATATATATA/GATATATATATATATATATA	TGTTTTGCTTGGTTTCACGA	GCACCGAAACATGACACAAC	60.3	500	INTERGENIC	--	--	--
Ca5_P3_31960994	Ca5	31960994	GATATATATATATATATAT/GATATATATATATATATAT	GTCCTCAAAATTTCATGCCC	GAAAATTTGAATTTGCCGTGA	59.4	601	INTERGENIC	--	--	--
Ca5_P3_32115199	Ca5	32115199	C/CCTTTTAAT	GGATTCTTGAAACCCCGTCT	AACCAATGACGATGAATCGAG	60.3	263	INTERGENIC	--	--	--
Ca5_P3_32137515	Ca5	32137515	GT/GTT	ACCACCAAACGTAAAACCCA	TTTCTTGTGTGAAGCCATGC	60.1	429	INTERGENIC	--	--	--
Ca5_P3_32139548	Ca5	32139548	TTATATATATATATATATATAT/TTATATATATATATATAT	GCGACTTCGCAATGTGTAGA	GTTAAGGAAGTCCGCGTTTG	60.0	531	INTERGENIC	--	--	--
Ca5_P3_32179322	Ca5	32179322	CTTTTTTTTTTT/CTTTTTTTTTT	TTTCGGAAGGGTTGAAGTTG	GGCTGTTCATGGTTGGTTTT	60.1	690	INTERGENIC	--	--	--
Ca5_P3_32279202	Ca5	32279202	CAATAATAATAATAATAATA/CAATAATAATAATAATAATA	CACGGCTCTGATCATAAGAAA	AACCAGCTTTTTAGTTCGTCCTT	58.0	868	INTERGENIC	--	--	--
Ca5_P3_32299791	Ca5	32299791	ATTTTTTTTTT/ATTTTTTTTTTT	CAAGCAAAACCCTCAAAAGC	GGCGGATCAACTCTTTGTGT	59.9	364	INTERGENIC	--	--	--

INDEL marker IDs	Chromosomes/ unanchored scaffolds	Physical positions (bp)	InDels (*Kabuli* reference genome-CDC Frontier/PII)	Forward primers (5'-3')	Reverse primers (5'-3')	Anneling temperature (0C)	Expected amplified product size (bp)	Structural annotation		Functional annotation	
								Sequence components of *kabuli* genome	*Kabuli* gene accession IDs	NCBI-KOG	TFs
Ca5_P3_32375018	Ca5	32375018	AATTATTATTATTATTATTATTATTATTATTATTATTATT/AATTATTATTATTATTATTATTATTATTATTATTATTATTATTATTATTATTATT	CACCTCTCCACAAGCAAACA	AACGGAAGCCCCTATTATGC	59.9	964	INTRON	Ca_04936	--	WRKY
Ca5_P3_32388360	Ca5	32388360	TATAATAATAATAATAATAATAATAATAATAATAATAAT/TATAATAATAATAATAATAATAATAATAATAATAAT	CAAATCGGGAGGAAGTTGTT	TTTTGCCCCAATTGCTTATC	59.0	715	DRR	Ca_04938	--	bZIP
Ca5_P3_32388967	Ca5	32388967	CTATTATTTTATTATTTTATTA/CTATTATTTTATTATTTTATTATTTTATTA	AATTGCGATAAGCAATTGGG	TAGGATTACACGGGGCAGAC	59.9	821	DRR	Ca_04938	--	bZIP
Ca5_P3_32467772	Ca5	32467772	AATATATATATATATATATATATA/AATATATATATATATATATA	AAGCCCAAGTTGCAAATGAC	GGCCCACATATGAAGGTGAA	60.1	729	INTERGENIC	--	--	--
Ca5_P3_32605650	Ca5	32605650	TAAAAAAAAAA/TAAAAAAAAA	TCAATCACAAATCAGGGTGC	ATCCAGATCCAATACGACGG	59.5	874	INTERGENIC	--	--	--
Ca5_P3_32610734	Ca5	32610734	TAAAAAAAAA/TAAAAAAAAAA	ATCCAGCCGTAAAAACAACG	GCTGGGGTTTGAAAAATCCT	60.0	832	INTERGENIC	--	--	--
Ca5_P3_32622195	Ca5	32622195	TCTCT/TCTCTCTCGCTCT	ATGGCTCTTTGGGCTAAGGT	GCCTCTGCTTGACTAATGCC	60.1	907	INTRON	Ca_04968	U	--
Ca5_P3_32673383	Ca5	32673383	TTGTGTGTGTGTGTGTGT/TTGTGTGTGTGTGTGTGTGT	TGAGGGTTAAGGGATAGGGG	CATGGAGTGTGACGAAAGGA	60.1	691	INTRON	Ca_04975	--	SBP
Ca5_P3_32694142	Ca5	32694142	AAATAATAATAATAATAATAATAATAA/AAATAATAATAATAATAATAATAATAATAA	GCCATGATATTTTGCCGATAA	GATCGCAACCTGAGGATGTT	59.8	553	DRR	Ca_04975	--	SBP
Ca5_P3_32736061	Ca5	32736061	TTT/TTTCCTT	AAAAGGGTGTCACAAGTGGG	TGAAAAGGATGAAAGTCGCA	59.9	250	INTRON	Ca_04978	T	--

INDEL marker IDs	Chromosomes/ unanchored scaffolds	Physical positions (bp)	InDels (*Kabuli* reference genome-CDC Frontier/PII)	Forward primers (5'-3')	Reverse primers (5'-3')	Anneling temperature (0C)	Expected amplified product size (bp)	Structural annotation		Functional annotation	
								Sequence components of *kabuli* genome	*Kabuli* gene accession IDs	NCBI-KOG	TFs
Ca5_P3_32745764	Ca5	32745764	TAAA/TAAAA	AGGCATCACATTGAGGGACT	CCTCGGTGACCCTGAATAAA	59.5	148	INTERGENIC	--	--	--
Ca5_P3_327512	Ca5	327512	ATTTTTTTTTT/ATTTTTTTTTTT	GCTTCTGCATGGTTTTGTCA	AGGATCATCCATTGGAGCAG	59.8	466	INTRON	Ca_18189	G	--
Ca5_P3_32848829	Ca5	32848829	GAAAAAAAAAA/GAAAAAAAAAAA	AACATTTACCAGTCCAGCGG	TCACCGTATGCAAAGACGAG	60.0	848	INTERGENIC	--	--	--
Ca5_P3_328796	Ca5	328796	ATTTTTTTTTTTTT/ATTTTTTTTTTTT	ATGAACAGGTGGCATGTGAA	TGCAAATGAGCAATCTCCAG	60.0	467	INTRON	Ca_18189	G	--
Ca5_P3_32879637	Ca5	32879637	AGTAAACAATGTAAACAA/AGTAAACAA	TCCTGCTAAAGATGGTCATTCA	TTGTTGTAGGATGGATGGAGTG	59.7	778	INTERGENIC	--	--	--
Ca5_P3_32891516	Ca5	32891516	TAAAAAAAAAAA/TAAAAAAAAAAAA	TCTCTCTCTCTCCATCCCCA	AAGGCATGGCTCGTGTGTAT	59.9	840	INTERGENIC	--	--	--
Ca5_P3_32912336	Ca5	32912336	AATATATATATATATATATATATATAT/AATATATATATATATATATATATATATATATATATATAT	ACCCCCTCACCTCCTTATTG	TTTTCTTGCGTCACTTGTTGA	60.2	499	INTERGENIC	--	--	--
Ca5_P3_3293717	Ca5	3293717	GTATATATATATATATATATATATATATA/GTATATATATATATATATATATATATA	GCCTCTCAGGCCTAACATCA	TTTGGGGACAAGATCCAACT	60.4	391	INTERGENIC	--	--	--
Ca5_P3_3295289	Ca5	3295289	CATATATATATATA/CATATATATATA	CCAACAAACTTTACGGAACCA	AAGGGGGAGAGGAATGATGT	59.9	217	INTERGENIC	--	--	--
Ca5_P3_33099227	Ca5	33099227	ACCCC/ACCCCC	GATCACAATGGACGGTAAAGC	CAAATGTGGAGGAAAGTGGG	59.4	784	INTERGENIC	--	--	--

INDEL marker IDs	Chromosomes/ unanchored scaffolds	Physical positions (bp)	InDels (*Kabuli* reference genome-CDC Frontier/PII)	Forward primers (5'-3')	Reverse primers (5'-3')	Anneling temperature (0C)	Expected amplified product size (bp)	Structural annotation		Functional annotation	
								Sequence components of *kabuli* genome	*Kabuli* gene accession IDs	NCBI-KOG	TFs
Ca5_P3_33115755	Ca5	33115755	G/GGCCTTCAGGGA	TTTGCGGTTCTCTTGCTTTT	TGTGTTATGATCCCCCGATT	60.0	554	INTRON	Ca_05007	--	MYB_related
Ca5_P3_3317625	Ca5	3317625	ATATTATTATTATTATTATTATTATTATTATTATTATTATTATTATTATTATTATTATTATTATTATTATTATT/ATATTATTATTATTATTATTATTATTATTATTATTATTATTATTATTATTATTATTATTATTATTATTATTATTATTATT	GAAATTGATCTTCCCCAGCA	GGGTGTTAATCATTCTACATCATCG	60.0	762	INTERGENIC	--	--	--
Ca5_P3_33354598	Ca5	33354598	TAAAAAAAAAA/TAAAAAAAAA	TGACAGTTACAAACCGCTGC	CAAATTTCTACCAATTCCCCA	59.9	363	INTERGENIC	--	--	--
Ca5_P3_33395960	Ca5	33395960	AATTATTATTATTATTATT/AATTATTATTATTATT	TTTATCGGCTAAGGGACACG	GTGCTGCCTAGACAATCGGT	60.1	768	INTERGENIC	--	--	--
Ca5_P3_33444959	Ca5	33444959	GATATA/GATATATA	TTATGGAGGCCACATTCACA	GAAATTAAGGCCTCTTGATCTTTTT	59.9	961	DRR	Ca_01912	O	MYB_related
Ca5_P3_33456475	Ca5	33456475	TTATGTTAAATATGTTAA/TTATGTTAAATATGTTAAATATGTTAA	GATGAAGTGCTGATCAGAGGC	ACCAGGAAACATTGCCAGAC	60.0	789	DRR	Ca_01911	O	MYB_related
Ca5_P3_33457924	Ca5	33457924	ATTTTTTT/ATTTTTTTT	CCGACACCCAAAATTTCAAC	GCCCTCAGATGAGAGTCAGG	60.2	680	DRR	Ca_01911	O	MYB_related
Ca5_P3_33493974	Ca5	33493974	TAAAAAAAAAAAAAA/TAAAAAAAAAAAAAA	CAACAGAACAATAACGGCCA	CTTGTGACTTCGTGCGGTAA	59.6	484	INTERGENIC	--	--	--
Ca5_P3_33542100	Ca5	33542100	GTTTATTTATTTATTTAT/GTTTATTTATTTATTTATTTATTTAT	AATTCGGTTTTTGGCGTGTA	GTTAAGGGACCACGAATGCT	60.4	569	INTERGENIC	--	--	--
Ca5_P3_33547836	Ca5	33547836	TTATATATATATATATATATATATAT/TTATATATATATATATATATATATATATAT	TTGGGCACATCTTGTGCTAC	GGCACACATTCACAATTCACA	59.7	411	INTERGENIC	--	--	--

INDEL marker IDs	Chromosomes/ unanchored scaffolds	Physical positions (bp)	InDels (*Kabuli* reference genome-CDC Frontier/PII)	Forward primers (5'-3')	Reverse primers (5'-3')	Anneling temperature (0C)	Expected amplified product size (bp)	Structural annotation		Functional annotation	
								Sequence components of *kabuli* genome	*Kabuli* gene accession IDs	NCBI-KOG	TFs
Ca5_P3_33549168	Ca5	33549168	TAAAAAAAAA/TAAAAAAAAAA	CAAAAACGGGTTTCACCAAC	GCTCTGCATCCAATTCCATT	60.2	447	INTERGENIC	--	--	--
Ca5_P3_33600042	Ca5	33600042	CT/C	TAACCATTCCACAGCCACAA	CTTAGAGGTCGCTTCAACGG	60.0	680	DRR	Ca_01896	--	--
Ca5_P3_33605865	Ca5	33605865	TC/T	TTTTTCTCAGTGATGCGTGC	AAATGGGAGGGAAGTGTGTG	60.0	775	INTERGENIC	--	--	--
Ca5_P3_33606817	Ca5	33606817	ATTTTACTTGTTTT/ATTTT	GACCTTGCCGGCTACTCTCT	ATATTTCTCATAGGGGCGGC	60.9	449	INTERGENIC	--	--	--
Ca5_P3_33615152	Ca5	33615152	CTATAT/CTATATAT	AACCAACACAGCCACATGAA	CCGCATTGACATCGTAAAAA	60.0	575	INTERGENIC	--	--	--
Ca5_P3_33632878	Ca5	33632878	ATTTTTTTTT/ATTTTTTTT	ACAATCGTCCAGCTTTGGAG	CATGCCACAAAAATTCAACG	60.3	723	INTERGENIC	--	--	--
Ca5_P3_338511	Ca5	338511	ATTTTTTTTT/ATTTTTTTTTT	TTTTGTGGTAGGGCATAAAAA	CGCACGTGTGAGATTTTGTT	57.3	826	DRR	Ca_18191	A	--
Ca5_P3_33918677	Ca5	33918677	TTCTCTCTCTCTCTCTCTCTCTCTCTCTCTC/TTCTCTCTCTCTCTCTCTCTCTCTCTCTCTCTCTC	TGTGCCTTGTGCTTAAGTGG	GGTTGCGCATAACAAACCTT	59.9	414	INTRON	Ca_01860	P	--
Ca5_P3_33967546	Ca5	33967546	GAAAAAAAAAAA/GAAAAAAAAAA	TGTCGCAAATGGCAACTAAA	TCCATCTTCGCCTAACCAAT	60.3	619	INTERGENIC	--	--	--
Ca5_P3_34160184	Ca5	34160184	TAAAAAAAAA/TAAAAAAAAAA	TTCCAGTTTTGTGTCCCCAT	TTGTGTTTTTGGAACACCGA	60.2	455	INTERGENIC	--	--	--

INDEL marker IDs	Chromosomes/ unanchored scaffolds	Physical positions (bp)	InDels (*Kabuli* reference genome-CDC Frontier/PII)	Forward primers (5'-3')	Reverse primers (5'-3')	Anneling temperature (0C)	Expected amplified product size (bp)	Structural annotation		Functional annotation	
								Sequence components of *kabuli* genome	*Kabuli* gene accession IDs	NCBI-KOG	TFs
Ca5_P3_34164848	Ca5	34164848	C/CA	CTTCCAGGGGATGCAAGATA	CGTAGGCAATAAGACACGCA	60.0	377	DRR	Ca_01832	--	NF-YB
Ca5_P3_34191964	Ca5	34191964	AAATAATAATAATAATAATAATAATAAT/AAATAATAATAATAATAATAATAATAATAAT	AGGCAAAATGTTTTTCACGG	TTTGTTTTGGCACATGCACT	60.0	561	DRR	Ca_01828	--	--
Ca5_P3_34218888	Ca5	34218888	GATATATATATATATATATATATATATATAT/GATATATATATATATATATATATATATATAT	TAAGTGCACAAGGCATCGAG	TCCCACCTTCTAAAACCCAA	60.0	298	INTERGENIC	--	--	--
Ca5_P3_34220162	Ca5	34220162	GAAAAAAAAAAA/GAAAAAAAAAA	CCACTAACCTATTCGGAATCCA	GGCAATTGTCGGCACTTAAT	60.2	462	INTERGENIC	--	--	--
Ca5_P3_34221308	Ca5	34221308	GC/GCC	GGTGGTCTACGCATGAGGTT	TACACTACCCAGCCCAGTCC	60.0	970	INTERGENIC	--	--	--
Ca5_P3_34228604	Ca5	34228604	GTTTTTTTTTT/GTTTTTTTTT	CGAACATCTCCAACGGTATTC	TTATCTATTTGGGGGAGGGG	59.4	415	INTERGENIC	--	--	--
Ca5_P3_34235777	Ca5	34235777	AATATATATATATATATATATAT/AATATATATATATATATATATATATAT	CAATGGTCCAACCTATCACAA	GCCAGTACACCAAGTGACGA	58.3	206	INTERGENIC	--	--	--
Ca5_P3_34263941	Ca5	34263941	TTCAGCTATAGTGATATCAGCTATAGTGATAT/TTCAGCTATAGTGATATCAGCTATAGTGATATCAGCTATAGTGATAT	CAAGTGGATTCTTCCCCTCA	CCTTTTGTGGATTGCGTTTT	60.0	825	INTRON	Ca_01820	--	--
Ca5_P3_34315432	Ca5	34315432	GTTATTTTTATTTTTATTTTTATTTTTATTTTTATTTT/GTTATTTTTATTTTTATTTTTATTTTTATTTT	TGACGCACCGTAGATGAGAG	CAAGTAATGGGCTTCATGGG	60.0	248	DRR	Ca_01816	PT	C3H
Ca5_P3_34317381	Ca5	34317381	TAAAAAAAAAA/TAAAAAAAAA	AAAAACTTGCATAATTGTTTTGGA	TTTCGTATGATTTGGGCGAT	59.0	495	INTERGENIC	--	--	--

INDEL marker IDs	Chromosomes/ unanchored scaffolds	Physical positions (bp)	InDels (*Kabuli* reference genome-CDC Frontier/PII)	Forward primers (5'-3')	Reverse primers (5'-3')	Anneling temperature (0C)	Expected amplified product size (bp)	Structural annotation		Functional annotation	
								Sequence components of *kabuli* genome	*Kabuli* gene accession IDs	NCBI-KOG	TFs
Ca5_P3_34323415	Ca5	34323415	TTAT/TT	CGTTTCATTTGTTTCTCTTTCTCA	GCACCAACAACTCTTGCTCA	59.8	599	INTERGENIC	--	--	--
Ca5_P3_34355845	Ca5	34355845	AATATATATATATATATATATATATATATATA/AATATATATATATATATATATATATATATATATATA	TGGGTTTTTCAATTGTTGGG	AGCCTAAATCCATGGTTATTTTT	60.6	736	DRR	Ca_01810	--	--
Ca5_P3_34466555	Ca5	34466555	A/ATAAT	TGTTGTTCCTCTTTTCACGTTG	ACCAAAGTAATGCGGTTGAA	60.2	762	INTERGENIC	--	--	--
Ca5_P3_34548859	Ca5	34548859	GAAAAAAAA/GAAAAAAA	ATTGATTTCGCAATAAGCGG	CCTAGCCGTCTCATTTGGTT	60.1	629	INTERGENIC	--	--	--
Ca5_P3_34771204	Ca5	34771204	AATATATATATATATATATATATATAT/AATATATATATATATATATATATATATATAT	TCTCATGTTTCCCAACCACA	CTGGACCACATTCTGGCTCT	59.9	487	DRR	Ca_01764	--	ERF
Ca5_P3_34835083	Ca5	34835083	GATAATAATAATAATAATAATAATAATAATAATAATAATAATAATAATAAT/GATAATAATAATAATAATAATAATAATAATAATAATAATAATAATAATAATAAT	TCCTCTATTTGCGTCCTTCG	CACCCTAATAACCCGAGCAA	60.3	586	INTERGENIC	--	--	--
Ca5_P3_34851417	Ca5	34851417	TA/TAA	GACAGAGAGATGACGGGGAA	AAACCGAAAAGTTGAAAAACTGA	60.2	569	INTERGENIC	--	--	--
Ca5_P3_34871969	Ca5	34871969	GA/GAAATGAA	TGACGTTGCATTCACCAAAT	AGGGAGGACGAAAATGACCT	60.0	609	INTERGENIC	--	--	--
Ca5_P3_35056527	Ca5	35056527	A/AT	AGTCGCAACTTTGGCTAGGA	TTCGACAATCATGGCTGAAA	60.0	459	DRR	Ca_01730	J	--
Ca5_P3_35070670	Ca5	35070670	TATTAATTAATTAATTAATT/TATTAATTAATTAATTAATTAATT	TGTGACCTGCAGTGATATTTTG	ATAATGTTGCGAATGGAGGC	58.7	617	INTERGENIC	--	--	--

INDEL marker IDs	Chromosomes/ unanchored scaffolds	Physical positions (bp)	InDels (*Kabuli* reference genome-CDC Frontier/PII)	Forward primers (5'-3')	Reverse primers (5'-3')	Anneling temperature (0C)	Expected amplified product size (bp)	Structural annotation		Functional annotation	
								Sequence components of *kabuli* genome	*Kabuli* gene accession IDs	NCBI-KOG	TFs
Ca5_P3_35149505	Ca5	35149505	CTTTTTTTTTT/CTTTTTTTTTTT	CAATCACATGGCTTGGATGA	CAGCCACACCGATTACCTTT	60.5	400	INTERGENIC	--	--	--
Ca5_P3_35157632	Ca5	35157632	AATATATATATATATATATAT/AATATATATATATATATATATATAT	GTGTCGTTTACCACTGGGCT	CATGCCATACAAGCCACAAC	60.0	652	INTERGENIC	--	--	--
Ca5_P3_35190711	Ca5	35190711	GAAAA/GAAAAA	TGTCACTTTTTGTTTCGTCCT	TGAAATGTTGCCCACTGTGT	57.4	454	DRR	Ca_01716	O	--
Ca5_P3_35231327	Ca5	35231327	ATTTTTTTT/ATTTTTTTTT	CACGAACCATAGCATGGGA	ATTGGATCGGGCCTAGTGTA	60.5	834	INTERGENIC	--	--	--
Ca5_P3_35246700	Ca5	35246700	GAAAAAAAA/GAAAAAAAAA	GCCAGGTAGCTGCTCCATAA	GCATGCAACAAAAGGCTAAA	60.4	761	INTERGENIC	--	--	--
Ca5_P3_35328160	Ca5	35328160	ATTTTTTTTT/ATTTTTTTTTT	ACCACGTGACCCCTTAACTT	TGATGAAATTACGTTATATTGAGGATG	58.4	204	INTERGENIC	--	--	--
Ca5_P3_35331425	Ca5	35331425	TAAA/TAA	CCTCGGGTTTGCATATGTCT	TTTGGATGGAAGAGTACCCG	60.0	711	DRR	Ca_01701	--	--
Ca5_P3_35485623	Ca5	35485623	TAAAAAAAAA/TAAAAAAAAAA	CCCAACTTCTTCATTGCCTC	GGACCCAGCAGAGTTGGTAA	59.7	639	INTERGENIC	--	--	--
Ca5_P3_35516951	Ca5	35516951	CTAAA/C	CTGGATGGCCTTTTTGTTGT	TTCCAAAGCAATGTGTCAGC	60.0	641	INTRON	Ca_01680	IE	B3
Ca5_P3_35527896	Ca5	35527896	ATTTTTTTT/ATTTTTTTTT	AAATAAAAGGGGAAGGTGGC	CGTTTAACTCTGCGTGGGAT	59.3	717	INTERGENIC	--	--	--

INDEL marker IDs	Chromosomes/ unanchored scaffolds	Physical positions (bp)	InDels (*Kabuli* reference genome-CDC Frontier/PII)	Forward primers (5'-3')	Reverse primers (5'-3')	Anneling temperature (0C)	Expected amplified product size (bp)	Structural annotation		Functional annotation	
								Sequence components of *kabuli* genome	*Kabuli* gene accession IDs	NCBI-KOG	TFs
Ca5_P3_35595695	Ca5	35595695	TTAATAATAATAATAATAATAATAATAATAATAATAATAATAAT/TTAATAATAATAATAATAATAATAATAATAATAATAATAATAATAATAAT	ACCATTCAAACGGCGAGTAG	ATGCAATCGTATCTCGTGGT	60.1	306	INTERGENIC	--	--	--
Ca5_P3_35668277	Ca5	35668277	CATATATATATATATA/CATATATATATATATATA	ATTGATTGTCTTGTCGGCCT	AATTAACTTCCACGGAGCCC	59.6	590	INTERGENIC	--	--	--
Ca5_P3_35689449	Ca5	35689449	TC/T	CGTTGCAACAGAAGCAGAAG	GATCCAAAGCCCTCAATGAA	59.8	566	DRR	Ca_01660	M	--
Ca5_P3_35751387	Ca5	35751387	AATATATATATATATATATATATATATAT/AATATATATATATATATATATATATATATATATAT	TCATTTTTCTTTCAATTTTGTCCTT	TTTTCACTTGGCATCCATCC	59.5	776	INTERGENIC	--	--	--
Ca5_P3_357876	Ca5	357876	A/AC	GTGAACAAGCCCATTGGAGT	GGATTGGCAATTTCATTTGG	60.0	161	INTERGENIC	--	--	--
Ca5_P3_35872148	Ca5	35872148	TAAAAAAAAAAA/TAAAAAAAAAA	ACCCTCACGTAAGCCTGATG	TCGCCTTCGTAAATAACGATT	60.1	357	INTERGENIC	--	--	--
Ca5_P3_35915901	Ca5	35915901	ATTTTTTTTTT/ATTTTTTTTT	AGCATGTGTTCTTGTGGCAG	TCGGAAGTTTCACATTCATCC	59.9	589	INTERGENIC	--	--	--
Ca5_P3_35919269	Ca5	35919269	ATTTATTGTTTATTGTTTATTGTTT/ATTTATTGTTTATTGTTT	TGGGATATTGCAAGGAGTTCT	GGGATTTCGTTTTCACTGGT	58.7	671	INTERGENIC	--	--	--
Ca5_P3_359833	Ca5	359833	GCT/GCTCT	CTACCACATTTGTTGTGCCG	TGGCAAGCAATTGAACAAAG	60.0	689	DRR	Ca_18192	P	--
Ca5_P3_360986	Ca5	360986	ATTTTTTTTTTT/ATTTTTTTTTT	TGTGAGTTTAAGGGAGGGGA	GCATCCAGGATCAGAGGTGT	59.5	390	DRR	Ca_18192	P	--

INDEL marker IDs	Chromosomes/ unanchored scaffolds	Physical positions (bp)	InDels (*Kabuli* reference genome-CDC Frontier/PII)	Forward primers (5'-3')	Reverse primers (5'-3')	Anneling temperature (0C)	Expected amplified product size (bp)	Structural annotation		Functional annotation	
								Sequence components of *kabuli* genome	*Kabuli* gene accession IDs	NCBI-KOG	TFs
Ca5_P3_36101390	Ca5	36101390	TAAAAAAAAA/TAAAAAAAA	AGAGCAAAAATCCTTTC GTGA	TAGTGTCAATGGGCCC TCTC	59.0	711	INTERGENIC	--	--	--
Ca5_P3_36285568	Ca5	36285568	TAAAAAAAA/TAAAAAAAAA	GGCTTAACGCTCTGTC GTCT	AGCAGCTGCCATTTGA GAAT	59.6	630	INTERGENIC	--	--	--
Ca5_P3_36308359	Ca5	36308359	TTTATT/TTTATTGGTATTATTATTATT	GTTTGTGGCGGATTTT GTTT	CCGATACAACGGAATT GTCC	59.8	423	DRR	Ca_01593	Z	--
Ca5_P3_36317817	Ca5	36317817	CTTTTTTTTTTT/CTTTTTTTTTT	TCTCACCTCGGACACC AAAT	TGGAAGGTACAAGTGA GGGG	60.5	598	INTERGENIC	--	--	--
Ca5_P3_36414199	Ca5	36414199	ATTTTTTT/ATTTTTT	AACTCGCAATGCATCC AATC	TGTGACCATGATTTTG TGGG	61.0	240	INTERGENIC	--	--	--
Ca5_P3_36452806	Ca5	36452806	TAAAAAAAA/TAAAAAAAAA	TCAATGAATTGACTGGC TTGA	GCTCACAATAATACCA ATGACCTTT	59.3	749	INTERGENIC	--	--	--
Ca5_P3_36583877	Ca5	36583877	CATATATATATATATATAT/CATATATAT ATATATATATAT	TCACTTTTAGGATCCGT TTGA	CACAATGGACCATGCA CTTC	57.4	809	INTERGENIC	--	--	--
Ca5_P3_36590424	Ca5	36590424	TAAAAAAAAAA/TAAAAAAAAAAA	TAAGCCCTCATTGATAC CCG	AAGATTTTTAATGATGC TTTTGGA	59.9	464	INTERGENIC	--	--	--
Ca5_P3_36668167	Ca5	36668167	ACCCC/ACCC	GGCAAACCTCTCTACG TTGC	TCTCAGCAGGCACACA AATC	59.9	866	INTRON	Ca_01555	AJ	NAC
Ca5_P3_36742914	Ca5	36742914	CTTTTTTTTTTT/CTTTTTTTTTT	CAAATGTTGGGCTTGG AGTC	ATTTCTTTGGGGATTTT GGG	60.5	351	INTERGENIC	--	--	--

INDEL marker IDs	Chromosomes/ unanchored scaffolds	Physical positions (bp)	InDels (*Kabuli* reference genome-CDC Frontier/PII)	Forward primers (5'-3')	Reverse primers (5'-3')	Anneling temperature (0C)	Expected amplified product size (bp)	Structural annotation		Functional annotation	
								Sequence components of *kabuli* genome	*Kabuli* gene accession IDs	NCBI-KOG	TFs
Ca5_P3_36804599	Ca5	36804599	GTTTTT/GTTTT	CCTACCTCATCACACGCAAA	GGTACAATTGTTGCACCACG	59.7	323	INTERGENIC	--	--	--
Ca5_P3_36893479	Ca5	36893479	TTATATATATATATATAT/TTATATATATATATATAT	GTAATTGTTCAGTTGGGCCG	GACACAAGCGCACGTAATTG	60.4	541	INTERGENIC	--	--	--
Ca5_P3_36904833	Ca5	36904833	GCTCTCTCTCTCTCTCTCTCTCTCTCTCTCTC/GCTCTCTCTCTCTCTCTCTCTCTCTCTCTCTCTCTCTC	TTCTGGAGAAAGAGGCGAAG	TGCGTAATTTGGTGCTGGTA	59.7	638	INTERGENIC	--	--	--
Ca5_P3_36963471	Ca5	36963471	ATAT/A	TGCCGCTGTGTCAGTTAGTT	GTACCGCCTCGGTCTCTAAA	59.5	704	INTERGENIC	--	--	--
Ca5_P3_37086090	Ca5	37086090	AGGGGGGGGGG/AGGGGGGGGG	CCGTGCAAAGGATGAAAAGT	TACTCGGCCCAAAATTAACG	60.1	614	INTRON	Ca_01507	--	G2-like
Ca5_P3_37101601	Ca5	37101601	CTTTTTTTTTT/CTTTTTTTTTTT	ATACCAACACCATCATGCGA	TTTGGTTCCAAAAGGAAAGC	59.8	595	INTERGENIC	--	--	--
Ca5_P3_37112216	Ca5	37112216	CTTATTTATTTATTTATTTATTTATTTAT/CTTATTTATTTATTTATTTATTTATTTATTTATTTAT	TTCTTCCAGGGACATCCAAG	AGGAGGTGCCCATTTAGGAT	60.0	648	INTRON	Ca_01505	R	--
Ca5_P3_37124729	Ca5	37124729	CAAAAAAAAAAAAA/CAAAAAAAAAAAA	TGGAAGACTCCACACAATGC	GGAGAAACAAAATGGAGGGA	59.7	166	INTERGENIC	--	--	--
Ca5_P3_37218586	Ca5	37218586	TATACATACATACATACATACATA/TATACATACATACATACATA	CACTCGTATCATGCTTGTCG	CTTTTTCGCCACCATTTGTT	57.9	680	INTERGENIC	--	--	--
Ca5_P3_37235701	Ca5	37235701	AATATATATATATATATATATATATATA/AATATATATATATATATATATATA	GTTGGAGCCAAAAACTAGCG	ACATTCATTGGCCCTTTTGA	59.9	577	INTERGENIC	--	--	--

INDEL marker IDs	Chromosomes/ unanchored scaffolds	Physical positions (bp)	InDels (*Kabuli* reference genome-CDC Frontier/PII)	Forward primers (5'-3')	Reverse primers (5'-3')	Anneling temperature (0C)	Expected amplified product size (bp)	Structural annotation		Functional annotation	
								Sequence components of *kabuli* genome	*Kabuli* gene accession IDs	NCBI-KOG	TFs
Ca5_P3_37236169	Ca5	37236169	TAAAAAAAAA/TAAAAAAAAAA	TCAAAAGGGCCAATGAATGT	TTGACACAGTTGAGATGCGA	60.3	546	INTERGENIC	--	--	--
Ca5_P3_37287992	Ca5	37287992	TA/TAA	AACGATAATCCAACAACCACAA	AAATTCATAAATGCGCGTGA	59.2	242	INTERGENIC	--	--	--
Ca5_P3_37319609	Ca5	37319609	AATATATATATATATATATATATA/AATATATATATATATATATATATATA	TGGCTGTGAAGGAGACAGTG	GCTTAAGTCACACACTTCCAAAAA	60.0	756	INTERGENIC	--	--	--
Ca5_P3_3742109	Ca5	3742109	TT/TTACATAT	ATTTACGAGCAGCGGAAAGA	TGGTCATGCAGAAACTCAGC	60.0	605	INTERGENIC	--	--	--
Ca5_P3_37603247	Ca5	37603247	ATATTTTATTTTATTTTATTTT/ATATTTTATTTTATTTT	CACTTGATCTTAGTTCCATTGTGG	TCTCCCTTGTTCGCTTCATT	59.9	828	INTERGENIC	--	--	--
Ca5_P3_37611622	Ca5	37611622	CAAA/CAA	ACGCGTATTTTTATGGGCAG	GGCAAGGTTCATTTTGCATT	60.0	236	INTRON	Ca_01461	T	FAR1
Ca5_P3_37633576	Ca5	37633576	TAAAAAAAAAA/TAAAAAAAAAAA	TGCAATGTTAAAGATGGTTGG	GTGCGAATTTTGTGATTACTGA	58.5	264	INTERGENIC	--	--	--
Ca5_P3_37670957	Ca5	37670957	GTTTTTTTTT/GTTTTTTTT	GCCTGCTCTTCTTTTTGGTG	TGAAAATATACATCTGTGGGGG	60.0	294	INTERGENIC	--	--	--
Ca5_P3_37763543	Ca5	37763543	TTATATATATATATATATATATATATATATATATAT/TTATATATATATATATATATATATATATATATAT	TGGCTGCAACGATAATCAAG	TGAAGCGACTGTCTCCAAAC	59.8	671	INTERGENIC	--	--	--
Ca5_P3_37868766	Ca5	37868766	CATTATATTATATTATATTATATTATAT/CATTATATTATATTATATTATATTATATTATAT	GCGAAAACTTTGGTGGTGTT	ACAAGATCCACACCGCTACC	60.0	541	INTRON	Ca_01437	H	--

INDEL marker IDs	Chromosomes/ unanchored scaffolds	Physical positions (bp)	InDels (*Kabuli* reference genome-CDC Frontier/PII)	Forward primers (5'-3')	Reverse primers (5'-3')	Anneling temperature (0C)	Expected amplified product size (bp)	Structural annotation		Functional annotation	
								Sequence components of *kabuli* genome	*Kabuli* gene accession IDs	NCBI-KOG	TFs
Ca5_P3_37905515	Ca5	37905515	ATTTTTTTTTT/ATTTTTTTTT	TGATCGAAGACAATTTGTGTCA	CCGATCTCACATGCTTCCTT	59.2	670	INTERGENIC	--	--	--
Ca5_P3_37980921	Ca5	37980921	TGGG/TGG	TGATTTATGATTGTGGGCGA	GTTTGGATGCGTGTTTCCTT	59.9	704	DRR	Ca_01423	U	bHLH
Ca5_P3_38006809	Ca5	38006809	G/GATCGTT	TGGAAGTGCATATGATGTTGG	CGAGGTGTTGCACATGACTT	59.4	585	INTERGENIC	--	--	--
Ca5_P3_38012392	Ca5	38012392	CAAAAA/C	TGTCGTCTTCTTCCTTTCGG	TTGGGCTAACCACCTTTTTG	60.4	446	DRR	Ca_01420	BK	NAC
Ca5_P3_38016624	Ca5	38016624	TAAGAAA/TAA	ATCCTCGAGAAGGAAGGAGC	CCTTTTCCCTCCAATGTCAA	59.9	276	INTRON	Ca_01419	A	--
Ca5_P3_38020094	Ca5	38020094	GTTTTT/GTTT	AGCAAGAGTCGGCACAAAGT	TTAAATCCCCTCAAACACCG	60.1	357	DRR	Ca_01419	A	--
Ca5_P3_38025101	Ca5	38025101	TC/T	TCCATAGCATTGCACTCAGC	TATGGTCTTCCTCGCTCCAG	60.0	660	INTERGENIC	--	--	--
Ca5_P3_38025852	Ca5	38025852	AT/A	ATGCCAAAAATAAACGCCAG	TGAGGTTGAGAAGCCAAACC	60.0	647	INTERGENIC	--	--	--
Ca5_P3_38027085	Ca5	38027085	AC/A	AAGCCTCTTGTTCTCCACCA	TGCACCACAAAAGTGTGTATCA	59.8	504	INTERGENIC	--	--	--
Ca5_P3_38027475	Ca5	38027475	T/TA	TGGAGTGATACACACTTTTGTGG	TGCTTACAAGGTATTCTTGCCA	59.9	446	INTERGENIC	--	--	--

INDEL marker IDs	Chromosomes/ unanchored scaffolds	Physical positions (bp)	InDels (*Kabuli* reference genome-CDC Frontier/PII)	Forward primers (5'-3')	Reverse primers (5'-3')	Anneling temperature (0C)	Expected amplified product size (bp)	Structural annotation		Functional annotation	
								Sequence components of *kabuli* genome	*Kabuli* gene accession IDs	NCBI-KOG	TFs
Ca5_P3_38033573	Ca5	38033573	GTTTTTTTTT/GTTTTTTTTTTT	AAAGCGGCATTATCTC GTTG	CGCGTCAAATAAAGCA TTGA	60.2	520	INTERGENIC	--	--	--
Ca5_P3_38034465	Ca5	38034465	AATTATTAT/AATTAT	TGCTATTGCGATCCCT CTCT	TTGTCGGTATAAATCG GACG	59.9	311	INTERGENIC	--	--	--
Ca5_P3_38034841	Ca5	38034841	TTTA/TTTAATTA	TCGTCCGATTTATACCG ACA	CGACGAGGTATATGCC AACA	59.0	856	INTERGENIC	--	--	--
Ca5_P3_38036769	Ca5	38036769	TAA/TA	TGATAACTCGTCGGCT TGTG	TTTTTCCTTCCAAATAA GCCTC	59.9	633	INTERGENIC	--	--	--
Ca5_P3_38038986	Ca5	38038986	AGG/AGGG	TTACCAATACCGGGTA CCGT	TCACACAACAAGGAAC CCAA	59.1	516	INTERGENIC	--	--	--
Ca5_P3_38048496	Ca5	38048496	CGAGTT/C	GTTGCATTTGGGCAATT TCT	CTCCTTCAGCAAAGCA TACG	59.9	527	INTERGENIC	--	--	--
Ca5_P3_38061782	Ca5	38061782	ATTTT/ATT	CCCTCCGTGTTTATTTA CCG	CACTCTAGCTTTGGGC TTGG	59.3	687	DRR	Ca_01416	--	--
Ca5_P3_38068613	Ca5	38068613	ATTTTTTT/ATTTT	CGCTTAGCAGGTAAGG GAAC	ATCCACAGCCTATGAC CACC	59.0	614	DRR	Ca_01413	--	--
Ca5_P3_38076142	Ca5	38076142	CATATATATATATATATATATAT/CAT ATATATATATATATATATATAT	ACCGAATTTGTTTGCTA CCG	TCGCAGAACCACAATG CTAC	60.0	237	DRR	Ca_01412	O	MYB_related
Ca5_P3_38080854	Ca5	38080854	A/AC	AATAGATCCTGATGGC CGTG	TCGATTCACCATGACA GCAT	59.9	813	INTRON	Ca_01411	D	WRKY

INDEL marker IDs	Chromosomes/ unanchored scaffolds	Physical positions (bp)	InDels (*Kabuli* reference genome-CDC Frontier/PII)	Forward primers (5'-3')	Reverse primers (5'-3')	Anneling temperature (0C)	Expected amplified product size (bp)	Structural annotation		Functional annotation	
								Sequence components of *kabuli* genome	*Kabuli* gene accession IDs	NCBI-KOG	TFs
Ca5_P3_38088810	Ca5	38088810	CTAAAATATAA/CTAA	ATTTACATGCGGTTCGGTTC	GACTCAATTGCGGCCATATT	59.8	566	DRR	Ca_01409	--	--
Ca5_P3_38099316	Ca5	38099316	GATTATTATTATTATTATTATTATTATTATTATTATTATTATTATTATTATTATTAT/GATTATTATTATTATTATTATTATTATTATTATTATTATTAT	GGAAAACAAATATGGTATGCCC	CACGGATAATCGGAGATTCCT	59.5	767	INTERGENIC	--	--	--
Ca5_P3_38101800	Ca5	38101800	G/GTACAAAGTGGACCGCT	GTCGAAGTACTCGGCGCTAC	ACGTCGAAGGGAGACTCAGA	60.0	610	INTERGENIC	--	--	--
Ca5_P3_38102985	Ca5	38102985	TA/T	GCTGGTGGCATACTTTGGTT	GTCCATGACAGCAGCAAAAA	60.0	122	CDS (FRAME SHIFT)	Ca_01408	P	--
Ca5_P3_38117706	Ca5	38117706	AAC/A	CACTTCCGAACTTGTAGAATTGTT	TGAACCAGATGTCTTTCACGA	58.8	823	INTERGENIC	--	--	--
Ca5_P3_38126896	Ca5	38126896	AATATAT/AATAT	GCATTACGACTTTGTTGCGA	TCACCACCTTCATTGTCAGC	59.9	483	INTERGENIC	--	--	--
Ca5_P3_38142286	Ca5	38142286	GACACA/GACA	TTGTTCCCTTTTGGATGAGG	ACTTTGCTGGCAATGTGTGT	59.9	526	DRR	Ca_01403	R	NAC
Ca5_P3_38149011	Ca5	38149011	TTATTATTATTATTATTATTATTATTATTATTATTATTATTATTATTATTATTATTATTATTATTATTAT/TTATTATTATTATTATTAATATTATTATTATTATTATTATTATTATTATTATTATTATTATTATTATTATTATTATTATT[illegible]	GCCGATATGTTCTTGGTGGT	GCCTCTCCCACAATTGACTC	59.8	940	DRR	Ca_01402	--	bHLH
Ca5_P3_38155679	Ca5	38155679	CTTA/CTTAATTA	TGTTGTTGTTGGATTGGCAT	AAAATGGAGCATTGGAAAAA	59.8	769	INTERGENIC	--	--	--
Ca5_P3_38159703	Ca5	38159703	GTT/GTTGGCCATTTT	CCAACTCGCAAACAAATCCT	ATTGGTTCACCCCTTCAAAA	60.1	559	INTERGENIC	--	--	--

INDEL marker IDs	Chromosomes/ unanchored scaffolds	Physical positions (bp)	InDels (*Kabuli* reference genome-CDC Frontier/PII)	Forward primers (5'-3')	Reverse primers (5'-3')	Anneling temperature (0C)	Expected amplified product size (bp)	Structural annotation		Functional annotation	
								Sequence components of *kabuli* genome	*Kabuli* gene accession IDs	NCBI-KOG	TFs
Ca5_P3_38255302	Ca5	38255302	AAATAATAATAATAAT/AAATAATAATAAT	TGCCTCAACTCAACACTTGC	CAATTTACCAATCGCACGAA	60.0	762	INTERGENIC	--	--	--
Ca5_P3_38256989	Ca5	38256989	AATA/AA	GGACTTCAGATGATCGCACA	GGGAGAAGAGGAAGGAGGAA	59.8	320	INTERGENIC	--	--	--
Ca5_P3_38407176	Ca5	38407176	AATATATATATATATATATATATATAT/AATATATATATATATATATATATATATATAT	TCTCAAAGTCTCAATGGTCCAA	TTGAAGTCAAATTTCGCCAG	59.7	658	INTERGENIC	--	--	--
Ca5_P3_38476780	Ca5	38476780	CA/CGTA	CACACTTGGAGAGCGCATTA	TCCTTGGGGTGATCTCGTAG	60.0	652	INTERGENIC	--	--	--
Ca5_P3_38516011	Ca5	38516011	CTATATATATATATATATATATATATATAT/CTATATATATATATATATATATATATATATATAT	AAAATTCTGCTCCATGTCGG	AACAAATTCAGCAACCTCCG	60.1	451	INTERGENIC	--	--	--
Ca5_P3_3865777	Ca5	3865777	CTTTTTTT/CTTTTTTTT	ATGTGACACCCTAAACCCCA	TTGGTTTAGAGGGGATGTGAA	60.1	605	INTERGENIC	--	--	--
Ca5_P3_3869268	Ca5	3869268	AAATAATAATAATAATAATAATAATA/AAATAATAATAATAATAATA	CCGCTACCAAGAAGATCCAT	CCAAATTTGATGACATGCAC	59.2	574	INTERGENIC	--	--	--
Ca5_P3_38755750	Ca5	38755750	TGGGG/TGGGGG	GAAGTTGAGCGCAATGTTGA	TTAAGACGGGTGATGGAAGC	60.0	528	INTERGENIC	--	--	--
Ca5_P3_38755924	Ca5	38755924	TAAAA/TAAA	GTCTGCCACATCACCCTTTT	CGGATCAACTTTTCTTTGTGG	60.0	661	INTERGENIC	--	--	--
Ca5_P3_38790519	Ca5	38790519	ATTTTTTATTTTTTTATTTTTTT/ATTTTTATTTTTT	ATTGACCAGTTGAAGCCTGG	CAGGTGTGGTGATGAAGTGG	60.1	708	DRR	Ca_07429	O	--

INDEL marker IDs	Chromosomes/ unanchored scaffolds	Physical positions (bp)	InDels (*Kabuli* reference genome-CDC Frontier/PII)	Forward primers (5'-3')	Reverse primers (5'-3')	Anneling temperature (0C)	Expected amplified product size (bp)	Structural annotation		Functional annotation	
								Sequence components of *kabuli* genome	*Kabuli* gene accession IDs	NCBI-KOG	TFs
Ca5_P3_3887629	Ca5	3887629	AATATATATATATATATATATATAT/AATATATATATATATATATAT	CACGTTTCAAGAGTGCGAAG	TTTGAGAGGCATTTTTGATGG	59.6	680	INTERGENIC	--	--	--
Ca5_P3_39188187	Ca5	39188187	ATATCT/AT	GCTTGCTTGGACAAATGACA	TCACAAAGTTTTGTTTTGTGTGG	59.8	655	INTERGENIC	--	--	--
Ca5_P3_39188975	Ca5	39188975	TTATATATATATATATATAT/TTATATATATATATAT	GAGGGAAAAATATGTGTCACGA	CCCAAAGTTGGTTGCAAAAC	59.0	580	INTERGENIC	--	--	--
Ca5_P3_39340722	Ca5	39340722	C/CT	CAATTTGCGTATGCATGGAG	AATGCGGACATTTTCTTTGG	60.1	496	INTERGENIC	--	--	--
Ca5_P3_39360163	Ca5	39360163	CNNNNNNNNNN/CNNNNNNN	TCCGAACGAAGCTAACATCA	TTGAACAAGAATTTGTGGCG	59.4	695	INTERGENIC	--	--	--
Ca5_P3_39362210	Ca5	39362210	TG/T	AAAAATCCGCAGGAACACAC	CATCAGGTTGTTGCATGGAG	60.0	747	INTERGENIC	--	--	--
Ca5_P3_39449601	Ca5	39449601	AATATATATATATATATATATATAT/AATATATATATATATATAT	AGCCTTTAGGAATGACCCGT	GTCAAACTTTGTGGTGCCAA	60.0	753	INTERGENIC	--	--	--
Ca5_P3_39537232	Ca5	39537232	AATATATATATATATATATATATATATATATA/AATATATATATATATATATATATATATATATATA	TTGGCAACCAGTCAACAAAA	CACGTGAGAAAACCTCGACA	60.1	473	INTERGENIC	--	--	--
Ca5_P3_39557215	Ca5	39557215	GATATATATATATATATATAT/GATATATATATATATATATATAT	GCAAAAAGGGGCACAATAAA	AATGTCAATCCTCCATTGCC	59.9	357	INTRON	Ca_07489	TBLD	--
Ca5_P3_39569272	Ca5	39569272	ACTCTTNNNNNNNNNNNNCTCTT/ACTCTT	TAGACAAGCCCATTTCCAGG	TTCATGCAAACATTGGTGCT	60.1	450	INTRON	Ca_07489	TBLD	--

INDEL marker IDs	Chromosomes/ unanchored scaffolds	Physical positions (bp)	InDels (*Kabuli* reference genome-CDC Frontier/PII)	Forward primers (5'-3')	Reverse primers (5'-3')	Anneling temperature (0C)	Expected amplified product size (bp)	Structural annotation		Functional annotation	
								Sequence components of *kabuli* genome	*Kabuli* gene accession IDs	NCBI-KOG	TFs
Ca5_P3_39630428	Ca5	39630428	TTATATATATATATATATATATATA/TTATATATATATATATATA	GCGACAAAAGTGCATTTGAA	TGCTTGGGTTTAACAAAGGG	59.9	500	INTERGENIC	--	--	--
Ca5_P3_39668251	Ca5	39668251	GTAATAATAATAATAATAATAA/GTAATAATAATAATAATAATAATAA	CGTTCCACTCCCAATACACC	TTTCGCACGTCTACTCAAACA	60.2	854	INTERGENIC	--	--	--
Ca5_P3_39783717	Ca5	39783717	AATATATATATATATATATAT/AATATATATATATATATAT	GCCTTTGTTTCAGTTGCCAT	CCGCTAATTTGACGGAAAAA	60.1	890	INTERGENIC	--	--	--
Ca5_P3_39793489	Ca5	39793489	CAAAAAAAA/CAAAAAAA	AGGGCACTTTGTCTCCTCAA	ACCAAATCGATGGAATGACC	59.8	529	DRR	Ca_07506	S	bZIP
Ca5_P3_39815597	Ca5	39815597	AATATATATATATATATATAT/AATATATATATATATATATATAT	TGGTCACATGGACATTTGCT	TCTTGGTTTTACTCCAACGACA	60.0	637	INTRON	Ca_07507	E	--
Ca5_P3_39918314	Ca5	39918314	TTTC/TTTCATATTCTTC	TCCATTTCAGTCTTCCACCC	AGTTGCACACGTTGTGGTGT	59.9	756	INTERGENIC	--	--	--
Ca5_P3_39929111	Ca5	39929111	AAATAATAATAATAATAATAATAATAA/AAATAATAATAATAATAATAATAATAATAA	GACCAAATGAAGGCCAAAAA	AGGTCGCTGGTCAGATATGC	59.9	701	INTERGENIC	--	--	--
Ca5_P3_39932205	Ca5	39932205	CTTTTTTTTTTTTT/CTTTTTTTTTTTT	AAGTACGAAGCCCATGTTGG	GGGGAGATGTGGTTCAAAAA	60.0	603	INTERGENIC	--	--	--
Ca5_P3_39936599	Ca5	39936599	GAAAAAAAAAAA/GAAAAAAAAAAAA	GAAGTGGATTGTTGGGTTGG	CATTTCCATGCCCCATAAAT	60.2	968	INTERGENIC	--	--	--
Ca5_P3_40297526	Ca5	40297526	GAGAAGAAGAAGA/GAGAAGAAGAAGAAGA	AAGGGTGATGGTGAGAAGGTT	TGCATCCCTGCAAATACAAA	59.8	261	INTERGENIC	--	--	--

INDEL marker IDs	Chromosomes/ unanchored scaffolds	Physical positions (bp)	InDels (*Kabuli* reference genome-CDC Frontier/PII)	Forward primers (5'-3')	Reverse primers (5'-3')	Anneling temperature (0C)	Expected amplified product size (bp)	Structural annotation		Functional annotation	
								Sequence components of *kabuli* genome	*Kabuli* gene accession IDs	NCBI-KOG	TFs
Ca5_P3_40391178	Ca5	40391178	TTATATATATATATATATATATAT/TTATATATATATATATATATAT	CCTAAGCACCGTTTCGTCAT	TGAAAGATTTTAATGCCCCAA	60.1	695	INTERGENIC	--	--	--
Ca5_P3_40392907	Ca5	40392907	GTATATATATATATATATATATATATATATATATAT/GTATATATATATATATATATATATATATAT	GCATTATCACGAGCAACACG	TCAATGTGGTTCGCAAAAGA	60.3	192	INTERGENIC	--	--	--
Ca5_P3_40434180	Ca5	40434180	A/ACATCTG	TTTCACCCAGTAGGAGGTCG	TGAACCGGAAGAACCTCAAC	60.1	500	INTERGENIC	--	--	--
Ca5_P3_40471737	Ca5	40471737	CTTTTTTTTTTT/CTTTTTTTTTT	TGATTTTCCCTGTGCATCAA	AATGTGGCAAAGGATTCCAG	60.0	757	DRR	Ca_07567	--	--
Ca5_P3_40512625	Ca5	40512625	GTATATATATATATATA/GTATATATATATATA	ATTTCCGTATCGTCAATGCC	TGCGGACAAAATTCAAAACA	59.8	626	INTERGENIC	--	--	--
Ca5_P3_40572884	Ca5	40572884	ATTTTTTTT/ATTTTTTT	TTAGAGGAGCCACGTAAGCC	CCCTAGCCACTTTCTCTCCC	59.5	584	INTERGENIC	--	--	--
Ca5_P3_40588242	Ca5	40588242	CAAAAAAAAAA/CAAAAAAAAAAA	ATCGCGAAAGTAGTCCTCCA	GCTCCCACACTCTTCCTCTG	59.8	642	DRR	Ca_07577	O	--
Ca5_P3_4064742	Ca5	4064742	G/GC	TATGTTTGTTTTGCCGTCCA	AGGATGTACCATTCTTGCGG	60.0	375	INTERGENIC	--	--	--
Ca5_P3_40655426	Ca5	40655426	GTTTTTTTTTTTTATTTTTTTTTTTT/GTTTTTTTTTTTTATTTTTTTTTTTTT	TGGCAGATGATGCTGTTAGC	CGTACCTACACCCATACCCG	60.0	185	INTERGENIC	--	--	--
Ca5_P3_40747443	Ca5	40747443	C/CT	TTGCAGAGAAAGAAAACTTGATATG	TGTGGAAGTTCCTAAAATACGC	59.0	237	INTERGENIC	--	--	--

INDEL marker IDs	Chromosomes/ unanchored scaffolds	Physical positions (bp)	InDels (*Kabuli* reference genome-CDC Frontier/PII)	Forward primers (5'-3')	Reverse primers (5'-3')	Anneling temperature (0C)	Expected amplified product size (bp)	Structural annotation		Functional annotation	
								Sequence components of *kabuli* genome	*Kabuli* gene accession IDs	NCBI-KOG	TFs
Ca5_P3_40781378	Ca5	40781378	TTATATATATATATATATATATATATATATATA/TTATATATATATATATATATATATATATA	TAAACAGGCCAGGCCATAAG	AGCCCGAAGACACTCGTCTA	60.1	416	INTRON	Ca_07597	R	--
Ca5_P3_40858234	Ca5	40858234	GATATATATATATATATATATATATATATATATATATATA/GATATATATATATATATATATATATATATATATATATATATATATA	AGGCATGGTGTTGGAGTCAT	TCCGACAAGGAGAATAACCG	60.4	650	DRR	Ca_07607	K	M-type
Ca5_P3_40868817	Ca5	40868817	TTTATTATTATTATTATTATTATTATTATTATTATTATTATTATTATTATTA/TTTATTATTATTATTATTATTATTATTATTATTATTATTATTA	CAGAGGCGTTTGTGTATGGA	GATTTAGATGGCTGTCGGGA	59.7	622	INTERGENIC	--	--	--
Ca5_P3_41056125	Ca5	41056125	TAAAAAAAAAAA/TAAAAAAAAAA	GCACTCAGCATAGGTTTCGC	GTAGCTCACGATTCGCTTCC	60.9	454	INTRON	Ca_07624	I	NF-YC
Ca5_P3_41108441	Ca5	41108441	GTTTTTTTTTTT/GTTTTTTTTTTTT	GGAGCGGAAAAGACTGAAAA	TTTTGTCTATGCGCACAAGG	59.4	586	INTERGENIC	--	--	--
Ca5_P3_41179556	Ca5	41179556	AAG/AAAGAGTGTTGAAAAGAG	GACTCAATGCAGATGGGGTT	GTCCCATACCTGCAAAAGGA	59.9	596	INTRON	Ca_07636	--	--
Ca5_P3_41190334	Ca5	41190334	A/ATT	ACAACAAATTTTTGCCCGAC	TGTCCGGAACTTTTCCTTTG	59.8	590	INTERGENIC	--	--	--
Ca5_P3_41207521	Ca5	41207521	CATAAATATA/CATA	TGGTTGGTAGGCAAGGTTGT	TGTTGGAGATGGAAGGAAGG	60.4	368	INTERGENIC	--	--	--
Ca5_P3_41267150	Ca5	41267150	TTAATAATAATAATAATAATAATAATAATAATAATAATAA/TTAATAATAATAATAATAATAATAATAATAATAATAATAATAATAA	GTGACGAGGTGTGTCACCG	AAGTCGCCATTTGCAAAAAC	61.3	456	INTERGENIC	--	--	--
Ca5_P3_41351134	Ca5	41351134	TNNNNNNNNNNN/TGGTTAGGGAAAGANNNNNNNNNN	TTGCGAAGAATGGTGTGAAG	CCGACAACCCTACGACAACT	59.8	587	DRR	Ca_19240	--	--

INDEL marker IDs	Chromosomes/ unanchored scaffolds	Physical positions (bp)	InDels (*Kabuli* reference genome-CDC Frontier/PII)	Forward primers (5'-3')	Reverse primers (5'-3')	Anneling temperature (0C)	Expected amplified product size (bp)	Structural annotation		Functional annotation	
								Sequence components of *kabuli* genome	*Kabuli* gene accession IDs	NCBI-KOG	TFs
Ca5_P3_41428807	Ca5	41428807	TTATATATATATATATATATATATATATATATATATATATATAT/TTATATATATATATATATATATATATATATATATATATATATATATATAT	TGAATTGAGTTTGGTTGATTTGA	AAAGGATGCAGTATGGGTTTTT	59.5	591	INTERGENIC	--	--	--
Ca5_P3_41455567	Ca5	41455567	AATATATATATATATATATATATATATA/AATATATATATATATATATATA	TTGCCACTTGATACAGCAAAA	TGCATGTTGAAACACCGACT	59.4	757	INTERGENIC	--	--	--
Ca5_P3_41463439	Ca5	41463439	CTT/CTTGATATT	CACGACATTTTGTGCCATCT	ATGTGTTTGCAAAATCGCAG	59.6	532	INTERGENIC	--	--	--
Ca5_P3_41467556	Ca5	41467556	TA/TAA	AAAGGATGCAGTATGGGTTTTT	TCACTTGGGTGGAAAAATGAG	58.9	453	INTERGENIC	--	--	--
Ca5_P3_41467895	Ca5	41467895	TA/TAA	CTCATTTTTCCACCCAAGTGA	AGCGACAAGATGAAAGCTCC	60.0	529	INTERGENIC	--	--	--
Ca5_P3_41467907	Ca5	41467907	AATTATTATTATTATTATTAT/AATTATTATTATTATTATTATTAT	CTCATTTTTCCACCCAAGTGA	AGCGACAAGATGAAAGCTCC	60.0	529	INTERGENIC	--	--	--
Ca5_P3_41477742	Ca5	41477742	CAA/CAAA	TGAGGCAAGCATGTGTAAGC	AATGTGCATGAGAGGCTGTG	60.0	223	DRR	Ca_19232	B	MYB_related
Ca5_P3_4164578	Ca5	4164578	GTATATATATATATATATATATATAT/GTATATATATATATATATATAT	GCCTTGGGTTTCAATTCTCA	AATCCCAAAACCCAAAACCT	60.1	520	INTERGENIC	--	--	--
Ca5_P3_41678103	Ca5	41678103	TCT/TCTTTCCCTTAAAGGGACT	CAGTGGGTCCTCTTCCACAT	CCCGAACTCCTGTTACTCCA	60.0	551	INTRON	Ca_19215	J	C3H
Ca5_P3_41765343	Ca5	41765343	GAAAAAAAAAAA/GAAAAAAAAAA	TCACGCTGTTCACCTTTCTG	GATGCCCAACCGTAGTTGTC	60.0	806	INTERGENIC	--	--	--

INDEL marker IDs	Chromosomes/ unanchored scaffolds	Physical positions (bp)	InDels (*Kabuli* reference genome-CDC Frontier/PII)	Forward primers (5'-3')	Reverse primers (5'-3')	Anneling temperature (0C)	Expected amplified product size (bp)	Structural annotation		Functional annotation	
								Sequence components of *kabuli* genome	*Kabuli* gene accession IDs	NCBI-KOG	TFs
Ca5_P3_41789237	Ca5	41789237	GAAAA/GAAAAA	TAAATCAAACGAACACCGCA	TTCCTTTAAATGGGTCACGC	60.1	625	INTERGENIC	--	--	--
Ca5_P3_4179599	Ca5	4179599	GATATATATATAT/GATATATATAT	CGGTTTAGGGATTGGGTTTT	TCGATTGAGTCTTTTGCACTT	60.0	166	INTERGENIC	--	--	--
Ca5_P3_4180242	Ca5	4180242	AGGTTTAGGGTTTAGGGTTT/AGGTTTAGGGTTTAGGGTTTAGGGTTT	GGGTTTGGGGTTTAAGGTTT	TCCATTTCGTCTTTTGCACTT	59.1	321	INTERGENIC	--	--	--
Ca5_P3_4182020	Ca5	4182020	AGTTTAGGGTTTAGGGTTTAGGGTTTAGAATTTAGGGTTTAGGTTTTAGGGTTTAGGGATTAGGGTTTAGGGTTTAAGGTTTAGGCTTTAAGGTTTAGGGTTTAGGGTTTAAGGTTTAGGGTTTAGGGTTTAGGGTTTAGG/AGTTTAGGGTTTAGGGTTT [illegible]	GGGTTTTGGGTTTACGGTTT	TCGAATCCATTTCGTCTTTTG	60.0	583	INTERGENIC	--	--	--
Ca5_P3_41858516	Ca5	41858516	TTATATATATATATATATATATATATATAT/TTATATATATATATATATATATATATATATATATAT	AGCAATGAACAACGCATCAA	AAGGCATCATCATTTGAGAAAGA	60.3	614	INTERGENIC	--	--	--
Ca5_P3_41860187	Ca5	41860187	TAAGAAAG/TAAGAAAGAAAG	ACAGAAACCACAGTCGCACA	ACTTTAGCAACCACCGCTTC	60.4	318	INTERGENIC	--	--	--
Ca5_P3_41955510	Ca5	41955510	ATATTATTATTATTATTATTATTATTATTATTATTATTATTATTATTATTATTAT/ATATTATTATTATTATTATTATTATTATTATTATTATTATTATTATTATTATTATTATTAT	TTAAATAGGGCGGTGGTTGA	TAATTCACCTCCACATGCCA	60.3	828	DRR	Ca_11417	R	--
Ca5_P3_41962522	Ca5	41962522	TA/TGATAACCAAA	ATGTTGGGCTTTGGAGTGTC	AGACACGCAAATTATTGCCC	60.0	381	INTERGENIC	--	--	--
Ca5_P3_4212019	Ca5	4212019	CATATATATATATATATATA/CATATATATATATATATA	GGTGCTTCTTTCTCGAACCA	AGAGAGGCAGCCAGAATCAA	60.4	760	INTERGENIC	--	--	--
Ca5_P3_4213008	Ca5	4213008	TTACTATACTAT/TTACTATACTATACTAT	TTTCAATTTTGCGTCTGCTG	AGGAGCAACGGAACTGCTAA	60.0	313	INTERGENIC	--	--	--

**INDEL marker IDs**	**Chromosomes/ unanchored scaffolds**	**Physical positions (bp)**	**InDels (*Kabuli* reference genome-CDC Frontier/PII)**	**Forward primers (5'-3')**	**Reverse primers (5'-3')**	**Anneling temperature (0C)**	**Expected amplified product size (bp)**	**Structural annotation**		**Functional annotation**	
								**Sequence components of *kabuli* genome**	***Kabuli* gene accession IDs**	**NCBI-KOG**	**TFs**
Ca5_P3_42231500	Ca5	42231500	CC/CCGAGAACGTAC	TCAAAATCCACACCCCACTT	GCCATCTCCAATAGCACCAT	60.2	888	INTERGENIC	--	--	--
Ca5_P3_42235939	Ca5	42235939	CATTATTATTATTATTATTATTATTATTATTATTATTATTATTATTATTATTATT/CATTATTATTATTATTATTATTATTATTATTATTATTATTATTATTATTATTATTATT	ATGAGTGAGGGCAAAGGTTG	TTCTTTACATGGGCCAAACC	60.1	431	INTERGENIC	--	--	--
Ca5_P3_422839	Ca5	422839	GTAATAATAATAATAATAATAATAATAATAATAATAATAATAATAA/GTAATAATAATAATAATAATAATAATAATAATAATAATAATAATAATAATAATA A	GCCATGGCTTCAAAATCTTC	TGAGAGCACGATTTCTCCCT	59.7	625	INTERGENIC	--	--	--
Ca5_P3_42303599	Ca5	42303599	TTATATATATATATATATATATATATAT/TTATATATATATATATATATATATATATATAT	GGTGGATATTGGTGTAGCCG	TGCACAATAATTTGGACGGT	60.2	422	INTERGENIC	--	--	--
Ca5_P3_42353503	Ca5	42353503	TAAAAAAAAAAA/TAAAAAAAAAA	TTTTTCAGCGACAAAATAATACTCA	CGGACAACAAAAATTGCTCA	59.3	741	INTERGENIC	--	--	--
Ca5_P3_42425779	Ca5	42425779	CATGTTTTATGTTTTA/CATGTTTTA	TCAATGGAATGGAGTCGTCA	CGACAATTTCATTATCCGTCC	60.0	749	INTERGENIC	--	--	--
Ca5_P3_42427467	Ca5	42427467	ATTATAT/A	CAACTGATCGGAGTGGGATT	AAATCCTTAAGATCGGCGGT	59.9	618	DRR	Ca_11375	--	--
Ca5_P3_42427494	Ca5	42427494	ATATATCT/AT	GTGTTGCTGGTGTCAAGGAA	AAATCCTTAAGATCGGCGGT	59.7	401	DRR	Ca_11375	--	--
Ca5_P3_4244924	Ca5	4244924	TTTAGGGTTTAGGGATTAGGGTTTAGGG/TTTAGGGTTTAGGGATTAGGGTTTAGGGATTAGGGTTTAGGG	TTAGGGTTTAGGGTTTGGGG	GCATTGATTTAAACAAAAACGGA	60.0	142	INTERGENIC	--	--	--
Ca5_P3_42557966	Ca5	42557966	TAAAAAA/TAAAAAAA	TGAAAGTGCATGTGACCGAT	CACAAACCAACGTGGAAGTG	60.1	613	INTERGENIC	--	--	--

INDEL marker IDs	Chromosomes/ unanchored scaffolds	Physical positions (bp)	InDels (*Kabuli* reference genome-CDC Frontier/PII)	Forward primers (5'-3')	Reverse primers (5'-3')	Anneling temperature (0C)	Expected amplified product size (bp)	Structural annotation		Functional annotation	
								Sequence components of *kabuli* genome	*Kabuli* gene accession IDs	NCBI-KOG	TFs
Ca5_P3_42720218	Ca5	42720218	T/TG	AAAGGTTTGGATGTCGATGC	AGCAAAATCCAGAAAAGCCA	59.9	675	DRR	Ca_11339	J	--
Ca5_P3_4273567	Ca5	4273567	CTATATATATATATATAT/CTATATATATATATATATAT	TTGGAAGGAGCCCTTACCTT	CGATTGAGGACCAAGGATGT	60.1	908	INTERGENIC	--	--	--
Ca5_P3_42822040	Ca5	42822040	AGCGNNNNNNNNNNGCG/AGCG	TCCATTGCAGTCAGCAACTC	GCTTGGTCCTAGAAGATGCG	60.0	433	INTRON	Ca_11327	R	MYB
Ca5_P3_42826985	Ca5	42826985	TAAAAAAAAAAA/TAAAAAAAAAA	AACAATTATGTGATGTGATGAACTTG	CGTTTAATCGCGACTAGGTTG	59.3	492	INTERGENIC	--	--	--
Ca5_P3_42827434	Ca5	42827434	CAAAAAAAATAAA/CAAAAAAAATAAAAAAAAATAAA	TCGCGATTAAACGACTAAATCA	GCACCAGAAAATCTTGAGGC	59.8	372	INTERGENIC	--	--	--
Ca5_P3_42901533	Ca5	42901533	TA/TAGGTAAGAAAAATGA	ATTGCCATAACACAGAGGGC	CGGTCCGACTTTCACATTTA	60.0	776	INTERGENIC	--	--	--
Ca5_P3_42975249	Ca5	42975249	CTTATTATTATTATTATTATTATTATTATTATT/CTTATTATTATTATTATTATTATTATTATT	TCTGATGAGTTGCTTGTGCC	GGCCGCAATTATTGTTTTGT	60.0	528	INTERGENIC	--	--	--
Ca5_P3_4302445	Ca5	4302445	TA/T	AGGCAATGCAATCCGATAAG	AGCTTACCATCCACAAACGG	60.1	502	INTERGENIC	--	--	--
Ca5_P3_432048	Ca5	432048	ACC/AC	AGAACCACCTGAAATGCCAC	CTTCCTCCTCCAAATCACCA	60.0	734	DRR	Ca_18195	R	HD-ZIP
Ca5_P3_43298677	Ca5	43298677	AATATATATATATATATATATATATATA/AATATATATATATATATATATATATATATA	TCAAATTTGCAAAGACAGATTTTC	TTTTGGTGTCACTTAAGAGTGGAA	59.7	893	DRR	Ca_12727	--	--

INDEL marker IDs	Chromosomes/ unanchored scaffolds	Physical positions (bp)	InDels (*Kabuli* reference genome-CDC Frontier/PII)	Forward primers (5'-3')	Reverse primers (5'-3')	Anneling temperature (0C)	Expected amplified product size (bp)	Structural annotation		Functional annotation	
								Sequence components of *kabuli* genome	*Kabuli* gene accession IDs	NCBI-KOG	TFs
Ca5_P3_43299130	Ca5	43299130	TC/T	TTGGTTTACGAATTTTATGGTGAA	CTGCATAGGCAGGGTGGTAG	59.7	385	DRR	Ca_12727	--	--
Ca5_P3_43319730	Ca5	43319730	GAAAAAAAAA/GAAAAAAAAAA	TGCGATTGTGGGAAATATCA	GCTTCAAGGTCAGGTCAAGC	59.9	860	INTERGENIC	--	--	--
Ca5_P3_43446181	Ca5	43446181	ATTTTTTTTT/ATTTTTTTTTT	TTGGGATTGGACTGTTGTCA	AGGACAATCATGCTGCCTCT	59.9	564	DRR	Ca_12710	--	--
Ca5_P3_43456554	Ca5	43456554	TTCTTGGGCAGTCTTGGGCAG/TTCTTGGGCAGTCTTGGGCAGTCTTGGGCAG	TCTGGATGATAATTTCGGGC	TTGAATTCACCTTTGACCCC	59.9	520	INTRON	Ca_12708	O	MYB
Ca5_P3_43459935	Ca5	43459935	TA/T	TGTTGTGTTGGGAACTGGAA	CCTTCCATTTTTAATGAATGAACC	60.0	680	INTERGENIC	--	--	--
Ca5_P3_43492544	Ca5	43492544	ATTTTTTTTT/ATTTTTTTTTT	GCCACAATTCCCGAAGTAAA	AGATGAAGTTGCACATGGTGA	59.9	408	INTERGENIC	--	--	--
Ca5_P3_435121	Ca5	435121	CA/CAAA	TGCAAATCAGGTGATCCAAA	CAGCAACCACAATTCAACTCA	60.0	497	DRR	Ca_18196	--	--
Ca5_P3_43517939	Ca5	43517939	AATATATATATATATATATATATA/AATATATATATATATATATATATA	TGAGATGTTCATTACTGCGTTG	AATTTTTGCGGCCAAGTATG	58.8	876	INTERGENIC	--	--	--
Ca5_P3_43659116	Ca5	43659116	TA/TAA	AATGGACCCACCTTTCTTCC	CTGGAGCGAAGGTTGAGAAC	60.2	303	INTRON	Ca_12686	G	--
Ca5_P3_43761974	Ca5	43761974	TT/TTGAT	GCCATCTCTAAATTGATTGGG	GATCCATGACATGATTGGCA	58.5	583	INTERGENIC	--	--	--

INDEL marker IDs	Chromosomes/ unanchored scaffolds	Physical positions (bp)	InDels (*Kabuli* reference genome-CDC Frontier/PII)	Forward primers (5'-3')	Reverse primers (5'-3')	Anneling temperature (0C)	Expected amplified product size (bp)	Structural annotation		Functional annotation	
								Sequence components of *kabuli* genome	*Kabuli* gene accession IDs	NCBI-KOG	TFs
Ca5_P3_43796946	Ca5	43796946	TTTATTATTATTATTATTATTATTATCATTATTATTATTATCATTATTATTATTATTATTATTATTATT/TTTATTATTATTATTATTATTATTATTATCATTATTATTATTATCATTATTATTATTATTATTATTATT	AGCCTGTACGCAAAAAGCAT	GGATAACGCGGAGGTATTGA	59.9	668	INTERGENIC	--	--	--
Ca5_P3_438333	Ca5	438333	T/TCGA	TTAAAGCTGGATGGTACCCG	GTGGTGGCTGCCATTCTTAT	60.0	110	INTRON	Ca_18196	--	--
Ca5_P3_43847991	Ca5	43847991	GT/GTGTGTGTGTCT	GGAAACTCGTTTTTGAGTGGA	TGTCAATGTGCCAAAGTATCG	59.2	679	INTERGENIC	--	--	--
Ca5_P3_4389089	Ca5	4389089	ATTTTTTTTTT/ATTTTTTTTTTTTTTTTTTT	TTTACCACTTGGAGTTGGGC	TCCTTTGCTAGCCCTTTTGA	60.0	392	INTERGENIC	--	--	--
Ca5_P3_43916587	Ca5	43916587	ATTTTTTTTTTTTT/ATTTTTTTTTTTTTT	TGAAGCTTTGGCAGATGATG	TTGAAGGGACTGGACGTAGC	59.9	622	INTERGENIC	--	--	--
Ca5_P3_43918951	Ca5	43918951	CTTTT/CTTT	TTGCAAGGTAAAAAGCACGA	ACGATGCCCCATACAATGAT	59.5	783	INTERGENIC	--	--	--
Ca5_P3_43919096	Ca5	43919096	ATTTT/ATTTTT	TGCTGCTTGATATACGGTGG	ACGATGCCCCATACAATGAT	59.7	441	INTERGENIC	--	--	--
Ca5_P3_43921147	Ca5	43921147	CTATA/CTATATA	TGGGACCAAACATGTGAAGA	TCTAATTGTTTTGATTTATCCCTCA	59.9	583	INTERGENIC	--	--	--
Ca5_P3_43925403	Ca5	43925403	TAAAAAAAAAA/TAAAAAAAAAAA	TCCCATCAACCATAGCCTTC	AGAACCATCAAATTGTCGCA	59.9	561	INTERGENIC	--	--	--
Ca5_P3_440231	Ca5	440231	ATTTTTTTTT/ATTTTTTTTTT	GGGATTTTGTTTGGCGTCTA	CATCGACACGGAAAATTGAA	59.9	868	INTERGENIC	--	--	--

INDEL marker IDs	Chromosomes/ unanchored scaffolds	Physical positions (bp)	InDels (*Kabuli* reference genome-CDC Frontier/PII)	Forward primers (5'-3')	Reverse primers (5'-3')	Anneling temperature (0C)	Expected amplified product size (bp)	Structural annotation		Functional annotation	
								Sequence components of *kabuli* genome	*Kabuli* gene accession IDs	NCBI-KOG	TFs
Ca5_P3_44086162	Ca5	44086162	CTTTTTTTTT/CTTTTTTTTTT	CGGGTCAACTTTGATAGACGA	GATGTGCCGTGAAAACAATG	60.1	594	INTERGENIC	--	--	--
Ca5_P3_44089107	Ca5	44089107	AAATAATAATAATAATAATAATAATAATAATAATAATAA/AAATAATAATAATAATAATAATAATAATAATAA	GCTTGTAGCTAGGCTCGACTC	TTTGGGGGCAAAGTTGATAA	58.5	630	INTERGENIC	--	--	--
Ca5_P3_44090754	Ca5	44090754	TAAAAAAAAA/TAAAAAAAA	TCGTTTCTCCATAAAAGAGCC	CACAAGTCCTATTGGTGGGG	58.5	349	INTERGENIC	--	--	--
Ca5_P3_44177714	Ca5	44177714	GTATATATATATATATAT/GTATATATATATATATATAT	GGTCACGAACATCCCAAAAT	CCATGGTGAGAGGGTCAAAT	59.7	377	INTERGENIC	--	--	--
Ca5_P3_44189157	Ca5	44189157	CATATATATATATATATATATATATAT/CATATATATATATATATATATATATATATATATAT	AATGGGAGAAAGGAGGGAGA	GTGTTTTGGATTTTGGCTCG	60.0	435	INTERGENIC	--	--	--
Ca5_P3_44194778	Ca5	44194778	TTTATT/TTT	TCCTAGAACATGCATCACGC	AGCCTCTTTGAGTCTTGCCA	59.8	539	INTERGENIC	--	--	--
Ca5_P3_44209005	Ca5	44209005	TC/T	TCAAATGATGATGTCTTATTTTTGAA	TCATTTCATCGATTCATTCTGTG	59.0	348	INTERGENIC	--	--	--
Ca5_P3_44212939	Ca5	44212939	CAA/CAAA	ATTCCAAACCAAAACCCTCC	AATGTTTTGCGAGAAAACCG	60.0	638	INTERGENIC	--	--	--
Ca5_P3_44239225	Ca5	44239225	GCC/G	AGCGGTTCTCTTGATTGGAA	TCCACAATGGGATGGAATTT	59.8	141	INTERGENIC	--	--	--
Ca5_P3_44244007	Ca5	44244007	A/AG	CTTGGTTGGTGATGCATTTG	TGTTGTTTGGTTTGTGCCAT	60.0	833	INTRON	Ca_03869	P	--

INDEL marker IDs	Chromosomes/ unanchored scaffolds	Physical positions (bp)	InDels (*Kabuli* reference genome-CDC Frontier/PII)	Forward primers (5'-3')	Reverse primers (5'-3')	Anneling temperature (0C)	Expected amplified product size (bp)	Structural annotation		Functional annotation	
								Sequence components of *kabuli* genome	*Kabuli* gene accession IDs	NCBI-KOG	TFs
Ca5_P3_44244203	Ca5	44244203	AA/AATTTAGA	CAATGAACACATTTGCCACC	GGACGATCAAGCTCAACCAT	59.8	747	INTRON	Ca_03869	P	--
Ca5_P3_44265725	Ca5	44265725	CAAAAAAAAAA/CAAAAAAAAAAA	AGGAGTTGGGTTGGATCCTT	CCGACCCTAAACCCGAAATA	59.8	326	DRR	Ca_03872	D	M-type
Ca5_P3_44407311	Ca5	44407311	ATTTTTTTTTTTT/ATTTTTTTTTTTTT	TATGGAACCATCTTCCTCGG	CTAATTATTGGCCCCAACCC	59.9	532	DRR	Ca_03888	O	MYB_related
Ca5_P3_44420124	Ca5	44420124	ATATTATTATTATTATTATTATTATTATTATTATTATTATTATTATTATT/ATATTATTATTATTATTATTATTATTATTATTATTATTATT	CACCCACATCCAGGTAGCTT	CAACTCACCCATGCATTACG	60.0	391	INTRON	Ca_03889	U	FAR1
Ca5_P3_445610	Ca5	445610	GCCC/GCCCC	ACAGTGGCTTCAATTCCCAC	CCAGCCAAATAGCTGGAATG	60.0	336	INTERGENIC	--	--	--
Ca5_P3_44565619	Ca5	44565619	TAAAAA/TAAAA	AGATGATGCTTTAGGGAAGAAA	TGGGACCGAATCAAAAAGAA	57.2	153	INTERGENIC	--	--	--
Ca5_P3_44581064	Ca5	44581064	CAGA/CA	AAAACCGGGTTGTAAGGTGA	GTCCAGATTGGGCAATCAAT	59.3	550	INTRON	Ca_03901	U	--
Ca5_P3_44596809	Ca5	44596809	ATT/ATTT	TTGGCACATGCATTTTTGTT	GACCAAATCTTGTTGTTGCAGA	60.0	582	INTERGENIC	--	--	--
Ca5_P3_44597015	Ca5	44597015	CAAAAA/CAAAAAA	TTGGCACATGCATTTTTGTT	AGCTATGTCCACTCGTTCGC	60.0	944	INTERGENIC	--	--	--
Ca5_P3_44597112	Ca5	44597112	CTATA/CTATATATA	GGGGTTAGTGCAAGCCAAAT	GTTCGCACGTGATGAACAAT	61.2	862	INTERGENIC	--	--	--

INDEL marker IDs	Chromosomes/ unanchored scaffolds	Physical positions (bp)	InDels (*Kabuli* reference genome-CDC Frontier/PII)	Forward primers (5'-3')	Reverse primers (5'-3')	Anneling temperature (0C)	Expected amplified product size (bp)	Structural annotation		Functional annotation	
								Sequence components of *kabuli* genome	*Kabuli* gene accession IDs	NCBI-KOG	TFs
Ca5_P3_44707178	Ca5	44707178	TCC/TCCC	CCCTAACTTTCCCATCTCCC	GTTGAGTTTGCCCCATTTGT	59.8	131	INTERGENIC	--	--	--
Ca5_P3_44914869	Ca5	44914869	TTATATATATATATATATATATATA/TTATATATATATATATATATATATATA	TTTCGAGCAATCTCATCCCT	CGGCAGCTTTTTACTACCCA	59.8	759	INTERGENIC	--	--	--
Ca5_P3_45041530	Ca5	45041530	ATTTTTTTTTT/ATTTTTTTTTTT	GCCCCAATTAAATGCAGAAA	AAAACCCTAGATGGGTTGGG	59.9	636	INTERGENIC	--	--	--
Ca5_P3_45074327	Ca5	45074327	TAT/TATAATAAT	GCCTAAGACGGTGAATCGAA	AGCTCGGTAGAAAGTCGCTG	60.2	585	INTERGENIC	--	--	--
Ca5_P3_45082437	Ca5	45082437	TAAAAAAAAAA/TAAAAAAAAA	TTTTTGTAGGTAATTCACGTCGTT	GCCCAATACTCATCACTCGC	59.1	796	INTERGENIC	--	--	--
Ca5_P3_45086826	Ca5	45086826	TA/TAA	CAAACGCAAAATTAAACACACAA	AATACAGCTGGGTTTGTGGC	60.0	367	INTERGENIC	--	--	--
Ca5_P3_45087098	Ca5	45087098	C/CG	ATGAAGGAGGAGGGGAGAAA	AATCCGTCACAAAGGCAGAC	60.0	237	INTERGENIC	--	--	--
Ca5_P3_45094101	Ca5	45094101	A/AG	TTCAATGAAAGCGCATGTGT	GAAAAGGGTTCGGATGGATT	60.3	379	INTERGENIC	--	--	--
Ca5_P3_45094437	Ca5	45094437	TG/T	AATCCATCCGAACCCTTTTC	TTGTCAATGTTGCATGTCTGAA	60.1	259	INTERGENIC	--	--	--
Ca5_P3_45103038	Ca5	45103038	AC/A	GTTCCCCAAATGCATCACTC	AAAAATGTTGGGGCAAAATG	60.3	243	INTERGENIC	--	--	--

INDEL marker IDs	Chromosomes/ unanchored scaffolds	Physical positions (bp)	InDels (*Kabuli* reference genome-CDC Frontier/PII)	Forward primers (5'-3')	Reverse primers (5'-3')	Anneling temperature (0C)	Expected amplified product size (bp)	Structural annotation		Functional annotation	
								Sequence components of *kabuli* genome	*Kabuli* gene accession IDs	NCBI-KOG	TFs
Ca5_P3_45132493	Ca5	45132493	CATATATATATATATATA/CATATATATATATATATATA	AATTTCCTGCTTCCGTTTGA	TCTACCCAAAACCGTCCTGA	59.7	522	INTERGENIC	--	--	--
Ca5_P3_45372527	Ca5	45372527	TTTATTATTATTATTATTATTATTATTATTATTATTATTATTATTATTATTATTA/TTTATTATTATTATTATTATTATTATTATTATTATTATTATTATTATTATTATTATTATTATTATTA	CCCAGTGTCACCGTCTTTTT	TTCACACAAATGTCCACCAA	60.0	537	INTERGENIC	--	--	--
Ca5_P3_4542302	Ca5	4542302	G/GC	GGGGTGTTGCAACCTTTAGA	GCTTGTCCGCGTAACTCTTC	60.0	336	INTERGENIC	--	--	--
Ca5_P3_4543921	Ca5	4543921	ATT/ATTT	TTCGCAACTCCACTCTTCCT	TCCTGGGCTTAATTCAATCG	60.0	588	INTERGENIC	--	--	--
Ca5_P3_45511101	Ca5	45511101	TA/TAA	CAAGGTTTCTCTTTCGCTGC	CTCAAATTGGTCCCCTGAAA	60.1	539	INTRON	Ca_03998	T	--
Ca5_P3_45518520	Ca5	45518520	TA/T	ATGGATGGTGACAAGCATGA	ACAATGAAACATTTGGGGGA	59.9	685	INTERGENIC	--	--	--
Ca5_P3_45548755	Ca5	45548755	TATAATAATAATAATAATAATAATAATAATAATAAT/TATAATAATAATAATAATAATAATAAT	TAACTGCAACAGATGGTCGC	TTCACCATTTTGACACATTTCTTT	59.9	436	INTERGENIC	--	--	--
Ca5_P3_45557067	Ca5	45557067	CATATATATATATATATATATATATATAT/CATATATATATATATATAT	CAATGAAAAAGTGTGGCCCT	ACGTTGATGGAACAATGCCT	60.0	873	INTERGENIC	--	--	--
Ca5_P3_45569629	Ca5	45569629	TAA/TAAAA	TCCCTCGACTACCTCCCTTC	GGCAAATGTTGATAAGTGCTCA	60.6	749	INTERGENIC	--	--	--
Ca5_P3_4557985	Ca5	4557985	AATATATATATATATATATATATATATATATATATATAT/AATATATATATATATATATATATATATATATATATATATATATATAT	TTTTTATGCGAGTGGCTTCA	TCCCCTTGGTTTGAGAGAGA	59.4	848	INTERGENIC	--	--	--

INDEL marker IDs	Chromosomes/ unanchored scaffolds	Physical positions (bp)	InDels (*Kabuli* reference genome-CDC Frontier/PII)	Forward primers (5'-3')	Reverse primers (5'-3')	Anneling temperature (0C)	Expected amplified product size (bp)	Structural annotation		Functional annotation	
								Sequence components of *kabuli* genome	*Kabuli* gene accession IDs	NCBI-KOG	TFs
Ca5_P3_45616614	Ca5	45616614	CTATATATATATATATATATATATA/CTATATATATATATATATATATATA	GAGGCCATGAAGAAGAATGC	TGAAACCATTTCACCACCAA	59.8	543	INTERGENIC	--	--	--
Ca5_P3_456204	Ca5	456204	TAAAAAAAAAAAAAA/TAAAAAAAAAAA	TGCCTACAACCACACATGCT	CGTTTTCCGATTCATGCTTT	60.2	685	INTERGENIC	--	--	--
Ca5_P3_45631363	Ca5	45631363	TAAAAAAAAAAAAA/TAAAAAAAAAAAAA	CAAAGTCGCACGAGAGTTCA	CGCCAAATTGCTGATTCTTT	60.2	848	INTERGENIC	--	--	--
Ca5_P3_45724530	Ca5	45724530	AATTATTATTATTATTATTATTATTATTATTATTAT/AATTATTATTATTATTATTATTATTATTATTATTAT	GCGGTTGAATAAATGTGACAAA	TAGTTGGAGGTTGCTGACCC	59.9	616	INTERGENIC	--	--	--
Ca5_P3_45746975	Ca5	45746975	ATT/ATTACACTCTTT	GGAGTTGCAAAAGAGCATGA	TGGTTAACACTGGAGAGGGG	59.0	136	DRR	Ca_04024	G	--
Ca5_P3_45779614	Ca5	45779614	GAAAAAAAAAA/GAAAAAAAAAAA	ACGCGGATCCATTCAATAAG	GATGTTGAGTGAAAACACAAACG	59.9	546	INTERGENIC	--	--	--
Ca5_P3_45925212	Ca5	45925212	TAAAA/TAAA	ACAGAGCCAAACCCTCCTTT	AGCATCCCTTGTGAATTTGG	60.1	489	CDS (FRAME SHIFT)	Ca_04037	--	S1Fa-like
Ca5_P3_45928990	Ca5	45928990	CGTG/C	GATTGCAGGAGCAAGATGGT	TGTATTTTGCAGCATTGGAG	60.2	615	INTERGENIC	--	--	--
Ca5_P3_45975576	Ca5	45975576	GA/GAAATTGTTGAAAA	CCAAATTCTCACCCGAAGAA	CAAAATGGGGCAGTTAAGGA	60.0	634	INTERGENIC	--	--	--
Ca5_P3_45989618	Ca5	45989618	TAAAAAAAAAA/TAAAAAAAAA	TTGCTCCTCTGGTCCTCACT	GTTGGCTTTCGTACCCTCAA	60.0	325	INTRON	Ca_04045	--	SAP

INDEL marker IDs	Chromosomes/ unanchored scaffolds	Physical positions (bp)	InDels (*Kabuli* reference genome-CDC Frontier/PII)	Forward primers (5'-3')	Reverse primers (5'-3')	Anneling temperature (0C)	Expected amplified product size (bp)	Structural annotation		Functional annotation	
								Sequence components of *kabuli* genome	*Kabuli* gene accession IDs	NCBI-KOG	TFs
Ca5_P3_46005575	Ca5	46005575	TTATATATATATATATATATATATATATA T/TTATATATATATATATATATATATAT	CTCCGCATTTCAGGATT GTT	GCAAACGGTGTGTAGT GGTG	60.1	606	INTERGENIC	--	--	--
Ca5_P3_460106	Ca5	460106	ATTT/ATTTT	ACACATACCCAAAGGG ATGC	CCCCAGCAGTCGGTAA AATA	59.7	541	INTRON	Ca_18199	--	--
Ca5_P3_46132848	Ca5	46132848	TTTATTATTATTATTATTATTATTATTAT TATTATTATT/TTTATTATTATTATTATTA TTATTATTATTATTATT	TGTACCGTCACATTGCA CCT	TACTGTATCAACGCCG CAAG	60.0	771	INTERGENIC	--	--	--
Ca5_P3_46208612	Ca5	46208612	AAT/AATAT	ATGCAGCACAAAATGC AAGT	TTCGAATCTTATTTTGA CAATTTTT	59.4	502	INTERGENIC	--	--	--
Ca5_P3_46212141	Ca5	46212141	AATGATG/AATG	AAAAATCAATCCTTTCT CCAATG	AGTTTCAAAAACGGTG GTGG	58.6	107	INTERGENIC	--	--	--
Ca5_P3_46228817	Ca5	46228817	TGGGCAGGGGCAG/TGGGCAG	CGGAATGGACAGCCTA ATTT	GTGGTCTTGGAGCATT GGTT	59.0	557	CDS (large-effect mutations)	Ca_04075	S	--
Ca5_P3_46231130	Ca5	46231130	ATTTTTTTT/ATTTTTTT	GGAAGCCAAGCATTCA AGAG	TGGAAAAAGAATGAAA TTGCTTA	60.0	849	INTERGENIC	--	--	--
Ca5_P3_46271953	Ca5	46271953	TTTTAATT/TTTTAATTTAATT	CCATCAAATGTTGGTCA CTCC	ATTCATCTCGCCTTTG CTGT	60.2	352	INTERGENIC	--	--	--
Ca5_P3_46305506	Ca5	46305506	GATATATATATATATATATATATATATA TATATATAT/GATATATATATATATATAT ATATATATATATATATATATATATAT	TGCCCTCAATCTGAAAA CAA	GAACAAGAGCGGTCTC ACAA	59.2	876	DRR	Ca_04084	--	--
Ca5_P3_463852	Ca5	463852	CTTTTTTTTTT/CTTTTTTTTT	AAGCTGATCTGAGGTT CCGA	TTCGATAATTGATTCGT TGTACG	60.0	572	INTERGENIC	--	--	--

INDEL marker IDs	Chromosomes/ unanchored scaffolds	Physical positions (bp)	InDels (*Kabuli* reference genome-CDC Frontier/PII)	Forward primers (5'-3')	Reverse primers (5'-3')	Anneling temperature (0C)	Expected amplified product size (bp)	Structural annotation		Functional annotation	
								Sequence components of *kabuli* genome	*Kabuli* gene accession IDs	NCBI-KOG	TFs
Ca5_P3_46393868	Ca5	46393868	AG/AGTGATG	ACAGTTCAAAGTCCCTGGCA	TCAATCATTTAAAATTACTCTGAATCG	60.7	561	INTERGENIC	--	--	--
Ca5_P3_46416403	Ca5	46416403	ATT/ATTT	ACCCTAGCAGCACCTCTTCA	CCCGTTAACAACGAAAGCAT	60.0	395	INTERGENIC	--	--	--
Ca5_P3_46417387	Ca5	46417387	GTTTTTTTTTT/GTTTTTTTTT	AACTCGCACCTGCACCTAAT	AATTACAAATCGCCACCTGC	59.8	684	INTERGENIC	--	--	--
Ca5_P3_46605106	Ca5	46605106	TAAAAAAAAAAA/TAAAAAAAAAA	GCCTTGGCACCACTATCATT	AAGCGTTTTCTCGTCAGGAA	60.0	697	INTRON	Ca_04118	J	NAC
Ca5_P3_46636015	Ca5	46636015	A/AGACGAAAGAGCGGTT	TGGTGGTCAGCGTATACCAA	TGTGGTCGACAGATACCCAA	60.0	346	INTERGENIC	--	--	--
Ca5_P3_46661039	Ca5	46661039	AATATATATATATATATATATAT/AATATATATATATATATATATATAT	TGGAATTCAAGGGACCAAAA	ACTTGCGAGATAATTTAGTAGGGA	60.3	863	INTERGENIC	--	--	--
Ca5_P3_46676212	Ca5	46676212	ATTTTTTTTTT/ATTTTTTTTTTT	GCCACCACACCTCCAACTAT	TTGAGATGGGACTTTGGAGC	59.9	880	INTERGENIC	--	--	--
Ca5_P3_46735071	Ca5	46735071	CATATATATATATATATATATATATATAT/CATATATATATATATATATAT	TTGATTGGGAATCACGCATA	TGGCATGCATAACATCTGCT	59.9	712	INTRON	Ca_04131	A	--
Ca5_P3_4681704	Ca5	4681704	ATTTTTTTTTTTT/ATTTTTTTTTTT	CCCCATTTTGTTTCTCTCCA	CTTTTATCATGCGATCCCCC	59.9	712	INTERGENIC	--	--	--
Ca5_P3_46946905	Ca5	46946905	AATATATATATATATATAT/AATATATATATATATATATAT	CATCCGTGGTCCTTTGACTT	TGGTCATTGCATTTTCCTCA	60.0	482	DRR	Ca_04149	K	ERF

INDEL marker IDs	Chromosomes/ unanchored scaffolds	Physical positions (bp)	InDels (*Kabuli* reference genome-CDC Frontier/PII)	Forward primers (5'-3')	Reverse primers (5'-3')	Anneling temperature (0C)	Expected amplified product size (bp)	Structural annotation		Functional annotation	
								Sequence components of *kabuli* genome	*Kabuli* gene accession IDs	NCBI-KOG	TFs
Ca5_P3_46985054	Ca5	46985054	AATTATTATTATTATTATTATTATTATTATTATTATTATTATTATT/AATTATTATTATTATTATTATTATTATTATTATTATTATTATT	TCGCATTTTAAGGGAGGAAA	AGCAAGGAAGCATCATCCAA	59.7	212	INTERGENIC	--	--	--
Ca5_P3_46995665	Ca5	46995665	TTTATTATTATTATTATTATTATTATTATTATTATTATTATTATTATTATTATTATTATTATT/TTTATTATTATTATTATTATTATTATTATTATTATTATTATTATTATT	TTGTTCCAAAGCTGCAAATCT	GCACAATATTTTCTTTTGCACATT	59.9	636	INTERGENIC	--	--	--
Ca5_P3_47064195	Ca5	47064195	CAAAAAAAA/CAAAAAAAAA	CCGCTTCTACCTCTAGCCCT	AAAATGCCCTCACACACACA	60.0	587	INTERGENIC	--	--	--
Ca5_P3_471069	Ca5	471069	GAAATTAAATTAAA/GAAATTAAATTAAATTAAA	GTGACACCGTCTCGTTGGTA	AACTGGGTGAGAGCCTGAGA	59.6	314	INTRON	Ca_18200	U	--
Ca5_P3_4733803	Ca5	4733803	GC/GTC	TGCATCCAATAGGTGTGTTTG	AGGGAGGACGAAAATGACCT	59.5	460	INTERGENIC	--	--	--
Ca5_P3_47476526	Ca5	47476526	CAAAAAAAAAAA/CAAAAAAAAAA	GGTTCTCAAATCTCCGACCA	TGGGTCAATCTGACCTCATTT	60.1	678	INTERGENIC	--	--	--
Ca5_P3_47637472	Ca5	47637472	AATATATATATATATAT/AATATATATATATATATAT	TGGTTCTGCTCTGCTCCTTT	TGGTGGTTAGTGGTGGTTGA	60.1	768	INTERGENIC	--	--	--
Ca5_P3_4768599	Ca5	4768599	GT/GTTGAGAAAT	TTTCTTCCCAACCTTCATCG	TGAAAAGCTCATGGCTGATG	60.0	179	INTERGENIC	--	--	--
Ca5_P3_47689133	Ca5	47689133	GTATATATATATATATATATATATA/GTATATATATATATATATATATA	AGGAAATCCTCAGCCTGGTT	TTTTACTTGCCCATACCCCA	60.1	219	INTRON	Ca_04228	--	--
Ca5_P3_47692025	Ca5	47692025	ATTTTTTTTT/ATTTTTTTT	CACCAAAAGCAGGGTCTCTC	GCCTTAAAGCTTTGGGTGAA	59.8	346	DRR	Ca_04228	--	--

INDEL marker IDs	Chromosomes/ unanchored scaffolds	Physical positions (bp)	InDels (*Kabuli* reference genome-CDC Frontier/PII)	Forward primers (5'-3')	Reverse primers (5'-3')	Anneling temperature (0C)	Expected amplified product size (bp)	Structural annotation		Functional annotation	
								Sequence components of *kabuli* genome	*Kabuli* gene accession IDs	NCBI-KOG	TFs
Ca5_P3_47714017	Ca5	47714017	GATATATATATATATATATATATATATA/ GATATATATATATATATATATATATA	ATGCGTGTGAAATTGT GCAT	TCAGCTCAGTGGATGA TTGG	60.0	402	INTRON	Ca_04232	G	C2H2
Ca5_P3_47765685	Ca5	47765685	GTACT/GT	TTATTTTTGAGTGTCCG GGG	GCCTTGTTTGGTGGAG TGAT	59.8	121	INTERGENIC	--	--	--
Ca5_P3_479066	Ca5	479066	TAAAAAAAAAA/TAAAAAAAAAAA	CCTGTTCTGTAGTGGG AGCC	TCCATGTTACTTCTCC GCCT	59.7	508	DRR	Ca_18200	U	--
Ca5_P3_479350	Ca5	479350	CATATATATATATATA/CATATATATATA TATATA	TTGTCACACAATAAGGC GGA	TCATCACGATCTGAAT CCCA	60.1	674	DRR	Ca_18200	U	--
Ca5_P3_47999247	Ca5	47999247	ATTTTTTTTTTT/ATTTTTTTTTTTT	TCCCTCGCTCTTTATTT CAA	TGACTGCATTTAATTTT CTCTTCAA	57.6	883	INTERGENIC	--	--	--
Ca5_P3_48017474	Ca5	48017474	ACCGCTACCCGCTAC/ACCGCTAC	CCTAGCCTTGGAAGTA GCGA	GGGCTCGACATACTTT GCAT	59.6	479	INTRON	Ca_04261	R	--
Ca5_P3_48120820	Ca5	48120820	TAAAAAAAAAA/TAAAAAAAAA	GAGAAACGTGAGGAAA AATTCA	TGTTGGGTTGGCCATA AAAT	58.4	959	INTERGENIC	--	--	--
Ca5_P3_48125139	Ca5	48125139	AATTATTATTATTATTATTATTATTATTA TTATTATTATTATTATT/AATTATTATTA TTATTATTATTATTATTATTATTATTATT ATTATTATT	GGGACCAAAACTGGAG ACAA	GCGATCAGCTGAAAAC CACT	59.9	847	INTERGENIC	--	--	--
Ca5_P3_48154714	Ca5	48154714	ATATTATTATTATTATTATTATTATTATT ATTATTATTATTATTATTATTATTAT/AT ATTATTATTATTATTATTATTATTATTAT TATTATTATTATTAT	AAGTAAGGGCCCGAAT GAAC	GTTGGGCCACAATTAT ACGG	60.3	543	INTERGENIC	--	--	--
Ca5_P3_481685	Ca5	481685	CAAAAAAAA/CAAAAAAAAA	TTGAGGGACCAAAACT GGAG	GAGTTCGCTGACAGAT GCAA	60.1	191	DRR	Ca_18201	--	--

INDEL marker IDs	Chromosomes/ unanchored scaffolds	Physical positions (bp)	InDels (*Kabuli* reference genome-CDC Frontier/PII)	Forward primers (5'-3')	Reverse primers (5'-3')	Anneling temperature (0C)	Expected amplified product size (bp)	Structural annotation		Functional annotation	
								Sequence components of *kabuli* genome	*Kabuli* gene accession IDs	NCBI-KOG	TFs
Ca5_P3_482095	Ca5	482095	GC/G	TTGCATCTGTCAGCGAACTC	TTTTTCGGGGGTCAAATGTA	60.1	800	DRR	Ca_18201	--	--
Ca5_P3_4844622	Ca5	4844622	CTATATATATATATATATATATATATATAT/CTATATATATATATATATATATATATAT	CTTGTCCACAAGCGCTGTAA	TTGAATCCCACCAGGAAAAG	60.1	641	INTERGENIC	--	--	--
Ca5_P3_4857245	Ca5	4857245	GAAAA/GAAAAA	CCCACATTTCTTTGGCAGTT	TGTGCCTAAACTTGCTGGTG	60.0	501	INTERGENIC	--	--	--
Ca5_P3_4860237	Ca5	4860237	GATAAATATATATATATATATATATATATATATATATATATATATAT/GATATATATATATATATATATATATATATATAT	CTGCGCTTTATGCGTGTTTA	ATGGACCACGAGGTGTCATC	60.0	368	INTERGENIC	--	--	--
Ca5_P3_4869565	Ca5	4869565	GAAAAAAA/GAAAAAAAA	ATGCAAAATCGCACAATGAA	TGCAAGCAACTTGAGTCCAC	60.1	447	INTERGENIC	--	--	--
Ca5_P3_490277	Ca5	490277	TAAAAAAAAAAAAA/TAAAAAAAAAA	TGGTTGAAACATTCCCTATGC	GAGCATATAGCCCAACCCAA	59.8	695	DRR	Ca_18202	R	--
Ca5_P3_4921108	Ca5	4921108	TTAT/TTATATAT	ACATATCATGCAACCTGCGA	GGAGGATGTGGCATAGAGGA	60.1	320	INTERGENIC	--	--	--
Ca5_P3_4970490	Ca5	4970490	T/TG	ATTCCTACGCGATTACCACG	CCCGTGATAGGCAGTACGTT	60.0	528	INTERGENIC	--	--	--
Ca5_P3_497099	Ca5	497099	GAAAA/GAAA	TTGAGTGCAGCTGGAGAAGA	TCACTCAAAAACCAAGCACG	59.9	110	INTERGENIC	--	--	--
Ca5_P3_4979941	Ca5	4979941	GTTTTTTTTTTT/GTTTTTTTTTT	GGCTGTTCATGGTTGGTTTT	GATGAGTGTTAACGGGTGGC	59.8	796	INTERGENIC	--	--	--

INDEL marker IDs	Chromosomes/ unanchored scaffolds	Physical positions (bp)	InDels (*Kabuli* reference genome-CDC Frontier/PII)	Forward primers (5'-3')	Reverse primers (5'-3')	Anneling temperature (0C)	Expected amplified product size (bp)	Structural annotation		Functional annotation	
								Sequence components of *kabuli* genome	*Kabuli* gene accession IDs	NCBI-KOG	TFs
Ca5_P3_4992401	Ca5	4992401	TTATATATATATATATATATATATA/TTATATATATATATATATATATATATA	ATCGATGTAGTCAAACCCCG	GCCCCCGTGTTATCCTTATT	59.8	284	INTRON	Ca_25545	A	bHLH
Ca5_P3_5061240	Ca5	5061240	CTTTTTTTTTT/CTTTTTTTTTTT	CATGCAAACCCATTGTGAAG	AAAAAGAAGGCGGTGACAAA	60.0	845	INTERGENIC	--	--	--
Ca5_P3_5190851	Ca5	5190851	CTATATATATATATATATATATAT/CTATATATATATATATATATATATAT	AGCGCTATGTGACCCTTTTG	AATGTGAATCCCACCAGGAA	60.3	555	INTERGENIC	--	--	--
Ca5_P3_5223462	Ca5	5223462	AATATATATATATATATATATATATATA/AATATATATATATATATATATATATATATATATA	TTTCGAGTTTTTCCGTCCAC	GTGCCAAGGCATAAAGGTGT	60.1	530	INTERGENIC	--	--	--
Ca5_P3_5262068	Ca5	5262068	CAAAAAA/CAAAAA	TGCCATAATTCTGCATCCAA	TTTTCGAACTTGGGGTTGTC	60.0	403	INTERGENIC	--	--	--
Ca5_P3_5318962	Ca5	5318962	TC/T	ACGTTGACCAATTCAGCTCC	CACTAGGGACCCTTTGGTGA	60.1	720	INTERGENIC	--	--	--
Ca5_P3_5492827	Ca5	5492827	TTTATTATTATTATTATTATTATTATTATTATTATTATTATTATTATTATTATTATTATTATTATTATTATTATTATTAT/TTTATTATTATTATTATTATTATTATTATTATTATTATTATTATTATTATTATTATTATTATTATTAT[illegible]	TATCCCCTCGATTTGCTTCA	TTTTGCCTCAAAAAGTTTCACA	60.5	603	INTERGENIC	--	--	--
Ca5_P3_5582691	Ca5	5582691	AATATATATATATATATATATATATATAT/AATATATATATATATATATATATAT	CAACACCGTTTTTCTCCGTT	GAATTGGGAATTGGAGAGCA	60.0	728	INTERGENIC	--	--	--
Ca5_P3_5602407	Ca5	5602407	CATATATATATATATATATATATATATAT/CATATATATATATATATATATATATATATATATAT	GTTGGTTCCCTAGCACCGTA	CAATTGAGTTGCGACGAGAG	60.0	552	INTERGENIC	--	--	--
Ca5_P3_5655437	Ca5	5655437	GTAATAAATTT/GTAATAAATTTAATTAATAATAAATTT	CGAAACATTCGAAATGCAGA	TTCTATCACCCCCACATTCG	59.8	628	INTERGENIC	--	--	--

INDEL marker IDs	Chromosomes/ unanchored scaffolds	Physical positions (bp)	InDels (*Kabuli* reference genome-CDC Frontier/PII)	Forward primers (5'-3')	Reverse primers (5'-3')	Anneling temperature (0C)	Expected amplified product size (bp)	Structural annotation		Functional annotation	
								Sequence components of *kabuli* genome	*Kabuli* gene accession IDs	NCBI-KOG	TFs
Ca5_P3_5705191	Ca5	5705191	GA/G	AATCGATTGGCTTTGATTCG	CTCTCATCTGAGGGCAAAGC	60.0	412	INTERGENIC	--	--	--
Ca5_P3_577875	Ca5	577875	CG/CGG	CGTGTGCTTGCAGTACGTTT	ACCTATGGAGGACGCAAATG	60.0	632	INTERGENIC	--	--	--
Ca5_P3_5798731	Ca5	5798731	TTATATATATATATATATATATATA/TTATATATATATATATATATATATATATATA	CTTCGACACTGTAGCACCCA	ATATTTCTGCGCATTCTCGC	59.9	217	INTERGENIC	--	--	--
Ca5_P3_5805706	Ca5	5805706	TAAAAAAAAAAA/TAAAAAAAAAAAA	AAACCAATTGTGTCTAGCCCAT	TTTTTCACCCCTCTTTCCAA	59.8	767	INTERGENIC	--	--	--
Ca5_P3_5836817	Ca5	5836817	TACCCTAAACC/TACCCTAAACCCTAAACCCTAAACC	GTATGAGGAGGATCTTGCGG	TCAAAATGCACCATAAACCG	59.7	594	INTERGENIC	--	--	--
Ca5_P3_5837067	Ca5	5837067	ACCTAAACCCTAAACCCTAAACCCTAA/ACCTAAACCCTAAACCCTAA	AAAGAAAAACACTCGCGCTG	AGCGTTTTGGGTTTAGGGTT	60.6	825	INTERGENIC	--	--	--
Ca5_P3_5888717	Ca5	5888717	AATATATATATATATATATATATATATATATA/AATATATATATATATATATATATATATATATATATATATATA	TCTTGAGCTACTGGGGCTTG	CAAGTTTGAAATGGAGGGGA	60.5	882	INTERGENIC	--	--	--
Ca5_P3_5912049	Ca5	5912049	TAAATATACATCAAATATA/TAAATATACATCAAATATACATCAAATATA	TTTTTGTTGTCTCACTCGATCA	CAGTGTTAATTGGCACGAAA	58.4	276	INTERGENIC	--	--	--
Ca5_P3_6074643	Ca5	6074643	GATATATATATATATA/GATATATATATATA	AAATTAAAGTTTCAAACCACGG	ATTCCCCCTTCATCTTGGAC	57.3	864	INTERGENIC	--	--	--
Ca5_P3_6200121	Ca5	6200121	CT/CTT	TTCGCAACTCCACTCTTCCT	GATCCATTTTCCCCCAAATC	60.0	342	INTERGENIC	--	--	--

INDEL marker IDs	Chromosomes/ unanchored scaffolds	Physical positions (bp)	InDels (*Kabuli* reference genome-CDC Frontier/PII)	Forward primers (5'-3')	Reverse primers (5'-3')	Anneling temperature (0C)	Expected amplified product size (bp)	Structural annotation		Functional annotation	
								Sequence components of *kabuli* genome	*Kabuli* gene accession IDs	NCBI-KOG	TFs
Ca5_P3_622630	Ca5	622630	GAAG/GAAGGAGTTTTGTTCAAG	CATGCTTACCCTCTGGCCTA	ACAGCAGAATGGGGTAGTGG	60.2	651	INTERGENIC	--	--	--
Ca5_P3_6269152	Ca5	6269152	AGGAAGTCGTTCCCTGGGGAAG/AGGAAG	GGGTTGCTTTTCTTCACTGC	ACGACCCCCTGTAAACATTG	59.9	360	INTERGENIC	--	--	--
Ca5_P3_6365922	Ca5	6365922	ATTTTTTTTTT/ATTTTTTTTT	CAGGAATTTCCAGCAACCAT	ATGCATGTGGTGGAGAAAAA	59.9	943	INTERGENIC	--	--	--
Ca5_P3_6369660	Ca5	6369660	TC/TCAC	GAGGAAACACGGTGAATGGT	CGCTCACTAGTCGAACGAGA	59.8	486	INTERGENIC	--	--	--
Ca5_P3_6432371	Ca5	6432371	TAAAAAAAAAA/TAAAAAAAAA	TTGCTTGCGAGATTTCTCCT	AGAGAGCGACCTAGTCTGCG	60.1	705	INTERGENIC	--	--	--
Ca5_P3_64660	Ca5	64660	CAAAAAA/CAAAA	TGGATCATCAAAGATCAACGG	ATTGAAAGTTGCGGGGACTA	60.8	438	INTERGENIC	--	--	--
Ca5_P3_6510151	Ca5	6510151	CTTTTTTTTTT/CTTTTTTTTTTT	TCGCGTTCATGATTTTGTGT	CGGAGTGATAATTGGTGCAA	60.1	816	INTERGENIC	--	--	--
Ca5_P3_6532288	Ca5	6532288	AAAAAGAACAAAA/AAAAA	TGTCTTCAACTTCTGCGGTG	TTGCAACGTGTCATTGTCCT	60.0	831	INTERGENIC	--	--	--
Ca5_P3_6545834	Ca5	6545834	GTTTTTTTTTTTT/GTTTTTTTTTTT	TTTCTGCCTCTGTCCCACTT	TTGATTTGCAGCAAGATTGG	59.8	509	INTERGENIC	--	--	--
Ca5_P3_6663718	Ca5	6663718	CAA/CAAA	AGGGAGGACGAAAATGACCT	TAACCAATCTTGTGCACCCA	59.9	171	INTERGENIC	--	--	--

INDEL marker IDs	Chromosomes/ unanchored scaffolds	Physical positions (bp)	InDels (*Kabuli* reference genome-CDC Frontier/PII)	Forward primers (5'-3')	Reverse primers (5'-3')	Anneling temperature (0C)	Expected amplified product size (bp)	Structural annotation		Functional annotation	
								Sequence components of *kabuli* genome	*Kabuli* gene accession IDs	NCBI-KOG	TFs
Ca5_P3_666553	Ca5	666553	TGTTC/TTCGTTC	TTGGTCGACAAGCAACTGAG	AAAATCCAACAATCTCCCCC	60.0	732	DRR	Ca_23223	--	--
Ca5_P3_6693	Ca5	6693	ACTCTCTCTCT/ACTCTCTCTCTCT	GGAGGTATCATCACGAGGGA	CCTCAGAGAGGAGAATAGGGTG	59.9	523	INTERGENIC	--	--	--
Ca5_P3_6727251	Ca5	6727251	CGG/CGGG	TTTGGATTTTGACTCGGAGG	AGGAAGAGTGGAGTTGCGAA	60.0	509	INTERGENIC	--	--	--
Ca5_P3_6763101	Ca5	6763101	AGGG/AGGGG	CAAATGTCGGGAGCTACCAT	GGATCCCCAATCGATAACCT	60.0	437	INTERGENIC	--	--	--
Ca5_P3_6763849	Ca5	6763849	AATATATATATATATATATA/AATATATATATATATATA	TGGGTGGGAGTAATTCTTCG	GAGTTGCGGCGAGAGTAGAT	59.9	484	INTERGENIC	--	--	--
Ca5_P3_6764095	Ca5	6764095	GTATATATATATATATATATATAT/GTATATATATATATATATATATATATATATATATATATAT	TCAATTTGGGTTCAGGGTGT	ATGATGGGCCAGACGTTAAG	60.2	841	INTERGENIC	--	--	--
Ca5_P3_6765423	Ca5	6765423	GCAACA/GCAACAACA	CGAAGCTGCCAGGTAAAAAG	TGAGTCCATTCTCCAAACCA	60.0	652	INTERGENIC	--	--	--
Ca5_P3_6765484	Ca5	6765484	CATATATATATATATATATATATAT/CATATATATATATATATATATATATATAT	CGAAGCTGCCAGGTAAAAAG	TGAGTCCATTCTCCAAACCA	60.0	652	INTERGENIC	--	--	--
Ca5_P3_6772456	Ca5	6772456	GCACACA/GCACA	TGCACAACAAAAGCACAACA	TGTACCATCATAGTGGCGGA	59.9	512	INTERGENIC	--	--	--
Ca5_P3_6773829	Ca5	6773829	G/GA	TCGCGAAGAGATAGAAGGGA	CGCGAGTAAAACCGGTAAAA	60.1	104	INTERGENIC	--	--	--

INDEL marker IDs	Chromosomes/ unanchored scaffolds	Physical positions (bp)	InDels (*Kabuli* reference genome-CDC Frontier/PII)	Forward primers (5'-3')	Reverse primers (5'-3')	Anneling temperature (0C)	Expected amplified product size (bp)	Structural annotation		Functional annotation	
								Sequence components of *kabuli* genome	*Kabuli* gene accession IDs	NCBI-KOG	TFs
Ca5_P3_6833150	Ca5	6833150	TTTATTATTATTATTATTATTATTATTATT/TTTATTATTATTATTATTATTATTATTATTATTATT	TGCTAGGAACCGAGCCTTTA	TTGGCATCGTGATTTTATGC	60.0	924	INTERGENIC	--	--	--
Ca5_P3_6833700	Ca5	6833700	ATATT/AT	GCATAAAATCACGATGCCAA	CGATTTTTCGTCTACATGTGC	59.5	211	INTERGENIC	--	--	--
Ca5_P3_6833833	Ca5	6833833	AATA/AATATA	AAATCACGATGCCAAAGAAAA	AAGAAAGTTGTAAATGTGTTTTATGGA	59.6	616	INTERGENIC	--	--	--
Ca5_P3_6833924	Ca5	6833924	ATTT/ATT	AAATCACGATGCCAAAGAAAA	AAGAAAGTTGTAAATGTGTTTTATGGA	59.6	616	INTERGENIC	--	--	--
Ca5_P3_6834075	Ca5	6834075	ATATTATTATTATTATTATTATTATTATTATTATTATTATTATTATTATTATTA/ATATTATTATTATTATTATTATTATTATTATTATTATTATTA	GCACATGTAGACGAAAAATCG	ATGGCATGGTGATTTTATGC	58.3	775	INTERGENIC	--	--	--
Ca5_P3_6834263	Ca5	6834263	CT/C	GCTTAATCCATAAAACACATTTACAA	TTCTCATGCGCTTGACTAATG	57.9	526	INTERGENIC	--	--	--
Ca5_P3_6834706	Ca5	6834706	TAAAAAA/TAAAAAAA	TGCATAAAATCACCATGCCA	TTGTACGTTTCTTTTCTATGATTTGC	60.9	360	INTERGENIC	--	--	--
Ca5_P3_6834978	Ca5	6834978	ATATTATTATTATTATTATTATT/ATATTATTATTATTATTATTATTATT	TAGTCAAGCGCATGAGAAAA	GGCATGATGATTTTATGCATATTT	57.3	748	INTERGENIC	--	--	--
Ca5_P3_6837967	Ca5	6837967	TAAAAAAAAAAAA/TAAAAAAAAA	TCGAGAACAACTCGTCTCGTG	AATTTGCAGTGTCGCAAAAA	61.6	254	INTERGENIC	--	--	--
Ca5_P3_6848979	Ca5	6848979	GTATATATATATATATATATA/GTATATATATATATATATATA	GGAGTACCATAGGCCAACGA	CGAAAATGAAAACGGGAGAA	60.0	534	INTERGENIC	--	--	--

INDEL marker IDs	Chromosomes/ unanchored scaffolds	Physical positions (bp)	InDels (*Kabuli* reference genome-CDC Frontier/PII)	Forward primers (5'-3')	Reverse primers (5'-3')	Anneling temperature (0C)	Expected amplified product size (bp)	Structural annotation		Functional annotation	
								Sequence components of *kabuli* genome	*Kabuli* gene accession IDs	NCBI-KOG	TFs
Ca5_P3_6851602	Ca5	6851602	TGG/TG	AATCGATTGATTCAGCCCAG	CTCTCATCTGAGGGCAAAGC	60.0	361	INTERGENIC	--	--	--
Ca5_P3_6872599	Ca5	6872599	ATTATTTATTTATT/ATTATTTATTTATTTATT	CGGCCATCACACAATGATAA	TCAGGATGTCACCACGAAGA	60.3	454	INTERGENIC	--	--	--
Ca5_P3_6907615	Ca5	6907615	A/AGTG	GGTTGTGTTATCCCTCGACA	AGGGTGCCACATTTACTCCA	58.4	539	INTERGENIC	--	--	--
Ca5_P3_6907875	Ca5	6907875	G/GAAA	GGTTGTGTTATCCCTCGACA	AGGGTGCCACATTTACTCCA	58.4	539	INTERGENIC	--	--	--
Ca5_P3_6909501	Ca5	6909501	TATATATAATTTAAATATATAATTTAAAT/TATATATAATTTAAAT	AATGATGGAGGGAGTGAAGC	AACTGTCCATTTGTGAGGGC	59.1	405	INTERGENIC	--	--	--
Ca5_P3_6909628	Ca5	6909628	ATTTTTTT/ATTTTTT	AATGATGGAGGGAGTGAAGC	AACTGTCCATTTGTGAGGGC	59.1	405	INTERGENIC	--	--	--
Ca5_P3_6909656	Ca5	6909656	TAAA/TAA	AATGATGGAGGGAGTGAAGC	AACTGTCCATTTGTGAGGGC	59.1	405	INTERGENIC	--	--	--
Ca5_P3_6909949	Ca5	6909949	CAAAGA/CA	CCTAAGCCCTCACAAATGGA	TAAAGGGAGAAAAGCAGCCA	60.1	304	INTERGENIC	--	--	--
Ca5_P3_6910352	Ca5	6910352	AA/AAAATA	TGGCTGCTTTTCTCCCTTTA	TCCTTTTTGCATGTGGTTGA	60.0	683	INTERGENIC	--	--	--
Ca5_P3_6914599	Ca5	6914599	ATT/ATTTT	TGGAAGAAGTCCTGCATTGA	TGGTCACGATTTGGGAACAT	59.4	610	INTERGENIC	--	--	--

INDEL marker IDs	Chromosomes/ unanchored scaffolds	Physical positions (bp)	InDels (*Kabuli* reference genome-CDC Frontier/PII)	Forward primers (5'-3')	Reverse primers (5'-3')	Anneling temperature (0C)	Expected amplified product size (bp)	Structural annotation		Functional annotation	
								Sequence components of *kabuli* genome	*Kabuli* gene accession IDs	NCBI-KOG	TFs
Ca5_P3_6981736	Ca5	6981736	GTTTTTT/GTTTTTTT	ATAGGGTGCCCAAACTTTCC	AGCCACCTAACCCTTGCTTT	60.2	470	INTERGENIC	--	--	--
Ca5_P3_7031251	Ca5	7031251	AATATATATATA/AATATATATA	TTGCAATCTCATCAACTCGG	TGGGAACCCTCATATCCTCA	59.8	613	INTERGENIC	--	--	--
Ca5_P3_7060900	Ca5	7060900	GAAAAAAA/GAAAAAAAA	GGGTGCCAGAGACTAGGACA	CATTGTTCACATAAAGGGCG	60.3	216	INTERGENIC	--	--	--
Ca5_P3_7107813	Ca5	7107813	G/GA	TGTCTCGAGTTCTATCCCGAA	TCACACACTTTCTTCCAACCTTT	59.8	569	INTERGENIC	--	--	--
Ca5_P3_7260086	Ca5	7260086	ATTTTTTTTTTT/ATTTTTTTTTTTTT	GAACCAGAGCTCTTTTTGCG	AAAAATTATGCGCAAACGCT	60.1	605	INTERGENIC	--	--	--
Ca5_P3_7365427	Ca5	7365427	ATTTATTTTTATTTTTAT/ATTTATTTTTATTTTTATTTTTTAT	AGAGGCAAACAAGAACCGAA	GATCGTGGGTGGAAATATGG	59.9	401	INTERGENIC	--	--	--
Ca5_P3_7366837	Ca5	7366837	G/GT	TTGAGGCTGTTGTTCGTCAG	AAAACCCCGGTGTATTTTCC	60.0	255	INTRON	Ca_21575	--	--
Ca5_P3_7374796	Ca5	7374796	CAAAAAAAAAAAAA/CAAAAAAAAAAAAAA	GGGGTTTAAGGGTTTTGGAA	TTGAATTAGGGGCTGATTGC	60.0	301	INTERGENIC	--	--	--
Ca5_P3_7560783	Ca5	7560783	ATTTTTTT/ATTTTTTTT	GAGAGTGGCGAGTGAATAGGA	ATGGGAGGTTCCCTTCAAGT	59.4	760	INTERGENIC	--	--	--
Ca5_P3_7594694	Ca5	7594694	ATTTTTTTTTT/ATTTTTTTTTTT	CGAATCAAATGAATTTTGGGA	TTGCTGAAACATTTTGGTGC	59.8	436	INTERGENIC	--	--	--

INDEL marker IDs	Chromosomes/ unanchored scaffolds	Physical positions (bp)	InDels (*Kabuli* reference genome-CDC Frontier/PII)	Forward primers (5'-3')	Reverse primers (5'-3')	Anneling temperature (0C)	Expected amplified product size (bp)	Structural annotation		Functional annotation	
								Sequence components of *kabuli* genome	*Kabuli* gene accession IDs	NCBI-KOG	TFs
Ca5_P3_7597727	Ca5	7597727	TAAAAAAAAAA/TAAAAAAAAAAA	GCACGATCATACTCTTGCCA	TTTTGAGTTCGGGAGTCGAT	59.8	241	INTERGENIC	--	--	--
Ca5_P3_7684766	Ca5	7684766	AATTATTATTATTATTATTATTATTATTATTATTATTATTATTATTATTATTATTATTATTATTATTATTAT/AATTATTATTATTATTATTATTATTATTATTATTATTATTATTATTATTATTATTATTATTATTATTATTATTATTATTATTAT	TTAGTGGCTGAAGTGGGAGAA	TCACTAAAACCACCGCATCA	59.9	801	INTRON	Ca_18733	K	MIKC
Ca5_P3_7752773	Ca5	7752773	AT/ATT	CAAACGCATGTCCTTGTCTG	ATGCCGATTATGCTGGAGAC	60.3	647	INTERGENIC	--	--	--
Ca5_P3_7774072	Ca5	7774072	TTATATATATATATATATATATATATATATATATA/TTATATATATATATATATATATATATA	TGGCGACTCCGTATTGTAAA	TGAGCATGATTTTACGCCAA	59.2	586	INTERGENIC	--	--	--
Ca5_P3_7809252	Ca5	7809252	A/ACGAGGATTTTT	CGATGTTAGTGGTGACCGTG	ATCTAAGGGCAAAGCCCAAT	60.0	398	INTERGENIC	--	--	--
Ca5_P3_7941049	Ca5	7941049	GA/GAA	TCTCATTTCCACTTAAACTTTCG	TAAGCAAGCCATTGCAAAAA	57.6	459	INTERGENIC	--	--	--
Ca5_P3_7941755	Ca5	7941755	GA/G	TCAATGGATTGCTTGTGCAT	TTTCATGAACCCACCAAACA	60.1	833	INTERGENIC	--	--	--
Ca5_P3_8054679	Ca5	8054679	CAAAAAAAAA/CAAAAAAAA	TTTTCTTCTTCGGCCTTGAG	TTCGTACGATCCACAACAAAA	59.6	695	DRR	Ca_18716	--	--
Ca5_P3_8232129	Ca5	8232129	ATTTTTT/ATTTTT	CGCTAATGTTGCATTGATGG	ACTCAATGTGCTTTGGGTCC	60.1	690	INTERGENIC	--	--	--
Ca5_P3_8298192	Ca5	8298192	TA/TAATAA	TCCTGCGAGGTTCTTCCTAA	CAAGAACACAACGAACCCAA	59.9	778	INTERGENIC	--	--	--

INDEL marker IDs	Chromosomes/ unanchored scaffolds	Physical positions (bp)	InDels (*Kabuli* reference genome-CDC Frontier/PII)	Forward primers (5'-3')	Reverse primers (5'-3')	Anneling temperature (0C)	Expected amplified product size (bp)	Structural annotation		Functional annotation	
								Sequence components of *kabuli* genome	*Kabuli* gene accession IDs	NCBI-KOG	TFs
Ca5_P3_8314333	Ca5	8314333	ATT/ATTT	TGGGGTTTAGGGTGTCACAT	TTCCATCATCAATTCTGCCA	60.1	375	INTERGENIC	--	--	--
Ca5_P3_8399938	Ca5	8399938	ATTTTTTTTTTT/ATTTTTTTTTT	CACTTCATTTAAAATTGATCACCG	TTGGTACGTATCTTTGGAGAGGA	59.8	355	INTERGENIC	--	--	--
Ca5_P3_8413319	Ca5	8413319	GAAAAAAAAAA/GAAAAAAA	CTTGCACGAAAATGCTTGAA	TCGATTTGTCAATCGCTTTG	60.0	706	INTERGENIC	--	--	--
Ca5_P3_8496342	Ca5	8496342	T/TA	CCTCCCCCTTTTTGTTGTTT	AGAGTAGCCGGCAAGGTCTC	60.2	428	INTERGENIC	--	--	--
Ca5_P3_8596558	Ca5	8596558	GATAATAATAATAATAATAATAATAATAATAATAATAATAATAA/GATAATAATAATAATAATAATAATAATAATAATAATAATAATAATAA	CGAAGCCGAGAAAATGACTC	AGAGGCAAACAAGAACCGAA	60.0	343	INTERGENIC	--	--	--
Ca5_P3_8735941	Ca5	8735941	AATATATATATATATATATATATATAT/AATATATATATATATATATATATAT	TCAAACTCTGCTGGCACATC	AAGCGACCTCCTACTGGGTT	60.0	646	INTERGENIC	--	--	--
Ca5_P3_8824148	Ca5	8824148	A/ATGT	GGAGCTGAGGTTGAGGTTGA	CAATATCGGCAAATTGAAGTGA	60.4	476	DRR	Ca_20336	A	--
Ca5_P3_8866859	Ca5	8866859	CATATATATATATATATATATATA/CATATATATATATATATATATA	TTTTGCACAAAGAAATCCAAC	TTGCTATGGTTGCTGGAGTG	57.8	901	INTERGENIC	--	--	--
Ca5_P3_8876553	Ca5	8876553	CATATATATATATATATATATATATATATA/CATATATATATATATATATATATATATA	GTCGTTACACCAAGACGCAA	CATGGAAGGAGAGGATGGAG	59.8	584	INTRON	Ca_20339	--	bZIP
Ca5_P3_8947962	Ca5	8947962	CATATTTATATTTATATTTAT/CATATTTATATTTATATTTATATTTAT	TGCGCATAACGTTCTTTTACC	TGCAAATTGTTTTGGTGCAT	60.1	507	INTRON	Ca_20342	--	--

INDEL marker IDs	Chromosomes/ unanchored scaffolds	Physical positions (bp)	InDels (*Kabuli* reference genome-CDC Frontier/PII)	Forward primers (5'-3')	Reverse primers (5'-3')	Anneling temperature (0C)	Expected amplified product size (bp)	Structural annotation		Functional annotation	
								Sequence components of *kabuli* genome	*Kabuli* gene accession IDs	NCBI-KOG	TFs
Ca5_P3_8998961	Ca5	8998961	A/AAT	TGCAATAATGGCATTTCCAA	TGAGCTTGAATGGCAAGATG	59.9	556	INTERGENIC	--	--	--
Ca5_P3_8999793	Ca5	8999793	CA/CAAA	CTCGATCAATGACCAGAGCA	TCACTTCTTCCCCCATTTTG	59.9	470	INTERGENIC	--	--	--
Ca5_P3_9014027	Ca5	9014027	GATATATATATATATATATATATATATA/GATATATATATATATATATATATATATATATA	GAAACACCCCCACATAATGC	AGGGTCACACGATAGAACGG	60.1	637	INTERGENIC	--	--	--
Ca5_P3_912739	Ca5	912739	TTATATATATATATATATATATAT/TTATATATATATATATATATAT	ACAGGAGACGAACGCAAACT	TAAAGGACCAAGTCGGGAAA	59.9	638	INTERGENIC	--	--	--
Ca5_P3_9198127	Ca5	9198127	ATATTATTATTATTATTATTATTATTATTATTAT/ATATTATTATTATTATTATTATTATTATTATTATTAT	CTCAATCGTGGGTGACCTTT	TGGTTTGACAAGGGGAAGAC	60.0	379	INTERGENIC	--	--	--
Ca5_P3_9200613	Ca5	9200613	ACCCC/ACCCCC	ATCCAGTACCGGCAGTTTTG	TCCCGTCAGCAGCTCTTAAT	60.0	812	INTERGENIC	--	--	--
Ca5_P3_9264601	Ca5	9264601	ATTTTTTTTTTT/ATTTTTTTTTTTT	TTCCAGAAAATTGCATTTTGAA	ACCTTCCACAAGATCGTCAA	59.6	756	INTERGENIC	--	--	--
Ca5_P3_927603	Ca5	927603	TTAATAATAATAATAATAATAATAATAATAATAA/TTAATAATAATAATAATAATAATAATAATAATAATAA	GGTCTTTTGAAAATAATCTTAACGC	AAGGGGGAAGTTAAGCCAGA	58.8	664	INTERGENIC	--	--	--
Ca5_P3_9309141	Ca5	9309141	CATATATATATATATATATATATA/CATATATATATATATATATATA	CCAAATCTCACATCGCTCAA	CCACATAGTAAGGGAGCCCA	59.8	747	INTERGENIC	--	--	--
Ca5_P3_9403421	Ca5	9403421	T/TTTAACCTT	GGCACTTTTCACGTCCATTT	ACAGCAGAATGGGGTAGTGG	60.0	836	INTERGENIC	--	--	--

INDEL marker IDs	Chromosomes/ unanchored scaffolds	Physical positions (bp)	InDels (*Kabuli* reference genome-CDC Frontier/PII)	Forward primers (5'-3')	Reverse primers (5'-3')	Anneling temperature (0C)	Expected amplified product size (bp)	Structural annotation		Functional annotation	
								Sequence components of *kabuli* genome	*Kabuli* gene accession IDs	NCBI-KOG	TFs
Ca5_P3_9416660	Ca5	9416660	TA/TAA	CCATCTTAAATCATGGTGTTGG	TTCAACTCCTTTTGTTCCCG	59.2	522	INTERGENIC	--	--	--
Ca5_P3_9441399	Ca5	9441399	AATTTTATTTT/AATTTTATTTTATTTT	TGCGATGGAGAAATCAAAGC	CTAGTTTGGCTTGACGGGAA	61.3	865	INTERGENIC	--	--	--
Ca5_P3_9527023	Ca5	9527023	TTATATATATATATATATATATATATATATATATA/TTATATATATATATATATATATATATATATATATATATA	CGGCATTGCGATTAATTTTT	TGAGGTGATGGGTGAAATGA	59.9	372	INTERGENIC	--	--	--
Ca5_P3_9625867	Ca5	9625867	GAAA/GAA	ACATCCAGTCCTGCAAATCC	TGGCAAATCGATTGACAAAA	59.9	730	INTERGENIC	--	--	--
Ca5_P3_9626102	Ca5	9626102	CCACA/CCA	AGTTTCCTCCCCGAAACCTA	TGGCAAATCGATTGACAAAA	59.9	657	INTERGENIC	--	--	--
Ca5_P3_96552	Ca5	96552	AATATATATATATATATATATA/AATATATATATATATATATA	TGTAAACGTGACTCAATGGCA	GGGCTTGTTATCTGCATCCT	60.2	585	DRR	Ca_18165	R	FAR1
Ca5_P3_9708216	Ca5	9708216	AATATATATATATATATATATATATA/AATATATATATATATATATATATATATATATATA	GGCAACGTATGTGTGTTTGG	AGAGTTGTCAATGGATCGGA	59.9	539	INTERGENIC	--	--	--
Ca5_P3_9718615	Ca5	9718615	TTAT/TT	AAGGCCACAAGGGTGTATGA	TTCCCCTCCCTCACTTTTCT	60.4	283	INTRON	Ca_20495	--	--
Ca5_P3_97364	Ca5	97364	T/TCAGCCTC	GGTGGGGATGGATGTAGATG	TGAACAAAACATTGGGCAAA	60.0	630	DRR	Ca_18165	R	FAR1
Ca5_P3_9820327	Ca5	9820327	TTGT/TT	CAGAACTCAACTTGCACCGA	TTGGCACCGAGAAGTTCTTT	60.0	701	DRR	Ca_20501	O	--

INDEL marker IDs	Chromosomes/ unanchored scaffolds	Physical positions (bp)	InDels (*Kabuli* reference genome-CDC Frontier/PII)	Forward primers (5'-3')	Reverse primers (5'-3')	Anneling temperature (0C)	Expected amplified product size (bp)	Structural annotation		Functional annotation	
								Sequence components of *kabuli* genome	*Kabuli* gene accession IDs	NCBI-KOG	TFs
Ca5_P3_9825211	Ca5	9825211	CTATATATATATATATATATATATATA/CTATATATATATATATATATATATATATA	TTTCAAACCAACAAGTGGCA	GGTGGGAAAATGGAGGAGAT	60.1	642	INTERGENIC	--	--	--
Ca5_P3_9912749	Ca5	9912749	ATTTTTTTTTT/ATTTTTTTTTTT	GCGTTTATGCTTTGCTCAGA	TTCGAATTTGGAACCTCACA	59.2	505	INTERGENIC	--	--	--
Ca5_P3_9954032	Ca5	9954032	TAAAAAAAAAAAAAAA/TAAAAAAAAAAAAAAAAA	ATTCCAAAGTGTTTGGCAGC	GGAAGTTGGAATGAGGTTGG	60.1	462	INTERGENIC	--	--	--
Ca5_P3_9966982	Ca5	9966982	AG/AGTCG	TTTTGAGCGGTTGTTATGCC	GACGGTTCGCAGTATGGTTT	61.0	315	INTERGENIC	--	--	--
Ca6_P3_10090816	Ca6	10090816	ATTATTTATTTATTTATTTATTTATTTATTTATTTATTTATT/ATTATTTATTTATTTATTTATTTATTTATTTATT	CCCGCAAAATTAAGTGGGTA	CCCATACCCGTTATCCACAC	59.8	560	DRR	Ca_08583	Z	Trihelix
Ca6_P3_10127292	Ca6	10127292	CAA/CA	TGGTTGCGACTTGTGTTGTT	CCGACTCAAATCAAAGGGAG	60.2	100	INTERGENIC	--	--	--
Ca6_P3_10138748	Ca6	10138748	AGACAATGACAATGACAATGACAATGAC/AGACAATGACAATGACAATGACAATGACAATGAC	CTCGTCCGAAGAGGACTACG	CAATCTGCTTCGTGGAGACA	60.0	550	CDS (large-effect mutations)	Ca_08579	--	FAR1
Ca6_P3_10141273	Ca6	10141273	TAAAAAAAAAAA/TAAAAAAAAAA	GGAAATATCAAATGTTGTTACCGA	TCCAAAAACGACTTAGACCTCA	59.2	736	INTERGENIC	--	--	--
Ca6_P3_10146529	Ca6	10146529	CATATATATATATATATATATAT/CATATATATATATATATATATATAT	TTCAGCCATTGATTATGCCA	GATGTTCATGAGGGGGAAAA	60.0	594	DRR	Ca_08578	--	--
Ca6_P3_10174986	Ca6	10174986	ATTTTTTTTTTT/ATTTTTTTTTT	CCGAACTCCAACAACTCATTC	TGTTTGGAGCAAAGGAAAAA	59.6	969	INTERGENIC	--	--	--

INDEL marker IDs	Chromosomes/ unanchored scaffolds	Physical positions (bp)	InDels (*Kabuli* reference genome-CDC Frontier/PII)	Forward primers (5'-3')	Reverse primers (5'-3')	Anneling temperature (0C)	Expected amplified product size (bp)	Structural annotation		Functional annotation	
								Sequence components of *kabuli* genome	*Kabuli* gene accession IDs	NCBI-KOG	TFs
Ca6_P3_10189127	Ca6	10189127	CTAT/CT	CCAAGGAGTTTTGTGC CATT	AGCTTGACCCCCACCT AACT	60.0	679	DRR	Ca_08571	O	MYB_related
Ca6_P3_10232573	Ca6	10232573	CGG/CG	AGGTCATTAGGTGGGG GAAC	CCCCCTCAAGCTGTTG AATA	60.1	792	INTRON	Ca_08566	T	FAR1
Ca6_P3_10274946	Ca6	10274946	TAT/TATCTATGAT	TGCATTCAGCTTTGTCT TGG	TCCATCCCTTAACCATA CCG	60.0	301	DRR	Ca_08560	R	--
Ca6_P3_10318872	Ca6	10318872	CTTTTTTTTTTTTT/CTTTTTTTTTTTT	TTAACCACCCGGTTCTC AAG	TGACATGGTTGGCTGT GAAT	60.0	724	INTERGENIC	--	--	--
Ca6_P3_10331799	Ca6	10331799	CTTTTTTTTTTTTTT/CTTTTTTTTTTTTT	CAAAGGAAATGCGCAC CTAT	AAATCCATCATGCCTA ACCG	60.1	388	INTERGENIC	--	--	--
Ca6_P3_10480197	Ca6	10480197	CTATATATATATATATATATATATATA TAT/CTATATATATATATATATATATA TATATATATAT	GATGGGATCAAGAAGC GAAA	AGCAGCTTTCTGCATC GAAT	60.2	492	INTRON	Ca_08548	E	CAMTA
Ca6_P3_10501710	Ca6	10501710	ACC/AC	TTGATTGCTTTTGTTGC TGC	GAACTGCAACTCCACC CAAT	60.0	461	INTRON	Ca_08546	--	--
Ca6_P3_10502342	Ca6	10502342	ATT/ATTTCAACTT	AAAATTGGGTGGAGTT GCAG	TGCAGTGGGTTGGTAT GAGA	60.0	510	INTERGENIC	--	--	--
Ca6_P3_10503058	Ca6	10503058	AATATATA/AATATA	AAACATGCTTTTGCATT TTTGTT	GACTTCAACCGGTAGC TGGA	59.9	407	INTERGENIC	--	--	--
Ca6_P3_10507057	Ca6	10507057	GA/GAA	CGATTCAGATTGCATC GAGA	GGCATGTCCACTGTGA TGTC	59.9	395	INTERGENIC	--	--	--

INDEL marker IDs	Chromosomes/ unanchored scaffolds	Physical positions (bp)	InDels (*Kabuli* reference genome-CDC Frontier/PII)	Forward primers (5'-3')	Reverse primers (5'-3')	Anneling temperature (0C)	Expected amplified product size (bp)	Structural annotation		Functional annotation	
								Sequence components of *kabuli* genome	*Kabuli* gene accession IDs	NCBI-KOG	TFs
Ca6_P3_10508206	Ca6	10508206	T/TTAAAGAGAGA	TGTCATTCGTGTATCTCCTCAA	GAAAACGGGCACCAAGAGTA	58.2	719	DRR	Ca_08544	S	--
Ca6_P3_10510710	Ca6	10510710	AGG/AG	CGACTAGATGTCGCTGACCA	CACATTTTTGGTGTGCTTGG	60.0	385	INTRON	Ca_08544	S	--
Ca6_P3_10513734	Ca6	10513734	C/CTATGATT	GATCATTCCAAACCACCACC	CGACTCAATGACGGGTTTTT	60.0	653	INTERGENIC	--	--	--
Ca6_P3_10514411	Ca6	10514411	CAAAAAAAAA/CAAAAAAAAAAA	TTCGCAATCTGTGTTTTTCG	CAAGAATTCGTATCGTCTAAATGC	59.8	642	INTERGENIC	--	--	--
Ca6_P3_10514563	Ca6	10514563	TAAAAAAAAAA/TAAAAAAAAAAAAA	TCACCAATCCTATGCTGTTGTC	ACGAAGTGACACATGACCCA	60.0	924	INTERGENIC	--	--	--
Ca6_P3_10556820	Ca6	10556820	ATTT/ATTTT	TATGACGGGCACACTTGAGA	CGTCATTTGCAAAAACGACTT	60.3	474	INTERGENIC	--	--	--
Ca6_P3_10572244	Ca6	10572244	TAAA/TAA	CCGATGTAAGACTCTCCCCA	TGTTTCAACCTTGCATTGGA	60.1	790	INTERGENIC	--	--	--
Ca6_P3_10572280	Ca6	10572280	TAAAAAAAAAAA/TAAAAAAAAAAAAA	CCGATGTAAGACTCTCCCCA	TGTTTCAACCTTGCATTGGA	60.1	790	INTERGENIC	--	--	--
Ca6_P3_10575764	Ca6	10575764	ATTTTTTT/ATTTTTTTT	TTTGTGTTGTGTGTTGTCGC	CCCCATGATACCCTTTCCTT	59.2	294	INTERGENIC	--	--	--
Ca6_P3_10575854	Ca6	10575854	TC/T	TTTGTGTTGTGTGTTGTCGC	CCCCATGATACCCTTTCCTT	59.2	294	INTERGENIC	--	--	--

INDEL marker IDs	Chromosomes/ unanchored scaffolds	Physical positions (bp)	InDels (*Kabuli* reference genome-CDC Frontier/PII)	Forward primers (5'-3')	Reverse primers (5'-3')	Anneling temperature (0C)	Expected amplified product size (bp)	Structural annotation		Functional annotation	
								Sequence components of *kabuli* genome	*Kabuli* gene accession IDs	NCBI-KOG	TFs
Ca6_P3_10576207	Ca6	10576207	TTGGTAAACT/TT	AAGGAAAGGGTATCATGGGG	AATTGGTGAGGTGTCCCTTG	60.0	701	INTERGENIC	--	--	--
Ca6_P3_10577482	Ca6	10577482	ATT/ATTT	GCTCCATTTCATTTGCGTTT	GCCACTCCGTAAGCCTATCA	60.1	834	INTERGENIC	--	--	--
Ca6_P3_10586207	Ca6	10586207	ATT/ATTT	TCATTGAAGGCCAAACTTCC	GAGCGACTGGGTGAAAGAAC	60.1	668	INTERGENIC	--	--	--
Ca6_P3_10586533	Ca6	10586533	ATTTCC/ATTTCCTAGTTCTTTTCC	TGGCGGGTGAGAAGTAAAAG	TAATTGTGAGCGACTGGGTG	60.2	254	INTERGENIC	--	--	--
Ca6_P3_10586822	Ca6	10586822	CAAAAAAA/CAAAAAAAA	TTCCCAGTTCTTTCACCCAG	AACTGTAACACACGTGGCGA	60.1	573	INTERGENIC	--	--	--
Ca6_P3_10588831	Ca6	10588831	AT/ATT	AACCGTAACAGACGCCAATC	GTTCCTCGGTCTTTTCCTCC	60.0	308	DRR	Ca_08539	--	--
Ca6_P3_10591465	Ca6	10591465	AT/ATT	CAAATGATGTTTTTCCCGTG	TGGTGCTCTTTTGCTTTGTG	58.9	435	INTRON	Ca_08538	E	--
Ca6_P3_10594459	Ca6	10594459	TAAAAA/TAAAAAA	GCGACCTCCAACTGACAACT	CAAAATAAATCGCGTGTCCC	60.3	563	INTERGENIC	--	--	--
Ca6_P3_10600474	Ca6	10600474	GAAAA/GAAAAA	TGACTCCACCACCAATGAAA	GACCAAATCTTAAACGCCCA	59.9	222	INTERGENIC	--	--	--
Ca6_P3_10606593	Ca6	10606593	CAAA/CA	CGTCGAATTCTTTGGTAGCC	TTTATAACTTTCCGCCCACG	59.7	526	DRR	Ca_08537	H	--

INDEL marker IDs	Chromosomes/ unanchored scaffolds	Physical positions (bp)	InDels (*Kabuli* reference genome-CDC Frontier/PII)	Forward primers (5'-3')	Reverse primers (5'-3')	Anneling temperature (0C)	Expected amplified product size (bp)	Structural annotation		Functional annotation	
								Sequence components of *kabuli* genome	*Kabuli* gene accession IDs	NCBI-KOG	TFs
Ca6_P3_10609017	Ca6	10609017	CGAGG/CGAGGAGG	AACATTTGCTGCCATTTGGT	GCAACACCTTGATGTTTCCA	60.4	689	INTERGENIC	--	--	--
Ca6_P3_10609448	Ca6	10609448	ATT/ATTTT	TTTGGAGCATCTCCCAGAAT	TGTGAAGAAGAAGGAGGAGGA	59.6	653	INTERGENIC	--	--	--
Ca6_P3_10609875	Ca6	10609875	TTCT/TT	TCAAAGTTCGATGATGATGAAGA	TGGTTTTGGATTAGTGGCTTC	59.7	419	INTERGENIC	--	--	--
Ca6_P3_10611247	Ca6	10611247	TAATAAATAAATAAATAAA/TAATAAATAAATAAATAAATAAA	TCTGACATGACAAAATGGTGG	TCGGAATTGGTCCGAAATTA	59.4	409	DRR	Ca_08536	K	HSF
Ca6_P3_10616353	Ca6	10616353	CAAT/CAATGAAAAT	TGAGATTGAGAGAGCAATTGTGA	CGAATTGCAGGAATGTGTTG	60.0	544	INTERGENIC	--	--	--
Ca6_P3_10617092	Ca6	10617092	C/CA	TTGTTTAATTGTTGGGGGAGA	AACGTAAGTTGGCACCATGC	59.3	222	INTERGENIC	--	--	--
Ca6_P3_10619329	Ca6	10619329	CAATAATA/CAATA	TGCGGTAGCCTATCTCAACC	ATCACCCGTAGATCTGTCGC	60.2	647	INTERGENIC	--	--	--
Ca6_P3_10620292	Ca6	10620292	TAAAAAACA/TAAAAAACAAAAAAACA	CAGTTGTGGCGATTGATTTG	GGATCTGAAATCCCCTCTCC	60.1	836	INTERGENIC	--	--	--
Ca6_P3_10620474	Ca6	10620474	A/AT	CCATGTGAAGCAAGTGGTTG	GGATCTGAAATCCCCTCTCC	60.2	714	INTERGENIC	--	--	--
Ca6_P3_10621679	Ca6	10621679	GTTTTTTTTT/GTTTTTTTT	TTGAAGCCATGTTGAAATCG	CAAATATGTTTGGTGGATTGTTG	59.7	550	INTERGENIC	--	--	--

INDEL marker IDs	Chromosomes/ unanchored scaffolds	Physical positions (bp)	InDels (*Kabuli* reference genome-CDC Frontier/PII)	Forward primers (5'-3')	Reverse primers (5'-3')	Anneling temperature (0C)	Expected amplified product size (bp)	Structural annotation		Functional annotation	
								Sequence components of *kabuli* genome	*Kabuli* gene accession IDs	NCBI-KOG	TFs
Ca6_P3_10623188	Ca6	10623188	TATAA/TA	CTGAGGCGGCAGAGTAATTC	TTTTGGCATCCACTTCAATTT	60.0	490	INTERGENIC	--	--	--
Ca6_P3_10625658	Ca6	10625658	AA/AATA	GCTCTCCAAGACTTCAACGG	TCCAACCTTCCCTCATCTTG	60.0	782	INTERGENIC	--	--	--
Ca6_P3_10631599	Ca6	10631599	ATTT/ATT	CTGACACAAAATCCACCACG	ACCACCACGGAAATGATTGT	60.0	812	DRR	Ca_08534	R	NAC
Ca6_P3_10640464	Ca6	10640464	A/AGCACGTAAGTATG	CCTTCAGCATTAGACTCCGC	AATCGCGAATATGGTCCAAG	60.0	787	INTERGENIC	--	--	--
Ca6_P3_10642500	Ca6	10642500	C/CCTATAGA	CGATTTGACAGCAAAAGCAA	GACAACCTTCGTTGCCCTAA	60.0	437	INTERGENIC	--	--	--
Ca6_P3_10649918	Ca6	10649918	GTTTGTTTTGTTTTGTTTT/GTTTGTTTTGTTTTGTTTTGTTTT	GACGGTTATGGAGCCTTCAA	TTCGATATCTTGTGCCTCCC	60.1	363	DRR	Ca_08531	I	--
Ca6_P3_10652240	Ca6	10652240	TA/T	TTCGAATTTTCATGGTGGGT	GATAAGCGACGTCTTGGAGC	60.2	804	INTRON	Ca_08531	I	--
Ca6_P3_10657533	Ca6	10657533	C/CTT	ACATCTCTCATTGCCCATCC	ATCAGGTGCTTTGCTGCTTT	59.9	769	INTRON	Ca_08531	I	--
Ca6_P3_10660543	Ca6	10660543	G/GCC	TGATTGGACTGCCTCACAAG	GGCCTCAGATCAGTGGAAAA	59.8	617	INTRON	Ca_08531	I	--
Ca6_P3_10662809	Ca6	10662809	ATT/ATTT	CAATTGTGCTCCTCCACCTT	AGCTCTAGGGGTATGGGTGG	60.1	648	INTERGENIC	--	--	--

INDEL marker IDs	Chromosomes/ unanchored scaffolds	Physical positions (bp)	InDels (*Kabuli* reference genome-CDC Frontier/PII)	Forward primers (5'-3')	Reverse primers (5'-3')	Anneling temperature (0C)	Expected amplified product size (bp)	Structural annotation		Functional annotation	
								Sequence components of *kabuli* genome	*Kabuli* gene accession IDs	NCBI-KOG	TFs
Ca6_P3_10665731	Ca6	10665731	ATTTTTTTTTTT/ATTTTTTTTT	TGAAAGTTTCGTATTGCTCGAA	CATTTGTCCTTGAAGCCACC	59.9	692	INTERGENIC	--	--	--
Ca6_P3_10667900	Ca6	10667900	ATTTTTT/ATTTTTTT	CAATGCGCTTTTCTCCTCTC	ATGCGGCACAACACTAACAC	60.1	438	INTRON	Ca_08530	--	bHLH
Ca6_P3_10673743	Ca6	10673743	TA/T	TCTGTATGCTTTGCTGTGCC	ACTGCCAGATGAAACCCATC	60.0	608	INTRON	Ca_08530	--	bHLH
Ca6_P3_10677110	Ca6	10677110	TCCCC/TCCCCC	TTCAACACGTGCATGGAAAT	GCTAACACATGATTTCCGCA	60.0	581	INTRON	Ca_08530	--	bHLH
Ca6_P3_10681474	Ca6	10681474	ACAAGTCA/ACA	AATTGCGAATTTGTGGGTTC	GGACAGATGGCAAGCATTTT	59.8	694	DRR	Ca_08530	--	bHLH
Ca6_P3_10689272	Ca6	10689272	CAAAAAAAAA/CAAAAAAAAAA	GGAAACATCAACCCCAAAAA	TTCCCCAATAACCAAACCAA	59.6	551	INTERGENIC	--	--	--
Ca6_P3_10695719	Ca6	10695719	C/CTA	CTCCGTAATGAAACCCGAGA	AGGAAGGGGAACGACCTAGA	60.1	744	DRR	Ca_08528	--	bHLH
Ca6_P3_10700691	Ca6	10700691	AG/A	ATAAAGAGAAGCGGAGGGGA	CAACGAAGAGCGTCGAGAAT	60.2	909	INTERGENIC	--	--	--
Ca6_P3_10708022	Ca6	10708022	GCT/GCTGAATCT	CCAAGGGTTGGCTAGAACTG	TGGAAAATATGTGTGGGATCAG	59.7	689	DRR	Ca_08527	GC	HSF
Ca6_P3_10708360	Ca6	10708360	CTGAT/CT	TGAAAATTCGTTGCAAAACAA	GTGTGGCTGAAAATGCATGA	59.2	940	DRR	Ca_08527	GC	HSF

INDEL marker IDs	Chromosomes/ unanchored scaffolds	Physical positions (bp)	InDels (*Kabuli* reference genome-CDC Frontier/PII)	Forward primers (5'-3')	Reverse primers (5'-3')	Anneling temperature (0C)	Expected amplified product size (bp)	Structural annotation		Functional annotation	
								Sequence components of *kabuli* genome	*Kabuli* gene accession IDs	NCBI-KOG	TFs
Ca6_P3_10794924	Ca6	10794924	AATATATATATATATATATATATATATATAT/AATATATATATATATATATATATATATATATAT	TTGGTTTTGAAACATTGATTGAT	CATTTACCTCGTGCACCACT	58.4	374	INTERGENIC	--	--	--
Ca6_P3_10848271	Ca6	10848271	TGGC/TGGCGGC	GGCCTCTTTCAACAAATGGA	ACAAATTTGGTGGTGGTGGT	60.1	311	CDS (large-effect mutations)	Ca_08513	K	MIKC
Ca6_P3_10857523	Ca6	10857523	CTTT/CTTTT	TTCGTGAAACTGTGCAAACC	AACGAAACGGGTTACACCAC	59.7	550	INTERGENIC	--	--	--
Ca6_P3_10968845	Ca6	10968845	AATATATATATATATATATATATATAT/AATATATATATATATATATATAT	TGAAAACTTCATTTTCTTTATTGAGTC	GCACATGGTTGCATGAAAAA	58.5	637	INTERGENIC	--	--	--
Ca6_P3_11035101	Ca6	11035101	TTAT/TTATTATTATTTGTAT	TGTTTCCGAAGTCTTTTGTTGA	TTTTTCCTCCCGATTTAGTCC	59.8	963	INTERGENIC	--	--	--
Ca6_P3_11111942	Ca6	11111942	CAGAG/CAG	ATACCCCATGAAGGTAGCCC	ACCGTGAACACCAAAAGGAG	60.0	383	INTRON	Ca_20478	G	--
Ca6_P3_11270649	Ca6	11270649	C/CA	AACAAGGGCCCCAAAATAGT	AAATACATGTCATTCGGGGC	59.7	496	INTERGENIC	--	--	--
Ca6_P3_112870	Ca6	112870	TG/T	AACTCCTTGGGACCGTGATT	ACCTTGCAACTGGAGCAAAT	60.7	242	INTERGENIC	--	--	--
Ca6_P3_11310241	Ca6	11310241	GTTTTGCATA/G	CATGTCGAAGAAGCTCCACA	TCCTCCGGAACATAACTTGG	60.0	417	INTERGENIC	--	--	--
Ca6_P3_11310757	Ca6	11310757	AAACCGAACCGAACCGAAC/AAACCGAACCGAAC	CATTATGCTGAATGATGCCG	TTTTTCCTTTTGGTGGATGG	60.1	742	INTERGENIC	--	--	--

INDEL marker IDs	Chromosomes/ unanchored scaffolds	Physical positions (bp)	InDels (*Kabuli* reference genome-CDC Frontier/PII)	Forward primers (5'-3')	Reverse primers (5'-3')	Anneling temperature (0C)	Expected amplified product size (bp)	Structural annotation		Functional annotation	
								Sequence components of *kabuli* genome	*Kabuli* gene accession IDs	NCBI-KOG	TFs
Ca6_P3_11316099	Ca6	11316099	AC/A	GTTTGTTGTGGTTGCAGTGG	GCTGATTGGTTGCCTCTCTC	60.0	399	INTERGENIC	--	--	--
Ca6_P3_11363335	Ca6	11363335	ATTTTTTTTTTTT/ATTTTTTTTTTT	TCCAATTCAGCGTAGAGAACAA	GGTGCAAAGAGATCAATGATGA	59.9	332	INTERGENIC	--	--	--
Ca6_P3_11367342	Ca6	11367342	CAAAAAAAAAA/CAAAAAAAAA	TAGATCCACCTTCAAACCCG	AAAAGCGGTGACAGATGGAC	59.9	667	INTRON	Ca_23422	K	ARR-B
Ca6_P3_11493441	Ca6	11493441	T/TC	GGTTGTCGGTAATCCTCCAA	CACCCGTTGTCCCTTAACTT	59.8	791	INTERGENIC	--	--	--
Ca6_P3_11546028	Ca6	11546028	CAAA/CAAAA	GATCGATTTGGGCTACGAAA	ATCAGATTGCTGCAGGCTTT	60.0	651	INTERGENIC	--	--	--
Ca6_P3_11546493	Ca6	11546493	TAAAAAAAAAAAAAAA/TAAAAAAAAAA	TCGGAAAGGTCATTCAAAGC	TTTGTGTCCAAAGCATTCTCC	60.2	472	INTERGENIC	--	--	--
Ca6_P3_11590997	Ca6	11590997	CTTTT/CTTTTT	ATGTGCTCCCCACAAAAGTC	CCCTCCAGTAGCATCACCAT	60.0	538	INTERGENIC	--	--	--
Ca6_P3_11592152	Ca6	11592152	A/AC	AGATGACAATCCCCGACAAC	TCACCAAGACATTGAGGCAA	59.8	349	INTERGENIC	--	--	--
Ca6_P3_11596627	Ca6	11596627	ATTTTTTTTT/ATTTTTTTT	CTCAAGGACCCAAGACCAAA	CCTTTGCACAAGCTTCACAT	60.1	596	INTERGENIC	--	--	--
Ca6_P3_11600068	Ca6	11600068	AC/A	GTCCTTTGCCAAGAATCCAA	CCTAAAGTGGGTTTCGTCCA	60.1	334	INTERGENIC	--	--	--

INDEL marker IDs	Chromosomes/ unanchored scaffolds	Physical positions (bp)	InDels (*Kabuli* reference genome-CDC Frontier/PII)	Forward primers (5'-3')	Reverse primers (5'-3')	Anneling temperature (0C)	Expected amplified product size (bp)	Structural annotation		Functional annotation	
								Sequence components of *kabuli* genome	*Kabuli* gene accession IDs	NCBI-KOG	TFs
Ca6_P3_11600363	Ca6	11600363	TAAAAAA/TAAAAAAA	TGGACGAAACCCACTTTAGG	AAATTGCAGATTGCCTCACC	60.0	286	INTERGENIC	--	--	--
Ca6_P3_11600751	Ca6	11600751	ATCAA/ATCAACAATTAGGTCAA	TGGACGAAACCCACTTTAGG	TGGGCCACTAGTGTTGATGA	60.0	622	INTERGENIC	--	--	--
Ca6_P3_11630320	Ca6	11630320	AAGTAGT/AAGT	TGCATATGTGTGGGGGAATA	GCTTGATACTTGGCAAAGCC	59.6	621	INTERGENIC	--	--	--
Ca6_P3_11631502	Ca6	11631502	TGAGA/TGAGAGA	CTGCTTCTATTCTTTCGCGG	GGAGGAAGGGCTAACTTTGG	60.1	562	DRR	Ca_25057	S	--
Ca6_P3_11631784	Ca6	11631784	ATTTTTTTT/ATTTTTTT	CTGCTTCTATTCTTTCGCGG	GGAGGAAGGGCTAACTTTGG	60.1	562	DRR	Ca_25057	S	--
Ca6_P3_11631813	Ca6	11631813	TAAAAAAAAAA/TAAAAAAAAAAA	CTGCTTCTATTCTTTCGCGG	GGAGGAAGGGCTAACTTTGG	60.1	562	DRR	Ca_25057	S	--
Ca6_P3_11631972	Ca6	11631972	CAAAAAAAA/CAAAAAAA	CCAAAGTTAGCCCTTCCTCC	AGCACTGACCTTTTGAGCGT	60.1	445	DRR	Ca_25057	S	--
Ca6_P3_11632067	Ca6	11632067	GGTGTG/GGTG	CCAAAGTTAGCCCTTCCTCC	AGCACTGACCTTTTGAGCGT	60.1	445	DRR	Ca_25057	S	--
Ca6_P3_11636913	Ca6	11636913	CTTCTATT/CTT	AGGTGCCCATTTGTTAACCC	CACTCACCAACAAATCAAACG	61.0	635	INTERGENIC	--	--	--
Ca6_P3_11654198	Ca6	11654198	GAAAAAAAAAA/GAAAAAAAA	GTTAACCTCGGTCAAGCTGC	TGTCGGCTCACGTTTTTGTA	59.9	891	INTERGENIC	--	--	--

**INDEL marker IDs**	**Chromosomes/ unanchored scaffolds**	**Physical positions (bp)**	**InDels (*Kabuli* reference genome-CDC Frontier/PII)**	**Forward primers (5'-3')**	**Reverse primers (5'-3')**	**Anneling temperature (0C)**	**Expected amplified product size (bp)**	**Structural annotation**		**Functional annotation**	
								**Sequence components of *kabuli* genome**	***Kabuli* gene accession IDs**	**NCBI-KOG**	**TFs**
Ca6_P3_11656581	Ca6	11656581	ACCGGCCGG/ACCGG	TCCCTCACTCATCACCACAA	AACAGCTCCATCGCCTAGAA	60.1	340	DRR	Ca_25060	--	FAR1
Ca6_P3_11658302	Ca6	11658302	A/AC	ATGCCAAACAAAGCCAAAAC	TGGAGCCACATTATCTGCTG	60.0	563	INTRON	Ca_25060	--	FAR1
Ca6_P3_11660800	Ca6	11660800	AA/AACA	GATGAGCACCTACCAAAGAACC	TCCTCTTCTCTCCTCCCACA	60.0	549	INTERGENIC	--	--	--
Ca6_P3_11667031	Ca6	11667031	AT/ATTT	TTCACCACAGAAACCACCAA	ACGCTTTTCCCTGAGCACTA	60.0	698	INTRON	Ca_25061	Q	G2-like
Ca6_P3_11685034	Ca6	11685034	TGGG/TGG	ATCGCCAGCAACTTTAGCAT	GGGAACCAAAGTGTTGACCA	59.9	508	INTERGENIC	--	--	--
Ca6_P3_11686059	Ca6	11686059	AATTATTATTATT/AATTATT	GGAGATGCGGTCTCTCAAAG	GTCCTAACGCTGCAGACACA	60.0	395	INTERGENIC	--	--	--
Ca6_P3_11706061	Ca6	11706061	TTGTATTCTTGTGTATT/TTGTATT	CCACGCTGTCATATGTGGTC	GCTCAAGTTGAACAGCACCA	60.0	734	INTERGENIC	--	--	--
Ca6_P3_11713842	Ca6	11713842	TTATAA/T	TTAGTGCTCTGGTGCGTTTG	GCTCGTGGAGATTTAGCGTC	60.1	433	DRR	Ca_25062	--	--
Ca6_P3_11718910	Ca6	11718910	A/AG	CTCCGCTTCAAAAGACATCC	AATGGTGCTTGGGTCGATAG	59.8	506	INTERGENIC	--	--	--
Ca6_P3_11719036	Ca6	11719036	TAAAAAAAAAA/TAAAAAAAAAAAA	TAAAATCGACCACAAGTGCG	AATCGAGCTTCAGCAAGAGG	59.7	662	INTERGENIC	--	--	--

INDEL marker IDs	Chromosomes/ unanchored scaffolds	Physical positions (bp)	InDels (*Kabuli* reference genome-CDC Frontier/PII)	Forward primers (5'-3')	Reverse primers (5'-3')	Anneling temperature (0C)	Expected amplified product size (bp)	Structural annotation		Functional annotation	
								Sequence components of *kabuli* genome	*Kabuli* gene accession IDs	NCBI-KOG	TFs
Ca6_P3_11726949	Ca6	11726949	CTTCAATTTCAAT/CTTCAAT	TTGAGCCATATGTTGACCCA	CTCCTCACTCACCACAAGCA	59.9	635	INTERGENIC	--	--	--
Ca6_P3_11727726	Ca6	11727726	AACACA/AACA	TGCTTGTGGTGAGTGAGGAG	TCCCGACGACTAACAACTCA	60.0	563	INTERGENIC	--	--	--
Ca6_P3_11738985	Ca6	11738985	TAAAAAAAA/TAAAAAAA	TTCAGCAGCTTCTTCGACAA	ATTACCGACATTGCAGAGGG	59.9	254	INTERGENIC	--	--	--
Ca6_P3_11746077	Ca6	11746077	AATATATATATATATATATA/AATATATATATATATATATATATA	TGCAAGTCGAAATTCACACG	TGGGTCAAGCCCAATAAAAC	60.8	538	INTERGENIC	--	--	--
Ca6_P3_11752280	Ca6	11752280	CAAAAAAAAAAAAA/CAAAAAAAAAAA	GCGAGTCCCAGATGAATTGT	GTCGTATTAATCGCGTTGGG	60.1	569	INTERGENIC	--	--	--
Ca6_P3_11755125	Ca6	11755125	GTCAAAGATTTTCTTCAA/GTCAA	CGATCATGTTCGTATTTTGTTCA	TGAAATTCCCTGAGCTGGTC	59.9	527	INTERGENIC	--	--	--
Ca6_P3_1175724	Ca6	1175724	CTTT/CTTTT	TGTGACCGTTGGAAAATTCA	GCCTCACGAGTTGGAAGAAA	59.9	715	INTERGENIC	--	--	--
Ca6_P3_11779723	Ca6	11779723	CGGT/C	CAACAAGGCCACATCAACAC	AAGTTGGCGGTAATCCACAC	60.0	863	INTERGENIC	--	--	--
Ca6_P3_11784060	Ca6	11784060	ATTTTTTTTT/ATTTTTTTTTT	TAAGGTTGTCTGGGGAGGTG	GGGTCTACGGTGCGATACTC	60.0	469	INTERGENIC	--	--	--
Ca6_P3_11791617	Ca6	11791617	ATTTT/ATTT	TGGGAGGATTTGCAACATTT	TTTCAATTGTCCGTCCAACA	60.3	814	INTERGENIC	--	--	--

INDEL marker IDs	Chromosomes/ unanchored scaffolds	Physical positions (bp)	InDels (*Kabuli* reference genome-CDC Frontier/PII)	Forward primers (5'-3')	Reverse primers (5'-3')	Anneling temperature (0C)	Expected amplified product size (bp)	Structural annotation		Functional annotation	
								Sequence components of *kabuli* genome	*Kabuli* gene accession IDs	NCBI-KOG	TFs
Ca6_P3_11792161	Ca6	11792161	AAT/A	TGTTGGACGGACAATTGAAA	CAAAATGCCATAAAATCGGC	59.9	408	INTERGENIC	--	--	--
Ca6_P3_11792622	Ca6	11792622	GTTCTTTT/GT	GCCGATTTTATGGCATTTTG	GGGCTATGAGACCCATCAGA	60.3	777	INTERGENIC	--	--	--
Ca6_P3_120405	Ca6	120405	TCA/TCAAATTCCA	GAGGCACAAAACCATCATCC	CCCAAAATGTGGCTTTCAAT	60.3	562	INTERGENIC	--	--	--
Ca6_P3_12061832	Ca6	12061832	AT/ATT	ACATAGCTCCAGTCAGGCGT	ACCCAATTTGTCGTGGTGAT	59.9	768	INTERGENIC	--	--	--
Ca6_P3_12089970	Ca6	12089970	AG/AGG	ATGCCAAACACAAACAACGA	TTCATTCGGGGCTTACTACG	60.0	789	INTERGENIC	--	--	--
Ca6_P3_12154986	Ca6	12154986	TAA/TA	TGAAGCATCAATGTGTGCAA	ATTTTCCATAATGCCCCTCC	59.8	600	INTERGENIC	--	--	--
Ca6_P3_12233101	Ca6	12233101	TG/TGG	CCTCAACGTGAGTTGCAGAA	TGCATGGCATTTGGATAAGA	60.0	344	CDS (FRAME SHIFT)	Ca_24847	--	--
Ca6_P3_12253208	Ca6	12253208	TA/TAA	CTCCGTTTATCTCCGTTGGA	CCACCCCAATATGATGGAGA	60.1	379	INTERGENIC	--	--	--
Ca6_P3_12253239	Ca6	12253239	ATAT/ATATATATATATCTAT	CTCCGTTTATCTCCGTTGGA	CGTGATGATTGATGCATTTCA	60.1	746	INTERGENIC	--	--	--
Ca6_P3_1230210	Ca6	1230210	CAAAAAAAAAA/CAAAAAAAAA	ATGATGAACGGTGACCACAA	TGGGAACCTACATGGTCACAT	59.8	318	INTERGENIC	--	--	--

INDEL marker IDs	Chromosomes/ unanchored scaffolds	Physical positions (bp)	InDels (*Kabuli* reference genome-CDC Frontier/PII)	Forward primers (5'-3')	Reverse primers (5'-3')	Anneling temperature (0C)	Expected amplified product size (bp)	Structural annotation		Functional annotation	
								Sequence components of *kabuli* genome	*Kabuli* gene accession IDs	NCBI-KOG	TFs
Ca6_P3_12312832	Ca6	12312832	TAAAAAAAAAAAA/TAAAAAAAAAAAAA	TCATGTTTGGGAGGAACCAT	TCTGTGACACCCTAAACCCC	60.2	352	INTERGENIC	--	--	--
Ca6_P3_12335628	Ca6	12335628	TGAAG/TG	GGAAGGGTGTTGGTGTGTTT	TTGGTCACTAAGGGGGTGAG	59.7	968	DRR	Ca_24849	--	--
Ca6_P3_12362007	Ca6	12362007	GTTTTTTTT/GTTTTTTTTT	ACTATGTGAAAGCGCAAGCC	CATTTTATAGCGCGCCTTTT	60.4	142	DRR	Ca_05021	R	--
Ca6_P3_12389573	Ca6	12389573	TACACACACACACAC/TACACACACACACACAC	TGTGCAATTGACATTTTGGAA	TTAATTTAAACCCCTCGCCC	60.0	505	DRR	Ca_05022	O	--
Ca6_P3_12528757	Ca6	12528757	AATATATATATATATATATATATATATATATATATAT/AATATATATATATATATATATATATATATATATATATAT	TGGGGGATGAGAGATCAAAG	GGAGATTTGCAACACCCTTC	60.0	741	INTERGENIC	--	--	--
Ca6_P3_12534444	Ca6	12534444	GTTTTTTTTTT/GTTTTTTTTT	GCTTTCTCTGCTGGAATTGG	TGGAGCCACGTGTTTGTATC	60.0	188	INTRON	Ca_05031	R	--
Ca6_P3_12535819	Ca6	12535819	TATT/TATTCTCATT	CGATCGATTTGGAAGGAAAA	ACCTTTCGAAACAACCAACG	60.0	467	CDS (large-effect mutations)	Ca_05031	R	--
Ca6_P3_12550846	Ca6	12550846	TAAAAAAAAAAAAAAAA/TAAAAAAAAAAAAAAAAAAA	TCCACAAGGGATTGTTAGCC	TGTCTCTTGTGTCGGGTCTTT	59.9	561	DRR	Ca_05032	R	--
Ca6_P3_12562114	Ca6	12562114	ATGTGTGTGTGT/ATATGTGTGTGTGTGT	GCGAGTCTTATGGTCACTTCAA	CACTTCAATGATGTTCGGTATGA	59.4	564	INTERGENIC	--	--	--
Ca6_P3_12565656	Ca6	12565656	TTTATTATTATTATTATTATTATTATTATTATTATTATTATT/TTTATTATTATTATTATTATTATTATTATTATTATTATTATTATT	ACATCCAATGAAATAGCCGC	AAAATAGCCTTTTCGCCACC	59.9	825	INTERGENIC	--	--	--

INDEL marker IDs	Chromosomes/ unanchored scaffolds	Physical positions (bp)	InDels (*Kabuli* reference genome-CDC Frontier/PII)	Forward primers (5'-3')	Reverse primers (5'-3')	Anneling temperature (0C)	Expected amplified product size (bp)	Structural annotation		Functional annotation	
								Sequence components of *kabuli* genome	*Kabuli* gene accession IDs	NCBI-KOG	TFs
Ca6_P3_1257304	Ca6	1257304	TTATATATATATATATATATAT/TTATATATATATATATATATAT	GGTGAGGATTGGTTCACTCG	ATGAGTGGGGATCGATCTTG	60.5	341	DRR	Ca_13331	--	NF-YB
Ca6_P3_12679710	Ca6	12679710	TAAAAAA/TAAAAA	TCCATCAAATCAATCCATTACC	ACTTCGGCAGTCACCACAG	58.6	422	INTERGENIC	--	--	--
Ca6_P3_12721633	Ca6	12721633	TAAAAAAAAAAA/TAAAAAAAAAA	CCGGAAAACTTTTACCTACCG	AAAATTTATCCTGCACCCCC	59.9	159	INTERGENIC	--	--	--
Ca6_P3_12756200	Ca6	12756200	GATATATATATATATATATATATATATATATATATATATATATATAT/GATATATATATATATATATATATATATATATATATATATATATATATATATAT	GCGCCTCAACAATAAAGAGG	CCAATCTCTTTCCCACCTCA	59.8	853	INTERGENIC	--	--	--
Ca6_P3_12787897	Ca6	12787897	AATATATATATATATATATATATAT/AATATATATATATATATATAT	ATCAAACAAATGTTGCGAGC	CAAACCCCTATTTTTGTGATTGA	58.8	749	INTERGENIC	--	--	--
Ca6_P3_12808800	Ca6	12808800	GTTTTTTTTTTT/GTTTTTTTTT	CATGGAATTGACGAACATCG	GAAGGTGTTTGTGACACTTCTAGC	59.9	360	INTERGENIC	--	--	--
Ca6_P3_12853638	Ca6	12853638	TAAAA/TAAA	CTCATCCATCACACCAAAGC	CTCAGATTCTGCGTCCATCA	59.1	875	INTRON	Ca_05058	T	TALE
Ca6_P3_12855032	Ca6	12855032	A/ATCT	TCGAAGATGGAGATGGAGAGA	CTCTGATATCGAACCCCTGC	59.9	181	INTRON	Ca_05058	T	TALE
Ca6_P3_12856029	Ca6	12856029	CCT/C	AGCCACATGGAAGAACCAAC	CAGTCGAACTTGCGATCTTG	60.0	532	DRR	Ca_05058	T	TALE
Ca6_P3_12860020	Ca6	12860020	A/AT	GGTTCGAGAAGGAGCAATCA	TGAATCCACCAGCAAAACAA	60.3	549	INTERGENIC	--	--	--

INDEL marker IDs	Chromosomes/ unanchored scaffolds	Physical positions (bp)	InDels (*Kabuli* reference genome-CDC Frontier/PII)	Forward primers (5'-3')	Reverse primers (5'-3')	Anneling temperature (0C)	Expected amplified product size (bp)	Structural annotation		Functional annotation	
								Sequence components of *kabuli* genome	*Kabuli* gene accession IDs	NCBI-KOG	TFs
Ca6_P3_12861842	Ca6	12861842	ATTTTTTT/ATTTTTT	TCAATCCCTTCATTCGGAAG	TCGATAGTGGGTTTCGTGTTC	60.0	612	INTERGENIC	--	--	--
Ca6_P3_12868319	Ca6	12868319	TTGCG/T	GCATGTCAAGATAATGGGCA	CCGCATAACCAAACACTCAC	59.5	637	INTERGENIC	--	--	--
Ca6_P3_12870795	Ca6	12870795	TAAAAAAAAA/TAAAAAAAAAA	CATCCCAAGCAAAAACGAAT	CATTGAGAGGATCCAAAATCAA	59.9	533	INTERGENIC	--	--	--
Ca6_P3_12877302	Ca6	12877302	CG/CGCTTTTTTTG	GTTCGTAAAGGACGGGATGA	CTTTTACAGCGCTTTCTGGG	59.9	235	INTERGENIC	--	--	--
Ca6_P3_12878483	Ca6	12878483	GATA/GATATATA	CCCCATCATGCAAATCTTCT	TCGAGGGCACTTTTCTTTGT	59.9	373	INTERGENIC	--	--	--
Ca6_P3_12879113	Ca6	12879113	ATTTTTTTTT/ATTTTTTTTTT	TTGCGTTAGGAAATTGATGAAA	TTTCCCGACTTGGTCCTTTA	59.6	175	INTERGENIC	--	--	--
Ca6_P3_12879345	Ca6	12879345	TAAAAAAAAAA/TAAAAAAAAA	TAAAGGACCAAGTCGGGAAA	ATCCAAAGGAAAGCATCGTG	59.5	599	INTERGENIC	--	--	--
Ca6_P3_12888429	Ca6	12888429	T/TC	CCGTCAATTTATATCCTCCCA	AATCAGGGTGCCATTGTAGC	58.8	733	INTERGENIC	--	--	--
Ca6_P3_12888838	Ca6	12888838	ATTTT/ATT	GCTACAATGGCACCCTGATT	AGGATTTGAACATTGGCCCT	60.0	334	INTERGENIC	--	--	--
Ca6_P3_12892058	Ca6	12892058	GTTTTTTTT/GTTTTTTTTT	GGTAAAAGCCCACTTGGTGA	TTGGATGTCTGTTGCATGGT	60.0	913	INTERGENIC	--	--	--

INDEL marker IDs	Chromosomes/ unanchored scaffolds	Physical positions (bp)	InDels (*Kabuli* reference genome-CDC Frontier/PII)	Forward primers (5'-3')	Reverse primers (5'-3')	Anneling temperature (0C)	Expected amplified product size (bp)	Structural annotation		Functional annotation	
								Sequence components of *kabuli* genome	*Kabuli* gene accession IDs	NCBI-KOG	TFs
Ca6_P3_12898258	Ca6	12898258	TAAAAAAAAAA/TAAAAAAAAAAA	ACTACCATGTTTGGTGGGGA	CACATTGAAAATCAACTTTACACCA	60.1	646	INTERGENIC	--	--	--
Ca6_P3_12904889	Ca6	12904889	C/CAG	ACATATGTCAGCGTAGCCCA	TTGCTGCCACAATAACTTGG	59.2	479	INTERGENIC	--	--	--
Ca6_P3_12905966	Ca6	12905966	T/TGA	AGAGTTGTTCGCGAAAAGGA	TTACGCACCGGACATAATGA	60.0	203	INTRON	Ca_05064	T	bHLH
Ca6_P3_12908239	Ca6	12908239	ATGCTGCTGCTGCTGCT/ATGCTGCTGCTGCTGCTGCT	CCACAAGGGACTCCAGGTTA	GGAGCCGGATTCTACCAAAT	60.0	477	CDS (large-effect mutations)	Ca_05064	T	bHLH
Ca6_P3_12921236	Ca6	12921236	T/TA	ATTAGAGGTAATGCCGCCG	GGACTTTGGAGGTGCTTGTC	60.1	415	INTERGENIC	--	--	--
Ca6_P3_12926732	Ca6	12926732	TTGTG/TTG	CCTTTTCGGTCACTTGGTGT	CCTTGGGAAACCAAAACAAA	60.0	490	DRR	Ca_05065	--	C2H2
Ca6_P3_12927504	Ca6	12927504	ATGTTAAT/A	GAACAGTCTCGCACCTCTCC	ACACCCCATATTCCCATTCA	60.0	336	DRR	Ca_05065	--	C2H2
Ca6_P3_12928504	Ca6	12928504	CAAAAAAA/CAAAAAA	CTTGCGAATGTCTCACATGG	GGCCCAACCCGTTATAATTT	60.3	664	DRR	Ca_05065	--	C2H2
Ca6_P3_12930659	Ca6	12930659	TAAAAAAAA/TAAAAAAA	TGCCCAGAACTACTACAAAAGC	CGATGCATTGTTAATGTGGG	58.6	535	INTERGENIC	--	--	--
Ca6_P3_12930965	Ca6	12930965	A/AC	AATGCATCGGTCAAACCAAC	ATTGCCCATGTAAACCGTGT	60.8	362	INTERGENIC	--	--	--

INDEL marker IDs	Chromosomes/ unanchored scaffolds	Physical positions (bp)	InDels (*Kabuli* reference genome-CDC Frontier/PII)	Forward primers (5'-3')	Reverse primers (5'-3')	Anneling temperature (0C)	Expected amplified product size (bp)	Structural annotation		Functional annotation	
								Sequence components of *kabuli* genome	*Kabuli* gene accession IDs	NCBI-KOG	TFs
Ca6_P3_12931395	Ca6	12931395	CAAAAAAAAAA/CAAAAAAAAAAAA	CACGGTTTACATGGGCAATA	ATCTGGTATCAAATGCCCCA	59.3	493	INTERGENIC	--	--	--
Ca6_P3_12940169	Ca6	12940169	GTAATTTGTT/GT	ACGTCAATCCAAAAAGCCAC	AAGGTTGGGAGGAGAGGCTA	60.0	279	DRR	Ca_05066	--	--
Ca6_P3_12940701	Ca6	12940701	TAAAAAAA/TAAAAAA	TAGCCTCTCCTCCCAACCTT	ATTCATGGATTGGCATTGGT	60.2	665	DRR	Ca_05066	--	--
Ca6_P3_12944263	Ca6	12944263	ATTTTTTT/ATTTTTTTT	CATTGGTGCTTCAAACCGAT	TCCCTCGTACTTCCAAAACG	60.9	533	INTERGENIC	--	--	--
Ca6_P3_12945433	Ca6	12945433	G/GATTCAGAT	TGGTGGTGTAGGTCCACAGA	TTCACGGTGTTCAAATTCGT	60.0	415	INTERGENIC	--	--	--
Ca6_P3_12946214	Ca6	12946214	TAAAAAAA/TAAAAAAAA	TGGGTTCATCACGTAAACAGA	GAATAGTCAGAGCCAAGCGG	59.0	440	INTERGENIC	--	--	--
Ca6_P3_12946396	Ca6	12946396	GAAAAAAAAAAAAAAAA/GAAAAAAAAAAA	ATTTTATTTCCCGCTTGGCT	CAAACAAATGAAGCCCAAAA	59.9	213	INTERGENIC	--	--	--
Ca6_P3_12950292	Ca6	12950292	C/CAGTCA	AGGTCAGACTACATGGCGCA	CACAACCTCCCAATGCTTCT	61.8	562	INTERGENIC	--	--	--
Ca6_P3_12953295	Ca6	12953295	GTTTTTTT/GTTTTTTTT	AAATTCATGGATCAATGGGAG	GGGGAGGGCGTTAGAAAATA	58.7	267	INTERGENIC	--	--	--
Ca6_P3_12954110	Ca6	12954110	GTTAA/GTTAAATTAA	CGAACAACCGGGGAATAATA	TGAATTGCATCTTGCTCGTC	59.7	438	INTERGENIC	--	--	--

INDEL marker IDs	Chromosomes/ unanchored scaffolds	Physical positions (bp)	InDels (*Kabuli* reference genome-CDC Frontier/PII)	Forward primers (5'-3')	Reverse primers (5'-3')	Anneling temperature (0C)	Expected amplified product size (bp)	Structural annotation		Functional annotation	
								Sequence components of *kabuli* genome	*Kabuli* gene accession IDs	NCBI-KOG	TFs
Ca6_P3_12954436	Ca6	12954436	TAAAAA/TAAAA	TGAAATGATGAGGGGG AGAC	CCCTTCATCCCTTACC CCTA	59.9	716	INTERGENIC	--	--	--
Ca6_P3_12956363	Ca6	12956363	GTGTT/GT	AATCACGAATGACCAAA CGAG	AGCAATGGTTGATGCA TTTG	60.0	804	INTERGENIC	--	--	--
Ca6_P3_12956531	Ca6	12956531	A/ACG	TTCTCTCCGTTGATCTC CAAA	TTTGTCCATCCTTGGT AGGG	59.8	869	INTERGENIC	--	--	--
Ca6_P3_12957525	Ca6	12957525	CAAA/CAAAA	CGGTTCACCTGTTTGC TTTT	TGAAAAGCTTGAGGAA GGGA	60.1	718	INTERGENIC	--	--	--
Ca6_P3_12963047	Ca6	12963047	TTATAAAT/TTATAAATAACTATAAAT	GCAGAAAACGACATCC ATGA	ACCTGGTTGGCAAACA AAAG	59.7	595	DRR	Ca_05068	--	MYB_related
Ca6_P3_12964776	Ca6	12964776	T/TA	TGAAGATTTAGGACGG GCTG	TGATGAAGAGAATAGT GTCCAGTTG	60.2	751	INTERGENIC	--	--	--
Ca6_P3_12964933	Ca6	12964933	TCATTA/T	CTTGTTGCCAAGTAGG GTCG	CCAAGGGAGAGGTGC TTAAA	60.7	976	INTERGENIC	--	--	--
Ca6_P3_12967633	Ca6	12967633	TAAAAAA/TAAAAAAA	TGCAAAAAATTAAGGGG CATT	TTTGATATTTTTGGACA AGCCA	59.4	583	INTERGENIC	--	--	--
Ca6_P3_12970794	Ca6	12970794	TTTTATTTATTTATTT/TTTTATTTATTTA TTTATTT	GTTGTGGTTGGCACTG TTTG	TTGGTTCTTGTTATGC GGTG	60.0	584	INTRON	Ca_05069	S	--
Ca6_P3_12972982	Ca6	12972982	GTTTTTTTTTTT/GTTTTTTTTTT	AGTTGGTGAGAGGAAG CGAA	GTCCAACCCAGTGGAG AAAA	60.0	487	INTRON	Ca_05069	S	--

INDEL marker IDs	Chromosomes/ unanchored scaffolds	Physical positions (bp)	InDels (*Kabuli* reference genome-CDC Frontier/PII)	Forward primers (5'-3')	Reverse primers (5'-3')	Anneling temperature (0C)	Expected amplified product size (bp)	Structural annotation		Functional annotation	
								Sequence components of *kabuli* genome	*Kabuli* gene accession IDs	NCBI-KOG	TFs
Ca6_P3_12977836	Ca6	12977836	ATCT/AT	TGTTCATTTCAAGCACTCGG	CGCCATCATAGATGTCCCTT	59.8	715	DRR	Ca_05071	O	E2F/DP
Ca6_P3_12978174	Ca6	12978174	ATTTT/ATTT	ATCTATGATGGCGCAAGAGC	TGTGCAGATCACAAACACCA	60.3	529	DRR	Ca_05071	O	E2F/DP
Ca6_P3_12979257	Ca6	12979257	TATAAAATATTTTCATAA/TATAAAATATTTTCATAAAATATTTTCATAA	CCAGCAGTTTTGATGGTGTG	AGGTGTGCTTCCTCGTGAAT	60.2	545	INTRON	Ca_05071	O	E2F/DP
Ca6_P3_12980658	Ca6	12980658	TAAAA/TAAA	GCAAAATCCCCAGCAATCTA	TGTCGTGGTGCATTTTCAAT	60.0	500	INTRON	Ca_05071	O	E2F/DP
Ca6_P3_12980846	Ca6	12980846	TAAAAAAAA/TAAAAAAAAAAA	ATTGAAAATGCACCACGACA	GGAATTTTGGTGGGGAGAAT	60.0	417	INTRON	Ca_05071	O	E2F/DP
Ca6_P3_12999133	Ca6	12999133	ATAA/ATAATGTAA	ACGAAAGGACATATCGCCAC	GGAAGGGTGCAAAACAATTC	60.0	943	INTERGENIC	--	--	--
Ca6_P3_13128943	Ca6	13128943	CTTTTTTTTTTT/CTTTTTTTTTT	CACCACGAGATCAAAACCCT	CCATTGGCAGTGGAACTCTT	60.0	482	INTERGENIC	--	--	--
Ca6_P3_13170216	Ca6	13170216	AATATATA/AATATATATA	TCAACCAAGAATTGCTGCTAAA	AGGGAGTATTCACGGCACAC	59.9	502	INTERGENIC	--	--	--
Ca6_P3_13178639	Ca6	13178639	TG/T	GTGGGGTGTCTCTGCTGATT	TCGGTCTCCAACACTGACAC	60.1	443	INTRON	Ca_05088	--	--
Ca6_P3_13180103	Ca6	13180103	TTTATTATTATTATTATTATTATTATTAT/TTTATTATTATTATTATTATTATTATTATTAT	TTTGAGTCAGTCAACGCAGG	TAAACCATTCAAACCCCCAA	60.0	830	INTERGENIC	--	--	--

INDEL marker IDs	Chromosomes/ unanchored scaffolds	Physical positions (bp)	InDels (*Kabuli* reference genome-CDC Frontier/PII)	Forward primers (5'-3')	Reverse primers (5'-3')	Anneling temperature (0C)	Expected amplified product size (bp)	Structural annotation		Functional annotation	
								Sequence components of *kabuli* genome	*Kabuli* gene accession IDs	NCBI-KOG	TFs
Ca6_P3_13206663	Ca6	13206663	AAGAGAGAGAGAGAGAGAGA/AAGAGAGAGAGAGAGAGAGAGA	TGATGGTTGACCAGACCAAA	TGGAAGCATTGTTTAAGGGG	59.9	501	INTERGENIC	--	--	--
Ca6_P3_13207368	Ca6	13207368	GTTTTTTTTTTT/GTTTTTTTTTTTT	TTGCTTTCCACTGTTTTGGA	AAAAACATGTTAATTGGGAGGAA	59.3	538	INTERGENIC	--	--	--
Ca6_P3_13246843	Ca6	13246843	AATATATATATATATATATATAT/AATATATATATATATATATAT	GGCTTGCCTCAAATTCAAGA	TTTGTCACAAGTGCGAGTTAAT	60.3	656	INTERGENIC	--	--	--
Ca6_P3_13381796	Ca6	13381796	TAAAAAAAAAAAA/TAAAAAAAAAAA	GGGTTCGTAGTTGTATGCGAA	CATGGATCAGAGTTGCCTTG	60.0	883	INTERGENIC	--	--	--
Ca6_P3_13394015	Ca6	13394015	GATATATATATATATATATATATATA/GATATATATATATATATATATATA	TTGCCACTTGATACAGCAAAA	TGCAACTTGCGTCTAGGATG	59.4	771	DRR	Ca_05112	Q	B3
Ca6_P3_13422865	Ca6	13422865	CAAAAAAAAAA/CAAAAAAAAAAA	CATCGTCGTCAACCAGTGTT	TGTGAGCCTGCTCATTGTTC	59.6	550	INTERGENIC	--	--	--
Ca6_P3_13428719	Ca6	13428719	CTATTTATTTATTTATTT/CTATTTATTTATTTATTTATTTATTTATTTATTT	GTCTGCGAAAAAGGAGTTGC	TTTCCCTATTGCACCTGGAG	60.0	329	INTERGENIC	--	--	--
Ca6_P3_1343859	Ca6	1343859	GAAAAA/GAAAAAA	CAACGAGTTTGATTTCTATTCTAACG	TTGGGTAGCCAAGGTTTCAC	59.7	721	INTERGENIC	--	--	--
Ca6_P3_13440291	Ca6	13440291	CATATATATATATATATATATATATATATATATATAT/CATATATATATATATATATATATATATATATATATATAT	GCAAACAACGTTGAGGTTCA	CGCTTTAGGTTTTGTTCGTT	59.7	355	INTERGENIC	--	--	--
Ca6_P3_1345603	Ca6	1345603	AACTAT/A	CAGCCACGATATCTCGTCAA	TTAAGTCCAATATGCGGGGA	59.8	560	INTERGENIC	--	--	--

INDEL marker IDs	Chromosomes/ unanchored scaffolds	Physical positions (bp)	InDels (*Kabuli* reference genome-CDC Frontier/PII)	Forward primers (5'-3')	Reverse primers (5'-3')	Anneling temperature (0C)	Expected amplified product size (bp)	Structural annotation		Functional annotation	
								Sequence components of *kabuli* genome	*Kabuli* gene accession IDs	NCBI-KOG	TFs
Ca6_P3_13475824	Ca6	13475824	AAAGAAGAAGAAGAAGAAGAA/AAAGAAGAAGAAGAAGAA	TGTGGTCCCAAACAATCTCA	TTTTGGGTCAAGGAAAATGG	59.9	330	INTERGENIC	--	--	--
Ca6_P3_13481060	Ca6	13481060	GAAAAAAAAAAA/GAAAAAAAAAA	AAAGGTGCTGCCTGTCCTTA	GGAGTTGGAACTCGGAACAA	59.9	483	INTRON	Ca_05119	S	--
Ca6_P3_13496923	Ca6	13496923	AATA/AA	GAAAGCAGTCGTGACCATCA	TTCAAAACATGTCACGTCGTC	59.8	419	INTERGENIC	--	--	--
Ca6_P3_1350878	Ca6	1350878	A/AGTCTGT	ACACATCAGCTTTTTGGCTG	AGCCCACTCACCTTCTTCCT	58.9	428	INTERGENIC	--	--	--
Ca6_P3_1351704	Ca6	1351704	TGT/TGTTAGT	CTCATCGCATGCTTTTTGAA	ATGAAATGAAGAAAGGGGCA	60.0	350	INTERGENIC	--	--	--
Ca6_P3_1354322	Ca6	1354322	GAA/GAACCAA	AATTTGCATAGGCGGTGAAG	GGTGTTACAGCTTTCATGAACAAA	60.1	420	INTERGENIC	--	--	--
Ca6_P3_13659732	Ca6	13659732	AT/AATTTT	GCATAACACGCAGGTTAGCA	CATGTTGAAAATCACGGCTG	59.9	584	INTERGENIC	--	--	--
Ca6_P3_1377550	Ca6	1377550	GATATA/GATATATA	GGCCACACTAAAGTGCCATT	TGTCCATTTTCTGGGACACA	60.0	551	INTERGENIC	--	--	--
Ca6_P3_13838996	Ca6	13838996	CCT/CCTTCT	TGTGTGATTGGTGTTGAGCA	GCAACAACGGGTGAAAATCT	59.7	563	INTERGENIC	--	--	--
Ca6_P3_13839034	Ca6	13839034	CAAAAAAAAA/CAAAAAA	ATGACTTTGTTTTGGCCCTG	GCAACAACGGGTGAAAATCT	60.0	166	INTERGENIC	--	--	--

INDEL marker IDs	Chromosomes/ unanchored scaffolds	Physical positions (bp)	InDels (*Kabuli* reference genome-CDC Frontier/PII)	Forward primers (5'-3')	Reverse primers (5'-3')	Anneling temperature (0C)	Expected amplified product size (bp)	Structural annotation		Functional annotation	
								Sequence components of *kabuli* genome	*Kabuli* gene accession IDs	NCBI-KOG	TFs
Ca6_P3_13839096	Ca6	13839096	AATATA/AATA	ATGACTTTGTTTTGGCCCTG	GCAACAACGGGTGAAAATCT	60.0	166	INTERGENIC	--	--	--
Ca6_P3_14061005	Ca6	14061005	AAGAGAGAGAGAGAGAGAGAGAGAGAGA/AAGAGAGAGAGAGAGAGAGAGAGAGA	GAGGGAAGCGTGTCAAAGAG	ATCCCCTCCGACATTTTCTC	60.0	126	INTERGENIC	--	--	--
Ca6_P3_14219953	Ca6	14219953	CAAAAAAAAAA/CAAAAAAAAAAA	TTTGTTTTTCCAACTTGGTCC	TTTGTTTCAAAGTCCCCGAG	59.0	543	INTERGENIC	--	--	--
Ca6_P3_14270626	Ca6	14270626	CATATATATATATATATATATATATATATATATATAT/CATATATATATATATATATATATATATAT	ACCGAGTGTCGAACTCAAGG	ATGCATTAATGGCCTCTCCA	60.3	710	INTERGENIC	--	--	--
Ca6_P3_14277957	Ca6	14277957	AATATATATATATAT/AATATATATATATATATAT	GGAGAAAATTAAGAGGGAGGGA	ATGGGGCCATCAAGTACTCA	59.9	261	INTERGENIC	--	--	--
Ca6_P3_14278804	Ca6	14278804	CCTTTAAATGC/CC	GACTTGGTCCAATCATACACCA	TTTTGTAACCGCTTTTCACG	59.7	636	INTERGENIC	--	--	--
Ca6_P3_14293231	Ca6	14293231	AATATATATATATATATATATAT/AATATATATATATATATATATATATAT	CCCCAACACACCAATAAACC	ACCAACAATGGGTATTCGGA	59.9	504	INTERGENIC	--	--	--
Ca6_P3_14362429	Ca6	14362429	G/GAAGAATTATC	AAGGGGAGTGGTCTGGAGAT	CTTAGGAAAAATCCTCCGCC	59.9	683	INTERGENIC	--	--	--
Ca6_P3_14410304	Ca6	14410304	TTTTATTTATTTATTTATTTATTTATTTATTTATTTATTTAT/TTTTATTTATTTATTTATTTATTTATTTAT	TTGTGCATGGTTGTGGATCT	TCTCAAGTTTGTAAGCGAGTCAA	60.0	193	INTERGENIC	--	--	--
Ca6_P3_14457838	Ca6	14457838	AA/AATA	GGAGAAAGGAGAAGAGGTCCA	AACAGACCTCCAAATGCCAC	59.8	464	INTERGENIC	--	--	--

INDEL marker IDs	Chromosomes/ unanchored scaffolds	Physical positions (bp)	InDels (*Kabuli* reference genome-CDC Frontier/PII)	Forward primers (5'-3')	Reverse primers (5'-3')	Anneling temperature (0C)	Expected amplified product size (bp)	Structural annotation		Functional annotation	
								Sequence components of *kabuli* genome	*Kabuli* gene accession IDs	NCBI-KOG	TFs
Ca6_P3_14534609	Ca6	14534609	AATATATATATATATATATA/AATATATATATATATATA	TGATGTGTAATAGGGCAATGGA	CCTATGGCCTGGCCTACATA	60.2	222	INTERGENIC	--	--	--
Ca6_P3_14614892	Ca6	14614892	AAG/A	GGATTCAAATTCTAAATCAGGGG	CACATCAATCAATCTCTGCTCA	60.0	189	INTERGENIC	--	--	--
Ca6_P3_14755534	Ca6	14755534	AATATATATATATATATATATATAT/AATATATATATATATATATATATATATAT	TTTTAACCGTGGTTTGAAACTT	AGTCAGACGCTACTCGGCAT	57.8	212	INTERGENIC	--	--	--
Ca6_P3_14755753	Ca6	14755753	ATT/ATTT	AACTTGATGCCGAGTAGCGT	TTTGTGAGGGCTTTTTCAGC	59.9	633	INTERGENIC	--	--	--
Ca6_P3_14909181	Ca6	14909181	ATTTTTTTTTTTTT/ATTTTTTTTTTT	CTGCATGCCCTTTCTTGTTT	TGATTTTGGAAGGGGAAAAA	60.2	887	INTRON	Ca_05265	Q	Nin-like
Ca6_P3_1491943	Ca6	1491943	CT/CTT	TGGTTTTCGTACCGGACTCT	CAATGTCATCAAATAGCGGC	59.6	772	INTRON	Ca_27033	H	--
Ca6_P3_1492206	Ca6	1492206	TAAGAAGAAAAG/TAAGAAGAAAAGAAGAAAAG	CCGTGCATATGACAGAAATGA	ATGCACCGTTGAAACAACAA	59.6	510	INTRON	Ca_27033	H	--
Ca6_P3_1492346	Ca6	1492346	ATAGC/ATAGCGCCGCTGTAGC	CCGTGCATATGACAGAAATGA	ATGCACCGTTGAAACAACAA	59.6	510	INTRON	Ca_27033	H	--
Ca6_P3_1492728	Ca6	1492728	AT/ATT	TTGTTGTTTCAACGGTGCAT	ATGGTTGGGGGATAAAAAGG	60.0	651	INTRON	Ca_27033	H	--
Ca6_P3_14938058	Ca6	14938058	TAAAAAAAAAA/TAAAAAAAAAAA	CCTCCTACATTGTGGGTGCT	ACATTTGGCCCATTTGGTTA	60.0	754	INTERGENIC	--	--	--

INDEL marker IDs	Chromosomes/ unanchored scaffolds	Physical positions (bp)	InDels (*Kabuli* reference genome-CDC Frontier/PII)	Forward primers (5'-3')	Reverse primers (5'-3')	Anneling temperature (0C)	Expected amplified product size (bp)	Structural annotation		Functional annotation	
								Sequence components of *kabuli* genome	*Kabuli* gene accession IDs	NCBI-KOG	TFs
Ca6_P3_14959810	Ca6	14959810	CTTTTTTTTTTT/CTTTTTTTTTT	GCGAAATGCTTCTCAAAACC	CGTTACAACGTTGCTGAATGA	59.8	745	INTERGENIC	--	--	--
Ca6_P3_15244446	Ca6	15244446	TAAAAAAAAAAA/TAAAAAAAAAA	TCGCGTGTGGGTATGTTTTA	TGAGCAAATGAAAATTAAAAACCA	60.0	520	DRR	Ca_05306	--	--
Ca6_P3_15277411	Ca6	15277411	AATTATTATTATTATTATTATTATTATTATTATTATTATTATTATT/AATTATTATTATTATTATTATTATTATTATTATTATTATT	CGTCTTTCTAATATTTTGCCGA	CATGAAAGTGGTCAGATGCG	58.5	700	INTERGENIC	--	--	--
Ca6_P3_15358737	Ca6	15358737	AT/ATTAAATATTT	GTTTCTGACCCAACTCGCAT	AAGATGAGAGGACGATATCCAAA	60.1	522	INTERGENIC	--	--	--
Ca6_P3_15358761	Ca6	15358761	A/ATTTTTC	GTTTCTGACCCAACTCGCAT	AAGATGAGAGGACGATATCCAAA	60.1	522	INTERGENIC	--	--	--
Ca6_P3_1539975	Ca6	1539975	AACA/AA	GCTCTCCAATGTGTAATCGCT	GCAATCAAACCACCATTTCC	59.4	748	INTERGENIC	--	--	--
Ca6_P3_1540568	Ca6	1540568	CTTTTTTTTTTT/CTTTTTTTTTT	GGAAATGGTGGTTTGATTGC	TTTTTGGGTAAGGTTTGAAATGA	60.2	496	INTERGENIC	--	--	--
Ca6_P3_1548359	Ca6	1548359	GTTTTTTTTTT/GTTTTTTTTT	AAGGAGTTGGTTGGTGTGTTTT	TGACTAGGAAATTCGAGGGG	59.8	601	INTERGENIC	--	--	--
Ca6_P3_15508917	Ca6	15508917	TTATATATATATATATATATATATAT/TTATATATATATATATATATATATATATAT	TGTTTGATTGGGTTGGTTCA	TGGGTGCTTTATTAAGATGGG	59.8	849	DRR	Ca_05334	--	--
Ca6_P3_15524701	Ca6	15524701	TA/TCCCTGAGTTCA	GAGATGTCAAGGGAAGGGAA	TTGTGATGCCAACTTAACGG	59.1	523	INTERGENIC	--	--	--

INDEL marker IDs	Chromosomes/ unanchored scaffolds	Physical positions (bp)	InDels (*Kabuli* reference genome-CDC Frontier/PII)	Forward primers (5'-3')	Reverse primers (5'-3')	Anneling temperature (0C)	Expected amplified product size (bp)	Structural annotation		Functional annotation	
								Sequence components of *kabuli* genome	*Kabuli* gene accession IDs	NCBI-KOG	TFs
Ca6_P3_15555599	Ca6	15555599	AATATATA/AATATA	TGTGGGCACATTAGTAGGCA	TCATCGTCGTTGCTTGAGTC	60.1	640	INTERGENIC	--	--	--
Ca6_P3_15559125	Ca6	15559125	CTTT/CTT	TCACCAAACAAAAACTGGGG	CTACATTCCGGTTAGGGCAA	60.8	480	INTERGENIC	--	--	--
Ca6_P3_1556126	Ca6	1556126	GA/G	ATTGTTCTCCGTTGTCAGGG	CTGAGATTAGCTTCGGTGGG	60.0	348	INTERGENIC	--	--	--
Ca6_P3_1556869	Ca6	1556869	TAAAAA/TAAAAAA	CAATTTGGTTGGGCGTAAAC	TTGCTTTATACTCATATTTTGGCCT	60.2	528	INTERGENIC	--	--	--
Ca6_P3_15577471	Ca6	15577471	TTCATTATCATTATCATTATCATTATCATTATCATTAT/TTCATTATCATTATCATTATCATTATCATTATCATTATCATTAT	ACATCCAGTCCTGCAAATCC	TGAACTCTGAGCGTGTGACC	59.9	852	INTERGENIC	--	--	--
Ca6_P3_1559285	Ca6	1559285	GTTA/GTTATTA	CAGAGCAGTGTGACAGCAAGA	TCAAAAATTTCAAACAAAATCCA	60.4	456	INTERGENIC	--	--	--
Ca6_P3_1559414	Ca6	1559414	CAAAAAAAAAAAA/CAAAAAAAAAAA	CAGAGCAGTGTGACAGCAAGA	TATTTTATTCCCACCGCTGC	60.4	879	INTERGENIC	--	--	--
Ca6_P3_1559495	Ca6	1559495	GAACAAA/GA	GGATTTTGTTTGAAATTTTTGATG	TATTTTATTCCCACCGCTGC	58.9	445	INTERGENIC	--	--	--
Ca6_P3_1559581	Ca6	1559581	ATTTTTTTTTTTT/ATTTTTTTTTT	TGAAGTTAAATTAAAGGATCGCA	TATTTTATTCCCACCGCTGC	58.0	283	INTERGENIC	--	--	--
Ca6_P3_15597965	Ca6	15597965	TAAA/TAAAA	CAGTTTCCACACGCACAGAT	TGGGAACAAGCATGGTGTAA	59.8	681	DRR	Ca_05341	--	bHLH

INDEL marker IDs	Chromosomes/ unanchored scaffolds	Physical positions (bp)	InDels (*Kabuli* reference genome-CDC Frontier/PII)	Forward primers (5'-3')	Reverse primers (5'-3')	Anneling temperature (0C)	Expected amplified product size (bp)	Structural annotation		Functional annotation	
								Sequence components of *kabuli* genome	*Kabuli* gene accession IDs	NCBI-KOG	TFs
Ca6_P3_1560016	Ca6	1560016	TTT/TTTAATT	AATATTACTGCAGCGGTGGG	GGACCACAAGAAATCCTCCA	60.0	719	INTERGENIC	--	--	--
Ca6_P3_15607994	Ca6	15607994	AACA/AACATCTACA	CAATCGATTCCCTCAGCACT	TTCCCAACTAGCTTTGTTCCA	60.2	463	INTERGENIC	--	--	--
Ca6_P3_15727278	Ca6	15727278	ATTTATTTATTTTATTTATTTTACTTATTTTATTTATTTT/ATTTATTTATTTTATTTATTTTATTTATTTTACTTATTTTATTTATTTT	TCCCCCGTCTAACTAGGGTT	TCGTGTCCCAAAACTCAAGG	59.8	766	INTERGENIC	--	--	--
Ca6_P3_15727514	Ca6	15727514	ATTTATTTTATTTTATTTTATTT/ATTTATTTTATTTTATTTTATTTTATTT	TTAGTTTGGGCTTGGGTTTT	TTGCTCCTACGTATCTCCCG	58.6	911	INTERGENIC	--	--	--
Ca6_P3_15728010	Ca6	15728010	CAAAA/CAAAAA	CGGGAGATACGTAGGAGCAA	TCGATTATGCGTAGGGAAGG	60.2	248	INTERGENIC	--	--	--
Ca6_P3_15730699	Ca6	15730699	TAAA/TAA	ATTTGAACCTGGCAGCAAAA	GGTTTGAAAAAGGGGTGATG	60.6	175	INTERGENIC	--	--	--
Ca6_P3_15730843	Ca6	15730843	T/TGA	ATTTGAACCTGGCAGCAAAA	GCTTGATGAATAAATCAATGGCT	60.6	525	INTERGENIC	--	--	--
Ca6_P3_15731057	Ca6	15731057	TAAAAA/TAA	ATTTGAACCTGGCAGCAAAA	GGGACAAAAGGCTAGTTTGG	60.6	909	INTERGENIC	--	--	--
Ca6_P3_1580380	Ca6	1580380	GAAAAAAAAA/GAAAAAAAA	GACTCAAGTTGGGCTCTTCG	TTGGCAAAATGATTGCATCT	60.0	922	INTERGENIC	--	--	--
Ca6_P3_15867174	Ca6	15867174	ATTATTTATTTATTTATTTATTTATTTATTT/ATTATTTATTTATTTATTTATTTATTT	TTTTAGACGAGGCCTAGGGA	TTTTGATGTAGCTCGACGCA	58.9	504	DRR	Ca_22021	K	MYB

INDEL marker IDs	Chromosomes/ unanchored scaffolds	Physical positions (bp)	InDels (*Kabuli* reference genome-CDC Frontier/PII)	Forward primers (5'-3')	Reverse primers (5'-3')	Anneling temperature (0C)	Expected amplified product size (bp)	Structural annotation		Functional annotation	
								Sequence components of *kabuli* genome	*Kabuli* gene accession IDs	NCBI-KOG	TFs
Ca6_P3_15946706	Ca6	15946706	GATATATATATATATAT/GATATATATATATATATAT	TGTTGCAGTCATGCTTTTGA	TGAATTCTGCTATGCGCAAC	59.0	306	DRR	Ca_22027	C	--
Ca6_P3_15966204	Ca6	15966204	GTTATTATTATTATTATTATTATTATTATTATTATTATTATTATTATTA/GTTATTATTATTATTATTATTATTATTATTATTATTATTA	CTGCCTGAAATTTGGTTGCT	AGATGCGACCTCCTACTTGC	60.2	229	INTERGENIC	--	--	--
Ca6_P3_15977742	Ca6	15977742	TTTATTATTATTATTATTATTATTATTATTATTATTATTATTATT/TTTATTATTATTATTATTATTATTATTATTATTATTATTATTATTATT	CAAGTTTTGGAGAAACCCCA	CCCATATAGAAAAATGATTGGTTCA	59.9	823	INTERGENIC	--	--	--
Ca6_P3_15999975	Ca6	15999975	A/AG	AAGTGTGGGCATTTCCTTTG	ATGGACCTCTGTATTCGCCA	60.0	137	INTERGENIC	--	--	--
Ca6_P3_16011007	Ca6	16011007	AATATATATATATATATATATATA/AATATATATATATATATATATATATATA	ACCAAAAAGGTTTTGCATCA	GGATGTCTGACTATTTTGCATCA	58.1	265	INTERGENIC	--	--	--
Ca6_P3_16012701	Ca6	16012701	TA/TAA	TAAGAGTTTGGTTCCGGTGG	TGTGCAGATAAATGGCCAAA	60.0	397	INTERGENIC	--	--	--
Ca6_P3_16023505	Ca6	16023505	GTTTTTTTTT/GTTTTTTTTTT	TGAGGATCAACCTTTGTTTTGA	TGGGCTAAATATCACTTGTGGTC	59.6	654	INTERGENIC	--	--	--
Ca6_P3_16052205	Ca6	16052205	AATTATTATTATTATTATTATTATTATTATTATTAT/AATTATTATTATTATTATTATTATTATTATTATTATTATTATTAT	TACGATCAATCCGCAACTGA	TTGGAGCATCACAAAGTAAAACA	60.2	728	INTERGENIC	--	--	--
Ca6_P3_16093896	Ca6	16093896	AAAAAATATATAAAAATATA/AAAAAATATA	TGCAAAAAGATGGCAATGAA	TTTCCTTAAATATCCATCCAGTCA	60.2	598	INTERGENIC	--	--	--
Ca6_P3_16253665	Ca6	16253665	AATATATATATATATATATATATATATAT/AATATATATATATATATATATATATATATATATATAT	CGTGGAATCGTATTCGTGTG	TTGAATCGACACTCAACCCA	60.0	490	INTERGENIC	--	--	--

INDEL marker IDs	Chromosomes/ unanchored scaffolds	Physical positions (bp)	InDels (*Kabuli* reference genome-CDC Frontier/PII)	Forward primers (5'-3')	Reverse primers (5'-3')	Anneling temperature (0C)	Expected amplified product size (bp)	Structural annotation		Functional annotation	
								Sequence components of *kabuli* genome	*Kabuli* gene accession IDs	NCBI-KOG	TFs
Ca6_P3_16339675	Ca6	16339675	AATATATATATATATATATATATATATATATATATA/AATATATATATATATATATATATATATATATATA	GGATGTGCCAATGATTTGGT	TCTCCCTTTCCTTGCTCTCA	60.6	700	INTERGENIC	--	--	--
Ca6_P3_163779	Ca6	163779	CGAGAGAGAGAGAGAGAGAGAGAGAGAGAGAGAGAGAGAGAGAGAGAG/CGAGAGAGAGAGAGAGAGAGAGAGAGAGAGAGAGAGAGAGAGAGAGAGAGAGAG	CACAGACCCTCATGTTCCCT	GGCGAGATACTTGGTCGAAA	60.0	563	INTERGENIC	--	--	--
Ca6_P3_16476384	Ca6	16476384	AGAGAA/AGAGAAATGACGAGAA	CTTGATGTTTCCGATGTGGA	TCTCTAAGGGGACACCATCG	59.5	671	INTERGENIC	--	--	--
Ca6_P3_16547931	Ca6	16547931	GA/GAGAGCTTA	TTCCTTCCTGCTCGTGCTAT	TTCTTTGGCTGAAATGGTCC	60.0	640	INTRON	Ca_06284	A	--
Ca6_P3_16693859	Ca6	16693859	TTATATATATATATATATATATATATATA/TTATATATATATATATATATATATA	TAAAACCCCTGCTTTGGTTG	TTCCACATGGGGTTTTCATT	60.0	237	INTERGENIC	--	--	--
Ca6_P3_16808061	Ca6	16808061	TTTATTATTATTATTATTATTATTATTATTATTATTATTATTATTATTATTATT/TTTATTATTATTATTATTATTATTATTATTATTATTATT	TGAGTGAAAGGTGTAGTGGGTG	GCGTGTTGAAACCCATTCTT	60.1	582	INTERGENIC	--	--	--
Ca6_P3_1682722	Ca6	1682722	GTGCATCTGTTT/G	GAGGCATTGTCTTTGGTGGT	GCAAATGTTTGGAATGGCTT	60.0	656	INTERGENIC	--	--	--
Ca6_P3_1683226	Ca6	1683226	CAA/CA	AATAAGTCCATCCGGCTCCT	TTGTGGATGGCGTTTGATAA	59.9	276	DRR	Ca_10299	--	HB-other
Ca6_P3_1683327	Ca6	1683327	AC/A	TTATCAAACGCCATCCACAA	CACTTGCGTGTGGTAGTGCT	59.9	504	DRR	Ca_10299	--	HB-other
Ca6_P3_1683470	Ca6	1683470	C/CCAAAACAATCATAAC	TTATCAAACGCCATCCACAA	CACTTGCGTGTGGTAGTGCT	59.9	504	DRR	Ca_10299	--	HB-other

INDEL marker IDs	Chromosomes/ unanchored scaffolds	Physical positions (bp)	InDels (*Kabuli* reference genome-CDC Frontier/PII)	Forward primers (5'-3')	Reverse primers (5'-3')	Anneling temperature (0C)	Expected amplified product size (bp)	Structural annotation		Functional annotation	
								Sequence components of *kabuli* genome	*Kabuli* gene accession IDs	NCBI-KOG	TFs
Ca6_P3_16854632	Ca6	16854632	GAAAAAAAAAA/GAAAAAAAAA	TTGCCCTATTATTGACTTTTGGA	AGATCGCTCTCCGACCCTAT	59.9	421	INTERGENIC	--	--	--
Ca6_P3_1694188	Ca6	1694188	AATATATATATATATATATATATATATATATATATATATATA/AATATATATATATATATATATATATATATATATATATA	GCGAATGACATATGTGACGG	TGTTCGTATTTTGTTCAGATTGC	60.0	826	INTERGENIC	--	--	--
Ca6_P3_16988108	Ca6	16988108	TTATATATATATATATATATATAT/TTATATATATATATATATAT	CCTCTGACGAAGAGTTTGTCC	AACTCGCAAGTTACGCGA	58.9	512	INTERGENIC	--	--	--
Ca6_P3_16990146	Ca6	16990146	TTATATATATATATATATATA/TTATATATATATATATATATA	TGGGCAAATTAAGGAACCAA	ACAGAGGGCCGGAATATGAT	60.3	619	INTERGENIC	--	--	--
Ca6_P3_16996867	Ca6	16996867	TTTCCCTNNNNNNNNNNTTCCCT/TTTCCCT	GACGTTCATAAAGGTTAGTCTTAAACC	ATAAACGGGACCGGGTAATC	59.1	547	INTERGENIC	--	--	--
Ca6_P3_17008571	Ca6	17008571	ATTTTTTTTTTTTTTTTTTT/ATTTTTTTTTTTTTTT	CGAAAACTAACCTACTGTCACCC	GTCCGTATGAAGTGATGGGG	59.1	591	INTERGENIC	--	--	--
Ca6_P3_17013081	Ca6	17013081	CTTTTTTTTTTTT/CTTTTTTTTTTT	CGAATCAAATGGAATTTGGG	TTTGTGATTGGGTGTTTGGA	60.1	366	DRR	Ca_06323	--	B3
Ca6_P3_17025431	Ca6	17025431	ATTTTTTTTTTTT/ATTTTTTTTTTT	TTTTGCTCCACGTTTTCCTT	GGCTTATAGGCCCACGTTTT	59.7	562	INTERGENIC	--	--	--
Ca6_P3_17025727	Ca6	17025727	AATA/AA	TTTTGCTCCACGTTTTCCTT	GGCTTATAGGCCCACGTTTT	59.7	562	INTERGENIC	--	--	--
Ca6_P3_17043003	Ca6	17043003	CTTTTTTTTT/CTTTTTTTTTT	CGAGATTTAAAACAAAATTGGGA	TATCTTGGTGGGACTGGGAG	59.4	533	DRR	Ca_06325	--	--

INDEL marker IDs	Chromosomes/ unanchored scaffolds	Physical positions (bp)	InDels (*Kabuli* reference genome-CDC Frontier/PII)	Forward primers (5'-3')	Reverse primers (5'-3')	Anneling temperature (0C)	Expected amplified product size (bp)	Structural annotation		Functional annotation	
								Sequence components of *kabuli* genome	*Kabuli* gene accession IDs	NCBI-KOG	TFs
Ca6_P3_17044026	Ca6	17044026	AAATAATAATAATAATAATAATAATAATAATAATAATAATAATAATAATAA/AAATAATAATAATAATAATAATAATAATAATAATAATAATAA	AATTGGGGCATGTGCTATTG	TGCTGCTGCAATTTGATTTC	60.7	874	DRR	Ca_06325	--	--
Ca6_P3_17076597	Ca6	17076597	GTTTTTTTTTT/GTTTTTTTTT	TCCAAAGTAATTGGACCCCA	TCGCATATCACAAAACAGCA	60.2	641	INTERGENIC	--	--	--
Ca6_P3_17082875	Ca6	17082875	A/ACCTTAT	GGACACAATTGCAACAGCAT	TGATTCGCGAAAATAGTCCC	59.6	817	INTERGENIC	--	--	--
Ca6_P3_17083127	Ca6	17083127	AGA/AGAATTTTGA	TTAGCTGAATCACGAACCCC	TGATTCGCGAAAATAGTCCC	60.1	271	INTERGENIC	--	--	--
Ca6_P3_17085352	Ca6	17085352	TTTTATTTATTT/TTTTATTTATTTATTT	CGCAAAAATGCAAATGACTG	TTCCCCATGCATCACTATCA	60.3	385	INTERGENIC	--	--	--
Ca6_P3_17087281	Ca6	17087281	TG/T	GTATGGGGAATGTGGCTTGT	CGGATCTTAAAAAGTGGACCC	59.7	715	INTERGENIC	--	--	--
Ca6_P3_17088760	Ca6	17088760	ATT/ATTT	TTTCGTGGCTTTCATTGTTT	CGATTCAATTTGTTGTCCCA	58.3	783	INTERGENIC	--	--	--
Ca6_P3_17092081	Ca6	17092081	A/AG	TGCACCCCTAATTTTAAGCAA	GGATTTTGGGGAGCTTGAA	59.6	468	DRR	Ca_06327	C	--
Ca6_P3_17094283	Ca6	17094283	GAAAAAAAA/GAAAAAAA	ATTATCACTTGAGGCCGTGG	CATCTTTTGTTGGGATGGCT	60.0	414	INTRON	Ca_06327	C	--
Ca6_P3_17095470	Ca6	17095470	A/AT	TGCTTCTGCAGCTTCACCTA	TCTCCACTGATAACTGCCCC	59.9	341	INTRON	Ca_06327	C	--

INDEL marker IDs	Chromosomes/ unanchored scaffolds	Physical positions (bp)	InDels (*Kabuli* reference genome-CDC Frontier/PII)	Forward primers (5'-3')	Reverse primers (5'-3')	Anneling temperature (0C)	Expected amplified product size (bp)	Structural annotation		Functional annotation	
								Sequence components of *kabuli* genome	*Kabuli* gene accession IDs	NCBI-KOG	TFs
Ca6_P3_17096641	Ca6	17096641	TAAAAA/TAAAA	CAATTCAGCTGCTACCGACA	ATGTTGCTGGCTTACTCGCT	60.0	542	INTRON	Ca_06327	C	--
Ca6_P3_17100524	Ca6	17100524	A/AC	CTCGAAACCAGCAAACCATT	TTGAAAAAGAAAGGCACAACTTT	60.1	687	INTERGENIC	--	--	--
Ca6_P3_17107590	Ca6	17107590	TAAA/TAA	TGTTTGGGACCAAAGTGAAA	CACATACACCAAACCGATGAA	59.0	796	INTERGENIC	--	--	--
Ca6_P3_17115611	Ca6	17115611	CAAA/CAA	TGATCATTGTTCGGAGTTGC	TGCTTCTTTTCCTCCCTCAA	59.7	466	INTERGENIC	--	--	--
Ca6_P3_17119631	Ca6	17119631	CTTTTTTTTT/CTTTTTTTT	TGCTCAAAATGTTGGACAGG	GCGGCAAGTAGGACATTCAT	59.7	462	INTRON	Ca_06328	O	--
Ca6_P3_17125164	Ca6	17125164	A/AATGTTATT	CTTGAAGCTATGGCAGGAGG	GAAGAGGGGTTGCTCTGTCA	60.0	344	DRR	Ca_06329	--	--
Ca6_P3_17128637	Ca6	17128637	CAAA/CAAAA	CGGATCTAAGCTATCCGTCG	CATTTTCACGTGACGTTTCC	59.8	487	INTERGENIC	--	--	--
Ca6_P3_17138667	Ca6	17138667	TAAAAAAA/TAAAAAAAAAA	GGGGTGGGAAACTATGTTCA	AATTAGGGGGATCAGTTCCG	59.6	496	INTERGENIC	--	--	--
Ca6_P3_17139073	Ca6	17139073	GTTTT/GTTTTT	ACGGAACTGATCCCCCTAAT	TTTGTGCCAAAACCTTTCGT	59.7	539	INTERGENIC	--	--	--
Ca6_P3_17234998	Ca6	17234998	TTATATATATATATATATATATATATATATAT/TTATATATATATATATATATATAT	AAGGGGATCCGTGGAAATAC	TTCAGCCTCTCCAAACTCGT	60.0	495	INTERGENIC	--	--	--

**INDEL marker IDs**	**Chromosomes/ unanchored scaffolds**	**Physical positions (bp)**	**InDels (*Kabuli* reference genome-CDC Frontier/PII)**	**Forward primers (5'-3')**	**Reverse primers (5'-3')**	**Anneling temperature (0C)**	**Expected amplified product size (bp)**	**Structural annotation**		**Functional annotation**	
								**Sequence components of *kabuli* genome**	***Kabuli* gene accession IDs**	**NCBI-KOG**	**TFs**
Ca6_P3_17289693	Ca6	17289693	AATATATATATATATATATATATATATAT /AATATATATATATATATATATATATATA TATATATATATAT	GAAGGCCTCCATTTGT GTGT	TTGAAATGGCCCAAAG ATTC	60.0	604	INTERGENIC	--	--	--
Ca6_P3_17391778	Ca6	17391778	TAAAAAAAAAAA/TAAAAAAAAAA	CGACAAGGGTGCCATA AACT	TGTCTAAGGGGAATTG CAGC	60.0	724	INTERGENIC	--	--	--
Ca6_P3_17478296	Ca6	17478296	TTCTCTCTCTCTCTCTCTCTCTCTCTCT CTCTCTCTCTCTCTCTCTCTC/TTCTCT CTCTCTCTCTCTCTCTCTCTCTCTCTC TCTCTCTCTCTC	CAGATGCTTCGTGAGT GGAA	ACACATTGCACTTTTTG GCA	60.0	880	INTRON	Ca_06361	--	VOZ
Ca6_P3_17488371	Ca6	17488371	A/AT	CCAAGGCTCCAATATTC CAA	TCATCCTTTTCCCGTG ATTC	59.9	779	INTERGENIC	--	--	--
Ca6_P3_17503155	Ca6	17503155	GATATATATATATAT/GATATATATATAT ATAT	ATTCCCATTAAGTGCCC ACA	TCCAAATGGCGACTTC CTAC	60.2	556	INTERGENIC	--	--	--
Ca6_P3_17626184	Ca6	17626184	ATTTTTTTTT/ATTTTTTTT	CGTTTCATGTTTCATCC GTG	CCACACAACGTTTTCG AACTT	60.0	677	INTERGENIC	--	--	--
Ca6_P3_17784290	Ca6	17784290	ATTTTTTT/ATTTTTT	TCATTTTGGGCTATGAA GGC	TGGAGGGAGGTTAAG GTGTG	60.0	393	INTERGENIC	--	--	--
Ca6_P3_17827994	Ca6	17827994	TATAATAATAATAATAATAAT/TATAATA ATAATAATAATAATAAT	AGGATCGCGGGTAGTA GGAT	TTTTCCCCGGCAACAT ATTA	59.9	568	INTRON	Ca_06399	S	--
Ca6_P3_17850166	Ca6	17850166	ATTTTTTTTTTT/ATTTTTTTTTTTT	AAAAGAAAGCCCCCAA CACT	CTGCCTCATCAACTCC CAAT	60.0	837	INTERGENIC	--	--	--
Ca6_P3_17930276	Ca6	17930276	ATTTTTTT/ATTTTTTTT	TCCTCCCTTAATTCATT CGTG	TCAGTTGGTGACCCAT GAAC	59.0	391	INTERGENIC	--	--	--

INDEL marker IDs	Chromosomes/ unanchored scaffolds	Physical positions (bp)	InDels (*Kabuli* reference genome-CDC Frontier/PII)	Forward primers (5'-3')	Reverse primers (5'-3')	Anneling temperature (0C)	Expected amplified product size (bp)	Structural annotation		Functional annotation	
								Sequence components of *kabuli* genome	*Kabuli* gene accession IDs	NCBI-KOG	TFs
Ca6_P3_17989965	Ca6	17989965	AAATGAATGAATGAATGAATGAATGAATGAATGAATGAA/AAATGAATGAATGAATGAATGAATGAATGAATGAATGAATGAA	AAGAAGCGTGCCTACAGCAT	TCAGCGAGGATGAGGTTTTT	60.0	137	INTRON	Ca_06421	T	YABBY
Ca6_P3_1801654	Ca6	1801654	TAT/TATTGTACAT	ACGGTGAACTGGTGGACTTC	TCACACATTCGCTCAAATCC	60.0	482	INTERGENIC	--	--	--
Ca6_P3_1802085	Ca6	1802085	TAAA/TAAAA	GGATTTGAGCGAATGTGTGA	TCAAAATTATCGGAGATTTGCAT	59.7	230	INTERGENIC	--	--	--
Ca6_P3_1803443	Ca6	1803443	GAAAAAAAAAA/GAAAAAAAAA	AACTGCCAGAGAGTCAGGGA	ATGGAACCAGAATTGCTTGC	60.0	876	INTERGENIC	--	--	--
Ca6_P3_1803749	Ca6	1803749	GTAAAAAAAAA/GTAAAAAAAAAAATAAAAAAAAA	GCATTTAAAAATAGAAATATCTGGTCA	AAATGGGTACGCAGAAAACG	58.3	529	INTERGENIC	--	--	--
Ca6_P3_1813601	Ca6	1813601	TTTTTATTTTAT/TTTTTAT	GGATCACATGAAACAACTGTGC	TGCAAACTTCACCTTAGCTTGA	60.4	229	INTERGENIC	--	--	--
Ca6_P3_1813683	Ca6	1813683	AA/AACA	GGATCACATGAAACAACTGTGC	TGCAAACTTCACCTTAGCTTGA	60.4	229	INTERGENIC	--	--	--
Ca6_P3_1813721	Ca6	1813721	GCCAATAAAAT/GCCAATAAAATCCAATAAAAT	GGATCACATGAAACAACTGTGC	GCAAAACATTGTACCAAGACGA	60.4	682	INTERGENIC	--	--	--
Ca6_P3_1815783	Ca6	1815783	A/AC	CGAATCATTCCAAACCGTCT	GAGGATTTGGGTTGGGATCT	59.9	129	DRR	Ca_10308	TZ	--
Ca6_P3_18181029	Ca6	18181029	ATTTTTTTTTT/ATTTTTTTTTTT	GAGGTCGATTGGTCATTATGG	TCATCATTCCACCACAGCAT	59.3	573	INTERGENIC	--	--	--

INDEL marker IDs	Chromosomes/ unanchored scaffolds	Physical positions (bp)	InDels (*Kabuli* reference genome-CDC Frontier/PII)	Forward primers (5'-3')	Reverse primers (5'-3')	Anneling temperature (0C)	Expected amplified product size (bp)	Structural annotation		Functional annotation	
								Sequence components of *kabuli* genome	*Kabuli* gene accession IDs	NCBI-KOG	TFs
Ca6_P3_18216735	Ca6	18216735	TTACTTAAGTTG/TTACTTAAGTTGATACTTAAGTTG	TGTTCAACCGCATTAATTTGA	ATAGCAACTTCCGAGATGCG	59.1	517	INTERGENIC	--	--	--
Ca6_P3_1824130	Ca6	1824130	GT/GTT	TTGCAGAATGCATGGGAATA	TCCAAACTCTCCGACCAAAC	60.0	779	INTERGENIC	--	--	--
Ca6_P3_1824803	Ca6	1824803	TCC/TC	GTTTGGTCGGAGAGTTTGGA	CCCTCCTTATCCTTACGGTTG	60.1	470	INTERGENIC	--	--	--
Ca6_P3_1824964	Ca6	1824964	ATTT/ATTTT	GTTTGGTCGGAGAGTTTGGA	TTTGTGCATGACGCAAGACT	60.1	786	INTERGENIC	--	--	--
Ca6_P3_1825208	Ca6	1825208	A/AGATATTG	CAACCGTAAGGATAAGGAGGG	TTTGTGCATGACGCAAGACT	59.8	337	INTERGENIC	--	--	--
Ca6_P3_18259252	Ca6	18259252	GAAAAAAA/GAAAAAA	TTCAAGGATGGGAAATGCTC	ACAGCCATCCATGCTCGTAT	60.0	980	DRR	Ca_06443	T	HSF
Ca6_P3_1825931	Ca6	1825931	C/CT	TCAAATCACATCACCGTTTG	AGCTCCCAATTTTGCACTTG	57.9	508	INTERGENIC	--	--	--
Ca6_P3_18298213	Ca6	18298213	TAAAAAAAAA/TAAAAAAAAAA	TGTGTAAGATCCGCCACTTG	GCAAAGTGTGGCTTTGTTCA	59.7	717	DRR	Ca_06446	--	ERF
Ca6_P3_1831021	Ca6	1831021	CAA/CA	GTGTACCTGTTGGGGCAACT	TTGGACCTTGTTCTTTTGCC	59.9	279	DRR	Ca_10310	G	--
Ca6_P3_1831168	Ca6	1831168	AATATA/AATATATAAATAAAAGATATA	GGCAAAAGAACAAGGTCCAA	CCTGGATGAACCAAATACACAA	60.1	354	DRR	Ca_10310	G	--

INDEL marker IDs	Chromosomes/ unanchored scaffolds	Physical positions (bp)	InDels (*Kabuli* reference genome-CDC Frontier/PII)	Forward primers (5'-3')	Reverse primers (5'-3')	Anneling temperature (0C)	Expected amplified product size (bp)	Structural annotation		Functional annotation	
								Sequence components of *kabuli* genome	*Kabuli* gene accession IDs	NCBI-KOG	TFs
Ca6_P3_18331022	Ca6	18331022	G/GTTCCGGGTATC	TATATTGCTGCCACAATCGC	TTTCCTCCTTGGAATCATGG	59.7	402	INTRON	Ca_06449	QI	B3
Ca6_P3_1833737	Ca6	1833737	ACC/ACCC	AAGGTGGGTTCAGACCTTCT	ATTCCTCGGTTGACGAGATG	57.7	450	INTERGENIC	--	--	--
Ca6_P3_18382715	Ca6	18382715	TAAAAAAAAA/TAAAAAAA	GTGGCAACCTTCTCTTCGAC	GGCTACCTCGCGTGTAACAT	59.9	664	DRR	Ca_06453	--	--
Ca6_P3_18426160	Ca6	18426160	A/ATATATATATATATAT	TCCACCTAATCATTCATAAATATCG	TAAATTGTTGTGGCATCGGA	58.4	319	INTERGENIC	--	--	--
Ca6_P3_1844963	Ca6	1844963	TAAAAA/TAAAAAA	TGATGGAAATGTCATATCACGG	CAGTGCGATGCACAACTTTT	60.6	426	INTERGENIC	--	--	--
Ca6_P3_1845280	Ca6	1845280	AA/AAGCA	AAAAGTTGTGCATCGCACTG	GGCAACCCTTTGTTGTTCAT	59.9	329	INTERGENIC	--	--	--
Ca6_P3_18457126	Ca6	18457126	TAAAAAAAA/TAAAAAAAAA	TTCAATCTCTCTCGTGTTGGAG	ACAAGTGTCGGGAACATTCA	59.5	861	DRR	Ca_06460	EI	--
Ca6_P3_18458309	Ca6	18458309	TA/TAATAATAGAA	CCAACGATAAAATTAATTGGGA	GACGGATGTGTGAAATCTTGAA	58.0	468	INTERGENIC	--	--	--
Ca6_P3_18463608	Ca6	18463608	GAAAAAAAAAA/GAAAAAAAAAAA	TTTCCCGACTTGGTCCTTTA	AAGAACGAATATCAAACTCCATCA	59.5	405	INTERGENIC	--	--	--
Ca6_P3_18464095	Ca6	18464095	ATTTTTTTT/ATTTTTTT	TGATGGAGTTTGATATTCGTTCTT	AGGGATAGTTTGGGAATGGG	59.1	765	INTERGENIC	--	--	--

INDEL marker IDs	Chromosomes/ unanchored scaffolds	Physical positions (bp)	InDels (*Kabuli* reference genome-CDC Frontier/PII)	Forward primers (5'-3')	Reverse primers (5'-3')	Anneling temperature (0C)	Expected amplified product size (bp)	Structural annotation		Functional annotation	
								Sequence components of *kabuli* genome	*Kabuli* gene accession IDs	NCBI-KOG	TFs
Ca6_P3_1847264	Ca6	1847264	CAGAGA/CAGA	GAAAGGCCACATGCAAGATT	GGACGTGATCTTTTGGCAAT	60.1	734	DRR	Ca_10312	R	--
Ca6_P3_18557871	Ca6	18557871	AAGAGAGAGAGAGAGAGAGAGAGAGAGAGAGAGAGAGAGAGAGAGA/AAGAGAGAGAGAGAGAGAGAGAGAGAGAGAGAGAGAGAGAGAGA	CGAAGAAAAACGCACAGTCA	GGGCACAACCACGATTCTTA	60.0	803	INTERGENIC	--	--	--
Ca6_P3_18601304	Ca6	18601304	G/GT	GATTCAGGAATCTGGGCTGA	AAAGGAAAACCAATGTTGCG	60.2	515	DRR	Ca_06471	--	--
Ca6_P3_18618271	Ca6	18618271	TTATTATATTAT/TTATTAT	GGAGAACGTTAAGGCAGCAG	TTCCGCACAAAAATGAAACA	60.0	720	INTERGENIC	--	--	--
Ca6_P3_1862123	Ca6	1862123	C/CAA	AAATCCATTTGGGACCGACT	GCTTGGGAGCTCAACCATTA	60.6	548	INTERGENIC	--	--	--
Ca6_P3_1863689	Ca6	1863689	ATTTTTTTTT/ATTTTTTT	TTTTTGGTTATTGATGTCCATTC	TTGTGTTGGTTGAGGGTTGA	57.9	800	INTERGENIC	--	--	--
Ca6_P3_1864547	Ca6	1864547	A/AG	TTCTTCCAATTTGTGGAGCC	AGATACCTCGTTGTCGTGCC	60.1	501	INTERGENIC	--	--	--
Ca6_P3_18649492	Ca6	18649492	AATTTATATTATTTATATTATTTATATTATTTATATTATTTATATTATTTATATTATTT/AATTTATATTATTTATATTATTTATATTATTTATATTATTTATATTATTTATATTATTT	TCACATCTCGACATCGTTTAATG	CGAAAGCTGACGTAAGCAAA	60.0	431	INTERGENIC	--	--	--
Ca6_P3_1865844	Ca6	1865844	AA/AATA	AAGAAAAGGCAGATGGCTCA	TGAAACGACCCCTTCAACTC	60.0	534	INTERGENIC	--	--	--
Ca6_P3_18675177	Ca6	18675177	TATATTAATATT/TATATTAATATTAATATT	GGGCAGTGGATTTTGTCTGT	CCGTATCATTACCCTCTGGG	60.0	689	INTERGENIC	--	--	--

INDEL marker IDs	Chromosomes/ unanchored scaffolds	Physical positions (bp)	InDels (*Kabuli* reference genome-CDC Frontier/PII)	Forward primers (5'-3')	Reverse primers (5'-3')	Anneling temperature (0C)	Expected amplified product size (bp)	Structural annotation		Functional annotation	
								Sequence components of *kabuli* genome	*Kabuli* gene accession IDs	NCBI-KOG	TFs
Ca6_P3_18780292	Ca6	18780292	TAAAAA/TAAAA	AAGGCACGTAACCAAATTGC	TGAATTAGGATGTGCTTCAAGG	60.0	771	INTERGENIC	--	--	--
Ca6_P3_18892944	Ca6	18892944	AATATATATATATATATA/AATATATATATATATA	TCATGTGCAGACAAAGTCACC	GCACCCTATACAATTGCACAAA	59.7	802	INTRON	Ca_06496	K	HD-ZIP
Ca6_P3_189837	Ca6	189837	AC/A	TTTGCGTGCAAAAACCTATC	TTCGTGGCACAATCAGTAGC	58.8	251	INTERGENIC	--	--	--
Ca6_P3_19093188	Ca6	19093188	ATATTATTATTATTATTATTATTATTATTATTATTATTATTATTATTATTATTATTATTAT/ATATTATTATTATTATTATTATTATTATTATTATTATTATTATTATTATTATTATTATTATTAT	CCAATGGGAAATTCCATGAT	TGTCTTGCCTCTCCCACTCT	59.4	596	INTERGENIC	--	--	--
Ca6_P3_19174154	Ca6	19174154	TTATATATATATATATATAT/TTATATATATATATATATATATATAT	GGTGAAATTCATCCGCATCT	ACATCCGACAAATCGGAAAA	59.9	321	INTERGENIC	--	--	--
Ca6_P3_19189189	Ca6	19189189	CG/C	TGAAGCACCGAAACAGACAG	TACCTGTGCCTTCCAAAAGC	60.0	452	INTRON	Ca_06527	G	--
Ca6_P3_19245503	Ca6	19245503	ATAGAATTGTCTAGAATTGTCTA/ATAGAATTGTCTA	GCAGAATAGAGGCAGGATCG	CATTGCTGCAGGAGATGAAA	59.9	497	DRR	Ca_06530	--	--
Ca6_P3_19381311	Ca6	19381311	CAGAGAGAGAGAGAGAGAGAGAGAGAGAGAGAGAGAGAGA/CAGAGAGAGAGAGAGAGAGAGAGAGAGAGAGAGAGAGAGA	GTGAATGAGGAAAAGGGCAA	TATTGTTGGGGCAAGGAATC	60.1	169	INTERGENIC	--	--	--
Ca6_P3_1939700	Ca6	1939700	AATATATATATATATAT/AATATATATATATATAT	TGATCAATTTACCAACGATTACAGA	ATTTTACATGCCCAAAAGCG	59.8	718	INTERGENIC	--	--	--
Ca6_P3_19421271	Ca6	19421271	TTTTA/TTTTATTTA	CTCATGCTTGGGCATCACTA	TGATCAATGGAAGCCTGAAA	59.8	483	INTERGENIC	--	--	--

INDEL marker IDs	Chromosomes/ unanchored scaffolds	Physical positions (bp)	InDels (*Kabuli* reference genome-CDC Frontier/PII)	Forward primers (5'-3')	Reverse primers (5'-3')	Anneling temperature (0C)	Expected amplified product size (bp)	Structural annotation		Functional annotation	
								Sequence components of *kabuli* genome	*Kabuli* gene accession IDs	NCBI-KOG	TFs
Ca6_P3_1948984	Ca6	1948984	TTATATATATATATATATATATATATATA T/TTATATATATATATATATATATATATA TATATATATATATAT	GGTGCACTCATCCGCT AAAT	AGCATAATCGCACCGA AAAC	60.1	509	INTERGENIC	--	--	--
Ca6_P3_1951178	Ca6	1951178	TTAATAATAATAATAATAATAATAATAA TAATAATAATAA/TTAATAATAATAATAA TAATAATAATAATAATAATAA	TTATTTACCGGTGGCTT TGG	GGCAGCTTTTATCGTG CAAT	59.8	439	DRR	Ca_10320	--	--
Ca6_P3_19524549	Ca6	19524549	GTTTTTTTTTTT/GTTTTTTTTTTTT	AAACTCGAAAAGCATG GTGG	GCCCAAAATTGCCTCT ATGA	60.1	623	DRR	Ca_06559	--	--
Ca6_P3_19678237	Ca6	19678237	TTATATATATATATATATATAT/TTATAT ATATATATATATATATAT	CGATTATAAATGGGATT GCCC	TTGTGATCTTGACGGT AAAACTG	60.4	649	INTERGENIC	--	--	--
Ca6_P3_1975397	Ca6	1975397	A/AAAGG	CACAAAGGCCTGATCC ATCT	GCTTTTGCGGCATCTA GTTC	60.1	653	INTERGENIC	--	--	--
Ca6_P3_1975492	Ca6	1975492	GATCCGCTG/GATCCGCTGCTACATCC GCTG	GTAGTTTGGCTTCCCTT CCC	GCTTTTGCGGCATCTA GTTC	59.9	458	INTERGENIC	--	--	--
Ca6_P3_1976018	Ca6	1976018	AAT/AATAGAT	GAACTAGATGCCGCAA AAGC	GTACGTGGTCCTTCCC TGAA	60.0	560	DRR	Ca_10322	--	--
Ca6_P3_1976736	Ca6	1976736	TC/TCCC	TTATTCTCCATTGGGCA TCC	TCCTGCGGGTAATGAT AAGC	59.7	963	DRR	Ca_10322	--	--
Ca6_P3_1976756	Ca6	1976756	AACATC/AACATCTACATC	TCACGCATCAAAGTGA AACC	TCCTGCGGGTAATGAT AAGC	59.7	843	DRR	Ca_10322	--	--
Ca6_P3_1977351	Ca6	1977351	TTT/TTTTTT	GCTTATCATTACCCGCA GGA	TAGCATAGTCCAAGCG GACC	60.1	465	DRR	Ca_10322	--	--

INDEL marker IDs	Chromosomes/ unanchored scaffolds	Physical positions (bp)	InDels (*Kabuli* reference genome-CDC Frontier/PII)	Forward primers (5'-3')	Reverse primers (5'-3')	Anneling temperature (0C)	Expected amplified product size (bp)	Structural annotation		Functional annotation	
								Sequence components of *kabuli* genome	*Kabuli* gene accession IDs	NCBI-KOG	TFs
Ca6_P3_1977557	Ca6	1977557	ATT/ATCCCTCATTGTTT	GCTTATCATTACCCGCAGGA	TCTGGAACCGGAGCAATAAC	60.1	761	DRR	Ca_10322	--	--
Ca6_P3_1978360	Ca6	1978360	A/ATCG	CGTGGAGCATTCCGAGTATT	GGAGTTAGGGCGTGTGATGT	60.1	575	INTRON	Ca_10322	--	--
Ca6_P3_1978522	Ca6	1978522	C/CCTGAGG	ACATCACACGCCCTAACTCC	CTTAGCCTTGCAAATGAGGC	60.0	197	INTRON	Ca_10322	--	--
Ca6_P3_20515518	Ca6	20515518	GAAAA/GAAAAA	AACCAAGAGCAGCCACAGTT	GGGCCACTCACTGGAAGTAG	59.9	572	DRR	Ca_16380	B	--
Ca6_P3_20531602	Ca6	20531602	CTTATTATTATTATTATTATTATTATTATTA/CTTATTATTATTATTATTATTATTATTATTATTATTA	AAAGGACCAAGTCGGAAAAA	GTGGGGGCAGACTGATTAAA	58.7	347	INTERGENIC	--	--	--
Ca6_P3_20533537	Ca6	20533537	TTAATAATAATAATAATAATAATAATAATAATAATAATAATAATAATAATAATAATAATAATA/TTAATAATAATAATAATAATAATAATAATAATAATAATAATAATAATAATAATAATA	TTTTCTCTCCGTTGACCATTC	ATTTTGCGGTTCATGAAGGA	59.2	828	INTERGENIC	--	--	--
Ca6_P3_20626328	Ca6	20626328	TAAAAAAA/TAAAAAAAA	AGACAAACGACCTTTCATTCG	CAATTTTGACATGAGCATTTGG	59.2	393	INTERGENIC	--	--	--
Ca6_P3_2063027	Ca6	2063027	GGAAAGAAAGAAAGAAAGAAAGAAAGAAA/GGAAAGAAAGAAAGAAAGAAAGAAAGAAA	AGGCAAAGAAAGGAGCAACA	GCATGACACGACAATGGAAG	60.0	807	INTRON	Ca_10326	A	--
Ca6_P3_2063379	Ca6	2063379	CAA/CAACCTGTATTAA	TGTTCCTCTTGTGGAGAGGG	TGACACGGCGTAAGTACTCG	60.2	909	INTERGENIC	--	--	--
Ca6_P3_20679328	Ca6	20679328	CATATATATATATAT/CATATATATATATATAT	CAAATTTGTCACGGAAAGCA	CAATACTTGTGTTGTGGCGG	59.7	605	INTERGENIC	--	--	--

INDEL marker IDs	Chromosomes/ unanchored scaffolds	Physical positions (bp)	InDels (*Kabuli* reference genome-CDC Frontier/PII)	Forward primers (5'-3')	Reverse primers (5'-3')	Anneling temperature (0C)	Expected amplified product size (bp)	Structural annotation		Functional annotation	
								Sequence components of *kabuli* genome	*Kabuli* gene accession IDs	NCBI-KOG	TFs
Ca6_P3_20799573	Ca6	20799573	CAAAA/CAAAAAA	AATGATGGGATGGGTTACGA	ATAAATCGCATGCCTTCGTC	60.0	601	INTERGENIC	--	--	--
Ca6_P3_20910168	Ca6	20910168	TATAATAATAATAATAATAATAATAATAATAA/TATAATAATAATAATAATAATAATAATAATAATAATAATAA	TTGCCAAACTGAAATGCAAG	GATAGCAGCCAAGCTGGAAG	59.8	817	INTERGENIC	--	--	--
Ca6_P3_20930771	Ca6	20930771	ATTT/ATTTT	TGCATTCGTAATTTGCAGGA	CCAACTTACACGCCACATTG	60.2	629	INTERGENIC	--	--	--
Ca6_P3_21021586	Ca6	21021586	CTATTTATTTATTTATTTATTTATTT/CTATTTATTTATTTATTTATTT	CACCGTCGTGTTCATCTGTC	TTATTTCACCGTCGTGTCCA	60.2	458	INTERGENIC	--	--	--
Ca6_P3_21052205	Ca6	21052205	TAAAAAAAAAA/TAAAAAAAAAAA	TGCCGTTAATTGAAACTCTCC	TGTACCAAGCGACACCTCTG	59.2	604	INTERGENIC	--	--	--
Ca6_P3_21057110	Ca6	21057110	CTTTTTTT/CTTTTTTTT	TTCACTCGATGATTGCCGTA	CGCAAATGGATTAATTGACAAA	60.2	535	DRR	Ca_16328	O	--
Ca6_P3_2109965	Ca6	2109965	AATTATTATTATTATTATTATTATTATTATTATTATTATTATTATTATTATTATTATTATT/AATTATTATTATTATTATTATTATTATTATTATTATTATTATTATTATTATTATTATTATTATTATTATTATT	CGCCTCAATGACATCACAAC	AGTCTCTCAGCCATGGACTACTT	60.1	806	INTERGENIC	--	--	--
Ca6_P3_21100809	Ca6	21100809	ATTTTTTTTTTTT/ATTTTTTTTTTTTT	TGAGGGATTCCTCGAAGTTG	TGTGATTTGTTGTGGGAAGAA	60.2	490	INTERGENIC	--	--	--
Ca6_P3_21110757	Ca6	21110757	TAA/TAAA	GGTTGCTTGTGAGCTTCCTC	TTGGTGCGTGTTGTGTTTTT	60.0	497	DRR	Ca_16322	G	--
Ca6_P3_21139884	Ca6	21139884	ATTTTTTTT/ATTTTTTTTT	CAGAAGGATTGCTCGTGTGA	TGCATAGGCAAAAATGATTGA	60.0	528	INTERGENIC	--	--	--

INDEL marker IDs	Chromosomes/ unanchored scaffolds	Physical positions (bp)	InDels (*Kabuli* reference genome-CDC Frontier/PII)	Forward primers (5'-3')	Reverse primers (5'-3')	Anneling temperature (0C)	Expected amplified product size (bp)	Structural annotation		Functional annotation	
								Sequence components of *kabuli* genome	*Kabuli* gene accession IDs	NCBI-KOG	TFs
Ca6_P3_21141563	Ca6	21141563	GTTTTTT/GTTTTT	TCCGATAGGAAGAGCCAATG	ACGTTTATTTTTCGGACCCC	60.2	697	INTERGENIC	--	--	--
Ca6_P3_21146689	Ca6	21146689	AT/A	CACACCTTTTGGATGCACAC	TGGTCATTTTCGTCATCCCT	60.0	379	INTERGENIC	--	--	--
Ca6_P3_21151309	Ca6	21151309	A/AT	TGCGTTGATCGACATCAAAT	AACGCATCGGTAACCAACTC	60.1	163	INTERGENIC	--	--	--
Ca6_P3_21196310	Ca6	21196310	TTTATTATTATTATTATTATTATTATTATTATTATTATTATT/TTTATTATTATTATTATTATTATTATTATTATTATTATTATTATTATT	GGGATGTCATTCAATCCACC	TCGGGTCGTGACACTTACAA	60.0	778	INTERGENIC	--	--	--
Ca6_P3_21199074	Ca6	21199074	CTATATATATATATATA/CTATATATATATATATATA	AAAATGGAGGAGGGGAGTGT	GAGTCAGTGGGGGAAATTGA	59.8	547	INTERGENIC	--	--	--
Ca6_P3_21204480	Ca6	21204480	GTATATATATATATATATATATATATATGTATATATATAT/GTATATATATATATATATATATATATATATATATATGTATATATATAT	CTGGGGACTTCGTGATTGAT	TGACAGCAGAAATGATTCGG	59.9	616	INTRON	Ca_19883	S	--
Ca6_P3_2128046	Ca6	2128046	CTTTTTTTTT/CTTTTTTTT	AATTTGCTTTGATTTTGCCG	GTGCTCGTGCGACTTGTAAA	60.1	562	INTERGENIC	--	--	--
Ca6_P3_21401312	Ca6	21401312	C/CT	AGCTCAGTCCTCACAACCGT	TTAGATCGGACAAGAAACCGA	59.9	344	INTERGENIC	--	--	--
Ca6_P3_21408015	Ca6	21408015	ATTTTTTTT/ATTTTTTT	AGGCCTTTTATTTCGTTGCC	TTGAATCGCAAAACAACCAA	60.4	897	DRR	Ca_19860	--	--
Ca6_P3_2145461	Ca6	2145461	G/GT	TCATGGATTGAAAAATGTTAAGGA	TGCTCAATTGATGCTAGGTGA	59.7	506	INTERGENIC	--	--	--

INDEL marker IDs	Chromosomes/ unanchored scaffolds	Physical positions (bp)	InDels (*Kabuli* reference genome-CDC Frontier/PII)	Forward primers (5'-3')	Reverse primers (5'-3')	Anneling temperature (0C)	Expected amplified product size (bp)	Structural annotation		Functional annotation	
								Sequence components of *kabuli* genome	*Kabuli* gene accession IDs	NCBI-KOG	TFs
Ca6_P3_21461728	Ca6	21461728	CAAAAAAAAAAAAAAA/CAAAAAAAAAAAAAAAAA	AAATGTGGACGATGTCGGTT	AATTCATTCGCATACGCCAT	60.2	662	INTERGENIC	--	--	--
Ca6_P3_21470474	Ca6	21470474	CAAAAAAAAAA/CAAAAAAAAAAA	TTTCACATATCCTATCAAACACATTTT	TTCGTTGCTCCAAAGACTCC	59.2	367	INTERGENIC	--	--	--
Ca6_P3_21485595	Ca6	21485595	GTTTTTTT/GTTTTTTTT	TGCGGGCTAGCTCACTTAAT	TCAAAACAAATAGGCAGGGG	60.0	115	INTERGENIC	--	--	--
Ca6_P3_21485767	Ca6	21485767	ACCCC/ACCCCC	TGCGGGCTAGCTCACTTAAT	TTTGATTCTCTGGCCGAGTT	60.0	263	INTERGENIC	--	--	--
Ca6_P3_21496505	Ca6	21496505	CATATATATATATATATATA/CATATATATATATATATATATA	TAAACTGTGCCGTACGATGC	TCGCCCAAGACAAAATCTTC	59.8	413	INTRON	Ca_11292	--	bZIP
Ca6_P3_21512779	Ca6	21512779	TA/TAA	GTGGTTTGAGAGCGGATGAT	CACCACTATTTTGCGGGTCT	60.1	230	INTERGENIC	--	--	--
Ca6_P3_21671378	Ca6	21671378	ATTTTTTTTT/ATTTTTTTTTT	ACCGTTGAAATTCACTTCGG	AGGTGGCGCAAATATGAAAC	60.0	174	INTERGENIC	--	--	--
Ca6_P3_21710318	Ca6	21710318	TTTTATTTATTT/TTTTATTTATTTATTT	TCCTAATGAAGTCGTGGCCT	TGACAAGATAATGGGTGGCA	59.7	743	INTERGENIC	--	--	--
Ca6_P3_21739441	Ca6	21739441	AATATATATATATATATATATATATATATATATATATAT/AATATATATATATATATATATATATATATATATATATATATATATATATAT	TGAAGAACCATGGTCATATTAGACA	AAACAAGGCTTCAACCCTGA	59.8	569	INTERGENIC	--	--	--
Ca6_P3_21773137	Ca6	21773137	ATTTTTTTTTTT/ATTTTTTTTTTTT	TGATGCAGAAGAATTTGTGGA	TTTCGACTCCTTTGAGCGAT	59.3	553	INTERGENIC	--	--	--

INDEL marker IDs	Chromosomes/ unanchored scaffolds	Physical positions (bp)	InDels (*Kabuli* reference genome-CDC Frontier/PII)	Forward primers (5'-3')	Reverse primers (5'-3')	Anneling temperature (0C)	Expected amplified product size (bp)	Structural annotation		Functional annotation	
								Sequence components of *kabuli* genome	*Kabuli* gene accession IDs	NCBI-KOG	TFs
Ca6_P3_21777762	Ca6	21777762	TCCTCT/TCCTCTTATAAGCCCCCTCT	GATTGTCAGCCCAAAAGAGC	TGAGGAACGACAGATCGAAA	59.8	753	DRR	Ca_11261	--	ERF
Ca6_P3_21780665	Ca6	21780665	TA/T	TTGGCGAAGGAGTTAACTTGA	AAAACAAATGACCAAATCGAAAA	59.9	246	INTERGENIC	--	--	--
Ca6_P3_21782757	Ca6	21782757	TAAA/TAAAA	TAATGAGTTGGGACGGGAGA	ATGCAAACGCTACCCAATTC	60.5	782	INTERGENIC	--	--	--
Ca6_P3_21790392	Ca6	21790392	GGAA/GGAAGAA	TGGAAAGAAAACGGATGAGG	TTTTAGGTAACGTTTTGGTCCTTT	60.0	138	INTERGENIC	--	--	--
Ca6_P3_21794552	Ca6	21794552	CGGG/CGG	AAGGGTTTGGAATGCTGATG	GGTGAGGGTAGTGGACCAAA	59.9	528	INTERGENIC	--	--	--
Ca6_P3_21794670	Ca6	21794670	TAAAAAAAA/TAAAAAAAAA	AAGGGTTTGGAATGCTGATG	GGTGAGGGTAGTGGACCAAA	59.9	528	INTERGENIC	--	--	--
Ca6_P3_21794740	Ca6	21794740	ATTTTTT/ATTTTTTT	AAGGGTTTGGAATGCTGATG	GGTGAGGGTAGTGGACCAAA	59.9	528	INTERGENIC	--	--	--
Ca6_P3_21797613	Ca6	21797613	GTTTTTTTTTT/GTTTTTTTT	TTTATACACTGTCATGTAATACGTCCT	AAAGATGACACAAATCGGGG	57.4	476	INTERGENIC	--	--	--
Ca6_P3_21801686	Ca6	21801686	GA/GAA	TCTTGAACCGATACCCTTTTT	GACGGATGAGCATCGGTTAT	57.7	311	INTERGENIC	--	--	--
Ca6_P3_21805198	Ca6	21805198	ACTCTCTCTCTCT/ACTCTCTCTCT	TGGACCCATGCACAAAGTTA	CAGTCGTTCAGCATGCAACT	60.0	682	INTERGENIC	--	--	--

INDEL marker IDs	Chromosomes/ unanchored scaffolds	Physical positions (bp)	InDels (*Kabuli* reference genome-CDC Frontier/PII)	Forward primers (5'-3')	Reverse primers (5'-3')	Anneling temperature (0C)	Expected amplified product size (bp)	Structural annotation		Functional annotation	
								Sequence components of *kabuli* genome	*Kabuli* gene accession IDs	NCBI-KOG	TFs
Ca6_P3_21810623	Ca6	21810623	TTAATAATAATAATAATAATAATAATAATAATAATAATAATAATAATAA/TTAATAATAATAATAATAATAATAATAATAATAATAATAATAATAATAATAATAA	TCAAAAACATTGATTGAGCTGC	TTTGACGTAGAAAAAGTGATATTTGT	60.3	159	INTERGENIC	--	--	--
Ca6_P3_21881499	Ca6	21881499	ATTTTTTTTTTTT/ATTTTTTTTTTT	ATTTTGTCATTCACCCTGCC	TTCTCCGTCATTTCTCACCC	59.8	546	INTERGENIC	--	--	--
Ca6_P3_21891223	Ca6	21891223	TTCTC/TTC	GGGAAGACCTTGGATTCACA	CAATACCGTCCTGCACATTG	59.9	517	INTRON	Ca_11250	S	--
Ca6_P3_21919231	Ca6	21919231	TTA/TTAATA	CTTGCATGGGTACGTTAGCA	ACTTAGCACGGGCTCGATAC	59.8	370	DRR	Ca_11246	O	--
Ca6_P3_2206319	Ca6	2206319	ACCCC/ACCC	GCTTAACCCGTTTAAAGCCC	ATTTGGTCCCCCAACTTACC	60.0	345	DRR	Ca_10348	J	--
Ca6_P3_22099449	Ca6	22099449	ATTTTTTTTT/ATTTTTTTTTTTT	TGTGTTGCTTCTTGGCTTTG	ACGTGTGGGTTTCAGGTCTC	60.0	620	INTERGENIC	--	--	--
Ca6_P3_22124013	Ca6	22124013	TTCTCTCTCTCTCTCTCTCTCT/TTCTCTCTCTCTCTCTCTCT	ATTCCAACGATCCAAGATGG	TGCTCCAATGATGTTAGAAACG	59.8	692	INTERGENIC	--	--	--
Ca6_P3_22128416	Ca6	22128416	CTTTTTTTTTT/CTTTTTTTTT	CACACACACACAAAGAGGGG	ATAGGACCTGCTGCCTGAGA	60.0	557	INTERGENIC	--	--	--
Ca6_P3_22438706	Ca6	22438706	ATTTTTTTTTT/ATTTTTTTTT	ATCATAATATCGCGGGATCG	TTCATTCAACCGCATAGAAGTG	59.7	382	INTERGENIC	--	--	--
Ca6_P3_22520390	Ca6	22520390	A/AC	TTGAGGAAGAAAATTCCCCAT	GCGAGAAAGATAGATGAGGAGG	59.8	125	DRR	Ca_11188	I	--

INDEL marker IDs	Chromosomes/ unanchored scaffolds	Physical positions (bp)	InDels (*Kabuli* reference genome-CDC Frontier/PII)	Forward primers (5'-3')	Reverse primers (5'-3')	Anneling temperature (0C)	Expected amplified product size (bp)	Structural annotation		Functional annotation	
								Sequence components of *kabuli* genome	*Kabuli* gene accession IDs	NCBI-KOG	TFs
Ca6_P3_2266195	Ca6	2266195	GTTT/GT	AGTGGGTTTGCGTAACCTTG	GCTTTCTTCAATGCGTGACA	60.0	367	DRR	Ca_10355	R	MYB
Ca6_P3_2272367	Ca6	2272367	T/TA	TCATCGCAGGTGTACCATGT	TGGGAATTTGGGAATTTTGA	60.0	654	INTERGENIC	--	--	--
Ca6_P3_2283477	Ca6	2283477	TTATATATATATATATATATATATATA/TTATATATATATATATATATATATATA	CTTGAGAGTGTCAGCCACCA	TGAGTTTCCACCTCCTGGAT	60.0	968	DRR	Ca_10356	--	--
Ca6_P3_22962831	Ca6	22962831	AATATATATATATATAT/AATATATATATATATATAT	AAATCCTTAATGGGAGTGGGA	TTGAGGGAGGGCAGCTACTA	59.7	837	INTERGENIC	--	--	--
Ca6_P3_23042823	Ca6	23042823	CATATATATATATAT/CATATATATATAT	CAACGATCTGCACCACACAT	GGTGCAGCAAGTGAGCAATA	60.6	665	INTRON	Ca_11143	--	ERF
Ca6_P3_23042981	Ca6	23042981	TAAA/TAA	CAACGATCTGCACCACACAT	GGTGCAGCAAGTGAGCAATA	60.6	665	INTRON	Ca_11143	--	ERF
Ca6_P3_23044574	Ca6	23044574	CTTTTTTTTTTT/CTTTTTTTTTT	CCTAATGATTGTGCAGCTGTG	GCCATTAACGACGTCACTCAC	59.4	891	INTERGENIC	--	--	--
Ca6_P3_23064332	Ca6	23064332	CCATGTGTGTTATTC/CC	TGGGTTGATTTTCCCACATT	TGGAGTTATAGAGACCATTCCG	60.0	391	INTERGENIC	--	--	--
Ca6_P3_23064745	Ca6	23064745	CA/C	TGGGTTGATTTTCCCACATT	TCTCGAGCAAATCACAAGTTAAA	60.0	571	INTERGENIC	--	--	--
Ca6_P3_23070968	Ca6	23070968	ATTTTTTT/ATTTTTTTT	TCACACTTCTTCGGGATCAA	AGTGATAAAGATGCGGTGGG	59.2	475	INTERGENIC	--	--	--

**INDEL marker IDs**	**Chromosomes/ unanchored scaffolds**	**Physical positions (bp)**	**InDels (*Kabuli* reference genome-CDC Frontier/PII)**	**Forward primers (5'-3')**	**Reverse primers (5'-3')**	**Anneling temperature (0C)**	**Expected amplified product size (bp)**	**Structural annotation**		**Functional annotation**	
								**Sequence components of *kabuli* genome**	***Kabuli* gene accession IDs**	**NCBI-KOG**	**TFs**
Ca6_P3_23078754	Ca6	23078754	TGG/TGGG	TGGACAATCGCCCAAATTAT	TCTCGAGCAAATCACGAGTTA	60.2	716	INTERGENIC	--	--	--
Ca6_P3_23078938	Ca6	23078938	T/TC	TGGACAATCGCCCAAATTAT	TCTCGAGCAAATCACGAGTTA	60.2	716	INTERGENIC	--	--	--
Ca6_P3_23089725	Ca6	23089725	A/AC	CGTAGGAGGGATCCAACTCA	CTGGGGATAATTCCACAGGA	60.1	582	INTERGENIC	--	--	--
Ca6_P3_23090578	Ca6	23090578	AC/A	GCCAAGCTTGTTTGTTGGTT	TGTGTGCACATGTTGTTCGT	60.2	490	INTERGENIC	--	--	--
Ca6_P3_23197578	Ca6	23197578	TTATATATATATATATATATATATATATATATATATAT/TTATATATATATATATATATATATATATATATATATATATATATATATATATATAT	TGAATCAAGGATGCCACAAA	CATTTCCCGTTACACAAGGA	60.0	726	DRR	Ca_11132	R	--
Ca6_P3_23238479	Ca6	23238479	GTTTTTTT/GTTTTTT	TCAACGGATTTCCACCCTTA	TCTTGTCTCCACCCGTTAAAA	60.3	368	INTERGENIC	--	--	--
Ca6_P3_23273647	Ca6	23273647	CATATATATATATATATATATATATATATATATATA/CATATATATATATATATATATATATATATATATATATATATA	TGGGTGCTATTTGCAATCAA	GTTTGGCGAGGGCACTATAA	60.1	383	DRR	Ca_11129	--	Trihelix
Ca6_P3_23338210	Ca6	23338210	AA/ATAATATA	CGAGTCAAAATAAAGGGGTCA	TGTGCAATGTCCATTTTTGC	59.1	678	INTERGENIC	--	--	--
Ca6_P3_23343422	Ca6	23343422	CTTTTTTT/CTTTTTTTT	ATATGCTTCACTTGCCCCAC	CCGGTCTTTCGTTTACCACA	60.0	514	DRR	Ca_11125	E	--
Ca6_P3_233726	Ca6	233726	CTATTTATTTATTTATTTATTTATTTATTTATTTATTTATTTATTTATTTAT/CTATTTATTTATTTATTTATTTATTTATTTATTTATTTATTTATTTATTTATTTATTTATTTATTTAT	CGGATCCCACCACTTGATAA	TTGATCGTTGGGTTGATGAAT	60.7	640	INTERGENIC	--	--	--

INDEL marker IDs	Chromosomes/ unanchored scaffolds	Physical positions (bp)	InDels (*Kabuli* reference genome-CDC Frontier/PII)	Forward primers (5'-3')	Reverse primers (5'-3')	Anneling temperature (0C)	Expected amplified product size (bp)	Structural annotation		Functional annotation	
								Sequence components of *kabuli* genome	*Kabuli* gene accession IDs	NCBI-KOG	TFs
Ca6_P3_23409253	Ca6	23409253	CTTTTT/CTTTTTT	TGCCGTATAAAATGGGCTTC	CTTGCTCTTCCTTCGTTGCT	59.9	533	INTERGENIC	--	--	--
Ca6_P3_23409707	Ca6	23409707	TAAAAAAAAAAAAAA/TAAAAAAAAAAAA	AGCAACGAAGGAAGAGCAAG	CCGAAAGCCTTGTCAGAAAT	59.8	219	INTERGENIC	--	--	--
Ca6_P3_23439828	Ca6	23439828	GTATATATATATATATATATATATATATATATATA/GTATATATATATATATATATATATATATATATATATA	AAGGATGGAGGAGGCAAAAT	TTTTCATTAATGGCAAAAGCA	59.9	882	INTERGENIC	--	--	--
Ca6_P3_2345807	Ca6	2345807	ATT/AT	TTTTTGTGGGACGACATTTG	TGACGTCGTGGCAATTACAT	59.4	682	INTERGENIC	--	--	--
Ca6_P3_2353000	Ca6	2353000	CTATATATATATATATATA/CTATATATATATATATATA	CCGGAAATTATGGATTCTTTTT	TGTCGAACATGACCAATACGA	58.4	364	DRR	Ca_10366	R	--
Ca6_P3_2353259	Ca6	2353259	A/ACCGC	CAAACAGCGTTTATTCGTCG	TTGTCGTCCTTTTCAGCTTT	59.4	352	DRR	Ca_10366	R	--
Ca6_P3_23562113	Ca6	23562113	AATATATATATATATATATATATATATA/AATATATATATATATATATATATATATATATATA	TAAGGTGGGTCAGGAATGGA	AGCTATGGAGTGAGCGGAAA	60.3	351	INTERGENIC	--	--	--
Ca6_P3_23642077	Ca6	23642077	T/TTTCC	GATTACCATGGTTGGGATCG	CAATTTCGGTGCTTGTTCCT	60.0	137	INTRON	Ca_11091	R	--
Ca6_P3_23755333	Ca6	23755333	TAC/T	GGAGGAGCAGAAGCAACAAC	CTGTGGGATCAATTGGCTCT	60.0	256	INTERGENIC	--	--	--
Ca6_P3_23755434	Ca6	23755434	TG/T	GGAGGAGCAGAAGCAACAAC	CTGTGGGATCAATTGGCTCT	60.0	256	INTERGENIC	--	--	--

INDEL marker IDs	Chromosomes/ unanchored scaffolds	Physical positions (bp)	InDels (*Kabuli* reference genome-CDC Frontier/PII)	Forward primers (5'-3')	Reverse primers (5'-3')	Anneling temperature (0C)	Expected amplified product size (bp)	Structural annotation		Functional annotation	
								Sequence components of *kabuli* genome	*Kabuli* gene accession IDs	NCBI-KOG	TFs
Ca6_P3_23764400	Ca6	23764400	GATATATATATATATATATATATATATATATATATATATATAT/GATATATATATATATATATATATATATATATATATATAT	ACTCCAACGTGCGATAGCTT	AACGAAGTAATTCAGCGCAAA	59.9	730	DRR	Ca_11079	K	--
Ca6_P3_2384865	Ca6	2384865	TTAATAATAATAATAATAATAATAATAATAATAATAA/TTAATAATAATAATAATAATAATAATAATAATAATAA	AGATTGCCCCACTTCAAAAA	TAACCTGCCAAAGAGGAGGA	59.5	866	INTERGENIC	--	--	--
Ca6_P3_2402829	Ca6	2402829	A/ATTGAAATTC	TATTTGCTCTGTCAGCACGC	CCCCTTTCTGCTCTTCCTTT	60.2	954	CDS (large-effect mutations)	Ca_10371	J	--
Ca6_P3_2427053	Ca6	2427053	AATATATATATATATATATATATATATAT/AATATATATATATATATATATATAT	CCGACAATTCCATTGCTTTT	GGAAGGTGTTGAGGTGCATT	59.9	566	INTERGENIC	--	--	--
Ca6_P3_24296638	Ca6	24296638	CATATATATATATATATATATATA/CATATATATATATATATATATATATA	GAGAAGGAGGGAAAAATGGC	ATGTTTTGGATTTTGGCTCG	60.0	421	INTERGENIC	--	--	--
Ca6_P3_244129	Ca6	244129	TATATATAA/T	TGGCTTATGGTTATGGGTGG	CGCCATCATAAGTAAGGGTGA	60.6	448	INTERGENIC	--	--	--
Ca6_P3_2441510	Ca6	2441510	ATT/AT	CGAATGCCACATCATTTACG	GACCCGAAATATGGCTTGAA	60.0	425	INTERGENIC	--	--	--
Ca6_P3_24582806	Ca6	24582806	CATATATATATATATATATATATATATATAT/CATATATATATATATATATATATATATATATAT	TCGTTTTGTTTATTGGGGGA	GTTAAATGCGGGGTTTATCG	60.2	565	DRR	Ca_24169	--	bHLH
Ca6_P3_24637049	Ca6	24637049	TTTCTTCTTCTTCTTCTTCTTCTTCT/TTTCTTCTTCTTCTTCTTCTTCTTCT	TGTTTTTGGTGAAACGTGGA	AGGATGAATGTGGTGTTGAAGA	60.0	376	INTERGENIC	--	--	--
Ca6_P3_24744388	Ca6	24744388	TACACACACA/TACACA	TTTGGATTTTGACTCGGAGG	AATGGAGGAAGGAGGGAGAA	60.0	384	INTERGENIC	--	--	--

INDEL marker IDs	Chromosomes/ unanchored scaffolds	Physical positions (bp)	InDels (*Kabuli* reference genome-CDC Frontier/PII)	Forward primers (5'-3')	Reverse primers (5'-3')	Anneling temperature (0C)	Expected amplified product size (bp)	Structural annotation		Functional annotation	
								Sequence components of *kabuli* genome	*Kabuli* gene accession IDs	NCBI-KOG	TFs
Ca6_P3_24958780	Ca6	24958780	GACAACAAC/GACAACAACAAC	AGAACGGTTTTCTGACCGTG	AACGGTTTTAAAGCATTGCC	60.1	216	INTERGENIC	--	--	--
Ca6_P3_2501071	Ca6	2501071	TAACAACAACAACAACAACAA/TAACAACAACAACAACAA	CCCACCAACCCAAATTAAAA	TCACCATCGTGTGATGGACT	59.5	305	INTERGENIC	--	--	--
Ca6_P3_25130773	Ca6	25130773	AT/ACACCTT	TGCATGCCTAACTCCAAGTG	AAGCTCAACATGCAAAACCC	59.9	187	DRR	Ca_26068	--	--
Ca6_P3_25130968	Ca6	25130968	A/AATAAAGCCT	CCAAAAAGGGTGGAGATTGA	CCTTTTACAGCGCTTTTCCA	59.9	275	DRR	Ca_26068	--	--
Ca6_P3_25131365	Ca6	25131365	TTT/TTTTATT	GGGTTTTGCATGTTGAGCTT	TTGCATTTGGTTTGACTCCA	60.1	649	DRR	Ca_26068	--	--
Ca6_P3_2518811	Ca6	2518811	TA/TAA	AATCTGGCTCAAAATGCAGC	AATGCATTTGCCTGACTCAA	60.4	723	DRR	Ca_10384	--	--
Ca6_P3_2523387	Ca6	2523387	TAAAA/TAAAAAAA	TTGCATTTGTTTCAGCAAGG	TTGCCATCATCAATTCATCC	59.8	822	DRR	Ca_10385	--	--
Ca6_P3_2524006	Ca6	2524006	A/AT	GGATGAATTGATGATGGCAA	CCATCAAAACATCATTTGAACA	59.3	600	DRR	Ca_10385	--	--
Ca6_P3_2525217	Ca6	2525217	TT/TTGCAT	AGTAACCGACCAGCCAATCA	CAAATTTTGGTCACATTCCG	60.5	741	INTERGENIC	--	--	--
Ca6_P3_2525428	Ca6	2525428	TAAAAAAAAAA/TAAAAAAAAAAAA	CCTGAAATTTGATTCTGCGT	CAAATTTTGGTCACATTCCG	57.8	578	INTERGENIC	--	--	--

INDEL marker IDs	Chromosomes/ unanchored scaffolds	Physical positions (bp)	InDels (*Kabuli* reference genome-CDC Frontier/PII)	Forward primers (5'-3')	Reverse primers (5'-3')	Anneling temperature (0C)	Expected amplified product size (bp)	Structural annotation		Functional annotation	
								Sequence components of *kabuli* genome	*Kabuli* gene accession IDs	NCBI-KOG	TFs
Ca6_P3_2528953	Ca6	2528953	AATAT/AAT	TGGGTCAATGTTAAGAGGGC	CGTCAACGTCGTGCATTTAC	59.9	515	INTERGENIC	--	--	--
Ca6_P3_2529860	Ca6	2529860	CA/CATA	TGATGTGTTTGCTGTTCGGT	GCGTCAAAGACATTGATGTGA	60.2	878	INTERGENIC	--	--	--
Ca6_P3_2533591	Ca6	2533591	AT/ATT	CTCTTATGTCGGTCGCCAAT	AACTTTGTGGAAGCTGTGGG	60.1	533	INTERGENIC	--	--	--
Ca6_P3_2542709	Ca6	2542709	CAA/CAAAA	GCAAAACCCTTTCCTGTGAG	TGTGATCTGTGCATCCCAAT	59.7	241	DRR	Ca_10386	--	--
Ca6_P3_25469197	Ca6	25469197	TAAAAAAAAAA/TAAAAAAAAA	GCCCTCAGATGAGAGTCAGG	CAAGGCTGTTTCAGCATTCA	59.9	511	INTERGENIC	--	--	--
Ca6_P3_25616921	Ca6	25616921	AGG/AGGG	GGGTTCAAGGTTTTGGGTTT	TGGCGAACTACAATGGATAACA	60.1	403	INTERGENIC	--	--	--
Ca6_P3_25630460	Ca6	25630460	CATATATATATATATATATATAT/CATATATATATATATATATATAT	AGGAAGAGTGGAGTTGCGAA	TATTTTGGATTTTGGCTCGG	60.0	392	INTERGENIC	--	--	--
Ca6_P3_25638625	Ca6	25638625	GTT/GT	TTGTCCGTGAGAGACTGCAC	GTATTGCAACCAAACATGCG	60.0	335	INTERGENIC	--	--	--
Ca6_P3_25669283	Ca6	25669283	GTTAGGGTTTAGGGTTTAGGGTTTAGGGTTTA/GTTAGGGTTTAGGGTTTAGGGTTTAGGGTTTAGGGTTTA	TTTGGGTTTACGCATTAGGG	ATTGGCGAACTACAACGGAT	59.8	505	INTERGENIC	--	--	--
Ca6_P3_25692901	Ca6	25692901	CTATATATATATATATATA/CTATATATATATATATATATA	ATTCCAAACCAAAACCCTCC	AAATTGGTCCACTAGCACCG	60.0	883	INTERGENIC	--	--	--

INDEL marker IDs	Chromosomes/ unanchored scaffolds	Physical positions (bp)	InDels (*Kabuli* reference genome-CDC Frontier/PII)	Forward primers (5'-3')	Reverse primers (5'-3')	Anneling temperature (0C)	Expected amplified product size (bp)	Structural annotation		Functional annotation	
								Sequence components of *kabuli* genome	*Kabuli* gene accession IDs	NCBI-KOG	TFs
Ca6_P3_257739	Ca6	257739	CATATATATATATATATATATATATATA/CATATATATATATATATATATATATATA	CGACACATGTGCTATTTAGCCT	ATGGGTTGCAGTTAGCTTGG	59.3	799	INTERGENIC	--	--	--
Ca6_P3_26092614	Ca6	26092614	GATATATATATATATATATATATATAT/GATATATATATATATATATATATATATAT	ATGTTTTGGATTTTGGCTCG	AATTCCTTCCCACACAACCA	59.9	716	INTERGENIC	--	--	--
Ca6_P3_26170580	Ca6	26170580	ATTT/ATT	TTAAAATTCCCTCCTCGGCT	TACCTTGTCCCATTTGCCTC	60.0	701	INTERGENIC	--	--	--
Ca6_P3_26277672	Ca6	26277672	T/TG	TTAGGTGCGCTTGACTGATG	GTTTTGGCGTTGGTTTCACT	60.0	558	INTERGENIC	--	--	--
Ca6_P3_26277860	Ca6	26277860	CG/CGG	TTAGGTGCGCTTGACTGATG	ACGTAGCGAGCCCTAGATGA	60.0	788	INTERGENIC	--	--	--
Ca6_P3_26278491	Ca6	26278491	ATGT/ATGTTGT	AGTGAAACCAACGCCAAAAC	GGTAGCTCCCGACATTTTGA	60.0	811	INTERGENIC	--	--	--
Ca6_P3_26354164	Ca6	26354164	GT/GTT	TGTTGATTTGGGGAAAGAGG	GGTGCCAACATCCAACTTCT	59.9	650	DRR	Ca_16678	--	GRAS
Ca6_P3_2639909	Ca6	2639909	CTT/CT	AGCTGAAGCAATTTTCGCAT	GCAATGCAGCAGCAATAAAA	60.0	479	INTERGENIC	--	--	--
Ca6_P3_26437457	Ca6	26437457	AT/ATAATT	AGGCATCCCCTTATACACCC	GCTTAGGCAGCCACAAAATC	60.0	520	INTERGENIC	--	--	--
Ca6_P3_26438751	Ca6	26438751	ATTTTTTTTT/ATTTTTTTTTTT	TCAGACACTACCCAGCATCAA	CTTAGGCCGCCACAAAATA	59.3	960	INTERGENIC	--	--	--

INDEL marker IDs	Chromosomes/ unanchored scaffolds	Physical positions (bp)	InDels (*Kabuli* reference genome-CDC Frontier/PII)	Forward primers (5'-3')	Reverse primers (5'-3')	Anneling temperature (0C)	Expected amplified product size (bp)	Structural annotation		Functional annotation	
								Sequence components of *kabuli* genome	*Kabuli* gene accession IDs	NCBI-KOG	TFs
Ca6_P3_26461192	Ca6	26461192	CTTTTTTTTT/CTTTTTTTT	AGTCGGTCTTCAGAGCAAGG	GGTTAAAATGGTGTTTATCGGAA	59.6	740	INTERGENIC	--	--	--
Ca6_P3_26666718	Ca6	26666718	CAAAAAAAAAAAA/CAAAAAAAAAAA	CCCTAAACCATCAAACTGAACA	AATTCCATCAAGCCTCAACG	59.0	550	INTERGENIC	--	--	--
Ca6_P3_26667186	Ca6	26667186	AGATGATGATGATGATGATGATGATGA/AGATGATGATGATGATGATGATGATGATGATGA	CGTTGAGGCTTGATGGAATTA	GCCGATCAACACGAAGGTAT	60.1	380	INTERGENIC	--	--	--
Ca6_P3_26668470	Ca6	26668470	T/TCTATATTTTTTTA	CGACGCAATGCATTAAGAGA	CCTTAATCGCCGAAAATCAT	60.0	490	DRR	Ca_16695	--	--
Ca6_P3_26686327	Ca6	26686327	TTATATATATATATATAT/TTATATATATATATATATAT	CTAGTGCGAATGGCAGACAA	GATTATGGCCCAGCTTTTGA	60.0	358	INTERGENIC	--	--	--
Ca6_P3_2674985	Ca6	2674985	ATGTT/AT	GAAACTTCGTGCCTTTTTGC	ACGATAGTAGATGTGGCGGC	59.9	152	INTRON	Ca_10401	V	--
Ca6_P3_26782160	Ca6	26782160	TAAAAAAAAAAAA/TAAAAAAAAAAA	TGAGGATCAATTTTGTGAATCG	TTTTGAGAACGGAGCACACA	59.9	806	INTERGENIC	--	--	--
Ca6_P3_26790864	Ca6	26790864	GAAAAAAAA/GAAAAAAA	CCGAAAATTTGAAAATCCGA	CAAGTACAGGTGGAGTGGCA	59.9	728	INTERGENIC	--	--	--
Ca6_P3_26867680	Ca6	26867680	CC/CCCTAAATATAAC	TGCAGAATCGTTCAAACCAA	CAAGTGGCCTCGAACTCAAT	60.2	720	INTERGENIC	--	--	--
Ca6_P3_26931819	Ca6	26931819	TTATATATATATATATAT/TTATATATATATATATATATAT	GTACCACATTGCCGAGGTTT	GCTTGCCAAAATAAATGGCT	59.9	580	INTERGENIC	--	--	--

INDEL marker IDs	Chromosomes/ unanchored scaffolds	Physical positions (bp)	InDels (*Kabuli* reference genome-CDC Frontier/PII)	Forward primers (5'-3')	Reverse primers (5'-3')	Anneling temperature (0C)	Expected amplified product size (bp)	Structural annotation		Functional annotation	
								Sequence components of *kabuli* genome	*Kabuli* gene accession IDs	NCBI-KOG	TFs
Ca6_P3_2708428	Ca6	2708428	ATTTTTTTTTTT/ATTTTTTTTTT	TGCTTAAGCTAGGGGT GTGC	TGGTCCAAAAACATTT CCAA	60.4	818	DRR	Ca_10404	--	--
Ca6_P3_27108382	Ca6	27108382	AT/ATGT	GCTGCACATGTGGGTA CATC	ATTGTCCTTTGCCACC TACG	60.0	738	DRR	Ca_14631	--	Dof
Ca6_P3_2711937	Ca6	2711937	TACGTTGA/TA	ATCGGCGATTATTTACA GCG	AGTCTGGTGCCTCGTG ACTT	60.1	501	INTERGENIC	--	--	--
Ca6_P3_27165011	Ca6	27165011	ATATTATTATTATTATTATTATTATTATT /ATATTATTATTATTATTATTATTATT	CATTGTTTGATCCATGT CGC	CCATCATTGACACTCG AAAGAA	59.9	675	INTERGENIC	--	--	--
Ca6_P3_27264510	Ca6	27264510	ATTTTTTTTT/ATTTTTTTT	AACGTCCCATTAAGTTC GCA	TGACAAATTGCCTTTG CTTG	60.5	465	INTERGENIC	--	--	--
Ca6_P3_27289106	Ca6	27289106	GATATATAT/GATATATATAT	CAATTCCCACAGGTAC GCTT	AATTTCAAGGATTGGC GAAG	60.0	612	INTERGENIC	--	--	--
Ca6_P3_27315680	Ca6	27315680	GAGTTTTCAATTTTAGTTTTCAATTTTA/ GAGTTTTCAATTTTA	CATGCATCGGAAGTTG TGTT	TCACTGTCAGGCAGAT TTCG	59.6	496	INTERGENIC	--	--	--
Ca6_P3_27348230	Ca6	27348230	ATTTTTTTTTT/ATTTTTTTTTTT	TTGATCCCATATCCTCC CAA	GACACCCAAATCAAAC CCAC	60.1	595	INTERGENIC	--	--	--
Ca6_P3_2735214	Ca6	2735214	AATATATATATATATATATATATAT/AAT ATATATATATATATATATATAT	TCCTGAATTCGATTTGG GTT	TGGGTGGAAACAGTGA ATGA	59.4	645	INTERGENIC	--	--	--
Ca6_P3_27447919	Ca6	27447919	ATTTTTTTT/ATTTTTTTTT	CACCATGTGCCACTTC ATTC	TGGGCGGCTATGTTAA AACT	60.0	359	INTERGENIC	--	--	--

INDEL marker IDs	Chromosomes/ unanchored scaffolds	Physical positions (bp)	InDels (*Kabuli* reference genome-CDC Frontier/PII)	Forward primers (5'-3')	Reverse primers (5'-3')	Anneling temperature (0C)	Expected amplified product size (bp)	Structural annotation		Functional annotation	
								Sequence components of *kabuli* genome	*Kabuli* gene accession IDs	NCBI-KOG	TFs
Ca6_P3_27448301	Ca6	27448301	TTATATATATATATATATATATATATATATAT/TTATATATATATATATATATATATATATATATATAT	CACCATGTGCCACTTCATTC	CTAGTGCAAGGTCGCTCCTC	60.0	629	INTERGENIC	--	--	--
Ca6_P3_27517577	Ca6	27517577	CCCAGCCAGC/CCCAGC	TTTAAAACCCCTTTGGGAGG	GACTAAAGAGTGGGGGAGGC	60.2	418	INTERGENIC	--	--	--
Ca6_P3_27529474	Ca6	27529474	ATTTTTTTTTTT/ATTTTTTTTTTTT	TGGTGTATTGGAAACCGTGA	CGTCTTTTGGAATGACGACTT	59.8	520	DRR	Ca_14605	--	LBD
Ca6_P3_2763820	Ca6	2763820	AA/AATAATAATAATA	ATCGCGTTCAATGTGTTTGA	TCCTTAATTGAAGTTTATGAACCA	60.1	725	INTERGENIC	--	--	--
Ca6_P3_2766218	Ca6	2766218	TAGAGAGAGAGAGAGAGAGAGAGAGAGAGAGAGA/TAGAGAGAGAGAGAGAGAGAGAGAGAGAGAGAGA	TGCCAATTACCATGTGCTGT	AAATCTCACCAATTGCGACC	60.0	594	INTERGENIC	--	--	--
Ca6_P3_27691258	Ca6	27691258	ATTTTTTTTTTT/ATTTTTTTTTTTT	CACTCGGTTGAGACGTGAGA	AGGTCGGCGTCATAAACATC	60.0	290	INTERGENIC	--	--	--
Ca6_P3_2783350	Ca6	2783350	CTCACGTT/CT	GTTGATGGAATTAGCCCGAA	TCGCAACGTGCGAATTATTA	59.9	276	DRR	Ca_10412	KR	--
Ca6_P3_27961769	Ca6	27961769	AAGAAAAATTAATGGAGAAAAATTAATGGAGAAAAAT/AAGAAAAATTAATGGAGAAAAAT	CTTTCGATTGCACGGTTTTT	CCTACGACTTTCCACTTCGC	60.1	583	INTERGENIC	--	--	--
Ca6_P3_28098136	Ca6	28098136	ATTTTTTTTTTT/ATTTTTTTTTT	TTTGGATTGTGTCACTGTAGCA	TGCGTCTTACAAATTTCAATCA	59.2	318	INTERGENIC	--	--	--
Ca6_P3_28190835	Ca6	28190835	ATATTATTATTATTATT/ATATTATTATTATTATTATT	AGGTCTCATACCAGAGATGCG	AAGTTAAGGGACCGCAAATG	59.3	871	INTERGENIC	--	--	--

INDEL marker IDs	Chromosomes/ unanchored scaffolds	Physical positions (bp)	InDels (*Kabuli* reference genome-CDC Frontier/PII)	Forward primers (5'-3')	Reverse primers (5'-3')	Anneling temperature (0C)	Expected amplified product size (bp)	Structural annotation		Functional annotation	
								Sequence components of *kabuli* genome	*Kabuli* gene accession IDs	NCBI-KOG	TFs
Ca6_P3_28193559	Ca6	28193559	AGGG/AGG	AAGGACTAAGTCGGGAAGAAAAA	GATGGTTTTTGTCCATTGAATTT	59.7	131	INTERGENIC	--	--	--
Ca6_P3_28194024	Ca6	28194024	ATTTTTT/ATTTTTTT	GGACCACGAATGTAATTTAGCC	TGCGAGCATTAGAAGTTGTTG	59.7	737	INTERGENIC	--	--	--
Ca6_P3_28243483	Ca6	28243483	CATATATATATATATATATA/CATATATATATATATATATA	TTCATATTAATGGGCGTGAAA	TTTTTGATGGGGCAACATTT	58.0	406	INTERGENIC	--	--	--
Ca6_P3_28326755	Ca6	28326755	AT/ATT	TTTGGGCTTGAAGATGAAAA	TTTAAAAGCAAGGGTGGTGG	58.3	336	DRR	Ca_25968	A	--
Ca6_P3_28327723	Ca6	28327723	CATATATATATAT/CATATATATATAT	CAAGAGCATCGTGTCAAGGA	GTCCTGGCGTGACAAGAAAT	60.0	424	DRR	Ca_25968	A	--
Ca6_P3_28332319	Ca6	28332319	ATTTT/ATTTTT	TTTCAAAAAGTCCCAATCGC	GGTTTGGAGAAGAAGACGAAA	60.1	751	INTERGENIC	--	--	--
Ca6_P3_28332462	Ca6	28332462	ATTT/AT	TTTCAAAAAGTCCCAATCGC	GGTTTGGAGAAGAAGACGAAA	60.1	751	INTERGENIC	--	--	--
Ca6_P3_28332579	Ca6	28332579	A/AG	GTCGGAGCTCTGCCATAAAA	GGTTTGGAGAAGAAGACGAAA	60.4	599	INTERGENIC	--	--	--
Ca6_P3_28332651	Ca6	28332651	C/CT	GTCGGAGCTCTGCCATAAAA	GGTTTGGAGAAGAAGACGAAA	60.4	599	INTERGENIC	--	--	--
Ca6_P3_28332758	Ca6	28332758	TAAA/TAAAA	TTTCGTCTTCTTCTCCAAACC	CCCAACATATTACCTGTTACCCA	58.4	502	INTERGENIC	--	--	--

INDEL marker IDs	Chromosomes/ unanchored scaffolds	Physical positions (bp)	InDels (*Kabuli* reference genome-CDC Frontier/PII)	Forward primers (5'-3')	Reverse primers (5'-3')	Anneling temperature (0C)	Expected amplified product size (bp)	Structural annotation		Functional annotation	
								Sequence components of *kabuli* genome	*Kabuli* gene accession IDs	NCBI-KOG	TFs
Ca6_P3_28332811	Ca6	28332811	A/AG	TTTCGTCTTCTTCTCCAAACC	CCCAACATATTACCTGTTACCCA	58.4	502	INTERGENIC	--	--	--
Ca6_P3_28332973	Ca6	28332973	A/AT	TTTCGTCTTCTTCTCCAAACC	CGTGCAAAGTCGAAGCGTAT	58.4	669	INTERGENIC	--	--	--
Ca6_P3_28354937	Ca6	28354937	GTAT/GT	TTTTATGTGACCCGCTTTGC	AGAACGGCATCCAGAGAAGA	61.0	600	INTERGENIC	--	--	--
Ca6_P3_28443338	Ca6	28443338	AAAAAAAAATA/AA	AGACCGACTTGGATGGTCAG	TGATCATTTTCCCCTTTTGG	60.1	548	INTERGENIC	--	--	--
Ca6_P3_28443583	Ca6	28443583	CTCTTTTGTTGTTA/CTCTTTTGTTGTTATATCTTTTGTTGTTA	AAAAACACCAAAAGCCCCTC	CATAAAATGCCATTGATCCCT	60.3	499	INTERGENIC	--	--	--
Ca6_P3_28503640	Ca6	28503640	AATA/AA	TGGCATGCTTCATTTTTGAAT	AAAATGCATTCAATGGACACA	60.5	947	DRR	Ca_25962	E	--
Ca6_P3_28513162	Ca6	28513162	TA/TCTTCAACTTTCCCTACCA	CCCACACCTTCTTACCAGGA	GGGACCCAAATTGTCGATAG	60.0	492	INTERGENIC	--	--	--
Ca6_P3_28513941	Ca6	28513941	ANNNNNNNNNN/ANNNNN	TGAAGAAAATTTAAAGGGACGAA	GTGTTGCATCTCATTGGTGG	59.2	569	INTERGENIC	--	--	--
Ca6_P3_28515482	Ca6	28515482	GAAAAAAA/GAAAAAAAA	GGCATAGGTGAGATGACCGT	GCTCCTTATCAAACCATTTGC	60.0	507	INTERGENIC	--	--	--
Ca6_P3_28520849	Ca6	28520849	A/AC	TTAGAAACAGGGACCCCTCC	TTGTCTGTGAACCGAAGCTG	60.3	577	INTERGENIC	--	--	--

INDEL marker IDs	Chromosomes/ unanchored scaffolds	Physical positions (bp)	InDels (*Kabuli* reference genome-CDC Frontier/PII)	Forward primers (5'-3')	Reverse primers (5'-3')	Anneling temperature (0C)	Expected amplified product size (bp)	Structural annotation		Functional annotation	
								Sequence components of *kabuli* genome	*Kabuli* gene accession IDs	NCBI-KOG	TFs
Ca6_P3_28520924	Ca6	28520924	CAA/CAATTAAA	TTAGAAACAGGGACCCCTCC	TTGTCTGTGAACCGAAGCTG	60.3	577	INTERGENIC	--	--	--
Ca6_P3_28520971	Ca6	28520971	TCTTGCCTTGCCTTG/TCTTGCCTTG	TTAGAAACAGGGACCCCTCC	TTGTCTGTGAACCGAAGCTG	60.3	577	INTERGENIC	--	--	--
Ca6_P3_28521027	Ca6	28521027	A/ACATAG	TCTCATTCTTGCCTTGCCTT	TTGTCTGTGAACCGAAGCTG	60.0	172	INTERGENIC	--	--	--
Ca6_P3_28521076	Ca6	28521076	AACTAT/A	TCTCATTCTTGCCTTGCCTT	TTGTCTGTGAACCGAAGCTG	60.0	172	INTERGENIC	--	--	--
Ca6_P3_28536872	Ca6	28536872	TAAAAAAAAAAAAAA/TAAAAAAAAAAA	TCCCCAAATTAAGGAGATTTTG	TTCCACTTCCTTTTCCACCA	59.3	477	INTERGENIC	--	--	--
Ca6_P3_28537234	Ca6	28537234	ATT/AT	ATTCCTCAAAGCCCTCACCT	TTCCACTTCCTTTTCCACCA	60.1	436	INTERGENIC	--	--	--
Ca6_P3_28575687	Ca6	28575687	TAAAAAAAA/TAAAAAAA	ATTCAGACGCCCTGAATCAC	AGCGAAATTAAAGGCCCAAT	60.1	674	INTRON	Ca_17492	--	--
Ca6_P3_28577470	Ca6	28577470	GATATATATATATATATAT/GATATATATATATATATAT	GGTGTTGGAGTCATGTCTTTGA	ATTGAACTAACCCGTGCGTC	60.0	235	DRR	Ca_17491	O	Trihelix
Ca6_P3_28608731	Ca6	28608731	CTTTTTTTTTTT/CTTTTTTTTTTTT	TGGTGCGACATAACTCAAGG	AGCCGACATTGCAAAAGACT	59.7	500	INTERGENIC	--	--	--
Ca6_P3_28610431	Ca6	28610431	CT/C	AGGATTTGGTTCAAGGGTCC	TGTCCTGACTCTTGGCAGTG	60.2	643	DRR	Ca_17488	K	--

INDEL marker IDs	Chromosomes/ unanchored scaffolds	Physical positions (bp)	InDels (*Kabuli* reference genome-CDC Frontier/PII)	Forward primers (5'-3')	Reverse primers (5'-3')	Anneling temperature (0C)	Expected amplified product size (bp)	Structural annotation		Functional annotation	
								Sequence components of *kabuli* genome	*Kabuli* gene accession IDs	NCBI-KOG	TFs
Ca6_P3_28646044	Ca6	28646044	GAAAAAAAAAA/GAAAAAAAAA	ATTTTTGGAGCCACTCTTGC	AATATCGCCCGTGAAAATGA	59.3	605	INTERGENIC	--	--	--
Ca6_P3_28696962	Ca6	28696962	ATTT/ATT	CCATAGTGAAGGTTCGCGTT	CAATTGAGTTGCGACGAGAG	60.1	563	INTRON	Ca_17480	Q	Nin-like
Ca6_P3_28844267	Ca6	28844267	CCAACA/CCAACAACA	ATGGCGTATCCATTCCAAAA	TGACACGCAAGTGTAGGAGC	60.2	422	INTERGENIC	--	--	--
Ca6_P3_28878487	Ca6	28878487	T/TCA	CTCCGTTTTCTGCACATCAA	TGATGTTGCCTCTGTTTTGC	59.8	396	INTERGENIC	--	--	--
Ca6_P3_28880869	Ca6	28880869	ATATTATTATTATTATTATTATTATTATTATT/ATATTATTATTATTATTATTATTATTATTATTATT	TTAACCGCATGTTCAGTTGG	ACCCTTGCTTACTCCTCGCT	59.6	254	INTERGENIC	--	--	--
Ca6_P3_28958770	Ca6	28958770	AAT/AATAT	GAGTAACCGTTGGCTGTGGT	TCGGTTCAGTTGAGTTTGGC	60.0	478	INTERGENIC	--	--	--
Ca6_P3_29085384	Ca6	29085384	CAAAAAAAA/CAAAAAAA	CGCTAGCAACAGAATTGCAG	TTCGACCGATTGAAGAAAGC	59.8	300	DRR	Ca_17464	U	LSD
Ca6_P3_29128097	Ca6	29128097	AGT/A	AAATCCATTTGAGGCACCTTT	CATTTCCACCATCATATTGCC	59.8	305	INTERGENIC	--	--	--
Ca6_P3_29231459	Ca6	29231459	AAAGTCTCAAGTCTCAAGTCTCAA/AAAGTCTCAAGTCTCAAGTCTCAAGTCTCAA	ACTCCCACACACCTCAAAGG	TTTCAAGTCTAGTCGAAGTTCATCA	60.0	834	INTERGENIC	--	--	--
Ca6_P3_29395219	Ca6	29395219	AT/A	GCGTTGGTGCTTCCATTATT	GCCAACTGTTCCACCTTGTT	60.0	487	INTERGENIC	--	--	--

INDEL marker IDs	Chromosomes/ unanchored scaffolds	Physical positions (bp)	InDels (*Kabuli* reference genome-CDC Frontier/PII)	Forward primers (5'-3')	Reverse primers (5'-3')	Anneling temperature (0C)	Expected amplified product size (bp)	Structural annotation		Functional annotation	
								Sequence components of *kabuli* genome	*Kabuli* gene accession IDs	NCBI-KOG	TFs
Ca6_P3_2939568	Ca6	2939568	CATATATATATATATATATA/CATATATATATATATATA	AGCTGAAACACCCACACACA	CTTGGCTTCTAAGGCACCAG	60.2	107	INTERGENIC	--	--	--
Ca6_P3_29478598	Ca6	29478598	CTATATATATATATATATATAT/CTATATATATATATATATATATAT	TGAAGAGGAAACTGAAGCGG	ATGTGAATCCCACCAGGAAA	60.5	837	INTERGENIC	--	--	--
Ca6_P3_29492158	Ca6	29492158	A/ATCTG	TGTGCCGGTCCTAAAAATTC	GGATAAACCGGTGACCATTG	59.9	289	INTERGENIC	--	--	--
Ca6_P3_29526669	Ca6	29526669	A/AAATATTAG	CACTGCCTATTTGTGGCAGA	TTATATCATGGCATCGGCAA	59.9	829	DRR	Ca_25977	O	B3
Ca6_P3_2960468	Ca6	2960468	CATATATATATATATAT/CATATATATATATATATATAT	AAGAAAGGGGTATTGGTGGC	CACAAATTTGACAATAAACTTTGACA	60.2	698	INTRON	Ca_10438	T	bHLH
Ca6_P3_29669305	Ca6	29669305	GAAAAAAAAAAAA/GAAAAAAAAA	GCCCTCAGATGAGAGTCAGG	AATGGCCAGATGAAATCGAG	59.9	596	INTERGENIC	--	--	--
Ca6_P3_29990272	Ca6	29990272	CAATAATAATAATAATAATAATAATAATAAT/CAATAATAATAATAATAATAATAATAATAATAAT	TGAACCCAAATTGATCCCAT	AAAATTTTGGGGTTGTCGTG	60.0	744	INTERGENIC	--	--	--
Ca6_P3_29991077	Ca6	29991077	TACACACACACACACACACA/TACACACACACACACACACA	CGACAACCCCAAAATTTTCTT	TTCAACACAAACAATAGCAATTCA	60.2	833	INTERGENIC	--	--	--
Ca6_P3_30062004	Ca6	30062004	TA/TACCCGCGA	CCGTGTTTGTCTTTCCAGGT	CCCAAGATGGTACTGACCCA	60.0	509	DRR	Ca_24052	GM	FAR1
Ca6_P3_30070176	Ca6	30070176	GAAAAAAAAA/GAAAAAAAAAA	GCGACCATTTACTGCTCCTC	AGCCTCGTAGGTTCGACTGA	59.8	835	INTERGENIC	--	--	--

INDEL marker IDs	Chromosomes/ unanchored scaffolds	Physical positions (bp)	InDels (*Kabuli* reference genome-CDC Frontier/PII)	Forward primers (5'-3')	Reverse primers (5'-3')	Anneling temperature (0C)	Expected amplified product size (bp)	Structural annotation		Functional annotation	
								Sequence components of *kabuli* genome	*Kabuli* gene accession IDs	NCBI-KOG	TFs
Ca6_P3_30157946	Ca6	30157946	GTTTT/GTTT	GCCAACATTGCATGAGAGAA	CATTTTCAAAGAGGGGTCCA	59.8	554	INTERGENIC	--	--	--
Ca6_P3_3021594	Ca6	3021594	GAATAATAATAATAATAATAATAATAATAATAATAATAATAATAA/GAATAATAATAATAATAATAATAATAATAATAATAATAATAATAA	CCTCCCCAAATACATGGTTG	GCCCGCTTCTGTGTATTGAT	60.0	394	INTERGENIC	--	--	--
Ca6_P3_30456247	Ca6	30456247	AATTAT/AAT	GCGTTTCGACACAATTTTCC	AAAATAACGGGCAAGCCTTT	60.5	882	INTERGENIC	--	--	--
Ca6_P3_30544976	Ca6	30544976	TTATATATATATATATATATATATATAT/TTATATATATATATATATATATATATATAT	TCCATGGCTAGGGTTTTGAG	GCAAGCTAAGCAAAGGATGG	60.1	885	INTERGENIC	--	--	--
Ca6_P3_30580681	Ca6	30580681	GAGAAGAAGAAGAAGAAGAAGAAGAAG/GAGAAGAAGAAGAAGAAGAAGAAG	GACCTCGAAATTGAGAAGCG	GCATCGTCATCTTTGCTTCA	60.0	109	CDS (large-effect mutations)	Ca_16460	--	--
Ca6_P3_30588229	Ca6	30588229	AATCGC/A	GGACGTCTACAACATTGGGG	CCACATGAGTGTGTTGAGGG	60.2	948	DRR	Ca_16461	--	SAP
Ca6_P3_30637059	Ca6	30637059	TTATATATATATATATATATATA/TTATATATATATATATATATATATA	ATTTGACGATGCCAGGAAAA	AACTTGCATTTCAACAGGGG	60.4	513	INTRON	Ca_16463	I	--
Ca6_P3_30646281	Ca6	30646281	TTATATATATATATATATATATATATAT/TTATATATATATATATATATATATATAT	TGGTTTTCTTTTCATTTTTCCC	TGCAACACTTACTCCAACCG	59.4	739	INTERGENIC	--	--	--
Ca6_P3_30698837	Ca6	30698837	GTTATTTATTTATTTATTTATTTATTTATTTATT/GTTATTTATTTATTTATTTATTTATTTATTTATTTATTTATT	CGTTTTCGGTTTCAGGGTAA	CCCATACCTATTACCCACGC	60.0	949	INTERGENIC	--	--	--
Ca6_P3_30724309	Ca6	30724309	AATATATATATATATATATATAT/AATATATATATATATATATATATAT	CCGTTGGGAGTTTGGAAGTA	TGCGTATGCTTCAACGTCAT	60.0	587	INTERGENIC	--	--	--

INDEL marker IDs	Chromosomes/ unanchored scaffolds	Physical positions (bp)	InDels (*Kabuli* reference genome-CDC Frontier/PII)	Forward primers (5'-3')	Reverse primers (5'-3')	Anneling temperature (0C)	Expected amplified product size (bp)	Structural annotation		Functional annotation	
								Sequence components of *kabuli* genome	*Kabuli* gene accession IDs	NCBI-KOG	TFs
Ca6_P3_3098575	Ca6	3098575	TAAAAAAAAA/TAAAAAAAAAA	TGGAGGAACTTCACCCTCTT	CTGCTTCTCCACTTCCACAA	58.7	818	INTERGENIC	--	--	--
Ca6_P3_3108108	Ca6	3108108	AT/ATAATAATAATAATATT	CACCAGTGTCCAATCACCCT	CGTCTCATGTTCCTCCAGGT	60.8	508	INTERGENIC	--	--	--
Ca6_P3_31096995	Ca6	31096995	AAATAA/AAA	GACGTGTTTTTAAGGATTGGAAA	CTTTGCTTGGACCAAACTGG	59.4	420	INTERGENIC	--	--	--
Ca6_P3_31097071	Ca6	31097071	TTAT/TT	AAGAGTCAATGACCACCTTCAAA	CTTTGCTTGGACCAAACTGG	60.0	318	INTERGENIC	--	--	--
Ca6_P3_31097180	Ca6	31097180	ATTT/ATTTTT	TCCCAATTGAGTGACATCTTTC	CTTATAGCCGAATGGCCAAA	59.0	587	INTERGENIC	--	--	--
Ca6_P3_31097254	Ca6	31097254	ATT/ATTTTT	CAACAATTTCCCAAACAAAACA	CTTATAGCCGAATGGCCAAA	59.8	492	INTERGENIC	--	--	--
Ca6_P3_31099504	Ca6	31099504	TA/T	TGGGATGATGTTGGGAAAGT	TTGCAAAGAAAACCTCGCTT	60.2	129	INTERGENIC	--	--	--
Ca6_P3_31132134	Ca6	31132134	TAAA/TA	CACTCCAGGTTCCATTCCTC	GCTGGTGGCTAATTATTCCATT	59.5	708	INTERGENIC	--	--	--
Ca6_P3_31146150	Ca6	31146150	TAAA/TAAAA	TGCACGAGATGATTTGTTTGA	TCAATGGTGGCTTGTGGTTA	60.3	374	INTERGENIC	--	--	--
Ca6_P3_31163168	Ca6	31163168	GAA/GA	TATCACCTATCAGAGCGGGG	GATCTATGAGGACAGGGCCA	60.1	241	INTERGENIC	--	--	--

INDEL marker IDs	Chromosomes/ unanchored scaffolds	Physical positions (bp)	InDels (*Kabuli* reference genome-CDC Frontier/PII)	Forward primers (5'-3')	Reverse primers (5'-3')	Anneling temperature (0C)	Expected amplified product size (bp)	Structural annotation		Functional annotation	
								Sequence components of *kabuli* genome	*Kabuli* gene accession IDs	NCBI-KOG	TFs
Ca6_P3_31288919	Ca6	31288919	AATATATATATATATATATATTTATATATATATATATTTATATATATATATATATATA/AATATATATATATATATATTTATATATATATATATTTATATATATATATATATATA	TGATTCTTGAAAGTTAGCAAACACA	ATCGACAAACCAACATGCAA	60.2	460	INTERGENIC	--	--	--
Ca6_P3_31301748	Ca6	31301748	TTTTTTTTAATTTTTTTT/TTTTTTTTAATTTTTTTAATTTTTTTT	AGTTGTGGGGGAGGCTTAGT	AGGCAGGGGCAACTAAAGAT	60.0	410	INTERGENIC	--	--	--
Ca6_P3_31312507	Ca6	31312507	GAAAA/GAAAAA	CACTGCAAGTCACACATGACA	TGAATGTTGTTTATACGCCG	59.3	744	INTERGENIC	--	--	--
Ca6_P3_31331884	Ca6	31331884	TTATATATATATATATATATA/TTATATATATATATATATA	TGACATTGTGTATTTCGTTGTTTT	GTTTGTTGAAATTGGGGGTG	58.5	874	DRR	Ca_15166	--	--
Ca6_P3_31471169	Ca6	31471169	TTATATATATATATATAT/TTATATATATATATATATATAT	CAACGTCGTCGAAGACAAGA	CCTTCGGCAAACTTCTTCAG	60.0	731	INTERGENIC	--	--	--
Ca6_P3_31528724	Ca6	31528724	AG/A	CCCCCAACCACTTTGTCATA	GCCCAATGACCCACATACTC	60.6	526	DRR	Ca_15182	--	--
Ca6_P3_31551380	Ca6	31551380	A/ATTG	ATGGCATCGTTGTCACAAAA	CAACTAAACTGCGCCACCTT	60.0	699	INTERGENIC	--	--	--
Ca6_P3_31632123	Ca6	31632123	A/ATATATT	TTCACACCTTACGTTGCTAAAA	TCCGCTTCTCTAACCTTTGC	57.6	294	DRR	Ca_15189	R	SBP
Ca6_P3_3166147	Ca6	3166147	TATCGGTTCGGCCATTTCCATCGGTTCGGCC/TATCGGTTCGGCC	TCAGATTCTATCGGTTCGGC	ATATGCTGCACCTTGGTTCC	60.2	202	INTERGENIC	--	--	--
Ca6_P3_31681100	Ca6	31681100	ATTTTTTTTT/ATTTTTTTTTT	GAGCCCCGATCCATATACCT	GTCACACAATCACTGACCGC	60.1	635	INTERGENIC	--	--	--

INDEL marker IDs	Chromosomes/ unanchored scaffolds	Physical positions (bp)	InDels (*Kabuli* reference genome-CDC Frontier/PII)	Forward primers (5'-3')	Reverse primers (5'-3')	Anneling temperature (0C)	Expected amplified product size (bp)	Structural annotation		Functional annotation	
								Sequence components of *kabuli* genome	*Kabuli* gene accession IDs	NCBI-KOG	TFs
Ca6_P3_3170767	Ca6	3170767	AT/ATT	AAGTAGCATGCTTGGCTCAAA	CACCTTCCATTGCCATTCTT	60.0	129	INTERGENIC	--	--	--
Ca6_P3_3170785	Ca6	3170785	ATTTTTTTTT/ATTTTTTTT	AAGTAGCATGCTTGGCTCAAA	CACCTTCCATTGCCATTCTT	60.0	129	INTERGENIC	--	--	--
Ca6_P3_3176026	Ca6	3176026	AGG/AGGG	GCAATGGAAGGTGAAAATGG	GCATAGGAGCGTGGAAATTG	60.3	522	INTERGENIC	--	--	--
Ca6_P3_3176136	Ca6	3176136	G/GAAAAATGTCTCGTC	TGGCAATGGAAGGTGAAAAT	TTTAACCCTTTTCAACCGCA	60.3	769	INTERGENIC	--	--	--
Ca6_P3_31789591	Ca6	31789591	GATATATATATATATATATATATATATATATA/GATATATATATATATATATATATATATATATATA	CCAGAAATGAATGTCTGCCA	GCTTATTGCCAAAACGCTGT	59.6	892	INTERGENIC	--	--	--
Ca6_P3_31939689	Ca6	31939689	G/GAT	CTGCGGTTCTTGCTGATGTA	AGGCAGCTTGATTTCTTGGA	60.0	382	INTERGENIC	--	--	--
Ca6_P3_31946423	Ca6	31946423	TAAAAAAAAAA/TAAAAAAAAA	ATCAAGCATTCAAGTTGGGG	TTTGTTCCCCGATCCTTATG	59.9	520	INTERGENIC	--	--	--
Ca6_P3_31950641	Ca6	31950641	ATTTTTTTT/ATTTTTTT	CCACTTCAGCGTGCAATCTA	TTCTTGGGTAGAAAATGCGG	60.0	612	INTERGENIC	--	--	--
Ca6_P3_32089022	Ca6	32089022	TAAAAAAAAAA/TAAAAAAAAAAAA	AAGATGTCGGGGTGTTACCTT	CCCTGTTGGTACGTTGGTCT	59.7	747	INTERGENIC	--	--	--
Ca6_P3_3215275	Ca6	3215275	ATTTTTTTT/ATTTTTTTTT	TTTGCTCATTGAAATTTTCGG	CGGTGCAACGTGATATTTTG	60.1	638	DRR	Ca_26315	A	--

**INDEL marker IDs**	**Chromosomes/ unanchored scaffolds**	**Physical positions (bp)**	**InDels (*Kabuli* reference genome-CDC Frontier/PII)**	**Forward primers (5'-3')**	**Reverse primers (5'-3')**	**Anneling temperature (0C)**	**Expected amplified product size (bp)**	**Structural annotation**		**Functional annotation**	
								**Sequence components of *kabuli* genome**	***Kabuli* gene accession IDs**	**NCBI-KOG**	**TFs**
Ca6_P3_32199865	Ca6	32199865	AAT/A	AGGGAGGCCTTGCTTTAGTG	CACGTTTTAGGGATGTGACG	60.8	553	DRR	Ca_15217	--	--
Ca6_P3_32300884	Ca6	32300884	AATATATATATATATATATATATATATATATATATAT/AATATATATATATATATATATATATATATAT	GGCAAAATGTGTTGTGACCA	TTGTGGTTTGGAATCATTTCTTT	60.4	757	INTERGENIC	--	--	--
Ca6_P3_32322921	Ca6	32322921	AC/A	GTGCACGCCACGAGTAACTA	TCACTTCACAACCCTCATCAA	59.9	283	INTERGENIC	--	--	--
Ca6_P3_3232392	Ca6	3232392	ATTTATTTTTATTT/ATTTATTTTTATTTTTATTT	ACGTGGCTCCTTATACCACG	TTTTTGAGCCTTTTTCACCAA	60.0	563	INTERGENIC	--	--	--
Ca6_P3_32325547	Ca6	32325547	C/CACATACCAAT	TTCCTCCATTTTATTTGCCG	TGTTTCCGCTTGAGGTTTTT	59.9	200	INTERGENIC	--	--	--
Ca6_P3_32372827	Ca6	32372827	CAA/CA	GAATACGCGGCTGATTCATT	CGCCCCGTTTTAACTCATTA	60.1	613	INTERGENIC	--	--	--
Ca6_P3_32372983	Ca6	32372983	TTTATT/TTT	GAATACGCGGCTGATTCATT	CGCCCCGTTTTAACTCATTA	60.1	613	INTERGENIC	--	--	--
Ca6_P3_32373493	Ca6	32373493	ATAATA/ATAATATTAATA	TAATGAGTTAAAACGGGGCG	TGGTCCAACACGAAGAGAAA	60.0	381	INTERGENIC	--	--	--
Ca6_P3_32373522	Ca6	32373522	AATA/AAAATATA	TAATGAGTTAAAACGGGGCG	TTGGTCCAACACGAAGAGA	60.0	382	INTERGENIC	--	--	--
Ca6_P3_32391786	Ca6	32391786	AATATATATATATATA/AATATATATATATATATA	CGCTATGCCCCATTAGATGT	TTTGATTGACTCATTGCGTG	59.9	294	INTERGENIC	--	--	--

INDEL marker IDs	Chromosomes/ unanchored scaffolds	Physical positions (bp)	InDels (*Kabuli* reference genome-CDC Frontier/PII)	Forward primers (5'-3')	Reverse primers (5'-3')	Anneling temperature (0C)	Expected amplified product size (bp)	Structural annotation		Functional annotation	
								Sequence components of *kabuli* genome	*Kabuli* gene accession IDs	NCBI-KOG	TFs
Ca6_P3_32394139	Ca6	32394139	GTTTTTTTTTTTTT/GTTTTTTTTTTTTTTT	ACGCTTTCGTCCCTGATAAA	CTGTGCCTTTTTGAATTGACC	59.7	672	INTERGENIC	--	--	--
Ca6_P3_32406729	Ca6	32406729	TCAAG/T	GCCCCCACTATTTCTCGATT	AAAAGTGAAACGCTTGGCAT	60.3	593	INTERGENIC	--	--	--
Ca6_P3_32519592	Ca6	32519592	AG/A	CGCATGCCACTCACTCTAAA	CGCTGCCACTTTCTACATCA	60.0	611	INTERGENIC	--	--	--
Ca6_P3_32521477	Ca6	32521477	GACATGATAC/GAC	TTGCCAACGTGTCAATCACT	TTTTCCCTACTATTTTGCAGCC	60.2	259	INTERGENIC	--	--	--
Ca6_P3_32521795	Ca6	32521795	ATTTTGTTTGTTTT/ATTTT	TTGCCAACGTGTCAATCACT	TGCTAGGACCAACGATCATGT	60.2	902	INTERGENIC	--	--	--
Ca6_P3_32522263	Ca6	32522263	CA/CAA	TTGTTCATTTTTGGTCGTTCA	CCTTGGCGGATATAGGGAAT	59.0	347	INTERGENIC	--	--	--
Ca6_P3_32539826	Ca6	32539826	TAAAAAAAAAA/TAAAAAAAAA	TATGAGCCTCTGCCAGCTTT	GTCCGCGTCCATTGATAAAC	60.1	562	INTERGENIC	--	--	--
Ca6_P3_32540257	Ca6	32540257	TACATAA/TACATAAGAAACATAA	GTTTATCAATGGACGCGGAC	TTCCCCTAAAAACGGTTGTG	60.3	341	INTERGENIC	--	--	--
Ca6_P3_32540480	Ca6	32540480	GTTTTTTTT/GTTTTTTT	CACAACCGTTTTTAGGGGAA	GAGGATTGCTATGGACAACCA	59.8	214	INTERGENIC	--	--	--
Ca6_P3_32540585	Ca6	32540585	AATATATAT/AATATAT	CACAACCGTTTTTAGGGGAA	AAGGAGAACCAGTCAACCACA	59.8	278	INTERGENIC	--	--	--

INDEL marker IDs	Chromosomes/ unanchored scaffolds	Physical positions (bp)	InDels (*Kabuli* reference genome-CDC Frontier/PII)	Forward primers (5'-3')	Reverse primers (5'-3')	Anneling temperature (0C)	Expected amplified product size (bp)	Structural annotation		Functional annotation	
								Sequence components of *kabuli* genome	*Kabuli* gene accession IDs	NCBI-KOG	TFs
Ca6_P3_3255826	Ca6	3255826	AATATATATATATATAT/AATATATATATATATATAT	TGGCTTGTTTGATTTAGGACA	ATTCGAATGATCAGTTGCCC	58.3	547	INTERGENIC	--	--	--
Ca6_P3_32617100	Ca6	32617100	AAA/AAATAA	GAACGGTCCATGCAAAAGTT	CCCCCGATCCTTATACGACT	60.0	554	INTERGENIC	--	--	--
Ca6_P3_32629991	Ca6	32629991	TAATGCA/TA	CGCTCTCAATCGTGCAATAA	TAGTGATTGGAAAGGACGGC	60.0	719	INTERGENIC	--	--	--
Ca6_P3_32630102	Ca6	32630102	TAAAAAAAAA/TAAA	TTTGAGGGAGGGAATTAGGG	TAGTGATTGGAAAGGACGGC	60.3	583	INTERGENIC	--	--	--
Ca6_P3_32630124	Ca6	32630124	TAAA/TAAAA	TTTGAGGGAGGGAATTAGGG	TAGTGATTGGAAAGGACGGC	60.3	583	INTERGENIC	--	--	--
Ca6_P3_32630376	Ca6	32630376	AGGGG/AGGGGGG	GCCGTCCTTTCCAATCACTA	ATCGGGAAGAGGTTAGGGAA	60.1	123	INTERGENIC	--	--	--
Ca6_P3_32646614	Ca6	32646614	ATTTTTTTTT/ATTTTTTTT	CATCACGTGCTTGATGATGA	TGAATTCAGAACAAAACGATCAA	59.2	503	INTERGENIC	--	--	--
Ca6_P3_32906720	Ca6	32906720	AATATATATATATATATATATATATAT/AATATATATATATATATATATATAT	CCTCGAAAAGGCAATGTGAT	TAATCATGACCTTCTCGCCC	60.1	575	INTERGENIC	--	--	--
Ca6_P3_330218	Ca6	330218	GATATATATATATATATATATA/GATATATATATATATATATATA	GCAGTTTGATCCTTAGGCTGAT	TATTGTGATCCTTGGGCTCC	59.8	880	INTERGENIC	--	--	--
Ca6_P3_33094621	Ca6	33094621	ATTT/ATT	TCAAAATGTAGCGGGTTTCC	AAGCCTTCCTCAAAATCCAA	59.9	398	INTERGENIC	--	--	--

INDEL marker IDs	Chromosomes/ unanchored scaffolds	Physical positions (bp)	InDels (*Kabuli* reference genome-CDC Frontier/PII)	Forward primers (5'-3')	Reverse primers (5'-3')	Anneling temperature (0C)	Expected amplified product size (bp)	Structural annotation		Functional annotation	
								Sequence components of *kabuli* genome	*Kabuli* gene accession IDs	NCBI-KOG	TFs
Ca6_P3_33094845	Ca6	33094845	CAAAAA/CAAAAAA	TCAAAATGTAGCGGGTTTCC	AAGTTGGACAAAATTGGGGT	59.9	950	INTERGENIC	--	--	--
Ca6_P3_33113807	Ca6	33113807	TTTATTATTATTATTATTATTATTATTATTATTATTATTATTATTATT/TTTATTATTATTATTATTATTATTATTATTATTATTATTATTATTATT	AGAGGCAAACAAGAACCGAA	TTGTGGCATTTTCCACTTCA	59.9	433	INTERGENIC	--	--	--
Ca6_P3_33229235	Ca6	33229235	CTT/CTTT	GCTTTTATTCCAAATTATGGCG	AAATTTTAGACCGCGACACG	59.8	758	INTERGENIC	--	--	--
Ca6_P3_33229272	Ca6	33229272	GAAAA/GAAA	TGTTGACGGTGTGTTTTTAAGG	AAATTTTAGACCGCGACACG	59.9	477	INTERGENIC	--	--	--
Ca6_P3_33229296	Ca6	33229296	TG/TGG	CGGTGTGTTTTTAAGGATTGG	AAATTTTAGACCGCGACACG	59.4	471	INTERGENIC	--	--	--
Ca6_P3_33229369	Ca6	33229369	ATTTTTTTTTTTTTT/AATGTTTTTTTTTTTTTTT	CGGTGTGTTTTTAAGGATTGG	AAATTTTAGACCGCGACACG	59.4	471	INTERGENIC	--	--	--
Ca6_P3_33229523	Ca6	33229523	AAAAACGAAAA/AAAAA	CGGTGTGTTTTTAAGGATTGG	GCGGATATCGTAGGGTGCTA	59.4	644	INTERGENIC	--	--	--
Ca6_P3_33229803	Ca6	33229803	TAAAAAAAA/TAAAAAA	AAGCAAGCCAAAAACGAAAA	GCGGATATCGTAGGGTGCTA	59.9	382	INTERGENIC	--	--	--
Ca6_P3_33229847	Ca6	33229847	T/TC	AAGCAAGCCAAAAACGAAAA	GCGGATATCGTAGGGTGCTA	59.9	382	INTERGENIC	--	--	--
Ca6_P3_33240261	Ca6	33240261	A/AAAGAG	GTTTGGTATTGGGCTCCCTT	CACAATTCCTCCCTCTCCAA	60.2	314	INTERGENIC	--	--	--

INDEL marker IDs	Chromosomes/ unanchored scaffolds	Physical positions (bp)	InDels (*Kabuli* reference genome-CDC Frontier/PII)	Forward primers (5'-3')	Reverse primers (5'-3')	Anneling temperature (0C)	Expected amplified product size (bp)	Structural annotation		Functional annotation	
								Sequence components of *kabuli* genome	*Kabuli* gene accession IDs	NCBI-KOG	TFs
Ca6_P3_33240380	Ca6	33240380	A/AG	GTTTGGTATTGGGCTCCCTT	CGAAACTCACCCCTTGTTGT	60.2	750	INTERGENIC	--	--	--
Ca6_P3_33240744	Ca6	33240744	AA/AAAATATATATA	TTGGAGAGGGAGGAATTGTG	CGAAACTCACCCCTTGTTGT	60.0	456	INTERGENIC	--	--	--
Ca6_P3_33293827	Ca6	33293827	TGGG/TGGGG	CCAAGGCTATGAGACCTATCTGA	TGAAAACCCTTTGAAGGTGC	59.8	510	INTERGENIC	--	--	--
Ca6_P3_33308806	Ca6	33308806	ATATCAGTATCAGTAT/ATATCAGTATCAGTATCAGTAT	TTTTGGAGCTTCAGCATTTG	CCTCTTTTCTTCGCATTTCG	59.0	518	INTERGENIC	--	--	--
Ca6_P3_3331064	Ca6	3331064	TAAAAAAA/TAAAAAA	CAAATTGAAAAGTGGGGCAT	TTAAAGTTGGGAGACCACCA	59.8	417	INTERGENIC	--	--	--
Ca6_P3_33340808	Ca6	33340808	GTTTTTTT/GTTTTTTTT	TTCAATGTCATACACAGGCCA	ATGATAACGCATGAATGGCA	60.0	841	INTERGENIC	--	--	--
Ca6_P3_3340164	Ca6	3340164	ATTTTTTTTT/ATTTTTTTTTT	AGGACAATGGGCAAAGACAC	TCATCACCTCAACCAATCCA	60.0	267	INTERGENIC	--	--	--
Ca6_P3_33428657	Ca6	33428657	GTATATATATAT/GTATATATATATATAT	CCGCGAGTAATTCCGATAAA	TTTTGGGATCGTTCGAAATC	60.1	764	INTERGENIC	--	--	--
Ca6_P3_3346076	Ca6	3346076	TATATAT/TATATATACATATAT	ATAAATCACGGCCCAACAAC	CCATTGCACATTTGGTTTTC	59.7	611	INTERGENIC	--	--	--
Ca6_P3_33506970	Ca6	33506970	AAAAGTTAA/AAA	CCATTTGATCTGCAACATTGA	GAAAAGGGGTGATTTGGGAT	59.5	273	INTERGENIC	--	--	--

INDEL marker IDs	Chromosomes/ unanchored scaffolds	Physical positions (bp)	InDels (*Kabuli* reference genome-CDC Frontier/PII)	Forward primers (5'-3')	Reverse primers (5'-3')	Anneling temperature (0C)	Expected amplified product size (bp)	Structural annotation		Functional annotation	
								Sequence components of *kabuli* genome	*Kabuli* gene accession IDs	NCBI-KOG	TFs
Ca6_P3_3351397	Ca6	3351397	GAT/GATAAAATTAT	CGAAAATGGAAAACACCACC	AAGTTCGCGGTGTAATTGTTG	60.2	278	INTERGENIC	--	--	--
Ca6_P3_3351662	Ca6	3351662	GATATAT/GATATATATAT	CGAAAATGGAAAACACCACC	TCGAGTTGCGTAACTGACATAG	60.2	687	INTERGENIC	--	--	--
Ca6_P3_3351745	Ca6	3351745	CATATTATATATA/CATA	GGTTGATTTAGGCTTCACGG	TCGAGTTGCGTAACTGACATAG	59.6	580	INTERGENIC	--	--	--
Ca6_P3_3352912	Ca6	3352912	AATTA/AA	AAAGTGGGTGGCAATTAACG	TTATCCAATTCCGTTGCACA	59.9	247	INTERGENIC	--	--	--
Ca6_P3_33533375	Ca6	33533375	TAA/TAAA	CAAAGAGCGTGATGTCAGATG	CCAAAATAGCCGTTTTGACA	59.5	598	INTERGENIC	--	--	--
Ca6_P3_3355103	Ca6	3355103	GCTATTGATCAATCTATTGAT/GCTATTGAT	TGAATAGATGTGCGCGAGAC	TCCACGGGAGTGAGAGAGAT	60.0	401	INTERGENIC	--	--	--
Ca6_P3_3355875	Ca6	3355875	CTTT/CTTTT	AATTTGGCCCTATTGGCTTT	AGAAACGGAATGAGTCACGG	59.8	709	INTERGENIC	--	--	--
Ca6_P3_3374756	Ca6	3374756	AT/ATGTGTGTACACATGT	GAAACTTCAGCAAAAACCGC	TGTGATATCCCAATCCGCTT	59.9	806	INTERGENIC	--	--	--
Ca6_P3_3377584	Ca6	3377584	CTTTTT/CTT	ATGCGGTGATGGTGATATTG	TATGGCGAATATGGATGGGT	59.2	745	INTERGENIC	--	--	--
Ca6_P3_3378118	Ca6	3378118	GAGAA/GA	ACCCATCCATATTCGCCATA	TTTGCCATCCTCTCTTTTTCA	60.0	653	INTERGENIC	--	--	--

INDEL marker IDs	Chromosomes/ unanchored scaffolds	Physical positions (bp)	InDels (*Kabuli* reference genome-CDC Frontier/PII)	Forward primers (5'-3')	Reverse primers (5'-3')	Anneling temperature (0C)	Expected amplified product size (bp)	Structural annotation		Functional annotation	
								Sequence components of *kabuli* genome	*Kabuli* gene accession IDs	NCBI-KOG	TFs
Ca6_P3_3382789	Ca6	3382789	CTTTTT/CTTT	ATCACCGACCACTCACCTTC	GTTCAATCAGTGCAGCGTGT	60.0	810	INTERGENIC	--	--	--
Ca6_P3_3382946	Ca6	3382946	CAAAAA/CAAAA	TAACCACCAACATTCTGCCA	TGTTCCGTCCCACTCATACA	60.0	927	INTERGENIC	--	--	--
Ca6_P3_3387209	Ca6	3387209	CA/CAAAATGGTGTA	ACCCCTGAACGATGTAAACG	CGCTCTTATCCTGTTGGCTC	59.9	547	INTERGENIC	--	--	--
Ca6_P3_3387853	Ca6	3387853	AGTACGTA/AGTA	GAGCCAACAGGATAAGAGCG	GAAAATTGAGTTCCATGACGTTT	60.0	477	INTERGENIC	--	--	--
Ca6_P3_3387937	Ca6	3387937	A/AAT	GAGCCAACAGGATAAGAGCG	GAAAATTGAGTTCCATGACGTTT	60.0	477	INTERGENIC	--	--	--
Ca6_P3_3388065	Ca6	3388065	GATATATATAT/GATATAT	GAGCCAACAGGATAAGAGCG	TTGTCTTTCACTTCTTTTTCCG	60.0	841	INTERGENIC	--	--	--
Ca6_P3_33886477	Ca6	33886477	GTTTAGGTT/GGTATTTAGGTT	CTGGGCAAGGGATCTGAATA	GCGCTTTTTAGCTTTTTAGCTTT	60.0	575	INTERGENIC	--	--	--
Ca6_P3_3393001	Ca6	3393001	AAATAATAATAATAATAATAATA/AAATAATAATAATAATA	AGATTACTCTGCCTTCCCGC	TTTTCGTTTTAGATGGCCAAA	60.7	707	INTERGENIC	--	--	--
Ca6_P3_3393568	Ca6	3393568	GGTGTAGTGTAG/GGTGTAG	CATTTTGGCCATCTAAAACGA	TGGAAAAATAAACGTTGCCC	60.0	603	INTERGENIC	--	--	--
Ca6_P3_3393624	Ca6	3393624	CAAAAA/CAAAAAA	CATTTTGGCCATCTAAAACGA	TGGAAAAATAAACGTTGCCC	60.0	603	INTERGENIC	--	--	--

INDEL marker IDs	Chromosomes/ unanchored scaffolds	Physical positions (bp)	InDels (*Kabuli* reference genome-CDC Frontier/PII)	Forward primers (5'-3')	Reverse primers (5'-3')	Anneling temperature (0C)	Expected amplified product size (bp)	Structural annotation		Functional annotation	
								Sequence components of *kabuli* genome	*Kabuli* gene accession IDs	NCBI-KOG	TFs
Ca6_P3_3393664	Ca6	3393664	CAAAAAAA/CCAAAAAAAAA	CATTTTGGCCATCTAAAACGA	TGGAAAAATAAACGTTGCCC	60.0	603	INTERGENIC	--	--	--
Ca6_P3_3394137	Ca6	3394137	AT/ATT	CCATCCAGAGCACCTGAAAT	TCAATTGCCTTGCATGTTGT	60.1	371	INTERGENIC	--	--	--
Ca6_P3_3395377	Ca6	3395377	GTA/GTAATTGTA	GGCAGGAACGATACCACAGT	TTGCCCGTACAAAAGAATCC	60.0	428	CDS (large-effect mutations)	Ca_05973	U	Trihelix
Ca6_P3_3395635	Ca6	3395635	GTT/GT	GGCAGGAACGATACCACAGT	TTGCCCGTACAAAAGAATCC	60.0	428	INTRON	Ca_05973	U	Trihelix
Ca6_P3_34164737	Ca6	34164737	CCCACCAC/CCCAC	CCACTTTGTTGGGAAAAACC	AGAAGAACGGTTGCCAAAGA	59.3	504	INTERGENIC	--	--	--
Ca6_P3_34169955	Ca6	34169955	GAAAAA/GAAAAAA	TTTTTAACGCGTGCGTTTTT	ACTGACCTGAATGGGACCTG	60.6	795	INTERGENIC	--	--	--
Ca6_P3_3420698	Ca6	3420698	TTATATATATATATATATATATATATAT/TTATATATATATATATATATATATATATATATAT	TAGCCGACAACATTGGAACA	TGAACTCGCACATTTGAGGA	60.1	386	INTERGENIC	--	--	--
Ca6_P3_34570118	Ca6	34570118	TTTTTTG/T	TGGTCGACAAGCAACTGAAG	AACAATCTCCCCCTTTTTGG	60.0	583	DRR	Ca_15816	--	--
Ca6_P3_34684644	Ca6	34684644	GC/GCC	TTGACAAAGGGGGAGAAAAA	GCCCAATCCAAAGCTCATAA	59.5	680	INTERGENIC	--	--	--
Ca6_P3_34728984	Ca6	34728984	TTATATATATATATATATATAT/TTATATATATATATATATATATAT	TAGGCACATCAAGTCAACCG	TCAGACCATCCGATAGAGGC	59.7	570	INTERGENIC	--	--	--

INDEL marker IDs	Chromosomes/ unanchored scaffolds	Physical positions (bp)	InDels (*Kabuli* reference genome-CDC Frontier/PII)	Forward primers (5'-3')	Reverse primers (5'-3')	Anneling temperature (0C)	Expected amplified product size (bp)	Structural annotation		Functional annotation	
								Sequence components of *kabuli* genome	*Kabuli* gene accession IDs	NCBI-KOG	TFs
Ca6_P3_3479783	Ca6	3479783	AA/AATTTTGTA	GTTGAGGTCAGCTCCAAAGC	TCTATGTGCTCTCTCCGATGAA	60.0	621	INTERGENIC	--	--	--
Ca6_P3_34816135	Ca6	34816135	ATTTT/ATTTTT	ATGCAATGCGTATATGCCAA	GTGTTTTGGTGTGGATCGTG	59.9	872	INTERGENIC	--	--	--
Ca6_P3_34827878	Ca6	34827878	CATATATATATATATATATATATATATATATA/CATATATATATATATATATATATATATATA	AAGTTTCGGGGTAGAGCGAT	TGTAGCATTCTCATGGCGAG	60.1	645	INTERGENIC	--	--	--
Ca6_P3_34838383	Ca6	34838383	TTAATAATAATAATAATAATAATAATAATAACAATAATAATAATAATAACAATAATAATAACAATAATAACAATAATAATAATAACAATAATAATAATAATAATAATAATAATAATAATAATAATAATAATAATAATAAC[illegible]	CACGTTTGATGTTGGATCAGA	TGCCACCAATTTTGTCATGT	59.6	537	INTERGENIC	--	--	--
Ca6_P3_34856533	Ca6	34856533	CATATATATATATATATATATATATA/CATATATATATATATATATATATA	CTGACTCTGGTGGGGAATGT	ACAACAATGGCTCCACATCA	60.0	487	INTRON	Ca_15803	G	--
Ca6_P3_34859064	Ca6	34859064	GATATATATATATATATATATATATA/GATATATATATATATATATATATATATATA	GAAAGAGTTTTGAGCCACGG	AAGTCCAATCTCGGTCAAACTT	59.9	625	INTERGENIC	--	--	--
Ca6_P3_35093227	Ca6	35093227	GAGTGTTGACTTTTTCA/GAGTGTTGACTTTTTCATAGTGTTGACTTTTTCA	AACCGCTATTGGGCTAGGTT	CTCTGATATGCGTGGCAAGA	60.0	646	INTERGENIC	--	--	--
Ca6_P3_35161798	Ca6	35161798	TA/TAAATATAAAA	AGGAAGAGTGGAGTTGCGAA	CGGTTGAGAACCAAGGATGT	60.0	649	INTERGENIC	--	--	--
Ca6_P3_35169658	Ca6	35169658	GTATATATATATATATATATATATATA/GTATATATATATATATATATATATATATATATATATA	AGGAAGAGTGGAGTTGCGAA	TATTTTGGATTTTGGCTCGG	60.0	524	INTERGENIC	--	--	--
Ca6_P3_3519049	Ca6	3519049	GTTTTTTTTTTT/GTTTTTTTTTT	AAGACTGCCCTGACCAGAGA	GTGGGGGACTAAGCTAAGGG	60.0	334	INTERGENIC	--	--	--

INDEL marker IDs	Chromosomes/ unanchored scaffolds	Physical positions (bp)	InDels (*Kabuli* reference genome-CDC Frontier/PII)	Forward primers (5'-3')	Reverse primers (5'-3')	Anneling temperature (0C)	Expected amplified product size (bp)	Structural annotation		Functional annotation	
								Sequence components of *kabuli* genome	*Kabuli* gene accession IDs	NCBI-KOG	TFs
Ca6_P3_35192974	Ca6	35192974	ATTTTTTTTTT/ATTTTTTTTTTTT	TCTGCTAGGTTGTCGTTCCC	CTCAGTCCTTGCCATTGTGA	60.3	396	INTERGENIC	--	--	--
Ca6_P3_35336378	Ca6	35336378	CAAAA/CAAA	GACTGATGGACAAAGAAGTAGACG	TTGCACCCTCCTTTGATTTC	59.3	546	INTRON	Ca_15781	R	G2-like
Ca6_P3_35376032	Ca6	35376032	GTTCAACTTTCAACTTT/GTTCAACTTT	TCCAGGACCAAACACATTGA	TGCGTGTTTGGGTTTGTTTA	59.9	731	INTERGENIC	--	--	--
Ca6_P3_35399356	Ca6	35399356	GTTTGCTTTATA/GAATTTGCTTTATA	TTGAAGCTCAGATGGCACAC	CTCCCCCTTTTTGGCATAAT	60.0	718	INTERGENIC	--	--	--
Ca6_P3_35616799	Ca6	35616799	C/CCTT	GTTTCTGAGGCAAAATCCCA	TGCATTTGTCTTCCCATTGA	60.1	537	INTERGENIC	--	--	--
Ca6_P3_356286	Ca6	356286	TTATATATATATATATATATATATATATATA/TTATATATATATATATATATATATATATATATATA	TTTGGTACCCCAATTGGTAA	CGATCCTTCGACTCATCTCA	57.7	628	INTRON	Ca_13261	O	--
Ca6_P3_35647062	Ca6	35647062	TTATATATATATAT/TTATATATATAT	TGCACAACAAATCTCTTCACAA	CAATGAAACCATGTGGCAAA	59.4	569	INTERGENIC	--	--	--
Ca6_P3_35678654	Ca6	35678654	TAAAAAAA/TAAAAAAAA	TTTTTCATGAGGATCCGAAA	AACTAATAGCCGTGCGCATT	58.1	633	INTERGENIC	--	--	--
Ca6_P3_35719325	Ca6	35719325	AATAGAATACTTTATTAT/AATAGAATACTTTATTATAGAATACTTTATTAT	TGCATCGTTTTTCATCTTTCA	GCGGCTAATTTCAACCAAAT	59.3	630	INTRON	Ca_15773	--	--
Ca6_P3_35802672	Ca6	35802672	TTTCT/TT	GAGCCTTAGAGTTGGGAGCA	ACCCAACTTGTCGAACCAAC	59.6	778	INTERGENIC	--	--	--

INDEL marker IDs	Chromosomes/ unanchored scaffolds	Physical positions (bp)	InDels (*Kabuli* reference genome-CDC Frontier/PII)	Forward primers (5'-3')	Reverse primers (5'-3')	Anneling temperature (0C)	Expected amplified product size (bp)	Structural annotation		Functional annotation	
								Sequence components of *kabuli* genome	*Kabuli* gene accession IDs	NCBI-KOG	TFs
Ca6_P3_35805652	Ca6	35805652	CAA/CA	AATGACATGTTTTCCGCCTC	TAAACTTGTGTCATTGGGCG	59.9	679	INTERGENIC	--	--	--
Ca6_P3_35805694	Ca6	35805694	CTTTAATTTTTTTTA/CTTTAATTTTTTTTAATTTTTTTTA	AATGACATGTTTTCCGCCTC	TAAACTTGTGTCATTGGGCG	59.9	679	INTERGENIC	--	--	--
Ca6_P3_35810337	Ca6	35810337	C/CT	TCATCGATTGCAACTTGCTT	CCCTAAAATTTTGTGGGCTT	59.4	672	INTERGENIC	--	--	--
Ca6_P3_35832130	Ca6	35832130	TTCTCT/TTCT	TCGCCTTCTTCGAAATTAAGTC	AAAAGATTGGTTGGATTAAAAAGA	59.9	147	INTERGENIC	--	--	--
Ca6_P3_35922388	Ca6	35922388	AT/ATT	TCTCCGTTTCATTCTCCACC	CCTGGGGGCTGTTTTACATA	60.1	450	INTERGENIC	--	--	--
Ca6_P3_35998479	Ca6	35998479	GA/G	CGCGTGTGAAATTGTGTTTC	TTCCTCTGCCAATCCATCTC	60.2	487	INTERGENIC	--	--	--
Ca6_P3_36029920	Ca6	36029920	CTTT/CTTTT	TTTGGAACCAAAAACTTCTTCAA	GGAGAAAAAGAATTAAAATCCTCTAAA	60.0	520	INTERGENIC	--	--	--
Ca6_P3_36039668	Ca6	36039668	CATAA/C	CAAGTCGTCGCTTCCAAAGT	AATTGCGGCGAGAGAAGATA	60.4	394	INTERGENIC	--	--	--
Ca6_P3_36247127	Ca6	36247127	A/ATTT	TTTTCGGTGATTCAAAAGCC	AGGGAGGGGAGAACTTCAAA	60.1	840	DRR	Ca_23959	S	--
Ca6_P3_3648114	Ca6	3648114	ATTTTTTTT/ATTTTTTTTT	TCTCACGGTTCAACTCGTTT	TTTCGCTCACCTCACTCAGA	58.3	394	INTERGENIC	--	--	--

INDEL marker IDs	Chromosomes/ unanchored scaffolds	Physical positions (bp)	InDels (*Kabuli* reference genome-CDC Frontier/PII)	Forward primers (5'-3')	Reverse primers (5'-3')	Anneling temperature (0C)	Expected amplified product size (bp)	Structural annotation		Functional annotation	
								Sequence components of *kabuli* genome	*Kabuli* gene accession IDs	NCBI-KOG	TFs
Ca6_P3_36491037	Ca6	36491037	GATATATATATATATATATATATATATATATATA/GATATATATATATATATATATATATATATATATATATATATA	TGGAAATTGGCACACAAGAA	TGCTTAACTTTCGGTGCCTC	60.1	667	INTERGENIC	--	--	--
Ca6_P3_36555408	Ca6	36555408	CCCGGACCGGACCGGACCGGACCGG/CCCGGACCGGACCGGACCGG	CCAGGATAAAACAGCCCAGA	GCAATTTTGTGAAGGAGGGA	60.1	498	INTERGENIC	--	--	--
Ca6_P3_36556485	Ca6	36556485	AC/A	TGTGTCTTGAGGGAATTGGTG	ACATCACCAGCCACTTAGGC	60.9	800	INTERGENIC	--	--	--
Ca6_P3_3659457	Ca6	3659457	GTATATATATATATATATATATATATATA/GTATATATATATATATATATATATATATA	TTCACGTTTGTCTGGGAACA	CCGCTGAACTTGTTAAATCG	60.1	547	INTERGENIC	--	--	--
Ca6_P3_36722414	Ca6	36722414	ACCC/ACC	CTTAGGTTTGCACTCAGCCC	TCGCGGATCTGCTCTTAAAT	59.9	685	INTERGENIC	--	--	--
Ca6_P3_36722630	Ca6	36722630	TA/TAA	TTGCTCCTACGTATCCCCAG	TCGCGGATCTGCTCTTAAAT	60.1	295	INTERGENIC	--	--	--
Ca6_P3_36898169	Ca6	36898169	AATATATATATAT/AATATATATATATATAT	AAGAGAGGTCACTTTGGCGA	CAATTCCCACAGGTACGCTT	60.0	692	INTERGENIC	--	--	--
Ca6_P3_37029786	Ca6	37029786	GA/GAA	TTATGTTTGGGGTGGTGGTT	CTTTTGGAGGATTTTCGGGT	59.9	273	INTERGENIC	--	--	--
Ca6_P3_37387169	Ca6	37387169	ATATTATTATTATTATTAT/ATATTATTATTATTATTATTAT	CCGTAGTGTTTTGCCCTTGT	TAAGGGCATCCAAGGATTTG	60.0	586	DRR	Ca_19797	--	E2F/DP
Ca6_P3_37518297	Ca6	37518297	AATATATATATATATATATATATATATATA/AATATATATATATATATATATATATATATATA	GGATGAGGTAGCTTGGGACA	ACCAAACCTCACCAAGAGGA	60.1	389	INTERGENIC	--	--	--

INDEL marker IDs	Chromosomes/ unanchored scaffolds	Physical positions (bp)	InDels (*Kabuli* reference genome-CDC Frontier/PII)	Forward primers (5'-3')	Reverse primers (5'-3')	Anneling temperature (0C)	Expected amplified product size (bp)	Structural annotation		Functional annotation	
								Sequence components of *kabuli* genome	*Kabuli* gene accession IDs	NCBI-KOG	TFs
Ca6_P3_37602341	Ca6	37602341	ATATCAATT/ATATCAATTTATCAATT	TTGAAGTTGCAATGCCAAAC	CACATTCGTTGTCCCAACAC	59.7	735	INTERGENIC	--	--	--
Ca6_P3_37631958	Ca6	37631958	TTATATATATATATATATATATATATATATATATATATATAT/TATATATATATATATATATATATATATATATATATATATATAT	CCGTACCGTTTCTGGGATAA	TGGGTGCTATTTGCAATCAA	59.8	496	INTRON	Ca_19791	--	--
Ca6_P3_37741039	Ca6	37741039	CAAA/CAA	AGGGTGAGTGATCCGTTGTC	CCACGAGTTCAGGGTTTCAT	60.0	558	INTERGENIC	--	--	--
Ca6_P3_37836711	Ca6	37836711	TGG/TG	TTCAAACGACAGCTTGATGC	CCTTGTCAGGATTGCCATTT	60.0	665	INTRON	Ca_19543	--	--
Ca6_P3_37849692	Ca6	37849692	CAAAA/CAAAAA	TCGAAACCCATGACGTTGTA	GAATCCTGGGCTGAAATCAA	60.0	547	INTERGENIC	--	--	--
Ca6_P3_37893728	Ca6	37893728	TTTTAGAATATAA/TTTTAGAATATAAGCTTTAGAATATAA	TGTTGTTTTGGCTGTGGTGT	GAACAAATGCAAGCAGCAAA	60.0	715	INTERGENIC	--	--	--
Ca6_P3_37935896	Ca6	37935896	CTATATATATATATATATATATATATAGATATATATATATATATAT/CTATATATATATATATATATATATATAGATATATATATATATATAT	TGCACTCTCGCAAAATTGAC	TTGTTGCAAATTCTCCCCTAA	60.0	301	INTERGENIC	--	--	--
Ca6_P3_37948457	Ca6	37948457	AATATATATATATATAT/AATATATATATATAT	CGATACTGAGGGCCAATGAT	TTCCAAATTTGTGCATTGGA	59.9	680	INTERGENIC	--	--	--
Ca6_P3_37971622	Ca6	37971622	GTATATATATATATATATATATAT/GTATATATATATATATATATATATAT	AGCTCTGGAGACCTCCTTCC	TTGTTTGTAAATGCTCCCCT	60.0	386	INTERGENIC	--	--	--
Ca6_P3_37974732	Ca6	37974732	TCTACTACTACTACTACTACTA/TCTACTACTACTACTACTACTACTA	TGTTGCTCCTTTCTTTGCCT	GAGCATGTGGTGAAGCAGAA	60.0	149	INTRON	Ca_19550	A	--

INDEL marker IDs	Chromosomes/ unanchored scaffolds	Physical positions (bp)	InDels (*Kabuli* reference genome-CDC Frontier/PII)	Forward primers (5'-3')	Reverse primers (5'-3')	Anneling temperature (0C)	Expected amplified product size (bp)	Structural annotation		Functional annotation	
								Sequence components of *kabuli* genome	*Kabuli* gene accession IDs	NCBI-KOG	TFs
Ca6_P3_37981019	Ca6	37981019	AATATATATATATATAT/AATATATATAT ATATATATATATAT	TTGACTTTCAAATGAAA ATGGC	AAAAGGGGCACAACAT TCCT	59.1	838	INTERGENIC	--	--	--
Ca6_P3_37981858	Ca6	37981858	TAAA/TAA	TGCAATGACCTTGAAAT TCG	CTTTTAGCCTTCATCG CCAG	59.7	817	INTERGENIC	--	--	--
Ca6_P3_37982172	Ca6	37982172	AATGAT/AAT	GGTACACTTTCCCATCA GCAA	CTTTTAGCCTTCATCG CCAG	60.0	468	INTERGENIC	--	--	--
Ca6_P3_37986173	Ca6	37986173	TAAAAGAAGTACAAAAAGAAGT/TAAAA GAAGT	TTCCTATTGCTCACCCC TTG	GAAAAACAAGACCCGC GATA	60.1	475	DRR	Ca_19551	--	--
Ca6_P3_38205786	Ca6	38205786	GAA/GA	TTGCAATCTCATCAACT CGG	GTAAAATGAAGCGCTT TCGC	59.8	824	INTERGENIC	--	--	--
Ca6_P3_38228228	Ca6	38228228	ATTTATTTTATTT/ATTTATTT	ACAAACAAGTTGAGCA CCCC	TTGGAAAAGCCCACCT ACAC	60.0	682	INTERGENIC	--	--	--
Ca6_P3_38257858	Ca6	38257858	A/AG	CTTCGAGTCCAACCTT GGTG	CCAGCATCTGCTACTG TCCA	60.7	706	INTERGENIC	--	--	--
Ca6_P3_38259089	Ca6	38259089	TC/T	TCCTAGAACATGCATCA CGC	TACCCTTTGCTAGCCC CTTT	59.8	541	INTERGENIC	--	--	--
Ca6_P3_38317090	Ca6	38317090	ACC/ACCAAAATGCC	CAATCACAACCTTTGCC CTT	GTGGGAAGTCGCCATT TAGA	60.0	531	INTERGENIC	--	--	--
Ca6_P3_38570002	Ca6	38570002	ATTTGATTTTGATTT/ATTTGATTTTGAT TTTGATTT	TCAGTCCGATAGTGGC AACA	TCCGAACAAGATTTTC TTTTTGA	60.3	824	INTERGENIC	--	--	--

INDEL marker IDs	Chromosomes/ unanchored scaffolds	Physical positions (bp)	InDels (*Kabuli* reference genome-CDC Frontier/PII)	Forward primers (5'-3')	Reverse primers (5'-3')	Anneling temperature (0C)	Expected amplified product size (bp)	Structural annotation		Functional annotation	
								Sequence components of *kabuli* genome	*Kabuli* gene accession IDs	NCBI-KOG	TFs
Ca6_P3_38695029	Ca6	38695029	ATTTTTT/ATTTTTTT	CCATTACAACCCGAAAGTCC	TCCTAGAACATGCATCACGC	59.3	103	INTERGENIC	--	--	--
Ca6_P3_38703536	Ca6	38703536	CATCTTATCTTATCTTATCTTATCT/CATCTTATCTTATCTTATCTTATCTTATCT	CGTGACTCGGAAAATTGTGA	GTCGGCCACTTTGTTTTGTT	59.7	649	INTERGENIC	--	--	--
Ca6_P3_38717646	Ca6	38717646	GAA/GAAA	GGATGCTCAAGACACGTGAA	GGCTAAACCAATCGATTCCA	59.8	226	INTERGENIC	--	--	--
Ca6_P3_38737253	Ca6	38737253	AAGA/AAGAGAGA	CGCTCCAAGAGTCAATGTCA	TTTCACCTCCTCGTGAGCTT	60.0	350	INTERGENIC	--	--	--
Ca6_P3_38746593	Ca6	38746593	GTGTTGTTGTTGTTG/GTGTTGTTGTTG	GTGGTGCTTATGGTGGCTTT	TCATAGCTCTCTCCACCGCT	60.0	557	INTERGENIC	--	--	--
Ca6_P3_39145289	Ca6	39145289	CAAAAAAAAAAAAAA/CAAAAAAAAAAAAAAAA	AGTGTTGCATCAGGACAACG	CCGCATAAGATGAGTTAGGGTT	59.8	385	DRR	Ca_15910	T	--
Ca6_P3_39182715	Ca6	39182715	GAAAAAAAAA/GAAAAAAAAAA	TGGACGAAGTCAATGGTCAA	TCAATGGAATTTTTCGAGTCAA	60.1	843	DRR	Ca_15911	P	ERF
Ca6_P3_3920180	Ca6	3920180	GTTGGAAATCTTTT/GTTGGAAATCTTTTGGAAATCTTTT	TGGCATCCAACAATTGTCAT	TTGTGCAACCAATCACTTGAA	59.8	899	DRR	Ca_05908	R	--
Ca6_P3_39585216	Ca6	39585216	CA/C	TCCCATCTCCATCAACCATT	TCGACTTGTCAATCGCTTTG	60.1	777	INTERGENIC	--	--	--
Ca6_P3_39717059	Ca6	39717059	ATT/ATTGTTGTTGTTGTTGTT	ATGTAGGGGGTGAGATGTGC	GCCGAGGCCATAATCAATAA	59.8	915	DRR	Ca_15925	--	MYB_related

INDEL marker IDs	Chromosomes/ unanchored scaffolds	Physical positions (bp)	InDels (*Kabuli* reference genome-CDC Frontier/PII)	Forward primers (5'-3')	Reverse primers (5'-3')	Anneling temperature (0C)	Expected amplified product size (bp)	Structural annotation		Functional annotation	
								Sequence components of *kabuli* genome	*Kabuli* gene accession IDs	NCBI-KOG	TFs
Ca6_P3_39767459	Ca6	39767459	AATATATATATATATATATATATATATATATAT/AATATATATATATATATATATATATAT	TCATTTCTACCACGAGTCGCT	TTCAATGTTTTGTGGCTTGC	59.9	499	INTERGENIC	--	--	--
Ca6_P3_39879844	Ca6	39879844	GA/GAA	ATCAACCCGCCCTTTACTTT	CGGCGGCTCTTTATTTAGTG	59.8	497	INTERGENIC	--	--	--
Ca6_P3_39963428	Ca6	39963428	CATATATATATATATATATATAT/CATATATATATATATATATATATAT	AGGACGAATGGGGTCTTTCT	TATTTTGGGTATTGGCTCGG	59.9	450	INTERGENIC	--	--	--
Ca6_P3_40036320	Ca6	40036320	CTT/CTTTATGTATTTT	ACCCAATTAGTGGAGATGCG	CGAGTAATTCCAAACGGCAT	60.0	219	INTERGENIC	--	--	--
Ca6_P3_4009339	Ca6	4009339	TAAAAAAAAAA/TAAAAAAAAAAA	ATCCAACTCGCAAACAGACC	CTCTCATCGGTCCCCTACAA	60.1	278	INTERGENIC	--	--	--
Ca6_P3_40429280	Ca6	40429280	TGAGAG/TGAG	CGATCCTTCACCGCTAACTC	TCCCTGCAATCATGTTGTGT	59.8	565	DRR	Ca_15953	R	--
Ca6_P3_40504576	Ca6	40504576	GTATATATATATATATATATATATATATATATATATATATATATAT/GTATATATATATATATATATATATATATATATATATATATATATATATATATAT	AACGGGGAAGTTTGTGTTTG	TATTTTGGATTTTGGCTCGG	59.9	604	INTERGENIC	--	--	--
Ca6_P3_40530522	Ca6	40530522	TCA/TGGAAGAGCGGTCCATGCA	AAATTGGCTCGCTAGGTTGA	CGAGGTGAAGCAAAGTCCAT	59.8	491	INTERGENIC	--	--	--
Ca6_P3_40600830	Ca6	40600830	TA/TAA	CTTTGAACTCGGAGCGAAAC	AAATTATGCTCGTGGGGATG	60.0	468	INTERGENIC	--	--	--
Ca6_P3_40723954	Ca6	40723954	CATATATATATATATATATATAT/CATATATATATATATATATATATATATATATAT	TGCGTTTTGAACGAAAATTG	CCTCAATTGAAGATTTCGCC	59.7	591	INTERGENIC	--	--	--

INDEL marker IDs	Chromosomes/ unanchored scaffolds	Physical positions (bp)	InDels (*Kabuli* reference genome-CDC Frontier/PII)	Forward primers (5'-3')	Reverse primers (5'-3')	Anneling temperature (0C)	Expected amplified product size (bp)	Structural annotation		Functional annotation	
								Sequence components of *kabuli* genome	*Kabuli* gene accession IDs	NCBI-KOG	TFs
Ca6_P3_40950297	Ca6	40950297	AATATATATATATATATATATATATATATATAT/AATATATATATATATATATATATATATATATATATAT	TTGGTCAGAAATTGGCAGTG	TCAATCTGCTCATAGATTTCGC	59.7	891	INTERGENIC	--	--	--
Ca6_P3_41168319	Ca6	41168319	ATATTATTATTATTATTATTATTATTATTATTATTATTATTATTATTATTATT/ATATTATTATTATTATTATTATTATTATTATTATTATTATT	TTGAATCGTGAGGGTGTCAG	TGATTTCAAAAGCAATGTCAAAA	59.7	600	INTERGENIC	--	--	--
Ca6_P3_41203445	Ca6	41203445	AT/A	GGATTTTGACCTAGTGCCCA	ATGGTGACAGCCTTGAGAGG	59.9	685	INTERGENIC	--	--	--
Ca6_P3_41247026	Ca6	41247026	ATTT/ATTTT	CTTTTATATGCGCTTGACTGG	GTTTTGGCGTTGGTTTCACT	57.6	803	INTERGENIC	--	--	--
Ca6_P3_41371679	Ca6	41371679	AATATATATATATATATATATATATATATATATATATATA/AATATATATATATATATATATATATATATATATATATATATATATA	GTGACAGCCTAAACCCCAAA	TGAAGTTCAACCTTGGGAGG	60.0	382	INTERGENIC	--	--	--
Ca6_P3_41386971	Ca6	41386971	GNNNNNNNNNN/GNNN	GATGTTGGAGGTGGAATTGG	GCATTGTGTGCAAGAAGCAT	60.2	560	INTRON	Ca_23152	R	--
Ca6_P3_41432949	Ca6	41432949	GAATTAAATTAA/GAATTAAATTAAATTAA	TCGAATTTTGAGACGGAAAGA	GGTTCGAGTGTGTGCCATAA	59.8	684	INTERGENIC	--	--	--
Ca6_P3_41464281	Ca6	41464281	CTTTGATTTGA/CTTTGA	AAGCAGAAGGTAGTGGCGAA	GTACGTGGTCCTTCCCTGAA	60.0	395	INTERGENIC	--	--	--
Ca6_P3_41574227	Ca6	41574227	CATAATAATAATAATAAT/CATAATAATAATAATAATAAT	TGATTAATCGCAAATTGTCCA	TTTTTGGGGTCGTTTTATGC	59.0	471	INTERGENIC	--	--	--
Ca6_P3_41574282	Ca6	41574282	TTTATTATTATTATTATT/TTTATTATTATTATTATTATT	TGATTAATCGCAAATTGTCCA	TTTTTGGGGTCGTTTTATGC	59.0	471	INTERGENIC	--	--	--

INDEL marker IDs	Chromosomes/ unanchored scaffolds	Physical positions (bp)	InDels (*Kabuli* reference genome-CDC Frontier/PII)	Forward primers (5'-3')	Reverse primers (5'-3')	Anneling temperature (0C)	Expected amplified product size (bp)	Structural annotation		Functional annotation	
								Sequence components of *kabuli* genome	*Kabuli* gene accession IDs	NCBI-KOG	TFs
Ca6_P3_41574883	Ca6	41574883	ATTTTTTTTT/ATTTTTTTTTTT	TAAAGGCATTGACGGCTCTT	GCATGCCAAAAAGAATTGGT	59.8	646	INTERGENIC	--	--	--
Ca6_P3_41628240	Ca6	41628240	T/TTATTA	CTCTCTCATGTGTGTGCTCCA	CAAAATCTCCCTTTGCATGA	60.0	858	INTERGENIC	--	--	--
Ca6_P3_41654561	Ca6	41654561	GAAAAAAAAAAA/GAAAAAAAAA	CCTTTTGTGAAAGAGGCAGC	CACGGGGAAAGTTGTTTTGT	60.0	623	INTERGENIC	--	--	--
Ca6_P3_41659483	Ca6	41659483	CAAAA/CAAAAA	CCATCAGAAACTGGGATGGT	TGGCCCTTCATTTTTCAATC	59.8	495	INTERGENIC	--	--	--
Ca6_P3_41669872	Ca6	41669872	ATT/ATTT	TGTAGAATCCACCAATGTCCA	ACCAATCATGTTTCGTGGAA	58.8	651	INTERGENIC	--	--	--
Ca6_P3_417004	Ca6	417004	TACACACACACACACA/TACACACACACACACACA	CATTATTATTGGTCGCCGGT	CGCTTGTGATGGAGTTCAAA	59.7	326	INTERGENIC	--	--	--
Ca6_P3_41875891	Ca6	41875891	ATTTTT/ATTTTTT	GGATCTATGATTTCCCTCTTGC	AGTCCCCATGTAATTGTCACG	59.1	568	INTERGENIC	--	--	--
Ca6_P3_41957220	Ca6	41957220	CTATATATATAT/CTATATATAT	TCCCCAACTTTTGAATTTGC	TGCTTCAGTCAATGGGACAG	59.9	877	DRR	Ca_21949	O	--
Ca6_P3_42073012	Ca6	42073012	TTTATTATTATTATTATTATTATTATTATTATTATTATTATTACTATTATTATTAT/TTTATTATTATTATTATTATTATTATTATTATTATTATTATTACTATTATTATTAT	TCAAATTGTGCTGCTCGTTC	CATAGGCAAACAAGGGGAAA	60.0	878	INTERGENIC	--	--	--
Ca6_P3_42229485	Ca6	42229485	TTATATATATATATATATATATAT/TTATATATATATATATATATATATAT	TGTCGCTTCCTAAGTTGGCT	AATTGCGGCGAGAGAAGATA	60.0	263	INTERGENIC	--	--	--

INDEL marker IDs	Chromosomes/ unanchored scaffolds	Physical positions (bp)	InDels (*Kabuli* reference genome-CDC Frontier/PII)	Forward primers (5'-3')	Reverse primers (5'-3')	Anneling temperature (0C)	Expected amplified product size (bp)	Structural annotation		Functional annotation	
								Sequence components of *kabuli* genome	*Kabuli* gene accession IDs	NCBI-KOG	TFs
Ca6_P3_42360168	Ca6	42360168	T/TCCAAAAA	AGAACAGAGGGAACGCAGAA	CAGCGCTTATTTGGGAAAAG	60.0	625	INTERGENIC	--	--	--
Ca6_P3_42360857	Ca6	42360857	CTTAATT/CTT	CAGCGCTTTTCCCAAATAAG	TACGCATCATGGGATTCAAA	59.8	601	INTERGENIC	--	--	--
Ca6_P3_42391110	Ca6	42391110	GTTATTATTATTATTATTATTATTATTATTATTATTATTATTATTATTATTATTA/GTTATTATTATTATTATTATTATTATTATTATTATTATTATTATTATTATTATTA	TGAGGAGCAATGATTGTCAAA	TTCCTTTTTCTCTCCCAAACC	59.3	849	INTERGENIC	--	--	--
Ca6_P3_42418850	Ca6	42418850	ATATTATTATTATTATTATT/ATATTATTATTATTATT	CCGACGTCTCTCATTCTCAA	AACGACAACTTTCAACCCATTT	59.0	686	INTERGENIC	--	--	--
Ca6_P3_4241955	Ca6	4241955	ATT/AT	CAATGTCGAGATGCAACACC	TGTTTCGACGACCTTGAATG	60.1	739	INTERGENIC	--	--	--
Ca6_P3_42464969	Ca6	42464969	AA/AGA	GGTGAGTTTTCCACAAGCGT	GCTTCAATGAAATTTGGCGT	60.2	982	INTERGENIC	--	--	--
Ca6_P3_42465017	Ca6	42465017	TA/TACAA	CTTCGTGGTATGCTTGACCC	GCTTCAATGAAATTTGGCGT	60.5	581	INTERGENIC	--	--	--
Ca6_P3_42467087	Ca6	42467087	TTATATATATATAT/TTATATATATATATAT	TCAAACCTGATTTGGGGGTA	TCGACACTCATTGGTCCTCA	60.2	771	INTERGENIC	--	--	--
Ca6_P3_42532875	Ca6	42532875	GT/GTCT	ATTTGAGGCTCGTGGTCTTG	CATCATCTACCCCCACCATC	60.3	373	INTERGENIC	--	--	--
Ca6_P3_42608452	Ca6	42608452	TCC/TC	ATACGCAGAGAGCTTGGCAT	TGGGAACGTGTATTCGACAA	60.0	301	INTERGENIC	--	--	--

INDEL marker IDs	Chromosomes/ unanchored scaffolds	Physical positions (bp)	InDels (*Kabuli* reference genome-CDC Frontier/PII)	Forward primers (5'-3')	Reverse primers (5'-3')	Anneling temperature (0C)	Expected amplified product size (bp)	Structural annotation		Functional annotation	
								Sequence components of *kabuli* genome	*Kabuli* gene accession IDs	NCBI-KOG	TFs
Ca6_P3_4290626	Ca6	4290626	ACATCATCATCATCAT/ACATCATCATCATCATCAT	CATTCCAGACATTGACGGTG	ACCTGGAAACATTTTGCTCG	60.0	684	CDS (large-effect mutations)	Ca_05873	--	C2H2
Ca6_P3_42907384	Ca6	42907384	T/TGAGGGA	ACTGGCTGACCACTCTCGTT	CAGCGCTTATTTGGGAAAAA	59.9	840	INTERGENIC	--	--	--
Ca6_P3_43042789	Ca6	43042789	AA/AAGA	TGCAAACAATGCTGCCTAAA	TAGCTGTTGCGACCAAATCA	60.4	655	INTERGENIC	--	--	--
Ca6_P3_43043105	Ca6	43043105	CA/C	TGATTTGGTCGCAACAGCTA	AAAAATTGTGCCAAAGTTATGTGA	60.4	556	INTERGENIC	--	--	--
Ca6_P3_43138332	Ca6	43138332	GAAAA/GAAA	GTTCCCTTTGGAATGCTTGA	TCTTTGGGCCAGTGAAAATC	60.1	741	INTERGENIC	--	--	--
Ca6_P3_43144384	Ca6	43144384	TTTCTTCTTCTTCTTCTTCTTCTTCT/TTTCTTCTTCTTCTTCTTCTTCTTCTTCTTCT	TCATGTGGACTAAATGGGCA	ACAGCTCAAGCGAAACCCTA	59.9	399	INTERGENIC	--	--	--
Ca6_P3_43144709	Ca6	43144709	TTTATTATTATTATTATTATTATTATTATTATTATTATTATAATTATAATTATTATTATTATTATTATTATTATTATTATTATTATTATT/TTTATTATTATTATTATTATTATTATTATTATTATTATTATTATTATAATTATAATTA[illegible]	TAGGGTTTCGCTTGAGCTGT	TCCGGATCCTTCAGATTCAC	60.0	656	INTERGENIC	--	--	--
Ca6_P3_43757197	Ca6	43757197	CTTTTTTTTTTT/CTTTTTTTTTTTTT	GGGACCAAAACATGGTGAAG	TTTCCCGACTTGGTCCTTTA	60.2	654	INTERGENIC	--	--	--
Ca6_P3_43845920	Ca6	43845920	T/TC	ACCCAACAAAAAGCAAAACG	TTGGTCAAATGGGAATGGTT	60.0	305	INTERGENIC	--	--	--
Ca6_P3_43909185	Ca6	43909185	TTGAAG/TTGAAGCAAGTACAACTCATGAAG	TGATTGGGTTTGGGTTCATT	ACATAGATGCCGCCTTCATT	60.0	445	INTERGENIC	--	--	--

INDEL marker IDs	Chromosomes/ unanchored scaffolds	Physical positions (bp)	InDels (*Kabuli* reference genome-CDC Frontier/PII)	Forward primers (5'-3')	Reverse primers (5'-3')	Anneling temperature (0C)	Expected amplified product size (bp)	Structural annotation		Functional annotation	
								Sequence components of *kabuli* genome	*Kabuli* gene accession IDs	NCBI-KOG	TFs
Ca6_P3_43909507	Ca6	43909507	CTTTTTTTTTT/CTTTTT	GAAGGCGGCATCTATG TGTT	GGCTTTTGAATCACCG AAAA	60.1	447	INTERGENIC	--	--	--
Ca6_P3_44072205	Ca6	44072205	TTATATATATATATATATATATATATATAT/ TTATATATATATATATATATATATATAT	GACATTTCGAACTTGAA TTTCCT	TTTGGAATTGCGACTT CCTT	58.7	363	INTERGENIC	--	--	--
Ca6_P3_44104056	Ca6	44104056	CTGT/CT	AATGCCCACCCATAATT TCA	CTGGGCAAGGGATCTG AATA	60.0	692	DRR	Ca_24230	--	Trihelix
Ca6_P3_4413696	Ca6	4413696	TAAA/TAA	TCACTCTCTTCCCAATC GCT	CATTCTTGCCTTGTTG CAGA	60.0	571	INTERGENIC	--	--	--
Ca6_P3_44251485	Ca6	44251485	AA/AATAATA	TGTTTTCTCGTGACAGT GGC	TTCTTTTCGGTGGCCA AATA	59.9	845	INTERGENIC	--	--	--
Ca6_P3_44263063	Ca6	44263063	TTATATATATATATATATATATATATATAT/ TTATATATATATATATATATATATATAT	CCCTTCCTCCACCATCA CTA	TTTAAAATTCGGCCGT GAAG	59.9	643	INTERGENIC	--	--	--
Ca6_P3_44266489	Ca6	44266489	AATATATATATATATATATATATATATATAT A/AATATATATATATATATATATATATA	CCGCATTATAATTCCGC TGT	GTGCCGAGAAAATGTG GATT	60.0	819	INTERGENIC	--	--	--
Ca6_P3_44301191	Ca6	44301191	CTTTTT/CTTTTTT	TGCACATTTTACAGCGC TTC	TGAAATTATGGGTGGG CATT	60.0	254	INTERGENIC	--	--	--
Ca6_P3_4432554	Ca6	4432554	ATTTTTTTTTT/ATTTTTTTTT	TTTCCTTTGCCAGAGA GACG	AAGTGTCTTTGGGGAC GTTG	60.5	424	INTERGENIC	--	--	--
Ca6_P3_44678754	Ca6	44678754	GTTTTTTTTTTT/GTTTTTTTT	GCTATGGCCCATTGAT AGGA	ATTTGCGTCGTTATGT GCAA	59.9	714	INTERGENIC	--	--	--

INDEL marker IDs	Chromosomes/ unanchored scaffolds	Physical positions (bp)	InDels (*Kabuli* reference genome-CDC Frontier/PII)	Forward primers (5'-3')	Reverse primers (5'-3')	Anneling temperature (0C)	Expected amplified product size (bp)	Structural annotation		Functional annotation	
								Sequence components of *kabuli* genome	*Kabuli* gene accession IDs	NCBI-KOG	TFs
Ca6_P3_44700288	Ca6	44700288	AA/AAAGGACA	GAGATTTAGAACCATCAAATTGTCA	ATCGAACCCAATCAGCAAAC	58.6	785	INTERGENIC	--	--	--
Ca6_P3_4488440	Ca6	4488440	GTTTTTTTT/GTTTTTTT	CCTATGGGTTGGGAGGGTAT	CAGTCTAGCAGGGTCAAGCC	59.9	571	DRR	Ca_05853	YU	--
Ca6_P3_44918810	Ca6	44918810	TTAATAATAATAATAAT/TTAATAATAATAATAATAAT	GAAATAAGTTTTTCACGCTGCAT	TTTGCTTTTCTTTTGGCACA	59.7	432	INTERGENIC	--	--	--
Ca6_P3_4498664	Ca6	4498664	TAAAA/TAA	TCAGCCACATTGTTTCAAGC	AATAGTGTCAGCGGGTAGTTTT	59.8	373	DRR	Ca_05852	--	BES1
Ca6_P3_45018304	Ca6	45018304	CA/CATCGTCAGA	TCAAGACGGGAATTGGAAAG	TTGCAAAGGATGTATTGGCA	60.0	433	INTERGENIC	--	--	--
Ca6_P3_45282323	Ca6	45282323	TCCTTTTTAGAAAGTAC/TC	TGTTGGAGAGGCTCTGCTTT	CGTTTTTCTGCACTCTGCAA	60.1	214	INTERGENIC	--	--	--
Ca6_P3_45290500	Ca6	45290500	AT/A	GGCACGCTATGGAAAGAGTC	TGAAATTCCATAGGAAGCGG	59.8	589	INTERGENIC	--	--	--
Ca6_P3_45329060	Ca6	45329060	T/TTTAAAA	TTTTTGCTATTTTATTCTCGTGG	TCGATTATGCGTAGGGAAGG	57.6	762	INTERGENIC	--	--	--
Ca6_P3_45329313	Ca6	45329313	TTCT/TT	GGGAGATACGTAGGAGCACG	TCGATTATGCGTAGGGAAGG	59.7	242	INTERGENIC	--	--	--
Ca6_P3_45336183	Ca6	45336183	TTTATTATTATTATTATTATTATTATTATTA/TTTATTATTATTATTATTATTATTATTATTATTATTA	TTGCCACAAACAGAACGTGT	CCAAAAACACCAACTGGCTT	60.2	688	INTRON	Ca_13834	T	--

INDEL marker IDs	Chromosomes/ unanchored scaffolds	Physical positions (bp)	InDels (*Kabuli* reference genome-CDC Frontier/PII)	Forward primers (5'-3')	Reverse primers (5'-3')	Anneling temperature (0C)	Expected amplified product size (bp)	Structural annotation		Functional annotation	
								Sequence components of *kabuli* genome	*Kabuli* gene accession IDs	NCBI-KOG	TFs
Ca6_P3_45484176	Ca6	45484176	GTTTTTTTTTTTT/GTTTTTTTTTTTTTT	CACCAAAAATTGTACCACTTGC	GAGGCAACGACTCAAACCTC	59.4	864	INTERGENIC	--	--	--
Ca6_P3_45675880	Ca6	45675880	A/ATTCCTTTTCGTGTAG	TCACAGGTGGACACAACAAAA	TCTTTCTTCAAAACACAATCGAA	60.0	505	INTERGENIC	--	--	--
Ca6_P3_45703285	Ca6	45703285	TTCTCTCTCTCTCTCTCTCTCTCTCTCTCTCTCTCTCTCTC/TTCTCTCTCTCTCTCTCTCTCTCTCTCTCTCTCTC	AGCAAGCAAGAAGAGACCCA	GCAACTTCGGAAGCAGAATC	60.1	414	INTERGENIC	--	--	--
Ca6_P3_45707996	Ca6	45707996	ATTTTTT/ATTTTT	AACTGAACCAAATCAACACGC	CAAGCCGATTATGCAAGGTT	60.0	491	INTRON	Ca_13854	--	WRKY
Ca6_P3_45747593	Ca6	45747593	CC/CCAGC	CAAAGCAGCCACAATAAGCA	TGACCGAAAAAGCTCTCACA	60.0	508	INTERGENIC	--	--	--
Ca6_P3_45797406	Ca6	45797406	TATTTGATG/TATTTGATGAATTTGATG	TCCACTTTCGAATTTGGGAC	GTGGATATCATCACCCCCAA	59.9	781	INTERGENIC	--	--	--
Ca6_P3_45892250	Ca6	45892250	TAAAAAAA/TAAAAA	GGAGCGCTAAAGTCTTCCAA	TTGAGGGACCAAAACTGGAG	59.6	426	INTERGENIC	--	--	--
Ca6_P3_45895264	Ca6	45895264	TTATATATATATATATATA/TTATATATATATATATA	TTGAAAGAACCAAACGAGGG	TTTGAGAAATCCCCATTGGA	60.1	470	INTERGENIC	--	--	--
Ca6_P3_4595550	Ca6	4595550	CAATAATAATAATAATAATAATAATAATAA/CAATAATAATAATAATAATAATAATAA	TGAAGAACAAATGAAGGGGG	CCACTCTCACCATTTTCCGT	59.9	140	INTERGENIC	--	--	--
Ca6_P3_46119282	Ca6	46119282	AATATATATATATATATA/AATATATATATATATATATA	CGCGTGGTTTGTACAGAGAG	AAAAGTCGAATCAAATGATCTTAAAAA	59.5	768	INTERGENIC	--	--	--

INDEL marker IDs	Chromosomes/ unanchored scaffolds	Physical positions (bp)	InDels (*Kabuli* reference genome-CDC Frontier/PII)	Forward primers (5'-3')	Reverse primers (5'-3')	Anneling temperature (0C)	Expected amplified product size (bp)	Structural annotation		Functional annotation	
								Sequence components of *kabuli* genome	*Kabuli* gene accession IDs	NCBI-KOG	TFs
Ca6_P3_46133711	Ca6	46133711	CATATATATATATATATATAT/CATATATATATATATATAT	GATTGGAACTTTGGCTTGGA	GTGACCATAATATTTGCCCCT	60.1	801	DRR	Ca_13879	--	MYB_related
Ca6_P3_46134674	Ca6	46134674	TAAAAAAAAA/TAAAAAAAA	CCTGAAATTCCTAACGCCAA	GCAGCGAAAAATCACCAAGT	60.1	446	DRR	Ca_13879	--	MYB_related
Ca6_P3_46311376	Ca6	46311376	T/TCGCGTGCC	TTTGCTGAGTTCCTGGCTTT	GGAGGGTTTTGGTTTGGAAT	60.0	452	INTERGENIC	--	--	--
Ca6_P3_46346627	Ca6	46346627	TATA/TATAAATA	TAACCCAAAAATCCCAACCA	ACTTGGGAACCCGAAGAAGT	60.0	466	INTERGENIC	--	--	--
Ca6_P3_4635371	Ca6	4635371	G/GAGAAAAATTCGGT	CCAAAGCCAACGGCTATAAT	ATGAATTGAGGGGGTAAGGG	59.1	302	INTERGENIC	--	--	--
Ca6_P3_46399842	Ca6	46399842	T/TCA	TAACCTTGCATGGGGAAAAG	TTCATGGTGTTTTCTTGGCA	59.9	619	INTERGENIC	--	--	--
Ca6_P3_4649762	Ca6	4649762	AATAAGT/AATAAGTATAAGT	GGTCGAACCACTTGAGGTGT	AATCCACTCCCACTCCACTG	60.0	389	INTERGENIC	--	--	--
Ca6_P3_46525125	Ca6	46525125	G/GGT	TCTTGCAATTCCCCTGAATC	GGCTGAATTGCTCAAACCAT	60.0	395	INTERGENIC	--	--	--
Ca6_P3_46703881	Ca6	46703881	AT/A	AATCACGTTGGGACAAAAGC	AAAGACGAGAATTGCATCTCTAAA	60.0	479	INTERGENIC	--	--	--
Ca6_P3_46772986	Ca6	46772986	A/ATT	GATTATTCTGCCTCCCGTCA	AAACGCAATCCTTGAGTTCG	60.0	633	INTERGENIC	--	--	--

INDEL marker IDs	Chromosomes/ unanchored scaffolds	Physical positions (bp)	InDels (*Kabuli* reference genome-CDC Frontier/PII)	Forward primers (5'-3')	Reverse primers (5'-3')	Anneling temperature (0C)	Expected amplified product size (bp)	Structural annotation		Functional annotation	
								Sequence components of *kabuli* genome	*Kabuli* gene accession IDs	NCBI-KOG	TFs
Ca6_P3_46779497	Ca6	46779497	ATTTTTT/ATTTTTTTT	AATTTTCTCGAGAGCGGACA	TATAGGTGGAGAGGGAAGCG	60.0	190	DRR	Ca_17736	Q	B3
Ca6_P3_46789811	Ca6	46789811	ATTTT/ATTT	TTTGCATTTGAAGGTGTGGA	TCGTGTCCTAAAACTCAAGGAA	60.1	890	INTERGENIC	--	--	--
Ca6_P3_46869916	Ca6	46869916	CGAAGAAGAAGAAGAAGAAGAAGAAGAAGAAGAAGAA/CGAAGAAGAAGAAGAAGAAGAAGAAGAAGAAGAAGAAGAAGAA	CGCATGATTGAAGGGAAAAT	AGAAAGGCTCAAACGACCAA	59.9	398	INTERGENIC	--	--	--
Ca6_P3_4694284	Ca6	4694284	ATCTTCTT/ATCTT	CTCACGAGCAACATCCTTCA	GTGCCCCAATTGACACTTCT	60.0	513	INTERGENIC	--	--	--
Ca6_P3_4699561	Ca6	4699561	C/CAT	TTCTCTTCAAAGCGTGAGCA	TTCCTTGTCCCATCTCCATC	59.9	304	INTERGENIC	--	--	--
Ca6_P3_4699610	Ca6	4699610	CG/CGG	TTCTCTTCAAAGCGTGAGCA	TTCCTTGTCCCATCTCCATC	59.9	304	INTERGENIC	--	--	--
Ca6_P3_47001052	Ca6	47001052	A/AG	GAAGCGGTACTTCCCATTGA	GGTTACAATGTGGGGACGAG	60.1	417	INTERGENIC	--	--	--
Ca6_P3_47002003	Ca6	47002003	TCCACCCAC/TCCAC	CCAATCCGAAGACGATCATT	CGGATTAATTACCGATGCGT	59.9	147	INTERGENIC	--	--	--
Ca6_P3_47011341	Ca6	47011341	TTATATATATATATATATATATAT/TTATATATATATATATATATATATAT	GAAGAGTTGTGTTCGGATGC	ATGCCATGCTCTCCTTTCAG	58.3	593	INTERGENIC	--	--	--
Ca6_P3_4712479	Ca6	4712479	AATATATATATATATATATATATATA/AATATATATATATATATATATA	TTATTTTTGGAATGCGACCA	TGAGGCACTCAATAGCAAAA	59.0	460	INTERGENIC	--	--	--

INDEL marker IDs	Chromosomes/ unanchored scaffolds	Physical positions (bp)	InDels (*Kabuli* reference genome-CDC Frontier/PII)	Forward primers (5'-3')	Reverse primers (5'-3')	Anneling temperature (0C)	Expected amplified product size (bp)	Structural annotation		Functional annotation	
								Sequence components of *kabuli* genome	*Kabuli* gene accession IDs	NCBI-KOG	TFs
Ca6_P3_47148957	Ca6	47148957	GA/GAA	AAACGGGAACTCCAGAATGA	TGATGTTGCCTCTGTTTTGC	59.5	388	INTERGENIC	--	--	--
Ca6_P3_4731176	Ca6	4731176	ATTTTTTTTTT/ATTTTTTTT	GACACGTGCAAACCACTAGC	AAGCAACGATGATAAAATACGACA	59.4	611	INTERGENIC	--	--	--
Ca6_P3_47356970	Ca6	47356970	TAAAAAAAAAA/TAAAAAAAAA	TTTGCGACGTTTGTTCGTTA	TCTGTTGTCGTACACGGGTC	60.3	381	INTERGENIC	--	--	--
Ca6_P3_47608974	Ca6	47608974	AATATATATATATATATATATATATATATATATAT/AATATATATATATATATATATATATATAT	CAATTTTCCCTGGGTTACCA	TGAAGTGCTACTCCAATCCCA	59.7	589	INTERGENIC	--	--	--
Ca6_P3_47697947	Ca6	47697947	GTTCTGATTAATTTATTTTCTG/GTTCTGATTAATTTATTTTCTGATTAATTTATTTTCTG	TATTTTGGGGTTATCGAGCG	TTGGTTTATTTGGGTTGGGA	59.9	801	INTERGENIC	--	--	--
Ca6_P3_4771733	Ca6	4771733	TTATATATATATATATATATAT/TTATATATATATATATAT	GGGCCACATGTATTTGTGTTT	GGAGGCAAGGCAATAAATCA	59.6	532	INTERGENIC	--	--	--
Ca6_P3_47745696	Ca6	47745696	TT/TTAT	GCCAATCGATTATCGCAAGT	ATGGCCTGTGACAAACACAA	60.1	433	INTERGENIC	--	--	--
Ca6_P3_47816277	Ca6	47816277	AATATATATATATATATATATATATATA/AATATATATATATATATATATATA	TGGGAATAGGCCAGACTTTG	TTCCGAGTTGTTTGAGAGGA	60.1	748	INTERGENIC	--	--	--
Ca6_P3_4795726	Ca6	4795726	AAACGT/A	AGTTGGCTTTTGTACCCCCT	AGTTGGCTTTTGTACCCCCT	59.9	940	INTERGENIC	--	--	--
Ca6_P3_48038283	Ca6	48038283	AATTATTATTATTATTATTATTATTATTATTATTATTATTATTATTATTATTATTATTATT/AATTATTATTATTATTATTATTATTATTATTATTATTATTATTATTATTATTATTATTATTATTATTATT	GTGGGTAATCCGTGCGTAAT	CCCAAATTTGTTTAAAGAAGGC	59.7	663	DRR	Ca_23440	--	bZIP

INDEL marker IDs	Chromosomes/ unanchored scaffolds	Physical positions (bp)	InDels (*Kabuli* reference genome-CDC Frontier/PII)	Forward primers (5'-3')	Reverse primers (5'-3')	Anneling temperature (0C)	Expected amplified product size (bp)	Structural annotation		Functional annotation	
								Sequence components of *kabuli* genome	*Kabuli* gene accession IDs	NCBI-KOG	TFs
Ca6_P3_48160520	Ca6	48160520	GTATATATATATATATATATATATAT/GTATATATATATATATATATATAT	TTTTTGGTTGGAGGTGAAGG	GGCTAGGTCGTTCATCAGGA	59.9	769	INTERGENIC	--	--	--
Ca6_P3_48189409	Ca6	48189409	ATTTT/ATTTTT	GTCAACGACTTGGTGGGACT	TTTTTCCCCATCATCCACAT	60.0	584	INTERGENIC	--	--	--
Ca6_P3_48189626	Ca6	48189626	TGGGG/TGG	TTGCTTTGGTTTGATGTGGA	AACGCCCCGAATTTTTAATC	60.1	448	INTERGENIC	--	--	--
Ca6_P3_4823442	Ca6	4823442	AATATATATATATATATATATATATAT/AATATATATATATATATATATATAT	CATCTTGCAAAACAAACAAATG	CGGTGTTCATCCACAACAAA	58.3	854	INTERGENIC	--	--	--
Ca6_P3_48247002	Ca6	48247002	TTTATTATTATTATTATTATTATTATTATTATTATTATTATTATTATTATTATTATCATTATTATTAT/TTTATTATTATTATTATTATTATTATTATTATTATTATTATTATTATTATTATTATTATCATTATTATTAT	GAATTTAGGGCAATGGCTCA	TTTTTGGCACCAGTAGGAGG	60.0	632	INTRON	Ca_24603	A	HD-ZIP
Ca6_P3_48256838	Ca6	48256838	AAT/AATAT	TTGGCCAACGTTAGTTAGGG	TCTATTCCGTTGCCGAGTTC	60.0	336	INTERGENIC	--	--	--
Ca6_P3_48260819	Ca6	48260819	GAAAATTAATAAAATA/GAAAATTAATAAAATAAAATTAATAAAATA	GTGGATGGTGGGGAATAGTG	CTGAGGTCCCAAGCTACGTC	60.1	504	INTERGENIC	--	--	--
Ca6_P3_48271494	Ca6	48271494	TATTATTATTATC/TATTATTATTATTATCATTATTATTATC	TGACTAATTGGTCGCCCTTC	GGATGCGATCATGTGAAGAA	60.1	685	INTERGENIC	--	--	--
Ca6_P3_48279188	Ca6	48279188	T/TA	TCAAGAACGTTTCGACCACA	GCAACTCGCGGGTTAGTATC	60.3	562	INTERGENIC	--	--	--
Ca6_P3_48279466	Ca6	48279466	G/GA	AACCAGATACTAACCCGCGA	TCTGGAAGTTTCTTGACCGTG	59.6	620	INTERGENIC	--	--	--

INDEL marker IDs	Chromosomes/ unanchored scaffolds	Physical positions (bp)	InDels (*Kabuli* reference genome-CDC Frontier/PII)	Forward primers (5'-3')	Reverse primers (5'-3')	Anneling temperature (0C)	Expected amplified product size (bp)	Structural annotation		Functional annotation	
								Sequence components of *kabuli* genome	*Kabuli* gene accession IDs	NCBI-KOG	TFs
Ca6_P3_48279626	Ca6	48279626	ATTTTTTT/ATTTTTT	AACCAGATACTAACCC GCGA	TCTGGAAGTTTCTTGA CCGTG	59.6	620	INTERGENIC	--	--	--
Ca6_P3_48280097	Ca6	48280097	GAAAAAAA/GAAAAAAAA	GACACGGTCAAGAAAC TTCCA	TGCGTTGACTCTTCAA TACACA	60.1	376	INTERGENIC	--	--	--
Ca6_P3_48280225	Ca6	48280225	GTAT/GT	GACACGGTCAAGAAAC TTCCA	TGTGGCACATTCCAAA TTTAT	60.1	601	INTERGENIC	--	--	--
Ca6_P3_48280286	Ca6	48280286	ATTTTTTTTTTTTTTT/ATTTTTTTTTTTT	GACACGGTCAAGAAAC TTCCA	TGTGGCACATTCCAAA TTTAT	60.1	601	INTERGENIC	--	--	--
Ca6_P3_48280545	Ca6	48280545	ATT/ATTT	TGTGTATTGAAGAGTCA ACGCAT	AAAAGTTGTGCCAAAG TTATGTGA	59.7	732	INTERGENIC	--	--	--
Ca6_P3_48280766	Ca6	48280766	TCTA/TCTAAAACTA	TGTATTGATGAGTCGTT GAATGAT	GGAGACGTTAAAGTCC CACA	58.5	902	INTERGENIC	--	--	--
Ca6_P3_48281570	Ca6	48281570	TA/TAATTAAA	TAGGGCAATTGTGCAC ATGA	CTCCAGGTAACGGTTG TGGT	61.1	335	INTERGENIC	--	--	--
Ca6_P3_48385345	Ca6	48385345	G/GA	GGCTTTTGCAATGGTT GTTT	TGCACTCACTCGCCAT TTAC	60.0	586	INTERGENIC	--	--	--
Ca6_P3_48788599	Ca6	48788599	CATATAT/CATATATATAT	TACAATTGATTGGCGG CATA	ATTGTGGTGGAGGTTG TGGT	59.9	738	INTERGENIC	--	--	--
Ca6_P3_48791033	Ca6	48791033	GATTAACTATTAACAATTAACTATTAAC /GATTAACTATTAACTATTAACAATTAA CTATTAAC	TTGTTTCTCCCCAGTTT TGG	CTTTTGGCCCTCACAA ATTC	59.9	863	DRR	Ca_20702	--	--

INDEL marker IDs	Chromosomes/ unanchored scaffolds	Physical positions (bp)	InDels (*Kabuli* reference genome-CDC Frontier/PII)	Forward primers (5'-3')	Reverse primers (5'-3')	Anneling temperature (0C)	Expected amplified product size (bp)	Structural annotation		Functional annotation	
								Sequence components of *kabuli* genome	*Kabuli* gene accession IDs	NCBI-KOG	TFs
Ca6_P3_48792073	Ca6	48792073	TAAATTACAAATTACAAAT/TAAATTACAAAT	TTTGCAGCCACAAAAGACTG	GAACTCTGCCTTCCAGCAAC	60.0	906	DRR	Ca_20702	--	--
Ca6_P3_48905266	Ca6	48905266	CTAAATAAATAAATAAATAA/CTAAATAAATAAATAAATAAATAAATAA	CACGGTCCCTAGAGCCAATA	ATCGACTCCCGAACTCAAAA	60.1	666	INTERGENIC	--	--	--
Ca6_P3_48905387	Ca6	48905387	GAAAAAA/GAAAAAAAA	CACGGTCCCTAGAGCCAATA	ATCGACTCCCGAACTCAAAA	60.1	666	INTERGENIC	--	--	--
Ca6_P3_48905781	Ca6	48905781	ATTTT/ATTTTT	TCGATTATGCGTAGGGAAGG	TTCTTGTCAAGCACATAAATAAAAA	60.1	225	INTERGENIC	--	--	--
Ca6_P3_48925	Ca6	48925	ATTCTTCTTCTTCTTCTTCTTCTTCTT/ATTCTTCTTCTTCTTCTTCTTCTTCTTCTT	ATGTGTTTTCGTTCTTCGGG	TCCATGGGTTTATTGTGGCT	60.0	485	DRR	Ca_26378	--	--
Ca6_P3_48986240	Ca6	48986240	CACAATACAA/CACAATACAATACAA	CTTGATCCGGTTCTCACGTT	CCTTTGGTTATACGGGCTCA	60.1	674	INTERGENIC	--	--	--
Ca6_P3_49125859	Ca6	49125859	CTTTTTTTTTTT/CTTTTTTTTTT	TTTTGAAACCAAAACATAGCTCAA	AACCACTTTGCCAGTCAACC	60.0	759	INTERGENIC	--	--	--
Ca6_P3_49183151	Ca6	49183151	TTTAAAATATTATTAAAATATTATTAAAA/TTTAAAATATTATTAAAA	TGTTGTTGTTGATGTTGTTGGA	GGGGTAGACACATGGTAAGAGG	59.9	751	INTERGENIC	--	--	--
Ca6_P3_49218883	Ca6	49218883	TAAAAAA/TAAAAAAAA	CCCCTTCATATGCTTCCAGA	GTGCTTGCTTTTACTTCGCC	60.0	446	INTERGENIC	--	--	--
Ca6_P3_49222205	Ca6	49222205	T/TG	CCTGCGGAATTTCCTAATGA	AGGTAACACGGCAGGTAACG	60.0	197	INTERGENIC	--	--	--

INDEL marker IDs	Chromosomes/ unanchored scaffolds	Physical positions (bp)	InDels (*Kabuli* reference genome-CDC Frontier/PII)	Forward primers (5'-3')	Reverse primers (5'-3')	Anneling temperature (0C)	Expected amplified product size (bp)	Structural annotation		Functional annotation	
								Sequence components of *kabuli* genome	*Kabuli* gene accession IDs	NCBI-KOG	TFs
Ca6_P3_49223631	Ca6	49223631	TTATATATATATATATATATATATATATA TA/TTATATATATATATATATATATATAT ATATATA	TGAATTTGAATTTTGAA ACGACA	TACGGGACCAAAGGAA TCAA	59.5	419	DRR	Ca_20725	--	--
Ca6_P3_49357563	Ca6	49357563	AATATATATATATATATATATATATATAT ATATATATAT/AATATATATATATATATA TATATATATATATATAT	TTTTAGCGAAACTCGCC ACT	ATGATTCACGGCCTTA AACG	60.0	233	INTERGENIC	--	--	--
Ca6_P3_49497708	Ca6	49497708	ATATTATTATTATTATTATTATTA/ATAT TATTATTATTATTATTA	GTGCGATCAGTTGAAG GTCA	TTTTCCAATCACACGAA TCAA	59.8	525	INTERGENIC	--	--	--
Ca6_P3_49611302	Ca6	49611302	AAG/AAGCTAG	CATTGAAGGCAACCAA TGTG	CTGTAGGCCACACAAA ATGC	60.0	951	INTERGENIC	--	--	--
Ca6_P3_4964508	Ca6	4964508	TAAAAAAAAAA/TAAAAAAAAA	GACAGAAGCGACATGG TTGA	AGTTGCTTAGTTGGCA ACCC	59.8	767	INTERGENIC	--	--	--
Ca6_P3_49733827	Ca6	49733827	TCC/TC	TTGCAATCTCATCAACT CGG	GTAAAATGAAGCGCTT TCGC	59.8	810	INTERGENIC	--	--	--
Ca6_P3_49738545	Ca6	49738545	AATATATATATATATAT/AATATATATAT ATAT	ATCCTAAGCCCGATCC ACTT	GAGTTGCAAAAGCGAT TTCC	59.9	326	INTERGENIC	--	--	--
Ca6_P3_49749941	Ca6	49749941	TATAATAATAATAATAATAATAATAAAA ATAATAATAATAATAATAATAATAATAA/ TATAATAATAATAATAATAATAATAATA ATAAAAATAATAATAATAATAATAATAA TAATAA	GACGCACGGGTTACAC ACTA	ATGTGGATGGTGCAGT ACGA	59.6	299	INTERGENIC	--	--	--
Ca6_P3_49861454	Ca6	49861454	AGTGTGTGTGTGT/AGTGTGTGTGT	ACCTTGCATTTTCTCCC CTT	CTTGCCAAGTTGCCAT TTTT	59.9	656	INTERGENIC	--	--	--
Ca6_P3_49868787	Ca6	49868787	AATATATATATATATATATATATATATAT ATA/AATATATATATATATATATATATAT ATATA	TGGGTATAGGGATCCT CACTG	CCATCCATTGGTAATTT GGTG	58.9	500	INTERGENIC	--	--	--

INDEL marker IDs	Chromosomes/ unanchored scaffolds	Physical positions (bp)	InDels (*Kabuli* reference genome-CDC Frontier/PII)	Forward primers (5'-3')	Reverse primers (5'-3')	Anneling temperature (0C)	Expected amplified product size (bp)	Structural annotation		Functional annotation	
								Sequence components of *kabuli* genome	*Kabuli* gene accession IDs	NCBI-KOG	TFs
Ca6_P3_49901408	Ca6	49901408	TAAAAAAA/TAAAAAA	TGTGGTTGTGGCATAGGAGA	TGAGATGTGACTTTGCACGA	60.1	972	INTERGENIC	--	--	--
Ca6_P3_50016281	Ca6	50016281	TA/TAA	GTTGAAGGGCCACAAGTGAT	AAGGTTATGGTGGCTGCAAA	60.0	435	INTERGENIC	--	--	--
Ca6_P3_50049167	Ca6	50049167	AAG/A	TCACCCAACTCGGGTTAGTC	AGCAAACTTCAGCTCTCTCCA	60.0	333	INTERGENIC	--	--	--
Ca6_P3_50254785	Ca6	50254785	GATATATATATATATATATAT/GATTGATATATATATATATATATATAT	ACGGCAATCTCAACACATGA	TTGATCGTGCAAAATTCCAA	60.1	642	DRR	Ca_13474	O	FAR1
Ca6_P3_5031791	Ca6	5031791	ATTTTTTT/ATTTTTT	TGATGTCGCATGGGTATTGT	AAAAAGTTGAGGCCCAAAGG	59.8	459	INTERGENIC	--	--	--
Ca6_P3_50516806	Ca6	50516806	AATATATATATATATATATATATATATATATATATATATATATATATATAT/AATATATATATATATATATATATATATATATATATATATATATATATATATAT	CTGCCAGTGACTCTCCATCA	TCACTTTGTTGGATGCTGAA	60.0	536	INTERGENIC	--	--	--
Ca6_P3_50517117	Ca6	50517117	A/AAAAGTGTTT	CTGCCAGTGACTCTCCATCA	TCACTTTGTTGGATGCTGAA	60.0	536	INTERGENIC	--	--	--
Ca6_P3_50669135	Ca6	50669135	TAAAA/TA	CATGAAAATTGCACAAGACAAA	TCCCAAATTTATTTTTAATGTCCC	58.7	162	INTERGENIC	--	--	--
Ca6_P3_50799518	Ca6	50799518	CAAAAAAAA/CAAAAAAAAAA	TTGCAGGGACAATTGTTTGA	TCCGATGCCACAACAACTTA	60.1	314	INTERGENIC	--	--	--
Ca6_P3_50818138	Ca6	50818138	AAAAATTTTATA/AA	CACATACTTCACATTTTGCATTGA	TGTAAAACCCTTACCGTGTGTC	59.9	682	INTERGENIC	--	--	--

**INDEL marker IDs**	**Chromosomes/ unanchored scaffolds**	**Physical positions (bp)**	**InDels (*Kabuli* reference genome-CDC Frontier/PII)**	**Forward primers (5'-3')**	**Reverse primers (5'-3')**	**Anneling temperature (0C)**	**Expected amplified product size (bp)**	**Structural annotation**		**Functional annotation**	
								**Sequence components of *kabuli* genome**	***Kabuli* gene accession IDs**	**NCBI-KOG**	**TFs**
Ca6_P3_50898755	Ca6	50898755	GTTATTATTATTATTATTATTATTATTATTATTATTATTATTATTATT/GTTATTATTATTATTATTATTATTATTATTATTATTATT	GGAACGCGTAACCATGTTCT	ACTCCCCCTAGGTGTTGCTT	60.0	617	INTERGENIC	--	--	--
Ca6_P3_51094981	Ca6	51094981	ATTTT/ATTTTCTTTT	TCACTTGCCGGAATAAAGAAA	GAAATCTTGCATGGCAGACA	59.7	734	INTERGENIC	--	--	--
Ca6_P3_51131297	Ca6	51131297	TA/T	AATCATCCAATTAGGCAGCG	AGCTGGTCTCTCTGTGCCAT	60.1	949	INTERGENIC	--	--	--
Ca6_P3_51176638	Ca6	51176638	TTTATTATTATTATTATTATTATTATTATTATTA/TTTATTATTATTATTATTATTATTATTATTA	AATGGAAGACGTCGGTTTTG	AGGGAGAGGCAAGTAAAGCC	60.0	496	INTERGENIC	--	--	--
Ca6_P3_51185360	Ca6	51185360	ACC/AC	CACTCCATGGCTTCTGATGA	AACACCGGACATGACACTGA	59.8	439	INTRON	Ca_19181	--	--
Ca6_P3_51244062	Ca6	51244062	CGAAAGTTTGAAAGTTT/CGAAAGTTT	GCACCCCCTCATTTTTACCT	TCGAGCATCAAACCATTTCA	60.2	364	DRR	Ca_19177	--	--
Ca6_P3_51291605	Ca6	51291605	AATATATATATATATATATATATATATATATATATATATATATATA/AATATATATATATATATATATATATATATATATATATATATATATATATA	TCGATGATCCAACAATTCCA	GTTTGGGTCAATGGAAAGCA	59.9	690	INTERGENIC	--	--	--
Ca6_P3_51398741	Ca6	51398741	GATATATATATATATATATATATATAT/GATATATATATATATATATATATATAT	AAAGAGTGGGGTTGGAGTCA	ACGGAAACAAACGACGATCT	59.5	230	INTERGENIC	--	--	--
Ca6_P3_51483268	Ca6	51483268	TTTATTATTATTATTATTATTATTATTATTATTATCATCATTATTATTATTATTAACATTATTATTATTATTATTATTATTATTATTATTATTATTATTATTATTATTATTATTAT/TTTATTATTATTATTATTATTATTATTA[illegible]	CTGGACGATTTTGGGAGTGT	TGCATAATCATTGCCCTCAA	60.0	758	INTERGENIC	--	--	--
Ca6_P3_51514779	Ca6	51514779	CTATATATATATATATATATATATAT/CTATATATATATATATATATAT	TTGTTTGGGTTATGCTGCAA	TTGAAAATGGTGGGGGATT	60.1	608	INTERGENIC	--	--	--

INDEL marker IDs	Chromosomes/ unanchored scaffolds	Physical positions (bp)	InDels (*Kabuli* reference genome-CDC Frontier/PII)	Forward primers (5'-3')	Reverse primers (5'-3')	Anneling temperature (0C)	Expected amplified product size (bp)	Structural annotation		Functional annotation	
								Sequence components of *kabuli* genome	*Kabuli* gene accession IDs	NCBI-KOG	TFs
Ca6_P3_51672786	Ca6	51672786	AATAGGTATA/AATAGGTATAGGTATA	GGTCAAAACTGGGTGGAAGA	CCCTCTGCAATGTCGGTAAT	59.9	542	INTERGENIC	--	--	--
Ca6_P3_51703884	Ca6	51703884	CATATATATATATATATATATAT/CATATATATATATATATATATATAT	AGGAAGAGTGGAGTTGCGAA	TATTTTGGATTTTGGCTCGG	60.0	475	INTERGENIC	--	--	--
Ca6_P3_51763905	Ca6	51763905	AAATAATAATAATAATAATAATAATAATAATAATAATAATAATAA/AAATAATAATAATAATAATAATAATAATAATAATAATAATAATAATAA	GAAAAAGCGCTGTAAATGGC	TCCCCCTTTGTCTTCTTGTG	59.9	646	INTERGENIC	--	--	--
Ca6_P3_51785881	Ca6	51785881	GTTTTTTTTTTT/GTTTTTTTTTT	AACATTGTTGCGGCCTAAAA	CAGAAGATCACTCGTGCCAA	60.5	382	INTERGENIC	--	--	--
Ca6_P3_5187973	Ca6	5187973	CATATAT/CATAT	AAGCGTGGTGCTGAATAGAAA	TTTGTACAGGACCCCTTCCA	59.9	155	INTERGENIC	--	--	--
Ca6_P3_51899244	Ca6	51899244	AATATATATATATATATATATATAT/AATATATATATATATATATATATATATAT	TGACGCGAGAGACATTTGAC	TTCAAGTTTGGCAGAAACCC	60.0	363	INTERGENIC	--	--	--
Ca6_P3_51959426	Ca6	51959426	CAA/CAAAA	TAATGGTTTTCACGCCAACA	ACCGGAAACGCATGTTAAAG	60.0	586	INTERGENIC	--	--	--
Ca6_P3_51967957	Ca6	51967957	AATATATATATATATATATATAT/AATATATATATATATATATATAT	CCAAGGTTGGGTTCGAAGTA	TAAGAATTCCCCACCAGACG	60.0	719	INTERGENIC	--	--	--
Ca6_P3_5202330	Ca6	5202330	TTG/TTGGTG	TTCCCTGCCAGAAGTTGTTT	GTTGCACGCTGAAAGATTGA	59.7	686	DRR	Ca_05776	J	MYB
Ca6_P3_52074906	Ca6	52074906	TTTATTAAGCGATTTATTATTCAGCGATTTATTATTA/TTTATTCAGCGATTTATTATTA	TCCTGAAGATGAGATGCGTG	GGCTATCGCTTATGCAGGAC	59.9	797	DRR	Ca_17514	--	SAP

INDEL marker IDs	Chromosomes/ unanchored scaffolds	Physical positions (bp)	InDels (*Kabuli* reference genome-CDC Frontier/PII)	Forward primers (5'-3')	Reverse primers (5'-3')	Anneling temperature (0C)	Expected amplified product size (bp)	Structural annotation		Functional annotation	
								Sequence components of *kabuli* genome	*Kabuli* gene accession IDs	NCBI-KOG	TFs
Ca6_P3_52075058	Ca6	52075058	GTTATTATTATT/GTTATTATTATTATT	GCGTGTCTTATTGGAGAGAGC	GGCTATCGCTTATGCAGGAC	59.1	782	DRR	Ca_17514	--	SAP
Ca6_P3_52089565	Ca6	52089565	ATATTTTTCT/ATATTTTTCTTATTATTTTTCT	CCGGCACGACAATACCTTAT	AGCGCATCTTTTCATCCAAC	59.8	568	DRR	Ca_17516	A	--
Ca6_P3_52407594	Ca6	52407594	GAAAAAAA/GAAAAAAAA	GAACAAATTTCCAGGGCAGA	GAATCGCGAAATGCTATGGT	60.1	303	INTERGENIC	--	--	--
Ca6_P3_52418069	Ca6	52418069	ACTCTCTCTCTCTCTCTCT/ACTCTCTCTCTCTCTCTCTCT	GCGACTCCGCAATCATATTT	ATTTGCTGGAAAGGACCTGA	60.1	383	INTERGENIC	--	--	--
Ca6_P3_52435335	Ca6	52435335	TTATATATATATATATATATATATATATATATATATATATA/TTATATATATATATATATATATATATATATATATATA	GCAAATCAAATTTTGGGCAT	GCTCCCACTTCCTTTTCCTC	59.8	819	INTERGENIC	--	--	--
Ca6_P3_52529525	Ca6	52529525	TAAAAAAAAAAA/TAAAAAAAAAAAAAA	CCCCAGGTGCAATAGAGAAA	TCGCCTACTCACGGATTTTT	60.1	278	INTERGENIC	--	--	--
Ca6_P3_52572155	Ca6	52572155	T/TG	GGGAGTTGTGTCTTGGCAAT	TCATCTTCGACACAGGCAAG	60.0	344	INTRON	Ca_17540	--	ARF
Ca6_P3_52670436	Ca6	52670436	AATATATATATATATATATATATAT/AATATATATATATATATATATAT	TTTGTTTCATAAAATAATCGCATGT	CTTGCAACCGATAGCCATTT	58.9	810	INTERGENIC	--	--	--
Ca6_P3_52676255	Ca6	52676255	GATATATATATATATATATATATAT/GATATATATATATATATATATATATAT	ATGGGTATAAGTACCCGCGA	TGTGTCCATCCCTAAAACGA	59.3	223	INTERGENIC	--	--	--
Ca6_P3_52783443	Ca6	52783443	AATATATATATATATATATATATATATATAT/AATATATATATATATATATATATATATATATATATAT	CGGTGAATCCTTTACGGAGA	GAAGAAGGAGGAGGAATGGG	60.1	290	INTERGENIC	--	--	--

INDEL marker IDs	Chromosomes/ unanchored scaffolds	Physical positions (bp)	InDels (*Kabuli* reference genome-CDC Frontier/PII)	Forward primers (5'-3')	Reverse primers (5'-3')	Anneling temperature (0C)	Expected amplified product size (bp)	Structural annotation		Functional annotation	
								Sequence components of *kabuli* genome	*Kabuli* gene accession IDs	NCBI-KOG	TFs
Ca6_P3_52787964	Ca6	52787964	CG/C	TTCTCACGAACGAACACGAG	GTGGCACCTCGGTAAACAGT	60.0	667	INTERGENIC	--	--	--
Ca6_P3_52789381	Ca6	52789381	CATATATATATATATATATATATATATATATATA/CATATATATATATATATATATATATATATATATATATATATATA	GTTGGTTCCCTAGCACCGTA	AAGAAGGACGGAAGCTCACA	60.0	867	INTERGENIC	--	--	--
Ca6_P3_5281987	Ca6	5281987	CTTTTTTTTTT/CTTTTTTTTT	CCTCCCCGTTGTTAAGTTCA	GAAGAAGACCCAGATCCGAA	60.0	426	DRR	Ca_05771	O	--
Ca6_P3_52848807	Ca6	52848807	ACTCGCT/A	CTGCCAAATACTGCGACTCA	TCAATTTTGCTTGTAGGATGGA	60.0	381	INTERGENIC	--	--	--
Ca6_P3_52850074	Ca6	52850074	ACTAATTTCTAATT/ACTAATTTCTAATTTCTAATT	TCTGATTCGTTAATGACAACCTTT	CGTGGGAGCAATTATGTCCT	59.1	218	INTERGENIC	--	--	--
Ca6_P3_52850184	Ca6	52850184	TA/T	AGGACATAATTGCTCCCACG	TCCATCTCCAACAACATCAAA	60.0	305	INTERGENIC	--	--	--
Ca6_P3_52858291	Ca6	52858291	AAAATAA/AAA	CACATGCTGCAAAATTGAATG	CCACAAAAATGGTGTTGCAG	60.1	190	INTERGENIC	--	--	--
Ca6_P3_52858643	Ca6	52858643	GTATCTATCTATCTAT/GTATCTATCTATCTATCTAT	CTGCAACACCATTTTTGTGG	TTTGCTGAAAGCATGTTTGTTT	60.0	689	INTERGENIC	--	--	--
Ca6_P3_52859293	Ca6	52859293	ATATATTATATT/ATATATT	AAGTGAATTCCACCTTCCCA	CGAGCCTCCAACTTCTCTTG	59.4	560	INTERGENIC	--	--	--
Ca6_P3_52862596	Ca6	52862596	T/TTATAA	ACATGAAGGCTTGGGTTGAC	GAGGAGCATGATAGCAAGGC	60.0	193	INTERGENIC	--	--	--

INDEL marker IDs	Chromosomes/ unanchored scaffolds	Physical positions (bp)	InDels (*Kabuli* reference genome-CDC Frontier/PII)	Forward primers (5'-3')	Reverse primers (5'-3')	Anneling temperature (0C)	Expected amplified product size (bp)	Structural annotation		Functional annotation	
								Sequence components of *kabuli* genome	*Kabuli* gene accession IDs	NCBI-KOG	TFs
Ca6_P3_52889876	Ca6	52889876	ATT/ATTT	TAAAGGACCAAGTCGGGAAA	TGCTCATGGAGTTAGAAGCAA	59.5	471	INTERGENIC	--	--	--
Ca6_P3_52897673	Ca6	52897673	CATATATATATATATATATATATATATATATATAT/CATATATATATATATATATATATATATATATAT	TTTTTGTCTTCATTTGTTGCG	TTGATCCTTCGATTTTTGAATG	58.9	838	INTERGENIC	--	--	--
Ca6_P3_52935026	Ca6	52935026	CATATATATATATATATATATATATAT/CATATATATATATATATATATATATATAT	CTTTGTGTGCTTCCCATCAA	TGCACGAACTTGTGTATGAAAA	59.7	888	INTERGENIC	--	--	--
Ca6_P3_5295925	Ca6	5295925	TTTATTATTTATTA/TTTATTATTTATTATTATTTATTA	TTGACATTTCAAACCGGTCTAT	TGGAAAAGACTCCTGAATATGGA	58.5	359	INTERGENIC	--	--	--
Ca6_P3_52996524	Ca6	52996524	CATTTT/CT	CTCCGTCATCGCCATAATTT	TTTTCGGTGATTCAAAAGCC	59.9	891	INTERGENIC	--	--	--
Ca6_P3_53010946	Ca6	53010946	CA/C	TGCATCGATAACCAATCCAA	TACATGCGCCCCTGTTTAAT	59.9	301	INTERGENIC	--	--	--
Ca6_P3_53045421	Ca6	53045421	GTATATATATATATATATATATATATATAT/GTATATATATATATATATATATATATATATATATAT	AAATGCGACCTAGGCAAAAA	AGTTGCAGGGACAACAAACG	59.7	155	INTERGENIC	--	--	--
Ca6_P3_53069266	Ca6	53069266	T/TATTA	CATCATCATGTGAGTTGGGG	AGCCGACACCTTTTGACTTC	59.8	499	INTERGENIC	--	--	--
Ca6_P3_53110822	Ca6	53110822	TTATATATATATATATATATAT/TTATATATATATATATATATATATAT	CGGTGAACGGCTAAGTGAAT	TTTCACCAATTCACAAAATTGA	60.1	680	INTERGENIC	--	--	--
Ca6_P3_53218190	Ca6	53218190	TCTTCCTTTTTCCTTCCTTCCTTT/TCTTCCTTT	AGGGTGAGCAATTTTCCCTT	GCTTTGACGGAGGATCTTTG	59.9	685	INTERGENIC	--	--	--

INDEL marker IDs	Chromosomes/ unanchored scaffolds	Physical positions (bp)	InDels (*Kabuli* reference genome-CDC Frontier/PII)	Forward primers (5'-3')	Reverse primers (5'-3')	Anneling temperature (0C)	Expected amplified product size (bp)	Structural annotation		Functional annotation	
								Sequence components of *kabuli* genome	*Kabuli* gene accession IDs	NCBI-KOG	TFs
Ca6_P3_53258128	Ca6	53258128	TAAAA/TAAA	TCTGATTTGTGGGGTC CTTC	CAGCCTTGCACTCAAG ATCA	59.9	360	INTERGENIC	--	--	--
Ca6_P3_53305336	Ca6	53305336	ATTTTTTTT/ATTTTTTTTT	CAAGATTTCGTGGTGC AAGA	TTGAGGACAAAAGCTG CTGA	59.8	660	INTRON	Ca_22925	G	--
Ca6_P3_53311252	Ca6	53311252	AT/ATT	GATGGTGGCCATTGAA GAGT	CAACAGTGTATGCACC TGCC	59.9	721	INTERGENIC	--	--	--
Ca6_P3_53312302	Ca6	53312302	AAAAATAAAATAAAATAAAATAAAA/AAA AATAAAATAAAATAAAATAAAATAAAA	ATAGCAATTGGTGCATC CCT	ATGACCAAGGCATTGA GGAC	59.4	761	INTERGENIC	--	--	--
Ca6_P3_5331868	Ca6	5331868	TAAAAAA/TAAAAAAA	AACAACCATTTGATTGA TAACCTTC	TCGGTCAAATTCTTAC CACAAA	59.6	546	INTERGENIC	--	--	--
Ca6_P3_5333071	Ca6	5333071	ATATC/A	AACTAGTGGGTAACCC GTGC	TGGAGTCATGTTTTTC ATACGTG	59.0	211	INTERGENIC	--	--	--
Ca6_P3_53344452	Ca6	53344452	TGGGG/TGGG	CTTGGGCTTATTCAATG CAA	TAACTCAAGCCAATGA GGGG	58.8	751	INTERGENIC	--	--	--
Ca6_P3_53411441	Ca6	53411441	GGA/G	GACCAAGGCAAGCAAG AAAG	GGTGGAAGCTAGAGTG ACGG	60.0	249	DRR	Ca_18883	GC	--
Ca6_P3_53413835	Ca6	53413835	ATT/ATTT	CGTCATTTGGGGGACT AAAA	GAGTTCGGGAGTAGG GAAGG	59.8	978	INTERGENIC	--	--	--
Ca6_P3_53414207	Ca6	53414207	AG/A	TCGCACCGGGAGATAC TTAG	GAGTTCGGGAGTAGG GAAGG	60.2	258	INTERGENIC	--	--	--

INDEL marker IDs	Chromosomes/ unanchored scaffolds	Physical positions (bp)	InDels (*Kabuli* reference genome-CDC Frontier/PII)	Forward primers (5'-3')	Reverse primers (5'-3')	Anneling temperature (0C)	Expected amplified product size (bp)	Structural annotation		Functional annotation	
								Sequence components of *kabuli* genome	*Kabuli* gene accession IDs	NCBI-KOG	TFs
Ca6_P3_53574745	Ca6	53574745	TAAAAAAAA/TAAAAAAA	AAAAGTGGTGTTGACTGGCA	AGCTCTCCCGAAATGTGTTT	59.2	365	INTERGENIC	--	--	--
Ca6_P3_53615531	Ca6	53615531	ATTTTTTTTTT/ATTTTTTT	GATGATTTTCTCGGGAAGCA	TGAGAAAGAAGCAGCATCCA	60.2	547	INTERGENIC	--	--	--
Ca6_P3_53628454	Ca6	53628454	ATTTTTTTT/ATTTTTTTTT	TGTGATGTCACTGCTCCCTC	TGCACATGCACACATGCTTA	59.8	545	INTERGENIC	--	--	--
Ca6_P3_53680637	Ca6	53680637	CATATATATATATATATATATATATAT/CATATATATATATATATATATATATATATAT	TCTATGGCACTTGGAGCCTT	AATTTGCATGTCTTTTGCCC	59.8	667	INTRON	Ca_18901	--	--
Ca6_P3_53799025	Ca6	53799025	ATTT/ATTTT	GGCCCAATATTTGAAACCCT	CAACTTGCAAAGACCCATCA	60.0	704	INTERGENIC	--	--	--
Ca6_P3_53889398	Ca6	53889398	TTATATATATATATATATAT/TTATATATATATATATATATATAT	ACAACCTTTGTGAAATCGGC	CCAAATGGCGACTACCAACT	60.0	749	INTERGENIC	--	--	--
Ca6_P3_5405978	Ca6	5405978	CATATATATATATATATATATATATATATATATAT/CATATATATATATATATATATATATAT	TCATATTACTTCCGGCGACC	AGCAACAAACAAGATTGCCC	59.9	681	INTERGENIC	--	--	--
Ca6_P3_54068748	Ca6	54068748	CCATCAT/CCAT	GTAAGTGAAAGCGGGAGTGC	ACCACCAACTTGCTTGCTCT	59.9	527	INTERGENIC	--	--	--
Ca6_P3_54068881	Ca6	54068881	TAATATAAAAA/TAATATAAAAATATAAAAA	GATCCCAACCATCATTGTCC	ACCACCAACTTGCTTGCTCT	60.0	258	INTERGENIC	--	--	--
Ca6_P3_54068968	Ca6	54068968	GA/GATA	GATCCCAACCATCATTGTCC	ACCACCAACTTGCTTGCTCT	60.0	258	INTERGENIC	--	--	--

INDEL marker IDs	Chromosomes/ unanchored scaffolds	Physical positions (bp)	InDels (*Kabuli* reference genome-CDC Frontier/PII)	Forward primers (5'-3')	Reverse primers (5'-3')	Anneling temperature (0C)	Expected amplified product size (bp)	Structural annotation		Functional annotation	
								Sequence components of *kabuli* genome	*Kabuli* gene accession IDs	NCBI-KOG	TFs
Ca6_P3_54069131	Ca6	54069131	ATTTT/ATTTTTT	GATCCCAACCATCATTG TCC	AATTACCACGGCCTCT TGTG	60.0	824	INTERGENIC	--	--	--
Ca6_P3_54069845	Ca6	54069845	ATTTTTTTT/ATTTTTTTTT	ACAAGAGGCCGTGGTA ATTG	TTTTGGTGGGTTCCAC TTTT	60.0	751	INTERGENIC	--	--	--
Ca6_P3_54070088	Ca6	54070088	ATT/ATTT	GCAATTGTCGTGATTTT ATTTTTG	TTTTGGTGGGTTCCAC TTTT	59.8	379	INTERGENIC	--	--	--
Ca6_P3_54070236	Ca6	54070236	TAAAA/TAA	TTTCTTTCCTCATGCTT TAGAGG	TTTTGGTGGGTTCCAC TTTT	59.1	123	INTERGENIC	--	--	--
Ca6_P3_54095986	Ca6	54095986	TCA/T	TTTTTCCGACTTGGTCC TTT	TTTTTCCACGTGAGCA TCAA	58.7	466	INTERGENIC	--	--	--
Ca6_P3_5410706	Ca6	5410706	ATTTTTTTT/ATTTTTTTTT	TTTGAAAAAGTGAGGG GTGG	CTTTGGTGGTGGTCAG AGGT	59.9	956	INTERGENIC	--	--	--
Ca6_P3_54143633	Ca6	54143633	AATATATA/AATATATATA	CCAAGAGATATACGGG GCAA	CGACAAAACAAACCGC ATAA	59.9	907	INTERGENIC	--	--	--
Ca6_P3_54144071	Ca6	54144071	ATTTT/ATTT	GCCGCCGAATCTTATAT TACC	CGTGCATTAACATCGA CAGG	59.8	580	INTERGENIC	--	--	--
Ca6_P3_54144443	Ca6	54144443	ATAAACTAA/ATAAACTAAACTAA	TGTTTTGTCGGGTGGA CATA	CGTGCATTAACATCGA CAGG	59.8	450	INTERGENIC	--	--	--
Ca6_P3_54160975	Ca6	54160975	AAATAT/AAATATTAATAT	TTGATTTGACAATTTTC ATTGCTT	TCAGTTTTCCGGTTTC TTGC	59.9	703	INTERGENIC	--	--	--

INDEL marker IDs	Chromosomes/ unanchored scaffolds	Physical positions (bp)	InDels (*Kabuli* reference genome-CDC Frontier/PII)	Forward primers (5'-3')	Reverse primers (5'-3')	Anneling temperature (0C)	Expected amplified product size (bp)	Structural annotation		Functional annotation	
								Sequence components of *kabuli* genome	*Kabuli* gene accession IDs	NCBI-KOG	TFs
Ca6_P3_54161011	Ca6	54161011	TGAGAGA/TGAGA	TTGATTTGACAATTTTC ATTGCTT	TCAGTTTTCCGGTTTC TTGC	59.9	703	INTERGENIC	--	--	--
Ca6_P3_54181298	Ca6	54181298	CTTTTTTTTTTT/CTTTTTTTTTT	TTTTCCGCATAAATGTC ACG	CAAAATTTTCAAACAAA ACCCA	59.6	835	INTERGENIC	--	--	--
Ca6_P3_54192734	Ca6	54192734	ATTTTTTTT/ATTTTTTTTT	GGACGGCGAAAATAAA ACAA	TTGAAAATGGCTAAGG CAAAA	59.9	642	INTERGENIC	--	--	--
Ca6_P3_54198768	Ca6	54198768	TAAAAAAAAAA/TAAAAAAAAA	TAATTTTGCTTTCCCCG ACA	CAAAACCACATACCGC AACA	60.4	761	INTERGENIC	--	--	--
Ca6_P3_54202309	Ca6	54202309	AATATATATATATATATATA/AATATATA TATATATATA	ATGTCATGCGGAGGGA CTAC	TCTGCACCAACCTCAA CACT	60.0	364	DRR	Ca_16276	G	--
Ca6_P3_54215448	Ca6	54215448	ATTTTTTTT/ATTTTTTT	TCGAACTGCTTGCACT GAAC	TGTGCGTGTGTCTAAA AGTTGTC	60.2	908	INTERGENIC	--	--	--
Ca6_P3_54259137	Ca6	54259137	TAAAAAAAAAA/TAAAAAAAAA	CGGGCATCATATTTAAC AACAA	TCTATCGGGGGACAGA TAAGT	59.7	660	INTERGENIC	--	--	--
Ca6_P3_54304338	Ca6	54304338	ATTTT/ATT	AATGGTATTGCCGACC ATGT	AAGTGTGGTGTTGAAA GTTGGA	60.1	594	INTERGENIC	--	--	--
Ca6_P3_54322946	Ca6	54322946	TATTG/TATTGATTG	TATGAATCTTCACCTCC GCC	CCAGTCTGACCTGACC ATGA	60.0	560	INTERGENIC	--	--	--
Ca6_P3_54324356	Ca6	54324356	GTTTTTTTTTTTT/GTTTTTTTTTTTTTTT	TAGGATGCTCTAAATCC GGC	CCTTGTGTACCAGGCC TACC	59.3	624	INTERGENIC	--	--	--

INDEL marker IDs	Chromosomes/ unanchored scaffolds	Physical positions (bp)	InDels (*Kabuli* reference genome-CDC Frontier/PII)	Forward primers (5'-3')	Reverse primers (5'-3')	Anneling temperature (0C)	Expected amplified product size (bp)	Structural annotation		Functional annotation	
								Sequence components of *kabuli* genome	*Kabuli* gene accession IDs	NCBI-KOG	TFs
Ca6_P3_54332702	Ca6	54332702	TAAA/TAA	ATGGACGACTTTGGGTTCTG	AGCCCCATAATGTCAAGCAC	60.0	456	INTRON	Ca_16289	O	WRKY
Ca6_P3_54338140	Ca6	54338140	TTTATTATT/TTTATT	ATACCCGACTCCCTCTTGCT	GCAGTTCGGAATAATGCACA	60.1	337	DRR	Ca_16289	O	WRKY
Ca6_P3_54339604	Ca6	54339604	GAGGATCA/GA	ATCATGCGTTTCTCACATGC	TACGGGGTTGGGGATAATTT	59.7	216	DRR	Ca_16289	O	WRKY
Ca6_P3_54339840	Ca6	54339840	CAAAAA/CAAAA	TAAATTATCCCCAACCCCGT	CTGATGAGCTTTCGGTGCTT	60.3	205	INTERGENIC	--	--	--
Ca6_P3_54339929	Ca6	54339929	TC/T	AAATTATCCCCAACCCCGTA	TCATTGGACTCTCATCCTCTCA	60.3	628	INTERGENIC	--	--	--
Ca6_P3_54356339	Ca6	54356339	T/TG	TTTCCTACAATGTCAGTCCAAAAA	CATGGCTTTATTTGGTTCCG	59.9	521	DRR	Ca_16293	--	GRAS
Ca6_P3_54369897	Ca6	54369897	CATATATATATATATATATATATATATATA/CATATATATATATATATATATATATATATATATA	CCTATCATGGTCTGGCCTGT	TTCCATTGCCTTATTGCACA	60.0	888	INTERGENIC	--	--	--
Ca6_P3_5441673	Ca6	5441673	CTATATATATATATATATATATATATATA/CTATATATATATATATATATATATATATATA	AAACCTGTGTGATGTGCTGC	CACCACAAAGAGGATAGTCGG	59.8	623	DRR	Ca_05757	QI	B3
Ca6_P3_5465652	Ca6	5465652	CATTATTATTATTATTATTATT/CATTATTATTATTATTATT	TGGAGCTGCTGGTACCTCTT	CCACATTTTATTCGCAAGCA	60.0	732	INTERGENIC	--	--	--
Ca6_P3_54674096	Ca6	54674096	A/AC	TGAAATGAGGGAATTAAAATCACA	AGGCTAGCCCCACAAAAGTT	59.7	679	DRR	Ca_21197	O	--

INDEL marker IDs	Chromosomes/ unanchored scaffolds	Physical positions (bp)	InDels (*Kabuli* reference genome-CDC Frontier/PII)	Forward primers (5'-3')	Reverse primers (5'-3')	Anneling temperature (0C)	Expected amplified product size (bp)	Structural annotation		Functional annotation	
								Sequence components of *kabuli* genome	*Kabuli* gene accession IDs	NCBI-KOG	TFs
Ca6_P3_54683653	Ca6	54683653	TTAATAATAATAATAATAATAATAATAATAATAATAATAATAATAATAATAATAATAA/TTAATAATAATAATAATAATAATAATAATAATAATAATAATAATAATAA	TAGTCTCATGCTGACACGCC	TGCGAGAGAGAAGAAGGAGG	60.0	219	INTERGENIC	--	--	--
Ca6_P3_54712565	Ca6	54712565	AAAACTAA/AAAACTAAACTAA	GCATACCCTTGGTAAATTGCTC	TGCTAGAAATAGAATTTGGCACA	59.9	352	INTERGENIC	--	--	--
Ca6_P3_54720503	Ca6	54720503	CTT/CT	GTCTACTCCCTCACCCCACA	CTGGACATCTATTGGTAGCCG	60.0	378	INTERGENIC	--	--	--
Ca6_P3_54754837	Ca6	54754837	GCCAAATCGATTT/GCCAAATCGATTTCCAAATCGATTT	GTAAAGGCCACCAATCGAAA	TTGTCCGTGAGAGACTGCAC	59.9	939	INTERGENIC	--	--	--
Ca6_P3_55145116	Ca6	55145116	C/CTAGCCCTCCT	GACAAACCGTGGAAACCAAT	GGAGCGACACGATCTAGAGG	59.7	328	INTERGENIC	--	--	--
Ca6_P3_55332973	Ca6	55332973	CAATAATAATAATAATAATAATAATAATAATAATAATA/CAATAATAATAATAATAATAATAATAATAATAATA	AGAAACAAGGCAAGCTCCAA	GGAACATTTGGCCTCCAATA	60.0	512	CDS (large-effect mutations)	Ca_21219	K	TALE
Ca6_P3_55437144	Ca6	55437144	AT/A	TGACTTTGATGTCGCAAAGC	AGGGATGCGACCATCTATTG	60.0	645	INTERGENIC	--	--	--
Ca6_P3_55445574	Ca6	55445574	GTTATTATTATTATTATTATTATTATTATTATTATTATTATTATTATTATT/GTTATTATTATTATTATTATTATTATTATTATTATTATTATTATTATTATTATTATTATT	AATCACGCGAATCCACAAAT	AATTCCTACCCTCCCCACAC	60.3	582	INTERGENIC	--	--	--
Ca6_P3_55584714	Ca6	55584714	AATTATTATTATTATTATTATTATTATTATTATTATTATTATTATTATTATTATTATTATTATT/AATTATTATTATTATTATTATTATTATTATTATTATTATTATTATTATTATTATTATTATTATTATT	GCTTCCACATAGGTACTGCG	TTCCTCCATTATAGCTCAAGTTTGT	58.4	842	INTERGENIC	--	--	--
Ca6_P3_55769150	Ca6	55769150	C/CT	TCGATTATGCGTAGGGAAGG	TTTTTCCTTTGTAGGCCCAA	60.1	943	INTERGENIC	--	--	--

INDEL marker IDs	Chromosomes/ unanchored scaffolds	Physical positions (bp)	InDels (*Kabuli* reference genome-CDC Frontier/PII)	Forward primers (5'-3')	Reverse primers (5'-3')	Anneling temperature (0C)	Expected amplified product size (bp)	Structural annotation		Functional annotation	
								Sequence components of *kabuli* genome	*Kabuli* gene accession IDs	NCBI-KOG	TFs
Ca6_P3_55771133	Ca6	55771133	GTC/GTCTTC	GGAAACGTGTGAACGGAAGT	GGTAACCAATCCGAAGACGA	60.0	575	INTERGENIC	--	--	--
Ca6_P3_55771190	Ca6	55771190	TAT/TATCGTCTTCGGAT	TTACCGATGCATTGATCGAC	GGTAACCAATCCGAAGACGA	59.5	143	INTERGENIC	--	--	--
Ca6_P3_55771649	Ca6	55771649	TAAAAAAAAA/TAAAAAAAAAAAAA	GGATTGATTATCCGATGCGT	GAACGCGAGATATTGGGTGT	59.8	544	INTERGENIC	--	--	--
Ca6_P3_55781099	Ca6	55781099	GTTTTT/GTTTT	AGAAGGCTACCACTACGCGA	ATCGACTCCCGAACTCAAAA	60.0	500	INTERGENIC	--	--	--
Ca6_P3_55987809	Ca6	55987809	AATATATATATATATATATATATATATATATATATAT/AATATATATATATATATATATATATATATATATATATATAT	TTCAAGGTTCATTAACATCAACG	AAAGTTGACTCATATCAAAACCCA	59.0	390	INTERGENIC	--	--	--
Ca6_P3_56048704	Ca6	56048704	CTTT/CTT	AAAAACAGGTTGTGCGGAAG	AATGGCCACGAATCTGAAAG	60.1	374	INTERGENIC	--	--	--
Ca6_P3_56087656	Ca6	56087656	CTTT/CTTTT	TTGTGAGCATGAGAAGTTGTTG	TGAAAAATTATTGAAAAGTTACTTCC	59.0	737	INTERGENIC	--	--	--
Ca6_P3_56087684	Ca6	56087684	CAGA/CA	TTGTGAGCATGAGAAGTTGTTG	CAATTACTCGGTAGTGTCAATCCA	59.0	872	INTERGENIC	--	--	--
Ca6_P3_56139090	Ca6	56139090	AGG/AAACGG	ACCCTCTCACCCCTCTTGAT	TCCTCATCATTCCCTCCTTG	59.9	387	INTERGENIC	--	--	--
Ca6_P3_56145952	Ca6	56145952	AAATAATAATAATAATAATAATAATAATAATAATAATAA/AAATAATAATAATAATAATAATAATAATAATAATAATAATAA	CCGGAGAGTGACCAATTGAA	TCAATTGCATAAGCCAAAACA	61.0	687	INTERGENIC	--	--	--

INDEL marker IDs	Chromosomes/ unanchored scaffolds	Physical positions (bp)	InDels (*Kabuli* reference genome-CDC Frontier/PII)	Forward primers (5'-3')	Reverse primers (5'-3')	Anneling temperature (0C)	Expected amplified product size (bp)	Structural annotation		Functional annotation	
								Sequence components of *kabuli* genome	*Kabuli* gene accession IDs	NCBI-KOG	TFs
Ca6_P3_563674	Ca6	563674	AATATATATATATATATATATATATTTATATATATATAT/AATATATATATATATATATATATTTATATATATATAT	GGATCAGATGGGTCGGTAGA	ATGCACGTTTGGTATGGTCA	59.9	441	DRR	Ca_13277	C	--
Ca6_P3_56413983	Ca6	56413983	ATTT/ATT	TTCAACAATCGCAACCACAT	CGCGGGTCATGTTCTAGTTT	60.0	376	INTERGENIC	--	--	--
Ca6_P3_5645292	Ca6	5645292	CTATATATATATATATATAT/CTATATATATATATATATATATAT	GTAAACCCAAGAGAGGTGCG	TTCTAAATGGCGACTTTCCA	59.7	923	INTERGENIC	--	--	--
Ca6_P3_5645464	Ca6	5645464	TTATATATATATATATATATATATATATATAT/TTATATATATATATATATATATATATATATATATAT	GGCCCACATATGAAGGTGAA	GGTCGCTTTAATTGCACACA	60.7	846	INTERGENIC	--	--	--
Ca6_P3_56462708	Ca6	56462708	AATATATATATATATATATATAT/AATATATATATATATATATATATAT	CATTCCCTCTGGCTTGTCAT	AGGAAGAGTGGAGTTGCGAA	60.1	605	INTERGENIC	--	--	--
Ca6_P3_56503979	Ca6	56503979	AAACAA/AAA	GTTGGCGCAAACTCTTCTTC	TGCAACTTTCAGGTCCCTTT	60.0	583	INTERGENIC	--	--	--
Ca6_P3_56504001	Ca6	56504001	ATTTTT/ATTTT	GTTGGCGCAAACTCTTCTTC	TGCAACTTTCAGGTCCCTTT	60.0	583	INTERGENIC	--	--	--
Ca6_P3_56504140	Ca6	56504140	AAAAGAAA/AAAA	AAATGCAAAGTCGGATGGTC	TGCGAAGAAGAATGATCACG	59.9	896	INTERGENIC	--	--	--
Ca6_P3_56504596	Ca6	56504596	CTATA/CTA	CGTGATCATTCTTCTTCGCA	ACGTCCGACTTGCGTCTTAT	59.9	174	INTERGENIC	--	--	--
Ca6_P3_56504753	Ca6	56504753	ATTTTTTTTTTTTT/ATTTTTTTTTT	CGTGATCATTCTTCTTCGCA	TCGGTCACCAAGTTACCACA	59.9	614	INTERGENIC	--	--	--

INDEL marker IDs	Chromosomes/ unanchored scaffolds	Physical positions (bp)	InDels (*Kabuli* reference genome-CDC Frontier/PII)	Forward primers (5'-3')	Reverse primers (5'-3')	Anneling temperature (0C)	Expected amplified product size (bp)	Structural annotation		Functional annotation	
								Sequence components of *kabuli* genome	*Kabuli* gene accession IDs	NCBI-KOG	TFs
Ca6_P3_56505598	Ca6	56505598	AC/A	TGGTAACTTGGTGACCGACA	AATTTTTGCAGAAGAAAGAAAAAG	60.0	485	INTERGENIC	--	--	--
Ca6_P3_56507452	Ca6	56507452	TATCA/TA	CGTGATCATTCTTCTTCGCA	ATCCAAACATTGGCCATTTC	59.9	685	INTERGENIC	--	--	--
Ca6_P3_56507592	Ca6	56507592	ATTTTTTTTTT/ATTTTTTTTT	CGTGATCATTCTTCTTCGCA	ATCCAAACATTGGCCATTTC	59.9	685	INTERGENIC	--	--	--
Ca6_P3_56507723	Ca6	56507723	GTTTTTTTTTTTTTT/GTTTTTTTTTTTTTTTTT	CGTGATCATTCTTCTTCGCA	CTCCCAATTTGCAGTGTCCT	59.9	966	INTERGENIC	--	--	--
Ca6_P3_56507798	Ca6	56507798	CA/CAA	AAATTCAAGTCTCGACGCAAA	CTCCCAATTTGCAGTGTCCT	59.9	511	INTERGENIC	--	--	--
Ca6_P3_5653829	Ca6	5653829	TAAAAAAAA/TAAAAAAA	ATTAAGCCGCGTTGGTTATG	TAGGCGGCCCAAGTACAATA	60.0	832	INTERGENIC	--	--	--
Ca6_P3_56554078	Ca6	56554078	GATAATAATAATAATAATAATAATAATAATAATCATAATAATCATAATAATAATAATAATAAT/GATAATAATAATAATAATAATAATAATAATCATAATAATCATAATAATAATAATAATAAT	TTGGACTCACGTCGTCAGAG	GTTTGCCCATAAGTGGCAGT	60.0	682	INTRON	Ca_17393	D	--
Ca6_P3_56593690	Ca6	56593690	CATGTG/C	GTCGAATCAATCTGCCCAAT	TGCTTACATGACACCCATGC	59.9	549	INTERGENIC	--	--	--
Ca6_P3_56634765	Ca6	56634765	CATATATATA/CATATATATATATATATATATATATA	GATCTGGCAGAGGAACTTCG	GAGCCTATGGGAAGGAGAGG	60.0	566	DRR	Ca_17399	--	Dof
Ca6_P3_56688837	Ca6	56688837	GTTTT/GTTTTT	TGTTGAGTCGTGGTTATGGG	TGAACATGCATTCCGAAAAA	59.4	725	DRR	Ca_17406	--	--

INDEL marker IDs	Chromosomes/ unanchored scaffolds	Physical positions (bp)	InDels (*Kabuli* reference genome-CDC Frontier/PII)	Forward primers (5'-3')	Reverse primers (5'-3')	Anneling temperature (0C)	Expected amplified product size (bp)	Structural annotation		Functional annotation	
								Sequence components of *kabuli* genome	*Kabuli* gene accession IDs	NCBI-KOG	TFs
Ca6_P3_56713561	Ca6	56713561	ATT/ATATTT	TTCACGTTGGAACTTGCTTG	TTTTTGTGATTCAGGTGGCA	59.9	833	INTERGENIC	--	--	--
Ca6_P3_56727392	Ca6	56727392	TAA/TAAA	AGGCAAAACATGTTCCCTCA	AATGTTAGTGCACCGTGTCG	60.5	706	INTERGENIC	--	--	--
Ca6_P3_56738738	Ca6	56738738	TAAAAAAAA/TAAAAAAAAA	TTGTCATCATTAACACGTGGC	GGTTCTAGGGGCTCGAGATT	59.5	337	INTERGENIC	--	--	--
Ca6_P3_56767873	Ca6	56767873	TAAAAAAAAAAAA/TAAAAAAAAAAAAA	TCATGGATTCAACCAGATGC	TTCATCTTTCAAACTTGAGACTCG	59.5	188	INTERGENIC	--	--	--
Ca6_P3_56782339	Ca6	56782339	AAATAATAATAATAATAATAATAATAATAATAATAATAATAATAGTAATAATAATAATAATAATAATAA/AAATAATAATAATAATAATAATAATAATAATAATAATAATAATAGTAATAATAATAATAATAATA[illegible]	TGAAGTGGTAACAAGGGCATT	ACAAATCCTCAAGAGGGTGC	59.5	409	INTERGENIC	--	--	--
Ca6_P3_56797374	Ca6	56797374	GTATTATTATTATTATTATTATTATTATTATTAT/GTATTATTATTATTATTATTATTATTATTATTAT	TGTCATCGTAGGGAGAAGGG	GGTGATTGTTCGAGTGACCA	60.1	629	INTERGENIC	--	--	--
Ca6_P3_56874982	Ca6	56874982	AAATAATAATAATAATAATAATAATAATAATAATAA/AAATAATAATAATAATAATAATAATAATAA	TGGAATGATGATTTTATGCATATTT	TTATTTTGACGTTTTCCCGC	58.7	725	INTERGENIC	--	--	--
Ca6_P3_56904639	Ca6	56904639	CATATATATATATATATATATATATATATATATATA/CATATATATATATATATATATATATATATATATATATATA	ATCGCTTGCAATAGGATTCG	GGAAATGGTTTGGGATGTTG	60.2	322	INTERGENIC	--	--	--
Ca6_P3_57039572	Ca6	57039572	TAAAAAAAA/TAAAAAAAAA	GGTGTAGTGGATCGGTCTTGA	ATAGTGCCGAAGGAAGCAAA	60.0	334	INTERGENIC	--	--	--
Ca6_P3_57068021	Ca6	57068021	TATAATAATAATAATAATAATAATAAT/TATAATAATAATAATAATAATAATAATAATAATAATAATAATAAT	GGGACTGGGGTCATTTAGGT	TCGTTCCTTCAGGAAGTCGT	60.1	741	INTERGENIC	--	--	--

INDEL marker IDs	Chromosomes/ unanchored scaffolds	Physical positions (bp)	InDels (*Kabuli* reference genome-CDC Frontier/PII)	Forward primers (5'-3')	Reverse primers (5'-3')	Anneling temperature (0C)	Expected amplified product size (bp)	Structural annotation		Functional annotation	
								Sequence components of *kabuli* genome	*Kabuli* gene accession IDs	NCBI-KOG	TFs
Ca6_P3_57072104	Ca6	57072104	TAAAAAAAAAAAA/TAAAAAAAAAAA	CTCGCTTCCTCATTCTCACC	GAGGTGGGCTCAAATGAGTT	60.0	457	INTERGENIC	--	--	--
Ca6_P3_57093681	Ca6	57093681	GTAATAATAATAATAATAATAATAATA/GTAATAATAATAATAATAATAATAATAATA	CCTAATTGAACGGCTATCGAA	GCAAAAGACGAGGAGTGGTC	59.2	585	INTERGENIC	--	--	--
Ca6_P3_57104113	Ca6	57104113	AT/ATATATATATATATTT	TCAACAAAATTGGAATGCAGA	TTCCTCGCAGAGACGAGAAT	59.1	763	INTERGENIC	--	--	--
Ca6_P3_57117976	Ca6	57117976	AATATATATATATATATATATATATATATATA/AATATATATATATATATATATATATATA	TTTCATGTGCGTGTGAGTGA	TCAACCAATTCAACAATTTGACA	59.9	519	INTERGENIC	--	--	--
Ca6_P3_57153972	Ca6	57153972	TTATATATATATA/TTATATATATATATA	TTCACCCTTGTGTTTGGTTTC	CATCCGTCGGTCATTTCATA	59.9	105	INTERGENIC	--	--	--
Ca6_P3_57167945	Ca6	57167945	AATATATATATATATATA/AATATATATATATATATATA	CGCACCCATACCTGCTTAAT	TGCAATAGGTATTTTGGTGAGC	60.0	881	INTERGENIC	--	--	--
Ca6_P3_57284439	Ca6	57284439	AATATATATATATATATATATATATATATATAT/AATATATATATATATATATATATATATATATATAT	CTTTACCACCTTCATTCGCC	GGGTGAACAGTGAATTAGAATCG	59.6	645	INTRON	Ca_13708	G	NAC
Ca6_P3_57388933	Ca6	57388933	TAAAAAAAAAAAAA/TAAAAAAAAAAAAAA	TTCATGAAGCACGGAAACTG	GGACGCATCCGTTACTCATT	59.8	533	INTERGENIC	--	--	--
Ca6_P3_57443271	Ca6	57443271	AATATATATATATATATATATA/AATATATATATATATATATA	TTGTGCATTCAACTTTTGCC	GGTAATTGGTCAACCATCGC	59.7	697	DRR	Ca_13699	R	C2H2
Ca6_P3_57482241	Ca6	57482241	TAAAAAAAAAAAAAA/TAAAAAAAAAAAAA	ATGATTATTTGCCCAGAGGC	TGTGTTAACTTGTCTGTCACATGG	59.0	530	INTERGENIC	--	--	--

INDEL marker IDs	Chromosomes/ unanchored scaffolds	Physical positions (bp)	InDels (*Kabuli* reference genome-CDC Frontier/PII)	Forward primers (5'-3')	Reverse primers (5'-3')	Anneling temperature (0C)	Expected amplified product size (bp)	Structural annotation		Functional annotation	
								Sequence components of *kabuli* genome	*Kabuli* gene accession IDs	NCBI-KOG	TFs
Ca6_P3_57490883	Ca6	57490883	ATTTTTT/ATTTTT	CAAGCATCATGTCGAGCCTA	CTTGTTCGGGTACCTCGTGT	60.0	621	INTERGENIC	--	--	--
Ca6_P3_5765272	Ca6	5765272	TTTTTTATTTTTATTTTTATTTTTATT/TTTTTTATTTTTATTTTTATT	CTCTTGGACATGTGGTGGTG	TCCCTTTCGTTTTGGTTGAC	60.0	541	INTERGENIC	--	--	--
Ca6_P3_57716318	Ca6	57716318	AATATATATATATATATATATATATATATATAT/AATATATATATATATATATATATATATATAT	GTCAACCACCGTTGAACCTT	TTGAACATTTGAAATCGGGA	59.9	485	INTERGENIC	--	--	--
Ca6_P3_58164188	Ca6	58164188	A/AGG	GCCCTGTTGCAGGTTATTGT	TCTTCAGCCTCACCAGAGGT	60.0	384	INTRON	Ca_13624	U	--
Ca6_P3_58195717	Ca6	58195717	AATATATATATATATATATATATATATAT/AATATATATATATATATATATATAT	TTTGGAAAAGTCCAAGCTGC	AATTGCTGCAACCGACTTCT	60.4	636	DRR	Ca_13621	T	bHLH
Ca6_P3_58302986	Ca6	58302986	AATATATATATATATATATATATATATATATATA/AATATATATATATATATATATATATATATATATATATA	TCTGGGCACTTATGCAACAC	TTTTTAGGATGTGGACGTTCG	59.7	616	INTERGENIC	--	--	--
Ca6_P3_58373671	Ca6	58373671	GATATATATATATATATATATATA/GATATATATATATATATATATATATA	TCACGCAAAAGATTCATAAAAGA	AGTTTGTAAGCTGTGCGTCG	58.9	500	INTERGENIC	--	--	--
Ca6_P3_58408707	Ca6	58408707	GATATATATATATATATATATATATATATATATATATATATAT/GATATATATATATATATATATATATATATAT	AAGACAGCAGCAAAAGCCAT	TGTTTTTGTGCGTTGACACAT	60.0	593	DRR	Ca_15377	U	--
Ca6_P3_5846506	Ca6	5846506	TAAAAAAAAAAAAA/TAAAAAAAAAA	TGATTAGGCCTGAATTGTGGT	GCAAGTTGCCAAGAAAAAGC	59.4	461	INTERGENIC	--	--	--
Ca6_P3_58539579	Ca6	58539579	CATATATATATATATATATATATATATATATATATATAT/CATATATATATATATATATATATATATATATATATATAT	CGAGCCTAATAGTCGACCCA	AACGGGTCAACCAATCACAT	60.2	258	INTERGENIC	--	--	--

**INDEL marker IDs**	**Chromosomes/ unanchored scaffolds**	**Physical positions (bp)**	**InDels (*Kabuli* reference genome-CDC Frontier/PII)**	**Forward primers (5'-3')**	**Reverse primers (5'-3')**	**Anneling temperature (0C)**	**Expected amplified product size (bp)**	**Structural annotation**		**Functional annotation**	
								**Sequence components of *kabuli* genome**	***Kabuli* gene accession IDs**	**NCBI-KOG**	**TFs**
Ca6_P3_58563480	Ca6	58563480	AT/A	AGCAAAAGGGGGATGAAAGT	GAAAGCATCGTGGGACATTT	59.9	160	INTERGENIC	--	--	--
Ca6_P3_58594670	Ca6	58594670	TAAAA/TAAAAA	GATGATGTGGCTGCTCAAGA	TTCATTTGTCCGTTGAGTGC	60.0	490	DRR	Ca_15401	--	--
Ca6_P3_58615933	Ca6	58615933	GTAGTT/G	CAAATTGTCCGTGCAATCAC	GAAGAATGGTAGGCGGAACA	60.0	227	INTERGENIC	--	--	--
Ca6_P3_58644726	Ca6	58644726	AATATATATATATATATATATATATA/AATATATATATATATATATATATA	TTCCGTACAATTGGGTTGGT	TTGATAACAAAGATGTGTCTTGTATTT	60.1	594	INTERGENIC	--	--	--
Ca6_P3_58647704	Ca6	58647704	CTTTTTTTTTTT/CTTTTTTTTTT	TGCGAGTTCGCTGTTGTAGT	CAGATTCATTTGCCACAAACA	59.7	351	DRR	Ca_15410	U	B3
Ca6_P3_58668815	Ca6	58668815	TTCTTAAAATAATTATCTTAAAATAATT/TTCTTAAAATAATT	AAACACTTGTGACGACACCG	ATCCTTGTCAAACTCGCACC	59.6	537	INTERGENIC	--	--	--
Ca6_P3_58691212	Ca6	58691212	GTTTTTTTTTTTT/GTTTTTTTTTTT	GGCACGTCGTAGGCTTACTT	GGGTTAGCCTAAGGTCCAGG	59.4	835	INTERGENIC	--	--	--
Ca6_P3_58709173	Ca6	58709173	AAATAATAATAATAATAATAATAATAATAATAATAATAATAATAATAATAA/AAATAATAATAATAATAATAATAATAATAATAATAATAATAATAATAATAATAATAATAATAATAATAA	TGAGTCCGTGTCAGTGCTTC	GACTGCACTGTTTGGCTTGA	60.0	848	DRR	Ca_15414	--	--
Ca6_P3_58758402	Ca6	58758402	ATTTTTTTTTT/ATTTTTTTTTTT	TGGTCTGGCCTATTCTCACC	GATCCGTCAAGTCCCTCGTA	60.1	449	INTERGENIC	--	--	--
Ca6_P3_58781089	Ca6	58781089	C/CGTA	TTCAACGGACGATTTTCTCC	CCTCCGACTAAATGAGGTCC	60.1	756	INTERGENIC	--	--	--

INDEL marker IDs	Chromosomes/ unanchored scaffolds	Physical positions (bp)	InDels (*Kabuli* reference genome-CDC Frontier/PII)	Forward primers (5'-3')	Reverse primers (5'-3')	Anneling temperature (0C)	Expected amplified product size (bp)	Structural annotation		Functional annotation	
								Sequence components of *kabuli* genome	*Kabuli* gene accession IDs	NCBI-KOG	TFs
Ca6_P3_58781204	Ca6	58781204	CTTTTTTT/CTTTTTT	TTCAACGGACGATTTTCTCC	CCTCCGACTAAATGAGGTCC	60.1	756	INTERGENIC	--	--	--
Ca6_P3_58785175	Ca6	58785175	TTCTCTCTCTCTCTCT/TTCTCTCTCTCTCT	TCAAATATTGATGCGACAAAAA	TTGTCAAATGCTGTTTGCGT	58.2	522	DRR	Ca_15422	K	MYB
Ca6_P3_58787039	Ca6	58787039	TAATAAATAAATAAATAAATAAA/TAATAAATAAATAAATAAATAAATAAATAAA	GCAAAGCATCTAGGTGTCCC	GCCAATCGATGAGATCCAGT	59.7	233	DRR	Ca_15422	K	MYB
Ca6_P3_58789488	Ca6	58789488	TC/T	CGATGTTTCCTCCTTCTTCG	AAACAAAAGCGCACACTCAA	59.8	769	INTERGENIC	--	--	--
Ca6_P3_58799381	Ca6	58799381	ATTTTTTTT/ATTTTTTTTT	TAGCAACCGGTCATGAGTGA	TGGAAAACACGTTTCAATGTT	60.3	802	INTERGENIC	--	--	--
Ca6_P3_58799717	Ca6	58799717	TAAAAAAAAA/TAAAAAAAA	TTCTTTGAGGAGAAAAATCATCA	TGGAAAACACGTTTCAATGTT	58.0	452	INTERGENIC	--	--	--
Ca6_P3_58808278	Ca6	58808278	T/TC	TGGTGCGTCCACTATTCAAA	ATTTTCCCACATGGACTGGT	60.1	622	INTERGENIC	--	--	--
Ca6_P3_58811718	Ca6	58811718	TAAAAAAA/TAAAAAAAA	AAGTATCCATGTCAAGGAATCAAA	TTTATGGAAGTCTTTTGCGGA	58.9	814	DRR	Ca_15423	K	GATA
Ca6_P3_58813046	Ca6	58813046	AATAT/AAT	AATAGAACCGCAAAACACGG	AAAAGGTTACCGACGACATATCA	60.0	267	INTERGENIC	--	--	--
Ca6_P3_58819093	Ca6	58819093	GAAAAAAA/GAAAAAA	GTTGAGCCGACAAAAATTGA	TAGTTGAACTCGTGCGTTGC	58.7	815	INTERGENIC	--	--	--

INDEL marker IDs	Chromosomes/ unanchored scaffolds	Physical positions (bp)	InDels (*Kabuli* reference genome-CDC Frontier/PII)	Forward primers (5'-3')	Reverse primers (5'-3')	Anneling temperature (0C)	Expected amplified product size (bp)	Structural annotation		Functional annotation	
								Sequence components of *kabuli* genome	*Kabuli* gene accession IDs	NCBI-KOG	TFs
Ca6_P3_58819395	Ca6	58819395	TAAAAAAA/TAAAAAAAA	TGCAAAAAGGTCAACAGATTT	TAGTTGAACTCGTGCGTTGC	57.4	381	INTERGENIC	--	--	--
Ca6_P3_58823708	Ca6	58823708	GTAA/GTAAATAA	AATTTTAGATCACGTGGCGT	GCACTCATTAGCCGTTCCAT	57.2	830	DRR	Ca_15424	S	--
Ca6_P3_58823752	Ca6	58823752	TAGGTCAGGTCAG/TAGGTCAGGTCAGGTCAG	GACTACTGAAGTTAGGGGTGGG	GCACTCATTAGCCGTTCCAT	59.0	314	DRR	Ca_15424	S	--
Ca6_P3_58823828	Ca6	58823828	A/ATCATAG	CTTTGAAAGGCCTGAACCTG	GCACTCATTAGCCGTTCCAT	59.8	276	DRR	Ca_15424	S	--
Ca6_P3_58827188	Ca6	58827188	GAAAAA/GAAAAAA	GTGCAAACCAGTGGAATCCT	TTGGTTTGGGTTGGAACATT	60.0	600	INTRON	Ca_15424	S	--
Ca6_P3_58834846	Ca6	58834846	GTTTTTTTTTT/GTTTTTTTTTTT	TGAAACGTCAACTTTTCGGA	AGGACTTCACCATCAATTCCA	59.3	292	INTERGENIC	--	--	--
Ca6_P3_58851448	Ca6	58851448	AG/A	CTTGATTTGCGGATGGATTT	TTAAAGCGCTCCCATCAACT	59.9	896	INTERGENIC	--	--	--
Ca6_P3_58858147	Ca6	58858147	TTTTATTTATTTATTTATTTATTTATTTATTTAT/TTTTATTTATTTATTTATTTATTTATTTATTTATTTATTTAT	AGCTTTTCTTCGCAAATCCA	AGCATTGGTCCCAAACAAAC	60.0	739	INTERGENIC	--	--	--
Ca6_P3_58873196	Ca6	58873196	GATATATATATATATATATATATATATATA/GATATATATATATATATATATATATATATATA	TTGAGCGAGAATGTAGAGAGAATG	AGAGCAAGCTTAAGCCGACA	60.0	233	INTERGENIC	--	--	--
Ca6_P3_58874492	Ca6	58874492	GTATATATATATATATATATATATATA/GTATATATATATATATATATATA	TCTCAATGTGATTTTAATGTTCCC	GCCTCCTCATGCTGATTCAC	59.3	671	INTERGENIC	--	--	--

INDEL marker IDs	Chromosomes/ unanchored scaffolds	Physical positions (bp)	InDels (*Kabuli* reference genome-CDC Frontier/PII)	Forward primers (5'-3')	Reverse primers (5'-3')	Anneling temperature (0C)	Expected amplified product size (bp)	Structural annotation		Functional annotation	
								Sequence components of *kabuli* genome	*Kabuli* gene accession IDs	NCBI-KOG	TFs
Ca6_P3_58875941	Ca6	58875941	GTTTTTTTTTTT/GTTTTTTTTTT	TGGTCCTTTCTGGGAATCTG	TCCGTCTGCCCACTAGTTTT	60.0	628	INTERGENIC	--	--	--
Ca6_P3_59035946	Ca6	59035946	TCCGCACCCGCACCCGCACCC/TCCGCACCCGCACCCGCACCCGCACCC	CTAATAATACTTGCGGGGCG	GGTTGTTCTTACGGGACCAA	59.6	394	INTERGENIC	--	--	--
Ca6_P3_59066187	Ca6	59066187	TAAAAAAAAA/TAAAAAAAAAA	TGTTTGTCACACTTTCAAATCCA	TGTGGGCTGATGTTGGTTTA	60.4	608	INTERGENIC	--	--	--
Ca6_P3_59067085	Ca6	59067085	AAAAACAGAGAA/AAAAACAGAGAAAACAGAGAA	GGCCGGTTTAACAAGAATGA	GCAACACATTCACCAGCAAC	59.9	493	INTERGENIC	--	--	--
Ca6_P3_5906777	Ca6	5906777	TCC/TC	AGGCACATTTAGTCGACCGT	CATGGAAATTGAACCAGGCT	59.6	584	INTERGENIC	--	--	--
Ca6_P3_59072034	Ca6	59072034	CTT/CT	TTGCTTGAAGGATGGAGACC	AGGGAGAAACAAGACTCGCA	60.2	775	INTERGENIC	--	--	--
Ca6_P3_59093194	Ca6	59093194	ATTTTTTTTTTT/ATTTTTTTTTT	GGAGGCAACCCAACTCTATG	TCGCATAATTGGAACGTCAA	59.6	271	INTERGENIC	--	--	--
Ca6_P3_5909643	Ca6	5909643	GTTACTTAC/GTTAC	CCCATTTCGGTTTTGATGAC	GATGATTTGGGTTTTGTGGG	60.2	479	INTERGENIC	--	--	--
Ca6_P3_5912125	Ca6	5912125	A/AG	GCGAGAAGATTGCTTCCAAA	GGCTTTTCAATTACCCGTCA	60.5	561	INTERGENIC	--	--	--
Ca6_P3_59141297	Ca6	59141297	CAACAATAATAA/CAA	TTGAATTTTGAAATTTTGTGCG	CATTCGCGGTCCCTTAACT	60.0	675	INTERGENIC	--	--	--

INDEL marker IDs	Chromosomes/ unanchored scaffolds	Physical positions (bp)	InDels (*Kabuli* reference genome-CDC Frontier/PII)	Forward primers (5'-3')	Reverse primers (5'-3')	Anneling temperature (0C)	Expected amplified product size (bp)	Structural annotation		Functional annotation	
								Sequence components of *kabuli* genome	*Kabuli* gene accession IDs	NCBI-KOG	TFs
Ca6_P3_59193085	Ca6	59193085	CTTTTTTTTT/CTTTTTTTTTT	ATGGGGTTTACCCATTTGAA	ACATGATGGCAAGACCAAGA	59.0	511	INTERGENIC	--	--	--
Ca6_P3_59252761	Ca6	59252761	TAGAGAGAGAGAGAG/TAGAGAGAGAGAGAGAG	AATATGCTTTTGGGTCGTGC	CCATTTTAGGCTCGGATTGA	60.0	633	DRR	Ca_18849	--	--
Ca6_P3_59273276	Ca6	59273276	TA/TAAA	TGAAGCTTGTGCCAAAGGTA	GGTGGAGAATGAAACGGAGA	59.5	754	INTERGENIC	--	--	--
Ca6_P3_59273311	Ca6	59273311	TA/T	TTTTTGGGCTGAAGCCATAC	GGTGGAGAATGAAACGGAGA	60.1	359	INTERGENIC	--	--	--
Ca6_P3_59349983	Ca6	59349983	AATATATATATATATATATATATATATATA/AATATATATATATATATATATATATATATATATATA	TTTCCATTGCACTCACTCCA	CCTATCATGGCCTGACCTGT	60.2	777	INTERGENIC	--	--	--
Ca6_P3_59370629	Ca6	59370629	TAAAAAAAAAAAA/TAAAAAAAAAAAAA	CCATGGTTCCACCTCAATCT	CAAGCGATATCCAACGTGAA	59.8	710	INTERGENIC	--	--	--
Ca6_P3_59384361	Ca6	59384361	AATATATATATATATATA/AATATATATATATATATATA	CGGCCTTATCCTAAAGACCC	AAAATAGGGAGACCAATTTCG	59.9	393	INTERGENIC	--	--	--
Ca6_P3_59388804	Ca6	59388804	ATTTTTTTTTTT/ATTTTTTTTTT	TAGTGGAAGGGAACAGAGCG	CAGGATCGACTATTGGATTGG	60.4	622	INTERGENIC	--	--	--
Ca6_P3_59428823	Ca6	59428823	TA/T	ATGATGTCACTTGATGCGCT	TGAATGCAACCAAATTCGAC	59.3	312	INTERGENIC	--	--	--
Ca6_P3_59434628	Ca6	59434628	CAAAAA/CAAAAAA	ACAGCAGCCTTGCATTCTCT	TGTTGCCTCTGTTTTTGCAG	60.2	374	INTERGENIC	--	--	--

INDEL marker IDs	Chromosomes/ unanchored scaffolds	Physical positions (bp)	InDels (*Kabuli* reference genome-CDC Frontier/PII)	Forward primers (5'-3')	Reverse primers (5'-3')	Anneling temperature (0C)	Expected amplified product size (bp)	Structural annotation		Functional annotation	
								Sequence components of *kabuli* genome	*Kabuli* gene accession IDs	NCBI-KOG	TFs
Ca6_P3_6036338	Ca6	6036338	ATCTTCTTCTTCTTCTTCTTCTTCTTCT TCTTCTTCTTCTTCTTCTTCTTCTTCTT/ ATCTTCTTCTTCTTCTTCTTCTTCTTCT TCTTCTTCTTCTT	TCTCAAGAGTCACACG CCAC	TGGATTGATGGTGATT GATGA	60.0	478	DRR	Ca_05692	R	MYB
Ca6_P3_6082681	Ca6	6082681	TTATATATATATATATATATAT/TTATAT ATATATATATATATATATAT	CGCACTAGACTAAATG CGTGC	GCCCTTTGACGTCCAA CTAA	61.0	442	INTERGENIC	--	--	--
Ca6_P3_6089953	Ca6	6089953	ATTTTTTTTT/ATTTTTTTTTT	CGAATATCAAGCACGC ACAT	GCAACGGCAAGGATTG TTAT	59.7	921	INTERGENIC	--	--	--
Ca6_P3_6099996	Ca6	6099996	AATGTATGTATGTATGTATG/AATGTAT GTATGTATGTATGTATG	CTTCACTGCCTGCAAAT TCA	CCAAAAGAACACTGCA GCAA	60.0	285	INTRON	Ca_05686	C	--
Ca6_P3_6131095	Ca6	6131095	GAAAAAAAAAAA/GAAAAAAAAA	AGTGAAAAAGTGGAGA AGCGA	AAAGAAAATGGCTGCG CTAA	59.1	693	INTERGENIC	--	--	--
Ca6_P3_6153000	Ca6	6153000	AT/ATGT	CCATTAGTTGACCATCG CCT	GAGGACTAGAGGGAG TGCCC	60.0	155	INTERGENIC	--	--	--
Ca6_P3_6158112	Ca6	6158112	GTATATATATATATAT/GTATATATATAT ATATAT	GATGAGTGGAAGGAGC CAAA	TGAAAGATAATCCAATT TTAAATGAGT	60.2	418	INTERGENIC	--	--	--
Ca6_P3_6163420	Ca6	6163420	ATTTTTTTTT/ATTTTTTTTTT	CCGTCCCTTAGCAAAAT TCA	AAGCAACTATGGCAAA TGGG	60.1	443	DRR	Ca_05680	T	G2-like
Ca6_P3_6204955	Ca6	6204955	ATTTTTTTTTTT/ATTTTTTTTTT	ATGTCACGGATGATGT GCTG	CATTTTCAAATCAAATT CGTGAAC	60.6	599	INTERGENIC	--	--	--
Ca6_P3_6210136	Ca6	6210136	CTTTTTTTTTTT/CTTTTTTTTTT	CTGCATTACATTGGTGA CCG	TTGGAAAAGTTGGAGG ACCA	60.0	762	INTERGENIC	--	--	--

INDEL marker IDs	Chromosomes/ unanchored scaffolds	Physical positions (bp)	InDels (*Kabuli* reference genome-CDC Frontier/PII)	Forward primers (5'-3')	Reverse primers (5'-3')	Anneling temperature (0C)	Expected amplified product size (bp)	Structural annotation		Functional annotation	
								Sequence components of *kabuli* genome	*Kabuli* gene accession IDs	NCBI-KOG	TFs
Ca6_P3_623704	Ca6	623704	TAAAAAAAA/TAAAAAAAAA	CGTGTTTCGTAGGTCTCCCT	CATGAAGCAAATGGAGATGG	59.2	722	INTERGENIC	--	--	--
Ca6_P3_6242461	Ca6	6242461	CATATATATATATATATATATATATATATAT/CATATATATATATATATATATATATATATATATATAT	GGCATCCATTGGCTTAGAGA	ATGCGATAGCAAGGGGAGTA	60.2	389	INTERGENIC	--	--	--
Ca6_P3_6409588	Ca6	6409588	TTATATATATATATATAT/TTATATATATATATATATATAT	TCCACGGCCTCTTGTGTTAT	AGAGCAAGCAAGTTGGTGGT	60.5	581	INTERGENIC	--	--	--
Ca6_P3_6410135	Ca6	6410135	CACCAACT/CACCAACTACCAACT	ACCACCAACTTGCTTGCTCT	GATCCCAACCATCATTGTCC	59.9	543	INTERGENIC	--	--	--
Ca6_P3_6411151	Ca6	6411151	ATAATTTTGTTAATTTTGTT/ATAATTTTGTTAATTTTGTTAATTTTGTT	GCACTCCCGCTTTCACTTAC	TTTGGGAAGGGTCAAGAAAA	59.9	929	INTERGENIC	--	--	--
Ca6_P3_6411336	Ca6	6411336	AAT/AATAT	ATCTCGTGGTGCAACAATCA	TTTGGGAAGGGTCAAGAAAA	60.1	628	INTERGENIC	--	--	--
Ca6_P3_6411391	Ca6	6411391	AATTTATTTATTTATTTAT/AATTTATTTATTTATTTATTTATTTAT	ATCTCGTGGTGCAACAATCA	TTTGGGAAGGGTCAAGAAAA	60.1	628	INTERGENIC	--	--	--
Ca6_P3_6498713	Ca6	6498713	ATTTTTTTTTTT/ATTTTTTTTTTTT	TTCCAAAAACAACCATAGTTTCA	TTGTTTTGCCCCATACAAAA	58.6	782	INTERGENIC	--	--	--
Ca6_P3_6534305	Ca6	6534305	CTTTTTTTTT/CTTTTTTTT	TGAGTCGGCTTATCATGTTTAAT	ACTCATTGCCCCATCATTTG	57.9	821	INTERGENIC	--	--	--
Ca6_P3_6583204	Ca6	6583204	TAAAAAAAAAA/TAAAAAAAAAAA	TACGCGGATACGGGTTATGT	AGGACAAATGTGGGAATTGG	60.2	421	INTERGENIC	--	--	--

INDEL marker IDs	Chromosomes/ unanchored scaffolds	Physical positions (bp)	InDels (*Kabuli* reference genome-CDC Frontier/PII)	Forward primers (5'-3')	Reverse primers (5'-3')	Anneling temperature (0C)	Expected amplified product size (bp)	Structural annotation		Functional annotation	
								Sequence components of *kabuli* genome	*Kabuli* gene accession IDs	NCBI-KOG	TFs
Ca6_P3_6584882	Ca6	6584882	ATTATTTTTATTTTTATTTTTATTTTTAT/ATTATTTTTATTTTTATTTTTATTTTTATTTTTAT	AAAGACAAAATTATCCTTGTGATAAAA	GACAAATGAATGAAAGCCCG	57.9	476	INTERGENIC	--	--	--
Ca6_P3_6613313	Ca6	6613313	GTTTTTTTTTTT/GTTTTTTTTTT	ATAACGCATCCATTCTTGCC	TGTTTTCTTCTTCTCCATGTCG	59.9	495	INTERGENIC	--	--	--
Ca6_P3_6622549	Ca6	6622549	CAAAAA/CAAAAAA	TTGGTTTAGGAATTATGGGCA	AATCACAAACCGGAGTGAGG	59.3	527	DRR	Ca_16922	--	--
Ca6_P3_67019	Ca6	67019	TTATATATATATATAT/TTATATATATATATATATAT	TCATCATCAGCAGAGCAAGG	ACGCATCGATAACCAATTCC	60.1	728	DRR	Ca_26377	--	--
Ca6_P3_6749373	Ca6	6749373	TTAATAATAATAATAATAATAATAATAATAATAATAATAATAATAATAATAATA/TTAATAATAATAATAATAATAATAATAATAATAATAATAATAATAATAATAATAATAATAATAATA	CCAGATCAATCGTGTTGCAC	TGGAAGTTTCGAAAAATGGG	60.1	568	INTERGENIC	--	--	--
Ca6_P3_6754190	Ca6	6754190	AATAATTATTAT/AAT	AAGATTGTCACGTGTTTGTCGT	TTGATTGACAAAGCACCGAA	59.6	688	INTERGENIC	--	--	--
Ca6_P3_6821610	Ca6	6821610	T/TTATTAC	TTTTCAACGTTTCCCGATTC	CAATTTTTCCGATACACGCC	59.9	434	INTERGENIC	--	--	--
Ca6_P3_6866662	Ca6	6866662	AT/A	CGTGTATGGCATTCAAGTGG	TGGTCTAGCTTGGCACTCCT	60.0	113	INTERGENIC	--	--	--
Ca6_P3_6914708	Ca6	6914708	TG/TGG	CCATGTCAACATAGCCTCCA	CATTAGTGGCACACCATTGC	59.5	652	INTRON	Ca_16954	KL	G2-like
Ca6_P3_6990532	Ca6	6990532	TTATATATATATATATATATATATATATATATATAT/TTATATATATATATATATATATATAT	GGCCACAAACCAAAGTGTTT	AGTTCACGGGTGCTTGTTTC	59.9	500	INTERGENIC	--	--	--

INDEL marker IDs	Chromosomes/ unanchored scaffolds	Physical positions (bp)	InDels (*Kabuli* reference genome-CDC Frontier/PII)	Forward primers (5'-3')	Reverse primers (5'-3')	Anneling temperature (0C)	Expected amplified product size (bp)	Structural annotation		Functional annotation	
								Sequence components of *kabuli* genome	*Kabuli* gene accession IDs	NCBI-KOG	TFs
Ca6_P3_706595	Ca6	706595	GATATATATATATATATATATATATATATATAT/GATATATATATATATATATATATATATATATATATATATATAT	GGTGCACGGTACATTCCTCT	AAAAAGTCACACCAGAGTTTGTCA	60.0	604	INTERGENIC	--	--	--
Ca6_P3_7250197	Ca6	7250197	AATATATATATATATATATATATATATATATATATAT/AATATATATATATATATATATATATATATATATATATATATATAT	TGAAAGAGGTTAGACTCCCACAA	TGACGAGTTTTAGATTGGCG	60.2	852	INTERGENIC	--	--	--
Ca6_P3_7301565	Ca6	7301565	TTTTATTATTTATTATTT/TTTTATTATTTATTATTTATTATTT	CAATGCGTCCACAGCTTCTA	TGATGGATGTAGCAACGGAA	60.0	878	INTERGENIC	--	--	--
Ca6_P3_7343632	Ca6	7343632	ATTTTTTTTTTT/ATTTTTTTTTT	CTGAGTCGGTATCGCCAAAT	AGGGCTTTCCAAAAGGTGTT	60.1	600	INTRON	Ca_09645	--	--
Ca6_P3_7350115	Ca6	7350115	TAAAAAAAAA/TAAAAAAAA	TGAGGGTGTTCACTTGTTCG	TTGGGAGGATTTGCAATTTT	59.7	735	INTRON	Ca_09644	--	GRAS
Ca6_P3_7386884	Ca6	7386884	AATATATATATATATATATA/AATATATATATATATATA	TGTACCGACACATAGTCGCC	TTGTGTCGTTGTCGTCGTTT	59.6	799	INTERGENIC	--	--	--
Ca6_P3_7459135	Ca6	7459135	TGG/TG	CCCGTCGAGTTAGATTGCAT	ACCTCATAAAATCCGGGTCC	60.1	394	INTERGENIC	--	--	--
Ca6_P3_7586339	Ca6	7586339	GTGATGATGATGA/GTGATGATGATGATGA	GCACCTCTTGGACCATCAAT	ACAGTGTCAGTGCCTACCCC	59.9	454	CDS (large-effect mutations)	Ca_09626	C	--
Ca6_P3_7644876	Ca6	7644876	ATGTGTGTGTGTGTGT/ATGTGTGTGTGTGT	GACTTCGTTCGTGCTTCCTC	GCCACCGTTGTGTTCCTTAT	60.0	495	INTERGENIC	--	--	--
Ca6_P3_7651324	Ca6	7651324	T/TACATTATTA	ATGTTTGAATTGGAAAATAGACAAT	TTTCGAAAACCGAAAATTAGC	57.3	415	DRR	Ca_09618	--	--

INDEL marker IDs	Chromosomes/ unanchored scaffolds	Physical positions (bp)	InDels (*Kabuli* reference genome-CDC Frontier/PII)	Forward primers (5'-3')	Reverse primers (5'-3')	Anneling temperature (0C)	Expected amplified product size (bp)	Structural annotation		Functional annotation	
								Sequence components of *kabuli* genome	*Kabuli* gene accession IDs	NCBI-KOG	TFs
Ca6_P3_7669236	Ca6	7669236	ATTTTTTTT/ATTTTTTT	TGGTGTATGGGTCTGTTTGTTT	GGCCATGCTCTAGCATTACAA	59.3	743	INTERGENIC	--	--	--
Ca6_P3_7679757	Ca6	7679757	TC/TCC	CAGGGGCTCCAAATACTGAA	CGTAGCGATAAAACCTGGGA	60.1	902	CDS (FRAME SHIFT)	Ca_09613	G	--
Ca6_P3_7686149	Ca6	7686149	TA/T	CAATTTTTGGTGGCTGCTTT	GCAAATTCAAAAGAATTCAAAGG	60.1	838	INTERGENIC	--	--	--
Ca6_P3_7708884	Ca6	7708884	AAGAG/AAG	CTCATCACACTGGGCAAATG	TGTTCATTTCCAATGCCAAA	60.1	983	INTERGENIC	--	--	--
Ca6_P3_7712583	Ca6	7712583	CATATATATATATATATATAT/CATATATATATATATATATATATAT	ATCGCAGGTATGAGGACAGG	GCCATGGTTGTAGGAGAAACA	60.1	256	INTERGENIC	--	--	--
Ca6_P3_7720318	Ca6	7720318	CTATTATTATTATTATTATTATTATTATTATTATTATT/CTATTATTATTATTATTATTATTATTATTATTATT	CTTTTGGCCTTGGTGTTGTT	AAAGGGAAAGGGCAAAGAAA	60.0	682	INTRON	Ca_09607	--	--
Ca6_P3_7734896	Ca6	7734896	TAAAAAAAAAAA/TAAAAAAAAAA	TCACAAGTCGGTGATTTTATTGA	TGACAGGGTGGAACTCATCA	59.5	710	INTERGENIC	--	--	--
Ca6_P3_7791689	Ca6	7791689	TAAAAAAAAA/TAAAAAAAAAAA	GGACTAGGACCGTCTTAAACCTT	TCCAATCCTCACATTTTACACAA	59.1	537	INTERGENIC	--	--	--
Ca6_P3_7806679	Ca6	7806679	TA/TACA	TCAAGGGATCCCCATAACAA	TTCACATTCCCCATTAAGCC	60.1	742	INTERGENIC	--	--	--
Ca6_P3_7841191	Ca6	7841191	ATTTTTTTTTT/ATTTTTTTTT	GGCTTTGTTTTTGGGTTTGA	CCTCGCCAAGCAAGATTAAG	60.0	891	INTERGENIC	--	--	--

INDEL marker IDs	Chromosomes/ unanchored scaffolds	Physical positions (bp)	InDels (*Kabuli* reference genome-CDC Frontier/PII)	Forward primers (5'-3')	Reverse primers (5'-3')	Anneling temperature (0C)	Expected amplified product size (bp)	Structural annotation		Functional annotation	
								Sequence components of *kabuli* genome	*Kabuli* gene accession IDs	NCBI-KOG	TFs
Ca6_P3_7841423	Ca6	7841423	AT/ATATGT	TTTGTAACCCTTTTCGTTATGC	CCTCGCCAAGCAAGATTAAG	58.2	628	INTERGENIC	--	--	--
Ca6_P3_7861016	Ca6	7861016	TAAAAAAAAAA/TAAAAAAAAA	CAAATATTCCAACTGCAACCAA	ACGCCTCCGCTTTTATTGAT	59.9	556	INTERGENIC	--	--	--
Ca6_P3_7866313	Ca6	7866313	TAAAA/TAAA	CCCACCCACTCAACTTTCTC	TGCATCTGAGGAAAACAACG	59.5	365	INTERGENIC	--	--	--
Ca6_P3_7972862	Ca6	7972862	GTATATATATAT/GTATATATATATATAT	CTCATTGACTTGGGATCCGT	GAGAAAAGGTGGATCCTACATACC	59.9	921	INTERGENIC	--	--	--
Ca6_P3_7985102	Ca6	7985102	TTT/TTTAATT	TCAACAGGATTTGCCAACTG	CACACCACCAAACGTTGAAG	59.7	473	INTRON	Ca_09577	G	--
Ca6_P3_7997168	Ca6	7997168	AAAAAATTAT/AAAAAATTATGAAAAATTAT	GCTTGCATTCAGTCAGACCA	GACACATGGTTGCACGATTC	60.0	625	INTERGENIC	--	--	--
Ca6_P3_8022121	Ca6	8022121	TAATTAAAAATTAAAAAT/TAATTAAAAATTAAAAATTAAAAAT	TTTGTTTGCGATGGCTCATA	TGTTTTTCCGAGCGTACACA	60.2	818	INTERGENIC	--	--	--
Ca6_P3_8045263	Ca6	8045263	ATTTTTTTTTT/ATTTTTTTTTTT	TGCGAATCTTCTTCTTCAACA	TTTTCCCCCACTAATTGCAT	58.6	492	INTERGENIC	--	--	--
Ca6_P3_8087365	Ca6	8087365	ATAATTAA/ATAA	CGTTCAACTGAAATTTGCCA	ATCCACACTTCCAGCCTGAT	59.7	450	INTRON	Ca_09562	S	CAMTA
Ca6_P3_8108556	Ca6	8108556	ATTTTTTTTTTT/ATTTTTTTTTT	GCGCGAAAGAATAACCAAAA	CCTCTAGGTGGCCAAACAAA	60.2	353	INTERGENIC	--	--	--

INDEL marker IDs	Chromosomes/ unanchored scaffolds	Physical positions (bp)	InDels (*Kabuli* reference genome-CDC Frontier/PII)	Forward primers (5'-3')	Reverse primers (5'-3')	Anneling temperature (0C)	Expected amplified product size (bp)	Structural annotation		Functional annotation	
								Sequence components of *kabuli* genome	*Kabuli* gene accession IDs	NCBI-KOG	TFs
Ca6_P3_8109504	Ca6	8109504	CTTTTTTTTT/CTTTTTTTT	TTTTTCTCGGAAATTGTTCTTAAA	TTTGAAAAATAAGAGGTGGCA	58.5	794	INTERGENIC	--	--	--
Ca6_P3_8174663	Ca6	8174663	ATT/ATTTT	TGACCTGACTTATTTCCACCC	GAGTGGTGAGCATGCGTTTA	58.9	302	DRR	Ca_09552	K	--
Ca6_P3_8240133	Ca6	8240133	ATTTTTTTT/ATTTTTTTTT	ATCTTCCCCTATTCGTTGGA	GCTAGAAGTGCAAGAGCCCA	58.5	575	INTERGENIC	--	--	--
Ca6_P3_8254638	Ca6	8254638	TTATATATATATATATATATATATATATATATA/TTATATATATATATATATATATATATATATATATATATATATA	TGTACCGGAAAACTATGTTGC	TTGTGACGATTCTCCGTTGA	57.7	810	INTERGENIC	--	--	--
Ca6_P3_8259286	Ca6	8259286	CATATATATATATATATATATATA/CATATATATATATATATATATATATA	GGAACCATGGGTGCTTTTTA	GACATCGGGAAGTGACAACA	59.8	610	INTRON	Ca_09544	T	YABBY
Ca6_P3_8313713	Ca6	8313713	GTTTTTTTTTT/GTTTTTTTTTTT	TTTGACAATCTCATGTTTTGCAG	GGGTCAAAAAGTGTGCAGGT	60.2	730	INTERGENIC	--	--	--
Ca6_P3_8364911	Ca6	8364911	CATATATATATATATAT/CATATATATATATAT	ACCGATGCACCGTTAGATTC	CGAATGGTGCGGATCTCTTA	60.0	612	DRR	Ca_09538	K	HD-ZIP
Ca6_P3_8395678	Ca6	8395678	AATATATATATATATATA/AATATATATATATATATATA	AACAAGACAAGGTGGGAACG	CACACATGCTTATGAACCCG	60.0	369	INTERGENIC	--	--	--
Ca6_P3_8410500	Ca6	8410500	CAA/CA	AATCGATTGATTCAGCCCAG	TAATGCCCCGTTTTTCAAAG	60.0	795	INTERGENIC	--	--	--
Ca6_P3_850916	Ca6	850916	TCC/TCCC	AATCAAGTGCAAAAGTGGGG	ACCACACCCATATCCAGCAT	60.0	704	INTERGENIC	--	--	--

INDEL marker IDs	Chromosomes/ unanchored scaffolds	Physical positions (bp)	InDels (*Kabuli* reference genome-CDC Frontier/PII)	Forward primers (5'-3')	Reverse primers (5'-3')	Anneling temperature (0C)	Expected amplified product size (bp)	Structural annotation		Functional annotation	
								Sequence components of *kabuli* genome	*Kabuli* gene accession IDs	NCBI-KOG	TFs
Ca6_P3_8539955	Ca6	8539955	CTGT/C	ATGCCAAGCTCTCTGCGTAT	TATGCAGGATGCAAAATGGA	60.0	431	INTERGENIC	--	--	--
Ca6_P3_8705473	Ca6	8705473	AAGAGAGAGAGAG/AAGAGAGAGAG	GCTTGTGGGGATGTTAAACC	GAAGGGAATGAAATGCGAAA	59.3	492	INTERGENIC	--	--	--
Ca6_P3_8746520	Ca6	8746520	TAAAAAAAAAAA/TAAAAAAAAAAAA	GCGGTGATGCCAGTTAGAAT	GGGAATGGTTTGCATTTGAC	60.1	627	DRR	Ca_16759	--	HD-ZIP
Ca6_P3_8796967	Ca6	8796967	A/AG	GCCAAACAATAGTTGAAACCAA	AACGTTACGCATGAACCTCC	59.0	588	INTRON	Ca_16754	E	--
Ca6_P3_8816206	Ca6	8816206	TAAAAAAAAAAAAA/TAAAAAAAAAAA	AAAAACAAACCGCAAAATGC	ACGCGCTGATAAATATTCCG	60.0	699	DRR	Ca_16752	--	--
Ca6_P3_8834153	Ca6	8834153	ATTTTTTTTT/ATTTTTTTT	TTGCAGATTTTTGAGAAGTTCA	TTTGGTGGGTGATAGGCAAT	57.7	401	INTERGENIC	--	--	--
Ca6_P3_8877553	Ca6	8877553	ATTTTTTTTTT/ATTTTTTTTTTT	CATAATGGGAAGCTGTTGCC	TGACACATCTATTCAACCGCA	60.5	353	INTERGENIC	--	--	--
Ca6_P3_8923181	Ca6	8923181	TAAAAAAAAA/TAAAAAAAAAA	AAAATTCAAGAGGTTCGCCA	TTTTATTCCTTCCCCAAGCC	59.7	546	INTERGENIC	--	--	--
Ca6_P3_8924425	Ca6	8924425	AATATATATATATATATATATATA/AATATATATATATATATATATATATA	ATTTGGCATATTCTCGGTCG	TTCATGGGTCTTGCATTTGA	59.9	419	INTERGENIC	--	--	--
Ca6_P3_9014214	Ca6	9014214	CA/CAA	TACTGGGGTGTCTGGTGTGA	AGCTGCACGCAGACTGTAAA	60.0	441	INTERGENIC	--	--	--

INDEL marker IDs	Chromosomes/ unanchored scaffolds	Physical positions (bp)	InDels (*Kabuli* reference genome-CDC Frontier/PII)	Forward primers (5'-3')	Reverse primers (5'-3')	Anneling temperature (0C)	Expected amplified product size (bp)	Structural annotation		Functional annotation	
								Sequence components of *kabuli* genome	*Kabuli* gene accession IDs	NCBI-KOG	TFs
Ca6_P3_9035166	Ca6	9035166	ATTTTTTTTT/ATTTTTTTTT	CCATCATATTTTTGAACCCCTT	AAGGCATAGACTACATTTGGCA	59.1	597	DRR	Ca_16737	--	--
Ca6_P3_9060428	Ca6	9060428	TTATAAAACATATAAAA/TTATAAAA	AAAGTACAATGAGTCAAACAATTATCC	ATATGCTTCACTTGCCCCAC	58.2	555	DRR	Ca_16733	E	--
Ca6_P3_9091565	Ca6	9091565	GTTATTATTATTATTATTATTATTACTATTATTATTACTATTATTATTATTGTTATTATTATTATTATTATT/GTTATTATTATTATTATTATTATTATTACTATTATTATTACTATTATTATTATTGTTATTATTATTATTATTATT	CTGAGCTCGAGGAAGCAGAT	GGCCGAAAATGATGTGAAAT	59.9	564	INTERGENIC	--	--	--
Ca6_P3_9101782	Ca6	9101782	G/GCCA	TTGATAACTTGAAAATGAAATGAAA	GTTTTGATCGGAGCAACCAT	57.1	521	INTERGENIC	--	--	--
Ca6_P3_9128509	Ca6	9128509	CTTTTTTTTTT/CTTTTTTTTTTT	AAACACCAAAGGTGAGGCAC	TTTGCGTTACGAATTTGATGA	60.0	411	INTERGENIC	--	--	--
Ca6_P3_9140484	Ca6	9140484	TAA/TAAA	TGTTTCATTTTTAAATGCGTCG	TCTACCCCAGGCCACTGTAG	60.0	476	DRR	Ca_08683	E	--
Ca6_P3_9203559	Ca6	9203559	GAA/GTTATATTAA	TCGTGAAATAGTGGACGGTG	TGAACATCAAAATGCCCAAA	59.6	763	DRR	Ca_08676	OT	--
Ca6_P3_9269804	Ca6	9269804	GGCGC/GGC	TATTGTGCAAAGCTCAGGGA	GGGGATAAAGTCCTTCCTGC	59.4	292	INTRON	Ca_08672	--	--
Ca6_P3_9279949	Ca6	9279949	TA/T	AGTTTAGGCGTGCGGTTTTT	GGATTTTGGGGATGTTTGAA	61.0	826	INTERGENIC	--	--	--
Ca6_P3_9369260	Ca6	9369260	ATTTTTTTTTTTT/ATTTTTTTTTTTTT	TGCGATTAGTTGGTCCCTTC	TTTGCGATGTTAATTTGCCA	60.1	856	INTERGENIC	--	--	--

**INDEL marker IDs**	**Chromosomes/ unanchored scaffolds**	**Physical positions (bp)**	**InDels (*Kabuli* reference genome-CDC Frontier/PII)**	**Forward primers (5'-3')**	**Reverse primers (5'-3')**	**Anneling temperature (0C)**	**Expected amplified product size (bp)**	**Structural annotation**		**Functional annotation**	
								**Sequence components of *kabuli* genome**	***Kabuli* gene accession IDs**	**NCBI-KOG**	**TFs**
Ca6_P3_9403873	Ca6	9403873	AATATATATATATATA/AATATATATATATATATATA	TTTTGTCGCAATGATAGTTTGA	TAGCGTTCCGAATCTTTCGT	58.4	891	INTERGENIC	--	--	--
Ca6_P3_9507466	Ca6	9507466	TTAATAATAATAATAATAATAATAATAATAATAATAATA/TTAATAATAATAATAATAATAATAATAATAATAATAATAATAATA	GGTCATTTGGACCCTAGGAA	TTACGCAGATTTGCTGTTTTT	58.8	592	INTERGENIC	--	--	--
Ca6_P3_9579497	Ca6	9579497	C/CCGA	GATCGATTGATGTATCCGCA	TTTTTCAATGGTTTAAACTCGC	59.5	607	INTERGENIC	--	--	--
Ca6_P3_9623905	Ca6	9623905	CT/C	TCCAAAAACGTCAAACGACA	AAAAACAAAATTCCATGCCG	60.1	599	INTERGENIC	--	--	--
Ca6_P3_9640750	Ca6	9640750	CAAAAAAAAAAA/CAAAAAAAAAA	CACACACAATTCACATGGCA	TTAAACCAGTCCGAGTTGGG	60.0	564	INTERGENIC	--	--	--
Ca6_P3_9666045	Ca6	9666045	CTATGATATTGGTGTGAATTATGATATTGGTGTGAATTAT/CTATGATATTGGTGTGAATTAT	TATAAGGGAGGATGGGGCTC	TGCATGTTGTCCTTCCAAAA	60.2	656	INTERGENIC	--	--	--
Ca6_P3_9740823	Ca6	9740823	GGT/GGTGATGGTGT	TATCCAGCAAGAATCTGCCC	ACCCGCACCGTGATATAAAA	60.2	205	INTERGENIC	--	--	--
Ca6_P3_9873238	Ca6	9873238	GTTCAAC/GTTCAACTTCAAC	AACTGTGGAACCAGACAGGG	AACATCGACCGGATTTAACG	60.0	544	INTRON	Ca_08602	F	--
Ca6_P3_9884658	Ca6	9884658	AT/ATGT	GCCTTTGAGCGGTAATGAAA	TCGGAAATTTGGAGTCAAGC	60.2	716	INTERGENIC	--	--	--
Ca6_P3_9916884	Ca6	9916884	CTTT/CTTTT	GGTTGAAAGGTGAATTCCGA	TTTTCGCGTAGTGACGTGAG	59.9	524	INTERGENIC	--	--	--

INDEL marker IDs	Chromosomes/ unanchored scaffolds	Physical positions (bp)	InDels (*Kabuli* reference genome-CDC Frontier/PII)	Forward primers (5'-3')	Reverse primers (5'-3')	Anneling temperature (0C)	Expected amplified product size (bp)	Structural annotation		Functional annotation	
								Sequence components of *kabuli* genome	*Kabuli* gene accession IDs	NCBI-KOG	TFs
Ca6_P3_9998146	Ca6	9998146	AATATATATATATATATA/AATATATATATATATATATA	AGTGCATGTTCCATGACGTG	CTCACTTACCCTCGCTCCTG	60.6	116	INTERGENIC	--	--	--
Ca7_P3_10102297	Ca7	10102297	ATTTTTTTTTT/ATTTTTTTTTTT	ATCGGATACGCAACCGTACT	CATGCGGTACACACCAAAAA	59.5	375	INTERGENIC	--	--	--
Ca7_P3_10118295	Ca7	10118295	GAAAAAAAAAA/GAAAAAAAAAAA	AGGCTGTCCGTTAGGGACTT	AGTGTGGTCTGACCTGAGCC	60.1	526	INTERGENIC	--	--	--
Ca7_P3_1018603	Ca7	1018603	TAAAAAAA/TAAAAAAAAAA	TTGCAAACATGATAGCTGCC	GCGATCTTTTACGGGAACAA	59.8	254	INTERGENIC	--	--	--
Ca7_P3_1018745	Ca7	1018745	C/CT	TTGCAAACATGATAGCTGCC	TTGTTGGCATTGTTGTGGTT	59.8	467	INTERGENIC	--	--	--
Ca7_P3_1018777	Ca7	1018777	ATTGCAT/ATTGCATATTTGCAT	TTGTTCCCGTAAAAGATCGC	TTGTTGGCATTGTTGTGGTT	60.1	233	INTERGENIC	--	--	--
Ca7_P3_10192729	Ca7	10192729	TTTTTTTCATATA/TTTTTTTCATATAATGTTTTTTCATATA	CCCATTCCGAATACATTGCT	GAGTTGGGTGCACGATTTTT	59.8	624	INTERGENIC	--	--	--
Ca7_P3_10231657	Ca7	10231657	TA/T	CTGATTTCACAAGCGAAGCA	AGAAGAAAGCAAGCCACCAA	60.1	309	CDS (FRAME SHIFT)	Ca_12785	V	--
Ca7_P3_10239926	Ca7	10239926	CATTATTATTATTATTATTATTATTATTATTATTATTATT/CATTATTATTATTATTATTATTATTATTATTATTATTATTATTATTATTATTATT	TCATCCTCAACACAAGGTCG	CGGCAAAATATAAGGAACACG	59.7	485	INTERGENIC	--	--	--
Ca7_P3_10240117	Ca7	10240117	ATTTTTTT/ATTTTTTTT	CCTCAGGATGCGACCATCTA	CCAGCTTGAACAATGTTTCTT	61.2	687	INTERGENIC	--	--	--

INDEL marker IDs	Chromosomes/ unanchored scaffolds	Physical positions (bp)	InDels (*Kabuli* reference genome-CDC Frontier/PII)	Forward primers (5'-3')	Reverse primers (5'-3')	Anneling temperature (0C)	Expected amplified product size (bp)	Structural annotation		Functional annotation	
								Sequence components of *kabuli* genome	*Kabuli* gene accession IDs	NCBI-KOG	TFs
Ca7_P3_10240372	Ca7	10240372	CTTATTATTATTATTATTATTATTATTA/ CTTATTATTATTATTATTATTATTATTAT TATTATTA	CGTGTTCCTTATATTTT GCCG	AATCTCAAAATGCCCC ACTT	59.5	629	INTERGENIC	--	--	--
Ca7_P3_10245354	Ca7	10245354	G/GATTTTACCAAGGTT	ACAGGGTCTGGTTGCA AGAT	GAATGGCCACTTTCTA CCCA	59.6	238	INTERGENIC	--	--	--
Ca7_P3_10291279	Ca7	10291279	GTTTTTTTTTTTTT/GTTTTTTTTTTTT	TCTGCTGCTACTGCTC CTGA	GAACTGCTCCCTCAAC AAGC	60.0	480	INTRON	Ca_12791	--	--
Ca7_P3_10293605	Ca7	10293605	GTTT/GTT	CCACGGGAGCAAACTT TCTA	TTGGGAAGCTTAAGGA GCAA	60.2	721	DRR	Ca_12792	E	--
Ca7_P3_10337607	Ca7	10337607	AATATATATATATATATATATATATATAT ATATATA/AATATATATATATATATATAT ATATATATATA	CACGGGCACCTGTTAA GAAT	AAAGATGGGCTTGGTA CGTG	60.0	881	INTRON	Ca_12794	A	--
Ca7_P3_10357948	Ca7	10357948	TTATATATATATATATATATATATATAT/ TTATATATATATATATATATATATATATATA TAT	GTGGTACTGGAGGCCA CATT	GAGACGAGTGTGGGG TGAAT	59.9	722	INTERGENIC	--	--	--
Ca7_P3_10371145	Ca7	10371145	ATTTTTTTTT/ATTTTTTTT	TTATTGGTGGAGAAGA CGGC	CCCAAACTAATTCGCC ACAT	60.1	346	INTERGENIC	--	--	--
Ca7_P3_1038892	Ca7	1038892	TTATATATATATATATATATATATATATA TAT/TTATATATATATATATATATATATA TATATATATATAT	GTACGAGATGACGACG CCTT	ATGAATGCAGTTACGT GGCA	60.3	717	INTERGENIC	--	--	--
Ca7_P3_10398617	Ca7	10398617	AATATATATATATATATATA/AATATATA TATATATATATATATA	TATTGAAACAAACGCAC CCA	TTGCACTCACTCCAAC GAAC	60.0	600	INTERGENIC	--	--	--
Ca7_P3_10399061	Ca7	10399061	TAAAAAAAAAA/TAAAAAAAAAAA	CAAACAAATCATCTCGT GCAA	AGGGGGCTTACCTAGT TGGA	59.7	826	INTERGENIC	--	--	--

INDEL marker IDs	Chromosomes/ unanchored scaffolds	Physical positions (bp)	InDels (*Kabuli* reference genome-CDC Frontier/PII)	Forward primers (5'-3')	Reverse primers (5'-3')	Anneling temperature (0C)	Expected amplified product size (bp)	Structural annotation		Functional annotation	
								Sequence components of *kabuli* genome	*Kabuli* gene accession IDs	NCBI-KOG	TFs
Ca7_P3_10433773	Ca7	10433773	TAAAAAAAAAAAA/TAAAAAAAAAAAAA	TACAACTTTTGCAATGGGCA	TGACCATCCAAATCGAATCA	60.1	498	INTERGENIC	--	--	--
Ca7_P3_10568493	Ca7	10568493	TTATATATATATATATATATATATATAT/TTATATATATATATATATATATAT	ACGATGGAGGCCAAAGTATG	AAAATAAGGCATGGTGTTGGA	60.0	487	INTERGENIC	--	--	--
Ca7_P3_10592238	Ca7	10592238	TTTTATT/TTTTATTGATTTATT	ACTGTCAGGAAGGAACGGTG	GTCCACTGTGCCACATCATC	60.2	569	INTERGENIC	--	--	--
Ca7_P3_10667777	Ca7	10667777	TA/T	AAACGTCATAGCAAATAGATGAAA	ACTCACTAGGCAACCGCAAT	57.2	713	INTERGENIC	--	--	--
Ca7_P3_10682834	Ca7	10682834	TTATATATATATATATAT/TTATATATATATATATAT	CAATGCTACCTCTTGCCGAT	AAAGGGCCCGGTTATAAAAA	60.2	514	INTERGENIC	--	--	--
Ca7_P3_10740094	Ca7	10740094	TAAAAATTT/TAAAAATTTTAAAAAAATTT	TTTTTGCATTGCCACTTCTG	TTCTGATTTAAATTCCAACTTGTGAG	59.8	492	INTERGENIC	--	--	--
Ca7_P3_10783409	Ca7	10783409	ATTTTTT/ATTTTTTTT	GGAGGGAGAACTTTTAAGAGAGAAA	GTCCATTTTCCAGTGGCTGT	59.8	454	INTERGENIC	--	--	--
Ca7_P3_10821468	Ca7	10821468	AAATAATAATAATAATAATAATAATAATAATAATAAT/AAATAATAATAATAATAATAATAATAATAATAATAATAAT	ACGTGACGTGGATGGGTTAT	TTTCGTCGTCTCAAATCGAA	60.1	816	INTERGENIC	--	--	--
Ca7_P3_10843337	Ca7	10843337	TTTATTATTATTATTATTATTATTATTATTA/TTTATTATTATTATTATTATTATTATTATTA	CCAAAAATCAGCCCGATCTA	AACAGACCCGCAGTTTTGAG	60.0	626	INTERGENIC	--	--	--
Ca7_P3_10850624	Ca7	10850624	ATTTTTTTTTTT/ATTTTTTTTTTTT	GCATTCAATTAAGTGCTCCCA	GGCAGTTTTGTCTCCTTCCA	60.1	552	DRR	Ca_12839	R	SBP

INDEL marker IDs	Chromosomes/ unanchored scaffolds	Physical positions (bp)	InDels (*Kabuli* reference genome-CDC Frontier/PII)	Forward primers (5'-3')	Reverse primers (5'-3')	Anneling temperature (0C)	Expected amplified product size (bp)	Structural annotation		Functional annotation	
								Sequence components of *kabuli* genome	*Kabuli* gene accession IDs	NCBI-KOG	TFs
Ca7_P3_10863846	Ca7	10863846	TGGGGGGGG/TGGGGGGG	GGACAGGGAAGAGGAAAAGG	ACAACCTACAACAGTCCCCG	60.0	466	INTERGENIC	--	--	--
Ca7_P3_10867933	Ca7	10867933	TA/T	GCAGCTGAGCTTGCACATAG	CATTGCGAACACATTGATCC	59.9	369	DRR	Ca_12841	--	Dof
Ca7_P3_10914028	Ca7	10914028	G/GTTTACGGT	TGGTTGGGCTAATCCTTCTTT	GGTGATAAGGTGATTGCATCC	60.0	139	DRR	Ca_12848	--	--
Ca7_P3_10982487	Ca7	10982487	ACTCTCTCTC/ACTCTC	TGGACCGTAGAAAACCGTTC	GCCCCTTCGATTTTTAGAGC	60.0	325	INTERGENIC	--	--	--
Ca7_P3_11004650	Ca7	11004650	T/TC	CGCAATCCAAGAAAAGAAGC	GCTACAACTGTTGCGTTGGA	60.0	910	INTERGENIC	--	--	--
Ca7_P3_11061823	Ca7	11061823	CAAAAAAAAAAA/CAAAAAAAAAAAAA	ACTCGGGTGATGATTGAACC	TCACCGTCCTATACATGTCTCC	59.8	250	INTERGENIC	--	--	--
Ca7_P3_11073789	Ca7	11073789	CTTTTTTTTTTT/CTTTTTTTTTT	TTGCAGCATCAACTAGGCAC	CCTCTCCCAAATCGCTACAA	60.0	649	INTERGENIC	--	--	--
Ca7_P3_11082142	Ca7	11082142	TTATATATATATATATATATATATA/TTATATATATATATATATATA	GCCCGAAATAGCTTTCCTTC	GACGTGGCACAGTCTAATGC	60.2	671	INTERGENIC	--	--	--
Ca7_P3_11083612	Ca7	11083612	TAAAAAAAAAAA/TAAAAAAAAAAAA	ACTGGTTTGTCATCAACCTGA	TTTTTAGGCAGGTGGACATATT	58.0	702	INTERGENIC	--	--	--
Ca7_P3_11332314	Ca7	11332314	AATATATATATATATATATA/AATATATATATATATATATATA	AACCCAAGTTCCAAATGACG	ACCCAAGAGAGGTGCGACTA	59.8	569	INTERGENIC	--	--	--

INDEL marker IDs	Chromosomes/ unanchored scaffolds	Physical positions (bp)	InDels (*Kabuli* reference genome-CDC Frontier/PII)	Forward primers (5'-3')	Reverse primers (5'-3')	Anneling temperature (0C)	Expected amplified product size (bp)	Structural annotation		Functional annotation	
								Sequence components of *kabuli* genome	*Kabuli* gene accession IDs	NCBI-KOG	TFs
Ca7_P3_11382209	Ca7	11382209	ATTTTTTTTTT/ATTTTTTTTTTT	TGTGCTACTTGCCATGCTCT	CGACCGACTCACTTTTCCAT	59.6	826	INTERGENIC	--	--	--
Ca7_P3_11401602	Ca7	11401602	TATAATAATAATAATAATAATAATAAT/TATAATAATAATAATAATAATAATAATAAT	GTGGAGAATTGTCGGGCTAA	CCTTAGACCGGTTCTGGACA	60.1	585	INTERGENIC	--	--	--
Ca7_P3_11436682	Ca7	11436682	CAATAATAATAATAATAATAATAATAATAATAATAATAATAATA/CAATAATAATAATAATAATAATAATAATAATAATAATAATA	TGTGCGTTTTAGAGTGCATTG	AATGTCACCAAAGGCCAAAG	59.9	578	INTERGENIC	--	--	--
Ca7_P3_11511673	Ca7	11511673	C/CCCGCGAAACTT	TGACCCTAAGAACCCACCAG	GGATGGGGTAAACATTGTCC	60.0	514	INTERGENIC	--	--	--
Ca7_P3_11603131	Ca7	11603131	TA/TATAGA	CACACGAGGAGGGATCAAAT	TGCTGTCATCGCTTTGTTTT	59.9	682	INTERGENIC	--	--	--
Ca7_P3_11616012	Ca7	11616012	TC/T	CCTAACAGTTTAGCAGGCCG	GCCTGGGTTCAATTGCTTTA	59.9	871	DRR	Ca_09338	--	--
Ca7_P3_11647837	Ca7	11647837	GATTTTATTTT/GATTTTATTTTAATTTTATTTT	GCACCAAACAAGAAGGGAAA	GGAGATTCGTGCGCATATTT	60.1	166	INTERGENIC	--	--	--
Ca7_P3_11655384	Ca7	11655384	CTTTTTTTTT/CTTTTTTTT	GTGCATGGTGGAATGGACTT	TATGTGGACTGCCACGTGTT	60.8	758	DRR	Ca_09334	TDK	C3H
Ca7_P3_11665923	Ca7	11665923	GTATATATATATATATA/GTATATATATATATATA	ACCAAACGAGAGAGCCTCAA	CCCAAGAAACGAAAACGAAA	60.0	426	INTERGENIC	--	--	--
Ca7_P3_11692112	Ca7	11692112	CTATATATATATATATATATATATATAT/CTATATATATATATATATATATATATATATATATATATAT	GTGGAAAAGGAAGTCGCAAG	GCTGAAGACACGTATGCGAA	59.9	872	INTERGENIC	--	--	--

INDEL marker IDs	Chromosomes/ unanchored scaffolds	Physical positions (bp)	InDels (*Kabuli* reference genome-CDC Frontier/PII)	Forward primers (5'-3')	Reverse primers (5'-3')	Anneling temperature (0C)	Expected amplified product size (bp)	Structural annotation		Functional annotation	
								Sequence components of *kabuli* genome	*Kabuli* gene accession IDs	NCBI-KOG	TFs
Ca7_P3_11697587	Ca7	11697587	ATAAATCGCTAA/ATAA	GGCTATCGCTTATGCAGGAC	TTCAGCGATTTATTCAACGATTT	59.8	709	DRR	Ca_09328	--	SAP
Ca7_P3_11722297	Ca7	11722297	CCTCTCTCTCTCTCTCTCTCTCT/CCTCTCTCTCTCTCTCTCTCTCTCT	TTGGCATAATCAGCGTCAAA	TGGAGGAGATGATTTGGGAG	60.2	312	INTERGENIC	--	--	--
Ca7_P3_11747378	Ca7	11747378	AAAAAT/AAAAATAATTAAAAT	TCTCTCTGGGAGTGCGACTT	ACTTCGTCAACCATCGATCA	60.1	439	INTERGENIC	--	--	--
Ca7_P3_11747910	Ca7	11747910	GTATTATTATTATTATTATTATTATT/GTATTATTATTATTATTATTATTATTATTATT	CAATTGATCGATGGTTGACG	TGTGCCACCCCTTTTTAGAG	59.9	704	INTERGENIC	--	--	--
Ca7_P3_11829063	Ca7	11829063	AATATATATATATATATATATATA/AATATATATATATATATATATATATATATA	ACATGTAGCCTGGCCTATGG	TTTCCATTGCACTCACTCCA	60.0	764	INTERGENIC	--	--	--
Ca7_P3_11835682	Ca7	11835682	CATATATATATATATATATATA/CATATATATATATATATATATA	GTCTCACCAGCAGCAACAAA	TTTTTGGCCGATGAGTACCT	60.0	485	INTERGENIC	--	--	--
Ca7_P3_11930884	Ca7	11930884	TAAAAAAAAA/TAAAAAAAAAA	TGGACATAAATAATTGTCATGTGTTG	TTGAAGAGGGTCCCAAAATG	59.9	726	INTERGENIC	--	--	--
Ca7_P3_12003043	Ca7	12003043	TT/TTTAT	GATGGCAAAGACACACATGG	CGGTTGTGAGCATTGGACTA	60.0	884	INTERGENIC	--	--	--
Ca7_P3_12204671	Ca7	12204671	ATTTTTTTTTTT/ATTTTTTTTTT	GCTTGGTTGGATAGCGAGAG	CACCTCAGCTACCGTTCACA	60.0	730	INTERGENIC	--	--	--
Ca7_P3_12291336	Ca7	12291336	TATAATAATAATAATAATAATAATAATAATAATAATAAT/TATAATAATAATAATAATAATAATAATAATAATAATAATAAT	TAAAGCCAACAACCACACCA	TTTTTCCCCATGTTTTCCG	60.0	523	INTERGENIC	--	--	--

INDEL marker IDs	Chromosomes/ unanchored scaffolds	Physical positions (bp)	InDels (*Kabuli* reference genome-CDC Frontier/PII)	Forward primers (5'-3')	Reverse primers (5'-3')	Anneling temperature (0C)	Expected amplified product size (bp)	Structural annotation		Functional annotation	
								Sequence components of *kabuli* genome	*Kabuli* gene accession IDs	NCBI-KOG	TFs
Ca7_P3_12365777	Ca7	12365777	TTATATATATATATATATAT/TTATATATATATATATATATATAT	TTCGGGAACAGATTTTGGAG	TTCAAAAGAATCGGAGGAAGA	60.0	434	INTRON	Ca_09277	K	HSF
Ca7_P3_12368556	Ca7	12368556	CAAAAAAAAAAAAA/CAAAAAAAAAAAA	TTTGTTTGTTTGTGCCGTGT	TCGAACGGAGGCTCTAAAAA	60.1	727	DRR	Ca_09277	K	HSF
Ca7_P3_12425707	Ca7	12425707	ATTTTTTT/ATTTTTTTT	GGTTCTGGTTCAAAGGTGGA	CATGAAAAATATTTATGCAACACACA	59.9	212	INTERGENIC	--	--	--
Ca7_P3_12447118	Ca7	12447118	TAAAAAAAA/TAAAAAAAAA	TTTGTCCCGAGTTCGAATGT	ATAAGGGGCAGCCTTAGGAA	60.5	301	INTERGENIC	--	--	--
Ca7_P3_1245279	Ca7	1245279	A/AATTTCAT	TGCACAAAACACACAACCCT	TCGCTCCTGTGTCAGTTTTG	60.0	468	INTERGENIC	--	--	--
Ca7_P3_12465968	Ca7	12465968	TTATATATATATATATATATATATATATATAT/TTATATATATATATATATATATATATATATATATATAT	TTATGAACTCGGTTGCTTCG	CAGATGTGAGTCAGTCCGAGA	58.9	331	DRR	Ca_09270	--	--
Ca7_P3_12483013	Ca7	12483013	G/GA	GCGCAAACTGCAAAGTCATA	CCTTTTCGGACAGGAAATCA	60.0	723	DRR	Ca_09268	R	bHLH
Ca7_P3_12522504	Ca7	12522504	TTATATATATATATATATAT/TTATATATATATATATATATATAT	GTCCAATTTGCTGTGTGGTG	TCAAAAGCATTGTCGGATGA	60.0	374	INTERGENIC	--	--	--
Ca7_P3_12539752	Ca7	12539752	TTTTATTTA/TTTTATTTTATTTATTTA	TCCTACTGAAGTCGTGGCCT	TGCATTTTCACCAACATCGT	59.9	596	INTERGENIC	--	--	--
Ca7_P3_12601954	Ca7	12601954	ATCTCTCTCTCTCTCTCTCTCTCTCT/ATCTCTCTCTCTCTCTCTCTCTCT	GAGATGGGTCGCACGTTATT	AGCATGCTTTGAAAGGGGTA	60.0	193	INTERGENIC	--	--	--

INDEL marker IDs	Chromosomes/ unanchored scaffolds	Physical positions (bp)	InDels (*Kabuli* reference genome-CDC Frontier/PII)	Forward primers (5'-3')	Reverse primers (5'-3')	Anneling temperature (0C)	Expected amplified product size (bp)	Structural annotation		Functional annotation	
								Sequence components of *kabuli* genome	*Kabuli* gene accession IDs	NCBI-KOG	TFs
Ca7_P3_1267304	Ca7	1267304	TAAAAAAAAAAA/TAAAAAAAAAAAA	AGATGCATCCAAAATGGGAG	TTCTTGGACTTTCGTTGTTTTG	59.9	361	INTERGENIC	--	--	--
Ca7_P3_1267778	Ca7	1267778	GAAAAAAAAAAA/GAAAAAAAAAAAA	ATGGAGGAGGAATGTTGACC	ATTACCCTTTGCGGCCTATT	58.8	576	INTERGENIC	--	--	--
Ca7_P3_12691360	Ca7	12691360	CAAAAATCATTTT/CAAAAATCATTTTATTAAAAAAATCATTTT	GTCAAGGGCCTATGGTCAGA	TTTGGTCATGGGATGTGCTA	60.1	909	INTERGENIC	--	--	--
Ca7_P3_12748420	Ca7	12748420	TAAAAAAAAAA/TAAAAAAAAA	TACTTTGTTGGATGGGAGCC	CTTTGCTTCCTCTCAGGTGG	59.9	421	INTRON	Ca_09244	T	YABBY
Ca7_P3_12758535	Ca7	12758535	ACC/AC	ATACCCAGAATTTGCGTTGC	AATGATCATGTGCCTCACCA	60.0	851	INTERGENIC	--	--	--
Ca7_P3_1277335	Ca7	1277335	CTTTTTTTTTTT/CTTTTTTTTTT	CACGATAGTGGGGAGCAACT	TAACCGTGGGATCCACAAAT	60.1	553	INTERGENIC	--	--	--
Ca7_P3_12809836	Ca7	12809836	AATATATATATATATATATATATATATAT/AATATATATATATATATATATATATATATATATAT	TTATTCTCCCACACAAGCCC	CGTTCTTGAGCCGATCTACC	59.9	857	DRR	Ca_09238	--	Dof
Ca7_P3_12844900	Ca7	12844900	CTTATTTTTATTTTTATTTTTATTTTTATTTTTATTTTTATTTTTATTTTTA/CTTATTTTTATTTTTATTTTTATTTTTATTTTTA	TGCTCTTCAAAAGTAATTGTTCC	AGATCGACTGCTCCCAAATG	57.6	458	INTRON	Ca_09236	--	--
Ca7_P3_12854739	Ca7	12854739	CATATATATATATATAT/CATATATATATATATATAT	GAACATGCACTTGCTCTCCA	TCTTTTATCCCCATTTCTCACA	60.0	426	INTERGENIC	--	--	--
Ca7_P3_13037732	Ca7	13037732	TA/TAA	AAATGTGCTGATCGGGTTTC	GGCCGATACGATAATGGGTA	59.9	715	INTERGENIC	--	--	--

INDEL marker IDs	Chromosomes/ unanchored scaffolds	Physical positions (bp)	InDels (*Kabuli* reference genome-CDC Frontier/PII)	Forward primers (5'-3')	Reverse primers (5'-3')	Anneling temperature (0C)	Expected amplified product size (bp)	Structural annotation		Functional annotation	
								Sequence components of *kabuli* genome	*Kabuli* gene accession IDs	NCBI-KOG	TFs
Ca7_P3_1326824	Ca7	1326824	T/TA	TCCAAAAATGCACTCAAATCA	ATGAGTGGGAGAACATTGCC	59.1	715	INTERGENIC	--	--	--
Ca7_P3_13317205	Ca7	13317205	AAT/AATAT	CGAAATGACAATGGTGTTGC	AGAGCCTGGCTTTTTGAACA	60.0	454	INTRON	Ca_16026	K	TALE
Ca7_P3_1339137	Ca7	1339137	ATTTTTTTTT/ATTTTTTTTTT	CTTCACAACCAGAGCACCAA	TGCATTAAATTTTACGGAGAAACA	59.9	638	INTERGENIC	--	--	--
Ca7_P3_13454799	Ca7	13454799	AT/A	GGCTCTTAACCTTGTGGTCG	TGGTTCTATCACTTTGGGGC	59.7	868	INTERGENIC	--	--	--
Ca7_P3_13555513	Ca7	13555513	C/CGTAAT	TTTCTAATCAGTGGCCTGGTC	TCAACGAGCATGTGGAAAAG	59.2	554	DRR	Ca_16040	--	bZIP
Ca7_P3_13588964	Ca7	13588964	AATATATATATATATAT/AATATATATATATATATAT	AAATCGATGAATGGGTGGAG	AGAAACGTGCGGTGTCTCTT	59.8	561	INTERGENIC	--	--	--
Ca7_P3_13651611	Ca7	13651611	GGATGATGATGA/GGATGA	GGGTTTCATGTTGCTCCTGT	TCCATCAATCTCCCCTTCAG	60.0	294	CDS (large-effect mutations)	Ca_16047	DR	--
Ca7_P3_13712605	Ca7	13712605	AAATAATAATAATAATAATAATAATAATAATAATAA/AAATAATAATAATAATAATAATAA	TTGTCTCGAGTTTGAACCCA	GGATGCCAATGGGTTAAGTG	59.3	476	INTERGENIC	--	--	--
Ca7_P3_13811117	Ca7	13811117	CTTTTTTTTTT/CTTTTTTTTTTT	TGGTCTCATCCACATGATGC	TGCTTGCTCGTTTATTTTGA	60.5	648	INTERGENIC	--	--	--
Ca7_P3_13825459	Ca7	13825459	AT/ATT	GGATGCCTGAATTTCTTCCA	AATGTTGGGGATGCTCTCTG	60.0	483	CDS (FRAME SHIFT)	Ca_16059	R	--

INDEL marker IDs	Chromosomes/ unanchored scaffolds	Physical positions (bp)	InDels (*Kabuli* reference genome-CDC Frontier/PII)	Forward primers (5'-3')	Reverse primers (5'-3')	Anneling temperature (0C)	Expected amplified product size (bp)	Structural annotation		Functional annotation	
								Sequence components of *kabuli* genome	*Kabuli* gene accession IDs	NCBI-KOG	TFs
Ca7_P3_13891702	Ca7	13891702	CTATATATATATATATAT/CTATATATATATATATAT	TGCCTTTTACTCTTGGTGGG	GAGTGGGATTTTGGAGGTGA	60.1	596	INTERGENIC	--	--	--
Ca7_P3_13909902	Ca7	13909902	AATATATATATATATATA/AATATATATATATATATATA	TCGTCATTTGAAACAGCGAC	GAAAACTTCGGCAATGTGGT	59.8	611	INTERGENIC	--	--	--
Ca7_P3_14008662	Ca7	14008662	A/AC	TGTTCAAACACGCAAACAAG	GTTGCGCTCTCGTAGCTCTT	58.4	441	INTERGENIC	--	--	--
Ca7_P3_14026857	Ca7	14026857	GAAAAA/GAAAA	TACAAATTGGATGCGGTTGA	TCTTCCACCCAGTTTTCACC	59.9	233	INTERGENIC	--	--	--
Ca7_P3_14062165	Ca7	14062165	GATATATATATATATATATATATATATAT/GATATATATATATATATATATATATATATATATATAT	AAGAGCGGTGTTGGAGTCAT	CGGAAACAAACGGACAATCT	59.7	231	INTERGENIC	--	--	--
Ca7_P3_14075293	Ca7	14075293	CATATATATATATATAT/CATATATATATATAT	TTTGTTTAGCCACAACTTTTGA	CGTGCTCGGGATTTATTTGT	57.6	862	INTERGENIC	--	--	--
Ca7_P3_14141705	Ca7	14141705	T/TG	CCTTGCTACGAATCCGATGT	CCGGAGATTGCAGAATTTGT	60.1	571	INTERGENIC	--	--	--
Ca7_P3_14198908	Ca7	14198908	GTAATAAATAATAAATAATAAATAATAAATAAT/GTAATAAATAATAAATAATAAATAAT	TAGCCCCACCTGGACTATTG	TCATCCAACACTTCAACCCTT	59.9	413	DRR	Ca_23036	J	--
Ca7_P3_14226740	Ca7	14226740	CAAAAAAAAAAA/CAAAAAAAAAA	CGTCATTGCAAAAAGAGACG	CATATTGGACTCCCGTTGCT	59.5	220	INTERGENIC	--	--	--
Ca7_P3_14235712	Ca7	14235712	ATGTTGTTGTTGTTGTTGT/ATGTTGTTGTTGTTGTTGTTGTTGT	CTTGGCTTTTGGTGTTGGTT	TCACCAATCCACCTTGTTCA	60.0	423	CDS (large-effect mutations)	Ca_23041	--	NF-YB

INDEL marker IDs	Chromosomes/ unanchored scaffolds	Physical positions (bp)	InDels (*Kabuli* reference genome-CDC Frontier/PII)	Forward primers (5'-3')	Reverse primers (5'-3')	Anneling temperature (0C)	Expected amplified product size (bp)	Structural annotation		Functional annotation	
								Sequence components of *kabuli* genome	*Kabuli* gene accession IDs	NCBI-KOG	TFs
Ca7_P3_1423751	Ca7	1423751	GTAATTAATTAA/GTAATTAATTAATTAA	CGAGCTTTAAGCCACCTTTG	GGATTCGACCCTTGTGCTTA	60.0	706	INTRON	Ca_03325	--	--
Ca7_P3_14255944	Ca7	14255944	AT/ATT	CGTCTTGTTGCATTGTTCGT	CAAATTTGTTTCTCATACCGCA	59.8	519	INTERGENIC	--	--	--
Ca7_P3_14311103	Ca7	14311103	AA/AACA	GAGGGCCAAATTCACTTCAA	CCAATTCCTCCCTGCATTTA	60.1	541	INTERGENIC	--	--	--
Ca7_P3_14350409	Ca7	14350409	CGA/CGAAGA	CGTCAAAAAGAGACGACACAA	ATATATACGCTCCGCCCAAC	59.0	180	INTERGENIC	--	--	--
Ca7_P3_14411410	Ca7	14411410	AATTATTATTATTATTATTATTATTATTAT/AATTATTATTATTATTATTATTATTAT	TCGTTCCTTCAGGAAGTCGT	TGGGGTCATTTAGGTGGACA	59.8	733	INTERGENIC	--	--	--
Ca7_P3_14443370	Ca7	14443370	ACC/ACCC	CCGGAAGAACATTCAAAGGA	GCTATGTTCCGATTGCCATT	60.0	352	INTERGENIC	--	--	--
Ca7_P3_14469399	Ca7	14469399	ACTCTCTCTCTCTCTCTCTCTCTCTCTCT/ACTCTCTCTCTCTCTCTCTCTCTCTCTCT	ATCGTTTACGCGAATATGCC	CTCAGTGTGAAGCCTCCTCC	60.0	774	CDS (FRAME SHIFT)	Ca_09992	KT	DBB
Ca7_P3_14691767	Ca7	14691767	ATCTTCTTCTTCTTC/ATCTTCTTC	GCATTCACTCTTGGCATGTG	AAGGTGTTGGAACCAAGTTGA	60.3	715	INTERGENIC	--	--	--
Ca7_P3_14761486	Ca7	14761486	TC/T	GAAGCCGCAACACATGACTA	TGTCTCACAGCTTGTCCGTC	59.9	678	DRR	Ca_09972	QR	B3
Ca7_P3_14896597	Ca7	14896597	CTTTTTTTTT/CTTTTTTTTTT	TCTTTTGAGGGGTAAACGGA	AAGCATCCGCTTTTTAACCA	59.5	351	DRR	Ca_09961	P	--

INDEL marker IDs	Chromosomes/ unanchored scaffolds	Physical positions (bp)	InDels (*Kabuli* reference genome-CDC Frontier/PII)	Forward primers (5'-3')	Reverse primers (5'-3')	Anneling temperature (0C)	Expected amplified product size (bp)	Structural annotation		Functional annotation	
								Sequence components of *kabuli* genome	*Kabuli* gene accession IDs	NCBI-KOG	TFs
Ca7_P3_14912277	Ca7	14912277	C/CCCCATTT	GGACTGGATCCGACTTTGAA	TCCCCTTTCGTTGTTTTCAG	60.1	624	INTERGENIC	--	--	--
Ca7_P3_14948950	Ca7	14948950	ATAGTTAGTTAGTTAGTTAGTT/ATAGTTAGTTAGTTAGTTAGTTAGTT	AAAAAGCCTTACAGCCCCAT	GTCCAAGTTTGACAGCCACA	60.0	685	INTRON	Ca_09958	--	bZIP
Ca7_P3_14992578	Ca7	14992578	TTATATATATATATATATATATAT/TTATATATATATATATATATAT	GACTTGCACAACGAAGCAAA	GCCCTATTTAATGCAGGCAA	60.0	345	INTRON	Ca_09954	A	--
Ca7_P3_15071788	Ca7	15071788	A/AAAAT	GGGGTGTGAATAGCTACCGA	TAGCCACGGCCCTAGTATTG	60.0	201	DRR	Ca_09947	--	--
Ca7_P3_15083591	Ca7	15083591	ATAATA/ATAATATTAATA	TAGTAGGTTGTTGGCCGAGG	CACCCGAAGAGCATCAATTT	60.1	433	INTERGENIC	--	--	--
Ca7_P3_15131960	Ca7	15131960	TTATATATATATATATATATAT/TTATATATATATATATATATATAT	ACCCAAATTCCAAATGAGCA	GTTCCAAATGACGACTTCCC	60.3	520	INTERGENIC	--	--	--
Ca7_P3_15401109	Ca7	15401109	ATTTTTT/ATTTTT	CCCATAACCGAAGCTTCAAA	GAAAACGATTTAAAGAATCAACTTCC	60.1	771	INTERGENIC	--	--	--
Ca7_P3_15418326	Ca7	15418326	AATATATATATATATATATAT/AATATATATATATATATATATATAT	CAGGGAAAGCGATCACCTAA	CCGAAATAACCGCAACTTGT	60.2	551	DRR	Ca_09919	R	FAR1
Ca7_P3_15467222	Ca7	15467222	TTTATTATTATTATTATTATTATTATTATTATTATTATTATTAT/TTTATTATTATTATTATTATTATTATTATTATTATTATTATTATTATTATTAT	TGGGAAACGGTGCTAAACTT	TCCATTTACGGCAAATTCAA	59.6	716	INTERGENIC	--	--	--
Ca7_P3_15507861	Ca7	15507861	AAT/AATGAT	AACACACTCCCTCCCTCCAT	CCACCAGCCTTCCCTAATTT	60.8	653	INTERGENIC	--	--	--

INDEL marker IDs	Chromosomes/ unanchored scaffolds	Physical positions (bp)	InDels (*Kabuli* reference genome-CDC Frontier/PII)	Forward primers (5'-3')	Reverse primers (5'-3')	Anneling temperature (0C)	Expected amplified product size (bp)	Structural annotation		Functional annotation	
								Sequence components of *kabuli* genome	*Kabuli* gene accession IDs	NCBI-KOG	TFs
Ca7_P3_15562093	Ca7	15562093	GAAAATAA/GAA	AATACACCACCTTCCACCCA	TTTTTAAGGGATCCGACGTG	60.1	161	INTERGENIC	--	--	--
Ca7_P3_15653714	Ca7	15653714	CTTTTTT/CTTTTTTT	TTTCTTGCAAGGGTAATTTGC	GCTTGTGTGGCCGACTTAAT	59.2	641	INTERGENIC	--	--	--
Ca7_P3_15731042	Ca7	15731042	CAAAAAAA/CAAAAAAAA	TCAAAGGTGAGGAAACTGGC	TGTACACCCAACGTGGATCA	60.2	451	DRR	Ca_09898	A	--
Ca7_P3_15772403	Ca7	15772403	GTT/GTTATTATTATT	TCGACCATGTTTCACAGACG	TGTTGATATTTTTGTGTGTGCG	60.7	122	INTERGENIC	--	--	--
Ca7_P3_15841363	Ca7	15841363	GC/GTGATC	CTTTCGGAAGTTCAGGCAAC	GGTGCTGTCTCGGATGAAAT	59.9	415	INTERGENIC	--	--	--
Ca7_P3_16001405	Ca7	16001405	AATTATTATTATTATTATTATTATTATTAT/AATTATTATTATTATTATTATTATTATTATTAT	AAAACACAACGTGCAAACGA	GGGTCACCAAGCACAGTTTT	60.2	679	INTERGENIC	--	--	--
Ca7_P3_16038352	Ca7	16038352	AATATATATATATATATATATATAT/AATATATATATATATATATAT	CACGCGGGATATTCTTCATT	AATGCTTTTTGCCTTGTTGG	59.9	612	INTERGENIC	--	--	--
Ca7_P3_16051093	Ca7	16051093	TAA/TAAA	TGGTTCGTCGGATATGTGAA	TTGAGTTCACGTACGCGTTT	59.9	600	INTERGENIC	--	--	--
Ca7_P3_16104383	Ca7	16104383	CATATATATATATATATATATATATATA/CATATATATATATATATATATATATATATATATA	GGTGATTGGCAGCAACTGTA	GAGGGGAGAAGTGTCATGGA	59.7	608	INTERGENIC	--	--	--
Ca7_P3_16126431	Ca7	16126431	TAAAAAAAAAAA/TAAAAAAAAAAAA	TCACTATTTCATGCAACCGC	TTTGAAGATTTGCGTTACGA	59.7	494	INTERGENIC	--	--	--

INDEL marker IDs	Chromosomes/ unanchored scaffolds	Physical positions (bp)	InDels (*Kabuli* reference genome-CDC Frontier/PII)	Forward primers (5'-3')	Reverse primers (5'-3')	Anneling temperature (0C)	Expected amplified product size (bp)	Structural annotation		Functional annotation	
								Sequence components of *kabuli* genome	*Kabuli* gene accession IDs	NCBI-KOG	TFs
Ca7_P3_16190416	Ca7	16190416	GATATATATATATATATATATATATAT/GATATATATATATATATATATATATATATATAT	AACCTTTTGCGTAGCCTCAA	CAAAAACCCAAAATGGGAAA	59.9	484	INTERGENIC	--	--	--
Ca7_P3_16191254	Ca7	16191254	ACCAT/A	TTTTCTTCTCCCACCGCATA	AAAAACGTTCTGCTTTTGTTGTC	60.6	456	INTERGENIC	--	--	--
Ca7_P3_16240640	Ca7	16240640	TCAACAACAA/TCAACAACAACAA	AACCCAAAGTTAGGGATCCAA	CACTTCACGTAGAGGACGCA	59.7	306	INTERGENIC	--	--	--
Ca7_P3_16251538	Ca7	16251538	ATTTTTTTTTT/ATTTTTTTTTTT	GGAGTCGATTATGCGTAGGG	TCAAACCAACTCATTTTGGCT	59.6	394	INTERGENIC	--	--	--
Ca7_P3_16259772	Ca7	16259772	AT/ATATT	GGAAAGCAACTGTTGGGGTA	AAGAAGACTTTGTTTTTGCAAGG	60.0	391	INTERGENIC	--	--	--
Ca7_P3_16297902	Ca7	16297902	ATTTTTTTTTTTT/ATTTTTTTTTTTTTT	ACAGAGGTGTGTGTGGGTGA	CCTTCACCAGATGGAAGAACA	60.0	716	INTERGENIC	--	--	--
Ca7_P3_16349610	Ca7	16349610	CTTTTTTTTTTT/CTTTTTTTTTT	GCAGTGCTGAATTTTGGTGA	TTGGTTTCATGACTTGCATCT	59.8	740	INTERGENIC	--	--	--
Ca7_P3_16367679	Ca7	16367679	TTATTAT/TTATTATATTAT	AATGCATTGTTCCCCTATCG	AACGACCCCACTTGACACTC	59.8	805	INTERGENIC	--	--	--
Ca7_P3_16438750	Ca7	16438750	A/ATAATAT	TCTCCGAGAAGTGGATGCTT	CACCATAATTTTGATGTGCCA	60.0	580	INTERGENIC	--	--	--
Ca7_P3_16452686	Ca7	16452686	TTATATATATATATATATAT/TTATATATATATATATATATAT	GCCGATCGCTGATTTAATGT	ATTGAAAACCACGAACCCAA	60.1	715	INTERGENIC	--	--	--

INDEL marker IDs	Chromosomes/ unanchored scaffolds	Physical positions (bp)	InDels (*Kabuli* reference genome-CDC Frontier/PII)	Forward primers (5'-3')	Reverse primers (5'-3')	Anneling temperature (0C)	Expected amplified product size (bp)	Structural annotation		Functional annotation	
								Sequence components of *kabuli* genome	*Kabuli* gene accession IDs	NCBI-KOG	TFs
Ca7_P3_1649022	Ca7	1649022	ATTTTTT/ATTTT	CCTTAGTGATTTGGAGGGCA	CGAGAAGGTCGTGAGGTAGC	60.1	564	INTERGENIC	--	--	--
Ca7_P3_16537182	Ca7	16537182	CTTTTTTTTTT/CTTTTTTTTTTT	AGCGCTATAAAGTGGTGCGT	AAGCCTTCCACAACAAAGGA	59.9	795	INTERGENIC	--	--	--
Ca7_P3_16687870	Ca7	16687870	CTTTTTTTTTTTTTT/CTTTTTTTTTTTTT	CCCGATCAAACATTGAGACC	TTGCAAGAATGGTGTAACAAGTG	60.3	773	INTERGENIC	--	--	--
Ca7_P3_16717763	Ca7	16717763	TCCCCCC/TCCCCC	CTCTCTCCCCTCATTTTCCC	TCTTCAACAACAAAAGAATTAGCAA	60.0	395	INTERGENIC	--	--	--
Ca7_P3_16923436	Ca7	16923436	G/GAAT	ATGATTTGAATCCTGGCAGC	GGGAAGGAACCGAGCTATTC	60.0	829	INTRON	Ca_15886	--	--
Ca7_P3_16941588	Ca7	16941588	TAAA/TAAAA	TGCCGTAGTATTCACCACCA	TGCCATGAAATTCTGCCATA	60.0	852	INTERGENIC	--	--	--
Ca7_P3_16970821	Ca7	16970821	TTATATATATATATATATATATATA/TTATATATATATATATATATA	CAAAGGAACATTGTGATCGAA	CAAAGCCAAAACCAACATCA	58.6	742	INTERGENIC	--	--	--
Ca7_P3_17003791	Ca7	17003791	AATATATATATATATATATATATATATATATATATATATATATATATATA/AATATATATATATATATATATATATATATATATATATA	GGTTCAAACCCAAGACCTCA	AAGTTGCCATCTTGAATCGG	59.9	660	INTRON	Ca_15881	--	--
Ca7_P3_17028658	Ca7	17028658	TAAAAAAA/TAAAAAAAA	AGCTACCGAAAAACTTCGCC	TGCCACTGATCTTTCTCGTG	60.7	249	INTRON	Ca_15881	--	--
Ca7_P3_17073394	Ca7	17073394	AATATATATATATATATATATA/AATATATATATATATATATATATATATATA	TCCTTAGCCAAAGAGCCAAA	GTGTGGGAACCTTGTTGCTT	60.0	726	INTERGENIC	--	--	--

INDEL marker IDs	Chromosomes/ unanchored scaffolds	Physical positions (bp)	InDels (*Kabuli* reference genome-CDC Frontier/PII)	Forward primers (5'-3')	Reverse primers (5'-3')	Anneling temperature (0C)	Expected amplified product size (bp)	Structural annotation		Functional annotation	
								Sequence components of *kabuli* genome	*Kabuli* gene accession IDs	NCBI-KOG	TFs
Ca7_P3_17118683	Ca7	17118683	AAATAATAATAATAATAATAATAATAATAA/AAATAATAATAATAATAATAATAATAATAATAA	CATAGTCGTTTATCCGTTTGTCA	TTTGCCATCGCAAGTAAAAA	59.1	761	INTERGENIC	--	--	--
Ca7_P3_17133790	Ca7	17133790	ATTTTTTT/ATTTTTT	GACACTACCCGGCATCAAGT	CCAACCCAAATAAATGAAAATCA	60.0	530	INTERGENIC	--	--	--
Ca7_P3_17172621	Ca7	17172621	AATCATAT/AATCATATCATAT	GGCTAAACCTTGTCTTTGGC	AGACTCTGCTTGACCGGAAA	58.8	541	INTERGENIC	--	--	--
Ca7_P3_17374590	Ca7	17374590	CTTTTTTTTTTTT/CTTTTTTTTT	GCATCTGTAAAACAAGACCCG	ACATCGATAAGAGGCAGCGT	59.6	525	INTERGENIC	--	--	--
Ca7_P3_1746463	Ca7	1746463	ATTTTTTTTTT/ATTTTTTTTT	TCCTTCACGATATCCAAGGAC	GGGCAGGTTAAAGCCACATA	59.0	463	INTERGENIC	--	--	--
Ca7_P3_17564589	Ca7	17564589	CTTTC/CTTTCCACCTTTTTC	CAACAAAGGGAACGAAGGAA	TCAAGAAAAACCAAAGAGCCA	60.1	738	INTERGENIC	--	--	--
Ca7_P3_17596965	Ca7	17596965	GACTTAACTTAA/GACTTAA	CATCTATGCCACCTCCACCT	GATCCAGAAGAAGGCTGCAC	60.0	789	INTRON	Ca_15849	--	--
Ca7_P3_17922655	Ca7	17922655	TGGGGGG/TGGGGG	AAGTCGAGTGCTTGAGCCAT	TCTTTGCGTGCGTGCTATAC	60.0	606	DRR	Ca_15831	--	bHLH
Ca7_P3_17949907	Ca7	17949907	AATATATATATATATATATATATATATA/AATATATATATATATATATATATATATATATATA	GTTGTCGTAGCATCGGGAGT	TTCCCCTTATGGTGAGTAATGA	60.1	265	INTERGENIC	--	--	--
Ca7_P3_18007229	Ca7	18007229	TAATAATAATAATAATAATAA/TAATAATAATAATAATAATAATAA	GGCGACACTTCAATGGACTT	TGCATTGCCATTTCCAGTTA	60.1	627	INTERGENIC	--	--	--

INDEL marker IDs	Chromosomes/ unanchored scaffolds	Physical positions (bp)	InDels (*Kabuli* reference genome-CDC Frontier/PII)	Forward primers (5'-3')	Reverse primers (5'-3')	Anneling temperature (0C)	Expected amplified product size (bp)	Structural annotation		Functional annotation	
								Sequence components of *kabuli* genome	*Kabuli* gene accession IDs	NCBI-KOG	TFs
Ca7_P3_18007668	Ca7	18007668	CTTATTATTATTATTATTAT/CTTATTAT TATTATTAT	CTCGTGTTTCTCGGTG GTTT	AAGTCCATTGAAGTGT CGCC	60.1	264	INTERGENIC	--	--	--
Ca7_P3_180427	Ca7	180427	C/CAGAGGCA	TCAACAGCAGCTTTGC ATTC	TTTCGCAGCTGGTATG TGAG	60.1	280	INTERGENIC	--	--	--
Ca7_P3_18085316	Ca7	18085316	ATTTTTATTTTTTATTTTTT/ATTTTTATT TTTT	ACAAACAAGTTGAGCA CCCC	AAACGCCTCAAGCTAT TCCA	60.0	682	INTERGENIC	--	--	--
Ca7_P3_18121209	Ca7	18121209	TAAAA/TAAAAA	TGAAAAGCAATCCCAAA TGT	AATGGGCATTCGTAAA TCGT	58.1	429	INTERGENIC	--	--	--
Ca7_P3_18122610	Ca7	18122610	AATATATATATATATATAT/AATATATAT ATATATATATAT	GCGAGAACAATTTCTTT GGG	CCACACACTAACACAA CGCC	59.7	305	INTERGENIC	--	--	--
Ca7_P3_18130662	Ca7	18130662	TAAAAAAAAAAA/TAAAAAAAAAA	TTCACCCCATTTTTCGA GAC	GGTTCGAACATTTTGA TGTTTTT	59.9	339	INTERGENIC	--	--	--
Ca7_P3_18144279	Ca7	18144279	AGTGTGTGTGTGTGTGTGTGTGTGT/A GTGTGTGTGTGTGT	GACAGCGGATCACTAC CTCC	GAAATGTGCATTTTCC GCTT	59.7	262	INTERGENIC	--	--	--
Ca7_P3_18188416	Ca7	18188416	CTTT/CTTTT	TGTCTTGAGCATGCCTT GTC	AAATGCAAACCCAAAT GCTC	60.0	525	INTERGENIC	--	--	--
Ca7_P3_18199412	Ca7	18199412	CAGA/CA	GCCCATTTGAGTGGAA GTGT	TCCACCTTGGCACCTT TAAT	60.0	355	DRR	Ca_22138	--	--
Ca7_P3_18234685	Ca7	18234685	ATTTTT/ATTTTTT	CCAAAATTTCACTCTCT CACCC	TCAACTTCTGTTGCCC ATGA	60.0	500	INTERGENIC	--	--	--

INDEL marker IDs	Chromosomes/ unanchored scaffolds	Physical positions (bp)	InDels (*Kabuli* reference genome-CDC Frontier/PII)	Forward primers (5'-3')	Reverse primers (5'-3')	Anneling temperature (0C)	Expected amplified product size (bp)	Structural annotation		Functional annotation	
								Sequence components of *kabuli* genome	*Kabuli* gene accession IDs	NCBI-KOG	TFs
Ca7_P3_18333235	Ca7	18333235	A/ATTAT	GCAAGTGTGTTTGTGGCACT	TTCAAGCACATAATGCAAGTCC	59.8	113	INTERGENIC	--	--	--
Ca7_P3_1835089	Ca7	1835089	CAA/CAAA	AACAAAAGTAGCGTGCGACA	ATTTGTCAACTCGGGGTTTG	59.5	632	INTERGENIC	--	--	--
Ca7_P3_18356812	Ca7	18356812	TAAAAAAAAAAAA/TAAAAAAAAAA	TTCCTCGATGGTGATTGACA	AATGCAATCAGACGCAAACA	60.0	497	INTERGENIC	--	--	--
Ca7_P3_18439833	Ca7	18439833	TAGAG/TAG	TATTTGGAGCAGCGGGTTAG	CCCACAACCACACACTCATC	60.2	813	INTERGENIC	--	--	--
Ca7_P3_18587623	Ca7	18587623	AAAGGCGGAAGGCGG/AAAGGCGG	CGATGATGACTGATTGGGGT	TGTTTGTGTTTTGAGGCGTT	60.7	374	INTERGENIC	--	--	--
Ca7_P3_18587996	Ca7	18587996	CTTTTTTTTT/CTTTTTTTT	GAGTGTACAAAGGCGGAAGG	GGGTGTTACAAGGAGTCTTGG	59.7	865	INTERGENIC	--	--	--
Ca7_P3_18588285	Ca7	18588285	ATTTTTTTT/ATTTTTTT	AACGCCTCAAAACACAAACA	GGGTGTTACAAGGAGTCTTGG	59.2	630	INTERGENIC	--	--	--
Ca7_P3_18588675	Ca7	18588675	TTATAT/TTATATAT	CCAAGACTCCTTGTAACACCC	GCACATATATACACGTAAAATCGCA	58.6	299	INTERGENIC	--	--	--
Ca7_P3_18773517	Ca7	18773517	TAAAAAAAAA/TAAAAAAAAAA	CCGTCTAAGATTGGGCTGAT	AGGGCATGCATGATGATTTT	59.2	709	INTERGENIC	--	--	--
Ca7_P3_18823370	Ca7	18823370	TTATATATATATATATATATATA/TTATATATATATATATATATATA	TGTTGTCTTGAAATTGTTGTGTTTT	GCACAATTTTCCAAACATGAAA	59.9	112	INTERGENIC	--	--	--

INDEL marker IDs	Chromosomes/ unanchored scaffolds	Physical positions (bp)	InDels (*Kabuli* reference genome-CDC Frontier/PII)	Forward primers (5'-3')	Reverse primers (5'-3')	Anneling temperature (0C)	Expected amplified product size (bp)	Structural annotation		Functional annotation	
								Sequence components of *kabuli* genome	*Kabuli* gene accession IDs	NCBI-KOG	TFs
Ca7_P3_18914671	Ca7	18914671	G/GCTGAAAAGT	GGTTGGAGCACTGAGAAAGC	TGTAGTTGGGGCCTTAGTGG	60.0	337	INTERGENIC	--	--	--
Ca7_P3_18945632	Ca7	18945632	GATATATATATATATATATATATATATATATATAT/GATATATGTATATATATATATATATATATATATATATATATATAT	TGTTCCACGAGTTTTTGTGAA	GGAAATTCGTCGACCTCAAA	59.2	697	DRR	Ca_12313	C	--
Ca7_P3_19006687	Ca7	19006687	T/TATTA	GGCAGTACCTCCCATTGTGT	ATGACCATTGAGTTCGCATC	59.9	809	DRR	Ca_12315	--	--
Ca7_P3_1902358	Ca7	1902358	GTTTTAATAATA/G	CAAGTTTATCTCTTCAATCGCGT	TCGCATATTGCTCTCCCTCT	59.8	318	INTERGENIC	--	--	--
Ca7_P3_19095896	Ca7	19095896	GTTTTTTTT/GTTTTTTT	GTTTTGCTTCCCACTTCCAA	CTCCTTGCAGATATAGCGCC	60.1	580	INTERGENIC	--	--	--
Ca7_P3_19229684	Ca7	19229684	GTTTTTTTTTTTTT/GTTTTTTTTTTTT	AATTGTTTGGTTGCGGAAAG	AAAATGGTGGTGTGCAAAGA	60.0	374	DRR	Ca_12330	T	MYB_related
Ca7_P3_19247078	Ca7	19247078	GTATATATATATATATATATATATATAT/GTATATATATATATATATATATATAT	GAGCAAACAAAATTTGAGAAACA	CTAATTTGCGCGGTCTTCAT	58.5	376	INTERGENIC	--	--	--
Ca7_P3_19257572	Ca7	19257572	AAATAATAATAATAATAATAATAATAATAATA/AAATAATAATAATAATAATAATAATAATA	TTTTTACAGGGAAGCGAGGA	AAAATTTGAATAAACCAATTTCACA	59.8	768	INTERGENIC	--	--	--
Ca7_P3_19310476	Ca7	19310476	AATATATATATATATATATATATATATAT/AATATATATATATATATATATATATATATATATAT	TGGTACACAACAAAATACCTGTG	CGTCTTTGTCTTTCTTCCATCA	57.6	813	INTERGENIC	--	--	--
Ca7_P3_19359944	Ca7	19359944	A/AGCCATTGAC	ACGAGTGCGGGTATCAATGT	GAGAAATGGAGGCAATGGAA	60.4	456	INTERGENIC	--	--	--

INDEL marker IDs	Chromosomes/ unanchored scaffolds	Physical positions (bp)	InDels (*Kabuli* reference genome-CDC Frontier/PII)	Forward primers (5'-3')	Reverse primers (5'-3')	Anneling temperature (0C)	Expected amplified product size (bp)	Structural annotation		Functional annotation	
								Sequence components of *kabuli* genome	*Kabuli* gene accession IDs	NCBI-KOG	TFs
Ca7_P3_19399714	Ca7	19399714	GTTTTTTTTT/GTTTTTTTTTT	TCGCCATCATCATCCAT<br>CTA	TCGGTTGGATAACCAC<br>TTATGTT	60.0	678	INTERGENIC	--	--	--
Ca7_P3_19456451	Ca7	19456451	AATATATATATATATATATATATATATATA/<br>AATATATATATATATATATATATATA	TTACTCCATCCGTTCCA<br>AAAA	CGACGTGGCATATTAC<br>CATC	59.4	411	INTERGENIC	--	--	--
Ca7_P3_19464912	Ca7	19464912	T/TAAC	GATGCGACGACTTCAA<br>ACAA	TTTTCTTAACAACGCC<br>CTTCT	59.8	470	INTERGENIC	--	--	--
Ca7_P3_19534402	Ca7	19534402	TTATATATATATATATATATATATATATATA<br>TAT/TTATATATATATATATATATATATATA<br>TAT	GCCCAAATTCTAAATGG<br>CAA	CACCCTCGGTCTAAAG<br>TCCA	59.9	448	INTRON	Ca_12348	--	--
Ca7_P3_19582568	Ca7	19582568	AATATATATATATATA/AATATATATATATA<br>TATATA	AAGTTCGAAATGCCGA<br>TTTG	CCCGTACGTGTGAGAA<br>AACC	60.1	584	INTERGENIC	--	--	--
Ca7_P3_19589014	Ca7	19589014	AATTTATCTATTTATCTATTTAT/AATTT<br>ATCTATTTATCTATTTATCTATTTAT	GGTCGTCCAAATAGTA<br>CCCG	CCGATTTCCCAAAGGT<br>TTTT	59.3	358	INTERGENIC	--	--	--
Ca7_P3_19589201	Ca7	19589201	TTATATATATATATATATATATATAT/TTAT<br>ATATATATATATATATATATATAT	GGTCGTCCAAATAGTA<br>CCCG	AGGAAGCCCAAGTTCC<br>AAAT	59.3	828	INTERGENIC	--	--	--
Ca7_P3_19594228	Ca7	19594228	CATTATTATTATTATTATTATTATTATTA<br>TTATTATTATTATTATTATTATTATTATT<br>ATTATT/CATTATTATTATTATTATTATT<br>ATTATTATTATTATTATTATTATTATTAT<br>TATTATTATTATTATTATTATTATT	AGTTGTATCGGCAAAA<br>CCGA	GACCACGTAATGTGTT<br>TGCCT	60.5	683	INTERGENIC	--	--	--
Ca7_P3_1962967	Ca7	1962967	AACCACC/AACC	GGTGGTTTAGCAGCCA<br>TGTT	TGCAAGCTCAAACACA<br>CACA	60.0	556	CDS (large-effect mutations)	Ca_03278	--	--
Ca7_P3_1964425	Ca7	1964425	AATTTAT/AAT	ATTCGATGTTCATGCCA<br>CAA	CAATTCCGTCATCCAT<br>ACCC	59.9	666	DRR	Ca_03278	--	--

INDEL marker IDs	Chromosomes/ unanchored scaffolds	Physical positions (bp)	InDels (*Kabuli* reference genome-CDC Frontier/PII)	Forward primers (5'-3')	Reverse primers (5'-3')	Anneling temperature (0C)	Expected amplified product size (bp)	Structural annotation		Functional annotation	
								Sequence components of *kabuli* genome	*Kabuli* gene accession IDs	NCBI-KOG	TFs
Ca7_P3_19694255	Ca7	19694255	TATAATAATAATAATAATA/TATAATAATAATAATAATAATA	CAAAAGGCACGAATCGAAAT	AACTGAGACCCTGCTGGCTA	60.1	285	INTERGENIC	--	--	--
Ca7_P3_19718791	Ca7	19718791	GTAATAATAATAATAATAATAATAATAATAATAATAATAATAATAA/GTAATAATAATAATAATAATAATAATAATAATAATAATAATAATAATAATAATAATAA	TTAGGGAGCTTACATCGCGT	TAGCTGTGAGTTGACGACGG	59.9	488	INTERGENIC	--	--	--
Ca7_P3_19731743	Ca7	19731743	ATTTTTTTTT/ATTTTTTTTTT	TCAAAACCTCCAAACGAACC	TTTGCATAGGAGCAAAGCCT	59.9	628	INTERGENIC	--	--	--
Ca7_P3_19857139	Ca7	19857139	AAATAATAATAATAATAATAATAATAATAATAATAA/AAATAATAATAATAATAATAATAATAATAATAATAATAA	CCATATGTCATAGTGCGGTCA	AGAGGCAAACAAGAACCGAA	59.4	314	INTERGENIC	--	--	--
Ca7_P3_19859015	Ca7	19859015	GAAAAAAAAA/GAAAAAAAAAA	TGAGATCTATACCCGGGCTG	GGGTCTTTCAGTTGATCCAGA	60.1	638	INTERGENIC	--	--	--
Ca7_P3_19860378	Ca7	19860378	GT/G	CCTCAATATGTTGGGACATGC	TGATTCTCAGGGAGGGTGTC	60.2	464	INTERGENIC	--	--	--
Ca7_P3_19968336	Ca7	19968336	TG/TGCGTAGGG	TCAAATTTGGAGTTCGGGAG	CCCAATACCCTAAACCCTAACC	60.0	405	INTERGENIC	--	--	--
Ca7_P3_19977840	Ca7	19977840	T/TA	ACCCAATTACTGGAGATGCG	AGGAAGAGTGGAGTTGCGAA	60.0	693	INTERGENIC	--	--	--
Ca7_P3_20059991	Ca7	20059991	TTGT/TTGTGT	TGATTCGCATCAAACCAATAA	TCATCATTTGAGTCATCTTGCC	59.0	487	INTERGENIC	--	--	--
Ca7_P3_20079503	Ca7	20079503	TTAAAGCCA/TTAAAGCCATAAAGCCA	GGGGAGGAATTTGTTGGAAT	GGTCATTGATGGGAGACATGA	60.0	674	INTERGENIC	--	--	--

INDEL marker IDs	Chromosomes/ unanchored scaffolds	Physical positions (bp)	InDels (*Kabuli* reference genome-CDC Frontier/PII)	Forward primers (5'-3')	Reverse primers (5'-3')	Anneling temperature (0C)	Expected amplified product size (bp)	Structural annotation		Functional annotation	
								Sequence components of *kabuli* genome	*Kabuli* gene accession IDs	NCBI-KOG	TFs
Ca7_P3_20217805	Ca7	20217805	TAT/TATATATTAAT	GCTTTGTGGAGGAAATGGAA	CGATGAGATCGACCCGTACT	60.1	558	INTERGENIC	--	--	--
Ca7_P3_20251980	Ca7	20251980	ATTTTTT/ATTTT	TAACGGCTGTTTGAGCAATG	TGGTAGGTCGAAGGGTTTTG	59.9	363	DRR	Ca_12384	A	--
Ca7_P3_20252580	Ca7	20252580	GTAGAATA/GTAGAATAGAATA	ACCTGTCAGCTAAGAGCCCA	CACATGTGTGAACCTCCTGG	60.0	641	DRR	Ca_12383	--	--
Ca7_P3_20296904	Ca7	20296904	TAAAAAAAAAAA/TAAAAAAAAAA	AATGATGTGCATGCATGTGT	GTGCAGGTGTCTGTCGAGAA	58.4	651	INTERGENIC	--	--	--
Ca7_P3_20323683	Ca7	20323683	CTATATATATATATATATATATATATATAT/CTATATATATATATATATATATATATATATATATAT	TAATTGAGGCCCCAAAACAG	GGGTGATTGATTGGGAATGA	59.9	726	INTERGENIC	--	--	--
Ca7_P3_20450539	Ca7	20450539	ATTTTTTT/ATTTTTTTT	TTTGTGATTTTGTGGTGTGGA	AAGCGGGGACACATGTTATT	59.8	618	INTERGENIC	--	--	--
Ca7_P3_20451371	Ca7	20451371	TAAAAAAAA/TAAAAAAAAA	TTGGTGCACCCAAAAGTAAA	TATTGCAGGGGAAACAGAGG	59.1	552	INTERGENIC	--	--	--
Ca7_P3_20455914	Ca7	20455914	AGTTTTGC/AGTTTTGCAATGTTTTGC	TGTGGACGAGAAGACTGACG	TCATAAATGGACGTTTGGCA	60.0	366	INTRON	Ca_12396	T	S1Fa-like
Ca7_P3_20458103	Ca7	20458103	ATTTTTT/ATTTTT	TCTCCATCAATGACATTTCAACA	CCGTTAGGGTCCAACTCAAA	60.4	562	DRR	Ca_12396	T	S1Fa-like
Ca7_P3_20463048	Ca7	20463048	GTAATAATAATAATAATA/GTAATAATAATAATAATAATA	GAACATAAATAAGAAAACAAAAGGCA	GATGCATTTTATGTATCGTGGAA	59.1	421	DRR	Ca_12397	R	--

INDEL marker IDs	Chromosomes/ unanchored scaffolds	Physical positions (bp)	InDels (*Kabuli* reference genome-CDC Frontier/PII)	Forward primers (5'-3')	Reverse primers (5'-3')	Anneling temperature (0C)	Expected amplified product size (bp)	Structural annotation		Functional annotation	
								Sequence components of *kabuli* genome	*Kabuli* gene accession IDs	NCBI-KOG	TFs
Ca7_P3_20480558	Ca7	20480558	GAAAA/GAA	TTCCGCTTATGGTGAG TAATGA	TTTGTTTGTGCAATTGT ATTGTAGTT	59.6	520	INTERGENIC	--	--	--
Ca7_P3_20482683	Ca7	20482683	TAAAAAAA/TAAAAAA	ACCAAAATTCTGATGTG GCA	TTTTATTCAATGCCGAA GGC	59.0	387	INTERGENIC	--	--	--
Ca7_P3_20486117	Ca7	20486117	AATATATATATATATATAT/AATATATAT ATATAT	TGCTCTACGACACAATA TCAACAA	GCGAGCAGTGTTGTTT TCAA	59.7	391	INTERGENIC	--	--	--
Ca7_P3_20488727	Ca7	20488727	TC/T	CAAACATGGTGTCCTT GTGC	ATTGCCCCATGTTTCA AGAG	60.0	731	CDS (FRAME SHIFT)	Ca_12399	T	M-type
Ca7_P3_20492867	Ca7	20492867	TGAAA/TGAAAGAAA	AAGGAACGAATGAACG AACG	CCCCTCTCAATCATCC TCAA	60.1	595	INTERGENIC	--	--	--
Ca7_P3_20493080	Ca7	20493080	AGG/AG	TTGAGGATGATTGAGA GGGG	ACGAACGTGTGCCAAC ATAA	60.0	265	INTERGENIC	--	--	--
Ca7_P3_20494272	Ca7	20494272	CAA/CAAA	AGACGTGTCGATCGAG CTTT	AGCTGAATGCAAACCC TTGA	60.0	497	DRR	Ca_12400	--	AP2
Ca7_P3_20494326	Ca7	20494326	CA/CATCGA	AGACGTGTCGATCGAG CTTT	AGCTGAATGCAAACCC TTGA	60.0	497	DRR	Ca_12400	--	AP2
Ca7_P3_20494626	Ca7	20494626	CTTTTTTTTT/CTTTTTTTTTTT	AGACGTGTCGATCGAG CTTT	GCACCTCAACACCACC CTAT	60.0	623	DRR	Ca_12400	--	AP2
Ca7_P3_20497715	Ca7	20497715	CAAAAAAAA/CAAAAAAAAA	TGTTTCACACCAACCAC GTT	CGTAGCAGCTATAAAG GGCG	59.9	606	INTRON	Ca_12400	--	AP2

INDEL marker IDs	Chromosomes/ unanchored scaffolds	Physical positions (bp)	InDels (*Kabuli* reference genome-CDC Frontier/PII)	Forward primers (5'-3')	Reverse primers (5'-3')	Anneling temperature (0C)	Expected amplified product size (bp)	Structural annotation		Functional annotation	
								Sequence components of *kabuli* genome	*Kabuli* gene accession IDs	NCBI-KOG	TFs
Ca7_P3_20503190	Ca7	20503190	TAAA/TAA	GGTCAAAAGAAAAGCTCAGGC	ATGAAGGAGTGGAGCGAGAA	60.4	935	INTERGENIC	--	--	--
Ca7_P3_20503526	Ca7	20503526	T/TCAC	TTGAAGGTTTTTGGGAATATGG	ATGAAGGAGTGGAGCGAGAA	60.0	141	INTERGENIC	--	--	--
Ca7_P3_20505258	Ca7	20505258	TAGAGAGAGA/TAGAGAGA	CGACAAACATCCAAACATGG	TTTTTCAGAATTTTCCATGCC	59.8	438	INTERGENIC	--	--	--
Ca7_P3_20505889	Ca7	20505889	ATA/ATAGAAATTA	GGCATGGAAAATTCTGAAAAA	TTCATTTCTCGATTGAGGGC	59.0	803	INTERGENIC	--	--	--
Ca7_P3_20505916	Ca7	20505916	GGA/G	GGCATGGAAAATTCTGAAAAA	TTCATTTCTCGATTGAGGGC	59.0	803	INTERGENIC	--	--	--
Ca7_P3_20510218	Ca7	20510218	GTAAT/GTAATAAT	TTGAACAATGCAAGAGCCAC	CTGCTCATGTTTCCCGTTTT	59.8	933	INTERGENIC	--	--	--
Ca7_P3_20517142	Ca7	20517142	ATTTTTTT/ATTTTTTTT	CCCAGTGACAGGTTTCAGGT	ACGGTTTGTTGGGTCAGTTC	60.0	579	INTERGENIC	--	--	--
Ca7_P3_20530900	Ca7	20530900	AACG/AACGACG	GCAAACTCCTAGTTGCTGAGG	CGAGTGTCACATAGATGGCAA	59.1	890	INTERGENIC	--	--	--
Ca7_P3_20532893	Ca7	20532893	ATTATTTATTTATTTATTTAT/ATTATTTATTTATTTAT	GATATATTGCAGGTGCCGGT	TGACACGTGGCTCAATTGTT	59.8	618	INTRON	Ca_12402	--	--
Ca7_P3_20533749	Ca7	20533749	TG/T	AGCCACGTGTCACTCAACAG	GATGGTTTTGCGAGCTTCAT	59.9	721	DRR	Ca_12402	--	--

INDEL marker IDs	Chromosomes/ unanchored scaffolds	Physical positions (bp)	InDels (*Kabuli* reference genome-CDC Frontier/PII)	Forward primers (5'-3')	Reverse primers (5'-3')	Anneling temperature (0C)	Expected amplified product size (bp)	Structural annotation		Functional annotation	
								Sequence components of *kabuli* genome	*Kabuli* gene accession IDs	NCBI-KOG	TFs
Ca7_P3_20537234	Ca7	20537234	CAAAAA/CAAAAAA	TCGCACAGGATGAGAACTTG	TCAGCCTCTCCATCAAACCT	60.0	179	INTERGENIC	--	--	--
Ca7_P3_20538991	Ca7	20538991	GTTTT/GTTT	GGCCATTGGATCTAGCAAAA	GCCATTCATCAAGGAAGGAA	60.0	635	DRR	Ca_12404	F	--
Ca7_P3_20553068	Ca7	20553068	GGTT/G	CCATGGCGGACTTTGTTATT	TGCACATTGTTGTTCCCAGT	59.8	637	CDS (large-effect mutations)	Ca_12405	K	--
Ca7_P3_20554410	Ca7	20554410	AA/AACCTA	CATCTGACCTGGACTTCGGT	TTTTCTGAAGGGTTGTTGCC	60.1	682	INTRON	Ca_12405	K	--
Ca7_P3_20555621	Ca7	20555621	TACACACA/TACACACACA	GTACGAGTGGCAAGAGAGGC	TACCTGGCAGATATCCCGTC	60.0	158	DRR	Ca_12405	K	--
Ca7_P3_20556517	Ca7	20556517	ATA/ATATTGATTTTA	TGCAGTGGCATATTCCAAAA	ATAGCAGAGGCGGCAAAGTA	60.1	457	DRR	Ca_12405	K	--
Ca7_P3_20560193	Ca7	20560193	CAAAAAAAA/CAAAAAAA	TGCATTTGAAATTTGCAGGA	TCAACCCCAATACACCCCTA	60.2	602	DRR	Ca_12406	--	M-type
Ca7_P3_20566104	Ca7	20566104	CCAC/CC	GTTTGCATGACATGAGCGAC	ATGTAAAAGCAAAAGGGGCA	60.3	103	INTERGENIC	--	--	--
Ca7_P3_20566359	Ca7	20566359	T/TA	GGTGACTGGTGGGATTGTTC	TGAAAGTTCTCTTTAATCTGTCACG	60.2	370	INTERGENIC	--	--	--
Ca7_P3_20567011	Ca7	20567011	TAAAAAAA/TAAAAAA	TGGAATTCATGTGTCTTTGGA	CACCTTCCTTGTGTTCCGAT	59.0	281	INTERGENIC	--	--	--

INDEL marker IDs	Chromosomes/ unanchored scaffolds	Physical positions (bp)	InDels (*Kabuli* reference genome-CDC Frontier/PII)	Forward primers (5'-3')	Reverse primers (5'-3')	Anneling temperature (0C)	Expected amplified product size (bp)	Structural annotation		Functional annotation	
								Sequence components of *kabuli* genome	*Kabuli* gene accession IDs	NCBI-KOG	TFs
Ca7_P3_20567982	Ca7	20567982	TAAAAAA/TAAAAAAA	CGGTGAGGACTATTTTTAGAACG	TCCCCCTCCAATTACAGCTT	59.2	778	INTERGENIC	--	--	--
Ca7_P3_20568263	Ca7	20568263	AT/A	TGGAAGAGTAAGATAGGGAGGAA	CCCCTCCAATTACAGCTTTTC	58.4	508	INTERGENIC	--	--	--
Ca7_P3_20569701	Ca7	20569701	ATT/AT	GGTTTTTGGGAGGTTTGCTT	CATCCAAAGGGAAGACGAAA	60.3	695	INTERGENIC	--	--	--
Ca7_P3_20570561	Ca7	20570561	TAA/TAAAAA	TTTCGTCTTCCCTTTGGATG	TTGCGCATGACGATATCAAT	60.0	757	DRR	Ca_12407	--	--
Ca7_P3_20573093	Ca7	20573093	GCAAGACAA/GCAAGACAAGACAA	AATGCAAGCAACCAAAAACC	ATAGACCTGGCTGCAAGGAA	60.0	617	INTRON	Ca_12407	--	--
Ca7_P3_20574766	Ca7	20574766	GTTT/GTTTT	GGGAGATCGGAATGCATAGA	ATGCGTGACGGTGCATAATA	60.0	276	INTERGENIC	--	--	--
Ca7_P3_20575471	Ca7	20575471	T/TA	AAATCCATTTGGTTGACACG	ACCCACCTCCAGATTTTTCC	58.3	743	INTERGENIC	--	--	--
Ca7_P3_20576017	Ca7	20576017	TC/TCC	GGAAAAATCTGGAGGTGGGT	TGCCTCAAATTCAACATGGA	60.2	489	INTERGENIC	--	--	--
Ca7_P3_20596404	Ca7	20596404	CATATATAT/CATATATATAT	CCTTTCACCTTCTTCACCCA	GGTGAAATAAAACACCCCCTT	60.1	480	INTERGENIC	--	--	--
Ca7_P3_20599872	Ca7	20599872	TGG/TG	TCCCCAAGTGGTTTCCATAA	TCTGCACTGTCTCAAGCACC	60.2	758	DRR	Ca_12410	--	bHLH

INDEL marker IDs	Chromosomes/ unanchored scaffolds	Physical positions (bp)	InDels (*Kabuli* reference genome-CDC Frontier/PII)	Forward primers (5'-3')	Reverse primers (5'-3')	Anneling temperature (0C)	Expected amplified product size (bp)	Structural annotation		Functional annotation	
								Sequence components of *kabuli* genome	*Kabuli* gene accession IDs	NCBI-KOG	TFs
Ca7_P3_20602595	Ca7	20602595	ATTCCTTTCCT/ATTCCT	CTACAGTGCTGGCAAGGTGA	ATTAATGGTCCAACACCCCA	60.0	196	CDS (FRAME SHIFT)	Ca_12411	--	--
Ca7_P3_20614138	Ca7	20614138	CTA/CTATA	CTTTGCAACAGCAACTGCAT	CCGACATCACAGCTTTCTGA	60.1	389	INTRON	Ca_12414	--	MIKC
Ca7_P3_20617780	Ca7	20617780	C/CAAT	GGCGAGGGATGGAATTTTAG	GAATTGGGACTTCTGCCGTA	60.8	777	INTERGENIC	--	--	--
Ca7_P3_20617963	Ca7	20617963	ATTTTTTT/ATTTTTT	TCAATTTGAATGTCAAACACACA	GAATTGGGACTTCTGCCGTA	59.0	393	INTERGENIC	--	--	--
Ca7_P3_20622427	Ca7	20622427	CTTTTTTTTTTTTTTT/CTTTTTTTTTT	TTTTGGAAACTCTTGCCGTC	GGGTTGGTCAAGAATGTGCT	60.2	183	INTERGENIC	--	--	--
Ca7_P3_20623409	Ca7	20623409	TAACAA/TAA	AGGTGTCGAAGGATCACCAC	CTCGAGGAGACGAGAAATGC	60.0	212	INTERGENIC	--	--	--
Ca7_P3_20626429	Ca7	20626429	CTAATA/CTAATAATA	TCAGTTATCGTCGTTGGTCG	CGTGCAACATTTTTCTTCCA	59.7	623	INTERGENIC	--	--	--
Ca7_P3_20629724	Ca7	20629724	AATGAT/AAT	ATGTGGACTCAGGGGATGAG	CGGAGCGCAACTTCATATTT	59.9	655	INTERGENIC	--	--	--
Ca7_P3_2066755	Ca7	2066755	CAAAAAAAAAAA/CAAAAAAAAAAAA	TCGTTGGAGAGTTGGTGATG	TGGGATGGTAAGCATTTGAA	59.7	563	INTERGENIC	--	--	--
Ca7_P3_2076110	Ca7	2076110	GAAAAAAAAAAAA/GAAAAAAAAAAA	TTAATGCAGCCTCTCGGACT	GGTCAGGTGTGGACGTCTTT	60.0	442	INTRON	Ca_03264	G	MYB_related

INDEL marker IDs	Chromosomes/ unanchored scaffolds	Physical positions (bp)	InDels (*Kabuli* reference genome-CDC Frontier/PII)	Forward primers (5'-3')	Reverse primers (5'-3')	Anneling temperature (0C)	Expected amplified product size (bp)	Structural annotation		Functional annotation	
								Sequence components of *kabuli* genome	*Kabuli* gene accession IDs	NCBI-KOG	TFs
Ca7_P3_20934834	Ca7	20934834	TAAAAAAAA/TAAAAAA	CCAAATTGGGTCAATAACGC	GTGACCGGACCTATTTGCAT	60.2	951	INTERGENIC	--	--	--
Ca7_P3_20947613	Ca7	20947613	TTCAAATCTTGATCAAATCTT/TTCAAATCTTGATCAAATCTTGATCAAATCTT	TAAACCACTATCGGGTTGGC	GAAAGTTGCTTTAGCGGCAC	59.8	824	INTERGENIC	--	--	--
Ca7_P3_21024512	Ca7	21024512	CTTTTTTTTTTTTT/CTTTTTTTTTTTT	CGAAGCAAATGGTGATTGTG	AGGTAACATCGTAGGGCGTG	60.1	437	INTERGENIC	--	--	--
Ca7_P3_21091701	Ca7	21091701	AATATATATATATATATATATATATA/AATATATATATATATATATA	TGAATTTTTGAGGCCTACGG	TTCCGAGTTGTTTGAGAGGA	60.1	644	INTERGENIC	--	--	--
Ca7_P3_21099731	Ca7	21099731	T/TGCGCAA	GAGAAATCAAAATTGCCCCA	AGTGCCTCCTGTTCCTGAGA	59.9	500	INTERGENIC	--	--	--
Ca7_P3_21103274	Ca7	21103274	TAAAAAAA/TAAAAAAAA	TAAAATGTCCCCAGTGGAGC	CGGGAGATACGTAGGAGCAA	59.9	359	INTERGENIC	--	--	--
Ca7_P3_21106311	Ca7	21106311	TAAA/TAA	TGAACGGAACAAGAGGCTTT	CAATTGGTTGATCCTCGTGA	59.9	595	INTERGENIC	--	--	--
Ca7_P3_21207750	Ca7	21207750	GCATCCCCA/GCATCCCCATCCCCA	TTTTATCCACCAAAAATTCAAAAA	GAATGAGCAAGAAAGACGGC	58.9	505	INTERGENIC	--	--	--
Ca7_P3_21234294	Ca7	21234294	AGTGTGTGTGTGTGTGTGTGTGTG/AGTGTGTGTGTGTGTGTGTGTG	TTCAGTGGCATTGAGACATTG	TAGTTTGGGAGCATGTCGTG	59.7	544	DRR	Ca_14495	S	--
Ca7_P3_21237171	Ca7	21237171	TAAAAAAAAAAA/TAAAAAAAAAA	TGAAGCGCTCTCCCATAGTT	CCATGGATGTTAGTCCGTGA	60.0	226	INTERGENIC	--	--	--

INDEL marker IDs	Chromosomes/ unanchored scaffolds	Physical positions (bp)	InDels (*Kabuli* reference genome-CDC Frontier/PII)	Forward primers (5'-3')	Reverse primers (5'-3')	Anneling temperature (0C)	Expected amplified product size (bp)	Structural annotation		Functional annotation	
								Sequence components of *kabuli* genome	*Kabuli* gene accession IDs	NCBI-KOG	TFs
Ca7_P3_21244805	Ca7	21244805	TAAAAAAAAAA/TAAAAAAAAAAA	GTCATCTCACTCTCGG CCTG	TGCGACTGTGGGAAAT ATCA	61.0	576	INTERGENIC	--	--	--
Ca7_P3_21386667	Ca7	21386667	AA/AAGTTAGGGA	AAAAAGGATTCTCGGG GAAA	CTCGCGTTCCCGTGTA TATT	59.9	622	INTERGENIC	--	--	--
Ca7_P3_21434053	Ca7	21434053	ATTTTTTTT/ATTTTTTTTT	TTTTTGTGGGCTAAGT GGAG	GAAGCACAAAGAATAA AAGCAACA	57.9	939	INTERGENIC	--	--	--
Ca7_P3_2143788	Ca7	2143788	GAAAAAAAAAAA/GAAAAAAAAAA	TTATTTTGCTGCACTTT GCG	TCCTCTCTCCTGCTCT GCTC	60.0	343	INTERGENIC	--	--	--
Ca7_P3_21670841	Ca7	21670841	A/AAGAT	TGACAAATCTTTGCAAC CCA	ATTGCTCATTTCATGC CCTC	60.1	217	INTERGENIC	--	--	--
Ca7_P3_21673889	Ca7	21673889	CTATTATTATTATTATTATTATTATTATT ATTATTATTATTATTAATATTAATATTAT TATTATTATTATTATTATTATTATTATTA CTATTATTATTATTATTATTATTATTATT ATTATTATTATTATTATTATTATTATTAT TATTATTAT/CTATTATTATTATTATTAT [illegible]	CCGTAAAGGAAAAGGA ACTTTG	CGATTTCCCTCACGAC AAAT	59.2	755	INTERGENIC	--	--	--
Ca7_P3_21675349	Ca7	21675349	CTAT/CTATTAT	AGCCAAAGACAAACAAA CCAG	CAACCTCTTCACGCAT CAAA	59.3	167	INTERGENIC	--	--	--
Ca7_P3_21678336	Ca7	21678336	CATATATATATATATATA/CATATATA TATATATATATATATA	TCTCTTTATCATTGCGT TTGC	ATAACCGTGCCAATAG GTGG	58.1	489	INTERGENIC	--	--	--
Ca7_P3_2168224	Ca7	2168224	CATA/CATATA	TTCTGAGATTGGCAAAA GGG	CGCGATCGTTCACTGA TATG	60.2	820	INTRON	Ca_03253	P	--
Ca7_P3_21685577	Ca7	21685577	TT/TTAT	TTTATAAATTGGCCCCA TCG	TAGGGGGACCAAGAAA CTGG	59.6	501	INTERGENIC	--	--	--

INDEL marker IDs	Chromosomes/ unanchored scaffolds	Physical positions (bp)	InDels (*Kabuli* reference genome-CDC Frontier/PII)	Forward primers (5'-3')	Reverse primers (5'-3')	Anneling temperature (0C)	Expected amplified product size (bp)	Structural annotation		Functional annotation	
								Sequence components of *kabuli* genome	*Kabuli* gene accession IDs	NCBI-KOG	TFs
Ca7_P3_21688080	Ca7	21688080	TAAAAAAAAAAAA/TAAAAAAAAAAAAA	TGACCGTGACGTTTGTTCAT	ACAAGCCACGTGAGTTTTCC	60.0	361	INTERGENIC	--	--	--
Ca7_P3_21688548	Ca7	21688548	TTTATTATTATTATTATTATTATTATTATTATTATTATTATTATTATT/TTTATTATTATTATTATTATTATTATTATTATTATTATTATT	GCAAATCCAACTGCGTGTAA	TGAACTTTTAAGACCCCACTAACC	59.7	189	INTERGENIC	--	--	--
Ca7_P3_2169903	Ca7	2169903	T/TA	CAGGGCCTTACTGCTACAGG	CAACGGAAAACATCGTTCAA	59.9	397	INTRON	Ca_03253	P	--
Ca7_P3_21728387	Ca7	21728387	CAAAA/CAAA	ATCTCTGCGCAATATCGCTT	AGCCGACTTTTCCTACCACA	60.0	674	INTERGENIC	--	--	--
Ca7_P3_21729097	Ca7	21729097	TAAAAA/TAAAA	TGCTCAAAAGGGGAAGAAAA	GCCACGTGGTTGAAAGAAGT	59.8	240	INTERGENIC	--	--	--
Ca7_P3_21729303	Ca7	21729303	T/TC	TTGCTCAAAAGGGGAAGAAA	TTGTTATTTTCTTTATGCCGTTG	59.8	426	INTERGENIC	--	--	--
Ca7_P3_21731192	Ca7	21731192	ATTTTTTTTT/ATTTTTTTT	TGATGGATACCCTTTTGAACG	ACCGCTCCAGCTGAGAATAA	59.8	838	INTERGENIC	--	--	--
Ca7_P3_21743270	Ca7	21743270	CAA/CA	TTGCCCCTCTAAATTCTTGG	TCCCTCGTATTTAGCCCTCA	59.2	686	DRR	Ca_14521	--	--
Ca7_P3_21743482	Ca7	21743482	TGGCTAAATATGAGGGCTAAATA/TGGCTAAATA	TCGAAGCATAAGGTTGATCC	GGGATTGAGCGCCTATACAA	57.8	500	DRR	Ca_14521	--	--
Ca7_P3_21743627	Ca7	21743627	ATTTTTTT/ATTTTTTTT	TGAGGGCTAAATACGAGGGA	GGGATTGAGCGCCTATACAA	59.7	461	DRR	Ca_14521	--	--

INDEL marker IDs	Chromosomes/ unanchored scaffolds	Physical positions (bp)	InDels (*Kabuli* reference genome-CDC Frontier/PII)	Forward primers (5'-3')	Reverse primers (5'-3')	Anneling temperature (0C)	Expected amplified product size (bp)	Structural annotation		Functional annotation	
								Sequence components of *kabuli* genome	*Kabuli* gene accession IDs	NCBI-KOG	TFs
Ca7_P3_21743947	Ca7	21743947	AA/AAATA	CCTGGTGTGCATTGAAAAGA	AATTGCGGTTTTGGTTCAAG	59.7	410	DRR	Ca_14521	--	--
Ca7_P3_21744212	Ca7	21744212	CT/CTCTTTTTTTTTT	TTGTATAGGCGCTCAATCCC	AATTGCGGTTTTGGTTCAAG	60.1	354	INTERGENIC	--	--	--
Ca7_P3_21745626	Ca7	21745626	TTTTATATTTAT/TTTTAT	ATAGGGCCCATTTTACAGCG	CCAGTTGCCCTGTCCATACT	61.2	455	INTRON	Ca_14522	O	MYB
Ca7_P3_21749692	Ca7	21749692	A/AG	AAGGTTTCGTGGAAGGCTCT	GACTCAAGCGACGATGATGA	60.2	430	INTERGENIC	--	--	--
Ca7_P3_21750508	Ca7	21750508	CTTTTT/CTTTTTT	GTATACTGTGTGCAGCCCGA	GAACCAACTGCCCTTGTCAT	59.7	382	INTERGENIC	--	--	--
Ca7_P3_21755502	Ca7	21755502	TG/TAAATAGAGATGAGG	TTTTGAGGTGGTGTATGGCA	TTTCCTGGCTGCAAAAGAGT	60.0	780	CDS (FRAME SHIFT)	Ca_14523	R	ARF
Ca7_P3_21784554	Ca7	21784554	GAAAAAAAAAA/GAAAAAAAAAAA	GGGTTGCTCATGGGATAACA	TCCTAGAACATGCATCACGC	60.7	390	INTERGENIC	--	--	--
Ca7_P3_21950073	Ca7	21950073	TCACACACACACACACACACACACACACACACAC/TCACACACACACACACACACACACACACAC	AGCAATCGAAAGTGGAGCAT	TTACCCGTTCACTCCCACTC	59.8	302	INTERGENIC	--	--	--
Ca7_P3_21977655	Ca7	21977655	TACA/TA	AAATCCCTCTTCCCTGAGGA	TAAGCCAGAACATGCCGAGT	60.0	629	INTRON	Ca_14535	D	LBD
Ca7_P3_22028394	Ca7	22028394	G/GA	TCATCAAATCCGTAACGCAA	TCCATGTTTTGCTCTATTGGC	60.1	271	DRR	Ca_14536	R	MYB

INDEL marker IDs	Chromosomes/ unanchored scaffolds	Physical positions (bp)	InDels (*Kabuli* reference genome-CDC Frontier/PII)	Forward primers (5'-3')	Reverse primers (5'-3')	Anneling temperature (0C)	Expected amplified product size (bp)	Structural annotation		Functional annotation	
								Sequence components of *kabuli* genome	*Kabuli* gene accession IDs	NCBI-KOG	TFs
Ca7_P3_22036269	Ca7	22036269	GCTC/G	GCTCAGCCTTGAAATC GAAC	TTCATCATCATCTTCAA ACAAAA	60.0	627	INTERGENIC	--	--	--
Ca7_P3_22038011	Ca7	22038011	C/CT	CTCCAAATTTCCAGCCA AAA	ACACGGACGGGTCTTG TATC	60.0	822	INTERGENIC	--	--	--
Ca7_P3_22039243	Ca7	22039243	ACC/AC	CGTTATTGCAACCACAA CCA	CAATTCAACTCCGACG TGTG	60.4	622	INTERGENIC	--	--	--
Ca7_P3_22043317	Ca7	22043317	T/TG	TGGTTCAAATGCTGTAA TCTTCTC	ATTCGCTCAATACACG GGTC	59.7	884	INTERGENIC	--	--	--
Ca7_P3_22045478	Ca7	22045478	ATTTTTTTT/ATTTTTTT	TGTGGTGTTCCCAAAA GACA	CAAAGCGAAGTGCAAA ATGA	60.0	246	INTERGENIC	--	--	--
Ca7_P3_22046891	Ca7	22046891	CATAATAATAATAATAATAATAATAATA ATAATAATAATAATAAT/CATAATAATAA TAATAATAATAATAATAATAATAATAAT AATAATAATAATAATAAT	ATAATTTGCCTTTGCGG TTG	TTCTGTGAGCAAGGCT GATG	60.0	319	INTERGENIC	--	--	--
Ca7_P3_22046957	Ca7	22046957	CTATATATATATATATATATATAT/CT ATATATATATATATATATAT	ATAATTTGCCTTTGCGG TTG	TTCTGTGAGCAAGGCT GATG	60.0	319	INTERGENIC	--	--	--
Ca7_P3_22046996	Ca7	22046996	TGCGCGC/TGC	ATAATTTGCCTTTGCGG TTG	ACCTGGTCGTAGATGC CACT	60.0	414	INTERGENIC	--	--	--
Ca7_P3_22091542	Ca7	22091542	GAAAAAAAAAAA/GAAAAAAAAAA	CCCCAACCCTCCATTCT ATT	AATTGCAAGATGAGGG CAAC	60.0	347	INTERGENIC	--	--	--
Ca7_P3_22126044	Ca7	22126044	TAAAAAAAAA/TAAAAAAAAAA	TGAAGCAACAAAGCTG ATGAA	TTGTTGCAACCACTTC CAAA	59.6	518	INTERGENIC	--	--	--

INDEL marker IDs	Chromosomes/ unanchored scaffolds	Physical positions (bp)	InDels (*Kabuli* reference genome-CDC Frontier/PII)	Forward primers (5'-3')	Reverse primers (5'-3')	Anneling temperature (0C)	Expected amplified product size (bp)	Structural annotation		Functional annotation	
								Sequence components of *kabuli* genome	*Kabuli* gene accession IDs	NCBI-KOG	TFs
Ca7_P3_22156943	Ca7	22156943	ATTTTTTTTTTTTT/ATTTTTTTTTTTTTTT	ATGCATTCCATTCACTTCCA	GCCAAAATGTTTGTCAGGGT	58.9	674	DRR	Ca_14544	G	G2-like
Ca7_P3_22327481	Ca7	22327481	TC/T	GATTCATCGCAGCTCAAACA	GATCAAAACCGGGGAAAAAT	60.0	638	DRR	Ca_14548	--	--
Ca7_P3_22377445	Ca7	22377445	TAAAAAAAAAA/TAAAAAAAAAAA	TCAATTTTTATTTAGAGGCACGC	CCAAAAACCAAACCAACCAT	59.7	322	INTERGENIC	--	--	--
Ca7_P3_22380060	Ca7	22380060	CATATATATATATATATATATATA/CATATATATATATATATATA	GCGAATTGCGATTAATGTGA	GCCACTTGTTTGTTGTTGATG	59.7	510	INTERGENIC	--	--	--
Ca7_P3_22440146	Ca7	22440146	TAATATA/TAATATAAAATATA	GAAGTTGTAATCGGAGGCCA	GGCAGAGATGGAGGTGATGT	60.1	542	INTERGENIC	--	--	--
Ca7_P3_22455025	Ca7	22455025	TCCCC/TCCCCC	GCCCAAAGGCATAAGTCTCA	CCCTTCCCGAAAATTTCATT	60.2	272	INTERGENIC	--	--	--
Ca7_P3_22457765	Ca7	22457765	GATCAAAATCAAAATCAAAATCAAA/GATCAAAATCAAAATCAAA	TTGTCTCCAGTTTTGGTCCC	GGGCTATCGCTTATGTTGGA	59.9	696	DRR	Ca_14558	--	--
Ca7_P3_22523169	Ca7	22523169	CCGGACGG/CCGGACGGACGG	ACCTTGTGGATGGCAGTTTC	TGGCATTGATGATGGCTTTA	60.0	328	INTERGENIC	--	--	--
Ca7_P3_22584474	Ca7	22584474	TTGTGT/TTGT	TTTGGTATAGCGATTTGAACCA	CCCGCAAGGATATTGTGAGT	59.5	724	INTERGENIC	--	--	--
Ca7_P3_22587400	Ca7	22587400	TTATATATATATATATATATATATATAT/TTATATATATATATATATATATATATATATAT	AAGGAACAAAACGTCATTTTCAA	AGTCAGACGCTACTCGGCAT	59.9	519	INTERGENIC	--	--	--

INDEL marker IDs	Chromosomes/ unanchored scaffolds	Physical positions (bp)	InDels (*Kabuli* reference genome-CDC Frontier/PII)	Forward primers (5'-3')	Reverse primers (5'-3')	Anneling temperature (0C)	Expected amplified product size (bp)	Structural annotation		Functional annotation	
								Sequence components of *kabuli* genome	*Kabuli* gene accession IDs	NCBI-KOG	TFs
Ca7_P3_22658192	Ca7	22658192	GTATATATATATATATATATATATAT/GTATATATATATATATATATATAT	TACACCTGAGTCCGCTACCC	TCCCAAGTTTCAAATGCTGA	60.1	494	INTERGENIC	--	--	--
Ca7_P3_22678046	Ca7	22678046	AG/AGG	ACCAGTCAACAAATGCCCTC	CCACCAACTTTAGCTCCGAA	60.0	314	INTERGENIC	--	--	--
Ca7_P3_22689398	Ca7	22689398	ATTT/ATT	AAAGCATGTTCCTCCAATGC	CTGGCATGCTCAAGTTCTGA	60.1	457	INTRON	Ca_19533	--	NAC
Ca7_P3_22745128	Ca7	22745128	G/GGTAGTTAGTGAT	GGAGTTGGTCGCTACACCAT	CGAGGCATTCCTTCACAAAT	60.0	634	INTRON	Ca_19530	R	--
Ca7_P3_22898346	Ca7	22898346	CAAAAAAAAA/CAAAAAAAAAA	CCCCGAATACATAAACATAACCA	TGGCAGTGCTTCAATTCTTG	59.9	932	INTERGENIC	--	--	--
Ca7_P3_22899812	Ca7	22899812	CTTATTATTATTATTATTATTATTATTA/CTTATTATTATTATTATTATTATTATTATTA	TTGATTGGGGTAGCTTCGAG	TATTCAAAAGGAAGGCACGC	60.2	681	INTERGENIC	--	--	--
Ca7_P3_22944728	Ca7	22944728	GAAAAAA/GAAAAAAA	GCATGCCATAATCTTTTTGGA	TCGTCGTTGAACCTCAAGTG	59.9	799	INTERGENIC	--	--	--
Ca7_P3_2294516	Ca7	2294516	CAGAGAGAGAGAGAGAGAGAGAGAGAGAGAGAGAG/CAGAGAGAGAGAGAGAGAGAGAGAGAGAGAGAGAG	CATAACGAATTCGCGGTTTT	GTCTGGGTTGGTAGGCGTTA	60.0	428	INTERGENIC	--	--	--
Ca7_P3_22960245	Ca7	22960245	AATATATATATATATATATATATATATATATATATATAT/AATATATATATATATATATATATATATATAT	TTGGTATTGAACTCGGAGGC	CTTTCACGTTCGGTTACGGT	60.1	700	INTERGENIC	--	--	--
Ca7_P3_22969908	Ca7	22969908	CTT/CTTT	AATGTGCTGATCTGTGAGCG	CCTTGGAGTATTCTCGGCAG	60.0	894	INTERGENIC	--	--	--

INDEL marker IDs	Chromosomes/ unanchored scaffolds	Physical positions (bp)	InDels (*Kabuli* reference genome-CDC Frontier/PII)	Forward primers (5'-3')	Reverse primers (5'-3')	Anneling temperature (0C)	Expected amplified product size (bp)	Structural annotation		Functional annotation	
								Sequence components of *kabuli* genome	*Kabuli* gene accession IDs	NCBI-KOG	TFs
Ca7_P3_23075370	Ca7	23075370	AAATAATAATAATAATAATAATAATAAT A/AAATAATAATAATAATAATAATAATAA TAATAATAATA	AACGGATGACCCTCTC CTCT	TTTTGGTTGGGATTTTT GGA	60.1	812	INTERGENIC	--	--	--
Ca7_P3_23166901	Ca7	23166901	TTATATATATATATATATATATATAT/TT ATATATATATATATATATATAT	TTTTGGTAGGGTCAGG TTCG	GAAATGTGAAACCATC GCCT	60.0	775	INTERGENIC	--	--	--
Ca7_P3_23191638	Ca7	23191638	CAA/CAAA	AACCCAGGCCCAAACT AACT	ACATGTTGCCGTTGTC AAAA	59.9	313	INTERGENIC	--	--	--
Ca7_P3_2319219	Ca7	2319219	TNNNNNNNNNNNNNNN/TNNNNNNNN NNNNN	GGTTTTGCATCTTCCCT CAA	CAGGTCCCAAGTCCAA GGTA	60.1	370	INTRON	Ca_03240	L	--
Ca7_P3_23269244	Ca7	23269244	TAAAAAAA/TAAAAAAAA	TAAATCTCGTCTCGCTG GCT	TTTTTGGCAGCAATTAA ATGAG	60.1	956	INTERGENIC	--	--	--
Ca7_P3_23487930	Ca7	23487930	ATTTTTTTTT/ATTTTTTTTTT	TTCCACCCTTTTGAATC TGC	TTGGTTTCATGACCTG CATC	60.1	505	INTERGENIC	--	--	--
Ca7_P3_23564944	Ca7	23564944	TTAAATAAATAAATAAATAAATAAATAA ATAAATAAA/TTAAATAAATAAATAAATA AATAAATAAATAAA	TTGGACCACAAGGAGT GTCA	CCGAAATCAATTTTGAA CCG	60.1	795	INTERGENIC	--	--	--
Ca7_P3_23595743	Ca7	23595743	GTTTTTTTTTT/GTTTTTTTTTTT	GCGATTTGAGATCGGA GAGA	TGCCATTTTTGTTGAG GTGA	60.4	718	INTERGENIC	--	--	--
Ca7_P3_23631733	Ca7	23631733	AG/A	TCACATTGACCTCTTTG GTCC	AAATTTATTCTCGCCC CCAC	60.0	414	INTERGENIC	--	--	--
Ca7_P3_23644052	Ca7	23644052	AAAGAAGAAGAAGAAGAAGAAGAAGA AGAAGAAGAA/AAAGAAGAAGAAGAAG AAGAAGAAGAAGAA	CCAGGATAAAACAGCC CAGA	GCCATCCACAAGGTCA AAGT	60.1	550	INTERGENIC	--	--	--

INDEL marker IDs	Chromosomes/ unanchored scaffolds	Physical positions (bp)	InDels (*Kabuli* reference genome-CDC Frontier/PII)	Forward primers (5'-3')	Reverse primers (5'-3')	Anneling temperature (0C)	Expected amplified product size (bp)	Structural annotation		Functional annotation	
								Sequence components of *kabuli* genome	*Kabuli* gene accession IDs	NCBI-KOG	TFs
Ca7_P3_23667025	Ca7	23667025	AAATAATAATAATAATAATA/AAATAATAATAATAATAATAATA	ACACTGATTTCCCAAAACGC	TTTCCTTCCCTTCTCATTGC	60.0	728	DRR	Ca_26877	G	FAR1
Ca7_P3_23679781	Ca7	23679781	AATATATATATATATATATAT/AATATATATATATATATAT	ACATCCACCCATACAGGCAT	GCCATTCTCATGAGGGAAAA	60.1	816	INTERGENIC	--	--	--
Ca7_P3_23688628	Ca7	23688628	TTAATAATAATAATAATAATAATAATA/TTAATAATAATAATAATAATAATAATAATAATAATA	CCACAAAGACAATCGAGCAA	CGTGCAATCTGCTTGGAGTA	59.8	308	INTERGENIC	--	--	--
Ca7_P3_23750922	Ca7	23750922	ATATTATTATTATTATTATTATTATTATTATTATTATTATTATTATTATTATTATTAT/ATATTATTATTATTATTATTATTATTATTATTATTATTATTATTATTATTATTATTATTATTATTATTATTATTAT	TGCCATGCACAGGTAGAAGT	TAGTGGGGTGCATGTAAGGG	59.3	570	INTERGENIC	--	--	--
Ca7_P3_23766427	Ca7	23766427	TAAAA/TAAAAA	GGGAATCGGGTTAAAGAAGG	TTTTGGTTTTCGTTTCCTGG	59.8	373	INTERGENIC	--	--	--
Ca7_P3_23780060	Ca7	23780060	TTATATATATATATAT/TTATATATATATAT	CGCTCGTCTATGCGATTGTA	TAAAATCTCCGCTCGCAACT	60.0	946	INTERGENIC	--	--	--
Ca7_P3_23804197	Ca7	23804197	CATATATATATATATATATAT/CATATATATATATATATAT	GCACCGTGAGTAAATTCGGT	ATGTTTTGGATTTTGGCTCG	60.0	704	INTERGENIC	--	--	--
Ca7_P3_23807464	Ca7	23807464	AA/AATACTCTTA	TGGGTCCTGGAAGGTTAGTG	TGTCTCATGGCAAATACCGA	60.0	482	INTERGENIC	--	--	--
Ca7_P3_23850887	Ca7	23850887	GGA/GGAGA	TAACGTTCCCACGGCTTAAC	ATTAAATTCTCACGCAGCGG	60.0	195	DRR	Ca_26881	--	--
Ca7_P3_23879653	Ca7	23879653	TCC/TC	AGGAAGGAACACTGCAAAGC	TTTCATCTGCCCTTCCAAGT	59.5	619	INTERGENIC	--	--	--

INDEL marker IDs	Chromosomes/ unanchored scaffolds	Physical positions (bp)	InDels (*Kabuli* reference genome-CDC Frontier/PII)	Forward primers (5'-3')	Reverse primers (5'-3')	Anneling temperature (0C)	Expected amplified product size (bp)	Structural annotation		Functional annotation	
								Sequence components of *kabuli* genome	*Kabuli* gene accession IDs	NCBI-KOG	TFs
Ca7_P3_23882859	Ca7	23882859	AATATATATATATATATATA/AATATATATATATATATATATATA	TATTTTGGATTTTGGCTCGG	ATGGGTTTCGCGTAGACAAC	59.9	400	INTERGENIC	--	--	--
Ca7_P3_23903739	Ca7	23903739	TAAATAA/TAAATAAAAATAA	TGAGCATGGAATTTGGTTGT	TGAATTCATGGCTAAGAAGCCT	59.0	592	INTERGENIC	--	--	--
Ca7_P3_23907440	Ca7	23907440	AATATATATAT/AATATATATATAT	TTGGAGAATTCAAAAATAAGGCA	CGTGACAAAGCACGAAAGAA	60.0	513	INTERGENIC	--	--	--
Ca7_P3_23909711	Ca7	23909711	CTTTATTTATTTATTTATTTATTT/CTTTATTTATTTATTTATTTATTTATTT	CATTTCTTCTCTAAGCGGCG	GCATGTAGCACAAAGGAGCA	60.1	658	INTERGENIC	--	--	--
Ca7_P3_23944596	Ca7	23944596	GAAAAAAAAAA/GAAAAAAAAAAA	AAATCATTGAGCATGAAATGGA	TTTCCATCCTCCCTCTTTCA	59.4	825	INTERGENIC	--	--	--
Ca7_P3_24027767	Ca7	24027767	GATAATAATAATAATAATAATAATAA/GATAATAATAATAATAATAATAATAATAA	ACAATGGATGCTCACGACAC	GTTTCCCATCCTCCCTTTGT	59.6	633	INTERGENIC	--	--	--
Ca7_P3_24034829	Ca7	24034829	CCTGT/C	GGGCTGAGTGCAAACCTAAG	TCGGTTCCTAGAGCACACAA	59.9	184	INTERGENIC	--	--	--
Ca7_P3_24096310	Ca7	24096310	TCCCCC/TCCCCCC	CTTGCCACCACTAATGCTCA	ACAACCGATTTCAAGCCATC	59.9	818	DRR	Ca_19914	--	--
Ca7_P3_2416214	Ca7	2416214	TTATATATATATATATATATATATATATATATATATATATATATAT/TTATATATATATATATATATATATATATATATATATATATATATAT	TGGGTCTGTTTGAAAGGTGA	TTTGCACCAACCACACAAAT	59.1	530	INTERGENIC	--	--	--
Ca7_P3_24165192	Ca7	24165192	ATTTTTTTT/ATTTTTTT	TGAAATATTTTTGGTCCATCCA	GAGCCCCACAAATCAAAGAA	59.2	646	INTERGENIC	--	--	--

INDEL marker IDs	Chromosomes/ unanchored scaffolds	Physical positions (bp)	InDels (*Kabuli* reference genome-CDC Frontier/PII)	Forward primers (5'-3')	Reverse primers (5'-3')	Anneling temperature (0C)	Expected amplified product size (bp)	Structural annotation		Functional annotation	
								Sequence components of *kabuli* genome	*Kabuli* gene accession IDs	NCBI-KOG	TFs
Ca7_P3_24209885	Ca7	24209885	AGAGT/AGAGTTTTGAGT	CTCGATGAGGGTGTGGAAAT	GGGTGACATCAAGCCTCATT	59.9	354	INTERGENIC	--	--	--
Ca7_P3_24347972	Ca7	24347972	TAAAA/TAAAAA	CTCTCATCTGAGGGCAAAGC	CGATTGAATTAAGCCCAGGA	60.1	356	INTERGENIC	--	--	--
Ca7_P3_24348355	Ca7	24348355	TAA/TAAA	TCCTGGGCTTAATTCAATCG	CGTTCCTGAGGGAATTGTGT	60.0	378	INTERGENIC	--	--	--
Ca7_P3_24348515	Ca7	24348515	CAA/CAAA	TCCTGGGCTTAATTCAATCG	CGTTCCTGAGGGAATTGTGT	60.0	378	INTERGENIC	--	--	--
Ca7_P3_24366097	Ca7	24366097	G/GGC	GGAGGGTTTTGGTTTGGAAT	TCTCCATTCCTGTCCGTAGG	60.0	760	INTERGENIC	--	--	--
Ca7_P3_24421160	Ca7	24421160	AATATATATATATATATATATA/AATATATATATATATATA	AGGAAGCCCAAGTTCCAAAT	ACAGCCAACAACCACATCAA	59.9	469	INTERGENIC	--	--	--
Ca7_P3_24485265	Ca7	24485265	CG/CAACTTTG	GTCCCACCCTAGGAGCTTTC	CGGACCAAAAATTAAAGCGA	60.1	986	INTERGENIC	--	--	--
Ca7_P3_24495058	Ca7	24495058	TTTATTATTATTATTATTATTATTATTATTATTATTATTATTATTATTATTATTATT/TTTATTATTATTATTATTATTATTATTATTATTATTATTATTATTATTATTATTATTATTATT	TCCGTCGTAGCTTTTTGACA	TCTGAATCCGACATAGTGCG	59.5	625	INTERGENIC	--	--	--
Ca7_P3_24502290	Ca7	24502290	T/TAAGCA	AATTTGGCAGAACAGGGATG	AGAAAAGCAGGGGAAGCAAT	59.9	575	INTERGENIC	--	--	--
Ca7_P3_24561191	Ca7	24561191	TAA/TA	CCCACCCCTATCTGTCATGT	TGACATTGCTGAGGAGCTTG	59.7	766	INTERGENIC	--	--	--

INDEL marker IDs	Chromosomes/ unanchored scaffolds	Physical positions (bp)	InDels (*Kabuli* reference genome-CDC Frontier/PII)	Forward primers (5'-3')	Reverse primers (5'-3')	Anneling temperature (0C)	Expected amplified product size (bp)	Structural annotation		Functional annotation	
								Sequence components of *kabuli* genome	*Kabuli* gene accession IDs	NCBI-KOG	TFs
Ca7_P3_24782937	Ca7	24782937	AGATGGTTAATGATAATGA/AGATGGTTAATGATAATGATGGTTAATGATAATGA	CGCGAACTCAATTTCACGTA	AAATGATGGTTGTTGGGCAT	59.9	696	INTERGENIC	--	--	--
Ca7_P3_24786712	Ca7	24786712	AATATATATATATATATATATATAT/AATATATATATATATATATATATATAT	ACAGACCCGACCAAGTCAAC	TCAAACATTTAACGATCACCATT	60.0	447	DRR	Ca_19894	GC	HSF
Ca7_P3_24845569	Ca7	24845569	CTTT/CTTTT	TAAACGGCTTGCCTCTGACT	TCCTTCCAAGACATCGGTTC	60.0	412	INTERGENIC	--	--	--
Ca7_P3_25060530	Ca7	25060530	GTTTTTTTT/GTTTTTTTTT	TTGTCGATTTGGCACTTCAG	TGAATGTGGATCCCCTCAAT	59.8	327	INTERGENIC	--	--	--
Ca7_P3_25111194	Ca7	25111194	AATATATATATATATA/AATATATATATATATATA	GGGATTGAGAGGAGAGGGTC	ATTTAAATGTCGTGACCGGC	60.0	489	INTERGENIC	--	--	--
Ca7_P3_25133201	Ca7	25133201	ATATTATTATTATTATTATTATTATTATTAT/ATATTATTATTATTATTATTATTATTATTATTATTAT	CTCAATCGTGGGTGACCTTT	TCCTACGTATCCCCAAGTGC	60.0	381	INTERGENIC	--	--	--
Ca7_P3_25157289	Ca7	25157289	CTTGTTGTTGTTGTTGTTGTTGTTGTTGTTGTTGTTGTTGTTGTT/CTTGTTGTTGTTGTTGTTGTTGTTGTTGTTGTTGTTGTT	CATCTTCATTGCCTCCCACT	CGTTTGCACGAAGACCATTA	60.1	214	INTERGENIC	--	--	--
Ca7_P3_2525335	Ca7	2525335	CAATACAATTGATTAATA/CAATACAATTGATTAATACAATTGATTAATA	TGGTGGAAAAATAGCATTTGG	CCTCTCCCGAATGACTCAAG	59.8	868	INTERGENIC	--	--	--
Ca7_P3_25302232	Ca7	25302232	ATTTTTTTTTTT/ATTTTTTTTTT	TTGGGTGATTTGTGTTTCCA	TGGCACAAAGATTACGACCA	59.8	750	INTERGENIC	--	--	--
Ca7_P3_25342809	Ca7	25342809	TAAAAAAAA/TAAAAAAAAA	CATTCAACAGCCACATGACC	CGGTACCGCATTCGAGTATT	60.0	642	INTERGENIC	--	--	--

INDEL marker IDs	Chromosomes/ unanchored scaffolds	Physical positions (bp)	InDels (*Kabuli* reference genome-CDC Frontier/PII)	Forward primers (5'-3')	Reverse primers (5'-3')	Anneling temperature (0C)	Expected amplified product size (bp)	Structural annotation		Functional annotation	
								Sequence components of *kabuli* genome	*Kabuli* gene accession IDs	NCBI-KOG	TFs
Ca7_P3_25436582	Ca7	25436582	GAAAAAAA/GAAAAAAAA	ATTTTTGAGAGCCAAGCCAA	GAACGTGTTCGGTAAGGGTG	59.8	786	INTERGENIC	--	--	--
Ca7_P3_25482768	Ca7	25482768	CATATATATATATATATATATATATA/CATATATATATATATATATATATATA	GCTTTAAGAAGGGCTTGGCT	CCCAAGCCCACAAATACTGT	60.0	278	INTERGENIC	--	--	--
Ca7_P3_25540307	Ca7	25540307	CTTTT/CTTT	CCTCAGATGAGAGCCAGGAC	GAGCGCTATTACAGTTCGGC	59.9	472	INTERGENIC	--	--	--
Ca7_P3_25563600	Ca7	25563600	AAGAGAGAGAGAGAGAGAGAGAGAGAGAGAGA/AAGAGAGAGAGAGAGAGAGAGAGAGAGAGAGA	CACAAACAAGAACGACGGTG	GAGGCTACGTGTTGTACGCA	60.2	603	CDS (FRAME SHIFT)	Ca_23609	KT	--
Ca7_P3_25613830	Ca7	25613830	TAAAAAAAAA/TAAAAAAAA	TCGATGGCTACCTCAAATCC	GGTGGTCCTCTGCTCTTACG	60.0	512	INTERGENIC	--	--	--
Ca7_P3_25626892	Ca7	25626892	CGG/CGGG	TTGGCCAAGTCCCTGTTTAC	CCCGTGATAGGCAGTACGTT	60.0	420	INTERGENIC	--	--	--
Ca7_P3_25659871	Ca7	25659871	TTATATATATATATATATATAT/TTATATATATATATATATATATATAT	TCCGATTAATTGGTTTTTGAGC	ATCGAAGCTAGCGGAGGATT	60.3	571	INTERGENIC	--	--	--
Ca7_P3_25724934	Ca7	25724934	ATTTTTTTTTT/ATTTTTTTTTTT	ATTCTGCGCGACTATCAACC	GTATTGACGTGTGGCGAATG	60.2	755	INTRON	Ca_23630	--	--
Ca7_P3_25854060	Ca7	25854060	CATATATATATATATATATATATATAT/CATATATATATATATATATATATATATATATAT	AGGAAGAGTGGAGTTGCGAA	TCGACCCTTGCACATATTGA	60.0	665	INTERGENIC	--	--	--
Ca7_P3_26035041	Ca7	26035041	TAAAAAAAA/TAAAAAAAAA	TAAAGAGATTCGCAGCGGTT	CATGTTTTCGAAGCTCACCA	60.0	407	INTERGENIC	--	--	--

INDEL marker IDs	Chromosomes/ unanchored scaffolds	Physical positions (bp)	InDels (*Kabuli* reference genome-CDC Frontier/PII)	Forward primers (5'-3')	Reverse primers (5'-3')	Anneling temperature (0C)	Expected amplified product size (bp)	Structural annotation		Functional annotation	
								Sequence components of *kabuli* genome	*Kabuli* gene accession IDs	NCBI-KOG	TFs
Ca7_P3_26056806	Ca7	26056806	CTTTTTT/CTTTTTTTTT	GTTGCCACTTGGCGTAAAAT	GGCCGATTTACTGCTCACAT	60.0	393	INTERGENIC	--	--	--
Ca7_P3_26075420	Ca7	26075420	TTATATATATAT/TTATATATATATAT	TATTTTGGATTTTGGCTCGG	TTTGGATCAAGGAGAAACGG	59.9	584	INTERGENIC	--	--	--
Ca7_P3_26118670	Ca7	26118670	TTATATATATATATATATATA/TTATATATATATATATATA	CGGGACTAGAAAGCAACGAG	TTTTATAGTGGAGGCCAGGG	60.0	691	INTERGENIC	--	--	--
Ca7_P3_26160184	Ca7	26160184	AG/A	GTGGCTACCTTCTTGGGTGA	TTTCATCGCCGAAACCTATC	60.1	141	INTERGENIC	--	--	--
Ca7_P3_26221720	Ca7	26221720	AATATATATATATATATATAT/AATATATATATATATAT	CTTGGGAATTGGAGAGCAAG	GGGTTTCTTTCCTTGAAGCC	59.8	671	INTERGENIC	--	--	--
Ca7_P3_26228410	Ca7	26228410	GAAAAAAAAAA/GAAAAAAAAAAAA	ATGGTGCAAAATGGTGACAA	GAAAGTCGGTGCATGGAAGT	59.8	304	INTERGENIC	--	--	--
Ca7_P3_26235537	Ca7	26235537	TTATATATATATA/TTATATATATATATATA	TGCGACCTCAGTTTCTCAAA	GGGGTCATAGCATCCCCTAT	59.6	157	INTERGENIC	--	--	--
Ca7_P3_26240583	Ca7	26240583	G/GTAACATATT	GTTTCCCACATCCAGAGGAA	GAGATCAACGTGAATTGGTCA	59.9	330	INTRON	Ca_23643	--	HD-ZIP
Ca7_P3_26248461	Ca7	26248461	G/GA	TGCATACCAGGGAAAAATGA	TTCAATCTCCGAAATCGGTC	59.0	596	INTERGENIC	--	--	--
Ca7_P3_2625656	Ca7	2625656	CATATATATATATATATATATATATATATATATATATAT/CATATATATATATATATATATATATAT	CCTACTACAAGGATCGGCCA	TTAGGCTCCGTATCGGATTG	60.1	618	DRR	Ca_03215	G	--

INDEL marker IDs	Chromosomes/ unanchored scaffolds	Physical positions (bp)	InDels (*Kabuli* reference genome-CDC Frontier/PII)	Forward primers (5'-3')	Reverse primers (5'-3')	Anneling temperature (0C)	Expected amplified product size (bp)	Structural annotation		Functional annotation	
								Sequence components of *kabuli* genome	*Kabuli* gene accession IDs	NCBI-KOG	TFs
Ca7_P3_26427819	Ca7	26427819	ATTTTTTTTT/ATTTTTTTTTT	TCTTCACACAATCTCCCCAA	CATTTGCTAGCCCCTTTGAG	59.1	622	INTERGENIC	--	--	--
Ca7_P3_2646640	Ca7	2646640	ATATTATTATTATTATTATTATTAT/ATATTATTATTATTATTATTATTATTAT	GGATGGAGAGCAATCCTCAA	CAAAAGCCAAGCACATCAAA	60.2	683	INTERGENIC	--	--	--
Ca7_P3_26468080	Ca7	26468080	GAAAAAAAA/GAAAAAAAAA	AAAAGCCCAAGCAATGAATG	TTTGGTCTTGGGTCCTTCAG	60.1	561	INTERGENIC	--	--	--
Ca7_P3_26563030	Ca7	26563030	GTTATTATTATTATTATTATTATTATTATTATTATTATTATTATTATTATTATTATTATTATTATTATTACTATTATTATTATTATTAT/GTTATTATTATTATTATTATTATTATTATTATTATTATTATTATTATTATTATTATTATTA[illegible]	GCAAACAAGGCTGAAGAAGAA	CGGCTACATCCAGTCCTCTC	59.6	460	INTERGENIC	--	--	--
Ca7_P3_26567174	Ca7	26567174	TAAAAAAAAA/TAAAAAAAAAA	TATGCATTCGCAAACACGAT	TTCATTTTCTTTCGTTGGTCA	60.1	430	INTERGENIC	--	--	--
Ca7_P3_26596101	Ca7	26596101	TAAAAAAAAAAAA/TAAAAAAAAAAA	TTTTTGTTTCTTCCATGCCA	TTCGTGTGCGTTTGACTACA	59.1	454	INTERGENIC	--	--	--
Ca7_P3_26662956	Ca7	26662956	CAA/CA	TCCAAGCTTCCTTCAATGGT	CTCAAAAGCGCTCCAATTTC	59.7	542	INTERGENIC	--	--	--
Ca7_P3_26691926	Ca7	26691926	C/CTATT	CGGTGTGGTGTTATTCGTTG	TGCAAGAGACCGAAGAAGGT	59.9	443	INTERGENIC	--	--	--
Ca7_P3_26694678	Ca7	26694678	TCCCCC/TCCCCCCCCCC	CTCGGCCATCCGAGATATAA	GCTCACTAACTCTGCCTGGG	60.0	881	CDS (FRAME SHIFT)	Ca_16250	--	--
Ca7_P3_26696024	Ca7	26696024	C/CAGATAAGGGGA	CGTACACATTCCGACGATTG	CCGGCTTAGCTACGATCTTG	60.0	635	INTERGENIC	--	--	--

INDEL marker IDs	Chromosomes/ unanchored scaffolds	Physical positions (bp)	InDels (*Kabuli* reference genome-CDC Frontier/PII)	Forward primers (5'-3')	Reverse primers (5'-3')	Anneling temperature (0C)	Expected amplified product size (bp)	Structural annotation		Functional annotation	
								Sequence components of *kabuli* genome	*Kabuli* gene accession IDs	NCBI-KOG	TFs
Ca7_P3_26699922	Ca7	26699922	A/AACTAG	AAACTCCAGCCCGGTAAACT	AACCCATTTGAGGTGACTGC	60.0	465	DRR	Ca_16249	--	--
Ca7_P3_26701106	Ca7	26701106	A/ACTAGGAATTACTCCC	CAATAGGGCGAGTCGAATGT	AAGTCCAGTAGCCGAAAGCA	60.1	838	DRR	Ca_16249	--	--
Ca7_P3_26701534	Ca7	26701534	C/CG	AAGCTTCATCCTGGTCATGG	AAGTCCAGTAGCCGAAAGCA	60.1	381	DRR	Ca_16249	--	--
Ca7_P3_26754740	Ca7	26754740	TTATAT/TTAT	TTCTTCTCCTCCAATGTGGG	CTCTTTAAGACGTTTCGCGG	60.0	766	INTERGENIC	--	--	--
Ca7_P3_26840972	Ca7	26840972	CAAAAAAAAAAAAAAAA/CAAAAAAAAAAAAAA	TGGGAAATTTTTGGCTTCAG	CACGTATTTGCATGGACCAA	60.0	779	INTERGENIC	--	--	--
Ca7_P3_26852121	Ca7	26852121	ATTTTTTTTTT/ATTTTTTTTTTT	GCAAGCTGAAATCGATAGGC	TGAACCGTCGCAGACCTAAT	60.0	656	INTERGENIC	--	--	--
Ca7_P3_26906603	Ca7	26906603	ATTTTT/ATTTT	TGGAGTTCCAACACGCACTA	CAAGGTAATTTGCATTGAAGGA	60.3	112	INTERGENIC	--	--	--
Ca7_P3_2695916	Ca7	2695916	CATATATATATATATATATATA/CATATATATATATATATATATATATA	GTGACTTAGGATTGCCTCGC	GGGGATGAACGTGAAAGAGA	59.8	632	INTERGENIC	--	--	--
Ca7_P3_27009693	Ca7	27009693	ACCC/ACC	CAGCAGCAAACTCCAAATCA	AGGCAGTTCTTGCTTGGAAA	60.0	378	INTERGENIC	--	--	--
Ca7_P3_27031473	Ca7	27031473	TTCTCTCTCTCTCTCTCTCTCTCTCTCTCTCTCTCTCTC/TTCTCTCTCTCTCTCTCTCTCTCTCTCTCTCTCTCTCTC	TCTTCCTTCCTTGCTCTCCA	GACTTGGCATGTCATGATGG	60.1	551	INTERGENIC	--	--	--

INDEL marker IDs	Chromosomes/ unanchored scaffolds	Physical positions (bp)	InDels (*Kabuli* reference genome-CDC Frontier/PII)	Forward primers (5'-3')	Reverse primers (5'-3')	Anneling temperature (0C)	Expected amplified product size (bp)	Structural annotation		Functional annotation	
								Sequence components of *kabuli* genome	*Kabuli* gene accession IDs	NCBI-KOG	TFs
Ca7_P3_27081077	Ca7	27081077	TATTTT/TATTTTAAATTTAAATTTT	ACCATGACGTCTCCAACACA	ACCGCCACAAATGTTAAAGC	60.0	613	INTERGENIC	--	--	--
Ca7_P3_27115436	Ca7	27115436	GATATATATATATATAT/GATATATATATATAT	CAATTCCCACAGGTACGCTT	CAAACCCTAATTTTTCAAACACG	60.0	347	INTERGENIC	--	--	--
Ca7_P3_27272092	Ca7	27272092	CTTT/CTT	ATTTAGAGGCGAAGTTGCGA	GATTCCCCATTCTTAACGCC	60.0	540	INTERGENIC	--	--	--
Ca7_P3_27341260	Ca7	27341260	A/AG	CACGATCCACACCAAAACAC	TCTCCCTCCTTCTCCCATTT	59.9	382	INTERGENIC	--	--	--
Ca7_P3_27346895	Ca7	27346895	TTATATATATATATATATATATAT/TTATATATATATATATATATATATAT	TGCTTGATTGCTTGAGTTCC	CGCGCAGATTGTCAAATAGA	59.0	641	INTERGENIC	--	--	--
Ca7_P3_27410630	Ca7	27410630	TTTATTATTATTATTATTATTATTA/TTATTATTATTATTATTATTATTATTA	GGAGGAGGGAAAAGATGAGG	AATTTGACACTTTCACCGGC	60.0	463	INTERGENIC	--	--	--
Ca7_P3_27521328	Ca7	27521328	CAAA/CAA	GCCAAATTCCCTCTTTCTCC	CCTTTCCACACAACCACAGA	60.0	393	INTERGENIC	--	--	--
Ca7_P3_27530025	Ca7	27530025	TG/TGAAAAG	TCAAATGAGGCAAGTGCAAG	TTGAAAAAGGGCCAACTGTC	60.0	259	INTERGENIC	--	--	--
Ca7_P3_27641750	Ca7	27641750	T/TAATGAC	AATGACGACGACGATGATGA	TCCTCCTGCAGATCCTCCTA	60.1	327	DRR	Ca_16215	--	--
Ca7_P3_27761690	Ca7	27761690	TTATATATATATATATATATATAT/TTATATATATATATATATATATAT	AAATTCGCAGCACTTACGCT	GTTTGTGAGTAAGCTCGCCC	60.0	873	INTERGENIC	--	--	--

INDEL marker IDs	Chromosomes/ unanchored scaffolds	Physical positions (bp)	InDels (*Kabuli* reference genome-CDC Frontier/PII)	Forward primers (5'-3')	Reverse primers (5'-3')	Anneling temperature (0C)	Expected amplified product size (bp)	Structural annotation		Functional annotation	
								Sequence components of *kabuli* genome	*Kabuli* gene accession IDs	NCBI-KOG	TFs
Ca7_P3_27767778	Ca7	27767778	TATTATTAT/TATTATTATGATTATTAT	TTTTGCGACACTTGATCGTT	GAAGTGTGTGTGATCCGGTG	59.3	431	INTERGENIC	--	--	--
Ca7_P3_27774210	Ca7	27774210	TAAAAAAAA/TAAAAAAA	TGGGGGAAGTTCTCATCAAG	TTGGCCTTCTCAAACAGACC	60.0	597	INTERGENIC	--	--	--
Ca7_P3_27819009	Ca7	27819009	GAAAAAAAAAAAAA/GAAAAAAAAAAAAAA	CAAAAGGGCAACATGCCTAT	GCAAGATCGCATTCTTGTCA	60.0	454	INTERGENIC	--	--	--
Ca7_P3_27903023	Ca7	27903023	TGG/TG	TTCCCAGTCGGACCTATGAG	AGGCCACATGTTGTGTGAGA	60.1	434	INTERGENIC	--	--	--
Ca7_P3_27916399	Ca7	27916399	ATT/ATTATTTTT	GGCGGCGATAATAACAAAAG	TCCTCTCCTAGAACACCCCA	59.6	688	INTERGENIC	--	--	--
Ca7_P3_28037378	Ca7	28037378	ATTTTTTT/ATTTTTTTT	TGGCAGACAATATCGTTGGA	CTGATGCTCGGATCTACCGT	60.1	334	INTERGENIC	--	--	--
Ca7_P3_2808679	Ca7	2808679	GAAAAAAAAAA/GAAAAAAAAAAAA	ACTTCCCTGTGGAACGACAC	ATATGCACGGAACGAAAACC	60.0	431	INTERGENIC	--	--	--
Ca7_P3_28089294	Ca7	28089294	GAAAAA/GAAAAAA	CGTAAAGTCCACTTCCGTTTC	CAACAGATGTTGTGCGTGTG	58.8	500	INTERGENIC	--	--	--
Ca7_P3_28094429	Ca7	28094429	GAAAAAAAAAA/GAAAAAAAAAAA	ATCGGAAATGGATGTATGCC	TGGGATGTTGCACATCTTGT	59.6	587	INTERGENIC	--	--	--
Ca7_P3_28129563	Ca7	28129563	CAAAAAAAAAAAAA/CAAAAAAAAAAAAAA	TCTTCACCAAAACAAACCCA	GCATCGTAAAGTTGCACATCA	59.0	835	INTERGENIC	--	--	--

INDEL marker IDs	Chromosomes/ unanchored scaffolds	Physical positions (bp)	InDels (*Kabuli* reference genome-CDC Frontier/PII)	Forward primers (5'-3')	Reverse primers (5'-3')	Anneling temperature (0C)	Expected amplified product size (bp)	Structural annotation		Functional annotation	
								Sequence components of *kabuli* genome	*Kabuli* gene accession IDs	NCBI-KOG	TFs
Ca7_P3_28185239	Ca7	28185239	CA/CATAAA	TGTTGCCCTCTTGAGTTTCA	TGGAGTGCATTTGTTTCCAA	59.4	560	INTRON	Ca_26608	--	Trihelix
Ca7_P3_28190197	Ca7	28190197	AGTTTATTTGG/AGTTTATTTGGTTTATTTGG	TTGGTTTATTTGGGTTGGGA	ACCAGGAAAAGAAGAAGGGC	60.0	519	INTERGENIC	--	--	--
Ca7_P3_2831340	Ca7	2831340	TAAAAAAAAA/TAAAAAAAAAA	TGATAATCTCGCGGGATAGG	GAATTGCCGAAAAATGATTGA	60.0	465	DRR	Ca_03193	K	GATA
Ca7_P3_28317021	Ca7	28317021	ATTTTTTTTTTTT/ATTTTTTTTTTT	GATCCCATGTATGGTGAGGG	CTGTCTGTTGCAGCTGGTGT	60.0	481	INTERGENIC	--	--	--
Ca7_P3_28353975	Ca7	28353975	AATATATATATATATATAT/AATATATATATATAT	ACGGAAACAAATGGACGATG	TGGTGTTGGAGTCATGCTTT	60.8	215	INTERGENIC	--	--	--
Ca7_P3_28373395	Ca7	28373395	TTTCTTCTTCTTCTTCTTCTTCTTC/TTTCTTCTTCTTCTTCTTCTTC	CGAAAGCACTTCTTGCACAC	GTAACGACGGCGATGAATTT	59.6	485	INTERGENIC	--	--	--
Ca7_P3_28381412	Ca7	28381412	GATATATATATATAT/GATATATATATATATAT	AATGTGCAGGGTGTGAAACA	CCTCAGTTGGATCGACTGGT	60.0	384	INTRON	Ca_18923	R	--
Ca7_P3_28400986	Ca7	28400986	CATATATATATATATATATATATATATAT/CATATATATATATATATATATATATATAT	TCGAGTTATTCGCATGTGTATTG	ACTTTATCCGTTGCGGTGAC	60.0	288	INTERGENIC	--	--	--
Ca7_P3_28428539	Ca7	28428539	AATATATATATATATATATATATATATATATATATATAT/AATATATATATATATATATATATATATATATATATATATATATATAT	CGTGCTTTTTGTGTTTCCCT	AACTAGACGACGTGATCGGC	60.1	506	INTERGENIC	--	--	--
Ca7_P3_28451984	Ca7	28451984	CAAAAAAAAAA/CAAAAAAAAAAA	GGTGACGACATTGAGAGGGT	CACGGGCAAGTAAGCAAGAT	60.0	652	INTERGENIC	--	--	--

INDEL marker IDs	Chromosomes/ unanchored scaffolds	Physical positions (bp)	InDels (*Kabuli* reference genome-CDC Frontier/PII)	Forward primers (5'-3')	Reverse primers (5'-3')	Anneling temperature (0C)	Expected amplified product size (bp)	Structural annotation		Functional annotation	
								Sequence components of *kabuli* genome	*Kabuli* gene accession IDs	NCBI-KOG	TFs
Ca7_P3_28462671	Ca7	28462671	ATATTATTATTATTATTATTATTATTATTATTATTATTATT/ATATTATTATTATTATTATTATTATTATTATTATTATT	GGCGTTGGTTTCACTCAAAT	GTTGCATGAAGGCAAAGGAT	60.0	600	INTERGENIC	--	--	--
Ca7_P3_28529637	Ca7	28529637	ATTTTTTT/ATTTTTTTT	CAGACCAGACCGTGCTTTTT	ATGTGGCCTGTCATAGGCTT	60.3	498	INTERGENIC	--	--	--
Ca7_P3_28557235	Ca7	28557235	TACTTA/T	TGCGATGGAAATGGTATTGA	CACAACACCGAGAGAACGAA	59.9	233	INTRON	Ca_18943	L	C3H
Ca7_P3_28561460	Ca7	28561460	CCAC/CCACAC	CACTGCTTTAGCAACCACCA	TTCTAGAAGCGGCGTTGTTT	59.9	678	DRR	Ca_18943	L	C3H
Ca7_P3_28562877	Ca7	28562877	AGTACAAGTACAAGT/AGTACAAGTACAAGTACATGTACAAGTACAAGT	AAGCAACACAGCACAACCAA	TTGAGCACCACATTCAGAGC	60.3	658	INTERGENIC	--	--	--
Ca7_P3_28615286	Ca7	28615286	TAAAAAAAAAAA/TAAAAAAAAAA	GAATATTGAATTGTGGCTGGC	AGCATGTGCACCATGTGTTT	59.4	706	INTERGENIC	--	--	--
Ca7_P3_28698838	Ca7	28698838	TTATATATATATATATATATATAT/TTATATATATATATATATAT	TCCACACAAAAACCCAATCA	CCTGTGCAGAAGTTGCTTGA	59.8	691	INTRON	Ca_18957	--	--
Ca7_P3_28702659	Ca7	28702659	ATCCTCC/ATCC	TCCATTAACCCTCTTGAGGAAA	TCAGGATGAAGAAGGGGAGA	59.9	531	INTERGENIC	--	--	--
Ca7_P3_28706973	Ca7	28706973	T/TTAAGGTAC	TCACCCATCTTACACTCTTGTCA	TTGGTTTTTGATGGTGGGAT	59.6	503	INTERGENIC	--	--	--
Ca7_P3_28707412	Ca7	28707412	GAAATAACAATAAAAACAAATAACAATAAAA/GAAATAACAATAAAAACAAATAACAATAAAAACAAATAACAATAAAA	ATCCCACCATCAAAAACCAA	GATATGTTCCGAGTGCCGTT	60.0	491	INTERGENIC	--	--	--

INDEL marker IDs	Chromosomes/ unanchored scaffolds	Physical positions (bp)	InDels (*Kabuli* reference genome-CDC Frontier/PII)	Forward primers (5'-3')	Reverse primers (5'-3')	Anneling temperature (0C)	Expected amplified product size (bp)	Structural annotation		Functional annotation	
								Sequence components of *kabuli* genome	*Kabuli* gene accession IDs	NCBI-KOG	TFs
Ca7_P3_28889070	Ca7	28889070	ATTTTTTTTTT/ATTTTTTTTT	AAGACCAAAGGCTTCAGCAA	TTCCGCTGCTCGTAAATTCT	60.0	541	INTERGENIC	--	--	--
Ca7_P3_2900774	Ca7	2900774	TTAATAATAATAATAATAATAATAATAATAA/TTAATAATAATAATAATAATAATAATAATAATAA	GTCTCGGGTCTCTTCCTTCC	TTGCCCCTGATTTTATACCAA	60.2	789	DRR	Ca_03186	L	bHLH
Ca7_P3_29076436	Ca7	29076436	GTTTTTTTTTTTT/GTTTTTTTTTTTTT	CCGGAGAATTATTACCCATACA	CCATTTTTGCTTTGGCGTAT	57.9	412	INTERGENIC	--	--	--
Ca7_P3_29119308	Ca7	29119308	GGAGAGAGAGAGAGAGAGAGAGAGAGA/GGAGAGAGAGAGAGAGAGAGAGAGA	CCACAAGGAAGTTCGGAAGA	CTCCCAAACGCTCTCGTAAG	60.2	190	INTRON	Ca_11796	FE	--
Ca7_P3_29215835	Ca7	29215835	T/TG	CAACACACACATTGTTTCCAA	CGGTGAAAATAGAGGGACCA	57.9	560	DRR	Ca_11784	--	--
Ca7_P3_29245826	Ca7	29245826	AATATATATATATATATATATA/AATATATATATATATATATATATATA	ATTTCCAGCATGCCAATTTC	GCAACATTGGGTGTTGTTTG	59.9	516	INTERGENIC	--	--	--
Ca7_P3_29280548	Ca7	29280548	ATATTATTATTATTATTATTATTATTATTATT/ATATTATTATTATTATTATTATTATTATTATT	TTGTTTGAAAAACCAATATGACA	TTGCAACTACCAAAATAAACAATTTC	57.2	875	INTERGENIC	--	--	--
Ca7_P3_29311995	Ca7	29311995	ATATTATTATTATTATTATTATTATTATTATTATTATTATTATT/ATATTATTATTATTATTATTATTATTATTATTATTATTATTATTATTATT	TCATCGAAAACTTCAAAAACTATGA	GATGCAGGTCATGAAACCAA	59.2	494	INTERGENIC	--	--	--
Ca7_P3_29326465	Ca7	29326465	TTATATATATATATATATATAT/TTATATATATATATATATATATAT	GAAGTCAACGGAATCTCATCC	TGAATAGAGGATGTCGTGTGAAA	58.6	392	INTERGENIC	--	--	--
Ca7_P3_29416457	Ca7	29416457	ACC/AC	AAAATCATTACTCACCATAAGTGGAA	TGCTCTCCTCCTTCAACAACT	59.3	436	INTERGENIC	--	--	--

INDEL marker IDs	Chromosomes/ unanchored scaffolds	Physical positions (bp)	InDels (*Kabuli* reference genome-CDC Frontier/PII)	Forward primers (5'-3')	Reverse primers (5'-3')	Anneling temperature (0C)	Expected amplified product size (bp)	Structural annotation		Functional annotation	
								Sequence components of *kabuli* genome	*Kabuli* gene accession IDs	NCBI-KOG	TFs
Ca7_P3_29416537	Ca7	29416537	ATATAAATTATAAATTATAAATTAT/ATATAAATTATAAATTATAAATTATAAATTAT	AAAATCATTACTCACCATAAGTGGAA	TGCTCTCCTCCTTCAACAACT	59.3	436	INTERGENIC	--	--	--
Ca7_P3_29416730	Ca7	29416730	AAGAAGTTGTTGAAGGAG/AAG	ATTGTAGAAGGAAGAGGAGAAGCA	AAAAAGCTGATGTGTACCTGTTGA	59.9	100	INTERGENIC	--	--	--
Ca7_P3_29417874	Ca7	29417874	ATTT/ATT	AGTTGTTGAAGGAGGAGAGCA	TTGTAGGCTGGACATCATGC	59.1	645	INTERGENIC	--	--	--
Ca7_P3_29418101	Ca7	29418101	C/CT	CATGTACACGTCACATCGGA	TTGTAGGCTGGACATCATGC	59.0	329	INTERGENIC	--	--	--
Ca7_P3_29433568	Ca7	29433568	T/TG	AGGCATGATTCAAGGTACGG	CAGGTGAAGGTCCATTTCGT	60.0	278	DRR	Ca_11776	--	--
Ca7_P3_29446201	Ca7	29446201	ATTTTTTTTTT/ATTTTTTTTTTT	CGTTGCGCATGTGTAATTTG	TCATTCCCAACATTATTTCGTG	61.1	639	DRR	Ca_11775	R	--
Ca7_P3_2946667	Ca7	2946667	CTTTTTTTTTT/CTTTTTTTTT	GATGGATCTGCTGGGACCTA	CGCACTTAATGCACACCAAC	60.0	485	INTRON	Ca_03180	T	E2F/DP
Ca7_P3_29470400	Ca7	29470400	ATTTTTTT/ATTTTTTTT	CGATTTTGTTGTGATTTGTGC	GCAAAATGTTTTCTGCGGTT	59.1	542	DRR	Ca_11771	--	ERF
Ca7_P3_29475338	Ca7	29475338	ATTT/ATT	ACGGAAGGTAGCAAAATTGG	TTGTGGCTCACTTATGGTGAA	59.1	801	INTERGENIC	--	--	--
Ca7_P3_29547777	Ca7	29547777	AT/ATT	TGCAGATTAAGACGCGATTG	TAGCTTTTCTGGGGTGGATG	60.0	419	INTRON	Ca_11767	--	SBP

INDEL marker IDs	Chromosomes/ unanchored scaffolds	Physical positions (bp)	InDels (*Kabuli* reference genome-CDC Frontier/PII)	Forward primers (5'-3')	Reverse primers (5'-3')	Anneling temperature (0C)	Expected amplified product size (bp)	Structural annotation		Functional annotation	
								Sequence components of *kabuli* genome	*Kabuli* gene accession IDs	NCBI-KOG	TFs
Ca7_P3_29588870	Ca7	29588870	CTTTTTTTTTTTT/CTTTTTTTTTTT	TTTATTGGGAAGAGGGGAAAA	GGTAGGACTTACTGTCCCAGGAT	59.8	349	INTERGENIC	--	--	--
Ca7_P3_29610109	Ca7	29610109	TCCC/TTCCCC	AACAGAAAGAAGGCCGTTTG	TGAGTTAAGCCCCGCTAGAA	59.4	134	DRR	Ca_11764	--	--
Ca7_P3_29682311	Ca7	29682311	GGTTTTTATATAAGTTGTTTTTATA/GGTTTTTATA	AAAATTCATTCTCGCATCGC	CACTTGGTCTGCTCGCATAA	60.2	639	INTRON	Ca_11755	--	--
Ca7_P3_2977293	Ca7	2977293	CTTTTTTTTTTTTT/CTTTTTTTTTTTT	CCCCAATATCTCTGCACTCC	GCAACTCGTCCAATCAGGTT	59.5	456	INTRON	Ca_03176	R	--
Ca7_P3_29776063	Ca7	29776063	TAAAAAAA/TAAAAAAAA	TGGTTACACTTCGTGGCAAA	CATGGGCACTACATCGTCAC	60.1	679	INTERGENIC	--	--	--
Ca7_P3_29885289	Ca7	29885289	TAAAAAAAAA/TAAAAAAAAAA	TCAGGGGGTAACGAGTTTTG	ATTGCGCCCAAGAATAAATG	60.0	713	INTERGENIC	--	--	--
Ca7_P3_29987440	Ca7	29987440	AAATAATAATAATAATAATAATAATA/AAATAATAATAATAATAATAATAATAATAATAATAATA	GAACATGGTTGATTGGATTGC	CTCCTCGTTGTGTTGGATCA	60.2	751	INTERGENIC	--	--	--
Ca7_P3_29996051	Ca7	29996051	GAAAAAA/GAAAAAAAA	GGCCAGCACTAATTTCAGGA	CGAACCGATAAAAACTAATCTGG	60.2	774	INTERGENIC	--	--	--
Ca7_P3_30001737	Ca7	30001737	ATC/ATCTC	TATGATTCGTTTGGGGTGGT	CAGAGATGGTGGTGGTTGTG	60.1	511	INTERGENIC	--	--	--
Ca7_P3_30009196	Ca7	30009196	A/AC	AATTGTGGTTGTGGTTCGGT	GATGGTGCTGGGGAATCTAA	60.1	881	INTRON	Ca_11728	--	bHLH

INDEL marker IDs	Chromosomes/ unanchored scaffolds	Physical positions (bp)	InDels (*Kabuli* reference genome-CDC Frontier/PII)	Forward primers (5'-3')	Reverse primers (5'-3')	Anneling temperature (0C)	Expected amplified product size (bp)	Structural annotation		Functional annotation	
								Sequence components of *kabuli* genome	*Kabuli* gene accession IDs	NCBI-KOG	TFs
Ca7_P3_30011389	Ca7	30011389	TC/T	AGAATTTTCGTGGGTCTTCG	CCACTGCCAACCTGGTATTC	59.2	898	DRR	Ca_11728	--	bHLH
Ca7_P3_30011617	Ca7	30011617	ATTTTTT/ATTTTTTT	TTTGGTTCACCAATTATTACAAGC	CCACTGCCAACCTGGTATTC	59.4	670	DRR	Ca_11728	--	bHLH
Ca7_P3_30015369	Ca7	30015369	GTTT/GTTTTT	CCAAAATTAGAAAAGCTTTGAACG	CCCCAAGCAAAAGAAACAAA	60.5	562	INTERGENIC	--	--	--
Ca7_P3_30015428	Ca7	30015428	TAA/TAAA	CCAAAATTAGAAAAGCTTTGAACG	CCCCAAGCAAAAGAAACAAA	60.5	562	INTERGENIC	--	--	--
Ca7_P3_30016977	Ca7	30016977	TATA/T	CAACGGTTGGAACAAATTGA	TCAGAATGAAGCGACGTGAC	59.4	767	INTERGENIC	--	--	--
Ca7_P3_30019715	Ca7	30019715	CATAT/CAT	CGCACTTCTCAAATGAACGA	CGACCCTTCTGCATTGATCT	60.0	648	INTERGENIC	--	--	--
Ca7_P3_30020338	Ca7	30020338	TG/T	AGATCAATGCAGAAGGGTCG	GAGCTTGCAAATGCCATAAA	60.2	324	INTERGENIC	--	--	--
Ca7_P3_30024153	Ca7	30024153	A/AT	GCCAAAGGAGAGAATGCTTG	GTGGGGATATAGGGTTGGCT	60.0	823	INTRON	Ca_11727	--	--
Ca7_P3_30026167	Ca7	30026167	C/CTTTAATTTGT	CGGAACTTGATTTCCGATGT	ATCCAATCAGCAATGCATCA	59.9	653	INTRON	Ca_11727	--	--
Ca7_P3_30035988	Ca7	30035988	GAAAAAAA/GAAAAAA	CAACTTCACTTGCGGATTCA	ACAATTCCATGTGCTGGTGA	59.8	623	DRR	Ca_11724	O	--

INDEL marker IDs	Chromosomes/ unanchored scaffolds	Physical positions (bp)	InDels (*Kabuli* reference genome-CDC Frontier/PII)	Forward primers (5'-3')	Reverse primers (5'-3')	Anneling temperature (0C)	Expected amplified product size (bp)	Structural annotation		Functional annotation	
								Sequence components of *kabuli* genome	*Kabuli* gene accession IDs	NCBI-KOG	TFs
Ca7_P3_30039331	Ca7	30039331	CAAA/CA	AACAGGTCGGCTTCAT CATC	GGAAGGATATGAGCGT CCAA	60.1	223	INTRON	Ca_11724	O	--
Ca7_P3_30042599	Ca7	30042599	ATGCG/A	TGCGAAGTAGTTTTGAA TGTGG	ACGGATGAAACTGCCG TAAC	60.2	617	INTERGENIC	--	--	--
Ca7_P3_30042952	Ca7	30042952	TAAAAAAA/TAAAAAA	GTTACGGCAGTTTCAT CCGT	CAAGCAAAAACTCCAA CTTCC	60.0	662	INTERGENIC	--	--	--
Ca7_P3_30046644	Ca7	30046644	GCC/GC	GCCTCAACACAGGTTC CATT	AGGGGGATGTGAAATT AGGG	60.0	108	INTERGENIC	--	--	--
Ca7_P3_30047737	Ca7	30047737	GTTTTTTTTTTT/GTTTTTTTTT	ACTTCATCCCCCGACTA CCT	TGCCATTTACACACAC ACACA	59.8	485	DRR	Ca_11723	O	GeBP
Ca7_P3_30053648	Ca7	30053648	CAAAGT/C	GGACTGAAAAGAAAAAT ATGCGA	GTGCTCTCTTTTGCCG ATTC	59.6	774	INTERGENIC	--	--	--
Ca7_P3_30057009	Ca7	30057009	AGG/AGGG	CAAGACCCATTGCCAA AAGT	AACCAACAAGCAGCCA TTCT	60.0	476	INTRON	Ca_11722	P	NF-YB
Ca7_P3_30059154	Ca7	30059154	AGG/AGGG	GTGCAGTGCTGTTTTC TGGA	CAAATTGGCAACCTGG TTCT	60.0	481	INTRON	Ca_11722	P	NF-YB
Ca7_P3_30059206	Ca7	30059206	AAAGAAG/AAAG	GTGCAGTGCTGTTTTC TGGA	CAAATTGGCAACCTGG TTCT	60.0	481	INTRON	Ca_11722	P	NF-YB
Ca7_P3_30059256	Ca7	30059256	GGTGTG/GGTGTGTG	GTGCAGTGCTGTTTTC TGGA	CAAATTGGCAACCTGG TTCT	60.0	481	INTRON	Ca_11722	P	NF-YB

INDEL marker IDs	Chromosomes/ unanchored scaffolds	Physical positions (bp)	InDels (*Kabuli* reference genome-CDC Frontier/PII)	Forward primers (5'-3')	Reverse primers (5'-3')	Anneling temperature (0C)	Expected amplified product size (bp)	Structural annotation		Functional annotation	
								Sequence components of *kabuli* genome	*Kabuli* gene accession IDs	NCBI-KOG	TFs
Ca7_P3_30059401	Ca7	30059401	CAAAA/CAAAAA	GTGCAGTGCTGTTTTCTGGA	CTTTAGCTAACGGGTGCTCG	60.0	624	INTRON	Ca_11722	P	NF-YB
Ca7_P3_30070754	Ca7	30070754	AT/A	AACAGGAACTCACCGACAGC	TCAAATGTTTCGGTCTTCTTCTC	60.3	565	INTERGENIC	--	--	--
Ca7_P3_30072048	Ca7	30072048	ACAAAG/A	GCCATGGGAATAAAGAGTGC	TAATGGCCAAACGTTTCAGG	59.5	139	INTERGENIC	--	--	--
Ca7_P3_30072900	Ca7	30072900	GTTTTTT/GTTTTTTT	TGAGATTTGGAACTAACGGTGA	TCAAAATTTTCAAGCAAATCCC	59.6	667	INTERGENIC	--	--	--
Ca7_P3_30073920	Ca7	30073920	CAA/CA	TTGATCAAAACAATACGTGTGC	GCCAATGGGTGCTGATTATT	58.6	544	DRR	Ca_11719	--	NAC
Ca7_P3_30078953	Ca7	30078953	GAAAA/GAAA	ATGCCAAAACGTAGCAAAGC	CGCACCACTTTCCATCATAA	60.3	749	INTERGENIC	--	--	--
Ca7_P3_30096152	Ca7	30096152	GTTTTTT/GTTTT	GGCTTGGACGAATCGTGTTA	AACAAACGCGTGAAATCTCC	61.0	333	INTERGENIC	--	--	--
Ca7_P3_30097365	Ca7	30097365	GTAATTAA/GTAA	CCGGCAAACAATTTTCTCAT	TCAAACCTCCCCCTTTTTCT	59.9	613	INTERGENIC	--	--	--
Ca7_P3_30098086	Ca7	30098086	AGGG/AGG	TGAGAATGAAGGGGATACGC	ACGACCTTCGACGAAAACAT	60.0	340	INTERGENIC	--	--	--
Ca7_P3_30099385	Ca7	30099385	AT/ATTT	TGGGCACATGTCACAAAACT	TGAGGTATGGGAAGGGTGAG	60.0	840	INTERGENIC	--	--	--

INDEL marker IDs	Chromosomes/ unanchored scaffolds	Physical positions (bp)	InDels (*Kabuli* reference genome-CDC Frontier/PII)	Forward primers (5'-3')	Reverse primers (5'-3')	Anneling temperature (0C)	Expected amplified product size (bp)	Structural annotation		Functional annotation	
								Sequence components of *kabuli* genome	*Kabuli* gene accession IDs	NCBI-KOG	TFs
Ca7_P3_30102321	Ca7	30102321	GTTTTTTTTT/GTTTTTTTTTTTT	GCTCTGATTCCGTCGAGAAC	TGTGTGATTGCCCCAGTTTA	60.0	577	DRR	Ca_11717	--	bZIP
Ca7_P3_30102864	Ca7	30102864	GA/G	TAAACTGGGGCAATCACACA	TGATTATGCAGCAACCAAAA	60.0	585	DRR	Ca_11717	--	bZIP
Ca7_P3_30107818	Ca7	30107818	TA/TAACCAGTTTATAA	GAAGGCTGCAACTCCTTGAC	AACGTGAAACCCACCAAAAC	60.0	764	INTRON	Ca_11716	T	WRKY
Ca7_P3_30107844	Ca7	30107844	TCCC/TCCCC	GAGCTCGGAGATGGTTCTTG	CCACCAGTAAACTCTGCCGT	60.0	680	INTRON	Ca_11716	T	WRKY
Ca7_P3_30129218	Ca7	30129218	C/CATT	ACAACGAGTGCCGGTTATTC	ATCGCAAATTCAAAGCCATC	60.0	360	INTRON	Ca_11715	U	--
Ca7_P3_30130016	Ca7	30130016	T/TA	CCACGTGAGTTACCATGCTG	TCCACGTACGTTTAGCCACC	60.2	674	INTRON	Ca_11715	U	--
Ca7_P3_30130333	Ca7	30130333	CTTT/CT	GGTACGGTACGGTGGCTAAA	TCTCCGATCCATAATAATTGACCTA	59.9	302	INTRON	Ca_11715	U	--
Ca7_P3_30131573	Ca7	30131573	ATTTTT/ATTT	TGTTTTCGGCTAACACATCG	TGCAATGTCCAACTCACTTGT	59.7	372	INTRON	Ca_11715	U	--
Ca7_P3_30131606	Ca7	30131606	CAAAAA/CAAAA	TGTTTTCGGCTAACACATCG	TGCAATGTCCAACTCACTTGT	59.7	372	INTRON	Ca_11715	U	--
Ca7_P3_30132345	Ca7	30132345	AC/A	GGGGGTCATCACCCTAAACT	TGATGACCGTGTAGGAACCA	60.1	139	INTRON	Ca_11715	U	--

INDEL marker IDs	Chromosomes/ unanchored scaffolds	Physical positions (bp)	InDels (*Kabuli* reference genome-CDC Frontier/PII)	Forward primers (5'-3')	Reverse primers (5'-3')	Anneling temperature (0C)	Expected amplified product size (bp)	Structural annotation		Functional annotation	
								Sequence components of *kabuli* genome	*Kabuli* gene accession IDs	NCBI-KOG	TFs
Ca7_P3_30154060	Ca7	30154060	TAAAAAAAAAA/TAAAAAAAA	AAATGACCAAATCGGCAAAA	TTTTAAAGTCGACAGTTTTAGCTTC	60.3	533	INTERGENIC	--	--	--
Ca7_P3_30159383	Ca7	30159383	CATATAT/CATAT	ACAAGCGAGTTCACTTCACG	TTCCCGCAGATATGTTAGGC	59.1	610	INTERGENIC	--	--	--
Ca7_P3_30161331	Ca7	30161331	GTTTTTTT/GTTTTTT	TTTCCTCAATTTGTCGGCTC	GACAAAGAGAAATGTGGACCAA	60.2	187	INTRON	Ca_11712	Q	B3
Ca7_P3_30161681	Ca7	30161681	TAA/TAAAA	TTTCCTCAATTTGTCGGCTC	TGCAATTGTAGAATCTCTCAAACA	60.2	723	INTRON	Ca_11712	Q	B3
Ca7_P3_30161768	Ca7	30161768	ATTTTTTTTT/ATTTTTTT	TTTCCTCAATTTGTCGGCTC	TGCAATTGTAGAATCTCTCAAACA	60.2	723	INTRON	Ca_11712	Q	B3
Ca7_P3_30161856	Ca7	30161856	TA/TATATAAA	TGGTCCACATTTCTCTTTGTCA	TGCAATTGTAGAATCTCTCAAACA	60.5	557	INTRON	Ca_11712	Q	B3
Ca7_P3_30162231	Ca7	30162231	TCCC/TCC	TTTTTACAAAACTTTGAACCTCCT	TGTTGTCCAGCTCTTGTTGG	57.6	617	INTRON	Ca_11712	Q	B3
Ca7_P3_30190640	Ca7	30190640	ATTTTATTTTTATTTT/ATTTTATTTTTATTTTTATTTT	GTCCACATTGCTGGCCTTAT	GACAATGTCCAACCACGAGA	60.0	424	INTRON	Ca_11709	U	--
Ca7_P3_30191256	Ca7	30191256	ATTT/ATTTT	CTCAATCTCGTGGTTGGACA	GCCAACAAGCATTTTCCCTA	59.7	560	INTRON	Ca_11709	U	--
Ca7_P3_30192681	Ca7	30192681	TAAAA/TAAA	TGTCCGAGTCATTTTTGCAT	TCCCCATGGTTCAAAGTTGT	59.1	541	INTRON	Ca_11709	U	--

INDEL marker IDs	Chromosomes/ unanchored scaffolds	Physical positions (bp)	InDels (*Kabuli* reference genome-CDC Frontier/PII)	Forward primers (5'-3')	Reverse primers (5'-3')	Anneling temperature (0C)	Expected amplified product size (bp)	Structural annotation		Functional annotation	
								Sequence components of *kabuli* genome	*Kabuli* gene accession IDs	NCBI-KOG	TFs
Ca7_P3_30201680	Ca7	30201680	CTTTT/CTTTTT	GGTGGGGTTTATGGTCTTGA	ACGCCTTCTCAAGCTTACCA	59.6	842	INTERGENIC	--	--	--
Ca7_P3_30205006	Ca7	30205006	G/GTTT	AAAGAGGGGGTTGAGGCTTA	TTGTGTTTGGTTCAAAGGAGG	60.1	721	DRR	Ca_11707	--	ERF
Ca7_P3_30208302	Ca7	30208302	CGA/C	TTGGACGATGTGGATTGAAA	CCATCTCAGCATGTGTTTGG	59.9	642	INTRON	Ca_11706	--	--
Ca7_P3_30208770	Ca7	30208770	ATTT/ATT	CCAAACACATGCTGAGATGG	CTTCCTTTGCACGACCGTAT	60.1	707	INTRON	Ca_11706	--	--
Ca7_P3_30218967	Ca7	30218967	CTTTTTTTTT/CTTTTTTT	CCGATTTTGCAGGTCAATTT	TGCGGTTCAAAGTTATGCAA	59.9	419	DRR	Ca_11706	--	--
Ca7_P3_30220020	Ca7	30220020	CA/C	CGCTCTAGCGCAAACTAAGG	AACACCGACACCGAATTCAT	60.3	163	DRR	Ca_11705	GC	HSF
Ca7_P3_30221695	Ca7	30221695	T/TGAA	CTTGTTGATGCAATGTTGGC	TTTGGCCACACAAAAACAAA	60.1	538	INTERGENIC	--	--	--
Ca7_P3_30230176	Ca7	30230176	GTTTTT/GTTTT	ACAATTGCTCATGCGGTACA	GGTGATTACGACACATTGGC	60.1	540	INTERGENIC	--	--	--
Ca7_P3_30240597	Ca7	30240597	GTTTTTTTTT/GTTTTTTTTTT	TAAATGCGGCAATGTCAAAA	TCTGAGGCATGCAATAATAACC	60.1	646	INTERGENIC	--	--	--
Ca7_P3_30249579	Ca7	30249579	ATTTTTTTTTT/ATTTTTTTTT	TTTTCAGAAAGTCGCATCCC	AGCTTCGGCTTAATTTGGCT	60.2	392	INTERGENIC	--	--	--

INDEL marker IDs	Chromosomes/ unanchored scaffolds	Physical positions (bp)	InDels (*Kabuli* reference genome-CDC Frontier/PII)	Forward primers (5'-3')	Reverse primers (5'-3')	Anneling temperature (0C)	Expected amplified product size (bp)	Structural annotation		Functional annotation	
								Sequence components of *kabuli* genome	*Kabuli* gene accession IDs	NCBI-KOG	TFs
Ca7_P3_30251129	Ca7	30251129	TG/TGCTCGTGG	ATCATTCAATGTTCGACGCA	AAGTCGTTTCAAATCGTGCC	60.1	419	INTERGENIC	--	--	--
Ca7_P3_3033651	Ca7	3033651	TAAAAAAAAAA/TAAAAAAAAA	TCCGAATACCATTGTTCAAAA	ACGGGAATCAAGAAGAGGGT	58.0	760	INTERGENIC	--	--	--
Ca7_P3_3034764	Ca7	3034764	AATATATATATATATATATAT/AATATATATATATATATAT	TGAGTAGCACAATTGGAGGTTT	TTCAACGATTCAATCTAACCGA	58.8	536	INTERGENIC	--	--	--
Ca7_P3_30394072	Ca7	30394072	AAAA/AAAAAATGATAAA	GCCCAAAAATTCAAAGTCCA	TGCAGGAGTTGTCACAGCTTA	59.9	506	INTRON	Ca_11690	--	MYB_related
Ca7_P3_30415200	Ca7	30415200	C/CG	CAATTCAAAAGAAACCCCAGA	GTTAAATCGCCTTTTCCACG	59.1	750	INTERGENIC	--	--	--
Ca7_P3_30415401	Ca7	30415401	ATG/A	GGAGTCATTCTCTCCCATGAA	GTCCTCGTCCCTCATGTTGT	59.1	683	INTERGENIC	--	--	--
Ca7_P3_30415603	Ca7	30415603	TG/TGG	GGAGTCATTCTCTCCCATGAA	GTCCTCGTCCCTCATGTTGT	59.1	683	INTERGENIC	--	--	--
Ca7_P3_30424159	Ca7	30424159	A/AT	TCATCTTCGATGCAAATCCA	AGGAGGTCGCATCCAATAAC	60.2	407	DRR	Ca_11688	--	MYB_related
Ca7_P3_3050532	Ca7	3050532	CGTTAAAGAG/CGTTAAAGAGTTAAAGAG	GCCATTTGCTTTGGAAACAT	ATGGCAAAGAGGGTACTCCA	59.9	537	INTERGENIC	--	--	--
Ca7_P3_3053020	Ca7	3053020	ATTTT/ATT	TATTCCCCTCAAACCTGACG	AACAACAAATTGCATGGGCT	59.9	219	INTERGENIC	--	--	--

INDEL marker IDs	Chromosomes/ unanchored scaffolds	Physical positions (bp)	InDels (*Kabuli* reference genome-CDC Frontier/PII)	Forward primers (5'-3')	Reverse primers (5'-3')	Anneling temperature (0C)	Expected amplified product size (bp)	Structural annotation		Functional annotation	
								Sequence components of *kabuli* genome	*Kabuli* gene accession IDs	NCBI-KOG	TFs
Ca7_P3_30679476	Ca7	30679476	CAAAAAAAAAAA/CAAAAAAAAAAAA	AGAGGGGCCAAGAAGATCAG	TCCTCATCCTCATGAATTGTTC	60.7	532	INTERGENIC	--	--	--
Ca7_P3_30720515	Ca7	30720515	CTTTTTT/CTTTTTTTTT	CGTTAAGGAGGGGGAATCTC	CTATCACGGCCCGTACTGTT	59.9	477	INTERGENIC	--	--	--
Ca7_P3_30737184	Ca7	30737184	TTATATATATATATATATATATATATATA/TTATATATATATATATATATATATA	TCCGCTTTTATTTGTGTGTCA	AATCTGAACAAGGGCCAATTA	59.2	717	INTERGENIC	--	--	--
Ca7_P3_3076524	Ca7	3076524	ATTTT/ATTT	GAAATGGATTGTGGAAGGGA	CCTCTTAGGTGTGCCTCTGC	59.7	742	INTERGENIC	--	--	--
Ca7_P3_30779876	Ca7	30779876	GAAAAAAAA/GAAAAAAA	TGATGAGGTGAATGGCATGT	AGGAGATGGTGATGGTCAGC	59.9	536	INTERGENIC	--	--	--
Ca7_P3_30874290	Ca7	30874290	TAA/TAAA	TTGTCTCAACAATCTCCCCC	ACCACCTCACTGCCCTACAC	59.9	734	DRR	Ca_23317	--	--
Ca7_P3_30967683	Ca7	30967683	ATTTT/ATTTTT	CCCCATGATCTTCCCTTTTT	GCAACAAACCTATGGCAGGT	60.1	355	DRR	Ca_23312	--	--
Ca7_P3_30981750	Ca7	30981750	TG/TGG	GGTAAGGGCTCCTTCCAAAC	GGGTCTGCACGATTTACAAAA	59.9	967	INTERGENIC	--	--	--
Ca7_P3_31087789	Ca7	31087789	GAAAAAAAAAAA/GAAAAAAAAAAAA	ATTGACACAATCTTGGGGGA	TCAAAGCACATCGTGAAAGC	60.2	400	INTERGENIC	--	--	--
Ca7_P3_31112471	Ca7	31112471	TGG/TG	GCAAAGGCACTCATTCCATT	GCAAAAACACGAGAAGGAGG	60.1	493	INTERGENIC	--	--	--

INDEL marker IDs	Chromosomes/ unanchored scaffolds	Physical positions (bp)	InDels (*Kabuli* reference genome-CDC Frontier/PII)	Forward primers (5'-3')	Reverse primers (5'-3')	Anneling temperature (0C)	Expected amplified product size (bp)	Structural annotation		Functional annotation	
								Sequence components of *kabuli* genome	*Kabuli* gene accession IDs	NCBI-KOG	TFs
Ca7_P3_31154823	Ca7	31154823	GATATATATATATATATATATATAT/GATATATATATATATATATATATAT	CTGCGATAATGACGATGACG	TGTAACTTCTTGACTTTGTCCTTTTG	60.2	627	INTERGENIC	--	--	--
Ca7_P3_31185689	Ca7	31185689	GAAAAAAAAAA/GAAAAAAAAA	TTGGCCCCTTTGAGATATTTT	TTCCGGGTCTCTCAAAAGTG	59.8	820	DRR	Ca_10140	T	--
Ca7_P3_31196591	Ca7	31196591	C/CATATTT	AAAAATTACCACCGTGAACAA	TGTTCTTAACCTAGCTGGCGA	57.1	897	DRR	Ca_10138	--	--
Ca7_P3_31197800	Ca7	31197800	GTT/GTTT	CTGCCACCCTGTCCATAAGT	ATCCACTTGTGGGTTTCAGC	60.0	474	DRR	Ca_10138	--	--
Ca7_P3_31201080	Ca7	31201080	C/CGGA	TGATCCACCATGTCCATCAT	TACGACCCGACATGAAACAA	59.6	346	DRR	Ca_10137	--	bHLH
Ca7_P3_31201587	Ca7	31201587	T/TG	TGTCCGACATCGACACATTT	CTCACCTGATTCCATGAGCA	60.0	445	INTRON	Ca_10137	--	bHLH
Ca7_P3_31207254	Ca7	31207254	GCACAC/GCAC	AGTGAGGATGGTTAGGGGCT	TACATGTGTCGGTTTGGGTG	60.0	235	INTERGENIC	--	--	--
Ca7_P3_31210939	Ca7	31210939	CTC/CTCAATC	CCTTTCTCAGCCCATATCCA	TCAACAATGCAAACGAAGGA	60.0	306	DRR	Ca_10136	--	ARF
Ca7_P3_31217391	Ca7	31217391	A/AT	GGGACAAAATTGAACTGGGA	CCCTGGTGAACAGGTGTCTT	59.8	938	INTRON	Ca_10135	G	--
Ca7_P3_31217521	Ca7	31217521	GATACAT/GATACATCATACAT	GACCAGTGCAAAGGGATGTT	CCCTGGTGAACAGGTGTCTT	60.0	460	INTRON	Ca_10135	G	--

INDEL marker IDs	Chromosomes/ unanchored scaffolds	Physical positions (bp)	InDels (*Kabuli* reference genome-CDC Frontier/PII)	Forward primers (5'-3')	Reverse primers (5'-3')	Anneling temperature (0C)	Expected amplified product size (bp)	Structural annotation		Functional annotation	
								Sequence components of *kabuli* genome	*Kabuli* gene accession IDs	NCBI-KOG	TFs
Ca7_P3_31219950	Ca7	31219950	TAAAAAAAAAAA/TAAAAAAAAA	TGTTTGGCGTCCATATTTGA	AGGAGATGGGGAAAAGCAAT	59.9	500	INTRON	Ca_10135	G	--
Ca7_P3_31220451	Ca7	31220451	A/AC	ATTGCTTTTCCCCATCTCCT	TGGCTTTAAGGGTACTTGGC	59.9	293	INTERGENIC	--	--	--
Ca7_P3_31225268	Ca7	31225268	GAAAAAAAAA/GAAAAAAAAAA	CATGAGGAACAATCAGGCAA	TGATGATGCAGGTTCATGGT	59.6	330	INTRON	Ca_10134	A	--
Ca7_P3_31230604	Ca7	31230604	GAAAAAAAAAAAAAA/GAAAAAAAAAAAAAAA	TGGGATGAGTGTGCATTTTC	TCAAAATTTAGCGCCTACACAA	59.5	647	INTERGENIC	--	--	--
Ca7_P3_31232674	Ca7	31232674	CTTTTTTTT/CTTTTTTTTT	GCAAAATAGCTCTTGCGGTC	CTCTTCTTCGTTGAAACGCC	60.0	565	INTERGENIC	--	--	--
Ca7_P3_31241126	Ca7	31241126	ATTTTTT/ATTTTT	TGGACAAAGCTCACAACTGC	TCAAACTCCCACATGCAAAA	60.0	753	INTERGENIC	--	--	--
Ca7_P3_31252776	Ca7	31252776	CTTT/CTTTT	TCCCATTGACTGCTCCTTTT	TTGCAACACTTTCTGGAGCA	59.7	567	INTERGENIC	--	--	--
Ca7_P3_31252979	Ca7	31252979	TTATTTATTTATTTA/TTATTTATTTATTTATTTA	TCCCATTGACTGCTCCTTTT	TTGCAACACTTTCTGGAGCA	59.7	567	INTERGENIC	--	--	--
Ca7_P3_31262018	Ca7	31262018	CATATATATATATATATATATATATATATATA/CATATATATATATATATATATATATATATATATATA	TTTGGTTGCCATCAAGTGAC	ATGGGTATATGCCGTTTCCA	59.5	733	INTERGENIC	--	--	--
Ca7_P3_31291456	Ca7	31291456	TTGATTTACTGATTTACTGATT/TTGATTTACTGATT	GAGCATGAGCAACACCAAGA	CCTCATGAATGGCCAAATCT	60.0	713	INTERGENIC	--	--	--

INDEL marker IDs	Chromosomes/ unanchored scaffolds	Physical positions (bp)	InDels (*Kabuli* reference genome-CDC Frontier/PII)	Forward primers (5'-3')	Reverse primers (5'-3')	Anneling temperature (0C)	Expected amplified product size (bp)	Structural annotation		Functional annotation	
								Sequence components of *kabuli* genome	*Kabuli* gene accession IDs	NCBI-KOG	TFs
Ca7_P3_31336610	Ca7	31336610	AATATATATATATATATATA/AATATATATATATATATATATATA	TGCCCACAATCAACTATTGG	ATCCAAATTCATGCTTCCCA	59.4	341	INTERGENIC	--	--	--
Ca7_P3_31385029	Ca7	31385029	CATTATTATTATTATTAT/CATTATTATTATTAT	GGGTGGAAATAAGTAACACTGTTTG	TGGGTAGAAACAAAATGGGG	60.1	516	INTERGENIC	--	--	--
Ca7_P3_31406570	Ca7	31406570	CAATAATA/CAATA	GACCATCTTAAATGACCGCAA	ATGCGTCGGTGCAATAATAA	60.0	449	INTERGENIC	--	--	--
Ca7_P3_31413055	Ca7	31413055	AATATATATATATATATA/AATATATATATATATATATA	ACCTGGAGGGTAAGGGTACG	GGTTTAATTTTCGGGCCATT	60.2	655	INTERGENIC	--	--	--
Ca7_P3_31467652	Ca7	31467652	AATATATATATATATATATATATATATAT/AATATATATATATATATATATATAT	CACGTGTAGTGGTCGGATGT	AATAGGGGGACCAAAACTGC	59.5	491	DRR	Ca_10111	--	--
Ca7_P3_31500210	Ca7	31500210	CCC/CCCGAGCC	GTTCGAGATCTTTCGGTCCA	CATTGCATTGCATCTAACCG	60.2	170	INTERGENIC	--	--	--
Ca7_P3_31507309	Ca7	31507309	GATATATATATATATATATATAT/GATATATATATATATATATAT	TAACTTGCAAGAGGAGCCGT	TGTGTCCTACCTGCGTCAAG	60.0	454	INTRON	Ca_10108	O	--
Ca7_P3_31630725	Ca7	31630725	ATTTTTTTTTTT/ATTTTTTTTTTTTT	GGCTAAATTACTTGTGGTCCCTT	TTTTATGGGTTTTGGTTGAAAA	59.8	193	INTERGENIC	--	--	--
Ca7_P3_31745904	Ca7	31745904	TCCCC/TCCC	TGCATGGAATGGTGAGTGAT	ATTGATGGGTTTACTTGGCG	59.9	219	INTERGENIC	--	--	--
Ca7_P3_31808937	Ca7	31808937	CTATATATATATATATATATATATATATA/CTATATATATATATATATATATATATATATA	AACTGTACCCGAGTGATGCC	GATACAAGGGGTTTCTGCCA	60.0	304	INTRON	Ca_10083	R	G2-like

INDEL marker IDs	Chromosomes/ unanchored scaffolds	Physical positions (bp)	InDels (*Kabuli* reference genome-CDC Frontier/PII)	Forward primers (5'-3')	Reverse primers (5'-3')	Anneling temperature (0C)	Expected amplified product size (bp)	Structural annotation		Functional annotation	
								Sequence components of *kabuli* genome	*Kabuli* gene accession IDs	NCBI-KOG	TFs
Ca7_P3_31862312	Ca7	31862312	ATATTATTATTATTATTATTATTATTATTATTATTATTATTATTATTATTATTATTATTATTA/ATATTATTATTATTATTATTATTATTATTATTATTATTATTATTATTATTATTATTATTATTATTATTATTA	CAAATACAATAACACCTGTCTCCG	ACTTTTCCCACCCAACTTCA	59.8	498	INTERGENIC	--	--	--
Ca7_P3_31863555	Ca7	31863555	CATATATATATATATATATATATA/CATATATATATATATATATATATATATA	TTGTTGGTGTGTCGGTCCTA	ACCTGTCCCTTTGTTTTGGT	60.0	563	INTERGENIC	--	--	--
Ca7_P3_31969575	Ca7	31969575	TAAAAAAAAA/TAAAAAAAAAA	ACATTTAATTGCCCACTGGC	GATTGATCCTCGTCACCGTT	59.8	536	INTRON	Ca_10067	--	FAR1
Ca7_P3_32104861	Ca7	32104861	TTAT/TTATCTAT	CCGGAGGACCTATGTTGAGA	AGGCCGAAGAAAGAGGTAGC	60.1	698	INTRON	Ca_10059	--	Dof
Ca7_P3_32308830	Ca7	32308830	AATGATG/AATGATGATG	AAACTCAGCTTGGACGTGCT	ATCAAGGGTTCCATCCTCCT	60.1	535	INTRON	Ca_10045	R	--
Ca7_P3_32358180	Ca7	32358180	TTGG/TTGGACAGGATGG	GTTTTGAGAGCAGCCTGGAG	GCGGGTCTTGGTTTTGATAA	60.1	246	INTERGENIC	--	--	--
Ca7_P3_32410444	Ca7	32410444	ATTTTTTT/ATTTTTT	GGCTTTGGTATTCAGGAGCA	GTCCTTTGTGACCCGATGTT	60.2	616	INTRON	Ca_10037	J	--
Ca7_P3_32428294	Ca7	32428294	TTTATTATTATTATTATTATTATTATTATTATTATTATTATTATTATTATTATTAT/TTTATTATTATTATTATTATTATTATTATTATTATTATTATTATTATTATTATTATTAT	GCTAGACTCCTCCGTGGTGA	TAGTGTGTTGGGCCATTCAA	60.4	291	INTERGENIC	--	--	--
Ca7_P3_32478186	Ca7	32478186	TATATA/TATATAATATA	CCCCGTAAACAATTTTGGTG	TAGCAGGCTTGAACATGCAC	60.1	368	INTERGENIC	--	--	--
Ca7_P3_32479028	Ca7	32479028	AATATATATATATATATATATATATAT/AATATATATATATATATATATATATATAT	AGGGAGTTTAGTGGTGGGCT	CGGATGCATGAAAGTGAAAA	60.0	636	INTERGENIC	--	--	--

INDEL marker IDs	Chromosomes/ unanchored scaffolds	Physical positions (bp)	InDels (*Kabuli* reference genome-CDC Frontier/PII)	Forward primers (5'-3')	Reverse primers (5'-3')	Anneling temperature (0C)	Expected amplified product size (bp)	Structural annotation		Functional annotation	
								Sequence components of *kabuli* genome	*Kabuli* gene accession IDs	NCBI-KOG	TFs
Ca7_P3_3256595	Ca7	3256595	CTCGTTCGT/CTCGT	AAAGAAAACGTCGCCT GAGA	CACGGACTTGGCTTGG TAAT	60.0	559	INTERGENIC	--	--	--
Ca7_P3_32601479	Ca7	32601479	A/AGGAATGCT	CAGCAACAGCGCAAAG ATTA	GGATGACAAAGAAAGG CCAA	60.2	543	INTRON	Ca_10025	TU	--
Ca7_P3_32603436	Ca7	32603436	TACA/TA	TTGCAGGATGCACAGA AAAG	TTCTCTCTCCCTCAGT CCCA	60.0	504	DRR	Ca_10025	TU	--
Ca7_P3_32613866	Ca7	32613866	AT/ATT	AAAAGGTGGTTAGCTC TTTGACA	GGGGGCGAAAGATTTT ATTT	59.4	377	INTERGENIC	--	--	--
Ca7_P3_32635727	Ca7	32635727	CTTTTTTTT/CTTTTTTTTT	GAGGGAGCTTGAGAAG GACA	CAATTACCATCACACC CCCT	59.5	249	INTERGENIC	--	--	--
Ca7_P3_32649545	Ca7	32649545	T/TC	TTGCAGGATGCACAGA AAAG	AGGTGCATCCCCTGTA ACTG	60.0	415	INTERGENIC	--	--	--
Ca7_P3_32650059	Ca7	32650059	TAATAATGTAAAAAA/T	ATGGGACTGTGGGAGA GAGA	GAAAAAGGCTTCAAAC ATCAAA	59.6	398	INTERGENIC	--	--	--
Ca7_P3_32654894	Ca7	32654894	AAATAATAATAATAATAAT/AAATAATAA TAATAATAATAATAAT	AACGAAGTGAGACGTG AGCA	TGATGGCTATGGTGGA ACAA	59.6	618	INTERGENIC	--	--	--
Ca7_P3_32655163	Ca7	32655163	TTAATA/TTA	AACGAAGTGAGACGTG AGCA	TGATGGCTATGGTGGA ACAA	59.6	618	INTERGENIC	--	--	--
Ca7_P3_3270944	Ca7	3270944	GTTAATTAATTAATTAATTAAT/GTTAAT TAATTAAT	CAACACATGATCAGCAA CCC	TTGTCCCCTTTGAAGG ACAG	60.0	376	INTRON	Ca_03141	--	--

INDEL marker IDs	Chromosomes/ unanchored scaffolds	Physical positions (bp)	InDels (*Kabuli* reference genome-CDC Frontier/PII)	Forward primers (5'-3')	Reverse primers (5'-3')	Anneling temperature (0C)	Expected amplified product size (bp)	Structural annotation		Functional annotation	
								Sequence components of *kabuli* genome	*Kabuli* gene accession IDs	NCBI-KOG	TFs
Ca7_P3_32778038	Ca7	32778038	CATTTATTTATTTATTTATTT/CATTTATTTATTTATTTATTTATTT	TTTTGACTCAATCAACATTGACA	AAATTGTCTTTGATTCAGCTCATTT	58.2	560	DRR	Ca_10016	L	--
Ca7_P3_32820886	Ca7	32820886	TAAAAAAAAAA/TAAAAAAAAA	CCCAACATGCTTCTTGCTCT	TCATAAGAAAGTGGCTTCCCA	60.4	789	INTERGENIC	--	--	--
Ca7_P3_32929529	Ca7	32929529	T/TCAATG	TCCCGAAATAGGGAAACTCC	GAAGAGAGGGAAAGGGAAGG	60.3	665	DRR	Ca_10005	--	--
Ca7_P3_33068248	Ca7	33068248	CTTTTTT/CTTTTT	CTTCTTCGTCATCCATTGGC	AAGCCTATCACTCATCCAATCAA	60.6	624	INTERGENIC	--	--	--
Ca7_P3_33069265	Ca7	33069265	AATTATT/AATT	TGACATGCATGATTCAACTGG	TTTGAATTGGTCCCTGATTTT	60.5	617	INTERGENIC	--	--	--
Ca7_P3_33070699	Ca7	33070699	TTAATAATAATAATAA/TTAATAATAATAATAATAA	TCACAAGAACATAAGCCATAAATCA	GGGTTTACCGTGCATCACTC	59.9	456	INTERGENIC	--	--	--
Ca7_P3_33102819	Ca7	33102819	ATT/AT	AACAGTCCCGCATTGGATAG	CAGCACTGTCCAAATTGTGC	60.0	447	INTERGENIC	--	--	--
Ca7_P3_33108892	Ca7	33108892	CTCT/CTCTGTCT	TTAGCTTTTGAGGCGTGTGA	AAAAGTGTTTGAGGGGCAAG	59.6	438	INTERGENIC	--	--	--
Ca7_P3_33124042	Ca7	33124042	AGGG/AGG	TAAAGGACCAAGTCGGGAAG	TGCCAACCCTAAACACAACA	59.2	501	INTERGENIC	--	--	--
Ca7_P3_33134722	Ca7	33134722	ATTTTTTTTTT/ATTTTTTTT	TCGTGTGTTCAAATTCCTTGA	CGGTTGGAACTTGGAAGTGA	59.2	523	DRR	Ca_16144	--	--

INDEL marker IDs	Chromosomes/ unanchored scaffolds	Physical positions (bp)	InDels (*Kabuli* reference genome-CDC Frontier/PII)	Forward primers (5'-3')	Reverse primers (5'-3')	Anneling temperature (0C)	Expected amplified product size (bp)	Structural annotation		Functional annotation	
								Sequence components of *kabuli* genome	*Kabuli* gene accession IDs	NCBI-KOG	TFs
Ca7_P3_33138413	Ca7	33138413	TGG/TG	CATCCTAAAAGGACAG CCCA	AGAAGAGGCGCATGAA CAAT	60.1	522	DRR	Ca_16145	P	NF-YC
Ca7_P3_33149769	Ca7	33149769	CGG/CGGG	TCCGGTACACAACTTTT CCC	TGGCATTTTTGTTGCA TTGT	59.8	624	INTERGENIC	--	--	--
Ca7_P3_33175105	Ca7	33175105	CTT/CT	TGAAGAAATCAATCATG CAAGT	AACTTGCGTTAGAGTC GGGA	57.4	598	INTERGENIC	--	--	--
Ca7_P3_33181868	Ca7	33181868	AG/A	GAGCTCTTTGGCCTTT GATG	ATATTGATGTTGGTCG GGGA	60.0	603	DRR	Ca_16149	IOT	--
Ca7_P3_33181956	Ca7	33181956	CACATA/CA	GAGCTCTTTGGCCTTT GATG	ATATTGATGTTGGTCG GGGA	60.0	603	DRR	Ca_16149	IOT	--
Ca7_P3_33189130	Ca7	33189130	TG/TGCAG	AGACATTTTGTTCAATG GATCTG	AGGAGAAATGCGAGGA AACA	58.1	462	INTERGENIC	--	--	--
Ca7_P3_33190565	Ca7	33190565	TAAAAAAAAAA/TAAAAAAAAAAA	ACTCGGGCTAGTCCAG TCCT	TTGACGAGTTGTGAGC AAGG	60.3	720	INTERGENIC	--	--	--
Ca7_P3_33194117	Ca7	33194117	TAAAAAAA/TAAAAAAAA	TTGACCTATATACAAAA CCCAAAGA	CCCACGTGTCTTTTCA AGGT	58.2	796	INTERGENIC	--	--	--
Ca7_P3_33194624	Ca7	33194624	ATT/ATTT	ACCTTGAAAAGACACG TGGG	GGATTTAATCGAAATA CTGCAACA	60.0	532	INTERGENIC	--	--	--
Ca7_P3_33212883	Ca7	33212883	GA/GATTTTTATTTTCA	TTCAAAAACTCCGCAAA AGG	GGCCAATCATGTACCA AAAGA	60.2	368	INTERGENIC	--	--	--

INDEL marker IDs	Chromosomes/ unanchored scaffolds	Physical positions (bp)	InDels (*Kabuli* reference genome-CDC Frontier/PII)	Forward primers (5'-3')	Reverse primers (5'-3')	Anneling temperature (0C)	Expected amplified product size (bp)	Structural annotation		Functional annotation	
								Sequence components of *kabuli* genome	*Kabuli* gene accession IDs	NCBI-KOG	TFs
Ca7_P3_33213668	Ca7	33213668	CT/CTT	TTCTTGTGGCAATTTTG TTGTC	TCTTTTTCGAGGAAAG TCCAA	60.0	225	INTERGENIC	--	--	--
Ca7_P3_33213765	Ca7	33213765	GTTTTTTTT/GTTTTTTTTTT	TTCTTGTGGCAATTTTG TTGTC	TCTCATCACTTAAACCC ACACAA	60.0	379	INTERGENIC	--	--	--
Ca7_P3_33213840	Ca7	33213840	ATTTT/ATT	TTCTTGTGGCAATTTTG TTGTC	GACTTATAAGCCCACC TTTTCAA	60.0	640	INTERGENIC	--	--	--
Ca7_P3_33219414	Ca7	33219414	GT/GTT	GGGGAAAGTTGTCGTT TTCA	AAGACCCAAAACAAAG AGCTAAAA	59.9	246	INTRON	Ca_16150	--	--
Ca7_P3_33219780	Ca7	33219780	CA/CAA	GGGGAAAGTTGTCGTT TTCA	GAGAAAAATTTGCATG CCGT	59.9	848	INTRON	Ca_16150	--	--
Ca7_P3_33220954	Ca7	33220954	CTATATATATATATAT/CTATATATATAT ATATATAT	CATAAGTCGCGCATTTT GAA	TGAAGGAAAGCAGAAG GGAA	59.8	507	INTRON	Ca_16150	--	--
Ca7_P3_33224999	Ca7	33224999	ATTTTTTTT/ATTTTTTTTT	AGTCACTGCTCAAGCC TGGT	TTGCTCACTTGGCTGT ATGC	60.1	909	INTRON	Ca_16150	--	--
Ca7_P3_33227269	Ca7	33227269	TTAT/TTATAT	CACACGTGCGAGGTAT GTCT	TATCGGCTGCAAGACA ACTG	59.8	916	INTRON	Ca_16150	--	--
Ca7_P3_33230173	Ca7	33230173	GTT/GT	TGGCTTGGTTGTTGTT GGTA	AGACCGTGTGGAGAAT TTGG	60.0	741	DRR	Ca_16150	--	--
Ca7_P3_33230587	Ca7	33230587	GA/GAA	CCAAATTCTCCACACG GTCT	TGCGTCCATGCTTGTG TTAT	60.0	113	DRR	Ca_16150	--	--

INDEL marker IDs	Chromosomes/ unanchored scaffolds	Physical positions (bp)	InDels (*Kabuli* reference genome-CDC Frontier/PII)	Forward primers (5'-3')	Reverse primers (5'-3')	Anneling temperature (0C)	Expected amplified product size (bp)	Structural annotation		Functional annotation	
								Sequence components of *kabuli* genome	*Kabuli* gene accession IDs	NCBI-KOG	TFs
Ca7_P3_33232491	Ca7	33232491	TTCATAATCATAATCAT/TTCATAATCAT	CCTTATGGGAGACATG TGGAA	CGTGGTCATGTATCGT CCAG	59.8	517	INTERGENIC	--	--	--
Ca7_P3_33237542	Ca7	33237542	CAT/CATGTTTAT	ACGTTGTCCTCTTCGCT GTC	TTGGGTGATAAGGCCA AAAC	60.5	433	INTERGENIC	--	--	--
Ca7_P3_33238784	Ca7	33238784	GTATATATATATATAT/GTATATATATAT	TTCCCATGCCAATTGTA GGT	TGCACAAGAAATCGAA TGGA	60.2	592	INTERGENIC	--	--	--
Ca7_P3_33241567	Ca7	33241567	TG/T	GAGCCCTCTTTTTCTTG GCT	TGTTCTCTTGTTTCCCA CCA	60.0	399	INTRON	Ca_16151	--	--
Ca7_P3_33243964	Ca7	33243964	GA/GAA	CAACGGTGCAGAGTGA AAAA	TGTTTCATAACTGAAG GGGGA	59.9	624	DRR	Ca_16151	--	--
Ca7_P3_33246780	Ca7	33246780	ATGATGTTTG/ATGATGTTTGATGTTTG	GCATAATGTCTTGGGC AGGT	TTCATGACATTTTCCCC ACA	60.0	471	INTERGENIC	--	--	--
Ca7_P3_33253365	Ca7	33253365	A/AAT	TTCATCCATCAAATTGG TTCC	TGAACACATCAATCAAA CACGA	59.6	529	INTERGENIC	--	--	--
Ca7_P3_33253907	Ca7	33253907	AATA/AA	TCAAGTTGATTCATTCG CTCA	ATGGACGACAAAGGCG TTAC	59.4	955	INTERGENIC	--	--	--
Ca7_P3_33269093	Ca7	33269093	TCAACAA/TCAA	TTTGACATCCTTTTGCA CCA	CCAAATTGTCTTTGGG TGCT	60.1	637	INTERGENIC	--	--	--
Ca7_P3_33272874	Ca7	33272874	TG/TGG	GGACCCATTTACTTTCC TTGTTT	TCGCTTGTTTATTTTCC CCA	59.7	375	INTERGENIC	--	--	--

INDEL marker IDs	Chromosomes/ unanchored scaffolds	Physical positions (bp)	InDels (*Kabuli* reference genome-CDC Frontier/PII)	Forward primers (5'-3')	Reverse primers (5'-3')	Anneling temperature (0C)	Expected amplified product size (bp)	Structural annotation		Functional annotation	
								Sequence components of *kabuli* genome	*Kabuli* gene accession IDs	NCBI-KOG	TFs
Ca7_P3_33278017	Ca7	33278017	TAAAAAAAAAAAAAAAAAAA/TAAAAAAAAAAAA	CGTTTGTCCAACCCAATAGC	TGGAAAATGAGATGTGCCAA	60.4	335	DRR	Ca_16155	--	S1Fa-like
Ca7_P3_3328423	Ca7	3328423	CCCTTCACC/CCC	GCAATTGAATAGACGCGGTT	ATTTGTTGCCGACTCTGACC	60.1	327	INTERGENIC	--	--	--
Ca7_P3_33284980	Ca7	33284980	T/TTA	GCTCTGCTTACCTTGTCGGT	ATAGCGTCGGCTAAACTCCA	59.5	740	DRR	Ca_16156	R	--
Ca7_P3_3332398	Ca7	3332398	GAAGAGCTTCTGTA/GA	GGCTCGACAAAGCAAAGTCT	TTTTCGGAACCACAAACCTC	59.6	456	INTERGENIC	--	--	--
Ca7_P3_33326344	Ca7	33326344	ATTTTTT/ATTTTTTT	TTGTCGTCGGTTAGACTCCA	CATTGAACCGTGTGAACTTTG	59.3	929	INTERGENIC	--	--	--
Ca7_P3_33327221	Ca7	33327221	TAAA/TAAAA	TGCACATAACTTGTGCGACC	TTCCATTTTCCCTTAGTGCG	60.7	658	INTERGENIC	--	--	--
Ca7_P3_33327595	Ca7	33327595	T/TG	GCATTGATCCGCCTAAAGAA	TGGGCTGTCTATCCCAATTC	60.2	882	INTERGENIC	--	--	--
Ca7_P3_33327850	Ca7	33327850	TTATATATA/TTATATA	CGCACTAAGGGAAAATGGAA	TGGGCTGTCTATCCCAATTC	60.1	438	INTERGENIC	--	--	--
Ca7_P3_33330043	Ca7	33330043	TAAAATAAA/TA	TGGCAAGAATGAAAAATGGA	TGTAGTCGGTGATGGGACAA	59.1	466	INTERGENIC	--	--	--
Ca7_P3_33331664	Ca7	33331664	ATTTTTTTT/ATTTTTTTTTTT	TATGAATAGTCGGCAAGGGG	ACAACCGTGATTCATGCAAA	59.9	607	INTRON	Ca_16158	R	G2-like

INDEL marker IDs	Chromosomes/ unanchored scaffolds	Physical positions (bp)	InDels (*Kabuli* reference genome-CDC Frontier/PII)	Forward primers (5'-3')	Reverse primers (5'-3')	Anneling temperature (0C)	Expected amplified product size (bp)	Structural annotation		Functional annotation	
								Sequence components of *kabuli* genome	*Kabuli* gene accession IDs	NCBI-KOG	TFs
Ca7_P3_33334558	Ca7	33334558	AGTGTTCATAAAGTTGTGTTCATAAAG/ AGTGTTCATAAAG	CTTTCAGGAAAGTGGCTTGG	ACCAAAAGGCACCAGAGATG	59.8	682	INTRON	Ca_16158	R	G2-like
Ca7_P3_33336366	Ca7	33336366	T/TA	AAGCCAGGCGATTTATGATG	ATCGGCAAATATCCGAGAAA	60.1	510	DRR	Ca_16158	R	G2-like
Ca7_P3_33336451	Ca7	33336451	GATTA/GATTATGGTTAATTA	AAGCCAGGCGATTTATGATG	ATCGGCAAATATCCGAGAAA	60.1	510	DRR	Ca_16158	R	G2-like
Ca7_P3_33350171	Ca7	33350171	TG/T	TGTGGCTGAAGTCAATGAGC	AATTTGTCCTCAACAACCCG	60.0	547	INTRON	Ca_16161	YU	--
Ca7_P3_33351722	Ca7	33351722	TAA/TAAA	AGAGGCATATGGTGAAACCG	TTAGGCGACATCAGCACTTG	60.0	258	INTRON	Ca_16161	YU	--
Ca7_P3_33366145	Ca7	33366145	CTG/CTGTG	TGGGGAAAAGGTGTTTTCTG	TCGTGACTGCTCAAAAATGG	59.9	811	INTRON	Ca_16162	YU	--
Ca7_P3_33368154	Ca7	33368154	CA/CAA	AAGTTTCGTTGGTCTCCGTG	AGGCCATGAATACAACTCGG	60.1	644	DRR	Ca_16162	YU	--
Ca7_P3_33372788	Ca7	33372788	CTTTTTT/CTTTTTTT	CCTGCTCAAACACACTCACAA	ACTCGAACTCCCTTCAGACG	59.9	546	INTERGENIC	--	--	--
Ca7_P3_3337424	Ca7	3337424	GTATATATATATATATATATATATATATATATATATATAT/GTATATATATATATATATATATATATATATATATATATAT	TTTCTTCCCTCATCCTCCCT	TCGGCCACATGATGATTTTA	60.0	738	DRR	Ca_03132	R	--
Ca7_P3_33384355	Ca7	33384355	A/AC	TTAAGGGACCACAAGTGGTATT	TGGATAACCATGTCCAGACAAA	57.5	460	INTERGENIC	--	--	--

INDEL marker IDs	Chromosomes/ unanchored scaffolds	Physical positions (bp)	InDels (*Kabuli* reference genome-CDC Frontier/PII)	Forward primers (5'-3')	Reverse primers (5'-3')	Anneling temperature (0C)	Expected amplified product size (bp)	Structural annotation		Functional annotation	
								Sequence components of *kabuli* genome	*Kabuli* gene accession IDs	NCBI-KOG	TFs
Ca7_P3_33385148	Ca7	33385148	GAAA/G	TTCATCGTTTTCCCGAA CTC	GCAAACAAGAACCGAA AACA	60.1	420	INTERGENIC	--	--	--
Ca7_P3_33385317	Ca7	33385317	AT/A	TTCATCGTTTTCCCGAA CTC	CCTCTTTGTCTTCTTGT GGCA	60.1	723	INTERGENIC	--	--	--
Ca7_P3_33409249	Ca7	33409249	GAATAATA/GAATA	AAGTGCATGGACGCAT GATA	TCCAACAATCCCAGTG AAAA	60.1	185	DRR	Ca_16164	--	C3H
Ca7_P3_33412820	Ca7	33412820	ACATATCGAATT/ACATATCGAATTTCC ATATCGAATT	TTTCTTGCTTTCAACTC GCA	CCAGTTTCTCAATCCC TTGG	59.7	548	INTRON	Ca_16164	--	C3H
Ca7_P3_33413011	Ca7	33413011	TTGTGTGTGT/TTGTGTGTGTGT	AATTTGACAACCTTGGC ACC	ATGGTGGAGGGGAGA AAAAG	59.8	508	INTRON	Ca_16164	--	C3H
Ca7_P3_33413065	Ca7	33413065	CA/CAAA	AATTTGACAACCTTGGC ACC	ATGGTGGAGGGGAGA AAAAG	59.8	508	INTRON	Ca_16164	--	C3H
Ca7_P3_33415578	Ca7	33415578	TAAC/T	CATCGTTGAATCGTTG GTTG	GTGCGATGTACGAATG ATGC	60.0	617	DRR	Ca_16165	U	Dof
Ca7_P3_33416224	Ca7	33416224	TTGAAAAAGTTAATATGAA/TTGAAAAA GTTAATATGAAAAAGTTAATATGAA	CAATTAGCCTATTAGTT TGCCCA	TTTTGTTTTTCCCCCTT CAA	59.6	462	DRR	Ca_16165	U	Dof
Ca7_P3_33420385	Ca7	33420385	TCCAAAAACCAAAA/TCCAAAA	CCAGTTTCAACCGACAT CCT	TTTATAGTTCGCCAGG GCAC	60.0	619	INTRON	Ca_16165	U	Dof
Ca7_P3_33420924	Ca7	33420924	TC/TCC	ACCTAATCAGGGCCGA TTCT	TTGGTGTGTTGTTTCC CTGA	59.9	292	INTRON	Ca_16165	U	Dof

INDEL marker IDs	Chromosomes/ unanchored scaffolds	Physical positions (bp)	InDels (*Kabuli* reference genome-CDC Frontier/PII)	Forward primers (5'-3')	Reverse primers (5'-3')	Anneling temperature (0C)	Expected amplified product size (bp)	Structural annotation		Functional annotation	
								Sequence components of *kabuli* genome	*Kabuli* gene accession IDs	NCBI-KOG	TFs
Ca7_P3_33421552	Ca7	33421552	AATATATATATATATATATA/AATATATATATATATATA	GTCTTGGGTGATGGCAAAGT	GATTCTGCCTACTCATGTTTCAA	60.0	479	INTRON	Ca_16165	U	Dof
Ca7_P3_33423876	Ca7	33423876	AGTTGGTT/AGTTGGTTGGTT	CCCTTAGTTGGTTCATATTATTGC	CTGAGAAAAACTGGGTCCGA	58.1	493	INTRON	Ca_16165	U	Dof
Ca7_P3_33425512	Ca7	33425512	A/AT	AACCAGGCTGCCCTTTTATT	ATGCGGTGTGTGAAATGTGT	60.0	520	INTRON	Ca_16165	U	Dof
Ca7_P3_33442845	Ca7	33442845	ATTAAAA/ATTAAAATTTAAAA	CAATGAGGAGGCTGAACCAT	CAAACTCGCAAAGGGGTTTA	60.1	509	INTERGENIC	--	--	--
Ca7_P3_33467078	Ca7	33467078	TTTGGGCTTGGGCTTGGGC/TTTGGGCTTGGGC	CGTCGTAGGGATGTGAAGGT	TAGGGATCTTCGTTCATCGG	60.0	156	CDS (large-effect mutations)	Ca_16168	--	--
Ca7_P3_33467659	Ca7	33467659	AGTATGTATGTAT/AGTATGTATGTATGTATGTAT	AGTGCTTTTCATGAGGGTGG	GGTTCCCATCGCTTGAAGTA	60.1	423	INTRON	Ca_16168	--	--
Ca7_P3_33485683	Ca7	33485683	GAAAT/GAAATTAAAT	AATTTGGGGCCTGTTTTCAC	ATCGGAACCCACTTCTCCTT	61.1	570	INTRON	Ca_16169	--	--
Ca7_P3_33485739	Ca7	33485739	ATATAT/ATATATCTTTTTATAT	TAGTTGCGAATGGATGGTCA	ATCGGAACCCACTTCTCCTT	60.1	246	INTRON	Ca_16169	--	--
Ca7_P3_33521232	Ca7	33521232	ATTTTTTTTTTTT/ATTTTTTTTTT	TCAAAATTTTCAACCAAAACCC	ATGAATCAACGATACGGCTG	60.1	548	INTERGENIC	--	--	--
Ca7_P3_33521345	Ca7	33521345	TA/TAGA	TGTTGAACATTGTAACAGCCG	GACGAAAAATGATTTAGACGTTCA	59.6	364	INTERGENIC	--	--	--

INDEL marker IDs	Chromosomes/ unanchored scaffolds	Physical positions (bp)	InDels (*Kabuli* reference genome-CDC Frontier/PII)	Forward primers (5'-3')	Reverse primers (5'-3')	Anneling temperature (0C)	Expected amplified product size (bp)	Structural annotation		Functional annotation	
								Sequence components of *kabuli* genome	*Kabuli* gene accession IDs	NCBI-KOG	TFs
Ca7_P3_33521411	Ca7	33521411	ATT/ATTT	TGTTGAACATTGTAACA GCCG	GACGAAAAATGATTTA GACGTTCA	59.6	364	INTERGENIC	--	--	--
Ca7_P3_33526924	Ca7	33526924	A/ACGTAG	TGGCATTGGAAAAGAA AAGG	GATGCCACCACCTGAA AGAT	60.0	721	INTRON	Ca_16172	S	M-type
Ca7_P3_33527250	Ca7	33527250	GAAAAAAAAAA/GAAAAAAAAA	GCATTCCACAAAATCCA CCT	GATGCCACCACCTGAA AGAT	59.8	319	INTRON	Ca_16172	S	M-type
Ca7_P3_33527602	Ca7	33527602	ATCTCTCTCTCTCTCTCTCT/ATCTCTC TCT	ATCTTTCAGGTGGTGG CATC	GGGCCAAACAAAACTA GCAA	59.9	326	INTERGENIC	--	--	--
Ca7_P3_33533856	Ca7	33533856	GAAAAAAA/GAAAAAAAAA	CCAAATTGGGAAGAGC TGAA	ATGCCGTGAAGATTCC ATTC	60.2	546	INTERGENIC	--	--	--
Ca7_P3_33534157	Ca7	33534157	AT/ATT	GAATGGAATCTTCACG GCAT	AATCATGCGCAAATAT GGGT	59.9	308	INTERGENIC	--	--	--
Ca7_P3_33535580	Ca7	33535580	AT/A	TGCAATGAAAACACTG GGAG	AAAGTTCTCATTCAGC ATTACGG	59.7	246	INTERGENIC	--	--	--
Ca7_P3_33535802	Ca7	33535802	TAAAAAAA/TAAAAAAAAA	TGCAATGAAAACACTG GGAG	GCTTCCCTGTAAAAAG GTTGG	59.7	712	INTERGENIC	--	--	--
Ca7_P3_33539042	Ca7	33539042	TA/TAA	GGTAAATATGTGCATG GCCC	GGATTAGCACCGTCCA ATTT	60.0	722	INTERGENIC	--	--	--
Ca7_P3_33539096	Ca7	33539096	AA/AATA	GGTAAATATGTGCATG GCCC	GGATTAGCACCGTCCA ATTT	60.0	722	INTERGENIC	--	--	--

INDEL marker IDs	Chromosomes/ unanchored scaffolds	Physical positions (bp)	InDels (*Kabuli* reference genome-CDC Frontier/PII)	Forward primers (5'-3')	Reverse primers (5'-3')	Anneling temperature (0C)	Expected amplified product size (bp)	Structural annotation		Functional annotation	
								Sequence components of *kabuli* genome	*Kabuli* gene accession IDs	NCBI-KOG	TFs
Ca7_P3_33539636	Ca7	33539636	GTTTT/GTTT	AAATTGGACGGTGCTAATCC	CACGAAAGGTTTCATCACAAA	58.9	243	INTERGENIC	--	--	--
Ca7_P3_33555164	Ca7	33555164	CAATTAAT/CAAT	TCATGGGATCAAACCCATATC	TAATGATTTTTGGACGTGCG	59.5	632	INTERGENIC	--	--	--
Ca7_P3_33556956	Ca7	33556956	ATTATAATTTTATAA/ATTATAA	TGCGGATTTTTACGCTACCT	CCATTTTCCAATGGTTTGATG	59.7	646	INTERGENIC	--	--	--
Ca7_P3_33558467	Ca7	33558467	A/AGCTT	TGAAGGACAACTCAGTGTCACC	TGCATTTTTCCTTCTTCCACTT	60.2	897	INTERGENIC	--	--	--
Ca7_P3_33558610	Ca7	33558610	CTTTG/CTTTGTTAGTTTTTG	TTCAAGGACATTGCCAATTTAT	TGCATTTTTCCTTCTTCCACTT	58.5	492	INTERGENIC	--	--	--
Ca7_P3_33560322	Ca7	33560322	CT/CTGT	TCACACCATTGTCACTTTGAGA	CCAAAGTCCCTCATTCTTTCC	59.2	773	DRR	Ca_16175	QI	B3
Ca7_P3_33560340	Ca7	33560340	AAAATAAA/AAAA	TCACACCATTGTCACTTTGAGA	CCAAAGTCCCTCATTCTTTCC	59.2	773	DRR	Ca_16175	QI	B3
Ca7_P3_33561157	Ca7	33561157	TATCATCATCAT/TATCATCAT	CGTGTACTCCCCTTTCCCTT	GGCCATTTTGTGCAGATTTT	60.4	431	DRR	Ca_16175	QI	B3
Ca7_P3_33562209	Ca7	33562209	CTG/CTGTG	CATCTGGCAACACATCATCC	TGTGACAAAGATGAGCCAGG	59.9	599	INTRON	Ca_16175	QI	B3
Ca7_P3_33564972	Ca7	33564972	TTATATATATA/TTATATATA	TGCTTCATTTGTTTTGTGCC	GGGATTGATTTTTCACTTTTCAA	59.7	287	INTERGENIC	--	--	--

INDEL marker IDs	Chromosomes/ unanchored scaffolds	Physical positions (bp)	InDels (*Kabuli* reference genome-CDC Frontier/PII)	Forward primers (5'-3')	Reverse primers (5'-3')	Anneling temperature (0C)	Expected amplified product size (bp)	Structural annotation		Functional annotation	
								Sequence components of *kabuli* genome	*Kabuli* gene accession IDs	NCBI-KOG	TFs
Ca7_P3_33568045	Ca7	33568045	CT/C	GGTTGGAGGAAGGTGCATTA	TGATCCACGACACAGAGAGC	59.9	637	DRR	Ca_16176	O	--
Ca7_P3_33575013	Ca7	33575013	TAAAAAA/TAAAAAAA	CAAATTGCATTTTCCTCCCT	AATAATTCACCACGAACGGG	59.0	516	DRR	Ca_16178	I	--
Ca7_P3_33575264	Ca7	33575264	TAAAAAAAAA/TAAAAAAAAAAAA	CAAATTGCATTTTCCTCCCT	TTGTTGGTGAGTGCTCTTGG	59.0	691	DRR	Ca_16178	I	--
Ca7_P3_33576122	Ca7	33576122	GTTTTTTTTTT/GTTTTTTTTT	TAATTTAACGAACGGCCGAG	TTGGGTGACTCATGGTGAGA	60.1	410	DRR	Ca_16178	I	--
Ca7_P3_33581462	Ca7	33581462	AGTATT/A	AATTCTTGGGATTGTGCTGC	TTAGCCTTTCAATATGGCGG	60.1	531	INTRON	Ca_16178	I	--
Ca7_P3_33597364	Ca7	33597364	CTAATTAATT/CTAATT	GATGTTAACGCACCTCCACC	CACACTTCGATCTCCGATCA	60.4	483	INTERGENIC	--	--	--
Ca7_P3_33597557	Ca7	33597557	TC/T	GATGTTAACGCACCTCCACC	CGGTTTGTCCACACTTCGAT	60.4	492	INTERGENIC	--	--	--
Ca7_P3_33627476	Ca7	33627476	GAAAAAA/GAAAAAAAA	TTTCCAAGCCATGACAATCA	ATCAAGCGTGAACCAAGGTC	60.0	322	INTERGENIC	--	--	--
Ca7_P3_33633265	Ca7	33633265	TAAA/TAAAA	GAAATGGTGGTACCAATGGC	TGAAGAAGACCAAAGCGAAGA	60.1	423	INTERGENIC	--	--	--
Ca7_P3_33633508	Ca7	33633508	ATTTTTTTT/ATTTTTTTTTT	TGGTTCAAATTTTTGGAGGAA	TGAATGAAGGGGAAGGAAGA	59.4	693	INTERGENIC	--	--	--

INDEL marker IDs	Chromosomes/ unanchored scaffolds	Physical positions (bp)	InDels (*Kabuli* reference genome-CDC Frontier/PII)	Forward primers (5'-3')	Reverse primers (5'-3')	Anneling temperature (0C)	Expected amplified product size (bp)	Structural annotation		Functional annotation	
								Sequence components of *kabuli* genome	*Kabuli* gene accession IDs	NCBI-KOG	TFs
Ca7_P3_33633816	Ca7	33633816	TCTTCCTTCCCCTTC/TCTTC	TCTTCGCTTTGGTCTTC TTCA	TTCAAGTTCCCAACGG GTAG	60.1	900	INTERGENIC	--	--	--
Ca7_P3_33636918	Ca7	33636918	CTT/CAATTTTTTT	TTTTTCACCTTTTCACA ACCC	GGCACTTAAGTTTGGG CTGA	59.0	633	INTERGENIC	--	--	--
Ca7_P3_33638562	Ca7	33638562	AATGAAT/AATGAATATGAAT	TCGCAAGAGTTTGGAC CTTT	TTCTAAAAACGGAAAG CTATTTTGA	59.9	643	INTERGENIC	--	--	--
Ca7_P3_33639029	Ca7	33639029	TAAAAAAAAA/TAAAAAAAAAA	TCAGAGCTTTGCAGTTA TGTGA	GCACATGGTTTCGAAT GTTG	58.7	555	DRR	Ca_16180	--	ERF
Ca7_P3_33639526	Ca7	33639526	T/TTGCCTTC	CAACATTCGAAACCATG TGC	TATTCACACCGACGAT TTGG	60.0	752	DRR	Ca_16180	--	ERF
Ca7_P3_33640029	Ca7	33640029	TTA/T	CCAAATCGTCGGTGTG AATA	TTTTGGAGGAGATGTT TGGG	59.4	526	DRR	Ca_16180	--	ERF
Ca7_P3_33640082	Ca7	33640082	TAA/TAAA	CCAAATCGTCGGTGTG AATA	CCTTTGTTTGCTGGGA TCAT	59.4	614	DRR	Ca_16180	--	ERF
Ca7_P3_33642641	Ca7	33642641	ATTTTTTTTTTT/ATTTTTTTTTT	CCAGGCAAAGCTAGGT GAAC	GGGTTCCTGCTGTCAA TTTT	59.9	166	INTRON	Ca_16180	--	ERF
Ca7_P3_33643277	Ca7	33643277	CAAAAAAAAAAA/CAAAAAAAAAA	CCCAATAAAATGTGGG GAAA	ATCTTGTGGCCGAATT TGAG	59.5	312	INTRON	Ca_16180	--	ERF
Ca7_P3_33648393	Ca7	33648393	TTCTCTCTCTCTCTCTCT/TTCTCTCTC TCTCTCTCTCT	GGTTTAAGCCCTCAATT GCT	GGGGGATATATTGAAT GAAGAAA	58.3	755	INTERGENIC	--	--	--

**INDEL marker IDs**	**Chromosomes/ unanchored scaffolds**	**Physical positions (bp)**	**InDels (*Kabuli* reference genome-CDC Frontier/PII)**	**Forward primers (5'-3')**	**Reverse primers (5'-3')**	**Anneling temperature (0C)**	**Expected amplified product size (bp)**	**Structural annotation**		**Functional annotation**	
								**Sequence components of *kabuli* genome**	***Kabuli* gene accession IDs**	**NCBI-KOG**	**TFs**
Ca7_P3_33648564	Ca7	33648564	A/AT	TCCCAAATAAGCATTAG GATGAA	GGGGGATATATTGAAT GAAGAAA	59.8	317	INTERGENIC	--	--	--
Ca7_P3_33653612	Ca7	33653612	GAAAAAAAAAAA/GAAAAAAAAA	TGCCACATCACCCTAAA TCA	TTGGAGTTGTGCCAAT GTGT	59.9	517	INTERGENIC	--	--	--
Ca7_P3_33659410	Ca7	33659410	AAAAAA/AAAAAAGAAATGAAAAA	AACGGAGTAGTAATGC AGCAGA	ATTCAATGATGGTGAT GCGA	59.1	242	INTERGENIC	--	--	--
Ca7_P3_33660304	Ca7	33660304	GAAAAA/GAAAAAA	TCGGCAGTACCAATTTC ACA	TACAACGGTTTGATCG GTGA	60.1	610	INTERGENIC	--	--	--
Ca7_P3_33660715	Ca7	33660715	GTACTA/GTACTACTA	TCACCGATCAAACCGTT GTA	GGCAATTCACGAATTA TGGG	60.0	556	INTERGENIC	--	--	--
Ca7_P3_33666530	Ca7	33666530	T/TTTTTA	GATTCCCCACTTTGCAT GAT	CAATCTTCGCTTGTTTT CCTTT	59.8	430	INTERGENIC	--	--	--
Ca7_P3_33670297	Ca7	33670297	ATTTTTTTTTTTTT/ATTTTTTTT	CCTAAAATTTGCCATTC GGA	TTGAACGGTCGAGTTC TAACA	59.9	979	INTERGENIC	--	--	--
Ca7_P3_33683250	Ca7	33683250	CAA/CA	AAAAACGCAAGTCATCA TCCT	GATTGAGCCGAAAGTG AAGC	58.7	427	INTERGENIC	--	--	--
Ca7_P3_33683634	Ca7	33683634	C/CCTT	GCTTCACTTTCGGCTC AATC	CACAATCAAGGCCATC AAAA	60.0	214	INTERGENIC	--	--	--
Ca7_P3_33706376	Ca7	33706376	TAAAA/TAAA	GAAAATTCCAAAGGTGT AATTGCT	GAGTGCCGGAACAGA CAAAT	59.8	395	INTERGENIC	--	--	--

INDEL marker IDs	Chromosomes/ unanchored scaffolds	Physical positions (bp)	InDels (*Kabuli* reference genome-CDC Frontier/PII)	Forward primers (5'-3')	Reverse primers (5'-3')	Anneling temperature (0C)	Expected amplified product size (bp)	Structural annotation		Functional annotation	
								Sequence components of *kabuli* genome	*Kabuli* gene accession IDs	NCBI-KOG	TFs
Ca7_P3_33719688	Ca7	33719688	AC/A	TCACATCAATGTGTTTGGACC	CATGCGTGACAGAAAGGAGA	59.3	653	INTERGENIC	--	--	--
Ca7_P3_33722781	Ca7	33722781	TAAAAAAA/TAAAA	AAACAAGGAACAAAATGCGG	TGCAGCTCCTAACGATGTTG	60.0	699	INTERGENIC	--	--	--
Ca7_P3_33724426	Ca7	33724426	A/ACAT	CCTCTTTTGGTGACCACCTT	TTTTTCTCATTGGGAGCTGG	59.0	863	INTERGENIC	--	--	--
Ca7_P3_33724685	Ca7	33724685	CAAAAA/CAAAAAA	TCTCAGCAAACTAACTGCCG	TTTTTCTCATTGGGAGCTGG	59.2	297	INTERGENIC	--	--	--
Ca7_P3_33732435	Ca7	33732435	ATTTTTTTTTT/ATTTTTTTTTTT	GTTGATCGGGAAAAAGGGAT	GGAAGCGATAAGCACCAAAG	60.1	190	INTERGENIC	--	--	--
Ca7_P3_33732961	Ca7	33732961	TT/TTGAAAGT	CTTTGGTGCTTATCGCTTCC	TGGTCCCCCTATTTTAGCTG	59.8	919	INTERGENIC	--	--	--
Ca7_P3_33734013	Ca7	33734013	ATTTT/ATTT	ACATCACCTTCGACCACTCC	AATGACCAAGTCGGGAAAAA	60.0	607	INTERGENIC	--	--	--
Ca7_P3_33739910	Ca7	33739910	TG/T	GCCACACCTTCTTGGTGACT	AGCTCTGAGTCCATCCTCCA	60.2	512	INTRON	Ca_16182	--	--
Ca7_P3_33740406	Ca7	33740406	ACT/ACTCT	TTGGAGGATGGACTCAGAGC	ATGGTGGCAACTGAGAATCC	60.3	470	INTRON	Ca_16182	--	--
Ca7_P3_33745537	Ca7	33745537	AA/AATA	CTTGCCTCAACCTCTTCCAC	CCGTTTCATTCAACGGAGAG	59.8	750	DRR	Ca_16182	--	--

INDEL marker IDs	Chromosomes/ unanchored scaffolds	Physical positions (bp)	InDels (*Kabuli* reference genome-CDC Frontier/PII)	Forward primers (5'-3')	Reverse primers (5'-3')	Anneling temperature (0C)	Expected amplified product size (bp)	Structural annotation		Functional annotation	
								Sequence components of *kabuli* genome	*Kabuli* gene accession IDs	NCBI-KOG	TFs
Ca7_P3_33745626	Ca7	33745626	CAAAAA/CAAAAAA	CTTGCCTCAACCTCTTCCAC	CCGTTTCATTCAACGGAGAG	59.8	750	DRR	Ca_16182	--	--
Ca7_P3_33746470	Ca7	33746470	T/TAA	CAAAATTAAAATGGCACAGGG	TTTCCCAATCCACCACTCTC	59.4	332	DRR	Ca_16182	--	--
Ca7_P3_33746942	Ca7	33746942	ATTTTTT/ATTTTTTT	GAGAGTGGTGGATTGGGAAA	GACATTTATTTTGTTTTCCGCC	59.9	623	DRR	Ca_16182	--	--
Ca7_P3_33747403	Ca7	33747403	T/TG	GGCGGAAAACAAAATAAATGTC	CCATGGGAAGTGGCATTATC	59.7	202	INTERGENIC	--	--	--
Ca7_P3_33749761	Ca7	33749761	ATTTTTTTT/ATTTTTTTTT	ACCAAACCATTCATCCTTGG	AAAATCGTGATGCGAACACA	59.6	417	DRR	Ca_16183	J	--
Ca7_P3_33753838	Ca7	33753838	AG/A	CCTCCAAAAACACTCTCCCA	TGACAAGAACCTCTGCGTTG	60.1	810	INTRON	Ca_16183	J	--
Ca7_P3_33766122	Ca7	33766122	ATTT/ATTTT	AATATTTGGCTCCACCACCA	GGGTTACAAACTTCCTGCCA	60.2	564	INTRON	Ca_16185	--	--
Ca7_P3_33768316	Ca7	33768316	CTA/CTATA	GCTTCATTGGTGCATCCTTT	TCAGGCTAAAAGTGAAAACTCG	60.1	303	INTERGENIC	--	--	--
Ca7_P3_33769034	Ca7	33769034	ATTTTTTTTT/ATTTTTTTT	TCACAAGGGTAGTTTTGTCTTTTT	CAACATTTTATTTTGTCATGTATCTGG	58.4	439	INTERGENIC	--	--	--
Ca7_P3_33772634	Ca7	33772634	AAGA/AA	TGGATTTTGTTTGGATGCAG	TTCCCAACTTTTCAAAATATCCA	59.5	512	INTERGENIC	--	--	--

INDEL marker IDs	Chromosomes/ unanchored scaffolds	Physical positions (bp)	InDels (*Kabuli* reference genome-CDC Frontier/PII)	Forward primers (5'-3')	Reverse primers (5'-3')	Anneling temperature (0C)	Expected amplified product size (bp)	Structural annotation		Functional annotation	
								Sequence components of *kabuli* genome	*Kabuli* gene accession IDs	NCBI-KOG	TFs
Ca7_P3_33775388	Ca7	33775388	TAAAAAAAA/TAAAAAAA	TTTTTGACGGAAAAACGACC	TCGTGCATCCTCACTCTCAC	60.0	368	INTERGENIC	--	--	--
Ca7_P3_3377629	Ca7	3377629	GTTTTTT/GTTTTTTTT	ATAACCGCAAGCAAAACCAA	GGGGATTTTGGAGTTGGAGT	60.5	649	INTRON	Ca_03127	T	C3H
Ca7_P3_33778992	Ca7	33778992	GAAA/GAAAA	CGTTGAGTGACGATGAAATGA	TTTTTCAGCGCGAGATTTTT	59.7	675	INTERGENIC	--	--	--
Ca7_P3_33781676	Ca7	33781676	ATT/ATTATTT	ATGGAATTCTGTGTGGTTCG	TCGTAAATACCAGCATCATCAA	58.4	508	INTERGENIC	--	--	--
Ca7_P3_33782058	Ca7	33782058	ATTTTTT/ATTTTTTT	CGTAGACATGTTTTTGCATTTCA	TTTGGAAAAGCATGGATCATT	60.0	656	INTERGENIC	--	--	--
Ca7_P3_33786813	Ca7	33786813	CCTC/CCTCACTC	TTTTGAAGGTTGCCCAAAAC	AATATCTCAAACATGCCCCG	60.0	370	INTRON	Ca_16186	T	bHLH
Ca7_P3_33786874	Ca7	33786874	CTTTT/CTTT	TGAGTTTACCACCCTCGTCC	AATATCTCAAACATGCCCCG	60.0	121	INTRON	Ca_16186	T	bHLH
Ca7_P3_33792984	Ca7	33792984	ATTTTTTT/ATTTTTT	CCTTGGAGGACCAAAGAACA	CAGAGACAAGAATCCCCCAA	60.1	578	DRR	Ca_16187	--	--
Ca7_P3_33795086	Ca7	33795086	T/TA	GGTGTAACAAAAGGCCCAAA	AAGGATATGATGAAGGGCCA	59.8	408	INTRON	Ca_16187	--	--
Ca7_P3_33795817	Ca7	33795817	AAAATGAAATGAAATG/AAAATGAAATGAAATGAAATG	GTGGGGCATAGCTTCGTTTA	TTCCCCGGGTAACACAATAA	60.1	359	INTERGENIC	--	--	--

INDEL marker IDs	Chromosomes/ unanchored scaffolds	Physical positions (bp)	InDels (*Kabuli* reference genome-CDC Frontier/PII)	Forward primers (5'-3')	Reverse primers (5'-3')	Anneling temperature (0C)	Expected amplified product size (bp)	Structural annotation		Functional annotation	
								Sequence components of *kabuli* genome	*Kabuli* gene accession IDs	NCBI-KOG	TFs
Ca7_P3_33795948	Ca7	33795948	TTAAT/TT	GTGGGGCATAGCTTCG TTTA	TTCCCCGGGTAACACA ATAA	60.1	359	INTERGENIC	--	--	--
Ca7_P3_33796459	Ca7	33796459	ATTT/ATTTT	TTACCCGGGGAACTCA AACT	GGATGTGAAAACAGCC CAAT	60.7	804	INTERGENIC	--	--	--
Ca7_P3_33798138	Ca7	33798138	GAAAAAAAAA/GAAAAAAAA	TCGCAGTTGGATATTC GAGA	GCACAGTCAGATGCAA TGCT	59.4	552	INTERGENIC	--	--	--
Ca7_P3_33802472	Ca7	33802472	TTATATATATTCAT/TTATATATATTCAT ATATATATTCAT	TACTGACCTCTATCGCC GGT	CGTATGCGACTCATCC ATTG	59.7	612	DRR	Ca_16188	P	GRAS
Ca7_P3_33803390	Ca7	33803390	TACAC/TAC	TGAGGATGGAATGGGT TTTG	CTTTGGTTTCACATTG GGCT	60.7	555	DRR	Ca_16188	P	GRAS
Ca7_P3_33803853	Ca7	33803853	T/TTTG	AGCCCAATGTGAAACC AAAG	CGATTTTAAAACCAAAA TGTTAAATG	60.0	865	INTERGENIC	--	--	--
Ca7_P3_33805672	Ca7	33805672	TAAAATGAAAAATGAAAA/TAAAATGAA AAATGAAAAATGAAAA	TCACCGCAACTGACAA AGTC	GCGTTATTAGACGAAT GACTTGAA	59.9	406	INTERGENIC	--	--	--
Ca7_P3_33806939	Ca7	33806939	AAAAAATTAAAAATTAAAA/AAAAAATTA AAAATTAAAAATTAAAA	AGGCATAGGCACAGGA AAGA	TTCGATAGTCGCACAA CCAC	59.8	842	INTERGENIC	--	--	--
Ca7_P3_33807259	Ca7	33807259	AT/A	TCCAATCCAAGAGCTAA ACCA	TTCGATAGTCGCACAA CCAC	59.7	465	INTERGENIC	--	--	--
Ca7_P3_33815682	Ca7	33815682	AATAT/AAT	ATGGCTATTCACTGCCA TGC	ATCATCCTTGGTCCCG AATA	61.0	434	DRR	Ca_16190	E	--

INDEL marker IDs	Chromosomes/ unanchored scaffolds	Physical positions (bp)	InDels (*Kabuli* reference genome-CDC Frontier/PII)	Forward primers (5'-3')	Reverse primers (5'-3')	Anneling temperature (0C)	Expected amplified product size (bp)	Structural annotation		Functional annotation	
								Sequence components of *kabuli* genome	*Kabuli* gene accession IDs	NCBI-KOG	TFs
Ca7_P3_33815769	Ca7	33815769	TAAA/TA	ATGGCTATTCACTGCCATGC	ATCATCCTTGGTCCCGAATA	61.0	434	DRR	Ca_16190	E	--
Ca7_P3_3381604	Ca7	3381604	CATATATATATATATATAT/CATATATATATATATAT	TTCTCATGGAGAAGGATGGG	CAGCGAAAGCAAAACAACAA	60.0	301	INTRON	Ca_03127	T	C3H
Ca7_P3_33820036	Ca7	33820036	GA/GATA	AGGGTATTGAGGGATGGGAC	ATAGAGCGAGCATGGCAGAT	60.0	452	INTRON	Ca_16190	E	--
Ca7_P3_33820121	Ca7	33820121	AAG/AAGTAG	AGGGTATTGAGGGATGGGAC	ATAGAGCGAGCATGGCAGAT	60.0	452	INTRON	Ca_16190	E	--
Ca7_P3_33821649	Ca7	33821649	CTATATAT/CTATAT	ATTCCCCATTTCCAACATGA	AAACACATGCCTTATTGCCC	60.0	553	INTRON	Ca_16190	E	--
Ca7_P3_33822073	Ca7	33822073	ATTTTTTTT/ATTTTTTT	GGGCAATAAGGCATGTGTTT	GAAGGTTGAGTCAATCGGGA	59.8	520	INTRON	Ca_16190	E	--
Ca7_P3_33823687	Ca7	33823687	AAATTTTTTTTT/A	CTAGAGGTTGCCCCACGTTA	TGGGAAATGATGCATGGTAA	60.1	857	INTERGENIC	--	--	--
Ca7_P3_33823773	Ca7	33823773	AATA/AA	CGCGTTTCGACATTTTATCC	TGGGAAATGATGCATGGTAA	60.5	632	INTERGENIC	--	--	--
Ca7_P3_33826188	Ca7	33826188	CTT/CT	TCGAATGTGGCTGAAAAGAA	TTGGTGCACCGATAGTTTGA	59.4	617	INTERGENIC	--	--	--
Ca7_P3_33834706	Ca7	33834706	AGG/AG	CAAGGCTAGTAGCAGGTCGG	GGGTTGGTCGGTTATGTTGA	60.0	371	DRR	Ca_16191	--	NAC

INDEL marker IDs	Chromosomes/ unanchored scaffolds	Physical positions (bp)	InDels (*Kabuli* reference genome-CDC Frontier/PII)	Forward primers (5'-3')	Reverse primers (5'-3')	Anneling temperature (0C)	Expected amplified product size (bp)	Structural annotation		Functional annotation	
								Sequence components of *kabuli* genome	*Kabuli* gene accession IDs	NCBI-KOG	TFs
Ca7_P3_33839241	Ca7	33839241	CAAA/CAAAA	CATCATAGTGTTTCGCGAATTT	CGGCTATTTACTCTGCCAGC	59.1	729	INTERGENIC	--	--	--
Ca7_P3_33846076	Ca7	33846076	CTATATA/CTATA	TTAAGATGAACCAAGCCCTCT	GAAAGAGGTTGGAGCACAGC	57.9	471	INTRON	Ca_16192	--	NAC
Ca7_P3_33846722	Ca7	33846722	T/TA	GCTGTGCTCCAACCTCTTTC	CAGCGTGCACATGGATTAGT	60.0	544	INTRON	Ca_16192	--	NAC
Ca7_P3_33850307	Ca7	33850307	TAA/TAAA	CATTAAGACGGTCCTAGTCGG	ACCCGTTCATTACTTGCACC	58.7	558	INTERGENIC	--	--	--
Ca7_P3_33852197	Ca7	33852197	CTATATATATAT/CTATATATATATAT	TGCATCAACTTTTTGGGTGA	TTGGCCATATTTGAACCACTC	60.1	303	INTERGENIC	--	--	--
Ca7_P3_33852463	Ca7	33852463	CAAA/CAAAAA	TGCATCAACTTTTTGGGTGA	GCCCTCTTGGACTTCTTTCC	60.1	733	INTERGENIC	--	--	--
Ca7_P3_33852684	Ca7	33852684	TAA/TAAA	CCAAAATGGGTTGCTATTTTCT	GCCCTCTTGGACTTCTTTCC	59.4	654	INTERGENIC	--	--	--
Ca7_P3_33852713	Ca7	33852713	ATTTTTTT/ATTTTTT	CCAAAATGGGTTGCTATTTTCT	GCCCTCTTGGACTTCTTTCC	59.4	654	INTERGENIC	--	--	--
Ca7_P3_33855544	Ca7	33855544	CT/C	TGCAACATTTTACTCAATCCG	ACTTGATTGCACATGGATCG	58.7	137	INTERGENIC	--	--	--
Ca7_P3_33855784	Ca7	33855784	TAAAAAAAAAAAAA/TAAAAAAAA	CGATCCATGTGCAATCAAGT	CACTCTAAGAGGGGCCTCAA	59.5	528	INTERGENIC	--	--	--

INDEL marker IDs	Chromosomes/ unanchored scaffolds	Physical positions (bp)	InDels (*Kabuli* reference genome-CDC Frontier/PII)	Forward primers (5'-3')	Reverse primers (5'-3')	Anneling temperature (0C)	Expected amplified product size (bp)	Structural annotation		Functional annotation	
								Sequence components of *kabuli* genome	*Kabuli* gene accession IDs	NCBI-KOG	TFs
Ca7_P3_33855831	Ca7	33855831	TAAA/TAA	CGATCCATGTGCAATCAAGT	CACTCTAAGAGGGGCCTCAA	59.5	528	INTERGENIC	--	--	--
Ca7_P3_33855896	Ca7	33855896	TTTTGTTT/TTTT	CGATCCATGTGCAATCAAGT	CACTCTAAGAGGGGCCTCAA	59.5	528	INTERGENIC	--	--	--
Ca7_P3_33856093	Ca7	33856093	AT/A	GCCCCTCTTAGAGTGTTTGC	AAGATGTCATTTAAATCGAACCAA	58.9	106	INTERGENIC	--	--	--
Ca7_P3_33856959	Ca7	33856959	GAAAAAAAA/GAAAAAA	TCCCATCATAAATCTTGTGCC	ACTTTTGCTCTGGCTTCCAA	59.8	161	INTERGENIC	--	--	--
Ca7_P3_33857243	Ca7	33857243	GAAAAA/GAAAAAA	TTGGAAGCCAGAGCAAAAGT	GCCTCGTTTCATTTAATCTGC	60.0	691	INTERGENIC	--	--	--
Ca7_P3_33857446	Ca7	33857446	GAAA/GAA	TTGGAAGCCAGAGCAAAAGT	GCCTCGTTTCATTTAATCTGC	60.0	691	INTERGENIC	--	--	--
Ca7_P3_33862970	Ca7	33862970	GAAAAAAA/GAAAAAAAA	TTGGACACCAGACCCAAAAT	GGGGAGGGGTCTTTACTGAG	60.2	887	INTRON	Ca_16193	U	--
Ca7_P3_33877621	Ca7	33877621	TACAATG/TACAATGTGTACTACAACAATG	TTGTGCATGTATTGGGCTTG	CGGGGGTATTTCTGTGAAGA	60.5	544	INTRON	Ca_16194	O	--
Ca7_P3_33878318	Ca7	33878318	TTGATG/TTG	TCTTCACAGAAATACCCCCG	TGTTGGGACAAGCAAAATCA	59.9	508	INTRON	Ca_16194	O	--
Ca7_P3_33880621	Ca7	33880621	GA/GACA	AAAAAGATGTCTGCGGCTGT	GAAAAGAACGTGTGCATCATTT	59.9	811	DRR	Ca_16194	O	--

INDEL marker IDs	Chromosomes/ unanchored scaffolds	Physical positions (bp)	InDels (*Kabuli* reference genome-CDC Frontier/PII)	Forward primers (5'-3')	Reverse primers (5'-3')	Anneling temperature (0C)	Expected amplified product size (bp)	Structural annotation		Functional annotation	
								Sequence components of *kabuli* genome	*Kabuli* gene accession IDs	NCBI-KOG	TFs
Ca7_P3_33880672	Ca7	33880672	TGAC/T	AAAAAGATGTCTGCGGCTGT	GAAAAGAACGTGTGCATCATTT	59.9	811	DRR	Ca_16194	O	--
Ca7_P3_33880711	Ca7	33880711	ATTTTTTTTT/ATTTTTTT	AAAAAGATGTCTGCGGCTGT	GAAAAGAACGTGTGCATCATTT	59.9	811	DRR	Ca_16194	O	--
Ca7_P3_33880787	Ca7	33880787	ATT/ATTT	AAACACTGGCTGTGGAAAAA	TCAGACCCAAATGAGAGGAA	58.3	985	DRR	Ca_16194	O	--
Ca7_P3_33880924	Ca7	33880924	TAAAAAAA/TAAAAAAAAAA	TTTTTGTAATGATTGCTTGAATTT	CCGTTTCACTTTACCTTCGC	57.1	711	DRR	Ca_16194	O	--
Ca7_P3_33881967	Ca7	33881967	TG/TGG	TCCGACAAAAGCTTCACACA	AGTTGGTGTGGAGATGGAGG	60.4	502	INTERGENIC	--	--	--
Ca7_P3_33894708	Ca7	33894708	ATTTTTT/ATTTTT	TCACTATGACAACGGGGTGA	CCCGTGAAATGTCTTGGAGT	60.0	286	INTERGENIC	--	--	--
Ca7_P3_33894736	Ca7	33894736	GTTTTTTTTTT/GTTTTTTTT	TCACTATGACAACGGGGTGA	CCCGTGAAATGTCTTGGAGT	60.0	286	INTERGENIC	--	--	--
Ca7_P3_33895177	Ca7	33895177	CTTTTT/CTTTT	ACTCCAAGACATTTCACGGG	GCATGCAATTCACGCATTAT	60.0	340	INTERGENIC	--	--	--
Ca7_P3_33917687	Ca7	33917687	ATTTTTTTTTTT/ATTTTTTTTTT	TCATGTTTTCTTTTGCTGCG	GAGCCATAATTACTCAAGATGCAA	60.0	703	INTERGENIC	--	--	--
Ca7_P3_3402006	Ca7	3402006	GTTTTTTTTTTTTT/GTTTTTTTTTTTTTTT	GATCTAACACGACCAAGCCC	TGGTGCTTTTCAATTTGCAG	59.6	571	DRR	Ca_03124	--	--

INDEL marker IDs	Chromosomes/ unanchored scaffolds	Physical positions (bp)	InDels (*Kabuli* reference genome-CDC Frontier/PII)	Forward primers (5'-3')	Reverse primers (5'-3')	Anneling temperature (0C)	Expected amplified product size (bp)	Structural annotation		Functional annotation	
								Sequence components of *kabuli* genome	*Kabuli* gene accession IDs	NCBI-KOG	TFs
Ca7_P3_34031619	Ca7	34031619	AATATATATATATATATAT/AATATATATATATATATATATATATATATATATATAT	AGCCCCACAAAGTTGTTCAC	GTTGGTTCCCTAGCACCGTA	60.0	801	INTERGENIC	--	--	--
Ca7_P3_34047376	Ca7	34047376	ATTTTTTTTTTT/ATTTTTTTTTT	TTAGATTTGTCGGCTGAGGC	AGAACCAAGAGGCGACTTCA	60.4	572	INTERGENIC	--	--	--
Ca7_P3_34080547	Ca7	34080547	AT/A	AGGCAGGGGCAACTAAAGAT	TTTGTGGGGGTGCTTAGTTC	60.1	446	INTRON	Ca_16200	--	--
Ca7_P3_34108298	Ca7	34108298	AT/ATTT	CAAAGGGTGTGCTGAGAGAA	CGAAAATATGGACGCAACAA	59.0	479	INTERGENIC	--	--	--
Ca7_P3_34108342	Ca7	34108342	TAAAAAAAAAA/TAAAAAAAA	CAAAGGGTGTGCTGAGAGAA	CGAAAATATGGACGCAACAA	59.0	479	INTERGENIC	--	--	--
Ca7_P3_34111135	Ca7	34111135	TAAAAAA/TAAAAA	AGCAACAAACATGACCACCA	AAAACACAAATTTGAGGCCG	60.0	511	INTERGENIC	--	--	--
Ca7_P3_34116846	Ca7	34116846	ATTTTTTT/ATTTTTTTT	GATTTGACGTCCTGTCTCGG	AGAATTGGCAGATTCGATGG	60.7	571	INTERGENIC	--	--	--
Ca7_P3_34143500	Ca7	34143500	CA/CAA	AATGAGATGCCGATTTTTGC	AATGTTACCTTGCAGGACCG	60.0	615	INTRON	Ca_21006	K	ERF
Ca7_P3_34144763	Ca7	34144763	CTT/CTTT	GGCCATAGAGTGCAAGAAGC	CTCACCCAAAACCCTGAAAA	60.0	388	INTRON	Ca_21006	K	ERF
Ca7_P3_34152194	Ca7	34152194	TAA/TAAAAGAA	AAGCTTGGAGCAGGACAGAA	CTGCAAGATCGCAGGTGTAA	60.1	775	INTRON	Ca_21006	K	ERF

INDEL marker IDs	Chromosomes/ unanchored scaffolds	Physical positions (bp)	InDels (*Kabuli* reference genome-CDC Frontier/PII)	Forward primers (5'-3')	Reverse primers (5'-3')	Anneling temperature (0C)	Expected amplified product size (bp)	Structural annotation		Functional annotation	
								Sequence components of *kabuli* genome	*Kabuli* gene accession IDs	NCBI-KOG	TFs
Ca7_P3_34157692	Ca7	34157692	CA/CAAA	TCCTCTTCCTGGGATTCCTT	GGCTACGGGTTTCAAACAAA	60.0	427	INTRON	Ca_21006	K	ERF
Ca7_P3_34165927	Ca7	34165927	ATTTT/ATTTTT	TCTTCTTTGGCCATTCCATC	ACACCTTCTTGACAGGGGTG	60.0	507	INTRON	Ca_21006	K	ERF
Ca7_P3_34167836	Ca7	34167836	CATTATTA/CATTA	CATGCAGCTGTCGAGTGAAT	CCTCCAGTCAAAGAAACCGA	60.0	709	DRR	Ca_21006	K	ERF
Ca7_P3_34171842	Ca7	34171842	TTT/TTTTATAATGTT	CCAATCCAATCACCCAATTC	AAGAACGTAGATGATGGCGG	60.0	238	INTERGENIC	--	--	--
Ca7_P3_34173546	Ca7	34173546	GT/GTATCT	TGGGGTCAAATGTTACGCAT	TTTGATCTTCTTCGGTCGCT	61.1	499	INTERGENIC	--	--	--
Ca7_P3_34174102	Ca7	34174102	CTTTTTTTT/CTTTTTTTTTT	AGCGACCGAAGAAGATCAAA	GCTTGCATTTTGGGTGAGAT	60.0	509	INTERGENIC	--	--	--
Ca7_P3_34178534	Ca7	34178534	CTATAT/CTATATAT	GGAGACAATTTTCGTTGGCT	GGTTGTGGTGGTCGTCTTCT	59.2	891	INTERGENIC	--	--	--
Ca7_P3_34178572	Ca7	34178572	TAAA/TAA	GGAGACAATTTTCGTTGGCT	GGTTGTGGTGGTCGTCTTCT	59.2	891	INTERGENIC	--	--	--
Ca7_P3_34187425	Ca7	34187425	GAA/GAAA	AGGTCTGGATTCGTGGTTTG	ACATCCGTGCAAGGAAAAAG	60.0	646	INTRON	Ca_21004	S	--
Ca7_P3_34190333	Ca7	34190333	AAG/AAGAG	TCGAACTACTTGCGTGAGAA	TTGGTTCTTTTGCATCTTCG	57.2	559	DRR	Ca_21004	S	--

INDEL marker IDs	Chromosomes/ unanchored scaffolds	Physical positions (bp)	InDels (*Kabuli* reference genome-CDC Frontier/PII)	Forward primers (5'-3')	Reverse primers (5'-3')	Anneling temperature (0C)	Expected amplified product size (bp)	Structural annotation		Functional annotation	
								Sequence components of *kabuli* genome	*Kabuli* gene accession IDs	NCBI-KOG	TFs
Ca7_P3_34195399	Ca7	34195399	CTGATGA/CTGA	GTTGAATTTGGCCTTCC TGA	AGGCTAACACCATTGC CAAC	60.1	837	DRR	Ca_21002	--	WRKY
Ca7_P3_34195712	Ca7	34195712	AT/ATTT	GAGGCTCCATATTGCG AAAA	AGGCTAACACCATTGC CAAC	60.2	189	DRR	Ca_21002	--	WRKY
Ca7_P3_34195793	Ca7	34195793	TAAAAAAAAAA/TAAAAAAAAA	GAGGCTCCATATTGCG AAAA	AGGCTAACACCATTGC CAAC	60.2	189	DRR	Ca_21003	O	FAR1
Ca7_P3_34196242	Ca7	34196242	GTTT/GTT	GTTGGCAATGGTGTTA GCCT	TTGAGGGTTGCTTTTG TTGTT	60.0	771	DRR	Ca_21002	--	WRKY
Ca7_P3_34196824	Ca7	34196824	ACC/ACCGCC	AACAACAAAAGCAACCC TCAA	GTTAGTGCAGCGAACC AACA	59.6	302	CDS (large-effect mutations)	Ca_21002	--	WRKY
Ca7_P3_34202611	Ca7	34202611	AAGAGAGAGAGA/AAGAGAGAGAGAG A	TATTGCGTTTGGTGTC GGTA	TAGCTAAATCGTGCTT GCCA	60.0	814	INTRON	Ca_21002	--	WRKY
Ca7_P3_34202826	Ca7	34202826	CTTTT/CTTTTT	CACCATTGATGACAAG AGCG	ATCGTGCTTGCCAGCT TTAC	60.3	248	INTERGENIC	--	--	--
Ca7_P3_34203381	Ca7	34203381	A/AATATATAGGTAG	GTAAAGCTGGCAAGCA CGAT	CCCAGAACAAAAGTTG GTGAA	60.4	725	INTERGENIC	--	--	--
Ca7_P3_34204751	Ca7	34204751	TTATATATATA/TTATATATATATA	AGCATTGCGCGTAAAG AGAT	TGATGTTACATGACGT GTTTGG	60.0	665	INTERGENIC	--	--	--
Ca7_P3_34205414	Ca7	34205414	ATTTT/ATTTTT	TTGAAATACACACACAA AATTGACA	TTGACCTTGAAATCAA CTCACC	59.4	267	INTERGENIC	--	--	--

INDEL marker IDs	Chromosomes/ unanchored scaffolds	Physical positions (bp)	InDels (*Kabuli* reference genome-CDC Frontier/PII)	Forward primers (5'-3')	Reverse primers (5'-3')	Anneling temperature (0C)	Expected amplified product size (bp)	Structural annotation		Functional annotation	
								Sequence components of *kabuli* genome	*Kabuli* gene accession IDs	NCBI-KOG	TFs
Ca7_P3_34213991	Ca7	34213991	TCAGAATTACAGAATTACAG/TCAGAATTACAG	TGCGCGATCTCTAATCAATG	CGGCAACCTTACCTCAACTC	59.9	763	INTERGENIC	--	--	--
Ca7_P3_34216629	Ca7	34216629	GTTTTTTTT/GTTTTTTT	TTTGTCCCTCCCAAGAAATG	ATCTCACCCCTGGTTGTCTG	59.9	427	DRR	Ca_20999	L	--
Ca7_P3_34217652	Ca7	34217652	TAAAAAAAA/TAAAAAAAAA	GCCACAAACCTCATGCCTAT	TGCAGCAGTCAATGTTCCTC	60.0	347	DRR	Ca_20999	L	--
Ca7_P3_34222973	Ca7	34222973	A/AAAATAT	GCAAAACTGGGGAGAAACAA	TCGCTTACTGGCTTCCATCT	60.1	792	INTRON	Ca_20999	L	--
Ca7_P3_34223513	Ca7	34223513	ACCCCC/ACCCCCC	AGATGGAAGCCAGTAAGCGA	ACAGAGCCTAAAACCAGGCA	60.0	244	INTERGENIC	--	--	--
Ca7_P3_34224527	Ca7	34224527	CAAAAAAAA/CAAAAAAA	GCATTTTGACACATCTATTCAACC	GGCGGACGAACTTATTTTCA	59.8	893	INTERGENIC	--	--	--
Ca7_P3_34226505	Ca7	34226505	AAATA/AA	AACACCAATCGCTCTTCCAC	GAGGGAACACCAACTCCAAA	60.1	889	INTERGENIC	--	--	--
Ca7_P3_34226865	Ca7	34226865	TTCTCTCTCTCTCTCTCTCTCT/TTCTCTCTCTCTCTCTCTCTCT	GCGAATCAATGTTCACAAGC	GAGGGAACACCAACTCCAAA	59.3	196	INTERGENIC	--	--	--
Ca7_P3_34229450	Ca7	34229450	GATAT/GAT	GCATTGGAAGCGAATGGTAT	AGCCAGCCAGCTAAAATTCA	59.9	435	INTRON	Ca_20998	--	--
Ca7_P3_34237238	Ca7	34237238	GGTTGTTGTTGTTGTTGTTGTTGTTGT/GGTTGTTGTTGTTGT	TTCCCAATTGCAACAATCAA	GGTCTCATCTCATCCTCCCA	59.9	437	CDS (large-effect mutations)	Ca_20997	--	S1Fa-like

INDEL marker IDs	Chromosomes/ unanchored scaffolds	Physical positions (bp)	InDels (*Kabuli* reference genome-CDC Frontier/PII)	Forward primers (5'-3')	Reverse primers (5'-3')	Anneling temperature (0C)	Expected amplified product size (bp)	Structural annotation		Functional annotation	
								Sequence components of *kabuli* genome	*Kabuli* gene accession IDs	NCBI-KOG	TFs
Ca7_P3_34237601	Ca7	34237601	TTG/TTGGTTTTGGTTATG	TGGGAGGATGAGATGAGACC	CAAGGAACCATCCATTTGCT	60.0	590	INTERGENIC	--	--	--
Ca7_P3_34238466	Ca7	34238466	TAAA/TAA	AGCAAATGGATGGTTCCTTG	AATGCAGCTCCACACGTTTA	59.9	915	INTERGENIC	--	--	--
Ca7_P3_34239748	Ca7	34239748	CAAAAA/CAAAA	GCTCATTCAAAAGTAAAACCGAA	TTTCAAACCGTCAATTATTAATGC	59.7	583	INTERGENIC	--	--	--
Ca7_P3_34251685	Ca7	34251685	TGA/TGAAGA	GGGTCGATTGGTGAAGAAGA	GGTTCAGAATCGTGGTCGTT	60.1	165	CDS (large-effect mutations)	Ca_20996	S	--
Ca7_P3_34278071	Ca7	34278071	T/TC	TCTTCAAGTGGTCGCTTCCT	CCTTCGCCATTTAATTTTGG	60.0	915	INTERGENIC	--	--	--
Ca7_P3_34278231	Ca7	34278231	ATTTTTT/ATTT	TGGACTGCTCGATTGTCAAA	CCTTCGCCATTTAATTTTGG	60.4	557	INTERGENIC	--	--	--
Ca7_P3_34278471	Ca7	34278471	T/TAAATAC	TTTGACCCATCAAATCATCAAA	CCTTCGCCATTTAATTTTGG	60.2	102	INTERGENIC	--	--	--
Ca7_P3_34279049	Ca7	34279049	ATT/ATTT	GGTCATGAGTTTTGATTCCCA	AGACAAATATCGGTACGGCG	59.8	642	INTERGENIC	--	--	--
Ca7_P3_34279316	Ca7	34279316	CCA/CCACCACA	CGCCGTACCGATATTTGTCT	TAGCAGGACGGAATTGCTCT	60.0	217	INTERGENIC	--	--	--
Ca7_P3_34282882	Ca7	34282882	TAAA/TAAAA	TGCAGCATGGAACATTTGAT	CCTCCGACTTGTGTCCATTT	60.1	199	INTRON	Ca_20990	A	--

INDEL marker IDs	Chromosomes/ unanchored scaffolds	Physical positions (bp)	InDels (*Kabuli* reference genome-CDC Frontier/PII)	Forward primers (5'-3')	Reverse primers (5'-3')	Anneling temperature (0C)	Expected amplified product size (bp)	Structural annotation		Functional annotation	
								Sequence components of *kabuli* genome	*Kabuli* gene accession IDs	NCBI-KOG	TFs
Ca7_P3_34283590	Ca7	34283590	ATTT/ATTTTT	TTTGTGCACTGATGCTGTCA	TTGCTCGCATATGGGTTATG	60.0	355	DRR	Ca_20990	A	--
Ca7_P3_34285857	Ca7	34285857	CATATATATA/CATATATA	AGCTTGAAAGCAAACGGTGT	AGCACATCCAATGTCTGACG	59.9	503	INTERGENIC	--	--	--
Ca7_P3_34286325	Ca7	34286325	A/AAATAT	ACGTCAGACATTGGATGTGC	TTGTTTTCAAGGAGTTGCGA	59.6	666	INTERGENIC	--	--	--
Ca7_P3_34290113	Ca7	34290113	T/TAG	TTTTGAACAGAACCAAGGGC	GAAGAGAAAAGCCGGTGTTG	60.1	362	INTRON	Ca_20989	K	NAC
Ca7_P3_34295146	Ca7	34295146	AATTTGT/AATTTGTTATCTATTTGT	CCAACTGGCCTAGGATGAAA	TGGGAGAAAAACAAGCAAGG	60.1	579	INTERGENIC	--	--	--
Ca7_P3_34309669	Ca7	34309669	GTTTTTTT/GTTTTTT	TGTGAGTCGATGAATCAGCC	TCGAATTTCGTGTCCACTCA	59.8	417	INTERGENIC	--	--	--
Ca7_P3_34309692	Ca7	34309692	TA/T	TGTGAGTCGATGAATCAGCC	TCGAATTTCGTGTCCACTCA	59.8	417	INTERGENIC	--	--	--
Ca7_P3_34309775	Ca7	34309775	ATTTTTTTTTT/ATTTTTTTTT	TGTGAGTCGATGAATCAGCC	TCGAATTTCGTGTCCACTCA	59.8	417	INTERGENIC	--	--	--
Ca7_P3_34311495	Ca7	34311495	TAAAA/TAAA	TCCAGCATGCAGCATAGTTT	TGCAGGACCAGATAGGGAAG	59.4	214	INTERGENIC	--	--	--
Ca7_P3_34311535	Ca7	34311535	TAAAAAAAA/TAAAAAAA	TCCAGCATGCAGCATAGTTT	TGCAGGACCAGATAGGGAAG	59.4	214	INTERGENIC	--	--	--

INDEL marker IDs	Chromosomes/ unanchored scaffolds	Physical positions (bp)	InDels (*Kabuli* reference genome-CDC Frontier/PII)	Forward primers (5'-3')	Reverse primers (5'-3')	Anneling temperature (0C)	Expected amplified product size (bp)	Structural annotation		Functional annotation	
								Sequence components of *kabuli* genome	*Kabuli* gene accession IDs	NCBI-KOG	TFs
Ca7_P3_34312118	Ca7	34312118	AAATTAAT/AAAT	TTGCTAATTGCTAATTG CTTTG	TGGAGTGAGTGTGAGA AAGGAG	57.4	388	INTERGENIC	--	--	--
Ca7_P3_34312381	Ca7	34312381	CTTT/CTTTTT	CTCCTTTCTCACACTCA CTCCA	TTGGTGGACTTCCCAA CTTC	59.5	515	INTERGENIC	--	--	--
Ca7_P3_34312433	Ca7	34312433	GATAAATA/GATA	CTCCTTTCTCACACTCA CTCCA	TTGGTGGACTTCCCAA CTTC	59.5	515	INTERGENIC	--	--	--
Ca7_P3_34312678	Ca7	34312678	ATTTTTTTTT/ATTTTTTTT	CTCCTTTCTCACACTCA CTCCA	TTGGTGGACTTCCCAA CTTC	59.5	515	INTERGENIC	--	--	--
Ca7_P3_34313194	Ca7	34313194	TTCACAATC/TTCACAATCTCACAATC	TGGGAAGTCCACCAAA GTTC	TTGATTGTGGTTGTTTT ACCG	59.9	618	INTERGENIC	--	--	--
Ca7_P3_34315074	Ca7	34315074	GATATATA/GATATATACATATATATATA	TTCTGATAATTGGAATC ACTTCTAACA	TTGCACCCGTAGTATT GTGG	59.5	561	INTERGENIC	--	--	--
Ca7_P3_34315993	Ca7	34315993	GTA/GTATTTA	AACAAAAGATTGAATTG AAAGACA	TCTTCTAGGGGTGAGA CCCA	57.1	555	INTERGENIC	--	--	--
Ca7_P3_34316435	Ca7	34316435	ATAATTAAT/ATAATTAATTAAT	CATGGGTCTCACCCCT AGAA	AAGGTTGCCATTGTAT TGGG	59.9	412	INTERGENIC	--	--	--
Ca7_P3_34317406	Ca7	34317406	ATATA/ATATAGTATA	TGGAATTTGTATGCGA GACG	GCAGGGTTTTTGTTTT TGGA	59.7	896	INTERGENIC	--	--	--
Ca7_P3_34317535	Ca7	34317535	AATATATATATATA/AATATATATATA	TTGTTTTTGGTGAAAAG TGTGTG	GCAGGGTTTTTGTTTT TGGA	60.0	429	INTERGENIC	--	--	--

INDEL marker IDs	Chromosomes/ unanchored scaffolds	Physical positions (bp)	InDels (*Kabuli* reference genome-CDC Frontier/PII)	Forward primers (5'-3')	Reverse primers (5'-3')	Anneling temperature (0C)	Expected amplified product size (bp)	Structural annotation		Functional annotation	
								Sequence components of *kabuli* genome	*Kabuli* gene accession IDs	NCBI-KOG	TFs
Ca7_P3_34318359	Ca7	34318359	AACAAATACAAATA/AACAAATA	TGTGTTGCTTTTGATTTCCA	GCTAGCAGCCACCTTCTTTG	57.7	917	INTERGENIC	--	--	--
Ca7_P3_34318595	Ca7	34318595	CATTATTACAGATTT/CATTATTACAGATTTATTATTACAGATTT	TCAATGACTGTGACAAAATCCA	GCTAGCAGCCACCTTCTTTG	59.0	503	INTERGENIC	--	--	--
Ca7_P3_34320519	Ca7	34320519	C/CTA	TTAACAAAACGGCTCCAACC	ACATGGCTTGGAGTTGGAAT	60.0	438	INTERGENIC	--	--	--
Ca7_P3_34321055	Ca7	34321055	AA/AATA	CTCCAAGCCATGTTGGATTT	CATTTTTCCCTCCCCAATTT	59.9	382	INTERGENIC	--	--	--
Ca7_P3_34321800	Ca7	34321800	TA/T	TGTAAGAGTCGGTGCCTTCA	TTTTCTCATGGATCTTTTGGA	59.4	820	INTERGENIC	--	--	--
Ca7_P3_34321844	Ca7	34321844	ATTTTTTTT/ATTTTTTTTT	TCGGTGCCTTCATATTTGACT	TTTTCTCATGGATCTTTTGGA	59.6	812	INTERGENIC	--	--	--
Ca7_P3_34328779	Ca7	34328779	AT/ATTCAGT	AGGGAGGCACTTCGGAGTAT	GTGCAACCGATTGGTATCCT	60.1	793	INTERGENIC	--	--	--
Ca7_P3_34331157	Ca7	34331157	GAAAAA/GAAAA	CTCCTTTGGCCATAAAACCA	ATTGCGTTCGAATCTTGGAG	59.9	368	INTERGENIC	--	--	--
Ca7_P3_34331908	Ca7	34331908	GAGTAGTTTA/G	GGACAATTTCCAACGACCAA	CCATTCACGAAGGGGAAGTA	60.7	800	INTERGENIC	--	--	--
Ca7_P3_34335084	Ca7	34335084	TTATATA/TTATA	CCAATAACAATGGGGGTTCA	CCTCTTTGTTGGATGAGGGA	60.4	259	INTERGENIC	--	--	--

INDEL marker IDs	Chromosomes/ unanchored scaffolds	Physical positions (bp)	InDels (*Kabuli* reference genome-CDC Frontier/PII)	Forward primers (5'-3')	Reverse primers (5'-3')	Anneling temperature (0C)	Expected amplified product size (bp)	Structural annotation		Functional annotation	
								Sequence components of *kabuli* genome	*Kabuli* gene accession IDs	NCBI-KOG	TFs
Ca7_P3_34336023	Ca7	34336023	AA/AAAAAAGA	TATTTGACCCGTGAGA CGTG	CAACCACAATTCAATTT ATGCAA	59.6	539	INTERGENIC	--	--	--
Ca7_P3_34336122	Ca7	34336122	T/TTTA	TATTTGACCCGTGAGA CGTG	CAACCACAATTCAATTT ATGCAA	59.6	539	INTERGENIC	--	--	--
Ca7_P3_34338542	Ca7	34338542	AACTTGTA/AA	CCCCAACAAAGGTCAT CAAT	TGAGCTCCACCTTTTG ATCC	59.6	503	INTERGENIC	--	--	--
Ca7_P3_34342253	Ca7	34342253	TTTTT/TTTTTATTTT	AATGATAGGTGGACGT TCAAA	GGGTAACCTCGCCACA ACTA	57.1	725	INTERGENIC	--	--	--
Ca7_P3_34347667	Ca7	34347667	A/AC	TCATTTAGCGGCTGGT TTCT	TGCCGCAATAAAATGA AACA	59.8	643	DRR	Ca_20986	C	--
Ca7_P3_34348983	Ca7	34348983	T/TA	TTACCCTCCAAAGACAC TTCG	CTTGTCGGCTTAAATC CCAA	59.2	321	DRR	Ca_20986	C	--
Ca7_P3_34348997	Ca7	34348997	ATTTTTTTT/ATTTTTTT	TTACCCTCCAAAGACAC TTCG	CTTGTCGGCTTAAATC CCAA	59.2	321	DRR	Ca_20986	C	--
Ca7_P3_34350373	Ca7	34350373	TAAA/TAA	AAAGAAGCGACGAATC TCCA	CATGCAGGTAAATTGT TGCC	60.0	599	INTRON	Ca_20986	C	--
Ca7_P3_34370452	Ca7	34370452	AA/AAGA	AGCCCAAATATGCGCT TAGA	CCGAAACTATTTCCAC GACC	59.8	522	INTERGENIC	--	--	--
Ca7_P3_34375146	Ca7	34375146	GTCCACTCCACTCCACTC/GTCCACTC CACTCCACTCCACTC	GAGGGGTTTGTGGAAT GGTA	GTAATCACCAACCAAT CGGG	59.6	271	INTERGENIC	--	--	--

INDEL marker IDs	Chromosomes/ unanchored scaffolds	Physical positions (bp)	InDels (*Kabuli* reference genome-CDC Frontier/PII)	Forward primers (5'-3')	Reverse primers (5'-3')	Anneling temperature (0C)	Expected amplified product size (bp)	Structural annotation		Functional annotation	
								Sequence components of *kabuli* genome	*Kabuli* gene accession IDs	NCBI-KOG	TFs
Ca7_P3_34383642	Ca7	34383642	TTCT/TT	TTAATTCAGTCGATGCCACG	TCTGGAAAATACACGTTGAATACAT	59.7	841	INTERGENIC	--	--	--
Ca7_P3_34392098	Ca7	34392098	ATTTTTTT/ATTTTTTTTT	GGCGAGCATGAAATACCAAT	TCTGCCAAAAACGAAGATCG	59.9	819	INTERGENIC	--	--	--
Ca7_P3_34394315	Ca7	34394315	AG/A	TATTTGGCTATCTGCGGTCC	TCGGATGTAAATGCGATGAA	60.1	726	INTERGENIC	--	--	--
Ca7_P3_34396059	Ca7	34396059	TATCTTATAAAA/TATCTTATAAAATCTTATAAAA	GTGCCAGAGGAGGTGGTAGA	TGACATTTGGGTCAGCATGT	60.3	944	INTRON	Ca_20981	QR	B3
Ca7_P3_34396656	Ca7	34396656	CTT/CT	CACATGCTGACCCAAATGTC	TTGAACAAAATCACGACCGA	60.0	216	INTRON	Ca_20981	QR	B3
Ca7_P3_34397123	Ca7	34397123	ATTTTTTTTT/ATTTTTTTT	TGTCGGTCGTGATTTTGTTC	ATGCAAGATGGGTATCGTGT	59.5	594	INTRON	Ca_20981	QR	B3
Ca7_P3_34397143	Ca7	34397143	AT/A	TGTCGGTCGTGATTTTGTTC	ATGCAAGATGGGTATCGTGT	59.5	594	INTRON	Ca_20981	QR	B3
Ca7_P3_34397419	Ca7	34397419	CTA/CTATA	ACACGATACCCATCTTGCAT	TCGATGCAGCACACTCTTGT	57.9	608	INTRON	Ca_20981	QR	B3
Ca7_P3_34407884	Ca7	34407884	TAAAAAAAAAAA/TAAAAAAAAAA	ATGCAACTTGGTGGATGCTT	GCATGCAGCCTTGTTGATTA	60.5	586	INTERGENIC	--	--	--
Ca7_P3_34412063	Ca7	34412063	ATTTTTTT/ATTTTTTTTT	AATGCGAGGAAAACTCGCTA	TCCCTGTTTCTACGCCAAAC	60.0	746	INTRON	Ca_20980	PT	FAR1

INDEL marker IDs	Chromosomes/ unanchored scaffolds	Physical positions (bp)	InDels (*Kabuli* reference genome-CDC Frontier/PII)	Forward primers (5'-3')	Reverse primers (5'-3')	Anneling temperature (0C)	Expected amplified product size (bp)	Structural annotation		Functional annotation	
								Sequence components of *kabuli* genome	*Kabuli* gene accession IDs	NCBI-KOG	TFs
Ca7_P3_34412369	Ca7	34412369	TACGCTA/TACGCTAACGCTA	ATTGTTGCTTCCCAATTTCG	GCTCAGCTGGTTTCATAGGC	59.9	741	INTRON	Ca_20980	PT	FAR1
Ca7_P3_34456097	Ca7	34456097	TTATATATATAT/TTATATATATATAT	ACAATTGTTGCACCACGAGA	GCATATAAGTGGCTAACCGCA	60.2	619	INTERGENIC	--	--	--
Ca7_P3_34509317	Ca7	34509317	AATATATATATATATATATATATATATATATATAT/AATATATATATATATATATATATATATATAT	GAGTCTCTTTGATTGATTGATAGGA	GGTCGAGGAAGAACCACAAA	58.0	945	INTERGENIC	--	--	--
Ca7_P3_34583170	Ca7	34583170	CATATATATATATATATATATATATATATATATAT/CATATATATATATATATATATATATATAT	TTCATCCACCAAGAAGCTCA	GAACTCGACCAGTGCAGACA	59.4	253	INTERGENIC	--	--	--
Ca7_P3_34587026	Ca7	34587026	TTATATATATATATATATATATATATATATATA/TTATATATATATATATATATATATATATATATATATA	CGTTTGATTTTGGTTGGTGA	TAGTGCGAACTTCCCTCGTT	59.4	455	INTERGENIC	--	--	--
Ca7_P3_34650181	Ca7	34650181	GAAAAAAAAAAAA/GAAAAAAAAAAA	CGACTAATCTTGACAACACGGA	TGGAACTCGGTTACATTTTGAA	60.2	505	INTERGENIC	--	--	--
Ca7_P3_34663130	Ca7	34663130	ATTTTTTTTTTT/ATTTTTTTTTTTT	AAATTTTGGTTGCGATTCCA	ACCCGGGTATAAGGTTCCAC	60.3	385	INTERGENIC	--	--	--
Ca7_P3_34672457	Ca7	34672457	ATATTATTATTATTATTATTATTATTATTATTATT/ATATTATTATTATTATTATTATTATTATT	TTGTGCATTATTGAGGTGGA	ATTTGCACTTGGTTTGACCC	57.5	897	INTERGENIC	--	--	--
Ca7_P3_34730133	Ca7	34730133	CTTTTTTTTTTT/CTTTTTTTTTTTTT	GAGTTTGAATGGTTGCCGTT	TTGGTTTTCCAAATTTGTGTCT	60.0	722	DRR	Ca_17610	Q	B3
Ca7_P3_34735432	Ca7	34735432	CAA/CAAA	GGTTTTGCCCCTATTTCCAT	TTGCAAGTTTAGATCGCTTGTC	60.0	435	INTERGENIC	--	--	--

INDEL marker IDs	Chromosomes/ unanchored scaffolds	Physical positions (bp)	InDels (*Kabuli* reference genome-CDC Frontier/PII)	Forward primers (5'-3')	Reverse primers (5'-3')	Anneling temperature (0C)	Expected amplified product size (bp)	Structural annotation		Functional annotation	
								Sequence components of *kabuli* genome	*Kabuli* gene accession IDs	NCBI-KOG	TFs
Ca7_P3_34800785	Ca7	34800785	AATATATATATATATATATATATAT/AATATATATATATATATATATATAT	AACACATCCGGGTTCCATTA	AGCGGCATAACCGTATGAAC	60.1	547	DRR	Ca_17614	--	--
Ca7_P3_34922873	Ca7	34922873	AATTATTATTATTATTATTATTATTATTATTATTATTATTATTA/AATTATTATTATTATTATTATTATTATTATTATTATTATTATTATTA	GCCACCACCTTCTTGAACAT	TTGTCCAAGGTGCAATTCAA	60.0	604	INTERGENIC	--	--	--
Ca7_P3_35048654	Ca7	35048654	TTATATAT/TTATATATAT	AGGTGGGCAACTTTGATGAC	GCATCTGGCTAGGAACTTGG	60.0	673	INTRON	Ca_17632	R	--
Ca7_P3_3505469	Ca7	3505469	TC/T	AAATCAAGAGAGGCCTGCAA	GGGAGAGCTAGGGAGGAAGA	60.0	507	INTRON	Ca_03117	Z	MYB_related
Ca7_P3_35065891	Ca7	35065891	AT/A	TTTTGAAACAATGCTCGTGG	CAACCATCTTAAATGACCACAAA	59.7	644	DRR	Ca_17634	--	--
Ca7_P3_3509080	Ca7	3509080	CGT/CGTTTGTGT	GCCTGTGTCACTCAAATGGA	TGCCTGCCTGCCTACTACTT	59.7	963	INTRON	Ca_03117	Z	MYB_related
Ca7_P3_35109014	Ca7	35109014	ATTTTTTT/ATTTTTTTTT	CCTGGGATGGTGATGAGAGT	TCCCAACCAGACTGAACTGG	59.9	806	INTERGENIC	--	--	--
Ca7_P3_35109351	Ca7	35109351	TAAAAAAA/TAAAAAA	TGTTTTAACGTTTGCGTTGC	TCCAATGAATCCCTTGCTTC	59.8	674	INTERGENIC	--	--	--
Ca7_P3_35110110	Ca7	35110110	AC/ACC	GAAGCAAGGGATTCATTGGA	CATCAGAGGCATGAGAAGCA	60.0	717	INTERGENIC	--	--	--
Ca7_P3_3512331	Ca7	3512331	ATTT/ATT	AATTCGCATGCATATTCAACA	GCAGAGATTTGATTCGAGGC	59.0	677	INTRON	Ca_03117	Z	MYB_related

INDEL marker IDs	Chromosomes/ unanchored scaffolds	Physical positions (bp)	InDels (*Kabuli* reference genome-CDC Frontier/PII)	Forward primers (5'-3')	Reverse primers (5'-3')	Anneling temperature (0C)	Expected amplified product size (bp)	Structural annotation		Functional annotation	
								Sequence components of *kabuli* genome	*Kabuli* gene accession IDs	NCBI-KOG	TFs
Ca7_P3_3512361	Ca7	3512361	AG/A	AATTCGCATGCATATTCAACA	GCAGAGATTTGATTCGAGGC	59.0	677	INTRON	Ca_03117	Z	MYB_related
Ca7_P3_35131412	Ca7	35131412	TAAAAAAA/TAAAAAAAA	TCGTGGTCGCATTGAACTTA	GAATCAAAATGGAGCGGAAA	60.3	376	INTERGENIC	--	--	--
Ca7_P3_35134144	Ca7	35134144	TAAA/TAAAA	ACCATTCCATTTCTCCTTGC	CCTTCAATTTTGCCGTCAGT	59.0	494	DRR	Ca_17638	--	ERF
Ca7_P3_35139699	Ca7	35139699	TG/TGAG	ATTCAAACAAACGCATGCAA	CAGCTGGCACCCTTACAAGT	60.1	535	INTERGENIC	--	--	--
Ca7_P3_35140766	Ca7	35140766	G/GA	TTTTAAGCCACATCATGCCA	GTTCCTCCCTCCATCCCTAA	60.1	585	INTERGENIC	--	--	--
Ca7_P3_35141972	Ca7	35141972	TA/TAA	TTGTCCCACGAGGAGAAAAC	CGACAAACACAATCACTGGG	60.1	824	INTERGENIC	--	--	--
Ca7_P3_3515316	Ca7	3515316	GGTGTG/GGTG	TCGGAGAGGGTTTGTACCAC	TTTATGTCCGCCTTTCTTCA	60.0	259	DRR	Ca_03116	I	--
Ca7_P3_35156256	Ca7	35156256	ATTTTTT/ATTTTTTT	TTCCTGTTTTGTAGCACCCC	CACCATTGTCCGATTGTCAG	60.0	757	INTERGENIC	--	--	--
Ca7_P3_35166389	Ca7	35166389	CATGT/CATGTTATTTTTATGT	ATCAAGGTGGGGATTGTTGA	TTGTAGTTTTTCACGTGGTGC	60.2	781	INTERGENIC	--	--	--
Ca7_P3_35166766	Ca7	35166766	TAAAAAAAA/TAAAAAAA	GGCGCTATATGGTATTTTGAGTT	GGTTCTTCGCCTTCACAAAC	58.7	948	INTERGENIC	--	--	--

INDEL marker IDs	Chromosomes/ unanchored scaffolds	Physical positions (bp)	InDels (*Kabuli* reference genome-CDC Frontier/PII)	Forward primers (5'-3')	Reverse primers (5'-3')	Anneling temperature (0C)	Expected amplified product size (bp)	Structural annotation		Functional annotation	
								Sequence components of *kabuli* genome	*Kabuli* gene accession IDs	NCBI-KOG	TFs
Ca7_P3_35166938	Ca7	35166938	AAATAA/AAA	GCACCACGTGAAAAACTACAA	GGTTCTTCGCCTTCACAAAC	58.7	437	INTERGENIC	--	--	--
Ca7_P3_35170170	Ca7	35170170	TAAAAA/TAAAA	TGACTTTTGGTGGCTAGGGT	TTTTGTCCATTTCGTATCCCA	59.6	362	INTERGENIC	--	--	--
Ca7_P3_35173494	Ca7	35173494	ATTTTTTTT/ATTTTTTTTTTTT	CTCCTTGCTCAGATTCTTGTGA	TTTTTAAATCAAGATTTCCTTCCTC	59.6	725	INTERGENIC	--	--	--
Ca7_P3_35175205	Ca7	35175205	GAAAAA/GAAAAAA	TACTTTGCGTGTTCTCGTCG	CCCATGAAGGAAAAATAGCG	60.1	657	INTERGENIC	--	--	--
Ca7_P3_35177660	Ca7	35177660	A/ATAAAAATAAAT	GCCAACCGACTCCATCTTTA	TTTAGCAGTTTTTCAATTTCCAA	60.1	690	INTERGENIC	--	--	--
Ca7_P3_35177885	Ca7	35177885	GAAAAA/GAAAAAA	ACTTGCAAATTGCCCACTGT	TTGAAGAGGGTACAATAGACATTGA	60.6	527	INTERGENIC	--	--	--
Ca7_P3_35178628	Ca7	35178628	TT/TTCT	TTTTCAAACTTTTCCCCTCG	TCGTTCATCAAATCACGAGAA	59.2	241	INTERGENIC	--	--	--
Ca7_P3_35190083	Ca7	35190083	TGG/TG	GCATTGTTGTTTTTGGAGGG	CATGGACAAAGTCGTTTCAA	60.3	506	INTERGENIC	--	--	--
Ca7_P3_35192395	Ca7	35192395	ATTT/ATTTTT	TCTCCACTTGCGGAAGAGAT	GAAAACAAGGCAAAGTCCCA	60.0	735	DRR	Ca_17640	--	Trihelix
Ca7_P3_35206628	Ca7	35206628	GT/GTAT	CGGACATCTGTTTGTCCTTG	TCCCATTTTATTTGTCCCCA	59.1	334	INTERGENIC	--	--	--

INDEL marker IDs	Chromosomes/ unanchored scaffolds	Physical positions (bp)	InDels (*Kabuli* reference genome-CDC Frontier/PII)	Forward primers (5'-3')	Reverse primers (5'-3')	Anneling temperature (0C)	Expected amplified product size (bp)	Structural annotation		Functional annotation	
								Sequence components of *kabuli* genome	*Kabuli* gene accession IDs	NCBI-KOG	TFs
Ca7_P3_35210755	Ca7	35210755	TAAAA/TAAAAA	TTGAGTTTTCCTTCATT GGTATG	ATATCCGACACTGCTC ACCC	58.2	824	INTERGENIC	--	--	--
Ca7_P3_3521546	Ca7	3521546	CTTT/CTTTTT	TAAGGCTATCCACGTC GTCC	ACCAGCTTCACTCTCT GGGA	60.1	785	INTRON	Ca_03116	I	--
Ca7_P3_35217838	Ca7	35217838	T/TAAAA	AAGGCTAGGATTCTCG GGAT	ATGCGGGATGTTCTTT TCAT	59.2	369	INTERGENIC	--	--	--
Ca7_P3_35217866	Ca7	35217866	ATTTTTT/ATTTTTTT	AAGGCTAGGATTCTCG GGAT	ATGCGGGATGTTCTTT TCAT	59.2	369	INTERGENIC	--	--	--
Ca7_P3_35225570	Ca7	35225570	GAAAAAAAAA/GAAAAAAAAAA	CACCTCCACTTTCTTTC CGA	TGAGAAAAATTAAATG GGGACG	60.2	537	INTERGENIC	--	--	--
Ca7_P3_35225622	Ca7	35225622	GTTTTTTTTTT/GTTTTTTTTT	CACCTCCACTTTCTTTC CGA	TGAGAAAAATTAAATG GGGACG	60.2	537	INTERGENIC	--	--	--
Ca7_P3_35227195	Ca7	35227195	GA/GACA	AAAGCGAACGCCATTTT AGA	TCAGAGCAACTGCACT TCAA	59.9	809	INTERGENIC	--	--	--
Ca7_P3_3522923	Ca7	3522923	CTT/CTTT	CCACTTTGCCTAGGAG CAAC	TTCCCCAGTGTCAAAG AAGG	59.9	723	INTRON	Ca_03116	I	--
Ca7_P3_35230084	Ca7	35230084	CTT/CTTT	TTTTGGTTGCAGTTTGG TTT	TCTTCCCATTTCCATTT TTGA	58.2	402	DRR	Ca_17641	--	--
Ca7_P3_35230846	Ca7	35230846	ATTTT/ATTT	AAGTAGCAAAGCCTCA CCCA	CGGATAATTTCAAATG GATCACT	59.9	401	INTERGENIC	--	--	--

INDEL marker IDs	Chromosomes/ unanchored scaffolds	Physical positions (bp)	InDels (*Kabuli* reference genome-CDC Frontier/PII)	Forward primers (5'-3')	Reverse primers (5'-3')	Anneling temperature (0C)	Expected amplified product size (bp)	Structural annotation		Functional annotation	
								Sequence components of *kabuli* genome	*Kabuli* gene accession IDs	NCBI-KOG	TFs
Ca7_P3_35232044	Ca7	35232044	AA/AAATTA	TTTGACCCTCAGCCATTTTC	GAAAAATGGGAATGCAATGG	60.1	244	INTERGENIC	--	--	--
Ca7_P3_35256420	Ca7	35256420	ATTTTTTTTTTTT/ATTTTTTTTTT	CATTGTGGACTTGTCGGAGA	CTCACACGGTGCTTCTCAAA	59.7	557	INTERGENIC	--	--	--
Ca7_P3_35273690	Ca7	35273690	C/CTTAA	GAGGGGCGTGAGTAACAAAA	AAAGAACCACATGAATCGCC	60.1	882	INTERGENIC	--	--	--
Ca7_P3_35275595	Ca7	35275595	ATTTT/ATTTTT	TTGAAGGTGCCTCTGAAACC	CTCATGAAGGATCGCGGTAT	60.2	479	DRR	Ca_17646	R	--
Ca7_P3_35282492	Ca7	35282492	CTT/CT	GAGCCAGCTCTGTGTGTTGA	ACAGCAGGAGCATACAGCCT	60.2	451	INTRON	Ca_17646	R	--
Ca7_P3_3528413	Ca7	3528413	GTT/GT	GGGGAACACATCAAAACCAC	TTTTCCGGTACAACCGAATC	60.1	266	INTERGENIC	--	--	--
Ca7_P3_35292837	Ca7	35292837	AGG/AGGG	TGCAGGATTTAGTGGTGGTG	GACCCCTCTTTGTAACGCAA	59.6	154	INTERGENIC	--	--	--
Ca7_P3_35293466	Ca7	35293466	TAAAAAAAAAA/TAAAAAAAAAAAAA	AATTCCACCAGCTCCAAGTG	TCGGTGCAACATTTTCTTCA	60.1	359	INTERGENIC	--	--	--
Ca7_P3_3529498	Ca7	3529498	TTATATATATATATA/TTATATATATATA	TGTACACACTTATTCGGTCTCATTT	CCATCCCTCCCCAAATTTTA	59.0	379	INTERGENIC	--	--	--
Ca7_P3_35299260	Ca7	35299260	ATTTTT/ATTTTTT	TTGGAGAGAAAGCAAAATGGA	AGAGTTTTGGTCCCGTTCCT	59.8	336	INTERGENIC	--	--	--

INDEL marker IDs	Chromosomes/ unanchored scaffolds	Physical positions (bp)	InDels (*Kabuli* reference genome-CDC Frontier/PII)	Forward primers (5'-3')	Reverse primers (5'-3')	Anneling temperature (0C)	Expected amplified product size (bp)	Structural annotation		Functional annotation	
								Sequence components of *kabuli* genome	*Kabuli* gene accession IDs	NCBI-KOG	TFs
Ca7_P3_35302573	Ca7	35302573	GTTTTT/GTTTTTT	TTTGAACAGCAATCCAACCA	AGTGAACAACCAACCCATTTT	60.1	299	INTERGENIC	--	--	--
Ca7_P3_35305750	Ca7	35305750	GAAAAAAAA/GAAAAAAA	AGGGAAGCTCCAAAGGTCAT	ATGGATAATTGCGACGGTTC	60.1	631	INTERGENIC	--	--	--
Ca7_P3_35313643	Ca7	35313643	TC/T	TTCGATAAAGCAAAAGCTCCA	AACTGAATATGGGCCAGTCG	60.0	805	INTERGENIC	--	--	--
Ca7_P3_35316497	Ca7	35316497	ATTTTTTTT/ATTTTTTTTT	TTTTGGTTCGTTTGATTCGAG	TTGGCGAAGGAGAGTCAGAT	60.1	757	INTERGENIC	--	--	--
Ca7_P3_35317068	Ca7	35317068	AT/ATT	TGACTCTCCTTCGCCAAGAT	CAATCACCATCGACCATCAA	60.0	444	INTERGENIC	--	--	--
Ca7_P3_35317088	Ca7	35317088	GTTTTTTTTTTT/GTTTTTTTTT	TGACTCTCCTTCGCCAAGAT	CAATCACCATCGACCATCAA	60.0	444	INTERGENIC	--	--	--
Ca7_P3_35319735	Ca7	35319735	TAAA/TAA	TGCGTAAACAAAATACGAGAAAAA	ACTCAATACAACGAACGGGC	60.1	239	INTERGENIC	--	--	--
Ca7_P3_35319763	Ca7	35319763	CAAAAAAAAAAAA/CAAAAAAAAAAAAAA	TGCGTAAACAAAATACGAGAAAAA	ACTCAATACAACGAACGGGC	60.1	239	INTERGENIC	--	--	--
Ca7_P3_35330032	Ca7	35330032	TTCT/TTCTCTCT	TCACGTGCACTATGCCTCTC	TCGGTGATGGGTATTTGACA	60.0	421	INTRON	Ca_17647	BK	FAR1
Ca7_P3_35331728	Ca7	35331728	A/AG	GTTGCTGATCTGAGGGAAGC	ATCTACATTGCAGGGGTCCA	60.0	727	INTRON	Ca_17647	BK	FAR1

INDEL marker IDs	Chromosomes/ unanchored scaffolds	Physical positions (bp)	InDels (*Kabuli* reference genome-CDC Frontier/PII)	Forward primers (5'-3')	Reverse primers (5'-3')	Anneling temperature (0C)	Expected amplified product size (bp)	Structural annotation		Functional annotation	
								Sequence components of *kabuli* genome	*Kabuli* gene accession IDs	NCBI-KOG	TFs
Ca7_P3_35331861	Ca7	35331861	TTATAT/TTAT	GTTGCTGATCTGAGGGAAGC	TGGCATGCACTGAAAAATGT	60.0	860	INTRON	Ca_17647	BK	FAR1
Ca7_P3_35332956	Ca7	35332956	CAAAAA/CAAAA	AGCGGGAGGAATTGAAAGTT	AAACCCGGACCAGATATTGA	60.1	483	DRR	Ca_17647	BK	FAR1
Ca7_P3_35334121	Ca7	35334121	A/ATTT	TATCCTCCGCTTGGCTTATG	TGTTCATTGTGTTTGGTTTGG	60.2	838	DRR	Ca_17647	BK	FAR1
Ca7_P3_35337950	Ca7	35337950	C/CT	CATGATTGCTTTCCATGCAG	GGCTAGGGATGAACTACCCC	60.2	345	INTRON	Ca_17648	--	TCP
Ca7_P3_35350676	Ca7	35350676	A/AT	CATACCGAAAATGCGACCTT	CCTCACCAAGCTTTGCTACC	60.0	565	DRR	Ca_17650	PT	FAR1
Ca7_P3_35357002	Ca7	35357002	CTTTTTTTTT/CTTTTTTTTTTT	TGGATGTCATGTTTCTTAATGGA	CACATACGGGGTTTTAGATTCG	59.3	657	INTERGENIC	--	--	--
Ca7_P3_35357787	Ca7	35357787	ATT/AT	AAGTTCGAATCTAAAACCCCG	CCCCATTTTCCAATCCTTTT	59.5	768	INTERGENIC	--	--	--
Ca7_P3_35364737	Ca7	35364737	AGATTAGGA/AGA	CGCATTCCTCTCGAAAAGTC	TTGGAGAAAGATCTCCGGTT	60.0	439	INTERGENIC	--	--	--
Ca7_P3_35365053	Ca7	35365053	T/TCC	CGCATTCCTCTCGAAAAGTC	CAAACACACTCTTGGGAAATCA	60.0	935	INTERGENIC	--	--	--
Ca7_P3_35369159	Ca7	35369159	CAAAAAAAA/CAAAAAAAAA	CCAAAGGCATTTGAGGATTC	TTGCCATTCTTTGACACACA	59.5	308	DRR	Ca_17651	--	--

INDEL marker IDs	Chromosomes/ unanchored scaffolds	Physical positions (bp)	InDels (*Kabuli* reference genome-CDC Frontier/PII)	Forward primers (5'-3')	Reverse primers (5'-3')	Anneling temperature (0C)	Expected amplified product size (bp)	Structural annotation		Functional annotation	
								Sequence components of *kabuli* genome	*Kabuli* gene accession IDs	NCBI-KOG	TFs
Ca7_P3_35370023	Ca7	35370023	CCACACA/CCACA	TTGACCGAATTTTGCATGTC	CCCGTCATGGCTCAAATTAT	59.5	621	DRR	Ca_17651	--	--
Ca7_P3_35370294	Ca7	35370294	TCTAC/TCTACTAC	ATAATTTGAGCCATGACGGG	TCTTGACAGGTTTGGTTGGA	59.8	128	DRR	Ca_17651	--	--
Ca7_P3_35376790	Ca7	35376790	AGTG/AGTGGTG	GTAAAAGGCGTATGACGGGA	CTATTTTCCGGAATCCACGA	60.0	459	INTERGENIC	--	--	--
Ca7_P3_35377784	Ca7	35377784	CAGAGAGAGAGAGAGAGA/CAGAGAGAGAGAGAGA	ACCCCACCACAAAGTTCAAG	TTCCCAGCAACAAACCTTTC	59.9	245	INTERGENIC	--	--	--
Ca7_P3_35378747	Ca7	35378747	ATT/AT	GGACCCAAATGTGACCTATTGA	CACCGATCATCATTGCTTTA	61.0	825	INTERGENIC	--	--	--
Ca7_P3_35379689	Ca7	35379689	AAAT/A	TCCTCATCACCTGCACAATC	TCCTCTACCCACCAAACCAA	59.6	445	INTERGENIC	--	--	--
Ca7_P3_35380408	Ca7	35380408	ATTT/ATTTT	TGGTTTGGTGGGTAGAGGAA	AAATATCTCCATGGGGGAGG	60.3	523	INTERGENIC	--	--	--
Ca7_P3_35382567	Ca7	35382567	G/GCTAA	CCGATCCAATTATATCGACAG	TTATCAAGGCTCCCACCATC	57.1	742	INTERGENIC	--	--	--
Ca7_P3_35387341	Ca7	35387341	GATATATATATAT/GATATATATAT	AATTTTTCACTCAACCCCCG	TTGTGCGATGTCTCAACCAT	61.1	512	INTERGENIC	--	--	--
Ca7_P3_3540137	Ca7	3540137	GTAT/GTATATAT	TTGTTGGTACGTGCATAGAGC	CATTCACTACGTGCAAACGG	58.9	762	INTERGENIC	--	--	--

INDEL marker IDs	Chromosomes/ unanchored scaffolds	Physical positions (bp)	InDels (*Kabuli* reference genome-CDC Frontier/PII)	Forward primers (5'-3')	Reverse primers (5'-3')	Anneling temperature (0C)	Expected amplified product size (bp)	Structural annotation		Functional annotation	
								Sequence components of *kabuli* genome	*Kabuli* gene accession IDs	NCBI-KOG	TFs
Ca7_P3_3540357	Ca7	3540357	CTCAAAAT/CT	TCAACAATGAAGCAACACATCA	CATTGCGCAAGAGAATACGA	60.2	634	INTERGENIC	--	--	--
Ca7_P3_35424480	Ca7	35424480	ATTTTTTT/ATTTTTTTT	ATGAATTTGGCGTGTTGGTT	GTGACCGAACTTGAGCCAAT	60.2	369	INTERGENIC	--	--	--
Ca7_P3_3545324	Ca7	3545324	ATT/AT	TTGACCCAAAAGATAAGCCG	TTGTTGGTTATCGAAGAAGGAAA	60.1	865	DRR	Ca_03114	S	--
Ca7_P3_35490894	Ca7	35490894	TAAAAAA/TAAAAAAA	AATGCACGAGTGCCATATTTC	CAACAACTTCTCATGTTCGCA	60.0	557	INTERGENIC	--	--	--
Ca7_P3_35578932	Ca7	35578932	CAAAAAAAAAAAA/CAAAAAAAAAAA	TGTAACGGCCACATAGGTCA	TTGATTGCTGGTTTGTTGGA	60.0	280	DRR	Ca_17765	U	--
Ca7_P3_35605462	Ca7	35605462	CATATATATATATATATATATATA/CATATATATATATATATATATATA	TCAACCATTCCGAGTGCATA	CGCCGTAAATGGCTTAAAAA	60.1	667	INTERGENIC	--	--	--
Ca7_P3_3564384	Ca7	3564384	ACT/A	TGCACTTCCAATGACCAGAG	TAGCCAAATGGGCTTAGGTG	59.8	670	DRR	Ca_03113	T	bHLH
Ca7_P3_35645685	Ca7	35645685	TTATATATATATATATATATATATATATATAT/TTATATATATATATATATATATATATAT	TTTGTCTCGTTTGAATTTGTGG	CATCATTTCATTCCAACCCC	60.0	468	INTERGENIC	--	--	--
Ca7_P3_3566099	Ca7	3566099	AATAT/AAT	GGCAGTTGCTCCTGAATAGC	GGATCAATTTGTGAATTCGTGA	60.0	519	INTERGENIC	--	--	--
Ca7_P3_35661773	Ca7	35661773	AATATATATATATATATATATAT/AATATATATATATATATAT	TTTGTGTTTGAGCGAAGACG	TGAACTTGTGTTGCTCCTGC	60.0	603	INTRON	Ca_17773	--	--

INDEL marker IDs	Chromosomes/ unanchored scaffolds	Physical positions (bp)	InDels (*Kabuli* reference genome-CDC Frontier/PII)	Forward primers (5'-3')	Reverse primers (5'-3')	Anneling temperature (0C)	Expected amplified product size (bp)	Structural annotation		Functional annotation	
								Sequence components of *kabuli* genome	*Kabuli* gene accession IDs	NCBI-KOG	TFs
Ca7_P3_3567096	Ca7	3567096	CAA/CA	TTGTGCAGGTCGTGGTACAT	TTTCACAACTCCCCATTTCC	60.0	450	INTERGENIC	--	--	--
Ca7_P3_357521	Ca7	357521	CTTT/CTTTT	TATTGGTTCCTTATGGGGCA	AGCTCTGGAGACCTCCTTCC	60.1	581	INTERGENIC	--	--	--
Ca7_P3_35786413	Ca7	35786413	TTATTAATATTA/TTATTA	GGGAAACCCACCTTTTGATT	GGAGGGTGTGAATTGGAAGA	60.0	679	INTERGENIC	--	--	--
Ca7_P3_35787388	Ca7	35787388	A/AG	CCTTCCAATGTAGCTGATGTTTT	TGGACGCAAATAGATGCAAA	59.6	540	INTERGENIC	--	--	--
Ca7_P3_3580383	Ca7	3580383	ATTT/ATTTT	TGTCAACAAAAACCCAAGCA	TTTGACTGCTGGCTTGACTG	60.1	339	INTRON	Ca_03112	U	MYB
Ca7_P3_35883375	Ca7	35883375	GTTTTTTTTTTTTT/GTTTTTTTTTTTT	TGATTGTTTTGGTAAGAAAATGGA	CAACCTCAAATTCCCTGCAT	59.8	527	INTERGENIC	--	--	--
Ca7_P3_35892068	Ca7	35892068	ACTTCTTCTTCTTCTTCTTCTTCTTCTTCTTCTTCTTCT/ACTTCTTCTTCTTCTTCTTCTTCTTCTTCTTCTTCTTCTTCT	CTGACGTGTTGGTGTGGAAC	ATAGTCAAGCCCTGCAGCAT	60.0	379	INTRON	Ca_17793	L	--
Ca7_P3_3594126	Ca7	3594126	GTTTTTTT/GTTTTTTTTT	ATTGCGGCTATACCAGATGC	AGAATGGAGCAACAGCGACT	60.1	261	DRR	Ca_03109	--	NAC
Ca7_P3_35962308	Ca7	35962308	CTATATATATATATATATATATA/CTATATATATATATATATATATATATATATA	ATTGGGCTGAGCTTTGCTTA	CAGACCGTTTCACAGGGATT	60.0	667	INTERGENIC	--	--	--
Ca7_P3_35962887	Ca7	35962887	TAAAAAAAAAA/TAAAAAAAAA	AATCCCTGTGAAACGGTCTG	TATGCAGGGGTAACACGTTG	60.0	893	INTERGENIC	--	--	--

INDEL marker IDs	Chromosomes/ unanchored scaffolds	Physical positions (bp)	InDels (*Kabuli* reference genome-CDC Frontier/PII)	Forward primers (5'-3')	Reverse primers (5'-3')	Anneling temperature (0C)	Expected amplified product size (bp)	Structural annotation		Functional annotation	
								Sequence components of *kabuli* genome	*Kabuli* gene accession IDs	NCBI-KOG	TFs
Ca7_P3_35977553	Ca7	35977553	AATGA/AATGATGA	TGAGTGCGATGGTTCAGAAG	GCGATTCATCATTCTCCGTT	60.0	608	INTERGENIC	--	--	--
Ca7_P3_3600073	Ca7	3600073	C/CATA	GCTTTTCTGGTCCTGCTTTG	GGTGTCTGGTCTTTGACCGT	60.0	424	INTRON	Ca_03108	--	NAC
Ca7_P3_3600373	Ca7	3600373	AC/ACTC	ACGGTCAAAGACCAGACACC	GTTGATGGCTGGACCACTTT	60.0	350	INTRON	Ca_03108	--	NAC
Ca7_P3_3602053	Ca7	3602053	CTTTTT/CTTTT	GGGGAAAATATCCAAAGGGA	GAAGGGCTCCAAAGTCACTG	60.0	547	INTERGENIC	--	--	--
Ca7_P3_3602121	Ca7	3602121	GTAT/GTATATAT	GGGGAAAATATCCAAAGGGA	GAAGGGCTCCAAAGTCACTG	60.0	547	INTERGENIC	--	--	--
Ca7_P3_3602980	Ca7	3602980	TTGAGCTCTG/TTG	AATTCTATGCAGTCGGTGGG	TGGCAGACTCTATGGAACCAA	60.0	157	DRR	Ca_03107	--	NAC
Ca7_P3_36051090	Ca7	36051090	AATATATATATATAT/AATATATATATATATAT	CAATGAAAACCCCCATTCTG	TCATGCAAATTCAAAAGAAATGA	60.2	911	INTERGENIC	--	--	--
Ca7_P3_3608175	Ca7	3608175	CA/CAA	TTTTCGTGGGATCAAATTCC	ATTCACGAACCTGCGATACC	59.7	495	INTERGENIC	--	--	--
Ca7_P3_3608219	Ca7	3608219	GT/GTCCCAATTT	TTTTCGTGGGATCAAATTCC	ATTCACGAACCTGCGATACC	59.7	495	INTERGENIC	--	--	--
Ca7_P3_3609653	Ca7	3609653	ATTTTTTT/ATTTTTT	TGCATGTGATGTGATGTGATG	CCCCGTGAGCTAGGCTAAAT	60.0	773	INTERGENIC	--	--	--

INDEL marker IDs	Chromosomes/ unanchored scaffolds	Physical positions (bp)	InDels (*Kabuli* reference genome-CDC Frontier/PII)	Forward primers (5'-3')	Reverse primers (5'-3')	Anneling temperature (0C)	Expected amplified product size (bp)	Structural annotation		Functional annotation	
								Sequence components of *kabuli* genome	*Kabuli* gene accession IDs	NCBI-KOG	TFs
Ca7_P3_36117059	Ca7	36117059	CTTT/CTTTTCTTTATTTT	TGGATTGGGCTTTCCA ATAA	TGCCTTAACGTACCCA AACA	60.3	435	DRR	Ca_13721	G	Trihelix
Ca7_P3_3612730	Ca7	3612730	G/GTTTAA	CTATGGCATCGACATT GCAT	ATCGAGAGATCGCGAG AGAG	59.5	803	INTERGENIC	--	--	--
Ca7_P3_3612993	Ca7	3612993	ATATAATT/AT	AACACTCCGTATGTCC CACC	ATCGAGAGATCGCGAG AGAG	59.7	148	INTERGENIC	--	--	--
Ca7_P3_3613058	Ca7	3613058	CAGAA/CAGAAGAA	AACACTCCGTATGTCC CACC	CAACCCAACACCCAAC TACA	59.7	433	INTERGENIC	--	--	--
Ca7_P3_3615926	Ca7	3615926	TAAAAAAA/TAAAAAA	CATGTTCTTGTGCCGTT TTG	GGCCCATTGATTTTGA TTTG	60.1	438	INTERGENIC	--	--	--
Ca7_P3_3621838	Ca7	3621838	GAAAAAA/GAAAAAAA	TTCTCGAGTCCCTTAAC TTGATTT	TTTGGTGAGTGAGGTT AAGAAGC	59.7	839	INTERGENIC	--	--	--
Ca7_P3_362262	Ca7	362262	TTAT/TT	TGATGTGTTGTACATTG ATTTGTG	TAGTCGGCCACAAGAA GACA	58.4	533	INTERGENIC	--	--	--
Ca7_P3_36243491	Ca7	36243491	TAAAAAAAA/TAAAAAAAAA	TGTATCGCGTTAATCTG CGT	TTTGGTTTCATGGATG TGGA	59.4	886	INTERGENIC	--	--	--
Ca7_P3_3632331	Ca7	3632331	GT/GTT	TTTGCAGGTGGACGTT GTTA	TACTTCCGATCGATTC GTCC	60.1	840	INTRON	Ca_03106	E	--
Ca7_P3_36358822	Ca7	36358822	AAGAGAGAGAGAGAGAGAGAGA/AAG AGAGAGAGAGAGAGAGA	AAAACCCCAACTCAACC GTA	TCGCGCTTTCTTTCTC TCTC	59.3	587	INTERGENIC	--	--	--

INDEL marker IDs	Chromosomes/ unanchored scaffolds	Physical positions (bp)	InDels (*Kabuli* reference genome-CDC Frontier/PII)	Forward primers (5'-3')	Reverse primers (5'-3')	Anneling temperature (0C)	Expected amplified product size (bp)	Structural annotation		Functional annotation	
								Sequence components of *kabuli* genome	*Kabuli* gene accession IDs	NCBI-KOG	TFs
Ca7_P3_3636919	Ca7	3636919	T/TTCACATGAGA	TTTTTGAGGGACACCAAATCTT	GTGGTAGTGGTAGGGTCGGA	59.9	745	DRR	Ca_03106	E	--
Ca7_P3_36439529	Ca7	36439529	ATTTTTTT/ATTTTTTTT	CGCTTTTTCCGTCTTAGTGC	TCATTTCGATAATTTCACACAAA	60.0	628	INTERGENIC	--	--	--
Ca7_P3_3649235	Ca7	3649235	GAATCCAATATCTA/GAATCCAATATCTAAAATCCAATATCTA	GTCCGAGTCCTAACAGCAGC	TCATGGTGAGATTGGCAAAA	60.0	722	DRR	Ca_03103	U	LBD
Ca7_P3_3650792	Ca7	3650792	A/AATATTAG	ATTACCTGGTCGCAGCAAAC	AGCTGCGTGCTCCTATCATT	60.1	616	INTRON	Ca_03103	U	LBD
Ca7_P3_3654337	Ca7	3654337	GTGGGAATCTTTT/GTGGGAATCTTTTCATGGGAATCTTTT	AAAGCAAACTCAGCAACGGT	TATCAGAGCCTTCCATTGGG	59.9	427	INTRON	Ca_03103	U	LBD
Ca7_P3_36548120	Ca7	36548120	TAAAA/TAAAAAA	ACAACCAAGCGTGTCGTGTA	TGTTTGTTGGAGAAATGCCA	60.2	213	DRR	Ca_13744	O	--
Ca7_P3_3659184	Ca7	3659184	A/AC	TTTAGGTGCGCATTCATCTG	TTAAAGGGTTTCGGCTTCCT	59.8	747	INTRON	Ca_03103	U	LBD
Ca7_P3_3662479	Ca7	3662479	TA/T	GTTTCGTGCAGTGTGGATTG	GGAGTGTTTCACAAGCCGAT	60.2	448	INTRON	Ca_03103	U	LBD
Ca7_P3_36641003	Ca7	36641003	CTTT/CTT	ACCCAAATTAAATCGAACGG	ATGTAAATGCTTTTTCGGCG	58.8	456	INTERGENIC	--	--	--
Ca7_P3_36665611	Ca7	36665611	AATATATATATATATATAT/AATATATATATATATATATAT	AGACCAATTTCGTGAGTCAACA	CAAGGGCTAAGGATCGAGTG	59.6	850	INTERGENIC	--	--	--

INDEL marker IDs	Chromosomes/ unanchored scaffolds	Physical positions (bp)	InDels (*Kabuli* reference genome-CDC Frontier/PII)	Forward primers (5'-3')	Reverse primers (5'-3')	Anneling temperature (0C)	Expected amplified product size (bp)	Structural annotation		Functional annotation	
								Sequence components of *kabuli* genome	*Kabuli* gene accession IDs	NCBI-KOG	TFs
Ca7_P3_3668391	Ca7	3668391	GC/GCC	GCGGGTTTTTGACAAT GTTT	TTACTTTTGACGGGAA ACGG	59.8	618	INTERGENIC	--	--	--
Ca7_P3_3670152	Ca7	3670152	TAGAAG/TAGAAGAAG	CACCAAAGCTGGTCAA AGGT	CCACCATCACCTATTG CCTT	60.1	293	DRR	Ca_03102	TU	--
Ca7_P3_36717770	Ca7	36717770	AATATATATATATATATA/AATATATATA TATATATATA	TGCCTTTAACAATAATC GAGAATG	TGGTTTGGTTCCATCA GGTT	59.6	803	DRR	Ca_13757	S	--
Ca7_P3_3671929	Ca7	3671929	AG/A	TGGAAGGGTAGAAGTG GTGG	TACAGTTTCAGCCTGG GTCC	60.0	700	DRR	Ca_03102	TU	--
Ca7_P3_3674765	Ca7	3674765	ATT/ATTT	AGCTCACATCCCATTCA ACC	CGATCCTGTGGGAGAA CATT	59.9	654	INTRON	Ca_03102	TU	--
Ca7_P3_36774662	Ca7	36774662	TTTATTATTATTATTATTATTATTATTAT/ TTTATTATTATTATTATTATTATTATTAT TATTATTATTATTAT	TGGCCTGACCTACTTAT GGC	GGCCTGAGTCTGACCT ACGA	60.1	580	DRR	Ca_13762	I	BES1
Ca7_P3_3679071	Ca7	3679071	T/TGGTTA	GTTGCGCCGCATTATTT TAT	TGAAAACTGAACCAAA CCGA	60.0	409	INTERGENIC	--	--	--
Ca7_P3_3679152	Ca7	3679152	AT/A	GTTGCGCCGCATTATTT TAT	TGAAAACTGAACCAAA CCGA	60.0	409	INTERGENIC	--	--	--
Ca7_P3_3679296	Ca7	3679296	GC/GCAAAAAAAATATC	GAAAGGGGCTCAGAGG TAGG	GGTAATCACCAAAGTC CATGC	60.2	940	INTERGENIC	--	--	--
Ca7_P3_36803710	Ca7	36803710	TTT/TTTTATT	AAGTGATCATTTGATGC ACAGG	TGACATGTGCAAATTG AAAGG	60.0	720	INTERGENIC	--	--	--

INDEL marker IDs	Chromosomes/ unanchored scaffolds	Physical positions (bp)	InDels (*Kabuli* reference genome-CDC Frontier/PII)	Forward primers (5'-3')	Reverse primers (5'-3')	Anneling temperature (0C)	Expected amplified product size (bp)	Structural annotation		Functional annotation	
								Sequence components of *kabuli* genome	*Kabuli* gene accession IDs	NCBI-KOG	TFs
Ca7_P3_3682108	Ca7	3682108	TCGATTC/T	CCATTTGCAGGATTCAAGGT	GGTGCTTTGCTACCTAGCCTT	59.9	629	INTRON	Ca_03101	--	--
Ca7_P3_36821484	Ca7	36821484	TATATATATATATATATATATATATATAT/TATATATATATATATATATATATATATATATAT	AACGGTGAACTGCCTTATGC	AACAGGCCCTAATGGGCTAC	60.1	646	INTERGENIC	--	--	--
Ca7_P3_3689483	Ca7	3689483	TTA/T	AACCAATGACCATCGAGAATC	ACTCCTCTTCCCCCTACCAT	58.9	640	DRR	Ca_03100	S	--
Ca7_P3_3689525	Ca7	3689525	GTTTTT/GTTTT	AACCAATGACCATCGAGAATC	TTTCCCAACGGTATCCACAT	58.9	914	DRR	Ca_03100	S	--
Ca7_P3_3689798	Ca7	3689798	TAGAGAG/TAGAG	ATGGTAGGGGGAAGAGGAGT	CTTTTCGTTTGGCCAATGTT	58.9	381	DRR	Ca_03100	S	--
Ca7_P3_37005703	Ca7	37005703	T/TAATAATAATAAA	GCATATCCAAGATGCGACCT	GCAAAAGCTGAAAAAGTGGC	60.1	550	INTERGENIC	--	--	--
Ca7_P3_3705330	Ca7	3705330	AATATATATATATATA/AATATATATATATATATA	CTTTGCAAATGGCGACTTCT	CTTTCGTGGAGCATGAGACA	60.4	218	INTERGENIC	--	--	--
Ca7_P3_37073262	Ca7	37073262	ATTTTTT/ATTTTTTT	CCCCAAAATCTTTAAGACAAACC	GACACGTTGACGGATTTTGA	60.1	345	INTERGENIC	--	--	--
Ca7_P3_37147869	Ca7	37147869	CATATATATATATATATATATATATATATATAT/CACACACATATATATATATATATATATATATATATATATAT	GGAGAAGGAGGGAGAAATGG	TGCGTTACGGTGAAATGGTA	60.0	283	INTERGENIC	--	--	--
Ca7_P3_37169083	Ca7	37169083	AAATAATAATAATAATAATAATAATAA/AAATAATAATAATAATAATAATAATAATAA	GCTTATCGCAACATGGGAAT	TTGACCTTGTGTTGCTCCAG	59.9	509	INTERGENIC	--	--	--

INDEL marker IDs	Chromosomes/ unanchored scaffolds	Physical positions (bp)	InDels (*Kabuli* reference genome-CDC Frontier/PII)	Forward primers (5'-3')	Reverse primers (5'-3')	Anneling temperature (0C)	Expected amplified product size (bp)	Structural annotation		Functional annotation	
								Sequence components of *kabuli* genome	*Kabuli* gene accession IDs	NCBI-KOG	TFs
Ca7_P3_37171389	Ca7	37171389	TAAAAAAAAAA/TAAAAAAAAAAA	ATGACCGCACCTCAAATAGG	CGACTTTTCAAGCATTGGGT	60.0	438	INTERGENIC	--	--	--
Ca7_P3_37177641	Ca7	37177641	TAAAAAAAAA/TAAAAAAAAAA	CCTGACATAGGGAGAGCGTT	TGTTTCGTTTTGCCAAAGATT	59.3	565	INTERGENIC	--	--	--
Ca7_P3_3732489	Ca7	3732489	TTATATATATAT/TTATATATATATAT	AAGTGGCATATTTTCTCTTTCCA	TACTAAAGCCAATGACCGGC	59.2	208	DRR	Ca_03096	T	--
Ca7_P3_37520348	Ca7	37520348	ACTTATTT/A	GGAAGTAGAAGGGTTGGGGA	GGGATTTGTTTGGAAGGGTT	60.3	376	INTERGENIC	--	--	--
Ca7_P3_37538296	Ca7	37538296	CTTTT/CTTT	TTTGAATCCCATGATGCGTA	TCCCAACCCAAATAAACCAA	59.9	283	INTERGENIC	--	--	--
Ca7_P3_3754420	Ca7	3754420	GTCTCTCTCTCTCTCTCTCTCTCTCTCT/GTCTCTCTCTCTCTCTCTCTCTCTCTCTCTCTCTCT	AAATCGACGGCCACAGATAC	CACAATGTCCATGCTCCAAC	60.0	703	INTRON	Ca_03092	BK	--
Ca7_P3_37556688	Ca7	37556688	TAAAAAAAAA/TAAAAAAAA	GGGCTGACATTTTCACCAAA	CCAACTTGGTTCCCAAAATC	60.9	400	INTERGENIC	--	--	--
Ca7_P3_37568375	Ca7	37568375	ATTTTTTTTTTT/ATTTTTTTTT	TGTGTTGCAAGTGTGCTTGA	GTTCGGGTTTGTGTTTGCTT	60.1	851	INTERGENIC	--	--	--
Ca7_P3_37569442	Ca7	37569442	TCTGATGCTGATGCTG/TCTGATGCTG	CCAACTGAGAAGCTTTTGGC	GATGCCTCCAACATGTTCCT	60.0	698	INTERGENIC	--	--	--
Ca7_P3_37586910	Ca7	37586910	TAAAAAAAAA/TAAAAAAAA	TCATTGGCCAAAACTTGTGA	CACCGCCATCAAGCTAAAGT	60.1	260	INTERGENIC	--	--	--

| INDEL marker IDs | Chromosomes/ unanchored scaffolds | Physical positions (bp) | InDels (*Kabuli* reference genome-CDC Frontier/PII) | Forward primers (5'-3') | Reverse primers (5'-3') | Anneling temperature (0C) | Expected amplified product size (bp) | Structural annotation | | Functional annotation | |
[truncated: 325,676 more chars]
