# Supplementary material for: A High-Resolution InDel (Insertion–Deletion) Markers-Anchored Consensus Genetic Map Identifies Major QTLs Governing Pod Number and Seed Yield in Chickpea
Source: Front Plant Sci. 2016 Sep 16;7:1362. doi: 10.3389/fpls.2016.01362 (PMC5025440; doi:10.3389/fpls.2016.01362)
Supplement: Supplementary file 5 [file Table5.PDF]

Table S5. InDel markers (17077) differentiating low pod number-containing parental accession (ILWC 46) and homozygous bulk (LPNB) of a mapping population (Pusa 256 x ILWC 46) from reference *kabuli* accession (CDC Frontier)

INDEL marker IDs	Chromosomes/ unanchored scaffolds	Physical positions (bp)	InDels (*Kabuli* reference genome-CDC Frontier/PII)	Forward primers (5'-3')	Reverse primers (5'-3')	Anneling temperature (0C)	Expected amplified product size (bp)	Structural annotation		Functional annotation	
								Sequence components of *kabuli* genome	*Kabuli* gene accession IDs	NCBI-KOG	TFs
Ca1_P4_19084336	Ca1	19084336	TGCAGG/TG	ACAACAAACCGTGCAATCAA	ACCCTGGATGCTTGTATGAA	60.0	631	CDS (FRAME SHIFT)	Ca_17356	--	NF-X1
Ca1_P4_2073961	Ca1	2073961	CTTTT/CTTT	AGAGGCACGAGATTTCCAGA	AAATATCCCCGCAATAAGGG	60.0	336	CDS (FRAME SHIFT)	Ca_00263	T	M-type
Ca1_P4_33781062	Ca1	33781062	CTTTTTTT/CTTTTTTTTT	AATTTCCCAATCACCCACAA	TCCACAATGTCTCCCAACAA	60.0	517	CDS (FRAME SHIFT)	Ca_19460	--	--
Ca1_P4_45244574	Ca1	45244574	TC/TCATAAAGCTCAC	TCGGGGTCTTGCAATAAAAG	GACAATGGGAATTCGCCTAA	60.1	532	CDS (FRAME SHIFT)	Ca_26020	--	--
Ca2_P4_17551687	Ca2	17551687	G/GC	ATGTGGTTGATAAGGCCTCG	AGCAAATGCAATGCTCTGAA	60.0	398	CDS (FRAME SHIFT)	Ca_16000	OE	--
Ca2_P4_3145520	Ca2	3145520	GAAGTAAGT/GAAGT	TCGAACTTCGCTTTGTTGTG	TCTTGAATTCCCCGTAATCG	60.0	385	CDS (FRAME SHIFT)	Ca_10533	--	AP2
Ca2_P4_31960983	Ca2	31960983	CA/C	TATCATCCTGCCAATGCAAA	CCCCCTCCTCCTCTCACTAC	60.0	322	CDS (FRAME SHIFT)	Ca_17822	E	--
Ca2_P4_32424473	Ca2	32424473	TGATGGAAGATA/T	TTGCAATTGTATGCAAGTTTCTTT	CGGATTTTTCAGTGTGAGGG	60.1	775	CDS (FRAME SHIFT)	Ca_10276	--	SAP
Ca2_P4_35715341	Ca2	35715341	CCNNNNNNNNNNC/CC	TCTGCATCACTCTGCTTGCT	GATCAATGGCACACAACAGC	59.9	100	CDS (FRAME SHIFT)	Ca_09742	O	--

INDEL marker IDs	Chromosomes/ unanchored scaffolds	Physical positions (bp)	InDels (*Kabuli* reference genome-CDC Frontier/PII)	Forward primers (5'-3')	Reverse primers (5'-3')	Anneling temperature (0C)	Expected amplified product size (bp)	Structural annotation		Functional annotation	
								Sequence components of *kabuli* genome	*Kabuli* gene accession IDs	NCBI-KOG	TFs
Ca3_P4_13185454	Ca3	13185454	AT/ATTT	GCACACGACCTTCTCGTACA	AATCTGGATCGGGGAAAATC	59.9	302	CDS (FRAME SHIFT)	Ca_22687	--	Trihelix
Ca3_P4_33243720	Ca3	33243720	CTT/CT	GATGTGGCTGCAACAGCTTA	TGACAGACTCGTCGTTCAGG	60.0	462	CDS (FRAME SHIFT)	Ca_12059	--	--
Ca3_P4_35768993	Ca3	35768993	TAAAAAAAAAAA/TAAAAAAAAAAAA	AAAAAGCAAGAGCCCCCTAA	TTTTCCTCAACCCACTCACC	60.2	477	CDS (FRAME SHIFT)	Ca_00863	U	bZIP
Ca4_P4_2032207	Ca4	2032207	TC/T	TGCTGAGTTCGAGGAAAGGT	CACATCTCTTGTTCGCTCCA	60.0	517	CDS (FRAME SHIFT)	Ca_07822	D	--
Ca4_P4_2035160	Ca4	2035160	CAA/CA	TAATGCATGGCCTGACAAAC	TGGGCAGCTTAGACTGGACT	59.5	692	CDS (FRAME SHIFT)	Ca_07822	D	--
Ca4_P4_25975114	Ca4	25975114	TGGGG/TGGGGG	ACACATGTTGGCCAATGAGA	GAGTCCGATTTCCATGAGGA	60.0	621	CDS (FRAME SHIFT)	Ca_16608	--	--
Ca4_P4_27855296	Ca4	27855296	CAAAAAAAA/CAAAAAAAAA	ATCTTCACAAAGCCATTGCC	CGTTCCATGGTGTCTTTGTG	60.1	927	CDS (FRAME SHIFT)	Ca_21302	--	--
Ca4_P4_39780105	Ca4	39780105	GACA/GA	TGAAGCTAAGGCATCCAACA	TTTCCATGTGTCGAGACCCT	59.4	295	CDS (FRAME SHIFT)	Ca_14927	--	--
Ca4_P4_5960166	Ca4	5960166	CTTGTGGTGATGTGT/CTTGTGGTGATGTGTTGTGGTGATGTGT	GAATCAGCCACTGAGTGCAA	AATGCCACCTACCTTCAACG	60.0	257	CDS (FRAME SHIFT)	Ca_03652	--	HD-ZIP
Ca4_P4_8342865	Ca4	8342865	T/TTAAG	GAGCTTCAACCACAAGCACA	GCTTTTTGAGCAACTCGTCC	60.0	691	CDS (FRAME SHIFT)	Ca_08321	--	--

INDEL marker IDs	Chromosomes/ unanchored scaffolds	Physical positions (bp)	InDels (*Kabuli* reference genome-CDC Frontier/PII)	Forward primers (5'-3')	Reverse primers (5'-3')	Anneling temperature (0C)	Expected amplified product size (bp)	Structural annotation		Functional annotation	
								Sequence components of *kabuli* genome	*Kabuli* gene accession IDs	NCBI-KOG	TFs
Ca5_P4_11169613	Ca5	11169613	C/CG	TGAAGCAGTTGTTTGGGTGA	GAGCCCAAATCCAAAATTCA	60.3	225	CDS (FRAME SHIFT)	Ca_23744	A	--
Ca5_P4_23672060	Ca5	23672060	TC/TCGCCC	TGAGTAAGGGGGCTGAAATG	TGAGAGACGGTGGTTTACCC	60.1	822	CDS (FRAME SHIFT)	Ca_17683	--	--
Ca5_P4_25856813	Ca5	25856813	CTTTT/CTTTTT	GAACCCGCGTATGACTTGTT	TTACACTGACGCAAACACCC	60.0	312	CDS (FRAME SHIFT)	Ca_09017	S	--
Ca5_P4_31824861	Ca5	31824861	CAA/CAAA	GTGGCATCGAGGAGATAAGC	CGACGTTAGGGGTCTTTTGA	59.8	596	CDS (FRAME SHIFT)	Ca_04889	--	SAP
Ca5_P4_38085194	Ca5	38085194	ATT/AT	TTTACTTGTTTCGCACGCAG	CAATTTACCGGCGTTTCCTA	60.1	233	CDS (FRAME SHIFT)	Ca_01410	--	--
Ca5_P4_41768893	Ca5	41768893	TGG/TG	ATGGCGTATATTCTGGTCCG	ACGCCCAAAATGATACAAGC	59.8	238	CDS (FRAME SHIFT)	Ca_19205	O	--
Ca6_P4_12233101	Ca6	12233101	TG/TGG	CCTCAACGTGAGTTGCAGAA	TGCATGGCATTTGGATAAGA	60.0	344	CDS (FRAME SHIFT)	Ca_24847	--	--
Ca6_P4_1349360	Ca6	1349360	A/ACTCT	ATGCCTCATTTCCCAACAAG	TTCTGAGTGGCACCCTATCC	59.9	165	CDS (FRAME SHIFT)	Ca_27264	S	--
Ca6_P4_13987966	Ca6	13987966	CG/CGTG	CAAACCTGCAAGAGTGACGA	TCCCCTGAAAAATGAGATGC	60.0	863	CDS (FRAME SHIFT)	Ca_05163	--	--
Ca6_P4_17183232	Ca6	17183232	ATTTT/ATTT	AATGGTTGAGTTGGCGAGTC	CCAGTTTGGTCACACCCTCT	60.1	680	CDS (FRAME SHIFT)	Ca_06338	L	--

INDEL marker IDs	Chromosomes/ unanchored scaffolds	Physical positions (bp)	InDels (*Kabuli* reference genome-CDC Frontier/PII)	Forward primers (5'-3')	Reverse primers (5'-3')	Anneling temperature (0C)	Expected amplified product size (bp)	Structural annotation		Functional annotation	
								Sequence components of *kabuli* genome	*Kabuli* gene accession IDs	NCBI-KOG	TFs
Ca6_P4_2379069	Ca6	2379069	T/TCAAG	GGTCTCAGTTGCAGCACAAA	CCGATTGGGTTAGCTGTGTT	60.0	305	CDS (FRAME SHIFT)	Ca_10369	T	MYB
Ca6_P4_32124189	Ca6	32124189	CTT/CTTT	TTTGCCAATGTTGCTAGCTG	CGAGCTAAGAAAAACCGTGC	60.0	662	CDS (FRAME SHIFT)	Ca_15213	--	--
Ca7_P4_14452319	Ca7	14452319	ACNNNNNNNNNNC/AC	TTGAAGCTGCAACAAGAGGA	GGGGAACCTGTAGTGGACAA	59.7	305	CDS (FRAME SHIFT)	Ca_09993	C	bHLH
Ca7_P4_20149771	Ca7	20149771	CTTTTT/CTTTT	GACGAAAGTCTCTTCCCGTG	TTGGTTTGTTTTTCCTTCGG	59.8	398	CDS (FRAME SHIFT)	Ca_12378	--	--
Ca7_P4_20488727	Ca7	20488727	TC/T	CAAACATGGTGTCCTTGTGC	ATTGCCCCATGTTTCAAGAG	60.0	731	CDS (FRAME SHIFT)	Ca_12399	T	M-type
Ca7_P4_22886024	Ca7	22886024	G/GCTAC	GCCCTTCTATGCTTGTCAGC	TTGGGAAGGTATGAAGGCAG	60.0	728	CDS (FRAME SHIFT)	Ca_19525	S	ARF
Ca7_P4_25563600	Ca7	25563600	AAGAGAGAGAGAGAGAGAGAGAGAGAGAGAGA/AAGAGAGAGAGAGAGAGAGAGAGAGAGAGAGAGA	CACAAACAAGAACGACGGTG	GAGGCTACGTGTTGTACGCA	60.2	603	CDS (FRAME SHIFT)	Ca_23609	KT	--
Ca7_P4_26694678	Ca7	26694678	TCCCCC/TCCCCCCCCCC	CTCGGCCATCCGAGATATAA	GCTCACTAACTCTGCCTGGG	60.0	881	CDS (FRAME SHIFT)	Ca_16250	--	--
Ca7_P4_30966048	Ca7	30966048	CT/C	CTTTGCCAATTCCAGTCCAT	TATTCAGATCCCTTGCCCAG	59.9	136	CDS (FRAME SHIFT)	Ca_23312	--	--
Ca7_P4_35480318	Ca7	35480318	CAAAA/CAAA	GCGTCCAGTCTGGTATTGGT	AGGGTTAGTGGTCGTTGGTG	60.0	201	CDS (FRAME SHIFT)	Ca_17756	--	--

INDEL marker IDs	Chromosomes/ unanchored scaffolds	Physical positions (bp)	InDels (*Kabuli* reference genome-CDC Frontier/PII)	Forward primers (5'-3')	Reverse primers (5'-3')	Anneling temperature (0C)	Expected amplified product size (bp)	Structural annotation		Functional annotation	
								Sequence components of *kabuli* genome	*Kabuli* gene accession IDs	NCBI-KOG	TFs
Ca7_P4_39347364	Ca7	39347364	AGGGG/AGGG	CGTGTCGAACTCGCTTATGA	TGTCGTCCAAGACACCACAT	60.0	674	CDS (FRAME SHIFT)	Ca_19589	R	bHLH
Ca7_P4_40430153	Ca7	40430153	AAGTAAGTA/AAGTAAGTAGTAAGTA	GTTGGTTCAAAAGCAACCGT	ACAAGGATACGTGCAGGGAC	60.0	493	CDS (FRAME SHIFT)	Ca_20258	--	Trihelix
Ca7_P4_43840665	Ca7	43840665	GC/GCCC	GCGTTTTTATGGTTGCTGGT	GAGTCACCCCAAGTGCAAAT	60.0	304	CDS (FRAME SHIFT)	Ca_21356	--	--
Ca7_P4_5911086	Ca7	5911086	TGGGG/TGGGGG	CGGTTAAGCTTCAAAACCCA	TATGCAGCAAAATGAACCCA	60.1	379	CDS (FRAME SHIFT)	Ca_06767	--	bHLH
Ca8_P4_14074282	Ca8	14074282	CCGCG/CCG	AAACAAAATCCACCGCACTC	TAGGTTCCAAAGGTGCCAAC	60.0	606	CDS (FRAME SHIFT)	Ca_22744	U	--
scaffold1202_P4_253511	scaffold1202	253511	GCGACTCACGA/GCGA	ACTCACGAGTCACCAGTCCC	GTGGTACGAAAATCTCCCGA	60.2	699	CDS (FRAME SHIFT)	Ca_24668	--	--
scaffold1252_P4_66355	scaffold1252	66355	AGGGGGG/AGGGGGGG	AGGTGGGTTGAGTTGTCAGG	TTGACTCTTGGGAAGTTGGG	60.0	808	CDS (FRAME SHIFT)	Ca_27777	--	--
scaffold1414_P4_190587	scaffold1414	190587	GAA/GAAA	TCGGATCACGGTCTGATACA	GAGGCAAATGGGACAAGGTA	60.1	611	CDS (FRAME SHIFT)	Ca_25542	R	--
scaffold1418_P4_15609	scaffold1418	15609	G/GC	GAACACTTGAAGGGCATGGT	GGATTTGGTGGAGGGAAAAT	60.0	590	CDS (FRAME SHIFT)	Ca_25260	R	bHLH
scaffold1521_P4_19418	scaffold1521	19418	CAAAA/CAAA	GAAGAATGGGAACAATGCGT	CAACGGGCAAAAATGAAACT	59.9	748	CDS (FRAME SHIFT)	Ca_26979	--	bHLH

INDEL marker IDs	Chromosomes/ unanchored scaffolds	Physical positions (bp)	InDels (*Kabuli* reference genome-CDC Frontier/PII)	Forward primers (5'-3')	Reverse primers (5'-3')	Anneling temperature (0C)	Expected amplified product size (bp)	Structural annotation		Functional annotation	
								Sequence components of *kabuli* genome	*Kabuli* gene accession IDs	NCBI-KOG	TFs
scaffold1548_P4_887	scaffold1548	887	TTT/TTTATACATT	CGTTTTCTTCTCCATTTGGG	CAGCCATAACCAAGCCATTT	59.5	600	CDS (FRAME SHIFT)	Ca_27706	QI	--
scaffold1580_P4_251123	scaffold1580	251123	CNNNNNNNNNN/C	TGTTCTCTTAGGGGGTGGTG	GGCCATCTTTTAAACCCCTT	60.0	569	CDS (FRAME SHIFT)	Ca_20799	B	--
scaffold167_P4_63349	scaffold167	63349	AA/AAAACCAAATGA	ATGGTCAAGACCTGGATTGC	TCATGGCTCTTGTTGCTCAC	59.9	709	CDS (FRAME SHIFT)	Ca_24147	R	MYB
scaffold1722_P4_66168	scaffold1722	66168	TG/TGG	CCAGAGACGATGGACAAGGT	CGAACGGGTAGTTGACGATT	60.1	649	CDS (FRAME SHIFT)	Ca_26630	G	--
scaffold1964_P4_11803	scaffold1964	11803	TAGAATTATGCAG/TAGAATTATGCAGAATTATGCAG	GCACCATTCCTCTTTTGGAA	GATGGACCACCTTCTCTCCA	60.1	553	CDS (FRAME SHIFT)	Ca_25209	G	--
scaffold273_P4_189091	scaffold273	189091	C/CCTAAGATTAA	TTTTGGTGACAACACCTGGA	GCAACTATGGTAGGGCGGTA	60.0	415	CDS (FRAME SHIFT)	Ca_26225	--	--
scaffold290_P4_675706	scaffold290	675706	AAGC/AAGCTGGCATAGTGCAGC	AAAATGATGGTCCAACGAGC	GGGCTACATGGTGATGGAAC	59.9	251	CDS (FRAME SHIFT)	Ca_19845	--	--
scaffold3837_P4_5910	scaffold3837	5910	ACCC/ACC	ACCTCGATCTGGAAATCACG	ATTGAGGGTAGGGGAGCAGT	60.1	467	CDS (FRAME SHIFT)	Ca_27570	--	--
scaffold543_P4_69059	scaffold543	69059	TGGGG/TGGGGG	TCGCTACTGAAGATGGGCTT	TTGATAACTTGAAAACGAAATGAAA	60.0	363	CDS (FRAME SHIFT)	Ca_24780	--	--
scaffold587_P4_17163	scaffold587	17163	AC/A	GGAAAATCCATGAGGCAAGA	AGGCCCCAAAGAAGAGTGAT	60.0	496	CDS (FRAME SHIFT)	Ca_27621	--	--

INDEL marker IDs	Chromosomes/ unanchored scaffolds	Physical positions (bp)	InDels (*Kabuli* reference genome-CDC Frontier/PII)	Forward primers (5'-3')	Reverse primers (5'-3')	Anneling temperature (0C)	Expected amplified product size (bp)	Structural annotation		Functional annotation	
								Sequence components of *kabuli* genome	*Kabuli* gene accession IDs	NCBI-KOG	TFs
scaffold599_P4_234597	scaffold599	234597	TC/T	TTGCTCCCTTCAACAAT TCC	ATTGCAGGAACATGGA AAGC	60.1	352	CDS (FRAME SHIFT)	Ca_25342	O	--
scaffold772_P4_152481	scaffold772	152481	GTT/GT	GGAGGATCTATGGTGC TGGA	GTGTTCTGCCGCTTTTT CTC	60.0	170	CDS (FRAME SHIFT)	Ca_26416	--	--
scaffold772_P4_23600	scaffold772	23600	G/GAGAGACATGTGTT	GGAAACAATGCCTCCA AGAA	GGAGGATGGTAACGAC GAAA	60.1	191	CDS (FRAME SHIFT)	Ca_26413	--	--
scaffold864_P4_247808	scaffold864	247808	TGGGG/TGGGGG	TTCTGCCCTCATGGAT TTTC	CATGGCTCGAGTGAAA CAGA	60.0	349	CDS (FRAME SHIFT)	Ca_24213	--	--
scaffold949_P4_3822	scaffold949	3822	CAAAAAA/CAAAAAAA	AAGAGGGGCGTGAACT TTTT	GCTTCCATACCTGTTTT GGG	60.1	646	CDS (FRAME SHIFT)	Ca_25481	--	--
Ca1_P4_14441702	Ca1	14441702	CNNNNNNNNNN/CNNNNNNN	TTTCCATTTTCCTCTGC CAC	TTGGTGTTGGAATTCG TTGA	60.1	408	CDS (large-effect mutations)	Ca_07092	--	--
Ca1_P4_5494812	Ca1	5494812	TTCA/T	GTAAAGCAGTTGCAGG GGAG	CAATTTTGGCCATGAC CTCT	59.9	551	CDS (large-effect mutations)	Ca_00634	--	--
Ca1_P4_6417631	Ca1	6417631	AAGGAGGAG/AAGGAG	TGTGATTGAGCCAGAA AAGC	CCATCCTTTGGCAGTG AAAT	59.0	454	CDS (large-effect mutations)	Ca_07905	P	MIKC
Ca1_P4_6489856	Ca1	6489856	AAAGAGCAAGAGCAAGAGCAAGAG CAAGAGCA/AAAGAGCAAGAGCAAG AGCAAGAGCA	CCTGGTTGGCTGATGA AAGT	CTCCGTTTAGGTGCTG GAAC	60.1	513	CDS (large-effect mutations)	Ca_07911	D	B3
Ca2_P4_30867703	Ca2	30867703	GCAACAACA/GCAACA	AAGACGAGACTGGCTG CAAT	AGGTCGTCAAGGTTTG TTGG	60.0	496	CDS (large-effect mutations)	Ca_12474	R	C3H

INDEL marker IDs	Chromosomes/ unanchored scaffolds	Physical positions (bp)	InDels (*Kabuli* reference genome-CDC Frontier/PII)	Forward primers (5'-3')	Reverse primers (5'-3')	Anneling temperature (0C)	Expected amplified product size (bp)	Structural annotation		Functional annotation	
								Sequence components of *kabuli* genome	*Kabuli* gene accession IDs	NCBI-KOG	TFs
Ca2_P4_35944964	Ca2	35944964	CAAAA/CA	TCCAACCATACCTGAAAGGC	CTCGTGCGAACTTGTGTGTT	59.9	313	CDS (large-effect mutations)	Ca_09769	--	--
Ca4_P4_44625405	Ca4	44625405	CATGATGATGATGATGATGA/CATGATGATGATGATGA	TTGAGGGAGTCCAATCCAAG	AGCTTTCAAGGGCTTCTTCC	60.0	744	CDS (large-effect mutations)	Ca_09133	--	WRKY
Ca5_P4_24097297	Ca5	24097297	AAGCTTTGGATAC/A	CAGAGCCAACACTACAGGCA	ATTTGAAGGGCCATGTGAAG	60.0	329	CDS (large-effect mutations)	Ca_17656	S	--
scaffold290_P4_848963	scaffold290	848963	AGCGCGCCCTGCGCGCC/AGCGCGCC	GCAAACTACATCCGACAGCA	CCGAGTTCCCAAGAGTCAAA	59.9	215	CDS (large-effect mutations)	Ca_19852	--	--
Ca1_P4_1801730	Ca1	1801730	ACACACAACGAGTC/ACACACAACGAGTCACACAACGAGTC	CAGGGAAAATTCCAAGCTGA	TGGACAAGAGCATCTGCAAC	60.2	565	CDS (large-effect mutations)	Ca_00228	F	MYB_related
Ca1_P4_6345643	Ca1	6345643	AATGATGATGATGATGATGATG/AATGATGATGATGATGATGATGATGATGATG	CACCTTGTTCACACACACCC	TTTGTTGCCAATGTTGTTTCA	59.9	450	CDS (large-effect mutations)	Ca_07895	--	--
Ca1_P4_7630412	Ca1	7630412	G/GACAAAA	TCGTTGCGAAGATAGTGTGG	AGCATGAGTTGCACAAGTGG	59.9	768	CDS (large-effect mutations)	Ca_08016	--	HD-ZIP
Ca2_P4_25228216	Ca2	25228216	T/TTGG	TGTCCTTTTCAAACCCCATC	ACAAGCAAAACCTCACCACC	59.8	351	CDS (large-effect mutations)	Ca_14254	R	--
Ca3_P4_30899185	Ca3	30899185	G/GACA	CTTTAGTGGGGAGCCATGAA	GAAATTCGCCATTTTCTGGA	60.1	520	CDS (large-effect mutations)	Ca_12186	O	--
Ca4_P4_11311023	Ca4	11311023	CATTATTATTATTATTATTAT/CATTATTATTATTATTATTATTAT	ATGTTTATGTGCCGTCCCTC	GGCCAACCATCAACCATATC	59.8	434	CDS (large-effect mutations)	Ca_04364	D	WRKY

INDEL marker IDs	Chromosomes/ unanchored scaffolds	Physical positions (bp)	InDels (*Kabuli* reference genome-CDC Frontier/PII)	Forward primers (5'-3')	Reverse primers (5'-3')	Anneling temperature (0C)	Expected amplified product size (bp)	Structural annotation		Functional annotation	
								Sequence components of *kabuli* genome	*Kabuli* gene accession IDs	NCBI-KOG	TFs
Ca4_P4_14398669	Ca4	14398669	GTCTTCTT/GTCTTCTTCTT	GGCAAGTACAAAGGGCATGT	GTTGCTGGAAGGCAGAGTTC	60.0	426	CDS (large-effect mutations)	Ca_05664	--	--
Ca4_P4_17663581	Ca4	17663581	CGTGGTGGTGGTGGTGGTGG/CGTGGTGGTGGTGGTGGTGGTGG	TGCCGTGTGGACATGAGTAT	CCAACTAGGCAAAAGCTTCG	60.0	338	CDS (large-effect mutations)	Ca_05362	O	--
Ca4_P4_26900710	Ca4	26900710	TTGGTCCTGGTC/TTGGTCCTGGTCCTGGTC	CCACATGAGTGTGTTGAGGG	TCTGATCTTCAGGACCACCC	60.0	241	CDS (large-effect mutations)	Ca_20442	--	SAP
Ca4_P4_28516642	Ca4	28516642	TTCTAA/TTCTAACTCTAA	TAATCTTCGGCGAAACTGCT	TAACAATCTGGTGCTGCTCG	60.0	897	CDS (large-effect mutations)	Ca_26231	--	--
Ca4_P4_30538166	Ca4	30538166	AAAGAAG/AAAGAAGAAG	TTGTTCTTGCATTTCCATTTTG	CGCCATCTTCCTCTTCTTTG	60.0	380	CDS (large-effect mutations)	Ca_14204	--	NAC
Ca4_P4_39183756	Ca4	39183756	A/ATGAAGTGGG	AATTTGCAGGTGGAATCGTC	GCAAGCGAACAACATTGAGA	59.9	348	CDS (large-effect mutations)	Ca_13064	T	bHLH
Ca4_P4_40578315	Ca4	40578315	G/GGTC	GCAGGTCTTTTTGGTGGAAA	AACCCTTCATCTTTCGCCTT	60.1	608	CDS (large-effect mutations)	Ca_14868	K	--
Ca4_P4_44968440	Ca4	44968440	GATAATAATAATAATAATAATAATAATAATAA/GATAATAATAATAATAATAATAATAATAATAATAATAA	TAGGCCCCACTTAAACATGC	TCACCATCATCATTTGCACC	60.0	348	CDS (large-effect mutations)	Ca_09160	R	C2H2
Ca4_P4_8712908	Ca4	8712908	GCAACAACAACAACAACA/GCAACAACAACAACAACAACA	TGGTGGACACAGAGCAAGTC	CCCCTTTTGCCTCTCTTTCT	59.9	836	CDS (large-effect mutations)	Ca_08367	R	C2H2
Ca5_P4_192213	Ca5	192213	AGA/AGATGATGA	TCTTCAGGGATCCCAACAAC	CAGTCGGAATCATCCTTGGT	59.9	394	CDS (large-effect mutations)	Ca_18174	L	--

INDEL marker IDs	Chromosomes/ unanchored scaffolds	Physical positions (bp)	InDels (*Kabuli* reference genome-CDC Frontier/PII)	Forward primers (5'-3')	Reverse primers (5'-3')	Anneling temperature (0C)	Expected amplified product size (bp)	Structural annotation		Functional annotation	
								Sequence components of *kabuli* genome	*Kabuli* gene accession IDs	NCBI-KOG	TFs
Ca6_P4_10848271	Ca6	10848271	TGGC/TGGCGGC	GGCCTCTTTCAACAAATGGA	ACAAATTTGGTGGTGGTGGT	60.1	311	CDS (large-effect mutations)	Ca_08513	K	MIKC
Ca6_P4_1212419	Ca6	1212419	TTCTG/TTCTGGATCTG	GCGTGCCATAGTGGAAGATT	CTCCCACCTTTACTTGCTGC	60.1	576	CDS (large-effect mutations)	Ca_13328	R	bHLH
Ca6_P4_5033352	Ca6	5033352	TGAAGAAGAAGAAGAAGAAGAAG/TGAAGAAGAAGAAGAAGAAGAAGAAG	ACCACCACTGATGATGACGA	GAGCGTTGGAGTGAGAGGAG	60.0	343	CDS (large-effect mutations)	Ca_05790	--	--
Ca7_P4_12415176	Ca7	12415176	ACACAATCACAATCACAATCACAATCACAATCA/ACACAATCACAATCACAATCACAATCACAATCACAATCA	TGAAATCTCATGGCATGCTT	ACCCTTTTGGAGGCCTAAGA	59.2	370	CDS (large-effect mutations)	Ca_09274	--	--
Ca7_P4_28454949	Ca7	28454949	CTCTTCTTCTTCTTCTTCTTCTTCTTCTTC/CTCTTCTTCTTCTTCTTCTTCTTCTTCTTCTTC	CAAAAATGGAATGGTTTGGG	TGGAAGAAGGTTTTGATCCG	60.0	688	CDS (large-effect mutations)	Ca_18933	S	--
Ca7_P4_3750531	Ca7	3750531	ATCATCTTCATCTTC/ATCATCTTCATCTTCATCTTC	TTGCCTTTGCTTTTGCTTTT	TCAACCAATGTGGTCCTCAA	60.0	474	CDS (large-effect mutations)	Ca_03093	--	ARF
Ca7_P4_6089038	Ca7	6089038	TAAAAAAAAAAA/TAAAAAAAAAAAAAA	TGTCCTTCGTATATTGGGGG	TCGAGCACATTCAACTCCTG	59.6	321	CDS (large-effect mutations)	Ca_06750	O	--
scaffold174_P4_379532	scaffold174	379532	T/TTAC	GCCGTTCATGTTCTACCGTT	GGTGGTCCTCGTAACCTTGA	60.0	503	CDS (large-effect mutations)	Ca_21720	--	--
scaffold2161_P4_7991	scaffold2161	7991	CAAG/CAAGAAG	CGGACATGTTGGAGACACAC	ACCACCTTGCACAAACTTCC	60.0	277	CDS (large-effect mutations)	Ca_26956	H	--
scaffold3015_P4_68656	scaffold3015	68656	A/AACCGGCACAGCC	TTCGCAGACAAAACTCATGC	AGCATTATGGGCCATCTGAG	60.0	283	CDS (large-effect mutations)	Ca_27318	--	--

INDEL marker IDs	Chromosomes/ unanchored scaffolds	Physical positions (bp)	InDels (*Kabuli* reference genome-CDC Frontier/PII)	Forward primers (5'-3')	Reverse primers (5'-3')	Anneling temperature (0C)	Expected amplified product size (bp)	Structural annotation		Functional annotation	
								Sequence components of *kabuli* genome	*Kabuli* gene accession IDs	NCBI-KOG	TFs
Ca1_P4_1849075	Ca1	1849075	AATTATTATTATTATTATTATTAT/AATTATTATTATTATTATTAT	GAATTCAACGGTGTGGGACT	TGTTTGAGCCAAATGTGGAA	59.8	271	CDS (large-effect mutations)	Ca_00232	K	MYB
Ca1_P4_38561475	Ca1	38561475	AGAAGTTGAAG/AGAAG	TGGTGTGCAAGAGCTGAAAC	CCACACTTGCAAAATTGGTG	60.0	160	CDS (large-effect mutations)	Ca_13957	--	--
Ca3_P4_21635884	Ca3	21635884	CGAAGACAAAGAAGAAGA/CGAAGA	TGTCATGTTGTGGAGGAGGA	GGAAGCAATAGAAAATGGCG	60.1	573	CDS (large-effect mutations)	Ca_09516	--	--
Ca3_P4_39963962	Ca3	39963962	AGATCAGGATC/AGATC	TTGCAGCCAACACTGAACTC	CGTTCTTCGCCTTTCTCAAC	60.0	578	CDS (large-effect mutations)	Ca_01380	--	--
Ca4_P4_17769895	Ca4	17769895	GCATCATCATCATCATCATCA/GCATCATCATCATCATCA	AAAAACTGGTCGAGGCTGAA	GCACTCACTTTGCCCTTCTC	59.9	560	CDS (large-effect mutations)	Ca_05348	--	--
Ca4_P4_8711997	Ca4	8711997	GAACAACAACAACAACAACAACAACAA/GAACAACAACAACAACAA	AAGGTTTGGTTGGCAAAGTG	CCCTTGATTGTCCTTGTGCT	60.0	751	CDS (large-effect mutations)	Ca_08367	R	C2H2
Ca5_P4_5284789	Ca5	5284789	AGAGGAG/AGAG	CACTGTCACTTTCCTCCGGT	AAATGTGCTCCAACGTTTCC	60.2	309	CDS (large-effect mutations)	Ca_22622	--	bHLH
Ca6_P4_31218413	Ca6	31218413	CAATGATAATGATAATGATAATGATAATGATAATGATAATGAT/CAATGATAATGATAATGATAATGATAATGATAATGAT	GCATTTGATAGACGCCCATT	TTTCACATTCCCATTCCCAT	59.9	301	CDS (large-effect mutations)	Ca_15160	--	NAC
Ca6_P4_39049911	Ca6	39049911	TTCCCTCTCCCTCT/TTCCCTCT	ATCCAAACATCACCCCAAAA	AGCGCTGTTTATCGCATTCT	60.0	752	CDS (large-effect mutations)	Ca_15907	--	--
Ca7_P4_20609902	Ca7	20609902	AAACATCGAC/A	AAACCTGACCATGAAGTCGG	ACAGGCCTAGATGGAGCAGA	60.0	248	CDS (large-effect mutations)	Ca_12413	--	--

INDEL marker IDs	Chromosomes/ unanchored scaffolds	Physical positions (bp)	InDels (*Kabuli* reference genome-CDC Frontier/PII)	Forward primers (5'-3')	Reverse primers (5'-3')	Anneling temperature (0C)	Expected amplified product size (bp)	Structural annotation		Functional annotation	
								Sequence components of *kabuli* genome	*Kabuli* gene accession IDs	NCBI-KOG	TFs
Ca7_P4_21698571	Ca7	21698571	ATCGTCGT/ATCGT	AAGGTAGGGAATCAATGGGG	CCGACGATGTTGCTTTCTCT	60.0	423	CDS (large-effect mutations)	Ca_14517	--	NAC
Ca7_P4_33278396	Ca7	33278396	TTATTATTAATATTATTA/TTATTATTA	ATTTATGCGTGGAGGAGGAA	CTCGTTGCGACGTGTACTGT	59.5	763	CDS (large-effect mutations)	Ca_16155	--	S1Fa-like
Ca7_P4_34237238	Ca7	34237238	GGTTGTTGTTGTTGTTGTTGTTGTTGT/GGTTGTTGTTGTTGT	TTCCCAATTGCAACAATCAA	GGTCTCATCTCATCCTCCCA	59.9	437	CDS (large-effect mutations)	Ca_20997	--	S1Fa-like
Ca8_P4_1185649	Ca8	1185649	TAGAAGAAGAA/TAGAAGAA	GACTCGCCAACTCAACCATT	ACACATGCTCCATACCACCA	60.1	282	CDS (large-effect mutations)	Ca_15061	--	--
Ca8_P4_15436905	Ca8	15436905	GAGAAGAAGAAGAAGAAG/GAGAAGAAGAAGAAG	TCTTCCTCACCCACAAGTCC	CCATCTCCACAAGCTTCTCC	60.1	561	CDS (large-effect mutations)	Ca_17897	--	bHLH
scaffold1006_P4_553062	scaffold1006	553062	GTGCTGCTGCTGCTGCTGCT/GTGCTGCTGCTGCTGCT	CTTGCACCAGTATGGGAGGT	AGATTTTCACCCGTTGTTGC	60.0	376	CDS (large-effect mutations)	Ca_18040	--	--
scaffold240_P4_7668	scaffold240	7668	GATCAT/GAT	CTGGAAGGCAGAGTTTGGAG	GAAAAGCAACTTGGATGGGA	60.0	773	CDS (large-effect mutations)	Ca_25652	--	--
Ca1_P4_3149193	Ca1	3149193	GA/GATTA	GATTTCGAACAGCAAGAGCC	ATTCCCGAATATGGTGGACA	60.0	512	CDS (large-effect mutations)	Ca_00385	--	--
Ca1_P4_6809796	Ca1	6809796	CAGAAGAAGAAGAAG/CAGAAGAAGAAGAAGAAG	CCTCTTGCTGCAACACAAAA	CCGAACAGAAAAAGCTCGAC	60.0	528	CDS (large-effect mutations)	Ca_07947	S	--
Ca1_P4_7992079	Ca1	7992079	ACTCCTCCTCCTCCT/ACTCCTCCTCCTCCTCCT	TGATTCTCTCCCATGCCATT	TTCTTCAGCTGGTGTTGCAC	60.4	450	CDS (large-effect mutations)	Ca_08049	--	WRKY

INDEL marker IDs	Chromosomes/ unanchored scaffolds	Physical positions (bp)	InDels (*Kabuli* reference genome-CDC Frontier/PII)	Forward primers (5'-3')	Reverse primers (5'-3')	Anneling temperature (0C)	Expected amplified product size (bp)	Structural annotation		Functional annotation	
								Sequence components of *kabuli* genome	*Kabuli* gene accession IDs	NCBI-KOG	TFs
Ca1_P4_7992471	Ca1	7992471	TGTAGTA/TGTAGTAGTA	GTGCAACACCAGCTGAAGAA	CAACATCATGATCAATGGCA	60.0	310	CDS (large-effect mutations)	Ca_08049	--	WRKY
Ca2_P4_29314373	Ca2	29314373	GATC/GATCATC	CCAAAGCAAATCACAGAGCA	AGGTGACATTCATGGGCAGT	60.0	433	CDS (large-effect mutations)	Ca_25184	TR	FAR1
Ca2_P4_29962574	Ca2	29962574	TCCGCCGCCG/TCCGCCGCCGCCG	CGGACTCCATAACCTCCTCA	CCTCCTAATCATGGCTTCCA	60.1	266	CDS (large-effect mutations)	Ca_16094	A	--
Ca2_P4_32243132	Ca2	32243132	TCAA/TCAACAA	TCTGAGGGGATTCGTTATGC	TCCATAACTCTTGCCAACCC	60.0	524	CDS (large-effect mutations)	Ca_10292	--	bHLH
Ca2_P4_36372982	Ca2	36372982	GATTATTATTATTATTAATATTATTATTA/GATTATTATTATTATTATTATTATTAATATTATTATTA	TGAGCTGAAATTGTTCGTCG	CCAATCCTCGTTGTCAAGGT	60.0	274	CDS (large-effect mutations)	Ca_09826	--	HD-ZIP
Ca3_P4_22099014	Ca3	22099014	CAGAAGAAGAAGAAGAAGAAGAAGAAGAAGAAGAAGAAGAAG/CAGAAGAAGAAGAAGAAGAAGAAGAAGAAGAAGAAGAAGAAGAAGAAGAAG	GGAACGAACGTGGAAGTGAT	TCGCGTTAGTTGTTGTGAGG	60.0	356	CDS (large-effect mutations)	Ca_20416	O	FAR1
Ca3_P4_24304854	Ca3	24304854	CAGCCT/CAGCCTTAGCCT	CCATAGGCGTCAAAGGAAAA	CCCGTCTCATATCTACCCGA	60.1	523	CDS (large-effect mutations)	Ca_06090	--	--
Ca3_P4_36119763	Ca3	36119763	ACTT/ACTTCTT	GCATATTCATCACCCCCATC	TCCTTCTTGCAGGTGCTTTT	60.0	376	CDS (large-effect mutations)	Ca_00911	D	--
Ca3_P4_36426774	Ca3	36426774	TAGATGAAGATGAAGATGAAGATGA/TAGATGAAGATGAAGATGAAGATGAAGATGA	TCAAAGGAAGCTTAGCCCAA	GCAAACGCTTCTCTGGTTTC	60.0	861	CDS (large-effect mutations)	Ca_00956	GM	WRKY
Ca3_P4_36429933	Ca3	36429933	TTCTTCTTCTTCTTCTTCT/TTCTTCTTCTTCTTCTTCTTCTTCT	AGGGATGCACCAGCTGTAAC	AGAGAAGAAACCTGGTGCGA	60.1	593	CDS (large-effect mutations)	Ca_00957	H	--

INDEL marker IDs	Chromosomes/ unanchored scaffolds	Physical positions (bp)	InDels (*Kabuli* reference genome-CDC Frontier/PII)	Forward primers (5'-3')	Reverse primers (5'-3')	Anneling temperature (0C)	Expected amplified product size (bp)	Structural annotation		Functional annotation	
								Sequence components of *kabuli* genome	*Kabuli* gene accession IDs	NCBI-KOG	TFs
Ca3_P4_37211475	Ca3	37211475	GAACAACAACA/GAACAACAACAACAACAACA	TGGTCAAATTCTTGGAGCAA	TGATCACACCCCAAGTTTTG	59.2	336	CDS (large-effect mutations)	Ca_01053	--	bHLH
Ca3_P4_6838667	Ca3	6838667	AGCA/AGCAGGATCCAATGCAATGGCA	GGAACCCCACAATCATCATC	GCAGCCAATTTTGTTGCATA	60.0	547	CDS (large-effect mutations)	Ca_16550	R	bHLH
Ca4_P4_19228202	Ca4	19228202	TATG/TATGATCGTGATG	GCAATCTGGAAAGATGGGAA	AAATTCGCCCTGTGTTTTTG	60.0	288	CDS (large-effect mutations)	Ca_14021	T	WRKY
Ca4_P4_4029055	Ca4	4029055	GATCATCATCATCAT/GATCATCATCATCATCATCAT	ACATTCCTGTTGGTGGTGGT	ACATTCCAGTCCAAAACCCA	60.1	612	CDS (large-effect mutations)	Ca_03849	--	Dof
Ca5_P4_31241305	Ca5	31241305	GAATTCCAATTCCAATTCC/GAATTCCAATTCCAATTCCAATTCC	CGATGAACGCAAACTCAAGA	TAATATTACGCGCGAAACCC	60.0	325	CDS (large-effect mutations)	Ca_04830	R	--
Ca5_P4_38030247	Ca5	38030247	TTCTTG/TTCTTGTTCATCTTG	TCAAAAACGGACATGAGCAA	TTTATGGACAGCTCCCGAAC	60.2	614	CDS (large-effect mutations)	Ca_01418	R	DBB
Ca6_P4_26884697	Ca6	26884697	CT/CTTGTGCTTTCCCTACTAAT	TCCTCCAATGATGGCAATTT	TCAATGCTCAAGTCGCATTC	60.3	446	CDS (large-effect mutations)	Ca_16720	--	--
Ca6_P4_7586339	Ca6	7586339	GTGATGATGATGA/GTGATGATGATGATGA	GCACCTCTTGGACCATCAAT	ACAGTGTCAGTGCCTACCCC	59.9	454	CDS (large-effect mutations)	Ca_09626	C	--
Ca7_P4_20562920	Ca7	20562920	CTCTTCTTCTT/CTCTTCTTCTTCTT	CATTTCTCGCCAATCAGGTT	TCGATAGCCTCTCGGAGAAA	60.1	443	CDS (large-effect mutations)	Ca_12406	--	M-type
Ca7_P4_30207547	Ca7	30207547	CCTTCTTCTTCT/CCTTCTTCTTCTTCTTCTTCT	TTTTGCAAGGAAATGGGTTC	TTTCAATCCACATCGTCCAA	59.9	380	CDS (large-effect mutations)	Ca_11706	--	--

**INDEL marker IDs**	**Chromosomes/ unanchored scaffolds**	**Physical positions (bp)**	**InDels (*Kabuli* reference genome-CDC Frontier/PII)**	**Forward primers (5'-3')**	**Reverse primers (5'-3')**	**Anneling temperature (0C)**	**Expected amplified product size (bp)**	**Structural annotation**		**Functional annotation**	
								**Sequence components of *kabuli* genome**	***Kabuli* gene accession IDs**	**NCBI-KOG**	**TFs**
Ca7_P4_31246533	Ca7	31246533	CTCAACATCATCA/CTCAACATCATCAACATCATCA	ATCTGGGTTGTTGATCCTGC	AGATGGAGGAGGATGTCCCT	59.9	108	CDS (large-effect mutations)	Ca_10133	--	--
Ca7_P4_33344144	Ca7	33344144	CTCTTCGTCTTC/CTCTTCGTCTTCGTCTTC	TAAATGATTTGCGGTGGTGA	CGTCGATTTCAAGCACTCAA	59.9	277	CDS (large-effect mutations)	Ca_16160	--	--
Ca7_P4_6148021	Ca7	6148021	ATGTT/ATGTTGTT	CTAAAGATCGCGGCAGAAAG	GGGATACGTTTGGGGAAAGT	60.1	471	CDS (large-effect mutations)	Ca_06742	--	--
Ca8_P4_1155325	Ca8	1155325	CTATTATTATTATTATTATTATTATTATTATTAT/CTATTATTATTATTATTATTATTATTATTATTATTAT	GCAAAGGAGACAGAATGGGA	CGCTAGGCTCAATTTTCCTG	60.2	608	CDS (large-effect mutations)	Ca_15064	--	ERF
Ca8_P4_15577896	Ca8	15577896	CCA/CCATCA	TTTTCAGCAAACCCCAAAAG	TAGAGGCTTGTCATTCGGCT	60.1	665	CDS (large-effect mutations)	Ca_17886	T	EIL
Ca8_P4_4628017	Ca8	4628017	GGCAGCAGCAGC/GGCAGCAGCAGCAGC	CACCACCAACACTGCAAAAC	CAAGAACTTGCTCGTCCTCC	60.0	787	CDS (large-effect mutations)	Ca_02095	K	TALE
Ca8_P4_470107	Ca8	470107	TTTCTTCTTCTTCTTCTTCTTCTTCTTCTTCTTCTTCTTC/TTTCTTCTTCTTCTTCTTCTTCTTCTTCTTCTTCTTCTTCTTCTTC	AAGTATTCCGTCCGTTGGTG	TCATGGCTTCAACTTAGCCC	59.9	922	CDS (large-effect mutations)	Ca_11888	--	--
scaffold1045_P4_35689	scaffold1045	35689	CCGATGTCT/CCGATGTCTGCGATGTCT	AACGAGGCTGGTCCTCACTA	AGCTGGACTTGGATTGTGCT	59.9	518	CDS (large-effect mutations)	Ca_27889	--	--
scaffold1067_P4_213896	scaffold1067	213896	ACA/ACAGCA	TTTCTTTCGGAGGTGCTGAC	TTCCACCGAATGCTCCTATC	60.4	370	CDS (large-effect mutations)	Ca_24426	--	--
scaffold174_P4_192046	scaffold174	192046	GGGTGGTGGTG/GGGTGGTGGTGGTG	AGCAGCACTAGGGCTTGGTA	ATCCCTCACACCATCACCAT	60.0	356	CDS (large-effect mutations)	Ca_21713	R	C2H2

INDEL marker IDs	Chromosomes/ unanchored scaffolds	Physical positions (bp)	InDels (*Kabuli* reference genome-CDC Frontier/PII)	Forward primers (5'-3')	Reverse primers (5'-3')	Anneling temperature (0C)	Expected amplified product size (bp)	Structural annotation		Functional annotation	
								Sequence components of *kabuli* genome	*Kabuli* gene accession IDs	NCBI-KOG	TFs
scaffold2763_P4_9760	scaffold2763	9760	C/CCTCACG	ATGCCAGTCGTTATTCCCAG	GAGATGCCATTGAAGCCATT	60.0	400	CDS (large-effect mutations)	Ca_24393	R	--
scaffold584_P4_12275	scaffold584	12275	A/AAAT	GCAACGTCGGTTTGAAAAGT	TCGTCGTTGTTGTCCATGTT	60.2	619	CDS (large-effect mutations)	Ca_25249	--	Trihelix
scaffold845_P4_66947	scaffold845	66947	GAACAACAACAACAACAACAA/GAACAACAACAACAACAACAA	CACCCTAACCTCAACCTCCA	TTGACGACGCATTGGTACAT	60.0	547	CDS (large-effect mutations)	Ca_22413	--	--
scaffold847_P4_232160	scaffold847	232160	T/TCCCTCTTTTCTA	TCCAGTCTCATCCCGAAATC	AAGAGTCAACGTCAATGGGG	60.0	450	CDS (large-effect mutations)	Ca_25695	--	--
C11140974_P4_511	C11140974	511	ATTTTTTTTTTTT/ATTTTTTTTTT	TTCATCACCAAACTGATGGG	CATGATTGGTGTTGGCAGTC	59.3	424	DRR	Ca_27911	--	--
C11154966_P4_887	C11154966	887	GAAAAAAAAAAA/GAAAAAAAAAA	TTCAAGAGTCTCCTAGGGCG	TTGCTTGTGTCGTTAGCTGC	59.6	669	DRR	Ca_28249	E	MYB
C11178328_P4_12753	C11178328	12753	CAT/CATTAT	CCATCTTTTAGGCCTCCTCC	GAAGGCAACCATTCTAGGCA	60.0	925	DRR	Ca_28266	T	M-type
Ca1_P4_10070211	Ca1	10070211	GAATAATAATAATAATAATAATAATAA/GAATAATAATAATAATAATAATAATAATAA	TGCTGATGCGTCTGTCCTAC	TTATATGGGAAACCGGAGGG	60.0	410	DRR	Ca_02782	--	--
Ca1_P4_10733080	Ca1	10733080	T/TG	CCGGTAACGAGTTGTGTTCA	AAAATGGCACGTTTTTCTGG	59.6	428	DRR	Ca_02689	--	DBB
Ca1_P4_11161524	Ca1	11161524	ATATATATATTT/AT	TGGAACAATCCGATACCGTT	AAAAACATGATTCCAACGCC	60.2	676	DRR	Ca_02643	R	--

INDEL marker IDs	Chromosomes/ unanchored scaffolds	Physical positions (bp)	InDels (*Kabuli* reference genome-CDC Frontier/PII)	Forward primers (5'-3')	Reverse primers (5'-3')	Anneling temperature (0C)	Expected amplified product size (bp)	Structural annotation		Functional annotation	
								Sequence components of *kabuli* genome	*Kabuli* gene accession IDs	NCBI-KOG	TFs
Ca1_P4_11195317	Ca1	11195317	TTCTCTCTCTCTCTCTCTCTCTCTC/TTCTCTCTCTCTCTCTCTCTC	AGGTGGTAAAGACACGTGGG	TGAACTCGTCACGAACCTTG	59.9	338	DRR	Ca_02638	--	--
Ca1_P4_11253885	Ca1	11253885	GTATATATATATATATATATATATATATATAT/GTATATATATATATATATATATATATATATATATATAT	AAGCCTCTTGGCTTTTGTGT	TGGTGGATGACTCAATCGAA	59.0	318	DRR	Ca_02630	P	--
Ca1_P4_11540175	Ca1	11540175	AATATATATATATATATATATATATATATATATATATATAT/AATATATATATATATATATATATATATATATATATAT	GCGAAGGAAAGAAGTTCACG	CATTTAAAAGTTCAAAGGCCTGA	60.0	728	DRR	Ca_02595	KB	--
Ca1_P4_12311892	Ca1	12311892	CTTT/CT	GTTGTATTGTGAGGACCGCC	TTGTGTACCGTAGCTCCAAAAT	60.4	199	DRR	Ca_02521	--	--
Ca1_P4_12678832	Ca1	12678832	GTTTTTTTTT/GTTTTTTTT	GGTGCGGTTTTCTCCAATTA	CCTCCAATCAAAGGTGAGGA	59.9	358	DRR	Ca_02486	R	--
Ca1_P4_12721902	Ca1	12721902	CATATATATATATATATATATATAT/CATATATATATATATATATATAT	CGCATGATTCAAACAAATGG	CCAACTCAGGTTCAGCAAGA	59.9	746	DRR	Ca_02480	--	--
Ca1_P4_12816686	Ca1	12816686	CAAAAAAAAAA/CAAAAAAAAAAA	CCTTCATTGGACTTGATGGG	TAAAATAGGACGGGTGCAGG	60.3	818	DRR	Ca_02467	U	WOX
Ca1_P4_12876413	Ca1	12876413	AAGGGTAGG/AAGG	AAGCACGGCCTTCAAACTTA	CCAAAACATGCTGCTAACCC	59.9	574	DRR	Ca_02459	R	C2H2
Ca1_P4_13212423	Ca1	13212423	TAATAAAAATAAAAATAAAAATAAAAATAAAAATAAAAATAAAAATAAAAATAAA/TAATAAAAATAAAAATAAAAATAAAAATAAAAATAAAAATAAAAATAAAAATAAAAATAAA	GGCAAAGATTCATTTGAGCG	TTTGATCCAACCCGAATACA	60.7	558	DRR	Ca_14083	J	--
Ca1_P4_13298440	Ca1	13298440	CATATATATATATATATATATATATATATAT/CATATATATATATATATATATATATATAT	TTGCAAAGTTGCTCATAGCC	GTGTCATGTTCCCATTTCCC	59.1	484	DRR	Ca_14092	--	--

INDEL marker IDs	Chromosomes/ unanchored scaffolds	Physical positions (bp)	InDels (*Kabuli* reference genome-CDC Frontier/PII)	Forward primers (5'-3')	Reverse primers (5'-3')	Anneling temperature (0C)	Expected amplified product size (bp)	Structural annotation		Functional annotation	
								Sequence components of *kabuli* genome	*Kabuli* gene accession IDs	NCBI-KOG	TFs
Ca1_P4_13676036	Ca1	13676036	CTTTTTTT/CTTTTTT	CCGCTACAAGGCGATAGAAG	TTCTGATGAACGGGGGATTA	60.0	248	DRR	Ca_14132	--	--
Ca1_P4_13676235	Ca1	13676235	T/TC	CCGCTACAAGGCGATAGAAG	GGGTGTCATCATAGGTGGCT	60.0	856	DRR	Ca_14132	--	--
Ca1_P4_13676261	Ca1	13676261	ACT/ACTCT	CCGCTACAAGGCGATAGAAG	GGGTGTCATCATAGGTGGCT	60.0	856	DRR	Ca_14132	--	--
Ca1_P4_13676496	Ca1	13676496	AT/A	TAATCCCCCGTTCATCAGAA	GGGTGTCATCATAGGTGGCT	60.3	628	DRR	Ca_14132	--	--
Ca1_P4_13676564	Ca1	13676564	AG/A	TAATCCCCCGTTCATCAGAA	CAAATTCCCCTGCCTCTACA	60.3	933	DRR	Ca_14132	--	--
Ca1_P4_13677888	Ca1	13677888	ATTTTTTTT/ATTTTTTTTT	CGGAATACCCTCTCAACCAA	GATTTTCCGAAAGGGAACAA	59.9	314	DRR	Ca_14133	--	ERF
Ca1_P4_13678173	Ca1	13678173	CTATATATATATATATATAT/CTATATATATATATATAT	CGGAATACCCTCTCAACCAA	CAAACCTCATTAGGAAAAGGGA	59.9	463	DRR	Ca_14133	--	ERF
Ca1_P4_13678320	Ca1	13678320	CATA/CATAAACGAGCCTATA	TTGTTCCCTTTCGGAAAATC	TCCAAAGTATTTCTGCATTGAA	59.0	675	DRR	Ca_14133	--	ERF
Ca1_P4_13679372	Ca1	13679372	CTTTT/CTTT	AGGTGCTCTCCTCTCCACAG	TTTGGCATGGATAGGGGTTA	59.6	429	DRR	Ca_14133	--	ERF
Ca1_P4_13697453	Ca1	13697453	TAAAAAAA/TAAAAAAAA	TCTGAAAACAATTTCCCCTTG	CTAGACCATGGCCGTTCAAT	59.1	865	DRR	Ca_14135	--	bHLH

INDEL marker IDs	Chromosomes/ unanchored scaffolds	Physical positions (bp)	InDels (*Kabuli* reference genome-CDC Frontier/PII)	Forward primers (5'-3')	Reverse primers (5'-3')	Anneling temperature (0C)	Expected amplified product size (bp)	Structural annotation		Functional annotation	
								Sequence components of *kabuli* genome	*Kabuli* gene accession IDs	NCBI-KOG	TFs
Ca1_P4_13939746	Ca1	13939746	TATTA/TACTTTATTAAAAAATTA	TCAGGTGTCTCATCCACTGC	TGGATTTTCAATGCCAGACA	59.8	755	DRR	Ca_07137	--	--
Ca1_P4_14385063	Ca1	14385063	CAA/CAAA	AATCGTCTCCGTCACACACA	GCGAATTGCAGACTGAAACA	60.2	662	DRR	Ca_07096	--	SAP
Ca1_P4_14591320	Ca1	14591320	AATATATATATATATATAT/AATATATATATATATATATAT	TTACCTCCGGTGGATGTAGC	TTGCATGATCAGTGGAAGGT	60.0	470	DRR	Ca_07075	--	--
Ca1_P4_14800179	Ca1	14800179	AATATATATATATATATATA/AATATATATATATATATATATA	TTGGATGTTGATAAATTGAAGCA	GAGGAATTGTTATTGGTTGTTGG	59.5	284	DRR	Ca_07050	R	MYB
Ca1_P4_16215217	Ca1	16215217	TTTATTATTATTATTATTATTATTATTATTATTATTATTATTATTATTATTATTATTATTATT/TTTATTATTATTATTATTATTATTATTATTATTATTATTATTATTATTATTATTATTATTATTATTAT[illegible]	GGAAAAAGAGAGAGTGGGGG	TGAGAGTTTGTGGTCCCCTC	60.0	787	DRR	Ca_06936	--	bHLH
Ca1_P4_16866662	Ca1	16866662	AATATATATATATAT/AATATATATATATATAT	CAATTTGGGTCGTCCATCTC	TTAAGACCAACCCGAACCTG	60.3	614	DRR	Ca_06887	--	--
Ca1_P4_16943629	Ca1	16943629	TTTTAAATTTA/TTTTAAATTTAAATTTA	TTTTCTTAAGACGGGCATGA	TGAAGGGTATACGGCACCTC	58.4	776	DRR	Ca_06876	O	--
Ca1_P4_1716073	Ca1	1716073	TTATATATATATATATATA/TTATATATATATATATA	GCCTGAATTTAGCAGATGGG	GATTATTGCACAAACTCCAAAAA	59.7	466	DRR	Ca_00214	--	bHLH
Ca1_P4_1785166	Ca1	1785166	AG/A	TCTACTTGCGGCAACTTGTG	CATCATTCCAATCAAGCACG	60.1	319	DRR	Ca_00224	S	--
Ca1_P4_1785215	Ca1	1785215	A/AAATG	TCTACTTGCGGCAACTTGTG	CATCATTCCAATCAAGCACG	60.1	319	DRR	Ca_00224	S	--

INDEL marker IDs	Chromosomes/ unanchored scaffolds	Physical positions (bp)	InDels (*Kabuli* reference genome-CDC Frontier/PII)	Forward primers (5'-3')	Reverse primers (5'-3')	Anneling temperature (0C)	Expected amplified product size (bp)	Structural annotation		Functional annotation	
								Sequence components of *kabuli* genome	*Kabuli* gene accession IDs	NCBI-KOG	TFs
Ca1_P4_1804269	Ca1	1804269	CTCAGAA/C	TCACTTCGAAAGCATGCAAC	TTGTGGCGTTTGCTAACTTG	60.0	281	DRR	Ca_00228	F	MYB_related
Ca1_P4_1805050	Ca1	1805050	TAAA/TAAAA	TTCAGTTGACCTTTCTGCATTT	GGTTTGGGATTTTTGCATCA	58.9	727	DRR	Ca_00228	F	MYB_related
Ca1_P4_1848130	Ca1	1848130	AC/A	ATCTTGGACTCACTGTCGGG	ACATGCCGTATTCAATCCAT	60.1	969	DRR	Ca_00232	K	MYB
Ca1_P4_1856274	Ca1	1856274	ACAT/A	TGTTGGCCACGATCTAATGA	TGCCTCGCTATTTTTCTAACG	60.1	390	DRR	Ca_00233	FP	bHLH
Ca1_P4_186939	Ca1	186939	TAA/TAAA	CTCTTGGGGGATCTTCATCA	GCATGCCATGTAGTGCATTT	60.0	329	DRR	Ca_00014	--	--
Ca1_P4_1907609	Ca1	1907609	ATTTCTTTT/ATTTCTTTTCTTTT	TGCAATGATGTCAATTGCTCT	AAATCAATTTTGGTCCGCTC	59.3	926	DRR	Ca_00239	--	--
Ca1_P4_1907983	Ca1	1907983	CTT/C	TTCCCGTGAATTTCATCCAT	GCATGGGAGGATAAAAGCAA	60.1	721	DRR	Ca_00239	--	--
Ca1_P4_1908061	Ca1	1908061	TTATATA/TTATA	TTCCCGTGAATTTCATCCAT	GCATGGGAGGATAAAAGCAA	60.1	721	DRR	Ca_00239	--	--
Ca1_P4_1964395	Ca1	1964395	GTTTTTTT/GTTTTTT	TCCCGCTATGCACATTGTAA	TGTTTTTCTTCTTGGGGTGG	60.1	672	DRR	Ca_00244	--	B3
Ca1_P4_1966252	Ca1	1966252	TTCTCT/TTCTCTCT	GGCAAGAGAGCATCAAAAGG	CAAGTGAAGTTTTGATTCAAGGG	60.0	703	DRR	Ca_00245	U	bZIP

INDEL marker IDs	Chromosomes/ unanchored scaffolds	Physical positions (bp)	InDels (*Kabuli* reference genome-CDC Frontier/PII)	Forward primers (5'-3')	Reverse primers (5'-3')	Anneling temperature (0C)	Expected amplified product size (bp)	Structural annotation		Functional annotation	
								Sequence components of *kabuli* genome	*Kabuli* gene accession IDs	NCBI-KOG	TFs
Ca1_P4_1966518	Ca1	1966518	CTTTT/CTTT	CATGCATGTTTACTGACAATGG	TTCGAGTTGATTTGATTTCGG	59.0	540	DRR	Ca_00245	U	bZIP
Ca1_P4_1986222	Ca1	1986222	TAAAAAAAAAAAA/TAAAAAAAAAA	TATTTTTGCTGTTTGCACCG	GAGAAAAGTTGAAACCATGACCA	59.7	394	DRR	Ca_00248	T	--
Ca1_P4_1987558	Ca1	1987558	ATAGTAG/ATAG	GTGGCATAGGCTCCAATCAT	GGAATGGTGCTGGACATTTT	59.9	922	DRR	Ca_00248	T	--
Ca1_P4_2001586	Ca1	2001586	TAAG/TAAGAAG	AGCACTGGGCAGAAAGAAAA	TATCCCTGGCTGATGTAGCA	60.0	538	DRR	Ca_00251	J	--
Ca1_P4_2001789	Ca1	2001789	ATGT/AT	AGCACTGGGCAGAAAGAAAA	CACACGTCATGATCCCCATA	60.0	751	DRR	Ca_00251	J	--
Ca1_P4_2006799	Ca1	2006799	CTTT/CTTTT	ATAGACCTCTTGTGGTGCGG	TGTGGTTTGGGTGTGAGAGA	60.1	400	DRR	Ca_00252	--	TCP
Ca1_P4_2015582	Ca1	2015582	TTGT/TT	CTCTAGAACCCCCAACTCCC	CTCCAATGTTGGGAGACTACG	59.9	499	DRR	Ca_00253	E	--
Ca1_P4_2023148	Ca1	2023148	A/AC	TGCAGTTTCGGTAGGGAAGT	CGACGAGATTTACTTTGACCG	59.7	541	DRR	Ca_00254	FP	bHLH
Ca1_P4_2023898	Ca1	2023898	TAA/TAAA	TAATGAGGGCTTGAATTCGC	TCCGTTGTTGAGGATGATGA	60.2	386	DRR	Ca_00255	--	--
Ca1_P4_2055811	Ca1	2055811	ATTTTTT/ATTTTTTT	TCGTTTTCCCTGTAATTCTTGG	CAACAACTTCTCATGCTCGC	60.3	573	DRR	Ca_00260	T	M-type

INDEL marker IDs	Chromosomes/ unanchored scaffolds	Physical positions (bp)	InDels (*Kabuli* reference genome-CDC Frontier/PII)	Forward primers (5'-3')	Reverse primers (5'-3')	Anneling temperature (0C)	Expected amplified product size (bp)	Structural annotation		Functional annotation	
								Sequence components of *kabuli* genome	*Kabuli* gene accession IDs	NCBI-KOG	TFs
Ca1_P4_2058722	Ca1	2058722	CAGAGAGA/CAGAGA	CGGTTCTACCGTCCTAACCA	CAATCCTCCTCCCCTTTCTC	60.0	204	DRR	Ca_00261	--	--
Ca1_P4_2058843	Ca1	2058843	CAAAA/CAA	GAGAAAGGGGAGGAGGATTG	AGACTCTGCAGCCACCTCAT	60.0	357	DRR	Ca_00261	--	--
Ca1_P4_2091338	Ca1	2091338	T/TA	GTGTGGAAAGTAGCCCCAAA	TCGAACTGTTGTTCTGGCTG	60.0	164	DRR	Ca_00266	--	MYB_related
Ca1_P4_2213930	Ca1	2213930	GATAATAATAATAATAATAATAATAATA/GATAATAATAATAATAATAATA	TGTGCAAACAGCAATGAATG	TACGGTTGCTGAATTTCGTG	59.3	341	DRR	Ca_00276	--	--
Ca1_P4_22204858	Ca1	22204858	TTATATATATATATATATATAT/TTATATATATATATATAT	TGACATTTCGAACTTGAGTTTCC	CACAAGTCGCCATTTGGAAT	60.5	532	DRR	Ca_20635	--	TALE
Ca1_P4_2248466	Ca1	2248466	AT/AATT	GCCGGATTCACGTGATAGAT	CGATGTCTGTTGACGGAGTG	59.9	593	DRR	Ca_00281	O	--
Ca1_P4_2299200	Ca1	2299200	TAAA/TAAAA	CAGCAACTGCGATGAGATGT	GAATCAAAGGACACGATCCAA	60.0	227	DRR	Ca_00288	R	--
Ca1_P4_2300704	Ca1	2300704	T/TG	CATTCCATTCCTCATTGCCT	GCAAGTGCTGATGATGCTGT	59.9	556	DRR	Ca_00288	R	--
Ca1_P4_2323997	Ca1	2323997	GTTAATTAATTAA/GTTAATTAA	GGGCCTCAACAAAGCTTACA	TCCACAAACATGCATTAATCAAC	60.2	431	DRR	Ca_00291	V	NAC
Ca1_P4_2324632	Ca1	2324632	ATTTTTTTT/ATTTTTTTTTTT	TTAAGCTGAGCACTTCCAAGA	GAATCCGAAACCACACGAAT	57.9	523	DRR	Ca_00291	V	NAC

INDEL marker IDs	Chromosomes/ unanchored scaffolds	Physical positions (bp)	InDels (*Kabuli* reference genome-CDC Frontier/PII)	Forward primers (5'-3')	Reverse primers (5'-3')	Anneling temperature (0C)	Expected amplified product size (bp)	Structural annotation		Functional annotation	
								Sequence components of *kabuli* genome	*Kabuli* gene accession IDs	NCBI-KOG	TFs
Ca1_P4_2341397	Ca1	2341397	ATCAGTT/AT	GTGACATGGTTGCAGAGGTG	GGTTTGCTGCCACTTGAAAT	60.2	852	DRR	Ca_00294	A	--
Ca1_P4_2341920	Ca1	2341920	G/GC	ATTTCAAGTGGCAGCAAACC	TCATTTTGCTCATGCTTTGG	60.1	521	DRR	Ca_00294	A	--
Ca1_P4_23426642	Ca1	23426642	TAAA/TAAAA	AAGTTAAGGAACCACGAGTGC	AGAGAAGTGAATGGTGCATACAA	58.4	790	DRR	Ca_19317	--	--
Ca1_P4_2347223	Ca1	2347223	TCCC/TCC	TGGCAATATCACACAGCCAT	CCCTATCACAAACCACCCAC	60.0	826	DRR	Ca_00295	--	Dof
Ca1_P4_2347337	Ca1	2347337	TTCT/TTCTCT	TGCCTGCAACAAGTAATGGA	TGGCACAGAAGCAAAGAATG	60.3	947	DRR	Ca_00295	--	Dof
Ca1_P4_2347502	Ca1	2347502	TAAAA/TAAA	CGTATTGAAAATCCGTGCAA	TGGCACAGAAGCAAAGAATG	59.6	779	DRR	Ca_00295	--	Dof
Ca1_P4_2347965	Ca1	2347965	C/CG	GTGGGTGGTTTGTGATAGGG	CTGTGGGGCAAACAAAAGAT	60.1	482	DRR	Ca_00295	--	Dof
Ca1_P4_2348870	Ca1	2348870	ATAAATTTTT/ATAAATTTTTTAAATTTTT	TTGCTTATAGGCATGGGGAC	AACCACGTTTCCGAAAAATC	59.9	437	DRR	Ca_00295	--	Dof
Ca1_P4_23496241	Ca1	23496241	CT/CTTT	AGGGTGGATCAGTTCGATTG	TTATATTAATTGGGTGCGCGT	59.9	376	DRR	Ca_19314	--	--
Ca1_P4_2358700	Ca1	2358700	GAA/GAAA	TAACGGATTTTGACGATCCA	CGCTTCGGCTTTTCTTACAG	59.0	847	DRR	Ca_00296	L	--

INDEL marker IDs	Chromosomes/ unanchored scaffolds	Physical positions (bp)	InDels (*Kabuli* reference genome-CDC Frontier/PII)	Forward primers (5'-3')	Reverse primers (5'-3')	Anneling temperature (0C)	Expected amplified product size (bp)	Structural annotation		Functional annotation	
								Sequence components of *kabuli* genome	*Kabuli* gene accession IDs	NCBI-KOG	TFs
Ca1_P4_2360177	Ca1	2360177	TGG/T	ACACCGTCGAAGAAGCTGTT	CGCATGGGCTTATGATTTTT	59.9	898	DRR	Ca_00297	--	--
Ca1_P4_2369335	Ca1	2369335	ATAGTACTACTCCATTAGTACTACTCCATTA/ATAGTACTACTCCATTA	ACATCACGATTTTTCCCAAA	CCTATTTAGCATGTTGCCGC	57.9	165	DRR	Ca_00301	DR	--
Ca1_P4_2369599	Ca1	2369599	TTG/TTGTG	CAACTAGCGGCAACATGCTA	AGTACAACCCCCTATTGCCC	60.0	496	DRR	Ca_00301	DR	--
Ca1_P4_2399709	Ca1	2399709	CCTTTT/C	TGCGAGACCAGAAGAAAGGT	GCCAGCTTGAGCTGATTTCT	60.0	417	DRR	Ca_00306	O	--
Ca1_P4_24030518	Ca1	24030518	C/CATGTT	CCAATTCAAAAGCTCCCAAA	CAGATCCTCCTTTCCCAACA	60.0	424	DRR	Ca_19291	OK	--
Ca1_P4_2443873	Ca1	2443873	CTTT/CTT	CAATATCCATGCAGTGGTCG	TAAGGTGTGTGGTGGCAGAA	59.9	224	DRR	Ca_00309	--	--
Ca1_P4_2452266	Ca1	2452266	CTTTTTTTTT/CTTTTTTT	TTGCAAAGCTGTTGAGGTTG	TTACCGTGAGACGAAGGGAC	60.0	347	DRR	Ca_00310	--	--
Ca1_P4_24636488	Ca1	24636488	ATTTT/ATTTATTTTT	GATGGTGTGAGTGGAGCTGA	AAACACGTTGGCATTTGTGA	59.8	906	DRR	Ca_18338	--	--
Ca1_P4_2470802	Ca1	2470802	ATTT/ATT	GACGTTATCGACTGGGCAAT	GCGTGAGTAGCAAACGAACA	60.0	320	DRR	Ca_00312	J	bHLH
Ca1_P4_2471521	Ca1	2471521	ATTCTT/ATT	TTCGTTTGCTACTCACGCAC	TGACTTATTCAGGCCCTGCT	60.1	310	DRR	Ca_00312	J	bHLH

INDEL marker IDs	Chromosomes/ unanchored scaffolds	Physical positions (bp)	InDels (*Kabuli* reference genome-CDC Frontier/PII)	Forward primers (5'-3')	Reverse primers (5'-3')	Anneling temperature (0C)	Expected amplified product size (bp)	Structural annotation		Functional annotation	
								Sequence components of *kabuli* genome	*Kabuli* gene accession IDs	NCBI-KOG	TFs
Ca1_P4_24797994	Ca1	24797994	GTTTT/GTTT	TGATTTTCGAAGACTTGGGG	TGCATCGTTCTCCTCTTCCT	60.0	894	DRR	Ca_18329	T	--
Ca1_P4_25539046	Ca1	25539046	CATATATATATATATATATATATATA/CATATATATATATATATATATATATATATATATA	CTGATGTTGGTCCTGGTCCT	AACCAACCCAACGAGAAGTG	60.0	684	DRR	Ca_25082	--	--
Ca1_P4_25781138	Ca1	25781138	GG/GGGAAATACTTG	GCTTCAAGAGACGAAATCCG	ACACGACCGATAGGGATCAG	60.0	709	DRR	Ca_18582	--	--
Ca1_P4_26473008	Ca1	26473008	CTAAATT/CTAAATTCTGTAAATT	CCATTGACCCTTGCAAACTT	CTGCAGGAAGCTAAAATCCG	60.0	368	DRR	Ca_18608	R	NAC
Ca1_P4_2669284	Ca1	2669284	TAAAAAAAAAAAAA/TAAAAAAAAAA	CCCCAAACTTTGAAGCAACT	TTTGTGCTGCTATGTGCCTC	59.2	344	DRR	Ca_00327	--	ERF
Ca1_P4_2670477	Ca1	2670477	AACA/AA	TAGCAGACTCCAAATCCCCA	AAACCAGCGGTTTCAATCTG	60.6	778	DRR	Ca_00327	--	ERF
Ca1_P4_2686396	Ca1	2686396	GTT/GTTT	AAACGCCATTGCACCTTAAC	CAATTCATATCGGCAATCCC	60.0	684	DRR	Ca_00328	--	MIKC
Ca1_P4_2686857	Ca1	2686857	TG/T	AATGTGATGCACGTTTTGGA	CAAATTTCACGCAACAATCG	60.0	444	DRR	Ca_00328	--	MIKC
Ca1_P4_2691980	Ca1	2691980	AAGAG/AAG	TTAGCTCAGCTTGCACAGGA	GCATTGTACATTCTCGTGCG	59.9	541	DRR	Ca_00330	--	--
Ca1_P4_2692135	Ca1	2692135	AC/ACCTC	ACAATGCCATTTTCAAGGCT	CGTGAAGACTAAAGGTCAGAAAAA	59.6	532	DRR	Ca_00330	--	--

INDEL marker IDs	Chromosomes/ unanchored scaffolds	Physical positions (bp)	InDels (*Kabuli* reference genome-CDC Frontier/PII)	Forward primers (5'-3')	Reverse primers (5'-3')	Anneling temperature (0C)	Expected amplified product size (bp)	Structural annotation		Functional annotation	
								Sequence components of *kabuli* genome	*Kabuli* gene accession IDs	NCBI-KOG	TFs
Ca1_P4_2693052	Ca1	2693052	CT/CTT	CAACCAGTAATAATGCGCCA	CGTGACAGAGGACGCTTGTA	59.6	468	DRR	Ca_00330	--	--
Ca1_P4_2693080	Ca1	2693080	TAAAAAAAA/TAAAAAAAAA	CAACCAGTAATAATGCGCCA	CGTGACAGAGGACGCTTGTA	59.6	468	DRR	Ca_00330	--	--
Ca1_P4_27597440	Ca1	27597440	ATT/AT	AAAATCCAACAATCTCCCCC	CCCTGAGACTCACGAAGAGG	60.0	559	DRR	Ca_25069	--	--
Ca1_P4_2802326	Ca1	2802326	GTTT/GTT	GGGTTACAGACCGACCTTGA	TCGGCAGTTGTGATCGAATA	60.0	242	DRR	Ca_00344	--	NAC
Ca1_P4_2802455	Ca1	2802455	ATTTTT/ATTTT	GGGTTACAGACCGACCTTGA	GCGGATTTGTCCATCTTTGT	60.0	765	DRR	Ca_00344	--	NAC
Ca1_P4_2808661	Ca1	2808661	TAAA/TAAAAA	TCATGTTGTTTGCCGTTTGT	TCAGCACAATCAACAAGGGA	60.0	233	DRR	Ca_00345	T	M-type
Ca1_P4_2831499	Ca1	2831499	GTTTTTTTTTTTTTT/GTTTTTTTTTTT	CCTCTGACCACATGGGACTT	TCAAAAACAAAAGCGCACTG	60.0	625	DRR	Ca_00347	--	ARF
Ca1_P4_2831879	Ca1	2831879	TAAAAA/TAAAAAA	TTTGAGTCTTCGAGCCACAG	CTGGTCCTTTCCTTTCCCTC	59.2	342	DRR	Ca_00347	--	ARF
Ca1_P4_2867118	Ca1	2867118	CA/CAA	AGGAGCCTTTGGTTTTGGTT	TCCCTCTTCCCCTTTCTCAT	60.0	555	DRR	Ca_00348	R	--
Ca1_P4_2867217	Ca1	2867217	ATTTTTTTT/ATTTTTTTTT	AGGAGCCTTTGGTTTTGGTT	TCCCTCTTCCCCTTTCTCAT	60.0	555	DRR	Ca_00348	R	--

INDEL marker IDs	Chromosomes/ unanchored scaffolds	Physical positions (bp)	InDels (*Kabuli* reference genome-CDC Frontier/PII)	Forward primers (5'-3')	Reverse primers (5'-3')	Anneling temperature (0C)	Expected amplified product size (bp)	Structural annotation		Functional annotation	
								Sequence components of *kabuli* genome	*Kabuli* gene accession IDs	NCBI-KOG	TFs
Ca1_P4_2875002	Ca1	2875002	CTTTTTTTTTT/CTTTTTTTTT	TGCCAAAGGTAATTTTTCCG	GCCCGCAAAAGTTAAAAGAA	59.9	149	DRR	Ca_00349	--	--
Ca1_P4_2877412	Ca1	2877412	CA/CAA	TTTGGTTCAGAAGCTGGACA	AATCGACGCCGATGACTAAC	59.4	565	DRR	Ca_00350	GC	SBP
Ca1_P4_2884045	Ca1	2884045	TAAA/TAA	GAGCTTCTCCACCTCACGTC	GTGGCATTCAGGTGAGGAAT	60.0	412	DRR	Ca_00351	T	M-type
Ca1_P4_2884405	Ca1	2884405	AGAGACATT/A	ATTCCTCACCTGAATGCCAC	TGAAAGCATCATGCACACAA	59.9	511	DRR	Ca_00351	T	M-type
Ca1_P4_2884853	Ca1	2884853	GTTTT/GTTT	TCTCCGTCGTGTTGTGTTGT	ATTGCGGAGTTAACAATGGC	60.2	743	DRR	Ca_00351	T	M-type
Ca1_P4_2885377	Ca1	2885377	ATTTTTTT/ATTTTTTTT	GCCATTGTTAACTCCGCAAT	CATTTGGAGAGGGCATAGGA	60.0	360	DRR	Ca_00351	T	M-type
Ca1_P4_2886871	Ca1	2886871	ATTTTTTTTTTTT/ATTTTTTTTTTTTT	ACAATGACACAGTCGGTGGA	CAATTGCATTTTTGAGTCTTTCA	60.0	167	DRR	Ca_00352	--	--
Ca1_P4_28910299	Ca1	28910299	CATT/CATTAATT	GAAAATGATCGGTGGAGGAA	CTTGCGTGTTGCACGAGTAT	59.9	620	DRR	Ca_18486	R	--
Ca1_P4_28910417	Ca1	28910417	CTTTTTTT/CTTTTTTTTTTT	GAGTCATTGGTGCCTTGGTT	CTTGCGTGTTGCACGAGTAT	60.0	385	DRR	Ca_18486	R	--
Ca1_P4_28910492	Ca1	28910492	GATTAA/GATTAATTAA	GAGTCATTGGTGCCTTGGTT	CTTGCGTGTTGCACGAGTAT	60.0	385	DRR	Ca_18486	R	--

INDEL marker IDs	Chromosomes/ unanchored scaffolds	Physical positions (bp)	InDels (*Kabuli* reference genome-CDC Frontier/PII)	Forward primers (5'-3')	Reverse primers (5'-3')	Anneling temperature (0C)	Expected amplified product size (bp)	Structural annotation		Functional annotation	
								Sequence components of *kabuli* genome	*Kabuli* gene accession IDs	NCBI-KOG	TFs
Ca1_P4_2904188	Ca1	2904188	TG/T	AGGAAGCAGGTGTAGGGGAT	ACACGGAAGTTGGTTTCCTG	60.0	602	DRR	Ca_00354	--	bHLH
Ca1_P4_2904668	Ca1	2904668	A/AAAAGAGTGG	CAGGAAACCAACTTCCGTGT	GGCAAAGAAAGCAGTTCCAG	60.0	849	DRR	Ca_00354	--	bHLH
Ca1_P4_2932731	Ca1	2932731	AA/AATAGGTTGA	GAATCTGGTTCCGCACTCAT	GATTCCGGTATTTGCCTCAA	60.1	771	DRR	Ca_00358	E	--
Ca1_P4_2933145	Ca1	2933145	AAGA/AAGAGA	CACACGGTCACAATCGAACT	AGTGCCTTGGACATTTTTGG	59.6	590	DRR	Ca_00358	E	--
Ca1_P4_29386975	Ca1	29386975	AATATATATATATATATATATATATATAT/AATATATATATATATATATATATATATATATATATAT	TCGTTTCCCAGAGAAGCAAC	AGAAAATGCGGGTTATGTCG	60.4	743	DRR	Ca_22561	R	HB-other
Ca1_P4_29422590	Ca1	29422590	TT/TTATGAT	AGTGGATCGGGTGAAACAAG	GTCGCGTCGAAAACTAGCTC	60.0	783	DRR	Ca_22560	--	--
Ca1_P4_2952450	Ca1	2952450	GTT/GT	ACCTCAAATCCGCAAAGATG	GTGCAAGAGTTTGGCTTGGT	60.1	571	DRR	Ca_00360	--	ERF
Ca1_P4_2952486	Ca1	2952486	CAATAAATAAATAAATA/CAATAAATAAATAAATAAATA	ACCTCAAATCCGCAAAGATG	GTGCAAGAGTTTGGCTTGGT	60.1	571	DRR	Ca_00360	--	ERF
Ca1_P4_2980062	Ca1	2980062	ATTTTTTTTT/ATTTTTTTTTT	TTGTTCAGGATTTGTTGGCA	AAGTTTGGGAGGCCATAACC	60.1	665	DRR	Ca_00362	PET	NAC
Ca1_P4_2981588	Ca1	2981588	GC/G	TGTTCGGCCATTAGACATCA	AATCGCCCATTTCTCCTCTT	60.1	345	DRR	Ca_00361	--	--

INDEL marker IDs	Chromosomes/ unanchored scaffolds	Physical positions (bp)	InDels (*Kabuli* reference genome-CDC Frontier/PII)	Forward primers (5'-3')	Reverse primers (5'-3')	Anneling temperature (0C)	Expected amplified product size (bp)	Structural annotation		Functional annotation	
								Sequence components of *kabuli* genome	*Kabuli* gene accession IDs	NCBI-KOG	TFs
Ca1_P4_29846530	Ca1	29846530	CTTTT/CTTTTT	TGGATTGGGGATCATTCATT	TCAACATTTGGAAGCACAAGA	60.0	897	DRR	Ca_24388	--	--
Ca1_P4_29846941	Ca1	29846941	TAAAAAAAAAA/TAAAAAAAA	TGTTGCCAACTGAAAATCGT	ATACAGACACCGAATGGGGA	59.2	828	DRR	Ca_24388	--	--
Ca1_P4_3030441	Ca1	3030441	AATATATAT/AATATAT	AATAGTGAGGGGTGGAGGCT	TGATGGATGAGACAAAATGCC	60.0	415	DRR	Ca_00367	--	--
Ca1_P4_3030828	Ca1	3030828	TAAAAAAA/TAAAAAA	TCACACACATGGTGCAGCTA	CTGCTTTGCACACGCATATT	59.9	710	DRR	Ca_00367	--	--
Ca1_P4_3033238	Ca1	3033238	CT/CTTT	CCGCTGAAGTTGAAGCAGTA	TCAGTTGCAAAGCCAAACAC	59.2	758	DRR	Ca_00369	--	--
Ca1_P4_3036457	Ca1	3036457	TC/T	TGAAGAGAGAATGAGGGGGA	TTGAGCCATTGATTTTGTGC	59.7	662	DRR	Ca_00370	--	ARF
Ca1_P4_3036576	Ca1	3036576	ATTTTTT/ATTTTTTT	TGAAGAGAGAATGAGGGGGA	CCTTATTCAACGGCTGGTGT	59.7	801	DRR	Ca_00370	--	ARF
Ca1_P4_3071285	Ca1	3071285	C/CAAAAAATTTAATTG	AGGCACAAATGGAGCAAATC	TCCATTCGACCATTTTCACA	60.1	623	DRR	Ca_00374	EH	bHLH
Ca1_P4_3079517	Ca1	3079517	A/AAATTTTTTG	TCAAATTCGTAACTCAAATCTTCAA	ATTGCCCCAAGACATTATCG	59.2	478	DRR	Ca_00375	--	bHLH
Ca1_P4_3087515	Ca1	3087515	CAAA/CAAAAA	GGGCAATTCTATCGCTCAAA	ACTGGTGGACGAGCAAGACT	60.2	681	DRR	Ca_00376	--	bHLH

INDEL marker IDs	Chromosomes/ unanchored scaffolds	Physical positions (bp)	InDels (*Kabuli* reference genome-CDC Frontier/PII)	Forward primers (5'-3')	Reverse primers (5'-3')	Anneling temperature (0C)	Expected amplified product size (bp)	Structural annotation		Functional annotation	
								Sequence components of *kabuli* genome	*Kabuli* gene accession IDs	NCBI-KOG	TFs
Ca1_P4_3088149	Ca1	3088149	AGG/AG	AGTCTTGCTCGTCCACCAGT	AGGAAGCACAAGCTCAAACG	59.9	654	DRR	Ca_00376	--	bHLH
Ca1_P4_3089287	Ca1	3089287	TTGTAAT/TT	GCAAAACGTCTTATCGGGAA	TGCAAACTATTTGACATTGACG	60.1	591	DRR	Ca_00378	I	--
Ca1_P4_3089369	Ca1	3089369	AT/ATT	AAAATCGGTCTTGGGCTCTT	CCGTGTTAGTCGTCTATTTTTACCA	60.1	713	DRR	Ca_00378	I	--
Ca1_P4_3089741	Ca1	3089741	ATTTTT/ATTTTTT	TCAAATAGTTTGCACTACGACG	GGAGACCACTGTTCACCCAT	58.1	691	DRR	Ca_00378	I	--
Ca1_P4_3104052	Ca1	3104052	TTT/TTTTCTT	TCAATACATCACCCGTGTTTTT	TTGAACACAGGAAGAACCCTC	59.2	474	DRR	Ca_00380	--	--
Ca1_P4_31086522	Ca1	31086522	TAAAA/TAAA	TATAGGTTGGCACCCAAAGC	GGTTCGCCATATACCAATGC	60.0	706	DRR	Ca_25826	S	--
Ca1_P4_3113861	Ca1	3113861	TA/TAA	TGATTACAATCCTTTAAGCACG	GGAATCGAACTCGAGACTGC	57.1	564	DRR	Ca_00381	--	--
Ca1_P4_3135689	Ca1	3135689	TCTACTACT/TCTACT	TTTGGTTGTGGTAGCAGCAG	TTTTTCCAGCAACTTGGAGC	59.9	413	DRR	Ca_00383	--	--
Ca1_P4_31454507	Ca1	31454507	TNNNNNNNNNNNNNNNNN/TNNNNNNNNN	TCGACAACAAAGTAATGCCAA	CAAACATCAAACTGCAACGG	59.2	737	DRR	Ca_21861	--	--
Ca1_P4_31498666	Ca1	31498666	TAAAAAA/TAAAAAAA	TTGGAGCTTCAGCATTTGTG	CCTCTTTTCTTCGCATTTCG	60.0	747	DRR	Ca_21863	--	M-type

INDEL marker IDs	Chromosomes/ unanchored scaffolds	Physical positions (bp)	InDels (*Kabuli* reference genome-CDC Frontier/PII)	Forward primers (5'-3')	Reverse primers (5'-3')	Anneling temperature (0C)	Expected amplified product size (bp)	Structural annotation		Functional annotation	
								Sequence components of ***kabuli*** genome	***Kabuli*** gene accession IDs	NCBI-KOG	TFs
Ca1_P4_31498685	Ca1	31498685	AGG/AG	TTGGAGCTTCAGCATTTGTG	CCTCTTTTCTTCGCATTTCG	60.0	747	DRR	Ca_21863	--	M-type
Ca1_P4_33105635	Ca1	33105635	TC/T	TGCCAAGAACGATAAAAGCA	TTCTGCATTTTCCTTGAGCA	59.4	101	DRR	Ca_19438	--	--
Ca1_P4_33388984	Ca1	33388984	ATTTTTTTTTTTTTTT/ATTTTTTTTTTTTTTTTT	TGTTGACGTTTCAACGGAGA	TCCCTATTCAACCCCCTTTC	60.3	136	DRR	Ca_19442	--	--
Ca1_P4_33722196	Ca1	33722196	AAGA/AA	GATTTTACGAGGATGGCGAA	ATGAGCGAGGTAGAAGCCAA	60.0	610	DRR	Ca_19456	--	--
Ca1_P4_338839	Ca1	338839	AACA/AA	CTTTGATTTGAATTTGGCCC	AGTTGGGTCCTTGCCTTTTT	59.4	399	DRR	Ca_00034	S	B3
Ca1_P4_34005765	Ca1	34005765	ATA/ATAGTA	AATCGTTTCGCAAGACCTTC	CGACGCTTGATCGTTAATTG	59.3	539	DRR	Ca_27634	--	--
Ca1_P4_34136656	Ca1	34136656	ATTTTTTTT/ATTTTTTT	TTTCTTGGTGACCCACATCA	AAGTCGAGGACAATGCTTGC	59.9	338	DRR	Ca_25472	QI	bHLH
Ca1_P4_34137295	Ca1	34137295	CTTT/CTT	GCAAGCATTGTCCTCGACTT	TGTTACCAGGCTGAACCACA	60.4	845	DRR	Ca_25472	QI	bHLH
Ca1_P4_34160353	Ca1	34160353	ATT/AT	GGTTTCGCCCTGACATAAAA	ACTCTGGACACTTGGGTTGC	59.9	773	DRR	Ca_25473	O	--
Ca1_P4_34730486	Ca1	34730486	TTTTA/TTTTAGTCCTTTA	ATATGACTCCTGAGCGGTGC	TTTTTCCGACTTGGTCCTTT	60.2	501	DRR	Ca_21419	--	C3H

INDEL marker IDs	Chromosomes/ unanchored scaffolds	Physical positions (bp)	InDels (*Kabuli* reference genome-CDC Frontier/PII)	Forward primers (5'-3')	Reverse primers (5'-3')	Anneling temperature (0C)	Expected amplified product size (bp)	Structural annotation		Functional annotation	
								Sequence components of *kabuli* genome	*Kabuli* gene accession IDs	NCBI-KOG	TFs
Ca1_P4_34865996	Ca1	34865996	TAATAAATAAATAAA/TAATAAATAAATAAATAAA	TTGTTCGAGAATTTGGAGGG	TGAGGTGGTGACCAATTGAA	60.0	462	DRR	Ca_21411	--	MYB_related
Ca1_P4_35240205	Ca1	35240205	ATAT/ATATTAT	GTAGCATTTTTGGCCTCCCT	GTCGAGAAGAAGGCTTGCAT	60.5	782	DRR	Ca_26097	QI	bHLH
Ca1_P4_35240820	Ca1	35240820	GTTTTTTTT/GTTTTTTTTT	TGCAAGCCTTCTTCTCGACT	TTGGTGGAATGTGTGCATGT	60.3	543	DRR	Ca_26098	QI	--
Ca1_P4_35317313	Ca1	35317313	CTTTTTTT/CTTTTTTTT	CCTGATCCTCATCGTCCATT	GCCAGGTTCACTCGATTCAT	59.9	464	DRR	Ca_26100	QI	bHLH
Ca1_P4_355593	Ca1	355593	TA/TAAAA	TTCACATTATCCGGCTCCTC	TGTGCCATTTAAGTTTGCCA	60.0	430	DRR	Ca_00037	E	--
Ca1_P4_35569645	Ca1	35569645	TAAAAAA/TAAAAA	TGAGTGCACCTTTTACAGCG	TTGCACGCTTAGTATGCACC	60.1	256	DRR	Ca_24107	--	--
Ca1_P4_355898	Ca1	355898	ATTATTTAT/ATTAT	TATTTGCAGGCCCACTATCC	TCGAATTTCTTACCATGACCAA	59.9	709	DRR	Ca_00037	E	--
Ca1_P4_3677454	Ca1	3677454	GTATATATATATATATATATA/GTATATATATATATATA	ACACATCGTCCAAACAATCG	AATATTTGTGGGTGGCCAAA	59.4	590	DRR	Ca_00448	--	FAR1
Ca1_P4_3708800	Ca1	3708800	GTATATATATATATATATATA/GTATATATATATATATATATA	GCCTGGTGACTTGTTTAGGC	TTGAGTGGGATAGATCGTTGG	59.7	651	DRR	Ca_00451	--	G2-like
Ca1_P4_37197766	Ca1	37197766	GTTT/GTT	TGAGAGGAAAGAAGACAACCATC	TTTTCTTTTTAAAGCGGCGA	59.7	605	DRR	Ca_21831	L	C3H

INDEL marker IDs	Chromosomes/ unanchored scaffolds	Physical positions (bp)	InDels (*Kabuli* reference genome-CDC Frontier/PII)	Forward primers (5'-3')	Reverse primers (5'-3')	Anneling temperature (0C)	Expected amplified product size (bp)	Structural annotation		Functional annotation	
								Sequence components of *kabuli* genome	*Kabuli* gene accession IDs	NCBI-KOG	TFs
Ca1_P4_37412282	Ca1	37412282	CCATG/CCATGCATG	CGTGGATGATGCTTTTCTCA	TGCTTCTCATCTGCTTGGTG	59.8	580	DRR	Ca_21842	R	GRAS
Ca1_P4_37693180	Ca1	37693180	AATTAT/AAT	TGTGGAACATTCTCTGCGTC	TTCCCGAGAGATTTGTCTTCA	59.8	593	DRR	Ca_23533	--	G2-like
Ca1_P4_37693633	Ca1	37693633	TAAAAAAAAAA/TAAAAAAAAAAA	TGAAGACAAATCTCTCGGGAA	TTTTTGCCTTTCTTCCATTCA	59.8	170	DRR	Ca_23533	--	G2-like
Ca1_P4_37721004	Ca1	37721004	TAAAAAAAA/TAAAAAAAAAA	AAGACCTTCTTAACTCACCAATCG	ATTTCTCTCACCCGCATTTG	60.1	481	DRR	Ca_23534	--	--
Ca1_P4_37721057	Ca1	37721057	ATTTTTTTTT/ATTTTTTTT	AAAAATGCTCCCAAAGTCATT	ATTTCTCTCACCCGCATTTG	57.7	260	DRR	Ca_23534	--	--
Ca1_P4_37721398	Ca1	37721398	TAAAAA/TAAA	CAAATGCGGGTGAGAGAAAT	CATTGGTGGGGAGGTGTATT	60.1	598	DRR	Ca_23534	--	--
Ca1_P4_37721826	Ca1	37721826	AAAA/AAAATAAA	TCCATAGCACCTCTTGATTTTG	CGTTGATGGCATCTTGATTG	59.2	420	DRR	Ca_23534	--	--
Ca1_P4_38184317	Ca1	38184317	ATTTT/ATTTTT	TGACTCGTGATCCTCACTGC	TTAAGCGTGCTCTCAACGTG	60.0	572	DRR	Ca_13975	--	--
Ca1_P4_38184769	Ca1	38184769	TAT/TATAAT	AAGGAGAGGCACGTTGAGAG	CCTACCACTCCTTCTGCACC	59.6	768	DRR	Ca_13975	--	--
Ca1_P4_38265402	Ca1	38265402	CAAAAAAAAAAAA/CAAAAAAAAAA	TGGTATTACTGACCCGCACA	ACAATTGTTGGAAAGCCCAG	60.0	589	DRR	Ca_13970	--	--

INDEL marker IDs	Chromosomes/ unanchored scaffolds	Physical positions (bp)	InDels (*Kabuli* reference genome-CDC Frontier/PII)	Forward primers (5'-3')	Reverse primers (5'-3')	Anneling temperature (0C)	Expected amplified product size (bp)	Structural annotation		Functional annotation	
								Sequence components of *kabuli* genome	*Kabuli* gene accession IDs	NCBI-KOG	TFs
Ca1_P4_38278656	Ca1	38278656	C/CG	TCCAGCATTGCCTTTTCTTT	GCATGGGTGAGAATGGAGTT	59.8	687	DRR	Ca_13968	O	--
Ca1_P4_38338075	Ca1	38338075	ATTTTTTTTT/ATTTTTTTT	TATGGGTCCATTTTAGGCCA	AATCCCCAATTCGAAAAACC	60.1	623	DRR	Ca_13967	--	--
Ca1_P4_38345242	Ca1	38345242	TA/TAAA	CACTCCCTCTTTGATGCACTT	TGGGGTGTTGGATTGTTTCT	59.3	487	DRR	Ca_13966	--	--
Ca1_P4_38487463	Ca1	38487463	ATTTTTTTTTT/ATTTTTTTTT	TGCTACCTGCAACCAGTGAG	GTCAACTTTAAGGGGGCGTC	60.0	872	DRR	Ca_13962	--	--
Ca1_P4_38535428	Ca1	38535428	CAAAAAAAA/CAAAAAAA	AGGGCAATATCACATCCGAG	CGCATCAACGACATATGGAC	59.9	668	DRR	Ca_13959	--	--
Ca1_P4_38597934	Ca1	38597934	CAAAAA/CAAAAAA	CATTTGCCTCCCTTGTTGTT	TGTAGTTGCTGCAAACGAGG	60.0	551	DRR	Ca_13956	--	--
Ca1_P4_38789465	Ca1	38789465	TTATA/TTA	GGTCATGCATCATACCCACA	TCATCCAATACACCACCCTT	60.2	691	DRR	Ca_13953	--	--
Ca1_P4_38882967	Ca1	38882967	GTTAGGA/GTTAGGACTTAGGA	ATTAGGAACCCTGGCATGTG	GGCAGCATTTGGTGAACTTT	59.8	464	DRR	Ca_13949	G	--
Ca1_P4_39073445	Ca1	39073445	AATATATATAT/AATATATATATAT	GAAAACATGCCCATGGATCT	GCATTAAGGTGCGATGAATG	59.8	578	DRR	Ca_13946	--	--
Ca1_P4_39073809	Ca1	39073809	A/AC	CATTCATCGCACCTTAATGC	TTGAAGAAGCATCCCTCTGA	59.1	309	DRR	Ca_13946	--	--

INDEL marker IDs	Chromosomes/ unanchored scaffolds	Physical positions (bp)	InDels (*Kabuli* reference genome-CDC Frontier/PII)	Forward primers (5'-3')	Reverse primers (5'-3')	Anneling temperature (0C)	Expected amplified product size (bp)	Structural annotation		Functional annotation	
								Sequence components of *kabuli* genome	*Kabuli* gene accession IDs	NCBI-KOG	TFs
Ca1_P4_39446823	Ca1	39446823	GATT/G	GTTGTAAGCTTGCAGGAGGG	TTTTCGCCCCAATAAAGTTG	59.9	713	DRR	Ca_13931	Z	NAC
Ca1_P4_39447104	Ca1	39447104	TTAATG/TTAATGTTGATCTAATG	CAACTTTATTGGGGCGAAAA	CCCTCGATTAATTCTCTTGAGGT	59.9	253	DRR	Ca_13931	Z	NAC
Ca1_P4_39673797	Ca1	39673797	ATTTTT/ATTTTTT	GCCTCAAAAGCGAAATTTGT	TTGGAACACATAAAAATTAATAATCCA	59.3	759	DRR	Ca_13921	--	--
Ca1_P4_39733036	Ca1	39733036	CTGAT/C	TGATTGAGAGGGGAGAAAGG	AAACAGACCCCGAATGTCAC	59.2	117	DRR	Ca_13916	R	bZIP
Ca1_P4_39743942	Ca1	39743942	TAAAA/TAAAAA	TTCCATTTCCTCCACAAAGC	TGCTACAACCGCATTGTCAT	60.1	563	DRR	Ca_13915	--	--
Ca1_P4_40234096	Ca1	40234096	ATTTTTTTTTTTTTT/ATTTTTTTTTTTT	GAACGTGGAGGTGAGGAAAA	AACAAAATTCAACAACCAAAGAA	60.1	575	DRR	Ca_13896	--	--
Ca1_P4_40234165	Ca1	40234165	TTTATTATTATTATTATTA/TTTATTATTATTA	GAACGTGGAGGTGAGGAAAA	AACAAAATTCAACAACCAAAGAA	60.1	575	DRR	Ca_13896	--	--
Ca1_P4_40373644	Ca1	40373644	C/CG	TACCAAAACTCAGCATTGCG	TTCTGTCGAAGAACTTGTGGAA	59.9	415	DRR	Ca_13892	--	Trihelix
Ca1_P4_40422016	Ca1	40422016	TAAAAAAAAAA/TAAAAAAAAAAAA	TATTTCCAAGCCAAAGACCG	CACGCTCAAACTTCCTCGAT	60.1	558	DRR	Ca_13890	A	--
Ca1_P4_40872000	Ca1	40872000	AAATATATATATATATA/AATATATATATA	GCAAACCCTGCCCTAACATA	CCAAGACAACTGAAAGATCCG	60.0	468	DRR	Ca_18460	--	--

INDEL marker IDs	Chromosomes/ unanchored scaffolds	Physical positions (bp)	InDels (*Kabuli* reference genome-CDC Frontier/PII)	Forward primers (5'-3')	Reverse primers (5'-3')	Anneling temperature (0C)	Expected amplified product size (bp)	Structural annotation		Functional annotation	
								Sequence components of *kabuli* genome	*Kabuli* gene accession IDs	NCBI-KOG	TFs
Ca1_P4_409913	Ca1	409913	GGAAAG/GG	GCACCACCTTGGCTCT AATC	GGCGAATTCAAAGTTC CGTA	59.7	550	DRR	Ca_00043	--	--
Ca1_P4_41167792	Ca1	41167792	AATATATATATATATATATATA/AATA TATATATATATATATA	TTTTGGTCGGTATTCC AATTT	GGGTCAAAATGTATGG TCGG	58.3	802	DRR	Ca_18480	--	--
Ca1_P4_413371	Ca1	413371	AAAGTAAGT/AAAGT	GAAGTCAGACGCGCTA ATCC	CTTTCCCAAAAGGCAA GTGA	60.0	533	DRR	Ca_00045	--	--
Ca1_P4_41774189	Ca1	41774189	CAAAA/CAA	TCCATTCACACTTGTTG GGA	GGCACATTCATGTTCA GACG	59.9	237	DRR	Ca_22517	G	--
Ca1_P4_41935770	Ca1	41935770	AGGG/AGGGG	TCGATCAGATAAAGCA ACCG	CAATGCTTCATACGTC CTGC	58.9	410	DRR	Ca_22531	--	--
Ca1_P4_425631	Ca1	425631	AT/A	TACCACGTGGCGTCAC TTTA	AGCTTGGAGGTTTGCG TAGA	60.2	705	DRR	Ca_00046	--	NF-YC
Ca1_P4_42687636	Ca1	42687636	ATTACAGAGTTACA/ATTACAGAGTT ACAGAGTTACA	CTGGGCAAGGGATCTG AATA	TGCATTTTACAGCGCTT TTG	60.0	500	DRR	Ca_13605	--	Trihelix
Ca1_P4_42822054	Ca1	42822054	AATATATATATATATATATATATAT/A ATATATATATATATATATATATATATA TAT	AAAGGCATGACCAACT CCAT	CACCTGATTCCTCGTT GGTT	59.4	671	DRR	Ca_13599	--	C2H2
Ca1_P4_43681169	Ca1	43681169	GAAAAAAA/GAAAAAA	GTGCAAAGATTGCAAC GAAA	AGCCATTAGGCTTTGA TTGG	59.9	327	DRR	Ca_13559	--	NAC
Ca1_P4_45003074	Ca1	45003074	AA/AAATAAAATTCCGAATCA	TCTTCCACATTGATTTT GTTGC	TGCTTATTCCCCAGATA CCG	60.0	988	DRR	Ca_25714	--	--

INDEL marker IDs	Chromosomes/ unanchored scaffolds	Physical positions (bp)	InDels (*Kabuli* reference genome-CDC Frontier/PII)	Forward primers (5'-3')	Reverse primers (5'-3')	Anneling temperature (0C)	Expected amplified product size (bp)	Structural annotation		Functional annotation	
								Sequence components of *kabuli* genome	*Kabuli* gene accession IDs	NCBI-KOG	TFs
Ca1_P4_45243775	Ca1	45243775	T/TATGCACTTCTTTTCTC	TGTGCCAAGGTTTCAGAACA	CTTTTATTGCAAGACCCCGA	60.3	605	DRR	Ca_26020	--	--
Ca1_P4_45607074	Ca1	45607074	AA/AAGCGCTA	TGCATCACTCCAAGAACCAA	ACTCACCTATTCCGACCGTG	60.2	344	DRR	Ca_24440	R	--
Ca1_P4_46531041	Ca1	46531041	T/TG	GCGAAAATAATGTTGATGCAGA	GTCTGCGGATAATTTCGCTT	60.1	139	DRR	Ca_21551	M	--
Ca1_P4_46728277	Ca1	46728277	ATTT/ATT	TTCTTTAACCTGGCCAAACG	CCGTCTTCGAATCACCGTAT	60.1	681	DRR	Ca_25109	--	--
Ca1_P4_46834640	Ca1	46834640	AATATATATATATATATATATATATATAT/AATATATATATATATATATATATATAT	TGAATTACAGCTATAAAACGAAGATAA	GATCAGGATGAACCGGAAGA	57.1	435	DRR	Ca_25116	U	--
Ca1_P4_46867289	Ca1	46867289	GCT/G	GCCCTCAGATGAGAGTCAGG	CGTTTTTCAAAGCGAGGGTA	59.9	439	DRR	Ca_25118	--	--
Ca1_P4_46867363	Ca1	46867363	CTTTT/CTTT	GCCCTCAGATGAGAGTCAGG	CAAACCTTTCATGCGGGTAT	59.9	836	DRR	Ca_25118	--	--
Ca1_P4_46916401	Ca1	46916401	GATATATATATATATATATA/GATATATATATATATATATATA	CAGCAAGCACCAACCACTTA	TTGCCAGTTATTTTTGCAAGG	59.9	363	DRR	Ca_12949	--	--
Ca1_P4_4725547	Ca1	4725547	GAAAAAAAAAA/GAAAAAAAAAAAAAAAA	TTGCCACTCATAGGTCACAAAG	TGGCTTCCTCTCTGTGTGTG	60.2	571	DRR	Ca_00556	--	--
Ca1_P4_47292040	Ca1	47292040	CAACAA/CAACAAACAA	GTATAGCTGCGGAAGCTTGG	CATAAACCCTCACCCCTCCT	60.0	669	DRR	Ca_12923	--	BES1

INDEL marker IDs	Chromosomes/ unanchored scaffolds	Physical positions (bp)	InDels (*Kabuli* reference genome-CDC Frontier/PII)	Forward primers (5'-3')	Reverse primers (5'-3')	Anneling temperature (0C)	Expected amplified product size (bp)	Structural annotation		Functional annotation	
								Sequence components of *kabuli* genome	*Kabuli* gene accession IDs	NCBI-KOG	TFs
Ca1_P4_47337298	Ca1	47337298	CT/CAAATATATACTT	ATTGGGCATAGGAAGCACTG	AGTTGACCACGGACCCATAG	60.1	533	DRR	Ca_12919	--	--
Ca1_P4_48246328	Ca1	48246328	CTT/CTTT	GACACCTCCTTTCCTCCACA	CAATTCCACCGCTTTCAAGT	60.1	911	DRR	Ca_12858	--	--
Ca1_P4_4899842	Ca1	4899842	CTTTTTT/CTTTTTTT	GCACATTTGGCAAACCTTCT	CAAAAAGCATGCTGCAAAGA	60.1	445	DRR	Ca_00576	R	M-type
Ca1_P4_4900100	Ca1	4900100	TATTCAAT/TATTCAATTCAAT	TCTTTGCAGCATGCTTTTTG	TTTCTGAAGCGACCAATTCC	60.1	561	DRR	Ca_00575	--	--
Ca1_P4_4900435	Ca1	4900435	C/CT	GGCTTCCCCCTTTCAACTAA	TCCATAAGCTGATCCCCAAG	60.4	920	DRR	Ca_00576	R	M-type
Ca1_P4_4900555	Ca1	4900555	AGAAGTAGACTCTGAAGTA/AGAAGTA	GGAATTGGTCGCTTCAGAAA	TCCATAAGCTGATCCCCAAG	60.2	428	DRR	Ca_00576	R	M-type
Ca1_P4_4957124	Ca1	4957124	CAAAAAAAA/CAAAAAAAAA	TATCACCTCAACGACCCCTC	CGTGTCTGCCTTGGAATTTT	59.9	482	DRR	Ca_00586	T	WRKY
Ca1_P4_5446706	Ca1	5446706	GTTTTT/GTTTTTTT	TGACGCAGTCTCATAGTCGG	GCAATTGTCAAGCACCATGT	60.0	336	DRR	Ca_00630	--	--
Ca1_P4_5482292	Ca1	5482292	A/AAG	TACAAATCCGGATCGAAACA	TGTGTTTGTGTTGGGATGAGA	59.0	301	DRR	Ca_00633	--	S1Fa-like
Ca1_P4_5498637	Ca1	5498637	AAATATTAATATTAATATTAATATTAATA/AAATATTAATATTAATATTAATA	TCCTATGATGGCTAGGGGTG	CCATTGCAAGTAAAGGCACA	59.9	568	DRR	Ca_00635	C	--

INDEL marker IDs	Chromosomes/ unanchored scaffolds	Physical positions (bp)	InDels (*Kabuli* reference genome-CDC Frontier/PII)	Forward primers (5'-3')	Reverse primers (5'-3')	Anneling temperature (0C)	Expected amplified product size (bp)	Structural annotation		Functional annotation	
								Sequence components of *kabuli* genome	*Kabuli* gene accession IDs	NCBI-KOG	TFs
Ca1_P4_5998961	Ca1	5998961	TATAATAATAATAATAATAATAATAATAATAATAATAATAATAATAATAATAATAATAATA/TATAATAATAATAATAATAATAATAATAATAATAATAATAATAATAATAATAATAATAATAATAATAATAATAATAATA	ATAGCGCACCGTCTCTCTGT	ATTTTGTGGCACATGAAGTCT	60.0	872	DRR	Ca_00687	--	--
Ca1_P4_6148078	Ca1	6148078	CAA/CA	TTGCTATTCCCTTTCCCCTT	CTCTCATCTCTGGCCTTTGG	59.9	558	DRR	Ca_00699	S	--
Ca1_P4_6205233	Ca1	6205233	CAAAAAAAAAAA/CAAAAAAAAAAAA	GGGTATTGGATAAAAATTTCAAAAA	TGAAAATTAAGGGACGGGTTT	58.7	604	DRR	Ca_07880	T	--
Ca1_P4_6385666	Ca1	6385666	TC/T	TACTGTCATGGCGGCAGTTA	CATGCGGTGTTTTCTTCAAA	60.3	419	DRR	Ca_07901	R	AP2
Ca1_P4_638582	Ca1	638582	ACTCT/ACT	TGCCTAACAAGGAGGAAACG	CTTAGCAGCATGCATTGACG	60.2	554	DRR	Ca_00074	--	--
Ca1_P4_6418036	Ca1	6418036	TATAATAATAAT/TATAATAAT	ATTTCACTGCCAAAGGATGG	TCGAGCCAAATTGTCCTTCT	59.9	544	DRR	Ca_07905	P	MIKC
Ca1_P4_6523042	Ca1	6523042	GAACAAACA/GAACA	GGAGCAGTTGTTCCCAAAAA	CGAGGAAGAAGCGGATATTG	60.1	444	DRR	Ca_07917	I	Trihelix
Ca1_P4_6538753	Ca1	6538753	AT/ATT	CAAGGAAAGGGAATGGCATA	AATTGAAGGCTGAAACGTGG	59.9	787	DRR	Ca_07919	R	bZIP
Ca1_P4_6538802	Ca1	6538802	CTTTTTT/CTTTTTTTT	CAAGGAAAGGGAATGGCATA	AATTGAAGGCTGAAACGTGG	59.9	787	DRR	Ca_07919	R	bZIP
Ca1_P4_6593836	Ca1	6593836	TT/TTATAT	AAGTGGGTTGTCGCTACCTC	CGTTCTTCTTCTTCCACATTCC	59.2	590	DRR	Ca_07923	R	--

INDEL marker IDs	Chromosomes/ unanchored scaffolds	Physical positions (bp)	InDels (*Kabuli* reference genome-CDC Frontier/PII)	Forward primers (5'-3')	Reverse primers (5'-3')	Anneling temperature (0C)	Expected amplified product size (bp)	Structural annotation		Functional annotation	
								Sequence components of *kabuli* genome	*Kabuli* gene accession IDs	NCBI-KOG	TFs
Ca1_P4_7649777	Ca1	7649777	ATATG/A	ATGTAGGGGTGGGAATAGGC	TTTCCATTGCACTCACTCCA	60.0	825	DRR	Ca_08019	Z	--
Ca1_P4_7748427	Ca1	7748427	CTTTT/CTTTTT	TAGTGGGTTCACTTGGGGAG	TTCTTTTTGTGCATTAAGTTGGAA	60.0	460	DRR	Ca_08026	--	--
Ca1_P4_7781791	Ca1	7781791	CAA/C	TGCAATCTCCTTCACACTCG	AAGTTGCTTTGTTTGAACGC	60.0	324	DRR	Ca_08030	--	GRAS
Ca1_P4_7810052	Ca1	7810052	TCTAA/TCTAACTAA	CTTTGTGGGCAAACCAAACT	GGGTGGTTGAAACTTCAGGA	60.0	521	DRR	Ca_08031	--	FAR1
Ca1_P4_784516	Ca1	784516	ATTTTTTTTTT/ATTTTTTTTTTT	TTGCTTCATGCTTTCCTTGA	TGCATCATGCGACCTCTTAT	59.5	837	DRR	Ca_00097	--	--
Ca1_P4_7926977	Ca1	7926977	TG/T	GCATTGGCATTCATTGTGAG	CCCTTACCTTGAGGCAAACA	60.1	758	DRR	Ca_08042	--	HB-other
Ca1_P4_7948690	Ca1	7948690	ATTTTTTT/ATTTTTTTT	TTTTGCATCTCTCCTCTTCCA	CCAACAGTTCCGACATCTCA	59.9	453	DRR	Ca_08045	QI	B3
Ca1_P4_7971447	Ca1	7971447	CTTTTTTTTT/CTTTTTTTTTTT	CCATATTAGGGTGCTGCGTT	TGGCAACCATCAACTACCAA	60.0	200	DRR	Ca_08047	U	MYB
Ca1_P4_7988333	Ca1	7988333	CTTAT/C	TCAATCCAAACAAGTCCAGAA	AATCCAGCAAAACGAGGTTG	58.2	517	DRR	Ca_08049	--	WRKY
Ca1_P4_7988388	Ca1	7988388	ATT/ATTTT	TCAATCCAAACAAGTCCAGAA	AATGCATGCTTTGGTTCCTC	58.2	761	DRR	Ca_08049	--	WRKY

INDEL marker IDs	Chromosomes/ unanchored scaffolds	Physical positions (bp)	InDels (*Kabuli* reference genome-CDC Frontier/PII)	Forward primers (5'-3')	Reverse primers (5'-3')	Anneling temperature (0C)	Expected amplified product size (bp)	Structural annotation		Functional annotation	
								Sequence components of *kabuli* genome	*Kabuli* gene accession IDs	NCBI-KOG	TFs
Ca1_P4_7989526	Ca1	7989526	TG/T	GCAACCTTCTGTTCCTCAGC	GGGGGAGAGATTTTCCCTTT	60.0	591	DRR	Ca_08049	--	WRKY
Ca1_P4_8039162	Ca1	8039162	AA/ACCATAAGCTGA	TAATGGCAAATGCTCAACCA	GGTGAATGGCCAAAGAAAAA	60.1	343	DRR	Ca_08051	M	FAR1
Ca1_P4_8101006	Ca1	8101006	GT/GTT	CCTCACCCGTGCATTCTTAT	GGCGGAGACAAAGAGTCAAG	60.0	587	DRR	Ca_08056	C	--
Ca1_P4_8101258	Ca1	8101258	AAAAAAAATAAAAAAA/AAAAAAAA	CCTCACCCGTGCATTCTTAT	GGCGGAGACAAAGAGTCAAG	60.0	587	DRR	Ca_08056	C	--
Ca1_P4_8115168	Ca1	8115168	TG/T	AGTTTGTTCCCGTCCAACAC	CACATGGGAGGTGAGTGGTA	59.9	435	DRR	Ca_08057	A	--
Ca1_P4_8124056	Ca1	8124056	AATATATATATATA/AATATATATATATATATATA	TGTTGAGAAGCATGCGAAAG	TCGAATCGAACACAATCCAA	60.1	918	DRR	Ca_08058	R	--
Ca1_P4_8433346	Ca1	8433346	AAAAAAAAAAATTAAAAAAA/AAAAAAA	GGATCGCGGAGACTATTTTG	AGATCCAAGAGCGGATGAGA	59.7	290	DRR	Ca_08078	--	--
Ca1_P4_8909387	Ca1	8909387	T/TCC	AGGCAAAAGGTTTTTGGGTT	GTGAGGCGTGAAGTGGTTTT	59.9	806	DRR	Ca_02914	--	--
Ca1_P4_9106412	Ca1	9106412	TAAAAAAAAA/TAAAAAAAA	GTGCTCGATGCTGTCAAAAA	CGGGTGCAAAATCAAAAGAT	60.0	667	DRR	Ca_02885	--	--
Ca1_P4_9371422	Ca1	9371422	TAAAAAAAA/TAAAAAAAAA	TCAATTTGGTGAATTGGTGAA	TGGAAACACAAACAATCAGGA	58.9	215	DRR	Ca_02861	--	C3H

INDEL marker IDs	Chromosomes/ unanchored scaffolds	Physical positions (bp)	InDels (*Kabuli* reference genome-CDC Frontier/PII)	Forward primers (5'-3')	Reverse primers (5'-3')	Anneling temperature (0C)	Expected amplified product size (bp)	Structural annotation		Functional annotation	
								Sequence components of *kabuli* genome	*Kabuli* gene accession IDs	NCBI-KOG	TFs
Ca1_P4_9492143	Ca1	9492143	CATATATATATATATATATATA/CATATATATATATATATATATA	TCCGATTGTTAGCGCTTTTT	GGCTGCAACCATTCTTCTTC	59.9	180	DRR	Ca_02848	Q	E2F/DP
Ca1_P4_987900	Ca1	987900	CAGGAAGGAA/CAGGAA	ACCATTTTGTGGGATGATGG	TGCCACATCACCTTAAATCAA	60.4	689	DRR	Ca_00122	T	--
Ca1_P4_9930936	Ca1	9930936	TAATAC/TAATACAACAATAC	TTTGGAATGCCATGCAGTTA	ATGGATGCTGTGGTGAATGA	60.1	424	DRR	Ca_02792	O	WRKY
Ca2_P4_10097433	Ca2	10097433	CG/C	GCTAAATTCGTCTTCGACGC	CATTCAAATGGCAATGCAAC	60.0	139	DRR	Ca_20377	J	--
Ca2_P4_10532302	Ca2	10532302	ATTTTTTTTT/ATTTTTTTT	CCGAAGAGAATGGAGAAAGTT	TCAAATTCGTAACTCAAATCTTCAA	57.5	381	DRR	Ca_17578	--	--
Ca2_P4_10594707	Ca2	10594707	CTTTTTTTTTTT/CTTTTTTTTTTTT	TGACATGATTCTTGCGATCC	CAATGTTCTGGTTGCACAGG	59.6	455	DRR	Ca_17574	O	bHLH
Ca2_P4_17046294	Ca2	17046294	AATAAATT/AAAATTTATAAATT	CTATTCCATCCACGAATGCC	TCCATGGAGGATATTTATGTCG	60.3	523	DRR	Ca_24768	--	G2-like
Ca2_P4_20761456	Ca2	20761456	A/AT	AAAATCCAACAATCTCCCCC	TGTTTTCTGCCTTTTTGCCT	60.0	342	DRR	Ca_23762	--	--
Ca2_P4_20761850	Ca2	20761850	CATA/CATATATA	AAAATCCAACAATCTCCCCC	ACTCCTCCACAAGCCTCTGA	60.0	939	DRR	Ca_23762	--	--
Ca2_P4_21783996	Ca2	21783996	ATTTTTT/ATTTTTTTT	ATCTCGTGGGTGGATCTGAC	GCTATGGGACCTATCAGGCA	59.9	381	DRR	Ca_23057	--	--

INDEL marker IDs	Chromosomes/ unanchored scaffolds	Physical positions (bp)	InDels (*Kabuli* reference genome-CDC Frontier/PII)	Forward primers (5'-3')	Reverse primers (5'-3')	Anneling temperature (0C)	Expected amplified product size (bp)	Structural annotation		Functional annotation	
								Sequence components of *kabuli* genome	*Kabuli* gene accession IDs	NCBI-KOG	TFs
Ca2_P4_22812516	Ca2	22812516	ATTTTTTT/ATTTT	GGGGGAGAAAATTTGGTGTT	TTGATCTTGCTGCATTGTCC	60.0	215	DRR	Ca_14986	--	--
Ca2_P4_2373367	Ca2	2373367	AATATATATATATATATATATATATA/AATATATATATATATATATATATATATATATATA	AAAATTGTCAGGTTCACCGC	GGTGGTGATTTGGTGCTCTT	60.0	896	DRR	Ca_12541	R	DBB
Ca2_P4_2382639	Ca2	2382639	T/TCATA	TTGGAAATGCCAGAGGTATTAGA	TGTGATTTGGTTTCGCAAAG	60.0	658	DRR	Ca_12540	R	--
Ca2_P4_25352886	Ca2	25352886	ATTTTTTTTTT/ATTTTTTTTTTT	AAAGAGACACATGACATAACTCCTC	ATGGGGGATGGACCTAGAAC	57.9	685	DRR	Ca_14268	--	--
Ca2_P4_25535051	Ca2	25535051	TTTTTTTTT/TTTTTTTTTTTTTTT	CCAGATTTGAACTGGGGAAA	AATCAAGTGGGTTCGAGTGG	59.9	551	DRR	Ca_14283	R	MYB
Ca2_P4_2726175	Ca2	2726175	TTATATATATATATATATATATATAT/TTATATATATATATATATATATAT	CGTTTCGGGTTTCGTAAAAA	TTGTTGACAATTCGATCCAAAG	60.0	302	DRR	Ca_10571	Q	B3
Ca2_P4_28723013	Ca2	28723013	GTTT/GCTTTTTT	ATCATCCCGTCCATTACGAA	ATGCCATTACTCCGTGCTTT	60.2	507	DRR	Ca_14359	R	--
Ca2_P4_29072040	Ca2	29072040	ATCTGCAGACTTT/ATCTGCAGACTTTCTGCAGACTTT	TTGCTGGCAGAACAATCAAG	TGTGGGAAGCACCATGTTTA	60.0	546	DRR	Ca_14332	--	--
Ca2_P4_29072081	Ca2	29072081	TTATATATATATATATATATATATATATATATA/TTATATATATATATATATATATATATATATA	TTGCTGGCAGAACAATCAAG	TGTGGGAAGCACCATGTTTA	60.0	546	DRR	Ca_14332	--	--
Ca2_P4_29195958	Ca2	29195958	CATATATATATATATATATA/CATATATATATATATATATATA	TTGTTTCGGAGGGTTGACTC	CCACCGATTGAAACATACCA	60.1	612	DRR	Ca_14321	PT	C3H

INDEL marker IDs	Chromosomes/ unanchored scaffolds	Physical positions (bp)	InDels (*Kabuli* reference genome-CDC Frontier/PII)	Forward primers (5'-3')	Reverse primers (5'-3')	Anneling temperature (0C)	Expected amplified product size (bp)	Structural annotation		Functional annotation	
								Sequence components of *kabuli* genome	*Kabuli* gene accession IDs	NCBI-KOG	TFs
Ca2_P4_29238503	Ca2	29238503	A/AGTT	TGTGGAACACCAACGAATGT	ACCTCCCGCTAATGTCAATG	59.9	746	DRR	Ca_14317	--	--
Ca2_P4_29238544	Ca2	29238544	AAATAATAATAATAATAATAATAATAATAATAATAATAATAATAATAATAA/AAATAATAATAATAATAATAATAATAATAATAATAATAATAA	TGTGGAACACCAACGAATGT	ACCTCCCGCTAATGTCAATG	59.9	746	DRR	Ca_14317	--	--
Ca2_P4_30259949	Ca2	30259949	GAAAAAAAAAA/GAAAAAAAAAAA	TCAAATTTTTCAAACAAAATCCA	AATATCACCCGTGGTCCCTT	58.6	403	DRR	Ca_12523	A	MYB
Ca2_P4_30349843	Ca2	30349843	GAGACAGACA/GAGACA	TGTCCAGAAACAGCTCATGG	AACTCCTCAAAGTGACGGACA	59.8	638	DRR	Ca_12518	GE	bZIP
Ca2_P4_30356368	Ca2	30356368	AATATATA/AATATATATA	CCACATGGCTGCTTATGATG	AGCGCATGGTAATGTTGAGA	60.1	459	DRR	Ca_12517	--	ERF
Ca2_P4_30369374	Ca2	30369374	TAAAA/TAAAAA	TGTACTTCCTCCGATTCCAAA	TGAATGACGACGACGGTAAA	59.6	632	DRR	Ca_12515	Q	--
Ca2_P4_30402091	Ca2	30402091	CAAAAAAA/CAAAAAA	TGATTTTAGAAATTGCGTGGC	TTGGGCTTTTATTGTCCACC	60.1	217	DRR	Ca_12511	--	--
Ca2_P4_30402988	Ca2	30402988	ATT/AT	TACCGGATTGGTAACGTGGT	GCGTTTGATGCATATTTTCG	60.1	419	DRR	Ca_12511	--	--
Ca2_P4_30403193	Ca2	30403193	ATTGAGTT/ATTGAGTTGAGTT	AAAGTTGCACCAAGAGGTGG	TCTCTGGCCTTCGTTAGTCAA	60.1	989	DRR	Ca_12511	--	--
Ca2_P4_30419368	Ca2	30419368	TTCATCAT/TTCAT	GTTGCAATCATCACCACACC	TGAGTGTTCTTGGTGATTTGG	59.8	568	DRR	Ca_12509	C	M-type

INDEL marker IDs	Chromosomes/ unanchored scaffolds	Physical positions (bp)	InDels (*Kabuli* reference genome-CDC Frontier/PII)	Forward primers (5'-3')	Reverse primers (5'-3')	Anneling temperature (0C)	Expected amplified product size (bp)	Structural annotation		Functional annotation	
								Sequence components of *kabuli* genome	*Kabuli* gene accession IDs	NCBI-KOG	TFs
Ca2_P4_30448281	Ca2	30448281	AT/A	AGCGACAATGCTGAGGAGAT	TTATGGGTCCGTTTGATGGT	60.0	262	DRR	Ca_12507	--	--
Ca2_P4_30448973	Ca2	30448973	GAAAAAA/GAAAAAAA	GCTTTTGATTTTCTAAAGGATGG	TCTTCCTTGTTGGTTCTTTGG	58.4	759	DRR	Ca_12507	--	--
Ca2_P4_30449164	Ca2	30449164	TAAAAAAA/TAAAAAAAA	TGTTGCAAAGAGAAAGAACTTCA	TGACGGTGGCAATCGATATT	59.2	557	DRR	Ca_12507	--	--
Ca2_P4_30465917	Ca2	30465917	GAAAAAAAAA/GAAAAAAAAAA	TCCATGATTGAGTGACTAGGGA	GCAGTCAACCAGCGACAATA	59.6	322	DRR	Ca_12503	J	--
Ca2_P4_30478151	Ca2	30478151	CAATAAATAAATAAATAAA/CAATAAATAAATAAA	GTGCGTGAAGGGATGAAAAT	CATCATAGCCCCGAGAAAAA	59.9	758	DRR	Ca_12502	G	--
Ca2_P4_30478867	Ca2	30478867	AATATATATAT/AATATATATATATAT	TTTTTCTCGGGGCTATGATG	GTAGGGCATGTGTGTGTTGC	60.0	640	DRR	Ca_12502	G	--
Ca2_P4_30510130	Ca2	30510130	ATTTTTTTTTTTTTTT/ATTTTTTTTTTTTT	AGACCCCACGGTTAACATGA	AGCACCTTACAAGGGGGAGT	60.2	700	DRR	Ca_12500	--	--
Ca2_P4_30510322	Ca2	30510322	G/GATAACT	CTAGCTTCGCTGCACAAATG	AGCACCTTACAAGGGGGAGT	59.8	200	DRR	Ca_12501	GMW	--
Ca2_P4_30573602	Ca2	30573602	CGGG/CGGGG	TGGCTCATGACCCCTTTATC	GGCCCCTTTTCATTTTCATT	59.9	709	DRR	Ca_12495	E	--
Ca2_P4_30573670	Ca2	30573670	TAAAAAAAAAA/TAAAAAAAAA	TGCATGCAAAAGAATTGTCC	GGCCCCTTTTCATTTTCATT	59.7	534	DRR	Ca_12495	E	--

INDEL marker IDs	Chromosomes/ unanchored scaffolds	Physical positions (bp)	InDels (*Kabuli* reference genome-CDC Frontier/PII)	Forward primers (5'-3')	Reverse primers (5'-3')	Anneling temperature (0C)	Expected amplified product size (bp)	Structural annotation		Functional annotation	
								Sequence components of *kabuli* genome	*Kabuli* gene accession IDs	NCBI-KOG	TFs
Ca2_P4_30576695	Ca2	30576695	TGTGTTGGTGTTGGTGTTGGTGT/TGTGTTGGTGTTGGTGT	GCATTCAACACTCACAAGGG	GAGTCCTGAGCCACGAGAAC	59.1	430	DRR	Ca_12493	--	--
Ca2_P4_30578105	Ca2	30578105	AC/A	GCCCCTGCACTAGTTTGAAG	GGCTTGTTAGTCTGCTTGCC	59.9	583	DRR	Ca_12493	--	--
Ca2_P4_30578122	Ca2	30578122	TAAAAA/TAAAAAA	GCCCCTGCACTAGTTTGAAG	GGCTTGTTAGTCTGCTTGCC	59.9	583	DRR	Ca_12493	--	--
Ca2_P4_30618375	Ca2	30618375	AT/A	TGGTCACCGGGATATTTTTG	ACCTTGAAGAGCGAATGAGC	60.6	506	DRR	Ca_12490	R	--
Ca2_P4_30671540	Ca2	30671540	TAAAAAA/TAAAAA	TTCGATTGATGGTTAAGAGACA	ATAACGTGTTGGATCAGCCC	57.4	697	DRR	Ca_12489	QI	bHLH
Ca2_P4_30681354	Ca2	30681354	ATTATTTATTTATTTAT/ATTATTTATTTAT	CACATTTCAATAATTGCATCCG	GCATTTTGTGTGGGTGGATT	60.2	805	DRR	Ca_12488	F	--
Ca2_P4_30737874	Ca2	30737874	CATCATGATCA/CATCATGATCATGATCA	AGGGTTGGCAATTTTCTTGA	TGTAAAGAGGAGGCCGTGAC	59.5	428	DRR	Ca_12484	T	RAV
Ca2_P4_30739062	Ca2	30739062	AC/ACC	TTGTGTGTGGGCTCAACTTC	TGGCCTACCTCTTGTGATGA	59.7	569	DRR	Ca_12483	T	FAR1
Ca2_P4_30767153	Ca2	30767153	TAAAAAAAAAAA/TAAAAAAAAA	TCAAGGATTCCAGATCCAGG	CAAAATGCGGTGTAATGCTG	60.0	142	DRR	Ca_12479	K	ERF
Ca2_P4_30840476	Ca2	30840476	AG/A	GCATGCGAACATGAATTTTG	CCGGTGGATTGGATGTTAAA	60.1	589	DRR	Ca_12475	--	--

INDEL marker IDs	Chromosomes/ unanchored scaffolds	Physical positions (bp)	InDels (*Kabuli* reference genome-CDC Frontier/PII)	Forward primers (5'-3')	Reverse primers (5'-3')	Anneling temperature (0C)	Expected amplified product size (bp)	Structural annotation		Functional annotation	
								Sequence components of *kabuli* genome	*Kabuli* gene accession IDs	NCBI-KOG	TFs
Ca2_P4_30840563	Ca2	30840563	TAAAAAA/TAAAAA	GCATGCGAACATGAATTTTG	CCGGTGGATTGGATGTTAAA	60.1	589	DRR	Ca_12475	--	--
Ca2_P4_30840723	Ca2	30840723	TC/T	GCATGCGAACATGAATTTTG	GGCCACTCATGTTGTCTCCT	60.1	788	DRR	Ca_12475	--	--
Ca2_P4_30869262	Ca2	30869262	AATATA/AATA	TCAAGGGCGACTTTTATTGG	CGAATTTAGGGGATCCAGGT	60.1	130	DRR	Ca_12473	U	--
Ca2_P4_30902428	Ca2	30902428	CTGT/CT	CCCCATATTGCAAAAGGCTA	CAAATGGGTTTGCAAGTGTG	59.9	385	DRR	Ca_12471	--	--
Ca2_P4_30957679	Ca2	30957679	ATTTTTT/ATTTTTTTT	TCGGTTGTAGCCGAGCTAGT	TTTTGCTTGGGTCGAGAGAT	60.0	791	DRR	Ca_12469	U	bHLH
Ca2_P4_30957722	Ca2	30957722	CCA/CCACA	TCGGTTGTAGCCGAGCTAGT	TTTTGCTTGGGTCGAGAGAT	60.0	791	DRR	Ca_12469	U	bHLH
Ca2_P4_30979139	Ca2	30979139	TTTTTATTT/TTTT	CTCGCCAACTTACCGATTGT	TCATTATTGTGAAATAGGGCCA	60.1	554	DRR	Ca_12466	O	TCP
Ca2_P4_31004687	Ca2	31004687	TCACA/TCA	TGGTCTTTGGATTCCCATGT	TATTGCGGAGTCTCGTGTGA	60.2	414	DRR	Ca_12464	--	LBD
Ca2_P4_31004710	Ca2	31004710	TG/T	TGGTCTTTGGATTCCCATGT	TATTGCGGAGTCTCGTGTGA	60.2	414	DRR	Ca_12464	--	LBD
Ca2_P4_31926358	Ca2	31926358	CCTTT/C	GTGGGAGATGCAATCCAAGT	TGTCACAAATGTGTCGTGAAAA	59.9	652	DRR	Ca_17824	--	ERF

INDEL marker IDs	Chromosomes/ unanchored scaffolds	Physical positions (bp)	InDels (*Kabuli* reference genome-CDC Frontier/PII)	Forward primers (5'-3')	Reverse primers (5'-3')	Anneling temperature (0C)	Expected amplified product size (bp)	Structural annotation		Functional annotation	
								Sequence components of *kabuli* genome	*Kabuli* gene accession IDs	NCBI-KOG	TFs
Ca2_P4_31939044	Ca2	31939044	ATTTTTTTT/ATTTTTTT	ATCACATTGCAAAGCA ACGA	TGTATTCACGGTTTGG GTTTT	60.3	586	DRR	Ca_17823	R	C3H
Ca2_P4_31939517	Ca2	31939517	ACT/ACTCT	TCTTAAAACCCAAACC GTGAA	GAGCTTTTGGGGGATA GAGG	59.5	490	DRR	Ca_17823	R	C3H
Ca2_P4_3201185	Ca2	3201185	ATTTTTTTTT/ATTTTTTTTTT	TTTGGCAAATGTTTCCT TCA	GAGTGGGGGTTGGAG GTAGT	59.1	405	DRR	Ca_10527	K	HD-ZIP
Ca2_P4_32048288	Ca2	32048288	AC/A	CCAAATCGAACTCTCT CCTCA	CGTGATGCACCGATAA ATTG	59.4	634	DRR	Ca_17817	R	--
Ca2_P4_32048344	Ca2	32048344	CTTTTTT/CTTTTTTT	CCAAATCGAACTCTCT CCTCA	CGTGATGCACCGATAA ATTG	59.4	634	DRR	Ca_17817	R	--
Ca2_P4_32069395	Ca2	32069395	TTATATATATATATAT/TTATATATAT ATATATATAT	ACCCCGTTGATCATAA GTCG	AAAATCCTGGCAGTCA TCGT	59.8	922	DRR	Ca_17815	U	--
Ca2_P4_32074725	Ca2	32074725	AGTGTGTGT/AGTGTGT	TTCCTATGTGCAAAAGT GCAT	TCCCTTGAGTGGCAGT AACC	58.3	474	DRR	Ca_17814	S	--
Ca2_P4_32118444	Ca2	32118444	GACGAACG/GACG	ACCATGCCACATCATC TTGA	TGCAATTGAGAAATTTG CGT	59.9	342	DRR	Ca_17813	--	ERF
Ca2_P4_32129360	Ca2	32129360	CATTGAATTGAATTGAATTGAATTG/ CATTGAATTGAATTGAATTG	CTCCTAATTTTGCGTTT CCG	GTTCAGCTCGCAACAA TTCA	59.7	368	DRR	Ca_17812	O	--
Ca2_P4_32173774	Ca2	32173774	TC/T	ATGTTTTGATTGGGGC TTTG	TAAATCAGCCCTTTTGG TGG	59.8	631	DRR	Ca_17806	--	WRKY

INDEL marker IDs	Chromosomes/ unanchored scaffolds	Physical positions (bp)	InDels (*Kabuli* reference genome-CDC Frontier/PII)	Forward primers (5'-3')	Reverse primers (5'-3')	Anneling temperature (0C)	Expected amplified product size (bp)	Structural annotation		Functional annotation	
								Sequence components of *kabuli* genome	*Kabuli* gene accession IDs	NCBI-KOG	TFs
Ca2_P4_32175488	Ca2	32175488	AAT/AATAT	CACAACATCATCAAGAAACCAAA	GGCATCGCTAACGCAATTAT	59.9	681	DRR	Ca_17806	--	WRKY
Ca2_P4_32199784	Ca2	32199784	ATT/ATTT	TCCCATTACCACCAAAGACA	TGCCATAACCACCAAAGACA	58.8	413	DRR	Ca_10295	--	G2-like
Ca2_P4_32241009	Ca2	32241009	GAAAAAAAAAAAA/GAAAAAAAAA	TGAGACGTCACCCTCAGTTG	GAGGCCAAAGGTCCAATATG	59.9	568	DRR	Ca_10293	--	--
Ca2_P4_32241234	Ca2	32241234	AATATATATATATATAT/AATATATATATATAT	CATATTGGACCTTTGGCCTC	TGCTTGCCAATTCATGTCTC	59.4	434	DRR	Ca_10293	--	--
Ca2_P4_32327707	Ca2	32327707	GT/GTAATGATAT	TTTCCCTTCACTATCGGTGC	CAAATTTGCAATTGTGCCTG	60.1	660	DRR	Ca_10285	R	--
Ca2_P4_32327737	Ca2	32327737	A/ATC	TTTCCCTTCACTATCGGTGC	CAAATTTGCAATTGTGCCTG	60.1	660	DRR	Ca_10285	R	--
Ca2_P4_32354755	Ca2	32354755	TAGTTGTCAA/TAGTTGTCAAAGTTGTCAA	TTCCAGCCAACTCCTGATTC	TTTAGTGGTCCCTTGCCAAC	60.2	548	DRR	Ca_10283	--	--
Ca2_P4_32412446	Ca2	32412446	ATTTTTTTTTT/ATTTTTTTTTTT	GGCTCATTACAGTGGAGGGA	TAATGGGACCAAAACTGCAC	60.1	688	DRR	Ca_10279	--	bHLH
Ca2_P4_32435073	Ca2	32435073	GTTTTTT/GTTTTTTT	TCGCTCAGTGATGTTGAAGG	GCCTCAAATTCTTTTTCGTTTC	60.0	695	DRR	Ca_10275	O	C2H2
Ca2_P4_32436418	Ca2	32436418	CG/C	TCACATGGTGGCATCCTAGA	CGAGTCATCCATGCTCTGTT	60.1	302	DRR	Ca_10275	O	C2H2

INDEL marker IDs	Chromosomes/ unanchored scaffolds	Physical positions (bp)	InDels (*Kabuli* reference genome-CDC Frontier/PII)	Forward primers (5'-3')	Reverse primers (5'-3')	Anneling temperature (0C)	Expected amplified product size (bp)	Structural annotation		Functional annotation	
								Sequence components of *kabuli* genome	*Kabuli* gene accession IDs	NCBI-KOG	TFs
Ca2_P4_32477117	Ca2	32477117	TTTGTT/TTTGTTGTT	CTGCAATGATAATTGGGGCT	CAACTGCAATGTTCCATATTCG	59.9	555	DRR	Ca_10273	R	C3H
Ca2_P4_32673561	Ca2	32673561	ATTTTTTTTTTT/ATTTTTTTTTT	TTCAACCAAGCCTACCCTTC	TGGCCAACAAAATTTAACCA	59.2	449	DRR	Ca_10254	DR	--
Ca2_P4_33058448	Ca2	33058448	CATATTAGTATATTAGTA/CATATTAGTA	CCTTACCATGACCCCAAATG	TGCCTGGGTTTCTTTAATGG	60.0	630	DRR	Ca_10218	R	B3
Ca2_P4_33076756	Ca2	33076756	CAAAAAAAAAA/CAAAAAAAAAAA	TGCACAATTACCATAAAACAATTT	TGGTGGTAATGGAGGCAGTT	57.7	558	DRR	Ca_10215	K	MYB
Ca2_P4_33147399	Ca2	33147399	AGT/A	CAACCCAAACATTGGACAAA	CGGTCACAGGATAGGGATGT	59.3	771	DRR	Ca_10205	D	M-type
Ca2_P4_33398226	Ca2	33398226	CTAGTA/CTAGTAGTA	AAACGTGCCTTGAAAATTGG	AAATGCAGGGTCACAACACA	60.0	727	DRR	Ca_10186	T	M-type
Ca2_P4_33398441	Ca2	33398441	TACACAC/TACAC	AGAGCCTACAGCAAGCCAAA	AAATGCAGGGTCACAACACA	60.2	205	DRR	Ca_10186	T	M-type
Ca2_P4_33661751	Ca2	33661751	ATTTTTTTTT/ATTTTTTTT	GTTTGTGGTAGGGGCTTCAA	TTGTCTATGCAAACATGCCC	60.0	388	DRR	Ca_10172	--	HB-other
Ca2_P4_34272124	Ca2	34272124	AATATATATATATATATATATATATA/AATATATATATATATATATATA	GGCACCTCATATGCAACCTT	TGGCACCAAACTTACACCAA	60.0	652	DRR	Ca_15251	--	WRKY
Ca2_P4_34864878	Ca2	34864878	GTATA/GTA	CTTCAGTCTGACGCTCTCCC	TTGCTGCCAAAATAAACAACA	60.1	651	DRR	Ca_16882	--	FAR1

INDEL marker IDs	Chromosomes/ unanchored scaffolds	Physical positions (bp)	InDels (*Kabuli* reference genome-CDC Frontier/PII)	Forward primers (5'-3')	Reverse primers (5'-3')	Anneling temperature (0C)	Expected amplified product size (bp)	Structural annotation		Functional annotation	
								Sequence components of *kabuli* genome	*Kabuli* gene accession IDs	NCBI-KOG	TFs
Ca2_P4_34945283	Ca2	34945283	GAAAAAA/GAAAAAAA	ACACAGTGAGCACACCCAAA	ACGATCGCACTTCGTCTTCT	60.2	443	DRR	Ca_16876	--	--
Ca2_P4_35559233	Ca2	35559233	CAA/CAAA	CCAAAATATCCACCGTGTTT	TTCCTGGACGAAAATGAACC	57.3	582	DRR	Ca_09721	K	NF-YB
Ca2_P4_35839544	Ca2	35839544	AATATATATATATATATATATATATATA/AATATATATATATATATATATATATATATATA	CTGCACAGGCTCTACAACCA	GGACCTGCAATCCAACCTAA	60.0	783	DRR	Ca_09758	O	MYB
Ca2_P4_35843737	Ca2	35843737	TATGATA/TATGATAATGATA	TTTGTTGTCTTTTTGGCCTT	GGAGAGAGAGACACGGTTGC	57.4	699	DRR	Ca_09759	E	MYB
Ca2_P4_35843945	Ca2	35843945	ATTTTTT/ATTT	TGTGATTTAACATTCACTTGGCA	GGAGAGAGAGACACGGTTGC	60.4	274	DRR	Ca_09759	E	MYB
Ca2_P4_35846531	Ca2	35846531	ATT/ATTT	TTATTTGTTCGGTGGCAACA	TGATCGGAATGCGAATAACA	60.0	455	DRR	Ca_09760	J	--
Ca2_P4_35857180	Ca2	35857180	GTATATATATATATATATATAT/GTATATATATATATATATAT	TTTGATTGCCTTCCTCCATC	TTCGGGATTAAAACATTCGG	60.0	509	DRR	Ca_09762	R	--
Ca2_P4_35858374	Ca2	35858374	AAAGCAAGCAA/AAAGCAA	CCCCTCAAACATTACCCCTT	TTTCGCCGTCAGAGAGAAAT	60.0	762	DRR	Ca_09762	R	--
Ca2_P4_35914172	Ca2	35914172	AAAATAAA/AAAA	ACAAATGCATGGGAATGACA	GGGGGAGACTCATCACTCAA	59.8	182	DRR	Ca_09766	--	ERF
Ca2_P4_35918468	Ca2	35918468	TAAAAAAA/TAAAAAAAAAAAA	AGCTGCAAGAGAAACAGATGG	TTTTCAACGCTACCTTCATCA	59.6	804	DRR	Ca_09767	M	--

INDEL marker IDs	Chromosomes/ unanchored scaffolds	Physical positions (bp)	InDels (*Kabuli* reference genome-CDC Frontier/PII)	Forward primers (5'-3')	Reverse primers (5'-3')	Anneling temperature (0C)	Expected amplified product size (bp)	Structural annotation		Functional annotation	
								Sequence components of *kabuli* genome	*Kabuli* gene accession IDs	NCBI-KOG	TFs
Ca2_P4_35965637	Ca2	35965637	CTT/CT	TGGAAGAATGCTTTCAAGGG	ACATTCCATGGGCTGCTTAG	60.2	636	DRR	Ca_09771	J	NAC
Ca2_P4_35965829	Ca2	35965829	CAAAAA/CA	ATTTTTCTGCTAGCGGGACC	ACATTCCATGGGCTGCTTAG	60.6	260	DRR	Ca_09771	J	NAC
Ca2_P4_35966301	Ca2	35966301	CT/CTT	AGCCCATGGAATGTGAAAAG	CCAAAACCTTTGCCTTGAAA	59.9	506	DRR	Ca_09771	J	NAC
Ca2_P4_35987956	Ca2	35987956	ATGTTGT/ATGT	GGATTGGCCTCCCATAATTT	GCGTTTTCAAGGTTGGATGT	60.0	851	DRR	Ca_09772	T	LBD
Ca2_P4_36401773	Ca2	36401773	TATAATAATAATAATA/TATAATAATAATA	GGATAAGGATCTGCATCGGA	ATCCATTTTTGTGGAACCAA	60.0	434	DRR	Ca_09829	R	--
Ca2_P4_3758160	Ca2	3758160	ACTCTCTCTCTCTCTCTCTCTCTCTCTCT/ACTCTCTCTCTCTCTCTCTCTCTCTCTCTCTCT	GGTGTTTGTGCTCCATAGGC	GAAACAGCACTGCCCAATTT	60.5	200	DRR	Ca_10485	J	--
Ca2_P4_3809184	Ca2	3809184	TTATATATATATATATATATATATATATATAT/TTATATATATATATATATATATATATATATATATATAT	TCTTCGAGCAATTGTGTGCT	CTAAAATAAGGCACGCCGAA	59.6	538	DRR	Ca_10479	--	--
Ca2_P4_3901584	Ca2	3901584	C/CGAG	TTCAAGTTCAGCAGTGACCG	ACAGCAAACTTATGGGCACC	60.0	565	DRR	Ca_10467	G	--
Ca2_P4_3901844	Ca2	3901844	AC/ACTTCACGAGC	GTCGCTATCTCCCCAGTGAA	CATTCTGTGCCGCTATCTGA	60.2	248	DRR	Ca_10466	GT	--
Ca2_P4_453297	Ca2	453297	TTATATATATATATATAT/TTATATATATATATATATAT	TTCAAACCAAATGATCCAATGA	ATAAATGTTGGGTGGCCAAA	60.2	685	DRR	Ca_16985	L	--

**INDEL marker IDs**	**Chromosomes/ unanchored scaffolds**	**Physical positions (bp)**	**InDels (*Kabuli* reference genome-CDC Frontier/PII)**	**Forward primers (5'-3')**	**Reverse primers (5'-3')**	**Anneling temperature (0C)**	**Expected amplified product size (bp)**	**Structural annotation**		**Functional annotation**	
								**Sequence components of *kabuli* genome**	***Kabuli* gene accession IDs**	**NCBI-KOG**	**TFs**
Ca2_P4_4812881	Ca2	4812881	ATTTTTTT/ATTTTTTTTT	AACCAGTTCCGTTCCAAGTG	TCCCCCAAACAGAATTTGAG	60.0	731	DRR	Ca_21090	--	MYB
Ca2_P4_5095861	Ca2	5095861	GC/G	GCCCGTTTGATTCACTTTTC	TCCAAACTGCACCACAAAAC	59.6	611	DRR	Ca_14700	K	--
Ca2_P4_5163788	Ca2	5163788	CAAAAAAAAA/CAAAAAAAAAA	TGCGAGAAAGTGAATTCCAG	GGAATGATGATGGGGTTTTG	59.0	658	DRR	Ca_14696	--	--
Ca2_P4_5373619	Ca2	5373619	CATATATATATATATATATATATATATATATATATATATATATATATAT/CATATATATATATATATATATATATATATATATATATATAT	CGGAGGCTTAAGGACAATCA	CGTTGCTCCAAATTTTATAATGG	60.2	784	DRR	Ca_14670	E	MYB
Ca2_P4_753916	Ca2	753916	CTTTTTTTTTT/CTTTTTTTTT	TTGCCCATTAACTTTACCCG	ACTTGTGCATTTGCATTAGTTC	59.8	555	DRR	Ca_17000	--	ARF
Ca2_P4_7626448	Ca2	7626448	AAATTATAATTATAATTA/AAATTATAATTATAATTATAATTA	AACAAGCATTTTGTGAGGGC	ATTCGACTAGTGGTGGTGCC	60.1	931	DRR	Ca_18119	--	--
Ca2_P4_7684658	Ca2	7684658	CAA/CAAA	GCGCTTTTGGGTGTACATTT	GGGAAATAGCATGTTGTGGG	60.0	553	DRR	Ca_18122	--	--
Ca2_P4_7684720	Ca2	7684720	ATTTTTTTT/ATTTTTTTTT	GCGCTTTTGGGTGTACATTT	GGGAAATAGCATGTTGTGGG	60.0	553	DRR	Ca_18122	--	--
Ca2_P4_7801863	Ca2	7801863	AA/AACA	TTCGGAGTTGCAGATGATTG	TCATTTCTTCACCTTGAACCA	59.8	203	DRR	Ca_18127	--	ERF
Ca2_P4_7818010	Ca2	7818010	GATA/GATATTCTAATA	TGGACATAGAAAAACCAAATGGA	ATGTGGAAGTTGAACCCCAA	60.6	598	DRR	Ca_18128	QR	B3

INDEL marker IDs	Chromosomes/ unanchored scaffolds	Physical positions (bp)	InDels (*Kabuli* reference genome-CDC Frontier/PII)	Forward primers (5'-3')	Reverse primers (5'-3')	Anneling temperature (0C)	Expected amplified product size (bp)	Structural annotation		Functional annotation	
								Sequence components of *kabuli* genome	*Kabuli* gene accession IDs	NCBI-KOG	TFs
Ca2_P4_7819525	Ca2	7819525	TAACAA/TAA	GCCACAAATCATGTGTTCCA	GCATCCTCCAAGGTATCGAA	60.4	539	DRR	Ca_18128	QR	B3
Ca2_P4_7821264	Ca2	7821264	CTTTTTTTTTT/CTTTTTTTTTTTT	TTCAGGAACACCATGGTTCA	GAGGTTTCTGAAAGCGTTGC	59.9	948	DRR	Ca_18129	R	CPP
Ca2_P4_7881740	Ca2	7881740	ATATTCC/ATATTCCTATTCC	TCATCTCCATGTGGTCTCCA	TGCAAATGACCACCCACTTA	60.0	642	DRR	Ca_18136	--	--
Ca2_P4_7886311	Ca2	7886311	CAAAA/CAA	TTGTGGTCAAATTGGAACAAGT	GGGAATCCCATAGGTAAAAGC	59.4	742	DRR	Ca_18137	E	--
Ca2_P4_7886536	Ca2	7886536	CTTTT/CTTT	AAAAGGCAGACAACATGGAT	GTCCTTTTGCTCCTCTGCAC	57.1	461	DRR	Ca_18137	E	--
Ca2_P4_7887518	Ca2	7887518	ATTTTTTT/ATTTTTTTT	AAGCAAAATACATCTCGAAGTTCT	TCCCAAGAAAAGCTTCAACC	57.8	550	DRR	Ca_18137	E	--
Ca2_P4_7896699	Ca2	7896699	TGGG/TGGGG	TTGGACGCATCCTGATTGTA	CCGGACGACGATTAGTGATT	60.1	847	DRR	Ca_18138	--	Dof
Ca2_P4_7896829	Ca2	7896829	GAAA/GAAAA	CTTTGAGCTAGTGTGTGCCG	GCTGAGCTTGTACGTAGGGC	59.7	477	DRR	Ca_18138	--	Dof
Ca2_P4_7913902	Ca2	7913902	GAAAAAAAAAA/GAAAAAAAAA	TCAAATCTACCTCTAACGATTCACA	AAGGACAGTTGTCCCAATGC	59.2	326	DRR	Ca_18139	FE	--
Ca2_P4_7935559	Ca2	7935559	TCTA/T	AACCGTTTGAGGAATTGACG	TCGAACTTGCATTGCCATAA	60.0	865	DRR	Ca_18144	--	--

INDEL marker IDs	Chromosomes/ unanchored scaffolds	Physical positions (bp)	InDels (*Kabuli* reference genome-CDC Frontier/PII)	Forward primers (5'-3')	Reverse primers (5'-3')	Anneling temperature (0C)	Expected amplified product size (bp)	Structural annotation		Functional annotation	
								Sequence components of *kabuli* genome	*Kabuli* gene accession IDs	NCBI-KOG	TFs
Ca2_P4_7935610	Ca2	7935610	TATTAT/TATTATTAT	GGTGATGGGTAGGTGGAATG	TCGAACTTGCATTGCCATAA	60.1	590	DRR	Ca_18144	--	--
Ca2_P4_7936230	Ca2	7936230	AT/ATT	TATGGCAATGCAAGTTCGAC	TCGCAAAACTTCAAGAGTGG	59.7	443	DRR	Ca_18144	--	--
Ca2_P4_8362914	Ca2	8362914	GAATGAAATGAAA/GAATGAAATGAAATGAAATGAAA	GTGGGTATCCGGAGGTATCA	TCCAAACACTCCCAAAAAGC	59.6	307	DRR	Ca_21465	--	--
Ca2_P4_8597078	Ca2	8597078	CCT/C	TCACGTTTTTGAATTTGCGA	GACCCATCCATGTGGAGTTT	60.2	514	DRR	Ca_22316	A	--
Ca3_P4_10398321	Ca3	10398321	G/GATTGATCTTTCCC	CGGATTGCCAAGTACAAGGT	CAAACCACAACCAGCACAAC	60.0	770	DRR	Ca_18819	J	--
Ca3_P4_11249483	Ca3	11249483	TAAA/TAA	GCAATTGTGGTTGCTGACTG	TTCATCTTCCAAAGGCATTTTA	60.3	607	DRR	Ca_19372	--	C2H2
Ca3_P4_11250270	Ca3	11250270	TT/TTAT	AACAATTGATTCGTGTGATTGG	TTGGGTCCAATAGGAAGTCG	59.7	806	DRR	Ca_19372	--	C2H2
Ca3_P4_11250530	Ca3	11250530	TTTATTATTATTATTATTATTATT/TTTATTATTATTATTATTATTATT	TGGTAACAAATTGATTAATCGCAA	TTGGGTCCAATAGGAAGTCG	60.6	609	DRR	Ca_19372	--	C2H2
Ca3_P4_11590236	Ca3	11590236	ATT/A	TTGCTCCTACGTATCTCCCG	CCTTTTGTCATTTTGGGCTC	60.2	800	DRR	Ca_19380	--	--
Ca3_P4_12402301	Ca3	12402301	T/TG	GAAAAGGTTGAGCAACGAGC	GAATGATTTTGAAGTGGGGG	60.0	880	DRR	Ca_21030	--	--

INDEL marker IDs	Chromosomes/ unanchored scaffolds	Physical positions (bp)	InDels (*Kabuli* reference genome-CDC Frontier/PII)	Forward primers (5'-3')	Reverse primers (5'-3')	Anneling temperature (0C)	Expected amplified product size (bp)	Structural annotation		Functional annotation	
								Sequence components of *kabuli* genome	*Kabuli* gene accession IDs	NCBI-KOG	TFs
Ca3_P4_15072147	Ca3	15072147	AATATATATATATATATATATATATAT A/AATATATATATATATATATATATAT A	GAGTGGTGTTGGAGTC ATGTTT	AGACATGTGTTTCCCTT CCG	59.0	424	DRR	Ca_17011	--	--
Ca3_P4_15072299	Ca3	15072299	ATT/ATTT	GCGAAATCCAACTTTT CCAA	AGACATGTGTTTCCCTT CCG	60.1	211	DRR	Ca_17011	--	--
Ca3_P4_15073326	Ca3	15073326	GAAAAAAAAAAA/GAAAAAAAAAA	TGTGTGTGCATCCAAA AGGT	TTTGGGAGTAGGCCTT CCTT	60.0	205	DRR	Ca_17012	R	--
Ca3_P4_15396841	Ca3	15396841	CATATATATATATATATATATATATA TATATATATATATATATA/CATATATA TATATATATATATATATATATATAT ATATATATATATATATATA	TCAATGCGGTCTCTGA AGTG	CACATGCTAATCGACA CCGT	60.0	412	DRR	Ca_17029	--	Trihelix
Ca3_P4_15658487	Ca3	15658487	TTCCA/TTCCACTCCA	AAAGCATTCCCACTAC GGTG	TTGTCACAGCCTAAAAC CCC	60.0	343	DRR	Ca_17043	--	--
Ca3_P4_15658525	Ca3	15658525	TC/TCATTCC	AAAGCATTCCCACTAC GGTG	TTGTCACAGCCTAAAAC CCC	60.0	343	DRR	Ca_17043	--	--
Ca3_P4_16094863	Ca3	16094863	CTTTTTT/CTTTTTTT	TCTCCGCATAAGCTTT CGAT	TGATTGCAAATAGCAC CCAA	59.9	248	DRR	Ca_17065	--	--
Ca3_P4_16879725	Ca3	16879725	T/TCCCACTGGTCA	AAGCGTACCTGTGGGA ATTG	GCGGATCCACTCACTA AAGG	60.0	818	DRR	Ca_18286	R	MYB
Ca3_P4_18089824	Ca3	18089824	AATATATATATATATATATATATATAT ATATA/AATATATATATATATATATAT ATATATATATATATATA	TGCGTGATTATCTGTTT AACGAA	CCGACAGCGGATGATT TTAT	59.7	791	DRR	Ca_21136	--	MYB_related
Ca3_P4_18345308	Ca3	18345308	TAAAAAAA/TAAAAAAAA	GGTTTCCCTCCTAAGT TAAGGGT	GCGGTGCAATCAGTAA ACCT	60.1	271	DRR	Ca_19619	O	--

INDEL marker IDs	Chromosomes/ unanchored scaffolds	Physical positions (bp)	InDels (*Kabuli* reference genome-CDC Frontier/PII)	Forward primers (5'-3')	Reverse primers (5'-3')	Anneling temperature (0C)	Expected amplified product size (bp)	Structural annotation		Functional annotation	
								Sequence components of *kabuli* genome	*Kabuli* gene accession IDs	NCBI-KOG	TFs
Ca3_P4_19366983	Ca3	19366983	ATTTTTTTTT/ATTTTTTTTTT	AGCTACTCGTCATCCTCCGA	TTAAAAGTGAACACACGCCG	60.0	807	DRR	Ca_19336	--	--
Ca3_P4_19368565	Ca3	19368565	GTTTTT/GTTTTTT	GGTGATGCATGATGATTTGC	TGGGTTAGCTTTTGGGATTG	59.9	630	DRR	Ca_19336	--	--
Ca3_P4_19383912	Ca3	19383912	CAAA/CAA	GTCAGGTCAGGCTGGAAAAA	TCGTCTTAATCCACTTGGTGC	60.2	436	DRR	Ca_19337	--	--
Ca3_P4_20011560	Ca3	20011560	GTTGACACTT/GTTGACACTTGACACTT	GCAATTGCAAGAGCGTGATA	TTTGGCATGCAATCAAACAT	60.0	277	DRR	Ca_09404	--	--
Ca3_P4_21276082	Ca3	21276082	AT/ATTATTATTATTATTTT	TTGATCTTTTGTTCGGTTGC	TCTTGGACATCATCAAAGCG	58.7	530	DRR	Ca_09490	--	S1Fa-like
Ca3_P4_22165237	Ca3	22165237	AG/A	TTTGAAATGCCAATAAAGTTTGAA	CATGCAATGCACGAGATCAT	59.9	502	DRR	Ca_20412	U	--
Ca3_P4_22553987	Ca3	22553987	CGAC/CGACGTATTCAGAC	CCACATGAGTGTGTTGAGGG	GGCTATCGCTTATGCAGGAC	60.0	588	DRR	Ca_06259	--	SAP
Ca3_P4_23016776	Ca3	23016776	TCCTACCT/TCCT	GCACACCTATGTGGTCTTGC	CCATTTTGTCGAAGTTTGGTT	59.2	615	DRR	Ca_06208	QR	B3
Ca3_P4_23356020	Ca3	23356020	GA/G	TCGTTTCCGGAGTCAAGTTC	TGGGCTGGTATGTGACTTTG	60.2	718	DRR	Ca_06185	O	--
Ca3_P4_23876448	Ca3	23876448	AATATATATATATATAT/AATATATATATATATATATATAT	CATAATCCCATCGCTCTCGT	TCGGTTAACTGGCCAAACTC	60.1	568	DRR	Ca_06137	--	--

INDEL marker IDs	Chromosomes/ unanchored scaffolds	Physical positions (bp)	InDels (*Kabuli* reference genome-CDC Frontier/PII)	Forward primers (5'-3')	Reverse primers (5'-3')	Anneling temperature (0C)	Expected amplified product size (bp)	Structural annotation		Functional annotation	
								Sequence components of *kabuli* genome	*Kabuli* gene accession IDs	NCBI-KOG	TFs
Ca3_P4_24187706	Ca3	24187706	AA/AACCA	ATTGCATGGAACGACAATGA	CCATGAGTGTTTCGTGGATG	59.9	607	DRR	Ca_06101	J	--
Ca3_P4_24956986	Ca3	24956986	TAAAAAAAAAA/TAAAAAAAAAAA	TAAAGGACCAAGTCGGGAAA	CGAGAAAAATGATTATCCCTCAA	59.5	315	DRR	Ca_06033	--	Trihelix
Ca3_P4_24969495	Ca3	24969495	CATATATATATATATATATATATATATATATA/CATATATATATATATATATATATATATATATATATATA	TTGAAAATGTGATTCGATTGC	TTGAGGATTATTGCCTTGGG	58.6	469	DRR	Ca_06032	--	ERF
Ca3_P4_25585812	Ca3	25585812	TAAAAAAAA/TAAAAAAA	TGTTTCGTTTTTGTTCCCTCT	TCCATTGCAATCGTACTTAATGA	58.7	735	DRR	Ca_05976	--	B3
Ca3_P4_26724618	Ca3	26724618	TCC/T	AGGAGTGTTTTTGGGGTTTG	GCCTCTGATGGTGTCAACTG	58.9	569	DRR	Ca_08235	J	--
Ca3_P4_26777304	Ca3	26777304	GTATATATATATATATAT/GTATATATATATATATAT	CACTCATCACAACGGGACAC	CAATGAATCGATGAAATGAATTG	60.0	754	DRR	Ca_08228	P	--
Ca3_P4_27073479	Ca3	27073479	TTTT/TTTTTTATTT	ATCATTTTCTTCAATGCGGA	ATTGGCGTCAACCTTGTTTC	58.2	727	DRR	Ca_08197	E	--
Ca3_P4_27616898	Ca3	27616898	ATTTTTTTTTTT/ATTTTTTTTTT	CTGCTTTGGAATAAGGACGG	GAAGTTAAATGCTAAATGGTGGAAA	59.7	905	DRR	Ca_08146	--	bHLH
Ca3_P4_28307367	Ca3	28307367	GTTATTATTATTATTATTATTATTATTATTATTATTATTACTATTATTATTATTATTATTCTTATTATTATTATTATTATTATT/GTTATTATTATTATTATTATTATTATTATTATTATTATTATTACTATTAT[illegible]	TGAGACAGATGTTTGAGGAAAGA	TATCGTGCATGCAAATGGTT	59.0	516	DRR	Ca_07146	D	M-type
Ca3_P4_28334138	Ca3	28334138	TTATATATATATATATATATATA/TTATATATATATATATATATATATATA	CGACCTCCTGAAGATCCAAA	TCGATGCTCCATGTTAATGC	60.2	853	DRR	Ca_07149	R	NAC

INDEL marker IDs	Chromosomes/ unanchored scaffolds	Physical positions (bp)	InDels (*Kabuli* reference genome-CDC Frontier/PII)	Forward primers (5'-3')	Reverse primers (5'-3')	Anneling temperature (0C)	Expected amplified product size (bp)	Structural annotation		Functional annotation	
								Sequence components of *kabuli* genome	*Kabuli* gene accession IDs	NCBI-KOG	TFs
Ca3_P4_29263411	Ca3	29263411	TAAAAAAAA/TAAAAAAA	TTCACAAATGGACCGT GAAA	TTGCAACTTTACGAAAC CCC	59.9	537	DRR	Ca_07235	IR	Nin-like
Ca3_P4_29263688	Ca3	29263688	A/AAAGGT	TTGAAGATCTCTGTGG CGAA	CTTCTCTCCCCAGCTT GTTG	59.5	762	DRR	Ca_07235	IR	Nin-like
Ca3_P4_30017715	Ca3	30017715	ATTTTTTTTT/ATTTTTTTTTT	TTCATTTTCATCTGCCC CTC	AACCCTGCCTCAGTGT CAAC	60.0	707	DRR	Ca_07315	T	--
Ca3_P4_30559364	Ca3	30559364	TTTATTATTATTATTATTATTATTATT ATTATTATTATTATTATTATTATTATT ATTATTATAATTATTATTATTATTAT/ TTTATTATTATTATTATTATTATTATT ATTATTATTATTATTATTATTATTATA [illegible]	TTATCGTAACGTGGGT GTCG	AATTTCCAAGTGGTCC CCTC	59.5	576	DRR	Ca_07376	--	--
Ca3_P4_31126519	Ca3	31126519	ATTTTTTTTT/ATTTTTTTT	TATCGCTTTAAGGTCG CGTT	AAAATTCATCACCCCAT TCG	59.9	406	DRR	Ca_12210	R	HD-ZIP
Ca3_P4_31206870	Ca3	31206870	GTTTTTTTTTTT/GTTTTTTTTTT	CAATTCATCTGTTGGG AGATCA	CCTGCATTACTTGCTC CACA	59.9	727	DRR	Ca_12217	O	TCP
Ca3_P4_31290388	Ca3	31290388	AG/A	TTTAAAGAAGGGAGGT CGCTC	TTGAAGCAGTGTTGTG GGAA	59.8	632	DRR	Ca_12225	P	ERF
Ca3_P4_31290755	Ca3	31290755	ATATT/AT	GATGGTCGATTTGAGA GCGT	AAAAACACACACGCAA CAACA	60.2	725	DRR	Ca_12225	P	ERF
Ca3_P4_31298029	Ca3	31298029	TAAAAAAAAAA/TAAAAAAAA	GGACTTTTGGCACGAT GTTT	TTTGCAACTTTTGACGG ACA	60.0	716	DRR	Ca_12226	K	MYB
Ca3_P4_31306026	Ca3	31306026	GAAAAA/GAAAAAA	GTCGCATCCCTCAAAA GTGT	ACTTGAAGCAACGGCT AGGA	60.1	521	DRR	Ca_12229	--	Dof

INDEL marker IDs	Chromosomes/ unanchored scaffolds	Physical positions (bp)	InDels (*Kabuli* reference genome-CDC Frontier/PII)	Forward primers (5'-3')	Reverse primers (5'-3')	Anneling temperature (0C)	Expected amplified product size (bp)	Structural annotation		Functional annotation	
								Sequence components of *kabuli* genome	*Kabuli* gene accession IDs	NCBI-KOG	TFs
Ca3_P4_31356584	Ca3	31356584	A/AG	TCAATCACCCAAGCATTTCA	TTTGAAGGCGGTACAAGTCC	60.0	896	DRR	Ca_12235	--	Whirly
Ca3_P4_31413249	Ca3	31413249	AA/AATA	TGTTGCACGTTGGACAAGTT	GACTGCTACCAACCCAGCAT	60.2	374	DRR	Ca_12238	A	--
Ca3_P4_31490470	Ca3	31490470	CTT/CTTT	GTCCCCTTCCATTTTGGATT	ACTTTTTGAAGGTTTGGGGG	60.0	369	DRR	Ca_12247	T	--
Ca3_P4_31498522	Ca3	31498522	GC/G	CTAGCTCGGGTTTTCTGACG	TTGGCCACAGTTTAACCACA	60.0	531	DRR	Ca_12248	T	--
Ca3_P4_31499264	Ca3	31499264	TAAGC/T	TTAAAGGGATTTTCGAGGCA	CACATGTTATTTGCCGCTGT	59.7	476	DRR	Ca_12249	I	--
Ca3_P4_31499806	Ca3	31499806	CATATCAATATCAATATCAATATCAATAT/CATATCAATATCAATATCAATAT	GCGGCAAATAACATGTGAAA	TCCTAGACTTGTTGGGGCAC	59.6	565	DRR	Ca_12248	T	--
Ca3_P4_31588239	Ca3	31588239	TACAAC/TAC	CTTCCAAATACCTCCCCCAT	CCGTGGCCTTCTCTACTCTG	60.0	346	DRR	Ca_12258	BK	G2-like
Ca3_P4_31588307	Ca3	31588307	T/TC	CTTCCAAATACCTCCCCCAT	CCGTGGCCTTCTCTACTCTG	60.0	346	DRR	Ca_12258	BK	G2-like
Ca3_P4_31609545	Ca3	31609545	T/TTA	GACAAAGCCAGAACCTTTGC	ACCCAAGCAGGCATACAAAC	59.9	538	DRR	Ca_12260	--	--
Ca3_P4_31635608	Ca3	31635608	GTTTTTTTTTTT/GTTTTTTT	AATGGGTTCCCTGGATTTTC	CTTTGGTGGGATCAGAGAGC	60.0	222	DRR	Ca_12262	T	--

INDEL marker IDs	Chromosomes/ unanchored scaffolds	Physical positions (bp)	InDels (*Kabuli* reference genome-CDC Frontier/PII)	Forward primers (5'-3')	Reverse primers (5'-3')	Anneling temperature (0C)	Expected amplified product size (bp)	Structural annotation		Functional annotation	
								Sequence components of *kabuli* genome	*Kabuli* gene accession IDs	NCBI-KOG	TFs
Ca3_P4_31637117	Ca3	31637117	TGA/TGAGGA	TGCGTAAAAGCCAAACAAAA	GCACAAGCAGAGCAAAATGA	59.4	943	DRR	Ca_12264	--	NF-YB
Ca3_P4_31672462	Ca3	31672462	AG/AGG	AAGGCCGATTAGCCAAAAAT	TAGGAGGTCGCTTCTCCAAA	59.9	211	DRR	Ca_12269	--	NF-YB
Ca3_P4_32486522	Ca3	32486522	AAATAATAATAATAATAATAATAATAATAATAATAAT/AAATAATAATAATAATAATAATAATAATAATAAT	AACCACTTTTAAATCGATGCG	TGCAGTAGTTTGCTGCCATC	59.1	498	DRR	Ca_11974	--	--
Ca3_P4_32582344	Ca3	32582344	CGTGT/CGT	CAGAACTGATTCTTCCCCCA	TCTCGGGTAAGTAAAGGGCA	60.0	408	DRR	Ca_11984	P	--
Ca3_P4_32671018	Ca3	32671018	CATATATATATATATATATATATATA/CATATATATATATATATATATATATATATATATA	TGGTTGGAGAGCAAAGAAGG	TTAGTGGATTGCTTCCCTGG	60.4	655	DRR	Ca_11996	--	--
Ca3_P4_34821669	Ca3	34821669	C/CGTTGAGTGA	TGTCCTTTGATGTGCAGGAC	CCAGCAACTCACGTGACAAT	59.7	521	DRR	Ca_00758	--	--
Ca3_P4_35006302	Ca3	35006302	CTCTTCTTCTT/CTCTTCTT	CGATTGTTGATCCTCCTCGT	CTGCACACTTTGAGGCTGAG	60.1	460	DRR	Ca_00774	R	ZF-HD
Ca3_P4_3524015	Ca3	3524015	AATATATATATATAT/AATATATATATATATATAT	GTTTGCAGGAAGAGGCAAAG	GCCAGCAGCCTTCAAGATAG	60.0	712	DRR	Ca_21350	J	--
Ca3_P4_35341749	Ca3	35341749	ATTTTTTTT/ATTTTTTT	CTGCCAGAAAGTTGAGAAGGA	AAGGTTGTGTTTGAAACCGC	59.6	779	DRR	Ca_00813	G	--
Ca3_P4_35466145	Ca3	35466145	TCACGAC/TC	CAAATTTTTGCCGCCATTAT	GGGCTGTCAGAGCAGAAAAG	59.8	743	DRR	Ca_00829	R	NAC

INDEL marker IDs	Chromosomes/ unanchored scaffolds	Physical positions (bp)	InDels (*Kabuli* reference genome-CDC Frontier/PII)	Forward primers (5'-3')	Reverse primers (5'-3')	Anneling temperature (0C)	Expected amplified product size (bp)	Structural annotation		Functional annotation	
								Sequence components of *kabuli* genome	*Kabuli* gene accession IDs	NCBI-KOG	TFs
Ca3_P4_35691450	Ca3	35691450	TTATATATATATATATA/TTATATATATATATA	TCGTTACCCCTCTCGTCATC	CATTTGCATACACTTGCGCT	60.1	594	DRR	Ca_00858	R	ZF-HD
Ca3_P4_35954208	Ca3	35954208	A/AT	ATCCGTCTCTATACCCGCAA	ATCTACCCCGCACCCATAAT	59.6	607	DRR	Ca_00890	V	--
Ca3_P4_35955479	Ca3	35955479	TAAAA/TAAAAAA	AAAATCGGCCCAACCTAATC	TCACAAATTCACAATCAACCG	60.2	238	DRR	Ca_00890	V	--
Ca3_P4_35959327	Ca3	35959327	GAAAAAAA/GAAAAAAAA	TGTTGACCAATAAGCAAAATTGA	TGACACACAACATACAAGCCA	59.5	360	DRR	Ca_00891	V	GRAS
Ca3_P4_35959933	Ca3	35959933	GTTTTTTT/GTTTTTTTT	TCTGAACAAATGATCGGGTG	ACATGCAATTTCCATCCACA	59.5	164	DRR	Ca_00891	V	GRAS
Ca3_P4_35959985	Ca3	35959985	CAAAAAAAAAAAAAAA/CAAAAAAAAAAAAA	TCTGAACAAATGATCGGGTG	ACATGCAATTTCCATCCACA	59.5	164	DRR	Ca_00891	V	GRAS
Ca3_P4_35967790	Ca3	35967790	GAAAATAGAAA/GAAAATAGAAAACAAAATAGAAA	TTGATTATGAGCACCAAGCG	TGTCTGGATTTTTGTTTTTGAGA	59.8	335	DRR	Ca_00892	C	--
Ca3_P4_35981261	Ca3	35981261	GT/G	TACCGGGAGAATTGACGACT	TTGGGTCCATTTGGTTTGAT	59.6	317	DRR	Ca_00893	--	--
Ca3_P4_35992938	Ca3	35992938	ATT/AT	AGAAGCACAAGTCCCCTGAA	GCTCATGTGTAAAGCCGTGA	59.8	710	DRR	Ca_00894	--	--
Ca3_P4_35993475	Ca3	35993475	CATATA/CATA	TCACGGCTTTACACATGAGC	TTGATTCGAACCCAAAATCT	59.9	515	DRR	Ca_00894	--	--

INDEL marker IDs	Chromosomes/ unanchored scaffolds	Physical positions (bp)	InDels (*Kabuli* reference genome-CDC Frontier/PII)	Forward primers (5'-3')	Reverse primers (5'-3')	Anneling temperature (0C)	Expected amplified product size (bp)	Structural annotation		Functional annotation	
								Sequence components of *kabuli* genome	*Kabuli* gene accession IDs	NCBI-KOG	TFs
Ca3_P4_36016163	Ca3	36016163	TAAAAAAAAAA/TAAAAAAAAAAA	CAACTGCACAGATGGATTGG	AATTTGCAATTTGATCCCCA	60.1	650	DRR	Ca_00900	O	CAMTA
Ca3_P4_36022347	Ca3	36022347	AC/ACC	AACATTTACAAACATAAACGACGAA	TGGAGATGGAAATGGAGAGG	59.0	524	DRR	Ca_00901	O	CAMTA
Ca3_P4_36022379	Ca3	36022379	TCC/TC	AACATTTACAAACATAAACGACGAA	TGGAGATGGAAATGGAGAGG	59.0	524	DRR	Ca_00901	O	CAMTA
Ca3_P4_36032382	Ca3	36032382	ATTTTTTTTT/ATTTTTT	GCTTTGCTCCCACATCTCTC	AACGCTGAACCAAAATGACC	60.0	217	DRR	Ca_00902	S	bZIP
Ca3_P4_36033507	Ca3	36033507	TAGAGA/TAGAGAGA	CTTCTCACCCCCTCAAAACA	AGGCTTGATGACACGTGATG	60.1	696	DRR	Ca_00902	S	bZIP
Ca3_P4_36098742	Ca3	36098742	TC/T	TATGCTGATTCGGCCATTTT	CTCGGTTGTTGTTGGAGGTT	60.4	379	DRR	Ca_00908	K	--
Ca3_P4_36116492	Ca3	36116492	T/TC	TTGTGCTCAAACAGAGTCGG	ATGTGTCTGCCATGCTTCTG	60.0	510	DRR	Ca_00911	D	--
Ca3_P4_36116714	Ca3	36116714	AATGGCAGAAGCATGGCAGAAGCATGGCAGA/AATGGCAGAAGCATGGCAGA	TTGTGCTCAAACAGAGTCGG	TGGCTCAATCATTCATCTGC	60.0	770	DRR	Ca_00911	D	--
Ca3_P4_36116816	Ca3	36116816	CT/CTCATTAT	CGAAAAACCGCCATAAACAT	TGGCTCAATCATTCATCTGC	59.8	337	DRR	Ca_00911	D	--
Ca3_P4_36129194	Ca3	36129194	GTTAAATTA/GTTAAATTAAATTA	GCAAAAAGCACCTCGACTTT	CACACCAATCAAACGAGCAA	59.5	696	DRR	Ca_00912	YU	--

INDEL marker IDs	Chromosomes/ unanchored scaffolds	Physical positions (bp)	InDels (*Kabuli* reference genome-CDC Frontier/PII)	Forward primers (5'-3')	Reverse primers (5'-3')	Anneling temperature (0C)	Expected amplified product size (bp)	Structural annotation		Functional annotation	
								Sequence components of *kabuli* genome	*Kabuli* gene accession IDs	NCBI-KOG	TFs
Ca3_P4_36152348	Ca3	36152348	ATTT/ATT	TGGCTGTCAATTTGGAAAAA	GGTTGATGCTGTCGTGTTTG	59.1	297	DRR	Ca_00916	A	GeBP
Ca3_P4_36152949	Ca3	36152949	TAAAAAAA/TAAAAAAAAA	CAAACACGACAGCATCAACC	GGTGCTTTGAAGATTGGAGG	60.2	578	DRR	Ca_00916	A	GeBP
Ca3_P4_36153695	Ca3	36153695	TT/TTAT	TTATTTTTCGGGCTTTTGTCA	ATTGATTGATTGGCATGCTG	59.6	514	DRR	Ca_00916	A	GeBP
Ca3_P4_36167042	Ca3	36167042	GTTTTTTTTT/GTTTTTTTTTT	TTTTGACCCATCCAAGAAGG	CCCCGTCTAATTAGCCAACA	59.9	459	DRR	Ca_00918	KTDL	--
Ca3_P4_36178800	Ca3	36178800	TATTTTAATTTTAATTT/TATTTTAATTTTAATTTTAATTT	TGGATAAGACCGGAGAATGC	TCTCGAAACTAGAAAAGAACAAAAA	60.0	812	DRR	Ca_00921	Q	B3
Ca3_P4_36178910	Ca3	36178910	AAT/A	TGGATAAGACCGGAGAATGC	TCTCGAAACTAGAAAAGAACAAAAA	60.0	812	DRR	Ca_00921	Q	B3
Ca3_P4_36188427	Ca3	36188427	TAA/TAAA	GCATAGGACTAGGCCCAAAA	GGGAAGCGTCACACTAAAGC	59.2	521	DRR	Ca_00922	Q	B3
Ca3_P4_36189478	Ca3	36189478	AATAT/AATATTCAACATTATAT	CAACCGTTCAAATCACATCG	GCCAAGGCCAAATCAACTTA	60.0	690	DRR	Ca_00922	Q	B3
Ca3_P4_36193918	Ca3	36193918	GATATA/GATATATA	TGAGGGACAAAAGGTTCAGC	TGAAATATGGCTTCCCTTCG	60.2	558	DRR	Ca_00923	--	bHLH
Ca3_P4_36194243	Ca3	36194243	TAT/TATATGTAAAAAAAT	TGCCATAAAGATGCGAAACA	TGAAATATGGCTTCCCTTCG	60.2	249	DRR	Ca_00923	--	bHLH

INDEL marker IDs	Chromosomes/ unanchored scaffolds	Physical positions (bp)	InDels (*Kabuli* reference genome-CDC Frontier/PII)	Forward primers (5'-3')	Reverse primers (5'-3')	Anneling temperature (0C)	Expected amplified product size (bp)	Structural annotation		Functional annotation	
								Sequence components of *kabuli* genome	*Kabuli* gene accession IDs	NCBI-KOG	TFs
Ca3_P4_36198404	Ca3	36198404	TAAAAAAAA/TAAAAAAA	TTCTTGCAATCAAAGCATCG	AACAGAGGCGGTGTCACTCT	60.0	469	DRR	Ca_00924	--	--
Ca3_P4_36198453	Ca3	36198453	AATA/AA	TTCTTGCAATCAAAGCATCG	AACAGAGGCGGTGTCACTCT	60.0	469	DRR	Ca_00924	--	--
Ca3_P4_36199218	Ca3	36199218	TTTCTTCTT/TTTCTTCTTCTT	CACTCAAGGGACTAGGAGCG	GGTTTATGGGTCTTGGGGAT	60.0	482	DRR	Ca_00924	--	--
Ca3_P4_36204205	Ca3	36204205	ATTT/ATTTT	ACATGCACAAATCCCTCACA	GGTAGCAAAGCAATTTCGGA	60.0	435	DRR	Ca_00925	--	--
Ca3_P4_36204911	Ca3	36204911	ATATTAAATTT/ATATTAAATTTATTAAATTT	GTCGTTGGAGTCTTTGCGAT	TTCACCAAATGTTGACCGAA	60.3	452	DRR	Ca_00925	--	--
Ca3_P4_36205222	Ca3	36205222	GA/G	AAAATTGGAATTGTCACGCC	CATTCTTGTTGATGCCATGC	59.8	548	DRR	Ca_00925	--	--
Ca3_P4_36205967	Ca3	36205967	GT/GTCT	GCATGGCATCAACAAGAATG	CATCAGAAGTCTGTTGCCGA	60.1	467	DRR	Ca_00925	--	--
Ca3_P4_36213829	Ca3	36213829	ATG/ATGAAAAAGTG	AAAATTATTGTGGCCGAGCA	ACAGGGGATGCAGACTTTTT	60.5	845	DRR	Ca_00926	--	LSD
Ca3_P4_36215526	Ca3	36215526	TA/TAA	TTGATTGCCGACAGTCTTTG	CGCAATTTTGAATGTCATCATC	59.8	622	DRR	Ca_00927	--	CO-like
Ca3_P4_36215688	Ca3	36215688	ATTTTT/ATTTT	TGCTGTAACGATGATGCCAC	CAATGTGGATGCCTTTGTTG	60.7	981	DRR	Ca_00927	--	CO-like

INDEL marker IDs	Chromosomes/ unanchored scaffolds	Physical positions (bp)	InDels (*Kabuli* reference genome-CDC Frontier/PII)	Forward primers (5'-3')	Reverse primers (5'-3')	Anneling temperature (0C)	Expected amplified product size (bp)	Structural annotation		Functional annotation	
								Sequence components of *kabuli* genome	*Kabuli* gene accession IDs	NCBI-KOG	TFs
Ca3_P4_36220876	Ca3	36220876	TAGA/TA	AATCCTAAAGCGCCGAATTT	GCTGGCACATTGTGAAAAGA	60.1	565	DRR	Ca_00928	--	--
Ca3_P4_36221155	Ca3	36221155	CAAAA/CAAA	TTTTCACAATGTGCCAGCTC	AAATCCATCCCCGTATCTCC	59.8	405	DRR	Ca_00928	--	--
Ca3_P4_36236843	Ca3	36236843	CATTCCA/CATTCCATATTCCA	GAGTGGCTGGGAATCAACAT	CACCAAGACTCATCTTGCGA	59.9	511	DRR	Ca_00931	O	--
Ca3_P4_36256005	Ca3	36256005	CTAAAGATA/CTAAAGATATAAAGATA	CCCTCCCTTGTTGACGAATA	ATTGGCGAAGAAATGCAATC	59.9	705	DRR	Ca_00935	--	--
Ca3_P4_36257974	Ca3	36257974	AATATATAT/AATAT	CGAGCATGGTGCAACTTCTA	CCTATTCCGTTTTCATGCGT	60.0	583	DRR	Ca_00936	--	MYB_related
Ca3_P4_36273608	Ca3	36273608	ATTT/ATTTT	TTCACTCTGCTTGGGCTTTT	GCGGGACATATTCAGGAGAA	60.0	405	DRR	Ca_00937	J	Dof
Ca3_P4_36274277	Ca3	36274277	T/TA	TGGGTTTTCATTGGTTTGGT	AAGCCGACTTCAAGAAGGAC	60.1	419	DRR	Ca_00937	J	Dof
Ca3_P4_36340133	Ca3	36340133	A/AGTAGAGATATAATTG	TAGCCCGTCACAATTTAGGG	CTCTTTCTTTCCCTCCGCTC	60.0	910	DRR	Ca_00942	--	HB-other
Ca3_P4_36343651	Ca3	36343651	TAAAAAAAAA/TAAAAAAAAAA	GGGGTGATCGAATTAGGGTT	CCTAGAGGATCAAAAGGCCC	60.0	608	DRR	Ca_00943	R	C2H2
Ca3_P4_36343819	Ca3	36343819	ATTTT/ATTT	GGCCTTTTGATCCTCTAGGC	TGCATTTGAATTGCCAACAT	60.2	320	DRR	Ca_00943	R	C2H2

INDEL marker IDs	Chromosomes/ unanchored scaffolds	Physical positions (bp)	InDels (*Kabuli* reference genome-CDC Frontier/PII)	Forward primers (5'-3')	Reverse primers (5'-3')	Anneling temperature (0C)	Expected amplified product size (bp)	Structural annotation		Functional annotation	
								Sequence components of *kabuli* genome	*Kabuli* gene accession IDs	NCBI-KOG	TFs
Ca3_P4_36344189	Ca3	36344189	GCCC/GCC	GGCCTTTTGATCCTCTAGGC	TGCAATTTTTATCACATCCCC	60.2	777	DRR	Ca_00943	R	C2H2
Ca3_P4_36363400	Ca3	36363400	ATTTTTTTTT/ATTTTTTT	CAATGCATATGGACGGAATG	GACGACAAAGGATATTCATGAGC	59.8	352	DRR	Ca_00945	--	NF-X1
Ca3_P4_36403564	Ca3	36403564	TCA/T	TGAGGTGCTAACACCGAAGA	AGTGACAACCCCTCAATTCG	59.4	223	DRR	Ca_00951	H	--
Ca3_P4_36404085	Ca3	36404085	GTT/GT	CGAATTGAGGGGTTGTCACT	TGAATCTTATTGCGTGAGCG	60.0	803	DRR	Ca_00951	H	--
Ca3_P4_36424396	Ca3	36424396	AA/AAAAATATA	GGACTGGGATTTGAACGAAA	TGCAAAGCCAAATGCTACAC	59.9	586	DRR	Ca_00955	--	--
Ca3_P4_36445861	Ca3	36445861	ATTTTTTTTTT/ATTTTTTTTT	CGAAGGTTGTTCCTCGACAT	ACCGACACGTCTACACACCA	60.1	267	DRR	Ca_00960	O	--
Ca3_P4_36446253	Ca3	36446253	CAG/CAGAG	ATCCCAATCACCTCACTTGC	CCCAGATTTCTATGCGGAAA	59.9	533	DRR	Ca_00960	O	--
Ca3_P4_36447139	Ca3	36447139	CAT/CATAT	GGATGAGTGCAATTTGGGAT	AATGAATGGCTGCGCTAGTT	59.8	473	DRR	Ca_00960	O	--
Ca3_P4_36526857	Ca3	36526857	CAAAAAAAAAAA/CAAAAAAAAAA	AAGTCGCCAACGCAATAAAC	CTCTTTGTGCAAACAGGCAA	60.1	827	DRR	Ca_00970	--	--
Ca3_P4_36932559	Ca3	36932559	GAAAAAAAAAAAAA/GAAAAAAAAAAA	GTTACGATTTTCATTGGCGA	CTTTGGTATGTTGGGCCTGT	58.6	917	DRR	Ca_01018	--	WRKY

INDEL marker IDs	Chromosomes/ unanchored scaffolds	Physical positions (bp)	InDels (*Kabuli* reference genome-CDC Frontier/PII)	Forward primers (5'-3')	Reverse primers (5'-3')	Anneling temperature (0C)	Expected amplified product size (bp)	Structural annotation		Functional annotation	
								Sequence components of *kabuli* genome	*Kabuli* gene accession IDs	NCBI-KOG	TFs
Ca3_P4_36986782	Ca3	36986782	AACT/A	TTTCAAGGTGTGTTCACCGA	TCTTACACCTCTTCGGGTGC	60.1	502	DRR	Ca_01027	U	C2H2
Ca3_P4_37030222	Ca3	37030222	AATATATATATATATATATATATATATA/AATATATATATATATATATATATATATATATA	GAAAAGCCCATTTTGTCTCG	AAAGGTTTGAAAATGGATGGTC	59.7	593	DRR	Ca_01031	G	C3H
Ca3_P4_3703442	Ca3	3703442	TAAAAAAAAAA/TAAAAAAAAA	GCGGAGATATTTGCTGCGTA	TGGGCCCATTTACGTCTAAG	61.3	649	DRR	Ca_21344	--	--
Ca3_P4_3704345	Ca3	3704345	TAAAAAA/TAAAAA	TGGCAAGGAAATGACTCACA	GCAGATTGTGGGTTTGATTG	60.2	944	DRR	Ca_21343	A	--
Ca3_P4_37736258	Ca3	37736258	AAATAATAATAATAATAATAATAATAATAATAATAATA/AAATAATAATAATAATAATAATAATAATAATAATAATAATAATAATA	TTTTGTCATTTGGTATTCATTTCA	TCTCATTTCACATCCGGACA	58.5	595	DRR	Ca_01110	O	--
Ca3_P4_37991623	Ca3	37991623	TAAAAAAAAA/TAAAAAAAAAA	TAGATTCCCCACCAGTCCAG	TGGTACGCGACAATTTTTGTT	59.9	793	DRR	Ca_01140	--	--
Ca3_P4_38073514	Ca3	38073514	CAAAAAAAAAAAAA/CAAAAAAAAAAAAAA	CTTCTGAATGGGTTTGGTGG	ATGGACTCAATTGGGAGTGG	60.3	439	DRR	Ca_01151	DL	--
Ca3_P4_38155393	Ca3	38155393	ATTTT/ATTT	GTTTTGCGCCAATTTTCATT	AGGGTGTACATTCCGTGCAT	60.0	484	DRR	Ca_01160	L	MYB_related
Ca3_P4_38174096	Ca3	38174096	AGG/AG	CGAAACACGTTACCATCATCC	CCGATGAGTGTGGATGTGAA	60.2	410	DRR	Ca_01161	--	NF-YA
Ca3_P4_38174447	Ca3	38174447	A/AT	TTCACATCCACACTCATCGG	CTGGCACTGGCATTCCTAAT	60.5	628	DRR	Ca_01161	--	NF-YA

INDEL marker IDs	Chromosomes/ unanchored scaffolds	Physical positions (bp)	InDels (*Kabuli* reference genome-CDC Frontier/PII)	Forward primers (5'-3')	Reverse primers (5'-3')	Anneling temperature (0C)	Expected amplified product size (bp)	Structural annotation		Functional annotation	
								Sequence components of *kabuli* genome	*Kabuli* gene accession IDs	NCBI-KOG	TFs
Ca3_P4_38184409	Ca3	38184409	TGAAAA/TGAAAAGAAAA	TCCACGAAACGAAGAAGGTT	AGTGGCAAACCCTTAATCCC	59.7	603	DRR	Ca_01162	--	--
Ca3_P4_38184714	Ca3	38184714	ATTTTTTTT/ATTTTTTTTTTT	GGGATTAAGGGTTTGCCACT	CTGGCTTGTGAACCCAAAAT	60.2	298	DRR	Ca_01162	--	--
Ca3_P4_38184945	Ca3	38184945	T/TG	AAGAAGAGAGGTTGGAGCCC	CTGGCTTGTGAACCCAAAAT	59.8	143	DRR	Ca_01162	--	--
Ca3_P4_38185351	Ca3	38185351	TAAAACAAAACAA/TAAAACAA	ATTTTGGGTTCACAAGCCAG	CAATTGGTGTTAGGCCACCT	60.0	497	DRR	Ca_01162	--	--
Ca3_P4_38206155	Ca3	38206155	TGGGGGG/TGGGGGGG	GCATGCTGCCCATACCTATT	CTCCCCCTTCTCCAGGATAG	60.0	691	DRR	Ca_01163	E	--
Ca3_P4_38215897	Ca3	38215897	CTT/CTTCTCTTCATT	TGTTGCCTGGAAATGAGTTG	TGCTGCATTGAAATCGAGTC	59.7	688	DRR	Ca_01165	R	--
Ca3_P4_38226965	Ca3	38226965	A/AC	GGCTGGAAAAGATGTGGAAA	CTATTTGGCACGTCTGGGTT	60.1	281	DRR	Ca_01167	R	--
Ca3_P4_38228316	Ca3	38228316	T/TC	ATGATCCACTGAGATTCCGC	CCAGAGTGTGACGCTTTTGA	60.0	472	DRR	Ca_01168	R	bHLH
Ca3_P4_38228566	Ca3	38228566	GAA/GAAA	AAATGTGAAAGCATGAGGGG	TGTCCACTTCCTCGGCTTAC	59.9	796	DRR	Ca_01168	R	bHLH
Ca3_P4_38230070	Ca3	38230070	CTTTATAAGCCGGTTTTTTATAAGCCGGTTTT/CTTTATAAGCCGGTTTT	CCCTGGCTACCAAGTACCAA	TCCGAGACGGGTAAATATGC	60.0	610	DRR	Ca_01168	R	bHLH

INDEL marker IDs	Chromosomes/ unanchored scaffolds	Physical positions (bp)	InDels (*Kabuli* reference genome-CDC Frontier/PII)	Forward primers (5'-3')	Reverse primers (5'-3')	Anneling temperature (0C)	Expected amplified product size (bp)	Structural annotation		Functional annotation	
								Sequence components of *kabuli* genome	*Kabuli* gene accession IDs	NCBI-KOG	TFs
Ca3_P4_38333156	Ca3	38333156	TTATATATATATATATATATATATAT/TTATATATATATATATATATATAT	CCTGCCTTGCACCTATCAGT	TAGGTGTGATTGCCCTTTTT	60.3	683	DRR	Ca_01182	R	--
Ca3_P4_38612369	Ca3	38612369	TAAAAA/TAAAA	TTTTGAAAGCTCTCGCTTGA	TGCGACCTCATGAAGACTAAAA	58.9	423	DRR	Ca_01216	--	GeBP
Ca3_P4_38690322	Ca3	38690322	AATATATATATATATATATATATA/AATATATATATATATATATATATATATATATATA	GAAGCAGGGAGGAGGAAAAC	TGTCAATGAGGATTATGCCAA	60.2	910	DRR	Ca_01224	K	Trihelix
Ca3_P4_38970415	Ca3	38970415	GTTTTTT/GTTTTTTT	CGCAACCTCGTAATCCATTT	CATCCCTAGAATGCGACCAT	60.0	565	DRR	Ca_01259	--	--
Ca3_P4_39547263	Ca3	39547263	CTTTATTTTTTTTTTTATTTTTTTTT/CTTTATTTTTTTTTTTATTTTTTTTTTATTTTTTTTT	GGGTGAGAAGTCTGACGGAG	TGGAAAAACCAAGAAATGTTGA	59.8	806	DRR	Ca_01325	--	ERF
Ca3_P4_39848936	Ca3	39848936	AATATATATATATATATATATATATATAT/AATATATATATATATATATATATATATATAT	GAAACAGTGAATGCCACCCT	GGTTTTGAATTGCCATGCTT	60.0	325	DRR	Ca_01370	P	GRAS
Ca3_P4_4121386	Ca3	4121386	CTTT/CTTTT	GACTGGACGTAACCCACGTT	AGCATTGTGCTTACGACGAA	59.9	379	DRR	Ca_22386	--	--
Ca3_P4_5070771	Ca3	5070771	ATTTTTTTTTTTTTTT/ATTTTTTTTTTTTT	AACCTTGATGCTCACATGAAA	TTACACTTGTTAGCCGGATGC	58.2	696	DRR	Ca_23878	--	--
Ca3_P4_5414883	Ca3	5414883	ATGTTTGG/ATGTTTGGTTTTTTGTTTGG	CTCACATATGCACCTGCTCG	TTCTGAAGGTCAAAACGTGC	60.4	470	DRR	Ca_20660	--	--
Ca3_P4_5692377	Ca3	5692377	GTT/GT	TGTGCAATTAAGAGAGGACACAA	CGAGAAATAGCTCACGAGGC	59.8	268	DRR	Ca_20648	--	--

INDEL marker IDs	Chromosomes/ unanchored scaffolds	Physical positions (bp)	InDels (*Kabuli* reference genome-CDC Frontier/PII)	Forward primers (5'-3')	Reverse primers (5'-3')	Anneling temperature (0C)	Expected amplified product size (bp)	Structural annotation		Functional annotation	
								Sequence components of *kabuli* genome	*Kabuli* gene accession IDs	NCBI-KOG	TFs
Ca3_P4_6345015	Ca3	6345015	CAAAAAAAAAAA/CAAAAAAAAAA	CAGCTGTTCCGATGTCTTCA	GCTCATGGGATGTCAACCTT	60.0	979	DRR	Ca_24832	--	B3
Ca3_P4_6772905	Ca3	6772905	GTTT/GTT	TCCCTTTGTCTTTTCGCACT	CACTGCCGTTCTTCACTTCA	59.9	383	DRR	Ca_16559	R	NAC
Ca3_P4_6774064	Ca3	6774064	T/TTAA	TTTGCTGGACTTTGAAGCCT	CCCGGTCCTAGAGAGAAAGA	60.0	640	DRR	Ca_16559	R	NAC
Ca3_P4_7228177	Ca3	7228177	GAGTTACATAGTTACATA/GAGTTACATA	CTGTTGGGCAAGGGATCTAA	ACAGCGCTTTTGTGCTTTTT	60.1	463	DRR	Ca_16534	--	--
Ca3_P4_7312652	Ca3	7312652	TAAAAAAAAA/TAAAAAAAAAA	CGCAAGATTTTACCTACCTGTG	TGATCAAGCTTCCAGTCCAA	58.8	720	DRR	Ca_16529	--	LFY
Ca3_P4_781722	Ca3	781722	CAA/CAAAA	AAAACGCGCCACTAATGACT	AGCAAGAGTGCAAAGTCGGT	59.8	546	DRR	Ca_22643	V	B3
Ca3_P4_8081601	Ca3	8081601	CATATATATATATATATATA/CATATATATATATATA	AAAGCGCTGTAAAATGCACA	GAAATAGCTTGCTGTTGGGC	59.5	580	DRR	Ca_24371	--	Trihelix
Ca3_P4_8730393	Ca3	8730393	AAA/AAACCAAACAA	GCCAAACAAATTGAAACCCA	ATCTCCGAACCCCTTCCTAA	60.7	414	DRR	Ca_23717	--	B3
Ca3_P4_8900151	Ca3	8900151	TAAAAAA/TAAAAAAA	GTGAAAATCCACCCTCGACT	TCAAACCTGCCATTTTCTCA	59.0	156	DRR	Ca_25279	R	--
Ca3_P4_9306348	Ca3	9306348	AATATATATATATATATATATATA/AATATATATATATATATATATA	TCCAGGTGAAAAGTAACGCC	TGCTGAGCAAAACACTCTGG	60.1	432	DRR	Ca_20961	--	Trihelix

INDEL marker IDs	Chromosomes/ unanchored scaffolds	Physical positions (bp)	InDels (*Kabuli* reference genome-CDC Frontier/PII)	Forward primers (5'-3')	Reverse primers (5'-3')	Anneling temperature (0C)	Expected amplified product size (bp)	Structural annotation		Functional annotation	
								Sequence components of *kabuli* genome	*Kabuli* gene accession IDs	NCBI-KOG	TFs
Ca3_P4_9649838	Ca3	9649838	ATTT/ATT	TAAGGCAGATTGCCCAAAAC	TTTCGGGAATGAAACTGGAC	60.1	462	DRR	Ca_20979	--	--
Ca3_P4_9651257	Ca3	9651257	CTTTTTT/CTTTTT	CTCCAAACTGGGGTGTCACT	CGGTCTCAACCTCTCCCATA	60.0	882	DRR	Ca_20979	--	--
Ca4_P4_10784287	Ca4	10784287	TAAAAAAAAAAA/TAAAAAAAAAA	TGGGCCGAATTTTGTGATAG	CGCACAATTGATACATCAACAC	60.8	486	DRR	Ca_04314	U	--
Ca4_P4_11070438	Ca4	11070438	T/TATTATTATTACG	TTTGCCCCAATAAAGAGCAC	AACAACTTTTGCGAACCACC	60.1	701	DRR	Ca_04340	J	--
Ca4_P4_11216218	Ca4	11216218	TAAAAAAAAA/TAAAAAAA	GCATAACCAATGCTCTTGGAA	TCAAACCAAGGATGATGCAA	60.1	853	DRR	Ca_04353	U	M-type
Ca4_P4_11222847	Ca4	11222847	A/AC	AATCTCCGAATCATTGTCGG	ACTGTCTCACGAGTCGGCTT	59.9	584	DRR	Ca_04354	A	--
Ca4_P4_11280849	Ca4	11280849	TC/TCC	GCTCAGCAGCTTTGACTTTG	TTCGAATTGAAACTCCTCCA	59.0	733	DRR	Ca_04359	--	--
Ca4_P4_11281622	Ca4	11281622	TG/T	TTTAAGGCATGTGAGTTCAGC	TGCAAAATCAATACATGGTTCG	58.0	641	DRR	Ca_04359	--	--
Ca4_P4_11285447	Ca4	11285447	C/CAA	TCCACATGCACCTGTGAACT	AGTAGTGCCTCAAACCACGG	60.2	382	DRR	Ca_04361	GE	GATA
Ca4_P4_11310573	Ca4	11310573	TAAAAAA/TAAAA	TACGAGAACATCCACGGTGA	GAGGGACGGCACATAAACAT	60.1	564	DRR	Ca_04363	--	--

INDEL marker IDs	Chromosomes/ unanchored scaffolds	Physical positions (bp)	InDels (*Kabuli* reference genome-CDC Frontier/PII)	Forward primers (5'-3')	Reverse primers (5'-3')	Anneling temperature (0C)	Expected amplified product size (bp)	Structural annotation		Functional annotation	
								Sequence components of *kabuli* genome	*Kabuli* gene accession IDs	NCBI-KOG	TFs
Ca4_P4_11311504	Ca4	11311504	A/ACAC	TGATGGTTGGCCTAAATGGT	TTGCTGAAGACTGATGCCAC	60.2	463	DRR	Ca_04363	--	--
Ca4_P4_11317243	Ca4	11317243	ATCTCTCTCTCTCTCTC/ATCTCTCTCTCTCTC	CCTTCCACTGCCAAAACATT	CAATTGGTCGGTTTCTGGTT	60.0	430	DRR	Ca_04364	D	WRKY
Ca4_P4_11331252	Ca4	11331252	TAAA/TAAAA	AGAGGGAAAAAGGGGTTGAA	TCTCTTCTCGCCCCAGTTTA	59.9	742	DRR	Ca_04367	U	FAR1
Ca4_P4_11346851	Ca4	11346851	GTTTTTTT/GTTTTTTTT	AACAGAAAAGATTGCATGCGT	TGCAATGACTCCCATTACGA	59.8	323	DRR	Ca_04368	R	--
Ca4_P4_11347660	Ca4	11347660	TTATA/TTA	CCGAATGAGCCTTCACAAGT	TTAGGGGTGGACAAAGTTCG	60.3	595	DRR	Ca_04368	R	--
Ca4_P4_11362167	Ca4	11362167	AAAAAATAAA/AAAAAATAAAATAAGAAAAATAAA	TCCCAAACCTAGACCAGGAA	TTGCATGCTGCTGGAACTAC	59.5	265	DRR	Ca_04370	--	ERF
Ca4_P4_11394293	Ca4	11394293	AATATATATATA/AATATATATATATATATATATA	TCAACACATCGCAAATATCCA	GCCGCAAAACCAAATACATC	59.9	522	DRR	Ca_04373	J	--
Ca4_P4_11395658	Ca4	11395658	GTAACATATTA/GTAACATATTAACATATTA	CCAAGTTCCAATGGCTTGAT	CGCTTCCAGACAACACAAGA	59.9	587	DRR	Ca_04373	J	--
Ca4_P4_11425739	Ca4	11425739	A/AT	CAAAAAGACATCGAATGGCA	CAACCAACGGATCATATGAATTT	59.7	778	DRR	Ca_04376	GC	bHLH
Ca4_P4_11437417	Ca4	11437417	TTATTAT/TT	AAGGGAGTCTGGTGAGCAAA	CATTGAAAAACCATTCGCCT	59.8	811	DRR	Ca_04378	S	--

INDEL marker IDs	Chromosomes/ unanchored scaffolds	Physical positions (bp)	InDels (*Kabuli* reference genome-CDC Frontier/PII)	Forward primers (5'-3')	Reverse primers (5'-3')	Anneling temperature (0C)	Expected amplified product size (bp)	Structural annotation		Functional annotation	
								Sequence components of *kabuli* genome	*Kabuli* gene accession IDs	NCBI-KOG	TFs
Ca4_P4_11440445	Ca4	11440445	ACTCT/ACT	CGATGCTGCCCATAATTCTT	AAAAACCGTTGTCGAATGCT	60.1	757	DRR	Ca_04380	J	--
Ca4_P4_11453608	Ca4	11453608	ATATATTAT/ATAT	CTCCATTTTGCACGAAGACA	CCCATGTTCACCCCTAATTG	59.8	556	DRR	Ca_04381	--	--
Ca4_P4_11462375	Ca4	11462375	AC/A	GCATGTGGGTGTTTGTTTGA	TCAAGCTTGGAAGTTGCTTG	60.4	612	DRR	Ca_04382	--	--
Ca4_P4_11555971	Ca4	11555971	CATATATATAT/CATATAT	CATTATTGGATTCGTTGGGG	GGGTGATAGATTCTTGATGCAAT	60.0	362	DRR	Ca_04385	K	Trihelix
Ca4_P4_11556235	Ca4	11556235	ATTTTTTTTTT/ATTTTTTTT	CATTATTGGATTCGTTGGGG	AAGCTCGCTTTTTGAAATGG	60.0	790	DRR	Ca_04385	K	Trihelix
Ca4_P4_1169667	Ca4	1169667	TTTATTATTATTATTATTATTATTATTATTATTATTATTATTATTATTAT/TTTATTATTATTATTTTTATTATTATTATTATTATTATTATTATTATTATTATTATTATTATTAT	TTTTAGCCCCGTTTTGTTCA	TAAGGTGTCCGACATCAGATT	60.5	701	DRR	Ca_07748	OU	--
Ca4_P4_11849287	Ca4	11849287	TTTAGTCATT/TTTAGTCATTAGTCATT	TTTAGCTGACTTGTGGGGCT	TGAAATTATCCACCCACCAA	59.9	777	DRR	Ca_04405	M	C2H2
Ca4_P4_12011085	Ca4	12011085	CTTATTATTATTATTATTATTATTATTATTATTATTATTATTATTATTATTATTA/CTTATTATTATTATTATTATTATTATTATTATTATTATTATTATTATTATTATTA	TCATGCGATTGTGATGACCT	TGATTGTGCTTGAGTTGGGA	60.1	671	DRR	Ca_04426	--	--
Ca4_P4_12174371	Ca4	12174371	T/TA	ACCAAACAGCGACCAAACTC	AAGGTAGAGATCGCGTCGAA	60.2	544	DRR	Ca_04442	--	GRAS
Ca4_P4_12225286	Ca4	12225286	G/GAGTCTC	TCATGGAGGCAAAGAAGGAG	TGACATGTGGATCGTGGTTT	60.3	724	DRR	Ca_04444	--	--

INDEL marker IDs	Chromosomes/ unanchored scaffolds	Physical positions (bp)	InDels (*Kabuli* reference genome-CDC Frontier/PII)	Forward primers (5'-3')	Reverse primers (5'-3')	Anneling temperature (0C)	Expected amplified product size (bp)	Structural annotation		Functional annotation	
								Sequence components of ***kabuli*** genome	***Kabuli*** gene accession IDs	NCBI-KOG	TFs
Ca4_P4_12393377	Ca4	12393377	TAAAAAAAAAAAAAAAA/TAAAAAAAAAAAAAA	TGTGCTGTTGTGATTAGGGG	AGCGTTCAAGGTGCAAAAGT	59.6	659	DRR	Ca_04463	QI	bHLH
Ca4_P4_12472732	Ca4	12472732	GAAAAAAAAAA/GAAAAAAAAAAAA	GGGGAGTTTGGTTCCTTATTTT	GATCAACGGCGAGAGAAAAG	59.6	465	DRR	Ca_04469	Z	bHLH
Ca4_P4_12493651	Ca4	12493651	ATTTTTT/ATTTTTTTT	ATTCCTCGTCCGTTTTCAGA	TTTTTCTTCCTGATTTGGTCC	59.7	293	DRR	Ca_04473	--	--
Ca4_P4_12528630	Ca4	12528630	TAAAAAA/TAAAAA	TAACGCTAGGGTCCCATGTC	AGCTTCTTGGAGCCATGTCA	60.0	211	DRR	Ca_04475	C	bZIP
Ca4_P4_12535193	Ca4	12535193	GAAAAAAAA/GAAAAAA	CGAAGCCTGCAACTTTTACC	CCAAACATGCAGGTTCCTCT	59.9	231	DRR	Ca_04476	--	--
Ca4_P4_12554607	Ca4	12554607	A/AC	CCAAAGCAAAACAAGCCATT	TGCTTCTTAGGGTAAGCCGA	60.1	508	DRR	Ca_04478	A	EIL
Ca4_P4_12571013	Ca4	12571013	GTATATA/GTATA	GGCTCACATCAGGAATGGAT	GGCTCTTGGTCATTGGATGT	59.9	968	DRR	Ca_04479	E	--
Ca4_P4_12583830	Ca4	12583830	ATTTTTTTT/ATTTTTTTTT	TGTTGTTGGATGCACCTGTT	GATACATGGGCATTCAGGCT	60.0	835	DRR	Ca_04480	L	--
Ca4_P4_12592291	Ca4	12592291	ATA/ATACAAATACTTGATGTA	TGACTGCCGCTTTGTCATTA	TTTCGATTCAAACCCAAACC	60.4	588	DRR	Ca_04481	R	--
Ca4_P4_12597000	Ca4	12597000	AT/ATCTAATGGT	CACATGGTTCCAGGTTCCTT	CTTATGGCGTGGCCTTAAAC	59.8	820	DRR	Ca_04482	R	--

INDEL marker IDs	Chromosomes/ unanchored scaffolds	Physical positions (bp)	InDels (*Kabuli* reference genome-CDC Frontier/PII)	Forward primers (5'-3')	Reverse primers (5'-3')	Anneling temperature (0C)	Expected amplified product size (bp)	Structural annotation		Functional annotation	
								Sequence components of *kabuli* genome	*Kabuli* gene accession IDs	NCBI-KOG	TFs
Ca4_P4_12866217	Ca4	12866217	GGG/GGGAGCTTTATTTTTTGG	TAGACTCCACTAAGCCCCCA	CCTTCCAACGCAACATTTCT	59.7	294	DRR	Ca_04506	T	FAR1
Ca4_P4_13546142	Ca4	13546142	TAAA/TAA	GAGATTCACGAAGACCGAGG	GCCAATGCTAATCTGCAACA	59.8	665	DRR	Ca_04571	T	--
Ca4_P4_13555418	Ca4	13555418	ATTTTTTTTTT/ATTTTTTTTT	CTTCTACTGGCATGTGGCTG	TTCGCATTGGAGATGACAAA	59.5	640	DRR	Ca_04572	--	--
Ca4_P4_13555921	Ca4	13555921	CCTCGTTCAATC/CCTCGTTCAATCTCGTTCAATC	AAGCATTCTTCTCGGGTCAA	CATCCCTAAGATGCGACCAT	59.8	333	DRR	Ca_04572	--	--
Ca4_P4_13586319	Ca4	13586319	CTT/CTTT	CCTTCAACGAGCAGCCTTAG	TTCACACCTTGCAGCTATCG	60.1	511	DRR	Ca_04576	--	--
Ca4_P4_13586598	Ca4	13586598	ATTTTTT/ATTTTT	CCTTCAACGAGCAGCCTTAG	AAATTGGGAAATTTGGAGGG	60.1	678	DRR	Ca_04576	--	--
Ca4_P4_13593543	Ca4	13593543	TA/T	TGGTTCTCATTGCTTGCTTG	ACCCAATGAATTTTCCCTCC	60.0	565	DRR	Ca_04577	--	HB-other
Ca4_P4_13617448	Ca4	13617448	TG/TGG	GGGCCTTTTCTTCAGCTTTT	TGCATGTTATCCGCAATCTC	59.8	713	DRR	Ca_04579	--	--
Ca4_P4_13618875	Ca4	13618875	C/CG	AATGCATTTCGCGTACCTTC	CAACATATGCATTTCCGTGG	60.1	329	DRR	Ca_04579	--	--
Ca4_P4_13624309	Ca4	13624309	CTTT/CTT	CTGGAAAAGAAGAGGGGGAC	CAGGTTGGTCGAATCATGTG	60.0	748	DRR	Ca_04580	--	--

INDEL marker IDs	Chromosomes/ unanchored scaffolds	Physical positions (bp)	InDels (*Kabuli* reference genome-CDC Frontier/PII)	Forward primers (5'-3')	Reverse primers (5'-3')	Anneling temperature (0C)	Expected amplified product size (bp)	Structural annotation		Functional annotation	
								Sequence components of *kabuli* genome	*Kabuli* gene accession IDs	NCBI-KOG	TFs
Ca4_P4_13624341	Ca4	13624341	CAAAA/CAAAAA	CTGGAAAAGAAGAGGGGGAC	CAGGTTGGTCGAATCATGTG	60.0	748	DRR	Ca_04580	--	--
Ca4_P4_13663032	Ca4	13663032	TAAAAA/TAAAA	AGTACCTTCGTCGTGTTCGC	GGACTTTTCAAAACGGCTCA	60.3	231	DRR	Ca_04585	R	bHLH
Ca4_P4_13663581	Ca4	13663581	T/TG	AACGAAGACCCTGTGACCTG	GCCGGCTTGTAGGTTGAATA	60.2	168	DRR	Ca_04585	R	bHLH
Ca4_P4_13667763	Ca4	13667763	T/TA	CTCAAGATTCAATGGGACGA	TTTGTGATGGTGATGGGTTG	58.6	275	DRR	Ca_04586	Q	B3
Ca4_P4_13667908	Ca4	13667908	TGG/TGGG	CAACCCATCACCATCACAAA	AAATCGACGCCACTTTCATC	60.2	148	DRR	Ca_04586	Q	B3
Ca4_P4_13678246	Ca4	13678246	C/CT	CCGACGAATTTCCTTCAAAA	TGTTCGAGTCGGTGAGACAG	60.0	583	DRR	Ca_04588	E	--
Ca4_P4_13697619	Ca4	13697619	AAAGAAGAAG/AAAGAAG	ACACACAAAGGCAAACCACA	ATCAGCTGACCCCAAAAATG	60.0	934	DRR	Ca_04591	--	--
Ca4_P4_13735535	Ca4	13735535	TAGAATTCAGAAT/TAGAATTCAGAATTCAGAAT	TTGTTCCTTGACCTTTTGGG	TGGTTCTGAAATCTGCCTGA	59.9	745	DRR	Ca_04597	U	--
Ca4_P4_13756219	Ca4	13756219	GTTT/GTTTT	TACCGGAAATTTCAGCAAGG	GGCATGGTATTCCCTCCTTT	60.1	599	DRR	Ca_04600	--	NF-YB
Ca4_P4_13788471	Ca4	13788471	ATGTTGT/ATGTTGTTGT	CAGGCCAGCTAACTTTTTCG	GCCAACTTTGAGTGGTGGAT	60.0	600	DRR	Ca_04603	Q	--

INDEL marker IDs	Chromosomes/ unanchored scaffolds	Physical positions (bp)	InDels (*Kabuli* reference genome-CDC Frontier/PII)	Forward primers (5'-3')	Reverse primers (5'-3')	Anneling temperature (0C)	Expected amplified product size (bp)	Structural annotation		Functional annotation	
								Sequence components of *kabuli* genome	*Kabuli* gene accession IDs	NCBI-KOG	TFs
Ca4_P4_13788854	Ca4	13788854	CA/CAAA	AAGTGCAATTAGGCCAGTGC	GCCAACTTTGAGTGGTGGAT	60.3	105	DRR	Ca_04603	Q	--
Ca4_P4_13789143	Ca4	13789143	CAA/CAAA	ATCCACCACTCAAAGTTGGC	TTGGTACGAAACATCCATCAA	60.0	517	DRR	Ca_04603	Q	--
Ca4_P4_13798628	Ca4	13798628	CGG/CGGG	GCGATTGTTTAGGTTGGGAA	TTTTCAACACGAGGGAATCA	59.9	443	DRR	Ca_04605	R	--
Ca4_P4_14090717	Ca4	14090717	TGAGAGAG/TGAGAG	GTTGGTTTGAATGGAGGGAA	AGTTCCAGCTCTTAACCGCA	59.8	660	DRR	Ca_04636	OR	YABBY
Ca4_P4_14405624	Ca4	14405624	ATGTTGTT/ATGTTGTTGTT	CGGTGGTTGCTCTCTCTTTC	GGGGTACAATGTGGAACCAA	60.0	440	DRR	Ca_05663	E	MYB
Ca4_P4_14405755	Ca4	14405755	ATCTCTCTCT/ATCTCTCT	ATGTGCATGCCAAGTTGTGT	ACCTATAAGCCCTTTGCGCT	60.0	963	DRR	Ca_05663	E	MYB
Ca4_P4_14406037	Ca4	14406037	GTCTTT/GT	TTGGTTCCACATTGTACCCC	TTGTTGCTGGAGTTGGATCA	60.5	639	DRR	Ca_05663	E	MYB
Ca4_P4_14406133	Ca4	14406133	CAAA/CAAAA	TTGGTTCCACATTGTACCCC	CCATTGGTTGGGATATGGAG	60.5	892	DRR	Ca_05663	E	MYB
Ca4_P4_15396694	Ca4	15396694	GATAATAATAATAATAATAATAATAATAATAATAATAATAATAATAA/GATAATAATAATAATAATAATAATAATAATAATAATAA	TCCCTCGTGAGAACTTGTCT	AGGGGACCAAAATTGTGAAT	57.4	514	DRR	Ca_05556	R	B3
Ca4_P4_15916952	Ca4	15916952	TA/TAAA	CCACCAGCGATTATCAACCT	TGTCTTCCTTCCCTGTCTGG	60.0	490	DRR	Ca_05516	--	bHLH

INDEL marker IDs	Chromosomes/ unanchored scaffolds	Physical positions (bp)	InDels (*Kabuli* reference genome-CDC Frontier/PII)	Forward primers (5'-3')	Reverse primers (5'-3')	Anneling temperature (0C)	Expected amplified product size (bp)	Structural annotation		Functional annotation	
								Sequence components of *kabuli* genome	*Kabuli* gene accession IDs	NCBI-KOG	TFs
Ca4_P4_15920083	Ca4	15920083	AGGTG/AGGTGGTG	TGATTTCATGCCTAATGCCA	AATGAAAATGGGGTCCAATG	60.0	495	DRR	Ca_05514	--	SBP
Ca4_P4_15920382	Ca4	15920382	TAAAAA/TAAAAAA	AATTGTCTTGCATTGGACCC	GTGCCGCTCCAGCTACTATC	59.8	324	DRR	Ca_05514	--	SBP
Ca4_P4_15921827	Ca4	15921827	GAAAAAAAAAA/GAAAAAAAA	GGGGGCCAATTGATTCTTAC	GGTCCACATCCAGCTCATTT	60.5	455	DRR	Ca_05515	--	ERF
Ca4_P4_15926036	Ca4	15926036	AAAGA/AAAGAAGA	GTTTCCCTTACGCCAGATGA	GCATTGCTGTATTGGTGCAT	60.1	532	DRR	Ca_05513	R	--
Ca4_P4_15958358	Ca4	15958358	CT/C	ACAAAGCTCAAACTCGCCAT	CCCCTATTCACCGCATAAGA	59.9	342	DRR	Ca_05511	--	C2H2
Ca4_P4_15958886	Ca4	15958886	CTT/C	CTTATGCGGTGAATAGGGGA	GCATGTTATTGGTCCCACCT	59.9	372	DRR	Ca_05511	--	C2H2
Ca4_P4_15969199	Ca4	15969199	TAA/TA	TAAGGCGTTAACTTGCACCC	TGGTTGTGGCGATTACAAGA	60.1	494	DRR	Ca_05510	J	NF-YA
Ca4_P4_16097791	Ca4	16097791	TTATATATAT/TTATATATATAT	GTGGAATGTAAGATCGCCGT	GCAAACAGAGGAAGAGAAGGAA	60.0	535	DRR	Ca_05500	--	--
Ca4_P4_16098339	Ca4	16098339	TAAAAAAAAA/TAAAAAAA	CCTTCTCTTCCTCTGTTTGCTC	CTCATACATGAGGGGAAAGCA	59.6	674	DRR	Ca_05500	--	--
Ca4_P4_16098449	Ca4	16098449	C/CT	CAATTCCAAAATATGACAACAACC	TCATACATGAGGGGAAAGCA	59.5	528	DRR	Ca_05500	--	--

INDEL marker IDs	Chromosomes/ unanchored scaffolds	Physical positions (bp)	InDels (*Kabuli* reference genome-CDC Frontier/PII)	Forward primers (5'-3')	Reverse primers (5'-3')	Anneling temperature (0C)	Expected amplified product size (bp)	Structural annotation		Functional annotation	
								Sequence components of *kabuli* genome	*Kabuli* gene accession IDs	NCBI-KOG	TFs
Ca4_P4_16146480	Ca4	16146480	CT/C	AAAGAAGCAGAGGAAGCGTG	GCAAACGTATGATGGGGACT	59.8	337	DRR	Ca_05497	I	bHLH
Ca4_P4_1641697	Ca4	1641697	AATATATATATA/AATATATATATATA	GTTGCTGGTTGAACAAGGGT	TGGCCTGTATGATGGAACAA	60.0	633	DRR	Ca_07792	B	NF-YC
Ca4_P4_16728592	Ca4	16728592	TAAAAAAAAAA/TAAAAAAAAA	CATGGGGATGCTGCTATTTT	CAACTTCCTCTTTTGGCACA	59.9	388	DRR	Ca_05454	O	MYB
Ca4_P4_16813063	Ca4	16813063	CTAGAAGCTTTAGAAGCTTTA/CTAGAAGCTTTA	GTAAGTGATGGGTGCGTGTG	TGTGAACCCAGCTGCTCTAA	60.0	700	DRR	Ca_05442	--	--
Ca4_P4_16826129	Ca4	16826129	A/AAATT	TTTTATCCACCAAAAATTCAAAAA	TGATTTTTCAGGAGGCCACT	58.9	521	DRR	Ca_05439	--	--
Ca4_P4_16826292	Ca4	16826292	CAAAAAAA/CAAAAAA	GCGACCATCTGTTGCAGTTA	TGATTTTTCAGGAGGCCACT	59.9	459	DRR	Ca_05439	--	--
Ca4_P4_16960625	Ca4	16960625	GTT/GTTT	GCGAATTGCAGACTGAAACA	CTGGAAAATCCTCCTCCTCC	60.0	925	DRR	Ca_05428	--	--
Ca4_P4_16997698	Ca4	16997698	GTT/GTTTTT	CAAACCTCAAGTCCCCATTG	TCCGAAGCCACCACTTCTAC	60.3	651	DRR	Ca_05426	U	--
Ca4_P4_17010005	Ca4	17010005	AAAAAATAATATC/AAAAAATAATATCATTTAAAAATAATATC	ACGGCCTAGCTTATTCCCAT	GTATGCAAATGGGTGCTGTG	60.0	690	DRR	Ca_05423	--	--
Ca4_P4_17010261	Ca4	17010261	GAGACA/GAGACAGACA	GTTAGCCTCACAGCACCCAT	GCTTCTAACTCGGCAAGTGG	60.1	342	DRR	Ca_05422	--	G2-like

INDEL marker IDs	Chromosomes/ unanchored scaffolds	Physical positions (bp)	InDels (*Kabuli* reference genome-CDC Frontier/PII)	Forward primers (5'-3')	Reverse primers (5'-3')	Anneling temperature (0C)	Expected amplified product size (bp)	Structural annotation		Functional annotation	
								Sequence components of *kabuli* genome	*Kabuli* gene accession IDs	NCBI-KOG	TFs
Ca4_P4_17030182	Ca4	17030182	TAA/TAAAA	AAGATTTTGTGTTTGAACCCTCA	TCTCGCCTTTTGTATCGTCA	59.9	426	DRR	Ca_05420	R	bHLH
Ca4_P4_17030352	Ca4	17030352	T/TTGGAAATC	AAGATTTTGTGTTTGAACCCTCA	CTTCATCGTTGAATTTGGGG	59.9	596	DRR	Ca_05420	R	bHLH
Ca4_P4_17036708	Ca4	17036708	GAAAAAAA/GAAAAAA	TTGTTGATGTGACGGGAAGA	CCGGAGGAGAGGTATGGATT	60.1	574	DRR	Ca_05419	C	bHLH
Ca4_P4_17047964	Ca4	17047964	GAAAAAAAAAAA/GAAAAAAAAA	AGAGGTCATTTGGGTGGACA	AAGCGACCAGTTGAAGGAAA	60.4	453	DRR	Ca_05418	DA	--
Ca4_P4_17048228	Ca4	17048228	CTTTTTTTTTTT/CTTTTTTTTTTTT	TTTCCTTCAACTGGTCGCTT	TCATCATCTTGCTCTCCACG	59.9	527	DRR	Ca_05418	DA	--
Ca4_P4_17082004	Ca4	17082004	TGAT/TGATCGAT	TTTTGACAATGGGCACTCAA	CTGCAGGTAAGCATCCCAAT	60.1	295	DRR	Ca_05415	--	--
Ca4_P4_17101658	Ca4	17101658	ATTTT/ATTTTTT	TGATGTTAACTGACAAATGCTGTTT	GTCGAGAGTTGGGTCATGGT	60.0	322	DRR	Ca_05414	--	--
Ca4_P4_17115270	Ca4	17115270	ATTTTT/ATTTT	GGGACCGTTCGTTTCAAATA	AACATGATTCGAATTCAATTTCTC	59.8	221	DRR	Ca_05412	CR	Nin-like
Ca4_P4_17141313	Ca4	17141313	T/TC	TTTCAATCGTATCAAGTTTTTGC	AAACTGAAAGGCCTAATTCCAA	58.4	682	DRR	Ca_05410	CR	Nin-like
Ca4_P4_17142439	Ca4	17142439	TAA/T	CACGAACCCTGCCAAATTAT	TTGTGCTTTAGAGGATGGGG	59.8	603	DRR	Ca_05410	CR	Nin-like

INDEL marker IDs	Chromosomes/ unanchored scaffolds	Physical positions (bp)	InDels (*Kabuli* reference genome-CDC Frontier/PII)	Forward primers (5'-3')	Reverse primers (5'-3')	Anneling temperature (0C)	Expected amplified product size (bp)	Structural annotation		Functional annotation	
								Sequence components of *kabuli* genome	*Kabuli* gene accession IDs	NCBI-KOG	TFs
Ca4_P4_17146199	Ca4	17146199	TTTTTTTTTATTATTATTTTTT/TTTTTT	ATTCCAGCTCCCACAATCAC	GGCTCTTGATGGGTTGTGTT	59.9	442	DRR	Ca_05409	K	--
Ca4_P4_17146270	Ca4	17146270	AGGGG/AGGG	ATTCCAGCTCCCACAATCAC	GGCTCTTGATGGGTTGTGTT	59.9	442	DRR	Ca_05409	K	--
Ca4_P4_17152700	Ca4	17152700	TAACAACA/TAACA	CTGCAAATGTCAAGGCAGAA	TGTCTTTGGTGCATCTCTGG	60.0	617	DRR	Ca_05408	K	ERF
Ca4_P4_17158347	Ca4	17158347	TAAAAAAAA/TAAAAAAA	CCAATCTTGTCCACACCATGT	TCACCTAATGCCAAACACACA	60.7	300	DRR	Ca_05407	--	--
Ca4_P4_17159336	Ca4	17159336	A/AC	GCCACGCTCTAGGTTGAGAT	CCTCAGGCCTGCAACTGTAT	59.5	222	DRR	Ca_05407	--	--
Ca4_P4_17193639	Ca4	17193639	ATTTT/ATTT	AAGCAGCAAGGATCACCAAT	CCCCATTAGTATTGCCGCTA	59.7	532	DRR	Ca_05405	T	--
Ca4_P4_17200546	Ca4	17200546	GTT/GTTT	GCATTAGGGGTGCTTTGTGT	ATGGGCGAATGCAAAAGTTA	60.0	763	DRR	Ca_05404	--	--
Ca4_P4_17214261	Ca4	17214261	GCTATC/G	GCTTTTTAGGCTGGGGAATC	AATAATTTGGTCAGGCGTGC	60.0	194	DRR	Ca_05402	C	--
Ca4_P4_17214540	Ca4	17214540	A/ATATAG	GCTTTTTAGGCTGGGGAATC	TTGGTCAATGCGGAAGTGTA	60.0	627	DRR	Ca_05402	C	--
Ca4_P4_17214599	Ca4	17214599	AC/ACTTC	GCTTTTTAGGCTGGGGAATC	TTGGTCAATGCGGAAGTGTA	60.0	627	DRR	Ca_05402	C	--

INDEL marker IDs	Chromosomes/ unanchored scaffolds	Physical positions (bp)	InDels (*Kabuli* reference genome-CDC Frontier/PII)	Forward primers (5'-3')	Reverse primers (5'-3')	Anneling temperature (0C)	Expected amplified product size (bp)	Structural annotation		Functional annotation	
								Sequence components of *kabuli* genome	*Kabuli* gene accession IDs	NCBI-KOG	TFs
Ca4_P4_17238317	Ca4	17238317	CTTTATTT/CTTTATTTATTT	AAAAATGGGGTGGATGAACA	TTCCAATGTTGCAGAAGCAG	60.0	439	DRR	Ca_05400	YU	--
Ca4_P4_17268685	Ca4	17268685	TGG/TGGG	CATCCCATCCAGGACCTTTA	CTGGTTCCCGGTTGAACTTA	59.7	308	DRR	Ca_05398	R	--
Ca4_P4_17293581	Ca4	17293581	GTAT/GTATAT	TCACATCCGTTGAACGAAGA	TTTGTTTTATTAGTTTTGGTGTGG	60.2	652	DRR	Ca_05392	R	--
Ca4_P4_17293735	Ca4	17293735	GGAGAGAG/GGAGAG	TCACATCCGTTGAACGAAGA	TTTGTTTTATTAGTTTTGGTGTGG	60.2	652	DRR	Ca_05392	R	--
Ca4_P4_17299142	Ca4	17299142	TTTA/TTTAATTA	GGCCCCAAAGAGGAAAAATA	AATCCTTGTTTGCGTTGGTC	60.3	171	DRR	Ca_05391	TBLD	--
Ca4_P4_17314848	Ca4	17314848	CAA/CAAACCGAACTAGAA	TTGTAAAAACTGCATCAATCGAA	CTTCCCCACTCACAGTCCAT	59.7	488	DRR	Ca_05390	M	--
Ca4_P4_17315192	Ca4	17315192	TAAA/TAAAA	ATGGACTGTGAGTGGGGAAG	TTGGTCCAACAGCTTGATGA	60.0	716	DRR	Ca_05390	M	--
Ca4_P4_17342101	Ca4	17342101	ATTTTT/ATTT	CTTCCTCTAGCACCAGCACC	CACGATGAGTTTTCGTGTCA	60.0	572	DRR	Ca_05389	A	--
Ca4_P4_17376716	Ca4	17376716	ATTTTTTTTT/ATTTTTTTTTTT	CCCCCATCTTCAAATGTGTT	AGATGTAGCAACGCAAACCA	59.6	698	DRR	Ca_05387	R	--
Ca4_P4_17471371	Ca4	17471371	TG/T	CACAGTGGCTGAGAGTGGAA	AGTAGGTGGTGGTTTGCCTG	60.0	625	DRR	Ca_05380	AB	--

INDEL marker IDs	Chromosomes/ unanchored scaffolds	Physical positions (bp)	InDels (*Kabuli* reference genome-CDC Frontier/PII)	Forward primers (5'-3')	Reverse primers (5'-3')	Anneling temperature (0C)	Expected amplified product size (bp)	Structural annotation		Functional annotation	
								Sequence components of *kabuli* genome	*Kabuli* gene accession IDs	NCBI-KOG	TFs
Ca4_P4_17494891	Ca4	17494891	GAAAAAAAAAAAA/GAAAAAAAAAA	AGATCTCCGAACGATGATGG	AAGCAAACCCCACTTCACAC	60.0	245	DRR	Ca_05377	R	B3
Ca4_P4_17495202	Ca4	17495202	GT/G	GTGTGAAGTGGGGTTTGCTT	CGAAATTCATGCATTCAACG	60.0	476	DRR	Ca_05377	R	B3
Ca4_P4_17495331	Ca4	17495331	TATACTCA/TA	GTGTGAAGTGGGGTTTGCTT	CGAAATTCATGCATTCAACG	60.0	476	DRR	Ca_05377	R	B3
Ca4_P4_17495385	Ca4	17495385	AAT/A	GTGTGAAGTGGGGTTTGCTT	CGAAATTCATGCATTCAACG	60.0	476	DRR	Ca_05377	R	B3
Ca4_P4_17495441	Ca4	17495441	AG/A	GTGTGAAGTGGGGTTTGCTT	CGAAATTCATGCATTCAACG	60.0	476	DRR	Ca_05377	R	B3
Ca4_P4_17495949	Ca4	17495949	C/CTT	TCATTGCTGAAGAGCACGAT	TGCTCAACAACAGGAAGTGG	59.6	728	DRR	Ca_05377	R	B3
Ca4_P4_17524638	Ca4	17524638	GTATTATTATTATTA/GTATTATTATTATTATTA	GGAGATGGAGATGGCTGTGT	GGGTATCAAGCCGAATCAAA	60.1	862	DRR	Ca_05374	R	NAC
Ca4_P4_17608586	Ca4	17608586	CAATTTTAATTTTA/CAATTTTAATTTTAATTTTA	CTGACAGTTGAGCACTCCCA	TGTCACCCCGTTCTTGTGTA	60.0	634	DRR	Ca_05370	O	--
Ca4_P4_17626592	Ca4	17626592	A/AG	TGTCCCTTTCTCGTGCTCTT	TGCACGGAAGAGTGTGAATC	60.0	813	DRR	Ca_05368	F	--
Ca4_P4_17640423	Ca4	17640423	CTTTTTTT/CTTTTTT	CAAGAAGAAGTGGTGGGGAA	GAACCCTTTCCATCGAGTCA	60.1	524	DRR	Ca_05367	Z	WRKY

INDEL marker IDs	Chromosomes/ unanchored scaffolds	Physical positions (bp)	InDels (*Kabuli* reference genome-CDC Frontier/PII)	Forward primers (5'-3')	Reverse primers (5'-3')	Anneling temperature (0C)	Expected amplified product size (bp)	Structural annotation		Functional annotation	
								Sequence components of *kabuli* genome	*Kabuli* gene accession IDs	NCBI-KOG	TFs
Ca4_P4_17645039	Ca4	17645039	ATCT/AT	GGGAGAGCCCTAGTTGGTTT	TGGTTGCTTTGAGCTTTTGA	59.6	262	DRR	Ca_05366	--	--
Ca4_P4_17649601	Ca4	17649601	TAAAAAAAA/TAAAAAA	GCCTTAGTTTTTCGGCTTCC	CAAAATAAGCACCAACCTCTCA	60.2	945	DRR	Ca_05365	H	--
Ca4_P4_17653817	Ca4	17653817	AATATA/AATA	GATGTGAGGGCTTGCAATTT	GGTGCAACTTTATTTGGTCG	60.1	870	DRR	Ca_05364	S	--
Ca4_P4_17654597	Ca4	17654597	TCACACAC/TCACACACAC	TCCAATTGACACCACTGCTC	TTTTCAACATTCTAAAAATGGGC	59.7	542	DRR	Ca_05364	S	--
Ca4_P4_17661717	Ca4	17661717	ATT/AT	TGATCCGGTCGGTTGTAAAT	AAGCTCGACATTTCAAAGGC	60.2	840	DRR	Ca_05363	R	WRKY
Ca4_P4_17661910	Ca4	17661910	ATTTTTT/ATTTTTTT	AACATGTGAAATGGGAAGGC	TTAACGGTTCGTTTTCCAGG	59.8	982	DRR	Ca_05363	R	WRKY
Ca4_P4_17672684	Ca4	17672684	GATA/GA	TGTAGTCCCTCTGTCCCAAAA	CGGAAGATACCAAGTCGCAT	59.6	433	DRR	Ca_05360	--	--
Ca4_P4_17704939	Ca4	17704939	CATATATAT/CATATATATATATAT	AAGTCGAACCCCAATAACCC	TTTAGGGATGATTGCTTGGG	60.1	600	DRR	Ca_05355	R	SBP
Ca4_P4_17705462	Ca4	17705462	GAAAAA/GAAAAAA	GTGCTTTCCCAAGCAATCAT	TCTGAGATGGGTTTGCCTTT	60.1	885	DRR	Ca_05355	R	SBP
Ca4_P4_17707066	Ca4	17707066	ATTTTTT/ATTTTTTT	TTGCATGCTTGGATTCAAAA	CCATCGAAAAGGAATGGCTA	60.2	413	DRR	Ca_05354	O	--

INDEL marker IDs	Chromosomes/ unanchored scaffolds	Physical positions (bp)	InDels (*Kabuli* reference genome-CDC Frontier/PII)	Forward primers (5'-3')	Reverse primers (5'-3')	Anneling temperature (0C)	Expected amplified product size (bp)	Structural annotation		Functional annotation	
								Sequence components of *kabuli* genome	*Kabuli* gene accession IDs	NCBI-KOG	TFs
Ca4_P4_17721218	Ca4	17721218	ATTCATG/A	CAAGACTTTGTTGGGGGAAA	TCAGTTCTGTCACAGTCGGC	59.9	413	DRR	Ca_05353	O	SRS
Ca4_P4_17758892	Ca4	17758892	GTTTTTTTT/GTTTTTTT	TTTGATGCAAACAAGTTCCTG	CGATGAAGGAGTTCTCAGCC	58.8	499	DRR	Ca_05349	O	--
Ca4_P4_17759068	Ca4	17759068	GAAAA/GAAA	GGCTGAGAACTCCTTCATCG	CGCACCACCTTTACATTCCT	60.0	431	DRR	Ca_05349	O	--
Ca4_P4_17766593	Ca4	17766593	TAAAA/TAAA	ATTGCATTGTGCTCCACTTG	AGGGGTTGGTATAGTAGAAGGAA	59.7	512	DRR	Ca_05348	--	--
Ca4_P4_17766645	Ca4	17766645	CTTTTTT/CTTTTTTT	ATTGCATTGTGCTCCACTTG	GGCTCTGTTTGATACAGCCA	59.7	744	DRR	Ca_05348	--	--
Ca4_P4_17766884	Ca4	17766884	A/AAAT	TGCACCAGACATATTTTCGC	AAAAGCCACACAGGATGGAC	59.7	873	DRR	Ca_05348	--	--
Ca4_P4_17775093	Ca4	17775093	A/AC	AAGAGTTGCCAAAGTGGTGC	ACGCGGAGAAATTTGCTATT	60.3	519	DRR	Ca_05347	R	--
Ca4_P4_17863849	Ca4	17863849	AA/AATAACCAATA	TTTGATTGTGGCTTTTGCAG	AAATCTGCCAATCATGCCTC	59.8	563	DRR	Ca_18623	--	WOX
Ca4_P4_17864168	Ca4	17864168	ACGTAACC/AC	GAGGCATGATTGGCAGATTT	TCGTCATTCCAAATAGCGAC	60.0	536	DRR	Ca_18623	--	WOX
Ca4_P4_17864334	Ca4	17864334	ATTTTTTT/ATTTTTTTT	GAGGCATGATTGGCAGATTT	TCGTCATTCCAAATAGCGAC	60.0	536	DRR	Ca_18623	--	WOX

INDEL marker IDs	Chromosomes/ unanchored scaffolds	Physical positions (bp)	InDels (*Kabuli* reference genome-CDC Frontier/PII)	Forward primers (5'-3')	Reverse primers (5'-3')	Anneling temperature (0C)	Expected amplified product size (bp)	Structural annotation		Functional annotation	
								Sequence components of *kabuli* genome	*Kabuli* gene accession IDs	NCBI-KOG	TFs
Ca4_P4_18295337	Ca4	18295337	A/ATT	TTCCTATTGCTCACCCCTTG	GAAAAACAAGACCCGCGATA	60.1	539	DRR	Ca_18662	--	--
Ca4_P4_1831456	Ca4	1831456	TTA/TTATA	TCCAATAAGGTTGCCTAAGGA	GAGATTAAGCCGGAAGACCC	58.7	256	DRR	Ca_07804	--	--
Ca4_P4_1840388	Ca4	1840388	C/CA	GCGAACCAAAGTGTGAGTGA	TTCTCTTCCATCTGCTGGCT	59.9	654	DRR	Ca_07805	--	--
Ca4_P4_1843722	Ca4	1843722	ATACTACTACTACTAC/ATACTACTACTACTACTACTAC	CAAGTGTGGGCATCTCAGAA	CTGCTGTTTCAAGCCAATCA	59.8	418	DRR	Ca_07806	R	WRKY
Ca4_P4_18883677	Ca4	18883677	AATATATATATATATATATATATATATATA/AATATATATATATATATATATATATATATATATATA	AAAATGTAGGGTGGACGGTG	TATTCCCCCAATGAAGGACA	59.7	750	DRR	Ca_13985	E	WRKY
Ca4_P4_18943390	Ca4	18943390	TATAATAATAATAATAATA/TATAATAATAATAATAATA	CGACCATCACATACTGTGCC	TTGAAATATTGGGCTCAGGC	60.0	700	DRR	Ca_13992	--	--
Ca4_P4_19276409	Ca4	19276409	AC/A	CAATGACGTAGGGAAATCGAA	CCTTGAGTTCGACACTCGGT	59.9	296	DRR	Ca_14024	--	--
Ca4_P4_19680920	Ca4	19680920	AATATATATATATATATATAT/AATATATATATATATATATAT	TTTGTTCACTTTTATGATGTGGTG	TTATATAGCCCCAGCCGTTG	59.0	610	DRR	Ca_14054	S	--
Ca4_P4_19708013	Ca4	19708013	TAAAAAAAAAAA/TAAAAAAAAAA	CAATTGCCAGAAGTTGTTGG	GATTGCTGGATTTGGAAGGA	59.2	842	DRR	Ca_14058	J	NF-YB
Ca4_P4_19757541	Ca4	19757541	GAAAAAAAAAAAA/GAAAAAAAAAAAAA	TATGAGGAAAAATGGGCCAG	AACCTATTGCGAACGTCGTC	59.9	163	DRR	Ca_14064	U	GATA

INDEL marker IDs	Chromosomes/ unanchored scaffolds	Physical positions (bp)	InDels (*Kabuli* reference genome-CDC Frontier/PII)	Forward primers (5'-3')	Reverse primers (5'-3')	Anneling temperature (0C)	Expected amplified product size (bp)	Structural annotation		Functional annotation	
								Sequence components of *kabuli* genome	*Kabuli* gene accession IDs	NCBI-KOG	TFs
Ca4_P4_20213739	Ca4	20213739	TCGTTAC/TC	TCAGCTTTTTGGCTGATGTG	GCGTGAATTTGAACACGAGA	60.0	698	DRR	Ca_17141	PT	FAR1
Ca4_P4_2035697	Ca4	2035697	ATTT/AT	AGTCCAGTCTAAGCTGCCCA	TGGAATCATGCCATGCTAGA	60.0	436	DRR	Ca_07823	R	MYB
Ca4_P4_2035798	Ca4	2035798	A/AGTGT	AGTCCAGTCTAAGCTGCCCA	TGGAATCATGCCATGCTAGA	60.0	436	DRR	Ca_07823	R	MYB
Ca4_P4_20870966	Ca4	20870966	ATTTTTTTT/ATTTTTTT	ACTCATTGTGTTGGCTTTCG	GTGGATGAAGGGGGTAGGTT	58.8	776	DRR	Ca_19043	--	--
Ca4_P4_21175270	Ca4	21175270	ATTTTT/ATTTTTT	GACACCAGGAGGCATGTTTT	GGAAGGCTTGTCAGCTTGAG	60.0	386	DRR	Ca_19056	--	--
Ca4_P4_21230030	Ca4	21230030	CTTTTT/CTTTTTT	TCACTTTGATTGGTTGCTGG	TGAAATTATGGGTGGGCATT	59.7	371	DRR	Ca_19058	--	Trihelix
Ca4_P4_21774546	Ca4	21774546	AATATATATATATATATATATATATATATATATA/AATATATATATATATATATATATATATATATATATATA	TCAACCAATATGAATCCGCA	AAAAGGAATGATTGATGCCG	59.9	557	DRR	Ca_19080	--	--
Ca4_P4_21933188	Ca4	21933188	GAAAAAAAAAAA/GAAAAAAAAAA	GTTGGGCAATGGATCTGAAT	TGCTCACCGTTTGATCTTTG	59.8	862	DRR	Ca_20146	--	--
Ca4_P4_22134995	Ca4	22134995	CATAT/CAT	GCACCTATGGTGGCAAAAAC	TTTGATGCCCACATTGAAGA	60.4	506	DRR	Ca_20134	J	--
Ca4_P4_22237363	Ca4	22237363	TAG/T	CCCTCTTGATTTGGCATGAT	AATAAACAGCATGGCGGAAG	59.9	568	DRR	Ca_20127	--	ERF

INDEL marker IDs	Chromosomes/ unanchored scaffolds	Physical positions (bp)	InDels (*Kabuli* reference genome-CDC Frontier/PII)	Forward primers (5'-3')	Reverse primers (5'-3')	Anneling temperature (0C)	Expected amplified product size (bp)	Structural annotation		Functional annotation	
								Sequence components of *kabuli* genome	*Kabuli* gene accession IDs	NCBI-KOG	TFs
Ca4_P4_22288876	Ca4	22288876	TTTCTACAAAATCAGTTCTACAAAATCAGT/TTTCTACAAAATCAGT	TGCACTCCACACCCTACAAA	CCATGCAAAACTCCATGTTG	60.2	685	DRR	Ca_20125	TR	--
Ca4_P4_22649708	Ca4	22649708	TTAATAATAATAATAATAATAATAATAA/TTAATAATAATAATAATAATAA	CGTACAACTTGGGGCTTGAT	CATTTACCATGTGCTTTTTATGC	60.0	725	DRR	Ca_14478	--	--
Ca4_P4_22792153	Ca4	22792153	GAAAAAAAAA/GAAAAAAAAAA	CTCATTTTCATTCACCGGCT	ACGAAGCATTAGGCATGGAT	60.1	931	DRR	Ca_14472	--	--
Ca4_P4_22808161	Ca4	22808161	A/AC	CATGGTTTTCGCTTACCGTT	TTGGGTGATGTGGCAAAATA	60.0	559	DRR	Ca_14470	H	--
Ca4_P4_22837725	Ca4	22837725	AAATAATAA/AAATAA	TTGAAATGGCAAAGAGGGTC	ATGCATGGGTTGAAATACCC	60.1	556	DRR	Ca_14468	--	--
Ca4_P4_22973152	Ca4	22973152	TTAAAATGTAAAATGTA/TTAAAATGTAAAATGTAAAATGTA	ATTTTATTGGCATCGGCATT	TGCGAATGTGCTTTCTGTCT	59.3	672	DRR	Ca_14460	--	--
Ca4_P4_22974338	Ca4	22974338	A/AAT	ATTTTGGGCGAAATCCTTTT	GAACCACTCATCCTCCCAAA	59.8	442	DRR	Ca_14460	--	--
Ca4_P4_23055017	Ca4	23055017	T/TG	GTGTGCTTGGAAAGTCGTTG	TTTGATGTTGTTGGAGATGAAAA	59.3	485	DRR	Ca_14459	--	--
Ca4_P4_23055098	Ca4	23055098	CTTTATTTTATTTTATTTTATTTT/CTTTATTTTATTTTATTTT	GTGTGCTTGGAAAGTCGTTG	TTTGATGTTGTTGGAGATGAAAA	59.3	485	DRR	Ca_14459	--	--
Ca4_P4_23055622	Ca4	23055622	TAAGAA/TAAGAAAGAA	TGAATTAAAGGATTGCATGAACA	CCGTTGAATGGAAAATCACC	59.5	530	DRR	Ca_14459	--	--

INDEL marker IDs	Chromosomes/ unanchored scaffolds	Physical positions (bp)	InDels (*Kabuli* reference genome-CDC Frontier/PII)	Forward primers (5'-3')	Reverse primers (5'-3')	Anneling temperature (0C)	Expected amplified product size (bp)	Structural annotation		Functional annotation	
								Sequence components of *kabuli* genome	*Kabuli* gene accession IDs	NCBI-KOG	TFs
Ca4_P4_23098190	Ca4	23098190	TC/T	GGCGGTCGAGAGTTTATGTG	TGAGTGCCAACAAGCTGAAC	60.7	495	DRR	Ca_14457	R	MYB
Ca4_P4_23098234	Ca4	23098234	GTTTT/GTTTTT	GGCGGTCGAGAGTTTATGTG	TGAGTGCCAACAAGCTGAAC	60.7	495	DRR	Ca_14457	R	MYB
Ca4_P4_23108687	Ca4	23108687	C/CT	GTGGGTGGAATGGAAATACG	GCATCGGGAGATACGTAGGA	60.1	659	DRR	Ca_14456	--	--
Ca4_P4_23340817	Ca4	23340817	TTGTGTGT/TTGTGT	TGTTTGGCAGCTAAGCAATG	AAGTTCCCTTACGGTCCTTG	60.0	396	DRR	Ca_14446	S	--
Ca4_P4_24438141	Ca4	24438141	CAAAAAA/CAAAAA	CCGCTCTGGAAATCATTCAT	AAGACTCGGGATAATGGCCT	60.0	551	DRR	Ca_20867	--	--
Ca4_P4_24608867	Ca4	24608867	GTTTTTTTTTT/GTTTTTTTTTTT	AGTGGATGGGTCGCATTTAG	GCTTTGCCACGTCAGTGTAA	60.0	570	DRR	Ca_20856	GE	HB-other
Ca4_P4_24786141	Ca4	24786141	CTTTTTTTTT/CTTTTTTTTTTT	CTTCACTGCGTCCCTTCTTC	GGGGTTGCAGATGATGTTGT	60.0	415	DRR	Ca_20850	--	--
Ca4_P4_24865397	Ca4	24865397	ATTTTTTT/ATTTTTT	TTCGGTCGCTTATTCTCTCC	CCAGCTAGAACAAAATGGCG	59.4	534	DRR	Ca_20847	T	bZIP
Ca4_P4_25252210	Ca4	25252210	GTT/GT	AAGGGGGCCCTAATAAGGTT	GATGTGCCTGGTTGGAAAGT	60.0	855	DRR	Ca_16567	CD	--
Ca4_P4_25282410	Ca4	25282410	CTTA/CTTATTA	AGCTTTAGCTTGAGGGGGAG	TGGTCACTGGTGCTTCTCTG	60.0	965	DRR	Ca_16569	B	--

INDEL marker IDs	Chromosomes/ unanchored scaffolds	Physical positions (bp)	InDels (*Kabuli* reference genome-CDC Frontier/PII)	Forward primers (5'-3')	Reverse primers (5'-3')	Anneling temperature (0C)	Expected amplified product size (bp)	Structural annotation		Functional annotation	
								Sequence components of *kabuli* genome	*Kabuli* gene accession IDs	NCBI-KOG	TFs
Ca4_P4_25457334	Ca4	25457334	ATTTTTTT/ATTTTTTTT	CATTTTACTCATCGATCTTTGTGG	TGGACAAAATCATGTCTCTTTGA	59.9	336	DRR	Ca_16577	--	--
Ca4_P4_25457701	Ca4	25457701	AATATATATATATATATATATATATA/AATATATATATATATATATATATATATATATA	TCAAAGAGACATGATTTTGTCCA	TCACTTTGCTTTGTGTGTCTCA	59.6	524	DRR	Ca_16577	--	--
Ca4_P4_25526065	Ca4	25526065	ATG/ATGTG	ATTCGCACCAAACAACAACA	CTTCCCAACTGAACTCCGAA	60.0	689	DRR	Ca_16580	--	--
Ca4_P4_25527044	Ca4	25527044	TAAAAAAAA/TAAAAAAAAA	ATTGAAGCGGCACCTATTGA	GCAAATTGTAAAAGTAGTCTTTGAGC	60.6	706	DRR	Ca_16580	--	--
Ca4_P4_25596215	Ca4	25596215	ATGTGTGTGTGTGT/ATGTGTGTGTGT	TTCTCACGAAAGCGCTGTAA	GGGCAAGGGGATCTGAATA	59.8	585	DRR	Ca_16586	--	Trihelix
Ca4_P4_25779217	Ca4	25779217	CTTAATTAATTAATTAATTAATTA/CTTAATTAATTAATTAATTA	ATACCGAGATGGACACCACC	TAACCCTTCAACATCTGGGC	59.7	367	DRR	Ca_16597	R	NAC
Ca4_P4_25779465	Ca4	25779465	AATA/AA	GCCCAGATGTTGAAGGGTTA	TTTGTCATCTCATAGGCCCC	59.9	209	DRR	Ca_16597	R	NAC
Ca4_P4_25916811	Ca4	25916811	CTTTTTTTTTTT/CTTTTTTTTTTTTT	ATGGTAACAGATATCGCCGC	ATTGTAGCATTGCCCAAAGG	60.0	627	DRR	Ca_16606	I	--
Ca4_P4_25962811	Ca4	25962811	GTATATATAT/GTATATATATAT	TGGTTGGAAGCTCCAGAAGT	GAAAAGGTGAGGAATCGCTG	59.8	275	DRR	Ca_16607	--	B3
Ca4_P4_25974119	Ca4	25974119	AG/A	AGCTCCGTGACCTTCTCAAA	TCTTTTCTGATCAATGGCCC	60.0	934	DRR	Ca_16608	--	--

INDEL marker IDs	Chromosomes/ unanchored scaffolds	Physical positions (bp)	InDels (*Kabuli* reference genome-CDC Frontier/PII)	Forward primers (5'-3')	Reverse primers (5'-3')	Anneling temperature (0C)	Expected amplified product size (bp)	Structural annotation		Functional annotation	
								Sequence components of *kabuli* genome	*Kabuli* gene accession IDs	NCBI-KOG	TFs
Ca4_P4_26011159	Ca4	26011159	AT/AACAGAACTCCT	GAAACCCCACAACAAAATGG	AGCATCATGGATTTCTTCCG	60.1	438	DRR	Ca_16611	C	--
Ca4_P4_2609181	Ca4	2609181	T/TGTGCTATA	GCTTCTGCTAGGCATCCATC	ATGCATGTGCTGATGCTTGT	59.9	907	DRR	Ca_12062	--	--
Ca4_P4_26173966	Ca4	26173966	CATAT/CAT	AGAGTGCATGTGGGATAGGG	TTCCATGATGCTCTTTTGGG	60.0	563	DRR	Ca_16612	--	HD-ZIP
Ca4_P4_26326139	Ca4	26326139	GAAAAAAAA/GAAAAAAA	CTCCAACAGCATCAATTCCA	GATCCTGGGATTGTGAGCAT	59.6	724	DRR	Ca_25306	R	--
Ca4_P4_26337485	Ca4	26337485	GTTTTTT/GTTTTTTT	CCTTTCCACCCAGAAAGGAG	TGGCAGAGCACTACCAACAA	61.0	362	DRR	Ca_25305	--	FAR1
Ca4_P4_26418036	Ca4	26418036	TCACACACACACACACA/TCACACACACACACACACA	CCAAAATTCAAACACCACCC	AACCCTGATTGACGGTGAAG	60.1	542	DRR	Ca_25299	--	--
Ca4_P4_26519771	Ca4	26519771	CAAA/CAAAAAA	AAACCTCTTCGCCTTTTTGC	TATTTTACGTACCCGCTGCC	60.7	351	DRR	Ca_25005	R	NAC
Ca4_P4_26524092	Ca4	26524092	GAA/GAAAA	TGAAATAGAGGGCCAGGTTG	TTCGTCCTTTGTCATCAAACA	60.1	317	DRR	Ca_25004	P	--
Ca4_P4_26524408	Ca4	26524408	C/CCTTAA	TGTTTGATGACAAAGGACGAA	AGAGGCACATTGAGAGCGTA	59.2	421	DRR	Ca_25004	P	--
Ca4_P4_26766370	Ca4	26766370	ATT/ATTT	AGTTTTGGCCCCTTTTGATT	TTGAGATGTTTCATGTTTCCATTT	59.8	678	DRR	Ca_20437	K	MYB

INDEL marker IDs	Chromosomes/ unanchored scaffolds	Physical positions (bp)	InDels (*Kabuli* reference genome-CDC Frontier/PII)	Forward primers (5'-3')	Reverse primers (5'-3')	Anneling temperature (0C)	Expected amplified product size (bp)	Structural annotation		Functional annotation	
								Sequence components of *kabuli* genome	*Kabuli* gene accession IDs	NCBI-KOG	TFs
Ca4_P4_26970354	Ca4	26970354	TAAAAAAA/TAAAA	ATTGTTTTGCGAGTTGAGGC	TTCTCGTTACAAAAATTTCCAAAA	60.3	696	DRR	Ca_20452	A	--
Ca4_P4_26970810	Ca4	26970810	TAAAAAAAA/TAAAAAAA	GAAAAGCTGAAACTGTATTTTGGAA	AACAGTGCCGATGAATAGTTTT	60.1	306	DRR	Ca_20452	A	--
Ca4_P4_27197342	Ca4	27197342	ATTTTTT/ATTTTTTT	CCTCAGACATCTTTGCCCAT	AAGTGTTGGACGGACCCTTA	60.1	656	DRR	Ca_20458	--	NF-YB
Ca4_P4_27197635	Ca4	27197635	TAAAAAAAAAAA/TAAAAAAAAAAAA	TTCAGGTTGCACCACAAAAC	TCAAATGCTACCGTTGGACA	59.6	691	DRR	Ca_20458	--	NF-YB
Ca4_P4_27267123	Ca4	27267123	TAAAAA/TAAAA	CCCTCAACTCAACTGAGATCC	TGCATTGGTTTGAATTTGGA	58.7	568	DRR	Ca_20461	R	--
Ca4_P4_27283830	Ca4	27283830	GTTT/GTTTT	TATTGGTCGTCAAGCAGCAG	TTTCCCAATCGATTTGCTTC	60.0	681	DRR	Ca_20463	--	--
Ca4_P4_27378491	Ca4	27378491	TAAAAAAAAAA/TAAAAAAAAA	GGATCCGTCTCCTGCATAAA	TTTGCACAGGTTGTTTGTTG	60.0	585	DRR	Ca_21323	--	--
Ca4_P4_27627265	Ca4	27627265	A/AGAAGAG	TTCTGCACTTGTGGTGAAGG	ATGCCATCGCTTTCTTCAAT	59.9	526	DRR	Ca_21312	--	--
Ca4_P4_27637047	Ca4	27637047	TAAAAAAA/TAAAAAA	CAACAAAGCGTCCACCTGTA	CCAATGATTTTCATGCCACA	59.8	810	DRR	Ca_21310	--	--
Ca4_P4_27637430	Ca4	27637430	TG/TGG	TTTGGAGTGGTGGTTCTCAA	CCAATGATTTTCATGCCACA	59.1	482	DRR	Ca_21310	--	--

INDEL marker IDs	Chromosomes/ unanchored scaffolds	Physical positions (bp)	InDels (*Kabuli* reference genome-CDC Frontier/PII)	Forward primers (5'-3')	Reverse primers (5'-3')	Anneling temperature (0C)	Expected amplified product size (bp)	Structural annotation		Functional annotation	
								Sequence components of *kabuli* genome	*Kabuli* gene accession IDs	NCBI-KOG	TFs
Ca4_P4_27638049	Ca4	27638049	CAAAAAAAAAA/CAAAAAAAAA	TTGTTTGATTCAGCAGCATGT	TTCAATTGGATTTGGATTTGAT	59.3	510	DRR	Ca_21310	--	--
Ca4_P4_28060298	Ca4	28060298	GATATATATATATATATAT/GATATATATATATATAT	TGCTGCACCCAAATTAACAA	TGTCAGCCGACAACAAAAA	60.1	318	DRR	Ca_23178	--	--
Ca4_P4_28234444	Ca4	28234444	AAAAA/AAAAATAAAA	GTTCCATCTGTTGCTGCTGA	CCTTTCTTTTGCCATCTTGC	60.0	818	DRR	Ca_23168	--	--
Ca4_P4_28282247	Ca4	28282247	ATTTTTT/ATTTTTTT	TCCAGAAGGCATTTCTTGGT	AGGTTAGTTGGAGCGTTTGAA	59.7	430	DRR	Ca_23166	TR	FAR1
Ca4_P4_28788052	Ca4	28788052	GTTTTTTTTTTT/GTTTTTTTTTT	AAGCATGAAGAGCCTCGGTA	GGCTCAAGTTGCTGTGGATT	60.0	923	DRR	Ca_23697	E	TALE
Ca4_P4_28949557	Ca4	28949557	CAAAAAAAA/CAAAAAAA	TTTCATCATTTCTGGAGGCA	GTGAGGATGAGCTGACACGA	59.2	528	DRR	Ca_23705	J	--
Ca4_P4_28996068	Ca4	28996068	CTTTTTTTTTTTT/CTTTTTTTTTT	TAACAGCATTGCATTGGGAA	TTCCAACACTAAAGGGGTGC	60.1	243	DRR	Ca_23706	--	NF-YB
Ca4_P4_29156546	Ca4	29156546	CTATA/CTA	AGTTGGTTTGGTGGTGAAGG	GCATGGTAAAGGCTGTTGGT	59.9	841	DRR	Ca_23711	--	--
Ca4_P4_29211121	Ca4	29211121	AT/A	GATGTTTAAGGTCTGTTTGGCA	AACTTCAGCCGTCATTGGTT	59.1	641	DRR	Ca_14151	--	--
Ca4_P4_29367942	Ca4	29367942	TTA/TTATATA	ATTTTGGCAACACATCACCA	TGGATCAAGTTGTTTGTCACG	59.8	634	DRR	Ca_14154	--	--

INDEL marker IDs	Chromosomes/ unanchored scaffolds	Physical positions (bp)	InDels (*Kabuli* reference genome-CDC Frontier/PII)	Forward primers (5'-3')	Reverse primers (5'-3')	Anneling temperature (0C)	Expected amplified product size (bp)	Structural annotation		Functional annotation	
								Sequence components of *kabuli* genome	*Kabuli* gene accession IDs	NCBI-KOG	TFs
Ca4_P4_29368197	Ca4	29368197	TTA/TTAGTA	ATTTTGGCAACACATCACCA	CCTTGGCATATTTTTGCTGG	59.8	737	DRR	Ca_14154	--	--
Ca4_P4_29646991	Ca4	29646991	GGATCG/GG	AGGATTCGGTCTGTGTTTGG	TCAGGACTGCAAATAACCCA	60.0	555	DRR	Ca_14165	S	--
Ca4_P4_29721716	Ca4	29721716	G/GA	CCTCACCTTCCCTTTCAACA	CGGTCGACGTGTGATATGAA	60.1	259	DRR	Ca_14167	--	--
Ca4_P4_29722401	Ca4	29722401	AAT/AATAT	TTCGCAGTGGAGTTTGAATG	CGATAAAAACAAAATTGTACACCG	59.8	550	DRR	Ca_14167	--	--
Ca4_P4_29904472	Ca4	29904472	T/TA	CCAAGTGTCGCAAACTTCAA	ATGTTTGACGAAGAGGGCAC	59.9	731	DRR	Ca_14176	--	M-type
Ca4_P4_30045432	Ca4	30045432	CATATAT/CATATATAT	GCGTGCATAAGTGGTTGATG	ACTCACGGTGATGAGTGTGG	60.1	345	DRR	Ca_14181	--	S1Fa-like
Ca4_P4_30206461	Ca4	30206461	TAAAAAAAAAAA/TAAAAAAAAA	TCCCTCGAGCTATAGGATGC	ATGCAATGGTTTATTCCCCA	59.4	643	DRR	Ca_14187	--	--
Ca4_P4_30230521	Ca4	30230521	A/AAAG	GGTATGCTGTTTCGGTTCGT	TTCGCTAATTTTGGCAAACC	60.0	406	DRR	Ca_14188	--	--
Ca4_P4_30230800	Ca4	30230800	ATTT/ATT	CCGTGAACCAGTACTCACGA	CATGACCATTTTGGATGTCG	59.7	397	DRR	Ca_14188	--	--
Ca4_P4_30543529	Ca4	30543529	T/TC	CACGAGATTCGTCGAGAACA	TGATGGGTCATTTAGTGCCA	60.0	196	DRR	Ca_14205	--	--

INDEL marker IDs	Chromosomes/ unanchored scaffolds	Physical positions (bp)	InDels (*Kabuli* reference genome-CDC Frontier/PII)	Forward primers (5'-3')	Reverse primers (5'-3')	Anneling temperature (0C)	Expected amplified product size (bp)	Structural annotation		Functional annotation	
								Sequence components of *kabuli* genome	*Kabuli* gene accession IDs	NCBI-KOG	TFs
Ca4_P4_30575118	Ca4	30575118	ATT/ATTT	TGTTCAACCATCAATTGTTTCC	GGCGGAATTTTTCATAGCAA	59.7	222	DRR	Ca_14206	A	--
Ca4_P4_30856676	Ca4	30856676	A/AT	TGCACTGTATTGGAATTGTTGA	GAAATGACTGAAAAATTTTGGACC	59.1	482	DRR	Ca_14212	--	bZIP
Ca4_P4_31029219	Ca4	31029219	GA/GATAA	TGCATCGTGAGTTTGAGGAG	GCCATTTGGAACTGGAAGAA	60.0	714	DRR	Ca_14217	Q	--
Ca4_P4_31195751	Ca4	31195751	GAAAAAAAA/GAAAAAAAAA	CAACACCATTCAAACCACCA	GCGGCAGAATTCAAATCAGT	60.3	500	DRR	Ca_14224	--	S1Fa-like
Ca4_P4_31248634	Ca4	31248634	ACC/AC	TGGGTCATTTCTCATTATGTCG	TGCTATGCCTTTTGATCGC	59.8	645	DRR	Ca_14228	--	Trihelix
Ca4_P4_31255409	Ca4	31255409	CA/CAA	CGCTTACCAGAGCAGTTTCC	GAGAGAAGTGTCTTTGCGGG	60.0	681	DRR	Ca_14229	R	bHLH
Ca4_P4_31261325	Ca4	31261325	AAAATAAA/AAAA	TTGAAGTTTGAGAGGACGGG	AATTCCAATTGTGCCACCTC	60.2	323	DRR	Ca_14230	R	MYB_related
Ca4_P4_31360317	Ca4	31360317	TAAAAAAA/TAAAAAAAA	ACTCATATGACGGGGTGCAT	GGCAGACGTTGGAAAGCTAA	60.2	715	DRR	Ca_14232	--	Trihelix
Ca4_P4_31653241	Ca4	31653241	GATAAA/GATAAAAAATAAA	TTCAAACGATGGCAACAAAA	TCATGGGATTAAGCCTCCAC	60.1	268	DRR	Ca_15442	U	EIL
Ca4_P4_31754313	Ca4	31754313	CTTTTTTTTTTT/CTTTTTTTTTT	GGTTGGTGCAGATTTTTGCT	AGCACAAAGGCAGAGAAGGA	60.1	266	DRR	Ca_15446	--	--

INDEL marker IDs	Chromosomes/ unanchored scaffolds	Physical positions (bp)	InDels (*Kabuli* reference genome-CDC Frontier/PII)	Forward primers (5'-3')	Reverse primers (5'-3')	Anneling temperature (0C)	Expected amplified product size (bp)	Structural annotation		Functional annotation	
								Sequence components of *kabuli* genome	*Kabuli* gene accession IDs	NCBI-KOG	TFs
Ca4_P4_31866813	Ca4	31866813	GAGAAAA/G	TCGCACAATAGTGCAAACTCA	TGGTAAGGCGACAAACAACA	60.5	512	DRR	Ca_15450	--	C2H2
Ca4_P4_31978850	Ca4	31978850	CAA/CAAA	ACATTCTTATGTCGGGGCAG	ACAGCCTTTTCTCCATCCCT	60.0	605	DRR	Ca_15457	S	--
Ca4_P4_31994404	Ca4	31994404	TAAAA/TAAAAA	ATCAGCGAGGATGCAGAACT	CATCGCTCAGTTTGGACTCA	60.0	796	DRR	Ca_15458	--	--
Ca4_P4_32045859	Ca4	32045859	T/TA	TGGGGTTTTGATCCATTTGT	TGATGTCTTTCGATCCACCT	60.0	607	DRR	Ca_15459	--	--
Ca4_P4_32046303	Ca4	32046303	GTTTTTT/GTTTTTTT	TTTGGGCTTTTCTTGATTGG	AGTCGGCTTCAAAGGCACTA	60.0	646	DRR	Ca_15459	--	--
Ca4_P4_32069410	Ca4	32069410	A/AC	CCAGTGGTGTCCCTCTTGAT	GAGACGGAATTTGGGACAGA	60.0	767	DRR	Ca_15461	R	--
Ca4_P4_32565833	Ca4	32565833	TAAAAAAAA/TAAAAAAA	CCGTTGACTAGACGTAAACAACA	ACCAACAAACCATGCACAAA	59.3	879	DRR	Ca_15483	T	YABBY
Ca4_P4_32602184	Ca4	32602184	CA/C	TGGGGATCAACAGACTCTAAAA	GTGAAAATCGAAGTGCGACA	58.7	456	DRR	Ca_15484	R	AP2
Ca4_P4_32603125	Ca4	32603125	TCC/TC	GTCAAAACATGTTCCCACCC	GCCCAACCACTAGGTTGAAA	60.1	219	DRR	Ca_15484	R	AP2
Ca4_P4_32635688	Ca4	32635688	TAAAAAAAAAAA/TAAAAAAAAA	TATGTGTGTGGCAGCATGAA	TGCATTTTGCCTGCTCTATG	59.7	639	DRR	Ca_15486	R	NAC

INDEL marker IDs	Chromosomes/ unanchored scaffolds	Physical positions (bp)	InDels (*Kabuli* reference genome-CDC Frontier/PII)	Forward primers (5'-3')	Reverse primers (5'-3')	Anneling temperature (0C)	Expected amplified product size (bp)	Structural annotation		Functional annotation	
								Sequence components of *kabuli* genome	*Kabuli* gene accession IDs	NCBI-KOG	TFs
Ca4_P4_32650019	Ca4	32650019	ATTTTTT/ATTTTT	TTGTGTTTTGGTTGGGATCA	TTTCATGGGTTTTGTTTGAAAT	59.8	451	DRR	Ca_15487	--	--
Ca4_P4_33308913	Ca4	33308913	GAAAA/GAAA	TCATTTTCCCCTCGTTTCTG	TTGGCCAAAACCTTTTGATG	60.0	183	DRR	Ca_24021	R	C2H2
Ca4_P4_33397720	Ca4	33397720	ATTTTTTTTTTT/ATTTTTTTTTT	CTCCATTTTGTTGGGGCTTA	GCCAACAAACAACCATGCTA	59.9	584	DRR	Ca_24022	--	--
Ca4_P4_3365913	Ca4	3365913	AATATATATATATATATATATATATAT/AATATATATATATATATATATAT	AAAGAGGGCATGACACCAAT	TCGGATCGGACACTATGACA	59.4	466	DRR	Ca_12139	--	--
Ca4_P4_3413984	Ca4	3413984	A/ACTTGG	CGTCATTGCCACATCATTTC	TTCAGAGAATCTGCATCCCA	59.9	508	DRR	Ca_12143	J	--
Ca4_P4_34458862	Ca4	34458862	CAAAA/CAAAAA	CCAAACGTTCTCCCACAACT	GAGGATGCCAAAATGCCTAA	60.0	744	DRR	Ca_20351	--	--
Ca4_P4_34492879	Ca4	34492879	TAAAAAAAAA/TAAAAAAAAAA	CTGATCAGTCGTTGGGAGGT	TGAGTTGATCGTTTCCACCA	60.1	538	DRR	Ca_20350	R	G2-like
Ca4_P4_35292340	Ca4	35292340	C/CTAAGATTGTTTTGA	TGGCCAAAGGAGTCATGTTT	GTGTTGATGTCCGACAATGC	60.5	496	DRR	Ca_20016	DZ	--
Ca4_P4_35681392	Ca4	35681392	TAAAAAAAAAAAA/TAAAAAAAAAAAAA	TGGTCCCGAGTACGTAGGAG	GCAGGTTCCACCTAGACCAA	60.1	548	DRR	Ca_22446	QI	B3
Ca4_P4_36245892	Ca4	36245892	TTATATATATATATATATATATAT/TTATATATATATATATATAT	TACATGCAAGCGAAATGGAA	AGGCATGGTGTTGGAGTCAT	60.2	287	DRR	Ca_14846	O	--

INDEL marker IDs	Chromosomes/ unanchored scaffolds	Physical positions (bp)	InDels (*Kabuli* reference genome-CDC Frontier/PII)	Forward primers (5'-3')	Reverse primers (5'-3')	Anneling temperature (0C)	Expected amplified product size (bp)	Structural annotation		Functional annotation	
								Sequence components of *kabuli* genome	*Kabuli* gene accession IDs	NCBI-KOG	TFs
Ca4_P4_36377514	Ca4	36377514	TCACACA/TCACA	AGCACGTGGTATCATG TCTCC	CGGCTTCATAATTGGG CTTA	60.0	792	DRR	Ca_14838	TU	C2H2
Ca4_P4_36404496	Ca4	36404496	TAAA/TAA	GGAATGGTATTGTCAA CCGC	AGGCTCAATGACGCAG AACT	60.2	701	DRR	Ca_14836	R	NAC
Ca4_P4_36450503	Ca4	36450503	TTAT/TT	GACACCAAATTGGGAA AGGA	GCGGTGACATGTACGA TTTG	59.8	710	DRR	Ca_14833	S	--
Ca4_P4_36458353	Ca4	36458353	ATTT/ATTTTT	AATCCGATTCAACCCA CAAA	CAAGGTCCATATTTCAA TGCG	60.2	438	DRR	Ca_14832	T	YABBY
Ca4_P4_36483891	Ca4	36483891	GTTTTTTTTTTT/GTTTTTTTTT	AATATGTTGGAGTGGC CCTG	CGGACCACAAAATTGT AAACG	59.8	552	DRR	Ca_14829	QR	B3
Ca4_P4_36487114	Ca4	36487114	TAAAATCTAACATAAAAAT/TAAAAT	AACAGCCATGGAATTT GTCA	GCCTTCTTCTCATGAG CCAC	59.0	713	DRR	Ca_14828	--	--
Ca4_P4_36487202	Ca4	36487202	CA/CAA	TCCTCCATTGTTTCCCT AAGAA	CGTCCCAGACCATCTT TTGT	59.9	947	DRR	Ca_14828	--	--
Ca4_P4_36565480	Ca4	36565480	T/TAAACCCC	CACGCTTTCTCCCAAA ACAT	GGCACTGAGATCAACA TGAGG	60.1	603	DRR	Ca_14822	--	--
Ca4_P4_36566713	Ca4	36566713	TAAAAA/TAAAAAA	TGATTTATAATGCCCAC CAAAA	GAGATGCAGAAAGGGG ATGA	59.2	686	DRR	Ca_14822	--	--
Ca4_P4_36601135	Ca4	36601135	ATT/ATTT	ATTGATTCCCCATTGTT GGA	TGAAGGTGGAATTGGG GTAG	60.0	341	DRR	Ca_14819	--	MYB

INDEL marker IDs	Chromosomes/ unanchored scaffolds	Physical positions (bp)	InDels (*Kabuli* reference genome-CDC Frontier/PII)	Forward primers (5'-3')	Reverse primers (5'-3')	Anneling temperature (0C)	Expected amplified product size (bp)	Structural annotation		Functional annotation	
								Sequence components of *kabuli* genome	*Kabuli* gene accession IDs	NCBI-KOG	TFs
Ca4_P4_36602493	Ca4	36602493	TTA/TTAATA	CGGTTTCAACGTTGTTGTTG	GCCTTTTTATGCTGATTTCTTTG	60.0	866	DRR	Ca_14819	--	MYB
Ca4_P4_36638657	Ca4	36638657	GAAAA/GAAAAA	CTGGGATTATGGGGTCATTG	ATTCAGCTTAGTGGCAAGGC	60.0	446	DRR	Ca_14816	H	--
Ca4_P4_36928403	Ca4	36928403	TAAAAAAAAAA/TAAAAAAAAA	TTCAAAAATTGAAAACAATACCCA	TTGGAAAGCTTATTCTCGCA	59.7	548	DRR	Ca_14798	--	--
Ca4_P4_37096660	Ca4	37096660	ATTTTTTTTTTT/ATTTTTTTT	GCGACCAATTCTATGATGAATAAA	GGGAGTGGTGATTTCCTTTTC	59.4	504	DRR	Ca_14791	--	B3
Ca4_P4_37155367	Ca4	37155367	ACCCC/ACCCCC	TGGTCTATCGTACGGTGACATAC	ATGTAAGAGTTGGAACGCCG	58.9	634	DRR	Ca_15155	G	--
Ca4_P4_3716263	Ca4	3716263	TTAATAATAATAATAATAATAATAATAATAATAA/TTAATAATAATAATAATAATAATAATAATAA	TGCTTTGGCCTGAAAGAAGT	GCACGATTTCTCCCTCTCTG	60.0	824	DRR	Ca_12170	R	--
Ca4_P4_37174812	Ca4	37174812	GAAAAAA/GAAAAAAA	AAATGGACCCCACATTCAAA	TCCCTCTCTCTCTGCCACAT	60.0	532	DRR	Ca_15153	A	bHLH
Ca4_P4_37187797	Ca4	37187797	TT/TTCT	AAAAATGCATGGTGGCATCT	TTTCATTCAGTTTGGCATCG	60.3	567	DRR	Ca_15152	I	bHLH
Ca4_P4_3734203	Ca4	3734203	ATTTTTTTTTTTTTT/ATTTTTTTTTTTTT	GAAAATGTTGACCGTATGCAA	TGTCTTTAATTCTCCAAACCCAA	58.5	183	DRR	Ca_12174	R	--
Ca4_P4_37366698	Ca4	37366698	AAA/AAACCAA	TTACCCTCTGTTGCTTTGCC	ACCATGGACACCGTTTGTTT	60.2	430	DRR	Ca_15139	R	NF-YA

INDEL marker IDs	Chromosomes/ unanchored scaffolds	Physical positions (bp)	InDels (*Kabuli* reference genome-CDC Frontier/PII)	Forward primers (5'-3')	Reverse primers (5'-3')	Anneling temperature (0C)	Expected amplified product size (bp)	Structural annotation		Functional annotation	
								Sequence components of *kabuli* genome	*Kabuli* gene accession IDs	NCBI-KOG	TFs
Ca4_P4_37367041	Ca4	37367041	TC/T	CCACCTTTGTCATTGCTTCA	GATCAACACCTTCGACCGTT	59.7	520	DRR	Ca_15140	S	--
Ca4_P4_37367413	Ca4	37367413	TCCC/TCC	CCACCTTTGTCATTGCTTCA	GATCAACACCTTCGACCGTT	59.7	520	DRR	Ca_15140	S	--
Ca4_P4_373755	Ca4	373755	AATATATATATATATATATATATATATATAT/AATATATATATATATATATATATATAT	ATTATGGGGCAACATGGAAA	GAACTCGATTTGGATTCGGA	60.0	227	DRR	Ca_07670	--	--
Ca4_P4_37385994	Ca4	37385994	TTACTA/TTA	TGTGCTTGCATAGTGCATTG	TGAATAATTTCCTGCTCCGC	59.5	221	DRR	Ca_15138	T	HSF
Ca4_P4_37396824	Ca4	37396824	TATTAAAATTAA/TATTAAAATTAAAATTAA	TGTCAATCAAACGGATCCAA	TACATTGCACCCACCATGAC	59.9	222	DRR	Ca_15137	R	MYB
Ca4_P4_37397137	Ca4	37397137	TA/T	CGGATCCAAAATGTTAACGG	TCCACCATTGGTTCCTCATT	60.2	884	DRR	Ca_15137	R	MYB
Ca4_P4_37408226	Ca4	37408226	TTTAAG/T	CAGAGCCAGTGTCTATGCCA	AGCCAAAGCTGTCCTCTGAA	60.0	534	DRR	Ca_15136	--	--
Ca4_P4_37544414	Ca4	37544414	TG/TGG	AAGAAATGTTGATGGAGGCG	TAATGTGTGCTCCATTCCCA	60.1	296	DRR	Ca_15126	--	--
Ca4_P4_37615197	Ca4	37615197	T/TAG	TATTGCACTCCAGCAACAGG	AACCTGAGCCAATCATTTCA	59.9	655	DRR	Ca_15120	--	--
Ca4_P4_37648089	Ca4	37648089	CA/CATA	GCTCATGCAAATGCTAGCTG	ACCCGTGCAAACACATAACA	59.7	617	DRR	Ca_15116	--	--

INDEL marker IDs	Chromosomes/ unanchored scaffolds	Physical positions (bp)	InDels (*Kabuli* reference genome-CDC Frontier/PII)	Forward primers (5'-3')	Reverse primers (5'-3')	Anneling temperature (0C)	Expected amplified product size (bp)	Structural annotation		Functional annotation	
								Sequence components of *kabuli* genome	*Kabuli* gene accession IDs	NCBI-KOG	TFs
Ca4_P4_37691102	Ca4	37691102	ATACAATTACAA/ATACAA	ATGCCCAAAAACAAAAGCAA	ATGAACTGCGATGTGGACAA	60.5	604	DRR	Ca_15112	--	--
Ca4_P4_37701400	Ca4	37701400	ATTTTTTTTTT/ATTTTTTTTTTT	TGAACACCGCATTAATTGGA	GGGTTTTTGTTCGAAGATTTTG	59.9	262	DRR	Ca_15111	F	--
Ca4_P4_37705943	Ca4	37705943	GA/GATAA	GCCGGGATGTTCTTTTTGTA	TTTACTGAATGCCATGCTGC	59.9	679	DRR	Ca_15110	C	MYB
Ca4_P4_37706091	Ca4	37706091	TTCT/TT	GCCGGGATGTTCTTTTTGTA	GAGGGGAGGCCTTCTCATAC	59.9	818	DRR	Ca_15110	C	MYB
Ca4_P4_37706158	Ca4	37706158	G/GA	GCCGGGATGTTCTTTTTGTA	GAGGGGAGGCCTTCTCATAC	59.9	818	DRR	Ca_15110	C	MYB
Ca4_P4_37744026	Ca4	37744026	ATAGAAAATTTATTTA/ATA	TCGATTCTCTTGTGGTTGGA	ACTTATGTTGCGGTTGGAGG	59.2	698	DRR	Ca_15105	--	--
Ca4_P4_37980075	Ca4	37980075	TCC/TC	AGGGAGAGGGATAAGGCGTA	TTGTGGCGACATAGCACAAT	60.1	588	DRR	Ca_13148	--	--
Ca4_P4_37981676	Ca4	37981676	AATATATATATATATATA/AATATATATATATATATATA	AAATGGCAACTTCCCACAAG	GGCATTTCGAACTTGGACTT	60.0	489	DRR	Ca_13148	--	--
Ca4_P4_38018994	Ca4	38018994	T/TG	TGGGCTAAAGCATCTCCATC	AATCGGCATTTGGATTTTTG	60.2	998	DRR	Ca_13143	E	--
Ca4_P4_38026240	Ca4	38026240	CTTTTTTT/CTTTT	GGATTCGAAGTCGGAACAAA	CAATGTGTTTTCACTCCATTTTT	60.1	277	DRR	Ca_13142	O	ERF

INDEL marker IDs	Chromosomes/ unanchored scaffolds	Physical positions (bp)	InDels (*Kabuli* reference genome-CDC Frontier/PII)	Forward primers (5'-3')	Reverse primers (5'-3')	Anneling temperature (0C)	Expected amplified product size (bp)	Structural annotation		Functional annotation	
								Sequence components of *kabuli* genome	*Kabuli* gene accession IDs	NCBI-KOG	TFs
Ca4_P4_38035645	Ca4	38035645	CTTA/CTTATTA	TTCCTGCCAAGCTTTCACTT	TCCCACCAAGGAAGAAAATG	60.0	652	DRR	Ca_13141	--	MYB_related
Ca4_P4_38051216	Ca4	38051216	AT/ATGT	GGAAATTAAATGGTGCCCCT	TTTCTTCTAACCACATTTTCAACC	60.0	807	DRR	Ca_13140	P	--
Ca4_P4_38051360	Ca4	38051360	GAAAA/GAAAAAA	GGAAATTAAATGGTGCCCCT	TTTCTTCTAACCACATTTTCAACC	60.0	807	DRR	Ca_13140	P	--
Ca4_P4_38175024	Ca4	38175024	CTATTATTATTATTATTATTATTATTATTACTATTATTATTATTGTTATTATTATTATTATTATTATTAT/CTATTATTATTATTATTATTATTATTACTATTATTATTATTGTTATTATTATTATTATTATTATTAT	GTGGACAACATATCCGGACC	AGGCTAGGGTGAGACAGCAA	60.1	558	DRR	Ca_13136	--	--
Ca4_P4_38367094	Ca4	38367094	T/TAGATTGGAA	TCGTTTAGCGGTGAAACTCA	CAAGCTCTTGCCATGTTTGA	59.5	158	DRR	Ca_13120	S	--
Ca4_P4_38414507	Ca4	38414507	CA/CAAAA	GGTTGAACGTCTTGTCTCGAA	TGATCAACTTCATGGCCAAA	60.3	407	DRR	Ca_13116	R	--
Ca4_P4_38446174	Ca4	38446174	AATTAT/AATTATTAT	CTCCTTGATTCACGTGGGTT	TTAAAAACACTGTTGGCGTGA	60.0	230	DRR	Ca_13113	O	--
Ca4_P4_38446443	Ca4	38446443	TAA/TAAGAA	CTCCTTGATTCACGTGGGTT	TGATCGAAATTTTATGGGGA	60.0	484	DRR	Ca_13113	O	--
Ca4_P4_38536722	Ca4	38536722	TTATATATAT/TTATATATATAT	GAAGGCGTGTTCACCTTGAT	TTCCAATCCATCCTCACACA	60.1	260	DRR	Ca_13110	G	--
Ca4_P4_38537057	Ca4	38537057	TTTAATT/TTT	TGTGTGAGGATGGATTGGAA	TCTAGCGGTATCAGCTTGGG	59.9	595	DRR	Ca_13110	G	--

INDEL marker IDs	Chromosomes/ unanchored scaffolds	Physical positions (bp)	InDels (*Kabuli* reference genome-CDC Frontier/PII)	Forward primers (5'-3')	Reverse primers (5'-3')	Anneling temperature (0C)	Expected amplified product size (bp)	Structural annotation		Functional annotation	
								Sequence components of *kabuli* genome	*Kabuli* gene accession IDs	NCBI-KOG	TFs
Ca4_P4_38537586	Ca4	38537586	ACGT/ACGTCGT	TTCGCAACATGGGAACATAA	TTGGTTTGGAATTTGGAAGG	59.9	257	DRR	Ca_13110	G	--
Ca4_P4_38547921	Ca4	38547921	CATT/CATTAATT	TCTGAAAAGAGGGGGTGAAA	CCGGAAAAGCTGACCAAATA	59.6	326	DRR	Ca_13108	T	bHLH
Ca4_P4_38620126	Ca4	38620126	TAAAAATAAAATAAAAAA/TAAAAA	CCCACCCAATCACCATTTAC	CCCCATTGCTTGTTTGACTT	59.9	434	DRR	Ca_13103	L	--
Ca4_P4_38620449	Ca4	38620449	CCTTCTT/CCTT	AAGTCAAACAAGCAATGGGG	GTTGCTGCCCTTGAGACTTC	60.0	461	DRR	Ca_13103	L	--
Ca4_P4_38816584	Ca4	38816584	AATTTATATTTA/AATTTATATTTATATTTA	ATGCCTTTACATGCACAAAAT	GCAAATATTGTCTGCCAAAAGTG	57.2	724	DRR	Ca_13093	K	NF-YB
Ca4_P4_38837626	Ca4	38837626	CTTTTT/CTTTT	TCATTGGATCGCGGTTTT	TGAAGGATTTAGGAGCTGACAA	60.0	814	DRR	Ca_13090	--	--
Ca4_P4_38891821	Ca4	38891821	CA/CAA	TTATTGCTTTTGCATGGTGA	TTGGACAAATTAGCCTATGAAACA	57.8	516	DRR	Ca_13087	--	ERF
Ca4_P4_38959403	Ca4	38959403	CGTGT/CGT	TCTGAAACGGAGGAAGTAGCA	ATCCTTTGGTTCCCGAAATC	60.0	879	DRR	Ca_13083	O	MYB_related
Ca4_P4_38963393	Ca4	38963393	AT/A	TGGTGCGTATTTGGAATTGA	TGATTGACGACCAACTAGCAA	59.9	513	DRR	Ca_13082	O	MYB_related
Ca4_P4_38963586	Ca4	38963586	TA/TAA	GGGGAGAGACAAATGCGTTA	TGTCTATTTCGGCCGTATGTT	60.1	770	DRR	Ca_13082	O	MYB_related

INDEL marker IDs	Chromosomes/ unanchored scaffolds	Physical positions (bp)	InDels (*Kabuli* reference genome-CDC Frontier/PII)	Forward primers (5'-3')	Reverse primers (5'-3')	Anneling temperature (0C)	Expected amplified product size (bp)	Structural annotation		Functional annotation	
								Sequence components of *kabuli* genome	*Kabuli* gene accession IDs	NCBI-KOG	TFs
Ca4_P4_38983278	Ca4	38983278	TAAAA/TAAA	GAGGTGGGGAAAGTAGCCTC	TTGTTGGTGTGTCGTGGTTT	60.1	814	DRR	Ca_13081	H	--
Ca4_P4_38993838	Ca4	38993838	AAAAATTTC/AAAAATTTCTCATAACGAAAATTTC	CCAAAAATTTCGAAAACTCGAC	AAGACCGGAAAAGGGTATGC	60.0	643	DRR	Ca_13080	--	bHLH
Ca4_P4_39038460	Ca4	39038460	ACTTGCTTG/ACTTG	CCGGTCACATTCAATAGCCT	ATTGCGTTGTTTCTTCCAGG	60.0	411	DRR	Ca_13078	A	--
Ca4_P4_39051123	Ca4	39051123	T/TC	TGTTTGCATTTAGCGGTTCA	CCCAAAGCAGAAAACCAAAA	60.3	285	DRR	Ca_13076	--	--
Ca4_P4_40348298	Ca4	40348298	AATATATATATATATATATATATATATATATATATA/AATATATATATATATATATATATATATATATATATATATA	CCATCCTTCGAATTTGGCTA	TGGTTAAAGGCCAGGATTTC	60.0	668	DRR	Ca_14889	PET	NAC
Ca4_P4_40354186	Ca4	40354186	TAAAAAAAA/TAAAAAAAAA	TAGCCTAAACCCGATGGATG	ATTGTTCCATCACTTGCGTG	59.9	424	DRR	Ca_14888	J	--
Ca4_P4_40483531	Ca4	40483531	ATT/ATTAGCGGTCTT	TCCCACATTGGTAGTTCCGT	AGCTGAACCCTTGCTGTGAT	60.2	500	DRR	Ca_14877	I	--
Ca4_P4_40534591	Ca4	40534591	TGG/TGGG	TAGGAGACTGCTTCCGCAAT	GGTGCTCTTGACAGCACAAA	60.0	109	DRR	Ca_14873	--	--
Ca4_P4_40559341	Ca4	40559341	TAA/TA	TCAGGCCATTAGGGTTTCTG	ATTGAATGCGGTCTCAGTGC	60.1	410	DRR	Ca_14870	--	--
Ca4_P4_40588010	Ca4	40588010	TAAA/TAAAA	CTTGCACACAATGCGATCAT	GGTCCGAAACTGACAAAAGC	60.7	641	DRR	Ca_14868	K	--

INDEL marker IDs	Chromosomes/ unanchored scaffolds	Physical positions (bp)	InDels (*Kabuli* reference genome-CDC Frontier/PII)	Forward primers (5'-3')	Reverse primers (5'-3')	Anneling temperature (0C)	Expected amplified product size (bp)	Structural annotation		Functional annotation	
								Sequence components of *kabuli* genome	*Kabuli* gene accession IDs	NCBI-KOG	TFs
Ca4_P4_40588106	Ca4	40588106	ATTTTTT/ATTTTTTT	CTTGCACACAATGCGATCAT	GGTCCGAAACTGACAAAAGC	60.7	641	DRR	Ca_14868	K	--
Ca4_P4_40647015	Ca4	40647015	GTTTTTTT/GTTTTTTTT	CCAAGCATCATTCCAAATCC	CACCACAAGCCAGAGCAATA	60.3	553	DRR	Ca_14865	--	--
Ca4_P4_40720882	Ca4	40720882	CTTTTTT/CTTTTTTT	TCATCAATGGGAACCTCACA	TGATGACTGTCAAAGAGCCG	59.9	143	DRR	Ca_23910	S	C2H2
Ca4_P4_40738323	Ca4	40738323	TAAAAAAAAA/TAAAAAAAA	TTTACCCGAGCCAATTTCAG	TCAAGGTCAGCAGCATGGTA	60.1	920	DRR	Ca_23908	R	NAC
Ca4_P4_40799742	Ca4	40799742	TCCCCC/TCCCCCC	ACAAACCCCATTGGTGACAT	TGGCTTGTAAGGCTGTGTTG	60.0	740	DRR	Ca_23902	R	--
Ca4_P4_40811635	Ca4	40811635	GAAAA/GAA	TTACCACCAAGGTCAGCTCC	CGGGAAGATCATTCACGAAG	60.1	704	DRR	Ca_23901	R	MYB
Ca4_P4_40853669	Ca4	40853669	AA/AAGA	TTCAATTTTATGGATTTTGTTTGAA	CGTGGGTACTTTCTGGGTGT	58.9	411	DRR	Ca_23897	--	--
Ca4_P4_40854093	Ca4	40854093	TAAAAAA/TAAAAA	ACACCCAGAAAGTACCCACG	TGAAATGCATTCGTCACACA	59.9	610	DRR	Ca_23897	--	--
Ca4_P4_40893508	Ca4	40893508	AG/A	TGAACTCGGCTAGGAGGAAA	CGCAAGTGTTGGACAATGAT	59.9	703	DRR	Ca_11020	R	G2-like
Ca4_P4_40924286	Ca4	40924286	ACAATATG/ACAATATGTAGCTTCAATATG	GTGATCTACGTGGTGCCCTT	ACCCAAAATGAGGTGGTTCA	60.0	548	DRR	Ca_11015	--	bZIP

INDEL marker IDs	Chromosomes/ unanchored scaffolds	Physical positions (bp)	InDels (*Kabuli* reference genome-CDC Frontier/PII)	Forward primers (5'-3')	Reverse primers (5'-3')	Anneling temperature (0C)	Expected amplified product size (bp)	Structural annotation		Functional annotation	
								Sequence components of *kabuli* genome	*Kabuli* gene accession IDs	NCBI-KOG	TFs
Ca4_P4_40940117	Ca4	40940117	TAAAAAAAAAAAAA/TAAAAAAAAAA	TCCAAGTTCTCATCACGCAG	GATCATGTGGAGCGGAAGAT	60.0	635	DRR	Ca_11014	O	MYB
Ca4_P4_40948599	Ca4	40948599	CAAAAAAAA/CAAAAAAAAAAA	AGGGTTGGGTTTGAGAGAAA	GGGAAGGGAAAAATGGATGT	58.6	702	DRR	Ca_11013	--	--
Ca4_P4_40996538	Ca4	40996538	GTTT/GTT	CGGTGTTAAAAATTGGGGTG	AAACGTGGAGGTGATTTTGC	60.1	445	DRR	Ca_11010	--	--
Ca4_P4_41010880	Ca4	41010880	CATTATATTATATT/CATTATATT	CTTCTCCCCTGTTAGTCCCC	AGGTCAAACCGTCAAAGTGC	59.9	730	DRR	Ca_11009	--	bHLH
Ca4_P4_41012180	Ca4	41012180	TAAAAA/TAAAA	GCTTGATGGGAGAGCAAAAA	TTTGGCAGCACTTCTATTGTG	60.3	738	DRR	Ca_11009	--	bHLH
Ca4_P4_41012767	Ca4	41012767	AAA/AAACAA	TTCCCAAATGGGTTTTTCTT	GGAGGAGGAGGAGGATCAAC	58.4	540	DRR	Ca_11009	--	bHLH
Ca4_P4_4103051	Ca4	4103051	CA/CAAA	AACATGGCAATTGATTGAAGG	AGTCCAACTTCATTGCCTGC	59.8	496	DRR	Ca_03844	--	--
Ca4_P4_4143078	Ca4	4143078	CTT/CTTCTGTT	ATCCATCCAATGATTCCCAA	TGCTGATCTGACTTTGCCAC	60.0	746	DRR	Ca_03842	--	--
Ca4_P4_41808321	Ca4	41808321	ATTT/ATTTT	TGCAAAACTAGCCGAGTGAA	GGGTCAGTGCCATTATTGCT	59.6	908	DRR	Ca_10934	QR	B3
Ca4_P4_41808826	Ca4	41808826	CAAAAA/CAAAAAA	TCCAACCAAATTGTGCAAGA	AAGGAGTTGAAAGTGTCATCCA	60.1	153	DRR	Ca_10934	QR	B3

INDEL marker IDs	Chromosomes/ unanchored scaffolds	Physical positions (bp)	InDels (*Kabuli* reference genome-CDC Frontier/PII)	Forward primers (5'-3')	Reverse primers (5'-3')	Anneling temperature (0C)	Expected amplified product size (bp)	Structural annotation		Functional annotation	
								Sequence components of *kabuli* genome	*Kabuli* gene accession IDs	NCBI-KOG	TFs
Ca4_P4_4196538	Ca4	4196538	ATTTT/ATTT	ATTGGCCGGAAATTGTACTG	TAACCCAAGGCCATATCCTG	59.8	841	DRR	Ca_03838	R	--
Ca4_P4_4196827	Ca4	4196827	AT/A	TGCACGTGATCACAAAACCT	AATGTGTGGGCACTGATTGA	60.2	349	DRR	Ca_03838	R	--
Ca4_P4_41984896	Ca4	41984896	ACTCT/ACTCTCT	TCCGCTTTCTTTTGGGTATG	AATCCTCACACCCTACGTGC	60.1	845	DRR	Ca_10923	B	NF-YC
Ca4_P4_42118929	Ca4	42118929	GTAT/GTATAT	GCAGAAGTTGGCTTCTTTGG	TCACGGCATAAGGTTTGTCA	60.0	677	DRR	Ca_10911	R	Nin-like
Ca4_P4_42159815	Ca4	42159815	TTATATATATATATATATATATATAT/TTATATATATATATATATATATAT	CGGGCATCGATTTGTCTATT	GCAAATTCCAAAATCTAAAAGCC	59.9	413	DRR	Ca_10909	L	--
Ca4_P4_4242821	Ca4	4242821	A/ATCTG	TCCGTTTCAATCATCGTTCA	CACAAATAAGCACCTCCCAAA	60.0	765	DRR	Ca_03833	I	--
Ca4_P4_42772443	Ca4	42772443	TATAATAATAATAATAATAATAATAATAATAATAATAATA/TATAATAATAATAATAATAATAATAATAATAATA	CGAGCAGTGCACCAATTCTA	GCCAAGCCAACCACTATCAT	60.0	753	DRR	Ca_10886	--	SRS
Ca4_P4_4324103	Ca4	4324103	TTTTAAAATTTAAAATTTAAAAT/TTTTAAAATTTAAAAT	CCAATGGCATCACTCTTTCA	ATCGGATCCCATTTTGAGGT	59.6	631	DRR	Ca_03827	K	--
Ca4_P4_43320740	Ca4	43320740	TCA/T	CCTGGAATGGTTGTTACGCT	TCCCTTGTTGGGTTTCAAAG	60.0	279	DRR	Ca_23683	D	M-type
Ca4_P4_43323630	Ca4	43323630	TATAA/TA	GCAACAACTATTTGTCTTGTGTGC	CAAATGATTGTCACGAAAGAGTT	61.0	326	DRR	Ca_23684	--	bHLH

INDEL marker IDs	Chromosomes/ unanchored scaffolds	Physical positions (bp)	InDels (*Kabuli* reference genome-CDC Frontier/PII)	Forward primers (5'-3')	Reverse primers (5'-3')	Anneling temperature (0C)	Expected amplified product size (bp)	Structural annotation		Functional annotation	
								Sequence components of *kabuli* genome	*Kabuli* gene accession IDs	NCBI-KOG	TFs
Ca4_P4_43346458	Ca4	43346458	CA/CAAACTATAA	GGGCAAGAGTCCCCATTATT	CTACATTTGCGCCTCTGTCA	60.2	657	DRR	Ca_23685	--	S1Fa-like
Ca4_P4_43450204	Ca4	43450204	A/AT	TTCGTGTCAGCAAGACCAAG	AGGGAGATTCTTGGAGAGAGC	60.0	194	DRR	Ca_23690	--	--
Ca4_P4_44522624	Ca4	44522624	GAAAAAA/GAAAAAAA	GAAGTTGCTTGCTCCGATTC	CCCTAGCAAGTGCTAACCCA	60.0	675	DRR	Ca_09124	--	ERF
Ca4_P4_44522868	Ca4	44522868	A/AG	ACAATCCCTTCGTTCCCTCT	CCCTAGCAAGTGCTAACCCA	59.9	516	DRR	Ca_09124	--	ERF
Ca4_P4_44532358	Ca4	44532358	TAAAAAAA/TAAAAAAAA	CTTCATGGCTGATGAGTGGA	CCGATGAACATTCACCGATT	59.8	143	DRR	Ca_09125	L	--
Ca4_P4_44568994	Ca4	44568994	ATTACTCTCA/ATTACTCTCACGTTTTTTTTTACTCTCA	CGGAATTGGGATGAGAAAGA	AGACTGCAGTTCCTGGGCTA	60.0	411	DRR	Ca_09127	P	--
Ca4_P4_4458327	Ca4	4458327	CAAAAAAAAAA/CAAAAAAAAA	GGAGTGATCGGATGTGTGTG	AATAGTGGGGAGGGAACCAG	60.0	709	DRR	Ca_03811	--	--
Ca4_P4_4459373	Ca4	4459373	TAA/TA	AGCGAAGGCCTCTGTAGTCA	GGCCAAGCATTGTCGTTATT	60.2	439	DRR	Ca_03811	--	--
Ca4_P4_44629912	Ca4	44629912	A/ACGTTAAAG	TTGGATTGGGACTGTCCTTC	ATCACAGCCACATAGAGGGG	59.9	613	DRR	Ca_09133	--	WRKY
Ca4_P4_44638085	Ca4	44638085	TC/T	CAATGATGGCACACATCACA	TTCTTGATCATTGTTTATGACGC	60.0	377	DRR	Ca_09135	R	MYB

INDEL marker IDs	Chromosomes/ unanchored scaffolds	Physical positions (bp)	InDels (*Kabuli* reference genome-CDC Frontier/PII)	Forward primers (5'-3')	Reverse primers (5'-3')	Anneling temperature (0C)	Expected amplified product size (bp)	Structural annotation		Functional annotation	
								Sequence components of *kabuli* genome	*Kabuli* gene accession IDs	NCBI-KOG	TFs
Ca4_P4_44654366	Ca4	44654366	G/GGA	CCCGTACACACCATGTATTCC	ATGGTGCTGGCTTTGGTTAT	60.0	813	DRR	Ca_09137	R	NF-YA
Ca4_P4_4465836	Ca4	4465836	CAAAAAAAAA/CAAAAAAAAAAA	TAGATTTCGAGGAGGGAGGG	GTCATACGTGTCACATCGCC	60.5	491	DRR	Ca_03810	GOT	--
Ca4_P4_44670060	Ca4	44670060	AC/ACC	TGCCTAAGACCCCAAATCAA	GCCACTTCTGGAATCACCAT	60.4	682	DRR	Ca_09140	E	--
Ca4_P4_4480380	Ca4	4480380	TC/TCC	TGGTCACATGGAGGATGAAA	TTTAATACCTCGTATTTCCTATCAAGA	59.9	609	DRR	Ca_03809	--	S1Fa-like
Ca4_P4_4480858	Ca4	4480858	AATGAAAGT/A	TTGGTTAAACAAAATTGACTTACTTGA	GGCCTTGAGAAAATTTGATTTC	59.5	574	DRR	Ca_03809	--	S1Fa-like
Ca4_P4_4481135	Ca4	4481135	TAA/TAAA	TGAAATCAAATTTTCTCAAGGC	AAACGACATTACGTTATTTCTTCATT	58.4	120	DRR	Ca_03809	--	S1Fa-like
Ca4_P4_4481876	Ca4	4481876	A/ATAT	GGAGGTAGAAGAGGCGGAAC	AGCAAACGGAAAGAGCAATC	60.2	520	DRR	Ca_03809	--	S1Fa-like
Ca4_P4_44830692	Ca4	44830692	TAGAAGAAG/TAGAAG	AAGACGCCAAAGTTCGAAGA	CCACACTGCTGAAGGTCAAA	60.0	311	DRR	Ca_09148	K	--
Ca4_P4_44856654	Ca4	44856654	GTGTTACAT/GTGTTACATCTCTGTTACAT	CGTTGTGGCCATAATGTTGA	CTGTCTCTGAAAAGACCGCC	60.4	491	DRR	Ca_09150	DT	--
Ca4_P4_44856846	Ca4	44856846	AT/ATGTTT	TCTAGCAGCTCCTTCCTTGC	TCATCTCAATTTGGTTGGCA	59.9	616	DRR	Ca_09150	DT	--

INDEL marker IDs	Chromosomes/ unanchored scaffolds	Physical positions (bp)	InDels (*Kabuli* reference genome-CDC Frontier/PII)	Forward primers (5'-3')	Reverse primers (5'-3')	Anneling temperature (0C)	Expected amplified product size (bp)	Structural annotation		Functional annotation	
								Sequence components of *kabuli* genome	*Kabuli* gene accession IDs	NCBI-KOG	TFs
Ca4_P4_4487000	Ca4	4487000	TAAA/TAAAA	GGCCAAATGAAGGACTCAAA	GAATCGTTCGGGAGAGTCTG	60.1	672	DRR	Ca_03808	--	--
Ca4_P4_4487086	Ca4	4487086	AT/ACAGAAATATATT	GGCCAAATGAAGGACTCAAA	CGTACGACGTCTTTCGTCAA	60.1	932	DRR	Ca_03808	--	--
Ca4_P4_4487144	Ca4	4487144	CAAAAA/CAAAAAA	GAAGTTCAACTGGCACCACA	CGTACGACGTCTTTCGTCAA	59.7	647	DRR	Ca_03808	--	--
Ca4_P4_4487412	Ca4	4487412	AT/ATTAATAAAGT	TCAGACTCTCCCGAACGATT	CGTACGACGTCTTTCGTCAA	59.8	281	DRR	Ca_03808	--	--
Ca4_P4_44901004	Ca4	44901004	GT/GTGTAT	AGCTTCAAGTGGCTGAAGGA	ATGAACACGGGCGTATCTTC	60.1	527	DRR	Ca_09152	--	C2H2
Ca4_P4_44902306	Ca4	44902306	ATTTTTT/ATTTTTTT	TTGGGCAATTTTAAGCCAAA	TTATTTGTGTGTTGCAGCGTC	60.4	765	DRR	Ca_09152	--	C2H2
Ca4_P4_44917117	Ca4	44917117	A/ACAACAACAACAC	TTCCACCTTCAATGCAACAA	TCAGGCACACATTTTTCACC	60.1	235	DRR	Ca_09155	Q	--
Ca4_P4_44917486	Ca4	44917486	AATATATATATA/AATATATATATATA	TTCCACCTTCAATGCAACAA	TTTGGAATTGGTCCAAGAGC	60.1	899	DRR	Ca_09155	Q	--
Ca4_P4_44944900	Ca4	44944900	TAAAA/TAAA	GGGAAACAAATTCGTGGAAA	ACATGCCAAATGCAATGTGT	59.8	388	DRR	Ca_09157	G	--
Ca4_P4_4500366	Ca4	4500366	ATTTTTTTT/ATTTTTTTTT	CTCGAGTGCACTGTCGTCAT	CGGCCAAGTCTTCATATCGT	60.1	783	DRR	Ca_03807	--	FAR1

INDEL marker IDs	Chromosomes/ unanchored scaffolds	Physical positions (bp)	InDels (*Kabuli* reference genome-CDC Frontier/PII)	Forward primers (5'-3')	Reverse primers (5'-3')	Anneling temperature (0C)	Expected amplified product size (bp)	Structural annotation		Functional annotation	
								Sequence components of *kabuli* genome	*Kabuli* gene accession IDs	NCBI-KOG	TFs
Ca4_P4_4500439	Ca4	4500439	AC/ACC	CTCGAGTGCACTGTCGTCAT	CGGCCAAGTCTTCATATCGT	60.1	783	DRR	Ca_03807	--	FAR1
Ca4_P4_4501473	Ca4	4501473	CT/CTT	TGTACGGAGCCTCCAAAAAT	TGTCGGAAAACATTGGTGAA	59.6	576	DRR	Ca_03807	--	FAR1
Ca4_P4_45024273	Ca4	45024273	AATTATTATTATTATTATTATTATTATTATTATTATTATT/AATTATTATTATTATTATTATTATTATTATTATTATT	CATGCACCTTGGTGACAACT	CGACACCTCCTTTTCCAATTT	59.6	373	DRR	Ca_09165	D	bHLH
Ca4_P4_45051417	Ca4	45051417	TAAAAAAAA/TAAAAAAA	CGTTCGAAGGAAACACACCT	CATTTGGTACAGAGGCATGG	60.1	795	DRR	Ca_09167	K	GATA
Ca4_P4_45107861	Ca4	45107861	CATAT/CAT	TGTGAGGGTGGGAAAAGAAC	TGCTTGGCTCACTGTTTCTG	59.9	512	DRR	Ca_09170	--	--
Ca4_P4_45114505	Ca4	45114505	AT/ATT	TGATTAATCTTTTTCGCCCG	AATGGTGCAAATGAATGCAA	60.0	388	DRR	Ca_09171	--	MYB_related
Ca4_P4_45122277	Ca4	45122277	TAA/TAAA	GCGAGCGTATTGGTATTGGT	ATGTGACAAATTGGGGGAAA	60.0	551	DRR	Ca_09172	T	--
Ca4_P4_45122635	Ca4	45122635	CG/C	CCCCCAATTTGTCACATTCT	GACCCTCTCTACACCCCCTC	59.6	379	DRR	Ca_09172	T	--
Ca4_P4_45135433	Ca4	45135433	GACACTAACACT/GACACT	CATTCTGCGAGACACGAAAA	CATGCTGTGACTCGAAAGGA	60.0	661	DRR	Ca_09175	S	--
Ca4_P4_45138231	Ca4	45138231	CATTATATTATATTATATT/CATTATATTATATT	ACCACACTCATGCAACGAAA	AATAAATCGCAGGGCTTGTG	60.2	654	DRR	Ca_09177	T	--

INDEL marker IDs	Chromosomes/ unanchored scaffolds	Physical positions (bp)	InDels (*Kabuli* reference genome-CDC Frontier/PII)	Forward primers (5'-3')	Reverse primers (5'-3')	Anneling temperature (0C)	Expected amplified product size (bp)	Structural annotation		Functional annotation	
								Sequence components of *kabuli* genome	*Kabuli* gene accession IDs	NCBI-KOG	TFs
Ca4_P4_45165137	Ca4	45165137	A/AC	TTGTTCTTCCTCCCCCTTCT	TTCAGCCCGCTAGCTATGAT	60.0	669	DRR	Ca_09179	P	--
Ca4_P4_45199986	Ca4	45199986	TAAAA/TAAAAA	TATGGGAATGAGGAGGGTGA	TGGGAGTTAGGAGCCAGAGA	60.3	199	DRR	Ca_09182	--	--
Ca4_P4_45210841	Ca4	45210841	CAAA/CA	TGGATCTTTTGTTGTCCCCT	ATGCTAAGCCACCAACCAAC	59.4	680	DRR	Ca_09183	O	--
Ca4_P4_45211124	Ca4	45211124	TAAAAAAAA/TAAAAAAAAAAAA	TCCAATCTAAATTATCCCCCA	ATGCTAAGCCACCAACCAAC	58.3	396	DRR	Ca_09183	O	--
Ca4_P4_45221189	Ca4	45221189	GTTTTT/GTTTT	GCACTCAAAGAGCACCATCA	CTGGCTCCGAAATCTATTGG	60.0	505	DRR	Ca_09184	A	C2H2
Ca4_P4_45222131	Ca4	45222131	TA/TAA	CCACCTTGTTCCCACTCCTA	TCCAATGAATTTGGAGAGCC	60.0	334	DRR	Ca_09184	A	C2H2
Ca4_P4_45226893	Ca4	45226893	TTC/TTCGTC	ATTAGTTCCTGGCCGGTCTC	GGAGAGAGGACCCACAAACA	60.5	868	DRR	Ca_09185	S	--
Ca4_P4_45245580	Ca4	45245580	TAAA/TAA	TGCTTGTTGCACAAGTTTCC	AAAAACGCAGTATCCATACAAAA	59.9	617	DRR	Ca_09188	--	B3
Ca4_P4_45245680	Ca4	45245680	TAAAAAAAAA/TAAAAAAA	TGCTTGTTGCACAAGTTTCC	AAAAACGCAGTATCCATACAAAA	59.9	617	DRR	Ca_09188	--	B3
Ca4_P4_45261134	Ca4	45261134	ATTTTTTTTT/ATTTTTTTT	TACTCTACCATGCCCAAGGC	CATAAAGCGGGTGAAGGGTA	60.1	479	DRR	Ca_09189	O	--

INDEL marker IDs	Chromosomes/ unanchored scaffolds	Physical positions (bp)	InDels (*Kabuli* reference genome-CDC Frontier/PII)	Forward primers (5'-3')	Reverse primers (5'-3')	Anneling temperature (0C)	Expected amplified product size (bp)	Structural annotation		Functional annotation	
								Sequence components of *kabuli* genome	*Kabuli* gene accession IDs	NCBI-KOG	TFs
Ca4_P4_45262079	Ca4	45262079	ACCCC/ACCC	TGCATAGGTACACAAAGCCC	TGAAAAATGGTGACCGTTGA	58.7	292	DRR	Ca_09189	O	--
Ca4_P4_45273886	Ca4	45273886	CATGTGA/CA	ATGGAAGCATTGTCGGTCTC	TGTGTGGATTGTCGTTCGAG	60.1	617	DRR	Ca_09191	O	--
Ca4_P4_45274571	Ca4	45274571	T/TG	TCGAACGACAATCCACACAT	CGTGGGGGAAATTTTTCTTT	60.0	518	DRR	Ca_09191	O	--
Ca4_P4_45274816	Ca4	45274816	ATTTTTTTT/ATTTTTTTTT	TCGAACGACAATCCACACAT	TTAACACTTGGAAGGGCGAC	60.0	811	DRR	Ca_09191	O	--
Ca4_P4_45274881	Ca4	45274881	CTTT/CTT	AAAGAAAAATTTCCCCCACG	TTAACACTTGGAAGGGCGAC	60.2	313	DRR	Ca_09191	O	--
Ca4_P4_45322213	Ca4	45322213	CTTT/CTTTT	TGGATACCGCTACTTGGGAG	CACAAGGCAAGGGACAATTT	60.1	394	DRR	Ca_09197	--	--
Ca4_P4_45322675	Ca4	45322675	ATTTT/ATTTTTT	AAATTGTCCCTTGCCTTGTG	CCAAGGCAATTTCGTTGTTT	60.0	595	DRR	Ca_09197	--	--
Ca4_P4_4533290	Ca4	4533290	GAAAAA/GAAAA	AAATGCCCGCTATGAGTTTG	GGTGAAAAGAGTACGAGGGG	60.1	360	DRR	Ca_03805	P	--
Ca4_P4_45341287	Ca4	45341287	CATAT/CAT	TGCTCAAATGGATTGTTGTCA	TCCTCCTCTTTGTTTGTCCG	60.1	798	DRR	Ca_09199	F	--
Ca4_P4_45341608	Ca4	45341608	AAGAGAATCGGA/AAGAGAATCGGAGAGAATCGGA	GCTGGACAAGTCTGCCAAG	CTAGTGTGCAGTGGTGCCAT	59.6	874	DRR	Ca_09198	L	--

INDEL marker IDs	Chromosomes/ unanchored scaffolds	Physical positions (bp)	InDels (*Kabuli* reference genome-CDC Frontier/PII)	Forward primers (5'-3')	Reverse primers (5'-3')	Anneling temperature (0C)	Expected amplified product size (bp)	Structural annotation		Functional annotation	
								Sequence components of *kabuli* genome	*Kabuli* gene accession IDs	NCBI-KOG	TFs
Ca4_P4_45377587	Ca4	45377587	ATTTTTTTT/ATTTTTTT	CTCCATTGGTGGTGTCCTTT	GTCTGAGCGTTTGCATGGTA	59.8	821	DRR	Ca_09201	--	--
Ca4_P4_45377807	Ca4	45377807	AC/A	TCCCATCATGTTTTGATAGCC	GTCTGAGCGTTTGCATGGTA	59.8	603	DRR	Ca_09201	--	--
Ca4_P4_4543916	Ca4	4543916	TAAAAAAAAA/TAAAAAAAAAA	TAGGTCTTTCCCTGGGTGTG	TTAGGCGTGCTTCCTTCACT	60.0	384	DRR	Ca_03804	--	--
Ca4_P4_45513340	Ca4	45513340	ATTTT/ATTT	TACCCACGTTGAAGTCCTGG	TGTTTGAGTTGGTGTGGTTCA	60.9	336	DRR	Ca_09211	E	--
Ca4_P4_4567851	Ca4	4567851	AAAAGTT/A	GTGGGGCATTACGATACCAC	GGGGAAGCCATATCACATCA	60.1	636	DRR	Ca_03802	UZ	--
Ca4_P4_4568058	Ca4	4568058	ATTTGATTTT/ATTTGATTTTTGATTTT	GTGGGGCATTACGATACCAC	GGGGAAGCCATATCACATCA	60.1	636	DRR	Ca_03802	UZ	--
Ca4_P4_45690560	Ca4	45690560	TAAAAAAAAAAAAAA/TAAAAAAAAAAAA	CCAATCCCGCAAAATTTCTA	TAAACCAACCCCCAAATGAA	59.9	701	DRR	Ca_18393	--	--
Ca4_P4_45858707	Ca4	45858707	AAATA/AA	ATGTTGGTCCAAGCAGGAAG	CGCAGTTTTATGGTGGGTTT	60.1	716	DRR	Ca_18375	R	--
Ca4_P4_4591902	Ca4	4591902	CA/CACAA	TCAGACCTGCGTGCATACTC	TCTAATTGGCCGACAAATCC	60.0	207	DRR	Ca_03798	--	--
Ca4_P4_45931997	Ca4	45931997	CT/CTATAAT	CCAATAATTAGGGCGGTGTC	GCTGGTTTGACTCTGGGGTA	59.3	574	DRR	Ca_18367	--	--

**INDEL marker IDs**	**Chromosomes/ unanchored scaffolds**	**Physical positions (bp)**	**InDels (*Kabuli* reference genome-CDC Frontier/PII)**	**Forward primers (5'-3')**	**Reverse primers (5'-3')**	**Anneling temperature (0C)**	**Expected amplified product size (bp)**	**Structural annotation**		**Functional annotation**	
								**Sequence components of *kabuli* genome**	***Kabuli* gene accession IDs**	**NCBI-KOG**	**TFs**
Ca4_P4_4593540	Ca4	4593540	CTAATTAATTAA/CTAATTAA	TTGAGGAATGTAAGGATGTCCA	TTAATGGCGGCTACGTTTTC	59.4	928	DRR	Ca_03798	--	--
Ca4_P4_46057636	Ca4	46057636	AC/ACGAAGATGATC	CGCATCGGTAAACAATCTGA	CGATGCGTAGATCGACAAAA	59.7	154	DRR	Ca_18363	--	--
Ca4_P4_46137643	Ca4	46137643	AG/AGAAGG	TTATTCAATGGCTTAGGCGG	CTCCCGTGCAAAGGAAATAA	60.1	665	DRR	Ca_18358	--	G2-like
Ca4_P4_46422010	Ca4	46422010	ATTTTTTT/ATTTTTTTT	CACTGAAGTCCACGCACCTA	TCTTTCTTTCCCTCCCGATT	59.9	768	DRR	Ca_19272	--	WOX
Ca4_P4_46732510	Ca4	46732510	G/GCA	CGGTATAGCCTGGGAAATGA	GATGGAAGGAATGCCAAAGA	59.9	766	DRR	Ca_19245	P	--
Ca4_P4_46738774	Ca4	46738774	GTGTT/GTGTTGTT	CTAGGCTCACCAGCACCTTC	GAGCCATTTGAGACGCTTTC	60.0	318	DRR	Ca_19244	--	bZIP
Ca4_P4_46767409	Ca4	46767409	GTT/GT	GCCCAGCTCCTCTTACAGTG	ATGCTTTTCAGTGAGGTGGG	60.0	593	DRR	Ca_19242	R	bZIP
Ca4_P4_47195956	Ca4	47195956	TCAATA/T	GCTTTTGAGTCACGAGGGAG	TTCAAACTGCATGAACCTCG	60.0	387	DRR	Ca_10862	--	--
Ca4_P4_47211846	Ca4	47211846	TG/TGG	TTGAAATGCAACCTTGTCCA	AAGCCCCTGCTTTGATTTCT	60.1	774	DRR	Ca_10861	--	LBD
Ca4_P4_47233866	Ca4	47233866	CAAA/CAAACAAAA	ACAGACAAAGCCCAAAATGC	CATCACGGAATAGGGCAACT	60.1	331	DRR	Ca_10858	--	--

INDEL marker IDs	Chromosomes/ unanchored scaffolds	Physical positions (bp)	InDels (*Kabuli* reference genome-CDC Frontier/PII)	Forward primers (5'-3')	Reverse primers (5'-3')	Anneling temperature (0C)	Expected amplified product size (bp)	Structural annotation		Functional annotation	
								Sequence components of *kabuli* genome	*Kabuli* gene accession IDs	NCBI-KOG	TFs
Ca4_P4_47247298	Ca4	47247298	ATTTT/ATTT	GCGTTGGGAAATACCAAGAA	GCCAAACAACACAAGCATGA	59.9	663	DRR	Ca_10857	--	B3
Ca4_P4_47277224	Ca4	47277224	TGGCTCGGCTCGGCTCGGCTC/TGGCTCGGCTCGGCTC	GGACCCATTTGGGACCTTAT	CTGCCCAGTAAGCACCAACT	59.9	657	DRR	Ca_10854	--	G2-like
Ca4_P4_47425741	Ca4	47425741	TTATATATATATATATATATAT/TTATATATATATATATATATATATAT	TGACACACACATGAAATGCG	CAGACACAAATTGCTTTCGC	60.2	312	DRR	Ca_10845	R	ERF
Ca4_P4_47597511	Ca4	47597511	TAA/TAAA	GGTACAATTGTTGCACCACG	TTTCAATGCGTCAAAAGCAG	59.9	666	DRR	Ca_10829	S	--
Ca4_P4_47852567	Ca4	47852567	TAAAAAAAAA/TAAAAAAAA	AGCAGAGCAAAATTTGGTGG	TGGTATTGGTCCAACAACCTG	60.2	483	DRR	Ca_10809	O	MYB_related
Ca4_P4_47966378	Ca4	47966378	CTTTTTTTTTT/CTTTTTTTT	AGCAAATTGGGAACCTTGTG	CGAGAAACCCATGAGGAAAA	60.0	361	DRR	Ca_10792	--	B3
Ca4_P4_47991117	Ca4	47991117	GTTTTTT/GTTTTTTTT	ATTAGCCACGACGATGGTTC	TGGTGTACATGTAGGCGTGC	60.0	564	DRR	Ca_10789	--	B3
Ca4_P4_47991306	Ca4	47991306	GCA/GTTCACA	TAATTGCAAGTCATTGCCCA	CGAGTTGATGTGATTGGTGG	60.1	805	DRR	Ca_10789	--	B3
Ca4_P4_48042400	Ca4	48042400	AATATATATATATATATATATATATATATAT/AATATATATATATATATATATATATATATATAT	TTTTGGTTCACATTCACCTGAT	GGGCAAACCCCTCAGAAATA	59.3	885	DRR	Ca_10784	--	MYB_related
Ca4_P4_48226194	Ca4	48226194	AGT/AGTGT	AAAGAAAAAGGGGGTGATGG	GGAAGCCATTACCAACCAAA	60.2	251	DRR	Ca_10766	--	DBB

INDEL marker IDs	Chromosomes/ unanchored scaffolds	Physical positions (bp)	InDels (*Kabuli* reference genome-CDC Frontier/PII)	Forward primers (5'-3')	Reverse primers (5'-3')	Anneling temperature (0C)	Expected amplified product size (bp)	Structural annotation		Functional annotation	
								Sequence components of *kabuli* genome	*Kabuli* gene accession IDs	NCBI-KOG	TFs
Ca4_P4_48265483	Ca4	48265483	ACTAAATTATTTGTTCTAAATTATTT/ ACTAAATTATTT	TCTTGTATTTACACACC CCAACA	GATGACGGAGCCACAA AGAT	59.3	438	DRR	Ca_10761	K	B3
Ca4_P4_48297107	Ca4	48297107	AAATTTTAAT/AAATTTTAATTTTAAT	AACAAACACATTTGTG GCGA	TTCTGACTGTTTAGGC CGCT	60.0	678	DRR	Ca_10759	--	--
Ca4_P4_48303620	Ca4	48303620	TA/TAA	AAGTGCAAAAAGTTGA GACGC	ATTGCATTGTGTGTTTG GGA	59.6	631	DRR	Ca_10757	B	C3H
Ca4_P4_48303926	Ca4	48303926	AT/ATT	AAAACCCGTACAACGG ATGA	CGAAGTTCCAAAACCA CTGC	60.2	756	DRR	Ca_10757	B	C3H
Ca4_P4_48314680	Ca4	48314680	ATTT/ATTTTT	GTAATGCGTTTGACCT GCAA	ATAGGCGCATCTCAAT CACC	59.7	649	DRR	Ca_10756	--	BBR-BPC
Ca4_P4_48314877	Ca4	48314877	TA/T	TGCGCCTATCTCTCTG TGTG	TTCGATCCATTTTACCA CCC	60.2	365	DRR	Ca_10756	--	BBR-BPC
Ca4_P4_48323095	Ca4	48323095	AACACACACACA/AACACACACACA CA	GCAGCCAAACCAGCTT AGAC	AATTTGCAAAAATTCGG CTG	60.0	515	DRR	Ca_10754	Q	GeBP
Ca4_P4_48331462	Ca4	48331462	TTATCTCCCTTAAGATAT/TTAT	CAGCTTGAAGTCCTTG CTCC	GTGGCCGTTTAATGCA CTTT	60.1	834	DRR	Ca_10753	J	--
Ca4_P4_48332874	Ca4	48332874	C/CAA	TTAGCTGTGGAAGGGC ATTT	AACTGGCAGATTCATC ACACC	59.7	590	DRR	Ca_10753	J	--
Ca4_P4_48365846	Ca4	48365846	GC/G	GTTGGCCTGTTAGGCA TTGT	GTGCTTGTTTCGGTGG AGAT	60.0	274	DRR	Ca_10750	A	bHLH

INDEL marker IDs	Chromosomes/ unanchored scaffolds	Physical positions (bp)	InDels (*Kabuli* reference genome-CDC Frontier/PII)	Forward primers (5'-3')	Reverse primers (5'-3')	Anneling temperature (0C)	Expected amplified product size (bp)	Structural annotation		Functional annotation	
								Sequence components of *kabuli* genome	*Kabuli* gene accession IDs	NCBI-KOG	TFs
Ca4_P4_4839789	Ca4	4839789	CTTT/CTTTTTTTT	ACGTAATACTTTGTGG CGGC	TCTCTCCTTTGTGACG GCTT	60.0	138	DRR	Ca_03775	--	--
Ca4_P4_48459296	Ca4	48459296	CATAAT/CATAATATAAT	CGAGTGGGTGAGGGA GATTA	GCCCTAAAGCCACCCA CTAT	60.1	381	DRR	Ca_10747	--	--
Ca4_P4_48459552	Ca4	48459552	AT/ATT	TGGTTGGCAGTAAGAT GCAA	CGTTTGTTTGACGAGC ACAA	60.3	649	DRR	Ca_10747	--	--
Ca4_P4_48500161	Ca4	48500161	TA/T	GAAGTACCTGCACGGG TGAT	TTTGGAGGGAAAGGGA AAGT	60.0	234	DRR	Ca_10742	V	GRAS
Ca4_P4_48673700	Ca4	48673700	GAAAAAAAAAA/GAAAAAAAAAAAA	TACCGAACATGCGGTG TAAG	TGACAAAAAGGAGAAG ATAATGC	59.6	736	DRR	Ca_23017	--	--
Ca4_P4_48674038	Ca4	48674038	ATTTT/ATTTTT	TCCTCTGTGTGTGACA ACCC	TGACCGAATCAAAAAG AAGAAAA	59.6	694	DRR	Ca_23017	--	--
Ca4_P4_48708348	Ca4	48708348	TAAAAAA/TAAAAAAA	GCGCTAGAGGAACTGG ATTG	GGCGATGAATTGGTTT GTTT	60.0	944	DRR	Ca_23020	Q	Nin-like
Ca4_P4_48708876	Ca4	48708876	TTTTATTT/TTTT	CGCCACTTGTATTTCAA GCA	GCATCAGCTCTTCCAG CTTC	59.9	517	DRR	Ca_23020	Q	Nin-like
Ca4_P4_48718705	Ca4	48718705	GATATATATATATATATATATATA TAT/GATATATATATATATATATAT ATATATAT	CGAGAGTGTCCAAAGT GCAA	GCTGGTGGAGAAGGTT TTGA	60.0	576	DRR	Ca_23022	--	--
Ca4_P4_48834023	Ca4	48834023	AATA/AATATA	ACTCTAGACCATCGAC GGCA	GAACACACCAAAAGCT CCGT	60.8	398	DRR	Ca_23026	--	FAR1

INDEL marker IDs	Chromosomes/ unanchored scaffolds	Physical positions (bp)	InDels (*Kabuli* reference genome-CDC Frontier/PII)	Forward primers (5'-3')	Reverse primers (5'-3')	Anneling temperature (0C)	Expected amplified product size (bp)	Structural annotation		Functional annotation	
								Sequence components of *kabuli* genome	*Kabuli* gene accession IDs	NCBI-KOG	TFs
Ca4_P4_4894873	Ca4	4894873	TAAAAAAAA/TAAAAAAA	GACTTGGAGCGAAGGTGAAA	GCAATGTTGCGAGAACAAGA	60.4	705	DRR	Ca_03768	K	--
Ca4_P4_4907090	Ca4	4907090	CTTT/CTTTT	ATTGCATCCCAATCCCATAA	TTAGTCGAGTGTTTGGTGCG	60.0	266	DRR	Ca_03767	F	--
Ca4_P4_4908744	Ca4	4908744	A/ACCGT	CACGAACAAAATCAAGCGAA	ATTGCGTGGGAAAAAGTTTG	59.8	529	DRR	Ca_03767	F	--
Ca4_P4_4908883	Ca4	4908883	T/TA	CAAACTTTTTCCCACGCAAT	TCATCATTGTGCCGTCATTT	60.0	318	DRR	Ca_03767	F	--
Ca4_P4_49089991	Ca4	49089991	AGC/AGCGC	GCTTCCATGCACAGAAGTGA	TGAGAGCACGAAAGGATGTG	60.0	190	DRR	Ca_20778	R	MYB
Ca4_P4_5182858	Ca4	5182858	TATAATAATAATAATAATAATAA/TATAATAATAATAATAATAA	AGGCCAGACCAGACTTTGAA	CAGTGGCTTATGGCCTGACT	59.8	606	DRR	Ca_03735	E	--
Ca4_P4_5469578	Ca4	5469578	CGTTTAGTTTAGTTTAGTTT/CGTTTAGTTTAGTTTAGTTTAGTTT	TCCATTGACCAAAGCATCAA	GCGAATCGAAAACAAGTGGT	60.0	290	DRR	Ca_03705	P	--
Ca4_P4_582760	Ca4	582760	T/TATGGTGCTACTC	CAGGTGACTCATCCCGATTT	CGCAATTTCAGTGAAGCAAA	59.9	591	DRR	Ca_07688	O	FAR1
Ca4_P4_6139965	Ca4	6139965	GTTTTTTTTTTTTT/GTTTTTTTTTTTT	AATCACATTCCAGTCCTCCG	GGGTTGTGGCTCTCCATTTA	59.9	788	DRR	Ca_03629	--	--
Ca4_P4_6790926	Ca4	6790926	AG/AGG	GAAGCACCCCATGTTCCTAA	AAATGACAAGTTCTACCGCGA	59.9	956	DRR	Ca_03562	QI	bHLH

INDEL marker IDs	Chromosomes/ unanchored scaffolds	Physical positions (bp)	InDels (*Kabuli* reference genome-CDC Frontier/PII)	Forward primers (5'-3')	Reverse primers (5'-3')	Anneling temperature (0C)	Expected amplified product size (bp)	Structural annotation		Functional annotation	
								Sequence components of *kabuli* genome	*Kabuli* gene accession IDs	NCBI-KOG	TFs
Ca4_P4_7180740	Ca4	7180740	TT/TTGT	TGAAGGTGCTCAACAAGGTG	GGCAATCATCCATGGAACTC	59.9	472	DRR	Ca_03517	--	GRAS
Ca4_P4_730137	Ca4	730137	TTATATATATATATATATATAT/TTATATATATATATATATAT	GTCAACCTTGGTGCTTGGTT	AAGTATTTCCGCCTGTCACG	60.0	775	DRR	Ca_07702	G	--
Ca4_P4_7584272	Ca4	7584272	AATATATATATATATATATATATATATAT/AATATATATATATATATATATATATATATATATATAT	AAAGTGCACCACACAAGTGC	TTTGTAAACGGTTAAGGGCAA	59.8	644	DRR	Ca_03478	--	--
Ca4_P4_7825515	Ca4	7825515	TAAAAAAAAAAA/TAAAAAAAAAAAA	TGCACAAAAATAAAAACATTTCAA	AAAGGATGGAAGCCCATTTT	58.7	959	DRR	Ca_03457	G	NAC
Ca4_P4_8167017	Ca4	8167017	CAAAA/CAAA	GGGTGGGTTTAGAGGACCAT	TTTCTTGAATGGGCAAAACC	60.1	259	DRR	Ca_08305	V	GRAS
Ca4_P4_8167238	Ca4	8167238	ATCTT/ATCTTCTT	GGGTGGGTTTAGAGGACCAT	TGAAGCTTTTTGCTACAAGGAA	60.1	737	DRR	Ca_08305	V	GRAS
Ca4_P4_8167347	Ca4	8167347	ATATTATTTTTA/ATATTATTTTTATTATTTTTA	GGGTGGGTTTAGAGGACCAT	TGAAGCTTTTTGCTACAAGGAA	60.1	737	DRR	Ca_08305	V	GRAS
Ca4_P4_8184653	Ca4	8184653	CTTTTTTTT/CTTTTTTT	GCTGTCTTTTTCGTCCTGCT	GTATTGGCCAACCAGACCAC	59.6	856	DRR	Ca_08308	T	--
Ca4_P4_8193266	Ca4	8193266	TAAAAAAAAA/TAAAAAAAAAA	GCATCTCTCCAAGATGTCTGC	TGGGAATTGGGAGCTATCAG	60.0	620	DRR	Ca_08309	R	--
Ca4_P4_8203373	Ca4	8203373	ATTTCTTTCTTTCTTTCT/ATTTCTTTCTTTCT	CGTGGAAGGATAGAAATCGG	GCAGCTTCTGAATGGAAACC	59.5	536	DRR	Ca_08310	T	MYB_related

INDEL marker IDs	Chromosomes/ unanchored scaffolds	Physical positions (bp)	InDels (*Kabuli* reference genome-CDC Frontier/PII)	Forward primers (5'-3')	Reverse primers (5'-3')	Anneling temperature (0C)	Expected amplified product size (bp)	Structural annotation		Functional annotation	
								Sequence components of *kabuli* genome	*Kabuli* gene accession IDs	NCBI-KOG	TFs
Ca4_P4_8221604	Ca4	8221604	TAAAAAA/TATTAAAAAAAA	AACACAAGGAGGGAAACACG	CAAGAAACTGCCAATGCAAA	60.0	644	DRR	Ca_08311	T	bHLH
Ca4_P4_8222289	Ca4	8222289	TAAAAAAAAAA/TAAAAAAAAAAA	TTTGCATTGGCAGTTTCTTG	CCGCAACAACAAAGACAGAA	59.8	609	DRR	Ca_08312	--	--
Ca4_P4_8244322	Ca4	8244322	TT/TTCT	GCATTTCTGGTCGGTTTGTT	ACACCACACAAAGGCAGTGA	60.0	164	DRR	Ca_08315	S	--
Ca4_P4_8245965	Ca4	8245965	GATATATATATATAT/GATATATATATATATATATATATATAT	TCAAATTTTCTTATGCGGGC	TGAGGAAACACCACCACGTA	60.0	790	DRR	Ca_08314	KB	--
Ca4_P4_8289104	Ca4	8289104	CAA/CA	GCGGGGTTCAAATCCTAAAT	ATAGACGCCCCAAGGTATCC	60.2	355	DRR	Ca_08316	--	NAC
Ca4_P4_8292742	Ca4	8292742	GATA/GATATATA	AGAGAAAGGGACGAGCATCA	AGGCATTTTCGTGACGAGTT	60.0	649	DRR	Ca_08317	--	--
Ca4_P4_8301399	Ca4	8301399	GCAACCTAATGTTCAACCTAA/GCAACCTAA	CACAATGATGTTTGGTTCGG	AGGGGTGTTTACGCATTGAG	59.8	483	DRR	Ca_08318	S	--
Ca4_P4_8301438	Ca4	8301438	AG/A	CACAATGATGTTTGGTTCGG	AGGGGTGTTTACGCATTGAG	59.8	483	DRR	Ca_08318	S	--
Ca4_P4_8335376	Ca4	8335376	ATTTT/ATTT	CGACAAAAAGTGAGGGGAAA	GCAATGCGTTGTCTGTGTCT	60.1	192	DRR	Ca_08321	--	--
Ca4_P4_8344606	Ca4	8344606	TAAAAAAA/TAAAAAA	GAGCAGAACGAGTGAAAGGG	CTCCTTCGCATTTCGTCTTC	60.0	545	DRR	Ca_08322	--	--

INDEL marker IDs	Chromosomes/ unanchored scaffolds	Physical positions (bp)	InDels (*Kabuli* reference genome-CDC Frontier/PII)	Forward primers (5'-3')	Reverse primers (5'-3')	Anneling temperature (0C)	Expected amplified product size (bp)	Structural annotation		Functional annotation	
								Sequence components of *kabuli* genome	*Kabuli* gene accession IDs	NCBI-KOG	TFs
Ca4_P4_8345457	Ca4	8345457	T/TA	TCTTTGCTTTCAACGCTGTTT	AGGAGGTGCATCGAGGTATG	60.0	915	DRR	Ca_08322	--	--
Ca4_P4_8351185	Ca4	8351185	TAAAAAAAA/TAAAAAAA	ATTTTCGGTTTCCCTTCCAC	CGATTTCCCTTGGTCCAAAT	60.2	960	DRR	Ca_08323	--	--
Ca4_P4_8354913	Ca4	8354913	ATTTTTTTTTT/ATTTTTTTT	TTGATTTGGGGGCTTTGATA	GGAGCAGTTGTCCCATTTGT	60.3	765	DRR	Ca_08324	T	bZIP
Ca4_P4_8355635	Ca4	8355635	T/TC	GGACAACTGCTCCCTCTTGA	TTGGGAATCGGTGAAAACTT	60.4	857	DRR	Ca_08324	T	bZIP
Ca4_P4_8377254	Ca4	8377254	ATTTTTTTTTTT/ATTTTTTTTTTTTT	CCACATATATCTGCCCCACC	TGACTGTGTGCACTGAAACAA	60.0	563	DRR	Ca_08327	KL	--
Ca4_P4_8377484	Ca4	8377484	ATTTTTTTTTT/ATTTTTTTT	TTCGTCCATTTGAATCCTGA	GGGTGAGACTGAAGCTGGTC	59.0	720	DRR	Ca_08327	KL	--
Ca4_P4_8377585	Ca4	8377585	ATTT/ATT	TTCGTCCATTTGAATCCTGA	CCTGCAAGCACTTACGACAA	59.0	891	DRR	Ca_08327	KL	--
Ca4_P4_8396244	Ca4	8396244	CAAA/CA	TTTACAACACATTGCGCACC	CGTTTATTGACGGAAGGCAT	60.6	681	DRR	Ca_08328	R	AP2
Ca4_P4_8417735	Ca4	8417735	ATTTTT/ATTTT	CACAGGGGAATAAGGTTGGA	ACAAGAGGCGGAGACAGGTA	59.8	862	DRR	Ca_08331	--	ERF
Ca4_P4_8430074	Ca4	8430074	AAT/AATAT	TTTTGGCATGAAAAAGGGAC	GAAATTTACCGCTTGACCGA	59.9	772	DRR	Ca_08334	--	--

INDEL marker IDs	Chromosomes/ unanchored scaffolds	Physical positions (bp)	InDels (*Kabuli* reference genome-CDC Frontier/PII)	Forward primers (5'-3')	Reverse primers (5'-3')	Anneling temperature (0C)	Expected amplified product size (bp)	Structural annotation		Functional annotation	
								Sequence components of *kabuli* genome	*Kabuli* gene accession IDs	NCBI-KOG	TFs
Ca4_P4_8449229	Ca4	8449229	ATTTTTTTTT/ATTTTTTTT	TGGAGTTTTAGCCACTTCCA	TCTCGCCAAATATCTACAATCG	58.4	337	DRR	Ca_08335	R	--
Ca4_P4_8449477	Ca4	8449477	TG/T	TGGAGTTTTAGCCACTTCCA	TCTCGCCAAATATCTACAATCG	58.4	337	DRR	Ca_08336	--	--
Ca4_P4_8454111	Ca4	8454111	T/TTTTC	TTCTTTGGGTCTCCCAACAC	TTATGAGCTTTGGATTGGGC	59.9	631	DRR	Ca_08337	R	MYB
Ca4_P4_8483192	Ca4	8483192	A/ACT	ATTTCGAGGAAAAGGGAGGA	GACCCTATCAGAGTGGCAGC	60.0	886	DRR	Ca_08340	R	--
Ca4_P4_8499889	Ca4	8499889	AG/A	TTCCCAAATTTCCTGCATCT	AGCCATACTTCAATGGTGCC	59.5	958	DRR	Ca_08342	C	--
Ca4_P4_8514977	Ca4	8514977	G/GT	CACACCCCTAGCTTTGCTTC	CACGAACGCCTGTGAACTTA	59.9	547	DRR	Ca_08343	YU	--
Ca4_P4_8515876	Ca4	8515876	AATG/A	GAAATGTTGGTGAGGGATGG	AACATTTTTAGTGCCCACGC	60.2	550	DRR	Ca_08344	--	--
Ca4_P4_8616583	Ca4	8616583	GATGT/GATGTATGT	GCGAGAATACAATTTCCAGCA	ACTCGATCGTGGAAGCAAGT	60.2	324	DRR	Ca_08355	O	--
Ca4_P4_8639881	Ca4	8639881	TA/TACAA	ACTCCCTCAATGGCAACATC	ACACCACGAACGCAACAATA	59.9	652	DRR	Ca_08357	--	--
Ca4_P4_8650272	Ca4	8650272	T/TTTGGATTACACAATAGAG	TTCCCTTTGAGTGGGATTTG	TCTTCCCGTTTAGCTTATATCCA	59.9	597	DRR	Ca_08358	--	G2-like

INDEL marker IDs	Chromosomes/ unanchored scaffolds	Physical positions (bp)	InDels (*Kabuli* reference genome-CDC Frontier/PII)	Forward primers (5'-3')	Reverse primers (5'-3')	Anneling temperature (0C)	Expected amplified product size (bp)	Structural annotation		Functional annotation	
								Sequence components of *kabuli* genome	*Kabuli* gene accession IDs	NCBI-KOG	TFs
Ca4_P4_8701352	Ca4	8701352	ATTTTTTT/ATTTTT	TTACGACTTTGAATGCCACG	TGTTTCGCAATTTAGGGGAG	59.7	503	DRR	Ca_08365	G	--
Ca4_P4_8716168	Ca4	8716168	AATTA/AA	GGTGCCATTAAAGAGCTTGC	CAACTAAATCACGTAGGTCCCA	59.9	566	DRR	Ca_08368	Q	--
Ca4_P4_8734624	Ca4	8734624	TAAAAAAAA/TAAAAAAAAAA	CCGGTCAATCTATTGCGACT	TGGGTCATGTGGAAGGAAGT	60.1	537	DRR	Ca_08370	R	WOX
Ca4_P4_8756554	Ca4	8756554	TT/TTGT	GCTAACCTTTCCCAATTCCC	TGAATTGGCCAAAAACTCAA	59.8	458	DRR	Ca_08371	--	NAC
Ca4_P4_8757082	Ca4	8757082	GATGGATATGGATA/GATGGATA	TGAGTTTTTGGCCAATTCAA	GCAGGTGCAGAAAGATTACCA	59.1	605	DRR	Ca_08371	--	NAC
Ca4_P4_8774335	Ca4	8774335	CT/C	GACGATTGCGAATTTGAGGT	TGATTTATGTTTGTGGCGGA	60.1	295	DRR	Ca_08373	C	--
Ca4_P4_8775167	Ca4	8775167	ATT/ATTT	TGTTCCCTGCAATTCACATC	ACGGGTCTCGATCAATGTGT	59.5	360	DRR	Ca_08373	C	--
Ca4_P4_8802549	Ca4	8802549	TA/T	TCACAAAGCGCCATTAAACA	TGTACGCAGTGTGCTTGTCA	60.3	370	DRR	Ca_08378	AR	MYB
Ca4_P4_8850307	Ca4	8850307	GAAAAA/GAAAAAA	CTCCGATTGTTGAGGTGGTT	AAATTAGCTTTGGCTGGGGT	60.0	548	DRR	Ca_08382	--	MIKC
Ca4_P4_8891981	Ca4	8891981	CAAA/CAA	ATACACCACAACGCGTCAAA	AAGCATGGTATTCGGGAGTG	60.0	204	DRR	Ca_08386	--	--

INDEL marker IDs	Chromosomes/ unanchored scaffolds	Physical positions (bp)	InDels (*Kabuli* reference genome-CDC Frontier/PII)	Forward primers (5'-3')	Reverse primers (5'-3')	Anneling temperature (0C)	Expected amplified product size (bp)	Structural annotation		Functional annotation	
								Sequence components of *kabuli* genome	*Kabuli* gene accession IDs	NCBI-KOG	TFs
Ca4_P4_8993223	Ca4	8993223	CATAAATAAATAAATAAATAAATAAATAAA/CATAAATAAATAAATAAATAAATAAA	TCACAAATCACATTCACCCC	GGCGGTTTGACAGTAGGAAA	59.2	314	DRR	Ca_08394	GMW	--
Ca4_P4_9017988	Ca4	9017988	TTATATATATATATATA/TTATATATATATATA	GCGTGGCATCAATATTCCTT	ATAGATTGATCGGGACGGTG	59.9	633	DRR	Ca_08396	AD	--
Ca4_P4_9049540	Ca4	9049540	TA/TACA	AAGAATTGTCAACCAAGTTCTACC	AACATGGCGTTTTGGTTGAT	57.8	667	DRR	Ca_08398	Q	B3
Ca4_P4_9197695	Ca4	9197695	AAAC/AAACAAC	TGCACCAATCGAACTAATCG	TTTCTCGTCTTGGTGCAGTG	59.7	369	DRR	Ca_08410	I	--
Ca4_P4_9321669	Ca4	9321669	GTATTATTAT/GTATTAT	GTTGTGTCCGTGTCAGATGC	GTCCTTGACATTGGTGGGAG	60.2	439	DRR	Ca_08420	A	bZIP
Ca4_P4_9463038	Ca4	9463038	ATTAATTTT/ATT	CAAGAGGAAACATGGAGGGA	CCAAAAAGCAACTTTCAAGCC	60.0	632	DRR	Ca_08435	R	C2H2
Ca4_P4_9704009	Ca4	9704009	AAGTTAGGAGTTAGGAGTTAGGAGTTAGGAGTTAGG/AAGTTAGGAGTTAGGAGTTAGGAGTTAGGAGTTAGGAGTTAGG	TTCCAAAAGCAAACACACCA	CTCTTTCTTGACCTGCCCTG	60.1	745	DRR	Ca_08447	Q	Nin-like
Ca4_P4_9727283	Ca4	9727283	AT/A	TGTCTGCGGTCACAAAACTC	TGGTGTCCATTTTTGCACAT	59.9	504	DRR	Ca_08452	E	--
Ca4_P4_9827543	Ca4	9827543	GAAAAAAAAAA/GAAAAAAAAA	CGGTCACCGTGTACCACTTT	CAATCTGCCATGTCAATGAA	60.9	332	DRR	Ca_08460	--	--
Ca5_P4_10511669	Ca5	10511669	TC/T	CACTACTTGGTTGGGGCCTA	TTGTTGGGATCCGACTTTGT	60.0	364	DRR	Ca_21230	--	--

INDEL marker IDs	Chromosomes/ unanchored scaffolds	Physical positions (bp)	InDels (*Kabuli* reference genome-CDC Frontier/PII)	Forward primers (5'-3')	Reverse primers (5'-3')	Anneling temperature (0C)	Expected amplified product size (bp)	Structural annotation		Functional annotation	
								Sequence components of *kabuli* genome	*Kabuli* gene accession IDs	NCBI-KOG	TFs
Ca5_P4_10668285	Ca5	10668285	TAAAAAAAAAA/TAAAAAAAAAAA	GGGCGATTATATACCAGCGA	TGGCAACTATTTTGAGCTTTTTC	59.9	564	DRR	Ca_21221	--	--
Ca5_P4_11983861	Ca5	11983861	GTCT/GATCTTCGATCCTTCT	CTGCTGTCAACTCCACTCCA	TCAAAATCCCCCAATAACCA	60.0	713	DRR	Ca_17106	--	--
Ca5_P4_12659145	Ca5	12659145	TTAGTCGTAGTCGTAGTCGT/TTAGTCGTAGTCGTAGTCGTAGTCGT	GTGACTGGGGACTGGTGACT	GACGGACGGTGTAGCAAAAT	60.0	451	DRR	Ca_17066	--	FAR1
Ca5_P4_1269046	Ca5	1269046	AAATT/A	TCCGGTACACAACTTTTCCC	ACCCATCATTTCCAACCAAC	59.8	376	DRR	Ca_26280	E	--
Ca5_P4_14059187	Ca5	14059187	CTTGTTAGTTG/CTTGTTAGTTGTTAGTTG	CGGTCTCAACCTCTCCCATA	TTTCCCAATCGATTTGCTTC	60.1	706	DRR	Ca_20824	R	--
Ca5_P4_143697	Ca5	143697	A/AT	AGCAAGAGGAGCAAAACTGG	CCACCGCAGACTTAAACCAT	59.6	706	DRR	Ca_18170	--	--
Ca5_P4_16765808	Ca5	16765808	TAGA/TA	CGATCGTGCTACGTCTTCAA	TGGTGGGAATGTTGTGATTG	60.0	677	DRR	Ca_17941	R	HSF
Ca5_P4_168246	Ca5	168246	GATTTATTTATTTATTTATTT/GATTTATTTATTTATTTATTTATTT	TCAGATACACGCATCCCTTT	CGAGACCATTGAGTCCAAAAA	58.2	467	DRR	Ca_18171	--	NAC
Ca5_P4_172991	Ca5	172991	TAAAAAAAAAA/TAAAAAAAAAAA	GCGAATGACAAGCAAAAAGA	ACCGTGAGATGCCACATTTT	59.0	907	DRR	Ca_18172	J	--
Ca5_P4_173324	Ca5	173324	ATTTTTTTTTTTT/ATTTTTTTTTTT	TGTTTATCCACAAGCTTTCCG	ACCGTGAGATGCCACATTTT	60.1	270	DRR	Ca_18172	J	--

INDEL marker IDs	Chromosomes/ unanchored scaffolds	Physical positions (bp)	InDels (*Kabuli* reference genome-CDC Frontier/PII)	Forward primers (5'-3')	Reverse primers (5'-3')	Anneling temperature (0C)	Expected amplified product size (bp)	Structural annotation		Functional annotation	
								Sequence components of *kabuli* genome	*Kabuli* gene accession IDs	NCBI-KOG	TFs
Ca5_P4_198698	Ca5	198698	AA/AAGA	CCGGAGCACGTAAAGCTTAG	GTGGCATCCACAATGACAAA	60.0	488	DRR	Ca_18174	L	--
Ca5_P4_20542450	Ca5	20542450	AT/ATT	ATGCATTCACCTGTGGCATA	ACTCCTTCCTTTCGTCCCAT	60.0	331	DRR	Ca_18213	--	HB-other
Ca5_P4_20781552	Ca5	20781552	GATATATATATATATATATATATATAT/GATATATATATATATATATATATATATATAT	GCTGAGGTGGGAAACATTTG	CCAAATCAGAACCAAAGGCA	60.5	598	DRR	Ca_18223	--	ARF
Ca5_P4_209072	Ca5	209072	GTTTTT/GTTTTTTT	TGGCATCACCATCACTTGTT	ACGTTTATGGGATGCTTCAA	60.0	607	DRR	Ca_18176	--	C2H2
Ca5_P4_209466	Ca5	209466	AAAAAACTTAAAAACTTAAAA/AAAAAACTTAAAAACTTAAAAACTTAAAA	AATTGAAGCATCCCATAAACG	TTAGACCATCCACAACACGG	59.0	698	DRR	Ca_18176	--	C2H2
Ca5_P4_209690	Ca5	209690	ATTTTTTT/ATTTTTTTT	AAAACATTCAACCAACCACCA	TTAGACCATCCACAACACGG	60.1	320	DRR	Ca_18176	--	C2H2
Ca5_P4_209767	Ca5	209767	GAAAAAAAA/GAAAAAAAAAA	AAAACATTCAACCAACCACCA	TTAGACCATCCACAACACGG	60.1	320	DRR	Ca_18176	--	C2H2
Ca5_P4_209793	Ca5	209793	AAATTAAT/AAATTAATTAAT	AAAACATTCAACCAACCACCA	TTAGACCATCCACAACACGG	60.1	320	DRR	Ca_18176	--	C2H2
Ca5_P4_210200	Ca5	210200	ATTTTTTTTT/ATTTTTTTTTT	TTAAACACCCGTGTTGTGGA	AAATGGCTTAGGGAGGGAGA	59.9	607	DRR	Ca_18176	--	C2H2
Ca5_P4_210510	Ca5	210510	ATT/ATTT	TCTCCCTCCCTAAGCCATTT	GAATTATCCGCTGCTTGCAT	60.0	201	DRR	Ca_18176	--	C2H2

INDEL marker IDs	Chromosomes/ unanchored scaffolds	Physical positions (bp)	InDels (*Kabuli* reference genome-CDC Frontier/PII)	Forward primers (5'-3')	Reverse primers (5'-3')	Anneling temperature (0C)	Expected amplified product size (bp)	Structural annotation		Functional annotation	
								Sequence components of *kabuli* genome	*Kabuli* gene accession IDs	NCBI-KOG	TFs
Ca5_P4_210754	Ca5	210754	TAAAAAAAAAAAA/TAAAAAAAAAAAAA	TCTCCCTCCCTAAGCCATTT	TGGCTCAGAGCTGATTCTGAT	60.0	746	DRR	Ca_18176	--	C2H2
Ca5_P4_21281623	Ca5	21281623	TC/T	CAGGCTGATGAAGATGACGA	TCATTGATCCCCCGTAATGT	59.9	613	DRR	Ca_18240	--	--
Ca5_P4_21853723	Ca5	21853723	TAA/TAAAAAAAATCAA	AGCCCTATGTCACGACCAAC	TTTTTGCATGACGACTCCAG	60.0	834	DRR	Ca_24465	--	--
Ca5_P4_22069880	Ca5	22069880	GATATA/GATA	TTCGTATGCGTGCCAATATC	GCATGTTCTTTCGTTCGTCA	59.5	129	DRR	Ca_21161	--	--
Ca5_P4_225405	Ca5	225405	TTCTCTC/TTCTC	CGGATATGCCATCACAGTTG	GGCCCCATTGATGGTTACTA	59.9	834	DRR	Ca_18177	--	ZF-HD
Ca5_P4_225505	Ca5	225505	ATTTTTTTTT/ATTTTTTTT	CGGATATGCCATCACAGTTG	GGCCCCATTGATGGTTACTA	59.9	834	DRR	Ca_18177	--	ZF-HD
Ca5_P4_22695785	Ca5	22695785	C/CGGAGAGGAGGG	CTAACCGAAAAAGGCAGCAG	ACAATATTCCCCGGGCTAAC	60.0	358	DRR	Ca_20069	C	--
Ca5_P4_22919631	Ca5	22919631	AG/A	GATTTGGTGGAATCTCCGAA	GCCACCGACTACTTCTCCCT	59.9	336	DRR	Ca_21962	--	--
Ca5_P4_22919722	Ca5	22919722	GTT/GT	GATTTGGTGGAATCTCCGAA	GCCACCGACTACTTCTCCCT	59.9	336	DRR	Ca_21962	--	--
Ca5_P4_230199	Ca5	230199	ATTTTT/ATTTT	CAGGTTGGATCTGATGGGTT	CAATTGTGGAATGCAACCAG	59.8	576	DRR	Ca_18178	S	--

INDEL marker IDs	Chromosomes/ unanchored scaffolds	Physical positions (bp)	InDels (*Kabuli* reference genome-CDC Frontier/PII)	Forward primers (5'-3')	Reverse primers (5'-3')	Anneling temperature (0C)	Expected amplified product size (bp)	Structural annotation		Functional annotation	
								Sequence components of *kabuli* genome	*Kabuli* gene accession IDs	NCBI-KOG	TFs
Ca5_P4_23945434	Ca5	23945434	AATATATATATATATATATATATATAT/AATATATATATATATATATATATAT	AAAACAGCTTGTAATTCCGTGA	TTTTTCGTCAATTAACTTTTTCCA	58.8	790	DRR	Ca_17664	O	bHLH
Ca5_P4_24267648	Ca5	24267648	AGGGGGG/AGGGGGGG	GCAAGATGATCCCGACTCTC	GACTGGACGTAACCCACGTT	59.8	528	DRR	Ca_15569	--	--
Ca5_P4_25186556	Ca5	25186556	GAAAAAAAA/GAAAAAAAAA	TCAGAGGTACATTCGCCAGA	GTCAATTTGATAGGCCCCCT	59.4	686	DRR	Ca_15627	L	--
Ca5_P4_25186591	Ca5	25186591	CAAAAAAAAAA/CAAAAAAAAAAA	TCAGAGGTACATTCGCCAGA	GTCAATTTGATAGGCCCCCT	59.4	686	DRR	Ca_15627	L	--
Ca5_P4_25803856	Ca5	25803856	TAAAAAAAAAAAAA/TAAAAAAAAAAA	ATCCGATAAATCGCATTGTG	CCCACAAGGTCCGAGAATTA	58.5	203	DRR	Ca_09020	--	--
Ca5_P4_25896623	Ca5	25896623	AT/ATAAAGT	TCCACGTTAGGGGGTTACCT	TCAGCACCGATCTTGATGAC	60.6	839	DRR	Ca_09015	S	--
Ca5_P4_260923	Ca5	260923	CATAT/CATATAT	CTTCGGCCTAAAGATTTTGC	ATTGCGGTTGTTCGGTTATC	59.0	358	DRR	Ca_18180	A	--
Ca5_P4_261320	Ca5	261320	TCAC/TC	GATAACCGAACAACCGCAAT	CGGAGCTTCGGTAGGACATA	59.8	754	DRR	Ca_18179	R	--
Ca5_P4_26559939	Ca5	26559939	AATATATATATATATAT/AATATATATATATATAT	TTTTTGGGTTGAGGACAAGG	GCACTATCCTATCAAATGGTCTCTC	59.9	145	DRR	Ca_08966	--	bHLH
Ca5_P4_26571749	Ca5	26571749	TTTCATGACTTCAT/TTTCAT	TCCTAAAACGTCGAAACCAAA	TCACAATCTTAAAATGCGAAAAA	59.6	371	DRR	Ca_08965	--	--

INDEL marker IDs	Chromosomes/ unanchored scaffolds	Physical positions (bp)	InDels (*Kabuli* reference genome-CDC Frontier/PII)	Forward primers (5'-3')	Reverse primers (5'-3')	Anneling temperature (0C)	Expected amplified product size (bp)	Structural annotation		Functional annotation	
								Sequence components of *kabuli* genome	*Kabuli* gene accession IDs	NCBI-KOG	TFs
Ca5_P4_26873199	Ca5	26873199	AATATATATATATATATATAT/AATATATATATATATATATATAT	TCAAAATTTTCGATCAAAACCC	TTGCGTAACATTCACACAAAAA	60.2	852	DRR	Ca_08941	K	M-type
Ca5_P4_26994998	Ca5	26994998	TC/TCC	GGAGCGGGAATATGACTGAA	GGGAGAGGTTGGGAGCTTAC	60.0	492	DRR	Ca_08931	R	MYB
Ca5_P4_26995171	Ca5	26995171	TAG/TAGCACAAGCACTAAG	GGAGCGGGAATATGACTGAA	GCGGCAGAAGAACCTATTTG	60.0	576	DRR	Ca_08931	R	MYB
Ca5_P4_26996169	Ca5	26996169	TG/TGATCG	ATGTTGGGAACTTGCACTCC	AACTATGTCCTGTGGACCGC	60.0	458	DRR	Ca_08931	R	MYB
Ca5_P4_27950124	Ca5	27950124	TATCCAAAAT/TATCCAAAATTATTTTGATCCAAAAT	TTGCAACAAAGCGCTGTAAC	CTTTGCCAATTCCAGTCCAT	60.1	640	DRR	Ca_13422	--	Trihelix
Ca5_P4_28012407	Ca5	28012407	AATATATATATATATAT/AATATATATATATAT	TTGAAGGAAGATGAATCGTTGA	ATGCGGATACTCTCTGCCAC	59.7	622	DRR	Ca_13417	S	--
Ca5_P4_280542	Ca5	280542	AATTATTATTATT/AATTATTATTATTATT	CAAACAAGCTTTTTCACTTTCTCA	AGGACCTCCCTTTTTCTCCA	60.0	636	DRR	Ca_18183	--	ARF
Ca5_P4_28150139	Ca5	28150139	TGG/TGGACAGG	ATTGTGCAATGAATGTCCGA	CACTTGTGCGCTACACCACT	59.9	199	DRR	Ca_13406	--	--
Ca5_P4_28241837	Ca5	28241837	TTATATATATATATATATATAT/TTATATATATATATATATAT	TCGACCATAAACTTATGCAGACA	TTCGTTCAAAGAACCCAACC	59.7	781	DRR	Ca_13400	--	ARF
Ca5_P4_28281944	Ca5	28281944	CTATATATATATATATATATAT/CTATATATATATATATATAT	GCAGAACAAGACGTCAGTATCG	AAATGTTCATCCGCCTTCTG	59.9	702	DRR	Ca_13397	D	--

INDEL marker IDs	Chromosomes/ unanchored scaffolds	Physical positions (bp)	InDels (*Kabuli* reference genome-CDC Frontier/PII)	Forward primers (5'-3')	Reverse primers (5'-3')	Anneling temperature (0C)	Expected amplified product size (bp)	Structural annotation		Functional annotation	
								Sequence components of *kabuli* genome	*Kabuli* gene accession IDs	NCBI-KOG	TFs
Ca5_P4_288048	Ca5	288048	CTT/CTTTT	GGACCAGCAAGAAGCAACTC	TGTGAGAAATGGGAATTGGA	60.0	933	DRR	Ca_18185	R	B3
Ca5_P4_288684	Ca5	288684	ATACTTACTT/ATACTT	CTTGGACTCCAGTGCCTCAT	GTGCATTTTGCAGGCAATTA	60.3	474	DRR	Ca_18185	R	B3
Ca5_P4_291688	Ca5	291688	ATTTTTT/ATTTTTTTT	AAAGAGCCAAAGAAGCACCA	AGCAAATGCGACTGTGATTG	60.0	722	DRR	Ca_18186	--	--
Ca5_P4_291943	Ca5	291943	GCGCAACCGCAA/GCGCAACCGCAACCGCAA	TTCGTGGCTTAATCCCACTC	GTCTGGTGCTCTTCCCAATC	60.1	517	DRR	Ca_18186	--	--
Ca5_P4_292149	Ca5	292149	A/AC	AAAGTGCCGAAGTTTGCAGT	TGCTTTCCCTTCAACAGTCA	59.9	485	DRR	Ca_18186	--	--
Ca5_P4_29735774	Ca5	29735774	CATATATATATATATATATATATATATATATATATA/CATATATATATATATATATATATATATATATATATA	GTTAATACCGCGGGGAAAAT	CTCTCCAACTCAATGCACCA	60.0	705	DRR	Ca_16655	R	--
Ca5_P4_29938246	Ca5	29938246	ATT/ATTT	GCAAAAACAGCGACACTGAA	TTGTGTGGGAAAGGTGACAA	60.0	764	DRR	Ca_16674	--	--
Ca5_P4_29938358	Ca5	29938358	ATT/AT	AGCTTTGAATGGCAATGGAC	TTGTGTGGGAAAGGTGACAA	60.1	380	DRR	Ca_16674	--	--
Ca5_P4_31133950	Ca5	31133950	A/AC	GAGGAGATTGGGGAGCAACT	CTCATTGGCCATGAAAGGAT	60.6	539	DRR	Ca_04815	--	--
Ca5_P4_31147221	Ca5	31147221	CG/CGG	CTTGGTCAGCAACCAACGTA	ACCCTGGATATTAGGGTGGC	59.8	378	DRR	Ca_04818	--	GRAS

INDEL marker IDs	Chromosomes/ unanchored scaffolds	Physical positions (bp)	InDels (*Kabuli* reference genome-CDC Frontier/PII)	Forward primers (5'-3')	Reverse primers (5'-3')	Anneling temperature (0C)	Expected amplified product size (bp)	Structural annotation		Functional annotation	
								Sequence components of *kabuli* genome	*Kabuli* gene accession IDs	NCBI-KOG	TFs
Ca5_P4_31161168	Ca5	31161168	TTATATATATATATATATATATATATAT/TTATATATATATATATATATATATATATAT	TTCTTTGAAAGCATGGACCC	TCTCGGGAATCTTTCCAAAC	60.1	469	DRR	Ca_04820	S	CPP
Ca5_P4_31183021	Ca5	31183021	ATTT/ATTTTTT	CCAAACACTCAAACCGACTC	AAAGACCAAATTGGACACTTCA	58.2	817	DRR	Ca_04823	--	WOX
Ca5_P4_31512273	Ca5	31512273	AT/ATATATCT	GCCATGATCAATGCAAGAGA	TTTCCAACCCACAAAATCAA	59.8	450	DRR	Ca_04853	Q	--
Ca5_P4_31691419	Ca5	31691419	TACATACAC/TAC	CCGTTCAACCTCTGCATCTT	TCTGTCATCCAATCTCAACTCG	60.3	216	DRR	Ca_04871	OJ	bZIP
Ca5_P4_31813449	Ca5	31813449	TTAT/TT	TTTTCAACCTTTTCGTTCCAA	GGTCAGCGTTATATCCCACAA	59.6	696	DRR	Ca_04886	Z	--
Ca5_P4_32005288	Ca5	32005288	CAATAACAAATAACAAA/CAATAACAAATAACAAATAACAAA	AAACCCGATAAAAAGCGTGA	ACAGAAAAAGCGGATAGGCA	59.6	526	DRR	Ca_04904	Z	MYB_related
Ca5_P4_32233	Ca5	32233	ATTTTTTTTTTT/ATTTTTTTTTTTT	TTTTGGTTTGGGATGGTTGT	GGAGATCACTCGGGTTGTGT	60.1	585	DRR	Ca_18158	--	--
Ca5_P4_32388967	Ca5	32388967	CTATTATTTTATTATTTTATTA/CTATTATTTTATTATTTTATTATTTTATTA	AATTGCGATAAGCAATTGGG	TAGGATTACACGGGGCAGAC	59.9	821	DRR	Ca_04938	--	bZIP
Ca5_P4_33146690	Ca5	33146690	TAAAAAAAAA/TAAAAAAAAAA	TACCCGAACAACTTTGACCC	TGCTTTCGTGATTCCAACAA	59.8	731	DRR	Ca_05009	D	YABBY
Ca5_P4_3337230	Ca5	3337230	CATATATATATAT/CATATATATATATATAT	TCATCCCGAATCAGTTCACA	GGCATGATGCAAGGGATAGT	60.0	291	DRR	Ca_18697	--	--

INDEL marker IDs	Chromosomes/ unanchored scaffolds	Physical positions (bp)	InDels (*Kabuli* reference genome-CDC Frontier/PII)	Forward primers (5'-3')	Reverse primers (5'-3')	Anneling temperature (0C)	Expected amplified product size (bp)	Structural annotation		Functional annotation	
								Sequence components of *kabuli* genome	*Kabuli* gene accession IDs	NCBI-KOG	TFs
Ca5_P4_33444959	Ca5	33444959	GATATA/GATATATA	TTATGGAGGCCACATTCACA	GAAATTAAGGCCTCTTGATCTTTTT	59.9	961	DRR	Ca_01912	O	MYB_related
Ca5_P4_33456475	Ca5	33456475	TTATGTTAAATATGTTAA/TTATGTTAAATATGTTAAATATGTTAA	GATGAAGTGCTGATCAGAGGC	ACCAGGAAACATTGCCAGAC	60.0	789	DRR	Ca_01911	O	MYB_related
Ca5_P4_33457459	Ca5	33457459	ATT/ATTT	TCTTCAAATTAAAATTCCACCCA	GTTGAAATTTTGGGTGTCGG	59.7	750	DRR	Ca_01911	O	MYB_related
Ca5_P4_33457924	Ca5	33457924	ATTTTTTT/ATTTTTTTT	CCGACACCCAAAATTTCAAC	GCCCTCAGATGAGAGTCAGG	60.2	680	DRR	Ca_01911	O	MYB_related
Ca5_P4_33525483	Ca5	33525483	TTATATATATATATATATATAT/TTATATATATATATATATATATAT	CAACATGCATCAGGCGTATC	TTGGATTGGACGTTGTTGTT	60.1	433	DRR	Ca_01906	--	--
Ca5_P4_33600042	Ca5	33600042	CT/C	TAACCATTCCACAGCCACAA	CTTAGAGGTCGCTTCAACGG	60.0	680	DRR	Ca_01896	--	--
Ca5_P4_33600975	Ca5	33600975	C/CAACCTA	CGTTGAGGACTTACCCGAAA	CCGTTGGTGTTAAAGGGAGA	60.1	351	DRR	Ca_01896	--	--
Ca5_P4_336785	Ca5	336785	CAAAAAAAAA/CAAAAAAAA	GCCTACGTCAGACCGATGTT	TCCTGGCAGAATTTTGGTTC	60.1	458	DRR	Ca_18190	H	--
Ca5_P4_337016	Ca5	337016	ATTT/ATTTT	CCAAAACTGGGGAGAAACAA	TGATATTGGAGGGGGCATAA	59.9	306	DRR	Ca_18190	H	--
Ca5_P4_3406072	Ca5	3406072	TAAAAA/TAAAA	TTCACCCATTGTATGCTTTGA	GCGGTGTTGAAAAGGAAAAG	59.0	832	DRR	Ca_18699	K	MYB

INDEL marker IDs	Chromosomes/ unanchored scaffolds	Physical positions (bp)	InDels (*Kabuli* reference genome-CDC Frontier/PII)	Forward primers (5'-3')	Reverse primers (5'-3')	Anneling temperature (0C)	Expected amplified product size (bp)	Structural annotation		Functional annotation	
								Sequence components of *kabuli* genome	*Kabuli* gene accession IDs	NCBI-KOG	TFs
Ca5_P4_34191964	Ca5	34191964	AAATAATAATAATAATAATAATAATAAT/AAATAATAATAATAATAATAATAATAATAAT	AGGCAAAATGTTTTTCACGG	TTTGTTTTGGCACATGCACT	60.0	561	DRR	Ca_01828	--	--
Ca5_P4_34332054	Ca5	34332054	AATATATATATATATATATATAT/AATATATATATATATATATATATATATATAT	AAATTGCAAAGACCAATGAACA	CAACAAAACTGGCACAATCC	59.5	659	DRR	Ca_01814	--	--
Ca5_P4_34771204	Ca5	34771204	AATATATATATATATATATATATATAT/AATATATATATATATATATATATATATAT	TCTCATGTTTCCCAACCACA	CTGGACCACATTCTGGCTCT	59.9	487	DRR	Ca_01764	--	ERF
Ca5_P4_34854970	Ca5	34854970	AAATAATAATAATAATAATA/AAATAATAATAATAATAATA	TCCCACCGTGATCGTTTTAT	CAAGGAACTTGGACCGTCAT	60.2	732	DRR	Ca_01754	--	ARF
Ca5_P4_35176516	Ca5	35176516	GATATATATATATATAT/GATATATATATATATATAT	TTTGCATAACAAGCCAGCAC	AGGCTGTCCCCATCATTGTA	59.9	490	DRR	Ca_01718	R	--
Ca5_P4_35689449	Ca5	35689449	TC/T	CGTTGCAACAGAAGCAGAAG	GATCCAAAGCCCTCAATGAA	59.8	566	DRR	Ca_01660	M	--
Ca5_P4_360986	Ca5	360986	ATTTTTTTTTTT/ATTTTTTTTTT	TGTGAGTTTAAGGGAGGGGA	GCATCCAGGATCAGAGGTGT	59.5	390	DRR	Ca_18192	P	--
Ca5_P4_36176137	Ca5	36176137	AATATATATATAT/AATATATATATATAT	TCATTGACATTTTGTCCCCA	TTGGTTGGAAAGGTTTGTCA	59.8	617	DRR	Ca_01608	--	--
Ca5_P4_36212540	Ca5	36212540	GTT/GTTAGATT	TTTCATTGTTTCCGTCACCA	TTGAGTCAATGCAAGAGTCCTT	59.9	874	DRR	Ca_01603	I	B3
Ca5_P4_36308359	Ca5	36308359	TTTATT/TTTATTGGTATTATTATTATT	GTTTGTGGCGGATTTTGTTT	CCGATACAACGGAATTGTCC	59.8	423	DRR	Ca_01593	Z	--

INDEL marker IDs	Chromosomes/ unanchored scaffolds	Physical positions (bp)	InDels (*Kabuli* reference genome-CDC Frontier/PII)	Forward primers (5'-3')	Reverse primers (5'-3')	Anneling temperature (0C)	Expected amplified product size (bp)	Structural annotation		Functional annotation	
								Sequence components of *kabuli* genome	*Kabuli* gene accession IDs	NCBI-KOG	TFs
Ca5_P4_36410469	Ca5	36410469	AG/A	TGGAAACGAGTCCAACAACA	ACCTCCCTGTTTGTCAATCG	60.1	452	DRR	Ca_01583	E	--
Ca5_P4_36432725	Ca5	36432725	AG/A	TCTCCAAAACACCGAGACAA	TGGATCAACATTTCCCAATTC	59.3	679	DRR	Ca_01577	--	--
Ca5_P4_37107723	Ca5	37107723	TAAAAAAAAAA/TAAAAAAAAA	TTACCCGCTGAAAACCTTTG	GAGCAGAGGCGGATGTTTTA	60.1	445	DRR	Ca_01506	D	LBD
Ca5_P4_37460031	Ca5	37460031	CATATATATATATATATAT/CATATATATATATAT	GGACAGCCCTGGTAATCAAA	CCTGCAAACCACATTTTCCT	59.9	958	DRR	Ca_01474	U	--
Ca5_P4_37799581	Ca5	37799581	CTTTTT/CTTTTTT	GCACCGTGATTTTGATGATG	ATCAAGTCAGTGCTGCATCG	59.9	594	DRR	Ca_01443	--	--
Ca5_P4_37981188	Ca5	37981188	TAAAA/TAAAAA	AAGGAAACACGCATCCAAAC	TGCGCAAAATCGTAGACAAG	60.0	311	DRR	Ca_01422	B	MYB_related
Ca5_P4_37981277	Ca5	37981277	ATT/AT	AAGGAAACACGCATCCAAAC	TGCGCAAAATCGTAGACAAG	60.0	311	DRR	Ca_01422	B	MYB_related
Ca5_P4_38028193	Ca5	38028193	AT/A	TGGCAAGAATACCTTGTAAGCA	TCAAATTCGTAACTCAAATCTTCAA	59.8	595	DRR	Ca_01418	R	DBB
Ca5_P4_38028753	Ca5	38028753	A/AC	TGGACCACGTGTACTATTTAACC	CCAAGTGTTATTGGAGGGAGA	58.0	524	DRR	Ca_01418	R	DBB
Ca5_P4_38028815	Ca5	38028815	TAAAAAAAA/TAAAAAAA	TCAATCACCTTAGAATCTCACCC	TCCAAGTGTTATTGGAGGGAG	59.5	287	DRR	Ca_01418	R	DBB

INDEL marker IDs	Chromosomes/ unanchored scaffolds	Physical positions (bp)	InDels (*Kabuli* reference genome-CDC Frontier/PII)	Forward primers (5'-3')	Reverse primers (5'-3')	Anneling temperature (0C)	Expected amplified product size (bp)	Structural annotation		Functional annotation	
								Sequence components of *kabuli* genome	*Kabuli* gene accession IDs	NCBI-KOG	TFs
Ca5_P4_38030046	Ca5	38030046	AAT/AATAT	TCAAAAACGGACATGAGCAA	TTTATGGACAGCTCCCGAAC	60.2	614	DRR	Ca_01418	R	DBB
Ca5_P4_38059062	Ca5	38059062	ATTTTTTTTT/ATTTTTTT	CGGCCTAAATAGTACCGCAA	TTTGTGGCACCACACAAGAT	60.1	728	DRR	Ca_01417	R	--
Ca5_P4_38061782	Ca5	38061782	ATTTT/ATT	CCCTCCGTGTTTATTTACCG	CACTCTAGCTTTGGGCTTGG	59.3	687	DRR	Ca_01416	--	--
Ca5_P4_38067755	Ca5	38067755	AGACAG/A	AGCAATGTGCACAATCCAAA	GCGACAGATTCAACTCACAGA	60.1	106	DRR	Ca_01413	--	--
Ca5_P4_38068074	Ca5	38068074	ATTTTTTTTT/ATTTTTTT	GCAATGTGCACAATCCAAAC	CCCTTACCTGCTAAGCGAAA	60.0	580	DRR	Ca_01413	--	--
Ca5_P4_38068190	Ca5	38068190	GAAAAGAAAAAGA/GAAAAGAAAAAGAAAAAGA	GCAATGTGCACAATCCAAAC	CCCTTACCTGCTAAGCGAAA	60.0	580	DRR	Ca_01413	--	--
Ca5_P4_38076271	Ca5	38076271	T/TTAACAAATG	CCGAATTTGTTTGCTACCGT	TTTTGACACATCTATCCAATCG	60.0	388	DRR	Ca_01412	O	MYB_related
Ca5_P4_38087876	Ca5	38087876	ACCTCAAC/ACCTCAACAGTCCTCAAC	CCTTGTTTTCGTGAATGCCT	GCATTTTGAATGCCTCCACT	60.1	567	DRR	Ca_01409	--	--
Ca5_P4_38104582	Ca5	38104582	TAAAAAAAA/TAAAAAAAAAA	TACTGGTGGATTGATCCGGT	TGGGCATCCCAAGTGTTAAT	60.2	641	DRR	Ca_01408	P	--
Ca5_P4_38110814	Ca5	38110814	AATATAT/AATATATAT	ACCCAATCCAGCAGACAATC	CAATCTGTTTTCGTGGTCTTTG	59.9	243	DRR	Ca_01407	S	--

INDEL marker IDs	Chromosomes/ unanchored scaffolds	Physical positions (bp)	InDels (*Kabuli* reference genome-CDC Frontier/PII)	Forward primers (5'-3')	Reverse primers (5'-3')	Anneling temperature (0C)	Expected amplified product size (bp)	Structural annotation		Functional annotation	
								Sequence components of *kabuli* genome	*Kabuli* gene accession IDs	NCBI-KOG	TFs
Ca5_P4_38128399	Ca5	38128399	GCAACACAACCA/GCA	GGGAAGTTGGCATTCA CTGT	GCTGGTTCTTTTCCCTT GTG	60.0	693	DRR	Ca_01405	A	--
Ca5_P4_38142286	Ca5	38142286	GACACA/GACA	TTGTTCCCTTTTGGAT GAGG	ACTTTGCTGGCAATGT GTGT	59.9	526	DRR	Ca_01403	R	NAC
Ca5_P4_38142542	Ca5	38142542	ATTTTTT/ATTTTTTTTT	TGCAACATGAAGGTCA CACA	ATCGATCGTCAGCTGA TTCC	59.7	466	DRR	Ca_01403	R	NAC
Ca5_P4_38149695	Ca5	38149695	TGATGTAGATGTAGA/TGATGTAGA TGTAGATGTAGA	ATGGCCAGTCATGTCA CAAA	TGGAGGAAGAACCGAA AATG	60.0	330	DRR	Ca_01402	--	bHLH
Ca5_P4_38364224	Ca5	38364224	CTATATATATATATATATATATATAT ATA/CTATATATATATATATATATATA TATA	TCGCAATTTGGAAAGA GTTTT	ACGTCGGTGGACTCAA ATTC	58.9	333	DRR	Ca_01388	--	--
Ca5_P4_38382482	Ca5	38382482	TATAATAATAATAATAATAATAATAA TAATAATAATAAT/TATAATAATAATA ATAATAATAATAATAATAATAATAAT AATAAT	GGTGGAGACGTGGAA CAACT	CCGACGGAAATGTACC AATC	60.0	806	DRR	Ca_01386	--	LFY
Ca5_P4_38726886	Ca5	38726886	AATCAATCTTCCATCA/AATCAATCT TCCATCATCAATCTTCCATCA	CGAGTTTGTGGGGGA GATTA	TGGAGGAGGCAAAATC TGTC	59.9	465	DRR	Ca_07423	O	--
Ca5_P4_39793489	Ca5	39793489	CAAAAAAAA/CAAAAAAA	AGGGCACTTTGTCTCC TCAA	ACCAAATCGATGGAAT GACC	59.8	529	DRR	Ca_07506	S	bZIP
Ca5_P4_39872057	Ca5	39872057	GATATATATATATATATATATATATA TA/GATATATATATATATATATATATA TATATA	AGGAAAACATTGGTGG GACA	ATCAAACAACCGAAAGT GCC	60.2	656	DRR	Ca_07512	F	--
Ca5_P4_40858234	Ca5	40858234	GATATATATATATATATATATATATA TATATATATATA/GATATATATATATA TATATATATATATATATATATA	AGGCATGGTGTTGGAG TCAT	TCCGACAAGGAGAATA ACCG	60.4	650	DRR	Ca_07607	K	M-type

INDEL marker IDs	Chromosomes/ unanchored scaffolds	Physical positions (bp)	InDels (*Kabuli* reference genome-CDC Frontier/PII)	Forward primers (5'-3')	Reverse primers (5'-3')	Anneling temperature (0C)	Expected amplified product size (bp)	Structural annotation		Functional annotation	
								Sequence components of *kabuli* genome	*Kabuli* gene accession IDs	NCBI-KOG	TFs
Ca5_P4_41377707	Ca5	41377707	TG/TGGTTAGGG	AAGAAACGGGTATGTGGCTG	TTGCAATCGCACTCCAATAG	60.0	575	DRR	Ca_19238	--	--
Ca5_P4_42076019	Ca5	42076019	GTTTTTTTTTTT/GTTTTTTTTTTTT	TGAGAATCCAATTTCGGGAC	TTGTTACGATTCTCGCAGGT	59.9	197	DRR	Ca_11404	R	G2-like
Ca5_P4_42657457	Ca5	42657457	AT/ATT	TGTCAAATTGGCCTGCATTA	GTGCAACAGCTGCAATAGGA	60.1	222	DRR	Ca_11347	B	C3H
Ca5_P4_42948243	Ca5	42948243	TTA/TTATA	GGGAAGGAGTTTTACGGAGG	GAACGACAAGTCAACAAAGTCC	59.9	679	DRR	Ca_11314	O	--
Ca5_P4_43007181	Ca5	43007181	AATATATATATATATATATATATATATATATA/AATATATATATATATATATATATATATA	ACCGATGGAATGCAACACTT	CCCGAAATTAAGTTAAGGGACC	60.4	657	DRR	Ca_11308	--	bHLH
Ca5_P4_43179356	Ca5	43179356	AAACAATAACAA/AAACAA	GGAAACATGGTCAGCTGCTT	GCATTTTCTTCACTAGGCCC	60.3	757	DRR	Ca_12741	--	--
Ca5_P4_43298677	Ca5	43298677	AATATATATATATATATATATATATATA/AATATATATATATATATATATATATATATA	TCAAATTTGCAAAGACAGATTTTC	TTTTGGTGTCACTTAAGAGTGGAA	59.7	893	DRR	Ca_12727	--	--
Ca5_P4_43299130	Ca5	43299130	TC/T	TTGGTTTACGAATTTTATGGTGAA	CTGCATAGGCAGGGTGGTAG	59.7	385	DRR	Ca_12727	--	--
Ca5_P4_43366856	Ca5	43366856	CAAC/CAACATAAATGATAAC	TGTAGATTGGGTGTGGCAGA	ATTGGGTGCTCAAGGACAAG	60.1	970	DRR	Ca_12719	--	SAP
Ca5_P4_43861667	Ca5	43861667	CAATAATAATAATAATAATAATAATAATAATAATAATAATAATA/CAATAATAATAATAATAATAATAATAATAATAATAATAATA	TCTTCCCATGTGCCCTTTAG	AAGAGGCAAGTTTGAACTCGTAA	60.1	467	DRR	Ca_12668	I	G2-like

INDEL marker IDs	Chromosomes/ unanchored scaffolds	Physical positions (bp)	InDels (*Kabuli* reference genome-CDC Frontier/PII)	Forward primers (5'-3')	Reverse primers (5'-3')	Anneling temperature (0C)	Expected amplified product size (bp)	Structural annotation		Functional annotation	
								Sequence components of *kabuli* genome	*Kabuli* gene accession IDs	NCBI-KOG	TFs
Ca5_P4_44522099	Ca5	44522099	TTTATTATTATTATTATTATTATTATTATTATTATTA/TTTATTATTATTATTATTATTATTATTATTATTA	TAGCCGCTACCAATTATCGC	CTTTTAATGGCAAATGGCGT	60.2	663	DRR	Ca_03897	R	--
Ca5_P4_45182561	Ca5	45182561	AATATATATATATATATATATATATAT/AATATATATATATATATATATATATATATAT	TAGATATCCGAATCGGCCTG	TGACTTGATTCGGGTGATGA	60.0	272	DRR	Ca_03960	--	Trihelix
Ca5_P4_45327318	Ca5	45327318	CGGTGATGGTGATGGTGATGGTGATGGTGATGGTGATG/CGGTGATGGTGATGGTGATGGTGATGGTGATGGTGATG	AGGAATGTAGTTTGGCGTGG	TCCATAGATAAAACAAAGGGGAAA	60.0	770	DRR	Ca_03975	QR	B3
Ca5_P4_45523588	Ca5	45523588	TAAAAATTAAAAAATTAAAAAATTAAAAAA/TAAAAATTAAAAAATTAAAAAA	TCCACATGTTTTCCGATAGTTG	TCGGTACAAAAGGTGTTTTCG	59.9	585	DRR	Ca_04000	YU	--
Ca5_P4_457302	Ca5	457302	CGT/CAAAGACCGTTGTGT	CACAAGAAAGGGAATGACACTG	ACCACACAACTTTGGGCTTC	59.6	643	DRR	Ca_18199	--	--
Ca5_P4_45746975	Ca5	45746975	ATT/ATTACACTCTTT	GGAGTTGCAAAAGAGCATGA	TGGTTAACACTGGAGAGGGG	59.0	136	DRR	Ca_04024	G	--
Ca5_P4_46305506	Ca5	46305506	GATATATATATATATATATATATATATATATATAT/GATATATATATATATATATATATATATATATATATATATATATATAT	TGCCCTCAATCTGAAAACAA	GAACAAGAGCGGTCTCACAA	59.2	876	DRR	Ca_04084	--	--
Ca5_P4_46946905	Ca5	46946905	AATATATATATATATATAT/AATATATATATATATATATATAT	CATCCGTGGTCCTTTGACTT	TGGTCATTGCATTTTCCTCA	60.0	482	DRR	Ca_04149	K	ERF
Ca5_P4_47632016	Ca5	47632016	CAGTGT/C	GGAGGTCGCTTGCTAAGTTG	AATGGCCCAACTCCTATTCC	60.0	695	DRR	Ca_04223	S	--
Ca5_P4_47692025	Ca5	47692025	ATTTTTTTTT/ATTTTTTTT	CACCAAAAGCAGGGTCTCTC	GCCTTAAAGCTTTGGGTGAA	59.8	346	DRR	Ca_04228	--	--

INDEL marker IDs	Chromosomes/ unanchored scaffolds	Physical positions (bp)	InDels (*Kabuli* reference genome-CDC Frontier/PII)	Forward primers (5'-3')	Reverse primers (5'-3')	Anneling temperature (0C)	Expected amplified product size (bp)	Structural annotation		Functional annotation	
								Sequence components of *kabuli* genome	*Kabuli* gene accession IDs	NCBI-KOG	TFs
Ca5_P4_479066	Ca5	479066	TAAAAAAAAAA/TAAAAAAAAAAA	CCTGTTCTGTAGTGGGAGCC	TCCATGTTACTTCTCCGCCT	59.7	508	DRR	Ca_18200	U	--
Ca5_P4_479350	Ca5	479350	CATATATATATATATA/CATATATATATATATATA	TTGTCACACAATAAGGCGGA	TCATCACGATCTGAATCCCA	60.1	674	DRR	Ca_18200	U	--
Ca5_P4_480514	Ca5	480514	AATAATAATAAT/AATAATAATAATTATAATAATAAT	AAGAAACTTGAACAATGAGGTGG	GATCAGGATGAACCGGAAGA	59.6	610	DRR	Ca_18201	--	--
Ca5_P4_482095	Ca5	482095	GC/G	TTGCATCTGTCAGCGAACTC	TTTTTCGGGGGTCAAATGTA	60.1	800	DRR	Ca_18201	--	--
Ca5_P4_5292404	Ca5	5292404	GA/G	GGGAGGATTGTAACAGCGAA	GCTGCAAGAATCAACGTCAA	60.1	177	DRR	Ca_22623	--	--
Ca5_P4_667833	Ca5	667833	GC/G	TGAGCCACTGTTTGGAACAC	TGTTGCTCCTCACGAATCTG	59.7	473	DRR	Ca_23223	--	--
Ca5_P4_668104	Ca5	668104	ATT/AT	CAGATTCGTGAGGAGCAACA	TGAATCCCCTCTGATCCTTG	60.0	331	DRR	Ca_23223	--	--
Ca5_P4_71060	Ca5	71060	GC/GCCTCTGTTATGAAAC	GGTGGGGATGGATGTAGATG	TGAACAAAACATTGGGCAAA	60.0	627	DRR	Ca_18161	R	FAR1
Ca5_P4_7639595	Ca5	7639595	AAGAGAGAGAGAGAGAGAGAGAGAGAGAGAGAGAGA/AAGAGAGAGAGAGAGAGAGAGAGAGAGAGAGAGAGA	AACCAAGCCAACCTCAACAG	CCCTTTCATGCCAACAAAAT	60.1	392	DRR	Ca_18738	--	B3
Ca5_P4_7726586	Ca5	7726586	GTT/GTTT	CTGGAAAATCCTCCTCCTCC	TTGTCTCAACAATCTCCCCC	60.0	339	DRR	Ca_18731	--	--

INDEL marker IDs	Chromosomes/ unanchored scaffolds	Physical positions (bp)	InDels (*Kabuli* reference genome-CDC Frontier/PII)	Forward primers (5'-3')	Reverse primers (5'-3')	Anneling temperature (0C)	Expected amplified product size (bp)	Structural annotation		Functional annotation	
								Sequence components of *kabuli* genome	*Kabuli* gene accession IDs	NCBI-KOG	TFs
Ca5_P4_7920333	Ca5	7920333	AATATA/AATATATA	ATGCTTAGCGGAGTGGAAAA	GATATTGTTGGCGGTTGGAG	59.8	193	DRR	Ca_18721	T	M-type
Ca5_P4_8824148	Ca5	8824148	A/ATGT	GGAGCTGAGGTTGAGGTTGA	CAATATCGGCAAATTGAAGTGA	60.4	476	DRR	Ca_20336	A	--
Ca5_P4_9360000	Ca5	9360000	TAAAAAA/TAAAAA	GGCTGATGTTATTGCCTGGA	TCGTCATCACTTGTGGTGGT	61.0	791	DRR	Ca_19977	T	--
Ca5_P4_9787406	Ca5	9787406	CT/CTTTATCTGT	CACGTCGAAAAGTCACATCG	TGCATTTACCTCCCCTCAAG	60.3	728	DRR	Ca_20498	S	--
Ca5_P4_9820324	Ca5	9820324	CTTTT/CTT	CAGAACTCAACTTGCACCGA	TTGGCACCGAGAAGTTCTTT	60.0	701	DRR	Ca_20501	O	--
Ca6_P4_10090816	Ca6	10090816	ATTATTTATTTATTTATTTATTTATTTATTTATTTATTTATT/ATTATTTATTTATTTATTTATTTATTTATTTATTTATTTATTTATTTATT	CCCGCAAAATTAAGTGGGTA	CCCATACCCGTTATCCACAC	59.8	560	DRR	Ca_08583	Z	Trihelix
Ca6_P4_10120649	Ca6	10120649	TAAAAAAAAAAA/TAAAAAAAAAA	TGTTATTTGAACGCAAATGG	AATCAGAAGGAGGGACCAAAA	57.2	786	DRR	Ca_08580	J	--
Ca6_P4_10566584	Ca6	10566584	C/CTT	TTTTGTCATTTGGTATTCATTTCA	GGCTTGCAACCCAATTATTTT	58.5	600	DRR	Ca_08541	--	--
Ca6_P4_10606087	Ca6	10606087	TTCT/TTCTCT	GAAGGTTTACGTTTTGCCCA	GGCTACCAAAGAATTCGACG	60.0	533	DRR	Ca_08537	H	--
Ca6_P4_10611238	Ca6	10611238	AG/A	TCTGACATGACAAAATGGTGG	TCGGAATTGGTCCGAAATTA	59.4	409	DRR	Ca_08536	K	HSF

INDEL marker IDs	Chromosomes/ unanchored scaffolds	Physical positions (bp)	InDels (*Kabuli* reference genome-CDC Frontier/PII)	Forward primers (5'-3')	Reverse primers (5'-3')	Anneling temperature (0C)	Expected amplified product size (bp)	Structural annotation		Functional annotation	
								Sequence components of *kabuli* genome	*Kabuli* gene accession IDs	NCBI-KOG	TFs
Ca6_P4_10611559	Ca6	10611559	AG/AGTTTACACTTCCGATG	ATATTTTTCTCCAAGGGCGG	TTGTTTGGTTCAAGGGAAGG	60.3	711	DRR	Ca_08536	K	HSF
Ca6_P4_10649780	Ca6	10649780	TAA/TAAA	GACGGTTATGGAGCCTTCAA	TTCGATATCTTGTGCCTCCC	60.1	363	DRR	Ca_08532	--	HSF
Ca6_P4_10649918	Ca6	10649918	GTTTGTTTTGTTTTGTTTT/GTTTGTTTTGTTTTGTTTTGTTTT	GACGGTTATGGAGCCTTCAA	TTCGATATCTTGTGCCTCCC	60.1	363	DRR	Ca_08531	I	--
Ca6_P4_10695609	Ca6	10695609	TAAAAAAAAAAAA/TAAAAAAAAAA	CTCCGTAATGAAACCCGAGA	AGGAAGGGGAACGACCTAGA	60.1	744	DRR	Ca_08528	--	bHLH
Ca6_P4_10708022	Ca6	10708022	GCT/GCTGAATCT	CCAAGGGTTGGCTAGAACTG	TGGAAAATATGTGTGGGATCAG	59.7	689	DRR	Ca_08527	GC	HSF
Ca6_P4_10708200	Ca6	10708200	TATTAATTA/TATTA	GTGGGTACAGAAACATGGCA	TGGAAAATATGTGTGGGATCAG	59.4	539	DRR	Ca_08527	GC	HSF
Ca6_P4_10708661	Ca6	10708661	TAAAAAAA/TAAAAAA	TGAAATTCAACTGATCCCACA	TCCTTTGGTGGTGTTTCCTC	59.0	811	DRR	Ca_08527	GC	HSF
Ca6_P4_1094043	Ca6	1094043	ATTTT/ATTTTT	TGTCATGGCAACATTCACAA	TCCTGCCACCATCTTTAACC	59.5	572	DRR	Ca_13316	--	--
Ca6_P4_10953097	Ca6	10953097	CTATTATTATTATTATTATTATTATTATTATTATTATTATTATTATTATTATTA/CTATTATTATTATTATTATTATTATTATTATTATTATTATTATTATTA	TTGGCTTCTCGTTGGATTTT	AGTTTCGGGGTTCCTTCTTT	59.7	298	DRR	Ca_20466	QR	B3
Ca6_P4_11513553	Ca6	11513553	ATGTGTGTGTGT/ATGTGTGTGT	ATGCCCACCCATAATTTTCA	CTTTGCCAATTCCAGTCCAT	60.0	404	DRR	Ca_23432	--	--

INDEL marker IDs	Chromosomes/ unanchored scaffolds	Physical positions (bp)	InDels (*Kabuli* reference genome-CDC Frontier/PII)	Forward primers (5'-3')	Reverse primers (5'-3')	Anneling temperature (0C)	Expected amplified product size (bp)	Structural annotation		Functional annotation	
								Sequence components of *kabuli* genome	*Kabuli* gene accession IDs	NCBI-KOG	TFs
Ca6_P4_11632067	Ca6	11632067	GGTGTG/GGTG	CCAAAGTTAGCCCTTCCTCC	AGCACTGACCTTTTGAGCGT	60.1	445	DRR	Ca_25057	S	--
Ca6_P4_11649630	Ca6	11649630	GACTA/GACTAACTA	TTGCTTGTGCAAAAGAATGC	TACTATGCGTGGACCCTTCC	60.0	522	DRR	Ca_25059	J	--
Ca6_P4_12335628	Ca6	12335628	TGAAG/TG	GGAAGGGTGTTGGTGTGTTT	TTGGTCACTAAGGGGGTGAG	59.7	968	DRR	Ca_24849	--	--
Ca6_P4_12509102	Ca6	12509102	AT/A	TTCCTCCTTTCTTCTCCCAA	AAAATGAAGGGCTCGGATTT	58.8	263	DRR	Ca_05029	A	--
Ca6_P4_1257304	Ca6	1257304	TTATATATATATATATATAT/TTATATATATATATATATATAT	GGTGAGGATTGGTTCACTCG	ATGAGTGGGGATCGATCTTG	60.5	341	DRR	Ca_13331	--	NF-YB
Ca6_P4_12775088	Ca6	12775088	TTATATATATATATAT/TTATATATATATATATAT	TGCTGCAAAGGTTTCAATTTT	TGCAACAATTTCCCTTCACA	59.8	899	DRR	Ca_05050	O	C2H2
Ca6_P4_12865354	Ca6	12865354	CT/CTTAATT	CGCGTAACGTGGTGAATAAA	CTTCCACCCATTATGCCACT	59.6	428	DRR	Ca_05059	O	MYB_related
Ca6_P4_12865567	Ca6	12865567	GAAAAAAAAAA/GAAAAAAAAA	CGCGTAACGTGGTGAATAAA	CTTCCACCCATTATGCCACT	59.6	428	DRR	Ca_05059	O	MYB_related
Ca6_P4_12866234	Ca6	12866234	TAA/TAAA	GGGTGGAAGTCATCCTTAGTGA	AAAGGGAGGGAATGCCTTAG	60.4	603	DRR	Ca_05059	O	MYB_related
Ca6_P4_12908477	Ca6	12908477	G/GGAATCCGAAT	AAATGATGAATGCAGTGGCA	GCAGAAGAAATGGAATGCAA	60.1	844	DRR	Ca_05064	T	bHLH

INDEL marker IDs	Chromosomes/ unanchored scaffolds	Physical positions (bp)	InDels (*Kabuli* reference genome-CDC Frontier/PII)	Forward primers (5'-3')	Reverse primers (5'-3')	Anneling temperature (0C)	Expected amplified product size (bp)	Structural annotation		Functional annotation	
								Sequence components of *kabuli* genome	*Kabuli* gene accession IDs	NCBI-KOG	TFs
Ca6_P4_12910113	Ca6	12910113	TTTAAAA/T	TGGACAGGTGAAGACCAACA	TTCCTTCCTCCAATCAATGC	60.1	696	DRR	Ca_05064	T	bHLH
Ca6_P4_12939201	Ca6	12939201	CAA/CA	GAGAATGGAGGCAACAAAGC	GCACGATGAAGTTGGGTTCT	59.8	496	DRR	Ca_05066	--	--
Ca6_P4_12962499	Ca6	12962499	TAAAAAAAA/TAAAAAAA	TTGAGAGGTCGTATCCCCTG	AAGAGACACATGGGGTTTGC	60.1	215	DRR	Ca_05068	--	MYB_related
Ca6_P4_1304145	Ca6	1304145	ATTTTTTTTTT/ATTTTTTTTTTT	CCGGTTCACTACTACCCCAC	GGGAACCAATTTCGTGAATC	59.3	722	DRR	Ca_13336	T	--
Ca6_P4_13118128	Ca6	13118128	TAAAAAAAAAAA/TAAAAAAAAAA	TTTGGCTGCTTCAACTCTCA	CTGCCCAAACAACCAACTTT	59.7	655	DRR	Ca_05085	U	FAR1
Ca6_P4_15456380	Ca6	15456380	AAA/AAACAA	GCTGTGTATTTCATGCCTGC	GAGATGAGGTGGTAGCGAGC	59.3	913	DRR	Ca_05328	O	C2H2
Ca6_P4_15508917	Ca6	15508917	TTATATATATATATATATATATATAT/TTATATATATATATATATATATATAT	TGTTTGATTGGGTTGGTTCA	TGGGTGCTTTATTAAGATGGG	59.8	849	DRR	Ca_05334	--	--
Ca6_P4_15597965	Ca6	15597965	TAAA/TAAAA	CAGTTTCCACACGCACAGAT	TGGGAACAAGCATGGTGTAA	59.8	681	DRR	Ca_05341	--	bHLH
Ca6_P4_15800387	Ca6	15800387	TGAACCTCTTTA/TGAACCTCTTTAGAACCTCTTTA	CGTGGTCAACAAAGTGGAAA	TTGAAGCATGTCCTCTCTTGAA	59.6	598	DRR	Ca_22015	--	TALE
Ca6_P4_15946706	Ca6	15946706	GATATATATATATATAT/GATATATATATATATAT	TGTTGCAGTCATGCTTTTGA	TGAATTCTGCTATGCGCAAC	59.0	306	DRR	Ca_22027	C	--

INDEL marker IDs	Chromosomes/ unanchored scaffolds	Physical positions (bp)	InDels (*Kabuli* reference genome-CDC Frontier/PII)	Forward primers (5'-3')	Reverse primers (5'-3')	Anneling temperature (0C)	Expected amplified product size (bp)	Structural annotation		Functional annotation	
								Sequence components of *kabuli* genome	*Kabuli* gene accession IDs	NCBI-KOG	TFs
Ca6_P4_1683094	Ca6	1683094	TATTAAC/TATTAACCATTGATTAAC	AATAAGTCCATCCGGCTCCT	TTGTGGATGGCGTTTGATAA	59.9	276	DRR	Ca_10299	--	HB-other
Ca6_P4_1683226	Ca6	1683226	CAA/CA	AATAAGTCCATCCGGCTCCT	TTGTGGATGGCGTTTGATAA	59.9	276	DRR	Ca_10299	--	HB-other
Ca6_P4_1683327	Ca6	1683327	AC/A	TTATCAAACGCCATCCACAA	CACTTGCGTGTGGTAGTGCT	59.9	504	DRR	Ca_10299	--	HB-other
Ca6_P4_1683470	Ca6	1683470	C/CCAAAACAATCATAAC	TTATCAAACGCCATCCACAA	CACTTGCGTGTGGTAGTGCT	59.9	504	DRR	Ca_10299	--	HB-other
Ca6_P4_17043003	Ca6	17043003	CTTTTTTTTT/CTTTTTTTTTT	CGAGATTTAAAACAAAATTGGGA	TATCTTGGTGGGACTGGGAG	59.4	533	DRR	Ca_06325	--	--
Ca6_P4_17044026	Ca6	17044026	AAATAATAATAATAATAATAATAATAATAATAATAATAATAATAATAATAA/AAATAATAATAATAATAATAATAATAATAATAATAATAATAATAA	AATTGGGGCATGTGCTATTG	TGCTGCTGCAATTTGATTTC	60.7	874	DRR	Ca_06325	--	--
Ca6_P4_17092081	Ca6	17092081	A/AG	TGCACCCCTAATTTTAAGCAA	GGATTTTGGGGAGCTTGAA	59.6	468	DRR	Ca_06327	C	--
Ca6_P4_17503727	Ca6	17503727	GAAAAAAAA/GAAAAAAA	GTAGGAAGTCGCCATTTGGA	TGTCTCGATTTGACTGAGCG	60.1	808	DRR	Ca_06365	--	WOX
Ca6_P4_17755625	Ca6	17755625	CTGTGT/CTGT	ATCATCCACGATCCACCAAT	ACGTGGGGGTGAAAAACATA	60.0	821	DRR	Ca_06394	E	--
Ca6_P4_17855203	Ca6	17855203	CATTTATTTATTTATTTATTTATTTATTTATTTATT/CATTTATTTATTTATTTATTTATTTATTTATTTATT	ATTTTGGCGAAATGGTATGC	TTTGGGTTACGAATTTGATGA	59.8	527	DRR	Ca_06403	--	--

INDEL marker IDs	Chromosomes/ unanchored scaffolds	Physical positions (bp)	InDels (*Kabuli* reference genome-CDC Frontier/PII)	Forward primers (5'-3')	Reverse primers (5'-3')	Anneling temperature (0C)	Expected amplified product size (bp)	Structural annotation		Functional annotation	
								Sequence components of *kabuli* genome	*Kabuli* gene accession IDs	NCBI-KOG	TFs
Ca6_P4_1831168	Ca6	1831168	AATATA/AATATATAAATAAAAGATATA	GGCAAAAGAACAAGGTCCAA	CCTGGATGAACCAAATACACAA	60.1	354	DRR	Ca_10310	G	--
Ca6_P4_1831390	Ca6	1831390	GATT/GATTGGAAAATT	GGCAAAAGAACAAGGTCCAA	TGTTAATCGGTGATGAATTTCG	60.1	745	DRR	Ca_10310	G	--
Ca6_P4_1831423	Ca6	1831423	TAAAAAA/TAAAAAAA	GGCAAAAGAACAAGGTCCAA	TGTTAATCGGTGATGAATTTCG	60.1	745	DRR	Ca_10310	G	--
Ca6_P4_1832442	Ca6	1832442	A/AC	AAAGGTACGTGAGGTGTGCC	CGTAATTAATGATGGTGGAGTGG	60.0	614	DRR	Ca_10310	G	--
Ca6_P4_1847229	Ca6	1847229	CGC/CGCAAGC	GAAAGGCCACATGCAAGATT	GGACGTGATCTTTTGGCAAT	60.1	734	DRR	Ca_10312	R	--
Ca6_P4_19172700	Ca6	19172700	TTTTTAAATTAATTTTTTATTTTAA/TTTTTAAATTAATTTTTTATTTTAAATTAATTTTTTATTTTAA	GTGGCACAGTAGACCCCATT	CGTGACATTAACCTTGGCCT	59.9	712	DRR	Ca_06524	R	--
Ca6_P4_19244093	Ca6	19244093	TTAGAGTTTG/T	AAATGAACCAAAACCGCAAG	TCAAAATCCCCCAATAACCA	60.0	325	DRR	Ca_06530	--	--
Ca6_P4_19524549	Ca6	19524549	GTTTTTTTTTTT/GTTTTTTTTTTTT	AAACTCGAAAAGCATGGTGG	GCCCAAAATTGCCTCTATGA	60.1	623	DRR	Ca_06559	--	--
Ca6_P4_1976018	Ca6	1976018	AAT/AATAGAT	GAACTAGATGCCGCAAAAGC	GTACGTGGTCCTTCCCTGAA	60.0	560	DRR	Ca_10322	--	--
Ca6_P4_1976736	Ca6	1976736	TC/TCCC	TTATTCTCCATTGGGCATCC	TCCTGCGGGTAATGATAAGC	59.7	963	DRR	Ca_10322	--	--

INDEL marker IDs	Chromosomes/ unanchored scaffolds	Physical positions (bp)	InDels (*Kabuli* reference genome-CDC Frontier/PII)	Forward primers (5'-3')	Reverse primers (5'-3')	Anneling temperature (0C)	Expected amplified product size (bp)	Structural annotation		Functional annotation	
								Sequence components of *kabuli* genome	*Kabuli* gene accession IDs	NCBI-KOG	TFs
Ca6_P4_1977350	Ca6	1977350	A/ACTT	GCTTATCATTACCCGCAGGA	TAGCATAGTCCAAGCGGACC	60.1	465	DRR	Ca_10322	--	--
Ca6_P4_21396710	Ca6	21396710	ATTTT/ATTTTT	GGTTCGCAAAACAAGGCTTA	TTGAATCGCAAAACAACCAA	60.2	867	DRR	Ca_19862	--	--
Ca6_P4_21408015	Ca6	21408015	ATTTTTTTT/ATTTTTTT	AGGCCTTTTATTTCGTTGCC	TTGAATCGCAAAACAACCAA	60.4	897	DRR	Ca_19860	--	--
Ca6_P4_21660027	Ca6	21660027	CTTTTTTTTTTT/CTTTTTTTTTTTT	TCCTCGTTCTGCTTCCTTGT	CTTTTCAGCCTCCATCAAGC	60.0	934	DRR	Ca_11274	R	C2H2
Ca6_P4_21777762	Ca6	21777762	TCCTCT/TCCTCTTATAAGCCCCCTCT	GATTGTCAGCCCAAAAGAGC	TGAGGAACGACAGATCGAAA	59.8	753	DRR	Ca_11261	--	ERF
Ca6_P4_21777889	Ca6	21777889	GTA/GTATA	AGCACCACCAATGGATGTTT	GTCGTACGCGTTGTGTATGG	60.2	966	DRR	Ca_11261	--	ERF
Ca6_P4_21919231	Ca6	21919231	TTA/TTAATA	CTTGCATGGGTACGTTAGCA	ACTTAGCACGGGCTCGATAC	59.8	370	DRR	Ca_11246	O	--
Ca6_P4_23295842	Ca6	23295842	CAAA/CAAAA	GGCATCGAATTCAAATTTTGTT	TTGTGGAACATGCAAAAGGA	60.2	654	DRR	Ca_11127	--	--
Ca6_P4_2341889	Ca6	2341889	AACA/AA	GTTTCCCCGTAGCAACAATC	CCCTCTTCAATCTTCTTCATTCA	59.4	448	DRR	Ca_10365	--	--
Ca6_P4_23573771	Ca6	23573771	ATATTATTATTATTATTATTATTATTATTATTA/ATATTATTATTATTATTATTATTATTATTA	CCCCCTAGTAAGAGGTCGCT	CTCGTTCAGAGGGCTAGACG	59.7	542	DRR	Ca_11098	--	--

INDEL marker IDs	Chromosomes/ unanchored scaffolds	Physical positions (bp)	InDels (*Kabuli* reference genome-CDC Frontier/PII)	Forward primers (5'-3')	Reverse primers (5'-3')	Anneling temperature (0C)	Expected amplified product size (bp)	Structural annotation		Functional annotation	
								Sequence components of *kabuli* genome	*Kabuli* gene accession IDs	NCBI-KOG	TFs
Ca6_P4_23995395	Ca6	23995395	CTTTTTTTTTT/CTTTTTTTTTTT	TGGGTTCTGGTTGTTG ATGA	GCGGACATTCTCTCCA GTTC	59.9	177	DRR	Ca_11062	--	--
Ca6_P4_24582806	Ca6	24582806	CATATATATATATATATATATATATA TATAT/CATATATATATATATATATATAT ATATATATATAT	TCGTTTTGTTTATTGGG GGA	GTTAAATGCGGGGTTT ATCG	60.2	565	DRR	Ca_24169	--	bHLH
Ca6_P4_25130968	Ca6	25130968	A/AATAAAGCCT	CCAAAAAGGGTGGAGA TTGA	CCTTTTACAGCGCTTTT CCA	59.9	275	DRR	Ca_26068	--	--
Ca6_P4_2516180	Ca6	2516180	GAAAA/GAAAAA	CGGAAAGGATACCAAC TGGA	AAGAACCCATTGGTGA CGAG	59.9	771	DRR	Ca_10383	A	--
Ca6_P4_2518811	Ca6	2518811	TA/TAA	AATCTGGCTCAAAATG CAGC	AATGCATTTGCCTGACT CAA	60.4	723	DRR	Ca_10384	--	--
Ca6_P4_2523036	Ca6	2523036	GGTAAT/G	TTGGTCTCTTGGTTTG AGGG	CATGCTAGCAAATGCC ATACA	60.1	791	DRR	Ca_10385	--	--
Ca6_P4_2523359	Ca6	2523359	ATT/ATTT	TTGCATTTGTTTCAGCA AGG	TTGCCATCATCAATTCA TCC	59.8	822	DRR	Ca_10385	--	--
Ca6_P4_2545558	Ca6	2545558	CTTTTTTT/CTTTTTTTT	AGCCCTTGAGGGATGT TTCT	TACTGAAGTGGCATCT CCCC	60.1	570	DRR	Ca_10387	O	MYB_related
Ca6_P4_25959668	Ca6	25959668	TACATCAAA/TACATCAAATATATGA CATCAAA	TGTCATCCCAAACACG TCAT	TTTTCCCCATGCAAGG TTAG	59.8	339	DRR	Ca_25499	R	C2H2
Ca6_P4_26113844	Ca6	26113844	TAAAAAAAAAA/TAAAAAAAAAAA	AGTTTGGTTCTGTGCT GCCT	TTGTAAGCCCATTCTCA CACA	59.9	769	DRR	Ca_25504	R	C2H2

INDEL marker IDs	Chromosomes/ unanchored scaffolds	Physical positions (bp)	InDels (*Kabuli* reference genome-CDC Frontier/PII)	Forward primers (5'-3')	Reverse primers (5'-3')	Anneling temperature (0C)	Expected amplified product size (bp)	Structural annotation		Functional annotation	
								Sequence components of *kabuli* genome	*Kabuli* gene accession IDs	NCBI-KOG	TFs
Ca6_P4_26354164	Ca6	26354164	GT/GTT	TGTTGATTTGGGGAAA GAGG	GGTGCCAACATCCAAC TTCT	59.9	650	DRR	Ca_16678	--	GRAS
Ca6_P4_26668470	Ca6	26668470	T/TCTATATTTTTTTA	CGACGCAATGCATTAA GAGA	CCTTAATCGCCGAAAAT CAT	60.0	490	DRR	Ca_16695	--	--
Ca6_P4_26704842	Ca6	26704842	GCC/GC	TTCAACTTGGCCTAACT CCG	ACATGGGAATCCCTTT CACA	60.2	580	DRR	Ca_16698	I	--
Ca6_P4_26749884	Ca6	26749884	ATTAAAAATTTAAAAA/ATTAAAAATT TAAAAATTTAAAAA	ATGGAGTGGAATGGAA CCAA	TTGCTTGCATTGTTTGG CTA	60.2	480	DRR	Ca_16705	B	--
Ca6_P4_26811709	Ca6	26811709	GTGTTATGTTA/GTGTTA	TTGGAGGACACACATG AGGA	TCAAAGATCGAACATC CGTTT	60.1	641	DRR	Ca_16710	K	MYB
Ca6_P4_27194999	Ca6	27194999	CA/CAA	TCACCGATAGGATTCG AACA	GACCAATTAAGAGGTT TGATGGA	59.1	538	DRR	Ca_14627	K	--
Ca6_P4_28326755	Ca6	28326755	AT/ATT	TTTGGGCTTGAAGATG AAAA	TTTAAAAGCAAGGGTG GTGG	58.3	336	DRR	Ca_25968	A	--
Ca6_P4_28327723	Ca6	28327723	CATATATATATATAT/CATATATATAT AT	CAAGAGCATCGTGTCA AGGA	GTCCTGGCGTGACAAG AAAT	60.0	424	DRR	Ca_25968	A	--
Ca6_P4_28577470	Ca6	28577470	GATATATATATATATATAT/GATATAT ATATATATATATAT	GGTGTTGGAGTCATGT CTTTGA	ATTGAACTAACCCGTG CGTC	60.0	235	DRR	Ca_17491	O	Trihelix
Ca6_P4_29085384	Ca6	29085384	CAAAAAAAA/CAAAAAAA	CGCTAGCAACAGAATT GCAG	TTCGACCGATTGAAGA AAGC	59.8	300	DRR	Ca_17464	U	LSD

**INDEL marker IDs**	**Chromosomes/ unanchored scaffolds**	**Physical positions (bp)**	**InDels (*Kabuli* reference genome-CDC Frontier/PII)**	**Forward primers (5'-3')**	**Reverse primers (5'-3')**	**Anneling temperature (0C)**	**Expected amplified product size (bp)**	**Structural annotation**		**Functional annotation**	
								**Sequence components of *kabuli* genome**	***Kabuli* gene accession IDs**	**NCBI-KOG**	**TFs**
Ca6_P4_30588229	Ca6	30588229	AATCGC/A	GGACGTCTACAACATTGGGG	CCACATGAGTGTGTTGAGGG	60.2	948	DRR	Ca_16461	--	SAP
Ca6_P4_30869145	Ca6	30869145	AATTATTATTATTATTATTATTATTATTATTATTATTATTATTATT/AATTATTATTATTATTATTATTATTATTATTATTATTATT	TGAAATTTTCTTAATTCAAATCGTGA	CCGCTTCATTACCAAAAGGA	60.2	735	DRR	Ca_16482	R	C2H2
Ca6_P4_31255770	Ca6	31255770	TAAGAAGAAGAAGAAGAAGAAGAAGAAGAAGAA/TAAGAAGAAGAAGAAGAAGAAGAAGAAGAAGAAGAA	TTACAAGAGGGAGGGCTTTTT	ATCAAATGGATTGGAGGCAG	59.3	780	DRR	Ca_15164	--	--
Ca6_P4_31331884	Ca6	31331884	TTATATATATATATATATATATA/TTATATATATATATATATATA	TGACATTGTGTATTTCGTTGTTTT	GTTTGTTGAAATTGGGGGTG	58.5	874	DRR	Ca_15166	--	--
Ca6_P4_31769480	Ca6	31769480	A/AT	CGGGAGATACGTAGGAGCAA	TCGATTATGCGTAGGGAAGG	60.2	250	DRR	Ca_15194	--	--
Ca6_P4_32150554	Ca6	32150554	ATTTTCCACTTTTCCACTTTTCCA/ATTTTCCACTTTTCCA	ATTTGAAATCACATGGGGGA	CTGCATGTTTTCCATGGTTG	60.0	292	DRR	Ca_15215	MW	--
Ca6_P4_34570110	Ca6	34570110	ATTTTTTTTTTTTT/ATTTTTTT	TGGTCGACAAGCAACTGAAG	AACAATCTCCCCCTTTTTGG	60.0	583	DRR	Ca_15816	--	--
Ca6_P4_3852175	Ca6	3852175	T/TACTA	GCGGTATTGGTCCGTGTAGT	GTTCTGTGATCCCTGGTGGT	59.9	479	DRR	Ca_05916	--	--
Ca6_P4_39781733	Ca6	39781733	CATATATATATATATATATATATATA/CATATATATATATATATATATATATATATA	TCTCCGCATAAGCTTTCGAT	TGCTTCGACACAGTAGCACC	59.9	478	DRR	Ca_15927	--	--
Ca6_P4_41118947	Ca6	41118947	CA/CAA	AAGAGTCCAAGTCCGCTGAG	ACGCTCTAAAAGCAGACCCA	59.6	635	DRR	Ca_23143	--	Trihelix

INDEL marker IDs	Chromosomes/ unanchored scaffolds	Physical positions (bp)	InDels (*Kabuli* reference genome-CDC Frontier/PII)	Forward primers (5'-3')	Reverse primers (5'-3')	Anneling temperature (0C)	Expected amplified product size (bp)	Structural annotation		Functional annotation	
								Sequence components of *kabuli* genome	*Kabuli* gene accession IDs	NCBI-KOG	TFs
Ca6_P4_41957220	Ca6	41957220	CTATATATATAT/CTATATATAT	TCCCCAACTTTTGAATTTGC	TGCTTCAGTCAATGGGACAG	59.9	877	DRR	Ca_21949	O	--
Ca6_P4_4498664	Ca6	4498664	TAAAA/TAA	TCAGCCACATTGTTTCAAGC	AATAGTGTCAGCGGGTAGTTTT	59.8	373	DRR	Ca_05852	--	BES1
Ca6_P4_4503593	Ca6	4503593	CTTTTTTTTTTTTTTT/CTTTTTTTTTTTTTT	ACCCTCAACATACCCTGCAT	GATTTGCGTTACCAATTTGATG	59.3	413	DRR	Ca_05851	--	--
Ca6_P4_46134674	Ca6	46134674	TAAAAAAAAA/TAAAAAAAA	CCTGAAATTCCTAACGCCAA	GCAGCGAAAAATCACCAAGT	60.1	446	DRR	Ca_13879	--	MYB_related
Ca6_P4_4800175	Ca6	4800175	A/ATGCATTAACATGG	ATGGGATTTGCGCATCTAAC	GCATCCACTACCGCTGAAAT	59.9	704	DRR	Ca_05819	--	--
Ca6_P4_48038283	Ca6	48038283	AATTATTATTATTATTATTATTATTATTATTATTATTATTATTATTATTATTATTATT/AATTATTATTATTATTATTATTATTATTATTATTATTATTATTATTATTATTATTATTATTATTATT	GTGGGTAATCCGTGCGTAAT	CCCAAATTTGTTTAAAGAAGGC	59.7	663	DRR	Ca_23440	--	bZIP
Ca6_P4_48792073	Ca6	48792073	TAAATTACAAATTACAAAT/TAAATTACAAAT	TTTGCAGCCACAAAAGACTG	GAACTCTGCCTTCCAGCAAC	60.0	906	DRR	Ca_20702	--	--
Ca6_P4_48925	Ca6	48925	ATTCTTCTTCTTCTTCTTCTTCTTCTT/ATTCTTCTTCTTCTTCTTCTTCTTCTTCTT	ATGTGTTTTCGTTCTTCGGG	TCCATGGGTTTATTGTGGCT	60.0	485	DRR	Ca_26378	--	--
Ca6_P4_49140036	Ca6	49140036	AA/AACA	AGAAGCATGCTGATCTCACG	CAATGCACCTGAGCTTTCAA	59.1	945	DRR	Ca_20719	--	ERF
Ca6_P4_4919152	Ca6	4919152	GTTTTTTTT/GTTTTTTTTT	AACGTGAGTGGAAAAATGGC	TCAGCTAAAATAAATGGACCAAAA	60.0	541	DRR	Ca_05801	C	--

INDEL marker IDs	Chromosomes/ unanchored scaffolds	Physical positions (bp)	InDels (*Kabuli* reference genome-CDC Frontier/PII)	Forward primers (5'-3')	Reverse primers (5'-3')	Anneling temperature (0C)	Expected amplified product size (bp)	Structural annotation		Functional annotation	
								Sequence components of *kabuli* genome	*Kabuli* gene accession IDs	NCBI-KOG	TFs
Ca6_P4_50525240	Ca6	50525240	A/AC	TGGTATGGGAGAGGGATGAG	TCCCAAAGATGCATTCCATT	59.9	817	DRR	Ca_13457	--	NAC
Ca6_P4_50867728	Ca6	50867728	AAAATAAATAAATAAA/AAAATAAATAAATAAATAAA	CCAAGAGTTATTGGGTGGAGA	TGAATTCACCTTTTACGACGC	59.0	787	DRR	Ca_19198	--	Trihelix
Ca6_P4_50977476	Ca6	50977476	TTAA/TA	TTACCTCTCACGTGCAATCG	AAGGGAAACGTTGTGTACCG	59.9	593	DRR	Ca_19195	--	--
Ca6_P4_51495570	Ca6	51495570	CTATTATTATTATTATTATT/CTATTATTATTATTATT	GCAAGCGAACAAACAACTGA	ACCTCAACCTAACCTGCCCT	60.0	837	DRR	Ca_19164	--	Nin-like
Ca6_P4_51504679	Ca6	51504679	TCA/T	ACCAACGAACGTCCTGAATC	TTTCTGCTTAAATCGGTGGG	60.0	342	DRR	Ca_19163	--	Nin-like
Ca6_P4_52075058	Ca6	52075058	GTTATTATTATT/GTTATTATTATTATT	GCGTGTCTTATTGGAGAGAGC	GGCTATCGCTTATGCAGGAC	59.1	782	DRR	Ca_17514	--	SAP
Ca6_P4_52481848	Ca6	52481848	ATTTTTTTTT/ATTTTTTTTTT	GAGCCATGGAGTTTGGTTGT	TGTGGCAACACTGTTCAGAAA	60.0	468	DRR	Ca_17538	--	--
Ca6_P4_53237470	Ca6	53237470	AATATATATATATATATATATATATA/AATATATATATATATATATATATATATATA	TCTGACTTTGGAGTTTTCACG	AGGGCACCTTCCTATTCTCC	58.0	628	DRR	Ca_22920	V	ERF
Ca6_P4_53481690	Ca6	53481690	ACT/ACTTGCATCT	GCATGCAAGTTCGAGATCAA	TAGACTTTGCGGGAGCAACT	60.0	733	DRR	Ca_18888	O	GRAS
Ca6_P4_53481750	Ca6	53481750	TAAAAAAAAAAAAA/TAAAAAAAAAAAAAAA	GCATGCAAGTTCGAGATCAA	TAGACTTTGCGGGAGCAACT	60.0	733	DRR	Ca_18888	O	GRAS

INDEL marker IDs	Chromosomes/ unanchored scaffolds	Physical positions (bp)	InDels (*Kabuli* reference genome-CDC Frontier/PII)	Forward primers (5'-3')	Reverse primers (5'-3')	Anneling temperature (0C)	Expected amplified product size (bp)	Structural annotation		Functional annotation	
								Sequence components of *kabuli* genome	*Kabuli* gene accession IDs	NCBI-KOG	TFs
Ca6_P4_54152599	Ca6	54152599	AT/ATGGTGTGCT	CGGGCAACTCTTTTTCACAT	AGCCGCTTGTCATCCTCTAA	60.1	151	DRR	Ca_16269	--	FAR1
Ca6_P4_54354788	Ca6	54354788	ATT/ATTT	TGTCTGTCAGGGTTCCATTG	ATACCAAACACGCGCCTAAA	59.5	786	DRR	Ca_16292	--	--
Ca6_P4_54354857	Ca6	54354857	AAT/A	TGTCTGTCAGGGTTCCATTG	GCATGCCTATCTCCTTCCAA	59.5	964	DRR	Ca_16292	--	--
Ca6_P4_54933465	Ca6	54933465	TTTTCT/TTTTCTTTCT	TTTACCACTTGGAGTTGGGC	TTGGTCGTTTGAGCCTTTCT	60.0	248	DRR	Ca_21211	--	B3
Ca6_P4_5546080	Ca6	5546080	TTATATATATATATATATATAT/TTATATATATATATATATATAT	GAAGCGTCGATTCACCATTT	GGGGATTCATAGAAGTCGCT	60.1	748	DRR	Ca_05744	L	C3H
Ca6_P4_5613994	Ca6	5613994	CTATATATATATATATATATATATAT/CTATATATATATATATATATATATAT	ATCTCAGCGGAGCTGGTAAA	CGGAATCATACACCTGCCTT	60.0	771	DRR	Ca_05735	O	--
Ca6_P4_563674	Ca6	563674	AATATATATATATATATATATATATATTTATATATATATAT/AATATATATATATATATATATATATTTATATATATATAT	GGATCAGATGGGTCGGTAGA	ATGCACGTTTGGTATGGTCA	59.9	441	DRR	Ca_13277	C	--
Ca6_P4_56688837	Ca6	56688837	GTTTT/GTTTTT	TGTTGAGTCGTGGTTATGGG	TGAACATGCATTCCGAAAAA	59.4	725	DRR	Ca_17406	--	--
Ca6_P4_5729834	Ca6	5729834	GAAAAAAAAA/GAAAAAAAAAA	TCTCCGCTCACCGATTACTT	ACAACCACCACGATCACTCA	59.8	630	DRR	Ca_05721	--	--
Ca6_P4_57786688	Ca6	57786688	ATTTTTTTTT/ATTTTTTTTTT	CCTTGAATGCGGGTTATGTT	GGGGTACTTGGGAGGGATTA	59.8	777	DRR	Ca_13662	R	C3H

INDEL marker IDs	Chromosomes/ unanchored scaffolds	Physical positions (bp)	InDels (*Kabuli* reference genome-CDC Frontier/PII)	Forward primers (5'-3')	Reverse primers (5'-3')	Anneling temperature (0C)	Expected amplified product size (bp)	Structural annotation		Functional annotation	
								Sequence components of *kabuli* genome	*Kabuli* gene accession IDs	NCBI-KOG	TFs
Ca6_P4_57992954	Ca6	57992954	TTATATATATATATATATAT/TTATATATATATATATATATAT	CTGATCCATCTCAAAGGGGA	GAGGAAGAAACCACGTTTGAA	60.0	434	DRR	Ca_13636	O	--
Ca6_P4_58594670	Ca6	58594670	TAAAA/TAAAAA	GATGATGTGGCTGCTCAAGA	TTCATTTGTCCGTTGAGTGC	60.0	490	DRR	Ca_15401	--	--
Ca6_P4_58785175	Ca6	58785175	TTCTCTCTCTCTCTCT/TTCTCTCTCTCTCT	TCAAATATTGATGCGACAAAAA	TTGTCAAATGCTGTTTGCGT	58.2	522	DRR	Ca_15422	K	MYB
Ca6_P4_58785791	Ca6	58785791	AAATAATAATAATAATAATAATAATAATA/AAATAATAATAATAATAATA	CGTACGCAAACAGCATTTGA	AAGAGGTCGCATCTCAAGGA	60.8	791	DRR	Ca_15422	K	MYB
Ca6_P4_58786039	Ca6	58786039	TT/TTAT	GAATACGTCAAAATTAATGCAGTTG	AAGAGGTCGCATCTCAAGGA	59.1	471	DRR	Ca_15422	K	MYB
Ca6_P4_58787039	Ca6	58787039	TAATAAATAAATAAATAAATAAA/TAATAAATAAATAAATAAATAAATAAATAAA	GCAAAGCATCTAGGTGTCCC	GCCAATCGATGAGATCCAGT	59.7	233	DRR	Ca_15422	K	MYB
Ca6_P4_58811254	Ca6	58811254	GTTTTTTTTT/GTTTTTTTTTT	GAGTGGATTTCCGTTGAGGA	TTTGATTCCTTGACATGGATACTT	60.1	388	DRR	Ca_15423	K	GATA
Ca6_P4_58823708	Ca6	58823708	GTAA/GTAAATAA	AATTTTAGATCACGTGGCGT	GCACTCATTAGCCGTTCCAT	57.2	830	DRR	Ca_15424	S	--
Ca6_P4_58845180	Ca6	58845180	AATATATATATATATATATAT/AATATATATATATAT	CTTCGCATGATTGGTTGTTG	TTTCCCCTCATGTTTGTTCC	60.1	375	DRR	Ca_15426	G	ERF
Ca6_P4_59081987	Ca6	59081987	TCG/T	TCTAATTGTGCACGCGAGTC	GATTCCCTTCAGTGGCATGT	60.0	395	DRR	Ca_18866	--	--

INDEL marker IDs	Chromosomes/ unanchored scaffolds	Physical positions (bp)	InDels (*Kabuli* reference genome-CDC Frontier/PII)	Forward primers (5'-3')	Reverse primers (5'-3')	Anneling temperature (0C)	Expected amplified product size (bp)	Structural annotation		Functional annotation	
								Sequence components of *kabuli* genome	*Kabuli* gene accession IDs	NCBI-KOG	TFs
Ca6_P4_59096470	Ca6	59096470	ACC/AC	TTTATTCGAGGTCCGCATTC	AGAGCCCTCCCACTGATTTT	60.0	455	DRR	Ca_18864	--	--
Ca6_P4_59254408	Ca6	59254408	GTATATATATATATATATATATAT/GTATATATATATATATATATATATATATATATAT	GTTGGGCACGGTTATCTGTT	TTTGATAACTTTAATTGCATTTTCG	59.9	851	DRR	Ca_18849	--	--
Ca6_P4_59399550	Ca6	59399550	GAGAAAGAAAGAAAGAAAGAAAGAAAGAAAG/GAGAAAGAAAGAAAGAAAGAAAGAAAGAAAGAAAG	GGCTATCGCTTATGCAGGAC	TTGTCTGTCAAAGTTCCCTCC	59.8	326	DRR	Ca_18842	--	SAP
Ca6_P4_595167	Ca6	595167	AATATATATATATATATATATGTATGTATATATATATATATATATATAT/AATATATATATATATATATATGTATGTATATATATATATATATATAT	TTTGCAATCATCAACCATTCA	CCTCTGACCAGGATTTTCAA	59.9	948	DRR	Ca_13281	Z	--
Ca6_P4_6163420	Ca6	6163420	ATTTTTTTTT/ATTTTTTTTTT	CCGTCCCTTAGCAAAATTCA	AAGCAACTATGGCAAATGGG	60.1	443	DRR	Ca_05680	T	G2-like
Ca6_P4_6311158	Ca6	6311158	GATATATATATATATATATATATATAT/GATATATATATATATATATATATATATAT	TGATTGACATTTCATCAAGACATAA	TGAACACGATAAGAATTGTGATAAAA	58.6	763	DRR	Ca_16901	--	--
Ca6_P4_7295988	Ca6	7295988	TTATATATATATATATATATATATATATA/TTATATATATATATATATATATATATATA	CAATTCATGTTGCACCCTTG	GGTCTGAGCACATTTGGGAG	60.0	824	DRR	Ca_09652	--	--
Ca6_P4_7321786	Ca6	7321786	A/AC	CACCCTGACCTACGGTGATT	CACCTCCAGTTGCTCTCACA	59.8	470	DRR	Ca_09649	O	TCP
Ca6_P4_7336213	Ca6	7336213	AGTTGTT/AGTT	TGTGTTTGTCAGTGTCGGGT	AACAGAATGCCTCACATCCA	60.0	477	DRR	Ca_09646	--	--
Ca6_P4_7373908	Ca6	7373908	TTATATATATATATATATATATAT/TTATATATATATATATATATATATAT	GGAAAGGCTTCACAAAGCTG	TAGCCCCTCCTGCTCATAGA	60.0	696	DRR	Ca_09643	Q	--

INDEL marker IDs	Chromosomes/ unanchored scaffolds	Physical positions (bp)	InDels (*Kabuli* reference genome-CDC Frontier/PII)	Forward primers (5'-3')	Reverse primers (5'-3')	Anneling temperature (0C)	Expected amplified product size (bp)	Structural annotation		Functional annotation	
								Sequence components of *kabuli* genome	*Kabuli* gene accession IDs	NCBI-KOG	TFs
Ca6_P4_7436843	Ca6	7436843	AA/AATTTCTTTTTGTATATCCA	TGCCAATATGAAACAAATCTCC	GTTATTTAGCTTCGCCGTGC	58.9	278	DRR	Ca_09637	O	--
Ca6_P4_7579531	Ca6	7579531	CTTTTTTTT/CTTTTTTTTT	AGCTCGTCTCACATTGCCTT	TCTTAACTCTGGCCCCTTCA	60.0	298	DRR	Ca_09627	S	--
Ca6_P4_7609224	Ca6	7609224	C/CGATTTATTCAG	TCCCTGTCTTGGTCTTGGTC	CCCGATATGTTGAGGGATTG	60.1	366	DRR	Ca_09624	R	--
Ca6_P4_7613784	Ca6	7613784	TTTCTTCTTCTTCTTCTTCTT/TTTCTTCTTCTTCTTCTT	TGATGATGACGATACGGTTGA	AAGATGACATTTCACAAATCAATCA	59.9	435	DRR	Ca_09622	--	--
Ca6_P4_7627660	Ca6	7627660	T/TG	GGCCGCAAAATTGAAAATAA	AAGTGTCCGCAAACTTTCTG	59.9	376	DRR	Ca_09619	R	WOX
Ca6_P4_7650831	Ca6	7650831	T/TAA	ACCTGCACACTGAATTGCAC	GAAACCAAAACTACTTCCAAGTGAA	59.8	738	DRR	Ca_09618	--	--
Ca6_P4_7651653	Ca6	7651653	ATCTT/ATCTTCTT	TTTAGCTAATTTTCGGTTTTCG	AAAGTCCGTTTGTTGTCGATT	57.8	355	DRR	Ca_09618	--	--
Ca6_P4_7652212	Ca6	7652212	TATAAATAAATAAATAAATAAA/TATAAATAAATAAATAAATAAATAAATAAATAAA	AATCGACAACAAACGGACTTT	TTTATTATTGAACCAATTAAACGCA	58.6	723	DRR	Ca_09617	J	FAR1
Ca6_P4_7664765	Ca6	7664765	ACCCC/ACCC	TGGACCCATACTTCAGCTCC	TCCCATAACAAAGCTCCGTC	60.1	253	DRR	Ca_09615	J	FAR1
Ca6_P4_7664932	Ca6	7664932	TA/TAA	GGACCCATACTTCAGCTCCA	GATAACAATGCGGAGATGGG	60.1	419	DRR	Ca_09615	J	FAR1

INDEL marker IDs	Chromosomes/ unanchored scaffolds	Physical positions (bp)	InDels (*Kabuli* reference genome-CDC Frontier/PII)	Forward primers (5'-3')	Reverse primers (5'-3')	Anneling temperature (0C)	Expected amplified product size (bp)	Structural annotation		Functional annotation	
								Sequence components of *kabuli* genome	*Kabuli* gene accession IDs	NCBI-KOG	TFs
Ca6_P4_7665642	Ca6	7665642	TACACACAC/TACACAC	AAAGTCCTCCCCTTCC TTGA	GGGAGACACATTATCC GGTG	60.0	254	DRR	Ca_09615	J	FAR1
Ca6_P4_7700010	Ca6	7700010	TA/T	CGGAGCGCTAAAGTTG AATC	CACGATGTCACGCATT AACC	60.0	382	DRR	Ca_09611	--	--
Ca6_P4_7705531	Ca6	7705531	CTATTATTA/CTATTATTATTA	CAGATGGGCCGTATGT TCTT	CAACCGACAATCACAT CACA	60.0	511	DRR	Ca_09610	--	--
Ca6_P4_7706133	Ca6	7706133	TAAAAA/TAAAA	TGTGATGTGATTGTCG GTTG	CGGTCGAATCTTTACC CTCA	58.9	664	DRR	Ca_09609	--	--
Ca6_P4_7723583	Ca6	7723583	TG/T	GCAATTTTCCTGGCTG GTTA	TGCATGCCGAACCATT ATAG	60.1	431	DRR	Ca_09607	--	--
Ca6_P4_7729316	Ca6	7729316	GATATATATATA/GATATATATA	TGAGATGGTCCGACAA AGAA	GGGTATCAGGAAATGA GCCA	59.2	431	DRR	Ca_09605	K	NF-YB
Ca6_P4_7760430	Ca6	7760430	CAAAAAAAAAA/CAAAAAAAAA	CAAGCATTTTTGGTCC CTGT	TCCTTGGTGGAACAAA ACATC	60.0	291	DRR	Ca_09602	R	SBP
Ca6_P4_7766962	Ca6	7766962	ATT/ATTT	GGAAGCCATTATTGGG GATT	CCCAGCAGAACATACA GGGT	60.0	505	DRR	Ca_09600	--	bZIP
Ca6_P4_7826714	Ca6	7826714	GT/GTGTTAAAT	CCAAAACCTTGCGATC AATAA	TCACACGCACATAAAT GATTAAGTT	60.0	387	DRR	Ca_09596	--	--
Ca6_P4_7839856	Ca6	7839856	AATGAT/AAT	GTTCCCTCCCCATTTC CTAA	TCCACGTGCTTTGACA CATT	60.1	341	DRR	Ca_09595	S	GRAS

INDEL marker IDs	Chromosomes/ unanchored scaffolds	Physical positions (bp)	InDels (*Kabuli* reference genome-CDC Frontier/PII)	Forward primers (5'-3')	Reverse primers (5'-3')	Anneling temperature (0C)	Expected amplified product size (bp)	Structural annotation		Functional annotation	
								Sequence components of *kabuli* genome	*Kabuli* gene accession IDs	NCBI-KOG	TFs
Ca6_P4_7847894	Ca6	7847894	A/AG	TTGCCTTTCATTGCACTCAG	TTTTGTACCCCGTTGTGTCA	60.0	461	DRR	Ca_09594	P	MIKC
Ca6_P4_7878598	Ca6	7878598	GT/GTT	GTTGGCAGGTCGAAAGAAAG	GGGAATTTGCAATCAGAGGA	59.9	739	DRR	Ca_09589	--	NAC
Ca6_P4_7892768	Ca6	7892768	AG/A	GGTGTCTCCGTCACGAGTTT	TCCGCTTCAAAAGTTCAACA	60.2	724	DRR	Ca_09586	I	--
Ca6_P4_7893085	Ca6	7893085	AAAGAA/AAA	CCTTACTTTGACGACTTGTTGG	CAATCCGCTTCAAAAGTTCA	58.8	526	DRR	Ca_09586	I	--
Ca6_P4_792792	Ca6	792792	ATTTGTTTTGTTTTGTTTTGTT/ATTTGTTTTGTTTTGTT	CGCAGCCCTTTTTGTTTGTA	CAATTGTTTTAAGGAATTCCAATG	61.1	421	DRR	Ca_13295	--	GRAS
Ca6_P4_7941617	Ca6	7941617	CTAGT/CTAGTAGT	TTGCTTCTAATGGGTCGGTC	ATAGGACTCGGTCACATGGC	60.1	154	DRR	Ca_09580	--	--
Ca6_P4_7947224	Ca6	7947224	TATATAAAATAATATAAAATAAT/TATATAAAATAAT	TGATCCTTCCGATGATAGCC	ACGGCAATTGCGTTAAAACT	60.0	678	DRR	Ca_09579	J	--
Ca6_P4_7948041	Ca6	7948041	AA/AAATA	AATTGCCGTTGAAAGTCGAG	GCCTATGGCCAAATTGCTTA	60.2	906	DRR	Ca_09579	J	--
Ca6_P4_7958310	Ca6	7958310	AATATATATA/AATATATA	CAAATTCAACCCAAACACCC	AATACCACATTGCGAGGCTT	60.1	352	DRR	Ca_09578	--	ERF
Ca6_P4_7958786	Ca6	7958786	AAATAT/AAATATCGACAATAT	AAGCCTCGCAATGTGGTATT	ATCCAACGGCGTCAAATAAA	59.6	675	DRR	Ca_09578	--	ERF

INDEL marker IDs	Chromosomes/ unanchored scaffolds	Physical positions (bp)	InDels (*Kabuli* reference genome-CDC Frontier/PII)	Forward primers (5'-3')	Reverse primers (5'-3')	Anneling temperature (0C)	Expected amplified product size (bp)	Structural annotation		Functional annotation	
								Sequence components of *kabuli* genome	*Kabuli* gene accession IDs	NCBI-KOG	TFs
Ca6_P4_7998659	Ca6	7998659	TATCATATTAT/TATCATATTATCAATCATATTAT	TGGTCCACGTGATACCAAAA	CCATTTAAACGCACAACCAA	59.8	709	DRR	Ca_09575	--	--
Ca6_P4_8061727	Ca6	8061727	CTT/CTTCAATCCTTT	TGTTTTGCAGGGATTTGTGA	GATGGATGGGAGACGAAAGA	60.1	631	DRR	Ca_09567	G	--
Ca6_P4_8062172	Ca6	8062172	T/TG	TTTCGTCTCCCATCCATCTC	CGTCACCCAAAGCAATTGTA	60.0	532	DRR	Ca_09567	G	--
Ca6_P4_8062636	Ca6	8062636	ATTTTTTTTTT/ATTTTTTTT	TACAATTGCTTTGGGTGACG	AGGCGCCTAAATCTTACGGT	59.6	774	DRR	Ca_09567	G	--
Ca6_P4_8062787	Ca6	8062787	CTTTT/CTTT	TACAATTGCTTTGGGTGACG	GAAGGGTACTGGGCAGATGA	59.6	864	DRR	Ca_09567	G	--
Ca6_P4_8066437	Ca6	8066437	CTA/CTACCTAAAATA	AAGTGGTTGGGATGAAAAAGAA	TTGATTTCGGTTTTGGAAGC	59.9	567	DRR	Ca_09565	--	MYB_related
Ca6_P4_8078323	Ca6	8078323	GATATATATATATATATATATATAT/GATATATATATATATAT	ATGTTGGTTCTTGTGCCCTC	CCAAAATCCACAATCATCCC	60.0	623	DRR	Ca_09564	U	Trihelix
Ca6_P4_8093284	Ca6	8093284	CTAT/CTATAT	GCCTTTTGCAGGTAAAGCAA	TCAACGCCCTGTATGAATGA	60.4	571	DRR	Ca_09562	S	CAMTA
Ca6_P4_8133267	Ca6	8133267	T/TATTTTTTTTA	CATGCAGATCTGGTGGTTGT	TTGCAGCAGTTGCACTTAGG	59.6	466	DRR	Ca_09555	P	GRAS
Ca6_P4_8174386	Ca6	8174386	TG/T	AACTCACCGGTTGACTTTGC	GAGTGGTGAGCATGCGTTTA	60.2	924	DRR	Ca_09552	K	--

INDEL marker IDs	Chromosomes/ unanchored scaffolds	Physical positions (bp)	InDels (*Kabuli* reference genome-CDC Frontier/PII)	Forward primers (5'-3')	Reverse primers (5'-3')	Anneling temperature (0C)	Expected amplified product size (bp)	Structural annotation		Functional annotation	
								Sequence components of *kabuli* genome	*Kabuli* gene accession IDs	NCBI-KOG	TFs
Ca6_P4_8594301	Ca6	8594301	ATTATAT/A	AAGCACCATTTGGCTACACC	GCCTCAACCGTTTTATTTGG	60.0	806	DRR	Ca_16779	--	--
Ca6_P4_9060428	Ca6	9060428	TTATAAAACATATAAAA/TTATAAAA	AAAGTACAATGAGTCAAACAATTATCC	ATATGCTTCACTTGCCCCAC	58.2	555	DRR	Ca_16733	E	--
Ca6_P4_9203559	Ca6	9203559	G/GTTATATT	TCGTGAAATAGTGGACGGTG	TGAACATCAAAATGCCCAAA	59.6	763	DRR	Ca_08676	OT	--
Ca6_P4_9952396	Ca6	9952396	ATTAATTTAATTTAATTTAATTTAATTTAATTTAATTTA/ATTAATTTAATTTAATTTAATTTAATTTAATTTAATTTAATTTA	GGAGCACAAGAAATCTCGGA	TTGGAAATGGTTTGCTTGAA	60.3	345	DRR	Ca_08595	--	--
Ca7_P4_10049659	Ca7	10049659	GTCTCTCTCTCTCT/GTCTCTCTCTCT	TGGATTTGTGTGTCGTTGGT	TATGCAGATGCCTCGTTCAG	59.9	679	DRR	Ca_12768	--	--
Ca7_P4_10690477	Ca7	10690477	GTT/GTTTT	CCTGGCTTGCAAACATACAA	CAGCCAAGTAACACGCAAAA	59.7	897	DRR	Ca_12825	K	ARR-B
Ca7_P4_10757230	Ca7	10757230	A/AT	GGTCTCATAGTTCTCATCGCGT	TCGCTTGGACAATTCTCGTT	60.7	533	DRR	Ca_12830	E	TALE
Ca7_P4_10831556	Ca7	10831556	GTTTTTTTT/GTTTTTTT	CACCTCCAAGTCCAACCAAT	TGCAGCGTTTTGTTTTGTTC	59.8	334	DRR	Ca_12836	S	--
Ca7_P4_10877853	Ca7	10877853	CTTTTT/CTTT	GATGCGGGTTCGGTCTAGTA	TTGCGTTGTGTTGTGATGTG	60.1	444	DRR	Ca_12842	R	CPP
Ca7_P4_11106661	Ca7	11106661	CAATTTTAAT/CAAT	AGTGTTTGGGAAATGCTTGG	TGAAATTATGCACGTGTGGG	60.0	689	DRR	Ca_09376	G	--

INDEL marker IDs	Chromosomes/ unanchored scaffolds	Physical positions (bp)	InDels (*Kabuli* reference genome-CDC Frontier/PII)	Forward primers (5'-3')	Reverse primers (5'-3')	Anneling temperature (0C)	Expected amplified product size (bp)	Structural annotation		Functional annotation	
								Sequence components of *kabuli* genome	*Kabuli* gene accession IDs	NCBI-KOG	TFs
Ca7_P4_11697550	Ca7	11697550	TAATAATAATAACTCTGAATAATAATAACTCTGAATAATAA/TAATAATAATAACTCTGAATAATAATAACTCTGAATAATAATAACTCTGAATAATAA	GGCTATCGCTTATGCAGGAC	TTCAGCGATTTATTCAACGATTT	59.8	709	DRR	Ca_09328	--	SAP
Ca7_P4_12465968	Ca7	12465968	TTATATATATATATATATATATATATATAT/TTATATATATATATATATATATATATATATATATATAT	TTATGAACTCGGTTGCTTCG	CAGATGTGAGTCAGTCCGAGA	58.9	331	DRR	Ca_09270	--	--
Ca7_P4_12483012	Ca7	12483012	T/TAAGGAG	GCGCAAACTGCAAAGTCATA	CCTTTTCGGACAGGAAATCA	60.0	723	DRR	Ca_09268	R	bHLH
Ca7_P4_12940315	Ca7	12940315	AA/AATA	TTCCTTCCCTCCCTCTCATT	TTTCATCTAGCGAACCCCTC	60.0	671	DRR	Ca_09227	--	--
Ca7_P4_13555513	Ca7	13555513	C/CGTAAT	TTTCTAATCAGTGGCCTGGTC	TCAACGAGCATGTGGAAAAG	59.2	554	DRR	Ca_16040	--	bZIP
Ca7_P4_13958576	Ca7	13958576	GTTATTTATTTATTTATTTATTTAT/GTTATTTATTTATTTATTTAT	TACATTGGCAACTCATCCCA	TTTTTAATTTGGTGTGGCCT	59.9	668	DRR	Ca_16068	--	--
Ca7_P4_1420274	Ca7	1420274	TAAAAAAAAAAA/TAAAAAAAAAAAA	TTAGATTAATGTTTTCTTTTTGTTCG	TCTAAAAGACTCATCGCGGC	57.4	466	DRR	Ca_03326	--	--
Ca7_P4_14529836	Ca7	14529836	TAAAAAAAAA/TAAAAAAAA	TAACCCAACGCAATTTGATG	ACGTCGAGGGGTGTGAATAG	59.4	317	DRR	Ca_09990	Q	B3
Ca7_P4_14904978	Ca7	14904978	GCCCC/GCCCCC	AGAATGAAGAGGAGCGACCA	TTTTAACTGGACCGAAACCG	60.0	534	DRR	Ca_09960	O	bZIP
Ca7_P4_16548666	Ca7	16548666	TG/T	TCCCTTCTACTTGGTGGTGG	GTGATTCATGCTCCAAGGCT	60.0	624	DRR	Ca_20555	R	--

INDEL marker IDs	Chromosomes/ unanchored scaffolds	Physical positions (bp)	InDels (*Kabuli* reference genome-CDC Frontier/PII)	Forward primers (5'-3')	Reverse primers (5'-3')	Anneling temperature (0C)	Expected amplified product size (bp)	Structural annotation		Functional annotation	
								Sequence components of *kabuli* genome	*Kabuli* gene accession IDs	NCBI-KOG	TFs
Ca7_P4_16861754	Ca7	16861754	AT/A	ATTTTGTGACGCTTGTTCCA	AATGGGCGCTGTTAAATGTC	59.2	532	DRR	Ca_15889	--	--
Ca7_P4_17550568	Ca7	17550568	TTATATATATATATATATATATATATATATATAT/TTATATATATATATATATATATATATATATATATATATATATATATATATAT	GCCGTTTCAATATTCTTTTCTCTT	GCATATGGGGCCTACAAGAA	59.3	547	DRR	Ca_15852	S	--
Ca7_P4_17551081	Ca7	17551081	CATTATTATTATTATTATTATTATTATTATTATTATTATTATTATTATTATTATTA/CATTATTATTATTATTATTATTATTATTATTATTATTATTATTATTATTATTATTATTATTA	CCTTTCTTGTAGGCCCCATA	TCACCCTTACCTTCAACAACAA	59.0	419	DRR	Ca_15852	S	--
Ca7_P4_17922655	Ca7	17922655	TGGGGGG/TGGGGG	AAGTCGAGTGCTTGAGCCAT	TCTTTGCGTGCGTGCTATAC	60.0	606	DRR	Ca_15831	--	bHLH
Ca7_P4_1807047	Ca7	1807047	CAAAAAAAAA/CAAAAAAAAAA	TGTTGTCACACCAACTCTTGC	GCTTGGTCCCATTTGAGAGA	59.8	846	DRR	Ca_03288	--	--
Ca7_P4_1807643	Ca7	1807643	ATTATTTTTTTTTATTTTTTTTTTATTTTT/ATTATTTTTTTTTTATTTTT	TCTCTCAAATGGGACCAAGC	TCATGTTTGCCGTACTCCAG	60.2	566	DRR	Ca_03288	--	--
Ca7_P4_18163966	Ca7	18163966	CTATATATATATATATATAT/CTATATATATATATATATATATAT	CCACTCCATTTTCCAACGAT	TCTTTAGTAGCCCGCCAAGA	59.8	594	DRR	Ca_22139	R	ZF-HD
Ca7_P4_18165031	Ca7	18165031	ATAGAATTGTCTAGAATTGTCTA/ATAGAATTGTCTA	GCTACGTACGGCTGAAGTCC	CGATTGTTGATCCTCCTCGT	59.9	744	DRR	Ca_22139	R	ZF-HD
Ca7_P4_19007034	Ca7	19007034	TAAA/TAA	TTATGGACGCTTTATTTAGTTTAAGA	ATGACCATTGAGTTCGCATC	57.1	347	DRR	Ca_12315	--	--
Ca7_P4_20252580	Ca7	20252580	GTAGAATA/GTAGAATAGAATA	ACCTGTCAGCTAAGAGCCCA	CACATGTGTGAACCTCCTGG	60.0	641	DRR	Ca_12383	--	--

INDEL marker IDs	Chromosomes/ unanchored scaffolds	Physical positions (bp)	InDels (*Kabuli* reference genome-CDC Frontier/PII)	Forward primers (5'-3')	Reverse primers (5'-3')	Anneling temperature (0C)	Expected amplified product size (bp)	Structural annotation		Functional annotation	
								Sequence components of *kabuli* genome	*Kabuli* gene accession IDs	NCBI-KOG	TFs
Ca7_P4_2047396	Ca7	2047396	TTATATATATATATATATATATATAT/TTATATATATATATATATATATATATAT	ATGGGACTAGGCAACACGTC	TTTCAACCGTAAATCATACTTGG	60.0	630	DRR	Ca_03268	GC	HSF
Ca7_P4_20494272	Ca7	20494272	CAA/CAAA	AGACGTGTCGATCGAGCTTT	AGCTGAATGCAAACCCTTGA	60.0	497	DRR	Ca_12400	--	AP2
Ca7_P4_20525384	Ca7	20525384	TAAAAAAAAAA/TAAAAAAAAA	TGTTCACTGCCAAACTCTGC	GGAAATAGTGATAGCGGCCA	60.0	640	DRR	Ca_12401	--	--
Ca7_P4_20539386	Ca7	20539386	CAAAAAAAAA/CAAAAAAAA	TTCCTTCCTTGATGAATGGC	GTAAGTCACCGACAAGGGGA	60.0	335	DRR	Ca_12403	--	C2H2
Ca7_P4_20557114	Ca7	20557114	ATCACTCA/ATCACTCACTCA	TACTTTGCCGCCTCTGCTAT	CAAACGCGCAGATCAGTAAA	60.0	589	DRR	Ca_12405	K	--
Ca7_P4_20571099	Ca7	20571099	AAAA/AAAATAAA	TCCCAAATAAATGAGGGCAA	ACAGACGGCGAAAATTGTTT	60.3	381	DRR	Ca_12407	--	--
Ca7_P4_20600329	Ca7	20600329	GAAAAAAAAAA/GAAAAAAAA	TGGTGCAGAGTAAAGAATGGAA	GTCCCCCATAAACATTCACG	59.7	579	DRR	Ca_12410	--	bHLH
Ca7_P4_20604250	Ca7	20604250	CTAATATAATATAATA/CTAATATAATATAATATAATA	ACCATTTCCCCTTTCGATTT	TGCTTTGTTCAATTGCTTGC	59.6	589	DRR	Ca_12412	YU	--
Ca7_P4_21743270	Ca7	21743270	CAA/CA	TTGCCCCTCTAAATTCTTGG	TCCCTCGTATTTAGCCCTCA	59.2	686	DRR	Ca_14521	--	--
Ca7_P4_21743482	Ca7	21743482	TGGCTAAATATGAGGGCTAAATA/TGGCTAAATA	TCGAAGCATAAGGTTGATCC	GGGATTGAGCGCCTATACAA	57.8	500	DRR	Ca_14521	--	--

INDEL marker IDs	Chromosomes/ unanchored scaffolds	Physical positions (bp)	InDels (*Kabuli* reference genome-CDC Frontier/PII)	Forward primers (5'-3')	Reverse primers (5'-3')	Anneling temperature (0C)	Expected amplified product size (bp)	Structural annotation		Functional annotation	
								Sequence components of *kabuli* genome	*Kabuli* gene accession IDs	NCBI-KOG	TFs
Ca7_P4_21743627	Ca7	21743627	ATTTTTTT/ATTTTTTTT	TGAGGGCTAAATACGAGGGA	GGGATTGAGCGCCTATACAA	59.7	461	DRR	Ca_14521	--	--
Ca7_P4_21743947	Ca7	21743947	AA/AAATA	CCTGGTGTGCATTGAAAAGA	AATTGCGGTTTTGGTTCAAG	59.7	410	DRR	Ca_14521	--	--
Ca7_P4_21876016	Ca7	21876016	GTTTTTTTT/GTTTTTTT	GTTTGGGGTTTTGGGTTCTT	CCACCATTGTTATGTGGACG	60.1	593	DRR	Ca_14530	S	--
Ca7_P4_22327481	Ca7	22327481	TC/T	GATTCATCGCAGCTCAAACA	GATCAAAACCGGGGAAAAAT	60.0	638	DRR	Ca_14548	--	--
Ca7_P4_22362135	Ca7	22362135	AG/A	TGTCAGTACCCACCCACTCA	CAAGGGAGGCTGTTGTGAAT	60.0	914	DRR	Ca_14551	--	SAP
Ca7_P4_22554279	Ca7	22554279	TC/TCCAAC	AGTGGAGGCAAACCATAACG	CACCCACCCACCAATAGTGT	60.0	578	DRR	Ca_19538	T	FAR1
Ca7_P4_23667025	Ca7	23667025	AAATAATAATAATAATAATA/AAATAATAATAATAATAATAATA	ACACTGATTTCCCAAAACGC	TTTCCTTCCCTTCTCATTGC	60.0	728	DRR	Ca_26877	G	FAR1
Ca7_P4_23851009	Ca7	23851009	G/GCA	TAACGTTCCCACGGCTTAAC	AAGGATGCGACGAAATTGAG	60.0	593	DRR	Ca_26881	--	--
Ca7_P4_24786712	Ca7	24786712	AATATATATATATATATATATATAT/AATATATATATATATATATATATATAT	ACAGACCCGACCAAGTCAAC	TCAAACATTTAACGATCACCATT	60.0	447	DRR	Ca_19894	GC	HSF
Ca7_P4_25118793	Ca7	25118793	AGG/AG	CTCTCACCCAAGAAGATCGC	CCCTGTGACAAACACAATCG	60.0	608	DRR	Ca_23601	--	--

INDEL marker IDs	Chromosomes/ unanchored scaffolds	Physical positions (bp)	InDels (*Kabuli* reference genome-CDC Frontier/PII)	Forward primers (5'-3')	Reverse primers (5'-3')	Anneling temperature (0C)	Expected amplified product size (bp)	Structural annotation		Functional annotation	
								Sequence components of *kabuli* genome	*Kabuli* gene accession IDs	NCBI-KOG	TFs
Ca7_P4_25984688	Ca7	25984688	CTTTTTTTTT/CTTTTTTTT	GCCATTTATGGCAGAGGTTT	TTTGATGTTGTTGGAGATGAAAA	59.0	571	DRR	Ca_23636	--	NAC
Ca7_P4_26700837	Ca7	26700837	C/CTT	ACTATCCGTTCTGTGGTGCC	CAGTGGCTTGGTTAAGGGAA	60.0	835	DRR	Ca_16249	--	--
Ca7_P4_26701106	Ca7	26701106	A/ACTAGGAATTACTCCC	CAATAGGGCGAGTCGAATGT	AAGTCCAGTAGCCGAAAGCA	60.1	838	DRR	Ca_16249	--	--
Ca7_P4_26701534	Ca7	26701534	C/CG	AAGCTTCATCCTGGTCATGG	AAGTCCAGTAGCCGAAAGCA	60.1	381	DRR	Ca_16249	--	--
Ca7_P4_2831340	Ca7	2831340	TAAAAAAAAA/TAAAAAAAAAA	TGATAATCTCGCGGGATAGG	GAATTGCCGAAAAATGATTGA	60.0	465	DRR	Ca_03193	K	GATA
Ca7_P4_29038300	Ca7	29038300	TAAAAAAAAAAAA/TAAAAAAAAAAA	TTCGAAATCTTAATGTGCTCAAA	GGGTAAATCGATTAGTCCGGT	58.9	646	DRR	Ca_11800	--	--
Ca7_P4_29433568	Ca7	29433568	T/TG	AGGCATGATTCAAGGTACGG	CAGGTGAAGGTCCATTTCGT	60.0	278	DRR	Ca_11776	--	--
Ca7_P4_29446201	Ca7	29446201	ATTTTTTTTTT/ATTTTTTTTTTT	CGTTGCGCATGTGTAATTTG	TCATTCCCAACATTATTTCGTG	61.1	639	DRR	Ca_11775	R	--
Ca7_P4_29610109	Ca7	29610109	T/TTC	AACAGAAAGAAGGCCGTTTG	TGAGTTAAGCCCCGCTAGAA	59.4	134	DRR	Ca_11764	--	--
Ca7_P4_30036720	Ca7	30036720	TCTACTA/TCTA	CACCTTTGCCAACCACTTTT	ACCACATTCATGTGCTTGGA	60.0	638	DRR	Ca_11724	O	--

INDEL marker IDs	Chromosomes/ unanchored scaffolds	Physical positions (bp)	InDels (*Kabuli* reference genome-CDC Frontier/PII)	Forward primers (5'-3')	Reverse primers (5'-3')	Anneling temperature (0C)	Expected amplified product size (bp)	Structural annotation		Functional annotation	
								Sequence components of *kabuli* genome	*Kabuli* gene accession IDs	NCBI-KOG	TFs
Ca7_P4_30102864	Ca7	30102864	GA/G	TAAACTGGGGCAATCACACA	TGATTATGCAGCAACCAAAA	60.0	585	DRR	Ca_11717	--	bZIP
Ca7_P4_30166196	Ca7	30166196	CAA/CTTAAA	TGAGCCCATAATGTGCAGAA	CTTTTTGAAAGGGCATCAGC	60.2	520	DRR	Ca_11711	Q	B3
Ca7_P4_30220020	Ca7	30220020	CA/C	CGCTCTAGCGCAAACTAAGG	AACACCGACACCGAATTCAT	60.3	163	DRR	Ca_11705	GC	HSF
Ca7_P4_30232337	Ca7	30232337	ATTTTTT/ATTTTT	GGAGCACAAGATCACTCATGTC	TTCGGGAGTCGACTATACGC	59.7	245	DRR	Ca_11704	GC	HSF
Ca7_P4_30233283	Ca7	30233283	CT/CTAAT	CCATTATGCACCCCTTTCAC	GGAAGATGGATCCTCCACAA	60.2	420	DRR	Ca_11704	GC	HSF
Ca7_P4_30424399	Ca7	30424399	AT/ATT	TCATCTTCGATGCAAATCCA	TCGTTCACGACTGATGTTGA	60.2	938	DRR	Ca_11688	--	MYB_related
Ca7_P4_30874290	Ca7	30874290	TAA/TAAA	TTGTCTCAACAATCTCCCCC	ACCACCTCACTGCCCTACAC	59.9	734	DRR	Ca_23317	--	--
Ca7_P4_30930614	Ca7	30930614	TCC/TC	CCACTCAAGGCCAGTGATTT	ATGAGCAGCACCAACATCAA	60.1	222	DRR	Ca_23314	--	--
Ca7_P4_30967683	Ca7	30967683	ATTTT/ATTTTT	CCCCATGATCTTCCCTTTTT	GCAACAAACCTATGGCAGGT	60.1	355	DRR	Ca_23312	--	--
Ca7_P4_31186109	Ca7	31186109	T/TG	CACTTTTGAGAGACCCGGAA	GAGCAAATCCCTTACCCACA	60.2	320	DRR	Ca_10140	T	--

INDEL marker IDs	Chromosomes/ unanchored scaffolds	Physical positions (bp)	InDels (*Kabuli* reference genome-CDC Frontier/PII)	Forward primers (5'-3')	Reverse primers (5'-3')	Anneling temperature (0C)	Expected amplified product size (bp)	Structural annotation		Functional annotation	
								Sequence components of *kabuli* genome	*Kabuli* gene accession IDs	NCBI-KOG	TFs
Ca7_P4_31196032	Ca7	31196032	ATTTT/ATT	CAACCAAGATTTTGATGAGTCG	TTGTTCACGGTGGTAATTTTT	59.6	287	DRR	Ca_10139	K	NAC
Ca7_P4_31196582	Ca7	31196582	TTTTATATT/TTTTATATTTATATT	AAAAATTACCACCGTGAACAA	TGTTCTTAACCTAGCTGGCGA	57.1	897	DRR	Ca_10138	--	--
Ca7_P4_31197530	Ca7	31197530	A/AGTGACACG	CCTTCGTTTAAACTTGTGACACC	GTGGCAGGTGCAGGTAAAGT	60.0	602	DRR	Ca_10138	--	--
Ca7_P4_31210939	Ca7	31210939	CTC/CTCAATC	CCTTTCTCAGCCCATATCCA	TCAACAATGCAAACGAAGGA	60.0	306	DRR	Ca_10136	--	ARF
Ca7_P4_31215378	Ca7	31215378	GAAAAAAAAA/GAAAAAAAAAA	GTTGTGCCGCTTAAGTTGGT	ATGGGACCACTTGGTGGTAA	60.2	854	DRR	Ca_10135	G	--
Ca7_P4_31216107	Ca7	31216107	TC/T	TTACCACCAAGTGGTCCCAT	CGAAATTTTAAAGGTCGCCA	60.1	741	DRR	Ca_10135	G	--
Ca7_P4_32230923	Ca7	32230923	TACA/TA	CTGGGCAAGGGATCTGAATA	ATTGCCCACCCATAATTTCA	60.0	540	DRR	Ca_10050	--	Trihelix
Ca7_P4_32901214	Ca7	32901214	AATTTTTTTATTTTTTTATTTTTTTA/AATTTTTTTATTTTTTTA	GTAGTGCAAACCCTGGTGCT	GTCGCTGGTGTGACTGAAAA	60.2	462	DRR	Ca_10009	--	bZIP
Ca7_P4_33060172	Ca7	33060172	GTTTTTTTTT/GTTTTTTTTTTTT	TGAATTGTTGAAGGGGCCTA	TGGGAATTTGGATCTTTTTGTC	60.4	635	DRR	Ca_09996	--	--
Ca7_P4_33060237	Ca7	33060237	ATTTTTTT/ATTTTTT	TGAATTGTTGAAGGGGCCTA	TGGGAATTTGGATCTTTTTGTC	60.4	635	DRR	Ca_09996	--	--

INDEL marker IDs	Chromosomes/ unanchored scaffolds	Physical positions (bp)	InDels (*Kabuli* reference genome-CDC Frontier/PII)	Forward primers (5'-3')	Reverse primers (5'-3')	Anneling temperature (0C)	Expected amplified product size (bp)	Structural annotation		Functional annotation	
								Sequence components of *kabuli* genome	*Kabuli* gene accession IDs	NCBI-KOG	TFs
Ca7_P4_33159162	Ca7	33159162	CT/C	TGTTCCAACTTCCAAC CTCC	GACAAATGATGCTTCT GGCA	59.9	207	DRR	Ca_16147	--	--
Ca7_P4_33230823	Ca7	33230823	TTATATATA/TTATATATATA	CCAAATTCTCCACACG GTCT	GAATTATGTCGATGGTT TTTGACA	60.0	622	DRR	Ca_16150	--	--
Ca7_P4_33243964	Ca7	33243964	GA/GAA	CAACGGTGCAGAGTGA AAAA	TGTTTCATAACTGAAGG GGGA	59.9	624	DRR	Ca_16151	--	--
Ca7_P4_33244102	Ca7	33244102	TT/TTTTAT	CGAATGTGGAACAATT CATGT	TGTTTCATAACTGAAGG GGGA	58.3	547	DRR	Ca_16151	--	--
Ca7_P4_33258061	Ca7	33258061	TTC/T	CCTGTGGGGGACACTT TCTA	ACTCCATCTGTTCCCC AATG	60.0	333	DRR	Ca_16153	S	--
Ca7_P4_33258253	Ca7	33258253	TAATGA/TAATGATCATTTAATAAAAT GA	CCTGTGGGGGACACTT TCTA	ACTCCATCTGTTCCCC AATG	60.0	333	DRR	Ca_16153	S	--
Ca7_P4_33336366	Ca7	33336366	T/TA	AAGCCAGGCGATTTAT GATG	ATCGGCAAATATCCGA GAAA	60.1	510	DRR	Ca_16158	R	G2-like
Ca7_P4_33336445	Ca7	33336445	TTG/TTGCTTGATTATG	AAGCCAGGCGATTTAT GATG	ATCGGCAAATATCCGA GAAA	60.1	510	DRR	Ca_16158	R	G2-like
Ca7_P4_33361035	Ca7	33361035	GATATATATATATATATA/GATATATA TATATATATATATA	TGCACGACGCTAATTC AGTC	TCCATGTACACTGGCA CTCC	60.0	410	DRR	Ca_16161	YU	--
Ca7_P4_33367476	Ca7	33367476	CT/CTT	GGGGGATGATCATATC TTGTG	CACGGAGACCAACGAA ACTT	59.1	677	DRR	Ca_16162	YU	--

INDEL marker IDs	Chromosomes/ unanchored scaffolds	Physical positions (bp)	InDels (*Kabuli* reference genome-CDC Frontier/PII)	Forward primers (5'-3')	Reverse primers (5'-3')	Anneling temperature (0C)	Expected amplified product size (bp)	Structural annotation		Functional annotation	
								Sequence components of *kabuli* genome	*Kabuli* gene accession IDs	NCBI-KOG	TFs
Ca7_P4_33367558	Ca7	33367558	ATTTTTTTT/ATTTTTTT	GGGGGATGATCATATCTTGTG	CACGGAGACCAACGAAACTT	59.1	677	DRR	Ca_16162	YU	--
Ca7_P4_33367648	Ca7	33367648	TTATATAT/TTATAT	CCCTATTAAGCATGAACATGGA	CACGGAGACCAACGAAACTT	59.0	507	DRR	Ca_16162	YU	--
Ca7_P4_33382900	Ca7	33382900	TCC/TC	TGACGTGACACATCTAACAAACA	TGAAAGCATCATGCAAAAAGA	59.2	379	DRR	Ca_16163	--	--
Ca7_P4_33409151	Ca7	33409151	TA/TAAATAACAA	GCATCATGAGTTTGAGGGGA	TATCATGCGTCCATGCACTT	61.0	430	DRR	Ca_16164	--	C3H
Ca7_P4_33409928	Ca7	33409928	CAAAAA/CAAAA	CCGAAAGAATTTGGATCATCA	TTCTTGCATGATTTGGGACA	59.9	405	DRR	Ca_16164	--	C3H
Ca7_P4_33483669	Ca7	33483669	TTATATA/TTATA	ATTCAGTGGGCAACAGAACC	CATGACTGTGCCAAACTGCT	60.0	731	DRR	Ca_16169	--	--
Ca7_P4_33537677	Ca7	33537677	CA/C	GTCAATGTTGTGGGAGGTGA	ATTTTGGCCAATCCAGCTAA	59.4	337	DRR	Ca_16173	--	--
Ca7_P4_33538151	Ca7	33538151	AAAAAA/AAAAAAGAAAAA	TTAGCTGGATTGGCCAAAAT	GGTCCATGGATGAGCTTGTT	59.5	396	DRR	Ca_16173	--	--
Ca7_P4_33560601	Ca7	33560601	TGG/TGGG	TCTTGAGATCTTGGGCTTTGA	AAGGGAAAGGGGAGTACACG	59.9	337	DRR	Ca_16175	QI	B3
Ca7_P4_33575183	Ca7	33575183	TAAAAAAAAAAA/TAAAAAAAAAAAA	CAAATTGCATTTTCCTCCCT	TTGTTGGTGAGTGCTCTTGG	59.0	691	DRR	Ca_16178	I	--

INDEL marker IDs	Chromosomes/ unanchored scaffolds	Physical positions (bp)	InDels (*Kabuli* reference genome-CDC Frontier/PII)	Forward primers (5'-3')	Reverse primers (5'-3')	Anneling temperature (0C)	Expected amplified product size (bp)	Structural annotation		Functional annotation	
								Sequence components of *kabuli* genome	*Kabuli* gene accession IDs	NCBI-KOG	TFs
Ca7_P4_33576122	Ca7	33576122	GTTTTTTTTTT/GTTTTTTTTT	TAATTTAACGAACGGCCGAG	TTGGGTGACTCATGGTGAGA	60.1	410	DRR	Ca_16178	I	--
Ca7_P4_33639029	Ca7	33639029	TAAAAAAAAA/TAAAAAAAAAA	TCAGAGCTTTGCAGTTATGTGA	GCACATGGTTTCGAATGTTG	58.7	555	DRR	Ca_16180	--	ERF
Ca7_P4_33640029	Ca7	33640029	TTA/T	CCAAATCGTCGGTGTGAATA	TTTTGGAGGAGATGTTTGGG	59.4	526	DRR	Ca_16180	--	ERF
Ca7_P4_33640082	Ca7	33640082	TAA/TAAA	CCAAATCGTCGGTGTGAATA	CCTTTGTTTGCTGGGATCAT	59.4	614	DRR	Ca_16180	--	ERF
Ca7_P4_33640133	Ca7	33640133	CAAA/CAAAAATAAAA	CCAAATCGTCGGTGTGAATA	CCTTTGTTTGCTGGGATCAT	59.4	614	DRR	Ca_16180	--	ERF
Ca7_P4_33746470	Ca7	33746470	T/TAA	CAAAATTAAAATGGCACAGGG	TTTCCCAATCCACCACTCTC	59.4	332	DRR	Ca_16182	--	--
Ca7_P4_33758707	Ca7	33758707	ACC/AC	CGAATTCAAACTCGGGTCAT	ACAATTTGGCGGTGAGAAAC	59.9	452	DRR	Ca_16184	--	B3
Ca7_P4_33802472	Ca7	33802472	TTATATATATTCAT/TTATATATATTCATATATATATTCAT	TACTGACCTCTATCGCCGGT	CGTATGCGACTCATCCATTG	59.7	612	DRR	Ca_16188	P	GRAS
Ca7_P4_33802677	Ca7	33802677	CT/CTT	TCATCCAATGAAATGGGTTTT	CGTATGCGACTCATCCATTG	59.1	201	DRR	Ca_16188	P	GRAS
Ca7_P4_33802690	Ca7	33802690	GAATAAATAAATAAATA/GAATAAATAAATAAATAAATA	TCATCCAATGAAATGGGTTTT	CGTATGCGACTCATCCATTG	59.1	201	DRR	Ca_16188	P	GRAS

INDEL marker IDs	Chromosomes/ unanchored scaffolds	Physical positions (bp)	InDels (*Kabuli* reference genome-CDC Frontier/PII)	Forward primers (5'-3')	Reverse primers (5'-3')	Anneling temperature (0C)	Expected amplified product size (bp)	Structural annotation		Functional annotation	
								Sequence components of *kabuli* genome	*Kabuli* gene accession IDs	NCBI-KOG	TFs
Ca7_P4_33803105	Ca7	33803105	CTTTTT/CTTTT	ACATCAATGGATGAGTCGCA	CTTTGGTTTCACATTGGGCT	60.1	767	DRR	Ca_16188	P	GRAS
Ca7_P4_33803283	Ca7	33803283	TTTA/T	TGAGGATGGAATGGGTTTTG	CTTTGGTTTCACATTGGGCT	60.7	555	DRR	Ca_16188	P	GRAS
Ca7_P4_33803390	Ca7	33803390	TACAC/TAC	TGAGGATGGAATGGGTTTTG	CTTTGGTTTCACATTGGGCT	60.7	555	DRR	Ca_16188	P	GRAS
Ca7_P4_33812222	Ca7	33812222	ATT/AT	TGGATGAAAGAGACAAGCAAAA	AGTGTACCCATGTGAGGTGCT	59.9	543	DRR	Ca_16189	--	C3H
Ca7_P4_33812418	Ca7	33812418	TC/T	TTGGATGAAAGAGACAAGCAAA	GTATCCATGAATTGGGGGTG	59.9	687	DRR	Ca_16189	--	C3H
Ca7_P4_33812533	Ca7	33812533	CTTTTT/CTTTT	TTGGATGAAAGAGACAAGCAAA	GTATCCATGAATTGGGGGTG	59.9	687	DRR	Ca_16189	--	C3H
Ca7_P4_33815491	Ca7	33815491	GT/GTT	TTTGTTTTCATGCATGTGTCC	TCTGCATGGCAGTGAATAGC	59.4	162	DRR	Ca_16190	E	--
Ca7_P4_33815769	Ca7	33815769	TAAA/TA	ATGGCTATTCACTGCCATGC	ATCATCCTTGGTCCCGAATA	61.0	434	DRR	Ca_16190	E	--
Ca7_P4_33843751	Ca7	33843751	TTTCTATTTTCTCCTCT/TT	TAGAGCATGGCATTTCACCA	GGCCTTTAAATTGGGCTTTC	60.2	229	DRR	Ca_16192	--	NAC
Ca7_P4_33881339	Ca7	33881339	GTT/GAATT	TTCCTCTCATTTGGGTCTGA	TCCTCCTCCTAAGGGACCAT	58.2	172	DRR	Ca_16194	O	--

INDEL marker IDs	Chromosomes/ unanchored scaffolds	Physical positions (bp)	InDels (*Kabuli* reference genome-CDC Frontier/PII)	Forward primers (5'-3')	Reverse primers (5'-3')	Anneling temperature (0C)	Expected amplified product size (bp)	Structural annotation		Functional annotation	
								Sequence components of *kabuli* genome	*Kabuli* gene accession IDs	NCBI-KOG	TFs
Ca7_P4_33890873	Ca7	33890873	ATTT/ATTTT	ATTGTTTCCCATCGTTTCCA	TTGCTATGGATGGCTAAGGG	60.2	537	DRR	Ca_16196	O	TCP
Ca7_P4_34183842	Ca7	34183842	GC/GCC	TCCCAAGAATGTCCACACAA	GGACGAATTTTCGGAGTGAA	59.9	628	DRR	Ca_21005	--	--
Ca7_P4_34189529	Ca7	34189529	CTTT/CTTTCTTTATTTT	TGTGCATCGTATTCACCTCG	GGGAAACAAATGCCTGAATG	60.7	511	DRR	Ca_21004	S	--
Ca7_P4_34189939	Ca7	34189939	ATTTTTTT/ATTTTTT	CATTCAGGCATTTGTTTCCC	TTGGTTCTTTTGCATCTTCG	60.3	700	DRR	Ca_21004	S	--
Ca7_P4_34190282	Ca7	34190282	GTTTTTTTT/GTTTTTTTTT	AACAATCGAACTACTTGCGTGA	TTGGTTCTTTTGCATCTTCG	59.8	564	DRR	Ca_21004	S	--
Ca7_P4_34190333	Ca7	34190333	AAG/AAGAG	TCGAACTACTTGCGTGAGAA	TTGGTTCTTTTGCATCTTCG	57.2	559	DRR	Ca_21004	S	--
Ca7_P4_34190508	Ca7	34190508	AT/A	TCGAAGATGCAAAAGAACCA	AAAGGTGACACATCCTTTGCTT	59.4	189	DRR	Ca_21004	S	--
Ca7_P4_34195712	Ca7	34195712	AT/ATTT	GAGGCTCCATATTGCGAAAA	AGGCTAACACCATTGCCAAC	60.2	189	DRR	Ca_21002	--	WRKY
Ca7_P4_34195793	Ca7	34195793	TAAAAAAAAAA/TAAAAAAAAA	GAGGCTCCATATTGCGAAAA	AGGCTAACACCATTGCCAAC	60.2	189	DRR	Ca_21003	O	FAR1
Ca7_P4_34196242	Ca7	34196242	GTTT/GTT	GTTGGCAATGGTGTTAGCCT	TTGAGGGTTGCTTTTGTTGTT	60.0	771	DRR	Ca_21002	--	WRKY

INDEL marker IDs	Chromosomes/ unanchored scaffolds	Physical positions (bp)	InDels (*Kabuli* reference genome-CDC Frontier/PII)	Forward primers (5'-3')	Reverse primers (5'-3')	Anneling temperature (0C)	Expected amplified product size (bp)	Structural annotation		Functional annotation	
								Sequence components of *kabuli* genome	*Kabuli* gene accession IDs	NCBI-KOG	TFs
Ca7_P4_34206613	Ca7	34206613	GTTTTTTTTT/GTTTTTTTTTT	ACCAACATCATAAAAG CGGC	TGCAACCTTTATGTCAA CGC	60.0	831	DRR	Ca_21001	A	--
Ca7_P4_34216629	Ca7	34216629	GTTTTTTTT/GTTTTTTT	TTTGTCCCTCCCAAGA AATG	ATCTCACCCCTGGTTG TCTG	59.9	427	DRR	Ca_20999	L	--
Ca7_P4_34256619	Ca7	34256619	TCA/TCACA	CGGAGTGGACTCTAAC CCAA	TTAGGTGATTCTATGC GCCC	60.1	532	DRR	Ca_20996	S	--
Ca7_P4_34274392	Ca7	34274392	TAAAA/TAAA	ACCACCGAGTCATCAG CTCT	AGCCCGTTTGCTACCT TTTT	59.9	552	DRR	Ca_20991	--	M-type
Ca7_P4_34283808	Ca7	34283808	CTTTTTTTTT/CTTTTTTTT	CATAACCCATATGCGA GCAA	TCCATGAATTTGTCAAC CGA	59.5	662	DRR	Ca_20990	A	--
Ca7_P4_34283992	Ca7	34283992	GAAAAAAAAA/GAAAAAAAA	CATAACCCATATGCGA GCAA	TCCATGAATTTGTCAAC CGA	59.5	662	DRR	Ca_20990	A	--
Ca7_P4_34303984	Ca7	34303984	A/ATT	AGGACAGTGACTTGGG ATGG	TCTTCATCTGTGGGAC GAAA	60.0	649	DRR	Ca_20988	--	NAC
Ca7_P4_34324551	Ca7	34324551	CT/CTGATT	TCGCAATTGTGTGTGA GATTC	ACCGATGAGAAAGCTT TGGA	59.7	977	DRR	Ca_20987	R	NAC
Ca7_P4_34347433	Ca7	34347433	CTTT/CTTTT	CCATCAAATCTCAATG CGTG	AGAAACCAGCCGCTAA ATGA	60.1	515	DRR	Ca_20986	C	--
Ca7_P4_34347667	Ca7	34347667	A/AC	TCATTTAGCGGCTGGT TTCT	TGCCGCAATAAAATGA AACA	59.8	643	DRR	Ca_20986	C	--

INDEL marker IDs	Chromosomes/ unanchored scaffolds	Physical positions (bp)	InDels (*Kabuli* reference genome-CDC Frontier/PII)	Forward primers (5'-3')	Reverse primers (5'-3')	Anneling temperature (0C)	Expected amplified product size (bp)	Structural annotation		Functional annotation	
								Sequence components of *kabuli* genome	*Kabuli* gene accession IDs	NCBI-KOG	TFs
Ca7_P4_34349210	Ca7	34349210	CTT/CTTT	TTGGGATTTAAGCCGACAAG	AATGAATTTTTGCCACCAGC	60.1	663	DRR	Ca_20986	C	--
Ca7_P4_34363960	Ca7	34363960	CAAA/CAAAA	TCATTGCTTCGTGGGTCATA	TTGTATTGGTGTTGCGGAGA	60.1	426	DRR	Ca_20984	--	Trihelix
Ca7_P4_34382063	Ca7	34382063	TAAAAAAA/TAAAAAA	TTCTTTTTAGGGCCTGAGCA	TCATTTCCTTGCATTTATGGG	60.0	553	DRR	Ca_20982	OUT	YABBY
Ca7_P4_34902553	Ca7	34902553	GATATATATATATATATATATATATATATATATATATATATAT/GATATATATATATATATATATATATATATATATATAT	ATATATCGGATCCAACGGCA	CTCGAGCCTTTAATTCCCAA	60.1	339	DRR	Ca_17624	--	ARF
Ca7_P4_35060418	Ca7	35060418	ATTTT/ATT	TATTTCCCGAAATGCCTCTG	TCTCGTGGTCATTTTAGGGC	60.0	802	DRR	Ca_17632	R	--
Ca7_P4_35134144	Ca7	35134144	TAAA/TAAAA	ACCATTCCATTTCTCCTTGC	CCTTCAATTTTGCCGTCAGT	59.0	494	DRR	Ca_17638	--	ERF
Ca7_P4_3515789	Ca7	3515789	TC/TCC	CGTTTATGAAGAAAGGCGGA	TGATCCACCGGGGATTAGTA	60.2	782	DRR	Ca_03116	I	--
Ca7_P4_35192514	Ca7	35192514	C/CT	TCTCCACTTGCGGAAGAGAT	GAAAACAAGGCAAAGTCCCA	60.0	735	DRR	Ca_17640	--	Trihelix
Ca7_P4_35230178	Ca7	35230178	TAAA/TAAAA	TTTTGGTTGCAGTTTGGTTT	TCTTCCCATTTCCATTTTTGA	58.2	402	DRR	Ca_17641	--	--
Ca7_P4_35332726	Ca7	35332726	TTATATATA/TTATATATATA	AGCGGGAGGAATTGAAAGTT	CATGCTGAACCAGAAAAGCA	60.1	400	DRR	Ca_17647	BK	FAR1

INDEL marker IDs	Chromosomes/ unanchored scaffolds	Physical positions (bp)	InDels (*Kabuli* reference genome-CDC Frontier/PII)	Forward primers (5'-3')	Reverse primers (5'-3')	Anneling temperature (0C)	Expected amplified product size (bp)	Structural annotation		Functional annotation	
								Sequence components of *kabuli* genome	*Kabuli* gene accession IDs	NCBI-KOG	TFs
Ca7_P4_35333033	Ca7	35333033	C/CT	TGCAAAGCACCCAATTGTAG	CTTTTCCATGCCACGGTACT	59.7	974	DRR	Ca_17647	BK	FAR1
Ca7_P4_3535267	Ca7	3535267	CCATCATCA/CCATCA	CGTGCGAGTTTGACACCTAA	AATGGCAATGGAAGGAAATG	59.9	222	DRR	Ca_03115	S	--
Ca7_P4_35369159	Ca7	35369159	CAAAAAAAA/CAAAAAAAAA	CCAAAGGCATTTGAGGATTC	TTGCCATTCTTTGACACACA	59.5	308	DRR	Ca_17651	--	--
Ca7_P4_35370294	Ca7	35370294	TCTAC/TCTACTAC	ATAATTTGAGCCATGACGGG	TCTTGACAGGTTTGGTTGGA	59.8	128	DRR	Ca_17651	--	--
Ca7_P4_3544380	Ca7	3544380	T/TA	TGAAGCATGAAGAGACACGG	AATGGCAAGATCACCCAAAG	60.0	160	DRR	Ca_03114	S	--
Ca7_P4_3567820	Ca7	3567820	AATATATATAT/AATATATAT	CGCGCTAACAACATAAAGCA	TGATGATGTGACGTGCAATG	60.0	448	DRR	Ca_03112	U	MYB
Ca7_P4_3598965	Ca7	3598965	GAAAATAATTAAAA/GAAAA	CACCTGCTAGCCTAACCACA	TCCAATGGGATGGACAAAGT	58.9	743	DRR	Ca_03108	--	NAC
Ca7_P4_36117059	Ca7	36117059	CTTTA/CTTTTCTTTATTTTA	TGGATTGGGCTTTCCAATAA	TGCCTTAACGTACCCAAACA	60.3	435	DRR	Ca_13721	G	Trihelix
Ca7_P4_3638430	Ca7	3638430	TTAT/TTATAT	AGGGCACATAAGCCACAAGT	TAATGTCGTTGCCGCCTATT	59.6	619	DRR	Ca_03105	--	--
Ca7_P4_3638546	Ca7	3638546	TAAAAAA/TAAAAAAA	AGGGCACATAAGCCACAAGT	TAATGTCGTTGCCGCCTATT	59.6	619	DRR	Ca_03105	--	--

INDEL marker IDs	Chromosomes/ unanchored scaffolds	Physical positions (bp)	InDels (*Kabuli* reference genome-CDC Frontier/PII)	Forward primers (5'-3')	Reverse primers (5'-3')	Anneling temperature (0C)	Expected amplified product size (bp)	Structural annotation		Functional annotation	
								Sequence components of *kabuli* genome	*Kabuli* gene accession IDs	NCBI-KOG	TFs
Ca7_P4_3643180	Ca7	3643180	GTTTT/GT	GTTGCCCAATAACGTAACCG	TTTTCGCCCAAATGAAAAAG	60.2	525	DRR	Ca_03104	--	ERF
Ca7_P4_3649235	Ca7	3649235	GAATCCAATATCTA/GAATCCAATATCTAAAATCCAATATCTA	GTCCGAGTCCTAACAGCAGC	TCATGGTGAGATTGGCAAAA	60.0	722	DRR	Ca_03103	U	LBD
Ca7_P4_36548120	Ca7	36548120	TAAAA/TAAAAAA	ACAACCAAGCGTGTCGTGTA	TGTTTGTTGGAGAAATGCCA	60.2	213	DRR	Ca_13744	O	--
Ca7_P4_36603437	Ca7	36603437	ATTTTTTTTT/ATTTTTTTT	AGCCAGTCAGCCAGTCATCT	GGACCAAATCGTGAATGCTT	60.0	684	DRR	Ca_13749	--	--
Ca7_P4_3670558	Ca7	3670558	A/AT	AAGGCAATAGGTGATGGTGG	TCACTATTCACACGCGGAAG	59.8	717	DRR	Ca_03102	TU	--
Ca7_P4_36717770	Ca7	36717770	AATATATATATATATATA/AATATATATATATATATATA	TGCCTTTAACAATAATCGAGAATG	TGGTTTGGTTCCATCAGGTT	59.6	803	DRR	Ca_13757	S	--
Ca7_P4_3672046	Ca7	3672046	AT/A	ACATAGGTCGGCATACTCGG	TACAGTTTCAGCCTGGGTCC	60.0	330	DRR	Ca_03102	TU	--
Ca7_P4_3683029	Ca7	3683029	A/AGAAAAG	ATTGAGGCTACTTGAGGGCA	GAAGCCAAAAAGTCTGCATAGAA	59.8	532	DRR	Ca_03101	--	--
Ca7_P4_3683088	Ca7	3683088	T/TA	ATTGAGGCTACTTGAGGGCA	GAAGCCAAAAAGTCTGCATAGAA	59.8	532	DRR	Ca_03101	--	--
Ca7_P4_3683696	Ca7	3683696	TA/T	CCATGAAATCACCTTAAATGACA	CCGACAAGGCGACAAGTAAT	58.9	451	DRR	Ca_03101	--	--

INDEL marker IDs	Chromosomes/ unanchored scaffolds	Physical positions (bp)	InDels (*Kabuli* reference genome-CDC Frontier/PII)	Forward primers (5'-3')	Reverse primers (5'-3')	Anneling temperature (0C)	Expected amplified product size (bp)	Structural annotation		Functional annotation	
								Sequence components of *kabuli* genome	*Kabuli* gene accession IDs	NCBI-KOG	TFs
Ca7_P4_3684559	Ca7	3684559	CAAAAA/CAAA	AAAATTGAAAACTGGAATGGTACA	GGTTGGCGCGATATAAACAG	58.9	472	DRR	Ca_03101	--	--
Ca7_P4_36871436	Ca7	36871436	AAGAGAGAGAGAGAGAGAGAGAGA/AAGAGAGAGAGAGAGAGAGAGAGAGA	TGAGCTGAAACAACATGCGT	TGTCGGGAACCATTTCCTTA	60.5	893	DRR	Ca_13774	R	--
Ca7_P4_3689131	Ca7	3689131	CATAT/CAT	AACCAATGACCATCGAGAATC	TGGGATTCTCTTGTACCTCACA	58.9	513	DRR	Ca_03100	S	--
Ca7_P4_3689211	Ca7	3689211	CTTTTTT/CTTTTTTT	AACCAATGACCATCGAGAATC	TGGGATTCTCTTGTACCTCACA	58.9	513	DRR	Ca_03100	S	--
Ca7_P4_3689317	Ca7	3689317	ATTTT/ATTT	AACCAATGACCATCGAGAATC	ACTCCTCTTCCCCCTACCAT	58.9	640	DRR	Ca_03100	S	--
Ca7_P4_3689798	Ca7	3689798	TAGAGAG/TAGAG	ATGGTAGGGGGAAGAGGAGT	CTTTTCGTTTGGCCAATGTT	58.9	381	DRR	Ca_03100	S	--
Ca7_P4_37047318	Ca7	37047318	ATTTTTTTTTTT/ATTTTTTTTTT	GAAAAAGCGCTGTAAATGGC	CATCCTCCTCTCTGGCTTTG	59.9	714	DRR	Ca_13788	--	Trihelix
Ca7_P4_37154177	Ca7	37154177	TTATATATATATATA/TTATATATATATATATA	TGTCATCAAAATGGGTCACAA	TCAAAGGTTTTCTGCCATCC	59.8	693	DRR	Ca_13797	--	--
Ca7_P4_37711486	Ca7	37711486	TG/T	TACCCTGTGGATGGGAATGT	TTGTCGATCCGAAATGTGAA	60.1	870	DRR	Ca_22800	--	--
Ca7_P4_3782917	Ca7	3782917	CATATATATATATAT/CATATAT	TGGGGACAAGCCTTATTTTG	GATTGAAGCCACCCAACATT	59.9	668	DRR	Ca_03087	O	--

INDEL marker IDs	Chromosomes/ unanchored scaffolds	Physical positions (bp)	InDels (*Kabuli* reference genome-CDC Frontier/PII)	Forward primers (5'-3')	Reverse primers (5'-3')	Anneling temperature (0C)	Expected amplified product size (bp)	Structural annotation		Functional annotation	
								Sequence components of *kabuli* genome	*Kabuli* gene accession IDs	NCBI-KOG	TFs
Ca7_P4_3785579	Ca7	3785579	TAAAAA/TAAAA	CCGGTTCAACCTCCGTACTA	ATTCTTGTGCGCTTTCGTTT	60.0	302	DRR	Ca_03086	--	--
Ca7_P4_38333257	Ca7	38333257	AAGA/AA	GTCATGGACGCAAGTCCTTT	TTAGATTTTCCGCCTTGGTG	60.1	585	DRR	Ca_16409	P	--
Ca7_P4_38757753	Ca7	38757753	TAAAAAAAAA/TAAAAAAAA	GCCATCTTTATTCCAACCGA	TTTGATGAATTTGATGTTGTTGG	59.9	776	DRR	Ca_16426	--	SBP
Ca7_P4_39361236	Ca7	39361236	CAA/CAAAA	CGATTGCAGAGTGAAAATCC	CCTAATGCGCTGACACAGAC	58.3	464	DRR	Ca_19590	--	BBR-BPC
Ca7_P4_39397852	Ca7	39397852	TCACAGAGGTAC/TC	CCTCCCTTTCAAATCGATCA	CAGTACCATTTGCCGAGGTT	60.0	401	DRR	Ca_19592	--	--
Ca7_P4_39830592	Ca7	39830592	AGGGGGGG/AGGGGGG	CCTGCTCAAAGCACACGTTA	TAATCATCCTCGGGTTGAGG	60.1	636	DRR	Ca_25100	--	--
Ca7_P4_40304518	Ca7	40304518	CAAA/CAA	CACCAATGATATTGGGCAAA	AGTCGGTCAACGGTCAAAAC	59.2	592	DRR	Ca_20249	S	--
Ca7_P4_4224423	Ca7	4224423	CCGCGC/CCGC	CAGTGGCATAGCTCCAGACA	CAGAGACAAACCCAAGGGAG	60.0	419	DRR	Ca_03038	--	--
Ca7_P4_42270355	Ca7	42270355	CTTTTTTTTTTT/CTTTTTTTTTT	TTAGGCGAAGGCTAGGTTGA	TTTTGGGAAAAACAAGTTCGAT	60.0	422	DRR	Ca_17965	G	--
Ca7_P4_42337005	Ca7	42337005	GAAAAAAAAAA/GAAAAAAAAA	CCAAACAAAATTGCCTCGAT	TGATCAGCGAGATGGACAAG	59.9	704	DRR	Ca_17962	--	--

INDEL marker IDs	Chromosomes/ unanchored scaffolds	Physical positions (bp)	InDels (*Kabuli* reference genome-CDC Frontier/PII)	Forward primers (5'-3')	Reverse primers (5'-3')	Anneling temperature (0C)	Expected amplified product size (bp)	Structural annotation		Functional annotation	
								Sequence components of *kabuli* genome	*Kabuli* gene accession IDs	NCBI-KOG	TFs
Ca7_P4_44150096	Ca7	44150096	T/TTTA	AGTCTTACCAGCGCCACACT	TCCCAACTACAACGAGGACC	59.9	567	DRR	Ca_21366	O	--
Ca7_P4_44150736	Ca7	44150736	TAAAAA/TAAAAAAA	GGTCCTCGTTGTAGTTGGGA	CCAAGGCATCAGCTCACATA	60.0	467	DRR	Ca_21366	O	--
Ca7_P4_44372091	Ca7	44372091	GAATAATAATAATAATAATAATAATAATA/GAATAATAATAATAATAATAATAATAATAATA	CGGAGAGTTGATTTCACACAA	CGTCAAAGACGTTCTTCAATCA	58.8	498	DRR	Ca_21371	--	--
Ca7_P4_4439880	Ca7	4439880	ATTTTTTTTTTT/ATTTTTTTTTTTT	TGCCTTGTGTTGAAAATGGA	TGGACGCTCCAATTAAGCAG	60.1	753	DRR	Ca_03014	R	--
Ca7_P4_44885868	Ca7	44885868	CA/CAGAAAAATA	TTTGGTTCAAACAAGGGGAG	ACGAAAATGTCAAAAACCGC	59.9	373	DRR	Ca_15709	--	FAR1
Ca7_P4_45248869	Ca7	45248869	AATATATATATATATATATATATATATATATATAT/AATATATATATATATATATATATATATATATATATATATATAT	GTTATCGCAATTTGGGGATG	TCAAAGTTAACGTGTTTGAGTAGATG	60.2	576	DRR	Ca_15725	I	bHLH
Ca7_P4_45731609	Ca7	45731609	AATTTATATTTA/AATTTA	TTTGCAGCCACAAAAGTCTG	TTGTGTGTTTAATTTTGCGTTTG	60.0	625	DRR	Ca_15750	--	--
Ca7_P4_45762039	Ca7	45762039	ATTTGT/ATTACTAGTTATTTGTTTGT	TTTTAAGGCGTGTTTTTGGG	TCAACAAGTCACAGGCGATT	60.0	893	DRR	Ca_15751	J	--
Ca7_P4_4592459	Ca7	4592459	TA/T	CGGATACGGTCTGGTCAAGT	CACATCACACCAAATCAACACA	60.0	517	DRR	Ca_02993	U	GATA
Ca7_P4_45933626	Ca7	45933626	GAAAAAAAAAAAA/GAAAAAAAAAAA	GCACATGCGTGTGTCTTCTC	TCTTTGGAGTATTCAAGTGGAGG	60.5	455	DRR	Ca_15757	T	E2F/DP

INDEL marker IDs	Chromosomes/ unanchored scaffolds	Physical positions (bp)	InDels (*Kabuli* reference genome-CDC Frontier/PII)	Forward primers (5'-3')	Reverse primers (5'-3')	Anneling temperature (0C)	Expected amplified product size (bp)	Structural annotation		Functional annotation	
								Sequence components of *kabuli* genome	*Kabuli* gene accession IDs	NCBI-KOG	TFs
Ca7_P4_47208313	Ca7	47208313	ATTTTTTTTT/ATTTTTTTTTT	TTCTGCAAATGTGGATCATTG	GCAGGCCAACCATAGTTCTG	59.5	524	DRR	Ca_19491	G	E2F/DP
Ca7_P4_47551388	Ca7	47551388	C/CCA	TGGCATTGATGATGGCTTTA	CCAGGATAAAACAGCCCAGA	60.0	622	DRR	Ca_19500	O	MYB_related
Ca7_P4_47551645	Ca7	47551645	ATTCTAGAACGGTTTTCTA/ATTCTAGAACGGTTTTCTAGAACGGTTTTCTA	GCAATTTTGTGAAGGAGGGA	CCAGGATAAAACAGCCCAGA	60.1	497	DRR	Ca_19500	O	MYB_related
Ca7_P4_48703820	Ca7	48703820	CAAAAAAAA/CAAAAAAAAAA	TCAGTCAAACGCACACAAAA	TTCGGTTTGAGGGAAGTCAC	58.9	496	DRR	Ca_26166	--	--
Ca7_P4_5254880	Ca7	5254880	CTTTT/CTTT	TGGGCTGGACTATACAAGGG	CACGGCCATTTTCATCTCTT	59.9	705	DRR	Ca_06846	C	--
Ca7_P4_5379802	Ca7	5379802	CAAAAAAA/CAAAAAAAA	TGGTCAGGATCACGCTAACA	AACGAATGGCCAACTTCAAT	60.3	688	DRR	Ca_06834	A	--
Ca7_P4_5379954	Ca7	5379954	CAAA/CAA	TCGGTTTGTTGTCAATGACTC	GAACCGAATTGGGCTACAAA	58.6	904	DRR	Ca_06834	A	--
Ca7_P4_5380199	Ca7	5380199	TAAAAAAA/TAAAAAA	ATTGAAGTTGGCCATTCGTT	TGTTGCCCACGTGTACCTTA	59.4	554	DRR	Ca_06834	A	--
Ca7_P4_5396891	Ca7	5396891	CAAAA/CAAA	CGGGGCAACAAATTATCAAG	TCCACTCTGACAATGTTCGG	60.3	737	DRR	Ca_06831	R	--
Ca7_P4_5416537	Ca7	5416537	TA/TAA	TGCAGGCACAATCTCTTTTG	ATTTTCCTCCGGTTTGCTTT	60.0	925	DRR	Ca_06827	T	WRKY

INDEL marker IDs	Chromosomes/ unanchored scaffolds	Physical positions (bp)	InDels (*Kabuli* reference genome-CDC Frontier/PII)	Forward primers (5'-3')	Reverse primers (5'-3')	Anneling temperature (0C)	Expected amplified product size (bp)	Structural annotation		Functional annotation	
								Sequence components of *kabuli* genome	*Kabuli* gene accession IDs	NCBI-KOG	TFs
Ca7_P4_5418013	Ca7	5418013	ACTA/ACTAATGTATAATCTA	TGTGAACTCACGTTTCACCA	GGGACCTTCTACTTACCCGC	58.7	517	DRR	Ca_06827	T	WRKY
Ca7_P4_5434792	Ca7	5434792	CTTTT/CTTT	GAAAGAGGATCAACCCGACA	CATGGTCCACTTCGAGGAAT	60.1	466	DRR	Ca_06825	--	--
Ca7_P4_5436143	Ca7	5436143	AAA/AAATATCACAA	TGTTTAGATGTGGTGGTGCTG	CAAGCAGGTATGTGAGGGGT	59.6	721	DRR	Ca_06825	--	--
Ca7_P4_5464695	Ca7	5464695	TTATATATATATATATATATATA/TTATATATATATATATATA	TTTTGTGGCTGCATTCTCTG	GCTGCTGAGGCTGTTATTCC	60.0	656	DRR	Ca_06820	--	--
Ca7_P4_5513596	Ca7	5513596	TTATATATATAT/TTATATATAT	AGGCTGCTGTAGGAGATGGA	AATCAACCTTGGAATGGTGC	60.0	504	DRR	Ca_06816	BK	G2-like
Ca7_P4_5517542	Ca7	5517542	GAAA/GAA	TGCAGCCTACCAAATTCAAA	TTGTTGGTGGTCTTTCCTGA	59.3	709	DRR	Ca_06815	R	MYB_related
Ca7_P4_5524205	Ca7	5524205	CATGGAAAAATGGAAAAATG/CATGGAAAAATG	AGGCCTGAACACAGTGGAAC	AAGGAACCGTGTGAACCAAG	60.2	248	DRR	Ca_06814	--	TCP
Ca7_P4_5525422	Ca7	5525422	T/TG	AATGTCAAGGGCATTTGGTC	TGTTTAGAGTTTTAGCAGCCACA	59.8	337	DRR	Ca_06814	--	TCP
Ca7_P4_5540435	Ca7	5540435	TTTAACAATATT/TTTAACAATATTAAATTAACAATATT	GCATCCAACTCCAAAGTGCT	TGCACAAACTCCAACATTGA	60.3	711	DRR	Ca_06810	R	G2-like
Ca7_P4_5540728	Ca7	5540728	TC/T	TCAATGTTGGAGTTTGTGCAT	CGCATATCAAACATTGCAGC	59.0	569	DRR	Ca_06810	R	G2-like

INDEL marker IDs	Chromosomes/ unanchored scaffolds	Physical positions (bp)	InDels (*Kabuli* reference genome-CDC Frontier/PII)	Forward primers (5'-3')	Reverse primers (5'-3')	Anneling temperature (0C)	Expected amplified product size (bp)	Structural annotation		Functional annotation	
								Sequence components of *kabuli* genome	*Kabuli* gene accession IDs	NCBI-KOG	TFs
Ca7_P4_5549714	Ca7	5549714	ACTCT/ACT	TCTGTGACTGCTTTGATGGC	TCATGATGGTTAAGGGCAAA	60.0	672	DRR	Ca_06809	--	--
Ca7_P4_5562230	Ca7	5562230	TTATATATA/TTATATATATA	ACACCGTTTGAAGCAAATCC	AGCTGAGACTTGTGGTGGCT	60.0	833	DRR	Ca_06807	E	MYB_related
Ca7_P4_5567293	Ca7	5567293	TC/T	GCACCATAGTGTTCACCAAAAA	TCTGATCCTCTCCCACCAAC	59.9	585	DRR	Ca_06806	--	--
Ca7_P4_5567501	Ca7	5567501	TAAAAAAAAA/TAAAAAAAAAA	TCATGAAATGTCATTCGGTTG	TTTTTGGGCCTGAAAACAAG	59.4	200	DRR	Ca_06806	--	--
Ca7_P4_5578208	Ca7	5578208	AG/AGAATGAATG	ATGGGAAATTGTGAATGGGA	TGCCCGTTAGTCAATGTTTG	60.0	435	DRR	Ca_06805	--	--
Ca7_P4_5633058	Ca7	5633058	T/TAAG	GCAAGCCAAACCACAAAAAT	ACAGGCGCTTCCTTCTTGTA	60.0	386	DRR	Ca_06796	GC	HSF
Ca7_P4_5658376	Ca7	5658376	CATAT/CATATAT	CTGCTGGATCTGGGAAATGT	TCTTGCTATTGGCTGCAGTG	60.1	334	DRR	Ca_06792	GC	HSF
Ca7_P4_5658804	Ca7	5658804	TTTTATTTA/T	CACTGCAGCCAATAGCAAGA	TCGTTTTCAACCTAAACCCA	60.2	495	DRR	Ca_06792	GC	HSF
Ca7_P4_5671646	Ca7	5671646	CAAAA/CAAAAA	TTTAAATGACACTGGGCGTG	TGGTGTAAATATGGCAGCGA	59.6	612	DRR	Ca_06790	--	C2H2
Ca7_P4_5671743	Ca7	5671743	ATT/AT	TTTAAATGACACTGGGCGTG	TGGTGTAAATATGGCAGCGA	59.6	612	DRR	Ca_06790	--	C2H2

INDEL marker IDs	Chromosomes/ unanchored scaffolds	Physical positions (bp)	InDels (*Kabuli* reference genome- CDC Frontier/PII)	Forward primers (5'-3')	Reverse primers (5'-3')	Anneling temperature (0C)	Expected amplified product size (bp)	Structural annotation		Functional annotation	
								Sequence components of *kabuli* genome	*Kabuli* gene accession IDs	NCBI-KOG	TFs
Ca7_P4_5683344	Ca7	5683344	TGC/T	TTTATTTGATGGGTGGGGAA	CAGACAGCAGTTGGAGTGGA	60.0	795	DRR	Ca_06788	Q	--
Ca7_P4_5684253	Ca7	5684253	ATTTTTTTTTT/ATTTTTTTTT	TGGAAGTGAAGCATCTGACG	TTTGTTTCTTGCATGTGGGA	60.0	830	DRR	Ca_06789	K	--
Ca7_P4_5685708	Ca7	5685708	T/TG	TTCGGTTTTAGCAGTTTGGG	GCGTCACACTGAGACTGGAA	60.1	788	DRR	Ca_06789	K	--
Ca7_P4_5686029	Ca7	5686029	CAA/CA	AACCGCTTGTTTTGAACTCC	GCGTCACACTGAGACTGGAA	59.2	365	DRR	Ca_06789	K	--
Ca7_P4_5713279	Ca7	5713279	CAAAA/CAAA	GCCTGGTTGCTCCAATTAAA	GGGCTTCAGAAGTTGCTCAC	60.1	488	DRR	Ca_06787	Q	--
Ca7_P4_5729677	Ca7	5729677	AT/ATGCTT	TCAGCTGGAGCTTTTCAACA	CGTCGACATAGAGCAACCAA	59.7	355	DRR	Ca_06785	R	FAR1
Ca7_P4_5790699	Ca7	5790699	CAATTTAA/CAA	TTTGGCAGTGTGACCAACAT	ATCGAAGAGCCTTAAAATGTGT	60.0	575	DRR	Ca_06779	S	--
Ca7_P4_5791015	Ca7	5791015	CTTTTTTT/CTTTTTTTTTT	TTTCGGTGGAATTTGAGTTTTT	CATGTGCGTGTAAACTCTGGA	59.9	861	DRR	Ca_06779	S	--
Ca7_P4_5791147	Ca7	5791147	AATTTTAAAGT/AATTTTAAAGTAATAATATTTTAAAGT	CGACATGAATAAATTGAGGATGA	TGATCTGGTTGGTGTTGTCC	59.0	988	DRR	Ca_06779	S	--
Ca7_P4_5799703	Ca7	5799703	CAAAAAAAAAA/CAAAAAAAAA	CCATCATACCCCTAGCGAAA	CATCACCATATGAATGCACCA	59.9	666	DRR	Ca_06778	R	--

INDEL marker IDs	Chromosomes/ unanchored scaffolds	Physical positions (bp)	InDels (*Kabuli* reference genome-CDC Frontier/PII)	Forward primers (5'-3')	Reverse primers (5'-3')	Anneling temperature (0C)	Expected amplified product size (bp)	Structural annotation		Functional annotation	
								Sequence components of *kabuli* genome	*Kabuli* gene accession IDs	NCBI-KOG	TFs
Ca7_P4_5799756	Ca7	5799756	TGAGA/TGAGAGA	CCATCATACCCCTAGCGAAA	CAGCCTATGTTTGGAAACCC	59.9	730	DRR	Ca_06778	R	--
Ca7_P4_5800832	Ca7	5800832	TTTCTT/TTT	AAAAGCGGGCCATTCTAGTT	TTCTAGCATTGTGGCTGTGC	60.1	375	DRR	Ca_06778	R	--
Ca7_P4_5841713	Ca7	5841713	CAA/CA	TTTTACCGGACTACGGTTGC	TGCCATTGTTTTTCCAAGTTT	60.0	918	DRR	Ca_06775	--	--
Ca7_P4_5841891	Ca7	5841891	CTTT/CTTTT	TGCAACTTTGAAGGTGCTTG	CAACAAAGCCACAAAAGCAA	60.0	731	DRR	Ca_06775	--	--
Ca7_P4_5842196	Ca7	5842196	T/TG	TTGTTTCGAACACCAATGGA	CAACAAAGCCACAAAAGCAA	59.9	511	DRR	Ca_06775	--	--
Ca7_P4_5842517	Ca7	5842517	GA/GAAA	TTGCTTTTGTGGCTTTGTTG	ATTAATTGCGCCGTCAAAGA	59.9	487	DRR	Ca_06775	--	--
Ca7_P4_5842987	Ca7	5842987	TAAAA/TAAAAA	TCATCGTTGACAGACCTGGA	TTTAGGCAATTTTGCCCTTG	60.2	352	DRR	Ca_06775	--	--
Ca7_P4_585059	Ca7	585059	TAAA/TAA	GTTTAGGCCGCCACAAAATA	GAACTCTGCCTTCCAGCAAC	60.0	871	DRR	Ca_24416	--	--
Ca7_P4_5850631	Ca7	5850631	ATGTGT/ATGT	CAATTCGGACGTGAACTTGA	TGTTCCCACTTTTTCATTCTTG	59.7	290	DRR	Ca_06773	O	--
Ca7_P4_5850664	Ca7	5850664	AATA/AA	CAATTCGGACGTGAACTTGA	TGTTCCCACTTTTTCATTCTTG	59.7	290	DRR	Ca_06773	O	--

INDEL marker IDs	Chromosomes/ unanchored scaffolds	Physical positions (bp)	InDels (*Kabuli* reference genome-CDC Frontier/PII)	Forward primers (5'-3')	Reverse primers (5'-3')	Anneling temperature (0C)	Expected amplified product size (bp)	Structural annotation		Functional annotation	
								Sequence components of *kabuli* genome	*Kabuli* gene accession IDs	NCBI-KOG	TFs
Ca7_P4_5850863	Ca7	5850863	TAAA/TAA	CAATTCGGACGTGAACTTGA	AAGTATCTTCCAGGCGCAAA	59.7	819	DRR	Ca_06773	O	--
Ca7_P4_5852220	Ca7	5852220	CTTGAATTTTGAATTTT/CTTGAATTTTGAATTTTGAATTTT	TTGCTGCGAGCGTTACTCTA	TTCTGGGTATTGAACTCGGC	59.9	527	DRR	Ca_06773	O	--
Ca7_P4_5867187	Ca7	5867187	ATATTATTATTATTATTAT/ATATTATTATTATTATTATTAT	ACAACAGATGATGGCTGCTG	AATGTACGCACTCCACCACA	59.9	606	DRR	Ca_06771	P	--
Ca7_P4_5867216	Ca7	5867216	TGTAGTAGTAGTAGTA/TGTAGTAGTAGTAGTAGTA	ACAACAGATGATGGCTGCTG	AATGTACGCACTCCACCACA	59.9	606	DRR	Ca_06771	P	--
Ca7_P4_5867639	Ca7	5867639	CAAAAAA/CAAAAAAAAA	TGTGGTGGAGTGCGTACATT	TGAACATTCATTCGTTCCCA	60.0	321	DRR	Ca_06771	P	--
Ca7_P4_5868409	Ca7	5868409	AATATATAT/AATATAT	ACTGTTGAACCAGCTGAGGC	TGGTGAGACAATGGGATGAA	60.5	673	DRR	Ca_06771	P	--
Ca7_P4_586986	Ca7	586986	AACA/AATTACA	TTCCATTTCCTCCACAAAGC	CAACCGCATTGTCATCATTC	60.1	531	DRR	Ca_24415	--	--
Ca7_P4_5889222	Ca7	5889222	CTTT/CTT	ATTGGCAAGAGTGTTACGGG	GTGTGACTTTGGCCACCTTA	60.0	520	DRR	Ca_06769	O	--
Ca7_P4_5890416	Ca7	5890416	TAAAAAA/TAAAAA	TCGCACTCTCAAATGACCAC	TCAAAATATCACCCGTATCAAA	59.8	630	DRR	Ca_06769	O	--
Ca7_P4_5945202	Ca7	5945202	AGGTTGGGTTG/AGGTTG	CACCGCAAGTTCTTCCAAAT	GCGAGAAAGGCGTGTTGTAT	60.1	534	DRR	Ca_06763	S	C2H2

INDEL marker IDs	Chromosomes/ unanchored scaffolds	Physical positions (bp)	InDels (*Kabuli* reference genome-CDC Frontier/PII)	Forward primers (5'-3')	Reverse primers (5'-3')	Anneling temperature (0C)	Expected amplified product size (bp)	Structural annotation		Functional annotation	
								Sequence components of *kabuli* genome	*Kabuli* gene accession IDs	NCBI-KOG	TFs
Ca7_P4_5978218	Ca7	5978218	GGAAGATGAT/GGAAGATGATGAAGATGAT	AAAATCCGTGCAACACACG	ACTGATGGGGCAAGCTACAC	60.6	448	DRR	Ca_06760	--	--
Ca7_P4_6010128	Ca7	6010128	CTTTGAGCTTTGTAGTAT/CTTTGAGCTTTGTAGTATTTGAGCTTTGTAGTAT	ATGTGCGTTAAGGGAACTGG	TTGGAGACCCACGTTCTACC	60.0	260	DRR	Ca_06759	--	--
Ca7_P4_6020433	Ca7	6020433	TAAAAAAA/TAAAAAAAA	AATCCGTTGAGATTGGGATG	TTTTTGTTCCATTGGTGTTTATTTT	59.8	677	DRR	Ca_06757	R	--
Ca7_P4_6034341	Ca7	6034341	ATTTT/ATTTTT	AACATGGAAATTGTTTGGCA	TTTTCGAAGGCAATTGAACC	58.9	638	DRR	Ca_06755	A	--
Ca7_P4_6063507	Ca7	6063507	ATT/ATTT	GCACGAGCTAAAACTATAGGCG	ATTGGTCATTTTCCCACGAA	60.4	660	DRR	Ca_06754	K	TALE
Ca7_P4_6065107	Ca7	6065107	ATTTTTTTTT/ATTTTTTTT	CCATGAAAATATCCTCGTGCT	TGCTCAGTGCTCACGAGTCT	59.1	948	DRR	Ca_06752	T	bZIP
Ca7_P4_6065331	Ca7	6065331	TAAAAA/TAAAAAA	TTGTCCACCAAAGTTTCATTTC	ATCATGCTTTTTCCCGTCTG	59.0	492	DRR	Ca_06752	T	bZIP
Ca7_P4_6065402	Ca7	6065402	ATTTTT/ATTTT	TTGTCCACCAAAGTTTCATTTC	ATCATGCTTTTTCCCGTCTG	59.0	492	DRR	Ca_06752	T	bZIP
Ca7_P4_6089737	Ca7	6089737	ATTTTTTTTTT/ATTTTTTTT	AGGATCAGCAAGAGAAGCCA	CGGCTTGATCCTACAAGAGG	60.1	324	DRR	Ca_06750	O	--
Ca7_P4_6106620	Ca7	6106620	TA/T	TTAGGGCATGTCTCCAGCTT	ACTTTCGACCACCACTTCCA	59.8	553	DRR	Ca_06747	--	--

INDEL marker IDs	Chromosomes/ unanchored scaffolds	Physical positions (bp)	InDels (*Kabuli* reference genome-CDC Frontier/PII)	Forward primers (5'-3')	Reverse primers (5'-3')	Anneling temperature (0C)	Expected amplified product size (bp)	Structural annotation		Functional annotation	
								Sequence components of *kabuli* genome	*Kabuli* gene accession IDs	NCBI-KOG	TFs
Ca7_P4_6107206	Ca7	6107206	AA/AACAATA	TGGAAGTGGTGGTCGAAAGT	TGAAAATGGTGAAAGTGGAACA	60.5	647	DRR	Ca_06747	--	--
Ca7_P4_6126281	Ca7	6126281	TAAAAA/TAAAA	TTTCTGCCTAGAAAAGTTCCAT	TGTAGCCACGTAAGGAAACCT	57.2	246	DRR	Ca_06744	O	--
Ca7_P4_6126376	Ca7	6126376	TAAAATAAAT/TAAAATAAATAAAAAATAAAT	CGGTCATTTTTCAACGTATTTT	TGTAGCCACGTAAGGAAACCT	58.1	151	DRR	Ca_06744	O	--
Ca7_P4_6145585	Ca7	6145585	ATTTTTTT/ATTTTTTTT	AAGGATCGTGCCAAAACAAC	AAAATTCATCGGATCAACGG	60.0	316	DRR	Ca_06742	--	--
Ca7_P4_6146295	Ca7	6146295	GAAAAAAA/GAAAAAA	CAAAGAGAAACCAAATCGCC	TCCCCATAAAGCTCTTCCCT	59.7	341	DRR	Ca_06742	--	--
Ca7_P4_6154870	Ca7	6154870	TT/TTGAT	CCCTTCAGAGTCCCATGCTA	TGTCATGTGCTGAGTCGTCA	60.2	625	DRR	Ca_06741	--	--
Ca7_P4_6195527	Ca7	6195527	GTATATATATATATATATAT/GTATATATATATATATATAT	GATCTAACGGCTGAGCGAAC	CAGAAGCTCCAAGAACTGCC	60.0	156	DRR	Ca_06738	--	--
Ca7_P4_760350	Ca7	760350	T/TGTA	TCTAGCGACGGGTCTGATCT	CAAATGCCAACAAGCGTTTA	60.0	365	DRR	Ca_03403	R	--
Ca7_P4_760856	Ca7	760856	GTTGAAGATTG/GTTGAAGATTGAAGATTG	TAAACGCTTGTTGGCATTTG	AGTTCGCAGGCTCTGTTGTT	59.7	445	DRR	Ca_03403	R	--
Ca7_P4_7685716	Ca7	7685716	AATATATATATATATATATATAT/AATATATATATATATATATATATAT	ACTCACAGAGAATCTCCGGC	TTACGATGCTGACGCCATAG	59.4	360	DRR	Ca_06591	J	--

INDEL marker IDs	Chromosomes/ unanchored scaffolds	Physical positions (bp)	InDels (*Kabuli* reference genome- CDC Frontier/PII)	Forward primers (5'-3')	Reverse primers (5'-3')	Anneling temperature (0C)	Expected amplified product size (bp)	Structural annotation		Functional annotation	
								Sequence components of *kabuli* genome	*Kabuli* gene accession IDs	NCBI-KOG	TFs
Ca7_P4_7751929	Ca7	7751929	G/GT	AAGGTATCCCCTTCCCAAGA	TTGTTCGCCATTGATTTTGA	59.8	821	DRR	Ca_13244	--	--
Ca7_P4_8096374	Ca7	8096374	ATATTATTATTATTATTATTAT/ATATTATTATTATTATTATTATTATTAT	GCTTGGGACCTGAGATCCTT	TCGGTGTGTCATTTATTTTGTG	60.6	650	DRR	Ca_13206	L	NAC
Ca7_P4_8097200	Ca7	8097200	AATTATTATTATTATTATTATTATTATTATTATT/AATTATTATTATTATTATTATTATTATTATT	CAACTCTGTTCGAAAGAAGATCAA	TGTTGTCGTAGAACCGGTGA	59.9	556	DRR	Ca_13206	L	NAC
Ca7_P4_816979	Ca7	816979	AAATAAAAATTAATAAAAAT/AAATAAAAATTAATAAAAATTAATAAAAAT	CGACCAATGTTTTTCTTCTTCA	CAGATCATGGCACGTGAAAG	59.2	496	DRR	Ca_03396	--	--
Ca7_P4_977714	Ca7	977714	GGAAGAAGAAGAAGAAGAAGAAGAAGAAGAAGAAGAAGAAGAAGAAGAAGAAGAAGAAGAA/GGAAGAAGAAGAAGAAGAAGAAGAAGAAGAAGAAGAAGAAGAAGAAGAAG[illegible]	GTCGAAGAGGCAAGAAAACG	GTCCCTCTCCATCTCCTTCC	60.0	255	DRR	Ca_03380	--	ERF
Ca7_P4_9828006	Ca7	9828006	CTATATATATATATATATATATATATAT/CTATATATATATATATATATATATATATAT	TTTAGAGGGAGGGAGGGAAA	AGGACGCAAAGGAAGACAGA	60.0	264	DRR	Ca_12748	U	--
Ca8_P4_10301817	Ca8	10301817	TTAATAATAATAATAATAA/TTAATAATAATAATAA	ACTGGGGAAGTTGGCTTTTT	ACGAGGTGTCAGATTTTGGC	60.0	689	DRR	Ca_18422	R	Trihelix
Ca8_P4_10509590	Ca8	10509590	GA/GAA	GCCATGTCAACCGAAGAGAT	ATGCCAAGCGTTTCACTTTT	60.1	596	DRR	Ca_18435	--	--
Ca8_P4_10934374	Ca8	10934374	AT/ATT	TCCGATTCATCCTCTTGGTC	AATGAAAAAGCGGGGAAACT	60.0	368	DRR	Ca_19747	R	NAC
Ca8_P4_11124508	Ca8	11124508	CATAT/CAT	CAGTGGGAAAAGTCAACGGT	GGTTCCGACATCACAGGAGT	60.0	536	DRR	Ca_16840	--	WRKY

**INDEL marker IDs**	**Chromosomes/ unanchored scaffolds**	**Physical positions (bp)**	**InDels (*Kabuli* reference genome-CDC Frontier/PII)**	**Forward primers (5'-3')**	**Reverse primers (5'-3')**	**Anneling temperature (0C)**	**Expected amplified product size (bp)**	**Structural annotation**		**Functional annotation**	
								**Sequence components of *kabuli* genome**	***Kabuli* gene accession IDs**	**NCBI-KOG**	**TFs**
Ca8_P4_11449628	Ca8	11449628	ATT/ATTT	GAAAGGGGGAAAATGGTGAT	TGGTCAAACAACATAAGCCG	60.0	833	DRR	Ca_16817	--	NAC
Ca8_P4_11536143	Ca8	11536143	CATTATTATTATTATTATT/CATTATTATTATTATTATTATT	TCTTACACTGCAGCAATAAAAATCA	TGGTTGATGCCCAAGTACAA	60.2	576	DRR	Ca_16812	R	MYB
Ca8_P4_13993624	Ca8	13993624	TTG/T	GCACAATTTGGCAAGAACAA	TTGGGATTGAGGTTGAGGAG	59.7	455	DRR	Ca_22739	K	--
Ca8_P4_13993907	Ca8	13993907	GTTTTT/GTTTT	CTCCTCAACCTCAATCCCAA	TCACCGGTCAATCAATTAAAAA	60.0	228	DRR	Ca_22739	K	--
Ca8_P4_14077330	Ca8	14077330	TAGAGAAAAGAAGAGAAA/TAGAGAAA	TTGATATGTCATCCCCGGTT	TTCTTTCCCACCCTTCTTCC	60.0	696	DRR	Ca_22744	U	--
Ca8_P4_14077398	Ca8	14077398	GA/GAAA	TTGATATGTCATCCCCGGTT	AGCTGCATCTTCCACGATTT	60.0	940	DRR	Ca_22744	U	--
Ca8_P4_14138599	Ca8	14138599	AA/AACACA	TTGAAATCCACAAAATTCCAAA	TTCATTTTCATCCTCTCGGC	59.3	730	DRR	Ca_13046	O	--
Ca8_P4_14138805	Ca8	14138805	TAAAAAAA/TAAAAAA	CAAAGAAAAGGAAGAACCCACA	TTCATTTTCATCCTCTCGGC	60.5	290	DRR	Ca_13046	O	--
Ca8_P4_14143645	Ca8	14143645	ATTTTTTTT/ATTTTTTTTT	ATGCATGTGAAAGGGACACA	AAGGGCCCCACTATCACTTC	60.0	497	DRR	Ca_13045	--	--
Ca8_P4_14248630	Ca8	14248630	TAAAAAAAAA/TAAAAAAA	ATGCTTCCAAACAATTTCCG	TTCCCTTGTTTTCTATGCCAA	59.9	797	DRR	Ca_13039	--	--

INDEL marker IDs	Chromosomes/ unanchored scaffolds	Physical positions (bp)	InDels (*Kabuli* reference genome-CDC Frontier/PII)	Forward primers (5'-3')	Reverse primers (5'-3')	Anneling temperature (0C)	Expected amplified product size (bp)	Structural annotation		Functional annotation	
								Sequence components of *kabuli* genome	*Kabuli* gene accession IDs	NCBI-KOG	TFs
Ca8_P4_14248780	Ca8	14248780	ATTTTTTTTTT/ATTTTTTTTTTTT	ATGCTTCCAAACAATTTCCG	TTCCCTTGTTTTCTATGCCAA	59.9	797	DRR	Ca_13039	--	--
Ca8_P4_14257165	Ca8	14257165	GTT/GTTCAGACCTTT	AGGCCGCAGATGAACTGTAT	TGGTTTGCTTTTAAGCGAGG	59.7	824	DRR	Ca_13038	--	--
Ca8_P4_14257769	Ca8	14257769	AG/A	CCTCGCTTAAAAGCAAACCA	GAGTGGCGAAACCCTAATGA	60.4	437	DRR	Ca_13037	O	MYB_related
Ca8_P4_14279667	Ca8	14279667	CAAAAAAA/CAAAAAA	CAAGTCTCCCAACTTCCCAA	ACGAGCCCGTCTTTAGTTGA	60.1	533	DRR	Ca_13036	S	--
Ca8_P4_14320669	Ca8	14320669	A/AG	CAATGGCAGTAACATGCAGC	TCCCAAACAACTCAATTCCA	60.3	700	DRR	Ca_13032	Z	MYB_related
Ca8_P4_14320978	Ca8	14320978	TG/T	TGATGCTTTGGTTTCATGTCA	TCCCAAACAACTCAATTCCA	60.1	359	DRR	Ca_13032	Z	MYB_related
Ca8_P4_14574261	Ca8	14574261	AGTG/AGTGTG	GTGGAATGGTGTGAAAGGGT	TAGCCAGAGAACAACCCTCC	59.7	520	DRR	Ca_13010	--	--
Ca8_P4_14576894	Ca8	14576894	ATTTTTTT/ATTTTTT	TTTTGTTTGGTGGTTGATCATT	AGCAGCTCCAGAATTGACTTG	59.2	923	DRR	Ca_13009	Z	MYB_related
Ca8_P4_14577677	Ca8	14577677	AATA/AA	CAAGTCAATTCTGGAGCTGCTA	AAAGACGAAGAAATCGAGCG	59.7	620	DRR	Ca_13009	Z	MYB_related
Ca8_P4_14603641	Ca8	14603641	GCA/GCACTATACCA	AATGCCGTTAAGTTGATGGG	CGTATTGCGGAACAACAATG	59.8	268	DRR	Ca_13007	C	--

INDEL marker IDs	Chromosomes/ unanchored scaffolds	Physical positions (bp)	InDels (*Kabuli* reference genome-CDC Frontier/PII)	Forward primers (5'-3')	Reverse primers (5'-3')	Anneling temperature (0C)	Expected amplified product size (bp)	Structural annotation		Functional annotation	
								Sequence components of *kabuli* genome	*Kabuli* gene accession IDs	NCBI-KOG	TFs
Ca8_P4_14615758	Ca8	14615758	GACAC/GAC	CCCGCATCAAAGATCAAACT	GGTGCAGCAATTGTTGAGAA	60.1	324	DRR	Ca_13006	K	C3H
Ca8_P4_14615814	Ca8	14615814	TAAA/TAAAA	CCCGCATCAAAGATCAAACT	GGTGCAGCAATTGTTGAGAA	60.1	324	DRR	Ca_13006	K	C3H
Ca8_P4_14617513	Ca8	14617513	AT/A	GCTCCAACCGTAAAACCAAA	GTGAGGACGGAGAGGAGACA	60.0	598	DRR	Ca_13006	K	C3H
Ca8_P4_14623977	Ca8	14623977	TAAAAA/TAAAAAAAA	GCAATTGTGCATTTGTCCAC	GTGAATGTAAATGGTGGGGG	60.0	695	DRR	Ca_13005	U	--
Ca8_P4_14625060	Ca8	14625060	CAAAAAAAA/CAAAAAAA	GCGTCAACACTTCCAACACA	CACTCCAACGAACCCAGTTT	60.8	426	DRR	Ca_13005	U	--
Ca8_P4_14625324	Ca8	14625324	TC/T	GCGTCAACACTTCCAACACA	TAAGGACTGGACGTAACCCG	60.8	682	DRR	Ca_13005	U	--
Ca8_P4_14625350	Ca8	14625350	CTTTTTTTT/CTTTTTTTTT	AAACTGGGTTCGTTGGAGTG	TAAGGACTGGACGTAACCCG	60.0	276	DRR	Ca_13005	U	--
Ca8_P4_14643292	Ca8	14643292	TCCC/TCCCC	AGCAAATTCTTGAAGGGACG	CAATGGGAGTTTGTGGTGTG	59.3	638	DRR	Ca_13004	--	--
Ca8_P4_14652708	Ca8	14652708	GGA/G	CCCTAATTACACGCCCTCAA	TGGTGTTAGCTTGGTTGGTG	60.0	152	DRR	Ca_13003	--	--
Ca8_P4_14693755	Ca8	14693755	TAATTAGAAT/TAATTAGAATTAGAAT	TCAAATCACCGTTGATGGTC	ATCATGGGAATGGTTGAGGA	59.3	515	DRR	Ca_13001	--	--

INDEL marker IDs	Chromosomes/ unanchored scaffolds	Physical positions (bp)	InDels (*Kabuli* reference genome-CDC Frontier/PII)	Forward primers (5'-3')	Reverse primers (5'-3')	Anneling temperature (0C)	Expected amplified product size (bp)	Structural annotation		Functional annotation	
								Sequence components of *kabuli* genome	*Kabuli* gene accession IDs	NCBI-KOG	TFs
Ca8_P4_1469473	Ca8	1469473	TTATATATATATATATATATATAT/TTATATATATATATATATATATAT	ACCAACCGGATTGTTTTCAG	AAAAACACATTGGTTGAGATGC	59.8	570	DRR	Ca_15025	D	--
Ca8_P4_14698427	Ca8	14698427	CATTATTATTATTATTATTATTATTATTATTATTAT/CATTATTATTATTATTATTATTATTATTATTATTAT	GGCAGTTTCATCCCTCAAGA	TTATGGGACCAACCCAGGTA	60.2	886	DRR	Ca_13000	--	--
Ca8_P4_14751499	Ca8	14751499	TGG/TG	ATGAAGACCAACCAAGTGCC	ACAAAGCTCGTTTGCTCACA	60.0	189	DRR	Ca_12994	--	--
Ca8_P4_14757174	Ca8	14757174	TAAAAAAAA/TAAAAAAAAA	CATGGAATCCAAATCGATGA	ACCTCCCCTCCAAACAAATC	59.3	477	DRR	Ca_12993	O	--
Ca8_P4_14768319	Ca8	14768319	ATTTTTTT/ATTTTTTTT	TAATCGATCACCCGACCATT	TTGGGACTGGTGAAAGGGTA	60.2	621	DRR	Ca_12991	O	SRS
Ca8_P4_14821171	Ca8	14821171	GAAAAAAAAAA/GAAAAAAAAAAA	ACATCCGTGATGGTCACACA	GATCAGCTGCAGACATTGGA	60.9	657	DRR	Ca_12987	D	bHLH
Ca8_P4_1568214	Ca8	1568214	AATATATATATATATATATATATATA/AATATATATATATATATATATATATA	TGGATCACTTGGCATTGGTA	AAGTAAAAGTTTGCCAGCCG	59.9	561	DRR	Ca_15013	T	FAR1
Ca8_P4_15821014	Ca8	15821014	CAAAAAAAAAA/CAAAAAAAAAAA	GTCGCGATCAAGAAAAACAA	TCAAGTGACAAAGAAAGGGAGTT	58.9	500	DRR	Ca_17863	--	NF-YB
Ca8_P4_15862033	Ca8	15862033	GTATATATATATATATATATATATATATATATATATATATATATATATATATAT/GTATATATATATATATATATATATATATATATATATATATAT	AGGGAATCAATGGCATCTTG	CTCACAGGCAACTCCAAACA	59.9	856	DRR	Ca_17858	T	--
Ca8_P4_16132504	Ca8	16132504	AATATATATATATATATATATATATAT/AATATATATATATATATATATATAT	CGTGGCGAGCATTATTTCAT	CTGGCTGGACTGACATAGCA	61.0	681	DRR	Ca_15525	J	--

INDEL marker IDs	Chromosomes/ unanchored scaffolds	Physical positions (bp)	InDels (*Kabuli* reference genome-CDC Frontier/PII)	Forward primers (5'-3')	Reverse primers (5'-3')	Anneling temperature (0C)	Expected amplified product size (bp)	Structural annotation		Functional annotation	
								Sequence components of *kabuli* genome	*Kabuli* gene accession IDs	NCBI-KOG	TFs
Ca8_P4_16135891	Ca8	16135891	GAAAAAAAAAAA/GAAAAAAAAAAAAA	TGTTGTGGTTCGGATTTTTG	TGCACATGCATCCAAATTTTA	59.4	484	DRR	Ca_15526	Q	--
Ca8_P4_1631334	Ca8	1631334	AA/ATAATAATAATA	GCGGCTAACCTAACCAAATTC	TGCTGATTGGCTTCACTTTTT	60.0	407	DRR	Ca_02445	--	WRKY
Ca8_P4_1777813	Ca8	1777813	AATTATTATTATTATT/AATTATTATTATTATTATT	TCAATTGATTTTCGGTATCCTTC	AGGCAAATGACCAATTGAGG	59.3	698	DRR	Ca_02430	--	GRF
Ca8_P4_1881652	Ca8	1881652	ATGTGTGTGTGTGTGTGTGTGTGT/ATGTGTGTGTGTGTGTGTGTGTGTGT	TGAAAAGTTTTGAATGAAAAGTGG	CCATCCAAATTACACATGCCT	59.6	549	DRR	Ca_02414	T	NAC
Ca8_P4_1993541	Ca8	1993541	A/AGGGAATAATAAT	ATTTCATCCCCAACCGTGTA	GGACTCCAAAGGAAATGCAA	60.1	666	DRR	Ca_02403	R	--
Ca8_P4_2289033	Ca8	2289033	CTATATATATATATATATATATAT/CTATATATATATATATATATATATAT	TGAGCTGCTCGTGAATATGG	TTCCAAATACACCCCTCACG	60.0	786	DRR	Ca_02360	F	bHLH
Ca8_P4_2325798	Ca8	2325798	AATATATATATATATATATATATATAT/AATATATATATATATATATATATATATATATAT	TTTTTGAGCGAGCCATTTCT	TTGTTGTTCTCCTAAGGTTTTGC	60.0	695	DRR	Ca_02357	C	--
Ca8_P4_2521414	Ca8	2521414	ATGG/ATGGTGG	TGAAGCACACTGGCAAAGTC	ATCCAAATTCCTGGCTTGC	60.0	613	DRR	Ca_02333	L	--
Ca8_P4_2522963	Ca8	2522963	ATTTTTTTTT/ATTTTTTTTTT	ACCAATGGGGAGGTTTTCAT	CTCAGTGACTTTCATTTGGCA	60.4	332	DRR	Ca_02333	L	--
Ca8_P4_3565360	Ca8	3565360	TTATATATATATATATATATATATATATATATATATA/TTATATATATATATATATATATATATATATATATATATATATATA	CGAGAATAAGAAATGAAGCCTGA	GGTCAATTTTGTTGTGGCCT	59.9	539	DRR	Ca_02220	P	GRAS

INDEL marker IDs	Chromosomes/ unanchored scaffolds	Physical positions (bp)	InDels (*Kabuli* reference genome-CDC Frontier/PII)	Forward primers (5'-3')	Reverse primers (5'-3')	Anneling temperature (0C)	Expected amplified product size (bp)	Structural annotation		Functional annotation	
								Sequence components of *kabuli* genome	*Kabuli* gene accession IDs	NCBI-KOG	TFs
Ca8_P4_3804574	Ca8	3804574	T/TC	AATGTTTGGGCACACATGAA	GATTTTCCTTCCCCACCAAT	59.8	337	DRR	Ca_02194	K	--
Ca8_P4_4307029	Ca8	4307029	GTTTATTTATTTATTTATTTATTTATTT/GTTTATTTATTTATTTATTTATTTATTTATTT	AACACTGCCTCGGTGTTTCT	CCGTCTTTGTAGCCCATTGT	59.8	576	DRR	Ca_02136	--	bZIP
Ca8_P4_4439470	Ca8	4439470	GAAAAAAAAAAA/GAAAAAAAAAA	GAGGAATTGTGTGTTGCTGC	TTGAAGTGAAGTGAAGCTGGAA	59.3	518	DRR	Ca_02122	--	G2-like
Ca8_P4_4983898	Ca8	4983898	AATATATATATAT/AATATATATAT	TATGTTCGTCTCTGCATGGC	TTGGAATGAGCAAATAGGCA	59.8	594	DRR	Ca_02055	Q	GeBP
Ca8_P4_5033226	Ca8	5033226	ATATATTTATATTTATATTTATATTTATATTT/ATATATTTATATTTATATTTATATTTATATTTATATTT	GCCCATTCTATCACCCATCA	GACGGAGCATCTCTCACACA	60.7	857	DRR	Ca_02051	QI	bHLH
Ca8_P4_5467804	Ca8	5467804	CATATATATATATATATATATATATA/CATATATATATATATATATATATATA	TCCATATATTTCACGCTGATCG	TCCTCCATTCCTTCAAATCG	60.0	847	DRR	Ca_01997	T	--
Ca8_P4_6311569	Ca8	6311569	CAATAATA/CAATA	TTTTTGGCCTAGTACCTGCAT	CCAGGCCAAGCCATAAGTAG	58.8	143	DRR	Ca_23580	BK	--
Ca8_P4_6775686	Ca8	6775686	TTATATATATATATA/TTATATATATATA	GATCCATCCTGACATGGCTT	CAACATGCACGCTTCTTCAT	59.9	272	DRR	Ca_10705	D	--
Ca8_P4_7455373	Ca8	7455373	AATTATTATTATTATTATTATTATTATTATTATTATTATTATTATTATT/AATTATTATTATTATTATTATTATTATTATTATTATTATTATTATTATTATTATT	ACTCTATTTGGGGGAGCGAT	TCAATTGATTTTCGGTATCCTTC	59.9	584	DRR	Ca_10646	K	C3H
Ca8_P4_7560262	Ca8	7560262	TAAAAA/TAAAAAA	GGGGGTTGAATAGGGATTTT	TGGAAGTAGCCATCTTTGGG	59.0	194	DRR	Ca_10632	--	--

INDEL marker IDs	Chromosomes/ unanchored scaffolds	Physical positions (bp)	InDels (*Kabuli* reference genome-CDC Frontier/PII)	Forward primers (5'-3')	Reverse primers (5'-3')	Anneling temperature (0C)	Expected amplified product size (bp)	Structural annotation		Functional annotation	
								Sequence components of *kabuli* genome	*Kabuli* gene accession IDs	NCBI-KOG	TFs
Ca8_P4_7798966	Ca8	7798966	CATATATATATATATATATATATA/CATATATATATATATATATATA	CCAAACATTGGCTGCAAAAT	AGGGGATAAGCATACCGTCC	60.9	734	DRR	Ca_10605	--	M-type
Ca8_P4_7927288	Ca8	7927288	CAAA/CAAAA	AGGACCGTGCAAATGTTTCT	TGGCCTACTTATGGTCTGGC	59.6	349	DRR	Ca_11431	J	--
Ca8_P4_7927539	Ca8	7927539	GTATTGCTTTTAT/GTATTGCTTTTATTGCTTTTAT	GCCAGACCATAAGTAGGCCA	GCAACTGGATTGCTGGATTT	60.1	509	DRR	Ca_11430	J	--
Ca8_P4_7927639	Ca8	7927639	ATT/AT	GCCAGACCATAAGTAGGCCA	GCAACTGGATTGCTGGATTT	60.1	509	DRR	Ca_11430	J	--
Ca8_P4_7956165	Ca8	7956165	GTTATATAATATTA/GTTATATAATATTATTATATAATATTA	TTGATTTGTCTAGAAGCAGCACA	CACTTCTTGGCTGAGTTGGA	60.1	476	DRR	Ca_11432	--	G2-like
Ca8_P4_7979395	Ca8	7979395	TTTAATAATAATTATTTTATTA/TTTA	CACCTGAACCCGTTCGTAAT	CCCCGTTTCATAGCAGATGT	59.9	291	DRR	Ca_11434	--	--
Ca8_P4_7987942	Ca8	7987942	AATATATATATATATATATATATA/AATATATATATATATATATATA	TTTATGTTTGCATTTGCGGA	ATTGCACACGTGTAAAGCCA	60.1	701	DRR	Ca_11437	I	G2-like
Ca8_P4_799203	Ca8	799203	ATT/A	AGTGTCTCGACCGTCATGAAT	GCAACTTGTGCTGTCTGCAT	59.6	836	DRR	Ca_11842	I	--
Ca8_P4_8287736	Ca8	8287736	ATTTTTTTTT/ATTTTTTTT	AACAAGCCATTCCATTCAGG	TCGACGTCTTTGCTTCTGTG	59.9	308	DRR	Ca_11462	J	--
Ca8_P4_8290043	Ca8	8290043	A/ACTCTCGGCAT	CAATGTGGCATGGAGATGAC	GTCGTGGTGTTGGAAGGACT	59.9	448	DRR	Ca_11461	J	--

INDEL marker IDs	Chromosomes/ unanchored scaffolds	Physical positions (bp)	InDels (*Kabuli* reference genome-CDC Frontier/PII)	Forward primers (5'-3')	Reverse primers (5'-3')	Anneling temperature (0C)	Expected amplified product size (bp)	Structural annotation		Functional annotation	
								Sequence components of *kabuli* genome	*Kabuli* gene accession IDs	NCBI-KOG	TFs
Ca8_P4_8366370	Ca8	8366370	ATTTTTTTTT/ATTTTTTTT	AACAGTCGTATTGTATGGGCCT	GTTTCAGGGTTGGTGCTTGT	59.8	705	DRR	Ca_11470	--	HD-ZIP
Ca8_P4_8774111	Ca8	8774111	TA/TAGCGAA	CCACATGAGTGTGTTGAGGG	CGCTAATAATGACACATTCGCT	60.0	655	DRR	Ca_11507	--	SAP
Ca8_P4_8854983	Ca8	8854983	TTAAAG/TTAAAGGAAAAATAAAG	GCCATATCCGGCTTACCTTT	CCCTTGACCAATTCCAGAGA	60.3	475	DRR	Ca_11515	--	--
Ca8_P4_8882090	Ca8	8882090	T/TTCCAAAC	ATCATTCCACGGATTTTCCA	TGAAAACAAAACAAGAAACAGAAA	60.1	108	DRR	Ca_11519	--	--
Ca8_P4_8885241	Ca8	8885241	TTTGTCACATTGT/TTTGTCACATTGTCACATTGT	GTGTGGAAGCACGAGACAGA	AAAGGAACATTGTTGGACCG	60.0	723	DRR	Ca_11520	C	--
Ca8_P4_8894265	Ca8	8894265	ATTTTTTTT/ATTTTTTT	GAGGCCCAAAATGAATAGCA	TTGTCGACGTCCTCGGTATT	60.0	485	DRR	Ca_11521	G	--
Ca8_P4_8909443	Ca8	8909443	TATATATATACA/TA	TTGCAAGAGCATGAAGAACG	TCATGGGATGGTGAATGCTA	60.1	457	DRR	Ca_11522	--	--
Ca8_P4_8922765	Ca8	8922765	ATCT/ATCTACTCT	GATTCGATAGGGGTGCAAGA	GGCAAGTGCCTGGAAGTTAG	60.0	335	DRR	Ca_11523	--	--
Ca8_P4_9057136	Ca8	9057136	TAAAAAAA/TAAAAAAAA	GCGGAGACGCAACTACTGTT	TGGCTTTGTGAACTCCAAGA	60.5	599	DRR	Ca_11528	--	--
Ca8_P4_9101623	Ca8	9101623	CT/CTTT	CAAGGTTCTCCAGAAACCCA	ACACGTGTGCGCTCAAAATA	60.1	671	DRR	Ca_11533	--	C2H2

INDEL marker IDs	Chromosomes/ unanchored scaffolds	Physical positions (bp)	InDels (*Kabuli* reference genome-CDC Frontier/PII)	Forward primers (5'-3')	Reverse primers (5'-3')	Anneling temperature (0C)	Expected amplified product size (bp)	Structural annotation		Functional annotation	
								Sequence components of *kabuli* genome	*Kabuli* gene accession IDs	NCBI-KOG	TFs
Ca8_P4_9101771	Ca8	9101771	ATTTTTTTT/ATTTTTTTTT	TCATGTTTTGAACGTAGGGGA	TACACGTGTGCGCTCAAAAT	60.4	526	DRR	Ca_11533	--	C2H2
Ca8_P4_9102845	Ca8	9102845	CTTTT/CTTTTT	TTGTTTGCATATTGGAAATTTTG	AAAGTGTTCTCGATTTCAATGC	58.9	523	DRR	Ca_11533	--	C2H2
Ca8_P4_9102997	Ca8	9102997	TTAAGT/TTAAGTAAGT	TTCGAAAACGGAAAAATTTGA	AGAATTGCTCCTTGCCAAAA	59.6	633	DRR	Ca_11533	--	C2H2
Ca8_P4_9103038	Ca8	9103038	CAAAAA/CAAAAAA	TTCGAAAACGGAAAAATTTGA	AGAATTGCTCCTTGCCAAAA	59.6	633	DRR	Ca_11533	--	C2H2
Ca8_P4_9103229	Ca8	9103229	GA/GATAAAAA	TTCGAAAACGGAAAAATTTGA	AGAATTGCTCCTTGCCAAAA	59.6	633	DRR	Ca_11533	--	C2H2
Ca8_P4_9110561	Ca8	9110561	TCTTATTTGCA/T	ACGTGGAACGTAACTTTGGC	TCATCTCAGATCTCCAATCCG	60.0	558	DRR	Ca_11534	--	--
Ca8_P4_9151161	Ca8	9151161	CAAAA/CAAA	ACAATGGGGGTTTCGATATG	TTCCGTGTCTTCACGTCTTG	59.5	288	DRR	Ca_11539	O	--
Ca8_P4_9151647	Ca8	9151647	AAAAA/AAAAAGCAAAA	CAAGACGTGAAGACACGGAA	CAGACACAATGGCCTGATTG	59.9	363	DRR	Ca_11539	O	--
Ca8_P4_9220067	Ca8	9220067	C/CTA	AAAATTAGGGGGAACACAGGA	GTATGTGTGGTGCCATTTGC	59.7	909	DRR	Ca_11542	--	--
Ca8_P4_9220216	Ca8	9220216	CTTTTT/CTTTTTT	ACCATTCAAGTAGCCGACCA	GTATGTGTGGTGCCATTTGC	60.5	368	DRR	Ca_11542	--	--

INDEL marker IDs	Chromosomes/ unanchored scaffolds	Physical positions (bp)	InDels (*Kabuli* reference genome-CDC Frontier/PII)	Forward primers (5'-3')	Reverse primers (5'-3')	Anneling temperature (0C)	Expected amplified product size (bp)	Structural annotation		Functional annotation	
								Sequence components of *kabuli* genome	*Kabuli* gene accession IDs	NCBI-KOG	TFs
Ca8_P4_9220602	Ca8	9220602	TAA/TAAA	GCAAATGGCACCACACATAC	GGCATCACCCTCGAAAACTA	59.9	303	DRR	Ca_11542	--	--
Ca8_P4_9323319	Ca8	9323319	ATCAAT/ATCAATCAAACACTTTCAAT	CAGTGGTTGAGACGAGCGTA	TTTAATTTCTTGAAACGGTTGG	60.0	585	DRR	Ca_11553	--	Dof
Ca8_P4_9951759	Ca8	9951759	TTATAT/TTATATAT	TGGAGGTCATGTGGATCAAA	ACTTGTGACGTGGAATGCTG	59.9	438	DRR	Ca_18394	--	--
Ca8_P4_9953316	Ca8	9953316	TTGTGT/TTGTGTGT	AGCAGCAACAACCGTCTTTT	AAAAATCGATCGGGTGCATA	59.9	312	DRR	Ca_18394	--	--
scaffold10_P4_15748	scaffold10	15748	TAAAAAAA/TAAAAAA	CCTCCCGTTGGTGTTAAAAA	CAAGGACTGGTTCGGTTCAT	59.8	478	DRR	Ca_27293	--	--
scaffold1003_P4_13636	scaffold1003	13636	GAAGTAAAG/GAAG	TGCCACTATCGGGCTAATTT	ATTTTCGCATCCAATTACCG	59.6	885	DRR	Ca_27802	QR	B3
scaffold1006_P4_520865	scaffold1006	520865	ATTTTTT/ATTTTTTT	TGGAGTGTGCCAAAAAGTTG	CAACCTCCCTTTGTTTTGCT	59.7	567	DRR	Ca_18036	M	HB-other
scaffold1006_P4_582985	scaffold1006	582985	GC/G	TCTGCTCCAAAAGGGAGAAA	TGATCCCTCCTTGTCTTTGG	59.9	197	DRR	Ca_18042	--	--
scaffold1047_P4_53093	scaffold1047	53093	AT/A	CAATTAGCGCATTTGTTTTAGTTT	TTGATTTTGCGTATTGGAATTG	58.9	608	DRR	Ca_17175	R	Trihelix
scaffold1055_P4_202942	scaffold1055	202942	GA/G	GAGGCATATGGACTGCCATT	CGATACTCACCCCTCGTTGT	59.9	400	DRR	Ca_25313	--	--

INDEL marker IDs	Chromosomes/ unanchored scaffolds	Physical positions (bp)	InDels (*Kabuli* reference genome-CDC Frontier/PII)	Forward primers (5'-3')	Reverse primers (5'-3')	Anneling temperature (0C)	Expected amplified product size (bp)	Structural annotation		Functional annotation	
								Sequence components of *kabuli* genome	*Kabuli* gene accession IDs	NCBI-KOG	TFs
scaffold1067_P4_203101	scaffold1067	203101	ATTTTTTTT/AATTTTTTTTT	TGAAACTGGGAGGAGGAAGA	CAGTGCGATGGAGAAATCAA	59.8	418	DRR	Ca_24425	--	--
scaffold1109_P4_148572	scaffold1109	148572	CAATTAATTAATTAAT/CAATTAATTAATTAATTAAT	TGGGTGGTTGTTTGAGTGAC	GCGCTGTAAAATGTGCAAAA	59.4	849	DRR	Ca_25534	--	--
scaffold1114_P4_55938	scaffold1114	55938	TT/TTAATAT	ACGAGAGCGAAAATGGAGAA	AGGTGGGAGGAAATGAGGTT	60.0	592	DRR	Ca_27287	--	--
scaffold1131_P4_12797	scaffold1131	12797	CAAAAAAAAAAAA/CAAAAAAAAAAAAA	AAAATGGTTATTGCCAGCTTTT	TTCCAAAGGCATAGTTTGGC	59.1	331	DRR	Ca_25661	K	--
scaffold1153_P4_24313	scaffold1153	24313	AAAGAAGAAGAAGAAGAAGAA/AAAGAAGAAGAAGAAGAAGAAGAA	GAAGGAGGGAGGGAATCAAG	ACACTGAATCCAATGGCACA	60.0	215	DRR	Ca_27057	--	--
scaffold1180_P4_36749	scaffold1180	36749	CTATTTATTTATTTATTTATTTATTTATTTT/CTATTTATTTATTTATTTATTTATTTT	TACGGTTCGATCTCATGCAA	TGCCTCTTCCCCTGTTTCTA	60.2	419	DRR	Ca_25737	--	G2-like
scaffold1196_P4_165815	scaffold1196	165815	TTATATATATATAT/TTATATATATATATAT	CCACCTTGGAGTGTTTCGAT	ATGCCTCGCCTTCTCACATA	60.0	705	DRR	Ca_26185	V	--
scaffold12_P4_101779	scaffold12	101779	TAAAAAAAAAA/TAAAAAAAAAAAA	CTGGGCAAGGGATCTGAATA	AAGCGTTGTAAAATGCCCAC	60.0	628	DRR	Ca_22971	--	Trihelix
scaffold1202_P4_177461	scaffold1202	177461	AG/ATTTAGAGG	CAGGCCTGGGTAATCTTTGA	CACACTTGGGTGTCCCTCTT	60.1	397	DRR	Ca_24666	--	--
scaffold1202_P4_6534	scaffold1202	6534	TAAAAAAA/TAAAAAA	AGTGCCCTCGTCAAACAAAC	TTACAGCGCGTGTTGAAAAG	60.2	824	DRR	Ca_24658	--	--

**INDEL marker IDs**	**Chromosomes/ unanchored scaffolds**	**Physical positions (bp)**	**InDels (*Kabuli* reference genome-CDC Frontier/PII)**	**Forward primers (5'-3')**	**Reverse primers (5'-3')**	**Anneling temperature (0C)**	**Expected amplified product size (bp)**	**Structural annotation**		**Functional annotation**	
								**Sequence components of *kabuli* genome**	***Kabuli* gene accession IDs**	**NCBI-KOG**	**TFs**
scaffold1240_P4_120572	scaffold1240	120572	CATATATATATATATATATATATA/CATATATATATATATATATATATATA	CACTGCAGCACTACCTCCAA	GCAGATAGCGTTGTGGTCAA	60.0	499	DRR	Ca_26525	I	--
scaffold127_P4_111345	scaffold127	111345	GAAAAAAAAAA/GAAAAAAAAA	TCATTGCTCCCCCGTAATAG	CGACACTCGCTTCACCAATA	59.9	504	DRR	Ca_26797	--	--
scaffold1278_P4_34631	scaffold1278	34631	GA/GAA	TGTTCCAAGCAATATGTTCCA	TGCCTTAACTGACAAAGGGG	59.0	433	DRR	Ca_27224	--	--
scaffold128_P4_363869	scaffold128	363869	AGGG/AGG	TTGTCATTGGCAAAATTGGA	TAAGGCAAGTAGGAGGTGGC	59.9	535	DRR	Ca_21442	--	--
scaffold128_P4_7129	scaffold128	7129	TAAAAAAAAA/TAAAAAAAA	TTTCAATTTGACATGTACGGG	ATAATTTCATGCGGAGCCAA	58.4	690	DRR	Ca_21428	K	--
scaffold1309_P4_58470	scaffold1309	58470	CTTTT/CTTTTT	TGGGTAACGTAACCTACGGC	ATCCGGTAAAAATCTCCGCT	59.9	951	DRR	Ca_26763	--	--
scaffold1309_P4_58539	scaffold1309	58539	A/ACCAT	CAGCTTGTCCGTCGGTAAAT	TTGGAAGGAGCCCTTACCTT	60.1	875	DRR	Ca_26763	--	--
scaffold1316_P4_13036	scaffold1316	13036	AATTTATTTATTTATTTATTTATT/AATTTATTTATTTATTTATTTATTTATT	ACATGCACAGAGAATGCCAA	GTGCCAGTCGATTGCCTAAT	60.3	631	DRR	Ca_28021	--	--
scaffold1324_P4_279339	scaffold1324	279339	ATTTT/ATTT	CATCATGAATTCGACCATGC	ATAGTTGCACCACTTTGGGC	59.9	722	DRR	Ca_19767	--	BES1
scaffold1324_P4_49560	scaffold1324	49560	AT/ATT	ATTTTTCGGTTTGGAAGCAA	CGATCAAGATTTCGTTCCTG	59.6	602	DRR	Ca_19753	P	--

INDEL marker IDs	Chromosomes/ unanchored scaffolds	Physical positions (bp)	InDels (*Kabuli* reference genome-CDC Frontier/PII)	Forward primers (5'-3')	Reverse primers (5'-3')	Anneling temperature (0C)	Expected amplified product size (bp)	Structural annotation		Functional annotation	
								Sequence components of *kabuli* genome	*Kabuli* gene accession IDs	NCBI-KOG	TFs
scaffold1324_P4_49921	scaffold1324	49921	AAAGAGAA/AAA	GATCGATGCCAGTTGTTTATGA	GCAACCTCAACTGGGTGAAT	60.0	468	DRR	Ca_19753	P	--
scaffold1331_P4_31763	scaffold1331	31763	ATTTTTT/ATTTTTTT	TTGTCACAAAGCGGTCTCAC	GTGACCCTTGCCTCAACACT	59.9	149	DRR	Ca_26940	--	--
scaffold1335_P4_45191	scaffold1335	45191	AAGCTAGCTA/AAGCTA	TGAAGAGTTGCACGAGATGG	ATGCCTCATTTCCCAACAAG	60.0	198	DRR	Ca_28106	S	--
scaffold1335_P4_46035	scaffold1335	46035	G/GC	TTGACCTCCTAGCATTGGCT	GGTTAAAAGTGGGGGTCGTT	59.8	411	DRR	Ca_28106	S	--
scaffold1335_P4_46272	scaffold1335	46272	TC/T	TTCGTGCAACTGACGCTAAC	CCATTTGGATTTAAAAGAAGCC	60.1	345	DRR	Ca_28106	S	--
scaffold1359_P4_74272	scaffold1359	74272	T/TC	CCAATTTGAATTGTCCGTCC	TTAAAGAGATTCGCTGCGGT	60.2	663	DRR	Ca_26683	KR	G2-like
scaffold1359_P4_74591	scaffold1359	74591	ATTTTTTTT/ATTTTTTTTT	TGGGTGGGAGTAATTTTTCG	TTAAAGAGATTCGCTGCGGT	59.8	297	DRR	Ca_26683	KR	G2-like
scaffold1365_P4_4805	scaffold1365	4805	GTTTTTTTT/GTTTTTTTTT	TGCATTTTACAGCGTTTTCG	TGGTGGATGAAAATGACCAA	59.9	367	DRR	Ca_27716	--	Trihelix
scaffold1418_P4_31681	scaffold1418	31681	G/GT	GTCGCAATTTTTGTCCCCTA	TTTCTTTCCGATTTGGTTCTTT	59.9	733	DRR	Ca_25262	S	--
scaffold1418_P4_33272	scaffold1418	33272	TAAAAAAA/TAAAAAA	TCTTTGTGATTGCTGCAGGT	CCAAAATGCAAATTTAACATGG	59.4	798	DRR	Ca_25262	S	--

INDEL marker IDs	Chromosomes/ unanchored scaffolds	Physical positions (bp)	InDels (*Kabuli* reference genome-CDC Frontier/PII)	Forward primers (5'-3')	Reverse primers (5'-3')	Anneling temperature (0C)	Expected amplified product size (bp)	Structural annotation		Functional annotation	
								Sequence components of *kabuli* genome	*Kabuli* gene accession IDs	NCBI-KOG	TFs
scaffold1419_P4_17488	scaffold1419	17488	A/ATATATAT	TGAATGAAATCGATTGGCTG	TAAACACATGCGCTTCCAAA	59.6	570	DRR	Ca_26980	--	--
scaffold1448_P4_99427	scaffold1448	99427	GAATTTAATATAAATTAATTTAATATAAATT/GAATTTAATATAAATTAATTTAATATAAATTAATTTAATATAAATT	CCAGGAAATGACCCCCTATT	TTTATTTTAGCGCGGTGTCC	60.0	774	DRR	Ca_26688	--	--
scaffold1455_P4_19091	scaffold1455	19091	CG/C	TATTGGGTGCACCGTTTACA	TTAGGTGGTACGGAAATCGC	59.8	438	DRR	Ca_27725	--	--
scaffold1467_P4_11911	scaffold1467	11911	AATATATATATATATATATATATATAT/AATATATATATATATATATATATATATAT	TCGCAGCTGTGATGCTAAGT	ATGGGAGGTCTTGTGCAGTT	59.8	359	DRR	Ca_26807	Q	GeBP
scaffold1483_P4_54945	scaffold1483	54945	CTTTT/CTTTTT	GCCAGTTCAATCATTCCCAT	GCAAAAAGGGCAAAAGAGAG	59.8	142	DRR	Ca_26255	R	MIKC
scaffold1504_P4_227426	scaffold1504	227426	TA/TAA	ATGCAGGGTTGGTAGATGCT	GGTTTGATATATGGCCGGTG	59.6	571	DRR	Ca_24095	O	MYB_related
scaffold1504_P4_314477	scaffold1504	314477	GAGCATCAA/GAGCATCAAAGCATCAA	GGAGGGTCAGAGGATGTGAA	TCTGGGAGATGGAGTTGTCC	60.0	615	DRR	Ca_24100	--	Dof
scaffold1504_P4_391760	scaffold1504	391760	CTATATATATATATATA/CTATATATATATATATATATATATA	ATCTGAGGACTCTCCAGGCA	CCCCTTTGTCAGTTAAGGCA	59.9	488	DRR	Ca_24103	--	--
scaffold151_P4_111639	scaffold151	111639	CTTTTTTTTTT/CTTTTTTTTTTT	TTCAAATTTTGTTTGGGGGA	TTCATCCATTCTGGTTGCAC	60.1	654	DRR	Ca_25625	--	--
scaffold1511_P4_116957	scaffold1511	116957	TAAAAA/TAAAAAA	AATTTGCATTGTCGACACCA	TCCAAGAAACTGCACATCCA	60.0	159	DRR	Ca_25145	--	--

INDEL marker IDs	Chromosomes/ unanchored scaffolds	Physical positions (bp)	InDels (*Kabuli* reference genome-CDC Frontier/PII)	Forward primers (5'-3')	Reverse primers (5'-3')	Anneling temperature (0C)	Expected amplified product size (bp)	Structural annotation		Functional annotation	
								Sequence components of *kabuli* genome	*Kabuli* gene accession IDs	NCBI-KOG	TFs
scaffold1511_P4_127158	scaffold1511	127158	TAAAAAA/TAAAAA	AAGACCGATGACCCTTCCTT	TTAGTCGGTCGATAAAGCCG	59.9	546	DRR	Ca_25146	--	--
scaffold1511_P4_191187	scaffold1511	191187	CTATAT/CTATATAT	AACAAAGCGCTGCAATAGGT	AGCGCTGTAAAAGGTGCATT	59.9	126	DRR	Ca_25148	--	Trihelix
scaffold1511_P4_191407	scaffold1511	191407	ATTTTTTTT/ATTTTTTT	TGTTTGCACCTTTTACAGCG	GCGGTAGTGTCTTGCCTTTC	59.9	525	DRR	Ca_25148	--	Trihelix
scaffold1521_P4_10780	scaffold1521	10780	CA/CATCGA	TCATGTGAACAATGCAACCA	CCACAGCTTGTCTGTCGGTA	59.5	296	DRR	Ca_26977	--	bHLH
scaffold1521_P4_19920	scaffold1521	19920	CT/CGTATTTCTGTAT	AGTTTCATTTTTGCCCGTTG	TTCCAACCGTGTGATTTTGA	60.0	464	DRR	Ca_26979	--	bHLH
scaffold1521_P4_21280	scaffold1521	21280	A/ACTTAAGGT	TTGAACCCGAAATTTTCCAC	TACGAGGGACGATGTTGTGA	59.8	264	DRR	Ca_26979	--	bHLH
scaffold153_P4_28056	scaffold153	28056	GAAA/GAA	AATGGCCACGAATCTGAAAG	TGTTTGAAATATGACGCGGA	60.1	518	DRR	Ca_27484	--	--
scaffold1545_P4_16569	scaffold1545	16569	TAAAAAA/TAAAAAAA	CCTGTTGTGGAGGTCATGTG	CAGCGATCCCAGAGTTCAAT	60.0	789	DRR	Ca_23852	--	--
scaffold1548_P4_24822	scaffold1548	24822	CAAAAAAA/CAAAAAA	GAGCATGCCAGTTCTTAGGC	GGAGAGAATTAAGGGGCAGG	60.0	620	DRR	Ca_27707	--	--
scaffold157_P4_11932	scaffold157	11932	AATATATATATATATATATATA/AATATATATATATATATATATA	TTCTTGTGCATCTTCGTTGG	CAAAATTTTCAAACAAAACCCA	59.8	576	DRR	Ca_21061	R	Trihelix

**INDEL marker IDs**	**Chromosomes/ unanchored scaffolds**	**Physical positions (bp)**	**InDels (*Kabuli* reference genome-CDC Frontier/PII)**	**Forward primers (5'-3')**	**Reverse primers (5'-3')**	**Anneling temperature (0C)**	**Expected amplified product size (bp)**	**Structural annotation**		**Functional annotation**	
								**Sequence components of *kabuli* genome**	***Kabuli* gene accession IDs**	**NCBI-KOG**	**TFs**
scaffold157_P4_307810	scaffold157	307810	ACAA/ACAAACAGACTCAA	CTTTTACAGCGCTCGATTCC	TTGAAACCGGTCCTTGGTTA	60.0	538	DRR	Ca_21076	--	--
scaffold157_P4_358753	scaffold157	358753	ATTTTT/ATTTT	ATTTAGATCGGCGGTTGAGA	AACGTAGCTTTTGTGCGCTA	59.7	216	DRR	Ca_21079	--	SBP
scaffold157_P4_359689	scaffold157	359689	CTATCTACATA/CTATCTACATATATCTACATA	AACAAATCAAACAGGCCAGG	TCTTGTTTTCCTGTATGTGTCCC	60.0	620	DRR	Ca_21079	--	SBP
scaffold157_P4_360030	scaffold157	360030	AATATATATATA/AATATATATA	GATTTTCCCTCCACCATCAA	TCAATCACATATATTTCTCCCGT	59.7	502	DRR	Ca_21079	--	SBP
scaffold157_P4_360093	scaffold157	360093	CAT/CATAT	TTTCACGACATTCTCTATGCCT	AGTGACAAAAATGTATCTCAATCG	58.9	766	DRR	Ca_21079	--	SBP
scaffold1614_P4_191446	scaffold1614	191446	CAAAAAAAAAAAAA/CAAAAAAAAAAAAAAA	AGGCTTGGAGAGTGGCTACA	GCGCTGTAAAATGTGCAAAA	60.0	588	DRR	Ca_27067	--	--
scaffold1632_P4_26463	scaffold1632	26463	AAGTAG/AAG	ACGTTTGTTTGTCGACGGTT	ATCCAATCCACTTCTTTCCG	60.5	554	DRR	Ca_26993	--	--
scaffold1632_P4_4760	scaffold1632	4760	TTATATATATATATATATATA/TTATATATATATATATATATATATATATA	TTTTGGGTGGGATTTTACGA	ACCAATGCATGGTGTTGAAA	60.2	465	DRR	Ca_26992	--	--
scaffold1633_P4_15743	scaffold1633	15743	AAATAATAATAATAATAATAATAATAATAATAAT/AAATAATAATAATAATAATAATAATAATAATAATAAT	AACGTTTTCGTCGAATGTCC	TCAATTTTGGATGCACCTGA	60.0	669	DRR	Ca_26202	UR	--
scaffold1659_P4_155048	scaffold1659	155048	TAAAAAAAAAA/TAAAAAAAAAAA	CGATTTCTTTCGAATGGTTTG	CGCGTAAATGTGAGATTTTGAA	59.6	620	DRR	Ca_24192	--	--

INDEL marker IDs	Chromosomes/ unanchored scaffolds	Physical positions (bp)	InDels (*Kabuli* reference genome-CDC Frontier/PII)	Forward primers (5'-3')	Reverse primers (5'-3')	Anneling temperature (0C)	Expected amplified product size (bp)	Structural annotation		Functional annotation	
								Sequence components of *kabuli* genome	*Kabuli* gene accession IDs	NCBI-KOG	TFs
scaffold1708_P4_68435	scaffold1708	68435	TCC/TCCC	GAAAAAGCGCTGTAAATGGC	CATTCGAGACGTGAGCAAAA	59.9	510	DRR	Ca_27642	--	--
scaffold1731_P4_49034	scaffold1731	49034	CATATATATATATATATATATA/CATATATATATATATATA	ATGGGAAGCAACAACGATTC	CCGTACCGTTTCTGGGATAA	59.9	784	DRR	Ca_27041	IR	--
scaffold1731_P4_85329	scaffold1731	85329	CATATATATATATATATATA/CATATATATATATATATATATA	TCTCCGCATAAGCTTTCGAT	GCTTCGACACTGTAGCACCTC	59.9	478	DRR	Ca_27043	--	--
scaffold1731_P4_86422	scaffold1731	86422	AATATATATATATATATATATAT/AATATATATATATATATATAT	TTTTGGGAATTCCTGCTCTG	GTGACACCCTAAACCCCAAA	60.2	299	DRR	Ca_27043	--	--
scaffold1755_P4_125108	scaffold1755	125108	TATTACATTATTAA/TATTACATTATTAATTACATTATTAA	TTTCCCAATCGATTTGCTTC	GTGAGGACCAGCCCACTTTA	60.0	740	DRR	Ca_27387	--	--
scaffold1771_P4_119568	scaffold1771	119568	A/AAAATGATGAAAG	TGTCATCAGCCAAGCAACTC	CATCGACACATCACCACACA	60.0	657	DRR	Ca_27324	Q	--
scaffold1804_P4_191033	scaffold1804	191033	GA/GACA	TTGGCAACACTTGGGTTACA	TCTCCGTAAAGGATTCACCG	60.0	806	DRR	Ca_25513	--	--
scaffold1844_P4_166750	scaffold1844	166750	TTTATTATTATTATTATTA/TTTATTATTATTATTA	GGCTAGACGAGTTCGACGAC	CTAGATGGTCGCATTCTGGG	60.0	418	DRR	Ca_24145	--	--
scaffold1844_P4_167253	scaffold1844	167253	CAAAAAA/CAAAA	CCCCAATTTGCGATCAATAA	TCAAAACTAAGAAACTCGCCAA	60.6	694	DRR	Ca_24145	--	--
scaffold1867_P4_211346	scaffold1867	211346	TTTATTATTATTATTATTATTATTATTATT/TTTATTATTATTATTATTATTATTATTATTATT	GCCATTGATTCATTTCCTTGA	TGCTTTTGAAGCATTTGCAG	59.9	503	DRR	Ca_25353	--	--

INDEL marker IDs	Chromosomes/ unanchored scaffolds	Physical positions (bp)	InDels (*Kabuli* reference genome-CDC Frontier/PII)	Forward primers (5'-3')	Reverse primers (5'-3')	Anneling temperature (0C)	Expected amplified product size (bp)	Structural annotation		Functional annotation	
								Sequence components of *kabuli* genome	*Kabuli* gene accession IDs	NCBI-KOG	TFs
scaffold1928_P4_78124	scaffold1928	78124	ATT/ATTT	GCATCAAGCTGTCGTTTGAA	CCCCCTTTTTGCATAAGTGA	60.0	663	DRR	Ca_26945	--	--
scaffold1943_P4_180228	scaffold1943	180228	TAAAAAAAAA/TAAAAAAAAAA	CCACAAGAAGACAAAGGGGA	TGGGGTATGTGAACGAGACA	60.1	599	DRR	Ca_21921	--	--
scaffold1943_P4_281573	scaffold1943	281573	ATTTTT/ATTT	TGGGTTGGGATGACATTAAAA	CCAACCCAAATAAACCAAACA	60.0	824	DRR	Ca_21930	--	M-type
scaffold1943_P4_282409	scaffold1943	282409	CATATA/CATATATATA	TTGGTTTATTTGGGTTGGGA	AGCGATGCATGTCTCAAGTG	60.0	912	DRR	Ca_21930	--	M-type
scaffold1959_P4_47247	scaffold1959	47247	GC/GCC	TCCTCCTCAAGTTGGAGCAT	ATATTGCATTGCAGTGGTGG	59.8	458	DRR	Ca_26462	K	G2-like
scaffold1964_P4_11011	scaffold1964	11011	A/ACTC	ATGTTCCTCGCCAATCATTC	TTCCAAAAGAGGAATGGTGC	59.9	771	DRR	Ca_25209	G	--
scaffold198_P4_267630	scaffold198	267630	A/AG	TCCTAGAACATGCATCACGC	AGCCACATTCCAAGCAAAAC	59.8	473	DRR	Ca_20909	--	--
scaffold198_P4_632725	scaffold198	632725	TAAAAAAAAAA/TAAAAAAAA	TTTTGCTGTTGTGTGCACTG	GCTGTAAACTGCACCCCATT	59.5	364	DRR	Ca_20924	--	--
scaffold198_P4_632887	scaffold198	632887	A/AAAATGTACAAC	TTTTGCTGTTGTGTGCACTG	GCTGTAAACTGCACCCCATT	59.5	364	DRR	Ca_20924	--	--
scaffold1981_P4_149526	scaffold1981	149526	ATTTTTTTT/ATTTTTTTTT	CTCAAATTTTGTTTGGGGGA	TGTGAGTACGGATAAGGGGG	59.8	443	DRR	Ca_21257	C	--

INDEL marker IDs	Chromosomes/ unanchored scaffolds	Physical positions (bp)	InDels (*Kabuli* reference genome-CDC Frontier/PII)	Forward primers (5'-3')	Reverse primers (5'-3')	Anneling temperature (0C)	Expected amplified product size (bp)	Structural annotation		Functional annotation	
								Sequence components of *kabuli* genome	*Kabuli* gene accession IDs	NCBI-KOG	TFs
scaffold1981_P4_23471	scaffold1981	23471	GATATATATATATATATATATAT/GATATATATATATATATATATATATATATAT	CTCTCAAGGCCCAAAGCATA	GATTCCTCACCTCGCTTTTG	60.3	512	DRR	Ca_21250	--	--
scaffold1981_P4_317439	scaffold1981	317439	TAAA/TAAAA	TCTCATTTGCTCCCGACTCT	TTGTCTCAACAATCTCCCCC	60.0	501	DRR	Ca_21265	--	--
scaffold1981_P4_341254	scaffold1981	341254	TGGG/TGG	ATGCATCATTTGTTGGGGAT	GCACCATTAGCCAACCTGAT	60.0	644	DRR	Ca_21267	R	bHLH
scaffold1997_P4_15458	scaffold1997	15458	AAAAATAGACAT/AAAAATAGACATTTAAAATAGACAT	CCTATACCGACGGGGTTTTT	TTGAAGACGGAATATTGGCA	60.1	531	DRR	Ca_27157	--	--
scaffold2005_P4_14836	scaffold2005	14836	CAGAA/CA	AAAATCCAACAATCTCCCCC	CGGTCTCAACCTCTCCCATA	60.0	515	DRR	Ca_28003	--	--
scaffold2010_P4_3634	scaffold2010	3634	AT/ATT	AATCGATTGATTCAGCCCAG	TGATTTCCAACGGTCATCAA	60.0	869	DRR	Ca_27976	--	--
scaffold2033_P4_183883	scaffold2033	183883	CGAAGAAGAAG/CGAAGAAGAAGAAG	CTTGGGGTGGCAAGTACAGT	TCGAATACATGAATTAACAAAAGGA	60.0	371	DRR	Ca_26143	--	bHLH
scaffold2040_P4_180917	scaffold2040	180917	TGTTCAGTTCAGTTCAGTTCAG/TATTCAGTTCAGTTCAGTTCAGTTCAG	TTGGTTTATTTGGGTTGGGA	ACCAGGAAAAGAAGAAGGGC	60.0	514	DRR	Ca_26621	O	--
scaffold205_P4_40671	scaffold205	40671	AT/ATT	CTACTCTCAATTTTCCGCCG	GCACAATCAGAAGCGCATTA	59.8	761	DRR	Ca_25491	--	--
scaffold207_P4_9636	scaffold207	9636	T/TAGGAAA	TGCCTTAACTGACAAAGGGG	GTGCCAGTCGATTGCCTAAT	60.1	491	DRR	Ca_28033	--	--

INDEL marker IDs	Chromosomes/ unanchored scaffolds	Physical positions (bp)	InDels (*Kabuli* reference genome-CDC Frontier/PII)	Forward primers (5'-3')	Reverse primers (5'-3')	Anneling temperature (0C)	Expected amplified product size (bp)	Structural annotation		Functional annotation	
								Sequence components of *kabuli* genome	*Kabuli* gene accession IDs	NCBI-KOG	TFs
scaffold2155_P4_39661	scaffold2155	39661	GAAAA/GTAAAAAAAA	CCCCCTTTTTGCATAA GTGA	CCCTGCTACTCCTGCT TTTG	59.9	826	DRR	Ca_27850	--	--
scaffold224_P4_186416	scaffold224	186416	A/AGGGTTG	TGAAGTGTTCGAATTG CAGC	TTGCGTTGAAAGACTC GATG	60.0	462	DRR	Ca_26345	--	--
scaffold224_P4_186566	scaffold224	186566	GTTTT/GTTTTT	TGAAGTGTTCGAATTG CAGC	TTGCGTTGAAAGACTC GATG	60.0	462	DRR	Ca_26345	--	--
scaffold224_P4_95002	scaffold224	95002	ATTT/ATTTT	CTTCACCTCCCACCAG ATGT	AAAAGTTGCTCCCCCG TAAT	60.0	365	DRR	Ca_26341	--	--
scaffold227_P4_144419	scaffold227	144419	C/CAG	CCTCAAACTTCCATGG CCTA	CAACTGGAATTTTCGG GAGA	60.1	641	DRR	Ca_25191	--	--
scaffold2348_P4_176388	scaffold2348	176388	TTTTATTTATTTATTT/TTTTATTTATT TATTTATTT	GGGAGGAAGTCTGTGC TGAG	CAACATGTTTGCAGTC CACC	60.0	564	DRR	Ca_25471	--	--
scaffold2356_P4_144020	scaffold2356	144020	CAAAAAAAAAAA/CAAAAAAAAAA	GGATAACAGCGATGGG AAGA	ATCCCTCATTTGCAATC CAC	60.0	590	DRR	Ca_24345	A	--
scaffold2368_P4_25942	scaffold2368	25942	CGTG/CGTGTG	TTGTTTGCATCCATGG GTAA	AAATCCACGTCATCCC AAAA	59.8	593	DRR	Ca_26718	--	--
scaffold2368_P4_53512	scaffold2368	53512	TGGG/TGGGG	AACCCTGGAGATGGGA AAGT	CTAGCCATTAGCTTCG ACCG	59.8	607	DRR	Ca_26720	P	--
scaffold2368_P4_54204	scaffold2368	54204	GTT/GTTT	CATCGGTCAAACACCA TCTG	GCTTTGCAGAACATGC GTAA	60.0	490	DRR	Ca_26720	P	--

INDEL marker IDs	Chromosomes/ unanchored scaffolds	Physical positions (bp)	InDels (*Kabuli* reference genome-CDC Frontier/PII)	Forward primers (5'-3')	Reverse primers (5'-3')	Anneling temperature (0C)	Expected amplified product size (bp)	Structural annotation		Functional annotation	
								Sequence components of *kabuli* genome	*Kabuli* gene accession IDs	NCBI-KOG	TFs
scaffold2371_P4_308163	scaffold2371	308163	CTTATTATTATTATTATTATTATTCTTATTATTATTATTA/CTTATTATTATTATTATTATTATTCTTATTATTATTATTA	GGCTAGACGAGTTCGACGAC	AATAGATGGTCGCATCCTGG	60.0	345	DRR	Ca_23997	--	--
scaffold2388_P4_19728	scaffold2388	19728	AATTATTATTATTATTATTATTATTATTATTATTATTATTAT/AATTATTATTATTATTATTATTATTATTATTAT	ACAGCATGGCGAGAACTTTT	CCTACCGATTGCACCAAGTT	59.9	284	DRR	Ca_27844	O	SRS
scaffold2392_P4_354336	scaffold2392	354336	AAACAA/AAA	TGTTGCGTTTTCATTCTTTCA	TACCAAATATCCCAGGCCAA	59.3	859	DRR	Ca_25138	KL	--
scaffold240_P4_148270	scaffold240	148270	G/GTAGTAGTAGTA	TGTGAGGCATGTCAAAAAGG	CGGCTGTGTTTTTCTGTGTC	59.7	822	DRR	Ca_25660	--	--
scaffold240_P4_148297	scaffold240	148297	TAG/TAGAG	TGTGAGGCATGTCAAAAAGG	CGGCTGTGTTTTTCTGTGTC	59.7	822	DRR	Ca_25660	--	--
scaffold242_P4_692807	scaffold242	692807	GAAA/GAAAA	TTGTCTTGCTTGCATCCTTG	TGTGTAAAGATTGGCGGTTG	60.0	399	DRR	Ca_22275	F	--
scaffold242_P4_706018	scaffold242	706018	TCTACT/TCT	TGCCGGTTTTACTTGTTTCTG	GTGCACCACCTCAAAAACCT	60.2	753	DRR	Ca_22276	T	M-type
scaffold2617_P4_234200	scaffold2617	234200	AATATATATATATATATATATATATAT/AATATATATATATATATATATATATATATAT	TTTGGGAAACTGAACCAACA	AAAATTGAAATTATCAAGAAAGTTGTG	59.0	322	DRR	Ca_21877	--	--
scaffold2617_P4_320997	scaffold2617	320997	TAAAA/TAAA	TGGGGTATTCCTTTACGTCTT	TGAAAGAGGGGAAAAGGAGC	57.6	296	DRR	Ca_21883	--	WRKY
scaffold2617_P4_321441	scaffold2617	321441	ATTTTTTTT/ATTTTTTTTTT	TGCTCCTTTTCCCCTCTTTC	TTATGTGCGCATGACCAATC	60.7	542	DRR	Ca_21883	--	WRKY

INDEL marker IDs	Chromosomes/ unanchored scaffolds	Physical positions (bp)	InDels (*Kabuli* reference genome-CDC Frontier/PII)	Forward primers (5'-3')	Reverse primers (5'-3')	Anneling temperature (0C)	Expected amplified product size (bp)	Structural annotation		Functional annotation	
								Sequence components of *kabuli* genome	*Kabuli* gene accession IDs	NCBI-KOG	TFs
scaffold2617_P4_321825	scaffold2617	321825	TAAAAA/TAAAAAAAAAA	TTGGTCATGCGCACATAATA	GATGGCTGTGTCGGAGACTT	58.6	529	DRR	Ca_21883	--	WRKY
scaffold271_P4_205973	scaffold271	205973	GAA/GA	GCACCGTGAGTAAATTCGGT	TATTTTGGATTTTGGCTCGG	60.0	692	DRR	Ca_25334	--	--
scaffold273_P4_190574	scaffold273	190574	CAAAAAAAAAA/CAAAAAAAAA	GAAGAAGGTCCCAAGGGTTC	CGCTTCTTCCCCTTCTCTTT	59.9	757	DRR	Ca_26226	--	--
scaffold275_P4_687972	scaffold275	687972	TTTATTATTATTATTATTATTATTATTATTATTATTATTATTAT/TTTATTATTATTATTATTATTATTATTATTATTATTAT	TCCCCTTTGTCTTCTTGTGG	TTGAAGCCGAGAAAACGACT	60.1	778	DRR	Ca_23469	R	--
scaffold2763_P4_57690	scaffold2763	57690	CTATATATATATATATATAT/CTATATATATATATATATAT	ATTTTAACTGGGCCAAAGCC	TGTTTCCCTCAAATTGAATGC	60.3	668	DRR	Ca_24398	--	bHLH
scaffold2763_P4_81370	scaffold2763	81370	T/TG	AACCAACTGGTCCTGACTGG	GAATGGAGAAGGTCGTGCAT	60.0	536	DRR	Ca_24399	--	--
scaffold277_P4_204924	scaffold277	204924	AAATTTTCTTAATTTTCT/AAATTTTCT	ATTCGCCGTCACTTTCAAAT	TCCACTCCCACACCTAAACC	59.6	609	DRR	Ca_25752	--	--
scaffold277_P4_205504	scaffold277	205504	T/TGATATATATATA	GGTTTAGGTGTGGGAGTGGA	TGATACAAGGTTCAACCGCA	59.8	444	DRR	Ca_25752	--	--
scaffold2780_P4_1451	scaffold2780	1451	GTTTTTTTT/GTTTTT	GGGGGAGAAAATTTGGTGTT	TTGATCTTGCTGCATTGTCC	60.0	233	DRR	Ca_27853	--	--
scaffold2871_P4_4009	scaffold2871	4009	ATT/AT	AGCTTGGGGCCAAGTCTATT	ACACCAATTTGCGATTGACA	60.1	404	DRR	Ca_27744	--	HB-other

INDEL marker IDs	Chromosomes/ unanchored scaffolds	Physical positions (bp)	InDels (*Kabuli* reference genome-CDC Frontier/PII)	Forward primers (5'-3')	Reverse primers (5'-3')	Anneling temperature (0C)	Expected amplified product size (bp)	Structural annotation		Functional annotation	
								Sequence components of *kabuli* genome	*Kabuli* gene accession IDs	NCBI-KOG	TFs
scaffold290_P4_298253	scaffold290	298253	GAAAAAA/GAAAAA	CACCGTGTTTCTTGATTGGA	GGCATCCGAGGATACGACTA	59.5	705	DRR	Ca_19835	R	C2H2
scaffold290_P4_299066	scaffold290	299066	TAAAAAAAAAAAAAAA/TAAAAAAAAAAA	GTAGGACGGAAATGCGAGAC	TGTGCATTGATTTGATGACATT	59.7	608	DRR	Ca_19835	R	C2H2
scaffold290_P4_451319	scaffold290	451319	GTTTTTTTTTTTT/GTTTTTTTTTTT	GCAAGCTTACGCGGTATTGT	TCAAATTGATGGAAACCTTCG	60.3	583	DRR	Ca_19837	S	--
scaffold290_P4_662838	scaffold290	662838	CTTTTTTTTTTT/CTTTTTTTTTT	AGGACCTAAGCAACACCCCT	TGTCGAATTGGGCAATAACA	60.0	232	DRR	Ca_19844	--	--
scaffold296_P4_261904	scaffold296	261904	CAAAAA/CAAAA	CCATACACATGAGAGTTGCGA	TTGACTCAGCCTTAAATTTTTATTGA	59.7	534	DRR	Ca_20542	--	--
scaffold296_P4_262823	scaffold296	262823	ATATTATTATTATTATTATTA/ATATTATTATTATTATTA	CACTTTTTAACCGGAGGAATG	GCAGTGGATAAGGCCGATAA	58.6	140	DRR	Ca_20542	--	--
scaffold296_P4_422245	scaffold296	422245	CTTTTTTTTT/CTTTTTTTTTT	GACAAAGGCAGGCAAGAAAG	TCGGGAAACAGGAAACAAAC	60.0	591	DRR	Ca_20548	--	--
scaffold3015_P4_69170	scaffold3015	69170	AG/A	GGCCCATAATGCTCAAGTGT	TCATAGGCATGGGATTTGAA	60.0	472	DRR	Ca_27318	--	--
scaffold3019_P4_17640	scaffold3019	17640	GTTTT/GTTTTT	TGCATTATGGGTAGGTGCAA	GATGTCTACCAGTTGCGGGT	60.0	570	DRR	Ca_28069	--	--
scaffold303_P4_162542	scaffold303	162542	AAGCGCTGT/AAGCGCTGTGGAAACGAGCGCTGT	GCCATTTACAGCGCTTTTTC	TGAAATTATGGGTGGGCATT	59.9	357	DRR	Ca_26661	--	Trihelix

INDEL marker IDs	Chromosomes/ unanchored scaffolds	Physical positions (bp)	InDels (*Kabuli* reference genome-CDC Frontier/PII)	Forward primers (5'-3')	Reverse primers (5'-3')	Anneling temperature (0C)	Expected amplified product size (bp)	Structural annotation		Functional annotation	
								Sequence components of *kabuli* genome	*Kabuli* gene accession IDs	NCBI-KOG	TFs
scaffold306_P4_13557	scaffold306	13557	TTATTACTATTA/TTATTACTATTACTATTA	TCCACGGAATTCAAATCTCA	TGATTTTATGATGATGGATAATTTTGA	59.0	280	DRR	Ca_22704	A	--
scaffold306_P4_186956	scaffold306	186956	CTTTTTTTTTTTT/CTTTTTTTTTT	CAACGCTCCATAACCAGCTT	AATCCCATGCATCCATCATC	60.3	674	DRR	Ca_22713	--	--
scaffold311_P4_131360	scaffold311	131360	A/ACAT	GAAGCTCAGATGGCACACAA	CAATCTCCCCCTTGAGTTGA	60.0	716	DRR	Ca_26332	--	--
scaffold3134_P4_12154	scaffold3134	12154	ACATC/A	TAAACGAGGTAAACCGGTGC	GAAAGTCGCATGTGCAACAC	60.0	437	DRR	Ca_27104	O	C2H2
scaffold3134_P4_12226	scaffold3134	12226	TTTTTTT/TTTTTTTGTTTTTT	TAAACGAGGTAAACCGGTGC	GAAAGTCGCATGTGCAACAC	60.0	437	DRR	Ca_27104	O	C2H2
scaffold3134_P4_12915	scaffold3134	12915	CAA/CAAA	TGACCGAATTCCGCTTATTC	CCAAACATTTTTCACCTGGC	60.0	523	DRR	Ca_27104	O	C2H2
scaffold3134_P4_13444	scaffold3134	13444	TA/TATTAAACAAA	CCAATCAGTTGGAGCCAGTT	CCAAGAGAAAGGCAGTCGAG	60.1	891	DRR	Ca_27104	O	C2H2
scaffold3134_P4_13740	scaffold3134	13740	ACTCCATC/AC	TTGATTCGTGCAAATCAGGA	CCAAGAGAAAGGCAGTCGAG	60.2	289	DRR	Ca_27104	O	C2H2
scaffold314_P4_404143	scaffold314	404143	AT/ATAACAGT	TTTTGAAACTTGGGTGGGAG	CATTGCACTGCATGCTTTTT	59.9	651	DRR	Ca_21806	KR	G2-like
scaffold317_P4_30584	scaffold317	30584	CAAAAAATAAAAAAAAA/CAAAAAATAAAAAAAAATAAAAAAAAA	CTCGCTCGGTTACCCTCATA	AGTTCGATTCAAAGACCCCA	60.2	682	DRR	Ca_25635	D	--

INDEL marker IDs	Chromosomes/ unanchored scaffolds	Physical positions (bp)	InDels (*Kabuli* reference genome-CDC Frontier/PII)	Forward primers (5'-3')	Reverse primers (5'-3')	Anneling temperature (0C)	Expected amplified product size (bp)	Structural annotation		Functional annotation	
								Sequence components of *kabuli* genome	*Kabuli* gene accession IDs	NCBI-KOG	TFs
scaffold3228_P4_47967	scaffold3228	47967	C/CAA	TGACAAAAGGGGGAGATTGA	TGCGTCTTTCTCAAGTGCAT	60.4	414	DRR	Ca_27761	--	--
scaffold331_P4_155436	scaffold331	155436	CATATATATATATATATATAT/CATATATATATATATAT	TTGGAAGGAGCCCTTACCTT	TTGGTATTGAACTCGGAGGC	60.1	716	DRR	Ca_25647	FH	--
scaffold3321_P4_25022	scaffold3321	25022	ATTTTTTTTT/ATTTTTTTT	TTTTCTGGTTGTAGGACGGG	CAACAACTTCTCATGCTCGC	60.0	711	DRR	Ca_25706	QR	B3
scaffold335_P4_133682	scaffold335	133682	CC/CCCTAC	ATGGCTCAAATCAGAATGGC	TGATAACCTTTCCTCGCGTT	60.0	578	DRR	Ca_26029	--	NAC
scaffold3397_P4_17764	scaffold3397	17764	AT/ATT	GATCAGATCTGGCAGCAACA	GAGGATGGAAATTGCTGCAT	60.0	366	DRR	Ca_26271	--	--
scaffold340_P4_18699	scaffold340	18699	CA/CAAA	GAATGCACACAGCAGAAGGA	CTAACTCTAATGGGCGGCAG	60.0	639	DRR	Ca_24954	DL	--
scaffold340_P4_274458	scaffold340	274458	ATTTTTTTTT/ATTTTTTTT	GCAGTGGGACGCACTTATTT	CAGCTAGCAACAATGGCAAA	60.1	702	DRR	Ca_24963	--	--
scaffold3404_P4_2056	scaffold3404	2056	TAAA/TAA	GCATCAAGCTGTCGTTTGAA	CCCCTTTGTCAGTTAAGGCA	60.0	319	DRR	Ca_27915	--	--
scaffold350_P4_68142	scaffold350	68142	TTTCTTCCGTTCTT/TTTCTTCCGTTCTTCCGTTCTT	CAACATGCGGGTCTTTTTCT	GGTCTCCCACCCATTTCTTT	60.1	722	DRR	Ca_26734	--	--
scaffold362_P4_15120	scaffold362	15120	ATATTT/AT	TCCTAGAACATGCATCACGC	TACCCTTTGCTAGCCCCTTT	59.8	521	DRR	Ca_24987	--	B3

INDEL marker IDs	Chromosomes/ unanchored scaffolds	Physical positions (bp)	InDels (*Kabuli* reference genome-CDC Frontier/PII)	Forward primers (5'-3')	Reverse primers (5'-3')	Anneling temperature (0C)	Expected amplified product size (bp)	Structural annotation		Functional annotation	
								Sequence components of *kabuli* genome	*Kabuli* gene accession IDs	NCBI-KOG	TFs
scaffold362_P4_166434	scaffold362	166434	TGA/T	CCCGGTATTGTTTGTGCTCT	CACATCAGCCACATTTGACC	60.0	774	DRR	Ca_24991	C	--
scaffold369_P4_10343	scaffold369	10343	CAAAAAAAAAAA/CAAAAAAAAAA	TGCATGCATGATTGTTATGG	TTGATCAATTTGACGGCTCA	58.9	405	DRR	Ca_25290	--	HD-ZIP
scaffold374_P4_205813	scaffold374	205813	CAT/CATCCTGAT	AGCTGTTTCTGCTAGGCTGG	TTTATTTACGCGAATGCCCT	59.8	941	DRR	Ca_26214	O	--
scaffold38_P4_239944	scaffold38	239944	ATTTTTTTTT/ATTTTTTTTTT	TTCCATGGCATTGAGAAGAA	TTAGGACCAACGGTGCACTT	59.2	635	DRR	Ca_23619	R	--
scaffold38_P4_248171	scaffold38	248171	CTTTTTTTTTTT/CTTTTTTTT	TAGCCTTGAGACCGAGCATT	CAAGGACTAAACTGAAGCCCA	60.0	372	DRR	Ca_23620	--	--
scaffold383_P4_77382	scaffold383	77382	ATTT/ATT	GTGTCTGCTTGTTTGCCTGA	CAAAAGGGGGAGATTGGTTT	60.0	507	DRR	Ca_27371	--	--
scaffold3947_P4_5994	scaffold3947	5994	CAAAAAAA/CAAAAA	AAAATCCAACAATCTCCCCC	TTGGTCGACAAGCAACTGAG	60.0	577	DRR	Ca_27632	--	--
scaffold398_P4_275366	scaffold398	275366	ATTTTT/ATTTTTT	GCACCACAGTCTTCCTCCTC	TCCGTGCCCATGATAAATAA	59.8	579	DRR	Ca_22289	B	--
scaffold398_P4_301322	scaffold398	301322	TTTTT/TTTTTATTTT	TCTAGATACGGGATGGGCAC	TCGCGAAGAAGGATAAAATG	59.9	523	DRR	Ca_22290	A	--
scaffold398_P4_339502	scaffold398	339502	T/TG	GGATGCTGGAATTTTGAAGG	TTTTACTTCCGCCAGCTTGT	59.5	810	DRR	Ca_22294	K	C3H

INDEL marker IDs	Chromosomes/ unanchored scaffolds	Physical positions (bp)	InDels (*Kabuli* reference genome-CDC Frontier/PII)	Forward primers (5'-3')	Reverse primers (5'-3')	Anneling temperature (0C)	Expected amplified product size (bp)	Structural annotation		Functional annotation	
								Sequence components of *kabuli* genome	*Kabuli* gene accession IDs	NCBI-KOG	TFs
scaffold398_P4_339939	scaffold398	339939	CAAAAAAAAAA/CAAAAAAAAAAA	ACAAGCTGGCGGAAGTAAAA	AATTCGACGTGATACCGAGG	59.9	203	DRR	Ca_22294	K	C3H
scaffold398_P4_340646	scaffold398	340646	TA/TAAA	ATTAGACCGTCCTGGTGTGG	AGGTCATGGGTTTGAGCAAC	59.8	496	DRR	Ca_22294	K	C3H
scaffold398_P4_372482	scaffold398	372482	TTGTGTG/TTGTGTGTG	TGGGAGTTTGTGAGGTATTTCC	GCCAAAGAAGAAGTGAACCG	60.2	772	DRR	Ca_22296	J	--
scaffold40_P4_1016318	scaffold40	1016318	CA/CAA	CCGAGGAGCAATGTGGTTAT	TGTGGGAACAGTGGAATGAA	60.0	478	DRR	Ca_18998	--	MYB_related
scaffold40_P4_1056072	scaffold40	1056072	G/GGT	ACCAGGTGCAGGAATTTACG	GCGGAGTCGGTCGACTAATA	60.0	720	DRR	Ca_19000	O	MYB
scaffold40_P4_466383	scaffold40	466383	AT/ATATCTTTATATT	ATGCAGGTTTTGGATTTTGC	TACCTCGGCATATTTCAGGC	59.9	714	DRR	Ca_18971	T	--
scaffold40_P4_496475	scaffold40	496475	ATATTATTATTATTATTATTATTATTATTATTATT/ATATTATTATTATTATTATTATTATTATTATTATTATTATTATT	AGTATCCTGGGTATGCGACG	TTTGATAACAATTTTCGCTCCTC	60.0	692	DRR	Ca_18973	R	--
scaffold40_P4_973137	scaffold40	973137	TTGTGTAACAAATCAT/TTGTGTAACAAATCATGTGTAACAAATCAT	GCCAAGAAGGGTGATTTTCA	CATATGGGTTTGGCACCTCT	60.1	314	DRR	Ca_18995	S	--
scaffold40_P4_998454	scaffold40	998454	GGCG/GGCGCG	CCAACCTCGTCGATAAAGGA	AAGTGCGAAAGGTAAGCTGC	60.1	684	DRR	Ca_18996	S	--
scaffold4011_P4_38659	scaffold4011	38659	TTAATAATAATAATAATAATAATAATAATAA/TTAATAATAATAATAATAATAATAATAATAATAA	CCTTCTTGTGGAAATTTGTGG	TTCAATTGCAATATGCGACC	59.5	796	DRR	Ca_26887	T	bHLH

INDEL marker IDs	Chromosomes/ unanchored scaffolds	Physical positions (bp)	InDels (*Kabuli* reference genome-CDC Frontier/PII)	Forward primers (5'-3')	Reverse primers (5'-3')	Anneling temperature (0C)	Expected amplified product size (bp)	Structural annotation		Functional annotation	
								Sequence components of *kabuli* genome	*Kabuli* gene accession IDs	NCBI-KOG	TFs
scaffold4057_P4_6605	scaffold4057	6605	TTT/TCTTTTATT	CCTATGTGCTCTTCGTGCAA	AAAGCTTATATCCCAAGCTCAAA	60.0	667	DRR	Ca_27488	--	--
scaffold408_P4_40537	scaffold408	40537	T/TATTATAATA	GCTTTTTCCAAGAAGCGATG	AAAAAGCGTTGTAAAGGGCA	60.0	457	DRR	Ca_25272	--	M-type
scaffold409_P4_2612	scaffold409	2612	GTAAAATTT/G	AAGTCACAGTGGAACCCCAG	TCGATTGCAACCAAATTGAA	60.0	151	DRR	Ca_27582	--	--
scaffold414_P4_21975	scaffold414	21975	GAAAAA/GAAAA	TTCCTATTGCTCACCCCTTG	GAAAAACAAGACCCGCGATA	60.1	516	DRR	Ca_27849	--	--
scaffold415_P4_9749	scaffold415	9749	T/TCTGA	TTCGAGCTCAAAGAGGTGCT	AGCGCTGTAAAATGTGCCTT	60.3	727	DRR	Ca_28252	--	Trihelix
scaffold418_P4_152839	scaffold418	152839	AACACACACACACACACA/AAACACACACACACACACACA	CTGGGCAAGGGATCTGAATA	TGCATTTTACAGCGCTTTTG	60.0	596	DRR	Ca_24681	--	--
scaffold418_P4_154154	scaffold418	154154	AATATATATATATATATATATA/AATATATATATATATATATATA	ACTCCTCAAAACTCGGCAAA	TTGAACTTGCATTCGTTCCA	59.9	719	DRR	Ca_24681	--	--
scaffold420_P4_224153	scaffold420	224153	ACC/ACCTCC	TCGATTGACATTACCCAGCA	TAGGTGGTGGTGAATGGGTT	60.1	269	DRR	Ca_22201	R	MYB
scaffold429_P4_179314	scaffold429	179314	CATATATATATATATATATATATATATATATATATA/CATATATATATATATATATATATATATATATA	AAGCTGCAACAATCAAAGCA	TAACCAATCTTGTGCACCCA	59.6	923	DRR	Ca_26240	--	--
scaffold433_P4_12663	scaffold433	12663	TAACATAAAAACATAAAAA/TAACATAAAAA	TGGTCTAAAGAATGCTGGGG	TGCCCCAAATGAAGTGTGTA	60.1	525	DRR	Ca_26817	--	--

INDEL marker IDs	Chromosomes/ unanchored scaffolds	Physical positions (bp)	InDels (*Kabuli* reference genome-CDC Frontier/PII)	Forward primers (5'-3')	Reverse primers (5'-3')	Anneling temperature (0C)	Expected amplified product size (bp)	Structural annotation		Functional annotation	
								Sequence components of *kabuli* genome	*Kabuli* gene accession IDs	NCBI-KOG	TFs
scaffold433_P4_165941	scaffold433	165941	A/AG	AAAATTGAAACATCCGACGC	ACTTGTCGCTGGACGAATCT	59.9	512	DRR	Ca_26821	--	--
scaffold44_P4_74426	scaffold44	74426	TG/T	GTGTCACTGGTCCGGAGATT	TTGGTCGTTTGAGCCTTTCT	60.0	325	DRR	Ca_25410	--	--
scaffold450_P4_205054	scaffold450	205054	AATTTATATT/AATTTATATTTATATT	TTGTGTGTTTAATTTTGCGTTTG	CTAAGGCAGCCAAAATCAGC	60.0	633	DRR	Ca_23795	--	--
scaffold451_P4_108506	scaffold451	108506	TAAAAAAAAAAAA/TAAAAAAAAAAAAA	ACATAGTTCGCAAAATGCCA	GGTTGGACATCATGTTATGGG	59.2	631	DRR	Ca_22605	R	SBP
scaffold4511_P4_22911	scaffold4511	22911	CAAAAA/CAAAA	CTCCCCCTGAGTTTTGGAAT	GACGACAATCACCCGAAGAT	60.3	753	DRR	Ca_28219	--	Trihelix
scaffold4511_P4_22953	scaffold4511	22953	TAAAA/TAAAAA	CTCCCCCTGAGTTTTGGAAT	GACGACAATCACCCGAAGAT	60.3	753	DRR	Ca_28219	--	Trihelix
scaffold46_P4_39790	scaffold46	39790	AGGGGG/AGGGG	TGTTGAGATTGGCAAGCAAG	TCCAAGCTTTTGTTCCAAGTG	60.0	744	DRR	Ca_27601	--	--
scaffold46_P4_40359	scaffold46	40359	ATTT/ATTTT	TGTGCCTGAGAAAGATGCAA	CAGAATCAATGTGCCAATCG	60.5	509	DRR	Ca_27600	--	--
scaffold469_P4_176029	scaffold469	176029	AATATATATATATA/AATATATATATATATA	AGAAGTTAGGCTTGGAGCCG	CGGTTTGGTCAATTTCCATC	60.9	142	DRR	Ca_25911	I	G2-like
scaffold480_P4_34490	scaffold480	34490	CA/CAGTTCAGAA	TTGGTTTATTTGGGTTGGGA	TGGGATACAAAGACGAGCCT	60.0	638	DRR	Ca_26867	--	M-type

INDEL marker IDs	Chromosomes/ unanchored scaffolds	Physical positions (bp)	InDels (*Kabuli* reference genome-CDC Frontier/PII)	Forward primers (5'-3')	Reverse primers (5'-3')	Anneling temperature (0C)	Expected amplified product size (bp)	Structural annotation		Functional annotation	
								Sequence components of *kabuli* genome	*Kabuli* gene accession IDs	NCBI-KOG	TFs
scaffold4902_P4_108605	scaffold4902	108605	CAAAAAAAAAAA/CAAAAAAAAAA	TACAACGCTCGTTTCCACAG	CGTGAACTGTTAGCGCACTT	59.9	507	DRR	Ca_26351	--	Trihelix
scaffold4902_P4_39096	scaffold4902	39096	TAAAAAA/TAAAAA	AAGCCGCTGCTTACGAAATA	AGTTAGTGGCTGGGGGAGAT	60.0	494	DRR	Ca_26348	TU	--
scaffold495_P4_131292	scaffold495	131292	ATTTTTTTTTT/ATTTTTTTTT	CGAGGAAATGTAATTTTTAGGGG	CCCATTTGTGGCTGCTATTT	60.1	701	DRR	Ca_26761	--	AP2
scaffold510_P4_13129	scaffold510	13129	CATATATATATATATATATATATA/CATATATATATATATATATATATATATA	TGGGTGCTATTTGCAATCAA	CCTTCATGAACCGCAATACA	60.1	406	DRR	Ca_26546	--	--
scaffold531_P4_83931	scaffold531	83931	CCGG/CCGGCCGGTTCGG	GAGAACGCAAGCTAACGGAC	ACCTTGTGGATGGCAGTTTC	60.0	180	DRR	Ca_22395	--	--
scaffold543_P4_229568	scaffold543	229568	ATTTTT/ATTTT	TTTGCCCCCAACTCTACATC	ATGGGCACATGCTATCATCA	59.9	755	DRR	Ca_24786	--	--
scaffold543_P4_250171	scaffold543	250171	TAAAAAAAA/TAAAAAAAAA	GTCTTGGGTGATGGCAAAGT	GAAGTTGAGCGCAATGTTGA	60.0	703	DRR	Ca_24788	--	--
scaffold543_P4_28493	scaffold543	28493	ATTTTTTTTTT/ATTTTTTTTTTT	GACAGCTCTCTCCAAATGCC	AAGGAATCCGCATGCTTAAA	60.0	507	DRR	Ca_24779	IR	--
scaffold543_P4_69595	scaffold543	69595	GATAATAATAATAATAATAATAATAATAAT/GATAATAATAATAATAATAATAATAAT	TTTCATTTCGTTTTCAAGTTATCAA	CCTCCTAATGCGTTGGTTTT	59.1	619	DRR	Ca_24780	--	--
scaffold544_P4_146396	scaffold544	146396	CTTTTTT/CTTT	TTCCTATTGCTCACCCCTTG	GAAAAACAAGACCCGCGATA	60.1	520	DRR	Ca_26540	--	--

INDEL marker IDs	Chromosomes/ unanchored scaffolds	Physical positions (bp)	InDels (*Kabuli* reference genome-CDC Frontier/PII)	Forward primers (5'-3')	Reverse primers (5'-3')	Anneling temperature (0C)	Expected amplified product size (bp)	Structural annotation		Functional annotation	
								Sequence components of *kabuli* genome	*Kabuli* gene accession IDs	NCBI-KOG	TFs
scaffold544_P4_147706	scaffold544	147706	CAAAAA/CAAAA	CCCCCTTTTTGCATAAGTGA	GAGGAGACATCTGCTGAGGG	59.9	582	DRR	Ca_26540	--	--
scaffold545_P4_217429	scaffold545	217429	TTTATTATTGTTATTATTATTATTGTTATTAT/TTTATTATTGTTATTATTATTATTGTTATTATTATTATTGTTATTAT	TGATTTTGATCGACGGATGA	TGGAAATTTGTGCCATGAAA	60.0	674	DRR	Ca_23926	S	--
scaffold548_P4_212010	scaffold548	212010	G/GA	GGAACACTTCGGTGTGGACT	CATGTCCCGAAAAGAGCAAT	60.0	563	DRR	Ca_21489	--	--
scaffold553_P4_291778	scaffold553	291778	GATAATAATAATAATAATAATAATAAT/GATAATAATAATAATAATAATAATAATAAT	GAACTGCCTTGTTGGGGTTA	TTGCAACCTGATCTTTTCCA	60.0	609	DRR	Ca_19933	R	MYB
scaffold553_P4_463470	scaffold553	463470	GAA/GAATAGAAAA	AACACCAAATTTTCTCCCCC	CGACACTCGCTTCACCACTA	60.0	364	DRR	Ca_19939	--	--
scaffold561_P4_25431	scaffold561	25431	CTCTTTCT/CTCT	TTGATTCATCAAACACCGGA	AAATTGGGTTCTGCCAATCA	59.9	399	DRR	Ca_26215	--	MYB_related
scaffold575_P4_65125	scaffold575	65125	G/GT	TTGAAGTTAAGGGGTGCTGC	TCCATCCAACCTTGTGGATT	60.2	531	DRR	Ca_27003	--	--
scaffold5839_P4_7420	scaffold5839	7420	GAAAA/GAAAAA	AAAATCCAACAATCTCCCCC	GGTTTCAACCCCAATGCTTA	60.0	327	DRR	Ca_28048	--	--
scaffold587_P4_18087	scaffold587	18087	T/TCA	GTCCTTGCGCTTATGCTTGT	TGCTTGATTGCTTGAGATCC	60.4	347	DRR	Ca_27621	--	--
scaffold5891_P4_5025	scaffold5891	5025	TTATATAT/TTATAT	GCCAATCGATTATCGCAAGT	CCCTGTGACAAACACAATCG	60.1	481	DRR	Ca_27904	--	--

INDEL marker IDs	Chromosomes/ unanchored scaffolds	Physical positions (bp)	InDels (*Kabuli* reference genome-CDC Frontier/PII)	Forward primers (5'-3')	Reverse primers (5'-3')	Anneling temperature (0C)	Expected amplified product size (bp)	Structural annotation		Functional annotation	
								Sequence components of *kabuli* genome	*Kabuli* gene accession IDs	NCBI-KOG	TFs
scaffold593_P4_209245	scaffold593	209245	AACA/AACACA	GGGTGAAATTTTAGGGGTGAA	GGAATAAAATATGCATCCGAGC	60.0	734	DRR	Ca_26412	--	C2H2
scaffold593_P4_209468	scaffold593	209468	TC/T	GCAAACCTTTGCAAAAAGAAA	GGAATAAAATATGCATCCGAGC	59.4	153	DRR	Ca_26412	--	C2H2
scaffold593_P4_209698	scaffold593	209698	CT/CTTT	GCAAACCTTTGCAAAAAGAAA	GGAATGGAAATAATTGAAAAGTTTG	59.4	847	DRR	Ca_26412	--	C2H2
scaffold593_P4_210090	scaffold593	210090	TT/TTATTTAAT	GAGCACAGTTTCAATTAACAGCA	GTCTCTGCTCAACCAGGAGG	59.5	862	DRR	Ca_26412	--	C2H2
scaffold598_P4_99722	scaffold598	99722	ATTTTTTT/ATTTTTT	CATCGCCTGAATGGCTTAAT	TGACATGTACGGTGATGCCT	60.1	795	DRR	Ca_21751	R	--
scaffold599_P4_351148	scaffold599	351148	ATAAAAAGC/ATAAAAAGCTAAAAAGC	CTGTTGGGCAAGGGATCTAA	CACGCTTGTTTTGCACATTT	60.1	641	DRR	Ca_25346	--	--
scaffold599_P4_352138	scaffold599	352138	TTATATATATATATATATATATATATATATATATATA/TTTTATATATATATATATATATATATATATATATATATATATATATA	GGTGGAATATTTGCATGAACC	TGATGACAAAAGTAAGGAAGAGGA	59.2	955	DRR	Ca_25346	--	--
scaffold599_P4_352330	scaffold599	352330	TAAAA/TAAA	AAAAATACGGCAAAGCAAAA	TCAATGGACATGAGTCTTCACA	57.5	887	DRR	Ca_25346	--	--
scaffold599_P4_373801	scaffold599	373801	TAAAAAAAAAAAA/TAAAAAAAAAAAAAAAA	CCCCCTAAGTTCATTCCACA	GGCTTGTCCGTCGGTAAATA	59.8	567	DRR	Ca_25347	--	--
scaffold6185_P4_2912	scaffold6185	2912	GA/G	GCAAAGTCGCATTATGGTATGA	AGCCCTCACATTAATTCGCA	60.0	740	DRR	Ca_27924	--	--

INDEL marker IDs	Chromosomes/ unanchored scaffolds	Physical positions (bp)	InDels (*Kabuli* reference genome-CDC Frontier/PII)	Forward primers (5'-3')	Reverse primers (5'-3')	Anneling temperature (0C)	Expected amplified product size (bp)	Structural annotation		Functional annotation	
								Sequence components of *kabuli* genome	*Kabuli* gene accession IDs	NCBI-KOG	TFs
scaffold6185_P4_3433	scaffold6185	3433	TAAAAA/TAAAA	GCAAGCCATTTGTCCATTTT	TCAGCTCACTCTCGCTTTGA	59.9	190	DRR	Ca_27924	--	--
scaffold626_P4_55533	scaffold626	55533	CTATT/CT	TGAACTCCTTTGTCCTTTGGA	TCATTGCTCCCCCGTAATAG	59.7	320	DRR	Ca_25170	--	--
scaffold642_P4_395178	scaffold642	395178	AT/ATTATT	TTTGCGACGCTTGATCATTA	CGCACACGGGTCAAATATAA	60.4	397	DRR	Ca_19107	--	--
scaffold645_P4_238730	scaffold645	238730	CATATATATATATATATATATATATATATAT/CATATATATATATATATATATATATATAT	TTGTCGAGTGTGACCTCCAG	CCAAAACTGGGGAGAAACAA	59.9	539	DRR	Ca_25158	I	Trihelix
scaffold647_P4_200535	scaffold647	200535	A/AT	CAAGCTGTCGGTTGAAGACA	AACAATCTCCCCCTTTTTGG	60.0	603	DRR	Ca_27495	--	--
scaffold653_P4_200790	scaffold653	200790	TTATA/TTA	GAGGAAGACTCAAACGCGAC	CTTGACAAAACCCCAAGCAT	60.0	400	DRR	Ca_24313	E	--
scaffold661_P4_293075	scaffold661	293075	TGA/T	TGGGCAAGGGATCTGAATAG	AATGCCCACCCATAATTTCA	60.0	651	DRR	Ca_23950	--	--
scaffold674_P4_155701	scaffold674	155701	TTTTTTTTT/TTTTTTTTTTTT	ACGTAGAGAGAGGCTCGGAA	CAAAAATTCGCGGGTACATT	59.2	725	DRR	Ca_20156	R	--
scaffold674_P4_156790	scaffold674	156790	CTA/CTATAATCAATTA	CCAACAAATTAGTTATTGCTCTCG	TTGGTGATGGCTAAGAGCCT	59.3	680	DRR	Ca_20156	R	--
scaffold674_P4_157426	scaffold674	157426	GAAAAAAAAAAA/GAAAAAAAAAA	AGGCTCTTAGCCATCACCAA	AGCACTTTCAAGCACCCAGT	59.8	635	DRR	Ca_20156	R	--

INDEL marker IDs	Chromosomes/ unanchored scaffolds	Physical positions (bp)	InDels (*Kabuli* reference genome-CDC Frontier/PII)	Forward primers (5'-3')	Reverse primers (5'-3')	Anneling temperature (0C)	Expected amplified product size (bp)	Structural annotation		Functional annotation	
								Sequence components of *kabuli* genome	*Kabuli* gene accession IDs	NCBI-KOG	TFs
scaffold674_P4_630014	scaffold674	630014	CATATATATATATATAT/CATATATATATATATATAT	GCGAGGATTGGTATCGTCAT	GCAAAACACATTCAAAATCCAA	59.9	263	DRR	Ca_20173	BD	--
scaffold674_P4_630332	scaffold674	630332	TAAAAAAA/TAAAAAAAA	GCGAGGATTGGTATCGTCAT	TGCACGAAAGGTATAATTGGG	59.9	922	DRR	Ca_20173	BD	--
scaffold682_P4_239210	scaffold682	239210	ACC/AC	TCCAAAGCATCACGTATCCA	TGCGTGACAGGAACAAGAAG	60.1	667	DRR	Ca_23188	R	ERF
scaffold682_P4_239323	scaffold682	239323	AGATGATG/AGATG	CAACTCCCCCTGCAAACTTA	TGCGTGACAGGAACAAGAAG	60.1	417	DRR	Ca_23188	R	ERF
scaffold720_P4_179447	scaffold720	179447	AACACACA/AACACA	CAGCCACAAAGTGCTTGAAA	ATTCGACTAGTGGTGGTGCC	60.0	637	DRR	Ca_24736	--	--
scaffold724_P4_100088	scaffold724	100088	ATTTTTTTT/ATTTTTTTTTT	ACAGGAGGGTGATCGAGAGA	TGCATACTTGTGTGGCATTTT	59.8	727	DRR	Ca_25051	Q	NAC
scaffold724_P4_100193	scaffold724	100193	TAAAAAAAA/TAAAAAAA	CTGTAAGCGGAGAAAATTGG	TTGCATACTTGTGTGGCATTT	57.5	319	DRR	Ca_25051	Q	NAC
scaffold724_P4_100328	scaffold724	100328	TAAAAAAA/TAAAAAA	CTGTAAGCGGAGAAAATTGG	AATGGTAGTCCCTCTGGCCT	57.5	690	DRR	Ca_25051	Q	NAC
scaffold724_P4_100591	scaffold724	100591	AATTTAACATAT/A	AAATGCCACACAAGTATGCAA	AATGGTAGTCCCTCTGGCCT	59.1	392	DRR	Ca_25051	Q	NAC
scaffold724_P4_60095	scaffold724	60095	TAAAAAAAA/TAAAAAAAAA	CTGCCACGTCACCCTTTTAT	ATACCATTGACTTGCCGGAG	60.0	401	DRR	Ca_25048	--	--

INDEL marker IDs	Chromosomes/ unanchored scaffolds	Physical positions (bp)	InDels (*Kabuli* reference genome-CDC Frontier/PII)	Forward primers (5'-3')	Reverse primers (5'-3')	Anneling temperature (0C)	Expected amplified product size (bp)	Structural annotation		Functional annotation	
								Sequence components of *kabuli* genome	*Kabuli* gene accession IDs	NCBI-KOG	TFs
scaffold731_P4_315903	scaffold731	315903	GATGA/GATGAATGA	AACTCGAAGTGCCATGCTTT	CAATACGCAATGGTGGTGAG	59.9	660	DRR	Ca_23814	J	--
scaffold731_P4_361613	scaffold731	361613	TGG/TGGG	TGTGCTATTGCTCAAATCCG	AGCAGTCGTTTCCAGCTGTT	59.8	640	DRR	Ca_23818	--	--
scaffold731_P4_94332	scaffold731	94332	ATTTTTTTTTT/ATTTTTTTT	GAGCGCTGTAAAAGACCTCC	TGAAGATGGTTTGCAATGGA	59.1	188	DRR	Ca_23810	--	B3
scaffold731_P4_94685	scaffold731	94685	CATATATATATATATATATATATATATATATATATA/CATATATATATATATATATATATATATATATATA	TCCATTGCAAACCATCTTCA	AAAAATGCGCTGCTTCTTGT	60.0	613	DRR	Ca_23810	--	B3
scaffold7330_P4_2275	scaffold7330	2275	AG/AAGTTTTTTTG	TTGGTCGACAAGCAACTGAG	AAAATCCAACAATCTCCCCC	60.0	699	DRR	Ca_28207	--	--
scaffold741_P4_6784	scaffold741	6784	AATATATATATATATATATATATATATAT/AATATATATATATATATATATATATATATATAT	TTGGGATGGTGATTCTCACA	TTTACCACTTGGAGTTGGGC	59.9	215	DRR	Ca_28074	--	B3
scaffold750_P4_360259	scaffold750	360259	GA/G	TGTTTGTCTTGCTGCTTTGC	AAAATCCAACAATCTCCCCC	60.2	626	DRR	Ca_25944	--	--
scaffold752_P4_227110	scaffold752	227110	ATAATA/ATAGTAATA	TTGATGTTTGTCGGGCAATA	AATGGCCACGAATCTGAAAG	59.9	601	DRR	Ca_25232	--	--
scaffold752_P4_495403	scaffold752	495403	CAAAAAAAAAA/CAAAAAAAAA	TCCGTTGTGAAGGCTTTTTC	ATTGGGCCTTTGTAGTGACG	60.2	175	DRR	Ca_25238	L	--
scaffold775_P4_449733	scaffold775	449733	CTTATCAATTGTGTTT/CTTATCAATTGTGTTTATCAATTGTGTTT	CTCTCTTCTGCGCATCAACT	TTGACCATGACACATGCCTT	58.3	459	DRR	Ca_23414	--	NAC

INDEL marker IDs	Chromosomes/ unanchored scaffolds	Physical positions (bp)	InDels (*Kabuli* reference genome-CDC Frontier/PII)	Forward primers (5'-3')	Reverse primers (5'-3')	Anneling temperature (0C)	Expected amplified product size (bp)	Structural annotation		Functional annotation	
								Sequence components of *kabuli* genome	*Kabuli* gene accession IDs	NCBI-KOG	TFs
scaffold775_P4_450377	scaffold775	450377	TG/TGG	AAGGCATGTGTCATGGTCAA	TTTACGGGAACGAGGAGAGA	60.0	738	DRR	Ca_23414	--	NAC
scaffold789_P4_18724	scaffold789	18724	GAAA/GAAAA	TTGTCAGGATTGCCATTTCA	ATACTCAGGAGGGGCTGGTT	60.0	558	DRR	Ca_27694	--	--
scaffold7923_P4_2123	scaffold7923	2123	AAGCTCA/AA	TCGGGGTCTTTAGTCGTCAT	GCGAGGAAGCAAAAGCTAGA	59.6	796	DRR	Ca_27506	O	--
scaffold7923_P4_22242	scaffold7923	22242	CAAAAAAAAAA/CAAAAAAAAA	TTTCCCGACTTGGTCCTTTA	TTTGCGTTACGAATTTGATGA	59.5	177	DRR	Ca_27507	QI	bHLH
scaffold7923_P4_2279	scaffold7923	2279	C/CAA	TTAGCAGGGGTTAGCTCTGAA	ACCACCTATTTTGCAGCACC	59.1	858	DRR	Ca_27506	O	--
scaffold801_P4_20920	scaffold801	20920	ATTTTTTTTT/ATTTTTTTT	TACCCATGTGCGTCACTGTT	GGACATGGTTGAGGAAATGG	60.0	223	DRR	Ca_26173	--	--
scaffold801_P4_72301	scaffold801	72301	CTTTTTTTTTTTT/CTTTTTTTTTTTTTT	TGTTAGCGTAACAAATGGGG	TGTGTTTTATGGATTGCGGA	58.6	557	DRR	Ca_26176	OE	WRKY
scaffold808_P4_3720	scaffold808	3720	CAAA/CAAAA	GCGTTTTCCAGAACTGCTTC	GTGTTGACAAAGGGGGAGAA	60.0	357	DRR	Ca_27975	--	--
scaffold812_P4_279194	scaffold812	279194	A/AG	AACAATAAAATCCGCACCCA	AATTTGTGGGCTTAGGAGGC	60.2	764	DRR	Ca_23374	Q	B3
scaffold845_P4_102448	scaffold845	102448	A/AG	CTGACCGTCAAAAGCACTGA	ACGCATAATACCTGAACGGC	60.0	480	DRR	Ca_22415	R	--

INDEL marker IDs	Chromosomes/ unanchored scaffolds	Physical positions (bp)	InDels (*Kabuli* reference genome-CDC Frontier/PII)	Forward primers (5'-3')	Reverse primers (5'-3')	Anneling temperature (0C)	Expected amplified product size (bp)	Structural annotation		Functional annotation	
								Sequence components of *kabuli* genome	*Kabuli* gene accession IDs	NCBI-KOG	TFs
scaffold845_P4_133709	scaffold845	133709	TCC/TC	TCACGTGGACCTAATCCTCC	TTGTTTCGCCCTTCAAATTC	59.9	776	DRR	Ca_22417	U	--
scaffold845_P4_134750	scaffold845	134750	CAA/CAAAA	CCGTTTGGGCCCTTAGTTAT	TCGTGTCCATTGCTATTTTCA	60.2	546	DRR	Ca_22417	U	--
scaffold845_P4_27150	scaffold845	27150	TAAA/TAA	ATCGACCGCGTAAATGAAAC	TGATCCTTTTTGGCCTTGAG	60.0	689	DRR	Ca_22410	--	bHLH
scaffold845_P4_44165	scaffold845	44165	T/TG	CGATCGCATGTTATTGCTTG	GACCCAAAAGAGGCCCTAAC	60.2	615	DRR	Ca_22411	P	--
scaffold845_P4_52018	scaffold845	52018	GAA/GAAA	GCAAGTGTGCAACAACCTTC	TTCTCCACCGAGTCATTTCC	59.3	296	DRR	Ca_22412	--	--
scaffold845_P4_69320	scaffold845	69320	TAAAAAA/TAAAAA	TGAGTGTGAGTCCGAATGGA	GAACCCCAACCTGAAAACAA	60.2	493	DRR	Ca_22414	R	--
scaffold873_P4_199356	scaffold873	199356	CTTATTATTATTATTATTATTATT/CTTATTATTATTATTATTATT	CCCTTACTTGGACCAAAGTTACC	CTGCGAACTGGACAAATGAA	60.1	585	DRR	Ca_23485	--	--
scaffold877_P4_10844	scaffold877	10844	TAAAAA/TAAAA	GCAGCAGTTGCAGCAGTTTA	TGATTTAGCGTCGGTGTCAG	60.3	601	DRR	Ca_28083	F	--
scaffold877_P4_11190	scaffold877	11190	CCTTTT/C	CTGACACCGACGCTAAATCA	AGTGCATAAGACCCGTTTGC	59.9	210	DRR	Ca_28083	F	--
scaffold886_P4_133033	scaffold886	133033	T/TATTATTATTA	TTGGGATTAAGCTGTTTGGG	CCGACAAAACATGATGGTCA	59.9	341	DRR	Ca_26670	R	--

INDEL marker IDs	Chromosomes/ unanchored scaffolds	Physical positions (bp)	InDels (*Kabuli* reference genome-CDC Frontier/PII)	Forward primers (5'-3')	Reverse primers (5'-3')	Anneling temperature (0C)	Expected amplified product size (bp)	Structural annotation		Functional annotation	
								Sequence components of *kabuli* genome	*Kabuli* gene accession IDs	NCBI-KOG	TFs
scaffold886_P4_151276	scaffold886	151276	AG/AGCTG	AGGGATGCTTGTAGTGGTTGA	TTTGACGTGCATCCAATGTT	59.6	162	DRR	Ca_26671	--	--
scaffold886_P4_151448	scaffold886	151448	TA/T	GACAACATTGGATGCACGTC	TCGTGGTATGCTTGACCTGT	60.0	259	DRR	Ca_26671	--	--
scaffold892_P4_33546	scaffold892	33546	TTTCTT/TTTCTTCTT	TCCCAATTTGGTTTTGGTGT	TGGAATCTTCACCCTGACAA	60.1	713	DRR	Ca_27455	R	--
scaffold892_P4_33776	scaffold892	33776	TC/T	TCAAAAGACACCAAATGGAAAA	TGGAATCTTCACCCTGACAA	59.5	251	DRR	Ca_27455	R	--
scaffold892_P4_34457	scaffold892	34457	T/TTTTATTATG	CATGCCTAACGCACGAATTA	CCGACATGGTCTTCTTCCTT	59.7	426	DRR	Ca_27455	R	--
scaffold892_P4_42751	scaffold892	42751	TGGGG/TGGGGG	GACGATGAAAAATATGCGGG	TGTTGATTGGCGAACACAAT	60.3	387	DRR	Ca_27456	--	--
scaffold916_P4_3252	scaffold916	3252	AATATA/AATA	AAAGCGCTGTAAAATGGGTG	TATAGGGTCAAAACGCCCAC	60.1	726	DRR	Ca_21034	G	E2F/DP
scaffold916_P4_3317	scaffold916	3317	TTTAATT/TTT	ACATCTCATGCCATTGTGGA	GAGACAGTCAGAAGGCCACC	59.9	520	DRR	Ca_21034	G	E2F/DP
scaffold916_P4_3519	scaffold916	3519	AATATATATATATATATATAT/AATATATATATATATAT	ACATCTCATGCCATTGTGGA	GAGACAGTCAGAAGGCCACC	59.9	520	DRR	Ca_21034	G	E2F/DP
scaffold919_P4_190377	scaffold919	190377	CAAAA/CAAAAA	GAATCCTGGGCTGAAATCAA	CACAGGTGTTTGTCCGTGAG	60.0	877	DRR	Ca_22832	--	--

**INDEL marker IDs**	**Chromosomes/ unanchored scaffolds**	**Physical positions (bp)**	**InDels (*Kabuli* reference genome-CDC Frontier/PII)**	**Forward primers (5'-3')**	**Reverse primers (5'-3')**	**Anneling temperature (0C)**	**Expected amplified product size (bp)**	**Structural annotation**		**Functional annotation**	
								**Sequence components of *kabuli* genome**	***Kabuli* gene accession IDs**	**NCBI-KOG**	**TFs**
scaffold93_P4_562448	scaffold93	562448	TA/TAA	TATAGCCACGGCAAAAATCC	CCGAAACACAAACCCAGACT	59.9	514	DRR	Ca_23082	--	--
scaffold953_P4_51991	scaffold953	51991	TAAAAAAAAA/TAAAAAAAAAA	AATACGCCCCATAAGGAACC	TTGCCTTCCTTTTCAGCACT	60.0	372	DRR	Ca_26909	--	--
scaffold953_P4_73770	scaffold953	73770	ATT/AT	TCGGGGTATTTAATTGGCAG	ACCACATGCAAATTGTTCCA	59.8	353	DRR	Ca_26910	--	--
C11060010_P4_683	C11060010	683	ATTATT/ATTATTATT	TCCGATATGCACCAATGAAA	AGGCGTCGGTTTTTCTTTCT	59.9	413	INTERGENIC	--	--	--
C11119398_P4_1062	C11119398	1062	TTAT/TTATAT	AGAAATGGAACACTCGCCAC	CCCTCAGCATGCATGATTTT	60.1	312	INTERGENIC	--	--	--
C11119398_P4_1161	C11119398	1161	TTATATATATA/TTATATATA	CATGCATGCTGAGGGATATG	TGTTTGTTTCGTCAATCACTTT	60.1	268	INTERGENIC	--	--	--
C11119398_P4_643	C11119398	643	CAAAAAA/CAAAAAAA	AGCTCCAAATTTGATGGCAC	GTGGCGAGTGTTCCATTTCT	60.1	281	INTERGENIC	--	--	--
C11122410_P4_1998	C11122410	1998	ATTTTTTT/ATTTTTTTT	AGCACACAACGAAACCATGA	GCAACGGGAATGTGTTTTTC	60.2	797	INTERGENIC	--	--	--
C11154862_P4_1914	C11154862	1914	ATTTT/ATTTTT	GTCCAATCCCCAAACAACAC	TGAAATTATGGAAAGGAAGCTCA	60.1	521	INTERGENIC	--	--	--
C11154862_P4_4400	C11154862	4400	CTTTTTT/CTTTTTTT	TGTCGATATATTTACCGAAACCC	GCATTAGAGCTGAGGCCAAC	59.2	296	INTERGENIC	--	--	--

**INDEL marker IDs**	**Chromosomes/ unanchored scaffolds**	**Physical positions (bp)**	**InDels (*Kabuli* reference genome-CDC Frontier/PII)**	**Forward primers (5'-3')**	**Reverse primers (5'-3')**	**Anneling temperature (0C)**	**Expected amplified product size (bp)**	**Structural annotation**		**Functional annotation**	
								**Sequence components of *kabuli* genome**	***Kabuli* gene accession IDs**	**NCBI-KOG**	**TFs**
C11174756_P4_5574	C11174756	5574	TAAAAAA/TAAAAAAA	TGGATTGAGAGCATTCCCAT	GTGAGCAGTTGAGAACCGTG	60.4	606	INTERGENIC	--	--	--
C11178116_P4_10748	C11178116	10748	CTTCAT/CTTCATTCAT	TTGGAGGGTGGTTTGAAAAA	TCACTTGGTCATGGATTGGA	60.3	646	INTERGENIC	--	--	--
C11178116_P4_4183	C11178116	4183	TTCTCTCTCTCTCTCTCTCTCTCTCTCTCTC/TTCTCTCTCTCTCTCTCTCTCTCTCTCTCTCTC	CGCACGATAAGAAAGCATCA	AGCGAGTGGTCCATGGTTAC	60.0	742	INTERGENIC	--	--	--
C11178532_P4_13478	C11178532	13478	TTATATATATATATATAT/TTATATATATATATATATAT	TTGATGCGTTTGTAGAAATTGTT	TGAGTGAGGAACGACACCAA	58.7	507	INTERGENIC	--	--	--
C11180336_P4_12584	C11180336	12584	CAAAAAAAAA/CAAAAAAAAAA	TCCAAGTTTTCTCCACGTGTT	GCATTTTTGAGTGATTTGCG	59.6	498	INTERGENIC	--	--	--
Ca1_P4_10017783	Ca1	10017783	GTATATATATATATATATA/GTATATATATATATATATATA	TCAATAATGGAAACTGGTACACAAA	CGGTTGGAGAGTAAACACGG	59.7	795	INTERGENIC	--	--	--
Ca1_P4_10057638	Ca1	10057638	CATATATATATATATATAT/CATATATATATATAT	TGATGGGAGCAATGAATGAA	TCAAATTCCGACTTGAGGGA	60.0	542	INTERGENIC	--	--	--
Ca1_P4_10064752	Ca1	10064752	TTATATATATATATATATA/TTATATATATATATATATATA	ATGCAAATTCTCCCCCTCTT	TCATTTGATTTTAATTGGACGA	59.9	750	INTERGENIC	--	--	--
Ca1_P4_10099545	Ca1	10099545	TTATATATATATATATATATATATAT/TTATATATATATATATATATATATATATAT	ATATTGCAAGTAGGCCGGTG	TCCTTCTCAAAACAATTGATCG	60.0	559	INTERGENIC	--	--	--
Ca1_P4_10104142	Ca1	10104142	TTATATATATATATATATATATAT/TTATATATATATATATATATATATAT	TTCCAATGAGGACATGTGTGA	GGAGCTTTTTGTTGATAACGG	60.0	383	INTERGENIC	--	--	--

INDEL marker IDs	Chromosomes/ unanchored scaffolds	Physical positions (bp)	InDels (*Kabuli* reference genome-CDC Frontier/PII)	Forward primers (5'-3')	Reverse primers (5'-3')	Anneling temperature (0C)	Expected amplified product size (bp)	Structural annotation		Functional annotation	
								Sequence components of ***kabuli*** genome	***Kabuli*** gene accession IDs	NCBI-KOG	TFs
Ca1_P4_10350471	Ca1	10350471	TTATATATATATATATATATATAT/TTATATATATATATATATATATATATATAT	TCTGAGTCCGCTACCCAGTT	ATGTCACATTGTGTGTGGGG	59.9	517	INTERGENIC	--	--	--
Ca1_P4_10428835	Ca1	10428835	GTTTTT/GTTTTTT	TCAAACAAAGCCTAAAACATCA	TCATGCCTTCTTTCACTTATTCA	57.5	361	INTERGENIC	--	--	--
Ca1_P4_10471260	Ca1	10471260	TTATATATATATATATATATATA/TTATATATATATATATATATATA	TTCTCCATTCGCACTCACAC	TCAAATTTGGAGGCTGGAAC	59.8	312	INTERGENIC	--	--	--
Ca1_P4_10510182	Ca1	10510182	CATTATTATTATTATTATTATTATTATTATTATTATTATTATTATTATTATT/CATTATTATTATTATTATTATTATTATTATTATTATTATTATT	TTCGCTGAAGTCAGTCAAACA	TTTGTTCCGACTTTGCAATTA	59.6	328	INTERGENIC	--	--	--
Ca1_P4_10514048	Ca1	10514048	AATATATATATATATATATATATATAT/AATATATATATATATATATATATAT	AGGACAACGCAATTACAATGA	CCATGCAAGAAAGACAGCAA	58.2	633	INTERGENIC	--	--	--
Ca1_P4_10664922	Ca1	10664922	TCCCC/TCCC	CGAACAAAAATATCGCCACA	GCTCCTTAATAGGTTGGTTTGG	59.6	606	INTERGENIC	--	--	--
Ca1_P4_10801339	Ca1	10801339	TTATATATATATATATATATATATAT/TTATATATATATATATATATATATATATATATATAT	ATATGGTAGTTTGGCGCGTT	TCTGCCCAACCAAACTTTTT	59.5	473	INTERGENIC	--	--	--
Ca1_P4_10803149	Ca1	10803149	AATATATATATATATATATAT/AATATATATATATATATATATATAT	GGCCCCCACATAATCTCTCT	ACCTGAGTTCAAGCCCGAGT	60.3	457	INTERGENIC	--	--	--
Ca1_P4_10846457	Ca1	10846457	CAAAAAA/CAAAAAAA	TTCCCACCTAAACGATCGAC	CAATGAACCTGAGTTTTGTCCC	59.9	865	INTERGENIC	--	--	--
Ca1_P4_11145101	Ca1	11145101	TTATATATATATATATATATATATAT/TTATATATATATATATATATAT	TTTGAGTCCAAATTGAAGGCT	CGGATCAAAATTCGCACAAT	58.8	837	INTERGENIC	--	--	--

INDEL marker IDs	Chromosomes/ unanchored scaffolds	Physical positions (bp)	InDels (*Kabuli* reference genome-CDC Frontier/PII)	Forward primers (5'-3')	Reverse primers (5'-3')	Anneling temperature (0C)	Expected amplified product size (bp)	Structural annotation		Functional annotation	
								Sequence components of *kabuli* genome	*Kabuli* gene accession IDs	NCBI-KOG	TFs
Ca1_P4_11151072	Ca1	11151072	CACTCTNNNNNNNNNNACTCT/CACTCT	CAAAGACAAGACGACCACCA	CCTTTGCAATCCAGCATTTT	59.7	464	INTERGENIC	--	--	--
Ca1_P4_11162119	Ca1	11162119	GATATATATATATATATATATATA/GATATATATATATATATATATA	GGCGTTGGAATCATGTTTTT	TGTAGCGGAAGCATCAACAG	59.8	556	INTERGENIC	--	--	--
Ca1_P4_11162292	Ca1	11162292	CAAA/CA	GGCGTTGGAATCATGTTTTT	TGTAGCGGAAGCATCAACAG	59.8	556	INTERGENIC	--	--	--
Ca1_P4_11382617	Ca1	11382617	AAATAACAATAACAATAACAATAACAATAACAA/AAATAACAATAACAATAACAATAACAA	TTGCCTCCATCATTCAAACA	ACCTTGTTGTTTTTGGCTGG	60.0	378	INTERGENIC	--	--	--
Ca1_P4_11425487	Ca1	11425487	TTATATATATATATA/TTATATATATATATATA	TTGTCTCACGTGAATCTGGG	GGAGTTTGGAGAGAAGGAGTGA	59.7	745	INTERGENIC	--	--	--
Ca1_P4_1145463	Ca1	1145463	AATATATATATATATATATATATATATATA/AATATATATATATATATATATATATATATATA	TTCACAAACAACACCACACAA	GTGTCTCCTCCTACCACCCA	58.5	485	INTERGENIC	--	--	--
Ca1_P4_11521081	Ca1	11521081	GTTATTATT/GTTATTATTATT	GGTCATGCTGATAGGTGGATG	ACCACAACCTTGGAATGGAA	60.4	908	INTERGENIC	--	--	--
Ca1_P4_11563390	Ca1	11563390	ATT/AT	AATGAACATGCGGACCAAAT	TTATACTGTCTCCCGCCACC	60.2	303	INTERGENIC	--	--	--
Ca1_P4_11735185	Ca1	11735185	GTATTATTATTATTATT/GTATTATTATTATTATTATTATT	TGACAGTCAGAGTGCGATCC	TCTCTGGCAACCGTTCTTCT	60.0	441	INTERGENIC	--	--	--
Ca1_P4_11788825	Ca1	11788825	AATATATATATATATA/AATATATATATATATATA	TGGGAATAGGTCAGACCAGG	CCAAGTTGTTTGAGAGAATTTGTTT	59.9	696	INTERGENIC	--	--	--

INDEL marker IDs	Chromosomes/ unanchored scaffolds	Physical positions (bp)	InDels (*Kabuli* reference genome-CDC Frontier/PII)	Forward primers (5'-3')	Reverse primers (5'-3')	Anneling temperature (0C)	Expected amplified product size (bp)	Structural annotation		Functional annotation	
								Sequence components of *kabuli* genome	*Kabuli* gene accession IDs	NCBI-KOG	TFs
Ca1_P4_11930560	Ca1	11930560	AG/A	CGACTTGCCAAAGAACCAGT	TAGTAGGAGGTCGCTGGACG	60.3	812	INTERGENIC	--	--	--
Ca1_P4_11948916	Ca1	11948916	AATATATATATATATATATATATATATA/AATATATATATATATATATATATA	AGGCAAACATGGCAAAATTC	TCTTTGCCTATGCCTATGAGC	59.9	746	INTERGENIC	--	--	--
Ca1_P4_12110141	Ca1	12110141	ATTTTTTTTTTTTTT/ATTTTTTTTTT	TGAATTGGGCCAAGACATTT	TGGCAGACCACAGATAACCA	60.3	943	INTERGENIC	--	--	--
Ca1_P4_12112319	Ca1	12112319	C/CTTGG	GCGAAATTATTTGACGGAGTTT	CATTTCAGAAGTGAAGAGCAACA	59.5	874	INTERGENIC	--	--	--
Ca1_P4_12142151	Ca1	12142151	ATTTTTTT/ATTTTTT	TGCTCCTCATGTTGTAGCAC	AAGGGATGGACATCAAATCG	57.4	522	INTERGENIC	--	--	--
Ca1_P4_12225439	Ca1	12225439	CTAGTTT/CTAGTTTTAGTTT	GCATAGGTGGCAGACGAAGT	GCTTTCGGTTTCCTGTTTCA	60.3	721	INTERGENIC	--	--	--
Ca1_P4_12352185	Ca1	12352185	AATATATATATATATATATATATATATATA/AATATATATATATATATATATATATA	GATGATGGCGATTGCATAGA	GACTAAACGATGCGAGGAGC	59.6	523	INTERGENIC	--	--	--
Ca1_P4_12420239	Ca1	12420239	CAAAAAAAAA/CAAAAAAAAAA	AAATGCAATCTTGGTTTCCA	TTTTGGTAATGTGCCCATGATA	58.1	595	INTERGENIC	--	--	--
Ca1_P4_12544202	Ca1	12544202	TTATATATATATATAT/TTATATATATATAT	TCGGTGTCTCCCTTCATTTC	TCATTCAAGGATGCAACTGG	60.1	656	INTERGENIC	--	--	--
Ca1_P4_12580190	Ca1	12580190	TAAAAAAAAAA/TAAAAAAAAAAA	TAATTCACTCACCATCGCCA	TTCATGTTTGGTGTGGCACT	60.1	476	INTERGENIC	--	--	--

INDEL marker IDs	Chromosomes/ unanchored scaffolds	Physical positions (bp)	InDels (*Kabuli* reference genome-CDC Frontier/PII)	Forward primers (5'-3')	Reverse primers (5'-3')	Anneling temperature (0C)	Expected amplified product size (bp)	Structural annotation		Functional annotation	
								Sequence components of *kabuli* genome	*Kabuli* gene accession IDs	NCBI-KOG	TFs
Ca1_P4_12603890	Ca1	12603890	GTTTTTTTTTTTTT/GTTTTTTTTTTTT	TTGGTAGCATTGTTTTGGCA	GCATCGGCTCTTTTTGCTAC	60.1	834	INTERGENIC	--	--	--
Ca1_P4_12644727	Ca1	12644727	TGTA/TGTACGTA	TTGATTTTCAATTGGGAGCC	TGCCAGGATGTAACCCTTTC	59.9	807	INTERGENIC	--	--	--
Ca1_P4_12644922	Ca1	12644922	AC/A	ACCCAACCGCAAAAACAATA	TGCCAGGATGTAACCCTTTC	60.2	440	INTERGENIC	--	--	--
Ca1_P4_12690899	Ca1	12690899	TTAAATAAATAAATAAATAA/TTAAATAAATAAATAAATAAATAA	TGCTTCATACCAACGTTCCA	CAGAGTTTTAAACGTATGACGGTG	60.1	516	INTERGENIC	--	--	--
Ca1_P4_12699932	Ca1	12699932	ACCCC/ACCC	ACCGAATGTCGAACTCAAGG	TTGGGAACTTGGGAGACTTG	60.1	768	INTERGENIC	--	--	--
Ca1_P4_12766880	Ca1	12766880	ATTTTT/ATTTT	TTTTCTGCAGCATTGCATTC	AGTTGGGTGCTTGTAGCGAT	60.0	419	INTERGENIC	--	--	--
Ca1_P4_12772337	Ca1	12772337	TAAAAA/TAAAA	GGAAGATGAGTGGGGATCAA	TTAATTTGGTCTTCCCGGTG	59.9	183	INTERGENIC	--	--	--
Ca1_P4_12773945	Ca1	12773945	GCATGCAT/GCATGCATCATGCAT	GAAGCAGCCTCAACCTCAAC	GAAAGGTGGAGATTGGTTGG	60.0	150	INTERGENIC	--	--	--
Ca1_P4_12776177	Ca1	12776177	T/TC	ATCTCCCCGTCAGATATCCC	GACAATAACCACCCAGTGCC	60.1	225	INTERGENIC	--	--	--
Ca1_P4_12783589	Ca1	12783589	TAGAAACAGAAACAGAA/TAGAAACAGAA	GCCACACAAAAAGGAAGGAA	ACAATGTGGCCTCCTTGAAC	60.1	287	INTERGENIC	--	--	--

INDEL marker IDs	Chromosomes/ unanchored scaffolds	Physical positions (bp)	InDels (*Kabuli* reference genome-CDC Frontier/PII)	Forward primers (5'-3')	Reverse primers (5'-3')	Anneling temperature (0C)	Expected amplified product size (bp)	Structural annotation		Functional annotation	
								Sequence components of *kabuli* genome	*Kabuli* gene accession IDs	NCBI-KOG	TFs
Ca1_P4_12791859	Ca1	12791859	GAT/GATCTGCAT	TGTAAAATGCACGCCCATAA	TGCAAAATGATTCAACGTGAG	60.0	520	INTERGENIC	--	--	--
Ca1_P4_12947634	Ca1	12947634	ATAAATTTAAA/ATAAATTTAAAATTTAAATTTAAA	AACTTTGTGGTCGTTTTGGC	TGCTTATTGTGGCTGCTTTG	60.0	647	INTERGENIC	--	--	--
Ca1_P4_12950561	Ca1	12950561	T/TC	CAAGCATGCAGCAAAAAGAA	GCGGTGCCAGTGAAATAAAT	60.1	609	INTERGENIC	--	--	--
Ca1_P4_12956191	Ca1	12956191	ATCTC/A	TGCTAACCCAACACAATTTCT	GTCAGATGTTTGGGGCATTT	57.3	469	INTERGENIC	--	--	--
Ca1_P4_12956423	Ca1	12956423	CAAAAAAA/CAAAAA	TTGTCGGATACCTGCTCAGT	TTGCAAATTCGGACAAACAA	58.3	455	INTERGENIC	--	--	--
Ca1_P4_12988931	Ca1	12988931	A/ACCGACTTTCGGC	ATCGGCATCCTTCCTTCTTT	GACGCTAAGCCCCTTTCTCT	60.0	354	INTERGENIC	--	--	--
Ca1_P4_13007507	Ca1	13007507	TTAT/TTATATAT	CACGGGTCCAGATGGTTAGT	CCAAGCATCTTGGTGCATAA	59.8	322	INTERGENIC	--	--	--
Ca1_P4_13012418	Ca1	13012418	TCC/TC	TTGATGAGGATCTTTCTATGGGA	TGCGATAATATTCATCCCGTG	59.9	306	INTERGENIC	--	--	--
Ca1_P4_13399858	Ca1	13399858	TAAAAGAAATTTTGAAAAGAAATT/TAAAAGAAATT	TCAACTCCGAGGCCTCTTTA	GCTCTCAACACTGAAAGCCC	59.9	368	INTERGENIC	--	--	--
Ca1_P4_13487363	Ca1	13487363	TAAAAAAAAAAAA/TAAAAAAAAAAAAA	GATGATTGCAGCAAGTTGGA	TCGTGTTGGCTAATTCAACG	59.8	558	INTERGENIC	--	--	--

INDEL marker IDs	Chromosomes/ unanchored scaffolds	Physical positions (bp)	InDels (*Kabuli* reference genome-CDC Frontier/PII)	Forward primers (5'-3')	Reverse primers (5'-3')	Anneling temperature (0C)	Expected amplified product size (bp)	Structural annotation		Functional annotation	
								Sequence components of *kabuli* genome	*Kabuli* gene accession IDs	NCBI-KOG	TFs
Ca1_P4_13578342	Ca1	13578342	GAAAAAAAAAAA/GAAAAAAAAAA	CCGGCTTGTAGGACGAGTAG	TCTGTTTTCGAGGGCACTTT	59.9	519	INTERGENIC	--	--	--
Ca1_P4_1366043	Ca1	1366043	CTATATATATATATATATATATATA/CTATATATATATATATATATATATA	TTTGGGACGAGACGGATAAC	CGAATTTTGTCAATCGAACCA	59.9	819	INTERGENIC	--	--	--
Ca1_P4_13672025	Ca1	13672025	TA/T	CCAAATTGAACCTATCATAAACCA	AAAAGACCAAATCGGGAAAAA	59.2	748	INTERGENIC	--	--	--
Ca1_P4_13680810	Ca1	13680810	ATTTTT/ATTTT	TGGTTGAACTGTGAACAGGC	CCAATTTCCCCTACCCAAAT	59.7	559	INTERGENIC	--	--	--
Ca1_P4_13690393	Ca1	13690393	GAAAAAAAAAA/GAAAAAAAAA	GCCTTTGGAAGCATATCATCA	GCCGTAGGACTGTTCATGGT	60.1	663	INTERGENIC	--	--	--
Ca1_P4_13718939	Ca1	13718939	AGATGAT/AGAT	GCCAAAATCTATTGTGGGGT	TGACTGGGATTCCATTTCTG	58.8	127	INTERGENIC	--	--	--
Ca1_P4_13731515	Ca1	13731515	ATTTTTTTTTTTTT/ATTTTTTTTTTTT	TTTCACTTCCAAAACACCCC	CTGTCCAGACGGGTTCGTAT	59.8	847	INTERGENIC	--	--	--
Ca1_P4_13748109	Ca1	13748109	TA/TAGCCATTCGTAAA	ATTCGGGATAAGTGGAACCC	TGCAAATGAGATAACCAGCG	60.0	650	INTERGENIC	--	--	--
Ca1_P4_13753170	Ca1	13753170	ATCTCTCTCTCTCTCT/ATCTCTCTCTCTCT	CGCTGCATATTATTGTGCGT	TTCTTATTAAAACACCCAATTCAGC	59.8	417	INTERGENIC	--	--	--
Ca1_P4_13800579	Ca1	13800579	GTTTTTTT/GTTTTTTTT	AGGGATAACCTGGTGCTGTG	TCTTTGAACGATACCGCCTC	60.0	622	INTERGENIC	--	--	--

INDEL marker IDs	Chromosomes/ unanchored scaffolds	Physical positions (bp)	InDels (*Kabuli* reference genome-CDC Frontier/PII)	Forward primers (5'-3')	Reverse primers (5'-3')	Anneling temperature (0C)	Expected amplified product size (bp)	Structural annotation		Functional annotation	
								Sequence components of *kabuli* genome	*Kabuli* gene accession IDs	NCBI-KOG	TFs
Ca1_P4_1395879	Ca1	1395879	ATATTATTATTATTATTATTATTATTATTATTATTATTATTATTATTATTATTATTAT/ATATTATTATTATTATTATTATTATTATTATTATTATTATTATTATTATTATTATTATTAT	CATTTCTCGTCGAAGACTTTTG	TGACGGCAATTTCGTATCTTC	59.0	784	INTERGENIC	--	--	--
Ca1_P4_1397435	Ca1	1397435	CA/C	TGGAAACAGCAACGTGGTAG	CAAAGGGTTGGATTTTCCAC	59.8	555	INTERGENIC	--	--	--
Ca1_P4_1408705	Ca1	1408705	GATATATATATATATATATAT/GATATATATATATATATATATAT	GGTTTGTCTAAGAGGATGGGA	TGAGTTGGGTTAGAAATCAACAA	58.1	697	INTERGENIC	--	--	--
Ca1_P4_14309917	Ca1	14309917	G/GAA	AACCATTGGAAGGCAAAATG	TCTTCCACCTCTTTCGCATC	59.8	106	INTERGENIC	--	--	--
Ca1_P4_14356029	Ca1	14356029	CA/CATATATA	CACCCACACACACACACACA	CATGAACAACCAATATTCCCA	61.1	440	INTERGENIC	--	--	--
Ca1_P4_14426009	Ca1	14426009	CATATATATATATATATATATATATATAT/CATATATATATATATATATATATATATATATAT	CAGCCAAGACCCAATTTCAT	ACTCTCCCAAAAAGGCGATT	59.9	432	INTERGENIC	--	--	--
Ca1_P4_14541002	Ca1	14541002	GATATATATATAT/GATATATATATATAT	CTAGTTCGTAAGCCGTGCGT	AGGCATGGTGTTGGAGTCAT	60.5	259	INTERGENIC	--	--	--
Ca1_P4_14606001	Ca1	14606001	TAAAAAAAAAAA/TAAAAAAAAAAAA	AATTTGACCAATCCCAACCA	CAACACACTATTCCCTTCCTCC	60.0	682	INTERGENIC	--	--	--
Ca1_P4_14649425	Ca1	14649425	CAATAATAATAATAATAATAATAATAATAATAATAA/CAATAATAATAATAATAATAATAATAATAATAATAATAA	ATGCATGACACATTCTCCGA	TTCCTTCAACCCATAACCCA	60.1	559	INTERGENIC	--	--	--
Ca1_P4_14832487	Ca1	14832487	TCTTACTTACT/TCTTACT	TTTTCCAGAAGGAATGGCAG	TTTTATTTATCCGCGGCAAG	60.2	554	INTERGENIC	--	--	--

INDEL marker IDs	Chromosomes/ unanchored scaffolds	Physical positions (bp)	InDels (*Kabuli* reference genome-CDC Frontier/PII)	Forward primers (5'-3')	Reverse primers (5'-3')	Anneling temperature (0C)	Expected amplified product size (bp)	Structural annotation		Functional annotation	
								Sequence components of *kabuli* genome	*Kabuli* gene accession IDs	NCBI-KOG	TFs
Ca1_P4_14891282	Ca1	14891282	AATATATATATATATATATATATATATAT/AATATATATATATATATATAT	TCACAAGATAGTTTTGCCTTTTT	CAAAATCATCGGAACGATTACA	57.2	360	INTERGENIC	--	--	--
Ca1_P4_14893455	Ca1	14893455	GTTTTTTTT/GTTTTTTT	AGGGACAAGGTTGGGTTTTT	TTAAAACTTTGCACGGAGGG	59.7	291	INTERGENIC	--	--	--
Ca1_P4_14995819	Ca1	14995819	TTGTTATGTTAT/TTGTTATGTTATGTTAT	CAGCGCTTTTCCCAAATAAG	CCAAAAAGGGTGGAGATTGA	59.8	359	INTERGENIC	--	--	--
Ca1_P4_14995847	Ca1	14995847	ACATGCATGCA/ACATGCA	CAGCGCTTTTCCCAAATAAG	CCAAAAAGGGTGGAGATTGA	59.8	359	INTERGENIC	--	--	--
Ca1_P4_14996344	Ca1	14996344	CGTGT/CGTGTGT	TCAATCTCCACCCTTTTTGG	GACATTTAACAGCGCCCATT	59.9	599	INTERGENIC	--	--	--
Ca1_P4_15037301	Ca1	15037301	CATATATATATATATA/CATATATATATATATATA	GTCACACGAGCATGGAAAGT	TAACCTGGCCAGTCACCATT	58.7	578	INTERGENIC	--	--	--
Ca1_P4_15186134	Ca1	15186134	AATATATATATATATATATAT/AATATATATATATATATATATAT	AGTGGCCCACGAGTAAGATG	CCACAGCACTCTTCTGTCCA	60.1	543	INTERGENIC	--	--	--
Ca1_P4_15188200	Ca1	15188200	TTATATATATATATATAT/TTATATATATATAT	TCAGCCAAACCACAATTTGA	TCCTAGTGCGAGGTCGTTTT	60.1	661	INTERGENIC	--	--	--
Ca1_P4_15188491	Ca1	15188491	TTAATAATAATAATAA/TTAATAATAATAA	AAAACGACCTCGCACTAGGA	CGCTCATGACCTTACTCGAA	59.9	408	INTERGENIC	--	--	--
Ca1_P4_15551966	Ca1	15551966	AGGGG/AGGG	TCAATTTTTAAAGTGGAGATCAAGC	ACTTGATTGACATGCGCAAA	60.0	491	INTERGENIC	--	--	--

INDEL marker IDs	Chromosomes/ unanchored scaffolds	Physical positions (bp)	InDels (*Kabuli* reference genome-CDC Frontier/PII)	Forward primers (5'-3')	Reverse primers (5'-3')	Anneling temperature (0C)	Expected amplified product size (bp)	Structural annotation		Functional annotation	
								Sequence components of *kabuli* genome	*Kabuli* gene accession IDs	NCBI-KOG	TFs
Ca1_P4_15821162	Ca1	15821162	ATT/AT	GGGGGAATTGGTGAAAAGTT	ATTAAACCAGCCACACAGGC	60.0	530	INTERGENIC	--	--	--
Ca1_P4_15849587	Ca1	15849587	AATATATATATATATATATATATATATATATATATATA/AATATATATATATATATATATATATATATATATA	CCCCATTTTGTTTCTCTCCA	ATTCTTGGAGGAGCGGGTAT	59.9	872	INTERGENIC	--	--	--
Ca1_P4_15922408	Ca1	15922408	ATTTTTTTTTTTTTTTT/ATTTTTTTTTT	GGGAAAAGGAAGGTCGAAAC	TTGGGTTGAATTGCTATTGTTG	59.9	783	INTERGENIC	--	--	--
Ca1_P4_16077599	Ca1	16077599	CAAAAAAA/CAAAAAA	GCAACATCGAACCCTCAAAT	TTGTCGTTTGAGCATTTAAAAAGT	59.9	494	INTERGENIC	--	--	--
Ca1_P4_16092158	Ca1	16092158	CTATATATATATATATAT/CTATATATATATATATATAT	AGCTACGGTGACTCGGTTTG	CAACATGTCCATGCAGCATAC	60.3	710	INTERGENIC	--	--	--
Ca1_P4_16118156	Ca1	16118156	AT/A	ATGCCATTTTGAAGACTGCC	TGGGGGCAGTTACCCTTATT	60.1	489	INTERGENIC	--	--	--
Ca1_P4_16119755	Ca1	16119755	T/TG	CTGCTCCAACCATCGGTACT	TTCCTAAAAGCGTCCCAAGA	60.1	547	INTERGENIC	--	--	--
Ca1_P4_16134448	Ca1	16134448	TTAT/TT	AATTGGGCCACATTTTTCTG	CGATTGATTTTTCGGTTCGT	59.8	761	INTERGENIC	--	--	--
Ca1_P4_16156646	Ca1	16156646	AATATATATATATATATATATATATA/AATATATATATATATATATATATATATATA	GCCAATAGCACCTTTGCTTC	AAGTGCCATCAAATTTGGCTA	59.9	606	INTERGENIC	--	--	--
Ca1_P4_16160389	Ca1	16160389	TT/TTCT	CATGTGGATTTGTCTTTGCG	GCACAAGATCCTCAAAACTGG	60.1	238	INTERGENIC	--	--	--

**INDEL marker IDs**	**Chromosomes/ unanchored scaffolds**	**Physical positions (bp)**	**InDels (*Kabuli* reference genome-CDC Frontier/PII)**	**Forward primers (5'-3')**	**Reverse primers (5'-3')**	**Anneling temperature (0C)**	**Expected amplified product size (bp)**	**Structural annotation**		**Functional annotation**	
								**Sequence components of *kabuli* genome**	***Kabuli* gene accession IDs**	**NCBI-KOG**	**TFs**
Ca1_P4_16161515	Ca1	16161515	GC/G	GGGGCTTTACGTATACAACCG	GGAATGAAAAGCCATGAGGA	60.6	253	INTERGENIC	--	--	--
Ca1_P4_16176422	Ca1	16176422	AAAATATAAATATAAA/AAAATATAAA	CCGTTTGATGGACATGATAAGA	ACCCTTGTGTAAAATCATAAACATT	59.8	397	INTERGENIC	--	--	--
Ca1_P4_16189552	Ca1	16189552	C/CATTTCGA	GGGAGAAAGTCCTCGTAGGG	TGTACGGATTGAGGTGAGGA	60.1	284	INTERGENIC	--	--	--
Ca1_P4_16198179	Ca1	16198179	TAAAAA/TAAAAAA	AAATTTTGTTGGTTGCCTCC	TAGCGATATCGATCAAGCCC	58.9	425	INTERGENIC	--	--	--
Ca1_P4_16206365	Ca1	16206365	AA/AATTA	CTCGCAAGTTGTAGGTTCAATC	GGGGCTTACGAGTACAGACTCA	58.9	128	INTERGENIC	--	--	--
Ca1_P4_16206852	Ca1	16206852	ATATTAT/ATAT	GAGTCTGTACTCGTAAGCCCCT	ATTTTGACAATGGCCACACA	58.9	697	INTERGENIC	--	--	--
Ca1_P4_16207830	Ca1	16207830	ATTTTT/ATTTTTT	CAACGCCCAACAACTAATCA	GAGAGGGTCTTGAGTGTCGC	59.6	657	INTERGENIC	--	--	--
Ca1_P4_16208160	Ca1	16208160	T/TA	TTGCGACGACCACAAAATAA	GCAGCATTTCCAACTGTGGT	60.1	245	INTERGENIC	--	--	--
Ca1_P4_16210891	Ca1	16210891	TA/T	CCAAGGTGGGAAAACACTTG	GCCTCTTCCAGCTGTTTGTT	60.4	280	INTERGENIC	--	--	--
Ca1_P4_16216789	Ca1	16216789	TTTATTATTATTATTATTATTA/TTTATTATTATTATTATTA	ACTCTGTCATTGGGCTCCAC	AGCATGATGTGGGCAATTTT	60.1	740	INTERGENIC	--	--	--

INDEL marker IDs	Chromosomes/ unanchored scaffolds	Physical positions (bp)	InDels (*Kabuli* reference genome- CDC Frontier/PII)	Forward primers (5'-3')	Reverse primers (5'-3')	Anneling temperature (0C)	Expected amplified product size (bp)	Structural annotation		Functional annotation	
								Sequence components of *kabuli* genome	*Kabuli* gene accession IDs	NCBI-KOG	TFs
Ca1_P4_16259144	Ca1	16259144	GATATATATATATATATATATA/GATATATATATATATATATA	AAGTGTTTTGGGGAGATGAGAA	GCGTGAACTCCTAAAGCCTG	60.0	386	INTERGENIC	--	--	--
Ca1_P4_16308001	Ca1	16308001	ATTTTTT/ATTTTTTT	TTCGGGCTTAATGAGTTTGG	GAAGCACCTTCTGGCAGTTC	60.1	425	INTERGENIC	--	--	--
Ca1_P4_16442399	Ca1	16442399	TATT/TATTAATT	AGTGTGTAACTCGTGCGTCG	AAAAAGTGGTGTTGGATGCC	60.0	259	INTERGENIC	--	--	--
Ca1_P4_16584419	Ca1	16584419	AG/A	GGTGCAACCAATTGAGTCCT	AGGAATGGATCCGGAGATTT	60.0	481	INTERGENIC	--	--	--
Ca1_P4_16629521	Ca1	16629521	TTTAATTAATTAATTAATT/TTTAATTAATTAATTAATTAATTAATT	AACGTTTGAGGATTCCGTTG	GAAACTCCAGCTCCAAAACG	60.0	370	INTERGENIC	--	--	--
Ca1_P4_16751256	Ca1	16751256	ATTTTTTTTTTTTTT/ATTTTTTTTTTTTT	GTCAACATCAGGGACGGACT	CTAGGCCTGACCTTGACCTG	60.0	610	INTERGENIC	--	--	--
Ca1_P4_16757631	Ca1	16757631	AATATATATATATATATATATATATAT A/AATATATATATATATATATATATATATATATATA	GATTCAAGACGAGACGCAAA	AAAAATGTTTTTCAATAAGCAAATC	59.0	562	INTERGENIC	--	--	--
Ca1_P4_16777733	Ca1	16777733	ATTTTTTTTT/ATTTTTTTT	GAGTGACAATTTTGTGCGGA	TGGAGGAAACTTAGCCCTCA	59.7	595	INTERGENIC	--	--	--
Ca1_P4_16835364	Ca1	16835364	TG/TGG	CGACAACGCATGATCAAATC	ATTTGGCGAAGCTTTTCAAC	60.1	652	INTERGENIC	--	--	--
Ca1_P4_16836777	Ca1	16836777	GTATAT/GTAT	TTAAAATGAATACTCCATTCGTCA	ACATAGGCGCACATGATTGA	57.8	646	INTERGENIC	--	--	--

INDEL marker IDs	Chromosomes/ unanchored scaffolds	Physical positions (bp)	InDels (*Kabuli* reference genome-CDC Frontier/PII)	Forward primers (5'-3')	Reverse primers (5'-3')	Anneling temperature (0C)	Expected amplified product size (bp)	Structural annotation		Functional annotation	
								Sequence components of *kabuli* genome	*Kabuli* gene accession IDs	NCBI-KOG	TFs
Ca1_P4_16982792	Ca1	16982792	CAAAAAAA/CAAAAAAAAAAAAA	GCATCAACAATACGCCACAC	CCCCATGGAAACCCTAACTT	60.0	316	INTERGENIC	--	--	--
Ca1_P4_1706990	Ca1	1706990	TATAATAATA/TATAATAATAATA	AATGGTATGGTTGCTGCATT	TGCGATGGTGTATTGAGAGC	58.0	570	INTERGENIC	--	--	--
Ca1_P4_17107416	Ca1	17107416	AATATATATATATATATATATATATATATAT/AATATATATATATATATATATATATATATATATATATATAT	GCTTTTCTCACCTCTCCCCT	TCCAAACCATGCTGTAGAGG	59.8	495	INTERGENIC	--	--	--
Ca1_P4_17158043	Ca1	17158043	A/AAGCAATT	TGATGTTGATCCAGTTCGGA	CCTCCAAATCACCATGAAGG	60.0	643	INTERGENIC	--	--	--
Ca1_P4_17185713	Ca1	17185713	TTTGATATTTGATTAT/TTTGATATTTGATTATTGATATTTGATTAT	GCGTTAAAGTTGGAAACCCA	TGACGTGACATCGAGAGTCTG	60.0	594	INTERGENIC	--	--	--
Ca1_P4_17187364	Ca1	17187364	AA/AATTTATA	CAACAACTTCTCATTCTCGCA	TGTTGCTACAAAAATGAATTGACA	59.1	966	INTERGENIC	--	--	--
Ca1_P4_17215249	Ca1	17215249	ATT/AT	GGTTGGGTTGAGGAGGAAAT	TGTGAACGAACACCTTCGAC	60.2	447	INTERGENIC	--	--	--
Ca1_P4_17234877	Ca1	17234877	AT/A	CCCTATAGGCCCACTAAGCC	ACCCCAAAAGAGAAAGCACA	59.9	695	INTERGENIC	--	--	--
Ca1_P4_17242129	Ca1	17242129	GATATATATATATATATATATATATATATATATATATA/GATATATATATATATATATATATATATATATATATA	TACGAACCCTCACAACCACA	AAAAATTTGGGGCGTTACAC	60.0	856	INTERGENIC	--	--	--
Ca1_P4_17275483	Ca1	17275483	AATATATATATATATATATATATATATATATATATATATATATATAT/AATATATATATATATATATATATATATATATATATAT	TGTGTGTAGGACGGATCCAA	CAATACTCTCTGCACGTGGTTT	60.0	503	INTERGENIC	--	--	--

INDEL marker IDs	Chromosomes/ unanchored scaffolds	Physical positions (bp)	InDels (*Kabuli* reference genome-CDC Frontier/PII)	Forward primers (5'-3')	Reverse primers (5'-3')	Anneling temperature (0C)	Expected amplified product size (bp)	Structural annotation		Functional annotation	
								Sequence components of *kabuli* genome	*Kabuli* gene accession IDs	NCBI-KOG	TFs
Ca1_P4_17436816	Ca1	17436816	A/AC	GACGAAAAGATCACCCTCACA	CATGTGCCACAGGATCATTC	60.1	512	INTERGENIC	--	--	--
Ca1_P4_17507058	Ca1	17507058	CAAAAAAAAAA/CAAAAAAAAAAA	AAATTGGACAGCAAAATCGG	GGCAACAAGCACCAGTGTTA	59.9	231	INTERGENIC	--	--	--
Ca1_P4_17566974	Ca1	17566974	ATTTTT/ATTTTTT	TAATAGCGACCAACCAAGGG	TGATGCGGTATACGATTTCAT	60.0	754	INTERGENIC	--	--	--
Ca1_P4_17624683	Ca1	17624683	AATATATATATATATATATATATATATATATAT/AATATATATATATATATATATATAT	ATACACCTCACTTCCGCCAC	TCACTGCTCTGCATCCATTC	60.0	239	INTERGENIC	--	--	--
Ca1_P4_17633701	Ca1	17633701	CAAA/CAA	TGCGCCGAAAGTTCTTTACT	ATTTTTGTGGAAAAATGCGG	60.0	364	INTERGENIC	--	--	--
Ca1_P4_17634013	Ca1	17634013	TTATATATATATATATAT/TTATATATATATATATATAT	TGATTGGATGTGGGTGTGAG	TTTCAAAGGCAACAACCCTC	60.4	529	INTERGENIC	--	--	--
Ca1_P4_1777586	Ca1	1777586	AGATGATGATGATGATGATGA/AGATGATGATGATGATGATGATGA	CGTCACAAAGCATCACAGGT	CTACGGTGCAAACAGAACCA	59.8	563	INTERGENIC	--	--	--
Ca1_P4_1777834	Ca1	1777834	CTCT/CTCTATCT	CGTCACAAAGCATCACAGGT	ATTGCACAACGGCTACAACA	59.8	906	INTERGENIC	--	--	--
Ca1_P4_1778696	Ca1	1778696	TATAATA/TATA	AGGGTATTTGTGGCTGCAAG	TCGATGCCAAATGGAGTGTA	60.1	237	INTERGENIC	--	--	--
Ca1_P4_1780243	Ca1	1780243	ATTATTTATT/ATTATTTATTTATT	GAGTGAAAGGACGATAGCACG	TAAAAGGTCGCATTTCGGTT	59.9	942	INTERGENIC	--	--	--

INDEL marker IDs	Chromosomes/ unanchored scaffolds	Physical positions (bp)	InDels (*Kabuli* reference genome-CDC Frontier/PII)	Forward primers (5'-3')	Reverse primers (5'-3')	Anneling temperature (0C)	Expected amplified product size (bp)	Structural annotation		Functional annotation	
								Sequence components of *kabuli* genome	*Kabuli* gene accession IDs	NCBI-KOG	TFs
Ca1_P4_17811471	Ca1	17811471	CTATATATATATATATATATAT/CTATATATATATATATATATAT	AGGAAAATGGATTGGCAGAA	TGTCTGCTTCCTTAAAGCCG	59.5	186	INTERGENIC	--	--	--
Ca1_P4_17812819	Ca1	17812819	AT/ATT	CACCGCACGGAATTCTAATC	GCAATGCCATTTCAATTTCT	60.5	392	INTERGENIC	--	--	--
Ca1_P4_17816780	Ca1	17816780	TCC/TC	TGGCCATCAAGTGTCTTCTC	TTTCCAGTCCTTCTGATGCC	58.8	568	INTERGENIC	--	--	--
Ca1_P4_17920630	Ca1	17920630	CATATATATATATATATATATATA/CATATATATATATATATATATA	CCAAAAATCAAAAGGGTGGA	TTTCTTCTTTCTGCTCCCCA	59.8	574	INTERGENIC	--	--	--
Ca1_P4_18054297	Ca1	18054297	GAAAAAAAA/GAAAAAAA	GTTTTTCCTGCGTAATGCGT	TTTCATCATATCGCTTCACCTTT	60.1	503	INTERGENIC	--	--	--
Ca1_P4_18088	Ca1	18088	TA/TAA	CCGCTTGTTTTTCTTTGCAG	TTGATTGCTTGTTGCTCCAG	60.9	543	INTERGENIC	--	--	--
Ca1_P4_18181716	Ca1	18181716	AATATATATATATATATATATATATA/AATATATATATATATATATATATATATATATA	CGAATACTCATGGTTTTTGATCTG	ACCCAACGTAGCACGTTACC	59.9	626	INTERGENIC	--	--	--
Ca1_P4_1822026	Ca1	1822026	TC/T	ATGTTTGGGGCTATCGACTG	GAGGAATTTTGCTCTCGTGC	60.0	449	INTERGENIC	--	--	--
Ca1_P4_1822426	Ca1	1822426	TG/T	TGTGCACGAGAGCAAAATTC	TCTTCATGGGCTGAAAAAGC	60.0	489	INTERGENIC	--	--	--
Ca1_P4_1822473	Ca1	1822473	AG/A	TGTGCACGAGAGCAAAATTC	TCTTCATGGGCTGAAAAAGC	60.0	489	INTERGENIC	--	--	--

INDEL marker IDs	Chromosomes/ unanchored scaffolds	Physical positions (bp)	InDels (*Kabuli* reference genome-CDC Frontier/PII)	Forward primers (5'-3')	Reverse primers (5'-3')	Anneling temperature (0C)	Expected amplified product size (bp)	Structural annotation		Functional annotation	
								Sequence components of *kabuli* genome	*Kabuli* gene accession IDs	NCBI-KOG	TFs
Ca1_P4_18298376	Ca1	18298376	CAAA/CAA	GGTAATGCCTCGTACAAAACAA	CATTCCTTATGCCTCCTACACC	59.1	751	INTERGENIC	--	--	--
Ca1_P4_18299880	Ca1	18299880	TTCCTATCCTATCC/TTCCTATCC	TGGATCACTCACCTCTCTTCC	TGGAAGTGGTGGTTTTGTTG	59.2	185	INTERGENIC	--	--	--
Ca1_P4_18300620	Ca1	18300620	GTTTTTTTTT/GTTTTTTT	TTTCCATGAGTTATATTTGTCACGA	TGGCCTTCAATTTTCTTGCT	59.8	134	INTERGENIC	--	--	--
Ca1_P4_18300800	Ca1	18300800	ATTT/ATT	AGCAAGAAAATTGAAGGCCA	GGAAGCTTTAAAGGGAGCAAA	59.8	161	INTERGENIC	--	--	--
Ca1_P4_18317638	Ca1	18317638	T/TG	TCATTTTCAACCCTTAGCAACA	TGGTAAGGTATACGCGGGAG	59.6	690	INTERGENIC	--	--	--
Ca1_P4_18319296	Ca1	18319296	T/TTAATAGATCACTC	CCCTTGAGTTTCATAGTCACCA	CGATCGACAGGAAGAGGAAG	59.1	159	INTERGENIC	--	--	--
Ca1_P4_18325896	Ca1	18325896	TC/T	ATTGAAAACATGGCTTTGGC	TTTTGGAGGAACTACGGCAC	59.9	648	INTERGENIC	--	--	--
Ca1_P4_18327589	Ca1	18327589	ATTTTTTT/ATTTTTTTT	TTCACAAAATGACACAACGG	TCCTAAGGGCTTTCTTTTGG	58.0	655	INTERGENIC	--	--	--
Ca1_P4_18328227	Ca1	18328227	GTCTATCT/GTCT	TTAAAACCACAAAATGGGGC	GAGGCAAAGCTTCCAAATCA	59.7	475	INTERGENIC	--	--	--
Ca1_P4_18328327	Ca1	18328327	TATTATTCAT/TAT	TTAAAACCACAAAATGGGGC	GAGGCAAAGCTTCCAAATCA	59.7	475	INTERGENIC	--	--	--

INDEL marker IDs	Chromosomes/ unanchored scaffolds	Physical positions (bp)	InDels (*Kabuli* reference genome-CDC Frontier/PII)	Forward primers (5'-3')	Reverse primers (5'-3')	Anneling temperature (0C)	Expected amplified product size (bp)	Structural annotation		Functional annotation	
								Sequence components of *kabuli* genome	*Kabuli* gene accession IDs	NCBI-KOG	TFs
Ca1_P4_1835247	Ca1	1835247	ATGAC/A	TCATAAGCCAGAACCCAAGC	GGTGGTGGAGATTCCAAGAA	60.2	448	INTERGENIC	--	--	--
Ca1_P4_18364053	Ca1	18364053	GAAA/GAAAA	CCCGTAGCAAGACTTGAAGC	TTGGAGAAACTTTTCCGCTG	60.0	627	INTERGENIC	--	--	--
Ca1_P4_1836414	Ca1	1836414	ATTTTTTTTTTTTTT/ATTTTTTTTT	AAACCCTAAGGCCCAAGAAA	CGTGAAACGGTATGCAAATG	59.9	594	INTERGENIC	--	--	--
Ca1_P4_1836769	Ca1	1836769	ATTT/ATTTT	CATTTGCATACCGTTTCACG	TTTGAGTTACGAATTTGACGAAA	60.0	360	INTERGENIC	--	--	--
Ca1_P4_18407793	Ca1	18407793	A/ACCGGGAGG	GGCAAGGTGAGAGAACAAGC	ATTTCTGGTTTTGGCAGTGG	60.0	162	INTERGENIC	--	--	--
Ca1_P4_1841034	Ca1	1841034	TTTCTTCT/TTTCT	TCAAGGTGTTGATTCGGTAGG	TTTTGCAACCCGGTCTATTC	60.0	595	INTERGENIC	--	--	--
Ca1_P4_1841424	Ca1	1841424	A/AT	GAATAGACCGGGTTGCAAAA	CCTCTTTCCGGACAACACAT	59.9	302	INTERGENIC	--	--	--
Ca1_P4_18441371	Ca1	18441371	CACAAGAGAC/CAC	CCCTCTGTATGCCAGTCGAT	TGGCATTGATGATGGCTTTA	60.1	771	INTERGENIC	--	--	--
Ca1_P4_1845260	Ca1	1845260	GATCAT/GAT	ACATTGTTCATTTGCAGCCA	GGTGTGTCTTCGCCGTTTAT	60.1	665	INTERGENIC	--	--	--
Ca1_P4_1846453	Ca1	1846453	AATATATATA/AATATATA	TGTGGCTAAAAAGTGCAGGA	TATGGGGAAAGTCCCATTGA	59.5	590	INTERGENIC	--	--	--

INDEL marker IDs	Chromosomes/ unanchored scaffolds	Physical positions (bp)	InDels (*Kabuli* reference genome-CDC Frontier/PII)	Forward primers (5'-3')	Reverse primers (5'-3')	Anneling temperature (0C)	Expected amplified product size (bp)	Structural annotation		Functional annotation	
								Sequence components of *kabuli* genome	*Kabuli* gene accession IDs	NCBI-KOG	TFs
Ca1_P4_1852604	Ca1	1852604	TA/TAA	ACACGGCGTTCATTTTCTTC	CAAACCATGGGAGAGAGAGC	60.1	843	INTERGENIC	--	--	--
Ca1_P4_18552012	Ca1	18552012	ATTTTTTTT/ATTTTTTTTT	TCTGATGCAGTGTTTGGCTC	GCTTTCTTTGTCTGCCTTGG	60.0	331	INTERGENIC	--	--	--
Ca1_P4_18688865	Ca1	18688865	TTGT/TT	TAGCAGCTGGAGGAACATGA	GCACCCAGTGTAAGACCCAT	59.5	301	INTERGENIC	--	--	--
Ca1_P4_1876420	Ca1	1876420	AT/ATACGTAGTTCGGTTGAGT	CGGGTCTTCAAAATGGAATG	CAAAGGAAACGTGCTTCCAG	60.3	861	INTERGENIC	--	--	--
Ca1_P4_1882571	Ca1	1882571	TC/T	ATATCGTTTTTCACACCCGC	GGTGCAAATGCAAGAGTCAA	59.8	282	INTERGENIC	--	--	--
Ca1_P4_18868658	Ca1	18868658	CGG/CGGGAATGG	AATCGATTGATTCAGCCCAG	CGTTTTTCAAAGCGAGGGTA	60.0	707	INTERGENIC	--	--	--
Ca1_P4_18868716	Ca1	18868716	AT/ATT	AATCGATTGATTCAGCCCAG	CGTTTTTCAAAGCGAGGGTA	60.0	707	INTERGENIC	--	--	--
Ca1_P4_18873	Ca1	18873	GAAAAAAAAAAAAAAAAA/GAAAAAAAAAAA	CAGAACCGAGGAATTAAGCG	GTTCATGTTGTGTGGCGAAT	59.8	133	INTERGENIC	--	--	--
Ca1_P4_19093958	Ca1	19093958	GTTATTATTATTATTATTATTATTATTAT/GTTATTATTATTATTATTATTATTATTATTAT	ATGCTAGGTCGTTCGTCAGG	AGTATGGCATGGCCTGAAAG	60.3	510	INTERGENIC	--	--	--
Ca1_P4_1914068	Ca1	1914068	AC/A	GCCACATCATTATTTGAGCG	TGGTTGAGTGGTGTTAATTTGTG	59.1	666	INTERGENIC	--	--	--

INDEL marker IDs	Chromosomes/ unanchored scaffolds	Physical positions (bp)	InDels (*Kabuli* reference genome-CDC Frontier/PII)	Forward primers (5'-3')	Reverse primers (5'-3')	Anneling temperature (0C)	Expected amplified product size (bp)	Structural annotation		Functional annotation	
								Sequence components of *kabuli* genome	*Kabuli* gene accession IDs	NCBI-KOG	TFs
Ca1_P4_19150973	Ca1	19150973	CTT/CT	AACTAGCCCGTCTTTG GGTT	GAAAACAAAAGCTTTAC GGTCA	60.0	488	INTERGENIC	--	--	--
Ca1_P4_1915229	Ca1	1915229	CTCAT/CTCATCTGTTTCAT	TGGATACACAAAAACC GAACA	CACCTTTTCCATCCATC GAC	58.9	709	INTERGENIC	--	--	--
Ca1_P4_19168	Ca1	19168	GCTCTCT/GCTCT	CAGAACCGAGGAATTA AGCG	AGGCTTGAAGCGTGGT AATG	59.8	637	INTERGENIC	--	--	--
Ca1_P4_1930262	Ca1	1930262	ATTTTTTTT/ATTTTTTTTTT	GGCAACATTTTACAAAA GTTAAGG	AGTTTGATTAATGTCGG GCG	58.3	318	INTERGENIC	--	--	--
Ca1_P4_19308411	Ca1	19308411	TATTAAAGATTAAAGATT/TATTAAA GATTAAAGATTAAAGATTAAAGATT	GCGAGCATGAGAAGTT GTTG	TCCTCCTTACCATTATA TGATTCCA	59.6	337	INTERGENIC	--	--	--
Ca1_P4_19422873	Ca1	19422873	CTTATTTATTT/CTTATTT	GCCCATAACCTAGCCC ATTT	TCGTGTCCCAAAACTC AATG	60.2	713	INTERGENIC	--	--	--
Ca1_P4_19423044	Ca1	19423044	TAAAAAAA/TAAAAAA	TCTTTGAGTTGTTAGG ATACAAGTGG	TCGATTATGCGTAGGG AAGG	60.0	728	INTERGENIC	--	--	--
Ca1_P4_19431535	Ca1	19431535	TCA/T	TCTGATGCAGTGTTTG GCTC	GTGATAAAGGCGTCAA CGGT	60.0	688	INTERGENIC	--	--	--
Ca1_P4_1944303	Ca1	1944303	CCAGCATCG/C	CCCTCCTTCCTCTCATT CCT	AAGACAATGTGGCTCT TGGC	59.6	113	INTERGENIC	--	--	--
Ca1_P4_19460421	Ca1	19460421	T/TTCA	CGTTGCAATTGATGAG GATG	TTCGCTCCTTCTTCTTG TCC	60.1	748	INTERGENIC	--	--	--

INDEL marker IDs	Chromosomes/ unanchored scaffolds	Physical positions (bp)	InDels (*Kabuli* reference genome-CDC Frontier/PII)	Forward primers (5'-3')	Reverse primers (5'-3')	Anneling temperature (0C)	Expected amplified product size (bp)	Structural annotation		Functional annotation	
								Sequence components of *kabuli* genome	*Kabuli* gene accession IDs	NCBI-KOG	TFs
Ca1_P4_19498678	Ca1	19498678	TTTTGATCCTTTGAT/TTTTGAT	GCCCGAGTTAACAACAAACG	ATCGATTTCCTCAGCACTGG	60.5	374	INTERGENIC	--	--	--
Ca1_P4_19536862	Ca1	19536862	TCCCC/TCCCCC	TAGCACCTTCCTCCCAGAAA	CTACCAACAGAAAGGCCCAA	59.8	352	INTERGENIC	--	--	--
Ca1_P4_19631803	Ca1	19631803	CATATATATATATATATATAT/CATATATATATATATATATATATATATAT	ATATTCGCAACGCGTATGTT	AAATGTGGCAAATTGGGACT	58.2	447	INTERGENIC	--	--	--
Ca1_P4_19661120	Ca1	19661120	TATATAAAT/TAT	GGCCATCACAAATGGGTAAG	AAGGGCCATAAGTAGGCCAG	60.2	662	INTERGENIC	--	--	--
Ca1_P4_19694253	Ca1	19694253	TATATAGATATA/TATATA	ATGTTTTGAATTTGGCCTCG	CGAAAGACGAAAATGGAGGA	59.9	454	INTERGENIC	--	--	--
Ca1_P4_1970052	Ca1	1970052	TTTAATTATATTAATTATA/TTTAATTATA	CGGATTTGGGCAGTGATATT	TTCGAATTTTCCCGTACCTG	59.8	266	INTERGENIC	--	--	--
Ca1_P4_19710704	Ca1	19710704	ATTTTTTTT/ATTTTTTT	GACTTTGCAAGGCCTGAGTC	AGGCTTGACAGACCGACCTA	60.0	556	INTERGENIC	--	--	--
Ca1_P4_1971572	Ca1	1971572	AA/AAGAAATGCA	ATGTGCATCACCTCTGACGA	GCTGCCATGGAGGTAGTCAT	60.3	807	INTERGENIC	--	--	--
Ca1_P4_1972044	Ca1	1972044	ACTTCTTCT/ACTTCTTCTTCT	GCCCTCAGCCTCACTTTATG	AGGGAGGCTACGAACGATTT	59.8	356	INTERGENIC	--	--	--
Ca1_P4_1981443	Ca1	1981443	G/GA	GGGTGACATGTGGACCATCT	TCAAGAATCAAAATGATACTGTCAAA	60.7	919	INTERGENIC	--	--	--

INDEL marker IDs	Chromosomes/ unanchored scaffolds	Physical positions (bp)	InDels (*Kabuli* reference genome-CDC Frontier/PII)	Forward primers (5'-3')	Reverse primers (5'-3')	Anneling temperature (0C)	Expected amplified product size (bp)	Structural annotation		Functional annotation	
								Sequence components of *kabuli* genome	*Kabuli* gene accession IDs	NCBI-KOG	TFs
Ca1_P4_19855108	Ca1	19855108	TTTTATTTATTTATTTATTTATTTATTT/TTTTATTTATTTATTTATTTATTTATTT	TTGTTGTTGCAATTTAAGCGA	CAAAAGCTTGCAATTTTGATTTT	59.4	786	INTERGENIC	--	--	--
Ca1_P4_1989852	Ca1	1989852	AAA/AAATAA	TTGGGCTTATTGGGTCACTC	TATCCAATCCCAACCCAAAA	59.9	115	INTERGENIC	--	--	--
Ca1_P4_19941125	Ca1	19941125	CAAAAAAAAAAAA/CAAAAAAAAAAAA	CGGGAAACAGGGGTTTAACT	AAGGAGAGCATGATATATTTTGTGA	60.2	591	INTERGENIC	--	--	--
Ca1_P4_1998915	Ca1	1998915	T/TTATAAAAATGA	TGCTTGTTTCACCACTTTGC	CGACCGATATTCGTCCACTC	59.9	481	INTERGENIC	--	--	--
Ca1_P4_1999397	Ca1	1999397	AGTGT/AGT	GACGAATATCGGTCGAATACAA	GTCCAAACATCGGAAAAGGA	59.0	706	INTERGENIC	--	--	--
Ca1_P4_2000025	Ca1	2000025	TAAAAAAA/TAAAAAA	TCCTTTTCCGATGTTTGGAC	TTAAGGACGTCGTTGGAAGG	59.9	778	INTERGENIC	--	--	--
Ca1_P4_2057177	Ca1	2057177	CACCATAA/C	AATGTGGAAGGACGTTGTCA	ACGATTCAAAGAAAGTGGCG	59.0	448	INTERGENIC	--	--	--
Ca1_P4_2085412	Ca1	2085412	AAGTTTTAT/AAGTTTTATAGAGTTTTAT	TTTTTGGGCTCACACAATTT	CAATTGTCCAAAACTCTTATCCAA	58.1	723	INTERGENIC	--	--	--
Ca1_P4_2085895	Ca1	2085895	GAAAAATAAAAA/GAAAAATAAAAATAAAAA	TGTTTTTGGATAAGAGTTTTGGA	TCCTCAATCGCGTTCTTTCT	57.9	626	INTERGENIC	--	--	--
Ca1_P4_2088597	Ca1	2088597	TTTTT/TTTTTTTAAAAACTTTT	ACAGCAACCAAGAGTTGTGC	GCATCCGGCCAAAATAAATA	58.9	654	INTERGENIC	--	--	--

INDEL marker IDs	Chromosomes/ unanchored scaffolds	Physical positions (bp)	InDels (*Kabuli* reference genome-CDC Frontier/PII)	Forward primers (5'-3')	Reverse primers (5'-3')	Anneling temperature (0C)	Expected amplified product size (bp)	Structural annotation		Functional annotation	
								Sequence components of *kabuli* genome	*Kabuli* gene accession IDs	NCBI-KOG	TFs
Ca1_P4_2088953	Ca1	2088953	ATATTTATTTATTT/ATATTTATTTATTTATTTATTT	GGTAAATTGATGCCACCTGC	TTTTGCTGCCACCATGATTA	60.3	469	INTERGENIC	--	--	--
Ca1_P4_2099948	Ca1	2099948	ATTTTT/ATTTT	TTGGTATATGTATGAATGTTGGCA	TGGTGTTAATTAATCTCGTCACTT	59.2	574	INTERGENIC	--	--	--
Ca1_P4_2113146	Ca1	2113146	TAAAAAAAAAA/TAAAAAAAA	TGTGGACACGTTAAGTTGTGG	TGATCTCGCCCTATTATTCAAAA	59.5	409	INTERGENIC	--	--	--
Ca1_P4_2115049	Ca1	2115049	TAAAAAAAAA/TAAAAAAAAAA	TGGACTAAAATTGCTTTTTGGAA	TTAGAAAGAGCCACACGCAA	60.0	897	INTERGENIC	--	--	--
Ca1_P4_2116565	Ca1	2116565	TT/TTGT	TTGCGTTCATTTACGTTTGA	TCAATTCACTTCATGCCCAA	57.8	636	INTERGENIC	--	--	--
Ca1_P4_2120541	Ca1	2120541	AATTAT/AAT	TGTATGGAAAGAGGTAAAATGGAA	AACGACAAATGTTGCCATGA	59.0	532	INTERGENIC	--	--	--
Ca1_P4_2129116	Ca1	2129116	GAAAA/GAAAAA	CAAGCAGAGAGAAAAACGGG	ATAGTGGACCCCACAATCCA	60.0	379	INTERGENIC	--	--	--
Ca1_P4_2131188	Ca1	2131188	AT/A	TGCTATTCATCCCACAAGCA	CTTGGATTCGATGCATTCCT	60.2	640	INTERGENIC	--	--	--
Ca1_P4_2131678	Ca1	2131678	TAT/TATTGAT	AGGAATGCATCGAATCCAAG	CCACAAGTGCCATTCAGCTA	60.0	516	INTERGENIC	--	--	--
Ca1_P4_2131832	Ca1	2131832	CTTT/CTTTT	AGGAATGCATCGAATCCAAG	CCACAAGTGCCATTCAGCTA	60.0	516	INTERGENIC	--	--	--

INDEL marker IDs	Chromosomes/ unanchored scaffolds	Physical positions (bp)	InDels (*Kabuli* reference genome-CDC Frontier/PII)	Forward primers (5'-3')	Reverse primers (5'-3')	Anneling temperature (0C)	Expected amplified product size (bp)	Structural annotation		Functional annotation	
								Sequence components of *kabuli* genome	*Kabuli* gene accession IDs	NCBI-KOG	TFs
Ca1_P4_2132012	Ca1	2132012	CTG/CTGTG	AGGAATGCATCGAATCCAAG	AATGACCAACCCACAAGTGC	60.0	526	INTERGENIC	--	--	--
Ca1_P4_2135161	Ca1	2135161	AGTGTG/AGTGTGTG	AGAATTTACTCCACGTCCCG	TGAACCTCGTGCATGTAATTCT	59.1	437	INTERGENIC	--	--	--
Ca1_P4_21653919	Ca1	21653919	TTATATATATATATATATATATATAT/TTATATATATATATATATATATATATAT	GCTTGCAAAACAGATGCAGA	ACGAAACGAAAGCGAGAAAA	60.1	536	INTERGENIC	--	--	--
Ca1_P4_21657413	Ca1	21657413	TTA/T	TGGAGTACGAGCTTGGGTCT	TGTCTACTGCAGAAGCCGAA	59.9	786	INTERGENIC	--	--	--
Ca1_P4_21845802	Ca1	21845802	GATATATATATATATATATATA/GATATATATATATATATATATATA	GGGTATGAAAGGTTGGGGTT	TCGAGTAATTTGATGGCAGAAA	59.9	713	INTERGENIC	--	--	--
Ca1_P4_22029011	Ca1	22029011	TTATATATATATATATATATATATATA TA/TTATATATATATATATATATA	CGAATCTTCACCCTCTTTGG	GCCGGATCCAAAATCAAAAT	59.7	839	INTERGENIC	--	--	--
Ca1_P4_22067149	Ca1	22067149	GGCT/GGCTAGCT	TTCTTTGACGTTCATCTGCG	TAACAAGGGCTTCAACCACC	60.0	353	INTERGENIC	--	--	--
Ca1_P4_22100599	Ca1	22100599	ATATATATATAT/AATTATATATATAT	ATGTGTGAGAAAACCTCGGC	TGTCCAGCTTCATTCAACCA	60.1	574	INTERGENIC	--	--	--
Ca1_P4_22131328	Ca1	22131328	AATATATAT/AATATATATAT	GTGTGGACATGCCTAAAACG	GTTGCAATTGAGGTGCTGAA	59.1	403	INTERGENIC	--	--	--
Ca1_P4_22131513	Ca1	22131513	GAAAAA/GAAAA	GTGTGGACATGCCTAAAACG	GTTGCAATTGAGGTGCTGAA	59.1	403	INTERGENIC	--	--	--

INDEL marker IDs	Chromosomes/ unanchored scaffolds	Physical positions (bp)	InDels (*Kabuli* reference genome-CDC Frontier/PII)	Forward primers (5'-3')	Reverse primers (5'-3')	Anneling temperature (0C)	Expected amplified product size (bp)	Structural annotation		Functional annotation	
								Sequence components of *kabuli* genome	*Kabuli* gene accession IDs	NCBI-KOG	TFs
Ca1_P4_22132039	Ca1	22132039	G/GC	GGATGGTAGTTCAGCACCTCA	GAGCATTAGTGCTTGGCACA	60.1	774	INTERGENIC	--	--	--
Ca1_P4_22133403	Ca1	22133403	CTTT/CTT	GCTGTAGCCAAGGAATAGCG	CATCCCTTGAGTGGGTTTTG	60.0	731	INTERGENIC	--	--	--
Ca1_P4_22195947	Ca1	22195947	CATATATATATATATATATATATATATAT/CATATATATATATATATATATATATATATATATATATATAT	TTTCATTGGCACTTGGTAGG	TCGTCGCGTCACTATGAATC	58.6	838	INTERGENIC	--	--	--
Ca1_P4_2220697	Ca1	2220697	TTAT/TTATTTATATATATATATATAT	GGGTGTTGCACCTAAGGGTA	TGGACTTCTTTGGCCAATTT	59.9	648	INTERGENIC	--	--	--
Ca1_P4_2221938	Ca1	2221938	TAAA/TAAAA	ACCCCAAACAATCATAGCCA	TCCATATTGGTCGTTGTCCA	60.2	926	INTERGENIC	--	--	--
Ca1_P4_2222614	Ca1	2222614	A/AATAACG	CGTGGACAACGACCAATATG	CTTTTACTCTCCTGGGGATGA	59.8	696	INTERGENIC	--	--	--
Ca1_P4_22260973	Ca1	22260973	CTAAAATAAAATAAAATAAAATAAA/CTAAAATAAAATAAAATAAAATAAAATAAA	AAAGCCGGTTGGTTTTTCTT	ACCATAGAAGTGCCCCACTG	60.0	425	INTERGENIC	--	--	--
Ca1_P4_22329728	Ca1	22329728	CAAAA/CAAAAA	GACAAAAGTCATGACGGCCT	TTTCCACCCAGAAAGCAATC	60.1	728	INTERGENIC	--	--	--
Ca1_P4_22384776	Ca1	22384776	G/GAACAAGTGTGT	CTTCAGATCCTTGAGCTGCC	TGAATCTTTGTGTCCCACCC	60.1	539	INTERGENIC	--	--	--
Ca1_P4_22406616	Ca1	22406616	ATTTTTTTT/ATTTTTTTTT	TTCTTCCCAACCCACAAGTT	TGATGGACCAAAACTGGAGA	59.4	584	INTERGENIC	--	--	--

INDEL marker IDs	Chromosomes/ unanchored scaffolds	Physical positions (bp)	InDels (*Kabuli* reference genome-CDC Frontier/PII)	Forward primers (5'-3')	Reverse primers (5'-3')	Anneling temperature (0C)	Expected amplified product size (bp)	Structural annotation		Functional annotation	
								Sequence components of *kabuli* genome	*Kabuli* gene accession IDs	NCBI-KOG	TFs
Ca1_P4_2244855	Ca1	2244855	AA/AATTA	TGCATGAGCAATAATG CCTAAA	GGCAATGCCAAAATAT GAGTT	60.6	158	INTERGENIC	--	--	--
Ca1_P4_2244906	Ca1	2244906	A/AG	TGCATGAGCAATAATG CCTAAA	GGCAATGCCAAAATAT GAGTT	60.6	158	INTERGENIC	--	--	--
Ca1_P4_2245864	Ca1	2245864	TAAA/TAA	TGGGACACATAATTTTC ACAGG	TTTTCCCGTGTTGTTTT TATG	59.7	764	INTERGENIC	--	--	--
Ca1_P4_2247683	Ca1	2247683	CTATA/CTA	GATGAAGAGATGGATG GGGA	TTGACGTTTTCAATGTT GCAG	59.8	611	INTERGENIC	--	--	--
Ca1_P4_2254683	Ca1	2254683	CAAAA/CAAA	TGCCTTGTTTTTAGGC CTTG	CCAACAATCCTCTTCAC CCT	60.2	574	INTERGENIC	--	--	--
Ca1_P4_2256401	Ca1	2256401	ATT/AT	CCGCCCGGTAATTAAG ATTT	TATGTGCCGCCGTAAA TACA	60.2	816	INTERGENIC	--	--	--
Ca1_P4_2257787	Ca1	2257787	AC/A	TCAAGCGAATCTCTTTT TGTGA	AAATTATGTCGCCCACA ACC	60.0	356	INTERGENIC	--	--	--
Ca1_P4_2257997	Ca1	2257997	GATATA/GATATATATA	GGTTGTGGGCGACATA ATTT	ATTCTTCCACTTGGGC AAAA	59.7	230	INTERGENIC	--	--	--
Ca1_P4_2258161	Ca1	2258161	CATT/CATTGATT	GGTTGTGGGCGACATA ATTT	CTCCCCTCCTCCTCAA ACTT	59.7	617	INTERGENIC	--	--	--
Ca1_P4_22621219	Ca1	22621219	TAAAAAAAAAA/TAAAAAAAAA	CATTTTTCCTCTGCACA ACG	GCCGTCTTTTAGAAAC ATCGT	59.3	409	INTERGENIC	--	--	--

INDEL marker IDs	Chromosomes/ unanchored scaffolds	Physical positions (bp)	InDels (*Kabuli* reference genome-CDC Frontier/PII)	Forward primers (5'-3')	Reverse primers (5'-3')	Anneling temperature (0C)	Expected amplified product size (bp)	Structural annotation		Functional annotation	
								Sequence components of *kabuli* genome	*Kabuli* gene accession IDs	NCBI-KOG	TFs
Ca1_P4_2264238	Ca1	2264238	T/TAG	CAAATGAGTCACCACATCCG	TGTGTTTTCTCGTCGAACCA	60.0	711	INTERGENIC	--	--	--
Ca1_P4_22737650	Ca1	22737650	TAAAAA/TAAAA	TTTGCGCCAAAACCATTTAT	AAGGGGATGCTCATTTTGTG	60.3	570	INTERGENIC	--	--	--
Ca1_P4_22885854	Ca1	22885854	TTA/TTATA	TGATTTGGGGTTTTGGGTAA	GGGTGAGTTCTTTGAGGGTG	60.0	531	INTERGENIC	--	--	--
Ca1_P4_22886112	Ca1	22886112	GAA/GAAA	TGATTTGGGGTTTTGGGTAA	GGGTGAGTTCTTTGAGGGTG	60.0	531	INTERGENIC	--	--	--
Ca1_P4_22896758	Ca1	22896758	CTTTTTTTTTTTTTTT/CTTTTTTTTTTT	GCACCGTTTACAACGCTTTT	GATGAAACCGAGGACCAGAA	60.2	400	INTERGENIC	--	--	--
Ca1_P4_23139745	Ca1	23139745	TTATATATATATATATATATATATATATA/TTATATATATATATATATATATATA	TTCGGAAAAATTCATCTGCC	GCAGCGTGACAATGAACAAC	60.0	368	INTERGENIC	--	--	--
Ca1_P4_2315915	Ca1	2315915	AACGTTACG/AACG	ACCGTCGGAGACAAAAACAC	AGCAAACAAGCAAAGCCAAC	60.0	412	INTERGENIC	--	--	--
Ca1_P4_2316762	Ca1	2316762	CTTTTTTTTTTTTTTTTTT/CTTTTTTTTTTT	TTGTGGTTGGTGGTGATGTC	TGCTGCAAAGTACGAAAGGA	60.3	315	INTERGENIC	--	--	--
Ca1_P4_2317093	Ca1	2317093	TG/T	TTGTGGTTGGTGGTGATGTC	CCTGGGTACATCATAACATTTTTC	60.3	664	INTERGENIC	--	--	--
Ca1_P4_2317888	Ca1	2317888	TTATATATATATATATATATATATATATATATATATATATATA/TTATATATATATATATATATATATATATATATATATATATATATATA	TTGAGTTAAAAGACCACTACCCA	GTTTTCATGACGGGGATGAC	57.9	631	INTERGENIC	--	--	--

INDEL marker IDs	Chromosomes/ unanchored scaffolds	Physical positions (bp)	InDels (*Kabuli* reference genome-CDC Frontier/PII)	Forward primers (5'-3')	Reverse primers (5'-3')	Anneling temperature (0C)	Expected amplified product size (bp)	Structural annotation		Functional annotation	
								Sequence components of ***kabuli*** genome	***Kabuli*** gene accession IDs	NCBI-KOG	TFs
Ca1_P4_23192284	Ca1	23192284	TTAATAATAATAATAATAATAATAATAATAATAATAATAATAATAATAATAATAATAA/TTAATAATAATAATAATAATAATAATAATAATAATAATAATAATAATAATAATAATAATAATAA	TTCGAAACTTTTGGCCACTT	TTGGATTTTGGCCACATTTT	59.7	527	INTERGENIC	--	--	--
Ca1_P4_23195497	Ca1	23195497	GTTT/GTTTTT	TCAAAATTTTCAAGCAAAACCC	ATGGTAGTTTCGCCACTGCT	60.3	825	INTERGENIC	--	--	--
Ca1_P4_2320237	Ca1	2320237	GAAGAAAGAAA/GAAGAAA	TCGTATTTATCCAACATAATGGGA	ATGAACGGTGTAGCCCTTTG	59.5	511	INTERGENIC	--	--	--
Ca1_P4_23222434	Ca1	23222434	AGTTATGTTATGTTATGTTATGTTAT/AGTTATGTTATGTTATGTTAT	CACTCGACAGTTGGGAGGTT	TCGAGCAACAAAGCATCAAG	60.2	423	INTERGENIC	--	--	--
Ca1_P4_23244199	Ca1	23244199	AAGAGAGAGA/AAGAGAGA	AAAGCACCCAAATTGTCCAC	ATGAACTCCTGCGTTTGAGG	59.8	533	INTERGENIC	--	--	--
Ca1_P4_23250478	Ca1	23250478	CAAGAAG/CAAG	TTGGTTTCATGACCTGCATC	CGGCCGTTTCTAACATCATT	59.5	351	INTERGENIC	--	--	--
Ca1_P4_23304825	Ca1	23304825	CATATATATATATATATAT/CATATATATATATATATATATAT	CCATTACGTGCAACCAGAAA	TGACCTGCGTCCATAAATCA	59.6	928	INTERGENIC	--	--	--
Ca1_P4_23319597	Ca1	23319597	CAAAAAAAAAAAA/CAAAAAAAAAAAAA	AAAGGCCATGTGATTTTTGC	GGCAGAGCTTTGGTGTCAAT	59.9	973	INTERGENIC	--	--	--
Ca1_P4_2332780	Ca1	2332780	CTT/CT	CAAAAATGGCACATGCAAAG	TTCACCAATTCCGTGTTTCA	60.1	399	INTERGENIC	--	--	--
Ca1_P4_2332823	Ca1	2332823	TA/TAA	CAAAAATGGCACATGCAAAG	TTCACCAATTCCGTGTTTCA	60.1	399	INTERGENIC	--	--	--

INDEL marker IDs	Chromosomes/ unanchored scaffolds	Physical positions (bp)	InDels (*Kabuli* reference genome-CDC Frontier/PII)	Forward primers (5'-3')	Reverse primers (5'-3')	Anneling temperature (0C)	Expected amplified product size (bp)	Structural annotation		Functional annotation	
								Sequence components of *kabuli* genome	*Kabuli* gene accession IDs	NCBI-KOG	TFs
Ca1_P4_23467419	Ca1	23467419	CTATATATATATATATATATATATATAT/ CTATATATATATATATATATATATAT	CGGGACTTACAACCCA GAAA	TGGTGTTAGAGTCATG CTTTTGA	60.0	248	INTERGENIC	--	--	--
Ca1_P4_23492453	Ca1	23492453	AATATATATATATATATATATATATATAT /AATATATATATATATATATATATATATA TATATATAT	TGTGTAGCGCCCCTTA ACTT	ACGGTAGCTTTAACGA GCGA	59.8	935	INTERGENIC	--	--	--
Ca1_P4_2351691	Ca1	2351691	ATTTT/ATTT	CGACGAAGCAAAATCA AAGC	GAAAAAGGTTGGATGA ATTTGTT	60.9	171	INTERGENIC	--	--	--
Ca1_P4_23565928	Ca1	23565928	AA/AACATCATATTA	GCGGTTAAGCCAATTT TGAG	TGGTGTAACGAGCATC AACAA	59.7	450	INTERGENIC	--	--	--
Ca1_P4_23569757	Ca1	23569757	AT/ATTATT	CGTTTGCTTTGAGAGA TTTGTG	CAAATTGTGGCGACTT TGAA	59.9	455	INTERGENIC	--	--	--
Ca1_P4_23571644	Ca1	23571644	AA/AAGTCTTCCGATCTTTTGA	TGTGTTGGCTTAAAAC ATTTGAT	GGGCAATGTTGGTGGT ACTC	58.6	810	INTERGENIC	--	--	--
Ca1_P4_23613804	Ca1	23613804	CTCTTTCTTTCTTTCTTTCTTTCTTT CTTT/CTCTTTCTTTCTTTCTTTCTTT CTTT	CCTAATCTTGCATGGG GAAA	CCCGAGGAAGAATCCA ATTT	59.9	402	INTERGENIC	--	--	--
Ca1_P4_23622145	Ca1	23622145	CTTTTTTTTTT/CTTTTTTTTT	ACGATGAAGGTGGAAG ATGG	TTGCAACTTTGATAACG AAAACA	59.9	767	INTERGENIC	--	--	--
Ca1_P4_236262	Ca1	236262	TAAAAAAAAA/TAAAAAAAA	TGAAAGAACAATATTCC ACTAAACCA	GCAGCAATTGATGGTG TGAT	60.2	129	INTERGENIC	--	--	--
Ca1_P4_23637540	Ca1	23637540	TTA/TTAAATA	TTCATCACCTCCTCCAA ACC	AATCAGGGCAGACGAC AATC	59.9	543	INTERGENIC	--	--	--

INDEL marker IDs	Chromosomes/ unanchored scaffolds	Physical positions (bp)	InDels (*Kabuli* reference genome-CDC Frontier/PII)	Forward primers (5'-3')	Reverse primers (5'-3')	Anneling temperature (0C)	Expected amplified product size (bp)	Structural annotation		Functional annotation	
								Sequence components of *kabuli* genome	*Kabuli* gene accession IDs	NCBI-KOG	TFs
Ca1_P4_23784964	Ca1	23784964	ATTTTTTTT/ATTTTTT	GTCAACCAATTGACCCCAAC	TGCCAAGAAAACACCATGAA	60.1	663	INTERGENIC	--	--	--
Ca1_P4_2379049	Ca1	2379049	T/TCA	AACCCAAAAACAGAAACCCC	TTTGCTTGCAAAGCCTTTTT	60.1	129	INTERGENIC	--	--	--
Ca1_P4_23838777	Ca1	23838777	ATGTGTGTGTGT/ATGTGTGT	AATGCCCACCCATAATTTCA	TGTACGAGAAGGTCGTGTGC	60.0	593	INTERGENIC	--	--	--
Ca1_P4_23892469	Ca1	23892469	TA/TAAA	CGTGCATACCCCATACACAA	GGTGCAGTTGTTCGTCAGAA	60.3	550	INTERGENIC	--	--	--
Ca1_P4_23966814	Ca1	23966814	GA/GAA	AACTGTGGAGGAATCCATGC	GCTTCAAGTCTTGCTACGGG	59.9	490	INTERGENIC	--	--	--
Ca1_P4_2405906	Ca1	2405906	T/TCAAATAAACC	TCTAAAGGGATTGCGACGTT	CCAAATTCAGTGTTTGCGTG	59.7	749	INTERGENIC	--	--	--
Ca1_P4_2407002	Ca1	2407002	ATTT/ATTTT	CACTTGCACATTCATGCAGTT	ACAAAAATGGGTCCCTCCTT	59.8	549	INTERGENIC	--	--	--
Ca1_P4_24079570	Ca1	24079570	AATATATATATATATATATATATATA/AATATATATATATATATATATATATATATATATATATA	CGAAGCCTTTGGTTTCATTT	GGAAAAATAAAATTGGGGAACA	59.2	541	INTERGENIC	--	--	--
Ca1_P4_2409275	Ca1	2409275	TA/T	GCATATGCCCAAGTTTTGGT	CTAGGCTCTGAACCGGTAAAA	59.8	188	INTERGENIC	--	--	--
Ca1_P4_24104335	Ca1	24104335	CGG/CG	TCAAACAAGCGTAATCAACCC	CCCTGGAGCTTTTAGAGCCT	60.0	447	INTERGENIC	--	--	--

INDEL marker IDs	Chromosomes/ unanchored scaffolds	Physical positions (bp)	InDels (*Kabuli* reference genome-CDC Frontier/PII)	Forward primers (5'-3')	Reverse primers (5'-3')	Anneling temperature (0C)	Expected amplified product size (bp)	Structural annotation		Functional annotation	
								Sequence components of *kabuli* genome	*Kabuli* gene accession IDs	NCBI-KOG	TFs
Ca1_P4_24109260	Ca1	24109260	CTTTTTTTTTTT/CTTTTTTTTTTTTT	TTGGGGGAACCAGGATAAAT	CCCTGCTTAAGGCACATGAT	60.4	599	INTERGENIC	--	--	--
Ca1_P4_2411747	Ca1	2411747	T/TA	TGTGTCTTGTTGGGGAAACA	TGACGCTTTTGAAGCAGTTG	60.0	547	INTERGENIC	--	--	--
Ca1_P4_2412470	Ca1	2412470	AATATAT/AATATATAT	AGGACAATAGGACGAGGCAA	AGTCACAGAGCATGCACCAC	59.7	436	INTERGENIC	--	--	--
Ca1_P4_2412541	Ca1	2412541	TTTATTA/TTTA	AGGACAATAGGACGAGGCAA	AGTCACAGAGCATGCACCAC	59.7	436	INTERGENIC	--	--	--
Ca1_P4_2415241	Ca1	2415241	CA/CAGA	TATATAGGTGGTGCCCGGTG	CCCCTCTCACAACTAAGGCA	60.6	657	INTERGENIC	--	--	--
Ca1_P4_2416072	Ca1	2416072	AAA/AAACAA	TATTTGCCATGCATCAAGGA	CCAACTTCATGGTGTCATCC	60.0	734	INTERGENIC	--	--	--
Ca1_P4_2416287	Ca1	2416287	T/TA	TTGCACATTGGTACCCAAAA	TAGCCTTTTTGTTGGTTGGC	59.8	543	INTERGENIC	--	--	--
Ca1_P4_2416502	Ca1	2416502	GA/G	CAAAATCAGTCGCATGTTGG	TAGCCTTTTTGTTGGTTGGC	60.1	248	INTERGENIC	--	--	--
Ca1_P4_2416894	Ca1	2416894	T/TC	GCCAACCAACAAAAAGGCTA	ACAGTCCAAAACCCGAAGTG	60.1	628	INTERGENIC	--	--	--
Ca1_P4_24220141	Ca1	24220141	TTAATTAATAATTAATAATTAATAATTAATAAT/TTAATTAATAATTAATAATTAATAATTAATAATTAATAAT	TGATTCAGTGCGAAAAGACG	GCAGTCGATTGGGAGAAGAG	60.0	473	INTERGENIC	--	--	--

INDEL marker IDs	Chromosomes/ unanchored scaffolds	Physical positions (bp)	InDels (*Kabuli* reference genome-CDC Frontier/PII)	Forward primers (5'-3')	Reverse primers (5'-3')	Anneling temperature (0C)	Expected amplified product size (bp)	Structural annotation		Functional annotation	
								Sequence components of *kabuli* genome	*Kabuli* gene accession IDs	NCBI-KOG	TFs
Ca1_P4_24290082	Ca1	24290082	A/AGAGAGAGAGAGAGG	AAATGCTTCAGCCAGACACC	CGAACCCTAAAGACGCAAAG	60.3	378	INTERGENIC	--	--	--
Ca1_P4_24301670	Ca1	24301670	AATATATATATATATATATATATA/AATATATATATATATATATATATA	TCAAGCATGTCAAATTCCCA	GGGGTGCTACAGTGTCGAAT	60.0	633	INTERGENIC	--	--	--
Ca1_P4_2431426	Ca1	2431426	TGTTT/TGTTTAAGTTT	TCACAAAGTTGCTTTCCGTG	TGAAAATGCCCCGATTAAAG	59.9	339	INTERGENIC	--	--	--
Ca1_P4_2431602	Ca1	2431602	GTCTATT/GT	CTTTAATCGGGGCATTTTCA	CAATCATTGTGAGATGGATGAAG	59.9	269	INTERGENIC	--	--	--
Ca1_P4_2434803	Ca1	2434803	ATTTTTTTTT/ATTTTTTT	AAGAGTGAGATGGGCACCAC	AGGGATTTCATTTGGAGAAAA	60.1	421	INTERGENIC	--	--	--
Ca1_P4_2436388	Ca1	2436388	CAAAA/CAAAAA	ACAATGATTTTCTCCACGCC	ATCGTCAATGTTGCACATCC	59.9	144	INTERGENIC	--	--	--
Ca1_P4_2440150	Ca1	2440150	ACCCC/ACCC	ATCTGCTGCTCGTAGGCATT	GAAGGGCGAAAACATGAAAA	60.0	677	INTERGENIC	--	--	--
Ca1_P4_2448249	Ca1	2448249	TAT/TATATAAAT	GGCTCCACAATATTATAGTCCTTCA	AAGGATCCACGTCAGTCCAG	59.8	776	INTERGENIC	--	--	--
Ca1_P4_2448896	Ca1	2448896	TATT/TATTAATT	CTGGACTGACGTGGATCCTT	TGTTGTTATGGTGGGTCCCT	60.1	508	INTERGENIC	--	--	--
Ca1_P4_2454328	Ca1	2454328	ACTCTCTCTCT/ACTCTCTCTCTCT	TTTTGAGATAACCCAGGCCA	GCATGGGACGCTTTTGTAAT	60.4	616	INTERGENIC	--	--	--

INDEL marker IDs	Chromosomes/ unanchored scaffolds	Physical positions (bp)	InDels (*Kabuli* reference genome-CDC Frontier/PII)	Forward primers (5'-3')	Reverse primers (5'-3')	Anneling temperature (0C)	Expected amplified product size (bp)	Structural annotation		Functional annotation	
								Sequence components of ***kabuli*** genome	***Kabuli*** gene accession IDs	NCBI-KOG	TFs
Ca1_P4_2454529	Ca1	2454529	CAGA/CA	GATAGCTCATGGATGGGTCC	GCATGGGACGCTTTTGTAAT	59.3	425	INTERGENIC	--	--	--
Ca1_P4_24623839	Ca1	24623839	ATTTTTTTT/ATTTTTTTTT	AAATTTGTTGCGAAGGCAGT	TTTTGTGATTGGGCACTTGA	59.8	446	INTERGENIC	--	--	--
Ca1_P4_24624813	Ca1	24624813	T/TTAGAAA	CATCATCATTTTCAATGGGCT	ATGGTTTTATTCGGCCACAA	59.8	801	INTERGENIC	--	--	--
Ca1_P4_24632359	Ca1	24632359	GATATATATATATATA/GATATATATATATATATA	GACGCCTGACGGTTATTGTT	CTTGGCCAGAAAATCGAAAA	60.0	703	INTERGENIC	--	--	--
Ca1_P4_2463656	Ca1	2463656	ATTTAAATTTTGTGTTTAAATTTTT/ATTTAAATTTT	GGAAACAAATTTTGCGCACT	TTCACTTGTATCGCACCCAT	60.1	495	INTERGENIC	--	--	--
Ca1_P4_24648509	Ca1	24648509	TTATATATATATATATATATATATA/TTATATATATATATATATATATA	CACTTGAATTTGTTTCTCTCGC	TGCTTTTTCTGGATTGGTCC	59.0	464	INTERGENIC	--	--	--
Ca1_P4_2475450	Ca1	2475450	ATTTT/ATTT	CCCCTATGCTCCATCTCAAA	CAAAGATTGCTCACATTTGTCC	60.0	581	INTERGENIC	--	--	--
Ca1_P4_24771219	Ca1	24771219	TCT/TCTCTTTCCCTCTTCCT	TTGCAAGCACGATTTGAAAC	TGGGTCCAAAGTGGATGAAT	59.9	528	INTERGENIC	--	--	--
Ca1_P4_24801961	Ca1	24801961	CTTTTTTTTT/CTTTTTTTTTT	TTTCAATGATGTTGGGTTGC	CAGCATATCCCCAACAAAAGA	59.4	998	INTERGENIC	--	--	--
Ca1_P4_24935055	Ca1	24935055	TTATATATATATATATATATAT/TTATATATATATATATATAT	TGTGTGTTTTCTACAATGTGCTAGTT	TTGGGTTTGACCCATGAAAT	59.3	730	INTERGENIC	--	--	--

INDEL marker IDs	Chromosomes/ unanchored scaffolds	Physical positions (bp)	InDels (*Kabuli* reference genome-CDC Frontier/PII)	Forward primers (5'-3')	Reverse primers (5'-3')	Anneling temperature (0C)	Expected amplified product size (bp)	Structural annotation		Functional annotation	
								Sequence components of *kabuli* genome	*Kabuli* gene accession IDs	NCBI-KOG	TFs
Ca1_P4_25064793	Ca1	25064793	AATATATATATATATATATATATATATATA/AATATATATATATATATATATATATATATATATA	GTGAGGGTGCTTGTCTCACA	AATGGGCGCTGTTAAATGTC	59.9	613	INTERGENIC	--	--	--
Ca1_P4_25133935	Ca1	25133935	ATTTTTTTTTTTTTTTT/ATTTTTTTTTTTTTT	TGTTGGTAGCACAATCACGAG	CTTAGGTCTTGCGATCACCC	59.8	155	INTERGENIC	--	--	--
Ca1_P4_25134278	Ca1	25134278	AAATAATAATAATAATAATAATAATAATAA/AAATAATAATAATAATAATAATAATAATAATAA	GATCGCAAGACCTAAGTGCC	AGATGTGCCAGCAGTGTTGT	59.8	554	INTERGENIC	--	--	--
Ca1_P4_2518243	Ca1	2518243	CATATATATATATATATATA/CATATATATATATATATATATA	AACGGGATAAATGTTTGTGCTT	GCGCTTTTACGGGACAACTA	59.8	794	INTERGENIC	--	--	--
Ca1_P4_252332	Ca1	252332	AATATATATATATATATATATATATATATAT/AATATATATATATATATATATATAT	ACACATGAAGCCCAACATCA	TGGTTGACTTTGTTAAATCGGA	60.0	132	INTERGENIC	--	--	--
Ca1_P4_25296432	Ca1	25296432	CTTT/CTTTT	GTTGGTGGCTGGACTCAGAT	TTCCCTAGTTGTTTGGGTGC	60.1	817	INTERGENIC	--	--	--
Ca1_P4_25303242	Ca1	25303242	CATATATATATATATATATATATATATATAT/CATATATATATATATATATATATATATATATAT	TAGGCAACCTTGGGTGTTGT	TCAGCATGTCAATTTCCCAA	60.4	813	INTERGENIC	--	--	--
Ca1_P4_25358096	Ca1	25358096	AACCA/AA	TCTGGAGCCCATAGGAGTTG	CCAACGGGATGGTGGTATAG	60.2	716	INTERGENIC	--	--	--
Ca1_P4_25382154	Ca1	25382154	GAA/GAAA	GAATCACTAGGCTTGCCAGG	GGGGTGAGAGAACGTTTGAA	59.8	714	INTERGENIC	--	--	--
Ca1_P4_25437512	Ca1	25437512	AATATATATATATATATATATATATATATATATATATATATATA/ATATATATATATATATATATATATATATATATATATATATATA	GACTTTGATGCAAGTCACCG	CTCCCATCAGTTTAATCGGC	59.3	875	INTERGENIC	--	--	--

INDEL marker IDs	Chromosomes/ unanchored scaffolds	Physical positions (bp)	InDels (*Kabuli* reference genome-CDC Frontier/PII)	Forward primers (5'-3')	Reverse primers (5'-3')	Anneling temperature (0C)	Expected amplified product size (bp)	Structural annotation		Functional annotation	
								Sequence components of *kabuli* genome	*Kabuli* gene accession IDs	NCBI-KOG	TFs
Ca1_P4_25437564	Ca1	25437564	T/TATATA	GCATGGCAGTGTTTTACGTC	CTCCCATCAGTTTAATCGGC	59.2	783	INTERGENIC	--	--	--
Ca1_P4_25482802	Ca1	25482802	GAAAAAAAAAA/GAAAAAAAAAAA	TGTAGTGGCATGTTTGGCAT	TTTGGAGGACTATCGGGTTG	60.0	638	INTERGENIC	--	--	--
Ca1_P4_25507843	Ca1	25507843	CAATAATAATAATAATAATAATAATAATAATAATAATA/CAATAATAATAATAATAATAATAATAATAATA	TCTCTGGCAACCGTTCTTCT	GAATGAAGTCCAAGGGTCCA	60.0	341	INTERGENIC	--	--	--
Ca1_P4_25509778	Ca1	25509778	AATATATATATATATATATATATATATATATATATATCTATATATATAT/AATATATATATATATATATATATATATATATATATATATATATATCTATATATATAT	ATTTTGGCTCGGAGGTTTTT	GTTTTCGAATCAACGAGGGA	59.9	550	INTERGENIC	--	--	--
Ca1_P4_2551219	Ca1	2551219	CATATATATATATATATATATAT/CATATATATATATATATATAT	TTCTGTTTCACCATTCCAACA	GGAGTGTGGTGATGTAAGGACTC	59.0	524	INTERGENIC	--	--	--
Ca1_P4_25572037	Ca1	25572037	GTTTT/GTTTTT	GTCAGGCATTCCCCTCTACA	CCCGAAACGCATTGAATTAT	60.1	781	INTERGENIC	--	--	--
Ca1_P4_2572725	Ca1	2572725	TAAAAAAAAAA/TAAAAAAAAAAA	TGGTTGTAAATCCTCTTCACAAAA	GATAGAGTGCATCGGGCATT	59.9	413	INTERGENIC	--	--	--
Ca1_P4_25802229	Ca1	25802229	ACCCC/ACCCCCCC	TGTCGAACTCAAAGATTGCG	TTGGGAAGTTGGGAGACTTG	60.0	764	INTERGENIC	--	--	--
Ca1_P4_25816089	Ca1	25816089	CT/CTT	TCCAAGAAAGTGAGGGTTGG	ACCGTTTATTTTCGCGGTTA	60.1	753	INTERGENIC	--	--	--
Ca1_P4_25851157	Ca1	25851157	TTATATATATATATATATATATATATATAT/TTATATATATATATATATATATATATATATATATATAT	ATTCTTCAACCCCTTTCGCT	TGTTGGAGTCATGTTTTTCATCA	60.1	567	INTERGENIC	--	--	--

INDEL marker IDs	Chromosomes/ unanchored scaffolds	Physical positions (bp)	InDels (*Kabuli* reference genome-CDC Frontier/PII)	Forward primers (5'-3')	Reverse primers (5'-3')	Anneling temperature (0C)	Expected amplified product size (bp)	Structural annotation		Functional annotation	
								Sequence components of *kabuli* genome	*Kabuli* gene accession IDs	NCBI-KOG	TFs
Ca1_P4_25853462	Ca1	25853462	TAAAAAAAAA/TAAAAAA	TCGCACGATTCTAACAACCA	AAGGCGAGATAATAGCCGGT	60.3	531	INTERGENIC	--	--	--
Ca1_P4_25948887	Ca1	25948887	TTATATATATATATATATATATA/TTATATATATATATATATATATATATA	TTTCTATAAAGCAAATCCGCA	GTCGCCGACAGTTTTAAGGA	57.6	855	INTERGENIC	--	--	--
Ca1_P4_26094587	Ca1	26094587	TAAAAAAAA/TAAAAAAA	CTCGAAGCCGAGAAAATGAC	TCCCCCTCCTATTGTCCTCT	60.0	723	INTERGENIC	--	--	--
Ca1_P4_2615903	Ca1	2615903	GAAAAAAAAAAA/GAAAAAAAAAAAA	TGTGATCAAATGTGTGAGTGAAA	TCCAAGATTCAATCCAACCC	59.1	221	INTERGENIC	--	--	--
Ca1_P4_26352919	Ca1	26352919	TAA/TA	TAAAGGACCAACTCGGGAAA	GCATTGGATCGCTTCAAGAT	59.5	786	INTERGENIC	--	--	--
Ca1_P4_26377123	Ca1	26377123	ATTTTTTT/ATTTTTTTT	TGTTCTAGATGGGGTTTGGC	GCCACTATCCAACAAATGGC	59.9	655	INTERGENIC	--	--	--
Ca1_P4_26447765	Ca1	26447765	ATATTATTATTATTATTATTATTATTATT/ATATTATTATTATTATTATTATTATTATTATTATT	AAATAGGGCCACACCAAAAA	TTAAATCGGAGGTATGGGGA	59.3	320	INTERGENIC	--	--	--
Ca1_P4_26447923	Ca1	26447923	CACATATCTG/C	AAATAGGGCCACACCAAAAA	GTAATGGGTATGGGTACGGG	59.3	420	INTERGENIC	--	--	--
Ca1_P4_2653521	Ca1	2653521	T/TC	GGTGGGTGACCCTCACTTTA	CATTCCAGTTAGTTGAACGGGT	59.8	525	INTERGENIC	--	--	--
Ca1_P4_2655421	Ca1	2655421	TGG/TGGG	TCCGTGGAAATGAACCTAGC	CGCGTGATGATTGCTAAAAA	60.1	228	INTERGENIC	--	--	--

INDEL marker IDs	Chromosomes/ unanchored scaffolds	Physical positions (bp)	InDels (*Kabuli* reference genome-CDC Frontier/PII)	Forward primers (5'-3')	Reverse primers (5'-3')	Anneling temperature (0C)	Expected amplified product size (bp)	Structural annotation		Functional annotation	
								Sequence components of *kabuli* genome	*Kabuli* gene accession IDs	NCBI-KOG	TFs
Ca1_P4_2656475	Ca1	2656475	A/AG	TTGCACAATTTCTGATTGGC	GATCATTGCATCCCCATTCT	59.7	569	INTERGENIC	--	--	--
Ca1_P4_2656961	Ca1	2656961	T/TAA	TCGTAGAATGGGGATGCAAT	TGACATCAACTGGAATCAACA	60.3	480	INTERGENIC	--	--	--
Ca1_P4_2659291	Ca1	2659291	GACCT/G	AGGATGGTGGAGAGCAAGTC	AAACCAAACCCAACTCCTCC	59.3	604	INTERGENIC	--	--	--
Ca1_P4_2659614	Ca1	2659614	TAAA/TAA	CAATCAGGATCGGCCAATTA	CGTGTTTAGCGGTGAGTTGA	60.8	642	INTERGENIC	--	--	--
Ca1_P4_26605020	Ca1	26605020	CT/C	AAAGGCATCCTACAATTCACA	TGGAGTTTGGTGGTGAAAAA	57.2	587	INTERGENIC	--	--	--
Ca1_P4_2661346	Ca1	2661346	TAAAA/TAAAAA	CGCCGTTTTTCTTCAAACTC	TGGTGTTCATTTTGATTCAGGA	59.9	840	INTERGENIC	--	--	--
Ca1_P4_26629862	Ca1	26629862	TAAAAAAA/TAAAAAA	TTTCATTCACTCAACCTTTTGG	GGGCCATCCTATATTCCCAC	59.1	645	INTERGENIC	--	--	--
Ca1_P4_2663715	Ca1	2663715	TGAAAAGAAAA/TGAAAA	GTTGCCATGCCTATAACGGT	TGAAGACCATGGACACCAAC	59.9	547	INTERGENIC	--	--	--
Ca1_P4_2664568	Ca1	2664568	GATGATGTGAATGATGTGAA/GATGATGTGAA	CCAAAAATTGATGAATTGACGTT	TGAATCATGAAGCGGAAAAA	60.1	324	INTERGENIC	--	--	--
Ca1_P4_26651379	Ca1	26651379	TTATATATATATATATATAT/TTATATATATATATATATAT	GAAGCATTGGAAGTTTGGGA	GGTCCCTGTTGGTGCAGTAT	60.1	767	INTERGENIC	--	--	--

INDEL marker IDs	Chromosomes/ unanchored scaffolds	Physical positions (bp)	InDels (*Kabuli* reference genome-CDC Frontier/PII)	Forward primers (5'-3')	Reverse primers (5'-3')	Anneling temperature (0C)	Expected amplified product size (bp)	Structural annotation		Functional annotation	
								Sequence components of *kabuli* genome	*Kabuli* gene accession IDs	NCBI-KOG	TFs
Ca1_P4_2666278	Ca1	2666278	ATTTTTT/ATTTTT	TCGGAATACACGACGAAACA	CTGCGGGTAGGATTGCTTTA	60.1	740	INTERGENIC	--	--	--
Ca1_P4_2668175	Ca1	2668175	AC/A	AATGCATCACGTGGGATTTT	CGATTGCACCCGTAGTTGTA	60.2	206	INTERGENIC	--	--	--
Ca1_P4_26683611	Ca1	26683611	TG/T	CAATTGTGTAAAACGTGCGG	TTGCATTTGGTTTGACTCCA	60.0	538	INTERGENIC	--	--	--
Ca1_P4_26725325	Ca1	26725325	CTATATATATATATATATATAT/CTATATATATATATATATAT	AATTAGCAGCGGAAGGAAAG	GCATCACAGTTAAATCATCGC	58.6	352	INTERGENIC	--	--	--
Ca1_P4_2674516	Ca1	2674516	CTAGATTGGAT/CTAGATTGGATTAGATTGGAT	TGTCGGAATATGGGAAGCTC	GCCGTTGTTTTCAGATCCAT	60.0	934	INTERGENIC	--	--	--
Ca1_P4_2679013	Ca1	2679013	CCTCT/CCTCTCT	CATCCTTATCCCTCATTCCTTG	AAAATATACCCCATCTGTCCCT	59.8	303	INTERGENIC	--	--	--
Ca1_P4_26876468	Ca1	26876468	C/CG	TTGTGCATCCGTCTATCCAC	TCGGTGGAAATTGTAATGAGAG	59.5	937	INTERGENIC	--	--	--
Ca1_P4_26877204	Ca1	26877204	TAAAAAAAAAA/TAAAAAAAAAAA	CTCTCATTACAATTTCCACCGA	GAAGGGTCATAAAATGGGGG	59.1	449	INTERGENIC	--	--	--
Ca1_P4_26894036	Ca1	26894036	TGTAATTATTG/TGTAATTATTGTAATTCGTAATTATTG	ATGCACACACTTTTGATGCC	TCATACAAATTCGAGGTTCCAA	59.6	670	INTERGENIC	--	--	--
Ca1_P4_26958642	Ca1	26958642	TT/TTTGAT	TGCTCCATTTGATTGCTGAC	CCGACCAATAGACTTTTGTTGA	59.8	663	INTERGENIC	--	--	--

INDEL marker IDs	Chromosomes/ unanchored scaffolds	Physical positions (bp)	InDels (*Kabuli* reference genome-CDC Frontier/PII)	Forward primers (5'-3')	Reverse primers (5'-3')	Anneling temperature (0C)	Expected amplified product size (bp)	Structural annotation		Functional annotation	
								Sequence components of *kabuli* genome	*Kabuli* gene accession IDs	NCBI-KOG	TFs
Ca1_P4_26958895	Ca1	26958895	CAAAAAAAAA/CAAAAAAAA	TGAGCGTGAAAAATTGTGGT	TTAGAGCGTTCAAATTATAGTTACACA	59.2	709	INTERGENIC	--	--	--
Ca1_P4_2704069	Ca1	2704069	GTTT/GTT	CACGCATGATTGAAATTGTTC	AAGCTTCTTCGAGCAACGAG	59.0	264	INTERGENIC	--	--	--
Ca1_P4_27143610	Ca1	27143610	AATATATATATATATATATATATATATATA/AATATATATATATATATATATATATATATATATA	GTTCAATCAATACTCGGCGG	GGCATCTTTGATCAATGGTCTT	60.5	681	INTERGENIC	--	--	--
Ca1_P4_27204677	Ca1	27204677	C/CT	GCCGAAGTACCAATTACCGA	TACGGTGCTAGGGAACCAAC	60.0	705	INTERGENIC	--	--	--
Ca1_P4_27232314	Ca1	27232314	AATATATATATATATATATATATATATAT/AATATATATATATATATATATATATATATATATATATAT	CTTGGGAACTGGAGAGCAAG	AGGGAGGTTTGTGTTTGACG	60.0	682	INTERGENIC	--	--	--
Ca1_P4_2725497	Ca1	2725497	TAA/TA	GTGTAACCTCACGGGAAGGA	GGGTCAATTTGAAACAACGG	60.0	660	INTERGENIC	--	--	--
Ca1_P4_2727220	Ca1	2727220	GAAAAAA/GAAAAAAA	CCACATATTAGCCACCCCAC	TATCGCTCAATTTGCGTTTG	60.1	906	INTERGENIC	--	--	--
Ca1_P4_27282747	Ca1	27282747	G/GCAGTT	AAAATCCAACAATCTCCCCC	CCCTGAGACTCACGAAGAGG	60.0	561	INTERGENIC	--	--	--
Ca1_P4_27282877	Ca1	27282877	AAAAAA/AAAAAAAAA	AAAATCCAACAATCTCCCCC	CCCTGAGACTCACGAAGAGG	60.0	561	INTERGENIC	--	--	--
Ca1_P4_27300538	Ca1	27300538	TTGTGGTGTGGTGTG/TTGTGGTGTG	CAGAAATCTCTGGGTGGGAA	CTGGTTCCCCTTATGACCAA	60.0	109	INTERGENIC	--	--	--

INDEL marker IDs	Chromosomes/ unanchored scaffolds	Physical positions (bp)	InDels (*Kabuli* reference genome-CDC Frontier/PII)	Forward primers (5'-3')	Reverse primers (5'-3')	Anneling temperature (0C)	Expected amplified product size (bp)	Structural annotation		Functional annotation	
								Sequence components of *kabuli* genome	*Kabuli* gene accession IDs	NCBI-KOG	TFs
Ca1_P4_27320145	Ca1	27320145	GAAAAAAAAAAA/GAAAAAAAAAA	GAAACTTCGGTGCCATCATT	CGGTCACAGAGAGGAAGAGG	59.9	616	INTERGENIC	--	--	--
Ca1_P4_2745007	Ca1	2745007	GTTTTTTTTTT/GTTTTTTTTT	GAACGAACGGTGTGATGATG	GGGCCCACAATAACACAAAC	60.0	513	INTERGENIC	--	--	--
Ca1_P4_2761120	Ca1	2761120	GA/GAATA	TCATCATGCTCCATGGTTTC	CTAGCCCCATTACAAGCCAA	59.5	609	INTERGENIC	--	--	--
Ca1_P4_2762433	Ca1	2762433	T/TC	TATTTGGACAAACGAAGGGC	TGGAAGGATGGATGCTTAGG	59.9	126	INTERGENIC	--	--	--
Ca1_P4_27640153	Ca1	27640153	AAAGAAGAAGAAGAAGAA/AAAGAAGAAGAAGAA	CCAACACCGTTTTCGAATCT	TGTGTGTGTTTGGAGGGAAA	60.0	297	INTERGENIC	--	--	--
Ca1_P4_27640312	Ca1	27640312	CATATATATATATAT/CATATATATATATATAT	TTTCCCTCCAAACACACACA	TGAGTCGCGACGAGAGTAGA	60.0	147	INTERGENIC	--	--	--
Ca1_P4_2765307	Ca1	2765307	C/CAATGTG	CAGCCTCTGATGTTCAATGG	CTATGTGCATATCGGCCCTT	59.2	423	INTERGENIC	--	--	--
Ca1_P4_2765494	Ca1	2765494	TAAAAAAAAA/TAAAAAAAAAAA	CAGCCTCTGATGTTCAATGG	CTATGTGCATATCGGCCCTT	59.2	423	INTERGENIC	--	--	--
Ca1_P4_2765671	Ca1	2765671	GTTTTTTTTT/GTTTTTTT	TAAAAAGGGCCGATATGCAC	CATGCACGATTGTGGTAATTG	59.9	564	INTERGENIC	--	--	--
Ca1_P4_2766077	Ca1	2766077	CTTATGTT/CTTATGTTATGTT	AAAACAATTGATTGGGCTCC	CCAATCATACGCAGGTGAAA	58.9	561	INTERGENIC	--	--	--

INDEL marker IDs	Chromosomes/ unanchored scaffolds	Physical positions (bp)	InDels (*Kabuli* reference genome-CDC Frontier/PII)	Forward primers (5'-3')	Reverse primers (5'-3')	Anneling temperature (0C)	Expected amplified product size (bp)	Structural annotation		Functional annotation	
								Sequence components of *kabuli* genome	*Kabuli* gene accession IDs	NCBI-KOG	TFs
Ca1_P4_2766176	Ca1	2766176	A/AG	AAAACAATTGATTGGGCTCC	CCAATCATACGCAGGTGAAA	58.9	561	INTERGENIC	--	--	--
Ca1_P4_2766346	Ca1	2766346	AATATA/AATATATA	TTTCACCTGCGTATGATTGG	TGACCTCTTATGCTTCTCGAAAT	59.5	501	INTERGENIC	--	--	--
Ca1_P4_2766600	Ca1	2766600	CTTTTTTTT/CTTTTTTT	TTTCACCTGCGTATGATTGG	TTGAAAGTTGAAACGTGGGA	59.5	779	INTERGENIC	--	--	--
Ca1_P4_2772818	Ca1	2772818	TAAAAAA/TAAAAAAA	TATTGGGCAACAATTCACGA	TCGCCTACTGTGTTGGATGA	59.9	222	INTERGENIC	--	--	--
Ca1_P4_27744633	Ca1	27744633	TCCC/TCC	CAAGACTGATGCAAACCCAA	CTCTCGAAGATCCACTTGCC	59.7	348	INTERGENIC	--	--	--
Ca1_P4_27845331	Ca1	27845331	AT/A	TTATTTTCCCCAATCTCCCC	TTTTGAAGATGGTGAACATGC	60.0	731	INTERGENIC	--	--	--
Ca1_P4_27850705	Ca1	27850705	CTTTTTTTTTTT/CTTTTTTTTT	CACCACCGAAACCAAACTCT	TGACAGTCAGAGTGCGATCC	60.0	444	INTERGENIC	--	--	--
Ca1_P4_2791561	Ca1	2791561	ATTT/AT	ATCGAAAGCAACCATTTTGG	TCATTCAAATGGTGACTCGTG	59.9	419	INTERGENIC	--	--	--
Ca1_P4_2791646	Ca1	2791646	TT/TTTTAAT	ATCGAAAGCAACCATTTTGG	TTGTGGTGGTTCTTTGTTGG	59.9	869	INTERGENIC	--	--	--
Ca1_P4_2796283	Ca1	2796283	T/TG	GGTGGACAGAGGCATCAACT	AAAAAGCGAGTTTCAGGGTG	60.1	530	INTERGENIC	--	--	--

INDEL marker IDs	Chromosomes/ unanchored scaffolds	Physical positions (bp)	InDels (*Kabuli* reference genome-CDC Frontier/PII)	Forward primers (5'-3')	Reverse primers (5'-3')	Anneling temperature (0C)	Expected amplified product size (bp)	Structural annotation		Functional annotation	
								Sequence components of *kabuli* genome	*Kabuli* gene accession IDs	NCBI-KOG	TFs
Ca1_P4_2797230	Ca1	2797230	A/AC	TGTCTTAAAATCTGTGCATTCAA	AAATAGTTGACAAACCGCCG	57.5	581	INTERGENIC	--	--	--
Ca1_P4_2797326	Ca1	2797326	A/AC	CCCTGTAAATTCAGACACAATCG	AAATAGTTGACAAACCGCCG	60.8	272	INTERGENIC	--	--	--
Ca1_P4_2797692	Ca1	2797692	TTTCT/TT	ACGGCGGTTTGTCAACTATT	AAGAAGCCAAACAACGAAGC	59.5	567	INTERGENIC	--	--	--
Ca1_P4_2797913	Ca1	2797913	ATTTTTTTTTTTT/ATTTTTTTTTT	ACGGCGGTTTGTCAACTATT	GGGTGTTTTTGTCATTTGGG	59.5	743	INTERGENIC	--	--	--
Ca1_P4_28015338	Ca1	28015338	T/TTAA	AATGACATTGAAGGGCGAAG	ATTGGTTTCCACGGTTTGTC	60.1	404	INTERGENIC	--	--	--
Ca1_P4_28024777	Ca1	28024777	TAGGCCAGACCA/TAGGCCAGACCAGGCCAGACCA	GGCTACCGAATATGGAACGA	GTGGTGAGTAGGGATGGGAA	59.9	330	INTERGENIC	--	--	--
Ca1_P4_2803383	Ca1	2803383	TAA/TA	ACAACGGAATTGGTCATGGT	AAGACCCATCATCACCTTGC	60.1	172	INTERGENIC	--	--	--
Ca1_P4_2803396	Ca1	2803396	GTGATGATG/GTGATG	ACAACGGAATTGGTCATGGT	TTCCTAATTCCAATCCCATGT	60.1	360	INTERGENIC	--	--	--
Ca1_P4_2810482	Ca1	2810482	CAT/CATAT	TGCATAACCAACTGAATGCC	GCGTCGTAAAAGACGTGCTA	59.5	813	INTERGENIC	--	--	--
Ca1_P4_2826983	Ca1	2826983	AGT/A	CGCCCTGCCTCAAATTATTA	TTTAATGTTGTTGTTGAAGATGAAAA	60.1	962	INTERGENIC	--	--	--

INDEL marker IDs	Chromosomes/ unanchored scaffolds	Physical positions (bp)	InDels (*Kabuli* reference genome-CDC Frontier/PII)	Forward primers (5'-3')	Reverse primers (5'-3')	Anneling temperature (0C)	Expected amplified product size (bp)	Structural annotation		Functional annotation	
								Sequence components of *kabuli* genome	*Kabuli* gene accession IDs	NCBI-KOG	TFs
Ca1_P4_2827577	Ca1	2827577	TTC/T	TCGAATTCATAACTCAAATCTTCA	AGATCTGTTGGAGGTGGTGG	58.0	505	INTERGENIC	--	--	--
Ca1_P4_2829715	Ca1	2829715	GATAT/GAT	ATCCGGGTCCAAAGTTTCTT	TGCATGCCTTCAATAAGTTCA	59.8	303	INTERGENIC	--	--	--
Ca1_P4_28339292	Ca1	28339292	GCTCTCTCTCTCTCTCTCTCTCTCTCTCTCTCTCTCTCTCTCTCT/GCTCTCTCTCTCTCTCTCTCTCTCTCTCTCTCTCTCTCTCT	CGCCTTTCTGTTTTCCACAT	TGGTGTGCTTGGTGATGAAT	60.1	305	INTERGENIC	--	--	--
Ca1_P4_2837552	Ca1	2837552	TTCTCTCTCTCTCTCTCTCTCTCT/TTCTCTCTCTCTCTCTCTCT	CAGCACAAGCATGCCATAGT	TGCATCTGTTCCTGCTTCAC	59.9	671	INTERGENIC	--	--	--
Ca1_P4_2840718	Ca1	2840718	GTTTTTTTTTT/GTTTTTTTTT	GCTACATGTAACGCAGCGAA	ATTTAAGATGGCGATGTGCC	60.0	512	INTERGENIC	--	--	--
Ca1_P4_2841174	Ca1	2841174	AAT/AATAT	TCAATTGCTTGCTACTGCCA	TTTTTCTCGACGAGTTTTGACA	60.5	506	INTERGENIC	--	--	--
Ca1_P4_2843480	Ca1	2843480	AC/A	AAGACGGAGAATCAAACGGA	CTGAGGCTGTGTTTTGGGTT	59.7	614	INTERGENIC	--	--	--
Ca1_P4_2843534	Ca1	2843534	GAAA/GAAAA	GACGGAGAATCAAACGGAAA	GTAGGGCCCACATTCACAAC	60.1	685	INTERGENIC	--	--	--
Ca1_P4_28436190	Ca1	28436190	TAAAAAAAAAAAAA/TAAAAAAAAAAAAAA	TCGATAGAACCCAATCTTCAAAA	TTAGCCGGACATTATTTGCC	60.0	586	INTERGENIC	--	--	--
Ca1_P4_2845108	Ca1	2845108	TAAAAA/TAAA	CCACCTTTCAAAGACGAAACA	GCTTGATTTCAAAATCTCCTCC	60.1	686	INTERGENIC	--	--	--

INDEL marker IDs	Chromosomes/ unanchored scaffolds	Physical positions (bp)	InDels (*Kabuli* reference genome-CDC Frontier/PII)	Forward primers (5'-3')	Reverse primers (5'-3')	Anneling temperature (0C)	Expected amplified product size (bp)	Structural annotation		Functional annotation	
								Sequence components of *kabuli* genome	*Kabuli* gene accession IDs	NCBI-KOG	TFs
Ca1_P4_2845159	Ca1	2845159	TAAAAAAAA/TAAAAAAA	CCACCTTTCAAAGACGAAACA	GCTTGATTTCAAAATCTCCTCC	60.1	686	INTERGENIC	--	--	--
Ca1_P4_2845363	Ca1	2845363	AT/A	TTTGTCATTTAATTCACGCATTC	GCTTGATTTCAAAATCTCCTCC	59.0	376	INTERGENIC	--	--	--
Ca1_P4_28458887	Ca1	28458887	AAATAATAATAATAATAATAATAATA/AAATAATAATAATAATAATAATAATAATAATA	CGTCGTTTGTTTTGACACATC	CGTTGTTTGTTTCGACACATCT	59.1	612	INTERGENIC	--	--	--
Ca1_P4_2846167	Ca1	2846167	AAT/A	GTTTGAACACGGGTGCTTTT	ATTTTAGACGCTTGCCCTCA	60.0	165	INTERGENIC	--	--	--
Ca1_P4_2846214	Ca1	2846214	TT/TTGT	GTTTGAACACGGGTGCTTTT	ATTTTAGACGCTTGCCCTCA	60.0	165	INTERGENIC	--	--	--
Ca1_P4_28550132	Ca1	28550132	ATTTTTTTTTT/ATTTTTTT	TGGAAGCACGCTTAAGGACT	TTTCCCAATCGATTTGCTTC	60.0	664	INTERGENIC	--	--	--
Ca1_P4_28550408	Ca1	28550408	AT/ATT	GAAGCAAATCGATTGGGAAA	ACGCTCAGAGTTCACTGGGT	60.0	538	INTERGENIC	--	--	--
Ca1_P4_2861050	Ca1	2861050	CGTAGTAG/CGTAG	ACATCTCAATTGATGTCTCAAAAA	GATGAAGCCACTAATGGGGA	57.8	505	INTERGENIC	--	--	--
Ca1_P4_28638034	Ca1	28638034	ATATTATTATTATTATTATTATTATTATTATTATTATTATTATT/ATATTATTATTATTATTATTATTATTATTATTATTATTATTATTATTATT	TGGCAGGAAATGTTGTGTGT	TTCGTTTTGCCAATCATCAA	60.0	597	INTERGENIC	--	--	--
Ca1_P4_2867984	Ca1	2867984	AAT/AATGAT	ATGAGAAAGGGGAAGAGGGA	AAGGGGTGAACAGTGAGGTG	60.0	646	INTERGENIC	--	--	--

INDEL marker IDs	Chromosomes/ unanchored scaffolds	Physical positions (bp)	InDels (*Kabuli* reference genome-CDC Frontier/PII)	Forward primers (5'-3')	Reverse primers (5'-3')	Anneling temperature (0C)	Expected amplified product size (bp)	Structural annotation		Functional annotation	
								Sequence components of *kabuli* genome	*Kabuli* gene accession IDs	NCBI-KOG	TFs
Ca1_P4_28692976	Ca1	28692976	TCC/TC	CGTTTTTCAAAGCGAGGGTA	GCCCTCAGATGAGAGTCAGG	60.2	450	INTERGENIC	--	--	--
Ca1_P4_28711868	Ca1	28711868	TAGAGAGAGAGAGAGAGAGAG/TAGAGAGAGAGAGAGAGAG	TGTTGGGTTGACAAAAACCA	TTAATGGGGAAGCACAAAGC	59.8	532	INTERGENIC	--	--	--
Ca1_P4_28713213	Ca1	28713213	TTA/TTAGAAAGAGTGTA	TTCATTTCTCTGATTCCCCG	TCCGCATTATAACCTCTGCC	60.0	425	INTERGENIC	--	--	--
Ca1_P4_28903274	Ca1	28903274	TAAAAAA/TAAAAAAA	GGGGGTTAGGATGTGGTAAAA	TTATTGGTTGCCCTCCATTC	59.9	862	INTERGENIC	--	--	--
Ca1_P4_28905129	Ca1	28905129	TCTCCT/TCT	TGTTGCATTGGCGTTTCTTA	CGTGGGTTGCCATCTAGTTT	60.3	220	INTERGENIC	--	--	--
Ca1_P4_28906702	Ca1	28906702	TAAAAAAA/TAAAAAA	TATGGAGAGCAGCGACTCAA	TGTTGGGTTGCACAACATTC	59.7	184	INTERGENIC	--	--	--
Ca1_P4_28929600	Ca1	28929600	GTTTTTTT/GTTTTTT	AAATTCGAACTGGGCATTTG	TGAAGGCACCATGTCTTTGA	59.9	750	INTERGENIC	--	--	--
Ca1_P4_28930406	Ca1	28930406	T/TA	TCAAAGACATGGTGCCTTCA	TTGGTGCCAATTTATGTATTTCA	60.2	605	INTERGENIC	--	--	--
Ca1_P4_28930954	Ca1	28930954	A/ATT	TGAAAAGTTTCTTTGTCCCTTTG	TGGTGTTGCCTTCAAATTCA	59.7	349	INTERGENIC	--	--	--
Ca1_P4_28935215	Ca1	28935215	ATTTTTTTT/ATTTTTTTTT	TTTTGTTGGCAGTTTGTTTG	CAGTCAACATTGATGTGACGTTAT	57.3	490	INTERGENIC	--	--	--

INDEL marker IDs	Chromosomes/ unanchored scaffolds	Physical positions (bp)	InDels (*Kabuli* reference genome-CDC Frontier/PII)	Forward primers (5'-3')	Reverse primers (5'-3')	Anneling temperature (0C)	Expected amplified product size (bp)	Structural annotation		Functional annotation	
								Sequence components of *kabuli* genome	*Kabuli* gene accession IDs	NCBI-KOG	TFs
Ca1_P4_28939471	Ca1	28939471	AAAATAAAACAAA/AAAA	CCATGTGTGAACCCTAATTGTT	GTTTTGGCGTTGGTTTCACT	58.7	226	INTERGENIC	--	--	--
Ca1_P4_28941503	Ca1	28941503	CAA/CAAAA	TGGGGTTTAGGGTGTCACAT	CTTTTGTAAAGGCAGCTCCG	60.1	207	INTERGENIC	--	--	--
Ca1_P4_28941872	Ca1	28941872	GAAAAAAA/GAAAAAAAAAA	TGGGGTTTAGGGTGTCACAT	TCAATTTGGATTTCTTGGTGG	60.1	847	INTERGENIC	--	--	--
Ca1_P4_28943560	Ca1	28943560	GG/GGTG	ACTTGGGACAAATCACATGG	TGATCACCAGCTTTCCAATG	58.3	252	INTERGENIC	--	--	--
Ca1_P4_28944045	Ca1	28944045	TAAAAAA/TAAAAA	TTGCCTTTAATGGACCAAGG	CAATATGAAGTCTGGTGCCAA	59.9	345	INTERGENIC	--	--	--
Ca1_P4_28944496	Ca1	28944496	TTATATATAT/TTATATAT	TTGGCACCAGACTTCATATTG	AAATGTACTTTGCGGCACAT	58.6	775	INTERGENIC	--	--	--
Ca1_P4_29124803	Ca1	29124803	ATTT/ATTTT	AGTGGCATTGGGCAAATTAC	ACCAAATATACGCGTCTGCC	59.8	350	INTERGENIC	--	--	--
Ca1_P4_2912876	Ca1	2912876	GGTAC/GGTACGTAC	TGTCATCCCTAGCAACACGA	TGTAATGCATTTCCCCTTGTC	60.3	192	INTERGENIC	--	--	--
Ca1_P4_29161382	Ca1	29161382	ATT/AT	TTCCACCAAGATGGAACAAA	TTAGAAGGAAGGGGAAACTCA	58.9	331	INTERGENIC	--	--	--
Ca1_P4_2918789	Ca1	2918789	GTTATTATTAT/GTTATTATTATTAT	TTGCAGGCAGGCTTAGACTT	TTCCATGTTCCCTTTGCTTC	60.2	770	INTERGENIC	--	--	--

INDEL marker IDs	Chromosomes/ unanchored scaffolds	Physical positions (bp)	InDels (*Kabuli* reference genome-CDC Frontier/PII)	Forward primers (5'-3')	Reverse primers (5'-3')	Anneling temperature (0C)	Expected amplified product size (bp)	Structural annotation		Functional annotation	
								Sequence components of *kabuli* genome	*Kabuli* gene accession IDs	NCBI-KOG	TFs
Ca1_P4_2919131	Ca1	2919131	CAAAAAAAAAAA/CAAAAAAAAAA	GTATCCCATCCCCTGCAATA	TTCCATGTTCCCTTTGCTTC	59.6	202	INTERGENIC	--	--	--
Ca1_P4_2920885	Ca1	2920885	TTTAAT/TTTAATTAAT	ATTGCCATGGATTTGACGTT	GAATTGAGGAACCAAAAATGAAA	60.2	542	INTERGENIC	--	--	--
Ca1_P4_2921722	Ca1	2921722	CAATA/CA	CAACTGGGCCAAATGCTTAT	TGATGATCACACGAGGGAGA	60.0	570	INTERGENIC	--	--	--
Ca1_P4_2921905	Ca1	2921905	ATTT/ATT	CAACTGGGCCAAATGCTTAT	TGATGATCACACGAGGGAGA	60.0	570	INTERGENIC	--	--	--
Ca1_P4_2922949	Ca1	2922949	CTTTTTTTTTTTT/CTTTTTTTTTTT	TTTGTGGATCGTATTGCGAA	AGCATATGGTCCCCTTTTCA	60.1	947	INTERGENIC	--	--	--
Ca1_P4_2923053	Ca1	2923053	TATGAA/TATGAATGAATGAA	CGCGAAATCTTATTTTCATGG	TGATGCGATTGTAGTCCCAA	59.6	606	INTERGENIC	--	--	--
Ca1_P4_2923274	Ca1	2923274	A/ATATTAAAATAAT	CGCGAAATCTTATTTTCATGG	TGATGCGATTGTAGTCCCAA	59.6	606	INTERGENIC	--	--	--
Ca1_P4_2923894	Ca1	2923894	TAAAAAAAAA/TAAAAAAAA	TTGGGACTACAATCGCATCA	ATTGACCCGATTTGCTGAAC	60.1	718	INTERGENIC	--	--	--
Ca1_P4_2923992	Ca1	2923992	TTA/T	TTGGGACTACAATCGCATCA	ATTGACCCGATTTGCTGAAC	60.1	718	INTERGENIC	--	--	--
Ca1_P4_2924550	Ca1	2924550	CTT/CTTT	GTTCAGCAAATCGGGTCAAT	TGAGTTAATGCGGCCTAACC	59.9	595	INTERGENIC	--	--	--

INDEL marker IDs	Chromosomes/ unanchored scaffolds	Physical positions (bp)	InDels (*Kabuli* reference genome-CDC Frontier/PII)	Forward primers (5'-3')	Reverse primers (5'-3')	Anneling temperature (0C)	Expected amplified product size (bp)	Structural annotation		Functional annotation	
								Sequence components of *kabuli* genome	*Kabuli* gene accession IDs	NCBI-KOG	TFs
Ca1_P4_2924687	Ca1	2924687	TAAAAAAAAAAAA/TAAAAAAAAAAA	GTTCAGCAAATCGGGTCAAT	TGAGTTAATGCGGCCTAACC	59.9	595	INTERGENIC	--	--	--
Ca1_P4_29343809	Ca1	29343809	AATA/AATATA	AACTTTCGGTGCAAACAACC	TGGAATAAACCACATGCGAG	60.0	392	INTERGENIC	--	--	--
Ca1_P4_2937855	Ca1	2937855	G/GT	CGGATGGAACAAGTGGAAGT	CGAACTCAATCTTCTATTCGCC	60.0	933	INTERGENIC	--	--	--
Ca1_P4_2939248	Ca1	2939248	GTCT/GT	TTGTCCCTCGAACTTTCCTT	GCAACCGTGTGGATATGTTG	58.8	112	INTERGENIC	--	--	--
Ca1_P4_29402165	Ca1	29402165	TG/TGTAG	GGCCGTAAACCAGACTCAAA	GGGGTAAGTTTCCTTGACCA	60.1	627	INTERGENIC	--	--	--
Ca1_P4_29511714	Ca1	29511714	GTATATATATATATATATATATATATATA/GTATATATATATATATATATATATATATATATA	CGGTCACCACTAACATCGTG	CGCACTTGAGGAGTTGGATT	60.0	812	INTERGENIC	--	--	--
Ca1_P4_29524255	Ca1	29524255	ATTTTTTTTTTT/ATTTTTTTTTTTTT	GGCTTCTTTCCAAAATGCAG	CACCATATTCCCCCAAAACA	59.8	669	INTERGENIC	--	--	--
Ca1_P4_2955757	Ca1	2955757	CTTTTTTTT/CTTTTTT	CCTCCACTCATTTTCTCCCA	TTATCATTCTTCCCCCACCA	60.0	454	INTERGENIC	--	--	--
Ca1_P4_2956307	Ca1	2956307	TAAA/TAA	TGGTGGGGGAAGAATGATAA	GCAACTTCCTTTGAGATGGG	60.1	348	INTERGENIC	--	--	--
Ca1_P4_2956674	Ca1	2956674	TTATATATAT/TTATAT	CCCATCTCAAAGGAAGTTGC	CCACTGCTGCGCTATTATGA	59.7	641	INTERGENIC	--	--	--

INDEL marker IDs	Chromosomes/ unanchored scaffolds	Physical positions (bp)	InDels (*Kabuli* reference genome-CDC Frontier/PII)	Forward primers (5'-3')	Reverse primers (5'-3')	Anneling temperature (0C)	Expected amplified product size (bp)	Structural annotation		Functional annotation	
								Sequence components of *kabuli* genome	*Kabuli* gene accession IDs	NCBI-KOG	TFs
Ca1_P4_2958127	Ca1	2958127	CTTTTTTTTT/CTTTTTTTT	GCTCTCAAAAGCTTCCCGTA	TTGAACAATCAACTTGTTGCG	59.6	656	INTERGENIC	--	--	--
Ca1_P4_29583858	Ca1	29583858	GAAAAAAAAAAA/GAAAAAAAAAA	GGGGTGCTCAACTTGTTTGT	TAACATGGCCAAGGTAAGGG	60.0	242	INTERGENIC	--	--	--
Ca1_P4_2959912	Ca1	2959912	T/TTTTTTTTA	TGTTATGATCCGTAAACGATGG	TTGCCGCCAATAAATATTAAAAA	59.7	554	INTERGENIC	--	--	--
Ca1_P4_2960581	Ca1	2960581	ATTTTTTTT/ATTTTTTT	TCTGTGATAAGGGTCTATCCCG	AAAACGTGTGGAAAATATTTATAATCG	60.3	374	INTERGENIC	--	--	--
Ca1_P4_2960620	Ca1	2960620	ATTTTTTTT/ATTTTTTTTT	TCTGTGATAAGGGTCTATCCCG	AAAACGTGTGGAAAATATTTATAATCG	60.3	374	INTERGENIC	--	--	--
Ca1_P4_2960960	Ca1	2960960	GTTTTTTTT/GTTTTTTTTT	TTGTCAAATATTGATTTCGTGTTTTT	TGGCACATATTTGTCACTCATAA	60.0	741	INTERGENIC	--	--	--
Ca1_P4_2961037	Ca1	2961037	ATTTTTTTTTTTTTTT/ATTTTTTTTTTTTTT	TTGTCAAATATTGATTTCGTGTTTTT	TGGCACATATTTGTCACTCATAA	60.0	741	INTERGENIC	--	--	--
Ca1_P4_2961596	Ca1	2961596	ATTTTTTT/ATTTTTT	CATGTGATTTTATGTGTCAGCG	TGGAACACATAATTTTCACCGA	59.1	156	INTERGENIC	--	--	--
Ca1_P4_2961815	Ca1	2961815	A/AAT	TCGGTGAAAATTATGTGTTCCA	TTATCTTACCCCTTGCACCG	60.2	629	INTERGENIC	--	--	--
Ca1_P4_2962103	Ca1	2962103	GTTTTTTTTT/GTTTTTTT	TCGGTGAAAATTATGTGTTCCA	TTATCTTACCCCTTGCACCG	60.2	629	INTERGENIC	--	--	--

INDEL marker IDs	Chromosomes/ unanchored scaffolds	Physical positions (bp)	InDels (*Kabuli* reference genome-CDC Frontier/PII)	Forward primers (5'-3')	Reverse primers (5'-3')	Anneling temperature (0C)	Expected amplified product size (bp)	Structural annotation		Functional annotation	
								Sequence components of *kabuli* genome	*Kabuli* gene accession IDs	NCBI-KOG	TFs
Ca1_P4_2963212	Ca1	2963212	TA/TACAA	CATCGACCATCAATCACAATG	GGGTGAAGTGGGATGATTTG	59.8	288	INTERGENIC	--	--	--
Ca1_P4_2966165	Ca1	2966165	A/AC	CCACGTCTGTAAATTCGCTTT	TGACGTTGTTTTGTCTGTCG	59.3	592	INTERGENIC	--	--	--
Ca1_P4_2966305	Ca1	2966305	T/TTC	CCACGTCTGTAAATTCGCTTT	GCTCGCCACTCTCTTTCTCT	59.3	785	INTERGENIC	--	--	--
Ca1_P4_29667241	Ca1	29667241	AACACACACACACACACA/AACACACACACACACACA	CCGAGCCAAAATCCAAAATA	TTGGGTGCACATTTTACAGC	59.9	436	INTERGENIC	--	--	--
Ca1_P4_2969456	Ca1	2969456	CATATATATAT/CATATATAT	GCTACCAATTTGGGTGGAGA	AGAAATGCACGAAAACAGGC	59.9	580	INTERGENIC	--	--	--
Ca1_P4_2970042	Ca1	2970042	TTATAT/TTAT	GCCTGTTTTCGTGCATTTCT	GTGAGGGGATGGTGCTCTAA	60.3	608	INTERGENIC	--	--	--
Ca1_P4_2970188	Ca1	2970188	TAAAAAAA/TAAAAAAAAA	GCCTGTTTTCGTGCATTTCT	GTGAGGGGATGGTGCTCTAA	60.3	608	INTERGENIC	--	--	--
Ca1_P4_29738380	Ca1	29738380	GTATATATATATATATATATAT/GTATATATATATATATATATAT	TATTTTGGATTTTGGCTCGG	AAACCGGTAAAATCTCCGCT	59.9	699	INTERGENIC	--	--	--
Ca1_P4_29791416	Ca1	29791416	GAAAA/GAAAAA	TCCGATAAAATCTCCGTTCG	AAAAACACGTTCCCCTCCTT	60.0	323	INTERGENIC	--	--	--
Ca1_P4_29801275	Ca1	29801275	TAAAAAAAAAAA/TAAAAAAAAAA	GTTTTTCCATCTTGGTGCGT	TCACCCAAATTTATGGCATGT	60.0	573	INTERGENIC	--	--	--

INDEL marker IDs	Chromosomes/ unanchored scaffolds	Physical positions (bp)	InDels (*Kabuli* reference genome-CDC Frontier/PII)	Forward primers (5'-3')	Reverse primers (5'-3')	Anneling temperature (0C)	Expected amplified product size (bp)	Structural annotation		Functional annotation	
								Sequence components of *kabuli* genome	*Kabuli* gene accession IDs	NCBI-KOG	TFs
Ca1_P4_29806954	Ca1	29806954	ATT/ATTT	GCATGCTGCAAGGGATAGTT	CGCGCTGTTAAAAATCAAGG	60.2	423	INTERGENIC	--	--	--
Ca1_P4_29817198	Ca1	29817198	GTTTTTT/GTTTTTTTTT	TGTTAGGAGGATCTCACGGG	TTGTACAGGTGTCTGCCGAG	60.1	522	INTERGENIC	--	--	--
Ca1_P4_29818737	Ca1	29818737	ACTA/ACTAAATTACCGAGGTGCTA	CTCGGAATTCCAAACCAAAA	ATCGATGGCTCAATTGGTTC	59.9	359	INTERGENIC	--	--	--
Ca1_P4_29825526	Ca1	29825526	ATTTTT/ATTT	CAAGACTTAAGGCTCCCCCT	CCCATTTGCCTAACCAAGAA	59.7	500	INTERGENIC	--	--	--
Ca1_P4_29825613	Ca1	29825613	GCTTT/G	CAAGACTTAAGGCTCCCCCT	CCCATTTGCCTAACCAAGAA	59.7	500	INTERGENIC	--	--	--
Ca1_P4_29842423	Ca1	29842423	AC/A	TCATCAAAGGCATGCAAATC	GACCAAAACACAACGCAATG	59.6	359	INTERGENIC	--	--	--
Ca1_P4_29843804	Ca1	29843804	G/GA	ATGGATCAAGAGAAGGGGCT	CCTATGCGAAGTGGTGGTTT	60.0	535	INTERGENIC	--	--	--
Ca1_P4_29844165	Ca1	29844165	TTTATT/TTT	ATGGATCAAGAGAAGGGGCT	CCTATGCGAAGTGGTGGTTT	60.0	535	INTERGENIC	--	--	--
Ca1_P4_29853119	Ca1	29853119	TAA/TA	TCCACGAGATTTTCCTTTGG	TGTCATCATTCTCTGCTGGC	60.0	357	INTERGENIC	--	--	--
Ca1_P4_29853730	Ca1	29853730	GTGGAAGTCT/GTGGAAGTCTGGAAGTCT	TTGCATTCCTAGGTTCAGCC	TATCGCTCGACCTCTGCTTT	60.2	602	INTERGENIC	--	--	--

INDEL marker IDs	Chromosomes/ unanchored scaffolds	Physical positions (bp)	InDels (*Kabuli* reference genome-CDC Frontier/PII)	Forward primers (5'-3')	Reverse primers (5'-3')	Anneling temperature (0C)	Expected amplified product size (bp)	Structural annotation		Functional annotation	
								Sequence components of *kabuli* genome	*Kabuli* gene accession IDs	NCBI-KOG	TFs
Ca1_P4_29856647	Ca1	29856647	CTTT/CTTTT	GCATTCCCTGCTCAAGACTC	AGACAAGGGAGAGTCCCACA	60.0	812	INTERGENIC	--	--	--
Ca1_P4_2994165	Ca1	2994165	TAAAAAAAAAA/TAAAAAAAAA	CCGTGACACTCACACAAACC	CGCCATTGTTCTCTCCTCTC	60.0	841	INTERGENIC	--	--	--
Ca1_P4_29994387	Ca1	29994387	GTTTT/GTTTTT	TCCAGCTTCCTCGAAACCTA	AACAAGACCTGCGAAAATCG	59.9	480	INTERGENIC	--	--	--
Ca1_P4_30125583	Ca1	30125583	CCAAATTCAAATTCAAA/CCAAATTCAAA	GCTTGGGGACAAGCAATAGA	CTGCTTGGGCATAAGTCTCC	60.2	623	INTERGENIC	--	--	--
Ca1_P4_3014145	Ca1	3014145	ATTTTTTTT/ATTTTTTTTT	TGCGGCTATTACATTGGTGA	GTGGGAACTTTTCATGCCAG	60.1	700	INTERGENIC	--	--	--
Ca1_P4_30152959	Ca1	30152959	ACATATACCATATACCATAT/ACATATACCATAT	CGAATCATGCGCAAGAACTA	CGATCAGGTGGACAATGACA	60.0	330	INTERGENIC	--	--	--
Ca1_P4_3016722	Ca1	3016722	ATAAAATTA/ATAAAATTACTAAAATTA	TGGAAATGGAGGTAAGTCGC	TTTGATTATGAAGCATTGTCAACAT	60.1	535	INTERGENIC	--	--	--
Ca1_P4_3016741	Ca1	3016741	AATATATATATATATATA/AATATATATATATATATATA	TGGAAATGGAGGTAAGTCGC	TTTGATTATGAAGCATTGTCAACAT	60.1	535	INTERGENIC	--	--	--
Ca1_P4_3017014	Ca1	3017014	TAAAAAAAAAA/TAAAAAAAAAAAA	GGAAATGGAGGTAAGTCGCA	GGCCACATTGTGCTTCATATT	60.1	729	INTERGENIC	--	--	--
Ca1_P4_3018574	Ca1	3018574	CAATTAATTAATT/CAATTAATT	AGCCAACAGCCCAACATAAA	AAGGTTCATGTCCAAATCGC	60.5	242	INTERGENIC	--	--	--

INDEL marker IDs	Chromosomes/ unanchored scaffolds	Physical positions (bp)	InDels (*Kabuli* reference genome-CDC Frontier/PII)	Forward primers (5'-3')	Reverse primers (5'-3')	Anneling temperature (0C)	Expected amplified product size (bp)	Structural annotation		Functional annotation	
								Sequence components of *kabuli* genome	*Kabuli* gene accession IDs	NCBI-KOG	TFs
Ca1_P4_30194339	Ca1	30194339	GATAAATAAATAAATAAATAAATAAATAA/GATAAATAAATAAATAA	TGCCTTCATCTTCACTGCAC	TAGCCATTGATTCCACCCAT	60.0	838	INTERGENIC	--	--	--
Ca1_P4_3020023	Ca1	3020023	ATAATATTAATAT/ATAATATTAATATTAATAT	CAAGCAACATGCTGAAGGAA	TTTAAGCACATCTGGTCAATCA	60.0	237	INTERGENIC	--	--	--
Ca1_P4_3020354	Ca1	3020354	AGG/AGGG	CAAGCAACATGCTGAAGGAA	ATGTCCTTAGTGTCACGGGG	60.0	808	INTERGENIC	--	--	--
Ca1_P4_30207170	Ca1	30207170	CTATATATATATA/CTATATATATATATA	TGATTTACACGCAGAAAGCG	CGGAAAAACACTAAGGTGCAA	60.0	266	INTERGENIC	--	--	--
Ca1_P4_3020722	Ca1	3020722	GGCTT/G	CCCCGTGACACTAAGGACAT	GAAGGTCGCATCTCCATGAT	59.8	513	INTERGENIC	--	--	--
Ca1_P4_3020788	Ca1	3020788	TTTTA/TTTTAATTTA	CCCCGTGACACTAAGGACAT	GAAGGTCGCATCTCCATGAT	59.8	513	INTERGENIC	--	--	--
Ca1_P4_30208977	Ca1	30208977	GAAA/GACGAAAAAATATAAAA	ATGGTGTCTTTAACGGTGGG	AGTTGAGAGGCACCCTGCTA	59.7	263	INTERGENIC	--	--	--
Ca1_P4_30243449	Ca1	30243449	AGG/AG	CCAAACCAAATAAAACATTCCAA	GAACTCTGCCTTCCAGCAAC	60.0	463	INTERGENIC	--	--	--
Ca1_P4_3025881	Ca1	3025881	ATTTTTTTT/ATTTTTTT	CCCCACCCACCTTTTATTCT	GCAACCCATGAAACATTTTG	60.0	608	INTERGENIC	--	--	--
Ca1_P4_30287511	Ca1	30287511	GTTTT/GTTT	CCATCAAACATCGTCATGGA	ACTCAGATTGGTCTGTGGGG	60.3	305	INTERGENIC	--	--	--

INDEL marker IDs	Chromosomes/ unanchored scaffolds	Physical positions (bp)	InDels (*Kabuli* reference genome-CDC Frontier/PII)	Forward primers (5'-3')	Reverse primers (5'-3')	Anneling temperature (0C)	Expected amplified product size (bp)	Structural annotation		Functional annotation	
								Sequence components of *kabuli* genome	*Kabuli* gene accession IDs	NCBI-KOG	TFs
Ca1_P4_30352443	Ca1	30352443	CACA/CACAACA	TCATCTTGGGAGGTCTCGAT	TTGTGGTCTAGGCGTCAAAA	59.6	673	INTERGENIC	--	--	--
Ca1_P4_30398618	Ca1	30398618	GTTTTTTTTTT/GTTTTTTTTTTTT	TTAGGCCCAGTACAGTTCGG	GGGGTGCTCAACTTGTTTGT	60.1	901	INTERGENIC	--	--	--
Ca1_P4_3039987	Ca1	3039987	C/CAT	TTCTCCGCTAATTTCTCAACC	ATCCCCCATCTGATTTGTCA	58.5	683	INTERGENIC	--	--	--
Ca1_P4_3042228	Ca1	3042228	ATTT/ATTTT	GAGCCAAGATCGATGGGAT	TTTGTATGAACACAACCCCAAA	60.0	502	INTERGENIC	--	--	--
Ca1_P4_3043888	Ca1	3043888	GAATAAATAAATAAATAAATAA/GAATAAATAAATAAATAA	GGACCGAAATGTTGCCTAAA	GACTTGGTCAATTTGCCGAT	59.9	531	INTERGENIC	--	--	--
Ca1_P4_30452929	Ca1	30452929	TT/TTAT	GAAGAGTTACGCGGACAAGC	AGGAAGAGTGGAGTTGCGAA	60.0	768	INTERGENIC	--	--	--
Ca1_P4_30475415	Ca1	30475415	A/AC	ACGGCAAGACCCTAAAACCT	AAACTTCTACTCGGGCCCAT	60.0	906	INTERGENIC	--	--	--
Ca1_P4_30475519	Ca1	30475519	ATTT/ATTTT	AGGATGCATGGGATGAAAAG	AAACTTCTACTCGGGCCCAT	59.9	766	INTERGENIC	--	--	--
Ca1_P4_3051235	Ca1	3051235	CTTCTGA/CTTCTGAGTTCTGA	TTGTGCACACAAGTCCAACA	TTGATCCAAATTCGTGGGTT	59.8	456	INTERGENIC	--	--	--
Ca1_P4_3051311	Ca1	3051311	CAAAAAA/CAAAA	TTGTGCACACAAGTCCAACA	TTGATCCAAATTCGTGGGTT	59.8	456	INTERGENIC	--	--	--

INDEL marker IDs	Chromosomes/ unanchored scaffolds	Physical positions (bp)	InDels (*Kabuli* reference genome-CDC Frontier/PII)	Forward primers (5'-3')	Reverse primers (5'-3')	Anneling temperature (0C)	Expected amplified product size (bp)	Structural annotation		Functional annotation	
								Sequence components of *kabuli* genome	*Kabuli* gene accession IDs	NCBI-KOG	TFs
Ca1_P4_30643052	Ca1	30643052	TAAAAAAAAAAA/TAAAAAAAAAA	ACCCATGTTGGGTTCACAGT	TAGGGGTCATCTACGATGGG	60.1	382	INTERGENIC	--	--	--
Ca1_P4_30682856	Ca1	30682856	CATATATATATATATATATATAT/CATATATATATATATAT	GTTGGTTCCCTAGCACCGTA	TCCGCCTAGGAGATAGCAGA	60.0	672	INTERGENIC	--	--	--
Ca1_P4_30745547	Ca1	30745547	C/CT	TTTTTGGGTTTCCTTTTCCC	TGCAATATGTATCGTGGATGG	60.1	817	INTERGENIC	--	--	--
Ca1_P4_30755262	Ca1	30755262	CAAAAAAAAAAA/CAAAAAAAAAA	AAGAGAGGTCACTTTGGCGA	TGACTGAGACAGCTCGATGG	60.0	489	INTERGENIC	--	--	--
Ca1_P4_30768111	Ca1	30768111	GAAAAA/GAAAAAA	TCCGGTATAAAAGGTTTCCG	CCGTTGAAAACAACACATGAA	58.9	482	INTERGENIC	--	--	--
Ca1_P4_30769862	Ca1	30769862	TAAAAAA/TAAAAAAAA	ATGGGTCTTGGAAACACTCG	ACCAACTCCTCCAAGCCACT	60.0	354	INTERGENIC	--	--	--
Ca1_P4_30770184	Ca1	30770184	TAAA/TAAAA	AGTGGCTTGGAGGAGTTGGT	CGTTCGGCTTTCTTCTTCTG	61.1	535	INTERGENIC	--	--	--
Ca1_P4_30789723	Ca1	30789723	AC/A	TGCTTTTCAAGGGAGGAATG	CTCCAGTTTTGGTCCCTCAA	60.2	695	INTERGENIC	--	--	--
Ca1_P4_30790839	Ca1	30790839	TGTAAC/T	TTTTTGATGGTTCAACGAGAA	GGTTGAATAATTGAAAAGAGGTGG	58.3	250	INTERGENIC	--	--	--
Ca1_P4_30808046	Ca1	30808046	GAA/GAAATAA	ACAACCCGAGTGATCTCCAC	TAGGGTGCTTGACACGTGAG	60.0	701	INTERGENIC	--	--	--

INDEL marker IDs	Chromosomes/ unanchored scaffolds	Physical positions (bp)	InDels (*Kabuli* reference genome-CDC Frontier/PII)	Forward primers (5'-3')	Reverse primers (5'-3')	Anneling temperature (0C)	Expected amplified product size (bp)	Structural annotation		Functional annotation	
								Sequence components of *kabuli* genome	*Kabuli* gene accession IDs	NCBI-KOG	TFs
Ca1_P4_30844081	Ca1	30844081	AATATATATATATATATA/AATATATATATATATATATA	TGAAAAGGGATGGAATGGTT	AAGCATTATGAAGATGACGCC	59.2	387	INTERGENIC	--	--	--
Ca1_P4_3089236	Ca1	3089236	ATTT/ATT	GCAAAACGTCTTATCGGGAA	TGCAAACTATTTGACATTGACG	60.1	591	INTERGENIC	--	--	--
Ca1_P4_3093464	Ca1	3093464	CATATATATATAT/CATATATATATATATAT	ATATATAGGCAGGGGCGGAC	AATTGAACCCAAGTGCAGGT	60.2	481	INTERGENIC	--	--	--
Ca1_P4_30938741	Ca1	30938741	CTTTTT/CTTTT	TCTTCCACCCAGTTTTCACC	AAAGCATCTCACACAGCACG	59.9	561	INTERGENIC	--	--	--
Ca1_P4_3096084	Ca1	3096084	T/TC	ACCACCTTATTTGTGGCTGC	CCAAAATGCTCCGCTATGTT	60.0	346	INTERGENIC	--	--	--
Ca1_P4_3096580	Ca1	3096580	TAAAAAA/TAAAAAAA	AACATAGCGGAGCATTTTGG	CATGGTAGCCCAAATCCAAT	60.1	511	INTERGENIC	--	--	--
Ca1_P4_30969929	Ca1	30969929	ATCAATTTCAATTT/ATCAATTT	CATGTTGGATACACATGCTTTG	TTCAGCAGATTTGCGACAAC	59.0	969	INTERGENIC	--	--	--
Ca1_P4_30993040	Ca1	30993040	AAAAATTAAAATTAAAATTAA/AAAAATTAAAATTAAAATTAAAATTAA	TCCTTCCGATCGAGTAGTGG	AAACATGAAATGGACAATACAAAAA	60.2	398	INTERGENIC	--	--	--
Ca1_P4_31003439	Ca1	31003439	GGGTCGGACCGGGCCGG/GGGTCGGACCGGGCCGGTCGGACCGGGCCGG	CCCCAGGATAAAACAGCTCA	AGCAAATTGACCACCACACA	60.1	220	INTERGENIC	--	--	--
Ca1_P4_31004479	Ca1	31004479	GTATATATATATATATATATA/GTATATATATATATATATA	ATAAAATGGGGGTTGGGAAA	TCAACGGTGGAGGATAGTGA	60.2	396	INTERGENIC	--	--	--

INDEL marker IDs	Chromosomes/ unanchored scaffolds	Physical positions (bp)	InDels (*Kabuli* reference genome-CDC Frontier/PII)	Forward primers (5'-3')	Reverse primers (5'-3')	Anneling temperature (0C)	Expected amplified product size (bp)	Structural annotation		Functional annotation	
								Sequence components of *kabuli* genome	*Kabuli* gene accession IDs	NCBI-KOG	TFs
Ca1_P4_31005058	Ca1	31005058	AC/A	CACTATCCTCCACCGTTGAAA	GCCTTAATTATCGCTCCTCAAA	60.0	722	INTERGENIC	--	--	--
Ca1_P4_31014910	Ca1	31014910	TTTTTTATTT/TTTTTTATTTTTATTTCATTTTTATTT	GGAGCATGAAGCAACCTCTC	TGATGAATCTGTGTGTCCATGA	60.0	199	INTERGENIC	--	--	--
Ca1_P4_31022339	Ca1	31022339	GTTTT/GTTT	GTTTCAACCGCATCCAATTT	TCCCAATCGAATCTACGAGG	59.8	442	INTERGENIC	--	--	--
Ca1_P4_31028139	Ca1	31028139	TAAAAAAAAAAAA/TAAAAAAAAAAAAAA	ACGCAAAATGGCTTACGTTC	CATGACCCTCCCTTTTCGTA	60.1	415	INTERGENIC	--	--	--
Ca1_P4_31072463	Ca1	31072463	ATT/AT	CGCATGTGACCTACATGGTT	CTCTATACCCGAGCTGAGCG	59.4	282	INTERGENIC	--	--	--
Ca1_P4_31072943	Ca1	31072943	TAAAAAAA/TAAAAAA	CGCTCAGCTCGGGTATAGAG	CAAACCGGATGTGACAGTTG	60.1	615	INTERGENIC	--	--	--
Ca1_P4_3107570	Ca1	3107570	ATGTGTGTG/ATGTGTGTGTG	TCCTTGCACGCTTTTAATCA	AAACGTGATCGAACGAATCC	59.4	440	INTERGENIC	--	--	--
Ca1_P4_3108095	Ca1	3108095	GAAAAAAAA/GAAAAAAAAAA	GGATTCGTTCGATCACGTTT	ACGGGCAAAGTCAACATAGG	59.9	592	INTERGENIC	--	--	--
Ca1_P4_31093242	Ca1	31093242	ATTTTTTTTTT/ATTTTTTTTTTT	TTTTTAGGAGAGCGGCGTTA	TTGATCAAGCTCCCAATTATCA	60.0	597	INTERGENIC	--	--	--
Ca1_P4_31095231	Ca1	31095231	TTTATTAT/TTTAT	GATACATTATGCACGCCTCG	TGACGATGGTTTTGGGTTTT	59.2	506	INTERGENIC	--	--	--

INDEL marker IDs	Chromosomes/ unanchored scaffolds	Physical positions (bp)	InDels (*Kabuli* reference genome-CDC Frontier/PII)	Forward primers (5'-3')	Reverse primers (5'-3')	Anneling temperature (0C)	Expected amplified product size (bp)	Structural annotation		Functional annotation	
								Sequence components of *kabuli* genome	*Kabuli* gene accession IDs	NCBI-KOG	TFs
Ca1_P4_3110033	Ca1	3110033	ATTTTTTTTTTT/ATTTTTTTTTT	TGTCTACAATGGTGTTTGGCA	TCGAGCAAGTGAGAAATGGA	60.0	877	INTERGENIC	--	--	--
Ca1_P4_31117965	Ca1	31117965	AATATA/AATA	GGGACATCAGAATGCTCACA	TGCGACACACGAATTACACA	59.6	536	INTERGENIC	--	--	--
Ca1_P4_3112331	Ca1	3112331	AC/A	AATCTTCCACGGAACAATCAA	GGGAGATGGAAATTGGGAAT	59.4	877	INTERGENIC	--	--	--
Ca1_P4_31141128	Ca1	31141128	C/CA	ATTATTGAGGTTGGCCCCTC	TCCACTTTTTGTGCTTTAGGC	60.2	376	INTERGENIC	--	--	--
Ca1_P4_31162887	Ca1	31162887	T/TG	CCGGGGATTGCCATATAACT	CTAATAGGCTGCATCGGAGG	60.9	342	INTERGENIC	--	--	--
Ca1_P4_31163466	Ca1	31163466	T/TAGA	ACTTTTGCGGAGAAGCACAT	CGTTCGTCTTTTCCTCTTGC	59.9	360	INTERGENIC	--	--	--
Ca1_P4_3118706	Ca1	3118706	GTTAC/GTTACTTAC	CCAAATGAATGCTTCGGTCT	AGCATTCACAGGCAAACAAA	60.1	799	INTERGENIC	--	--	--
Ca1_P4_31212342	Ca1	31212342	CTTTTTTTTTTTT/CTTTTTTTTTT	GCCAAAAATGCCATTAAGGA	TGCCTCCAAATATCCTCCAA	59.9	514	INTERGENIC	--	--	--
Ca1_P4_3126822	Ca1	3126822	AATAT/AATATATTATTGATAT	TGTTGCTTGGTGTAGGGACA	CCTTTAGCCCGTGAGTCTTG	60.2	987	INTERGENIC	--	--	--
Ca1_P4_3130356	Ca1	3130356	TAAAAAAA/TAAAAAAAA	TCGTTTATCCAACCCCTTTG	CTGGTCTGGCTTGAAAACCT	59.8	736	INTERGENIC	--	--	--

INDEL marker IDs	Chromosomes/ unanchored scaffolds	Physical positions (bp)	InDels (*Kabuli* reference genome-CDC Frontier/PII)	Forward primers (5'-3')	Reverse primers (5'-3')	Anneling temperature (0C)	Expected amplified product size (bp)	Structural annotation		Functional annotation	
								Sequence components of *kabuli* genome	*Kabuli* gene accession IDs	NCBI-KOG	TFs
Ca1_P4_3131301	Ca1	3131301	TATA/TATACTCATA	GGCCAGACTCAGACCTTTCA	ACCCATTTTTCTGACAACCG	60.4	506	INTERGENIC	--	--	--
Ca1_P4_31324079	Ca1	31324079	AAA/AAATAA	TATTCCGTTGTCGAGTTCCC	AGGGATAACCTGGTGCTGTG	59.9	604	INTERGENIC	--	--	--
Ca1_P4_3133490	Ca1	3133490	AATTATATATTATA/AATTATA	CCACATTTTGCCACTTTTCA	TCGCCTTTTAAACCAACCAG	59.6	853	INTERGENIC	--	--	--
Ca1_P4_31375439	Ca1	31375439	GAAAAAAAAAAAA/GAAAAAAAAAA	GCCGTTTAGCAACAAAGAAAA	TCATCGAGAACGCATCCATA	59.4	265	INTERGENIC	--	--	--
Ca1_P4_3138107	Ca1	3138107	A/ATT	ATCGGGGTGGACAAAACATA	GGGCTTTAAATTGAGTGAGGC	60.1	683	INTERGENIC	--	--	--
Ca1_P4_31386654	Ca1	31386654	TG/TGAAG	TTTCAACCGCTCAACATCAA	TGTCGCGCTACCTTTCTTCT	60.2	445	INTERGENIC	--	--	--
Ca1_P4_31401462	Ca1	31401462	CTATATTATATA/CTATA	ATTCGTAGTAGGCCATGCCA	TTTGCGGCCTTCAAAATATC	60.5	501	INTERGENIC	--	--	--
Ca1_P4_31422768	Ca1	31422768	ATTTTTTTTTT/ATTTTTTTTT	CTCCTTCAAAAGCACAAGCC	GTTGCTCCGACGAAACAAGT	60.0	243	INTERGENIC	--	--	--
Ca1_P4_31477691	Ca1	31477691	CAAAA/CAAAAA	AAGGGAAACGGGTACCAAAC	ACCCGTAAACCCCAAATCTC	60.1	629	INTERGENIC	--	--	--
Ca1_P4_31481152	Ca1	31481152	GT/G	GCAAATGCAACTCTGAAAACTG	GTTATACCGCCAGCTGCATT	59.9	535	INTERGENIC	--	--	--

INDEL marker IDs	Chromosomes/ unanchored scaffolds	Physical positions (bp)	InDels (*Kabuli* reference genome-CDC Frontier/PII)	Forward primers (5'-3')	Reverse primers (5'-3')	Anneling temperature (0C)	Expected amplified product size (bp)	Structural annotation		Functional annotation	
								Sequence components of ***kabuli*** genome	***Kabuli*** gene accession IDs	NCBI-KOG	TFs
Ca1_P4_31505143	Ca1	31505143	GTATATATATATATATATATA/GTATATATATATATATATA	TCCCATTCATATTTATTGCGG	CATTTGACTATGCCACTAAATGC	59.6	831	INTERGENIC	--	--	--
Ca1_P4_3156637	Ca1	3156637	GTTTTTT/GTTTTTTT	GCATGTGAGACCCAGCCTAT	CCTTCATGTCCACATACAATTTC	60.1	829	INTERGENIC	--	--	--
Ca1_P4_3156701	Ca1	3156701	ATTTTTTT/ATTTTTT	TGGCTGTGATATTGTCGCAT	CCTTCATGTCCACATACAATTTC	60.1	696	INTERGENIC	--	--	--
Ca1_P4_3156764	Ca1	3156764	TAA/TAAA	TGGCTGTGATATTGTCGCAT	CCTTCATGTCCACATACAATTTC	60.1	696	INTERGENIC	--	--	--
Ca1_P4_31597098	Ca1	31597098	TAAAAAAAA/TAAAAAAAAA	GCCAAAGCCTTTCACCTTCT	CGAAAAGTTTGGAGAGTGGAA	60.7	626	INTERGENIC	--	--	--
Ca1_P4_31600847	Ca1	31600847	TG/T	AAGGTAGTGAAAGAGGGGCTG	AGGATCATGTTTGTGGTTGC	59.8	669	INTERGENIC	--	--	--
Ca1_P4_31602245	Ca1	31602245	TTATATATATATATATATATATA/TTATATATATATATATATA	TTTCGTGTGCGTCAAATCAT	AGAGGCACACGGGTCTAATG	60.1	756	INTERGENIC	--	--	--
Ca1_P4_31627142	Ca1	31627142	TAAAAAAAA/TAAAAAAA	CTTCGAATCCAACCTTGGTG	CCGGAACCACATTCAGACTT	60.5	892	INTERGENIC	--	--	--
Ca1_P4_31645800	Ca1	31645800	GTTTTTTTTTTT/GTTTTTTTTTTTTTTT	ATGGAGAAATCCCCCAAATC	TGTTTCAACCAATGTGGGAA	60.0	501	INTERGENIC	--	--	--
Ca1_P4_31716302	Ca1	31716302	A/AC	TCGGTATTTGCCATGAGACA	AAACAACCTTCATGCCTTGG	60.1	482	INTERGENIC	--	--	--

INDEL marker IDs	Chromosomes/ unanchored scaffolds	Physical positions (bp)	InDels (*Kabuli* reference genome-CDC Frontier/PII)	Forward primers (5'-3')	Reverse primers (5'-3')	Anneling temperature (0C)	Expected amplified product size (bp)	Structural annotation		Functional annotation	
								Sequence components of *kabuli* genome	*Kabuli* gene accession IDs	NCBI-KOG	TFs
Ca1_P4_31768486	Ca1	31768486	TT/TTTTTTAT	ACACATTATATTAAATGCGCGA	TGCCAAACGATAGGTTGTTG	57.4	646	INTERGENIC	--	--	--
Ca1_P4_31783488	Ca1	31783488	AATATATATATATATATATATATAT/AATATATATATATATAT	TGGATTGGGCTGGAATTAAA	TATAACCGACGAGGGACGAG	60.3	466	INTERGENIC	--	--	--
Ca1_P4_31783811	Ca1	31783811	TTAATAATAATAATAATAATAATAATAATAATAAT/TTAATAATAATAATAATAATAATAATAATAATAAT	ATACTGCCTTCGATCCGTTG	CGGGAAAAAGGGTGGTAAAT	60.1	477	INTERGENIC	--	--	--
Ca1_P4_31823620	Ca1	31823620	ATTT/ATT	ATGCATGTTTGGCATATGGA	TTGGAAGGAGCCCTTACCTT	59.8	854	INTERGENIC	--	--	--
Ca1_P4_31995444	Ca1	31995444	ATCTTC/ATCTTCTTC	TGCTTTGAAGACCATGTGGA	AAAAGGATTCATGCGTGTCC	60.2	354	INTERGENIC	--	--	--
Ca1_P4_31995500	Ca1	31995500	CTTT/CTTTT	TGCTTTGAAGACCATGTGGA	AAAAGGATTCATGCGTGTCC	60.2	354	INTERGENIC	--	--	--
Ca1_P4_32037334	Ca1	32037334	CTTTTT/CTTTT	TCCAGTCATGTGGCTTCTCA	CTGAGCTTGTGACCAAATCG	60.4	559	INTERGENIC	--	--	--
Ca1_P4_32037353	Ca1	32037353	CTTCTTTTT/CTTCTTTTTCTTTTT	TCCAGTCATGTGGCTTCTCA	CTGAGCTTGTGACCAAATCG	60.4	559	INTERGENIC	--	--	--
Ca1_P4_3213987	Ca1	3213987	TTATATATATATATATATATATA/TTATATATATATATATATATA	TCGGCAATGTGGTACTGAAA	TTGCAAATGCGACTTTCTTG	60.1	344	INTERGENIC	--	--	--
Ca1_P4_32140100	Ca1	32140100	TT/TTATTAT	GGCCAAAACCCTAATTGACA	TGGCAAAGGGGAAGTCAGTA	59.8	505	INTERGENIC	--	--	--

INDEL marker IDs	Chromosomes/ unanchored scaffolds	Physical positions (bp)	InDels (*Kabuli* reference genome-CDC Frontier/PII)	Forward primers (5'-3')	Reverse primers (5'-3')	Anneling temperature (0C)	Expected amplified product size (bp)	Structural annotation		Functional annotation	
								Sequence components of *kabuli* genome	*Kabuli* gene accession IDs	NCBI-KOG	TFs
Ca1_P4_32147050	Ca1	32147050	T/TA	CGGCCTCCAAATCTTTAACA	TTGTCATGAAAGTCTCGCCT	60.1	670	INTERGENIC	--	--	--
Ca1_P4_32151710	Ca1	32151710	TAAAAAAAAAAA/TAAAAAAAAAAAAAAAA	TGAAGGTGTGAGCAGATTCG	TCGGGTGGAGAATTCCTATG	60.0	593	INTERGENIC	--	--	--
Ca1_P4_32153452	Ca1	32153452	TTTATTTTAT/TTTATTTTATATTATTTTAT	GTTTTGGCGTTGGTTTCACT	TGGGTTTGACCCTATGTGTG	60.0	353	INTERGENIC	--	--	--
Ca1_P4_32155872	Ca1	32155872	TTATATATATATATATATATATATA/TTATATATATATATATATATATATATATATATATA	TTACCCAAGATGGAGGAAAAA	TGTTGTCCCTTGTGCTGGTA	58.5	554	INTERGENIC	--	--	--
Ca1_P4_32176200	Ca1	32176200	CAAAA/CAAAAA	CCCGTCACTTGGAGACAAAT	ACTCTCCACTGGAATGGTGG	60.0	636	INTERGENIC	--	--	--
Ca1_P4_32203101	Ca1	32203101	CAAA/CAA	CCACGTTCTTGACCCATTTC	CCTCTTTTGCAACCTCCTTG	60.4	352	INTERGENIC	--	--	--
Ca1_P4_32203178	Ca1	32203178	TATGA/TATGAATGA	GTTCCAAAGGTAATGCCCAA	CCTCTTTTGCAACCTCCTTG	59.8	137	INTERGENIC	--	--	--
Ca1_P4_32244195	Ca1	32244195	A/AG	TGAATAGAGGGAAACGGGTG	CAGTGACCTTGTGGAGAGCA	59.9	640	INTERGENIC	--	--	--
Ca1_P4_32244737	Ca1	32244737	CCACAA/CCACAAACCCATACACAA	AATGGATCATTCCTTGCAGC	TTCAGAGATCGTGCGTGAAC	60.0	422	INTERGENIC	--	--	--
Ca1_P4_32246863	Ca1	32246863	TGG/TG	CCTGGGATCATGTTGCTTCT	AAACCTCAACCAACGTCCTG	60.1	575	INTERGENIC	--	--	--

INDEL marker IDs	Chromosomes/ unanchored scaffolds	Physical positions (bp)	InDels (*Kabuli* reference genome-CDC Frontier/PII)	Forward primers (5'-3')	Reverse primers (5'-3')	Anneling temperature (0C)	Expected amplified product size (bp)	Structural annotation		Functional annotation	
								Sequence components of *kabuli* genome	*Kabuli* gene accession IDs	NCBI-KOG	TFs
Ca1_P4_32254955	Ca1	32254955	ATT/ATTT	TAATTTTCAGCAACCCGAGG	GGGCTGGTTCCCATTAGTTA	60.1	841	INTERGENIC	--	--	--
Ca1_P4_32265930	Ca1	32265930	GATA/GA	TCCAGCATCTGTTGATTCCA	AAAGTTTTGCCCCTCCTCAT	60.2	665	INTERGENIC	--	--	--
Ca1_P4_32266454	Ca1	32266454	GAAAAAAAA/GAAAAAAAAAA	TAATGATGAGGAGGGGCAAA	GGGAGTCCGAGTGGTACAAA	60.4	450	INTERGENIC	--	--	--
Ca1_P4_32267410	Ca1	32267410	T/TTTAA	TTCGTGTTAAAATAGTGTTAGCCA	AAAAATCACATTCCTTGACATAGC	58.0	635	INTERGENIC	--	--	--
Ca1_P4_32395659	Ca1	32395659	ATTTTTTTTTTTT/ATTTTTTTTTTT	TTCGCATCAGCCATATTTTG	TCTCGGATTGCTCCGATATT	59.7	486	INTERGENIC	--	--	--
Ca1_P4_32430396	Ca1	32430396	TG/T	TCGTTTGAGCCTTTCGAACT	GCAATCACAATGCAGCAAAC	60.0	473	INTERGENIC	--	--	--
Ca1_P4_32437062	Ca1	32437062	CTATATATATATATATATATATACATATATATATATAT/CTATATATATATATATATATATATACATATATATATATAT	AGTGGAAGTGATGGGAGGTG	ACGGTAGCTTTAACGAGCGA	60.0	560	INTERGENIC	--	--	--
Ca1_P4_32454365	Ca1	32454365	A/AG	AGGAAACACATGAAGGCACC	TCTTCGTTGTCACTCCATGC	60.0	572	INTERGENIC	--	--	--
Ca1_P4_32551161	Ca1	32551161	GGAGAGAGAGAGAGAGAGAGAGAGAGAGAGAGAGAGAGA/GGAGAGAGAGAGAGAGAGAGAGAGAGAGAGAGAGAGAGAGA	TTGAACTGAAAACTTGGGCA	TTCGGCACTAGTATTGCGTG	59.3	345	INTERGENIC	--	--	--
Ca1_P4_32758604	Ca1	32758604	G/GAAAAA	TCGTTTTCGACGCTTTCTTT	TCACGGGTCAACACGAATTA	60.0	810	INTERGENIC	--	--	--

INDEL marker IDs	Chromosomes/ unanchored scaffolds	Physical positions (bp)	InDels (*Kabuli* reference genome-CDC Frontier/PII)	Forward primers (5'-3')	Reverse primers (5'-3')	Anneling temperature (0C)	Expected amplified product size (bp)	Structural annotation		Functional annotation	
								Sequence components of *kabuli* genome	*Kabuli* gene accession IDs	NCBI-KOG	TFs
Ca1_P4_32804656	Ca1	32804656	ATTTTTTTTTTT/ATTTTTTTTTTTTT	AGACCGAGCTCTAATGCCAA	GCCCTCAGATGAGAGTCAGG	60.0	875	INTERGENIC	--	--	--
Ca1_P4_32822582	Ca1	32822582	TTATATATATATATAT/TTATATATATATATATATAT	TGGATGCAAATTGGACAGAA	CGTAGCGTGGTAGTGAGCAA	60.0	745	INTERGENIC	--	--	--
Ca1_P4_32857397	Ca1	32857397	ATTTTT/ATTTT	GCGAAAAATCTCGTTCGTGT	AGAATCGAGATCGTTGGCAC	60.3	519	INTERGENIC	--	--	--
Ca1_P4_32905999	Ca1	32905999	AATATAT/AATAT	TTGCTTCGTCAGCACAATTC	TCGTATGTCATAGCGGTGCT	60.0	920	INTERGENIC	--	--	--
Ca1_P4_32906329	Ca1	32906329	TTATATATATATATATAT/TTATATATATATAT	TCATTAAATCAAAGGCACACAAA	TCGTATGTCATAGCGGTGCT	59.5	488	INTERGENIC	--	--	--
Ca1_P4_32911601	Ca1	32911601	AAATAATAATAATAA/AAATAATAATAATAATAA	CAAAATGGAGCAAGAAACAGG	CAAGTTCAACGAGCGCCTAT	59.7	892	INTERGENIC	--	--	--
Ca1_P4_32917904	Ca1	32917904	TAAAAAAAAAAAA/TAAAAAAAAAAAAAA	TCAACCGCATTAAGTGTGATG	TTGATGAGGATGAAGTTTTTCAA	59.6	711	INTERGENIC	--	--	--
Ca1_P4_32919001	Ca1	32919001	TAAAAA/TAAAAAA	AACTGCTCATGCGGTACCTT	AAGAAGGGACATTTGCGATG	59.8	714	INTERGENIC	--	--	--
Ca1_P4_32937588	Ca1	32937588	AA/AATCCCCCA	TCCCTCCTTCACAAAATTGC	TGGAGTGGGGTACCTAGTGG	60.1	498	INTERGENIC	--	--	--
Ca1_P4_32970710	Ca1	32970710	A/ATCCATCT	AACCTAGCCATCGGTGTTTG	CTCTTCGCAAACCGAAAAAG	60.0	304	INTERGENIC	--	--	--

INDEL marker IDs	Chromosomes/ unanchored scaffolds	Physical positions (bp)	InDels (*Kabuli* reference genome-CDC Frontier/PII)	Forward primers (5'-3')	Reverse primers (5'-3')	Anneling temperature (0C)	Expected amplified product size (bp)	Structural annotation		Functional annotation	
								Sequence components of *kabuli* genome	*Kabuli* gene accession IDs	NCBI-KOG	TFs
Ca1_P4_33018948	Ca1	33018948	CTT/CT	TTCGCAACTCCACTCTTCCT	CACATCATCACAACATGCCA	60.0	481	INTERGENIC	--	--	--
Ca1_P4_33034774	Ca1	33034774	AATATATATATATATA/AATATATATATA	ACCCCAACTTGTAAAGGCAA	TCACGTTGATTGAGGGATGA	59.5	618	INTERGENIC	--	--	--
Ca1_P4_33054577	Ca1	33054577	ATTTTTTTT/ATTTTTTTTT	GAGCATTGGCTGCAGTATCA	AGAATTTTGGGTTCATGTCAA	60.0	505	INTERGENIC	--	--	--
Ca1_P4_33055263	Ca1	33055263	CTTTTTTTTT/CTTTTTTTTTT	CAACAATGTTCAAGTTCAATGC	TTTTCATGCACGGATTATGC	58.2	342	INTERGENIC	--	--	--
Ca1_P4_33102523	Ca1	33102523	TTT/TTTATT	AAGGCTAAATCACATTCGTGG	TCACTTCCGTCCATGTCAAA	59.1	835	INTERGENIC	--	--	--
Ca1_P4_33134129	Ca1	33134129	CTATA/CTATATATA	CGTTGACTATTTCAATCGTTGG	CACCTGTAGTCCAACACTCGTT	59.5	265	INTERGENIC	--	--	--
Ca1_P4_33143190	Ca1	33143190	AATATATATATA/AATATATATATATA	GAGGAGGGAGGGATTTTGAG	CGGTGAATCCTTTACGGAGA	60.0	319	INTERGENIC	--	--	--
Ca1_P4_3322440	Ca1	3322440	CAAAAAAAAAAA/CAAAAAAAAAAAA	GAGCGGTACATGGTTGGTTT	ATGAACCGGTTGAATACCCA	59.9	834	INTERGENIC	--	--	--
Ca1_P4_33237244	Ca1	33237244	GATGTATGTATGTATGTATGTATGT/ GATGTATGTATGTATGTATGT	TGCTGCGAGAAATAACATCG	ACGTTGCTTTCAGAGGGAAA	60.0	550	INTERGENIC	--	--	--
Ca1_P4_33240319	Ca1	33240319	A/AT	AAACGCAATCCTTGAGTTCG	CTATCTAGCGTCGGAGTCGG	60.2	280	INTERGENIC	--	--	--

INDEL marker IDs	Chromosomes/ unanchored scaffolds	Physical positions (bp)	InDels (*Kabuli* reference genome-CDC Frontier/PII)	Forward primers (5'-3')	Reverse primers (5'-3')	Anneling temperature (0C)	Expected amplified product size (bp)	Structural annotation		Functional annotation	
								Sequence components of *kabuli* genome	*Kabuli* gene accession IDs	NCBI-KOG	TFs
Ca1_P4_33244510	Ca1	33244510	ATTTTTT/ATTTTTTTT	AATTCAGACGCGAAAGAGGA	CAAGTCTCCCAACTTCCCAA	60.0	221	INTERGENIC	--	--	--
Ca1_P4_33247147	Ca1	33247147	TA/T	GTGTTGGACCGGTTCTTGAT	GTCTGATGACAAAAGGGGGA	59.8	643	INTERGENIC	--	--	--
Ca1_P4_33247360	Ca1	33247360	GTTTTTATTT/GTTTTTATTTTTTATTT	TCCCCCTTTTGTCATCAGAC	CATGCCAACAACAGAAGTTGA	59.9	499	INTERGENIC	--	--	--
Ca1_P4_33269540	Ca1	33269540	AGGTTTTTGTGGGTTTTTG/AGGTTTTTGTGGGTTTTTGTGGGTTTTTG	CTCCACGTCAAAACCACCTT	CTGTCTGCACTCCACTCCAA	60.0	629	INTERGENIC	--	--	--
Ca1_P4_33286471	Ca1	33286471	ATTT/ATTTT	ATTATCAGAGAGTCCCCGGC	CAGATTGGCACCTATGAGCA	60.4	879	INTERGENIC	--	--	--
Ca1_P4_3329136	Ca1	3329136	CATAAAATAAAATAAAA/CATAAAATAAAATAAAATAAAATAAAA	TTTGATTAATGTTTTCATACGCA	CATTAATGGGTTCATTGGGC	57.4	334	INTERGENIC	--	--	--
Ca1_P4_33300564	Ca1	33300564	ATTTTTTTTTTTT/ATTTTTTTTTT	CTCAACCATCAGAGCACCAA	TTATACGGCGGAGGAATGAG	59.8	382	INTERGENIC	--	--	--
Ca1_P4_33379812	Ca1	33379812	GTTTTTTTT/GTTTTTTT	AGAATTGGTGGTTGAGGACG	TTGGGAAAGAAATCGAAAACA	60.0	588	INTERGENIC	--	--	--
Ca1_P4_33379930	Ca1	33379930	ATTTTTTT/ATTTTTTTT	GTACTGGCCAAAGTGATGGG	TTGGGAAAGAAATCGAAAACA	60.4	529	INTERGENIC	--	--	--
Ca1_P4_33401507	Ca1	33401507	AAGAGAGAG/AAGAGAGAGAG	AGCTCTTTGATGACCGTTGG	GGAGGATGGCAGACCTATGA	60.3	303	INTERGENIC	--	--	--

INDEL marker IDs	Chromosomes/ unanchored scaffolds	Physical positions (bp)	InDels (*Kabuli* reference genome-CDC Frontier/PII)	Forward primers (5'-3')	Reverse primers (5'-3')	Anneling temperature (0C)	Expected amplified product size (bp)	Structural annotation		Functional annotation	
								Sequence components of *kabuli* genome	*Kabuli* gene accession IDs	NCBI-KOG	TFs
Ca1_P4_33407768	Ca1	33407768	TA/TAA	TGGCCATCAATTAATCCACTC	GATCTGCAACTGGGGGTAAA	59.8	230	INTERGENIC	--	--	--
Ca1_P4_33408081	Ca1	33408081	TATAATAATAATAATAATAATAAT/TATAATAATAATAATAATAATAATAAT	ACTAGATGGTCGCATCCTGG	TCACAATCCACTAATGCGGA	60.1	551	INTERGENIC	--	--	--
Ca1_P4_33442658	Ca1	33442658	TAA/TA	CGTTGTACGCATGCTCAAGA	TGTTTACCGAGGACCAAAGG	61.0	824	INTERGENIC	--	--	--
Ca1_P4_33451777	Ca1	33451777	TG/T	TGCCACTTCTGTTTCTGCTG	TGCAAATTTGAAAACTGGTATTG	60.2	710	INTERGENIC	--	--	--
Ca1_P4_33559859	Ca1	33559859	GCC/GC	CAACGCCAAGAGAAATGGAT	GAAGTCGGGGTGTTACTCCA	60.1	913	INTERGENIC	--	--	--
Ca1_P4_33560144	Ca1	33560144	TAAAAAA/TAAAAA	CTCAAATTCTGTTTGGGGGA	GAAGTCGGGGTGTTACTCCA	59.9	515	INTERGENIC	--	--	--
Ca1_P4_33566643	Ca1	33566643	CCTTCTTCTTCTTCTTCTT/CCTTCTTCTTCTTCTTCTTCTT	TACCTTTGGCACATGCTTCA	TTCACAAAAACGTTGTGCATC	60.3	753	INTERGENIC	--	--	--
Ca1_P4_33597493	Ca1	33597493	ATT/ATTT	CGGCTTTGGTCCCTTCTTA	CTGGATCATGAAAGCCCTGT	60.2	498	INTERGENIC	--	--	--
Ca1_P4_33620705	Ca1	33620705	T/TC	TTCATGAACCAATGTTTTAGCC	AACAGCGGTTAACACAACACC	59.0	916	INTERGENIC	--	--	--
Ca1_P4_33653494	Ca1	33653494	TTTATTATTATTATTATTATTA/TTTATTATTATTATTATTATTATTA	TGGGCAAAATGTTAAGAACACA	AGAACAAATGCATGATTCTTGG	60.4	573	INTERGENIC	--	--	--

INDEL marker IDs	Chromosomes/ unanchored scaffolds	Physical positions (bp)	InDels (*Kabuli* reference genome-CDC Frontier/PII)	Forward primers (5'-3')	Reverse primers (5'-3')	Anneling temperature (0C)	Expected amplified product size (bp)	Structural annotation		Functional annotation	
								Sequence components of *kabuli* genome	*Kabuli* gene accession IDs	NCBI-KOG	TFs
Ca1_P4_33653780	Ca1	33653780	TAAAAAAAAAAA/TAAAAAAAAA	AAGGTGGTTTGTTTGTTCAATTT	AGAACAAATGCATGATTCTTGG	58.9	381	INTERGENIC	--	--	--
Ca1_P4_33658726	Ca1	33658726	ATTT/ATT	ATGTGAATCCCACCAGGAAA	TTGCACAGCCAAGTTCGTAG	60.2	778	INTERGENIC	--	--	--
Ca1_P4_33692847	Ca1	33692847	CATATATATATATATA/CATATATATATATATATA	AAAAAGTGGGGTGCAAACAG	TGATAAGGCAGTCGAGATCGT	60.0	536	INTERGENIC	--	--	--
Ca1_P4_33728733	Ca1	33728733	TA/TAA	ATTGGGAGAAATTGGTGACG	TGTTCATCCAACCGAGTTCA	59.8	815	INTERGENIC	--	--	--
Ca1_P4_33777775	Ca1	33777775	ATTT/ATT	CATGCTCAATACACGGATGG	AACGTTCTTGAGTGGCTTGG	59.9	870	INTERGENIC	--	--	--
Ca1_P4_33834255	Ca1	33834255	TAAA/TA	ACCAGTTGCCGACCAATAAG	TAGTACCATTGGAGAGGCGG	60.0	482	INTERGENIC	--	--	--
Ca1_P4_33916734	Ca1	33916734	TAAAAA/TCAAAAAA	TCACCTATCACGGGTCAACA	ATTCGTCGTATTTGGCGTGT	60.0	468	INTERGENIC	--	--	--
Ca1_P4_3396002	Ca1	3396002	AATATATATATATATATATATATATATATATATATATATAT/AATATATATATATATATATATATATATATATAT	TTTCCGCGTTGTTGTAGTTG	TTTTGGGAGTGTATACGGTTGA	59.8	876	INTERGENIC	--	--	--
Ca1_P4_33980217	Ca1	33980217	ATTTAGGGTTTA/ATTTAGGGTTTAGGGTTTA	TGATTGGATTAAGCATCCCA	TTGATCATAGCATTTGGGCA	58.9	685	INTERGENIC	--	--	--
Ca1_P4_34014248	Ca1	34014248	GAAAAAAAAAAA/GAAAAAAAAAA	GGGGTGCTCAACTTGTTTGT	TCTTTGCTCACAACACGACC	60.0	126	INTERGENIC	--	--	--

INDEL marker IDs	Chromosomes/ unanchored scaffolds	Physical positions (bp)	InDels (*Kabuli* reference genome-CDC Frontier/PII)	Forward primers (5'-3')	Reverse primers (5'-3')	Anneling temperature (0C)	Expected amplified product size (bp)	Structural annotation		Functional annotation	
								Sequence components of *kabuli* genome	*Kabuli* gene accession IDs	NCBI-KOG	TFs
Ca1_P4_34057382	Ca1	34057382	C/CA	GGGGATTAAGTGCTTGGACA	AAAGAAAGGCACTAAGGCCC	59.9	156	INTERGENIC	--	--	--
Ca1_P4_34132085	Ca1	34132085	TTTA/TTTATCAATTA	ACCTCCTTTGTTATCGGCAA	CGAATGACAAACATCCGTTG	59.6	349	INTERGENIC	--	--	--
Ca1_P4_34132998	Ca1	34132998	CTA/CTAATTA	GAGGGGGAGATTGTTGGATT	GTTTTGGCGTTGGTTTCACT	60.1	656	INTERGENIC	--	--	--
Ca1_P4_34143148	Ca1	34143148	ATTTT/ATTT	TGAAGGGGGTGCTATTTGTT	TGTCGAATTTGTTTAGGGGG	59.4	566	INTERGENIC	--	--	--
Ca1_P4_34143398	Ca1	34143398	CTTTATTTTATTTTATTTT/CTTTATTTTATTTTATTTTATTTT	CCCCCTAAACAAATTCGACA	TTAGGGTGCTTTGTCACACG	59.8	643	INTERGENIC	--	--	--
Ca1_P4_34160007	Ca1	34160007	C/CAT	TGGAGACACTTGCCCTCAAT	TTTTATGTCAGGGCGAAACC	60.7	501	INTERGENIC	--	--	--
Ca1_P4_34164170	Ca1	34164170	TC/T	GCACTCATGCCACAACACTT	TAAAAAGCGGATAAATGGGC	59.8	456	INTERGENIC	--	--	--
Ca1_P4_34169923	Ca1	34169923	GAA/GAAA	CCATTTCCTTTGTCCTCGTG	AAGCCTTGGAAGGAGAAAGC	60.5	538	INTERGENIC	--	--	--
Ca1_P4_34193507	Ca1	34193507	TAT/TATAGAT	ATTCTCAACGCCTTTCATGG	ACGAAGTTTCTTGCGATGCT	60.1	602	INTERGENIC	--	--	--
Ca1_P4_34199097	Ca1	34199097	CAAAAAAAAA/CAAAAAAAA	GACACTCGGCACTACCGTTT	GTCCATTTGGTTATGGGTGC	60.2	468	INTERGENIC	--	--	--

INDEL marker IDs	Chromosomes/ unanchored scaffolds	Physical positions (bp)	InDels (*Kabuli* reference genome-CDC Frontier/PII)	Forward primers (5'-3')	Reverse primers (5'-3')	Anneling temperature (0C)	Expected amplified product size (bp)	Structural annotation		Functional annotation	
								Sequence components of *kabuli* genome	*Kabuli* gene accession IDs	NCBI-KOG	TFs
Ca1_P4_34200799	Ca1	34200799	CTTTTTTTTTTT/CTTTTTTTTTTTT	AGGTTGGTGCCATTATGAGG	AGAGGAAGCCCTACGTGGAT	59.8	425	INTERGENIC	--	--	--
Ca1_P4_34203117	Ca1	34203117	CAAAAA/CAAAA	CGTATGTGCCCTGCTTACAA	GAAGAAGACCCTTTTTGCCC	59.8	432	INTERGENIC	--	--	--
Ca1_P4_34250780	Ca1	34250780	CTT/CTTCATTTTTATTTT	TTGGAGTCGAAGTGAAACCC	ACATTCAGGTGGTGGTGGAT	60.1	561	INTERGENIC	--	--	--
Ca1_P4_34286273	Ca1	34286273	ATATTTTTTATTTTTTATTTTTT/ATATTTTTTATTTTTT	TATGCATGCGGACAAGAGTC	TTGGAAAAGCCCACCTACAC	59.8	448	INTERGENIC	--	--	--
Ca1_P4_34311038	Ca1	34311038	ATT/ATTT	GGTGTGGGAAGCAAAAGTGT	GATGAGATCAACGAAGGGGA	60.0	388	INTERGENIC	--	--	--
Ca1_P4_34332058	Ca1	34332058	CATATAT/CATATATAT	AATTGGAAGCAGGGGTTTTT	TGAACCCGTTATAAATATTTTTCCT	59.8	356	INTERGENIC	--	--	--
Ca1_P4_343407	Ca1	343407	CATA/CA	ATCAACTTCAGGTTGGGTGG	CCAAAGATGAGCCCAAGAAA	59.8	704	INTERGENIC	--	--	--
Ca1_P4_34345340	Ca1	34345340	ATTT/ATTTT	CTACACGAATTCCCGTTCACT	TGAAATAATTGGTCGGAGCC	59.1	456	INTERGENIC	--	--	--
Ca1_P4_34362589	Ca1	34362589	GATAAATA/GA	GGAGGAAACAAGCGTACGAA	TAAGGAGGCCTTTTACAGCG	60.2	859	INTERGENIC	--	--	--
Ca1_P4_34363515	Ca1	34363515	GCC/GCCC	GCGTTGTAAAAGACGCACTG	TGGAAATGATGAGCTTTTGG	59.5	463	INTERGENIC	--	--	--

INDEL marker IDs	Chromosomes/ unanchored scaffolds	Physical positions (bp)	InDels (*Kabuli* reference genome-CDC Frontier/PII)	Forward primers (5'-3')	Reverse primers (5'-3')	Anneling temperature (0C)	Expected amplified product size (bp)	Structural annotation		Functional annotation	
								Sequence components of *kabuli* genome	*Kabuli* gene accession IDs	NCBI-KOG	TFs
Ca1_P4_34386272	Ca1	34386272	GTTT/GTTTT	AATCTGGAGGGCGGTAAGTT	ATATCGGTCAAACGCACACA	60.0	413	INTERGENIC	--	--	--
Ca1_P4_34396578	Ca1	34396578	CAAAAAAAAAAA/CAAAAAAAAAA	TTCGCATATGGTACTTGGCA	GGCTCAATGCATTTTCCACT	60.1	600	INTERGENIC	--	--	--
Ca1_P4_34413066	Ca1	34413066	TTCTCTCTCTCTCTCTCTC/TTCTCTCTCTCTCTCTCTC	GACGGCACTTGGTGGATACT	GTGGTTTTGATTCGAGCCAT	60.0	452	INTERGENIC	--	--	--
Ca1_P4_34469736	Ca1	34469736	AT/ATT	CACATAAAGGCATCATCCCC	ATTAAAGGGGCAACCGATTC	60.2	553	INTERGENIC	--	--	--
Ca1_P4_34509630	Ca1	34509630	TTATATATATATATATATATATATATAT/TTATATATATATATATATATATATATATATATATATATATATAT	GTGCATTTGCGACTGAAGAA	AAGAAGCCGTCTCCGTCTTA	60.0	391	INTERGENIC	--	--	--
Ca1_P4_34565446	Ca1	34565446	GGAGAGAGAGAGAGAGAGAGAGAGAGAGAGAGAGAGAGAGAGAGA/GGAGAGAGAGAGAGAGAGAGAGAGAGAGAGAGA	CGAGCCAAAATCCAAAACAT	AGACCACCATCGACAACCAT	59.9	341	INTERGENIC	--	--	--
Ca1_P4_34593389	Ca1	34593389	CTA/CTATA	AAACTTTTGTCGGACGGTGT	CTAGGAAGGCCTAAAGCGGT	59.5	526	INTERGENIC	--	--	--
Ca1_P4_34634594	Ca1	34634594	TTCTCTCTCTCTCTCTCTCTCTCTCTCT/TTCTCTCTCTCTCTCTCT	CTAACAATCACCCGCAATGA	AAAAGCAGAGGGAGAGGGAG	59.5	342	INTERGENIC	--	--	--
Ca1_P4_34667053	Ca1	34667053	ATTTTTTTTTT/ATTTTTTTTT	AGACTGCTCGGTCCAAAGAA	GTTACCGCAGAGGGACGTAA	60.0	264	INTERGENIC	--	--	--
Ca1_P4_34694319	Ca1	34694319	AGCAAGTG/AGCAAGTGTATTGATCGTTGCAAGTG	GTCGGCATTTTCGCAATTAT	CGCAATCTTTGAGTTCGACA	59.9	507	INTERGENIC	--	--	--

INDEL marker IDs	Chromosomes/ unanchored scaffolds	Physical positions (bp)	InDels (*Kabuli* reference genome-CDC Frontier/PII)	Forward primers (5'-3')	Reverse primers (5'-3')	Anneling temperature (0C)	Expected amplified product size (bp)	Structural annotation		Functional annotation	
								Sequence components of *kabuli* genome	*Kabuli* gene accession IDs	NCBI-KOG	TFs
Ca1_P4_34694405	Ca1	34694405	TAAAAAAAAAAA/TAAAAAAAAAA	TGACAATTTCTGGCAACAGC	CCAAGGTTGGATTCGAAGTG	59.8	247	INTERGENIC	--	--	--
Ca1_P4_34697560	Ca1	34697560	GAAAAAAAAA/GAAAAAAAA	ATGTTTCATCAAAGTCGCCC	TGGGTGTAAATGCTTTGTGC	59.9	685	INTERGENIC	--	--	--
Ca1_P4_34768259	Ca1	34768259	AAAATAAATA/AAAATAAATAAATA	ATCGCCAGTGTCAATTTTCC	TGCGATTTTACGTGTATATGTGAG	59.9	677	INTERGENIC	--	--	--
Ca1_P4_34810237	Ca1	34810237	GTTTTT/GTTTT	ATTGCCTCGCTTGAAGAATG	TTTGGATGAGCAACAAGCAG	60.4	598	INTERGENIC	--	--	--
Ca1_P4_34814277	Ca1	34814277	T/TG	GCGTGAACAAAAATCAAGCA	TCGAAGACGTCTCGTTGTTG	59.9	452	INTERGENIC	--	--	--
Ca1_P4_34822198	Ca1	34822198	ATTTTTTTTT/ATTTTTTTT	AACATTCTGGTATCGCGTCC	TTCAATGAGGTGGAAGGAGG	60.0	935	INTERGENIC	--	--	--
Ca1_P4_34845186	Ca1	34845186	CTGTATATTGCTTAGAAGTGTA/CTGTA	TTGGGTGGAAGGTTAGTCCA	TTGTTCAAACGGTTATGCCA	60.3	391	INTERGENIC	--	--	--
Ca1_P4_34853862	Ca1	34853862	CAAAAAAAAAAA/CAAAAAAAAA	AGTTTGGCTAGATGGGCCTT	TAATTCAAACCGCACTGCAA	60.1	256	INTERGENIC	--	--	--
Ca1_P4_34886508	Ca1	34886508	AAAATAATAAAT/AAAATAATAAATAATAAAT	AGTGAACTTGAACCAACGCA	CACCACCTTCACCTCCAGTT	59.3	573	INTERGENIC	--	--	--
Ca1_P4_34913480	Ca1	34913480	AAT/AATAT	TTGTGTTCACCGAGGTTGTC	CAACAACCAACTTCGTGCAT	59.6	572	INTERGENIC	--	--	--

INDEL marker IDs	Chromosomes/ unanchored scaffolds	Physical positions (bp)	InDels (*Kabuli* reference genome-CDC Frontier/PII)	Forward primers (5'-3')	Reverse primers (5'-3')	Anneling temperature (0C)	Expected amplified product size (bp)	Structural annotation		Functional annotation	
								Sequence components of *kabuli* genome	*Kabuli* gene accession IDs	NCBI-KOG	TFs
Ca1_P4_34921085	Ca1	34921085	TAAAAAAAAAAAA/TAAAAAAAAAAA	TCAATCGCATCTCGAA ACAC	AAGGCTTTTATAGAGG CGGC	59.8	626	INTERGENIC	--	--	--
Ca1_P4_34935340	Ca1	34935340	GAAAAAAA/GAAAAAAAA	CCGTATGAATCATGTAT GGGA	TGCGTCCGTACAAATC TAGG	58.2	515	INTERGENIC	--	--	--
Ca1_P4_34971020	Ca1	34971020	CAAAAAAA/CAAAAA	AAATACCCCTTTTTCCC ACG	TGAAAACGCTCAATGC CATA	60.0	689	INTERGENIC	--	--	--
Ca1_P4_34981671	Ca1	34981671	CAACATAACATAA/CAACATAA	TGGTCTTGCTTTTCCG TCTC	TGATTGACATGTGCAA CAAAA	60.4	415	INTERGENIC	--	--	--
Ca1_P4_35044743	Ca1	35044743	AG/A	GTACCGAATAGCCCGC ATAA	CCAGTCCATATCGGGT CAGT	60.0	654	INTERGENIC	--	--	--
Ca1_P4_35059495	Ca1	35059495	TAAAAAAAAA/TAAAAAAAAAAAAA	CTGACCACAAAGAAAA AGCCT	ATGGGTTTCCAACCAC AAAA	58.5	480	INTERGENIC	--	--	--
Ca1_P4_35060041	Ca1	35060041	CAAAAAAA/CAAAAAA	TTTTGTGGTTGGAAAC CCAT	TCCCAACATTGAAAGTT CCC	60.1	613	INTERGENIC	--	--	--
Ca1_P4_35061932	Ca1	35061932	TAAA/TAA	GGCCTTGAGAAGCATT TGTT	TTCTAAGGCCTCTCAC CATGA	59.3	660	INTERGENIC	--	--	--
Ca1_P4_35072166	Ca1	35072166	CTA/CTATATA	AGTTGGCTCACTAGCA CCGT	CACAAGTGGATGAACA ACCG	59.9	696	INTERGENIC	--	--	--
Ca1_P4_35091571	Ca1	35091571	CTAT/CT	CGAGCTGTTGAGGAAC ACAA	GTTGCTCTTGCATCTG GTCA	60.0	578	INTERGENIC	--	--	--

INDEL marker IDs	Chromosomes/ unanchored scaffolds	Physical positions (bp)	InDels (*Kabuli* reference genome-CDC Frontier/PII)	Forward primers (5'-3')	Reverse primers (5'-3')	Anneling temperature (0C)	Expected amplified product size (bp)	Structural annotation		Functional annotation	
								Sequence components of *kabuli* genome	*Kabuli* gene accession IDs	NCBI-KOG	TFs
Ca1_P4_35096364	Ca1	35096364	AT/A	TTGATGTGGTCGGAGATTGA	ACAATTTTCAGCAACCCGAG	60.0	588	INTERGENIC	--	--	--
Ca1_P4_35141865	Ca1	35141865	T/TAA	GCCCACTTCGGTACATATTTC	TTTCAGTTCAAAATCCGCAA	58.5	173	INTERGENIC	--	--	--
Ca1_P4_35155669	Ca1	35155669	AATTTGGTGATTTGGTGATTT/AATTTGGTGATTT	TCATTCCCCTTCTCCCTTCT	TTAATCCGCAAATCACCCAT	60.0	838	INTERGENIC	--	--	--
Ca1_P4_35156057	Ca1	35156057	GAA/GAAA	TTGTGATTGAGGGGTGGTTT	GTGCAGTCTCTCACGGACAA	60.2	630	INTERGENIC	--	--	--
Ca1_P4_35167911	Ca1	35167911	GTT/GT	CAAATGTGTGGAGATCGGTG	ATTGGGACGTGCTTTTCTTG	60.0	511	INTERGENIC	--	--	--
Ca1_P4_35180632	Ca1	35180632	CAAAA/CAAAAA	ATGCCCCAATGAATTGTGAT	CCTTCTCGTGTTTGCACCTT	60.0	405	INTERGENIC	--	--	--
Ca1_P4_35186204	Ca1	35186204	ATTTTTTTTT/ATTTTTTT	CCAAGAAACCCTAGCACCAA	AGCCTCGAAAATGAACTCCA	60.1	287	INTERGENIC	--	--	--
Ca1_P4_35186475	Ca1	35186475	ATTTT/ATTTTT	GGTCAGTGAGCCAAGGGATA	CCGTTTAAACCCACCTGCTA	60.1	729	INTERGENIC	--	--	--
Ca1_P4_35221996	Ca1	35221996	T/TGCTGAAATAAGTC	CACGTGCCCAAATGTTATGA	TGATTCTCCTTGGGATCTGG	60.4	540	INTERGENIC	--	--	--
Ca1_P4_35227202	Ca1	35227202	TAAAAA/TAAAAAA	ATCAGTGGTCCCGTGAGAAG	GTCAAGAGTGCGGTCACATC	60.1	764	INTERGENIC	--	--	--

INDEL marker IDs	Chromosomes/ unanchored scaffolds	Physical positions (bp)	InDels (*Kabuli* reference genome-CDC Frontier/PII)	Forward primers (5'-3')	Reverse primers (5'-3')	Anneling temperature (0C)	Expected amplified product size (bp)	Structural annotation		Functional annotation	
								Sequence components of *kabuli* genome	*Kabuli* gene accession IDs	NCBI-KOG	TFs
Ca1_P4_35227782	Ca1	35227782	AGG/AGGG	AGATGTGACCGCACTCTTGA	TCAACCACAACTGACCAACG	59.4	671	INTERGENIC	--	--	--
Ca1_P4_35233074	Ca1	35233074	AAT/A	TTGGTTCCTTTGGGGTGTAA	CACAAAACAAAGCCAACCCT	60.2	218	INTERGENIC	--	--	--
Ca1_P4_35235861	Ca1	35235861	CATTATTATTATT/CATTATTATTATTATT	GGGATGCGACCTTGTACTGT	TCCTCCTCTTTTGGCACATT	60.0	463	INTERGENIC	--	--	--
Ca1_P4_35236185	Ca1	35236185	ATTTTT/ATTTT	ATGTGCCAAAAGAGGAGGAA	GGGATGCGACCTTGTACTGT	59.7	704	INTERGENIC	--	--	--
Ca1_P4_35236504	Ca1	35236504	ATTTTTTT/ATTTTTTTT	CAACTTCCTCCTCTTTTGGC	GGGATGCGACCTTGTACTGT	58.9	522	INTERGENIC	--	--	--
Ca1_P4_35246080	Ca1	35246080	AT/ATT	AAGAGGGATTTGGGGAAGAG	TGGTTCAACATACGGAAGAAAA	59.5	289	INTERGENIC	--	--	--
Ca1_P4_35252010	Ca1	35252010	GAAAA/GAAAAA	CGTTGCACTAATGGCATCAC	CCGACACATAATGCCCTTTT	60.1	628	INTERGENIC	--	--	--
Ca1_P4_35260458	Ca1	35260458	ATT/ATTT	ATGGCTCATACTGGGATTGC	AAGACTGGGTTTGCCTGCTA	59.9	592	INTERGENIC	--	--	--
Ca1_P4_35261821	Ca1	35261821	ATT/AT	TGGATTGTCATGGTCTGGAA	AATGGTCTTGAATTTTGCCG	59.9	640	INTERGENIC	--	--	--
Ca1_P4_35264601	Ca1	35264601	TAAAAAAAAA/TAAAAAAAAAA	TTCTTTGAGTTTTTGAGACACAAG	TCATGAATGTCGCGGTCTAA	57.8	574	INTERGENIC	--	--	--

INDEL marker IDs	Chromosomes/ unanchored scaffolds	Physical positions (bp)	InDels (*Kabuli* reference genome-CDC Frontier/PII)	Forward primers (5'-3')	Reverse primers (5'-3')	Anneling temperature (0C)	Expected amplified product size (bp)	Structural annotation		Functional annotation	
								Sequence components of *kabuli* genome	*Kabuli* gene accession IDs	NCBI-KOG	TFs
Ca1_P4_35264715	Ca1	35264715	CTTTTTTTTTTTT/CTTTTTTTT	CGGGAGATACGTAGGAGCAA	TCATGAATGTCGCGGTCTAA	60.2	379	INTERGENIC	--	--	--
Ca1_P4_35265473	Ca1	35265473	TAAAAAAA/TAAAAAA	TTCCCTGAATCGAGTGAACC	TTTTCCAATAACTCTACCGCGT	60.1	674	INTERGENIC	--	--	--
Ca1_P4_35266220	Ca1	35266220	ATAT/ATATTAT	CGCGGTAGAGTTATTGGAAAA	TGGGGATACGTAGGAGCAAG	59.3	682	INTERGENIC	--	--	--
Ca1_P4_35282790	Ca1	35282790	TCCCC/TCCC	AATTTGCACGGTTCAGTTCC	CATACATGCCAAAACAAATGC	60.0	535	INTERGENIC	--	--	--
Ca1_P4_35283797	Ca1	35283797	TGGGGG/TGGGG	GGGTGTGTTTGATAGGAGGG	CTCCCCTTCCATCTCCTCTT	59.3	139	INTERGENIC	--	--	--
Ca1_P4_35284017	Ca1	35284017	ATTT/ATT	GAGATGGAAGGGGAGGAAAG	GCACAAAAGAAAAATACAAGTCCA	60.0	273	INTERGENIC	--	--	--
Ca1_P4_35284253	Ca1	35284253	TT/TTGT	GAGATGGAAGGGGAGGAAAG	TCTGATCAAATATGTGGCAGAAA	60.0	657	INTERGENIC	--	--	--
Ca1_P4_35284860	Ca1	35284860	CAAAAA/CAA	TGAAGGGAGTGCTATTTGGT	TCACCTTTTGACTTCCTCATGTT	57.3	108	INTERGENIC	--	--	--
Ca1_P4_35285068	Ca1	35285068	GACACA/GACA	TGAAGGGAGTGCTATTTGGT	GCGAGCATTAATGTTGGAAAA	57.3	688	INTERGENIC	--	--	--
Ca1_P4_35285526	Ca1	35285526	ATTTTTT/ATTTTT	CGAGTCCTCCAAAACTAATGC	GCCAGAGTGCAATAGGAAGG	58.8	640	INTERGENIC	--	--	--

INDEL marker IDs	Chromosomes/ unanchored scaffolds	Physical positions (bp)	InDels (*Kabuli* reference genome-CDC Frontier/PII)	Forward primers (5'-3')	Reverse primers (5'-3')	Anneling temperature (0C)	Expected amplified product size (bp)	Structural annotation		Functional annotation	
								Sequence components of *kabuli* genome	*Kabuli* gene accession IDs	NCBI-KOG	TFs
Ca1_P4_35285573	Ca1	35285573	TG/T	CGAGTCCTCCAAAACTAATGC	GCCAGAGTGCAATAGGAAGG	58.8	640	INTERGENIC	--	--	--
Ca1_P4_35285638	Ca1	35285638	TATT/TATTAATT	GACCTTATGCTCGTGCAATGT	GCCAGAGTGCAATAGGAAGG	60.2	419	INTERGENIC	--	--	--
Ca1_P4_35288350	Ca1	35288350	ATTTT/ATTTTT	CAACAAGTCAGTGGCAAAACA	GTGGGCATTGTTCCAAGAGT	59.8	219	INTERGENIC	--	--	--
Ca1_P4_35290921	Ca1	35290921	ATTT/ATTTT	CACACCTAATGCCTGTCACG	TGATGGACCAAACAAGTCCA	60.2	760	INTERGENIC	--	--	--
Ca1_P4_35300452	Ca1	35300452	AAAGTAA/AAA	TTGGAAAAGCCCACCTACAC	TATGCATGCGGACAAGAGTC	60.0	488	INTERGENIC	--	--	--
Ca1_P4_35310736	Ca1	35310736	ATTTTTTTTT/ATTTTTTTT	TTGATGCAACCTGTGACTCC	TTGGATCCATCCCCTAGATG	59.7	572	INTERGENIC	--	--	--
Ca1_P4_35310853	Ca1	35310853	ATTTTTTTT/ATTTTTTTTT	TTGATGCAACCTGTGACTCC	AATGCCCAGTTAGGGAGGAT	59.7	690	INTERGENIC	--	--	--
Ca1_P4_35335691	Ca1	35335691	ATTTT/ATTT	TTCTAGGCATGTTTCCCCTG	GAGGCTGTGCATTTTGGAAT	60.1	615	INTERGENIC	--	--	--
Ca1_P4_35337257	Ca1	35337257	CTTTTTT/CTTTTTTT	TACATGCACACACTCCACCA	TGGGAGCCTCAATTGCTTAG	59.6	290	INTERGENIC	--	--	--
Ca1_P4_35349139	Ca1	35349139	GAAAAAAAA/GAAAAAAAAA	TCTGTTGGCACAAAATGGAA	CAAGGCTTCTTGAACTTGGG	60.1	608	INTERGENIC	--	--	--

INDEL marker IDs	Chromosomes/ unanchored scaffolds	Physical positions (bp)	InDels (*Kabuli* reference genome-CDC Frontier/PII)	Forward primers (5'-3')	Reverse primers (5'-3')	Anneling temperature (0C)	Expected amplified product size (bp)	Structural annotation		Functional annotation	
								Sequence components of *kabuli* genome	*Kabuli* gene accession IDs	NCBI-KOG	TFs
Ca1_P4_35353052	Ca1	35353052	G/GTTGA	TCAGCAGCAAATAGGCTTCA	AGCCCATTCAGATTTTGTGG	59.7	352	INTERGENIC	--	--	--
Ca1_P4_35353239	Ca1	35353239	GAA/GAAA	CCAACAACTTCCTTTCGAGC	AGCCCATTCAGATTTTGTGG	59.9	147	INTERGENIC	--	--	--
Ca1_P4_35362478	Ca1	35362478	TTATATATATA/TTATATATATATA	TTCAGCTTGCCCGTTAGTCT	AAACTCTTGACCAGAGGCGA	60.0	975	INTERGENIC	--	--	--
Ca1_P4_35377236	Ca1	35377236	TGG/TGGAGG	TGAAAATTGTAGCAAGTCGCTTA	CAAAGGAAACATGCCCAAAT	59.1	841	INTERGENIC	--	--	--
Ca1_P4_35422117	Ca1	35422117	TAAAAAAAAAA/TAAAAAAAAAAA	TGTAGTGGCATGTTTGGCAT	CACTTTTGGAGGACTTTCGG	60.0	677	INTERGENIC	--	--	--
Ca1_P4_35430389	Ca1	35430389	GATAT/GATATAT	TCTTGCTTTCGCACAATCTTT	CGCCGAACCAAGAACATTAT	60.0	547	INTERGENIC	--	--	--
Ca1_P4_35442355	Ca1	35442355	GGTGTGTGTGTGTGTGTGTGTGTG/GGTGTGTGTGTGTGTGTGTGTGTGTGTGTGTG	AGGCCGAGAAACCCTAGAAG	CATTTTGGATTCAACTCGGG	59.8	309	INTERGENIC	--	--	--
Ca1_P4_35500602	Ca1	35500602	TGACCTTTCCTATGGTAG/TGACCTTTCCTATGGTAGGACCTTTCCTATGGTAG	TTTTGTTGGGTCCTGGTTTC	GGCCATATCGAGATGCAGAT	59.8	488	INTERGENIC	--	--	--
Ca1_P4_35505624	Ca1	35505624	GTTTTTTTTTTT/GTTTTTTTTTT	GGGATTTCCCCTGTTTTCAT	TGAAGCGCTCTCCCATAGTT	60.0	448	INTERGENIC	--	--	--
Ca1_P4_35537314	Ca1	35537314	T/TG	ACATGGAACCCATCCAAAGA	AATCGATTGATTCAGCCCAG	60.2	475	INTERGENIC	--	--	--

INDEL marker IDs	Chromosomes/ unanchored scaffolds	Physical positions (bp)	InDels (*Kabuli* reference genome-CDC Frontier/PII)	Forward primers (5'-3')	Reverse primers (5'-3')	Anneling temperature (0C)	Expected amplified product size (bp)	Structural annotation		Functional annotation	
								Sequence components of *kabuli* genome	*Kabuli* gene accession IDs	NCBI-KOG	TFs
Ca1_P4_35540202	Ca1	35540202	AT/ATT	TTGTTTATGCCCCTTTGAGC	ACAAACAAGTTGAGCACCCC	60.1	146	INTERGENIC	--	--	--
Ca1_P4_35553587	Ca1	35553587	CA/CAA	TATGGACCGGAAATGGATGT	TTTGAATCGTCTTCACTGCG	60.0	779	INTERGENIC	--	--	--
Ca1_P4_35553773	Ca1	35553773	GTATCCTA/GTA	GTCGAGGAGATTGCAAAAGC	TTTGAATCGTCTTCACTGCG	60.0	469	INTERGENIC	--	--	--
Ca1_P4_3556309	Ca1	3556309	TTATATATATACATATATATAT/TTATATATAT	TGACCTCATGGATACCCACA	AAGGTCGCATCTTCATCCTG	59.8	371	INTERGENIC	--	--	--
Ca1_P4_35563563	Ca1	35563563	TAAAAAAAAAAAAA/TAAAAAAAAAA	ATGGGAATGCTTGACTGGAG	CGACTGTAACACCCCGTTTT	60.1	390	INTERGENIC	--	--	--
Ca1_P4_35565045	Ca1	35565045	TTAAT/TTAATAAT	AGCGATGCATGTCTCAAGTG	GCCCTTCTTCTTTTCCTGGT	60.0	427	INTERGENIC	--	--	--
Ca1_P4_35767551	Ca1	35767551	GTGAT/GT	TGGGAGATGCGATATGTTTT	ACAACTTGTGATTTTCGGGG	58.0	867	INTERGENIC	--	--	--
Ca1_P4_35798308	Ca1	35798308	GTTGGTTTTAATT/GT	CACTTCTTCCCGGCTGATAA	TTGGAAGGAGCCCTTACCTT	60.2	753	INTERGENIC	--	--	--
Ca1_P4_35832819	Ca1	35832819	CAAAAAA/CAAAAA	GGGTTTTCTAACACCCCGAT	TGGTCATGCAGAAACTCAGC	60.1	769	INTERGENIC	--	--	--
Ca1_P4_35842524	Ca1	35842524	GAAAAAAAAAAAA/GAAAAAAAAAA	TCATGACCTTATGACAACGCTT	CAGGGGCACTACACCAAAAT	59.6	247	INTERGENIC	--	--	--

INDEL marker IDs	Chromosomes/ unanchored scaffolds	Physical positions (bp)	InDels (*Kabuli* reference genome-CDC Frontier/PII)	Forward primers (5'-3')	Reverse primers (5'-3')	Anneling temperature (0C)	Expected amplified product size (bp)	Structural annotation		Functional annotation	
								Sequence components of *kabuli* genome	*Kabuli* gene accession IDs	NCBI-KOG	TFs
Ca1_P4_35899211	Ca1	35899211	TTTTAGACAC/TTTTAGACACATTTAGACAC	CACCTCCCCTGAGTTCGTTA	GCAAGCTCCAAATGGATTACA	60.1	404	INTERGENIC	--	--	--
Ca1_P4_35946765	Ca1	35946765	AG/A	GCGCCATTTTGTTCTTTGTT	ATTTCTTGCCCTTTGTCCCT	60.1	738	INTERGENIC	--	--	--
Ca1_P4_35946848	Ca1	35946848	AT/ATTTT	GTTTCGTAGCGCCGTTTATC	ATTTCTTGCCCTTTGTCCCT	59.7	531	INTERGENIC	--	--	--
Ca1_P4_35973449	Ca1	35973449	TTATATATATATATATATATATATATATATATATAT/TTATATATATATATATATATATATATATATATAT	GCCCACCCAACAAATACATC	TTGGCACCAGAGAAAAACAA	60.1	733	INTERGENIC	--	--	--
Ca1_P4_35977319	Ca1	35977319	TA/TAA	AACTCGTTGGAACTTCCCTCT	GCAAGCAGCCTTGTTGACTA	59.2	540	INTERGENIC	--	--	--
Ca1_P4_36035161	Ca1	36035161	AAGAGGGAGAGGGAGAGGGAGAGG/AAGAGGGAGAGGGAGAGGGAGAGGGAGAGG	TTCGTGATCCGTGAATTTGA	AACGAAACAATGCCGAACAT	60.0	518	INTERGENIC	--	--	--
Ca1_P4_36044248	Ca1	36044248	CGG/CG	CGATTTCCCTGCATGTTTTT	GCCTGTGATAGGCGGTACAT	59.9	540	INTERGENIC	--	--	--
Ca1_P4_36063199	Ca1	36063199	T/TTTTTCC	CGTTCGTGGTCCCTTAACTT	TTGCGTTACGGATTTGATGA	59.1	227	INTERGENIC	--	--	--
Ca1_P4_36100635	Ca1	36100635	CAATAATAATAATAATAATAATAATAATAATAATAATAA/CAATAATAATAATAATAATAATAATAATAATAATAATAATAATAATAA	TGCATACACGTAGTGGCACA	GTGACGGCACATTTGTGTTC	59.8	854	INTERGENIC	--	--	--
Ca1_P4_36124030	Ca1	36124030	GTTTTT/GTTTT	TTTTCAGCTTTAAGTTCCGCA	TTTAGGAAGACTGGCTCGGA	60.0	683	INTERGENIC	--	--	--

INDEL marker IDs	Chromosomes/ unanchored scaffolds	Physical positions (bp)	InDels (*Kabuli* reference genome-CDC Frontier/PII)	Forward primers (5'-3')	Reverse primers (5'-3')	Anneling temperature (0C)	Expected amplified product size (bp)	Structural annotation		Functional annotation	
								Sequence components of *kabuli* genome	*Kabuli* gene accession IDs	NCBI-KOG	TFs
Ca1_P4_36154229	Ca1	36154229	ACTGCTGCT/ACTGCT	TTGCATTGATAATTGGCTGC	TGTTCCTTTTCCTGCAATCA	59.7	849	INTERGENIC	--	--	--
Ca1_P4_36249439	Ca1	36249439	TTTATTATTATTATTATTATTATTATTATTATTATTATTATTATT/TTTATTATTATTATTATTATTATTATTATTATTATTATTATTATT	TCCCCCTTTGTCTTCTTGTG	TCCATCTAATTGCTACCGGC	60.1	930	INTERGENIC	--	--	--
Ca1_P4_3636721	Ca1	3636721	TAA/T	TTCATGGAACATTCTGCCAA	CGACAAGTTTGAGGGGTTGT	60.0	350	INTERGENIC	--	--	--
Ca1_P4_3642296	Ca1	3642296	ATTTTTTTTTTT/ATTTTTTTTTT	AACCTCCCTAGTGCCGAAAT	AAGTTGATGCCGAACAAAATG	60.0	538	INTERGENIC	--	--	--
Ca1_P4_36467494	Ca1	36467494	AGGGG/AGGGGG	AGAGCACGGAGAAGGAACAA	CCCGAGTTGGGTGAACTATG	60.0	571	INTERGENIC	--	--	--
Ca1_P4_36603071	Ca1	36603071	CTTTTT/CTTTT	TGCTTTGTCCCCTTAGTTGG	CATAGAGCTGTGCGTGGAAA	60.1	596	INTERGENIC	--	--	--
Ca1_P4_36642222	Ca1	36642222	TTATATATATATATATATATATATATAT/TTATATATATATATATATATATAT	GATTTGGATTGGCAAAATGC	AGCAGAGTCACATGTCCACG	60.3	785	INTERGENIC	--	--	--
Ca1_P4_36724754	Ca1	36724754	CTTTTTTTT/CTTTTTTTTT	TTGCACCACACTTCTTCACC	CTTATTTCGACGCTTTTCGC	59.7	279	INTERGENIC	--	--	--
Ca1_P4_36726460	Ca1	36726460	GAAA/GAAAA	ACAGGAACGACCGAGAGAAA	ATAGTTTTTGGGCGGGCTAC	59.8	502	INTERGENIC	--	--	--
Ca1_P4_36746332	Ca1	36746332	ATTTTTTTTTTTTT/ATTTTTTTTTTT	GCTCAAACTCCTAACTCGGC	TGGTGATGAAGGGAGAAATGA	59.1	411	INTERGENIC	--	--	--

INDEL marker IDs	Chromosomes/ unanchored scaffolds	Physical positions (bp)	InDels (*Kabuli* reference genome-CDC Frontier/PII)	Forward primers (5'-3')	Reverse primers (5'-3')	Anneling temperature (0C)	Expected amplified product size (bp)	Structural annotation		Functional annotation	
								Sequence components of *kabuli* genome	*Kabuli* gene accession IDs	NCBI-KOG	TFs
Ca1_P4_36869128	Ca1	36869128	CA/CAAA	TCCACAACAACCTACGTCCA	CCCAGAAATCGATTAGGCAA	60.0	555	INTERGENIC	--	--	--
Ca1_P4_36876798	Ca1	36876798	AGGG/AGGGG	GTTTCCAGCCATCCAACACT	AATTGGTTTCATGACCTGCAC	60.0	503	INTERGENIC	--	--	--
Ca1_P4_36962435	Ca1	36962435	TA/TATAA	CAAGTCTCCCAACTTCCCAA	CAAGCATGATCCAGGGAAAT	60.1	646	INTERGENIC	--	--	--
Ca1_P4_370295	Ca1	370295	AAAGAAGAAGAAGA/AAAGAAGAAGAAGAAGA	TTGCATGTGAAAAACAGAAAGAA	ACTCCATTGTCGATTCCCTG	59.8	195	INTERGENIC	--	--	--
Ca1_P4_37078918	Ca1	37078918	A/AAATTAATG	TGTGACACCCTAAACCCTAAGAA	TTGATTGCTTGCGTGTTCTC	59.9	499	INTERGENIC	--	--	--
Ca1_P4_37079213	Ca1	37079213	CTCACAT/CTTCACAT	TAAATATGGTCCCAACCCGA	TTGATTGCTTGCGTGTTCTC	60.0	257	INTERGENIC	--	--	--
Ca1_P4_37092021	Ca1	37092021	CTTTTTTTTTT/CTTTTTTT	TATCAGAGTTCTCCCCACCG	TTGAGGTGTGGTTTGTGGTG	60.1	759	INTERGENIC	--	--	--
Ca1_P4_37122105	Ca1	37122105	AAAAAAAAAA/AAAAAAAAAAAA	CGTATCTCGTATCACCGACG	AGTTGCGCTCCTCCTAATCA	59.2	873	INTERGENIC	--	--	--
Ca1_P4_37122520	Ca1	37122520	AGG/AG	TGCAATCATTGGTGCTTAGG	AAAAAGCGCCGTAAAATGAG	59.7	710	INTERGENIC	--	--	--
Ca1_P4_37122699	Ca1	37122699	GTTT/GTT	GGAGGAGCGCAACTTAATCA	AAAAAGCGCCGTAAAATGAG	60.4	372	INTERGENIC	--	--	--

**INDEL marker IDs**	**Chromosomes/ unanchored scaffolds**	**Physical positions (bp)**	**InDels (*Kabuli* reference genome-CDC Frontier/PII)**	**Forward primers (5'-3')**	**Reverse primers (5'-3')**	**Anneling temperature (0C)**	**Expected amplified product size (bp)**	**Structural annotation**		**Functional annotation**	
								**Sequence components of *kabuli* genome**	***Kabuli* gene accession IDs**	**NCBI-KOG**	**TFs**
Ca1_P4_37122810	Ca1	37122810	TACAAC/TACAACAAC	GGAGGAGCGCAACTTAATCA	AAAAAGCGCCGTAAAATGAG	60.4	372	INTERGENIC	--	--	--
Ca1_P4_37125192	Ca1	37125192	GATGGAGATGGTTTGCAATGGAGATG/GATGGAGATG	AGCGGTTTAGGGTTTAGGGA	CCAAATGTGCATACGAACCA	60.0	113	INTERGENIC	--	--	--
Ca1_P4_37128515	Ca1	37128515	ATTTTTTT/ATTTT	CAATTCCCACAGGTACGCTT	TTGGTCTCATCAATGATTGTAAAAA	60.0	568	INTERGENIC	--	--	--
Ca1_P4_3717542	Ca1	3717542	ATT/AT	AGTGATGGTGTTGGGTGGAT	CAATAATGTCCTCTCTATGCACCTT	60.1	597	INTERGENIC	--	--	--
Ca1_P4_37176254	Ca1	37176254	TATA/TATACATA	TCAAGTTTGGAGGAACCCAG	CTGGACGTAGCCCGAGTTAG	60.1	651	INTERGENIC	--	--	--
Ca1_P4_37207886	Ca1	37207886	TA/T	GGACCGACTAAAATCCCACA	TTTTGCTTTGTGTCCCATCA	59.8	529	INTERGENIC	--	--	--
Ca1_P4_37243078	Ca1	37243078	AAATTAT/AAATTATAGAATTAT	TCATCCACATCAGCCAAAAA	TTTGTGCATTTTAATAATTGGAAG	60.0	490	INTERGENIC	--	--	--
Ca1_P4_37410363	Ca1	37410363	GAAAAAA/GAAAAAAA	TGAATGAGGAGCACGTCTTG	CTGTTGTTGCCAACGGTTTT	60.0	445	INTERGENIC	--	--	--
Ca1_P4_37420494	Ca1	37420494	ATTTTTTTTT/ATTTTTTT	GGGAAAAGGAAACCCAAAAA	TAGTGGAGTCCCCGTCAAGT	60.1	630	INTERGENIC	--	--	--
Ca1_P4_37420598	Ca1	37420598	ATTTT/ATTT	GGGAAAAGGAAACCCAAAAA	TGAGTGAGGGAGACAAAGGG	60.1	950	INTERGENIC	--	--	--

INDEL marker IDs	Chromosomes/ unanchored scaffolds	Physical positions (bp)	InDels (*Kabuli* reference genome-CDC Frontier/PII)	Forward primers (5'-3')	Reverse primers (5'-3')	Anneling temperature (0C)	Expected amplified product size (bp)	Structural annotation		Functional annotation	
								Sequence components of *kabuli* genome	*Kabuli* gene accession IDs	NCBI-KOG	TFs
Ca1_P4_37421082	Ca1	37421082	CCTCACTCACTCACTCACTCAC/CCTCACTCACTCACTCAC	ACTTGACGGGGACTCCACTA	ACATGTTCCTTGTGCTCGTG	59.6	637	INTERGENIC	--	--	--
Ca1_P4_37422521	Ca1	37422521	TAAAAAAAAA/TAAAAAAAAAAA	CATCGTACTTCGTTGCCCTT	AGGTGTGCTAGCTAGGGGTG	60.1	573	INTERGENIC	--	--	--
Ca1_P4_37424329	Ca1	37424329	CATATATATATATATATATATAT/CATATATATATATATATAT	AAAAAGTATTGAACAAACACGCT	TGAAATCCAAATTAAATTGCTGAA	57.2	549	INTERGENIC	--	--	--
Ca1_P4_37424604	Ca1	37424604	CTTTTT/CTTTT	AAAAAGTATTGAACAAACACGCT	TGAAATCCAAATTAAATTGCTGAA	57.2	549	INTERGENIC	--	--	--
Ca1_P4_37424983	Ca1	37424983	AC/A	GCAATTTAATTTGGATTTCATGG	TCACATCCATCCACGGTACA	59.6	761	INTERGENIC	--	--	--
Ca1_P4_37425070	Ca1	37425070	ATTTTTTTTTT/ATTTTTTTT	GCAATTTAATTTGGATTTCATGG	TCACATCCATCCACGGTACA	59.6	761	INTERGENIC	--	--	--
Ca1_P4_37425153	Ca1	37425153	G/GAT	GCAATTTAATTTGGATTTCATGG	TCACATCCATCCACGGTACA	59.6	761	INTERGENIC	--	--	--
Ca1_P4_37425439	Ca1	37425439	TG/T	TGTACCGTGGATGGATGTGA	TGACACGTATCAATTTAGGGAGAA	60.8	222	INTERGENIC	--	--	--
Ca1_P4_37425620	Ca1	37425620	ACG/A	TGTACCGTGGATGGATGTGA	TATGCATCCTCACGGTTTTG	60.8	675	INTERGENIC	--	--	--
Ca1_P4_37433380	Ca1	37433380	CAA/CAACGTAAA	AATAAATTAGTGCAGCAAACCAA	GGCAAAGAGAGGAAAGTGGA	58.0	756	INTERGENIC	--	--	--

INDEL marker IDs	Chromosomes/ unanchored scaffolds	Physical positions (bp)	InDels (*Kabuli* reference genome-CDC Frontier/PII)	Forward primers (5'-3')	Reverse primers (5'-3')	Anneling temperature (0C)	Expected amplified product size (bp)	Structural annotation		Functional annotation	
								Sequence components of *kabuli* genome	*Kabuli* gene accession IDs	NCBI-KOG	TFs
Ca1_P4_37433540	Ca1	37433540	TATAATAGATAATAGATAATA/TATAATAGATAATA	TGAAAAACATCTCTCTAATAGGCG	ACCACTGCACCAAGGGTAAG	59.0	532	INTERGENIC	--	--	--
Ca1_P4_37489932	Ca1	37489932	CAAAAA/CAAAA	ATTGTCCAGTTTCCTCACCG	CGATTGGTAAATCGATTGGG	60.0	594	INTERGENIC	--	--	--
Ca1_P4_37489959	Ca1	37489959	AT/ATT	ATTGTCCAGTTTCCTCACCG	CGATTGGTAAATCGATTGGG	60.0	594	INTERGENIC	--	--	--
Ca1_P4_37490066	Ca1	37490066	A/AATGTAT	ATTGTCCAGTTTCCTCACCG	TGAACTCTGAGCGTGTGACC	60.0	775	INTERGENIC	--	--	--
Ca1_P4_37567284	Ca1	37567284	CATATATATATATATATATATATATAT/CATATATATATATATATATATATATATATAT	AGGTGCCCCTAACCAAGTTT	TTTGAGCAGAGAGAACCCGT	59.9	651	INTERGENIC	--	--	--
Ca1_P4_3764450	Ca1	3764450	GATATATATATATATATATATATA/GATATATATATATATATATATATA	TTTTGGAGGTGTGATTAATGTATTTT	TGGTTGATGTGTCGAGTGTCT	59.2	515	INTERGENIC	--	--	--
Ca1_P4_37686545	Ca1	37686545	TCCCC/TCCCCC	GAACTCCCTTCATGACCCAA	TTTGTTGGTGGTGGAGAACA	59.9	527	INTERGENIC	--	--	--
Ca1_P4_37694287	Ca1	37694287	AAAATTTTATAAA/AAAA	TTTGATTCTTCAATCCAGCAA	TTGATATCGGGTGGTCCAAT	58.3	371	INTERGENIC	--	--	--
Ca1_P4_37701839	Ca1	37701839	AT/A	ATGCAGTCGCAATTGTGGTA	TGTCACTTGTGGGGACAAAA	60.1	498	INTERGENIC	--	--	--
Ca1_P4_37710484	Ca1	37710484	AATTATTATTAT/AATTATTAT	GCGCTCTTTTGCTTGTTGAT	GGATACAGTGGTCGCCAAAT	60.5	740	INTERGENIC	--	--	--

INDEL marker IDs	Chromosomes/ unanchored scaffolds	Physical positions (bp)	InDels (*Kabuli* reference genome-CDC Frontier/PII)	Forward primers (5'-3')	Reverse primers (5'-3')	Anneling temperature (0C)	Expected amplified product size (bp)	Structural annotation		Functional annotation	
								Sequence components of *kabuli* genome	*Kabuli* gene accession IDs	NCBI-KOG	TFs
Ca1_P4_37712787	Ca1	37712787	A/ACATGTG	GGTGTTGGAGGTGTAATGGG	CATGTCCCGTATTCGCTTTT	60.1	639	INTERGENIC	--	--	--
Ca1_P4_37714757	Ca1	37714757	ATTT/ATTTT	TCGCTCACATATAAGGTTGCAT	TTCGCCCATATTTCCTTTTG	59.6	640	INTERGENIC	--	--	--
Ca1_P4_37716222	Ca1	37716222	T/TTA	ATGGACAAACTACATGGGGC	TCCAACAACAACAACAACATCA	59.7	499	INTERGENIC	--	--	--
Ca1_P4_37716984	Ca1	37716984	CTTT/CTT	TGATGTTGTTGTTGTTGTTGGA	GTTTTGGGTACACCCCATTG	59.9	473	INTERGENIC	--	--	--
Ca1_P4_37728195	Ca1	37728195	TAAAA/TAAA	CTCAAACTCGTTCCTGCCTC	CCCGCACCCTTACTACACAT	60.0	687	INTERGENIC	--	--	--
Ca1_P4_37747301	Ca1	37747301	TAAA/TAAAA	ATGGGTTTGATGGCGATTAG	GGAAACAACATGTGGCATAAAA	59.8	125	INTERGENIC	--	--	--
Ca1_P4_37747367	Ca1	37747367	GTTTTTTTTT/GTTTTTTTTTTTT	ATGGGTTTGATGGCGATTAG	TGCCACGTCTTAGTTTAATTAGCA	59.8	497	INTERGENIC	--	--	--
Ca1_P4_37747411	Ca1	37747411	C/CGTTTATGT	ATGGGTTTGATGGCGATTAG	TGCCACGTCTTAGTTTAATTAGCA	59.8	497	INTERGENIC	--	--	--
Ca1_P4_37754968	Ca1	37754968	CAAAAA/CAAAA	ATTGTTCACCAGGGCACTTC	CGCGCACAAAAGTAATTGAA	60.0	852	INTERGENIC	--	--	--
Ca1_P4_37759391	Ca1	37759391	GT/G	CCATCTTGATCCTTTGCCTC	TCAAACGGTCAAATTGGATG	59.6	268	INTERGENIC	--	--	--

INDEL marker IDs	Chromosomes/ unanchored scaffolds	Physical positions (bp)	InDels (*Kabuli* reference genome-CDC Frontier/PII)	Forward primers (5'-3')	Reverse primers (5'-3')	Anneling temperature (0C)	Expected amplified product size (bp)	Structural annotation		Functional annotation	
								Sequence components of *kabuli* genome	*Kabuli* gene accession IDs	NCBI-KOG	TFs
Ca1_P4_37761657	Ca1	37761657	TCC/TC	GTTGGGCAACAATTGCTTTT	TAATCCTTGTGGAGGCGATT	60.0	375	INTERGENIC	--	--	--
Ca1_P4_37773157	Ca1	37773157	CTT/CT	GACGAGGAGTGGTTTGGTGT	AGCCGACATTGGAAAAACTG	60.0	687	INTERGENIC	--	--	--
Ca1_P4_37775837	Ca1	37775837	TAAAAAAAAA/TAAAAAAAAAAA	GCGAGGTATGGTGATTTCGT	GAGGTATGCTTTTTAACCCCTTT	60.0	283	INTERGENIC	--	--	--
Ca1_P4_377760	Ca1	377760	TAAAAAA/TAAAAAAA	TCGGCTTTTAATACAAGAGTGATTC	GCAACGCTCCTTTCTTTCAC	60.0	553	INTERGENIC	--	--	--
Ca1_P4_37776874	Ca1	37776874	A/AC	CTCCTTTCGTCCCATAACGA	TGGAATTTTGAGTTTGCGTTC	60.1	622	INTERGENIC	--	--	--
Ca1_P4_37781056	Ca1	37781056	TCA/T	CCGGTAATCTGAAGTTTGGC	ACAGAGGCAAAACAGGTTGG	59.6	796	INTERGENIC	--	--	--
Ca1_P4_37782834	Ca1	37782834	AATATATATATATATATATATA/AATATATATATATATATATATATA	CTTTGGATCTACGCATCGGT	CAAGGCTTGACAGTGAGCAA	60.1	699	INTERGENIC	--	--	--
Ca1_P4_37783462	Ca1	37783462	TTT/TTTCTT	ATACCAGACGCACCGAAGAG	CAAACTCAAAAGAGAGGGCG	60.3	106	INTERGENIC	--	--	--
Ca1_P4_37800107	Ca1	37800107	TA/TATTTAAA	TGGGTCCTAAACCGCAATAC	CAAAAATGATGCAACCATCG	59.8	371	INTERGENIC	--	--	--
Ca1_P4_37800689	Ca1	37800689	ATATTATTATTATT/ATATTATTATT	ACCGATGGTTGCATCATTTT	CAACATGCAAACATGCACAA	60.2	731	INTERGENIC	--	--	--

**INDEL marker IDs**	**Chromosomes/ unanchored scaffolds**	**Physical positions (bp)**	**InDels (*Kabuli* reference genome-CDC Frontier/PII)**	**Forward primers (5'-3')**	**Reverse primers (5'-3')**	**Anneling temperature (0C)**	**Expected amplified product size (bp)**	**Structural annotation**		**Functional annotation**	
								**Sequence components of *kabuli* genome**	***Kabuli* gene accession IDs**	**NCBI-KOG**	**TFs**
Ca1_P4_37802286	Ca1	37802286	CAAAAAAAAAAA/CAAAAAAAAAAAAAAA	GAAAGGAGTTCGTTCTCGGA	TTGCGTCAAATCCACACAAT	59.4	625	INTERGENIC	--	--	--
Ca1_P4_37811315	Ca1	37811315	A/ATC	GCACTCAAATGCATGAAAGC	TGGCAAAAGAATGGATGATG	59.4	213	INTERGENIC	--	--	--
Ca1_P4_37811375	Ca1	37811375	TAAAAAA/TAAAAAAA	CATCATCCATTCTTTTGCCA	CACACCGAGCCAAATTTTAAG	59.5	479	INTERGENIC	--	--	--
Ca1_P4_37811640	Ca1	37811640	GTTTTTTT/GTTTTTTTT	CATCATCCATTCTTTTGCCA	AGTTCCCATATGTTGCAGCC	59.5	654	INTERGENIC	--	--	--
Ca1_P4_37812183	Ca1	37812183	GTTTTTTT/GTTTTTT	GGCTGCAACATATGGGAACT	ACCTGAGTTTAACGGGGATG	60.0	706	INTERGENIC	--	--	--
Ca1_P4_3783626	Ca1	3783626	TATTTGCATTTG/TATTTG	TTCACAATTACCACAAAGATCG	GCGACTATCCAGGAAATCCA	57.7	433	INTERGENIC	--	--	--
Ca1_P4_37836904	Ca1	37836904	CAGTTAGTTAGTTAGTTAGTTAGTTA/CAGTTAGTTAGTTAGTTAGTTAGTTAGTTAGTTA	ATGGATGTAGCGATTACGCC	CCAATACTCCACCTGCGATT	60.0	442	INTERGENIC	--	--	--
Ca1_P4_3784964	Ca1	3784964	TTAAATAAATAAATAAATAAATAAATAAATAAATAAA/TTAAATAAATAAATAAATAAATAAATAAATAAA	CTCCTTCTATGGGGCATCAA	ATAAGCCCCTTCCAAAGCAT	60.0	272	INTERGENIC	--	--	--
Ca1_P4_37852569	Ca1	37852569	TAAAAAAA/TAAAAAA	TGTGGTATGTGCGGTTCCTA	TTTGATGGGTTCATGTTCCA	60.0	814	INTERGENIC	--	--	--
Ca1_P4_37913622	Ca1	37913622	AATATATATATATA/AATATATATATATATA	ACGTTCGCCACCTATCTCAT	CCTTATGCGGTAATCCCTGA	59.6	563	INTERGENIC	--	--	--

INDEL marker IDs	Chromosomes/ unanchored scaffolds	Physical positions (bp)	InDels (*Kabuli* reference genome-CDC Frontier/PII)	Forward primers (5'-3')	Reverse primers (5'-3')	Anneling temperature (0C)	Expected amplified product size (bp)	Structural annotation		Functional annotation	
								Sequence components of *kabuli* genome	*Kabuli* gene accession IDs	NCBI-KOG	TFs
Ca1_P4_37917967	Ca1	37917967	ATATGACAGTTA/ATA	TCCACAAGTTATTCGACGAAAC	TTCCATTGATCTACGTTAATGTTTG	59.1	558	INTERGENIC	--	--	--
Ca1_P4_37927331	Ca1	37927331	TAAA/TAAATTGTATCAAA	ATTTTGATCGCGGAAGTTTG	TCAATCAACCAGATTTTGCATT	60.1	553	INTERGENIC	--	--	--
Ca1_P4_37927862	Ca1	37927862	G/GTA	TGCAAAATCTGGTTGATTGAG	TCCCAGATCAAAATTGGGTT	58.8	745	INTERGENIC	--	--	--
Ca1_P4_37928853	Ca1	37928853	CT/CTGAAT	CCGTGCAACAATGTCAATTC	GACCACCATATTAACCGTCACA	60.0	571	INTERGENIC	--	--	--
Ca1_P4_37929646	Ca1	37929646	TAAAAAAAA/TAAAAAAAAAA	TGTGACGGTTAATATGGTGGTC	TTGACTTGTTTTGCGGATTG	59.6	817	INTERGENIC	--	--	--
Ca1_P4_37929711	Ca1	37929711	ATTTTTTT/ATTTTTTTT	TGTGACGGTTAATATGGTGGTC	TTGACTTGTTTTGCGGATTG	59.6	817	INTERGENIC	--	--	--
Ca1_P4_37929753	Ca1	37929753	ATTTTTTTTT/ATTTTTTTTTTT	TGTGACGGTTAATATGGTGGTC	TTGACTTGTTTTGCGGATTG	59.6	817	INTERGENIC	--	--	--
Ca1_P4_37935823	Ca1	37935823	TAAAAAAA/TAAAAAAAA	TTGTGGATAGTTTTCTCCCTGAA	CTCAAACTTCCCCGATCAAA	60.0	767	INTERGENIC	--	--	--
Ca1_P4_37954337	Ca1	37954337	GTTTTT/GTTT	TCTTTTCTCCGATCCCATTG	CGACTCCCGAACTCAAAATC	60.0	868	INTERGENIC	--	--	--
Ca1_P4_37980254	Ca1	37980254	GTAATAATAATAATAATAATAATAATAATAATAATAATAATAATAATAATAATA/GTAATAATAATAATAATAATAATAATAATAATAATAATAATAATAATAATAATAATA	CCATTTTGTCCATGTTTCCC	AATTGGGGTGTCCATTTTGA	60.0	348	INTERGENIC	--	--	--

INDEL marker IDs	Chromosomes/ unanchored scaffolds	Physical positions (bp)	InDels (*Kabuli* reference genome-CDC Frontier/PII)	Forward primers (5'-3')	Reverse primers (5'-3')	Anneling temperature (0C)	Expected amplified product size (bp)	Structural annotation		Functional annotation	
								Sequence components of *kabuli* genome	*Kabuli* gene accession IDs	NCBI-KOG	TFs
Ca1_P4_37993665	Ca1	37993665	CAAAAAA/CAAAAA	GTGAGCCCTTTACCAATCCA	CAATTCCCACAGGTACGCTT	59.9	654	INTERGENIC	--	--	--
Ca1_P4_37993738	Ca1	37993738	TG/T	GTGAGCCCTTTACCAATCCA	CAATTCCCACAGGTACGCTT	59.9	654	INTERGENIC	--	--	--
Ca1_P4_38043395	Ca1	38043395	GATATATATATATATATATATATATATATATATATATATATATATAT/GATATATATATATATATATATATATATATATATATATAT	TCTGAATTTGGCAACATCATT	CTTTCGCGATTCAGTCAAAA	58.1	846	INTERGENIC	--	--	--
Ca1_P4_38064927	Ca1	38064927	AT/ATT	GTGGATTCCTCCTTCATCCA	CCCGACGTTAAAGCAGAAAG	59.9	392	INTERGENIC	--	--	--
Ca1_P4_38067157	Ca1	38067157	ATT/AT	TTCGTAAATCATTCGCCCAT	ACAAACAGGGTCCTTTCCAA	60.3	655	INTERGENIC	--	--	--
Ca1_P4_38070666	Ca1	38070666	T/TA	AACCATTGGGGGAGAATAGAA	TTGTTAGAACCATTGGGGGA	59.7	362	INTERGENIC	--	--	--
Ca1_P4_38070895	Ca1	38070895	ATGTGTGT/ATGTGT	CTCCCCCAATGGTTCTAACA	CACTGGAATCCGTTTCTTTTG	59.8	587	INTERGENIC	--	--	--
Ca1_P4_38071534	Ca1	38071534	TACACACAC/TACACAC	CTCCTCCTCATTCACCCAAC	ACACACCCACTCTCCTTTGC	59.5	189	INTERGENIC	--	--	--
Ca1_P4_38090626	Ca1	38090626	CTTTTTTT/CTTTTTTTT	TATTCGTGGCCCCTTAACTT	TGATTTGATGCCGTGAGTGT	58.6	821	INTERGENIC	--	--	--
Ca1_P4_38090984	Ca1	38090984	CAT/CATATATAT	GGAATAAAGGACTAAATCGGGAA	TGATTTGATGCCGTGAGTGT	59.7	545	INTERGENIC	--	--	--

INDEL marker IDs	Chromosomes/ unanchored scaffolds	Physical positions (bp)	InDels (*Kabuli* reference genome-CDC Frontier/PII)	Forward primers (5'-3')	Reverse primers (5'-3')	Anneling temperature (0C)	Expected amplified product size (bp)	Structural annotation		Functional annotation	
								Sequence components of *kabuli* genome	*Kabuli* gene accession IDs	NCBI-KOG	TFs
Ca1_P4_38106060	Ca1	38106060	TATA/T	AGGAGAAAATTGCAACACCC	TGCATATACACGTAAATTCGCC	59.0	291	INTERGENIC	--	--	--
Ca1_P4_38108337	Ca1	38108337	ATTTTTT/ATTTTTTT	CTTTTGAGCCGTAAGGCAAG	CCAAGCCAGTCAAAACACAA	60.0	551	INTERGENIC	--	--	--
Ca1_P4_38130303	Ca1	38130303	C/CG	AAAGGGGGAGAACCATCTGT	TGCATTTGCTTGACTTGGAG	59.8	680	INTERGENIC	--	--	--
Ca1_P4_38131605	Ca1	38131605	C/CT	CGTAACTCGTGTTGGGTGAA	GAAGCAAGAGGCAACTCCAC	59.6	379	INTERGENIC	--	--	--
Ca1_P4_38141538	Ca1	38141538	GGAA/GGAAGAA	TCATCAAATCCGTAATGCAAA	TGTTTTATAAGCATCATGTGTCACTC	59.0	166	INTERGENIC	--	--	--
Ca1_P4_38143864	Ca1	38143864	GAAAAAAAA/GAAAAAAAAA	TTTGTTCTCTCACCCGCTTT	TGATAGGTCTCATAGCCCCG	59.9	610	INTERGENIC	--	--	--
Ca1_P4_38147657	Ca1	38147657	TC/TCC	TGTTGACACATCACATCCTTAACA	TTTCATGTCTCATTTCCCCA	60.3	858	INTERGENIC	--	--	--
Ca1_P4_38148096	Ca1	38148096	CAAAAAAAAAA/CAAAAAAAA	TGGGGAAATGAGACATGAAA	ACGTTGCGCAGGTGTAATTT	58.9	567	INTERGENIC	--	--	--
Ca1_P4_38148667	Ca1	38148667	GAAA/GAAAA	GCAACGTGCGGGTTATATTT	GAAAAAGCGCTGTAAATGGC	59.9	228	INTERGENIC	--	--	--
Ca1_P4_38170055	Ca1	38170055	C/CT	GAAAAAGGGAGCTTCATCCC	TTGGAGCTCAAAGAGGAGGA	60.0	935	INTERGENIC	--	--	--

**INDEL marker IDs**	**Chromosomes/ unanchored scaffolds**	**Physical positions (bp)**	**InDels (*Kabuli* reference genome-CDC Frontier/PII)**	**Forward primers (5'-3')**	**Reverse primers (5'-3')**	**Anneling temperature (0C)**	**Expected amplified product size (bp)**	**Structural annotation**		**Functional annotation**	
								**Sequence components of *kabuli* genome**	***Kabuli* gene accession IDs**	**NCBI-KOG**	**TFs**
Ca1_P4_38185784	Ca1	38185784	ACC/ACCC	TCCATGCTCCATGCATTAAA	GGAAGTGTGATTCAGTTGGTGA	60.0	686	INTERGENIC	--	--	--
Ca1_P4_38193113	Ca1	38193113	CAAAAAAAAAA/CAAAAAAAAAAAA	CGAACAAACTCACCGGATCT	CCAATGGAGTCCCAAAAAGA	60.1	724	INTERGENIC	--	--	--
Ca1_P4_38223459	Ca1	38223459	GAA/GAAA	ATTGCTTTCCCAAATCAACG	GCCCTTTTTCATCATGCTTC	59.9	573	INTERGENIC	--	--	--
Ca1_P4_3822628	Ca1	3822628	AATATATATATATATATATATATAT/AATATATATATATATATATATATATAT	GCACGTTTTTAAAAGGAGTCAAA	TGGAGAAATTTGTTCGTGAGC	59.7	529	INTERGENIC	--	--	--
Ca1_P4_38227247	Ca1	38227247	GT/GTT	CTGAGAATGGCAGCACAAAA	TTGATGTATTTTGGCGATTCA	60.0	348	INTERGENIC	--	--	--
Ca1_P4_38236366	Ca1	38236366	A/AGAT	AGCAAAGAGTCTGCCTTGGA	TTCTTGCTTTGCTCCTGGAT	60.1	630	INTERGENIC	--	--	--
Ca1_P4_38242184	Ca1	38242184	AGTGTG/AGTG	CCTTGAGTTCGACACTCGGT	CCATTTCGAACGAAGAAAGC	60.3	768	INTERGENIC	--	--	--
Ca1_P4_38243964	Ca1	38243964	ATTTTTTT/ATTTTTT	GCCTCCTTTTAATGGCACAA	GCCCAGAAGTCCTGTGTAGC	60.1	791	INTERGENIC	--	--	--
Ca1_P4_38281902	Ca1	38281902	TTCT/TT	GAAACACGCTCGAACTCCTC	TGGAAGAAAGAACCTTTGGC	60.0	888	INTERGENIC	--	--	--
Ca1_P4_38283164	Ca1	38283164	T/TCA	ATTCGTAGCAACGCATCCTT	GTGGGCCAAAAGCTACACAT	59.7	482	INTERGENIC	--	--	--

INDEL marker IDs	Chromosomes/ unanchored scaffolds	Physical positions (bp)	InDels (*Kabuli* reference genome-CDC Frontier/PII)	Forward primers (5'-3')	Reverse primers (5'-3')	Anneling temperature (0C)	Expected amplified product size (bp)	Structural annotation		Functional annotation	
								Sequence components of *kabuli* genome	*Kabuli* gene accession IDs	NCBI-KOG	TFs
Ca1_P4_38288491	Ca1	38288491	ATGTTGTTGT/ATGTTGT	CATCAAGAAAATTGCCCAGAA	TGAGTTGGCAAAATATCCATGA	60.1	529	INTERGENIC	--	--	--
Ca1_P4_38291576	Ca1	38291576	CA/C	TTGGGCATTGCAACTTACAC	AGGGCTTATGGCCCTAGGTA	59.6	964	INTERGENIC	--	--	--
Ca1_P4_38303577	Ca1	38303577	TT/TTGT	GGTCGTGCTGCAAGGATTAT	GTGTGGTACTCATGCGATGC	60.1	873	INTERGENIC	--	--	--
Ca1_P4_38393926	Ca1	38393926	CAAAAAAA/CAAAAAAAA	GTTGAGGCAGTAGCCTTTGC	GGTCACATTAAGGAAGCCGA	60.0	730	INTERGENIC	--	--	--
Ca1_P4_38450592	Ca1	38450592	ATGTGTGTGTGTGTGTG/ATGTGTGTGTGTGTG	TGCTTGACAATCTCCTGCAC	TTATCTTCGATCCCACCAGG	60.0	454	INTERGENIC	--	--	--
Ca1_P4_38483352	Ca1	38483352	TAAAAAAAAAAAA/TAAAAAAAAAA	AGACCTCAATGTTGCGAACC	TCGGTGAATGATGGAGTGAA	60.1	551	INTERGENIC	--	--	--
Ca1_P4_38493608	Ca1	38493608	ATTTTTTTT/ATTTTTTT	TCTGGGGAAGCGACTTCTAA	TGTGATTGGTAATGTGGCAAA	59.9	533	INTERGENIC	--	--	--
Ca1_P4_38494341	Ca1	38494341	TAAGAAGAAGAAGAAGAAGAAGAA/TAAGAAGAAGAAGAAGAAGAA	ATCCGTCTTCAAGTTGTGGC	ACGCCCCTTGAGTGAGATTA	60.1	462	INTERGENIC	--	--	--
Ca1_P4_38514075	Ca1	38514075	GTTTTTTT/GTTTTTTTT	GACGTGGTTACAAGGGAGGA	TGTGGGAATCTTCTGCAAAC	60.0	604	INTERGENIC	--	--	--
Ca1_P4_38568165	Ca1	38568165	GTTTTTTT/GTTTTTTTT	GGTTCCGTTTAGATTGAACTCG	GCAACGGTCATTTCAAGGTT	60.0	229	INTERGENIC	--	--	--

INDEL marker IDs	Chromosomes/ unanchored scaffolds	Physical positions (bp)	InDels (*Kabuli* reference genome-CDC Frontier/PII)	Forward primers (5'-3')	Reverse primers (5'-3')	Anneling temperature (0C)	Expected amplified product size (bp)	Structural annotation		Functional annotation	
								Sequence components of *kabuli* genome	*Kabuli* gene accession IDs	NCBI-KOG	TFs
Ca1_P4_38577185	Ca1	38577185	GTTTTT/GTTTT	CCATTTTCTGTCCCCTTTCA	GCATTGGCTAAGCATGGATT	59.9	760	INTERGENIC	--	--	--
Ca1_P4_38613531	Ca1	38613531	GAAAAAA/GAAAAA	CCTTCCTTCATGGTGATTTGA	TCCCTGATCACACTTCCTCTG	59.9	610	INTERGENIC	--	--	--
Ca1_P4_38623512	Ca1	38623512	ATTTTTT/ATTTT	AGCAACTGCTTCAACCCACT	CGTCACGGCTTCCATTTATT	59.9	858	INTERGENIC	--	--	--
Ca1_P4_38636457	Ca1	38636457	AAT/AATAT	AATATCCCAACAGCCTCACG	TTGATTGAAACATTGATTTCCG	60.0	710	INTERGENIC	--	--	--
Ca1_P4_38639253	Ca1	38639253	ATTTATTTTTT/ATT	TGCCCTTCATGTACCAATCA	ATGTGCGCATGATTGATGTT	59.9	436	INTERGENIC	--	--	--
Ca1_P4_38656327	Ca1	38656327	T/TG	AATGGGTTTTGGAGTGCAAG	ACAAAGAGAGAACATGGCCC	60.0	261	INTERGENIC	--	--	--
Ca1_P4_38657360	Ca1	38657360	TAAAAAAA/TAAAAAA	CCATCAGTGATAACTTGTCCCA	CACAGGACAAGTGCAATCTCA	59.9	451	INTERGENIC	--	--	--
Ca1_P4_38671088	Ca1	38671088	CTTGTGATT/CTTGTGATTATTTTGTTGTGATT	TGTTTGGGTCCTTGATGGTT	TTAGAGCGGAGCTTCTTCCA	60.2	825	INTERGENIC	--	--	--
Ca1_P4_38708599	Ca1	38708599	AATATATATATATATATAT/AATATATATATAT	TTTGGTTGCAAATTGTGTGG	GAAGGCATGAAACATCACGA	60.4	435	INTERGENIC	--	--	--
Ca1_P4_38708894	Ca1	38708894	AATTATTA/AATTA	TTTGGTTGCAAATTGTGTGG	CAGCTCTTGGGATCGAGAAG	60.4	781	INTERGENIC	--	--	--

INDEL marker IDs	Chromosomes/ unanchored scaffolds	Physical positions (bp)	InDels (*Kabuli* reference genome-CDC Frontier/PII)	Forward primers (5'-3')	Reverse primers (5'-3')	Anneling temperature (0C)	Expected amplified product size (bp)	Structural annotation		Functional annotation	
								Sequence components of *kabuli* genome	*Kabuli* gene accession IDs	NCBI-KOG	TFs
Ca1_P4_38744149	Ca1	38744149	CTTTTTT/CTTTTT	GAACAGTCTCATTAATGTCGCAA	TGTGCTTGTCCCACACACTT	59.3	283	INTERGENIC	--	--	--
Ca1_P4_38746497	Ca1	38746497	A/AAATC	TCAAGCCACAAAATTGGAAA	CCCGTGTAAAGCATTGATGG	59.1	454	INTERGENIC	--	--	--
Ca1_P4_38757124	Ca1	38757124	TAAAAAAAA/TAAAAAAA	CACACACCTCAGCAAAATGG	TGGAATTCCCTCCTTCCTCT	60.2	895	INTERGENIC	--	--	--
Ca1_P4_38759589	Ca1	38759589	TG/T	TGTACTCCCCCAGACGTAGG	TGTGCTTGTCCCACACACTT	60.0	883	INTERGENIC	--	--	--
Ca1_P4_38766572	Ca1	38766572	CTT/CTTT	GCTTGAGGCATTGTCCATTT	GAGCCACCGACTTGAAGAAG	60.1	443	INTERGENIC	--	--	--
Ca1_P4_38771127	Ca1	38771127	GTTATTATTATTAT/GTTATTATTATTATTATTAT	CCTTGTGGGAAGAAGCTTGA	ATATTGAGCGTCGTAACCCG	60.4	657	INTERGENIC	--	--	--
Ca1_P4_38773402	Ca1	38773402	AT/A	GTTTTGGCGTTGGTTTCACT	TGGGTGCTTTCTATAATGTGC	60.0	977	INTERGENIC	--	--	--
Ca1_P4_38800314	Ca1	38800314	TTCTCTC/TTCTCTCTCTC	TGGCACAAAAGAGTTGGTCA	AAATATTTGGTTTTCGGCCC	60.3	530	INTERGENIC	--	--	--
Ca1_P4_38852863	Ca1	38852863	A/AAT	ATGACCCGGAGGCTATAGGT	TATGGCTCAACACAAACCCA	59.8	187	INTERGENIC	--	--	--
Ca1_P4_38852972	Ca1	38852972	AATTAGGAT/AAT	TGGGTTTGTGTTGAGCCATA	TTTGAGTTCCTAAGGTCCCG	60.0	440	INTERGENIC	--	--	--

INDEL marker IDs	Chromosomes/ unanchored scaffolds	Physical positions (bp)	InDels (*Kabuli* reference genome-CDC Frontier/PII)	Forward primers (5'-3')	Reverse primers (5'-3')	Anneling temperature (0C)	Expected amplified product size (bp)	Structural annotation		Functional annotation	
								Sequence components of *kabuli* genome	*Kabuli* gene accession IDs	NCBI-KOG	TFs
Ca1_P4_38853658	Ca1	38853658	AA/AATAATA	CGGGACCTTAGGAACTCAAA	GGTGTGTTTTTGGGGTTTGT	59.2	549	INTERGENIC	--	--	--
Ca1_P4_38872978	Ca1	38872978	TAAAAAAA/TAAAAAA	TGGCCACTTCTTATCACAAAA	TGAAGCAAGAAAATTGTGCTGT	58.3	519	INTERGENIC	--	--	--
Ca1_P4_38874695	Ca1	38874695	TTA/TTATA	AAAGTTTTTGCCAAGTCCCA	AGGTCGTTCAACTGGATTCG	59.6	661	INTERGENIC	--	--	--
Ca1_P4_38875592	Ca1	38875592	TCA/T	ACCGAATCCAGTTGAACGAC	GGTCAATCCATCCATAGCCA	60.0	516	INTERGENIC	--	--	--
Ca1_P4_38875978	Ca1	38875978	CAAA/CAA	GCCGGCAACTCTTTTCAATA	AGACTGCGAACTGTCATCCA	60.2	679	INTERGENIC	--	--	--
Ca1_P4_38886569	Ca1	38886569	TAAAAAAAA/TAAAAAAAAAAA	GCAACACTAGAACAACAGACGG	ATTTATCCATCCATGTCGCC	59.8	786	INTERGENIC	--	--	--
Ca1_P4_38887147	Ca1	38887147	GTTTTTTT/GTTTTTTTT	GGCGACATGGATGGATAAAT	TTTCCGGTGGTACGAGAGAG	59.6	438	INTERGENIC	--	--	--
Ca1_P4_38890999	Ca1	38890999	AAGT/A	CTGGGGGCCATAAACCTAAT	TAAATGAGTCGGATAGCCCG	60.0	329	INTERGENIC	--	--	--
Ca1_P4_38895300	Ca1	38895300	ATTTTTTTTT/ATTTTTTTTTTTT	AATGGGTGTGTTTGTTTGAGA	TTTGAAAATCCCAAATTGCC	58.0	480	INTERGENIC	--	--	--
Ca1_P4_38896192	Ca1	38896192	CAAAA/CAAA	ATATGCAGCGGCTCTTGTTC	CAATGCTTCTTGCTTGTTTCA	60.4	378	INTERGENIC	--	--	--

INDEL marker IDs	Chromosomes/ unanchored scaffolds	Physical positions (bp)	InDels (*Kabuli* reference genome-CDC Frontier/PII)	Forward primers (5'-3')	Reverse primers (5'-3')	Anneling temperature (0C)	Expected amplified product size (bp)	Structural annotation		Functional annotation	
								Sequence components of *kabuli* genome	*Kabuli* gene accession IDs	NCBI-KOG	TFs
Ca1_P4_38897537	Ca1	38897537	TATAATAATAATAATAATAA/TATAATAATAATAA	GCTGCTCGTAGAGCCAAATAA	GGACTACTTTTGCGATTCAGC	59.6	486	INTERGENIC	--	--	--
Ca1_P4_38915417	Ca1	38915417	CATATATTATATATTATATATTAT/CATATATTATATATTAT	TTTTCACACAAATAACGCGG	AATGACGGTGGCAGAGTTTC	59.6	489	INTERGENIC	--	--	--
Ca1_P4_38922989	Ca1	38922989	GCCCC/GCCC	GAACCGGCATTTCTTGTGTT	ATGGAAAGGTTCGAATGTGC	60.0	496	INTERGENIC	--	--	--
Ca1_P4_38941583	Ca1	38941583	AAAAA/AAAAAATATATAAAA	TCACATTAACTGCAAAAGCACA	TCGACTTCTTGGTGGAGTTTG	59.4	510	INTERGENIC	--	--	--
Ca1_P4_38943432	Ca1	38943432	TA/TAA	GCTCTTGTGAGTTGGTGTTGC	TGGCCTCTTCGGTACTGATT	60.9	571	INTERGENIC	--	--	--
Ca1_P4_38944490	Ca1	38944490	T/TC	TGAAGGGGATCCATCATATAAC	GCGATATCCAGAATGGGCTA	57.8	628	INTERGENIC	--	--	--
Ca1_P4_38945208	Ca1	38945208	ATTTTTTTT/ATTTTTTTTTT	CCCATTCTGGATATCGCACT	CCCGAAAACTTCATTCCAAA	59.9	825	INTERGENIC	--	--	--
Ca1_P4_390566	Ca1	390566	TATATCATATCATA/TATATCATATCATATCATA	TTGGTGGATCGTTTAGGAGG	TACGGTCCCCACTCATTCTC	59.9	782	INTERGENIC	--	--	--
Ca1_P4_39105122	Ca1	39105122	GTAATTAATTA/GTAATTA	CATTTTCTGACGCACCTTCA	TCTATGTTGGCTTACGCACG	59.8	483	INTERGENIC	--	--	--
Ca1_P4_39110990	Ca1	39110990	ATT/ATTT	TTTTATGGGTCTGTGTAATGCG	ACACGGGGCACTTATTTTGA	59.9	273	INTERGENIC	--	--	--

INDEL marker IDs	Chromosomes/ unanchored scaffolds	Physical positions (bp)	InDels (*Kabuli* reference genome-CDC Frontier/PII)	Forward primers (5'-3')	Reverse primers (5'-3')	Anneling temperature (0C)	Expected amplified product size (bp)	Structural annotation		Functional annotation	
								Sequence components of *kabuli* genome	*Kabuli* gene accession IDs	NCBI-KOG	TFs
Ca1_P4_39141870	Ca1	39141870	AATATAATATATATAATATATATAATAT/AATATAATATATATAATAT	CAGCAAAACAACGCACATTC	AACAAGTGGGTGACCTGCAT	60.3	644	INTERGENIC	--	--	--
Ca1_P4_39143008	Ca1	39143008	CTTTTTTTTT/CTTTTTTTTTT	TTCACAAATGTTGTTTTCCTCG	TCCATCTCCAACAACATCAAA	60.0	422	INTERGENIC	--	--	--
Ca1_P4_392320	Ca1	392320	CA/CAGAA	TGAGTTGCATCAAATGGTGTT	CCTAACGCAGAGAGGGTTTG	59.0	851	INTERGENIC	--	--	--
Ca1_P4_39254298	Ca1	39254298	AAATAATAAT/AAATAAT	TTCAATTTTTCCGCCCTATG	ATGGCTACACCAATGCATCC	59.9	589	INTERGENIC	--	--	--
Ca1_P4_39261977	Ca1	39261977	A/AT	GGTTTGCTGTCGAGCCTTAG	AAACACAAGTCCAATCAAAAGG	60.0	421	INTERGENIC	--	--	--
Ca1_P4_39265068	Ca1	39265068	GTTTTT/GTTTTTT	TTGGAAGCTAAGAAGCCGAA	ATAATGGCCAAGGAACATGC	60.1	566	INTERGENIC	--	--	--
Ca1_P4_39303782	Ca1	39303782	ATTTT/ATTTTT	CCATCCACATTTTCAAGTGATTT	TGAATCGTGAGGGTGTTATGA	60.1	533	INTERGENIC	--	--	--
Ca1_P4_39319575	Ca1	39319575	TTTATTTATTTGCTTATTTATTT/TTTATTTATTTGCTTATTTATTTGCTTATTTATTT	TTTGCCTCTTCTCCTCCAGA	CCACCAAATACAAATAAATTACTTACG	60.1	970	INTERGENIC	--	--	--
Ca1_P4_39382557	Ca1	39382557	AC/A	ATACGGTAGCAAGACCGCAC	ATCGCCGATTGATCATTTTC	60.2	915	INTERGENIC	--	--	--
Ca1_P4_39387109	Ca1	39387109	TC/TCC	GAAAGTAAAGACGCGTCCCA	CTGGGCCAAGGTTGAGACTA	60.2	548	INTERGENIC	--	--	--

INDEL marker IDs	Chromosomes/ unanchored scaffolds	Physical positions (bp)	InDels (*Kabuli* reference genome-CDC Frontier/PII)	Forward primers (5'-3')	Reverse primers (5'-3')	Anneling temperature (0C)	Expected amplified product size (bp)	Structural annotation		Functional annotation	
								Sequence components of *kabuli* genome	*Kabuli* gene accession IDs	NCBI-KOG	TFs
Ca1_P4_39390988	Ca1	39390988	TA/TAA	GCCAGCTCACGTCTAATGAA	GCCTAATTTTGCCACCGATA	59.0	566	INTERGENIC	--	--	--
Ca1_P4_39392138	Ca1	39392138	ATTTTTTTT/ATTTTTTTTT	CGCCCCTGGGTAATATGTTT	AATCTATCAGGCACCCATGC	60.9	713	INTERGENIC	--	--	--
Ca1_P4_39399195	Ca1	39399195	CAT/CATAT	CATTCGCCCAAGAATCATCT	GGATCTTTTGGCAGGTTGAA	60.0	278	INTERGENIC	--	--	--
Ca1_P4_39400591	Ca1	39400591	TATTAATT/TATT	GTCGTGTGTCAGCCCCTATT	CAAATGACCAAATCGGAAAAA	60.0	599	INTERGENIC	--	--	--
Ca1_P4_39400871	Ca1	39400871	CTTTTTTTTTT/CTTTTTTTTTTT	TGGTAAGCATGAAGAAGGCA	TTTCTTCTCGATTTGGTCCTG	59.4	813	INTERGENIC	--	--	--
Ca1_P4_39403148	Ca1	39403148	CTTTTTTTT/CTTTTTTT	TCTCTAATCAATGGTTGCAAAA	CAGTGCGAAATGCTGACCTA	57.5	806	INTERGENIC	--	--	--
Ca1_P4_39406657	Ca1	39406657	CA/CAAA	ACCATGAAACCTTCGACCTG	AAAAAGGTGACGTGGCAGAC	60.0	634	INTERGENIC	--	--	--
Ca1_P4_39428106	Ca1	39428106	CTTTT/CTTT	AGAGCGGAGATAATGGGTCA	GTGCCAGTCGATTGCCTAAT	59.7	593	INTERGENIC	--	--	--
Ca1_P4_39428398	Ca1	39428398	TAAATCGATTGGGAAATCGATT/TAAATCGATT	CGGAACCAATTGTTGGACTT	GTGCCAGTCGATTGCCTAAT	59.8	284	INTERGENIC	--	--	--
Ca1_P4_39430506	Ca1	39430506	ATT/ATTTT	TGCATCTCCCTCAAGCTTTT	TGGAAAATTTTGGGGTTCAA	60.0	976	INTERGENIC	--	--	--

INDEL marker IDs	Chromosomes/ unanchored scaffolds	Physical positions (bp)	InDels (*Kabuli* reference genome-CDC Frontier/PII)	Forward primers (5'-3')	Reverse primers (5'-3')	Anneling temperature (0C)	Expected amplified product size (bp)	Structural annotation		Functional annotation	
								Sequence components of *kabuli* genome	*Kabuli* gene accession IDs	NCBI-KOG	TFs
Ca1_P4_39431107	Ca1	39431107	A/AC	TTGAACCCCAAAATTTTCCA	TCATTACCCCCTTCCACTTG	60.1	583	INTERGENIC	--	--	--
Ca1_P4_39431958	Ca1	39431958	CAAA/CAA	TCAAGTGGAAGGGGGTAATG	ACCCCCAATCTGCATAAAAA	59.8	902	INTERGENIC	--	--	--
Ca1_P4_39432122	Ca1	39432122	CTTTTTTTT/CTTTTTTTTTTTTT	CAATTGCACAAATCATTGTTGA	CAGGTGCATTATTCGGTGTG	59.5	870	INTERGENIC	--	--	--
Ca1_P4_39450720	Ca1	39450720	TAAAAAA/TAAAAAAA	CCTGTGCCTTTGCTTTATTTG	TTGCATAAATGGGAGGGAAG	59.8	705	INTERGENIC	--	--	--
Ca1_P4_39460851	Ca1	39460851	ACCCCC/ACCCCCC	GTGCCTTAGAAACCCCATCA	CCAACATTGCATTGAACCTG	59.9	820	INTERGENIC	--	--	--
Ca1_P4_39463854	Ca1	39463854	AAATTTAAT/AAAT	ATATTGAGAGGTTGCGTGGG	ACCATCTCGATGCAACACAA	60.0	556	INTERGENIC	--	--	--
Ca1_P4_39463964	Ca1	39463964	TAAA/TAAAAA	ATATTGAGAGGTTGCGTGGG	ACCATCTCGATGCAACACAA	60.0	556	INTERGENIC	--	--	--
Ca1_P4_39472871	Ca1	39472871	CAAAAAAAAAA/CAAAAAAAA	CATCAAACTCTAGACGGGGG	TTTTAGGCGAATATGGAGCAA	59.5	587	INTERGENIC	--	--	--
Ca1_P4_39504560	Ca1	39504560	T/TTATTA	CAACTCGGTTCACACCTTCA	CAGCTAAAATACGGGGACCA	59.7	418	INTERGENIC	--	--	--
Ca1_P4_39508256	Ca1	39508256	GAAAAAAAAA/GAAAAAAAAAAA	ACCTTTAGACTTGGGGCACA	CGGTTACCATTGTAAAAACAATCA	59.6	254	INTERGENIC	--	--	--

INDEL marker IDs	Chromosomes/ unanchored scaffolds	Physical positions (bp)	InDels (*Kabuli* reference genome-CDC Frontier/PII)	Forward primers (5'-3')	Reverse primers (5'-3')	Anneling temperature (0C)	Expected amplified product size (bp)	Structural annotation		Functional annotation	
								Sequence components of *kabuli* genome	*Kabuli* gene accession IDs	NCBI-KOG	TFs
Ca1_P4_39511343	Ca1	39511343	CA/CAA	CGACGAAAGTGATGTGCATT	TGGATTTCACAATCATGGGTT	59.7	136	INTERGENIC	--	--	--
Ca1_P4_39574370	Ca1	39574370	T/TA	CACACATCTATACAACCGCGA	TCAAAGCGTTGTTGAGTGCT	59.6	164	INTERGENIC	--	--	--
Ca1_P4_39584232	Ca1	39584232	CAAAAAAAAAA/CAAAAAAAA	GCAGCTGGCAGACATAATGA	ATTTGCTTCAGCGCTGCTAT	60.0	519	INTERGENIC	--	--	--
Ca1_P4_39595566	Ca1	39595566	ATTTTTTTTT/ATTTTTTTTTTTTTTT	GAGCTCACTCAAGCATGTCG	TTTGAGATCTTATTTTTGGTTTAATGA	59.7	573	INTERGENIC	--	--	--
Ca1_P4_39605985	Ca1	39605985	CTTTT/CTTTTT	TTGGCCAAATTTTCACAACA	TTGGTACTATGCACGGCTTG	59.9	439	INTERGENIC	--	--	--
Ca1_P4_39606006	Ca1	39606006	CTT/CT	TTGGCCAAATTTTCACAACA	TTGGTACTATGCACGGCTTG	59.9	439	INTERGENIC	--	--	--
Ca1_P4_39608275	Ca1	39608275	AGGG/AGGGG	GGTCGACACATGGAGAGTTG	TTATTACCAAACTGCCCCCA	59.1	637	INTERGENIC	--	--	--
Ca1_P4_39609113	Ca1	39609113	AATAT/AAT	ATGGCCTTTCATTTTCAACG	TTGTCTCAAGCGTTCAATTTTT	59.9	725	INTERGENIC	--	--	--
Ca1_P4_39621400	Ca1	39621400	TC/T	TGATCGTTGATCCAATCAAAA	TCTTCCAGCCTCTTTACCGA	59.0	537	INTERGENIC	--	--	--
Ca1_P4_39652228	Ca1	39652228	ACCC/ACCCC	GGAGTGACAATGCCCTCAAT	CGGGAGATACGTAGGAGCAA	59.9	774	INTERGENIC	--	--	--

INDEL marker IDs	Chromosomes/ unanchored scaffolds	Physical positions (bp)	InDels (*Kabuli* reference genome-CDC Frontier/PII)	Forward primers (5'-3')	Reverse primers (5'-3')	Anneling temperature (0C)	Expected amplified product size (bp)	Structural annotation		Functional annotation	
								**Sequence components of *kabuli* genome**	***Kabuli* gene accession IDs**	**NCBI-KOG**	**TFs**
Ca1_P4_39654601	Ca1	39654601	CT/CTGTT	CAGGGTTTGACAGTGAACAAAG	CCAAATTGCCTCAAACACCT	59.7	696	INTERGENIC	--	--	--
Ca1_P4_39682708	Ca1	39682708	GCT/G	TCAATTGGCATTTTGCACAT	AAATTCAGCACTGCACCAAA	59.9	802	INTERGENIC	--	--	--
Ca1_P4_39718500	Ca1	39718500	TTCATAGTCATA/TTCATA	TTTGAGAAAAATCTTGACATCCAA	CCAAACGTCGTGATTTGTTG	60.0	656	INTERGENIC	--	--	--
Ca1_P4_39773308	Ca1	39773308	TTTTAAAATATTAAATTTAAAATATTAA/TTTTAAAATATTAA	AAATGTATTTGTTGTGGGGTGA	TTGGTTGTAAGGGGAACCAG	59.1	958	INTERGENIC	--	--	--
Ca1_P4_39782012	Ca1	39782012	TA/T	TTTTCCTAGACCATCTGCGG	CGATCAAATATTTTTCACCGAA	60.2	678	INTERGENIC	--	--	--
Ca1_P4_39783200	Ca1	39783200	ATT/ATTGACCTT	TGGACGGATGGTTATTGTTG	GTCGTTGTCAGGTTGTGTGG	59.2	453	INTERGENIC	--	--	--
Ca1_P4_39793723	Ca1	39793723	CAAA/CAAAA	TGTGAGGCATGTTTGAAAGG	TGCGGGTTCAATTTTAGACA	59.7	930	INTERGENIC	--	--	--
Ca1_P4_39837467	Ca1	39837467	GT/GTCCTTT	TTCATGGTGTTTTCTTGGCA	CGAGAAATAGCTCACGAGGC	60.1	534	INTERGENIC	--	--	--
Ca1_P4_39847433	Ca1	39847433	AAATA/AAATAATA	GAGGAAATTATTCCCGCAAA	TTTGGACTTCGTCGTTTTGA	59.0	735	INTERGENIC	--	--	--
Ca1_P4_39853129	Ca1	39853129	AACAGACA/AACAGACAATTAGAAAAACACAGACA	ATTGCCCACTTGGCTATGAC	CGTGGGAACTTGAACCTTGT	60.0	813	INTERGENIC	--	--	--

INDEL marker IDs	Chromosomes/ unanchored scaffolds	Physical positions (bp)	InDels (*Kabuli* reference genome-CDC Frontier/PII)	Forward primers (5'-3')	Reverse primers (5'-3')	Anneling temperature (0C)	Expected amplified product size (bp)	Structural annotation		Functional annotation	
								Sequence components of *kabuli* genome	*Kabuli* gene accession IDs	NCBI-KOG	TFs
Ca1_P4_39871055	Ca1	39871055	TAAA/TAAAA	CATTGGCCACCTTTAG GAAA	TGCATTGCTTCAGTCAT GGT	59.9	329	INTERGENIC	--	--	--
Ca1_P4_39906834	Ca1	39906834	CATGAGTA/CATGAGTATGAGTA	TTAAAATGCGCTCATG GTGT	CACGCTAATCTGAGCC AACA	59.2	677	INTERGENIC	--	--	--
Ca1_P4_39949239	Ca1	39949239	GAAAAAA/GAAAAAAA	TGAAGAATTTACAAATG CAATCTGA	TAGTCTGACAACCCCC GACT	60.0	475	INTERGENIC	--	--	--
Ca1_P4_39955215	Ca1	39955215	GTA/GTATA	CAAATGGATGAAGAGG AAAAGG	AACGACTTGCAATTGT CCCT	59.9	435	INTERGENIC	--	--	--
Ca1_P4_40010790	Ca1	40010790	TCCCCC/TCCCC	CCAACAATCTCCCCAT TTGT	TGTCAACAACAGAGGA TGGG	59.6	591	INTERGENIC	--	--	--
Ca1_P4_40021624	Ca1	40021624	AC/A	TGAAGCATGCAATTAG CAAAA	CATACCCCCATTAGCG GATA	59.5	435	INTERGENIC	--	--	--
Ca1_P4_40035747	Ca1	40035747	T/TG	AACTGGAGCCTACCGG AAAT	GAACCCAAGGGGTAAG GGTA	60.0	473	INTERGENIC	--	--	--
Ca1_P4_40056652	Ca1	40056652	ATTTTTT/ATTTTTTT	TTGGTCCCATCAAAAC CACT	TTTGCCCAGATTGTTCT TCC	60.2	680	INTERGENIC	--	--	--
Ca1_P4_40081687	Ca1	40081687	AATTTATATAT/AAT	TTTCCACCATTGGATG ATGA	TTGCCTGAAAAAGAGA TGTCAA	59.7	475	INTERGENIC	--	--	--
Ca1_P4_40086233	Ca1	40086233	GAAAA/GAAA	ACAACTCCTCACACGG GAAA	TTGACTCAATCCACGA GCAG	60.5	407	INTERGENIC	--	--	--

INDEL marker IDs	Chromosomes/ unanchored scaffolds	Physical positions (bp)	InDels (*Kabuli* reference genome-CDC Frontier/PII)	Forward primers (5'-3')	Reverse primers (5'-3')	Anneling temperature (0C)	Expected amplified product size (bp)	Structural annotation		Functional annotation	
								Sequence components of *kabuli* genome	*Kabuli* gene accession IDs	NCBI-KOG	TFs
Ca1_P4_40091424	Ca1	40091424	GAAAAAAAAAA/GAAAAAAAAAAA	AACTTGGATTTGGCACGAAC	CAACACCTTCTCTGATTTGAACA	60.0	705	INTERGENIC	--	--	--
Ca1_P4_40121038	Ca1	40121038	AATATATATATATATATATATAT/AATATATATATATATAT	ATTTCGGTGATCAGCGGTT	TCGAACCAAGGATTTTCCAC	60.5	683	INTERGENIC	--	--	--
Ca1_P4_40126750	Ca1	40126750	CATA/CA	AGGCGCCACAATCTAGAAGA	TTCTCCATCACAGCGTTCAG	60.0	544	INTERGENIC	--	--	--
Ca1_P4_40147055	Ca1	40147055	CGTTTCAAATTAAGT/CGT	ACGAAACAATCATTTGAACGTA	AGCGTTGCCAAAAATAATGC	57.3	532	INTERGENIC	--	--	--
Ca1_P4_40149739	Ca1	40149739	TCAACCA/TCA	GAAAGGGAGACATTGATCGG	TCTGCAACAACATTAAGAAGCA	59.5	697	INTERGENIC	--	--	--
Ca1_P4_401568	Ca1	401568	ATT/AT	CAAAATTACTGCAGGCCCAT	CCACGCTAAATCACATTCCA	60.0	580	INTERGENIC	--	--	--
Ca1_P4_40176468	Ca1	40176468	GTTTT/GTTT	AAACGCAATCTATGTGAAAACAA	TTTGTCATCACCACCCAAGA	58.7	618	INTERGENIC	--	--	--
Ca1_P4_40177252	Ca1	40177252	TAAAAAAAAAAAA/TAAAAAAAAA	TCTTGGGTGGTGATGACAAA	GCATCATGAGATATGGAAAGGA	59.9	764	INTERGENIC	--	--	--
Ca1_P4_40181142	Ca1	40181142	AATGGTGATGCGATGATGGTGATGCGATG/AATGGTGATGCGATG	GATGTGATGACGGTGATTCG	CAAATGGAGCTGTGATGTGG	59.9	273	INTERGENIC	--	--	--
Ca1_P4_40214195	Ca1	40214195	CT/C	ACAAAGGCAGGATTTTGTGG	AAGCTCTCACAAAGGGCAAA	60.0	158	INTERGENIC	--	--	--

INDEL marker IDs	Chromosomes/ unanchored scaffolds	Physical positions (bp)	InDels (*Kabuli* reference genome-CDC Frontier/PII)	Forward primers (5'-3')	Reverse primers (5'-3')	Anneling temperature (0C)	Expected amplified product size (bp)	Structural annotation		Functional annotation	
								Sequence components of *kabuli* genome	*Kabuli* gene accession IDs	NCBI-KOG	TFs
Ca1_P4_40216460	Ca1	40216460	GTT/GTTCTT	GTCTCTTCTGATTGGGGCAA	TGGGTGGAAAGCAATAGAGG	60.2	738	INTERGENIC	--	--	--
Ca1_P4_40219666	Ca1	40219666	TG/T	GACAAACTTCCACCTCCGTG	CCTTCCCTGCAAAGATACCA	60.5	701	INTERGENIC	--	--	--
Ca1_P4_40224219	Ca1	40224219	TAAAAAAAAAA/TAAAAAAAA	CATTTGCATCTGATTGGTGG	TCTTTGCCTCATAGGAGCGT	59.9	664	INTERGENIC	--	--	--
Ca1_P4_40231747	Ca1	40231747	GT/GTT	CGGTGTAATAAATAAGACGGAGTG	CTCAAATCCAAACACCTTGG	59.0	613	INTERGENIC	--	--	--
Ca1_P4_40232307	Ca1	40232307	CAAAAA/CAAA	TCCAAGGTGTTTGGATTTGAG	CAGAAATTTGGTTGAGGGGA	60.0	717	INTERGENIC	--	--	--
Ca1_P4_40246424	Ca1	40246424	TACA/TA	CAATGGAAATGTCCGAAACC	TGGTTCCCAAGATTCGAAAA	60.2	186	INTERGENIC	--	--	--
Ca1_P4_4026071	Ca1	4026071	CAAAAAAAAAAAAA/CAAAAAAAAAAAAA	GCATCATGTTTGGTTATGCG	CAAGGTCTCGAGCAATAGCC	60.0	692	INTERGENIC	--	--	--
Ca1_P4_40264315	Ca1	40264315	CAAAAAAAA/CAAAAAAA	TCAAATCATTGGTGAAGCCA	CCACCAGTCCAAATCAACCT	60.0	712	INTERGENIC	--	--	--
Ca1_P4_40281112	Ca1	40281112	AGGG/AGG	CGCATAATTGACTCCCGAAC	TTGTTTTGTTCTTCGCCCTT	60.5	214	INTERGENIC	--	--	--
Ca1_P4_40298582	Ca1	40298582	TAAAAAAAAAAA/TAAAAAAAAA	TTTTAAAGATGCTGAAACAAACAAA	AGCCGTGTTGATTTTCGCTA	59.3	251	INTERGENIC	--	--	--

INDEL marker IDs	Chromosomes/ unanchored scaffolds	Physical positions (bp)	InDels (*Kabuli* reference genome-CDC Frontier/PII)	Forward primers (5'-3')	Reverse primers (5'-3')	Anneling temperature (0C)	Expected amplified product size (bp)	Structural annotation		Functional annotation	
								Sequence components of *kabuli* genome	*Kabuli* gene accession IDs	NCBI-KOG	TFs
Ca1_P4_40300289	Ca1	40300289	GA/GAA	TGGGCTAGCAAAGGAGAGAA	CACCTGAAACCCCTTTTGAA	60.1	464	INTERGENIC	--	--	--
Ca1_P4_40313158	Ca1	40313158	TCT/TCTAACT	CGAATTGAGTTTACGCGGTT	GGATGCTTCTGAATGAATGGA	60.1	466	INTERGENIC	--	--	--
Ca1_P4_40313177	Ca1	40313177	CTCTCTCTTCTCTCTTCTCTCT/CTCTCTCTTCTCTCT	CGAATTGAGTTTACGCGGTT	GGATGCTTCTGAATGAATGGA	60.1	466	INTERGENIC	--	--	--
Ca1_P4_403262	Ca1	403262	TA/TAA	TGCAATGACAAAATAGGGCA	ACCGTTAATTTGGGTGGAAA	60.1	572	INTERGENIC	--	--	--
Ca1_P4_40326561	Ca1	40326561	GTT/GTTT	CCGGCAAATATGACGAACTT	AAACCAAGAACAAAATGGCG	60.0	540	INTERGENIC	--	--	--
Ca1_P4_40394902	Ca1	40394902	ATT/AT	GGTAGCCTTCATTGGGTGAA	GAGAACCTTGCTCTGATGCC	59.9	235	INTERGENIC	--	--	--
Ca1_P4_40398387	Ca1	40398387	CT/C	TGAAAAATACTTTTGGGCCG	AAAACGCCATGGATTGTCAT	59.9	858	INTERGENIC	--	--	--
Ca1_P4_403989	Ca1	403989	CT/CTCGTATTGACAGT	TTGCACCTTATGCTTCACCA	CAATGGGAGCGCAGTTTTAT	60.3	228	INTERGENIC	--	--	--
Ca1_P4_40400180	Ca1	40400180	TG/T	TTGGGGGAATTGGTGTAAAA	ATCACACAAATCACGAGCCA	60.0	801	INTERGENIC	--	--	--
Ca1_P4_40407895	Ca1	40407895	GAAAAAA/GAAAAAAA	TGGATGTAGCCTTCATTGGG	TTCAAAAGAAGCAAAACCGA	60.9	473	INTERGENIC	--	--	--

INDEL marker IDs	Chromosomes/ unanchored scaffolds	Physical positions (bp)	InDels (*Kabuli* reference genome-CDC Frontier/PII)	Forward primers (5'-3')	Reverse primers (5'-3')	Anneling temperature (0C)	Expected amplified product size (bp)	Structural annotation		Functional annotation	
								Sequence components of *kabuli* genome	*Kabuli* gene accession IDs	NCBI-KOG	TFs
Ca1_P4_40408466	Ca1	40408466	CTTATTTATTTATTTA/CTTATTTATTTATTTATTTA	TCACAAACAATTGCGAAAACA	CCACCTCCATGCCTCTAAAA	60.1	657	INTERGENIC	--	--	--
Ca1_P4_40408801	Ca1	40408801	ATT/ATTGAGCCAACTT	TTTTAGAGGCATGGAGGTGG	TTTTGCAAATGTTGGGTTCA	60.1	343	INTERGENIC	--	--	--
Ca1_P4_40424012	Ca1	40424012	GTTTTTTTT/GTTTTTTTTTT	TTTTAGAGGCATGGAGGTGG	TCCAAGAGGTCAAACGTCAA	60.1	432	INTERGENIC	--	--	--
Ca1_P4_40424034	Ca1	40424034	TTATATATATAT/TTATATAT	TTTTAGAGGCATGGAGGTGG	TCCAAGAGGTCAAACGTCAA	60.1	432	INTERGENIC	--	--	--
Ca1_P4_40424994	Ca1	40424994	C/CCAAAATATTATAA	TGCAAAACTTTGTTCATCGAG	GCATCATTCTCCTTCGAGACA	59.0	748	INTERGENIC	--	--	--
Ca1_P4_40425232	Ca1	40425232	AAA/AAATAA	CATTTTTAAGCTTTTGTTGCCA	GCATCATTCTCCTTCGAGACA	59.3	513	INTERGENIC	--	--	--
Ca1_P4_40425317	Ca1	40425317	GTCTC/GTCTCTC	CATTTTTAAGCTTTTGTTGCCA	TTTCATTAAGGTGAATCGTTCAGA	59.3	608	INTERGENIC	--	--	--
Ca1_P4_40425540	Ca1	40425540	ATTTTTTTT/ATTTTTTTTT	TGTCTCGAAGGAGAATGATGC	AAACCGGTTCAAAAATGCAC	60.4	600	INTERGENIC	--	--	--
Ca1_P4_40482336	Ca1	40482336	GAAAAAAAAAAA/GAAAAAAAAAA	CAAAGTCTAGTTCGGCCCAG	TATGCATAAGGGTGTGGCAA	59.9	500	INTERGENIC	--	--	--
Ca1_P4_40516551	Ca1	40516551	CATATATATATATATATATATATATATA/CATATATATATATATATATATATATA	TGGTTGGCATGTTGTTCCTA	TCTAAGCAAAGCTCCACTTTTT	60.0	455	INTERGENIC	--	--	--

INDEL marker IDs	Chromosomes/ unanchored scaffolds	Physical positions (bp)	InDels (*Kabuli* reference genome-CDC Frontier/PII)	Forward primers (5'-3')	Reverse primers (5'-3')	Anneling temperature (0C)	Expected amplified product size (bp)	Structural annotation		Functional annotation	
								Sequence components of *kabuli* genome	*Kabuli* gene accession IDs	NCBI-KOG	TFs
Ca1_P4_40535465	Ca1	40535465	TTAATAATAATAATAATAATA/TTAATAATAATAATAATAATA	CATGCGTTCCTTGTGGTAGA	ATCACAGTTTGGCCAAGTCC	59.7	599	INTERGENIC	--	--	--
Ca1_P4_40535882	Ca1	40535882	AATATATATATATATATATATAT/AATATATATATATATATATATATATAT	GATGATGATGATGTCGTGCC	ATCACAGTTTGGCCAAGTCC	59.9	311	INTERGENIC	--	--	--
Ca1_P4_40611283	Ca1	40611283	GATATATATATATATATATATATATATATATATATATATATATAT/GATATATATATATATATATATATATATATATATATATATATATATATAT	ATGCATGTGTGTCGAGTGGT	TGTCCGAATTGCATTCTCAA	60.0	632	INTERGENIC	--	--	--
Ca1_P4_40665843	Ca1	40665843	TTATATATATATATATATAT/TTATATATATATATATATATAT	CCACGTCAATTTTGACATACATC	ACCAAGATCCGCTTACATGG	59.3	290	INTERGENIC	--	--	--
Ca1_P4_40735911	Ca1	40735911	AATATATATATATATATATATATATATATATATATATATATATAT/AATATATATATATATATATATATATATATATAT	AAATGCAAAATTTTAAGCGTGA	CCATTGGAAAGTGTGTAGTGC	58.8	533	INTERGENIC	--	--	--
Ca1_P4_40814692	Ca1	40814692	TA/T	TGGGGAATCTCTTAATGTTTCG	CTATCACAAGCCCGTACCGT	60.3	100	INTERGENIC	--	--	--
Ca1_P4_40889769	Ca1	40889769	TTATATA/TTA	GCATCTCTTGATGCCCTTTT	AACATACGCCATACCCAAGC	59.3	289	INTERGENIC	--	--	--
Ca1_P4_40890559	Ca1	40890559	ATTTTT/ATTTTTT	CGGATTTGCAAGTGTTCGTA	TGGGGTTTAGGATATCACATCA	59.7	707	INTERGENIC	--	--	--
Ca1_P4_40890662	Ca1	40890662	CAAA/CA	CAAGAAACAAATTGCAATGGA	TCAAGTTTGGGAGGAACCAC	58.7	704	INTERGENIC	--	--	--
Ca1_P4_40899161	Ca1	40899161	GTTTTTTTTTTTTT/GTTTTTTTTT	ATTTGGTTCCATTTGATCCG	TTATGTTAGGGCAGGGTTTGA	59.6	360	INTERGENIC	--	--	--

INDEL marker IDs	Chromosomes/ unanchored scaffolds	Physical positions (bp)	InDels (*Kabuli* reference genome-CDC Frontier/PII)	Forward primers (5'-3')	Reverse primers (5'-3')	Anneling temperature (0C)	Expected amplified product size (bp)	Structural annotation		Functional annotation	
								Sequence components of *kabuli* genome	*Kabuli* gene accession IDs	NCBI-KOG	TFs
Ca1_P4_40966090	Ca1	40966090	A/AG	CTTTGGTTTCAAAGTTGCCC	CGTGTTTATACGCACACCCA	59.6	571	INTERGENIC	--	--	--
Ca1_P4_41040587	Ca1	41040587	TA/TATAAA	CAGGACCTGGGAATGTCAAT	TTTCCCGACTTGGTCCTTTA	59.8	658	INTERGENIC	--	--	--
Ca1_P4_41103687	Ca1	41103687	TACACACA/TACACA	GGACGGACAACCGCTATTTA	TTGTTCGTGTTGCGTTTAGC	60.0	278	INTERGENIC	--	--	--
Ca1_P4_41248476	Ca1	41248476	GAAAAAAA/GAAAA	CCAAAAAGAAGGACGACCAA	AAACAATTGGATAGCGGCAC	60.1	547	INTERGENIC	--	--	--
Ca1_P4_41275062	Ca1	41275062	TTATATATATATATATATATATATATATATATATATATATATATATATATA/TTATATATATATATATATATATATATATATATATATATATATATATATATATA	CACGATGCATTGGTGTAGGT	TTTGACTGTTGGATTGGAAGA	59.4	554	INTERGENIC	--	--	--
Ca1_P4_4144763	Ca1	4144763	CTTTTTTTT/CTTTTTTT	TTAGGGTCCATCCAACAAGC	TTCAACATAGAAGGCCCACC	59.9	564	INTERGENIC	--	--	--
Ca1_P4_41492072	Ca1	41492072	AATATATATATATATATATATATATATATATATATATATAT/AATATATATATATATATATATATATATATATAT	AACATGCAAAAGGGTTCTGG	TGGAAAAATGAACAAACATGAAA	60.0	704	INTERGENIC	--	--	--
Ca1_P4_41493960	Ca1	41493960	TAAAAAAAA/TAAAAAAAAA	TCGGCATAAAAATGAAAACAA	GAAGATGCCTCTGGCCAATA	58.2	388	INTERGENIC	--	--	--
Ca1_P4_41504734	Ca1	41504734	ACTAGCTAGC/ACTAGCTAGCTAGC	GAATAAGAAATATAAATGGCAAGGC	TGCTCAGCTACGGTTCCTTT	58.6	177	INTERGENIC	--	--	--
Ca1_P4_41695402	Ca1	41695402	CTA/CTATA	TACTTCCATGCAAACCAGCA	CCAACTCTGAAATACAAAAGCCA	60.3	486	INTERGENIC	--	--	--

INDEL marker IDs	Chromosomes/ unanchored scaffolds	Physical positions (bp)	InDels (*Kabuli* reference genome-CDC Frontier/PII)	Forward primers (5'-3')	Reverse primers (5'-3')	Anneling temperature (0C)	Expected amplified product size (bp)	Structural annotation		Functional annotation	
								Sequence components of *kabuli* genome	*Kabuli* gene accession IDs	NCBI-KOG	TFs
Ca1_P4_41695561	Ca1	41695561	ATTTTTTTTTT/ATTTTTTTTTTTT	TTCCAAAGTATTGTGGGACACTT	TTGTTGGAGATAATTGAAGCTGA	59.8	964	INTERGENIC	--	--	--
Ca1_P4_41704419	Ca1	41704419	CATAATAAT/CATAAT	GGGGAATTTTTCATTTTGTGG	TGTTTTGGTTTCAAGGGACA	60.4	311	INTERGENIC	--	--	--
Ca1_P4_41704503	Ca1	41704503	CTTTTTTT/CTTTTTT	GGGGAATTTTTCATTTTGTGG	TGTTTTGGTTTCAAGGGACA	60.4	311	INTERGENIC	--	--	--
Ca1_P4_41705653	Ca1	41705653	ATTTTTTTT/ATTTTTTT	CATCACCGTAACATGACTTACCA	TCCCAATAATTTTTCCTTTTCG	59.8	723	INTERGENIC	--	--	--
Ca1_P4_41709578	Ca1	41709578	AGGGGG/AGGGG	TTGTGGAAGCAAGAGGTGAA	TCCCCTCATCATATTTTGCC	59.4	136	INTERGENIC	--	--	--
Ca1_P4_4171296	Ca1	4171296	T/TCATTTTTTAGCTTA	CAACAAAACGCGCTAATGAC	CGAGATTTTGAGGCATGTTG	59.4	522	INTERGENIC	--	--	--
Ca1_P4_41718814	Ca1	41718814	T/TG	GGGTCGAATGTCGATTCTGT	TCATCTCTCAGCCCCACTCT	59.9	156	INTERGENIC	--	--	--
Ca1_P4_41721873	Ca1	41721873	ATTTT/ATTT	TGGTGAGTAAGAAATTCAAGGGA	TGACAATCAAAGTAAAATGAGTCAAA	60.0	533	INTERGENIC	--	--	--
Ca1_P4_417847	Ca1	417847	CCAAAGCAA/CCAA	TAAGGGTAAGGCCACCAAAG	AACCGTTATACGGCAACGAC	59.1	594	INTERGENIC	--	--	--
Ca1_P4_417899	Ca1	417899	AAAAAG/A	ACCAAAGCAAGCGTTTTAGG	GTTTTGGCGAGTCCATCATT	59.4	510	INTERGENIC	--	--	--

INDEL marker IDs	Chromosomes/ unanchored scaffolds	Physical positions (bp)	InDels (*Kabuli* reference genome-CDC Frontier/PII)	Forward primers (5'-3')	Reverse primers (5'-3')	Anneling temperature (0C)	Expected amplified product size (bp)	Structural annotation		Functional annotation	
								Sequence components of *kabuli* genome	*Kabuli* gene accession IDs	NCBI-KOG	TFs
Ca1_P4_41852137	Ca1	41852137	ATTTTTT/ATTTTTTT	TGACCGAAATGTTTAAAGCG	AACATTGTGAATGATCTCGTCA	58.8	300	INTERGENIC	--	--	--
Ca1_P4_41949447	Ca1	41949447	ATATTTTTATTTTTATTTTT/ATATTTTTATTTTTATTTTTATTTTT	TGTTTTTGTTAAAAGTGTGCAGAA	TTGTTTCCACTCATGCGAAG	58.9	851	INTERGENIC	--	--	--
Ca1_P4_4196063	Ca1	4196063	TCTACT/TCT	GCTGCAAAATGCAAAACTGA	TGGAACGAGGAATTGGAGTC	60.0	694	INTERGENIC	--	--	--
Ca1_P4_41998443	Ca1	41998443	CG/CGG	CCATTTTTCTCACGTGCTTTC	ATCCCTCTATTGGTGGGTCC	59.7	339	INTERGENIC	--	--	--
Ca1_P4_42053807	Ca1	42053807	CGTTGTTGTTGTTGTTGTTGTTGTTGT/CGTTGTTGTTGTTGTTGTTGTTGT	GGGATCTTTTTGGCTGATGA	CTCATCCTCGCCATTGTTTT	60.0	452	INTERGENIC	--	--	--
Ca1_P4_42057802	Ca1	42057802	ATTTTTTTTT/ATTTTTTTTTT	CGGTTAAAAACGTCGCAAAT	TTTGATACGTGTACTCAACCGAA	60.0	841	INTERGENIC	--	--	--
Ca1_P4_4214755	Ca1	4214755	CTTTTTTTTTTTT/CTTTTTTTTTTT	TTTTGGTCCACATCAAGCTG	GTATAATTGGTGGCCCGATG	59.7	512	INTERGENIC	--	--	--
Ca1_P4_42168404	Ca1	42168404	TCC/TCCC	CCAATCGATTTCCGTCAGTT	TGGTGATTGTGTGGGAATTG	59.9	207	INTERGENIC	--	--	--
Ca1_P4_42206966	Ca1	42206966	G/GATGCT	AGACTCCGATACCACACCAA	GGGAAAATCATGTTTATCACAATC	58.0	363	INTERGENIC	--	--	--
Ca1_P4_42209181	Ca1	42209181	GAAA/GAAAA	TTTTTCCCCCTTATGGTGAG	GCGAGCATGAGAAGTTGTTG	58.9	319	INTERGENIC	--	--	--

INDEL marker IDs	Chromosomes/ unanchored scaffolds	Physical positions (bp)	InDels (*Kabuli* reference genome-CDC Frontier/PII)	Forward primers (5'-3')	Reverse primers (5'-3')	Anneling temperature (0C)	Expected amplified product size (bp)	Structural annotation		Functional annotation	
								Sequence components of *kabuli* genome	*Kabuli* gene accession IDs	NCBI-KOG	TFs
Ca1_P4_42382215	Ca1	42382215	GTATATATATATATATATATATATATATATATATATATAT/GTATATATATATATATATATATATATATATATATATATATAT	AGAAAGGAGCATGGTGAGCA	GCTCAATCCTCATTCACCTCA	60.9	815	INTERGENIC	--	--	--
Ca1_P4_4241058	Ca1	4241058	TC/T	TTTCCAACGCCTTTTCAAAC	ATTGGGACCCACTGAGATGT	60.1	957	INTERGENIC	--	--	--
Ca1_P4_42493816	Ca1	42493816	TA/TAACA	GGGTCTTTCCTTCGCCTATC	CGGCATCCATAAAGTGAAAAA	60.0	968	INTERGENIC	--	--	--
Ca1_P4_42546201	Ca1	42546201	CAAA/CAA	TGAAGAAGAGCCGTAACGAA	TGCACTACCAACACATGAAACA	58.6	408	INTERGENIC	--	--	--
Ca1_P4_42740504	Ca1	42740504	TTATATATATATATATATATATATATATATATATATATATATATATA/TTACATATATATATATATATATATATATATATATATATATATATATA	TGTCCTTCTCATTGGGAACC	AAAAAGCGCTGAAAATCCCT	59.9	697	INTERGENIC	--	--	--
Ca1_P4_42767298	Ca1	42767298	AAAAAAATTTCAAAAAA/AAAAAAATTTCAAAAAATTTCAAAAAA	ACATGTTGGGGGAGTCAAAT	TACACAAAAGCCCCCAACTC	59.1	513	INTERGENIC	--	--	--
Ca1_P4_42900410	Ca1	42900410	ATTTTTTTTT/ATTTTTTTTTT	CAAAAGGATGTGTGGGAACA	GACGTCTTGTCTCACGTTCG	59.4	459	INTERGENIC	--	--	--
Ca1_P4_4296528	Ca1	4296528	GATAATAATAATAATAATAAT/GATAATAATAATAATAATAAT	GACTGCCATTAACCCACGTT	AATGGGCGTTGGTTGTAGAG	59.9	787	INTERGENIC	--	--	--
Ca1_P4_4316128	Ca1	4316128	CTCTAT/CT	TCATGTCAAATGGGTCAAGC	ATGGGGCCAAATATGGAGTT	59.5	931	INTERGENIC	--	--	--
Ca1_P4_43438410	Ca1	43438410	TGCGTCG/TG	CATGAACGACGTTTGATTGG	TGGCCACCAAATTAATGACA	60.0	575	INTERGENIC	--	--	--

INDEL marker IDs	Chromosomes/ unanchored scaffolds	Physical positions (bp)	InDels (*Kabuli* reference genome-CDC Frontier/PII)	Forward primers (5'-3')	Reverse primers (5'-3')	Anneling temperature (0C)	Expected amplified product size (bp)	Structural annotation		Functional annotation	
								Sequence components of *kabuli* genome	*Kabuli* gene accession IDs	NCBI-KOG	TFs
Ca1_P4_43467858	Ca1	43467858	CTTTTT/CTTTTTT	GAGATTTAGAACCATCAAATTGTCA	GCAACCTGCCATCCATACTT	58.6	603	INTERGENIC	--	--	--
Ca1_P4_43534972	Ca1	43534972	CTTTTTTTTTT/CTTTTTTTTTTTT	GTTGTTTGCGTTTTCCCCTA	TGTGTTAAATCCACCGCAAA	60.0	460	INTERGENIC	--	--	--
Ca1_P4_43550360	Ca1	43550360	ATTT/ATT	TGCAGAGTAATTCGGTTTGTTTT	GGCATTAAGCCTCAAATCCA	60.1	458	INTERGENIC	--	--	--
Ca1_P4_43595794	Ca1	43595794	AAAGAAGAAGAAGAAGAAGAAGAAGAAGAA/AAAGAAGAAGAAGAAGAAGAAGAAGAAGAAGAA	GGAAGCCTGGAATGACACAT	TTCAATTCGGATTTTGACCC	59.9	555	INTERGENIC	--	--	--
Ca1_P4_43662427	Ca1	43662427	TCAC/TCACAC	TCACGTCACAACACTCCACA	CGCGTCAGAGGTAGGAAAAG	59.7	382	INTERGENIC	--	--	--
Ca1_P4_43687147	Ca1	43687147	TAA/TA	TGTGGCTAAGATTAGAATCCCAT	TTGATGCAAACGTAATTCATAAAGA	59.0	586	INTERGENIC	--	--	--
Ca1_P4_43690044	Ca1	43690044	AATATATATATATATATATATATATAT/AATATATATATATATATATATATAT	AGACATATGTTCGCTGGCCT	TGAGTTTGTTTGAGAGGATTTGTT	59.7	781	INTERGENIC	--	--	--
Ca1_P4_43911901	Ca1	43911901	GTTATTATTATTATTATTATTATTATTATTATTATTATTATTATTATTATTATT/GTTATTATTATTATTATTATTATTATTATTATTATTATTATTATTATTATTATTATT	TGAGGGACTGGATTCAGGAG	GGCGTGTTCTTTGTTTGGTC	60.2	578	INTERGENIC	--	--	--
Ca1_P4_43986145	Ca1	43986145	AA/AATA	CCTCTTTGGTTCCATGTGCT	TGATGTGTTTGTCTTGGGGA	60.1	385	INTERGENIC	--	--	--
Ca1_P4_44247311	Ca1	44247311	GTTTTTTTTTT/GTTTTTTTTT	TTCTCCAGACTCTAGCCCGA	AGCTTCTCAGTAGGGGGCTC	60.1	592	INTERGENIC	--	--	--

INDEL marker IDs	Chromosomes/ unanchored scaffolds	Physical positions (bp)	InDels (*Kabuli* reference genome-CDC Frontier/PII)	Forward primers (5'-3')	Reverse primers (5'-3')	Anneling temperature (0C)	Expected amplified product size (bp)	Structural annotation		Functional annotation	
								Sequence components of *kabuli* genome	*Kabuli* gene accession IDs	NCBI-KOG	TFs
Ca1_P4_44248190	Ca1	44248190	AATATATATATATATATATATAT/AATATATATATATATATATAT	GCTCACTGACAATGGCTTCA	CAACAATGGAGGAAGGAGGA	60.0	536	INTERGENIC	--	--	--
Ca1_P4_44368738	Ca1	44368738	GATAATAATAATAATAATAATAATAATAATAATAATAATAATAATAA/GATAATAATAATAATAATAATAATAATAATAATAATAATAA	TTGTAACTCCCCGAATTGTTG	AGAGGCAAACAAGAACCGAA	59.8	534	INTERGENIC	--	--	--
Ca1_P4_44406344	Ca1	44406344	TAA/TA	TGCAGAAATCTCATCAAGCG	GTGCCACTGACTTGTGCAAT	60.1	533	INTERGENIC	--	--	--
Ca1_P4_44467151	Ca1	44467151	GTTT/GTTTT	ATGCAGCTCAAAAACAGCCT	GGTTCACCCAAAGATGGCTA	60.0	537	INTERGENIC	--	--	--
Ca1_P4_44650256	Ca1	44650256	T/TC	TCGTTTGCGTCATTGGATTA	AGGGAGGACGAAAATGACCT	60.1	444	INTERGENIC	--	--	--
Ca1_P4_44745225	Ca1	44745225	A/AC	TCCGGATATAGTCCTGCCAC	TCCTTGTGAACGACTTGGTG	59.9	632	INTERGENIC	--	--	--
Ca1_P4_44776081	Ca1	44776081	AATATATATATATATATATATATATATATATATATATATA/AATATATATATATATATATATATATATATATATATATATATATATA	TGGAATACCATATATTTTACGTTTTCA	TGACGGATCCTTTCTTGACA	59.1	795	INTERGENIC	--	--	--
Ca1_P4_44808505	Ca1	44808505	GAAAAAAAAA/GAAAAAAAAAA	GAAATGAGCTGCACACCAGA	TAAGGCATGCAAAGGTTGGT	60.0	383	INTERGENIC	--	--	--
Ca1_P4_44847252	Ca1	44847252	ATCTGATGTTCTGATG/ATCTGATGTTCTGATGTTCTGATG	TGTTTTCTGCCTTTTTGCCT	AAAATCCAACAATCTCCCCC	59.9	274	INTERGENIC	--	--	--
Ca1_P4_44847313	Ca1	44847313	TATAATAATAATAATAATAATA/TATAATAATAATAATAATA	TGTTTTCTGCCTTTTTGCCT	AAAATCCAACAATCTCCCCC	59.9	274	INTERGENIC	--	--	--

INDEL marker IDs	Chromosomes/ unanchored scaffolds	Physical positions (bp)	InDels (*Kabuli* reference genome-CDC Frontier/PII)	Forward primers (5'-3')	Reverse primers (5'-3')	Anneling temperature (0C)	Expected amplified product size (bp)	**Structural annotation**		**Functional annotation**	
								**Sequence components of *kabuli* genome**	***Kabuli* gene accession IDs**	**NCBI-KOG**	**TFs**
Ca1_P4_4485491	Ca1	4485491	CATATATATATATATATA/CATATATATATATATATATA	TTTGATGTTGTTGGAGATGAAA	TGCCATGCATCAGTTGTTTT	58.1	792	INTERGENIC	--	--	--
Ca1_P4_44921329	Ca1	44921329	ACTT/AGGATGCTGATCTTCTT	CCTCAAACGATTTTCTGGGA	CTTTCCCAATGCGTGAAGAT	60.0	478	INTERGENIC	--	--	--
Ca1_P4_44943657	Ca1	44943657	CTATATATATATATATATATATATATATATATATATATATATATATAT/CTATATATATATATATATATATATATATATATATATATATATATAT	CATACCATGCATCCCAACAA	TACTCGTTGCTCTCGTTCCC	60.2	567	INTERGENIC	--	--	--
Ca1_P4_45057162	Ca1	45057162	CATAAA/CATAAATAAA	TTGCTTGAATAGGCAATGGA	ACGTCGACGACATCAAAGTG	59.3	645	INTERGENIC	--	--	--
Ca1_P4_45057964	Ca1	45057964	CAAA/CAA	AAACTAGATTATGGCCTGCG	TGCTCGCAATTAAAACATGC	57.0	426	INTERGENIC	--	--	--
Ca1_P4_45098064	Ca1	45098064	GTTTTTTTTTT/GTTTTTTTTT	AAGCCACTGTTAATCCACGC	TGCTAGGGCACAACTCTCCT	60.1	125	INTERGENIC	--	--	--
Ca1_P4_45124635	Ca1	45124635	GTATATATATATATATATATATATA/GTATATATATATATATATATATATATA	TCCGAGACACAAGACCAACA	TTACGAACACAATTGTTGCCA	60.3	836	INTERGENIC	--	--	--
Ca1_P4_45232358	Ca1	45232358	T/TAGCAATAATG	GTTAAGCCCCGATCAATGAA	CATCCCCCTCTTTAACCCAT	59.9	515	INTERGENIC	--	--	--
Ca1_P4_45238172	Ca1	45238172	T/TGACA	ATTCTTTTTGGAGCGCAGAA	CCAATTAGGTTTACGGCGAA	60.0	401	INTERGENIC	--	--	--
Ca1_P4_45246034	Ca1	45246034	C/CGTCTTGTT	CGATTTCGCTTTTAGCATCC	CAATTGCGATATTATGGGGG	59.8	253	INTERGENIC	--	--	--

INDEL marker IDs	Chromosomes/ unanchored scaffolds	Physical positions (bp)	InDels (*Kabuli* reference genome-CDC Frontier/PII)	Forward primers (5'-3')	Reverse primers (5'-3')	Anneling temperature (0C)	Expected amplified product size (bp)	Structural annotation		Functional annotation	
								Sequence components of *kabuli* genome	*Kabuli* gene accession IDs	NCBI-KOG	TFs
Ca1_P4_45251376	Ca1	45251376	C/CCTATGA	CTAGCTCTGGGTTCGAGTGG	ATCCGACTAGTTCCGGGTTC	60.0	247	INTERGENIC	--	--	--
Ca1_P4_45392704	Ca1	45392704	ATTT/ATTTT	GGGGGCTCTTCATTTTTCTC	CACAGTTTCATCGCTCCTCA	60.0	476	INTERGENIC	--	--	--
Ca1_P4_45395070	Ca1	45395070	AT/A	CACGAACGACAGAAGAACCA	CCTCAGATGAGAGCCAGGAC	59.9	481	INTERGENIC	--	--	--
Ca1_P4_45443277	Ca1	45443277	GTAATAATAATAATAATAATAATAATAATAATAATAATAATAATAATAATAATAATAATAGTAATAGTAATAATAATAATAATAATAATAACAATAATAATAATAATAATAATAATAATAGTAATAGTAA[illegible]	CCCACATTCATGACGTGTTC	CTTAGAGGTGGGGAACGTGA	59.8	741	INTERGENIC	--	--	--
Ca1_P4_45497101	Ca1	45497101	TTTTTTTTTTT/TTTTTTTTTTTTTT	AGTATAAGTGGGGCCGTGTG	TGGATTGTGCCAACTTTTCA	59.9	237	INTERGENIC	--	--	--
Ca1_P4_45535603	Ca1	45535603	GAAAAAAAAAA/GAAAAAAAAAAA	GTTTAGGGTTGCTCATGGGA	TTTGGAAGACTTTCGGGTTG	59.9	318	INTERGENIC	--	--	--
Ca1_P4_45609121	Ca1	45609121	C/CT	TCGCGTGAGAAAATAATCCC	CGAACCCTCGGTAAACAAAA	60.0	295	INTERGENIC	--	--	--
Ca1_P4_45609375	Ca1	45609375	A/ATTACAAT	TTTTGTTTACCGAGGGTTCG	CAATGATCTTGAAGGCACCA	60.0	142	INTERGENIC	--	--	--
Ca1_P4_45833155	Ca1	45833155	CTTTTTTTTTT/CTTTTTTTTT	TGTGGCATTGGACCAACTTA	GGGTAAAAACGGGTTGGACT	60.0	981	INTERGENIC	--	--	--
Ca1_P4_45859437	Ca1	45859437	G/GAATTTGGA	AGACCTGTGGTGTGATGCTG	AAGAGAGGTCACTTTGGCGA	59.7	772	INTERGENIC	--	--	--

INDEL marker IDs	Chromosomes/ unanchored scaffolds	Physical positions (bp)	InDels (*Kabuli* reference genome-CDC Frontier/PII)	Forward primers (5'-3')	Reverse primers (5'-3')	Anneling temperature (0C)	Expected amplified product size (bp)	Structural annotation		Functional annotation	
								Sequence components of *kabuli* genome	*Kabuli* gene accession IDs	NCBI-KOG	TFs
Ca1_P4_46088799	Ca1	46088799	CTATATATATATATATATATATATATATAT/CTATATATATATATATATATATATATATATATATAT	TAAGTCCGTAAGTGGTGCCC	GGGCTTTCCAAAGTGTTATCC	60.0	586	INTERGENIC	--	--	--
Ca1_P4_4612204	Ca1	4612204	AATATATATATATATATATATATATATATATATATA/AATATATATATATATATATATATATATATATATATATATATATATATATA	GCCATTTGGAATGACGACTT	CGTTTTTGCAACTTCCCAAC	59.9	327	INTERGENIC	--	--	--
Ca1_P4_46178255	Ca1	46178255	AATTTATAATTTAT/AAT	TCAAACATGCTCTGAATGCC	AACCAATACATAACATCACAGTGC	59.8	570	INTERGENIC	--	--	--
Ca1_P4_46186559	Ca1	46186559	AGTGATTGGTTTGTTTGTGATTGGTTTGTTTG/AGTGATTGGTTTGTTTGTGATTGGTTTGTTTGTGATTGGTTTGTTTG	AAAGCACACGATTTTCACCC	TTCGGCATCTAGCACTTCCT	60.0	775	INTERGENIC	--	--	--
Ca1_P4_46268212	Ca1	46268212	TA/T	AAACGCAATCCTTGAGTTCG	CATGACTTGGATTGGTCGTG	60.2	226	INTERGENIC	--	--	--
Ca1_P4_46278771	Ca1	46278771	ATGGCCTGGCCTGGCCTGGCCTG/ATGGCCTGGCCTG	ACCTTCAGTCAGGGGCCTAT	AAGTCTGGTCTGGCCTGAAA	60.0	538	INTERGENIC	--	--	--
Ca1_P4_46283204	Ca1	46283204	CA/CATA	AGCTGCCATGCCTAATGAAT	CAATGGGCTTCTTACCTGGA	59.7	425	INTERGENIC	--	--	--
Ca1_P4_46286614	Ca1	46286614	CTTTT/CTTT	CCAAGTTATGCTTGATCGCA	CGAGAACCATCCTGCATTTT	59.8	820	INTERGENIC	--	--	--
Ca1_P4_46286661	Ca1	46286661	GTAGGGATA/GTA	CCAAGTTATGCTTGATCGCA	CGAGAACCATCCTGCATTTT	59.8	820	INTERGENIC	--	--	--
Ca1_P4_46352138	Ca1	46352138	CACAAC/CACAACAAC	AGCGATGCATGTCTCAAGTG	TGCCCTTCTTCTTTTCCTAGTG	60.0	396	INTERGENIC	--	--	--

INDEL marker IDs	Chromosomes/ unanchored scaffolds	Physical positions (bp)	InDels (*Kabuli* reference genome-CDC Frontier/PII)	Forward primers (5'-3')	Reverse primers (5'-3')	Anneling temperature (0C)	Expected amplified product size (bp)	Structural annotation		Functional annotation	
								Sequence components of *kabuli* genome	*Kabuli* gene accession IDs	NCBI-KOG	TFs
Ca1_P4_4647010	Ca1	4647010	AATATATATATATATATATATATATA/AATATATATATATATATATATATATATATA	AAAAATACCACAACTTGAATTAATGA	CATGTGAAATGTAGCCAGTTGC	57.4	700	INTERGENIC	--	--	--
Ca1_P4_46491737	Ca1	46491737	CAAG/CAAGAAG	GATGCATCTCACCAAGGGTT	CGGTGCAAACTCTACGTTCA	59.9	420	INTERGENIC	--	--	--
Ca1_P4_46500141	Ca1	46500141	GCC/GC	GTAGCCAAAGGATTGCCAAA	ATGTGCAAGGAACCCGTTAG	60.1	586	INTERGENIC	--	--	--
Ca1_P4_46507085	Ca1	46507085	ATTTTTTTTTTTT/ATTTTTTTTTTT	TCAATTTCATGCTCCGGAAT	TTCCTTTGGGTTGCGTTTAC	60.4	386	INTERGENIC	--	--	--
Ca1_P4_46523548	Ca1	46523548	CAAAAAAAAAAAAAAAA/CAAAAAAAAAAAAA	GTGGACAAACCTGCATTCCT	ACCATGCACAGTTGCTGAGA	60.0	675	INTERGENIC	--	--	--
Ca1_P4_46525859	Ca1	46525859	GAAAAAA/GAAAAAAA	CCCACCTGGATACAAGGCTA	AGTAGAGGGAGTTCGGGTGC	59.9	711	INTERGENIC	--	--	--
Ca1_P4_46526481	Ca1	46526481	CAAAAAAA/CAAAAAAAA	TTAAAATTCCGAGGCGAAAA	ACATGGTTGTGGTGGTGATG	59.7	698	INTERGENIC	--	--	--
Ca1_P4_46528558	Ca1	46528558	CT/CTT	TTAAAATTCCCTCCTCGGCT	AACTCTGGGAACCCTCCTGT	60.0	398	INTERGENIC	--	--	--
Ca1_P4_46528735	Ca1	46528735	GTGACAGCCTGACAG/GTGACAG	TTAAAATTCCCTCCTCGGCT	GGGGAAAACATCCCGTTATT	60.0	865	INTERGENIC	--	--	--
Ca1_P4_46539303	Ca1	46539303	TTCAAATCTTCATCAAATCTTC/TTCAAATCTTC	TCAAGCCAATCTTCCCAAAC	TCTGATGACAAAAGGGGGAG	60.1	757	INTERGENIC	--	--	--

INDEL marker IDs	Chromosomes/ unanchored scaffolds	Physical positions (bp)	InDels (*Kabuli* reference genome-CDC Frontier/PII)	Forward primers (5'-3')	Reverse primers (5'-3')	Anneling temperature (0C)	Expected amplified product size (bp)	Structural annotation		Functional annotation	
								Sequence components of *kabuli* genome	*Kabuli* gene accession IDs	NCBI-KOG	TFs
Ca1_P4_46539360	Ca1	46539360	CAAA/CAAAA	TCCCAAACAGCCTGATAACC	CTGATGACAAAAGGGGGAGA	59.9	744	INTERGENIC	--	--	--
Ca1_P4_46633354	Ca1	46633354	GTATATATATATATATATATAT/GTATATATATATATATATAT	CGACATGGCTCTGATACCAA	TCAGACCGCCTTTACCAATC	59.7	615	INTERGENIC	--	--	--
Ca1_P4_46662012	Ca1	46662012	AAT/AATAT	GCATTTACGTCGATATTTATGAGTG	TCAGCCAAAAAGCTGATGTG	59.1	617	INTERGENIC	--	--	--
Ca1_P4_46798111	Ca1	46798111	CCCTAAACCTAAACC/CCCTAAACC	AAACCTGAAGCCCTACACCA	TTAGGGTTTCGGGATAAGGG	59.6	682	INTERGENIC	--	--	--
Ca1_P4_46896717	Ca1	46896717	AATATATATATATATATATA/AATATATATATATATATATATATA	CACGATGCATTGGTGTAGGT	TCGTTCAATAACCTTTTTCTTGG	59.4	555	INTERGENIC	--	--	--
Ca1_P4_46900592	Ca1	46900592	ATTT/ATTTT	GATGCCTTGACCATCCTTGT	TGGGTACACGCATATTAACCAA	59.9	555	INTERGENIC	--	--	--
Ca1_P4_47016631	Ca1	47016631	ACTCTCTCTCTCTCT/ACTCTCTCTCTCTCTCT	ATTGCCTCTCAGTCGCGTTA	AAAATTCGACATCGTGAGGG	60.9	531	INTERGENIC	--	--	--
Ca1_P4_47048638	Ca1	47048638	TG/T	TAGCACCAGACCATTCTCCC	CAATTGAAGGCCCAAAACAT	60.1	735	INTERGENIC	--	--	--
Ca1_P4_47161229	Ca1	47161229	TAGAG/TAG	TCTGCAATGAAAGCAAACACA	AGTTTTGGCTCCCTATCGGT	60.4	829	INTERGENIC	--	--	--
Ca1_P4_47186820	Ca1	47186820	ATTTTTTTTT/ATTTTTTTT	CTCACTCTTAAACCAGCCGC	GTCTGCCACGTCACCTTTTT	60.0	480	INTERGENIC	--	--	--

INDEL marker IDs	Chromosomes/ unanchored scaffolds	Physical positions (bp)	InDels (*Kabuli* reference genome-CDC Frontier/PII)	Forward primers (5'-3')	Reverse primers (5'-3')	Anneling temperature (0C)	Expected amplified product size (bp)	Structural annotation		Functional annotation	
								Sequence components of *kabuli* genome	*Kabuli* gene accession IDs	NCBI-KOG	TFs
Ca1_P4_47346271	Ca1	47346271	TA/TAA	CAGTCACACTAGCGACCGAA	GAACCCAAGGCTTTGTCGTA	60.0	706	INTERGENIC	--	--	--
Ca1_P4_47373127	Ca1	47373127	T/TTGTTGC	CAGCAGCAAGAGCCATGTTA	GCCATTCCTCTACCAACCAA	60.2	475	INTERGENIC	--	--	--
Ca1_P4_4742142	Ca1	4742142	TAAA/TAA	GACCGCGTAGATGTGGAACT	GGATGAGACCTCCTACTTGCC	60.1	677	INTERGENIC	--	--	--
Ca1_P4_47455633	Ca1	47455633	GAAAAAAAA/GAAAAAAAAA	TTGGGGTCAGTCAAACACAA	CGGTGAAATAGGTATGCCAAA	60.0	600	INTERGENIC	--	--	--
Ca1_P4_47580436	Ca1	47580436	ATTTTTTTTTTTTT/ATTTTTTTTTT	AAATGGTGAGGGGTGAAGTG	CGACCCTTGGCTAATTCAAA	59.8	376	INTERGENIC	--	--	--
Ca1_P4_47580698	Ca1	47580698	ATTTTTTTTTTT/ATTTTTTTTTT	TTTGAATTAGCCAAGGGTCG	TCGATTCGAAATGGATGTCA	60.1	615	INTERGENIC	--	--	--
Ca1_P4_47626582	Ca1	47626582	ATTT/ATTTT	CATGATGATGGTTGCTTTGC	CTTGCGCTACCATGTGAAGA	60.1	360	INTERGENIC	--	--	--
Ca1_P4_47630703	Ca1	47630703	TAAAAAGAAAAAGAAAAAGAAA/TAAAAAGAAAAAGAAAAAGAAAAAGAAA	CGTGTCCGGCAAAGTAAAAT	GTGATGCACGCAGTAATTGG	60.0	517	INTERGENIC	--	--	--
Ca1_P4_47630977	Ca1	47630977	AC/ACC	GGCCTTCCATCGTGAATAAA	TTTCGAATTCCAGAACTGCC	59.9	916	INTERGENIC	--	--	--
Ca1_P4_47730180	Ca1	47730180	GATATATATATATATATATA/GATATATATATATATATATA	TGCGTATCTTAGGAATGCGA	GGCTAGACGAGTTCGACGAC	59.4	321	INTERGENIC	--	--	--

INDEL marker IDs	Chromosomes/ unanchored scaffolds	Physical positions (bp)	InDels (*Kabuli* reference genome-CDC Frontier/PII)	Forward primers (5'-3')	Reverse primers (5'-3')	Anneling temperature (0C)	Expected amplified product size (bp)	Structural annotation		Functional annotation	
								Sequence components of *kabuli* genome	*Kabuli* gene accession IDs	NCBI-KOG	TFs
Ca1_P4_4776428	Ca1	4776428	GAAAAAAAA/GAAAAAAA	CTAACTCGCCAACAATGCAA	TTTCACAAAGAAGGAACCGC	59.9	829	INTERGENIC	--	--	--
Ca1_P4_47908081	Ca1	47908081	CCTCTCTCTCTCTCTCTCTCT/CCTCTCTCTCTCTCTCTCTCTCT	ACCTTTTTCCTTCACTTGCG	CCAACCCTTTTTCCCAAAAT	59.4	478	INTERGENIC	--	--	--
Ca1_P4_47969192	Ca1	47969192	GTTTTTTTT/GTTTTTTTTT	AAAATGAAGATGCGACCAACT	TTTCGTCTTGTTGCAATGTTT	58.7	485	INTERGENIC	--	--	--
Ca1_P4_4801141	Ca1	4801141	GAAAAAAAAA/GAAAAAA	AATCAAAGTGGCCAAAATGC	GCTATTCACACCCCCTCAAA	59.9	902	INTERGENIC	--	--	--
Ca1_P4_48055075	Ca1	48055075	GTATATATATATATATATATATATATATATATA/GTATATATATATATATATATATATATATATATATATATA	TGACACTCGCATCTTTGTGA	GGCCATATCCGAAAACATTG	59.0	432	INTERGENIC	--	--	--
Ca1_P4_48114146	Ca1	48114146	TTATATATATATATATATATATATAT/TTATATATATATATATATATATAT	TGATTGTTGTTGGGCCACTA	TTTGTTCTCCATTTTGCTTTTG	60.0	807	INTERGENIC	--	--	--
Ca1_P4_48114666	Ca1	48114666	TTATATATATATATATATATATATATATATATATA/TTATATATATATATATATATATATATATATATATATA	CAAAAGCAAAATGGAGAACAAA	GGTCTTGCGATGACTTCCAT	59.3	365	INTERGENIC	--	--	--
Ca1_P4_48201393	Ca1	48201393	AATATATATATATATATATATATATATATAT/AATATATATATATATATATATATATATATATATATATATATATAT	TTCCTCCGTGTTTTCATTCC	TGGCGTTTGAGAATTATACGTG	59.9	407	INTERGENIC	--	--	--
Ca1_P4_48206332	Ca1	48206332	GTATATATATATATATAT/GTATATATATATATATATAT	TAGTGTGTAACCCGTGCGTC	TCATGCAAATTCAAAAGAAATGA	59.6	530	INTERGENIC	--	--	--
Ca1_P4_48236817	Ca1	48236817	TAA/TA	TGGGTGTTGCCCATATATCA	TTTGGATCCATTTCATTTGATT	59.6	626	INTERGENIC	--	--	--

INDEL marker IDs	Chromosomes/ unanchored scaffolds	Physical positions (bp)	InDels (*Kabuli* reference genome-CDC Frontier/PII)	Forward primers (5'-3')	Reverse primers (5'-3')	Anneling temperature (0C)	Expected amplified product size (bp)	Structural annotation		Functional annotation	
								Sequence components of *kabuli* genome	*Kabuli* gene accession IDs	NCBI-KOG	TFs
Ca1_P4_4971192	Ca1	4971192	T/TC	CGCCATGTACAATGAAATGC	GGAGTTGGGTCTTTGGTGAA	60.0	401	INTERGENIC	--	--	--
Ca1_P4_4974908	Ca1	4974908	ATTTTTTTTTTT/ATTTTTTTTTT	ATGGGTTAACGAAAAAGCCC	TTAATCCCAAGCTCCACCAG	60.2	641	INTERGENIC	--	--	--
Ca1_P4_5139845	Ca1	5139845	TTTCTTCTTCTTCTTCTTCTTCTTCTTCTTCTTCTT/TTTCTTCTTCTTCTTCTTCTTCTTCTTCTTCTTCTT	ACGTCTGAATCGGATTTTGC	CCCAGAAGAAGATGGAGCTG	60.1	306	INTERGENIC	--	--	--
Ca1_P4_5180264	Ca1	5180264	CTAATAATAATAATAATAATAATAATAATAATAATAAT/CTAATAATAATAATAATAATAATAATAATAATAATAATAATAAT	ATGCATGGAGCTATAAGCCG	TTCAAAGTTGGAATAACCCTTCTC	60.2	684	INTERGENIC	--	--	--
Ca1_P4_5273611	Ca1	5273611	TTATATATATATATATATATATATAT/TTATATATATATATATATATATATATATATATATAT	TGCATTAATTTTGAAAACGTCAA	TGTTTGCCACGGTTGTTAAA	59.5	593	INTERGENIC	--	--	--
Ca1_P4_5351086	Ca1	5351086	CAAA/CAA	TGTGGCAGACAGCCAACTAA	TTTGGCAATCGAAAATTGTG	60.4	607	INTERGENIC	--	--	--
Ca1_P4_5393125	Ca1	5393125	AGTGT/AGT	GGCAATTCAGAATGGTGTGTT	GAGGCCATGTCAAGGAAGTT	59.9	564	INTERGENIC	--	--	--
Ca1_P4_5417726	Ca1	5417726	GTTTTTTTTTT/GTTTTTTTT	GGAGTGGTGAAGTGTGGCTT	TTCCCCATCAATTATTTGCC	60.2	521	INTERGENIC	--	--	--
Ca1_P4_5417880	Ca1	5417880	GATCA/GA	GGAGTGGTGAAGTGTGGCTT	TTTCAACTCACTATGGCCCC	60.2	692	INTERGENIC	--	--	--
Ca1_P4_5420246	Ca1	5420246	GTTAATT/GTT	CCCATATGCAACTCCTTGGT	CACCAACAACATTTAGAGGCAA	59.8	213	INTERGENIC	--	--	--

INDEL marker IDs	Chromosomes/ unanchored scaffolds	Physical positions (bp)	InDels (*Kabuli* reference genome- CDC Frontier/PII)	Forward primers (5'-3')	Reverse primers (5'-3')	Anneling temperature (0C)	Expected amplified product size (bp)	Structural annotation		Functional annotation	
								Sequence components of *kabuli* genome	*Kabuli* gene accession IDs	NCBI-KOG	TFs
Ca1_P4_5426267	Ca1	5426267	TTATATATATATATATATATATAT/TTATATATATATATATATATATATATAT	ACCAACCGACCCACTTGATA	TCAAACACACGAGATGGTCC	60.2	317	INTERGENIC	--	--	--
Ca1_P4_5428904	Ca1	5428904	TCACA/TCA	TTAATGGGTAGGTGGGTGGA	TTTTCAGCGGTGTCACTGAG	60.0	839	INTERGENIC	--	--	--
Ca1_P4_5428960	Ca1	5428960	GTT/GTTT	AGAATGAGGTTCTGAGCCGA	TTTTCAGCGGTGTCACTGAG	60.0	807	INTERGENIC	--	--	--
Ca1_P4_5437282	Ca1	5437282	CATATATATATATATATATATATA/CATATATATATATATATATA	TGCAATGTTAATTAAGCGGG	GAACTGCATTGTGATGGGTG	58.7	456	INTERGENIC	--	--	--
Ca1_P4_5454590	Ca1	5454590	TA/TATAA	GTGTTCGGAAAAGGACGTGT	AATGGTCCCAGTTCAACCAC	60.0	655	INTERGENIC	--	--	--
Ca1_P4_5455204	Ca1	5455204	TTGTT/TTGTTAATGTT	TGGTTGAACTGGGACCATTA	GGCATCAACAACAGCAAGAA	58.8	300	INTERGENIC	--	--	--
Ca1_P4_5484309	Ca1	5484309	ATATTATTATTATTATTATTATTATTATT/ATATTATTATTATTATTATTATTATTATTATT	CGAAATTTTGATTGAGAAAGTGG	CGCGCGATGCATAGTATAAA	60.0	605	INTERGENIC	--	--	--
Ca1_P4_5500920	Ca1	5500920	CTAT/CTATAAACGTCATAT	GGAAGGGAATCGATGAGAAA	TATTCATTGGTTGGGGGAAA	59.1	456	INTERGENIC	--	--	--
Ca1_P4_5530672	Ca1	5530672	CTTTTTTTTTTTTTT/CTTTTTTTTT	TGGATTTCTCAATGCCTTCC	GGAAAAAGCCAAGAGAAGCA	60.0	482	INTERGENIC	--	--	--
Ca1_P4_5872810	Ca1	5872810	TTATATATATATATATATAT/TTATATATATATATATAT	GTAAGCTTCTTGCGACCACC	TGAAATGACTATAATTTTGATCGAATG	59.9	454	INTERGENIC	--	--	--

INDEL marker IDs	Chromosomes/ unanchored scaffolds	Physical positions (bp)	InDels (*Kabuli* reference genome-CDC Frontier/PII)	Forward primers (5'-3')	Reverse primers (5'-3')	Anneling temperature (0C)	Expected amplified product size (bp)	Structural annotation		Functional annotation	
								Sequence components of *kabuli* genome	*Kabuli* gene accession IDs	NCBI-KOG	TFs
Ca1_P4_5875747	Ca1	5875747	TTAATTTAAATT/TTAATTTAAATTAAATAATTTAAATT	TGATGGATGTCCTGGAAACA	CATCCATGCAAAAGCAGAGA	59.9	504	INTERGENIC	--	--	--
Ca1_P4_5948606	Ca1	5948606	AA/AATA	GCGTATAAAAAGGAACCAAAGC	TAGAGCCGCAGCTGATTTTT	59.2	450	INTERGENIC	--	--	--
Ca1_P4_6071071	Ca1	6071071	TTAATAATAATAATAATAATAATAATAATAATAATAATAATAATAATAATAATAA/TTAATAATAATAATAATAATAATAATAATAATAATAA	AAGAGAGATCGTGACCGTTACA	GCGGTTAACGAATACGCAAT	58.9	853	INTERGENIC	--	--	--
Ca1_P4_6129000	Ca1	6129000	CTTTTT/CTTT	CGTCATGTATCTTGGTCGGA	TGTTTAACACATTAACATGACCCA	59.5	556	INTERGENIC	--	--	--
Ca1_P4_6129831	Ca1	6129831	TTATATATATATATATATATATAT/TTATATATATATATATATATATAT	TCTTGGACGAGTTTTTCAACA	TTAAAACCGACACGGAAAGG	58.4	579	INTERGENIC	--	--	--
Ca1_P4_6134198	Ca1	6134198	ATAT/ATATAAAATTAT	TGGCAAATATTGCGAGTGAA	TGTGGGGCCAAGACATAAAT	60.2	600	INTERGENIC	--	--	--
Ca1_P4_6138299	Ca1	6138299	AT/ATT	GGCACGATGTTCTTGATTTTT	CCGATTGTTGATCGAGGTTT	59.1	846	INTERGENIC	--	--	--
Ca1_P4_6149449	Ca1	6149449	CTTTT/CTTTTT	CTGAATATTGAGGCCCATCC	CTTCGGTTGCTAAAAGAGAGC	59.3	393	INTERGENIC	--	--	--
Ca1_P4_6157606	Ca1	6157606	A/AT	TGGAGTGTCATTGAGGACCA	GGAACGTTACATGACCGGAA	60.1	789	INTERGENIC	--	--	--
Ca1_P4_6157953	Ca1	6157953	AC/A	TGGTTTGTCATTCCGGTCAT	ATGTCTCATTCATGCTCCCC	61.2	359	INTERGENIC	--	--	--

INDEL marker IDs	Chromosomes/ unanchored scaffolds	Physical positions (bp)	InDels (*Kabuli* reference genome-CDC Frontier/PII)	Forward primers (5'-3')	Reverse primers (5'-3')	Anneling temperature (0C)	Expected amplified product size (bp)	Structural annotation		Functional annotation	
								Sequence components of *kabuli* genome	*Kabuli* gene accession IDs	NCBI-KOG	TFs
Ca1_P4_6158400	Ca1	6158400	TAAAA/TAAAAA	GGGGAGCATGAATGAGACAT	AAACCGCGACAATTTAATCTC	59.9	387	INTERGENIC	--	--	--
Ca1_P4_6163758	Ca1	6163758	G/GTTTCTTAAATACAAA	TCTTTCATGTAGGATGCGATAAA	ATGGAACCTTCCACTCATGG	58.8	767	INTERGENIC	--	--	--
Ca1_P4_6164122	Ca1	6164122	TAG/T	GTTGTGTATGGGTCTTCCCG	TGACGGAATTACAACGCAAA	60.2	473	INTERGENIC	--	--	--
Ca1_P4_6178179	Ca1	6178179	ATTTTAATTTTTAATT/ATTTTAATT	AATTTTGGCATGCGACTACC	TTTCCTTCCCAATATCTGCC	60.0	514	INTERGENIC	--	--	--
Ca1_P4_6202713	Ca1	6202713	CTTTTT/CTTTTTT	CGCTTCCTTCTTCACACACA	AAAAGGCAATACATCCAATCAA	60.0	640	INTERGENIC	--	--	--
Ca1_P4_6203281	Ca1	6203281	GTTTTTTTTT/GTTTTTTTTTT	CTCCTTGAGTTTTGAGACACGA	TCGATTATGCGTAGGGAAGG	59.5	453	INTERGENIC	--	--	--
Ca1_P4_6216689	Ca1	6216689	CT/CTAT	TTTGGAATGCACATCCTCCT	GGGATCAAGGGTTGAGTGAA	60.5	254	INTERGENIC	--	--	--
Ca1_P4_6267078	Ca1	6267078	GAAA/GA	TTTCTTGCACTGGTTTGTGC	TGGGTCCCTCATTGTACTCC	59.9	560	INTERGENIC	--	--	--
Ca1_P4_6267684	Ca1	6267684	GT/GTTT	GGAGTACAATGAGGGACCCA	GCCGCGGTATTATTCCTACA	59.8	384	INTERGENIC	--	--	--
Ca1_P4_6279594	Ca1	6279594	ACAACCTCAACCTCAACCTCAAC/ACAACCTCAAC	ATCCGACGGTAACAGAAACG	CAAAGGAGAAATGAAGCCCA	60.0	327	INTERGENIC	--	--	--

INDEL marker IDs	Chromosomes/ unanchored scaffolds	Physical positions (bp)	InDels (*Kabuli* reference genome-CDC Frontier/PII)	Forward primers (5'-3')	Reverse primers (5'-3')	Anneling temperature (0C)	Expected amplified product size (bp)	Structural annotation		Functional annotation	
								Sequence components of *kabuli* genome	*Kabuli* gene accession IDs	NCBI-KOG	TFs
Ca1_P4_6293009	Ca1	6293009	TAAAAA/TAAAAAAA	TGTGGTTCAAAGCAGATTCG	CATCGATCAGTGTGTGTTTGG	59.8	675	INTERGENIC	--	--	--
Ca1_P4_6307014	Ca1	6307014	ATTTTT/ATTTTTT	ATTGATGACACGTGGAGGGT	CGAAAATCACATTCGAAGAGC	60.2	826	INTERGENIC	--	--	--
Ca1_P4_6311323	Ca1	6311323	T/TCACGA	CGGAGAACCAATCACTCACA	GTTGAGCCGAGGCAACTATG	59.7	601	INTERGENIC	--	--	--
Ca1_P4_6313076	Ca1	6313076	CG/C	CCGCAAATGAAATTAAGGGA	GGGAAACATTCTCACATTTGC	59.9	628	INTERGENIC	--	--	--
Ca1_P4_6313907	Ca1	6313907	T/TC	GGAGTCTTGTGTCATGTGCAA	TTCACGTGCTTTTTAATTTTGTG	59.7	529	INTERGENIC	--	--	--
Ca1_P4_6342773	Ca1	6342773	TG/T	AGGGTCATGGGTGTCACAAT	AGCACATCCACCTTCCATTC	60.1	693	INTERGENIC	--	--	--
Ca1_P4_63669	Ca1	63669	TTATATATATATATATATATAT/TTATATATATATATATATATAT	AAGGGCGAAGATAAATCGGT	AAACCTGACGGGAAACATTG	59.9	610	INTERGENIC	--	--	--
Ca1_P4_6374330	Ca1	6374330	AAAGAAGAAGAAGAA/AAAGAAGAAGAA	CAAAATGATGGACACCCTTG	TCCATCTTGGACCAATCTCA	58.8	291	INTERGENIC	--	--	--
Ca1_P4_6376706	Ca1	6376706	TTGGATAAATTGTGGATAAATTGTG/TTGGATAAATTGTG	GCTCGTCCAAATATTTACGCA	CCCAGCAGTGAAGCAAACTT	60.1	379	INTERGENIC	--	--	--
Ca1_P4_6404378	Ca1	6404378	ATTTTTTTT/ATTTTTTTTT	AATTGCATTTTATGTCCGCC	TGTTGCAGGTTCTGAGATCG	59.8	858	INTERGENIC	--	--	--

INDEL marker IDs	Chromosomes/ unanchored scaffolds	Physical positions (bp)	InDels (*Kabuli* reference genome-CDC Frontier/PII)	Forward primers (5'-3')	Reverse primers (5'-3')	Anneling temperature (0C)	Expected amplified product size (bp)	Structural annotation		Functional annotation	
								Sequence components of *kabuli* genome	*Kabuli* gene accession IDs	NCBI-KOG	TFs
Ca1_P4_6472948	Ca1	6472948	TAA/TAAA	TGGCAGTTTCATTTGTCGTC	TCTTCATCCCCAACCTTCAC	59.7	830	INTERGENIC	--	--	--
Ca1_P4_6559705	Ca1	6559705	CATATATATATATATATATA/CATATATATATATATATATATA	GGGTCAAAACCCTTGGAAAT	TCCTCGTGTTCACTTTCGC	60.0	910	INTERGENIC	--	--	--
Ca1_P4_6714588	Ca1	6714588	ATTTTTTTTTTT/ATTTTTTTTTTTT	TTTGGGGCCCTCTTCTTTAT	GCAGTGAAGCTCCCTGATTC	59.9	678	INTERGENIC	--	--	--
Ca1_P4_6972063	Ca1	6972063	CTATTATTATTATTATTATTATTATTATTATTATTATTATTATTATTATTATTATTATTATTATTATTATTAT/CTATTATTATTATTATTATTATTATTATTATTATTATTATTATTATTATTATTATTATTATTATTATTATTATTATTAT	GTTTTCGTTCTCTCCTCCCC	CCGGTGCATTTTAACCTTGT	60.1	578	INTERGENIC	--	--	--
Ca1_P4_7017413	Ca1	7017413	ATTTT/ATTT	GCTTTGCCTTAAAAACCACG	CGAGCATGGTCAAGAGTTTT	59.8	734	INTERGENIC	--	--	--
Ca1_P4_7103961	Ca1	7103961	AAATAATAATAATAATAATAATAATAATAATAATAATAATAATAATAATAATAATAAT/AAATAATAATAATAATAATAATAATAATAATAATAATAATAATAATAATAATAATAATAATAAT	CAAGGCCCAAACCAAACTAA	CTTATGGACATTGGGGTTGG	60.0	642	INTERGENIC	--	--	--
Ca1_P4_7215414	Ca1	7215414	AA/AACA	AACCCCCAACTACAAGGTCC	CAATTTGACAAGCCCCCTAA	60.1	639	INTERGENIC	--	--	--
Ca1_P4_7274145	Ca1	7274145	AATATATATATATATATATATATATATA/AATATATATATATATATATATATATATA	TGAATGAACTTTTATCCTGCCA	CAGCGTGAACGACTTCAAAA	59.6	539	INTERGENIC	--	--	--
Ca1_P4_7423109	Ca1	7423109	TTCTCTCTCTCTCTCTTTCTCTCTCTCTCTCT/TTCTCTCTCTCTCTCTCTTTCTCTCTCTCTCTCT	CGGTTTTTGACAAATGGTTT	ATGGCCATGTCTGACTCGTT	57.5	345	INTERGENIC	--	--	--
Ca1_P4_7582813	Ca1	7582813	CATATATATATATATATATATATATATATATATATATATATAT/CATATATATATATATATATATATATATATATATATATAT	CTCCAGTCGATGAGATTCAAA	TGTTGAAATTCCATTCATTAGCA	57.9	769	INTERGENIC	--	--	--

INDEL marker IDs	Chromosomes/ unanchored scaffolds	Physical positions (bp)	InDels (*Kabuli* reference genome-CDC Frontier/PII)	Forward primers (5'-3')	Reverse primers (5'-3')	Anneling temperature (0C)	Expected amplified product size (bp)	Structural annotation		Functional annotation	
								Sequence components of *kabuli* genome	*Kabuli* gene accession IDs	NCBI-KOG	TFs
Ca1_P4_7618300	Ca1	7618300	AGGTGTCTAG/A	GAAGAGGCCACATGGTTCAT	CCAAGCATCATCACCAATG	59.9	743	INTERGENIC	--	--	--
Ca1_P4_7635339	Ca1	7635339	ATGTCATCCCT/AT	GCACGCGTTTTCAATGTAGA	AAATGTCATCCCTCAGCGAC	59.9	207	INTERGENIC	--	--	--
Ca1_P4_7635551	Ca1	7635551	GAAAAA/GAAAA	TGTCATGTCATCCCTCTGGA	GCCCTTTCACATGGATCAAG	60.0	250	INTERGENIC	--	--	--
Ca1_P4_7642434	Ca1	7642434	TA/TAA	TGCCACACAAAAATCTTGGA	TAACCTACGCTTGTGGGGTC	60.1	219	INTERGENIC	--	--	--
Ca1_P4_7648479	Ca1	7648479	ATTTTT/ATTTTTT	TGCCAAGACAGCTGAAAGTG	GACAAATGGAAGTGCTGGGT	60.2	399	INTERGENIC	--	--	--
Ca1_P4_7657426	Ca1	7657426	ATT/AT	CAGCCTCCTCAAATCAGCTT	AATGATTGGGGAACATTGGA	59.6	765	INTERGENIC	--	--	--
Ca1_P4_7661306	Ca1	7661306	CTT/CTTT	AGTGTGAGCTTCTTTTCCGC	CACGAAATGAATAAACAGACATTTG	59.6	266	INTERGENIC	--	--	--
Ca1_P4_7668769	Ca1	7668769	TAAAA/TAAAAA	TTGACCATCCTCTTCGATCC	TAAGCAACACGCAAAGAACG	60.0	297	INTERGENIC	--	--	--
Ca1_P4_7672846	Ca1	7672846	AAACACTCCTACCTCCAACACTCCTACCTCCAACAC/AAACACTCCTACCTCCAACAC	TAAGCCCCATGTTTGAGACC	GGCCTGATGCTGTCAACTTT	59.9	583	INTERGENIC	--	--	--
Ca1_P4_7677005	Ca1	7677005	TCA/T	TTGCGAATGTCGACCTTAAA	TGAATTTTGTTCGCATTTCATT	59.3	870	INTERGENIC	--	--	--

INDEL marker IDs	Chromosomes/ unanchored scaffolds	Physical positions (bp)	InDels (*Kabuli* reference genome-CDC Frontier/PII)	Forward primers (5'-3')	Reverse primers (5'-3')	Anneling temperature (0C)	Expected amplified product size (bp)	Structural annotation		Functional annotation	
								Sequence components of *kabuli* genome	*Kabuli* gene accession IDs	NCBI-KOG	TFs
Ca1_P4_7678204	Ca1	7678204	C/CATTT	TCACGCACTTCATACCAATCA	CATGAGCAATCACGGTTTTG	60.1	776	INTERGENIC	--	--	--
Ca1_P4_7679519	Ca1	7679519	TCAC/TCACCAC	ACCCACACAAACCTACCTCG	GGAAAAAGCCAAGGACATGA	59.9	762	INTERGENIC	--	--	--
Ca1_P4_7679708	Ca1	7679708	AGGG/AGG	ACCCACACAAACCTACCTCG	GGAAAAAGCCAAGGACATGA	59.9	762	INTERGENIC	--	--	--
Ca1_P4_7702136	Ca1	7702136	ACA/ACATGCA	TTGAGATATTGATTTGCATCACA	ATGCCCTTGCAGAGCTAGAA	58.2	382	INTERGENIC	--	--	--
Ca1_P4_7705518	Ca1	7705518	A/AG	AAGGTCAAGTTGAGTTGGGG	TTCATTGGCCCCTAAAATTG	59.0	559	INTERGENIC	--	--	--
Ca1_P4_7713699	Ca1	7713699	TAAAAAAAA/TAAAAAAAAA	TGTGACAATCATCGTCGTCT	TGTTTTGGATGGAAGATTTGC	57.6	652	INTERGENIC	--	--	--
Ca1_P4_7720463	Ca1	7720463	C/CT	TCAAAATTTTCAACCAAAACCC	ATCGGTCCAACGGTTATGTC	60.1	845	INTERGENIC	--	--	--
Ca1_P4_7721034	Ca1	7721034	CTTTT/CTTT	CATAACCGTTGGACCGATTC	GTTTTGCCATTGGTGCTTTT	60.2	713	INTERGENIC	--	--	--
Ca1_P4_7726229	Ca1	7726229	CAAAAAAAA/CAAAAAAAAA	TTTAGTGCCCGAGAGGAAGA	TTTGAGTCCCAACCAAAGGA	59.9	394	INTERGENIC	--	--	--
Ca1_P4_7726361	Ca1	7726361	GAATAAATAAA/GAATAAA	TTTAGTGCCCGAGAGGAAGA	TTTGAGTCCCAACCAAAGGA	59.9	394	INTERGENIC	--	--	--

INDEL marker IDs	Chromosomes/ unanchored scaffolds	Physical positions (bp)	InDels (*Kabuli* reference genome-CDC Frontier/PII)	Forward primers (5'-3')	Reverse primers (5'-3')	Anneling temperature (0C)	Expected amplified product size (bp)	Structural annotation		Functional annotation	
								Sequence components of *kabuli* genome	*Kabuli* gene accession IDs	NCBI-KOG	TFs
Ca1_P4_7726541	Ca1	7726541	AT/ATATATATATATGT	GCGTGATCCATCATTTGACA	GGAAATTGCTCAAAACTCACG	60.5	566	INTERGENIC	--	--	--
Ca1_P4_7745366	Ca1	7745366	A/ATAT	TGGAGAAACTCTTCCGCTGT	ACTTTGGGGAGAGGCTTTGT	60.0	175	INTERGENIC	--	--	--
Ca1_P4_7750166	Ca1	7750166	ATTT/ATTTT	TTGCAATAGGCGTTGAAGTTT	AGGTCAAGGGTTGGGCTACT	59.8	649	INTERGENIC	--	--	--
Ca1_P4_7750284	Ca1	7750284	AAAGAA/AAA	TTGACAATAGAATCGATCAAAAACA	AGGTCAAGGGTTGGGCTACT	59.9	312	INTERGENIC	--	--	--
Ca1_P4_7763628	Ca1	7763628	ATGAAA/ATGAAAATCTGTGAAA	CAGCTCCTCCCAACATCATT	AGCTGCAGTGTTGTATGCCA	60.1	364	INTERGENIC	--	--	--
Ca1_P4_7775058	Ca1	7775058	C/CCTTAAT	AGCCCAACCCTTGACCTAAT	GCACGGAAACAACATGTGAC	59.8	838	INTERGENIC	--	--	--
Ca1_P4_7782299	Ca1	7782299	AG/A	TTGTCGAAAAGAGGAAGTGCT	CAAGACACACCCTTGGGATT	59.1	448	INTERGENIC	--	--	--
Ca1_P4_7783146	Ca1	7783146	TAAA/TAA	AATTCAAGCCAAATGCCATC	CTGTTGTTTTTGCCTCAACG	59.9	834	INTERGENIC	--	--	--
Ca1_P4_7783374	Ca1	7783374	GAAAAAAAA/GAAAAAAAAAA	ACAAAATGCTGAAATTGCCA	CTGTTGTTTTTGCCTCAACG	59.2	334	INTERGENIC	--	--	--
Ca1_P4_7786618	Ca1	7786618	CAAAAAAAA/CAAAAAAAAA	CCAATGAGGGAGAAGGTTCA	TTAAAATTGCTACCCACGCC	60.0	312	INTERGENIC	--	--	--

INDEL marker IDs	Chromosomes/ unanchored scaffolds	Physical positions (bp)	InDels (*Kabuli* reference genome-CDC Frontier/PII)	Forward primers (5'-3')	Reverse primers (5'-3')	Anneling temperature (0C)	Expected amplified product size (bp)	Structural annotation		Functional annotation	
								Sequence components of *kabuli* genome	*Kabuli* gene accession IDs	NCBI-KOG	TFs
Ca1_P4_7824017	Ca1	7824017	AT/ATT	AAGAATGGTGGACACTTGCC	ACCCATGTGGCTACTGGAAG	60.0	433	INTERGENIC	--	--	--
Ca1_P4_7824410	Ca1	7824410	TTTTTT/TTTTTTCTTTTT	TGGTTCATGGGTTTTTGGAT	AAAATTGGCCCACAAATTAGG	60.0	123	INTERGENIC	--	--	--
Ca1_P4_7858563	Ca1	7858563	AATATATATATAT/AATATATAT	AAAAACAAATGCAATCGATCA	TTTTCATGCGTTCCCTCTTT	58.1	233	INTERGENIC	--	--	--
Ca1_P4_7865713	Ca1	7865713	C/CT	TGCACTCACTCCAACAAACA	CAAAGCATACCAACCACAGC	58.8	799	INTERGENIC	--	--	--
Ca1_P4_7888288	Ca1	7888288	TC/TCC	GCTTTCAAGCCAGACCAGAC	TTTGGAGCGGAGAAGTAATGA	60.0	274	INTERGENIC	--	--	--
Ca1_P4_7913832	Ca1	7913832	GAGAAGAAGAAGAAGAAG/GAGAAGAAGAAGAAGAAGAAG	ACACTTGCAAAAATGAGGGG	TCACTCCTCTTCTCCCCAAA	60.0	370	INTERGENIC	--	--	--
Ca1_P4_7914166	Ca1	7914166	T/TG	TGCATAGGTTGTTGTGGGAA	GACGAGAGGTTGGAGACTCG	60.0	939	INTERGENIC	--	--	--
Ca1_P4_7914415	Ca1	7914415	T/TC	TTTGGGGAGAAGAGGAGTGA	GACGAGAGGTTGGAGACTCG	59.8	697	INTERGENIC	--	--	--
Ca1_P4_7929432	Ca1	7929432	T/TTA	ATATCCGCAAGCTGAATCGT	GGTTTGAACCCTCCACAAGA	59.7	677	INTERGENIC	--	--	--
Ca1_P4_7929572	Ca1	7929572	CCATCTCATC/CCATCTCATCTCATC	ACTTACTGTTTCAACGCGGG	GGTTTGAACCCTCCACAAGA	60.2	325	INTERGENIC	--	--	--

INDEL marker IDs	Chromosomes/ unanchored scaffolds	Physical positions (bp)	InDels (*Kabuli* reference genome-CDC Frontier/PII)	Forward primers (5'-3')	Reverse primers (5'-3')	Anneling temperature (0C)	Expected amplified product size (bp)	Structural annotation		Functional annotation	
								Sequence components of *kabuli* genome	*Kabuli* gene accession IDs	NCBI-KOG	TFs
Ca1_P4_7933054	Ca1	7933054	TTTAATTA/TTTA	TCCTGCATAACACCATTGGA	CCTTCACAAACTTGAAGGACC	59.9	883	INTERGENIC	--	--	--
Ca1_P4_7933130	Ca1	7933130	TG/TGG	TCCTGCATAACACCATTGGA	CCTTCACAAACTTGAAGGACC	59.9	883	INTERGENIC	--	--	--
Ca1_P4_7947164	Ca1	7947164	T/TA	AACGATTTTTCACGCTTGCT	CAATGATTGGAATTTGGAGGA	59.9	693	INTERGENIC	--	--	--
Ca1_P4_7959222	Ca1	7959222	TAAAAAAAAA/TAAAAAAAAAA	AGTGCCAAGAAGACTTCCGA	TAATTGACACCAAGCCACGA	60.0	444	INTERGENIC	--	--	--
Ca1_P4_7959979	Ca1	7959979	GTTT/GTTTATTAAAAAGGATTT	TCGTGGCTTGGTGTCAATTA	GGTCGCCAATTTTGGTTATG	60.1	666	INTERGENIC	--	--	--
Ca1_P4_7960391	Ca1	7960391	ATTT/ATTTT	CCTAGGGTAAACCGTGCTCA	CCTCTCCTGCCTTAGTCCCT	60.1	480	INTERGENIC	--	--	--
Ca1_P4_7961218	Ca1	7961218	CAAA/CAAAGAAAAAA	TTGAGTTTTGGAGGGGAGAA	GAAGGGAAGGAAGGAGATGG	59.6	171	INTERGENIC	--	--	--
Ca1_P4_7965606	Ca1	7965606	CTTTT/CTTT	TGTCTTGTGAATAAATCATCGCA	TGGTCCATTCATTAGGTGGG	60.5	344	INTERGENIC	--	--	--
Ca1_P4_7981639	Ca1	7981639	AATATATA/AATATA	GAAGCCATGGAAGTGTTGGT	TCCTCCCGAGGGATATTACC	60.0	499	INTERGENIC	--	--	--
Ca1_P4_7984637	Ca1	7984637	TAAAAAA/TAAAAAAAA	TTGATTGCTTGCCATTATGC	TGGTTTTGCCCATATGGTTT	59.7	509	INTERGENIC	--	--	--

INDEL marker IDs	Chromosomes/ unanchored scaffolds	Physical positions (bp)	InDels (*Kabuli* reference genome-CDC Frontier/PII)	Forward primers (5'-3')	Reverse primers (5'-3')	Anneling temperature (0C)	Expected amplified product size (bp)	Structural annotation		Functional annotation	
								Sequence components of *kabuli* genome	*Kabuli* gene accession IDs	NCBI-KOG	TFs
Ca1_P4_7994670	Ca1	7994670	ACT/A	TTAACGTGCCACCTTCACAA	ACTTCATGGGCATGGGTTAG	60.1	613	INTERGENIC	--	--	--
Ca1_P4_7995701	Ca1	7995701	ATTAAATGAGTGTTAAAT/ATTAAAT	GCGTAAGTTATGCATGCCAAT	GGTGCAAGGGAAGAAAATGA	60.0	902	INTERGENIC	--	--	--
Ca1_P4_7995766	Ca1	7995766	CAAAAAAA/CAAAAAAAA	GCCAATTGCGTGTAGCTGT	GGTGCAAGGGAAGAAAATGA	59.9	887	INTERGENIC	--	--	--
Ca1_P4_7995820	Ca1	7995820	CTATA/CTATATATA	TTGTTTTGTGAATAAAAATCCCTT	GGTGCAAGGGAAGAAAATGA	58.1	492	INTERGENIC	--	--	--
Ca1_P4_7996602	Ca1	7996602	CTTTT/CTTTTT	TCATTTTCTTCCCTTGCACC	TTCTCAAGCACCAAGCACAC	60.1	926	INTERGENIC	--	--	--
Ca1_P4_8000567	Ca1	8000567	TT/TTAT	CCAACCCTTCACACTCGTTT	TGACACCAAACTCAGTCCTTG	60.0	489	INTERGENIC	--	--	--
Ca1_P4_8003639	Ca1	8003639	AC/A	TTGGTCATCTCCAAAGGTCC	GGACACAAAATATAATTGAGCCA	59.9	631	INTERGENIC	--	--	--
Ca1_P4_8003853	Ca1	8003853	TAT/TATATATAAAT	TGATAATTTCTTGCCCATTTTC	TGACATTTTAACTCAAGAGACCGA	58.1	628	INTERGENIC	--	--	--
Ca1_P4_8003954	Ca1	8003954	CCCTATGTT/C	TGATAATTTCTTGCCCATTTTC	TGACATTTTAACTCAAGAGACCGA	58.1	628	INTERGENIC	--	--	--
Ca1_P4_8004049	Ca1	8004049	AAT/AATATATAT	GGCTCAATTATATTTTGTGTCCC	TCATGATATTATGCACCGGG	58.8	551	INTERGENIC	--	--	--

INDEL marker IDs	Chromosomes/ unanchored scaffolds	Physical positions (bp)	InDels (*Kabuli* reference genome-CDC Frontier/PII)	Forward primers (5'-3')	Reverse primers (5'-3')	Anneling temperature (0C)	Expected amplified product size (bp)	Structural annotation		Functional annotation	
								Sequence components of *kabuli* genome	*Kabuli* gene accession IDs	NCBI-KOG	TFs
Ca1_P4_8004353	Ca1	8004353	AAAT/AAATAAT	TCGGTCTCTTGAGTTAAAATGTCA	TGGAAGGGATCTCAATTTGC	60.2	635	INTERGENIC	--	--	--
Ca1_P4_8017826	Ca1	8017826	AAAGA/AAAGAAGAAGAAGAAGA	TGTGCTATATTCGGTTGGCA	TCAGTCAAACCATCGAACCA	60.1	156	INTERGENIC	--	--	--
Ca1_P4_8018022	Ca1	8018022	G/GAAACAT	TGTTTGGTTCGATGGTTTGA	TGTATTGGGCCAGTTTCACA	59.9	544	INTERGENIC	--	--	--
Ca1_P4_8018283	Ca1	8018283	GAAA/GAA	TGTTTGGTTCGATGGTTTGA	TGTATTGGGCCAGTTTCACA	59.9	544	INTERGENIC	--	--	--
Ca1_P4_8020277	Ca1	8020277	ATTT/ATTTGTTT	TCGCGGTGAATATCACAAGA	GTCGATTCAAGTTGGCGAAT	60.2	756	INTERGENIC	--	--	--
Ca1_P4_8021607	Ca1	8021607	AT/ATT	CGTCCACCCTCACCTACCTA	TGCCAACAAATCTTCTGAAAAA	60.0	939	INTERGENIC	--	--	--
Ca1_P4_8031450	Ca1	8031450	TAAAAAA/TAAAAAAA	ATCTTCTTTTTGTGGGGCCT	CTTGGCAGATCTCGAGTATGAA	59.9	397	INTERGENIC	--	--	--
Ca1_P4_8031692	Ca1	8031692	TTA/TTATA	ATCTTCTTTTTGTGGGGCCT	CTTGGCAGATCTCGAGTATGAA	59.9	397	INTERGENIC	--	--	--
Ca1_P4_8031727	Ca1	8031727	CG/C	ATCTTCTTTTTGTGGGGCCT	CTTGGCAGATCTCGAGTATGAA	59.9	397	INTERGENIC	--	--	--
Ca1_P4_8033091	Ca1	8033091	TTCTCTCTCTCTCTCTCTCTCTCTC/TTCTCTCTCTCTCTCTCTCTCTCTCTCTCTCTCTC	GCTTTTACAACATGGACGCA	GGAAGGGAATGAAGGTGTGA	59.7	171	INTERGENIC	--	--	--

INDEL marker IDs	Chromosomes/ unanchored scaffolds	Physical positions (bp)	InDels (*Kabuli* reference genome-CDC Frontier/PII)	Forward primers (5'-3')	Reverse primers (5'-3')	Anneling temperature (0C)	Expected amplified product size (bp)	Structural annotation		Functional annotation	
								Sequence components of *kabuli* genome	*Kabuli* gene accession IDs	NCBI-KOG	TFs
Ca1_P4_8033455	Ca1	8033455	CTTTTTTTTT/CTTTTTTTTTTTT	TCACACCTTCATTCCCTTCC	CCCAGGAGAAGAAAATCGTG	59.9	766	INTERGENIC	--	--	--
Ca1_P4_8041307	Ca1	8041307	T/TG	CGGCGGCAATTGTTATAAGT	CAATTTCAAGCAAAATCGCA	60.0	890	INTERGENIC	--	--	--
Ca1_P4_8041662	Ca1	8041662	TAAC/TAACATTAAAC	CCCTTCATTGGAGATGTTCC	TTGGGGACACTAAGAGGTGG	59.3	866	INTERGENIC	--	--	--
Ca1_P4_8053901	Ca1	8053901	TCTCTG/TCTCTGCTCTG	CTCCAATGTGTCCCAACCTT	GTTGTAAATTGCACGGGTCC	59.8	497	INTERGENIC	--	--	--
Ca1_P4_8062841	Ca1	8062841	T/TGATGATAA	TCGGTTCACGTACTACCAATGA	TGTTGCTCTTGCCATACTGC	60.4	608	INTERGENIC	--	--	--
Ca1_P4_8090497	Ca1	8090497	ATTTTT/ATTTTTT	GAGGGGCTACCACCAAATTC	TGCATCCAACATCTTCATCC	60.7	608	INTERGENIC	--	--	--
Ca1_P4_8091150	Ca1	8091150	CA/CAA	TGGCTAACGTTCATTTGCAG	TCGAAACAACCTCCACACAA	59.9	202	INTERGENIC	--	--	--
Ca1_P4_8091521	Ca1	8091521	TTTAAAAA/TTTAAAAATTAAAAA	GTTTGTTGTCATGTCACCGC	TGAAACTCTGGCGAAGATGA	60.0	485	INTERGENIC	--	--	--
Ca1_P4_8094210	Ca1	8094210	AAAT/AAATAAT	GCCAACTTGTCCCAAGACAC	TGGCTTTGTGGTACATTGGA	60.6	340	INTERGENIC	--	--	--
Ca1_P4_8107780	Ca1	8107780	T/TTGAAATC	CTTTTGTGCAATTGGGTGTG	CTGCTGAGTTGCCTGTACCA	60.0	496	INTERGENIC	--	--	--

INDEL marker IDs	Chromosomes/ unanchored scaffolds	Physical positions (bp)	InDels (*Kabuli* reference genome-CDC Frontier/PII)	Forward primers (5'-3')	Reverse primers (5'-3')	Anneling temperature (0C)	Expected amplified product size (bp)	Structural annotation		Functional annotation	
								Sequence components of *kabuli* genome	*Kabuli* gene accession IDs	NCBI-KOG	TFs
Ca1_P4_8116992	Ca1	8116992	ATTTTTTTTTT/ATTTTTTTT	TGTGTGTGTAAGGGATTTCTAACC	CAATCGAACGAGACGAGTCA	59.3	600	INTERGENIC	--	--	--
Ca1_P4_8248772	Ca1	8248772	ATTTTTTTTTT/ATTTTTTTTTTT	TGCAAACATGAATTGAAAGGA	ATAGTCGGGAATGCAACCTG	59.1	486	INTERGENIC	--	--	--
Ca1_P4_8319139	Ca1	8319139	TGGGCGCG/TGGGCGCGCTCGGGCGCG	CCCAAGTTCCCAAGAGTCAA	CAAAGACACCGGAACCAAAT	60.1	292	INTERGENIC	--	--	--
Ca1_P4_8327619	Ca1	8327619	AATATATATATATATATATATATATATATATATATA/AATATATATATATATATATATATATATATATATATATATATATATATA	AATAGCTCCCGCAAGTTTGA	CATGACATCTTTCAATCCAATCA	59.8	425	INTERGENIC	--	--	--
Ca1_P4_8340429	Ca1	8340429	TAAAAAAAA/TAAAAAAAAA	TGGACAAATCCATATCGTCG	GTGCACCGACGTGTCTAAAA	59.4	334	INTERGENIC	--	--	--
Ca1_P4_8415684	Ca1	8415684	TA/TAA	AGAAATGTGATGCAAGAGGAAA	GGGTCAAGATGAGAGAGCCA	58.8	740	INTERGENIC	--	--	--
Ca1_P4_8430467	Ca1	8430467	TT/TTATTATTATTAT	AGGTCATGGGCTGGATGTAG	ACCCTTTCACTGCGCTCTTA	60.0	612	INTERGENIC	--	--	--
Ca1_P4_8590076	Ca1	8590076	TAAAAAAAAA/TAAAAAAAA	TCCTTGAAGCTTTGCCACTT	TCCATATCTTGCGACATGCT	60.0	664	INTERGENIC	--	--	--
Ca1_P4_8836170	Ca1	8836170	ATTTTTTTTTTTT/ATTTTTTTTTTTTT	GTAGAGCCTGACTTTTGCCG	GGGGTTTAGCACGACTGTGT	60.0	722	INTERGENIC	--	--	--
Ca1_P4_89785	Ca1	89785	ATTTTTTTTTTTTT/ATTTTTTTTTTTTTT	CACGAGTTGACATTTCACGG	TCCATCAAGATCATTTCCAGC	60.2	510	INTERGENIC	--	--	--

INDEL marker IDs	Chromosomes/ unanchored scaffolds	Physical positions (bp)	InDels (*Kabuli* reference genome-CDC Frontier/PII)	Forward primers (5'-3')	Reverse primers (5'-3')	Anneling temperature (0C)	Expected amplified product size (bp)	Structural annotation		Functional annotation	
								Sequence components of *kabuli* genome	*Kabuli* gene accession IDs	NCBI-KOG	TFs
Ca1_P4_9130103	Ca1	9130103	TTATATATATATATATATATAT/TTATATATATATATATATAT	GGGTCAAAATTCGTTGACCAT	TACGGGTCAAGACAGGTTCG	61.0	550	INTERGENIC	--	--	--
Ca1_P4_9223996	Ca1	9223996	TAAAAAAAAAA/TAAAAAAAAAAA	AATGGATTCTTCCGCTCAAA	ACAGAACGGTGCGTTGTAAA	59.6	489	INTERGENIC	--	--	--
Ca1_P4_9225279	Ca1	9225279	GTTTTTTT/GTTTTTT	AGGATTCCACAGATCAGCGT	TCAATTCACAAAGTTCGAGGG	59.7	646	INTERGENIC	--	--	--
Ca1_P4_9233850	Ca1	9233850	GTTTTTTTTTTTTT/GTTTTTTTTTT	TGAGTTCTTTTGAATTTGCGTG	GTTGCGTCTAGCAACTTTCTG	60.3	637	INTERGENIC	--	--	--
Ca1_P4_9286959	Ca1	9286959	GAAAAAAAAAA/GAAAAAAAAAAA	TAGTTGGGGCCATATCCAAA	TGGAAACGCTACACGCTATG	60.1	631	INTERGENIC	--	--	--
Ca1_P4_9322441	Ca1	9322441	TTATATATATATATATATATATATATATATCTATATATATATATAT/TTATATATATATATATATATATATATATCTATATATATATAT	TGCCTTTTCGTTTTCAATTC	AACGGTTGAGCAATGAATCC	57.9	442	INTERGENIC	--	--	--
Ca1_P4_9516400	Ca1	9516400	AATATATATATATATATATATATATATATATATATATAT/AATATATATATATATATATATATATATATATATATATATATAT	ACATTGAACCCTAATCGCGT	TGATGATAAACCGAGGGGAG	59.5	412	INTERGENIC	--	--	--
Ca1_P4_9681529	Ca1	9681529	CTATATATATATATATA/CTATATATATATATATATA	GGATGTTATGACGTGCCAAA	TTGAACAATATCAATGCACAACA	59.4	310	INTERGENIC	--	--	--
Ca1_P4_9686315	Ca1	9686315	TTATTATTATTATTA/TTATTATTATTATTAATATTATTATTATTA	TCAAATGCTTTAATGGCTCAGA	TTTTTAGTTTGAAGACCTTTGTCA	59.9	446	INTERGENIC	--	--	--
Ca1_P4_9830366	Ca1	9830366	AATATATATATATATATATA/AATATATATATATATATATATATA	TCCAGGATGTGTTACCATGAA	CTATTCCGGCTTTTGGATCA	58.8	575	INTERGENIC	--	--	--

INDEL marker IDs	Chromosomes/ unanchored scaffolds	Physical positions (bp)	InDels (*Kabuli* reference genome-CDC Frontier/PII)	Forward primers (5'-3')	Reverse primers (5'-3')	Anneling temperature (0C)	Expected amplified product size (bp)	Structural annotation		Functional annotation	
								Sequence components of *kabuli* genome	*Kabuli* gene accession IDs	NCBI-KOG	TFs
Ca1_P4_9867623	Ca1	9867623	AATATATATATATATATATATATAT/AATATATATATATATATATATATAT	GCCAAATATGTGTGTCTTTTCG	TTCTGTCGTCACAGAGGACG	59.5	566	INTERGENIC	--	--	--
Ca1_P4_9871519	Ca1	9871519	ATT/A	CTTAGTCCACGTGCAAACGA	AAAGTGCGGGCCAATATCTA	59.9	600	INTERGENIC	--	--	--
Ca1_P4_9965386	Ca1	9965386	CATATATATATATATAT/CATATATATATATATAT	TGGAATCAACTAATCATGGTGG	GATGAAAAGAGGGAGGAGGG	59.7	824	INTERGENIC	--	--	--
Ca1_P4_9965836	Ca1	9965836	CAAAAAAAAAAA/CAAAAAAAAAAAA	CCCTCCTCCCTCTTTTCATC	CACCACCTCATCATCCTGTG	60.0	496	INTERGENIC	--	--	--
Ca1_P4_9995156	Ca1	9995156	C/CG	TTGTATTTTGGGGCCATTGT	TGCAAGTGACAATTTGATCG	60.1	934	INTERGENIC	--	--	--
Ca2_P4_10013835	Ca2	10013835	ATTTTTTTTT/ATTTTTTTT	TACTCACCCAAATCCGCTTC	TAGACCCTCATCCTCATCCG	60.1	393	INTERGENIC	--	--	--
Ca2_P4_10081600	Ca2	10081600	CAAAAAAAAAAAAAAA/CAAAAAAAAAAAAAA	TTTTGATTGATTGATGATGGG	TGCAAATTCAACCTCACGAA	58.3	552	INTERGENIC	--	--	--
Ca2_P4_10149284	Ca2	10149284	AATTATTATTATTATTATTATTATTATTATTATTATTATTATTATTATTATTATTATTATTA/AATTATTATTATTATTATTATTATTATTATTATTATTATTATTATTATTATTATTATTA	GGCTAAAAAGTCTCCGAGTTCA	TTCCTACGTATCTCCCGGTG	59.9	822	INTERGENIC	--	--	--
Ca2_P4_10169628	Ca2	10169628	CAAAAAAA/CAAAAAAAA	TCATAATGAGCAGCGTATCTTCA	TGCCTAATCGATTGCACATT	59.9	691	INTERGENIC	--	--	--
Ca2_P4_1023421	Ca2	1023421	TTATATATATATATATATA/TTATATATATATATATATA	TCCACGTCCTAAAGCCATTC	ACAAGACAGACCCTTTTGACA	60.1	639	INTERGENIC	--	--	--

INDEL marker IDs	Chromosomes/ unanchored scaffolds	Physical positions (bp)	InDels (*Kabuli* reference genome-CDC Frontier/PII)	Forward primers (5'-3')	Reverse primers (5'-3')	Anneling temperature (0C)	Expected amplified product size (bp)	Structural annotation		Functional annotation	
								Sequence components of *kabuli* genome	*Kabuli* gene accession IDs	NCBI-KOG	TFs
Ca2_P4_1024227	Ca2	1024227	CAATAAATAAATAAATAAATAAATAAATAAATAAATAAAT/CAATAAATAAATAAATAAATAAATAAATAAATAAATAAATAAATAAATAAAT	TTGTCAAAAGGGTCTGTCTTG	TGACTGCCGCACATTTAGAG	57.8	411	INTERGENIC	--	--	--
Ca2_P4_1033751	Ca2	1033751	CATTTATTA/C	TGTGTGATTTTCAATAGTCACCG	AAGTTAAGGGACCACGGGAT	59.9	845	INTERGENIC	--	--	--
Ca2_P4_10362435	Ca2	10362435	TAA/TA	GAGGGGTAAGCGAGGGTAAG	CCCGACACAAAGTCATGAAA	60.1	440	INTERGENIC	--	--	--
Ca2_P4_10388305	Ca2	10388305	TTTTAAATTTATTTAAATTTATT/TTTTAAATTTATT	TCTCCTTAGCGGCTATTCCA	TTGACCGAATGAGATTTGTTAGA	59.9	564	INTERGENIC	--	--	--
Ca2_P4_10411790	Ca2	10411790	TCGAACTTAC/TC	TAGCTGCAGCAAGAAGCAAA	GGTTTCATATTCGAACCCGAG	60.0	882	INTERGENIC	--	--	--
Ca2_P4_1074412	Ca2	1074412	ATTT/ATTTTTTT	CCTCCTCGGTCTCTTCTCCT	AAATTGAGTTTCAAAACCACCAA	59.9	397	INTERGENIC	--	--	--
Ca2_P4_1074830	Ca2	1074830	CATAT/CAT	CTTGGTGGTTTTGAAACTCAAT	TCGATTATGCGTAGGGAAGG	58.2	504	INTERGENIC	--	--	--
Ca2_P4_1075762	Ca2	1075762	ATTTTTTTTTTT/ATTTTTTTTTT	TTAGGCCGCGACAATAATTC	CTGTACTCACGTCAGGCTGC	60.1	707	INTERGENIC	--	--	--
Ca2_P4_10763562	Ca2	10763562	CAAAAA/CAAAA	CTATAGCATCGAAAAGCGGC	AGTGTTGGCTAGGTCGATGC	60.0	121	INTERGENIC	--	--	--
Ca2_P4_10777745	Ca2	10777745	TGAA/TGAATTGTCGAA	CCTCGAGTTTGCATCCTCTC	TTACATTGTTCAATCGTCGAAA	60.0	725	INTERGENIC	--	--	--

INDEL marker IDs	Chromosomes/ unanchored scaffolds	Physical positions (bp)	InDels (*Kabuli* reference genome-CDC Frontier/PII)	Forward primers (5'-3')	Reverse primers (5'-3')	Anneling temperature (0C)	Expected amplified product size (bp)	Structural annotation		Functional annotation	
								Sequence components of *kabuli* genome	*Kabuli* gene accession IDs	NCBI-KOG	TFs
Ca2_P4_10779124	Ca2	10779124	TTA/TTACATA	CACTTTTCTTATGCACCGCA	GCAGCGAGTAGGAGAAATGC	59.9	663	INTERGENIC	--	--	--
Ca2_P4_10890760	Ca2	10890760	CTTTTTT/CTTTTT	TGGCAAATCGATTGAAACAA	TACATCAACGAAGATCGCCA	60.1	697	INTERGENIC	--	--	--
Ca2_P4_10914293	Ca2	10914293	GAAAAAAA/GAAAAAAAA	GCACTCAAAGAATGCGATCA	GCATTTCCATGTTGTGTTGC	60.0	579	INTERGENIC	--	--	--
Ca2_P4_10928556	Ca2	10928556	ATTTTTTTT/ATTTTTTTTT	CATCCGTGGTCCCTTAACTT	GCTAAATTACATTCGTGGTCCC	58.9	370	INTERGENIC	--	--	--
Ca2_P4_10948001	Ca2	10948001	ATTA/ATTACACTGACGTTA	TGATTCTTGATCATGTTGTTTTCA	CCATCCTCACTCCAAAGACAA	59.5	702	INTERGENIC	--	--	--
Ca2_P4_1107319	Ca2	1107319	ATAATA/ATAATAATATTAATA	AAAAGTTTTGTGCACCTCCA	ATGCCATGGTTGTGGAACTT	58.3	459	INTERGENIC	--	--	--
Ca2_P4_11085781	Ca2	11085781	AATATATATATATATATATATATATATATATATATATTTATATATATATATAT/AATATATATATATATATATATATATATATTTATATATATATAT	TTTGGTTTTGAACTCGGAGG	CACCGTAGTGTGGTGGTGAG	60.1	660	INTERGENIC	--	--	--
Ca2_P4_11294573	Ca2	11294573	CAAAAAAAAAAA/CAAAAAAAAAAAA	AGCACATTGATCACGTCCAA	TCTTATTCAACCGCACACCA	60.1	343	INTERGENIC	--	--	--
Ca2_P4_11320069	Ca2	11320069	A/AC	ACCAAGGCATCGTCAAAATC	CCGATGCACTTATACTTCAAAGA	59.9	497	INTERGENIC	--	--	--
Ca2_P4_11347624	Ca2	11347624	TTATATATATATATATATATA/TTATATATATATATATATA	AATACCTCCCACGGTCCTTT	CAACAAATTGCTGCCTTGTG	59.7	461	INTERGENIC	--	--	--

INDEL marker IDs	Chromosomes/ unanchored scaffolds	Physical positions (bp)	InDels (*Kabuli* reference genome- CDC Frontier/PII)	Forward primers (5'-3')	Reverse primers (5'-3')	Anneling temperature (0C)	Expected amplified product size (bp)	Structural annotation		Functional annotation	
								Sequence components of *kabuli* genome	*Kabuli* gene accession IDs	NCBI-KOG	TFs
Ca2_P4_11497600	Ca2	11497600	AG/A	TGATGAATGGTTGTGGCACT	ACGGGGTGTTACATTGTGGT	60.0	480	INTERGENIC	--	--	--
Ca2_P4_11671380	Ca2	11671380	G/GT	CCACTAGGTACCCCACTCCA	ATAGCCGGTGGTGCTGTATC	59.8	579	INTERGENIC	--	--	--
Ca2_P4_11716435	Ca2	11716435	TTATATATATATATATATATATATATAT/TTATATATATATATATATATATATATATATATATAT	CACAGTTCCGCATGTGCTAT	TGCACTGACAGGGAAATCAA	59.7	857	INTERGENIC	--	--	--
Ca2_P4_11722953	Ca2	11722953	AATATATATATATATA/AATATATATATATATATA	TTTGAAATGGCGACTTTCTT	AATTGGCATTTCGAACTTGG	57.5	534	INTERGENIC	--	--	--
Ca2_P4_11829316	Ca2	11829316	TTATATATATATATATATATATATA/TTATATATATATATATATATATA	TTCCCCTCAACAATTTTCAA	CTCAAGGGCCTTCACCTTTT	58.0	549	INTERGENIC	--	--	--
Ca2_P4_11998730	Ca2	11998730	AA/AATAATA	AGCTCCACGTCTGCTTTTGT	CAACGCTTGCCATCAATTAG	60.1	474	INTERGENIC	--	--	--
Ca2_P4_12040519	Ca2	12040519	ATT/AT	CCACATCAACCAAAAAGCTG	GTGAGGTGGCATTCGAGAGT	59.2	665	INTERGENIC	--	--	--
Ca2_P4_12041702	Ca2	12041702	GTTTTTTT/GTTTTTT	TCATCCTTTCTCCCCTCTCA	GAGGAGAGGTCCACATCAGC	59.7	768	INTERGENIC	--	--	--
Ca2_P4_12048675	Ca2	12048675	AATAATTTATAATTTATA/AATAATTTATA	CAAACATGCTGATGTGGACC	CCATTGGTAAATGGCTAACAA	60.0	278	INTERGENIC	--	--	--
Ca2_P4_12167727	Ca2	12167727	GTATATATATATATATATATA/GTATATATATATATATATATATA	AAGGTCGTAAGCTTGTCGATG	TAGCACCGTAGCGGAGAGAT	59.4	715	INTERGENIC	--	--	--

INDEL marker IDs	Chromosomes/ unanchored scaffolds	Physical positions (bp)	InDels (*Kabuli* reference genome-CDC Frontier/PII)	Forward primers (5'-3')	Reverse primers (5'-3')	Anneling temperature (0C)	Expected amplified product size (bp)	Structural annotation		Functional annotation	
								Sequence components of *kabuli* genome	*Kabuli* gene accession IDs	NCBI-KOG	TFs
Ca2_P4_12307967	Ca2	12307967	GAAA/GAAAA	TGTCTTTGGTCCCTGCAAAT	TCAAACCCCCATTTTTGTGT	60.5	755	INTERGENIC	--	--	--
Ca2_P4_12384702	Ca2	12384702	TAAAA/TAAA	CCTACGTATCTCCTGGTGCG	TTAATCGTTGCGTTGCTGAG	60.7	649	INTERGENIC	--	--	--
Ca2_P4_12475607	Ca2	12475607	TTGTATTTGATGTAT/TTGTAT	TCAAAGAGAAATGACTTTGTCCA	TCACTTTGCTTTGTGTGTCTCA	58.9	852	INTERGENIC	--	--	--
Ca2_P4_12483187	Ca2	12483187	AATATATATATATATATATATATATATATATATATATATATATATATATATATA/AATATATATATATATATATATATATATATATATATATATATATATA	TTTGGTGACCACTCATTTGC	TTGAAATTAACTTTTTCAACACAACA	59.5	451	INTERGENIC	--	--	--
Ca2_P4_12495891	Ca2	12495891	GATATATATATATATATATATATATATATATATATATATATATAT/GATATATATATATATATATATATATATATATATATATAT	TTTTGGGTCTTGGATTTTGG	TTGCAAAACAAATTCACGATG	59.8	530	INTERGENIC	--	--	--
Ca2_P4_12553140	Ca2	12553140	ATTTTTTTTTT/ATTTTTTTTTTT	CCCCCTCCAACCTTTCATAC	ATCCAAATTCAATGCATCCC	60.5	811	INTERGENIC	--	--	--
Ca2_P4_12567970	Ca2	12567970	ATTTTTTTTTTT/ATTTTTTTTTTTT	GGAAGTTGCCTTTCCCTTTC	TCGCAAGGATCTACACACTGA	60.1	743	INTERGENIC	--	--	--
Ca2_P4_12634541	Ca2	12634541	CTTTTTTTT/CTTTTTTT	GTTCAAAGCAGCTGCAACAA	CCACAACTACCCTTGGCATT	60.2	236	INTERGENIC	--	--	--
Ca2_P4_12652728	Ca2	12652728	TTCTCTCTCTCTCTCTCTCTCTCTCT/TTCTCTCTCTCTCTCTCTCTCTCTCTCT	GCGGTGGTTTTCTTGTGATT	AACATCCCCTTCAATCCTCC	60.0	625	INTERGENIC	--	--	--
Ca2_P4_1292040	Ca2	1292040	GAAAAAAAAAAAA/GAAAAAAAAAAA	TTCCCGATTTAGTCCTTTATTCC	AAAACGGCACAACAAACACA	59.7	370	INTERGENIC	--	--	--

INDEL marker IDs	Chromosomes/ unanchored scaffolds	Physical positions (bp)	InDels (*Kabuli* reference genome-CDC Frontier/PII)	Forward primers (5'-3')	Reverse primers (5'-3')	Anneling temperature (0C)	Expected amplified product size (bp)	Structural annotation		Functional annotation	
								Sequence components of *kabuli* genome	*Kabuli* gene accession IDs	NCBI-KOG	TFs
Ca2_P4_12933106	Ca2	12933106	A/AT	AGGTTGAAAGAAGCCCCACT	ATCGGTTGGTTGGTGAAATG	60.1	834	INTERGENIC	--	--	--
Ca2_P4_12933573	Ca2	12933573	CACCAACCAACC/CACCAACC	TCATTGACAAGGAAAGAACATGA	CGAATTGGTTTTGGGATTTG	59.6	413	INTERGENIC	--	--	--
Ca2_P4_12934202	Ca2	12934202	AAA/AAACAA	AGAAAGCAAGCAAATCCCAA	TTCCAACACCTCACGATTCA	59.8	488	INTERGENIC	--	--	--
Ca2_P4_12934953	Ca2	12934953	TTCTATCTATCTATCTATCTATCTATC/TTCTATCTATCTATCTATCTATCTATCTATCTATC	TGCCTCAAAAATCTGGTGTG	CTGCTCCAAACTGATCACGA	59.7	516	INTERGENIC	--	--	--
Ca2_P4_12937651	Ca2	12937651	ATTTT/ATTTTT	GATTGCTTTCCTCTATGCCG	GTTTTGGCGTTGGTTTCACT	59.8	353	INTERGENIC	--	--	--
Ca2_P4_12939688	Ca2	12939688	AATATATA/AATATA	TTTTGGTGTTGAGGGTGTCA	TTAATCAAAGCGGCAAAAGAA	60.0	564	INTERGENIC	--	--	--
Ca2_P4_12941200	Ca2	12941200	CAAAA/CAAA	TGTGCTTCTTCCTGATTTTGG	GGCCAAAGACGGATATGGTA	60.2	888	INTERGENIC	--	--	--
Ca2_P4_12941800	Ca2	12941800	T/TA	CATATCCGTCTTTGGCCATT	CCTTGAACAATTTGGGCAGT	59.8	643	INTERGENIC	--	--	--
Ca2_P4_12946880	Ca2	12946880	A/AC	AACTACGGCATAACAACCGC	TTGAAGTCCCACATTGGAAAG	60.0	416	INTERGENIC	--	--	--
Ca2_P4_12947970	Ca2	12947970	G/GT	TGCTTTTATGAGCATTACGGG	CGACTTCAAATCCCTTCGAG	60.1	777	INTERGENIC	--	--	--

INDEL marker IDs	Chromosomes/ unanchored scaffolds	Physical positions (bp)	InDels (*Kabuli* reference genome-CDC Frontier/PII)	Forward primers (5'-3')	Reverse primers (5'-3')	Anneling temperature (0C)	Expected amplified product size (bp)	Structural annotation		Functional annotation	
								Sequence components of *kabuli* genome	*Kabuli* gene accession IDs	NCBI-KOG	TFs
Ca2_P4_12952557	Ca2	12952557	ACATATCATATCA/ACATATCA	AAAGCATTTTATCCCGACCA	TTTTTCATCTCACTCTCTCCCA	59.4	787	INTERGENIC	--	--	--
Ca2_P4_12952944	Ca2	12952944	TGTCATGATT/TGTCATGATTGTAGTCATGATT	TGGGAGAGAGTGAGATGAAAAA	ACGGATGTCTAATCCGTTGG	58.9	308	INTERGENIC	--	--	--
Ca2_P4_12960292	Ca2	12960292	GT/GTTAT	TTCATACATCCAATCGTGGTTT	CCACAAGAAGCAACACAAGC	59.2	527	INTERGENIC	--	--	--
Ca2_P4_12963805	Ca2	12963805	ATTTTTTTTTTT/ATTTTTTTTTT	TGATTGTGCTATCTTACCTTTGGA	GATCCCATCAAAACCACCAG	60.0	867	INTERGENIC	--	--	--
Ca2_P4_13230836	Ca2	13230836	GTATATATATATAT/GTATATATATATATAT	TTCGGAAGTCCATCGGTATC	AAAAGTTTCAAACACATGTAAAACA	59.9	891	INTERGENIC	--	--	--
Ca2_P4_13284989	Ca2	13284989	TGG/TGGG	GTCGGCTGTCGGTTGTTATT	GGTCTGAGGTGGTTTAACGC	60.0	803	INTERGENIC	--	--	--
Ca2_P4_13639596	Ca2	13639596	TAATTAAATAAAAT/TAAT	AAGCCAGCCGCATGTAAATA	TTCACTTCCTCTGCATCTTTCA	60.6	788	INTERGENIC	--	--	--
Ca2_P4_13748106	Ca2	13748106	GTATTATTATTATTATT/GTATTATTATTATTATTATT	TTATGGAGCCACCAAAGGAC	TCTAATCAAGCAAGCACCCC	59.9	550	INTERGENIC	--	--	--
Ca2_P4_13757790	Ca2	13757790	AATATATATATATATATAT/AATATATATATATATATATAT	TTTTCTCGTATGGGTAGTTTTGTT	CAAGATGCTGTTCTCGTTCG	58.3	496	INTERGENIC	--	--	--
Ca2_P4_13853423	Ca2	13853423	CAAAAAAAA/CAAAAAAAAA	CATTGCCAGTATGCGACATC	AAGGTCCACAAAGAGCCTCC	60.1	529	INTERGENIC	--	--	--

INDEL marker IDs	Chromosomes/ unanchored scaffolds	Physical positions (bp)	InDels (*Kabuli* reference genome-CDC Frontier/PII)	Forward primers (5'-3')	Reverse primers (5'-3')	Anneling temperature (0C)	Expected amplified product size (bp)	Structural annotation		Functional annotation	
								Sequence components of *kabuli* genome	*Kabuli* gene accession IDs	NCBI-KOG	TFs
Ca2_P4_13923559	Ca2	13923559	ATGCAGCTTTCTAAA/ATGCAGCTTTCTAAATTCTGCAGCTTTCTAAA	TTTGACCAAAACCTTGGAGG	CAGGGGCGTACAAACCTTTA	59.9	436	INTERGENIC	--	--	--
Ca2_P4_13956607	Ca2	13956607	ATT/ATTATTT	TGAAATTTGTGATTTAGTTGCTTAAAA	AAACAAGTTGGTGGTGGAGC	59.6	686	INTERGENIC	--	--	--
Ca2_P4_13957739	Ca2	13957739	TGG/TG	ATTATCCCCACCCCCACTAC	TTTCAAAAACGGTGGTGTGA	59.8	755	INTERGENIC	--	--	--
Ca2_P4_13957855	Ca2	13957855	TACA/TACACA	GCTCCACCACCAACTTGTTT	CAGCTACGCACAATGGCTAA	60.0	196	INTERGENIC	--	--	--
Ca2_P4_14069182	Ca2	14069182	GAAAAAAAAAAAAA/GAAAAAAAAAAA	TTCATAAGGGATTTTTCTTCAACA	TGTTCAACCCTCGAAGATCA	59.1	436	INTERGENIC	--	--	--
Ca2_P4_14192522	Ca2	14192522	GAAAAAAAAAA/GAAAAAAAAAAA	TACAAATTGGATGCGGTTGA	CCCTCTGCAATGTCGGTAAT	59.9	755	INTERGENIC	--	--	--
Ca2_P4_15154581	Ca2	15154581	ATTT/ATT	GTTAGAACTGGGCCCAACAA	AAATTTTCCCTGTACCCCCA	60.0	884	INTERGENIC	--	--	--
Ca2_P4_15157427	Ca2	15157427	AGAGC/AGAGCGACGAGC	CAGAGCAATCGGAGAAGAGG	GGGGTTCTTCAAAATTGGGT	60.1	203	INTERGENIC	--	--	--
Ca2_P4_15168662	Ca2	15168662	ACGCGCGCGCGCGC/ACGCGCGCGCGC	GGGGATTGTTGGAAATGATG	CAGATTCGGAGAGCTTTTGG	60.0	502	INTERGENIC	--	--	--
Ca2_P4_15190617	Ca2	15190617	TTTTATCTTTATC/TTTTATC	AGCCTCCAACTATTAGGCCC	AACGGTGGATGCATGTATGA	59.6	799	INTERGENIC	--	--	--

INDEL marker IDs	Chromosomes/ unanchored scaffolds	Physical positions (bp)	InDels (*Kabuli* reference genome-CDC Frontier/PII)	Forward primers (5'-3')	Reverse primers (5'-3')	Anneling temperature (0C)	Expected amplified product size (bp)	Structural annotation		Functional annotation	
								Sequence components of *kabuli* genome	*Kabuli* gene accession IDs	NCBI-KOG	TFs
Ca2_P4_15307192	Ca2	15307192	AATATATATATATATATATAT/AATATATATATATATATATATATAT	ACGGTGGGCTGTATTTTTGA	GCAACCCTTTCATTGTGGAT	60.4	638	INTERGENIC	--	--	--
Ca2_P4_15313880	Ca2	15313880	ATATTATTATTATTATTATTATTATTATTATTATTATTATTATTATTATTATTATTATTATTATTATTATT/ATATTATTATTATTATTATTATTATTATTATTATTATTATTATTATTATTATT	GCAAAAAGCAGCGCAATAAT	ACGAATCTAACGGTGGTTGC	60.4	360	INTERGENIC	--	--	--
Ca2_P4_15343692	Ca2	15343692	CTTTTTTTTT/CTTTTTTTTTT	AAGGGGAAACTTTGACCGAC	ACAATTTTAAACCGTTGCGG	60.3	725	INTERGENIC	--	--	--
Ca2_P4_15363492	Ca2	15363492	AGCAG/AGCAGCACAGAATAATGCAG	GTTTTGGTCCATTTGATGGC	CTGCATTTTGATGTGCTGCT	60.2	594	INTERGENIC	--	--	--
Ca2_P4_15589036	Ca2	15589036	ATTT/ATTTT	ATACGCCTCAACAAAATGGC	CTGCTTTGCTTGATTCCGTT	60.0	376	INTERGENIC	--	--	--
Ca2_P4_15598813	Ca2	15598813	TA/TAA	GGAGGCGCATTAGATATGGA	GCCTCGTGAGTTGCATCATA	60.0	518	INTERGENIC	--	--	--
Ca2_P4_15653418	Ca2	15653418	TAAAAAAAAAA/TAAAAAAAAAAA	CGTCACTGTTTTCTCCCCAT	GGTCATGGGTTCACGAATCT	60.0	869	INTERGENIC	--	--	--
Ca2_P4_15700651	Ca2	15700651	ATTTTTTTTTTT/ATTTTTTTTTT	GTGACTCCAACATGACCCCT	CCAATGCATTCCATTCAACA	59.8	743	INTERGENIC	--	--	--
Ca2_P4_15778128	Ca2	15778128	G/GGATGGA	GATGGATGGAAAAGAGGCAA	ATCTTAAGCCCCTTCCCAAA	60.0	788	INTERGENIC	--	--	--
Ca2_P4_15812866	Ca2	15812866	TAAAAAAAAA/TAAAAAAAAAA	CATTGGCGGAGCTTTACAAT	CCATGGCCACTTCAAGATTT	60.1	298	INTERGENIC	--	--	--

INDEL marker IDs	Chromosomes/ unanchored scaffolds	Physical positions (bp)	InDels (*Kabuli* reference genome-CDC Frontier/PII)	Forward primers (5'-3')	Reverse primers (5'-3')	Anneling temperature (0C)	Expected amplified product size (bp)	Structural annotation		Functional annotation	
								Sequence components of *kabuli* genome	*Kabuli* gene accession IDs	NCBI-KOG	TFs
Ca2_P4_15920758	Ca2	15920758	GTATATATATATATATATAT/GTATATATATATATAT	TTCGGAAGTCCATCGGTATC	GCAAATGCTCCCCTAATTGA	59.9	546	INTERGENIC	--	--	--
Ca2_P4_16003270	Ca2	16003270	AAT/A	GGTACACTTCCGGCAAGAAA	AAGAGAGGTCACTTTGGCGA	60.1	482	INTERGENIC	--	--	--
Ca2_P4_16296988	Ca2	16296988	TAAAAAAAAAA/TAAAAAAAAAAA	GAGGGAGGTGTGTAGTCCCA	GCGTTGTTGTATCCTCCGTT	60.0	623	INTERGENIC	--	--	--
Ca2_P4_1640062	Ca2	1640062	ATTTTTTTTT/ATTTTTT	AGATCATGTGCAAAAAGGGG	AGTAACCGTGAGGGAACGTG	59.9	509	INTERGENIC	--	--	--
Ca2_P4_16419363	Ca2	16419363	GCT/GCTCT	TGAACTTCTCAGCCGAACCT	TCACACGACTTTTGCTGGAG	60.0	135	INTERGENIC	--	--	--
Ca2_P4_16496636	Ca2	16496636	GTCAAAC/GTCAAACTAAAAGGACTATTCAAAC	TATGGACTTTGTGGTGGCAA	TTGGCACGTACTCCCATACA	60.0	441	INTERGENIC	--	--	--
Ca2_P4_16514436	Ca2	16514436	AC/A	TGTGCTGGAAAGGCACATAG	GTCTCCCCCACTACAGTCCA	59.9	812	INTERGENIC	--	--	--
Ca2_P4_16650133	Ca2	16650133	ATTTTTTTTTTT/ATTTTTTTTTTTT	ACAGGCTCCCGTTTTATGTG	AAACGCAATCCTTGAGTTCG	60.0	558	INTERGENIC	--	--	--
Ca2_P4_16938931	Ca2	16938931	CTATTATTATTATTATTATTATTATTATTATTATTATTATTATTATTATTATTATTATT/CTATTATTATTATTATTATTATTATTATTATTATTATTATTATTATTATTATTATTATTATTATTATT	GAAAGCGCTTCCGTTTACAG	ACAGCCACTGCCCAAGTATC	60.0	532	INTERGENIC	--	--	--
Ca2_P4_17061321	Ca2	17061321	TAATATATA/TAATATATAAATATATA	TTTGGCTCGGAAGCTTTTTA	AGGAAGAGTGGAGTTGCGAA	60.0	498	INTERGENIC	--	--	--

INDEL marker IDs	Chromosomes/ unanchored scaffolds	Physical positions (bp)	InDels (*Kabuli* reference genome-CDC Frontier/PII)	Forward primers (5'-3')	Reverse primers (5'-3')	Anneling temperature (0C)	Expected amplified product size (bp)	Structural annotation		Functional annotation	
								Sequence components of ***kabuli*** genome	***Kabuli*** gene accession IDs	NCBI-KOG	TFs
Ca2_P4_17368826	Ca2	17368826	A/ATTTATTTTTAGGG	TGACATATCCGCTATGAACGA	ATCCATCAAACGCTTTCACC	59.2	592	INTERGENIC	--	--	--
Ca2_P4_17388033	Ca2	17388033	GTT/GTTT	TGGACACGACGGTGAAATAA	GTCGGCCACAAGAAGACAAT	60.0	578	INTERGENIC	--	--	--
Ca2_P4_17491578	Ca2	17491578	CATATATATATATATATATATATATATA/CATATATATATATATATATATATATATA	AGTTGGTCCCCTAGAACCGT	CGGTGAATCCTTTACGGAGA	59.9	541	INTERGENIC	--	--	--
Ca2_P4_17994837	Ca2	17994837	AATATATATATATATATATATATATAT/AATATATATATATATATATATATATATAT	TGTGGCCCTATGTACTGCTG	TTGGAAGGAGCCCTTACCTT	59.7	799	INTERGENIC	--	--	--
Ca2_P4_18223174	Ca2	18223174	TTAATAATAATAATAATAATAATAATAATAATAATAATAA/TTAATAATAATAATAATAATAATAATAATAATAA	CTCAGGATGCGACTTGTTGA	AAGTGATTTGTGGGTGATTGG	60.0	558	INTERGENIC	--	--	--
Ca2_P4_18225715	Ca2	18225715	A/ATATAT	GCCATTCCAAATGTTTGCTT	TGGCTAATAACGATTTCCCG	59.9	502	INTERGENIC	--	--	--
Ca2_P4_18343132	Ca2	18343132	T/TGGCGACAACATA	TCAAATGAATGGTTTGAGCG	TCCCTCCTTCACAAAATTGG	59.7	657	INTERGENIC	--	--	--
Ca2_P4_18496873	Ca2	18496873	AATATATATATATATATATATATATATATATATATATAT/AATATATATATATATATATATATATATATATATATATATAT	AAAAATCCCACGTTTGTCCA	TAAGGTTGTGCGAGAAGTGC	60.2	914	INTERGENIC	--	--	--
Ca2_P4_1853896	Ca2	1853896	AATATATATATATATATATATATAT/AATATATATATATATATATATATATATAT	TGAGTCCGCTTCCCAATTAC	TTGAGCACGACTTTTTGCAC	60.1	536	INTERGENIC	--	--	--
Ca2_P4_1856734	Ca2	1856734	ATT/AT	TCCCACTTTCCACGTACACA	AACCATTTGAAGCAACCGTC	60.0	709	INTERGENIC	--	--	--

INDEL marker IDs	Chromosomes/ unanchored scaffolds	Physical positions (bp)	InDels (*Kabuli* reference genome-CDC Frontier/PII)	Forward primers (5'-3')	Reverse primers (5'-3')	Anneling temperature (0C)	Expected amplified product size (bp)	Structural annotation		Functional annotation	
								Sequence components of *kabuli* genome	*Kabuli* gene accession IDs	NCBI-KOG	TFs
Ca2_P4_18833380	Ca2	18833380	TAAAAAAAAAAA/TAAAAAAAAAAAA	TCGAAAAATCCCTTGAAAAAGA	GGACCTATCAAGTGAGCTTGTG	60.0	551	INTERGENIC	--	--	--
Ca2_P4_18859929	Ca2	18859929	TTATATATATATATATATAT/TTATATATATATATATAT	AAAACAAGCGATCCATTTGC	GGTGCTTTGGGGTGTAAAGA	60.1	500	INTERGENIC	--	--	--
Ca2_P4_18870465	Ca2	18870465	GAAAAAAAAA/GAAAAAAAAAA	TTGCTCCGATTCTATCCTTCA	TTGATCCTGCCATCTTGTCA	59.8	766	INTERGENIC	--	--	--
Ca2_P4_18957648	Ca2	18957648	CNNNNNNNNNN/CNNNNNNNNN	CACAAAAACCAACCTGCCTT	CGTTGTCTCTTTTATTGACGATG	60.0	623	INTERGENIC	--	--	--
Ca2_P4_19132671	Ca2	19132671	AATATATATATATATATATATAT/AATATATATATATATATAT	CGGTGAATCCTTTACGGAGA	CTCCTTTTCCCTCCACACAA	60.1	142	INTERGENIC	--	--	--
Ca2_P4_1919646	Ca2	1919646	TC/TCTCC	TTGAAGCGATGTGGTTGAAG	CCAAAACAACTATGCCCGTT	59.8	339	INTERGENIC	--	--	--
Ca2_P4_19219016	Ca2	19219016	ATTTT/ATTT	CCCGTGAGATCCTCCTAACA	TGAATCTACGGCTTTCTGGG	60.1	498	INTERGENIC	--	--	--
Ca2_P4_19384298	Ca2	19384298	GGAGAGAGAGAGAGAGAGAGAGAGAGAGAGAGAGAG/GGAGAGAGAGAGAGAGAGAGAGAGAGAGAGAG	TGGTTTGGTCAATTTGAGGC	GAAGCCCAAGTTCCAAATGA	60.9	679	INTERGENIC	--	--	--
Ca2_P4_19387244	Ca2	19387244	TTATATATATATATATAT/TTATATATATATATATAT	CGACATTGTAGCACCCCTTT	TTTAGATCCCTTGCACGACC	60.0	191	INTERGENIC	--	--	--
Ca2_P4_1941868	Ca2	1941868	TTATATATATATATATATAT/TTATATATATATATATATAT	CCGTTACTGCGTCCTATGGT	CAATGTCAAAAGTTGGTCTCTGA	60.0	581	INTERGENIC	--	--	--

INDEL marker IDs	Chromosomes/ unanchored scaffolds	Physical positions (bp)	InDels (*Kabuli* reference genome- CDC Frontier/PII)	Forward primers (5'-3')	Reverse primers (5'-3')	Anneling temperature (0C)	Expected amplified product size (bp)	Structural annotation		Functional annotation	
								Sequence components of *kabuli* genome	*Kabuli* gene accession IDs	NCBI-KOG	TFs
Ca2_P4_19437057	Ca2	19437057	TTATATATATATATAT/TTATATATATATATATAT	GAATTTAAGGCGTTTCGGGT	CTTGGTCTAACCCTCACCCA	60.3	351	INTERGENIC	--	--	--
Ca2_P4_19589028	Ca2	19589028	GATATATATATATATATATATATATA/GATATATATATATATATATATATA	AATGTGAATCCCACCAGGAA	GTCTTTTACAGCGCTTTGGC	60.2	533	INTERGENIC	--	--	--
Ca2_P4_19603206	Ca2	19603206	ACC/AC	GAGTCCGTAGGAGTAGGGGG	AATTGAAACTTGACGTCGCC	60.0	537	INTERGENIC	--	--	--
Ca2_P4_19616982	Ca2	19616982	TC/TCC	AGCTTACCATCCACAAACGG	AAAAGTCTGCGACGCTTGTT	60.0	347	INTERGENIC	--	--	--
Ca2_P4_19705772	Ca2	19705772	GAAAA/GAAAAA	TATTTTGGATTTTGGCTCGG	AGGAAGAGTGGAGTTGCGAA	59.9	475	INTERGENIC	--	--	--
Ca2_P4_19727969	Ca2	19727969	AATTTATTTATTTATTTATTTATTT/AATTTATTTATTTATTTATTT	GGCGACTCCATTGAATTTGA	TTCCTTAGCATTGGGATTGC	61.0	833	INTERGENIC	--	--	--
Ca2_P4_19791685	Ca2	19791685	C/CG	TAGGCCATTTTCGTCCTCAC	AATCCAATGACACAAACGCA	60.1	392	INTERGENIC	--	--	--
Ca2_P4_20175480	Ca2	20175480	GATATATATATATATATATA/GATATATATATATATATATATA	TTTCAGAGTGTCTGTGGTTAGCA	GATCTGAGGTGTCATGGGCT	60.0	669	INTERGENIC	--	--	--
Ca2_P4_20348649	Ca2	20348649	AATATATATATATATATATAT/AATATATATATATATATATAT	TTCGTGGTTTACAAAATTCAATACA	GGTCGAAATTGGTGTTGGTC	59.7	664	INTERGENIC	--	--	--
Ca2_P4_20353586	Ca2	20353586	T/TG	TCTCCCTCCTTCCTCCATTT	CATTAAGAGGTTCCCCCTCC	60.0	844	INTERGENIC	--	--	--

INDEL marker IDs	Chromosomes/ unanchored scaffolds	Physical positions (bp)	InDels (*Kabuli* reference genome-CDC Frontier/PII)	Forward primers (5'-3')	Reverse primers (5'-3')	Anneling temperature (0C)	Expected amplified product size (bp)	Structural annotation		Functional annotation	
								Sequence components of *kabuli* genome	*Kabuli* gene accession IDs	NCBI-KOG	TFs
Ca2_P4_20474785	Ca2	20474785	GAAAA/GAAAAA	ATACTGGCTCGGAGGCTTTT	CGTAGCGTGGTAGTGAGCAA	60.2	614	INTERGENIC	--	--	--
Ca2_P4_20689303	Ca2	20689303	AATATATATATATATATATATATATATATAT/AATATATATATATATATATATATATAT	CCCGTAAATTTCGAGGACAA	TTCACACATGGGTCAAACTCA	59.9	740	INTERGENIC	--	--	--
Ca2_P4_20715795	Ca2	20715795	T/TCATCA	GACATTGCATTTTGGACACG	AAATGGATTTCAGCCAATCG	60.0	539	INTERGENIC	--	--	--
Ca2_P4_21001173	Ca2	21001173	CATATATATATATATATATATATATATA/CATATATATATATATATATATATATATATATATA	TCAATGCGGTCTCTGAAGTG	CCCACAGTCGATGTTGTTTG	60.0	303	INTERGENIC	--	--	--
Ca2_P4_210507	Ca2	210507	TATAATAATAATAATAATAATAATAATAATAATAATAATAA/TATAATAATAATAATAATAATAATAATAATAATAATAATAATAATA	CATTGTCAAACCTTCGCTCA	CCATGGTTTCTTTGCTTGTT	59.8	542	INTERGENIC	--	--	--
Ca2_P4_21312312	Ca2	21312312	GTTTTTT/GTTTTTTTT	TATCAGAGTTCTCCCCACCG	AGAAACCACAACGCAACACA	60.1	356	INTERGENIC	--	--	--
Ca2_P4_21314611	Ca2	21314611	AG/AGG	CAAACCCTTTCAAGGTGCAT	GCACTGCCCCAAGAATTTTA	60.0	443	INTERGENIC	--	--	--
Ca2_P4_21330368	Ca2	21330368	AAATAATAATAATAATAATAATAATAATAATAATAATAATAATAATAATAA/AAATAATAATAATAATAATAATAATAATAATAATAATAATAATAATAATAATA	ACAAACAAGTTGAGCACCCC	ATGAACGAAAGACGTGACGG	60.0	399	INTERGENIC	--	--	--
Ca2_P4_21631180	Ca2	21631180	CATATATATATATATATATATATATAT/CATATATATATATATATATATATAT	ACGCACCATCTTGTAAAGGG	ATCGACGCGTAGTTTCAAGG	60.0	711	INTERGENIC	--	--	--
Ca2_P4_21672716	Ca2	21672716	ATT/AT	TGATGCCGTGCCATAAATAG	TAACCTTGCCTTTGGGTGAG	59.5	595	INTERGENIC	--	--	--

INDEL marker IDs	Chromosomes/ unanchored scaffolds	Physical positions (bp)	InDels (*Kabuli* reference genome-CDC Frontier/PII)	Forward primers (5'-3')	Reverse primers (5'-3')	Anneling temperature (0C)	Expected amplified product size (bp)	Structural annotation		Functional annotation	
								Sequence components of *kabuli* genome	*Kabuli* gene accession IDs	NCBI-KOG	TFs
Ca2_P4_2169056	Ca2	2169056	C/CG	AACCTCTCCAAGAGGATCGG	GGAGAGTGAAACGGATGGAA	60.6	648	INTERGENIC	--	--	--
Ca2_P4_21695019	Ca2	21695019	CAAAAAAAAAA/CAAAAAAAAAAA	TGCCTCTGATTCCTTTTCAGA	TCTGTGCTCTCAAATGGTGC	59.9	389	INTERGENIC	--	--	--
Ca2_P4_21695844	Ca2	21695844	GTTATTATTATTATTATTATTATTATTATTAATATTATTATTATTATTA/GTTATTATTATTATTATTATTATTATTATTAATATTATTATTATTATTA	GATGAAATTCAGGCTCGCTC	TTGACAAGGGAAAACCTTGC	59.9	444	INTERGENIC	--	--	--
Ca2_P4_21717164	Ca2	21717164	TA/T	GGTAGAGGCAGGCCATATCA	GATTTGTGAGGGATTTTTCAGC	60.1	841	INTERGENIC	--	--	--
Ca2_P4_2174282	Ca2	2174282	ATTTTTTTTTTTTT/ATTTTTTTTTTT	TGACATCCATCTTATGTGGGTC	GGTCTACTCATGTTGAAAGACGC	59.7	674	INTERGENIC	--	--	--
Ca2_P4_2178973	Ca2	2178973	CATAAAATAAAATAAAATAAAA/CATAAAATAAAATAAAATAAAATAAAATAAAA	CCTTATTTCACGGCAAGTCG	TTGGGTTAGGGAAAGTCGTG	60.6	534	INTERGENIC	--	--	--
Ca2_P4_21804816	Ca2	21804816	AT/ATAAGT	CGCCATAGACCATTGGAAAC	TCCATTGATCACACAACGGT	60.3	860	INTERGENIC	--	--	--
Ca2_P4_21943275	Ca2	21943275	AGCGCG/AGCG	GCAGATTTGGAAGGTGTGGT	ACAATCAGCACGCAAGAATG	60.0	461	INTERGENIC	--	--	--
Ca2_P4_22196471	Ca2	22196471	TTGAGTTTGGGGTTGTGA/TTGA	CCAACAAGGACCCAAAGCTA	ATTTTTCGCATCTTTTGCGT	60.1	871	INTERGENIC	--	--	--
Ca2_P4_22301472	Ca2	22301472	CCAATCGATTGGG/CCAATCGATTGGGCAATCGATTGGG	CCCGTGAGATCCTCCTAACA	TTAAGTCAGGGGCTCAGGAA	60.1	588	INTERGENIC	--	--	--

INDEL marker IDs	Chromosomes/ unanchored scaffolds	Physical positions (bp)	InDels (*Kabuli* reference genome-CDC Frontier/PII)	Forward primers (5'-3')	Reverse primers (5'-3')	Anneling temperature (0C)	Expected amplified product size (bp)	Structural annotation		Functional annotation	
								Sequence components of *kabuli* genome	*Kabuli* gene accession IDs	NCBI-KOG	TFs
Ca2_P4_22468520	Ca2	22468520	CAAAAAAAAAA/CAAAAAAAAAAA	TGGCCATGTGCATTGTTTAT	AGGCATCAACGCCTAAATTG	59.8	674	INTERGENIC	--	--	--
Ca2_P4_22483205	Ca2	22483205	AT/A	CGTGCCACTTTTCTCTAGGC	AGATCATGTGCAAAAAGGGG	60.0	369	INTERGENIC	--	--	--
Ca2_P4_22495551	Ca2	22495551	TAAAAAAAAAA/TAAAAAAAAAAA	TGTTTTGCCGTTCTCTCCTC	AAGAAACTTCTCCAATGCGG	60.4	446	INTERGENIC	--	--	--
Ca2_P4_22533042	Ca2	22533042	CATATATATATATATATAT/CATATATATATATATATAT	GGTTGTCGCACCAAATTTCT	GCAAAAGTGTCCCCACAAGT	60.0	395	INTERGENIC	--	--	--
Ca2_P4_228183	Ca2	228183	GTTTTTTTTTT/GTTTTTTTTT	TTCGAAACGTTGGATGAATG	GCAACCATCTGACCAAGTGA	59.5	418	INTERGENIC	--	--	--
Ca2_P4_22939976	Ca2	22939976	TATTTAAATTTAA/TATTTAAATTTAAATTTAA	GCACCCACAAAGTAGGCATT	CTCGGCCTCAATACTTGCAT	60.0	893	INTERGENIC	--	--	--
Ca2_P4_2301289	Ca2	2301289	CATATATATATA/CATATATATATATA	TGCAAAATCAAGCGAATGAG	TTTTTGCAGGTCTACCCAGG	60.0	418	INTERGENIC	--	--	--
Ca2_P4_23030877	Ca2	23030877	ATATTATTATTATTATTATTATTATTATTATTATTATTATTATTAT/ATATTATTATTATTATTATTATTATTATTATTATTATTATTATTAT	GCGAGTGTTGGAGGTTTGAT	CACGCTTGAGTCCTGTTTCA	60.1	851	INTERGENIC	--	--	--
Ca2_P4_23050911	Ca2	23050911	AATG/AATGGTCTATTGGCTTATG	TATGGGGATCGGAAATCAAA	ACAATCGAAATTTTAGGCCG	60.1	907	INTERGENIC	--	--	--
Ca2_P4_23192749	Ca2	23192749	TCA/TCACAACA	GCCGAATAAAACCCTCACAA	AGCTGTATTGGGCCTCCTTT	59.9	319	INTERGENIC	--	--	--

INDEL marker IDs	Chromosomes/ unanchored scaffolds	Physical positions (bp)	InDels (*Kabuli* reference genome-CDC Frontier/PII)	Forward primers (5'-3')	Reverse primers (5'-3')	Anneling temperature (0C)	Expected amplified product size (bp)	Structural annotation		Functional annotation	
								Sequence components of *kabuli* genome	*Kabuli* gene accession IDs	NCBI-KOG	TFs
Ca2_P4_23193466	Ca2	23193466	TA/T	CCACAAATGCTCATCGTGAC	TTTGCAGCCACAAAAGACAG	60.1	561	INTERGENIC	--	--	--
Ca2_P4_23243854	Ca2	23243854	AAATAATAATAATAATAATAATAATAATAATAAT/AAATAATAATAATAATAATAATAATAATAATAATAAT	TTTCATGATAATTTTGGAGGGA	CGGCTTTACACCACCTGTTT	58.4	252	INTERGENIC	--	--	--
Ca2_P4_2324553	Ca2	2324553	GATAATAATAATAATAATAATAATAATAATAATAATAATAATAA/GATAATAATAATAATAATAATAATAATAATAATAATAATAATAATAA	GTTCAAAAGGAGCTTGACCG	AGAGGCAAACAAGAACCGAA	59.9	315	INTERGENIC	--	--	--
Ca2_P4_23360931	Ca2	23360931	ATTTTTTTTTT/ATTTTTTTTTTT	GAGTTGGAATGGCGGTAAAA	TGTCAGCCAACCCTTCTTCT	59.9	417	INTERGENIC	--	--	--
Ca2_P4_23496145	Ca2	23496145	TTATATATATATATATATAT/TTATATATATATATATATATAT	GTTTTGTAGTGGCCCCAAAG	CTTTTCAAACGGCGACTTTC	59.5	557	INTERGENIC	--	--	--
Ca2_P4_23509535	Ca2	23509535	AAAAACTTAAAACTTAAAACTTA/AAAAACTTAAAACTTA	CCCTCTTGAATTTTTGTTTTCC	CCCACACCCTCGACTAAAGA	59.0	542	INTERGENIC	--	--	--
Ca2_P4_2354894	Ca2	2354894	ATT/ATTT	CCTTCCCCAGATCCTTAACC	TGTAGTGGGTAATTTGAGCCG	59.8	866	INTERGENIC	--	--	--
Ca2_P4_2356056	Ca2	2356056	ATTCAAC/ATTCAACTTTTCAAC	GAAAGCGCAGCTACTTTTCG	ACGATTCCCACACTCACACA	60.3	918	INTERGENIC	--	--	--
Ca2_P4_23695824	Ca2	23695824	ATT/AT	AATGGTTTTCACGCCAAAGT	TCTCCCTCCCTCCTTCATTT	59.5	817	INTERGENIC	--	--	--
Ca2_P4_23792320	Ca2	23792320	GGAGTAAGAGAGTAA/GGAGTAAGAGAGTAAGAGAGTAA	TCAGAATCCCTGATCCCAAG	GCAACCAGGAGGCAAGTCTA	60.0	490	INTERGENIC	--	--	--

INDEL marker IDs	Chromosomes/ unanchored scaffolds	Physical positions (bp)	InDels (*Kabuli* reference genome- CDC Frontier/PII)	Forward primers (5'-3')	Reverse primers (5'-3')	Anneling temperature (0C)	Expected amplified product size (bp)	Structural annotation		Functional annotation	
								Sequence components of *kabuli* genome	*Kabuli* gene accession IDs	NCBI-KOG	TFs
Ca2_P4_2383872	Ca2	2383872	CTTTTTTTTTTT/CTTTTTTTTTT	TGGCCGCAAAGAAAATAATC	TTCCACCAACAAAGGAAAGG	60.0	660	INTERGENIC	--	--	--
Ca2_P4_2388005	Ca2	2388005	GTTTTTTTTT/GTTTTTTTTTT	GCCAACAACGTTACGAGCTA	CTTGCTAAATGCCCCACTTC	59.0	581	INTERGENIC	--	--	--
Ca2_P4_23887857	Ca2	23887857	TG/T	TCTAAAGTGGTTGCCCTTGG	TCATGGCAAATGCAGATGAT	60.1	425	INTERGENIC	--	--	--
Ca2_P4_23932608	Ca2	23932608	TAAATTAATGAATAAAAATTAAT/TAAATTAAT	AAGGCATCCAATCAAAGCAC	CTCAAGATTTTAGCGCCGTC	60.1	460	INTERGENIC	--	--	--
Ca2_P4_23986096	Ca2	23986096	TTATATATATATATATATATA/TTATATATATATATATATATATA	CAAAATTTTCAAAACAAACCCG	GCACGACAAACACCTCAAAA	60.6	574	INTERGENIC	--	--	--
Ca2_P4_23989802	Ca2	23989802	AAAAACAAAACAAAA/AAAAACAAAACAAAACAAAA	AATTCGTTGAGCCGGTCTAGT	AGCACGGGGAGTATGAGAAA	60.1	421	INTERGENIC	--	--	--
Ca2_P4_23990182	Ca2	23990182	AAAAACAAAACAAAA/AAAAACAAAACAAAACAAAA	TCTCATACTCCCCGTGCTTT	ATCCCCTACACCAGATGCAC	59.7	565	INTERGENIC	--	--	--
Ca2_P4_24078993	Ca2	24078993	ATATTATTATTATTATTATTATTATTATT/ATATTATTATTATTATTATTATTATTATTATT	CGCATGAGAAATTGTGCCTA	ATTGCAACCGCAACCATACT	59.8	883	INTERGENIC	--	--	--
Ca2_P4_24124699	Ca2	24124699	ATTTTTTTTTTTTTTT/ATTTTTTTTTTTTT	ATATATCGGGGTTCATGGCA	CCATGCTTCACCCTTGGTAT	60.0	603	INTERGENIC	--	--	--
Ca2_P4_24251584	Ca2	24251584	ACCCCC/ACCCCCC	GATTGCTTTCTGGGTGGAAA	TGATCAACCCTTGGTGTGAA	60.1	652	INTERGENIC	--	--	--

INDEL marker IDs	Chromosomes/ unanchored scaffolds	Physical positions (bp)	InDels (*Kabuli* reference genome-CDC Frontier/PII)	Forward primers (5'-3')	Reverse primers (5'-3')	Anneling temperature (0C)	Expected amplified product size (bp)	Structural annotation		Functional annotation	
								Sequence components of ***kabuli* genome**	***Kabuli* gene accession IDs**	**NCBI-KOG**	**TFs**
Ca2_P4_24252139	Ca2	24252139	TAA/TAAGAAA	GATCCTCAAATCGACCTCCA	TGTCACCATTGGTCTCCAAA	60.0	465	INTERGENIC	--	--	--
Ca2_P4_24349766	Ca2	24349766	AATTTAATT/AATTTAATTATATTTAATT	TCTTCACGCCAAGAAGAAAAA	AAAAACGATTGCGAATGGAG	60.0	614	INTERGENIC	--	--	--
Ca2_P4_24393200	Ca2	24393200	G/GAAGATA	TCCTAGAACATGCATCACGC	TACCCTTTGCTAGCCCCTTT	59.8	505	INTERGENIC	--	--	--
Ca2_P4_24393621	Ca2	24393621	CAAAAA/CAAAA	AGGGGCTAGCAAAGGGTAAA	GCTCAAGGTGACAAGCCTTC	60.1	344	INTERGENIC	--	--	--
Ca2_P4_24538889	Ca2	24538889	ATATTATTATTATTATTATTATTATTAT/ATATTATTATTATTATTATTATTATTATTATTATTATTAT	GGAGGACATGCAAATGAGGT	GTGGTTTGCAGGCTATGGAT	59.9	829	INTERGENIC	--	--	--
Ca2_P4_24597882	Ca2	24597882	C/CAGAAAAATCA	TTATTCGGGCCTGTCAAAAC	ATGGCGTTTACCCAAGACTG	59.9	564	INTERGENIC	--	--	--
Ca2_P4_24943194	Ca2	24943194	A/AATT	AACCCCTCCAAAGAACGACT	AGGGCCCCACTAACTCCTTA	60.0	680	INTERGENIC	--	--	--
Ca2_P4_24943252	Ca2	24943252	CTTT/CTT	AACCCCTCCAAAGAACGACT	AGGGCCCCACTAACTCCTTA	60.0	680	INTERGENIC	--	--	--
Ca2_P4_25241576	Ca2	25241576	GATATATATATATATA/GATATATATATATATATA	TATTTTGGATTTTGGCTCGG	AAATGGAGGAAGGAGGGAGA	59.9	433	INTERGENIC	--	--	--
Ca2_P4_25427657	Ca2	25427657	TTATATATATATATATATATATATATATAT/TTATATATATATATATATATATATATAT	TGTTGTTGAAGATGAAATATTGGC	TTTGGCGTAGTGAAGTTGACA	60.2	723	INTERGENIC	--	--	--

INDEL marker IDs	Chromosomes/ unanchored scaffolds	Physical positions (bp)	InDels (*Kabuli* reference genome-CDC Frontier/PII)	Forward primers (5'-3')	Reverse primers (5'-3')	Anneling temperature (0C)	Expected amplified product size (bp)	Structural annotation		Functional annotation	
								Sequence components of *kabuli* genome	*Kabuli* gene accession IDs	NCBI-KOG	TFs
Ca2_P4_25458560	Ca2	25458560	ATTTTTTTTTT/ATTTTTTTTTTT	CAAATTTAATGGCACAGGGG	AATTGGAGTGTGTGATTCGC	60.2	957	INTERGENIC	--	--	--
Ca2_P4_25527401	Ca2	25527401	GATAATAATAATAATAATAATAATAATAATAATAA/GATAATAATAATAATAATAATAATAATAATAATAATAATAATAA	ACCCTTCAATTTCGACATGC	CCAATTGGAATTTGCAACAA	59.9	598	INTERGENIC	--	--	--
Ca2_P4_25529860	Ca2	25529860	CTTT/CTTTTTT	AGCAGCCCAAGCAAGACTTA	TCATGCCGATTGATAGAGGTT	60.2	553	INTERGENIC	--	--	--
Ca2_P4_25554310	Ca2	25554310	TTGTGTGTGTGTGT/TTGTGTGTGTGT	ATGCGGCAGGTATGAGAACA	ATATCAACGCCCTTTTGCAT	61.6	473	INTERGENIC	--	--	--
Ca2_P4_25593755	Ca2	25593755	TTATATATATATATATATATATATATAT/TTATATATATATATATATATATATATATATATAT	AACCCGTTTTGATTGAGTGC	TGCGAAATTAGGCTTCCATC	60.0	466	INTERGENIC	--	--	--
Ca2_P4_25663538	Ca2	25663538	TAAAAAAAAAAAAA/TAAAAAAAAAAAAA	TGCTCTGACTCTCCAATTTCTTC	GGGATTGGACTGCCTAGCTT	60.0	587	INTERGENIC	--	--	--
Ca2_P4_25737290	Ca2	25737290	TAAAAAAAAAA/TAAAAAAAAAAAA	GGTCGTCTGTTGGGTTGTCT	GTCGACGGCGAGAAGAATAA	60.0	464	INTERGENIC	--	--	--
Ca2_P4_25795167	Ca2	25795167	GAATTTTTAAATTTTTAAA/GAATTTTTAAATTTTTAAATTTTTAAA	AGCCACGGCTCTAAAGATCA	TTGGTTTCATTGTTTTGCATTT	60.0	674	INTERGENIC	--	--	--
Ca2_P4_25911445	Ca2	25911445	GAAAAAAAAAAA/GAAAAAAAAAA	TTTCCCGACTTGGTCCTTTA	TGCGCTTAAAGACAGGGAGT	59.5	417	INTERGENIC	--	--	--
Ca2_P4_2607354	Ca2	2607354	AATATATATATATATATATATATAT/AATATATATATATATATATATATATAT	GGATATTGGGGATGGCCTAC	CCCATGTGATGAGCTACCCT	60.4	648	INTERGENIC	--	--	--

INDEL marker IDs	Chromosomes/ unanchored scaffolds	Physical positions (bp)	InDels (*Kabuli* reference genome-CDC Frontier/PII)	Forward primers (5'-3')	Reverse primers (5'-3')	Anneling temperature (0C)	Expected amplified product size (bp)	Structural annotation		Functional annotation	
								Sequence components of *kabuli* genome	*Kabuli* gene accession IDs	NCBI-KOG	TFs
Ca2_P4_26102860	Ca2	26102860	TAAAAAAAAAA/TAAAAAAAAAAA	ACGTATGGTTACCGCTCACA	AGCGATCCCTCCGTTTATTT	59.1	648	INTERGENIC	--	--	--
Ca2_P4_26111863	Ca2	26111863	AATATATATATATATATATATATAT/AATATATATATATATATATATATAT	ATGCGTACATCAAGGGTGGT	GGCTCATATATTTGCATTCACG	60.3	560	INTERGENIC	--	--	--
Ca2_P4_26504489	Ca2	26504489	CAA/CAAAA	CCGACGTCCAGTCAGAATTT	CTCAATTGGACACCGTTCCT	60.1	772	INTERGENIC	--	--	--
Ca2_P4_26531031	Ca2	26531031	TA/T	AGAGATCTGTGCAGCCGAGT	GCACTCAATTTGGGGAAAAA	60.2	746	INTERGENIC	--	--	--
Ca2_P4_26693813	Ca2	26693813	TAAAAAAAAAAAAA/TAAAAAAAAAAAAAA	GCACACATGCAGAAAGGAGA	TCCAATCTCGGAAAAACACTG	60.0	629	INTERGENIC	--	--	--
Ca2_P4_2671437	Ca2	2671437	TAAAAAAAAAA/TAAAAAAAAAAA	TAATTCAGCCAAGGAAGCCA	GGAAAACGCAAAAGGAATCA	60.7	235	INTERGENIC	--	--	--
Ca2_P4_26811414	Ca2	26811414	TAAAAAAA/TAAAAAAAA	GGGCTTGTTTGATTGTGTTTT	GGCTCCATTCTTTGCTCTTG	59.0	584	INTERGENIC	--	--	--
Ca2_P4_26823619	Ca2	26823619	AATA/AA	TGGCGACATATTTTTGGTGA	TATGCGGCTGCCTATTTTTC	59.9	502	INTERGENIC	--	--	--
Ca2_P4_26914055	Ca2	26914055	ATTTTTTTTTT/ATTTTTTTTTTTT	AGGTTTGAACCGTCACCAAC	TTTGCATGTTCAATCACTCCA	59.9	255	INTERGENIC	--	--	--
Ca2_P4_2692293	Ca2	2692293	ATTTTTTTT/ATTTTTTT	AAAGGTGATGAAGCAGATTTTGA	TGGTGTCCTCTCTTCTCTAATCG	60.1	478	INTERGENIC	--	--	--

INDEL marker IDs	Chromosomes/ unanchored scaffolds	Physical positions (bp)	InDels (*Kabuli* reference genome-CDC Frontier/PII)	Forward primers (5'-3')	Reverse primers (5'-3')	Anneling temperature (0C)	Expected amplified product size (bp)	Structural annotation		Functional annotation	
								Sequence components of *kabuli* genome	*Kabuli* gene accession IDs	NCBI-KOG	TFs
Ca2_P4_27143098	Ca2	27143098	AA/AACA	CGTTCATTTATTGTTGGGGC	GGGTTTTGGTAGCCAACTCA	60.2	200	INTERGENIC	--	--	--
Ca2_P4_27261925	Ca2	27261925	AATGCA/AA	CCAGTCCTCACACCCTTGTT	TGAATTTTCCAACGGTCACA	60.0	356	INTERGENIC	--	--	--
Ca2_P4_2744252	Ca2	2744252	TAAAAAAAAAAA/TAAAAAAAAAAAA	TTCCAAATACGCAACCACAA	TTCTTGCCTTTCATGTTCCA	60.0	552	INTERGENIC	--	--	--
Ca2_P4_27506078	Ca2	27506078	AATATATATAT/AATATATATATAT	GGCGTAAGGATGGAAAAACA	ACCTCAATTTTCTCGGCTCA	59.9	567	INTERGENIC	--	--	--
Ca2_P4_27544148	Ca2	27544148	CATATATATATATATATATATATAT/CATATATATATATATATATATATATAT	CCAGCAAATTCCTGCATTCT	TGCACGGATGGATCATAAAA	60.2	412	INTERGENIC	--	--	--
Ca2_P4_27612199	Ca2	27612199	TA/T	CCACATGATCACTCATAAATATCG	AAAAATGGATTGACATAAATTGAAA	58.4	650	INTERGENIC	--	--	--
Ca2_P4_27642027	Ca2	27642027	TAA/TA	ACCCCATTTTGTTGTGGTGT	TGCTTGGCTATGGCATAAGA	60.0	530	INTERGENIC	--	--	--
Ca2_P4_27679281	Ca2	27679281	AATATATATATATATATA/AATATATATATATATATATATATATATA	TGGTCATTTTGTCTTGCTGC	AAGGGTCAATTCAACGGTGT	59.8	413	INTERGENIC	--	--	--
Ca2_P4_27776478	Ca2	27776478	GTTTTTTTTTT/GTTTTTTTTTTT	CAATGAACTTGGTCAACCCC	GCCCATGTCTGCATAGTAACAA	60.2	407	INTERGENIC	--	--	--
Ca2_P4_27776538	Ca2	27776538	TAAAAAAA/TAAAAAA	CAATGAACTTGGTCAACCCC	GCCCATGTCTGCATAGTAACAA	60.2	407	INTERGENIC	--	--	--

INDEL marker IDs	Chromosomes/ unanchored scaffolds	Physical positions (bp)	InDels (*Kabuli* reference genome-CDC Frontier/PII)	Forward primers (5'-3')	Reverse primers (5'-3')	Anneling temperature (0C)	Expected amplified product size (bp)	Structural annotation		Functional annotation	
								Sequence components of *kabuli* genome	*Kabuli* gene accession IDs	NCBI-KOG	TFs
Ca2_P4_27800362	Ca2	27800362	ATTTTTT/ATTTTTTT	ATAACCCCACCTTTCATCCC	CCCTTGATTATCCATGCACC	59.9	821	INTERGENIC	--	--	--
Ca2_P4_27802687	Ca2	27802687	TTATATATATATATATATATATATATATATAT/TTATATATATATATATATATATATATAT	TTTGTCAAGCACTTCCATCG	TCACACCAAAATGAAATCACG	59.8	540	INTERGENIC	--	--	--
Ca2_P4_27896998	Ca2	27896998	GAAAAAA/GAAAAAAA	CTGCCACTTCTTTCCCTCAC	GGCTGACACACACACACACA	59.8	531	INTERGENIC	--	--	--
Ca2_P4_28009782	Ca2	28009782	TTATATATATATATATATAT/TTATATATATATATATATATAT	TGGCAATGTGGTACTGGAGA	GGAAGTCCGAATTTGCAATG	60.1	206	INTERGENIC	--	--	--
Ca2_P4_28037628	Ca2	28037628	AATATATATATATATATATATATATATAT/AATATATATATATATATATATATATAT	CTAGTGTTGCACGGGTCAAA	CATAACGAATTCTCCCACCG	59.8	768	INTERGENIC	--	--	--
Ca2_P4_28235085	Ca2	28235085	GTTTTTTTTTT/GTTTTTTTTTTT	TCCAAATAGCGACTTCCCAC	TCCAAATGTTACAGCCAGCTC	60.1	567	INTERGENIC	--	--	--
Ca2_P4_28235280	Ca2	28235280	AATATATATATATATATATA/AATATATATATATATATATATA	TCCAAATAGCGACTTCCCAC	ACAATCCTCAAATGCGGAGA	60.1	701	INTERGENIC	--	--	--
Ca2_P4_28235432	Ca2	28235432	AATATATATATATATATATATA/AATATATATATATATATATATATA	TCCAAATAGCGACTTCCCAC	GCTGCATGTTCTTATGCTCG	60.1	849	INTERGENIC	--	--	--
Ca2_P4_28262473	Ca2	28262473	GAA/GAAAA	CTGAGACACGGCGACAATAA	ATCGGAGCAATTCTGCATTC	59.9	305	INTERGENIC	--	--	--
Ca2_P4_28365177	Ca2	28365177	AATATATATATATATATATATATAT/AATATATATATATATATATATAT	TCGGTCTCCTCATTTGATCC	GGGCCACATGTAATGCTTCT	60.0	768	INTERGENIC	--	--	--

INDEL marker IDs	Chromosomes/ unanchored scaffolds	Physical positions (bp)	InDels (*Kabuli* reference genome-CDC Frontier/PII)	Forward primers (5'-3')	Reverse primers (5'-3')	Anneling temperature (0C)	Expected amplified product size (bp)	Structural annotation		Functional annotation	
								Sequence components of *kabuli* genome	*Kabuli* gene accession IDs	NCBI-KOG	TFs
Ca2_P4_28396846	Ca2	28396846	ATATTTTTT/ATTATTTTTT	TGGGCCCAACTTAATC CATA	CATGTCCAAAACAAAAT CAATCA	60.1	337	INTERGENIC	--	--	--
Ca2_P4_28464989	Ca2	28464989	AAAAA/AAAAATAAAA	TCATCGCAGGTGTACC ATGT	AACATTGCACCGGTTT GATT	60.0	414	INTERGENIC	--	--	--
Ca2_P4_28465231	Ca2	28465231	CAAAAA/CAAAA	TCATCGCAGGTGTACC ATGT	GGTATCAGAGCCCACC ATGT	60.0	908	INTERGENIC	--	--	--
Ca2_P4_28465620	Ca2	28465620	A/AT	AGCTCCATTGCAAAAA CACC	GCAATTCCCACAGGTA TGCT	60.1	553	INTERGENIC	--	--	--
Ca2_P4_28482218	Ca2	28482218	CATA/CATATA	TCCATAGAATTAATGGT CCCC	TTTGCTTTATCAAGCCA CCC	57.7	198	INTERGENIC	--	--	--
Ca2_P4_28556774	Ca2	28556774	GAAAAAAAA/GAAAAAAA	TCCTAGCAAATTCCAC ACGA	GACAATGAAATTGGGG TTGG	59.3	450	INTERGENIC	--	--	--
Ca2_P4_28569281	Ca2	28569281	T/TC	CATCTGTAATGATGCG CCAC	GACTGGACGTAACCCA CGTT	60.1	503	INTERGENIC	--	--	--
Ca2_P4_28614815	Ca2	28614815	AATATATATATATATATA/AATATATA TATATATATATA	ATTCCACATCCACCAA GGAA	GCAGTGCAGCAACAGA AAAA	60.2	441	INTERGENIC	--	--	--
Ca2_P4_286502	Ca2	286502	TAAAAAAA/TAAAAAAAA	AATAGGCTGCCACAAG AGGA	TGGACACGACGGTGAA ATAA	59.8	610	INTERGENIC	--	--	--
Ca2_P4_2881711	Ca2	2881711	CTTTTGTTACTCTTT/CTTTTGTTACT CTTTTTGTTACTCTTT	TTCATTTCATCAATTCG ATCCTT	GGCAATGCCACCGTAA AATA	59.8	776	INTERGENIC	--	--	--

INDEL marker IDs	Chromosomes/ unanchored scaffolds	Physical positions (bp)	InDels (*Kabuli* reference genome-CDC Frontier/PII)	Forward primers (5'-3')	Reverse primers (5'-3')	Anneling temperature (0C)	Expected amplified product size (bp)	Structural annotation		Functional annotation	
								Sequence components of *kabuli* genome	*Kabuli* gene accession IDs	NCBI-KOG	TFs
Ca2_P4_28873803	Ca2	28873803	GTTTT/GTTTTT	GGCAGGCAACAAGACAAAAT	TCCGTGCATGCCATAATCTA	60.1	656	INTERGENIC	--	--	--
Ca2_P4_28981150	Ca2	28981150	TAAAAAA/TAAAAAAA	TGTTTAGGGATCAAAACTGAGGA	CAAAGGTATGTATAAGTGGGGAAA	60.0	454	INTERGENIC	--	--	--
Ca2_P4_29089904	Ca2	29089904	CTAAATTTTTTAAATTTT/CTAAATTTTTTAAATTTTTTAAATTTT	TCATAAATCACCCCCTCCTTT	TGGTGAAACAATTCAAACGC	59.7	644	INTERGENIC	--	--	--
Ca2_P4_29142241	Ca2	29142241	TAAAAAAAAAAAA/TAAAAAAAAAA	CAACTCGATTCAACCAGGGTA	TCCCTACAAGATGGCATGAA	60.0	670	INTERGENIC	--	--	--
Ca2_P4_29156803	Ca2	29156803	ACG/A	ACATGACATTTTGCCGATGA	GTCATGTGGCTTTGGTGTTG	59.9	544	INTERGENIC	--	--	--
Ca2_P4_29184413	Ca2	29184413	GAAAAAAAAA/GAAAAAAAAAA	TGGTGGGTGATTGCTGATTA	AAGATTGGAACTTGGGCCTT	59.9	435	INTERGENIC	--	--	--
Ca2_P4_29212961	Ca2	29212961	TTATATATATATATATATATATATATATATATATAT/TTATATATATATATATATATATATATATATATATAT	TAGTTTGTAACCCGTGCGTC	CAAAAATAAGACATGGTGTTGGA	58.7	273	INTERGENIC	--	--	--
Ca2_P4_29215176	Ca2	29215176	T/TTTGATATTCTCTC	ACGGTTGAGAATTGAGTGGC	CGCACACCTCTAACAACCAA	60.1	508	INTERGENIC	--	--	--
Ca2_P4_29220253	Ca2	29220253	GATATATATATATATATATATATA/GATATATATATATATATATATA	AGGCATGGTGTTGGAGTCAT	CTAGTTCGTAACCCGTGCGT	60.4	253	INTERGENIC	--	--	--
Ca2_P4_29220546	Ca2	29220546	GATAATAATAATAATAATAATAATAATAATAAT/GATAATAATAATAATAATAATAATAATAATAAT	CACGTGTCAAACTTGCCAAT	GTAACCGTTGGAGCACCACT	59.6	534	INTERGENIC	--	--	--

INDEL marker IDs	Chromosomes/ unanchored scaffolds	Physical positions (bp)	InDels (*Kabuli* reference genome-CDC Frontier/PII)	Forward primers (5'-3')	Reverse primers (5'-3')	Anneling temperature (0C)	Expected amplified product size (bp)	Structural annotation		Functional annotation	
								Sequence components of *kabuli* genome	*Kabuli* gene accession IDs	NCBI-KOG	TFs
Ca2_P4_29239999	Ca2	29239999	AAT/A	GGCTGCTGATCCTGTTCTTC	CTGACGGACGGACTCATTTT	60.0	482	INTERGENIC	--	--	--
Ca2_P4_29240150	Ca2	29240150	TC/TCACC	CTTAGAGCATCTCCAACGGC	GAAGCATCGTTCCTTCCTGA	60.0	134	INTERGENIC	--	--	--
Ca2_P4_29240273	Ca2	29240273	ATTTTTTTT/ATTTTTTT	CTTAGAGCATCTCCAACGGC	CAACGGTGAACTCAATATGGG	60.0	671	INTERGENIC	--	--	--
Ca2_P4_29240382	Ca2	29240382	TCCC/TCC	CTTAGAGCATCTCCAACGGC	CCTTGCACTATCATTTTCGGA	60.0	803	INTERGENIC	--	--	--
Ca2_P4_29240470	Ca2	29240470	T/TCAC	CTTAGAGCATCTCCAACGGC	TGTCCTTGTTCCCTTGCACT	60.0	838	INTERGENIC	--	--	--
Ca2_P4_292468	Ca2	292468	ATTTTTTTTTT/ATTTTTTTTT	GGAAGGAACGGTGCTTCATA	TTTTGCTCCAACGGTGAACT	60.1	471	INTERGENIC	--	--	--
Ca2_P4_29252004	Ca2	29252004	GTT/GT	TCAAAGGTGCACTCTTGCTATT	TGGCTTCCGGAAAGACTAAA	59.0	502	INTERGENIC	--	--	--
Ca2_P4_29348083	Ca2	29348083	TTATATATATATATATATATATAT/TTATATATATATATATATATATAT	TTTTCCAACACCAGAGTCCC	GATGCCTCATCGTGGAGATT	59.9	629	INTERGENIC	--	--	--
Ca2_P4_29356033	Ca2	29356033	CTTTTTTTTTTT/CTTTTTTTTTT	TGAAGTCAACCCGTCAACTG	AGGTGCAATTCCATCTACCG	59.7	638	INTERGENIC	--	--	--
Ca2_P4_2941126	Ca2	2941126	AATATATATATA/AATATATATATATA	CAAACCCATTGGTCCCTTTA	CGTGTATATACGCACACCCAA	59.7	503	INTERGENIC	--	--	--

INDEL marker IDs	Chromosomes/ unanchored scaffolds	Physical positions (bp)	InDels (*Kabuli* reference genome-CDC Frontier/PII)	Forward primers (5'-3')	Reverse primers (5'-3')	Anneling temperature (0C)	Expected amplified product size (bp)	Structural annotation		Functional annotation	
								Sequence components of *kabuli* genome	*Kabuli* gene accession IDs	NCBI-KOG	TFs
Ca2_P4_29417303	Ca2	29417303	TACA/TAACA	CCCAGAAATCGATTAGGCAA	TCAACGTTCGTTTTGGTTCA	60.0	522	INTERGENIC	--	--	--
Ca2_P4_29423510	Ca2	29423510	TAA/TAAAA	TGCCAACAAAAATACATGCAA	TGGAGGCTATCACCAATGTTC	60.0	846	INTERGENIC	--	--	--
Ca2_P4_29444244	Ca2	29444244	ATTTT/ATTTTT	AAATCCGACTGTCTAACCCG	TTTTCTCCTCCAATTTAGTCCAA	59.1	826	INTERGENIC	--	--	--
Ca2_P4_29445825	Ca2	29445825	TGTCTAACCCGTCTAACCCG/TGTCTAACCCG	TCACCCACACTCTCAATCCA	CCCTACCTAGGGGTGGACAT	60.1	419	INTERGENIC	--	--	--
Ca2_P4_29445927	Ca2	29445927	AACCCGAACCC/AACCCGAACCCACCCGAACCC	TGTCTAACCCGTCTAACCCG	CCCTACCTAGGGGTGGACAT	60.0	138	INTERGENIC	--	--	--
Ca2_P4_29466835	Ca2	29466835	CC/CCTC	TAAAACAGATCCCGAATGCC	GCATGTTTGAGAGGGGAGAA	59.9	130	INTERGENIC	--	--	--
Ca2_P4_2955850	Ca2	2955850	GGGCCGG/GGG	CCGTCCAACTTCATTCCATT	CACCACAAATTTCGATGACG	59.8	661	INTERGENIC	--	--	--
Ca2_P4_29677109	Ca2	29677109	ATTTTTT/ATTTTTTT	TGCAACGAGTTAAGTGTCCAA	CAAATTTAACGACCAGATGTGTG	59.4	362	INTERGENIC	--	--	--
Ca2_P4_29722067	Ca2	29722067	TTATATATATATATATATATATATATATATATATATATATATATAT/TTATATATATATATATATATATATATATATATATATATATATATATATATATAT	CCTCCCTCGTGTGAGATGTT	TCGTGCAACTCTTTAATCCAA	60.1	672	INTERGENIC	--	--	--
Ca2_P4_2975267	Ca2	2975267	GAAAAAAAA/GAAAAAAA	TTCACGACTTGGAAAGGACC	TGATCGCCATTGATTGAGAA	60.1	750	INTERGENIC	--	--	--

INDEL marker IDs	Chromosomes/ unanchored scaffolds	Physical positions (bp)	InDels (*Kabuli* reference genome-CDC Frontier/PII)	Forward primers (5'-3')	Reverse primers (5'-3')	Anneling temperature (0C)	Expected amplified product size (bp)	Structural annotation		Functional annotation	
								Sequence components of *kabuli* genome	*Kabuli* gene accession IDs	NCBI-KOG	TFs
Ca2_P4_29787158	Ca2	29787158	CATATATATATATATATATATA/CATATATATATATATATATATA	TATTGCACTTGCCGTACCAA	TTAGCAAAACGTTCGGTTCC	60.1	677	INTERGENIC	--	--	--
Ca2_P4_2982838	Ca2	2982838	TAAAAA/TAAAA	TCCCGAACGTTCAGATTAGC	CCCCATTTGATTATCGAACAA	60.2	272	INTERGENIC	--	--	--
Ca2_P4_29829814	Ca2	29829814	AAT/AAATAT	CTTTTCGTCGGAGTTTGTCC	TCGTCCTCTGGTTTTCTCGT	59.7	476	INTERGENIC	--	--	--
Ca2_P4_29829940	Ca2	29829940	TT/TTTCGAAAAAAT	CTTTTCGTCGGAGTTTGTCC	TCGTCCTCTGGTTTTCTCGT	59.7	476	INTERGENIC	--	--	--
Ca2_P4_2984623	Ca2	2984623	CATTA/CA	TGCCAAACCAATGTCTTCTT	GAGTCTCATGTCTCAACGAGGA	58.2	663	INTERGENIC	--	--	--
Ca2_P4_29847360	Ca2	29847360	CTTTTTTTTTTTT/CTTTTTTTTTT	AACGTTCTCCGTTTTTCAGC	GTGCGTCATGCAAATATGGT	59.4	838	INTERGENIC	--	--	--
Ca2_P4_2984806	Ca2	2984806	T/TG	TTTGCTCCGGTCTAAGAAAGA	GAGTCTCATGTCTCAACGAGGA	59.1	342	INTERGENIC	--	--	--
Ca2_P4_29850746	Ca2	29850746	TTATATATATATATATATATATAT/TTATATATATATATATAT	TCCACCTAAGACCCTAAAAGAAA	TTCTTTGGCTCCTCCTCGTA	58.4	449	INTERGENIC	--	--	--
Ca2_P4_29855866	Ca2	29855866	AATAATTTATAATTTAT/AATAATTTAT	TCAATATTCCGATTGCGTGA	CGACGAAACAAAACATGGAA	60.0	651	INTERGENIC	--	--	--
Ca2_P4_29884511	Ca2	29884511	ATGTGTGT/ATGTGTGTGT	GCTCAAGTATGGGCAAAAGC	TGGGGTCATCCAAATTCAGT	59.9	457	INTERGENIC	--	--	--

INDEL marker IDs	Chromosomes/ unanchored scaffolds	Physical positions (bp)	InDels (*Kabuli* reference genome-CDC Frontier/PII)	Forward primers (5'-3')	Reverse primers (5'-3')	Anneling temperature (0C)	Expected amplified product size (bp)	Structural annotation		Functional annotation	
								Sequence components of *kabuli* genome	*Kabuli* gene accession IDs	NCBI-KOG	TFs
Ca2_P4_29887425	Ca2	29887425	AATATATATATATATATATATATATAT A/AATATATATATATATATATATATAT ATATA	TTCGTTTGAAAACTCCT AGCAA	TACGACCTACCGAAGC AACA	59.1	708	INTERGENIC	--	--	--
Ca2_P4_2995772	Ca2	2995772	AAAGTA/AA	TGTGAACTTTTATGCAT GGC	TGTTGCGTTGGTCCTT AGAA	57.2	279	INTERGENIC	--	--	--
Ca2_P4_2996172	Ca2	2996172	ATTT/ATT	TTCTAAGGACCAACGC AACA	GTATGATCGATTATGC GCGA	59.3	434	INTERGENIC	--	--	--
Ca2_P4_29962779	Ca2	29962779	G/GC	AGCCAACGGACCACTA ACAC	TTCGCCATTCTTCTTCG TCT	60.0	237	INTERGENIC	--	--	--
Ca2_P4_29962869	Ca2	29962869	TAACAACAA/TAACAA	TGGAAGCCATGATTAG GAGG	TCTCCCATTCGTTGTTG ATG	60.0	182	INTERGENIC	--	--	--
Ca2_P4_29963169	Ca2	29963169	TGG/TGGG	TGGAAGCCATGATTAG GAGG	TGGAATCAGAGAGGGA CGTT	60.0	876	INTERGENIC	--	--	--
Ca2_P4_2996523	Ca2	2996523	ATTT/ATT	CTCGAGTCGGTGTTGT GATG	TTCTTCACGATCATCCA TGC	60.3	633	INTERGENIC	--	--	--
Ca2_P4_2996631	Ca2	2996631	CAAAAAAAAA/CAAAAAAAA	CTCGAGTCGGTGTTGT GATG	TTCTTCACGATCATCCA TGC	60.3	633	INTERGENIC	--	--	--
Ca2_P4_3000587	Ca2	3000587	ATTTTTTT/ATTTTTT	CCTGTCATTCTAATTAA CGTGTTTTG	TGTGAGACCATTTAAC CCATCA	60.2	999	INTERGENIC	--	--	--
Ca2_P4_3000644	Ca2	3000644	ATTTTTT/ATTTT	TGATTCAATTGTGAAGT TTTAATGTG	GGAGAAACATCACGTT GACC	59.4	949	INTERGENIC	--	--	--

INDEL marker IDs	Chromosomes/ unanchored scaffolds	Physical positions (bp)	InDels (*Kabuli* reference genome-CDC Frontier/PII)	Forward primers (5'-3')	Reverse primers (5'-3')	Anneling temperature (0C)	Expected amplified product size (bp)	Structural annotation		Functional annotation	
								Sequence components of *kabuli* genome	*Kabuli* gene accession IDs	NCBI-KOG	TFs
Ca2_P4_3002705	Ca2	3002705	TGG/TG	TGCAACGATTACCACATGAA	TTTGTCTTTTGGGTCTGCCT	58.5	340	INTERGENIC	--	--	--
Ca2_P4_3004734	Ca2	3004734	CA/CAA	GGTTGTTCATCCAACCCCTA	GAACGAGTTCATGTGGCCTT	59.6	871	INTERGENIC	--	--	--
Ca2_P4_30050486	Ca2	30050486	CAGAAGAAGAAGAAGAAGAAGAAGAA/CAGAAGAAGAAGAAGAAGAAGAAGAAGAAGAA	AATCGGAGAAATGAAGCCCT	TTGAAAATGAAGGTGGGGTC	60.0	157	INTERGENIC	--	--	--
Ca2_P4_3005264	Ca2	3005264	AATATACATATA/AATATA	ATCATTAATCGTCGACCCCA	AAAGCGGTTTTTGTCACACC	60.2	797	INTERGENIC	--	--	--
Ca2_P4_3005316	Ca2	3005316	TAAAAAAAAA/TAAAAAAAA	ATTGATGGGCCAACAGATTT	AAAGCGGTTTTTGTCACACC	59.2	692	INTERGENIC	--	--	--
Ca2_P4_3005699	Ca2	3005699	C/CT	AAACCACAATGGAGGCCTAA	CGAGAATTAATTGGAGGCCA	59.4	729	INTERGENIC	--	--	--
Ca2_P4_30095996	Ca2	30095996	TAAAAAAA/TAAAAAAAAA	CATTCCCGCTACATTTTGGT	GGAGAGAGGCCAATTTTTCA	59.8	226	INTERGENIC	--	--	--
Ca2_P4_30096276	Ca2	30096276	TA/TAAA	CATTCCCGCTACATTTTGGT	GAAGCTGCTTGTGTGCCATA	59.8	876	INTERGENIC	--	--	--
Ca2_P4_30096869	Ca2	30096869	CATATATATATATATATATATATATATATATATATAT/CATATATATATATATATATATATATATATATATATATATATATATATAT	CATTGCAGGTGCTATGGTTG	TTCTCTTATGAAGCGGAGGA	60.1	759	INTERGENIC	--	--	--
Ca2_P4_30125989	Ca2	30125989	TAAAAAA/TAAAAAAA	TTCCACTTATGGTGAGTAATGATTTT	TTGTGAGCATGAGAAGTTGTTG	59.3	641	INTERGENIC	--	--	--

INDEL marker IDs	Chromosomes/ unanchored scaffolds	Physical positions (bp)	InDels (*Kabuli* reference genome-CDC Frontier/PII)	Forward primers (5'-3')	Reverse primers (5'-3')	Anneling temperature (0C)	Expected amplified product size (bp)	Structural annotation		Functional annotation	
								Sequence components of *kabuli* genome	*Kabuli* gene accession IDs	NCBI-KOG	TFs
Ca2_P4_3016406	Ca2	3016406	AC/ACGAGC	TCGTTGTGTGCATCTATCCG	GCAGCGTTTTATCTCTCCCA	60.7	366	INTERGENIC	--	--	--
Ca2_P4_30165012	Ca2	30165012	T/TGA	TTGTTGTGACATCGAACGTCT	CAACAATTTCTCATGCTCGC	59.2	560	INTERGENIC	--	--	--
Ca2_P4_3016524	Ca2	3016524	GAAAAAAAA/GAAAAAAAAA	TGGGAGAGATAAAACGCTGC	TCTTTCACAACCTCAAAAGTGC	60.4	353	INTERGENIC	--	--	--
Ca2_P4_30173035	Ca2	30173035	GAAAAAAAAA/GAAAAAAAAAAA	CGAGTCTCAAAACGGTTGAA	TCCCCCTGATTTTTGTTTTG	58.9	589	INTERGENIC	--	--	--
Ca2_P4_30174020	Ca2	30174020	CTT/CTTT	TTATCCCCGCATATTGGACT	AGTCCTTGGGACCGAAAAGT	59.3	227	INTERGENIC	--	--	--
Ca2_P4_3019431	Ca2	3019431	CTT/CTTT	TTTCAACCATCAACATTTGACC	TGCTTTGTTTGCAAAATGGT	59.7	541	INTERGENIC	--	--	--
Ca2_P4_3020730	Ca2	3020730	CAAAAAAAA/CAAAAAAA	GTGAGGATTTGCAAGACGTG	ATGTTGGTATGCGTTGTTGA	59.3	625	INTERGENIC	--	--	--
Ca2_P4_3021004	Ca2	3021004	TAAAAAAAAA/TAAAAAAAAAA	ATCAACAACGCATACCAACA	ATTTCGGAAACGTTTTGCAG	57.5	380	INTERGENIC	--	--	--
Ca2_P4_3021370	Ca2	3021370	CACTA/CA	TGCAAAACGTTTCCGAAATA	TCAAAAATTGATTTCCACCCA	59.2	241	INTERGENIC	--	--	--
Ca2_P4_3022598	Ca2	3022598	AGTGGTC/A	TCCTGAAACAAATGCACCAA	TGAGAAACTTTCGAGTCGGG	60.1	614	INTERGENIC	--	--	--

INDEL marker IDs	Chromosomes/ unanchored scaffolds	Physical positions (bp)	InDels (*Kabuli* reference genome-CDC Frontier/PII)	Forward primers (5'-3')	Reverse primers (5'-3')	Anneling temperature (0C)	Expected amplified product size (bp)	Structural annotation		Functional annotation	
								Sequence components of *kabuli* genome	*Kabuli* gene accession IDs	NCBI-KOG	TFs
Ca2_P4_3025547	Ca2	3025547	GAAAAAAAAAA/GAAAAAAAAA	TCAAAATAATACCGACCCCG	AAAAACGAGGCGATGACAAT	59.7	589	INTERGENIC	--	--	--
Ca2_P4_30282909	Ca2	30282909	GAAAAAAAAAA/GAAAAAAAAAAA	TGCATAATTGTCCATGCGAT	CCATAATTGTTTTTGCAGTCCA	59.9	378	INTERGENIC	--	--	--
Ca2_P4_3028297	Ca2	3028297	TAAAAA/TAAAA	TCAGTTAATCCCAAACTGCAAA	ATGCACCAAGTGCAACTGAG	59.6	565	INTERGENIC	--	--	--
Ca2_P4_3029534	Ca2	3029534	ATAAT/A	ACCGCTCTATTAAGGGCGAG	TCCATCGCAATTGAATAATGA	60.7	765	INTERGENIC	--	--	--
Ca2_P4_3029555	Ca2	3029555	TAAAA/TAA	ACCGCTCTATTAAGGGCGAG	TCCATCGCAATTGAATAATGA	60.7	765	INTERGENIC	--	--	--
Ca2_P4_3031259	Ca2	3031259	ATTTTT/ATTTTTT	TCAATTGCGATGGATAGAGAAA	AATTGGTGTACCGATTGCGT	59.7	647	INTERGENIC	--	--	--
Ca2_P4_3031554	Ca2	3031554	C/CCAAAATATCT	CATCTTGTCCATCAAACGGA	GGTGTACCGATTGCGTATCC	59.5	279	INTERGENIC	--	--	--
Ca2_P4_3031875	Ca2	3031875	AG/A	TTGGATACGCAATCGGTACA	ATGCACTTGCAAGACCTTTT	60.0	440	INTERGENIC	--	--	--
Ca2_P4_3032103	Ca2	3032103	T/TTTAA	TTGGATACGCAATCGGTACA	ATGCACTTGCAAGACCTTTT	60.0	440	INTERGENIC	--	--	--
Ca2_P4_3033174	Ca2	3033174	CTTTTTTT/CTTTTTT	GATCCAATGCTCAGTCATGC	GCACCATCATTTGACCCTTC	59.2	619	INTERGENIC	--	--	--

INDEL marker IDs	Chromosomes/ unanchored scaffolds	Physical positions (bp)	InDels (*Kabuli* reference genome-CDC Frontier/PII)	Forward primers (5'-3')	Reverse primers (5'-3')	Anneling temperature (0C)	Expected amplified product size (bp)	Structural annotation		Functional annotation	
								Sequence components of *kabuli* genome	*Kabuli* gene accession IDs	NCBI-KOG	TFs
Ca2_P4_3033231	Ca2	3033231	TAAAA/TAAAAA	GATCCAATGCTCAGTCATGC	GCACCATCATTTGACCCTTC	59.2	619	INTERGENIC	--	--	--
Ca2_P4_3033295	Ca2	3033295	CAAAAAAAAA/CAAAAAAAAAAA	GATCCAATGCTCAGTCATGC	GCACCATCATTTGACCCTTC	59.2	619	INTERGENIC	--	--	--
Ca2_P4_3033478	Ca2	3033478	CAAAAAAAA/CAAAAAAA	GATCCAATGCTCAGTCATGC	CCTCCATTCCTGGTCAATGT	59.2	927	INTERGENIC	--	--	--
Ca2_P4_30340535	Ca2	30340535	TAAAAA/TAAAA	TTTGCATCGATGATCGTAATTT	GATCTCGAGTTTGAGTTCAGGA	59.5	553	INTERGENIC	--	--	--
Ca2_P4_30340851	Ca2	30340851	TA/TAA	TGTCTTATCCTGAACTCAAACTCG	ATTTGGTCAACCACCCAAAA	59.8	537	INTERGENIC	--	--	--
Ca2_P4_30341034	Ca2	30341034	TAA/TAAA	TGTCTTATCCTGAACTCAAACTCG	ATTTGGTCAACCACCCAAAA	59.8	537	INTERGENIC	--	--	--
Ca2_P4_30343224	Ca2	30343224	CT/CTT	TGGATGCGTAGCAGATTAGAGA	CGTCACTTGGTCACTTGGTG	60.0	581	INTERGENIC	--	--	--
Ca2_P4_30344280	Ca2	30344280	AAATGAA/AAA	TTTAAGAGCCCCGTGAAATG	GGTCCCCAACCAAGTATGAA	60.1	789	INTERGENIC	--	--	--
Ca2_P4_30352286	Ca2	30352286	TG/TAACG	TAAAAAGTGGTTCGGTTCGG	TTATTTTTCCCTTTTGGCCC	60.0	826	INTERGENIC	--	--	--
Ca2_P4_30352364	Ca2	30352364	ATTTTTTTT/ATTTTTT	TAAAAAGTGGTTCGGTTCGG	TTATTTTTCCCTTTTGGCCC	60.0	826	INTERGENIC	--	--	--

INDEL marker IDs	Chromosomes/ unanchored scaffolds	Physical positions (bp)	InDels (*Kabuli* reference genome-CDC Frontier/PII)	Forward primers (5'-3')	Reverse primers (5'-3')	Anneling temperature (0C)	Expected amplified product size (bp)	Structural annotation		Functional annotation	
								Sequence components of *kabuli* genome	*Kabuli* gene accession IDs	NCBI-KOG	TFs
Ca2_P4_30359357	Ca2	30359357	A/ATTC	TCACAAGGTTCCCTCTCCAG	TCCTTCATCTTCAACCAAAACA	60.2	401	INTERGENIC	--	--	--
Ca2_P4_30375925	Ca2	30375925	GTTT/GTT	TAGCCACCCCAAAAATCAAA	AAGAGGGTGAAAGAAAGGGTG	60.3	288	INTERGENIC	--	--	--
Ca2_P4_30384877	Ca2	30384877	TTATAT/TTAT	TGCATTGCAGCATAACAACTT	GTGTTGGGCCAAATCAAAAT	59.4	185	INTERGENIC	--	--	--
Ca2_P4_30385121	Ca2	30385121	A/AAT	TGATTTGGCCCAACACCTAT	GCAAATACAAAGGACCGTCAA	60.2	381	INTERGENIC	--	--	--
Ca2_P4_30385586	Ca2	30385586	CAA/CAAA	TTGACGGTCCTTTGTATTTGC	ACAGAAAATGTCGTCCCTGC	60.0	384	INTERGENIC	--	--	--
Ca2_P4_30393573	Ca2	30393573	CT/CTT	TCCCAAACAGGAATGAAACC	AGTGCAAGCATCATGGAGAA	59.8	714	INTERGENIC	--	--	--
Ca2_P4_30393592	Ca2	30393592	ATTTTTTT/ATTTTTT	TCCCAAACAGGAATGAAACC	AGTGCAAGCATCATGGAGAA	59.8	714	INTERGENIC	--	--	--
Ca2_P4_30393638	Ca2	30393638	TAAAAAAAAAA/TAAAAAAAAAAA	TCCCAAACAGGAATGAAACC	AGTGCAAGCATCATGGAGAA	59.8	714	INTERGENIC	--	--	--
Ca2_P4_30393886	Ca2	30393886	TC/T	ATTCTCCGATTTTTCCCCAT	TGTTTTCAGTGCAAGCATCA	59.6	475	INTERGENIC	--	--	--
Ca2_P4_30394906	Ca2	30394906	CAAAA/CAAAAA	GAGAGAGGGGCAAGGTCAC	AGGCTTTTGTTGTTGTTGGG	59.8	392	INTERGENIC	--	--	--

INDEL marker IDs	Chromosomes/ unanchored scaffolds	Physical positions (bp)	InDels (*Kabuli* reference genome-CDC Frontier/PII)	Forward primers (5'-3')	Reverse primers (5'-3')	Anneling temperature (0C)	Expected amplified product size (bp)	Structural annotation		Functional annotation	
								Sequence components of *kabuli* genome	*Kabuli* gene accession IDs	NCBI-KOG	TFs
Ca2_P4_30394922	Ca2	30394922	AA/AATATA	GAGAGAGGGGCAAGGTCAC	AGGCTTTTGTTGTTGTTGGG	59.8	392	INTERGENIC	--	--	--
Ca2_P4_30395203	Ca2	30395203	CATATATATA/CATATATATATA	CCCAACAACAACAAAAGCCT	AAACCAGCATGACAAAAGCA	60.0	289	INTERGENIC	--	--	--
Ca2_P4_30397553	Ca2	30397553	TAAAA/TAAAAA	TCTTTTTCATTCCAACCCTCA	CCGAAAGTGTGGGTCTCTTC	59.5	475	INTERGENIC	--	--	--
Ca2_P4_30399984	Ca2	30399984	CATATATATATATATAT/CATATATATATATAT	AGGTGGGCCCATTACTTGAT	AGTGGAGGACCACTTCCTCA	60.6	836	INTERGENIC	--	--	--
Ca2_P4_30401507	Ca2	30401507	TT/TTGT	GCATCACAAAGGGTTTTTGG	TCGATGCGAATACAACTAAATCA	60.3	610	INTERGENIC	--	--	--
Ca2_P4_30401609	Ca2	30401609	AACAAAAATA/AA	GCATCACAAAGGGTTTTTGG	CATATGAAAGTTTGGCCACG	60.3	828	INTERGENIC	--	--	--
Ca2_P4_30401833	Ca2	30401833	ATTT/ATT	GGTCGAGTTTGCAATGATGA	TGACCACAAGTTGCCAAAAA	59.7	761	INTERGENIC	--	--	--
Ca2_P4_30431126	Ca2	30431126	AATATATATATATATATATATATA/AATATATATATATATATATA	TTGACAATAATTTTTCTCGAGTTGA	ACCAACGTTGCACCAAGATT	59.2	638	INTERGENIC	--	--	--
Ca2_P4_30433128	Ca2	30433128	TAAAAAAAAA/TAAAAAAAA	GAAATTGCATTTATCTCCCCC	TTACAGTTAAGGGCCAACCG	59.6	737	INTERGENIC	--	--	--
Ca2_P4_30433806	Ca2	30433806	CTTTT/CTTTTT	CGGTTGGCCCTTAACTGTAA	TCCTCAAACCCAAAATGGTC	60.0	674	INTERGENIC	--	--	--

INDEL marker IDs	Chromosomes/ unanchored scaffolds	Physical positions (bp)	InDels (*Kabuli* reference genome-CDC Frontier/PII)	Forward primers (5'-3')	Reverse primers (5'-3')	Anneling temperature (0C)	Expected amplified product size (bp)	Structural annotation		Functional annotation	
								Sequence components of *kabuli* genome	*Kabuli* gene accession IDs	NCBI-KOG	TFs
Ca2_P4_30439365	Ca2	30439365	GAAAAAAAAAA/GAAAAAAAAA	CCAAGTTCAACACTCATATGCC	ATGGAGAATTCAGCCCAACA	59.5	406	INTERGENIC	--	--	--
Ca2_P4_30444678	Ca2	30444678	CATATAT/CATATATAT	CACTAGTGATGCCGTGATGC	GCGCTAGATGGCAAGGTAAG	60.3	457	INTERGENIC	--	--	--
Ca2_P4_30445438	Ca2	30445438	TTTTAAAATTTA/TTTTAAAATTTAAAATTTA	CTGAACCGAACCGGTTTTTA	TTGTTGCGATCGATATATTTTTG	60.0	646	INTERGENIC	--	--	--
Ca2_P4_30445916	Ca2	30445916	TAAAAAA/TAAAAAAA	CAAAAATATATCGATCGCAACAA	AATTGAAAGCTGCATTGGCT	59.0	406	INTERGENIC	--	--	--
Ca2_P4_30447122	Ca2	30447122	CATATATATATATAT/CATATATATATATATATATATATATAT	TGGTGCACACAATACAATCAAA	CAAGGCCGAGAAGACAAAAG	59.9	225	INTERGENIC	--	--	--
Ca2_P4_30447611	Ca2	30447611	A/AT	CTTTTGTCTTCTCGGCCTTG	CGTGCTCATCTCCATTGTTG	60.0	891	INTERGENIC	--	--	--
Ca2_P4_30447653	Ca2	30447653	AC/A	CTTTTGTCTTCTCGGCCTTG	ATCTCCTCAGCATTGTCGCT	60.0	943	INTERGENIC	--	--	--
Ca2_P4_30459901	Ca2	30459901	A/AT	GGAGTGGGCACTCAACTTTT	CACACTTCTCACCCCCACTT	59.2	219	INTERGENIC	--	--	--
Ca2_P4_30460218	Ca2	30460218	AATATAT/AATAT	ATCATGAAAGTGGGGGTGAG	TGAAACCGATCAAATCCACA	59.8	596	INTERGENIC	--	--	--
Ca2_P4_30461229	Ca2	30461229	AG/A	TCCCATTCAATCAAGACGTG	TTTTTGTCTCACATTGATTGTCTTT	59.5	666	INTERGENIC	--	--	--

INDEL marker IDs	Chromosomes/ unanchored scaffolds	Physical positions (bp)	InDels (*Kabuli* reference genome-CDC Frontier/PII)	Forward primers (5'-3')	Reverse primers (5'-3')	Anneling temperature (0C)	Expected amplified product size (bp)	Structural annotation		Functional annotation	
								Sequence components of *kabuli* genome	*Kabuli* gene accession IDs	NCBI-KOG	TFs
Ca2_P4_30461396	Ca2	30461396	CTTTTTTT/CTTTTTTTTT	AAAAAGAAGTGGTTGTCCGC	AAAACGATGCTTATCCGTGAA	59.2	929	INTERGENIC	--	--	--
Ca2_P4_30462677	Ca2	30462677	ATAAATT/ATAAATTGACTATTAAATT	GAAATTTAAAAAGCGTGCGG	AACAAACCCTAGTGCCATGC	59.7	505	INTERGENIC	--	--	--
Ca2_P4_30463432	Ca2	30463432	TAAAAAAAAAA/TAAAAAAAA	ATTCTCAACCCTTGGTGTGC	AGTGTTTGTTTGGGAGGTGG	60.0	670	INTERGENIC	--	--	--
Ca2_P4_30464150	Ca2	30464150	CTT/CTTT	CCACCTCCCAAACAAACACT	GGCAAGTTGCTTTTACCAAAAC	59.9	602	INTERGENIC	--	--	--
Ca2_P4_30464334	Ca2	30464334	ATCT/AT	CCACCTCCCAAACAAACACT	GGGTGCATGGCTTCTACATT	59.9	792	INTERGENIC	--	--	--
Ca2_P4_30476467	Ca2	30476467	CAAAAAAAAAA/CAAAAAAAAAAA	AGCAATGTGGCATGCATAAA	GGGAATGTTTGTTGAATGTCG	60.1	601	INTERGENIC	--	--	--
Ca2_P4_30481523	Ca2	30481523	TTTAAAAAAT/TTTAAAAAAATATGTTAAAAAAT	GTTGGCTCCCAAAGACACAT	TCGATTTTCATTAAAGTGTTGGA	60.0	631	INTERGENIC	--	--	--
Ca2_P4_30481983	Ca2	30481983	ATATTATTT/ATATTATTTAAATTATTATTT	TCAATGCATGGAAAATAATCTCT	TGGTCCTCATCCATCAAACA	57.8	401	INTERGENIC	--	--	--
Ca2_P4_30484744	Ca2	30484744	GG/GGACG	ACATGAGACCGTCAATGCAA	CGAAAAATCAAAATAACATCGC	60.1	386	INTERGENIC	--	--	--
Ca2_P4_30484802	Ca2	30484802	A/ATT	ACATGAGACCGTCAATGCAA	CGAAAAATCAAAATAACATCGC	60.1	386	INTERGENIC	--	--	--

INDEL marker IDs	Chromosomes/ unanchored scaffolds	Physical positions (bp)	InDels (*Kabuli* reference genome-CDC Frontier/PII)	Forward primers (5'-3')	Reverse primers (5'-3')	Anneling temperature (0C)	Expected amplified product size (bp)	Structural annotation		Functional annotation	
								Sequence components of *kabuli* genome	*Kabuli* gene accession IDs	NCBI-KOG	TFs
Ca2_P4_30485065	Ca2	30485065	AC/A	CACAACTTCTTCAGTCCTGTGG	TAAGAAAATTGGGGTGGCAG	59.8	889	INTERGENIC	--	--	--
Ca2_P4_30490084	Ca2	30490084	ATTTTTTTTTTTTTTTT/ATTTTTTTTTTT	CCCCCTACTACTTGACGATCC	TTCCCAAATGCAATTTTTCC	59.8	393	INTERGENIC	--	--	--
Ca2_P4_30490349	Ca2	30490349	TA/TTAA	GGAAAAATTGCATTTGGGAA	GTTGGCTTACACATTGGGCT	59.8	578	INTERGENIC	--	--	--
Ca2_P4_30490413	Ca2	30490413	TTAAAATAAAA/TTAAAA	GGAAAAATTGCATTTGGGAA	GTTGGCTTACACATTGGGCT	59.8	578	INTERGENIC	--	--	--
Ca2_P4_30494831	Ca2	30494831	GAAAAAAAAA/GAAAAAAAAAA	TGAGAACGCCCATGTCAATA	TCTCTGGCAACCGTTCTTCT	60.1	404	INTERGENIC	--	--	--
Ca2_P4_3049734	Ca2	3049734	AGAT/A	CGATCTCATATGTGGGGACC	TGCAATCGAAAGAAGTGTGG	60.2	536	INTERGENIC	--	--	--
Ca2_P4_30514044	Ca2	30514044	TAAAAAAA/TAAAAAA	TTGAATGGTTTGTGATCCGA	TCCATTGGAAGCATTCACTTC	59.9	483	INTERGENIC	--	--	--
Ca2_P4_30516125	Ca2	30516125	GATATA/GATA	GAGATGAAAACGGATGGGTG	CATGACAATCATTTGCAGCC	60.3	831	INTERGENIC	--	--	--
Ca2_P4_30516532	Ca2	30516532	GT/G	CACAAGGTTTGTGCACTGAAA	CATGACAATCATTTGCAGCC	59.8	440	INTERGENIC	--	--	--
Ca2_P4_30520425	Ca2	30520425	GAAAAAAAAA/GAAAAAAAAAAA	TTGACGTAGGGTCTTGGGTC	ATGTCCTGCTTCCATTCCAC	60.0	745	INTERGENIC	--	--	--

INDEL marker IDs	Chromosomes/ unanchored scaffolds	Physical positions (bp)	InDels (*Kabuli* reference genome-CDC Frontier/PII)	Forward primers (5'-3')	Reverse primers (5'-3')	Anneling temperature (0C)	Expected amplified product size (bp)	Structural annotation		Functional annotation	
								Sequence components of *kabuli* genome	*Kabuli* gene accession IDs	NCBI-KOG	TFs
Ca2_P4_30521356	Ca2	30521356	TTC/TTCGCTC	TAACAATTCCTTCCGCCAAG	GCAGCCGTCTATGCTATTCA	60.1	361	INTERGENIC	--	--	--
Ca2_P4_30522267	Ca2	30522267	AATTAT/AAT	GAACAGTGGCAATTCATCCA	TTTTGGTGCCAAATTCTTCTC	59.5	556	INTERGENIC	--	--	--
Ca2_P4_30522321	Ca2	30522321	ATTTTTTTT/ATTTTTT	GAACAGTGGCAATTCATCCA	CGATGTGTGCATGCTCTCTAA	59.5	865	INTERGENIC	--	--	--
Ca2_P4_30523254	Ca2	30523254	AATAT/AATATGAATATAT	TCATCGTTTGCAAGGTTTTG	TAATGCACTTCATTGCTGGC	59.7	249	INTERGENIC	--	--	--
Ca2_P4_30525393	Ca2	30525393	TTA/TTATA	CAGCCACAGCATAGCTTTCA	GACGTGTGTTGCTTTAAGGG	60.2	516	INTERGENIC	--	--	--
Ca2_P4_30526988	Ca2	30526988	GAAAA/GAAA	CTCGTGAAGGATTAGGTCGG	TTGCAACGCATCTCAATCTC	59.7	353	INTERGENIC	--	--	--
Ca2_P4_30527039	Ca2	30527039	TAAAAA/TAAAA	CTCGTGAAGGATTAGGTCGG	TTGCAACGCATCTCAATCTC	59.7	353	INTERGENIC	--	--	--
Ca2_P4_30527100	Ca2	30527100	TAAAAAAAAAA/TAAAAAAAA	CTCGTGAAGGATTAGGTCGG	TTGCAACGCATCTCAATCTC	59.7	353	INTERGENIC	--	--	--
Ca2_P4_30549976	Ca2	30549976	ATTTTTTT/ATTTTTT	TTTCTTTATTCTCGGTGGCG	GCTTGGATTAGGAAAAATCGAC	60.2	397	INTERGENIC	--	--	--
Ca2_P4_30550114	Ca2	30550114	TAAAAAAA/TAAAAAA	TTTCTTTATTCTCGGTGGCG	TGCATTCGATCAAGCTCAAC	60.2	640	INTERGENIC	--	--	--

INDEL marker IDs	Chromosomes/ unanchored scaffolds	Physical positions (bp)	InDels (*Kabuli* reference genome-CDC Frontier/PII)	Forward primers (5'-3')	Reverse primers (5'-3')	Anneling temperature (0C)	Expected amplified product size (bp)	Structural annotation		Functional annotation	
								Sequence components of *kabuli* genome	*Kabuli* gene accession IDs	NCBI-KOG	TFs
Ca2_P4_30550167	Ca2	30550167	ACA/ACAGTCA	TTTCTTTATTCTCGGTGGCG	TGCATTCGATCAAGCTCAAC	60.2	640	INTERGENIC	--	--	--
Ca2_P4_30550309	Ca2	30550309	ATTTTT/ATTTTTT	TTTCTTTATTCTCGGTGGCG	TGCATTCGATCAAGCTCAAC	60.2	640	INTERGENIC	--	--	--
Ca2_P4_30553119	Ca2	30553119	T/TTAGAC	TTTTTATGAGGCGTGGAAGG	AAAACTAACACACCCCCGTG	60.1	223	INTERGENIC	--	--	--
Ca2_P4_30553379	Ca2	30553379	GAAAA/GAAAAA	TTTTTATGAGGCGTGGAAGG	CCATGAACACGCCAAACATA	60.1	874	INTERGENIC	--	--	--
Ca2_P4_30553679	Ca2	30553679	TAAAAAAA/TAAAAAA	TGCACTTTATCACTTGGTAATCG	TGTTTGCGAGCTTCAATGAC	59.2	561	INTERGENIC	--	--	--
Ca2_P4_30554237	Ca2	30554237	AC/A	GTCATTGAAGCTCGCAAACA	ATGTTCCAAGGTTGGTCTGC	60.0	538	INTERGENIC	--	--	--
Ca2_P4_30559073	Ca2	30559073	AG/A	TGCATACAAAAGTCACCCCA	AAAAATTATGGGCTTTGGGC	60.0	982	INTERGENIC	--	--	--
Ca2_P4_30559202	Ca2	30559202	AT/ATT	CCCAACCACTTCATCAATCC	AAAAATTATGGGCTTTGGGC	60.2	750	INTERGENIC	--	--	--
Ca2_P4_30559409	Ca2	30559409	CT/CTT	GGAGAACTAAATTTCACATGAGTGG	AAAAATTATGGGCTTTGGGC	60.3	258	INTERGENIC	--	--	--
Ca2_P4_30566132	Ca2	30566132	AT/ATT	CAGAGACAAAAGAGGGAACCA	TTGTGTTGTTGTTGTGTGCG	59.3	884	INTERGENIC	--	--	--

INDEL marker IDs	Chromosomes/ unanchored scaffolds	Physical positions (bp)	InDels (*Kabuli* reference genome-CDC Frontier/PII)	Forward primers (5'-3')	Reverse primers (5'-3')	Anneling temperature (0C)	Expected amplified product size (bp)	Structural annotation		Functional annotation	
								Sequence components of *kabuli* genome	*Kabuli* gene accession IDs	NCBI-KOG	TFs
Ca2_P4_30567133	Ca2	30567133	TAAAAAAAA/TAAAAAAA	AAATTACCGCAACAATTGGC	GAAGCGCAACTAAACCAAGC	59.8	539	INTERGENIC	--	--	--
Ca2_P4_30574328	Ca2	30574328	TAAAAA/TAAAAAAA	CCATTGAGGAGTGGAGAACTTT	CTTGTGTGTTCCCAATGCAA	59.6	783	INTERGENIC	--	--	--
Ca2_P4_30575439	Ca2	30575439	ATCT/ATCTCT	GGTTGATTATGGTTTTGGCG	ATCCAAAGCAGCATCCATTC	60.2	862	INTERGENIC	--	--	--
Ca2_P4_30575554	Ca2	30575554	T/TC	TGCAAACGAGGTGTGTGTCT	ATCCAAAGCAGCATCCATTC	60.4	457	INTERGENIC	--	--	--
Ca2_P4_30585501	Ca2	30585501	CTTTTTTTTTT/CTTTTTTTTTTTT	GAAACGGTTGGAAGGTTGAA	ATCAAAATATCTTTGCGCGG	59.9	305	INTERGENIC	--	--	--
Ca2_P4_30623473	Ca2	30623473	T/TGAA	TGACAAAAAGGGTCAACGTG	CCGCAATCAATTTAGCGAAT	59.6	686	INTERGENIC	--	--	--
Ca2_P4_30624454	Ca2	30624454	C/CT	TCATGAATGCAACGTGGTTT	TTTTGCTGTTTGTTTTACGTCAT	60.0	646	INTERGENIC	--	--	--
Ca2_P4_30624742	Ca2	30624742	GTTTTTT/GTTTTTTT	TTGCTTGCAACACAAAACTTC	TGAACCATTCATCACAAAAAGAG	59.0	837	INTERGENIC	--	--	--
Ca2_P4_30626190	Ca2	30626190	T/TCA	TTGTCATTTTGATCATTGATTCG	GGTGGTTTGTAACAACAGCAAA	59.8	515	INTERGENIC	--	--	--
Ca2_P4_30635014	Ca2	30635014	ATGTGTGTG/ATGTGTGTGTG	TCGTGTGAGACGAAAAATCG	CTCACCAGCTGCATCACATT	59.8	367	INTERGENIC	--	--	--

INDEL marker IDs	Chromosomes/ unanchored scaffolds	Physical positions (bp)	InDels (*Kabuli* reference genome-CDC Frontier/PII)	Forward primers (5'-3')	Reverse primers (5'-3')	Anneling temperature (0C)	Expected amplified product size (bp)	Structural annotation		Functional annotation	
								Sequence components of *kabuli* genome	*Kabuli* gene accession IDs	NCBI-KOG	TFs
Ca2_P4_30642500	Ca2	30642500	GAAAAAAAAA/GAAAAAAAAAAA	TGCAGTTGCCGACAATTTTAT	TCCTCGGTAACCAAACAAGC	60.5	128	INTERGENIC	--	--	--
Ca2_P4_30647034	Ca2	30647034	CAT/CATAT	TTGACGTACCCAACCATTGA	CCCTCACTTAAGCCCACAAA	59.8	443	INTERGENIC	--	--	--
Ca2_P4_30647302	Ca2	30647302	AGGGGG/AGGGG	TTGACGTACCCAACCATTGA	GGGCCTGTGGGTCTACTTTT	59.8	759	INTERGENIC	--	--	--
Ca2_P4_30647346	Ca2	30647346	A/AT	TTTGTGGGCTTAAGTGAGGG	GGGCCTGTGGGTCTACTTTT	60.1	336	INTERGENIC	--	--	--
Ca2_P4_30647396	Ca2	30647396	ATTTTTTTTTT/ATTTTTTTTT	TTTGTGGGCTTAAGTGAGGG	GGGCCTGTGGGTCTACTTTT	60.1	336	INTERGENIC	--	--	--
Ca2_P4_30672819	Ca2	30672819	CAAAAAAAA/CAAAAAAAAAAA	ATGGCATATCCGTGGGTAAT	TAGGCCCTAGGGACAATGTG	59.0	834	INTERGENIC	--	--	--
Ca2_P4_30676386	Ca2	30676386	GAAAAAAAAAA/GAAAAAAAAAAA	ATCCACCAACGTCCAAGTTC	CCTTGGTATGACCCTAATTCCTT	59.8	532	INTERGENIC	--	--	--
Ca2_P4_30676605	Ca2	30676605	GAAAAA/GAAAA	GCTCCAGCAAAAATAGAAATGG	TCATCAATCAAACTACTTACGCAGA	60.1	504	INTERGENIC	--	--	--
Ca2_P4_30676782	Ca2	30676782	ACTAGC/ACTAGCTAGC	GCTCCAGCAAAAATAGAAATGG	TCATCAATCAAACTACTTACGCAGA	60.1	504	INTERGENIC	--	--	--
Ca2_P4_30676877	Ca2	30676877	CAAAAAAAA/CAAAAAAA	AAGGAATTAGGGTCATACCAAGG	TCATCTACAACAAAAATTGAACAAA	59.6	606	INTERGENIC	--	--	--

INDEL marker IDs	Chromosomes/ unanchored scaffolds	Physical positions (bp)	InDels (*Kabuli* reference genome- CDC Frontier/PII)	Forward primers (5'-3')	Reverse primers (5'-3')	Anneling temperature (0C)	Expected amplified product size (bp)	Structural annotation		Functional annotation	
								Sequence components of *kabuli* genome	*Kabuli* gene accession IDs	NCBI-KOG	TFs
Ca2_P4_30677085	Ca2	30677085	TAAAAAAAA/TTATTAAAAAAAAA	TCTGCGTAAGTAGTTTGATTGATGA	TTGCGCCTTTTCAGTATTTTC	60.2	668	INTERGENIC	--	--	--
Ca2_P4_30678202	Ca2	30678202	TA/T	AGCTAATTGTGGGCGAAAAA	TGGAAAAGGTTTTGGGTTTG	59.7	922	INTERGENIC	--	--	--
Ca2_P4_30679907	Ca2	30679907	GTTTTTT/GTTTT	AAAGCGAAATGATCAAACGG	TTCCCTTCAAATTGTAGCATGA	60.1	692	INTERGENIC	--	--	--
Ca2_P4_30680775	Ca2	30680775	AATATATAT/AATATAT	GGGCTGACTATGGATTTGGA	TATTTGGCTGACGTGGAACA	59.9	225	INTERGENIC	--	--	--
Ca2_P4_30690091	Ca2	30690091	CT/CTTT	GCGGTGTTTGTTTGGGTAGT	GGAGCACACAGATGCGAATA	59.9	335	INTERGENIC	--	--	--
Ca2_P4_30690274	Ca2	30690274	A/AT	TTAGGGGAACGTGAAATTGG	CGGTCATACTGCACACACAA	59.8	444	INTERGENIC	--	--	--
Ca2_P4_30690304	Ca2	30690304	ACC/AC	GCGGTGTTTGTTTGGGTAGT	TGTACATTCAAATCAAACAAAATTCA	59.9	825	INTERGENIC	--	--	--
Ca2_P4_30690368	Ca2	30690368	TAAAAAAA/TAAAAAAAA	TATTCGCATCTGTGTGCTCC	CGTGGTTGAGTAGATGTGCC	59.8	670	INTERGENIC	--	--	--
Ca2_P4_30690413	Ca2	30690413	AT/ATAAATT	TATTCGCATCTGTGTGCTCC	CGTGGTTGAGTAGATGTGCC	59.8	670	INTERGENIC	--	--	--
Ca2_P4_30690532	Ca2	30690532	CTATTTTATT/CTATT	TATTCGCATCTGTGTGCTCC	CGTGGTTGAGTAGATGTGCC	59.8	670	INTERGENIC	--	--	--

INDEL marker IDs	Chromosomes/ unanchored scaffolds	Physical positions (bp)	InDels (*Kabuli* reference genome-CDC Frontier/PII)	Forward primers (5'-3')	Reverse primers (5'-3')	Anneling temperature (0C)	Expected amplified product size (bp)	Structural annotation		Functional annotation	
								Sequence components of *kabuli* genome	*Kabuli* gene accession IDs	NCBI-KOG	TFs
Ca2_P4_30691545	Ca2	30691545	CTAATAAA/CTAATAAATAATAAA	CCACATTAGTCCATCACCGA	ATCAAGGTCGGGGAATAACC	59.4	436	INTERGENIC	--	--	--
Ca2_P4_30703095	Ca2	30703095	TT/TTATAAACT	AACGGGGGATTGAAGAGATT	CGCATACATACGGTGATTGC	59.8	882	INTERGENIC	--	--	--
Ca2_P4_30703318	Ca2	30703318	AC/A	ACCCGCAGAATTTGCTAAGA	CGCATACATACGGTGATTGC	59.8	389	INTERGENIC	--	--	--
Ca2_P4_30703947	Ca2	30703947	ATTTTTTTTTT/ATTTTTTTTT	CCGTATGTATGCGTGACACC	TCGAATTGGATTTCACATATTTTC	59.9	423	INTERGENIC	--	--	--
Ca2_P4_30704638	Ca2	30704638	TA/TAA	AAATTCAGTGCAACGCACAT	CCCACTTTGGATAACAATTCC	59.2	445	INTERGENIC	--	--	--
Ca2_P4_30705361	Ca2	30705361	GATATATATA/GATATATATATA	TGCCACATTTTCCTTTCTTG	GGATGAGGCTTTCAATTCTACG	58.7	264	INTERGENIC	--	--	--
Ca2_P4_30731994	Ca2	30731994	AATGATGAT/AATGAT	GGCCAAACTTGATTCAAACC	CGTCGACGATAGCATAAGCA	59.4	754	INTERGENIC	--	--	--
Ca2_P4_30742309	Ca2	30742309	CAGAGAGAG/CAGAGAGAGAGAGAG	CGTTGCGGTAACAAACAATG	TTGGAGAAAAGGCAGGCTTA	60.0	349	INTERGENIC	--	--	--
Ca2_P4_30742679	Ca2	30742679	TAAAAAA/TAAAAAAAA	TGTTTGCTTCATTGGCAGAG	AAATTTGATGTAGAATATAACCCAAAA	60.0	674	INTERGENIC	--	--	--
Ca2_P4_30744544	Ca2	30744544	TT/TTAT	CACCTATACATGCAAGAGCAAAA	TCAACGAGCATGTGGAAAAG	59.3	920	INTERGENIC	--	--	--

INDEL marker IDs	Chromosomes/ unanchored scaffolds	Physical positions (bp)	InDels (*Kabuli* reference genome-CDC Frontier/PII)	Forward primers (5'-3')	Reverse primers (5'-3')	Anneling temperature (0C)	Expected amplified product size (bp)	Structural annotation		Functional annotation	
								Sequence components of *kabuli* genome	*Kabuli* gene accession IDs	NCBI-KOG	TFs
Ca2_P4_30765377	Ca2	30765377	TAAA/TAAAAA	CATGCCATCTTGTAGGGACA	TCATTTTTCTCGCACGACAC	59.5	678	INTERGENIC	--	--	--
Ca2_P4_30783813	Ca2	30783813	CTTTTTTTT/CTTTTTTT	AAGGACATGGTACCACCAGC	TGTGAACTATGCCGATTAACTGA	59.9	710	INTERGENIC	--	--	--
Ca2_P4_30794341	Ca2	30794341	ATTTTT/ATTTTTT	GAGTTGAATGCAGCTGACCA	CCAAAAACAAATTTTTCGGGA	60.0	678	INTERGENIC	--	--	--
Ca2_P4_30794400	Ca2	30794400	CTTTTTTT/CTTTTTT	GAGTTGAATGCAGCTGACCA	TGCATTTCACTGGAGACGTT	60.0	741	INTERGENIC	--	--	--
Ca2_P4_30794960	Ca2	30794960	TTTTTATTTTATT/TTTTTATTTTATTTTATTTTATT	ACGTCTCCAGTGAAATGCAA	AGCCTTGATGTTGTTTGCG	59.3	506	INTERGENIC	--	--	--
Ca2_P4_30795414	Ca2	30795414	TAAAA/TAAA	GCAAACAACATCAAGGCTCA	TGAAAATAGTTTTTGGTCCCTG	59.8	558	INTERGENIC	--	--	--
Ca2_P4_30795526	Ca2	30795526	TTAATATAATAT/TTAATAT	GCAAACAACATCAAGGCTCA	TGAAAATAGTTTTTGGTCCCTG	59.8	558	INTERGENIC	--	--	--
Ca2_P4_30805003	Ca2	30805003	T/TA	TGCTGAATTGGATAGAGGGG	TTTTTGGTTTGTGTACATTTGTTTT	60.0	838	INTERGENIC	--	--	--
Ca2_P4_30805135	Ca2	30805135	CATTAATTA/CATTA	CCTCATGGTGTGTGATTTGC	GCGAAAGCAAAATAGAGTGGA	60.0	880	INTERGENIC	--	--	--
Ca2_P4_30806790	Ca2	30806790	T/TA	GAAGTCAACATGTGCATGGG	TTTAAAATTATGCGACGGCA	60.0	606	INTERGENIC	--	--	--

INDEL marker IDs	Chromosomes/ unanchored scaffolds	Physical positions (bp)	InDels (*Kabuli* reference genome-CDC Frontier/PII)	Forward primers (5'-3')	Reverse primers (5'-3')	Anneling temperature (0C)	Expected amplified product size (bp)	Structural annotation		Functional annotation	
								Sequence components of *kabuli* genome	*Kabuli* gene accession IDs	NCBI-KOG	TFs
Ca2_P4_30841102	Ca2	30841102	TCC/TCCC	TGACACTAGCTTGTGCGGTC	CCTAGCCAACATTGAAGGGA	60.1	460	INTERGENIC	--	--	--
Ca2_P4_30843280	Ca2	30843280	AAATTAA/AAA	CCACAATCATTGGGAAGTGA	TGGATGAATAGGGGATTGCT	59.3	305	INTERGENIC	--	--	--
Ca2_P4_30844156	Ca2	30844156	TTTAA/TTTAATTAA	TCCACACTCTTGTAGGTTGCAT	CAGAATCAGCACGCAAACAT	59.7	854	INTERGENIC	--	--	--
Ca2_P4_30844625	Ca2	30844625	A/AG	ATGTTTGCGTGCTGATTCTG	ATGTCATGACCGCATGATTG	59.9	387	INTERGENIC	--	--	--
Ca2_P4_30846242	Ca2	30846242	T/TG	CCTTTGCGAACTTGAAGACC	TCCTCCCTCTTGCAGGTTTA	59.9	815	INTERGENIC	--	--	--
Ca2_P4_30846291	Ca2	30846291	CAAA/CAA	CCACCTGGATCTGAGTGTGA	TCCTCCCTCTTGCAGGTTTA	59.7	761	INTERGENIC	--	--	--
Ca2_P4_30847431	Ca2	30847431	AA/AATAGTTA	CCAATCTTGTGGAGTGCTCA	TCCTCCATCATCTCATGTTACC	59.8	693	INTERGENIC	--	--	--
Ca2_P4_30848508	Ca2	30848508	TTGATAAT/TTGATAATAATGATAAT	TTGAAGGACCAAAATCATGAAC	CATACAACGCAAGCTAGGCA	58.9	533	INTERGENIC	--	--	--
Ca2_P4_30849743	Ca2	30849743	ATT/ATTT	TCAACTTAGATATGCCGTCCG	TGGGTTGGTTTGAATCATCA	60.1	410	INTERGENIC	--	--	--
Ca2_P4_30860005	Ca2	30860005	CAAAAA/CAAAA	AGACAACATGTACCCCGGAC	TGAAGTGGCAAATTCGCATA	59.7	601	INTERGENIC	--	--	--

INDEL marker IDs	Chromosomes/ unanchored scaffolds	Physical positions (bp)	InDels (*Kabuli* reference genome-CDC Frontier/PII)	Forward primers (5'-3')	Reverse primers (5'-3')	Anneling temperature (0C)	Expected amplified product size (bp)	Structural annotation		Functional annotation	
								Sequence components of *kabuli* genome	*Kabuli* gene accession IDs	NCBI-KOG	TFs
Ca2_P4_30860259	Ca2	30860259	AG/A	TGAACAAAAAGGTTGGGTGA	TGCTCAACTTTGACCCATTG	59.0	487	INTERGENIC	--	--	--
Ca2_P4_30860320	Ca2	30860320	TAA/TAAA	TGAACAAAAAGGTTGGGTGA	TGCTCAACTTTGACCCATTG	59.0	487	INTERGENIC	--	--	--
Ca2_P4_30860618	Ca2	30860618	ATTTT/ATTTTTT	TGGGTCAAAGTTGAGCATGA	CACCTTTGGAAAGAGCTGGA	60.2	557	INTERGENIC	--	--	--
Ca2_P4_30860700	Ca2	30860700	AC/A	TGGGTCAAAGTTGAGCATGA	CACCTTTGGAAAGAGCTGGA	60.2	557	INTERGENIC	--	--	--
Ca2_P4_30860764	Ca2	30860764	T/TG	TGGGTCAAAGTTGAGCATGA	CACCTTTGGAAAGAGCTGGA	60.2	557	INTERGENIC	--	--	--
Ca2_P4_30861287	Ca2	30861287	AA/AATA	CCAAAGGTGGGTGAAGAAGA	CAGGCCCAATTCACAAAGTT	60.1	554	INTERGENIC	--	--	--
Ca2_P4_30861518	Ca2	30861518	GAAA/GAAAA	CCAAAGGTGGGTGAAGAAGA	CAGGCCCAATTCACAAAGTT	60.1	554	INTERGENIC	--	--	--
Ca2_P4_30865173	Ca2	30865173	GAAAAAAAA/GAAAAAAA	GCTGAGGGATTTCGAAAGTAA	ATAATACCCCTCAGGGTGGC	58.5	576	INTERGENIC	--	--	--
Ca2_P4_30887613	Ca2	30887613	AT/A	TCAAGGGAGGATTACATGGG	AAAGGGAAAGTTGCATCACG	59.7	826	INTERGENIC	--	--	--
Ca2_P4_30887676	Ca2	30887676	AA/AAGTA	TCAAGGGAGGATTACATGGG	AAAGGGAAAGTTGCATCACG	59.7	826	INTERGENIC	--	--	--

INDEL marker IDs	Chromosomes/ unanchored scaffolds	Physical positions (bp)	InDels (*Kabuli* reference genome-CDC Frontier/PII)	Forward primers (5'-3')	Reverse primers (5'-3')	Anneling temperature (0C)	Expected amplified product size (bp)	Structural annotation		Functional annotation	
								Sequence components of ***kabuli*** genome	***Kabuli*** gene accession IDs	NCBI-KOG	TFs
Ca2_P4_30890128	Ca2	30890128	AAAATAAATAAATAAATAAA/AAAATAAATAAATAAA	GATGATGTAAATGGGAGCCAA	CATTTGTTGAATGTTGCCCA	59.8	697	INTERGENIC	--	--	--
Ca2_P4_30890182	Ca2	30890182	AAA/AAAGAA	GATGATGTAAATGGGAGCCAA	GGAATTGATCCACAACTCGG	59.8	958	INTERGENIC	--	--	--
Ca2_P4_30890210	Ca2	30890210	TG/T	GATGATGTAAATGGGAGCCAA	GGAATTGATCCACAACTCGG	59.8	958	INTERGENIC	--	--	--
Ca2_P4_30897629	Ca2	30897629	TAAAAAAAA/TAAAAAAA	CCAACAGATCAAGGGGAATG	ATCGAGATCTCACCGTGGAA	60.3	548	INTERGENIC	--	--	--
Ca2_P4_30897792	Ca2	30897792	AAT/AATATAT	CCAACAGATCAAGGGGAATG	ATTGGGGTTTGGTGAAATGA	60.3	708	INTERGENIC	--	--	--
Ca2_P4_30933751	Ca2	30933751	TC/T	TCGAAACGTGTGGTGATGAT	ACAATTCCACGCAATGTGAG	60.0	895	INTERGENIC	--	--	--
Ca2_P4_30936877	Ca2	30936877	TAAAAAAA/TAAAAAAAA	TTGCACACAAGCGTTAGAGC	ATCAACCGCACACAATTCAA	60.2	393	INTERGENIC	--	--	--
Ca2_P4_30936957	Ca2	30936957	ATTTTTTTTTT/ATTTTTTTTTTTT	TTGCACACAAGCGTTAGAGC	ATCAACCGCACACAATTCAA	60.2	393	INTERGENIC	--	--	--
Ca2_P4_30937234	Ca2	30937234	CTTTTTT/CTTTTTTTT	TTGAATTGTGTGCGGTTGAT	CGTTGATGTGTCACACGTTTC	60.0	467	INTERGENIC	--	--	--
Ca2_P4_30937778	Ca2	30937778	TAAAAAAAA/TAAAAAAAAA	TGTGAAACGTGTGACACATCA	CCTCCATTCTATTTTGCATCA	59.6	665	INTERGENIC	--	--	--

INDEL marker IDs	Chromosomes/ unanchored scaffolds	Physical positions (bp)	InDels (*Kabuli* reference genome-CDC Frontier/PII)	Forward primers (5'-3')	Reverse primers (5'-3')	Anneling temperature (0C)	Expected amplified product size (bp)	Structural annotation		Functional annotation	
								Sequence components of *kabuli* genome	*Kabuli* gene accession IDs	NCBI-KOG	TFs
Ca2_P4_30937965	Ca2	30937965	CAAAAAAAA/CAAAAAAAAA	TGTGAAACGTGTGACACATCA	CATTTGGTCCATCCCGATA	59.6	961	INTERGENIC	--	--	--
Ca2_P4_30939209	Ca2	30939209	ATTTTTTTTT/ATTTTTTTTTT	TACTAGGGCCTTGCATGGTT	CCATCTAGGTGTTGGAAGCAA	59.6	630	INTERGENIC	--	--	--
Ca2_P4_30940510	Ca2	30940510	ATTTTTTTTTT/ATTTTTTTTT	CATAAGTGGGTATCCAACCGA	CATTTGCAAGCAAAATTGACA	59.7	620	INTERGENIC	--	--	--
Ca2_P4_30963597	Ca2	30963597	GTTT/GTT	CGATGGTGTAGGCAATTGTG	CAACTTTGTGTTGCTAAGGCA	60.0	110	INTERGENIC	--	--	--
Ca2_P4_30963897	Ca2	30963897	TCAG/T	CGATGGTGTAGGCAATTGTG	TGGAGTGTGAAATATGGGTGA	60.0	380	INTERGENIC	--	--	--
Ca2_P4_30968038	Ca2	30968038	CAAA/CAA	GTGTGCGAAAGGTTGGTTTT	CGAACCTACCCGATCAAGAA	60.0	168	INTERGENIC	--	--	--
Ca2_P4_30968401	Ca2	30968401	CAAAAAAAA/CAAAAAAAAA	TTCTTGATCGGGTAGGTTCG	ATTTTGCTCCCGTCTCCTTT	60.1	507	INTERGENIC	--	--	--
Ca2_P4_30968434	Ca2	30968434	ATT/ATTT	TTCTTGATCGGGTAGGTTCG	ATTTTGCTCCCGTCTCCTTT	60.1	507	INTERGENIC	--	--	--
Ca2_P4_30968501	Ca2	30968501	A/AT	TTCTTGATCGGGTAGGTTCG	ATTTTGCTCCCGTCTCCTTT	60.1	507	INTERGENIC	--	--	--
Ca2_P4_30983014	Ca2	30983014	AGTGT/AGT	ACAAAATGGGATGGATGGAA	TAATCCCAAGGCGATGCTAC	60.0	517	INTERGENIC	--	--	--

INDEL marker IDs	Chromosomes/ unanchored scaffolds	Physical positions (bp)	InDels (*Kabuli* reference genome-CDC Frontier/PII)	Forward primers (5'-3')	Reverse primers (5'-3')	Anneling temperature (0C)	Expected amplified product size (bp)	Structural annotation		Functional annotation	
								Sequence components of *kabuli* genome	*Kabuli* gene accession IDs	NCBI-KOG	TFs
Ca2_P4_30983172	Ca2	30983172	GAAAA/GAAA	GTAGCATCGCCTTGGGATTA	TCGACATTCCCATTTTATTGC	60.1	267	INTERGENIC	--	--	--
Ca2_P4_30983322	Ca2	30983322	GTTTTT/GTTTT	GTAGCATCGCCTTGGGATTA	TGTCCACCCATAAGTAATCATTTG	60.1	744	INTERGENIC	--	--	--
Ca2_P4_30983649	Ca2	30983649	CTAT/CTATAT	GCAATAAAATGGGAATGTCG	TCGTTTTGGTGCAATATTTGTT	58.0	802	INTERGENIC	--	--	--
Ca2_P4_30983718	Ca2	30983718	GAAAA/GAAA	GCAATAAAATGGGAATGTCG	ATTTCTTCGTCTGAGTGCGG	58.0	872	INTERGENIC	--	--	--
Ca2_P4_30993636	Ca2	30993636	TAAAAAAA/TAAAAAAAA	TCATTTTCCATTGTTGTCTCCTT	GCTTGCCAAAATAACCAACG	59.9	369	INTERGENIC	--	--	--
Ca2_P4_30999439	Ca2	30999439	T/TC	CCCAAACCTTTTTGGAGTTG	TAGCTTGAGCGTGGTTTCCT	59.4	625	INTERGENIC	--	--	--
Ca2_P4_30999481	Ca2	30999481	GTT/GT	CCCAAACCTTTTTGGAGTTG	TAGCTTGAGCGTGGTTTCCT	59.4	625	INTERGENIC	--	--	--
Ca2_P4_31003095	Ca2	31003095	CTCATCA/CTCATCATCA	CGACATATTATTTGGGACAAACAT	GCAATACTTCCTCTTTGGCG	59.1	368	INTERGENIC	--	--	--
Ca2_P4_31012061	Ca2	31012061	ATTTTTTTTTTTT/ATTTTTTTTTTTTTT	GAGTCGGGTCAACCAGGATA	GCGAACTTTCGAGGTTGTTG	59.9	484	INTERGENIC	--	--	--
Ca2_P4_31130636	Ca2	31130636	TAAAAAAAAA/TAAAAAAAA	TCCGTGTTTCCTCAACATCA	AAATGATAAAAGCCCAGCCC	60.1	419	INTERGENIC	--	--	--

INDEL marker IDs	Chromosomes/ unanchored scaffolds	Physical positions (bp)	InDels (*Kabuli* reference genome-CDC Frontier/PII)	Forward primers (5'-3')	Reverse primers (5'-3')	Anneling temperature (0C)	Expected amplified product size (bp)	Structural annotation		Functional annotation	
								Sequence components of *kabuli* genome	*Kabuli* gene accession IDs	NCBI-KOG	TFs
Ca2_P4_31149462	Ca2	31149462	CTTTTTT/CTTTTTTTT	CCTCATTTTCCTCCTTTCCC	GCTGTACAACTCGTGTCCCA	59.9	877	INTERGENIC	--	--	--
Ca2_P4_31149520	Ca2	31149520	TAAAAA/TAAAAAA	ATGGGTAAGGAAGGAGGTGG	GCTGTACAACTCGTGTCCCA	60.2	802	INTERGENIC	--	--	--
Ca2_P4_31240228	Ca2	31240228	TCAA/T	CTGCCCCCACTATTTCTCAA	GAAACACTTGGCATGTTAAGGA	60.1	479	INTERGENIC	--	--	--
Ca2_P4_31271510	Ca2	31271510	TAAAAAAAAAAA/TAAAAAAAAAAAAAAA	TGGGTGGGAGTAATTTTTCG	TTAAAGAGATTCGCAGCGGT	59.8	329	INTERGENIC	--	--	--
Ca2_P4_31323113	Ca2	31323113	GATATATATATATATATATA/GATATATATATATATATATA	TGTTCACCACCAAACTGGAA	TTCATTTTAAAGGCATCGTGA	60.0	901	INTERGENIC	--	--	--
Ca2_P4_31329635	Ca2	31329635	TATAATAATAATAATAATAATAATAATAATAATAATAAT/TATAATAATAATAATAATAATAATAATAATAATAATAATAAT	CGTACCGCTTGGTGGTTTTA	CACCACCACCACCATTAAACT	60.9	740	INTERGENIC	--	--	--
Ca2_P4_31450111	Ca2	31450111	TCC/TC	TTCCCAATCGATCCTACGAG	ATTGTGGAGAATCTGCAGGG	60.0	313	INTERGENIC	--	--	--
Ca2_P4_31545785	Ca2	31545785	TGG/TG	TGTGGCATGAATATTAGGTTGG	CCTAAAGTTGAGTGAAGCGAAA	59.7	526	INTERGENIC	--	--	--
Ca2_P4_31648369	Ca2	31648369	AATAT/AATATCTCATATAT	TTCACGTTGGAACTTGCTTG	ACCCGTTGGAAGATGAAATG	59.9	897	INTERGENIC	--	--	--
Ca2_P4_31745963	Ca2	31745963	AATATATATATATATATATAT/AATATATATATATATATAT	TCCATAGAGCCATGGAAAGG	ATAATGTCAGCATGTATGCAAAA	60.0	448	INTERGENIC	--	--	--

INDEL marker IDs	Chromosomes/ unanchored scaffolds	Physical positions (bp)	InDels (*Kabuli* reference genome-CDC Frontier/PII)	Forward primers (5'-3')	Reverse primers (5'-3')	Anneling temperature (0C)	Expected amplified product size (bp)	Structural annotation		Functional annotation	
								Sequence components of *kabuli* genome	*Kabuli* gene accession IDs	NCBI-KOG	TFs
Ca2_P4_31758836	Ca2	31758836	TTAATAATAATAATAATAATAATAATAATAATAATAA/TTAATAATAATAATAATAATAATAATAATAATAATAA	CGACGTTTAATTAATTCATCCCA	GGAGTCATGCCCCTTATCAA	60.1	735	INTERGENIC	--	--	--
Ca2_P4_31841304	Ca2	31841304	GATATATATATATATATATAT/GATATATATATATATATATAT	GGAGAACTCAAGTTCATACCGTG	AGGTCAGTAGTTTGGCGTGT	60.0	534	INTERGENIC	--	--	--
Ca2_P4_31846061	Ca2	31846061	TTTATTATTATTATTATTATTATTATTATTATTATT/TTTATTATTATTATTATTATTATTATTATTATTATT	GATTGGAGCACCATCCTCTC	TTCGAGACAAAACGTTCAACC	59.6	805	INTERGENIC	--	--	--
Ca2_P4_31854667	Ca2	31854667	T/TTC	GTGTTCCGCCTGATTTCATT	TCAAGGAGAAAACACAACCAAA	59.9	349	INTERGENIC	--	--	--
Ca2_P4_31856079	Ca2	31856079	ATTT/AT	TCTATCGAGAAGCAAAATGATGA	ACAAGGCTTTCAATTGTGGG	59.0	529	INTERGENIC	--	--	--
Ca2_P4_31858178	Ca2	31858178	TTTAATATT/TTT	TGTGTTTCAGAGCTGCTAGTTGA	ATGAAAGGCATCCCAAAACA	60.2	368	INTERGENIC	--	--	--
Ca2_P4_31858424	Ca2	31858424	AATTA/AA	TGTTTTGGGATGCCTTTCAT	TGTTTTTCAAATTTGGGAGGA	60.3	428	INTERGENIC	--	--	--
Ca2_P4_31858882	Ca2	31858882	CAA/CAAA	TCCTCCCAAATTTGAAAAACA	TTTTCTTATAACGGGGCAAAAA	59.4	246	INTERGENIC	--	--	--
Ca2_P4_31860805	Ca2	31860805	AC/ACC	TCTCATTCACAAATTGGCCTC	TGTGCGACATACGACATACG	60.1	241	INTERGENIC	--	--	--
Ca2_P4_31864253	Ca2	31864253	TCA/TCAACA	GCAAATCAAACAGGCCCTAA	TGACGTTTTGTTTTATGCCG	60.1	763	INTERGENIC	--	--	--

INDEL marker IDs	Chromosomes/ unanchored scaffolds	Physical positions (bp)	InDels (*Kabuli* reference genome-CDC Frontier/PII)	Forward primers (5'-3')	Reverse primers (5'-3')	Anneling temperature (0C)	Expected amplified product size (bp)	Structural annotation		Functional annotation	
								Sequence components of *kabuli* genome	*Kabuli* gene accession IDs	NCBI-KOG	TFs
Ca2_P4_31864424	Ca2	31864424	T/TA	TGGCAACAAGAAGTTAGGCA	TGACGTTTTGTTTTATGCCG	59.5	473	INTERGENIC	--	--	--
Ca2_P4_31898826	Ca2	31898826	ATTTTTTTTTTTT/ATTTTTTTTTT	TTTCCATGTCTACCCCTTGG	CAATTGACAAAAAGGTGGTTCA	59.8	517	INTERGENIC	--	--	--
Ca2_P4_3190348	Ca2	3190348	CTTT/CT	CCGATCGGGTCTATTTGTGA	TGGTTGTCATACAATGCGTG	60.9	773	INTERGENIC	--	--	--
Ca2_P4_31929843	Ca2	31929843	ACC/AC	ACGGTTTGCAGCATTGTTTT	AAAAATCCTTTGGGAGGTCG	60.5	469	INTERGENIC	--	--	--
Ca2_P4_31931093	Ca2	31931093	TT/TTAT	TCGTAACGCAAATTTTCCAA	TCCATCTCCAATTTTGTTTTCA	59.2	377	INTERGENIC	--	--	--
Ca2_P4_31931439	Ca2	31931439	TGAGAGAGA/TGAGAGAGAGA	TCGTAACGCAAATTTTCCAA	TTTTTCACTTCTTTCATCTCTCG	59.2	611	INTERGENIC	--	--	--
Ca2_P4_31933761	Ca2	31933761	AATATATA/AATA	CTTTAGCCATGCCAATTCGT	TGGTTGTTTCCTTGCTTTTT	60.1	751	INTERGENIC	--	--	--
Ca2_P4_31935770	Ca2	31935770	GTTTTTTT/GTTTTTTTTT	CACCAATGGTCTTTAATTTTTCTG	TTTTGGGACGTAGACCTGTTG	59.0	370	INTERGENIC	--	--	--
Ca2_P4_31937114	Ca2	31937114	ATTTTTTTTT/ATTTTTTTT	TTGAACCCAAGACTTTGTAATGAA	GGTTAAAGACGTAACAAGAAAAACG	59.9	614	INTERGENIC	--	--	--
Ca2_P4_31937481	Ca2	31937481	TTTC/T	AATGCCATATTGGATTTTGTTAC	TCCGATCGTTCAAATTTTCC	57.1	661	INTERGENIC	--	--	--

INDEL marker IDs	Chromosomes/ unanchored scaffolds	Physical positions (bp)	InDels (*Kabuli* reference genome-CDC Frontier/PII)	Forward primers (5'-3')	Reverse primers (5'-3')	Anneling temperature (0C)	Expected amplified product size (bp)	Structural annotation		Functional annotation	
								Sequence components of *kabuli* genome	*Kabuli* gene accession IDs	NCBI-KOG	TFs
Ca2_P4_31945715	Ca2	31945715	GAAAAAAA/GAAAAAAAA	CACATGGAAGGCTAAAGGAA	TTGAGTACCAAGGCATTGACC	57.8	614	INTERGENIC	--	--	--
Ca2_P4_31945753	Ca2	31945753	TAT/TATTTAAT	CACATGGAAGGCTAAAGGAA	TTGAGTACCAAGGCATTGACC	57.8	614	INTERGENIC	--	--	--
Ca2_P4_31950818	Ca2	31950818	GTTATAC/GTTATACGAAAATAAATTATAC	CCAATTAATAAGTTAGCGGGTGA	TGTGTGAGGAGGACAACAAAA	59.4	870	INTERGENIC	--	--	--
Ca2_P4_31950929	Ca2	31950929	GAAAAAAAA/GAAAAAAAAAAA	GCACGCTAGTGTCCGTCTTT	TGTGTGAGGAGGACAACAAAA	60.5	388	INTERGENIC	--	--	--
Ca2_P4_31953699	Ca2	31953699	ATATA/ATATAGTATA	TTCGTTTGATGAACAAGTGACA	GTGGATTGTGGGAACCTCAT	59.2	838	INTERGENIC	--	--	--
Ca2_P4_31955680	Ca2	31955680	TAAAA/TAAA	GGTTTTAAACTTGTGAGGACCG	TGTCGTTGTTTGCATTGTGA	59.9	508	INTERGENIC	--	--	--
Ca2_P4_31955771	Ca2	31955771	CTTTTTT/CTTTTT	TCACAATGCAAACAACGACA	AAAAGCGGTGGCAAATGTTA	59.7	532	INTERGENIC	--	--	--
Ca2_P4_31963928	Ca2	31963928	GTACTT/GTACTTACTT	CCTCGAGAAACCCAGCTAGA	GCATTCATGAGTTTGCCCTT	59.6	187	INTERGENIC	--	--	--
Ca2_P4_31964191	Ca2	31964191	AT/ATT	AAGGGCAAACTCATGAATGC	CAAGGAAGGAGGAAAAGGAGA	60.1	609	INTERGENIC	--	--	--
Ca2_P4_31964265	Ca2	31964265	TG/T	AAGGGCAAACTCATGAATGC	GGTGTGACAAAGGAAACGTG	60.1	674	INTERGENIC	--	--	--

INDEL marker IDs	Chromosomes/ unanchored scaffolds	Physical positions (bp)	InDels (*Kabuli* reference genome-CDC Frontier/PII)	Forward primers (5'-3')	Reverse primers (5'-3')	Anneling temperature (0C)	Expected amplified product size (bp)	Structural annotation		Functional annotation	
								Sequence components of *kabuli* genome	*Kabuli* gene accession IDs	NCBI-KOG	TFs
Ca2_P4_31981326	Ca2	31981326	AT/ATTT	ACGTTGTGTGGACAATGAGG	TCCGGAGGGTTTTTCTACCT	59.4	353	INTERGENIC	--	--	--
Ca2_P4_31983759	Ca2	31983759	ATTTTTTTTT/ATTTTTTTTTTT	GACACTTGTTGCGTATGGGA	CAATGGAGATTCCAACCTGT	59.6	555	INTERGENIC	--	--	--
Ca2_P4_31991643	Ca2	31991643	ATTTTTTTTTTT/ATTTTTTTTTTTTT	TGGTCAATGAAATTTCCGGT	GGTTGTGTGTTTTCCATCCC	60.2	230	INTERGENIC	--	--	--
Ca2_P4_32003491	Ca2	32003491	TT/TTCCT	TCCAAATAGGTAAGGTTGGCA	CCATCTATCACTTGCAGCAGTC	59.5	530	INTERGENIC	--	--	--
Ca2_P4_32007729	Ca2	32007729	AT/A	CTCTGCCGCAGTTTGATACA	AAAGTTCCGGAGAAACTTCG	60.0	337	INTERGENIC	--	--	--
Ca2_P4_32009592	Ca2	32009592	GTTTTTTT/GTTTTTTTT	TTGGCATTCTCTAGCACACG	CGAGTAGTTTCAACCGCCAT	60.0	572	INTERGENIC	--	--	--
Ca2_P4_32012870	Ca2	32012870	A/AG	AGGGCTTTACTCCTTTTGGG	TTGACAAATGAAAGGAGGTGG	59.6	471	INTERGENIC	--	--	--
Ca2_P4_32013591	Ca2	32013591	TCCC/TCC	TCTCCCTCCAAACACCAAAC	ATGATTGAACGAAGGCCAAG	59.9	480	INTERGENIC	--	--	--
Ca2_P4_32022318	Ca2	32022318	CTTT/CTT	CATGTGGTTTGAGCATCCAG	TGCAAAATTCATTGGAAGCA	60.1	443	INTERGENIC	--	--	--
Ca2_P4_32032083	Ca2	32032083	ATATTTTTATTTTT/ATATTTTTATTTTTATTTTT	TGACAACGCATACGTGGATT	AAATAACACGAAATTCTAGCCCA	60.0	312	INTERGENIC	--	--	--

INDEL marker IDs	Chromosomes/ unanchored scaffolds	Physical positions (bp)	InDels (*Kabuli* reference genome-CDC Frontier/PII)	Forward primers (5'-3')	Reverse primers (5'-3')	Anneling temperature (0C)	Expected amplified product size (bp)	Structural annotation		Functional annotation	
								Sequence components of ***kabuli*** genome	***Kabuli*** gene accession IDs	NCBI-KOG	TFs
Ca2_P4_32070956	Ca2	32070956	TAAAAAAAAAAAA/TAAAAAAAAAAAAAAA	TTTTTCCGACTTGGTCCTTT	GGAGCTGAAACTAACGCAGC	58.7	814	INTERGENIC	--	--	--
Ca2_P4_32076569	Ca2	32076569	TTAAGT/TT	GGGGAACTAATTTTGTGAATCC	GGCTTCATCTAGCAATGGTTG	58.7	598	INTERGENIC	--	--	--
Ca2_P4_32080788	Ca2	32080788	TAAAAAAAAAAAAAA/TAAAAAAAAA	TGTGCATTAAAGATGCTCTTGG	AAGCGTTGAAACCACTGTGA	60.3	441	INTERGENIC	--	--	--
Ca2_P4_32080846	Ca2	32080846	TAAAAAAAA/TAAAAAAA	TGTGCATTAAAGATGCTCTTGG	AAGCGTTGAAACCACTGTGA	60.3	441	INTERGENIC	--	--	--
Ca2_P4_32098174	Ca2	32098174	C/CAATATCT	TTAGCGCAAATGATTGGTTG	TGATGTGAAGCCAAATTCCA	59.7	742	INTERGENIC	--	--	--
Ca2_P4_32102387	Ca2	32102387	TAAA/TAAAAAAA	TTCCAAACGGAATCCATCAT	TGTGGTCTTCCTTTGGCACT	60.1	308	INTERGENIC	--	--	--
Ca2_P4_32110176	Ca2	32110176	AAAACAAA/AAAA	GCACCCTTCAAGAAGCAAAC	TTGGTCCATCGAATCACTTG	59.9	221	INTERGENIC	--	--	--
Ca2_P4_32112721	Ca2	32112721	T/TAAA	TTGTCTTTCCTATGCCACCC	TAAAGGACATGTGCAAACGC	59.9	508	INTERGENIC	--	--	--
Ca2_P4_32116015	Ca2	32116015	CTTTTTTTTT/CTTTTTTTTTTT	CCCAAAGGCCCAAATTTTAT	TGGTCCTCCAACACCAATTT	60.0	599	INTERGENIC	--	--	--
Ca2_P4_32116482	Ca2	32116482	CTAT/CTATAT	CAAATTGGTGTTGGAGGACC	ATAGGAAAAGGCGAAAAGCC	60.2	153	INTERGENIC	--	--	--

INDEL marker IDs	Chromosomes/ unanchored scaffolds	Physical positions (bp)	InDels (*Kabuli* reference genome-CDC Frontier/PII)	Forward primers (5'-3')	Reverse primers (5'-3')	Anneling temperature (0C)	Expected amplified product size (bp)	Structural annotation		Functional annotation	
								Sequence components of *kabuli* genome	*Kabuli* gene accession IDs	NCBI-KOG	TFs
Ca2_P4_32117375	Ca2	32117375	C/CTGAGGAA	GGACGTGGGTAACATAAACCAT	TTCTAGAGGCGAGTAATGTAGAAAAA	59.9	644	INTERGENIC	--	--	--
Ca2_P4_32122608	Ca2	32122608	GAC/GACAC	TTTTGTGGGAATGTGGGTTT	CACACGTTCATCCCATTCAC	60.1	324	INTERGENIC	--	--	--
Ca2_P4_32152012	Ca2	32152012	TAAAAAAAAA/TAAAAAAAAAA	CCAACTCACCACACATCACC	CCGCTACCGATTGTTTTTGT	59.8	163	INTERGENIC	--	--	--
Ca2_P4_32152633	Ca2	32152633	T/TA	GAGTTGGGTAGGGTAGGGGA	TTTCCCCTTTTGCTTGTCAT	60.2	393	INTERGENIC	--	--	--
Ca2_P4_32160769	Ca2	32160769	TCTTCACT/TCT	ACATCAATGAGATGAAGCCG	GGCCGAACACGAAAACTAAA	58.7	695	INTERGENIC	--	--	--
Ca2_P4_32167519	Ca2	32167519	TGG/TG	AGAAATGGGAGCTTGTTCCA	GAGGATGGGTGAAAAAGCAA	59.7	588	INTERGENIC	--	--	--
Ca2_P4_32168741	Ca2	32168741	ATA/ATAGTAAATTA	CCTGGCCATTGTTCCTTAGA	AAATGAGTAGATGGTGCAACGA	60.1	687	INTERGENIC	--	--	--
Ca2_P4_32209131	Ca2	32209131	T/TG	GCAAAGATAGACACCACCCC	AACCATTCCCATGGAACAAA	59.4	577	INTERGENIC	--	--	--
Ca2_P4_32209334	Ca2	32209334	CTATATATA/CTATA	TCCATGGGAATGGTTTTGAT	TTGAAGAGGAAGTTTTGGCA	60.0	342	INTERGENIC	--	--	--
Ca2_P4_32209671	Ca2	32209671	TC/TCC	TCCATGGGAATGGTTTTGAT	TTTTAGTGTGCACCAATGTCAA	60.0	899	INTERGENIC	--	--	--

INDEL marker IDs	Chromosomes/ unanchored scaffolds	Physical positions (bp)	InDels (*Kabuli* reference genome-CDC Frontier/PII)	Forward primers (5'-3')	Reverse primers (5'-3')	Anneling temperature (0C)	Expected amplified product size (bp)	Structural annotation		Functional annotation	
								Sequence components of *kabuli* genome	*Kabuli* gene accession IDs	NCBI-KOG	TFs
Ca2_P4_32210099	Ca2	32210099	CA/CAAATA	TCAGCTTCAATTATCTCCAACAA	TCGGCGACAAAAATCACATA	58.9	644	INTERGENIC	--	--	--
Ca2_P4_32213171	Ca2	32213171	GTGAT/GTGATGAT	CAACATGACAAGGACTTTCCAA	TTACCGGTATCGCAAGGAAC	60.0	529	INTERGENIC	--	--	--
Ca2_P4_32213424	Ca2	32213424	CAAA/CAAAA	AGGGATAGATAACGGCACGA	AAGGCCCTGCTAGAATGATG	59.6	504	INTERGENIC	--	--	--
Ca2_P4_32218161	Ca2	32218161	GTTTTTTTTTTT/GTTTTTTTTTT	ATTGTGCCGGAGCAACTACT	TGTTACGGCTGTGAATCTGG	59.8	138	INTERGENIC	--	--	--
Ca2_P4_32220236	Ca2	32220236	TAAAAAAAAAAA/TAAAAAAAAAAAAA	GGTGCACGGTACAAACCTCT	TCAAGTGAGCAAATGAGCAAA	60.0	305	INTERGENIC	--	--	--
Ca2_P4_32223160	Ca2	32223160	GTT/GT	AATGGACGTACCATGCATCA	GGGTTGAATCGCAGACTAGG	59.8	438	INTERGENIC	--	--	--
Ca2_P4_32245950	Ca2	32245950	TA/TAGA	AACCGACAAAAGATTGCATGT	GCAGTTGCACAAGGTGGTAA	59.5	586	INTERGENIC	--	--	--
Ca2_P4_32253447	Ca2	32253447	ACAC/A	GCTGTATGTACGCACCCCTT	TGAAATTTGCCTGTGTTGCT	60.0	468	INTERGENIC	--	--	--
Ca2_P4_32260990	Ca2	32260990	GAGAA/GA	TGTTGTTTCCTCCAAACCCT	TTTTACCTTCTTTTCCGGGG	59.4	157	INTERGENIC	--	--	--
Ca2_P4_32261025	Ca2	32261025	AAAGAGAAGAGA/AAAGAGAAGAGAAGAGA	CCCCGGAAAAGAAGGTAAAA	CCTCTCTGTCTCCACCTGCT	60.3	126	INTERGENIC	--	--	--

INDEL marker IDs	Chromosomes/ unanchored scaffolds	Physical positions (bp)	InDels (*Kabuli* reference genome-CDC Frontier/PII)	Forward primers (5'-3')	Reverse primers (5'-3')	Anneling temperature (0C)	Expected amplified product size (bp)	Structural annotation		Functional annotation	
								Sequence components of *kabuli* genome	*Kabuli* gene accession IDs	NCBI-KOG	TFs
Ca2_P4_32263139	Ca2	32263139	A/AAAAATGG	GAAAACAAAATGTCACCGGC	ATTGCCAACAACACACCAAA	60.4	282	INTERGENIC	--	--	--
Ca2_P4_32263680	Ca2	32263680	CAATA/CAATAAAAAATA	TGCAACACCAAGTCACCATT	GTCAATGATTCGCGTAGGGT	60.0	738	INTERGENIC	--	--	--
Ca2_P4_32289345	Ca2	32289345	AT/ATGT	CCTCCTAGTTGCATGGCATT	AGGCATGGTCATCGATCAGT	60.1	563	INTERGENIC	--	--	--
Ca2_P4_32289886	Ca2	32289886	GTTTTTTTT/GTTTTTTTTTTTTT	ACTGATCGATGACCATGCCT	GCAAACTGACATGCCTCACA	60.5	550	INTERGENIC	--	--	--
Ca2_P4_32291117	Ca2	32291117	CA/C	TGTGCCAATAATTTGGTTGC	AAAGCTTTGGAGGCATGCTA	59.4	532	INTERGENIC	--	--	--
Ca2_P4_32293632	Ca2	32293632	AAGAAATTCAGAAATTCA/AAGAAATTCAGAAATTCAGAAATTCA	AAACCCTACGAAGTTTGGCA	TTGTCACCCGTCCAAATACA	59.6	237	INTERGENIC	--	--	--
Ca2_P4_32293814	Ca2	32293814	CT/CTTTGT	TGTATTTGGACGGGTGACAA	TACACCCACGAAACGTGAAA	59.8	571	INTERGENIC	--	--	--
Ca2_P4_32300236	Ca2	32300236	AA/AAATA	CCCATTAAATGAAGCTCGGA	CGCATTTAGTCTTTCCCTTTTG	60.0	550	INTERGENIC	--	--	--
Ca2_P4_32325491	Ca2	32325491	T/TA	TCGTGTTTCGCGTGGTAATA	CGTATCCAGTGTCCAACACG	60.1	601	INTERGENIC	--	--	--
Ca2_P4_32332565	Ca2	32332565	AAAATAAATAAATAAATA/AAAATAAATAAATAAATAAATA	CGCCAAAGTCATTGATTGAA	CATCTTGTCAGCCATAGCCA	59.7	338	INTERGENIC	--	--	--

INDEL marker IDs	Chromosomes/ unanchored scaffolds	Physical positions (bp)	InDels (*Kabuli* reference genome-CDC Frontier/PII)	Forward primers (5'-3')	Reverse primers (5'-3')	Anneling temperature (0C)	Expected amplified product size (bp)	Structural annotation		Functional annotation	
								Sequence components of *kabuli* genome	*Kabuli* gene accession IDs	NCBI-KOG	TFs
Ca2_P4_3233452	Ca2	3233452	A/AT	ACCCTTTAACCCCAAATTGC	TGCAAAATTGCAACAAACCT	60.1	458	INTERGENIC	--	--	--
Ca2_P4_32346783	Ca2	32346783	CAAAAAACA/CAAAAAACATAAAAAACA	TCCACACCAGAAGCATTCAA	GTTCTGGAGGGAACAGCTTG	60.2	628	INTERGENIC	--	--	--
Ca2_P4_32356323	Ca2	32356323	CAAA/CAAAA	GCTAGTTGCTGTGAATGGCA	ACGTGTGGATGATTGGATGA	60.0	711	INTERGENIC	--	--	--
Ca2_P4_32356462	Ca2	32356462	T/TG	GCTAGTTGCTGTGAATGGCA	ACGTGTGGATGATTGGATGA	60.0	711	INTERGENIC	--	--	--
Ca2_P4_32356770	Ca2	32356770	CCACAC/CCAC	AGCCTCACCTCATACGGAAA	GAATTGTTGAAGGATGAGAGCA	59.7	345	INTERGENIC	--	--	--
Ca2_P4_32358785	Ca2	32358785	AAT/AATATAT	AACAACTTCTCATCCTCGCAA	ATGTATGGGCTGCTTTGCTT	59.9	700	INTERGENIC	--	--	--
Ca2_P4_32359679	Ca2	32359679	GACACA/GACACACA	TTGATGAGTTCGGTCATGGA	AGCCGCTTAATTGATCGTTG	60.0	195	INTERGENIC	--	--	--
Ca2_P4_32360594	Ca2	32360594	CTTT/CTT	TTGGATCCACCTCATTATCCA	CATGATCTCATCGGCTGCT	60.1	418	INTERGENIC	--	--	--
Ca2_P4_32361822	Ca2	32361822	ATTT/ATT	ACGTTGTATTCGCAAGAGGG	TCACCTTTTCATCAATGCTCA	60.1	263	INTERGENIC	--	--	--
Ca2_P4_32365433	Ca2	32365433	TA/T	TCATTTTATCATCCATCTTTGTGG	GAAATGATGGACAAAGTCACGA	60.1	747	INTERGENIC	--	--	--

INDEL marker IDs	Chromosomes/ unanchored scaffolds	Physical positions (bp)	InDels (*Kabuli* reference genome-CDC Frontier/PII)	Forward primers (5'-3')	Reverse primers (5'-3')	Anneling temperature (0C)	Expected amplified product size (bp)	Structural annotation		Functional annotation	
								Sequence components of *kabuli* genome	*Kabuli* gene accession IDs	NCBI-KOG	TFs
Ca2_P4_32366421	Ca2	32366421	ATCAAAATTCAAAATT/ATCAAAATT	TCGTGACTTTGTCCATCATTTC	GCTTGGCAACATTCTTCACA	60.0	739	INTERGENIC	--	--	--
Ca2_P4_32369105	Ca2	32369105	GAAAAAAAAA/GAAAAAAAA	ACGTCCCTCCACGGTAATTT	GCCACTTTCTTTGGATCGAG	60.6	539	INTERGENIC	--	--	--
Ca2_P4_32370223	Ca2	32370223	AAAAAATTAATGAAAAATTAATGAAAAATTAA/AAAAAATTAATGAAAAATTAA	TCAAGCAAGTCACAACATTTCA	TGCTATTTTAATTGTGAGCATGAGA	59.4	637	INTERGENIC	--	--	--
Ca2_P4_32378287	Ca2	32378287	GAAAAAAA/GAAAAAAAA	AGCCTCTTCCAGAATGCAAA	TAAGACCACCGACCTTCCAC	60.0	485	INTERGENIC	--	--	--
Ca2_P4_32378634	Ca2	32378634	GTTTTT/GTTTT	GTGGAAGGTCGGTGGTCTTA	ACCACACCCAATCCTTTCAA	60.0	144	INTERGENIC	--	--	--
Ca2_P4_32379021	Ca2	32379021	CATTATTATATCATTGATT/CATT	TGGAAGGTCGGTGGTCTTAG	TACCGCAATAAACTCGCACA	60.1	616	INTERGENIC	--	--	--
Ca2_P4_32379585	Ca2	32379585	AATTAATTTT/AATTAATTTTGATTAATTTT	TGTGCGAGTTTATTGCGGTA	CATTTGGTTCAAAATGTCATGG	60.3	624	INTERGENIC	--	--	--
Ca2_P4_32379797	Ca2	32379797	AGC/A	GAAACAGAAAATTGGAGCTTTCA	TGGGCTGCGAAAAGTTAGTT	59.8	566	INTERGENIC	--	--	--
Ca2_P4_32392527	Ca2	32392527	GTTT/GTTTTT	CTGAAGGCTAGGCAGGTTTG	TCGAGCCACCATCAACATTA	60.0	327	INTERGENIC	--	--	--
Ca2_P4_32393084	Ca2	32393084	T/TA	TGCCCATCCTTATGCTCTTC	ATCACGTGCAACCACAAGTC	60.2	626	INTERGENIC	--	--	--

INDEL marker IDs	Chromosomes/ unanchored scaffolds	Physical positions (bp)	InDels (*Kabuli* reference genome-CDC Frontier/PII)	Forward primers (5'-3')	Reverse primers (5'-3')	Anneling temperature (0C)	Expected amplified product size (bp)	Structural annotation		Functional annotation	
								Sequence components of *kabuli* genome	*Kabuli* gene accession IDs	NCBI-KOG	TFs
Ca2_P4_32393117	Ca2	32393117	T/TC	TGCCCATCCTTATGCTCTTC	ATCACGTGCAACCACAAGTC	60.2	626	INTERGENIC	--	--	--
Ca2_P4_32393187	Ca2	32393187	AATAT/A	TGCCCATCCTTATGCTCTTC	ATCACGTGCAACCACAAGTC	60.2	626	INTERGENIC	--	--	--
Ca2_P4_32393441	Ca2	32393441	TGG/TGTGGG	TCGCACTTTTTCGTGTAGGA	GGGCTTGTTTGACGCATTAT	59.5	588	INTERGENIC	--	--	--
Ca2_P4_32393633	Ca2	32393633	TAACATAATAA/TA	GACTTGTGGTTGCACGTGAT	GGGCTTGTTTGACGCATTAT	59.6	445	INTERGENIC	--	--	--
Ca2_P4_32395584	Ca2	32395584	AT/ATCACT	AGTGACTAGGGGCAATGGTG	AGGAAGAATATGGGTTGGGG	60.0	724	INTERGENIC	--	--	--
Ca2_P4_32396302	Ca2	32396302	GTTTTTTTTTTTT/GTTTTTATTTTTTTTTTTT	CCCCAACCCATATTCTTCCT	TTTTTGGTGGCAAAATCCAT	60.0	740	INTERGENIC	--	--	--
Ca2_P4_32399316	Ca2	32399316	CTTTTTTTT/CTTTTTTTTTTTT	TGGGGCACTCTTCAAATAGG	GCCTCTTCAAAAGTCGGTCA	60.1	215	INTERGENIC	--	--	--
Ca2_P4_32406998	Ca2	32406998	GTATATATATATATATATATATATAT/GTATATATATATAT	CAGACCCTTTCCAAGCTGAC	CGATCAAGCTTTGGTTTTGG	59.8	406	INTERGENIC	--	--	--
Ca2_P4_32424283	Ca2	32424283	TAAAAAAAAAAA/TAAAAAAAAAAAA	GCGTGATTGATGACAACACA	TTCTCCAATCAAATGAAGTCCA	59.1	866	INTERGENIC	--	--	--
Ca2_P4_32453109	Ca2	32453109	ATTTTTTT/ATTTTTTTTT	TCTTCACACAATCCCACCAA	CTCAAGTCGACAAGCCTTCC	59.9	659	INTERGENIC	--	--	--

**INDEL marker IDs**	**Chromosomes/ unanchored scaffolds**	**Physical positions (bp)**	**InDels (*Kabuli* reference genome-CDC Frontier/PII)**	**Forward primers (5'-3')**	**Reverse primers (5'-3')**	**Anneling temperature (0C)**	**Expected amplified product size (bp)**	**Structural annotation**		**Functional annotation**	
								**Sequence components of *kabuli* genome**	***Kabuli* gene accession IDs**	**NCBI-KOG**	**TFs**
Ca2_P4_3245422	Ca2	3245422	GATATATATATATATATATATATA/GATATATATATATATATATA	TTTTCAACGGAACCACAACA	CATGTCAGGCAGTTACCCCT	60.0	541	INTERGENIC	--	--	--
Ca2_P4_32478862	Ca2	32478862	TAAAAAAAAA/TAAAAAAAA	AAATTGGTAATCGGACGCTG	TGTCCACATGGGAAGTTTGA	60.0	471	INTERGENIC	--	--	--
Ca2_P4_32478897	Ca2	32478897	CTT/CTTT	AAATTGGTAATCGGACGCTG	TGTCCACATGGGAAGTTTGA	60.0	471	INTERGENIC	--	--	--
Ca2_P4_32479454	Ca2	32479454	AG/A	CAAACTTCCCATGTGGACAA	TCCTATACCACATTTAAAGCAAAA	59.4	656	INTERGENIC	--	--	--
Ca2_P4_32520939	Ca2	32520939	ATTTTTTT/ATTTTTT	AACATGGAAGATCGGTTGGA	GATCAATGCGAGTTTGTTGG	60.3	318	INTERGENIC	--	--	--
Ca2_P4_32713699	Ca2	32713699	C/CG	AACGCATCGGTAAACACTCC	GAGGTACTACAGCTTGCCCC	60.0	234	INTERGENIC	--	--	--
Ca2_P4_32713890	Ca2	32713890	AAAAACAAAA/AAAAACAAAACAAAA	AACGCATCGGTAAACACTCC	TGTTTTGCAGCAAGATGTCC	60.0	695	INTERGENIC	--	--	--
Ca2_P4_32998084	Ca2	32998084	AC/A	TCAATCCCCATCAATCATCA	ACCAACACCCCCAATTGTTA	59.7	705	INTERGENIC	--	--	--
Ca2_P4_33023522	Ca2	33023522	GATATATATATATATATATA/GATATATATATATATATATATA	TTCAAGTGGTCGTGACCGTA	CATCAAAATTTTCAAACAAAACCA	60.2	885	INTERGENIC	--	--	--
Ca2_P4_3312282	Ca2	3312282	TGAAGAAGAAGAAGAAGAAGAAGAA/TGAAGAAGAAGAAGAAGAAGAAGAAGAA	AATTGAGCCTTTTGCCATTG	TCCCTCCCTTCACTCTCCTT	60.1	215	INTERGENIC	--	--	--

INDEL marker IDs	Chromosomes/ unanchored scaffolds	Physical positions (bp)	InDels (*Kabuli* reference genome-CDC Frontier/PII)	Forward primers (5'-3')	Reverse primers (5'-3')	Anneling temperature (0C)	Expected amplified product size (bp)	Structural annotation		Functional annotation	
								Sequence components of *kabuli* genome	*Kabuli* gene accession IDs	NCBI-KOG	TFs
Ca2_P4_33253608	Ca2	33253608	GATTAAAAAAATAATTAAAA/GATTAAAAAAATAATTAAAAAAATAATTAAAA	TGATTATGGTTTTCACCTTTTCTG	TGTCAGCCACTCAAGAATCG	59.4	383	INTERGENIC	--	--	--
Ca2_P4_33257195	Ca2	33257195	ATTTTTTTTT/ATTTTTTTTTT	ATCGCATTTTGCCTTTGTTT	GCTGTCGTTTGCAGCATAAA	59.6	801	INTERGENIC	--	--	--
Ca2_P4_33278516	Ca2	33278516	ATGTTGTTGTTGTTGTT/ATGTTGTTGTTGTTGTTGTT	AGGGGTCACATGGTTTTGAA	AGGTGGTTGCTGATAGGTGG	60.2	335	INTERGENIC	--	--	--
Ca2_P4_33379763	Ca2	33379763	T/TA	GGGGAGGTTGTTTCAAAGGT	CTCTTCTCGGCCATATCAGC	60.2	284	INTERGENIC	--	--	--
Ca2_P4_33389624	Ca2	33389624	TTATATATATATATATATA/TTATATATATATATATATATA	CGGGACTTACAACCCAGAAA	GCGCAAAGCTTTTGATTCTT	60.0	498	INTERGENIC	--	--	--
Ca2_P4_33405227	Ca2	33405227	AATATATATATA/AATATATATA	AAAGAGACATGACTTTGTCCATCA	TTTCGCTACCCATCATACCC	60.0	586	INTERGENIC	--	--	--
Ca2_P4_33463714	Ca2	33463714	TAAAA/TAAAAA	TCGTTCCATATTCGGTAGCC	ACAACCGTTCCCTCTGTGAC	59.9	574	INTERGENIC	--	--	--
Ca2_P4_33553516	Ca2	33553516	TAAAAAAAAA/TAAAAAAAAAA	ATTATCGGTTCAACGGTCCA	GATGTTAATGGAGTGTCCGAGA	60.2	752	INTERGENIC	--	--	--
Ca2_P4_33610275	Ca2	33610275	GATATATATATATATATATAT/GATATATATATATATATATATAT	CTAACCCTTTGCTGGTCCAC	ACTCCCTCCCGTCAATTTTT	59.6	677	INTERGENIC	--	--	--
Ca2_P4_33642680	Ca2	33642680	TATAATAATAATAA/TATAATAATAATAATAA	TGGATAGCCAGTGACAGGAA	TTTTTGCCGATAATGGGAAC	59.2	290	INTERGENIC	--	--	--

INDEL marker IDs	Chromosomes/ unanchored scaffolds	Physical positions (bp)	InDels (*Kabuli* reference genome-CDC Frontier/PII)	Forward primers (5'-3')	Reverse primers (5'-3')	Anneling temperature (0C)	Expected amplified product size (bp)	Structural annotation		Functional annotation	
								Sequence components of *kabuli* genome	*Kabuli* gene accession IDs	NCBI-KOG	TFs
Ca2_P4_33984310	Ca2	33984310	CTATATATATATATATATATATATATA/CCATATATATATATATATATATATATATATA	TGGCTATGATCACAATTGGC	CCCTTTGCAAGAGACTCCAA	59.5	869	INTERGENIC	--	--	--
Ca2_P4_33984629	Ca2	33984629	CAAAA/CAAA	AACAAAGCAGAAATATCCTGTCG	AGCATAGCCAAAACCCCTTT	59.7	389	INTERGENIC	--	--	--
Ca2_P4_33986085	Ca2	33986085	TAC/TTTTTCCCAAAATTGAC	CGAGACAAGTTTTCCAAGCA	ACTCCAAGATCATCATCGGC	59.0	818	INTERGENIC	--	--	--
Ca2_P4_33986265	Ca2	33986265	TT/TTATAT	AACTTTGGATCTACACGCGA	ACTCCAAGATCATCATCGGC	58.4	623	INTERGENIC	--	--	--
Ca2_P4_3400176	Ca2	3400176	TA/TAAA	TCATCCACCATGAAAGCAAA	TGTCTCGTCTCAATCACTTTCAG	60.0	490	INTERGENIC	--	--	--
Ca2_P4_34028835	Ca2	34028835	AC/A	AAAGGGGTTTTGGCTATGCT	CATGATCATAATTGCATGGAAAA	60.0	229	INTERGENIC	--	--	--
Ca2_P4_34054996	Ca2	34054996	GTT/GTTT	GGTAAGGGCTCCTTCCAAAC	ATCACGCAACATAACACCCA	59.9	207	INTERGENIC	--	--	--
Ca2_P4_34073061	Ca2	34073061	TTATATATATATATATATATATATATATAT/TTATATATATATATATATATATATATATAT	TGAATGTTTTCGATGATGTGC	GATAAGTCCATTGGTCCCCTT	59.5	758	INTERGENIC	--	--	--
Ca2_P4_34087701	Ca2	34087701	CATATATATATATATATAT/CATATATATATATATATATATAT	ATGGTGGTGAGCAACTTTCC	AACAAACAGCCATTCCAAGG	60.0	707	INTERGENIC	--	--	--
Ca2_P4_34327217	Ca2	34327217	GA/GAGAA	TATGCAAGCAGCACCATAGC	GGTGGTGTTTCCAAGAAGGA	60.0	659	INTERGENIC	--	--	--

INDEL marker IDs	Chromosomes/ unanchored scaffolds	Physical positions (bp)	InDels (*Kabuli* reference genome-CDC Frontier/PII)	Forward primers (5'-3')	Reverse primers (5'-3')	Anneling temperature (0C)	Expected amplified product size (bp)	Structural annotation		Functional annotation	
								Sequence components of *kabuli* genome	*Kabuli* gene accession IDs	NCBI-KOG	TFs
Ca2_P4_34353539	Ca2	34353539	CATATATATATATATATATATATATATATAT/CATATATATATATATATATATATATATAT	CATGAGCTTGAGTCGCAAAA	TAGGAGGCATATGTGAGGGG	60.1	760	INTERGENIC	--	--	--
Ca2_P4_34353805	Ca2	34353805	GTTTTTTTT/GTTTTTTTTT	AGTTGAAGGAGTGCATGAGGA	TAGGAGGCATATGTGAGGGG	59.9	633	INTERGENIC	--	--	--
Ca2_P4_34355206	Ca2	34355206	CTTGGGCT/CTTGGGCTTTGTTGGGCT	TCTTTGATGCAATGCCAGAG	ATCATTCGAGGTGCCATCAT	59.9	975	INTERGENIC	--	--	--
Ca2_P4_34355457	Ca2	34355457	TAAAAAA/TAAAAAAA	ATTCTTGGGCTGTGTTTTGG	GGGTGATGAGGACTGGTTGT	60.0	725	INTERGENIC	--	--	--
Ca2_P4_34355554	Ca2	34355554	CAAAAAAAAAA/CAAAAAAAAAAAA	ATTCTTGGGCTGTGTTTTGG	GGGTGATGAGGACTGGTTGT	60.0	725	INTERGENIC	--	--	--
Ca2_P4_34357732	Ca2	34357732	CATATATATATATATATATATATATATATAT/CATATATATATATATATATATATATATATATATATATATATAT	CATGAGCTTGAGTCGCAAAA	GCAACAAGATGGGGACAAGT	60.1	654	INTERGENIC	--	--	--
Ca2_P4_34450150	Ca2	34450150	TTATATATATATATATATATATATATATAT/TTATATATATATATATATATATATAT	AGCTAGCTCAGAATGCAGCC	CCCACAAAACTTGCTTCGTT	59.9	324	INTERGENIC	--	--	--
Ca2_P4_34562775	Ca2	34562775	TCTTTA/TCTTTACTTTA	ATAGCGTGCAACGATCCTCT	CATAGCGCCCATAAAAGGAA	59.9	515	INTERGENIC	--	--	--
Ca2_P4_34576817	Ca2	34576817	TTATATATATATATATATATATA/TTATATATATATATATATATATA	CCTCTGCTGCATGAATTCTC	TGAGTGAAGATTGAATGATGACG	58.5	242	INTERGENIC	--	--	--
Ca2_P4_34584559	Ca2	34584559	TAAAAAAAAAA/TAAAAAAAAA	TGGCGATTTCAATTTGTGTT	CCACGAAATTATTTTTGTTAGGC	59.0	160	INTERGENIC	--	--	--

INDEL marker IDs	Chromosomes/ unanchored scaffolds	Physical positions (bp)	InDels (*Kabuli* reference genome- CDC Frontier/PII)	Forward primers (5'-3')	Reverse primers (5'-3')	Anneling temperature (0C)	Expected amplified product size (bp)	Structural annotation		Functional annotation	
								Sequence components of *kabuli* genome	*Kabuli* gene accession IDs	NCBI-KOG	TFs
Ca2_P4_34683764	Ca2	34683764	TC/TCC	GTTTTTCGCAACCACCTTTC	CGCTTTTTGCACTAACCTCC	59.6	682	INTERGENIC	--	--	--
Ca2_P4_34684386	Ca2	34684386	CAACGTTAAC/CAAC	ACAATACTGCGGCCTGTTTC	GATTCCGATCGTTTTTCACG	60.1	830	INTERGENIC	--	--	--
Ca2_P4_34684591	Ca2	34684591	TTATATATATATATATATATATATATATA/TTATATATATATATATATATATATATA	TGGAGTTTTGTGTGATGGACA	GATTCCGATCGTTTTTCACG	60.0	758	INTERGENIC	--	--	--
Ca2_P4_34684688	Ca2	34684688	CATCTT/C	CGTACGTAGCAAATGCAACAA	ATTCCGATCGTTTTTCACGA	59.8	229	INTERGENIC	--	--	--
Ca2_P4_34684923	Ca2	34684923	ATTTTTTT/ATTTTTTTT	TCGTGAAAAACGATCGGAAT	TGCTTGATTTTAATGGATGCC	60.4	550	INTERGENIC	--	--	--
Ca2_P4_34685252	Ca2	34685252	CATAAATAAATAAATAAA/CATAAATAAA	TCGTGAAAAACGATCGGAAT	TGGAGAGAAACAAAATGGGG	60.4	816	INTERGENIC	--	--	--
Ca2_P4_34685695	Ca2	34685695	ATTTTTT/ATTTTTTT	CCCCATTTTGTTTCTCTCCA	TTGTTTCTCCCCAGTTTTGG	59.9	234	INTERGENIC	--	--	--
Ca2_P4_34775299	Ca2	34775299	AATATATATATATATATATATA/AATATATATATATATATATA	CCCAAGTGGTGGTTCTGAGT	CATGCTTCACTCTTAGGGCA	60.0	473	INTERGENIC	--	--	--
Ca2_P4_34854539	Ca2	34854539	ANNNNNNNNNN/ANNNNNNNNN	GTTCTTTTGCCCGTCATCAT	TCAAATTGAATGCGGGTTTT	59.9	387	INTERGENIC	--	--	--
Ca2_P4_34983521	Ca2	34983521	AATATATATATATATATATATATATATATATATATATATA/AATATATATATATATATATATATATATATATATATATATATATATATA	CAAATGCATGCTCCCCTAAT	CTAAAAGCCCAAAGCTGCAC	59.9	450	INTERGENIC	--	--	--

INDEL marker IDs	Chromosomes/ unanchored scaffolds	Physical positions (bp)	InDels (*Kabuli* reference genome-CDC Frontier/PII)	Forward primers (5'-3')	Reverse primers (5'-3')	Anneling temperature (0C)	Expected amplified product size (bp)	Structural annotation		Functional annotation	
								Sequence components of *kabuli* genome	*Kabuli* gene accession IDs	NCBI-KOG	TFs
Ca2_P4_35125192	Ca2	35125192	CT/CTTT	GATGGCTTTGAGGCAGAGAG	AAGAAGCAAGCAGTGGAAGC	60.1	138	INTERGENIC	--	--	--
Ca2_P4_35149862	Ca2	35149862	TTATATATATATATATATAT/TTATATATATATATATATATAT	ACTCATCATCGTGCCCTTTC	AACCACACACCACCCTTCTC	60.1	428	INTERGENIC	--	--	--
Ca2_P4_35227098	Ca2	35227098	AAATAATAATAATAATAATAATAATAATAATAA/AAATAATAATAATAATAATAATAATAATAATAATAA	AGAAAATGACCCGCATCTTG	TCATTTCTTCGGTCGGTTTC	60.1	549	INTERGENIC	--	--	--
Ca2_P4_35228308	Ca2	35228308	TTTATTATTATTATTATTATTATTATCATTATTATTATTATTATTATTATTATTATTATTA/TTTATTATTATTATTATTATTATTATTATTATCATTATTATTATTATTATTATTATTATTATTA	TTATTTCACCGTCGTGTCCA	GGAAAAGGAGTCACCACCAA	60.0	624	INTERGENIC	--	--	--
Ca2_P4_35258590	Ca2	35258590	A/ATT	AAAAAGTTTTAACACGGAGGTTTTT	TGTTGTTGGATTGGACATGG	59.8	487	INTERGENIC	--	--	--
Ca2_P4_35258736	Ca2	35258736	ATTTTTT/ATTTTT	AAAAAGTTTTAACACGGAGGTTTTT	TGTTGTTGGATTGGACATGG	59.8	487	INTERGENIC	--	--	--
Ca2_P4_35259201	Ca2	35259201	ATTTTTTT/ATTTTTTTTTT	CCATGTCCAATCCAACAACA	GTTGCGAAAAAGTTCATCCG	60.2	744	INTERGENIC	--	--	--
Ca2_P4_35288109	Ca2	35288109	ATTTTTT/ATTTTTTT	TGCTTTATAGTGCTATTATGTGCCA	CGTCATTAAAAGGGAAACCAA	60.1	757	INTERGENIC	--	--	--
Ca2_P4_35288455	Ca2	35288455	TT/TTAT	TTCGATGGTATGCAGTAATTGG	TTCCCATGAAAGAGAGGTGG	59.9	433	INTERGENIC	--	--	--
Ca2_P4_35291896	Ca2	35291896	GTTTT/GTTTTT	AAAAAGTTCCGAAGCGTATTC	TTCATTTAAATTGCACAAACCAA	57.6	579	INTERGENIC	--	--	--

INDEL marker IDs	Chromosomes/ unanchored scaffolds	Physical positions (bp)	InDels (*Kabuli* reference genome-CDC Frontier/PII)	Forward primers (5'-3')	Reverse primers (5'-3')	Anneling temperature (0C)	Expected amplified product size (bp)	Structural annotation		Functional annotation	
								Sequence components of *kabuli* genome	*Kabuli* gene accession IDs	NCBI-KOG	TFs
Ca2_P4_35297760	Ca2	35297760	AACTGAACTG/AACTGAACTGTACTGAACTG	ACCAGGAAAAGAAGAAGGGC	TTTTGGGTTGGGATGACATT	59.7	535	INTERGENIC	--	--	--
Ca2_P4_35297846	Ca2	35297846	TTGA/TTGATTCAACTGAACTGA	ACCAGGAAAAGAAGAAGGGC	TTTTGGGTTGGGATGACATT	59.7	535	INTERGENIC	--	--	--
Ca2_P4_35298057	Ca2	35298057	AT/AATAGCGCTT	AATGTCATCCCAACCCAAAA	TCGCAATAAAAACATATAAAACCA	60.0	190	INTERGENIC	--	--	--
Ca2_P4_35303488	Ca2	35303488	ATTTTTT/ATTTTTTT	TTCCGAATAAGGATTTCTTGG	ATGGTTTGGGGAAAAGAACC	58.2	137	INTERGENIC	--	--	--
Ca2_P4_35304698	Ca2	35304698	C/CG	GCTTGGAAACAGCCTCAGAC	CAGGGTGGTACTGCAATGTG	60.0	536	INTERGENIC	--	--	--
Ca2_P4_35329169	Ca2	35329169	AATATATATATATATATATATATATAT/AATATATATATATATATATATAT	CAATTTTGGAAGACGATTTTGA	TGTCCCGAAAAAGTTCATCC	59.1	330	INTERGENIC	--	--	--
Ca2_P4_35359085	Ca2	35359085	TTATATATATATATATATATATATATATATAT/TTATATATATATATATATATATATATAT	CGTCGAATCCTAGAGCGAAT	TGGTGTTGGAGTCATACTTTTCA	59.4	306	INTERGENIC	--	--	--
Ca2_P4_354010	Ca2	354010	TTCTCTCTCTCTCTCTCTCTCTCTCTCTC/TTCTCTCTCTCTCTCTCTCTCTCTCTC	GAGGAATTGGAACGCGTAAA	TGATTCAGGGCTTTTGATCC	60.1	675	INTERGENIC	--	--	--
Ca2_P4_35425064	Ca2	35425064	GAAAAAAAAAA/GAAAAAAAAAAA	TGTCGAAATGATTAGCAAAAGAA	ACATTTTCCCTCCCTTGGTC	58.9	257	INTERGENIC	--	--	--
Ca2_P4_35524033	Ca2	35524033	CTTTTTTTTT/CTTTTTTTTTT	TGTCGACCACTAAAATTTCAAAAA	ACAAAAATCCATACCTGCGG	59.9	647	INTERGENIC	--	--	--

INDEL marker IDs	Chromosomes/ unanchored scaffolds	Physical positions (bp)	InDels (*Kabuli* reference genome-CDC Frontier/PII)	Forward primers (5'-3')	Reverse primers (5'-3')	Anneling temperature (0C)	Expected amplified product size (bp)	Structural annotation		Functional annotation	
								Sequence components of *kabuli* genome	*Kabuli* gene accession IDs	NCBI-KOG	TFs
Ca2_P4_35559534	Ca2	35559534	ATTTTTTTTTTT/ATTTTTTTTTTTT	CCAAAATATCCACCGTGTTT	TTCCTGGACGAAAATGAACC	57.3	582	INTERGENIC	--	--	--
Ca2_P4_35581740	Ca2	35581740	AATATATATATATATATATATATATATA/AATATATATATATATATATATATA	TTGCCACTTGATACAGCAAAA	TTGAAAGGATAACATGGCCG	59.4	690	INTERGENIC	--	--	--
Ca2_P4_35648294	Ca2	35648294	GC/G	TGATACACGGTCCACACTTCA	TCGCTTTCCACCAAATTTTC	60.0	463	INTERGENIC	--	--	--
Ca2_P4_35656426	Ca2	35656426	AATATATATATATATATATATATATA/AATATATATATATATATATATA	TGGTGTTGGAGTCATATTTTTCA	AACTAGTGGTAACCCGTGCG	59.4	231	INTERGENIC	--	--	--
Ca2_P4_35657396	Ca2	35657396	AAGAGAGAGA/AAGAGAGAGAGA	GCCGTGATTTGGTGGTAAAT	CCTCCAACAAAAAGGAACCA	59.7	295	INTERGENIC	--	--	--
Ca2_P4_35737843	Ca2	35737843	TG/T	TACCGGAAATTTCAACGAGG	TTTATGGTTGGGCAACTTTTT	59.9	821	INTERGENIC	--	--	--
Ca2_P4_35789864	Ca2	35789864	AATTTGATTT/AATTTGATTTGATTT	TGCCTGCGAGATCTTAATCA	CCAAATCGAACCCAAATCAC	59.5	365	INTERGENIC	--	--	--
Ca2_P4_35815027	Ca2	35815027	ATTTTTTTT/ATTTTTTT	TAAGACCATGCATCTCACGC	CGTGCATGGATCAAACTAGG	59.8	650	INTERGENIC	--	--	--
Ca2_P4_35822980	Ca2	35822980	AATATATATATATATATATA/AATATATATATATATATATATATATA	TTAATCGCAGCGTGCAATAG	GAAAATTAGATTGCCGGAAGAA	60.0	261	INTERGENIC	--	--	--
Ca2_P4_35853696	Ca2	35853696	AAATAA/AAA	GTTGCCCAGCTTGTTCTTTC	GGGAGAGTGGGTGTTTATGC	59.9	404	INTERGENIC	--	--	--

INDEL marker IDs	Chromosomes/ unanchored scaffolds	Physical positions (bp)	InDels (*Kabuli* reference genome-CDC Frontier/PII)	Forward primers (5'-3')	Reverse primers (5'-3')	Anneling temperature (0C)	Expected amplified product size (bp)	Structural annotation		Functional annotation	
								Sequence components of *kabuli* genome	*Kabuli* gene accession IDs	NCBI-KOG	TFs
Ca2_P4_35854793	Ca2	35854793	TTTAAAGTATGTAAGAA/T	CAAAAGGTAGATGATG CCGAA	CCAATGCATTTTAGTTG TGGTG	60.1	526	INTERGENIC	--	--	--
Ca2_P4_35855144	Ca2	35855144	AAACTAAAATCACCACAACTAAAAT/ AAACTAAAAT	CAAAAGGTAGATGATG CCGAA	AGCCTCTGAAGCAAAA ACCA	60.1	962	INTERGENIC	--	--	--
Ca2_P4_35855722	Ca2	35855722	C/CG	TGGTTTTTGCTTCAGA GGCT	CTCAACATGTCCACCA GCAC	60.0	592	INTERGENIC	--	--	--
Ca2_P4_35855864	Ca2	35855864	ACAAT/A	TGGTTTTTGCTTCAGA GGCT	CTCAACATGTCCACCA GCAC	60.0	592	INTERGENIC	--	--	--
Ca2_P4_35866504	Ca2	35866504	CTATTATTATTATTATTA/CTATTATT ATTATTATTATTA	GAAGAGGAGGGTAATT TGGGA	AGACTTTCATGCATGC CCTT	59.4	594	INTERGENIC	--	--	--
Ca2_P4_35871808	Ca2	35871808	AACTC/A	GATGAAATCCAATCCA TGCC	TTTCCATTCCATGGGAA AAA	60.1	871	INTERGENIC	--	--	--
Ca2_P4_35872708	Ca2	35872708	T/TC	CCCTGACCTTGAAATA TGCC	TTTGTTTGCAAATCATT CACG	59.4	645	INTERGENIC	--	--	--
Ca2_P4_35883864	Ca2	35883864	TCCCCC/TCCCCCC	ATGCCACAGCCAAATT CATA	TTGTTCCACCAATTCCC AGT	59.0	536	INTERGENIC	--	--	--
Ca2_P4_35885474	Ca2	35885474	TACAC/TAC	TGGTCAAACACATGAC CAAA	ACATGAGGAAGGAGAC CACG	58.4	382	INTERGENIC	--	--	--
Ca2_P4_35892205	Ca2	35892205	AAAAATAAAAT/AAAAATAAAATAAAA T	CAACGACGAGTTTCAA GCAA	CATGTGTTTGGCTTGG TTTG	60.0	878	INTERGENIC	--	--	--

INDEL marker IDs	Chromosomes/ unanchored scaffolds	Physical positions (bp)	InDels (*Kabuli* reference genome-CDC Frontier/PII)	Forward primers (5'-3')	Reverse primers (5'-3')	Anneling temperature (0C)	Expected amplified product size (bp)	Structural annotation		Functional annotation	
								Sequence components of *kabuli* genome	*Kabuli* gene accession IDs	NCBI-KOG	TFs
Ca2_P4_35892945	Ca2	35892945	CAAA/CAA	CTCCAAATGGCAACCAAAAT	TTTAGGGGTGGACAAAATTCA	59.8	510	INTERGENIC	--	--	--
Ca2_P4_35893811	Ca2	35893811	ATTTTTTT/ATTTTTTTT	GGTTAGCTTGGCCCAAAAGT	GGGTAGAGTGGAGGAAAGGG	60.5	628	INTERGENIC	--	--	--
Ca2_P4_35924188	Ca2	35924188	TTAATGTAATGTAA/TTAATGTAA	TCCAACCTCAATCACCAATG	ACAACATCAGCCATGTCCAA	59.3	907	INTERGENIC	--	--	--
Ca2_P4_35925409	Ca2	35925409	CCTGT/CCTGTGGCTGT	TTGTTCAGTGACCTGCAAGC	TGGTGGGTTATCCCAATAAA	60.0	750	INTERGENIC	--	--	--
Ca2_P4_35926054	Ca2	35926054	AATATATA/AATATA	ATTGGGATAACCCACCAACA	GAGAAGATTGAAGGAGGGGC	59.9	624	INTERGENIC	--	--	--
Ca2_P4_35926372	Ca2	35926372	T/TA	ATTGGGATAACCCACCAACA	ATGCGCAAAGAGCCTTGTAT	59.9	691	INTERGENIC	--	--	--
Ca2_P4_35939441	Ca2	35939441	TTTTGTT/TTT	TCTCCGGTTTCTAAGGCAAA	GGGCTCCACAAAAATCTCAA	59.8	413	INTERGENIC	--	--	--
Ca2_P4_35940345	Ca2	35940345	GTTT/G	GCCGCCAAAGATTAAAATTG	AAGGAGGATTAGGGATGCGT	59.6	385	INTERGENIC	--	--	--
Ca2_P4_35957274	Ca2	35957274	ATTTTTTTT/ATTTTTTTTT	TCACCTCATTTTTGTTGGCA	TTTATTATATCACGGATTCACGGA	60.1	526	INTERGENIC	--	--	--
Ca2_P4_35961541	Ca2	35961541	ATTTTTTTT/ATTTTTTTTTT	TCATCAAAGACATTCGTGCC	TTCGACAATATGGCCAATGA	59.7	573	INTERGENIC	--	--	--

INDEL marker IDs	Chromosomes/ unanchored scaffolds	Physical positions (bp)	InDels (*Kabuli* reference genome- CDC Frontier/PII)	Forward primers (5'-3')	Reverse primers (5'-3')	Anneling temperature (0C)	Expected amplified product size (bp)	Structural annotation		Functional annotation	
								Sequence components of *kabuli* genome	*Kabuli* gene accession IDs	NCBI-KOG	TFs
Ca2_P4_35961843	Ca2	35961843	TAAA/TAA	TCATCAAAGACATTCGTGCC	TTCGACAATATGGCCAATGA	59.7	573	INTERGENIC	--	--	--
Ca2_P4_35961921	Ca2	35961921	TAAAA/TAAA	AGACGCTCATCACTCAATTTCA	TTCGACAATATGGCCAATGA	59.9	251	INTERGENIC	--	--	--
Ca2_P4_35962691	Ca2	35962691	ATTTTTTT/ATTTTTTTT	TGAGTCATGAGATCGACGAAA	TTCAAATTTAACGGTTGGATCA	59.4	367	INTERGENIC	--	--	--
Ca2_P4_35962726	Ca2	35962726	GAAAA/GAAAAA	TGAGTCATGAGATCGACGAAA	TTCAAATTTAACGGTTGGATCA	59.4	367	INTERGENIC	--	--	--
Ca2_P4_35971845	Ca2	35971845	TAT/TATATCACAT	GCGGAGCAGAACCCTTATTA	TGACTTTTTATTTTTCCGCACTC	59.3	955	INTERGENIC	--	--	--
Ca2_P4_35995557	Ca2	35995557	TAAAAAAAA/TAAAAAAAAAAAA	CGTGTGAGTTTCGTCGCTAA	TGGTTTGGGTGGTTCATTTT	60.1	499	INTERGENIC	--	--	--
Ca2_P4_35995631	Ca2	35995631	TCAAAAAAACAAAAA/TCAAAAAAACAAAAAAACAAAAA	CGTGTGAGTTTCGTCGCTAA	TGGTTTGGGTGGTTCATTTT	60.1	499	INTERGENIC	--	--	--
Ca2_P4_35996261	Ca2	35996261	TTCTCT/TTCT	CCATTCGTCAACGGCATAAT	AAAATCAACGACGATGAATAAGAA	60.7	330	INTERGENIC	--	--	--
Ca2_P4_36001001	Ca2	36001001	AAA/AAATAAATAA	CGCGTTGGTACTTTCATGTG	TCCCAAGTTAGGGGTAAGGC	60.2	386	INTERGENIC	--	--	--
Ca2_P4_36001500	Ca2	36001500	T/TC	GCCTTACCCCTAACTTGGGA	AAAAAGTCGTCGGGTCAATG	60.3	333	INTERGENIC	--	--	--

INDEL marker IDs	Chromosomes/ unanchored scaffolds	Physical positions (bp)	InDels (*Kabuli* reference genome-CDC Frontier/PII)	Forward primers (5'-3')	Reverse primers (5'-3')	Anneling temperature (0C)	Expected amplified product size (bp)	Structural annotation		Functional annotation	
								Sequence components of *kabuli* genome	*Kabuli* gene accession IDs	NCBI-KOG	TFs
Ca2_P4_36004626	Ca2	36004626	CAAAAAAAA/CAAAAAAA	CATTGTGGTGGTTTTCCTCC	AAGATACCTATCAACGGGAAAGA	60.2	539	INTERGENIC	--	--	--
Ca2_P4_36004750	Ca2	36004750	TAAAAAAAAAA/TAAAAAAAA	TCTTTCCCGTTGATAGGTATCTT	CACACACAAATCAAATGAGACAA	58.2	473	INTERGENIC	--	--	--
Ca2_P4_36005345	Ca2	36005345	TTTTAAAATTTAAAAT/TTTTAAAATTTAAAATTTAAAAT	TTGTCTCATTTGATTTGTGTGTG	TTAGTTGACATAGCGGTTGAAA	58.6	641	INTERGENIC	--	--	--
Ca2_P4_36047163	Ca2	36047163	G/GTGC	GGAAAAATCCGGCAGTGTTA	TCTTGGAGAGCGAGAGAAGC	59.9	589	INTERGENIC	--	--	--
Ca2_P4_36065466	Ca2	36065466	TTCTCTCTCTCTCTCTCT/TTCTCTCTCTCTCTCTCTCT	TGGGCACTCTCTTCCACTCT	GAACCACCAAACCAAACACC	60.0	500	INTERGENIC	--	--	--
Ca2_P4_36077479	Ca2	36077479	ATT/AT	TGAATGCTCACATCAACAATGA	TTAGCATCCGTCAAAACCAA	60.1	334	INTERGENIC	--	--	--
Ca2_P4_36093943	Ca2	36093943	CATTTTGAAATTT/CATTTTGAAATTTTGAAATTT	CAGTGAAAGGTTCGGGTCAT	TTGCAAGAAGCTGGAATGTG	60.0	674	INTERGENIC	--	--	--
Ca2_P4_36097983	Ca2	36097983	GTATATATATATATATATATATATATATATATATATATA/GTATATATATATATATATATA	TAAAGGACCAAGTCGGGAAA	ACCGTACGTGGTTCATTTTT	59.5	803	INTERGENIC	--	--	--
Ca2_P4_36146623	Ca2	36146623	GAAAAAA/GAAAAA	AAACAGCAACAAAAATCCCAA	TTTGGAAATCAAGAGGTGGG	59.5	201	INTERGENIC	--	--	--
Ca2_P4_36146925	Ca2	36146925	TTTATTATTATTAT/TTTATTATTATTATTAT	AGGGAAACCCACCTCTTGAT	AAATCCAAAGTGCTTGCGTT	59.8	623	INTERGENIC	--	--	--

INDEL marker IDs	Chromosomes/ unanchored scaffolds	Physical positions (bp)	InDels (*Kabuli* reference genome-CDC Frontier/PII)	Forward primers (5'-3')	Reverse primers (5'-3')	Anneling temperature (0C)	Expected amplified product size (bp)	Structural annotation		Functional annotation	
								Sequence components of *kabuli* genome	*Kabuli* gene accession IDs	NCBI-KOG	TFs
Ca2_P4_36146995	Ca2	36146995	ACAACCCCA/ACA	AGGGAAACCCACCTCTTGAT	AAATCCAAAGTGCTTGCGTT	59.8	623	INTERGENIC	--	--	--
Ca2_P4_36177749	Ca2	36177749	ATTTTTT/ATTTTTTTT	TAGTTGAAGGCCATGCAAGA	AGTACTCCAGACCCCCAACA	59.4	566	INTERGENIC	--	--	--
Ca2_P4_36290906	Ca2	36290906	ACCCCCCCCCCC/ACCCCCCCCC	TGCGATGTGCTGACAGTGTA	CGTGCGTCCACTGAAAATTA	60.1	884	INTERGENIC	--	--	--
Ca2_P4_36463610	Ca2	36463610	GTTTTTTTTT/GTTTTTTTTTT	GCCCTGATATACCAACCCCT	TACACACCACACGCTTCACA	60.0	912	INTERGENIC	--	--	--
Ca2_P4_36464328	Ca2	36464328	CTCGAGTT/CTCGAGTTCGAGTT	TGTGAAGCGTGTGGTGTGTA	AGGACTAACGGGAATTGGAG	59.8	754	INTERGENIC	--	--	--
Ca2_P4_36493996	Ca2	36493996	CATATATATATATATATATATAT/CATATATATATATATATATATAT	TCTTTCACTCACTCCCCTTTG	GAGAGATACGCGGATGGAAA	59.3	335	INTERGENIC	--	--	--
Ca2_P4_366324	Ca2	366324	ATTTTTTTTTTT/ATTTTTTTTTTTT	CAAGGGGCATTACCTTACCA	CTCCTCTCTTGCTTTTGGCA	59.8	727	INTERGENIC	--	--	--
Ca2_P4_3687741	Ca2	3687741	AT/ATT	GGACAAAATGGCAAGGAAGA	TTCCTACGCTTTTGAAACAATTT	60.1	223	INTERGENIC	--	--	--
Ca2_P4_3726404	Ca2	3726404	CTTTTTTTTTTTT/CTTTTTTTTTTT	TCTCCAGTTTTGGTCCTCCA	AATCGCAAAAGTAATCCCCC	60.6	252	INTERGENIC	--	--	--
Ca2_P4_37373	Ca2	37373	TTATATATATATATATATATATAT/TTATATATATATATATATAT	TCATGGTCTGGGTTTGAACT	TGAATCGCGAAATTAGTCCC	58.0	708	INTERGENIC	--	--	--

INDEL marker IDs	Chromosomes/ unanchored scaffolds	Physical positions (bp)	InDels (*Kabuli* reference genome-CDC Frontier/PII)	Forward primers (5'-3')	Reverse primers (5'-3')	Anneling temperature (0C)	Expected amplified product size (bp)	Structural annotation		Functional annotation	
								Sequence components of *kabuli* genome	*Kabuli* gene accession IDs	NCBI-KOG	TFs
Ca2_P4_3857450	Ca2	3857450	TTTTATTTATTTATTTATTTATTTATTT/TTTTATTTATTTATTTATTTATTTATTTATTTATTTATTT	GGGAAATTGTGGTGTGAGGT	TTCGTGCAACGCTATAATTCC	59.7	513	INTERGENIC	--	--	--
Ca2_P4_3937629	Ca2	3937629	GATAAATAAATAAATAAATAAATAAATAAATAA/GATAAATAAATAAATAAATAAATAAATAAATAAATAA	AAGTGTTTTGGTGCTGTCCC	GACTTCTATTGCCCCCACAA	60.0	154	INTERGENIC	--	--	--
Ca2_P4_3949908	Ca2	3949908	TAAAAAAAAAAAA/TAAAAAAAAAAAAA	TCAAAGAATAGAGGTATTGTCTCCG	CTAAAGCCTGCCCATTTCTG	60.0	795	INTERGENIC	--	--	--
Ca2_P4_3955516	Ca2	3955516	TATAATAATAATAATAATAATAA/TATAATAATAATAATAATAATAATAATAA	TTTCCGATTTCGAGGTGTTC	TGAATAGTGGAAAACACAACAATG	60.1	385	INTERGENIC	--	--	--
Ca2_P4_4057856	Ca2	4057856	AATATATATATATATATA/AATATATATATATATA	TGCAAAATGGAGATTTGTGG	TGATGGGAGCATGTGACACT	59.5	712	INTERGENIC	--	--	--
Ca2_P4_4060085	Ca2	4060085	AATATATATATATATATATATATATATATA/AATATATATATATATATATATATATATA	GACCTTGCATGGAGTGTTGA	GGCATAGCCATGAAGAACTTG	59.7	690	INTERGENIC	--	--	--
Ca2_P4_416248	Ca2	416248	TTATATATATATATATATATATATATATATAT/TTATATATATATATATATATATATAT	ATAAATGAAGGGGTGGGAGG	GTTTCCCATAAACAGCACCC	60.0	882	INTERGENIC	--	--	--
Ca2_P4_42172	Ca2	42172	TTATATATATATATATATATATATATA/TTATATATATATATATATA	TGGCAGTCTGTTTCACGTTT	CAAGGGACCAGATTCTGCAT	59.3	553	INTERGENIC	--	--	--
Ca2_P4_4266265	Ca2	4266265	A/ATTAATCCGAAAG	TGGACGTTTAACGGTTGACA	CCCAGTAGAAAAGAAGGGGC	60.0	705	INTERGENIC	--	--	--
Ca2_P4_428237	Ca2	428237	GAAAA/GAAAAA	GTGAAACCAACCCCAAAATG	GACTCTTTGCCCTTCAATCG	60.1	181	INTERGENIC	--	--	--

INDEL marker IDs	Chromosomes/ unanchored scaffolds	Physical positions (bp)	InDels (*Kabuli* reference genome-CDC Frontier/PII)	Forward primers (5'-3')	Reverse primers (5'-3')	Anneling temperature (0C)	Expected amplified product size (bp)	Structural annotation		Functional annotation	
								Sequence components of ***kabuli*** genome	***Kabuli*** gene accession IDs	NCBI-KOG	TFs
Ca2_P4_4285697	Ca2	4285697	TAAAA/TAAA	CCAGTCCCGAAATTAAACGA	ACTTGCGATAATCGATTGGG	59.9	613	INTERGENIC	--	--	--
Ca2_P4_4383503	Ca2	4383503	GTTTTTTTTT/GTTTTTTTT	GTTGGATTTATGGTGGCGAT	CTTTCAAAGCCTTGTCTGGC	59.7	221	INTERGENIC	--	--	--
Ca2_P4_4502043	Ca2	4502043	ANNNNNNNNNN/ANNNNNN	GCTTTGGGTGAACAAATGCT	AGGCACCCTTCAACAAAATG	60.1	258	INTERGENIC	--	--	--
Ca2_P4_4527479	Ca2	4527479	GAAGACAAAAAAAACATAAGACAAAAAAAACAT/GAAGACAAAAAAAAACATAAGACAAAAAAAAACATAAGACAAAAAAAACAT	GCAATTTCACCTCTTCTGCC	TGACGAAGCCCACAATAACA	59.8	523	INTERGENIC	--	--	--
Ca2_P4_4557115	Ca2	4557115	GTTTTT/GTTTTTT	ACGGTTGAGAATTGAGTGGC	AAAAGCTCGCGAATGTCAGT	60.1	677	INTERGENIC	--	--	--
Ca2_P4_4639205	Ca2	4639205	TAAAAAAAAAAA/TAAAAAAAAAA	AACACTCGTACCTTTATTTCTGGG	ATGCAGCAATTTCCATCCTC	59.8	950	INTERGENIC	--	--	--
Ca2_P4_4655023	Ca2	4655023	AATATATATATATATATATATATATATATA/AATATATATATATATATATATATATATA	AATATGCGGAATTTGGTCCT	TTGTGTTTCCGTAGCACGAG	58.4	891	INTERGENIC	--	--	--
Ca2_P4_4655248	Ca2	4655248	CAAAAAAAAAAA/CAAAAAAAAAA	CAAAAGGACCACAAAATTGG	TTGTGTTTCCGTAGCACGAG	58.0	630	INTERGENIC	--	--	--
Ca2_P4_4655453	Ca2	4655453	TTATATATATATATATATATATATATATATATATATATA/TTATATATATATATATATATATATATATATATATATATATATA	TCGTGCTACGGAAACACAAC	TCCTTTTCACGAGGTCATCC	59.8	370	INTERGENIC	--	--	--
Ca2_P4_4793170	Ca2	4793170	GATAATAATAATAATAATAATAATAAT/GATAATAATAATAATAATAATAATAATAAT	GCCATGTGGAAGAACAAGGT	CCAAAACTGGGGAGAAACAA	60.0	669	INTERGENIC	--	--	--

INDEL marker IDs	Chromosomes/ unanchored scaffolds	Physical positions (bp)	InDels (*Kabuli* reference genome-CDC Frontier/PII)	Forward primers (5'-3')	Reverse primers (5'-3')	Anneling temperature (0C)	Expected amplified product size (bp)	Structural annotation		Functional annotation	
								Sequence components of *kabuli* genome	*Kabuli* gene accession IDs	NCBI-KOG	TFs
Ca2_P4_4850252	Ca2	4850252	GTTATTATTATTATTATTATTATTATTATTATTATTATTATTATTATTATT/GTTATTATTATTATTATTATTATTATTATTATTATTATTATTATTATTATT	AAATTCATCCTCGCTCGCTA	TTTCCCGACTTGGTCCTTTA	59.9	658	INTERGENIC	--	--	--
Ca2_P4_4891048	Ca2	4891048	TTTTATTTATTTATTTATTTATTTATTT/TTTTATTTATTTATTTATTTATTTATTTATTTATTT	AACGGTGTGAAAGGGAAATG	TGAATTGAATTTGGAAGGGG	59.8	515	INTERGENIC	--	--	--
Ca2_P4_4891335	Ca2	4891335	AAATTGAAG/A	AACGGTGTGAAAGGGAAATG	TGAATTGAATTTGGAAGGGG	59.8	515	INTERGENIC	--	--	--
Ca2_P4_4892794	Ca2	4892794	CATATATATATATATATATATATATATATATAT/CATATATATATATATATATATATATATATATATATATATATATATATATATAT	GCGGACATAAGGAAGATGTGA	AGGTCTGCGAAGATGGAAAA	60.1	606	INTERGENIC	--	--	--
Ca2_P4_4894086	Ca2	4894086	TA/TAGACAACTCAA	TTGAACTACAACCAGCGCAC	TTTTGTTCAATTTCTTGCTCCA	59.9	493	INTERGENIC	--	--	--
Ca2_P4_4894836	Ca2	4894836	ATGATT/AT	AAAGGGAAGAAATTTCGGGA	AACCGGACGATCATTTGAAG	59.9	498	INTERGENIC	--	--	--
Ca2_P4_4895677	Ca2	4895677	AA/AATATGA	CCAGGATAAAACAGCCCAGA	TGGATGCTTTCCCAACTTTC	60.1	467	INTERGENIC	--	--	--
Ca2_P4_4915954	Ca2	4915954	AAATAATAATAATAATAATAATAATAATAATAATAATAATAATAATAATAA/AAATCATAATAATAATAATAATAATAATAATAATAATAATAATAATAATAATAATAA	TTATCGGACATTTTCCCGAG	CTAGGTCGCCCTTTCAGGTT	59.9	504	INTERGENIC	--	--	--
Ca2_P4_4966099	Ca2	4966099	A/ATGTTTTGCGCGGC	GGATGACATGGCTTTCAACA	GATGTTTCTTCTTAGCGGCG	59.5	455	INTERGENIC	--	--	--
Ca2_P4_5112695	Ca2	5112695	CGAATGAAT/CGAAT	TTGCTGTTTTCCTGTTGCTG	GGACAAAGCCCAGATTTTCA	60.0	381	INTERGENIC	--	--	--

INDEL marker IDs	Chromosomes/ unanchored scaffolds	Physical positions (bp)	InDels (*Kabuli* reference genome-CDC Frontier/PII)	Forward primers (5'-3')	Reverse primers (5'-3')	Anneling temperature (0C)	Expected amplified product size (bp)	Structural annotation		Functional annotation	
								Sequence components of *kabuli* genome	*Kabuli* gene accession IDs	NCBI-KOG	TFs
Ca2_P4_5139260	Ca2	5139260	AATATATATATATATATATATATATATATATATATAT/AATATATATATATATATATATATATATATATATATATATATAT	GTAATTGACGCGTGTGATGC	GATAAAATGCAGAGGCGGAG	60.2	537	INTERGENIC	--	--	--
Ca2_P4_5154866	Ca2	5154866	AAAATTG/A	TGTGTCGTGGTTTCTGTCTTG	TGAATTCATGGCTAAGAAGCCT	59.8	516	INTERGENIC	--	--	--
Ca2_P4_5181511	Ca2	5181511	ATATTATTATTATTATTATTATTATTATTATTATTATTATTATTATT/ATATTATTATTATTATTATTATTATTATTATTATTATTATTATTATTATTATTATTATTATT	GGTGGTTCCCTTTGCCTAAT	AGGAAAAATTGGCTTCCCAT	60.2	208	INTERGENIC	--	--	--
Ca2_P4_5312458	Ca2	5312458	TCC/TC	TTTGAGGGATCAGATTTGCC	AGCTTGCCAACTTCTGGGTA	60.0	531	INTERGENIC	--	--	--
Ca2_P4_5447710	Ca2	5447710	ATACATACATACTTAC/ATAC	ACAACCCCCATAGTGTGCAT	TTTCCACTTTCAATTTCGGG	60.1	619	INTERGENIC	--	--	--
Ca2_P4_5456582	Ca2	5456582	ATTTTTTT/ATTTTTTTT	CCTTCGTCTTTTGTATCCGC	CTTCTTGTTGCCTCCTCCTG	59.7	504	INTERGENIC	--	--	--
Ca2_P4_5502541	Ca2	5502541	TTCTCTCTCTCTCTCTCTCTCTCT/TTCTCTCTCTCTCTCTCTCTCTCTCT	CCAACCCTATTAATGGCAGC	TGGAATGAACAAACACCATGA	59.4	633	INTERGENIC	--	--	--
Ca2_P4_5595728	Ca2	5595728	AATATATATATATATATATATA/AATATATATATATATATATATATA	TTGCCACTTGATACAGCAAAA	CGCGTATTTATCGTGTGGAA	59.4	652	INTERGENIC	--	--	--
Ca2_P4_6082309	Ca2	6082309	AATATATATATATATATATA/AATATATATATATATATA	AGTTGGCCTAACACGACGAC	GGATGAAGAAAACCTTCGCA	60.2	368	INTERGENIC	--	--	--
Ca2_P4_6306996	Ca2	6306996	GTT/GT	CTGTTCAGCTTTTTCGTCCC	CTCCCCACCCTTAACTTGGT	59.9	922	INTERGENIC	--	--	--

INDEL marker IDs	Chromosomes/ unanchored scaffolds	Physical positions (bp)	InDels (*Kabuli* reference genome-CDC Frontier/PII)	Forward primers (5'-3')	Reverse primers (5'-3')	Anneling temperature (0C)	Expected amplified product size (bp)	Structural annotation		Functional annotation	
								Sequence components of *kabuli* genome	*Kabuli* gene accession IDs	NCBI-KOG	TFs
Ca2_P4_6505178	Ca2	6505178	TA/TAA	CGTAATAACGGCGAGGAAAA	TGAGCAGGGGGAAGAGTAGA	60.1	548	INTERGENIC	--	--	--
Ca2_P4_6513260	Ca2	6513260	AAATAATAATAATAATAATAATAATAATAATAAT/AAATAATAATAATAATAATAATAATAATAAT	ATGGTCCAAGGAAATTGTGC	AGTCGTCGAGGCCAAATCTA	59.8	265	INTERGENIC	--	--	--
Ca2_P4_661366	Ca2	661366	G/GGGGTTAA	AAGCTCAACATGCAAAACCC	TCCCAACCCAAATAAACCAA	60.1	412	INTERGENIC	--	--	--
Ca2_P4_6640316	Ca2	6640316	TAAAAAA/TAAAAAAA	ATGGCCACGAATCTGAAGAG	ACGGTTGTTTTGGCATTGAT	60.2	890	INTERGENIC	--	--	--
Ca2_P4_6640452	Ca2	6640452	GAAAA/GAAAAA	AAAAAGGTCCTAGGGTGGGA	ACGGTTGTTTTGGCATTGAT	59.8	473	INTERGENIC	--	--	--
Ca2_P4_6641737	Ca2	6641737	CATATAT/CATATATATAT	GTATTCAGGGGCGAAGGAAT	AATGCAAACGACAAAGGAGG	60.3	500	INTERGENIC	--	--	--
Ca2_P4_6641916	Ca2	6641916	TG/T	GTTCTATGGGGACAGGGTCA	TATCAACTACCAGAGCGGGG	59.8	476	INTERGENIC	--	--	--
Ca2_P4_6642487	Ca2	6642487	ATTTT/ATTTTTTTTT	CCCCGCTCTGGTAGTTGATA	ACCCACTTTGAGAAACGTGC	60.1	479	INTERGENIC	--	--	--
Ca2_P4_6642515	Ca2	6642515	TTATATATATATATATA/TATATATATATATATATATATATATA	CCCCGCTCTGGTAGTTGATA	ACCCACTTTGAGAAACGTGC	60.1	479	INTERGENIC	--	--	--
Ca2_P4_6643755	Ca2	6643755	G/GGA	ATGGAACAAGCAGCGAAGTT	GTTGATACGAGGGGGTTGAA	59.9	537	INTERGENIC	--	--	--

INDEL marker IDs	Chromosomes/ unanchored scaffolds	Physical positions (bp)	InDels (*Kabuli* reference genome-CDC Frontier/PII)	Forward primers (5'-3')	Reverse primers (5'-3')	Anneling temperature (0C)	Expected amplified product size (bp)	Structural annotation		Functional annotation	
								Sequence components of *kabuli* genome	*Kabuli* gene accession IDs	NCBI-KOG	TFs
Ca2_P4_6643843	Ca2	6643843	CTTTTTTTTTTT/CTTTTTTTT	ATGGAACAAGCAGCGAAGTT	GTTGATACGAGGGGGTTGAA	59.9	537	INTERGENIC	--	--	--
Ca2_P4_6648530	Ca2	6648530	TAAAAAA/TAAAAAAAA	TTATCGATCGTCGGTGTCAA	TTCCATATGCACCTCCCATT	60.1	612	INTERGENIC	--	--	--
Ca2_P4_6657720	Ca2	6657720	AATG/AATGATG	AAGAATTGTTGTCCCCCTCC	GGAACCAAAACAAAAGGCAA	60.2	635	INTERGENIC	--	--	--
Ca2_P4_6659290	Ca2	6659290	GTTT/GTTTT	TGTTTGACAAATGTTAAGTGCG	GGTGTGACTGGCCGTATTTT	58.8	621	INTERGENIC	--	--	--
Ca2_P4_66642	Ca2	66642	TTAATAATAATAATAATAATAATAATAATAATAATAATAATAATAATAATAATAATAATAA/TTAATAATAATAATAATAATAATAATAATAATAATAATAATAATAATAATAATAATAATAATAATAA	TGAAAATTTTGGGGTGTTACAA	GGTGACTTAATTTTGAAGAAGACGA	59.2	813	INTERGENIC	--	--	--
Ca2_P4_6692032	Ca2	6692032	AAATAATAATAATAAT/AAATAATAATAAT	GAACGAAAATGTGTTTTATGGG	CTCTCAAGGTGTGACTGCCA	58.4	157	INTERGENIC	--	--	--
Ca2_P4_6753729	Ca2	6753729	ATATAT/ATATATGTATAT	TGATGCCATGATATGAATGCT	GGCCAAGAGAAGTTCGAGTG	59.0	415	INTERGENIC	--	--	--
Ca2_P4_6815005	Ca2	6815005	ATTATTTATTTATTTATTTATTTA/ATTATTTATTTATTTATTTA	GAAATTTTAGGCGCAACAGC	TCCAATCCCATTGTCCACTT	59.9	703	INTERGENIC	--	--	--
Ca2_P4_6865791	Ca2	6865791	ATTTTTTTT/ATTTTTTT	ACCCACCCCATAAAAAGAGG	TTAAAAAGGAGATGTGCGGG	60.0	533	INTERGENIC	--	--	--
Ca2_P4_700339	Ca2	700339	ATTTTTTTTT/ATTTTTTTTTT	AAACTAGGTCAGAAACCGATAGAA	CCAACACAAACCTAAACCGA	57.7	896	INTERGENIC	--	--	--

INDEL marker IDs	Chromosomes/ unanchored scaffolds	Physical positions (bp)	InDels (*Kabuli* reference genome-CDC Frontier/PII)	Forward primers (5'-3')	Reverse primers (5'-3')	Anneling temperature (0C)	Expected amplified product size (bp)	Structural annotation		Functional annotation	
								Sequence components of *kabuli* genome	*Kabuli* gene accession IDs	NCBI-KOG	TFs
Ca2_P4_7041526	Ca2	7041526	T/TC	GCCCTCAGATGAGAGTCAGG	CAGCGGAAAAGTTTCTCCAA	59.9	324	INTERGENIC	--	--	--
Ca2_P4_7043443	Ca2	7043443	AATATATATATATATATATATATAT/AATATATATATATATATATATATATATAT	ATGGGTCGTGTTCGATTGTT	AACCAACTATTGACCCCAAAAA	60.2	267	INTERGENIC	--	--	--
Ca2_P4_7171869	Ca2	7171869	TTAT/TT	CGGCCATTAAATTTCTGACA	TTGCATTATGTTTCTGGCAAA	58.6	320	INTERGENIC	--	--	--
Ca2_P4_7171912	Ca2	7171912	TATTTAATTTAATTTAATT/TATTTAATTTAATT	CGGCCATTAAATTTCTGACA	TCATTTCATAACGAGTGACCCA	58.6	584	INTERGENIC	--	--	--
Ca2_P4_7172121	Ca2	7172121	CTATATATATATAT/CTATATATATAT	TTTGCCAGAAACATAATGCAA	TCATTTCATAACGAGTGACCCA	59.2	285	INTERGENIC	--	--	--
Ca2_P4_7172172	Ca2	7172172	ATTTTTTTTT/ATTTTTTTTTT	TTTGCCAGAAACATAATGCAA	TTTTGGAAAAATGAAGGACCA	59.2	774	INTERGENIC	--	--	--
Ca2_P4_7173102	Ca2	7173102	GAAAAAA/GAAAAA	CTCAATCCAAACACACGCTT	AATGACCACCTCTTGTTGCC	58.8	787	INTERGENIC	--	--	--
Ca2_P4_7220242	Ca2	7220242	TGA/T	GCCCTCAGATGAGAGTCAGG	TATTGTGCGACGTCTTCTCG	59.9	738	INTERGENIC	--	--	--
Ca2_P4_7276294	Ca2	7276294	CAAAA/CAAAAA	TCCACCACAATCGTTAAGCA	AGCGGAGATTTTACCGGATT	60.1	676	INTERGENIC	--	--	--
Ca2_P4_7295625	Ca2	7295625	GA/GAA	GAGGCAAATTACGTGGCAGT	CTCAAATCATGCTCTTGGCA	60.1	355	INTERGENIC	--	--	--

INDEL marker IDs	Chromosomes/ unanchored scaffolds	Physical positions (bp)	InDels (*Kabuli* reference genome-CDC Frontier/PII)	Forward primers (5'-3')	Reverse primers (5'-3')	Anneling temperature (0C)	Expected amplified product size (bp)	Structural annotation		Functional annotation	
								Sequence components of *kabuli* genome	*Kabuli* gene accession IDs	NCBI-KOG	TFs
Ca2_P4_7407914	Ca2	7407914	TTCTAGTTTTGGGATCCTCTAGTTTT/TTCTAGTTTTGGGATCCTCTAGTTTTGGGATCCTCTAGTTTT	TCCCTTTGGTTCAGGTTGTC	GGAAAAGCAAGGATGCTGAA	59.9	372	INTERGENIC	--	--	--
Ca2_P4_7528105	Ca2	7528105	CAAAAAAAA/CAAAAAAA	AGAGGCCAACCTCTGTCTCA	TCGTCAGGAGGTTGATGATG	60.0	313	INTERGENIC	--	--	--
Ca2_P4_7557453	Ca2	7557453	TTATATATATATATATAT/TTATATATATATATATATAT	CGCACACACGAATATTCACC	ACGACAACATGGTGGAACAC	60.0	465	INTERGENIC	--	--	--
Ca2_P4_7565416	Ca2	7565416	CATATATATATATATA/CATATATATATATATATA	AACATATGCAAACTTCGCCC	ATCTCTCTTCCGCCATTTTT	60.0	476	INTERGENIC	--	--	--
Ca2_P4_7586948	Ca2	7586948	AAATAATAATAATAATAATAATAATAATAATAATAA/AAATAATAATAATAATAATAATAATAATAATAATAATAATAATAA	GCGACCTGGTGATTTTGTTT	CCGCGTAGATGTGAAACTGA	60.0	348	INTERGENIC	--	--	--
Ca2_P4_7587644	Ca2	7587644	TTTATTATTATTATTATTATTATTATT/TTTATTATTATTATTATTATTATTATTATT	GGCTAGACGAGTTCGACGAC	AATAGATGGTCGCATCCTGG	60.0	495	INTERGENIC	--	--	--
Ca2_P4_7589173	Ca2	7589173	GATGTAA/GA	CAACACCTTGTGCAATCAGG	AGGCCATGAGGTTGTTTGAG	60.2	403	INTERGENIC	--	--	--
Ca2_P4_7607086	Ca2	7607086	TAAAAAAAAAAAA/TAAAAAAAAAAAAA	TGTCAAAGGAGCAACACAACT	ACCCCGACGTACACACTCTC	58.4	855	INTERGENIC	--	--	--
Ca2_P4_7617731	Ca2	7617731	CTAAAATAAAATAAAA/CTAAAATAAAATAAAATAAAATAAAA	CCAAGATCTCGTAGTGCAACA	TGGGTTTAGTCCGTTTTTCTTC	58.9	468	INTERGENIC	--	--	--
Ca2_P4_7635741	Ca2	7635741	CAAAAAAAAAA/CAAAAAAAAA	CCTCGATCTTGTCCTGGTTC	TGTAGAAAAAGAGGGCCACG	59.7	515	INTERGENIC	--	--	--

INDEL marker IDs	Chromosomes/ unanchored scaffolds	Physical positions (bp)	InDels (*Kabuli* reference genome-CDC Frontier/PII)	Forward primers (5'-3')	Reverse primers (5'-3')	Anneling temperature (0C)	Expected amplified product size (bp)	Structural annotation		Functional annotation	
								Sequence components of *kabuli* genome	*Kabuli* gene accession IDs	NCBI-KOG	TFs
Ca2_P4_7655513	Ca2	7655513	GTTTTTTTTTTT/GTTTTTTTTT	CCCACAATAGCACCACTCTTG	TCAAGTTGAAGCATCATGGTC	60.6	596	INTERGENIC	--	--	--
Ca2_P4_7657147	Ca2	7657147	CTTTTT/CTTTTTT	TTATGTGGGGGATTTGGATG	TTCCACGTCCTTTTCATTCC	60.4	705	INTERGENIC	--	--	--
Ca2_P4_7668484	Ca2	7668484	CC/CCCAC	CCAACACAAGCACCAACATC	GGAGGGGTAGAAGTGGAAGG	60.0	188	INTERGENIC	--	--	--
Ca2_P4_7669241	Ca2	7669241	TAA/TAAA	TGTTCGCCTCTTCATCAGTG	TGTGTTTTGGGTTCGACTAGG	60.0	475	INTERGENIC	--	--	--
Ca2_P4_7669371	Ca2	7669371	TAAAAAAAA/TAAAAAAAAAAA	TGTTCGCCTCTTCATCAGTG	TGTGTTTTGGGTTCGACTAGG	60.0	475	INTERGENIC	--	--	--
Ca2_P4_7669611	Ca2	7669611	A/ACACTCATT	CCTAGTCGAACCCAAAACACA	TTGCTCACGAAGGAAATGACT	60.0	450	INTERGENIC	--	--	--
Ca2_P4_7671677	Ca2	7671677	ATGATT/A	TACACCCACCATGGGAATCT	ACCCCCATAACCCTCCATTA	60.1	227	INTERGENIC	--	--	--
Ca2_P4_7678380	Ca2	7678380	AAA/AAATAA	GGTGAGGATGCATGAGATGA	ACCAAGATGACCAGCAAAGG	59.6	949	INTERGENIC	--	--	--
Ca2_P4_7678453	Ca2	7678453	GAAAAAAAAA/GAAAAAAAA	TTTTCGCATTTGATTAGAGAGATT	ACCAAGATGACCAGCAAAGG	58.5	515	INTERGENIC	--	--	--
Ca2_P4_7696798	Ca2	7696798	AAAA/AAAACAAA	TGTTTCCACGATTCAAAGTTTC	AAAGCCAATAATCTAATTTGGGG	59.1	582	INTERGENIC	--	--	--

INDEL marker IDs	Chromosomes/ unanchored scaffolds	Physical positions (bp)	InDels (*Kabuli* reference genome-CDC Frontier/PII)	Forward primers (5'-3')	Reverse primers (5'-3')	Anneling temperature (0C)	Expected amplified product size (bp)	Structural annotation		Functional annotation	
								Sequence components of *kabuli* genome	*Kabuli* gene accession IDs	NCBI-KOG	TFs
Ca2_P4_7705341	Ca2	7705341	ATAGTA/ATA	CTGCGGTACAACCATCTCCT	CGCCGTCGTTACTGTTCATA	60.1	355	INTERGENIC	--	--	--
Ca2_P4_7706402	Ca2	7706402	ATTTTTTTTTT/ATTTTTTTTT	GACCCATTTCCACAAACACC	CATCCACTTTCTTGCCTGTATTT	60.1	956	INTERGENIC	--	--	--
Ca2_P4_7708059	Ca2	7708059	ATTTTTTTT/ATTTTTTT	GCCTTGGGACCTCCTAAAAG	AGAGGGAGAGAGGAAGGCTG	60.1	638	INTERGENIC	--	--	--
Ca2_P4_7714112	Ca2	7714112	TG/TTGG	CTCACTCGATCGATGGTTCA	TGACAATGCTGGCTGTTAGC	59.8	588	INTERGENIC	--	--	--
Ca2_P4_7718955	Ca2	7718955	TGAAA/T	GAGCTTCATTGAAATCGGGA	TCATATAGGAGGTCGCACCC	60.2	821	INTERGENIC	--	--	--
Ca2_P4_7722723	Ca2	7722723	ATTTTTTTT/ATTTTTTT	AAAGCAGTGTCCTTCGGAGA	TCCTACCCTTTTGGCTTAAAAA	60.0	408	INTERGENIC	--	--	--
Ca2_P4_7723173	Ca2	7723173	ATTTT/ATTTTT	TTTTTAAGCCAAAAGGGTAGGA	AACCGCAATTTTTATAAGACACG	59.2	321	INTERGENIC	--	--	--
Ca2_P4_7723572	Ca2	7723572	ATTTTTTTTTT/ATTTTTTTTT	CGTGTCTTATAAAAATTGCGGTT	TTTGCAAGCCAAGCACATAC	59.5	558	INTERGENIC	--	--	--
Ca2_P4_7724864	Ca2	7724864	TA/TAAAGAGATTGAGA	ACCAGAGACACTGCAGCAAA	AACAAGATTCTCGAATGCTCC	59.6	464	INTERGENIC	--	--	--
Ca2_P4_7731377	Ca2	7731377	GAAAAA/GAA	GTGGTTGTGATGTTGAACGC	CTCCCGCTAACTCCTCTTCC	60.0	697	INTERGENIC	--	--	--

INDEL marker IDs	Chromosomes/ unanchored scaffolds	Physical positions (bp)	InDels (*Kabuli* reference genome-CDC Frontier/PII)	Forward primers (5'-3')	Reverse primers (5'-3')	Anneling temperature (0C)	Expected amplified product size (bp)	Structural annotation		Functional annotation	
								Sequence components of *kabuli* genome	*Kabuli* gene accession IDs	NCBI-KOG	TFs
Ca2_P4_7739105	Ca2	7739105	ACTTTGT/A	GGTTCTACGGCAACGTCAAT	TTACGCGTGTCATTTTCTCG	60.0	790	INTERGENIC	--	--	--
Ca2_P4_7743405	Ca2	7743405	CG/C	ATCCATCATGTTCATGCCCT	ATCTCCACCCGTTCTCTTGA	60.2	579	INTERGENIC	--	--	--
Ca2_P4_7744794	Ca2	7744794	CATGACATGAATGA/CATGA	CATTTTGATTGGGAATGTGC	CAACAACAGTCCAAAGTAGGCA	58.8	585	INTERGENIC	--	--	--
Ca2_P4_7745257	Ca2	7745257	CAAAA/CAAAAAA	TGGCATGACATGAATGAAGAA	GAGCAATTTGTGTGGACCCT	60.1	760	INTERGENIC	--	--	--
Ca2_P4_7749962	Ca2	7749962	TT/TTAT	GGAGCTGCAACCATCTTCTC	TGGATTTGGGCTGAATTAGG	60.0	321	INTERGENIC	--	--	--
Ca2_P4_7756359	Ca2	7756359	CAAA/CAAAA	AACCAGCATGTGTTGTTGGA	TGTGGTGGGGGAATTAAAAA	60.0	399	INTERGENIC	--	--	--
Ca2_P4_7771490	Ca2	7771490	ATTTTTTTTT/ATTTTTTTTTTT	ACCATTTATTATTTTCGTGTTAATGA	GGTACCATGGATGATATTTAGCC	57.3	188	INTERGENIC	--	--	--
Ca2_P4_7772575	Ca2	7772575	AGG/AGGG	TTAAGGGACCACGGGTGTTA	TCCCACCTCTCAATTTTTCAA	60.2	898	INTERGENIC	--	--	--
Ca2_P4_7772843	Ca2	7772843	C/CG	CAATTGGTGGCCGTTATTTT	GGGGTTGTAAAATGTGTGTGG	59.7	811	INTERGENIC	--	--	--
Ca2_P4_7772892	Ca2	7772892	ATTTT/ATTT	CAATTGGTGGCCGTTATTTT	TTGGGGGAAGAAAATTGAGA	59.7	958	INTERGENIC	--	--	--

INDEL marker IDs	Chromosomes/ unanchored scaffolds	Physical positions (bp)	InDels (*Kabuli* reference genome-CDC Frontier/PII)	Forward primers (5'-3')	Reverse primers (5'-3')	Anneling temperature (0C)	Expected amplified product size (bp)	Structural annotation		Functional annotation	
								Sequence components of *kabuli* genome	*Kabuli* gene accession IDs	NCBI-KOG	TFs
Ca2_P4_7773617	Ca2	7773617	TTAAATAAAAT/TTAAATAAAATAAAT AAAAT	GCTGAGCCCAAACAAG CTAA	CCACTGTGGAAAGGTA AATGC	60.5	845	INTERGENIC	--	--	--
Ca2_P4_7774634	Ca2	7774634	AA/AAGA	TTCGATGCTACCCATA ACACA	TCCTTACGTTCCACGG TAATTT	59.1	663	INTERGENIC	--	--	--
Ca2_P4_7774796	Ca2	7774796	TAAAAAAAAAA/TAAAAAAAA	TTGGATCATTTTGAGTT TGAGG	TCCTTACGTTCCACGG TAATTT	59.1	358	INTERGENIC	--	--	--
Ca2_P4_7774902	Ca2	7774902	ATTAATATAATTTTTTT/ATT	AAATTACCGTGGAACG TAAGGA	TGCTTTGTGTAATGCG TGGT	59.8	546	INTERGENIC	--	--	--
Ca2_P4_7780730	Ca2	7780730	ATTTTTTTTTTT/ATTTTTTTTTT	TTGTCTCCATCTCCAG CCTT	TGTTGTTTGAGTTGGG ACACA	59.8	995	INTERGENIC	--	--	--
Ca2_P4_7792210	Ca2	7792210	CAAAA/CAAA	GCGAAAACTTAAAGGA CTAAACCA	CAACCCGAAGTTGCTC ATTT	60.1	362	INTERGENIC	--	--	--
Ca2_P4_7792884	Ca2	7792884	TTC/T	ATGAGCAACTTCGGGT TGAC	CTAGTTCCTCGTTGGA GGGG	60.1	478	INTERGENIC	--	--	--
Ca2_P4_7793328	Ca2	7793328	GAAAAA/GAAAA	ATATCCCCTCCAACGA GGAA	TTTATTGCGGTTTCCCT TCA	60.7	877	INTERGENIC	--	--	--
Ca2_P4_7795279	Ca2	7795279	CAT/C	AAAAGAAACCCGCAGA ACAA	AGCAAGCACAAACCGT TCTT	59.7	515	INTERGENIC	--	--	--
Ca2_P4_7810805	Ca2	7810805	GAAA/GAAAA	GCTCTTTGAGAAGAGG GGGT	TGGTATCCTTCCTTCC GTTC	59.8	782	INTERGENIC	--	--	--

INDEL marker IDs	Chromosomes/ unanchored scaffolds	Physical positions (bp)	InDels (*Kabuli* reference genome-CDC Frontier/PII)	Forward primers (5'-3')	Reverse primers (5'-3')	Anneling temperature (0C)	Expected amplified product size (bp)	Structural annotation		Functional annotation	
								Sequence components of *kabuli* genome	*Kabuli* gene accession IDs	NCBI-KOG	TFs
Ca2_P4_7811963	Ca2	7811963	CAAAAAAA/CAAAAAAAA	ACTAACTCGGTGGGGAACAA	TCCCTTGTTCTTGCTGTGAA	59.4	330	INTERGENIC	--	--	--
Ca2_P4_7815016	Ca2	7815016	TAATAACAAATAACAAA/TAATAACAAATAACAAATAACAAA	TTTTCACCAACACCGTCAAA	AAATAATCGTGGCCTTGCAC	60.0	901	INTERGENIC	--	--	--
Ca2_P4_7893331	Ca2	7893331	CAG/CAGAG	AAACAAATTCGCGGTGCTAC	TGATGAAACCACCCAGTTGA	60.1	690	INTERGENIC	--	--	--
Ca2_P4_7894501	Ca2	7894501	ATT/AT	GGCTCTGGCTTTGAGTTGAG	GGGGATGCTACAACCATGAC	60.1	440	INTERGENIC	--	--	--
Ca2_P4_7894638	Ca2	7894638	ATTTTT/ATTTTTT	GTCATGGTTGTAGCATCCCC	GCCAAATGAGGTTAATGAAGGA	60.2	278	INTERGENIC	--	--	--
Ca2_P4_7894801	Ca2	7894801	TTTGCAATTGCA/TTTGCA	GTCATGGTTGTAGCATCCCC	GATGGGGATGGAAGATGAAA	60.2	639	INTERGENIC	--	--	--
Ca2_P4_7899077	Ca2	7899077	TAAA/TAAATAAAA	TCAGGCTCAACAACATCAGC	AGGTTTGGAAGTTTGGCCTT	60.0	436	INTERGENIC	--	--	--
Ca2_P4_7899449	Ca2	7899449	GAAAAAA/GAAAAA	AAGGCCAAACTTCCAAACCT	TGGATTGTGGGTTTGACTCC	60.0	604	INTERGENIC	--	--	--
Ca2_P4_7925215	Ca2	7925215	TTTTTTA/T	TTTTATGCCGCAAAGAATTG	ATACGGCCTTTGATGTCGAG	58.8	425	INTERGENIC	--	--	--
Ca2_P4_792603	Ca2	792603	ATTTTTTTTTT/ATTTTTTTTTTT	TCAATGATTTGCACGGAGAA	AACCAACACTTCCATGCCTC	60.2	772	INTERGENIC	--	--	--

INDEL marker IDs	Chromosomes/ unanchored scaffolds	Physical positions (bp)	InDels (*Kabuli* reference genome-CDC Frontier/PII)	Forward primers (5'-3')	Reverse primers (5'-3')	Anneling temperature (0C)	Expected amplified product size (bp)	Structural annotation		Functional annotation	
								Sequence components of *kabuli* genome	*Kabuli* gene accession IDs	NCBI-KOG	TFs
Ca2_P4_7932313	Ca2	7932313	ATTTTTTTT/ATTTTTTT	ATTTTCTTTGACGGACGGAA	GGAGAAATTTGCCAAAGGAA	59.5	596	INTERGENIC	--	--	--
Ca2_P4_7933051	Ca2	7933051	AC/A	CCCCAAAAAGTGCTTCAAAA	TGACAAGCACGCTTAAGGAA	60.1	694	INTERGENIC	--	--	--
Ca2_P4_7933075	Ca2	7933075	ATTTTATTTTTTTAT/ATTTTAT	CCCCAAAAAGTGCTTCAAAA	TGACAAGCACGCTTAAGGAA	60.1	694	INTERGENIC	--	--	--
Ca2_P4_7940035	Ca2	7940035	TA/TAA	CAACATTGACAACGGAATCG	AAAATGGACAAAATTGGAGCA	60.0	927	INTERGENIC	--	--	--
Ca2_P4_7940111	Ca2	7940111	ATTTTTTT/ATTTTTT	TCTTGACCGTTAGATTGAACCA	CAAAATGGACAAAATTGGAGC	59.6	714	INTERGENIC	--	--	--
Ca2_P4_7940136	Ca2	7940136	ATTTTTTT/ATTTTTTTT	TCTTGACCGTTAGATTGAACCA	CAAAATGGACAAAATTGGAGC	59.6	714	INTERGENIC	--	--	--
Ca2_P4_7940384	Ca2	7940384	CTTAATTTAAATATT/C	TGACCAAAATCTTCTCTTTCCA	GTGCCTCAACCATGACACTG	58.8	818	INTERGENIC	--	--	--
Ca2_P4_7940860	Ca2	7940860	ATTTTTT/ATTTTTTT	CAGTGTCATGGTTGAGGCAC	GAAGTTGGAACGACGGTGAT	60.2	437	INTERGENIC	--	--	--
Ca2_P4_7949331	Ca2	7949331	CAAATAAAATAAAATAAAATAAAATAAAATAA/CAAATAAAATAAAATAAAATAAAATAA	TTGCTAACTCTGTTGGCCCT	ACGAGGATCACCCAGTTCAC	59.9	496	INTERGENIC	--	--	--
Ca2_P4_7966683	Ca2	7966683	ATTTTTTTTTTT/ATTTTTTTTTTTT	GATGTCACTCCCTGGCTCAT	TTTTCACATTTTCATGTCAAATACA	60.1	397	INTERGENIC	--	--	--

INDEL marker IDs	Chromosomes/ unanchored scaffolds	Physical positions (bp)	InDels (*Kabuli* reference genome-CDC Frontier/PII)	Forward primers (5'-3')	Reverse primers (5'-3')	Anneling temperature (0C)	Expected amplified product size (bp)	Structural annotation		Functional annotation	
								Sequence components of *kabuli* genome	*Kabuli* gene accession IDs	NCBI-KOG	TFs
Ca2_P4_8029116	Ca2	8029116	ATTTTTTTTT/ATTTTTTTTTT	AATGTATCGGGACCTCACAA	TTTTTCTTGGTGCGCTTTTT	57.9	565	INTERGENIC	--	--	--
Ca2_P4_8062546	Ca2	8062546	CATATATATATATATATATATAT/CATATATATATATATATAT	ACGGGTGCGAATTTTCTATG	CACCTCTTCTACTCCCGCAC	60.0	389	INTERGENIC	--	--	--
Ca2_P4_8166930	Ca2	8166930	AAATAATAATAATAATAATAATAATAAT/AAATAATAATAATAATAATAATAATAATAAT	CCTAGGGATGCGACCATCTA	TCCTCCTCTTTTGGCACATT	60.1	385	INTERGENIC	--	--	--
Ca2_P4_8308316	Ca2	8308316	TAAAAAAAA/TAAAAAAA	AATTGGGACCATTTGATAGGC	GCCTCTACCATCTTTGACAGC	60.0	171	INTERGENIC	--	--	--
Ca2_P4_8374636	Ca2	8374636	AATATATATATATATATATATAT/AATATATATATATATATATAT	AAAGGGACAATGAAGGCAAG	TTGAAAATGTGATGTTTCTTCCA	59.2	636	INTERGENIC	--	--	--
Ca2_P4_8420149	Ca2	8420149	AATATATATATATATATATATATATA/AATATATATATATATATATATATATATATA	CACCCTTACAAGTCGCCATT	AATCGGCATTTCAAACTTGG	60.0	547	INTERGENIC	--	--	--
Ca2_P4_8420270	Ca2	8420270	AACA/AACACA	CCAAGTTTGAAATGCCGATT	GCTGCATGATCTTATGCTCG	59.9	451	INTERGENIC	--	--	--
Ca2_P4_8478800	Ca2	8478800	AATTT/A	CCCGTATGAATGAACGGAAC	CTACCCCCACATTTCAATCG	60.2	519	INTERGENIC	--	--	--
Ca2_P4_8478910	Ca2	8478910	ACT/A	CCCGTATGAATGAACGGAAC	CTACCCCCACATTTCAATCG	60.2	519	INTERGENIC	--	--	--
Ca2_P4_854136	Ca2	854136	TAAAAAAAAAA/TAAAAAAAAA	CAAGTGGAACCCACCAATTT	CTTGCCTGTGTCGAAGATGA	59.7	543	INTERGENIC	--	--	--

INDEL marker IDs	Chromosomes/ unanchored scaffolds	Physical positions (bp)	InDels (*Kabuli* reference genome-CDC Frontier/PII)	Forward primers (5'-3')	Reverse primers (5'-3')	Anneling temperature (0C)	Expected amplified product size (bp)	Structural annotation		Functional annotation	
								Sequence components of *kabuli* genome	*Kabuli* gene accession IDs	NCBI-KOG	TFs
Ca2_P4_8541841	Ca2	8541841	TAAAAAAAAAA/TAAAAAAAAAAA	TTCATATCATTCAAATGCCCA	ACACCTAGTTCACCCCCTCC	58.8	643	INTERGENIC	--	--	--
Ca2_P4_8708914	Ca2	8708914	ATTTTTTTT/ATTTTTTTTT	GCCCACACCGAAGAAATAAA	TTCATTTTGCTCCATCACTTTC	59.9	714	INTERGENIC	--	--	--
Ca2_P4_8714762	Ca2	8714762	TAAAAAAAAAAAA/TAAAAAAAAAAAAA	AGGATGCGCTCTTCCAACTA	TTGAACCATGTGCGACCTAA	60.0	222	INTERGENIC	--	--	--
Ca2_P4_8820736	Ca2	8820736	ATTTTTTTTTTT/ATTTTTTTTTT	TTACCATGATGTCGGTGGTG	GTGAGCAAATGTTTCGGGAT	60.2	690	INTERGENIC	--	--	--
Ca2_P4_9233338	Ca2	9233338	TAGAGAGAGAGAGAGAGAGAGAGAGAGA/TAGAGAGAGAGAGAGAGAGAGAGAGA	TCGACCGAATCGTAGGAAAC	TCATTCCATCTTTCATGGCA	60.1	557	INTERGENIC	--	--	--
Ca2_P4_9281281	Ca2	9281281	TTATATATATATATATATATATATA/TTATATATATATATATATATATATATATATATA	CAAATTGAGGCCCAACAACT	CGAATTCTTTTTGGGGGATT	60.0	601	INTERGENIC	--	--	--
Ca2_P4_9408358	Ca2	9408358	CTT/CTTTCTATT	CATCAAAAACAGCAGCGAAA	TCGTGTCTTCTTTCCATCCA	60.0	934	INTERGENIC	--	--	--
Ca2_P4_9427423	Ca2	9427423	TAAAAAAAAA/TAAAAAAAAAA	AATATTGCGACAACGCATGT	TGAAATGTTTAATTGTAGGTTCGG	59.1	598	INTERGENIC	--	--	--
Ca2_P4_9494071	Ca2	9494071	T/TA	GAACTGCAACAACAACGACAA	GGTCTCGAAATCCCTGATGA	59.8	609	INTERGENIC	--	--	--
Ca2_P4_9494155	Ca2	9494155	CAAA/CAAAA	GAACTGCAACAACAACGACAA	GGTCTCGAAATCCCTGATGA	59.8	609	INTERGENIC	--	--	--

INDEL marker IDs	Chromosomes/ unanchored scaffolds	Physical positions (bp)	InDels (*Kabuli* reference genome-CDC Frontier/PII)	Forward primers (5'-3')	Reverse primers (5'-3')	Anneling temperature (0C)	Expected amplified product size (bp)	Structural annotation		Functional annotation	
								Sequence components of *kabuli* genome	*Kabuli* gene accession IDs	NCBI-KOG	TFs
Ca2_P4_9500229	Ca2	9500229	CGTATGCTAGACCTGG/CGTATGCTAGACCTGGGTATGCTAGACCTGG	AGAGGTTGGGGGAGAAGAAA	GCAACAACAGCAGAGACAATG	60.0	213	INTERGENIC	--	--	--
Ca2_P4_9594399	Ca2	9594399	GTTTTTTTTTTTT/GTTTTTTTTTTTTT	CAACTCAGGAATGCGTGAAA	TTCACGCCAAACATTAAACG	59.8	726	INTERGENIC	--	--	--
Ca2_P4_9690407	Ca2	9690407	TAAAAAAAAAAAAA/TAAAAAAAAAAAAA	TGATGCAAATATCTACCGCAA	TTCAAGGGACGTAAATGCAA	59.2	558	INTERGENIC	--	--	--
Ca2_P4_9694226	Ca2	9694226	TTATATATATATATATATATATA/TTATATATATATATATATATA	TGTGCGTTGTAAAGGAGTGC	CGTCGGAATCCAAAAGTCAT	59.9	504	INTERGENIC	--	--	--
Ca2_P4_9709233	Ca2	9709233	ATTTTTTTTTTT/ATTTTTTTTTTTTT	TTCAATGCACTTCTGAACCG	TGTGAACACAAGAGCAAGGG	59.8	644	INTERGENIC	--	--	--
Ca2_P4_981417	Ca2	981417	ACTCTCTCTCTCTCTCTCTCTCTCTCTCTCTCT/ACTCTCTCTCTCTCTCTCTCTCTCTCTCTCTCTCTCTCTCTCT	ACATAATTCCCAACCGACCA	CCAAAGGGGTGAGTTTTTGA	60.1	328	INTERGENIC	--	--	--
Ca2_P4_9877069	Ca2	9877069	TTATATATATATATATATATATATATATATATA/TTATATATATATATATATATATATATATATATA	CCGACTTTCCTTCATTAACCA	GAAGCAATTTGGGATGCAAT	59.1	724	INTERGENIC	--	--	--
Ca2_P4_9922792	Ca2	9922792	CAT/CATTAT	ATCAATGGAGCAAGGACACC	CATGTTTTGCATTCGCAACT	59.9	636	INTERGENIC	--	--	--
Ca2_P4_9997900	Ca2	9997900	AAATAATAATAATAATAATAATAATAATAATA/AAATAATAATAATAATAATAATAATAATAATA	TAAATACGCGACGACCAACA	TTTCTGTCATAAAAAGTGGCACA	60.1	407	INTERGENIC	--	--	--
Ca3_P4_100899	Ca3	100899	AATATATATATATATATATATATATATATATAT/AATATATATATATATATATATATATATAT	AACAAACGACGACGTGACAT	ACGGTAAATGCATAGCCACC	59.1	746	INTERGENIC	--	--	--

INDEL marker IDs	Chromosomes/ unanchored scaffolds	Physical positions (bp)	InDels (*Kabuli* reference genome-CDC Frontier/PII)	Forward primers (5'-3')	Reverse primers (5'-3')	Anneling temperature (0C)	Expected amplified product size (bp)	Structural annotation		Functional annotation	
								Sequence components of *kabuli* genome	*Kabuli* gene accession IDs	NCBI-KOG	TFs
Ca3_P4_10091088	Ca3	10091088	ACCCCC/ACCCC	CCCCAAATTCACCAAATTGA	GGCGTGAACCAAAAGGTAAA	60.5	467	INTERGENIC	--	--	--
Ca3_P4_10128061	Ca3	10128061	TT/TTATTATTATTAT	TCCTCCCCCTTCCTCTTTTA	CACCACTCAATGCATCTTGG	60.0	274	INTERGENIC	--	--	--
Ca3_P4_10159368	Ca3	10159368	GA/G	CTAGGCTCACCAACCGGATA	CTAAGGGCGCTACAACAAGC	60.1	354	INTERGENIC	--	--	--
Ca3_P4_10159468	Ca3	10159468	T/TA	TGGGGCAAACAAAGCTATTC	CTAAGGGCGCTACAACAAGC	60.1	107	INTERGENIC	--	--	--
Ca3_P4_10159672	Ca3	10159672	AGGGGGGG/AGGGGGGGG	CACACCAACCCTTTCGTTCT	TGCTCCCACACACACAAACT	60.0	277	INTERGENIC	--	--	--
Ca3_P4_10169650	Ca3	10169650	GT/GTT	CAGATGGATTCCCCTCTTCA	CTTCCCGATGAGTACCAGGA	60.0	573	INTERGENIC	--	--	--
Ca3_P4_10194898	Ca3	10194898	ATTTTTTTTTTT/ATTTTTTTTTT	TGGAGAAGAGAGGAGCCAAA	CCGTTTTCTGTTTTCACAATCA	60.1	759	INTERGENIC	--	--	--
Ca3_P4_1038019	Ca3	1038019	AAT/AATAATCAT	GGAGCAAAATGTGTTTTATGGA	GGAAACGGTCTTCCCTTTTT	59.0	173	INTERGENIC	--	--	--
Ca3_P4_10406481	Ca3	10406481	CTTTTTTT/CTTTTTTTTTTTTT	GGTACGCCACACATGCATAC	TTGAAGTTGTGGCAATGGAA	59.9	233	INTERGENIC	--	--	--
Ca3_P4_10417401	Ca3	10417401	ATTTTTT/ATTTTTTT	TAAGCTTGAACACGTGCCTT	TGAGAGCATTCAAGGAAGCA	58.6	222	INTERGENIC	--	--	--

INDEL marker IDs	Chromosomes/ unanchored scaffolds	Physical positions (bp)	InDels (*Kabuli* reference genome-CDC Frontier/PII)	Forward primers (5'-3')	Reverse primers (5'-3')	Anneling temperature (0C)	Expected amplified product size (bp)	Structural annotation		Functional annotation	
								Sequence components of *kabuli* genome	*Kabuli* gene accession IDs	NCBI-KOG	TFs
Ca3_P4_10570421	Ca3	10570421	GATATATATATATA/GATATATATATATATA	ACCCGAATACCTCCAAGGTC	GATCTGCAGTTGCTGATGGA	60.2	816	INTERGENIC	--	--	--
Ca3_P4_10636003	Ca3	10636003	CAAAAAAAAAAA/CAAAAAAAAAA	AAGACCGATTGAGATGCGAC	GGGAAAACATTGGAAAACCC	60.2	584	INTERGENIC	--	--	--
Ca3_P4_10846962	Ca3	10846962	AATATATATATATATATATATATATATATA/AATATATATATATATATATATATATATATATATA	CCAAATGACGACTTCCCACT	CAAGACGTCGTCAAAATCCC	60.0	683	INTERGENIC	--	--	--
Ca3_P4_10859593	Ca3	10859593	TATATA/TATATACATATA	TGACCTTCTATTTTGCGATCA	CACGACCAACATGTCCTTTG	58.4	536	INTERGENIC	--	--	--
Ca3_P4_10923237	Ca3	10923237	T/TCC	GCTGATTGAAATCGAGCACA	TTAAAACAAGCACCAAGGGC	60.0	347	INTERGENIC	--	--	--
Ca3_P4_10973797	Ca3	10973797	TTAATAATAATAATAATAATAATAATAATAATAATAATAATAATAATA/TTAATAATAATAATAATAATAATAATAATAATAATAATAATA	TCTCCGGATCAAATCCAAAT	TCTTTCCGACTAAGATACGATGTTC	59.3	394	INTERGENIC	--	--	--
Ca3_P4_11041298	Ca3	11041298	AT/A	TCGTGTCCCAAAACTCAAGG	TTAAGTTGGTTTGGGCTTGG	61.1	830	INTERGENIC	--	--	--
Ca3_P4_11044475	Ca3	11044475	CAAAAAAAAAA/CAAAAAAAAA	GCATCCATCCAAACGACTTT	CATTACATCTTATTTGAGGGAGAGC	59.9	286	INTERGENIC	--	--	--
Ca3_P4_11104695	Ca3	11104695	GTTTTTTTT/GTTTTTTT	TGTGGGGCTTAGAGCTTTGT	AAACTTGAGTTCCCACTCATCC	59.9	522	INTERGENIC	--	--	--
Ca3_P4_11213656	Ca3	11213656	GTAATAATAATAATAATAATAATAATAATAATAATAATAATAATAATAATAATAATAATAA/GTAATAATAATAATAATAATAATAATAATAATAATAATAATAATAATAATAATAATAATAATAATAATAATAA	CTCAAATCCTTCACCCTCCA	CGTTAACCAAGAAGTGGGGA	60.0	419	INTERGENIC	--	--	--

INDEL marker IDs	Chromosomes/ unanchored scaffolds	Physical positions (bp)	InDels (*Kabuli* reference genome-CDC Frontier/PII)	Forward primers (5'-3')	Reverse primers (5'-3')	Anneling temperature (0C)	Expected amplified product size (bp)	Structural annotation		Functional annotation	
								Sequence components of *kabuli* genome	*Kabuli* gene accession IDs	NCBI-KOG	TFs
Ca3_P4_11247051	Ca3	11247051	AAAATAAATAAATAAATAAATA/AAAATAAATAAATAAATAAATA	CTGGGGATACGTAGGAGCAA	TCCCTTAAAACGGCTACCCT	60.1	180	INTERGENIC	--	--	--
Ca3_P4_11307149	Ca3	11307149	TTATATATATATATATAT/TTATATATATATATATAT	GTGGTACTGGAGGCCACATT	CCAAAATGCCCCCTTTTATT	59.9	792	INTERGENIC	--	--	--
Ca3_P4_11315222	Ca3	11315222	TTATATATATATATATATATATATATATATATAT/TATATATATATATATATATATATATATATATATATATAT	CTCATGATCGGGAAAATGGT	GTTGAGAGGCATGAAGAGGC	59.8	217	INTERGENIC	--	--	--
Ca3_P4_11462251	Ca3	11462251	ATTTTTTTTT/ATTTTTTTTTT	ATGATTCCCCAACATCCAAA	CACGAGTCCGATAAGGGTGT	60.0	149	INTERGENIC	--	--	--
Ca3_P4_1149110	Ca3	1149110	TAAAAAAAAAAAA/TAAAAAAAAAAA	TGTGCAATCGTTTTAACCCA	TGAGGGGTAATTTGAACCGA	60.0	272	INTERGENIC	--	--	--
Ca3_P4_11497481	Ca3	11497481	ATTATTTATTTATTTATTTATTTATTTATGTATTTATGTATTTATGTATTTATTTATTCATTTATTTATTTATT/ATTATTTATTTATTTATTTATTTATTTATTTATGTATTTATGTATTTATGTATTTATT[illegible]	GGGTTTGCACTTGAAGGTGT	CCTCCGAGCCAAAATACAAA	60.0	617	INTERGENIC	--	--	--
Ca3_P4_11519916	Ca3	11519916	AT/ACAGACTTATACTGTTTT	CCGAGCCAAAATCCAAAATA	AATTTCAAACGCAAACCAGG	59.9	198	INTERGENIC	--	--	--
Ca3_P4_11521142	Ca3	11521142	TAAA/TAA	TCCATCGTACCGGGAGATAC	TCGATTATGCGTAGGGAAGG	59.8	235	INTERGENIC	--	--	--
Ca3_P4_11537812	Ca3	11537812	CTTTTTTT/CTTTTTTTT	GATGCAACATCTTTTTAATCTTTGA	CCTACGTATCTCCTGGTGCG	58.8	688	INTERGENIC	--	--	--
Ca3_P4_11628961	Ca3	11628961	TTATA/TTATATA	CTCTTCCTTCACGTAGGCCA	TGGAACCAATGCTTGTTGAA	60.4	800	INTERGENIC	--	--	--

INDEL marker IDs	Chromosomes/ unanchored scaffolds	Physical positions (bp)	InDels (*Kabuli* reference genome-CDC Frontier/PII)	Forward primers (5'-3')	Reverse primers (5'-3')	Anneling temperature (0C)	Expected amplified product size (bp)	Structural annotation		Functional annotation	
								Sequence components of *kabuli* genome	*Kabuli* gene accession IDs	NCBI-KOG	TFs
Ca3_P4_11649200	Ca3	11649200	TAAAAAA/TAAAAA	ATTCATCGCTGTCTTCCACC	GGTCCCTTAGCTTAATTTCCG	60.1	656	INTERGENIC	--	--	--
Ca3_P4_11889131	Ca3	11889131	GTTT/GTTTTT	AGGTTGGCAACATTCAGGTC	AGGAGGAGCAAAAGCAACAA	60.0	263	INTERGENIC	--	--	--
Ca3_P4_11919605	Ca3	11919605	GAA/GA	CGGAAATTGAAATCGGAAAA	ATCGCGAAAGTAGTCTCCCA	59.9	633	INTERGENIC	--	--	--
Ca3_P4_11919676	Ca3	11919676	CNNNNNNNNNNNNNNNNNNN/CNNNNNNNNN	CGGAAATTGAAATCGGAAAA	ATCGCGAAAGTAGTCTCCCA	59.9	633	INTERGENIC	--	--	--
Ca3_P4_11991649	Ca3	11991649	CTTTTTTTTTT/CTTTTTTTTT	TTTCCCGACTTGGTCCTTTA	TCATCAAATTCGTAACGCAAA	59.5	503	INTERGENIC	--	--	--
Ca3_P4_12051620	Ca3	12051620	GATATATATATATATATATAT/GATATATATATATATATATAT	GCAAAAGGGAATGGATCAAA	CAGGACCTTTCATGGGAAAA	59.9	202	INTERGENIC	--	--	--
Ca3_P4_1206045	Ca3	1206045	C/CTTT	GGCCGCCACAAAATATTAAC	TTTGAATTGGGGAGAGATGG	59.3	683	INTERGENIC	--	--	--
Ca3_P4_12167077	Ca3	12167077	GGTTCGCAGAAA/GGTTCGCAGAAAGTTCGCAGAAA	AACATGGAGCTGTTTGGGTC	TTTCCACGATCATCTCCCTC	60.0	594	INTERGENIC	--	--	--
Ca3_P4_12255918	Ca3	12255918	TTATATATATATATATATATATATATATATATATATATATA/TTATATATATATATATATATATATATATATATATATATATATATATA	TTTCACCGCAATGCAAATAA	TCCAAACAAACCCTTCGTATT	60.1	246	INTERGENIC	--	--	--
Ca3_P4_12288956	Ca3	12288956	TAAAAAAAAAAA/TAAAAAAAAAA	TAGCAGGGTGCATTGGTGTA	GTCGCACAAGGCTACAGTCA	60.1	605	INTERGENIC	--	--	--

INDEL marker IDs	Chromosomes/ unanchored scaffolds	Physical positions (bp)	InDels (*Kabuli* reference genome-CDC Frontier/PII)	Forward primers (5'-3')	Reverse primers (5'-3')	Anneling temperature (0C)	Expected amplified product size (bp)	Structural annotation		Functional annotation	
								Sequence components of *kabuli* genome	*Kabuli* gene accession IDs	NCBI-KOG	TFs
Ca3_P4_12392107	Ca3	12392107	TAT/TATCAT	AAGGAATCATTGTCCCTCCC	CACTCATCATTTACCTTGGCA	60.1	419	INTERGENIC	--	--	--
Ca3_P4_12426718	Ca3	12426718	AAAACCAAA/AAAACCAAACCAAA	GGTTGTGTGTTTTCCATCCC	TTCACTAGAGGGCCATCGTT	60.1	528	INTERGENIC	--	--	--
Ca3_P4_12659757	Ca3	12659757	CATATATATATATATATATATATATATAT/CATATATATATATATATATATATATATATAT	TATTTTGGATTTTGGCTCGG	CACCGTAGTGTGGTGGTGAG	59.9	672	INTERGENIC	--	--	--
Ca3_P4_12715466	Ca3	12715466	T/TACACATTAAAG	TATTCCCGATTAATGCAGCC	GTCCCGAATGCAATGTCTCT	59.9	667	INTERGENIC	--	--	--
Ca3_P4_12911294	Ca3	12911294	C/CCT	TATCGAGTGTTTCCGGTTCC	CAGGTTCACGTGAGCAAGAA	59.9	337	INTERGENIC	--	--	--
Ca3_P4_12969818	Ca3	12969818	TAAAAAAA/TAAAAAAAA	TTACACCAATTCCCCCAAGA	CATGCAAGGTTGGGCTAGAT	60.2	400	INTERGENIC	--	--	--
Ca3_P4_13096897	Ca3	13096897	TCT/TCTGGAACT	TCTGGTCGACTTCTGCCTTT	TCATCAGTATCGTGGGTGGA	60.0	775	INTERGENIC	--	--	--
Ca3_P4_13097036	Ca3	13097036	A/AT	CCTTCTTTACGGGACCCAAT	TCATCAGTATCGTGGGTGGA	60.2	364	INTERGENIC	--	--	--
Ca3_P4_13169603	Ca3	13169603	ATT/ATTTTTTTT	TAGCTGGCAGGGTCTCAGTT	CGAAAGAAAGTTTCCCCTCC	60.0	419	INTERGENIC	--	--	--
Ca3_P4_13236644	Ca3	13236644	AATATATATATATATATATATAT/AATATATATATATATATATATATATAT	AAGACCTGTGGGATCATTGC	TTTTGTGATAGGTTCCTGATGC	59.9	928	INTERGENIC	--	--	--

INDEL marker IDs	Chromosomes/ unanchored scaffolds	Physical positions (bp)	InDels (*Kabuli* reference genome-CDC Frontier/PII)	Forward primers (5'-3')	Reverse primers (5'-3')	Anneling temperature (0C)	Expected amplified product size (bp)	Structural annotation		Functional annotation	
								Sequence components of *kabuli* genome	*Kabuli* gene accession IDs	NCBI-KOG	TFs
Ca3_P4_13359074	Ca3	13359074	TAAAAAAAAAAA/TAAAAAAAAAAAA	AGTGATGCAATCTTCCACCC	ACGAGGATGCGATCTCCTAA	59.9	847	INTERGENIC	--	--	--
Ca3_P4_13364914	Ca3	13364914	GT/GTTGCT	AAACCCTAACGCACAGATGG	CGTTCTCATATGGTAGAAAAGGC	60.0	540	INTERGENIC	--	--	--
Ca3_P4_13417490	Ca3	13417490	TAAAAAAAA/TAAAAAAAAA	TGGGAATAGGTCAGGCTTTG	TGCACGAGAGGATTTGTTTG	60.1	339	INTERGENIC	--	--	--
Ca3_P4_13647076	Ca3	13647076	AATATATATATATATATATATAT/AATATATATATATATATATATATAT	TTGCACCTTATTTGATGCGT	TCTCTCTCTCATGCCACCCT	59.2	183	INTERGENIC	--	--	--
Ca3_P4_13647375	Ca3	13647375	AAGAGAGAGAGAGAGAGAGAGAGA/AAGAGAGAGAGAGAGAGAGAGAGAGAGA	AGGGTGGCATGAGAGAGAGA	TCATAATGGTGTGTTTGATTTGC	59.9	518	INTERGENIC	--	--	--
Ca3_P4_13732656	Ca3	13732656	TTATATATATATATATATATATATATA/TTATATATATATATATATATATA	GGACTCGCAATTTATTTTAGGAA	TGAGAATTGCAGAAAAAGCA	58.7	544	INTERGENIC	--	--	--
Ca3_P4_13802266	Ca3	13802266	TAAAAAAA/TAAAAAA	TGATAAAGCTCTCAAACAGCAGA	GAATTCCGATCAGATTTGACA	59.3	830	INTERGENIC	--	--	--
Ca3_P4_13899750	Ca3	13899750	CTTTT/CTTTTT	CTAGCCCAAGCACGCTTAAC	GATCCCATTAGAACTCCGCA	60.0	345	INTERGENIC	--	--	--
Ca3_P4_1390976	Ca3	1390976	TAAAAAAAAAA/TAAAAAAAAAAA	AGTGGCAACTGCACCTTTTT	CCAACTTGGTTCCACCAATC	59.8	741	INTERGENIC	--	--	--
Ca3_P4_13935710	Ca3	13935710	GAT/GATAAT	CAGTACCCCTTTGGAGCTTG	ATTTTCGGGCAATTTCACTG	59.7	127	INTERGENIC	--	--	--

INDEL marker IDs	Chromosomes/ unanchored scaffolds	Physical positions (bp)	InDels (*Kabuli* reference genome-CDC Frontier/PII)	Forward primers (5'-3')	Reverse primers (5'-3')	Anneling temperature (0C)	Expected amplified product size (bp)	Structural annotation		Functional annotation	
								Sequence components of *kabuli* genome	*Kabuli* gene accession IDs	NCBI-KOG	TFs
Ca3_P4_13996887	Ca3	13996887	TTATATATATATATATATATATATAT/ TTATATATATATATATATATATATATAT	TTGCTTCGATGGTCATTGTC	GCATAGTGCAACACCCCTCT	59.7	431	INTERGENIC	--	--	--
Ca3_P4_14018560	Ca3	14018560	CTTTTTTTTTTT/CTTTTTTTTTTTT	AGGGCAATCACGAGATTTTG	CACGACTTGAGTGGTCCAAG	60.1	635	INTERGENIC	--	--	--
Ca3_P4_14072375	Ca3	14072375	TTATATATATATATATATATATATAT/TTATATATATATATATATATATATATATAT	TTCACCCCATGCCATCTAAT	TTCTCCTTGAAGCTGTCGGT	60.2	417	INTERGENIC	--	--	--
Ca3_P4_14138350	Ca3	14138350	TTTATTATTATTATTATTATTATTATTATTATTATTATTA/TTTATTATTATTATTATTATTATTATTATTA	TCGCACATCTACTCAAACACG	ATAGATGATCGCATACCGGG	59.9	557	INTERGENIC	--	--	--
Ca3_P4_14205152	Ca3	14205152	AATATATATATATATATA/AATATATATATATATATATA	ACGGAACACAACCTTTGGAG	TGCTGCCTCTCATTCTCACA	60.0	452	INTERGENIC	--	--	--
Ca3_P4_14232110	Ca3	14232110	GAAAAAAAAAA/GAAAAAAAAA	TCCGTAACGCAAATTTTCAA	TCAAACCTTGTGCCTGAGTG	59.2	312	INTERGENIC	--	--	--
Ca3_P4_14428244	Ca3	14428244	CTTT/CTTTT	TGATTTGTGTTGGTTGTTGCT	CCATAGCAGCTAGACGGTCC	59.1	434	INTERGENIC	--	--	--
Ca3_P4_14429203	Ca3	14429203	ATTTTTTTTTTT/ATTTTTTTTTT	TGCGATGTTATCCAAAACCA	TGCAAATATCAATTGGGGGT	59.9	610	INTERGENIC	--	--	--
Ca3_P4_14441213	Ca3	14441213	AATAATAATAATAATAATAATAATAATAATAATAAT/AATAATAATAATGATAATAATAATAATAATAATAATAATAATAAT	CGGCATACAAATAACATGCG	TGTTTGTTTGTGGTGCCATT	60.0	626	INTERGENIC	--	--	--
Ca3_P4_1446842	Ca3	1446842	GCC/GCACC	TTGAAACATTTGAAGTATCCGC	TGCATTTGAGGAATTTCACG	59.1	488	INTERGENIC	--	--	--

INDEL marker IDs	Chromosomes/ unanchored scaffolds	Physical positions (bp)	InDels (*Kabuli* reference genome-CDC Frontier/PII)	Forward primers (5'-3')	Reverse primers (5'-3')	Anneling temperature (0C)	Expected amplified product size (bp)	Structural annotation		Functional annotation	
								Sequence components of *kabuli* genome	*Kabuli* gene accession IDs	NCBI-KOG	TFs
Ca3_P4_14574960	Ca3	14574960	CA/C	GGTTGGTAGGTGACAGGATCA	TGTTGTGGAGTTCTAGGGGC	59.8	457	INTERGENIC	--	--	--
Ca3_P4_14615841	Ca3	14615841	CAAAAAACAAA/CAAAAAACAAAAAAACAAA	AAGACAACCATGCCTTTTGG	GGGTTGGGCTATCAAATCAA	60.0	334	INTERGENIC	--	--	--
Ca3_P4_14686306	Ca3	14686306	T/TC	ATTGCATTAGGGTGTTTGGC	CCCCGTGAGATCTTCCTACA	59.8	835	INTERGENIC	--	--	--
Ca3_P4_14703766	Ca3	14703766	TATTCA/T	GGTCTTCAGGTTCATTCCCA	CCACCAGGAAAAGAAGGACA	59.9	821	INTERGENIC	--	--	--
Ca3_P4_14843003	Ca3	14843003	AATATATATATATATATATATATATATA/AATATATATATATATATATATATATATATATA	GAGAAAGAAGATATCGGATTCTCAA	AAAAATCAACGCAGTGGGAG	59.3	356	INTERGENIC	--	--	--
Ca3_P4_14874833	Ca3	14874833	GCC/GCCC	AGGTCATTTTCGTCCTCCCT	CAAACACACCTGTTGGATGC	59.9	679	INTERGENIC	--	--	--
Ca3_P4_14880871	Ca3	14880871	TG/TGTCG	TTTCGGTCATTTTCGTCCTC	ACACACCTATCGGATGCACA	60.1	616	INTERGENIC	--	--	--
Ca3_P4_1488532	Ca3	1488532	TTATATATATATATATATATATA/TTATATATATATATATATA	TCCAAACACCTTCACCATGA	CTGATCAGTGCTGTGGCATT	59.9	507	INTERGENIC	--	--	--
Ca3_P4_14902851	Ca3	14902851	GAA/GAAA	CGGTGGGGAGAACTCTGATA	TTCACATGACATCATCCCGT	60.1	474	INTERGENIC	--	--	--
Ca3_P4_14919264	Ca3	14919264	GTTT/GTTTT	CGATTGTGTTTGTCACAGGG	GAAGCAAATCGATTGGGAAA	60.0	303	INTERGENIC	--	--	--

INDEL marker IDs	Chromosomes/ unanchored scaffolds	Physical positions (bp)	InDels (*Kabuli* reference genome-CDC Frontier/PII)	Forward primers (5'-3')	Reverse primers (5'-3')	Anneling temperature (0C)	Expected amplified product size (bp)	Structural annotation		Functional annotation	
								Sequence components of *kabuli* genome	*Kabuli* gene accession IDs	NCBI-KOG	TFs
Ca3_P4_15038933	Ca3	15038933	TG/T	CAACACGATCATGACAAGCA	CCAAAGATCCCTACCAATACCA	59.2	131	INTERGENIC	--	--	--
Ca3_P4_15039270	Ca3	15039270	TC/T	CAACACGATCATGACAAGCA	GCATTTTGCATTTGTCAACG	59.2	677	INTERGENIC	--	--	--
Ca3_P4_15047295	Ca3	15047295	TA/T	TACTTTGTGCCATGCTCTGC	ACACAAGCCAACACATCCAA	60.0	117	INTERGENIC	--	--	--
Ca3_P4_15095814	Ca3	15095814	GAAAAAAAAAAAA/GAAAAAAAAAAA	TTGGTGCAAGCCACAAATAA	ATGATGGTGGTGATGGAGGT	60.1	547	INTERGENIC	--	--	--
Ca3_P4_1533357	Ca3	1533357	TCAAAA/TCAAAACTCCAAAA	ACCCAAAAGCTAACTTGCCA	GCATCAACAAAGCAGCAGAA	59.7	533	INTERGENIC	--	--	--
Ca3_P4_1534003	Ca3	1534003	AAACTGCAACTAACT/AAACTGCAACTAACTGCAACTAACT	TTCTGCTGCTTTGTTGATGC	GTGGATGGGTGTTTTCCATC	60.1	774	INTERGENIC	--	--	--
Ca3_P4_15374725	Ca3	15374725	ATTTTTTTTTT/ATTTTTTTTTTT	TTTGAAAGACCTGCAAACCC	GCGTAATTCCAATGATTGCC	60.1	614	INTERGENIC	--	--	--
Ca3_P4_15660475	Ca3	15660475	TA/TAA	AAGTGAGCTAACGGGGGTTT	TGGACAGAGCAAGTGAGGTG	60.0	140	INTERGENIC	--	--	--
Ca3_P4_15660692	Ca3	15660692	ATT/AT	AAGTGAGCTAACGGGGGTTT	TGGGGTTGTCAGGAGAAGAC	60.0	658	INTERGENIC	--	--	--
Ca3_P4_15844609	Ca3	15844609	C/CTAGG	ATGGGTAGTAAGCCCCGTCT	TCTTTCCAAAGGATGGATGG	59.8	831	INTERGENIC	--	--	--

INDEL marker IDs	Chromosomes/ unanchored scaffolds	Physical positions (bp)	InDels (*Kabuli* reference genome-CDC Frontier/PII)	Forward primers (5'-3')	Reverse primers (5'-3')	Anneling temperature (0C)	Expected amplified product size (bp)	Structural annotation		Functional annotation	
								Sequence components of *kabuli* genome	*Kabuli* gene accession IDs	NCBI-KOG	TFs
Ca3_P4_15868825	Ca3	15868825	ATATTATTATTATTATTATTATTATTATTATTATTATTATTATTATTATTATTATTAT/ATATTATTATTATTATTATTATTATTATTATTATTATTATTATTATTATTATTATTATTATTAT	AATCGTGCGTTTTTGCTTTT	AGGATTCTCAGTGCAAAGCG	59.8	472	INTERGENIC	--	--	--
Ca3_P4_16078474	Ca3	16078474	ATTTTTTTTT/ATTTTTTTTTT	TGAAGTCCCGTTACAATAAAACC	TTTGCTTTTGGATGAAACCC	59.3	594	INTERGENIC	--	--	--
Ca3_P4_16108101	Ca3	16108101	TAAAAAAAAAAA/TAAAAAAAAA	AAAACAGGGCAAGAAAGCAA	AACAGCAAAAGAGAAGGCGA	59.9	292	INTERGENIC	--	--	--
Ca3_P4_16133157	Ca3	16133157	GTTTTATTTTATTTTATTT/GTTTTATTTTATTTTATTTTATTT	CGTCTCTTGAAGCGATGAAA	CATTGTTGGTCACTAGCAAATCA	59.1	698	INTERGENIC	--	--	--
Ca3_P4_16133384	Ca3	16133384	CAATAATAATAATAATAATAATAATAATAATAATA/CAATAATAATAATAATAATAATAATAATAATAATAATAATA	CGTCTCTTGAAGCGATGAAA	ATAAGCCGTCAATCTGCCAC	59.1	807	INTERGENIC	--	--	--
Ca3_P4_16133499	Ca3	16133499	TA/TACACAAAGAA	TTTGCCCCATTATAAACAAAA	ATAAGCCGTCAATCTGCCAC	57.2	625	INTERGENIC	--	--	--
Ca3_P4_16137267	Ca3	16137267	CATATATATATATATATATA/CATATATATATATATATATA	TGGCACATCTAAAAATAGGAGGA	AGAAAGTCGCATCCCAGAGA	60.0	544	INTERGENIC	--	--	--
Ca3_P4_16166714	Ca3	16166714	TTTTG/TTGCTTTG	TCAACCATCTGGGTTGGAAT	CCCCTAGCAAAGGAAACAAA	60.2	674	INTERGENIC	--	--	--
Ca3_P4_16171083	Ca3	16171083	T/TG	TACAGCGCTTCATTCCACAG	CGTGCATGAGCAAAAGAAAA	60.0	468	INTERGENIC	--	--	--
Ca3_P4_16182804	Ca3	16182804	ATTTTTTTT/ATTTTTTTTTT	CCGGCATCACCTATCAGAGT	TCCGACCTCCTTTGAGAAGA	60.1	583	INTERGENIC	--	--	--

INDEL marker IDs	Chromosomes/ unanchored scaffolds	Physical positions (bp)	InDels (*Kabuli* reference genome-CDC Frontier/PII)	Forward primers (5'-3')	Reverse primers (5'-3')	Anneling temperature (0C)	Expected amplified product size (bp)	Structural annotation		Functional annotation	
								Sequence components of *kabuli* genome	*Kabuli* gene accession IDs	NCBI-KOG	TFs
Ca3_P4_16190775	Ca3	16190775	CA/C	TCGCGGTTCTTGATGATGTA	AGCTCGACTTATCCTTCGCA	60.2	423	INTERGENIC	--	--	--
Ca3_P4_16195131	Ca3	16195131	AT/ATT	TGATAAAAGACTAACGATGCAAACA	TAAACCATCAATCTGCCACG	60.1	655	INTERGENIC	--	--	--
Ca3_P4_16195709	Ca3	16195709	AC/A	ACGTGGCAGATTGATGGTTT	GAACGTACGGAAGAGAAGCG	60.4	754	INTERGENIC	--	--	--
Ca3_P4_16212096	Ca3	16212096	TAA/TAAA	AATGCACTTTGCCACTGTTG	CCAAGATTTTGCCAGATCAA	59.8	550	INTERGENIC	--	--	--
Ca3_P4_16212378	Ca3	16212378	A/AACATTC	ATGCACTTTGCCACTGTTGT	GAGGGGATGATGCTTCAAAA	59.2	879	INTERGENIC	--	--	--
Ca3_P4_16251959	Ca3	16251959	ATTCA/ATTCAGTTTCA	TTAATGAGGGGCTGAATTGG	AAGCGTACCTGTGGGAATTG	59.9	253	INTERGENIC	--	--	--
Ca3_P4_1632200	Ca3	1632200	GATATATATATATATATATATATAT/GATATATATATATATATATATATATATAT	GCGACCTTCTATTGACCTCG	CCCATTTGTTTGTGTTTCTTCC	59.8	363	INTERGENIC	--	--	--
Ca3_P4_16441388	Ca3	16441388	GAAA/GAAAA	CGCTCGATAACCCCAAAATA	ATGTCTCGTCATTCGGGAAG	59.9	441	INTERGENIC	--	--	--
Ca3_P4_16448740	Ca3	16448740	TATT/TATTATTATTATTATCATT	GAGGACATTGACGGGAGAGA	CTTCTTTGTGCGTCCACTGA	60.2	568	INTERGENIC	--	--	--
Ca3_P4_16471674	Ca3	16471674	CA/CAA	GTGAGGACTGGACGTAACCC	TCCTTGCGGAGTTGTTCTTT	59.4	505	INTERGENIC	--	--	--

INDEL marker IDs	Chromosomes/ unanchored scaffolds	Physical positions (bp)	InDels (*Kabuli* reference genome-CDC Frontier/PII)	Forward primers (5'-3')	Reverse primers (5'-3')	Anneling temperature (0C)	Expected amplified product size (bp)	Structural annotation		Functional annotation	
								Sequence components of *kabuli* genome	*Kabuli* gene accession IDs	NCBI-KOG	TFs
Ca3_P4_16476513	Ca3	16476513	TNNNNNNNNNN/TNNN	GGCTCACATGTGGAGGCTAT	CCAATGCCATACCATAGAGGA	60.1	344	INTERGENIC	--	--	--
Ca3_P4_1653851	Ca3	1653851	TTTATTATTATTATTATTATTATTATTATTATTATT/TTTATTATTATTATTATTATTATTATTATTATTATT	TTTCTAGGGTGCGACCATGT	ACGCGTTTGGGTAGATATGC	60.5	627	INTERGENIC	--	--	--
Ca3_P4_16544552	Ca3	16544552	GTTATTATTATTATTATTATTATTATTATTATTATTA/GTTATTATTATTATTATTATTATTATTATTATTA	TGCTCGACATGTTTCTCCTT	CCACACAAACTGACAACTCCA	58.4	605	INTERGENIC	--	--	--
Ca3_P4_16548843	Ca3	16548843	TAAAAAAAAAAAAAA/TAAAAAAAAAAAA	TAGCCCTCCCAATCATTTTG	CTTTAGGGGTTTTGATCGCA	59.9	561	INTERGENIC	--	--	--
Ca3_P4_16674646	Ca3	16674646	ATTTT/ATTTTT	GGAGTGGAGCTGATCAGAGG	GGCCCACAAAGTTTGAAAAA	59.9	685	INTERGENIC	--	--	--
Ca3_P4_16793468	Ca3	16793468	TTATA/TTATATA	TAATCAAATGCCCCCAATGT	TTGAGTTCAAGCGTGAGTGG	60.0	851	INTERGENIC	--	--	--
Ca3_P4_1685924	Ca3	1685924	TTATATATATATATATATATATATATATA/TTATATATATATATATATATATATATATATATATA	CTCGCTCTCCTATATCCCCC	GGGTGAAAAATTAACCAAACCA	60.0	508	INTERGENIC	--	--	--
Ca3_P4_17033658	Ca3	17033658	ATT/AT	ATGCGATCATGTGGATGAGA	TGGCTAATGCAGTTCACACA	60.0	648	INTERGENIC	--	--	--
Ca3_P4_17039245	Ca3	17039245	CAAAAAA/CAAAAAAAA	TTCTAACTTTGCTGTCCCATTTT	GGGGTAAAACCAGGAGGAAG	59.2	750	INTERGENIC	--	--	--
Ca3_P4_17039468	Ca3	17039468	C/CA	TGTTTTGAGGACAAATGTTTTGA	GGGGTAAAACCAGGAGGAAG	59.5	301	INTERGENIC	--	--	--

INDEL marker IDs	Chromosomes/ unanchored scaffolds	Physical positions (bp)	InDels (*Kabuli* reference genome-CDC Frontier/PII)	Forward primers (5'-3')	Reverse primers (5'-3')	Anneling temperature (0C)	Expected amplified product size (bp)	Structural annotation		Functional annotation	
								Sequence components of *kabuli* genome	*Kabuli* gene accession IDs	NCBI-KOG	TFs
Ca3_P4_17135834	Ca3	17135834	TCTC/TCTCTTTCCCTC	CAACGCGCTTTTAGATGAGA	AAAGCTTTGAAGCAGGGTCA	59.2	655	INTERGENIC	--	--	--
Ca3_P4_1719359	Ca3	1719359	ATCT/AT	TTTCCCTCCAAACACACACA	CTTTTGATGCCCCGATTAAA	60.0	351	INTERGENIC	--	--	--
Ca3_P4_17217773	Ca3	17217773	TCC/TC	ACAATCGGACACCGTTTCAT	TCATGTGGCAACACTGTTCA	60.2	253	INTERGENIC	--	--	--
Ca3_P4_17284618	Ca3	17284618	TTATATATATATATATATAT/TTATATATATATATAT	CCCTTCAAAACTCCCAAACA	TCTTCTTCTTCACCAAGTCCAA	59.9	369	INTERGENIC	--	--	--
Ca3_P4_173424	Ca3	173424	TAAAAAAAAAAAAA/TAAAAAAAAAAAA	GAGAGCGACAACACTGTCAAA	GTGGGAGGCGTGTTGTTATT	59.1	646	INTERGENIC	--	--	--
Ca3_P4_17413597	Ca3	17413597	TTTAATTAATTA/TTTAATTA	AAGTTGGTTCGTCCACCAAG	GAAATCGGAGAGAGGAAATCG	60.0	830	INTERGENIC	--	--	--
Ca3_P4_17447451	Ca3	17447451	TAAAAAAA/TAAAAAAAA	TTGAGGCCGAGATTACAAAAA	GGACAAAGGTGCAAGCTCTC	59.7	538	INTERGENIC	--	--	--
Ca3_P4_17491839	Ca3	17491839	AATATATATATATATA/AATATATATATATA	TGAATTCATGGCTAAAAGCCT	GCAAAGATGATAATTCGGCAA	60.1	654	INTERGENIC	--	--	--
Ca3_P4_17706970	Ca3	17706970	AATTATTATTATTATTATTATTATTATTATTATTATTA/AATTATTATTATTATTATTATTATTATTATTATTATTA	ACGGAAGCAGATGGTAGTGG	ATTTTAACAGATGGCCGCAT	60.1	517	INTERGENIC	--	--	--
Ca3_P4_17712986	Ca3	17712986	T/TA	TGCAACAACTTTAGATTCGGA	ATGGAGATAATCGACGTGCC	58.4	505	INTERGENIC	--	--	--

INDEL marker IDs	Chromosomes/ unanchored scaffolds	Physical positions (bp)	InDels (*Kabuli* reference genome-CDC Frontier/PII)	Forward primers (5'-3')	Reverse primers (5'-3')	Anneling temperature (0C)	Expected amplified product size (bp)	Structural annotation		Functional annotation	
								Sequence components of *kabuli* genome	*Kabuli* gene accession IDs	NCBI-KOG	TFs
Ca3_P4_17725458	Ca3	17725458	CATATATATATATATATATAT/CATATATATATATATATATAT	AGAAGAAGGAGGATTCGCGT	GCGATTTGGGAGAGAAACAG	60.3	572	INTERGENIC	--	--	--
Ca3_P4_17798477	Ca3	17798477	AAGT/A	ACACTCATTTTCGCCTCCAC	CTGGCATAGTGGTGGACCTT	60.1	623	INTERGENIC	--	--	--
Ca3_P4_17819480	Ca3	17819480	AATATATATATATATATATATATATATATATA/AATATATATATATATATATATATATA	AACGATCTGATTGTAATGTGTTTCTT	GTCGTGAACGGTGCTACATC	59.4	884	INTERGENIC	--	--	--
Ca3_P4_1786985	Ca3	1786985	AAAAATTAAAATTAAAATTAAAATT/AAAAATTAAAATTAAAATT	TTTTAATTGTAGCTTTGGTCCCTC	CTCACGAAACCGGTCTTTCTA	59.9	404	INTERGENIC	--	--	--
Ca3_P4_18108730	Ca3	18108730	GCTCTCTCTCTCTCTCTCTCTCTCTCTCTCTCT/GCTCTCTCTCTCTCTCTCTCTCTCTCTCTCTCT	CCACAAATCATCCCACTTCA	CAAACGACCCTTTTCCTTCA	59.3	929	INTERGENIC	--	--	--
Ca3_P4_18251815	Ca3	18251815	GAAAAAAAAAA/GAAAAAAAAAAA	TTACCCTTTGCTAGCCCCTT	TCGGGTTGTAATGGGGTTTA	60.1	431	INTERGENIC	--	--	--
Ca3_P4_18253562	Ca3	18253562	TGGGG/TGGG	TACGATGTTTCCGATGTGGA	GGGACTGATTCGTCCTTTCA	59.9	594	INTERGENIC	--	--	--
Ca3_P4_18360628	Ca3	18360628	GAAAAAAAAAAA/GAAAAAAAAAA	GGCCTATGGCCTGACCTACT	GCCAAACCATAGACCCTTGA	60.5	679	INTERGENIC	--	--	--
Ca3_P4_18536264	Ca3	18536264	GAAAAAAAAAA/GAAAAAAAAAAA	TTACGGTGCACCAATTCAAA	TTCATTTGCAATTCTTTTCACAA	60.0	574	INTERGENIC	--	--	--
Ca3_P4_18632022	Ca3	18632022	AATATATATATATATATATAT/AATATATATATATATATATAT	TGGGGCTGGTCCAATATAAA	AGATGTGGGGACAACGACTC	60.1	399	INTERGENIC	--	--	--

INDEL marker IDs	Chromosomes/ unanchored scaffolds	Physical positions (bp)	InDels (*Kabuli* reference genome-CDC Frontier/PII)	Forward primers (5'-3')	Reverse primers (5'-3')	Anneling temperature (0C)	Expected amplified product size (bp)	Structural annotation		Functional annotation	
								Sequence components of *kabuli* genome	*Kabuli* gene accession IDs	NCBI-KOG	TFs
Ca3_P4_18663228	Ca3	18663228	A/AGAGTTTAAACAAATT	TGTGCACAACTCATCGGATT	AAAAGCGTGATTTTTGTGGG	60.1	260	INTERGENIC	--	--	--
Ca3_P4_18943181	Ca3	18943181	AAT/A	TGGAGTGTTGATGTCGTTGTC	AAATCGAAGATCGCAACAGT	59.6	428	INTERGENIC	--	--	--
Ca3_P4_19011433	Ca3	19011433	TAAA/TAA	GGCATTGCCTCACCTTTTTA	TTAGGTTGTACGTCAGGGGC	60.1	632	INTERGENIC	--	--	--
Ca3_P4_19058867	Ca3	19058867	CNNNNNNNNNN/CN	TGAAAATAATCCCCTTCCCC	TTCCATCAAGCCAAAGGTTC	60.0	516	INTERGENIC	--	--	--
Ca3_P4_19062819	Ca3	19062819	AATATATATATATATATATATATATATATATATATATATATATA/AATATATATATATATATATATATATATATATATATATATATATATATA	CATGCTAAATAACATGAAGAATGC	GCAGGTTTGACAGTGAGCAA	57.9	637	INTERGENIC	--	--	--
Ca3_P4_19089322	Ca3	19089322	T/TATC	CACCGTTGACCAGTTTGATG	CACGGGAAACTCGATGATCT	60.0	713	INTERGENIC	--	--	--
Ca3_P4_19096971	Ca3	19096971	CTTTATTTATTTATTTATTTATTTATTTATTTATTTATTTATTT/CTTTATTTATTTATTTATTTATTTATTTATTTATTTATTTATTTATTT	AGCTGTCAAACTCAGCCCAT	TCGTGTCCCAAAACTCAAGA	59.9	652	INTERGENIC	--	--	--
Ca3_P4_19139838	Ca3	19139838	CATATATATATATATATATAT/CATATATATATATATATAT	TACCGCGTACCTTGCTCTTC	AGGATGGAACCCATCAACAC	60.4	754	INTERGENIC	--	--	--
Ca3_P4_19141746	Ca3	19141746	TTTTATTTATTTATTTATTTATTTATTTATTTATTTATTTATTTATTT/TTTTATTTATTTATTTATTTATTTATTTATTTATTTATTT	GGAGCTCCTGGTTACAAAACA	GGGTGTACATTGGTTGGGTC	59.2	484	INTERGENIC	--	--	--
Ca3_P4_19198715	Ca3	19198715	A/AC	TCTTCCACCCAGTTTTCACC	ACCGAGGAATGATATCGACG	59.9	385	INTERGENIC	--	--	--

INDEL marker IDs	Chromosomes/ unanchored scaffolds	Physical positions (bp)	InDels (*Kabuli* reference genome-CDC Frontier/PII)	Forward primers (5'-3')	Reverse primers (5'-3')	Anneling temperature (0C)	Expected amplified product size (bp)	Structural annotation		Functional annotation	
								Sequence components of *kabuli* genome	*Kabuli* gene accession IDs	NCBI-KOG	TFs
Ca3_P4_19269160	Ca3	19269160	GT/GTT	CAATTGCAATGTTGTTTGCC	CCGTCGTTTTGCAGTATTTG	60.0	592	INTERGENIC	--	--	--
Ca3_P4_19269186	Ca3	19269186	CAAAAAAAA/CAAAAAAAAA	CAATTGCAATGTTGTTTGCC	CCGTCGTTTTGCAGTATTTG	60.0	592	INTERGENIC	--	--	--
Ca3_P4_19299532	Ca3	19299532	ATTTTTTTTTT/ATTTTTTTTT	CGCATTACAACTAAAATGGACG	TGTGCTTCCACCTTTCATCA	59.6	936	INTERGENIC	--	--	--
Ca3_P4_19359038	Ca3	19359038	TAAAA/TAAA	CGTCCTTTGTTCATTCCCAC	TGAGGGCCTTTAGAATCGTG	60.4	670	INTERGENIC	--	--	--
Ca3_P4_19359334	Ca3	19359334	CCA/C	AGGGAAATCAACGACAGAGC	TGAGGGCCTTTAGAATCGTG	59.3	365	INTERGENIC	--	--	--
Ca3_P4_19362553	Ca3	19362553	TAAAAA/TAAAA	CCAAAATGATGTTGCAGGGT	TCATCATCATTGAAAATCCGTT	60.8	361	INTERGENIC	--	--	--
Ca3_P4_19369520	Ca3	19369520	TAAAAAAAAA/TAAAAAAA	AGATGTTGGCGCAGATTCTT	CAGCAATGGTTTTGTTGTGG	59.8	659	INTERGENIC	--	--	--
Ca3_P4_19373696	Ca3	19373696	TAA/TA	GCTGAATGCGTGAAGAACAA	GGGTAGAAAACACGCGGAAT	60.0	339	INTERGENIC	--	--	--
Ca3_P4_19375049	Ca3	19375049	AAAAGAAA/AAAAGAAAGAAA	TCTCCGTTCCATTTACACGA	AACTCCAAATCCCTGCTCAA	59.1	365	INTERGENIC	--	--	--
Ca3_P4_19375109	Ca3	19375109	GAGAAGAAGAAGAAGAAGA/GAGAGAGAGAGAGAAGAAGAAGAAGAAGAAGA	TCTCCGTTCCATTTACACGA	AACTCCAAATCCCTGCTCAA	59.1	365	INTERGENIC	--	--	--

INDEL marker IDs	Chromosomes/ unanchored scaffolds	Physical positions (bp)	InDels (*Kabuli* reference genome-CDC Frontier/PII)	Forward primers (5'-3')	Reverse primers (5'-3')	Anneling temperature (0C)	Expected amplified product size (bp)	Structural annotation		Functional annotation	
								Sequence components of *kabuli* genome	*Kabuli* gene accession IDs	NCBI-KOG	TFs
Ca3_P4_19376077	Ca3	19376077	A/ATTT	TGACCTTGTGCATTGTTTTGA	GCAAACTCAACCTTGCCAAT	60.1	333	INTERGENIC	--	--	--
Ca3_P4_19380131	Ca3	19380131	TG/T	TTGGAGAGAATACTCCCCGTT	TGACGATCAGGAACACCAAA	59.9	413	INTERGENIC	--	--	--
Ca3_P4_19381557	Ca3	19381557	GTTATTATTAT/GTTATTATTATTAT	GGAGAAAAGCATCGAAAAGC	ACCTAACCCTCCAAGCCAGT	59.0	338	INTERGENIC	--	--	--
Ca3_P4_19382845	Ca3	19382845	TTT/TTTATT	TAGGGGCATATGGTCTCGTC	ACCACATGATGCTCTCCACA	59.9	782	INTERGENIC	--	--	--
Ca3_P4_19403411	Ca3	19403411	TG/TTCGGGG	AGGGTAATCCGCCAGTCTTT	GCAACATTCGTCCTCTCCAT	60.0	674	INTERGENIC	--	--	--
Ca3_P4_19496769	Ca3	19496769	CTATAT/CTATATGTATATATATATATAT	CGGAAAATAAGTTAAGGGACCA	AAAATACATCAAAAGTTCATCATTCG	59.4	383	INTERGENIC	--	--	--
Ca3_P4_1955332	Ca3	1955332	CAAA/CAA	TCATGGTTCTTTTCGAAGTGG	CACCCACTCATCCTTCACAA	60.1	930	INTERGENIC	--	--	--
Ca3_P4_19557962	Ca3	19557962	AC/A	TCGGTAACCAATCCGAAGAC	ATTACCCGATGCGTAAATCG	59.9	225	INTERGENIC	--	--	--
Ca3_P4_19560633	Ca3	19560633	TAAAAAAAAA/TAAAAAAAAAA	TCAGTGCGATGGAGAAATCA	TGACATTTTAAACCTTGAGTTGC	60.4	453	INTERGENIC	--	--	--
Ca3_P4_19626677	Ca3	19626677	GATATATATATATATATATATATA/GATATATATATATATATATATATA	TTCCCCATGCAAGGTTAGAC	TCATCGCAGGTGTACCATGT	59.9	797	INTERGENIC	--	--	--

INDEL marker IDs	Chromosomes/ unanchored scaffolds	Physical positions (bp)	InDels (*Kabuli* reference genome-CDC Frontier/PII)	Forward primers (5'-3')	Reverse primers (5'-3')	Anneling temperature (0C)	Expected amplified product size (bp)	Structural annotation		Functional annotation	
								Sequence components of *kabuli* genome	*Kabuli* gene accession IDs	NCBI-KOG	TFs
Ca3_P4_1964709	Ca3	1964709	A/AT	TCTCTTTTCTCGAACCGCAT	AAACGCAATCCTTGAGTTCG	60.0	317	INTERGENIC	--	--	--
Ca3_P4_19676099	Ca3	19676099	GTTTTTTTTTTT/GTTTTTTTTTTTTT	CAAACCCAAGCCATCTTCAT	TAAACTTTTTGGCGGTTTGG	59.9	380	INTERGENIC	--	--	--
Ca3_P4_19790164	Ca3	19790164	TTATATATATATATATATATATATATATA/TTATATATATATATATATATATATATATATA	GATGGAAGTGTTGAGCCTCC	AATTTAAGCGGTGGCAAATG	59.7	698	INTERGENIC	--	--	--
Ca3_P4_19960902	Ca3	19960902	TAAAAAAAAAAA/TAAAAAAAAAA	AGCTTTGATTTCTCCATCGC	GGTATTAGCACCCTACGGCA	59.4	243	INTERGENIC	--	--	--
Ca3_P4_20052639	Ca3	20052639	AATTATTATTATTATTATTATTATTATTATTATTATTATTAT/AATTATTATTATTATTATTATTATTATTAT	ATCGTGTGCAAAGGAAGGAT	TGTGCTCAAATAAATAACGTGTCTC	59.6	856	INTERGENIC	--	--	--
Ca3_P4_20074437	Ca3	20074437	CTTTTTTTTTT/CTTTTTTTTT	CAAATACATGTGCCACACAAA	TTTAACCAAATTGTGCAAGGAA	58.0	525	INTERGENIC	--	--	--
Ca3_P4_20159047	Ca3	20159047	AATATATATATATATATATATATATATATA/AATATATATATATATATATATATATATATATATATA	GATTGTTTATGCGCAAGGGT	TGCTTATTCCCCTCCTTCCT	60.0	489	INTERGENIC	--	--	--
Ca3_P4_20315844	Ca3	20315844	ATTTTTT/ATTTTTTT	CGCAAACACACTTGAACTGC	AAAGCATGTCACCAAATAAGCA	60.5	184	INTERGENIC	--	--	--
Ca3_P4_20339565	Ca3	20339565	TAA/TAAA	GGCAAAATGAATTTCCCTCA	AAACGCAATCCTTGAGTTCG	59.9	417	INTERGENIC	--	--	--
Ca3_P4_20349202	Ca3	20349202	TTATATATATATATATATATAT/TTATATATATATATATATATATAT	ACTGCCTAGCTCACTTCCCA	GGCGATTTTCATCGTGAGTT	60.0	417	INTERGENIC	--	--	--

INDEL marker IDs	Chromosomes/ unanchored scaffolds	Physical positions (bp)	InDels (*Kabuli* reference genome-CDC Frontier/PII)	Forward primers (5'-3')	Reverse primers (5'-3')	Anneling temperature (0C)	Expected amplified product size (bp)	Structural annotation		Functional annotation	
								Sequence components of *kabuli* genome	*Kabuli* gene accession IDs	NCBI-KOG	TFs
Ca3_P4_20378345	Ca3	20378345	CAGAGA/CAGA	AGGTCAATGCCTCTTCTCCA	ACCTCATGAGTTGGAAACGG	59.8	226	INTERGENIC	--	--	--
Ca3_P4_20390519	Ca3	20390519	TTTATTATTATTATTATTATTATTATTATTATTATTATTATTATTATTATTAT/TTTATTATTATTATTATTATTATTATTATTATTATTATTATTATTAT	CAATGGCACAAAGATGGAAA	CAGAAATTCAACATGAACCCAA	59.5	749	INTERGENIC	--	--	--
Ca3_P4_20424638	Ca3	20424638	CAAAAAAAAA/CAAAAAAAA	TACATTGGTTTGTGCGATGC	TGACTATGGGAGGTCAGGAAA	60.5	636	INTERGENIC	--	--	--
Ca3_P4_20709467	Ca3	20709467	CATATATATATATATATATATATATATA/CATATATATATATATATATATATATATATATA	ATGGGCTTGGTAGGGACTTT	CAATCCAAGCATGCAATCC	59.8	638	INTERGENIC	--	--	--
Ca3_P4_20940933	Ca3	20940933	GATATATATATATATATATATATATA/GATATATATATATATATATATATATATA	TTTTGTCACTTGATACAGCAAAA	TGAGCATTTAAAAGCCCTGC	57.6	798	INTERGENIC	--	--	--
Ca3_P4_20993915	Ca3	20993915	AATATATATATATATATATATATATATATAAATATATATATATATATATATATA/AATATATATATATATATATATATATATATATATATATATAAATATATATATATATATATATATA	GATTTCACGTGTTGCCGTAG	TCCGTTCGTTTTATTTTCCAA	59.2	298	INTERGENIC	--	--	--
Ca3_P4_21028328	Ca3	21028328	CAAAAAAAAAA/CAAAAAAAAA	TGTCGTAGAAACATTTGTGGG	AGCATTCTCCGAAGATACACG	58.5	685	INTERGENIC	--	--	--
Ca3_P4_21088951	Ca3	21088951	TTATATATATATATATATA/TTATATATATATATATATATATA	AAGCGATGACATAAGAACAACTACC	AAACGTGTATGCACAAGCCA	60.0	453	INTERGENIC	--	--	--
Ca3_P4_21092785	Ca3	21092785	CATATATATATATATATATATATATAT/CATATATATATATATATATATATATATATATATAT	GTGCGTGTGTGGAGAGAAGA	ATTGATTGTCGCCATGTGAA	60.0	512	INTERGENIC	--	--	--
Ca3_P4_21097843	Ca3	21097843	ATTTTTTTTT/ATTTTTTTTTT	GGCATTTTGATTTGATTCGC	CAAATTTTGTGTTTCAAAAACCATT	60.4	653	INTERGENIC	--	--	--

INDEL marker IDs	Chromosomes/ unanchored scaffolds	Physical positions (bp)	InDels (*Kabuli* reference genome-CDC Frontier/PII)	Forward primers (5'-3')	Reverse primers (5'-3')	Anneling temperature (0C)	Expected amplified product size (bp)	Structural annotation		Functional annotation	
								Sequence components of *kabuli* genome	*Kabuli* gene accession IDs	NCBI-KOG	TFs
Ca3_P4_21114306	Ca3	21114306	CTAGTA/CTAGTAGTA	TCATGCGATCATCCATTCTAA	ATTTGCAACGATGTGAACCA	59.1	594	INTERGENIC	--	--	--
Ca3_P4_2114353	Ca3	2114353	TCCCCC/TCCCCCC	AGTTTGTGTACACCCGAGGC	GGAGCCTTAAGTCAGGTGGA	60.0	505	INTERGENIC	--	--	--
Ca3_P4_21192862	Ca3	21192862	AATATATATATATATATATATATATATATATATATAT/AATATATATATATATATATATATATATATATATAT	TGTCTTTCCTCGTTTTTCAATG	TTTGTAGTTCACCAGCAGGG	59.2	414	INTERGENIC	--	--	--
Ca3_P4_21311568	Ca3	21311568	GAAAAAAAAA/GAAAAAAAAAA	CACGTCACATCGCAATTTTT	CATGTCATTTCAACCCACGA	59.6	542	INTERGENIC	--	--	--
Ca3_P4_21363714	Ca3	21363714	CTATATATATATATATATATATA/CTATATATATATATATATATA	AACGGACCGTGGTCTAACAG	AAGGTGCGTACAAGGAATGG	60.0	401	INTERGENIC	--	--	--
Ca3_P4_21481670	Ca3	21481670	CTATTATTATTATTATTATTATTATTATTATTATTATTATTATTATTATTAT/CTATTATTATTATTATTATTATTATTATTATTATTATTATTATTATTATTAT	GCGTAGTAAATCCTTCTTCCACA	TGAGACAAAGAATAACACCCGA	59.7	568	INTERGENIC	--	--	--
Ca3_P4_21696134	Ca3	21696134	CTATTATTATTATTATTATTATTATTATTATTATTATTATTATTATT/CTATTATTATTATTATTATTATTATTATTATTATTATTATTATT	TTTTTCAACAACTCTCTCCGA	GCAAATGCAGACCAGAATCA	57.6	532	INTERGENIC	--	--	--
Ca3_P4_21892232	Ca3	21892232	T/TG	TGTCATCTGGGATCGTTTCA	AATTCCATCTCCTCTGGGCT	60.0	784	INTERGENIC	--	--	--
Ca3_P4_21897869	Ca3	21897869	ATATTATTATTATTATTATTATTATTA/ATATTATTATTATTATTATTATTA	GAGTTGAGTGTTGCCAGGGT	AGCTACCAAGCTGATGGTGAA	60.2	296	INTERGENIC	--	--	--
Ca3_P4_21899233	Ca3	21899233	AATCATCATCATCATCATCATCAT/AATCATCATCATCATCATCATCATCAT	CGTTGCATGATAGCTCCTGA	TGCTCATGGAATCGTACTCG	60.0	735	INTERGENIC	--	--	--

**INDEL marker IDs**	**Chromosomes/ unanchored scaffolds**	**Physical positions (bp)**	**InDels (*Kabuli* reference genome-CDC Frontier/PII)**	**Forward primers (5'-3')**	**Reverse primers (5'-3')**	**Anneling temperature (0C)**	**Expected amplified product size (bp)**	**Structural annotation**		**Functional annotation**	
								**Sequence components of *kabuli* genome**	***Kabuli* gene accession IDs**	**NCBI-KOG**	**TFs**
Ca3_P4_21976340	Ca3	21976340	CATATATATATATATATATATATATATATAT/CATATATATATATATATATATATATATATATATATATAT	TCTTGTGTCGACCTTAGCGT	TTGATGGGGCTTGTCTTAGG	58.5	402	INTERGENIC	--	--	--
Ca3_P4_22085724	Ca3	22085724	GTTTTT/GTTTT	TGGACTTTCATGTGTGAGTCCTAT	CTGCAACCAACCTTTTCCAT	59.9	251	INTERGENIC	--	--	--
Ca3_P4_22114232	Ca3	22114232	ATTTTTTTTT/ATTTTTTTT	AAAAGTTGACGTGGCATTGA	ACTCGTATTTTACCCGTGCG	59.2	579	INTERGENIC	--	--	--
Ca3_P4_22137436	Ca3	22137436	CATATATATATATATATATATATATATATATA/CATATATATATATATATATATATATATATATATATATATATATA	ATCCGCACCTGTTTCATAGC	ATTAAATTTCGGGGACGGAG	60.1	339	INTERGENIC	--	--	--
Ca3_P4_22160995	Ca3	22160995	CAAAAAAA/CAAAAAA	CCTTTTCCTTCCCTTTCCTG	CAATGTGCTCACTTTTGTCTCA	60.0	527	INTERGENIC	--	--	--
Ca3_P4_2225131	Ca3	2225131	GAAAAAAAAA/GAAAAAAAAAA	CACACAATACATGTAGAGTCGCC	GGGAGAATTGTAACACCCCA	59.6	296	INTERGENIC	--	--	--
Ca3_P4_22369157	Ca3	22369157	TTATATATATATATATATATATATATATATATATATATATATATA/TTATATATATATATATATATATATATATATATATA	AAACATTGAGTTGCGATGTCA	CAATTGAGTTGCATTGGTGG	59.2	733	INTERGENIC	--	--	--
Ca3_P4_22376746	Ca3	22376746	ATATTATTATTATTATTATTATTATTATTATTATTATTATTA/ATATTATTATTATTATTATTATTATTATTATTATTATTATTATTA	TTTCTTAATCCCCGTGATTTG	TCTCTTGGGAGGATTGGTTG	58.9	175	INTERGENIC	--	--	--
Ca3_P4_22424661	Ca3	22424661	ATTTTTTT/ATTTTTTTT	GACCAAGCCCATCTTTACGA	AATGTGCCACAAACAAAACG	60.1	779	INTERGENIC	--	--	--
Ca3_P4_22478435	Ca3	22478435	AATATATATATATATATATATATATATATATATATAT/AATATATATATATATATATATATATATATAT	AATTATTGCGTGCATGTGGA	CAAAGAAACAGAAATGTGGAGG	60.0	638	INTERGENIC	--	--	--

INDEL marker IDs	Chromosomes/ unanchored scaffolds	Physical positions (bp)	InDels (*Kabuli* reference genome-CDC Frontier/PII)	Forward primers (5'-3')	Reverse primers (5'-3')	Anneling temperature (0C)	Expected amplified product size (bp)	Structural annotation		Functional annotation	
								Sequence components of *kabuli* genome	*Kabuli* gene accession IDs	NCBI-KOG	TFs
Ca3_P4_22487747	Ca3	22487747	TACAGTGA/TA	GTAATGCCCCGTGAGTAGGA	CCCAATTGATTCCAAAAGAAA	60.0	778	INTERGENIC	--	--	--
Ca3_P4_22534501	Ca3	22534501	TTTATTATTATTATTATTAT/TTTATTATTATTATTAT	CAACGAGGGATGGGAATAGA	GTGCACGGTACAAACCTCCT	59.9	594	INTERGENIC	--	--	--
Ca3_P4_2260100	Ca3	2260100	TAAAA/TAAAAA	TGGACTTGTTTGGTCCATCA	ACCCCATTTTGTTGTGGTGT	59.9	293	INTERGENIC	--	--	--
Ca3_P4_22809344	Ca3	22809344	TTTATTATTAT/TTTATTAT	GGATTGGGGGTCATTTAGGT	AGCGACCTCCCACTAGGTTT	59.9	670	INTERGENIC	--	--	--
Ca3_P4_22932908	Ca3	22932908	TATAATAATAATAATAATAATAATAATAA/TATAATAATAATAATAATAATAATAATAATAA	TCAGCAGAAAATGCAAAACG	CGACCGACCCATACAAGTTT	60.0	570	INTERGENIC	--	--	--
Ca3_P4_23014028	Ca3	23014028	CAAAAAAAAAAAAAAA/CAAAAAAAAAAAAA	GGATGTTGCACAATCAAGGA	GGCAGTGGTAGTGAAGCACA	59.5	370	INTERGENIC	--	--	--
Ca3_P4_23063614	Ca3	23063614	TTATATATATATATATATATATATA/TTATATATATATATATATATATATATATA	GTAACTTGCACCGACCCAAT	ATGACGGTGTTTGGAGACAA	59.9	599	INTERGENIC	--	--	--
Ca3_P4_23136591	Ca3	23136591	ATTT/ATTTT	GGGTGAGTTTCTGGGTCTCA	CTTCAAACAATGGTCCCTCAA	60.1	723	INTERGENIC	--	--	--
Ca3_P4_23166097	Ca3	23166097	TATAATAATAATAATAATGATAATAATAATAATAATAAT/TATAATAATAATAATAATGATAATAATAATAATAATAAT	TTTCATTTCGTTTTCAAGTTATCAA	TTAATGCTAACAGGGGGTCG	59.1	599	INTERGENIC	--	--	--
Ca3_P4_23219721	Ca3	23219721	CATTATTATTATTATTATTATTATTAT/CATTATTATTATTATTATTATTATTAT	GATTAGAGCGGCCAAAATGA	TTTTCAATTAGTCGGGTGGC	60.2	593	INTERGENIC	--	--	--

INDEL marker IDs	Chromosomes/ unanchored scaffolds	Physical positions (bp)	InDels (*Kabuli* reference genome-CDC Frontier/PII)	Forward primers (5'-3')	Reverse primers (5'-3')	Anneling temperature (0C)	Expected amplified product size (bp)	Structural annotation		Functional annotation	
								Sequence components of *kabuli* genome	*Kabuli* gene accession IDs	NCBI-KOG	TFs
Ca3_P4_23253882	Ca3	23253882	GAAAAAAAAAAA/GAAAAAAAAAA	TGGGTTGCGATCGTGTATTA	TCAGTCAGTTGCAACATAGGAGA	60.0	327	INTERGENIC	--	--	--
Ca3_P4_23463912	Ca3	23463912	CATATATATATATATATATATATATATATA/CATATATATATATATATATATATATA	TCAAATTTGGAGGAAACAACG	TCGGAAAACTTGTGGATGAA	60.0	716	INTERGENIC	--	--	--
Ca3_P4_23530917	Ca3	23530917	AATATATATATATATATATATATAT/AATATATATATATATATATATATATAT	ACCCATTAGCGTAGGAGTGC	AACGTAAATGCACTTGAACCG	59.2	786	INTERGENIC	--	--	--
Ca3_P4_23548884	Ca3	23548884	AATATATATATATATATATATATA/AATATATATATATATA	ATGTGCATGCGTGGTACTGT	AAAAAGTAATTGAGGCCGGG	60.1	704	INTERGENIC	--	--	--
Ca3_P4_23606563	Ca3	23606563	AATATATATATATATATATATATATATATA/AATATATATATATATATATATATATATATATATATA	TTGGTTTAATCACCCTTTTCAA	TTCAACCAATTGTGTCTGGC	58.5	660	INTERGENIC	--	--	--
Ca3_P4_23653507	Ca3	23653507	GTTTTTTTTTT/GTTTTTTTTTTT	AAAATAAAATGCGGTGAGCG	CCAAATGTTGTCAAAAAGGGA	60.1	656	INTERGENIC	--	--	--
Ca3_P4_23830553	Ca3	23830553	ACTCTCTCTCTCTCTCTCTCTCTCTCTC/ACTCTCTCTCTCTCTCTCTCTCTCTCTCTCTCTC	TCACGAAATTGGTCCCCTTA	GGGACCAAAACTGGAGAGAA	60.3	878	INTERGENIC	--	--	--
Ca3_P4_23881356	Ca3	23881356	TTATATATATATATATAT/TTATATATATATATATATATATATAT	ACAGCCAACAACCACATCAA	CGTAAGTGTGACCGACGCTA	60.0	864	INTERGENIC	--	--	--
Ca3_P4_23993355	Ca3	23993355	AATAAATTATAAATTATAAATTA/AATAAATTATAAATTA	CCGAGCCAAAATCCAAAATA	GTTTGGGGCACAATTGAGAT	59.9	599	INTERGENIC	--	--	--
Ca3_P4_24004974	Ca3	24004974	GAAAAAAAA/GAAAAAAAAAA	TCAGGCCATCGATCATGTTA	CTCACGACCAAGTGCAAGAA	60.0	564	INTERGENIC	--	--	--

INDEL marker IDs	Chromosomes/ unanchored scaffolds	Physical positions (bp)	InDels (*Kabuli* reference genome-CDC Frontier/PII)	Forward primers (5'-3')	Reverse primers (5'-3')	Anneling temperature (0C)	Expected amplified product size (bp)	Structural annotation		Functional annotation	
								Sequence components of ***kabuli*** genome	***Kabuli*** gene accession IDs	NCBI-KOG	TFs
Ca3_P4_240123	Ca3	240123	GCGCCGCCGCC/GCGCCGCCGCCGCC	GCAATTTTGTGAAGGAGGGA	GACCTTGTGGATGGCAGTTT	60.1	189	INTERGENIC	--	--	--
Ca3_P4_24035775	Ca3	24035775	TAAAAAAAAAAAAA/TAAAAAAAAAAAA	TTGTGAGGTATGGACGTGTGA	GAATTACATGCGTTTGTGCG	60.0	292	INTERGENIC	--	--	--
Ca3_P4_24156923	Ca3	24156923	TTATATATATATATATATATATAT/TTATATATATATATATATATATATAT	CAATAGCCCAATTTTGAAAGAA	TGCTTGGGTGAGTGAGAGTG	58.3	397	INTERGENIC	--	--	--
Ca3_P4_24199958	Ca3	24199958	ACACTCA/ACA	CACATGAAGCGTGCAATTTT	GGTGGGGTGATGACAAATTC	59.7	472	INTERGENIC	--	--	--
Ca3_P4_24272959	Ca3	24272959	GAATAATAA/GAATAATAATAA	GCATGTCCGTTACCTCCACT	GCGATTTATTATTCAGAGTTATTAGCG	60.0	646	INTERGENIC	--	--	--
Ca3_P4_24439636	Ca3	24439636	ATTTTTTTTTT/ATTTTTTTTT	ACTTTGCAAGCTTTTGCGAT	AACAATGTATGCACAGGCAAG	60.0	503	INTERGENIC	--	--	--
Ca3_P4_24441612	Ca3	24441612	AATATATA/AATATATATA	GGTGCCACATCATACCATTG	TCAAACAAAATTGGTTGCTTTTT	59.7	333	INTERGENIC	--	--	--
Ca3_P4_24550785	Ca3	24550785	AATATATATATATATATATAT/AATATATATATATATATATATATAT	TCGTGCATATCAATTAAACTTAGGA	CCGGTTCAATGATGTGACCT	59.2	664	INTERGENIC	--	--	--
Ca3_P4_24554701	Ca3	24554701	ATGTG/ATG	GGAGTATTGTGAACTCCACACG	AGATTTTGATTGATTGGCGG	59.5	764	INTERGENIC	--	--	--
Ca3_P4_24585169	Ca3	24585169	ATTTTTTTTTTT/ATTTTTTTTTT	TATGTTAGACCGTCCGTGGG	TTGTACTGGTGATCATCGAAAAG	60.8	888	INTERGENIC	--	--	--

INDEL marker IDs	Chromosomes/ unanchored scaffolds	Physical positions (bp)	InDels (*Kabuli* reference genome-CDC Frontier/PII)	Forward primers (5'-3')	Reverse primers (5'-3')	Anneling temperature (0C)	Expected amplified product size (bp)	Structural annotation		Functional annotation	
								Sequence components of *kabuli* genome	*Kabuli* gene accession IDs	NCBI-KOG	TFs
Ca3_P4_24685752	Ca3	24685752	GTTGTTATGACTTTGTTATG/GTTGTTATG	GGCTTTGATGCTTATGAAGGTT	GTTCGGTTCGGTTCTTTGAA	59.6	746	INTERGENIC	--	--	--
Ca3_P4_24731209	Ca3	24731209	ATTTTTTTTTTTT/ATTTTTTTTTTTTT	GGGGTTTCACTGGTTGAAGA	GCTTTGACCCCAGAATTCAA	59.9	467	INTERGENIC	--	--	--
Ca3_P4_24790252	Ca3	24790252	AGG/AATAAATGG	TCAGCCAAAAAGCTGATGTG	AAGTTAAGGGACCACGGATG	60.0	324	INTERGENIC	--	--	--
Ca3_P4_24791040	Ca3	24791040	GAAAAAA/GAAAAA	GGGACCACGAATGTAATTTAGC	TGCGAGCATAAGAAGTTGTTG	59.7	740	INTERGENIC	--	--	--
Ca3_P4_24796858	Ca3	24796858	AA/AACATTATCACAAACTA	AAAACCAAATGCTTCACATGC	GGCAATGACTTGTCCCTGTT	60.0	784	INTERGENIC	--	--	--
Ca3_P4_24850043	Ca3	24850043	T/TA	TGACGTGACATGCATACGATT	CAGCTACCAACACTCGACCA	60.0	823	INTERGENIC	--	--	--
Ca3_P4_24888413	Ca3	24888413	AATATATATATATAT/AATATATATATATATAT	CATGCATTCAATCAAATCACA	TGGGACAAAAACAAGCTAACAA	58.0	857	INTERGENIC	--	--	--
Ca3_P4_24927543	Ca3	24927543	ATATTATTATTATTATTATTATT/ATATTATTATTATTATTATTATTATTATT	TGTTGGATTCGATGTTTGTGA	GTGTCACTTTAATGGACAGCAA	60.0	260	INTERGENIC	--	--	--
Ca3_P4_2494761	Ca3	2494761	TAAA/TAAAA	GCATCTTTGTGGATGCCTTT	GTGCCAGTCGATTGCCTAAT	60.1	591	INTERGENIC	--	--	--
Ca3_P4_2496760	Ca3	2496760	A/AT	GGTTTCGAGGAAACTGGACA	GGCTTTTAGGTGGACGGATT	60.1	971	INTERGENIC	--	--	--

INDEL marker IDs	Chromosomes/ unanchored scaffolds	Physical positions (bp)	InDels (*Kabuli* reference genome-CDC Frontier/PII)	Forward primers (5'-3')	Reverse primers (5'-3')	Anneling temperature (0C)	Expected amplified product size (bp)	Structural annotation		Functional annotation	
								Sequence components of *kabuli* genome	*Kabuli* gene accession IDs	NCBI-KOG	TFs
Ca3_P4_25030440	Ca3	25030440	ATTTTTTTTTTT/ATTTTTTTTTT	TTGATTCCACGTCAATGTCA	TACACACACCTTGCTCGCTC	58.5	514	INTERGENIC	--	--	--
Ca3_P4_25195854	Ca3	25195854	AAGAATACTCTATGTT/AAGAATACTCTATGTTAGAATACTCTATGTT	TGCCCTAATTAAGGGAATAAATG	GTGGTTTGGGCTACTTTCCA	58.5	267	INTERGENIC	--	--	--
Ca3_P4_25199115	Ca3	25199115	TTTATTATTATTATTATTATTATTATTATTATTATTATT/TTTATTATTATTATTATTATTATTATTATT	AATGCACGAAGAGAGAGGGA	GAGGGAGTTGTGAAGAGACAAAA	60.0	562	INTERGENIC	--	--	--
Ca3_P4_25314505	Ca3	25314505	GAAAAAAAAAAA/GAAAAAAAAAAAA	CCCTCGAATTTGTTTTGGAA	TGGAAAATGATTGTTTTTGGG	59.9	711	INTERGENIC	--	--	--
Ca3_P4_25419094	Ca3	25419094	ATTTTTTTTTTT/ATTTTTTTTTT	TCACAGTTTGGATTGGTGGA	TTTCAATTTTGACCATTTGTGC	59.9	473	INTERGENIC	--	--	--
Ca3_P4_25519863	Ca3	25519863	AATATATATATATATATATATATATATATATAT/AATATATATATATATATATATATATAT	TCAGTCAACCGCACATAAAAG	TCATCTATTCAAAAAGTCTCACCG	58.8	541	INTERGENIC	--	--	--
Ca3_P4_25677601	Ca3	25677601	A/ATTATTATTATT	TGCTTAACTGAAAATGACAAGACA	CACCACACCACTTTCCCTCT	59.0	540	INTERGENIC	--	--	--
Ca3_P4_25798470	Ca3	25798470	AATATATATATATATATATATATATATATATA/AATATATATATATATATATATATATATATATATATATATATA	TTAAAAGGCTGCCGTTATCC	CCAAACTTCTTTAGGTCGAATTG	59.2	702	INTERGENIC	--	--	--
Ca3_P4_25872845	Ca3	25872845	ACTAAATTGGTCGCTAAAT/ACTAAAT	TGTGTACAAGATTGTGAAGGCA	CACGTAAGATCGCCAAAATC	59.2	680	INTERGENIC	--	--	--
Ca3_P4_25888060	Ca3	25888060	AT/ATT	TGTGGAAGACATCCTTTACTGC	ACCTGACCTATTTCCACCCC	59.2	551	INTERGENIC	--	--	--

INDEL marker IDs	Chromosomes/ unanchored scaffolds	Physical positions (bp)	InDels (*Kabuli* reference genome-CDC Frontier/PII)	Forward primers (5'-3')	Reverse primers (5'-3')	Anneling temperature (0C)	Expected amplified product size (bp)	Structural annotation		Functional annotation	
								Sequence components of *kabuli* genome	*Kabuli* gene accession IDs	NCBI-KOG	TFs
Ca3_P4_25888907	Ca3	25888907	GTTTTTTT/GTTTTTTTT	CAGGTCATGCCAGACTTTGA	TCTGACCCGACCTGGTCTAC	59.8	578	INTERGENIC	--	--	--
Ca3_P4_25889145	Ca3	25889145	ATTTTTTTT/ATTTTTTTTT	GTAGACCAGGTCGGGTCAGA	TGATATGTCACATTGTCGCAC	60.1	403	INTERGENIC	--	--	--
Ca3_P4_25890296	Ca3	25890296	TAA/TA	CGGGACTTGATGGAACCTTA	TTGAAACGATCGTGATGACTG	59.9	799	INTERGENIC	--	--	--
Ca3_P4_25906200	Ca3	25906200	CTATATATATATATATATATATATA/CTATATATATATATATATATATA	TTCATTTTTCACTCATTCAGATTTAGA	TGCAATTATCAATGACAAGGAA	59.6	868	INTERGENIC	--	--	--
Ca3_P4_25937655	Ca3	25937655	TTATATATATATATATATATATAT/TTATATATATATATATATATAT	TCGAGTATGACCAATTAACCGA	ATAAACTTTTAGGCGCCGGT	59.5	785	INTERGENIC	--	--	--
Ca3_P4_25950181	Ca3	25950181	AGATGGGGATGGGGATG/AGATGGGGATG	ACTTGCACCCCATCAGTTTC	TCCCCATCTTCATCTTCGTC	60.0	410	INTERGENIC	--	--	--
Ca3_P4_26020458	Ca3	26020458	CATATATATATATATATATAT/CATATATATATATATATATATAT	CCCACTAAAGGCCCATATCA	TTCAAATTCACGCCCCTAAC	59.8	789	INTERGENIC	--	--	--
Ca3_P4_26159882	Ca3	26159882	CATATATAT/CATATAT	TTGACCAGACCGAGATCACA	GGGGCATTATGGACTTGAAA	60.2	779	INTERGENIC	--	--	--
Ca3_P4_26215938	Ca3	26215938	TTATATATATATATATATATATATAT/TTATATATATATATATATATATATATATATATATATATAT	TTTATGGGAGCTCTTGCCAC	TTGACCCCTCAAAGAGATGG	60.2	742	INTERGENIC	--	--	--
Ca3_P4_26437635	Ca3	26437635	AATATATATATATATATATATATATATA/AATATATATATATATATATATATATA	TTTTTGAAAACTCGTCGCCT	GCTTGAGCACACAGCCATTA	59.9	593	INTERGENIC	--	--	--

INDEL marker IDs	Chromosomes/ unanchored scaffolds	Physical positions (bp)	InDels (*Kabuli* reference genome-CDC Frontier/PII)	Forward primers (5'-3')	Reverse primers (5'-3')	Anneling temperature (0C)	Expected amplified product size (bp)	Structural annotation		Functional annotation	
								Sequence components of *kabuli* genome	*Kabuli* gene accession IDs	NCBI-KOG	TFs
Ca3_P4_2653586	Ca3	2653586	GTTTTTTT/GTTTTTTTT	TGTTTTGCAGCAAGATGTCC	TGGAGATGGTTAGCAATGGA	59.8	536	INTERGENIC	--	--	--
Ca3_P4_26695279	Ca3	26695279	AATATATATATATATATATATATATATAT/AATATATATATATATATATATATAT	AAGTCATCATCACTCATTTGAAGA	GTACCGGAGCAGTGGATTGT	57.9	486	INTERGENIC	--	--	--
Ca3_P4_26823353	Ca3	26823353	AAATACAATAC/AAATAC	CGAAAATGTTGAGGGGCTAA	GTTTTGGTGCGGTTTCTTGT	60.1	979	INTERGENIC	--	--	--
Ca3_P4_26872317	Ca3	26872317	CAGAGAGAGAGAGAGAGAGAGAGAG/CAGAGAGAGAGAGAGAGAGAGAGAGAG	CAATGTGCTCGATTTCTCCA	TTACATTCCTTGCCTCGTCA	59.8	389	INTERGENIC	--	--	--
Ca3_P4_27188795	Ca3	27188795	AATATATATATATATATATAT/AATATATATATATATATATATAT	CCAAATGACGACTTCCCACT	TTACTACATTTCTGGCCCCG	60.0	865	INTERGENIC	--	--	--
Ca3_P4_27221901	Ca3	27221901	AATTAT/AATAATTATTAT	TGGCCTAAAAATGACCGACT	TGTGCGCTGATAGGCATTAG	59.6	929	INTERGENIC	--	--	--
Ca3_P4_2724739	Ca3	2724739	AATATATATATATATATATATA/AATATATATATATATATATA	GCGCAATACTCAAATGCAGA	GTGCACTTGCGGTCTTATCA	60.0	338	INTERGENIC	--	--	--
Ca3_P4_27277791	Ca3	27277791	CATATATATATATATATATATATA/CATATATATATATATATATATATATATATATATA	GCGAAGCGCCATACTAACTC	AAGCCCAAACTCCCTTTTTC	60.0	858	INTERGENIC	--	--	--
Ca3_P4_27378702	Ca3	27378702	CAA/CAAA	GTCGGGGACTAGTGGACTCA	CGGGGAAGTTCTAAGGTGCT	60.1	388	INTERGENIC	--	--	--
Ca3_P4_27551834	Ca3	27551834	TTATATATATATATATATATA/TTATATATATATATATATATATA	TCTTTGGATGGGTTCCATGT	TTGTTGACACCGAATTAGCG	60.2	672	INTERGENIC	--	--	--

INDEL marker IDs	Chromosomes/ unanchored scaffolds	Physical positions (bp)	InDels (*Kabuli* reference genome-CDC Frontier/PII)	Forward primers (5'-3')	Reverse primers (5'-3')	Anneling temperature (0C)	Expected amplified product size (bp)	Structural annotation		Functional annotation	
								Sequence components of *kabuli* genome	*Kabuli* gene accession IDs	NCBI-KOG	TFs
Ca3_P4_27572261	Ca3	27572261	TTATATATATATATATATATAT/TTATATATATATATATATATATATATATAT	ACATGCCTACTCGTGTCGTG	TTTTCCTTTTTATTTTCTTTTGTGA	59.8	604	INTERGENIC	--	--	--
Ca3_P4_27573773	Ca3	27573773	ATATATATCTATATAT/ATATATAT	TGAATTTTCATGTGCGTTTGA	TTCTCTTGCACATTAGGCTCC	60.1	514	INTERGENIC	--	--	--
Ca3_P4_2762503	Ca3	2762503	GTTTTT/GTTTTTT	CTCTCACCCAAGAAGATCGC	TTGGAACAAAACACCTGCAA	60.0	590	INTERGENIC	--	--	--
Ca3_P4_27743850	Ca3	27743850	TAAAAAAAAA/TAAAAAAAA	TAATGGACCACAATTGCACC	AATTGCACCGCTGAGTTTTT	59.3	216	INTERGENIC	--	--	--
Ca3_P4_27844143	Ca3	27844143	TTTATTATTATTATTATTATTATTATTATTATTATTATTATTATTATTA/TTTATTATTATTATTATTATTATTATTATTATTA	TCCACACAGTCCCACTACCA	TGTTACCACCACCAGAACCA	60.0	479	INTERGENIC	--	--	--
Ca3_P4_27853892	Ca3	27853892	A/ATTT	TGGATTGACATAAATTGAAAGAAA	AAAATAGACCCCCAAATGCC	57.8	608	INTERGENIC	--	--	--
Ca3_P4_27854154	Ca3	27854154	TGG/TG	TCGATAATGAGTGATTAGGTGGG	ACCTCACCCTCGAATGTCAC	60.2	459	INTERGENIC	--	--	--
Ca3_P4_27854804	Ca3	27854804	T/TA	GGATTGAGAGGGGAGAAAGG	TTTGAAAGCAAGAGCGTCAA	60.0	516	INTERGENIC	--	--	--
Ca3_P4_27854997	Ca3	27854997	ATTTTTTTTTTT/ATTTTTTTTT	GGATTGAGAGGGGAGAAAGG	TTTGAAAGCAAGAGCGTCAA	60.0	516	INTERGENIC	--	--	--
Ca3_P4_27856755	Ca3	27856755	TAAAAAAAAA/TAAAAAAAA	CGAAGATGAAGAGGCTTGAAA	CAACAACTTCTGATGCTCGC	59.6	593	INTERGENIC	--	--	--

INDEL marker IDs	Chromosomes/ unanchored scaffolds	Physical positions (bp)	InDels (*Kabuli* reference genome-CDC Frontier/PII)	Forward primers (5'-3')	Reverse primers (5'-3')	Anneling temperature (0C)	Expected amplified product size (bp)	Structural annotation		Functional annotation	
								Sequence components of *kabuli* genome	*Kabuli* gene accession IDs	NCBI-KOG	TFs
Ca3_P4_27867074	Ca3	27867074	TCAC/TCACAC	TGAGATAACCAGCGAATCCC	ATTCGGGATAAGTGGAACCC	60.0	550	INTERGENIC	--	--	--
Ca3_P4_27867135	Ca3	27867135	AT/ATT	TGAGATAACCAGCGAATCCC	ATTCGGGATAAGTGGAACCC	60.0	550	INTERGENIC	--	--	--
Ca3_P4_27882658	Ca3	27882658	TAAAAAAAAA/TAAAAAAAA	TTTGTGCAAGGACTCTGACG	TGGAAAACCTGTGTGCAAAA	60.0	747	INTERGENIC	--	--	--
Ca3_P4_28113252	Ca3	28113252	ATTTTTTTT/ATTTTTTT	TCCTTCTCCTCTTCCTTCCA	TGTTTGTGCAATTGAATTATAGTTATG	58.9	849	INTERGENIC	--	--	--
Ca3_P4_28188438	Ca3	28188438	CAAAA/CAAAAA	ACCCATAACGCACTCGTTTC	TCCTCCATTTTTCGTTTTCG	60.0	434	INTERGENIC	--	--	--
Ca3_P4_28193661	Ca3	28193661	AT/ATGTGT	GGTAAGGGCTCCTTCCAAAC	ATTCCCCATTCTTAACGCCT	59.9	588	INTERGENIC	--	--	--
Ca3_P4_28262436	Ca3	28262436	ATTATTTATTT/ATTATTT	TACTTTTACCCCCATTGCCA	AAACACCCATCCCACGTATG	60.2	414	INTERGENIC	--	--	--
Ca3_P4_28262509	Ca3	28262509	GTTTTTTTT/GTTTTTTTTTT	TACTTTTACCCCCATTGCCA	AAACACCCATCCCACGTATG	60.2	414	INTERGENIC	--	--	--
Ca3_P4_28293830	Ca3	28293830	TAAAAAAAAAA/TAAAAAAAAA	GATTACTGCTAGGGCCCAAAT	ATCCCACTTGCAACACTTCC	59.5	147	INTERGENIC	--	--	--
Ca3_P4_28294648	Ca3	28294648	AATATATATATATATATATAT/AATATATATATATATATATATAT	TACCAGTACCAGGAGGCAGG	TGGTGGGTAATCCGATGAAT	60.1	670	INTERGENIC	--	--	--

INDEL marker IDs	Chromosomes/ unanchored scaffolds	Physical positions (bp)	InDels (*Kabuli* reference genome-CDC Frontier/PII)	Forward primers (5'-3')	Reverse primers (5'-3')	Anneling temperature (0C)	Expected amplified product size (bp)	Structural annotation		Functional annotation	
								Sequence components of *kabuli* genome	*Kabuli* gene accession IDs	NCBI-KOG	TFs
Ca3_P4_28330863	Ca3	28330863	TTATATATATATATATATATATATAT/TTATATATATATATATATATATATATATAT	GATTTACGTTGCCGGATCTT	TCAGAAAGCCCAGAAAGCAT	59.0	526	INTERGENIC	--	--	--
Ca3_P4_28366500	Ca3	28366500	GTTTAATAATTTAATAA/GTTTAATAA	GGCCACGACATTCTCATTTT	TGCATTGCATCACCATTTTT	59.9	329	INTERGENIC	--	--	--
Ca3_P4_28428270	Ca3	28428270	GGTGTGTGTGTGTGTGT/GGTGTGTGTGTGTGTGTGT	TTGGACCCTACAAAACCAGC	CAAACCGATCTGTAGGGGAA	60.0	381	INTERGENIC	--	--	--
Ca3_P4_28535226	Ca3	28535226	CATATATATATATATATATATATATATA/CATATATATATATATATATATA	AAGCGCCAATCTAGTTTCATTT	AAAATGAGTCGTTGGATTCAAAA	59.3	411	INTERGENIC	--	--	--
Ca3_P4_2855780	Ca3	2855780	G/GA	TGGGAAACAAAATGATGTCAA	CACATGCAATTCCAACCAAG	58.9	591	INTERGENIC	--	--	--
Ca3_P4_2855993	Ca3	2855993	TAAA/TAA	CTTGGTTGGAATTGCATGTG	CGTTTTGGAAGGTAGGGTGT	60.0	514	INTERGENIC	--	--	--
Ca3_P4_28787069	Ca3	28787069	TTATATATATATATATATATATAT/TTATATATATATATATATATATATAT	TCCCTCATATTCAATTTCCCA	TCAACTGACAAAATACCGATGTG	59.2	569	INTERGENIC	--	--	--
Ca3_P4_28797135	Ca3	28797135	A/ATT	CAAGGAGCTGTTTTGCTTCA	GTAAAATGGCACGCGGTAGT	59.2	609	INTERGENIC	--	--	--
Ca3_P4_28797825	Ca3	28797825	A/AG	CATAGTCTGCGAAACCAGCA	GGTGGAATTGATCGGAGAGA	60.0	454	INTERGENIC	--	--	--
Ca3_P4_28799402	Ca3	28799402	AATATA/AATA	TCTAGCAAGTTCGCGCAATA	TTTGCTGCTGCAATTTAACAA	59.7	795	INTERGENIC	--	--	--

INDEL marker IDs	Chromosomes/ unanchored scaffolds	Physical positions (bp)	InDels (*Kabuli* reference genome- CDC Frontier/PII)	Forward primers (5'-3')	Reverse primers (5'-3')	Anneling temperature (0C)	Expected amplified product size (bp)	Structural annotation		Functional annotation	
								Sequence components of *kabuli* genome	*Kabuli* gene accession IDs	NCBI-KOG	TFs
Ca3_P4_2884020	Ca3	2884020	AA/AATAATAATAATTA	TCGAGAAAATTGAACGGACA	AACAAAATAAAGCGGACAGTCA	59.2	565	INTERGENIC	--	--	--
Ca3_P4_29028379	Ca3	29028379	TTATATATATATATATATATATATATATATAT/TTATATATATATATATAT	CACAGGCTCGAAACACACAT	TTATTTTTCTTGATGCAATGGA	59.8	302	INTERGENIC	--	--	--
Ca3_P4_29051833	Ca3	29051833	AATTATTATTATTATTATTATTAT/AATTATTATTATTATTATTAT	CCCACCTCTACCACCGACTA	TCTGAACCGTCTTGATTCGAT	60.0	387	INTERGENIC	--	--	--
Ca3_P4_29087544	Ca3	29087544	GAAATATATA/GA	GCTGGAAGCTTCTAATGGCA	AAACCGCCTTTTCAGAACAT	60.5	471	INTERGENIC	--	--	--
Ca3_P4_29087580	Ca3	29087580	TATATATAA/T	GCTGGAAGCTTCTAATGGCA	AAACCGCCTTTTCAGAACAT	60.5	471	INTERGENIC	--	--	--
Ca3_P4_29089899	Ca3	29089899	TTATATATATATATATATATATATATAT/TTATATATATATATATATATATATATATATATAT	CAACCCACATGCAAACACTC	CCTTTGGCAATCGAGGTAAA	60.0	399	INTERGENIC	--	--	--
Ca3_P4_29091788	Ca3	29091788	TAAAAAA/TAAAAAAA	TTTTGGAAAAGCGCTGTAAAA	CTTCGACACTGTAGCACCCA	59.9	852	INTERGENIC	--	--	--
Ca3_P4_29174780	Ca3	29174780	TTATATATATATATAT/TTATATATATATATATATATAT	ATCCACCCCACATTCAAATC	GGAGAGAAATACGCGTGGAG	59.5	439	INTERGENIC	--	--	--
Ca3_P4_29224433	Ca3	29224433	ATTTTTTTTTT/ATTTTTTTTTTT	TGCAATTTGTTGGAGCGTTA	TTGCTCCAAATTTCAAAGGC	60.3	394	INTERGENIC	--	--	--
Ca3_P4_29334875	Ca3	29334875	TTATATATATATATATATATAT/TTATATATATATATATATATATATATAT	GGGTGGCTCATGAAGCTTAG	TCTCAACCGTTTTGGTGACA	59.8	576	INTERGENIC	--	--	--

INDEL marker IDs	Chromosomes/ unanchored scaffolds	Physical positions (bp)	InDels (*Kabuli* reference genome-CDC Frontier/PII)	Forward primers (5'-3')	Reverse primers (5'-3')	Anneling temperature (0C)	Expected amplified product size (bp)	Structural annotation		Functional annotation	
								Sequence components of *kabuli* genome	*Kabuli* gene accession IDs	NCBI-KOG	TFs
Ca3_P4_29362184	Ca3	29362184	AAGAGAGAGAGAGAGAGAGAGAGAGAGAGAGAGAGAGAGAGAGAGAGAGAGAGAGAG/AAGAGAGAGAGAGAGAGAGAGAGAGAGAGAGAGAGAGAGAGAGAGAGAGAGAGAGAGAGAG	ACCACAATCTGAAGCCAACC	GTTGCACTGTGGGGATCTTT	60.0	679	INTERGENIC	--	--	--
Ca3_P4_29512683	Ca3	29512683	AATATATATATATATATATAT/AATATATATATATATATAT	TGCAATGAATGGAATCTTGG	TCCAAAACAACAAGGACACG	59.5	380	INTERGENIC	--	--	--
Ca3_P4_29534996	Ca3	29534996	CAAAAAAAAAA/CAAAAAAAAAAA	TCAATGACCTATTGTCGCCTT	TGGATAGCATCACATGAATAAAAA	59.6	736	INTERGENIC	--	--	--
Ca3_P4_29552129	Ca3	29552129	AATATATATATATATATATATATATATATA/AATATATATATATATATATATATATATATATA	AAAACACAGTTGGAGGTGGC	TATCAGTCGGCTGTGTTTGG	60.0	627	INTERGENIC	--	--	--
Ca3_P4_29636957	Ca3	29636957	TTAATAATAATAATAATAATAATAATAATAATAATAATAATAATAATAATAATAATAATAATAATAA/TTAATAATAATAATAATAATAATAATAATAATAATAATAATAATAATAATAATAATAATAATAATA[illegible]	CCTTTGAATTATCACGGAAGC	TTTTAGAGTTGAGAGGGGTGG	58.7	522	INTERGENIC	--	--	--
Ca3_P4_29809142	Ca3	29809142	ATAT/AATTAT	GCTCATGCCTCCTTCAAAAA	AAGCAGCCATAATGACAGGG	60.3	856	INTERGENIC	--	--	--
Ca3_P4_29829081	Ca3	29829081	CAAAAA/CAAAA	TTCATTCATCGCATTGAGGA	CTTTGAATCCTTGCCCAAAA	60.2	337	INTERGENIC	--	--	--
Ca3_P4_29849306	Ca3	29849306	AATTATTATTATTATTATTATTATTATTATTATTATTATTATTATTATTATTATTATT/AATTATTATTATTATTATTATTATTATTATTATTATTATTATTATTATTATTATTATTATTATT	TGGCAATCATTTGCTTTTTG	AGAGGCATTGACTTTGGTGG	59.7	228	INTERGENIC	--	--	--
Ca3_P4_29919321	Ca3	29919321	AATATATATATATATATATATATATATATATAT/AATATATATATATATATATATATATATATATATAT	TCAGCCCTCGCCTTACTTAG	TTTCAACATTTTCTCCCCATT	59.6	585	INTERGENIC	--	--	--
Ca3_P4_29949453	Ca3	29949453	TAAAAAAA/TAAAAAA	TGTGGAGGTTATTTTTGGGC	AAGCCGGCAGTGTAGTGTCT	59.8	692	INTERGENIC	--	--	--

INDEL marker IDs	Chromosomes/ unanchored scaffolds	Physical positions (bp)	InDels (*Kabuli* reference genome-CDC Frontier/PII)	Forward primers (5'-3')	Reverse primers (5'-3')	Anneling temperature (0C)	Expected amplified product size (bp)	Structural annotation		Functional annotation	
								Sequence components of *kabuli* genome	*Kabuli* gene accession IDs	NCBI-KOG	TFs
Ca3_P4_30061370	Ca3	30061370	CTTT/CT	CCAAAAATGACATTAATCTTGGC	ATCACAATTTGGTGCAGTTTT	59.7	194	INTERGENIC	--	--	--
Ca3_P4_30072887	Ca3	30072887	AGGTG/AGGTGTGTGGTG	CACCTCACGCACCACATTAC	CACCAATTATGCCCCTTGTC	60.0	267	INTERGENIC	--	--	--
Ca3_P4_30114026	Ca3	30114026	AAGA/AA	CCGTTGAGAATGGTGAGGTT	AAAAAGAAGAGAGTTCCCCCT	60.0	681	INTERGENIC	--	--	--
Ca3_P4_30291152	Ca3	30291152	CAAAAAAAA/CAAAAAAA	GCTGAATTTTGTGGTAGCCC	CCGATTAGAAAGCGGTTGAA	59.6	531	INTERGENIC	--	--	--
Ca3_P4_30339047	Ca3	30339047	AAATAATAATAATAATAATAATA/AAATAATAATAATAATAATAATA	CCTCCTCCAACCATGAAATG	CCTGTGTGGTCAGAAAGGGT	60.3	496	INTERGENIC	--	--	--
Ca3_P4_30420653	Ca3	30420653	TTATATATATATATATAT/TTATATATATATATATATAT	TGATCAAAAGAAAAGGTCTGAGAT	TGCACGTAAGTCAAATGCAA	58.5	813	INTERGENIC	--	--	--
Ca3_P4_3050975	Ca3	3050975	AATATATATATATATATATATATATATATATATATA/ATATATATATATATATATATATATATATATATATATATA	TGGCCTAAGCTTCTCCCTTT	TGTGCTCCAATTACTTCTTTAGCA	60.3	598	INTERGENIC	--	--	--
Ca3_P4_30564227	Ca3	30564227	TTA/TTATA	GCTGACGTGGACCTCTTCTC	TGTCGATATTTATGAGTGATTAGGTGA	60.0	333	INTERGENIC	--	--	--
Ca3_P4_30602332	Ca3	30602332	CTTTT/CTTT	GCCCTCAGATGAGAGTCAGG	AAATTAAACCCTCATCCCCG	59.9	842	INTERGENIC	--	--	--
Ca3_P4_30646834	Ca3	30646834	ATTTTTTTTTTT/ATTTTTTTTTT	TTTCCGGTACACAATTTCCA	CGCCTTCTACGACATTCACA	58.9	260	INTERGENIC	--	--	--

INDEL marker IDs	Chromosomes/ unanchored scaffolds	Physical positions (bp)	InDels (*Kabuli* reference genome-CDC Frontier/PII)	Forward primers (5'-3')	Reverse primers (5'-3')	Anneling temperature (0C)	Expected amplified product size (bp)	Structural annotation		Functional annotation	
								Sequence components of ***kabuli*** **genome**	***Kabuli*** **gene accession IDs**	**NCBI-KOG**	**TFs**
Ca3_P4_30729039	Ca3	30729039	TTATATATATATATAT/TTATATATATATAT	CGAAGTTTTATGGTCCCCAA	CCTCGCGCGATAAAAAGTTA	59.8	763	INTERGENIC	--	--	--
Ca3_P4_30760574	Ca3	30760574	TTATATATATATATATATATATATAT/TTATATATATATATATATATATAT	GCACCAAGTTCTTCTTTGCC	GGGAATTGGAGCACTTGAAA	59.9	258	INTERGENIC	--	--	--
Ca3_P4_30784930	Ca3	30784930	TAAA/TAAAA	CACACACAATTAACATTTGCCA	TTGTAACCAATGAGGTGTTGAAA	59.4	599	INTERGENIC	--	--	--
Ca3_P4_30811197	Ca3	30811197	ATTTTTTTTT/ATTTTTTTT	TCACGCGTCTTCTTAGGGTT	TTCAATTGGCCATATCACTTTG	59.9	798	INTERGENIC	--	--	--
Ca3_P4_3082418	Ca3	3082418	TTT/TTTATT	CCGTTTATCATTTTTGGGGTT	TTTGACAAAATGATGGGTTCA	59.9	465	INTERGENIC	--	--	--
Ca3_P4_30875852	Ca3	30875852	TTATATATATATATATATAT/TTATATATATATATATAT	CATGTACTATTGCGGCAAACA	CCAAACCAACCATGAGTTCAC	59.6	385	INTERGENIC	--	--	--
Ca3_P4_30890215	Ca3	30890215	ATTTTTTTTTTT/ATTTTTTTTTT	CATCAACAATATTCGCACGG	CAATGCAGACCGTCAATCAC	60.0	603	INTERGENIC	--	--	--
Ca3_P4_30930504	Ca3	30930504	GTTTTTATTTTTATTTTTA/GTTTTTATTTTTA	GCATGGACCGTTTAAAATTG	TTTAGGTGGAAAATTCATTAGAAAA	58.0	818	INTERGENIC	--	--	--
Ca3_P4_30936561	Ca3	30936561	ATTTTTTTT/ATTTTTTTTT	TGAGGCAGAACTTCTCGTTG	TGTTACGGGGTATTTAGCGG	59.2	427	INTERGENIC	--	--	--
Ca3_P4_30939663	Ca3	30939663	GTATATATATATATATAT/GTATATATATATATAT	TTGTTTATTTGGGGTACACGG	ATTTGTCGGGATGTCAACCA	59.6	271	INTERGENIC	--	--	--

INDEL marker IDs	Chromosomes/ unanchored scaffolds	Physical positions (bp)	InDels (*Kabuli* reference genome-CDC Frontier/PII)	Forward primers (5'-3')	Reverse primers (5'-3')	Anneling temperature (0C)	Expected amplified product size (bp)	Structural annotation		Functional annotation	
								Sequence components of *kabuli* genome	*Kabuli* gene accession IDs	NCBI-KOG	TFs
Ca3_P4_30942170	Ca3	30942170	ATTTTTTTTT/ATTTTTTTTTT	ACACTCAAGAGGTGGCATCC	TGTCTTGCGACTATTTCCAATTT	60.1	570	INTERGENIC	--	--	--
Ca3_P4_31026529	Ca3	31026529	TG/TGG	ATTCGCTATAGGCCGACACA	CGAGTGAGGGGATTTATGGA	60.6	708	INTERGENIC	--	--	--
Ca3_P4_31059721	Ca3	31059721	TAAAAAAAAA/TAAAAAAAA	CGTTAACCTGTGACCCGTTT	CAGTGATGGCAGGCAAACTA	59.9	710	INTERGENIC	--	--	--
Ca3_P4_31105400	Ca3	31105400	AATATATATATATATATA/AATATATATATATATA	AGGAAAAATGATGGAGGGGT	TTTTTGGTGAAATTGACGTG	59.6	485	INTERGENIC	--	--	--
Ca3_P4_3113742	Ca3	3113742	TATAATAATAAT/TATAATAATAATAAT	ACGGTGATGAAAGGAAAACG	TGGGATAAGCTTTCAAATCCA	60.0	564	INTERGENIC	--	--	--
Ca3_P4_31183194	Ca3	31183194	AATATATATATATATATATATATATATATATATATATATATATAT/AATATATATATATATATATATATATATATAT	CACCGTATTACACCAAAACGA	TTTCGACGACAACAACAAGG	58.5	599	INTERGENIC	--	--	--
Ca3_P4_31201179	Ca3	31201179	TTAATAATAATAATAATAATAATAATAATAATA/TTAATAATAATAATAATAATAATAATAATAATAATA	AAGCTATTTTTAGTGGGAGAACTTTT	TTGGTGACACTCTAGCAAAGGA	58.6	371	INTERGENIC	--	--	--
Ca3_P4_31253675	Ca3	31253675	CTTTTTTTTT/CTTTTTTTT	AATTGCAGGTATGAGGACGG	GTTTCGGAGTGTGGCATTTT	60.0	458	INTERGENIC	--	--	--
Ca3_P4_31263318	Ca3	31263318	ATGTTGTTGT/ATGTTGT	GACGCAAAATGTTGACCCTT	AATCCGGTTGTTGTCTGGAA	60.0	395	INTERGENIC	--	--	--
Ca3_P4_31264246	Ca3	31264246	TTATATATATA/TTATATATATATA	AAAAACGAGTGGATGCGTTC	AGAAGACCATCTCTCGTTAGGC	60.1	479	INTERGENIC	--	--	--

INDEL marker IDs	Chromosomes/ unanchored scaffolds	Physical positions (bp)	InDels (*Kabuli* reference genome-CDC Frontier/PII)	Forward primers (5'-3')	Reverse primers (5'-3')	Anneling temperature (0C)	Expected amplified product size (bp)	Structural annotation		Functional annotation	
								Sequence components of *kabuli* genome	*Kabuli* gene accession IDs	NCBI-KOG	TFs
Ca3_P4_31266098	Ca3	31266098	TC/T	TGCGTGCGTCTTTGATTAAC	TAGCCCAAGTCAACCGAATC	59.9	446	INTERGENIC	--	--	--
Ca3_P4_31291540	Ca3	31291540	ATTATTTTTATTTT/ATTATTTT	ATTTGCCGTGACCCTATGAA	TGGTCTTAAAATATGAAGAAAAGAAAA	60.3	743	INTERGENIC	--	--	--
Ca3_P4_31292520	Ca3	31292520	TGATGAGTGACGGA/TGATGAGTGACGGATGAGTGACGGA	TTGGGACATCAAGCCAATTT	TCGTTTATTTCAACCGCCTT	60.3	286	INTERGENIC	--	--	--
Ca3_P4_31293455	Ca3	31293455	TG/TGG	TGGGATGTTTGAGAAAAGCA	ATCCCATCCCACCAACAATA	59.2	522	INTERGENIC	--	--	--
Ca3_P4_31293569	Ca3	31293569	AT/ATT	TGGGATGTTTGAGAAAAGCA	CCCACATCGTTTATCCCATC	59.2	534	INTERGENIC	--	--	--
Ca3_P4_31293673	Ca3	31293673	G/GA	TGGGATGTTTGAGAAAAGCA	CCCACATCGTTTATCCCATC	59.2	534	INTERGENIC	--	--	--
Ca3_P4_31294489	Ca3	31294489	GTTTTTTTTTTTTT/GTTTTTTTT	CGTTAAATGTCACGCTGTCG	CGCTGTTTTAAGAATTGTGTACG	60.3	521	INTERGENIC	--	--	--
Ca3_P4_31325736	Ca3	31325736	ATTT/ATTTT	ATTAATTGGGCTGACGTGGA	GTCCACATTAGGGACACGCT	60.3	426	INTERGENIC	--	--	--
Ca3_P4_31326387	Ca3	31326387	GTTTT/GTTT	CGGGTACAGCCGGTAAGTAA	GGGTGCCTTTAAAGTGTGGA	60.0	639	INTERGENIC	--	--	--
Ca3_P4_31336604	Ca3	31336604	T/TC	AGCAGGCCTGGACCTTTACT	GGAATGCCAACGAATGAAAA	60.3	555	INTERGENIC	--	--	--

INDEL marker IDs	Chromosomes/ unanchored scaffolds	Physical positions (bp)	InDels (*Kabuli* reference genome-CDC Frontier/PII)	Forward primers (5'-3')	Reverse primers (5'-3')	Anneling temperature (0C)	Expected amplified product size (bp)	Structural annotation		Functional annotation	
								Sequence components of *kabuli* genome	*Kabuli* gene accession IDs	NCBI-KOG	TFs
Ca3_P4_31338630	Ca3	31338630	ATTT/ATT	TATCGTTCGTTGCTCTCGTG	GGCCGAAAGTTATTACGTGG	60.0	870	INTERGENIC	--	--	--
Ca3_P4_31339236	Ca3	31339236	T/TC	CCAACCCATGTACCGACATT	AGAGACCGTTTCATGAGATTTG	60.5	525	INTERGENIC	--	--	--
Ca3_P4_31339678	Ca3	31339678	ATT/ATTT	CAAATCTCATGAAACGGTCTCT	ACGTTATCCTCACCGTGGAC	58.3	266	INTERGENIC	--	--	--
Ca3_P4_31341112	Ca3	31341112	GTATATATATATATAT/GTATATATATATATATAT	TCACTCTGATCACAGGAACCC	AATCACGGCGTTTCTGATTC	60.1	312	INTERGENIC	--	--	--
Ca3_P4_31344262	Ca3	31344262	ATTTTTT/ATTTTTTTT	CGAACCTAGACTTGAGCATGG	TCTCTTCATCCACCCAGAGG	59.9	441	INTERGENIC	--	--	--
Ca3_P4_31345376	Ca3	31345376	AAATGGAAT/AAAT	ATGGAAAGCCAGCTGAAGAA	CTATGTTGTGGGAAGGTGGG	60.0	592	INTERGENIC	--	--	--
Ca3_P4_31348024	Ca3	31348024	AC/A	GGTCAACAACATCAATTGCG	TAGGGGACCAAAACTGAGGA	60.0	924	INTERGENIC	--	--	--
Ca3_P4_31350700	Ca3	31350700	TCATG/TCATGCATG	TCTTAGCGTAGATTCGCCGT	AACATCTGGGCTAATCGGTG	60.0	280	INTERGENIC	--	--	--
Ca3_P4_31364505	Ca3	31364505	CTTTTTTTT/CTTTTTTTTT	ACCAATGTCTCGCAAGCTCT	GGTAAACATTTGCATCGTTGG	60.0	458	INTERGENIC	--	--	--
Ca3_P4_31365869	Ca3	31365869	GTTTT/GTTT	AATTAAACCCGTGCAAATTCC	AAATGACATGCGATCCAACA	60.1	209	INTERGENIC	--	--	--

INDEL marker IDs	Chromosomes/ unanchored scaffolds	Physical positions (bp)	InDels (*Kabuli* reference genome-CDC Frontier/PII)	Forward primers (5'-3')	Reverse primers (5'-3')	Anneling temperature (0C)	Expected amplified product size (bp)	Structural annotation		Functional annotation	
								Sequence components of *kabuli* genome	*Kabuli* gene accession IDs	NCBI-KOG	TFs
Ca3_P4_31368075	Ca3	31368075	TA/T	TGATTAAGGCAAGGTGATCCA	AGCCGCTTCATGTAACCAAT	60.5	219	INTERGENIC	--	--	--
Ca3_P4_31375853	Ca3	31375853	TAATAAATAAATAA/TAATAAATAAATAAATAA	TGGTTGTCAGAGCAGGTTCA	CATATGTGAACCATGGCAGC	60.4	334	INTERGENIC	--	--	--
Ca3_P4_31389756	Ca3	31389756	C/CG	AAATATTCAGTCGGAGGGGG	TTTTATCTACGTTCACAAAAACACC	60.1	690	INTERGENIC	--	--	--
Ca3_P4_31392191	Ca3	31392191	A/ATAAAT	CACCCCATTTTGTATCGAGTT	TCTTGCTCATGGTGCTTCTG	58.8	351	INTERGENIC	--	--	--
Ca3_P4_31392713	Ca3	31392713	TGGG/TGGGG	CAGAAGCACCATGAGCAAGA	TGTCGGCAGTTAAGTTGGTG	60.1	439	INTERGENIC	--	--	--
Ca3_P4_31399757	Ca3	31399757	A/AC	TGTGTGAGCACGATTTCTCC	TTTGTTTCACAGCGCATTTC	59.8	680	INTERGENIC	--	--	--
Ca3_P4_31402077	Ca3	31402077	TAAAA/TAAAAA	TGTACACGTCGGTGAACAACT	TGTGGCGACAACTAACTTTGA	59.1	634	INTERGENIC	--	--	--
Ca3_P4_31415275	Ca3	31415275	T/TG	GTAGCTAACCCAACCGGTGA	GGGCCCGTTATATATTGGCT	60.0	565	INTERGENIC	--	--	--
Ca3_P4_31448365	Ca3	31448365	TTTATT/TTTATTATT	GACCTTACATGCTCCAACGC	TGGCCACACAAAAAGTACCA	60.7	440	INTERGENIC	--	--	--
Ca3_P4_31448937	Ca3	31448937	ATTT/ATTTAGAAAGTTT	TGGCCACCACTAACACAAAA	ACAGGTGATCCTCTTTCCCC	60.0	757	INTERGENIC	--	--	--

INDEL marker IDs	Chromosomes/ unanchored scaffolds	Physical positions (bp)	InDels (*Kabuli* reference genome-CDC Frontier/PII)	Forward primers (5'-3')	Reverse primers (5'-3')	Anneling temperature (0C)	Expected amplified product size (bp)	Structural annotation		Functional annotation	
								Sequence components of *kabuli* genome	*Kabuli* gene accession IDs	NCBI-KOG	TFs
Ca3_P4_31467437	Ca3	31467437	ACC/AC	GCCCTTGAAATCTCATCCAA	GCTCTCGCAAGAACAAAACC	60.0	177	INTERGENIC	--	--	--
Ca3_P4_31475167	Ca3	31475167	ATTAATTTAATTTAATT/ATTAATTTAATT	AACACCGCAACAAGTCACTG	AAATGACGGCACGACAAGAT	59.8	497	INTERGENIC	--	--	--
Ca3_P4_31476091	Ca3	31476091	TAAT/TAATCATTAGTTTTCAAAT	TGGTTCAGATTTGTTTGGTGA	CAAACGTGAGTGAAACAACCA	59.0	568	INTERGENIC	--	--	--
Ca3_P4_31476641	Ca3	31476641	TACAA/T	TGGTTGTTTCACTCACGTTTG	AGGGTGTCACAAATGGAAGC	59.6	612	INTERGENIC	--	--	--
Ca3_P4_31482581	Ca3	31482581	CTAAT/CTAATTAAT	TGCTCATGTGCCAAGAGTTG	TAGGGCCCACAATTCAAAAG	61.0	425	INTERGENIC	--	--	--
Ca3_P4_31494186	Ca3	31494186	ACTCTCTC/ACTCTCTCTC	TTCAGTCTCGATCATGAAGGG	GAGTGGCTACGATTTACGGC	60.2	666	INTERGENIC	--	--	--
Ca3_P4_31519164	Ca3	31519164	C/CT	TACACGTGGCCTCAAACAAA	TCAAGCATGGTTCCAATCAG	60.1	364	INTERGENIC	--	--	--
Ca3_P4_31535563	Ca3	31535563	GAAAAA/GAAAAAAAAA	ACACCCCAATCCCAAAAATA	CCTTGGAACGATGACGTTTT	59.0	761	INTERGENIC	--	--	--
Ca3_P4_31560705	Ca3	31560705	A/AAG	TGTTCACTGCCAAACTCTGC	GCAGTGCACAACTGATGGTT	60.0	585	INTERGENIC	--	--	--
Ca3_P4_31578353	Ca3	31578353	CTTTTTTTT/CTTTTTTTTTT	TGTGGGGGTCACATGTATTT	CCCAAATCTTCATGTCCTGC	58.5	681	INTERGENIC	--	--	--

INDEL marker IDs	Chromosomes/ unanchored scaffolds	Physical positions (bp)	InDels (*Kabuli* reference genome-CDC Frontier/PII)	Forward primers (5'-3')	Reverse primers (5'-3')	Anneling temperature (0C)	Expected amplified product size (bp)	Structural annotation		Functional annotation	
								Sequence components of *kabuli* genome	*Kabuli* gene accession IDs	NCBI-KOG	TFs
Ca3_P4_31604401	Ca3	31604401	TCTTACTTA/TCTTA	GATGAGCAAAGGGCATGATT	CAAATCAGCAGATTCCAGCA	60.0	393	INTERGENIC	--	--	--
Ca3_P4_31664819	Ca3	31664819	TGA/TGAGA	CAAGAGAATGTTGGACGGGT	TTCTTTCCAATTTTGCCCAC	60.0	441	INTERGENIC	--	--	--
Ca3_P4_31775359	Ca3	31775359	TAAAAAAAAAA/TAAAAAAAAAAA	CGAATGCGACTGTTGAGAAA	GTACCACCCGGAAATTTGAA	60.0	639	INTERGENIC	--	--	--
Ca3_P4_3185857	Ca3	3185857	TAAAAAAAAAA/TAAAAAAAA	CGCCTCTCTTTTTCTATGCG	TGTTGCTGTTGTTGCCTCTC	60.1	468	INTERGENIC	--	--	--
Ca3_P4_31895120	Ca3	31895120	CTAATATCTTAA/CTAATATCTTAATATCTTAA	GCCCATCAAACTCTGTGGAT	ATCGGGTGTCAATTGTCGAT	59.9	771	INTERGENIC	--	--	--
Ca3_P4_31918341	Ca3	31918341	TAATCTGTGAAAT/TAATCTGTGAAATCTGTGAAAT	TGGTCCAAAAACCACTCACA	TGAATCACCATTTGCCAGAA	60.0	761	INTERGENIC	--	--	--
Ca3_P4_31939842	Ca3	31939842	T/TG	GCACGGAAACAGCGATTTTA	CGCAAATTCAAAAGAATTCAAA	61.1	607	INTERGENIC	--	--	--
Ca3_P4_31970085	Ca3	31970085	ATTTTTTT/ATTTTTT	TCTCGTATCAGCCATTCCAA	ATTTCCAACGTGCCTTTGTC	59.2	743	INTERGENIC	--	--	--
Ca3_P4_3235341	Ca3	3235341	AGCTTCAGTCGCT/AGCT	TGGAGTGAGCTGTGGTTGAG	TGGTCACCACTTGGTGTTTG	60.0	840	INTERGENIC	--	--	--
Ca3_P4_3235471	Ca3	3235471	TAAA/TAA	ATAGGCCAAGCAACCAAAGA	GACGATGGTGAAGATGACGA	59.7	663	INTERGENIC	--	--	--

INDEL marker IDs	Chromosomes/ unanchored scaffolds	Physical positions (bp)	InDels (*Kabuli* reference genome-CDC Frontier/PII)	Forward primers (5'-3')	Reverse primers (5'-3')	Anneling temperature (0C)	Expected amplified product size (bp)	Structural annotation		Functional annotation	
								Sequence components of *kabuli* genome	*Kabuli* gene accession IDs	NCBI-KOG	TFs
Ca3_P4_3235549	Ca3	3235549	GCACACA/GCACACACA	ATAGGCCAAGCAACCAAAGA	GACGATGGTGAAGATGACGA	59.7	663	INTERGENIC	--	--	--
Ca3_P4_32387176	Ca3	32387176	TAAAAAAAAAAA/TAAAAAAAAAAAA	TCTCGCATTTATCTTCTATCCTCA	AAGGTAAACTTCCGAAATGCAA	59.4	484	INTERGENIC	--	--	--
Ca3_P4_32506988	Ca3	32506988	TG/T	ATTGCGAGAATTGTGGGATG	TTTGAAAAATGCACATAGCCC	60.9	495	INTERGENIC	--	--	--
Ca3_P4_32541478	Ca3	32541478	ATTGT/ATTGTTGT	TCCAATACCGAATTCAACACTT	TCACTAAGGCCTCTCATGCC	58.5	471	INTERGENIC	--	--	--
Ca3_P4_32559515	Ca3	32559515	TAAAAAA/TAAAAAAA	TGGTTCGGGTTCGAATTTAT	TTCTGAGTTTCGACTGCCCT	59.3	576	INTERGENIC	--	--	--
Ca3_P4_32560640	Ca3	32560640	ATTTTT/ATTTT	TTTAATAGTTCAGTGTGTTGATTTGC	TCACGTGGTAAGTCCGCATA	58.8	139	INTERGENIC	--	--	--
Ca3_P4_32563568	Ca3	32563568	TTATATATATATATATATAT/TTATATATATATATATATATATATAT	CGAAAGAAAAAGAAAACATCGC	GAAGGTGGCTCGGCATATAA	60.2	574	INTERGENIC	--	--	--
Ca3_P4_32591698	Ca3	32591698	CTT/CTTTT	CTTTATGGGTTGGAGATGGG	TCCAATACACCTGTCTTCCAAAT	59.2	314	INTERGENIC	--	--	--
Ca3_P4_32616318	Ca3	32616318	GTTTTTTTTTTTTT/GTTTTTTTTTTT	TGTGGTTGTTGTGGGCTTAG	CGAATGTGAGGTTTGGGACT	59.6	512	INTERGENIC	--	--	--
Ca3_P4_32651806	Ca3	32651806	TAAAAAAAAAAAAAAA/TAAAAAAAAAAAAAAAAA	GCCAGCACTGCACTAAAACA	TCCTTTAGCAAGGGCTGAGA	60.1	256	INTERGENIC	--	--	--

INDEL marker IDs	Chromosomes/ unanchored scaffolds	Physical positions (bp)	InDels (*Kabuli* reference genome-CDC Frontier/PII)	Forward primers (5'-3')	Reverse primers (5'-3')	Anneling temperature (0C)	Expected amplified product size (bp)	Structural annotation		Functional annotation	
								Sequence components of *kabuli* genome	*Kabuli* gene accession IDs	NCBI-KOG	TFs
Ca3_P4_32683395	Ca3	32683395	ACTTTC/ACTTTCAATGCATCTTTATCTTTC	GGTGCATAGTATCACGTGCG	TTTGGGTAAATCAGGACCCA	60.2	550	INTERGENIC	--	--	--
Ca3_P4_32691639	Ca3	32691639	A/ACCT	AATACCCACATGGCCAAAAA	CCGACAACTATGTGCACCTTT	60.1	498	INTERGENIC	--	--	--
Ca3_P4_32795871	Ca3	32795871	ATTTTTT/ATTTTTTT	TTGATGGGTCTGGATGTTTT	TTGAAATATCCAAAAACTGTTACAAA	57.8	297	INTERGENIC	--	--	--
Ca3_P4_32836823	Ca3	32836823	AATATATATATATATATATATATATATATATATATAT/AATATATATATATATATATATATATATATATATATATATATATAT	TATGGATGAAGCTTTCGCCT	TGGTTATTTAATTTGGGCCATT	59.8	610	INTERGENIC	--	--	--
Ca3_P4_32871592	Ca3	32871592	TTATATATATATATATATATA/TTATATATATATATATATATATA	AGGAAATTTTGGTTCGGCTT	TTTCAACCCTGTCCTTTTGG	59.9	644	INTERGENIC	--	--	--
Ca3_P4_32921141	Ca3	32921141	AT/A	AGCAGTTGTAATGAGGCAGGA	TCCTCAAACAAACCCCTCAC	59.9	726	INTERGENIC	--	--	--
Ca3_P4_33043917	Ca3	33043917	TAAAAAAAAAAA/TAAAAAAAAAA	GAAGCGAAAACTTTTGCAGG	GGCCTGTTTGAGGAATTCAA	60.0	695	INTERGENIC	--	--	--
Ca3_P4_33062899	Ca3	33062899	ATTTTTTTTTT/ATTTTTTTTTTT	TAATGGTTGGTTGGTGGTTG	TTGGGACTAAATCACTATGCCA	59.1	321	INTERGENIC	--	--	--
Ca3_P4_33099785	Ca3	33099785	TAAAAAAA/TAAAAAAAA	GCTTTTTCACTCTCGGTTGC	ATATGAGTTACGGGATGCGG	60.0	683	INTERGENIC	--	--	--
Ca3_P4_3317901	Ca3	3317901	AATATATATATATATATATATA/AATATATATATATATATATA	TGTTTTGAATTGCACCGTTTA	GGGGATCAGTTGACTTCAAGA	59.1	773	INTERGENIC	--	--	--

INDEL marker IDs	Chromosomes/ unanchored scaffolds	Physical positions (bp)	InDels (*Kabuli* reference genome-CDC Frontier/PII)	Forward primers (5'-3')	Reverse primers (5'-3')	Anneling temperature (0C)	Expected amplified product size (bp)	Structural annotation		Functional annotation	
								Sequence components of *kabuli* genome	*Kabuli* gene accession IDs	NCBI-KOG	TFs
Ca3_P4_33192861	Ca3	33192861	CATATATATATATATATATATA/CATATATATATATATATATA	CCTCACTTCACCCCCAACTA	CACTCTTAAACCCGCCTCAC	60.0	274	INTERGENIC	--	--	--
Ca3_P4_33224456	Ca3	33224456	TA/TAA	TGCAATAGGGCAATGGAAAT	GGCCTGGCCTACTTATAGCC	60.3	216	INTERGENIC	--	--	--
Ca3_P4_3339537	Ca3	3339537	TTT/TTTTCTT	GCATTAAGAGGTTCCCCCTC	TATCGATTTGGGGAAAAACG	59.9	872	INTERGENIC	--	--	--
Ca3_P4_3340030	Ca3	3340030	AG/A	AATTCCTTCCCACACAACCA	ACCACACTACGGTGCTAGGG	60.2	173	INTERGENIC	--	--	--
Ca3_P4_33418554	Ca3	33418554	GTAATAATAATAATAATAATAATAAT/GTAATAATAATAATAATAATAATAATAATAAT	GGTTTGTCAGCGAAAAAGGA	TTATTTCACCGTCGTGTCCA	60.2	605	INTERGENIC	--	--	--
Ca3_P4_3354325	Ca3	3354325	CGAGAGAGAGAGAGAGAGAGAGAGAGAGAGAG/CGAGAGAGAGAGAGAGAGAGAGAGAGAGAGAGAG	TAGGATAAGGTGAGACGGCG	CAACCTGTCCAAGGCATTTT	60.2	246	INTERGENIC	--	--	--
Ca3_P4_33559268	Ca3	33559268	TAAAAAAAAAAAA/TAAAAAAAAAAA	CCGAGCCAAAATCCAAAATA	GAAGCCACAAATGGACAGGT	59.9	801	INTERGENIC	--	--	--
Ca3_P4_33576979	Ca3	33576979	AATATATATATATATATATATATATATATATATA/AATATATATATATATATATATATATATATATATATATATA	GCTTAGGCAGCCACAAAATC	TCTTGTTGCCTTGTCACTCG	59.9	909	INTERGENIC	--	--	--
Ca3_P4_33579150	Ca3	33579150	AAAGNNNNNNNNNNNAAG/AAAG	TGTGGGAGCTGAAAGTGTTG	TTCCTTGCCATGTCACCATA	59.9	175	INTERGENIC	--	--	--
Ca3_P4_33693334	Ca3	33693334	AATATATATATATATATATATAT/AATATATATATATATATAT	CTCACCATAAACCACCCACG	CGTCCCGTCCAAATCTTCTA	61.2	464	INTERGENIC	--	--	--

INDEL marker IDs	Chromosomes/ unanchored scaffolds	Physical positions (bp)	InDels (*Kabuli* reference genome-CDC Frontier/PII)	Forward primers (5'-3')	Reverse primers (5'-3')	Anneling temperature (0C)	Expected amplified product size (bp)	Structural annotation		Functional annotation	
								Sequence components of *kabuli* genome	*Kabuli* gene accession IDs	NCBI-KOG	TFs
Ca3_P4_33695196	Ca3	33695196	TAAAAAAAAAAAAA/TAAAAAAAAAAA	CTACGGAGGAAAGGGGATGT	CGTTTTGAATTGATGGAGCA	60.3	819	INTERGENIC	--	--	--
Ca3_P4_33748791	Ca3	33748791	TAGTTTCAG/TAGTTTCAGTTTCAG	AGGTGCCAAAATTGATGAGG	CCTAGAAGGTCGCATCTCCA	59.9	417	INTERGENIC	--	--	--
Ca3_P4_33827405	Ca3	33827405	GATAATAATAATAATAATAATAATA/GATAATAATAATAATAATAATAATAATAATA	CTCATATCCCCAGGTGCAAT	CTCAATCGTGGGTGACCTTT	59.8	359	INTERGENIC	--	--	--
Ca3_P4_33828272	Ca3	33828272	TTTATTATTATTATTATTATTATTATTATTATTATTATTATTATTATTATT/TTTATTATTATTATTATTATTATTATTATTATTATTATTATTATTATT	AGAGGCAAACAAGAACCGAA	TGCATATAAGGAATTGACGCC	59.9	318	INTERGENIC	--	--	--
Ca3_P4_33877257	Ca3	33877257	AATATATATATATATAT/AATATATATATATATATAT	CTCACTTCCGCTCATGTCAA	ATATGGCAAGGATGGGAAAA	60.0	287	INTERGENIC	--	--	--
Ca3_P4_33884499	Ca3	33884499	TAAAAAAA/TAAAAAA	CAATTTTCGCAAGTCACACAA	CGAACCTTAATGGTCGGAAA	59.8	704	INTERGENIC	--	--	--
Ca3_P4_3392479	Ca3	3392479	TTG/TTGAAAGAGTG	AATGCATTTGGGTTTTCCAA	GGCAATCCTCGTTCACACTT	60.2	542	INTERGENIC	--	--	--
Ca3_P4_33954378	Ca3	33954378	TTATATATATATATATATATAT/TTATATATATATATATATATATAT	GGCTTCAAAGCCAACATCAT	TTTGTGCTGCCAACATTTTC	60.1	440	INTERGENIC	--	--	--
Ca3_P4_33954528	Ca3	33954528	ATATTATTATTATTATTATTATTATTATTATTATTATTATTATTATTATTATTATTATT/ATATTATTATTATTATTATTATTATTATTATTATTATTATTATTATTATTATTATTATTATTATT	GGCTTCAAAGCCAACATCAT	TCATACAAAAACCAAACGGG	60.1	622	INTERGENIC	--	--	--
Ca3_P4_33996297	Ca3	33996297	TAAAAAAAAAA/TAAAAAAAAA	TATTACACCAGAGCCCCCAA	CCAGCAAATGCAACAACAAT	60.3	593	INTERGENIC	--	--	--

INDEL marker IDs	Chromosomes/ unanchored scaffolds	Physical positions (bp)	InDels (*Kabuli* reference genome-CDC Frontier/PII)	Forward primers (5'-3')	Reverse primers (5'-3')	Anneling temperature (0C)	Expected amplified product size (bp)	Structural annotation		Functional annotation	
								Sequence components of *kabuli* genome	*Kabuli* gene accession IDs	NCBI-KOG	TFs
Ca3_P4_34032860	Ca3	34032860	CCAAAGC/CC	TAAATGTTGCGAACCTGCTG	CGTCGCGGTTATCACTTACA	59.9	678	INTERGENIC	--	--	--
Ca3_P4_34055571	Ca3	34055571	AATATATATATATATATATATATATATATATATATATATAT/AATATATATATATATATATATATATATATATATAT	TTTCGTGTGTGTTTGACTAAATGA	TTGGGCCAAATTGAAGAAAA	59.6	670	INTERGENIC	--	--	--
Ca3_P4_3417806	Ca3	3417806	TTATATATATATATATATATAT/TTATATATATATATATATATATATATAT	TGACCAGATGTCTTTTCAATATTTTC	GCTAATACATGTGTTTTGGGTTGA	59.8	556	INTERGENIC	--	--	--
Ca3_P4_34212501	Ca3	34212501	AATTATCTCTATTATCTC/AATTATCTC	TCCGAAAACGTTCGTACACA	AACGTTGTCACTTTCTTGGAGA	60.1	371	INTERGENIC	--	--	--
Ca3_P4_34219011	Ca3	34219011	TTCAATATCAATATCAATATCAATATCAATATCAATAT/TTCAATATCAATATCAATATCAATATCAATAT	CAATGAACATTGCCAACACA	CATCATCCTCCAATGATCCA	59.0	478	INTERGENIC	--	--	--
Ca3_P4_34264150	Ca3	34264150	TAAAAAAAAA/TAAAAAAAA	AATGGAAAGAGCATTGGGTG	GCAAACAAACAAAAACACATTGA	59.9	709	INTERGENIC	--	--	--
Ca3_P4_34267533	Ca3	34267533	CTTTTTTTTTT/CTTTTTTTTT	GCGGATACGAATTTTCTCCA	AAATTGTCGCTACCTTCCACA	60.0	633	INTERGENIC	--	--	--
Ca3_P4_34307680	Ca3	34307680	ATTTTTTTTTT/ATTTTTTTTTTT	TGAATTTGCAATTGGTCGAA	TGTCTTTGGTCTTTGCAATTATTA	60.1	607	INTERGENIC	--	--	--
Ca3_P4_34448761	Ca3	34448761	GATATATATATATATATATATATA/GATATATATATATATATATATATATA	TTTCCACACATGATCCAAAAA	GTGGAGAAAGAGTGGCCTTG	58.9	543	INTERGENIC	--	--	--
Ca3_P4_34456382	Ca3	34456382	AGG/AG	TCCCCTGTTTGGTTGTTGAT	GTAGTGTTGGTTTGTGGGGG	60.2	418	INTERGENIC	--	--	--

INDEL marker IDs	Chromosomes/ unanchored scaffolds	Physical positions (bp)	InDels (*Kabuli* reference genome-CDC Frontier/PII)	Forward primers (5'-3')	Reverse primers (5'-3')	Anneling temperature (0C)	Expected amplified product size (bp)	Structural annotation		Functional annotation	
								Sequence components of *kabuli* genome	*Kabuli* gene accession IDs	NCBI-KOG	TFs
Ca3_P4_34474422	Ca3	34474422	TAAAAAAAA/TAAAAAAA	AAAAATCAATTTATGCCGTTGT	TTATGCCGGATTGACACTCTT	57.7	478	INTERGENIC	--	--	--
Ca3_P4_34578013	Ca3	34578013	AGAGG/AGAGGAGG	CCAAACCGCAAGATTGATTT	TTTCCCAATATTCTGCCTCG	59.9	480	INTERGENIC	--	--	--
Ca3_P4_34813314	Ca3	34813314	GTTTTTTTTTTT/GTTTTTTTTTTTTT	AAAAATGCAAAAACACCATGAA	AAGTCGCCATTTGAAACTCG	59.4	340	INTERGENIC	--	--	--
Ca3_P4_34879718	Ca3	34879718	ATTTTTTT/ATTTTTT	GGTAAAAGGTGTAACCGAAGAAA	GCAATTAGATGGGTCACGCT	58.7	447	INTERGENIC	--	--	--
Ca3_P4_34943152	Ca3	34943152	TTATATATATATATATATA/TTATATATATATATATATATA	TGCATTTTTGTGATTGTGTGTG	GCTGGCTCTTTGGGTATGAG	60.5	454	INTERGENIC	--	--	--
Ca3_P4_35016413	Ca3	35016413	ATTTTTTTTTT/ATTTTTTTTT	AGGCTTAAGAGAGGGGTCCA	CCCTAAAATTTTGTGGGCTT	60.2	342	INTERGENIC	--	--	--
Ca3_P4_35171574	Ca3	35171574	TTATATATATATATATATATAT/TTATATATATATATATAT	GTGCACGGTACAAAACCCTT	CAGCACCATACTCAATTGTCA	59.9	965	INTERGENIC	--	--	--
Ca3_P4_35172088	Ca3	35172088	CATATATATATATATATATATATATATATA/CATATATATATATATATATATATATATATATA	TGACAATTGAGTATGGTGCTG	TTCTTCCAGAAACCCGATACA	57.2	184	INTERGENIC	--	--	--
Ca3_P4_35179411	Ca3	35179411	TAAAAAAAAAAA/TAAAAAAAAA	AATGGTGGCAACTACATCCC	TGGTATTGCGATAATGTCCCT	59.7	309	INTERGENIC	--	--	--
Ca3_P4_35250926	Ca3	35250926	TAAAAAAAAAA/TAAAAAAAAAAA	TTCGGTGGCCAATAGATTTC	TGTGTATGGTGAGGGACGAA	59.9	487	INTERGENIC	--	--	--

INDEL marker IDs	Chromosomes/ unanchored scaffolds	Physical positions (bp)	InDels (*Kabuli* reference genome-CDC Frontier/PII)	Forward primers (5'-3')	Reverse primers (5'-3')	Anneling temperature (0C)	Expected amplified product size (bp)	Structural annotation		Functional annotation	
								Sequence components of *kabuli* genome	*Kabuli* gene accession IDs	NCBI-KOG	TFs
Ca3_P4_35254569	Ca3	35254569	CAAAAAAAAAAAA/CAAAAAAAAAAA	TGAGCAAGTTGTCCGATTCA	GTACTCGCGCGGTAGGTAAG	60.4	909	INTERGENIC	--	--	--
Ca3_P4_35264037	Ca3	35264037	GAAAAAAAA/GAAAAAAA	GAATCCAAGGTTGGTTGGTG	ATGCTTAGGCATGCTGGATT	60.2	566	INTERGENIC	--	--	--
Ca3_P4_35302305	Ca3	35302305	ATTTTTTTTT/ATTTTTTTTTT	GAACTCCATGGGCACTTGTT	TTGGATTTGGGACATGGATT	60.0	708	INTERGENIC	--	--	--
Ca3_P4_35440962	Ca3	35440962	CTTTTTTTTTT/CTTTTTTTTTTT	TGTCAGGAGACCAGGAGAGG	CCGGGGTCTTTGATGACTAC	60.4	540	INTERGENIC	--	--	--
Ca3_P4_35533352	Ca3	35533352	TTATATATATATATATATAT/TTATATATATATATATAT	AATCCAGCCACAACAAGTCC	CGAATTTGAATGGCTTAGCTC	60.0	378	INTERGENIC	--	--	--
Ca3_P4_35584605	Ca3	35584605	TAATACAA/TAATACAAAATACAA	CATCCCTTTCATTTTCCCCT	TTTTTATGGGTTATTGGCCG	60.1	230	INTERGENIC	--	--	--
Ca3_P4_35629460	Ca3	35629460	TTATATATATATATATATAT/TTATATATATATATATATATAT	ACTGTGGGGCATATTGGTGT	TGCCCCTTGCTATTTGGTTA	60.1	660	INTERGENIC	--	--	--
Ca3_P4_35662143	Ca3	35662143	GTTTTTTTTT/GTTTTTTTT	CATCATGGTGTGTCCACTTGA	TTTTTGAAGGCATAGGACCG	60.4	676	INTERGENIC	--	--	--
Ca3_P4_35662446	Ca3	35662446	GAATAATAATAATAATAATAATAATAATAATAATAATAATAATAATA/GAATAATAATAATAATAATAATAATAATAATAATAATAATAATAATAATAATA	TTCGATTATAAATGGGACCACA	TTTTTGAAGGCATAGGACCG	59.2	274	INTERGENIC	--	--	--
Ca3_P4_35665629	Ca3	35665629	TAAAAAAAAAA/TAAAAAAAAA	TTCACATGAATCCCGTCAAA	TCATGGCTTGTGAGAAGGTTT	59.9	712	INTERGENIC	--	--	--

INDEL marker IDs	Chromosomes/ unanchored scaffolds	Physical positions (bp)	InDels (*Kabuli* reference genome-CDC Frontier/PII)	Forward primers (5'-3')	Reverse primers (5'-3')	Anneling temperature (0C)	Expected amplified product size (bp)	Structural annotation		Functional annotation	
								Sequence components of *kabuli* genome	*Kabuli* gene accession IDs	NCBI-KOG	TFs
Ca3_P4_35699071	Ca3	35699071	CTGTAATGTAAT/CTGTAAT	ACGAATCTCTCAACCATCGG	TGAGGCTCTCTTTGTGACTTTG	60.1	711	INTERGENIC	--	--	--
Ca3_P4_35703302	Ca3	35703302	TAAAAAAAAAA/TAAAAAAAAA	CGGATATGCGGACAAAAACT	TTAGCTTTGGGGTTTGATGG	60.0	600	INTERGENIC	--	--	--
Ca3_P4_35787583	Ca3	35787583	AATATATATATA/AATATATATA	ATGGAGAAGCATGTCCCTTG	TCAATGGCCTAAGGTGAAGG	60.1	305	INTERGENIC	--	--	--
Ca3_P4_35792530	Ca3	35792530	CTT/CT	GCTTCATGCATATACGCCAA	CCACCAAAAACACATCACCA	59.7	601	INTERGENIC	--	--	--
Ca3_P4_35834316	Ca3	35834316	GATATATATATATATATAT/GATATATATATATATATAT	GCTCCCCCTTCATGTGACTA	CCTTTTCGCCATCTTTGTGT	60.1	595	INTERGENIC	--	--	--
Ca3_P4_35861277	Ca3	35861277	ATTTTTTTTT/ATTTTTTTTTT	TGCAAACTTGGAGAAAAGGG	ATGGAGACAAATGGCTGTCA	60.2	417	INTERGENIC	--	--	--
Ca3_P4_35863496	Ca3	35863496	A/AG	CGTGAAGATATTGTTGCTTTGG	CCTTTCTACGACCAATTCCG	59.6	383	INTERGENIC	--	--	--
Ca3_P4_35863677	Ca3	35863677	CACATTAAACATTAAAC/CACATTAAAC	CGGAATTGGTCGTAGAAAGG	TGCAATTTCAGGTCTGATGC	59.6	428	INTERGENIC	--	--	--
Ca3_P4_35863925	Ca3	35863925	CTT/CT	CGGAATTGGTCGTAGAAAGG	TGCAATTTCAGGTCTGATGC	59.6	428	INTERGENIC	--	--	--
Ca3_P4_35878677	Ca3	35878677	TAAAAAAAAAA/TAAAAAAAAA	TCCCTTCCCTTTGTTGTGTC	TTTTATGTTTCGCGGGGATA	59.9	753	INTERGENIC	--	--	--

INDEL marker IDs	Chromosomes/ unanchored scaffolds	Physical positions (bp)	InDels (*Kabuli* reference genome-CDC Frontier/PII)	Forward primers (5'-3')	Reverse primers (5'-3')	Anneling temperature (0C)	Expected amplified product size (bp)	Structural annotation		Functional annotation	
								Sequence components of *kabuli* genome	*Kabuli* gene accession IDs	NCBI-KOG	TFs
Ca3_P4_35886280	Ca3	35886280	GAAAAAAAA/GAAAAAAA	CATGATGTGGTCGAAAAGGA	TGCAAGCAATGATGAAAACTC	59.5	209	INTERGENIC	--	--	--
Ca3_P4_35950711	Ca3	35950711	ATTTA/ATTTAAAATTACATGCAATTTTA	CATGCGATGGAACTGAATTG	GTAAAGTTGGCACAGGGGAA	60.1	479	INTERGENIC	--	--	--
Ca3_P4_35963610	Ca3	35963610	G/GA	AGCTGTCTCAAGCACCGAAT	TGCTCTGAATACTTTGTCCGC	60.0	823	INTERGENIC	--	--	--
Ca3_P4_35963663	Ca3	35963663	CT/CTAT	AGCTGTCTCAAGCACCGAAT	TGCTCTGAATACTTTGTCCGC	60.0	823	INTERGENIC	--	--	--
Ca3_P4_35979815	Ca3	35979815	TT/TTAT	TAGTGGCTGGGTAACGGAAC	CTTGGGACCCACGAAATAGA	60.0	296	INTERGENIC	--	--	--
Ca3_P4_36000041	Ca3	36000041	CTCATG/CTCATGTCATG	TCCAAATCATCCGCACATAA	TGATATGGGCCTTCACATTG	59.9	182	INTERGENIC	--	--	--
Ca3_P4_36000193	Ca3	36000193	AATATATATA/AATATATATATA	TCCAAATCATCCGCACATAA	TCGGTAAATAAGTTTTTGGACACA	59.9	662	INTERGENIC	--	--	--
Ca3_P4_36019725	Ca3	36019725	GAAA/GAA	ACCTTTGGATTCATGGTTGG	CCGATAGCCATTTGGACACT	59.6	614	INTERGENIC	--	--	--
Ca3_P4_36025831	Ca3	36025831	TAAAA/TAAAAA	ACGCCAGTCACACAGACAGA	CATTTAGGGGCGTAGCAAAT	60.5	875	INTERGENIC	--	--	--
Ca3_P4_36027335	Ca3	36027335	A/AT	GCTGATTCAAAAAGCCCTCA	TGTGCATTTTATTTTTGTTGGAA	60.3	375	INTERGENIC	--	--	--

INDEL marker IDs	Chromosomes/ unanchored scaffolds	Physical positions (bp)	InDels (*Kabuli* reference genome-CDC Frontier/PII)	Forward primers (5'-3')	Reverse primers (5'-3')	Anneling temperature (0C)	Expected amplified product size (bp)	Structural annotation		Functional annotation	
								Sequence components of *kabuli* genome	*Kabuli* gene accession IDs	NCBI-KOG	TFs
Ca3_P4_36028102	Ca3	36028102	TGAGCTTTAAAA/T	TCACAAATTTTCCAAAAATAACG	TGCCTCAAATTTCTTTTTCCA	58.2	447	INTERGENIC	--	--	--
Ca3_P4_36031623	Ca3	36031623	TAAAAAAAA/TAAAAAAA	CATGATATTTGCAGGTCGGA	TGGTTACCAATACCGAGGAAA	59.5	918	INTERGENIC	--	--	--
Ca3_P4_36032000	Ca3	36032000	TAATTTGAAATTTGAAATT/TAATTTGAAATT	TGGTAAGGACAAAAAGAGTTCCA	AACGCTGAACCAAAATGACC	60.0	801	INTERGENIC	--	--	--
Ca3_P4_36041204	Ca3	36041204	ATT/AT	TTCACACTCTCCAGTTCGGA	AAGTCGACGTTTGTGAAGAGTTC	59.4	699	INTERGENIC	--	--	--
Ca3_P4_36041601	Ca3	36041601	A/AAAC	CACGTGTAAAAATTCTTGTAAATCAAA	CGAGTGTTGCAAAGGATCAA	59.8	620	INTERGENIC	--	--	--
Ca3_P4_36042558	Ca3	36042558	ATTTTTT/ATTTTTTT	CTCCAGTTTTGGACCCTCAA	TTTCCAAAATAAACTCGACGC	60.1	430	INTERGENIC	--	--	--
Ca3_P4_36044328	Ca3	36044328	CAT/CATAT	TGTCAAATATCAAGGGGGATG	TGATGCTTTAAGCCGTCACA	59.6	541	INTERGENIC	--	--	--
Ca3_P4_36052612	Ca3	36052612	GTCACTTTCACTTT/GTCACTTT	CATCAATGCACGACCCATAC	TAAGTGAGCGCATCCATTTG	59.8	639	INTERGENIC	--	--	--
Ca3_P4_36076512	Ca3	36076512	AGAATT/A	GCATTTGTTTCAACAAGGTAAGG	TTTGGAGGACAAAAACTGGG	59.9	395	INTERGENIC	--	--	--
Ca3_P4_36089210	Ca3	36089210	CAAAAAAAAA/CAAAAAAAA	GGGCTTTGTTTTGTTTTGGA	AATAAAGGGGAAAAGGCCAA	60.0	185	INTERGENIC	--	--	--

INDEL marker IDs	Chromosomes/ unanchored scaffolds	Physical positions (bp)	InDels (*Kabuli* reference genome-CDC Frontier/PII)	Forward primers (5'-3')	Reverse primers (5'-3')	Anneling temperature (0C)	Expected amplified product size (bp)	Structural annotation		Functional annotation	
								Sequence components of *kabuli* genome	*Kabuli* gene accession IDs	NCBI-KOG	TFs
Ca3_P4_36089976	Ca3	36089976	AC/A	AATACGAATCATTGGAGCTTTTT	AAACTGGTTTGAGTGACGGC	58.3	167	INTERGENIC	--	--	--
Ca3_P4_36090173	Ca3	36090173	GTTTTTTTT/GTTTTTTT	ATGAATCCATTTGGCGCTAC	GAAGCGTCAAGTATCCTCCG	59.9	541	INTERGENIC	--	--	--
Ca3_P4_36093211	Ca3	36093211	CGG/CG	TTTTGGAAGGTTGCAGCATA	TGAAACCATCAATATCCTTATTCG	59.3	658	INTERGENIC	--	--	--
Ca3_P4_36105355	Ca3	36105355	CTTTTTT/CTTTTT	GAGTGTGGGAAGGATCGAAA	CCTGGAGAAGGCTGTCAAAG	60.1	372	INTERGENIC	--	--	--
Ca3_P4_36105726	Ca3	36105726	TC/T	CTTTGACAGCCTTCTCCAGG	AGGCTCATAAAACGCCTCAA	60.0	440	INTERGENIC	--	--	--
Ca3_P4_36107618	Ca3	36107618	GA/GAA	AGATCGGACGCTCCTCACTA	TGGCGAAGTTAAAGGGATTG	60.0	325	INTERGENIC	--	--	--
Ca3_P4_36126613	Ca3	36126613	A/AT	GACCCATCAATGAAGCGACT	GTAGGCATGTGGAGCCAAGT	60.1	612	INTERGENIC	--	--	--
Ca3_P4_3613355	Ca3	3613355	TAAATTTAAAATTTAAAATTTAAAAT/TAAATTTAAAATTTAAAAT	GCCCATTACTCGCTGGTTAG	TTTCTCCACCACTCCACTCC	59.7	178	INTERGENIC	--	--	--
Ca3_P4_36139586	Ca3	36139586	CT/CTGT	ATGCAAATTACCCAAGACGC	TTATACCGGCTTCGTTGACC	60.0	580	INTERGENIC	--	--	--
Ca3_P4_36139755	Ca3	36139755	TTAT/TTATATAT	ATGCAAATTACCCAAGACGC	TTATACCGGCTTCGTTGACC	60.0	580	INTERGENIC	--	--	--

INDEL marker IDs	Chromosomes/ unanchored scaffolds	Physical positions (bp)	InDels (*Kabuli* reference genome-CDC Frontier/PII)	Forward primers (5'-3')	Reverse primers (5'-3')	Anneling temperature (0C)	Expected amplified product size (bp)	Structural annotation		Functional annotation	
								Sequence components of *kabuli* genome	*Kabuli* gene accession IDs	NCBI-KOG	TFs
Ca3_P4_36140094	Ca3	36140094	CAA/CAAA	GGTCAACGAAGCCGGTATAA	CGAAAAATAACACCAATGCAAA	60.0	551	INTERGENIC	--	--	--
Ca3_P4_36140610	Ca3	36140610	CTCTTCCTTCTT/CTCTTCCTTCTTCCTTCTT	TTTGCATTGGTGTTATTTTTCG	TTGGATCAGCCTTGCTCTTT	59.9	431	INTERGENIC	--	--	--
Ca3_P4_36151877	Ca3	36151877	T/TG	TCCAATGTGTGGTTGGATAAAA	TTTTTCCAAATTGACAGCCA	60.1	600	INTERGENIC	--	--	--
Ca3_P4_36163828	Ca3	36163828	GTTTTTTTTTTT/GTTTTTTTTTT	AATCGTTTGAAAGACGTCGAA	TCAAACGGACCTCTCCATAAA	59.7	573	INTERGENIC	--	--	--
Ca3_P4_36184874	Ca3	36184874	AAAAA/AAAAAGAAAA	AACTGTCAACACGTTGAGTATCG	GGCGTACACATGTCTTTGGA	59.2	395	INTERGENIC	--	--	--
Ca3_P4_36185797	Ca3	36185797	AT/A	CGGAATGTAGTGTTGACCCC	CAGTGACAGTGAGACGTGGG	60.2	535	INTERGENIC	--	--	--
Ca3_P4_36187853	Ca3	36187853	AT/ATTGAGATTAAAATT	GCTAAAGTCAGCCCAAACCA	TCAATGGAACATCCAAAGCTC	60.2	790	INTERGENIC	--	--	--
Ca3_P4_36201223	Ca3	36201223	ATTTTT/ATTTT	CTAATTGTTGAAGCCGGGAA	CCCTTTTGTTGAAGTGGCAT	60.1	558	INTERGENIC	--	--	--
Ca3_P4_36201355	Ca3	36201355	ATTTTTT/ATTTTTTT	CTAATTGTTGAAGCCGGGAA	CCCTTTTGTTGAAGTGGCAT	60.1	558	INTERGENIC	--	--	--
Ca3_P4_36219342	Ca3	36219342	GTTT/GTT	CATGTGGCGAAGAGAGTGAA	GTATTTCCTTTGGGCGTGAA	60.0	531	INTERGENIC	--	--	--

INDEL marker IDs	Chromosomes/ unanchored scaffolds	Physical positions (bp)	InDels (*Kabuli* reference genome-CDC Frontier/PII)	Forward primers (5'-3')	Reverse primers (5'-3')	Anneling temperature (0C)	Expected amplified product size (bp)	Structural annotation		Functional annotation	
								Sequence components of *kabuli* genome	*Kabuli* gene accession IDs	NCBI-KOG	TFs
Ca3_P4_36224870	Ca3	36224870	AAGAGAGAGAGAGAGAGAGAG/AAGAGAGAGAGAGAGAGAGAGAGAGAGAG	ACCTGTGTGACTCTGAGGGG	CCCAGTAACCGCTCTCTCTG	60.2	721	INTERGENIC	--	--	--
Ca3_P4_36227106	Ca3	36227106	AATATATA/AATATATATA	CACGAAATCCGAAATGGAAC	CTTTATGTCGCACAAGCTGC	60.3	758	INTERGENIC	--	--	--
Ca3_P4_36240433	Ca3	36240433	TAAAAAAAAA/TAAAAAAAA	TTGTTTTGAATTTGGGCCAG	TTCCAAAATCTCACCATTCATT	60.8	859	INTERGENIC	--	--	--
Ca3_P4_36241503	Ca3	36241503	AAT/AATGAT	TAATGAAGGGGACTGCAACC	TTCGACTCCGAGATATAACATTCA	59.9	789	INTERGENIC	--	--	--
Ca3_P4_36241653	Ca3	36241653	CC/CCAC	TTCGCAGAAAAACTATGCCC	TCCGTATTCGAATTTGAGACAA	60.2	808	INTERGENIC	--	--	--
Ca3_P4_36242951	Ca3	36242951	GTTTTTTT/GTTTTTTTT	TGCTGCAAAAGACAATTTCG	AAGTGGTGGTGCTGTTTTCC	60.0	907	INTERGENIC	--	--	--
Ca3_P4_36251816	Ca3	36251816	TTATAT/TTAT	CGTCAAAAACAACAGGCAGA	CAGAAGCTTGCAGCTACCCT	59.9	718	INTERGENIC	--	--	--
Ca3_P4_3625461	Ca3	3625461	CTT/CTTT	CGGCCTAGCCTACGTTAGAGT	TCCTTCACTAGCTGGTTTTCG	59.9	785	INTERGENIC	--	--	--
Ca3_P4_36264735	Ca3	36264735	TA/TAA	ATCACCTGCAACATGGATCA	TTAATTAGGCACACCACGCA	59.9	706	INTERGENIC	--	--	--
Ca3_P4_36286597	Ca3	36286597	ATTTTTTT/ATTTTTTTT	TCATTTTGGCAACTTACCACC	TGCCTGAATTTATTGGGAATG	59.9	754	INTERGENIC	--	--	--

INDEL marker IDs	Chromosomes/ unanchored scaffolds	Physical positions (bp)	InDels (*Kabuli* reference genome-CDC Frontier/PII)	Forward primers (5'-3')	Reverse primers (5'-3')	Anneling temperature (0C)	Expected amplified product size (bp)	Structural annotation		Functional annotation	
								Sequence components of *kabuli* genome	*Kabuli* gene accession IDs	NCBI-KOG	TFs
Ca3_P4_36287175	Ca3	36287175	TAAAAAAAAA/TAAAAAAAAAAAA	CATTCCCAATAAATTCAGGCA	CGGGCATTAGAGATCCACAT	59.8	202	INTERGENIC	--	--	--
Ca3_P4_36289361	Ca3	36289361	TTATATATATATATATATAT/TTATATATATATATAT	GTGCTTTTGCGATTTCATCA	TGTAAGAAAATGCATGCGATAGA	59.8	759	INTERGENIC	--	--	--
Ca3_P4_3628980	Ca3	3628980	TAAAAAA/TAAAAAAA	CCACCATTAGTTAACGGAGTCA	TTGTCTAAAGAAATTGAAATCATGTTG	59.0	267	INTERGENIC	--	--	--
Ca3_P4_36289807	Ca3	36289807	T/TTGATATACCTAGATC	CGCATGCATTTTCTTACAACA	GCGTTTTTGTGTTTCCGTCT	59.8	446	INTERGENIC	--	--	--
Ca3_P4_36290753	Ca3	36290753	CA/C	AAAATCTCAAAATTCGCCTTTG	TGAGTGGGGTAAATAAACTCTGAA	59.6	416	INTERGENIC	--	--	--
Ca3_P4_36303360	Ca3	36303360	ATTTTTT/ATTTTTTT	GCAAACATGGTAAATTGGCA	CAAAAATTGTCACGTCGGTG	59.4	469	INTERGENIC	--	--	--
Ca3_P4_36303469	Ca3	36303469	GTTTTTTTTTT/GTTTTTTTTT	GCAAACATGGTAAATTGGCA	CAAAAATTGTCACGTCGGTG	59.4	469	INTERGENIC	--	--	--
Ca3_P4_36303688	Ca3	36303688	AT/A	GCAAACATGGTAAATTGGCA	CAAAAATTGTCACGTCGGTG	59.4	469	INTERGENIC	--	--	--
Ca3_P4_36308195	Ca3	36308195	T/TC	TTTGCCACTTGATACAGCAGA	TTGAACCTAAACGATGACACCTT	59.5	309	INTERGENIC	--	--	--
Ca3_P4_36324828	Ca3	36324828	T/TG	TCCATGAAACATTCCCCATT	CGCACTAAAACGACGAACAA	60.0	454	INTERGENIC	--	--	--

INDEL marker IDs	Chromosomes/ unanchored scaffolds	Physical positions (bp)	InDels (*Kabuli* reference genome-CDC Frontier/PII)	Forward primers (5'-3')	Reverse primers (5'-3')	Anneling temperature (0C)	Expected amplified product size (bp)	Structural annotation		Functional annotation	
								Sequence components of *kabuli* genome	*Kabuli* gene accession IDs	NCBI-KOG	TFs
Ca3_P4_36326063	Ca3	36326063	TAAAAAAAAAA/TAAAAAAAAA	CGCTGAGCAAAAATGACAAA	CTCAAACCAATCAAGCCCAT	60.0	692	INTERGENIC	--	--	--
Ca3_P4_36326214	Ca3	36326214	ATTTTTT/ATTTTTTT	TTGCAAAATGGTCCATCAAA	CTCAAACCAATCAAGCCCAT	59.9	419	INTERGENIC	--	--	--
Ca3_P4_36326321	Ca3	36326321	ACT/ACTCT	TTGCAAAATGGTCCATCAAA	GCAGTGTGATGCAAAAATGG	59.9	680	INTERGENIC	--	--	--
Ca3_P4_36327195	Ca3	36327195	ATTTTTTTTT/ATTTTTTTTTT	CACTTTCTCCTTGCAACCAA	TGGAGAAACAATGCAAATGG	58.9	512	INTERGENIC	--	--	--
Ca3_P4_36328591	Ca3	36328591	GAAA/GAA	CACGATGAAATCACTATGAAAAGC	CGTTCCAGACCTCCATTGTT	60.0	440	INTERGENIC	--	--	--
Ca3_P4_36342426	Ca3	36342426	GAAAAAA/GAAAAAAA	AGTATTGAAGCATCACCGCC	TCGAGGCATTTGTCTGAGAG	60.1	195	INTERGENIC	--	--	--
Ca3_P4_36347468	Ca3	36347468	CTTTTTTTT/CTTTTTTT	AAGCACCAGAAGGTCCTGAA	TGGAAAAGGGAAATTCGTTG	59.8	470	INTERGENIC	--	--	--
Ca3_P4_36348447	Ca3	36348447	GTTT/GTTTTTT	TCAAGAATGCCAACAACAAAA	TTCGACCTTGGAAAATGTCA	59.2	565	INTERGENIC	--	--	--
Ca3_P4_36349322	Ca3	36349322	TTCTC/TTCTCTC	TGACATTTTCCAAGGTCGAA	GTTGACGGTCGATTGAAACA	59.1	594	INTERGENIC	--	--	--
Ca3_P4_36350064	Ca3	36350064	TAT/TATAAT	GGTTTCCCCAATATTCCGTT	GATGGCAAGCTTTTGTCCTT	59.9	492	INTERGENIC	--	--	--

INDEL marker IDs	Chromosomes/ unanchored scaffolds	Physical positions (bp)	InDels (*Kabuli* reference genome-CDC Frontier/PII)	Forward primers (5'-3')	Reverse primers (5'-3')	Anneling temperature (0C)	Expected amplified product size (bp)	Structural annotation		Functional annotation	
								Sequence components of *kabuli* genome	*Kabuli* gene accession IDs	NCBI-KOG	TFs
Ca3_P4_36350864	Ca3	36350864	TA/TAA	AAGGACAAAAGCTTGC CATC	TTGTCCCCAATGTTGG TCTT	59.3	663	INTERGENIC	--	--	--
Ca3_P4_36351279	Ca3	36351279	TA/TAA	AAGACCAACATTGGGG ACAA	CGATTCAAGTGTCGCA TGTC	60.2	473	INTERGENIC	--	--	--
Ca3_P4_36351563	Ca3	36351563	TAAAAAAA/TAAAAAA	AAGACCAACATTGGGG ACAA	GCCTGCAATGAACCGT AAGT	60.2	873	INTERGENIC	--	--	--
Ca3_P4_36351613	Ca3	36351613	AA/AACA	GACATGCGACACTTGA ATCG	GCCTGCAATGAACCGT AAGT	60.3	420	INTERGENIC	--	--	--
Ca3_P4_36361613	Ca3	36361613	GACA/GA	ACTCATCTCCAACACG AGGG	TTGCTGCTGATTTTCTC GTC	60.1	769	INTERGENIC	--	--	--
Ca3_P4_36362897	Ca3	36362897	A/AC	AGGAAATTCTCCGACG TTGA	TTCCGTCCATATGCATT GATT	59.7	920	INTERGENIC	--	--	--
Ca3_P4_36363081	Ca3	36363081	CG/C	TGTGTAGCGATGTCAC ACTTG	TTCCGTCCATATGCATT GATT	58.3	426	INTERGENIC	--	--	--
Ca3_P4_36370183	Ca3	36370183	GA/GAAA	TGGAGAAAATGCGAGA TTCC	AAAACGGATCCCCTAC CAAC	60.2	541	INTERGENIC	--	--	--
Ca3_P4_36371742	Ca3	36371742	ATTTTTTTT/ATTTTTTT	GGGTTTTCTAGTCCCC CAAG	TGTTTTTCATTATGTGC CCG	59.8	483	INTERGENIC	--	--	--
Ca3_P4_36371828	Ca3	36371828	GT/GTTAT	GGGTTTTCTAGTCCCC CAAG	TGTTTTTCATTATGTGC CCG	59.8	483	INTERGENIC	--	--	--

INDEL marker IDs	Chromosomes/ unanchored scaffolds	Physical positions (bp)	InDels (*Kabuli* reference genome-CDC Frontier/PII)	Forward primers (5'-3')	Reverse primers (5'-3')	Anneling temperature (0C)	Expected amplified product size (bp)	Structural annotation		Functional annotation	
								Sequence components of *kabuli* genome	*Kabuli* gene accession IDs	NCBI-KOG	TFs
Ca3_P4_36381118	Ca3	36381118	T/TTTTTGAAAGGGCA	AAAAAGCGCGGGTTAATTTT	CCATGCTTCCCACGTCTAAT	60.0	544	INTERGENIC	--	--	--
Ca3_P4_36392224	Ca3	36392224	GTACTA/GTACTACTA	TTTTGGACTGAGGGAGGTTG	TGCCAATTGGAATCACAAGA	60.1	698	INTERGENIC	--	--	--
Ca3_P4_36392375	Ca3	36392375	ATTTTTT/ATTTTTTT	AACGTGATTCTCAGAACGGC	TCCCCAAACAAGTTTTCAGC	60.3	736	INTERGENIC	--	--	--
Ca3_P4_36419771	Ca3	36419771	ATTTTTTT/ATTTTTT	GTCGTCTTGGGCTCTTTCAC	TTGCCGTTGAGAAAATGAGA	59.9	477	INTERGENIC	--	--	--
Ca3_P4_36420190	Ca3	36420190	ATTTTTTTT/ATTTTTTT	TGTTTTTCGCTCATCCATTG	TGGTAGAAATCACGATGCCA	59.7	710	INTERGENIC	--	--	--
Ca3_P4_36420971	Ca3	36420971	AATATATATATATAT/AATATATATAT	TGGCATCGTGATTTCTACCA	GGCCATGGTGATTGTAAAGG	60.1	746	INTERGENIC	--	--	--
Ca3_P4_36481853	Ca3	36481853	TA/TAA	TCCTCGAGATCCAAAAGCAT	GTCCAATTCCCCCTTTCATT	59.8	272	INTERGENIC	--	--	--
Ca3_P4_36508230	Ca3	36508230	ATTTTTTTTTTT/ATTTTTTTTTT	AAAAATTGATTCGTGTGATTGG	GGCCAACGACTTCCTATTGA	58.9	135	INTERGENIC	--	--	--
Ca3_P4_36518439	Ca3	36518439	AATATATATATATATATATATATATATATATAT/AATATATATATATATATATATATATATATATATATATATAT	TTTTTCATGTGGCCGTGTAA	CCCGTGATACTCCTTGATCACT	60.0	672	INTERGENIC	--	--	--
Ca3_P4_36639180	Ca3	36639180	TTATATATATATATATATATATATAT/TTATATATATATATATATATATAT	CAACGCGTGAAAGAAACAAA	TCGGATGGGAAAACTACTCAA	59.9	626	INTERGENIC	--	--	--

INDEL marker IDs	Chromosomes/ unanchored scaffolds	Physical positions (bp)	InDels (*Kabuli* reference genome-CDC Frontier/PII)	Forward primers (5'-3')	Reverse primers (5'-3')	Anneling temperature (0C)	Expected amplified product size (bp)	Structural annotation		Functional annotation	
								Sequence components of *kabuli* genome	*Kabuli* gene accession IDs	NCBI-KOG	TFs
Ca3_P4_36668334	Ca3	36668334	GTTTT/GTTTTT	TGGGTAAAAGCTCGAGTTCC	CAGTGGTGGAAAAAGGCAAT	59.3	499	INTERGENIC	--	--	--
Ca3_P4_36740169	Ca3	36740169	GATATATATATAT/GATATATATATATAT	TGCGTTGCGACACAATAAAT	CCACACCTTATAGTTTCCACCC	60.1	783	INTERGENIC	--	--	--
Ca3_P4_36829016	Ca3	36829016	TAAAAAAAA/TAAAAAAA	CGTTCTTTGAATGAGTGACCAA	AGTGCAAAGAGGTTGCAAGG	60.1	321	INTERGENIC	--	--	--
Ca3_P4_37017817	Ca3	37017817	AATATATATATATATATATATATATATATATATATATA/AATATATATATATATATATATATATATATATATATATATATATATATATA	CAGAAGGCCAGGAGAAGAAA	TGTGCGTGAAGTTTCATGGT	59.5	707	INTERGENIC	--	--	--
Ca3_P4_37150587	Ca3	37150587	GAAAAAAAAAAA/GAAAAAAAAAA	TGCTTGCGTGAAAATTGAAA	TCCCATGTTCCGTATATCCAA	60.4	554	INTERGENIC	--	--	--
Ca3_P4_37191129	Ca3	37191129	TAAAAAAAAAA/TAAAAAAAAA	TGGTTTCATTCATCTTTTGCAG	GCCCAAATCAACCCATTTTA	60.1	445	INTERGENIC	--	--	--
Ca3_P4_37192959	Ca3	37192959	T/TCA	TCAAATTTCATGTGCGCTGT	TAATAGTCCGGCCCACTTTG	60.3	478	INTERGENIC	--	--	--
Ca3_P4_37245566	Ca3	37245566	GTATATATATATATATATATATATAT/GTATATATATATATATATATATAT	TCAAATGTCTCCGTCTACGCT	GGGTGACATGCATCTTTGTG	59.9	542	INTERGENIC	--	--	--
Ca3_P4_3725109	Ca3	3725109	AC/ACC	TGTGGTGGTGGAGAACAGAG	CGGTCTTTGTGGTGATTCCT	59.7	357	INTERGENIC	--	--	--
Ca3_P4_37378120	Ca3	37378120	GAGAAGAAGAAGAAGAAGAAGAA/GAGAAGAAGAAGAAGAAGAA	ACCTCAGATTCCAAACGTGC	GCTGCATTGTGGATAAGGGT	60.1	425	INTERGENIC	--	--	--

**INDEL marker IDs**	**Chromosomes/ unanchored scaffolds**	**Physical positions (bp)**	**InDels (*Kabuli* reference genome-CDC Frontier/PII)**	**Forward primers (5'-3')**	**Reverse primers (5'-3')**	**Anneling temperature (0C)**	**Expected amplified product size (bp)**	**Structural annotation**		**Functional annotation**	
								**Sequence components of *kabuli* genome**	***Kabuli* gene accession IDs**	**NCBI-KOG**	**TFs**
Ca3_P4_37384200	Ca3	37384200	GAAAAAAAAAA/GAAAAAAAAA	GGTTGACCGACAAAATCGAG	TCTTGTAGCCAATCCAACCA	60.5	826	INTERGENIC	--	--	--
Ca3_P4_37396352	Ca3	37396352	CAATTGGGAATTGG/CAATTGGGAATTGGGAATTGG	CCTTTGCCTTTCTGAGATGG	GCGGTGAATATCCAAAGGAC	59.8	481	INTERGENIC	--	--	--
Ca3_P4_37428682	Ca3	37428682	T/TGGA	TTGAGGATCAGCTACACCCC	TGGAACAATTTTGGTGGTGA	60.1	546	INTERGENIC	--	--	--
Ca3_P4_37484993	Ca3	37484993	TATAATAATAATAATAATAATAATATTAATAATAATA/TATAATAATAATAATAATAATATTAATAATAATA	CCCGCGTAAATATCAATGCT	GGTGGTGGTTTGGACGTAAC	60.0	482	INTERGENIC	--	--	--
Ca3_P4_37594905	Ca3	37594905	TTATATATATATATATATATATATA/TTATATATATATATATATATATA	GTCCGCCCTAAAAAGGTAGC	TCAAAACACCACATCAAAACG	60.1	188	INTERGENIC	--	--	--
Ca3_P4_37719535	Ca3	37719535	TGG/TGGG	TTGGGTCAAGAATTCATCTCG	CCCAAAATGTGGCTTGAAAT	60.1	581	INTERGENIC	--	--	--
Ca3_P4_37756750	Ca3	37756750	ATTTTTTTTT/ATTTTTTTTTT	AATAGGCCAGGTCAGGCTTT	GGATTCGTTGATGATGGCTT	60.1	656	INTERGENIC	--	--	--
Ca3_P4_37828643	Ca3	37828643	CCAGAC/CC	GGGAATAGGCTAGACCGTCC	GCTTGGCCTACGACCTATCA	59.9	512	INTERGENIC	--	--	--
Ca3_P4_37875029	Ca3	37875029	GT/GTT	ATGGCGTGACTGAACATGAA	AAGGGCAAGATCCCAAAAAC	60.1	672	INTERGENIC	--	--	--
Ca3_P4_38007387	Ca3	38007387	TTCTCTCTCTCTCTCTCTCTCTCTCT/TTCTCTCTCTCTCTCTCTCTCTCTCTCTCT	AAACAATACGCAAACCGGAG	CTTCTCCAACAAGAGCGACC	60.0	245	INTERGENIC	--	--	--

INDEL marker IDs	Chromosomes/ unanchored scaffolds	Physical positions (bp)	InDels (*Kabuli* reference genome-CDC Frontier/PII)	Forward primers (5'-3')	Reverse primers (5'-3')	Anneling temperature (0C)	Expected amplified product size (bp)	Structural annotation		Functional annotation	
								Sequence components of *kabuli* genome	*Kabuli* gene accession IDs	NCBI-KOG	TFs
Ca3_P4_3804824	Ca3	3804824	TAAA/TAA	AGTCAGACGCTACTCGGCAT	ATTAGGGGAGCATGCATTTG	60.0	479	INTERGENIC	--	--	--
Ca3_P4_38129457	Ca3	38129457	CATATATATATATATA/CATATATATATATATATA	CAAAAGCCGGACCAAAATTA	GGGCATGGGCAATAATCTAA	59.9	398	INTERGENIC	--	--	--
Ca3_P4_38142353	Ca3	38142353	TCGACGAC/TCGAC	ATCAATTGGTCGCACCTTTC	CATGATGGCAAATTCATAGGC	59.9	387	INTERGENIC	--	--	--
Ca3_P4_3818047	Ca3	3818047	G/GGA	ATGCTCTGCACTTGCAGCTA	TTCATCGCCTCAAAGTGTTG	59.9	707	INTERGENIC	--	--	--
Ca3_P4_38182761	Ca3	38182761	TTGATATGATAT/TTGATAT	AAACAACGTCATGAAGAAAATTGA	GGTTTTGCCAACATCTGCTT	59.9	606	INTERGENIC	--	--	--
Ca3_P4_38182839	Ca3	38182839	AGTGTGTGTGTGTGTGT/AGTGTGTGTGTGT	AAAGCGTCTTTCATTAATAGTGTGTG	GGTTTTGCCAACATCTGCTT	60.0	319	INTERGENIC	--	--	--
Ca3_P4_38183210	Ca3	38183210	TTAAATTAATAAATTAATAAA/TTAAATTAATAAA	AATCAAGCAAGCCGTTGACT	TAAAAGGTTTGTGCGTGTCG	59.9	356	INTERGENIC	--	--	--
Ca3_P4_38240907	Ca3	38240907	ATTTTTTTTTTTTTTT/ATTTTTTTTTTT	GGCTCCTCTCCTCTAACGGT	GACAAGTGCAACAATTTTCATCA	59.8	527	INTERGENIC	--	--	--
Ca3_P4_38241297	Ca3	38241297	TTTATT/TTT	TTGATGAAAATTGTTGCACTTG	TTGCTAACACAAAAGTATTTGAATGTC	58.7	189	INTERGENIC	--	--	--
Ca3_P4_38322519	Ca3	38322519	GTGAT/GT	CCTTGGCAGTCGTTGTTTCT	GCCATACACGTGTCTCCCTT	60.3	122	INTERGENIC	--	--	--

INDEL marker IDs	Chromosomes/ unanchored scaffolds	Physical positions (bp)	InDels (*Kabuli* reference genome-CDC Frontier/PII)	Forward primers (5'-3')	Reverse primers (5'-3')	Anneling temperature (0C)	Expected amplified product size (bp)	Structural annotation		Functional annotation	
								Sequence components of *kabuli* genome	*Kabuli* gene accession IDs	NCBI-KOG	TFs
Ca3_P4_38406107	Ca3	38406107	AATATATATATATATATATATAT/AATATATATATATATATATATATATAT	CTCTCAAAGTGCTGAAGAGACC	GGGACTGCATACCAATAGCTG	58.3	775	INTERGENIC	--	--	--
Ca3_P4_38693651	Ca3	38693651	TAA/T	GCCTTTCACATCAAACCGAT	GAGAGAGCATCCCGAAAACA	59.9	581	INTERGENIC	--	--	--
Ca3_P4_38729935	Ca3	38729935	CTTTTTTTT/CTTTTTTTTT	AAAAGGCAAATGTGTGTCATTG	CATTTGGCCACAGAACATTG	59.9	856	INTERGENIC	--	--	--
Ca3_P4_38735945	Ca3	38735945	TTTTATTTATTTATTTAT/TTTTATTTATTTATTTATTTATTTAT	GGGTTTGCTTATTTGAGAATACTG	GAATCTAGCAGCAGGAGCCA	58.3	674	INTERGENIC	--	--	--
Ca3_P4_38900161	Ca3	38900161	GACAACA/GACA	GCCATTCCATTAAAGGGTCA	TCTGCCTAATCAAACATGCC	59.8	891	INTERGENIC	--	--	--
Ca3_P4_39010533	Ca3	39010533	AG/AGG	CGGGCTTTCCTGTTACACAT	GCAGAGAAAGGAACAGAGCG	60.0	309	INTERGENIC	--	--	--
Ca3_P4_39248121	Ca3	39248121	CATATATATATATATATATATATATATATA/CATATATATATATATATATATATATATATATATATATA	CCAAGTTGGCTAAACCTTGC	CATACCACCATTCTTTCCCG	59.7	783	INTERGENIC	--	--	--
Ca3_P4_39355870	Ca3	39355870	TAAAAAAAAAA/TAAAAAAAAAAA	TGCGAGATCGAAGATTTGG	CGACGCGACTGGATTGTAA	59.9	725	INTERGENIC	--	--	--
Ca3_P4_39401096	Ca3	39401096	ATTTTTTTTTT/ATTTTTTTTT	ATTTGGGTCAAGGATGATGC	GCTTTGCTTGCCCATTTTTA	59.8	940	INTERGENIC	--	--	--
Ca3_P4_39417852	Ca3	39417852	AAT/A	TGAGTTGTGAGATGCTTCCCT	CCTTTGCAATTTTCTTTGCC	59.9	692	INTERGENIC	--	--	--

INDEL marker IDs	Chromosomes/ unanchored scaffolds	Physical positions (bp)	InDels (*Kabuli* reference genome-CDC Frontier/PII)	Forward primers (5'-3')	Reverse primers (5'-3')	Anneling temperature (0C)	Expected amplified product size (bp)	Structural annotation		Functional annotation	
								Sequence components of *kabuli* genome	*Kabuli* gene accession IDs	NCBI-KOG	TFs
Ca3_P4_39419535	Ca3	39419535	C/CAATAAAATA	CCTAACTCAGCATGGGGAAA	TTAATTAATTGTTGGGGTTTTGTT	60.1	431	INTERGENIC	--	--	--
Ca3_P4_39419619	Ca3	39419619	TAATATGAATATGAATAT/TAATATGAATAT	CCTAACTCAGCATGGGGAAA	GCGTGACAATAGTAAGATAATTGTTCC	60.1	506	INTERGENIC	--	--	--
Ca3_P4_3963300	Ca3	3963300	CAAAAAAAAA/CAAAAAAAAAAAAA	TCCATTGTGTGCCACAGTCT	TCTAGTCCGCGACTCAACCT	60.2	505	INTERGENIC	--	--	--
Ca3_P4_39670282	Ca3	39670282	TTATATATATATATATATATAT/TTATATATATATATATATATATAT	TGGTGCGACAATCATTAACC	TGGATATCGAACCCTCACCT	59.4	720	INTERGENIC	--	--	--
Ca3_P4_39706199	Ca3	39706199	TTATATATATATATATATA/TTATATATATATATATA	TGCTCAAACCAAATATGGCA	TTGGGCAAAACTCCATCTTC	60.1	770	INTERGENIC	--	--	--
Ca3_P4_39813917	Ca3	39813917	AATATATATATATATATATAT/AATATATATATATATATATATAT	GAGGTTGGTCACCGGTAAGA	TTGTAGGCGGTGTGTCAAAT	60.0	550	INTERGENIC	--	--	--
Ca3_P4_39988476	Ca3	39988476	GTTTT/GTTT	AGAACAGAGCTGGCCTCAAA	ATTGCAAGAAAACCAGCACC	60.1	148	INTERGENIC	--	--	--
Ca3_P4_4017573	Ca3	4017573	TAA/TAAA	ACCACAGATCACCAGTCGCT	ACGTCCAAGGTAAGGGCTTT	60.7	683	INTERGENIC	--	--	--
Ca3_P4_4073796	Ca3	4073796	CT/CTT	CTCAACATCCACCATGCAAT	TGCAAATTTCATCAAATGCATAA	59.4	746	INTERGENIC	--	--	--
Ca3_P4_4076583	Ca3	4076583	ATATTATTATTATTATTATTATTATTATTATTATTATTATTATTATTATT/ATATTATTATTATTATTATTATTATTATTATTATTATTATTATTATTATT	ATAGTCCTCACCCACGCATC	AAGCGTACCTGTGGGAATTG	60.0	607	INTERGENIC	--	--	--

INDEL marker IDs	Chromosomes/ unanchored scaffolds	Physical positions (bp)	InDels (*Kabuli* reference genome-CDC Frontier/PII)	Forward primers (5'-3')	Reverse primers (5'-3')	Anneling temperature (0C)	Expected amplified product size (bp)	Structural annotation		Functional annotation	
								Sequence components of *kabuli* genome	*Kabuli* gene accession IDs	NCBI-KOG	TFs
Ca3_P4_4132392	Ca3	4132392	TTATATATATATATATATAT/TTATATATATATATATATATATATATAT	CGTGTGAGAAAACCTCGACA	GAAGGCCAACTTCCAAATGA	59.9	738	INTERGENIC	--	--	--
Ca3_P4_4137886	Ca3	4137886	TTATATATATATATATATATATATATATATATATATATA/TATATATATATATATATATATATATATATATATATATATA	ATGTTCTCCTCCCCTTTTCA	GTTCCGACGAAAACGACATT	58.6	783	INTERGENIC	--	--	--
Ca3_P4_4152638	Ca3	4152638	CTTTTTTTTTT/CTTTTTTTTT	TGATGCAAAGTGTGCTGTGA	TTTTTGGTTGGGTGGGTAAA	60.0	675	INTERGENIC	--	--	--
Ca3_P4_4180993	Ca3	4180993	AATATATATATATATATAT/AATATATATATATATAT	TTCAATTTACTTGTTTTGATGTAGCA	AAATTTTGATGACCGCATCC	59.2	505	INTERGENIC	--	--	--
Ca3_P4_4194452	Ca3	4194452	GTTTTTTT/GTTTTTT	TGGCCTCTTTAGACAATGGC	TCGGTGGTTTGGAATCATTT	60.2	159	INTERGENIC	--	--	--
Ca3_P4_4280022	Ca3	4280022	CTTTTTTTT/CTTTTTTT	GCAAACCAACTCGAATGACA	ATCTTGGTGGCATTGGAAAG	59.7	145	INTERGENIC	--	--	--
Ca3_P4_4365472	Ca3	4365472	TC/TCC	ATCCAATGCGAGCAGTTCTT	TGAGGGACAAAACTATGGGAA	59.8	792	INTERGENIC	--	--	--
Ca3_P4_4491629	Ca3	4491629	TGAAAATGAGAAA/TGAAAATGAGAAAGTAGAAAATGAGAAA	ACAGGCACCGTTCAAATAGG	GATGTCGTGAGTGACATGGG	60.0	613	INTERGENIC	--	--	--
Ca3_P4_451560	Ca3	451560	ATAACGTGATCTTA/ATA	AGCAGAAAGGGAGAATGGGT	ACGGTGAAGCTCCGAAAATA	60.1	734	INTERGENIC	--	--	--
Ca3_P4_454494	Ca3	454494	AGGG/AGG	GGATTGGAGTGTTCAGCCAT	TTGTCATTAGTTTCAATCTCACCAA	59.9	460	INTERGENIC	--	--	--

INDEL marker IDs	Chromosomes/ unanchored scaffolds	Physical positions (bp)	InDels (*Kabuli* reference genome-CDC Frontier/PII)	Forward primers (5'-3')	Reverse primers (5'-3')	Anneling temperature (0C)	Expected amplified product size (bp)	Structural annotation		Functional annotation	
								Sequence components of *kabuli* genome	*Kabuli* gene accession IDs	NCBI-KOG	TFs
Ca3_P4_454633	Ca3	454633	GATATATATATATAT/GATATATATATAT	GGATTGGAGTGTTCAGCCAT	TTGTCATTAGTTTCAATCTCACCAA	59.9	460	INTERGENIC	--	--	--
Ca3_P4_458081	Ca3	458081	TTATATATATATATAT/TTATATATATATAT	GCGGTTTCCTTTAATTTTGGA	GAGGGAACGCAGAAAGTTCA	60.3	543	INTERGENIC	--	--	--
Ca3_P4_4689138	Ca3	4689138	TAAAAAA/TAAAAAAA	CAGCTTGCATTTGCTTTTGA	TTGCTCCTACGTATCTCCCG	60.1	888	INTERGENIC	--	--	--
Ca3_P4_47457	Ca3	47457	ATAGTGTAGTGTA/ATAGTGTA	GCGTACTTGGGCTGAGATGT	TTTTAGATGCATGTGTGCGG	60.3	385	INTERGENIC	--	--	--
Ca3_P4_488374	Ca3	488374	ATTTTTTTTTTT/ATTTTTTTTTTTT	TTTGCGTTACGAATTTGATGA	ACAAAATACCAAGTGCCCCA	59.2	361	INTERGENIC	--	--	--
Ca3_P4_4929398	Ca3	4929398	ATTTTTTTTT/ATTTTTTTTTT	GGCTTGTCACATCAGCTCAA	TCAGAACCATGTCAACGAGTTC	60.0	681	INTERGENIC	--	--	--
Ca3_P4_4965548	Ca3	4965548	ATTTT/ATTTTT	CGGGGAATGTGATTGATAGG	GTTGGAGAGATCGGTGGAAA	60.1	948	INTERGENIC	--	--	--
Ca3_P4_4965624	Ca3	4965624	AATT/AATTGAGGATT	TGCAAATCTGGAGCAGAGAA	GTTGGAGAGATCGGTGGAAA	59.7	510	INTERGENIC	--	--	--
Ca3_P4_4966098	Ca3	4966098	CTTTTTTTT/CTTTTTTT	TTTCCACCGATCTCTCCAAC	AAACCCGCTCCTTTCAAAAT	60.1	514	INTERGENIC	--	--	--
Ca3_P4_4975195	Ca3	4975195	AATATATATATATATA/AATATATATATATATATATATATA	CAAAGTTCAATACGCCAGCA	AGGTTTTGGCGTAACTCCCT	59.9	455	INTERGENIC	--	--	--

INDEL marker IDs	Chromosomes/ unanchored scaffolds	Physical positions (bp)	InDels (*Kabuli* reference genome-CDC Frontier/PII)	Forward primers (5'-3')	Reverse primers (5'-3')	Anneling temperature (0C)	Expected amplified product size (bp)	Structural annotation		Functional annotation	
								Sequence components of *kabuli* genome	*Kabuli* gene accession IDs	NCBI-KOG	TFs
Ca3_P4_5092682	Ca3	5092682	TTTT/TTTTGTTT	TCTGGATTCAAACCCCACTC	GTGGAAGGGTGCAAAGACAT	59.9	260	INTERGENIC	--	--	--
Ca3_P4_5269970	Ca3	5269970	CAAACAATAA/CAA	GATTGGCACGTCGATTAACA	TTCTCGCTTTCGTTTCGTTT	59.5	563	INTERGENIC	--	--	--
Ca3_P4_5270123	Ca3	5270123	GAA/GA	ACGAAACGAAAGCGAGAAAA	CGACCTCGAAATAATCGGAA	60.0	521	INTERGENIC	--	--	--
Ca3_P4_5270478	Ca3	5270478	TATCAATC/TATC	ACGAAACGAAAGCGAGAAAA	CGACCTCGAAATAATCGGAA	60.0	521	INTERGENIC	--	--	--
Ca3_P4_5286221	Ca3	5286221	ATTT/ATT	CACCCCTCCCAAAACATCT	TTGTGAGTGCATTTGATTTTGTT	59.8	876	INTERGENIC	--	--	--
Ca3_P4_5286324	Ca3	5286324	TAAAAAAAAAAAA/TAAAAAAAAAAA	CCTGAACCCAAAATCAAAACA	TTGACAAAGTTGAAATATGGGAAA	59.8	569	INTERGENIC	--	--	--
Ca3_P4_5288032	Ca3	5288032	AATATATATATATATATATATATAT/AATATATATATATATATATAT	AGAAGGTCGCATCTCCAAGA	TGGCACTTGTATTCACTCGC	60.0	456	INTERGENIC	--	--	--
Ca3_P4_5288343	Ca3	5288343	ATT/ATTGTTGTT	GCGAGTGAATACAAGTGCCA	TAGAAGATCGCATCCCCAAG	59.9	549	INTERGENIC	--	--	--
Ca3_P4_5347423	Ca3	5347423	CAATAATAATAATAATAATAATAATAATAATAATAA/CAATAATAATAATAATAATAATAATAATAATAA	TTGCTCCTACGTATCTCCCG	TTGTGACAATTTGATGGTTCTAAA	60.2	563	INTERGENIC	--	--	--
Ca3_P4_5389213	Ca3	5389213	AATTATTATTATTATTATT/AATTATTATTATTATTATTATT	AAGAGACTTCGCGTCCAAAA	CAAGTTTCAACAAATAAATGCACA	60.0	229	INTERGENIC	--	--	--

INDEL marker IDs	Chromosomes/ unanchored scaffolds	Physical positions (bp)	InDels (*Kabuli* reference genome-CDC Frontier/PII)	Forward primers (5'-3')	Reverse primers (5'-3')	Anneling temperature (0C)	Expected amplified product size (bp)	Structural annotation		Functional annotation	
								Sequence components of *kabuli* genome	*Kabuli* gene accession IDs	NCBI-KOG	TFs
Ca3_P4_540167	Ca3	540167	CTAATAATAATAATAATAATAATAATAATAATAATAATAATAATAATAATAATAATAATAATAATAATAATAATAA/CTAATAATAATAATAATAATAATAATAATAATAATAATAATAATAATA[illegible]	CAGAGGCAGCAAACAATGAA	GGAAAATCATCGAAAAGCAGA	60.0	511	INTERGENIC	--	--	--
Ca3_P4_5502243	Ca3	5502243	TG/T	TGCGTTTTTCTGCATTTCTG	ATTCTGCGCGACTATCAACC	60.0	661	INTERGENIC	--	--	--
Ca3_P4_5520403	Ca3	5520403	GTATATATATATATATATATATATA/GTATATATATATATATATATATA	CAAATGACGACGACGATGAC	GAATGATGACAATGCGGTTG	60.1	503	INTERGENIC	--	--	--
Ca3_P4_5520464	Ca3	5520464	TTATATATATATATAT/TTATATATATATATAT	CAAATGACGACGACGATGAC	GAATGATGACAATGCGGTTG	60.1	503	INTERGENIC	--	--	--
Ca3_P4_5643374	Ca3	5643374	TCCC/TCC	GAGAAATGGAGGCAATGGAA	GCAACACTCATTTGCCCTTT	60.0	420	INTERGENIC	--	--	--
Ca3_P4_5689033	Ca3	5689033	CT/CTT	TTGGTCAATGTGGCTTCTCA	TGAACTCTGAGCGTGTGACC	60.2	439	INTERGENIC	--	--	--
Ca3_P4_5689239	Ca3	5689239	GA/GATTATGTTA	TTGGTCAATGTGGCTTCTCA	TGAACTCTGAGCGTGTGACC	60.2	439	INTERGENIC	--	--	--
Ca3_P4_5689325	Ca3	5689325	ATTT/ATT	TTGGTCAATGTGGCTTCTCA	TGAACTCTGAGCGTGTGACC	60.2	439	INTERGENIC	--	--	--
Ca3_P4_5689863	Ca3	5689863	GTTT/GTTTT	GGTCACACGCTCAGAGTTCA	GAAGCAAATCGATTGGGAAA	60.0	543	INTERGENIC	--	--	--
Ca3_P4_5698499	Ca3	5698499	TTATAT/TTATATAT	GAGGATTTGGGGGAGAAGAG	AGAAGGGAGGAGATGGAAGC	60.0	397	INTERGENIC	--	--	--

INDEL marker IDs	Chromosomes/ unanchored scaffolds	Physical positions (bp)	InDels (*Kabuli* reference genome-CDC Frontier/PII)	Forward primers (5'-3')	Reverse primers (5'-3')	Anneling temperature (0C)	Expected amplified product size (bp)	Structural annotation		Functional annotation	
								Sequence components of *kabuli* genome	*Kabuli* gene accession IDs	NCBI-KOG	TFs
Ca3_P4_5729374	Ca3	5729374	TAAAAAAAAAAAAA/TAAAAAAAAAAAA	TCGTGTTTCACTATCCAACCC	TTCCAGCACATTGTCTTCCA	59.8	539	INTERGENIC	--	--	--
Ca3_P4_5769480	Ca3	5769480	ACC/AC	TAATGGTTTTCACGCCAACA	TACGGTGCTAGGGAACCAAC	60.0	512	INTERGENIC	--	--	--
Ca3_P4_5805263	Ca3	5805263	GT/GTT	CAATTGGTCGAAAGACGGAT	TTCGAATGAACATAATTGAACTTT	59.9	164	INTERGENIC	--	--	--
Ca3_P4_5805325	Ca3	5805325	TAAAAAA/TAAAAAAA	CAATTGGTCGAAAGACGGAT	TTCATCTTCGTCAACAATGAAAA	59.9	554	INTERGENIC	--	--	--
Ca3_P4_5921705	Ca3	5921705	GT/GAGAT	GGCGTTGGTTTCACTCAAAT	TCAGTCAAACGCACACAAAA	60.0	407	INTERGENIC	--	--	--
Ca3_P4_6057834	Ca3	6057834	TTATATATATATATAT/TTATATATATATATATAT	AATTCAATAGAAAGCAGGGGG	TCTTTGCCTGGTCTGACCTT	59.5	734	INTERGENIC	--	--	--
Ca3_P4_6352791	Ca3	6352791	AATATATATATATATATATAT/AATATATATATATATAT	TTTTTATGTTGGGCCCATGT	ATGCGCTTGACAAGAATGTG	60.1	655	INTERGENIC	--	--	--
Ca3_P4_6432046	Ca3	6432046	TAAAAAAAAAAA/TAAAAAAAAAA	TCCTGATGGCAATGCAAATA	AATAAGGTTGTCGGTTTGCG	60.0	172	INTERGENIC	--	--	--
Ca3_P4_6466373	Ca3	6466373	TTAATAATAATAATAATAATAA/TTAATAATAATAATAATAATAATAA	GAACCCCTGTAACACCCAGA	GGAGAACGTTAAGGCAGCAG	59.8	443	INTERGENIC	--	--	--
Ca3_P4_6548012	Ca3	6548012	CT/CTT	TTATGCCGTTTGGAGTTTCC	TTCAATTGCCTTGTGGCATA	59.9	615	INTERGENIC	--	--	--

INDEL marker IDs	Chromosomes/ unanchored scaffolds	Physical positions (bp)	InDels (*Kabuli* reference genome-CDC Frontier/PII)	Forward primers (5'-3')	Reverse primers (5'-3')	Anneling temperature (0C)	Expected amplified product size (bp)	Structural annotation		Functional annotation	
								Sequence components of *kabuli* genome	*Kabuli* gene accession IDs	NCBI-KOG	TFs
Ca3_P4_6664320	Ca3	6664320	TAAAAAA/TAAAAAAA	TGAAATTATGGGTGGGCATT	GCTGAAAAAGTGCCGAAGAA	60.0	569	INTERGENIC	--	--	--
Ca3_P4_6723619	Ca3	6723619	T/TAATAATAATA	TGGAAAGCTTGTTAGCAAATG	AACGCACGAGCAAATTCTTT	58.1	670	INTERGENIC	--	--	--
Ca3_P4_6730727	Ca3	6730727	CTTTTTTTTTTTT/CTTTTTTTTTTTTTTT	TTTAGGATTTTCCAACCACCA	TGGGGAGATAGGGTTGATTTT	59.3	655	INTERGENIC	--	--	--
Ca3_P4_6790470	Ca3	6790470	T/TA	GGCTGGCTACTTAGGGATCA	GACCTCGTCCAGCTTTTCAG	59.3	582	INTERGENIC	--	--	--
Ca3_P4_6814196	Ca3	6814196	AATATATATATATATATATATATAT/AATATATATATATATATATATATATAT	CATGAACCTCATCACATCCG	AACCATCCTAATTTGACACCAA	59.9	841	INTERGENIC	--	--	--
Ca3_P4_6821813	Ca3	6821813	TCCC/TCC	GAAACTTGTAGGGGAGGGGA	ATTGTAGCTTGGGATCACGC	60.3	304	INTERGENIC	--	--	--
Ca3_P4_6921816	Ca3	6921816	AATATATATATATATATATATATATATATAT/AATATATATATATATATATATATATATATATATATATATAT	TGTTTATGTTTGTTACGCCTGC	GGAATTGGGAAAAATGGGAT	60.1	168	INTERGENIC	--	--	--
Ca3_P4_6961692	Ca3	6961692	TTAATAATAATAATAATAATAATAA/TTAATAATAATAATAATAATAATAATAA	GCCAAAACAAATTGGAGAGG	TCCTAGATCTGGAACTAGAGGTCA	59.5	645	INTERGENIC	--	--	--
Ca3_P4_7118164	Ca3	7118164	AATATATATATATATATATATATATATATAT/AATATATATATATATATATATATATATATATAT	AAAGCATGTCTTCGGTTTTCA	TGAATATCAAACAATCAACTATCAACA	59.7	849	INTERGENIC	--	--	--
Ca3_P4_7330455	Ca3	7330455	GTTTTTTTTTTTTT/GTTTTTTTTTTTT	CCAACAAAAGGACGGAAGAA	TTTTGTCCCAACAATCTCCC	60.1	605	INTERGENIC	--	--	--

INDEL marker IDs	Chromosomes/ unanchored scaffolds	Physical positions (bp)	InDels (*Kabuli* reference genome-CDC Frontier/PII)	Forward primers (5'-3')	Reverse primers (5'-3')	Anneling temperature (0C)	Expected amplified product size (bp)	Structural annotation		Functional annotation	
								Sequence components of *kabuli* genome	*Kabuli* gene accession IDs	NCBI-KOG	TFs
Ca3_P4_7377309	Ca3	7377309	TAAAAAA/TAAAAAAA	CATATTCGCATTCCCAAACC	ACCTGCAAACCCAACTCAAC	60.2	306	INTERGENIC	--	--	--
Ca3_P4_7456958	Ca3	7456958	TTATATATATATATATATA/TTATATATATATATATATATA	CACATACCGCGGTTAGAACA	ACCATTTCCACGTGATTGCT	59.6	389	INTERGENIC	--	--	--
Ca3_P4_7494196	Ca3	7494196	T/TC	TTGCTTTGGTTTGATGTGGA	GAAGTTGCCTTAGTGGCAGG	60.1	694	INTERGENIC	--	--	--
Ca3_P4_7636434	Ca3	7636434	TTATTATTA/TTATTATTACTATTATTA	TGGCCTTGTGTGTATTGCAT	CTGAATTGCGTGTGTCTGGT	60.0	566	INTERGENIC	--	--	--
Ca3_P4_7749695	Ca3	7749695	G/GACT	GCTTGGTTTTCTGGGTTTGA	ACCTTGAATCGCTGCCTCTA	60.1	870	INTERGENIC	--	--	--
Ca3_P4_7773838	Ca3	7773838	AATATATATATATATAT/AATATATATATATAT	CATGCGAGTTGTAACACCCT	CGTGCATACTTAATATCAGTCAAGTG	58.7	365	INTERGENIC	--	--	--
Ca3_P4_7792015	Ca3	7792015	TAT/TATTATTCTTTCCCTAAT	TAGGTAAAGGTGGTTTGCCG	TGAGGGATAGATGCAGGTCA	60.0	611	INTERGENIC	--	--	--
Ca3_P4_7959750	Ca3	7959750	TTAATTTGATTATGTTAAGTAATTTGATTATGTTAA/TTAATTTGATTATGTTAAGTAATTTGATTATGTTAAGTAATTTGATTATGTTAA	TTGGCATAGGTCATGGAAGTT	TTTGCCAATGTTGCTAGCTG	59.4	848	INTERGENIC	--	--	--
Ca3_P4_805419	Ca3	805419	CTTTATTTATTTATTTATTTATTTATTTATTTATTTATTTATTTATTTATTT/CTTTATTTATTTATTTATTTATTTATTTATTTATTTATTTATTTATTTATTTATTTATTTATTT	GAACGTATGAGTTGTGCGGA	GGAGGTGATTTCAAAGGCAA	59.7	260	INTERGENIC	--	--	--
Ca3_P4_8061977	Ca3	8061977	T/TC	CTGAAAAAGTTCGACGAGCC	AATTGTGCTTGCTCCCATTC	60.0	468	INTERGENIC	--	--	--

INDEL marker IDs	Chromosomes/ unanchored scaffolds	Physical positions (bp)	InDels (*Kabuli* reference genome-CDC Frontier/PII)	Forward primers (5'-3')	Reverse primers (5'-3')	Anneling temperature (0C)	Expected amplified product size (bp)	Structural annotation		Functional annotation	
								Sequence components of *kabuli* genome	*Kabuli* gene accession IDs	NCBI-KOG	TFs
Ca3_P4_8088284	Ca3	8088284	CT/CTCTCTTCACAT	TCTTCCACCCAGTTTTGACC	ACATAACACGTGGCCTGTGA	59.9	477	INTERGENIC	--	--	--
Ca3_P4_8146397	Ca3	8146397	AT/A	TCAAGAAGGATGGAGGATCG	TCCTTTTGCACATGCTTCAC	60.2	448	INTERGENIC	--	--	--
Ca3_P4_8245494	Ca3	8245494	TCT/TCTTCTGCT	TGCGCCTCTTCTAAAATCGT	TATCGCAATCAGAGGAACCC	60.0	670	INTERGENIC	--	--	--
Ca3_P4_8393314	Ca3	8393314	GTATATATATATATATATAT/GTATATATATATATATATATATAT	GTTGGTTCCCTAGCACCGTA	TATTTTGGATTTTGGCTCGG	60.0	602	INTERGENIC	--	--	--
Ca3_P4_8523296	Ca3	8523296	ATTTTTTTTTT/ATTTTTTTT	CACATGACAAAATTGGTGGAA	CGTTAAGGCACATCTCACTCC	59.3	508	INTERGENIC	--	--	--
Ca3_P4_8544653	Ca3	8544653	CG/C	ACCTGCGGTTACTTCCACAG	TTTCTGCACTCTGCCACATC	60.2	600	INTERGENIC	--	--	--
Ca3_P4_8602266	Ca3	8602266	AATATATATATATA/AATATATATATATATA	GCTCAACAGAGACCACCACA	CGTATTCGTGTGTGAAAGCG	59.9	835	INTERGENIC	--	--	--
Ca3_P4_8611127	Ca3	8611127	ATATTATTATTATTATTATTATTATTATTATTATTATTAT/ATATTATTATTATTATTATTATTATTATTATTATTAT	AGAATTAATGGTTGCGGTCG	CTCCAGGTGCAATAGGGAAA	60.0	434	INTERGENIC	--	--	--
Ca3_P4_8775439	Ca3	8775439	GTATATATATATATATATATATATATATATATAT/GTATATATATATATATATATATATATATATATATATAT	AGTCGCATCTTCGTCGTTTC	GGCATCGGGAGTCTGTTTTA	60.4	537	INTERGENIC	--	--	--
Ca3_P4_8942193	Ca3	8942193	AT/ATT	TGGGGAAAAACCCTTACCTT	CACATGATTTCAATTGGCTTTG	59.7	326	INTERGENIC	--	--	--

INDEL marker IDs	Chromosomes/ unanchored scaffolds	Physical positions (bp)	InDels (*Kabuli* reference genome-CDC Frontier/PII)	Forward primers (5'-3')	Reverse primers (5'-3')	Anneling temperature (0C)	Expected amplified product size (bp)	Structural annotation		Functional annotation	
								Sequence components of *kabuli* genome	*Kabuli* gene accession IDs	NCBI-KOG	TFs
Ca3_P4_9186815	Ca3	9186815	CTATATATATATATATATATATATA/CTATATATATATATATATATATATA	TCCACAAACACGATAACATTGA	TGTAACAAGCATTTGTCGGG	58.9	693	INTERGENIC	--	--	--
Ca3_P4_9215609	Ca3	9215609	TAAAAAAAA/TAAAAAAAAA	AAAATCCCAAGTTGTACGGC	TTGGATGTGTTGGCTTGTGT	59.0	675	INTERGENIC	--	--	--
Ca3_P4_9289555	Ca3	9289555	TTATATATATATATATATATATAT/TTATATATATATATATATATATATAT	GAACCAAGTTGCATGGGAAT	CCTAGGGACTTCGTGGTTGA	59.8	651	INTERGENIC	--	--	--
Ca3_P4_939073	Ca3	939073	GTT/GTTTT	ATTGGTTCTGCTCTGGGTTG	GGGACCACAGCACCTATGTT	60.1	296	INTERGENIC	--	--	--
Ca3_P4_9416399	Ca3	9416399	AG/AGG	CCCAAGAGAACGCAATTCAT	TCTCACGGTTGTGAGGACTG	60.1	811	INTERGENIC	--	--	--
Ca3_P4_9426071	Ca3	9426071	CAAAAAAAAA/CAAAAAAAAAA	CCATAAAACCATTTCCATTGC	TACTCTAGCAACCACCGCCT	59.2	262	INTERGENIC	--	--	--
Ca3_P4_9427257	Ca3	9427257	TTTA/T	CATGCGTTCCTTGTGGTAGA	GGCCTAAGATCATAGCAATTCAA	59.7	165	INTERGENIC	--	--	--
Ca3_P4_9500843	Ca3	9500843	CAATCGATTTCC/CAATCGATTTCCAAATCGATTTCC	CCCGTGAGATCCTCCTAACA	CATGCAGGAATTCAGCAAAA	60.1	498	INTERGENIC	--	--	--
Ca3_P4_9735715	Ca3	9735715	TC/T	GGAATATTGATCCACTCGCC	GAAAAAGCGCTGTAAATGGC	59.3	410	INTERGENIC	--	--	--
Ca3_P4_9827185	Ca3	9827185	TAAAAAAAAAAAA/TAAAAAAAAAAA	GAAGGCTACCGTTACGCAAT	CGGGAGATACGTAGGAGCAA	59.3	756	INTERGENIC	--	--	--

INDEL marker IDs	Chromosomes/ unanchored scaffolds	Physical positions (bp)	InDels (*Kabuli* reference genome-CDC Frontier/PII)	Forward primers (5'-3')	Reverse primers (5'-3')	Anneling temperature (0C)	Expected amplified product size (bp)	Structural annotation		Functional annotation	
								Sequence components of *kabuli* genome	*Kabuli* gene accession IDs	NCBI-KOG	TFs
Ca3_P4_9846827	Ca3	9846827	AATATATATATATATATATATATATATATATATA/AATATATATATATATATATATATATATATATATATATATA	GTGGCTTTCGTTGAATGGTT	CAAATGTCCACAAACTCTGATG	60.0	280	INTERGENIC	--	--	--
Ca3_P4_9940560	Ca3	9940560	A/AT	ACCGGTATTGTAACACCCCA	CCTTATGCGGTAATCCCTGA	60.0	475	INTERGENIC	--	--	--
Ca3_P4_9953160	Ca3	9953160	GAAAAAA/GAAAAAAA	TGCCACGTTTCTATTTCCCT	ATCACGCCATAAGCACCTTC	59.6	511	INTERGENIC	--	--	--
Ca4_P4_10033685	Ca4	10033685	TTATATATATATATATATAT/TTATATATATATATATATATATAT	CTAGTTCGTAACCCGTGCGT	AGGCATGGTGTTGGAGTCAT	60.2	248	INTERGENIC	--	--	--
Ca4_P4_10035522	Ca4	10035522	GT/GTT	AAGGGAGACGCAAGAAATGA	CATCACGATCTTTGTCCACG	59.8	666	INTERGENIC	--	--	--
Ca4_P4_10092984	Ca4	10092984	GTTTTTTTT/GTTTTTTTTT	CGCACGAGTAACACGTCATT	ACGCGGAACAAATATTGTCA	59.8	432	INTERGENIC	--	--	--
Ca4_P4_10147217	Ca4	10147217	GTTTTTTTTT/GTTTTTTTT	TGAATGCGCGTATTGTGAAT	AAAAATGACAAACGAAACATAAAA	60.1	728	INTERGENIC	--	--	--
Ca4_P4_10173137	Ca4	10173137	ATTTTTTTT/ATTTTTTTTT	CCCGTTATTTTTGACGAAACA	TGTGTTGAGTGGTGGCATTT	59.9	791	INTERGENIC	--	--	--
Ca4_P4_10208528	Ca4	10208528	TAAAAAAAAAAAA/TAAAAAAAAAAA	CCACCAAATCCCAAAAATTG	CCCAACACCTTTGGCTTTTA	60.0	527	INTERGENIC	--	--	--
Ca4_P4_10223331	Ca4	10223331	AATATATATATATATATATATATATATATAT/AATATATATATATATATATAT	ACGTGAAGTCCAAATCTGCC	TTCGACTGTCCACGTATATCTCA	60.1	586	INTERGENIC	--	--	--

INDEL marker IDs	Chromosomes/ unanchored scaffolds	Physical positions (bp)	InDels (*Kabuli* reference genome-CDC Frontier/PII)	Forward primers (5'-3')	Reverse primers (5'-3')	Anneling temperature (0C)	Expected amplified product size (bp)	Structural annotation		Functional annotation	
								Sequence components of *kabuli* genome	*Kabuli* gene accession IDs	NCBI-KOG	TFs
Ca4_P4_10233239	Ca4	10233239	GACAATTATTTGGTACAATTAT/GACAATTATTTGGTACAATTATTTGGTACAATTAT	TGTCGGTTGTTGCTTGAAGA	ATACAATCATTTTCCCGCCA	60.4	403	INTERGENIC	--	--	--
Ca4_P4_10241994	Ca4	10241994	TAAAAAAAAAAA/TAAAAAAAAAAAA	CCTGCACCATTTCTCTTGGT	TTTTCTGGTAACTCGGACCC	60.1	649	INTERGENIC	--	--	--
Ca4_P4_10247608	Ca4	10247608	CAATATTGTAAAATA/CAATATTGTAAAATAATATTGTAAAATA	GAGTTAGGGTTGGTGAGGCA	TTTTTCACGTCGCATAAATCT	60.1	236	INTERGENIC	--	--	--
Ca4_P4_10253630	Ca4	10253630	GAAAAAAAA/GAAAAAAA	TGTGTTTGAATGGGCAAAAA	CTTCATTTAAACGCCCCAAA	59.9	312	INTERGENIC	--	--	--
Ca4_P4_10261671	Ca4	10261671	ATTTTTT/ATTTTT	TGGATTTTGGACTTGGTCTTG	AACAACACGTGATTTGAATTGC	60.0	654	INTERGENIC	--	--	--
Ca4_P4_10329029	Ca4	10329029	AC/A	GAGTTACGGATCCACAACCAA	TTTTTAACTTTCGGGGTGGT	59.8	414	INTERGENIC	--	--	--
Ca4_P4_10456572	Ca4	10456572	ATTTTTTTT/ATTTTTTT	ATCGATCAAAACCCGTGAAG	CGTTGTTGGTAAGGGAATCG	59.9	454	INTERGENIC	--	--	--
Ca4_P4_10460005	Ca4	10460005	ATTTTTTTTTTTT/ATTTTTTTTTTT	TCAAATCAAATCCAAAGGGC	TCGTCTCTTTGTGGTAATCGTG	59.9	687	INTERGENIC	--	--	--
Ca4_P4_10555606	Ca4	10555606	TTA/TTAATA	TGTTGAAATGTGGTGTGGCT	CAGCTTTAGCTCGGGTTTTG	60.0	764	INTERGENIC	--	--	--
Ca4_P4_10586036	Ca4	10586036	CATATATATATATATATAT/CATATATATATATATATATAT	GTAGCCGAGCAGGAAATGAG	CATGCGAGTTAATGACGGAG	60.0	860	INTERGENIC	--	--	--

INDEL marker IDs	Chromosomes/ unanchored scaffolds	Physical positions (bp)	InDels (*Kabuli* reference genome-CDC Frontier/PII)	Forward primers (5'-3')	Reverse primers (5'-3')	Anneling temperature (0C)	Expected amplified product size (bp)	Structural annotation		Functional annotation	
								Sequence components of *kabuli* genome	*Kabuli* gene accession IDs	NCBI-KOG	TFs
Ca4_P4_10612304	Ca4	10612304	TTTATTATTATTATTATTATTATTATTATTATTATTATTATTATTATT/TTTATTATTATTATTATTATTATTATTATTATTATTATT	AATCACGCAAACAAGTGTCG	AGCGACATAAATGGCTGCTA	59.8	408	INTERGENIC	--	--	--
Ca4_P4_10648356	Ca4	10648356	CTTTTTTTTTT/CTTTTTTTTT	GGCCATCTATGGGTCCTACA	GGTTGATCCAACAAGTTCAAAA	59.8	614	INTERGENIC	--	--	--
Ca4_P4_10649251	Ca4	10649251	TTATATATATATATATATATATAT/TTATATATATATATATATATATATATATATTAT	TGAGTCACTTAGAGGGAGGGA	CCCAACAAATAAACCCCCTT	58.9	295	INTERGENIC	--	--	--
Ca4_P4_10793822	Ca4	10793822	AT/ATGGT	TGGTGAAAATAGATTAGGACGAGA	CTGAACATTTGCATGCTCTCA	59.2	943	INTERGENIC	--	--	--
Ca4_P4_10879888	Ca4	10879888	ATATTATTATTATTATTATTATTATTATTATTATTATTATTATTATTA/ATATTATTATTATTATTATTATTATTATTATTATTATTATTATTATTA	TTGAGAAAGAAGGGGAAGACA	AATCCTGCCCAGCTAGAGTT	58.9	390	INTERGENIC	--	--	--
Ca4_P4_10954430	Ca4	10954430	AATATATATATATATATATATATA/AATATATATATATATATATATATA	TTCGTGAATTAGTTTTCCGTGA	TAACGTAACATGCCATTGCC	59.6	554	INTERGENIC	--	--	--
Ca4_P4_10993124	Ca4	10993124	CAAAAAAA/CAAAAAAAA	CCTAAAACCCTAACCCCCAA	AGCACCCCCAAAAAGAAAGT	60.0	613	INTERGENIC	--	--	--
Ca4_P4_11083084	Ca4	11083084	ATTTTTTT/ATTTTTT	GTTTTGGTGTTGAAGCGGAT	TGCAATTTTGACCGATACCA	60.0	905	INTERGENIC	--	--	--
Ca4_P4_11216489	Ca4	11216489	TAAAAAA/TAAAAAAA	CTTCGCGCCAGTGTCTTAAT	TCAAACCAAGGATGATGCAA	60.4	350	INTERGENIC	--	--	--
Ca4_P4_11216964	Ca4	11216964	TAAAAAA/TAAAAA	TTGCATCATCCTTGGTTTGA	TCATTCCCTACGTACTCGGC	60.0	603	INTERGENIC	--	--	--

INDEL marker IDs	Chromosomes/ unanchored scaffolds	Physical positions (bp)	InDels (*Kabuli* reference genome-CDC Frontier/PII)	Forward primers (5'-3')	Reverse primers (5'-3')	Anneling temperature (0C)	Expected amplified product size (bp)	Structural annotation		Functional annotation	
								Sequence components of *kabuli* genome	*Kabuli* gene accession IDs	NCBI-KOG	TFs
Ca4_P4_11225146	Ca4	11225146	GTG/GTGGCTTTG	TGGGGAGAAACAAAATGGAG	AGTGTGCTTTCGGCTAAGGA	59.9	607	INTERGENIC	--	--	--
Ca4_P4_11263983	Ca4	11263983	CAAAAAAAAA/CAAAAAAAAAAAA	CGCATGTGTGCTTGAATCTC	TCCACCAATTTTGCATTGAA	60.4	786	INTERGENIC	--	--	--
Ca4_P4_11264215	Ca4	11264215	TA/TAA	TGTGGGATCAATTGGGAAGT	TCCACCAATTTTGCATTGAA	60.2	171	INTERGENIC	--	--	--
Ca4_P4_11268024	Ca4	11268024	AAAGAA/AAA	TAGTGCCCTCCAAAAGGATG	GCGTTGAGGTGTCAACTGTG	60.1	733	INTERGENIC	--	--	--
Ca4_P4_11270494	Ca4	11270494	CATATAT/CATAT	TGTTGACAAACACAAAGACCG	ATCCACCATCAATGCCATCT	59.6	645	INTERGENIC	--	--	--
Ca4_P4_11282146	Ca4	11282146	T/TA	TCGAACCATGTATTGATTTTGC	ACGCCACTTTGTCAAACACA	59.8	695	INTERGENIC	--	--	--
Ca4_P4_11291506	Ca4	11291506	GAAACAAACTT/G	GATGAAAGTTTTTGGTGTGGC	CATTCGGATGGACACTCTCA	59.5	599	INTERGENIC	--	--	--
Ca4_P4_11298187	Ca4	11298187	CTTTTTTTTTTTT/CTTTTTTTTT	AAATGCTCCAAAAACCAACG	ATTAAAATGCCAGCAGTGGG	60.0	471	INTERGENIC	--	--	--
Ca4_P4_11300403	Ca4	11300403	ATATTATT/ATATT	GTGTTACTTCCGCCGTTGAT	TGTCAAGTTGTTTCATGCTGC	60.0	185	INTERGENIC	--	--	--
Ca4_P4_11300962	Ca4	11300962	C/CG	GCAGCATGAAACAACTTGACA	TTTCTTAATGTGCGCAATGG	59.9	553	INTERGENIC	--	--	--

INDEL marker IDs	Chromosomes/ unanchored scaffolds	Physical positions (bp)	InDels (*Kabuli* reference genome-CDC Frontier/PII)	Forward primers (5'-3')	Reverse primers (5'-3')	Anneling temperature (0C)	Expected amplified product size (bp)	Structural annotation		Functional annotation	
								Sequence components of *kabuli* genome	*Kabuli* gene accession IDs	NCBI-KOG	TFs
Ca4_P4_11318155	Ca4	11318155	ATTTTTTTTT/ATTTTTTT	ATTCTCATGGCAAGGGATTG	TGCGTAATGGACCAATTCAA	59.9	909	INTERGENIC	--	--	--
Ca4_P4_11319252	Ca4	11319252	GGGAG/GG	ATGACCTTTTTCCTGCCCTT	CGCACACAGACGCAATACTT	59.9	313	INTERGENIC	--	--	--
Ca4_P4_11319307	Ca4	11319307	TGCG/TG	ATGACCTTTTTCCTGCCCTT	CCACCACCAAAGTCCAAAGT	59.9	733	INTERGENIC	--	--	--
Ca4_P4_11321165	Ca4	11321165	AT/A	ATGATGATGATGCTACCGCA	TGGTTAATTGAAACGGTGAAGTT	60.1	522	INTERGENIC	--	--	--
Ca4_P4_11321437	Ca4	11321437	ATTTT/ATTTTT	AACTTCACCGTTTCAATTAACCA	CAGAGAATCCGACTCCGAAA	59.8	494	INTERGENIC	--	--	--
Ca4_P4_11321691	Ca4	11321691	ATT/ATTT	AACATGTGGCTAACAACCCTT	CAGAGAATCCGACTCCGAAA	58.5	316	INTERGENIC	--	--	--
Ca4_P4_11322366	Ca4	11322366	ATTT/ATTTTT	TTGTGAGGAATGCAAACTGC	ACAATCATCATGCCATCGAA	59.8	501	INTERGENIC	--	--	--
Ca4_P4_11322920	Ca4	11322920	AAGCTCCTCTTC/A	TCGATGGCATGATGATTGTT	AAGAGGATTGGAGATAGGAGAGC	59.9	371	INTERGENIC	--	--	--
Ca4_P4_11338339	Ca4	11338339	AAGA/AAGAGA	ACGGATAAAATGCCCATTGA	ATTGGTGTGAGTGCTGTGGA	60.2	821	INTERGENIC	--	--	--
Ca4_P4_11341314	Ca4	11341314	TAAA/TAA	TGCATTGCCTTCTCAAACTG	TCAGCTTATCACTGGGGTCC	60.0	784	INTERGENIC	--	--	--

INDEL marker IDs	Chromosomes/ unanchored scaffolds	Physical positions (bp)	InDels (*Kabuli* reference genome-CDC Frontier/PII)	Forward primers (5'-3')	Reverse primers (5'-3')	Anneling temperature (0C)	Expected amplified product size (bp)	Structural annotation		Functional annotation	
								Sequence components of *kabuli* genome	*Kabuli* gene accession IDs	NCBI-KOG	TFs
Ca4_P4_11341357	Ca4	11341357	AATATATATATATATATATATAT/AATATATATATAT	TGCATTGCCTTCTCAAACTG	TCAGCTTATCACTGGGGTCC	60.0	784	INTERGENIC	--	--	--
Ca4_P4_11348371	Ca4	11348371	ATG/A	CGAACTTTGTCCACCCCTAA	TTTGTTGGCATCGCTTATTG	60.0	849	INTERGENIC	--	--	--
Ca4_P4_11352038	Ca4	11352038	GAGAGAGAGA/GAGAGAGAGAAAGAGAGAGA	TGCCCATGTTTACCACAAAA	CCCTCTCTCTCTCTCTCTCGG	59.8	898	INTERGENIC	--	--	--
Ca4_P4_11354394	Ca4	11354394	CAACAATAACAATAACAATAACAATAACA/CAACAATAACAATAACAATAACA	GATGTTCCTTTGCCCCATAA	AGAGACAAATTCAGGGTGGG	59.8	720	INTERGENIC	--	--	--
Ca4_P4_11354532	Ca4	11354532	ATGTTGTTGTTG/ATGTTGTTGTTGTTGTTG	GATGTTCCTTTGCCCCATAA	AGAGACAAATTCAGGGTGGG	59.8	720	INTERGENIC	--	--	--
Ca4_P4_11357023	Ca4	11357023	C/CA	GCCTCGTTGATTACCCAAAA	GGCATGGACGTAGGCTAGAG	59.9	809	INTERGENIC	--	--	--
Ca4_P4_11359837	Ca4	11359837	ACCC/ACC	TGTTGCGGTTGTATGGTGTC	TGACATTGGGGGATTCATTT	60.4	455	INTERGENIC	--	--	--
Ca4_P4_11368276	Ca4	11368276	CG/C	TGATGAATTTTCATTCACCAATC	TCCCAAGGTCAATTAGGCAG	58.8	116	INTERGENIC	--	--	--
Ca4_P4_11370496	Ca4	11370496	TAAAAAAAAAA/TAAAAAAA	GAGATGGTGAAATCCAACGC	TTTGGAAGGGTTTAGAGGGG	60.5	538	INTERGENIC	--	--	--
Ca4_P4_11370815	Ca4	11370815	TAAA/TAAAAA	GAGATGGTGAAATCCAACGC	AGGGAGGGGATGAGAGAAAA	60.5	641	INTERGENIC	--	--	--

INDEL marker IDs	Chromosomes/ unanchored scaffolds	Physical positions (bp)	InDels (*Kabuli* reference genome-CDC Frontier/PII)	Forward primers (5'-3')	Reverse primers (5'-3')	Anneling temperature (0C)	Expected amplified product size (bp)	Structural annotation		Functional annotation	
								Sequence components of *kabuli* genome	*Kabuli* gene accession IDs	NCBI-KOG	TFs
Ca4_P4_11376655	Ca4	11376655	TGGGG/TGGGGG	ACTGTGTTGAAGTGAGGGGC	CGTTTGATAAGATTTCCGCA	60.2	753	INTERGENIC	--	--	--
Ca4_P4_11377196	Ca4	11377196	TAAAAAAA/TAAAAAAAA	TTGCGGAAATCTTATCAAACG	GATGAAGTTCACCCACCACA	60.1	331	INTERGENIC	--	--	--
Ca4_P4_11377416	Ca4	11377416	CAAAAAA/CAAAA	TTGCGGAAATCTTATCAAACG	TGCTGGACCATAAACCCATT	60.1	783	INTERGENIC	--	--	--
Ca4_P4_11383319	Ca4	11383319	GTTTTTT/GTTTTTTT	CAAGATTAACGGTGACTGCAT	GATCCACCTTTGGACAGAGC	57.8	514	INTERGENIC	--	--	--
Ca4_P4_11393203	Ca4	11393203	GCAT/GCATCAT	ACAAACATGCCACCAACAAA	TCCATTGCTCACATTTGGAA	59.9	585	INTERGENIC	--	--	--
Ca4_P4_11443015	Ca4	11443015	AATT/A	GGCTTGGTGTGGGTTTACAT	AGGCCCCTTAGTTGTCTCGT	59.7	614	INTERGENIC	--	--	--
Ca4_P4_11444419	Ca4	11444419	GAAAAAAA/GAAAAAAAA	AAACGGGCGATTTGTTTTTA	GGAAGGAACCATCAAGCAAA	59.5	553	INTERGENIC	--	--	--
Ca4_P4_11446405	Ca4	11446405	A/ACAT	TTAATGCGGTTGAAATGGTG	AATATGGCATTCCCATCCAA	59.4	438	INTERGENIC	--	--	--
Ca4_P4_11447422	Ca4	11447422	TTAAT/TT	GCCACAGCTACATCCAAAAA	GCATTGCTATCATTACTGTGCG	58.8	671	INTERGENIC	--	--	--
Ca4_P4_11447651	Ca4	11447651	AAAAAAGTAAAAAGTAAA/AAAAAAGTAAA	TCGCACAGTAATGATAGCAATG	TGGATAATATGGGCGGTGAT	58.9	461	INTERGENIC	--	--	--

INDEL marker IDs	Chromosomes/ unanchored scaffolds	Physical positions (bp)	InDels (*Kabuli* reference genome-CDC Frontier/PII)	Forward primers (5'-3')	Reverse primers (5'-3')	Anneling temperature (0C)	Expected amplified product size (bp)	Structural annotation		Functional annotation	
								Sequence components of *kabuli* genome	*Kabuli* gene accession IDs	NCBI-KOG	TFs
Ca4_P4_11449130	Ca4	11449130	C/CT	TCCTTGTGTCAATCACCTTCA	CACAATCAAACATGCACTCG	59.1	542	INTERGENIC	--	--	--
Ca4_P4_11449776	Ca4	11449776	ATTTTTTT/ATTTTTT	CTTGATCGAGTGCATGTTTGA	AGCTCGATGGTAAAGGCGTA	59.9	524	INTERGENIC	--	--	--
Ca4_P4_11459960	Ca4	11459960	CTAAGT/CTAAGTAAGT	GAGATCCAAAATCAAGGGCA	TTCATGCATCTGCAGGGTAG	60.0	698	INTERGENIC	--	--	--
Ca4_P4_11468609	Ca4	11468609	AAATTGAAAATAATT/AAATT	TTTGAATGGACGGATGTGTAA	TTGACGAGGAACTTTGAATGAA	58.9	264	INTERGENIC	--	--	--
Ca4_P4_11472250	Ca4	11472250	ATTTT/ATTTTT	TGTCATCACTCGTGTGGTATCT	CCAACCGAGGAGACAAGAAA	58.1	499	INTERGENIC	--	--	--
Ca4_P4_11473446	Ca4	11473446	GTTTTTTT/GTTTTTTTT	ACATTTGGAAGGCTTGAACG	CCCATCAACGGAAATTTGAC	60.1	572	INTERGENIC	--	--	--
Ca4_P4_11473509	Ca4	11473509	CA/CAAA	ACATTTGGAAGGCTTGAACG	CCCATCAACGGAAATTTGAC	60.1	572	INTERGENIC	--	--	--
Ca4_P4_11478077	Ca4	11478077	CTT/CT	CCGCTTCTCTTCAATGCTCT	AACACACCCCAATTTGCATT	59.7	463	INTERGENIC	--	--	--
Ca4_P4_11478441	Ca4	11478441	TTATTAATTGTATATTAATTGTATATTAATT/TTATTAATTGTATATTAATT	AATGCAAATTGGGGTGTGTT	TCACATATTTATAATTTCTGCACCG	60.1	359	INTERGENIC	--	--	--
Ca4_P4_11479120	Ca4	11479120	AT/ATTTT	AATTGAAGGTTATTTTTGGAATCA	AGTGTCATGCGCACACAAAT	58.1	372	INTERGENIC	--	--	--

INDEL marker IDs	Chromosomes/ unanchored scaffolds	Physical positions (bp)	InDels (*Kabuli* reference genome-CDC Frontier/PII)	Forward primers (5'-3')	Reverse primers (5'-3')	Anneling temperature (0C)	Expected amplified product size (bp)	Structural annotation		Functional annotation	
								Sequence components of *kabuli* genome	*Kabuli* gene accession IDs	NCBI-KOG	TFs
Ca4_P4_11482459	Ca4	11482459	TA/TAA	TGGGGAGACAAGGGA GTATG	TGGGATTAAAGCCCTT GACA	59.9	252	INTERGENIC	--	--	--
Ca4_P4_11483405	Ca4	11483405	AC/A	AAAGATTGGCCTGCTT TTGA	TTCAATGCCTCTCAAGT AATTGTT	59.8	490	INTERGENIC	--	--	--
Ca4_P4_11492296	Ca4	11492296	CTTTTTT/CTTTTT	GCAACTTATCCAGGGC TCAA	TGCTTCTTTGGGTGTG TGAG	60.2	174	INTERGENIC	--	--	--
Ca4_P4_11492735	Ca4	11492735	CAAAAAAAAA/CAAAAAAAAAAA	CTCACACACCCAAAGA AGCA	AACATCATGGTCAGCA CCAA	59.9	543	INTERGENIC	--	--	--
Ca4_P4_11492800	Ca4	11492800	AAAACAA/AAAACAATAAACAAACAA	CTCACACACCCAAAGA AGCA	AACATCATGGTCAGCA CCAA	59.9	543	INTERGENIC	--	--	--
Ca4_P4_11493423	Ca4	11493423	ATTTTTT/ATTTTTTT	CAAAGAAGCTGACCCT GGAG	CCCATAACTGGATGGG AGAA	60.0	679	INTERGENIC	--	--	--
Ca4_P4_11493841	Ca4	11493841	AT/ATT	GTTGCGACACCTACCA ATGA	CAGAAATGCTTGCGTC GTTA	59.6	242	INTERGENIC	--	--	--
Ca4_P4_11494266	Ca4	11494266	GAA/GAAAAA	TAACGACGCAAGCATT TCTG	GGTGCGAGAGAGAAAA CCTG	60.0	598	INTERGENIC	--	--	--
Ca4_P4_11494600	Ca4	11494600	ATAAATTTAAATTTAAATTTA/ATAAA TTTAAATTTA	CAGGTTTTCTCTCTCG CACC	CAACACAACCCGTGAA CATC	60.0	398	INTERGENIC	--	--	--
Ca4_P4_11494725	Ca4	11494725	TAAAAAAA/TAAAAAAAA	CAGGTTTTCTCTCTCG CACC	CAACACAACCCGTGAA CATC	60.0	398	INTERGENIC	--	--	--

INDEL marker IDs	Chromosomes/ unanchored scaffolds	Physical positions (bp)	InDels (*Kabuli* reference genome-CDC Frontier/PII)	Forward primers (5'-3')	Reverse primers (5'-3')	Anneling temperature (0C)	Expected amplified product size (bp)	Structural annotation		Functional annotation	
								Sequence components of *kabuli* genome	*Kabuli* gene accession IDs	NCBI-KOG	TFs
Ca4_P4_11505808	Ca4	11505808	GACA/GACAAAAACA	GCAATGATGACGGTGTATCG	CATGAACCATGATGTCGAGG	60.0	470	INTERGENIC	--	--	--
Ca4_P4_11506583	Ca4	11506583	CTTA/CTTAATTA	CTCGACATCATGGTTCATGG	CGGATCTATGCATGGGATAA	59.9	507	INTERGENIC	--	--	--
Ca4_P4_11514114	Ca4	11514114	AAAACA/AAAACAAACA	TGGATTTATGTAATGGGCTGG	TCGCCGAGTTTCAACTCTTT	59.7	509	INTERGENIC	--	--	--
Ca4_P4_11514377	Ca4	11514377	GATT/G	AAAAAGGATCCAACGGTTCA	TCGCCGAGTTTCAACTCTTT	59.4	102	INTERGENIC	--	--	--
Ca4_P4_11536367	Ca4	11536367	T/TA	GTGAGGGAAATGCATGTGTG	TTGATTTTTATCTGCAATTACCGT	60.0	811	INTERGENIC	--	--	--
Ca4_P4_11539392	Ca4	11539392	TA/TATGA	TGCGGTTTTCACAAAATGAC	CAACGGTTCATGATCCAATCT	59.6	192	INTERGENIC	--	--	--
Ca4_P4_11544878	Ca4	11544878	TAAAAAAA/TAAAAAA	CAGATTCCTTGCCATCGATTA	AAGTTGCCTTGCCATCCTAA	60.0	131	INTERGENIC	--	--	--
Ca4_P4_11545907	Ca4	11545907	GAATTTTTTT/GAATTTTTTTAAATTTTTTT	TGCACCGCTTTTACTTTTACC	GGATTTTGAGGTGTAGGGCA	59.3	713	INTERGENIC	--	--	--
Ca4_P4_11549203	Ca4	11549203	CTTTTTTTTT/CTTTTTTTTTTT	AGAAGGACGAGGAGGAGGAG	CGGTGGACCTTTACGAATTT	59.9	432	INTERGENIC	--	--	--
Ca4_P4_11549653	Ca4	11549653	TAA/TAAAA	AAATTCGTAAAGGTCCACCG	CCAACGGTGTGCATAATGAG	58.9	729	INTERGENIC	--	--	--

INDEL marker IDs	Chromosomes/ unanchored scaffolds	Physical positions (bp)	InDels (*Kabuli* reference genome-CDC Frontier/PII)	Forward primers (5'-3')	Reverse primers (5'-3')	Anneling temperature (0C)	Expected amplified product size (bp)	Structural annotation		Functional annotation	
								Sequence components of *kabuli* genome	*Kabuli* gene accession IDs	NCBI-KOG	TFs
Ca4_P4_11550605	Ca4	11550605	TAAAAAAAAAAAA/TAAAAAAAA	CTCATTATGCACACCGTTGG	GTGGCAAAGGGAATTTTGAA	60.0	991	INTERGENIC	--	--	--
Ca4_P4_11579781	Ca4	11579781	AATATATATATATATATATATA/AATATATATATATATATATA	AGGGTCCTACCCCGATTATG	ATATTCAACCGTGGTTCCCA	60.0	472	INTERGENIC	--	--	--
Ca4_P4_11585549	Ca4	11585549	TAAAAAAAA/TAAAAAAAAA	AACACTCACAAAAGCCACGA	TTGGTTTCTGGATTTGTCCC	59.3	516	INTERGENIC	--	--	--
Ca4_P4_11602402	Ca4	11602402	TTATATATATATATATATATATATATA/TTATATATATATATATATATATATATATATA	GCTTCAAGACAATCTTTGACCA	AATTAAAATGGCGGAGGACC	59.4	393	INTERGENIC	--	--	--
Ca4_P4_11611162	Ca4	11611162	AATATATATATATATATATATATATATATATATATATATATATAT/AATATATATATATATATATATATATATATATATATATATATAT	TCACACATACACCACACACACA	GAACAAAAGTTCGTGAACCACA	59.4	190	INTERGENIC	--	--	--
Ca4_P4_11615556	Ca4	11615556	AATATATATATATATATATATATATATA/AATATATATATATATATATATATATA	TCACACATACACCACACACACA	TCGAATAAGTTTTCGTGAACCA	59.4	187	INTERGENIC	--	--	--
Ca4_P4_11769762	Ca4	11769762	TA/T	TCCATAGTGGAGATGCGACA	GGAACATGCGAATCACAAAA	60.2	666	INTERGENIC	--	--	--
Ca4_P4_11818956	Ca4	11818956	CAAA/CAAAA	CCAAAACATGAAAGCAACAAAA	TAACATTGATGGCAGAGGCA	60.0	170	INTERGENIC	--	--	--
Ca4_P4_11988786	Ca4	11988786	TAAAATTAAATAAAAAA/TAAAATTAAATAAAAAAATTAAATAAAAAA	TCTTTAACAAGTGTCGTGCCA	GGCAACTGGGAAGCAAATAA	59.4	654	INTERGENIC	--	--	--
Ca4_P4_12015084	Ca4	12015084	CATATATATATATATATATATATAT/CATATATATATATATATATATATATATAT	ACCATAAAACAGCCTCCACG	GTGTTGCGATTTTGGTGTTG	60.0	499	INTERGENIC	--	--	--

**INDEL marker IDs**	**Chromosomes/ unanchored scaffolds**	**Physical positions (bp)**	**InDels (*Kabuli* reference genome-CDC Frontier/PII)**	**Forward primers (5'-3')**	**Reverse primers (5'-3')**	**Anneling temperature (0C)**	**Expected amplified product size (bp)**	**Structural annotation**		**Functional annotation**	
								**Sequence components of *kabuli* genome**	***Kabuli* gene accession IDs**	**NCBI-KOG**	**TFs**
Ca4_P4_12082492	Ca4	12082492	AATATATATAT/AATATATATATAT	GGACTCAGCATCGTGTTCCT	TTTCTTTAGGTTAGGCCCTCC	60.3	561	INTERGENIC	--	--	--
Ca4_P4_12090377	Ca4	12090377	TTATATATATATATATATATATAT/TTATATATATATATATATATATATATAT	ATTTGGTTCTGCGTTTGACC	ACTAGGTTGGAGCATGAGGC	60.0	567	INTERGENIC	--	--	--
Ca4_P4_12092916	Ca4	12092916	GTCTCTCTCTCTCTCTCTCTCTCTCTC/GTCTCTCTCTCTCTCTCTCTCTCTCTC	ACTAAACCGACCACCACACC	TGTACCTCCATTTGCCATGA	59.7	534	INTERGENIC	--	--	--
Ca4_P4_12127716	Ca4	12127716	AAATAATAATAATAATAATAATAATAAT/AAATAATAATAATAATAATAATAAT	CATTCGGTGGACCTTTTACG	TGAAGAGACGAAGCTGCAAA	60.4	343	INTERGENIC	--	--	--
Ca4_P4_12197670	Ca4	12197670	AAATAATAATAATAATAATAATAATAATGATAATGATAATGATAATAATAATAATAATAATAATAATAATAATAATAATAATAATA/AAATAATAATAATAATAATAATAATAATAATGATAATGATAATG[illegible]	CGGTGGTGGAGGAAGTAAAA	TGCATCTGCTTTACCCATGA	60.0	329	INTERGENIC	--	--	--
Ca4_P4_1236798	Ca4	1236798	AACACACACACACACACACA/AACACACACACACACACACACA	TGATGGGTGGTGAGATGAAA	AAAGAGGGGATGTTCCTTGG	59.9	673	INTERGENIC	--	--	--
Ca4_P4_1237884	Ca4	1237884	AATATATATATATATATATATATA/AATATATATATATATATA	GTGGTCTGGAACATCGTCCT	TTGCAGTTGCAATATCGTTG	60.0	863	INTERGENIC	--	--	--
Ca4_P4_12477637	Ca4	12477637	TAAAAAAAA/TAAAAAAAAAAAA	GGATTCAAATCATTCACCCG	TGGTTTAAGGTTTTGGGTTGA	60.1	536	INTERGENIC	--	--	--
Ca4_P4_12479950	Ca4	12479950	AGTA/AGTAGTA	GATGCCCCACAACCACTACT	CGACCATGTAAAGCAAAACTTG	59.9	599	INTERGENIC	--	--	--
Ca4_P4_12480535	Ca4	12480535	CAT/CATACAAAAT	CAAGTTTTGCTTTACATGGTCG	AAGTTGTGCTTTTTGTGCCC	59.7	438	INTERGENIC	--	--	--

INDEL marker IDs	Chromosomes/ unanchored scaffolds	Physical positions (bp)	InDels (*Kabuli* reference genome-CDC Frontier/PII)	Forward primers (5'-3')	Reverse primers (5'-3')	Anneling temperature (0C)	Expected amplified product size (bp)	Structural annotation		Functional annotation	
								Sequence components of *kabuli* genome	*Kabuli* gene accession IDs	NCBI-KOG	TFs
Ca4_P4_12483472	Ca4	12483472	CAA/CA	TCTACGGTCCAATACCCGAG	GGTCCGTGGAGAAATTTGA	59.9	532	INTERGENIC	--	--	--
Ca4_P4_12505498	Ca4	12505498	ATATATATAT/ATATATATATGTATATATAT	GCAAGGACAAGGGATGTAGG	TCAACCGCAATTGAGATTTTC	59.6	677	INTERGENIC	--	--	--
Ca4_P4_12507863	Ca4	12507863	AAAAGAAA/AAAA	ATTCAGCACATGCCACTGTC	CCTTCAATGTGGTGGTAGGG	59.7	827	INTERGENIC	--	--	--
Ca4_P4_12520618	Ca4	12520618	AATATATATATATAT/AATATATATATATATAT	TCAAAGCTGCATTTTGTTGC	TTCGTATGCTATGCACGCTC	60.0	441	INTERGENIC	--	--	--
Ca4_P4_12528813	Ca4	12528813	CATA/CATAATA	TAACGCTAGGGTCCCATGTC	TCGTTTTGATCATTTATCGTTTTT	60.0	720	INTERGENIC	--	--	--
Ca4_P4_12528995	Ca4	12528995	CTTTT/CTTT	TAACGCTAGGGTCCCATGTC	TCGTTTTGATCATTTATCGTTTTT	60.0	720	INTERGENIC	--	--	--
Ca4_P4_12529201	Ca4	12529201	TAAAAAAAAAA/TAAAAAAAA	CATGGCTCCAAGAAGCTTAAA	TTCGTTTTGATCATTTATCGTTTT	59.5	527	INTERGENIC	--	--	--
Ca4_P4_12561884	Ca4	12561884	AATAT/AAT	TCGTCTATGCGAGTCGTAACA	TTCAAACCTAAGCCACTGCAT	59.5	727	INTERGENIC	--	--	--
Ca4_P4_12563798	Ca4	12563798	GTTTTT/GTTTT	TGTGATATTGGGTCCATTGGT	TCACAAATCGAAATGCGGTA	59.9	672	INTERGENIC	--	--	--
Ca4_P4_12566243	Ca4	12566243	TTTATTATTATTATTATTATTATTATT/TTTATTATTATTATTATTATTATTATTATT	GTTGTGAATCAACTTGGCCC	GGGACTGCACCTATTTTATGC	60.4	834	INTERGENIC	--	--	--

INDEL marker IDs	Chromosomes/ unanchored scaffolds	Physical positions (bp)	InDels (*Kabuli* reference genome-CDC Frontier/PII)	Forward primers (5'-3')	Reverse primers (5'-3')	Anneling temperature (0C)	Expected amplified product size (bp)	Structural annotation		Functional annotation	
								Sequence components of *kabuli* genome	*Kabuli* gene accession IDs	NCBI-KOG	TFs
Ca4_P4_12566497	Ca4	12566497	CTTTTTTT/CTTTTTTTTT	CCGTCCGTCATGACCTATAA	CATCTCAAACAAACTCGGACA	58.4	718	INTERGENIC	--	--	--
Ca4_P4_12566654	Ca4	12566654	AG/A	TCAGTCTTTCCATATTCTCTCCG	AAAACCCAGGAGGTCAGCTT	59.7	619	INTERGENIC	--	--	--
Ca4_P4_12567928	Ca4	12567928	AATATATATAT/AATATATAT	ATTCGGCAATTCTACCCCTT	GAGGTAGAGCTCGACGATGG	59.8	446	INTERGENIC	--	--	--
Ca4_P4_12571781	Ca4	12571781	ACCCC/ACCCCC	ACATCCAATGACCAAGAGCC	CCACTTTCGGAACAATTTTGA	59.9	394	INTERGENIC	--	--	--
Ca4_P4_12571941	Ca4	12571941	AA/AATTTA	ACATCCAATGACCAAGAGCC	TTGTTTTGCATGCTCTTCCA	59.9	820	INTERGENIC	--	--	--
Ca4_P4_12586849	Ca4	12586849	TTAATATAATATAAT/TTAATATAATATAATATAAT	AACCTAATAAGCCCGGAAGC	GACAAAAGTGTTGGCGGTTT	59.6	916	INTERGENIC	--	--	--
Ca4_P4_12588357	Ca4	12588357	TAAAAAAAAAA/TAAAAAAAAAAAAA	ACAATTCCGAAGATACCCCC	GATGTGGTCGTTGTCGAGTG	60.0	506	INTERGENIC	--	--	--
Ca4_P4_12589362	Ca4	12589362	CTTTTTTTTT/CTTTTTT	CAATGGTTCTTTTGGGGTTC	ACAACGTCTTCCATTACGCC	59.3	615	INTERGENIC	--	--	--
Ca4_P4_12604453	Ca4	12604453	ATTTT/ATT	AGTCCATGCTTTCTGCTGCT	ATTTCACAAATGGGGCTGAG	60.2	669	INTERGENIC	--	--	--
Ca4_P4_12606654	Ca4	12606654	CAT/CATAT	GGGTGCTAATGCCATTGTTT	GGGATTGATTTGTAAGGAAGGA	59.8	618	INTERGENIC	--	--	--

INDEL marker IDs	Chromosomes/ unanchored scaffolds	Physical positions (bp)	InDels (*Kabuli* reference genome-CDC Frontier/PII)	Forward primers (5'-3')	Reverse primers (5'-3')	Anneling temperature (0C)	Expected amplified product size (bp)	Structural annotation		Functional annotation	
								Sequence components of *kabuli* genome	*Kabuli* gene accession IDs	NCBI-KOG	TFs
Ca4_P4_12607155	Ca4	12607155	ATTTTTTT/ATTTTTTTTTTT	TCCTTCCTTACAAATCAATCCC	AATTTTTGCGACTTTGACCG	59.3	194	INTERGENIC	--	--	--
Ca4_P4_12700885	Ca4	12700885	GAAAAAAAA/GAAAAAAAAA	TTGCAAACAAACTCACAACATTC	TCCACTCAATTCAATACCATCA	60.1	503	INTERGENIC	--	--	--
Ca4_P4_12788740	Ca4	12788740	TTATATATATATATATATATATA/TTATATATATATATATATATATATATATATA	TCACATGCGTCCAATTTTTG	AAAACGTGTGGCCAGCTAAC	60.5	845	INTERGENIC	--	--	--
Ca4_P4_12795892	Ca4	12795892	TTATATATATATATATATATATATATATATATATATATAT/TTATATATATATATATATATATATATATAT	TTTTTCCCTTTTCACCCAAG	TCTGAAAAAGCGCCGTAAAT	59.0	291	INTERGENIC	--	--	--
Ca4_P4_12797194	Ca4	12797194	GAAGAGAAAGAG/GAAGAG	ACAATAGGGTTATGGCGCAG	AATCATCTCCATTGCGAACC	60.0	347	INTERGENIC	--	--	--
Ca4_P4_12836741	Ca4	12836741	AATATATATATATATATATATATATATATAT/AATATATATATATATATATATATATATAT	TCGTTCAATCGAGGAAAACC	CTTTGTTGGGCTAATCACGG	60.1	886	INTERGENIC	--	--	--
Ca4_P4_12852660	Ca4	12852660	AATATATATAT/AATATATATATAT	CATTCTCTCTATGCCACGCA	AACTTGTGTCCTTCAAGGGG	60.0	593	INTERGENIC	--	--	--
Ca4_P4_12918326	Ca4	12918326	GAAAAAAAAAA/GAAAAAAAAAAAA	TGATTCAAAACATGCGAGAAA	TATCCATAAACACCCCTCCG	59.3	793	INTERGENIC	--	--	--
Ca4_P4_12918976	Ca4	12918976	GATATATATATATATATATATATATATAT/GATATATATATATATATATATATATATAT	TTAATGCACGGTCATGTGCT	GCTATTTGCAAAGGTGGCTT	60.1	698	INTERGENIC	--	--	--
Ca4_P4_1310245	Ca4	1310245	ATTTTTTTTTTT/ATTTTTTTTTT	TGAAATTGCTTGATTTTGCTTT	CGACCGATTTTATCCGTCTC	58.9	825	INTERGENIC	--	--	--

INDEL marker IDs	Chromosomes/ unanchored scaffolds	Physical positions (bp)	InDels (*Kabuli* reference genome-CDC Frontier/PII)	Forward primers (5'-3')	Reverse primers (5'-3')	Anneling temperature (0C)	Expected amplified product size (bp)	Structural annotation		Functional annotation	
								Sequence components of *kabuli* genome	*Kabuli* gene accession IDs	NCBI-KOG	TFs
Ca4_P4_13133292	Ca4	13133292	AT/ATATATTT	CAGCCGCCTCCAACTATTAC	CCATCCATGCAATTGAACAC	59.7	408	INTERGENIC	--	--	--
Ca4_P4_13133678	Ca4	13133678	CTTTTT/CTTTTTT	TGTTCAATTGCATGGATGGT	TTTAGGGCACCGTTTGAAGT	59.8	553	INTERGENIC	--	--	--
Ca4_P4_13149119	Ca4	13149119	CATTATTATTATTATTATTATTATTATTATTATTATT/CATTATTATTATTATTATTATTATTATTATTATT	AGCAGCAGTTGTATGGGCTT	GCCATGCATTCCGAAAATAA	59.9	211	INTERGENIC	--	--	--
Ca4_P4_13191736	Ca4	13191736	AAATAATAATAATAATAATAATAATAATAATAATAATAATAATAA/AAATAATAATAATAATAATAATAATAATAATAATAATAATAATAATAATAA	CAAGTCGTTTACTTTGGCACA	TTTTCGCACATCTACCCATT	58.9	418	INTERGENIC	--	--	--
Ca4_P4_13210889	Ca4	13210889	GA/GCCATCTGATTCTA	TTGTCTTTTTGGGCTGCTCT	GGTCACACACAAATGCCAAG	60.0	388	INTERGENIC	--	--	--
Ca4_P4_13352277	Ca4	13352277	CAG/CAGGAAG	TTTTCCAAATATGGTCGCAA	AATTTCAAGCCATCGACCAC	59.0	797	INTERGENIC	--	--	--
Ca4_P4_13433311	Ca4	13433311	TA/T	CGAGCAAACACAATGAATGG	TTGGTTGGTTCATTGGTTGA	60.1	569	INTERGENIC	--	--	--
Ca4_P4_13446467	Ca4	13446467	CAAAAAAATAAAAAAAAATAAAAA/CAAAAAAATAAAAA	TGAGATGCTGCATTACCTGC	ATCCAATGTTCTGGACGGTG	60.0	484	INTERGENIC	--	--	--
Ca4_P4_13478987	Ca4	13478987	ATTA/ATTTGAAATTACCTTA	CACTGGGATCTTCAATGTTCAG	CGTAGTTTAAATTTTGGGGGTG	59.6	733	INTERGENIC	--	--	--
Ca4_P4_13494856	Ca4	13494856	GGTAGT/GGTAGTAGT	CGAAGGCATGGGATAAAAAT	TTTCTAGGCCACCCATCTTG	58.9	707	INTERGENIC	--	--	--

INDEL marker IDs	Chromosomes/ unanchored scaffolds	Physical positions (bp)	InDels (*Kabuli* reference genome-CDC Frontier/PII)	Forward primers (5'-3')	Reverse primers (5'-3')	Anneling temperature (0C)	Expected amplified product size (bp)	Structural annotation		Functional annotation	
								Sequence components of *kabuli* genome	*Kabuli* gene accession IDs	NCBI-KOG	TFs
Ca4_P4_13509527	Ca4	13509527	ATTTT/ATTTTATTAAAAAATTTATTTTT	CCTCTTCACGGTGCTCATTT	CCTCGTTTTAAGCCCAATCA	60.3	158	INTERGENIC	--	--	--
Ca4_P4_13556277	Ca4	13556277	TTAATAATAATAATAA/TTAATAATAATAATAATAATAATAA	GTCGCAAGTGTTTATCGCAA	GGCTAGACGAGTTCGACGAC	59.9	459	INTERGENIC	--	--	--
Ca4_P4_13577543	Ca4	13577543	GTATATAT/GTATATATAT	TTGGCATAGCAAGACAACCA	TGAACCAGTGATGATTGCAGA	60.3	358	INTERGENIC	--	--	--
Ca4_P4_13578691	Ca4	13578691	TTACAATTAATAA/T	TTTTCCCGAAATTTCTGCTG	CTTAAGAAGGGCAAGGGCAT	60.2	364	INTERGENIC	--	--	--
Ca4_P4_13579909	Ca4	13579909	ATTT/AT	TTCCCAATTTTTCTTTCAATTTT	GCACTCACCAAGCAATAGCA	58.2	666	INTERGENIC	--	--	--
Ca4_P4_13582008	Ca4	13582008	GTTTATTTATTTATTT/GTTTATTTATTT	TTGTGATCGCCGAAAATACA	CAATCATGGTGCTGCGATAC	60.1	407	INTERGENIC	--	--	--
Ca4_P4_13582915	Ca4	13582915	GAAATAAAAA/GAAATAAAAAAAAATAAAAA	CCATTGCTGAGATCATGGTG	TGCATGCATCAGACAACAGA	60.1	535	INTERGENIC	--	--	--
Ca4_P4_13604124	Ca4	13604124	GA/G	TTCAGACCCCAGTTTCATCA	GACATGTATCGTGTGCGGTC	59.1	631	INTERGENIC	--	--	--
Ca4_P4_13608282	Ca4	13608282	ATTTTTTTT/ATTTTTTTTTT	TCTTTGTCCAAAGGCCAATC	CACATTTTGCAAGGTTCGTG	60.1	795	INTERGENIC	--	--	--
Ca4_P4_13608641	Ca4	13608641	ATT/ATTT	GGTGACGTGGCTTACTCTCC	CCCTTTCTGCATGGACAAGT	59.7	413	INTERGENIC	--	--	--

INDEL marker IDs	Chromosomes/ unanchored scaffolds	Physical positions (bp)	InDels (*Kabuli* reference genome-CDC Frontier/PII)	Forward primers (5'-3')	Reverse primers (5'-3')	Anneling temperature (0C)	Expected amplified product size (bp)	Structural annotation		Functional annotation	
								Sequence components of *kabuli* genome	*Kabuli* gene accession IDs	NCBI-KOG	TFs
Ca4_P4_13610617	Ca4	13610617	AAAGCCA/AAAGCCAAGCCA	GATGAAGCCAAACTACCCCA	ACGCTGAAGGAAGAAAGCTG	59.9	327	INTERGENIC	--	--	--
Ca4_P4_13610669	Ca4	13610669	AG/A	GATGAAGCCAAACTACCCCA	ACGCTGAAGGAAGAAAGCTG	59.9	327	INTERGENIC	--	--	--
Ca4_P4_13612409	Ca4	13612409	AACAATCAACACAATCA/AACAATCA	GCACCCTAACACTTCTTGCAT	CACCAAATTCACGAGAACCA	59.3	329	INTERGENIC	--	--	--
Ca4_P4_13619118	Ca4	13619118	AC/A	TTGTAATGCCAAAATTGCGA	TTGCCTCCAAAATCAACCTC	60.1	476	INTERGENIC	--	--	--
Ca4_P4_13621014	Ca4	13621014	GTTTTT/GTTTTTT	TTTGCTAACTTGCTCCCACC	GCGAGAGGCCCATGTAACTA	60.2	723	INTERGENIC	--	--	--
Ca4_P4_13627308	Ca4	13627308	A/AT	TTGGCGTGAAAGACAACATT	TGTTGGGTGAGTTTTCCACA	59.2	938	INTERGENIC	--	--	--
Ca4_P4_13630632	Ca4	13630632	AA/AAGAGTCTA	TATGCAGGCCCAAACTCTCT	TTTTCACAATCAATGCCCAA	59.8	370	INTERGENIC	--	--	--
Ca4_P4_13631062	Ca4	13631062	GCC/GC	TTGGGCATTGATTGTGAAAA	TCGCAACTCGTATAGGACCA	59.9	271	INTERGENIC	--	--	--
Ca4_P4_13633364	Ca4	13633364	TAAAAA/TAAAA	TGATTTACGAAATTGGTTCTCC	TTTCAAATTTCGCAGTTGTGT	58.1	812	INTERGENIC	--	--	--
Ca4_P4_13633711	Ca4	13633711	CTTATGTTAT/CTTAT	TTCAATCATTTGTCCAATTTCC	CTCGCGGTAAAATTCATGCT	58.8	432	INTERGENIC	--	--	--

INDEL marker IDs	Chromosomes/ unanchored scaffolds	Physical positions (bp)	InDels (*Kabuli* reference genome-CDC Frontier/PII)	Forward primers (5'-3')	Reverse primers (5'-3')	Anneling temperature (0C)	Expected amplified product size (bp)	Structural annotation		Functional annotation	
								Sequence components of *kabuli* genome	*Kabuli* gene accession IDs	NCBI-KOG	TFs
Ca4_P4_13643836	Ca4	13643836	ATTTTTT/ATTTTTTT	ATAGTTGTTGAAGGGGCACG	GGGTGAAGAATGTATGCCAAA	60.0	798	INTERGENIC	--	--	--
Ca4_P4_13643938	Ca4	13643938	AA/AATATA	GCTTCCGACAAAGGTCAGAG	GGGTGAAGAATGTATGCCAAA	60.0	641	INTERGENIC	--	--	--
Ca4_P4_13643954	Ca4	13643954	T/TA	GCTTCCGACAAAGGTCAGAG	GGGTGAAGAATGTATGCCAAA	60.0	641	INTERGENIC	--	--	--
Ca4_P4_13649620	Ca4	13649620	AG/A	TTGTCCCCCTAACAAATGGA	GACTTTCAAGCCCGATACCA	60.2	360	INTERGENIC	--	--	--
Ca4_P4_13650519	Ca4	13650519	TTTATT/TTT	AGACAGTTGCTCAATGCACG	AATGCAGTGCTTTGCAGTTG	60.1	610	INTERGENIC	--	--	--
Ca4_P4_13651001	Ca4	13651001	TC/T	AAGCAGCGAGTTGTTCAGGT	GGAAAACTGCTTTCGCTTTG	60.1	755	INTERGENIC	--	--	--
Ca4_P4_13657394	Ca4	13657394	CAAAAAAA/CAAAAAAAA	TCGTTTTTCAACCATCAGTCA	TCTCCCAATGTGATTGCTCA	59.2	823	INTERGENIC	--	--	--
Ca4_P4_13658364	Ca4	13658364	TTTTATTTATTT/TTTTATTTATTTATTT	CATCAACTGACTAACGACATGGA	TGAAGATGGTGGAATGGGAT	60.0	516	INTERGENIC	--	--	--
Ca4_P4_13659547	Ca4	13659547	CCAAACAAAC/CCAAAC	CCAAAACTGGGGAGAAACAA	AAAACGACGGTTATGAACGC	59.9	642	INTERGENIC	--	--	--
Ca4_P4_13669625	Ca4	13669625	TAAGAAG/TAAG	TCAAAACACAAACACAAGCCA	CCCTTTGTGGGTTCCCTTAT	60.2	800	INTERGENIC	--	--	--

INDEL marker IDs	Chromosomes/ unanchored scaffolds	Physical positions (bp)	InDels (*Kabuli* reference genome-CDC Frontier/PII)	Forward primers (5'-3')	Reverse primers (5'-3')	Anneling temperature (0C)	Expected amplified product size (bp)	Structural annotation		Functional annotation	
								Sequence components of *kabuli* genome	*Kabuli* gene accession IDs	NCBI-KOG	TFs
Ca4_P4_13669889	Ca4	13669889	TTTCTT/TTT	AACGTTATTTAAACGAGGATTTCA	CCCTTTGTGGGTTCCCTTAT	58.3	555	INTERGENIC	--	--	--
Ca4_P4_13671186	Ca4	13671186	TCAA/T	CGTCCATTGTGGTTATGCTC	TTCCTAAAAAGTTAAACCGGCA	59.0	685	INTERGENIC	--	--	--
Ca4_P4_13671252	Ca4	13671252	ATTTTTTTT/ATTTTTTTTT	CGTCCATTGTGGTTATGCTC	TTCCTAAAAAGTTAAACCGGCA	59.0	685	INTERGENIC	--	--	--
Ca4_P4_13671299	Ca4	13671299	ATTTTTTTT/AATTTTTTTTTT	CGTCCATTGTGGTTATGCTC	TTCCTAAAAAGTTAAACCGGCA	59.0	685	INTERGENIC	--	--	--
Ca4_P4_13676197	Ca4	13676197	AG/A	ACGTTTCCACGATTGGTGAT	GATGATCACGCTTCCACAAG	60.2	462	INTERGENIC	--	--	--
Ca4_P4_13676268	Ca4	13676268	AGTTT/AGTTTGTTT	ACGTTTCCACGATTGGTGAT	GATGATCACGCTTCCACAAG	60.2	462	INTERGENIC	--	--	--
Ca4_P4_13689699	Ca4	13689699	CTAAAAATACAATAAAAATACAATAA/CTAAAAATACAATAA	GTCGTGTTCCCGCTTAAGAT	GCAAATGGATAAAAGTCACATCA	59.2	684	INTERGENIC	--	--	--
Ca4_P4_13712400	Ca4	13712400	G/GC	TGGGGCTTATAGCCTCCAAT	AGGAAATGAAGGAAACGGGT	60.8	594	INTERGENIC	--	--	--
Ca4_P4_13731926	Ca4	13731926	ATTTT/ATTT	CAAGGGATTGTGAGTGTTTGAA	GTCCAAGTAATCTGGCGCAT	60.0	532	INTERGENIC	--	--	--
Ca4_P4_13732813	Ca4	13732813	TCC/TC	TCCGCGAGCTTAGTTTTGTT	CACGGAGATGAGAAGAGGGA	60.0	521	INTERGENIC	--	--	--

INDEL marker IDs	Chromosomes/ unanchored scaffolds	Physical positions (bp)	InDels (*Kabuli* reference genome-CDC Frontier/PII)	Forward primers (5'-3')	Reverse primers (5'-3')	Anneling temperature (0C)	Expected amplified product size (bp)	Structural annotation		Functional annotation	
								Sequence components of *kabuli* genome	*Kabuli* gene accession IDs	NCBI-KOG	TFs
Ca4_P4_13739989	Ca4	13739989	TA/T	TTTCTCCGTCTTAGTG CGGT	AAATGATCAAATCGAAA AGAAAAA	59.9	432	INTERGENIC	--	--	--
Ca4_P4_13750785	Ca4	13750785	ATTTTTTT/ATTTTTTTTT	GAGCATGTTTGGATTT ACTTTGC	TTTTTATGTAAAGGACG AGGATGA	60.0	721	INTERGENIC	--	--	--
Ca4_P4_13761691	Ca4	13761691	ATTTT/ATTT	AAGGTGTGTGAAAGGA TGCC	TGCAAACAGCTCAATC CTTG	60.0	544	INTERGENIC	--	--	--
Ca4_P4_13770396	Ca4	13770396	T/TG	AAGCACCCATCCTGAA ATTG	TTTCACCGCGATAAAAA TCA	59.9	586	INTERGENIC	--	--	--
Ca4_P4_13770434	Ca4	13770434	AGTAC/AGTACGTAC	AAGCACCCATCCTGAA ATTG	TTTCACCGCGATAAAAA TCA	59.9	586	INTERGENIC	--	--	--
Ca4_P4_13785898	Ca4	13785898	CA/CACAA	CCGCCGGGAATAAAGT AGTA	TGAGTCTCACCTTGGG CTCT	59.1	442	INTERGENIC	--	--	--
Ca4_P4_13793804	Ca4	13793804	GTTATCAAA/GTTATCAAACCCAATT ATCAAA	CACGAGGACAATGAAG AGCA	ATTGACGACGAAAATG AGGC	60.0	531	INTERGENIC	--	--	--
Ca4_P4_13795678	Ca4	13795678	ATGTTGTTGTT/ATGTTGTTGTTGTT	GCCGGAAATACACCGA CTAA	AAAGGGTAGGAACAAC CGCT	60.0	599	INTERGENIC	--	--	--
Ca4_P4_13807055	Ca4	13807055	AT/ATCT	ATAGAATTCGGGAGGG GACA	GGTGCCTGTGCTTTTC TTGT	60.7	248	INTERGENIC	--	--	--
Ca4_P4_13808690	Ca4	13808690	TA/TAGAAA	ATTCCACGTTCACTTCT CCG	CGCCACTCTTCTACGG TTCT	60.1	376	INTERGENIC	--	--	--

INDEL marker IDs	Chromosomes/ unanchored scaffolds	Physical positions (bp)	InDels (*Kabuli* reference genome-CDC Frontier/PII)	Forward primers (5'-3')	Reverse primers (5'-3')	Anneling temperature (0C)	Expected amplified product size (bp)	Structural annotation		Functional annotation	
								Sequence components of *kabuli* genome	*Kabuli* gene accession IDs	NCBI-KOG	TFs
Ca4_P4_13809545	Ca4	13809545	ATT/ATTT	AAAAGGGGATGAGTTTTTCCT	TCGTAATTAATCCACATGAATCTGA	58.1	923	INTERGENIC	--	--	--
Ca4_P4_13813294	Ca4	13813294	TG/T	TTTTTGCCCGTTAGACGATT	TGGGTGAATTGGGTAGGAGA	59.6	508	INTERGENIC	--	--	--
Ca4_P4_13826117	Ca4	13826117	ATTTTT/ATTTTTT	AGGTGGTTGTTGCACTTTGA	CAAATTTGTTCCGCTTCACA	59.2	474	INTERGENIC	--	--	--
Ca4_P4_13828147	Ca4	13828147	G/GA	GTAATTGCCGGGGTCCTTAT	CAATAGTGTTTGCACGTGGG	60.0	430	INTERGENIC	--	--	--
Ca4_P4_13829467	Ca4	13829467	AT/ATT	TGAGAATTTGCATGCCTCAC	AGCGAAGTGCTAGCAACACA	59.8	671	INTERGENIC	--	--	--
Ca4_P4_13830179	Ca4	13830179	AT/ATATTT	TGTGTTGCTAGCACTTCGCT	GTGGTTGCACACATTTCGAC	59.8	922	INTERGENIC	--	--	--
Ca4_P4_13831456	Ca4	13831456	GATATAT/GAT	GATATTTTGTTTGGCCCCCT	GCAGGAAATGTGTTGTGGTG	60.0	154	INTERGENIC	--	--	--
Ca4_P4_13835215	Ca4	13835215	AT/A	GAGACGGCATCTAATTGGGA	ACCCCCATTACCATCTTGTG	60.0	647	INTERGENIC	--	--	--
Ca4_P4_13847680	Ca4	13847680	CTTTTTTTT/CTTTTTTTTTT	TGTTCGAAGTTTCCATCGAG	CCTCCTCTTCTCCCCATCTC	58.8	756	INTERGENIC	--	--	--
Ca4_P4_13850847	Ca4	13850847	TAACAA/TAACAACAACAA	CCAAATTTTTGGAGGACATAAA	TGAAAGTGGAGAATTCAAAGC	58.0	401	INTERGENIC	--	--	--

INDEL marker IDs	Chromosomes/ unanchored scaffolds	Physical positions (bp)	InDels (*Kabuli* reference genome-CDC Frontier/PII)	Forward primers (5'-3')	Reverse primers (5'-3')	Anneling temperature (0C)	Expected amplified product size (bp)	Structural annotation		Functional annotation	
								Sequence components of *kabuli* genome	*Kabuli* gene accession IDs	NCBI-KOG	TFs
Ca4_P4_13852149	Ca4	13852149	ACTACCAACGGAGTTAAATCTA/ACTA	CATTGAATATGCAACCCACG	TCGATGCGTCCAATGATAAA	59.8	507	INTERGENIC	--	--	--
Ca4_P4_13853238	Ca4	13853238	TTTT/TTTTACTTT	TGGAGAATTCGTATGAAAGGG	AACGTGCATGAATCCAAAAA	59.0	790	INTERGENIC	--	--	--
Ca4_P4_13853568	Ca4	13853568	TGG/TG	TTCCCACAATGTTGAGTTTCA	TGCATATAATGGTCAGTGATCG	59.0	315	INTERGENIC	--	--	--
Ca4_P4_13854087	Ca4	13854087	TAAAAAAAAA/TAAAAAAA	CGATCACTGACCATTATATGCAC	CCTTCTTTCCCACAATGTTGA	59.4	611	INTERGENIC	--	--	--
Ca4_P4_13856503	Ca4	13856503	A/AT	TCCTGATAACATTCCTTTCACC	GGGTTCGAAATTGGTGTGTT	58.0	594	INTERGENIC	--	--	--
Ca4_P4_13908536	Ca4	13908536	TC/T	CTCCTCCGATGTCTGGAAAA	CGCGGTTGCTTCAATTCTAT	60.2	232	INTERGENIC	--	--	--
Ca4_P4_13909254	Ca4	13909254	CA/CAA	CATCACGATTTGACGAGTTGA	AAAAACATGACTCCAAACACCA	59.7	636	INTERGENIC	--	--	--
Ca4_P4_1395408	Ca4	1395408	TAAATA/T	TAATCGACATCAGCTTGCGT	TGCTGCGACTAGCTGAAAAA	59.4	670	INTERGENIC	--	--	--
Ca4_P4_14011652	Ca4	14011652	TAATAATAA/TAATAATAACAATAATAA	GCACCGACTTGCTGATGTAA	GCCTCATGCTCCAATTTTGT	59.9	755	INTERGENIC	--	--	--
Ca4_P4_14187232	Ca4	14187232	TTATATATATATATATATATATATAT/TTATATATATATATATATATATAT	GACTCACGTCTGAATCGCAA	GCACAAGGAACTCGAGAACA	60.0	865	INTERGENIC	--	--	--

INDEL marker IDs	Chromosomes/ unanchored scaffolds	Physical positions (bp)	InDels (*Kabuli* reference genome-CDC Frontier/PII)	Forward primers (5'-3')	Reverse primers (5'-3')	Anneling temperature (0C)	Expected amplified product size (bp)	Structural annotation		Functional annotation	
								Sequence components of *kabuli* genome	*Kabuli* gene accession IDs	NCBI-KOG	TFs
Ca4_P4_14280638	Ca4	14280638	T/TATATATATA	GTCTAAGGCTTGCTACCCCC	AGAGGCTTCTCGTTGGGTTT	60.1	596	INTERGENIC	--	--	--
Ca4_P4_14355071	Ca4	14355071	AT/ATGGT	ACCTGCAATTTTGGCTATGG	TGTGACGATCTCTTATGGCG	60.0	413	INTERGENIC	--	--	--
Ca4_P4_14392979	Ca4	14392979	G/GA	ATTGAAACTTGTCGCAGCCT	GCAAGTTTTCGTTTTGTTCG	59.9	551	INTERGENIC	--	--	--
Ca4_P4_14396557	Ca4	14396557	TTAGTAGT/TTAGT	AGTCAGACGCTACTCGGCAT	GTGTGTGTGTGTGTGTGTGTGT	60.0	590	INTERGENIC	--	--	--
Ca4_P4_14397300	Ca4	14397300	GATATATATATATATATATATGTATATATATATATA/GATATATATATATATATATATATATATATGTATATATATATATA	TAAATGCATGCTCCCCTGAT	AACACATCCGGGATCCATTA	60.4	273	INTERGENIC	--	--	--
Ca4_P4_14416755	Ca4	14416755	TTATATATATATA/TTATATATATATATA	TCATTTCAAACCCTTAAACTCCA	TTGCGGTCAATTGTATGAAGAC	59.9	392	INTERGENIC	--	--	--
Ca4_P4_1463289	Ca4	1463289	TAAAAAAAAAAA/TAAAAAAAAAA	CCCACTTTTACGGAAGACGA	CCCCAACCACTTCCTCACTA	60.1	511	INTERGENIC	--	--	--
Ca4_P4_14677950	Ca4	14677950	CAAAAAAA/CAAAAAAAA	TGTTGGGCAAAATAAATTGAGA	CAAACCTTACCACCGTCTTG	59.5	424	INTERGENIC	--	--	--
Ca4_P4_14800285	Ca4	14800285	CAAAA/CAA	TTGTACACGTAGTAGCGCCG	AAGATGGAATTCAAGGGGCT	60.0	475	INTERGENIC	--	--	--
Ca4_P4_14999642	Ca4	14999642	CATATATATATATATATATATATATATATATATATATAT/CATACATATATATATATATATATATATATATATATATATATATATATATATAT	ACTATAGTACCCACCCCGCC	GGAGGATCCGAAAATTATGAAA	60.1	635	INTERGENIC	--	--	--

INDEL marker IDs	Chromosomes/ unanchored scaffolds	Physical positions (bp)	InDels (*Kabuli* reference genome-CDC Frontier/PII)	Forward primers (5'-3')	Reverse primers (5'-3')	Anneling temperature (0C)	Expected amplified product size (bp)	Structural annotation		Functional annotation	
								Sequence components of *kabuli* genome	*Kabuli* gene accession IDs	NCBI-KOG	TFs
Ca4_P4_15005039	Ca4	15005039	AACA/AA	TTCCAACGGTCATAAAAGGC	CCCAAAGATGGCTACTTCCA	59.9	395	INTERGENIC	--	--	--
Ca4_P4_15048181	Ca4	15048181	TA/TAA	CAAAAGCTGTAACACCCCGT	AATCGATTGATTCAGCCCAG	60.0	528	INTERGENIC	--	--	--
Ca4_P4_15081049	Ca4	15081049	ATTT/ATTTT	TTTGCAACTCCATTCTTCCC	GATTCCCCATTCTTAACGCC	60.1	688	INTERGENIC	--	--	--
Ca4_P4_15111382	Ca4	15111382	CATAATAATAATAATAATAATAATAATAATAATAATAATAATAA/CATAATAATAATAATAATAATAATAATAATAATAATAATAATAA	TTGCACTTTTGAGAAGTCACG	GGCACTTGAGTAGATGTAGCAAAA	59.1	659	INTERGENIC	--	--	--
Ca4_P4_15465052	Ca4	15465052	T/TG	GCAACTCATTCGTCCCATTT	GGAAACGAAAAATTTGCAGC	59.9	712	INTERGENIC	--	--	--
Ca4_P4_15488514	Ca4	15488514	GTATATATATATATATATATATATATATAT/GTATATATATATATATATATATATATATATAT	CATGTTTCTCATCGTTCCAAA	TGAAGATATTTTTCGCTGTTTTT	58.6	653	INTERGENIC	--	--	--
Ca4_P4_1550381	Ca4	1550381	TTATATATATATATATATATATATAT/TTATATATATATATATATATATATATATATAT	CTTCCTCATCAATTTTGGCA	CAAAGTGTCGACAGGCTCTCT	58.7	494	INTERGENIC	--	--	--
Ca4_P4_15621540	Ca4	15621540	AATATATATATATATATATATATATATATAT/AATATATATATATATATATATATAT	CGACGGAAGTTGTTTCGAGT	AATTGTGGGGAAATCTGTGC	60.3	399	INTERGENIC	--	--	--
Ca4_P4_15676203	Ca4	15676203	ATTTTTTTTTT/ATTTTTTTTT	ACAAAACGGTGAAGTTTCCG	TTGCAAGCGTTTGAAAAGTG	60.0	552	INTERGENIC	--	--	--
Ca4_P4_15681893	Ca4	15681893	CAATAATAATAATAATAATAATAATAATAATAATAATAATAATAATAATAATAA/CAATAATAATAATAATAATAATAATAATAATAATAATAATAATAATAATAATAA	AAAAAGCGAGGTTCGGATTA	AAAGGGGAAATCCAATTTCTAA	58.8	445	INTERGENIC	--	--	--

INDEL marker IDs	Chromosomes/ unanchored scaffolds	Physical positions (bp)	InDels (*Kabuli* reference genome-CDC Frontier/PII)	Forward primers (5'-3')	Reverse primers (5'-3')	Anneling temperature (0C)	Expected amplified product size (bp)	Structural annotation		Functional annotation	
								Sequence components of *kabuli* genome	*Kabuli* gene accession IDs	NCBI-KOG	TFs
Ca4_P4_15764982	Ca4	15764982	AATATATATATATATATATATATATATATAT/AATATATATATATATATATATATATAT	GGGGCTAAGGAGTTTTGTGA	CGTCGCTGTGGCATTAAATA	59.2	250	INTERGENIC	--	--	--
Ca4_P4_1592013	Ca4	1592013	GATATATATATATATATA/GATATATATATATATATATA	CCCTAATGGGTGAGCAGTTC	GTTGGTCAATCCACTCGGTC	59.6	706	INTERGENIC	--	--	--
Ca4_P4_15937811	Ca4	15937811	TC/T	TCAGTTGATGTTCTGCCGAC	AACTATGTGCACCTACCGCC	59.8	401	INTERGENIC	--	--	--
Ca4_P4_15939075	Ca4	15939075	AT/ATT	GTGCACCGGTAACGAAGTTT	TTCGTTGGAGGTTTGGATTC	60.0	452	INTERGENIC	--	--	--
Ca4_P4_15952606	Ca4	15952606	AATTTATTTA/AATTTATTTATTTA	ATTTCAACGTCAATCACGCA	CCCATGTTAGACACGCATGA	60.1	580	INTERGENIC	--	--	--
Ca4_P4_15960155	Ca4	15960155	AGG/AG	ACGATTTTCATCCAAGCCAC	CGAGCAAAAGGTAAAACCCA	59.9	882	INTERGENIC	--	--	--
Ca4_P4_15960864	Ca4	15960864	TAGAGA/TAGA	TGGGTTTTACCTTTTGCTCG	ATTCGGTGAGGCCAATTACA	60.1	695	INTERGENIC	--	--	--
Ca4_P4_15962571	Ca4	15962571	ACTT/ACTTCTCTT	CAGGAAATTGTGCATCATGG	TGGTGCATGACTGAATGGTT	59.9	723	INTERGENIC	--	--	--
Ca4_P4_15981160	Ca4	15981160	AGAGAG/AGAGAGGGAGAG	TGGCCCTCTATTCGTGATTC	ATTGTTGCTTTGGCTCACCT	60.0	807	INTERGENIC	--	--	--
Ca4_P4_15988053	Ca4	15988053	ATTATTTATTTTATTTATTTTATT/ATTATTTATTTTATTTATTTTATTTATTTTATT	TTCGGGTGGTTTTAGTCCAG	GTTTCAATGAGGTTGGCACC	60.0	484	INTERGENIC	--	--	--

INDEL marker IDs	Chromosomes/ unanchored scaffolds	Physical positions (bp)	InDels (*Kabuli* reference genome-CDC Frontier/PII)	Forward primers (5'-3')	Reverse primers (5'-3')	Anneling temperature (0C)	Expected amplified product size (bp)	Structural annotation		Functional annotation	
								Sequence components of *kabuli* genome	*Kabuli* gene accession IDs	NCBI-KOG	TFs
Ca4_P4_15990734	Ca4	15990734	ATTTTTTTT/ATTTTTTTTTTTT	TGGCAAAACGGACTACCATT	TGCACTTACATGTATCCAATATTCC	60.4	740	INTERGENIC	--	--	--
Ca4_P4_15990786	Ca4	15990786	AA/AACTGTGA	TGGCAAAACGGACTACCATT	TGCACTTACATGTATCCAATATTCC	60.4	740	INTERGENIC	--	--	--
Ca4_P4_16015560	Ca4	16015560	ACTCT/ACT	GGTGGAGAATGAAACGGAGA	TTTGGGCTGAAGCCATAAAC	60.1	402	INTERGENIC	--	--	--
Ca4_P4_16028033	Ca4	16028033	CT/C	ATGCAACCTCCATTAGCCTG	TTGGTTTCTAACCCAACTCATATTC	60.1	322	INTERGENIC	--	--	--
Ca4_P4_16028817	Ca4	16028817	CGTGTGT/CGTGTGTGT	TGATTTTACACAAGGGTAATTTTG	CGCCGCATACTGTAATGGTA	57.3	951	INTERGENIC	--	--	--
Ca4_P4_16035272	Ca4	16035272	GTT/GTTT	TTCATCCTTGTTGGCATGTG	GGAATCCCTTTGTTGTTGGA	60.5	879	INTERGENIC	--	--	--
Ca4_P4_16056793	Ca4	16056793	TAAAAAA/TAAAAAAA	CTGCAATGCAACAACCAATC	TCAAAAAGAATCCCGACCTG	60.1	657	INTERGENIC	--	--	--
Ca4_P4_16057308	Ca4	16057308	CG/C	CAGGTCGGGATTCTTTTTGA	CATACTTACCCGTGCAGCAA	60.0	705	INTERGENIC	--	--	--
Ca4_P4_16064554	Ca4	16064554	T/TTA	TTGCAAGTAGCTTCCCCATT	AGGTGAGGTGAGGTGATTGA	59.7	820	INTERGENIC	--	--	--
Ca4_P4_16066119	Ca4	16066119	GTTGAAATTGAAATT/GTTGAAATTGAAATTGAAATT	GATGAAGCAGGGTTTTGCAT	TCCAATGCACTTCAATCGAG	60.1	211	INTERGENIC	--	--	--

INDEL marker IDs	Chromosomes/ unanchored scaffolds	Physical positions (bp)	InDels (*Kabuli* reference genome-CDC Frontier/PII)	Forward primers (5'-3')	Reverse primers (5'-3')	Anneling temperature (0C)	Expected amplified product size (bp)	Structural annotation		Functional annotation	
								Sequence components of *kabuli* genome	*Kabuli* gene accession IDs	NCBI-KOG	TFs
Ca4_P4_16067505	Ca4	16067505	CAAAAAAAA/CAAAAAAA	GGCTAAATTAAATCACTGGTCCC	TACATCTGCGGCCCTTTAAC	60.1	572	INTERGENIC	--	--	--
Ca4_P4_16080244	Ca4	16080244	GTTTTTTT/GTTTTTTTT	TTCCTCACGTGATCCCTTTC	TTTCCATCTCCAATAACATCAAA	60.1	303	INTERGENIC	--	--	--
Ca4_P4_16080441	Ca4	16080441	TAAAAAAAAAAAA/TAAAAAAAAAA	TTCCTCACGTGATCCCTTTC	ACAATTGCTCCAACGGTCAT	60.1	567	INTERGENIC	--	--	--
Ca4_P4_16086000	Ca4	16086000	CAAAA/CAAAAAA	TGAACACGAGTCCTTCCCTT	TCCTCCAACTCCAAACAACC	59.7	423	INTERGENIC	--	--	--
Ca4_P4_16088405	Ca4	16088405	ATTTATTTT/ATTTATTTTTCTTTATTTT	AGCGTTGATTAACTGCGTGA	TCCGAAAAGCAGCAATTCTC	59.5	860	INTERGENIC	--	--	--
Ca4_P4_16088431	Ca4	16088431	GAT/GATCAT	AGCGTTGATTAACTGCGTGA	CCCTCACTCACGATCTTTCC	59.5	906	INTERGENIC	--	--	--
Ca4_P4_16101561	Ca4	16101561	GT/G	CATCCACTCCATTGTCATGC	ACCTTCAAATCACGCGAAAC	59.9	617	INTERGENIC	--	--	--
Ca4_P4_16120236	Ca4	16120236	AATATATATATATATATATA/AATATATATATATATATA	TCGAAAGCATTCTTCTCCAAA	TGGTTTGACCTATTTATGGTTGG	59.9	550	INTERGENIC	--	--	--
Ca4_P4_16129990	Ca4	16129990	TTATATATATATATATATAT/TTATATATATATATAT	AGCTCTAACAAGCGGGAACA	GAGGTGTCAAATAAATAAAGAGGAA	60.0	575	INTERGENIC	--	--	--
Ca4_P4_16138106	Ca4	16138106	TTTATTATTATTATTATTATTATTATTATT/TTTATTATTATTATTATTATTATTATTATT	CAATGACATTACTTGGGCCTT	GGGAGAAGAAAAGAATGGGAA	59.0	391	INTERGENIC	--	--	--

INDEL marker IDs	Chromosomes/ unanchored scaffolds	Physical positions (bp)	InDels (*Kabuli* reference genome-CDC Frontier/PII)	Forward primers (5'-3')	Reverse primers (5'-3')	Anneling temperature (0C)	Expected amplified product size (bp)	Structural annotation		Functional annotation	
								Sequence components of *kabuli* genome	*Kabuli* gene accession IDs	NCBI-KOG	TFs
Ca4_P4_16175560	Ca4	16175560	CATATATATATATA/CATATATATATA	TGTTTGCTTGCTTTTGATGC	GGTGTGTGTAGGGGAAGGAA	60.0	269	INTERGENIC	--	--	--
Ca4_P4_16227307	Ca4	16227307	CTTTTTTTTT/CTTTTTTTTTT	TGAGGCTAAATAGAACCTGGTCA	TTTGGCTAACTAATATCGCAGTTC	60.1	530	INTERGENIC	--	--	--
Ca4_P4_16238878	Ca4	16238878	TTATATATATATATATATATATATA/TTATATATATATATATATATATA	TGTTGTTGCGTTATTCCAGC	CACCCATCGTTTCCCTCTTA	59.7	555	INTERGENIC	--	--	--
Ca4_P4_16257918	Ca4	16257918	ACATCAAAT/ACATCAAATTCATCAAAT	CATCGATGCATTTATGCCAG	CGAACATCGCAGCAAGATAA	60.1	891	INTERGENIC	--	--	--
Ca4_P4_16289827	Ca4	16289827	TAAAAAAAA/TAAAAAAAAA	GGCCGATGGGTAACTCAGTA	TTTCGCCACTCAAGATCGTA	60.0	625	INTERGENIC	--	--	--
Ca4_P4_16481759	Ca4	16481759	CA/CAAA	TCGAGACACCAACACCGTAA	AATTCGTCAAACCCACAACC	60.2	732	INTERGENIC	--	--	--
Ca4_P4_16494222	Ca4	16494222	AATTATTATTATTATTATTATTATTATTATTATTATTATTATTAT/AATTATTATTATTATTATTATTATTATTATTATTAT	CTCCCCAGTATTAGCCCCTC	TTAATTAATCGCAAATTGTCCA	59.9	766	INTERGENIC	--	--	--
Ca4_P4_16785536	Ca4	16785536	CATATATATATATATATATATATATA/CATATATATATATATATATA	AATATGGGCCCACTAAACCC	TTTTGGCCTAACTGGCCTAC	59.9	611	INTERGENIC	--	--	--
Ca4_P4_16793556	Ca4	16793556	AG/AGG	ACACTTGCTATGAAACGGGG	ACGCTTTGCAAGGTCTGAAT	60.0	590	INTERGENIC	--	--	--
Ca4_P4_16803738	Ca4	16803738	CATATATATATATATATATATATATATATATATATATA/CATATATATATATATATATATATATATATATATATATATATATATATA	CTACCGGAAATGTTGGCAAT	AGTGGCAGGTGGATACTTCG	59.8	869	INTERGENIC	--	--	--

INDEL marker IDs	Chromosomes/ unanchored scaffolds	Physical positions (bp)	InDels (*Kabuli* reference genome-CDC Frontier/PII)	Forward primers (5'-3')	Reverse primers (5'-3')	Anneling temperature (0C)	Expected amplified product size (bp)	Structural annotation		Functional annotation	
								Sequence components of *kabuli* genome	*Kabuli* gene accession IDs	NCBI-KOG	TFs
Ca4_P4_16824557	Ca4	16824557	TCCCC/TCCC	GCGTAGAGGAATGGAATGGA	GCGACCATCTGTTGCAGTTA	60.0	526	INTERGENIC	--	--	--
Ca4_P4_16862465	Ca4	16862465	ATTTTTTTTTTT/ATTTTTTTTTT	AACCAAACCAACATTTTGTCG	CTCGGTCATTTGTGTTTGACA	59.8	898	INTERGENIC	--	--	--
Ca4_P4_16922622	Ca4	16922622	TAA/TAAA	GAAACTTTGTCCAAGCTGGC	AATCTGGGATGCGACTTGTT	59.9	507	INTERGENIC	--	--	--
Ca4_P4_16943222	Ca4	16943222	TATGAATGAAT/TATGAATGAATGAAT	CTCAAGCTCAGTCCCAGTCC	TGCGTTTTGTTTGGTGTAGC	60.0	668	INTERGENIC	--	--	--
Ca4_P4_17001198	Ca4	17001198	TAAAAAAAA/TAAAAAA	CGATGGTGGTTGTGAAAGTG	TTGGGAAGAGAAAAGCCAAA	60.0	359	INTERGENIC	--	--	--
Ca4_P4_17001569	Ca4	17001569	ATTTTTT/ATTTTTTT	TTTGGCTTTTCTCTTCCCAA	AAAAATGGTTCGAAGGGGAG	59.8	217	INTERGENIC	--	--	--
Ca4_P4_17001899	Ca4	17001899	TAAAAAAAAAA/TAAAAAAAAA	CCCCTTCGAACCATTTTTCT	TTCCCCGAGTTTTCTAACCA	60.3	696	INTERGENIC	--	--	--
Ca4_P4_17015655	Ca4	17015655	GTCT/GT	CCACGCTTTTACCTCTCTGC	TTCATTTCTTGCCTTTGGCT	60.0	768	INTERGENIC	--	--	--
Ca4_P4_17015921	Ca4	17015921	TAAA/TAAAA	GGTTGCTTTTAATTCAACAGTGG	GCATCACCAGGTCTGGATTT	59.9	575	INTERGENIC	--	--	--
Ca4_P4_17019455	Ca4	17019455	CTT/CTTT	GAAACCGTCCAAAACTGCAT	AGTGAGTGATTGTGGGCACT	60.0	115	INTERGENIC	--	--	--

INDEL marker IDs	Chromosomes/ unanchored scaffolds	Physical positions (bp)	InDels (*Kabuli* reference genome-CDC Frontier/PII)	Forward primers (5'-3')	Reverse primers (5'-3')	Anneling temperature (0C)	Expected amplified product size (bp)	Structural annotation		Functional annotation	
								Sequence components of *kabuli* genome	*Kabuli* gene accession IDs	NCBI-KOG	TFs
Ca4_P4_17023946	Ca4	17023946	ATGATTAATTG/ATGATTAATTGATTAATTG	TCGAAGGATGTCTCAAGGCT	TCGGAGTTGATCAAACGAATC	60.0	462	INTERGENIC	--	--	--
Ca4_P4_17041835	Ca4	17041835	TAAAAAAAAAAA/TAAAAAAAAAA	AACGTTTGGGTCTCTCGTTG	TCAATCATTATTAAAACTGCCGAA	60.1	491	INTERGENIC	--	--	--
Ca4_P4_17071722	Ca4	17071722	ATTTTTTTTT/ATTTTTTTTTT	GGGCTGCTACATAAACCGAA	TCCGACATGCCATTTTGTTA	60.1	189	INTERGENIC	--	--	--
Ca4_P4_17095718	Ca4	17095718	TAAAAAAA/TAAAAAA	CGGTCATCATTGAGGGCTAT	TCCATATGTGCCCATTGCTA	59.9	694	INTERGENIC	--	--	--
Ca4_P4_17096036	Ca4	17096036	CTTTCAA/CTTTCAAGTTTCAA	CTGCAATTGTCAAGCTCCAA	TCCATATGTGCCCATTGCTA	60.0	276	INTERGENIC	--	--	--
Ca4_P4_17157304	Ca4	17157304	CAAAAAAAAAA/CAAAAAAAAAAAA	ATCTCCATCTCTGGACCGTG	ACGACGCATAGCAAGGTTCT	60.1	691	INTERGENIC	--	--	--
Ca4_P4_17168334	Ca4	17168334	GTA/GTATATGATATAATA	CTCTACCCTTGAGAGTGTGTGG	TGGACGGAGTATGCATTATTGA	58.9	581	INTERGENIC	--	--	--
Ca4_P4_17171401	Ca4	17171401	AGTGTGT/AGTGTGTGT	GATGGAGGGCAGTAACGTGT	GTGCTCTCAAAGTCGCACAA	60.0	465	INTERGENIC	--	--	--
Ca4_P4_17186848	Ca4	17186848	TC/T	CCGGCATCACTCGTTAAGAT	TGATTTGATCAGAGGTATGTGTCA	60.1	549	INTERGENIC	--	--	--
Ca4_P4_17186998	Ca4	17186998	AAATTA/AA	TTTGACACATACCTCTGATCAAATC	TCATCCCCTTAAGATTCCGA	59.4	521	INTERGENIC	--	--	--

INDEL marker IDs	Chromosomes/ unanchored scaffolds	Physical positions (bp)	InDels (*Kabuli* reference genome-CDC Frontier/PII)	Forward primers (5'-3')	Reverse primers (5'-3')	Anneling temperature (0C)	Expected amplified product size (bp)	Structural annotation		Functional annotation	
								Sequence components of *kabuli* genome	*Kabuli* gene accession IDs	NCBI-KOG	TFs
Ca4_P4_17196085	Ca4	17196085	TTCTCTCTCTCTCTCTCTCTCTCTCTCT/TTCTCTCTCTCTCTCTCTCTCTCTCTCTCTCT	CCTCAACCATGGCCTGTACT	AGTTGCATTGGAGACAAGGG	60.0	118	INTERGENIC	--	--	--
Ca4_P4_17206399	Ca4	17206399	ATTTTTT/ATTTTT	AACCGTTTGATGCGAAAAAT	ATCTATTGCCATCTGCCAGG	59.5	925	INTERGENIC	--	--	--
Ca4_P4_17206661	Ca4	17206661	T/TA	AATCAATCAACCAAGAACGGA	ATCTATTGCCATCTGCCAGG	59.4	598	INTERGENIC	--	--	--
Ca4_P4_17240626	Ca4	17240626	ATGTGT/ATGTGTGT	TGGTCTAAAAAGATTGGCCG	TACGTGGCAAACATCCAAAA	60.1	537	INTERGENIC	--	--	--
Ca4_P4_17242170	Ca4	17242170	GTCAT/GT	CTACATGAAGGGGCTCAACC	TCCAAGGACCGAGCTCATTA	59.6	515	INTERGENIC	--	--	--
Ca4_P4_17243538	Ca4	17243538	AT/A	TTCACACATGCATTGTTTACCA	TGCAAAACTGCTCTACCCAA	59.9	422	INTERGENIC	--	--	--
Ca4_P4_17247653	Ca4	17247653	GAAAAAAAA/GAAAAAAAAAA	TTTATGTTTGGCCAACTCCC	TTTTTAGGGACGCAATTCAAA	59.8	539	INTERGENIC	--	--	--
Ca4_P4_17248126	Ca4	17248126	GATTAAATTAAATTAAATTAAATTAAATTAAATTAAATTAAATTAAATT/GATTAAATTAAATTAAATTAAATTAAATTAAATTAAATTAAATT	TTTGAATTGCGTCCCTAAAAA	TTTTGGCATGAATTTGGGAT	59.6	653	INTERGENIC	--	--	--
Ca4_P4_17248915	Ca4	17248915	T/TC	ATCCCAAATTCATGCCAAAA	GCCCTCTTTTGAGTTGGAAA	60.1	396	INTERGENIC	--	--	--
Ca4_P4_17250760	Ca4	17250760	A/AC	GCCGAAAAACCAAAGTGTGT	CGTTGGATTCCTTTCCTCTC	60.0	514	INTERGENIC	--	--	--

INDEL marker IDs	Chromosomes/ unanchored scaffolds	Physical positions (bp)	InDels (*Kabuli* reference genome-CDC Frontier/PII)	Forward primers (5'-3')	Reverse primers (5'-3')	Anneling temperature (0C)	Expected amplified product size (bp)	Structural annotation		Functional annotation	
								Sequence components of *kabuli* genome	*Kabuli* gene accession IDs	NCBI-KOG	TFs
Ca4_P4_17251218	Ca4	17251218	TTTT/TTTTACATCTCGATTT	GAGAGGAAAGGAATCCAACG	CAAATTGTCGAACCATGTCG	58.7	653	INTERGENIC	--	--	--
Ca4_P4_17251690	Ca4	17251690	TAAT/TAATCAAT	GCTCGATAAAGCAGTCGTCC	GCCAAATCGAACCAACTTTT	60.0	577	INTERGENIC	--	--	--
Ca4_P4_17252021	Ca4	17252021	ATTT/ATT	GCTCGATAAAGCAGTCGTCC	CAACGTAACACGCCTCTTGA	60.0	661	INTERGENIC	--	--	--
Ca4_P4_17254750	Ca4	17254750	GTAATAA/GTAA	CAAATATTGTGCCAGCCAAA	TCTTAATCTTGACCAGCCCG	59.6	898	INTERGENIC	--	--	--
Ca4_P4_17257974	Ca4	17257974	AATA/AATATA	ACATGGCAGGTTGGATTCAT	TCATGGGTACCCCACCTTAG	60.2	605	INTERGENIC	--	--	--
Ca4_P4_17258294	Ca4	17258294	ATTTTTT/ATTTTTTT	ACATGGCAGGTTGGATTCAT	ATTGGTAGGCATGGCAAGAC	60.2	634	INTERGENIC	--	--	--
Ca4_P4_17259048	Ca4	17259048	CATATATATATATATATAT/CATATATATATATATAT	GCAAACTTTTTCACTTGCCC	TTCTGAAACAAATGCACCCA	59.7	604	INTERGENIC	--	--	--
Ca4_P4_17259358	Ca4	17259358	TAAA/TAAAA	ATGGGTGCATTTGTTTCAGA	GCCTGAGTTTGGCATACGAT	59.0	338	INTERGENIC	--	--	--
Ca4_P4_17290057	Ca4	17290057	TAAAAAA/TAAAAAAA	ATGCAGTATTCCCATCAGCC	TGTTTCATTGACACCATCCAA	59.9	843	INTERGENIC	--	--	--
Ca4_P4_17290739	Ca4	17290739	A/AC	TGAAAGCCAAGAATGGCATA	CGTCACGCGATAATTTTGTG	59.3	163	INTERGENIC	--	--	--

INDEL marker IDs	Chromosomes/ unanchored scaffolds	Physical positions (bp)	InDels (*Kabuli* reference genome-CDC Frontier/PII)	Forward primers (5'-3')	Reverse primers (5'-3')	Anneling temperature (0C)	Expected amplified product size (bp)	Structural annotation		Functional annotation	
								Sequence components of *kabuli* genome	*Kabuli* gene accession IDs	NCBI-KOG	TFs
Ca4_P4_17313148	Ca4	17313148	TC/T	AGAGGATGCAGCTATGCGTT	CAACATTTTGGGAGGGAAGA	60.0	170	INTERGENIC	--	--	--
Ca4_P4_17313521	Ca4	17313521	TAAA/TAA	GCTAGACAGGTTTGCCTTCG	AAGGCTCCTGCAAGCATTTA	60.0	966	INTERGENIC	--	--	--
Ca4_P4_17321954	Ca4	17321954	TA/TATATATATATAAA	CTGCCGACCTGAATACCAAT	TGCCTTTAATGAAACACACCA	60.0	541	INTERGENIC	--	--	--
Ca4_P4_17323828	Ca4	17323828	CATATA/CATA	CTTGGTACAGGTTTGGCGAT	TCTTGTGGTTGTGGGTTTCA	60.0	490	INTERGENIC	--	--	--
Ca4_P4_17324366	Ca4	17324366	TAAAA/TAAA	TGAAACCCACAACCACAAGA	CCCGACTTCTTTCTCAGCAC	60.0	567	INTERGENIC	--	--	--
Ca4_P4_17327959	Ca4	17327959	CATGTTTTTAAAATGTTTTTAAA/CATGTTTTTAAA	GGTGTTACTGTTGAAGCGGG	ATATTGCACGTGTCGCACTC	60.6	454	INTERGENIC	--	--	--
Ca4_P4_17328665	Ca4	17328665	GAA/GAAAA	ACACGTGCAATATCGGTGAA	TCCTCCCTCTTTATCATGCC	60.0	750	INTERGENIC	--	--	--
Ca4_P4_17328879	Ca4	17328879	GTTTTTT/GTTTTTTT	CGTGATAAAACGTGCATCAA	TCCTCCCTCTTTATCATGCC	58.2	244	INTERGENIC	--	--	--
Ca4_P4_17329310	Ca4	17329310	AC/A	GGCATGATAAAGAGGGAGGA	TAACCTCTCACGTGCAATCG	59.1	386	INTERGENIC	--	--	--
Ca4_P4_17332259	Ca4	17332259	GAAAAAAAAAAAA/GAAAAAAAAAA	CGGCAAAATTACATCTGTGG	GCGCATTGTCTAGCATTGAA	59.0	704	INTERGENIC	--	--	--

INDEL marker IDs	Chromosomes/ unanchored scaffolds	Physical positions (bp)	InDels (*Kabuli* reference genome-CDC Frontier/PII)	Forward primers (5'-3')	Reverse primers (5'-3')	Anneling temperature (0C)	Expected amplified product size (bp)	Structural annotation		Functional annotation	
								Sequence components of *kabuli* genome	*Kabuli* gene accession IDs	NCBI-KOG	TFs
Ca4_P4_17332738	Ca4	17332738	TAATAAATAAATAAA/TAATAAATAAATAAATAAA	GATGCAACTTCTTGGGCAAT	TTGTCACCGAACTGCATCAT	60.1	589	INTERGENIC	--	--	--
Ca4_P4_17339892	Ca4	17339892	ATTT/ATT	TTTTGGGGAACACAACCTTT	GACATTTCTCATAGGGGCGA	59.3	297	INTERGENIC	--	--	--
Ca4_P4_17348770	Ca4	17348770	TCCATATATTGAATC/T	TGGATTTCACAACTTTGCCA	ATTTGGACCATTGGCTGAAC	60.1	405	INTERGENIC	--	--	--
Ca4_P4_17367149	Ca4	17367149	AATAT/AAT	GCAGCTAAAGGAGAGTGCCA	AGACAGTTCGCTCGCAAATC	60.7	506	INTERGENIC	--	--	--
Ca4_P4_17367667	Ca4	17367667	AC/ACC	TAAATGTGATTTGCGAGCGA	ATGCATGGAGGTGATGATGA	60.4	284	INTERGENIC	--	--	--
Ca4_P4_17367739	Ca4	17367739	A/AAAATGTAA	TAAATGTGATTTGCGAGCGA	ATGCATGGAGGTGATGATGA	60.4	284	INTERGENIC	--	--	--
Ca4_P4_17421137	Ca4	17421137	GT/GTT	CCCGTGAGTTGTTGGAAGTT	ATGCCCTCATTGGAACTTGA	60.0	351	INTERGENIC	--	--	--
Ca4_P4_17421248	Ca4	17421248	G/GTTTAA	CCCGTGAGTTGTTGGAAGTT	TCGATAATTTGAAACCGCCT	60.0	569	INTERGENIC	--	--	--
Ca4_P4_17424073	Ca4	17424073	CATATATAT/CATATATATAT	GAGTGAGTGATGAGGGATGGA	GGGGACAAAGACATAATGGAGA	60.1	321	INTERGENIC	--	--	--
Ca4_P4_17445543	Ca4	17445543	GTTTTTTTTTTTT/GTTTTTTTTTT	CCCCTTTCAACATTCCTCAA	CCAGAACATGCCTTCGATTT	59.9	891	INTERGENIC	--	--	--

INDEL marker IDs	Chromosomes/ unanchored scaffolds	Physical positions (bp)	InDels (*Kabuli* reference genome-CDC Frontier/PII)	Forward primers (5'-3')	Reverse primers (5'-3')	Anneling temperature (0C)	Expected amplified product size (bp)	Structural annotation		Functional annotation	
								Sequence components of *kabuli* genome	*Kabuli* gene accession IDs	NCBI-KOG	TFs
Ca4_P4_17460394	Ca4	17460394	TAAA/TAA	TGAAGGGCCATATTTG ACAAT	GAACGTATGGACCACC GATT	59.3	574	INTERGENIC	--	--	--
Ca4_P4_17463034	Ca4	17463034	A/AT	AGAAATAAAATCACGA GTAACCAAA	TTGCGGATTCCCAGTT ACTC	57.6	734	INTERGENIC	--	--	--
Ca4_P4_17475440	Ca4	17475440	GT/GTTGTTATT	CGGGCAGTAGCATTAG ATGG	GGATGAATGGGAGGTG GTTA	60.6	210	INTERGENIC	--	--	--
Ca4_P4_17532051	Ca4	17532051	AT/A	ACTGGACTGGTCATCG AACC	GTCGCAGTTGCACCCT TTAT	60.0	114	INTERGENIC	--	--	--
Ca4_P4_17532611	Ca4	17532611	ATAAGTAAG/ATAAG	CCGATGGAGGTGAAAG AGAG	GCACGGTACAACTCAC CAGA	59.8	691	INTERGENIC	--	--	--
Ca4_P4_17533077	Ca4	17533077	CAGTG/CAGTGCATAAGTG	TGGTGAGTTGTACCGT GCTC	AAAATGGCACCAGCAA AAAG	59.8	358	INTERGENIC	--	--	--
Ca4_P4_17533461	Ca4	17533461	AA/AATTA	CTTTTTGCTGGTGCCA TTTT	TGCAAGAAGAAGGAAG CCAT	60.1	713	INTERGENIC	--	--	--
Ca4_P4_17533529	Ca4	17533529	GT/G	CTTTTTGCTGGTGCCA TTTT	TGCAAGAAGAAGGAAG CCAT	60.1	713	INTERGENIC	--	--	--
Ca4_P4_17533740	Ca4	17533740	TAAAAAAA/TAAAAAAAA	TGTCCATAATAAATATC GCATTTCA	TGCAAGAAGAAGGAAG CCAT	59.6	377	INTERGENIC	--	--	--
Ca4_P4_17534710	Ca4	17534710	ATATT/AT	CTGGATCCCCTCGGAA GTAT	AGAAACCGCACACACA CAAA	60.3	316	INTERGENIC	--	--	--

INDEL marker IDs	Chromosomes/ unanchored scaffolds	Physical positions (bp)	InDels (*Kabuli* reference genome-CDC Frontier/PII)	Forward primers (5'-3')	Reverse primers (5'-3')	Anneling temperature (0C)	Expected amplified product size (bp)	Structural annotation		Functional annotation	
								Sequence components of *kabuli* genome	*Kabuli* gene accession IDs	NCBI-KOG	TFs
Ca4_P4_17534970	Ca4	17534970	AAAA/AAAAACATATTTCAAA	TTTTTGTGTGTGTGCGGTTT	TGTGTCAAAGATGGTATTTCAATG	60.1	410	INTERGENIC	--	--	--
Ca4_P4_17545144	Ca4	17545144	TATTAAAATAATAATTA/TATTAAAATAATAATTAAAATAATAATTA	ATCCAAAAGGTCGTCCACAG	AAATCGGTTTCCCCAGTACC	60.0	652	INTERGENIC	--	--	--
Ca4_P4_17588721	Ca4	17588721	CTTT/CTT	TAACGATGATGACACGCTCC	TCACATTTTGTCACCTCCGA	59.7	455	INTERGENIC	--	--	--
Ca4_P4_17600797	Ca4	17600797	TC/T	AAGGGTGATGGACGAAAAAC	TTAAATGGTCGCATAGAGCAA	58.9	682	INTERGENIC	--	--	--
Ca4_P4_17600811	Ca4	17600811	TAAAAAA/TAAAAAAA	AAGGGTGATGGACGAAAAAC	TCCAACAACATCAAATTCATCC	58.9	951	INTERGENIC	--	--	--
Ca4_P4_17601611	Ca4	17601611	AATTAACTATTAACTATTAACTATT/AATTAACTATTAACTATTAACTATTAACTATT	ACACAATCACCCTTGTTCCC	CCGTGTCTATCCCCATTTTG	59.7	211	INTERGENIC	--	--	--
Ca4_P4_17601888	Ca4	17601888	TAAA/TAAAA	CAAAATGGGGATAGACACGG	TTTCAAATGAACGTTTGCTACA	60.2	440	INTERGENIC	--	--	--
Ca4_P4_17602358	Ca4	17602358	GT/GTT	TTTGAATGTGTATTGTAGCAAACG	CCTATTCTCGAGAGCAACCG	59.2	322	INTERGENIC	--	--	--
Ca4_P4_17628350	Ca4	17628350	CATATATATATATA/CATATATATA	GCAGACCACTTCATGCAAAA	TGGATCACCGATTGCTTGTA	59.8	250	INTERGENIC	--	--	--
Ca4_P4_1763098	Ca4	1763098	ATTTTTTTT/ATTTTTTTTTT	TGATAAGGGTATGTGATTTTGGTT	ACATTTTGGAAGGGCATCTG	58.7	477	INTERGENIC	--	--	--

INDEL marker IDs	Chromosomes/ unanchored scaffolds	Physical positions (bp)	InDels (*Kabuli* reference genome-CDC Frontier/PII)	Forward primers (5'-3')	Reverse primers (5'-3')	Anneling temperature (0C)	Expected amplified product size (bp)	Structural annotation		Functional annotation	
								Sequence components of *kabuli* genome	*Kabuli* gene accession IDs	NCBI-KOG	TFs
Ca4_P4_1764559	Ca4	1764559	TAAAAAAAAAA/TAAAAAAAAAAA	GCCAACTCATGTCTTTGTCG	ATTCTCGTGAGTGGGGTTTG	59.3	758	INTERGENIC	--	--	--
Ca4_P4_17650207	Ca4	17650207	AAGA/AA	CCAAGAGTTCAATACGAGCCA	TGCGGTTCCTATAAAATGGG	60.3	251	INTERGENIC	--	--	--
Ca4_P4_17650571	Ca4	17650571	TTATATATATAT/TTATATATATATATAT	CCCATTTTATAGGAACCGCA	TTGGTTTTTGGAACAACGTG	59.8	355	INTERGENIC	--	--	--
Ca4_P4_17650657	Ca4	17650657	GAA/GAAA	CCCATTTTATAGGAACCGCA	TGCTCCAGAAAACGAACTCA	59.8	809	INTERGENIC	--	--	--
Ca4_P4_17675509	Ca4	17675509	AAATAATAATAATAATA/AAATAATAATAATA	AAACTTGCCATGTGGTTGGT	CTAGCACATGATGCCCTCAA	60.3	471	INTERGENIC	--	--	--
Ca4_P4_17677705	Ca4	17677705	AT/A	GGGGGCCAAAAATTAAGAGA	ATATTGGGGATGATGTGGGA	60.3	528	INTERGENIC	--	--	--
Ca4_P4_17713411	Ca4	17713411	TAA/TA	TCCGTCGTTTATTCTTTGGC	TGAATCGTTTTGGAAAATTCG	60.1	673	INTERGENIC	--	--	--
Ca4_P4_17715354	Ca4	17715354	AATAA/AATAACAATATTCCATAA	TCAATTTGGAGGGTGACTCC	TACCAGCTTTGGTTTCTGCC	59.9	250	INTERGENIC	--	--	--
Ca4_P4_17716045	Ca4	17716045	ACT/ACTCT	TTTAGTGTGACGGGATGGTG	GGTTTGGAGAAACAAAACTTGG	59.4	624	INTERGENIC	--	--	--
Ca4_P4_17717044	Ca4	17717044	TGG/TG	TCTGTCAAAGTCGGTGCAAG	ATAATGGCGCCACAGAAAAG	60.0	821	INTERGENIC	--	--	--

INDEL marker IDs	Chromosomes/ unanchored scaffolds	Physical positions (bp)	InDels (*Kabuli* reference genome-CDC Frontier/PII)	Forward primers (5'-3')	Reverse primers (5'-3')	Anneling temperature (0C)	Expected amplified product size (bp)	Structural annotation		Functional annotation	
								Sequence components of *kabuli* genome	*Kabuli* gene accession IDs	NCBI-KOG	TFs
Ca4_P4_17734744	Ca4	17734744	ATTTAAAAAAAATT/AT	AACAAAAATCGCAATC CACC	GAAGTTAACTCCAATC GGGC	59.8	660	INTERGENIC	--	--	--
Ca4_P4_17735574	Ca4	17735574	T/TA	TCACGATCGAAAAACC TATTTG	AAACATGACAAAAGTTG ATGTTGAA	59.1	916	INTERGENIC	--	--	--
Ca4_P4_17735792	Ca4	17735792	TC/TCC	AAGAATTTTGATGACGT GACAATC	TGGGAGTGCATAATGT TTGA	59.4	787	INTERGENIC	--	--	--
Ca4_P4_17738882	Ca4	17738882	AC/A	TTCTTTCTCTTGGTGTT GCG	TTGCTGTATCAAGTGG CAAAA	59.0	745	INTERGENIC	--	--	--
Ca4_P4_17739507	Ca4	17739507	CAAAAAAA/CAAAAA	TCGGGATAGTGGATGA GGTAA	CGCTTGACCTCTAATC AGGC	59.4	539	INTERGENIC	--	--	--
Ca4_P4_1774381	Ca4	1774381	CCTTCTT/CCTTCTTCTT	CACACGAGTGTCGCCT AAGA	TGGTATCCAGAGCCAT CACA	60.0	437	INTERGENIC	--	--	--
Ca4_P4_17744056	Ca4	17744056	TAATAAATAAATAAA/TAATAAATAAA	TGCGAGTCTAACATTA AAACCTC	GCGGAGACTTTGACCT TTTG	57.7	745	INTERGENIC	--	--	--
Ca4_P4_17753614	Ca4	17753614	CTTTTTTT/CTTTTTTTT	TGGACGGACAATGCAA TAGA	TTTCAAAATGTTGCTTT AAAAATCT	60.1	751	INTERGENIC	--	--	--
Ca4_P4_17778807	Ca4	17778807	TG/T	TGATTGGGAACCTGGT GAAT	CATGCCAAAATCAACA GCAC	60.2	671	INTERGENIC	--	--	--
Ca4_P4_17786473	Ca4	17786473	TTTTTGT/TT	CGCGCCAACTTTACTT CTTC	TCCAAAATTTAGTGGCA GCA	60.0	621	INTERGENIC	--	--	--

INDEL marker IDs	Chromosomes/ unanchored scaffolds	Physical positions (bp)	InDels (*Kabuli* reference genome-CDC Frontier/PII)	Forward primers (5'-3')	Reverse primers (5'-3')	Anneling temperature (0C)	Expected amplified product size (bp)	Structural annotation		Functional annotation	
								Sequence components of *kabuli* genome	*Kabuli* gene accession IDs	NCBI-KOG	TFs
Ca4_P4_1780181	Ca4	1780181	GAAAAA/GAAAA	AATTCAAGAAGACTGGGGGAA	TTTTTGCCGGTTGAACTGAG	59.9	444	INTERGENIC	--	--	--
Ca4_P4_17821086	Ca4	17821086	AGGGGG/AGGGG	GGAACTGTCGTATTGTTCCCA	AGATGTGGGCATGTGTTTCA	59.8	805	INTERGENIC	--	--	--
Ca4_P4_17823516	Ca4	17823516	TTTCTT/TTT	ATATGCCCCTGACGAACAAC	TGAATGGTTTTAATTCTGCCA	59.8	671	INTERGENIC	--	--	--
Ca4_P4_17823791	Ca4	17823791	TAAAAAAAAAAAA/TAAAAAAAAAA	TCTCATGTCTTGCAAAATTCTTCT	TTTGTGATTGATATTTGCCGTT	59.4	354	INTERGENIC	--	--	--
Ca4_P4_17825306	Ca4	17825306	TAAAA/TAAA	AATCCATCAAACTCGGTTGC	TGCTATTTGGTTGGCCTTTT	59.9	301	INTERGENIC	--	--	--
Ca4_P4_17825457	Ca4	17825457	CGTTATGA/CGTTATGAGTTATGA	AATCCATCAAACTCGGTTGC	GGGCCCCGTAAGTATTCAGT	59.9	679	INTERGENIC	--	--	--
Ca4_P4_17825598	Ca4	17825598	ACC/ACCC	AATCCATCAAACTCGGTTGC	GGGCCCCGTAAGTATTCAGT	59.9	679	INTERGENIC	--	--	--
Ca4_P4_17825906	Ca4	17825906	ATTTTTTT/ATTTTTT	AAAAGGCCAACCAAATAGCA	CCTCAATTCTCGATTTCCCA	59.6	555	INTERGENIC	--	--	--
Ca4_P4_17825981	Ca4	17825981	ATTTTTTTT/ATTTTTTTTT	GGGGCCCCATAATCATATTT	CCTCAATTCTCGATTTCCCA	59.7	164	INTERGENIC	--	--	--
Ca4_P4_17843324	Ca4	17843324	TA/T	GGGGGAGACCTACCGTATCT	AGAAACGGGTTGTGCCTATG	59.3	224	INTERGENIC	--	--	--

INDEL marker IDs	Chromosomes/ unanchored scaffolds	Physical positions (bp)	InDels (*Kabuli* reference genome-CDC Frontier/PII)	Forward primers (5'-3')	Reverse primers (5'-3')	Anneling temperature (0C)	Expected amplified product size (bp)	Structural annotation		Functional annotation	
								Sequence components of *kabuli* genome	*Kabuli* gene accession IDs	NCBI-KOG	TFs
Ca4_P4_17853769	Ca4	17853769	CAAAAAAAAAAA/CAAAAAAAAAA	AATGGACAACATTCATCCCA	GAAACCCTAAAACCCTTCGC	58.6	586	INTERGENIC	--	--	--
Ca4_P4_17865327	Ca4	17865327	TTAAGAGTA/TTA	ATATGGAAAGTCGCCGTTTG	CCGAGGCAATAAAACACCTC	60.0	494	INTERGENIC	--	--	--
Ca4_P4_17865546	Ca4	17865546	TAAAAAA/TAAAAAAA	GAGGTGTTTTATTGCCTCGG	TGTTAATCCCAATTCAATATCCC	59.6	133	INTERGENIC	--	--	--
Ca4_P4_17873043	Ca4	17873043	TAAA/TA	CATTGAAATGCATGGACCAA	TTTTCTCCATGCACCCTACC	60.3	885	INTERGENIC	--	--	--
Ca4_P4_17942733	Ca4	17942733	CTATATATATATATAT/CTATATATATATATATAT	CATCGTCAGCACATCACAAA	CTTAGGAGGTCTTGCCCCTC	59.2	447	INTERGENIC	--	--	--
Ca4_P4_17959700	Ca4	17959700	C/CATTCCCTT	AGTGGCATGTGGGGATTAAA	TTTGGTGGAAATGGCATGTA	60.2	168	INTERGENIC	--	--	--
Ca4_P4_18005269	Ca4	18005269	TA/TAACGAA	CAACGTTGCTACGAGGACAA	CATTCGTCCGTCAGAGTGTG	59.9	663	INTERGENIC	--	--	--
Ca4_P4_18041622	Ca4	18041622	CTTTTTTT/CTTTTTTTT	CCTGTTTTCCTCCACCCTTT	ATGGGGACTTGGGGAGTAAC	60.3	660	INTERGENIC	--	--	--
Ca4_P4_18061059	Ca4	18061059	TATAATAATAATAATAATAATAATAA/TATAATAATAATAATAATAATAATAATAATAA	GAAGCGACCACCTGAAGAAC	CAGAACAAATGACGTGTCCC	59.9	536	INTERGENIC	--	--	--
Ca4_P4_18091373	Ca4	18091373	TCATCAC/T	CCACGGATGTGATTTAGCCT	GAAGCACCGTTCCTTCTTGA	60.0	401	INTERGENIC	--	--	--

INDEL marker IDs	Chromosomes/ unanchored scaffolds	Physical positions (bp)	InDels (*Kabuli* reference genome-CDC Frontier/PII)	Forward primers (5'-3')	Reverse primers (5'-3')	Anneling temperature (0C)	Expected amplified product size (bp)	Structural annotation		Functional annotation	
								Sequence components of *kabuli* genome	*Kabuli* gene accession IDs	NCBI-KOG	TFs
Ca4_P4_18110946	Ca4	18110946	ATTTTTTTTTT/ATTTTTTTTT	TTCTTTGGAGACTGAGTTGGTTC	GACTCGATGAAAATGATGGTGA	59.8	377	INTERGENIC	--	--	--
Ca4_P4_18152849	Ca4	18152849	CTTTTTTTT/CTTTTTTTTT	GTGAGCAGCATTGTGCAGTT	CTCGAGCTTGGTACGAGAGG	60.1	776	INTERGENIC	--	--	--
Ca4_P4_1818935	Ca4	1818935	GAAAAAA/GAAAAAAA	TTGCACTGTTTGGTGAAAGG	TTGTTGGCAATTGAAAAGGAC	59.7	634	INTERGENIC	--	--	--
Ca4_P4_1819613	Ca4	1819613	ATATTA/ATA	TCCTTTTCAATTGCCAACAAC	ATTGCATTTGGAACCCTGAC	60.0	808	INTERGENIC	--	--	--
Ca4_P4_18196212	Ca4	18196212	ATTT/ATTTAATCTTTTT	GTGGTCCAGATTCTCCCAAA	TGTCGCTTCAGCAAAAAGAA	59.9	549	INTERGENIC	--	--	--
Ca4_P4_1819707	Ca4	1819707	ATTTTTTTT/ATTTTTT	CAATTGCCAACAACAAAATCC	ATTGCATTTGGAACCCTGAC	60.2	801	INTERGENIC	--	--	--
Ca4_P4_1820022	Ca4	1820022	CAAA/CAAAA	AAAGTCAGGGTTCCAAATGC	CGATTCCCAATCGCTATCTT	59.0	131	INTERGENIC	--	--	--
Ca4_P4_1825344	Ca4	1825344	TAAAAAAA/TAAAAAA	ATTTGTACGGTTTTGGCTCG	TTTTGGCCCTCGTAAGTTTTT	60.0	612	INTERGENIC	--	--	--
Ca4_P4_1825724	Ca4	1825724	ATTTTTTTT/ATTTTTTTTT	AATGAAATCAGTCGACCCCA	TGTTGCAATGTTGGTTCTTCA	60.3	785	INTERGENIC	--	--	--
Ca4_P4_18265518	Ca4	18265518	CTTTTTTTTTT/CTTTTTTTTTTT	TTCAAAACCAATATCAAAACGC	TTGATTGAAACATTGATTTCCG	59.0	318	INTERGENIC	--	--	--

INDEL marker IDs	Chromosomes/ unanchored scaffolds	Physical positions (bp)	InDels (*Kabuli* reference genome-CDC Frontier/PII)	Forward primers (5'-3')	Reverse primers (5'-3')	Anneling temperature (0C)	Expected amplified product size (bp)	Structural annotation		Functional annotation	
								Sequence components of *kabuli* genome	*Kabuli* gene accession IDs	NCBI-KOG	TFs
Ca4_P4_1826824	Ca4	1826824	TTTTAACAGTTTA/TTTTA	AAAGACCGCATATGTAATTTAGCC	GGAGAGATATGCGGGTTGAA	59.8	577	INTERGENIC	--	--	--
Ca4_P4_1827077	Ca4	1827077	ATTTTTTTT/ATTTTTTT	AAAGACCGCATATGTAATTTAGCC	GGAGAGATATGCGGGTTGAA	59.8	577	INTERGENIC	--	--	--
Ca4_P4_1827115	Ca4	1827115	TA/TAA	AAAGACCGCATATGTAATTTAGCC	GGAGAGATATGCGGGTTGAA	59.8	577	INTERGENIC	--	--	--
Ca4_P4_18388650	Ca4	18388650	ATATTA/ATA	AGTTATCGATCACGGATGCC	CGTGTATATTCGCGCACTCA	59.9	514	INTERGENIC	--	--	--
Ca4_P4_18462391	Ca4	18462391	GTTTTTTTTTTT/GTTTTTTTTTT	AGGAAGGTGGATGCAGATTG	TGGAAGAAAAGGAATCCGAA	60.1	455	INTERGENIC	--	--	--
Ca4_P4_1848187	Ca4	1848187	TATAATAATAA/TATAATAATAATAA	AGAAGCAAAAGAGCCCTTCC	ATAGGAATATAGGCGCGGGT	60.0	323	INTERGENIC	--	--	--
Ca4_P4_18515805	Ca4	18515805	TATAAATAAATAAATAAATAAATAAAT/TATAAATAAATAAATAAATAAATAAATAAAT	TTTTTGAGATTGGTACGGGA	TCAGCGTTTGTTTGTTGACTT	58.1	738	INTERGENIC	--	--	--
Ca4_P4_18530309	Ca4	18530309	TTAATAATAATAATAATAAT/TTAATAATAATAATAATAATAATAAT	TGCGTTGATAATCCAGAGGA	AGGTAACAAGGGCAATGACG	59.2	403	INTERGENIC	--	--	--
Ca4_P4_18554841	Ca4	18554841	TTATATATATATATATATATATATATATATAT/TTATATATATATATATATATATATATATAT	TGGACCATATGTGTTGGGTC	CCCGGATAATTGTTCACTTTG	59.1	809	INTERGENIC	--	--	--
Ca4_P4_1858416	Ca4	1858416	GAAAAA/GAAAA	ATGGTGCACACAAAACAGGA	AAACCGATATGCCCTTTGAA	60.0	788	INTERGENIC	--	--	--

INDEL marker IDs	Chromosomes/ unanchored scaffolds	Physical positions (bp)	InDels (*Kabuli* reference genome-CDC Frontier/PII)	Forward primers (5'-3')	Reverse primers (5'-3')	Anneling temperature (0C)	Expected amplified product size (bp)	Structural annotation		Functional annotation	
								Sequence components of *kabuli* genome	*Kabuli* gene accession IDs	NCBI-KOG	TFs
Ca4_P4_1861853	Ca4	1861853	AT/ATGAACCCT	TGAGGACAAAAGATCCCCTG	AGGGTCCACAGAAAATCGTG	60.0	870	INTERGENIC	--	--	--
Ca4_P4_1861881	Ca4	1861881	GTTTATTTATTTATTTATTTATT/GTTTATTTATTTATTTATTTATTTATT	TGAGGACAAAAGATCCCCTG	AGGGTCCACAGAAAATCGTG	60.0	870	INTERGENIC	--	--	--
Ca4_P4_1863218	Ca4	1863218	TAAAAAAAAAA/TAAAAAAAAAAA	CCCGGGTTTAACTACCCATT	CAGCTACTCGCTGCACACTC	59.9	592	INTERGENIC	--	--	--
Ca4_P4_18651098	Ca4	18651098	AATATATATATATATATATAT/AATATATATATATATATATATAT	TGCTTTCTTTGATGTGCTGG	TTCCCCCAAACATCATTTAGTT	60.0	385	INTERGENIC	--	--	--
Ca4_P4_18703975	Ca4	18703975	GACACA/GACA	CCACATTGACATAAACATCTTCTCA	TCAACAACGTTGCTCTTTTTG	60.3	173	INTERGENIC	--	--	--
Ca4_P4_18705110	Ca4	18705110	AAT/AATTAT	TTGTGCATTCGACAACATCC	ATGCACAATCAAAAACAGCG	60.5	553	INTERGENIC	--	--	--
Ca4_P4_19010119	Ca4	19010119	ATTTTTTTTTTT/ATTTTTTTTTTTTT	TGTCAAGTACCAAGCAAGACCT	CAATCGGATCAATGGCTACC	58.9	134	INTERGENIC	--	--	--
Ca4_P4_19045820	Ca4	19045820	A/AT	GGGCTTGCTTGGTTTGAATA	TCAACTGCACGTGTTAAATGC	60.1	825	INTERGENIC	--	--	--
Ca4_P4_1907150	Ca4	1907150	TATAAAAAATAAAAA/TATAAAAAATAAAAAATAAAAA	AAAGAGTTTAAAGGCGGGGA	GCGGTCATATTCCGAATGAA	60.1	437	INTERGENIC	--	--	--
Ca4_P4_19081618	Ca4	19081618	CAATAATAATAATAATAATAATAA/CAATAATAATAATAATAATAATAATAA	CGACACTTCAACACAAAATTCA	TGTTGAAAACAACATGTTCCC	58.7	893	INTERGENIC	--	--	--

INDEL marker IDs	Chromosomes/ unanchored scaffolds	Physical positions (bp)	InDels (*Kabuli* reference genome-CDC Frontier/PII)	Forward primers (5'-3')	Reverse primers (5'-3')	Anneling temperature (0C)	Expected amplified product size (bp)	Structural annotation		Functional annotation	
								Sequence components of *kabuli* genome	*Kabuli* gene accession IDs	NCBI-KOG	TFs
Ca4_P4_19082750	Ca4	19082750	AGG/AG	GCCTTAACGTTCACTCCTGC	TAAGCCATAAGCGGAGAACG	59.9	134	INTERGENIC	--	--	--
Ca4_P4_19147123	Ca4	19147123	AT/ATACATCT	TTTCAACACTCCCCCTCAAG	GCTGAAGCTGAATACAGGGC	60.1	352	INTERGENIC	--	--	--
Ca4_P4_1928030	Ca4	1928030	AATATATATATATATATATATATATATA/AATATATATATATATATATATATATATATATA	CAATTCCTTCATCGATCCACT	ACAACCCATTCACCCTGGTA	59.0	475	INTERGENIC	--	--	--
Ca4_P4_19342121	Ca4	19342121	TAAAAAAAA/TAAAAAAAAA	TTCCGAAAATTCACATGCAC	GAATTTGGATTATTCGATTCTCTGA	59.5	388	INTERGENIC	--	--	--
Ca4_P4_19369951	Ca4	19369951	CAAAAA/CAAAAAA	CCCATGTTTAAGGGTGGTTG	AATTTTTCACGACCTTCTTCAA	60.1	561	INTERGENIC	--	--	--
Ca4_P4_19396924	Ca4	19396924	TGAAGAAGAAGAAGAAGAAGAAGAAGAAGAAGAAGAAGAAGAAGAAGAAGAAGAAGAAG/TGAAGAAGAAGAAGAAGAAGAAGAAGAAGAAGAAGAAGAAGAAGAAGAAGAAGAA[illegible]	TTTGGAGACCAATGGTGACA	TTGATTGAAACCAAGACCAGC	59.9	938	INTERGENIC	--	--	--
Ca4_P4_19397426	Ca4	19397426	GAAAA/GAAA	GCCAACATATGCTTGATGGA	TTGAACTTTCTCCGTTTGGG	59.5	667	INTERGENIC	--	--	--
Ca4_P4_19415220	Ca4	19415220	TAAAA/TAA	TCATCAAATCCGTAACGCAA	TCTTAAACATGCGGTCCTCG	60.1	575	INTERGENIC	--	--	--
Ca4_P4_19445900	Ca4	19445900	GA/GAA	ACAGATTCGTGGAGGTGGAG	ATGCCAAGCGTTTCACTTTT	60.1	429	INTERGENIC	--	--	--
Ca4_P4_1958352	Ca4	1958352	TAAAAAA/TAAAAA	AACAAGCCCAATTTTGCAGT	TGGAGATTGTTCACCCATCA	59.6	463	INTERGENIC	--	--	--

INDEL marker IDs	Chromosomes/ unanchored scaffolds	Physical positions (bp)	InDels (*Kabuli* reference genome-CDC Frontier/PII)	Forward primers (5'-3')	Reverse primers (5'-3')	Anneling temperature (0C)	Expected amplified product size (bp)	Structural annotation		Functional annotation	
								Sequence components of *kabuli* genome	*Kabuli* gene accession IDs	NCBI-KOG	TFs
Ca4_P4_19643688	Ca4	19643688	CTTTTT/CTTTTTT	GGTGCAGTTGTTTGATGCAC	ATGCGATTTACATGGTTGGC	60.2	255	INTERGENIC	--	--	--
Ca4_P4_19678061	Ca4	19678061	C/CGGCTGGTACAA	ATTTTGGCAACGGCTGATAC	CCACAATCCTCCGTTGTCTT	60.0	492	INTERGENIC	--	--	--
Ca4_P4_19789343	Ca4	19789343	A/AG	CTAGGGGATTGCATTCCAAA	TTTTCCTGCCAAACACCTTC	59.9	177	INTERGENIC	--	--	--
Ca4_P4_19886492	Ca4	19886492	CATTTCTTATTTCTT/CATTTCTT	GACCCCCTGACAAAGACAGA	AGAGGGTTGTGCACTTGAGC	60.1	415	INTERGENIC	--	--	--
Ca4_P4_19958903	Ca4	19958903	AAATAATAATAATAATAATAATAATAATAATAATAATAATAATAATAATAATAATAATAATAATAA/AAATAATAATAATAATAATAATAATAATAATAATAATAATAATAATAATAATAATAATAATAA	GGCAAACCTAGGAATCACCA	TGTGGTTTGGTTTAGATTCCG	59.9	433	INTERGENIC	--	--	--
Ca4_P4_20051846	Ca4	20051846	GTAT/GTATGTGATTAGTGACTAT	CACGAAGCGTATTGACTCCA	AAATTTGAAGGCGCTTTTTG	59.9	432	INTERGENIC	--	--	--
Ca4_P4_20068492	Ca4	20068492	TAAAA/TAAA	AACTGGATGCCCGACATAAG	CCGATTGGATTGTCAGGACT	60.0	499	INTERGENIC	--	--	--
Ca4_P4_20108947	Ca4	20108947	TTATATATATA/TTATATATA	GGCTAACATATGGACTCGCC	TGGTCATTTTGACCTCCACA	59.6	572	INTERGENIC	--	--	--
Ca4_P4_20170219	Ca4	20170219	GTTTATTTATTTATTTATTTATTTATTTATTTATTTATTTATTTATTTATTTATTTATT/GTTTATTTATTTATTTATTTATTTATTTATTTATTTATTTATTTATTTATT	AAAAGGCGTCTCCTCTCTCC	TCGCACATGAAACACGAAAT	60.0	233	INTERGENIC	--	--	--
Ca4_P4_20186435	Ca4	20186435	AT/A	GAGCTGTCTCGGTCAGGAGT	CAATTCCCACAGGTACGCTT	59.6	419	INTERGENIC	--	--	--

INDEL marker IDs	Chromosomes/ unanchored scaffolds	Physical positions (bp)	InDels (*Kabuli* reference genome-CDC Frontier/PII)	Forward primers (5'-3')	Reverse primers (5'-3')	Anneling temperature (0C)	Expected amplified product size (bp)	Structural annotation		Functional annotation	
								Sequence components of *kabuli* genome	*Kabuli* gene accession IDs	NCBI-KOG	TFs
Ca4_P4_2022623	Ca4	2022623	AAATAATAATAATAATAATAATAATAATAATAATAA/AAATAATAATAATAATAATAATAATAATAA	AACGTGTTTGAGTGCGAAAA	TCGAGATGCGATTATGTGGA	59.4	819	INTERGENIC	--	--	--
Ca4_P4_2030157	Ca4	2030157	GAAAAAAA/GAAAAAAAA	TGAAAGTGCCAGAAAACCCT	CGAATGTTTATGGTTGGGGT	59.7	354	INTERGENIC	--	--	--
Ca4_P4_20327473	Ca4	20327473	CATATATATATATATATATATATATATATATATATATATATATA/CATATATATATATATATATATATATATATATATATATATATATA	TAAGGTCATGAGGGATTGCC	CACCCCCTTTCCTTTTATGC	59.9	521	INTERGENIC	--	--	--
Ca4_P4_20346298	Ca4	20346298	ATTATTTATTTATTTATTTATTTATTTATTTATTTATTTAT/ATTATTTATTTATTTATTTATTTATTTATTTAT	TGTTTTGTTCCATGCATTTCTT	GATGCTCATCCCTCCAAAAA	59.5	334	INTERGENIC	--	--	--
Ca4_P4_20421380	Ca4	20421380	TAAAAAAAAAAA/TAAAAAAAAAA	TTCGAACTAAAAACTTGGGCA	CCTCTTGAGAATGTTGGGGA	59.7	560	INTERGENIC	--	--	--
Ca4_P4_20496333	Ca4	20496333	CTTTTTTTTTTTT/CTTTTTTTTTTT	TGAGGACCAAACGTAACCCT	GAGACGTTTTCAGAAACCCC	59.4	397	INTERGENIC	--	--	--
Ca4_P4_20500766	Ca4	20500766	GAAAAAAAAAAA/GAAAAAAAAAA	TCGTAAATTTGAGAACGTGAGG	AACCCAACCCATTATAGGGC	59.3	347	INTERGENIC	--	--	--
Ca4_P4_2052943	Ca4	2052943	GATATATATATATATATATATATATAT/GATATATATATATATATATATATATATATAT	ACAAAATGGTCAGTGGTGGC	TCAAAATTGTGAGCATCAAACA	60.8	693	INTERGENIC	--	--	--
Ca4_P4_20615648	Ca4	20615648	ATTTTTTTTTTTTTT/ATTTTTTTTTTTTT	CCATTGTCGTTGTGTTTTGC	TGAATAATCTGCACGTCCCA	60.0	460	INTERGENIC	--	--	--
Ca4_P4_20709240	Ca4	20709240	ATTTTTTT/ATTTTTTTT	ACTTTGCCCATCAGTTTTGC	TCCGTTGGTGAAAGTTTGAA	60.1	251	INTERGENIC	--	--	--

**INDEL marker IDs**	**Chromosomes/ unanchored scaffolds**	**Physical positions (bp)**	**InDels (*Kabuli* reference genome-CDC Frontier/PII)**	**Forward primers (5'-3')**	**Reverse primers (5'-3')**	**Anneling temperature (0C)**	**Expected amplified product size (bp)**	**Structural annotation**		**Functional annotation**	
								**Sequence components of *kabuli* genome**	***Kabuli* gene accession IDs**	**NCBI-KOG**	**TFs**
Ca4_P4_20738577	Ca4	20738577	GGCTTATG/GG	CTCCCCTCCATGAGGTTACA	ACGCCACAAGGATTAACAGC	59.9	818	INTERGENIC	--	--	--
Ca4_P4_20798832	Ca4	20798832	TTTTATTTATTTATTTATT/TTTTATTTATTTATT	TAGTGCTTTTGCATGCCTCA	TTTGTCCTGGGAAAATGCTC	60.5	316	INTERGENIC	--	--	--
Ca4_P4_20919644	Ca4	20919644	ATTTTTTTTT/ATTTTTTTTTT	TCAGAAATTTGTCATTTGTCCA	TTCGTTGAAATCGGTTATTTGA	58.1	719	INTERGENIC	--	--	--
Ca4_P4_20954508	Ca4	20954508	CTATTATTATTATTATTATTATCATTATTATTATCATTAATATTATTATTATTATTATTATTATTATGATTATTATTATTATTATTATTATTATTATTATTATTATTATTATTATTATTATTATTATTATTA[illegible]	TGAGAAAATGTTCCCTCTGATTC	TCGTTTATGTTAAATTACACCATGC	59.6	248	INTERGENIC	--	--	--
Ca4_P4_20955373	Ca4	20955373	CTATATATATATATATATATATAT/CTATATATATATATATATATATATAT	AGGCTTTATGCAAGGGGTTT	TCACAATTGGTGGTACCGTG	60.0	191	INTERGENIC	--	--	--
Ca4_P4_2102792	Ca4	2102792	ATAATGT/ATAATGTAATGT	AATGCATTATTGGTGGGTCAA	TTCTGTTCTCTTCCACCAAAGA	60.1	582	INTERGENIC	--	--	--
Ca4_P4_21082826	Ca4	21082826	AAATAATAATAATAATAATAATAA/AAATAATAATAATAATAATAA	GTGCGTGCTTTGGGATTTTA	CGAACTGAAGAACGATTAGGTG	61.0	332	INTERGENIC	--	--	--
Ca4_P4_21226050	Ca4	21226050	AT/AAAGGAACT	CACGGCTCTACGAAGGAAAG	TGAACACCCCCAAGGTTAAG	60.0	287	INTERGENIC	--	--	--
Ca4_P4_21248655	Ca4	21248655	TAAAAAAAAAAA/TAAAAAAAAAA	CAGGTTTAATTGTGGAGGTGC	TGCATTGCAGTGTGAAACG	59.5	102	INTERGENIC	--	--	--
Ca4_P4_21261688	Ca4	21261688	AATATATATATATATATATATATATATATATATATA/AATATATATATATATATATATATATATATATATATATATATATATATA	GAAAAATTTGCTGAGCTGGTG	TGCGACAGAATCTTAATGTGC	59.9	485	INTERGENIC	--	--	--

INDEL marker IDs	Chromosomes/ unanchored scaffolds	Physical positions (bp)	InDels (*Kabuli* reference genome-CDC Frontier/PII)	Forward primers (5'-3')	Reverse primers (5'-3')	Anneling temperature (0C)	Expected amplified product size (bp)	Structural annotation		Functional annotation	
								Sequence components of *kabuli* genome	*Kabuli* gene accession IDs	NCBI-KOG	TFs
Ca4_P4_2140233	Ca4	2140233	AATATATATATATATATATATAT/AATATATATATATATATATATAT	AACGTATTGGAAATTTGAATGG	TAGTACTCGTACCGGTGCCC	57.9	655	INTERGENIC	--	--	--
Ca4_P4_2141158	Ca4	2141158	T/TATTACCGACACGG	CTATAGTACTCGCCCCGCAC	TGCGATTGAGCTGGTACTTG	59.8	956	INTERGENIC	--	--	--
Ca4_P4_21529168	Ca4	21529168	CAAAA/CAA	TGGGGGATTGTATTCCAAAA	TATGGAATGGACCTCCTGCT	60.0	499	INTERGENIC	--	--	--
Ca4_P4_21529316	Ca4	21529316	T/TA	TGGGGGATTGTATTCCAAAA	TGAAATCGTCACCGTCTCAA	60.0	886	INTERGENIC	--	--	--
Ca4_P4_21622605	Ca4	21622605	TAATGTTAAAA/TAATGTTAAAAAAATGTTAAAA	CGTGTATATACGCACACCCAA	GTTTTGGCGTTGGTTTCACT	59.4	805	INTERGENIC	--	--	--
Ca4_P4_21640123	Ca4	21640123	GT/GTGTCTTTCCCT	TTTGGTATTGCATCCCCTTC	AGCCCATCAACGTCTTTGTC	59.8	650	INTERGENIC	--	--	--
Ca4_P4_21657242	Ca4	21657242	GTGAAAGAAAAT/GT	CGAGAGCTTTGAAGACTTTGC	CGGAGTTCCAATCTCACAGA	59.4	211	INTERGENIC	--	--	--
Ca4_P4_21726235	Ca4	21726235	TAAAAAAA/TAAAAAAAA	CGACATTCATTGTTACCCCA	TGGAGGAAGAACCGTTATCAA	59.2	739	INTERGENIC	--	--	--
Ca4_P4_21728459	Ca4	21728459	TAA/TAATAATAATAAAAA	CCATTTCGTTGCTCCATCTT	TGGTTTTTGTTGTTTGGTTTCT	60.1	267	INTERGENIC	--	--	--
Ca4_P4_21804779	Ca4	21804779	CTGT/CTGTTATGT	CCGCATCATAAACAGCCTTT	CTAAAGCTGGTGCTTTTGGC	60.1	159	INTERGENIC	--	--	--

INDEL marker IDs	Chromosomes/ unanchored scaffolds	Physical positions (bp)	InDels (*Kabuli* reference genome-CDC Frontier/PII)	Forward primers (5'-3')	Reverse primers (5'-3')	Anneling temperature (0C)	Expected amplified product size (bp)	Structural annotation		Functional annotation	
								Sequence components of *kabuli* genome	*Kabuli* gene accession IDs	NCBI-KOG	TFs
Ca4_P4_21838798	Ca4	21838798	ATTTTTTT/ATTTTTTTT	CCGTCTCGTGTTCTGTGCTA	CGTGACGACTTCCAAGATGA	60.0	663	INTERGENIC	--	--	--
Ca4_P4_21849002	Ca4	21849002	CAAAAAA/CAAAAAAA	AAGAGAGGTCACTTTGGCGA	TGGAGAATTTCAGCCTGCTT	60.0	613	INTERGENIC	--	--	--
Ca4_P4_21928505	Ca4	21928505	CTTTT/CTTTTT	ACCAGGAAAAGAAGAAGGGC	AAGCGCTGTAAAAGGCGTTA	59.7	900	INTERGENIC	--	--	--
Ca4_P4_21984745	Ca4	21984745	ATATATTTATAT/ATATAT	AATAAAAGGCGCATGGAAAA	TCAAAAGTTTGGTGGCATGA	59.6	507	INTERGENIC	--	--	--
Ca4_P4_22052600	Ca4	22052600	AATTATTATTATTATTATTA/AATTATTATTATTATTA	GGATGCGACCTGCTACTGAT	GGATGCGACCTCCTACTACG	60.2	801	INTERGENIC	--	--	--
Ca4_P4_22062454	Ca4	22062454	CT/CTT	TGCAACAGAACACGACAAAA	AGTCTACGGTCGGTGTAGCG	58.9	566	INTERGENIC	--	--	--
Ca4_P4_22327879	Ca4	22327879	TAAAAAAAAAAA/TAAAAAAAAAA	ACCGGAAATTTCACGCCTTA	GATTGGCGAGATTGACGAAT	61.7	608	INTERGENIC	--	--	--
Ca4_P4_2233798	Ca4	2233798	CT/C	TTCATTCTCGGGGAACTAGC	TTGTTATTTAGTCTTGCTTGCCA	59.3	264	INTERGENIC	--	--	--
Ca4_P4_22441886	Ca4	22441886	CTA/CTATA	TCCAATGGTGGAAAATAAACG	TCAATCACAAATTTCAAGGAGG	59.7	848	INTERGENIC	--	--	--
Ca4_P4_22460898	Ca4	22460898	GTT/GTTT	TTAATGCCCTCGTCATGTTT	TAATTTCCGCTGTGCACTTG	58.1	480	INTERGENIC	--	--	--

INDEL marker IDs	Chromosomes/ unanchored scaffolds	Physical positions (bp)	InDels (*Kabuli* reference genome-CDC Frontier/PII)	Forward primers (5'-3')	Reverse primers (5'-3')	Anneling temperature (0C)	Expected amplified product size (bp)	Structural annotation		Functional annotation	
								Sequence components of *kabuli* genome	*Kabuli* gene accession IDs	NCBI-KOG	TFs
Ca4_P4_22535036	Ca4	22535036	ATTTT/ATTTTT	TTTGAGTGTCGCATCCCATA	TCACGGCTTCATGGATGTTA	60.1	722	INTERGENIC	--	--	--
Ca4_P4_22546535	Ca4	22546535	AATATATATATATA/AATATATATATATATA	CATTTTGGTTTCAAAGCCGT	CCAGGGGATGAGATCATAGG	60.0	714	INTERGENIC	--	--	--
Ca4_P4_22601946	Ca4	22601946	AAATAATAATAATAATAATAATAATAATAATAATA/AAATAATAATAATAATAATAATAATAATAATAATAATAATAATA	TCGCGTCTTCCATTCATTTC	CGATTTCATGAATTGCAAATGT	61.1	626	INTERGENIC	--	--	--
Ca4_P4_22622741	Ca4	22622741	ACA/ACATGTGAATCCA	ACTTGTTGCCCTGTTTTTGG	ATGCACTGTGTGTTTGGGAA	60.0	520	INTERGENIC	--	--	--
Ca4_P4_22649924	Ca4	22649924	A/AT	CCGTCCAAATCAAATGAAAAC	CATTTACCATGTGCTTTTTATGC	59.3	491	INTERGENIC	--	--	--
Ca4_P4_22650061	Ca4	22650061	TAAAAA/TAAAA	TGATGCATAAAAAGCACATGG	TCACTTTAAAATTACGCTGGCA	59.6	503	INTERGENIC	--	--	--
Ca4_P4_22663072	Ca4	22663072	CCTTCTT/CCTT	ACCCCCTCTTCTCGTGTTTT	AGGCTCAGGCAACTAGGGAT	60.0	536	INTERGENIC	--	--	--
Ca4_P4_22664879	Ca4	22664879	AA/AAGA	TCGCGTTCGATTTGTTCATA	GGTCATTCTTATTGATTGACCCA	60.2	570	INTERGENIC	--	--	--
Ca4_P4_22668695	Ca4	22668695	TGG/TG	GTTTCCCGTCCTTTCATTCA	CAGTGACAAAAGGCCAATCA	59.9	712	INTERGENIC	--	--	--
Ca4_P4_22677817	Ca4	22677817	AC/ACC	TCTCCCCCTTTTTGATGATG	TCCTAAGGCTGAACCCTCAA	59.9	546	INTERGENIC	--	--	--

INDEL marker IDs	Chromosomes/ unanchored scaffolds	Physical positions (bp)	InDels (*Kabuli* reference genome-CDC Frontier/PII)	Forward primers (5'-3')	Reverse primers (5'-3')	Anneling temperature (0C)	Expected amplified product size (bp)	Structural annotation		Functional annotation	
								Sequence components of *kabuli* genome	*Kabuli* gene accession IDs	NCBI-KOG	TFs
Ca4_P4_22706706	Ca4	22706706	TCC/TC	CCAACAAATTGGGTGGAAAA	TCGCTGCAACCAATAAATGA	60.6	776	INTERGENIC	--	--	--
Ca4_P4_22719475	Ca4	22719475	A/AT	ACACGTCATTTGTTTTGGCA	TGTTGCAATAGAAGGTCGCA	60.0	390	INTERGENIC	--	--	--
Ca4_P4_22720347	Ca4	22720347	GAAAAAAAAA/GAAAAAAAAAA	ATGCCAAAACAAATGACTGTT	TGTTTGGTTTTGATTGCGAA	57.1	587	INTERGENIC	--	--	--
Ca4_P4_22728327	Ca4	22728327	ATTTTTTTTT/ATTTTTTTT	GCAAAAACGTTTGTCCCTTG	CCATCGTAGTTGACAAATGGC	60.5	315	INTERGENIC	--	--	--
Ca4_P4_22728505	Ca4	22728505	GTTG/GTTGTTTTTG	GCAAAAACGTTTGTCCCTTG	TCAGTGTTGTTCAGCACGTT	60.5	459	INTERGENIC	--	--	--
Ca4_P4_22744609	Ca4	22744609	ACCC/ACC	GCCATCTCGAGTCAACAACA	CATTGGTGGCTAGCATGAGA	59.8	343	INTERGENIC	--	--	--
Ca4_P4_22755158	Ca4	22755158	ATTTTTTT/ATTTTTTTT	CTATGTGCGTCGTCAGGAAA	CCAAAAACGGTTTCACCAGT	59.9	595	INTERGENIC	--	--	--
Ca4_P4_22774524	Ca4	22774524	TTCTCTCTC/TTCTCTCTCTC	CATCATCATCACGCCTCATC	AATCGGTGTAGAGGCAATGG	60.0	458	INTERGENIC	--	--	--
Ca4_P4_22774585	Ca4	22774585	TTTCTT/TTT	CATCATCATCACGCCTCATC	AATCGGTGTAGAGGCAATGG	60.0	458	INTERGENIC	--	--	--
Ca4_P4_22780799	Ca4	22780799	TTAAAATAAAATAAAATA/TTAAAATAAAATA	TCTATTTTTGATGATGTTGCGTT	TCCACACCGTTTTTGATATTG	58.7	495	INTERGENIC	--	--	--

INDEL marker IDs	Chromosomes/ unanchored scaffolds	Physical positions (bp)	InDels (*Kabuli* reference genome-CDC Frontier/PII)	Forward primers (5'-3')	Reverse primers (5'-3')	Anneling temperature (0C)	Expected amplified product size (bp)	Structural annotation		Functional annotation	
								Sequence components of *kabuli* genome	*Kabuli* gene accession IDs	NCBI-KOG	TFs
Ca4_P4_22782656	Ca4	22782656	TTATAAATATACAT/TTATAAATATACATATAAATATACAT	AAGAGCAGGACAAATTAATGAGA	TTTGGCATTGATGAATTGGA	57.2	733	INTERGENIC	--	--	--
Ca4_P4_22794336	Ca4	22794336	CAA/C	TGACGAGTGGCAGAACTTCA	TATGGACTAGAGCAACCCGC	60.6	655	INTERGENIC	--	--	--
Ca4_P4_22796646	Ca4	22796646	TGGG/TGGGG	ATTGCGATGGAGGAATTGAG	GCGCCAAATATCTTGGAAAA	60.0	805	INTERGENIC	--	--	--
Ca4_P4_22799910	Ca4	22799910	AAAA/AAAAAATTAAA	TCGGTGTGATTTGTGTGTGA	GTGCAATCATGCAATCAAGG	59.5	672	INTERGENIC	--	--	--
Ca4_P4_22800253	Ca4	22800253	CTAAAACTCAAT/C	CCTTGATTGCATGATTGCAC	CCTAAATTTTTAAGGGCTAAAATGG	60.1	165	INTERGENIC	--	--	--
Ca4_P4_22801771	Ca4	22801771	GATCA/GA	GCATACCTCAATTCCCTACCA	ACACAAGTGGGGGAGACAAC	58.9	389	INTERGENIC	--	--	--
Ca4_P4_22818473	Ca4	22818473	GTATATATATAT/GTATATATATATAT	CAATTGCATGTTGAAATGGTG	TGGCCATATTGTTTTTGCAT	59.8	821	INTERGENIC	--	--	--
Ca4_P4_22824281	Ca4	22824281	AAA/AAAGTAA	ACATGCACCAGAGTTAATGTTTT	GCTTCAACACCGGGAATAGA	57.8	167	INTERGENIC	--	--	--
Ca4_P4_22825336	Ca4	22825336	TTT/TTTGTT	TGGCTTCCATAGTAAGAGAGTGAA	AAATAACGTGTCACGGGGAT	59.5	546	INTERGENIC	--	--	--
Ca4_P4_22848738	Ca4	22848738	AATACATATA/AATA	AGTATTGAAATTTGCCGCGT	ACGACTGGCTAAGGTGCTGT	59.6	665	INTERGENIC	--	--	--

INDEL marker IDs	Chromosomes/ unanchored scaffolds	Physical positions (bp)	InDels (*Kabuli* reference genome-CDC Frontier/PII)	Forward primers (5'-3')	Reverse primers (5'-3')	Anneling temperature (0C)	Expected amplified product size (bp)	Structural annotation		Functional annotation	
								Sequence components of *kabuli* genome	*Kabuli* gene accession IDs	NCBI-KOG	TFs
Ca4_P4_22853847	Ca4	22853847	ATTTTTTTTTT/ATTTTTTTTTTT	TCAAAACACATTGGAG GCAC	AAGCGTAAATGGCAGA TGTGT	59.5	344	INTERGENIC	--	--	--
Ca4_P4_22854290	Ca4	22854290	GAAAAAAAAA/GAAAAAAAA	CACATCTGCCATTTAC GCTT	AAAATGGCACAAGGCA AACT	58.8	534	INTERGENIC	--	--	--
Ca4_P4_22855998	Ca4	22855998	TATCCGAT/TAT	CCTGTCAACTCCAAGA ATCTCC	AAAGCCATATTTTAACC CAAAAA	60.1	666	INTERGENIC	--	--	--
Ca4_P4_22856126	Ca4	22856126	GTTTATTT/GTTT	CCTGTCAACTCCAAGA ATCTCC	AAAGCCATATTTTAACC CAAAAA	60.1	666	INTERGENIC	--	--	--
Ca4_P4_22856342	Ca4	22856342	CTTTTTTTTTTT/CTTTTTTTTT	TTTTTGTACTTCTCGTG TTAAGAGTTT	AAAAATGGAATTACGTC CAATGA	58.3	701	INTERGENIC	--	--	--
Ca4_P4_22867161	Ca4	22867161	ATTTT/ATTTTT	TCACCACTGACCAACC CTTT	CACCACCACCACAAAA CACT	60.4	485	INTERGENIC	--	--	--
Ca4_P4_22867292	Ca4	22867292	TAAAAAAAAA/TAAAAAAAAAA	TCACCACTGACCAACC CTTT	CACCACCACCACAAAA CACT	60.4	485	INTERGENIC	--	--	--
Ca4_P4_22867454	Ca4	22867454	GAAAAAAAAAA/GAAAAAAAAA	GATCTTCATCCCCTGC AGAC	CACCACCACCACAAAA CACT	59.6	292	INTERGENIC	--	--	--
Ca4_P4_22869907	Ca4	22869907	TAAAAAAAA/TAAAAAAA	GAGAAGGGGCAAGAG AGTGA	TTTTGGTTTTCACCGAT TCC	59.5	590	INTERGENIC	--	--	--
Ca4_P4_22869940	Ca4	22869940	TAAAAAAAA/TAAAAAA	GAGAAGGGGCAAGAG AGTGA	TTTTGGTTTTCACCGAT TCC	59.5	590	INTERGENIC	--	--	--

INDEL marker IDs	Chromosomes/ unanchored scaffolds	Physical positions (bp)	InDels (*Kabuli* reference genome-CDC Frontier/PII)	Forward primers (5'-3')	Reverse primers (5'-3')	Anneling temperature (0C)	Expected amplified product size (bp)	Structural annotation		Functional annotation	
								Sequence components of *kabuli* genome	*Kabuli* gene accession IDs	NCBI-KOG	TFs
Ca4_P4_22879215	Ca4	22879215	A/AAT	TCATCTGCGTTTTTCTGCAC	ACCAATCCGAAGACGATCAC	60.0	828	INTERGENIC	--	--	--
Ca4_P4_22889580	Ca4	22889580	AC/A	GGGTCAGGATACTTACAACCCA	TCCACCCCCAAATTTTATCA	60.1	717	INTERGENIC	--	--	--
Ca4_P4_22890323	Ca4	22890323	GG/GGAGTCCATTATG	TGATAAAATTTGGGGGTGGA	AGGTAGGAGCAAAATCGCAA	60.0	622	INTERGENIC	--	--	--
Ca4_P4_22890495	Ca4	22890495	GAAAAAAAAAA/GAAAAAAAAA	TCATGGTCCCCCAAATTTTA	AGGTAGGAGCAAAATCGCAA	60.0	443	INTERGENIC	--	--	--
Ca4_P4_22890677	Ca4	22890677	AG/A	TTGCGATTTTGCTCCTACCT	AGGCGCAACTTGCATTTACT	59.8	526	INTERGENIC	--	--	--
Ca4_P4_22895304	Ca4	22895304	ATTTTTTT/ATTTTTTTT	TGGTATCAAATAAGTTTCCGGT	GTTGGGTTGAGCCTGTTTGT	57.6	576	INTERGENIC	--	--	--
Ca4_P4_22902727	Ca4	22902727	ATT/ATTT	CACTATGCAAAATGGCCTCA	TCTCTTATGTGGGGTGCCTC	59.7	186	INTERGENIC	--	--	--
Ca4_P4_22906009	Ca4	22906009	ATTT/ATTTT	TGTTGAAGCGAACTGCACAT	GGGGAAACAAAAACCCAGAT	60.5	785	INTERGENIC	--	--	--
Ca4_P4_22922451	Ca4	22922451	GA/GAA	CACCGTCCGAATACCAAAAC	AGTCATTTGCATGTGGGACA	60.2	391	INTERGENIC	--	--	--
Ca4_P4_2293129	Ca4	2293129	ATGT/A	CGGTCATCAATCCGACTAGG	AATGATTTATTGCGGCGAAG	60.5	521	INTERGENIC	--	--	--

INDEL marker IDs	Chromosomes/ unanchored scaffolds	Physical positions (bp)	InDels (*Kabuli* reference genome-CDC Frontier/PII)	Forward primers (5'-3')	Reverse primers (5'-3')	Anneling temperature (0C)	Expected amplified product size (bp)	Structural annotation		Functional annotation	
								Sequence components of *kabuli* genome	*Kabuli* gene accession IDs	NCBI-KOG	TFs
Ca4_P4_22974622	Ca4	22974622	TGG/TGGG	ATTTTGGGCGAAATCCTTTT	GAACCACTCATCCTCCCAAA	59.8	442	INTERGENIC	--	--	--
Ca4_P4_22985821	Ca4	22985821	TTTA/TTTAGTTA	ATAAGCCTTGCCATCTTCCA	CTTAGATGGGCCACCCAATA	59.7	642	INTERGENIC	--	--	--
Ca4_P4_22988357	Ca4	22988357	AG/A	CTCCAAAGGACGCATCTCAT	TGGGGTTTAGGGTGTCACAT	60.2	623	INTERGENIC	--	--	--
Ca4_P4_23002548	Ca4	23002548	ATTATTTTATTTTATTTTATT/ATTATTTTATTTTATTTTATTTTATT	TCGATCGCTGATGTCAAACT	TGGGCTTGACCTATGTGTGT	59.4	147	INTERGENIC	--	--	--
Ca4_P4_23002623	Ca4	23002623	TTTACTATTACTATT/TTTACTATTACTATTACTATT	TTTATGGCGGCTGGTTTTAC	ACGATGCATTTGATTGATGTT	60.0	546	INTERGENIC	--	--	--
Ca4_P4_23013499	Ca4	23013499	CTA/CTATA	TCGGTTGGTACACACCAATTT	AATTGGAGATAGCATCCCCTC	60.1	506	INTERGENIC	--	--	--
Ca4_P4_23014862	Ca4	23014862	CATATATA/CATATATATA	GAACATGTGCGTGCTACGTT	GCTGTTCAAAGGGCTAGACG	59.8	756	INTERGENIC	--	--	--
Ca4_P4_23018298	Ca4	23018298	AAAT/A	ATTTCGGTTTGTCCTTCGAG	TTTTAAAAAGATTCACATGCACTAA	59.2	549	INTERGENIC	--	--	--
Ca4_P4_23018550	Ca4	23018550	GAAAAAAA/GAAAAAAAA	TCAGAGAGAATGCACGACATTT	GGCCTGACGACACAGCTAAT	59.9	774	INTERGENIC	--	--	--
Ca4_P4_23019641	Ca4	23019641	G/GT	CCAATTGGCCCTCATTAGAC	GGAACCGTTGTTTCTTGGTC	59.4	445	INTERGENIC	--	--	--

INDEL marker IDs	Chromosomes/ unanchored scaffolds	Physical positions (bp)	InDels (*Kabuli* reference genome-CDC Frontier/PII)	Forward primers (5'-3')	Reverse primers (5'-3')	Anneling temperature (0C)	Expected amplified product size (bp)	Structural annotation		Functional annotation	
								Sequence components of *kabuli* genome	*Kabuli* gene accession IDs	NCBI-KOG	TFs
Ca4_P4_23037799	Ca4	23037799	AC/ACC	TCGACAAAAGCAAAGCAATG	GCGTTCTTGGTTTTCCAGTC	60.0	349	INTERGENIC	--	--	--
Ca4_P4_23039824	Ca4	23039824	CAAAAA/CAAAAAA	TAACACGAGGGCAAACTTCC	CGAGCCATAACATTGAAGAGG	60.1	361	INTERGENIC	--	--	--
Ca4_P4_23048460	Ca4	23048460	ATTTTTTTTTTTT/ATTTTTTTTTT	TAAGGAGCCCACAAACAAGG	TGGTTGCTCTTCTCGCCTAT	60.1	536	INTERGENIC	--	--	--
Ca4_P4_23058519	Ca4	23058519	ATCT/AT	CGGATATGAGTTTTATGGACGA	CTCCCTTTTTGCAACACACA	59.0	434	INTERGENIC	--	--	--
Ca4_P4_23060171	Ca4	23060171	AATG/AATGCATG	TGCATTTGGCAATTTGAAGA	TCAACGCATGTGACTGCATA	60.2	254	INTERGENIC	--	--	--
Ca4_P4_23076109	Ca4	23076109	CTTT/CTTTT	TGGCATTGCAAGAAGAAACA	CCTCTTTCAACACCTTTGGC	60.4	760	INTERGENIC	--	--	--
Ca4_P4_23077647	Ca4	23077647	ATT/AT	AAGAAAAGGGTTGCGTTTGA	TGAATTCATGTGTATTGTCGGA	59.7	644	INTERGENIC	--	--	--
Ca4_P4_23078135	Ca4	23078135	ATTTTTTTT/ATTTTTTTTT	TTTGAAGAACCAGAGTTTGAAGAA	TTCAATGCCCATAATCGTGA	59.5	656	INTERGENIC	--	--	--
Ca4_P4_23078167	Ca4	23078167	TC/T	TTTGAAGAACCAGAGTTTGAAGAA	TTCAATGCCCATAATCGTGA	59.5	656	INTERGENIC	--	--	--
Ca4_P4_23078243	Ca4	23078243	TAAAA/TAAA	AAAAATTCCGACAATACACATGAA	GAATGCGTACACCTTGACGA	59.7	897	INTERGENIC	--	--	--

INDEL marker IDs	Chromosomes/ unanchored scaffolds	Physical positions (bp)	InDels (*Kabuli* reference genome-CDC Frontier/PII)	Forward primers (5'-3')	Reverse primers (5'-3')	Anneling temperature (0C)	Expected amplified product size (bp)	Structural annotation		Functional annotation	
								Sequence components of *kabuli* genome	*Kabuli* gene accession IDs	NCBI-KOG	TFs
Ca4_P4_23079294	Ca4	23079294	ATCTTCTT/ATCTT	TAAATAGCATTTGCGGTCCC	TTTTTCTTCCGATTTGGTCC	59.9	508	INTERGENIC	--	--	--
Ca4_P4_23079344	Ca4	23079344	TCC/TCCC	AATTGCAAGTGGACTGGACC	CCGAGCCAAAATCCAAAATA	60.0	787	INTERGENIC	--	--	--
Ca4_P4_23100528	Ca4	23100528	AT/ATT	GTTGCTCGTGTTGCCCTTAT	TGCAAAAGTGGTTTTAGCTACC	60.1	465	INTERGENIC	--	--	--
Ca4_P4_23134800	Ca4	23134800	TG/T	ATGCTGCGAAATTGTTGAAA	CGGACCTCTCATTGCTGAAT	59.3	739	INTERGENIC	--	--	--
Ca4_P4_23145296	Ca4	23145296	TAAAAAAA/TAAAAAAAA	TGCATGTTTCATGGTGATCC	CACCAATTACAGTGGCATCAA	60.3	680	INTERGENIC	--	--	--
Ca4_P4_23145659	Ca4	23145659	ATTTTTTT/ATTTTTT	TGGGAACTTAGGATTCCGTTC	CAAATTTGAATCCGCCTACTG	60.3	587	INTERGENIC	--	--	--
Ca4_P4_23145891	Ca4	23145891	ATTTTTTTTTT/ATTTTTTTT	GCTTGAGGATTCAGTAGGCG	GAGTGGAGGACCACATGTCA	60.0	408	INTERGENIC	--	--	--
Ca4_P4_23192116	Ca4	23192116	CTA/CTATA	TTCGAACAAAATGATTCCCA	TAAACCAATGCCACACATGG	58.9	640	INTERGENIC	--	--	--
Ca4_P4_23217567	Ca4	23217567	AATATATATATATATATAT/AATATATATATATATATATATAT	CTCTAGGGAAGCGACCTCCT	CCAACAACCATTGTGGGAGT	60.0	633	INTERGENIC	--	--	--
Ca4_P4_23220387	Ca4	23220387	ATTT/ATT	GCCGCCACAAAATATTCACT	ATGCCTATACCGGCCTCTTT	60.0	357	INTERGENIC	--	--	--

INDEL marker IDs	Chromosomes/ unanchored scaffolds	Physical positions (bp)	InDels (*Kabuli* reference genome-CDC Frontier/PII)	Forward primers (5'-3')	Reverse primers (5'-3')	Anneling temperature (0C)	Expected amplified product size (bp)	Structural annotation		Functional annotation	
								Sequence components of *kabuli* genome	*Kabuli* gene accession IDs	NCBI-KOG	TFs
Ca4_P4_23272983	Ca4	23272983	ATTTTTTTTT/ATTTTTTTT	ACCACATCGGAAGAAACGAC	GGACGAAGTAGAACGGACCA	60.0	596	INTERGENIC	--	--	--
Ca4_P4_23408499	Ca4	23408499	TAAAAAAA/TAAAAAAAA	ACCAAAACGGTTGATACCCA	ACTACCGTCAATCGTGCTCC	60.1	362	INTERGENIC	--	--	--
Ca4_P4_23453202	Ca4	23453202	AATATATATATATATATATATATATATATATATA/AATATATATATATATATATATATATATATATATATATA	GCTTCCCCAGTCACACTCAT	TCACTGAATTGACACATAATAAATCA	60.1	419	INTERGENIC	--	--	--
Ca4_P4_23500297	Ca4	23500297	AG/A	GAACCCCAAGCAAGAATCAA	TGCACAAAAGCACTTCTGAAA	60.1	379	INTERGENIC	--	--	--
Ca4_P4_23517542	Ca4	23517542	GAAAAAAAAAAA/GAAAAAAAAAA	ATTGGATCTCGCTGAATCGT	GAAGGTAAAATGGTGGGGGT	59.7	351	INTERGENIC	--	--	--
Ca4_P4_23575550	Ca4	23575550	TAAAAAAAAAAAAA/TAAAAAAAAAAAAA	CATTGGTAAAAGTGAAAAATCCG	AACCGAAACAAATCGCACAT	59.8	363	INTERGENIC	--	--	--
Ca4_P4_23721197	Ca4	23721197	TAAAACA/TAAAACAAAACA	ATTTGTGTGCATTTGCCTGA	TGGGCAATCGACTGACTAAA	60.1	353	INTERGENIC	--	--	--
Ca4_P4_23756363	Ca4	23756363	TTATATATATATATATATA/TTATATATATATATATATATA	TTGGAACGTAATTTGATGCG	CCCGTTGCTTCTGTATGCTT	59.6	872	INTERGENIC	--	--	--
Ca4_P4_23760339	Ca4	23760339	CATATATATATATATATATATATATATATATATATATATATATATATATA/CATATATATATATATATATATATATATATATATATATATA	GGATGGGTGCTACCCCTAAT	AACTCGTGCCTTCGACAAAT	60.0	778	INTERGENIC	--	--	--
Ca4_P4_23762953	Ca4	23762953	GAAAAAAAAAAAAA/GAAAAAAAAAAAA	GATCATCCAGCAACAGCAGA	GGCCTTATTTTCGACTCAATG	60.0	635	INTERGENIC	--	--	--

INDEL marker IDs	Chromosomes/ unanchored scaffolds	Physical positions (bp)	InDels (*Kabuli* reference genome-CDC Frontier/PII)	Forward primers (5'-3')	Reverse primers (5'-3')	Anneling temperature (0C)	Expected amplified product size (bp)	Structural annotation		Functional annotation	
								Sequence components of *kabuli* genome	*Kabuli* gene accession IDs	NCBI-KOG	TFs
Ca4_P4_23824374	Ca4	23824374	CATATATATATATATATATATATA/CATATATATATATATATATATATATA	TCATTCACAAACAACCCCCT	TGCTATTAGAATGCTTATCGCTTA	60.2	551	INTERGENIC	--	--	--
Ca4_P4_23864193	Ca4	23864193	CTTTTTTTTTT/CTTTTTTTTT	GCAAATGTTCATTGGTGTGC	ACCATGCTTAGGGGGAAGAG	60.0	248	INTERGENIC	--	--	--
Ca4_P4_23891239	Ca4	23891239	CATATATATATATATATAT/CATATATATATATAT	TGTCCGTCAATTGGTCTTGA	TGTGTGGTTGTTATTGAACGC	60.1	469	INTERGENIC	--	--	--
Ca4_P4_2390421	Ca4	2390421	ATTTTTT/ATTTTTTT	GCACTTTTGGTCCCCCTATT	TATTCCACGTATGCGTCTGC	60.2	168	INTERGENIC	--	--	--
Ca4_P4_24140557	Ca4	24140557	CATTATTATTATTATTATTATTA/CATTATTATTATTATTATTATTATTA	TTATTTCACCGTCGTGTCCA	TTGTGTGTTGGCCGATTTTA	60.0	234	INTERGENIC	--	--	--
Ca4_P4_24147426	Ca4	24147426	AAATAATAATAATAATAATAATAATAAT/AAATAATAATAATAATAATAATAATAATAAT	GGAAAACGACTTACACCCGA	TTCCTCGGCTTTCTTTGCTA	60.0	654	INTERGENIC	--	--	--
Ca4_P4_24228039	Ca4	24228039	AATATTGATATATTG/AATATTGATATATTGATATATTG	TGATCCTCCCATCCTACCAA	TTGCGTCTTCTGTTTGATGC	60.3	560	INTERGENIC	--	--	--
Ca4_P4_24269057	Ca4	24269057	CTTTT/CTTTTT	CGTTGCACGGAATACTCACT	TCCTGCATGCAATCTGCTTA	58.8	490	INTERGENIC	--	--	--
Ca4_P4_24273906	Ca4	24273906	TTATATATATATATATATATATATATA/TTATATATATATATATATATATATA	GGGGGAGTTGTTATTCGGTT	GAAAAGTGTTGTCGTGTGTGC	60.1	646	INTERGENIC	--	--	--
Ca4_P4_24356217	Ca4	24356217	TCCC/TCC	CGCTTTGTTCTTTACCGCAT	CATGGAACAACTGCACCATAA	60.3	350	INTERGENIC	--	--	--

INDEL marker IDs	Chromosomes/ unanchored scaffolds	Physical positions (bp)	InDels (*Kabuli* reference genome-CDC Frontier/PII)	Forward primers (5'-3')	Reverse primers (5'-3')	Anneling temperature (0C)	Expected amplified product size (bp)	Structural annotation		Functional annotation	
								Sequence components of *kabuli* genome	*Kabuli* gene accession IDs	NCBI-KOG	TFs
Ca4_P4_24359275	Ca4	24359275	TAAAAAAAA/TAAAAAAAAA	GGGAGGGTGGGAGTAAATGT	TGTGGCTTTCCAATGTACGA	60.1	571	INTERGENIC	--	--	--
Ca4_P4_24362931	Ca4	24362931	TAA/TA	GGTTTAGATGGGGGATCGTT	CCCCGAGTTGGTCCTTTATT	60.0	947	INTERGENIC	--	--	--
Ca4_P4_24425317	Ca4	24425317	GTTT/GTTTT	TGGGGTGTTTCATAGCATCA	ACCGTTTAATTGGCGAACAG	59.9	267	INTERGENIC	--	--	--
Ca4_P4_24427573	Ca4	24427573	GTT/GTTT	CTGGAAAATCCTCCTCCTCC	TTCGTCTCAACAATCTCCCC	60.0	346	INTERGENIC	--	--	--
Ca4_P4_2445427	Ca4	2445427	TATAATA/TATA	GGAGGTCACAACCACTCCAA	ATTTGTGACGTTCCCCTCAA	61.0	504	INTERGENIC	--	--	--
Ca4_P4_24478956	Ca4	24478956	CAA/CAAA	CGGTTCTACACAAGTTGCGA	TCGATTATGCGTAGGGAAGG	59.9	746	INTERGENIC	--	--	--
Ca4_P4_24479035	Ca4	24479035	ATTTTTTT/ATTTTTTTTTT	CGGTTCTACACAAGTTGCGA	TCGATTATGCGTAGGGAAGG	59.9	746	INTERGENIC	--	--	--
Ca4_P4_24488859	Ca4	24488859	TAAA/TAAAA	TTGGAACTCATGCAAATGGA	CGATACTCACCCCTCGTTGT	60.0	557	INTERGENIC	--	--	--
Ca4_P4_24553735	Ca4	24553735	GATTAT/GAT	TGGTGCGATTGTGGAAAATA	GAGTAGATGTGCCAACAGCG	59.9	361	INTERGENIC	--	--	--
Ca4_P4_24554220	Ca4	24554220	T/TA	GTGCGGATTATGTCGGGTAT	ACTAGATGGTCGCATCCTGG	59.7	751	INTERGENIC	--	--	--

INDEL marker IDs	Chromosomes/ unanchored scaffolds	Physical positions (bp)	InDels (*Kabuli* reference genome-CDC Frontier/PII)	Forward primers (5'-3')	Reverse primers (5'-3')	Anneling temperature (0C)	Expected amplified product size (bp)	Structural annotation		Functional annotation	
								Sequence components of *kabuli* genome	*Kabuli* gene accession IDs	NCBI-KOG	TFs
Ca4_P4_24608449	Ca4	24608449	GTTTT/GTT	GGAATGAGGAGAGTGAAGCG	CTAAATGCGACCCATCCACT	60.0	684	INTERGENIC	--	--	--
Ca4_P4_24628360	Ca4	24628360	AT/ATATATATGTGT	TATTTTGGATTTTGGCTCGG	AGGAAGAGTGGAGTTGCGAA	59.9	540	INTERGENIC	--	--	--
Ca4_P4_24677817	Ca4	24677817	ATTTTTTTTT/ATTTTTTTTTT	CACGAAAACCTTAAACCCCA	TCTTAAAAGCGATGTAAAGGGC	59.8	526	INTERGENIC	--	--	--
Ca4_P4_2470923	Ca4	2470923	TTATATATATATATATATATAT/TTATATATATATATATATAT	CGGCCCTGATTTTAGTTGAA	TCGCTGCCACCTCTAAATCT	60.1	298	INTERGENIC	--	--	--
Ca4_P4_24737329	Ca4	24737329	AATTATTATTA/AATTATTATTATTATTATTATTA	CCCCTAGTTCTACGTTGGCA	AGGAAAGTTGGGTGTATGCG	60.1	798	INTERGENIC	--	--	--
Ca4_P4_24748886	Ca4	24748886	GAAAAAAAAA/GAAAAAAAA	CACGACCACATCTGTGAGAAA	TTCCAAAATTTCTTCAACACTCAA	59.7	629	INTERGENIC	--	--	--
Ca4_P4_24824930	Ca4	24824930	CTTTTTTT/CTTTTTTTT	TGAATCTTCGAGAAGCCTAACC	CCTTTTTAACGTCGTCCCAA	59.9	461	INTERGENIC	--	--	--
Ca4_P4_24828297	Ca4	24828297	ATTTTTTTTTT/ATTTTTTTTTTT	TGGAATTTCAGACTGCCTTG	GGGCACGAAGAAGCACTATC	58.8	716	INTERGENIC	--	--	--
Ca4_P4_24919292	Ca4	24919292	TAAA/TAAAA	GGAACCTTCGAAAATCCCTC	CTATGGCTTTGTCAGACGCA	59.9	603	INTERGENIC	--	--	--
Ca4_P4_24925584	Ca4	24925584	CAAAAAAAAAA/CAAAAAAAAAAA	TCGATTCTTTGAACCATCTCTTC	GACCATTTGAAAATAAAGTTAGGCA	59.7	464	INTERGENIC	--	--	--

INDEL marker IDs	Chromosomes/ unanchored scaffolds	Physical positions (bp)	InDels (*Kabuli* reference genome-CDC Frontier/PII)	Forward primers (5'-3')	Reverse primers (5'-3')	Anneling temperature (0C)	Expected amplified product size (bp)	Structural annotation		Functional annotation	
								Sequence components of *kabuli* genome	*Kabuli* gene accession IDs	NCBI-KOG	TFs
Ca4_P4_24956663	Ca4	24956663	GAAAAAAAAAA/GAAAAAAAAAAA	AAATTGGTGCTATTGCTGGG	GATTCTAACCGTCGTTTCCG	60.0	159	INTERGENIC	--	--	--
Ca4_P4_25011504	Ca4	25011504	GA/GAAGAAGAAAGAGAAAA	TCCTCGTCAAAATTGCTTCC	CATGTTGGAGTTGTTGGCTG	60.2	405	INTERGENIC	--	--	--
Ca4_P4_25034090	Ca4	25034090	GAAAAAAA/GAAAAAAAA	GTTGGTCATGCAGAAAGCAA	GCAGACGTTATTGGGAAGGA	59.8	603	INTERGENIC	--	--	--
Ca4_P4_25036917	Ca4	25036917	T/TA	ATCCAAGTTGAGCAAGCATT	CATTGAGACTTTGCCACGAA	57.4	605	INTERGENIC	--	--	--
Ca4_P4_25046968	Ca4	25046968	TGCTGAGCTGA/TGCTGA	ATGTTTCGTGCAATGGTCAA	AAGGTCAAGGGCCAAAAACT	60.0	601	INTERGENIC	--	--	--
Ca4_P4_25053641	Ca4	25053641	TAAAAAAAA/TAAAAAAAAA	CTGAGGCTTATGATTCCCCA	CGATAAGGGTGTCGAGGAAA	60.0	154	INTERGENIC	--	--	--
Ca4_P4_25061838	Ca4	25061838	GTTTTTTTT/GTTTTTTTTT	GGTAACCAATCCGAAGACGA	TCGATTATGCGTAGGGAAGG	59.9	666	INTERGENIC	--	--	--
Ca4_P4_25063885	Ca4	25063885	AAA/AAATAA	CCCCAACGATTACACTTGCT	ACCGTTTTGGTGTTTCTTGC	60.0	899	INTERGENIC	--	--	--
Ca4_P4_25064267	Ca4	25064267	AATATATATATATATATA/AATATATATATATATATATA	AATTCCATCCCCTCTCAACC	ACCGTTTTGGTGTTTCTTGC	60.1	561	INTERGENIC	--	--	--
Ca4_P4_25071336	Ca4	25071336	CA/C	TGAGACTGCTTGGCTACCCT	ATCTGAATCATCCCAAGCCA	60.0	488	INTERGENIC	--	--	--

INDEL marker IDs	Chromosomes/ unanchored scaffolds	Physical positions (bp)	InDels (*Kabuli* reference genome-CDC Frontier/PII)	Forward primers (5'-3')	Reverse primers (5'-3')	Anneling temperature (0C)	Expected amplified product size (bp)	Structural annotation		Functional annotation	
								Sequence components of *kabuli* genome	*Kabuli* gene accession IDs	NCBI-KOG	TFs
Ca4_P4_25088043	Ca4	25088043	CCT/CCTCCTTCTTCT	AACCTTCGCAAGAACCCTTC	ATCAATCTCGTTTTCCTGGG	60.6	219	INTERGENIC	--	--	--
Ca4_P4_25139153	Ca4	25139153	TCC/TC	TTGTGTTGCAATAGTGCTTGC	TGCTCCACATAAAGCGAAGA	59.9	642	INTERGENIC	--	--	--
Ca4_P4_25146347	Ca4	25146347	GAAAAAAAA/GAAAAAAA	TCATCCTTGCCTCGATTAGG	GGAAATGCAAATCAAAACCAA	60.2	566	INTERGENIC	--	--	--
Ca4_P4_25159546	Ca4	25159546	TTGATG/TTG	TTTATACGGTGGGGGAATGA	TCCAAATCCCACATACACACA	60.0	406	INTERGENIC	--	--	--
Ca4_P4_25168771	Ca4	25168771	AA/AATA	GACCAAGGCAAGCAAGAAAG	GTGGAAGTTGAAACGACGGT	60.0	178	INTERGENIC	--	--	--
Ca4_P4_25193095	Ca4	25193095	GAAAAAAAAAA/GAAAAAAAAA	CATCACCCACCATTGACAAG	CATCCACGAAACACTCATGG	59.8	587	INTERGENIC	--	--	--
Ca4_P4_25205443	Ca4	25205443	TAGATTTGGCACAAAAGATTTGG/TAGATTTGG	GATGGAGGATCAGCCACATT	ATGGTGGAATGCCTCAAAAG	59.9	648	INTERGENIC	--	--	--
Ca4_P4_25213751	Ca4	25213751	GAG/GAGATCATAG	TCATTCAACGTCATCGTATCAA	TGTGTTTGTATCAATATCGAAGGC	59.0	562	INTERGENIC	--	--	--
Ca4_P4_25235982	Ca4	25235982	ATTTTTTTTTTTT/ATTTTTTTTTT	TTTCAAATTCTCATCGGGGA	GGTTGCATGCATCATTTGAG	60.4	641	INTERGENIC	--	--	--
Ca4_P4_25243065	Ca4	25243065	A/AC	AATCCCCTTGATTGACAACG	TGAGACTCGTGTCCTCCACA	59.8	449	INTERGENIC	--	--	--

INDEL marker IDs	Chromosomes/ unanchored scaffolds	Physical positions (bp)	InDels (*Kabuli* reference genome-CDC Frontier/PII)	Forward primers (5'-3')	Reverse primers (5'-3')	Anneling temperature (0C)	Expected amplified product size (bp)	Structural annotation		Functional annotation	
								Sequence components of *kabuli* genome	*Kabuli* gene accession IDs	NCBI-KOG	TFs
Ca4_P4_25246291	Ca4	25246291	ATTTTT/ATTTTTTT	GAAAGCGCCTAAGGTAAGGG	ATTAAACCAGCCACACAGGC	60.2	677	INTERGENIC	--	--	--
Ca4_P4_25258427	Ca4	25258427	GAAAAAAAAA/GAAAAAAAAAA	TTTGTGCGCAAACAACAGA	TGTGGGGAGCAAAAGTGTTT	60.0	626	INTERGENIC	--	--	--
Ca4_P4_25280052	Ca4	25280052	CTTT/CTT	CTCACTTTTGTTCCCCCAAA	TGGTGCAAGGTCTAAAGGAGA	59.9	292	INTERGENIC	--	--	--
Ca4_P4_25303619	Ca4	25303619	GTTTTT/GTTTTTT	TTGAGGCAGGAACCTCAACT	CATTTCCTTAAGCCCCATGA	59.8	601	INTERGENIC	--	--	--
Ca4_P4_25348827	Ca4	25348827	AGGGGGG/AGGGGG	GGCAAATGCTAACCCATGTT	TTGCTTACAATCCGAGACGA	59.8	224	INTERGENIC	--	--	--
Ca4_P4_25352441	Ca4	25352441	AAATAATAATAAT/AAATAATAAT	CACTGCTATAAGCCGCACAA	AGCCTTACGCATGTATTGCC	60.0	906	INTERGENIC	--	--	--
Ca4_P4_25352610	Ca4	25352610	T/TA	GGAAATGGTTCTTTGAAAGCC	AGCCTTACGCATGTATTGCC	59.9	719	INTERGENIC	--	--	--
Ca4_P4_25357817	Ca4	25357817	ATTTTTTTT/AATTTTTTTTT	AAAATTCTGTTTGGGGGACC	ACATGCATGCAATTCAATAGAG	60.0	677	INTERGENIC	--	--	--
Ca4_P4_25358106	Ca4	25358106	TTAAATAAATAAATAAATAAATAA/TTAAATAAATAAATAAATAA	GGGACTACTTTCGCGATTCA	ATTACACGCTTGCAGACGAA	60.2	842	INTERGENIC	--	--	--
Ca4_P4_25359522	Ca4	25359522	GATATAT/GATATATATAT	CGACAAAGCCTCACGAGTTA	GGAGCCACAAACGATTGACT	59.1	660	INTERGENIC	--	--	--

INDEL marker IDs	Chromosomes/ unanchored scaffolds	Physical positions (bp)	InDels (*Kabuli* reference genome-CDC Frontier/PII)	Forward primers (5'-3')	Reverse primers (5'-3')	Anneling temperature (0C)	Expected amplified product size (bp)	Structural annotation		Functional annotation	
								Sequence components of *kabuli* genome	*Kabuli* gene accession IDs	NCBI-KOG	TFs
Ca4_P4_25365837	Ca4	25365837	GTTTTTTT/GTTTTTTTT	AATTTTGTTGCCCCTCCAAT	TGCTATGGCACAAGTCTCCA	60.5	547	INTERGENIC	--	--	--
Ca4_P4_25365904	Ca4	25365904	TTTTTT/TTTTTTTTTTATTTTT	AATTTTGTTGCCCCTCCAAT	TGCTATGGCACAAGTCTCCA	60.5	547	INTERGENIC	--	--	--
Ca4_P4_25366618	Ca4	25366618	AAATCCACTAAGGAAT/AAAT	TCTTGGAAAGACGCGTGTAA	CACCTTGAATCCAACCTTGA	59.5	227	INTERGENIC	--	--	--
Ca4_P4_25399861	Ca4	25399861	TA/TAAA	TTTAAAATGGAATGGGCTGTG	TTTCCGTGTGTCTCGTATGC	59.8	322	INTERGENIC	--	--	--
Ca4_P4_25439114	Ca4	25439114	TAA/TA	GAGGTGGAGAATGATTGGTCA	TCGAGACTTGGCTCATGTTG	59.9	674	INTERGENIC	--	--	--
Ca4_P4_25466708	Ca4	25466708	CATATATATATATAT/CATATATATATAT	TATAGGGAGCGGAACACACC	GCTTCACACAAGTCACTTCCA	60.0	367	INTERGENIC	--	--	--
Ca4_P4_25467890	Ca4	25467890	ATTTTTTTTTTTT/ATTTTTTTTTTT	TTGGGAATTAACATGAGAGCAA	TGTGTTGATAAAAACAAGATTTCAA	59.6	802	INTERGENIC	--	--	--
Ca4_P4_25468010	Ca4	25468010	TTATATATATATATATATATATAT/TTATATATATATATATATATATATATATAT	TTCAAAATACAAATGTGATGGAAAA	TTTTTGTTTCCTTATTGTTTGACA	59.7	650	INTERGENIC	--	--	--
Ca4_P4_25478172	Ca4	25478172	TAAAA/TAAAAA	TGTGATGCAGTTGCTCCATT	GACCTTGCTCTCTTGATGGG	60.3	703	INTERGENIC	--	--	--
Ca4_P4_25519864	Ca4	25519864	GAAAAAAAA/GAAAAAAAAA	GGGTCCTCGATCAAAGAGGT	TGTCAGTTGAGCTCCTGTCG	60.5	417	INTERGENIC	--	--	--

INDEL marker IDs	Chromosomes/ unanchored scaffolds	Physical positions (bp)	InDels (*Kabuli* reference genome-CDC Frontier/PII)	Forward primers (5'-3')	Reverse primers (5'-3')	Anneling temperature (0C)	Expected amplified product size (bp)	Structural annotation		Functional annotation	
								Sequence components of *kabuli* genome	*Kabuli* gene accession IDs	NCBI-KOG	TFs
Ca4_P4_25524341	Ca4	25524341	CAAAA/CAA	CTTTGGACGAGAGCCAAAAC	TTGGAAGGAATTTGAATGGC	59.9	764	INTERGENIC	--	--	--
Ca4_P4_25535456	Ca4	25535456	ACACCTTGAGTTTGGTCCA/ACACCTTGAGTTTGGTCCACCTTGAGTTTGGTCCA	TGTGGGATGCATGTCCTTTA	TTGAAATTGAACCAATCCGA	59.9	682	INTERGENIC	--	--	--
Ca4_P4_25543812	Ca4	25543812	TTTTCTTCTTTCTTCTTTC/TTTTCTTCTTTC	AGGACTTTCGACCACCACAC	TACCCTTTGCTAGCCCCTTT	60.0	415	INTERGENIC	--	--	--
Ca4_P4_25589839	Ca4	25589839	ATTTTTTTTT/ATTTTTTTT	AATTACGCCAAAGGATGCAC	CCCTGTGACGAACTGGAGAT	60.0	789	INTERGENIC	--	--	--
Ca4_P4_25596032	Ca4	25596032	TTAT/TTATAT	AGTGATGTGGGCAACAATGA	TGCATTTTACAGCGCTTTTG	60.0	515	INTERGENIC	--	--	--
Ca4_P4_25630012	Ca4	25630012	TG/T	AGTAGACTTGCCTCCCGGTT	CCTTGTTGTGGAGTGTGGTG	60.1	788	INTERGENIC	--	--	--
Ca4_P4_25642263	Ca4	25642263	CAAAAAA/CAAAAA	TTTGTTGTGGTGTTCTGGGA	TACAACCAAACCCCTTGAGC	60.0	318	INTERGENIC	--	--	--
Ca4_P4_25671434	Ca4	25671434	CAAGAAG/CAAG	CTCCGACAGGCTACTTCCAG	TGAGATGAAGCAAATGCTCG	60.0	347	INTERGENIC	--	--	--
Ca4_P4_25694964	Ca4	25694964	CAAAAAAAAA/CAAAAAAAAAAAA	CCACTTCGCTTAGGGATCAA	TATGGGCATTCTTCTTTGGG	60.2	675	INTERGENIC	--	--	--
Ca4_P4_25720958	Ca4	25720958	AAAGAAAGAGATA/AA	ACCACGCCATTCAAAATAGG	AGGACTTGACGTAACCCACG	59.8	738	INTERGENIC	--	--	--

INDEL marker IDs	Chromosomes/ unanchored scaffolds	Physical positions (bp)	InDels (*Kabuli* reference genome-CDC Frontier/PII)	Forward primers (5'-3')	Reverse primers (5'-3')	Anneling temperature (0C)	Expected amplified product size (bp)	Structural annotation		Functional annotation	
								Sequence components of *kabuli* genome	*Kabuli* gene accession IDs	NCBI-KOG	TFs
Ca4_P4_25754372	Ca4	25754372	AAAAATAAAATAAAATAAA/AAAAATAAAATAAA	AATGGGCGCTGTTAAATGTC	TCATTTAAACCACTGCGTCAA	60.0	750	INTERGENIC	--	--	--
Ca4_P4_25758155	Ca4	25758155	CAAGTAAGT/CAAGT	TTGGTCCTCGTCATTTCCTC	TCCAAACTTCCATGTGATTCC	60.1	422	INTERGENIC	--	--	--
Ca4_P4_25760041	Ca4	25760041	ATTTTTTT/ATTTTTT	CACAACGGAATTTGGGTTTC	TTGAGCTTGCAACTTGATCACT	60.2	565	INTERGENIC	--	--	--
Ca4_P4_25760083	Ca4	25760083	ATTTTTT/ATTTTTTT	CGGATTTTGTTGTCATCCCT	TTGAGCTTGCAACTTGATCACT	59.8	181	INTERGENIC	--	--	--
Ca4_P4_25761284	Ca4	25761284	ATTTTTTTTT/ATTTTTTTTTT	TTGTTGTGTGCAAGAGGATTG	GGAGATCATGGACGAAGGTG	59.7	366	INTERGENIC	--	--	--
Ca4_P4_25763103	Ca4	25763103	CTT/CT	ATCCTTGCGGGGATTTAGAG	ATTGCCTCATCCAAACGAAC	60.4	295	INTERGENIC	--	--	--
Ca4_P4_25774052	Ca4	25774052	TAAAAAAAAAAAA/TAAAAAAAAAAA	TGTCAAATCCAACGCAAAGA	TCGCAACCAAACAAAGCATA	60.2	661	INTERGENIC	--	--	--
Ca4_P4_25776089	Ca4	25776089	G/GAC	TGGAATGAGAGGGGAAGATG	TCCGAGACAGGTATTTTGGC	60.0	366	INTERGENIC	--	--	--
Ca4_P4_25790471	Ca4	25790471	CAAACGAAACGAAACGAAAC/CAAACGAAAC	CCTCAAATTCCAATTGTGCC	TCATCCTCACCCCCAGTTAG	60.3	646	INTERGENIC	--	--	--
Ca4_P4_25822369	Ca4	25822369	CAAAAAAA/CAAAAAA	TGTTTGAACCCGAGACTTCAC	CGTGGCATTTGAAAAAGCAT	60.1	611	INTERGENIC	--	--	--

INDEL marker IDs	Chromosomes/ unanchored scaffolds	Physical positions (bp)	InDels (*Kabuli* reference genome-CDC Frontier/PII)	Forward primers (5'-3')	Reverse primers (5'-3')	Anneling temperature (0C)	Expected amplified product size (bp)	Structural annotation		Functional annotation	
								Sequence components of *kabuli* genome	*Kabuli* gene accession IDs	NCBI-KOG	TFs
Ca4_P4_25830993	Ca4	25830993	CTTTTTTTTTTTT/CTTTTTTTTTT	CCCAGAAGGAGCAATTTGAA	TTGCGGTTTCCTTAATGGTC	60.2	315	INTERGENIC	--	--	--
Ca4_P4_25834950	Ca4	25834950	AAGACGTTCCGTA/AA	TCGCTGCTTATTCATGTTCA	ACCACCAGTGAGGATTTTGC	58.0	336	INTERGENIC	--	--	--
Ca4_P4_25847698	Ca4	25847698	T/TG	CATGCTACCAAAGCGATTGA	ATCGATTTCGCGAGTCTTGT	59.8	370	INTERGENIC	--	--	--
Ca4_P4_25848017	Ca4	25848017	AA/AAGA	CATGCTACCAAAGCGATTGA	CCCAAGAAAGTAGCCACCAG	59.8	806	INTERGENIC	--	--	--
Ca4_P4_25860833	Ca4	25860833	GAAAAAAAAAAA/GAAAAAAAAAA	CAGCTCGCATAAACCAATCA	AAAGCATTGTCGTAGGTGGC	59.8	630	INTERGENIC	--	--	--
Ca4_P4_25869082	Ca4	25869082	ATTTTT/ATTTT	TATTTGTCGATTTGCGTGGA	TTATAAGTGCGGTTAGCGGG	60.1	693	INTERGENIC	--	--	--
Ca4_P4_25925060	Ca4	25925060	GTTTTTTT/GTTTTTTTT	TTTCGCACAGCATAGGACAG	CAACGGCGCTGAGATTTATT	60.0	661	INTERGENIC	--	--	--
Ca4_P4_25933731	Ca4	25933731	ACGCGCGCGCG/ACGCGCGCGCGCG	TGGCATTGATGATGGCTTTA	ACCTTGTGGATGGCAGTTTC	60.0	340	INTERGENIC	--	--	--
Ca4_P4_25933783	Ca4	25933783	CTCGGTTCGGTTCGG/CTCGGTTCGGTTCGGTTCGG	TGGCATTGATGATGGCTTTA	ACCTTGTGGATGGCAGTTTC	60.0	340	INTERGENIC	--	--	--
Ca4_P4_25936338	Ca4	25936338	GTTTTTTTTTT/GTTTTTTTTT	GAGGTTTCGATTCCTCCACA	AAATTGCAATAGGTCGTCCG	60.1	705	INTERGENIC	--	--	--

INDEL marker IDs	Chromosomes/ unanchored scaffolds	Physical positions (bp)	InDels (*Kabuli* reference genome-CDC Frontier/PII)	Forward primers (5'-3')	Reverse primers (5'-3')	Anneling temperature (0C)	Expected amplified product size (bp)	Structural annotation		Functional annotation	
								Sequence components of *kabuli* genome	*Kabuli* gene accession IDs	NCBI-KOG	TFs
Ca4_P4_25936921	Ca4	25936921	CTTTTTTTTTTTT/CTTTTTTTTTTT	AGGTGCGAATATCCACCTTG	AAATGGACAACGTCGAAAGG	60.0	445	INTERGENIC	--	--	--
Ca4_P4_25937535	Ca4	25937535	GC/GCTTTTGCTC	TCGACGTTGTCCATTTGTGT	ACACGAGCGTTTGAGCTTTT	60.0	621	INTERGENIC	--	--	--
Ca4_P4_25941584	Ca4	25941584	A/AG	CGATCGTTGTTGGTATGTCG	AGCTGACCAAATGCGTAAGG	60.0	244	INTERGENIC	--	--	--
Ca4_P4_25941878	Ca4	25941878	CAA/CAAAA	CGATCGTTGTTGGTATGTCG	CCGTTTTTCTTGCGATTCTC	60.0	899	INTERGENIC	--	--	--
Ca4_P4_25949029	Ca4	25949029	CTTTTT/CTTTTTT	TTCATTCAGATTGATTATGCTCG	GCTAAACACATGGAGATCAAACA	59.2	754	INTERGENIC	--	--	--
Ca4_P4_25950770	Ca4	25950770	CAAAAAAAAAAAAAA/CAAAAAAAAAAAAA	GATCTTTGCTTAAGCGCTGG	TTTCCAATCAAAATGGCACA	60.1	505	INTERGENIC	--	--	--
Ca4_P4_25958010	Ca4	25958010	TTATATATATAT/TTATATATATATAT	TCGGGGAAATCAATTCGTTA	TGGAAGTCATCGACCCATTT	60.3	673	INTERGENIC	--	--	--
Ca4_P4_25959074	Ca4	25959074	GCC/GC	TGAAGATGAAGAAGAGGGCAA	TGTGGCATAAGGAGGAGTTTC	59.9	376	INTERGENIC	--	--	--
Ca4_P4_25966069	Ca4	25966069	ATTT/ATTTT	GGCATGGACAACTTCACCTT	CGAAATCCTCAAAATCGGAA	60.0	484	INTERGENIC	--	--	--
Ca4_P4_25967972	Ca4	25967972	ATTTTTTTT/ATTTTTTTTT	TTGTACCCTTGTGCAACCAA	TGGTTGGATACTTGCAATGG	60.0	180	INTERGENIC	--	--	--

INDEL marker IDs	Chromosomes/ unanchored scaffolds	Physical positions (bp)	InDels (*Kabuli* reference genome-CDC Frontier/PII)	Forward primers (5'-3')	Reverse primers (5'-3')	Anneling temperature (0C)	Expected amplified product size (bp)	Structural annotation		Functional annotation	
								Sequence components of *kabuli* genome	*Kabuli* gene accession IDs	NCBI-KOG	TFs
Ca4_P4_25968820	Ca4	25968820	GTTT/GTTTT	TTCTTCTGCCCAAAAATGCT	CCTCAAGCAGACCTTTGGAG	59.8	412	INTERGENIC	--	--	--
Ca4_P4_25969511	Ca4	25969511	ACC/AC	TCCCTTGCCTCTAGCCAGTA	GCAGATGTTCTCAACAGCCA	60.0	350	INTERGENIC	--	--	--
Ca4_P4_25970159	Ca4	25970159	AC/A	TGGTTGGGTCTTGGTTTGTT	CACCTGCTATGGATGCAATG	60.2	709	INTERGENIC	--	--	--
Ca4_P4_25971278	Ca4	25971278	GATTGAAGAA/GA	GCTTCGCCAAGCTTACAATC	CCAACGATCATCAAAGGGAT	60.0	268	INTERGENIC	--	--	--
Ca4_P4_25976449	Ca4	25976449	CTTTTTT/CTTTTT	CCCCTTTTTGCACATGATCT	GGGAACTCCTATCCACCCAT	59.9	510	INTERGENIC	--	--	--
Ca4_P4_25978445	Ca4	25978445	CT/CTTT	TGCCTCAAAGGCTTCATCTT	AGCACTCATCCTCGGGTAGA	60.0	435	INTERGENIC	--	--	--
Ca4_P4_25979689	Ca4	25979689	TG/TGG	GTCCTCGTCAGGAGGTTGAG	CCACTGTTGCTGGCAGATAA	59.8	707	INTERGENIC	--	--	--
Ca4_P4_25982514	Ca4	25982514	CAATAATA/CAATAATAATA	GCGCAAAAACAAGAAGGAAG	GGTCCAGGTTGTTTGGAGAA	60.0	290	INTERGENIC	--	--	--
Ca4_P4_25984201	Ca4	25984201	AG/A	CGATTTTTAACAGCGTGGGT	TTTGGGCTTTGCTCTCAAAT	60.0	539	INTERGENIC	--	--	--
Ca4_P4_25984442	Ca4	25984442	TGAGAG/TGAGAGAG	AGATCCTGTTGACACGGCTT	CGGTTGTTGTTGTGATTTGC	59.7	524	INTERGENIC	--	--	--

INDEL marker IDs	Chromosomes/ unanchored scaffolds	Physical positions (bp)	InDels (*Kabuli* reference genome-CDC Frontier/PII)	Forward primers (5'-3')	Reverse primers (5'-3')	Anneling temperature (0C)	Expected amplified product size (bp)	Structural annotation		Functional annotation	
								Sequence components of *kabuli* genome	*Kabuli* gene accession IDs	NCBI-KOG	TFs
Ca4_P4_25987622	Ca4	25987622	TCC/TC	TTCAACTGGGGTAAATGCCT	TGTTCTAGTCCAGATCAAAACGA	59.4	323	INTERGENIC	--	--	--
Ca4_P4_25987851	Ca4	25987851	CTTT/CT	TTCCTTCCTAATGTTCGTTTTGA	CGTGTCTCATCTTTGTGCTTT	60.0	732	INTERGENIC	--	--	--
Ca4_P4_25988978	Ca4	25988978	AAAGAAGAAGAAG/AAAGAAGAAG	AAGCTGGATGTGGACTTTGC	AGTTCCAATTGGCCTCCTTT	60.3	404	INTERGENIC	--	--	--
Ca4_P4_25989281	Ca4	25989281	TCCCC/TCCC	AAGCTGGATGTGGACTTTGC	AGTTCCAATTGGCCTCCTTT	60.3	404	INTERGENIC	--	--	--
Ca4_P4_25989322	Ca4	25989322	GTTTTT/GTTTTTT	GTACGCGATCTAGTCCCCAA	CCGGGGAGAAAGTTCTAGTG	60.1	168	INTERGENIC	--	--	--
Ca4_P4_25994447	Ca4	25994447	G/GT	ATCCACAACCGAGTTAGCGT	TACCCACACCCCTCAATCTT	59.6	389	INTERGENIC	--	--	--
Ca4_P4_25994543	Ca4	25994543	GTTTTTT/GTTTTTTT	TTAAGATTGAGGGGTGTGGG	CACCACTTCATTCGGGACTT	59.8	516	INTERGENIC	--	--	--
Ca4_P4_25994721	Ca4	25994721	AC/A	TTAAGATTGAGGGGTGTGGG	CACCACTTCATTCGGGACTT	59.8	516	INTERGENIC	--	--	--
Ca4_P4_25995802	Ca4	25995802	ATTTT/ATTT	CCCTAAATCAAAGCAGCGTC	TGCACTGTTGTTGTTGCTGA	59.8	470	INTERGENIC	--	--	--
Ca4_P4_26009737	Ca4	26009737	TC/T	GGAACTTCTCTTGTGGCAGC	ACCCTTTGCATCCCTTTCTT	60.0	617	INTERGENIC	--	--	--

INDEL marker IDs	Chromosomes/ unanchored scaffolds	Physical positions (bp)	InDels (*Kabuli* reference genome-CDC Frontier/PII)	Forward primers (5'-3')	Reverse primers (5'-3')	Anneling temperature (0C)	Expected amplified product size (bp)	Structural annotation		Functional annotation	
								Sequence components of *kabuli* genome	*Kabuli* gene accession IDs	NCBI-KOG	TFs
Ca4_P4_26018351	Ca4	26018351	TAAAAAAAA/TAAAAAAAAA	AGAACATGGTGGATGCACAG	TCCAAGAGCTTGGTCTGACA	59.6	373	INTERGENIC	--	--	--
Ca4_P4_26020559	Ca4	26020559	CTTTTTTTTTTT/CTTTTTTTTTT	CGTTTGCAATTTCTTTTGGG	TGTTCCAGTTCCACGTCAGA	60.5	518	INTERGENIC	--	--	--
Ca4_P4_26058753	Ca4	26058753	TAAA/TAAAA	AGGAACCAATCCTACCAGCC	TCTGGTCATGCATCCTTTGA	60.3	433	INTERGENIC	--	--	--
Ca4_P4_26071794	Ca4	26071794	CCACA/CCA	ATCGAGTGGCCAATTGAAAG	ACAGAAACCACATTCGCACA	60.1	729	INTERGENIC	--	--	--
Ca4_P4_26073975	Ca4	26073975	TAAAAAAAAAAA/TAAAAAAAAAA	GCTAAATAGCACTCACGATCCC	TTTGCTTGCTGAGTGATTGG	60.1	873	INTERGENIC	--	--	--
Ca4_P4_26074517	Ca4	26074517	GTTTA/GTTTAAATAAAATATTTTA	CCAATCACTCAGCAAGCAAA	CGAAGCATCCTGCATTTAAG	60.0	354	INTERGENIC	--	--	--
Ca4_P4_26075846	Ca4	26075846	TAAAAAA/TAAAAAAA	TTTAACAAAATCAAACAAACAGGA	AAGTGAAAAATATGTGTCCCGTACT	57.9	220	INTERGENIC	--	--	--
Ca4_P4_26082693	Ca4	26082693	TG/T	TTGATGGCTACATGGGATGA	TTTGGTTTGGTTCTGGTTTG	59.9	572	INTERGENIC	--	--	--
Ca4_P4_26108429	Ca4	26108429	GAAAAAAAAA/GAAAAAAAA	TTTGCGTTACGAAATTGGTG	TGCACTGCTAATTGCCTCTG	59.6	328	INTERGENIC	--	--	--
Ca4_P4_26110333	Ca4	26110333	TCCC/TCCCC	CCAACAATCTCCCCCTTTTT	GCATCTGGTTGCAGTTCTCA	60.2	583	INTERGENIC	--	--	--

INDEL marker IDs	Chromosomes/ unanchored scaffolds	Physical positions (bp)	InDels (*Kabuli* reference genome-CDC Frontier/PII)	Forward primers (5'-3')	Reverse primers (5'-3')	Anneling temperature (0C)	Expected amplified product size (bp)	Structural annotation		Functional annotation	
								Sequence components of *kabuli* genome	*Kabuli* gene accession IDs	NCBI-KOG	TFs
Ca4_P4_26110396	Ca4	26110396	CTTTTTTTTTT/CTTTTTTTTTTT	CCAACAATCTCCCCCTTTTT	GCATCTGGTTGCAGTTCTCA	60.2	583	INTERGENIC	--	--	--
Ca4_P4_26121192	Ca4	26121192	CTTTTTTTTT/CTTTTTTTTTT	GCTGGTTTTTGGCATGATTT	GAGGCATGCATTTGTCTCATT	59.9	525	INTERGENIC	--	--	--
Ca4_P4_26123152	Ca4	26123152	CTTTTTTTTTT/CTTTTTTTTTTT	CAAGCATGCAAATAAGCGAA	GCAGCAAGATGCAACTCTCA	60.0	640	INTERGENIC	--	--	--
Ca4_P4_26136319	Ca4	26136319	TTTTATTT/TTTT	TTTGACGCATTTTGTTCACAT	TGCTCTCTCTCCCTCACCAT	59.1	600	INTERGENIC	--	--	--
Ca4_P4_26140041	Ca4	26140041	TAAAAAAA/TAAAAAAAA	GAAGCCAACATGGCTCAAAT	AGCAGGGATTTTTGGCTTTT	60.1	754	INTERGENIC	--	--	--
Ca4_P4_26166307	Ca4	26166307	TAAAAAAAAAAAAA/TAAAAAAAAAAAA	GCATGCCATGAATCACACTT	GCACGTGGGTACAGTGATTG	59.5	803	INTERGENIC	--	--	--
Ca4_P4_26199834	Ca4	26199834	A/AT	GGGCAAGGTGAACACAAGAT	GCATCGTCCTTCCATGATTT	60.0	383	INTERGENIC	--	--	--
Ca4_P4_26205632	Ca4	26205632	TAAAAAAAAAAAAA/TAAAAAAAAAAAA	TGTGTCATGAAAAATTTAAGGGTT	TTTTCACTTTAGGTCCTTGATTTT	58.9	876	INTERGENIC	--	--	--
Ca4_P4_26236698	Ca4	26236698	TGGGGG/TGGGG	GTTACGATTGTGTTCGGCCT	TAATCTCACTGCGCACCTTG	60.0	368	INTERGENIC	--	--	--
Ca4_P4_26259040	Ca4	26259040	ATTTTTTTTTT/ATTTTTTTTT	TTCTCGAATCAATTCCGTCC	CTCAGAGGCCGACCTATACA	60.0	888	INTERGENIC	--	--	--

INDEL marker IDs	Chromosomes/ unanchored scaffolds	Physical positions (bp)	InDels (*Kabuli* reference genome-CDC Frontier/PII)	Forward primers (5'-3')	Reverse primers (5'-3')	Anneling temperature (0C)	Expected amplified product size (bp)	Structural annotation		Functional annotation	
								Sequence components of *kabuli* genome	*Kabuli* gene accession IDs	NCBI-KOG	TFs
Ca4_P4_26301785	Ca4	26301785	GAAAAAAAAA/GAAAAAAAAAA	ACCACTACGCCACACATCAA	GTGACAGTACTGGGGGAAGC	60.0	428	INTERGENIC	--	--	--
Ca4_P4_26313553	Ca4	26313553	TAAAAAAAAAA/TAAAAAAAAAAAA	TGAGGTATGCCCAAAGAAGG	TTTTGAAACATGAATTTCGGA	60.1	319	INTERGENIC	--	--	--
Ca4_P4_26313656	Ca4	26313656	ATTTTTTTTTTT/ATTTTTTTTTT	TGAGGTATGCCCAAAGAAGG	TTAGGCCGTAGTCAGGCTGT	60.1	837	INTERGENIC	--	--	--
Ca4_P4_26314670	Ca4	26314670	TAAAAAAAAAAA/TAAAAAAAAAA	ATGTTGTGCACCGGAAAACT	CCCAATATCCGAGTGTGTTCT	60.4	362	INTERGENIC	--	--	--
Ca4_P4_26339363	Ca4	26339363	CAAGAAGAAGAAGAAGAAGAAGAA/ CAAGAAGAAGAAGAAGAAGAAGAAGAAGAA	TCAAGACGTTGATCTCGTGC	CATCTTCATTGCCTCCCATT	60.0	397	INTERGENIC	--	--	--
Ca4_P4_26361460	Ca4	26361460	CTTTTTTT/CTTTTTT	GAAACTCGCAGACTTCGGAC	TATTGGGCGAATCAAGGAAG	60.0	520	INTERGENIC	--	--	--
Ca4_P4_26363618	Ca4	26363618	T/TTTAC	GCCAAAATTTCCTTCGTTGA	ATTCCCCAAGAACATCACCA	60.1	613	INTERGENIC	--	--	--
Ca4_P4_26363648	Ca4	26363648	CCTC/CCTCTC	GCCAAAATTTCCTTCGTTGA	ATTCCCCAAGAACATCACCA	60.1	613	INTERGENIC	--	--	--
Ca4_P4_26389681	Ca4	26389681	ATTTTTTTTTT/ATTTTTTTTT	CCGAGCCAAAATCCAAAATA	AGGGGCCTATGACTTGGTCT	59.9	643	INTERGENIC	--	--	--
Ca4_P4_26398945	Ca4	26398945	AATTTAATTT/AATTTAATTTATCTATTTAATTT	GTGGCGAATTGATTTGTCCT	CGACGTAATCTGGGTTGGTT	59.9	413	INTERGENIC	--	--	--

INDEL marker IDs	Chromosomes/ unanchored scaffolds	Physical positions (bp)	InDels (*Kabuli* reference genome-CDC Frontier/PII)	Forward primers (5'-3')	Reverse primers (5'-3')	Anneling temperature (0C)	Expected amplified product size (bp)	Structural annotation		Functional annotation	
								Sequence components of *kabuli* genome	*Kabuli* gene accession IDs	NCBI-KOG	TFs
Ca4_P4_26411287	Ca4	26411287	GTTT/GTTTT	TGTTCTGCTTAAGGGGCCTA	GATCAAGAGGTCACCGGTTC	59.8	841	INTERGENIC	--	--	--
Ca4_P4_26456492	Ca4	26456492	T/TG	GCGAGCATGAGAAGTTGTTG	AATTCAATGGGAAAAACCACA	59.6	713	INTERGENIC	--	--	--
Ca4_P4_26459581	Ca4	26459581	GTATTGATGTT/GT	CCAACTTATGAGCCCCTCAA	TAATTGAATCAGGGAAGGCG	60.1	597	INTERGENIC	--	--	--
Ca4_P4_26488208	Ca4	26488208	GAAACA/GA	AAATTGAACCGTCAAATCGG	TCCTCGACCGTGTAATTTCC	59.8	785	INTERGENIC	--	--	--
Ca4_P4_26492251	Ca4	26492251	ACTC/AC	CAAATGCACATGAGAACCGT	AATGATCCGGAACCATTTCA	59.6	519	INTERGENIC	--	--	--
Ca4_P4_26495554	Ca4	26495554	GAAAGTTGTTC/GAAAGTTGTTCAAAGTTGTTC	AACGATTTATCCTGGTCCCC	CCCGAATACAAACCCCAAGTC	60.0	844	INTERGENIC	--	--	--
Ca4_P4_26498057	Ca4	26498057	GTTTTTT/GTTTTT	ATGCTCTTGTGTGGCTAGGG	TGTTTGACGACAAGCAAAGG	60.3	850	INTERGENIC	--	--	--
Ca4_P4_26514892	Ca4	26514892	GAAAAAAAA/GAAAAAAA	TGCTCTTGGGGATGTCAAAT	TGGTCCGGTCTAGAAGCCTA	60.5	687	INTERGENIC	--	--	--
Ca4_P4_26525402	Ca4	26525402	GTTTTTTTTTT/GTTTTTTTTTTTT	TGTAGCTACCGCATTGATGG	GCTGCAAGGGATGATTTGAT	59.7	436	INTERGENIC	--	--	--
Ca4_P4_26526816	Ca4	26526816	GTTTTTT/GTTTTTTT	TGGAAATGGTTCAGGTGTCA	TTCATGAAAGGCCATTCTCC	59.9	255	INTERGENIC	--	--	--

INDEL marker IDs	Chromosomes/ unanchored scaffolds	Physical positions (bp)	InDels (*Kabuli* reference genome-CDC Frontier/PII)	Forward primers (5'-3')	Reverse primers (5'-3')	Anneling temperature (0C)	Expected amplified product size (bp)	Structural annotation		Functional annotation	
								Sequence components of *kabuli* genome	*Kabuli* gene accession IDs	NCBI-KOG	TFs
Ca4_P4_26529457	Ca4	26529457	ATCTCTCTCTCTCTC/ATCTCTCTCTCTCTCTC	ATAGCAGCAATTTCCATCCG	GTCCCACGAAGTAAGTCCCA	60.1	286	INTERGENIC	--	--	--
Ca4_P4_26542242	Ca4	26542242	TAAAAA/TAAAAAA	GCACGGAAGAAGACGAGTTT	GGCACACGTAAATGCACATC	59.5	185	INTERGENIC	--	--	--
Ca4_P4_26547528	Ca4	26547528	AT/A	TCCAAAACTTGTATATCCTCAAGC	TTTGTGCTTCTGCAACAAGG	58.8	815	INTERGENIC	--	--	--
Ca4_P4_26552272	Ca4	26552272	TAAAAAAA/TAAAAAA	TCCTGGTTCACTCAATGTGG	CTCAAGTTGAAGGCCCAAAG	59.5	818	INTERGENIC	--	--	--
Ca4_P4_26620833	Ca4	26620833	GTT/GT	AAAGAAGGAAGGGTCTTGCC	TCAAAATCGAACCAAATCACA	59.7	593	INTERGENIC	--	--	--
Ca4_P4_26622922	Ca4	26622922	GAAAAAAAAA/GAAAAAAAA	TGGCTGTTGTCCATTGTGTT	TCAGACACAGTTGGTCGGTT	60.0	604	INTERGENIC	--	--	--
Ca4_P4_26662668	Ca4	26662668	ATTTTTTTTTT/ATTTTTTTTT	GTGACGTGGGGGAGTTAAGA	CAACGACCTTAACATTTGGTG	60.0	799	INTERGENIC	--	--	--
Ca4_P4_26728004	Ca4	26728004	TG/T	ATGGTCGTGTTCCCACATTT	TTGTTGATTAAAGTGGCCGA	60.1	832	INTERGENIC	--	--	--
Ca4_P4_26733428	Ca4	26733428	TAAAAAAAAA/TAAAAAAAA	AGTACCATCCAAGTGCGGTC	TCTTTTTCACCATCCCGACT	60.0	302	INTERGENIC	--	--	--
Ca4_P4_26739311	Ca4	26739311	TAAAAAAAAAA/TAAAAAAAAA	TCCATGATTTGGTGGTTCCT	TTCTTCAGCAATGGTCCTCC	60.2	492	INTERGENIC	--	--	--

INDEL marker IDs	Chromosomes/ unanchored scaffolds	Physical positions (bp)	InDels (*Kabuli* reference genome-CDC Frontier/PII)	Forward primers (5'-3')	Reverse primers (5'-3')	Anneling temperature (0C)	Expected amplified product size (bp)	Structural annotation		Functional annotation	
								Sequence components of *kabuli* genome	*Kabuli* gene accession IDs	NCBI-KOG	TFs
Ca4_P4_26740657	Ca4	26740657	TAAT/TAATCAAT	TTGACCACAGGTCCACAAGA	TTTTAGGTGCGCATTTACCC	60.1	250	INTERGENIC	--	--	--
Ca4_P4_26750453	Ca4	26750453	GTATA/GTATATA	AAAAAGCAAATGATCCCATCC	TGGATCCTCTCAATTCAAATCTC	60.1	672	INTERGENIC	--	--	--
Ca4_P4_26753796	Ca4	26753796	G/GATTTA	AGAGCGGCAACTCAAACAAT	TGCAGGGGACATCTCTTACC	59.9	613	INTERGENIC	--	--	--
Ca4_P4_26775300	Ca4	26775300	TAAAAAAAAAAA/TAAAAAAAAAAAA	ACGGCGCTAAACCAACATAG	TTGTGTGCTTAACCTTTGCG	60.2	320	INTERGENIC	--	--	--
Ca4_P4_26778486	Ca4	26778486	GTTTTTTTTT/GTTTTTTTT	GCCCATCCAAGTGATGAGAT	TGAATCGCGAAAGTAGTCCC	59.9	643	INTERGENIC	--	--	--
Ca4_P4_26778550	Ca4	26778550	CTTT/CTTTT	GCCCATCCAAGTGATGAGAT	TTCCTTTCGGCAAAAATCAC	59.9	811	INTERGENIC	--	--	--
Ca4_P4_26792383	Ca4	26792383	CTTTT/CTTTTT	AAGGCAAATGAGGGTGTTTG	TTGGGCGTATAGTGATGCAA	60.0	612	INTERGENIC	--	--	--
Ca4_P4_26842237	Ca4	26842237	GAAAAAAAAAA/GAAAAAAAAA	TAGCATGCCTCAAATCCCTC	AGCAACACCGTATTGACATCC	60.2	726	INTERGENIC	--	--	--
Ca4_P4_26847079	Ca4	26847079	AAAAATAAAATAAAATAAAATA/AAAAATAAAATAAAATAAAATAAAATA	GGAGAACGTTAAGGCAGCAG	GTGGTGTAGGTTTCGATCCG	60.0	689	INTERGENIC	--	--	--
Ca4_P4_26847495	Ca4	26847495	A/AC	AGCTTCAAGTCGGATCGAAA	AAAAATCTTGTGGGAATAATTCAA	60.0	216	INTERGENIC	--	--	--

INDEL marker IDs	Chromosomes/ unanchored scaffolds	Physical positions (bp)	InDels (*Kabuli* reference genome-CDC Frontier/PII)	Forward primers (5'-3')	Reverse primers (5'-3')	Anneling temperature (0C)	Expected amplified product size (bp)	Structural annotation		Functional annotation	
								Sequence components of *kabuli* genome	*Kabuli* gene accession IDs	NCBI-KOG	TFs
Ca4_P4_26850722	Ca4	26850722	TAA/TAAA	GGAGAACGTTAAGGCAGCAG	GTGGTGTAGGTTTCGATCCG	60.0	726	INTERGENIC	--	--	--
Ca4_P4_26903304	Ca4	26903304	T/TG	ACTCGATTGGTCCATGCTTC	ATGTTTTGGACGCACACAGA	60.1	532	INTERGENIC	--	--	--
Ca4_P4_2690387	Ca4	2690387	ATTTTTTTTTTTTT/ATTTTTTTTTTTT	TCCTTTTTAAGTGTCGTCTTTCTG	TCAGACACAGATGCGAGGTC	59.0	728	INTERGENIC	--	--	--
Ca4_P4_26936840	Ca4	26936840	CAAAAAAAA/CAAAAAAAAA	TCAAGGCTTTAGGCGTGAGT	GTAAATGCGTTGTGCGATCA	60.0	615	INTERGENIC	--	--	--
Ca4_P4_26941918	Ca4	26941918	ATTTTTTTTTT/ATTTTTTTTT	GAGGGTTTAGTTCGGAAGGG	CAAAAATTGCATAAAATGAAACAA	59.9	851	INTERGENIC	--	--	--
Ca4_P4_26954877	Ca4	26954877	GTTTTTTTTTT/GTTTTTTTTTTTT	TCAATTTTGATGATGTGGCAA	CGGTCAAACAATCAGCTTCA	59.9	405	INTERGENIC	--	--	--
Ca4_P4_26957417	Ca4	26957417	GTTTTGATTTTTTTTTT/GTTTTGATTTTTTTTTATTTTTGATTTTTTTTTT	GTTTGTCCCCAAGCAACAAT	CTAGCCCATTTGAGCTTTGC	59.8	642	INTERGENIC	--	--	--
Ca4_P4_26959252	Ca4	26959252	TAAA/TAAAA	ACCACCAGCACACGACATTA	GGGGTTAGGGGTTACTTTGG	60.0	529	INTERGENIC	--	--	--
Ca4_P4_26967509	Ca4	26967509	TAAAAAAAA/TAAAAAAAAA	TTGGTGGGTTGTGTGTAATGA	TGAATGGGAAATGGAGGAAG	59.7	582	INTERGENIC	--	--	--
Ca4_P4_26992631	Ca4	26992631	T/TTAA	CACATCATCCTGAAACAACTCA	CAATTCAAAGGGGACTCGAA	58.6	650	INTERGENIC	--	--	--

INDEL marker IDs	Chromosomes/ unanchored scaffolds	Physical positions (bp)	InDels (*Kabuli* reference genome-CDC Frontier/PII)	Forward primers (5'-3')	Reverse primers (5'-3')	Anneling temperature (0C)	Expected amplified product size (bp)	Structural annotation		Functional annotation	
								Sequence components of *kabuli* genome	*Kabuli* gene accession IDs	NCBI-KOG	TFs
Ca4_P4_27017251	Ca4	27017251	GTTTTTTTTT/GTTTTTTTTTT	CCTTTGCATCTCAGGGACAT	TGAGACCGGGTTTGAGTACC	60.1	453	INTERGENIC	--	--	--
Ca4_P4_27052671	Ca4	27052671	ATTTTTTTTTT/ATTTTTTTTT	GGCATGGTTTAACTTGACACG	TGAGAAAATCCAAATCTTGCG	60.4	416	INTERGENIC	--	--	--
Ca4_P4_27067931	Ca4	27067931	ATTTTTTTT/ATTTTTTTTT	AACCTCCATTAATTTTTGCCA	CATTTGCAATTCAAGCGAAC	58.5	438	INTERGENIC	--	--	--
Ca4_P4_27074666	Ca4	27074666	GAAAAAAAAA/GAAAAAAAAAA	ATTGGCATCAGAGCCAGTCT	TTTTCCAAGAGAGCGCATTT	59.8	566	INTERGENIC	--	--	--
Ca4_P4_27087434	Ca4	27087434	TA/TAAAA	TTATTGCGATGCCGACTATG	AATGATGTGGCTGGGTTAGC	59.7	656	INTERGENIC	--	--	--
Ca4_P4_27096077	Ca4	27096077	CTTTTTT/CTTTTTTT	TGAAATTAAGTTAAGGAACCGCA	GCTCAGTCGCTCACCTAACC	60.0	429	INTERGENIC	--	--	--
Ca4_P4_27096186	Ca4	27096186	GAAAAAAAAA/GAAAAAAAAAA	GGTTAGGTGAGCGACTGAGC	GCTCTTTTCTTTTTCCCGGT	60.0	122	INTERGENIC	--	--	--
Ca4_P4_27138934	Ca4	27138934	GAAAAAAAAAAAAAA/GAAAAAAAAAA	GGAAGGCTTGTCGACTTGAG	AAGACGCATTGAGAAGCGAT	60.0	450	INTERGENIC	--	--	--
Ca4_P4_27152769	Ca4	27152769	ATTTTTTTTTTT/ATTTTTTTTTT	TGTGCAGTTATGTCCCTTCG	GACAGGGATGGAGATGGAAA	59.7	456	INTERGENIC	--	--	--
Ca4_P4_27159634	Ca4	27159634	ATTTTTTTTTTT/ATTTTTTTTTT	CGAAACTTTGAACGAACGTG	TCCACCTCAAAACACACGAA	59.4	408	INTERGENIC	--	--	--

INDEL marker IDs	Chromosomes/ unanchored scaffolds	Physical positions (bp)	InDels (*Kabuli* reference genome-CDC Frontier/PII)	Forward primers (5'-3')	Reverse primers (5'-3')	Anneling temperature (0C)	Expected amplified product size (bp)	Structural annotation		Functional annotation	
								Sequence components of *kabuli* genome	*Kabuli* gene accession IDs	NCBI-KOG	TFs
Ca4_P4_27165248	Ca4	27165248	A/AG	AGGGGCAGAAATGACATGAA	TTCAAAATCAGCATTATCCACG	60.5	582	INTERGENIC	--	--	--
Ca4_P4_27168549	Ca4	27168549	CAAAAAA/CAAAAA	AGGGCCAAGGGACAATAAGT	TGGTTTGTGCAATGTCCCTA	59.8	523	INTERGENIC	--	--	--
Ca4_P4_27185776	Ca4	27185776	TAAAAAAAAA/TAAAAAAAAAA	GCCCAAAACGGGAATAATTT	GGGCAAAAGATGGAACAAGA	60.0	712	INTERGENIC	--	--	--
Ca4_P4_27205960	Ca4	27205960	TAAAA/TAAA	CGAGTTGATTTGGTTCGGTT	AGCACAGCTGAATGAAGTGC	60.0	754	INTERGENIC	--	--	--
Ca4_P4_27260077	Ca4	27260077	GAAA/GAAAA	GCGTCCAAGTCTTTGAGGAA	TTTGGGGTTGCATAATTTGA	60.4	498	INTERGENIC	--	--	--
Ca4_P4_27261340	Ca4	27261340	CAAAAAAA/CAAAAAAAA	ACTCCAACACACAACCCCTC	AATTGAGCCCAGAAAACCCT	59.9	366	INTERGENIC	--	--	--
Ca4_P4_27285679	Ca4	27285679	G/GAA	TATTTCGAGGGCGTACCAAC	CCAGCCTACCCCTTTTATCTG	60.0	549	INTERGENIC	--	--	--
Ca4_P4_27318870	Ca4	27318870	TAAA/TAAAA	GACGAGCACCAATGGAGTTT	TGGTCTTCAAAAGTCACCCC	60.1	741	INTERGENIC	--	--	--
Ca4_P4_27319031	Ca4	27319031	TT/TTATAT	CAAAAAGGGGGAGATTATTGG	GCTTTAGCCATTTCTGGCTTT	59.7	710	INTERGENIC	--	--	--
Ca4_P4_27326085	Ca4	27326085	TTCT/TT	CAGAAAACAAAGAACGCTCG	TACAACTCCCAACGGACACA	58.7	656	INTERGENIC	--	--	--

INDEL marker IDs	Chromosomes/ unanchored scaffolds	Physical positions (bp)	InDels (*Kabuli* reference genome-CDC Frontier/PII)	Forward primers (5'-3')	Reverse primers (5'-3')	Anneling temperature (0C)	Expected amplified product size (bp)	Structural annotation		Functional annotation	
								Sequence components of *kabuli* genome	*Kabuli* gene accession IDs	NCBI-KOG	TFs
Ca4_P4_27357106	Ca4	27357106	TCC/TC	GAGCGAGAGGTGTTGTGTGA	AGTGCTTCACCCCAAAATGA	60.0	498	INTERGENIC	--	--	--
Ca4_P4_27364858	Ca4	27364858	TAAAAAAA/TAAAAAA	TTTTGGGGAGAAGCAATGTC	TAGCCATCTAACCATTCGGC	60.1	710	INTERGENIC	--	--	--
Ca4_P4_27365149	Ca4	27365149	AT/ATT	TTGCAATGGAAAAGGAGCTT	TAGCCATCTAACCATTCGGC	59.8	138	INTERGENIC	--	--	--
Ca4_P4_27366853	Ca4	27366853	TG/T	TGAGACAATGAGGCTTGTGG	TGCACTTGGTCAATGTGTGA	59.8	460	INTERGENIC	--	--	--
Ca4_P4_27387851	Ca4	27387851	CACAAAAACAACAAAAA/CACAAAAA	AACAAACAATTCCAAACCGC	GTCGTTCAGTGGCATAGGGT	59.8	769	INTERGENIC	--	--	--
Ca4_P4_27395838	Ca4	27395838	TT/TTGT	AAGTTTTGGGTTGCTCATGG	TTGTCCCCAAGCAACAATTA	60.0	627	INTERGENIC	--	--	--
Ca4_P4_27400557	Ca4	27400557	ACCCCC/ACCCC	AGCCTGATACTCAAGGAGGG	CATTTTCCTTCTCACCCATCA	58.4	659	INTERGENIC	--	--	--
Ca4_P4_27402844	Ca4	27402844	TAAAAAAAA/TAAAAAAA	CGAGTAGCGAAATCGACCAT	TATTTTGCTTCGTTGTTGCG	60.2	447	INTERGENIC	--	--	--
Ca4_P4_27403176	Ca4	27403176	GTTTTTTT/GTTTTTTTT	CGCAACAACGAAGCAAAATA	GCACGGTTGAGTGAAAAAGG	59.9	579	INTERGENIC	--	--	--
Ca4_P4_27405634	Ca4	27405634	TTATATATATATAT/TTATATATATAT	GAGTTGGTATTTCATTTGTTCGG	GTGGTTAAGATGGACCACCG	59.8	531	INTERGENIC	--	--	--

INDEL marker IDs	Chromosomes/ unanchored scaffolds	Physical positions (bp)	InDels (*Kabuli* reference genome-CDC Frontier/PII)	Forward primers (5'-3')	Reverse primers (5'-3')	Anneling temperature (0C)	Expected amplified product size (bp)	Structural annotation		Functional annotation	
								Sequence components of *kabuli* genome	*Kabuli* gene accession IDs	NCBI-KOG	TFs
Ca4_P4_27405693	Ca4	27405693	TAAAA/TAAAAAA	GAGTTGGTATTTCATTTGTTCGG	GTGGTTAAGATGGACCACCG	59.8	531	INTERGENIC	--	--	--
Ca4_P4_27406421	Ca4	27406421	ACTCTC/ACTC	TTTGATGGTTCGAAATCGTT	CGCAAAATAAGAGAAAAGACGC	58.1	593	INTERGENIC	--	--	--
Ca4_P4_27406459	Ca4	27406459	TTATATATATATAT/TTATATATATAT	TTTGATGGTTCGAAATCGTT	CGCAAAATAAGAGAAAAGACGC	58.1	593	INTERGENIC	--	--	--
Ca4_P4_27424528	Ca4	27424528	ATTTTTTTT/ATTTTT	CGAACCAAAAGTGGATTTGA	AAAGTTTTGTGGCCGTCTTT	58.6	733	INTERGENIC	--	--	--
Ca4_P4_27432873	Ca4	27432873	ATTTT/ATTT	ACGACAAGAAAGGCAGAGGA	AGCTCGTGTTCCTGCAATTT	60.0	713	INTERGENIC	--	--	--
Ca4_P4_27435493	Ca4	27435493	CAAAAAAAAAAAAAA/CAAAAAAAAAAA	GACACCAAGCCACAAACCTT	GCATGTTGGTTGAAGGAGGT	60.0	334	INTERGENIC	--	--	--
Ca4_P4_27484671	Ca4	27484671	TG/T	ACACATGTTGGTGGCTTTGA	CCAATGCTTGAGTTATGCCA	60.0	679	INTERGENIC	--	--	--
Ca4_P4_27496407	Ca4	27496407	CTG/CTGTG	TCGGAAACGAATTGGGAATA	GGAGTTTGAGAAAGGGGTCC	60.3	750	INTERGENIC	--	--	--
Ca4_P4_27498412	Ca4	27498412	GAAAA/GAAAAA	GACTCCATGGCAAAGAGGAG	CTTCAACAGCCATGCTCAAA	59.8	925	INTERGENIC	--	--	--
Ca4_P4_27498504	Ca4	27498504	CTCTTT/CT	AAGTTCGAATATTGACTGGCA	CTTCAACAGCCATGCTCAAA	57.4	739	INTERGENIC	--	--	--

INDEL marker IDs	Chromosomes/ unanchored scaffolds	Physical positions (bp)	InDels (*Kabuli* reference genome-CDC Frontier/PII)	Forward primers (5'-3')	Reverse primers (5'-3')	Anneling temperature (0C)	Expected amplified product size (bp)	Structural annotation		Functional annotation	
								Sequence components of *kabuli* genome	*Kabuli* gene accession IDs	NCBI-KOG	TFs
Ca4_P4_27503266	Ca4	27503266	AG/AGG	GGGGTTCTCCTTGAACCACT	CCTGCCATTTTCTTCCAAAA	60.3	343	INTERGENIC	--	--	--
Ca4_P4_27504292	Ca4	27504292	AG/A	CATTCATGCCACAGGTATCG	AACACCAACACACCAAAGCA	59.9	353	INTERGENIC	--	--	--
Ca4_P4_27506038	Ca4	27506038	CAA/CAAA	GAGATCATTGCATCCCAGGT	CACCTGAAACCCCTTTTGAA	59.9	819	INTERGENIC	--	--	--
Ca4_P4_27508639	Ca4	27508639	T/TC	TCCAATGGAGATCAATGCAA	AACATAATGCCATGCCCAAT	60.0	233	INTERGENIC	--	--	--
Ca4_P4_27509778	Ca4	27509778	TTAT/TT	AACATTCCTTCCATCCAACG	CATGGTTTTTCCGTGGTTTT	59.8	654	INTERGENIC	--	--	--
Ca4_P4_27513599	Ca4	27513599	ATTTTTTT/ATTTTTTTT	TGATGGGGTTTTGTGTGAGA	ATGCATTACACGACCGCATA	59.9	612	INTERGENIC	--	--	--
Ca4_P4_27514430	Ca4	27514430	CTTTTTTTTT/CTTTTTTTTTT	TGGTGCTCTCAACCATTGAA	TTATCCATCGCCTAAGTCGG	60.2	754	INTERGENIC	--	--	--
Ca4_P4_27537181	Ca4	27537181	AGTGTGTGTGTGT/AGTGTGTGTGTGTGT	TTTCATCCTCTCTCACGGCT	TCTTCATGGCAACACTTCCA	60.0	478	INTERGENIC	--	--	--
Ca4_P4_27539521	Ca4	27539521	TAA/TAACATAGTGAA	CTTGTGAAAGCCTCAAAGCC	TTGATTTCGTGAGTTCATTTCAA	60.0	776	INTERGENIC	--	--	--
Ca4_P4_27539986	Ca4	27539986	ATA/ATAGATTA	TTGAAATGAACTCACGAAATCAA	CACCCTCGCTTAGAAAAACG	59.6	542	INTERGENIC	--	--	--

INDEL marker IDs	Chromosomes/ unanchored scaffolds	Physical positions (bp)	InDels (*Kabuli* reference genome-CDC Frontier/PII)	Forward primers (5'-3')	Reverse primers (5'-3')	Anneling temperature (0C)	Expected amplified product size (bp)	Structural annotation		Functional annotation	
								Sequence components of *kabuli* genome	*Kabuli* gene accession IDs	NCBI-KOG	TFs
Ca4_P4_27548804	Ca4	27548804	TAAAA/TAAA	TTCCCCATTCTTAACACCCA	TTCGCAACTCCACTCTTCCT	60.2	672	INTERGENIC	--	--	--
Ca4_P4_27555037	Ca4	27555037	GCTC/GCTCATCCTC	CCAACAACACAGATGCAACC	TGCGCTTTCCTTTGTTATGA	60.0	527	INTERGENIC	--	--	--
Ca4_P4_27636569	Ca4	27636569	A/AAGGTAGAT	TGCCTCATAAATTGACCCCT	TACAGGTGGACGCTTTGTTG	59.4	331	INTERGENIC	--	--	--
Ca4_P4_27639256	Ca4	27639256	GAAAAAAAA/GAAAAAAAAA	TACCACCAAGGTTTGGCTTC	CGTGGAAATCACTTGCAAAA	60.0	435	INTERGENIC	--	--	--
Ca4_P4_27647713	Ca4	27647713	TAAAAAAA/TAAAAAA	TTGTCCACGATACATGCGTT	GCCACTCAATTCTCAACCGT	60.0	261	INTERGENIC	--	--	--
Ca4_P4_27682659	Ca4	27682659	C/CCTTAAGA	AGACGGCGCAAGTAGAGAAC	TGTTTTCTTTGCGTTGCTTG	59.6	262	INTERGENIC	--	--	--
Ca4_P4_27689404	Ca4	27689404	GATAT/GAT	TGCGTCATAAAATGGCTTGA	GGATGAGCTGACCCCTTACA	60.2	419	INTERGENIC	--	--	--
Ca4_P4_27746278	Ca4	27746278	CTTTTTTTTTTT/CTTTTTTTT	AGTTTTCTGTGGGTTCACCG	TGCCACATCACCCTAAATCA	60.0	546	INTERGENIC	--	--	--
Ca4_P4_27764770	Ca4	27764770	T/TA	AACCTCAACGAAATCTTTCCAA	GCGTGCGATAGGTTGGTATT	60.0	452	INTERGENIC	--	--	--
Ca4_P4_27767764	Ca4	27767764	TA/TAGGGGTGA	CGAACGGGGATTGAGACTTA	AAAACGGGAAAGTTTGACGA	60.1	324	INTERGENIC	--	--	--

INDEL marker IDs	Chromosomes/ unanchored scaffolds	Physical positions (bp)	InDels (*Kabuli* reference genome-CDC Frontier/PII)	Forward primers (5'-3')	Reverse primers (5'-3')	Anneling temperature (0C)	Expected amplified product size (bp)	Structural annotation		Functional annotation	
								Sequence components of *kabuli* genome	*Kabuli* gene accession IDs	NCBI-KOG	TFs
Ca4_P4_27796696	Ca4	27796696	AATATATATATATATATATATATATAT/AATATATATATATATATATATAT	GGCAGAAGTGAAACAATGATGA	TAATAAAGGTGGTGGTCGGC	60.1	731	INTERGENIC	--	--	--
Ca4_P4_27822471	Ca4	27822471	TTAT/TT	TTGATGCTTGCTCAACTTGG	TATCGCGGGTCTTGTTTTTC	60.0	470	INTERGENIC	--	--	--
Ca4_P4_27847035	Ca4	27847035	ATTTCCTTTCCTTTCCTTTC/ATTTCCTTTCCTTTC	AGGAAGAGTGGAGTTGCGAA	TTGATTTTGCTGCTCAATGC	60.0	628	INTERGENIC	--	--	--
Ca4_P4_27851087	Ca4	27851087	ATTTTTTT/ATTTTTTTT	TGACATTGACTCTTGGAGCG	CTTTCTTTTGCGGTTGTGGT	60.0	622	INTERGENIC	--	--	--
Ca4_P4_27865338	Ca4	27865338	AAATAATAATAATAATAATAATAATAATAATAATAATAATAA/AAATAATAATAATAATAATAATAATAATAATAATAA	TCCTAAAACAAATGCACCCA	TTAGGCTTGATATGCCGACC	59.0	557	INTERGENIC	--	--	--
Ca4_P4_27873116	Ca4	27873116	TTTTATTTATTTATTTATTTATTTATTTATTT/TTTTATTTATTTATTTATTTATTTATTT	GCATGCGAACAAGAGTGAAG	GTTCAACTCCCGTTGGAAAA	59.6	693	INTERGENIC	--	--	--
Ca4_P4_27878476	Ca4	27878476	ATTTTTTTTTTTTT/ATTTTTTTTTTT	ATCCTCACCTGCAAGACCAT	TGTCTCGAATTACCCCGAAG	59.5	640	INTERGENIC	--	--	--
Ca4_P4_2788113	Ca4	2788113	TTTATTATTATTATTATTATTATTATTATTATTATTATTATTATTATT/TTTATTATTATTATTATTATTATTATTATTATTATTATTATT	TGTGGCAAAACAAATAAGCG	ATATCCCGGGAACCAATTCT	59.7	734	INTERGENIC	--	--	--
Ca4_P4_27902426	Ca4	27902426	T/TC	CTGATTTGGCTCTGATGGCT	ATCATAGATCGCGAGCATCC	60.4	686	INTERGENIC	--	--	--
Ca4_P4_27928820	Ca4	27928820	CACAAC/CACAACAAC	AGCGATGCATGTCTCAAGTG	AGCGCTTTGTGACCTCCTTA	60.0	754	INTERGENIC	--	--	--

INDEL marker IDs	Chromosomes/ unanchored scaffolds	Physical positions (bp)	InDels (*Kabuli* reference genome-CDC Frontier/PII)	Forward primers (5'-3')	Reverse primers (5'-3')	Anneling temperature (0C)	Expected amplified product size (bp)	Structural annotation		Functional annotation	
								Sequence components of *kabuli* genome	*Kabuli* gene accession IDs	NCBI-KOG	TFs
Ca4_P4_27929931	Ca4	27929931	GTTTT/GTTTTT	CCTTCTCATTGCGAACCCTA	TGGTTGCACCACATTTGACT	60.2	427	INTERGENIC	--	--	--
Ca4_P4_27956988	Ca4	27956988	TCCCCCCC/TCCCCCCCC	ACTCCACTTCAAACAACCGC	TTTTAAGCCACACCACACCA	60.2	603	INTERGENIC	--	--	--
Ca4_P4_27998494	Ca4	27998494	CTTTTTTTTT/CTTTTTTTT	AAGGAGGAGGAGGTGAAGGA	GAAAAGGGAGCCACAAATGA	60.2	533	INTERGENIC	--	--	--
Ca4_P4_28018591	Ca4	28018591	A/ACTCACTGAACT	CAAAGAGAGACGTGCAACCA	TCCGATTCAAAAACGTTGAAG	60.0	708	INTERGENIC	--	--	--
Ca4_P4_28019655	Ca4	28019655	CAAAAAAAAA/CAAAAAAA	CGCATTAAGTTGGATTGGGT	TTGAGGTTGAACATGGTTGG	59.8	439	INTERGENIC	--	--	--
Ca4_P4_28020575	Ca4	28020575	TG/T	GGGAGCAAAAGCTTTGAATG	AATGAGACCTACGTCACGGG	59.8	427	INTERGENIC	--	--	--
Ca4_P4_28023593	Ca4	28023593	GAAAAAAAAAA/GAAAAAAAA	CTCAAACCATTGAGGCCAGT	AATCTGCATCCCAAAATTGC	60.1	487	INTERGENIC	--	--	--
Ca4_P4_28026871	Ca4	28026871	TAAGAA/TAAGAAGAA	GACATCACACCAATTCGACG	TTTTGAAGGAGATGGGGTTG	60.0	885	INTERGENIC	--	--	--
Ca4_P4_28042758	Ca4	28042758	TC/T	GTCCTTTTCCTTTGGCCTTC	CACCCTCCTCCATATCCATT	60.1	361	INTERGENIC	--	--	--
Ca4_P4_28058610	Ca4	28058610	TAAAAAAAAA/TAAAAAAAA	CTACCAGTTGGAGCCACCAT	CTTTCGAGAGCCACACAACA	60.0	250	INTERGENIC	--	--	--

INDEL marker IDs	Chromosomes/ unanchored scaffolds	Physical positions (bp)	InDels (*Kabuli* reference genome-CDC Frontier/PII)	Forward primers (5'-3')	Reverse primers (5'-3')	Anneling temperature (0C)	Expected amplified product size (bp)	Structural annotation		Functional annotation	
								Sequence components of *kabuli* genome	*Kabuli* gene accession IDs	NCBI-KOG	TFs
Ca4_P4_28062048	Ca4	28062048	GTTTTTTTT/GTTTTTTTTT	TCAACAATTGTAATTGACTATCAACG	TATAGGCCGATCAGTCAGGC	59.4	771	INTERGENIC	--	--	--
Ca4_P4_28086943	Ca4	28086943	GTAGAA/GTAGAATAGAA	TTTTGCATACGTCGGAATCA	ACGACGTTGATTGATCCAAA	60.1	577	INTERGENIC	--	--	--
Ca4_P4_28108572	Ca4	28108572	TAAAAAAAA/TAAAAAAAAA	GCAAATGTTGGCGAAGTACA	AGTGTGTGCTTGTGGGATCA	59.7	301	INTERGENIC	--	--	--
Ca4_P4_28120280	Ca4	28120280	TAAAAAAAA/TAAAAAAAAA	TAGGGTGAAGGAGAACGGTG	TTTTTGTGCGTTTGACTGATG	60.1	822	INTERGENIC	--	--	--
Ca4_P4_28120543	Ca4	28120543	TAAAAAA/TAAAAAAA	CCAACTCCCTTCCCCTTTTA	CAATACGAGCACCTGGCTCT	60.3	994	INTERGENIC	--	--	--
Ca4_P4_28121722	Ca4	28121722	TAAAAAAAAAAAAAAA/TAAAAAAAAAAAA	TGAACTCTTTCTTGGGTGGG	ATACGCATAATCGACTCCCG	60.1	643	INTERGENIC	--	--	--
Ca4_P4_28131693	Ca4	28131693	TT/TTCGGTGGCT	TAGCAAGAACACCATCGCAA	TTAACCCTCAATTGCCGTTC	60.4	463	INTERGENIC	--	--	--
Ca4_P4_28143040	Ca4	28143040	ATTTTTTTTTTTTTTTT/ATTTTTTTTTTTTT	TCAAACAATGCCAAGTCCAA	TGGATATTGTGTGGGAATGC	60.1	479	INTERGENIC	--	--	--
Ca4_P4_28148545	Ca4	28148545	ATACTTACT/ATACT	TGGTCATAATCGACGTTGGA	CTGCAAGCAGCAGAAGAACA	59.9	956	INTERGENIC	--	--	--
Ca4_P4_28150064	Ca4	28150064	GTTTTTTTTTTT/GTTTTTTTTT	GCAGCCCTATGAGGTGAAGA	ATTCAAGATATGGGCGCAAC	60.4	484	INTERGENIC	--	--	--

INDEL marker IDs	Chromosomes/ unanchored scaffolds	Physical positions (bp)	InDels (*Kabuli* reference genome-CDC Frontier/PII)	Forward primers (5'-3')	Reverse primers (5'-3')	Anneling temperature (0C)	Expected amplified product size (bp)	Structural annotation		Functional annotation	
								Sequence components of *kabuli* genome	*Kabuli* gene accession IDs	NCBI-KOG	TFs
Ca4_P4_28153242	Ca4	28153242	CAA/C	TTGGCAGTGGTTCATTTTCA	AAAAGCTTGCGAACATCACC	60.1	365	INTERGENIC	--	--	--
Ca4_P4_28159857	Ca4	28159857	TAAAAAAAAA/TAAAAAAAAAAA	TTTGGGGGTACAAGGGAAAT	TGCAACCTCATACTGGGAAA	60.4	645	INTERGENIC	--	--	--
Ca4_P4_28162271	Ca4	28162271	TAAAAAAAA/TAAAAAAA	AAACAAGTGGTTCTGGGCAC	CGAAACTTTGCATGCATCTC	60.0	553	INTERGENIC	--	--	--
Ca4_P4_28163369	Ca4	28163369	TGTTTATTTATGTTTAAGTTTATTTA/TGTTTATTTATGTTTAAGTTTATTTATGTTTAAGTTTATTTA	GAGGAGGTCCAGGATGATGA	AGCTGGTCTCTCTGTGCCAT	60.0	731	INTERGENIC	--	--	--
Ca4_P4_28169092	Ca4	28169092	GC/G	TTTTTGCCCATTCAAACACA	AAATGCGTCAGCCCAGTAAG	59.9	829	INTERGENIC	--	--	--
Ca4_P4_28169343	Ca4	28169343	TAAAAAAAA/TAAAAAAA	GGGGTGAGAAAAGAGTGTGG	AAATGCGTCAGCCCAGTAAG	59.5	180	INTERGENIC	--	--	--
Ca4_P4_28177449	Ca4	28177449	ATTTTTTTTT/ATTTTTTTTTTT	TCATAAAATTGCATGGCACA	TTCTATGGGACAGCCCAATC	58.6	364	INTERGENIC	--	--	--
Ca4_P4_28180440	Ca4	28180440	TAAAAAAA/TAAAAAA	GGAAGAGCGAGAAGGGAGAT	ACCAATGTCCAACGGGAATA	59.9	325	INTERGENIC	--	--	--
Ca4_P4_28183509	Ca4	28183509	GC/G	ACTGGCAGCCATTCACTCTT	TGACAACCCCAAACTTGTGA	59.9	745	INTERGENIC	--	--	--
Ca4_P4_28184015	Ca4	28184015	A/ACG	TCACAAGTTTGGGGTTGTCA	ACGCAAGGTCAACAAAGGTC	60.0	641	INTERGENIC	--	--	--

INDEL marker IDs	Chromosomes/ unanchored scaffolds	Physical positions (bp)	InDels (*Kabuli* reference genome-CDC Frontier/PII)	Forward primers (5'-3')	Reverse primers (5'-3')	Anneling temperature (0C)	Expected amplified product size (bp)	Structural annotation		Functional annotation	
								Sequence components of *kabuli* genome	*Kabuli* gene accession IDs	NCBI-KOG	TFs
Ca4_P4_28205282	Ca4	28205282	CAAAAAAA/CAAAAAA	GGCAACAATGTCTCAAAGCA	CACGTTGAATTCCACCTTCA	59.8	539	INTERGENIC	--	--	--
Ca4_P4_28208528	Ca4	28208528	TAAAATTAA/TAAAATTAAAAATTAA	CGATAGCACGTTGGTGAGAA	TTCCCTCCAAAGTTGGACAC	59.9	242	INTERGENIC	--	--	--
Ca4_P4_28209971	Ca4	28209971	T/TA	ATTGATGGAGCGAATCGAAC	AATTCCTCTTTTGGTGGGCT	60.0	891	INTERGENIC	--	--	--
Ca4_P4_28213499	Ca4	28213499	CTTTTTTTT/CTTTTTTTTT	ACTGACCTGACCTAGGCGTG	GTTGCAAAATTGGTGTCTGC	60.3	646	INTERGENIC	--	--	--
Ca4_P4_28214353	Ca4	28214353	GACAA/GACAACAA	TCCGAGTCTAATCGGGCTAA	TGGAAAAGGGAGCCAATATG	59.8	539	INTERGENIC	--	--	--
Ca4_P4_28237092	Ca4	28237092	C/CA	TCGGTTTTTGCCTCACTTTT	GTGGAGGCTCAGTTGGAGAG	59.7	758	INTERGENIC	--	--	--
Ca4_P4_28237880	Ca4	28237880	TG/T	CTCTCCAACTGAGCCTCCAC	GCAGTTTCGTGCTAAGGAGG	60.0	528	INTERGENIC	--	--	--
Ca4_P4_28254483	Ca4	28254483	CAACAA/CAACAAGACAAACAA	GGGGAACTCTCCAAGTCACA	CGTGGAAACCAACCTTGTCT	60.1	703	INTERGENIC	--	--	--
Ca4_P4_28275732	Ca4	28275732	GAAAAAAAAA/GAAAAAAAAAA	TTGGACGGTATCCTTTTTGAA	TGCAAAACAACAGAGTTGGC	59.4	631	INTERGENIC	--	--	--
Ca4_P4_28277078	Ca4	28277078	AC/A	TCAACATTTTACCGGTTGAGC	TCTCATTGTTGCGTGTAGGC	60.0	348	INTERGENIC	--	--	--

INDEL marker IDs	Chromosomes/ unanchored scaffolds	Physical positions (bp)	InDels (*Kabuli* reference genome-CDC Frontier/PII)	Forward primers (5'-3')	Reverse primers (5'-3')	Anneling temperature (0C)	Expected amplified product size (bp)	Structural annotation		Functional annotation	
								Sequence components of *kabuli* genome	*Kabuli* gene accession IDs	NCBI-KOG	TFs
Ca4_P4_28277405	Ca4	28277405	GTTTTTTTTTT/GTTTTTTTTTTTT	GCCTACACGCAACAATGAGA	AAGTTTTTCTAAACCGCAATTTTT	59.9	423	INTERGENIC	--	--	--
Ca4_P4_28278132	Ca4	28278132	GAAAAAAAAA/GAAAAAAAAAA	AAAAGGAATTTGAATGACCACAA	AAAGTGGAGTGCGACCAAGT	59.7	403	INTERGENIC	--	--	--
Ca4_P4_28290158	Ca4	28290158	TAACAACAA/TAACAACAACAA	ACCATTACACTACCGCTGCC	TTAATTAATATCCGCGCAACG	60.0	688	INTERGENIC	--	--	--
Ca4_P4_28290227	Ca4	28290227	A/AG	ACCATTACACTACCGCTGCC	TTAATTAATATCCGCGCAACG	60.0	688	INTERGENIC	--	--	--
Ca4_P4_28293593	Ca4	28293593	GTTTTTTTTT/GTTTTTTTT	TCATTACTCGCCATAAGTGGAA	ACTCTCGATGCCACCTCATC	59.6	432	INTERGENIC	--	--	--
Ca4_P4_28296583	Ca4	28296583	TCC/TCCC	TAGCTGCTCACACTGCCAAC	GGTTGTTGTGCCCAGAGATT	60.2	527	INTERGENIC	--	--	--
Ca4_P4_28305975	Ca4	28305975	AGG/AG	TTTCCCCACATGTCTCACAA	TTCTCGCAAAAGACAAACCC	59.9	391	INTERGENIC	--	--	--
Ca4_P4_28306538	Ca4	28306538	CTTTTTTTT/CTTTTTTTTT	GGGTTTGTCTTTTGCGAGAA	AATTGTCCCATCCCTTACCC	60.2	772	INTERGENIC	--	--	--
Ca4_P4_28320855	Ca4	28320855	ATTTTTTTT/ATTTTTTT	TGAGAAACGGCAAAACACAA	AAATATTGGGCATGTGAGCC	60.3	450	INTERGENIC	--	--	--
Ca4_P4_28322339	Ca4	28322339	A/ACCGT	TGCGTACACGAAAAAGCAAC	TGTTGTTGCCGACTGAGAAG	59.9	333	INTERGENIC	--	--	--

INDEL marker IDs	Chromosomes/ unanchored scaffolds	Physical positions (bp)	InDels (*Kabuli* reference genome-CDC Frontier/PII)	Forward primers (5'-3')	Reverse primers (5'-3')	Anneling temperature (0C)	Expected amplified product size (bp)	Structural annotation		Functional annotation	
								Sequence components of *kabuli* genome	*Kabuli* gene accession IDs	NCBI-KOG	TFs
Ca4_P4_28322577	Ca4	28322577	C/CCTTTTTTTTTT	CTTCTCAGTCGGCAACAACA	CTCCCAGAGTAGCAAGCAGG	60.0	431	INTERGENIC	--	--	--
Ca4_P4_28331028	Ca4	28331028	CAAAAAAAA/CAAAAAAAAA	CTTGAAGGGACGGCTAGATG	TTGCCATAATGTTGAGAAGGG	59.8	458	INTERGENIC	--	--	--
Ca4_P4_28333301	Ca4	28333301	TAAAAAAAAAA/TAAAAAAAAAAA	CAGGTCTTCCCAACGTTATCA	TTTGTGATTGCTTGCTTTAATTTC	60.0	393	INTERGENIC	--	--	--
Ca4_P4_28336543	Ca4	28336543	TAAAAAAAAAAA/TAAAAAAAAA	CAAAGCAGGGAGTTTCCATC	TGGTTTAAGTCCTCGGCTGT	59.7	723	INTERGENIC	--	--	--
Ca4_P4_28338388	Ca4	28338388	TAAAAA/TAAAAAA	TGACTCGGGCTGCAGATTAT	CACCGGTGGTCTTTCCTTTA	60.8	432	INTERGENIC	--	--	--
Ca4_P4_28338753	Ca4	28338753	TGGGGCT/TGGGGCTTAAGAGAGGGGGCT	AAGACCACCGGTGTTTATGG	TCAATTTGACAAGCCCCTTA	59.7	724	INTERGENIC	--	--	--
Ca4_P4_28338890	Ca4	28338890	CAAAAAAAAA/CAAAAAAAA	TATTTCATGGGGCTCAAAGC	TAACCGCCTATTTTCTTGCG	60.0	555	INTERGENIC	--	--	--
Ca4_P4_28346696	Ca4	28346696	A/AT	ACCACAGGTGAAAGAAACCG	TTTTCAACTCTATCCGTAGCCC	60.0	340	INTERGENIC	--	--	--
Ca4_P4_28349135	Ca4	28349135	ACAT/ACATATCAT	CTTTGCGATCAAATGGTCAA	TCAAGCTCAAAACGTGATGC	59.7	592	INTERGENIC	--	--	--
Ca4_P4_28352450	Ca4	28352450	AAATGAA/AAA	TTTTCTTCGGTTTTCATTTATGG	AAAAACCTCAAGGTTCCCAA	59.4	636	INTERGENIC	--	--	--

INDEL marker IDs	Chromosomes/ unanchored scaffolds	Physical positions (bp)	InDels (*Kabuli* reference genome-CDC Frontier/PII)	Forward primers (5'-3')	Reverse primers (5'-3')	Anneling temperature (0C)	Expected amplified product size (bp)	Structural annotation		Functional annotation	
								Sequence components of ***kabuli*** genome	***Kabuli*** gene accession IDs	NCBI-KOG	TFs
Ca4_P4_28358208	Ca4	28358208	GTTTTTTTTTT/GTTTTTTTTT	TGCGCTAAAATTTAACCCTTC	TCATGCACCAATCAGTTTCC	58.5	362	INTERGENIC	--	--	--
Ca4_P4_28390462	Ca4	28390462	TAA/TAAA	GTCATCAAAAGCCGACGAAT	GGCCCACAATTTTGAAGAGA	60.1	786	INTERGENIC	--	--	--
Ca4_P4_28401988	Ca4	28401988	TAATTAA/TAATTAATTAAATAAAATTAA	ACTTGGATGAAGGGAAAGGG	GCATTTCGCAAAAATTTGAA	60.3	561	INTERGENIC	--	--	--
Ca4_P4_28403002	Ca4	28403002	TTATATATAT/TTATATAT	CATTCTTCTTCATTAAGTGGGCA	TTGACGTGTCAACCTTGCTC	60.5	650	INTERGENIC	--	--	--
Ca4_P4_28407567	Ca4	28407567	CAAAAAAAA/CAAAAAAA	TGGTCATAAAGGGAACCAGG	GGAGGGGGTGAATTAGGTGT	59.8	146	INTERGENIC	--	--	--
Ca4_P4_28407643	Ca4	28407643	AA/AAGA	ACACCTAATTCACCCCCTCC	TCCCTTTTCCAACCACTTTG	60.1	517	INTERGENIC	--	--	--
Ca4_P4_28410297	Ca4	28410297	GAAAAA/GAAAA	TTTTGGGATCTCAAGGTGAAA	ATCGTGGCAAAGATGACACA	59.5	613	INTERGENIC	--	--	--
Ca4_P4_28414876	Ca4	28414876	CAAT/CAATAAT	GCCCCATGTTTGTGCTTAAT	GGATTCAGCATCCGTCAAGT	59.8	741	INTERGENIC	--	--	--
Ca4_P4_28417839	Ca4	28417839	CAA/CAATAGAA	TGCCTTGGAAGAGGCTTTTA	TGAAAAATAGCCCAATGGTGA	60.0	715	INTERGENIC	--	--	--
Ca4_P4_28421576	Ca4	28421576	TAAAAAAAAA/TAAAAAAAAAA	CCAATCCATCGGTTTTTCAC	TGCATGGAAAGCTGTGGTAG	60.2	472	INTERGENIC	--	--	--

INDEL marker IDs	Chromosomes/ unanchored scaffolds	Physical positions (bp)	InDels (*Kabuli* reference genome-CDC Frontier/PII)	Forward primers (5'-3')	Reverse primers (5'-3')	Anneling temperature (0C)	Expected amplified product size (bp)	Structural annotation		Functional annotation	
								Sequence components of *kabuli* genome	*Kabuli* gene accession IDs	NCBI-KOG	TFs
Ca4_P4_28422388	Ca4	28422388	TA/TAA	TGAAGGAAACTTTGATGCCA	TTGGGTGATGGTAGTTTCAATG	59.2	534	INTERGENIC	--	--	--
Ca4_P4_28457671	Ca4	28457671	TCTCTA/TCTCTATTTAAACTCTA	TACAAAGCACCTGACCCTCC	CCCAAATCAAAGCAAGCAAT	60.1	765	INTERGENIC	--	--	--
Ca4_P4_28504169	Ca4	28504169	CAA/CA	GCTGGCGGGAATTAGTATGA	GCTTCCCCACTATCACTCCA	60.1	132	INTERGENIC	--	--	--
Ca4_P4_28513752	Ca4	28513752	ATTTTT/ATTTTTT	CTATTCCGTGAACAACCCGT	TCACTATGGTGTAAAACAATTGGTAA	59.9	738	INTERGENIC	--	--	--
Ca4_P4_28519648	Ca4	28519648	TAAAAAAAAA/TAAAAAAAA	GCCATGAAGTGGCCTAACAT	TCACGGTTTTGTAGAGCGGT	60.0	620	INTERGENIC	--	--	--
Ca4_P4_28519731	Ca4	28519731	AA/AAGA	GCCATGAAGTGGCCTAACAT	TCACGGTTTTGTAGAGCGGT	60.0	620	INTERGENIC	--	--	--
Ca4_P4_28520308	Ca4	28520308	TAAAAAAAAA/TAAAAAAAA	ACCGCTCTACAAAACCGTGA	AGCAGCTAAACTTTTGGGCA	60.7	870	INTERGENIC	--	--	--
Ca4_P4_28536428	Ca4	28536428	ATTTTTTTTTTT/ATTTTTTTTTTTTT	TTGTAACACCCCGTTTTTCG	CCTCAGATGAGAGCCAGGAC	60.8	433	INTERGENIC	--	--	--
Ca4_P4_28541959	Ca4	28541959	TAAAAAAAA/TAAAAAAA	TTGCATCGGCCTACTTTCTT	CGGATTCATCCAAAGTTTCAA	59.8	637	INTERGENIC	--	--	--
Ca4_P4_28556813	Ca4	28556813	TAAAAAAAAA/TAAAAAAAAAA	GCAAAAATTCATTCAAAAATTCG	TGGTGGTTGGGTGAAGAAGT	59.9	295	INTERGENIC	--	--	--

INDEL marker IDs	Chromosomes/ unanchored scaffolds	Physical positions (bp)	InDels (*Kabuli* reference genome-CDC Frontier/PII)	Forward primers (5'-3')	Reverse primers (5'-3')	Anneling temperature (0C)	Expected amplified product size (bp)	Structural annotation		Functional annotation	
								Sequence components of *kabuli* genome	*Kabuli* gene accession IDs	NCBI-KOG	TFs
Ca4_P4_28558898	Ca4	28558898	AAAGTAAG/AAAG	GGCAGGCAATGGTATTGTTT	GTGGTTGTTTGCTCCTCGAT	59.8	840	INTERGENIC	--	--	--
Ca4_P4_28559061	Ca4	28559061	GAAAAAAAAAAA/GAAAAAAAAAA	AGGTCCGTTTGGAGGATTTT	GTGGTTGTTTGCTCCTCGAT	59.8	437	INTERGENIC	--	--	--
Ca4_P4_28581947	Ca4	28581947	TAAAAAAAAAAA/TAAAAAAAAAA	CATTTCCCCACACCAATTTC	AAGGATGATGATTGTGCATGT	60.0	379	INTERGENIC	--	--	--
Ca4_P4_28583861	Ca4	28583861	ATT/AT	AGAGGGAGGGACCAAAACTT	CTGGAGCAAGCTTTCCAAAC	59.0	524	INTERGENIC	--	--	--
Ca4_P4_28586798	Ca4	28586798	ATTTTT/ATTTT	GCCCAAAGCATACGAGACAT	TTGGGGGATAATTGTTTGGA	60.1	516	INTERGENIC	--	--	--
Ca4_P4_28597058	Ca4	28597058	GATATATATATATATATAT/GATATATATATAT	CGAATATCCGTGAATGTTGAA	ATTTCTTTTTGCCGTTTCCA	58.5	714	INTERGENIC	--	--	--
Ca4_P4_28622371	Ca4	28622371	CAAAAAAAAAAA/CAAAAAAAAAA	TAAACATCGCAACAAAACGG	GTGAGGTGATTTCGGTCCAT	59.6	183	INTERGENIC	--	--	--
Ca4_P4_28623236	Ca4	28623236	ATTTTTTTT/ATTTTTTT	GAAATGTTGAATGTGTGCCG	TGTCCGAAACATTAAACCCA	60.0	249	INTERGENIC	--	--	--
Ca4_P4_28714992	Ca4	28714992	GATAT/GAT	AGAGCCAACGCTCATAGGAA	TTAAAATTCCCTCCTCGGCT	60.0	531	INTERGENIC	--	--	--
Ca4_P4_28783066	Ca4	28783066	TAAA/TAA	AGGAGAGAAGTGGTTTGGCA	ATGAAGGAGCAAGCTTTCCA	59.8	711	INTERGENIC	--	--	--

INDEL marker IDs	Chromosomes/ unanchored scaffolds	Physical positions (bp)	InDels (*Kabuli* reference genome-CDC Frontier/PII)	Forward primers (5'-3')	Reverse primers (5'-3')	Anneling temperature (0C)	Expected amplified product size (bp)	Structural annotation		Functional annotation	
								Sequence components of *kabuli* genome	*Kabuli* gene accession IDs	NCBI-KOG	TFs
Ca4_P4_28793395	Ca4	28793395	TAA/TAAAAACAA	CCAAAATTACACCAAC GGTTC	TTGTTCGCATGCTCATT CTC	59.2	275	INTERGENIC	--	--	--
Ca4_P4_28797185	Ca4	28797185	AT/ATT	ATGGGGCTTAAGTAAT GGGG	TTAAAGAAACGCTTCGT GGG	60.0	891	INTERGENIC	--	--	--
Ca4_P4_28801392	Ca4	28801392	ATTT/ATT	TGGCATAGAGCTTGGT CTCA	CCCACTCATTCATGTTT TCCA	59.5	940	INTERGENIC	--	--	--
Ca4_P4_28802275	Ca4	28802275	AT/A	TTGTGGTTTGGACTGT GGAA	TTAGGTGCAAGTGTGG ATGG	60.0	645	INTERGENIC	--	--	--
Ca4_P4_28822156	Ca4	28822156	TC/T	AAGCGCAAAAGTGCAT GTTA	AACACCATTTGCGGAA GAAG	59.5	361	INTERGENIC	--	--	--
Ca4_P4_28822376	Ca4	28822376	ATTTT/ATTT	CTTCTTCCGCAAATGG TGTT	TGAATTGGAGAGTTCG TTGAAG	60.1	155	INTERGENIC	--	--	--
Ca4_P4_28823023	Ca4	28823023	ATTTTTTT/ATTTTTTTT	AAGCCTCTTTTCAAATT ACCAGA	TTCTCAAGATGGTCAA CAACTCA	58.5	907	INTERGENIC	--	--	--
Ca4_P4_28823256	Ca4	28823256	CAAAAAA/CAAAAAAA	TGGTTTTAATGTGTGC ATTTTATG	TTCTCAAGATGGTCAA CAACTCA	58.9	266	INTERGENIC	--	--	--
Ca4_P4_28823535	Ca4	28823535	AAATAATAATAATAATAATAATAATA ATA/AAATAATAATAATAATAATAATA	TGAGTTGTTGACCATC TTGAGAA	ACCAAGGGCAGATCTT AGGG	59.8	399	INTERGENIC	--	--	--
Ca4_P4_28826108	Ca4	28826108	CA/C	GCAGCAATTAGAACCG TCGT	TGGTCATGGAGCAACA TCAT	60.3	439	INTERGENIC	--	--	--

INDEL marker IDs	Chromosomes/ unanchored scaffolds	Physical positions (bp)	InDels (*Kabuli* reference genome-CDC Frontier/PII)	Forward primers (5'-3')	Reverse primers (5'-3')	Anneling temperature (0C)	Expected amplified product size (bp)	Structural annotation		Functional annotation	
								Sequence components of *kabuli* genome	*Kabuli* gene accession IDs	NCBI-KOG	TFs
Ca4_P4_28832888	Ca4	28832888	TGGTGTATA/T	CAGTTGTGCACTTTGAATGTTG	AAAATCACATGGAGGAACAAAAA	59.3	665	INTERGENIC	--	--	--
Ca4_P4_28846796	Ca4	28846796	ATTTTTTTTTTTTT/ATTTTTTTTTTTTTT	CCAACATTCGGGGACTTAAA	TGGTGATCGGTAGACACGAA	59.8	505	INTERGENIC	--	--	--
Ca4_P4_28848503	Ca4	28848503	A/AG	CGAAAAACCGTCCTCTTTCA	CATTGTGGCATCATTTCCTCT	60.2	151	INTERGENIC	--	--	--
Ca4_P4_28850439	Ca4	28850439	ATTATTTTTATTTT/ATTATTTTTATTTTTATTTT	GTCAGAGGCAGTGAGCCTTC	TTTTAGCTTTCCAACGCCTG	60.1	500	INTERGENIC	--	--	--
Ca4_P4_28854169	Ca4	28854169	GGTTCAGTT/GGTTCAGTTCAGTT	CCGAACTGAACCGGTTTTTA	ATACTCCTGCCGGATACACG	60.0	529	INTERGENIC	--	--	--
Ca4_P4_28909527	Ca4	28909527	ACC/ACCC	TGAAAATTGTTTTTAACGCCA	TCGTTTCCACAAATACCAACA	58.3	378	INTERGENIC	--	--	--
Ca4_P4_28913802	Ca4	28913802	TTTATTATTATTATTATTATTATTATTATTATTATTATT/TTTATTATTATTATTATTATTATTATT	AAATAGAAAGTCGCATCCCG	CAACGTCCTCCCATTTGTTT	59.2	510	INTERGENIC	--	--	--
Ca4_P4_28915766	Ca4	28915766	AATATATATATAT/AATATATATATATAT	TTGGCAAAACGTTATTTTTGG	ATGTTGAAAATGGTGCCTCC	59.9	525	INTERGENIC	--	--	--
Ca4_P4_28920238	Ca4	28920238	GTTAGGACTTAGGACTTAGGACT/GTTAGGACTTAGGACT	TCCCACGTCCTAATTTTTGC	ACTACATGCGACCGTTCTCC	59.9	732	INTERGENIC	--	--	--
Ca4_P4_28930811	Ca4	28930811	CATTATTATTATTATT/CATTATTATTATTATTATTATT	CTCGTTCAAAGGGCTAGACG	TAATTTGAAATGGCGCACAA	60.0	722	INTERGENIC	--	--	--

INDEL marker IDs	Chromosomes/ unanchored scaffolds	Physical positions (bp)	InDels (*Kabuli* reference genome-CDC Frontier/PII)	Forward primers (5'-3')	Reverse primers (5'-3')	Anneling temperature (0C)	Expected amplified product size (bp)	Structural annotation		Functional annotation	
								Sequence components of *kabuli* genome	*Kabuli* gene accession IDs	NCBI-KOG	TFs
Ca4_P4_28933902	Ca4	28933902	GTT/GT	GATGGACACTCATGTCAATGAAG	TGGCCAAACTGATGATTCAA	59.5	574	INTERGENIC	--	--	--
Ca4_P4_28938203	Ca4	28938203	ACCCCC/ACCCC	CTGATTTCCAACACGAGGGT	CATTTCTCCACCCAAATGCT	60.0	523	INTERGENIC	--	--	--
Ca4_P4_28989260	Ca4	28989260	TAAAAA/TAAAA	GAGGCAATTGACAACCCAGT	GTCCCATTGTTGAGGTCGTT	60.0	695	INTERGENIC	--	--	--
Ca4_P4_29008246	Ca4	29008246	GTTTT/GTTT	CCTACAATCCCAAGACAAGGA	ATTAATGCCCATGAAGGCAC	59.0	252	INTERGENIC	--	--	--
Ca4_P4_29034169	Ca4	29034169	TA/TAA	TGAATGGAAAGAACCTGGACA	TGTGAGCATCCACTCTCAGG	60.5	758	INTERGENIC	--	--	--
Ca4_P4_29035883	Ca4	29035883	ATTTTTTT/ATTTTTTTT	TTGGACAGAGGCATGCAATA	GGACCAACTCTGGAAGAAAAA	60.2	735	INTERGENIC	--	--	--
Ca4_P4_29037899	Ca4	29037899	TAA/TAAA	GAGCTGAAACATGCGAACAA	TGATACCTTGCATTGAACAAATTC	60.0	716	INTERGENIC	--	--	--
Ca4_P4_29052614	Ca4	29052614	CTTTTTTT/CTTTTTT	GGTTCCAACACGTGTCCTCT	CAATCAACATTGCAACAGGG	60.0	536	INTERGENIC	--	--	--
Ca4_P4_29070111	Ca4	29070111	TAA/TAAA	CGCGAGTCTGCTCTTGAACT	CCACCTATCGCGTCTGGTAT	60.9	671	INTERGENIC	--	--	--
Ca4_P4_29073548	Ca4	29073548	ATTTTTTTTT/ATTTTTTTTTT	TGTCAAAAATGAGGGTGCAA	CACCTATTTTGTGCCCCAAC	60.1	546	INTERGENIC	--	--	--

INDEL marker IDs	Chromosomes/ unanchored scaffolds	Physical positions (bp)	InDels (*Kabuli* reference genome-CDC Frontier/PII)	Forward primers (5'-3')	Reverse primers (5'-3')	Anneling temperature (0C)	Expected amplified product size (bp)	Structural annotation		Functional annotation	
								Sequence components of *kabuli* genome	*Kabuli* gene accession IDs	NCBI-KOG	TFs
Ca4_P4_29091211	Ca4	29091211	T/TAA	TTTTAAAATCTATCCAAAATGAAAAGC	GCGTGTTATTTTGTTGACGC	59.4	351	INTERGENIC	--	--	--
Ca4_P4_29091323	Ca4	29091323	TAAA/TAA	TTTTAAAATCTATCCAAAATGAAAAGC	GCGTGTTATTTTGTTGACGC	59.4	351	INTERGENIC	--	--	--
Ca4_P4_29100812	Ca4	29100812	GGTAGTA/GGTA	TCAAGAAATAGTGGGGGCAG	TGGAGAAACTCTTCCGCTGT	60.1	840	INTERGENIC	--	--	--
Ca4_P4_29110502	Ca4	29110502	ATTTTT/ATTTT	TGCAAACATGGATGGGAATA	CACGGTTTCTAACACCATTTGA	59.7	617	INTERGENIC	--	--	--
Ca4_P4_29122319	Ca4	29122319	TAAAAAAAA/TAAAAAAAAAAA	TTGTGTGCCACATCATCACA	TACCAGCGACATGCTTTCAG	60.6	832	INTERGENIC	--	--	--
Ca4_P4_29158154	Ca4	29158154	T/TG	TTTGGGGAGACTGAAGATGG	GCTCACGTTTTTGTAATGTCCA	60.0	713	INTERGENIC	--	--	--
Ca4_P4_29160471	Ca4	29160471	GATTATTATTATTATTATTATTATTATTAT/GATTATTATTATTATTATTATTATTATTATTATTATTATTAT	CAGTTTCAATTCATGCATTTCC	GTTTGAAATCGCACCCAAAT	59.5	334	INTERGENIC	--	--	--
Ca4_P4_29161765	Ca4	29161765	CTTTTTTT/CTTTTTTTT	CACATACACATTGCTTTCCGA	TTGCAATCAGAAGTCCCAAA	59.6	256	INTERGENIC	--	--	--
Ca4_P4_29162014	Ca4	29162014	TCC/TC	CTTTCCCGATTTCGTCCTTT	TGGACAAATCGTTTCTTTTGTG	60.4	550	INTERGENIC	--	--	--
Ca4_P4_29162382	Ca4	29162382	AATA/AA	CTTTCCCGATTTCGTCCTTT	GGCCAGCGAAAATTACTACC	60.4	804	INTERGENIC	--	--	--

INDEL marker IDs	Chromosomes/ unanchored scaffolds	Physical positions (bp)	InDels (*Kabuli* reference genome-CDC Frontier/PII)	Forward primers (5'-3')	Reverse primers (5'-3')	Anneling temperature (0C)	Expected amplified product size (bp)	Structural annotation		Functional annotation	
								Sequence components of *kabuli* genome	*Kabuli* gene accession IDs	NCBI-KOG	TFs
Ca4_P4_29191237	Ca4	29191237	CTTTTTTTT/CTTTTTTTTT	CCAACTTGGTTCCAACAATCT	GGAGAAAAGTTAGGGGGTGC	58.9	119	INTERGENIC	--	--	--
Ca4_P4_29208577	Ca4	29208577	CAAAA/CAA	CGGTCAACCTAATCGGAGAA	TTCCTCTTGTTCACTGTGCG	60.1	760	INTERGENIC	--	--	--
Ca4_P4_29209441	Ca4	29209441	AATATATATATATATA/AATATATATATA	AGGTTGCTCGCAGTTGAGAT	GCCGTCTCAACTCTCTGTCC	60.0	689	INTERGENIC	--	--	--
Ca4_P4_29220873	Ca4	29220873	AACAGCGCTT/AACAGCGCTTTTTTCACAGCGCTT	GTAGATGGCCGCACACTACA	CTGAATTCACAAGGCACCCT	59.7	612	INTERGENIC	--	--	--
Ca4_P4_29221849	Ca4	29221849	CTTTTTTT/CTTTTTT	TTGTTCGTACATCCGGGAAT	CGCACATAGAGAGGTGCATT	60.2	735	INTERGENIC	--	--	--
Ca4_P4_29221975	Ca4	29221975	ATTTTTTTTTTTTTT/ATTTTTTTTTT	TTGTTCGTACATCCGGGAAT	CGCACATAGAGAGGTGCATT	60.2	735	INTERGENIC	--	--	--
Ca4_P4_29222326	Ca4	29222326	ATTTTTTT/ATTTTTTTT	TGCACCTCTCTATGTGCGTG	TTCGTTCCGGACAAATCTTC	61.0	342	INTERGENIC	--	--	--
Ca4_P4_29228849	Ca4	29228849	T/TG	CATTTATTTCTTCCCCCGGT	CACGAAGACGTTGGAACAGA	60.0	362	INTERGENIC	--	--	--
Ca4_P4_29230365	Ca4	29230365	TAAAAAA/TAAAAAAAAA	TTGTCAACTTTGTTGGGGAA	GTTTGTTTTTGGCAAGACCG	59.0	156	INTERGENIC	--	--	--
Ca4_P4_29231627	Ca4	29231627	TA/TAGGA	TTCCTCCCATGAAACGATGT	TCTGGGGACAAAATGAGGAG	60.3	590	INTERGENIC	--	--	--

INDEL marker IDs	Chromosomes/ unanchored scaffolds	Physical positions (bp)	InDels (*Kabuli* reference genome-CDC Frontier/PII)	Forward primers (5'-3')	Reverse primers (5'-3')	Anneling temperature (0C)	Expected amplified product size (bp)	Structural annotation		Functional annotation	
								Sequence components of *kabuli* genome	*Kabuli* gene accession IDs	NCBI-KOG	TFs
Ca4_P4_29233852	Ca4	29233852	A/AT	AGGAAAATGATGCAAGGCTG	TAGAAGCAAAGGCGGAAAAA	60.2	726	INTERGENIC	--	--	--
Ca4_P4_29238831	Ca4	29238831	A/AC	CCATGAGATCCACATAATCCG	TTGCAAGAATCCATCCATCA	60.2	513	INTERGENIC	--	--	--
Ca4_P4_29243916	Ca4	29243916	TAAAAAAAAAAAAAAA/TAAAAAAAA	ATTCCTTGTGCGAGGTCAAC	TCACCTGCAAAGTCAGCATC	60.1	561	INTERGENIC	--	--	--
Ca4_P4_29245578	Ca4	29245578	T/TA	TTACCAACCAAATTGCACCA	CCCTAATGGGCCAAATTACA	59.8	583	INTERGENIC	--	--	--
Ca4_P4_29262137	Ca4	29262137	AAT/AATTAT	TTCAAAGGTGCATTGTCGAT	CACTGCGACGAAACTTACCA	59.1	619	INTERGENIC	--	--	--
Ca4_P4_29280125	Ca4	29280125	ATTTTTTTTTTTTT/ATTTTTTTTTTT	AGGTCGTCCAAAATCAAACG	TTCATCGCTCGCATTCATAC	60.0	328	INTERGENIC	--	--	--
Ca4_P4_29292512	Ca4	29292512	TCC/TC	CACATGTTTCAACTTGGTTGC	GATCCTCATGGGAACGTGAT	59.1	631	INTERGENIC	--	--	--
Ca4_P4_29310770	Ca4	29310770	TAAAAAAAAA/TAAAAAAAA	GTGGTGTCCATCTCGGTCTT	TAAGCCAGCAACTCCAGGAT	60.0	658	INTERGENIC	--	--	--
Ca4_P4_29315886	Ca4	29315886	CTTTTTTTTT/CTTTTTTTTTT	CGTGGCAACCCAAGACTTAT	TCAATGTTCAAGAATCGGTCC	60.0	733	INTERGENIC	--	--	--
Ca4_P4_29320476	Ca4	29320476	CTTTTTTTT/CTTTTTTT	TGGCTTTCAAGGAAGGGTAA	TTGGCAAACCTCGAAATAGG	59.7	376	INTERGENIC	--	--	--

INDEL marker IDs	Chromosomes/ unanchored scaffolds	Physical positions (bp)	InDels (*Kabuli* reference genome-CDC Frontier/PII)	Forward primers (5'-3')	Reverse primers (5'-3')	Anneling temperature (0C)	Expected amplified product size (bp)	Structural annotation		Functional annotation	
								Sequence components of *kabuli* genome	*Kabuli* gene accession IDs	NCBI-KOG	TFs
Ca4_P4_29321184	Ca4	29321184	ATTTTTTT/ATTTTTTTT	AACACATCAGCAAATGGCAA	TGGGTTCGGGTACAAGCTAC	60.1	682	INTERGENIC	--	--	--
Ca4_P4_29331432	Ca4	29331432	CAAAAAAAAAAAAA/CAAAAAAAAAA	TGCCCTTAGTCAAACGATCA	GCATTTGCTGTTGGAACCTT	59.3	575	INTERGENIC	--	--	--
Ca4_P4_29350232	Ca4	29350232	AC/A	GTTAAATTTGCTGGAGCACG	ACCAAGATTGGGCATCTGAA	58.4	160	INTERGENIC	--	--	--
Ca4_P4_29358565	Ca4	29358565	CAAAAAAAAA/CAAAAAAAAAA	AACCAATTCAATGCAAACCG	ATGCGAACCATCATCAGTCA	60.7	511	INTERGENIC	--	--	--
Ca4_P4_29364474	Ca4	29364474	CAAA/CAA	GGGCATGAACTCCAACTTGT	AACTTGATCAATCGCTGAGTGT	60.0	759	INTERGENIC	--	--	--
Ca4_P4_29369378	Ca4	29369378	ACATCAAATCCATCAAAT/ACATCAAAT	TTTCCATCTCCAACAACAACA	AACCACTTTGCAAGCAGTTG	59.0	476	INTERGENIC	--	--	--
Ca4_P4_29370210	Ca4	29370210	ATTTTT/ATTTT	GCATGCCTGTTCTCTCAACA	AAAGGGCCATAGGAGAGTCC	60.0	643	INTERGENIC	--	--	--
Ca4_P4_29379238	Ca4	29379238	CAAAAAAA/CAAAAAA	ATGCAAGTGGGCCTAGACAG	CACTTCGTTTTGGAATCGGT	60.3	630	INTERGENIC	--	--	--
Ca4_P4_29393253	Ca4	29393253	ATTATTTATTTATTTATTTATTTATTTATTT/ATTATTTATTTATTTATTTATTTATTTATTTATTTATTTATTT	GCTTGCAGCTAGGAAGCAGT	TTTGTTGATCTACGCATCGG	59.9	404	INTERGENIC	--	--	--
Ca4_P4_29415553	Ca4	29415553	C/CCG	AATATGCATAACTCGACGGAGA	ACAGGTGTCGAAGACCGACT	58.7	481	INTERGENIC	--	--	--

INDEL marker IDs	Chromosomes/ unanchored scaffolds	Physical positions (bp)	InDels (*Kabuli* reference genome-CDC Frontier/PII)	Forward primers (5'-3')	Reverse primers (5'-3')	Anneling temperature (0C)	Expected amplified product size (bp)	Structural annotation		Functional annotation	
								Sequence components of *kabuli* genome	*Kabuli* gene accession IDs	NCBI-KOG	TFs
Ca4_P4_29418531	Ca4	29418531	TT/TTAT	GGTGGTGTACGGTGGAAGTT	GTCAAGGCAGACCAGAAAGC	59.7	383	INTERGENIC	--	--	--
Ca4_P4_29420218	Ca4	29420218	ATTT/ATT	TCACAATGCAAGCTTATGCC	TTGAAACAAAAACCACGATGA	59.8	742	INTERGENIC	--	--	--
Ca4_P4_29420847	Ca4	29420847	T/TA	CTTCGCCGTTGCTTTTCTAC	GGCTCAAATGAAAAAGGCAT	60.0	526	INTERGENIC	--	--	--
Ca4_P4_29422304	Ca4	29422304	CA/CATA	ATGCGTTGCCAAAATGTATG	TCCCTCACATGATGGTAAAAAG	59.4	766	INTERGENIC	--	--	--
Ca4_P4_29428218	Ca4	29428218	ATTTTTTTTTT/ATTTTTTTTT	TCCATCTTCTTCATTGATCACG	TGCCAAAAATACTCTTGTAAACAAAT	60.1	539	INTERGENIC	--	--	--
Ca4_P4_29431394	Ca4	29431394	TAAAAAAAAAAAAA/TAAAAAAAAAA	TCGACCTTGTTAGGAGCGAT	CCATAGCCAATGGTTCCTTC	59.8	629	INTERGENIC	--	--	--
Ca4_P4_29442652	Ca4	29442652	T/TG	CGTGACTGATCCTCCATCCT	AAAGTTGGTTTGTTGGCAGG	60.1	630	INTERGENIC	--	--	--
Ca4_P4_29443305	Ca4	29443305	CTTTTTTTTTT/CTTTTTTTT	CCTGCCAACAAACCAACTTT	GGCTGGAGGAAAACAAACAA	60.0	643	INTERGENIC	--	--	--
Ca4_P4_29498412	Ca4	29498412	ATTTTTTTT/ATTTTTTT	TTGGATTGATTTTTGGATTTGTC	AACTTGATTTGAATATTGCCATTG	60.0	779	INTERGENIC	--	--	--
Ca4_P4_29501722	Ca4	29501722	TGG/TGGG	ATGGAGGTGCAGGGAAGAAT	TGAGTGGAAAATGGCATCAA	60.9	790	INTERGENIC	--	--	--

INDEL marker IDs	Chromosomes/ unanchored scaffolds	Physical positions (bp)	InDels (*Kabuli* reference genome-CDC Frontier/PII)	Forward primers (5'-3')	Reverse primers (5'-3')	Anneling temperature (0C)	Expected amplified product size (bp)	Structural annotation		Functional annotation	
								Sequence components of *kabuli* genome	*Kabuli* gene accession IDs	NCBI-KOG	TFs
Ca4_P4_29511821	Ca4	29511821	CTTTTTTTTT/CTTTTTTTTTT	AAAGGGCTAAATAGCTCCCG	TCCCGATTTGGTCATTTGTT	59.7	506	INTERGENIC	--	--	--
Ca4_P4_29531556	Ca4	29531556	G/GA	CAAGGTCGGGGATTTGACTA	GTTATGCGCAAAATAGCGGT	59.9	578	INTERGENIC	--	--	--
Ca4_P4_29532848	Ca4	29532848	TAAAAAAA/TAAAAAAAA	TCCTTGCAAAGGACATTAAACA	TGCCAAGTCATGGATAATATGGT	59.6	544	INTERGENIC	--	--	--
Ca4_P4_29532946	Ca4	29532946	TAAAAAAAAAAA/TAAAAAAAAAAAAA	CCATATTATCCATGACTTGGCA	CCGTTAGAAATCAAATGGGC	59.7	562	INTERGENIC	--	--	--
Ca4_P4_29534339	Ca4	29534339	CAAAAAAA/CAAAAAAAA	GGTCCGTCAAAAGAGTGTGAG	CCTCGAGCTATGCAATATGATG	59.8	564	INTERGENIC	--	--	--
Ca4_P4_29544316	Ca4	29544316	GATAT/GAT	CCATCAAACCACAACAAACA	CCCAATTTCCTTCCGTTTTT	57.8	706	INTERGENIC	--	--	--
Ca4_P4_29544653	Ca4	29544653	ATTTTTTTTTTTT/ATTTTTTTTTTT	TTTCAAGTGCAACTAAGGGCT	CACCCAGCGTATCACGTATG	59.0	362	INTERGENIC	--	--	--
Ca4_P4_29545135	Ca4	29545135	AATTATTTATTATTTAT/AATTATTTATTATTTATTATTTAT	CATACGTGATACGCTGGGTG	GTGAGGGAGGACCAAAACAA	60.0	659	INTERGENIC	--	--	--
Ca4_P4_29564954	Ca4	29564954	ATTTTTTTTTTT/ATTTTTTTTTT	GCATAAGGATCGGGGAAACT	GATCGTCTTCGGGTTGGTTA	60.3	396	INTERGENIC	--	--	--
Ca4_P4_29582409	Ca4	29582409	TAAAA/TAAA	AGCTGTATTGGGCCTCCTTT	GCCGAATAAAACCCTCACAA	60.1	212	INTERGENIC	--	--	--

INDEL marker IDs	Chromosomes/ unanchored scaffolds	Physical positions (bp)	InDels (*Kabuli* reference genome-CDC Frontier/PII)	Forward primers (5'-3')	Reverse primers (5'-3')	Anneling temperature (0C)	Expected amplified product size (bp)	Structural annotation		Functional annotation	
								Sequence components of *kabuli* genome	*Kabuli* gene accession IDs	NCBI-KOG	TFs
Ca4_P4_29583344	Ca4	29583344	ATGT/AAACATGTGT	TTGGCAAGATTGAAAAACGA	TAAGAAGGGAGCAATCACGC	59.3	780	INTERGENIC	--	--	--
Ca4_P4_29596950	Ca4	29596950	TTTTATTTATTTATTTATTTATTTATTTATTTAT/TTTTATTTATTTATTTATTTATTTATTTATTTATTTATTTATTTATTTAT	CCTGTTTTTGAACAAGCCAAA	CGGTCACCATATGTCACTTCA	60.1	399	INTERGENIC	--	--	--
Ca4_P4_29604902	Ca4	29604902	TAAAAAAA/TAAAAAA	TCAAAAATGCTACCTACACCCC	TTAAGACCGGTGATGAAGGG	60.2	128	INTERGENIC	--	--	--
Ca4_P4_29606095	Ca4	29606095	AG/AGGG	AGCACCCACATGATGTTTGA	CGCGACATTGACGATAAAAA	60.0	826	INTERGENIC	--	--	--
Ca4_P4_29618061	Ca4	29618061	CTTTTTTTTTT/CTTTTTTTTT	AGTGATTTCGGGACTGGATG	CCCCCACCGTATAAAGTTGT	59.9	382	INTERGENIC	--	--	--
Ca4_P4_29618138	Ca4	29618138	C/CAATGTTGT	AGTGATTTCGGGACTGGATG	CATCATCAACAACAACAACAACA	59.9	443	INTERGENIC	--	--	--
Ca4_P4_29622774	Ca4	29622774	ACGACCCACTGCGAC/ACGAC	TTGTTGTTGCGATCATCGTT	TGTTCCAAACATTGTTGCGT	60.1	361	INTERGENIC	--	--	--
Ca4_P4_29623951	Ca4	29623951	AT/A	GCGAGTCCATTTGGTTTGAT	GGGTTGGGAACCCTAGTTGT	59.9	821	INTERGENIC	--	--	--
Ca4_P4_29628090	Ca4	29628090	TAA/TAAA	TGACACCATTACCTAGGCCC	CTTCGAGAGTTGTTCCTCCG	59.8	281	INTERGENIC	--	--	--
Ca4_P4_29649360	Ca4	29649360	TAT/TATAAT	CTCGGTATGGCCTATTTCCA	TTGCTGGGCTAAATACCGAC	59.9	527	INTERGENIC	--	--	--

INDEL marker IDs	Chromosomes/ unanchored scaffolds	Physical positions (bp)	InDels (*Kabuli* reference genome-CDC Frontier/PII)	Forward primers (5'-3')	Reverse primers (5'-3')	Anneling temperature (0C)	Expected amplified product size (bp)	Structural annotation		Functional annotation	
								Sequence components of *kabuli* genome	*Kabuli* gene accession IDs	NCBI-KOG	TFs
Ca4_P4_29670240	Ca4	29670240	TAAAAAA/TAAAAAAA	TGGGGGATACCATTCTTTGA	TGCCCTTGAGCTTTGAGTTT	60.1	479	INTERGENIC	--	--	--
Ca4_P4_29701374	Ca4	29701374	G/GTTTAAATTTC	AGATCTTTCCCCTCATGGCT	CCTTGACAAGAAGCAACACG	60.0	474	INTERGENIC	--	--	--
Ca4_P4_29705345	Ca4	29705345	ACCCCCC/ACCCCC	CGGTAAGGTGAAGGCTGAAA	CCAATTTCGAGTGCAGATCA	60.2	357	INTERGENIC	--	--	--
Ca4_P4_29712689	Ca4	29712689	CAAA/CAA	TTTGCTTACTCAAACCCCCA	AGTTCGGTAGTGGCTTCCCT	60.5	837	INTERGENIC	--	--	--
Ca4_P4_29712921	Ca4	29712921	TA/TTATATTTAA	TCATGTTGAGCAAAGCCAAA	TCAGTTCGGTAGTGGCTTCC	60.4	406	INTERGENIC	--	--	--
Ca4_P4_29717512	Ca4	29717512	GAAATAAATA/GAAATAAATAAATA	GTTGGCATTTCTCCACTCGT	GTGCCCAACAAACAAGGACT	60.1	458	INTERGENIC	--	--	--
Ca4_P4_29717836	Ca4	29717836	CTCACATTTTACTC/CTCACATTTTACTCATCACATTTTACTC	AGTCCTTGTTTGTTGGGCAC	TAACAGCTCAACTGCCCTCC	60.0	373	INTERGENIC	--	--	--
Ca4_P4_29730514	Ca4	29730514	CTTTTTT/CTTTTTTT	AAAACTTGTTTGGAAGTTTTCATTTT	TGAATGTGAAGTGGATGGTCA	59.8	247	INTERGENIC	--	--	--
Ca4_P4_29769001	Ca4	29769001	CTTTTTTTTT/CTTTTTTTT	CCGATGAAGAAGAAAAAGCAA	TTGCAGTTGGATTTTGTAGGG	59.5	479	INTERGENIC	--	--	--
Ca4_P4_29769710	Ca4	29769710	ATTTT/ATTT	CCCTACAAAATCCAACTGCAA	GCTCCCAAGACAAAACCTGA	60.0	686	INTERGENIC	--	--	--

INDEL marker IDs	Chromosomes/ unanchored scaffolds	Physical positions (bp)	InDels (*Kabuli* reference genome-CDC Frontier/PII)	Forward primers (5'-3')	Reverse primers (5'-3')	Anneling temperature (0C)	Expected amplified product size (bp)	Structural annotation		Functional annotation	
								Sequence components of *kabuli* genome	*Kabuli* gene accession IDs	NCBI-KOG	TFs
Ca4_P4_29811970	Ca4	29811970	AGGCGCGC/AGGCGCGCAGGGCGCGC	GCGTTTTTATGGTTGCTGGT	ACTTTCATGAAGACACCGGG	60.0	152	INTERGENIC	--	--	--
Ca4_P4_29821605	Ca4	29821605	CTTTTTTTT/CTTTTTTT	GACTCATCCAAGGTTGCCAT	TTTGATGGCTCAGAAAGCCT	59.9	524	INTERGENIC	--	--	--
Ca4_P4_29823817	Ca4	29823817	AG/AGGG	CCAAAATTGGTTGCAAACTTC	TCATGTTTTGTTGGTCTGCC	59.5	233	INTERGENIC	--	--	--
Ca4_P4_29826827	Ca4	29826827	GTGTTGTT/GTGTT	CGAACCTACGAGGCTAAACG	GGGTGTCGAAACATTCCATC	59.9	290	INTERGENIC	--	--	--
Ca4_P4_29848231	Ca4	29848231	TGAAGA/TGAAGAAGA	GCATTGTTGAAACCAAACTCG	TTTGGGAGACAGACCCTCAC	60.5	907	INTERGENIC	--	--	--
Ca4_P4_29863037	Ca4	29863037	GTTATTATTATTAT/GTTATTATTATTATTAT	TCGGGGTGTTACAGTATGGA	ACATCCAAACCCCTCATTCA	58.8	605	INTERGENIC	--	--	--
Ca4_P4_29874025	Ca4	29874025	TAAAAAAA/TAAAAAA	GGCCCTATGCAGCGTATAAA	ATCATCTCAGAGTCGGCCC	60.1	103	INTERGENIC	--	--	--
Ca4_P4_29910860	Ca4	29910860	TAAAA/TAAAAA	TATCCGCAATCAAGGGAGAG	ACAGCATTTTGTGCCATTGA	60.2	447	INTERGENIC	--	--	--
Ca4_P4_29911237	Ca4	29911237	TATGTTGA/TATGTTGATGTTGA	GTCAATGGCACAAAATGCTG	GCAGAAGGGTGTTGGAAAAA	60.1	738	INTERGENIC	--	--	--
Ca4_P4_29911402	Ca4	29911402	CTTTTTTTTTTT/CTTTTTTTTTT	GTCAATGGCACAAAATGCTG	GCAGAAGGGTGTTGGAAAAA	60.1	738	INTERGENIC	--	--	--

INDEL marker IDs	Chromosomes/ unanchored scaffolds	Physical positions (bp)	InDels (*Kabuli* reference genome-CDC Frontier/PII)	Forward primers (5'-3')	Reverse primers (5'-3')	Anneling temperature (0C)	Expected amplified product size (bp)	Structural annotation		Functional annotation	
								Sequence components of *kabuli* genome	*Kabuli* gene accession IDs	NCBI-KOG	TFs
Ca4_P4_29911958	Ca4	29911958	TAAAAAAAAAA/TAAAAAAAAA	TTTTTCCAACACCCTTCTGC	TAGATTTGAGTGCCCATCGC	60.1	684	INTERGENIC	--	--	--
Ca4_P4_29912693	Ca4	29912693	GATGTC/GATGTCATGTC	TGAGATCCCAACAGGCCTAA	TGGTCTTTGGAAAGCCTCAC	60.6	483	INTERGENIC	--	--	--
Ca4_P4_29930444	Ca4	29930444	CAAAAAAAAAA/CAAAAAAAAAAAA	CCGACACGGTCTTTTTCCTA	AATGACCCGCAATTATGAGC	60.1	392	INTERGENIC	--	--	--
Ca4_P4_29930687	Ca4	29930687	TTTGATCTT/TTT	GCTCATAATTGCGGGTCATT	AGGTATGGACAAAATTGCGG	59.9	547	INTERGENIC	--	--	--
Ca4_P4_29931076	Ca4	29931076	A/AGTAACAATAAGT	TGTGCACCAATATCTGCCAT	GAAGAAAACCCGGGATCAGT	60.0	670	INTERGENIC	--	--	--
Ca4_P4_29931458	Ca4	29931458	CTTTTT/C	CCGCAATTTTGTCCATACCT	CCAAAAAGTAGGGGGAGAGAA	59.8	545	INTERGENIC	--	--	--
Ca4_P4_29933754	Ca4	29933754	ATTTTTTT/ATTTTTTTT	AGCCACACGATGTTGATTGA	TGATTAGGGAATCACCCGTT	60.1	845	INTERGENIC	--	--	--
Ca4_P4_29939743	Ca4	29939743	CAA/CA	TCCTTTTCGAGACTCAGGGA	GCACCACCTGTTTGATTTCC	59.9	718	INTERGENIC	--	--	--
Ca4_P4_29943561	Ca4	29943561	AATC/A	GTGAAAGGTGAGGAGAAGCG	TCAACCCTTTTATTCATGCCA	60.0	131	INTERGENIC	--	--	--
Ca4_P4_29945038	Ca4	29945038	TA/TAA	AAATGCAATAGCAGGTTGGC	CGATGAGGATAAAAGGAGCG	60.1	904	INTERGENIC	--	--	--

INDEL marker IDs	Chromosomes/ unanchored scaffolds	Physical positions (bp)	InDels (*Kabuli* reference genome-CDC Frontier/PII)	Forward primers (5'-3')	Reverse primers (5'-3')	Anneling temperature (0C)	Expected amplified product size (bp)	Structural annotation		Functional annotation	
								Sequence components of *kabuli* genome	*Kabuli* gene accession IDs	NCBI-KOG	TFs
Ca4_P4_29957864	Ca4	29957864	TAAAAAAAAAA/TAAAAAAAAA	AGGACTGAACGTAACCCACG	ACCTAAGTTCATTCGCCCCT	60.0	590	INTERGENIC	--	--	--
Ca4_P4_29962177	Ca4	29962177	AC/A	CACTCCTACGTACCCCCAAG	TTTTACCACCGGCTACTTCG	59.5	261	INTERGENIC	--	--	--
Ca4_P4_29962305	Ca4	29962305	GA/G	CACTCCTACGTACCCCCAAG	TTTTACCACCGGCTACTTCG	59.5	261	INTERGENIC	--	--	--
Ca4_P4_29962434	Ca4	29962434	GTAT/GTATATAT	CTAGTCGAAGTAGCCGGTGG	TTTGGAAGCATTGTGGTTGA	59.9	486	INTERGENIC	--	--	--
Ca4_P4_29986497	Ca4	29986497	GTGCGAATACATGCG/GTGCG	AAAAAGGATATAAATACCCGCAA	AAAAATCTTCTCCACAAGATGAA	58.1	449	INTERGENIC	--	--	--
Ca4_P4_29986966	Ca4	29986966	A/AGT	CAACCAGTGCGAATACATGC	CACCTGGAGCAGAGGTGATT	60.1	923	INTERGENIC	--	--	--
Ca4_P4_30040492	Ca4	30040492	TC/T	TAGCCGCGGTTAAGTGAAGT	TGGAACAAGAGAGGGATTGG	59.9	551	INTERGENIC	--	--	--
Ca4_P4_30046973	Ca4	30046973	GAAAAAAAAAA/GAAAAAAAAA	TGAATGAAATTTCTGGCAGG	CGTTTTGTTTGTGGCACATT	58.7	280	INTERGENIC	--	--	--
Ca4_P4_30049126	Ca4	30049126	G/GAA	TGAGGGCAAACAACTATTCAA	TTTGACCAATGTGCAATTCAA	58.3	771	INTERGENIC	--	--	--
Ca4_P4_30049460	Ca4	30049460	C/CTAGT	GAGTGGGAGAGAAATGTTTAGTGAA	TGACAAATCCCCTTTTCCAA	60.1	233	INTERGENIC	--	--	--

INDEL marker IDs	Chromosomes/ unanchored scaffolds	Physical positions (bp)	InDels (*Kabuli* reference genome-CDC Frontier/PII)	Forward primers (5'-3')	Reverse primers (5'-3')	Anneling temperature (0C)	Expected amplified product size (bp)	Structural annotation		Functional annotation	
								Sequence components of *kabuli* genome	*Kabuli* gene accession IDs	NCBI-KOG	TFs
Ca4_P4_30052378	Ca4	30052378	TG/T	TAAGCACAAATGACGTTGGG	TCCATTGGGTATTCAGCACA	59.6	963	INTERGENIC	--	--	--
Ca4_P4_30072648	Ca4	30072648	TAAAAAAA/TAAAAAAAA	TTTTCATGTTTCCGCATTCA	CGGGGTGAGTTAAATGTACGA	60.1	560	INTERGENIC	--	--	--
Ca4_P4_30075059	Ca4	30075059	CTAA/CTAATTGTTTTTATAA	TCGAAACCAACCACCTTTTC	GCAATTCGACAATGCACAAC	59.9	547	INTERGENIC	--	--	--
Ca4_P4_30076786	Ca4	30076786	GTTTTTTTT/GTTTTTTTTTT	GCACTTCCGCTAAAAGCATC	AAGCGCTCCCAAATATGCTA	60.0	432	INTERGENIC	--	--	--
Ca4_P4_30077034	Ca4	30077034	TAAATTAAAATTAAAAT/TAAATTAAAATTAAAATTAAAAT	GCATATTTGGGAGCGCTTTA	TGGATGATTGTGACAAAATTGC	60.2	508	INTERGENIC	--	--	--
Ca4_P4_30077287	Ca4	30077287	CTTTTTTTT/CTTTTTTTTTT	GCATATTTGGGAGCGCTTTA	TGGATGATTGTGACAAAATTGC	60.2	508	INTERGENIC	--	--	--
Ca4_P4_30081791	Ca4	30081791	CCTT/CCTTTCTT	TTGTCGTTGCAATGGTTCAT	TTCACAAAAGGGGGTGAAAG	60.0	467	INTERGENIC	--	--	--
Ca4_P4_30083166	Ca4	30083166	ATTTTTTTTTTTT/ATTTTTTTTT	AACGGAGGAACAAACCAGAA	CAACCTCGATCTCGACCAAT	59.6	790	INTERGENIC	--	--	--
Ca4_P4_30084699	Ca4	30084699	GTTTTTTTTT/GTTTTTTTT	CGAAATTTTGTAGCCGGAAA	AAGTTGCATTGTGGTGGTCA	60.1	277	INTERGENIC	--	--	--
Ca4_P4_30086898	Ca4	30086898	ACTTTCT/ACT	ACGCCGAAAAACACTTTCAC	TCCATTCGACTGTCCTTCAA	60.2	630	INTERGENIC	--	--	--

INDEL marker IDs	Chromosomes/ unanchored scaffolds	Physical positions (bp)	InDels (*Kabuli* reference genome-CDC Frontier/PII)	Forward primers (5'-3')	Reverse primers (5'-3')	Anneling temperature (0C)	Expected amplified product size (bp)	Structural annotation		Functional annotation	
								Sequence components of *kabuli* genome	*Kabuli* gene accession IDs	NCBI-KOG	TFs
Ca4_P4_30092930	Ca4	30092930	GTTTTT/GTTTTTTT	GATGCGACCATCTACTGCAA	TGATGTGTCCCTAATATTTTCCG	59.8	529	INTERGENIC	--	--	--
Ca4_P4_30098960	Ca4	30098960	ATTTTTTTT/ATTTTTTTTT	TGGCGAATCGTTGATCATAA	ATATGGCTTGAAGCAAACGC	60.0	697	INTERGENIC	--	--	--
Ca4_P4_30102122	Ca4	30102122	CTCTTCCAAT/CTCTTCCAATTCTTCCAAT	TGATTCTTCAATTGCCACCA	ACGAGAGACAAGTGAGGGGA	60.0	580	INTERGENIC	--	--	--
Ca4_P4_30102440	Ca4	30102440	TG/T	TCCCCTCACTTGTCTCTCGT	GTGTTTGGGATTGGGAAATG	59.8	609	INTERGENIC	--	--	--
Ca4_P4_30102999	Ca4	30102999	CC/CCCCTC	ATTTCCCAATCCCAAACACA	ATCCTTGCTAGCCACGAAAA	60.0	662	INTERGENIC	--	--	--
Ca4_P4_30114713	Ca4	30114713	TAAAAAAAA/TAAAAAAA	GCTCAAGTTATAGCCTACAAAGGG	CAGTTTTGGTCAATCGCTCA	59.7	974	INTERGENIC	--	--	--
Ca4_P4_30116632	Ca4	30116632	ATTTTTTTTTT/ATTTTTTTT	GATCACGCCAGTTGTTGGTA	TGTCGATTGCGAAGAGTTGT	59.6	896	INTERGENIC	--	--	--
Ca4_P4_30118773	Ca4	30118773	TG/TGG	CTCCTTCATTTCCTCCCTCC	AAGGTGGGTTAAAGAACCGC	60.0	778	INTERGENIC	--	--	--
Ca4_P4_30120661	Ca4	30120661	TAAAA/TAAA	TTTGACATCCATCTTATGCAGC	CCCTTGGGTGCGTTAGTTAT	60.1	379	INTERGENIC	--	--	--
Ca4_P4_30122538	Ca4	30122538	ATT/AT	ATGTCAATGGGCCACTTAGC	TTGTTTGTTGTGCTTTGACCA	60.0	764	INTERGENIC	--	--	--

INDEL marker IDs	Chromosomes/ unanchored scaffolds	Physical positions (bp)	InDels (*Kabuli* reference genome-CDC Frontier/PII)	Forward primers (5'-3')	Reverse primers (5'-3')	Anneling temperature (0C)	Expected amplified product size (bp)	Structural annotation		Functional annotation	
								Sequence components of *kabuli* genome	*Kabuli* gene accession IDs	NCBI-KOG	TFs
Ca4_P4_30131966	Ca4	30131966	CTT/CTTT	TCATGTACGCACTGATGGAAA	CTCTTTAAGACGTTTCGCGG	60.1	778	INTERGENIC	--	--	--
Ca4_P4_30138209	Ca4	30138209	CAAAAAAAA/CAAAAAAA	GCATGCAAACATGGCTCTAA	CAAATTTGGTTGGGTCTTGG	59.8	553	INTERGENIC	--	--	--
Ca4_P4_30142911	Ca4	30142911	C/CTTG	TGATGCATTGATGTTGTCAAAG	AAAGGAAAGGGGACGTGTCT	59.6	701	INTERGENIC	--	--	--
Ca4_P4_30172032	Ca4	30172032	G/GA	CTCAACAGCCTTATGTGCGA	GCATGGAGACTCAAAAATCCA	60.0	673	INTERGENIC	--	--	--
Ca4_P4_30172265	Ca4	30172265	TC/T	CTCAACAGCCTTATGTGCGA	TGCGATGGAATAATGTGTCG	60.0	752	INTERGENIC	--	--	--
Ca4_P4_3017713	Ca4	3017713	ATTATTTTAAATTTATTTTAAATTTATTTTAAATTTATTTTAAATTTATTTTAAATT/ATTATTTTAAATTTATTTTAAATTTATTTTAAATTTATTTTAAATTTATTTTAAATTTATTTTAAATT	GACAAATTAGTGGGGCAACC	TTGTATTGTTGGTGAGGCCA	59.3	902	INTERGENIC	--	--	--
Ca4_P4_30184775	Ca4	30184775	TG/T	ACAACGGAAACAAATCTCGC	GGGGGTTAGGAGTAAGAAAGGA	60.1	116	INTERGENIC	--	--	--
Ca4_P4_30196665	Ca4	30196665	CTTTTT/CTTTT	TCAAACCATGATTTTGGTGC	TGGATGGGAAAAAGGACAAG	59.4	665	INTERGENIC	--	--	--
Ca4_P4_30197320	Ca4	30197320	CAAAAA/CAAAAAA	CTTGTCCTTTTTCCCATCCA	GGCCTAATTAAACATGGGCA	59.9	793	INTERGENIC	--	--	--
Ca4_P4_30211443	Ca4	30211443	T/TA	ACATCAAACAAACAGCGCAC	CCGCTAAAATTGCCCAATAA	59.8	714	INTERGENIC	--	--	--

INDEL marker IDs	Chromosomes/ unanchored scaffolds	Physical positions (bp)	InDels (*Kabuli* reference genome-CDC Frontier/PII)	Forward primers (5'-3')	Reverse primers (5'-3')	Anneling temperature (0C)	Expected amplified product size (bp)	Structural annotation		Functional annotation	
								Sequence components of *kabuli* genome	*Kabuli* gene accession IDs	NCBI-KOG	TFs
Ca4_P4_30212032	Ca4	30212032	ATGCATTGCAT/ATGCAT	TTATTGGGCAATTTTAGCGG	AGCTTTGTGGGCAAGACACT	59.9	655	INTERGENIC	--	--	--
Ca4_P4_30225198	Ca4	30225198	T/TC	AGGCAAACAAGAACCGAAAA	ATGTGCTTTTAGCACCAGGG	59.7	424	INTERGENIC	--	--	--
Ca4_P4_30237136	Ca4	30237136	GTTTTTTTTTTT/GTTTTTTTTTT	CGGGAGTATTTTGGTCACTTT	CCTCTTGTTGCTTCCTCTGG	58.1	589	INTERGENIC	--	--	--
Ca4_P4_30240512	Ca4	30240512	ATTTTTTTTTT/ATTTTTTTTT	TTGGGCATTTTGATGATTGA	GACAACCGATTGAGCCTGAT	59.9	539	INTERGENIC	--	--	--
Ca4_P4_30252991	Ca4	30252991	TAAAAAAAAAAAAA/TAAAAATAAAAAAAAAAAAAAA	GGGCAGCGAACTTAGTCAAG	CGGTGGAAAAGTTTCGGATA	60.0	410	INTERGENIC	--	--	--
Ca4_P4_30266952	Ca4	30266952	CAAAAAAAAAA/CAAAAAAAAAAA	ATTTTTCCTTCTTGCGGGTT	GTTGAGGATGTCCAGGAGGA	59.9	389	INTERGENIC	--	--	--
Ca4_P4_30327391	Ca4	30327391	ATTTTTTT/ATTTTTTTT	CTCACGACTAAGGGTGGGAA	TTTTTCCTTCCTTGGGCTCT	60.1	497	INTERGENIC	--	--	--
Ca4_P4_30337279	Ca4	30337279	ATTTTTTTTTT/ATTTTTTTTTTT	CGAACTTAAATCCTTGCACGA	TTTTGGTCCTCCAAACAAAAT	60.3	726	INTERGENIC	--	--	--
Ca4_P4_30351459	Ca4	30351459	CTTTTTTT/CTTTTTTTT	TCTTTGAAACACATCACTTTCG	AAAATTTGATCGGTGGTGGA	57.9	314	INTERGENIC	--	--	--
Ca4_P4_30373191	Ca4	30373191	AATATATATATATAT/AATATATATATATATATATAT	ATTGGAGCAGTGGGGTACAG	ATATCGCTGTGCCTTTCCAC	60.0	532	INTERGENIC	--	--	--

INDEL marker IDs	Chromosomes/ unanchored scaffolds	Physical positions (bp)	InDels (*Kabuli* reference genome-CDC Frontier/PII)	Forward primers (5'-3')	Reverse primers (5'-3')	Anneling temperature (0C)	Expected amplified product size (bp)	Structural annotation		Functional annotation	
								Sequence components of *kabuli* genome	*Kabuli* gene accession IDs	NCBI-KOG	TFs
Ca4_P4_30383364	Ca4	30383364	CAAAAAAAAAAAAA/CAAAAAAAAAAA	CGGCAAGACATTTTACGACA	TCAAGTGGTGGATTGTTGGA	59.7	561	INTERGENIC	--	--	--
Ca4_P4_30404870	Ca4	30404870	AATAACAT/AAT	TTAACTGTTGGTGCGCAGTC	CGTCACATTTAACACGCCAT	59.9	798	INTERGENIC	--	--	--
Ca4_P4_30408440	Ca4	30408440	TAAAA/TAAA	TGCCTACGTCGTGGGATTAT	TCATCAAATCCGTAACGCAA	60.4	361	INTERGENIC	--	--	--
Ca4_P4_30408471	Ca4	30408471	TAAAAAAAA/TAAAAAAAAA	TGCCTACGTCGTGGGATTAT	TCATCAAATCCGTAACGCAA	60.4	361	INTERGENIC	--	--	--
Ca4_P4_30408624	Ca4	30408624	TC/T	TGCCTACGTCGTGGGATTAT	TCATCAAATCCGTAACGCAA	60.4	361	INTERGENIC	--	--	--
Ca4_P4_30408872	Ca4	30408872	AA/AAAAGA	TTGCGTTACGGATTTGATGA	AGACTTTCGACCGACGCTAA	60.1	485	INTERGENIC	--	--	--
Ca4_P4_30426524	Ca4	30426524	AC/ACC	TCGTTCAACCTCCCAATTTC	TTGCACCCATGTTAACCAAA	59.9	317	INTERGENIC	--	--	--
Ca4_P4_30429188	Ca4	30429188	T/TGA	TGATCGCCATATCCTTAGCC	GTACCGCATGAGCAATTGTG	60.0	315	INTERGENIC	--	--	--
Ca4_P4_30433017	Ca4	30433017	CAAAAAAA/CAAAAAA	GACTGACATTCCGATGAGCA	TGCAACACGATAAGGGAACA	59.8	721	INTERGENIC	--	--	--
Ca4_P4_30445654	Ca4	30445654	CTATATATATATATA/CTATATATATATA	AGGTCAGGTCAGGCTTTGAA	TGCACTCACTCCAACAAACA	59.8	802	INTERGENIC	--	--	--

INDEL marker IDs	Chromosomes/ unanchored scaffolds	Physical positions (bp)	InDels (*Kabuli* reference genome-CDC Frontier/PII)	Forward primers (5'-3')	Reverse primers (5'-3')	Anneling temperature (0C)	Expected amplified product size (bp)	Structural annotation		Functional annotation	
								Sequence components of *kabuli* genome	*Kabuli* gene accession IDs	NCBI-KOG	TFs
Ca4_P4_30445761	Ca4	30445761	GTATATATATATATAT/GTATATATATATAT	AGGTCAGGTCAGGCTTTGAA	TTTCCATTGCACTCACTCCA	59.8	809	INTERGENIC	--	--	--
Ca4_P4_30452386	Ca4	30452386	ATTTTTTT/ATTTTTTTT	TGAAGCAGATACCCAGACGA	TGCTATTGTTGTTGATGATGGA	59.4	705	INTERGENIC	--	--	--
Ca4_P4_30453970	Ca4	30453970	TAAAA/TAAGTACTTTAAAAA	GTGTTCGCGATATCTGGGTT	ATTCTATCACACAGCGCACG	60.0	545	INTERGENIC	--	--	--
Ca4_P4_30456018	Ca4	30456018	AT/A	GGCGAGAAAAGTTTGTTGATG	TCGTTGATTTTCAACCACCA	59.7	377	INTERGENIC	--	--	--
Ca4_P4_30458112	Ca4	30458112	C/CCTT	CAGGCAATGGGACATGACTT	AAGATTCGCTTTGATACGTGG	60.9	830	INTERGENIC	--	--	--
Ca4_P4_30459877	Ca4	30459877	GTTTTTTTTTTTT/GTTTTTTTTTTT	AAACCCTTTGCAACCAATGA	TGCCTGTTGAAATTGTTGGA	60.3	446	INTERGENIC	--	--	--
Ca4_P4_30463898	Ca4	30463898	ATTTTTTTTT/ATTTTTTTT	GGGGGAAAAGTGATAATGGG	AATGGCATGCAAAAACATCA	60.4	572	INTERGENIC	--	--	--
Ca4_P4_30464438	Ca4	30464438	CTTTTTTT/CTTTTTT	TGTGTTAGTGCGACCCACAT	TGTGAAGGGAGTGAGTTGCTT	60.0	407	INTERGENIC	--	--	--
Ca4_P4_30464759	Ca4	30464759	GACAACAACAA/GACAACAA	TGATGTTTTTGCATGCCATT	TTGGATCATCACACTCCAACA	59.9	612	INTERGENIC	--	--	--
Ca4_P4_30470866	Ca4	30470866	GAT/GATAT	GGCTTCGTTCAAAGTGGTTT	ACGCACCTCTTACCACATCC	59.2	786	INTERGENIC	--	--	--

INDEL marker IDs	Chromosomes/ unanchored scaffolds	Physical positions (bp)	InDels (*Kabuli* reference genome-CDC Frontier/PII)	Forward primers (5'-3')	Reverse primers (5'-3')	Anneling temperature (0C)	Expected amplified product size (bp)	Structural annotation		Functional annotation	
								Sequence components of *kabuli* genome	*Kabuli* gene accession IDs	NCBI-KOG	TFs
Ca4_P4_30496363	Ca4	30496363	CTTTTTTTTTT/CTTTTTTTTT	TCTCTGGCAACCGTTCTTCT	GACTCACTCCCCAAGTGGAA	60.0	177	INTERGENIC	--	--	--
Ca4_P4_30497066	Ca4	30497066	GAAAAAA/GAAAAAAA	AACATGTTGGCGAGAACCTT	TCCCAATGTGGAGGTCTTTC	59.6	189	INTERGENIC	--	--	--
Ca4_P4_3049939	Ca4	3049939	CATATATATATATATA/CATATATATATATATATA	GCCGAATGGACCAAACATAC	GGCAACAAAATAGAATCGGG	60.2	516	INTERGENIC	--	--	--
Ca4_P4_30535832	Ca4	30535832	TATAAATAAATAAATAAA/TATAAATAAATAAATAAATAAATAAATAAA	CCATTCAGAATTAGAAAATCACCA	CCAAATTTCCCAAGGGTTCT	59.4	582	INTERGENIC	--	--	--
Ca4_P4_30552355	Ca4	30552355	TG/T	TGTCGCATGCAACCTCTTAG	ACCTGTGTGTTCCCTTCCAC	60.0	528	INTERGENIC	--	--	--
Ca4_P4_30552695	Ca4	30552695	ATTTTTTT/ATTTTTTTT	CCAATTGAGAAGGGAATGGA	AATGACCCAACAAAATTGCC	59.9	646	INTERGENIC	--	--	--
Ca4_P4_30560828	Ca4	30560828	T/TC	CAGATTGTTCTTCCTCCCCA	CCACATCATCTCCAACCAAA	60.0	704	INTERGENIC	--	--	--
Ca4_P4_30570798	Ca4	30570798	TAAAAAAA/TAAAAAAAA	TGCTTCTCAATGACTAACACCAA	TGTTGGTGTGACTTTTCCTTCT	59.8	881	INTERGENIC	--	--	--
Ca4_P4_30582999	Ca4	30582999	AC/ACC	GGAGGGATGCAATATTAAGCA	ATTTCATAGACGCACCCGAG	59.0	436	INTERGENIC	--	--	--
Ca4_P4_30585117	Ca4	30585117	CT/CTTT	GCAATGAATGTGTCCTCCAA	TGTTTGGGATGACTGACCTTT	59.5	524	INTERGENIC	--	--	--

INDEL marker IDs	Chromosomes/ unanchored scaffolds	Physical positions (bp)	InDels (*Kabuli* reference genome-CDC Frontier/PII)	Forward primers (5'-3')	Reverse primers (5'-3')	Anneling temperature (0C)	Expected amplified product size (bp)	Structural annotation		Functional annotation	
								Sequence components of *kabuli* genome	*Kabuli* gene accession IDs	NCBI-KOG	TFs
Ca4_P4_30585901	Ca4	30585901	CTTTTTCTAATTTTTCTA/CTTTTTCTA	AAAGGTCAGTCATCCCAAACA	GAAACAGAAAACGGGAACCA	59.4	381	INTERGENIC	--	--	--
Ca4_P4_30605971	Ca4	30605971	AATATATATATATATATATAT/AATATATATATATATATATATATATATATATATATAT	TCCGACATCACACACCAAAC	TCGGTGTTAAAAATCGGAGTG	60.4	850	INTERGENIC	--	--	--
Ca4_P4_30619694	Ca4	30619694	CAAAAA/CAAAA	ACTCACTCTGCCCCTGTGTT	CCAACTTGGTTCCAACAAAAA	59.8	566	INTERGENIC	--	--	--
Ca4_P4_30620479	Ca4	30620479	GTTATTATTATTATTAT/GTTATTATTATTAT	GCCTGAAGAAGATGATCAACAA	CTAGGGTTGTCAGGATGGGA	59.3	566	INTERGENIC	--	--	--
Ca4_P4_30621604	Ca4	30621604	ATTTTTTTTTTT/ATTTTTTTTTTTT	TGGGCTTCAATTTAGTCCTTG	CTGATGACAAAAGGGGGAGA	59.2	516	INTERGENIC	--	--	--
Ca4_P4_30628637	Ca4	30628637	ATTCTGGTTCTG/ATTCTG	ATCGGGTTTGTCTGATGGTG	ACAAACCAAGACCCAACCAG	60.8	363	INTERGENIC	--	--	--
Ca4_P4_30641293	Ca4	30641293	CTGTTTT/CT	TGCATTTTTGGTGCATTTGT	TGTGTGCAGTGCTGCTATGA	60.0	171	INTERGENIC	--	--	--
Ca4_P4_3065355	Ca4	3065355	CATA/CATATA	ACAAAGGTCACCCTCGTCAC	CCAAAACTGGGGAAAAACAA	60.0	622	INTERGENIC	--	--	--
Ca4_P4_30662877	Ca4	30662877	CTTTTTTTTTTTTT/CTTTTTTTTTTT	TCGACATGTTGCATCACTCA	TCGCCGCCAATTTTATTTTA	59.8	659	INTERGENIC	--	--	--
Ca4_P4_30684801	Ca4	30684801	GGT/GGTGT	TGTTAGGGATTGTGTGGGGT	GAAGTTGGGGATGAGGACAA	60.1	605	INTERGENIC	--	--	--

**INDEL marker IDs**	**Chromosomes/ unanchored scaffolds**	**Physical positions (bp)**	**InDels (*Kabuli* reference genome-CDC Frontier/PII)**	**Forward primers (5'-3')**	**Reverse primers (5'-3')**	**Anneling temperature (0C)**	**Expected amplified product size (bp)**	**Structural annotation**		**Functional annotation**	
								**Sequence components of *kabuli* genome**	***Kabuli* gene accession IDs**	**NCBI-KOG**	**TFs**
Ca4_P4_30687261	Ca4	30687261	TA/TATCA	TCATCATTCATAGCTTGGGCT	AAATTTCCCAAGCAAAAGGG	59.7	393	INTERGENIC	--	--	--
Ca4_P4_30690853	Ca4	30690853	TAAAAAAAA/TAAAAAAAAAA	GAAACGAGTTGTTGCACTTGA	TTGAGTTGCACGAGAATTGC	59.0	565	INTERGENIC	--	--	--
Ca4_P4_30715317	Ca4	30715317	AAT/AATTAT	CCACTTCAAGCAATCGTTCA	TTTGCTTCGAGCACATGATT	59.8	564	INTERGENIC	--	--	--
Ca4_P4_30723844	Ca4	30723844	CTAATTAA/CTAA	TGCTCATGAAGTAGCAAAATTGA	TCATCTTTGGCATGAGAATCA	59.9	837	INTERGENIC	--	--	--
Ca4_P4_30730238	Ca4	30730238	ATTGTGAAA/ATTGTGAAACTTTTTGTGAAA	TGTGGATTTTCAAGGTTCTCG	TCGAAAAAGCGGGATGTTAC	60.1	667	INTERGENIC	--	--	--
Ca4_P4_307324	Ca4	307324	ATATTATTATTATTATTATTATTATTATTATTATTATTATTATTATTATTATTATTATTATTA/ATATTATTATTATTATTATTATTATTATTATTATTATTATTATTATTATTATTA	TATGGAACCCCATTCCTTCA	GTTGATGCATGCCCTAAACA	60.1	558	INTERGENIC	--	--	--
Ca4_P4_30773724	Ca4	30773724	A/AT	ATCCGATTGGACGGTTCATA	GCAAAATCAATTTATGCTGGAA	60.2	855	INTERGENIC	--	--	--
Ca4_P4_30775212	Ca4	30775212	CTTTTTTTTTT/CTTTTTTTTT	TGAACTCGTAACATAAGCCCAA	TGGGAATGCTTTCTTATGGG	59.6	931	INTERGENIC	--	--	--
Ca4_P4_30783657	Ca4	30783657	TTT/TTTATT	GCCAATAATTAGGGGGCTTG	GGATGGTAAGTCCATTAGAGCG	60.6	905	INTERGENIC	--	--	--
Ca4_P4_30790861	Ca4	30790861	TAAAAAAAAA/TAAAAAAAAAA	GAATTTTGGCCATCACCACT	TGTCTCGCACACCCATTTTA	59.8	530	INTERGENIC	--	--	--

INDEL marker IDs	Chromosomes/ unanchored scaffolds	Physical positions (bp)	InDels (*Kabuli* reference genome-CDC Frontier/PII)	Forward primers (5'-3')	Reverse primers (5'-3')	Anneling temperature (0C)	Expected amplified product size (bp)	Structural annotation		Functional annotation	
								Sequence components of *kabuli* genome	*Kabuli* gene accession IDs	NCBI-KOG	TFs
Ca4_P4_30791078	Ca4	30791078	AATATATATATATATATATA/AATATATATATATATATA	TAAAATGGGTGTGCGAGACA	ATAGGGCCCCAATTTAGCAG	60.1	199	INTERGENIC	--	--	--
Ca4_P4_30823011	Ca4	30823011	GAAAAAAAAAAAAA/GAAAAAAAAAAA	CGGAAAGAAACAGGACAAACA	CACCAGGAGGCGTGTTTTAT	60.1	165	INTERGENIC	--	--	--
Ca4_P4_30824879	Ca4	30824879	AT/A	ACCGAGTGTCGAACTCAAGG	ACCAGCAACCATAAAAACGC	60.3	823	INTERGENIC	--	--	--
Ca4_P4_30857682	Ca4	30857682	ATTTTTTTT/ATTTTTTTTTT	ATTTTCCACCGCATATCCAA	GTGAACCGGCCTATTTAGCA	60.2	220	INTERGENIC	--	--	--
Ca4_P4_30870230	Ca4	30870230	GAAAAAA/GAAAAA	TCAAGAAATGGCATGAACCA	TTGAGTTCCTTTTGGGATGC	60.0	434	INTERGENIC	--	--	--
Ca4_P4_30872683	Ca4	30872683	TG/T	GATGACAAAGGCGGAGAAAA	TGTTGTGCCACATCTGTTGA	60.2	458	INTERGENIC	--	--	--
Ca4_P4_30872761	Ca4	30872761	GAAAAAAAAA/GAAAAAAAAAAA	GATGACAAAGGCGGAGAAAA	AAGCCCTCAGAGGCAAATCT	60.2	710	INTERGENIC	--	--	--
Ca4_P4_30873753	Ca4	30873753	GAAA/GAAAAA	CAAGCTCAAACTTCCAAATTCA	ACCCCAAATTGTTAAAGGCA	59.4	190	INTERGENIC	--	--	--
Ca4_P4_30883211	Ca4	30883211	TGG/TG	TTGAAGCATGCATTTGAAGC	ATTGATTGGAGCGTGAATGA	60.0	428	INTERGENIC	--	--	--
Ca4_P4_30928717	Ca4	30928717	TAAAAAAA/TAAAAAAAA	CAATGTCCATGTCGACCAAA	TCTGTCGCACCATCTCAGTC	60.4	949	INTERGENIC	--	--	--

INDEL marker IDs	Chromosomes/ unanchored scaffolds	Physical positions (bp)	InDels (*Kabuli* reference genome-CDC Frontier/PII)	Forward primers (5'-3')	Reverse primers (5'-3')	Anneling temperature (0C)	Expected amplified product size (bp)	Structural annotation		Functional annotation	
								Sequence components of *kabuli* genome	*Kabuli* gene accession IDs	NCBI-KOG	TFs
Ca4_P4_30955630	Ca4	30955630	TAAAAAAAAA/TAAAAAAAA	CAAAGCCATGTTTCTTTCCA	TGAGCAAATGAGGCAAAATG	58.7	579	INTERGENIC	--	--	--
Ca4_P4_30956675	Ca4	30956675	CAATTTT/CAATTTTAAATTTT	TTGTTGGTGAAATTTCCGGT	AGTGCATCGTTTCCTTGCTT	60.2	661	INTERGENIC	--	--	--
Ca4_P4_30957181	Ca4	30957181	CAAAAAAAAAA/CAAAAAAAA	AAGCAAGGAAACGATGCACT	AGTTCCAATGTCACTTTCAATCA	59.9	303	INTERGENIC	--	--	--
Ca4_P4_30957575	Ca4	30957575	AC/A	AAGCAAGGAAACGATGCACT	CCAATCAACTGGTGTTGCTG	59.9	854	INTERGENIC	--	--	--
Ca4_P4_30958639	Ca4	30958639	GAAAAAAA/GAAAAAA	GCCAAACCAACTCATTCTTACA	CCCATCATAAATCTTTCAAAACAA	59.1	664	INTERGENIC	--	--	--
Ca4_P4_30965662	Ca4	30965662	GCAAAAAATCAAA/GCAAAAAATCAAAAAATCAAA	ATTGGCTCGTTGATCCATCT	TTTGCCTTCTTGTTTTGAATTG	59.5	419	INTERGENIC	--	--	--
Ca4_P4_31001884	Ca4	31001884	GAAAAAAAAA/GAAAAAAAA	GCTGTTGAAAGATGCAACCA	CCTTTGCTCCTTGTGTCTCC	59.8	395	INTERGENIC	--	--	--
Ca4_P4_31016017	Ca4	31016017	AG/A	TCAACCCTGATTCGACCTTC	TGAACCAAAACTCACGCAAG	60.1	551	INTERGENIC	--	--	--
Ca4_P4_31023487	Ca4	31023487	TAAAAAAAAAAAA/TAAAAAAAAAAAAAA	GGAATCCAGACTTTCCACCA	TTGCACAATCAAATGGCAAG	59.9	401	INTERGENIC	--	--	--
Ca4_P4_31055489	Ca4	31055489	TT/TTCAT	CATGCACAACTTGGTTAATGG	TGTAATTGGTGGCGTTTGAA	59.0	460	INTERGENIC	--	--	--

INDEL marker IDs	Chromosomes/ unanchored scaffolds	Physical positions (bp)	InDels (*Kabuli* reference genome-CDC Frontier/PII)	Forward primers (5'-3')	Reverse primers (5'-3')	Anneling temperature (0C)	Expected amplified product size (bp)	Structural annotation		Functional annotation	
								Sequence components of *kabuli* genome	*Kabuli* gene accession IDs	NCBI-KOG	TFs
Ca4_P4_31055592	Ca4	31055592	TAAAAACAAAAACAAAAA/TAAAAACAAAAACAAAAACAAAAA	CATGCACAACTTGGTTAATGG	TGTAATTGGTGGCGTTTGAA	59.0	460	INTERGENIC	--	--	--
Ca4_P4_31101573	Ca4	31101573	TGTCATTCCC/T	AATCGGACAAGAAACCGATCT	AGTTGTGACCCTCCGATACG	60.0	260	INTERGENIC	--	--	--
Ca4_P4_31106310	Ca4	31106310	CTTTTTTTTTTTT/CTTTTTTTTTT	ATAAGAGTGTGGCGGTTTGG	TGGTTGAAATGGACGACAAA	60.0	584	INTERGENIC	--	--	--
Ca4_P4_31106521	Ca4	31106521	CCTT/CCTTCTT	ATAAGAGTGTGGCGGTTTGG	TGGTTGAAATGGACGACAAA	60.0	584	INTERGENIC	--	--	--
Ca4_P4_31107634	Ca4	31107634	GTTTT/GTTTTT	GCCTGATTTCTTGGTTGAGC	GGGACTGAATCCGACTTGAA	59.8	394	INTERGENIC	--	--	--
Ca4_P4_31113293	Ca4	31113293	AT/ATT	AATGTGTTTAACGGGCAAGC	AGGGGGACTACTTTTGCGAT	60.0	577	INTERGENIC	--	--	--
Ca4_P4_31114996	Ca4	31114996	CT/CTGT	CTTCTTCAAGTCGGTGGCTC	TTTGCAGGTCTGGAATACCC	60.0	213	INTERGENIC	--	--	--
Ca4_P4_31116686	Ca4	31116686	GC/GCC	CAATCTTGGCAGGATCCACT	TAAAACCAGTCAGGCAACCC	60.1	709	INTERGENIC	--	--	--
Ca4_P4_31128772	Ca4	31128772	GATA/GATACATA	CAACCTACGTTCTCGTGTTTGT	TCTTTAAATGCGGGGATGTT	59.2	619	INTERGENIC	--	--	--
Ca4_P4_31129446	Ca4	31129446	ATTTTTTTT/ATTTTTTTTT	TGGGTATCCAAACCATATCCA	TGATTCACGTGGATGGAGAA	59.9	741	INTERGENIC	--	--	--

INDEL marker IDs	Chromosomes/ unanchored scaffolds	Physical positions (bp)	InDels (*Kabuli* reference genome-CDC Frontier/PII)	Forward primers (5'-3')	Reverse primers (5'-3')	Anneling temperature (0C)	Expected amplified product size (bp)	Structural annotation		Functional annotation	
								Sequence components of *kabuli* genome	*Kabuli* gene accession IDs	NCBI-KOG	TFs
Ca4_P4_31150025	Ca4	31150025	ATTTTTTTTTTTT/ATTTTTTTTTTT	TGAACGAAAAGCTTATGGATATGT	AATCGACCCAATCAAGAACG	59.1	412	INTERGENIC	--	--	--
Ca4_P4_31172721	Ca4	31172721	TG/TGG	TCTTTGGATGGGTTCCATGT	TTACACCAATTCCCCCAAGA	60.2	663	INTERGENIC	--	--	--
Ca4_P4_31172943	Ca4	31172943	TA/T	TCGCCTAAGGTAAGGGCTTT	TTACACCAATTCCCCCAAGA	60.2	421	INTERGENIC	--	--	--
Ca4_P4_31192815	Ca4	31192815	TGG/TG	TTGAAGGAGAAGCCAGAGTCA	TGAGGCATGCAGAAACGTAG	60.1	238	INTERGENIC	--	--	--
Ca4_P4_31220879	Ca4	31220879	G/GTTAA	GCCACATAGTTCCAACCTTCA	TTGTGAAATTCTGCCACGAG	60.0	228	INTERGENIC	--	--	--
Ca4_P4_31221206	Ca4	31221206	TAAAAAA/TAAAAAAA	CTCGTGGCAGAATTTCACAA	CAGGTACAAAACCCCGTCAC	59.8	614	INTERGENIC	--	--	--
Ca4_P4_31235171	Ca4	31235171	GTATATATATAT/GTATATATATATATAT	GCCACCCTTAGGAGGAAAAG	ATTTAAATGTCGTGACCGGC	60.1	607	INTERGENIC	--	--	--
Ca4_P4_31250934	Ca4	31250934	ATT/ATTTT	AGTTTTCGCCATTTGTCACC	AACCACTTGAGCATGGGTTC	60.0	196	INTERGENIC	--	--	--
Ca4_P4_31251511	Ca4	31251511	GTTTTTTT/GTTTTTTTT	AACCCATGCTCAAGTGGTTC	CAAGCGCATTTGTCCCTTAT	60.0	894	INTERGENIC	--	--	--
Ca4_P4_31270910	Ca4	31270910	GTTTTTTTTT/GTTTTTTTTTT	TGCATTCCTTTTAATTTCCTTGT	TTTGAGAAACATGCATCAAGAA	59.1	565	INTERGENIC	--	--	--

INDEL marker IDs	Chromosomes/ unanchored scaffolds	Physical positions (bp)	InDels (*Kabuli* reference genome-CDC Frontier/PII)	Forward primers (5'-3')	Reverse primers (5'-3')	Anneling temperature (0C)	Expected amplified product size (bp)	Structural annotation		Functional annotation	
								Sequence components of *kabuli* genome	*Kabuli* gene accession IDs	NCBI-KOG	TFs
Ca4_P4_31284485	Ca4	31284485	ATT/ATTT	ATGGCTACTCCAATGCATCC	ACATGGCTCCCCCTTACTCT	59.9	962	INTERGENIC	--	--	--
Ca4_P4_31316566	Ca4	31316566	GAAA/GAA	CATTGTTTTGCTGTTGTGGG	CCTCACGAGCTGCTTTAACC	60.0	664	INTERGENIC	--	--	--
Ca4_P4_31322872	Ca4	31322872	GAAAAAAAAA/GAAAAAAAA	CTCCTAAGGGTGGCCTTTTC	GGGTTGTTCTTGGACCTTCA	60.1	623	INTERGENIC	--	--	--
Ca4_P4_31332105	Ca4	31332105	ATTTT/ATTT	GCACATGTGTGGGTTGACTC	TTGAACCAACATGATCCAATTC	60.0	557	INTERGENIC	--	--	--
Ca4_P4_31334123	Ca4	31334123	CTTTGT/CTTTGTTTGT	TGCTCTCCCACTGAGAGTCAT	AAGGTTGTTGCCACTATCGG	60.0	214	INTERGENIC	--	--	--
Ca4_P4_31392404	Ca4	31392404	AT/A	CTCAAATCGAACCCTCCAAA	AAGCCAGGTGGTTGTTTGAC	60.0	403	INTERGENIC	--	--	--
Ca4_P4_31414937	Ca4	31414937	GTTTTTTT/GTTTTTTTT	GGAATACCGCATGCAAATCT	TGGTATCGGGAGAGAGGAGA	59.9	847	INTERGENIC	--	--	--
Ca4_P4_31445256	Ca4	31445256	ATT/ATTT	AGATCTATGCATCGGCAACC	AGGGTACTACAGCTGCCCCT	60.1	338	INTERGENIC	--	--	--
Ca4_P4_31453756	Ca4	31453756	G/GC	CGCTAAATTCATCTCATGTTGG	CCCTTTGGAGCAATAATTTGA	59.6	465	INTERGENIC	--	--	--
Ca4_P4_31463756	Ca4	31463756	CTTTTTTTT/CTTTTTTTTT	GGCTCCTTGCATCAGTTAGC	GAAAACCGATTAGGGATTCG	60.0	683	INTERGENIC	--	--	--

INDEL marker IDs	Chromosomes/ unanchored scaffolds	Physical positions (bp)	InDels (*Kabuli* reference genome-CDC Frontier/PII)	Forward primers (5'-3')	Reverse primers (5'-3')	Anneling temperature (0C)	Expected amplified product size (bp)	Structural annotation		Functional annotation	
								Sequence components of *kabuli* genome	*Kabuli* gene accession IDs	NCBI-KOG	TFs
Ca4_P4_31514043	Ca4	31514043	CAAAAAAAAAAA/CAAAAAAAAAA	AGCCAAAGCAGGTTGGTTTA	ACCAATCCGAAGACGATCAC	59.7	590	INTERGENIC	--	--	--
Ca4_P4_31523716	Ca4	31523716	AAATAATAATAATAATAATAATAATAATAATAATAATAATAATAATAATAA/AAATAATAATAATAATAATAATAATAATAATAATAATAATAATAATAATAATAATAATAATAA	GAAACTCTCACAAGGGTGGC	TGGCTTTAGGAACCGTGTTT	59.7	832	INTERGENIC	--	--	--
Ca4_P4_31528243	Ca4	31528243	GACACCTATTATAAACAC/GACACCTATTATAAACACCTATTATAAACAC	ATCCAATCCTTGCTCACGAT	TCTGCTTTCCGTCAACCTTT	59.5	396	INTERGENIC	--	--	--
Ca4_P4_31534458	Ca4	31534458	CAAAAAAA/CAAAAAAAAA	CAAGCCAACGTGTGTTTGTT	TGTGGGGGATCAAATTGTTT	59.7	371	INTERGENIC	--	--	--
Ca4_P4_31549191	Ca4	31549191	TAAAAAAA/TAAAAAA	TGTTTTTATGAATGGTCAGGGA	CCAAAACTGCAATTGAAAGGA	59.3	884	INTERGENIC	--	--	--
Ca4_P4_31558178	Ca4	31558178	GTT/GT	CCCTAATTTGTGTGAAGCATGA	TATCACTTCGACCCGTACCC	60.0	613	INTERGENIC	--	--	--
Ca4_P4_31560045	Ca4	31560045	ATTTTTTTTT/ATTTTTTTT	GGATATCCCACTGCAAGGAA	AACCTGTATTCCCACAAGCG	59.9	608	INTERGENIC	--	--	--
Ca4_P4_31611840	Ca4	31611840	GTTTTTTTTTT/GTTTTTTTTTTTT	CGTTTTCAGCTGAGCAAATTC	TTGATCCATTGGGATTGTGA	60.0	392	INTERGENIC	--	--	--
Ca4_P4_31611867	Ca4	31611867	AAT/AATAT	CGTTTTCAGCTGAGCAAATTC	TTGATCCATTGGGATTGTGA	60.0	392	INTERGENIC	--	--	--
Ca4_P4_31612473	Ca4	31612473	TAAAAAAA/TAAAAAAAA	TAAAATCCAAAACCGCAAGC	CACTTAAGCAAACGGGGTGT	60.1	462	INTERGENIC	--	--	--

INDEL marker IDs	Chromosomes/ unanchored scaffolds	Physical positions (bp)	InDels (*Kabuli* reference genome-CDC Frontier/PII)	Forward primers (5'-3')	Reverse primers (5'-3')	Anneling temperature (0C)	Expected amplified product size (bp)	Structural annotation		Functional annotation	
								Sequence components of *kabuli* genome	*Kabuli* gene accession IDs	NCBI-KOG	TFs
Ca4_P4_31620059	Ca4	31620059	ATTTTTTT/ATTTTTTTT	TATGAATTGGGTGGGG AATC	TGATCTTCTGGAAGT ATTTCAA	59.4	578	INTERGENIC	--	--	--
Ca4_P4_31629266	Ca4	31629266	TAAAAAAAAA/TAAAAAAAA	ATGCGTGCTCGCTTCT TTAC	CCCAAAGATATTCCAG GCAA	60.6	712	INTERGENIC	--	--	--
Ca4_P4_31632708	Ca4	31632708	ATTTTTTT/ATTTTTTTT	AATTGCTCCTTCATCAA CCG	TGGTATGTGGTTGCAG CATT	60.1	502	INTERGENIC	--	--	--
Ca4_P4_31634027	Ca4	31634027	CAAA/CAA	CACTTGGCTTGGAGGA GAAG	TTCCCACCAAGTGTGA GTGA	60.0	315	INTERGENIC	--	--	--
Ca4_P4_31636819	Ca4	31636819	CAA/CAAA	TCTCAACATCTCGTAG CGCA	TTCATGATTCATTGGGT CATCT	60.7	771	INTERGENIC	--	--	--
Ca4_P4_31639620	Ca4	31639620	GTTTT/GTTTTT	ATTTGGAGAATGATTT GGCG	GTGTTTGGTTCAAGGG GAGA	59.9	412	INTERGENIC	--	--	--
Ca4_P4_31640017	Ca4	31640017	AATCACTCATATAT/AATCACTCATA TATATCACTCATATAT	TGATTTGGCGATTGTT TTGA	CTAGATGTCTCCAGAC GCCC	60.1	617	INTERGENIC	--	--	--
Ca4_P4_31714419	Ca4	31714419	ATTTTTTTTTTT/ATTTTTTTTTT	CAAAGAGAGAGTGGGC AAGG	TGAGTCCCAAAACTCA AGGAA	60.0	883	INTERGENIC	--	--	--
Ca4_P4_31752393	Ca4	31752393	TC/T	GGTAACCAATCCGAAG ACGA	GACCAAGTCGGGAAAA CAAA	59.9	351	INTERGENIC	--	--	--
Ca4_P4_31753116	Ca4	31753116	AAATAATAATAATAATAATAATAATA ATAATAATAATAATA/AAATAATAATA ATAATAATAATAATAATAATAATAAT AATAATAATAATAATA	TTTCATCAAATTCGTCA CGC	AGCCATATGTTGTGTG CCAA	59.7	673	INTERGENIC	--	--	--

INDEL marker IDs	Chromosomes/ unanchored scaffolds	Physical positions (bp)	InDels (*Kabuli* reference genome-CDC Frontier/PII)	Forward primers (5'-3')	Reverse primers (5'-3')	Anneling temperature (0C)	Expected amplified product size (bp)	Structural annotation		Functional annotation	
								Sequence components of *kabuli* genome	*Kabuli* gene accession IDs	NCBI-KOG	TFs
Ca4_P4_31763705	Ca4	31763705	TAAAAAAAAAAA/TAAAAAAAAAA	TGCGAGCATGAGAATTTGTT	TTTGATAAACATGATTTTCCCTCA	59.4	518	INTERGENIC	--	--	--
Ca4_P4_31806633	Ca4	31806633	ATGTGACTAATTTT/ATGTGACTAATTTTGTGACTAATTTT	ACAAAAGCCTGGATAGCGAA	TCACATATACCCTTAAAATCGCA	59.8	410	INTERGENIC	--	--	--
Ca4_P4_31822803	Ca4	31822803	ATTTTTTT/ATTTTTTTT	CACAGATTTCTCGAGGCACA	TCTAAAGCTCATGTCCCGCT	60.0	841	INTERGENIC	--	--	--
Ca4_P4_31837452	Ca4	31837452	ATTTTTTTT/ATTTTTTTTT	TGGAGACCATGGAAATGCTT	TTTTGTGAATAGTCCATGCTTCA	60.5	456	INTERGENIC	--	--	--
Ca4_P4_31852769	Ca4	31852769	TAAAAAAAAAAA/TAAAAAAAAA	TTTGTCATGGCGAAACAAAC	GGTTTGTCTTTGCACCCATT	59.6	415	INTERGENIC	--	--	--
Ca4_P4_31859597	Ca4	31859597	TAAAAAAAAA/TAAAAAAAAAAA	GTTGGAGCCCCCACTTTATT	TGCAAGCAGCATCAGAGAAC	60.2	793	INTERGENIC	--	--	--
Ca4_P4_31873869	Ca4	31873869	TTATATATATATATATATATATATAT/TTATATATATATATATAT	CCCTGTTCATTTCATCCTTTTT	TCCCTTCAGTTCTTGGATGC	59.4	388	INTERGENIC	--	--	--
Ca4_P4_31885649	Ca4	31885649	TTA/TTATAGTA	GTGATTGATTTGGGATTGGG	TTGGTTGTTGGAAATGCAAA	60.0	541	INTERGENIC	--	--	--
Ca4_P4_31886261	Ca4	31886261	TTTAAAATTAAAATTAAAATTAAAATTAAAAT/TTTAAAATTAAAATTAAAATTAAAATTAAAATTAAAATTAAAAT	TGCATTTCCAACAACCAAGA	TGGAGATCCCCAATAGACCA	60.1	964	INTERGENIC	--	--	--
Ca4_P4_31886599	Ca4	31886599	CATTAT/CATTATTAT	AGCTAGTTGCATAGCGAGGG	TGGAGATCCCCAATAGACCA	59.6	210	INTERGENIC	--	--	--

INDEL marker IDs	Chromosomes/ unanchored scaffolds	Physical positions (bp)	InDels (*Kabuli* reference genome-CDC Frontier/PII)	Forward primers (5'-3')	Reverse primers (5'-3')	Anneling temperature (0C)	Expected amplified product size (bp)	Structural annotation		Functional annotation	
								Sequence components of *kabuli* genome	*Kabuli* gene accession IDs	NCBI-KOG	TFs
Ca4_P4_31892250	Ca4	31892250	CTTTTTTTT/CTTTTTTTTTT	AACCGTTAACCACCGAAACA	TGCGGTCAACACACTTAGGA	60.3	520	INTERGENIC	--	--	--
Ca4_P4_31892313	Ca4	31892313	TTTTTCTT/TTT	AACCGTTAACCACCGAAACA	TGCGGTCAACACACTTAGGA	60.3	520	INTERGENIC	--	--	--
Ca4_P4_31916653	Ca4	31916653	AATTACATTACATTACA/AATTACATTACA	GGTGATGAGGGAGAAACACC	CAGGTCGAAGGTTTCATGGT	59.4	945	INTERGENIC	--	--	--
Ca4_P4_31918515	Ca4	31918515	ATTTTTTTTT/ATTTTTTTT	ATTCCACATGAAACTTCCGC	GAAAGTAGGGGGTGGGAAAC	59.9	568	INTERGENIC	--	--	--
Ca4_P4_31948251	Ca4	31948251	AAAA/AAAATAAA	AAAATGCATATATTAATTCATCCCAA	CTGCATGTGCAATTGTGTCA	59.0	385	INTERGENIC	--	--	--
Ca4_P4_31973044	Ca4	31973044	GTTTTTTTT/GTTTTTTT	ATGTCCTATGCTGCTGGACC	AAACAACCGTGCTATTTAGCCT	60.1	279	INTERGENIC	--	--	--
Ca4_P4_31973405	Ca4	31973405	TGG/TG	GTGTCCAATGTGTTGCTCCA	ACCACACCCACTTTTTCAGC	60.6	911	INTERGENIC	--	--	--
Ca4_P4_31973453	Ca4	31973453	AA/AATA	GTGTCCAATGTGTTGCTCCA	ACCACACCCACTTTTTCAGC	60.6	911	INTERGENIC	--	--	--
Ca4_P4_31974935	Ca4	31974935	ATTT/ATT	TCACAGCCAACAGCACTCTC	TCGTGCAATGTCCATTCATT	60.2	633	INTERGENIC	--	--	--
Ca4_P4_31977289	Ca4	31977289	T/TG	CATCGAACTCGTCCCGTTAT	CATGTCGCTACCTGCACAGT	60.0	666	INTERGENIC	--	--	--

INDEL marker IDs	Chromosomes/ unanchored scaffolds	Physical positions (bp)	InDels (*Kabuli* reference genome-CDC Frontier/PII)	Forward primers (5'-3')	Reverse primers (5'-3')	Anneling temperature (0C)	Expected amplified product size (bp)	Structural annotation		Functional annotation	
								Sequence components of *kabuli* genome	*Kabuli* gene accession IDs	NCBI-KOG	TFs
Ca4_P4_31989643	Ca4	31989643	CTTTTTTTT/CTTTTTTTTTTT	GAAAGATTCCTCCCAC TCCC	TGCACATTGGAAAATT GGTG	59.9	246	INTERGENIC	--	--	--
Ca4_P4_3199026	Ca4	3199026	TTATATATATATATATATATAT/TTAT ATATATATATATATATATATAT	TCAAAACCCCAAACCA CAAT	CCAAAGTTACTTCGCAT GATGA	60.1	490	INTERGENIC	--	--	--
Ca4_P4_32001207	Ca4	32001207	TC/T	CTTGTTGCATCCAAAA CACG	GCCAAGCAGAAAGTTC ATCC	60.1	207	INTERGENIC	--	--	--
Ca4_P4_32006547	Ca4	32006547	CTTT/CT	CAAAAGAAGGGGGAAT GACC	TCCTTCTCCAACTTTTT CCAA	60.7	433	INTERGENIC	--	--	--
Ca4_P4_32016200	Ca4	32016200	TGAGATACC/TGAGATACCGAGATA CC	TCTAAATGTGGGTCGT GCAA	GAAGGATAAACATTGG GGCA	60.1	279	INTERGENIC	--	--	--
Ca4_P4_32021071	Ca4	32021071	ATT/ATTT	TCATGTGGCGTAAGTT CCAA	GAGTTGGGATGAAGGG ACAA	60.1	744	INTERGENIC	--	--	--
Ca4_P4_32038234	Ca4	32038234	G/GA	ACAATGCCCGACTTTA CTGG	ACTATGAGGTTTTGGT GGCG	60.0	812	INTERGENIC	--	--	--
Ca4_P4_32048513	Ca4	32048513	TAAAAAAAA/TAAAAAAAAA	CGATTTTGTCGGTCAA TCTCT	TCACCGTAGGAAAGCT GGAT	59.2	355	INTERGENIC	--	--	--
Ca4_P4_32048901	Ca4	32048901	ATTTTTTTTT/ATTTTTTTT	CGAAATCCAGCTTTCC TACG	TTTGGGCCTCCTTGAG ATAA	59.8	670	INTERGENIC	--	--	--
Ca4_P4_32049087	Ca4	32049087	ATTTTTTTT/ATTTTTTTTTT	CGAAATCCAGCTTTCC TACG	TTTGGGCCTCCTTGAG ATAA	59.8	670	INTERGENIC	--	--	--

INDEL marker IDs	Chromosomes/ unanchored scaffolds	Physical positions (bp)	InDels (*Kabuli* reference genome-CDC Frontier/PII)	Forward primers (5'-3')	Reverse primers (5'-3')	Anneling temperature (0C)	Expected amplified product size (bp)	Structural annotation		Functional annotation	
								Sequence components of *kabuli* genome	*Kabuli* gene accession IDs	NCBI-KOG	TFs
Ca4_P4_32049749	Ca4	32049749	AAA/AAAGAA	CCAAATCATTGGCATTCACA	TTCCTATCACATTGCATATCAAAAA	60.3	693	INTERGENIC	--	--	--
Ca4_P4_32051935	Ca4	32051935	ATTTTTTTTTTT/ATTTTTTTTT	TTGAAGATGGTGAATGTGGAA	TATGACCCGTTGGATTCGAT	59.0	797	INTERGENIC	--	--	--
Ca4_P4_32053695	Ca4	32053695	ATTTTTTTT/ATTTTTTTTT	AGGTCAACATCCACTCCAGG	ATGCTCCCCTGACATTCTTG	60.0	706	INTERGENIC	--	--	--
Ca4_P4_32053743	Ca4	32053743	GTTTTTTTTTTT/GTTTTTTTTTTTTT	AGGTCAACATCCACTCCAGG	ATGCTCCCCTGACATTCTTG	60.0	706	INTERGENIC	--	--	--
Ca4_P4_32057189	Ca4	32057189	AATATAT/AATAT	CGCTGCTCTTCCTTGTTCTC	AAAGCGAAGCGAAGCAATAA	60.3	294	INTERGENIC	--	--	--
Ca4_P4_32057338	Ca4	32057338	TG/T	TATTGCTTCGCTTCGCTTTT	GGGAAACTGCCCCTAATTCT	60.1	451	INTERGENIC	--	--	--
Ca4_P4_32057442	Ca4	32057442	TAAAAAAA/TAAAAAAAA	TATTGCTTCGCTTCGCTTTT	GGGAAACTGCCCCTAATTCT	60.1	451	INTERGENIC	--	--	--
Ca4_P4_32058252	Ca4	32058252	CTG/C	GGCAAAGCCTCTCATTACCA	TGACCAATGTTGCCATGTCT	60.2	557	INTERGENIC	--	--	--
Ca4_P4_32118876	Ca4	32118876	TAAAAAAAAAAA/TAAAAAAAAA	CCGATGCAATGAAGAAATCA	GGGCCAAGACACCATTTTT	59.6	589	INTERGENIC	--	--	--
Ca4_P4_32121497	Ca4	32121497	TAAAAAAAA/TAAAAAAAAAA	TGGCATACGACCAACTCAAA	TTTGCTTTACAAGCTTGGGG	60.1	440	INTERGENIC	--	--	--

INDEL marker IDs	Chromosomes/ unanchored scaffolds	Physical positions (bp)	InDels (*Kabuli* reference genome-CDC Frontier/PII)	Forward primers (5'-3')	Reverse primers (5'-3')	Anneling temperature (0C)	Expected amplified product size (bp)	Structural annotation		Functional annotation	
								Sequence components of *kabuli* genome	*Kabuli* gene accession IDs	NCBI-KOG	TFs
Ca4_P4_32121752	Ca4	32121752	ACCC/ACC	CCCCAAGCTTGTAAAG CAAA	AATGAAAGCGTTGATT GGGT	60.2	588	INTERGENIC	--	--	--
Ca4_P4_32121836	Ca4	32121836	AATA/AATATA	CCCCAAGCTTGTAAAG CAAA	AATGAAAGCGTTGATT GGGT	60.2	588	INTERGENIC	--	--	--
Ca4_P4_32137832	Ca4	32137832	CAAAAAAAA/CAAAAAAAAAA	CGACAGCACATTAGAT AAACCG	ATGGATGATGGGCAGT AAGC	59.7	806	INTERGENIC	--	--	--
Ca4_P4_32138975	Ca4	32138975	CATATATAT/CATATAT	TGGTGTGTGTGTGTGT GAGG	TGAAAAACCCAATCCAA ACC	60.1	892	INTERGENIC	--	--	--
Ca4_P4_32141276	Ca4	32141276	GTG/GTGGCACGTTG	ACCAACCTGTTGAAGG AAGC	CCCTCATGATATCCAC GACC	59.2	636	INTERGENIC	--	--	--
Ca4_P4_32143759	Ca4	32143759	GATATATA/GATATA	CGTTATCATTGCTTGAA CGG	TTGAGCACTTACTGGT GGGA	59.2	941	INTERGENIC	--	--	--
Ca4_P4_32147675	Ca4	32147675	AATATAT/AATAT	TTGCACAAAAGAAATC GAAAAA	ACAAATTGGCATTCACC CAT	59.7	471	INTERGENIC	--	--	--
Ca4_P4_32149191	Ca4	32149191	CTTTTTTT/CTTTTTT	CGGTGAAGGGACCACA CTAT	TGCCCATAACTGCACA ACAT	59.8	404	INTERGENIC	--	--	--
Ca4_P4_32149314	Ca4	32149314	TAGAAGAAGAAG/TAGAAGAAG	GCATGCTCACATGATT CCAC	TGCCCATAACTGCACA ACAT	60.1	238	INTERGENIC	--	--	--
Ca4_P4_32149597	Ca4	32149597	TC/T	ATGTTGTGCAGTTATG GGCA	TCATGAATCAAATCAAC AAACAAA	60.0	224	INTERGENIC	--	--	--

INDEL marker IDs	Chromosomes/ unanchored scaffolds	Physical positions (bp)	InDels (*Kabuli* reference genome-CDC Frontier/PII)	Forward primers (5'-3')	Reverse primers (5'-3')	Anneling temperature (0C)	Expected amplified product size (bp)	Structural annotation		Functional annotation	
								Sequence components of *kabuli* genome	*Kabuli* gene accession IDs	NCBI-KOG	TFs
Ca4_P4_32156545	Ca4	32156545	A/AC	TTCACTTGGTTGCTTTGCAG	CTTAAACCATAATCCCCGCA	60.0	692	INTERGENIC	--	--	--
Ca4_P4_32159010	Ca4	32159010	ATTTTTTTT/ATTTTTTTTTT	TCTCTTTTAATGGGCCATGC	ATGCATGGAATCCAGCTTTC	60.0	688	INTERGENIC	--	--	--
Ca4_P4_32159052	Ca4	32159052	TAAAA/TAAA	TCTCTTTTAATGGGCCATGC	ATGCATGGAATCCAGCTTTC	60.0	688	INTERGENIC	--	--	--
Ca4_P4_32167743	Ca4	32167743	A/AG	GCAGCATATGATCGCAAAAA	AAAGTCCCACTTGATGGTGG	59.8	565	INTERGENIC	--	--	--
Ca4_P4_32174509	Ca4	32174509	ATCT/AT	ACAATTGGTTGAAATGGGGA	CCGCACTTCGCATTTTTAAT	60.0	525	INTERGENIC	--	--	--
Ca4_P4_32179682	Ca4	32179682	CAATAATCAAACAAA/CAATAATCAAACAAATAATCAAACAAA	TTGCATACCTCATACCATCCC	TTGATGCATTACCTCCCACA	59.7	731	INTERGENIC	--	--	--
Ca4_P4_32181880	Ca4	32181880	ATTTT/ATTTTT	AGATTTAAGGGAGGGGGCTC	CGAACAATTCGTCCTTGGTT	60.8	401	INTERGENIC	--	--	--
Ca4_P4_32216199	Ca4	32216199	ATTTTTTTT/ATTTTTTT	ATAAAGGTCAACGCACGCTT	GAAATCAACGGATCTCCATCA	59.8	384	INTERGENIC	--	--	--
Ca4_P4_32220024	Ca4	32220024	G/GCC	CGTGGGGGTATTTTTCATTG	GCCTCCCAGAATATGGATCA	60.0	749	INTERGENIC	--	--	--
Ca4_P4_32224229	Ca4	32224229	A/AT	TCTTCTTCCACAGGGGATTG	ATATGGGCCAAAAGCAACAA	60.0	519	INTERGENIC	--	--	--

INDEL marker IDs	Chromosomes/ unanchored scaffolds	Physical positions (bp)	InDels (*Kabuli* reference genome-CDC Frontier/PII)	Forward primers (5'-3')	Reverse primers (5'-3')	Anneling temperature (0C)	Expected amplified product size (bp)	Structural annotation		Functional annotation	
								Sequence components of *kabuli* genome	*Kabuli* gene accession IDs	NCBI-KOG	TFs
Ca4_P4_32224834	Ca4	32224834	ATTTTTTTTTT/ATTTTTTTTT	ATATTTGTTGCTTTTGGCCC	GCGGAAAAATTTGATGAGTTG	58.9	328	INTERGENIC	--	--	--
Ca4_P4_32225442	Ca4	32225442	TTATATAT/TTAT	TCAATTCCAATCATTCAACCA	ACATTACACCAATGCGACGA	58.8	349	INTERGENIC	--	--	--
Ca4_P4_32225516	Ca4	32225516	TTA/TTATA	TCAATTCCAATCATTCAACCA	ACATTACACCAATGCGACGA	58.8	349	INTERGENIC	--	--	--
Ca4_P4_32227293	Ca4	32227293	AAAAA/AAAAATTAAAA	TCTTCACAACCCCTTGAACC	TTCTTCCAAACGAGCCTCTC	59.9	868	INTERGENIC	--	--	--
Ca4_P4_32227396	Ca4	32227396	GT/GTT	TCTTCACAACCCCTTGAACC	TTCTTCCAAACGAGCCTCTC	59.9	868	INTERGENIC	--	--	--
Ca4_P4_32241433	Ca4	32241433	ATT/A	ACAATTGTTGCACCACGAGA	ATCGCAAAAGTAGTCCCCCT	60.2	148	INTERGENIC	--	--	--
Ca4_P4_32242240	Ca4	32242240	TATTAAATTAAA/TATTAAA	TGCTAATGCTTTTCAAGGGC	GCGATGCACAGGTAAACAAA	60.3	482	INTERGENIC	--	--	--
Ca4_P4_32256317	Ca4	32256317	A/AC	AAACAAAACTCATCCTTCCCAA	CGTGAGTCTCGTTGAAGCAA	59.9	336	INTERGENIC	--	--	--
Ca4_P4_32257454	Ca4	32257454	GTTTTTTTTTTT/GTTTTTTTTTT	TGCACTTGTTAACCACACAATG	TGTGGGAGACCGATTTCTCT	59.6	567	INTERGENIC	--	--	--
Ca4_P4_32263608	Ca4	32263608	ATTTTTT/ATTTTTTT	ACACATGTCCCAGTTGACGA	TGACGATGGAGGCTTTTAGG	60.0	682	INTERGENIC	--	--	--

INDEL marker IDs	Chromosomes/ unanchored scaffolds	Physical positions (bp)	InDels (*Kabuli* reference genome-CDC Frontier/PII)	Forward primers (5'-3')	Reverse primers (5'-3')	Anneling temperature (0C)	Expected amplified product size (bp)	Structural annotation		Functional annotation	
								Sequence components of *kabuli* genome	*Kabuli* gene accession IDs	NCBI-KOG	TFs
Ca4_P4_32263960	Ca4	32263960	TAAAAAAA/TAAAAAAAAAA	GATCAAATCCCCTATG TATGTGC	TGACGATGGAGGCTTT TAGG	59.6	191	INTERGENIC	--	--	--
Ca4_P4_32264283	Ca4	32264283	TAAAAAA/TAAAAAAA	CCTAAAAGCCTCCATC GTCA	TGCTTGTCAGCTTGTT GTGTC	60.2	682	INTERGENIC	--	--	--
Ca4_P4_32264601	Ca4	32264601	TA/TAA	GATCAAATTCTCTATGC ATGTGCT	CCAATTGAAGGGTAAG TGCC	59.7	608	INTERGENIC	--	--	--
Ca4_P4_32264980	Ca4	32264980	AAT/AATAT	GCACTTACCCTTCAATT GGG	AGGAGAGGTCCATTTC TCAGG	59.4	262	INTERGENIC	--	--	--
Ca4_P4_32290820	Ca4	32290820	AAGA/AAGAGA	GGATCAGAGAAGAGAC GGGA	TTACTTGTAGGGCAGG TCCG	59.3	703	INTERGENIC	--	--	--
Ca4_P4_32297893	Ca4	32297893	CTGAAT/CTGAATGAAT	GCGGTGCGATATTCTT TGTT	TGGAAAATGTTGGAGC ATGA	60.1	471	INTERGENIC	--	--	--
Ca4_P4_32298040	Ca4	32298040	CT/CTTGATAAAGAT	GCGGTGCGATATTCTT TGTT	TGGAAAATGTTGGAGC ATGA	60.1	471	INTERGENIC	--	--	--
Ca4_P4_32335179	Ca4	32335179	AAAGAAGAAGAAGAAGAAGAA/AAA GAAGAAGAAGAAGAA	AAAGTCCGTTTGTGAA TCCG	TGGGCATTCAAATCAAA CAA	60.0	507	INTERGENIC	--	--	--
Ca4_P4_32351537	Ca4	32351537	TCTTTTTTTA/T	CAAAGGTAACCGATCC TCCA	GCAACGAAAACGGAGA ACAT	59.9	645	INTERGENIC	--	--	--
Ca4_P4_32373608	Ca4	32373608	ATTTTTTTT/ATTTTTTT	TCCACTTTTCAAACGA GATGG	AAGATCACGTTTCACAT CGG	60.1	564	INTERGENIC	--	--	--

INDEL marker IDs	Chromosomes/ unanchored scaffolds	Physical positions (bp)	InDels (*Kabuli* reference genome-CDC Frontier/PII)	Forward primers (5'-3')	Reverse primers (5'-3')	Anneling temperature (0C)	Expected amplified product size (bp)	Structural annotation		Functional annotation	
								Sequence components of *kabuli* genome	*Kabuli* gene accession IDs	NCBI-KOG	TFs
Ca4_P4_32373685	Ca4	32373685	TAAAAAAAAAA/TAAAAAAAAA	AGACGGGCAATTTTAGTCTCA	CGATGTGATCTCTTGTTTCCC	58.8	532	INTERGENIC	--	--	--
Ca4_P4_32380091	Ca4	32380091	GAAAAA/GAAAA	CAAACACAATTCAATCCCCC	CCTTTGCTTCTTGACCTTGC	60.0	514	INTERGENIC	--	--	--
Ca4_P4_32388627	Ca4	32388627	CTTTTTTTTTT/CTTTTTTTT	CGGCCCTTTGTTATGTCTCT	AGACCGTGAACGGCTTATAGA	59.2	611	INTERGENIC	--	--	--
Ca4_P4_32388983	Ca4	32388983	AAAT/A	CGGCCCTTTGTTATGTCTCT	TTTGGGCGAATTCCATTTTA	59.2	692	INTERGENIC	--	--	--
Ca4_P4_32395281	Ca4	32395281	TA/TAA	GGTTAACAAATCTCCTAAAGGCA	TTTTGTTAAAGAATTGCGACCTC	58.7	523	INTERGENIC	--	--	--
Ca4_P4_32395719	Ca4	32395719	TAAAAAAAAAAA/TAAAAAAAAAA	GGTTAACAAATCTCCTAAAGGCA	CCAAACAAAGAAATGACGAACA	58.7	665	INTERGENIC	--	--	--
Ca4_P4_32409897	Ca4	32409897	T/TTTTTATATTTTA	CACGCGAGCAAGATGTTCTA	TGGTTTGCATCTTGTGGTCT	60.2	674	INTERGENIC	--	--	--
Ca4_P4_32410631	Ca4	32410631	AT/ATT	TTATTCCACCCCACCTCAAA	CTCAGCTCATTGGTTTGGTG	60.2	262	INTERGENIC	--	--	--
Ca4_P4_32413475	Ca4	32413475	AAT/AATAT	TTGTGCAACCCTTGAAATGA	CAATTTTCCCATTCTAAGCTTCTC	60.1	722	INTERGENIC	--	--	--
Ca4_P4_32432200	Ca4	32432200	CATAATTTA/CA	TGATAAATTTGGGTCATGTATTTTT	TTGGATCATAGATTTCTGAATGATAA	57.9	564	INTERGENIC	--	--	--

INDEL marker IDs	Chromosomes/ unanchored scaffolds	Physical positions (bp)	InDels (*Kabuli* reference genome-CDC Frontier/PII)	Forward primers (5'-3')	Reverse primers (5'-3')	Anneling temperature (0C)	Expected amplified product size (bp)	Structural annotation		Functional annotation	
								Sequence components of *kabuli* genome	*Kabuli* gene accession IDs	NCBI-KOG	TFs
Ca4_P4_32450619	Ca4	32450619	ATTTTTTTT/ATTTTTTTTT	TCCCCCTTCGTCTTTTTCTT	AAAATAAGGGACACATTTTTGTCAT	60.0	539	INTERGENIC	--	--	--
Ca4_P4_32457295	Ca4	32457295	AC/A	TGTGCTCAACCCAAATTGAA	ACAAGTTGTGGGAGTGGGAC	60.1	278	INTERGENIC	--	--	--
Ca4_P4_32464896	Ca4	32464896	CTTTTTTTTT/CTTTTTTTT	CCTGTTTTGTGGGAACCTGT	TGGAGGAAGCAAGGAAAGAA	59.9	684	INTERGENIC	--	--	--
Ca4_P4_32466403	Ca4	32466403	ATT/ATTT	GCCAGTAGGAAAATCCTCCA	ACCCAAACAAGAACAAACGC	59.1	539	INTERGENIC	--	--	--
Ca4_P4_32470160	Ca4	32470160	GCTATGTTTGC/G	TGAAACACATTGGAGACCCA	GACAACCCTCCGCAATAAAA	59.9	336	INTERGENIC	--	--	--
Ca4_P4_32485754	Ca4	32485754	CAAAAAAAAAA/CAAAAAAAAA	GAAGAGGAATGAGTCTGAGTGGA	GGAAACTTCTTTCATGTGGACC	59.9	465	INTERGENIC	--	--	--
Ca4_P4_32487077	Ca4	32487077	GAAA/GAAATAAA	CGTCAATTTGAGACGACGAA	ATTTCATCAATTCGGGGTGA	59.8	618	INTERGENIC	--	--	--
Ca4_P4_32489750	Ca4	32489750	C/CA	GGGTTTTATTATTATTGATGGAGGG	GCAAACTCCAACTATTTTCGC	60.1	614	INTERGENIC	--	--	--
Ca4_P4_32491529	Ca4	32491529	GTT/GTTT	GGTGATGGTTTCGCCTAAAA	TCAAACACACACGAAAATAGCA	59.9	446	INTERGENIC	--	--	--
Ca4_P4_32494024	Ca4	32494024	TAAAAAAAAA/TAAAAAAAAAA	CGTCATTACCCGTTGAAACC	TCATCCATCCATATCCCCAT	60.2	418	INTERGENIC	--	--	--

INDEL marker IDs	Chromosomes/ unanchored scaffolds	Physical positions (bp)	InDels (*Kabuli* reference genome-CDC Frontier/PII)	Forward primers (5'-3')	Reverse primers (5'-3')	Anneling temperature (0C)	Expected amplified product size (bp)	Structural annotation		Functional annotation	
								Sequence components of *kabuli* genome	*Kabuli* gene accession IDs	NCBI-KOG	TFs
Ca4_P4_32498425	Ca4	32498425	CAAAAAAAAA/CAAAAAAAAAA	TAGCACGGACCCCAATTAAG	GGGAAATTTCAAGGCACAAA	60.0	368	INTERGENIC	--	--	--
Ca4_P4_32509855	Ca4	32509855	TAAAAAA/TAAAAA	GCATTCCCTCTGGACTTTGA	GAAACTTTGGCCCCTATGGT	60.2	454	INTERGENIC	--	--	--
Ca4_P4_32511208	Ca4	32511208	GAAAAAAAAA/GAAAAAAAAAA	ACTGTTTTGCCCAGAAATGC	TGATGCATGTCAGAAAGGGA	60.1	475	INTERGENIC	--	--	--
Ca4_P4_32511542	Ca4	32511542	A/AT	TCCCTTTCTGACATGCATCA	TCTAGACTAGGCTGCTCCCG	60.2	468	INTERGENIC	--	--	--
Ca4_P4_32511906	Ca4	32511906	T/TC	TTTCTCCCGTTATGTGTCGG	TGAGTGCTGGGAACTAACCC	60.9	704	INTERGENIC	--	--	--
Ca4_P4_32513097	Ca4	32513097	TGGGGGG/TGGGGG	GATTTGCATTGGCCTCTTGT	ACAGTGGTTGCCATTTCCTC	60.1	479	INTERGENIC	--	--	--
Ca4_P4_32514320	Ca4	32514320	GACATTGACTTTTT/GACATTGACTTTTTTCAACATTGACTTTTT	ATTACTGGACCAGAGGCACG	AATGTCCATTCTGGGACGAG	60.1	510	INTERGENIC	--	--	--
Ca4_P4_32522290	Ca4	32522290	GAAAAAAAAA/GAAAAAAAAAA	ACTCATTCATTCCACAGCCC	TTGCTAACGGTAACACAACGA	59.9	851	INTERGENIC	--	--	--
Ca4_P4_32523002	Ca4	32523002	GTT/GTTTT	CGCATGCTAATTCAATTTCAGA	CTCCTTACATTGAGGGGGCT	60.2	257	INTERGENIC	--	--	--
Ca4_P4_32523554	Ca4	32523554	TAAAAAAAAAAAAAA/TAAAAAAAAAA	TGATTTGCGTCGTGTTCAAT	AGAGAGGAGGAGAGCCCAAG	60.1	530	INTERGENIC	--	--	--

INDEL marker IDs	Chromosomes/ unanchored scaffolds	Physical positions (bp)	InDels (*Kabuli* reference genome-CDC Frontier/PII)	Forward primers (5'-3')	Reverse primers (5'-3')	Anneling temperature (0C)	Expected amplified product size (bp)	Structural annotation		Functional annotation	
								Sequence components of *kabuli* genome	*Kabuli* gene accession IDs	NCBI-KOG	TFs
Ca4_P4_32525507	Ca4	32525507	CAAAA/CAAAAA	GTCATGCATATGTTGGACGC	AAGTTTTCTTCCCGCTCACA	60.0	460	INTERGENIC	--	--	--
Ca4_P4_32531473	Ca4	32531473	TAA/T	TAAAATGGATGCATCGAGCC	ACAAATCAACCAGTGTCGCA	60.9	662	INTERGENIC	--	--	--
Ca4_P4_32547296	Ca4	32547296	AA/AACATTTATCAATTA	TGCGAGATTTTGAGTTCAACC	TGCTTATCTTGAACGGGACC	60.2	325	INTERGENIC	--	--	--
Ca4_P4_32554394	Ca4	32554394	A/ATTC	AAATTAAGTGCGGAGGGGTC	ACCCATTATGGTGAAGAGCG	60.3	832	INTERGENIC	--	--	--
Ca4_P4_32559742	Ca4	32559742	AAAGAAAGAAGAAAGAAGA/AAAGAAAGAAGA	TTCATTTCCAGTCTGTGGCA	ACGGCCGTTATAGGTTTCCT	60.2	669	INTERGENIC	--	--	--
Ca4_P4_32559814	Ca4	32559814	A/AC	TTCATTTCCAGTCTGTGGCA	TGAGAAACTTTTCCGCTGCT	60.2	872	INTERGENIC	--	--	--
Ca4_P4_32562355	Ca4	32562355	CTTTTT/CTTTTTT	AAATGGAGGAGAAGGAGGGA	CGGTGAATCCTTTACGGAGA	60.0	286	INTERGENIC	--	--	--
Ca4_P4_32567747	Ca4	32567747	TAAAAA/TAAAA	GAAGGTGAGACAGCAGAGGG	TGACGTGTGTAGTTTCCCCA	60.0	589	INTERGENIC	--	--	--
Ca4_P4_32586827	Ca4	32586827	GAAAAAAAA/GAAAAAAAAA	TATTCTTTGCCGCTCCAATC	AAGGACCAAAACCAATTGTCTC	60.2	750	INTERGENIC	--	--	--
Ca4_P4_32615628	Ca4	32615628	CAAA/CA	GCACTTGGCAAGGAACAAAC	TTTTGCTCGAACTTGGCTTT	60.7	641	INTERGENIC	--	--	--

INDEL marker IDs	Chromosomes/ unanchored scaffolds	Physical positions (bp)	InDels (*Kabuli* reference genome-CDC Frontier/PII)	Forward primers (5'-3')	Reverse primers (5'-3')	Anneling temperature (0C)	Expected amplified product size (bp)	Structural annotation		Functional annotation	
								Sequence components of *kabuli* genome	*Kabuli* gene accession IDs	NCBI-KOG	TFs
Ca4_P4_32631880	Ca4	32631880	CTTTCTTTTTCTTTTTCT/CTTTCTTTTTCTTTTTCTTTTTCT	TTACGTGGAGAGGGAAATGG	CACACATCACTTCAACCGTG	59.9	542	INTERGENIC	--	--	--
Ca4_P4_32634672	Ca4	32634672	CTTTATCT/CTTTATCTTTTATCT	CAGACCCGTGTGAACACAAA	AGCAGATGCCTCTATCGCAT	60.6	937	INTERGENIC	--	--	--
Ca4_P4_32663516	Ca4	32663516	TTTATTATTATTATTATTATTATT/TTTATTATTATTATTATT	TGCGTTTCACTCTTCCTTCC	TGTCAAGCCCAAAAACAAAA	60.4	257	INTERGENIC	--	--	--
Ca4_P4_32694376	Ca4	32694376	ATTTTTT/ATTTTTTT	AATCATTTTTGGAAGACGCC	TGAAAACTGTGCAGCATGAA	59.0	527	INTERGENIC	--	--	--
Ca4_P4_32696742	Ca4	32696742	AGG/AGGG	TGTATTTGCAGGGACGTGAA	CTCATGAGAGGCGATTGTGA	60.1	467	INTERGENIC	--	--	--
Ca4_P4_32696837	Ca4	32696837	TAAAAAAAAAA/TAAAAAAAAAAA	TGTATTTGCAGGGACGTGAA	CTCATGAGAGGCGATTGTGA	60.1	467	INTERGENIC	--	--	--
Ca4_P4_32697443	Ca4	32697443	TAAAAAAAAA/TAAAAAAAA	TCAAAGGCTTACCACCTTCG	CATCGGCCTTGAAAACAAAT	60.2	942	INTERGENIC	--	--	--
Ca4_P4_32714134	Ca4	32714134	GCTCTCTCTCTCTCTCTCTCTCTCTCTC/GCTCTCTCTCTCTCTCTCTCTCTCTCTCTCTC	AAGAACCCACGTTCACATCC	AACAATGATGACGAGAGGGG	59.8	150	INTERGENIC	--	--	--
Ca4_P4_32747280	Ca4	32747280	GAAAA/GAAA	TCTTCCACCCAGTTTTGACC	GGAGACAGGTGTGGACGTTT	59.9	386	INTERGENIC	--	--	--
Ca4_P4_32795346	Ca4	32795346	AATATATATATATATATATATATATATATAT/AATATATATATATATATATATATATATATATATATATATATATAT	CCTGATATGAGTTGAGTTAGAAACCA	AATTCATGGACTGCACCCTC	60.0	796	INTERGENIC	--	--	--

INDEL marker IDs	Chromosomes/ unanchored scaffolds	Physical positions (bp)	InDels (*Kabuli* reference genome-CDC Frontier/PII)	Forward primers (5'-3')	Reverse primers (5'-3')	Anneling temperature (0C)	Expected amplified product size (bp)	Structural annotation		Functional annotation	
								Sequence components of *kabuli* genome	*Kabuli* gene accession IDs	NCBI-KOG	TFs
Ca4_P4_32873081	Ca4	32873081	ATTTT/ATTTTTT	GTCATTGACCGGGGATATTG	ACGGTTGCACCGAATAAAAA	60.0	162	INTERGENIC	--	--	--
Ca4_P4_32886390	Ca4	32886390	AC/ACC	AATCATCCCATGTTGCCATT	CGAGATTTCACACCAAGGGT	60.0	519	INTERGENIC	--	--	--
Ca4_P4_32907762	Ca4	32907762	ATTTTTTTTTTTT/ATTTTTTTTTTT	TCTGATGGACGAGTTCAAACA	TTGCGTCCTTTTTACCCAAC	59.3	344	INTERGENIC	--	--	--
Ca4_P4_32913108	Ca4	32913108	TAAATTAAAATTAAAAT/TAAATTAAAATTAAAATTAAAAT	GGGTTTTTCAGAAAGAAACGG	AACGGTGAACTTAACACGGG	60.0	438	INTERGENIC	--	--	--
Ca4_P4_33053978	Ca4	33053978	ATGTT/AT	TGCTTTCATCTTTGGAACCC	TTTGCATTACGGATTTGATGA	60.1	654	INTERGENIC	--	--	--
Ca4_P4_33054018	Ca4	33054018	TAA/TAAA	GGAATGGCAAACTTTCATGC	TTTGCATTACGGATTTGATGA	60.5	520	INTERGENIC	--	--	--
Ca4_P4_33054232	Ca4	33054232	TAAAAAAAA/TAAAAAAAAA	TCATCAAATCCGTAATGCAAA	GCACATGGGGTACCAGACTT	59.0	445	INTERGENIC	--	--	--
Ca4_P4_33064079	Ca4	33064079	ATTGTTGTTGTTGTTGTTGTTG/ATTGTTGTTGTTGTTGTTGTTGTTG	TCGGGCCACTATCTGGATTA	GTGTGCCGTCACAAACAATC	60.4	810	INTERGENIC	--	--	--
Ca4_P4_33083922	Ca4	33083922	CATATATATATATATATATATATAT/CATATATATATATATATATATATATATAT	AGGAAGAAGAAGCTGGGAGC	GCTGTTAGCCCAGTTTTTGG	60.1	523	INTERGENIC	--	--	--
Ca4_P4_33145619	Ca4	33145619	TAAAAAAA/TAAAAAA	AAACCGTCGTTCCAGTTGTC	AGGCAACAAACATAGAATCGC	60.0	550	INTERGENIC	--	--	--

INDEL marker IDs	Chromosomes/ unanchored scaffolds	Physical positions (bp)	InDels (*Kabuli* reference genome-CDC Frontier/PII)	Forward primers (5'-3')	Reverse primers (5'-3')	Anneling temperature (0C)	Expected amplified product size (bp)	Structural annotation		Functional annotation	
								Sequence components of *kabuli* genome	*Kabuli* gene accession IDs	NCBI-KOG	TFs
Ca4_P4_33176390	Ca4	33176390	A/AC	ATCCACGGACAATCCATTTG	TGGATGTCTTGTTCTGGGTTC	60.6	478	INTERGENIC	--	--	--
Ca4_P4_33186066	Ca4	33186066	A/AG	GCGCACAACAACAAGAAAAA	CGGGTTGTGAAACACCTTCT	59.9	620	INTERGENIC	--	--	--
Ca4_P4_33204821	Ca4	33204821	AATATATATATATATATATATATAT/AATATATATATATATATAT	GCCACACTCCGAAACAAAAT	ATGGAAGTAGGTGGCAGTGG	60.0	823	INTERGENIC	--	--	--
Ca4_P4_33212027	Ca4	33212027	TT/TTAT	TCACAATCCACTAATGCGGA	TTGATAACATGAAAATGAAACGAAA	60.1	519	INTERGENIC	--	--	--
Ca4_P4_33212339	Ca4	33212339	AATATATATATATATATATATATATAT/AATATATATATATAT	CAAACACTGCTGGCACATCT	GAAATGCCACACTCCGAAAT	59.9	790	INTERGENIC	--	--	--
Ca4_P4_33213558	Ca4	33213558	CTTTTT/CTTTTTT	AATGATACGGACCGCATAGC	GCCACTAGGCATGTGGAAAT	60.0	353	INTERGENIC	--	--	--
Ca4_P4_33256637	Ca4	33256637	ATTTTTTTTT/ATTTTTTTT	CATTGATCATGGTTCTTGCC	GGCTTGTGAAAACAAATGGAA	58.9	329	INTERGENIC	--	--	--
Ca4_P4_33260832	Ca4	33260832	TGAAGAAGAAGAA/TGAAGAAGAAGAAGAA	ACATCTGGAATTTGCTTGCC	CGACTTTGGCACCATATCCT	60.1	968	INTERGENIC	--	--	--
Ca4_P4_33283356	Ca4	33283356	G/GAATTAATTTA	TTGGGATGGTTGACCTTTGT	ATCGGGCCAATTTACAAGAA	60.2	681	INTERGENIC	--	--	--
Ca4_P4_33293560	Ca4	33293560	CTA/C	GGAGGATCCATCGAAGATGA	TTGGAACAGACATTGACCCA	60.0	584	INTERGENIC	--	--	--

INDEL marker IDs	Chromosomes/ unanchored scaffolds	Physical positions (bp)	InDels (*Kabuli* reference genome-CDC Frontier/PII)	Forward primers (5'-3')	Reverse primers (5'-3')	Anneling temperature (0C)	Expected amplified product size (bp)	Structural annotation		Functional annotation	
								Sequence components of *kabuli* genome	*Kabuli* gene accession IDs	NCBI-KOG	TFs
Ca4_P4_33315743	Ca4	33315743	CTT/CTTT	ACATGAGTCTTTGTTGCCCC	TGGCACGTGATTTGTTTGAT	60.0	477	INTERGENIC	--	--	--
Ca4_P4_33321015	Ca4	33321015	AATATA/AATATATA	TATTGTTGCACCCCCAATTT	ATGTCTGCCCCATTTTCTTG	60.1	229	INTERGENIC	--	--	--
Ca4_P4_33327000	Ca4	33327000	CA/C	AATATTCCCGTCGAATGTGC	TTTATTGCAACATCGCCTTTT	59.8	662	INTERGENIC	--	--	--
Ca4_P4_33336961	Ca4	33336961	ATTTTTTTT/ATTTTTTT	CACGGAGTTTAACGATGTGAGA	CATCCCCTAACCCACTCAAA	60.2	911	INTERGENIC	--	--	--
Ca4_P4_33342440	Ca4	33342440	TTTTTTCTTCTT/TTTTTTCTTCTTCTTTTTCTTCTT	TGAAATTCCACCTCACCTCC	TTGGTTGAGGTTTATGCGTG	59.9	155	INTERGENIC	--	--	--
Ca4_P4_33346959	Ca4	33346959	TT/TTAT	TTTTCGGTTTGTTCAAAGGC	GGAAAACATTGGGAAACCCT	60.1	766	INTERGENIC	--	--	--
Ca4_P4_33348179	Ca4	33348179	ATT/AT	AGGTTGAGCGATTGGAGAAA	TCCTAAAAGACTGCCCGCTA	59.8	378	INTERGENIC	--	--	--
Ca4_P4_33362903	Ca4	33362903	TCATACACA/TCATACACAACATACACA	CGTTTGGTTGCACTGATGTC	CCAGACGCAACAGTCAAATG	60.2	119	INTERGENIC	--	--	--
Ca4_P4_33386822	Ca4	33386822	ATAAAATTAAAATTAAA/ATAAAATTAAAATTAAAATTAAA	CCCTCCTTTGTGACGGTTTA	TTCGCACCTACACGATCAAG	60.0	763	INTERGENIC	--	--	--
Ca4_P4_33392310	Ca4	33392310	ATTTTTTTTTTTT/ATTTTTTTTTTT	CGACCCAAGTCATTGGATTT	CTCGAAAAATTGAAACCCCC	59.8	177	INTERGENIC	--	--	--

INDEL marker IDs	Chromosomes/ unanchored scaffolds	Physical positions (bp)	InDels (*Kabuli* reference genome-CDC Frontier/PII)	Forward primers (5'-3')	Reverse primers (5'-3')	Anneling temperature (0C)	Expected amplified product size (bp)	Structural annotation		Functional annotation	
								Sequence components of *kabuli* genome	*Kabuli* gene accession IDs	NCBI-KOG	TFs
Ca4_P4_33413394	Ca4	33413394	ATTTTTTTTTT/ATTTTTTTTT	TAGAGCGGGGCTCACTTAAA	GCGGAGATTGCCATATCACT	60.0	447	INTERGENIC	--	--	--
Ca4_P4_33431316	Ca4	33431316	GAAAAA/GAAAAATAAAAAAAA	TTTATGCTCCGTTTGTGCTG	CACACACCACCTCTCCCTTT	59.9	435	INTERGENIC	--	--	--
Ca4_P4_3344088	Ca4	3344088	TAAAAAAAAAA/TAAAAAAAAA	GCCGTACAAAGGTTTACATGGT	CTTATCTCCCAATCTCCCCC	60.2	515	INTERGENIC	--	--	--
Ca4_P4_3345838	Ca4	3345838	TTATATATATATATATATATATATATATATATATA/TTATATATATATATATATATATATATATATATA	TTTCATCCCCTTCACACTCC	TTTGACAAAATGACCCCAACT	59.9	456	INTERGENIC	--	--	--
Ca4_P4_33467899	Ca4	33467899	ATTTTTT/ATTTTTTT	TCATATCATCAAATTGCAACTCA	TGATGTTAAAAGCTACAGCAACAA	58.2	455	INTERGENIC	--	--	--
Ca4_P4_33485131	Ca4	33485131	T/TAGGTGA	CATTCGTGAGTGGTGGTTTG	GATATTGTGGCCTCTGCTCC	60.0	314	INTERGENIC	--	--	--
Ca4_P4_33500201	Ca4	33500201	CAAAAAAAAAAA/CAAAAAAAAAA	TGAGAGAAACCTAGCTCGATGA	GGTGCTGAAAAACACAAGAGC	59.2	917	INTERGENIC	--	--	--
Ca4_P4_33500547	Ca4	33500547	TTAT/TT	TGCAATTCGAGTCAAAGTGATAA	GGAGCATAATTGTTGACGCA	59.8	391	INTERGENIC	--	--	--
Ca4_P4_33554788	Ca4	33554788	CAAAAAATAAAAAATAAAAAATAAAA/CAAAAAATAAAAAATAAAA	CCTTGAGTTCGACACTCGGT	CAAGCAGCCTTTCTAGGTGG	60.3	733	INTERGENIC	--	--	--
Ca4_P4_3355845	Ca4	3355845	CGA/CGATAAGGA	GTTGGGTATTGGTGTCCCAC	GAGGGTGATCAGCTTTCTCG	60.0	589	INTERGENIC	--	--	--

INDEL marker IDs	Chromosomes/ unanchored scaffolds	Physical positions (bp)	InDels (*Kabuli* reference genome-CDC Frontier/PII)	Forward primers (5'-3')	Reverse primers (5'-3')	Anneling temperature (0C)	Expected amplified product size (bp)	Structural annotation		Functional annotation	
								Sequence components of *kabuli* genome	*Kabuli* gene accession IDs	NCBI-KOG	TFs
Ca4_P4_3356082	Ca4	3356082	A/AGCCAAGCGTAACG	TCACCGGAAAGTAACTCGCT	ATCGATATTCTCCGCACCTG	59.9	622	INTERGENIC	--	--	--
Ca4_P4_33566311	Ca4	33566311	ATTTTTTTTTTT/ATTTTTTTTTTTTT	TCCATCCTCCTCATCTGACC	TTGTCTCAACAATCTCCCCC	60.0	362	INTERGENIC	--	--	--
Ca4_P4_33615467	Ca4	33615467	ATT/ATTT	GGTCCTTGTTGAGTCCAACG	CTCAAAAACCTGAGCATGGC	60.5	646	INTERGENIC	--	--	--
Ca4_P4_33617340	Ca4	33617340	CTTTTTTTTTT/CTTTTTTTTT	TTTTCCTAAATTTCGAGGACGA	TTTTCCCGATTTGGTCCTTT	60.1	798	INTERGENIC	--	--	--
Ca4_P4_33617796	Ca4	33617796	AAAAGAA/AAA	CCCCGATTTGGTCCTTTATT	TGCTAGTTGTTGCGACCAAA	60.0	765	INTERGENIC	--	--	--
Ca4_P4_33619155	Ca4	33619155	CGTGT/CGTGTGT	GAACATGCTGAAAACGGGAT	TGAGGGCAATGACAAACAAA	59.9	122	INTERGENIC	--	--	--
Ca4_P4_33619321	Ca4	33619321	TAAAAAAAA/TAAAAA	GAACATGCTGAAAACGGGAT	CCAATTCCAAGGTCATCTGG	59.9	448	INTERGENIC	--	--	--
Ca4_P4_33627666	Ca4	33627666	TATTTAAAATTTAAAATTTAAAATTTAAAAT/TATTTAAAATTTAAAATTTAAAAT	AGTGACATGACGGAAGTAGCA	TTGGTTCCAGTGTGCATGTT	58.4	432	INTERGENIC	--	--	--
Ca4_P4_33633372	Ca4	33633372	GTTTTTTT/GTTTTTT	ACGAGGAAACATATCGTCCG	ATGTCCACACATTGGGTGTC	60.0	726	INTERGENIC	--	--	--
Ca4_P4_33714267	Ca4	33714267	T/TCTTAATC	GAACACGTTGTTAGACCGGC	TGACAAAATGGGGAGAAAGG	60.6	713	INTERGENIC	--	--	--

INDEL marker IDs	Chromosomes/ unanchored scaffolds	Physical positions (bp)	InDels (*Kabuli* reference genome-CDC Frontier/PII)	Forward primers (5'-3')	Reverse primers (5'-3')	Anneling temperature (0C)	Expected amplified product size (bp)	Structural annotation		Functional annotation	
								Sequence components of *kabuli* genome	*Kabuli* gene accession IDs	NCBI-KOG	TFs
Ca4_P4_33714529	Ca4	33714529	ATT/ATTGTT	CGTTGTTAGACCGGCTCTTG	TGGGCTGACATAATCACCAA	60.8	940	INTERGENIC	--	--	--
Ca4_P4_33715942	Ca4	33715942	TAAAAAAAAAAAA/TAAAAAAAAAAAAAAAAAA	TTGGGTACGTTGGTGTAGCA	ATTGACTGGGCACCATGATT	60.0	634	INTERGENIC	--	--	--
Ca4_P4_33717821	Ca4	33717821	TAAAAAAAAAAAAAAAAAA/TAAAAAAAAAAA	CCAAAAACCCCTCTTTTTCC	CCACCAAAATTTGTCCAACC	59.8	730	INTERGENIC	--	--	--
Ca4_P4_33733792	Ca4	33733792	AATATTTGTTTATATT/AATATTTGTTTATATTTGTTTATATT	CGTTATTCCCATATGAGAGGC	TGTGATGTTGGACGTGTGTG	58.5	280	INTERGENIC	--	--	--
Ca4_P4_33739294	Ca4	33739294	ATTTTTTTTTT/ATTTTTTTTT	CGTCTTGTGATTTGGTGGTG	GTAGTGGGTGAACGTGGCTT	60.0	456	INTERGENIC	--	--	--
Ca4_P4_33746638	Ca4	33746638	CAAAAAAAA/CAAAAAAAAAA	CGTGGACAAAGATCGTGATG	AAGCCACGTTCACCCACTAC	60.1	289	INTERGENIC	--	--	--
Ca4_P4_33773434	Ca4	33773434	AC/ACC	GGATGTCTCCATGGGAATTG	GCATACAATTGCTTCTCGCA	60.1	528	INTERGENIC	--	--	--
Ca4_P4_33777332	Ca4	33777332	GAAAAAAAAAA/GAAAAAAAAAAA	TGGGTTACCAAACAATAGTGG	TCGTGCTGACACCTTCAATC	57.4	749	INTERGENIC	--	--	--
Ca4_P4_33795008	Ca4	33795008	TTATATATATATATATAT/TTATATATATATAT	GTCGTGTCAATGCAACAACC	TCGACTAAGGACATTGGGCT	60.0	514	INTERGENIC	--	--	--
Ca4_P4_33804679	Ca4	33804679	CAT/CATAT	TCAGAATGTTTGGAATCGCA	TCGTATGCGTCAATCCGATA	60.2	262	INTERGENIC	--	--	--

INDEL marker IDs	Chromosomes/ unanchored scaffolds	Physical positions (bp)	InDels (*Kabuli* reference genome-CDC Frontier/PII)	Forward primers (5'-3')	Reverse primers (5'-3')	Anneling temperature (0C)	Expected amplified product size (bp)	Structural annotation		Functional annotation	
								Sequence components of *kabuli* genome	*Kabuli* gene accession IDs	NCBI-KOG	TFs
Ca4_P4_33813940	Ca4	33813940	ATTT/ATTTTT	TGAAGCATGGACCAATCAAA	GTCCAATCACATGCACAAGG	60.0	483	INTERGENIC	--	--	--
Ca4_P4_33833551	Ca4	33833551	ATTTTTTT/ATTTTTTTTT	TCGGAGAGGAGATTGATGCT	TCCAACTCGCAAGGCTATCT	59.9	803	INTERGENIC	--	--	--
Ca4_P4_33833847	Ca4	33833847	TA/TAACTGCA	GCGTCATCGTCCCTACAAAT	TCCAACTCGCAAGGCTATCT	60.0	375	INTERGENIC	--	--	--
Ca4_P4_33835105	Ca4	33835105	CAAAAAAA/CAAAAAAAAA	TTTTCACCAAATCACTTGAAAGAA	TCGAGCCTTATTGTGGATGA	60.0	654	INTERGENIC	--	--	--
Ca4_P4_33835153	Ca4	33835153	AAGCT/A	TTTTCACCAAATCACTTGAAAGAA	TCGAGCCTTATTGTGGATGA	60.0	654	INTERGENIC	--	--	--
Ca4_P4_33835234	Ca4	33835234	TAAAAAAA/TAAAAAAAA	CCAATCAAATTGAGCATACCC	TGTGTTTGCTTTGCATGCTT	59.3	529	INTERGENIC	--	--	--
Ca4_P4_33835524	Ca4	33835524	TAA/TAAA	TGCATCCAGACATGAGTCAAA	TGTGTTTGCTTTGCATGCTT	60.3	309	INTERGENIC	--	--	--
Ca4_P4_33836766	Ca4	33836766	GAAAAAAAAA/GAAAAAAAAAAA	GTTTTGTGATGGGGGATGAC	CCATTGAAAAACGAACGACC	60.0	205	INTERGENIC	--	--	--
Ca4_P4_33837766	Ca4	33837766	CAAAAA/CAAAA	TCATTGTCTATGCCCGATCA	GGTTTGATCTGTGTTTGCGA	60.0	431	INTERGENIC	--	--	--
Ca4_P4_33856380	Ca4	33856380	CTTTTTTTTTT/CTTTTTTTTTTTTT	CGGGAATTAAGAGGAGAGGG	AAAATTTAATGCCGTGACCG	60.0	719	INTERGENIC	--	--	--

INDEL marker IDs	Chromosomes/ unanchored scaffolds	Physical positions (bp)	InDels (*Kabuli* reference genome-CDC Frontier/PII)	Forward primers (5'-3')	Reverse primers (5'-3')	Anneling temperature (0C)	Expected amplified product size (bp)	Structural annotation		Functional annotation	
								Sequence components of *kabuli* genome	*Kabuli* gene accession IDs	NCBI-KOG	TFs
Ca4_P4_33858628	Ca4	33858628	ATTT/ATTTT	GCAAACGCAGATCAAGTCAA	ATTATTGGGGAAATCTGGGC	60.0	841	INTERGENIC	--	--	--
Ca4_P4_33865093	Ca4	33865093	GAAAAAAAAAA/GAAAAAAAAA	TTTGATGTTGTTGGAGATGAAAA	CGTTTCAGAGGTGAACCGTAA	59.5	407	INTERGENIC	--	--	--
Ca4_P4_33865441	Ca4	33865441	GTTTTTTTT/GTTTTTTTTT	TGTTTACGGTTCACCTCTGAAA	ATAACCCAGCTGAAAGGTGC	59.6	587	INTERGENIC	--	--	--
Ca4_P4_33873292	Ca4	33873292	CTTTTTTTTTTTTTTT/CTTTTTTT	GCGAGGTTGAGAAAACAAGC	TTGAGGGACCAAAACTGAAGA	60.0	631	INTERGENIC	--	--	--
Ca4_P4_33878589	Ca4	33878589	CGTTTTCTGTTT/CGTTT	CTTTCAACAGCCTCCAAAGC	CGTGAATGGATCAGATGTGC	60.0	748	INTERGENIC	--	--	--
Ca4_P4_33884406	Ca4	33884406	GTTTTTTTTT/GTTTTTTTTTT	AGGCTAGTGCATCATCCAGG	GTACCCCAATTTTTCCCGTT	60.2	303	INTERGENIC	--	--	--
Ca4_P4_33953368	Ca4	33953368	CAT/CATATCATAT	TATTTTGGGATTGTGGGCTC	AGATTAGGGCCTCAGGAACC	59.8	523	INTERGENIC	--	--	--
Ca4_P4_33954309	Ca4	33954309	TAAAAAAAAAAA/TAAAAAAAAAA	CACGACCAAATCCATTCTTG	TGCCAAGATTCGTGAAACAA	59.0	747	INTERGENIC	--	--	--
Ca4_P4_33960188	Ca4	33960188	TAA/TAAA	AAATAAAGGACCAGGTCGGG	TTTGGCCAGACCACCTTTAC	60.2	662	INTERGENIC	--	--	--
Ca4_P4_33960521	Ca4	33960521	GTATATATATATATAT/GTATATATATATATATAT	TCATGTTCCTCCAGCTGCTA	TTTGGCCAGACCACCTTTAC	59.5	290	INTERGENIC	--	--	--

INDEL marker IDs	Chromosomes/ unanchored scaffolds	Physical positions (bp)	InDels (*Kabuli* reference genome-CDC Frontier/PII)	Forward primers (5'-3')	Reverse primers (5'-3')	Anneling temperature (0C)	Expected amplified product size (bp)	Structural annotation		Functional annotation	
								Sequence components of ***kabuli*** genome	***Kabuli*** gene accession IDs	NCBI-KOG	TFs
Ca4_P4_33972054	Ca4	33972054	AAACAACA/AAACAACAACA	TGAGTTCCAAAATCGGGAAA	AATTGGCTAAGCAAGCTCCA	60.4	500	INTERGENIC	--	--	--
Ca4_P4_34056670	Ca4	34056670	ATAAT/ATAATTAAT	AGGATGCAACAAACCCAAAG	GTCCAAATGAAAACGCCAAT	60.0	461	INTERGENIC	--	--	--
Ca4_P4_34136044	Ca4	34136044	AATATATATATATATATA/AATATATATATATATATATATATA	TTTTTGTGGAAAGGCCTACG	GCAAAGCCTCTTCAGTCTGG	60.1	522	INTERGENIC	--	--	--
Ca4_P4_34169705	Ca4	34169705	CAATGA/CAATGATAATGA	TCAAAGTGGCTCACAAGTGC	TCGACTCAAGAAGGCAAGTG	60.0	781	INTERGENIC	--	--	--
Ca4_P4_34229083	Ca4	34229083	GTTTTAACTT/GTTTTAACTTTTTAACTT	AATTAAACCCCCACGCCTAC	CTCATCCCACGCTAGGAAAA	60.1	494	INTERGENIC	--	--	--
Ca4_P4_34247514	Ca4	34247514	TTATATATATATATATATATATA/TTATATATATATATATATATATATATATA	TCATTAGCTCAATCAATTACGCA	TTGATAAGATTGATCCGTTGGA	59.8	434	INTERGENIC	--	--	--
Ca4_P4_34262481	Ca4	34262481	TAAAAAAAAA/TAAAAAAAAAA	GACCACCTACGCCACACTTT	TGTTCCAATTCCTCGTGTCA	60.0	807	INTERGENIC	--	--	--
Ca4_P4_34262689	Ca4	34262689	AATAAATATATATATATATATATATATATATATATATATAA/AATATATATATATATATATATATATATAA	TCTTGCCACCAACAAAACAA	TGTTCCAATTCCTCGTGTCA	60.1	223	INTERGENIC	--	--	--
Ca4_P4_34308809	Ca4	34308809	TAAAAA/TAAAAAAA	GTTGAGCATACCGACCAACC	ATGGTCGTTTTCGGAACTTG	60.4	870	INTERGENIC	--	--	--
Ca4_P4_34330740	Ca4	34330740	TAAAAAAAAAAA/TAAAAAAAAAAAA	TGCACACATCTACCCCTGAA	GGCATGGCAACTTCACTTTT	60.1	502	INTERGENIC	--	--	--

INDEL marker IDs	Chromosomes/ unanchored scaffolds	Physical positions (bp)	InDels (*Kabuli* reference genome-CDC Frontier/PII)	Forward primers (5'-3')	Reverse primers (5'-3')	Anneling temperature (0C)	Expected amplified product size (bp)	Structural annotation		Functional annotation	
								Sequence components of *kabuli* genome	*Kabuli* gene accession IDs	NCBI-KOG	TFs
Ca4_P4_34333563	Ca4	34333563	TAAAAAAAA/TAAAAAAA	ACGGTGACAAATTGGAGAGG	GGTCGCTCAACCTTTACGAG	60.0	744	INTERGENIC	--	--	--
Ca4_P4_34371605	Ca4	34371605	CAA/CAAA	ATGCCTTTGATTACGTTGCC	AACCATGCTTGGAAAACCTG	60.0	353	INTERGENIC	--	--	--
Ca4_P4_34384933	Ca4	34384933	CAAAA/CAAA	TACATTCCCATCCACAGGGT	TTCGGTTGGTGACCCTTTAC	60.1	103	INTERGENIC	--	--	--
Ca4_P4_34388082	Ca4	34388082	CATATATATATATAT/CATATATATATATATATATAT	GTTGGTTCCCTAGCACCGTA	CTGTCAATTGAGTTGCGACG	60.0	484	INTERGENIC	--	--	--
Ca4_P4_34388181	Ca4	34388181	GAAA/GAAAA	GTTGGTTCCCTAGCACCGTA	CTGTCAATTGAGTTGCGACG	60.0	484	INTERGENIC	--	--	--
Ca4_P4_34410371	Ca4	34410371	ATGTGTGTGTGTGTGTGTGTGTGT/ATGTGTGTGTGTGTGTGTGTGTGTGTGT	TGAAACAGGATCCAAAAGCC	GCGCATACACACACACACAC	60.1	848	INTERGENIC	--	--	--
Ca4_P4_34417460	Ca4	34417460	CATTTCTTGTATTTCTTGTA/CATTTCTTGTA	CCCAATAGTGGTTGCATTCC	CCAAGGATGTGTTTCCCTGT	60.2	371	INTERGENIC	--	--	--
Ca4_P4_34432600	Ca4	34432600	ATTTTTTT/ATTTTTT	GGGTGAGGATTGGTCAGTCA	ACCTTTGGGTCTTGCCTTTT	60.9	407	INTERGENIC	--	--	--
Ca4_P4_34462526	Ca4	34462526	TAAAAAAAAA/TAAAAAAAA	GTGGGAAAAGATGTGGGAGA	TCGAAAGGATTTTTCCATGC	59.9	584	INTERGENIC	--	--	--
Ca4_P4_34468802	Ca4	34468802	GTTTTTTT/GTTTTTTTT	GGGAAGCTTGTTTGCTTCTG	TAAGAGTGAGTGGGTTGGGC	60.0	353	INTERGENIC	--	--	--

INDEL marker IDs	Chromosomes/ unanchored scaffolds	Physical positions (bp)	InDels (*Kabuli* reference genome-CDC Frontier/PII)	Forward primers (5'-3')	Reverse primers (5'-3')	Anneling temperature (0C)	Expected amplified product size (bp)	Structural annotation		Functional annotation	
								Sequence components of *kabuli* genome	*Kabuli* gene accession IDs	NCBI-KOG	TFs
Ca4_P4_34470233	Ca4	34470233	TAAAAAAAAA/TAAAAAAAA	GCTCAATTTTTGTTTAGGTCCTTC	GCGTTGTGGGTTCACTCAAT	59.6	606	INTERGENIC	--	--	--
Ca4_P4_34471336	Ca4	34471336	ATTATTTATTTATTTATTTAT/ATTATTTATTTATTTATTTAT	AACGTGACATTTTCGGAAGC	TCAAGTTTTCCGATATAGCCGT	60.1	577	INTERGENIC	--	--	--
Ca4_P4_34503551	Ca4	34503551	G/GAACTCA	GGAGGGTTTTGGTTTGGAAT	GTAAAGGGTCACCAACCGAA	60.0	672	INTERGENIC	--	--	--
Ca4_P4_34515993	Ca4	34515993	TC/T	CAGTATACCACCCAGGCCAT	TCACATGAATAAATTTACAAGCGAA	59.7	155	INTERGENIC	--	--	--
Ca4_P4_34566172	Ca4	34566172	GTTTT/GTTTTT	CGCCTAAGGACTATGCAAGC	AAAATCCAACAATCTCCCCC	60.0	761	INTERGENIC	--	--	--
Ca4_P4_34566424	Ca4	34566424	ATT/AT	CCCTGAGACTCACGAAGAGG	AAAATCCAACAATCTCCCCC	60.0	612	INTERGENIC	--	--	--
Ca4_P4_34605005	Ca4	34605005	G/GT	AACCAATCTTGTGCATCCAA	TTGGACCAGAATAAGCCGAG	59.0	317	INTERGENIC	--	--	--
Ca4_P4_34884584	Ca4	34884584	T/TCCTCAG	CCCATGGTTGGGAACTAATG	AGCGACAGAGATACGCGATT	60.0	140	INTERGENIC	--	--	--
Ca4_P4_34889805	Ca4	34889805	AGGGGGGG/AGGGGGG	TGCAGTGCAATGTCCATTTT	GTAAGGCATGCCACCTCCTA	60.1	655	INTERGENIC	--	--	--
Ca4_P4_3492406	Ca4	3492406	TATCAAATCAAATCAAATC/TATCAAATCAAATC	CGATGATGTCCAGTTCACGA	TTTTCTGTTTGCATTGGCAG	60.7	365	INTERGENIC	--	--	--

INDEL marker IDs	Chromosomes/ unanchored scaffolds	Physical positions (bp)	InDels (*Kabuli* reference genome-CDC Frontier/PII)	Forward primers (5'-3')	Reverse primers (5'-3')	Anneling temperature (0C)	Expected amplified product size (bp)	Structural annotation		Functional annotation	
								Sequence components of *kabuli* genome	*Kabuli* gene accession IDs	NCBI-KOG	TFs
Ca4_P4_34944690	Ca4	34944690	ACTTC/ACTTCCGGCTTC	GCTTCTTGGTGCGAGTCATT	CGTGTTGAAGTCGGGAAAAT	60.4	114	INTERGENIC	--	--	--
Ca4_P4_34965051	Ca4	34965051	ATTTTTT/ATTTTTTT	TTGGGATTAGAGCAAGGTCG	CATGAGACCTTCATGTTGTCG	60.2	212	INTERGENIC	--	--	--
Ca4_P4_35114842	Ca4	35114842	CAAAA/CAAAAA	TTGATTGTACCCTTGTGCCA	AACTGTGGAGGAATCCATGC	60.0	495	INTERGENIC	--	--	--
Ca4_P4_35405054	Ca4	35405054	ATTTTTTTT/ATTTTTTT	AGCCTGTCAACACAAATCCA	TCACTCTCCCAATTTTTGGC	59.1	296	INTERGENIC	--	--	--
Ca4_P4_35431597	Ca4	35431597	CCAA/C	CCCTCACAATTTGACATTTGG	TTTTTGAATGCTTGTTTTGGA	60.2	477	INTERGENIC	--	--	--
Ca4_P4_35432013	Ca4	35432013	TTAT/TTATAT	TGTTCAACATCCACGAACAA	TGCCATACTCATGTCGGAAA	58.5	669	INTERGENIC	--	--	--
Ca4_P4_35432836	Ca4	35432836	TTATATATATATATATATATAT/TTATATATATATATATATATATAT	TTTCCGACATGAGTATGGCA	TTTGGAAAGTAACACTGCACG	60.1	785	INTERGENIC	--	--	--
Ca4_P4_35432970	Ca4	35432970	CATATATATATATATATATATATATATATA/CATATATATATATATATATATATATATATATATA	AAAAATGTGCATAAATTCGGTT	TTTGGAAAGTAACACTGCACG	57.7	428	INTERGENIC	--	--	--
Ca4_P4_35433291	Ca4	35433291	TTATATATATATATATATATATATAT/TTATATATATATATATATATATATATATATATATATATAT	CACGTGCAGTGTTACTTTCCA	TGACGAATTTCAAAAACCTGC	59.8	491	INTERGENIC	--	--	--
Ca4_P4_35445772	Ca4	35445772	AATATATATATATATATATAT/AATATATATATATATAT	TGCATTCAATTAAGCTTGCG	ATGTGGGCAAGTGACAAACA	60.0	654	INTERGENIC	--	--	--

INDEL marker IDs	Chromosomes/ unanchored scaffolds	Physical positions (bp)	InDels (*Kabuli* reference genome-CDC Frontier/PII)	Forward primers (5'-3')	Reverse primers (5'-3')	Anneling temperature (0C)	Expected amplified product size (bp)	Structural annotation		Functional annotation	
								Sequence components of *kabuli* genome	*Kabuli* gene accession IDs	NCBI-KOG	TFs
Ca4_P4_35457008	Ca4	35457008	TTGC/T	AATTGCAGAAAAATGCGGTC	TTGTACCAGATATTGGCCCG	60.1	776	INTERGENIC	--	--	--
Ca4_P4_35818039	Ca4	35818039	AAATAATAATAATAATA/AAATAATAATAATA	GTTCATAACGAAGATGACAATAACA	TGCAGTCAAACTTTAAAAGGAAG	57.4	728	INTERGENIC	--	--	--
Ca4_P4_35822348	Ca4	35822348	TGG/TG	GATCAAATCGAGGACCGAAA	CGAACAAAACGATGGGAAGT	60.0	695	INTERGENIC	--	--	--
Ca4_P4_35912616	Ca4	35912616	CAAAAAAAAAAA/CAAAAAAAAAA	ACGAAGCTCGGTCTGTTGTT	GATGGATTTAACTTTCGGTCG	59.9	287	INTERGENIC	--	--	--
Ca4_P4_35936788	Ca4	35936788	TA/TAA	TTGGGAAAGGCGATTTGTAG	CGGCCTGGTCAAAGTATGAT	60.1	696	INTERGENIC	--	--	--
Ca4_P4_35975112	Ca4	35975112	TTTATTATTATTATTATTATTATTATTATTATTATTATTATTAT/TTTATTATTATTATTATTATTATTATTATTATTATTATTATTATTAT	AGCCAAAAGTGGGTAACGTG	TTTTGTGTAACGCCCAGTCA	60.0	193	INTERGENIC	--	--	--
Ca4_P4_36079126	Ca4	36079126	TTGTG/TTGTGTG	ATTTTGATGCTCACGGTTCC	CGGAGATGATGGTTCAGGTT	59.9	595	INTERGENIC	--	--	--
Ca4_P4_36088961	Ca4	36088961	GNNNNNNNNNN/GNNNNNNN	TACCAACGAATCCCCTTCAG	TTTGGGATAAGTGGAACCCA	59.9	484	INTERGENIC	--	--	--
Ca4_P4_36138277	Ca4	36138277	ATT/AT	ACCATGAAAGACATGGGAGC	CGGAGTACCCGACCAAACTA	59.9	436	INTERGENIC	--	--	--
Ca4_P4_36204013	Ca4	36204013	AATATATATATATATATA/AATATATATATATATATATA	GAATGAGATTGCACATAATTAGGAA	CAAACACGTAAACACGCAAAA	58.6	693	INTERGENIC	--	--	--

INDEL marker IDs	Chromosomes/ unanchored scaffolds	Physical positions (bp)	InDels (*Kabuli* reference genome-CDC Frontier/PII)	Forward primers (5'-3')	Reverse primers (5'-3')	Anneling temperature (0C)	Expected amplified product size (bp)	Structural annotation		Functional annotation	
								Sequence components of *kabuli* genome	*Kabuli* gene accession IDs	NCBI-KOG	TFs
Ca4_P4_36335313	Ca4	36335313	ATTTTTT/ATTTTTTT	TTGCGACAAGATCAAGCAAC	TCAACACCAAACACAAGGGA	60.0	519	INTERGENIC	--	--	--
Ca4_P4_36336918	Ca4	36336918	TAAA/TATAAAA	ACAACAAGGGTGACCTCCAC	TGCTTGTCTATTCTGCGTCAA	59.9	736	INTERGENIC	--	--	--
Ca4_P4_36337139	Ca4	36337139	GC/G	TGCAATGTCAATATGAACCAAA	CCACAACCACTCATCACGAC	58.9	479	INTERGENIC	--	--	--
Ca4_P4_36337264	Ca4	36337264	AT/ATT	TGACGCAGAATAGACAAGCAA	CCACAACCACTCATCACGAC	59.6	341	INTERGENIC	--	--	--
Ca4_P4_36337395	Ca4	36337395	A/ATTATTATT	TGACGCAGAATAGACAAGCAA	CCACAACCACTCATCACGAC	59.6	341	INTERGENIC	--	--	--
Ca4_P4_36390176	Ca4	36390176	TATAATAATAA/TATAATAATAATAA	TATGCACGCTCTCTCTCCCT	AGGAGGAACCAAAACCGTCT	60.1	611	INTERGENIC	--	--	--
Ca4_P4_36392790	Ca4	36392790	TT/TTGACT	CCCTCAACAAGCATTCAACA	TTCTTTTGCTCGTGTCGTTG	59.7	711	INTERGENIC	--	--	--
Ca4_P4_36408080	Ca4	36408080	ATTTTTT/ATTTTT	CAACGATCCTTCATTGATGC	ACTATTTTCGCGAAGGCTGA	59.1	456	INTERGENIC	--	--	--
Ca4_P4_36419271	Ca4	36419271	A/ATTTTTTTTTT	CGTCCAAGGTCACCAAACTT	GGAAAATCCTATGTCTACCCCA	60.0	275	INTERGENIC	--	--	--
Ca4_P4_36420752	Ca4	36420752	CTTTATTTTTATTTTTATTTTT/CTTTATTTTTATTTTTATTTTTATTTTT	TGACTTTTGTCTTAACATGTAGGATG	TTTCTTTTCCTTCCTGATTTGG	58.7	657	INTERGENIC	--	--	--

INDEL marker IDs	Chromosomes/ unanchored scaffolds	Physical positions (bp)	InDels (*Kabuli* reference genome-CDC Frontier/PII)	Forward primers (5'-3')	Reverse primers (5'-3')	Anneling temperature (0C)	Expected amplified product size (bp)	Structural annotation		Functional annotation	
								Sequence components of *kabuli* genome	*Kabuli* gene accession IDs	NCBI-KOG	TFs
Ca4_P4_36438574	Ca4	36438574	TA/TAA	GGATAATTTTTCGCCTATTCAAA	CTCCTTTTCACGATTCTCGC	58.6	577	INTERGENIC	--	--	--
Ca4_P4_36453533	Ca4	36453533	TATATTTATAATATTTATAATATTTA/TATATTTATAATATTTA	TGTTGGGTGGTTAGCATACG	GGGGTTAGAGTAAGAGGCCA	59.5	597	INTERGENIC	--	--	--
Ca4_P4_36471974	Ca4	36471974	TG/T	CCTCGGCTGTAAGTAACGGA	ATGGTTCCAAAACCAATGCT	60.3	827	INTERGENIC	--	--	--
Ca4_P4_36472245	Ca4	36472245	TTGGTAC/TTGGTACAATGGTAC	TTGCTGTTGTTGAATTTCCATC	ACCACGGTTTCTGTCCTACG	60.0	618	INTERGENIC	--	--	--
Ca4_P4_36474350	Ca4	36474350	ACATAATTCA/ACATAATTCATAATTCA	CAAACCACATCACCCTCCTT	GCTACACCGACCACCTGAAT	59.8	585	INTERGENIC	--	--	--
Ca4_P4_36495365	Ca4	36495365	CGTTTTTGTTTTTGTT/CGTTTTTGTT	TTTTCTCCTATTGATTGGATATTGG	CCCAAACTCTTCTCTTCTTCCA	59.6	888	INTERGENIC	--	--	--
Ca4_P4_36502639	Ca4	36502639	TCCCC/TC	GTGGCAAAATGGATCGAACT	GCCGTCTAGTGTTTCTTTAATGACT	59.9	551	INTERGENIC	--	--	--
Ca4_P4_36510171	Ca4	36510171	T/TC	CGGGCTAATTGACTCTCAGC	CCGACGAAACGAAAAACAAT	60.0	454	INTERGENIC	--	--	--
Ca4_P4_36510514	Ca4	36510514	GTTTTTTTT/GTTTTTTTTTT	ATTGTTTTTCGTTTCGTCGG	GCAATGCCATAGAAGCCAAT	60.0	453	INTERGENIC	--	--	--
Ca4_P4_36527807	Ca4	36527807	TTATAT/TTAT	AATCCCATGCATTTAGGCAG	TTTGTATGCCGTTCACCTCA	59.9	346	INTERGENIC	--	--	--

INDEL marker IDs	Chromosomes/ unanchored scaffolds	Physical positions (bp)	InDels (*Kabuli* reference genome-CDC Frontier/PII)	Forward primers (5'-3')	Reverse primers (5'-3')	Anneling temperature (0C)	Expected amplified product size (bp)	Structural annotation		Functional annotation	
								Sequence components of *kabuli* genome	*Kabuli* gene accession IDs	NCBI-KOG	TFs
Ca4_P4_36560970	Ca4	36560970	TAAAAAAAAAA/TAAAAAAAA	CCGCATTTGTTTCCCTCTTA	TTGCAAAAATCAATCCAAACA	60.1	746	INTERGENIC	--	--	--
Ca4_P4_36561766	Ca4	36561766	CTTTTTTT/CTTTTTTTTT	GAACATGTTAATTTGGTTCATTCG	TTCAACTCAATGGAATTTTGGA	59.7	491	INTERGENIC	--	--	--
Ca4_P4_36622848	Ca4	36622848	ACCCC/ACCC	CTGTGAGTTGCAGGGAATGA	GCCAAGTGGCTTAACTGTCTG	59.8	698	INTERGENIC	--	--	--
Ca4_P4_36623599	Ca4	36623599	T/TA	GAAAATATTTGGGGGCATGA	TCCTGAAAATGTCTTTGTTGCT	59.6	292	INTERGENIC	--	--	--
Ca4_P4_36631595	Ca4	36631595	TC/TCC	CCCCCACTATGAAACACACC	CTGAATGTTTAATCCCCGGT	60.1	486	INTERGENIC	--	--	--
Ca4_P4_36633157	Ca4	36633157	AAATAATAAT/AAATAATAATAAT	GTCCCTTCCAATAACGTGGA	GCTTTGATGGTGTAGCCACTT	59.8	411	INTERGENIC	--	--	--
Ca4_P4_36693901	Ca4	36693901	ACTCT/ACT	CCGCTTTCGTCAATTTTCAT	TCCAGCTGTTTCTTCGAGGT	60.1	470	INTERGENIC	--	--	--
Ca4_P4_3683925	Ca4	3683925	ATTTTTTTTT/ATTTTTTTTTT	CGACTGACCACATGGATTGT	CGAGTCATCTTATTGTTTGCCA	59.4	734	INTERGENIC	--	--	--
Ca4_P4_36854161	Ca4	36854161	GCA/GCATCA	TGTTCATGATGCTTCCTCTCC	CCAAACGATTATCCAACCCT	60.2	437	INTERGENIC	--	--	--
Ca4_P4_36902625	Ca4	36902625	GTTTTTTTTTTT/GTTTTTTTTTT	TTTTCTCCTTCTCCGGTTCA	CACATAGAATTTGTGCGCGT	59.8	562	INTERGENIC	--	--	--

INDEL marker IDs	Chromosomes/ unanchored scaffolds	Physical positions (bp)	InDels (*Kabuli* reference genome-CDC Frontier/PII)	Forward primers (5'-3')	Reverse primers (5'-3')	Anneling temperature (0C)	Expected amplified product size (bp)	Structural annotation		Functional annotation	
								Sequence components of *kabuli* genome	*Kabuli* gene accession IDs	NCBI-KOG	TFs
Ca4_P4_36957693	Ca4	36957693	GTT/GTTT	GCCGAAAAAGTCCTCACAAA	GCGGCTAGAAGTATCAACGG	60.2	381	INTERGENIC	--	--	--
Ca4_P4_36959669	Ca4	36959669	CAAAA/CAA	CGGCAGACTTTTTCGTCAAT	AAAAATCACAACGTGGGGTC	60.2	433	INTERGENIC	--	--	--
Ca4_P4_36960688	Ca4	36960688	ATATCTTTGTTATTAT/ATATCTTTGTTATTATCTTTGTTATTAT	TTGATTTTGATGCAGGTGGA	CAAATACCTGTTGCCGAGGT	60.0	818	INTERGENIC	--	--	--
Ca4_P4_36966828	Ca4	36966828	ATTTTTTTTTT/ATTTTTTTTTTT	AGACGACGAATAGGGGTGTG	TTCGTGGTTTCATTGTTAAACG	60.0	710	INTERGENIC	--	--	--
Ca4_P4_36968132	Ca4	36968132	ATTTTTTTT/ATTTTTTTTT	GGCGAAAGTACCCAAACAAA	GACTTCTGCAAAGGACCTCG	60.0	426	INTERGENIC	--	--	--
Ca4_P4_36968194	Ca4	36968194	TAAAAAAAA/TAAAAAAAAA	GGCGAAAGTACCCAAACAAA	GACTTCTGCAAAGGACCTCG	60.0	426	INTERGENIC	--	--	--
Ca4_P4_36969491	Ca4	36969491	TAAAAAAAAAAA/TAAAAAAAAAA	CATGGGCCAAAAATAAAAAGA	GAGCTGTGGAGAAGAAAAGCA	59.0	814	INTERGENIC	--	--	--
Ca4_P4_36972358	Ca4	36972358	CTTT/CTT	ACAACCAACAAAATGCGACA	AGCCTTGAGCGACCACTTTA	60.0	484	INTERGENIC	--	--	--
Ca4_P4_36972540	Ca4	36972540	AATATATATATATA/AATATATATATATATATATATA	TAAAGTGGTCGCTCAAGGCT	TCTACATTCAAATTGGGGGC	60.0	608	INTERGENIC	--	--	--
Ca4_P4_37011834	Ca4	37011834	GTAACTAACTAACTAA/GTAACTAACTAACTAACTAA	GGCCAAGCATTGTAAAGGAA	ACCATTGAACCCAAACCGTA	60.1	816	INTERGENIC	--	--	--

INDEL marker IDs	Chromosomes/ unanchored scaffolds	Physical positions (bp)	InDels (*Kabuli* reference genome-CDC Frontier/PII)	Forward primers (5'-3')	Reverse primers (5'-3')	Anneling temperature (0C)	Expected amplified product size (bp)	Structural annotation		Functional annotation	
								Sequence components of *kabuli* genome	*Kabuli* gene accession IDs	NCBI-KOG	TFs
Ca4_P4_37034691	Ca4	37034691	ATTTTT/ATTTT	CTCCATTCCTTTCCACTCCA	AGGGAAAACAAGGGTTTTGG	60.0	522	INTERGENIC	--	--	--
Ca4_P4_37038560	Ca4	37038560	TGAGA/TGAGAGA	TGGTTTATAACACCGCTTATATTCA	AGGATTCAACCACAAAGGACA	59.0	242	INTERGENIC	--	--	--
Ca4_P4_37039222	Ca4	37039222	TGGG/TG	CAATCTTTCTTTCAATTCTCTACAAA	TGATTCTTGACACGGTGGAA	57.5	644	INTERGENIC	--	--	--
Ca4_P4_37040426	Ca4	37040426	TAAAAAAA/TAAAAAAAA	GCGCTACAGAGAGCCTTCAC	GGTCATAACAAAGGGCCAGA	60.3	648	INTERGENIC	--	--	--
Ca4_P4_37043985	Ca4	37043985	TAAAAA/TAAAAAAAA	CTAGTCCGGTGGACCCGTAT	TCACGAAATTAGCCCTCCAA	61.1	861	INTERGENIC	--	--	--
Ca4_P4_37045715	Ca4	37045715	AAT/A	CCAGCTGGCCATTTGTAAAG	TACCCCATATGGACAAGGGA	60.6	595	INTERGENIC	--	--	--
Ca4_P4_37047002	Ca4	37047002	TAAAAAAAAAA/TAAAAAAAAA	TTGACAATGAATTAAAGAAACTGGA	TCAAACCCATTTCCCACTTC	59.1	227	INTERGENIC	--	--	--
Ca4_P4_37047718	Ca4	37047718	GTTT/GTT	ATACTCTGCACCACACACGG	ATGATTGTGCCTCGTGAACA	59.6	241	INTERGENIC	--	--	--
Ca4_P4_37055356	Ca4	37055356	TAAAAAA/TAAAAAAAAA	TTCAAACACACTCCCTCCAA	AAACACAATACACTGTCCGGC	59.1	772	INTERGENIC	--	--	--
Ca4_P4_37055534	Ca4	37055534	ATTTTT/ATTTT	TGCTTTAAAAGAGTGAGAGGGAA	AAACACAATACACTGTCCGGC	59.5	598	INTERGENIC	--	--	--

INDEL marker IDs	Chromosomes/ unanchored scaffolds	Physical positions (bp)	InDels (*Kabuli* reference genome-CDC Frontier/PII)	Forward primers (5'-3')	Reverse primers (5'-3')	Anneling temperature (0C)	Expected amplified product size (bp)	Structural annotation		Functional annotation	
								Sequence components of *kabuli* genome	*Kabuli* gene accession IDs	NCBI-KOG	TFs
Ca4_P4_37057125	Ca4	37057125	CTTTTTT/CTTTTTTT	TGCACAGTCAATCCTC TTCTG	TCATGTGTGTATGGAG CACAAA	59.0	474	INTERGENIC	--	--	--
Ca4_P4_37057389	Ca4	37057389	ATTTTTTTTTT/ATTTTTTTTT	TGCACAGTCAATCCTC TTCTG	TCATGTGTGTATGGAG CACAAA	59.0	474	INTERGENIC	--	--	--
Ca4_P4_37059654	Ca4	37059654	CAAAAAA/CAAAAAAA	ATTGATCCACATCGTC CCAT	TGGGGACTGGAAAATG GTTA	60.0	345	INTERGENIC	--	--	--
Ca4_P4_37070263	Ca4	37070263	TC/TCAACTTTAC	TCAACTCCAATTCAAGT GCG	TGTTGCGTAGCCTCTG AAAA	59.8	206	INTERGENIC	--	--	--
Ca4_P4_37092034	Ca4	37092034	TGGGG/TGGG	CCAAGGTTTTGTGCAT TCCT	CACCACAAATTTCAACA ACGA	60.0	642	INTERGENIC	--	--	--
Ca4_P4_37099831	Ca4	37099831	TAAAAAAA/TAAAAAA	TGAATAATTCGCGTAC GGCT	TGTGAGGAGCTCTTTG TGGA	60.6	589	INTERGENIC	--	--	--
Ca4_P4_37104817	Ca4	37104817	ATATTAT/ATATTATTAT	ACTACGCCATCATTTG GAGG	GGGGTGAGAGACCATT TGAA	60.0	613	INTERGENIC	--	--	--
Ca4_P4_37105871	Ca4	37105871	TGA/T	AAGATTCTGTCTTTCCA CCGA	ACGAAGGTCAAGCTCA AGGA	58.8	367	INTERGENIC	--	--	--
Ca4_P4_37107564	Ca4	37107564	TAAAAAAAA/TAAAAAAA	CGACGAATTTGCAGAG TCAA	TCCCGCATTCAAATCCT ATC	60.0	751	INTERGENIC	--	--	--
Ca4_P4_37108408	Ca4	37108408	A/AATAT	GATAGGATTTGAATGC GGGA	TCATGCTGGCATGTTAT GCT	59.9	745	INTERGENIC	--	--	--

INDEL marker IDs	Chromosomes/ unanchored scaffolds	Physical positions (bp)	InDels (*Kabuli* reference genome-CDC Frontier/PII)	Forward primers (5'-3')	Reverse primers (5'-3')	Anneling temperature (0C)	Expected amplified product size (bp)	Structural annotation		Functional annotation	
								Sequence components of *kabuli* genome	*Kabuli* gene accession IDs	NCBI-KOG	TFs
Ca4_P4_37110290	Ca4	37110290	GC/G	TCCAACCCCGTACCTATTCA	AATGCCAAAGGGTGATGAAG	60.2	804	INTERGENIC	--	--	--
Ca4_P4_37119880	Ca4	37119880	CTT/CTTCTTT	TGGGAAAAATTTGCACAACA	ACGAAAATTCATGGCTGTCC	59.9	718	INTERGENIC	--	--	--
Ca4_P4_37119914	Ca4	37119914	TTATA/TTATATA	TGGGAAAAATTTGCACAACA	ACGAAAATTCATGGCTGTCC	59.9	718	INTERGENIC	--	--	--
Ca4_P4_37121983	Ca4	37121983	ATTTTTTT/ATTTTTTTT	TCAACATGACCTCCTTATGCTTT	TCCAAACACTCCAAACACCA	60.0	407	INTERGENIC	--	--	--
Ca4_P4_37128859	Ca4	37128859	G/GC	TTTGACGATGGATGCATAAGA	TTTAGGTTGGCTTTGGATGC	59.1	593	INTERGENIC	--	--	--
Ca4_P4_37128959	Ca4	37128959	TGAG/TGAGAGAG	TTTGACGATGGATGCATAAGA	TTTAGGTTGGCTTTGGATGC	59.1	593	INTERGENIC	--	--	--
Ca4_P4_37131217	Ca4	37131217	TC/T	TTGTGACCAAAGGTTGGAGG	AATTTGCGAGCATTACCTGC	60.9	710	INTERGENIC	--	--	--
Ca4_P4_37131327	Ca4	37131327	TAA/TAATAGTTTAAAAA	CAACTCAAACCACCTTCATTCA	AATTTGCGAGCATTACCTGC	60.0	633	INTERGENIC	--	--	--
Ca4_P4_37131533	Ca4	37131533	TAA/TAAA	GACTCGCAGGTAATGCTCG	TTCCACGATTTTCCCTTCAC	59.6	472	INTERGENIC	--	--	--
Ca4_P4_37132221	Ca4	37132221	CTAT/CTATAT	GTGAAGGGAAAATCGTGGAA	GGGCTCTCATAATCTTGGAGG	59.9	418	INTERGENIC	--	--	--

INDEL marker IDs	Chromosomes/ unanchored scaffolds	Physical positions (bp)	InDels (*Kabuli* reference genome-CDC Frontier/PII)	Forward primers (5'-3')	Reverse primers (5'-3')	Anneling temperature (0C)	Expected amplified product size (bp)	Structural annotation		Functional annotation	
								Sequence components of *kabuli* genome	*Kabuli* gene accession IDs	NCBI-KOG	TFs
Ca4_P4_37149399	Ca4	37149399	CTATAACTATAATAATATAACTATAATAA/CTATAACTATAATAA	TTGGGAGGAGACAATATCCAA	TCCATAACCGACCATGAAGA	59.4	337	INTERGENIC	--	--	--
Ca4_P4_37156938	Ca4	37156938	AG/AGG	CCATTGTATTTCCCCAATGC	AAGAATGAATGACGTTGAATTTGA	60.0	210	INTERGENIC	--	--	--
Ca4_P4_37194041	Ca4	37194041	GAAAA/GAAA	GACACCAAAAGTAACCATGTCAA	TCCATGCACCTACATCTTGC	58.9	787	INTERGENIC	--	--	--
Ca4_P4_37196987	Ca4	37196987	AC/A	TTTTCATCGCCAACAACATC	AATTTCACACCCAACGAACC	59.5	856	INTERGENIC	--	--	--
Ca4_P4_37202310	Ca4	37202310	CAAAAAAAAAAAAAAAA/CAAAAAAAAAAAAA	AGAGTTTTGATTTGCACCCG	AACCGTGACAAGGACACACA	60.1	424	INTERGENIC	--	--	--
Ca4_P4_37202681	Ca4	37202681	TACA/TA	TGTGTGTCCTTGTCACGGTT	GGGATCGATTTTCAAACAAAA	60.0	335	INTERGENIC	--	--	--
Ca4_P4_37203651	Ca4	37203651	ATTT/ATT	TGATTCTGCATTGCTTCTGC	ATGGCAAGTGCAAGTCAACA	60.1	504	INTERGENIC	--	--	--
Ca4_P4_37204010	Ca4	37204010	A/AAG	TGCCATGTACAGAGGACACAA	TAGTGAAAAGGCATGTGCAA	60.2	458	INTERGENIC	--	--	--
Ca4_P4_37221016	Ca4	37221016	A/AG	TGGTTGTCGACTTGTCTTGC	ATGCGGTAGCTTTTGATTCG	59.9	468	INTERGENIC	--	--	--
Ca4_P4_37221300	Ca4	37221300	GAAAAAA/GAAAAAAA	CGAATCAAAAGCTACCGCAT	TGCAACAATTTTCACATAGCG	60.2	150	INTERGENIC	--	--	--

INDEL marker IDs	Chromosomes/ unanchored scaffolds	Physical positions (bp)	InDels (*Kabuli* reference genome-CDC Frontier/PII)	Forward primers (5'-3')	Reverse primers (5'-3')	Anneling temperature (0C)	Expected amplified product size (bp)	Structural annotation		Functional annotation	
								Sequence components of *kabuli* genome	*Kabuli* gene accession IDs	NCBI-KOG	TFs
Ca4_P4_37245007	Ca4	37245007	GTAGCATA/GTA	AAGCGAGAGAGATTTGGGACT	TTTTCATCCCCCATTTTGAA	59.5	569	INTERGENIC	--	--	--
Ca4_P4_37249911	Ca4	37249911	GAAAAA/GAAAAAA	TCGTCGATGAGATCAAATATGC	GGCACAAAAGAGCTGGTCAT	60.1	251	INTERGENIC	--	--	--
Ca4_P4_37270731	Ca4	37270731	G/GTA	GCGGGGATTGAGGAATAAAT	CCATTTCCTTAAACACTGCCA	60.1	682	INTERGENIC	--	--	--
Ca4_P4_37283555	Ca4	37283555	GT/GTT	TGAATGAAATATTGGATACGTTTATGA	GCAGGGCATTTGTCTCTAGC	59.9	530	INTERGENIC	--	--	--
Ca4_P4_37293607	Ca4	37293607	GTATAT/GTATATAT	TCGAACGACAATTATGCTGC	TACGAAGTTGACGCCACAAA	59.8	259	INTERGENIC	--	--	--
Ca4_P4_37305722	Ca4	37305722	TGGG/TGGGG	TGCTGCAAAATATCAACGTACA	CTTGTTCACGTGGGACTTCA	59.3	634	INTERGENIC	--	--	--
Ca4_P4_37306375	Ca4	37306375	CTTT/CCTTTT	TCAATGAAGTCCCACGTGAA	TGCAGTCGAAACCAAAAGTG	60.1	381	INTERGENIC	--	--	--
Ca4_P4_37308252	Ca4	37308252	ATT/A	TCGCAAACAACCATTTACCA	TGCATGCAGAAAAGACACAA	60.0	547	INTERGENIC	--	--	--
Ca4_P4_37308462	Ca4	37308462	GAGTA/GA	TTTTCTGCATGCAATATTTGG	TGCAACCACATTCAACGACT	58.7	658	INTERGENIC	--	--	--
Ca4_P4_37331887	Ca4	37331887	CAA/CAAAA	TGACCGACGACTACAATTTCA	AATGGATCACAAAAATCATGTGT	59.2	782	INTERGENIC	--	--	--

INDEL marker IDs	Chromosomes/ unanchored scaffolds	Physical positions (bp)	InDels (*Kabuli* reference genome-CDC Frontier/PII)	Forward primers (5'-3')	Reverse primers (5'-3')	Anneling temperature (0C)	Expected amplified product size (bp)	Structural annotation		Functional annotation	
								Sequence components of *kabuli* genome	*Kabuli* gene accession IDs	NCBI-KOG	TFs
Ca4_P4_37332359	Ca4	37332359	TC/T	CACACATGATTTTTGTGATCCAT	ATTGGGCGATCAGCAAATAG	59.6	243	INTERGENIC	--	--	--
Ca4_P4_37332662	Ca4	37332662	TAA/TA	TCTGTAATCGGTTTGGGCTC	TCGACCAATGAACACGCTAA	60.1	520	INTERGENIC	--	--	--
Ca4_P4_37344294	Ca4	37344294	AATTTATT/AATTTATTTATT	TGTTGAATGTGTGTTGCTTCTATG	GAACCGGCTCAACGTGTTAT	60.1	569	INTERGENIC	--	--	--
Ca4_P4_37344783	Ca4	37344783	TATTTAA/TA	AGCCGGTTCTGACCATAAGA	GGTGCAAGTCCTAAAAGCAAA	59.7	513	INTERGENIC	--	--	--
Ca4_P4_37346206	Ca4	37346206	TTTTATTTATTTATTT/TTTTATTTATTTATTTATTT	GCCCCAACGTGAACATTATT	TCAATAATGCTGCCATCCAA	59.7	208	INTERGENIC	--	--	--
Ca4_P4_37390225	Ca4	37390225	TC/T	CGCATCCATTAAAGACGGTT	ATTTCTTTGGCGGAATGATG	60.0	697	INTERGENIC	--	--	--
Ca4_P4_3739769	Ca4	3739769	TACCTCCGGAGGAAACCTCCGGAGGAAACCTCCGGAGGAA/TACCTCCGGAGGAAACCTCCGGAGGAA	GTTCCAAATGCGGTTGACTT	CGAACGTTAACTCGTCGTCA	60.0	906	INTERGENIC	--	--	--
Ca4_P4_37403650	Ca4	37403650	CTTTT/CTTTTT	CTTATCCAAAAGCGAGGCAC	TGAGTTGGACTACAAGCCATCA	59.8	597	INTERGENIC	--	--	--
Ca4_P4_37403954	Ca4	37403954	AC/A	TGATGGCTTGTAGTCCAACTC	TTCTCTCAGCCAATTACACCC	57.8	456	INTERGENIC	--	--	--
Ca4_P4_37406028	Ca4	37406028	CGG/C	ATTCATCCCGACAAAGATCG	CTGTAATCCACAATGCCGTG	59.9	660	INTERGENIC	--	--	--

INDEL marker IDs	Chromosomes/ unanchored scaffolds	Physical positions (bp)	InDels (*Kabuli* reference genome-CDC Frontier/PII)	Forward primers (5'-3')	Reverse primers (5'-3')	Anneling temperature (0C)	Expected amplified product size (bp)	Structural annotation		Functional annotation	
								Sequence components of *kabuli* genome	*Kabuli* gene accession IDs	NCBI-KOG	TFs
Ca4_P4_37443931	Ca4	37443931	CT/CTAT	CCGCATTTTCTTCATTAGGG	ACGTTAAGGCATGAGTTGGG	59.5	364	INTERGENIC	--	--	--
Ca4_P4_37480497	Ca4	37480497	CAAA/CA	GATCTTCCTTCAATGAGATACCAGA	TTGTTGGCGAAACAAACAAA	60.0	470	INTERGENIC	--	--	--
Ca4_P4_37483206	Ca4	37483206	TAAAAAAAAAAAAAA/TAAAAAAAAAAA	AAATTGGGTGATGTGGTGGT	CCTAGAAGATGAGGGTGCCA	60.0	424	INTERGENIC	--	--	--
Ca4_P4_37508257	Ca4	37508257	ATTTTTTTTT/ATTTTTTTTTT	GGAGCTTTATGTTTAGATGCATTG	TCGTATCTCCACGATAAGACCA	59.2	707	INTERGENIC	--	--	--
Ca4_P4_37540554	Ca4	37540554	ATAATTAATTAATTAATTA/ATAATTAATTAATTAATTAATTA	CGTGCAACCATCAAATACAGA	TGACTGAGTCCACTGCCTTTT	59.6	671	INTERGENIC	--	--	--
Ca4_P4_37608001	Ca4	37608001	ACTCTCTCTCTCTCTC/ACTCTCTCTCTCTCTCTC	TTGGATTCCTGACACACACA	TAAAAATTTGGAGGGAGGGG	58.4	568	INTERGENIC	--	--	--
Ca4_P4_37618447	Ca4	37618447	ATAT/A	CATATGCCCATGTTGGTTTG	TTTCACACATCATCTTTCACGA	59.7	785	INTERGENIC	--	--	--
Ca4_P4_37671559	Ca4	37671559	CAA/CA	TTTCCACCCAGAAAGCAATC	GCAAAACAGAGGCAACATCA	60.1	250	INTERGENIC	--	--	--
Ca4_P4_37695722	Ca4	37695722	TTGTGTGTGTGTGT/TTGTGTGTGTGT	TCGATCCTCACTCTCAACCA	TTTTCAATGCATCTTCAGCG	59.3	398	INTERGENIC	--	--	--
Ca4_P4_37696000	Ca4	37696000	TTGGCAATGGCA/TTGGCA	CGCTGAAGATGCATTGAAAA	CGGATGAATCAAAACAATCCA	60.0	273	INTERGENIC	--	--	--

INDEL marker IDs	Chromosomes/ unanchored scaffolds	Physical positions (bp)	InDels (*Kabuli* reference genome-CDC Frontier/PII)	Forward primers (5'-3')	Reverse primers (5'-3')	Anneling temperature (0C)	Expected amplified product size (bp)	Structural annotation		Functional annotation	
								Sequence components of *kabuli* genome	*Kabuli* gene accession IDs	NCBI-KOG	TFs
Ca4_P4_37696707	Ca4	37696707	TA/TATAGACATAA	AATGTTTTTGGTAATCCCGGT	AACATTGGTGGTGCAAGACA	59.5	607	INTERGENIC	--	--	--
Ca4_P4_37703958	Ca4	37703958	C/CAAG	TAGTGGGGAAGGTCTGTTGG	TGTTGCTGCGTCTTGAAATC	60.0	733	INTERGENIC	--	--	--
Ca4_P4_37704077	Ca4	37704077	AG/AAGAAGTCG	TAGTGGGGAAGGTCTGTTGG	TGTTGCTGCGTCTTGAAATC	60.0	733	INTERGENIC	--	--	--
Ca4_P4_37704228	Ca4	37704228	T/TG	AGGAGGGATCAAACCGAACT	TGTTGCTGCGTCTTGAAATC	59.9	472	INTERGENIC	--	--	--
Ca4_P4_37704299	Ca4	37704299	T/TCAAA	AGGAGGGATCAAACCGAACT	TGTTGCTGCGTCTTGAAATC	59.9	472	INTERGENIC	--	--	--
Ca4_P4_37707068	Ca4	37707068	C/CTGGCAAGGAGCTACT	TGAGGCCTTAGCTTCGTGAT	CACAAGGACCTGACGGTTTT	60.0	555	INTERGENIC	--	--	--
Ca4_P4_37707135	Ca4	37707135	C/CGCCCCTCCCC	TGAGGCCTTAGCTTCGTGAT	CACAAGGACCTGACGGTTTT	60.0	555	INTERGENIC	--	--	--
Ca4_P4_37707688	Ca4	37707688	T/TTC	AAAACCGTCAGGTCCTTGTG	TGAGCGTTTTCTCGGTCTTT	60.0	386	INTERGENIC	--	--	--
Ca4_P4_37710631	Ca4	37710631	CTTTT/CTTTTT	ACGTGTTGAGCTAACCCTGC	TCTACCTGCCATTTCCGATT	60.3	275	INTERGENIC	--	--	--
Ca4_P4_37746382	Ca4	37746382	GTTTTT/GTTTT	TTGCAATAGGCATAGGACCC	TTGTTTCTCCCCAGTTTTGG	59.9	448	INTERGENIC	--	--	--

INDEL marker IDs	Chromosomes/ unanchored scaffolds	Physical positions (bp)	InDels (*Kabuli* reference genome-CDC Frontier/PII)	Forward primers (5'-3')	Reverse primers (5'-3')	Anneling temperature (0C)	Expected amplified product size (bp)	Structural annotation		Functional annotation	
								Sequence components of *kabuli* genome	*Kabuli* gene accession IDs	NCBI-KOG	TFs
Ca4_P4_37751701	Ca4	37751701	TTCTCTCTCTCTCTCTCT/TTCTCTCTCTCTCTCT	GCGTGGAGTGAAAGAGGAAG	AGGAAAATCACAATGCAGCC	60.0	513	INTERGENIC	--	--	--
Ca4_P4_37751797	Ca4	37751797	ATT/ATTT	GCGTGGAGTGAAAGAGGAAG	AGGAAAATCACAATGCAGCC	60.0	513	INTERGENIC	--	--	--
Ca4_P4_37760565	Ca4	37760565	ACAAC/A	AAACCAAAAGAACACCGCAC	CCGAACCGGTTTTCAGTTTA	60.0	485	INTERGENIC	--	--	--
Ca4_P4_37788269	Ca4	37788269	TT/TTAT	AAAATTGGTCCCAAGACTTCAA	TGTTTGTTGTTCATCGGCAT	59.9	246	INTERGENIC	--	--	--
Ca4_P4_3779072	Ca4	3779072	ATT/ATTTT	TTCCTAACAAACGTGTTGATGC	ACGTGCCTTTCGATTGTTTC	60.0	672	INTERGENIC	--	--	--
Ca4_P4_37817066	Ca4	37817066	AATCTCTCTCTAT/AATCTCTCTCTATCTCTCTCTAT	CCCTTCAAAAGGTGGTTCAA	ACAACACTCACCCTCAAGCC	59.9	816	INTERGENIC	--	--	--
Ca4_P4_37850626	Ca4	37850626	TG/T	CTGTATTTGCTCGCATCCAA	TGTCCCTCTCTTGGAATTTTG	59.8	722	INTERGENIC	--	--	--
Ca4_P4_37871651	Ca4	37871651	ATTTTTT/ATTTTT	ACCCTACCCATTGTCAACCA	TGGATGGTTATGATTTGCTGA	60.1	668	INTERGENIC	--	--	--
Ca4_P4_37872697	Ca4	37872697	CAAA/CAAAAA	TGATTTGGTTTGAGTATTATGCG	TTCCTATTCTCAAAGGCGGA	59.1	672	INTERGENIC	--	--	--
Ca4_P4_37881000	Ca4	37881000	ATTT/ATT	ACTTCGGGAAGAACTCGTGA	CATCGTGTTTCTTGTGTGGG	59.8	172	INTERGENIC	--	--	--

INDEL marker IDs	Chromosomes/ unanchored scaffolds	Physical positions (bp)	InDels (*Kabuli* reference genome-CDC Frontier/PII)	Forward primers (5'-3')	Reverse primers (5'-3')	Anneling temperature (0C)	Expected amplified product size (bp)	Structural annotation		Functional annotation	
								Sequence components of *kabuli* genome	*Kabuli* gene accession IDs	NCBI-KOG	TFs
Ca4_P4_37891409	Ca4	37891409	TA/TAAAAACA	GGATGTCACACAGGCTTTCA	AGTCCCCATGTAATTGTCACG	59.7	764	INTERGENIC	--	--	--
Ca4_P4_37895622	Ca4	37895622	TATAGGAGATAGGAGAT/TATAGGAGAT	GCCAAACTTTGTCATTGTATATGG	TGTTAGACGGAGAACATGCG	59.7	415	INTERGENIC	--	--	--
Ca4_P4_37909050	Ca4	37909050	GTTTTTTT/GTTTTTTTTTT	TGCATTTTAGGTCCATGATGA	ATTTGACCACCGAAAGATGC	59.0	190	INTERGENIC	--	--	--
Ca4_P4_37977728	Ca4	37977728	CGTGGTGGT/CGTGGT	GTTCCCAACTTGTGTGCCTT	TGCATTTCATCACAGACATTGA	60.0	543	INTERGENIC	--	--	--
Ca4_P4_37989911	Ca4	37989911	TTATATATA/TTATA	CGTTGACTCGTTAATGGAGC	TCAATTACCAAAAGGGCCAG	58.4	353	INTERGENIC	--	--	--
Ca4_P4_37992000	Ca4	37992000	TTATATATATA/TTATATA	ATCTCGAACATTTCCCACCC	AGTGGAGGATTTTCCTGGCT	61.1	669	INTERGENIC	--	--	--
Ca4_P4_37993885	Ca4	37993885	CTATATATATATATATATATATATATATATATA/CTATATATATATATATATATATATATATATA	GAAGGTCTTGCAAGTGAGTGC	CAAACGTGTGATGGATCTTCC	60.1	446	INTERGENIC	--	--	--
Ca4_P4_38015157	Ca4	38015157	A/AG	CAACGATGCTACTTACAAACGG	TCATTTCATTGAGGTGGCAA	59.7	723	INTERGENIC	--	--	--
Ca4_P4_38017134	Ca4	38017134	AAAACTTATAAA/AAAA	TGACAATCAAAGTACAATGAGTCAAA	AAAAGTTGTTGTGGCATCGG	60.0	673	INTERGENIC	--	--	--
Ca4_P4_38024785	Ca4	38024785	GTATATATATATATATATATATATATA/GTATATATATATATATATATATATA	AAGTGAAAACCCCAAAGGCT	GCATGTGCCTCATTTTGTTG	60.0	230	INTERGENIC	--	--	--

INDEL marker IDs	Chromosomes/ unanchored scaffolds	Physical positions (bp)	InDels (*Kabuli* reference genome-CDC Frontier/PII)	Forward primers (5'-3')	Reverse primers (5'-3')	Anneling temperature (0C)	Expected amplified product size (bp)	Structural annotation		Functional annotation	
								Sequence components of *kabuli* genome	*Kabuli* gene accession IDs	NCBI-KOG	TFs
Ca4_P4_38040589	Ca4	38040589	CTAATA/CTAATATAATA	CAAACAAATATGTGCCGTGC	TGGTGGTTGCCTTTCCTATC	60.0	734	INTERGENIC	--	--	--
Ca4_P4_38074525	Ca4	38074525	AC/A	TCCTTAAGCGTTGGTTTGCT	GAGTGGGGCATGTTGGTACT	59.9	470	INTERGENIC	--	--	--
Ca4_P4_38085487	Ca4	38085487	AT/A	TCCCCTTTCTTACGAATCCC	CACTTGGAAGAGGGCAACAT	60.3	476	INTERGENIC	--	--	--
Ca4_P4_38108114	Ca4	38108114	ATC/A	TCCACAACACACACAAAAAGG	AATGCTATTGGGGCAGTTCA	59.5	742	INTERGENIC	--	--	--
Ca4_P4_38108391	Ca4	38108391	CATATATATATATATATATATATATA/CATATATATATATATATATATATATATATATATA	GGAAGAATGAAAATTGCAGAAAA	CAATGCTATTGGGGCAGTTC	59.6	311	INTERGENIC	--	--	--
Ca4_P4_38123299	Ca4	38123299	TGTCTTC/T	ACGATGAGAATCGCATTTCC	CCATTGCAAAACCAAGCATA	60.0	690	INTERGENIC	--	--	--
Ca4_P4_38124061	Ca4	38124061	AT/ATT	TTCACATTTTCATGGCCTCA	TGCTACCAAGGGAGCAAAAT	60.0	410	INTERGENIC	--	--	--
Ca4_P4_38137988	Ca4	38137988	TG/T	TCGAGACCCAATATGCAAGA	GCTCTTCCGGACTTTTCAAT	59.2	455	INTERGENIC	--	--	--
Ca4_P4_38139279	Ca4	38139279	CTA/CTATA	GTCGGAGGTGGTGATGAGTT	TCAAGGTAATCCATCTAGTCGC	60.0	686	INTERGENIC	--	--	--
Ca4_P4_38169564	Ca4	38169564	ATCTCT/ATCT	CCCAAAGGATATGCGTGAAT	ACGATCTTGTCCTGCAAAGC	59.8	504	INTERGENIC	--	--	--

INDEL marker IDs	Chromosomes/ unanchored scaffolds	Physical positions (bp)	InDels (*Kabuli* reference genome-CDC Frontier/PII)	Forward primers (5'-3')	Reverse primers (5'-3')	Anneling temperature (0C)	Expected amplified product size (bp)	Structural annotation		Functional annotation	
								Sequence components of *kabuli* genome	*Kabuli* gene accession IDs	NCBI-KOG	TFs
Ca4_P4_38204868	Ca4	38204868	AATATA/AATA	CTGGTATCGTAGAAGGAAGGAA	GGCGGCGCTATTTATATTGA	57.5	501	INTERGENIC	--	--	--
Ca4_P4_38284143	Ca4	38284143	GAA/GA	AGACAATGTACGGTGCCTCC	TGATTTGGTTAAAGTGGATGTTG	60.0	602	INTERGENIC	--	--	--
Ca4_P4_38297090	Ca4	38297090	CAA/CAAA	TGATCTGTTTGATGACCAAGATAAA	AAAAACAAAATCATGACTAACCAAA	59.9	519	INTERGENIC	--	--	--
Ca4_P4_38297845	Ca4	38297845	GACCCTTAAAGTAC/GAC	AAAGGTTTCCAGTCCACATTTT	CATGATTCTTCGGTGTCGTG	58.9	425	INTERGENIC	--	--	--
Ca4_P4_38311670	Ca4	38311670	TAA/TA	TGTAGGAACAAGGGCCAAAG	AGATCATGGCTATTCACCCAA	60.1	560	INTERGENIC	--	--	--
Ca4_P4_38317091	Ca4	38317091	AAA/AAACAA	TTTTAAATTTTCCGAAACACAATG	TCCAAACACACTTGATCCCA	59.4	211	INTERGENIC	--	--	--
Ca4_P4_38317513	Ca4	38317513	GTATATAAATATA/GTATA	TGGGATCAAGTGTGTTTGGA	GGTTGCTCACTCCTTTGGAC	59.9	575	INTERGENIC	--	--	--
Ca4_P4_3832898	Ca4	3832898	ATTT/ATTTTTT	CGGAGGGTACGAACCTGTAA	ACTGTTGTGGAAATCTGGGC	60.0	484	INTERGENIC	--	--	--
Ca4_P4_38354604	Ca4	38354604	AT/ATT	CAATTTTTCCTCCGAACAGAA	CAGGGACATTTAATTGAACTTGA	59.2	690	INTERGENIC	--	--	--
Ca4_P4_38368647	Ca4	38368647	A/ATTCTACTTTCAATT	TATGGTTGGATTTTCCGCTC	TTGTCTTCCCCGTTTAGTGG	59.9	751	INTERGENIC	--	--	--

INDEL marker IDs	Chromosomes/ unanchored scaffolds	Physical positions (bp)	InDels (*Kabuli* reference genome-CDC Frontier/PII)	Forward primers (5'-3')	Reverse primers (5'-3')	Anneling temperature (0C)	Expected amplified product size (bp)	Structural annotation		Functional annotation	
								Sequence components of *kabuli* genome	*Kabuli* gene accession IDs	NCBI-KOG	TFs
Ca4_P4_38376891	Ca4	38376891	ATT/AT	CCTAGAAAAAGGGGCTGATG	TTTTACGGTGCATCACAATCA	58.8	435	INTERGENIC	--	--	--
Ca4_P4_38377327	Ca4	38377327	TTT/TTTGAATT	TGATTGTGATGCACCGTAAAA	AGCGAAAGTTCTTGGAGGGT	60.0	470	INTERGENIC	--	--	--
Ca4_P4_38378071	Ca4	38378071	TATATA/T	TTCTCATTCAACAACGGTGG	CTTTCTGTGCTGTGCTTTGG	59.5	342	INTERGENIC	--	--	--
Ca4_P4_38379738	Ca4	38379738	ATTTAAATTAT/ATTTAAATTATTTTAAATTAT	AATTTCATCCACATCTTCAAAAA	CTCCGTGTTAAGCCGAAAAA	57.7	712	INTERGENIC	--	--	--
Ca4_P4_38457252	Ca4	38457252	CTGTGT/CTGT	CGATCACCAAGTCCCCTAAA	CCACTCCCTCCAATCTTCAA	59.9	344	INTERGENIC	--	--	--
Ca4_P4_38479454	Ca4	38479454	CAAAA/CAAA	GGTGGGATCCATTTGAGTTG	CCGAAGGCTTTCTTCAACAG	60.2	676	INTERGENIC	--	--	--
Ca4_P4_38480745	Ca4	38480745	TGG/TG	GTGGAGCTTCTTTGGGTGAC	ATCACCACCGAAAAACTTGG	59.7	844	INTERGENIC	--	--	--
Ca4_P4_38482887	Ca4	38482887	CAAAAA/CAAAA	CAAATGAGAGCGTTAAGGCAG	TGTCCTTCAATTTCATTTCCAA	60.0	501	INTERGENIC	--	--	--
Ca4_P4_38482961	Ca4	38482961	A/AC	TTGGAAATGAAATTGAAGGACA	TTGTTGATTTCTAACCTTGCTCA	59.4	193	INTERGENIC	--	--	--
Ca4_P4_38483289	Ca4	38483289	AATATATATA/AATATATATATA	TTGGAAATGAAATTGAAGGACA	TTGGTTTCCTTTGCACACTG	59.4	636	INTERGENIC	--	--	--

INDEL marker IDs	Chromosomes/ unanchored scaffolds	Physical positions (bp)	InDels (*Kabuli* reference genome-CDC Frontier/PII)	Forward primers (5'-3')	Reverse primers (5'-3')	Anneling temperature (0C)	Expected amplified product size (bp)	Structural annotation		Functional annotation	
								Sequence components of *kabuli* genome	*Kabuli* gene accession IDs	NCBI-KOG	TFs
Ca4_P4_38483484	Ca4	38483484	AATA/AATATATA	TGAGCAAGGTTAGAAATCAACAA	ACCATTGGTTTCCTTTGCAC	58.9	470	INTERGENIC	--	--	--
Ca4_P4_38567221	Ca4	38567221	GAATAAAA/GAA	GACAAAAATGAGAAAGACCTGGA	AAATGCTGCCGAAGAAGCTA	59.6	824	INTERGENIC	--	--	--
Ca4_P4_38568371	Ca4	38568371	TAAAAAAAAAAAA/TAAAAAAAA	TCTCGACTTTTAAGCAAGCCA	TGTCCATGAAGAAAGAGCCC	60.1	741	INTERGENIC	--	--	--
Ca4_P4_38568495	Ca4	38568495	ATATT/ATATTATT	CGAAATTGACAAAATCACAATCA	TGTCCATGAAGAAAGAGCCC	59.9	340	INTERGENIC	--	--	--
Ca4_P4_38573939	Ca4	38573939	TAAA/TAA	TGGGAATTGGAAATTGGAAA	TCATTTGCTTTGTGAGGAACA	60.1	767	INTERGENIC	--	--	--
Ca4_P4_38585075	Ca4	38585075	ATTCTTCTTCTTCTTCTTCTTC/ATTCTTCTTCTTCTTCTTC	AGGCCCAAAATTTGTTGTTG	TCGATTTTGCAATACCACGA	59.8	457	INTERGENIC	--	--	--
Ca4_P4_38585148	Ca4	38585148	ATTTTTTTTT/ATTTTTTT	AGGCCCAAAATTTGTTGTTG	GGGAGTGGTGTGTTACATCG	59.8	587	INTERGENIC	--	--	--
Ca4_P4_38604351	Ca4	38604351	TA/TAA	GCGAACGTATTGGACTGGTT	GAAACCCAATTTCTCCGTGA	60.0	619	INTERGENIC	--	--	--
Ca4_P4_38615844	Ca4	38615844	TAAGAAGA/TAAGA	GGTGAGCCACGGAAAAATAA	TTTGGAGACACCAAGCCTCT	59.9	568	INTERGENIC	--	--	--
Ca4_P4_38618852	Ca4	38618852	CATAA/CA	GCCGACAGAGAAAATCCAAA	ATTGGGGAAGGCCTTGATAA	60.2	532	INTERGENIC	--	--	--

INDEL marker IDs	Chromosomes/ unanchored scaffolds	Physical positions (bp)	InDels (*Kabuli* reference genome-CDC Frontier/PII)	Forward primers (5'-3')	Reverse primers (5'-3')	Anneling temperature (0C)	Expected amplified product size (bp)	Structural annotation		Functional annotation	
								Sequence components of *kabuli* genome	*Kabuli* gene accession IDs	NCBI-KOG	TFs
Ca4_P4_38619240	Ca4	38619240	TAAA/TAAAA	TTATCAAGGCCTTCCCCAAT	CCAGCAGGTTTCATTTGGTT	60.6	545	INTERGENIC	--	--	--
Ca4_P4_38626043	Ca4	38626043	AATTATTATT/AATTATT	TTGATTCTCTTTGTGTGTGTGCT	GACCGCAGATGTAATTTAGCC	59.8	447	INTERGENIC	--	--	--
Ca4_P4_38644984	Ca4	38644984	GTTT/GTT	TAGAGGCCCAACAACGTAGC	ACCTCGACAACCACTTGCTC	60.3	635	INTERGENIC	--	--	--
Ca4_P4_38648334	Ca4	38648334	GTACAA/GTACAATACAA	AACGAAAACGAGGAGAGCAA	GAGACACGGTTTTTGGCAAT	60.0	258	INTERGENIC	--	--	--
Ca4_P4_38649330	Ca4	38649330	AG/AGG	TGTGCTGCATTCCAACTCTC	CGATGATTAATCCCTGCCAA	60.0	327	INTERGENIC	--	--	--
Ca4_P4_38656963	Ca4	38656963	TAAAA/TAAAAA	TTGACTTGCATTGATGTGCC	TGGTGAAAATCGACGAATCA	60.7	440	INTERGENIC	--	--	--
Ca4_P4_38668986	Ca4	38668986	CCA/CCACA	AAAAACCAACATTGGCAAGC	GCTGTCCCTGAAGCAAGTTC	60.0	307	INTERGENIC	--	--	--
Ca4_P4_38682900	Ca4	38682900	TACAAACATAA/TA	TTTAAATTTGAATTGTCCCCG	GTCATTTCGCTTGCACTTGA	58.8	435	INTERGENIC	--	--	--
Ca4_P4_38686412	Ca4	38686412	AA/AAGGTGGAAGA	AGGTCAAGCACCCACTTCAG	TTCACAATCTTCGGGTCCTC	60.3	323	INTERGENIC	--	--	--
Ca4_P4_38691498	Ca4	38691498	CA/CATGTGACTA	TTGGCCCTATTCGAAAGATG	ATGGTGCACACCCCCTACTA	60.0	568	INTERGENIC	--	--	--

INDEL marker IDs	Chromosomes/ unanchored scaffolds	Physical positions (bp)	InDels (*Kabuli* reference genome-CDC Frontier/PII)	Forward primers (5'-3')	Reverse primers (5'-3')	Anneling temperature (0C)	Expected amplified product size (bp)	Structural annotation		Functional annotation	
								Sequence components of *kabuli* genome	*Kabuli* gene accession IDs	NCBI-KOG	TFs
Ca4_P4_38705713	Ca4	38705713	ATTTTTTT/ATTTTTTTTT	TTGGGTACAACTCCGTCACA	TCAAAATCTTCCTTCAATGCAA	60.0	715	INTERGENIC	--	--	--
Ca4_P4_38710772	Ca4	38710772	TCACAAAA/TCACAAAAAATAAAATACACAAAA	TGGTGACACAAATGGCAAAC	TCCCTTTCAATTGCCAAATC	60.4	309	INTERGENIC	--	--	--
Ca4_P4_38714491	Ca4	38714491	TTTGAACT/TTTGAACTTGAACT	AAGGAGAAACCAAAAATGTCAA	TTTTTCGTTTTGAATGGAAGATT	57.8	444	INTERGENIC	--	--	--
Ca4_P4_38726989	Ca4	38726989	TA/T	TACCTGACTACCCCACTGGC	TGTTTACAATTTGGACGAGCA	60.0	501	INTERGENIC	--	--	--
Ca4_P4_38733548	Ca4	38733548	TT/TTTAACTTCGTGT	TTTGTCCTGCCTAACTCGCT	CGCTAGATGCAAATGTGGAA	60.0	360	INTERGENIC	--	--	--
Ca4_P4_38734510	Ca4	38734510	T/TTCATGCACCTAAGA	GAGACCAAATCTCATCCCCA	AAGTTGGAAAAATCGGAGGT	59.9	180	INTERGENIC	--	--	--
Ca4_P4_38739330	Ca4	38739330	CATA/CA	ACTTCAACATCCAACTCCCG	GGAAAAATGAGAGAAGGGGC	60.0	112	INTERGENIC	--	--	--
Ca4_P4_38742074	Ca4	38742074	TTATATATATATATATAT/TTATATATATATATAT	GCCATAAACACAACCCTTTCTT	AAAATTTCAGCGAGATTCTTTG	59.4	954	INTERGENIC	--	--	--
Ca4_P4_38743790	Ca4	38743790	ACC/AC	TTCCCATAAATGGAATGCAA	GCCAACCCTATGTGCATTTT	58.8	205	INTERGENIC	--	--	--
Ca4_P4_38744817	Ca4	38744817	CAAAAA/CAAAA	GAAGCACCATCGACCACTTT	TGGTGGCACTAGGTGTATTGA	60.1	194	INTERGENIC	--	--	--

INDEL marker IDs	Chromosomes/ unanchored scaffolds	Physical positions (bp)	InDels (*Kabuli* reference genome-CDC Frontier/PII)	Forward primers (5'-3')	Reverse primers (5'-3')	Anneling temperature (0C)	Expected amplified product size (bp)	Structural annotation		Functional annotation	
								Sequence components of *kabuli* genome	*Kabuli* gene accession IDs	NCBI-KOG	TFs
Ca4_P4_38750092	Ca4	38750092	GAAA/GAAAGAGAGAAAA	GGAGATGAGGACAGGGACAA	CAAGTGGGGTGGGAGTAAGA	60.0	155	INTERGENIC	--	--	--
Ca4_P4_38772029	Ca4	38772029	GTTT/GTT	AATAGGTGGCGATGAGTTCG	GCTCTGCAATAGTTCTCCCG	60.1	490	INTERGENIC	--	--	--
Ca4_P4_38772279	Ca4	38772279	ATT/ATTT	CGGGAGAACTATTGCAGAGC	AAATGCCACGTAGGTGAAAA	60.0	159	INTERGENIC	--	--	--
Ca4_P4_38772463	Ca4	38772463	TTATATATATATATATATATATAT/TTATATATATATATATATAT	CGGGAGAACTATTGCAGAGC	CTGATAGAAGGTTGGATTTGGA	60.0	572	INTERGENIC	--	--	--
Ca4_P4_38806144	Ca4	38806144	AA/AATGTTA	TGTTGGTCTCCGTAACTTCTCA	CCATGACCCGAGTCTATTGAA	59.8	546	INTERGENIC	--	--	--
Ca4_P4_38806212	Ca4	38806212	TA/TAA	TGTTGGTCTCCGTAACTTCTCA	GGATGCCATGAATGAAGCAT	59.8	655	INTERGENIC	--	--	--
Ca4_P4_38822967	Ca4	38822967	TTATATATATATATATATATATATAT/TTATATATATATATATATATATATATATAT	TCTATGGCAACCTAAGCCGT	CATGTGCAATGTACTTGGTTCC	59.7	622	INTERGENIC	--	--	--
Ca4_P4_38830492	Ca4	38830492	A/ATTGTTG	CAACGCCACAACTCTTCTCA	AGCTCGCGTCATTTTCAACT	60.0	580	INTERGENIC	--	--	--
Ca4_P4_38859836	Ca4	38859836	GATAT/GAT	TGTTGCTTCATCTGGTTTATACG	TGATAATGGTGGAAGTGGCA	59.2	299	INTERGENIC	--	--	--
Ca4_P4_38874560	Ca4	38874560	ATTATTTAAAAT/ATTATTTAAAATTTATTTAAAAT	TGATTCATTACACCGCGAAA	CCAAGCATGAACCTACTCCC	60.1	412	INTERGENIC	--	--	--

INDEL marker IDs	Chromosomes/ unanchored scaffolds	Physical positions (bp)	InDels (*Kabuli* reference genome-CDC Frontier/PII)	Forward primers (5'-3')	Reverse primers (5'-3')	Anneling temperature (0C)	Expected amplified product size (bp)	Structural annotation		Functional annotation	
								Sequence components of *kabuli* genome	*Kabuli* gene accession IDs	NCBI-KOG	TFs
Ca4_P4_38885489	Ca4	38885489	ATT/AT	CATAGCATAGGGTGGTGCCT	GCACGCAATTCCTCCATATT	60.0	557	INTERGENIC	--	--	--
Ca4_P4_38886119	Ca4	38886119	ATT/ATTTT	ATTGGTTACGCGACCAACTC	AAATTTTGAGCGGTTGATGC	60.0	449	INTERGENIC	--	--	--
Ca4_P4_38886399	Ca4	38886399	AATATATATATATATATATA/AATATATATATATATATATATATA	TATTGCATCAACCGCTCAAA	TACACATGCATAGGTGGCCT	60.2	416	INTERGENIC	--	--	--
Ca4_P4_38887313	Ca4	38887313	CG/C	TCATTGCTATGACCCCAGTTT	GGGGAGGATCACGGACTATT	59.4	470	INTERGENIC	--	--	--
Ca4_P4_38887574	Ca4	38887574	AAAATAAA/AAAA	AATAGTCCGTGATCCTCCCC	TTTGTTTGTTTCCCCAATCA	60.2	240	INTERGENIC	--	--	--
Ca4_P4_38895087	Ca4	38895087	AT/ATT	CACGGATGTAATTTAGCCGAA	TGATGGACCTGGGTTTTGAT	60.0	654	INTERGENIC	--	--	--
Ca4_P4_38895946	Ca4	38895946	AGTGT/AGT	TGCAATCGATCTAGTTTAAGCC	GCATAAAGATGGTGGTGCCT	58.5	549	INTERGENIC	--	--	--
Ca4_P4_38915071	Ca4	38915071	AAGAGAGAGA/AAGAGAGA	ATCTCTTCATTCACCACCCG	CCCTCATTGAAAGTGCCATT	59.9	155	INTERGENIC	--	--	--
Ca4_P4_38937830	Ca4	38937830	ATTT/ATT	GTTTTTCTCAAAAATTTCCCAA	TCAAAACAAGATTACGGGGC	57.4	377	INTERGENIC	--	--	--
Ca4_P4_38943074	Ca4	38943074	ATTTTTTTTT/ATTTTTTTT	ATGCGGGTATGGAGGTGATA	AGAAAAGGGAGAAGATCGGC	60.2	604	INTERGENIC	--	--	--

INDEL marker IDs	Chromosomes/ unanchored scaffolds	Physical positions (bp)	InDels (*Kabuli* reference genome-CDC Frontier/PII)	Forward primers (5'-3')	Reverse primers (5'-3')	Anneling temperature (0C)	Expected amplified product size (bp)	Structural annotation		Functional annotation	
								Sequence components of *kabuli* genome	*Kabuli* gene accession IDs	NCBI-KOG	TFs
Ca4_P4_38944216	Ca4	38944216	AACGTG/A	TGATTCCGTGCATGTGTTTT	CAAACATCCATCATTCTCCCA	60.0	246	INTERGENIC	--	--	--
Ca4_P4_38949129	Ca4	38949129	GTGGTTATGGT/GTGGTTATGGTTATGGT	GAAGCGACGGTCTCAGTTTC	CGCTTCGCATACTGATTTGA	60.0	979	INTERGENIC	--	--	--
Ca4_P4_38949892	Ca4	38949892	GT/G	TCAAATCAGTATGCGAAGCG	ACCCACACAACACACATGCT	60.0	360	INTERGENIC	--	--	--
Ca4_P4_38950263	Ca4	38950263	CA/CAA	AGCATGTGTGTTGTGTGGGT	TTAACATCCATGAAATCGCAA	59.9	743	INTERGENIC	--	--	--
Ca4_P4_38955357	Ca4	38955357	GATAAAATA/GATA	GAGTTTTGGGGTGACATGCT	CATCCTGCACAATGCTGTTC	60.0	251	INTERGENIC	--	--	--
Ca4_P4_38970599	Ca4	38970599	CCTT/C	AGCGTACGAGAAGGAGTCCA	ACCAAAATGGCAATGAGGAC	60.0	510	INTERGENIC	--	--	--
Ca4_P4_38987085	Ca4	38987085	GTTTTTT/GTTTTTTTT	GAGCGGCTAAATGGATCAAA	AAAGTTTGGAGGGGAGGAAA	60.2	617	INTERGENIC	--	--	--
Ca4_P4_38994620	Ca4	38994620	ATGTATT/ATGTATTGTATT	GCATACCCTTTTCCGGTCTT	GCATGCTCTAATCCATTCCAA	60.3	769	INTERGENIC	--	--	--
Ca4_P4_38997590	Ca4	38997590	TCAA/T	ACCCACCACTGATTCCATGT	TGTCTTCAACTAGCACTCGGG	60.1	480	INTERGENIC	--	--	--
Ca4_P4_39001801	Ca4	39001801	AG/A	TTGGGTCTGTAAATTGCAAGC	TTCAAATGCAAAACGAATGAA	60.1	664	INTERGENIC	--	--	--

INDEL marker IDs	Chromosomes/ unanchored scaffolds	Physical positions (bp)	InDels (*Kabuli* reference genome-CDC Frontier/PII)	Forward primers (5'-3')	Reverse primers (5'-3')	Anneling temperature (0C)	Expected amplified product size (bp)	Structural annotation		Functional annotation	
								Sequence components of *kabuli* genome	*Kabuli* gene accession IDs	NCBI-KOG	TFs
Ca4_P4_39002456	Ca4	39002456	ATT/AT	TTCATTCGTTTTGCATTTGAA	TTTGACGGAAGTTTTCCACTTT	59.2	611	INTERGENIC	--	--	--
Ca4_P4_39013296	Ca4	39013296	GTATATATATATAT/GTATATATATAT	ACCAACGGAGAGTTTGCCTA	TTCACTTCGACAGCAAAACG	59.7	613	INTERGENIC	--	--	--
Ca4_P4_39020294	Ca4	39020294	GTCACGA/GTCACGACTCACGA	ACTCCGTGCAAGGAATTTGA	TTTGCCACATTTGAGGAAATAG	60.6	278	INTERGENIC	--	--	--
Ca4_P4_39020809	Ca4	39020809	TAAAA/TAAAAA	TCCCAATTACCAATAACAAATGC	CCCCAATGTATTGGAACCAC	60.0	643	INTERGENIC	--	--	--
Ca4_P4_39027557	Ca4	39027557	CCAAACA/CCA	CGGTGTTTTGAGTGAGAGAGTT	CAGCCACCAAACAAACATTG	58.5	360	INTERGENIC	--	--	--
Ca4_P4_39062674	Ca4	39062674	TA/TAATGCA	GGTCCGTGTGGTTAATTTGG	AATTGCATGTTGGTTCATGG	60.1	494	INTERGENIC	--	--	--
Ca4_P4_39074802	Ca4	39074802	AGGGGG/AGGGG	AGAAGCCCTGAAAGCAACAA	AATTTATGCCATTGCTTCGG	60.0	602	INTERGENIC	--	--	--
Ca4_P4_39078155	Ca4	39078155	ATTTTTT/ATTTTTTT	TGTGTGCAATAGAAGGTCGC	CATCACTAGGATGCGATCACC	59.9	347	INTERGENIC	--	--	--
Ca4_P4_39078527	Ca4	39078527	AAATAA/AAA	GTGTGCAATAGAAGGTCGCA	CGAAGTAACAATTGTGTAAGGGG	59.9	860	INTERGENIC	--	--	--
Ca4_P4_39079360	Ca4	39079360	TATTTCAC/TATTTCACTCACATTTCAC	TGACGCCTGTTTGTCAATGT	GTTCGAGGAAAAAGAGTGCG	60.2	449	INTERGENIC	--	--	--

INDEL marker IDs	Chromosomes/ unanchored scaffolds	Physical positions (bp)	InDels (*Kabuli* reference genome-CDC Frontier/PII)	Forward primers (5'-3')	Reverse primers (5'-3')	Anneling temperature (0C)	Expected amplified product size (bp)	Structural annotation		Functional annotation	
								Sequence components of *kabuli* genome	*Kabuli* gene accession IDs	NCBI-KOG	TFs
Ca4_P4_39156446	Ca4	39156446	TAAAAAAAAAA/TAAAAAAAAA	GAGCATCGCAGGTTGAATTT	CCGCATGAGATATTTTGCATT	60.2	362	INTERGENIC	--	--	--
Ca4_P4_39165742	Ca4	39165742	TAAAAATAAAAAATAAAAAA/TAAAAATAAAAAATAAAAAATAAAAAA	CGTATACCGCTGCTCATCAA	TGATGTGATTTGTGCCATGA	59.9	259	INTERGENIC	--	--	--
Ca4_P4_39249438	Ca4	39249438	CTTTTTTTT/CTTTTTTT	ATTGTTTGGCTCCACACCTC	TTTCAACAGGAGTATTTTTATGCG	60.0	245	INTERGENIC	--	--	--
Ca4_P4_39257138	Ca4	39257138	ATTTTTTTTTT/ATTTTTTTTTTT	TTTGCGTTACGAATTTGATGA	TGAATTTTCCCGACTTGGTC	59.2	181	INTERGENIC	--	--	--
Ca4_P4_39356109	Ca4	39356109	CCTCTC/CCTCTCTC	TCTCTACCTGTGGGTTGCCT	ACTTTTGCGGAGAAGCACAT	59.7	611	INTERGENIC	--	--	--
Ca4_P4_3951674	Ca4	3951674	TTATATATATATATATATATAT/TTATATATATATATATATATATATATAT	GCACAACCAGTCACCACTCA	AGGAAGCAAGGGATCAAACA	60.8	918	INTERGENIC	--	--	--
Ca4_P4_39641904	Ca4	39641904	CATATATATATATATATATATATAT/CATATATATATATATATATATATAT	CCTTTAGCGTATGCACTGAGC	TAACCATCTCCACCGACACA	60.1	582	INTERGENIC	--	--	--
Ca4_P4_39642176	Ca4	39642176	CAAAAAA/CAAAA	CCTTTAGCGTATGCACTGAGC	TAACCATCTCCACCGACACA	60.1	582	INTERGENIC	--	--	--
Ca4_P4_39643073	Ca4	39643073	CA/CACAA	TCGTACCTTGTATCACCGCA	AACGCGCGGGTTATATTCTA	60.1	276	INTERGENIC	--	--	--
Ca4_P4_39644534	Ca4	39644534	GAA/GA	TGAGCTTTTAGGTGGCACAA	TGACACTCATTGCTCCTCCA	59.5	700	INTERGENIC	--	--	--

INDEL marker IDs	Chromosomes/ unanchored scaffolds	Physical positions (bp)	InDels (*Kabuli* reference genome-CDC Frontier/PII)	Forward primers (5'-3')	Reverse primers (5'-3')	Anneling temperature (0C)	Expected amplified product size (bp)	Structural annotation		Functional annotation	
								Sequence components of *kabuli* genome	*Kabuli* gene accession IDs	NCBI-KOG	TFs
Ca4_P4_39689187	Ca4	39689187	AC/ACC	ATTACCGACATTGCAGAGGG	CTCCCATTCTGCCCTTGATA	60.0	329	INTERGENIC	--	--	--
Ca4_P4_3991086	Ca4	3991086	ATTCTC/ATTCTCATTTCTC	AGCCTTCTCCAGCAAAAACA	AGCATGCAAATGCAAACAAA	60.0	217	INTERGENIC	--	--	--
Ca4_P4_39987654	Ca4	39987654	ATTTTTTTTTTT/ATTTTTTTTTT	TAAATTCCGCTGTGCACTTG	AACTTACCGATCAATTCAAATCAA	59.9	191	INTERGENIC	--	--	--
Ca4_P4_40056699	Ca4	40056699	AATATATATATATATATATATATATATATATATATATATATATATAT/AATATATATATATATATATATATATATATATATATATATATATATAT	CAGGACAGGGATGGTGTTTT	TTGCAATTTCATGAATGTTACTCA	59.8	639	INTERGENIC	--	--	--
Ca4_P4_4008216	Ca4	4008216	TTATATA/TTATA	GCTTCCTAAGAAACCTCATTCAA	TGGTTTTAATTTAGAGTTGATCCAAG	58.9	796	INTERGENIC	--	--	--
Ca4_P4_4008273	Ca4	4008273	TTTTAAAATATATTTAAAATA/TTTTAAAATATATTTAAAATATATTTAAAATA	GCTTCCTAAGAAACCTCATTCAA	TGGTTTTAATTTAGAGTTGATCCAAG	58.9	796	INTERGENIC	--	--	--
Ca4_P4_40111947	Ca4	40111947	GAATAATAATAATAATAATAATAATAATA/GAATAATAATAATAATAATAATAATA	TGCAAGTTGCTATCCCATTG	TTGCAATACGGATCACAAAAA	59.7	753	INTERGENIC	--	--	--
Ca4_P4_4011481	Ca4	4011481	ACC/AC	TTTTGGGCTCCAAATTTACG	TGAATGGTTAATGGATGACAGTG	59.9	348	INTERGENIC	--	--	--
Ca4_P4_4013042	Ca4	4013042	T/TGA	GCATATTGTGTATTTGGTGCAA	CACAAACACTAATTTGTTGGCA	58.5	539	INTERGENIC	--	--	--
Ca4_P4_4013283	Ca4	4013283	GTA/GTAAAGTGTGATGCTATA	TTGCCAACAAATTAGTGTTTGTG	CTTTGCCAAGCAAATCAACA	60.0	508	INTERGENIC	--	--	--

INDEL marker IDs	Chromosomes/ unanchored scaffolds	Physical positions (bp)	InDels (*Kabuli* reference genome-CDC Frontier/PII)	Forward primers (5'-3')	Reverse primers (5'-3')	Anneling temperature (0C)	Expected amplified product size (bp)	Structural annotation		Functional annotation	
								Sequence components of *kabuli* genome	*Kabuli* gene accession IDs	NCBI-KOG	TFs
Ca4_P4_4016329	Ca4	4016329	T/TA	CGATGTGACCGACCAGTTTA	TTGCCCACACTTACTTGTGAA	59.6	452	INTERGENIC	--	--	--
Ca4_P4_4025049	Ca4	4025049	CT/C	CGAAACATATCCAAATCGGG	ACCGAGCCATAGTGACCAAC	60.1	175	INTERGENIC	--	--	--
Ca4_P4_40251301	Ca4	40251301	TTATATATATATATATATATATATATATATATA/TTATATATATATATATATATATATATATA	ATAATTGAGATGGGATGGCG	CAATGCTAGGCCACAACCTT	59.7	693	INTERGENIC	--	--	--
Ca4_P4_40265020	Ca4	40265020	GTTTTTTTT/GTTTTTTT	AATCTGTGTTGACGCACGAG	TTTTCCTCCTCATTTTTCGTT	59.9	681	INTERGENIC	--	--	--
Ca4_P4_4031680	Ca4	4031680	AT/ATAACAGT	AAAATTTCTCTCCCCCTCTCC	AACCACGACTAAAACGACGC	59.9	859	INTERGENIC	--	--	--
Ca4_P4_4032682	Ca4	4032682	TAA/TTTAATAAGAGAA	ATCAGTAAAGCAACCCCAGC	CAACGGTGGAGTTTGGATCT	59.2	346	INTERGENIC	--	--	--
Ca4_P4_4034664	Ca4	4034664	ATTT/ATTTT	CAAACGTGGACGACTTCTCA	AGGCGGCCCTTAATAGAAAA	59.9	352	INTERGENIC	--	--	--
Ca4_P4_4035364	Ca4	4035364	AT/A	TTTTCTATTAAGGGCCGCCT	GTAACCTTTGCACGCTCCAT	60.1	772	INTERGENIC	--	--	--
Ca4_P4_4037177	Ca4	4037177	GTTTTTTTTTTTT/GTTTTTTTTT	AAAGTGAAAGCCACTCCCAA	AATGTTCGCCACCACTTTTC	59.7	369	INTERGENIC	--	--	--
Ca4_P4_4037315	Ca4	4037315	ATTTATTTT/ATTTATTTTATTTT	AAAGTGAAAGCCACTCCCAA	AATGTTCGCCACCACTTTTC	59.7	369	INTERGENIC	--	--	--

INDEL marker IDs	Chromosomes/ unanchored scaffolds	Physical positions (bp)	InDels (*Kabuli* reference genome-CDC Frontier/PII)	Forward primers (5'-3')	Reverse primers (5'-3')	Anneling temperature (0C)	Expected amplified product size (bp)	Structural annotation		Functional annotation	
								Sequence components of *kabuli* genome	*Kabuli* gene accession IDs	NCBI-KOG	TFs
Ca4_P4_4039327	Ca4	4039327	TACTC/T	TTTTGGTTTGGTAATTGTGGG	TGATGGAGAAATCCTCACCC	59.6	456	INTERGENIC	--	--	--
Ca4_P4_4041384	Ca4	4041384	A/AC	GGGTTTTGGTTGAAATTTTTG	TAATGACGACCAGCGAATGA	58.4	540	INTERGENIC	--	--	--
Ca4_P4_4045322	Ca4	4045322	CAA/CAAAAA	CACGATGTTCTACACGTACCAA	AGGAGGAAACAAACCCAACA	58.6	447	INTERGENIC	--	--	--
Ca4_P4_40460183	Ca4	40460183	AATATATATATATATATATA/AATATATATATATATATATA	CAAGCGTTTTGATAGGGTCA	CCGTAAATAACAGTACAATGGTGAA	58.8	630	INTERGENIC	--	--	--
Ca4_P4_40487175	Ca4	40487175	AAT/AATAT	TTTTCCGCGATTTGATCATT	GGCTAAATAGCATTCGCAATC	60.4	504	INTERGENIC	--	--	--
Ca4_P4_40503609	Ca4	40503609	TA/TACTCTCAA	GTGGTGTGTGCAAGAATTGG	CCAACAAGTAGGGTGGAGGA	60.0	800	INTERGENIC	--	--	--
Ca4_P4_40506274	Ca4	40506274	CAGTATAATAGTA/CAGTATAATAGTATAATAGTA	GTGGCTGTTTATGGATTGGG	TGATCCCCTTCATTCCTTCA	60.2	239	INTERGENIC	--	--	--
Ca4_P4_40514871	Ca4	40514871	AATA/AA	GGAGAACCAGCTTCCTCCTT	CGCTCTTGCTTCCTTTCACT	59.8	278	INTERGENIC	--	--	--
Ca4_P4_40515212	Ca4	40515212	CG/C	GGAGAACCAGCTTCCTCCTT	TTCTCGGTTCTCCTTTCTGC	59.8	916	INTERGENIC	--	--	--
Ca4_P4_40526310	Ca4	40526310	CATATATAT/CATATATATAT	CACGGAATTGATCCCCTTATT	TGCATGCCACTAATCGTTGT	60.0	706	INTERGENIC	--	--	--

INDEL marker IDs	Chromosomes/ unanchored scaffolds	Physical positions (bp)	InDels (*Kabuli* reference genome-CDC Frontier/PII)	Forward primers (5'-3')	Reverse primers (5'-3')	Anneling temperature (0C)	Expected amplified product size (bp)	Structural annotation		Functional annotation	
								Sequence components of *kabuli* genome	*Kabuli* gene accession IDs	NCBI-KOG	TFs
Ca4_P4_40560588	Ca4	40560588	TAAAAAA/TAAAAAAA	TCATTCTCCCGATCTTCCAC	TTTCTGTGGACTCACGCATC	60.0	636	INTERGENIC	--	--	--
Ca4_P4_40571712	Ca4	40571712	T/TG	CACTCATCATAGCCCCCTGT	AGATGCACGACAATGCTTCA	60.0	711	INTERGENIC	--	--	--
Ca4_P4_40573539	Ca4	40573539	AG/A	TATTGCACCGTTGCCTTACA	TCAACTTCCTTGGTTATATTTTTGC	60.1	721	INTERGENIC	--	--	--
Ca4_P4_40574527	Ca4	40574527	TA/T	TGCAGATGAGTTTTTGTCCA	TTTAAATGATTTTTCGCAATGA	57.8	426	INTERGENIC	--	--	--
Ca4_P4_40575366	Ca4	40575366	ATTTTTTTTTT/ATTTTTTT	CAATGTTGAACTTCCTTTTGTCTT	TCACCAGTGACACGTTCGAT	58.8	768	INTERGENIC	--	--	--
Ca4_P4_40601986	Ca4	40601986	TTATATATATATATATATATATATATATATATATATAT/TTATATATATATATATATATATATATATAT	GGTGCCTTATGGGTTAGGAA	TGTTTGTTGAGTTGGAAGACTTG	58.9	935	INTERGENIC	--	--	--
Ca4_P4_40602662	Ca4	40602662	GAAAAA/GAAAAAA	CCATTACGATGCACATTGCT	TGAATTGTTCTTACGAGCCG	59.6	257	INTERGENIC	--	--	--
Ca4_P4_40604039	Ca4	40604039	TGGGGGG/TGGGGG	TTTGTCGTAGTCACTCTCCGT	GTGCCCCTAAAAAGAGGGTC	57.5	719	INTERGENIC	--	--	--
Ca4_P4_40608520	Ca4	40608520	GTTTTTTTT/GTTTTTTT	TTTTTGCAAACGACAACTTTCT	TGTGAATACGTGACTTTTCTTTTCA	59.0	432	INTERGENIC	--	--	--
Ca4_P4_40615032	Ca4	40615032	TAA/TA	GAGCCCAACGATGTTCAAAT	TGAACCCCACCAGCATTATT	59.9	793	INTERGENIC	--	--	--

INDEL marker IDs	Chromosomes/ unanchored scaffolds	Physical positions (bp)	InDels (*Kabuli* reference genome-CDC Frontier/PII)	Forward primers (5'-3')	Reverse primers (5'-3')	Anneling temperature (0C)	Expected amplified product size (bp)	Structural annotation		Functional annotation	
								Sequence components of *kabuli* genome	*Kabuli* gene accession IDs	NCBI-KOG	TFs
Ca4_P4_40621809	Ca4	40621809	AAGA/AA	TCTTTTGCACGGAGAATCAA	TTCCCGTGACGTGTGTATGT	59.4	320	INTERGENIC	--	--	--
Ca4_P4_40624782	Ca4	40624782	TAAAAA/TAAAA	TCAAAAGAATGGAGCAAGTTGT	GGGGGCATTAAGAAGGAAAT	58.9	841	INTERGENIC	--	--	--
Ca4_P4_4063264	Ca4	4063264	GTTTTTT/GTTTTT	GAACCCGCGTCATTTGTAA	TTGTGTGCGTGTGTGTATGTG	59.5	713	INTERGENIC	--	--	--
Ca4_P4_40633755	Ca4	40633755	GTTTTTTTTTTT/GTTTTTTTTTT	GTGTGTCCAAAAGTGCGAGA	CCCATAATATTTCTTGGGGC	59.9	726	INTERGENIC	--	--	--
Ca4_P4_40638727	Ca4	40638727	AATATATATAT/AATATATATATAT	TCCTTCTAGCACATGGTCCC	TTGGAAAAGCCGCAATCTAT	60.1	863	INTERGENIC	--	--	--
Ca4_P4_40639226	Ca4	40639226	AGG/AG	AATAGATTGCGGCTTTTCCA	CAAGAAACCGTTGTGGTCCT	59.7	158	INTERGENIC	--	--	--
Ca4_P4_40639974	Ca4	40639974	AATATATATAT/AATATATATATATAT	CAACGCTATTTACCCACCCA	TCCTATGGAACCACCAAAGC	60.7	200	INTERGENIC	--	--	--
Ca4_P4_40649020	Ca4	40649020	ATTTTTT/ATTTTTTTT	TGTGATTATCCGGTACCACAAC	TGTATTCAGGACCTCGCAGA	59.6	207	INTERGENIC	--	--	--
Ca4_P4_4068418	Ca4	4068418	ATTTTTTTTTTT/ATTTTTTTTT	ATAGATGGTCGCATTCTGGG	TCATCAAATCCGTAACGCAA	59.9	698	INTERGENIC	--	--	--
Ca4_P4_4068531	Ca4	4068531	T/TTTC	CGTGCCAAAACAGACAAAAG	TCATCAAATCCGTAACGCAA	59.4	333	INTERGENIC	--	--	--

INDEL marker IDs	Chromosomes/ unanchored scaffolds	Physical positions (bp)	InDels (*Kabuli* reference genome-CDC Frontier/PII)	Forward primers (5'-3')	Reverse primers (5'-3')	Anneling temperature (0C)	Expected amplified product size (bp)	Structural annotation		Functional annotation	
								Sequence components of *kabuli* genome	*Kabuli* gene accession IDs	NCBI-KOG	TFs
Ca4_P4_40700886	Ca4	40700886	AGAC/A	TCTTGATGTAACGTTTTGTTTCAGA	TGAATATTTGAGTTACGAATTTGATGA	60.1	478	INTERGENIC	--	--	--
Ca4_P4_40702737	Ca4	40702737	ACTCT/ACT	CCCCCATCATAAAATTGTGC	AAGATTAGTGGGCGACCCTT	60.0	392	INTERGENIC	--	--	--
Ca4_P4_40704362	Ca4	40704362	TA/T	GCGTAGAATGGGAAACGAAA	GAATGCAGTTAGCACGAGCA	60.1	230	INTERGENIC	--	--	--
Ca4_P4_40729399	Ca4	40729399	TAAAAAA/TAAAAAAA	TTGTGAAATTGAAAAGGTTCCA	TTGTTTTCAATGAGAAGGTGGTT	59.5	256	INTERGENIC	--	--	--
Ca4_P4_40731137	Ca4	40731137	TC/T	TCATGGATAAGGTGGAAGACG	TGAATTCGAAAATCCATTTCATC	59.9	471	INTERGENIC	--	--	--
Ca4_P4_40743608	Ca4	40743608	TTCAAT/TT	GAATTTTGTGTGTTGCGGTG	GCGGTGTTTTTCAACACATTT	60.0	527	INTERGENIC	--	--	--
Ca4_P4_40762174	Ca4	40762174	ATTTTT/ATTTTTT	AATGGCGTATGTGTGAGGGT	TCACCAAATTGAAAGCATCC	60.3	543	INTERGENIC	--	--	--
Ca4_P4_40763607	Ca4	40763607	CAAAAA/CAAAA	GCTCTGCTTGATTGAAAATGC	CCAATGCATGAAATTTGTTCTT	60.0	696	INTERGENIC	--	--	--
Ca4_P4_40763691	Ca4	40763691	ATTTT/ATTT	GCTCTGCTTGATTGAAAATGC	GGGTCCCTCTAACTTGTGAGC	60.0	826	INTERGENIC	--	--	--
Ca4_P4_40778747	Ca4	40778747	T/TA	GGGAGTTTGGAGAAGAAGGG	AATAAAGCAACAGCCCCAAT	60.0	776	INTERGENIC	--	--	--

INDEL marker IDs	Chromosomes/ unanchored scaffolds	Physical positions (bp)	InDels (*Kabuli* reference genome-CDC Frontier/PII)	Forward primers (5'-3')	Reverse primers (5'-3')	Anneling temperature (0C)	Expected amplified product size (bp)	Structural annotation		Functional annotation	
								Sequence components of *kabuli* genome	*Kabuli* gene accession IDs	NCBI-KOG	TFs
Ca4_P4_4078034	Ca4	4078034	CA/CAGTA	AGCACGTGTGGAAGACATTG	CCCCTTCCACAAAAAGTGAA	59.8	654	INTERGENIC	--	--	--
Ca4_P4_40783665	Ca4	40783665	TTTAT/TTTATTAT	CCTCTAGTGGGTTTCTTTCATCA	AATGATGCCTCCATGCTTTC	59.6	853	INTERGENIC	--	--	--
Ca4_P4_40785871	Ca4	40785871	ATTTTTTTTT/ATTTTTT	GAGCTATTGTCTTCGGCACC	CCTTCAAGAGGTCGCATCAT	59.8	514	INTERGENIC	--	--	--
Ca4_P4_40790018	Ca4	40790018	A/AT	GCCCATTTTGTTCTTTGTTGA	TCATTGTCCAGTGAAGGCAA	60.0	667	INTERGENIC	--	--	--
Ca4_P4_40821058	Ca4	40821058	ACA/ACATGCCTCA	CCGTTAAAACGATGGTCGAT	TTAATTTTCCAGCCATGCAA	59.8	338	INTERGENIC	--	--	--
Ca4_P4_40833473	Ca4	40833473	ATTTTTTTT/ATTTTTTTTTT	TTTTTATTTGTCAACCCGCA	TTCAAACCCCCATTTCTTGT	59.1	766	INTERGENIC	--	--	--
Ca4_P4_40838372	Ca4	40838372	GTTTT/GTTT	AGTTATTTTGTTGAGACAATTACCAA	GCCTTTTCCACGCATTTTTA	57.5	755	INTERGENIC	--	--	--
Ca4_P4_40842793	Ca4	40842793	TC/T	CTGGATTGAGTTGAGGCCAT	TGGACCAAACCAAGCACATA	60.1	568	INTERGENIC	--	--	--
Ca4_P4_40842971	Ca4	40842971	AGTGTGT/AGTGTGTGT	AAAATTTGTGCATCAAGGGC	TCAAGATTGGGCCAAGAATC	59.9	551	INTERGENIC	--	--	--
Ca4_P4_40845072	Ca4	40845072	TG/TGG	CGAAAACAAATGTGTAGAGGCA	GGCAAATTAAAGGAGGCACA	60.2	870	INTERGENIC	--	--	--

INDEL marker IDs	Chromosomes/ unanchored scaffolds	Physical positions (bp)	InDels (*Kabuli* reference genome-CDC Frontier/PII)	Forward primers (5'-3')	Reverse primers (5'-3')	Anneling temperature (0C)	Expected amplified product size (bp)	Structural annotation		Functional annotation	
								Sequence components of ***kabuli*** genome	***Kabuli*** gene accession IDs	NCBI-KOG	TFs
Ca4_P4_40846133	Ca4	40846133	ATTT/ATTTT	CCATCAATTGGGTGGT TGTT	GGGAGAACATCATGCT CCAT	60.5	449	INTERGENIC	--	--	--
Ca4_P4_4084759	Ca4	4084759	CAG/CAGAAG	TAACCATGGCCATCAA GACA	CGTCTTAACTTTTGCTA TCGGC	59.9	644	INTERGENIC	--	--	--
Ca4_P4_40880303	Ca4	40880303	GA/GAA	GGAAGTTTCCAAAGCA CGAG	AAGTTCCATTGCTTGCT GGT	59.9	411	INTERGENIC	--	--	--
Ca4_P4_40881410	Ca4	40881410	CAAAA/CAAA	AAGACCCACATTTGGT GAAAA	ATGAGTTCTTCATCGGT CGG	59.3	506	INTERGENIC	--	--	--
Ca4_P4_40895378	Ca4	40895378	TAAAAA/TAAAAAA	TGTGTCCCTAATATTTT GCCG	TGACACATAGTTTGTTT TGGCA	59.8	441	INTERGENIC	--	--	--
Ca4_P4_40896370	Ca4	40896370	CAATAATA/CAATA	AGGTACTTTCAAATGG GTGTGA	GAACCTGATGAACTGT GGCA	58.5	258	INTERGENIC	--	--	--
Ca4_P4_40933977	Ca4	40933977	GTTTT/GTTTTT	GGATCATCCACCATTT ATCTGAA	TTCTCACGTTGTTGAC GAGC	60.0	618	INTERGENIC	--	--	--
Ca4_P4_40935936	Ca4	40935936	T/TC	TTTTCACGAATGATCCA CCA	GTCGTTCAATCCAACC TCGT	59.9	350	INTERGENIC	--	--	--
Ca4_P4_40955483	Ca4	40955483	ATTTTTT/ATTTTT	TTCGCATTCGTCGAAC TACA	TCCGTTTTTATTTTGGT CGC	60.4	674	INTERGENIC	--	--	--
Ca4_P4_40964102	Ca4	40964102	TT/TTCT	ATTTGGATAGGCCTGG CTTT	GCACTTTTTGATACGG GAGC	59.9	622	INTERGENIC	--	--	--

INDEL marker IDs	Chromosomes/ unanchored scaffolds	Physical positions (bp)	InDels (*Kabuli* reference genome-CDC Frontier/PII)	Forward primers (5'-3')	Reverse primers (5'-3')	Anneling temperature (0C)	Expected amplified product size (bp)	Structural annotation		Functional annotation	
								Sequence components of *kabuli* genome	*Kabuli* gene accession IDs	NCBI-KOG	TFs
Ca4_P4_40966159	Ca4	40966159	GCATTTG/GCATTTGATGCCATTGTCATTTG	AACTATGGCTTTGTCGGGTG	CTCAAAAACGAGCCAAAAGC	60.0	399	INTERGENIC	--	--	--
Ca4_P4_40968955	Ca4	40968955	TC/TCC	CACTGGAATGATTGCCAAAA	TTCTACCCAAGATCGGATGG	59.5	398	INTERGENIC	--	--	--
Ca4_P4_40997865	Ca4	40997865	TAAAAAAA/TAAAAAA	GATGAATGGAGACGACCGTT	AACCAATGATGCAGTTGTGC	59.9	534	INTERGENIC	--	--	--
Ca4_P4_41010535	Ca4	41010535	CTT/CTTT	TAGTTGGTCAATGCGGATCA	GGACTAACAGGGGAGAAGGG	60.1	278	INTERGENIC	--	--	--
Ca4_P4_41015513	Ca4	41015513	ATTTTTTTT/ATTTTTTT	CGTCATTTGAAAAAGCGACA	GCCCCATAAAAAGATGCTCA	59.8	623	INTERGENIC	--	--	--
Ca4_P4_41015733	Ca4	41015733	TAAAAAA/TAAAAAAA	CAAATTTTGATGATCTCACACGA	GCCCCATAAAAAGATGCTCA	60.0	504	INTERGENIC	--	--	--
Ca4_P4_41027713	Ca4	41027713	AATATATATATATATATATATATATATATAT/AATATATATATATATATATATATATATATATATATAT	TGAATCCCAAAATTTGAACCA	TTTGATCGCAAGGGTTTAGG	60.2	637	INTERGENIC	--	--	--
Ca4_P4_4115988	Ca4	4115988	ATTTTTT/ATTTTT	TGCAATATGAAGGTCCACCC	CCCCCTTTTTGGTCGTATCT	60.7	665	INTERGENIC	--	--	--
Ca4_P4_41172721	Ca4	41172721	AC/A	TCAAGTTAAAGGTGGAGCGG	TTCCTCCCTTTCTTTGACAAC	60.2	761	INTERGENIC	--	--	--
Ca4_P4_4118065	Ca4	4118065	CTTTTTTTTTTTTT/CTTTTTTTTTT	AAAAGGAATTTGTGGCAACG	TTTTGCAATCTATCGAGGGG	60.0	580	INTERGENIC	--	--	--

INDEL marker IDs	Chromosomes/ unanchored scaffolds	Physical positions (bp)	InDels (*Kabuli* reference genome-CDC Frontier/PII)	Forward primers (5'-3')	Reverse primers (5'-3')	Anneling temperature (0C)	Expected amplified product size (bp)	Structural annotation		Functional annotation	
								Sequence components of *kabuli* genome	*Kabuli* gene accession IDs	NCBI-KOG	TFs
Ca4_P4_41182654	Ca4	41182654	CCTCTCTCTCTCTCTCTCTCTCTCTCTCTCTCTCTC/CCTCTCTCTCTCTCTCTCTCTCTC	GCATTTCTCTGCCTCCTGTC	GCAACTAACAGAACCGGAGC	60.0	537	INTERGENIC	--	--	--
Ca4_P4_41268887	Ca4	41268887	AATATATATATATATATATA/AATATATATATATATATA	AAACCTCGTCAATTGGCTCA	CAATTGGTGGAACAATCGTG	60.6	299	INTERGENIC	--	--	--
Ca4_P4_41272417	Ca4	41272417	GATTATTATTATTATTATTATTATTATTATT/GATTATTATTATTATTATTATTATTATT	GGTTCACATGGAACTCCAACA	AGCCTTTTGCTTCCTTCCTC	60.8	737	INTERGENIC	--	--	--
Ca4_P4_4127483	Ca4	4127483	AG/A	TGTGCATGACCAAATACTCCA	ATATACGGCCGCTCATCCTA	60.0	248	INTERGENIC	--	--	--
Ca4_P4_4129506	Ca4	4129506	T/TTA	TTGACGAACCAACTCATCCA	CCACGCACTACTTCAACGAC	60.1	851	INTERGENIC	--	--	--
Ca4_P4_4130075	Ca4	4130075	TCA/T	TTTGGCCAATAAGAAAACCG	CAACGATAACACATCAACCACA	59.9	544	INTERGENIC	--	--	--
Ca4_P4_41306366	Ca4	41306366	CTTTTTTTTTT/CTTTTTTTTTTTT	GGTAGCATCCAACCATCTAGACA	GGGGATGTGAACACTTAAGCA	60.4	157	INTERGENIC	--	--	--
Ca4_P4_4133931	Ca4	4133931	GAAAAAAAAAA/GAAAAAAAAAAA	TTGGCTGAGCTTGTGCATAC	GGGAAACAACGACCTAATCC	60.0	417	INTERGENIC	--	--	--
Ca4_P4_41438091	Ca4	41438091	TATAATAATAATAATAATAATAATAATAATAATAACAATAATAATAATAATAA/TATAATAATAATAATAATAATAATAATAATAATAATAATAATAACAATAATAATAATAATAA	CCAGGATGCGACCATCTAGT	ATTCCTTAGTTACATCCATGTTGTT	60.1	446	INTERGENIC	--	--	--
Ca4_P4_4149368	Ca4	4149368	ATTTT/ATTTTT	CCACTGGTTCAGGTTCTGGT	TTTCCGGCAAAAACAAATTC	60.0	635	INTERGENIC	--	--	--

INDEL marker IDs	Chromosomes/ unanchored scaffolds	Physical positions (bp)	InDels (*Kabuli* reference genome-CDC Frontier/PII)	Forward primers (5'-3')	Reverse primers (5'-3')	Anneling temperature (0C)	Expected amplified product size (bp)	Structural annotation		Functional annotation	
								Sequence components of *kabuli* genome	*Kabuli* gene accession IDs	NCBI-KOG	TFs
Ca4_P4_41581170	Ca4	41581170	GA/GATCA	ACAAAAACTCCCAATGGTCG	TAGCCAACCACACATGGAAA	59.8	737	INTERGENIC	--	--	--
Ca4_P4_4158984	Ca4	4158984	CTT/CTTCAAGTT	ATATAAGAGGGTGGGGGCAT	TGAGGAAGGTACGAGGAGGA	59.5	625	INTERGENIC	--	--	--
Ca4_P4_41612820	Ca4	41612820	ATTG/ATTGTACATAAATACGGTGTTG	TGAATTGCGTTAGATGAGCA	GCAATATACGCCAAATGCAA	58.0	486	INTERGENIC	--	--	--
Ca4_P4_4162107	Ca4	4162107	AATTAAATT/AATTAAATTTATTAAATT	AGGGAGAGAGGCATGAGACA	ATGTGCGACATGAAAACCAA	59.9	735	INTERGENIC	--	--	--
Ca4_P4_4162204	Ca4	4162204	GTTTTTTTT/GTTTTTTTTTT	AGGGAGAGAGGCATGAGACA	ATGTGCGACATGAAAACCAA	59.9	735	INTERGENIC	--	--	--
Ca4_P4_4162985	Ca4	4162985	ATTTT/ATTT	TATTGCAGCAACACATGCAA	CAAATTTGTGAAAGACGTGGA	59.9	361	INTERGENIC	--	--	--
Ca4_P4_4163265	Ca4	4163265	TTAAAT/TT	TGACCACTTTAGAAAGCATGTGA	TGCAAATCCATAGACTCCAAGA	59.8	284	INTERGENIC	--	--	--
Ca4_P4_41634605	Ca4	41634605	CATTATTATTATTATTATTATTATTATTATTATTATTATTATTATTATTATTAT/CATTATTATTATTATTATTATTATTATTATTATTATTATTATTATTATTATTATTAT	TTGATGGAAGTTTACATCGGC	TGTTTGTTTGAGGGGAAAGG	60.0	890	INTERGENIC	--	--	--
Ca4_P4_41637679	Ca4	41637679	ATTTATTTTTATTTTT/ATTTATTTTTATTTTTATTTTT	TTCAAGGAGGAAGGGTGATG	GGACGAAAGCCACAAAACAT	60.0	517	INTERGENIC	--	--	--
Ca4_P4_4166322	Ca4	4166322	T/TAAG	GCGTTGAGAATCTTGAAATGC	TCCTCTCACAGGGCTCACTT	59.8	164	INTERGENIC	--	--	--

INDEL marker IDs	Chromosomes/ unanchored scaffolds	Physical positions (bp)	InDels (*Kabuli* reference genome-CDC Frontier/PII)	Forward primers (5'-3')	Reverse primers (5'-3')	Anneling temperature (0C)	Expected amplified product size (bp)	Structural annotation		Functional annotation	
								Sequence components of ***kabuli*** genome	***Kabuli*** gene accession IDs	NCBI-KOG	TFs
Ca4_P4_41666178	Ca4	41666178	ATTTTTTTTTT/ATTTTTTTTT	AGCCCATACTTGACGCAGAT	GGAGCCAATAAAAAGAAAATCG	59.7	487	INTERGENIC	--	--	--
Ca4_P4_4173930	Ca4	4173930	GAAAAAA/GAAAAAAA	GGACACCCCAATAACAAGGA	TCTTGACATAAAACTATTTTTCAGCG	59.6	699	INTERGENIC	--	--	--
Ca4_P4_41799900	Ca4	41799900	CATATATATATATATATATATAT/CATATATATATATATATAT	CACAAAACACAAGGTGTGGC	GAAGATTGTATATCCGCTCTACGAC	60.0	297	INTERGENIC	--	--	--
Ca4_P4_4180652	Ca4	4180652	AT/A	CACCATGTCATAACCAATGGA	TGCTTGCCAACACATATAACA	59.1	186	INTERGENIC	--	--	--
Ca4_P4_41810917	Ca4	41810917	C/CA	ACACAATTTGTCGTCCACCA	TGTAAGTAAAAATTCATCAGCACCA	59.9	262	INTERGENIC	--	--	--
Ca4_P4_4181784	Ca4	4181784	GAGAAGAAG/GAGAAG	GGGAGGGTTTGCAATTTTCT	TCGGATTGTGCTCACAACAT	60.3	477	INTERGENIC	--	--	--
Ca4_P4_41823980	Ca4	41823980	CATCAGA/CA	CATCAAGACCATTTCCAGCA	CAATTGTTCGTGTTGCGTCT	59.6	114	INTERGENIC	--	--	--
Ca4_P4_4183045	Ca4	4183045	TAAAAAAAAA/TAAAAAAA	TTACTTGTTGTGCGGCTGAA	TTGAGCATGTGGTTGTTGGT	60.4	216	INTERGENIC	--	--	--
Ca4_P4_41832968	Ca4	41832968	AATATATATATATA/AATATATATATA	TTTCCATTGCACTCACTCCA	CCTATCATGGCCTGACCTGT	60.2	758	INTERGENIC	--	--	--
Ca4_P4_4188614	Ca4	4188614	TAAAAAAAA/TAAAAAAA	TTGCAGAAATTTAGCGGAAA	TTGAATTGGAATTGTTTTGCAT	58.6	407	INTERGENIC	--	--	--

INDEL marker IDs	Chromosomes/ unanchored scaffolds	Physical positions (bp)	InDels (*Kabuli* reference genome-CDC Frontier/PII)	Forward primers (5'-3')	Reverse primers (5'-3')	Anneling temperature (0C)	Expected amplified product size (bp)	Structural annotation		Functional annotation	
								Sequence components of *kabuli* genome	*Kabuli* gene accession IDs	NCBI-KOG	TFs
Ca4_P4_42025518	Ca4	42025518	AATATATATATATATATATATATATA/ AATATATATATATATATATATATA	CACGGATCTTGTGAGTGACC	TCACAAGTCACACAATTTTCACA	59.1	504	INTERGENIC	--	--	--
Ca4_P4_42101261	Ca4	42101261	CGAGAGAGAGAGAGAGAGAGAGAGAGAGAGAGAGAGA/CGAGAGAGAGAGAGAGAGAGAGAGAGAGAGAGAGAGAGA	CCTCATTCCGTTGTTTCGTT	GCCGAACCGTTTCGTACTTA	60.0	565	INTERGENIC	--	--	--
Ca4_P4_42332097	Ca4	42332097	GAAAAAAAAA/GAAAAAAAAAA	TCAGGGCCAACTGAAAGTCT	CTGGATGTGCATCCTGTTTG	59.8	628	INTERGENIC	--	--	--
Ca4_P4_42412637	Ca4	42412637	GA/GAA	CTTCTCAAGGGACGCATCAT	AATTTGGCAAGTTTGACACG	60.2	170	INTERGENIC	--	--	--
Ca4_P4_4242385	Ca4	4242385	TTATATATATATATATATATATATATAT/TTATATATATATATATATATATATATATATATAT	GAACCATTGAGAGGCCGTT	TCGGTTTCCGAGTAGTGGAC	60.1	359	INTERGENIC	--	--	--
Ca4_P4_4248584	Ca4	4248584	CTTTTTTTTTT/CTTTTTTTTTTT	GCTGTAACAAGGTGCCCAAT	TTTGCAGCTCAATGAAATCC	60.0	386	INTERGENIC	--	--	--
Ca4_P4_42534372	Ca4	42534372	GTTTTTTTT/GTTTTTTT	TGGGGCAGTGCAATGTATTA	CATCGGCAAGAGTTGGGTAT	60.0	574	INTERGENIC	--	--	--
Ca4_P4_42548370	Ca4	42548370	ATTTTTTTT/ATTTTTTTTT	CGTGCCTTTGTCGTCTCTTT	AGAGGCCCAATATCCTTGCT	60.4	528	INTERGENIC	--	--	--
Ca4_P4_42574723	Ca4	42574723	AGGG/AGGGG	TCGTCATCACACAAGGTGGT	CAGGTTGTAAGCAAAAGCCC	60.0	227	INTERGENIC	--	--	--
Ca4_P4_4259095	Ca4	4259095	CT/C	GTTGGCTGAAAAGGGATTGA	CGATTTCCACTAACACCTTGG	60.1	383	INTERGENIC	--	--	--

INDEL marker IDs	Chromosomes/ unanchored scaffolds	Physical positions (bp)	InDels (*Kabuli* reference genome-CDC Frontier/PII)	Forward primers (5'-3')	Reverse primers (5'-3')	Anneling temperature (0C)	Expected amplified product size (bp)	Structural annotation		Functional annotation	
								Sequence components of *kabuli* genome	*Kabuli* gene accession IDs	NCBI-KOG	TFs
Ca4_P4_42633986	Ca4	42633986	TATAATAATAATAATAATAATAATAATAATAATA/TATAATAATAATAATAATAATAATAATAATAATAATAATA	TTGCTTTAGATGAATCAAATCCC	CGACAGGCTTAGGTTAGACCA	59.5	362	INTERGENIC	--	--	--
Ca4_P4_4269269	Ca4	4269269	TTATATATATATATATATATATATAT/TTATATATATATATATATATATATATAT	ACACGGTGATCGTCAGTGAA	ACACGTGACACTTACCTAGCAA	60.2	596	INTERGENIC	--	--	--
Ca4_P4_42693293	Ca4	42693293	TC/TCC	AAATGCTCGAACGAAAATGC	TCGAAGTTGGATGCTTTTGA	60.2	503	INTERGENIC	--	--	--
Ca4_P4_4272031	Ca4	4272031	CAAATAATTAAAAATAATTAAAA/CAAATAATTAAAAATAATTAAAAATAATTAAAA	TGAAGGGTTCAAAATGTTCTGTT	CTCGCATCTTCAAGCGTTTT	59.9	843	INTERGENIC	--	--	--
Ca4_P4_4273619	Ca4	4273619	ATTTTTTTTT/ATTTTTTTT	GCGCCCGTTGAAAATAATAA	ACACGTGTGGACGTGATTTG	59.9	408	INTERGENIC	--	--	--
Ca4_P4_42961696	Ca4	42961696	CAAAAAA/CAAAAA	CCCGGTGTCTTCATCAAAGT	GCTTGGGGACAAGCAATAGA	60.0	370	INTERGENIC	--	--	--
Ca4_P4_4296519	Ca4	4296519	ATTTTTTTTT/ATTTTTTTTTT	CAAAGCCCCCACACTAAAAA	TATGGCGGTTGGACTGTGTA	60.0	525	INTERGENIC	--	--	--
Ca4_P4_43195252	Ca4	43195252	TA/T	TGTTGGATATTGTCTCCTCCC	AAGTTCTGTGCCTCTATGTGGA	58.9	573	INTERGENIC	--	--	--
Ca4_P4_43219549	Ca4	43219549	GTATTATTATTATTATTATTATTATTATTATTATTATTATTA/GTATTATTATTATTATTATTATTATTATTATTATTATTA	TCATGAAGATTTGGAAGAAAAAGA	CCATCAACCCTAGCCTTCAA	59.3	364	INTERGENIC	--	--	--
Ca4_P4_43222555	Ca4	43222555	TTATATATATATATATATATATATAT/TTATATATATATATATATAT	AAAGATTTAAAGGGCAAAAAGTG	CTGGCAGCTGGGAAAGATT	58.0	908	INTERGENIC	--	--	--

INDEL marker IDs	Chromosomes/ unanchored scaffolds	Physical positions (bp)	InDels (*Kabuli* reference genome-CDC Frontier/PII)	Forward primers (5'-3')	Reverse primers (5'-3')	Anneling temperature (0C)	Expected amplified product size (bp)	Structural annotation		Functional annotation	
								Sequence components of *kabuli* genome	*Kabuli* gene accession IDs	NCBI-KOG	TFs
Ca4_P4_43243761	Ca4	43243761	GCTTCTTCTTCTTCTTCTTC/GCTTCTTCTTCTTCTTC	TCTGTTTTTGCCATCAAGCA	TTTGGAGACCAATGGTGACA	60.4	432	INTERGENIC	--	--	--
Ca4_P4_43291624	Ca4	43291624	TAA/TAAAA	GAGCGATTACCGGTGTTGTT	GCCTTCAAATGTAACCCCCT	60.0	946	INTERGENIC	--	--	--
Ca4_P4_43298328	Ca4	43298328	TTATATATATATATATATATAT/TTATATATATATATATATATATATAT	TGCGTTTGCAAAATCACAGT	TTCTAATCGGCACAATGTTCA	60.3	600	INTERGENIC	--	--	--
Ca4_P4_43301091	Ca4	43301091	ATAA/ATAATGTAA	ACCATAAAGGGAAACAGGGG	TCATGGTCTGCAATCTCGTC	60.0	714	INTERGENIC	--	--	--
Ca4_P4_43312802	Ca4	43312802	CAAA/CAA	GCCCCATTAAGCACACAAAA	ATATGGCTTGGTCCCACGTA	60.9	853	INTERGENIC	--	--	--
Ca4_P4_43328071	Ca4	43328071	ACA/ACATCA	TCCATTATATCCAAAAGGCGA	TTTCGTTCCGTTTCAGATCC	59.4	517	INTERGENIC	--	--	--
Ca4_P4_43333325	Ca4	43333325	ATTTTTTTT/ATTTTTT	TGTGGGGTAAAATGACTACAAAAA	AAAATGATTGCCGCATTTGT	59.7	617	INTERGENIC	--	--	--
Ca4_P4_43339918	Ca4	43339918	ATT/ATTT	TCATTCTCTTCATCGAAGATTTCA	TGGAGAATAATTAACAGAGCAGCA	60.2	565	INTERGENIC	--	--	--
Ca4_P4_43342892	Ca4	43342892	TAAAA/TAAAAA	AGCAGCAGCACAAGCAACTA	TCTTCATCATTGGCCAGAAA	60.0	698	INTERGENIC	--	--	--
Ca4_P4_43350385	Ca4	43350385	AACA/AA	GGACTAAATGTTTTAAAGCACGG	TTCATCATTTTGGAGAAAAGAAA	59.1	312	INTERGENIC	--	--	--

INDEL marker IDs	Chromosomes/ unanchored scaffolds	Physical positions (bp)	InDels (*Kabuli* reference genome-CDC Frontier/PII)	Forward primers (5'-3')	Reverse primers (5'-3')	Anneling temperature (0C)	Expected amplified product size (bp)	Structural annotation		Functional annotation	
								Sequence components of *kabuli* genome	*Kabuli* gene accession IDs	NCBI-KOG	TFs
Ca4_P4_43401153	Ca4	43401153	GAAAAAAAAAAAAA/GAAAAAAAAAAA	GCTAAATTACATCCGCGGTC	GTGCAAGTCCACACATGGTC	59.6	702	INTERGENIC	--	--	--
Ca4_P4_43422683	Ca4	43422683	GAAGCTAAG/GAAG	CACACTAGCCCTCATTTCCC	CACCACCAACCTCTTTGGTT	59.6	847	INTERGENIC	--	--	--
Ca4_P4_43426940	Ca4	43426940	CCACACACACACACACA/CCACACACACA	TCCAAAATAGATGCATCGGAC	TGGACATGTTAGGTGAGACCTG	59.9	271	INTERGENIC	--	--	--
Ca4_P4_43458722	Ca4	43458722	TAAAA/TAAAAA	CTTGGCTCTTTGCATTCGTT	GCGTCGGATACACCTTTGTT	60.4	365	INTERGENIC	--	--	--
Ca4_P4_43459528	Ca4	43459528	TA/T	TTCGCCCTCTTTTTATTCCA	TTCGCTCGAGTTTTGATCG	59.7	459	INTERGENIC	--	--	--
Ca4_P4_43460190	Ca4	43460190	ACC/AC	CTCGAGCGAAAATATCCCAA	TGAAACACAACAACAGGGGA	60.2	619	INTERGENIC	--	--	--
Ca4_P4_43475384	Ca4	43475384	AAA/AAAGAA	TCTCCTTCAAAATCGCCAAT	TCCAGTTGGCCATTTCTTTC	59.6	842	INTERGENIC	--	--	--
Ca4_P4_43486688	Ca4	43486688	GGGCCGG/GGG	TGAACTCTCATGTGCTTCGG	CCATCAACAAAAACACCACG	60.0	315	INTERGENIC	--	--	--
Ca4_P4_43536933	Ca4	43536933	T/TAAGA	TTGGAAGGGAGGTTCACGTA	ATTTTTGTTTCTCCGCAACG	60.5	502	INTERGENIC	--	--	--
Ca4_P4_43544345	Ca4	43544345	CAAAAAAA/CAAAAAAAA	GACCTAGTCCGCGACTCAAC	AAACCAGACTGCGTGTAGGG	59.9	781	INTERGENIC	--	--	--

**INDEL marker IDs**	**Chromosomes/ unanchored scaffolds**	**Physical positions (bp)**	**InDels (*Kabuli* reference genome-CDC Frontier/PII)**	**Forward primers (5'-3')**	**Reverse primers (5'-3')**	**Anneling temperature (0C)**	**Expected amplified product size (bp)**	**Structural annotation**		**Functional annotation**	
								**Sequence components of *kabuli* genome**	***Kabuli* gene accession IDs**	**NCBI-KOG**	**TFs**
Ca4_P4_43549490	Ca4	43549490	TA/TAA	ATGGAAGTGGAAGCCAACAG	AAAGAAGCGCGAATAATCCA	60.1	393	INTERGENIC	--	--	--
Ca4_P4_43549820	Ca4	43549820	TC/T	TGGATTATTCGCGCTTCTTT	TTCCGCTGCTCGTAAATTCT	59.8	674	INTERGENIC	--	--	--
Ca4_P4_43553592	Ca4	43553592	TG/T	AACGGGAGAAGGAGGAAAAA	CGGTTTCACACTCTGATCCA	60.0	843	INTERGENIC	--	--	--
Ca4_P4_43564232	Ca4	43564232	CTTTTTTT/CTTTTTT	TCAAACAAAATTGGGTTACAACA	TTTGGTAATCACAAAAATCCAAAA	59.3	664	INTERGENIC	--	--	--
Ca4_P4_43566104	Ca4	43566104	ATTGTGAGTTTTTTGTG/ATTGTG	AGATCCTCTCCCTTGCAAAA	TTCATTCATTGCCATTTCCA	58.9	581	INTERGENIC	--	--	--
Ca4_P4_43570031	Ca4	43570031	C/CG	GAACAGGAAAAGAACACCGC	TGGTGTGAGATTTGTTGGGA	59.7	383	INTERGENIC	--	--	--
Ca4_P4_43659877	Ca4	43659877	ATTTTTTTTT/ATTTTTTTT	TTTTTGCCACAGCTAGGGAG	TGGCACATCTATCCAATCACA	60.4	423	INTERGENIC	--	--	--
Ca4_P4_4378608	Ca4	4378608	CATATATATATATATATATATATAT/CATATATATATATATATATATATATATATATAT	TAAATTCACGCGCACGTATC	GGAGTCCGTGCAATACACCT	59.7	747	INTERGENIC	--	--	--
Ca4_P4_43895697	Ca4	43895697	GATATATATATATATATATATATATAT/GATATATATATATATATATATATAT	CCCGAGTAGTCCTCCATCAA	CTCCACAGCCTAACTCCGAC	60.1	920	INTERGENIC	--	--	--
Ca4_P4_43990162	Ca4	43990162	GTTTTTTT/GTTTTTT	TGTTGGCAAGTTTGCTTGAG	GTTCGATTTCCGCAAATGTT	60.0	511	INTERGENIC	--	--	--

INDEL marker IDs	Chromosomes/ unanchored scaffolds	Physical positions (bp)	InDels (*Kabuli* reference genome-CDC Frontier/PII)	Forward primers (5'-3')	Reverse primers (5'-3')	Anneling temperature (0C)	Expected amplified product size (bp)	Structural annotation		Functional annotation	
								Sequence components of *kabuli* genome	*Kabuli* gene accession IDs	NCBI-KOG	TFs
Ca4_P4_43999007	Ca4	43999007	TTATATATATATATATATATATATATACATATATATATATATATAT/TTATATATATATATATATATATATATATATACATATATATATATATATAT	ACGTGTATATTTGCGCACCC	TTTGTTTCAGCCCATCCAAT	60.8	625	INTERGENIC	--	--	--
Ca4_P4_44332657	Ca4	44332657	TAAATAAAATAAAATAAAA/TAAATAAAATAAAATAAAATAAAA	ACCGAAGTCAACCGCTTACA	AGTCACTTGCGAATGTCGTG	60.7	508	INTERGENIC	--	--	--
Ca4_P4_44344897	Ca4	44344897	ATTTTTTTTTTT/ATTTTTTTTTT	TTGATTATTGATTACCCACGGA	TACCCATTGATCCGATTCGT	59.2	510	INTERGENIC	--	--	--
Ca4_P4_44428431	Ca4	44428431	CATATATATATATATATATAT/CATATATATATATATATATATAT	TGTTGCGGAAATGTTAGTTCA	TTCATCCATGCAATATTCAAAAA	59.2	699	INTERGENIC	--	--	--
Ca4_P4_44486854	Ca4	44486854	ATAAA/ATAAATTAAA	CTCAGGATGAACAACTGCCA	TTCGTTTATCTCTTCAATCGCA	59.8	419	INTERGENIC	--	--	--
Ca4_P4_44488487	Ca4	44488487	T/TC	AAACCCAATTGTTGATGGGA	TTGACGGTTGTCAAAATCCA	60.0	715	INTERGENIC	--	--	--
Ca4_P4_44489358	Ca4	44489358	CTTTT/CTTT	TTTTGTTTCTCCACCCCAAA	TGACTGCACACAATAAACATTGA	60.3	400	INTERGENIC	--	--	--
Ca4_P4_44489639	Ca4	44489639	CTTAATT/CTT	TTCTGGACTGCTCCAAAACA	TCAATCGATCAGTTGCTTTTT	59.4	611	INTERGENIC	--	--	--
Ca4_P4_44490020	Ca4	44490020	AT/A	CAACTGATCGATTGATAGAAGCA	TGCTTCGCAAGATTAGTTAGGA	59.4	570	INTERGENIC	--	--	--
Ca4_P4_44493122	Ca4	44493122	CT/CTT	CAATCTTGGGAGATAGTTGTTGG	CTCACATGATGCTCGTTGCT	59.9	558	INTERGENIC	--	--	--

INDEL marker IDs	Chromosomes/ unanchored scaffolds	Physical positions (bp)	InDels (*Kabuli* reference genome-CDC Frontier/PII)	Forward primers (5'-3')	Reverse primers (5'-3')	Anneling temperature (0C)	Expected amplified product size (bp)	Structural annotation		Functional annotation	
								Sequence components of *kabuli* genome	*Kabuli* gene accession IDs	NCBI-KOG	TFs
Ca4_P4_44493805	Ca4	44493805	GACA/GA	TTGAATGCACACACAACCCT	CCATACCGAGATTATAACGCC	60.0	237	INTERGENIC	--	--	--
Ca4_P4_44507935	Ca4	44507935	CT/CTT	AGATATTTGGCGGTGGTCAG	TTAAGGGAGTGCATGAGCAA	60.0	780	INTERGENIC	--	--	--
Ca4_P4_44510870	Ca4	44510870	GT/GTTT	GCAAAACTTGCGTCTTAGGC	TTGGTTTCATGACCTGCATC	60.0	524	INTERGENIC	--	--	--
Ca4_P4_44524444	Ca4	44524444	TTTACGAATTACGA/TTTACGA	TTCTTCCCTCCTCCTTCGTT	TGTCCAATCACCAACGTCAT	60.2	490	INTERGENIC	--	--	--
Ca4_P4_44525079	Ca4	44525079	AGTATTTTTCTTA/AGTATTTTTCTTATGTATTTTTCTTA	ATGACGTTGGTGATTGGACA	TGGAGTAATCCCTAATCATTTCA	59.8	357	INTERGENIC	--	--	--
Ca4_P4_44525230	Ca4	44525230	TAAAAAAAAAAAA/TAAAAAAAA	ATGACGTTGGTGATTGGACA	GCGCTAGCAAAAATAGCCAC	59.8	884	INTERGENIC	--	--	--
Ca4_P4_44526873	Ca4	44526873	ACTCCAAA/ACTCCAAATGCTCCTCCAAA	TTCCATTCTTTTTGCCAAGG	TTGGCTCAAGTGGGATAAGC	60.0	397	INTERGENIC	--	--	--
Ca4_P4_44527209	Ca4	44527209	TG/TGG	CCATCGCTTAGCCAATTGTT	AGGCAGAAAGTCGCAGTGAT	60.1	852	INTERGENIC	--	--	--
Ca4_P4_44540601	Ca4	44540601	TAAAA/TAAAAA	AACAATGGGGTCTATCGTGTG	GGACCCATGGTCAAGAAAGA	59.7	678	INTERGENIC	--	--	--
Ca4_P4_44540632	Ca4	44540632	TAAA/TAAAA	AACAATGGGGTCTATCGTGTG	GATTGGAACCATCGAGGAGA	59.7	896	INTERGENIC	--	--	--

INDEL marker IDs	Chromosomes/ unanchored scaffolds	Physical positions (bp)	InDels (*Kabuli* reference genome-CDC Frontier/PII)	Forward primers (5'-3')	Reverse primers (5'-3')	Anneling temperature (0C)	Expected amplified product size (bp)	Structural annotation		Functional annotation	
								Sequence components of *kabuli* genome	*Kabuli* gene accession IDs	NCBI-KOG	TFs
Ca4_P4_44545143	Ca4	44545143	AATAT/AAT	TTTTCAGCCCATTCCTTTTG	TAATATGCAAGTGGAGGCCC	60.0	910	INTERGENIC	--	--	--
Ca4_P4_44555024	Ca4	44555024	T/TGAGATGGAGA	TTTCTCAAGGATCAAAATGGG	TGACCTGACCTATTCCCACC	59.0	629	INTERGENIC	--	--	--
Ca4_P4_44555673	Ca4	44555673	TA/T	AGGTCAGACCAGGCTTTGAA	TAAGTTAGGCCGTCCGTCAG	59.8	623	INTERGENIC	--	--	--
Ca4_P4_44561281	Ca4	44561281	ATTTTTTT/ATTTTTTTT	TCAAATCAAAGAATCAACAACAAAA	TTCGGTTAATTTTGCCAGGA	59.9	561	INTERGENIC	--	--	--
Ca4_P4_44563494	Ca4	44563494	CTTCTTTTT/CTTT	GGCAAATCACATGCTAGGCT	TCGTCCAATCTTGTGGTTCA	60.2	513	INTERGENIC	--	--	--
Ca4_P4_44580709	Ca4	44580709	TTAAT/TT	TGAGAGGTGTTGGGTTCAAA	CACAATATGTGGACGGCAAG	59.1	650	INTERGENIC	--	--	--
Ca4_P4_44585709	Ca4	44585709	GAATTAAATATAAATTAAATATA/GAATTAAATATAAATTAAATATAAATTAAATATA	TTTCTATTTGGGGCCTAGCA	TCTCATTGTCACCGTTGCAT	59.7	587	INTERGENIC	--	--	--
Ca4_P4_44601700	Ca4	44601700	TAAA/TAA	TGCAGGGATGAAATTCAAAAA	AGGACACGGATTCATTTGGA	60.4	380	INTERGENIC	--	--	--
Ca4_P4_44603106	Ca4	44603106	ATTTTTTT/ATTTTTT	TCAAAAACAATCAACATGGCTC	CCGTCTCAGTTGCGATCTCT	60.0	485	INTERGENIC	--	--	--
Ca4_P4_44607011	Ca4	44607011	ACTCT/ACT	GGGGAATGTAAGGGTAGTATCG	AATGCCAAACCTAAAGCGTG	58.8	579	INTERGENIC	--	--	--

INDEL marker IDs	Chromosomes/ unanchored scaffolds	Physical positions (bp)	InDels (*Kabuli* reference genome-CDC Frontier/PII)	Forward primers (5'-3')	Reverse primers (5'-3')	Anneling temperature (0C)	Expected amplified product size (bp)	Structural annotation		Functional annotation	
								Sequence components of *kabuli* genome	*Kabuli* gene accession IDs	NCBI-KOG	TFs
Ca4_P4_44607037	Ca4	44607037	ATTTT/ATTT	GGGGAATGTAAGGGTAGTATCG	AATGCCAAACCTAAAGCGTG	58.8	579	INTERGENIC	--	--	--
Ca4_P4_44611506	Ca4	44611506	TAAA/TAA	TTTTTGCCCCTATCACCAAG	TCCAATGAAGACGACAACGA	59.9	807	INTERGENIC	--	--	--
Ca4_P4_44613140	Ca4	44613140	T/TA	CCATCGTTGAAACGTTGTGAT	TGCGAGTTTCTTTATCATTTCC	60.8	428	INTERGENIC	--	--	--
Ca4_P4_44613867	Ca4	44613867	AACT/AACTGACT	TGATCAAGAAGTCACGAGTTCAA	TTAATGTTTGGGGAAGGCAA	59.9	312	INTERGENIC	--	--	--
Ca4_P4_44622646	Ca4	44622646	CT/CTGTCAGTTT	TGTTGAGTCGCCGTTAAATG	ACGCAAAGCACACACAAGAG	59.7	701	INTERGENIC	--	--	--
Ca4_P4_44631148	Ca4	44631148	GTCT/GT	CCCTATTTGGGTGCGTCTTA	CCACTTGCACAAGACTTGGA	60.0	484	INTERGENIC	--	--	--
Ca4_P4_44631480	Ca4	44631480	T/TTTA	TCCAAGTCTTGTGCAAGTGG	AGGGGTCTTTGGTGCAAAAT	59.9	683	INTERGENIC	--	--	--
Ca4_P4_44632456	Ca4	44632456	GAAAAAA/GAAAAAAA	CTTTTCGATTTTGGCTGACC	GAAGGAGAGGAGGAGGAGGA	59.7	575	INTERGENIC	--	--	--
Ca4_P4_44632709	Ca4	44632709	CAAA/CAA	TCCTCCTCCTCCTCTCCTTC	TTGAAACTTTAACGGTGCCAT	59.9	418	INTERGENIC	--	--	--
Ca4_P4_44661008	Ca4	44661008	TAAAAAA/TAAAAAAAAAA	AACTGTTGACCCAACTGACG	CTCCTTGGTTGCCATTTGTT	58.6	564	INTERGENIC	--	--	--

INDEL marker IDs	Chromosomes/ unanchored scaffolds	Physical positions (bp)	InDels (*Kabuli* reference genome-CDC Frontier/PII)	Forward primers (5'-3')	Reverse primers (5'-3')	Anneling temperature (0C)	Expected amplified product size (bp)	Structural annotation		Functional annotation	
								Sequence components of *kabuli* genome	*Kabuli* gene accession IDs	NCBI-KOG	TFs
Ca4_P4_44661599	Ca4	44661599	CAATAAT/CAATAATAAT	AACAAATGGCAACCAAGGAG	AAAATTGCGTTGAGTGGTCC	60.0	642	INTERGENIC	--	--	--
Ca4_P4_44662443	Ca4	44662443	TAAAAAAA/TAAAAAA	ACTCATTAGGGTTGTTGCCG	CACATAGCACTTGGCCTTGA	60.0	583	INTERGENIC	--	--	--
Ca4_P4_44662485	Ca4	44662485	TAAAA/TAAAAA	ACTCATTAGGGTTGTTGCCG	CACATAGCACTTGGCCTTGA	60.0	583	INTERGENIC	--	--	--
Ca4_P4_44665101	Ca4	44665101	ACCC/ACC	GGATATCCCAAATTGAGGCA	GGTGTTGAATTTGCAAAGGG	59.7	223	INTERGENIC	--	--	--
Ca4_P4_44668816	Ca4	44668816	TTGGAGAAA/TTGGAGAAAATGGAGAAA	GTTCCTCATTTGGCACACCT	TCAAGTTTCAAAATGCAACCC	60.0	212	INTERGENIC	--	--	--
Ca4_P4_44678377	Ca4	44678377	TC/T	CATTTTGCCGATCAGAGTCA	GCAGGAACATGTGGGCTAGT	59.8	694	INTERGENIC	--	--	--
Ca4_P4_44679413	Ca4	44679413	CATA/CATATATA	AATCCTCTATGGATGCCGTG	TTGTGGGATTAAAAGTCGAAAAA	59.9	707	INTERGENIC	--	--	--
Ca4_P4_44679485	Ca4	44679485	TCATG/TCATGCATG	AATCCTCTATGGATGCCGTG	TTGTGGGATTAAAAGTCGAAAAA	59.9	707	INTERGENIC	--	--	--
Ca4_P4_44679748	Ca4	44679748	ATTTTTTTT/ATTTTTTTTTT	TTTTTGTTTGCAATGGATAATCA	TACTCCGCAGTCACCAAAAG	59.4	784	INTERGENIC	--	--	--
Ca4_P4_44681210	Ca4	44681210	ATTTTT/ATTTTTT	TTTGCAATTCACCACCACTC	CGGCACATTACCCAAAAGTT	59.5	827	INTERGENIC	--	--	--

INDEL marker IDs	Chromosomes/ unanchored scaffolds	Physical positions (bp)	InDels (*Kabuli* reference genome-CDC Frontier/PII)	Forward primers (5'-3')	Reverse primers (5'-3')	Anneling temperature (0C)	Expected amplified product size (bp)	Structural annotation		Functional annotation	
								Sequence components of *kabuli* genome	*Kabuli* gene accession IDs	NCBI-KOG	TFs
Ca4_P4_44681328	Ca4	44681328	AAA/AAAGTTAA	TCTGATCCGTCTGTACCCCT	CGGCACATTACCCAAAAGTT	59.5	335	INTERGENIC	--	--	--
Ca4_P4_44681450	Ca4	44681450	TA/T	CCGTCTGTACCCCTTATCCA	CGGCACATTACCCAAAAGTT	59.8	329	INTERGENIC	--	--	--
Ca4_P4_44681620	Ca4	44681620	CTTTT/CTTTTT	CCGTCTGTACCCCTTATCCA	AAGTGGCTTGCTTTTGCATT	59.8	811	INTERGENIC	--	--	--
Ca4_P4_44685636	Ca4	44685636	CTTT/CTTTT	TGTTCAAGCGATTATGTCAAAGA	TATGTATGCAGGGAAGCACG	59.8	533	INTERGENIC	--	--	--
Ca4_P4_44685700	Ca4	44685700	T/TC	TTTCCCACACTAAATCCACTTG	TATGTATGCAGGGAAGCACG	59.0	385	INTERGENIC	--	--	--
Ca4_P4_44688565	Ca4	44688565	TAA/TA	CCTCTCACGACATTTCCACA	GGAGAGATCGCAGACTTGCT	59.7	201	INTERGENIC	--	--	--
Ca4_P4_44693876	Ca4	44693876	TAAA/TAAAA	TGGGCTAGGCTTTAGCTCTG	TCATCAATGCAAGCTCGTTC	59.7	425	INTERGENIC	--	--	--
Ca4_P4_44694609	Ca4	44694609	AC/A	ATCAGCACTCCTGAAGGGAA	CCCAGATGATCGAATCTGAA	59.8	445	INTERGENIC	--	--	--
Ca4_P4_44694688	Ca4	44694688	TAGA/TAGAGA	GATTCGATCATCTGGGGAAA	AGGCTTCTGGTGCTGTTCTT	59.8	539	INTERGENIC	--	--	--
Ca4_P4_44707356	Ca4	44707356	A/AT	CGGAATCTGAGCCATGTTTT	AGACTCGGGTTACGAGCAAA	60.1	394	INTERGENIC	--	--	--

INDEL marker IDs	Chromosomes/ unanchored scaffolds	Physical positions (bp)	InDels (*Kabuli* reference genome-CDC Frontier/PII)	Forward primers (5'-3')	Reverse primers (5'-3')	Anneling temperature (0C)	Expected amplified product size (bp)	Structural annotation		Functional annotation	
								Sequence components of *kabuli* genome	*Kabuli* gene accession IDs	NCBI-KOG	TFs
Ca4_P4_44709686	Ca4	44709686	TAAAAAAAAAAA/TAAAAAAAA	TGTTGTTGGTGTGGAATGCT	TTGACGGTTCAAATGCAAAA	60.0	482	INTERGENIC	--	--	--
Ca4_P4_44710249	Ca4	44710249	AAAGATAAGATAA/AAAGATAA	AGGAAGTGTCGCGGTTAAGA	TTTTTGTTGAATGGTGGAAAT	59.9	517	INTERGENIC	--	--	--
Ca4_P4_44710491	Ca4	44710491	GTA/GTATCATTTTTA	AGGAGTGGATGAGCCAAAAA	GGTGCTAAGTTCAAACACACAAA	59.7	822	INTERGENIC	--	--	--
Ca4_P4_44710559	Ca4	44710559	GAAAAAA/GAAAAAAA	AGGAGTGGATGAGCCAAAAA	GGTGCTAAGTTCAAACACACAAA	59.7	822	INTERGENIC	--	--	--
Ca4_P4_44711226	Ca4	44711226	TT/TTAT	TTTGTGTGTTTGAACTTAGCACC	TCTCCCCATCAACATCAGTG	59.2	478	INTERGENIC	--	--	--
Ca4_P4_44718587	Ca4	44718587	GAAA/GAA	CAAAGATCGCGTCAATGAGA	ATCTTCCTTCCCAAAGCCAT	59.9	969	INTERGENIC	--	--	--
Ca4_P4_44725573	Ca4	44725573	AC/A	GGCTCAATTGGTCCAACCTA	ATTGGTTGTGATGCCATGAA	59.9	803	INTERGENIC	--	--	--
Ca4_P4_44728647	Ca4	44728647	T/TA	TTCACCACGAGATCAAAATCA	GCTTGTGAATTTTCAAACCAAA	59.1	917	INTERGENIC	--	--	--
Ca4_P4_44738537	Ca4	44738537	ATTTTTTTTTT/ATTTTTTTT	TCATGCACCAACGTGCTTAT	AAGAAATTCCATGCAGGTGG	60.1	666	INTERGENIC	--	--	--
Ca4_P4_44738779	Ca4	44738779	TT/TTAT	GGTTGCCTCTTTTACATGGG	TCATGCATGGTTGGAAAAGA	59.4	649	INTERGENIC	--	--	--

INDEL marker IDs	Chromosomes/ unanchored scaffolds	Physical positions (bp)	InDels (*Kabuli* reference genome-CDC Frontier/PII)	Forward primers (5'-3')	Reverse primers (5'-3')	Anneling temperature (0C)	Expected amplified product size (bp)	Structural annotation		Functional annotation	
								Sequence components of *kabuli* genome	*Kabuli* gene accession IDs	NCBI-KOG	TFs
Ca4_P4_44739765	Ca4	44739765	ATTT/ATTTT	ACTCCTTTCCTTCCCACGAT	TCAAGGTCGTTTGTTGCTGT	59.9	490	INTERGENIC	--	--	--
Ca4_P4_44742473	Ca4	44742473	TAAAA/TAAAAA	TGAAAGATGGCACATGGAGA	AGATTCATTTTGGGATGCGA	60.2	851	INTERGENIC	--	--	--
Ca4_P4_44746122	Ca4	44746122	ACC/AC	TGGCCCATACTACATCTTCACA	TTGCGTCGGTTACAAATGAA	60.4	751	INTERGENIC	--	--	--
Ca4_P4_44747851	Ca4	44747851	ATTTTTTT/ATTTTTTTTT	TGGACTTGTGAAGCAAGCCT	CTTTCCCCGTGTGCTTACAT	61.0	580	INTERGENIC	--	--	--
Ca4_P4_44750498	Ca4	44750498	TT/TTACATATAT	AACTTCAATCCGCAACAAGC	TCATTTAGCCACTCGTGTGAT	60.3	602	INTERGENIC	--	--	--
Ca4_P4_44750857	Ca4	44750857	TAAAAAA/TAAAA	TCACACGAGTGGCTAAATGA	AAAACTTAATGTTTGACGAGTTGG	57.8	558	INTERGENIC	--	--	--
Ca4_P4_44764290	Ca4	44764290	GTTTT/GTTT	TCTGTGCACGTGTAAATAGACAAA	ACGACTACCGAGTGATTGCC	59.7	516	INTERGENIC	--	--	--
Ca4_P4_44764786	Ca4	44764786	ATTTTT/ATTTTTT	GGCAATCACTCGGTAGTCGT	AGGTTAGGCCATGCTTTGAA	60.1	627	INTERGENIC	--	--	--
Ca4_P4_44767237	Ca4	44767237	CT/CTATT	GCTGAACAATGCCCTAGCTC	TGAGTGACAGAAAAACGGAGAA	60.0	479	INTERGENIC	--	--	--
Ca4_P4_44771286	Ca4	44771286	CT/C	ATCGGATGTAAAATTCCCCC	TTGCTTCAGTTGGTGCATTC	59.9	411	INTERGENIC	--	--	--

INDEL marker IDs	Chromosomes/ unanchored scaffolds	Physical positions (bp)	InDels (*Kabuli* reference genome-CDC Frontier/PII)	Forward primers (5'-3')	Reverse primers (5'-3')	Anneling temperature (0C)	Expected amplified product size (bp)	Structural annotation		Functional annotation	
								Sequence components of *kabuli* genome	*Kabuli* gene accession IDs	NCBI-KOG	TFs
Ca4_P4_44772447	Ca4	44772447	AT/A	ACACCGCACGTAATGCTATG	CGAAATGTGTTTTGCTTTCG	59.6	149	INTERGENIC	--	--	--
Ca4_P4_44773132	Ca4	44773132	GAA/GA	TTGTTTCACTTTTTCCATTCCA	TAGGGTTCTTGATCCATCGC	59.5	596	INTERGENIC	--	--	--
Ca4_P4_44773439	Ca4	44773439	ATTTTTT/ATTTTTTTT	TTGTTTCACTTTTTCCATTCCA	TAGGGTTCTTGATCCATCGC	59.5	596	INTERGENIC	--	--	--
Ca4_P4_44773518	Ca4	44773518	TAAAAAAAAAAA/TAAAAAAAA	GACGATGACAATCTTTTATTGCAG	TAGGGTTCTTGATCCATCGC	60.0	106	INTERGENIC	--	--	--
Ca4_P4_44773874	Ca4	44773874	G/GA	GCGATGGATCAAGAACCCTA	GGATTTTGGCAACAGATGCT	60.0	737	INTERGENIC	--	--	--
Ca4_P4_44777732	Ca4	44777732	TAAAA/TAAA	GCTGGGTATGGTGATTTTGC	CCTTCTTAGATGCAACCCCA	60.3	476	INTERGENIC	--	--	--
Ca4_P4_44780377	Ca4	44780377	AC/A	AACCCCTCCTTCTCCTTTGA	AAGGCTCTCACGTGGAATTG	60.0	562	INTERGENIC	--	--	--
Ca4_P4_44781972	Ca4	44781972	CTT/CTTT	AGCAGAAATGGTGGCAGATT	AATCCAAATGCCATAGCTGC	59.7	538	INTERGENIC	--	--	--
Ca4_P4_44782327	Ca4	44782327	GT/GTT	TTTTGTGGCAGAAACAACGA	AATCCAAATGCCATAGCTGC	60.3	449	INTERGENIC	--	--	--
Ca4_P4_44836604	Ca4	44836604	A/AC	TGCATTAACTTGCCTCCAAA	GTTATTTTCTTGCCTCCCCG	59.3	139	INTERGENIC	--	--	--

INDEL marker IDs	Chromosomes/ unanchored scaffolds	Physical positions (bp)	InDels (*Kabuli* reference genome-CDC Frontier/PII)	Forward primers (5'-3')	Reverse primers (5'-3')	Anneling temperature (0C)	Expected amplified product size (bp)	Structural annotation		Functional annotation	
								Sequence components of *kabuli* genome	*Kabuli* gene accession IDs	NCBI-KOG	TFs
Ca4_P4_44837147	Ca4	44837147	G/GAGAT	CGGGGAGGCAAGAAAATAAC	TGCAATTTTACGTCCAAATCAT	60.8	815	INTERGENIC	--	--	--
Ca4_P4_44837783	Ca4	44837783	GTTTTT/GTTTT	CATGTTATCAATAGCGGTTCG	CTCAAACCATGCGTCAAATG	58.2	294	INTERGENIC	--	--	--
Ca4_P4_44838015	Ca4	44838015	CTT/CTTT	GTCATTTGACGCATGGTTTG	TTTCGTTGATAATTTTTGGCA	60.0	499	INTERGENIC	--	--	--
Ca4_P4_44838352	Ca4	44838352	GAAAAA/GAAAA	GTCATTTGACGCATGGTTTG	CCAAGGATTGGTGTGAAAAA	60.0	875	INTERGENIC	--	--	--
Ca4_P4_44843097	Ca4	44843097	C/CCCG	TAGGGTTGTTCATGGGCATT	TTCCACACCACATTCCATTG	60.2	468	INTERGENIC	--	--	--
Ca4_P4_44854235	Ca4	44854235	ATTTTTTTTT/ATTTTTTTT	GATGCGAGAATTGTATGCGA	TGAAATGATTAAGTGGCGAGTTC	59.8	865	INTERGENIC	--	--	--
Ca4_P4_44854537	Ca4	44854537	ATTTTTTT/ATTTTTT	CCAACTTTTTCATACACAAACACA	TGTTGCACTCGAACAAACTCA	59.0	567	INTERGENIC	--	--	--
Ca4_P4_44854776	Ca4	44854776	ATTT/ATTTT	AACTCGCCACTTAATCATTTCA	TGTTGCACTCGAACAAACTCA	58.8	400	INTERGENIC	--	--	--
Ca4_P4_44855429	Ca4	44855429	TTAG/TTAGCTAG	CACGAAAAATCAAATTCGGC	TTTTGTTTTGCAACCATCCA	60.4	417	INTERGENIC	--	--	--
Ca4_P4_44863007	Ca4	44863007	GAAAAAAA/GAAAAAA	TGAGCATGCAAAGTCCAATC	CAACCCTCCCAGTTTCACAT	59.8	285	INTERGENIC	--	--	--

INDEL marker IDs	Chromosomes/ unanchored scaffolds	Physical positions (bp)	InDels (*Kabuli* reference genome-CDC Frontier/PII)	Forward primers (5'-3')	Reverse primers (5'-3')	Anneling temperature (0C)	Expected amplified product size (bp)	Structural annotation		Functional annotation	
								Sequence components of *kabuli* genome	*Kabuli* gene accession IDs	NCBI-KOG	TFs
Ca4_P4_44865492	Ca4	44865492	TA/TAGA	TGACCATTGTGTGAAAATGAGA	TGGTCGTAAAGTGCATTGGA	59.0	685	INTERGENIC	--	--	--
Ca4_P4_44878187	Ca4	44878187	TG/TGG	CAATCGTGCAAACAATTTGG	ATGAAGCGATTGGTTGGAAG	60.0	738	INTERGENIC	--	--	--
Ca4_P4_44886483	Ca4	44886483	TTCT/TT	TAATGGAGGGCAATTTCGAG	AAAACACCCAACCCCCTAAT	60.0	349	INTERGENIC	--	--	--
Ca4_P4_44889469	Ca4	44889469	AATT/A	AACCGATATGTGTTGCCCTT	TCATCACATTGTAATCTTGGAGG	59.3	533	INTERGENIC	--	--	--
Ca4_P4_44889516	Ca4	44889516	ATT/ATTTGTT	AAACCGATATGTGTTGCCCT	TTCAAAATAGCCCTTCCAAAAA	59.3	711	INTERGENIC	--	--	--
Ca4_P4_44897539	Ca4	44897539	T/TA	ATTTGTTGGAGGATGGGTCA	CACTCTTGTTCACGGCAAAA	60.2	623	INTERGENIC	--	--	--
Ca4_P4_44900080	Ca4	44900080	T/TC	CCCCATTCGACCAAGATTTA	AGAAAGTGGGAATGTGGGGT	59.8	526	INTERGENIC	--	--	--
Ca4_P4_44922267	Ca4	44922267	CTTTTTT/CTTTTTTT	TGATTTGAGGGAGTTGGAGTG	CCCATAATGTCCCGATTCTG	60.1	676	INTERGENIC	--	--	--
Ca4_P4_44922583	Ca4	44922583	TTTTTT/TTTTTTATTTTT	CGCGAGAAATAAGTGCACAA	ACAAAACAATGGACGGAAGC	60.0	327	INTERGENIC	--	--	--
Ca4_P4_44927225	Ca4	44927225	TTT/TTTCTATTTTTATT	CGGGAGATACGTAGGAGCAA	GCTTGTGCATATCCCAACCT	60.2	438	INTERGENIC	--	--	--

INDEL marker IDs	Chromosomes/ unanchored scaffolds	Physical positions (bp)	InDels (*Kabuli* reference genome-CDC Frontier/PII)	Forward primers (5'-3')	Reverse primers (5'-3')	Anneling temperature (0C)	Expected amplified product size (bp)	Structural annotation		Functional annotation	
								Sequence components of *kabuli* genome	*Kabuli* gene accession IDs	NCBI-KOG	TFs
Ca4_P4_44934122	Ca4	44934122	T/TCC	TCAAATCGTTGAAACATGAGAGA	TCTCGTTCACCCATAGTCCC	59.7	533	INTERGENIC	--	--	--
Ca4_P4_44937033	Ca4	44937033	TAAAAAA/TAAAAA	TATCTCGCTCTTTCCTCCCA	GCTGTTTGATGGCTATGGGT	59.9	888	INTERGENIC	--	--	--
Ca4_P4_44937214	Ca4	44937214	T/TA	CGTGTGTGGTATTCCAATTTGT	GCTGTTTGATGGCTATGGGT	59.7	539	INTERGENIC	--	--	--
Ca4_P4_44939636	Ca4	44939636	TTA/TTATA	CTCGCTGAAATTGCCAAAGT	TTTTCCTAAAATAAACAACTCCCTG	60.4	460	INTERGENIC	--	--	--
Ca4_P4_44943130	Ca4	44943130	TAAAA/TAAAAA	GCTAAAACAGGGTGCAAAGG	AATAGCAACGCATTACGGCT	59.7	798	INTERGENIC	--	--	--
Ca4_P4_44949173	Ca4	44949173	AA/AAATTA	TGACGTGTCCCGTAACCTTT	ATAAGCCCATGTTGCGACTC	60.4	565	INTERGENIC	--	--	--
Ca4_P4_44971582	Ca4	44971582	TAAAAAA/TAAAAAAA	AAATTGACTAAAAATCGGGAAAAA	TTCGTCAACTCGCAACTCAC	59.3	687	INTERGENIC	--	--	--
Ca4_P4_45054175	Ca4	45054175	AA/AACA	GCACTTCTTGCTGGTTCCTC	ATGGCATCACGTGAATGTGT	60.0	933	INTERGENIC	--	--	--
Ca4_P4_4505602	Ca4	4505602	T/TA	CATTGAGAATTTGGGGACCA	TCATGTTGAAGGCTCTGTCG	60.7	891	INTERGENIC	--	--	--
Ca4_P4_45057895	Ca4	45057895	CATGGA/CATGGATGGA	GGATTGCGTATCAGTCGTCA	CGCGAAATGCAAACAAACTA	59.7	344	INTERGENIC	--	--	--

INDEL marker IDs	Chromosomes/ unanchored scaffolds	Physical positions (bp)	InDels (*Kabuli* reference genome-CDC Frontier/PII)	Forward primers (5'-3')	Reverse primers (5'-3')	Anneling temperature (0C)	Expected amplified product size (bp)	Structural annotation		Functional annotation	
								Sequence components of *kabuli* genome	*Kabuli* gene accession IDs	NCBI-KOG	TFs
Ca4_P4_45058561	Ca4	45058561	AAACAA/AAA	ATACTTTTCGACCCCGTTCC	TCACAGAAATGCTGGCAGTC	60.2	752	INTERGENIC	--	--	--
Ca4_P4_45062190	Ca4	45062190	GAA/GAATAATAA	AAGGGACGTGGTGTTTGAAG	ACCCCGTTGGTAAAAACTCC	60.0	588	INTERGENIC	--	--	--
Ca4_P4_45064783	Ca4	45064783	TAAA/TAA	GAACCAACTTCACCTCCCAA	ATCGAGTTGAGTCGTGGTCC	59.9	737	INTERGENIC	--	--	--
Ca4_P4_45065586	Ca4	45065586	TAAAA/TAAAAA	CGACTCAACTCGATCGTCAA	AAAACCTTAATCACCCGTGC	60.0	500	INTERGENIC	--	--	--
Ca4_P4_45065685	Ca4	45065685	ATGT/AT	GGCTCGAATGATGCCTAAAA	AAGATGTGATCGCAAGGAATG	60.2	899	INTERGENIC	--	--	--
Ca4_P4_4507561	Ca4	4507561	AA/AAATA	TTTGGGGACCAAAACTGAAG	TCCGAGCAACCATACTTTCA	59.9	698	INTERGENIC	--	--	--
Ca4_P4_45076676	Ca4	45076676	GTTT/GTTTT	CATTGACTTTCGGGCAAGAT	TCCACAAATAACAGGTTTTCCC	60.1	741	INTERGENIC	--	--	--
Ca4_P4_45076892	Ca4	45076892	CG/CGG	GGTCCAACATTCCTCCTTGA	TCCACAAATAACAGGTTTTCCC	59.9	606	INTERGENIC	--	--	--
Ca4_P4_45093112	Ca4	45093112	AAAAATAAAA/AAAAA	CAACCCTTTTTATCCAATAATGC	AGCACATGAATTGCAAGCAG	58.8	528	INTERGENIC	--	--	--
Ca4_P4_45098286	Ca4	45098286	TAAAAAA/TAAAAAAA	CCCAATTTCAATCCTCCAGA	GAGGCGGGGTGTATCACTAA	59.9	454	INTERGENIC	--	--	--

INDEL marker IDs	Chromosomes/ unanchored scaffolds	Physical positions (bp)	InDels (*Kabuli* reference genome-CDC Frontier/PII)	Forward primers (5'-3')	Reverse primers (5'-3')	Anneling temperature (0C)	Expected amplified product size (bp)	Structural annotation		Functional annotation	
								Sequence components of *kabuli* genome	*Kabuli* gene accession IDs	NCBI-KOG	TFs
Ca4_P4_45100378	Ca4	45100378	TTATATATATATATATATATA/TTATATATATATATATATATATATATATATATATATA	TTGCGTTGATCTTGTTGGAA	TTTGTTGACTGGTTTTGGGA	60.2	879	INTERGENIC	--	--	--
Ca4_P4_45108878	Ca4	45108878	CAAAAA/CAAAAAA	AAACCCAAACTTTTTCATGGT	GCTTTGGAGGTTGGTTTTGA	57.5	393	INTERGENIC	--	--	--
Ca4_P4_45115420	Ca4	45115420	TAAAAAAAAAAAAAAA/TAAAAAAAAAAAA	TCGCAATGAGTTGATTGTTCA	TCATCATCCGACTTTGCTTG	60.3	701	INTERGENIC	--	--	--
Ca4_P4_4512130	Ca4	4512130	GAAAAAAAAA/GAAAAAAAA	TAGACTCAACTCTGGCGGCT	CCGGCGAGAATTTTTCAATA	60.2	266	INTERGENIC	--	--	--
Ca4_P4_45148376	Ca4	45148376	TAAA/TAA	GGGGTTGGTTTTGCTTGTTA	ATGCGGCTGTTTTGTTCACT	59.8	711	INTERGENIC	--	--	--
Ca4_P4_45150211	Ca4	45150211	TTTCTTTTTATT/TTT	ACCATTGTGGTTGTGTGCAT	TGGTGGTGAAAGATGGATGA	59.7	831	INTERGENIC	--	--	--
Ca4_P4_45168651	Ca4	45168651	TAAAAAA/TAAAAAAAA	TTGATGGCCTTCTCGAAGAT	ATGCAGCTTTTGGATGTGTG	59.8	520	INTERGENIC	--	--	--
Ca4_P4_45169109	Ca4	45169109	CATATATA/CATA	CACACATCCAAAAGCTGCAT	TCAAAGAAAATCGTCGGAGG	59.7	632	INTERGENIC	--	--	--
Ca4_P4_4517051	Ca4	4517051	ATTT/ATTTT	AAAATGCGTTTCACTCGGAC	GCACACTTGTGCTTGGAAGA	60.1	304	INTERGENIC	--	--	--
Ca4_P4_4517104	Ca4	4517104	GATGAATTTATTAAT/GATGAATTTATTAATGAATTTATTAAT	AAAATGCGTTTCACTCGGAC	GCACACTTGTGCTTGGAAGA	60.1	304	INTERGENIC	--	--	--

INDEL marker IDs	Chromosomes/ unanchored scaffolds	Physical positions (bp)	InDels (*Kabuli* reference genome-CDC Frontier/PII)	Forward primers (5'-3')	Reverse primers (5'-3')	Anneling temperature (0C)	Expected amplified product size (bp)	Structural annotation		Functional annotation	
								Sequence components of *kabuli* genome	*Kabuli* gene accession IDs	NCBI-KOG	TFs
Ca4_P4_45174430	Ca4	45174430	CTACGTAC/CTAC	GTCAAACCCCAAATTCAACG	CAATGCTCATTGGTGCTTCA	60.2	579	INTERGENIC	--	--	--
Ca4_P4_45174707	Ca4	45174707	AGTTGTT/AGTTGTTGTT	GGCTGCAAAAAGTTCACGAC	AGAAGGTCTAACCGCTGCAA	60.8	702	INTERGENIC	--	--	--
Ca4_P4_45177041	Ca4	45177041	ACCCCCC/ACCCCC	CCAAGTTTTTGCAACCCAGT	TGTGTACCGGAAAACTTCAAAA	60.0	363	INTERGENIC	--	--	--
Ca4_P4_45177094	Ca4	45177094	ATT/AT	CCAAGTTTTTGCAACCCAGT	TGTGTACCGGAAAACTTCAAAA	60.0	363	INTERGENIC	--	--	--
Ca4_P4_45178018	Ca4	45178018	GAAAAAA/GAAAAA	TGAAATGGGTTTGTACTTTTCG	CCATTCGAGTGGTTGTGTTG	59.0	738	INTERGENIC	--	--	--
Ca4_P4_45182355	Ca4	45182355	AATATATATATATATATATATATAT/AATATATATATATATATATAT	TCATTGTCTTGGTCTTCCCC	GTGGTATTTAGTAAAATGAATGTGTCA	59.9	428	INTERGENIC	--	--	--
Ca4_P4_45182772	Ca4	45182772	AATATATATATATATAT/AATATATATAT	TGAAAAATTAAATAAAAAGCTGACGA	CAGTATTTATTCGGCGGGAA	59.6	629	INTERGENIC	--	--	--
Ca4_P4_45182831	Ca4	45182831	TTA/TTATGAAATA	AAAATTAAATAAAAAGCTGACGAAA	CAGTATTTATTCGGCGGGAA	57.1	626	INTERGENIC	--	--	--
Ca4_P4_45183168	Ca4	45183168	CTCACAATAAATATTATCACAATAAA/CTCACAATAAA	TTCCCGCCGAATAAATACTG	AGGGCCCATCTTTTCACTCT	59.9	671	INTERGENIC	--	--	--
Ca4_P4_45194041	Ca4	45194041	CAATA/CA	GAGCGAGTCAAAACCGAGTC	GCAAGCAATCAAGTAAAAATGG	60.0	597	INTERGENIC	--	--	--

INDEL marker IDs	Chromosomes/ unanchored scaffolds	Physical positions (bp)	InDels (*Kabuli* reference genome-CDC Frontier/PII)	Forward primers (5'-3')	Reverse primers (5'-3')	Anneling temperature (0C)	Expected amplified product size (bp)	Structural annotation		Functional annotation	
								Sequence components of *kabuli* genome	*Kabuli* gene accession IDs	NCBI-KOG	TFs
Ca4_P4_45196611	Ca4	45196611	GATATATATATATA/GATATATATATATATATATA	CCAAGTTTTGTCCAAGCAAA	TAGTGTCATTCGTGGGTTGC	58.8	662	INTERGENIC	--	--	--
Ca4_P4_4520382	Ca4	4520382	CG/C	CATGCAATTGAACAATATCCG	GTGGGCTTTCAAGTGTCCAT	58.9	520	INTERGENIC	--	--	--
Ca4_P4_45205031	Ca4	45205031	AG/AGGG	ACCCCATCTACCAAACCCTC	TATTCCCTCCTCCCATTTCC	60.1	592	INTERGENIC	--	--	--
Ca4_P4_45205185	Ca4	45205185	TC/T	GGAAATGGGAGGAGGGAATA	TCGTGTTTTGAATAAACAGGAAAA	60.1	300	INTERGENIC	--	--	--
Ca4_P4_45205721	Ca4	45205721	ACC/AC	CGTATTTTGGCTTAAATATTTCCAA	CCTCCAACCCACTGATCCTA	59.7	451	INTERGENIC	--	--	--
Ca4_P4_4521076	Ca4	4521076	AATATATATATATATATATA/AATATATATATATATA	CCACAGAAAATGTTGGGCTT	TCGTAAAATAGTTGGCCGGA	60.0	568	INTERGENIC	--	--	--
Ca4_P4_45211328	Ca4	45211328	CATATAT/CATATATAT	GTTGGTTGGTGGCTTAGCAT	TCGTTTCGCAATGACTTCTG	60.0	552	INTERGENIC	--	--	--
Ca4_P4_4521903	Ca4	4521903	ATTTTTTTTTTT/ATTTTTTTTTT	TGCTTGTATGGTTTTAATACGGG	AAACATAAAACAACACAGGACAAA	60.1	690	INTERGENIC	--	--	--
Ca4_P4_45223304	Ca4	45223304	ATTACT/ATGAATTACTTACT	TGAAGAGGGGAAATATGCAA	GAAAACGCTTTTATCATCATCCA	58.2	442	INTERGENIC	--	--	--
Ca4_P4_4522341	Ca4	4522341	ATTTTTTTTTT/ATTTTTTTTT	TTTGTCCTGTGTTGTTTTATGTTT	GGGCCGAAAATTTGTCATT	57.7	276	INTERGENIC	--	--	--

INDEL marker IDs	Chromosomes/ unanchored scaffolds	Physical positions (bp)	InDels (*Kabuli* reference genome-CDC Frontier/PII)	Forward primers (5'-3')	Reverse primers (5'-3')	Anneling temperature (0C)	Expected amplified product size (bp)	Structural annotation		Functional annotation	
								Sequence components of *kabuli* genome	*Kabuli* gene accession IDs	NCBI-KOG	TFs
Ca4_P4_4522910	Ca4	4522910	ATTTTTTTT/ATTTTTTT	AATGACAAATTTTCGGCCC	TACCCTCCTTCGCATCAATC	59.8	775	INTERGENIC	--	--	--
Ca4_P4_45247490	Ca4	45247490	AAAA/AAAAGAAA	GTTGGAAGCCATGGTGAACT	TTTTGGTTTTCAAGTATTGGCA	60.0	430	INTERGENIC	--	--	--
Ca4_P4_45247621	Ca4	45247621	TA/TAA	GTTGGAAGCCATGGTGAACT	TTTTGGTTTTCAAGTATTGGCA	60.0	430	INTERGENIC	--	--	--
Ca4_P4_45247654	Ca4	45247654	TAAAAAAAAAA/TAAAAAAAA	GTTGGAAGCCATGGTGAACT	TTTTGGTTTTCAAGTATTGGCA	60.0	430	INTERGENIC	--	--	--
Ca4_P4_45249900	Ca4	45249900	TAG/TAGAG	CCAAACAACAGGGGTTGAAG	CCCGGCCCATGTAACTAATA	60.4	949	INTERGENIC	--	--	--
Ca4_P4_45250132	Ca4	45250132	T/TATTA	ACGTCCACGTCAGCAAGATA	TGGTCGATCAGGTGTCTTCA	59.3	557	INTERGENIC	--	--	--
Ca4_P4_45271673	Ca4	45271673	TGGG/TGG	TTGTTGTGGTTTGGCTTCAA	CCCACCAATCTCTAAAGGCA	60.1	449	INTERGENIC	--	--	--
Ca4_P4_45271723	Ca4	45271723	TATTTA/T	TTGTTGTGGTTTGGCTTCAA	CCCACCAATCTCTAAAGGCA	60.1	449	INTERGENIC	--	--	--
Ca4_P4_4527520	Ca4	4527520	TTATATATATATATATATATATATATATATATAT/TTATATATATATATATATATATATATATAT	AAGGTATGCTGTTTCAAGGGAA	AATGTGCATCTGCCACCATA	60.0	892	INTERGENIC	--	--	--
Ca4_P4_4527820	Ca4	4527820	GAAAAAAAA/GAAAAAAA	TTTTCACTCGATACGGCTTTT	AATGTGCATCTGCCACCATA	58.9	163	INTERGENIC	--	--	--

INDEL marker IDs	Chromosomes/ unanchored scaffolds	Physical positions (bp)	InDels (*Kabuli* reference genome-CDC Frontier/PII)	Forward primers (5'-3')	Reverse primers (5'-3')	Anneling temperature (0C)	Expected amplified product size (bp)	Structural annotation		Functional annotation	
								Sequence components of *kabuli* genome	*Kabuli* gene accession IDs	NCBI-KOG	TFs
Ca4_P4_45289876	Ca4	45289876	GAAAAAAAAAA/GAAAAAAAA	AAGCGAGCATCAGAGAAAGC	CTTGATGGCAAAACCCAAGT	59.9	269	INTERGENIC	--	--	--
Ca4_P4_45290418	Ca4	45290418	GAAA/GAAAA	CTTGGGTTTTGCCATCAAGT	GAAGCAATCGTGTGTAGCCA	60.0	604	INTERGENIC	--	--	--
Ca4_P4_45295340	Ca4	45295340	TAAAAAAAAAA/TAAAAAAAA	GACGTATCCACTGTTCTTTTCG	CGGACAGGTTCCACTCCTAA	58.8	737	INTERGENIC	--	--	--
Ca4_P4_45296395	Ca4	45296395	TAAAAAA/TAAAAAAA	TTAAATTAAGCCGAAGGCGA	AAGATTCTCCAAAGAGGCAAA	59.8	800	INTERGENIC	--	--	--
Ca4_P4_45297365	Ca4	45297365	A/AT	CTTGTGTCCTCTCACACGTCA	ACCCTCGCCTTGATACACAC	59.9	343	INTERGENIC	--	--	--
Ca4_P4_45297508	Ca4	45297508	A/AGT	CAACAAGGTCGCTCATCGTA	ACCCTCGCCTTGATACACAC	59.9	147	INTERGENIC	--	--	--
Ca4_P4_45297724	Ca4	45297724	TG/T	GTGTGTATCAAGGCGAGGGT	AGTTAAGAGAGCGCGTGTCC	60.0	579	INTERGENIC	--	--	--
Ca4_P4_45297957	Ca4	45297957	TAAAAAAAA/TAAAAAAAAA	GTGTGTATCAAGGCGAGGGT	AGTTAAGAGAGCGCGTGTCC	60.0	579	INTERGENIC	--	--	--
Ca4_P4_45318040	Ca4	45318040	CAA/CAAA	GCAAACACAACTATATGCAGCG	CAGGGGTAAGAGACGATTCAA	60.7	190	INTERGENIC	--	--	--
Ca4_P4_45319208	Ca4	45319208	T/TGATAAAACA	TGGTTCACGGAATAGAGGAA	AAAAGAAGGACCAACCACCA	58.1	688	INTERGENIC	--	--	--

INDEL marker IDs	Chromosomes/ unanchored scaffolds	Physical positions (bp)	InDels (*Kabuli* reference genome-CDC Frontier/PII)	Forward primers (5'-3')	Reverse primers (5'-3')	Anneling temperature (0C)	Expected amplified product size (bp)	Structural annotation		Functional annotation	
								Sequence components of *kabuli* genome	*Kabuli* gene accession IDs	NCBI-KOG	TFs
Ca4_P4_45334419	Ca4	45334419	ATT/ATTT	TTTAATGTGGAAAGAGAGACATGAA	AAACACATTTGACCGCCATT	59.2	357	INTERGENIC	--	--	--
Ca4_P4_45334674	Ca4	45334674	TA/T	ATCATTCTTGAATGGCGGTC	CAAGACTTCGGGCACTTTTC	59.9	513	INTERGENIC	--	--	--
Ca4_P4_45334856	Ca4	45334856	AACG/AACGACG	ATCATTCTTGAATGGCGGTC	AGAAGCCATGTGATCGGAAC	59.9	684	INTERGENIC	--	--	--
Ca4_P4_45334966	Ca4	45334966	ATT/ATTT	ATCATTCTTGAATGGCGGTC	AGAAGCCATGTGATCGGAAC	59.9	684	INTERGENIC	--	--	--
Ca4_P4_45359294	Ca4	45359294	A/AATGCG	TGACCGATAATCGTGACAAAA	GGGGATCCAAAACTAAAGGC	59.0	720	INTERGENIC	--	--	--
Ca4_P4_45359395	Ca4	45359395	AG/A	TCAGTTAGCAATGACCCTCAAA	GGGGATCCAAAACTAAAGGC	59.7	429	INTERGENIC	--	--	--
Ca4_P4_45368880	Ca4	45368880	TA/TTAA	TGAATTGGTTGAGTGAAAGGAA	GAGTTATGTTAATTTTCGAGTTTTGAA	59.6	120	INTERGENIC	--	--	--
Ca4_P4_4537624	Ca4	4537624	TAATAAAATAAAATAAAAT/TAATAAAATAAAATAAAATAAAAT	TGCATCGCCCATATCAATAA	TATTGGACCGACCCTGAAAA	59.9	511	INTERGENIC	--	--	--
Ca4_P4_45384926	Ca4	45384926	CTCGACTCT/C	TCGATTGGTTGGTGACAAAA	CAAAGTTCGTGTAGGAGGGA	59.9	205	INTERGENIC	--	--	--
Ca4_P4_45385545	Ca4	45385545	ACT/A	CACCCATAATACTTTATGAGATGGAA	CACAAATTAAGGGAAGGCGA	59.6	363	INTERGENIC	--	--	--

INDEL marker IDs	Chromosomes/ unanchored scaffolds	Physical positions (bp)	InDels (*Kabuli* reference genome-CDC Frontier/PII)	Forward primers (5'-3')	Reverse primers (5'-3')	Anneling temperature (0C)	Expected amplified product size (bp)	Structural annotation		Functional annotation	
								Sequence components of *kabuli* genome	*Kabuli* gene accession IDs	NCBI-KOG	TFs
Ca4_P4_45386230	Ca4	45386230	ATATTTATTTATTTATT/ATATTTATTTATTTATTTATT	CGCCTTCCCTTAATTTGTGA	TACATTGAGATTGACCGCCA	60.1	827	INTERGENIC	--	--	--
Ca4_P4_45387909	Ca4	45387909	CCT/CCTTCT	CTTCCTCCTCCAAATGACCA	AGGACCACTTGAAATGCCAC	60.0	730	INTERGENIC	--	--	--
Ca4_P4_45390678	Ca4	45390678	ACCCC/ACCCCC	CTCGAAGTGATGGCAGTCAA	CAGTAGGGTTTGGGACTCCA	60.0	791	INTERGENIC	--	--	--
Ca4_P4_45391070	Ca4	45391070	GAAAAAA/GAAAAAAA	GGATTTCAACTTTGAGGGCA	GAGCAGTAGCTCAGTGTGGG	60.1	724	INTERGENIC	--	--	--
Ca4_P4_45391856	Ca4	45391856	CTTTTT/CTTTT	CCACACTGAGCTACTGCTCC	TCTCCGTGGGCTTAACAATC	58.6	634	INTERGENIC	--	--	--
Ca4_P4_45396716	Ca4	45396716	ATT/AT	CGGAAAGGTTTTTGGTTGTG	GTCACACTCACGTGACCCAC	60.4	748	INTERGENIC	--	--	--
Ca4_P4_45397276	Ca4	45397276	ATTTTTTT/ATTTTTTTT	CACGTGAGTGTGACATGGTG	TAAATGGGAACGCAAAAGGT	59.6	372	INTERGENIC	--	--	--
Ca4_P4_45399331	Ca4	45399331	ATTTTTTTTTTTTTTT/ATTTTTTTTTT	ACCACTTCACCACAAGAGGG	ATTCCGCTTTGCTGTCAAAC	60.0	445	INTERGENIC	--	--	--
Ca4_P4_45401996	Ca4	45401996	TAAAAAAAAA/TAAAAAAAA	TTGATAGTGGCTGGCACAAA	GGGGTTTTGGTTTTGGTTTT	60.3	480	INTERGENIC	--	--	--
Ca4_P4_4541237	Ca4	4541237	ATTT/ATT	TATTCAACAGCGCCAAAGTG	CCGCTACTTTCAACATCCCT	59.9	964	INTERGENIC	--	--	--

INDEL marker IDs	Chromosomes/ unanchored scaffolds	Physical positions (bp)	InDels (*Kabuli* reference genome-CDC Frontier/PII)	Forward primers (5'-3')	Reverse primers (5'-3')	Anneling temperature (0C)	Expected amplified product size (bp)	Structural annotation		Functional annotation	
								Sequence components of *kabuli* genome	*Kabuli* gene accession IDs	NCBI-KOG	TFs
Ca4_P4_45414589	Ca4	45414589	CTT/CTTT	TGCATAAATGCACGATGAAAA	GTTCAAACCGGCAATGTCTT	60.1	740	INTERGENIC	--	--	--
Ca4_P4_4541506	Ca4	4541506	TATACATACATACA/TATACATACA	CACTATTTAAAGTATGTCACGTCGC	GGACCACACTGCGGTAACTT	59.2	695	INTERGENIC	--	--	--
Ca4_P4_45431315	Ca4	45431315	CTT/CTTT	GAGGGCAAGACCAAGATCAA	CCACCAAAAATCTCCCTATGA	60.2	248	INTERGENIC	--	--	--
Ca4_P4_45431331	Ca4	45431331	GATATAT/GAT	GAGGGCAAGACCAAGATCAA	CCACCAAAAATCTCCCTATGA	60.2	248	INTERGENIC	--	--	--
Ca4_P4_45432247	Ca4	45432247	TTTGTT/TTT	TTTGTTGTGTGCATTCATGTG	GGTAAACACATGACCCGTCC	59.0	746	INTERGENIC	--	--	--
Ca4_P4_45433971	Ca4	45433971	TTA/TTAATA	ATGGGATTGGGTATTGTTGC	TTAATCGCCCTCAAGGTCAC	59.5	406	INTERGENIC	--	--	--
Ca4_P4_45434747	Ca4	45434747	A/ATATAT	AGACCATGTTTTGATAACGCC	GGTCCAATTTGCCTACCACA	59.0	582	INTERGENIC	--	--	--
Ca4_P4_45436047	Ca4	45436047	TAAAAAAAAAAAAA/TAAAAAAAAAA	TGTTTGGCTTTTCCACATCA	TCACATCATGCAAATCCAATG	60.1	397	INTERGENIC	--	--	--
Ca4_P4_45436331	Ca4	45436331	TT/TTCATAT	TGCTAATAACGTGTCACGCC	CTCTCCCAACATATGATGCCT	59.8	819	INTERGENIC	--	--	--
Ca4_P4_45436462	Ca4	45436462	AC/A	AACCATGCACATGACTCACAA	CGAGCGTGACGAAATAATCA	60.0	850	INTERGENIC	--	--	--

INDEL marker IDs	Chromosomes/ unanchored scaffolds	Physical positions (bp)	InDels (*Kabuli* reference genome-CDC Frontier/PII)	Forward primers (5'-3')	Reverse primers (5'-3')	Anneling temperature (0C)	Expected amplified product size (bp)	Structural annotation		Functional annotation	
								Sequence components of *kabuli* genome	*Kabuli* gene accession IDs	NCBI-KOG	TFs
Ca4_P4_45437836	Ca4	45437836	AA/AATA	TCAACTTGGCTTCATCCTCC	TTCGAAACGCACAATTAATCA	60.2	435	INTERGENIC	--	--	--
Ca4_P4_45439383	Ca4	45439383	GTTTTTTTTTTTT/GTTTTTTTTTTTTTT	TTTGGATGTGGTTGTGATGAA	ACCCTCGTAACTCCAAGCCT	59.8	338	INTERGENIC	--	--	--
Ca4_P4_45440447	Ca4	45440447	AT/A	GGAGGTCGAACTCGAGAATG	AAGTGGCGCTTAATCCAAAA	59.8	784	INTERGENIC	--	--	--
Ca4_P4_45441219	Ca4	45441219	GTATATAT/GTAT	TTTGGATTAAGCGCCACTTT	ATGGGTTCATGTTTGACCGT	59.7	949	INTERGENIC	--	--	--
Ca4_P4_45444757	Ca4	45444757	ACTCTCTCTCTCTCTCTCTCTC/ACTCTCTCTCTCTCTCTCTCTC	TTGCTGGATAAAGTTGGCAG	GGACCTTTCTTCACGTTTGC	58.9	634	INTERGENIC	--	--	--
Ca4_P4_45488073	Ca4	45488073	TTATATATATATATATATATAT/TTATATATATATATATAT	TCGCCATTTCGAACTTGAAT	GGCGACTTTCCACATGATTT	60.6	284	INTERGENIC	--	--	--
Ca4_P4_45556140	Ca4	45556140	AATAATATAATAT/AATAATATAATATAATAT	TCATAATCCACAATCGCCCT	TCTTTTAAGATGTCAGTTGGAATACG	60.3	936	INTERGENIC	--	--	--
Ca4_P4_45567618	Ca4	45567618	TTATATATATATATATATAT/TTATATATATATATAT	TCACAAATGGCAGTTTTGTCTT	CCAATCCTAGCTTCTGAAAATG	59.7	635	INTERGENIC	--	--	--
Ca4_P4_45580465	Ca4	45580465	CTTTTT/CT	GGAAGGAACGGTGCTTCATA	TGCCACATCACCCTAAATCA	60.1	453	INTERGENIC	--	--	--
Ca4_P4_45591547	Ca4	45591547	CTTTTTTT/CTTTTTTTT	TTTGGAAGGCAACTAATGTCAA	CGCCAAACGGTCTCTAAAAA	59.6	499	INTERGENIC	--	--	--

INDEL marker IDs	Chromosomes/ unanchored scaffolds	Physical positions (bp)	InDels (*Kabuli* reference genome-CDC Frontier/PII)	Forward primers (5'-3')	Reverse primers (5'-3')	Anneling temperature (0C)	Expected amplified product size (bp)	Structural annotation		Functional annotation	
								Sequence components of *kabuli* genome	*Kabuli* gene accession IDs	NCBI-KOG	TFs
Ca4_P4_45592829	Ca4	45592829	ATTTTTTTTTT/ATTTTTTTTTTT	TGCAAAGACATCATCTCCCA	GGCAATGTAGCATTTTGGGT	60.2	660	INTERGENIC	--	--	--
Ca4_P4_45645932	Ca4	45645932	GT/G	AACTTGATGCCGAGTAGCGT	GCCGAATAAAACCCTCACAA	59.9	713	INTERGENIC	--	--	--
Ca4_P4_45740702	Ca4	45740702	TTATATATATATATATATATATATATATATA/TTATATATATATATATATATATATATATATA	TGTGATGCGGATTTAAAACAA	AACAAAATACGCGTGCATGA	59.1	672	INTERGENIC	--	--	--
Ca4_P4_45756786	Ca4	45756786	GAAA/GAA	CAACTCCACACCCCTCTGTT	ATCACGCAACATAACACCCA	60.0	386	INTERGENIC	--	--	--
Ca4_P4_45844295	Ca4	45844295	AAATAATAATAATAATAATAATAATAATAATAATAACAATAATAATAATAATAA/AAATAATAATAATAATAATAATAATAATAATAATAATAATAACAATAATAATAATAATAA	TTCACTGCAACACCCTTCTG	TCCAACACTTCTTTCCCATGT	59.9	847	INTERGENIC	--	--	--
Ca4_P4_4589864	Ca4	4589864	CTTT/CTT	TTTCGCTCAATGTGAAAAGC	AACGGAGGCAGTAATTGTCG	59.0	322	INTERGENIC	--	--	--
Ca4_P4_4590029	Ca4	4590029	C/CAT	TTTCGCTCAATGTGAAAAGC	CCACGATAACGATGTGATGC	59.0	749	INTERGENIC	--	--	--
Ca4_P4_4591345	Ca4	4591345	TAAAAAAA/TAAAAAAAA	TTCGCATATCAATGGTGAATTT	GTTGGTGTTCGACCTTCCAG	59.3	752	INTERGENIC	--	--	--
Ca4_P4_4594832	Ca4	4594832	GAAA/GAAAAATAAA	TATGAGTGGGGATGATGGGT	CCTTGTTTCCCGCACATAAT	60.0	403	INTERGENIC	--	--	--
Ca4_P4_45949230	Ca4	45949230	AATTATTATTATTATTATTATTATTATTATTATTATGATTATTATTATTAT/AATTATTATTATTATTATTATTATTATTATTATTATTATTATTATGATTATTATTATTAT	TCGAAAGAACCTCGACTCGT	TCAATATGAGGTCGCTTCCC	60.0	508	INTERGENIC	--	--	--

INDEL marker IDs	Chromosomes/ unanchored scaffolds	Physical positions (bp)	InDels (*Kabuli* reference genome-CDC Frontier/PII)	Forward primers (5'-3')	Reverse primers (5'-3')	Anneling temperature (0C)	Expected amplified product size (bp)	Structural annotation		Functional annotation	
								Sequence components of *kabuli* genome	*Kabuli* gene accession IDs	NCBI-KOG	TFs
Ca4_P4_45959235	Ca4	45959235	TTTATTATTATTATTATTATTATTATTAT/TTTATTATTATTATTATTATTATTATTATTAT	GGCCTATGGCCTAGCCTACT	ACGGCCTACCATAGGCTTTT	59.7	555	INTERGENIC	--	--	--
Ca4_P4_46001807	Ca4	46001807	GATAATAATAATAATAATAATAATAATAATAATAATAATAATA/GATAATAATAATAATAATAATAATAATAATAATAATAATAATAATA	TTGCTCCTACGTATCCCCAG	CTCAATCGTGGGTGACCTTT	60.1	385	INTERGENIC	--	--	--
Ca4_P4_46004838	Ca4	46004838	TAAAAAA/TAAAAAAA	TGATTTCAATGAGAGGATTTTGTT	GCTCGATTGATGTTAAGGGC	59.0	425	INTERGENIC	--	--	--
Ca4_P4_46006002	Ca4	46006002	TATATAATATA/TATATA	TTTGTGTGCCGGAAAATATG	GGTGAAATGAAAGGACCCAA	59.4	793	INTERGENIC	--	--	--
Ca4_P4_46035567	Ca4	46035567	ATTTTTTTTT/ATTTTTTTTTT	GCAATCAATTATGAGTGGGGA	CAACAAAATCCTGTCATTGAAATC	59.8	452	INTERGENIC	--	--	--
Ca4_P4_46040811	Ca4	46040811	T/TG	TGCGCTTCTCATTTACGTTG	AGCAAGTTCTCCCAAAAGCA	60.0	423	INTERGENIC	--	--	--
Ca4_P4_46060270	Ca4	46060270	CATATATATATATATATATATATATATATATATATAT/CATATATATATATATATATATATATATATAT	ATTTTAACGGATGTCGCAGG	TGTGGTGAATTTTTCTTCTTCTGA	60.0	742	INTERGENIC	--	--	--
Ca4_P4_46189254	Ca4	46189254	GTTATTATTATTATTATTATTATTATTATTATTATT/GTTATTATTATTATTATTATTATTATTATTATTATTATTATTATTATTATTATT	AACAACTTGATCCTATTTTGGAGA	TTTTAGGTTGAATCACCGCC	58.3	838	INTERGENIC	--	--	--
Ca4_P4_46377594	Ca4	46377594	TTTTACAT/TT	ATGGGAACCCACAAACAATC	CCACATGCTATTTTAATCTTTGGA	59.5	732	INTERGENIC	--	--	--
Ca4_P4_46384693	Ca4	46384693	T/TCCCTATAAAAA	GAGGAGGGTGGAGAATGGAT	GGGTGCCTGGTACAAAACTC	60.3	708	INTERGENIC	--	--	--

INDEL marker IDs	Chromosomes/ unanchored scaffolds	Physical positions (bp)	InDels (*Kabuli* reference genome-CDC Frontier/PII)	Forward primers (5'-3')	Reverse primers (5'-3')	Anneling temperature (0C)	Expected amplified product size (bp)	Structural annotation		Functional annotation	
								Sequence components of *kabuli* genome	*Kabuli* gene accession IDs	NCBI-KOG	TFs
Ca4_P4_46493029	Ca4	46493029	AATATATATATATATATATATATATA/ AATATATATATATATATATATA	GGGAAAGTGATATTGG GGGT	CAAAGCTCCTTCAATTG TCTTG	59.9	407	INTERGENIC	--	--	--
Ca4_P4_46493521	Ca4	46493521	TAAAAAAAAAAAA/TAAAAAAAAAAAA AA	CAAGACAATTGAAGGA GCTTTG	CTTGTTTGGAACCCATT GCT	59.0	841	INTERGENIC	--	--	--
Ca4_P4_46494987	Ca4	46494987	TTGATGAT/TTGAT	TGAATTCGACTTGATTT CCAA	CTTGTTTGGAACCCATT GCT	58.2	622	INTERGENIC	--	--	--
Ca4_P4_46495777	Ca4	46495777	ATATTATTATTATTATTATTATTATTA TTATTATTATTATTATTATTATTATTA T/ATATTATTATTATTATTATTATTAT TATTATTATTATTATTATTATTATTAT TATTATTAT	GAGGCTTCATTCCTCT CGTG	TGAATTGGTGGATCAA TGAAAA	60.0	478	INTERGENIC	--	--	--
Ca4_P4_46508599	Ca4	46508599	AATATATATATATATATATATATA/AA TATATATATATATATATA	AAAGGGGGAGTATTGT TGGG	CAAAGCTCTTTCATAAT TGTCTTGA	60.0	492	INTERGENIC	--	--	--
Ca4_P4_46560817	Ca4	46560817	CAAAAAAAAAAA/CAAAAAAAAAA	ACCATTTCCTGATTTTT GCG	ATGAGGAGGATGTCAG CCC	59.9	838	INTERGENIC	--	--	--
Ca4_P4_46581610	Ca4	46581610	CAATAATAATAATAATAATAATAATA ATAATAATAATAATAATA/CAATAATA ATAATAATAATAATAATAATAATAAT AATAATAATAATA	CGTCCTCGAAACTAAG AGGAAA	TTTCGAAGAAGGCGAT AACAA	59.9	993	INTERGENIC	--	--	--
Ca4_P4_46674801	Ca4	46674801	A/AG	GGATGACTCGAAACGG TGTT	TTACCCAAAACCCCAAA TCA	60.0	667	INTERGENIC	--	--	--
Ca4_P4_46683003	Ca4	46683003	GAAGA/GAAGAGAAAGA	GGTGGTGGAATGAGGA GAAA	ATCACTTCTTGCCATGT CCC	59.9	106	INTERGENIC	--	--	--
Ca4_P4_46708395	Ca4	46708395	CAT/CATAT	CACACACACACCACCA CAAA	CACAAAAATAGATGCCA ACCA	59.9	480	INTERGENIC	--	--	--

INDEL marker IDs	Chromosomes/ unanchored scaffolds	Physical positions (bp)	InDels (*Kabuli* reference genome-CDC Frontier/PII)	Forward primers (5'-3')	Reverse primers (5'-3')	Anneling temperature (0C)	Expected amplified product size (bp)	Structural annotation		Functional annotation	
								Sequence components of *kabuli* genome	*Kabuli* gene accession IDs	NCBI-KOG	TFs
Ca4_P4_46711628	Ca4	46711628	GTTATAG/GTTATAGACTTATAG	TCATTTACTCGTGCATGAAAGTT	ATGCTTTTTCACCATCCTCG	58.8	685	INTERGENIC	--	--	--
Ca4_P4_46729372	Ca4	46729372	ACTAATTCTAATTCTAATTCTAAT/ACTAATTCTAATTCTAAT	ATTGCGATGTGGTTAACGGT	CCTCAAGTACGCATCTGCAA	60.3	495	INTERGENIC	--	--	--
Ca4_P4_46746914	Ca4	46746914	GATTATTATTATTATTATTA/GATTATTATTATTA	ACAGTGCAACACAACAAGGG	TCGCTCCATCTTTTCCATTC	59.6	539	INTERGENIC	--	--	--
Ca4_P4_46747194	Ca4	46747194	AATATA/AATA	ACAGTGCAACACAACAAGGG	TCGCTCCATCTTTTCCATTC	59.6	539	INTERGENIC	--	--	--
Ca4_P4_46753094	Ca4	46753094	AT/ATT	AAACAATCAACACATCCGCA	CGAAAGAAGTTCAATCAAACCC	60.0	563	INTERGENIC	--	--	--
Ca4_P4_46757820	Ca4	46757820	ATTTTTTT/ATTTTTTTTT	ATTTCCTCAGGGAACAATCG	TCATTCAACGGTAACTCCCA	59.0	561	INTERGENIC	--	--	--
Ca4_P4_46759694	Ca4	46759694	AATA/AATATA	AGTTAAGGCACCGCAAAAGA	GGAATCACGCGGAAATTTTA	59.9	520	INTERGENIC	--	--	--
Ca4_P4_46760540	Ca4	46760540	T/TAA	TAAAATTTCCGCGTGATTCC	GCAAAGCAAAGGAAGATACACC	59.9	543	INTERGENIC	--	--	--
Ca4_P4_46761044	Ca4	46761044	CTTT/CTTTT	GGTGTATCTTCCTTTGCTTTGC	CATTCGTTACCCTCGCTCTC	60.1	546	INTERGENIC	--	--	--
Ca4_P4_47185891	Ca4	47185891	A/AG	CGAAACCCTAGCCACCATTA	CTGCCACTTAGGCAACTTCC	60.0	510	INTERGENIC	--	--	--

INDEL marker IDs	Chromosomes/ unanchored scaffolds	Physical positions (bp)	InDels (*Kabuli* reference genome-CDC Frontier/PII)	Forward primers (5'-3')	Reverse primers (5'-3')	Anneling temperature (0C)	Expected amplified product size (bp)	Structural annotation		Functional annotation	
								Sequence components of *kabuli* genome	*Kabuli* gene accession IDs	NCBI-KOG	TFs
Ca4_P4_47206093	Ca4	47206093	TAAAAAAA/TAAAAAAAA	TCACCATTCAATCATTTTTGG	ATGCATGCTTGCCCTTTTAT	58.3	354	INTERGENIC	--	--	--
Ca4_P4_47206262	Ca4	47206262	A/ATAT	TCACCATTCAATCATTTTTGG	CCCGAGAAAGATTTTACGCA	58.3	550	INTERGENIC	--	--	--
Ca4_P4_47206910	Ca4	47206910	TATATATACATATATA/TATATATA	TGCGTAAAATCTTTCTCGGG	GGACCATAATGCCCTTGCTA	60.2	784	INTERGENIC	--	--	--
Ca4_P4_47219947	Ca4	47219947	TAAATAAAA/T	AAACTCCTCTCAAACGGGGT	CCATCGTGCTTCCAGAAAAT	60.0	595	INTERGENIC	--	--	--
Ca4_P4_47230296	Ca4	47230296	TG/TGG	GGAGCCCCACAAAATCTATG	CACCCATCTTGTGTTTGATTT	59.4	810	INTERGENIC	--	--	--
Ca4_P4_47232992	Ca4	47232992	ATTTT/ATTTTT	AGTGGTCATGTGTCGCATAAA	TGTGAAACGTCACAAATTCAA	59.1	791	INTERGENIC	--	--	--
Ca4_P4_47238699	Ca4	47238699	ATTT/ATT	TTTTGATCGAATCTGACCCA	TCGGCCATTTTTCTTCAATC	59.0	659	INTERGENIC	--	--	--
Ca4_P4_47238913	Ca4	47238913	TAAA/TAA	GATTGAAGAAAAATGGCCGA	ATAATCCAAAATGCGCGAAG	60.0	186	INTERGENIC	--	--	--
Ca4_P4_47240338	Ca4	47240338	AATATATATATA/AATATATATA	AAAGCTTAATTGTCGTGCATCA	TAGTTTGGAGCGAGGAGGAA	59.8	352	INTERGENIC	--	--	--
Ca4_P4_47242175	Ca4	47242175	AAATAATAATAATAATAATAATAA/AAATAATAATAATAATAATAA	CGCTCCTAATTCCCACACAT	TTTCTCGCATGCATTCTCAC	60.0	835	INTERGENIC	--	--	--

INDEL marker IDs	Chromosomes/ unanchored scaffolds	Physical positions (bp)	InDels (*Kabuli* reference genome-CDC Frontier/PII)	Forward primers (5'-3')	Reverse primers (5'-3')	Anneling temperature (0C)	Expected amplified product size (bp)	Structural annotation		Functional annotation	
								Sequence components of *kabuli* genome	*Kabuli* gene accession IDs	NCBI-KOG	TFs
Ca4_P4_47286913	Ca4	47286913	ACCCCCCCC/ACCCCCCCCC	GTTGTTGGTTTGAGCCCAGT	TGGAGATCACTTTCTCCGCT	60.0	619	INTERGENIC	--	--	--
Ca4_P4_47401934	Ca4	47401934	TC/TCC	TTGGGGACCATAGTCTTTCG	CAGAGAACCAGCCTTTGGAG	59.9	483	INTERGENIC	--	--	--
Ca4_P4_47457546	Ca4	47457546	CATTATTATTATTATTATTA/CATTATTATTATTATTATTATTA	GGTTGGGATGAAAACTGGAA	CCTGGAAAACATTAATTCCCC	59.8	543	INTERGENIC	--	--	--
Ca4_P4_47685776	Ca4	47685776	A/AGGTTTG	AGTCAGACGCTACTCGGCAT	CCGACGAAATAAGTCCCTCA	60.0	837	INTERGENIC	--	--	--
Ca4_P4_47685878	Ca4	47685878	AATATATATATATATA/AATATATATATATATATATATATA	AAATTAAAGTTTCAAACCACGG	CCGACGAAATAAGTCCCTCA	57.3	667	INTERGENIC	--	--	--
Ca4_P4_4769266	Ca4	4769266	TTATATATATATATATATAT/TTATATATATATATATATAT	ATACCGATGCAGTACCCGAG	TGTTTGCCAACCATATGTGAA	60.0	375	INTERGENIC	--	--	--
Ca4_P4_47842857	Ca4	47842857	GAAA/GAAAA	GGTTATCCCATTAATGCATCTG	TGTGTTCATTGTTTTTCGCTTT	58.3	606	INTERGENIC	--	--	--
Ca4_P4_48048628	Ca4	48048628	CATATATATATATATATATATATATATATA/CATATATATATATATATATATATATA	CGAAACCGTAACCCTACGAA	TTGTTAGTTTGACGAGGGCA	60.0	328	INTERGENIC	--	--	--
Ca4_P4_48074355	Ca4	48074355	TG/T	GGTCGACATGACATCACTAAATG	AGTGTCAAAGGGGGAGGAAG	59.4	601	INTERGENIC	--	--	--
Ca4_P4_48078751	Ca4	48078751	AATATATATATATATATATATAT/AATATATATATATATATATATATAT	TGGAATGTTCTCACCATTTTCA	TGATTTCCACTCATGGGGAT	60.3	941	INTERGENIC	--	--	--

INDEL marker IDs	Chromosomes/ unanchored scaffolds	Physical positions (bp)	InDels (*Kabuli* reference genome-CDC Frontier/PII)	Forward primers (5'-3')	Reverse primers (5'-3')	Anneling temperature (0C)	Expected amplified product size (bp)	Structural annotation		Functional annotation	
								Sequence components of *kabuli* genome	*Kabuli* gene accession IDs	NCBI-KOG	TFs
Ca4_P4_48115647	Ca4	48115647	AATATATATATATATATATAT/AATATATATATATATATAT	TCCTTCATATCCCACGAACC	TGGTGTTGGAGTCATGCTTT	59.8	290	INTERGENIC	--	--	--
Ca4_P4_48202910	Ca4	48202910	AAATAA/AAA	TCGTCCTTACACCGGAAAAC	CAAGTTTCGGTTGACTATACACG	60.0	638	INTERGENIC	--	--	--
Ca4_P4_48204434	Ca4	48204434	CT/CTT	CTCGGCATCTCAAAGTTTCTC	CCTTTGCCCTCTTCCTCTTT	59.1	391	INTERGENIC	--	--	--
Ca4_P4_48206793	Ca4	48206793	CAT/CATAT	TATTTGGCCAACAGGAAAGG	AGAGCTGATTTGGTTCCTCG	59.9	893	INTERGENIC	--	--	--
Ca4_P4_48207152	Ca4	48207152	CTTT/CTTTT	ATCGAATGTGCATCGTGAAG	CAATAAGGATAAGGGGCGGT	59.7	631	INTERGENIC	--	--	--
Ca4_P4_48208619	Ca4	48208619	A/AG	GTAGACCGATTCGAAGGTCG	GCGTAACGTGTAGTTCAGTGC	59.7	809	INTERGENIC	--	--	--
Ca4_P4_48208929	Ca4	48208929	TC/T	AAGTTTTTGAATACCGCAAAAT	AAGCTGCGATGGGACAATAC	57.0	490	INTERGENIC	--	--	--
Ca4_P4_48210066	Ca4	48210066	AAAGTAA/AAA	AATTCACTTGACGCGAAACC	TGGGTGTATCAGAGACCGAA	60.1	572	INTERGENIC	--	--	--
Ca4_P4_48210310	Ca4	48210310	TAA/TAAA	CCATAAGTCCACAACGGACC	TGGGTGTATCAGAGACCGAA	60.2	400	INTERGENIC	--	--	--
Ca4_P4_48211280	Ca4	48211280	TA/T	TGATGTCCGATGAACTTTGC	CAAGTTATCGGCTGAGAGGC	59.7	552	INTERGENIC	--	--	--

INDEL marker IDs	Chromosomes/ unanchored scaffolds	Physical positions (bp)	InDels (*Kabuli* reference genome-CDC Frontier/PII)	Forward primers (5'-3')	Reverse primers (5'-3')	Anneling temperature (0C)	Expected amplified product size (bp)	Structural annotation		Functional annotation	
								Sequence components of *kabuli* genome	*Kabuli* gene accession IDs	NCBI-KOG	TFs
Ca4_P4_48212003	Ca4	48212003	AATATATAT/AATATATATATAT	GCCTCTCAGCCGATAACTTG	ATTCCTTTACGGGGAAAACG	60.0	590	INTERGENIC	--	--	--
Ca4_P4_48214681	Ca4	48214681	TTTAT/TTTATTAT	GAAATGACCCCTCGAACTCA	ATGGATTTGAAAGGCGATGA	60.1	426	INTERGENIC	--	--	--
Ca4_P4_48214982	Ca4	48214982	GAAT/GAATTAAT	ATGCTTTCAAACTCATCGCC	CCATCGGATAGGTCAGAACC	60.2	525	INTERGENIC	--	--	--
Ca4_P4_48215838	Ca4	48215838	TTCT/TT	AGTTCAAAGCAAAAGCCCAA	TCCTCCATACCCACATCCAT	59.9	643	INTERGENIC	--	--	--
Ca4_P4_48217970	Ca4	48217970	ATAT/A	AGTGCATACAGCTCATGGGG	TGGCAGGATATGGTGGACTT	61.1	755	INTERGENIC	--	--	--
Ca4_P4_48218216	Ca4	48218216	TATTA/TATTAATTA	CAACTTGAATTTGGTTTGGCT	TCTGTCGGCAGATAACCCTT	59.1	885	INTERGENIC	--	--	--
Ca4_P4_48223941	Ca4	48223941	TTAT/TTATATTGAAAGGTGGCATAT	TCTTGAATTGTTGCAAAGAAACAT	CCAGCGAGAAGAAACCTGTC	60.0	735	INTERGENIC	--	--	--
Ca4_P4_48230328	Ca4	48230328	A/AC	TCCTTGAGGGACCAGGATAA	ATTGTGGATTTTGTGTCGCA	59.5	431	INTERGENIC	--	--	--
Ca4_P4_48278655	Ca4	48278655	ATTTTTTTT/ATTTTTTTTTTT	AATAGCAATGCCCAGGTTTG	ACCAACGACGAGATAGGGTG	60.0	297	INTERGENIC	--	--	--
Ca4_P4_48279056	Ca4	48279056	TAAAAA/TAAAAAA	CACCCTATCTCGTCGTTGGT	GTCTCGGGTGTTGGAGGATA	60.0	657	INTERGENIC	--	--	--

INDEL marker IDs	Chromosomes/ unanchored scaffolds	Physical positions (bp)	InDels (*Kabuli* reference genome-CDC Frontier/PII)	Forward primers (5'-3')	Reverse primers (5'-3')	Anneling temperature (0C)	Expected amplified product size (bp)	Structural annotation		Functional annotation	
								Sequence components of *kabuli* genome	*Kabuli* gene accession IDs	NCBI-KOG	TFs
Ca4_P4_48280011	Ca4	48280011	ATTT/ATT	TCCCAATAATCAAAATG AGCG	GCGTCGTTGCTAGTTC ACAA	59.9	878	INTERGENIC	--	--	--
Ca4_P4_48299604	Ca4	48299604	A/AT	TCAATTGCACCAAAGG ATGA	ATGAAAATCATGGCAG ACCC	60.0	536	INTERGENIC	--	--	--
Ca4_P4_48300219	Ca4	48300219	TAAAAAAA/TAAAAAAAAAA	ACTTATTTTGGGGTCT GCCA	GCCATTGAGAAGCTCC TTTG	59.4	660	INTERGENIC	--	--	--
Ca4_P4_4830250	Ca4	4830250	A/ATTATTATAGTTAT	CAACATGGGCAACAAG ACAC	CCCTTTCCCCTTCATCA TTT	60.0	273	INTERGENIC	--	--	--
Ca4_P4_48306501	Ca4	48306501	TAAAAA/TA	TTCATTGGACTTTGGG TTGA	TTTCCACAACGTTGAGT TTCA	58.9	835	INTERGENIC	--	--	--
Ca4_P4_48307832	Ca4	48307832	ATTTTTT/ATTTTTTT	CTGCAGTCAATCGTCA TCGT	CGTTTATCTCTTCAATC GCGT	59.9	203	INTERGENIC	--	--	--
Ca4_P4_4830820	Ca4	4830820	TTTATACATT/TTT	AAATGATGAAGGGGAA AGGG	CAACGGTTTACATGAAT TGGG	60.1	815	INTERGENIC	--	--	--
Ca4_P4_48309328	Ca4	48309328	AAAAGAAA/AAAA	TTCGTAACCTGTTGAC GTTGA	TAATGACGTTTGTGGA GGCA	59.2	451	INTERGENIC	--	--	--
Ca4_P4_48310812	Ca4	48310812	GTTTTT/GTTTTTT	TCGAAAATTTGGGGTT TTGA	ATGCAAACATGCTGAA GTGC	60.3	773	INTERGENIC	--	--	--
Ca4_P4_48316349	Ca4	48316349	TAA/TA	ACGAGCACCAAAGAGG ACAT	TTTTACCAGGTTGCATC CTAAA	59.7	493	INTERGENIC	--	--	--

INDEL marker IDs	Chromosomes/ unanchored scaffolds	Physical positions (bp)	InDels (*Kabuli* reference genome-CDC Frontier/PII)	Forward primers (5'-3')	Reverse primers (5'-3')	Anneling temperature (0C)	Expected amplified product size (bp)	Structural annotation		Functional annotation	
								Sequence components of *kabuli* genome	*Kabuli* gene accession IDs	NCBI-KOG	TFs
Ca4_P4_48335941	Ca4	48335941	TA/TAAA	CATGCACTCCATGTTTTTGG	CAGTCATGTGAACCACCCAC	60.0	493	INTERGENIC	--	--	--
Ca4_P4_48340280	Ca4	48340280	T/TGATAATATTCGGCCCAAA	TCCAAACAAAGTGGAGATGC	TTGGTCCCTGGTAGCTTACG	58.7	369	INTERGENIC	--	--	--
Ca4_P4_48340605	Ca4	48340605	GTTATTTTATTAT/GTTAT	CGTAAGCTACCAGGGACCAA	AAAAATGACACACCATTTGACA	60.1	598	INTERGENIC	--	--	--
Ca4_P4_48341983	Ca4	48341983	T/TTTATTTTTTA	CATTAGTGTGCTCCCCACCT	AATGGGTATCAAAAAGCCCC	60.0	904	INTERGENIC	--	--	--
Ca4_P4_48342297	Ca4	48342297	GC/G	TAAAGACCGTCGCCAAATTC	AATGGGTATCAAAAAGCCCC	60.1	419	INTERGENIC	--	--	--
Ca4_P4_48351278	Ca4	48351278	TAATTCCAATTCCAATT/TAATTCCAATTCCAATTCCAATT	GACACGTGATGTCTCTGTAATGG	AATGTATGTATCGAGAGCGCC	59.5	651	INTERGENIC	--	--	--
Ca4_P4_48368240	Ca4	48368240	T/TC	TCACTTTTATTGCAAGTGTTGGA	AACCTTGTCGGTTTCGATTG	59.7	328	INTERGENIC	--	--	--
Ca4_P4_48386323	Ca4	48386323	ATTATTTTTTATTTTTTATTTTTTT/ATTATTTTTTATTTTTTATTTTTTTATTTTTT	ACACCACCCCATTTTGTTGT	TTGGTGATTGTGCCTTGAGA	60.0	433	INTERGENIC	--	--	--
Ca4_P4_48389863	Ca4	48389863	TC/T	CCGTTTTCCACATTTTCGAT	CCATCAAATTCATCCACCAA	59.8	522	INTERGENIC	--	--	--
Ca4_P4_48401392	Ca4	48401392	GTTT/GT	GACCAAGGCAAGCAAGAAAG	GGAAAAGGAGTGCAGTCACG	60.0	573	INTERGENIC	--	--	--

INDEL marker IDs	Chromosomes/ unanchored scaffolds	Physical positions (bp)	InDels (*Kabuli* reference genome-CDC Frontier/PII)	Forward primers (5'-3')	Reverse primers (5'-3')	Anneling temperature (0C)	Expected amplified product size (bp)	Structural annotation		Functional annotation	
								Sequence components of *kabuli* genome	*Kabuli* gene accession IDs	NCBI-KOG	TFs
Ca4_P4_48404093	Ca4	48404093	GATTAA/GATTAATTAA	TTTGTTGCCGAGAGTCTTGA	GTGCATGGCAATTGAATGAT	59.6	410	INTERGENIC	--	--	--
Ca4_P4_48404724	Ca4	48404724	ACCCCC/ACCCCCC	TTGCATGACTGCTTTTGGAC	TTGTCATTGTTGCGGTTTGT	59.8	514	INTERGENIC	--	--	--
Ca4_P4_48404926	Ca4	48404926	ATTTTTTT/ATTTTTT	TTGCATGACTGCTTTTGGAC	TTGTCATTGTTGCGGTTTGT	59.8	514	INTERGENIC	--	--	--
Ca4_P4_48404970	Ca4	48404970	GTTTAATTT/GTTTAATTTTAATTT	TTGCATGACTGCTTTTGGAC	TTGTCATTGTTGCGGTTTGT	59.8	514	INTERGENIC	--	--	--
Ca4_P4_48405207	Ca4	48405207	TTCTCTCTC/TTCTCTC	ACAAACCGCAACAATGACAA	CTCACTCACCCGAAACCATT	60.0	485	INTERGENIC	--	--	--
Ca4_P4_48410108	Ca4	48410108	CTAGATATATAGATATATAGA/CTAGATATATAGA	TTTGGATTGTTTGGTGGGTT	GGCTGGCATATGGAGCTTAT	60.1	145	INTERGENIC	--	--	--
Ca4_P4_48410907	Ca4	48410907	ATTT/ATT	CACCGTCCAAATTATGCCTC	TGAGAACGTGCGACCTGTAG	60.3	518	INTERGENIC	--	--	--
Ca4_P4_48411214	Ca4	48411214	TG/T	CACCCCAAAACTACAGGTCG	CCACTTTTCTACTTTTGGCAGTT	60.4	104	INTERGENIC	--	--	--
Ca4_P4_48414921	Ca4	48414921	GTTT/GTTTT	TTGAATGTGAATGGGTGCAT	AAACCGAACAAATCAATGCC	59.8	513	INTERGENIC	--	--	--
Ca4_P4_48431277	Ca4	48431277	T/TAA	TTTGTTTTGTCCCATCCCAT	TCGCCATACCTTTCGAACTT	60.0	762	INTERGENIC	--	--	--

INDEL marker IDs	Chromosomes/ unanchored scaffolds	Physical positions (bp)	InDels (*Kabuli* reference genome-CDC Frontier/PII)	Forward primers (5'-3')	Reverse primers (5'-3')	Anneling temperature (0C)	Expected amplified product size (bp)	Structural annotation		Functional annotation	
								Sequence components of *kabuli* genome	*Kabuli* gene accession IDs	NCBI-KOG	TFs
Ca4_P4_48434785	Ca4	48434785	AG/A	GGTGAGAGTAGGTGGGGACA	TTCTTTCGAAATTCAACTGCAA	60.0	700	INTERGENIC	--	--	--
Ca4_P4_48436603	Ca4	48436603	AATA/AA	TGTGACCACCACCTTGAAAA	TGGAACTCTGGTGCAAGTCA	60.0	383	INTERGENIC	--	--	--
Ca4_P4_48440600	Ca4	48440600	GTT/GT	TTTGTTTACAAGGTATTCTTGCCA	GTCAATTGCACCTTTCCCAT	59.9	567	INTERGENIC	--	--	--
Ca4_P4_48443370	Ca4	48443370	T/TA	CCCAACCATCCATCCTATTG	TGATGATATGATATTCCCATTAATTTT	60.0	560	INTERGENIC	--	--	--
Ca4_P4_48445196	Ca4	48445196	GAGAATTTAGAATTT/GAGAATTT	GTAATAACGGCGGTGCAAAT	TCGCATGCAAAACTAACGTC	59.9	838	INTERGENIC	--	--	--
Ca4_P4_48445750	Ca4	48445750	CTT/CT	GTAGTTGGTGGGGTGGTGTC	ATGAGGCCCAACAAACTCAC	60.1	280	INTERGENIC	--	--	--
Ca4_P4_48446112	Ca4	48446112	A/ATT	GTGAGTTTGTTGGGCCTCAT	TTCCGACAATTCCATTGTGA	60.0	764	INTERGENIC	--	--	--
Ca4_P4_48446392	Ca4	48446392	CTTTTT/CTTT	CGCCCTCATTATGGTATGCT	GTGTTTTTCGAGTGAGGGGA	59.9	547	INTERGENIC	--	--	--
Ca4_P4_48447377	Ca4	48447377	T/TA	GTTGAGGTCGCGTACTGGTT	CCATGATGCATTGTGGATTT	60.2	592	INTERGENIC	--	--	--
Ca4_P4_48451241	Ca4	48451241	TTTT/TTTTATCTTT	TGTTTGGACCCCCTACTGAC	TTCGTCTCGCGGTATTTATTG	59.8	617	INTERGENIC	--	--	--

INDEL marker IDs	Chromosomes/ unanchored scaffolds	Physical positions (bp)	InDels (*Kabuli* reference genome-CDC Frontier/PII)	Forward primers (5'-3')	Reverse primers (5'-3')	Anneling temperature (0C)	Expected amplified product size (bp)	Structural annotation		Functional annotation	
								Sequence components of *kabuli* genome	*Kabuli* gene accession IDs	NCBI-KOG	TFs
Ca4_P4_48452771	Ca4	48452771	CTTTT/CTTT	ACAAGCTTTCACCAACGGAA	ATGGTAGTGGTGGTGGTGGT	60.7	926	INTERGENIC	--	--	--
Ca4_P4_48453751	Ca4	48453751	A/AT	GTTCCACCACCGCAAATATC	GGTGGTGGTAGGAAAAGCAA	60.2	742	INTERGENIC	--	--	--
Ca4_P4_48481643	Ca4	48481643	ATTTTT/ATTTT	TGACCAGTCATGACAAAAATTAAA	GCTATTGGACTTCACTTGTACCAC	58.6	487	INTERGENIC	--	--	--
Ca4_P4_4848593	Ca4	4848593	ATTTTTTTTT/ATTTTTTTT	TTTTGGCCCATGTCTTTTGT	TCAATTTGTTGAAAGTCACGATG	60.3	616	INTERGENIC	--	--	--
Ca4_P4_4848650	Ca4	4848650	GTTTTTTTT/GTTTTTTT	TTTTGGCCCATGTCTTTTGT	TCAATTTGTTGAAAGTCACGATG	60.3	616	INTERGENIC	--	--	--
Ca4_P4_48494467	Ca4	48494467	TTTAATT/TTTAATTATGTTAATT	TGGTTGACAAAAACTGCCAA	ACTGGACCCCAAACCTCTCT	60.1	664	INTERGENIC	--	--	--
Ca4_P4_48494594	Ca4	48494594	CA/CAA	GCATTCTGGCACAACAAAGA	ACTGGACCCCAAACCTCTCT	59.8	357	INTERGENIC	--	--	--
Ca4_P4_48509401	Ca4	48509401	GAAAA/GAAAAA	ACTGTCGAGCATAAATGGGG	CGATCGACCCGAACTAAAAC	60.0	472	INTERGENIC	--	--	--
Ca4_P4_4851248	Ca4	4851248	TAA/TA	GGAATGCATGGTATCCAACC	CAGCACTTCTCCCTTTTCCTT	60.0	678	INTERGENIC	--	--	--
Ca4_P4_48513254	Ca4	48513254	CA/CAA	CGAAACTTTCAACGCTCTCC	TTTGAAATTGCCCAAAGGAC	60.0	604	INTERGENIC	--	--	--

INDEL marker IDs	Chromosomes/ unanchored scaffolds	Physical positions (bp)	InDels (*Kabuli* reference genome-CDC Frontier/PII)	Forward primers (5'-3')	Reverse primers (5'-3')	Anneling temperature (0C)	Expected amplified product size (bp)	Structural annotation		Functional annotation	
								Sequence components of *kabuli* genome	*Kabuli* gene accession IDs	NCBI-KOG	TFs
Ca4_P4_48528463	Ca4	48528463	TTATATATATATATATATATATATATA TA/TTATATATATATATATATATATAT ATATATA	GCCGATTTAACAATTC CCCT	TGATTTGGGAAATTCG GAAG	60.2	590	INTERGENIC	--	--	--
Ca4_P4_48541135	Ca4	48541135	TAAAAAAAAAAAA/TAAAAAAAAA	AATTCCCACACGTACG CTTC	GATGTTCTTGGCTTGT GGCT	60.0	723	INTERGENIC	--	--	--
Ca4_P4_48577228	Ca4	48577228	A/AC	AACACATCCGGGATCC ATTA	TGATGACGAAGACGAA GACG	60.0	303	INTERGENIC	--	--	--
Ca4_P4_48577264	Ca4	48577264	CATATATATATA/CATATATATATATA	AACACATCCGGGATCC ATTA	TGATGACGAAGACGAA GACG	60.0	303	INTERGENIC	--	--	--
Ca4_P4_4859662	Ca4	4859662	ATCTCTCTCTCTCTCTCTCTCTCTC TCTCTC/ATCTCTCTCTCTCTCTC	ACACACAACGGTGATG CCTA	AAACTGAGATAGAGCC CGCA	60.0	243	INTERGENIC	--	--	--
Ca4_P4_48663015	Ca4	48663015	AAAA/AAAATTAAAAAATTGAAA	ATTAGACATTTCGGGT GCCA	TATGTTAGTGCCACTG CCCC	60.3	571	INTERGENIC	--	--	--
Ca4_P4_48663138	Ca4	48663138	ACATG/A	ATTAGACATTTCGGGT GCCA	TATGTTAGTGCCACTG CCCC	60.3	571	INTERGENIC	--	--	--
Ca4_P4_48663228	Ca4	48663228	A/AT	ATTAGACATTTCGGGT GCCA	TATGTTAGTGCCACTG CCCC	60.3	571	INTERGENIC	--	--	--
Ca4_P4_48666607	Ca4	48666607	TTATATATATATATATATATATATATA TATATATATATATATA/TTATATATAT ATATATATATATATA	TGGATTTTTCAATTAAC ACGGA	CCGCTACCTGCATAAC CAAT	59.3	456	INTERGENIC	--	--	--
Ca4_P4_48670108	Ca4	48670108	AAATATCT/A	TATCTCCATGGCGTCA CAAA	CTGCGCCGACTAACAA TACA	60.1	589	INTERGENIC	--	--	--

INDEL marker IDs	Chromosomes/ unanchored scaffolds	Physical positions (bp)	InDels (*Kabuli* reference genome-CDC Frontier/PII)	Forward primers (5'-3')	Reverse primers (5'-3')	Anneling temperature (0C)	Expected amplified product size (bp)	Structural annotation		Functional annotation	
								Sequence components of *kabuli* genome	*Kabuli* gene accession IDs	NCBI-KOG	TFs
Ca4_P4_48670468	Ca4	48670468	GATA/GATAAAATCTTATA	CTGTATTGTTAGTCGGCGCA	CCGACCCAACACGTTATGA	59.9	620	INTERGENIC	--	--	--
Ca4_P4_48671006	Ca4	48671006	ATTTTTTTT/ATTTTTTTTTTT	GGTCGGCCCTGACTTTAGAT	TGACAATGTGGAATCGGAAA	60.5	198	INTERGENIC	--	--	--
Ca4_P4_4868200	Ca4	4868200	TAAA/TAAAA	ATTGGTTTTGCTTTGACCCA	CACCAATATTCCATTTCCCG	60.3	423	INTERGENIC	--	--	--
Ca4_P4_48690534	Ca4	48690534	TCAA/TCAACAA	TCCAATTCCAAATCCCAAAA	TGTGGATATCAAGTGGCTGG	60.1	395	INTERGENIC	--	--	--
Ca4_P4_48694227	Ca4	48694227	ATATTTT/AT	TGCTGCCACACCTAAACTTG	TAATCCATTGGCCCACATTT	59.9	678	INTERGENIC	--	--	--
Ca4_P4_48721738	Ca4	48721738	TTATATATATATATATATATATATATATATATATATAT/TTATATATATATATATATATATATATATATATATATATATATATAT	GACTCTCAGCATCACACGGA	AACTCCTCCTAATTTCCGTTCT	60.0	545	INTERGENIC	--	--	--
Ca4_P4_48723130	Ca4	48723130	TTAATAATAATAATAATAATAA/TTAATAATAATAATAATAA	TTCTTGGGAAAGCGACATCT	GCAACTCCCTTTTATCGTCAA	59.8	435	INTERGENIC	--	--	--
Ca4_P4_48723538	Ca4	48723538	AATTATTATTA/AATTATTA	TTGACGATAAAAGGGAGTTGC	TCCTGGAGAAGCGACCTCTA	59.2	580	INTERGENIC	--	--	--
Ca4_P4_48726209	Ca4	48726209	GTTTTT/GTTTT	CCCAAAACAAGTTGTCCCAG	TGGAAAGAAAACGGATGAGG	60.4	653	INTERGENIC	--	--	--
Ca4_P4_48739702	Ca4	48739702	ATTTTTTTTT/ATTTTTTTTTT	ACACTCCACCAGCATCAACA	AGATTCTGCCAAATGCCAAC	60.2	577	INTERGENIC	--	--	--

**INDEL marker IDs**	**Chromosomes/ unanchored scaffolds**	**Physical positions (bp)**	**InDels (*Kabuli* reference genome-CDC Frontier/PII)**	**Forward primers (5'-3')**	**Reverse primers (5'-3')**	**Anneling temperature (0C)**	**Expected amplified product size (bp)**	**Structural annotation**		**Functional annotation**	
								**Sequence components of *kabuli* genome**	***Kabuli* gene accession IDs**	**NCBI-KOG**	**TFs**
Ca4_P4_48792373	Ca4	48792373	ATCT/AT	AAACTGCGGGTATGAGGATG	AATGAGGGATCGTTTTCACG	60.0	973	INTERGENIC	--	--	--
Ca4_P4_48793555	Ca4	48793555	TTCTCTCTCTCTCTCTCTCTC/TTCTCTCTCTCTCTCTCTCTC	ACGGAGAGTTTGGGTGTGAC	GTTATTCCACCGCGAGAAAA	60.0	451	INTERGENIC	--	--	--
Ca4_P4_48794099	Ca4	48794099	TAA/TA	TTACGGTCGAAAATGAAGGG	TCGGGTAGCACAATCTTCAA	59.9	640	INTERGENIC	--	--	--
Ca4_P4_48824681	Ca4	48824681	ATTTTTTT/ATTTTTT	TTTTGCTCGTTCTGTCATGG	GAATTTGGGTCCGAATCTTG	59.8	416	INTERGENIC	--	--	--
Ca4_P4_48831208	Ca4	48831208	GTATATA/GTATATATA	TATTTCCCAAGACCTCGTGC	ATGTGGTTGTTGTTGCCTGA	60.1	255	INTERGENIC	--	--	--
Ca4_P4_48837715	Ca4	48837715	ATT/ATTT	TGTGTAACGGGTAAGAAATACGG	CAAAATACATCATCACGCGG	60.2	630	INTERGENIC	--	--	--
Ca4_P4_48847654	Ca4	48847654	TTTGTT/TTT	GAAATTTGGACGAAAACGGA	CAATGCGTTTTACCAACCCT	59.9	183	INTERGENIC	--	--	--
Ca4_P4_48847771	Ca4	48847771	ATTTTTTT/ATTTTTTTT	GAAATTTGGACGAAAACGGA	GTGTATATACGCGCACCCAA	59.9	707	INTERGENIC	--	--	--
Ca4_P4_48848304	Ca4	48848304	AATAT/AAT	TTATATTAATTGGGTGCGCGT	TTTAGGGTGGAACAGTTCGG	59.4	379	INTERGENIC	--	--	--
Ca4_P4_48942067	Ca4	48942067	GTTTTTTTTTT/GTTTTTTTTTTT	TGATCTTTCTGCAATGAAACG	GATGGAAAATGAAGGTCCGA	58.9	528	INTERGENIC	--	--	--

INDEL marker IDs	Chromosomes/ unanchored scaffolds	Physical positions (bp)	InDels (*Kabuli* reference genome-CDC Frontier/PII)	Forward primers (5'-3')	Reverse primers (5'-3')	Anneling temperature (0C)	Expected amplified product size (bp)	Structural annotation		Functional annotation	
								Sequence components of *kabuli* genome	*Kabuli* gene accession IDs	NCBI-KOG	TFs
Ca4_P4_4899207	Ca4	4899207	T/TATTTA	TTCCCAAAACTGTCTG CTCA	CTCACGCGTTTGCTTA ACTT	59.4	810	INTERGENIC	--	--	--
Ca4_P4_4903904	Ca4	4903904	CTTTT/CTTTTTT	CCCTTGGATTGTTGTT GACC	TGACACACCCTAACCA TCCA	60.2	684	INTERGENIC	--	--	--
Ca4_P4_49100863	Ca4	49100863	GAAAAAAAAA/GAAAAAAAAAA	TCATCAAATTCGTAACG CAAA	GGAGGAAGGAGAGAG GGAGA	59.2	542	INTERGENIC	--	--	--
Ca4_P4_49100951	Ca4	49100951	GA/GAAGATA	TCATCAAATTCGTAACG CAAA	GGAGGAAGGAGAGAG GGAGA	59.2	542	INTERGENIC	--	--	--
Ca4_P4_49121831	Ca4	49121831	TTATATATATATATATAT/TTATATAT ATATATATATAT	CCAAAAATCTGCCAAG AACG	TGGGGTGTAAAGTGTG TGGA	60.6	491	INTERGENIC	--	--	--
Ca4_P4_49137090	Ca4	49137090	GTATATATATATATATATATA/GTATA TATATATATATATATATA	AACATGCAGTTGGAGG CTCT	TTTCAACTTGTAACATC CCGA	59.9	298	INTERGENIC	--	--	--
Ca4_P4_49149348	Ca4	49149348	TCC/TCCC	GCACCCTTGTATTTTG CTCC	GGGAAAACTTGTGTAC CGGA	59.6	493	INTERGENIC	--	--	--
Ca4_P4_4917514	Ca4	4917514	TAAAAAAA/TAAAAAA	TCTTTCGCACAATTTTT GTCA	GGACATGTTGTGATGA CATTGA	59.3	851	INTERGENIC	--	--	--
Ca4_P4_4941823	Ca4	4941823	TAGACCATTCAG/TAG	GCCTCACGTTTGAAAA CCAT	CGCTTAAGATTGGACA TTTCG	60.0	416	INTERGENIC	--	--	--
Ca4_P4_4943577	Ca4	4943577	TAAAAAAA/TAAAAAA	CCTCCTTTTCAAGCCA ACAA	ACAAACCGCAAGCTTC ATTC	60.2	940	INTERGENIC	--	--	--

INDEL marker IDs	Chromosomes/ unanchored scaffolds	Physical positions (bp)	InDels (*Kabuli* reference genome-CDC Frontier/PII)	Forward primers (5'-3')	Reverse primers (5'-3')	Anneling temperature (0C)	Expected amplified product size (bp)	Structural annotation		Functional annotation	
								Sequence components of *kabuli* genome	*Kabuli* gene accession IDs	NCBI-KOG	TFs
Ca4_P4_5010175	Ca4	5010175	GGAGAGAGAGAGAGAGAGAGAGAGAGAGAGAG/GGAGAGAGAGAGAGAGAGAGAGAGAGAGAGAGAGAGAG	AAATTCAACCACCAAAAGCG	GGCAACTTAGTTTTGCCGTT	60.0	314	INTERGENIC	--	--	--
Ca4_P4_5010839	Ca4	5010839	GATATATATATATATATATATATA/GATATATATATATATATATATA	ATTATTAAGGCGGACGACCC	GAACCCAACAACTTTTCTTAAAGG	60.2	125	INTERGENIC	--	--	--
Ca4_P4_5023916	Ca4	5023916	TTATATATATATATATATATATAT/TTATATATATATATATATATATATAT	AAAACAGAGGGCAAGGGACT	CCATTGTGGGCTCAACTTTT	60.1	892	INTERGENIC	--	--	--
Ca4_P4_5028595	Ca4	5028595	GTTTTTTTTTT/GTTTTTTTTTTT	TGATAAAAGGGGGAAAATCC	CGCATCCCCAGATCTAACAT	57.8	163	INTERGENIC	--	--	--
Ca4_P4_5029838	Ca4	5029838	ATTTTTTT/ATTTTTTTT	TTTGTTTTGCTAACATTCCCC	GGCAAAATAAATAATTGGTCCC	59.0	715	INTERGENIC	--	--	--
Ca4_P4_5040391	Ca4	5040391	AATATATATATATATATATATAT/AATATATATATATATATATATAT	GTGAAAAGAGACAACCGCCT	GGCCACACAAGACTTTGTCA	59.3	777	INTERGENIC	--	--	--
Ca4_P4_526483	Ca4	526483	TGAGAGAGAGAGAGAGAGAGAGAGAGAGAG/TGAGAGAGAGAGAGAGAGAGAGAGAG	GGGATTTTGAAAAACGCTCA	AAACGGTGTGGTCCAAAAAG	60.1	524	INTERGENIC	--	--	--
Ca4_P4_5300098	Ca4	5300098	TAAAAAAAAAA/TAAAAAAAAA	GGCTGGAGAAAGAATATGCG	AAAAGGAGTTTGGAAGGGGA	59.8	598	INTERGENIC	--	--	--
Ca4_P4_5307622	Ca4	5307622	TTTATTATTATTATTATTATTATT/TTTATTATTATTATTATTATTATT	AACGGGAGTTGGTATTGCTG	CTTTTTAGCCCCACTTGCTG	60.0	689	INTERGENIC	--	--	--
Ca4_P4_5334029	Ca4	5334029	TAAAAAAAA/TAAAAAAAAA	TGTGTGTGAGGGTATCTGCAT	TGAAATCAAGTTAAAGGGCCA	59.0	368	INTERGENIC	--	--	--

INDEL marker IDs	Chromosomes/ unanchored scaffolds	Physical positions (bp)	InDels (*Kabuli* reference genome-CDC Frontier/PII)	Forward primers (5'-3')	Reverse primers (5'-3')	Anneling temperature (0C)	Expected amplified product size (bp)	Structural annotation		Functional annotation	
								Sequence components of *kabuli* genome	*Kabuli* gene accession IDs	NCBI-KOG	TFs
Ca4_P4_5376318	Ca4	5376318	GTTTTTTTTTTTT/GTTTTTTTTTTTTT	GCCGCCTTGTATGTTTCATT	ATACCAAACACGAAGCGGAC	60.0	759	INTERGENIC	--	--	--
Ca4_P4_5420471	Ca4	5420471	TTATATATATATATATATATATATATATATATATATAT/TTATATATATATATATATATATATATATATAT	CTTTAACGTGGCAGCCTCTC	ACAACAGCGCTTGTCAACTC	60.0	299	INTERGENIC	--	--	--
Ca4_P4_548151	Ca4	548151	AA/AATAATAATTA	GCTGATCTATTTTGCCTGCC	TATGCCATTGCATGACCCTA	59.8	570	INTERGENIC	--	--	--
Ca4_P4_5484877	Ca4	5484877	GAAT/G	TCGATCCACCACCACTAACA	GGATACCCAAGTTGCTGCAT	60.0	299	INTERGENIC	--	--	--
Ca4_P4_5546894	Ca4	5546894	ATTT/ATT	ATCACTCTGGCCTTGTTTGG	AGCCATTAGTTTACTGGCGG	60.1	596	INTERGENIC	--	--	--
Ca4_P4_5701044	Ca4	5701044	GAAAAAA/GAAAAAAA	GGAAATCAGGAATCTTGGCA	TCCCACCCCTACCTTTAACC	60.0	222	INTERGENIC	--	--	--
Ca4_P4_5749738	Ca4	5749738	ATTTTTTTTTT/ATTTTTTTTTTT	TGTCCGAGTTCAAGATCAGG	TGAGTTTTGGGTGATGGGTT	58.8	511	INTERGENIC	--	--	--
Ca4_P4_5798679	Ca4	5798679	GTATA/GTATATA	TGCACCATAGAATTTGGCCT	TCGGAGGAAACAAAGAGGAA	60.5	208	INTERGENIC	--	--	--
Ca4_P4_5818841	Ca4	5818841	CATATATATATATATATAT/CATATATATATATATATATAT	TTAATGCGGGGAGTAGGTTG	TGGACTCTAAATCCAGCACG	60.0	541	INTERGENIC	--	--	--
Ca4_P4_5931184	Ca4	5931184	ATTATTATTATT/ATTATTATTATTTTTATTATTATT	CCAGCCCAATTTACATGCTT	CCGCCCATTTCAACTTTTTA	60.0	527	INTERGENIC	--	--	--

INDEL marker IDs	Chromosomes/ unanchored scaffolds	Physical positions (bp)	InDels (*Kabuli* reference genome-CDC Frontier/PII)	Forward primers (5'-3')	Reverse primers (5'-3')	Anneling temperature (0C)	Expected amplified product size (bp)	Structural annotation		Functional annotation	
								Sequence components of *kabuli* genome	*Kabuli* gene accession IDs	NCBI-KOG	TFs
Ca4_P4_5961971	Ca4	5961971	T/TGTG	CCTCTAATATCGTCGCTCCAA	ATAATCGGTGCGGATTTCCT	59.3	668	INTERGENIC	--	--	--
Ca4_P4_6091215	Ca4	6091215	AATACAT/AAT	GTGGTTTGTTACGGCCACTT	GATTAATTGTTGGATGCGACG	59.9	551	INTERGENIC	--	--	--
Ca4_P4_6146003	Ca4	6146003	ATTTTTTTTTT/ATTTTTTTTTTT	GCTCACATCTTCTTGCCACA	GGGAAAACGCACGTGTAACT	60.0	534	INTERGENIC	--	--	--
Ca4_P4_6185177	Ca4	6185177	TTATATATATATATATATATATAT/TTATATATATATATATATATATATAT	CCAAGCAGTGAAAGCACAAA	AATCGGCGTAACGAACTTCA	60.0	759	INTERGENIC	--	--	--
Ca4_P4_6209314	Ca4	6209314	TTATATATATATATATATATA/TTATATATATATATATATA	AAACTGTTAGACTTTGCAGGATGA	TCGCCCCTTAAATTTGACAC	59.4	205	INTERGENIC	--	--	--
Ca4_P4_626706	Ca4	626706	CGAGAGAGAGAGAGAGAGAGAGAGAGAGAGAGAGAGAGAGAGAGA/CGAGAGAGAGAGAGAGAGAGAGAGAGAGAGA	GAAGCTTAAAGTGATCGCCG	CCTTCATCTTCTTCTCCCCC	60.0	613	INTERGENIC	--	--	--
Ca4_P4_630475	Ca4	630475	TAAAAAAAAAA/TAAAAAAAAA	TTGGGATTAAGGGTGATGGA	TCTCTCAAACCAAATAATCGAACA	60.1	197	INTERGENIC	--	--	--
Ca4_P4_6324755	Ca4	6324755	ATTAATAATTTAATAATTTAATAATT/ATTAATAATTTAATAATT	TGGGTGGAACATGACAGAGA	ATTTTGCCATTTGGGATTGA	60.1	588	INTERGENIC	--	--	--
Ca4_P4_6371861	Ca4	6371861	AAATAATAATAATAATAATAATAATAATA/AAATAATAATAATAATAATAATAATA	GCAAGTCGAAAATGCAATCA	ATGGTGGGTTGTATGAGGGA	59.8	484	INTERGENIC	--	--	--
Ca4_P4_6519306	Ca4	6519306	TAAAAAAAAA/TAAAAAAAAAA	TGTGGAGTGATTTTGAGGGA	CCCTCGAAGTTACTAAAGCGG	59.1	720	INTERGENIC	--	--	--

INDEL marker IDs	Chromosomes/ unanchored scaffolds	Physical positions (bp)	InDels (*Kabuli* reference genome-CDC Frontier/PII)	Forward primers (5'-3')	Reverse primers (5'-3')	Anneling temperature (0C)	Expected amplified product size (bp)	Structural annotation		Functional annotation	
								Sequence components of *kabuli* genome	*Kabuli* gene accession IDs	NCBI-KOG	TFs
Ca4_P4_6528163	Ca4	6528163	TG/T	TCCAAGTAAAGGATCGTGGG	AAAAATTCGAATGAAGAATCAAAA	59.9	690	INTERGENIC	--	--	--
Ca4_P4_6538330	Ca4	6538330	ATTTTTTTTTTTTT/ATTTTTTTTTTTT	ATGCGACCTCCTGAAGAAGA	CGACCTAAACATACATTTTGCG	60.0	317	INTERGENIC	--	--	--
Ca4_P4_6542618	Ca4	6542618	TAAAAAAAAAA/TAAAAAAAAA	CCACTTTGGTCTGCCATTCT	GCTCACGCGGTTAAAGACTC	60.1	706	INTERGENIC	--	--	--
Ca4_P4_6609098	Ca4	6609098	TTTATTATTATTATTATTATTATTATTATTATT/TTTATTATTATTATTATTATTATTATT	AGCAATAAATGGTCGCATCC	CGTTCCACTCCCAACAACTT	59.9	594	INTERGENIC	--	--	--
Ca4_P4_6796797	Ca4	6796797	TGAGAGAGAGAGAGAGAGAGA/TGAGAGAGAGAGAGAGAGAGAGA	AGTCAGTGCATATGGCCTCC	GAACATGGCCACTTTCAACA	60.1	241	INTERGENIC	--	--	--
Ca4_P4_6899661	Ca4	6899661	ATTTTTTTT/ATTTTTTT	AACCTTTGCAGAAATTCCGA	AGAAATTGTCCCACCATTCG	59.7	395	INTERGENIC	--	--	--
Ca4_P4_690240	Ca4	690240	AACTGAATGACT/AACT	TGATGCCCTAAGGGAGACAT	GTGAAATGAGGGGAAAGCAA	59.5	480	INTERGENIC	--	--	--
Ca4_P4_6907181	Ca4	6907181	TTATATATATATATATAT/TTATATATATATATAT	TTTGAAATGGCGACTTCCTT	CCAAATGGCAACTTCCAACT	59.7	569	INTERGENIC	--	--	--
Ca4_P4_6907343	Ca4	6907343	TTATATATATATATATATATAT/TTATATATATATATATATAT	TTTGAAATGGCGACTTCCTT	CCAAATGGCAACTTCCAACT	59.7	569	INTERGENIC	--	--	--
Ca4_P4_7001379	Ca4	7001379	ATTTCTTTTTTTCTTTTTTTCTTTT/ATTTCTTTTTTTCTTTT	GGTACGATCTGCACAGGGTC	CAGGTCAAACTTCCTCCCAA	60.5	999	INTERGENIC	--	--	--

INDEL marker IDs	Chromosomes/ unanchored scaffolds	Physical positions (bp)	InDels (*Kabuli* reference genome-CDC Frontier/PII)	Forward primers (5'-3')	Reverse primers (5'-3')	Anneling temperature (0C)	Expected amplified product size (bp)	Structural annotation		Functional annotation	
								Sequence components of *kabuli* genome	*Kabuli* gene accession IDs	NCBI-KOG	TFs
Ca4_P4_7007714	Ca4	7007714	ATTTTTTTTTTTTT/ATTTTTTTTTTTTTT	ACGCCTCAAATCTGGAAGAA	CGACCCTTTGCATGGATATT	59.8	421	INTERGENIC	--	--	--
Ca4_P4_7029739	Ca4	7029739	AATATATATATATATATATATATAT/AATATATATATATATATATATATATATATAT	GCGAAAGTAGTCCCTCCATTT	AATCAAATGCAAGGTGTGGC	59.6	669	INTERGENIC	--	--	--
Ca4_P4_7031730	Ca4	7031730	GATATATATATATATATA/GATATATATATATATATA	GGATGTCGCTTGCAAGAAGT	TAAACTAACGTGTCGGCGTG	60.4	476	INTERGENIC	--	--	--
Ca4_P4_7081636	Ca4	7081636	AATATATATATATATATATATATATA/AATATATATATATATATATATATATATATATA	AATTGGCCACACTTCTTTCG	GGCTTTGTGAAAAACGAGGA	60.1	579	INTERGENIC	--	--	--
Ca4_P4_7089091	Ca4	7089091	GA/GAACAAAAGAGATTCACA	CTTTGCATACCCCTCTCGAC	TTGGGAGGATTGGTTTCAAG	59.7	644	INTERGENIC	--	--	--
Ca4_P4_7132134	Ca4	7132134	TAAAAAAAAAAAAA/TAAAAAAAAAAAA	CACACCAGGGGAGACTCACT	GTCTCCCTACCCCCACATTT	60.2	595	INTERGENIC	--	--	--
Ca4_P4_7299840	Ca4	7299840	T/TGTG	AGCTCTGTCCTTTGCCCATA	GCATTGATACCGATTCACCA	59.8	276	INTERGENIC	--	--	--
Ca4_P4_7319426	Ca4	7319426	TTATATATATATATATATATATATATATAT/TTATATATATATATATATATATATAT	TGGGAGTGAATGAAGGGTTC	TTTGAGTGGGCACAAATAAATG	59.9	677	INTERGENIC	--	--	--
Ca4_P4_7360660	Ca4	7360660	TTTTATTTATTTATTTATTTATTTATTTATTTA/TTTTATTTATTTATTTATTTATTTATTTA	ACTACGAAAACGGTCATCCG	CAAGCATGCTCTATCTCTTTACG	60.0	380	INTERGENIC	--	--	--
Ca4_P4_7452921	Ca4	7452921	C/CCTCAACTTATTTG	TGCCAACTCGATTGAACAAG	GCACGCTAAAAGCCTGAAAC	59.8	364	INTERGENIC	--	--	--

INDEL marker IDs	Chromosomes/ unanchored scaffolds	Physical positions (bp)	InDels (*Kabuli* reference genome-CDC Frontier/PII)	Forward primers (5'-3')	Reverse primers (5'-3')	Anneling temperature (0C)	Expected amplified product size (bp)	Structural annotation		Functional annotation	
								Sequence components of *kabuli* genome	*Kabuli* gene accession IDs	NCBI-KOG	TFs
Ca4_P4_7638568	Ca4	7638568	GTTTTTTTTT/GTTTTTTT	TAGGCCATCATCATTTGGGT	TTCATGCAACCCTCAAATAAAA	60.2	797	INTERGENIC	--	--	--
Ca4_P4_7638687	Ca4	7638687	AATATATATATAT/AATATATATATATAT	TAGGCCATCATCATTTGGGT	TTCGAATCAGGGATAAGACGTT	60.2	896	INTERGENIC	--	--	--
Ca4_P4_7937193	Ca4	7937193	TAAAAAAAAAAA/TAAAAAAAAAA	GCTCCGGTCAAACACAATTT	GAAAATTGAAAGGCATGGGA	60.0	403	INTERGENIC	--	--	--
Ca4_P4_7938199	Ca4	7938199	TTTTTATAA/TTTTTATAATTATTTTATAA	TGCAACACACAATTGCAAAA	TGAGGGGTGGAGTTTGGTAG	59.7	532	INTERGENIC	--	--	--
Ca4_P4_8003681	Ca4	8003681	GTATATATATATATATATATATATAT/GTATATATATATATATATATATATATATAT	AATGTGTCCTCGCCGTTAAA	TAGGGGGATGATGTGGAAAA	60.5	565	INTERGENIC	--	--	--
Ca4_P4_80132	Ca4	80132	G/GA	CCGTTTGGATTGTCAGGACT	TCGTTGAAAGCTGGAGGTCT	60.0	382	INTERGENIC	--	--	--
Ca4_P4_8032351	Ca4	8032351	GTTTTTTTT/GTTTTTTTTT	CTATGATCCCTCGAACGGAA	TCAACAAGAGCACGAGCAAG	60.0	669	INTERGENIC	--	--	--
Ca4_P4_8109855	Ca4	8109855	GTTTTTT/GTTTTTTT	GCGACCTTAAATTTTGGGATT	AAGTGATGCACTAAGACGGGA	59.3	274	INTERGENIC	--	--	--
Ca4_P4_8169626	Ca4	8169626	TAAATAAAAA/TAAATAAAAAATAAAAA	TATGGCACCTCCAGTTGAAA	TGTTAAATCCTCCAGACGGC	59.1	858	INTERGENIC	--	--	--
Ca4_P4_8170540	Ca4	8170540	TAAAAAA/TAAAAA	TCAACCCCTTTGTAGTTTCACA	CTGCCAACTCCAAAAGATACC	59.5	713	INTERGENIC	--	--	--

INDEL marker IDs	Chromosomes/ unanchored scaffolds	Physical positions (bp)	InDels (*Kabuli* reference genome-CDC Frontier/PII)	Forward primers (5'-3')	Reverse primers (5'-3')	Anneling temperature (0C)	Expected amplified product size (bp)	Structural annotation		Functional annotation	
								Sequence components of *kabuli* genome	*Kabuli* gene accession IDs	NCBI-KOG	TFs
Ca4_P4_8172016	Ca4	8172016	T/TC	CTAGTGTTCGGCAACAAACG	GCCTCAATTGGAGTTTGCTC	59.4	204	INTERGENIC	--	--	--
Ca4_P4_8173963	Ca4	8173963	TTAT/TTATAT	GAAGCTCGCTTTATCCAACG	TCCACGGAATTTTCTGCTTC	60.0	147	INTERGENIC	--	--	--
Ca4_P4_8183765	Ca4	8183765	TA/TAA	AACTCAATCCAACCAAGCCA	TCACCACACCAACCTCAAAA	60.5	548	INTERGENIC	--	--	--
Ca4_P4_8209527	Ca4	8209527	ATTTTTTT/ATTTTTT	TGGCTACATCCGGTTTCTTC	GGGCCTCTCTCTCTTGGTCT	60.1	779	INTERGENIC	--	--	--
Ca4_P4_8209784	Ca4	8209784	TAA/TA	TGAACCAGTGTTTCTCTCTGTGA	GGGCCTCTCTCTCTTGGTCT	59.9	480	INTERGENIC	--	--	--
Ca4_P4_8210483	Ca4	8210483	GATAT/GATATAT	GGCCAGGAAATCAACTTCAA	ATGAATTGCATGCATGGTGA	60.1	439	INTERGENIC	--	--	--
Ca4_P4_8210730	Ca4	8210730	TCC/TC	GGGGATACTTGCCTCAATCA	TGATGCTATTCATTTTGCGG	59.9	814	INTERGENIC	--	--	--
Ca4_P4_8211793	Ca4	8211793	AG/A	CCAATATGTGATGGTTGTTTGG	CTACAATGGAATGAGGCGCT	60.0	473	INTERGENIC	--	--	--
Ca4_P4_8214750	Ca4	8214750	TAAAAAAAAAAAA/TAAAAAAAA	AAAAGAAGGCGAGTGCAGAG	TTGGCTCTCTTCTTCAAGGC	59.8	858	INTERGENIC	--	--	--
Ca4_P4_8215117	Ca4	8215117	GTAA/GTAATAA	TTTTTGTGTAAGGATATTTTTGTCA	CAATTGACGCAAAGGAAACA	57.1	485	INTERGENIC	--	--	--

INDEL marker IDs	Chromosomes/ unanchored scaffolds	Physical positions (bp)	InDels (*Kabuli* reference genome-CDC Frontier/PII)	Forward primers (5'-3')	Reverse primers (5'-3')	Anneling temperature (0C)	Expected amplified product size (bp)	Structural annotation		Functional annotation	
								Sequence components of *kabuli* genome	*Kabuli* gene accession IDs	NCBI-KOG	TFs
Ca4_P4_8215566	Ca4	8215566	CTTT/CTTTT	TTTCCTTTGCGTCAATTGTG	TGCTAATCTCGTGCATTTTGA	59.7	398	INTERGENIC	--	--	--
Ca4_P4_8217219	Ca4	8217219	AG/A	ACCACATATTAAGCGGGTCG	ATGTTGGTGTTGGTGGAGGT	59.8	781	INTERGENIC	--	--	--
Ca4_P4_8237892	Ca4	8237892	TAAAAAAAAAA/TAAAAAAAAA	TCCAATCCTCAACCTCGTTC	CTTGCGTGTTCCCAATTTTT	60.1	364	INTERGENIC	--	--	--
Ca4_P4_8239012	Ca4	8239012	CTTTT/CTTT	TTCTCGATCCACAAAAACAAAA	CAACCTCCTCAAATTGGACA	59.6	636	INTERGENIC	--	--	--
Ca4_P4_8239700	Ca4	8239700	GTTTTTTT/GTTTTTTTT	TGTCCAATTTGAGGAGGTTG	CATGGTCATAACCTCACTAATGC	58.5	822	INTERGENIC	--	--	--
Ca4_P4_8240019	Ca4	8240019	TTTTTATTTAATTTTATTT/TTTTTATTT	CGTGGAATATGATAAAATAAATCGAA	CCAACCCATTAACATTTGCC	59.6	313	INTERGENIC	--	--	--
Ca4_P4_8281020	Ca4	8281020	T/TA	TTGGTGTCATTCGCCTTTTT	GGGAGACGGGTACAAGAGTG	60.5	656	INTERGENIC	--	--	--
Ca4_P4_8281041	Ca4	8281041	ATTTTTTT/ATTTTTTTT	TTTGGTGTCATTCGCCTTTT	TCGCAAGGATAATGGCTTTT	60.5	709	INTERGENIC	--	--	--
Ca4_P4_8283120	Ca4	8283120	TAAAAAAA/TAAAAAAAA	TTTTTGAGAAGCCATTTAATTTTT	TTTTGTGTTCGTTTATTGTAACCAA	57.7	270	INTERGENIC	--	--	--
Ca4_P4_8297213	Ca4	8297213	TAAAAAAA/TAAAAAAAA	GGGTTTTGTTTGAAAAATTTGAT	TGGTGCTAACATGTGGCTAA	58.4	676	INTERGENIC	--	--	--

INDEL marker IDs	Chromosomes/ unanchored scaffolds	Physical positions (bp)	InDels (*Kabuli* reference genome-CDC Frontier/PII)	Forward primers (5'-3')	Reverse primers (5'-3')	Anneling temperature (0C)	Expected amplified product size (bp)	Structural annotation		Functional annotation	
								Sequence components of *kabuli* genome	*Kabuli* gene accession IDs	NCBI-KOG	TFs
Ca4_P4_8307784	Ca4	8307784	ATTT/ATT	TACACCCATTGACGTGGCTA	TCAATCATGTCTGCATACCG	60.0	694	INTERGENIC	--	--	--
Ca4_P4_8308503	Ca4	8308503	GTTTTTT/GTTTTT	TAACAGGTGAGGGCATGTCA	TATGCAAACCCAATTGACCA	60.1	321	INTERGENIC	--	--	--
Ca4_P4_8309234	Ca4	8309234	TCAACACAA/TCAACACAACACAA	GAATTTAAGAGGAAGGCGATGA	TTCACCCTAATTCGTAGGCG	59.7	450	INTERGENIC	--	--	--
Ca4_P4_8309498	Ca4	8309498	A/ACC	CATCCCAGGATCAACACTCA	TTCACCCTAATTCGTAGGCG	59.5	272	INTERGENIC	--	--	--
Ca4_P4_8311787	Ca4	8311787	A/AG	TTACTCCCTCCTTCCCATGA	ATGGATGGGGGATAAAAAGG	59.5	433	INTERGENIC	--	--	--
Ca4_P4_8311844	Ca4	8311844	TG/T	TTACTCCCTCCTTCCCATGA	ATGGATGGGGGATAAAAAGG	59.5	433	INTERGENIC	--	--	--
Ca4_P4_8313290	Ca4	8313290	ATTG/ATTGTTGTTG	GGGTCACAGGGACAAGGTAA	CACAACAACCAGTCCACCAG	59.8	154	INTERGENIC	--	--	--
Ca4_P4_8313629	Ca4	8313629	ATTTTTTTTTTTT/ATTTTTTTTTTT	CTGGTGGACTGGTTGTTGTG	GGCAGACAGGAGAGGGAGTA	60.0	563	INTERGENIC	--	--	--
Ca4_P4_8315933	Ca4	8315933	CTT/CTTATT	GGACCCGTGATGCTGTTTAT	TCTCGGTTGATGTCATTATGCT	59.8	586	INTERGENIC	--	--	--
Ca4_P4_8316304	Ca4	8316304	GTTTTTTT/GTTTTTTTT	GCATAATGACATCAACCGAGAG	AAAAGAAATGGTTGTTTCTCGTTC	59.6	235	INTERGENIC	--	--	--

INDEL marker IDs	Chromosomes/ unanchored scaffolds	Physical positions (bp)	InDels (*Kabuli* reference genome-CDC Frontier/PII)	Forward primers (5'-3')	Reverse primers (5'-3')	Anneling temperature (0C)	Expected amplified product size (bp)	Structural annotation		Functional annotation	
								Sequence components of *kabuli* genome	*Kabuli* gene accession IDs	NCBI-KOG	TFs
Ca4_P4_8318067	Ca4	8318067	T/TA	GCATGGTAGCCAAAGAAAGC	TTCCGTTAAACTCAACTTCCG	59.9	758	INTERGENIC	--	--	--
Ca4_P4_8320140	Ca4	8320140	C/CG	CTTTGCCCCCATCCATATAA	AGGCTGAAGACTTCGACGAG	59.6	358	INTERGENIC	--	--	--
Ca4_P4_8320682	Ca4	8320682	AAGAGAGA/AAGA	AAAATCTTCCGGTGTCTCCA	TTTTCCCAAGTTGGAAGTGG	59.5	668	INTERGENIC	--	--	--
Ca4_P4_8321099	Ca4	8321099	A/ATTAATTTCTT	CCACTTCCAACTTGGGAAAA	GGACAATGCCGTAAAACGTC	59.9	484	INTERGENIC	--	--	--
Ca4_P4_8325707	Ca4	8325707	TA/T	ACGGGGCTGTTACTTTACCC	TTCTCAATGGGTGCGTACAA	60.2	739	INTERGENIC	--	--	--
Ca4_P4_8326100	Ca4	8326100	TAAAAAAAA/TAAAAAAA	TGTACGCACCCATTGAGAAA	TAATGGCTGGTTTCGTTTGG	60.1	547	INTERGENIC	--	--	--
Ca4_P4_8326843	Ca4	8326843	TTCT/TTCTCT	TAAAGAGCACGAATTGCACG	CCAATTCCAAAACCCATACG	60.0	601	INTERGENIC	--	--	--
Ca4_P4_8332622	Ca4	8332622	GTA/GTACCATA	GCCTTCTGATGTGGTTTGGT	TGTGCCAAACAAGTTAGCGT	60.0	916	INTERGENIC	--	--	--
Ca4_P4_8333108	Ca4	8333108	GAAA/GAAAA	TCCCACTTTCATGTCTCCAA	TGATCACCTTTGAAAAGCCC	59.1	147	INTERGENIC	--	--	--
Ca4_P4_8333830	Ca4	8333830	ATTTTTTTTTT/ATTTTTTTTTTT	ACGTAGTGTTTTGGCAAGGG	TCACACATCACCGCAAACTT	60.0	342	INTERGENIC	--	--	--

INDEL marker IDs	Chromosomes/ unanchored scaffolds	Physical positions (bp)	InDels (*Kabuli* reference genome-CDC Frontier/PII)	Forward primers (5'-3')	Reverse primers (5'-3')	Anneling temperature (0C)	Expected amplified product size (bp)	Structural annotation		Functional annotation	
								Sequence components of *kabuli* genome	*Kabuli* gene accession IDs	NCBI-KOG	TFs
Ca4_P4_8362831	Ca4	8362831	TT/TTAT	AGGTTCGAACGGATAT GTCG	CCATGTGCAAACCTAG GAAAA	60.0	749	INTERGENIC	--	--	--
Ca4_P4_8364920	Ca4	8364920	GT/GTTGTCACTGTTTTGCT	GCTGGAACTTGGTTAC GAGG	TTAGCATGCATTGGGA AACA	59.7	378	INTERGENIC	--	--	--
Ca4_P4_8376162	Ca4	8376162	CT/CTT	TCGGGCTTGAGCTCTA TCAT	TGGCCGAAATTGAGAG AATC	59.9	541	INTERGENIC	--	--	--
Ca4_P4_8376439	Ca4	8376439	TA/TAA	GATTCTCTCAATTTCG GCCA	TGCGATTGATTTCAGTT TTTG	60.2	373	INTERGENIC	--	--	--
Ca4_P4_8376670	Ca4	8376670	CAAAAAAAA/CAAAAAAA	GATTCTCTCAATTTCG GCCA	TGCCCCAAACACGTAC ACTA	60.2	575	INTERGENIC	--	--	--
Ca4_P4_8409923	Ca4	8409923	AAGAG/AAG	CGCATCAATACGACGA ACAA	ACCACCACTTTGTAGC CACC	60.7	509	INTERGENIC	--	--	--
Ca4_P4_8410561	Ca4	8410561	ATTTTTTTT/ATTTTTTTTT	GGTGGCTACAAAGTGG TGGT	ATAGCGTAAGGGCACA TGCT	59.9	603	INTERGENIC	--	--	--
Ca4_P4_8413764	Ca4	8413764	TA/T	AATGGTGAACAAGGAG CAGG	TCAAATATTAACTCCAA GCGCA	60.1	714	INTERGENIC	--	--	--
Ca4_P4_8414626	Ca4	8414626	C/CATTT	TCTTGGTCAGAACCAT GTGC	AAATGGCCAAATTGCAT GAT	59.7	616	INTERGENIC	--	--	--
Ca4_P4_8415263	Ca4	8415263	T/TAA	CACCTGCACCGCTAAG TTTT	TTGTTGAACACCCACAT GCT	60.3	274	INTERGENIC	--	--	--

INDEL marker IDs	Chromosomes/ unanchored scaffolds	Physical positions (bp)	InDels (*Kabuli* reference genome-CDC Frontier/PII)	Forward primers (5'-3')	Reverse primers (5'-3')	Anneling temperature (0C)	Expected amplified product size (bp)	Structural annotation		Functional annotation	
								Sequence components of *kabuli* genome	*Kabuli* gene accession IDs	NCBI-KOG	TFs
Ca4_P4_842960	Ca4	842960	AATTATTATTATTATTATTATTATTATTATTATTATTA/AATTATTATTATTATTATTATTATTATTATTATTATTATTA	AACATCAACAACTTCTTTTTGCC	AGGCCTATTGGGCTGAAAAT	59.6	673	INTERGENIC	--	--	--
Ca4_P4_8457992	Ca4	8457992	ATTTTTT/ATTTTTTT	CATGTGTGCCTCTAATCCCA	GAGCCCTTCAAAGAACTCACA	59.5	457	INTERGENIC	--	--	--
Ca4_P4_8458330	Ca4	8458330	A/ATC	GAGTTCTTTGAAGGGCTCACA	TCCCCTACCCAAAATTGGAA	59.5	569	INTERGENIC	--	--	--
Ca4_P4_8490694	Ca4	8490694	TTATATATATATATA/TTATATATATATATATATATA	TATGCCTTAGCGGTTTTTGG	GGTCCTTTGATCCCACGTTA	60.1	498	INTERGENIC	--	--	--
Ca4_P4_8491035	Ca4	8491035	AAGA/AA	TAACGTGGGATCAAAGGACC	ATATGAAACCACCAACCCCA	59.8	385	INTERGENIC	--	--	--
Ca4_P4_8491570	Ca4	8491570	GTTTTTTTTT/GTTTTTTTTTT	GCCAACGGTTGTTTGTTTTT	TGAATGAGCCAACACGTTTC	59.9	344	INTERGENIC	--	--	--
Ca4_P4_8504484	Ca4	8504484	ATTTTTTT/ATTTTTTTT	TTTGGAGTCAAATGATAAAAGTGG	TTTTCGAGTGCCGCTTACTT	59.4	522	INTERGENIC	--	--	--
Ca4_P4_8576551	Ca4	8576551	GTATATATATATATATATATATATATATAT/GTATATATATATATATATATATATAT	AAATGAAAAATAATGAATTCTCGTGAT	TTGTTATTTTTGTCTCCCACTCA	59.6	607	INTERGENIC	--	--	--
Ca4_P4_8576946	Ca4	8576946	GTTTT/GTTTTT	TGTCATAAGAAACAATTGGACG	TTGCAGACTAACCCAGATCG	57.7	440	INTERGENIC	--	--	--
Ca4_P4_8609734	Ca4	8609734	AGGGGG/AGGGG	GTAGGAAGTCGCCATTTGGA	CACCAAAATACCCCCATTTG	60.1	126	INTERGENIC	--	--	--

INDEL marker IDs	Chromosomes/ unanchored scaffolds	Physical positions (bp)	InDels (*Kabuli* reference genome-CDC Frontier/PII)	Forward primers (5'-3')	Reverse primers (5'-3')	Anneling temperature (0C)	Expected amplified product size (bp)	Structural annotation		Functional annotation	
								Sequence components of *kabuli* genome	*Kabuli* gene accession IDs	NCBI-KOG	TFs
Ca4_P4_8612545	Ca4	8612545	AA/AAATTAAAGGTGTA	TTTCTTTCTGCTGTCGGCTT	CATGCCATCCACTATGTTGC	60.1	393	INTERGENIC	--	--	--
Ca4_P4_8613689	Ca4	8613689	TTTTTCT/TT	TGCATTTGGTTGAAAATGAAA	TGGTGGGCCTACGAGTAAAG	59.0	374	INTERGENIC	--	--	--
Ca4_P4_8616163	Ca4	8616163	ATTTT/ATTTTT	TTCAAAAGGCCGTAGGTTTC	TAGTTTGGAATGGTCGCACA	59.2	933	INTERGENIC	--	--	--
Ca4_P4_8621719	Ca4	8621719	GAATAATAATAATAATAATAATAA/GAATAATAATAATAATAA	GTGCCTTTCTTGTCAGGGAG	TGGAAGCCCCATAAAGACTG	59.8	765	INTERGENIC	--	--	--
Ca4_P4_8626094	Ca4	8626094	TTATA/T	GGTTTGAATGTCAACACCCT	TGCATGTGTGTAACAACAACAAA	57.3	482	INTERGENIC	--	--	--
Ca4_P4_8633576	Ca4	8633576	TAAAA/TAAA	TTGCACGAAAAATTGTGAGC	TAAATGGACGGAGTTGGGAA	59.9	527	INTERGENIC	--	--	--
Ca4_P4_8633989	Ca4	8633989	CA/CAA	TTCCCAACTCCGTCCATTTA	TTGATCAAACGCGCAAATTA	60.3	271	INTERGENIC	--	--	--
Ca4_P4_8636225	Ca4	8636225	GTT/GTTT	GGGAAACCAAGGAGTGTGAA	GCATGCGATCGTAATTCCTT	59.9	771	INTERGENIC	--	--	--
Ca4_P4_8636291	Ca4	8636291	AAT/AATAT	GGGAAACCAAGGAGTGTGAA	GCATGCGATCGTAATTCCTT	59.9	771	INTERGENIC	--	--	--
Ca4_P4_8644121	Ca4	8644121	AATAT/AATATAT	TGAGAATATAGCATACCGACCG	GGGGGTTGAGTTTAGGGTTC	59.1	651	INTERGENIC	--	--	--

INDEL marker IDs	Chromosomes/ unanchored scaffolds	Physical positions (bp)	InDels (*Kabuli* reference genome-CDC Frontier/PII)	Forward primers (5'-3')	Reverse primers (5'-3')	Anneling temperature (0C)	Expected amplified product size (bp)	Structural annotation		Functional annotation	
								Sequence components of *kabuli* genome	*Kabuli* gene accession IDs	NCBI-KOG	TFs
Ca4_P4_8650892	Ca4	8650892	AATATA/AATATATA	TTTAAATTGAATGTGTTCGCGT	ACCACACATGATTTCTGCCA	59.5	785	INTERGENIC	--	--	--
Ca4_P4_8679645	Ca4	8679645	CAAAA/CAAAAA	TAGTACCCGTACTGGGCTGG	GCGTTTTGGAATCTGCATTT	60.0	289	INTERGENIC	--	--	--
Ca4_P4_8688751	Ca4	8688751	AACA/AA	AGGACCCACAACATCCACAT	ACCAATCCATGCACGCTACT	60.1	505	INTERGENIC	--	--	--
Ca4_P4_8689137	Ca4	8689137	ATTTTTT/ATTTTTTT	CCCATCTTGGGTGAAGAAAG	GGTGGTTTAGACGAATAGAGACG	59.5	670	INTERGENIC	--	--	--
Ca4_P4_8689670	Ca4	8689670	A/ATCTC	TGCAAATTTGGTGAAAACAGA	TGTCACATTTGCAGAAGAAAAA	59.2	300	INTERGENIC	--	--	--
Ca4_P4_8690768	Ca4	8690768	TAAAAAAA/TAAAAAAAAA	GTTTTAATGCAAGCCATGTAGA	GGTGTTTTGATGCGAAGTGA	57.1	397	INTERGENIC	--	--	--
Ca4_P4_8690798	Ca4	8690798	G/GA	AATTCCAGATCACCGCGA	GGTGTTTTGATGCGAAGTGA	60.2	313	INTERGENIC	--	--	--
Ca4_P4_8707105	Ca4	8707105	AAAT/A	CGAGAGGTATTTATGCTTCACTG	ATCTCTCCGACCATGTGGAA	58.1	712	INTERGENIC	--	--	--
Ca4_P4_8708859	Ca4	8708859	A/AC	CAAAGCGAATTTAGGAAAGTAAAA	GCATATTTATGCCCACCCAC	58.2	484	INTERGENIC	--	--	--
Ca4_P4_8715030	Ca4	8715030	ATTTTTT/ATTTTTTT	GAGAGGCGTTGAAGAAAACC	AGTGGACCACATGACACCAA	58.9	685	INTERGENIC	--	--	--

INDEL marker IDs	Chromosomes/ unanchored scaffolds	Physical positions (bp)	InDels (*Kabuli* reference genome-CDC Frontier/PII)	Forward primers (5'-3')	Reverse primers (5'-3')	Anneling temperature (0C)	Expected amplified product size (bp)	Structural annotation		Functional annotation	
								Sequence components of *kabuli* genome	*Kabuli* gene accession IDs	NCBI-KOG	TFs
Ca4_P4_8723077	Ca4	8723077	T/TC	GCGTGTTTGGTTGAAAGGAT	GGTCGAGTTCCAAATTCCAA	60.0	619	INTERGENIC	--	--	--
Ca4_P4_8727347	Ca4	8727347	TAAAAAAAAAAAA/TAAAAAAAAA	CGGGTCTCTTTGACTCTTCG	TCATCTTATTTTGGGCATTCA	60.0	563	INTERGENIC	--	--	--
Ca4_P4_8727913	Ca4	8727913	T/TCA	TGAATGCCCAAAATAAGATGA	TCACCAATCCAGCTGGTACA	58.1	425	INTERGENIC	--	--	--
Ca4_P4_8727978	Ca4	8727978	A/AG	TGAATGCCCAAAATAAGATGA	TGATTGAAAGAGCGTTGGATT	58.1	651	INTERGENIC	--	--	--
Ca4_P4_8728353	Ca4	8728353	TTTATT/TTTATTATT	AATCCAACGCTCTTTCAATCA	GGAACCAAACCAGCATGAAT	59.7	343	INTERGENIC	--	--	--
Ca4_P4_8741293	Ca4	8741293	TAA/TA	TCTTCCTCTGTCAACCAAACG	TCGGTTGAAAATGTTACTGGC	60.3	106	INTERGENIC	--	--	--
Ca4_P4_8744304	Ca4	8744304	TA/TAA	TCTTTCCTTGGTCAGCGTCT	TTTGTCGTGAAAATGCATTAGG	60.0	825	INTERGENIC	--	--	--
Ca4_P4_8745750	Ca4	8745750	C/CATT	ACTTGTCTCGTACCCGCAGT	TGCAACTTCTTATCAATTGCGT	59.8	420	INTERGENIC	--	--	--
Ca4_P4_8748044	Ca4	8748044	ACCCCCC/ACCCCC	CACGATCCCATCAAAATGAA	TCACAACATGAGAATGGGACA	59.3	577	INTERGENIC	--	--	--
Ca4_P4_8748580	Ca4	8748580	GAAAAAAAAAA/GAAAAAAAA	TGTCCCATTCTCATGTTGTGA	CAAAATTGCATTCAAAGTCCC	60.0	424	INTERGENIC	--	--	--

INDEL marker IDs	Chromosomes/ unanchored scaffolds	Physical positions (bp)	InDels (*Kabuli* reference genome-CDC Frontier/PII)	Forward primers (5'-3')	Reverse primers (5'-3')	Anneling temperature (0C)	Expected amplified product size (bp)	Structural annotation		Functional annotation	
								Sequence components of *kabuli* genome	*Kabuli* gene accession IDs	NCBI-KOG	TFs
Ca4_P4_8748930	Ca4	8748930	TAAAAAAA/TAA	GGGACTTTGAATGCAATTTTGT	CGAGGATCTGTGACCAATCA	60.2	482	INTERGENIC	--	--	--
Ca4_P4_8749252	Ca4	8749252	T/TTA	ATACGGATTGGATGAGGTGC	GGCGTAATTACTTCAAGGCAA	59.8	376	INTERGENIC	--	--	--
Ca4_P4_8749651	Ca4	8749651	ATATATTTAT/ATAT	ATACGGATTGGATGAGGTGC	CCGGCATTAAAATTTGGAAAA	59.8	878	INTERGENIC	--	--	--
Ca4_P4_8758399	Ca4	8758399	TAA/TA	CATAGCAGCTTCGATGCAAA	TCACAATTTGACAACCACGG	60.1	297	INTERGENIC	--	--	--
Ca4_P4_8766483	Ca4	8766483	TAAG/TAAGAAG	GGTTGCGGTCGAAGTTACAT	GATGGGCCAAACTGCTTTTA	60.0	575	INTERGENIC	--	--	--
Ca4_P4_8766663	Ca4	8766663	TAAAAAAAA/TAAAAAAA	GGTTGCGGTCGAAGTTACAT	GATGGGCCAAACTGCTTTTA	60.0	575	INTERGENIC	--	--	--
Ca4_P4_8780881	Ca4	8780881	TTAT/TT	AACCCAGCAAACATCAAAGA	CGGGGCAGAGTAGAGCTATG	58.2	881	INTERGENIC	--	--	--
Ca4_P4_8781421	Ca4	8781421	GAAAAA/GAAAAAAA	CATAGCTCTACTCTGCCCCG	GCAAGTTTGCCGGTTATGTT	60.0	114	INTERGENIC	--	--	--
Ca4_P4_8799497	Ca4	8799497	TAATAGAGTTAAAAA/TAA	TCAAAGTGCGATACAGGAAGC	TGGGTTTAATACATGGAGATGG	60.4	922	INTERGENIC	--	--	--
Ca4_P4_8800933	Ca4	8800933	TATAAGGACAAT/TAT	TGATTGGCATCCATTTTCCT	TTAGAAACGTGTTGGAGGGG	60.3	193	INTERGENIC	--	--	--

INDEL marker IDs	Chromosomes/ unanchored scaffolds	Physical positions (bp)	InDels (*Kabuli* reference genome-CDC Frontier/PII)	Forward primers (5'-3')	Reverse primers (5'-3')	Anneling temperature (0C)	Expected amplified product size (bp)	Structural annotation		Functional annotation	
								Sequence components of *kabuli* genome	*Kabuli* gene accession IDs	NCBI-KOG	TFs
Ca4_P4_8810790	Ca4	8810790	G/GTGA	AAAAATGGGTTATTAAGTGAGGAAAA	TGTTACCGGAATTAGCAAACG	59.6	549	INTERGENIC	--	--	--
Ca4_P4_8812070	Ca4	8812070	AT/ATGTT	TGGTGCACTTTTTGTGTCCA	TCAAACACACAATAATGGCGA	61.2	648	INTERGENIC	--	--	--
Ca4_P4_8812812	Ca4	8812812	ATTTTTTTT/ATTTTTTTTT	GTTTTTAATTCGCACCCCAA	GCAGTTTAAGCAATAAAATGATGC	59.8	427	INTERGENIC	--	--	--
Ca4_P4_8816656	Ca4	8816656	AT/A	ATCGTTGTCGTAGATTGGGC	TTGGATCAAAATCTGAAGCG	60.0	789	INTERGENIC	--	--	--
Ca4_P4_8828382	Ca4	8828382	GTT/GTTT	GCGACTACCACATTTGGGTT	TGGCACTCCTTCTAAATGGG	59.9	354	INTERGENIC	--	--	--
Ca4_P4_8829389	Ca4	8829389	C/CG	TTCTCTACCGATTTGGCACA	TGAAACATGTTGGTACGTGGA	59.3	287	INTERGENIC	--	--	--
Ca4_P4_8829890	Ca4	8829890	ACTAAAAAAATC/AC	TCCACGTACCAACATGTTTCA	AAAGGCGCGGTTTAGATTTT	59.9	526	INTERGENIC	--	--	--
Ca4_P4_8830201	Ca4	8830201	AATATA/AATATATA	AGAAAATCTAAACCGCGCCT	TGGACTTTCTTATTTGCCGC	60.2	303	INTERGENIC	--	--	--
Ca4_P4_8836705	Ca4	8836705	TTAAT/TTAATAAT	AGCTTCGATTTGGTGAGGTG	TGATGTATACCACAATGATTCCG	60.3	444	INTERGENIC	--	--	--
Ca4_P4_8836902	Ca4	8836902	T/TG	AGCTTCGATTTGGTGAGGTG	TTGAGTTTCAATATGGTGATGTGA	60.3	854	INTERGENIC	--	--	--

INDEL marker IDs	Chromosomes/ unanchored scaffolds	Physical positions (bp)	InDels (*Kabuli* reference genome-CDC Frontier/PII)	Forward primers (5'-3')	Reverse primers (5'-3')	Anneling temperature (0C)	Expected amplified product size (bp)	Structural annotation		Functional annotation	
								Sequence components of *kabuli* genome	*Kabuli* gene accession IDs	NCBI-KOG	TFs
Ca4_P4_8838334	Ca4	8838334	ATTTATCAAATATTTTATCA/ATTTATCA	TTTTCAACCCTTCTTTCCCA	GAGGTTTGCAAATTGTCGCT	59.5	242	INTERGENIC	--	--	--
Ca4_P4_8840917	Ca4	8840917	GTT/GTTTTT	GATACTCTAAGAATACCTCCGACAAA	TTTTTGCGCACATATATAAAACA	58.4	174	INTERGENIC	--	--	--
Ca4_P4_8864736	Ca4	8864736	TGGGG/TGG	TTTTTGGCTTATGGGGTGAG	TCAGCGTATTGCGAAACAAC	59.9	912	INTERGENIC	--	--	--
Ca4_P4_8871044	Ca4	8871044	CATAAAAT/CATAAAATTTATAAAAT	AAAACATCGGAGTCCACGTC	AATTTCATTGCGGCAAATTC	60.0	699	INTERGENIC	--	--	--
Ca4_P4_887746	Ca4	887746	ATTTTTTT/ATTTTTT	GCCGAATCAATTCAAATCAA	ATGCACAACCAATTTCACCA	58.6	412	INTERGENIC	--	--	--
Ca4_P4_8878932	Ca4	8878932	CAATAATAATAATAATAAT/CAATAATAATAATAAT	ATGGACGGATTGGATGGATA	ATACGATGGTGGTTGGTGGT	60.0	581	INTERGENIC	--	--	--
Ca4_P4_8884777	Ca4	8884777	TAAT/TAATATATAAAT	ACACGTGGCTTGTTGGTGTA	GTCGATCTCGCTCACTCCAC	60.1	675	INTERGENIC	--	--	--
Ca4_P4_8885675	Ca4	8885675	A/ATTAC	TGCTACATAATCAAGTGTTTTAGGC	AAGAATGCAGATCAATCGCC	58.9	744	INTERGENIC	--	--	--
Ca4_P4_8885843	Ca4	8885843	CTGT/CTGTGT	TGAGACCCCGTAAGATCCAA	AAGAATGCAGATCAATCGCC	60.5	194	INTERGENIC	--	--	--
Ca4_P4_8887604	Ca4	8887604	GTATTATT/GTATT	GTTCGTCCCTCGATCACAAT	CCAACCACCCAAAATCCAT	59.9	308	INTERGENIC	--	--	--

INDEL marker IDs	Chromosomes/ unanchored scaffolds	Physical positions (bp)	InDels (*Kabuli* reference genome-CDC Frontier/PII)	Forward primers (5'-3')	Reverse primers (5'-3')	Anneling temperature (0C)	Expected amplified product size (bp)	Structural annotation		Functional annotation	
								Sequence components of *kabuli* genome	*Kabuli* gene accession IDs	NCBI-KOG	TFs
Ca4_P4_8889782	Ca4	8889782	A/AG	TTAAGAAGTATCACGCGGGC	TCCTTTCTCCGTGGTGAGTT	60.2	472	INTERGENIC	--	--	--
Ca4_P4_8890585	Ca4	8890585	GTA/GTATA	CATGCCTCGTTAGAGTGCAA	TCCACCACACACTCCTCAAA	60.0	256	INTERGENIC	--	--	--
Ca4_P4_8894416	Ca4	8894416	CTATATATATATAT/CTATATATATATATATATAT	TGATATCCGGTTTGGGTGTT	CTCCCGTTATCCGAAACAGA	60.1	569	INTERGENIC	--	--	--
Ca4_P4_8897355	Ca4	8897355	TA/T	GATCCACTCAGGGTCGTGTT	ACTTTTGCATCTGGATGGCT	60.0	461	INTERGENIC	--	--	--
Ca4_P4_8904309	Ca4	8904309	CTTTTTATATTTTCATTTTTTTTTATATTTTCATT/CTTTTTATATTTTCATT	AAGAAAACCCACCACACTCG	CAACCCATAGAACAAAAATCCC	60.0	518	INTERGENIC	--	--	--
Ca4_P4_8946430	Ca4	8946430	TGAGAGAGAGAGAGA/TGAGAGAGAGAGAGAGA	ACAGCAACCGAAACTGCTTT	GGGGAGCATTGGTCACTAAA	59.9	432	INTERGENIC	--	--	--
Ca4_P4_9028821	Ca4	9028821	TTATATATATATATATATAT/TTATATATATATATATATATAT	ATGGAACAATCGGACACCAT	CTGTCAACTCACTCATCAAATCG	60.1	422	INTERGENIC	--	--	--
Ca4_P4_9066423	Ca4	9066423	ACC/AC	GCAACCCGTAGGTGTAGGAA	TGTGCGTTGGTAGTCAATGTG	60.0	546	INTERGENIC	--	--	--
Ca4_P4_9066518	Ca4	9066518	ATTTTTTTTT/ATTTTTTT	GCAACCCGTAGGTGTAGGAA	TGTGCGTTGGTAGTCAATGTG	60.0	546	INTERGENIC	--	--	--
Ca4_P4_9078995	Ca4	9078995	TAAAAAAAAA/TAAAAAAAAAA	GTTATTTTGGCCCACTACCG	ATTTTCTTTGGCGACTTCCA	59.3	480	INTERGENIC	--	--	--

INDEL marker IDs	Chromosomes/ unanchored scaffolds	Physical positions (bp)	InDels (*Kabuli* reference genome-CDC Frontier/PII)	Forward primers (5'-3')	Reverse primers (5'-3')	Anneling temperature (0C)	Expected amplified product size (bp)	Structural annotation		Functional annotation	
								Sequence components of *kabuli* genome	*Kabuli* gene accession IDs	NCBI-KOG	TFs
Ca4_P4_9229798	Ca4	9229798	TGGG/TGGGG	AAGGCTCAACATGCAAAACC	TGCCAATTCGATTGGAATAA	60.1	522	INTERGENIC	--	--	--
Ca4_P4_9230116	Ca4	9230116	TA/T	GTTGCGAAAAATGGGTTTGT	ACCAGGAAAAGAAGAAGGGC	59.8	608	INTERGENIC	--	--	--
Ca4_P4_9371255	Ca4	9371255	GAAAAAAAA/GAAAAAAAAA	GGAGCATATGCCTCGTTGTT	TGGAAATTCAACTTTTTGGACA	60.1	549	INTERGENIC	--	--	--
Ca4_P4_9386505	Ca4	9386505	AATATATATATATATATATATAT/AATATATATATATATATATATATAT	TTTGCAATTGTGGCAAGTGT	CGTGGACAAAGATCGTGATG	60.2	685	INTERGENIC	--	--	--
Ca4_P4_9453892	Ca4	9453892	GTATATATATATATATATATATA/GTATATATATATATATATATATATA	TTGATGGTGCTAGGAGAAGACA	AAACCCACAATTCATCAGGTT	59.9	433	INTERGENIC	--	--	--
Ca4_P4_9504124	Ca4	9504124	CTACTATTA/CTA	TTGTTGAGTATAAGGTTGTCTTGTG	GACTGGAACGCTTCGTCTTC	57.5	791	INTERGENIC	--	--	--
Ca4_P4_9574784	Ca4	9574784	TTATTATTATTATTATTATTATTATTATTATTATTATTATTATTATTATTATTATTATTA/TTATTATTATTATTATTATTATTATTATTATTATTATTATTATTATTATTATTATTATTATTATTATTATTATTATTATTATTA	ATTGACAAAAGTTGGAGGCG	TGCATGCTAATTTGCGCTAT	60.1	502	INTERGENIC	--	--	--
Ca4_P4_9586747	Ca4	9586747	CTTTTT/CTTGTATATTTTTTTT	AATTAACTGAGACGGCACGG	CGTTCATAAAGATTTGCCCA	60.1	827	INTERGENIC	--	--	--
Ca4_P4_9625757	Ca4	9625757	ATATTATTATTATTATTATTATTATTATTATT/ATATTATTATTATTATTATTATTATTATTATT	ATGTGCGAGAAAGGGAGAGA	CACTTCGGTCATTCTCTTCAAA	60.0	543	INTERGENIC	--	--	--
Ca4_P4_9629867	Ca4	9629867	ATTTTTTTTTT/ATTTTTTTT	CAACCGTAAACAAACCAAAAA	CAACCGAAGATTTGTTTGGAA	57.7	483	INTERGENIC	--	--	--

INDEL marker IDs	Chromosomes/ unanchored scaffolds	Physical positions (bp)	InDels (*Kabuli* reference genome-CDC Frontier/PII)	Forward primers (5'-3')	Reverse primers (5'-3')	Anneling temperature (0C)	Expected amplified product size (bp)	Structural annotation		Functional annotation	
								Sequence components of ***kabuli*** genome	***Kabuli*** gene accession IDs	NCBI-KOG	TFs
Ca4_P4_9630152	Ca4	9630152	GAA/GA	CCTCTTCCAAACAAATCTTCG	TCAAAATATGGCATGTGGGA	58.8	455	INTERGENIC	--	--	--
Ca4_P4_9630289	Ca4	9630289	ATTAGTTAGTT/ATTAGTTAGTTAGTT	CCTCTTCCAAACAAATCTTCG	CCAGCTCATCAAAGCCATTT	58.8	550	INTERGENIC	--	--	--
Ca4_P4_9631458	Ca4	9631458	GTTTCCTCCTCCGTCTTT/GTTTCCTCCTCCGTCTTTCCTCCTCCGTCTTT	TTCTTCATCTTCCTCCTCCG	TTATCGGATGTATGGGAGGG	59.4	148	INTERGENIC	--	--	--
Ca4_P4_9632653	Ca4	9632653	TG/T	TGGATTGTCGTGATCTCAAA	CACACCCGTACTCAGCATTG	57.6	630	INTERGENIC	--	--	--
Ca4_P4_9633059	Ca4	9633059	TAAAAAAAAAAA/TAAAAAAAAA	CAATGCTGAGTACGGGTGTG	AACACAAGGAGGCCATGTTC	60.2	503	INTERGENIC	--	--	--
Ca4_P4_9658674	Ca4	9658674	TAAAAAAA/TAAAA	TGCAATTTGATACCGAGCCT	TCCCCTTCTTTGAGGGATTT	60.6	914	INTERGENIC	--	--	--
Ca4_P4_9677869	Ca4	9677869	CATATATATATATATATATA/CATATATATATATATATATA	TTGCACATTTTGAATGCTGA	ATGCGATCCATTGAATTTGA	58.8	405	INTERGENIC	--	--	--
Ca4_P4_9736707	Ca4	9736707	ATCTCTCTCTCTCTCTCTCTCTCT/ATCTCTCTCTCTCTCTCTCTCT	CACTTCACCCACCTTCCCTA	ATCCAACAATCTGGGGACAA	60.0	256	INTERGENIC	--	--	--
Ca4_P4_9764739	Ca4	9764739	GTTTTTTT/GTTTTTTTT	TCAGGCGGGTTTTATAGACA	CTCTGCTCACAACCCAACAA	58.2	701	INTERGENIC	--	--	--
Ca4_P4_9857350	Ca4	9857350	TGTGCGAGTGCGAGTGCGAGT/TGTGCGAGTGCGAGT	TGAGACGTTTTTCACACCCA	CTGCAACAATGAATCTTGAAGT	60.1	469	INTERGENIC	--	--	--

INDEL marker IDs	Chromosomes/ unanchored scaffolds	Physical positions (bp)	InDels (*Kabuli* reference genome-CDC Frontier/PII)	Forward primers (5'-3')	Reverse primers (5'-3')	Anneling temperature (0C)	Expected amplified product size (bp)	Structural annotation		Functional annotation	
								Sequence components of *kabuli* genome	*Kabuli* gene accession IDs	NCBI-KOG	TFs
Ca4_P4_9967472	Ca4	9967472	ATTTTT/ATTTTTT	CCCCTTTTATGAAATGGTTGAA	TGAGACTGAAATCGGGTCTTTT	60.0	945	INTERGENIC	--	--	--
Ca5_P4_1000531	Ca5	1000531	AATATATATATATATATAT/AATATATATATATATATATAT	GGGTTTTGGAAGGAGGAATG	CTTGTGTTCGATACGCATCAT	60.7	874	INTERGENIC	--	--	--
Ca5_P4_10191815	Ca5	10191815	ATATTATTATTATTATTATTATTATTATTATT/ATATTATTATTATTATTATTATTATTATT	AGCACACACACGCAAAAATG	TGGGAAAGGACATTTGCAT	60.8	409	INTERGENIC	--	--	--
Ca5_P4_1020806	Ca5	1020806	TTTATTATTATTATTATTATTATTATTATTATTATTATTATTATTATT/TTTATTATTATTATTATTATTATTATTATTATTATTATTATTATTATTATTATT	AATTATGCTCCGACTTCCCC	GAAGCCGAGAAAACGACTTG	60.3	778	INTERGENIC	--	--	--
Ca5_P4_10213713	Ca5	10213713	TTTATTATTATTATTATTATTATTATTATTATTATTATTATTATTAT/TTTATTATTATTATTATTATTATTATTATTATTATTATTATTATTATTAT	AGAGGCAAACAAGAACCGAA	TGTGCGTTTGACTGTGTTGA	59.9	209	INTERGENIC	--	--	--
Ca5_P4_10270439	Ca5	10270439	TTATATATATATATATATATATATATAT/TTATATATATATATATATATATATATATATAT	CCATGTTCCCATCATATATCATTTT	CTAAGGCATTACCCACTGGC	60.1	565	INTERGENIC	--	--	--
Ca5_P4_10305520	Ca5	10305520	ATTTTTT/ATTTTTTT	CGAACCTGAGACCCTACCAA	ACATACTGTTCCTCACCGCC	60.1	695	INTERGENIC	--	--	--
Ca5_P4_10311736	Ca5	10311736	CGAGAGAGAGAGAGAGAGAGAGAGAGAGAGAGAGAGAGAGAGAGA/CGAGAGAGAGAGAGAGAGAGAGAGAGAGAGAGAGAGAGAGAGAGAGAGAGAGA	ACACGACAAACACTTTCGCA	GCACACTTTTTCTTCATTCGTC	60.3	553	INTERGENIC	--	--	--
Ca5_P4_10420287	Ca5	10420287	GTTTTTTTTT/GTTTTTTTT	GGTACAATTGTTGCACCACG	TTTGGGAGTAGGCCTTCCTT	59.9	702	INTERGENIC	--	--	--
Ca5_P4_10420664	Ca5	10420664	AATATATATATATATATATATA/AATATATATATATATATATATATA	AAGGAAGGCCTACTCCCAAA	TACCAAGGCGTCGTCAAAAT	60.1	597	INTERGENIC	--	--	--

INDEL marker IDs	Chromosomes/ unanchored scaffolds	Physical positions (bp)	InDels (*Kabuli* reference genome-CDC Frontier/PII)	Forward primers (5'-3')	Reverse primers (5'-3')	Anneling temperature (0C)	Expected amplified product size (bp)	Structural annotation		Functional annotation	
								Sequence components of *kabuli* genome	*Kabuli* gene accession IDs	NCBI-KOG	TFs
Ca5_P4_10513256	Ca5	10513256	AATATATATATATATATATA/AATATATATATATATATATA	TTGACCGACACCAATCAGAA	CTTGCATCCCACAGGAAAAT	60.1	544	INTERGENIC	--	--	--
Ca5_P4_105147	Ca5	105147	AATATATATATATATATATATATATATATA/AATATATATATATATATATATATATATATA	CAGAACAAACACGAGAGCCA	TTTGTACCCGAGGTTATCCG	60.0	650	INTERGENIC	--	--	--
Ca5_P4_10572522	Ca5	10572522	AATATATATATATATATATATAT/AATATATATATATATATAT	CGTGGACAAAGATCGTGATG	TCATTGTTAAAGAGCGGTGC	60.1	382	INTERGENIC	--	--	--
Ca5_P4_10634338	Ca5	10634338	AC/ACC	AATTTTTCAGTCCCGTGTCG	ATCCCTCAAAGGGCTACTGC	60.0	229	INTERGENIC	--	--	--
Ca5_P4_10686981	Ca5	10686981	TAAAAAAAA/TAAAAAAAAAAA	GCTCCTTCTGCATCAACACA	TGCCCCACATTTTAGACCTC	60.0	509	INTERGENIC	--	--	--
Ca5_P4_10687869	Ca5	10687869	TAAAAAAAA/TAAAAAAAAA	CGAGGAACGACAAGGGTAAA	TGAGTTGAGTTAAAAACCAGCAGA	60.1	673	INTERGENIC	--	--	--
Ca5_P4_10688557	Ca5	10688557	AGTTGTT/AGTT	TCCATCAAACTGATCCTAAGGG	TGGGGTTTAGATGATGGCTC	60.3	179	INTERGENIC	--	--	--
Ca5_P4_10689001	Ca5	10689001	GAAA/GAAAAA	GAGCCATCATCTAAACCCCA	TGCCAAAGAAAAAGGTTTCG	59.9	677	INTERGENIC	--	--	--
Ca5_P4_10689102	Ca5	10689102	TAAAA/TAA	ATGGGCCACCTATTGAGACA	TGCCAAAGAAAAAGGTTTCG	60.3	623	INTERGENIC	--	--	--
Ca5_P4_10689236	Ca5	10689236	ATT/ATTT	TTTTCCGAATTCCAAGTTGA	GGATTGTCCCTTCATTGGTG	58.2	631	INTERGENIC	--	--	--

INDEL marker IDs	Chromosomes/ unanchored scaffolds	Physical positions (bp)	InDels (*Kabuli* reference genome-CDC Frontier/PII)	Forward primers (5'-3')	Reverse primers (5'-3')	Anneling temperature (0C)	Expected amplified product size (bp)	Structural annotation		Functional annotation	
								Sequence components of *kabuli* genome	*Kabuli* gene accession IDs	NCBI-KOG	TFs
Ca5_P4_10689622	Ca5	10689622	AGAAGCATG/AG	CGAAACCTTTTTCTTTGGCA	AACGGATTGTCCCTTCATTG	60.2	418	INTERGENIC	--	--	--
Ca5_P4_10693789	Ca5	10693789	CTTGTGATTGTTTTGTTGTGATTG/CTTGTGATTG	CCAATTTGAATTGTCCGTCC	TTGCAAAAGCTGGGTCAAGT	60.2	406	INTERGENIC	--	--	--
Ca5_P4_1083059	Ca5	1083059	ATTTTTTTTTTTTTT/ATTTTTTTTTTTT	TCAATCCGAACAAAACATGAA	CGATGATCTTCTTCTTCACCC	59.0	338	INTERGENIC	--	--	--
Ca5_P4_10852097	Ca5	10852097	AATATATATATAT/AATATATATAT	CACAGTTGTCCACGTACGAAA	AAATGGAGGTTTCAATTGCG	59.7	499	INTERGENIC	--	--	--
Ca5_P4_10854983	Ca5	10854983	AA/AATA	TGGATTGACATTAATGAGTGATTG	GGATGTGATCCTTATGCGCT	58.9	487	INTERGENIC	--	--	--
Ca5_P4_10931505	Ca5	10931505	AG/A	TTGATGAATTTGATGTCGTTGA	AAATACCTTACGCGGTCCCT	59.0	344	INTERGENIC	--	--	--
Ca5_P4_11093470	Ca5	11093470	TTATATATATATATATATAT/TTATATATATATATATATAT	CAGCGCTTGCTAAACACAAG	TCAAAACAGAACAGATCATGTGG	59.8	617	INTERGENIC	--	--	--
Ca5_P4_11132723	Ca5	11132723	TTTATTATTATTATTATTA/TTTATTATTATTATTA	AAAAACCAACAATCTCCCCC	TGTTTTCTGCCTTTTTGCCT	60.0	262	INTERGENIC	--	--	--
Ca5_P4_11138840	Ca5	11138840	GT/G	TCGACTTGTCAATCGCTTTG	TCGCATTAACTACGAACGCA	60.0	676	INTERGENIC	--	--	--
Ca5_P4_11167847	Ca5	11167847	CAAAAAAAAAAAAA/CAAAAAAAA	GGTGGACTCGAGCAAGTAGC	AAAGCATCAGTTCCGTTTGAA	60.0	459	INTERGENIC	--	--	--

INDEL marker IDs	Chromosomes/ unanchored scaffolds	Physical positions (bp)	InDels (*Kabuli* reference genome-CDC Frontier/PII)	Forward primers (5'-3')	Reverse primers (5'-3')	Anneling temperature (0C)	Expected amplified product size (bp)	Structural annotation		Functional annotation	
								Sequence components of *kabuli* genome	*Kabuli* gene accession IDs	NCBI-KOG	TFs
Ca5_P4_11168411	Ca5	11168411	AATA/AA	TTCAAACGGAACTGATGCTTT	TTGGTTGGGGGAGTTATTGA	59.7	954	INTERGENIC	--	--	--
Ca5_P4_11168800	Ca5	11168800	CACTATTAG/C	TCGTTGAGGAAAAACAAACAAA	TTGGTTGGGGGAGTTATTGA	59.6	557	INTERGENIC	--	--	--
Ca5_P4_11168904	Ca5	11168904	GTCTCTCT/GTCTCT	TCAATAACTCCCCCAACCAA	TCACAATTGGGTTTGGATCA	60.2	227	INTERGENIC	--	--	--
Ca5_P4_11169035	Ca5	11169035	CG/C	TCAATAACTCCCCCAACCAA	TGACGCCGTGAAAAACATTA	60.2	626	INTERGENIC	--	--	--
Ca5_P4_111805	Ca5	111805	TTAT/TTATAT	CAACGACGGCATGACTCTAA	ATTTATCATTCAACCGGCCA	59.9	613	INTERGENIC	--	--	--
Ca5_P4_11233017	Ca5	11233017	TAAAAA/TAAA	ATTGGACGTATCCCGAGTTG	GTTGTGGGATGAACTGACCC	59.8	633	INTERGENIC	--	--	--
Ca5_P4_11324561	Ca5	11324561	T/TTGCTCAG	GCCATGAAATAGAGGTTGGC	TATTGCTGGCTGACAAACCA	59.5	405	INTERGENIC	--	--	--
Ca5_P4_11376503	Ca5	11376503	AATATATATATATATATATATATATATATATATA/AATATATATATATATATATATATATATATA	CGAACTCGTGATTCTCCACA	TAAAAAGCGTCGCACTCAGA	59.8	718	INTERGENIC	--	--	--
Ca5_P4_11414411	Ca5	11414411	A/AACCT	TCTGAATCGGAGTACCCCAC	AATGGCTTTCCTTCGTGATG	59.9	770	INTERGENIC	--	--	--
Ca5_P4_11424234	Ca5	11424234	CAAAAAAA/CAAAAAA	TAAAACCGGATCCAAATCCA	GAGGTCTATGAACCCCCTCC	60.1	399	INTERGENIC	--	--	--

INDEL marker IDs	Chromosomes/ unanchored scaffolds	Physical positions (bp)	InDels (*Kabuli* reference genome-CDC Frontier/PII)	Forward primers (5'-3')	Reverse primers (5'-3')	Anneling temperature (0C)	Expected amplified product size (bp)	Structural annotation		Functional annotation	
								Sequence components of *kabuli* genome	*Kabuli* gene accession IDs	NCBI-KOG	TFs
Ca5_P4_11438610	Ca5	11438610	TTATATATATATATATATATAT/TTATATATATATATATATATAT	GCCAAAACCCTCAGAAACAA	ACTTTTGCATTGAACGAGGG	60.1	773	INTERGENIC	--	--	--
Ca5_P4_11505972	Ca5	11505972	TTTCTTC/TTTC	GGACCTAAATTTTGCAACAACA	GAACGCGAGATATTGGGTGT	59.0	199	INTERGENIC	--	--	--
Ca5_P4_11551348	Ca5	11551348	ATTT/ATT	TTGACGGTTGAGATTGATTGA	TAGGGTTTATGCTCCATCGC	59.1	477	INTERGENIC	--	--	--
Ca5_P4_11582454	Ca5	11582454	TT/TTTAT	TGACTCATCAATTCGAACGC	GAATGCCACCAGGAAAAGAA	59.8	643	INTERGENIC	--	--	--
Ca5_P4_11658471	Ca5	11658471	GAAAAAAAAAA/GAAAAAAAAA	CTAGGTGCGGTGTTCATGTG	TTGTGGTGAACCGGGATAAT	60.2	491	INTERGENIC	--	--	--
Ca5_P4_11729570	Ca5	11729570	AAATAATAATAA/AAATAATAA	CACGGTCCCTAGAGCCAATA	CGGGAGATACGTAGGAGCAA	60.1	672	INTERGENIC	--	--	--
Ca5_P4_11879108	Ca5	11879108	TAA/TAAA	AAGATTTCCTCCCTCCTCCA	CTTTAACATGCGTTTCCGGT	60.0	327	INTERGENIC	--	--	--
Ca5_P4_12095777	Ca5	12095777	CAAAAAAAAA/CAAAAAA	CTCTTTAAGACGTTTCGCGG	TGCTCGCCTAATTTTCGAGT	60.0	461	INTERGENIC	--	--	--
Ca5_P4_12161825	Ca5	12161825	CAA/CA	GCGGATAATCAATTTCCCAA	CCTTGTTGTTTTTGGCTGGT	59.7	247	INTERGENIC	--	--	--
Ca5_P4_12161940	Ca5	12161940	AG/A	GCGGATAATCAATTTCCCAA	CCTTGTTGTTTTTGGCTGGT	59.7	247	INTERGENIC	--	--	--

INDEL marker IDs	Chromosomes/ unanchored scaffolds	Physical positions (bp)	InDels (*Kabuli* reference genome-CDC Frontier/PII)	Forward primers (5'-3')	Reverse primers (5'-3')	Anneling temperature (0C)	Expected amplified product size (bp)	Structural annotation		Functional annotation	
								Sequence components of *kabuli* genome	*Kabuli* gene accession IDs	NCBI-KOG	TFs
Ca5_P4_1230643	Ca5	1230643	TT/TTCCGT	ACGAAATCGAAGAGAAGCGA	TCCAGGACACAAACAATCCA	60.1	455	INTERGENIC	--	--	--
Ca5_P4_12325595	Ca5	12325595	GTA/G	ATGTATTGGGCCAGTCCTGA	GGCGCCGCTAATTAAAGTTAT	60.3	553	INTERGENIC	--	--	--
Ca5_P4_1239625	Ca5	1239625	AATATATATATATATATATATA/AATATATATATATATATATATATA	GGTTGATTTTGAATAGGTGTCG	ATTAAATTAAGGGGCCGCAG	58.5	107	INTERGENIC	--	--	--
Ca5_P4_12532663	Ca5	12532663	A/AT	TGCAATTGGTTGGAATGTGT	CCAATGAGTTTGAGCCTTCC	59.8	493	INTERGENIC	--	--	--
Ca5_P4_12544184	Ca5	12544184	AATATATATATATATATATATATATATATATATATATATA/AATATATATATATATATATATATATATATATATATATATATATATATA	TCTTCCACCATCCATCCTTC	GGCGATTTTACGTGTATATGTGAG	59.9	676	INTERGENIC	--	--	--
Ca5_P4_12672542	Ca5	12672542	AATATATATATATATATAT/AATATATATATATATATATATAT	AATGGAGGAAGGAGGGAGAA	CTTGGGAACTGGAGAGCAAG	60.0	515	INTERGENIC	--	--	--
Ca5_P4_131876	Ca5	131876	GTTTT/GTTTTT	ATCCAAGTATCCAACGGCTG	CACCTCTGCCCACTTCATTT	60.0	493	INTERGENIC	--	--	--
Ca5_P4_132937	Ca5	132937	CAA/CAAA	CCAAAGACACAGCAAAGCAA	CACTTCCACCAGTTGCAAAA	60.0	569	INTERGENIC	--	--	--
Ca5_P4_133061	Ca5	133061	GGTG/GG	GCCATGGAGTGCTGGTTATT	GCGTTGTGAAAACAGGACAA	60.0	709	INTERGENIC	--	--	--
Ca5_P4_133085	Ca5	133085	ACC/AC	GCCATGGAGTGCTGGTTATT	GCGTTGTGAAAACAGGACAA	60.0	709	INTERGENIC	--	--	--

INDEL marker IDs	Chromosomes/ unanchored scaffolds	Physical positions (bp)	InDels (*Kabuli* reference genome-CDC Frontier/PII)	Forward primers (5'-3')	Reverse primers (5'-3')	Anneling temperature (0C)	Expected amplified product size (bp)	Structural annotation		Functional annotation	
								Sequence components of *kabuli* genome	*Kabuli* gene accession IDs	NCBI-KOG	TFs
Ca5_P4_133283	Ca5	133283	AATATAT/AATAT	ACCTTTACATGGTGCCAAGC	GCGTTGTGAAAACAGGACAA	60.0	433	INTERGENIC	--	--	--
Ca5_P4_133428	Ca5	133428	GAACA/GAACAATTTTTATAATAACA	ACCTTTACATGGTGCCAAGC	GCGTTGTGAAAACAGGACAA	60.0	433	INTERGENIC	--	--	--
Ca5_P4_133648	Ca5	133648	GAAAAA/GAAAA	TTGTCCTGTTTTCACAACGC	TCAACAATTTGTGGGCTTCA	59.7	574	INTERGENIC	--	--	--
Ca5_P4_133972	Ca5	133972	CAAAAAA/CAAAAA	TTGTCCTGTTTTCACAACGC	TCAACAATTTGTGGGCTTCA	59.7	574	INTERGENIC	--	--	--
Ca5_P4_135216	Ca5	135216	GTTCTT/GTTCTTCTT	CCATCATTGGTTTGTGGTGA	AAATCCTAATCATGGCGCAG	60.2	459	INTERGENIC	--	--	--
Ca5_P4_13624288	Ca5	13624288	ATTTT/ATTTTTT	TGAAAATGTTGTTGGTAGTGGC	CGACGGTGCCAATTTTCTAT	59.9	948	INTERGENIC	--	--	--
Ca5_P4_136326	Ca5	136326	A/AT	TCGAATGTCAACAAACAAGCA	ATTCCTGCACACCACTTTCC	60.3	433	INTERGENIC	--	--	--
Ca5_P4_13636401	Ca5	13636401	A/AC	ATCGACAAACCAACATGCAA	GGGAAACTCACTTGCGGTTA	60.0	735	INTERGENIC	--	--	--
Ca5_P4_13636921	Ca5	13636921	CTGT/CT	TAACCGCAAGTGAGTTTCCC	GCGGTGGCTGAGTTTCTTAC	60.1	387	INTERGENIC	--	--	--
Ca5_P4_13754064	Ca5	13754064	CATTATTATTATTATTATTATTATTATTATTATTATTATTAT/CATTATTATTATTATTATTATTATTATTATTATTATTAT	TCGATTTTGGCTTTAACCAG	CCCACACCTTAAAAGCCACT	57.9	538	INTERGENIC	--	--	--

INDEL marker IDs	Chromosomes/ unanchored scaffolds	Physical positions (bp)	InDels (*Kabuli* reference genome-CDC Frontier/PII)	Forward primers (5'-3')	Reverse primers (5'-3')	Anneling temperature (0C)	Expected amplified product size (bp)	Structural annotation		Functional annotation	
								Sequence components of *kabuli* genome	*Kabuli* gene accession IDs	NCBI-KOG	TFs
Ca5_P4_1377883	Ca5	1377883	TCC/TC	GACTCTTTCCATAGCGTGCC	CACCTTGTAGCCAACGGTTT	59.8	720	INTERGENIC	--	--	--
Ca5_P4_13801981	Ca5	13801981	TAA/TAAGACCTCTCCACCGAA	TTTGCTCATGTGCTTTTTGC	TTCTCATTTGTGTTGGGTGAA	60.0	538	INTERGENIC	--	--	--
Ca5_P4_1390115	Ca5	1390115	CATATATATATATATATATAT/CATATATATATATATATAT	AGGCTCAAACGTCGAAGAAA	TGAGTTGAGTGCAAACCTGG	60.0	792	INTERGENIC	--	--	--
Ca5_P4_13970765	Ca5	13970765	TA/TGA	TATTTGGACAAACGAAGGGC	CCCCTTTTGCTTGTTGGTTA	59.9	282	INTERGENIC	--	--	--
Ca5_P4_13971864	Ca5	13971864	AATATATATATATATATATATATATATA/AATATATATATATATATATATATATATATATA	ATGGAGACTTTCTGCAGCGT	TCCTCCGGCTGCTAAATAAA	60.0	691	INTERGENIC	--	--	--
Ca5_P4_14022502	Ca5	14022502	ATTT/ATTTT	GGAAATGGATGTCTGCCAAT	TTGCAATCTCATCAACTCGG	59.8	318	INTERGENIC	--	--	--
Ca5_P4_14263455	Ca5	14263455	AATATATATATATATATATATATA/AATATATATATATATATATATATATATA	ACTTGCAGCAATTTCCATCC	GGATGCTTCCCATAATCTGC	60.1	716	INTERGENIC	--	--	--
Ca5_P4_14300751	Ca5	14300751	G/GA	TTTGCGTTACGAATTTGATGA	AAATGAACGAATTTGCACTCG	59.2	595	INTERGENIC	--	--	--
Ca5_P4_14316093	Ca5	14316093	TTATATATATA/TTATATATATATATATATATATATATA	TTTACGGAGGCCCCTAATTC	TGAAGAGGGATTGGTATGGC	60.3	627	INTERGENIC	--	--	--
Ca5_P4_14330970	Ca5	14330970	ATTTTTTTTTT/ATTTTTTTTTTT	TGATTAAGAAGTTTTGATTTGCG	GGTTTGGACAATCTGCCTTG	58.5	795	INTERGENIC	--	--	--

INDEL marker IDs	Chromosomes/ unanchored scaffolds	Physical positions (bp)	InDels (*Kabuli* reference genome-CDC Frontier/PII)	Forward primers (5'-3')	Reverse primers (5'-3')	Anneling temperature (0C)	Expected amplified product size (bp)	Structural annotation		Functional annotation	
								Sequence components of *kabuli* genome	*Kabuli* gene accession IDs	NCBI-KOG	TFs
Ca5_P4_14394852	Ca5	14394852	CTTTTT/CTTTT	TCTTCCACCCAGTTTTCACC	TTGAGGGCTATGTTTTTCGG	59.9	337	INTERGENIC	--	--	--
Ca5_P4_1453148	Ca5	1453148	CATATATATATATATATATAT/CATATATATATATATATATATAT	AGGAAGAGTGGAGTTGCGAA	TGCGTTACGGTGAAATGGTA	60.0	368	INTERGENIC	--	--	--
Ca5_P4_15176756	Ca5	15176756	AATATATATATATATATATATATATATATAT/AATATATATATATATATATATATATATATATATATATATAT	ACATTTAGGGCCACTTGGTG	GTTGGTTCCCTAGCACCGTA	59.9	725	INTERGENIC	--	--	--
Ca5_P4_1521350	Ca5	1521350	TT/TTAT	TTGGCCAAGTCCCTGTTTAC	CTCTCATCTGAGGGCAAAGC	60.0	350	INTERGENIC	--	--	--
Ca5_P4_152255	Ca5	152255	CATATATATATATATATAT/CATATATATATATATAT	TTTTGGCCCCTAAGACACAC	TTTAATTGCACGCTCACGTC	60.0	331	INTERGENIC	--	--	--
Ca5_P4_15339602	Ca5	15339602	GAAAAAAAAAA/GAAAAAAAAA	AGGAGAGAAAAAGGGGGAAA	GGGAAACCCACCTCTTGATT	59.1	211	INTERGENIC	--	--	--
Ca5_P4_153461	Ca5	153461	GTTTAATTTA/GTTTA	GCCTCGCAATTCCTGAAAGT	ATCCGGAACTTTTTGCCTTT	61.6	456	INTERGENIC	--	--	--
Ca5_P4_15462644	Ca5	15462644	CAAAAAAAAAA/CAAAAAAAAA	CTTGAAATGGTGCTGCAAGA	GGGGGAGTCTTGCTGTGTTA	60.0	733	INTERGENIC	--	--	--
Ca5_P4_15481624	Ca5	15481624	CTTTTTTTTTTTT/CTTTTTTTTTTT	TTTTGACACGCAGTAGGCAC	AGTTCGGGAGTCACGTATGG	59.9	781	INTERGENIC	--	--	--
Ca5_P4_155187	Ca5	155187	AA/AATA	TGTGACGTGGGAGGATTGTA	GAGGCTGCAGCATGTTCATA	60.0	355	INTERGENIC	--	--	--

INDEL marker IDs	Chromosomes/ unanchored scaffolds	Physical positions (bp)	InDels (*Kabuli* reference genome-CDC Frontier/PII)	Forward primers (5'-3')	Reverse primers (5'-3')	Anneling temperature (0C)	Expected amplified product size (bp)	Structural annotation		Functional annotation	
								Sequence components of *kabuli* genome	*Kabuli* gene accession IDs	NCBI-KOG	TFs
Ca5_P4_15543535	Ca5	15543535	CATATATATATATATATATATATATATATATATATATATAT/CATATATATATATATATATATATATATAT	AATCCGGTAAAATCTCCGCT	GTTTTCTTCCTTTGGGCCTT	59.9	731	INTERGENIC	--	--	--
Ca5_P4_1558914	Ca5	1558914	TAAAAAAAAA/TAAAAAAAAAAA	TCAAGATTGCAAACCTGCAT	TCTTCAGGTTCCAGCAAACC	59.3	452	INTERGENIC	--	--	--
Ca5_P4_15716362	Ca5	15716362	CTTTTTTTTTT/CTTTTTTTTT	TTTGTGCAGACTGCGTTTGT	TTGATAAAGTGGCGGAGAAAA	60.5	327	INTERGENIC	--	--	--
Ca5_P4_15716555	Ca5	15716555	ATTTTTTTTTTTT/ATTTTTTTTTTTTTT	TTTGTGCAGACTGCGTTTGT	TTGCAATGTCAACAACAGAGG	60.5	859	INTERGENIC	--	--	--
Ca5_P4_15756736	Ca5	15756736	GACAC/GAC	GCGCGATTTACCAAAAATGT	TTTGAGTGGTGGGTTGTGAA	60.0	431	INTERGENIC	--	--	--
Ca5_P4_15772842	Ca5	15772842	AT/ATATCT	GCCTTAAAATTTGGCAATGG	CCCTTAAAAACCAATAAAGAAACA	59.4	231	INTERGENIC	--	--	--
Ca5_P4_158353	Ca5	158353	AT/ATT	TTTTGCATACCAAAACACCC	ATGTCCATGACGCATCAAAA	58.4	463	INTERGENIC	--	--	--
Ca5_P4_15835478	Ca5	15835478	CAAAA/CAAA	TGCATGATTCCAGAATTCCA	AGCGGTGAGGAGAACTCTGA	60.0	690	INTERGENIC	--	--	--
Ca5_P4_1586054	Ca5	1586054	TAAAA/TAAA	CTGTTTTCTACCCCCACCCT	AGCGTTGTAAAAGATGCGCT	60.2	727	INTERGENIC	--	--	--
Ca5_P4_15877436	Ca5	15877436	GC/G	GGGCTACATCCAGTCCTCAC	TTTGGCTGTAGAGGTTCCAAA	59.5	474	INTERGENIC	--	--	--

INDEL marker IDs	Chromosomes/ unanchored scaffolds	Physical positions (bp)	InDels (*Kabuli* reference genome-CDC Frontier/PII)	Forward primers (5'-3')	Reverse primers (5'-3')	Anneling temperature (0C)	Expected amplified product size (bp)	Structural annotation		Functional annotation	
								Sequence components of *kabuli* genome	*Kabuli* gene accession IDs	NCBI-KOG	TFs
Ca5_P4_158794	Ca5	158794	TAAAAAA/TAAAAA	TGATGCGTCATGGACATAAA	TTTTAAAAATGTCCATTTACACCG	58.5	498	INTERGENIC	--	--	--
Ca5_P4_159164	Ca5	159164	TA/TAAA	TCAAAATTGTTTATTTACCGGTGT	TTGGGAACAAAAAGTGGGAG	58.8	554	INTERGENIC	--	--	--
Ca5_P4_15918981	Ca5	15918981	TTTATTATTATTATTATTATTATTATTATTATTATTATTATTATTATTATTATT/TTTATTATTATTATTATTATTATTATTATTATTATTATTATTATTATTATTATTATTATT	CCTCCCCCATTTGTCTTCTT	GCCTGATGTTCGAATTTTTCA	60.3	462	INTERGENIC	--	--	--
Ca5_P4_15929247	Ca5	15929247	AACCAC/AAC	TGATGACGAAGACGAAGACG	AACACATCCGGGATCCATTA	60.0	416	INTERGENIC	--	--	--
Ca5_P4_15929348	Ca5	15929348	ACCC/ACC	TGATGACGAAGACGAAGACG	AACACATCCGGGATCCATTA	60.0	416	INTERGENIC	--	--	--
Ca5_P4_15993780	Ca5	15993780	TTATATATATATATATATATATATATATATATAT/TTATATATATATATATATATATATAT	AGGAAGAGTGGAGTTGCGAA	ACCCCCGTTCCTTGTAAATC	60.0	716	INTERGENIC	--	--	--
Ca5_P4_16029850	Ca5	16029850	AAATAATAATAATAATAATAATAATAATGATAATAATAAT/AAATAATAATAATAATAATAATAATAATGATAATAATAAT	TTGAACAACCGAACTTCAAAGA	CAATATTGTGATGACATTGGTATGA	59.8	790	INTERGENIC	--	--	--
Ca5_P4_160572	Ca5	160572	TAAAAAAAAA/TAAAAAAAA	AATCCAATGGCTTTCAATCAA	CACACGACAAAGTGGAGTATCAG	59.4	570	INTERGENIC	--	--	--
Ca5_P4_16083320	Ca5	16083320	CAATTAATTAATTAAT/CAATTAATTAATTAATTAAT	TGAATGGAGACGTGAAGCTG	ACGCAGTGTAACACCCCAAT	60.0	710	INTERGENIC	--	--	--
Ca5_P4_161153	Ca5	161153	CTT/CTTAAATT	CTGATACTCCACTTTGTCGTGTG	GCATAATCCGCTCACCCTAA	59.7	819	INTERGENIC	--	--	--

INDEL marker IDs	Chromosomes/ unanchored scaffolds	Physical positions (bp)	InDels (*Kabuli* reference genome-CDC Frontier/PII)	Forward primers (5'-3')	Reverse primers (5'-3')	Anneling temperature (0C)	Expected amplified product size (bp)	Structural annotation		Functional annotation	
								Sequence components of *kabuli* genome	*Kabuli* gene accession IDs	NCBI-KOG	TFs
Ca5_P4_161487	Ca5	161487	GTTTTTTT/GTTTTTTTTT	ACGTGCAACGTATCCCAAAT	GCATAATCCGCTCACCCTAA	60.3	312	INTERGENIC	--	--	--
Ca5_P4_16319965	Ca5	16319965	TGGG/TGGGG	TTGTGTGCAGGATCCACATT	TTGACCATTGCACGAAACAT	60.0	502	INTERGENIC	--	--	--
Ca5_P4_164139	Ca5	164139	A/ATAAATGG	GGTAAAAGAGACTCGCGTGG	CTGATGTGCTCGTTGTTGCT	59.9	695	INTERGENIC	--	--	--
Ca5_P4_16469239	Ca5	16469239	GTATATATATATATATATATATATATAT/GTATATATATATATATATATATAT	TCGGTGTCAAATGAAGACCTC	CGATTTGACAACATCACGCT	60.1	561	INTERGENIC	--	--	--
Ca5_P4_16519732	Ca5	16519732	ATTTTTTT/ATTTT	ATCTGAATTTTTGTGGCGGT	GGACCAAATTGACAACGACA	59.4	573	INTERGENIC	--	--	--
Ca5_P4_16522289	Ca5	16522289	TTATATATATATATATATAT/TTATATATATATATATATATAT	CTTTTCCCTCTCGAGCTCAT	ATCCTTTGCATGTCCCACTC	58.6	238	INTERGENIC	--	--	--
Ca5_P4_16523502	Ca5	16523502	CTATATATATATATATATATATAT/CTATATATATATATATATATATATATAT	GCATGCCACTTAGCTTCCAT	GGGGAGGTTAAAGGATCCAA	60.2	296	INTERGENIC	--	--	--
Ca5_P4_16557774	Ca5	16557774	TTTATTATTATTATTATTATTATTATTATTATTATTA/TTTATTATTATTATTATTATTATTATTATTATTATTA	GTGCTCCTCCCTTCTTCCTT	TGTCCCTGATTCCGTTTTTC	59.8	331	INTERGENIC	--	--	--
Ca5_P4_16637694	Ca5	16637694	AATA/AATATA	GACACGTCCGTGCCTTTATT	ACAAGAAATTGGGGAGGGAG	60.0	284	INTERGENIC	--	--	--
Ca5_P4_16650628	Ca5	16650628	GTTTTTT/GTTTTTTT	CCCGAAAGTCCTCCAAAAAT	CCAAGTTCCCCAGAGTCAAA	60.3	583	INTERGENIC	--	--	--

INDEL marker IDs	Chromosomes/ unanchored scaffolds	Physical positions (bp)	InDels (*Kabuli* reference genome-CDC Frontier/PII)	Forward primers (5'-3')	Reverse primers (5'-3')	Anneling temperature (0C)	Expected amplified product size (bp)	Structural annotation		Functional annotation	
								Sequence components of *kabuli* genome	*Kabuli* gene accession IDs	NCBI-KOG	TFs
Ca5_P4_16715155	Ca5	16715155	CTTTTTTTTTT/CTTTTTTTTTTT	TGGGTTCACCCCAAATTAAA	TCATCAAATCCGTAACGCAA	60.0	459	INTERGENIC	--	--	--
Ca5_P4_16715406	Ca5	16715406	GAAAAAAAAA/GAAAAAAAAAA	TTGCGTTACGGATTTGATGA	GCTAAATTACATTCGTGGTCCC	60.1	240	INTERGENIC	--	--	--
Ca5_P4_1678181	Ca5	1678181	TTATATATATAT/TTATATATATATATAT	CGGTGAATCCTTTACGGAGA	AGGAAGAGTGGAGTTGCGAA	60.1	384	INTERGENIC	--	--	--
Ca5_P4_16881029	Ca5	16881029	GGTTGTTGTTGTTGTT/GGTTGTTGTTGTTGTTGTT	TGCATGCTTAATTTGACAGTGA	TGATTTAACGGTTGTGATTGATT	59.4	779	INTERGENIC	--	--	--
Ca5_P4_16948578	Ca5	16948578	CATATATATATATATATAT/CATATATATATATATATAT	GTTTTCGAATCAACGAGGGA	GCTCAGAGGCTTTTTAGCCA	60.1	509	INTERGENIC	--	--	--
Ca5_P4_17007480	Ca5	17007480	AATATATATATATATATA/AATATATATATATATATATA	TGCAATCAATTTTTGAGCCA	TGGTGGTTGTTGTCTCCTCA	60.2	626	INTERGENIC	--	--	--
Ca5_P4_17020837	Ca5	17020837	TAGAGAGA/TAGAGA	CCTACGGATGACCTCTCTCG	GGTTTAACATCCCCACAAGC	59.8	773	INTERGENIC	--	--	--
Ca5_P4_17048541	Ca5	17048541	AATATATATATATATATATATAT/AATATATATATATATATATATAT	GGTCAGCATTTACCCCATGA	TTTCTCCACCTTCCTTGGTG	60.7	534	INTERGENIC	--	--	--
Ca5_P4_17120322	Ca5	17120322	AATATATATATATATAT/AATATATATATATATATAT	CGGTCACCATCGACACTATG	TCCCTGCTCCTCGTCTTCTA	60.0	816	INTERGENIC	--	--	--
Ca5_P4_17244450	Ca5	17244450	AG/A	GTACCCTCCTCCTCGGTCTC	TTGTGTTTCAACAACTCCCAA	60.1	729	INTERGENIC	--	--	--

INDEL marker IDs	Chromosomes/ unanchored scaffolds	Physical positions (bp)	InDels (*Kabuli* reference genome-CDC Frontier/PII)	Forward primers (5'-3')	Reverse primers (5'-3')	Anneling temperature (0C)	Expected amplified product size (bp)	Structural annotation		Functional annotation	
								Sequence components of *kabuli* genome	*Kabuli* gene accession IDs	NCBI-KOG	TFs
Ca5_P4_17314539	Ca5	17314539	TAAAAAAAAAA/TAAAAAAAAAAA	TCGATTATGCGTAGGGAAGG	TTGCGACAACTTGGTGATTC	60.1	824	INTERGENIC	--	--	--
Ca5_P4_17448710	Ca5	17448710	CACTATACAA/CACTATACAACTATACAA	TGTGGATGTTCAAGGGACAA	ACAAATGGCGTATGCCTCTC	59.9	864	INTERGENIC	--	--	--
Ca5_P4_17494887	Ca5	17494887	CTGA/CTGACCTCCTATATGTGA	CTACGCCAAGAAACTGCTCC	GTACGCGTCCAGACATAGCA	60.0	245	INTERGENIC	--	--	--
Ca5_P4_17526738	Ca5	17526738	T/TG	GATTGGGGGAAGATGAGGAT	TTTGCGTTACGAATTTGATGA	60.1	465	INTERGENIC	--	--	--
Ca5_P4_17534242	Ca5	17534242	AATATATATATATATATATATATATATATAT/AATATATATATATATATATATATATATATATAT	TCATTGAGGATGTTCCAAAGTT	ACAAAAGGCATCTCTGGTCG	58.6	536	INTERGENIC	--	--	--
Ca5_P4_17549591	Ca5	17549591	TTATATATATATATATAT/TTATATATATATATATATATAT	GTGGGGGTGCAATGTAAAAT	TTGCATAAACTACACAATGGAAAAA	59.6	357	INTERGENIC	--	--	--
Ca5_P4_17733319	Ca5	17733319	A/ATCGGTTCAGC	CGCGATGTCGTCTAAAGTGA	GAGGGGAGGGTTTTTGCTAC	60.0	583	INTERGENIC	--	--	--
Ca5_P4_17760753	Ca5	17760753	TAATAAAAATAAAAATAAAAATAAAA/TAATAAAAATAAAAATAAAAATAAAAATAAAA	TGAAACCGCGTTTGATTGTA	GGTTGGGACCACTTCTCAAA	60.1	168	INTERGENIC	--	--	--
Ca5_P4_17876530	Ca5	17876530	CAA/CAAA	TTTCATCCATCACCGACAAA	CTCAACGAATGCCTGAAACA	59.9	573	INTERGENIC	--	--	--
Ca5_P4_17876692	Ca5	17876692	ACAC/A	TGTTTCAGGCATTCGTTGAG	TCAATACCATTTTCACCGCA	59.8	471	INTERGENIC	--	--	--

INDEL marker IDs	Chromosomes/ unanchored scaffolds	Physical positions (bp)	InDels (*Kabuli* reference genome-CDC Frontier/PII)	Forward primers (5'-3')	Reverse primers (5'-3')	Anneling temperature (0C)	Expected amplified product size (bp)	Structural annotation		Functional annotation	
								Sequence components of *kabuli* genome	*Kabuli* gene accession IDs	NCBI-KOG	TFs
Ca5_P4_17876974	Ca5	17876974	ATTTTT/ATTTTTTT	TGTTTCAGGCATTCGTTGAG	TCAATACCATTTTCACCGCA	59.8	471	INTERGENIC	--	--	--
Ca5_P4_1794712	Ca5	1794712	AATTATTATTATTATTATTATTATTATTATTATTATTATTATTATTATT/AATTATTATTATTATTATTATTATTATTATTATTATTATTATT	GAGAAGGCGAGAAGGAAGGT	GCAAGGTTTAGGTGGGTCAA	60.0	605	INTERGENIC	--	--	--
Ca5_P4_18049660	Ca5	18049660	CATATATATATAT/CATATATATAT	GCACGTTGTAAAGAGTGGACTG	AAAAGGCCCACAAGAGTTGA	59.8	694	INTERGENIC	--	--	--
Ca5_P4_18072367	Ca5	18072367	TAAAAAAAAAA/TAAAAAAAAA	CGAAAAGAAAATGGTGCAGTT	GTTCAACTGACGGGGAGAAA	59.3	613	INTERGENIC	--	--	--
Ca5_P4_18136111	Ca5	18136111	CTTTTTTTTTTT/CTTTTTTTTTT	CGGGTCATTTTCTTTCTCCA	TTGTGGTCCTTTAAGATGGTTG	60.0	327	INTERGENIC	--	--	--
Ca5_P4_18179865	Ca5	18179865	CAAAAAAAAAAAA/CAAAAAAAAAAA	GGCAAAATATCTTTCATTCCG	TCTGAACAGTGTTGCCACAGA	58.6	352	INTERGENIC	--	--	--
Ca5_P4_18589694	Ca5	18589694	TATAATAATAATAATAATAATAATAATAATAATAATAATAATAA/TAATAATAATAATAATAATAATAATAATAATAATAATAATAATAATAA	CACAAGCATCTTGTTCGTCC	TGTGGTTTTGCTAATTGGGG	59.3	661	INTERGENIC	--	--	--
Ca5_P4_18593873	Ca5	18593873	TTA/T	GGTCTGTCCAATCAGACCGT	TCCATTAAGCCCCTCAACAA	60.0	600	INTERGENIC	--	--	--
Ca5_P4_18686080	Ca5	18686080	G/GACAAATAAATCCT	GTCAGGTCGACCCACAAGAT	AGGTAAGGGCTCCTTCCAAA	60.0	808	INTERGENIC	--	--	--
Ca5_P4_18808476	Ca5	18808476	TATAAATAAATAAATAAATAAATAAATAAATAAA/TATAAATAAATAAATAAATAAATAAA	CAACGTTGATGCAAAAATAGG	CAAATTGCAAGGAAACCACA	58.2	564	INTERGENIC	--	--	--

INDEL marker IDs	Chromosomes/ unanchored scaffolds	Physical positions (bp)	InDels (*Kabuli* reference genome-CDC Frontier/PII)	Forward primers (5'-3')	Reverse primers (5'-3')	Anneling temperature (0C)	Expected amplified product size (bp)	Structural annotation		Functional annotation	
								Sequence components of *kabuli* genome	*Kabuli* gene accession IDs	NCBI-KOG	TFs
Ca5_P4_18944656	Ca5	18944656	CAAAA/CAA	TCCTGTCATGTGCTTTGAGC	CTAACCTCCCAGGACCCAAT	60.0	131	INTERGENIC	--	--	--
Ca5_P4_18962064	Ca5	18962064	AAATAATAATAATAATAATAATAATAATAATAATAATAATAA/AAATAATAATAATAATAATAATAATAATAATAATAA	GCCCTCTCCTCGTGTTACAA	CCGAAAATAGCGATGAGAGC	60.3	394	INTERGENIC	--	--	--
Ca5_P4_1903216	Ca5	1903216	GA/GAA	TGCATAGGCCTCTTGTCCTT	AGTTCCGCGCTAGATTTCAA	59.8	553	INTERGENIC	--	--	--
Ca5_P4_191284	Ca5	191284	GAATTCCAAATTCCAAATTCCAAATTCCAAATTCC/GAATTCCAAATTCCAAATTCC	GCAAGTGATGATGTAGCGGA	GCTCCAAAGTGCAAGTGTGA	59.8	605	INTERGENIC	--	--	--
Ca5_P4_191704	Ca5	191704	CGGA/C	TCACACTTGCACTTTGGAGC	TTGTTGGGATCCCTGAAGAG	60.0	484	INTERGENIC	--	--	--
Ca5_P4_19591188	Ca5	19591188	CATATATATATATATATATATATATATATAT/CATATATATATATATATATATATATATATATATATATATATATATAT	CCACAGCTTCAACAAAAGCA	TGGATGATTTGATGTCACATAGG	60.0	525	INTERGENIC	--	--	--
Ca5_P4_19705316	Ca5	19705316	TA/TACA	TTGGAAGGAGCCCTTACCTT	CTGCCACGTCACCCTTTTAT	60.1	742	INTERGENIC	--	--	--
Ca5_P4_19725939	Ca5	19725939	CTGA/CTGATGA	GCCCAGGAAAAAGAAAGGAC	TCGGTATTTGCCATGAGACA	60.1	405	INTERGENIC	--	--	--
Ca5_P4_19762990	Ca5	19762990	AAATAATAATAATAATAATAATAATAATAATAATAATAATAA/AAATAATAATAATAATAATAATAATAATAATAATAA	TCAGTCTGCCCTGACCTTTT	GGCCAATGGCATGATCTACT	59.8	598	INTERGENIC	--	--	--
Ca5_P4_19778378	Ca5	19778378	A/AAGAGAGTTAGTAGT	ATCAAGTTTTTGGCGCTGTT	CATACTCGCCGAGGGTACAT	59.8	393	INTERGENIC	--	--	--

INDEL marker IDs	Chromosomes/ unanchored scaffolds	Physical positions (bp)	InDels (*Kabuli* reference genome-CDC Frontier/PII)	Forward primers (5'-3')	Reverse primers (5'-3')	Anneling temperature (0C)	Expected amplified product size (bp)	Structural annotation		Functional annotation	
								Sequence components of *kabuli* genome	*Kabuli* gene accession IDs	NCBI-KOG	TFs
Ca5_P4_19815812	Ca5	19815812	A/AC	TTTTGGGTTGGGATGACATT	ACCAGGAAAAGAAGAAGGGC	60.0	378	INTERGENIC	--	--	--
Ca5_P4_19816092	Ca5	19816092	CATATA/CATATATATA	TTTTGGGTTGGGATGACATT	TGTCGATTTTAATAGGGCGG	60.0	682	INTERGENIC	--	--	--
Ca5_P4_19914483	Ca5	19914483	TTA/TTTAGATTGACTA	TGTTCCCAAATCAACACCAA	GCCCTCTATGGGTTGAAACA	59.8	584	INTERGENIC	--	--	--
Ca5_P4_1995521	Ca5	1995521	ATGTT/ATGTTGTT	TCTTCGTTTTGCAGTCAAACTT	TCTTCGTTTTATGGTTTTATTTCA	59.1	279	INTERGENIC	--	--	--
Ca5_P4_19959975	Ca5	19959975	TAAAAAAA/TAAAAAAAA	CAATGACAAATTTTTGTTTCGTG	TTGTTGGGTCATTAGCGATG	59.4	638	INTERGENIC	--	--	--
Ca5_P4_2000745	Ca5	2000745	T/TGC	TATGAAAATCGCCCGAAAAG	TTCTGGGTCCTCGATTTCAC	60.0	380	INTERGENIC	--	--	--
Ca5_P4_2008256	Ca5	2008256	G/GC	GGTCCTCGATTTCATCTGGA	TAGGCTTCAAGGAAGCTGGA	60.0	556	INTERGENIC	--	--	--
Ca5_P4_20107894	Ca5	20107894	ATTTTTTTTTT/ATTTTTTTTTTT	TTTGAAGCCATTGAGTCGTG	CTCACCCACCTTCACCATTT	59.8	207	INTERGENIC	--	--	--
Ca5_P4_20438947	Ca5	20438947	AT/A	AGTTTGGAACCAAGCCACTG	TAAGCCGTGGGAACGTTAAG	60.1	343	INTERGENIC	--	--	--
Ca5_P4_20531337	Ca5	20531337	ATTTTTTTTTT/ATTTTTTTTT	AGTCGCTGAAAATTCGGTTG	TGATTCAATGTCATAAGTGACGG	60.2	500	INTERGENIC	--	--	--

INDEL marker IDs	Chromosomes/ unanchored scaffolds	Physical positions (bp)	InDels (*Kabuli* reference genome-CDC Frontier/PII)	Forward primers (5'-3')	Reverse primers (5'-3')	Anneling temperature (0C)	Expected amplified product size (bp)	Structural annotation		Functional annotation	
								Sequence components of *kabuli* genome	*Kabuli* gene accession IDs	NCBI-KOG	TFs
Ca5_P4_20547334	Ca5	20547334	AACCAACACCAACACCAACA/AACCAACACCAACACCAACACCAACA	AGGAGATGGAGCGTTGTTTG	TTGGTTAGCGTGCATCTGAG	60.3	462	INTERGENIC	--	--	--
Ca5_P4_20717387	Ca5	20717387	TAAAAAAAAA/TAAAAAAAAAA	CGTCGTTAAAGTGGAGACCAA	TGAGTTCACTGTTGGAGATGCT	60.2	804	INTERGENIC	--	--	--
Ca5_P4_20740792	Ca5	20740792	GAAAAAAAA/GAAAAAAAAA	TCTCACAACACTCCAAAATAAACA	ATTCCGTGGCTGAGCTAAAA	58.7	633	INTERGENIC	--	--	--
Ca5_P4_208132	Ca5	208132	TAATGAATGAATGAA/TAATGAATGAATGAATGAA	TGTTTGGGGAACTTGCTTTC	GCCAACATGCGACTCTGTTA	60.1	578	INTERGENIC	--	--	--
Ca5_P4_20910377	Ca5	20910377	AATATATATATATATATATATATATATATA/AATATATATATATATATATATATA	CTGGGATACAGTGGTTGCCT	AAATGGAGGAAGGAGGGAGA	60.0	520	INTERGENIC	--	--	--
Ca5_P4_21005933	Ca5	21005933	CAAAAAAAAAAA/CAAAAAAAAAA	GCTTCCAAGCAAAGCAAATC	CCTTCATTTCCCTCTCCCTC	60.0	151	INTERGENIC	--	--	--
Ca5_P4_2100607	Ca5	2100607	ATTTTTTTT/ATTTTTTTTTTT	TGGACACGACGGTGAAATAA	TTCGGTTCTTGTTTGCCTCT	60.0	642	INTERGENIC	--	--	--
Ca5_P4_21085449	Ca5	21085449	A/AT	TTAGGCGACCACTGTATCCC	TGAGCCACTTTGATCAGCAC	60.0	758	INTERGENIC	--	--	--
Ca5_P4_21176907	Ca5	21176907	AT/ATTTT	AGGCTCGACTTGTAGCCAAA	CACGGTGTTGAATCGGAAC	60.0	784	INTERGENIC	--	--	--
Ca5_P4_212468	Ca5	212468	CCTCCCCTCTC/CC	TGTTCGACGCTTTGTTGAAG	CCCAACCAAGTTTTGTTGTAAA	60.0	922	INTERGENIC	--	--	--

INDEL marker IDs	Chromosomes/ unanchored scaffolds	Physical positions (bp)	InDels (*Kabuli* reference genome-CDC Frontier/PII)	Forward primers (5'-3')	Reverse primers (5'-3')	Anneling temperature (0C)	Expected amplified product size (bp)	Structural annotation		Functional annotation	
								Sequence components of *kabuli* genome	*Kabuli* gene accession IDs	NCBI-KOG	TFs
Ca5_P4_21289753	Ca5	21289753	CTATATATATATATATATATATATATATA/CTATATATATATATATATATATATATA	GGTACCGCGAGTAATTCCAA	TGTGGAAATTTGTGGGTGAA	60.0	521	INTERGENIC	--	--	--
Ca5_P4_21290533	Ca5	21290533	ATT/ATTT	GTGCAGGTGTCTGTCGAGAA	TGGATGAACTACATTGCCCA	60.0	595	INTERGENIC	--	--	--
Ca5_P4_21458602	Ca5	21458602	GATATATATATATATATATATATATATATATATAT/GATATATATATATATATATATATATATATATATATATAT	AAAATCAGAATGGCCCTTGA	TGGACATTTGGTTTTCATTGAG	59.5	576	INTERGENIC	--	--	--
Ca5_P4_21460979	Ca5	21460979	AATATATATATATATATATATATATA/AATATATATATATATATATATA	TGAGGTTTTGATTATCGCCC	CAACTTTAGGCTTGTCACCTCA	59.9	390	INTERGENIC	--	--	--
Ca5_P4_21503150	Ca5	21503150	AT/A	CCTCTGTTCTGCGGTTAAGG	AAAGTGGATGGCACAACACA	59.9	574	INTERGENIC	--	--	--
Ca5_P4_21598205	Ca5	21598205	TA/TAA	TTGGTGTGGATTGTCGATTG	GGGGAAATCGATTGAATGAA	60.4	953	INTERGENIC	--	--	--
Ca5_P4_21623515	Ca5	21623515	AAATTAATT/AAATT	AGGTCATTTTCATCCACCCA	ACACACCTATGGGATGCACA	60.2	678	INTERGENIC	--	--	--
Ca5_P4_21754940	Ca5	21754940	AAATAATAATAATAATAATAATAATAATAATAATAATAATAATA/AAATAATAATAATAATAATAATAATAATAATAATAATAATAATAATAATAATAATA	TAAACATGTGTTGCATCCGC	TTGCAGCAAAAACAAGCAAC	60.5	396	INTERGENIC	--	--	--
Ca5_P4_21759113	Ca5	21759113	AATATATATATATATATATATAT/AATATATATATATATATATATAT	AATGACAAAAAGGGGGAGAAA	TTGTCTCGCGTATGAACTGG	59.8	610	INTERGENIC	--	--	--
Ca5_P4_218483	Ca5	218483	ATCTCTC/ATCTCTCTC	TAGCGTGGTGGTGTTGTGAT	CCTTTCAAGGTCAGCCACAT	60.0	739	INTERGENIC	--	--	--

INDEL marker IDs	Chromosomes/ unanchored scaffolds	Physical positions (bp)	InDels (*Kabuli* reference genome-CDC Frontier/PII)	Forward primers (5'-3')	Reverse primers (5'-3')	Anneling temperature (0C)	Expected amplified product size (bp)	Structural annotation		Functional annotation	
								Sequence components of *kabuli* genome	*Kabuli* gene accession IDs	NCBI-KOG	TFs
Ca5_P4_2185075	Ca5	2185075	ATTCC/ATTCCTTTCC	TTGGGGGAATGGAGGTAAAT	TCCCCAAAACCCCTAAATTC	60.4	677	INTERGENIC	--	--	--
Ca5_P4_218592	Ca5	218592	TATAATAATAATAATAATA/TATAATAATAATAATAATAATA	TAGCGTGGTGGTGTTGTGAT	CCTTTCAAGGTCAGCCACAT	60.0	739	INTERGENIC	--	--	--
Ca5_P4_21866481	Ca5	21866481	GA/GAA	TCCGATTGGATTTTCAGGAC	GCTTCGTTCTTCGTTCTTCG	59.9	437	INTERGENIC	--	--	--
Ca5_P4_21899576	Ca5	21899576	TAAAAAAAAAAAA/TAAAAAAAAAAA	TGACCTGACCTGGCCTATTC	CCCAAACAAAGCCTTATGGA	60.1	519	INTERGENIC	--	--	--
Ca5_P4_21953528	Ca5	21953528	TATAAATAAATAA/TATAAATAA	AAGTTGCCGATGGAAAAATG	TTGCAAAAGTCAAACCCTCTT	59.9	513	INTERGENIC	--	--	--
Ca5_P4_21965060	Ca5	21965060	AG/AGG	ACGCTCAGAGTTCACTGGGT	GGCAAATCGATTGGACAAGT	59.9	365	INTERGENIC	--	--	--
Ca5_P4_22101626	Ca5	22101626	TTATATATAT/TTATATATATATATATATAT	TTTTGCGGTTGTAGTCCCTC	GGTGACGGATCGAAAAACAT	60.1	717	INTERGENIC	--	--	--
Ca5_P4_22105938	Ca5	22105938	CTATTTATTTATTTATTTAT/CTATTTATTTATTTATTTATTTAT	CACGCCACTAATGTAAATAATGTT	TCGCTCAAGTAATTTTTGAATTT	57.4	549	INTERGENIC	--	--	--
Ca5_P4_22149309	Ca5	22149309	TAAAAAAAAA/TAAAAAAAA	TGTGGTGCGGTAGTGGATTA	CAAACCTGCGATCCAAAAGT	60.0	440	INTERGENIC	--	--	--
Ca5_P4_22150301	Ca5	22150301	GTATATATATATATATATATATATATATATATATAT/GTATATATATATATATATATATATATATATATATATATATATATATAT	CTCAACCGTACATACACGAGC	TGGCAAGACAAAATGACTGTT	58.3	345	INTERGENIC	--	--	--

INDEL marker IDs	Chromosomes/ unanchored scaffolds	Physical positions (bp)	InDels (*Kabuli* reference genome-CDC Frontier/PII)	Forward primers (5'-3')	Reverse primers (5'-3')	Anneling temperature (0C)	Expected amplified product size (bp)	Structural annotation		Functional annotation	
								Sequence components of *kabuli* genome	*Kabuli* gene accession IDs	NCBI-KOG	TFs
Ca5_P4_222234	Ca5	222234	TT/TTAAT	ACCGCTTTACAGAACAACCG	GGTGAGGACAAAACATGGCT	60.2	538	INTERGENIC	--	--	--
Ca5_P4_22232670	Ca5	22232670	TAAAAAAA/TAAAAAAAA	AAAGTGGGGTCCTTATCATTTTT	CAGTTTTCTGGGACCTCCTG	59.2	930	INTERGENIC	--	--	--
Ca5_P4_22362053	Ca5	22362053	G/GA	CATGCGCAACTTCTTGGTAA	CGTATGCGACAACGAATCAC	59.9	948	INTERGENIC	--	--	--
Ca5_P4_22389657	Ca5	22389657	AT/ATT	TGCCTTTGCTAAGTCCCACT	TAAATAGCACCTGCGGTTCC	59.9	432	INTERGENIC	--	--	--
Ca5_P4_22485566	Ca5	22485566	TAATAAATAAATAAATAAAT/TAATAAATAAATAAATAAATAAAT	TTTTCCTGGGTTTGAACTCG	CCCACAAAACAAAGGAGGAA	60.1	624	INTERGENIC	--	--	--
Ca5_P4_22604396	Ca5	22604396	AC/A	GTTTTCGCCGTATTTGATCG	TATCAGAGTTCTCCCCACCG	60.5	803	INTERGENIC	--	--	--
Ca5_P4_22659444	Ca5	22659444	AATATATATATATATATATATATATATATA/AATATATATATATATATATATATATATATATATATA	CCAAGTTTGAAATGCCGATT	GCTGCATGTTCTTATGCTCG	59.9	485	INTERGENIC	--	--	--
Ca5_P4_22693454	Ca5	22693454	CGGG/CGGGG	CCTGCTTTGTTTTAGCTCCG	TGGGATGGATACCCGTTAAA	60.0	387	INTERGENIC	--	--	--
Ca5_P4_22734448	Ca5	22734448	TATA/TATACATA	CCAACTTTTCTCGCCCATAA	TCATGTTTTTACCCCTAAGTCCA	60.1	591	INTERGENIC	--	--	--
Ca5_P4_22735708	Ca5	22735708	ATATTATTATTATTATTATTATTATTATTATTATTATTATTAT/ATATTATTATTATTATTATTATTATTATTATTATTATTATTATTATTATTAT	GCAAACTTGCAATACCCCAA	TGGTGGATACATTGATGGGA	60.9	379	INTERGENIC	--	--	--

INDEL marker IDs	Chromosomes/ unanchored scaffolds	Physical positions (bp)	InDels (*Kabuli* reference genome-CDC Frontier/PII)	Forward primers (5'-3')	Reverse primers (5'-3')	Anneling temperature (0C)	Expected amplified product size (bp)	Structural annotation		Functional annotation	
								Sequence components of *kabuli* genome	*Kabuli* gene accession IDs	NCBI-KOG	TFs
Ca5_P4_2277714	Ca5	2277714	ATTTTTTTTTTT/ATTTTTTTTTT	TTTTCGGGAGAAGCCACTTA	TCGATTCACGAAATTGGTCAT	59.8	512	INTERGENIC	--	--	--
Ca5_P4_22788346	Ca5	22788346	ATACTA/ATA	TGATTGACATTTTTCAATTGGT	CAGCAAAATAAATATCATCTGCAA	57.0	539	INTERGENIC	--	--	--
Ca5_P4_22918447	Ca5	22918447	T/TG	TTATCGGCCTTATCCACTGC	GGATTCCACCAAATTCAGGA	60.1	721	INTERGENIC	--	--	--
Ca5_P4_22918526	Ca5	22918526	AT/ATT	TTATCGGCCTTATCCACTGC	TGAAAATCCATGAACCGTGA	60.1	915	INTERGENIC	--	--	--
Ca5_P4_22970811	Ca5	22970811	GATATATATATATA/GATATATATATATATA	AATGTGAATCCCACCAGGAA	CAAGCGCTATGTGACCCTTT	60.2	833	INTERGENIC	--	--	--
Ca5_P4_23081226	Ca5	23081226	ATTTT/ATTTTT	CATTTTAACGTTGGGGCAGT	ACCAGCAACCATAAAAACGC	59.9	677	INTERGENIC	--	--	--
Ca5_P4_23111759	Ca5	23111759	ACCCCC/ACCCCCC	TCTCACGGTTGTGAGGACTG	TAGAGAAGTGTGCACCGTGG	59.9	600	INTERGENIC	--	--	--
Ca5_P4_23166274	Ca5	23166274	AA/AACTAGTA	GAGAAGCGAAAGCGAAGAGA	GATATTAGGGGCAACTCGCA	60.0	658	INTERGENIC	--	--	--
Ca5_P4_23184362	Ca5	23184362	AATTATTATTATTATTATTATTA/AATTATTATTATTATTATTATTATTA	GAAAAAGCGCTGTAAATGGC	ATCCCACCAACCAAGACAAA	59.9	725	INTERGENIC	--	--	--
Ca5_P4_2323461	Ca5	2323461	GAAAA/GAAAAA	GCGCCAGATTTCAAACACTA	ACCAGCAGACCAAAATGGAC	58.9	262	INTERGENIC	--	--	--

INDEL marker IDs	Chromosomes/ unanchored scaffolds	Physical positions (bp)	InDels (*Kabuli* reference genome-CDC Frontier/PII)	Forward primers (5'-3')	Reverse primers (5'-3')	Anneling temperature (0C)	Expected amplified product size (bp)	Structural annotation		Functional annotation	
								Sequence components of *kabuli* genome	*Kabuli* gene accession IDs	NCBI-KOG	TFs
Ca5_P4_2323758	Ca5	2323758	TGA/TGGAGGA	GTCCATTTTGGTCTGCTGGT	TCCCTGTCCTCGATTTCATC	60.0	425	INTERGENIC	--	--	--
Ca5_P4_2327252	Ca5	2327252	TAA/TAAA	TCGAAATGAAATCGAGGACC	ACAGACGAGGGCTAGGGATT	60.0	721	INTERGENIC	--	--	--
Ca5_P4_23275268	Ca5	23275268	TAAAAAAAAA/TAAAAAAAAAA	AAGCCTAATTTCAGCCAGCA	TGATAGCCTCCAACGACAAA	60.0	482	INTERGENIC	--	--	--
Ca5_P4_23557611	Ca5	23557611	GGCCAGCCAGCC/GGCCAGCCAGCCAGCC	GTGACTGGGGACTGGTGACT	CTGTGACTAGGGAAGGCTCG	60.0	122	INTERGENIC	--	--	--
Ca5_P4_236073	Ca5	236073	CGAAGAAAAT/CGAAGAAAATGAAGAAAATGAAGAAAAT	TTGAATTGACCGGAGGAATC	GGGATAGGGAGGGTTGTGTT	59.9	680	INTERGENIC	--	--	--
Ca5_P4_23643411	Ca5	23643411	ATTTTTTT/ATTTTTTTT	AAGCAATCAACGCATTCACA	TTGACTCTCGAAATGTCCCC	60.3	462	INTERGENIC	--	--	--
Ca5_P4_23663124	Ca5	23663124	C/CGA	GAGGGTTTTGGGTCAGTGAA	AGTTTTCAAGGGTAGCGGGT	59.9	789	INTERGENIC	--	--	--
Ca5_P4_23664574	Ca5	23664574	TT/TTCCAACT	CAAGAAGGAACGAGGTGCTC	TAGCCTAGAGCAGGGGTGAA	60.0	626	INTERGENIC	--	--	--
Ca5_P4_23675051	Ca5	23675051	GT/G	TCCATGCACATTGCTCTCTC	TGGGTTATTTTCATGGGCTT	60.0	514	INTERGENIC	--	--	--
Ca5_P4_236894	Ca5	236894	CCACA/CCA	CCCTCACAAATTACAATCCCA	CCAACAAATGCATGTCCATC	59.7	763	INTERGENIC	--	--	--

INDEL marker IDs	Chromosomes/ unanchored scaffolds	Physical positions (bp)	InDels (*Kabuli* reference genome-CDC Frontier/PII)	Forward primers (5'-3')	Reverse primers (5'-3')	Anneling temperature (0C)	Expected amplified product size (bp)	Structural annotation		Functional annotation	
								Sequence components of *kabuli* genome	*Kabuli* gene accession IDs	NCBI-KOG	TFs
Ca5_P4_237162	Ca5	237162	TGCATTTGTTGGACG/TG	TCGTCTGATATTTTGGATGGA	TGCAACATTATATTTCCCGGT	58.0	143	INTERGENIC	--	--	--
Ca5_P4_237297	Ca5	237297	CTGTCAT/CTGTCATGTCAT	TCATTGCAATACCGGGAAAT	TGAATTTGGATAGGTGCAAAAA	60.2	233	INTERGENIC	--	--	--
Ca5_P4_23770283	Ca5	23770283	ATTTTT/ATTTTTT	TTGGCCTTCCTTTTACATGC	CCATCAAGCGTCTGTTGAGA	60.1	882	INTERGENIC	--	--	--
Ca5_P4_238011	Ca5	238011	GTTTTTT/GTTTTT	CATGCACGATTCACACATGA	GTGTCGTCCAACCTCTCCAT	60.1	486	INTERGENIC	--	--	--
Ca5_P4_23841713	Ca5	23841713	GA/G	TTTTCTCCCATCGTTGACAT	TTCTCAGCTATTCATCCGGG	58.0	712	INTERGENIC	--	--	--
Ca5_P4_23893834	Ca5	23893834	AATATATATATATATATATATATATATATATATATATATA/AATATATATATATATATATATATATATATATATATATA	CCAAATTTTACCTTCATCCACA	TGGATGCGATAAACCAACTTT	58.9	242	INTERGENIC	--	--	--
Ca5_P4_239546	Ca5	239546	AATA/AATATA	CTCAACGCTGCCACAATAGA	TGGATTCCCAAACACTAGCC	60.0	473	INTERGENIC	--	--	--
Ca5_P4_23976521	Ca5	23976521	GT/GTT	TTGGTGAAAGATTTTGTGCG	TTCAATGACAAAGGGGGAGA	59.7	759	INTERGENIC	--	--	--
Ca5_P4_23995501	Ca5	23995501	CATATATATATATATATATA/CATATATATATATATATA	TTGCGAGCATGAGAAGTTGT	AAAAACCACCTAATCGCTCA	59.6	714	INTERGENIC	--	--	--
Ca5_P4_240288	Ca5	240288	AAAAAAATAAAAA/AAAAAAATAAAAAATAAAAAA	GGCTAGTGTTTGGGAATCCA	ACGGTGTGTTGGGTTAAAGG	59.9	820	INTERGENIC	--	--	--

**INDEL marker IDs**	**Chromosomes/ unanchored scaffolds**	**Physical positions (bp)**	**InDels (*Kabuli* reference genome-CDC Frontier/PII)**	**Forward primers (5'-3')**	**Reverse primers (5'-3')**	**Anneling temperature (0C)**	**Expected amplified product size (bp)**	**Structural annotation**		**Functional annotation**	
								**Sequence components of *kabuli* genome**	***Kabuli* gene accession IDs**	**NCBI-KOG**	**TFs**
Ca5_P4_24054306	Ca5	24054306	CTATTATTATTATTATTATTATTATTATTATTATTAT/CTATTATTATTATTATTATTATTATTATTATTATTATTATTAT	TCAATAGAAGGTCGCATCCC	TCGCACATCTACCTAAACGC	60.0	759	INTERGENIC	--	--	--
Ca5_P4_240662	Ca5	240662	T/TCAATCCA	CCTTTAACCCAACACACCGT	TTGGTGCCTACAACATATTTTCC	59.8	268	INTERGENIC	--	--	--
Ca5_P4_241183	Ca5	241183	ATTTTTTT/ATTTTTTTT	AAAACACAACCCTTCAAGAGACA	GGGACGTCTTCGCATACATT	60.1	129	INTERGENIC	--	--	--
Ca5_P4_24297832	Ca5	24297832	AATATATATATATATA/AATATATATATATATATA	CCCAAGTTTTAAATGGGCAA	GGAGGCCACCTTCAACATTA	59.8	556	INTERGENIC	--	--	--
Ca5_P4_243143	Ca5	243143	GAT/GATAT	CAGAAAGTAAACGGGCCAAG	TGAAAATATACAACACCCATGAAAA	59.7	241	INTERGENIC	--	--	--
Ca5_P4_244132	Ca5	244132	TAAAAAAAAAA/TAAAAAAAAA	TTTTGCGCGAGAATTGATAA	TTTCAGAGGTTTGTTGCATTTTT	59.4	360	INTERGENIC	--	--	--
Ca5_P4_244256	Ca5	244256	CAAAAAAA/CAAAAAAAA	TTTTGCGCGAGAATTGATAA	TCGCTTTTTATTTCAGAGGTTTG	59.4	370	INTERGENIC	--	--	--
Ca5_P4_244348	Ca5	244348	TA/TAATACAAA	TTTTGCGCGAGAATTGATAA	GCACTTACCACCGCTCTCTT	59.4	918	INTERGENIC	--	--	--
Ca5_P4_2444633	Ca5	2444633	GACTAACTAA/GACTAA	GAAGCATCCAAAATGGGAGA	GTGAGTTTCATGATGCCACG	60.0	737	INTERGENIC	--	--	--
Ca5_P4_244612	Ca5	244612	ATT/ATTT	AAAAATGCAACAAACCTCTGAAA	GCACTTACCACCGCTCTCTT	60.0	581	INTERGENIC	--	--	--

INDEL marker IDs	Chromosomes/ unanchored scaffolds	Physical positions (bp)	InDels (*Kabuli* reference genome-CDC Frontier/PII)	Forward primers (5'-3')	Reverse primers (5'-3')	Anneling temperature (0C)	Expected amplified product size (bp)	Structural annotation		Functional annotation	
								Sequence components of *kabuli* genome	*Kabuli* gene accession IDs	NCBI-KOG	TFs
Ca5_P4_244659	Ca5	244659	C/CT	AAAAATGCAACAAACCTCTGAAA	AGCGTCTTCTGTGCTGTGAA	60.0	876	INTERGENIC	--	--	--
Ca5_P4_24625027	Ca5	24625027	GT/G	GGTCCAAACAATGGCAAATC	ACCCACAACTCCGTATGTCA	60.2	503	INTERGENIC	--	--	--
Ca5_P4_24701450	Ca5	24701450	CCATACATACATACATACATACATACATACATACATACATACATACATACATACA/CCATACATACATACATACATACATACATACATACATACATACATACATACATACATACATACA	CGTGTCCGAGTTAACAGCAA	TGTGTGTGGGTTATCACGCT	59.9	621	INTERGENIC	--	--	--
Ca5_P4_24750519	Ca5	24750519	ATTTTTT/ATTTT	GGACTAGGTCCCGTGGTTTT	AGAGGAGAGGAGGATGGGAG	60.2	233	INTERGENIC	--	--	--
Ca5_P4_24785187	Ca5	24785187	CTATATATATATATATATATAT/CTATATATATATATATATATATATAT	TTGGACCAATTTTGTGGCTT	CCAAACCAAATTGAATCAGGTAG	60.3	658	INTERGENIC	--	--	--
Ca5_P4_24822652	Ca5	24822652	TTCTCTCTCTCTCTCTCTCTCTCTCTCTCTCTCTCTCTCTCTC/TTCTCTCTCTCTCTCTCTCTCTCTCTCTCTCTCTCTCTCTCTC	AGGCAAAGAATCACATGCGT	GAAAATGAAGTGCAGCGTGA	60.7	237	INTERGENIC	--	--	--
Ca5_P4_24881793	Ca5	24881793	TAAAAAAAAAAAA/TAAAAAAAAAAA	CCGAAAGAATAGCTGGTGGA	TTCAAAAATCTGACGGAGGG	60.2	681	INTERGENIC	--	--	--
Ca5_P4_24932738	Ca5	24932738	ATTTTTTTT/ATTTTTTT	TGAAGAAACTTTTCCGCTGC	TGGAAATTTTACGGTGCAGA	60.5	710	INTERGENIC	--	--	--
Ca5_P4_24963058	Ca5	24963058	TAAAAA/TAAAA	ACCGAGACCAAATTCAGACG	CCCAAGTTCCCAAGAGTCAA	60.1	233	INTERGENIC	--	--	--
Ca5_P4_24994863	Ca5	24994863	ATTT/ATT	TGATTTGGCAATGAGCTGAA	TGAGTGGTTGGTTCTGTGGA	60.3	330	INTERGENIC	--	--	--

INDEL marker IDs	Chromosomes/ unanchored scaffolds	Physical positions (bp)	InDels (*Kabuli* reference genome-CDC Frontier/PII)	Forward primers (5'-3')	Reverse primers (5'-3')	Anneling temperature (0C)	Expected amplified product size (bp)	Structural annotation		Functional annotation	
								Sequence components of *kabuli* genome	*Kabuli* gene accession IDs	NCBI-KOG	TFs
Ca5_P4_25027142	Ca5	25027142	TAAAAAAAAAAAAAA/TAAAAAAAAA	AAATGGAATAACTTTGAAAATGGAA	CTCCATCGCACACTTTCTCA	59.2	602	INTERGENIC	--	--	--
Ca5_P4_25132630	Ca5	25132630	CTATATATATATATATATATATAT/CTATATATATATATATATAT	GCAAAAAGGAAGTTTGTCCC	TGCATGCATATTTGGAACATC	58.7	603	INTERGENIC	--	--	--
Ca5_P4_25232481	Ca5	25232481	T/TC	CCACCCTGATTTTTGGAGAA	AAAATCCAACAATCTCCCCC	59.9	790	INTERGENIC	--	--	--
Ca5_P4_25381692	Ca5	25381692	TTTATTATTATTATTATTATTATTATTATTATTATTATTATTATTATTATTATTATTA/TTATTATTATTATTATTATTATTATTATTATTATTATTATTATTATTATTATTATTA	ACGCGACTCAGCCAAGTAAT	AACCAAATGATATGCATCACG	59.9	376	INTERGENIC	--	--	--
Ca5_P4_2550837	Ca5	2550837	AC/ACC	TGAGACGTGGCATTGATGAT	CGAACTCGTCTAGCCCTTTG	60.1	567	INTERGENIC	--	--	--
Ca5_P4_25544088	Ca5	25544088	TAAAAAAAAAA/TAAAAAAAAAAA	TTCACAACCATTCAATTCCAA	TGGTTATGCATGTTCGTCGT	58.9	175	INTERGENIC	--	--	--
Ca5_P4_25574427	Ca5	25574427	CATATATATATATATATATATATATAT/CATATATATATATATATATATATATATAT	TTTTTGCAATCCACTCTTGAA	GATTGTTACACAATTCTTCACGC	58.4	301	INTERGENIC	--	--	--
Ca5_P4_25624396	Ca5	25624396	CATATATATATATATATATATATATATA/CATATATATATATATATATATATA	GGGTTGGACAACACTTGGTT	CGACGGGAGTTAACACCATA	59.7	682	INTERGENIC	--	--	--
Ca5_P4_25656601	Ca5	25656601	TCTT/TCTTACTT	CTACCCAAAAAGCCACGAAA	TGGGGCAACCTATCAAAGAG	60.1	317	INTERGENIC	--	--	--
Ca5_P4_25657788	Ca5	25657788	CAAA/CAAAA	GACTCGCAATCGTCTTCACA	AGATTCCTCGAGTTTCCCGT	60.0	343	INTERGENIC	--	--	--

INDEL marker IDs	Chromosomes/ unanchored scaffolds	Physical positions (bp)	InDels (*Kabuli* reference genome-CDC Frontier/PII)	Forward primers (5'-3')	Reverse primers (5'-3')	Anneling temperature (0C)	Expected amplified product size (bp)	Structural annotation		Functional annotation	
								Sequence components of *kabuli* genome	*Kabuli* gene accession IDs	NCBI-KOG	TFs
Ca5_P4_25661861	Ca5	25661861	TAAAAAAAAA/TAAAAAAAAAA	GGAGTGACAATGCCCTCAAT	TCCGCTTGGGACAATTTTAG	59.9	432	INTERGENIC	--	--	--
Ca5_P4_25756891	Ca5	25756891	ATATATATT/A	TGACATGTATTTTGAGAAACCAAA	TTCGAATCAATTGCGAGAGTT	58.6	693	INTERGENIC	--	--	--
Ca5_P4_25773766	Ca5	25773766	CAAAAAAAA/CAAAAAAA	CACTTGTACCTCACACCCCC	AACCAATCTTGTGCATCCAA	60.3	138	INTERGENIC	--	--	--
Ca5_P4_25785107	Ca5	25785107	CATA/CA	TGCCCGTGCTAATAATTTTG	AAGCACGGAAACAACGATTT	58.7	580	INTERGENIC	--	--	--
Ca5_P4_25802252	Ca5	25802252	ATTTTT/ATTTTTTT	TGATGGACTGGAATTGTCAAAG	TGCCAAGAAAACACCATGAA	60.0	846	INTERGENIC	--	--	--
Ca5_P4_25832671	Ca5	25832671	TAAA/TAAAA	AGCTTGCCAACTTCTGGGTA	GAAAACGTGTGGGAGAAGGA	59.9	835	INTERGENIC	--	--	--
Ca5_P4_25836482	Ca5	25836482	AG/A	AAGGCAACAACAAACAAGCC	ATGCCATTCAGCACATTTGA	60.2	344	INTERGENIC	--	--	--
Ca5_P4_25876394	Ca5	25876394	ATTTTTTTTTT/ATTTTTTTTTTT	TTGCCTCATATCGAGACTTTCA	GGGTAATCGTGCTACATGGG	59.8	899	INTERGENIC	--	--	--
Ca5_P4_25890397	Ca5	25890397	ACCCCCC/ACCCCC	GATTGCTTTCTGGGTGGAAA	TGCAGGGTTTAACGATAGCC	60.1	812	INTERGENIC	--	--	--
Ca5_P4_25902948	Ca5	25902948	CATATATATATATATATATATATATATATATAT/CATATATATATATATATATATATATATATATATATAT	GTGGAACCAAGTTCAACCGT	TTGGAAGATGAAAACCCTGAA	59.9	217	INTERGENIC	--	--	--

INDEL marker IDs	Chromosomes/ unanchored scaffolds	Physical positions (bp)	InDels (*Kabuli* reference genome-CDC Frontier/PII)	Forward primers (5'-3')	Reverse primers (5'-3')	Anneling temperature (0C)	Expected amplified product size (bp)	Structural annotation		Functional annotation	
								Sequence components of *kabuli* genome	*Kabuli* gene accession IDs	NCBI-KOG	TFs
Ca5_P4_26141778	Ca5	26141778	AG/AGG	ATGGGTAGGGTAAAAACCCG	TCTTTCCCCACCCTCCTACT	59.9	767	INTERGENIC	--	--	--
Ca5_P4_26159871	Ca5	26159871	TATAATAATAATAATAATAATAATAATAATAATAATAATAA/TATAATAATAATAATAATAATAATAATAATAATAATAATAATAATAA	TCCCAATGAATTTCGTTTTAGA	TGGATTAAACCAACTGTCCCA	58.6	389	INTERGENIC	--	--	--
Ca5_P4_26280416	Ca5	26280416	CTTTTTTTTTT/CTTTTTTTTTTT	GGGGAAAAAGGTACAAGCCT	AGCGACCTCCTACTTGGTGA	59.5	251	INTERGENIC	--	--	--
Ca5_P4_2628560	Ca5	2628560	AT/ATATATT	TTGTTTCTCCCCAGTTTTGG	TTTTAGCATTGACCGCTCCT	59.9	357	INTERGENIC	--	--	--
Ca5_P4_26325634	Ca5	26325634	T/TATATATAC	CACTTCCCTCTCCTTGCTCA	ATATAAGCGGCGCTATCCCT	60.5	164	INTERGENIC	--	--	--
Ca5_P4_26398930	Ca5	26398930	AGAAATTG/AGAAATTGAAATTG	TCGGTGGAGTGATGACAAAA	CCACCACTTAGAACCTTCGG	60.1	366	INTERGENIC	--	--	--
Ca5_P4_26457056	Ca5	26457056	AG/AGTAACATGGG	GCACTAGGCTTCAAACAGGC	CACATTCTTGCACGGTTCAT	60.0	450	INTERGENIC	--	--	--
Ca5_P4_26537164	Ca5	26537164	AC/A	CAGAGCTGGGTGCAGTCATA	TCGTTCCATATTCGGTAGCC	60.0	482	INTERGENIC	--	--	--
Ca5_P4_26856153	Ca5	26856153	AATATATATATATATATATATATATATATA/AATATATATATATATATATATATATATATATATATA	AAAAAGTTCATCATTCGAAATGTTAGT	TTACCCGACGACAAAAGTCC	59.8	650	INTERGENIC	--	--	--
Ca5_P4_26862103	Ca5	26862103	AT/ATTGTT	GAAAGTGAAAAGAAGAAAAAGGAAA	CCATGGTAATGCATCGTGAG	58.6	630	INTERGENIC	--	--	--

INDEL marker IDs	Chromosomes/ unanchored scaffolds	Physical positions (bp)	InDels (*Kabuli* reference genome-CDC Frontier/PII)	Forward primers (5'-3')	Reverse primers (5'-3')	Anneling temperature (0C)	Expected amplified product size (bp)	Structural annotation		Functional annotation	
								Sequence components of *kabuli* genome	*Kabuli* gene accession IDs	NCBI-KOG	TFs
Ca5_P4_26889455	Ca5	26889455	T/TTA	TCATCGCAGGTGTACC ATGT	GCGCTGACATGATCCC TATT	60.0	792	INTERGENIC	--	--	--
Ca5_P4_26989148	Ca5	26989148	AG/A	CCAAAGCAAGCCAGAA AGAC	GCGAGGTGGAACCACA CTAT	60.0	609	INTERGENIC	--	--	--
Ca5_P4_26996806	Ca5	26996806	A/AAAAGCAAAATTA	TCGTTTTGGGTGAATT GTGA	TGCCTCCTCGTTCCTTT TTA	59.9	410	INTERGENIC	--	--	--
Ca5_P4_26998600	Ca5	26998600	CGGGGGG/CGGGGGGG	TCAGTCGATACAGAGG TGCG	ATGTTTGATGCTTCTTG GGG	60.0	413	INTERGENIC	--	--	--
Ca5_P4_26999969	Ca5	26999969	GA/G	ATCCTGGCTTTTCCAC CTTT	CGAGCACTATTCAGTG CCAA	59.9	508	INTERGENIC	--	--	--
Ca5_P4_27000123	Ca5	27000123	ATCTCTCTC/ATCTC	ATCCTGGCTTTTCCAC CTTT	CGAGCACTATTCAGTG CCAA	59.9	508	INTERGENIC	--	--	--
Ca5_P4_27000261	Ca5	27000261	A/AG	ATCCTGGCTTTTCCAC CTTT	CGAGCACTATTCAGTG CCAA	59.9	508	INTERGENIC	--	--	--
Ca5_P4_27000469	Ca5	27000469	T/TATC	TTGGCACTGAATAGTG CTCG	TCGAAGCTGTGCTAAT GGTG	60.0	361	INTERGENIC	--	--	--
Ca5_P4_27000513	Ca5	27000513	A/AGTCG	TTGGCACTGAATAGTG CTCG	TCGAAGCTGTGCTAAT GGTG	60.0	361	INTERGENIC	--	--	--
Ca5_P4_27000752	Ca5	27000752	CTTTTTTTTTTTTTTT/CTTTTTTTTTT TTT	TTGGCACTGAATAGTG CTCG	CGTCCCGAATAGACGA ACAT	60.0	698	INTERGENIC	--	--	--

INDEL marker IDs	Chromosomes/ unanchored scaffolds	Physical positions (bp)	InDels (*Kabuli* reference genome-CDC Frontier/PII)	Forward primers (5'-3')	Reverse primers (5'-3')	Anneling temperature (0C)	Expected amplified product size (bp)	Structural annotation		Functional annotation	
								Sequence components of *kabuli* genome	*Kabuli* gene accession IDs	NCBI-KOG	TFs
Ca5_P4_27000834	Ca5	27000834	ATCTCT/ATCT	TTGGCACTGAATAGTGCTCG	CTCAGCGGGTAGAGTATCGC	60.0	940	INTERGENIC	--	--	--
Ca5_P4_27103158	Ca5	27103158	ANNNNNNNNNN/ANNNNN	CAAGTGGAGCTTGCCAATTT	CAGAGCGAAAATCAAGGAGG	60.2	685	INTERGENIC	--	--	--
Ca5_P4_2719069	Ca5	2719069	GTTTTTTTTTTTTT/GTTTTTTTTTTTT	TGAACTGCCGTTTGGTTAGA	TTTACCCTCTTCGTTCCCCT	59.3	616	INTERGENIC	--	--	--
Ca5_P4_27225961	Ca5	27225961	TG/T	ACTCATTTTCTGCGCAATCA	TGTGGAGGCACATACGACAT	59.4	880	INTERGENIC	--	--	--
Ca5_P4_27389155	Ca5	27389155	A/AAAAAAT	CACGAGCTTCAAGAGAGCCT	ATGAGATCGTTGTTGGGAGC	59.9	507	INTERGENIC	--	--	--
Ca5_P4_27397872	Ca5	27397872	AATATATATATATATATA/AATATATATATATATATA	CCTGCAACAATTTCACCAAA	TTCGTCGTGTTGCGTCTAAC	59.6	885	INTERGENIC	--	--	--
Ca5_P4_27458530	Ca5	27458530	TTATATATATATATATATATATATATATAT/TTATATATATATATATATATATATATATATAT	CAATTGGTCGGCAGAGTTTT	CCCACAACCATGCAATAGAA	60.1	531	INTERGENIC	--	--	--
Ca5_P4_27472549	Ca5	27472549	AG/A	GCATTATGCCGTCATCATTTT	TTCGCATTCAAAATTGTTGTT	59.8	491	INTERGENIC	--	--	--
Ca5_P4_27510424	Ca5	27510424	AATATATATATATATATAT/AATATATATATATATATATAT	CCAACACGGCAAAAAGAGAT	GCCATTTGGAATGACGACTT	60.1	799	INTERGENIC	--	--	--
Ca5_P4_27718024	Ca5	27718024	TAAAAAAA/TAAAAAAAA	TGGCAACAGAAACCAAACAA	TTCTTTTAGCCCGTTGCTGT	60.1	573	INTERGENIC	--	--	--

INDEL marker IDs	Chromosomes/ unanchored scaffolds	Physical positions (bp)	InDels (*Kabuli* reference genome-CDC Frontier/PII)	Forward primers (5'-3')	Reverse primers (5'-3')	Anneling temperature (0C)	Expected amplified product size (bp)	Structural annotation		Functional annotation	
								Sequence components of *kabuli* genome	*Kabuli* gene accession IDs	NCBI-KOG	TFs
Ca5_P4_27759321	Ca5	27759321	TATAATAATAATAATAATAATAATAATAATAATAATAATAA/TATAATAATAATAATAATAATAATAA	AGCCCAACTAAGCTGGAAAA	TCACCCTCTTTCATGCCAAT	59.0	702	INTERGENIC	--	--	--
Ca5_P4_27776620	Ca5	27776620	ATTTTTTTTTT/ATTTTTTTTTTT	TCCAAACTTGGGTTGGTTTC	CATGAGCATGCAAGATGGAT	59.8	603	INTERGENIC	--	--	--
Ca5_P4_27779493	Ca5	27779493	GTTATATA/GTTATATATTATATA	CATACTCCACGTCGTCGCTA	TTACAATTGTCACCGCCTCA	59.9	278	INTERGENIC	--	--	--
Ca5_P4_27784195	Ca5	27784195	AATATATATATATATATATATATATAT/AATATATATATATATATATATATATATATATAT	TCATGTCATGTTGGTTCATCA	GAGTGTTGGTGGCAATTGTG	58.3	776	INTERGENIC	--	--	--
Ca5_P4_27800920	Ca5	27800920	AC/A	TCTCAACAAGCACAATTGCC	TTTATTTGAGCCAAGGGGTG	59.8	112	INTERGENIC	--	--	--
Ca5_P4_27906217	Ca5	27906217	AATATATATATATATATATATATATAT/AATATATATATATATATATATAT	TCCAACAACATCAAATTCATCA	TGTCAACTCTTCCAGCACCA	58.9	630	INTERGENIC	--	--	--
Ca5_P4_27953689	Ca5	27953689	TA/T	AACAACAGCCCCAATGTAGC	ACGCTTTTCAAACAAACGCT	60.0	794	INTERGENIC	--	--	--
Ca5_P4_27964664	Ca5	27964664	TTTATTATTATTATTATTATTATTATTATTATTATTATTATTATTATTATTAT/TTTATTATTATTATTATTATTATTATTATTATTATTATTATTAT	GGGGTGTCGATGTTCACTCT	AACATGTCATTGCCTCCCTC	60.0	400	INTERGENIC	--	--	--
Ca5_P4_28061616	Ca5	28061616	TTATATATATATATATATATATATAT/TTATATATATATATATATAT	TAGGGGGATCAAAACTGCAA	GTCAGACCCGTTCAATTGCT	60.4	610	INTERGENIC	--	--	--
Ca5_P4_28101883	Ca5	28101883	AAATAATAATAATAATAATAATAATAATAATAATAATAATAATAATAATAAT/AAATAATAATAATAATAATAATAATAATAATAATAATAATAATAATAATAATAATAATAAT	TTGAGGAAGCGACTTATAACGA	CGTGTGATTGGTAATGTGGC	59.0	829	INTERGENIC	--	--	--

INDEL marker IDs	Chromosomes/ unanchored scaffolds	Physical positions (bp)	InDels (*Kabuli* reference genome-CDC Frontier/PII)	Forward primers (5'-3')	Reverse primers (5'-3')	Anneling temperature (0C)	Expected amplified product size (bp)	Structural annotation		Functional annotation	
								Sequence components of *kabuli* genome	*Kabuli* gene accession IDs	NCBI-KOG	TFs
Ca5_P4_28187994	Ca5	28187994	AATATATATATATAT/AATATATATATATATATAT	CAAGAAAAGTAACCATCGTAATGA	TGTGGATTCATGATCCTTGTG	57.5	494	INTERGENIC	--	--	--
Ca5_P4_28188039	Ca5	28188039	T/TC	CAAGAAAAGTAACCATCGTAATGA	TGTGGATTCATGATCCTTGTG	57.5	494	INTERGENIC	--	--	--
Ca5_P4_28192036	Ca5	28192036	CATATATATATATATATATATAT/CATATATATATATATATATATATAT	CCACATGCTCATTGTGTGACT	CCATTCTCCTTCACCCAGAC	59.6	653	INTERGENIC	--	--	--
Ca5_P4_28243344	Ca5	28243344	CG/CTGCGTATTTCTGAG	GCCTAATGGGTACGAAAAGGA	GGTTGGGATCCCTTTGTTTT	60.3	578	INTERGENIC	--	--	--
Ca5_P4_28291153	Ca5	28291153	CAAAAAAAAAAA/CAAAAAAAAAA	CACGTCCCTAACATTTTGCC	TGTTCCTGATATTTTGCCGA	60.4	400	INTERGENIC	--	--	--
Ca5_P4_28305464	Ca5	28305464	GTGTATATATG/GTGTATATATGTATATATG	AATATGTGGCGATTAATCTATTGTT	TGGTTGATCATTTCGTTAGTACG	57.3	960	INTERGENIC	--	--	--
Ca5_P4_28310357	Ca5	28310357	AAATAATAATAATAATAATAATAATAATAATAATAATAATAATAATAATAATAATAATAAT/AAATAATAATAATAATAATAATAATAATAATAATAATAATAATAATAATAATAATAATAATAAT	CCAACATGTGGGATAACTTGTG	TGGCCTGACCTATTCTCACC	60.1	635	INTERGENIC	--	--	--
Ca5_P4_28370910	Ca5	28370910	TTATATATATATATATATATATATATATATAT/TTATATATATATATATATATATATATATAT	CTAGTTCGTAACCCGTGCGT	AGGCATGGTGTTGGAGTCAT	60.2	260	INTERGENIC	--	--	--
Ca5_P4_28399764	Ca5	28399764	GT/GTACTAGCATCCT	AGAGTCGCCAGCTGAACAAT	TGATTTCTCCATAGCACCGA	60.0	502	INTERGENIC	--	--	--
Ca5_P4_28413401	Ca5	28413401	A/AT	TTCTCCTTGAAGCTGTCGGT	ATTGCCAACATTTCCGGTAG	60.0	252	INTERGENIC	--	--	--

INDEL marker IDs	Chromosomes/ unanchored scaffolds	Physical positions (bp)	InDels (*Kabuli* reference genome-CDC Frontier/PII)	Forward primers (5'-3')	Reverse primers (5'-3')	Anneling temperature (0C)	Expected amplified product size (bp)	Structural annotation		Functional annotation	
								Sequence components of *kabuli* genome	*Kabuli* gene accession IDs	NCBI-KOG	TFs
Ca5_P4_28463884	Ca5	28463884	TAAAAAAAAA/TAAAAAAAAAA	TGTTTCCTTTCGACACATTGA	TGTTGAGCCCAAAATACAAGG	59.2	590	INTERGENIC	--	--	--
Ca5_P4_28483546	Ca5	28483546	ATTTTTTTTTT/ATTTTTTTTT	TGAAGCGTATGGAACAATCG	TGAATCATTTGAATTTGCACG	59.7	674	INTERGENIC	--	--	--
Ca5_P4_28588926	Ca5	28588926	TGGG/TGG	CAACTGCTCCTGTTCTCCAA	CTAGGAGCATGTCGCTCTCC	59.0	412	INTERGENIC	--	--	--
Ca5_P4_286593	Ca5	286593	AGTTTGCGT/AGTTTGCGTGTTTGCGT	CACACTCCCATTTTTCCACC	TACGTGTATCCAACGGGGTT	60.2	573	INTERGENIC	--	--	--
Ca5_P4_28734209	Ca5	28734209	TTATATATATATATATATATATATATATAT/TTATATATATATATATATATATATATATATAT	TGTGGGATTCTTTCCCCATA	TTTTGATGATTCGACGGTTC	60.1	785	INTERGENIC	--	--	--
Ca5_P4_28869871	Ca5	28869871	TAAAA/TAAAAA	TGCTGGTGAACTTGGTGGTA	AAGTGGTCGACCGTCATTTC	60.2	912	INTERGENIC	--	--	--
Ca5_P4_28977874	Ca5	28977874	CAATGCAGAA/CAATGCAGAATAATGCAGAA	TGCAGAACCAGAACAAGCAC	TCTGGATTTTGATGTGCTGC	60.0	137	INTERGENIC	--	--	--
Ca5_P4_29001853	Ca5	29001853	TAAAAAAAAA/TAAAAAAAAAA	TGCACGGATAATGAAGTCGT	TCTCACATTTTATGCACACAACC	59.2	897	INTERGENIC	--	--	--
Ca5_P4_29097154	Ca5	29097154	ATTTTTTTTT/ATTTTTTTTTT	GCTGCATGCGTTACGAGATA	AAATTCACGGTGTCATCTTCG	60.0	271	INTERGENIC	--	--	--
Ca5_P4_29098754	Ca5	29098754	ATTTTTTTTTTT/ATTTTTTTTTT	CATCATGTTCAATGAAAGCCA	CCATCCATTATTCCAACCCA	59.5	822	INTERGENIC	--	--	--

INDEL marker IDs	Chromosomes/ unanchored scaffolds	Physical positions (bp)	InDels (*Kabuli* reference genome-CDC Frontier/PII)	Forward primers (5'-3')	Reverse primers (5'-3')	Anneling temperature (0C)	Expected amplified product size (bp)	Structural annotation		Functional annotation	
								Sequence components of *kabuli* genome	*Kabuli* gene accession IDs	NCBI-KOG	TFs
Ca5_P4_29099232	Ca5	29099232	CATTATTATTATTATTATTATTATTATTATTATTATTATTATTATTATTATTATTTATT/CATTATTATTATTATTATTATTATTATTATTATTATTATTATTATTATTATTATTATTATTATTATTATT	GGCATGACATGTGAAGATGG	TAGCCCCACAACACAAACAA	59.9	413	INTERGENIC	--	--	--
Ca5_P4_29144882	Ca5	29144882	AATTATTATTATTATTATTATTATTATTATTATTATTATTATTATTA/AATTATTATTATTATTATTATTATTATTATTATTATTATTATTATTATTA	AGTGCTGATTGATATGCCGT	ATTAGGGCTGCACTTGGACA	58.2	583	INTERGENIC	--	--	--
Ca5_P4_29351977	Ca5	29351977	CTATTATTATTATTATTATTATTATTATTATTATTATT/CTATTATTATTATTATTATTATTATTATTATTATT	GAGCCGAAAACGTATCCTAA	TTAGGCGCGTTTCAAGAACT	57.0	701	INTERGENIC	--	--	--
Ca5_P4_29384323	Ca5	29384323	CTTTTTTTTTTT/CTTTTTTTTT	CTTGGACAATTGAGGCATTTC	ATAGACAAACGTCCGCCTTC	59.6	545	INTERGENIC	--	--	--
Ca5_P4_29394828	Ca5	29394828	ATTATATATATATATATATATATATATATATATATAT/AATATATATATATATATATATATAT	GCGGTGAAACAATTCCAAAT	TGTTGCACACTCATTATCACCA	59.8	532	INTERGENIC	--	--	--
Ca5_P4_29418196	Ca5	29418196	CAAAAAAAAA/CAAAAAAAA	TGAAAAACACGGAGTCAAACA	CGTGACACTTCTAGCTTGGGA	59.2	512	INTERGENIC	--	--	--
Ca5_P4_29462599	Ca5	29462599	AATATATATATATATATATATATATATAT/AATATATATATATATATATATATAT	ATTGCATCATGGCTAGAGGG	CACATGCAATCAACCTCCAC	60.1	702	INTERGENIC	--	--	--
Ca5_P4_29515244	Ca5	29515244	TAAAAAAAAA/TAAAAAAAA	CAACTCCACAACACAACGCT	CGGACAATTTCAACGTCCTT	59.8	673	INTERGENIC	--	--	--
Ca5_P4_29614403	Ca5	29614403	ATTTTTTTT/ATTTTTTT	TGGAAGTTACAATCACGTGTAAAAA	CGTGTTCCAGAAATTTTTGTTT	59.9	641	INTERGENIC	--	--	--
Ca5_P4_29663443	Ca5	29663443	AATATATATATATATATA/AATATATATATATATATA	CTCCTCCACTAAAAATAATTCCAA	GGGGAGGAGCTTCCTATATCC	57.9	756	INTERGENIC	--	--	--

INDEL marker IDs	Chromosomes/ unanchored scaffolds	Physical positions (bp)	InDels (*Kabuli* reference genome-CDC Frontier/PII)	Forward primers (5'-3')	Reverse primers (5'-3')	Anneling temperature (0C)	Expected amplified product size (bp)	Structural annotation		Functional annotation	
								Sequence components of *kabuli* genome	*Kabuli* gene accession IDs	NCBI-KOG	TFs
Ca5_P4_29664249	Ca5	29664249	GAAAAAAAAAAA/GAAAAAAAAAA	ATTCATAAACACATGGCCCC	TAGGGTTAGGTGAGGGGCTT	59.5	255	INTERGENIC	--	--	--
Ca5_P4_29665140	Ca5	29665140	ATCACATTTCACATTTCACATTT/ATCACATTTCACATTTCACATTTCACATTT	TGGAAAGCGATTTATCCTGG	AGAGATCTTCTTGCGGTGGA	60.0	579	INTERGENIC	--	--	--
Ca5_P4_29725622	Ca5	29725622	TTATATATATATATATATATATATATATATATATATAT/TTATATATATATATATATATATATATAT	AAGACATCGTCAAAATCCCG	GAGATGAGAAATGAAGCGGC	59.9	675	INTERGENIC	--	--	--
Ca5_P4_29725806	Ca5	29725806	A/ATATAATAT	AAGACATCGTCAAAATCCCG	TCCACAGACGACGAGATGAG	59.9	687	INTERGENIC	--	--	--
Ca5_P4_29778022	Ca5	29778022	CAAAAAAAAAAAAA/CAAAAAAAAAAAAAAAA	GCTGCTGGCACATCTACTCA	TAACTGCAACAGATGGTCGC	60.2	547	INTERGENIC	--	--	--
Ca5_P4_29858771	Ca5	29858771	ATTTTTTTTTTTTT/ATTTTTTTTTTTT	TTTCAATTCTCTCACCGTGCT	CGCATCGAAAACAGTGGTAA	59.9	789	INTERGENIC	--	--	--
Ca5_P4_29858988	Ca5	29858988	GATATATATATATATATATA/GATATATATATATATATATATATA	TTTTGCCACTTTATACACCAAAA	TCAGGCCATGTGAGTGTGAT	58.6	878	INTERGENIC	--	--	--
Ca5_P4_29904685	Ca5	29904685	GAAAAAAAAA/GAAAAAAAA	GCTTCCACGTTTGATTTGGT	TGGGAAGATTACGCACTTCA	60.0	631	INTERGENIC	--	--	--
Ca5_P4_29920694	Ca5	29920694	CTATATATATATATATATA/CTATATATATATATA	AATTGTTCATGCGACATTTG	ATCTTCCTCACTCCCACCCT	57.1	453	INTERGENIC	--	--	--
Ca5_P4_29933647	Ca5	29933647	GATATATATATATA/GATATATATATATATA	GACCTTTTCAAAGGATGCATTA	CGTGATGACAGACCAACACC	58.3	713	INTERGENIC	--	--	--

INDEL marker IDs	Chromosomes/ unanchored scaffolds	Physical positions (bp)	InDels (*Kabuli* reference genome-CDC Frontier/PII)	Forward primers (5'-3')	Reverse primers (5'-3')	Anneling temperature (0C)	Expected amplified product size (bp)	Structural annotation		Functional annotation	
								Sequence components of *kabuli* genome	*Kabuli* gene accession IDs	NCBI-KOG	TFs
Ca5_P4_30106907	Ca5	30106907	TAAAAAAAA/TAAAAAAAAAA	GCCAGCAACCTCCAATTAAG	TGCACATTGACCCAAATTGT	59.7	779	INTERGENIC	--	--	--
Ca5_P4_30114759	Ca5	30114759	TCGGCGG/TCGGCGGCGG	TTGCCTGAAACTCGAGTAAAAA	ACGGGACTTTTTCAATTCCG	59.1	306	INTERGENIC	--	--	--
Ca5_P4_30121237	Ca5	30121237	TTATATATATATATATATATATATATATATATATA/TTATATATATATATATATATATATATATATATATATATATATATA	TTGCTTTTGAAAAATTGTGGA	TTGTGCAACCAAAAATGGAA	58.3	654	INTERGENIC	--	--	--
Ca5_P4_30121835	Ca5	30121835	TTATATATATATATATATATATATATAT/TTATATATATATATATATATATATATATATATATATAT	CATGCGTGAATTTGATCGTC	AAACGCGTCACTTTACCTTCTC	60.1	224	INTERGENIC	--	--	--
Ca5_P4_30126893	Ca5	30126893	ATTTTTTTTT/ATTTTTTTTTT	GTGGCTTCCAAAGAGAAACG	TTCGGTACACAATTTTCCCC	59.9	612	INTERGENIC	--	--	--
Ca5_P4_30128968	Ca5	30128968	GATTATAATTAAAAATA/GATTATAATTAAAAATAATTATAATTAAAAATA	TGTTTCTCTCTCCGCTCACA	CCGCCGACTATACCTGTTTT	59.7	681	INTERGENIC	--	--	--
Ca5_P4_30131050	Ca5	30131050	AATATATATATATATATATATATA/AATATATATATATATATATATA	CAAATTTCGAGCAACAATGC	AACACAAATGTTAAATTGCGAAGA	59.3	571	INTERGENIC	--	--	--
Ca5_P4_30284983	Ca5	30284983	ATATTATTATTATTATTATTATTATTATTATTATTGTTATTGTTATTATTATTATTAT/ATATTATTATTATTATTATTATTATTATTATTATTATTATTATTATTGTTATTGTTATTATTATTATTAT	TGTTGTCACATGCTTTGGGT	TTGTGCACATTTTATTCTTCAAA	60.0	651	INTERGENIC	--	--	--
Ca5_P4_30337231	Ca5	30337231	TTTGTGTTTAAAATTGTGTTTAAAATTGTGTT/TTTGTGTTTAAAATTGTGTT	TTGCCCAAGTTTTCAGTTCG	TCATACGATGTGGGGGTGTA	61.2	679	INTERGENIC	--	--	--
Ca5_P4_30612967	Ca5	30612967	TAAAAAAAAA/TAAAAAAAAAA	CGGCATATCATCACCCTTTT	ACCCCCGAAAAAGTGCTAGT	59.8	378	INTERGENIC	--	--	--

INDEL marker IDs	Chromosomes/ unanchored scaffolds	Physical positions (bp)	InDels (*Kabuli* reference genome-CDC Frontier/PII)	Forward primers (5'-3')	Reverse primers (5'-3')	Anneling temperature (0C)	Expected amplified product size (bp)	Structural annotation		Functional annotation	
								Sequence components of *kabuli* genome	*Kabuli* gene accession IDs	NCBI-KOG	TFs
Ca5_P4_30613533	Ca5	30613533	CATATATATATATATATATATATA/CATATATATATATATATATATATATA	ACTAGCACTTTTTCGGGGGT	AAATCCATAATTTGGTTCACTCA	60.0	746	INTERGENIC	--	--	--
Ca5_P4_3063134	Ca5	3063134	TAT/TATCAT	TTGTCTTCTTGTGGCCGACT	TTGAAGCCGAGAAAACGACT	60.8	812	INTERGENIC	--	--	--
Ca5_P4_30712358	Ca5	30712358	G/GGATAGAAA	TATTGGATTGGGCCAAACAT	TTCGTTGGTTTTGGGTTCTC	60.0	534	INTERGENIC	--	--	--
Ca5_P4_30917049	Ca5	30917049	CAAA/CAAAA	GTGCGCTAATGCTTCCATTT	CTCTCCAAGGTTCTACCCCC	60.2	617	INTERGENIC	--	--	--
Ca5_P4_30990176	Ca5	30990176	TTTTATTTATTTATTTATTTATTTATTTATTTATTTATTTAT/TTTTATTTATTTATTTATTTATTTATTTATTTATTTATTTAT	GAATCCCCAAACAAAACCCT	CACACCACCAGCATTTTGAC	60.0	540	INTERGENIC	--	--	--
Ca5_P4_31051193	Ca5	31051193	AATATATATATATATATATATAT/AATATATATATATATATATATAT	GCCCGAAAACAATTAAGACC	GCAATTTCTCAGTCAATTTCAA	58.6	686	INTERGENIC	--	--	--
Ca5_P4_3106419	Ca5	3106419	CACA/CACATAACTACA	CAATTTTCGTGATGTGGCAG	TGAGTGATGCGAGTTGTTCG	60.1	749	INTERGENIC	--	--	--
Ca5_P4_31068432	Ca5	31068432	TTTATTATTATTATTATTATTATTATTATT/TTTATTATTATTATTATTATTATTATTATTATT	AGCAAATTGATTAATCGCAAA	TTCAATAGGAGGTCGAGTTTTC	57.5	508	INTERGENIC	--	--	--
Ca5_P4_31144433	Ca5	31144433	CAA/CA	ATGGGAACACAAATCACCGT	TTGGGAACTTCGTAACAGCA	60.1	259	INTERGENIC	--	--	--
Ca5_P4_31153866	Ca5	31153866	ATTT/ATT	TGAACAATATTTCTCTTCCAGCA	TTGTGTGGAGGTAAAAGCCC	58.9	885	INTERGENIC	--	--	--

INDEL marker IDs	Chromosomes/ unanchored scaffolds	Physical positions (bp)	InDels (*Kabuli* reference genome-CDC Frontier/PII)	Forward primers (5'-3')	Reverse primers (5'-3')	Anneling temperature (0C)	Expected amplified product size (bp)	Structural annotation		Functional annotation	
								Sequence components of *kabuli* genome	*Kabuli* gene accession IDs	NCBI-KOG	TFs
Ca5_P4_31154228	Ca5	31154228	TTGTGTGT/TTGTGTGTGT	GACCATACTTCAATTTTATGCCAA	TTGTGTGGAGGTAAAAGCCC	59.3	432	INTERGENIC	--	--	--
Ca5_P4_31154924	Ca5	31154924	CAA/CA	GTGGGTGTACCGGAACAAAT	GCCATATTCGAGTTCAACGG	59.6	115	INTERGENIC	--	--	--
Ca5_P4_3115746	Ca5	3115746	CAAAAAAAA/CAAAAAAA	GCAACATGGGATGATTCAGA	TCCAAGTCGTTCAGCTTGTG	59.5	628	INTERGENIC	--	--	--
Ca5_P4_31166296	Ca5	31166296	TGAGAGAGAGAGAGA/TGAGAGAGAGAGAGAGA	ATTGAAGGAGCAGAAGCGAA	GTCAACCTCTCTCTCGCTGG	60.1	144	INTERGENIC	--	--	--
Ca5_P4_31187017	Ca5	31187017	A/AAT	GTGTCTTTCGGTGGAGGAAA	TGACGTTGATAGAAACGAAGACA	60.1	478	INTERGENIC	--	--	--
Ca5_P4_31187958	Ca5	31187958	AAATAATAATAAT/AAATAATAATAATAAT	CATTCCCCTTAAAGCACGAG	TTTCTCGCCTCGAACACTTT	59.7	197	INTERGENIC	--	--	--
Ca5_P4_31237915	Ca5	31237915	TAAAAAAA/TAAAAAAAA	ACACAAATTCGCCAAAATCC	CATCATGCCTCAAGATCACG	59.8	768	INTERGENIC	--	--	--
Ca5_P4_31291175	Ca5	31291175	CTATTTATTTATTTATTTATTTATTT/ CTATTTATTTATTTATTT	GAGTTCTGATATTTTGATGCCAA	CCCACAACTCCTTACAACCC	58.3	426	INTERGENIC	--	--	--
Ca5_P4_31324450	Ca5	31324450	ATTTTTTTTTTT/ATTTTTTTTTT	GAAATCAACGTGGATGAGTCAA	TAGCAAGCAAGCAGCAAAAA	60.0	693	INTERGENIC	--	--	--
Ca5_P4_31342028	Ca5	31342028	TTATATATATATATATATATATAT/TTATATATATATATATATATATAT	TTTCCCGGCTCAAAACTTTA	CGATCACAATTGAAAGGGGT	59.7	653	INTERGENIC	--	--	--

INDEL marker IDs	Chromosomes/ unanchored scaffolds	Physical positions (bp)	InDels (*Kabuli* reference genome-CDC Frontier/PII)	Forward primers (5'-3')	Reverse primers (5'-3')	Anneling temperature (0C)	Expected amplified product size (bp)	Structural annotation		Functional annotation	
								Sequence components of *kabuli* genome	*Kabuli* gene accession IDs	NCBI-KOG	TFs
Ca5_P4_31420220	Ca5	31420220	ATTTTTTTTTT/ATTTTTTTTT	TTGCTTGCTGGCTTCTTTTT	ATTTCTCCTCCATTCCTCCC	60.1	707	INTERGENIC	--	--	--
Ca5_P4_31599333	Ca5	31599333	AATATATATATATATATATATATATAT/AATATATATATATATATATATAT	CACAGTATGAAACGTCACCCA	ATATGCCTCTGACGGACGAC	59.5	359	INTERGENIC	--	--	--
Ca5_P4_31621509	Ca5	31621509	CT/CTGTATATCTACTTT	ATCCGAGTTTGCGTATGTCC	GGGACGTGGAGATTGTTGAT	60.0	635	INTERGENIC	--	--	--
Ca5_P4_31663882	Ca5	31663882	GATATATATATATATATATA/GATATATATATATATATATATA	TCGATCATGATGGGAAGACA	GTTCATGCATGGTGACCAAA	60.0	486	INTERGENIC	--	--	--
Ca5_P4_317066	Ca5	317066	TAAA/TAAAA	GAGCATTGATCCGGATTGTAA	TTTGACGATGCAACGGTTTA	59.9	544	INTERGENIC	--	--	--
Ca5_P4_31790895	Ca5	31790895	GTTTTTTTTTTTT/GTTTTTTTTTTTTT	TGAACAAAATAACGTCGAACTCA	ACTTGAGTCTGGCGCCTTAG	59.7	637	INTERGENIC	--	--	--
Ca5_P4_31821908	Ca5	31821908	ATTTTTTTTTTT/ATTTTTTTTTT	TGGGTGGTATCCAAGAAAGC	TGCTCTTTGCGACCCTTACT	59.9	631	INTERGENIC	--	--	--
Ca5_P4_31835054	Ca5	31835054	CTTT/CTTTCCTTTTTTT	GCAAGGAACAAGGTAACCCA	CCAACAAAAGAGGGGGAGTT	60.0	657	INTERGENIC	--	--	--
Ca5_P4_31858708	Ca5	31858708	GTCAA/G	CTCCAGCTTTTGGGGACATA	TCTTCTGCTTGGAGACAAAACA	60.1	267	INTERGENIC	--	--	--
Ca5_P4_31941021	Ca5	31941021	AATATATATATATATATATATATATATA/AATATATATATATATATATATATATATATA	CGTAAACAAGGCCCAAACAC	GCCCCCTTCAATAAATCCAT	60.4	568	INTERGENIC	--	--	--

INDEL marker IDs	Chromosomes/ unanchored scaffolds	Physical positions (bp)	InDels (*Kabuli* reference genome-CDC Frontier/PII)	Forward primers (5'-3')	Reverse primers (5'-3')	Anneling temperature (0C)	Expected amplified product size (bp)	Structural annotation		Functional annotation	
								Sequence components of *kabuli* genome	*Kabuli* gene accession IDs	NCBI-KOG	TFs
Ca5_P4_31961640	Ca5	31961640	TTATATATATATATATATATAT/TTATATATATATATATATAT	AAAAATCACGGCAAATTCAAA	TAAGATGTGCATGGGATGGA	59.5	549	INTERGENIC	--	--	--
Ca5_P4_32095261	Ca5	32095261	GAAAAAAAA/GAAAAAAA	CTCTCAAGTTCAACCACGCC	ACTGAAGAGTGACGCCAACA	60.8	677	INTERGENIC	--	--	--
Ca5_P4_32139547	Ca5	32139547	ATTAT/A	GCGACTTCGCAATGTGTAGA	GTTAAGGAAGTCCGCGTTTG	60.0	531	INTERGENIC	--	--	--
Ca5_P4_32290531	Ca5	32290531	ACCCCCC/ACCCCCCC	TTGGAAAATGGGCCACTTAG	CACCACCGTCAGATTTTCCT	59.9	545	INTERGENIC	--	--	--
Ca5_P4_32299791	Ca5	32299791	ATTTTTTTTTT/ATTTTTTTTTTT	CAAGCAAAACCCTCAAAAGC	GGCGGATCAACTCTTTGTGT	59.9	364	INTERGENIC	--	--	--
Ca5_P4_32302712	Ca5	32302712	AATATATATATATATATATATATA/AATATATATATATATATATA	CCATCATCTTTTCGTCCGAT	GTGCTCAGCAATTCAGGGA	59.9	263	INTERGENIC	--	--	--
Ca5_P4_323043	Ca5	323043	CTTTATTTATTTAT/CTTTATTTATTTATTTAT	ATGGTCTCAAAGATGCAGGG	TGTGGCGAAAAACAAATCAA	60.1	795	INTERGENIC	--	--	--
Ca5_P4_32364771	Ca5	32364771	TAAAAAAAAAA/TAAAAAAAAA	TCCTCAGGTTTCGAAGAGGA	CCCGCCATCACTCACTATTT	59.9	760	INTERGENIC	--	--	--
Ca5_P4_32396570	Ca5	32396570	TATAATAATAATAATAAT/TATAATAATAATAAT	CAGCTGTTCAGATCCGGTTT	TGGATCGAGAAGAAAGTGGC	60.3	810	INTERGENIC	--	--	--
Ca5_P4_32471582	Ca5	32471582	ACAACCT/A	AGTCGCCATTTGAAACTTGG	AATGCATTTTGGCCTGAGAA	60.1	560	INTERGENIC	--	--	--

INDEL marker IDs	Chromosomes/ unanchored scaffolds	Physical positions (bp)	InDels (*Kabuli* reference genome-CDC Frontier/PII)	Forward primers (5'-3')	Reverse primers (5'-3')	Anneling temperature (0C)	Expected amplified product size (bp)	Structural annotation		Functional annotation	
								Sequence components of *kabuli* genome	*Kabuli* gene accession IDs	NCBI-KOG	TFs
Ca5_P4_32522095	Ca5	32522095	TTATATATATATATA/TTATATATATATA	GCTGTTATGTTTGGTTCGCA	GGGTTTTGCCTTCTCCACTC	59.7	614	INTERGENIC	--	--	--
Ca5_P4_32605650	Ca5	32605650	TAAAAAAAAAA/TAAAAAAAAA	TCAATCACAAATCAGGGTGC	ATCCAGATCCAATACGACGG	59.5	874	INTERGENIC	--	--	--
Ca5_P4_32610734	Ca5	32610734	TAAAAAAAAA/TAAAAAAAAAA	ATCCAGCCGTAAAAACAACG	GCTGGGGTTTGAAAAATCCT	60.0	832	INTERGENIC	--	--	--
Ca5_P4_32649227	Ca5	32649227	ATTTTTTTTTTT/ATTTTTTTTTT	TTCTTCCTGCTCGCATTTTT	TTGTTTTTGCTAACTTGTCCGA	60.0	279	INTERGENIC	--	--	--
Ca5_P4_32739425	Ca5	32739425	GTTTTTTTTTTTTTT/GTTTTTTTTTTTTT	TTGGCGTGTGAGACAGAAAA	AATCCGCACGAGCATAATTC	60.4	307	INTERGENIC	--	--	--
Ca5_P4_32739512	Ca5	32739512	ATTTTTTT/ATTTTTTTT	TTGGCGTGTGAGACAGAAAA	AATCCGCACGAGCATAATTC	60.4	307	INTERGENIC	--	--	--
Ca5_P4_32745764	Ca5	32745764	TAAA/TAAAA	AGGCATCACATTGAGGGACT	CCTCGGTGACCCTGAATAAA	59.5	148	INTERGENIC	--	--	--
Ca5_P4_32769672	Ca5	32769672	GATATATATATATATATATATATATATATATA/GATATATATATATATATATATATATATA	CATGGCGTGAGTGAGTGAGT	TCACGGTTCCATGAAGAACA	59.9	760	INTERGENIC	--	--	--
Ca5_P4_32771430	Ca5	32771430	ATTTTTTTTTT/ATTTTTTTTT	GGGACAGTAGGGTTCCCAAT	GGAGACCTTTCACATCGCTC	60.1	519	INTERGENIC	--	--	--
Ca5_P4_32771738	Ca5	32771738	AT/ATT	GAGCGCTGTAAAAGACCACC	CGTTGTAAAAGGATTGCGCT	59.9	419	INTERGENIC	--	--	--

INDEL marker IDs	Chromosomes/ unanchored scaffolds	Physical positions (bp)	InDels (*Kabuli* reference genome-CDC Frontier/PII)	Forward primers (5'-3')	Reverse primers (5'-3')	Anneling temperature (0C)	Expected amplified product size (bp)	Structural annotation		Functional annotation	
								Sequence components of *kabuli* genome	*Kabuli* gene accession IDs	NCBI-KOG	TFs
Ca5_P4_32849143	Ca5	32849143	TAAAAAAAAAA/TAAAAAAAAA	CCCAGTTAACAAAAGGGGAA	TCACCGTATGCAAAGACGAG	58.9	426	INTERGENIC	--	--	--
Ca5_P4_32849892	Ca5	32849892	TTATATATATATATATATAT/TTATATATATATATATATATAT	GATGTACCAAGACATCGCCA	TCGAATAAGGGATGAATAACAGG	59.5	581	INTERGENIC	--	--	--
Ca5_P4_32879637	Ca5	32879637	AGTAAACAATGTAAACAA/AGTAAACAA	TCCTGCTAAAGATGGTCATTCA	TTGTTGTAGGATGGATGGAGTG	59.7	778	INTERGENIC	--	--	--
Ca5_P4_32912336	Ca5	32912336	AATATATATATATATATATATATATAT/AATATATATATATATATATATATATATATATATATAT	ACCCCCTCACCTCCTTATTG	TTTTCTTGCGTCACTTGTTGA	60.2	499	INTERGENIC	--	--	--
Ca5_P4_32950457	Ca5	32950457	AAATAATAATAATAATAATAATAATAATAATAATAATAATAATAATAATAAT/AAATAATAATAATAATAATAATAATAATAATAATAATAATAATAATAATAATAATAAT	ATTGCCACATTTCATGCTCA	GAATTCCCTACAATCGGCAA	60.1	345	INTERGENIC	--	--	--
Ca5_P4_33047107	Ca5	33047107	AAATAATAATAATAATAATAATAATAATAATAATAATAATAATA/AAATAATAATAATAATAATAATAATAATAATAATAATAATAATA	TAGAAAGGGCCAGAAAAGCA	CGATACAAGTCGTAGGAAGGG	60.0	236	INTERGENIC	--	--	--
Ca5_P4_33223744	Ca5	33223744	ACGAACATATTC/AC	GGAGAGGAGCCAAGAGAACC	CCAATGACGAATATTGCACG	60.3	303	INTERGENIC	--	--	--
Ca5_P4_33354598	Ca5	33354598	TAAAAAAAAAA/TAAAAAAAAA	TGACAGTTACAAACCGCTGC	CAAATTTCTACCAATTCCCCA	59.9	363	INTERGENIC	--	--	--
Ca5_P4_33355834	Ca5	33355834	TAAAAAAAAAAA/TAAAAAAAAAAAA	ATTCCCGAGAATCCACACAA	AAGCCGGAGAATTGAATGTG	60.3	553	INTERGENIC	--	--	--
Ca5_P4_33395960	Ca5	33395960	AATTATTATTATTATTATT/AATTATTATTATTATT	TTTATCGGCTAAGGGACACG	GTGCTGCCTAGACAATCGGT	60.1	768	INTERGENIC	--	--	--

INDEL marker IDs	Chromosomes/ unanchored scaffolds	Physical positions (bp)	InDels (*Kabuli* reference genome-CDC Frontier/PII)	Forward primers (5'-3')	Reverse primers (5'-3')	Anneling temperature (0C)	Expected amplified product size (bp)	Structural annotation		Functional annotation	
								Sequence components of *kabuli* genome	*Kabuli* gene accession IDs	NCBI-KOG	TFs
Ca5_P4_33438225	Ca5	33438225	CTTTTTTTTTTTT/CTTTTTTTTTTT	ACTTAAGCGGCGTCAACTGT	CACTTGTGCGTTGCATATGAG	59.9	535	INTERGENIC	--	--	--
Ca5_P4_33439122	Ca5	33439122	ATT/ATTT	AAATCGATCAAACTCGCACC	CGAAATTCGATACTCGACCG	60.1	347	INTERGENIC	--	--	--
Ca5_P4_33441420	Ca5	33441420	ATTT/ATTTT	GTGTGTGAGAGGTTGTTGGG	TAACTCGAACCAACCGATCC	59.0	204	INTERGENIC	--	--	--
Ca5_P4_33541448	Ca5	33541448	ATAATTTTTTTTTTAATTTTTTT/ATAATTTTTT	GCTTTGATTTGGGCTTTGAG	CCAAAAACCGAATTTCACCA	59.8	877	INTERGENIC	--	--	--
Ca5_P4_33542100	Ca5	33542100	GTTTATTTATTTATTTAT/GTTTATTTATTTATTTATTTATTTAT	AATTCGGTTTTTGGCGTGTA	GTTAAGGGACCACGAATGCT	60.4	569	INTERGENIC	--	--	--
Ca5_P4_33549168	Ca5	33549168	TAAAAAAAAA/TAAAAAAAAAA	CAAAAACGGGTTTCACCAAC	GCTCTGCATCCAATTCCATT	60.2	447	INTERGENIC	--	--	--
Ca5_P4_33567356	Ca5	33567356	GATAATAATAATAATAATAATAATAATAATAATAATAATAATAATAATAATAATAA/GATAATAATAATAATAATAATAATAATAATAATAATAATAATAATAATAATAA	AGCAAGTCACCTTGTCCGTT	TGGGTTTTGCATAGTCGGTT	59.8	516	INTERGENIC	--	--	--
Ca5_P4_33606817	Ca5	33606817	ATTTTACTTGTTTT/ATTTT	GACCTTGCCGGCTACTCTCT	ATATTTCTCATAGGGGCGGC	60.9	449	INTERGENIC	--	--	--
Ca5_P4_33682329	Ca5	33682329	GATATATATATATATATATATATAT/GATATATATATATATATATAT	AACGGTGAGGTGTGGGTAAA	TGAGGATTTTTGTGCACTCG	60.3	529	INTERGENIC	--	--	--
Ca5_P4_33797968	Ca5	33797968	AT/A	GCGGAATGACCTACCGAATA	ATAGGCTAGGTCGTCCGTCA	59.9	263	INTERGENIC	--	--	--

INDEL marker IDs	Chromosomes/ unanchored scaffolds	Physical positions (bp)	InDels (*Kabuli* reference genome-CDC Frontier/PII)	Forward primers (5'-3')	Reverse primers (5'-3')	Anneling temperature (0C)	Expected amplified product size (bp)	Structural annotation		Functional annotation	
								Sequence components of *kabuli* genome	*Kabuli* gene accession IDs	NCBI-KOG	TFs
Ca5_P4_33798523	Ca5	33798523	ATTTTTT/ATTTTT	TGACGGACGACCTAGCCTAT	TCATTTTCCCAATCTCTCCG	59.7	618	INTERGENIC	--	--	--
Ca5_P4_33883232	Ca5	33883232	AATTTAACTATATTT/AATTT	TGACATAAAGCACCACTGTTCA	TCCTACGGTCTTGTTTTGGG	59.2	559	INTERGENIC	--	--	--
Ca5_P4_33908663	Ca5	33908663	TTTTTATTTTATTTTATTTTATTTT/TTTTTATTTTATTTTATTTTATTTTATTTT	ATCGAAATGTGCGTGAACAA	GCAGCTTCTTTTCATTCGCT	60.1	373	INTERGENIC	--	--	--
Ca5_P4_33967986	Ca5	33967986	AATATATATATATATATATATA/AATATATATATATATATA	ATTGGTTAGGCGAAGATGGA	TTCCCTCCCCTTTCCTAAAA	59.5	506	INTERGENIC	--	--	--
Ca5_P4_34220162	Ca5	34220162	GAAAAAAAAAAA/GAAAAAAAAAA	CCACTAACCTATTCGGAATCCA	GGCAATTGTCGGCACTTAAT	60.2	462	INTERGENIC	--	--	--
Ca5_P4_34466553	Ca5	34466553	TAAAAAAAAA/TAAAAAAAA	TGTTGTTCCTCTTTTCACGTTG	ACCAAAGTAATGCGGTTGAA	60.2	762	INTERGENIC	--	--	--
Ca5_P4_34523237	Ca5	34523237	GTTTTTTTTTT/GTTTTTTTTTTT	ATTGAACTCGGAGACGGAAA	CCTTGACCCTGAACCAAATC	59.7	457	INTERGENIC	--	--	--
Ca5_P4_34546420	Ca5	34546420	TAGAGAGAGAGAGA/TAGAGAGAGAGA	TGTCGGCCACTCAAACATAA	TTGGGTTTTCATACAATCATGTG	60.1	330	INTERGENIC	--	--	--
Ca5_P4_34716959	Ca5	34716959	GA/GAGGGAATTGTAGGGAAA	AATTCGCAAGAAATCTCAGCA	AGGGGCTGCATGAACAATTA	60.0	323	INTERGENIC	--	--	--
Ca5_P4_34851417	Ca5	34851417	TA/TAA	GACAGAGAGATGACGGGGAA	AAACCGAAAAGTTGAAAAACTGA	60.2	569	INTERGENIC	--	--	--

INDEL marker IDs	Chromosomes/ unanchored scaffolds	Physical positions (bp)	InDels (*Kabuli* reference genome-CDC Frontier/PII)	Forward primers (5'-3')	Reverse primers (5'-3')	Anneling temperature (0C)	Expected amplified product size (bp)	Structural annotation		Functional annotation	
								Sequence components of *kabuli* genome	*Kabuli* gene accession IDs	NCBI-KOG	TFs
Ca5_P4_34981999	Ca5	34981999	ATTTTTTTTT/ATTTTTTTT	ATGTACCCGCACCAGAACTC	TTCAGGTTTCTCCACCCAAC	60.0	249	INTERGENIC	--	--	--
Ca5_P4_35032056	Ca5	35032056	AAATAATAATAATAATAATAATAATAATAATAATAATAATAATAATAATAATAATAATA/AAATAATAATAATAATAATAATAATAATAATAATAATAATAATAATAATAATAATAATAATAATAATA	GTGGAGTTGGGGAGAGTCTG	TTTCATGAGCAAACCAAGCA	59.7	841	INTERGENIC	--	--	--
Ca5_P4_35048447	Ca5	35048447	AATATATATATATA/AATATATATATATATA	TGGAGTTAGGAGGATTCATGG	TCAACCGTTGTTCACATGTTC	59.0	952	INTERGENIC	--	--	--
Ca5_P4_35058244	Ca5	35058244	TGA/T	TACACCGGTGGGATTTGACT	CCTTTACCTCCCATTTTCCA	60.2	798	INTERGENIC	--	--	--
Ca5_P4_35070670	Ca5	35070670	TATTAATTAATTAATTAATT/TATTAATTAATTAATTAATTAATT	TGTGACCTGCAGTGATATTTTG	ATAATGTTGCGAATGGAGGC	58.7	617	INTERGENIC	--	--	--
Ca5_P4_35091533	Ca5	35091533	TATAATAATAATAATAATAATAATAATAATAATAATAATAATAATAATAATAATAATAA/TATAATAATAATAATAATAATAATAATAATAATAATAATAA	TGGTGTGTAAATAATAAAAAGCTAAAA	TGATAATGCATGGAAAGCAAA	57.0	237	INTERGENIC	--	--	--
Ca5_P4_35115270	Ca5	35115270	TA/T	CCACCAATTCCGCAAGTATT	AAAGAGGCAGGTAGGGTCGT	59.8	482	INTERGENIC	--	--	--
Ca5_P4_35149505	Ca5	35149505	CTTTTTTTTTT/CTTTTTTTTTTT	CAATCACATGGCTTGGATGA	CAGCCACACCGATTACCTTT	60.5	400	INTERGENIC	--	--	--
Ca5_P4_35247530	Ca5	35247530	TAAAAAAAAA/TAAAAAAAAAA	GCTCACACGAGTGTTAATGGAA	GAAAAGTAGCAAGTACGGGTCG	60.2	584	INTERGENIC	--	--	--
Ca5_P4_3526464	Ca5	3526464	AATATATATATATATATATATATA/AATATATATATATATA	TTAAGGAAGCCCAAGTTCCA	AAAAAGCCTTTGGGAAATCG	59.7	521	INTERGENIC	--	--	--

INDEL marker IDs	Chromosomes/ unanchored scaffolds	Physical positions (bp)	InDels (*Kabuli* reference genome-CDC Frontier/PII)	Forward primers (5'-3')	Reverse primers (5'-3')	Anneling temperature (0C)	Expected amplified product size (bp)	Structural annotation		Functional annotation	
								Sequence components of *kabuli* genome	*Kabuli* gene accession IDs	NCBI-KOG	TFs
Ca5_P4_35328160	Ca5	35328160	ATTTTTTTTT/ATTTTTTTTTT	ACCACGTGACCCCTTAACTT	TGATGAAATTACGTTATATTGAGGATG	58.4	204	INTERGENIC	--	--	--
Ca5_P4_3535001	Ca5	3535001	ATTTTTTTTTT/ATTTTTTTTT	TACCTTCAAGTGGGCCAAAC	TGACCCCATCAAGGCTTTAG	60.0	300	INTERGENIC	--	--	--
Ca5_P4_35397670	Ca5	35397670	AATATATATATATAT/AATATATATATATATATAT	GGGGTGAATATCCTTTTCATTG	CATACACACGCATTAGAGCATT	59.6	577	INTERGENIC	--	--	--
Ca5_P4_35425903	Ca5	35425903	GATAATAATAATAATAATAATAATAATAATAATAATAATAATAATAATAATAATAATAA/GATAATAATAATAATAATAATAATAATAATAATAATAATAATAATAATAATAATAATAATAATAATAATAA	CACCAAAGTTCGTTGGACCT	CCTCATCATCTTCGTTCGTTC	60.0	837	INTERGENIC	--	--	--
Ca5_P4_35462508	Ca5	35462508	CATTATTATTATTATTATTATTATTATTATTATTATTATTATTATTATTATTATT/CTTTATTATTATTATTATTATTATTATTATTATTATTATTATTATTATTATTATTATTATTATTATTATT	AGTAAAACAAGCACCACCGC	CTTTTGCTCACACCCAACAA	60.2	459	INTERGENIC	--	--	--
Ca5_P4_35463186	Ca5	35463186	AATATATATATATATATATATATATATA/AATATATATATATATATATATA	GTGAGCAAAAGCATCATTCG	TCTTGCTTATTTCTCTCAAAGCA	59.4	987	INTERGENIC	--	--	--
Ca5_P4_35485623	Ca5	35485623	TAAAAAAAAA/TAAAAAAAAAA	CCCAACTTCTTCATTGCCTC	GGACCCAGCAGAGTTGGTAA	59.7	639	INTERGENIC	--	--	--
Ca5_P4_35495079	Ca5	35495079	AATATATATATATATATATATATAT/AATATATATATATATATATATAT	ATTTCTAGCATGGTGGGACG	CGTGTTGATTGAAATGAGCG	60.0	854	INTERGENIC	--	--	--
Ca5_P4_35595695	Ca5	35595695	TTAATAATAATAATAATAATAATAATAATAATAATAATAATAAT/TTAATAATAATAATAATAATAATAATAATAATAATAATAATAATAATAAT	ACCATTCAAACGGCGAGTAG	ATGCAATCGTATCTCGTGGT	60.1	306	INTERGENIC	--	--	--
Ca5_P4_35599782	Ca5	35599782	TTATATATATATATATATATATA/TTATATATATATATATATATA	ATTTCCAACTGTTTGGGCAG	TACTAGATCGCGTCCCACG	60.0	665	INTERGENIC	--	--	--

**INDEL marker IDs**	**Chromosomes/ unanchored scaffolds**	**Physical positions (bp)**	**InDels (*Kabuli* reference genome-CDC Frontier/PII)**	**Forward primers (5'-3')**	**Reverse primers (5'-3')**	**Anneling temperature (0C)**	**Expected amplified product size (bp)**	**Structural annotation**		**Functional annotation**	
								**Sequence components of *kabuli* genome**	***Kabuli* gene accession IDs**	**NCBI-KOG**	**TFs**
Ca5_P4_35701781	Ca5	35701781	T/TTAA	TCCGACACATCTCGAATAGAA	TTAAAAACATGGCGATTCTGC	58.3	459	INTERGENIC	--	--	--
Ca5_P4_35751387	Ca5	35751387	AATATATATATATATATATATATATATATAT/AATATATATATATATATATATATATATATATATATAT	TCATTTTTCTTTCAATTTTGTCCTT	TTTTCACTTGGCATCCATCC	59.5	776	INTERGENIC	--	--	--
Ca5_P4_3575663	Ca5	3575663	A/AC	TTTGACGAAGAGCCATGTTG	CGGGAATCAGTTTTGAGGAA	59.8	254	INTERGENIC	--	--	--
Ca5_P4_35783091	Ca5	35783091	ATATATTTATATTTATATTT/ATATATTTATATTTATATTTATATTT	TGACATCTCGTCGACATTTTATC	TGAATTAGATGATTGTGGTGAAGG	59.1	819	INTERGENIC	--	--	--
Ca5_P4_35836224	Ca5	35836224	ATTTTTTTTTTT/ATTTTTTTTTTTT	TAGGGAAAAGGGTGGTGTGA	GAGACATTTTGTGCTGGCCT	60.3	749	INTERGENIC	--	--	--
Ca5_P4_35872148	Ca5	35872148	TAAAAAAAAAAA/TAAAAAAAAAA	ACCCTCACGTAAGCCTGATG	TCGCCTTCGTAAATAACGATT	60.1	357	INTERGENIC	--	--	--
Ca5_P4_35899388	Ca5	35899388	CTTTTTTTTTT/CTTTTTTTTTTT	TTTGAGCTAGCATGCACACC	CGATCTCCATTTGCGTTTTT	60.0	688	INTERGENIC	--	--	--
Ca5_P4_35915901	Ca5	35915901	ATTTTTTTTTT/ATTTTTTTTT	AGCATGTGTTCTTGTGGCAG	TCGGAAGTTTCACATTCATCC	59.9	589	INTERGENIC	--	--	--
Ca5_P4_36023155	Ca5	36023155	TCCCC/TCCC	CAATCCGAGAGATTTAGGTGG	ATTTTTGTAATGACGGCCCA	58.7	364	INTERGENIC	--	--	--
Ca5_P4_36031906	Ca5	36031906	A/ATCTATTATTATT	TTGAATGCAGGCAAACCATA	AAGGATGCACCATTGCTACC	60.1	914	INTERGENIC	--	--	--

INDEL marker IDs	Chromosomes/ unanchored scaffolds	Physical positions (bp)	InDels (*Kabuli* reference genome-CDC Frontier/PII)	Forward primers (5'-3')	Reverse primers (5'-3')	Anneling temperature (0C)	Expected amplified product size (bp)	Structural annotation		Functional annotation	
								Sequence components of *kabuli* genome	*Kabuli* gene accession IDs	NCBI-KOG	TFs
Ca5_P4_36101390	Ca5	36101390	TAAAAAAAAA/TAAAAAAAA	AGAGCAAAAATCCTTTCGTGA	TAGTGTCAATGGGCCCTCTC	59.0	711	INTERGENIC	--	--	--
Ca5_P4_36317817	Ca5	36317817	CTTTTTTTTTTT/CTTTTTTTTTT	TCTCACCTCGGACACCAAAT	TGGAAGGTACAAGTGAGGGG	60.5	598	INTERGENIC	--	--	--
Ca5_P4_36414102	Ca5	36414102	AATATATATATATATATA/AATATATATATATATATATA	TGTTGCAAAGCATAACTTGACC	TGTGACCATGATTTTGTGGG	60.2	550	INTERGENIC	--	--	--
Ca5_P4_36583877	Ca5	36583877	CATATATATATATATATAT/CATATATATATATATATATAT	TCACTTTTAGGATCCGTTTGA	CACAATGGACCATGCACTTC	57.4	809	INTERGENIC	--	--	--
Ca5_P4_36657829	Ca5	36657829	CTTATTATTATTATTATTATTATTATTATTATTATTATTATTATTATT/CTTATTATTATTATTATTATTATTATTATTATTATTATTATTATTATTATT	CGTGAGATTTTCTGCTCGGT	AGAGGTCTTCCGTGTATGGC	60.4	877	INTERGENIC	--	--	--
Ca5_P4_36702458	Ca5	36702458	AATATATATATATATATATATATATATAT/AATATATATATATATATATATATATATATAT	CACCCGACTAATCGGAGAAA	TTTGGCCCCAGCAAATATAC	60.1	582	INTERGENIC	--	--	--
Ca5_P4_36814159	Ca5	36814159	TTATATATATATATATATATATATATA/TTATATATATATATATATATATA	GGAGTAAATTTGCAAGTCAAAGC	TTTCACGCACTCAACTCGTC	59.3	640	INTERGENIC	--	--	--
Ca5_P4_36904833	Ca5	36904833	GCTCTCTCTCTCTCTCTCTCTCTCTCTCTCTC/GCTCTCTCTCTCTCTCTCTCTCTCTCTCTCTCTC	TTCTGGAGAAAGAGGCGAAG	TGCGTAATTTGGTGCTGGTA	59.7	638	INTERGENIC	--	--	--
Ca5_P4_36963374	Ca5	36963374	CTTTATTTATTTATTTATTTATT/CTTTATTTATTTATT	TGCCGCTGTGTCAGTTAGTT	GACAATTATTTTGTCAATGAACGC	59.5	360	INTERGENIC	--	--	--
Ca5_P4_36963472	Ca5	36963472	TATAATAATAATAATAATAATAATAATAATAATAATAATAATAA/TATAATAATAATAATAATAATAATAATAATAATAATAA	TGCCGCTGTGTCAGTTAGTT	GTACCGCCTCGGTCTCTAAA	59.5	704	INTERGENIC	--	--	--

INDEL marker IDs	Chromosomes/ unanchored scaffolds	Physical positions (bp)	InDels (*Kabuli* reference genome-CDC Frontier/PII)	Forward primers (5'-3')	Reverse primers (5'-3')	Anneling temperature (0C)	Expected amplified product size (bp)	Structural annotation		Functional annotation	
								Sequence components of *kabuli* genome	*Kabuli* gene accession IDs	NCBI-KOG	TFs
Ca5_P4_37010323	Ca5	37010323	TAT/TAGTATATTGAT	TGCGTGAAAGGTTGAAAATG	TTGGTTTCATTGTGGGGAAT	59.7	540	INTERGENIC	--	--	--
Ca5_P4_37188296	Ca5	37188296	TT/TTAT	TCAAACCCCCATTTTTCTTG	CGAAGCTGGCCGTTTATTTA	59.8	809	INTERGENIC	--	--	--
Ca5_P4_37200733	Ca5	37200733	CATATATATATATATATATA/CATATATATATATATATATA	CATGCATGGTTTCTTCTCCA	TCCGTCTTTAATGGGATTGG	59.6	161	INTERGENIC	--	--	--
Ca5_P4_37218586	Ca5	37218586	TATACATACATACATACATACATA/TATACATACATACATACATA	CACTCGTATCATGCTTGTCG	CTTTTTCGCCACCATTTGTT	57.9	680	INTERGENIC	--	--	--
Ca5_P4_37222608	Ca5	37222608	CTATATATATATATATATATATAT/CTATATATATATATATATATAT	AAACTCGGTTGACCTTGCAC	ACGAGCGTTGAAGCTTGATT	60.2	348	INTERGENIC	--	--	--
Ca5_P4_37235701	Ca5	37235701	AATATATATATATATATATATATATA/AATATATATATATATATATATATA	GTTGGAGCCAAAAACTAGCG	ACATTCATTGGCCCTTTTGA	59.9	577	INTERGENIC	--	--	--
Ca5_P4_37278974	Ca5	37278974	CTATTTATTTATTTATTTATTTATTTATTTATT/CTATTTATTTATTTATTTATTTATTTATTTATT	AGTGAGTGGAAATCGGGTTG	AACGTCCCATCAAGTTCACA	60.0	199	INTERGENIC	--	--	--
Ca5_P4_3742109	Ca5	3742109	TT/TTACATAT	ATTTACGAGCAGCGGAAAGA	TGGTCATGCAGAAACTCAGC	60.0	605	INTERGENIC	--	--	--
Ca5_P4_37633576	Ca5	37633576	TAAAAAAAAAA/TAAAAAAAAAAA	TGCAATGTTAAAGATGGTTGG	GTGCGAATTTTGTGATTACTGA	58.5	264	INTERGENIC	--	--	--
Ca5_P4_37670957	Ca5	37670957	GTTTTTTTTT/GTTTTTTTT	GCCTGCTCTTCTTTTTGGTG	TGAAAATATACATCTGTGGGGG	60.0	294	INTERGENIC	--	--	--

INDEL marker IDs	Chromosomes/ unanchored scaffolds	Physical positions (bp)	InDels (*Kabuli* reference genome-CDC Frontier/PII)	Forward primers (5'-3')	Reverse primers (5'-3')	Anneling temperature (0C)	Expected amplified product size (bp)	Structural annotation		Functional annotation	
								Sequence components of *kabuli* genome	*Kabuli* gene accession IDs	NCBI-KOG	TFs
Ca5_P4_37690036	Ca5	37690036	TAAAAAAAAAA/TAAAAAAAAAAA	GGAGACGAGAAGGTCGTGAG	TTTTTATTCAAGTTGGCCCG	60.0	270	INTERGENIC	--	--	--
Ca5_P4_37851777	Ca5	37851777	CATTTATTTATTTATTTATTTATTT/CATTTATTTATTTATTTATTTATTTATTT	TGCTTGTAATCACAACAAGACCT	GCCAACTGCTTTGGGTAGAA	58.8	446	INTERGENIC	--	--	--
Ca5_P4_37905515	Ca5	37905515	ATTTTTTTTTT/ATTTTTTTTT	TGATCGAAGACAATTTGTGTCA	CCGATCTCACATGCTTCCTT	59.2	670	INTERGENIC	--	--	--
Ca5_P4_37930695	Ca5	37930695	TT/TTTTCATAGGT	TCCTTGCTCCACTTTTCCAT	TGGTTGATTGGCTGATTAATTG	59.7	644	INTERGENIC	--	--	--
Ca5_P4_37953081	Ca5	37953081	ATATTTTAATTATTTTAA/ATATTTTAA	TCAAATTGCCACACTCTAAAAA	TGCAAAATATGGTTGGATTGTC	57.5	834	INTERGENIC	--	--	--
Ca5_P4_37955990	Ca5	37955990	ATTTTTT/ATTTTTTT	GGCAAGACAAAAAGACAACGA	CCGTGATTGCAACTAAGTGAA	60.3	564	INTERGENIC	--	--	--
Ca5_P4_38005397	Ca5	38005397	CTCT/C	TGAGAGACCTTGATTGATACAAATG	TCAATTTGGTTCCAACGTCAT	59.6	540	INTERGENIC	--	--	--
Ca5_P4_38006809	Ca5	38006809	G/GATCGTT	TGGAAGTGCATATGATGTTGG	CGAGGTGTTGCACATGACTT	59.4	585	INTERGENIC	--	--	--
Ca5_P4_38023980	Ca5	38023980	AACACAC/AACAC	CCGCAGTAAGAGTTTGACCA	TTCGGAGGAATGGTTGTTTC	58.9	548	INTERGENIC	--	--	--
Ca5_P4_38025852	Ca5	38025852	AT/A	ATGCCAAAAATAAACGCCAG	TGAGGTTGAGAAGCCAAACC	60.0	647	INTERGENIC	--	--	--

INDEL marker IDs	Chromosomes/ unanchored scaffolds	Physical positions (bp)	InDels (*Kabuli* reference genome-CDC Frontier/PII)	Forward primers (5'-3')	Reverse primers (5'-3')	Anneling temperature (0C)	Expected amplified product size (bp)	Structural annotation		Functional annotation	
								Sequence components of *kabuli* genome	*Kabuli* gene accession IDs	NCBI-KOG	TFs
Ca5_P4_38027475	Ca5	38027475	T/TA	TGGAGTGATACACACTTTTGTGG	TGCTTACAAGGTATTCTTGCCA	59.9	446	INTERGENIC	--	--	--
Ca5_P4_38033573	Ca5	38033573	GTTTTTTTTT/GTTTTTTTTTTT	AAAGCGGCATTATCTCGTTG	CGCGTCAAATAAAGCATTGA	60.2	520	INTERGENIC	--	--	--
Ca5_P4_38034465	Ca5	38034465	AATTATTAT/AATTAT	TGCTATTGCGATCCCTCTCT	TTGTCGGTATAAATCGGACG	59.9	311	INTERGENIC	--	--	--
Ca5_P4_38034605	Ca5	38034605	CG/CGG	CTGCTATTGCGATCCCTCTC	TTTTGAACGCAAGAAAACAGAA	59.9	800	INTERGENIC	--	--	--
Ca5_P4_38034644	Ca5	38034644	GTAT/GTATATAT	CTGCTATTGCGATCCCTCTC	TTTTGAACGCAAGAAAACAGAA	59.9	800	INTERGENIC	--	--	--
Ca5_P4_38034841	Ca5	38034841	TTTA/TTTAATTA	TCGTCCGATTTATACCGACA	CGACGAGGTATATGCCAACA	59.0	856	INTERGENIC	--	--	--
Ca5_P4_38036769	Ca5	38036769	TAA/TA	TGATAACTCGTCGGCTTGTG	TTTTTCCTTCCAAATAAGCCTC	59.9	633	INTERGENIC	--	--	--
Ca5_P4_38042717	Ca5	38042717	CAAAAAAAAA/CAAAAAAAA	TTGGACCAAAACTGATGAAAAA	CCAAAGTTGCCTTGTCCTTC	59.5	716	INTERGENIC	--	--	--
Ca5_P4_38043767	Ca5	38043767	GTTTTTTT/GTTTTTTTTTTTTTTT	AGGAGGCTAATTGAGGGAGC	TGTGGGTGCATGGTAGAAAA	59.8	519	INTERGENIC	--	--	--
Ca5_P4_38043824	Ca5	38043824	ATTTTT/ATTTTTT	AGGAGGCTAATTGAGGGAGC	TGTGGGTGCATGGTAGAAAA	59.8	519	INTERGENIC	--	--	--

INDEL marker IDs	Chromosomes/ unanchored scaffolds	Physical positions (bp)	InDels (*Kabuli* reference genome-CDC Frontier/PII)	Forward primers (5'-3')	Reverse primers (5'-3')	Anneling temperature (0C)	Expected amplified product size (bp)	Structural annotation		Functional annotation	
								Sequence components of *kabuli* genome	*Kabuli* gene accession IDs	NCBI-KOG	TFs
Ca5_P4_38046137	Ca5	38046137	GT/GTAT	GCCCCAAATATGAATAACCG	TGAAATGCGAAAGAGGTGAA	59.1	190	INTERGENIC	--	--	--
Ca5_P4_38049889	Ca5	38049889	TAAAAAAA/TAAAAA	TCAAATGCATAATCATGAAAGGA	TCTCATTCACAAACGACCCA	59.4	190	INTERGENIC	--	--	--
Ca5_P4_38051854	Ca5	38051854	TTTTTCTTT/TTTT	AAAAGTTGGCCTTTCCTAACAA	TCAATTGTGTGCATCAGTTTCA	59.2	534	INTERGENIC	--	--	--
Ca5_P4_38091321	Ca5	38091321	CA/C	TCTGTTGCTTCGCTGACATC	GCTGGAATGGCTTTTGGTTA	60.1	591	INTERGENIC	--	--	--
Ca5_P4_38093452	Ca5	38093452	TCTAG/TCTAGCTAG	GCCTGCAGTCTCACTCTTCC	TGCTAAATTCTGCGACCCAT	60.1	499	INTERGENIC	--	--	--
Ca5_P4_38094477	Ca5	38094477	ATTTTTTTTTT/ATTTTTTTT	TCAATTGCACAAACAATTAGTTAAAA	ATTGCGAGCATGAGAAGTTG	59.1	147	INTERGENIC	--	--	--
Ca5_P4_38100297	Ca5	38100297	AC/ACGC	GTTGCCAGTTTTGGTCCACT	ATCGGATTAGGAAGGTTGGG	60.0	855	INTERGENIC	--	--	--
Ca5_P4_38100833	Ca5	38100833	TA/T	CGAATTTGAACCCGAGACAT	CATCATAACCGAGCACATGG	59.9	635	INTERGENIC	--	--	--
Ca5_P4_38122220	Ca5	38122220	A/AGTT	AGCCTAACCCCTAAACCCAA	TGTGCAAAAGTTTCACGACAT	59.8	588	INTERGENIC	--	--	--
Ca5_P4_38126712	Ca5	38126712	TAA/TAAAGGCTCAA	GCATTACGACTTTGTTGCGA	TACGGAGGCCTAAGACCAAA	59.9	234	INTERGENIC	--	--	--

INDEL marker IDs	Chromosomes/ unanchored scaffolds	Physical positions (bp)	InDels (*Kabuli* reference genome-CDC Frontier/PII)	Forward primers (5'-3')	Reverse primers (5'-3')	Anneling temperature (0C)	Expected amplified product size (bp)	Structural annotation		Functional annotation	
								Sequence components of *kabuli* genome	*Kabuli* gene accession IDs	NCBI-KOG	TFs
Ca5_P4_38133701	Ca5	38133701	TAAA/TAAAA	AAAGTTTCGAATGTGGGGTG	CGAATTGACCGAATTTGTCC	59.8	821	INTERGENIC	--	--	--
Ca5_P4_38140361	Ca5	38140361	GTATTATTATTATTATTATTAT/GTATTATTATTATTATTAT	TCACTCCACATCGGCATAAC	TGCATATGACGTGAGATTTGG	59.5	782	INTERGENIC	--	--	--
Ca5_P4_38140913	Ca5	38140913	AT/ATT	CGTCATATGCAGATTTTGCG	AAGCCCAAAAAGAAATGAGG	60.2	552	INTERGENIC	--	--	--
Ca5_P4_38141802	Ca5	38141802	AC/ACC	TGGTCAGTTGAGGACAAACG	GAAGACAAGGTGGTGCCAAT	59.7	162	INTERGENIC	--	--	--
Ca5_P4_38152938	Ca5	38152938	TTTAGATTAAATTAGATTAAAT/TTTAGATTAAAT	TTGTGAAGGAGGAGGACCAG	GCGGAAAACGAGAAATTTTG	60.2	950	INTERGENIC	--	--	--
Ca5_P4_38154241	Ca5	38154241	AAGA/AA	GCTTTTGGGTTGTTCGTGTT	TTTCCCCCTATTTTACGGCT	60.0	425	INTERGENIC	--	--	--
Ca5_P4_38154626	Ca5	38154626	AG/A	TTGTGGGCAAGGTTGACATA	AGGCACCACTTTTGATCGAC	60.0	659	INTERGENIC	--	--	--
Ca5_P4_38155355	Ca5	38155355	TTT/TTTATTTTTATT	TGTTGTTGTTGGATTGGCAT	AAACGGTATCGCGTTTAACTT	59.8	350	INTERGENIC	--	--	--
Ca5_P4_38155410	Ca5	38155410	TAAAAAAAAA/TAAAAAAAA	TGTTGTTGTTGGATTGGCAT	AAACGGTATCGCGTTTAACTT	59.8	350	INTERGENIC	--	--	--
Ca5_P4_38155679	Ca5	38155679	CTTA/CTTAATTA	TGTTGTTGTTGGATTGGCAT	AAAATGGAGCATTGGAAAAA	59.8	769	INTERGENIC	--	--	--

INDEL marker IDs	Chromosomes/ unanchored scaffolds	Physical positions (bp)	InDels (*Kabuli* reference genome-CDC Frontier/PII)	Forward primers (5'-3')	Reverse primers (5'-3')	Anneling temperature (0C)	Expected amplified product size (bp)	Structural annotation		Functional annotation	
								Sequence components of *kabuli* genome	*Kabuli* gene accession IDs	NCBI-KOG	TFs
Ca5_P4_38165078	Ca5	38165078	GTATA/GTATATA	AGCAGGAGCAAGGAAACAAA	CTGAAGGGGTGTGGTTGAGT	60.0	678	INTERGENIC	--	--	--
Ca5_P4_38229056	Ca5	38229056	GCAT/GCATACAT	CAAATCTGATCGTGGTGGTG	GGCTCTTGAGGCACTTGTTC	60.0	672	INTERGENIC	--	--	--
Ca5_P4_38255302	Ca5	38255302	AAATAATAATAATAAT/AAATAATAATAAT	TGCCTCAACTCAACACTTGC	CAATTTACCAATCGCACGAA	60.0	762	INTERGENIC	--	--	--
Ca5_P4_38407056	Ca5	38407056	CT/C	TCTCAAAGTCTCAATGGTCCAA	TTGAAGTCAAATTTCGCCAG	59.7	658	INTERGENIC	--	--	--
Ca5_P4_38407176	Ca5	38407176	AATATATATATATATATATATATATAT/AATATATATATATATATATATATATATATAT	TCTCAAAGTCTCAATGGTCCAA	TTGAAGTCAAATTTCGCCAG	59.7	658	INTERGENIC	--	--	--
Ca5_P4_3862372	Ca5	3862372	TAAA/TAA	TTCACATCCCCTCTAAACCAA	TCCCAATGACTCATCGTCAA	59.4	349	INTERGENIC	--	--	--
Ca5_P4_3865777	Ca5	3865777	CTTTTTTT/CTTTTTTTT	ATGTGACACCCTAAACCCCA	TTGGTTTAGAGGGGATGTGAA	60.1	605	INTERGENIC	--	--	--
Ca5_P4_3869500	Ca5	3869500	ATACAAAATTACAA/ATACAA	CATTAAATGTGTGCAAGGATCAA	GAGAGGTTGAAGCCTCAAAAGA	59.9	471	INTERGENIC	--	--	--
Ca5_P4_38704218	Ca5	38704218	CATATATATATATATATATATATATA/CATATATATATATATATATATATA	TTAACCTCACCCACTCCACC	ACCTTTCGCAGCCAATATCA	59.8	395	INTERGENIC	--	--	--
Ca5_P4_38718876	Ca5	38718876	AATCAATTTATCAATTTAT/AATCAATTTATCAATTTATCAATTTAT	CTTCGAAAAACCAAACCGAA	TGATTGCATAACCAGCCAAA	60.1	429	INTERGENIC	--	--	--

INDEL marker IDs	Chromosomes/ unanchored scaffolds	Physical positions (bp)	InDels (*Kabuli* reference genome-CDC Frontier/PII)	Forward primers (5'-3')	Reverse primers (5'-3')	Anneling temperature (0C)	Expected amplified product size (bp)	Structural annotation		Functional annotation	
								Sequence components of *kabuli* genome	*Kabuli* gene accession IDs	NCBI-KOG	TFs
Ca5_P4_3874588	Ca5	3874588	AC/A	GACCTTGCTTTGGGGTTGTA	CACATCCCCTCTAAACCAACC	60.0	409	INTERGENIC	--	--	--
Ca5_P4_38755876	Ca5	38755876	TA/TATGA	GAAGTTGAGCGCAATGTTGA	TTAAGACGGGTGATGGAAGC	60.0	528	INTERGENIC	--	--	--
Ca5_P4_38755924	Ca5	38755924	TAAAA/TAAA	GTCTGCCACATCACCCTTTT	CGGATCAACTTTTCTTTGTGG	60.0	661	INTERGENIC	--	--	--
Ca5_P4_38829826	Ca5	38829826	CTTATTATTATTATTATTATTATTATTATTATTATTATTATTATTATTATTATTATTATTATTATTATTATTAT/CTTATTATTATTATTATTATTATTATTATTATTATTATTATTATTATTATTATTATTATTATTATTATTATTATTATTATTATTAT	TGCAAACAAAGTAACGCCTG	TGCTACAACCAAACGATGGA	59.9	627	INTERGENIC	--	--	--
Ca5_P4_38866965	Ca5	38866965	AATTATTATTATTATTATTATTATTATTATTATTAT/AATTATTATTATTATTATTATTATTATTAT	CGCCTTCGATCAACATCTTT	GGTGCGAGGAAGAAGAAGAA	60.2	730	INTERGENIC	--	--	--
Ca5_P4_38880462	Ca5	38880462	ATATT/AT	ACACGTGAGAGGCACAAATG	CAATTGCACGTGGAGTGTTC	59.8	618	INTERGENIC	--	--	--
Ca5_P4_38902709	Ca5	38902709	TAAAAAA/TAAAAAAA	GAAGGGGTGAATGCTGACAT	ATGACCCCCTTCACTTTGTG	59.9	729	INTERGENIC	--	--	--
Ca5_P4_38912622	Ca5	38912622	AATATATATATATATATA/AATATATATATATATATATA	GTGGACCCCAATTTTCAATG	TCTCTCAAACCCTCCACCAC	60.0	706	INTERGENIC	--	--	--
Ca5_P4_38998221	Ca5	38998221	GAAAAAAAAA/GAAAAAAAAAA	ATGCGTGCGATAGGGAATAC	TGCCAATACACATTTGGGAC	60.0	549	INTERGENIC	--	--	--
Ca5_P4_39159883	Ca5	39159883	CAAAAAAAAA/CAAAAAAAAAA	TTGTCAAATAAAAGAAAGGGGG	TCTACCATTGCACCAAACCA	59.4	837	INTERGENIC	--	--	--

INDEL marker IDs	Chromosomes/ unanchored scaffolds	Physical positions (bp)	InDels (*Kabuli* reference genome-CDC Frontier/PII)	Forward primers (5'-3')	Reverse primers (5'-3')	Anneling temperature (0C)	Expected amplified product size (bp)	Structural annotation		Functional annotation	
								Sequence components of *kabuli* genome	*Kabuli* gene accession IDs	NCBI-KOG	TFs
Ca5_P4_39219117	Ca5	39219117	TAAAAAA/TAAAAA	TCACATTGACCAGGAATGGA	TGTTGGAAAGGCTCTGCTTT	59.9	566	INTERGENIC	--	--	--
Ca5_P4_39404451	Ca5	39404451	AAAATATA/AA	TGATTGACACTTAGGCCTGC	TGCGTGCTGAATACCGTTTA	58.9	263	INTERGENIC	--	--	--
Ca5_P4_39448578	Ca5	39448578	TTATATATATATATATATATATATAT/TTATATATATATATATATATATATATATATAT	TGGTTAAGAACGAACGGGAC	GCAACAAAGCACGGTGAATA	60.0	513	INTERGENIC	--	--	--
Ca5_P4_39484033	Ca5	39484033	GATATATATATATATATATATATATAT/GATATATATATATATATATATATAT	TTGATATCTTAAACAATGCGTCG	ACCAATGGTGGAGCTTGAAC	59.2	561	INTERGENIC	--	--	--
Ca5_P4_39533615	Ca5	39533615	TTATATATATATATATATATATATATATATATATAT/TTATATATATATATATATATATATATATATATATAT	TTCATGCATGGCAAGAGTTT	GTTTTTCGTCCCGTCTCAAA	59.3	686	INTERGENIC	--	--	--
Ca5_P4_39537232	Ca5	39537232	AATATATATATATATATATATATATATATATA/AATATATATATATATATATATATATATATA	TTGGCAACCAGTCAACAAAA	CACGTGAGAAAACCTCGACA	60.1	473	INTERGENIC	--	--	--
Ca5_P4_39744759	Ca5	39744759	AATATATATATATATATATATATATAT/AATATATATATATATATATATATATATATATATATAT	AACTTCTTCCGTTGGCAAAA	AAAAATCTCAAATGCGGATCA	59.7	611	INTERGENIC	--	--	--
Ca5_P4_39762076	Ca5	39762076	ATTTTTTTTTTT/ATTTTTTTTTT	CCTCAGTTTTGGTCCCTCAA	TTGTTTCTCCCCACTTCTGG	60.1	222	INTERGENIC	--	--	--
Ca5_P4_39804095	Ca5	39804095	TAAAAAAAAA/TAAAAAAAA	TGTGTTCAGTGCACATCTCATC	TCCAACACATAAGATGGAATTATTTA	59.8	267	INTERGENIC	--	--	--
Ca5_P4_3988569	Ca5	3988569	A/AT	CCAATTTTGTCCGACCATTT	TCGTGGTGGTAGTTTGTGGA	59.7	689	INTERGENIC	--	--	--

INDEL marker IDs	Chromosomes/ unanchored scaffolds	Physical positions (bp)	InDels (*Kabuli* reference genome-CDC Frontier/PII)	Forward primers (5'-3')	Reverse primers (5'-3')	Anneling temperature (0C)	Expected amplified product size (bp)	Structural annotation		Functional annotation	
								Sequence components of *kabuli* genome	*Kabuli* gene accession IDs	NCBI-KOG	TFs
Ca5_P4_3993030	Ca5	3993030	CA/C	CAATTTTTCATGCTCGCAAC	CAGGATAATTGTTTGACTCCTTG	59.3	250	INTERGENIC	--	--	--
Ca5_P4_39937669	Ca5	39937669	TTATATATATATATATATATATATATA T/TTATATATATATATATATATATATA TATAT	ATGGTGCAGCAATTGTCAAA	AATCCACTTCAAAAGTCTCTCG	60.1	559	INTERGENIC	--	--	--
Ca5_P4_39990866	Ca5	39990866	TC/T	AGCCTTTTCCGGGTTAAGAA	CAGCGTGACAACGATCAAGT	60.1	506	INTERGENIC	--	--	--
Ca5_P4_40190697	Ca5	40190697	CAAAA/CAAA	ACTCAGTCGCCAATTCAAGG	CCACCCACTCATGAATAAATGTT	60.3	651	INTERGENIC	--	--	--
Ca5_P4_40259990	Ca5	40259990	AATATATATATATATATATATATATAT ATATATATAT/AATATATATATATATA TATATATATATATATATATATATATAT AT	TGTGTGGAGATGCTCCATATTC	TCCGATGTCACAAAGCCATA	60.0	236	INTERGENIC	--	--	--
Ca5_P4_40274404	Ca5	40274404	TA/TAAAA	CGGTTGACTAACTGTGCCAA	TGCTTTAGTACAATGCCCCC	59.8	446	INTERGENIC	--	--	--
Ca5_P4_40319759	Ca5	40319759	ACATTTTATAGTGCTCATTTTATAG/ ACATTTTATAG	GGCTTCTTCAACAAAAGCGT	TTTCCCAATCGATTTGCTTC	59.5	722	INTERGENIC	--	--	--
Ca5_P4_40361243	Ca5	40361243	TAAAAAAAAAAAA/TAAAAAAAAAAA	CAATTCAACAGAAGGTCGCA	TGCCAAAACAAATGACGTGT	59.8	341	INTERGENIC	--	--	--
Ca5_P4_40392907	Ca5	40392907	GTATATATATATATATATATATATAT ATATATAT/GTATATATATATATATAT ATATATATATAT	GCATTATCACGAGCAACACG	TCAATGTGGTTCGCAAAAGA	60.3	192	INTERGENIC	--	--	--
Ca5_P4_40417439	Ca5	40417439	AATATATATATATATATATATATATAT ATATATATATA/AATATATATATATAT ATATATATATATATATATATA	TGAAATATGCACGGGACAAA	TGATTGCTCTAATTTTTATGTAGTCCA	59.9	556	INTERGENIC	--	--	--

INDEL marker IDs	Chromosomes/ unanchored scaffolds	Physical positions (bp)	InDels (*Kabuli* reference genome-CDC Frontier/PII)	Forward primers (5'-3')	Reverse primers (5'-3')	Anneling temperature (0C)	Expected amplified product size (bp)	Structural annotation		Functional annotation	
								Sequence components of *kabuli* genome	*Kabuli* gene accession IDs	NCBI-KOG	TFs
Ca5_P4_40434180	Ca5	40434180	A/ACATCTG	TTTCACCCAGTAGGAGGTCG	TGAACCGGAAGAACCTCAAC	60.1	500	INTERGENIC	--	--	--
Ca5_P4_40512133	Ca5	40512133	GAAAAAAAAAA/GAAAAAAAAA	TGTTGTAGCCCCGTCAATTT	CCACATGGCATTGACGATAC	60.4	448	INTERGENIC	--	--	--
Ca5_P4_40512625	Ca5	40512625	GTATATATATATATATA/GTATATATATATATA	ATTTCCGTATCGTCAATGCC	TGCGGACAAAATTCAAAACA	59.8	626	INTERGENIC	--	--	--
Ca5_P4_40572884	Ca5	40572884	ATTTTTTTT/ATTTTTTT	TTAGAGGAGCCACGTAAGCC	CCCTAGCCACTTTCTCTCCC	59.5	584	INTERGENIC	--	--	--
Ca5_P4_40615956	Ca5	40615956	AATATATATATATATATATATAT/AATATATATATATATATATAT	ATTAAACCAAACGCACGGAA	TGTGAACACCCTACGTGACAA	60.4	925	INTERGENIC	--	--	--
Ca5_P4_40616297	Ca5	40616297	AATATATATATATATATATATATATATATA/AATATATATATATATATATATATATATATATATATATATATA	TTCCTCAACAAATAAGCTCGAA	AACATTTTACGGCGGACAAC	59.0	734	INTERGENIC	--	--	--
Ca5_P4_4064742	Ca5	4064742	G/GC	TATGTTTGTTTTGCCGTCCA	AGGATGTACCATTCTTGCGG	60.0	375	INTERGENIC	--	--	--
Ca5_P4_40692265	Ca5	40692265	AATATTAAATATAT/AATATTAAATATATTAAATATAT	TACGTCCTCACTCCCAATCC	CGTAAAAGTGTCATTGCCTCC	59.9	517	INTERGENIC	--	--	--
Ca5_P4_40747443	Ca5	40747443	C/CT	TTGCAGAGAAAGAAAACTTGATATG	TGTGGAAGTTCCTAAAATACGC	59.0	237	INTERGENIC	--	--	--
Ca5_P4_40792737	Ca5	40792737	TAAAAAAAAAAAA/TAAAAAAAAAAA	CGACCTTCTTTGAACTTTGAGG	GACAACAGTCCAGCCCATTT	60.3	184	INTERGENIC	--	--	--

INDEL marker IDs	Chromosomes/ unanchored scaffolds	Physical positions (bp)	InDels (*Kabuli* reference genome-CDC Frontier/PII)	Forward primers (5'-3')	Reverse primers (5'-3')	Anneling temperature (0C)	Expected amplified product size (bp)	Structural annotation		Functional annotation	
								Sequence components of *kabuli* genome	*Kabuli* gene accession IDs	NCBI-KOG	TFs
Ca5_P4_40819075	Ca5	40819075	TATATA/TATACATATA	TGAGCTCGTAAAAACCCCAG	TGCCACAATTATCGGACAAA	60.2	874	INTERGENIC	--	--	--
Ca5_P4_41108441	Ca5	41108441	GTTTTTTTTTTT/GTTTTTTTTTTTT	GGAGCGGAAAAGACTGAAAA	TTTTGTCTATGCGCACAAGG	59.4	586	INTERGENIC	--	--	--
Ca5_P4_41219552	Ca5	41219552	AATATATATATATATATATAT/AATATATATATATATATATATAT	AGGAGAAGTGGCGAGAAAAA	CCATCAAATGCCTAATGGTTC	59.1	160	INTERGENIC	--	--	--
Ca5_P4_41226745	Ca5	41226745	CAAAAAAAAAA/CAAAAAAAAAAAA	GCCCAAAATCAAAAACTCCA	TGTGTCGTAAAAAGATGTCTAAGCA	59.9	611	INTERGENIC	--	--	--
Ca5_P4_41423998	Ca5	41423998	CTTTTT/CTTT	TCCCAAATGATTTTGCATATTTC	GGACGAAGCAGTATGCCATT	60.0	468	INTERGENIC	--	--	--
Ca5_P4_41428729	Ca5	41428729	T/TA	TGAATTGAGTTTGGTTGATTTGA	AAAGGATGCAGTATGGGTTTTT	59.5	591	INTERGENIC	--	--	--
Ca5_P4_41428807	Ca5	41428807	TTATATATATATATATATATATATATATATATATATATATAT/TTATATATATATATATATATATATATATATATATATATATATATATATATAT	TGAATTGAGTTTGGTTGATTTGA	AAAGGATGCAGTATGGGTTTTT	59.5	591	INTERGENIC	--	--	--
Ca5_P4_41428920	Ca5	41428920	TAAGGAAG/TAAG	TGAATTGAGTTTGGTTGATTTGA	AAAGGATGCAGTATGGGTTTTT	59.5	591	INTERGENIC	--	--	--
Ca5_P4_41429223	Ca5	41429223	TAGAGAGAGAG/TAGAGAGAG	AAAAACCCATACTGCATCCTTT	GATGAAAGCTCCAAAAGTTCAAA	58.9	509	INTERGENIC	--	--	--
Ca5_P4_414590	Ca5	414590	GAAAAAA/GAAAAAAA	TTGCACATGATTTTCCAAACA	CGTAAAAAGGTGCAGCAACA	60.0	517	INTERGENIC	--	--	--

INDEL marker IDs	Chromosomes/ unanchored scaffolds	Physical positions (bp)	InDels (*Kabuli* reference genome-CDC Frontier/PII)	Forward primers (5'-3')	Reverse primers (5'-3')	Anneling temperature (0C)	Expected amplified product size (bp)	Structural annotation		Functional annotation	
								Sequence components of *kabuli* genome	*Kabuli* gene accession IDs	NCBI-KOG	TFs
Ca5_P4_41460731	Ca5	41460731	ATTTTTGTTTTTTTGTTT/ATTTTTGTTT	GGTGGATCAGGAAGTTGCAT	GCAGCCAAAATCCCAAAATA	59.9	414	INTERGENIC	--	--	--
Ca5_P4_41467725	Ca5	41467725	CTT/CTTT	CTCATTTTTCCACCCAAGTGA	AGCGACAAGATGAAAGCTCC	60.0	529	INTERGENIC	--	--	--
Ca5_P4_41467977	Ca5	41467977	TTTATTATTATTATTATTATTATTATTATTATTATTATTATTATTATTATT/TTTATTATTATTATTATTATTATTATTATTATTATTATTATTATTATTATTATT	CTCATTTTTCCACCCAAGTGA	AGCGACAAGATGAAAGCTCC	60.0	529	INTERGENIC	--	--	--
Ca5_P4_41495328	Ca5	41495328	GTATAAAGTTA/GTA	CTCAGCAGTCAATCAGCTCG	CCATAAAGAACACGTACAGACGA	59.9	352	INTERGENIC	--	--	--
Ca5_P4_41509481	Ca5	41509481	GTTTTTTTTTTT/GTTTTTTTTTTTT	CCTGAAATTGCCAAACCTTC	GCCACGTGGTGTTCATACTG	59.5	710	INTERGENIC	--	--	--
Ca5_P4_41603707	Ca5	41603707	GTATATATATATATATATATATATAT/GTATATATATATATATATATATATATATATATATAT	ACGTCCATTTTCCATGTGGT	TTGCCATTTCCATCTTCCTC	60.1	444	INTERGENIC	--	--	--
Ca5_P4_41614182	Ca5	41614182	AATATATATATATATATATATAT/AATATATATATATATATATATATAT	TGTGGATTCATGATCCTTGTG	TTTAACGTCAATGCCAACTTT	59.4	916	INTERGENIC	--	--	--
Ca5_P4_41753735	Ca5	41753735	CTTTTTTTTTTTT/CTTTTTTTTTTT	CCGGGTGAGACTCATGAATAA	AACGCAAAAGCTAAGAGGCA	59.9	650	INTERGENIC	--	--	--
Ca5_P4_41795988	Ca5	41795988	ATT/AT	TGGAATGCTACACAAGCTGC	TTGAGGTGCATCGATCAAAA	60.0	317	INTERGENIC	--	--	--
Ca5_P4_4179599	Ca5	4179599	GATATATATATAT/GATATATATAT	CGGTTTAGGGATTGGGTTTT	TCGATTGAGTCTTTTGCACTT	60.0	166	INTERGENIC	--	--	--

INDEL marker IDs	Chromosomes/ unanchored scaffolds	Physical positions (bp)	InDels (*Kabuli* reference genome-CDC Frontier/PII)	Forward primers (5'-3')	Reverse primers (5'-3')	Anneling temperature (0C)	Expected amplified product size (bp)	Structural annotation		Functional annotation	
								Sequence components of *kabuli* genome	*Kabuli* gene accession IDs	NCBI-KOG	TFs
Ca5_P4_4180242	Ca5	4180242	AGGTTTAGGGTTTAGGGTTT/AGGTTTAGGGTTTAGGGTTTAGGGTTT	GGGTTTGGGGTTTAAGGTTT	TCCATTTCGTCTTTTGCACTT	59.1	321	INTERGENIC	--	--	--
Ca5_P4_41990694	Ca5	41990694	TTATATATATATATATATATATATATATATA/TTATATATATATATATATATATATATATA	AATTTTGTGGCGGATTGACT	TCAACTGATTTGACGAGAATGTG	59.4	725	INTERGENIC	--	--	--
Ca5_P4_42071548	Ca5	42071548	CATATATATATATATATATATATATATATATATAT/CATATATATATATATATATATATATATATATAT	TGAATTTTTCTGCTGCTGACTC	GGGTCGTGTGATGTTCTTTTT	59.6	361	INTERGENIC	--	--	--
Ca5_P4_42133013	Ca5	42133013	TTATATATATATATATATATATATATATATATATATATAT/TTATATATATATATATATATATATAT	GCATACGAAGGAAGCGAGTC	AAACGCCAAAAAGATAATGGA	60.0	328	INTERGENIC	--	--	--
Ca5_P4_42219742	Ca5	42219742	GTATATATATATATATATATATATATATATATATATATATATATA/GTATATATATATATATATATATATATATATATATATATATATATATA	AGAAAACAAGCGTGTGTGTG	GCAACACACAAGGAAGCAAG	57.4	734	INTERGENIC	--	--	--
Ca5_P4_42231500	Ca5	42231500	CC/CCGAGAACGTAC	TCAAAATCCACACCCCACTT	GCCATCTCCAATAGCACCAT	60.2	888	INTERGENIC	--	--	--
Ca5_P4_42363731	Ca5	42363731	CTATATATATATATATATATAT/CTATATATATATATATATATATATAT	AATTGTGATGGGAGAGGGTT	TGATGAAAAATATCTGAGGAACCA	58.3	426	INTERGENIC	--	--	--
Ca5_P4_42425779	Ca5	42425779	CATGTTTTATGTTTTA/CATGTTTTA	TCAATGGAATGGAGTCGTCA	CGACAATTTCATTATCCGTCC	60.0	749	INTERGENIC	--	--	--
Ca5_P4_42488342	Ca5	42488342	AATATATATATATATATATATATATATATATATAT/AATATATATATATATATATATATATATATAT	TTACCAAATGGCATCCAACA	CCCCCAACAAAAGTTGAAGA	59.8	687	INTERGENIC	--	--	--
Ca5_P4_42535531	Ca5	42535531	AT/A	GAAATTCACCCTCCAATCCA	TTGGCCGTAGGTAGATTTGC	59.7	269	INTERGENIC	--	--	--

INDEL marker IDs	Chromosomes/ unanchored scaffolds	Physical positions (bp)	InDels (*Kabuli* reference genome-CDC Frontier/PII)	Forward primers (5'-3')	Reverse primers (5'-3')	Anneling temperature (0C)	Expected amplified product size (bp)	Structural annotation		Functional annotation	
								Sequence components of *kabuli* genome	*Kabuli* gene accession IDs	NCBI-KOG	TFs
Ca5_P4_42557966	Ca5	42557966	TAAAAAA/TAAAAAAA	TGAAAGTGCATGTGACCGAT	CACAAACCAACGTGGAAGTG	60.1	613	INTERGENIC	--	--	--
Ca5_P4_42578018	Ca5	42578018	ATTTTTTTTTT/ATTTTTTTTT	GTTGAAACCGGTTGCGTAAT	AACACTTTTCGCTGCCAACT	59.9	451	INTERGENIC	--	--	--
Ca5_P4_42600585	Ca5	42600585	TAAAAAAAAA/TAAAAAAAA	CACCCCCATATCCCCTAGTT	TTGATTTGGTCCAACCCACT	59.9	681	INTERGENIC	--	--	--
Ca5_P4_42627539	Ca5	42627539	GAAAAA/GAAAAAAAGATAAAAAAA	CTTCCGAATCCGTACATCGT	CACCTCCGTTACGCGTATTT	60.0	417	INTERGENIC	--	--	--
Ca5_P4_4273567	Ca5	4273567	CTATATATATATATATAT/CTATATATATATATATAT	TTGGAAGGAGCCCTTACCTT	CGATTGAGGACCAAGGATGT	60.1	908	INTERGENIC	--	--	--
Ca5_P4_4275629	Ca5	4275629	GC/G	TTAGGGTTTAGGGTTTCGGG	TGACTTTTGCACTAATTGGACG	60.2	516	INTERGENIC	--	--	--
Ca5_P4_42902485	Ca5	42902485	TTATATATATATATATATAT/TTATATATATATATATAT	GTGGTTTTGCTTCGACCAGT	CATTTGCAACTATTTGTCATTGAT	60.2	326	INTERGENIC	--	--	--
Ca5_P4_42989134	Ca5	42989134	TCC/TC	GGCTTTGTTGAGTGGGGTAA	TCCCTGAGCAACTCGCTTAT	60.0	434	INTERGENIC	--	--	--
Ca5_P4_43481826	Ca5	43481826	TA/TACCTAAAATATCA	TGCAATGCTGTTATCGACTCA	CACTCCCTCCCTCTAATCTTGA	60.4	425	INTERGENIC	--	--	--
Ca5_P4_43495179	Ca5	43495179	GAAAAAAAAA/GAAAAAAAA	TGCGGCAAATCAGTTTACAA	TCCGTTTATCTTAATGTGTGCAA	60.3	502	INTERGENIC	--	--	--

INDEL marker IDs	Chromosomes/ unanchored scaffolds	Physical positions (bp)	InDels (*Kabuli* reference genome-CDC Frontier/PII)	Forward primers (5'-3')	Reverse primers (5'-3')	Anneling temperature (0C)	Expected amplified product size (bp)	Structural annotation		Functional annotation	
								Sequence components of *kabuli* genome	*Kabuli* gene accession IDs	NCBI-KOG	TFs
Ca5_P4_43517939	Ca5	43517939	AATATATATATATATATATATATA/AATATATATATATATATATATATATATATATATATA	TGAGATGTTCATTACTGCGTTG	AATTTTTGCGGCCAAGTATG	58.8	876	INTERGENIC	--	--	--
Ca5_P4_43575232	Ca5	43575232	GTAATAAATTAATAATAATAAATTAATAATAATAAATTTATAGTAATAAATTAATAATAATAAATT/GTAATAAATTAATAATAATAAATTAATAATAATAAATTAATAATAATAAATTTATAGTAATAAATTAATAATAATAAATT	TCGGAAGTTTGGTCTTCAATG	CGAAATGTAATGCTCGAAACA	60.1	338	INTERGENIC	--	--	--
Ca5_P4_43615551	Ca5	43615551	TTATATATATATATATATATATATATATAT/TTATATATATATATATATATATATATATATATATATAT	AAGAAAGGATCACCTCAAATCC	GCCTATTTCAATGTAGCCCC	58.6	456	INTERGENIC	--	--	--
Ca5_P4_43733246	Ca5	43733246	CTTTTTTT/CTTTTTTTT	TGATTTGTCGGTCTAGCTCATC	TGGAAGAAAGGGAACATGAA	59.3	290	INTERGENIC	--	--	--
Ca5_P4_43796946	Ca5	43796946	TTTATTATTATTATTATTATTATTATCATTATTATTATTATCATTATTATTATTATTATTATTATTATT/TTTATTATTATTATTATTATTATTATCATTATTATTATTATCATTATTATTATTATTATTATTATTATT	AGCCTGTACGCAAAAAGCAT	GGATAACGCGGAGGTATTGA	59.9	668	INTERGENIC	--	--	--
Ca5_P4_43798003	Ca5	43798003	AATATATATATATATATATATATGTATATATATAT/AATATATATATATATATATATATATATATATATATGTATATATATAT	ATCGACTCCCGAACTCAAAA	CTCCTATATGTGATGCGCGA	59.7	751	INTERGENIC	--	--	--
Ca5_P4_43825873	Ca5	43825873	TAATAAATAAATAAATAAATAAATAAATA/TAATAAATAAATAAATAAATAAATA	ACGCCGTACTTGATTGATGA	TCGGTACACATCGCTACAACA	59.2	227	INTERGENIC	--	--	--
Ca5_P4_43839431	Ca5	43839431	ATTTTTTTTTTT/ATTTTTTTTTTTT	TTCCGCCCTTTTAACAACAC	ATTTCAGCCGTCGCATATTT	60.0	455	INTERGENIC	--	--	--
Ca5_P4_43847991	Ca5	43847991	GT/GTGTGTGTGTCT	GGAAACTCGTTTTTGAGTGGA	TGTCAATGTGCCAAAGTATCG	59.2	679	INTERGENIC	--	--	--
Ca5_P4_4389073	Ca5	4389073	CTTTTTATTTTTTTTTATTTTTTTTT/CTTTTTATTTTTTTTTATTTTTTTTTATTTTTTTTT	TTTACCACTTGGAGTTGGGC	TCCTTTGCTAGCCCTTTTGA	60.0	392	INTERGENIC	--	--	--

**INDEL marker IDs**	**Chromosomes/ unanchored scaffolds**	**Physical positions (bp)**	**InDels (*Kabuli* reference genome-CDC Frontier/PII)**	**Forward primers (5'-3')**	**Reverse primers (5'-3')**	**Anneling temperature (0C)**	**Expected amplified product size (bp)**	**Structural annotation**		**Functional annotation**	
								**Sequence components of *kabuli* genome**	***Kabuli* gene accession IDs**	**NCBI-KOG**	**TFs**
Ca5_P4_43897317	Ca5	43897317	TTATATATATATATATAT/TTATATATATATATATATAT	GTGTCAAGGTGTCATCACGG	TGTTCAGTCCGGAATCACAAT	60.0	615	INTERGENIC	--	--	--
Ca5_P4_43900579	Ca5	43900579	TTATATATATATATATATATATATAT/TTATATATATATATATATAT	AGGCTCCCTATCAACCACAA	CACCTAATTGGGACAACAACTG	59.6	517	INTERGENIC	--	--	--
Ca5_P4_43918951	Ca5	43918951	CTTTT/CTTT	TTGCAAGGTAAAAAGCACGA	ACGATGCCCCATACAATGAT	59.5	783	INTERGENIC	--	--	--
Ca5_P4_43967867	Ca5	43967867	AATT/AATCTAATTATT	TATGCTCCGAGTTAGACGCA	CCATGCGTCGCACATAAATA	59.6	471	INTERGENIC	--	--	--
Ca5_P4_44086162	Ca5	44086162	CTTTTTTTTT/CTTTTTTTTTT	CGGGTCAACTTTGATAGACGA	GATGTGCCGTGAAAACAATG	60.1	594	INTERGENIC	--	--	--
Ca5_P4_44171535	Ca5	44171535	TATAATAATAATAATAATAATAATAATAATAATAATAATAATAATAATAATAATAA/TATAATAATAATAATAATAATAATAATAATAATAATAATAATAATAATAATAATAATAATAATAATAATAATAATAATAA TAATAA	ATGAACTTGCCGTTTTGGAC	TCCCCCATTAATAATTCAGTGATAA	60.0	776	INTERGENIC	--	--	--
Ca5_P4_44177714	Ca5	44177714	GTATATATATATATATAT/GTATATATATATATATATAT	GGTCACGAACATCCCAAAAT	CCATGGTGAGAGGGTCAAAT	59.7	377	INTERGENIC	--	--	--
Ca5_P4_44215833	Ca5	44215833	TAAAAAAAAAA/TAAAAAAAAA	TTGCTTTGGTTTGATGTGGA	GGGGGAAAACAAGGAAGAAG	60.1	788	INTERGENIC	--	--	--
Ca5_P4_445610	Ca5	445610	GCCC/GCCCC	ACAGTGGCTTCAATTCCCAC	CCAGCCAAATAGCTGGAATG	60.0	336	INTERGENIC	--	--	--
Ca5_P4_44596809	Ca5	44596809	ATT/ATTT	TTGGCACATGCATTTTTGTT	GACCAAATCTTGTTGTTGCAGA	60.0	582	INTERGENIC	--	--	--

INDEL marker IDs	Chromosomes/ unanchored scaffolds	Physical positions (bp)	InDels (*Kabuli* reference genome-CDC Frontier/PII)	Forward primers (5'-3')	Reverse primers (5'-3')	Anneling temperature (0C)	Expected amplified product size (bp)	Structural annotation		Functional annotation	
								Sequence components of *kabuli* genome	*Kabuli* gene accession IDs	NCBI-KOG	TFs
Ca5_P4_44597112	Ca5	44597112	CTATA/CTATATATA	GGGGTTAGTGCAAGCCAAAT	GTTCGCACGTGATGAACAAT	61.2	862	INTERGENIC	--	--	--
Ca5_P4_44729729	Ca5	44729729	CATATATATATATATATATATATATATATATATATATATAT/CATATATATATATATATATATATATAT	GGGGATTTGGAGGGTAACAT	GTGTTGGAACGGGTTTTGAT	59.9	663	INTERGENIC	--	--	--
Ca5_P4_44783564	Ca5	44783564	ATTTTTTTTTT/ATTTTTTTTTTT	ATTCACGGTAGCGGATCAAC	AATCCGTGGAACCAATTTTT	60.0	487	INTERGENIC	--	--	--
Ca5_P4_44784631	Ca5	44784631	TTTTCTGTCTA/T	TCTGCCACGTTATTTCATCG	GAGGCGTCGGTGTTGTTATT	59.7	293	INTERGENIC	--	--	--
Ca5_P4_44844449	Ca5	44844449	A/AGCCTGG	GGCTACCGAATATGGAACGA	ACCGACTGTTGCTGGTTTTC	59.9	520	INTERGENIC	--	--	--
Ca5_P4_44911043	Ca5	44911043	CAAAAAAAAAAA/CAAAAAAAAAA	ACATCACCTTTTGCTGACCC	CGCAATCTTGTTCCTGGTTT	60.0	409	INTERGENIC	--	--	--
Ca5_P4_44914869	Ca5	44914869	TTATATATATATATATATATATATATATA/TTATATATATATATATATATATATATATATA	TTTCGAGCAATCTCATCCCT	CGGCAGCTTTTTACTACCCA	59.8	759	INTERGENIC	--	--	--
Ca5_P4_45086826	Ca5	45086826	TA/TAA	CAAACGCAAAATTAAACACACAA	AATACAGCTGGGTTTGTGGC	60.0	367	INTERGENIC	--	--	--
Ca5_P4_45093671	Ca5	45093671	AATATATATATATATATATATATATATATATATATATATA/AATATATATATATATATATATATATATATATATATATATATATA	AAAGGAGAAGAAAGGTCCAAGG	AAACACATGCGCTTTCATTG	60.1	727	INTERGENIC	--	--	--
Ca5_P4_45094437	Ca5	45094437	TG/T	AATCCATCCGAACCCTTTTC	TTGTCAATGTTGCATGTCTGAA	60.1	259	INTERGENIC	--	--	--

INDEL marker IDs	Chromosomes/ unanchored scaffolds	Physical positions (bp)	InDels (*Kabuli* reference genome-CDC Frontier/PII)	Forward primers (5'-3')	Reverse primers (5'-3')	Anneling temperature (0C)	Expected amplified product size (bp)	Structural annotation		Functional annotation	
								Sequence components of *kabuli* genome	*Kabuli* gene accession IDs	NCBI-KOG	TFs
Ca5_P4_45097547	Ca5	45097547	ACTTTGAAACCTTTGAAAC/ACTTTGAAAC	CCGCTCTTTCGAAACATACA	AAAACCCCGGATTTGGATAG	58.9	591	INTERGENIC	--	--	--
Ca5_P4_45098220	Ca5	45098220	ATTTTTT/ATTTTTTT	ATGTTCGAAACGCTGATTCC	TTGCCAAGCCTTTTGAAACT	60.1	121	INTERGENIC	--	--	--
Ca5_P4_45277984	Ca5	45277984	ATTAGT/ATTAGTTAGT	TGTTGCGTTAAATGAGTTGGA	ATATTAGCGCGACCCAAAAA	59.2	285	INTERGENIC	--	--	--
Ca5_P4_45309218	Ca5	45309218	AATATATATATATATATAT/AATATATATATATATAT	GTCGTTGCCTTTTGTGGTTT	GCAAATTGACTACGGTCGGT	60.0	644	INTERGENIC	--	--	--
Ca5_P4_45321319	Ca5	45321319	TTATATATATATATATATATATATAT/TTATATATATATATATATATATATATATATATAT	TGGTATCCCTATTTTAACCGATTT	TACGGAAGAACCGACAAGTG	59.1	902	INTERGENIC	--	--	--
Ca5_P4_45371143	Ca5	45371143	CTTTTTTTTTTTTT/CTTTTTTTTTTTTT	TTGAACGTGGACACCCATAA	GTTGTGCATGTACCTTCGCT	59.8	574	INTERGENIC	--	--	--
Ca5_P4_45372527	Ca5	45372527	TTTATTATTATTATTATTATTATTATTATTATTATTATTATTATTATTATTATTA/TTTATTATTATTATTATTATTATTATTATTATTATTATTATTATTATTATTATTATTATTATTATTATTA	CCCAGTGTCACCGTCTTTTT	TTCACACAAATGTCCACCAA	60.0	537	INTERGENIC	--	--	--
Ca5_P4_45557067	Ca5	45557067	CATATATATATATATATATATATATATAT/CATATATATATATATATATAT	CAATGAAAAAGTGTGGCCCT	ACGTTGATGGAACAATGCCT	60.0	873	INTERGENIC	--	--	--
Ca5_P4_456196	Ca5	456196	CTTTTTTTT/CTTTTTTTTTTT	TGCCTACAACCACACATGCT	CGTTTTCCGATTCATGCTTT	60.2	685	INTERGENIC	--	--	--
Ca5_P4_456689	Ca5	456689	GGAAAACGAAAACG/GGAAAACG	TGTTGAGTTTTTGCACAGGC	TGAAAGGAGTCGGTTTTGTG	59.9	257	INTERGENIC	--	--	--

INDEL marker IDs	Chromosomes/ unanchored scaffolds	Physical positions (bp)	InDels (*Kabuli* reference genome-CDC Frontier/PII)	Forward primers (5'-3')	Reverse primers (5'-3')	Anneling temperature (0C)	Expected amplified product size (bp)	Structural annotation		Functional annotation	
								Sequence components of *kabuli* genome	*Kabuli* gene accession IDs	NCBI-KOG	TFs
Ca5_P4_45724530	Ca5	45724530	AATTATTATTATTATTATTATTATTATTATTATTAT/AATTATTATTATTATTATTATTATTATTATTATTATTAT	GCGGTTGAATAAATGTGACAAA	TAGTTGGAGGTTGCTGACCC	59.9	616	INTERGENIC	--	--	--
Ca5_P4_45792229	Ca5	45792229	TAAAA/TAAAAA	TCTCCATTTACGTTTGTGTTTTT	TTTTGTACCAAGTCCTCCGC	57.8	386	INTERGENIC	--	--	--
Ca5_P4_45795356	Ca5	45795356	GTTTTTTTTTT/GTTTTTTTTTTT	ACGGCTTTGGAAATGATGAA	CCATCGATTTCACTGTGTCA	60.4	404	INTERGENIC	--	--	--
Ca5_P4_45967395	Ca5	45967395	GTATATATATATATATATATAT/GTATATATATATATATATATATAT	TTTGATGCTTTGTCTTTGCG	TTCTTTCGGTGTCCATGTCA	60.0	479	INTERGENIC	--	--	--
Ca5_P4_46005130	Ca5	46005130	AATATATATATATATATA/AATATATATATATATA	AGTCGCCATTTGGAATTACG	AACAATCCTGAAATGCGGAG	60.0	335	INTERGENIC	--	--	--
Ca5_P4_46086372	Ca5	46086372	ATTTTTTTTT/ATTTTTTTTTT	GGCATTTATTGTGAAACGGG	TCCTTGTCTTCTTGCCCAAT	60.2	726	INTERGENIC	--	--	--
Ca5_P4_46091748	Ca5	46091748	CTTTTTTTTTT/CTTTTTTTTTTT	ATTCGTCACCACACACCAGA	CTAGCTGTGCAACATCGCAT	60.0	550	INTERGENIC	--	--	--
Ca5_P4_46180569	Ca5	46180569	A/ATAATAATAATAAT	AGCGGCTGCTGTGTTTAGTT	CCAAATCGAAGAAGAACAAGG	60.1	614	INTERGENIC	--	--	--
Ca5_P4_46208612	Ca5	46208612	AAT/AATAT	ATGCAGCACAAAATGCAAGT	TTCGAATCTTATTTTGACAATTTTT	59.4	502	INTERGENIC	--	--	--
Ca5_P4_46390107	Ca5	46390107	GAAAAAAAAAAAA/GAAAAAAAAAAA	GCAAGAATAGCACAAACAAGCA	ACGTCGCGCAACTGTGATA	60.4	239	INTERGENIC	--	--	--

INDEL marker IDs	Chromosomes/ unanchored scaffolds	Physical positions (bp)	InDels (*Kabuli* reference genome-CDC Frontier/PII)	Forward primers (5'-3')	Reverse primers (5'-3')	Anneling temperature (0C)	Expected amplified product size (bp)	Structural annotation		Functional annotation	
								Sequence components of *kabuli* genome	*Kabuli* gene accession IDs	NCBI-KOG	TFs
Ca5_P4_46393868	Ca5	46393868	AG/AGTGATG	ACAGTTCAAAGTCCCTGGCA	TCAATCATTTAAAATTACTCTGAATCG	60.7	561	INTERGENIC	--	--	--
Ca5_P4_464166	Ca5	464166	ATGT/AT	TCTTCAACGCTGAAATTGAAA	AACTTGTGTATTAATGTTTGCTGC	58.5	716	INTERGENIC	--	--	--
Ca5_P4_46429100	Ca5	46429100	GAAAAAAAAAAA/GAAAAAAAAAA	TGCTCACGTTCTTTTGTTGG	TTGGTCTTGCTTCTTTCAAATG	59.9	501	INTERGENIC	--	--	--
Ca5_P4_464299	Ca5	464299	TAAAAAAAA/TAAAAAAA	TCAAAGTGTTTGAATTTGGTCA	AACTTGTGTATTAATGTTTGCTGC	58.2	290	INTERGENIC	--	--	--
Ca5_P4_46552360	Ca5	46552360	TTTATTATTATTATTATTATTATTATTATTATTATTATTA/TTTATTATTATTATTATTATTATTATTATTA	CGAATAGCATGTCACGTTTG	TGGGTGGCTATCAAAGTTGC	57.8	521	INTERGENIC	--	--	--
Ca5_P4_46561532	Ca5	46561532	TTATATATATATATATATATATATA/TTATATATATATATATATATATATATATATATATATA	TAGACTGAGGTCCACCATGC	TGCAATCAATTTTTACTCTTCATCC	58.7	506	INTERGENIC	--	--	--
Ca5_P4_46661039	Ca5	46661039	AATATATATATATATATATATAT/AATATATATATATATATATATATAT	TGGAATTCAAGGGACCAAAA	ACTTGCGAGATAATTTAGTAGGGA	60.3	863	INTERGENIC	--	--	--
Ca5_P4_46676212	Ca5	46676212	ATTTTTTTTTT/ATTTTTTTTTTT	GCCACCACACCTCCAACTAT	TTGAGATGGGACTTTGGAGC	59.9	880	INTERGENIC	--	--	--
Ca5_P4_46879029	Ca5	46879029	ATTTTTTTT/ATTTTTTTTT	GGGGTCAATCGACAAGAGAA	CGGTTGTCACGTCAGAAAAA	60.1	663	INTERGENIC	--	--	--
Ca5_P4_46886435	Ca5	46886435	TA/TTAACTATACTCAAACA	CAGCCATGCTCGTATGCTAA	AGAACCCCATCCAAACAATG	60.0	444	INTERGENIC	--	--	--

INDEL marker IDs	Chromosomes/ unanchored scaffolds	Physical positions (bp)	InDels (*Kabuli* reference genome-CDC Frontier/PII)	Forward primers (5'-3')	Reverse primers (5'-3')	Anneling temperature (0C)	Expected amplified product size (bp)	Structural annotation		Functional annotation	
								Sequence components of *kabuli* genome	*Kabuli* gene accession IDs	NCBI-KOG	TFs
Ca5_P4_46910930	Ca5	46910930	CATATATATATATATA/CATATATATATATA	TTCATTGCGAGCCCTACTCT	TGCGCTGTAATAGGTGCATT	60.0	477	INTERGENIC	--	--	--
Ca5_P4_46985054	Ca5	46985054	AATTATTATTATTATTATTATTATTATTATTATTATTATTATT/AATTATTATTATTATTATTATTATTATTATTATTATTATTATTATT	TCGCATTTTAAGGGAGGAAA	AGCAAGGAAGCATCATCCAA	59.7	212	INTERGENIC	--	--	--
Ca5_P4_47133305	Ca5	47133305	ATTTTTTTTTT/ATTTTTTTTTTT	TAATGATATCCACCCCGCAT	AGGACCACCTGCTGAAACAT	60.0	935	INTERGENIC	--	--	--
Ca5_P4_47138535	Ca5	47138535	CTATTATTATTATTATTATTATTATTA/CTATTATTATTATTATTATTATTATTATTATTA	TGAATCTTTCCGCATTTGTG	CCTGAAAGACTTAGACGGCG	59.7	651	INTERGENIC	--	--	--
Ca5_P4_47213382	Ca5	47213382	CAAAAAAAAA/CAAAAAAAAAA	GGATGTTGCACAATCAAGGA	GCTCATGCACTTGTCTCCAA	59.5	507	INTERGENIC	--	--	--
Ca5_P4_47226447	Ca5	47226447	TTTATATTATATTATATTATAT/TTTATATTATATTATATTATATTATAT	ACCGTTACCTTTTGTGGCTG	GAAGCTTTCACGTACGGAGC	60.0	449	INTERGENIC	--	--	--
Ca5_P4_47478750	Ca5	47478750	AATATATATATATATATATATATATATATATAT/AATATATATATATATATATATATATATATATATATATAT	TGGGATGTGTCTAACGTTTGTT	GTAAGAGTTGGTGCCGATCC	59.4	983	INTERGENIC	--	--	--
Ca5_P4_47512183	Ca5	47512183	GTTTTTTTTTT/GTTTTTTTTTTT	TGGCATATTCCCATCCCATA	TGTGGGTATAAATGAGGGGC	60.9	404	INTERGENIC	--	--	--
Ca5_P4_47676757	Ca5	47676757	GAAAAAAA/GAAAAAA	GACCAAAACTGCACTTAAGCC	CTTCCCCATCTCTCTCACCA	58.9	665	INTERGENIC	--	--	--
Ca5_P4_47677095	Ca5	47677095	TTATATATATATATATA/TTATATATATATATA	TGGTGAGAGAGATGGGGAAG	TTGAATTATTGGAGGTTGACACA	60.2	496	INTERGENIC	--	--	--

INDEL marker IDs	Chromosomes/ unanchored scaffolds	Physical positions (bp)	InDels (*Kabuli* reference genome-CDC Frontier/PII)	Forward primers (5'-3')	Reverse primers (5'-3')	Anneling temperature (0C)	Expected amplified product size (bp)	Structural annotation		Functional annotation	
								Sequence components of *kabuli* genome	*Kabuli* gene accession IDs	NCBI-KOG	TFs
Ca5_P4_47683586	Ca5	47683586	TAAA/TAA	AAACACGTTGCCATTTGTGA	GAGCCTTTGCATTTTGTGGT	60.0	965	INTERGENIC	--	--	--
Ca5_P4_47808500	Ca5	47808500	AATATATATATATATATATATATATATATATATATATA/AATATATATATATATATATATATATATATATATATATATA	CCACTCAACAATGATCGGAA	AAACTCCCAAGGAGTCCCAC	59.5	785	INTERGENIC	--	--	--
Ca5_P4_47837680	Ca5	47837680	AATATATATATATATATATATA/AATATATATATATATATA	ATCAACAAAAGTGGGGAACG	GCTGGGCCTACAATGAGGTA	59.8	844	INTERGENIC	--	--	--
Ca5_P4_47945458	Ca5	47945458	TATAA/T	ACATCAAAGGCGGATCCATA	TCCTCGCAACACACACATTT	60.3	392	INTERGENIC	--	--	--
Ca5_P4_47972846	Ca5	47972846	AA/AATGA	CGCGAATCCACCTATCAAAA	TTGCTAAGTCTGCCGAACCT	61.0	589	INTERGENIC	--	--	--
Ca5_P4_47998896	Ca5	47998896	TTATATATATATATATATATATATATATATATATATATATAT/TTATATATATATATATATATATATATATATATATATATATATATATATATAT	ATGCGACTCTTCGCAACTTT	TCAAAATTTTCAAACAAAAACCA	60.0	705	INTERGENIC	--	--	--
Ca5_P4_48069008	Ca5	48069008	GNNNNNNNNNNNNNNN/GNNNNNNN	GCAAAACTCACATCCAACTCC	TTGTCACACAAGCATTCGGT	59.6	448	INTERGENIC	--	--	--
Ca5_P4_48121332	Ca5	48121332	TTTTATTTATTTATTTATTTATTTATTTATTTAT/TTTTATTTATTTATTTATTTATTTATTTAT	ATTTTATGGCCAACCCAACA	ACTCGGCTTCCATTGATGAG	60.1	178	INTERGENIC	--	--	--
Ca5_P4_48122737	Ca5	48122737	CTTTTTTTT/CTTTTTTT	ATGTGACGGTTAGCCTGGAC	TAGCAAAAGGTCGCCTCCTA	60.0	561	INTERGENIC	--	--	--
Ca5_P4_48123223	Ca5	48123223	GATAATAATAATAATAATAATAATAATAATAATAATAAT/GATAATAATAATAATAATAATAATAATAATAATAAT	TAGGAGGCGACCTTTTGCTA	GACCAAAGTGCAATTAAGCCA	60.0	735	INTERGENIC	--	--	--

INDEL marker IDs	Chromosomes/ unanchored scaffolds	Physical positions (bp)	InDels (*Kabuli* reference genome-CDC Frontier/PII)	Forward primers (5'-3')	Reverse primers (5'-3')	Anneling temperature (0C)	Expected amplified product size (bp)	Structural annotation		Functional annotation	
								Sequence components of *kabuli* genome	*Kabuli* gene accession IDs	NCBI-KOG	TFs
Ca5_P4_48164734	Ca5	48164734	AGG/AG	GGTTGCAATTATGGGAAATGTT	AACTCTAAATCCCCGCAAGG	60.0	485	INTERGENIC	--	--	--
Ca5_P4_4829908	Ca5	4829908	AATATATATATATATATATATATATAT/AATATATATATATATATATATATATATATAT	GTTGGTTCCCTAGCACCGTA	TGGATATTGAACTCGGAGGC	60.0	608	INTERGENIC	--	--	--
Ca5_P4_4857245	Ca5	4857245	GAAAA/GAAAAA	CCCACATTTCTTTGGCAGTT	TGTGCCTAAACTTGCTGGTG	60.0	501	INTERGENIC	--	--	--
Ca5_P4_4870580	Ca5	4870580	GG/GGGTCAGTAATG	CCAATCCTCCTTTCACCTTC	ACGGTCACCACTTTCTTTGG	58.6	653	INTERGENIC	--	--	--
Ca5_P4_492638	Ca5	492638	AT/ATAGCAAGTTT	ATTCTTGACCCGAAATGTGG	TCATTTTTGGCATCTAGGGC	59.8	693	INTERGENIC	--	--	--
Ca5_P4_4970490	Ca5	4970490	T/TG	ATTCCTACGCGATTACCACG	CCCGTGATAGGCAGTACGTT	60.0	528	INTERGENIC	--	--	--
Ca5_P4_497099	Ca5	497099	GAAAA/GAAA	TTGAGTGCAGCTGGAGAAGA	TCACTCAAAAACCAAGCACG	59.9	110	INTERGENIC	--	--	--
Ca5_P4_5013490	Ca5	5013490	TATAAATAAATAAATAAATAAATAAATAAA/TATAAATAAATAAATAAATAAATAAATAAATAAATAAA	GAGGGATGTCATTCATTTTCG	TGTGATCATTGGGGAACTCA	59.4	515	INTERGENIC	--	--	--
Ca5_P4_5038314	Ca5	5038314	AAATAATAATAATAATAATAATAATAATAATAATAATAATAATAATAATAA/AAATAATAATAATAATAATAATAATAATAATAATAATAATAATAATAATAATAATAATAA	ATTTGTGGTGTTGTGCTGGA	CATCAAACTCAAGTGTCAAAACAA	60.0	869	INTERGENIC	--	--	--
Ca5_P4_5038531	Ca5	5038531	TTTATTATTATTATTATTATTATTATTATTATTATTATTATT/TTTATTATTATTATTATTATTATTATTATTATTATTATTATTATTATTATTATTATTATT	TGTGGTTGAGTAGTTGTGCCA	TGCGGTTGATTAGTTGTGTCA	60.2	664	INTERGENIC	--	--	--

INDEL marker IDs	Chromosomes/ unanchored scaffolds	Physical positions (bp)	InDels (*Kabuli* reference genome-CDC Frontier/PII)	Forward primers (5'-3')	Reverse primers (5'-3')	Anneling temperature (0C)	Expected amplified product size (bp)	Structural annotation		Functional annotation	
								Sequence components of ***kabuli*** genome	***Kabuli*** gene accession IDs	NCBI-KOG	TFs
Ca5_P4_5190851	Ca5	5190851	CTATATATATATATATATATATATAT/CTATATATATATATATATATATATAT	AGCGCTATGTGACCCTTTTG	AATGTGAATCCCACCAGGAA	60.3	555	INTERGENIC	--	--	--
Ca5_P4_5318962	Ca5	5318962	TC/T	ACGTTGACCAATTCAGCTCC	CACTAGGGACCCTTTGGTGA	60.1	720	INTERGENIC	--	--	--
Ca5_P4_5408571	Ca5	5408571	TTATATATATATATATATATA/TTATATATATATATATATATATA	CCATTTTCCCTCGCTCATAA	CGTTGGTCATGATCCTTCCT	60.0	680	INTERGENIC	--	--	--
Ca5_P4_5410568	Ca5	5410568	A/ACC	TGCTTTGACTCGCACCATAG	TCTTCATCAATGAGCGTTGC	60.0	704	INTERGENIC	--	--	--
Ca5_P4_5492827	Ca5	5492827	TTTATTATTATTATTATTATTATTATTATTATTATTATTATTATTATTATTATTATTATTATTATTATTATTATTATTAT/TTTATTATTATTATTATTATTATTATTATTATTATTATTATTATTATTATTATTATTATTATTATTATTAT	TATCCCCTCGATTTGCTTCA	TTTTGCCTCAAAAAGTTTCACA	60.5	603	INTERGENIC	--	--	--
Ca5_P4_5618412	Ca5	5618412	ACCCC/ACC	TTTGTCGTGGGATGAACTGA	TGGGTCCATGTTCCATTTTT	60.1	605	INTERGENIC	--	--	--
Ca5_P4_5705191	Ca5	5705191	GA/G	AATCGATTGGCTTTGATTCG	CTCTCATCTGAGGGCAAAGC	60.0	412	INTERGENIC	--	--	--
Ca5_P4_5798731	Ca5	5798731	TTATATATATATATATATATATATA/TTATATATATATATATATATATATATATATATA	CTTCGACACTGTAGCACCCA	ATATTTCTGCGCATTCTCGC	59.9	217	INTERGENIC	--	--	--
Ca5_P4_5836817	Ca5	5836817	TACCCTAAACC/TACCCTAAACCCTAAACCCTAAACC	GTATGAGGAGGATCTTGCGG	TCAAAATGCACCATAAACCG	59.7	594	INTERGENIC	--	--	--
Ca5_P4_5837067	Ca5	5837067	ACCTAAACCCTAAACCCTAAACCCTAA/ACCTAAACCCTAAACCCTAA	AAAGAAAAACACTCGCGCTG	AGCGTTTTGGGTTTAGGGTT	60.6	825	INTERGENIC	--	--	--

INDEL marker IDs	Chromosomes/ unanchored scaffolds	Physical positions (bp)	InDels (*Kabuli* reference genome-CDC Frontier/PII)	Forward primers (5'-3')	Reverse primers (5'-3')	Anneling temperature (0C)	Expected amplified product size (bp)	Structural annotation		Functional annotation	
								Sequence components of *kabuli* genome	*Kabuli* gene accession IDs	NCBI-KOG	TFs
Ca5_P4_5888717	Ca5	5888717	AATATATATATATATATATATATATATATA/AATATATATATATATATATATATATATATATATATA	TCTTGAGCTACTGGGGCTTG	CAAGTTTGAAATGGAGGGGA	60.5	882	INTERGENIC	--	--	--
Ca5_P4_5906369	Ca5	5906369	TA/TACTCA	AACATCATGCTTGGGACAAA	TCTTGTGGATGACAATTATTTCG	59.0	271	INTERGENIC	--	--	--
Ca5_P4_5912049	Ca5	5912049	TAAATATACATCAAATATA/TAAATATACATCAAATATACATCAAATATA	TTTTTGTTGTCTCACTCGATCA	CAGTGTTAATTGGCACGAAA	58.4	276	INTERGENIC	--	--	--
Ca5_P4_5969929	Ca5	5969929	CG/C	GAGGACGAAAATGACCCAAA	TGCATCCGATACGTGTGTTT	59.9	853	INTERGENIC	--	--	--
Ca5_P4_6074415	Ca5	6074415	ATATTATT/ATATT	AGTCAGACGCTACTCGGCAT	AGCATGCATCTGCACACAAT	60.0	625	INTERGENIC	--	--	--
Ca5_P4_6199138	Ca5	6199138	TTTATTATTATTATTATTAT/TTTATTATTATTATTATTATTATTATTAT	GCATTCCCGTATGCGACTAT	GGCACATGTTATTTGTTTTGG	60.0	511	INTERGENIC	--	--	--
Ca5_P4_6200121	Ca5	6200121	CT/CTT	TTCGCAACTCCACTCTTCCT	GATCCATTTTCCCCCAAATC	60.0	342	INTERGENIC	--	--	--
Ca5_P4_6200571	Ca5	6200571	AT/ACT	GGTAAGGGCTCCTTCCAAAC	CCCCTCCTTAACGTCCAGTT	59.9	990	INTERGENIC	--	--	--
Ca5_P4_6227147	Ca5	6227147	ATT/ATTT	GGTTTAAACTCCACCGGGAT	CGGTAACCGGAAAAAGTCAA	60.1	408	INTERGENIC	--	--	--
Ca5_P4_6369660	Ca5	6369660	TC/TCAC	GAGGAAACACGGTGAATGGT	CGCTCACTAGTCGAACGAGA	59.8	486	INTERGENIC	--	--	--

INDEL marker IDs	Chromosomes/ unanchored scaffolds	Physical positions (bp)	InDels (*Kabuli* reference genome-CDC Frontier/PII)	Forward primers (5'-3')	Reverse primers (5'-3')	Anneling temperature (0C)	Expected amplified product size (bp)	Structural annotation		Functional annotation	
								Sequence components of *kabuli* genome	*Kabuli* gene accession IDs	NCBI-KOG	TFs
Ca5_P4_64660	Ca5	64660	CAAAAAA/CAAAA	TGGATCATCAAAGATCAACGG	ATTGAAAGTTGCGGGGACTA	60.8	438	INTERGENIC	--	--	--
Ca5_P4_6510151	Ca5	6510151	CTTTTTTTTT/CTTTTTTTTTT	TCGCGTTCATGATTTTGTGT	CGGAGTGATAATTGGTGCAA	60.1	816	INTERGENIC	--	--	--
Ca5_P4_6532288	Ca5	6532288	AAAAAGAACAAAA/AAAAA	TGTCTTCAACTTCTGCGGTG	TTGCAACGTGTCATTGTCCT	60.0	831	INTERGENIC	--	--	--
Ca5_P4_655741	Ca5	655741	CA/C	AGGTCATTTTCGTCCTCCCT	TTGGATGCACACGATTTGTT	59.9	767	INTERGENIC	--	--	--
Ca5_P4_6663718	Ca5	6663718	CAA/CAAA	AGGGAGGACGAAAATGACCT	TAACCAATCTTGTGCACCCA	59.9	171	INTERGENIC	--	--	--
Ca5_P4_6754121	Ca5	6754121	TA/T	AAAGAGGCAAAGCTGATGGA	CACGTGTCGTCTTCTGCTTC	60.0	546	INTERGENIC	--	--	--
Ca5_P4_6763101	Ca5	6763101	AGGG/AGGGG	CAAATGTCGGGAGCTACCAT	GGATCCCCAATCGATAACCT	60.0	437	INTERGENIC	--	--	--
Ca5_P4_6763849	Ca5	6763849	AATATATATATATATATATA/AATATATATATATATATA	TGGGTGGGAGTAATTCTTCG	GAGTTGCGGCGAGAGTAGAT	59.9	484	INTERGENIC	--	--	--
Ca5_P4_6765484	Ca5	6765484	CATATATATATATATATATATATAT/CATATATATATATATATATATATATAT	CGAAGCTGCCAGGTAAAAAG	TGAGTCCATTCTCCAAACCA	60.0	652	INTERGENIC	--	--	--
Ca5_P4_6772280	Ca5	6772280	CTTTTTTTTTTTT/CTTTTTTTTTT	CCAATGGCTTCGACATTTCT	AAGTCACCCCTCGTTGTTTG	60.1	726	INTERGENIC	--	--	--

INDEL marker IDs	Chromosomes/ unanchored scaffolds	Physical positions (bp)	InDels (*Kabuli* reference genome-CDC Frontier/PII)	Forward primers (5'-3')	Reverse primers (5'-3')	Anneling temperature (0C)	Expected amplified product size (bp)	Structural annotation		Functional annotation	
								Sequence components of *kabuli* genome	*Kabuli* gene accession IDs	NCBI-KOG	TFs
Ca5_P4_6772456	Ca5	6772456	GCACACA/GCACA	TGCACAACAAAAGCACAACA	TGTACCATCATAGTGGCGGA	59.9	512	INTERGENIC	--	--	--
Ca5_P4_6832771	Ca5	6832771	ATTA/ATTATATGATTA	TGCTAGGAACCGAGCCTTTA	CGTGTGCGTTTGTAATTGATG	60.0	217	INTERGENIC	--	--	--
Ca5_P4_6833175	Ca5	6833175	TT/TTATTATTAT	GAGAGCTCACAGCCTTGTCC	TTGGCATCGTGATTTTATGC	60.1	873	INTERGENIC	--	--	--
Ca5_P4_6833700	Ca5	6833700	ATATT/AT	GCATAAAATCACGATGCCAA	CGATTTTTCGTCTACATGTGC	59.5	211	INTERGENIC	--	--	--
Ca5_P4_6833833	Ca5	6833833	AATA/AATATA	AAATCACGATGCCAAAGAAAA	AAGAAAGTTGTAAATGTGTTTTATGGA	59.6	616	INTERGENIC	--	--	--
Ca5_P4_6833924	Ca5	6833924	ATTT/ATT	AAATCACGATGCCAAAGAAAA	AAGAAAGTTGTAAATGTGTTTTATGGA	59.6	616	INTERGENIC	--	--	--
Ca5_P4_6834263	Ca5	6834263	CT/C	GCTTAATCCATAAAACACATTTACAA	TTCTCATGCGCTTGACTAATG	57.9	526	INTERGENIC	--	--	--
Ca5_P4_6834706	Ca5	6834706	TAAAAAA/TAAAAAAA	TGCATAAAATCACCATGCCA	TTGTACGTTTCTTTTCTATGATTTGC	60.9	360	INTERGENIC	--	--	--
Ca5_P4_6834978	Ca5	6834978	ATATTATTATTATTATTATTATT/ATATTATTATTATTATTATTATTATT	TAGTCAAGCGCATGAGAAAA	GGCATGATGATTTTATGCATATTT	57.3	748	INTERGENIC	--	--	--
Ca5_P4_6837967	Ca5	6837967	TAAAAAAAAAAAA/TAAAAAAAAA	TCGAGAACAACTCGTCTCGTG	AATTTGCAGTGTCGCAAAAA	61.6	254	INTERGENIC	--	--	--

INDEL marker IDs	Chromosomes/ unanchored scaffolds	Physical positions (bp)	InDels (*Kabuli* reference genome-CDC Frontier/PII)	Forward primers (5'-3')	Reverse primers (5'-3')	Anneling temperature (0C)	Expected amplified product size (bp)	Structural annotation		Functional annotation	
								Sequence components of *kabuli* genome	*Kabuli* gene accession IDs	NCBI-KOG	TFs
Ca5_P4_6872599	Ca5	6872599	ATTATTTATTTATT/ATTATTTATTTATTTATT	CGGCCATCACACAATGATAA	TCAGGATGTCACCACGAAGA	60.3	454	INTERGENIC	--	--	--
Ca5_P4_6906256	Ca5	6906256	AT/A	CATCGGAATGAACGAGTGTG	TTTAGCGTGTCAAACCAACG	60.1	471	INTERGENIC	--	--	--
Ca5_P4_6907615	Ca5	6907615	A/AGTG	GGTTGTGTTATCCCTCGACA	AGGGTGCCACATTTACTCCA	58.4	539	INTERGENIC	--	--	--
Ca5_P4_6907876	Ca5	6907876	GA/GAATA	GGTTGTGTTATCCCTCGACA	AGGGTGCCACATTTACTCCA	58.4	539	INTERGENIC	--	--	--
Ca5_P4_6909501	Ca5	6909501	TATATATAATTTAAATATATAATTTAAAT/TATATATAATTTAAAT	AATGATGGAGGGAGTGAAGC	AACTGTCCATTTGTGAGGGC	59.1	405	INTERGENIC	--	--	--
Ca5_P4_6909628	Ca5	6909628	ATTTTTTT/ATTTTTT	AATGATGGAGGGAGTGAAGC	AACTGTCCATTTGTGAGGGC	59.1	405	INTERGENIC	--	--	--
Ca5_P4_6909656	Ca5	6909656	TAAA/TAA	AATGATGGAGGGAGTGAAGC	AACTGTCCATTTGTGAGGGC	59.1	405	INTERGENIC	--	--	--
Ca5_P4_6909949	Ca5	6909949	CAAAGA/CA	CCTAAGCCCTCACAAATGGA	TAAAGGGAGAAAAGCAGCCA	60.1	304	INTERGENIC	--	--	--
Ca5_P4_6910554	Ca5	6910554	A/AC	CTTTCAGGAACCAACAACCA	CCAAATTACCAAACGCCAAC	58.6	328	INTERGENIC	--	--	--
Ca5_P4_7019330	Ca5	7019330	T/TGCAC	GCTCACTTATCCCGCCAATA	TGGCTATGAGTGGTTCCCTT	60.1	996	INTERGENIC	--	--	--

INDEL marker IDs	Chromosomes/ unanchored scaffolds	Physical positions (bp)	InDels (*Kabuli* reference genome-CDC Frontier/PII)	Forward primers (5'-3')	Reverse primers (5'-3')	Anneling temperature (0C)	Expected amplified product size (bp)	Structural annotation		Functional annotation	
								Sequence components of *kabuli* genome	*Kabuli* gene accession IDs	NCBI-KOG	TFs
Ca5_P4_7092837	Ca5	7092837	AT/A	TCCCAGTAACTAGCCGTTGG	GGGCCATATCAACAATTTGG	60.1	221	INTERGENIC	--	--	--
Ca5_P4_7100474	Ca5	7100474	ACACCACCACCACCACCACCACCACCACCAC/ACACCACCACCACCACCACCAC	TGGACATTCCTAGATTCAGCC	TCATCAATCCGACACCTTCA	59.1	299	INTERGENIC	--	--	--
Ca5_P4_7107813	Ca5	7107813	G/GA	TGTCTCGAGTTCTATCCCGAA	TCACACACTTTCTTCCAACCTTT	59.8	569	INTERGENIC	--	--	--
Ca5_P4_7116888	Ca5	7116888	CAAAAAAA/CAAAAAA	TTAACAACTCCTGGTTCGGG	GTGGACTGTCGGTTCGATTT	60.0	544	INTERGENIC	--	--	--
Ca5_P4_7261145	Ca5	7261145	ATTT/ATT	CGCTGTAAAAGACATGCGCT	ACATCCATTTCCGGTCCATA	61.5	486	INTERGENIC	--	--	--
Ca5_P4_7293090	Ca5	7293090	AATATATATATATAT/AATATATATATATATAT	GCGAACATGAGAAATTGTCG	AACCAAGCAAGTCATAACATTTCA	59.3	684	INTERGENIC	--	--	--
Ca5_P4_7541226	Ca5	7541226	AATATATATATATATATATATATATATATAT/AATATATATATATATATATATATATATATAT	GCCTCATACTCAAGACATTGGA	GCTTGGAGAGAATGAGTCCG	59.2	649	INTERGENIC	--	--	--
Ca5_P4_7780304	Ca5	7780304	TTTATTATTATTATTATTATTATTATTATTATTATTATTA/TTTATTATTATTATTATTATTATTATTATTA	TCGTAAATATCCATCGGGGA	TGACCTTCATGCTTTTGTCG	60.1	674	INTERGENIC	--	--	--
Ca5_P4_7874012	Ca5	7874012	AAGAGAGAG/AAGAG	TCCTCCCTGCCAGACATAAC	TGTCCCTTGCTCAAGCTTTT	60.1	966	INTERGENIC	--	--	--
Ca5_P4_7898685	Ca5	7898685	TAAAAAAA/TAAAAAAAA	TCACTGTTCAAGATGTTTGTGC	GATGAGGAAAAAGTGGATTTTCA	58.8	398	INTERGENIC	--	--	--

INDEL marker IDs	Chromosomes/ unanchored scaffolds	Physical positions (bp)	InDels (*Kabuli* reference genome-CDC Frontier/PII)	Forward primers (5'-3')	Reverse primers (5'-3')	Anneling temperature (0C)	Expected amplified product size (bp)	Structural annotation		Functional annotation	
								Sequence components of *kabuli* genome	*Kabuli* gene accession IDs	NCBI-KOG	TFs
Ca5_P4_7937565	Ca5	7937565	GT/GTT	ATCGTTTTGTTTCGGGACTG	ATGCCCATGCATGTTTGTAA	60.0	404	INTERGENIC	--	--	--
Ca5_P4_7941049	Ca5	7941049	GA/GAA	TCTCATTTCCACTTAAACTTTCG	TAAGCAAGCCATTGCAAAAA	57.6	459	INTERGENIC	--	--	--
Ca5_P4_8035650	Ca5	8035650	T/TTA	GCGTTTCATGGTGTTGTTTG	CACGAGTCCGATAAGGGTGT	60.0	555	INTERGENIC	--	--	--
Ca5_P4_8098662	Ca5	8098662	ATT/AT	TCGTCAATGTTTGTGCCATT	TTGTATGTCCCCACAAAGCTC	60.0	567	INTERGENIC	--	--	--
Ca5_P4_812188	Ca5	812188	AATATATATATATATATATATATATAT/AATATATATATATATATATAT	TGTCAATTTCCTGTTTTGCTG	CGGGACCCATTTGAACATTA	58.8	806	INTERGENIC	--	--	--
Ca5_P4_8128213	Ca5	8128213	A/ATAT	CCAATTTGAATTGTCCGTCC	CCAATTGGTTGCACCTTTCT	60.2	576	INTERGENIC	--	--	--
Ca5_P4_8241804	Ca5	8241804	ATTTT/ATTT	TGACAGGAGCTTCCCTGAGT	GTACGAACTTCCTCCCACGA	60.0	678	INTERGENIC	--	--	--
Ca5_P4_8259506	Ca5	8259506	AATATATATATATATATATA/AATATATATATATATATATATATA	TCGTTCTGATCAATGGTGGA	AATTTTAAAACCTGCCGCCT	60.0	581	INTERGENIC	--	--	--
Ca5_P4_8314073	Ca5	8314073	GTATATATATATATATAT/GTATATATATATATATATAT	CCCAAACCCACATATAGCCA	TTCCATCATCAATTCTGCCA	60.6	537	INTERGENIC	--	--	--
Ca5_P4_8314333	Ca5	8314333	ATT/ATTT	TGGGGTTTAGGGTGTCACAT	TTCCATCATCAATTCTGCCA	60.1	375	INTERGENIC	--	--	--

INDEL marker IDs	Chromosomes/ unanchored scaffolds	Physical positions (bp)	InDels (*Kabuli* reference genome-CDC Frontier/PII)	Forward primers (5'-3')	Reverse primers (5'-3')	Anneling temperature (0C)	Expected amplified product size (bp)	Structural annotation		Functional annotation	
								Sequence components of *kabuli* genome	*Kabuli* gene accession IDs	NCBI-KOG	TFs
Ca5_P4_8345571	Ca5	8345571	CTTTTTTTTT/CTTTTTTTT	GCAGCCGTTCTCTTTCATTC	TGTGGTCTAACGGCTCCTTC	60.0	474	INTERGENIC	--	--	--
Ca5_P4_8596558	Ca5	8596558	GATAATAATAATAATAATAATAATAATAATAATAATAATAATAATAA/GATAATAATAATAATAATAATAATAATAATAATAATAATAATAATAA	CGAAGCCGAGAAAATGACTC	AGAGGCAAACAAGAACCGAA	60.0	343	INTERGENIC	--	--	--
Ca5_P4_8735941	Ca5	8735941	AATATATATATATATATATATATATTTA/AATATATATATATATATATATATATTTA	TCAAACTCTGCTGGCACATC	AAGCGACCTCCTACTGGGTT	60.0	646	INTERGENIC	--	--	--
Ca5_P4_8759374	Ca5	8759374	ATTTTTATTTTTTATTTTTTA/ATTTTTATTTTTTATTTTTTATTTTTTA	TTGTTCCCCTACCTCAACAAG	AAAAATTAAGAACAACAGGGGAA	59.1	405	INTERGENIC	--	--	--
Ca5_P4_8780838	Ca5	8780838	AATATATATATATATATATATATATATATATATATATATA/AATATATATATATATATATATATATATATATATATATATATATATATATATA	CAACACATCACAACCATCCA	TTCGACCTTTATGGATTTGAGA	58.8	374	INTERGENIC	--	--	--
Ca5_P4_885224	Ca5	885224	ATATATATATAT/ATATATATATATGTATATATATAT	AGAGGGGTTTGTGTTTGACG	TGAATTTTGACTCGGAGGCT	60.0	403	INTERGENIC	--	--	--
Ca5_P4_8975436	Ca5	8975436	AT/ATCT	GCTGTTGGAGATGCTTCAAGT	TTGACTTTCTCCCCGTTGAC	59.5	423	INTERGENIC	--	--	--
Ca5_P4_8998961	Ca5	8998961	A/AAT	TGCAATAATGGCATTTCCAA	TGAGCTTGAATGGCAAGATG	59.9	556	INTERGENIC	--	--	--
Ca5_P4_8999793	Ca5	8999793	CA/CAAA	CTCGATCAATGACCAGAGCA	TCACTTCTTCCCCCATTTTG	59.9	470	INTERGENIC	--	--	--
Ca5_P4_9250070	Ca5	9250070	AAAGAAGAAGAAGAAGAA/AAAGAAGAAGAAGAAGAAGAA	CCAGGATAAAACAGCCCAGA	TTCTGAGGCACTCTCCTGGT	60.1	317	INTERGENIC	--	--	--

INDEL marker IDs	Chromosomes/ unanchored scaffolds	Physical positions (bp)	InDels (*Kabuli* reference genome-CDC Frontier/PII)	Forward primers (5'-3')	Reverse primers (5'-3')	Anneling temperature (0C)	Expected amplified product size (bp)	Structural annotation		Functional annotation	
								Sequence components of *kabuli* genome	*Kabuli* gene accession IDs	NCBI-KOG	TFs
Ca5_P4_9264601	Ca5	9264601	ATTTTTTTTTTT/ATTTTTTTTTTTT	TTCCAGAAAATTGCATTTTGAA	ACCTTCCACAAGATCGTCAA	59.6	756	INTERGENIC	--	--	--
Ca5_P4_9264762	Ca5	9264762	CATATATATATATATATATATATAT/CATATATATATATATATATATATATATATAT	TTCCAGAAAATTGCATTTTGAA	GTGCCAATGCCAAAGAAAAT	59.6	802	INTERGENIC	--	--	--
Ca5_P4_927603	Ca5	927603	TTAATAATAATAATAATAATAATAATAATAATAA/TTAATAATAATAATAATAATAATAATAATAATAATAATAA	GGTCTTTTGAAAATAATCTTAACGC	AAGGGGGAAGTTAAGCCAGA	58.8	664	INTERGENIC	--	--	--
Ca5_P4_927667	Ca5	927667	TTAATAATAATAATAATAA/TTAATAATAATAATAATAATAA	AAAAGGAACGGTTAAAAATGTCTT	AAGGGGGAAGTTAAGCCAGA	58.7	224	INTERGENIC	--	--	--
Ca5_P4_9384842	Ca5	9384842	GT/GTT	TCAACAGTTTTGGTTCATCCC	TGCTTGATTTCCGTTTTGAG	59.8	372	INTERGENIC	--	--	--
Ca5_P4_9384889	Ca5	9384889	GAAAAAAAAAA/GAAAAAAAA	TCAACAGTTTTGGTTCATCCC	TGCTTGATTTCCGTTTTGAG	59.8	372	INTERGENIC	--	--	--
Ca5_P4_9403420	Ca5	9403420	CT/CAACCTT	GGCACTTTTCACGTCCATTT	ACAGCAGAATGGGGTAGTGG	60.0	836	INTERGENIC	--	--	--
Ca5_P4_9441399	Ca5	9441399	AATTTTATTTT/AATTTTATTTTATTTT	TGCGATGGAGAAATCAAAGC	CTAGTTTGGCTTGACGGGAA	61.3	865	INTERGENIC	--	--	--
Ca5_P4_9441477	Ca5	9441477	CAAAAAAAA/CAAAAAA	TCGTGTCTCAAAATTCAAGGAG	CTAGTTTGGCTTGACGGGAA	59.4	675	INTERGENIC	--	--	--
Ca5_P4_9586243	Ca5	9586243	ATATTATTATTATTATTATTATTATTATTATTATTATTA/ATATTATTATTATTATTATTATTATTATTATTATTATTATTATTATTA	GAAACGCGTTCCGTGTATTT	TTAGGCCTCCATGCAAGAGT	60.0	131	INTERGENIC	--	--	--

INDEL marker IDs	Chromosomes/ unanchored scaffolds	Physical positions (bp)	InDels (*Kabuli* reference genome-CDC Frontier/PII)	Forward primers (5'-3')	Reverse primers (5'-3')	Anneling temperature (0C)	Expected amplified product size (bp)	Structural annotation		Functional annotation	
								Sequence components of *kabuli* genome	*Kabuli* gene accession IDs	NCBI-KOG	TFs
Ca5_P4_966920	Ca5	966920	TTTATTATTATTATTATTATTATTATT/TTTATTATTATTATTATTATTATTATTATT	CAAGTTAAGCACCCCCACAT	TGGGTGAGAAACTTCGATCC	59.9	773	INTERGENIC	--	--	--
Ca5_P4_9708216	Ca5	9708216	AATATATATATATATATATATATATA/AATATATATATATATATATATATATATA	GGCAACGTATGTGTGTTTGG	AGAGTTGTCAATGGATCGGA	59.9	539	INTERGENIC	--	--	--
Ca5_P4_9741710	Ca5	9741710	GTTTTTTTTTTT/GTTTTTTTTTTTT	TTCAACTCAACTTCATTAGACCG	CATTTGGTGCGTTTGTTGTC	58.4	434	INTERGENIC	--	--	--
Ca5_P4_9890157	Ca5	9890157	GAAAAAAAAAAAA/GAAAAAAAAAAA	TTTCCTTCAATTTGAGCGGT	ACAGGGGGAGGAAATGAGAT	59.7	582	INTERGENIC	--	--	--
Ca6_P4_10003572	Ca6	10003572	CTTTTTTTTTT/CTTTTTTTTT	GCTGATTATCGCATTGAGGC	TGTCTAGAGGCTGGAGCAAAA	60.7	739	INTERGENIC	--	--	--
Ca6_P4_10127292	Ca6	10127292	CAA/CA	TGGTTGCGACTTGTGTTGTT	CCGACTCAAATCAAAGGGAG	60.2	100	INTERGENIC	--	--	--
Ca6_P4_10141273	Ca6	10141273	TAAAAAAAAAAA/TAAAAAAAAAA	GGAAATATCAAATGTTGTTACCGA	TCCAAAAACGACTTAGACCTCA	59.2	736	INTERGENIC	--	--	--
Ca6_P4_10174889	Ca6	10174889	GTTTTTTTT/GTTTTTTT	CCGAACTCCAACAACTCATTC	AAAACAAAGTGTAACGTTAAATGCAC	59.6	709	INTERGENIC	--	--	--
Ca6_P4_10244839	Ca6	10244839	TATAATA/TATAATAATA	GGAAGAAGCCCTGTTGTTTTT	CATGGCTTCAAACACCCTTT	59.6	577	INTERGENIC	--	--	--
Ca6_P4_10326205	Ca6	10326205	CTATATATATA/CTATATATATATA	CAAACATGTACCGTGAACTCAAA	CATGGGTACATCACCACCAA	59.9	463	INTERGENIC	--	--	--

INDEL marker IDs	Chromosomes/ unanchored scaffolds	Physical positions (bp)	InDels (*Kabuli* reference genome-CDC Frontier/PII)	Forward primers (5'-3')	Reverse primers (5'-3')	Anneling temperature (0C)	Expected amplified product size (bp)	Structural annotation		Functional annotation	
								Sequence components of *kabuli* genome	*Kabuli* gene accession IDs	NCBI-KOG	TFs
Ca6_P4_10335306	Ca6	10335306	TG/TGG	CAATTCCCACAGGTACGCTT	CAAACCCTAATTTTTCAACACG	60.0	305	INTERGENIC	--	--	--
Ca6_P4_10351153	Ca6	10351153	GTTATTATTATTATTATTAT/GTTATTATTATTATTAT	CAATTCCCACAGGTACGCTT	ACAACCATAGACAGCCGGAG	60.0	593	INTERGENIC	--	--	--
Ca6_P4_10503058	Ca6	10503058	AATATATA/AATATA	AAACATGCTTTTGCATTTTTGTT	GACTTCAACCGGTAGCTGGA	59.9	407	INTERGENIC	--	--	--
Ca6_P4_10503227	Ca6	10503227	TG/T	AAACATGCTTTTGCATTTTTGTT	GACTTCAACCGGTAGCTGGA	59.9	407	INTERGENIC	--	--	--
Ca6_P4_10513785	Ca6	10513785	AT/A	CCTCCAGTGGCTGAACAAAT	CGACTCAATGACGGGTTTTT	60.1	610	INTERGENIC	--	--	--
Ca6_P4_10514411	Ca6	10514411	CAAAAAAAAA/CAAAAAAAAAAA	TTCGCAATCTGTGTTTTTCG	CAAGAATTCGTATCGTCTAAATGC	59.8	642	INTERGENIC	--	--	--
Ca6_P4_10514563	Ca6	10514563	TAAAAAAAAAA/TAAAAAAAAAAAAA	TCACCAATCCTATGCTGTTGTC	ACGAAGTGACACATGACCCA	60.0	924	INTERGENIC	--	--	--
Ca6_P4_10516096	Ca6	10516096	TA/T	ATTTGCGTGTGTGCCTACAA	CACACACATTGCCTTAACGA	60.2	489	INTERGENIC	--	--	--
Ca6_P4_10517494	Ca6	10517494	GTT/GT	ACTCACGTGTTGGGGGTAAA	ACCATGCCCAAATTTGAAGT	60.3	417	INTERGENIC	--	--	--
Ca6_P4_10552453	Ca6	10552453	GTT/GTTT	ATAAGCAACGACAACCACCA	CTCAACAAGTCATTGGCCCT	59.1	312	INTERGENIC	--	--	--

INDEL marker IDs	Chromosomes/ unanchored scaffolds	Physical positions (bp)	InDels (*Kabuli* reference genome-CDC Frontier/PII)	Forward primers (5'-3')	Reverse primers (5'-3')	Anneling temperature (0C)	Expected amplified product size (bp)	Structural annotation		Functional annotation	
								Sequence components of *kabuli* genome	*Kabuli* gene accession IDs	NCBI-KOG	TFs
Ca6_P4_10552509	Ca6	10552509	CTTTTT/CTTTTTT	GCCCGCATTAAATTTGTCAC	TGAACATGAGGCTGCAAAAG	60.3	509	INTERGENIC	--	--	--
Ca6_P4_10554117	Ca6	10554117	TTATA/TTA	TAACTATCCCATCGGCCAAA	GGTGTTATATTTAGCATGGGGG	60.3	708	INTERGENIC	--	--	--
Ca6_P4_10554507	Ca6	10554507	AT/ATTT	TGGGTTAACGGAGAGTAGCC	ACTAGGACGACGAAAACGCA	59.2	741	INTERGENIC	--	--	--
Ca6_P4_10555535	Ca6	10555535	GT/GTATTATT	AGCATGTGGAGCAGTTGTTG	CCCCGTTAATCCACTTGCTA	59.9	445	INTERGENIC	--	--	--
Ca6_P4_10556820	Ca6	10556820	ATTT/ATTTT	TATGACGGGCACACTTGAGA	CGTCATTTGCAAAAACGACTT	60.3	474	INTERGENIC	--	--	--
Ca6_P4_10560990	Ca6	10560990	TAA/TAAA	CTTCCACAATTCAAACTCTCCA	TGAAGAAAAATGCGGGTCTT	59.2	631	INTERGENIC	--	--	--
Ca6_P4_10563725	Ca6	10563725	TA/T	TCGAAAAAGCGGGATGTTAC	GGGTGTCACATGTTGATGGT	60.1	916	INTERGENIC	--	--	--
Ca6_P4_10564033	Ca6	10564033	AATATAT/AAT	GAAAATCCCAATCCAAATTTCA	GCAGCACACAGCATAGGAAA	60.0	567	INTERGENIC	--	--	--
Ca6_P4_10564611	Ca6	10564611	TAGAGA/TAGA	TTTCCTATGCTGTGTGCTGC	CTTTTCACCCTTGATCGGAA	60.0	611	INTERGENIC	--	--	--
Ca6_P4_10572244	Ca6	10572244	TAAA/TAA	CCGATGTAAGACTCTCCCCA	TGTTTCAACCTTGCATTGGA	60.1	790	INTERGENIC	--	--	--

INDEL marker IDs	Chromosomes/ unanchored scaffolds	Physical positions (bp)	InDels (*Kabuli* reference genome-CDC Frontier/PII)	Forward primers (5'-3')	Reverse primers (5'-3')	Anneling temperature (0C)	Expected amplified product size (bp)	Structural annotation		Functional annotation	
								Sequence components of *kabuli* genome	*Kabuli* gene accession IDs	NCBI-KOG	TFs
Ca6_P4_10575764	Ca6	10575764	ATTTTTTT/ATTTTTTTT	TTTGTGTTGTGTGTTGTCGC	CCCCATGATACCCTTTCCTT	59.2	294	INTERGENIC	--	--	--
Ca6_P4_10576490	Ca6	10576490	CTTTTT/CTTTTTT	TTGCCAAAGGTTAATGCACA	ATGGTGGTTGCTAGGTTTGC	60.1	888	INTERGENIC	--	--	--
Ca6_P4_10586822	Ca6	10586822	CAAAAAAA/CAAAAAAAA	TTCCCAGTTCTTTCACCCAG	AACTGTAACACACGTGGCGA	60.1	573	INTERGENIC	--	--	--
Ca6_P4_10592926	Ca6	10592926	AAACTCATA/AAACTCATAACTCATA	GGCTAGTGCTAAACCGTTGG	AAAATCTGGCTGGATTTGCTT	59.8	955	INTERGENIC	--	--	--
Ca6_P4_10596898	Ca6	10596898	TTATTATATATATT/TTATT	CCGACATAACAAGCACATGG	GCCCTCCTCCATGACATAAA	60.0	312	INTERGENIC	--	--	--
Ca6_P4_10608315	Ca6	10608315	TG/TGG	ACCCGCTTAATTGTGGCTAA	ACATTACCCGCATCCACCTA	59.6	383	INTERGENIC	--	--	--
Ca6_P4_10608634	Ca6	10608634	TA/TACA	ACGAAGCTGGTACAAATGGG	CGAACCAAACCTACCATGAGA	60.0	773	INTERGENIC	--	--	--
Ca6_P4_10609160	Ca6	10609160	AATTA/AA	TGGTTAAACGGCTCAATCAA	TTCTGGGAGATGCTCCAAAT	59.2	162	INTERGENIC	--	--	--
Ca6_P4_10616106	Ca6	10616106	TC/T	TATTCCCCCTTTCGTCTCAG	CGAATTGCAGGAATGTGTTG	59.1	801	INTERGENIC	--	--	--
Ca6_P4_10616549	Ca6	10616549	ATTTTTTT/ATTTTTT	CAACACATTCCTGCAATTCG	AACTGTGAGGAGAGAGAAGCAAA	60.1	219	INTERGENIC	--	--	--

INDEL marker IDs	Chromosomes/ unanchored scaffolds	Physical positions (bp)	InDels (*Kabuli* reference genome-CDC Frontier/PII)	Forward primers (5'-3')	Reverse primers (5'-3')	Anneling temperature (0C)	Expected amplified product size (bp)	Structural annotation		Functional annotation	
								Sequence components of *kabuli* genome	*Kabuli* gene accession IDs	NCBI-KOG	TFs
Ca6_P4_10617092	Ca6	10617092	C/CA	TTGTTTAATTGTTGGGGGAGA	AACGTAAGTTGGCACCATGC	59.3	222	INTERGENIC	--	--	--
Ca6_P4_10618337	Ca6	10618337	TAAAA/TAAAAAA	TGGAGAAGAAACATTCACGG	AAAGATCGGCTCAAATGAACA	58.7	698	INTERGENIC	--	--	--
Ca6_P4_10618951	Ca6	10618951	ATTTTT/ATTTTTT	TGTTCATTTGAGCCGATCTTT	GAGATAGGCTACCGCACAGG	59.7	564	INTERGENIC	--	--	--
Ca6_P4_1061969	Ca6	1061969	TAAAAAAA/TAAAAAAAA	AAATGAATGTGGAGTGACCAAA	TCAAAAGGGCTTTTCATTGG	59.3	877	INTERGENIC	--	--	--
Ca6_P4_10620292	Ca6	10620292	TAAAAAACA/TAAAAAACAAAAAAACA	CAGTTGTGGCGATTGATTTG	GGATCTGAAATCCCCTCTCC	60.1	836	INTERGENIC	--	--	--
Ca6_P4_10622254	Ca6	10622254	AATATATATATAT/AATATATATAT	TCTTCTCGAAAACAATTTGTCA	AATTGTGCACATCGCTGATT	57.5	517	INTERGENIC	--	--	--
Ca6_P4_10622699	Ca6	10622699	TCACGCTAGCAAATAGC/TC	ATCAGCGATGTGCACAATTT	CAACCGTTGTTCGAGGCTAT	59.2	569	INTERGENIC	--	--	--
Ca6_P4_10623512	Ca6	10623512	TTTTCTT/TTT	TGGATGCCAAAATAATCTCCA	CCAAACTCCCTCACTACCCA	60.3	428	INTERGENIC	--	--	--
Ca6_P4_10623994	Ca6	10623994	AAAGAAGAA/AAAGAA	TGGGTAGTGAGGGAGTTTGG	TTAAAGGGGCCTTGATGTTG	60.0	749	INTERGENIC	--	--	--
Ca6_P4_10625658	Ca6	10625658	AAA/AATAA	GCTCTCCAAGACTTCAACGG	TCCAACCTTCCCTCATCTTG	60.0	782	INTERGENIC	--	--	--

INDEL marker IDs	Chromosomes/ unanchored scaffolds	Physical positions (bp)	InDels (*Kabuli* reference genome-CDC Frontier/PII)	Forward primers (5'-3')	Reverse primers (5'-3')	Anneling temperature (0C)	Expected amplified product size (bp)	Structural annotation		Functional annotation	
								Sequence components of *kabuli* genome	*Kabuli* gene accession IDs	NCBI-KOG	TFs
Ca6_P4_10625726	Ca6	10625726	GTTTTTTTTTTTT/GTTTTTTTTT	TTTCCCCTAACTACCACCCC	TCCAACCTTCCCTCATCTTG	60.0	592	INTERGENIC	--	--	--
Ca6_P4_10640465	Ca6	10640465	C/CGTAAGTATGA	CCTTCAGCATTAGACTCCGC	AATCGCGAATATGGTCCAAG	60.0	787	INTERGENIC	--	--	--
Ca6_P4_10642500	Ca6	10642500	C/CCTATAGA	CGATTTGACAGCAAAAGCAA	GACAACCTTCGTTGCCCTAA	60.0	437	INTERGENIC	--	--	--
Ca6_P4_10661984	Ca6	10661984	A/AGT	TCCCTTCGCTACAAGAAGGA	TTTGCGACAAACTTGAGCTG	59.9	752	INTERGENIC	--	--	--
Ca6_P4_10664293	Ca6	10664293	AATATATAT/AATATAT	TACACCTGCAAAAGGTGCAA	TTTGTGGTACCAAGCACACAC	60.3	293	INTERGENIC	--	--	--
Ca6_P4_10664476	Ca6	10664476	GAAAAAAAAAAAAA/GAAAAAAAAAA	TACACCTGCAAAAGGTGCAA	TCAATGTATTGGAAGGTGAGACA	60.3	700	INTERGENIC	--	--	--
Ca6_P4_10664595	Ca6	10664595	TAAAA/TAAA	ACCACAATCACGGTGAACAG	TCAATGTATTGGAAGGTGAGACA	59.4	631	INTERGENIC	--	--	--
Ca6_P4_10689201	Ca6	10689201	AAT/A	TGTTGCATCAATGCCGTTAT	TTCCCCAATAACCAAACCAA	60.0	758	INTERGENIC	--	--	--
Ca6_P4_10700691	Ca6	10700691	AG/A	ATAAAGAGAAGCGGAGGGGA	CAACGAAGAGCGTCGAGAAT	60.2	909	INTERGENIC	--	--	--
Ca6_P4_10701503	Ca6	10701503	GTTATAA/GTTATAACTTATAA	TCGACGCTCTTCGTTGACTA	GTGCACGGTACATTTCTCCC	59.7	487	INTERGENIC	--	--	--

INDEL marker IDs	Chromosomes/ unanchored scaffolds	Physical positions (bp)	InDels (*Kabuli* reference genome-CDC Frontier/PII)	Forward primers (5'-3')	Reverse primers (5'-3')	Anneling temperature (0C)	Expected amplified product size (bp)	Structural annotation		Functional annotation	
								Sequence components of *kabuli* genome	*Kabuli* gene accession IDs	NCBI-KOG	TFs
Ca6_P4_10702347	Ca6	10702347	CTTTCGAAAGTT/CTTTCGAAAGTTGAATTTTCGAAAGTT	TTGATGAATTTGAAAGTTCCGA	TCCAGATTTTGAAGCTTTTGC	59.6	714	INTERGENIC	--	--	--
Ca6_P4_10795007	Ca6	10795007	AT/A	TTGGTTTTGAAACATTGATTGAT	CATTTACCTCGTGCACCACT	58.4	374	INTERGENIC	--	--	--
Ca6_P4_10968845	Ca6	10968845	AATATATATATATATATATATATATAT/AATATATATATATATATATATAT	TGAAAACTTCATTTTCTTTATTGAGTC	GCACATGGTTGCATGAAAAA	58.5	637	INTERGENIC	--	--	--
Ca6_P4_11031611	Ca6	11031611	AGTCAGCAATAGCACTT/A	GTGAAGCTGTTGGGGTTGAT	GACCGGCAACCAGAGAATTA	60.0	238	INTERGENIC	--	--	--
Ca6_P4_11035330	Ca6	11035330	ATTTTTTTTTT/ATTTTTTTTTTT	TGGCTAAATTACATTTGTGGTCC	AGACGCATTCTTCTGTCCGT	60.1	476	INTERGENIC	--	--	--
Ca6_P4_11084875	Ca6	11084875	ATTTTTTTTTT/ATTTTTTTTTTT	GCGAATGTGTTAAAATGGGG	ACGTCTTTTGAAATGTCGGC	60.2	257	INTERGENIC	--	--	--
Ca6_P4_112870	Ca6	112870	TG/T	AACTCCTTGGGACCGTGATT	ACCTTGCAACTGGAGCAAAT	60.7	242	INTERGENIC	--	--	--
Ca6_P4_11310241	Ca6	11310241	GTTTTGCATA/G	CATGTCGAAGAAGCTCCACA	TCCTCCGGAACATAACTTGG	60.0	417	INTERGENIC	--	--	--
Ca6_P4_11310976	Ca6	11310976	AT/ATT	TTTTGCCCAGAAGAATTTGG	TTTTTCCTTTTGGTGGATGG	60.0	175	INTERGENIC	--	--	--
Ca6_P4_11316099	Ca6	11316099	AC/A	GTTTGTTGTGGTTGCAGTGG	GCTGATTGGTTGCCTCTCTC	60.0	399	INTERGENIC	--	--	--

INDEL marker IDs	Chromosomes/ unanchored scaffolds	Physical positions (bp)	InDels (*Kabuli* reference genome-CDC Frontier/PII)	Forward primers (5'-3')	Reverse primers (5'-3')	Anneling temperature (0C)	Expected amplified product size (bp)	Structural annotation		Functional annotation	
								Sequence components of *kabuli* genome	*Kabuli* gene accession IDs	NCBI-KOG	TFs
Ca6_P4_11362880	Ca6	11362880	ATTTTTTTT/ATTTTTTT	CCCCACCATCCATGATAAAC	TTGTTCTCTACGCTGAATTGGA	59.9	873	INTERGENIC	--	--	--
Ca6_P4_11391053	Ca6	11391053	ATTTTTTT/ATTTTTT	CGACGAAAATAGAGGGACCA	TGTGGGAGCCACATGTATTT	60.1	370	INTERGENIC	--	--	--
Ca6_P4_11490708	Ca6	11490708	AATATATATATATATATATATATA/AATATATATATATATATATA	CGTGAATGTTTGTGTTTGGC	TGTTATCCAGTCATGGTGTCAA	60.0	496	INTERGENIC	--	--	--
Ca6_P4_11497940	Ca6	11497940	ATTTTTTTT/ATTTTTTTTT	TTATTGCCTATTGCCACACA	GTGTGAGTGTGAGACCCGTG	57.7	666	INTERGENIC	--	--	--
Ca6_P4_11546028	Ca6	11546028	CAAA/CAAAA	GATCGATTTGGGCTACGAAA	ATCAGATTGCTGCAGGCTTT	60.0	651	INTERGENIC	--	--	--
Ca6_P4_11546493	Ca6	11546493	TAAAAAAAAAAAAAAA/TAAAAAAAAA	TCGGAAAGGTCATTCAAAGC	TTTGTGTCCAAAGCATTCTCC	60.2	472	INTERGENIC	--	--	--
Ca6_P4_11565007	Ca6	11565007	CTTTTT/CTTTTTTT	TGATCATTTGATGAACGTTTGA	CAACCACTCCTTGAAAACCAA	59.0	480	INTERGENIC	--	--	--
Ca6_P4_11565258	Ca6	11565258	AATATATATATATATATATATATAT/AATATATATATATATATATATATATATATATATAT	TTGGTTTTCAAGGAGTGGTTG	TTGCTGCCTCAACCATACAG	60.0	522	INTERGENIC	--	--	--
Ca6_P4_11568345	Ca6	11568345	AA/AACA	ATTGCCCAACCAATTATGGA	GAGGGTGCGATCAGCTAAAG	60.0	593	INTERGENIC	--	--	--
Ca6_P4_11569101	Ca6	11569101	T/TAAGTAA	CAAAAGGGGGTATTTTGGTTC	CGTTCAATTTCCTGCCAAAT	59.6	499	INTERGENIC	--	--	--

INDEL marker IDs	Chromosomes/ unanchored scaffolds	Physical positions (bp)	InDels (*Kabuli* reference genome-CDC Frontier/PII)	Forward primers (5'-3')	Reverse primers (5'-3')	Anneling temperature (0C)	Expected amplified product size (bp)	Structural annotation		Functional annotation	
								Sequence components of *kabuli* genome	*Kabuli* gene accession IDs	NCBI-KOG	TFs
Ca6_P4_11569249	Ca6	11569249	ATATTT/AT	ATTTGGCAGGAAATTGAACG	TCGGTGGTTGCACATTCTTA	59.9	570	INTERGENIC	--	--	--
Ca6_P4_11590997	Ca6	11590997	CTTTT/CTTTTT	ATGTGCTCCCCACAAAAGTC	CCCTCCAGTAGCATCACCAT	60.0	538	INTERGENIC	--	--	--
Ca6_P4_11592242	Ca6	11592242	GTTA/GTTATTGAATTCCTTTA	TGTTGAACTCAAGGATTGCG	ACCCAATTTGTTTGGGATGA	59.8	611	INTERGENIC	--	--	--
Ca6_P4_11599026	Ca6	11599026	TATTA/T	AAAATGGATTTTCCCCAAGG	GCAAGGATTGACATGGGAGT	60.0	607	INTERGENIC	--	--	--
Ca6_P4_11610694	Ca6	11610694	T/TA	CTCGACATGGGGTAGGTTGT	GCGCCCAATTAATGGATACT	59.8	508	INTERGENIC	--	--	--
Ca6_P4_11654526	Ca6	11654526	ATTTTTTTTTTTT/ATTTTTTTTTTTTT	GCGGCTAATAGTTGGTCGTG	TGTCGGCTCACGTTTTTGTA	60.7	543	INTERGENIC	--	--	--
Ca6_P4_11661530	Ca6	11661530	GAAAAA/GAAAAAAA	GTTGAGTTGCGGGTGTTTTT	ATATTCCCCTCCCAACATCC	60.0	421	INTERGENIC	--	--	--
Ca6_P4_11663719	Ca6	11663719	CAAAA/CAAA	GCATTACCGATCATGGGTGT	CAGTCACGTCAAACTGCTCG	60.6	676	INTERGENIC	--	--	--
Ca6_P4_11671266	Ca6	11671266	TAAAAA/TAAAA	TTGAATGGATATGCTTTTGGTG	TGCCCTAGCAAAGGTAGTCA	59.8	936	INTERGENIC	--	--	--
Ca6_P4_11679999	Ca6	11679999	T/TA	CTCACCCAAACACACACTCA	CATGGAAAGCAGAAGCACAA	58.1	462	INTERGENIC	--	--	--

INDEL marker IDs	Chromosomes/ unanchored scaffolds	Physical positions (bp)	InDels (*Kabuli* reference genome-CDC Frontier/PII)	Forward primers (5'-3')	Reverse primers (5'-3')	Anneling temperature (0C)	Expected amplified product size (bp)	Structural annotation		Functional annotation	
								Sequence components of *kabuli* genome	*Kabuli* gene accession IDs	NCBI-KOG	TFs
Ca6_P4_11685034	Ca6	11685034	TGGG/TGG	ATCGCCAGCAACTTTAGCAT	GGGAACCAAAGTGTTGACCA	59.9	508	INTERGENIC	--	--	--
Ca6_P4_11687884	Ca6	11687884	GTTTTTTTTTT/GTTTTTTTTT	CCTTTGAGTTTGGTCCTCCA	GGAGGAGGAGGAGAAGGATG	60.1	716	INTERGENIC	--	--	--
Ca6_P4_11693016	Ca6	11693016	TTAATAATAATAATAATAAT/TTAATAATAATAATAAT	TGACCTGCTGAATGATTTGG	TCGACGAAATCCTAAATGTGG	59.6	589	INTERGENIC	--	--	--
Ca6_P4_11706831	Ca6	11706831	AT/ATT	TCATCCACATTGGTGCTGTT	TGGAATTGGGCCTAAAGATG	60.0	643	INTERGENIC	--	--	--
Ca6_P4_11722419	Ca6	11722419	TATATT/TATATTAATATT	CCTTCGCCGTATATGATTCC	AAGCAAAGGTCCTGAGCAAA	59.4	185	INTERGENIC	--	--	--
Ca6_P4_11728216	Ca6	11728216	T/TG	TGAGTTGTTAGTCGTCGGGA	TCATGAGAATGCCTTTGTGA	59.3	412	INTERGENIC	--	--	--
Ca6_P4_11738985	Ca6	11738985	TAAAAAAAA/TAAAAAAA	TTCAGCAGCTTCTTCGACAA	ATTACCGACATTGCAGAGGG	59.9	254	INTERGENIC	--	--	--
Ca6_P4_11741312	Ca6	11741312	GCAGTACA/GCA	TGGAAACCAAGGTCCAAGTC	GCACTGCAAAATCATTCCCT	59.9	308	INTERGENIC	--	--	--
Ca6_P4_11742023	Ca6	11742023	ATTTTTTT/ATTTTTTTT	GCGCTTCAATTTACAACGCT	TTTTCGCACGAAATCAATCA	60.4	614	INTERGENIC	--	--	--
Ca6_P4_11744525	Ca6	11744525	AAAAAAAA/AAAAAAAATAAAAAAA	TTTGCGGAATGGTGATTCTT	TTGTGAACGAAATGCGAGTC	60.4	370	INTERGENIC	--	--	--

INDEL marker IDs	Chromosomes/ unanchored scaffolds	Physical positions (bp)	InDels (*Kabuli* reference genome-CDC Frontier/PII)	Forward primers (5'-3')	Reverse primers (5'-3')	Anneling temperature (0C)	Expected amplified product size (bp)	Structural annotation		Functional annotation	
								Sequence components of *kabuli* genome	*Kabuli* gene accession IDs	NCBI-KOG	TFs
Ca6_P4_11746077	Ca6	11746077	AATATATATATATATATATA/AATATATATATATATATATATATA	TGCAAGTCGAAATTCACACG	TGGGTCAAGCCCAATAAAAC	60.8	538	INTERGENIC	--	--	--
Ca6_P4_11746285	Ca6	11746285	TTA/TTAGTATTAGTA	TGCAAGTCGAAATTCACACG	TTTGTTGGGAAAACCCAAAG	60.8	930	INTERGENIC	--	--	--
Ca6_P4_11755125	Ca6	11755125	GTCAAAGATTTTCTTCAA/GTCAA	CGATCATGTTCGTATTTTGTTCA	TGAAATTCCCTGAGCTGGTC	59.9	527	INTERGENIC	--	--	--
Ca6_P4_11762104	Ca6	11762104	A/AG	TAAGCATTGGGGGTTGAAAG	ACGTTGAGGAGACACCTGCT	59.9	259	INTERGENIC	--	--	--
Ca6_P4_11788716	Ca6	11788716	TTTGGATAATTGGATAATTGGAT/TTTGGATAATTGGAT	GGTTCAGCAACCGAGAGTTC	TGATAATGCGACGAAAACCA	59.9	363	INTERGENIC	--	--	--
Ca6_P4_11996420	Ca6	11996420	CATATATATATATATATATATATATATATA/CATATATATATATATATATATATATATATATATA	GGGCGACTTTTCCTTATTCC	TATTTTTCTTGGTTTGCGGG	59.9	233	INTERGENIC	--	--	--
Ca6_P4_12253200	Ca6	12253200	AATATATATATATATATATATATATATATATATATATATAT/AATATATATATATATATATATATATATATATAT	CTCCGTTTATCTCCGTTGGA	CGTGATGATTGATGCATTTCA	60.1	746	INTERGENIC	--	--	--
Ca6_P4_12325715	Ca6	12325715	T/TAC	TTGTGAATGGCATGTTTTGG	TGAGAAACTTTTCCGCTGCT	60.4	868	INTERGENIC	--	--	--
Ca6_P4_12565656	Ca6	12565656	TTTATTATTATTATTATTATTATTATTATTATTATTATTATT/TTTATTATTATTATTATTATTATTATTATTATTATTATTATTATT	ACATCCAATGAAATAGCCGC	AAAATAGCCTTTTCGCCACC	59.9	825	INTERGENIC	--	--	--
Ca6_P4_12680067	Ca6	12680067	CATTATTATTATTATTATTATTATTATTATTAT/CATTATTATTATTATTATTATTATTATTAT	CCGGTCTCTGTGGTGACTG	AAGGACATGTTGGTTGAAACAC	60.3	643	INTERGENIC	--	--	--

INDEL marker IDs	Chromosomes/ unanchored scaffolds	Physical positions (bp)	InDels (*Kabuli* reference genome-CDC Frontier/PII)	Forward primers (5'-3')	Reverse primers (5'-3')	Anneling temperature (0C)	Expected amplified product size (bp)	Structural annotation		Functional annotation	
								Sequence components of *kabuli* genome	*Kabuli* gene accession IDs	NCBI-KOG	TFs
Ca6_P4_12680394	Ca6	12680394	CATATATATAT/CATATATATATATAT	TGTGTTTCAACCAACAT GTCC	GGAAACATGCAATTCG GACT	59.3	475	INTERGENIC	--	--	--
Ca6_P4_12705859	Ca6	12705859	ATTTTTT/ATTTTT	AATAACACACCAAACC CCCA	CAACTTAGGGTTAACTT ATGCATTTTT	59.9	659	INTERGENIC	--	--	--
Ca6_P4_12787897	Ca6	12787897	AATATATATATATATATATATAT/AAT ATATATATATATATATATAT	ATCAAACAAATGTTGC GAGC	CAAACCCCTATTTTTGT GATTGA	58.8	749	INTERGENIC	--	--	--
Ca6_P4_12861842	Ca6	12861842	ATTTTTTT/ATTTTTT	TCAATCCCTTCATTCG GAAG	TCGATAGTGGGTTTCG TGTTC	60.0	612	INTERGENIC	--	--	--
Ca6_P4_12868319	Ca6	12868319	TTGCG/T	GCATGTCAAGATAATG GGCA	CCGCATAACCAAACAC TCAC	59.5	637	INTERGENIC	--	--	--
Ca6_P4_12870795	Ca6	12870795	TAAAAAAAAA/TAAAAAAAAAAA	CATCCCAAGCAAAAAC GAAT	CATTGAGAGGATCCAA AATCAA	59.9	533	INTERGENIC	--	--	--
Ca6_P4_12872062	Ca6	12872062	AGG/AG	TTTTCTTTCATCTGTGG GGC	CCCAAACACAAAATCAT GGA	60.1	677	INTERGENIC	--	--	--
Ca6_P4_12877302	Ca6	12877302	CG/CGCTTTTTTTG	GTTCGTAAAGGACGGG ATGA	CTTTTACAGCGCTTTCT GGG	59.9	235	INTERGENIC	--	--	--
Ca6_P4_12888429	Ca6	12888429	T/TC	CCGTCAATTTATATCCT CCCA	AATCAGGGTGCCATTG TAGC	58.8	733	INTERGENIC	--	--	--
Ca6_P4_12898258	Ca6	12898258	TAAAAAAAAAA/TAAAAAAAAAAA	ACTACCATGTTTGGTG GGGA	CACATTGAAAATCAACT TTACACCA	60.1	646	INTERGENIC	--	--	--

INDEL marker IDs	Chromosomes/ unanchored scaffolds	Physical positions (bp)	InDels (*Kabuli* reference genome-CDC Frontier/PII)	Forward primers (5'-3')	Reverse primers (5'-3')	Anneling temperature (0C)	Expected amplified product size (bp)	Structural annotation		Functional annotation	
								Sequence components of *kabuli* genome	*Kabuli* gene accession IDs	NCBI-KOG	TFs
Ca6_P4_12899367	Ca6	12899367	ATTTTTTTT/ATTTTTTT	TTGGATGTATACGGCGAACA	TCCTACGAAAGTTTTAGTCATTGC	60.0	546	INTERGENIC	--	--	--
Ca6_P4_12910939	Ca6	12910939	CG/CGG	ATCAATGAAAGCCCCAAGTG	TCTTCCTCTTTCTTCCTCTTTTCA	59.9	852	INTERGENIC	--	--	--
Ca6_P4_12919764	Ca6	12919764	TTAATAAT/TTAAT	TTGTTCCGACATTGTTTTGG	CCACACACTCCACGAGAGAA	59.4	609	INTERGENIC	--	--	--
Ca6_P4_12920238	Ca6	12920238	CAAAAA/CAAAAAA	TTCTCTCGTGGAGTGTGTGG	TTGAAGAACCTCCTTCCTTCC	59.9	377	INTERGENIC	--	--	--
Ca6_P4_12922857	Ca6	12922857	A/AC	TTTTACCGCGGAAACAAATC	CAGAAACAGCAGAGACAGCG	59.9	686	INTERGENIC	--	--	--
Ca6_P4_12923575	Ca6	12923575	TTTACATATCATTACA/TTTACA	TGTTGGCAGAGATGAAGTGG	ATGGGGATGCAGAAGAGAGA	59.8	772	INTERGENIC	--	--	--
Ca6_P4_12923712	Ca6	12923712	AT/ATT	TGTTGGCAGAGATGAAGTGG	ATGGGGATGCAGAAGAGAGA	59.8	772	INTERGENIC	--	--	--
Ca6_P4_12931395	Ca6	12931395	CAAAAAAAAAA/CAAAAAAAAAAAA	CACGGTTTACATGGGCAATA	ATCTGGTATCAAATGCCCCA	59.3	493	INTERGENIC	--	--	--
Ca6_P4_12931779	Ca6	12931779	TAAAA/TAAA	CTTGGGGCATTTGATACCAG	TTGTTTGTGGCGATAGTTGC	60.3	576	INTERGENIC	--	--	--
Ca6_P4_12933054	Ca6	12933054	CTTTTTTTTTT/CTTTTTTTTTTTTTT	TTTCACAAATTAAATGACCCAAA	TGATTCAGAGCCATTAAACCA	58.5	442	INTERGENIC	--	--	--

INDEL marker IDs	Chromosomes/ unanchored scaffolds	Physical positions (bp)	InDels (*Kabuli* reference genome-CDC Frontier/PII)	Forward primers (5'-3')	Reverse primers (5'-3')	Anneling temperature (0C)	Expected amplified product size (bp)	Structural annotation		Functional annotation	
								Sequence components of *kabuli* genome	*Kabuli* gene accession IDs	NCBI-KOG	TFs
Ca6_P4_12933312	Ca6	12933312	AT/ATT	TTTCACAAATTAAATGACCCAAA	TCAACCCACTTAAATTAAATCTTTTT	58.5	481	INTERGENIC	--	--	--
Ca6_P4_12933675	Ca6	12933675	TG/T	AGTGGTTTAATGGCTCTGAATC	TGCAAGTTTGGAGTCTATAATTCATC	57.4	717	INTERGENIC	--	--	--
Ca6_P4_12933836	Ca6	12933836	TAAAAAAAAAA/TAAAAAAAAA	GTTAATCGAAAAAGAAAAGAGTAAAAA	CCCACTCCAAATAAATTTCACAA	57.3	622	INTERGENIC	--	--	--
Ca6_P4_12934479	Ca6	12934479	ATTTTTTGTTTTT/ATTTTTTGTTTTTTGTTTTT	CGGTTTGAAGATGAGGTTCAC	TTCCTCCCTCTAGATTGTTTGC	59.6	495	INTERGENIC	--	--	--
Ca6_P4_12944504	Ca6	12944504	TCCC/TCC	CATTGGTGCTTCAAACCGAT	TCCCTCGTACTTCCAAAACG	60.9	533	INTERGENIC	--	--	--
Ca6_P4_12945433	Ca6	12945433	G/GATTCAGAT	TGGTGGTGTAGGTCCACAGA	TTCACGGTGTTCAAATTCGT	60.0	415	INTERGENIC	--	--	--
Ca6_P4_12948486	Ca6	12948486	ATTTT/ATTT	CCGATGATTTCAAAAATTTAACC	CAAGTTTGTGTACCAGGCCA	58.8	534	INTERGENIC	--	--	--
Ca6_P4_12949470	Ca6	12949470	GTATATAT/GTATATATAT	TTGGATGGACCCAACTCAAT	GTCACCCATCAACGCCTATT	60.2	305	INTERGENIC	--	--	--
Ca6_P4_12952484	Ca6	12952484	TTATATATATATATATATATATATATA/TTATATATATATATATA	TCGGAAAACAATGCAAATCA	GATTTGAGTTCCAAATTTGATGA	60.1	587	INTERGENIC	--	--	--
Ca6_P4_12955482	Ca6	12955482	A/AATT	TGTGCAATTGGATGTTGTTG	AACCCCATCAAAATGATGAA	59.0	215	INTERGENIC	--	--	--

INDEL marker IDs	Chromosomes/ unanchored scaffolds	Physical positions (bp)	InDels (*Kabuli* reference genome-CDC Frontier/PII)	Forward primers (5'-3')	Reverse primers (5'-3')	Anneling temperature (0C)	Expected amplified product size (bp)	Structural annotation		Functional annotation	
								Sequence components of *kabuli* genome	*Kabuli* gene accession IDs	NCBI-KOG	TFs
Ca6_P4_12955724	Ca6	12955724	TG/T	TGTGCAATTGGATGTTGTTG	ACTCGTTTGGTCATTCGTGA	59.0	660	INTERGENIC	--	--	--
Ca6_P4_12957166	Ca6	12957166	T/TC	TGAATCCCGAAATTGACCAT	AAAAGCAAACAGGTGAACCG	60.1	404	INTERGENIC	--	--	--
Ca6_P4_12957525	Ca6	12957525	CAAA/CAAAA	CGGTTCACCTGTTTGCTTTT	TGAAAAGCTTGAGGAAGGGA	60.1	718	INTERGENIC	--	--	--
Ca6_P4_12957709	Ca6	12957709	T/TC	CGGTTCACCTGTTTGCTTTT	TGAAAAGCTTGAGGAAGGGA	60.1	718	INTERGENIC	--	--	--
Ca6_P4_12958210	Ca6	12958210	ATTTTTTTT/ATTTTTTTTTT	CTATTCCGCTTGGTCGAAAA	GAAGCGATAGAGAGAGGCGA	60.2	567	INTERGENIC	--	--	--
Ca6_P4_12958383	Ca6	12958383	TAAAAAA/TAAAAA	TCCCTTCCTCAAGCTTTTCA	TTTCTGGAAGAGGCAGAAGC	59.9	880	INTERGENIC	--	--	--
Ca6_P4_12958420	Ca6	12958420	TTATATATATATATAT/TTATATATATATATATAT	TCCCTTCCTCAAGCTTTTCA	TTTCTGGAAGAGGCAGAAGC	59.9	880	INTERGENIC	--	--	--
Ca6_P4_12964004	Ca6	12964004	ATAT/A	CTCGTGGCATGGTTCTCTTT	CAGCCCGTCCTAAATCTTCA	60.3	506	INTERGENIC	--	--	--
Ca6_P4_12964933	Ca6	12964933	TCATTA/T	CTTGTTGCCAAGTAGGGTCG	CCAAGGGAGAGGTGCTTAAA	60.7	976	INTERGENIC	--	--	--
Ca6_P4_12965919	Ca6	12965919	ATTTTTTTT/ATTTTTTTTTT	GCACACAATCAAAATGTATCACC	ACATTTTTGCCCTCATTTCG	59.3	439	INTERGENIC	--	--	--

INDEL marker IDs	Chromosomes/ unanchored scaffolds	Physical positions (bp)	InDels (*Kabuli* reference genome-CDC Frontier/PII)	Forward primers (5'-3')	Reverse primers (5'-3')	Anneling temperature (0C)	Expected amplified product size (bp)	Structural annotation		Functional annotation	
								Sequence components of *kabuli* genome	*Kabuli* gene accession IDs	NCBI-KOG	TFs
Ca6_P4_12967108	Ca6	12967108	TA/TAA	ATAAGCCCCAAGACTGTCCA	CCCCTTAATTTTTGCATGGTT	59.6	664	INTERGENIC	--	--	--
Ca6_P4_12982915	Ca6	12982915	GTAATAA/GTAA	TCATGAAATGTTGCCTGGAA	TGACATACGGCTGGAATGAA	60.0	850	INTERGENIC	--	--	--
Ca6_P4_13001289	Ca6	13001289	GACACA/GACACACA	CGTTTTGTTGGGCATAATCA	CCTAGGCCAAAATTCAAGCA	59.4	773	INTERGENIC	--	--	--
Ca6_P4_13003025	Ca6	13003025	TTATATATATATATATATATAT/TTATATATATATATATATAT	CTCCCACTTGCATCCATCTT	TCTTCTTCTGGTGCAATCCC	60.1	770	INTERGENIC	--	--	--
Ca6_P4_13016905	Ca6	13016905	TCC/TCCC	TGGATCACCACAAACCACAC	GAACGGCACAGAGAGGAAAC	60.3	500	INTERGENIC	--	--	--
Ca6_P4_13033375	Ca6	13033375	CTATATATATATATATATAT/CTATATATATATATATATATATATAT	TCCTAGAGTCAATGGATCCCC	GGGAGGTGGTTCAGAGTCAT	60.3	473	INTERGENIC	--	--	--
Ca6_P4_13061150	Ca6	13061150	CTAT/CTATAT	TCCTTATGGTCCAAGCGAAC	CAAGTTATGCCCCTTTTGACA	60.1	758	INTERGENIC	--	--	--
Ca6_P4_13128943	Ca6	13128943	CTTTTTTTTTTT/CTTTTTTTTTT	CACCACGAGATCAAAACCCT	CCATTGGCAGTGGAACTCTT	60.0	482	INTERGENIC	--	--	--
Ca6_P4_13170216	Ca6	13170216	AATATATA/AATATATATA	TCAACCAAGAATTGCTGCTAAA	AGGGAGTATTCACGGCACAC	59.9	502	INTERGENIC	--	--	--
Ca6_P4_13180103	Ca6	13180103	TTTATTATTATTATTATTATTATTATTAT/TTTATTATTATTATTATTATTATTATTATTAT	TTTGAGTCAGTCAACGCAGG	TAAACCATTCAAACCCCCAA	60.0	830	INTERGENIC	--	--	--

INDEL marker IDs	Chromosomes/ unanchored scaffolds	Physical positions (bp)	InDels (*Kabuli* reference genome-CDC Frontier/PII)	Forward primers (5'-3')	Reverse primers (5'-3')	Anneling temperature (0C)	Expected amplified product size (bp)	Structural annotation		Functional annotation	
								Sequence components of *kabuli* genome	*Kabuli* gene accession IDs	NCBI-KOG	TFs
Ca6_P4_13381796	Ca6	13381796	TAAAAAAAAAAAA/TAAAAAAAAAAA	GGGTTCGTAGTTGTATGCGAA	CATGGATCAGAGTTGCCTTG	60.0	883	INTERGENIC	--	--	--
Ca6_P4_1343859	Ca6	1343859	GAAAAA/GAAAAAA	CAACGAGTTTGATTTCTATTCTAACG	TTGGGTAGCCAAGGTTTCAC	59.7	721	INTERGENIC	--	--	--
Ca6_P4_13447479	Ca6	13447479	TTATATATATATATAT/TTATATATATATATATAT	TCATGATGAGTCGGCAACAT	TCTCCGACCTTTAAAATCAACAA	60.1	552	INTERGENIC	--	--	--
Ca6_P4_13450334	Ca6	13450334	CAAAAAA/CAAAAA	TGATGCGCTTGCATATGTTT	TGTCAGTACCCACCCACTCA	60.2	483	INTERGENIC	--	--	--
Ca6_P4_13469022	Ca6	13469022	CAATTAA/CAA	CTCAAATTCTGTTTGGGGGA	TTGTGGTCCCTCAGAGAAGG	59.9	170	INTERGENIC	--	--	--
Ca6_P4_13475824	Ca6	13475824	AAAGAAGAAGAAGAAGAAGAA/AAAGAAGAAGAAGAAGAA	TGTGGTCCCAAACAATCTCA	TTTTGGGTCAAGGAAAATGG	59.9	330	INTERGENIC	--	--	--
Ca6_P4_1350878	Ca6	1350878	A/AGTCTGT	ACACATCAGCTTTTTGGCTG	AGCCCACTCACCTTCTTCCT	58.9	428	INTERGENIC	--	--	--
Ca6_P4_13536206	Ca6	13536206	TAAAAAAAAAA/TAAAAAAAAAAA	TTAACAAATAAGCCGGCGAC	AGGGGTTGTGAAGTCGACAC	60.1	302	INTERGENIC	--	--	--
Ca6_P4_1354322	Ca6	1354322	GAA/GAACCAA	AATTTGCATAGGCGGTGAAG	GGTGTTACAGCTTTCATGAACAAA	60.1	420	INTERGENIC	--	--	--
Ca6_P4_13578932	Ca6	13578932	AATATATATATATATATATATAT/AATATATATATATATATATAT	TCAGCAATGGCAAATATGGA	AAAAATTCCATCTATGCTTCACA	60.0	848	INTERGENIC	--	--	--

INDEL marker IDs	Chromosomes/ unanchored scaffolds	Physical positions (bp)	InDels (*Kabuli* reference genome-CDC Frontier/PII)	Forward primers (5'-3')	Reverse primers (5'-3')	Anneling temperature (0C)	Expected amplified product size (bp)	Structural annotation		Functional annotation	
								Sequence components of *kabuli* genome	*Kabuli* gene accession IDs	NCBI-KOG	TFs
Ca6_P4_13654782	Ca6	13654782	GTTT/GT	CATCAAAACTCAAACGCATCA	CACGTCAATTTTCGACATGA	59.7	721	INTERGENIC	--	--	--
Ca6_P4_13838996	Ca6	13838996	CCT/CCTTCT	TGTGTGATTGGTGTTGAGCA	GCAACAACGGGTGAAAATCT	59.7	563	INTERGENIC	--	--	--
Ca6_P4_13959907	Ca6	13959907	CTAATAATAATAATAATAATAATAATAATAATAATAATAATAATAATAATAATAATAATAA/CTAATAATAATAATAATAATAATAATAATAATAATAATAATAATAATAATAA	ATCTCCTTCTTTCGCACCAA	TTTTTCTGGCACGTATTGGA	59.8	722	INTERGENIC	--	--	--
Ca6_P4_14163783	Ca6	14163783	CATATATATATATATATATATATATATA/CATATATATATATATATATATATATATATATATATATA	TAATCGGGAAAAACCTGCAC	AATTTAATATCCCGTCCGGC	59.9	565	INTERGENIC	--	--	--
Ca6_P4_14171375	Ca6	14171375	TGAAAAATTTCGAAA/TGAAA	CATGCAACTCCACAGGAAAA	GGTCTTTGGTTTGAGATGGC	59.7	526	INTERGENIC	--	--	--
Ca6_P4_14206895	Ca6	14206895	CCATCATCAATC/CCATCATCAATCATCATCAATC	TGCAACCAGAACCAATGTGT	TGACCATACCTCACCAACCC	60.0	930	INTERGENIC	--	--	--
Ca6_P4_14222488	Ca6	14222488	GATATATATATATATATATATATA/GATATATATATATATATATA	TTTCAATGGGGGAAAGAATG	CTGAACAACCTTGTGCAACC	59.7	362	INTERGENIC	--	--	--
Ca6_P4_14260813	Ca6	14260813	AATATATATATATATATATATATATATATATATAT/AATATATATATATATATATATATATATATATATATAT	GGAGAAAATTAAGAGGGAGGGA	TTGATTTTGAAATGCACCCA	59.9	411	INTERGENIC	--	--	--
Ca6_P4_14410304	Ca6	14410304	TTTTATTTATTTATTTATTTATTTATTTATTTATTTATTTAT/TTTTATTTATTTATTTATTTATTTATTTAT	TTGTGCATGGTTGTGGATCT	TCTCAAGTTTGTAAGCGAGTCAA	60.0	193	INTERGENIC	--	--	--
Ca6_P4_14519498	Ca6	14519498	TG/T	TTGGTGTCACAATCAACGAAA	TTTTAAACCACATCGCACCA	60.0	442	INTERGENIC	--	--	--

INDEL marker IDs	Chromosomes/ unanchored scaffolds	Physical positions (bp)	InDels (*Kabuli* reference genome-CDC Frontier/PII)	Forward primers (5'-3')	Reverse primers (5'-3')	Anneling temperature (0C)	Expected amplified product size (bp)	Structural annotation		Functional annotation	
								Sequence components of *kabuli* genome	*Kabuli* gene accession IDs	NCBI-KOG	TFs
Ca6_P4_14553627	Ca6	14553627	TTATATATATAT/TTATATATAT	TGTTCATCAAGACCTG CACTG	CAAAAATTTGTGAGAGA CCAAAA	59.9	232	INTERGENIC	--	--	--
Ca6_P4_14661772	Ca6	14661772	ATTTTAATTT/ATTTTAATTTCACTTT TAATTT	TGGCTTTTCAAACTAAA GCAAA	TCTGATGTTGTCAATTG TGAAGG	59.1	561	INTERGENIC	--	--	--
Ca6_P4_14676799	Ca6	14676799	TTCTCTCTCTCTCTCTCTCT/TTCTC TCTCTCTCTCTCTCT	AGTGGGTGGTGCCAAT AGAG	ACCCAGGCTATGGAAT GTCA	60.0	491	INTERGENIC	--	--	--
Ca6_P4_14755534	Ca6	14755534	AATATATATATATATATATATATAT/A ATATATATATATATATATATATATATA TATATAT	TTTTAACCGTGGTTTGA AACTT	AGTCAGACGCTACTCG GCAT	57.8	212	INTERGENIC	--	--	--
Ca6_P4_14884145	Ca6	14884145	ATTTTTTTTTTTTT/ATTTTTTTTTTT	AGCACCGATTAGATGA GTTGAA	CAAGCTCGACTAGCAG CTCA	58.9	719	INTERGENIC	--	--	--
Ca6_P4_14938058	Ca6	14938058	TAAAAAAAAAA/TAAAAAAAAAAA	CCTCCTACATTGTGGG TGCT	ACATTTGGCCCATTTG GTTA	60.0	754	INTERGENIC	--	--	--
Ca6_P4_14959810	Ca6	14959810	CTTTTTTTTTTT/CTTTTTTTTTT	GCGAAATGCTTCTCAA AACC	CGTTACAACGTTGCTG AATGA	59.8	745	INTERGENIC	--	--	--
Ca6_P4_1506907	Ca6	1506907	CTTTTT/CTTTT	CTGGAAAAAGCACTTC AGGC	GCAAGAGGAAAAGACG AACG	60.0	461	INTERGENIC	--	--	--
Ca6_P4_15073082	Ca6	15073082	CATATATATATATATATATATATATA TATATATATA/CATATATATATATATA TATATATATATA	CACTCACTCTCCGCCA CTTA	CATTCTTCGGGTGGAG AAAA	59.0	780	INTERGENIC	--	--	--
Ca6_P4_15097608	Ca6	15097608	AATATATATATATATATATATATAT A/AATATATATATATATATATATATAT ATATA	TGTTTGAAAATTTTGAT GACTTTTT	AACAAGTTTTGATTGCA GGAA	58.3	543	INTERGENIC	--	--	--

INDEL marker IDs	Chromosomes/ unanchored scaffolds	Physical positions (bp)	InDels (*Kabuli* reference genome-CDC Frontier/PII)	Forward primers (5'-3')	Reverse primers (5'-3')	Anneling temperature (0C)	Expected amplified product size (bp)	Structural annotation		Functional annotation	
								Sequence components of *kabuli* genome	*Kabuli* gene accession IDs	NCBI-KOG	TFs
Ca6_P4_15277411	Ca6	15277411	AATTATTATTATTATTATTATTATTATTATTATTATTATTATT/AATTATTATTATTATTATTATTATTATTATTATTATT	CGTCTTTCTAATATTTTGCCGA	CATGAAAGTGGTCAGATGCG	58.5	700	INTERGENIC	--	--	--
Ca6_P4_1540640	Ca6	1540640	ATATTATTATTATTATTATTATTATTATTATTATTATTATTATTATTATTATTATTATTATTATTATTATTATTAT/ATATTATTATTATTATTATTATTATTATTATTATTATTATTATTATTATTAT[illegible]	GGAAATGGTGGTTTGATTGC	TGATAAGGAGTTGGTTGGTGTG	60.2	780	INTERGENIC	--	--	--
Ca6_P4_15412187	Ca6	15412187	CAAAAAAAAA/CAAAAAAAAAA	TATAACCACGGCGAAAATCC	GCACCGAATACCGACATCTT	59.8	460	INTERGENIC	--	--	--
Ca6_P4_1542114	Ca6	1542114	ATT/ATTT	TGCACTTTGTGCACTCCTTA	GTAATGCTGCGTGCAAAACA	58.1	334	INTERGENIC	--	--	--
Ca6_P4_15429919	Ca6	15429919	TAAA/TAAAA	TCGACCACATCAGCAACTTC	CCGATGGCTCTATGTCTTCC	59.8	737	INTERGENIC	--	--	--
Ca6_P4_15528104	Ca6	15528104	ATTTTTT/ATTTTTTT	TGCACCAAATGAGTCACATTATTA	TGTTGTGGATTTGAGCACTTCT	59.4	374	INTERGENIC	--	--	--
Ca6_P4_15555599	Ca6	15555599	AATATATA/AATATA	TGTGGGCACATTAGTAGGCA	TCATCGTCGTTGCTTGAGTC	60.1	640	INTERGENIC	--	--	--
Ca6_P4_15559125	Ca6	15559125	CTTT/CTT	TCACCAAACAAAAACTGGGG	CTACATTCCGGTTAGGGCAA	60.8	480	INTERGENIC	--	--	--
Ca6_P4_15559149	Ca6	15559149	AG/A	TCACCAAACAAAAACTGGGG	CTACATTCCGGTTAGGGCAA	60.8	480	INTERGENIC	--	--	--
Ca6_P4_1556325	Ca6	1556325	TAAA/TAAAAAAAAAAAAA	ATTGTTCTCCGTTGTCAGGG	GTGATCGACATACTTCGGCA	60.0	864	INTERGENIC	--	--	--

INDEL marker IDs	Chromosomes/ unanchored scaffolds	Physical positions (bp)	InDels (*Kabuli* reference genome-CDC Frontier/PII)	Forward primers (5'-3')	Reverse primers (5'-3')	Anneling temperature (0C)	Expected amplified product size (bp)	Structural annotation		Functional annotation	
								Sequence components of *kabuli* genome	*Kabuli* gene accession IDs	NCBI-KOG	TFs
Ca6_P4_1556869	Ca6	1556869	TAAAAA/TAAAAAA	CAATTTGGTTGGGCGTAAAC	TTGCTTTATACTCATATTTTGGCCT	60.2	528	INTERGENIC	--	--	--
Ca6_P4_1557117	Ca6	1557117	TAAAAAAAAA/TAAAAAAAAAA	GCTTTTCTTTCATATTGCCGTT	TTTTGAGCGGAAAGAGTCGT	59.6	674	INTERGENIC	--	--	--
Ca6_P4_1559094	Ca6	1559094	TATGA/TTTTAATTTTTAAGTAATGA	CAGAGCAGTGTGACAGCAAGA	TCAAAAATTTCAAACAAAATCCA	60.4	456	INTERGENIC	--	--	--
Ca6_P4_1559285	Ca6	1559285	GTTA/GTTATTA	CAGAGCAGTGTGACAGCAAGA	TCAAAAATTTCAAACAAAATCCA	60.4	456	INTERGENIC	--	--	--
Ca6_P4_1559581	Ca6	1559581	ATTTTTTTTTTTT/ATTTTTTTTTT	TGAAGTTAAATTAAAGGATCGCA	TATTTTATTCCCACCGCTGC	58.0	283	INTERGENIC	--	--	--
Ca6_P4_1559995	Ca6	1559995	AT/A	AATATTACTGCAGCGGTGGG	ATGCCAATTTTACCCTGCAC	60.0	552	INTERGENIC	--	--	--
Ca6_P4_1560016	Ca6	1560016	TTT/TTTAATT	AATATTACTGCAGCGGTGGG	GGACCACAAGAAATCCTCCA	60.0	719	INTERGENIC	--	--	--
Ca6_P4_15727094	Ca6	15727094	ATTTATTTTATTTT/ATTTATTTTATTTTATTTT	TCCCCCGTCTAACTAGGGTT	CCACTTGTGTCCTAACAATTCAA	59.8	268	INTERGENIC	--	--	--
Ca6_P4_15727514	Ca6	15727514	ATTTATTTTATTTTATTTTATTT/ATTTATTTTATTTTATTTTATTTTATTT	TTAGTTTGGGCTTGGGTTTT	TTGCTCCTACGTATCTCCCG	58.6	911	INTERGENIC	--	--	--
Ca6_P4_15728024	Ca6	15728024	AA/AAATA	CGGGAGATACGTAGGAGCAA	TCGATTATGCGTAGGGAAGG	60.2	248	INTERGENIC	--	--	--

INDEL marker IDs	Chromosomes/ unanchored scaffolds	Physical positions (bp)	InDels (*Kabuli* reference genome-CDC Frontier/PII)	Forward primers (5'-3')	Reverse primers (5'-3')	Anneling temperature (0C)	Expected amplified product size (bp)	Structural annotation		Functional annotation	
								Sequence components of *kabuli* genome	*Kabuli* gene accession IDs	NCBI-KOG	TFs
Ca6_P4_15730699	Ca6	15730699	TAAA/TAA	ATTTGAACCTGGCAGCAAAA	GGTTTGAAAAAGGGGTGATG	60.6	175	INTERGENIC	--	--	--
Ca6_P4_15730843	Ca6	15730843	T/TGA	ATTTGAACCTGGCAGCAAAA	GCTTGATGAATAAATCAATGGCT	60.6	525	INTERGENIC	--	--	--
Ca6_P4_15731057	Ca6	15731057	TAAAAA/TAA	ATTTGAACCTGGCAGCAAAA	GGGACAAAAGGCTAGTTTGG	60.6	909	INTERGENIC	--	--	--
Ca6_P4_15897034	Ca6	15897034	AATATTATATTATA/AATATTATA	TGCACCCACCTCTTTTTAGA	CGGAAAACTTGAGGGTTTCAA	58.4	245	INTERGENIC	--	--	--
Ca6_P4_15966204	Ca6	15966204	GTTATTATTATTATTATTATTATTATTATTATTATTATTATTATTATTA/GTTATTATTATTATTATTATTATTATTATTATTATTATTATTATTA	CTGCCTGAAATTTGGTTGCT	AGATGCGACCTCCTACTTGC	60.2	229	INTERGENIC	--	--	--
Ca6_P4_15999975	Ca6	15999975	A/AG	AAGTGTGGGCATTTCCTTTG	ATGGACCTCTGTATTCGCCA	60.0	137	INTERGENIC	--	--	--
Ca6_P4_16012701	Ca6	16012701	TA/TAA	TAAGAGTTTGGTTCCGGTGG	TGTGCAGATAAATGGCCAAA	60.0	397	INTERGENIC	--	--	--
Ca6_P4_16063357	Ca6	16063357	AT/ATT	CGTGGAAAACTTCCCTCAAA	TGAGTGGTTAGTCCCACATTGA	60.1	473	INTERGENIC	--	--	--
Ca6_P4_16093896	Ca6	16093896	AAAAAATATATAAAAATATA/AAAAAATATA	TGCAAAAAGATGGCAATGAA	TTTCCTTAAATATCCATCCAGTCA	60.2	598	INTERGENIC	--	--	--
Ca6_P4_16121564	Ca6	16121564	ACATCAATCTCATCAAT/ACATCAAT	TCGGTTGCCTATGGTACTCC	ACGGCAGTAGCCATTTGAGT	60.0	487	INTERGENIC	--	--	--

INDEL marker IDs	Chromosomes/ unanchored scaffolds	Physical positions (bp)	InDels (*Kabuli* reference genome-CDC Frontier/PII)	Forward primers (5'-3')	Reverse primers (5'-3')	Anneling temperature (0C)	Expected amplified product size (bp)	Structural annotation		Functional annotation	
								Sequence components of *kabuli* genome	*Kabuli* gene accession IDs	NCBI-KOG	TFs
Ca6_P4_16191754	Ca6	16191754	GAAAAAAAAAA/GAAAAAAAAAAA	GGAAAGAGAGGAAACCAGGG	GCAGCTGAGTCATCCCCTAT	60.0	717	INTERGENIC	--	--	--
Ca6_P4_16259714	Ca6	16259714	GTTATTATTATTATTATTATTATTATTATTATT/GTTATTATTATTATTATTATTATTATTATT	GGGGGCAAGTTATATCCACC	TGGAAAATGTAACACCCCAAA	60.4	782	INTERGENIC	--	--	--
Ca6_P4_16286125	Ca6	16286125	CTTACTTTTT/CTTACTTTTTACTTTTT	TGTTGGGATATTTCCTTTGGA	CTTGAAAGAGCCGCAAGAAG	59.3	576	INTERGENIC	--	--	--
Ca6_P4_1629307	Ca6	1629307	TTACTACTACTACTACTACTA/TTACTACTACTACTACTA	GGAGTGAGTACCGTGGGAGA	TGTGACACATCCCGTGTCTT	60.1	229	INTERGENIC	--	--	--
Ca6_P4_16370279	Ca6	16370279	AATATATATATATATATATATATA/AATATATATATATATATATATATATATATATATA	CAGATTCATGCATATGGTCAAAA	GGAAAGCTTGCTTGATAAATACG	59.8	568	INTERGENIC	--	--	--
Ca6_P4_16400010	Ca6	16400010	TTATATATATATATATATATA/TTATATATATATATATATA	GGATGATCAAACTCGCAGGT	TTGACGCTTTTAGAGGCAGAA	60.1	785	INTERGENIC	--	--	--
Ca6_P4_16522140	Ca6	16522140	GAAAAAAAA/GAAAAAAA	CAAAAGCAAAAGAGGTTGAACA	TGGATTGACGATGAAGGAAA	59.4	907	INTERGENIC	--	--	--
Ca6_P4_16557006	Ca6	16557006	GTTTTTTTTTT/GTTTTTTTTTTT	TTGCTTCAGGGCCTTACATT	CAGTTTCACGCACTCAATCG	59.7	700	INTERGENIC	--	--	--
Ca6_P4_16579245	Ca6	16579245	ATTTTATGT/ATTTTATGTTTTATGT	AAGGAATACGTTATCACCGATAAAA	CCTTTGAAATAAACATGCTGC	59.3	714	INTERGENIC	--	--	--
Ca6_P4_16693405	Ca6	16693405	GAAAAAAAAAA/GAAAAAAAAAAA	TCGGACGACTAGTCCCAATC	CAACCAAAGCAGGGGTTTTA	60.1	767	INTERGENIC	--	--	--

INDEL marker IDs	Chromosomes/ unanchored scaffolds	Physical positions (bp)	InDels (*Kabuli* reference genome-CDC Frontier/PII)	Forward primers (5'-3')	Reverse primers (5'-3')	Anneling temperature (0C)	Expected amplified product size (bp)	Structural annotation		Functional annotation	
								Sequence components of *kabuli* genome	*Kabuli* gene accession IDs	NCBI-KOG	TFs
Ca6_P4_16710889	Ca6	16710889	AATATATATATATATATATATATA/AATATATATATATATATATATATATATA	TGTAACCAACACAAGGGGGT	TCTCGGCGTCTAAAAGTACAC	60.1	615	INTERGENIC	--	--	--
Ca6_P4_1682722	Ca6	1682722	GTGCATCTGTTT/G	GAGGCATTGTCTTTGGTGGT	GCAAATGTTTGGAATGGCTT	60.0	656	INTERGENIC	--	--	--
Ca6_P4_16911701	Ca6	16911701	ATTTTTTTTTTTT/ATTTTTTTTTTTTT	GTTTGGTGGGGATGAGAAGA	CACATGTTGAATGCATTGACTAAA	59.9	794	INTERGENIC	--	--	--
Ca6_P4_16942985	Ca6	16942985	ATTTTTTTTTTT/ATTTTTTTTTT	TGGCAGGTGAACTCGTGATA	TGAATCATTTTGTCATGTCCTCA	60.3	544	INTERGENIC	--	--	--
Ca6_P4_16949745	Ca6	16949745	GATATATATATATATATATATAT/GATATATATATATATATATAT	GGACGACATTGATGTGGAAA	TTGTTGGAAGTTTGTTGAGGC	59.3	448	INTERGENIC	--	--	--
Ca6_P4_16995027	Ca6	16995027	A/AT	TTAAAGGCAGATGGGCAAAC	TCAACAAACTGGTAGCGTCG	60.1	293	INTERGENIC	--	--	--
Ca6_P4_16999043	Ca6	16999043	CTTATTATTATTATTATTATTATTATTAT/CTTATTATTATTATTATTATTATTATTATTATTAT	TTTTCCACCACTTTTTCTTCTC	TTCATTCTTTGTGCCACCAG	57.5	196	INTERGENIC	--	--	--
Ca6_P4_17025329	Ca6	17025329	ATTTTTTTTT/ATTTTTTTTTT	TTTTGCTCCACGTTTTCCTT	GGCTTATAGGCCCACGTTTT	59.7	562	INTERGENIC	--	--	--
Ca6_P4_17025431	Ca6	17025431	ATTTTTTTTTTTT/ATTTTTTTTTTT	TTTTGCTCCACGTTTTCCTT	GGCTTATAGGCCCACGTTTT	59.7	562	INTERGENIC	--	--	--
Ca6_P4_17025729	Ca6	17025729	TAAAAAAAAAAAA/TAAAAAAAAAA	TTTTGCTCCACGTTTTCCTT	GGCTTATAGGCCCACGTTTT	59.7	562	INTERGENIC	--	--	--

INDEL marker IDs	Chromosomes/ unanchored scaffolds	Physical positions (bp)	InDels (*Kabuli* reference genome-CDC Frontier/PII)	Forward primers (5'-3')	Reverse primers (5'-3')	Anneling temperature (0C)	Expected amplified product size (bp)	Structural annotation		Functional annotation	
								Sequence components of *kabuli* genome	*Kabuli* gene accession IDs	NCBI-KOG	TFs
Ca6_P4_17056993	Ca6	17056993	ATTTTTTTT/ATTTTTTT	AAAAGCTAGTTTTATTATGGAGCAGT	TGTGGCACGCACTTTCTAAC	57.6	817	INTERGENIC	--	--	--
Ca6_P4_17057027	Ca6	17057027	TAAAAAAAA/TAAAAAAA	TGGATACAACCTAAAATGCCG	TGTGGCACGCACTTTCTAAC	59.8	337	INTERGENIC	--	--	--
Ca6_P4_17058914	Ca6	17058914	AT/A	TTGGTAAGGAAAGGGGAAAAA	TGAGATCAACGCTTTTTGGTT	59.8	598	INTERGENIC	--	--	--
Ca6_P4_17059083	Ca6	17059083	CGA/C	TGAGTAGATTCACGTCACCCA	ATAAAATGGACACAGGCGCT	59.2	683	INTERGENIC	--	--	--
Ca6_P4_17063685	Ca6	17063685	CA/CAA	CAGAAGATCAAGATGCCCGT	AAATTTTCGGGAGTTCCACC	60.2	344	INTERGENIC	--	--	--
Ca6_P4_17076415	Ca6	17076415	TT/TTAT	ACATCCCCTCACTCTCAACG	ATCGTTGCACTCAAACGACA	60.1	551	INTERGENIC	--	--	--
Ca6_P4_17083127	Ca6	17083127	AGA/AGAATTTTGA	TTAGCTGAATCACGAACCCC	TGATTCGCGAAAATAGTCCC	60.1	271	INTERGENIC	--	--	--
Ca6_P4_17085039	Ca6	17085039	GAAAAAAAA/GAAAAAAA	GGTTTATTGCAAGAGTGGGC	CAGTCATTTGCATTTTTGCG	59.6	560	INTERGENIC	--	--	--
Ca6_P4_17085352	Ca6	17085352	TTTTATTTATTT/TTTTATTTATTTATTT	CGCAAAAATGCAAATGACTG	TTCCCCATGCATCACTATCA	60.3	385	INTERGENIC	--	--	--
Ca6_P4_17088463	Ca6	17088463	TAAAAA/TAAAAAA	ACAAAGAGGGTGGTAAGGGG	AAATGTTGCCAAATTCCATTG	60.2	839	INTERGENIC	--	--	--

INDEL marker IDs	Chromosomes/ unanchored scaffolds	Physical positions (bp)	InDels (*Kabuli* reference genome-CDC Frontier/PII)	Forward primers (5'-3')	Reverse primers (5'-3')	Anneling temperature (0C)	Expected amplified product size (bp)	Structural annotation		Functional annotation	
								Sequence components of *kabuli* genome	*Kabuli* gene accession IDs	NCBI-KOG	TFs
Ca6_P4_17089209	Ca6	17089209	AT/ATCT	TTCAATGGAATTTGGCAACA	TTCCGAAGACGATGAAAATTG	59.9	454	INTERGENIC	--	--	--
Ca6_P4_17097536	Ca6	17097536	TGAG/TG	TAACCATCGCAAACCATGTG	AGGTTGCAACTTTTGGTCAT	60.4	915	INTERGENIC	--	--	--
Ca6_P4_17101167	Ca6	17101167	AT/A	AAAAGTTGTGCCTTTCTTTTTCA	AGCAAGAAATGTGTGACGCTT	59.4	776	INTERGENIC	--	--	--
Ca6_P4_17101873	Ca6	17101873	AG/A	AAGCGTCACACATTTCTTGCT	TCGCATTATTTAAAGCATCTCA	59.9	638	INTERGENIC	--	--	--
Ca6_P4_17102055	Ca6	17102055	AG/A	AAAACATAAAGTGGGGAAATCAA	AAGCGTTGGATGGAGAGAGA	58.8	707	INTERGENIC	--	--	--
Ca6_P4_17102897	Ca6	17102897	TGG/TG	CTCTCTCCATCCAACGCTTC	ATCTCGATCCAGCGATCATT	60.0	513	INTERGENIC	--	--	--
Ca6_P4_17103746	Ca6	17103746	TAAAAAA/TAAAAA	CCCTCTTTTACGAGTACCGTGT	GGTTCATGCAGGTGGAAAAT	59.6	928	INTERGENIC	--	--	--
Ca6_P4_17103901	Ca6	17103901	CATGTATATGTATAT/CATGTATAT	CAAAACAAAAACCCATCAAGAA	GGTTCATGCAGGTGGAAAAT	59.0	311	INTERGENIC	--	--	--
Ca6_P4_17109515	Ca6	17109515	GTATATATATAT/GTATATATATATATATAT	TGCAGAGTGCGAGATTTGTC	TCGATGTCTCGTGCATGTTT	60.1	433	INTERGENIC	--	--	--
Ca6_P4_17136094	Ca6	17136094	GAAA/GAA	AGACAAGCACGTCATTGGTG	TTTGCTTGTTTGGTCCATGA	59.8	219	INTERGENIC	--	--	--

INDEL marker IDs	Chromosomes/ unanchored scaffolds	Physical positions (bp)	InDels (*Kabuli* reference genome-CDC Frontier/PII)	Forward primers (5'-3')	Reverse primers (5'-3')	Anneling temperature (0C)	Expected amplified product size (bp)	Structural annotation		Functional annotation	
								Sequence components of *kabuli* genome	*Kabuli* gene accession IDs	NCBI-KOG	TFs
Ca6_P4_17136205	Ca6	17136205	CAAAAA/CAAAAAA	TCATGGACCAAACAAGCAAA	ATTTCTCGCGACATGCATTA	60.1	238	INTERGENIC	--	--	--
Ca6_P4_17138585	Ca6	17138585	TC/T	GGGGTGGGAAACTATGTTCA	AATTAGGGGGATCAGTTCCG	59.6	496	INTERGENIC	--	--	--
Ca6_P4_17139579	Ca6	17139579	TA/T	TTACGAAAGGTTTTGGCACA	AACGAGGTTGAATAATGCGG	59.2	583	INTERGENIC	--	--	--
Ca6_P4_17170239	Ca6	17170239	GTATTATTATTATTATTAT/GTATTATTATTATTAT	TTTGTTTGGGGGATGGTAAA	GGGTTTTTAGTGTGGGGGTT	60.0	547	INTERGENIC	--	--	--
Ca6_P4_17170994	Ca6	17170994	TTCTCTCTCTCTCTCTCT/TTCTCTCTCTCTCTCTCT	AACCCCCACACTAAAAACCC	AGGGAATTCGGACTTACGGT	60.0	747	INTERGENIC	--	--	--
Ca6_P4_17191873	Ca6	17191873	GTTTTTTTTT/GTTTTTTTTTT	TTGATATTGTGTTTGATTTCGTTT	TTCGAGAGAAATTGGGGTTG	57.7	653	INTERGENIC	--	--	--
Ca6_P4_17199265	Ca6	17199265	TTATATATATATAT/TTATATATATATATAT	TTCCCACCCCTACTGCTATG	TTTCATATTCAAATTTCTCACATTG	59.9	707	INTERGENIC	--	--	--
Ca6_P4_1729337	Ca6	1729337	GTATTATTATTATTATTATTATTATTATTATTATTATTATTATTATTAT/GTATTATTATTATTATTATTATTATTATTATTATTATTATTAT	TGGCTTAATTGTCCTTTTTGATG	TGCCGTCTTCAATGCTACAG	60.3	654	INTERGENIC	--	--	--
Ca6_P4_17333891	Ca6	17333891	TTATATATATATATATAT/TTATATATATATATATAT	TGCATAAGTTTCTCGTTGCG	CTTGCTGCAAAAATCGTTGA	60.0	683	INTERGENIC	--	--	--
Ca6_P4_17380756	Ca6	17380756	GTTTTTTTTT/GTTTTTTTTTT	CAAGTCATTTGCCGTTGAGA	TGGCAAGCAACATAAATGGA	59.8	841	INTERGENIC	--	--	--

INDEL marker IDs	Chromosomes/ unanchored scaffolds	Physical positions (bp)	InDels (*Kabuli* reference genome-CDC Frontier/PII)	Forward primers (5'-3')	Reverse primers (5'-3')	Anneling temperature (0C)	Expected amplified product size (bp)	Structural annotation		Functional annotation	
								Sequence components of *kabuli* genome	*Kabuli* gene accession IDs	NCBI-KOG	TFs
Ca6_P4_17398675	Ca6	17398675	ATTTTTTTTTTTTT/ATTTTTTTTTTTT	TCCATTTGATCGATGCTTCA	AACTTGGCGACACCAAAATC	60.2	305	INTERGENIC	--	--	--
Ca6_P4_17414159	Ca6	17414159	TTATATATATATATATATATATATAT/TTATATATATATATATATATATAT	CATGCAATTGGAAGCTCTGA	ATTTGGCAACTCTTGGGATG	59.9	599	INTERGENIC	--	--	--
Ca6_P4_17503155	Ca6	17503155	GATATATATATATAT/GATATATATATATATAT	ATTCCCATTAAGTGCCCACA	TCCAAATGGCGACTTCCTAC	60.2	556	INTERGENIC	--	--	--
Ca6_P4_1768942	Ca6	1768942	CTTTT/CTTT	GCATGCAGCTAAATGAATCAA	GTTCTTGGCCTGTTGTGGTT	58.9	212	INTERGENIC	--	--	--
Ca6_P4_17710771	Ca6	17710771	CAATAATAATAATAATAATAATAATAATAATAATAAT/CAATAATAATAATAATAATAATAATAATAATAATAAT	CAGCTAAGAGCGACTCATTGAC	GTGTTGTCTGGGCCTTCATT	59.3	618	INTERGENIC	--	--	--
Ca6_P4_17744108	Ca6	17744108	CTTTTTTTTTTTT/CTTTTTTTTTTT	TTATTGCACGATTACATGTCG	ATGCGTTAACGGAACATCAA	57.2	531	INTERGENIC	--	--	--
Ca6_P4_17844765	Ca6	17844765	GTATATATATATAT/GTATATATATATATAT	GCAAATATGCAACAACCTTCC	TTAATTTGGGTTAGACGCGG	59.5	467	INTERGENIC	--	--	--
Ca6_P4_17847880	Ca6	17847880	AAATAATAATAATAATAATAATAATAATAATAATAATAATAATAATAATAA/AAATAATAATAATAATAATAATAATAATAATAATAATAATAATAA	TAGCCGGCTTTTAGTTGACC	TTCATGGTTGAAAAGTGATTGTG	59.4	357	INTERGENIC	--	--	--
Ca6_P4_1788325	Ca6	1788325	CTTTTTTTTTTT/CTTTTTTTTTT	AGGGTGTAAGGCATGGATGA	CCATCAATCAAAGCAAACCA	60.3	330	INTERGENIC	--	--	--
Ca6_P4_17930276	Ca6	17930276	ATTTTTTT/ATTTTTTTT	TCCTCCCTTAATTCATTCGTG	TCAGTTGGTGACCCATGAAC	59.0	391	INTERGENIC	--	--	--

INDEL marker IDs	Chromosomes/ unanchored scaffolds	Physical positions (bp)	InDels (*Kabuli* reference genome-CDC Frontier/PII)	Forward primers (5'-3')	Reverse primers (5'-3')	Anneling temperature (0C)	Expected amplified product size (bp)	Structural annotation		Functional annotation	
								Sequence components of *kabuli* genome	*Kabuli* gene accession IDs	NCBI-KOG	TFs
Ca6_P4_1801492	Ca6	1801492	GTTTTTTTT/GTTTTTTTTT	ACGGTGAACTGGTGGACTTC	TCACACATTCGCTCAAATCC	60.0	482	INTERGENIC	--	--	--
Ca6_P4_181004	Ca6	181004	TAAAAAA/TAAAAAAA	AAACCTATCCGCGTCAACAC	TCGATGAATTATAAAGAGTCTTCAGTG	60.0	856	INTERGENIC	--	--	--
Ca6_P4_1814002	Ca6	1814002	TTCTCTCTCTCTCT/TTCTCTCTCTCT	TCGAAGTGACTCTGAAGGATGA	TTGAACCAAAGAACATCGCA	60.0	324	INTERGENIC	--	--	--
Ca6_P4_18175044	Ca6	18175044	ATATATATAT/ATATATATATCTATATATAT	TTTCTTTCTTGCGTGACCATT	GCCATGCACTGTCAAGAGAA	59.7	650	INTERGENIC	--	--	--
Ca6_P4_18180870	Ca6	18180870	TTATATATATATATATATATATATATATATATATATA/TTATATATATATATATATATATATATATATATATATA	GAGGTCGATTGGTCATTATGG	TCATCATTCCACCACAGCAT	59.3	573	INTERGENIC	--	--	--
Ca6_P4_18216681	Ca6	18216681	AATTATTATTATTATTATTATTATTATTA/AATTATTATTATTATTATTATTATTATTATTATTATTATTA	TGTTCAACCGCATTAATTTGA	ATAGCAACTTCCGAGATGCG	59.1	517	INTERGENIC	--	--	--
Ca6_P4_1824314	Ca6	1824314	C/CTCACAAA	TTGCAGAATGCATGGGAATA	TCCAAACTCTCCGACCAAAC	60.0	779	INTERGENIC	--	--	--
Ca6_P4_1824646	Ca6	1824646	AG/A	GTTTGGTCGGAGAGTTTGGA	CCCTCCTTATCCTTACGGTTG	60.1	470	INTERGENIC	--	--	--
Ca6_P4_1824728	Ca6	1824728	ATTTTT/ATTTT	GTTTGGTCGGAGAGTTTGGA	CCCTCCTTATCCTTACGGTTG	60.1	470	INTERGENIC	--	--	--
Ca6_P4_1833817	Ca6	1833817	AAGG/A	AAGGTGGGTTCAGACCTTCT	ATTCCTCGGTTGACGAGATG	57.7	450	INTERGENIC	--	--	--

**INDEL marker IDs**	**Chromosomes/ unanchored scaffolds**	**Physical positions (bp)**	**InDels (*Kabuli* reference genome-CDC Frontier/PII)**	**Forward primers (5'-3')**	**Reverse primers (5'-3')**	**Anneling temperature (0C)**	**Expected amplified product size (bp)**	**Structural annotation**		**Functional annotation**	
								**Sequence components of *kabuli* genome**	***Kabuli* gene accession IDs**	**NCBI-KOG**	**TFs**
Ca6_P4_1834196	Ca6	1834196	TT/TTTTATTTTAT	CATCTCGTCAACCGAGGAAT	ATTTGAGACTTGCGGGAGAA	60.1	302	INTERGENIC	--	--	--
Ca6_P4_18414573	Ca6	18414573	TTATATATATATATATATATATATATATATATAT/TTATATATATATATATATATATATAT	TCAACAAACTTCGTCACATTCA	TCCTCACGGACGACTTAACC	59.2	382	INTERGENIC	--	--	--
Ca6_P4_18423210	Ca6	18423210	GTATATATATATATATATATATATA/GTATATATATATATATATATA	CGCGGGATGTATTTTACCAA	TACCCGACGAACTCGAAAAC	60.7	286	INTERGENIC	--	--	--
Ca6_P4_1843596	Ca6	1843596	C/CCTAACG	AGCTGAGAGATGTGGCGTTT	TCTGCGAATCCGTCTTAACC	60.0	594	INTERGENIC	--	--	--
Ca6_P4_18471682	Ca6	18471682	AATATATATATATATATATA/AATATATATATATATATATATA	TCCTTTGTTGGATTCTTTGCT	ACACCCGAACTGAACTCACC	58.8	844	INTERGENIC	--	--	--
Ca6_P4_18508690	Ca6	18508690	CTA/C	AACATGTGGTGGTGGGTTTT	GCCGAGCCCATCTAAACATA	60.0	852	INTERGENIC	--	--	--
Ca6_P4_18567135	Ca6	18567135	AAATAATAATAATAATAATAATAATAATAATAATAATAA/AAATAATAATAATAATAATAATAATAATAATAATAATAATAATAATAA	TCAACGAGGTTGCATACCAG	TGATCCGGATAAAAATAGGAGG	59.7	426	INTERGENIC	--	--	--
Ca6_P4_18570186	Ca6	18570186	ATTTTTTTT/ATTTTTTTTT	GGCTCAGGGGATTTATAGGC	CCCACCAAACCGATCTAAAA	59.9	517	INTERGENIC	--	--	--
Ca6_P4_18625376	Ca6	18625376	TTAATAATAATAATAATAATAATAATAATAATAATAA/TTAATAATAATAATAATAATAATAA	CGAGTACATCGCTAATTTTGGA	AGCGGTTTGGTTCAGTTTTG	59.3	449	INTERGENIC	--	--	--
Ca6_P4_18626197	Ca6	18626197	TAGTCCAA/TA	CCGACTATCTTTTTGTCTCATAACC	CATCACCCATCAGGAGACCT	59.5	407	INTERGENIC	--	--	--

INDEL marker IDs	Chromosomes/ unanchored scaffolds	Physical positions (bp)	InDels (*Kabuli* reference genome-CDC Frontier/PII)	Forward primers (5'-3')	Reverse primers (5'-3')	Anneling temperature (0C)	Expected amplified product size (bp)	Structural annotation		Functional annotation	
								Sequence components of *kabuli* genome	*Kabuli* gene accession IDs	NCBI-KOG	TFs
Ca6_P4_1866112	Ca6	1866112	G/GCAA	TGAGTTGAAGGGGTCGTTTC	AATTGACCCTTAAGCATGGC	60.1	506	INTERGENIC	--	--	--
Ca6_P4_1866939	Ca6	1866939	TT/TTCT	ATAGGAACAATGCACCCCAC	GGAAGTTTGGTAACGGTGGA	59.7	538	INTERGENIC	--	--	--
Ca6_P4_1866990	Ca6	1866990	A/AT	ATAGGAACAATGCACCCCAC	GGAAGTTTGGTAACGGTGGA	59.7	538	INTERGENIC	--	--	--
Ca6_P4_18780292	Ca6	18780292	TAAAAA/TAAAA	AAGGCACGTAACCAAATTGC	TGAATTAGGATGTGCTTCAAGG	60.0	771	INTERGENIC	--	--	--
Ca6_P4_18790835	Ca6	18790835	TCCGTTGAG/T	TGCATGGTACGCCAAATAAA	AGCGGGTGGTTGCTAAAGTA	60.0	869	INTERGENIC	--	--	--
Ca6_P4_1898836	Ca6	1898836	GAAAAAAAAA/GAAAAAAAAAA	GGTTTGCAAGATGGTCGAAT	AAACACAAACCACCGACCTC	59.9	415	INTERGENIC	--	--	--
Ca6_P4_19063456	Ca6	19063456	CATATATATATATATATATATATATATATAT/CATATATATATATATATATATATATATATAT	GGTAAGAAATTTCCATCCATGTT	TGTTATGCATTGGTACTGTTCTGA	58.3	544	INTERGENIC	--	--	--
Ca6_P4_19093521	Ca6	19093521	TTATATATATATATATATATAT/TTATATATATATATATATAT	TTTGCGATCAAGTCTCATCAA	TGTCTTGCCTCTCCCACTCT	59.4	541	INTERGENIC	--	--	--
Ca6_P4_1939700	Ca6	1939700	AATATATATATATATAT/AATATATATATATATAT	TGATCAATTTACCAACGATTACAGA	ATTTTACATGCCCAAAAGCG	59.8	718	INTERGENIC	--	--	--
Ca6_P4_19421254	Ca6	19421254	GAATTTTTATTTATAATTTTTA/GAATTTTTATTTATAATTTTTATTTATAATTTTTA	CTCATGCTTGGGCATCACTA	TGATCAATGGAAGCCTGAAA	59.8	483	INTERGENIC	--	--	--

INDEL marker IDs	Chromosomes/ unanchored scaffolds	Physical positions (bp)	InDels (*Kabuli* reference genome-CDC Frontier/PII)	Forward primers (5'-3')	Reverse primers (5'-3')	Anneling temperature (0C)	Expected amplified product size (bp)	Structural annotation		Functional annotation	
								Sequence components of *kabuli* genome	*Kabuli* gene accession IDs	NCBI-KOG	TFs
Ca6_P4_1955247	Ca6	1955247	GAAAAAAAA/GAAAAAAA	ATCATGCCATTCATGACAGC	CACACGCCTTGACTACCTCA	59.5	405	INTERGENIC	--	--	--
Ca6_P4_19591689	Ca6	19591689	CATATATATATATATATATATATATAT/CATATATATATATATATATATATATATATATATAT	TGGAACACACATTTGTCATTGAT	TGTGCATATCTGCAGTTTCATTT	60.1	709	INTERGENIC	--	--	--
Ca6_P4_19606481	Ca6	19606481	TAAAAAA/TAAAAAAA	TGCATGGTCAAGGTTCCATA	CACCCCTCCAAATCCTTACA	59.9	931	INTERGENIC	--	--	--
Ca6_P4_19616619	Ca6	19616619	TATAATAATAATAATAATA/TATAATAATAATAATAATAATA	TGAGGATGCTCTATGGACCC	GCCCAATTCAAACGCATATT	60.0	593	INTERGENIC	--	--	--
Ca6_P4_19657592	Ca6	19657592	CTATATATATATATATATATATATAT/CTATATATATATATATATATATATAT	CAAGCGCTATGTGACCCTTT	AATGTGAATCCCACCAGGAA	60.3	611	INTERGENIC	--	--	--
Ca6_P4_19693307	Ca6	19693307	CTTTTTTTTTTTTT/CTTTTTTTTTTTTT	AACCTAGCAGCGGTGAAAAA	CCGGAAGTGTTTTGTGTGTG	59.9	637	INTERGENIC	--	--	--
Ca6_P4_19700932	Ca6	19700932	AAATAATAATAATAATAATAATAATAATAATAATAATAATAATAATAATAATAATA/AAATAATAATAATAATAATAATAATAATAATAATAATAATAATAATAATAATAATAATAATAATAATA	CTTAGAGCATCTCCAACGGC	CGTCCACAAACTTTTCTTGC	60.0	510	INTERGENIC	--	--	--
Ca6_P4_1975496	Ca6	1975496	CGCTG/CGCTGCTACATCTGCTG	GTAGTTTGGCTTCCCTTCCC	GCTTTTGCGGCATCTAGTTC	59.9	458	INTERGENIC	--	--	--
Ca6_P4_197574	Ca6	197574	GATAATAATAATAATAATAATAATAAT/GATAATAATAATAATAATAATAATAATAATAAT	GGGACTGGGGTCATTTAGGT	TCAATTTGCTGTCCCATTCA	60.1	993	INTERGENIC	--	--	--
Ca6_P4_2024308	Ca6	2024308	GTTTTTTTTT/GTTTTTTTT	TTGTCGTGCCATTAGTGACC	TCAATCGGAAATTTTATTTTTGAA	59.6	773	INTERGENIC	--	--	--

INDEL marker IDs	Chromosomes/ unanchored scaffolds	Physical positions (bp)	InDels (*Kabuli* reference genome-CDC Frontier/PII)	Forward primers (5'-3')	Reverse primers (5'-3')	Anneling temperature (0C)	Expected amplified product size (bp)	Structural annotation		Functional annotation	
								Sequence components of *kabuli* genome	*Kabuli* gene accession IDs	NCBI-KOG	TFs
Ca6_P4_2032078	Ca6	2032078	CATTATTATTATTATTATTATTATT/CATTATTATTATTATTATTATT	CCTCTCAACCCCTCTCCTCT	GTTGGTGAACAATGCACAGG	59.8	524	INTERGENIC	--	--	--
Ca6_P4_20659055	Ca6	20659055	AC/A	TGGAAATGAAAACAACACCG	TGAAAATCGATACTGCCCCT	59.4	303	INTERGENIC	--	--	--
Ca6_P4_20679328	Ca6	20679328	CATATATATATATATAT/CATATATATATATATAT	CAAATTTGTCACGGAAAGCA	CAATACTTGTGTTGTGGCGG	59.7	605	INTERGENIC	--	--	--
Ca6_P4_20697646	Ca6	20697646	CATATATATATATATATATATATATATAT/CATATATATATATATATATATATATATATATAT	CAAACAAGAAACATGGACCTCA	TGACGTTAACTTGCAGCCAC	60.0	504	INTERGENIC	--	--	--
Ca6_P4_2086027	Ca6	2086027	CAATAATAATAATAATAATAATAATAATAATAATAA/CAATAATAATAATAATAATAATAATAATAATAATAATAA	CGACCATCACAACAAAACAA	TTGAAATTGAAAATCGGTACACA	58.0	216	INTERGENIC	--	--	--
Ca6_P4_20883267	Ca6	20883267	ACCTAATCCTA/ACCTA	TGAAGGGACACTTGCACAAA	AATGTGTCGATGTGCGCTAA	60.3	362	INTERGENIC	--	--	--
Ca6_P4_20995016	Ca6	20995016	TAA/TA	GCTTGGGGACAAGCAATAGA	CCCAAGTTCCCAAGAGTCAA	60.2	566	INTERGENIC	--	--	--
Ca6_P4_21013539	Ca6	21013539	ATTTTTTTTTTT/ATTTTTTTTTT	GATGCATAAATTACTTTGCCACC	TCCTACACTCAGCCCAGTCC	59.8	256	INTERGENIC	--	--	--
Ca6_P4_21021586	Ca6	21021586	CTATTTATTTATTTATTTATTTATTT/CTATTTATTTATTTATTTATTT	CACCGTCGTGTTCATCTGTC	TTATTTCACCGTCGTGTCCA	60.2	458	INTERGENIC	--	--	--
Ca6_P4_2108083	Ca6	2108083	A/ATTATGT	CTGGAATTCTTCTGCTTGGC	CCATGATTTGGATTTTGGTG	60.0	807	INTERGENIC	--	--	--

INDEL marker IDs	Chromosomes/ unanchored scaffolds	Physical positions (bp)	InDels (*Kabuli* reference genome-CDC Frontier/PII)	Forward primers (5'-3')	Reverse primers (5'-3')	Anneling temperature (0C)	Expected amplified product size (bp)	Structural annotation		Functional annotation	
								Sequence components of *kabuli* genome	*Kabuli* gene accession IDs	NCBI-KOG	TFs
Ca6_P4_21100809	Ca6	21100809	ATTTTTTTTTTTT/ATTTTTTTTTTTTT	TGAGGGATTCCTCGAAGTTG	TGTGATTTGTTGTGGGAAGAA	60.2	490	INTERGENIC	--	--	--
Ca6_P4_21139884	Ca6	21139884	ATTTTTTTT/ATTTTTTTTT	CAGAAGGATTGCTCGTGTGA	TGCATAGGCAAAAATGATTGA	60.0	528	INTERGENIC	--	--	--
Ca6_P4_21141563	Ca6	21141563	GTTTTTT/GTTTTT	TCCGATAGGAAGAGCCAATG	ACGTTTATTTTTCGGACCCC	60.2	697	INTERGENIC	--	--	--
Ca6_P4_21154360	Ca6	21154360	GATAATAATAATAATAATAATAATAATAATAATAATAATAATAATAATAA/GATAATAATAATAATAATAATAATAATAATAATAATAATAATAATAA	TTTCGTCTGATGGATGACCA	GCGTTGGTTCTCTTTTGCTC	60.0	657	INTERGENIC	--	--	--
Ca6_P4_21245145	Ca6	21245145	T/TAATAATAA	TCAAGACGAGCAGCCAACTA	ATGTGTGGGACCCGATAAAA	59.7	686	INTERGENIC	--	--	--
Ca6_P4_21266964	Ca6	21266964	AATTATTATTATTATTATTATTATTATT/AATTATTATTATTATTATTATTATTATTATTATT	GAAGGAGAGGAGGAGGAGGA	TCCATTAGGGGTGGAAATGA	59.9	826	INTERGENIC	--	--	--
Ca6_P4_2128046	Ca6	2128046	CTTTTTTTTT/CTTTTTTTT	AATTTGCTTTGATTTTGCCG	GTGCTCGTGCGACTTGTAAA	60.1	562	INTERGENIC	--	--	--
Ca6_P4_21340779	Ca6	21340779	AT/ATT	GTTGAGGTGATGGGTATGGG	GACAATGAATCAATCACCATGA	60.1	654	INTERGENIC	--	--	--
Ca6_P4_21388665	Ca6	21388665	GTATATATATATATATATATAT/GTATATATATATATATAT	CGCTAGTCTTTTATCTCAATTTCG	TTCTTTTGCACCATGCAGTC	58.7	366	INTERGENIC	--	--	--
Ca6_P4_21399662	Ca6	21399662	AAATAATAATAATAATAATAA/AAATAATAATAATAA	TCCCTAGTTGGAGGTCGATG	GTCCACCTAAATGCCCTTGA	60.1	447	INTERGENIC	--	--	--

INDEL marker IDs	Chromosomes/ unanchored scaffolds	Physical positions (bp)	InDels (*Kabuli* reference genome-CDC Frontier/PII)	Forward primers (5'-3')	Reverse primers (5'-3')	Anneling temperature (0C)	Expected amplified product size (bp)	Structural annotation		Functional annotation	
								Sequence components of *kabuli* genome	*Kabuli* gene accession IDs	NCBI-KOG	TFs
Ca6_P4_21400148	Ca6	21400148	ATATTATTATTATTATTATTATT/ATATTATTATTATTATTATT	GATGTGGAACTCAAGGGCAT	AGCCAGCTGAAGACCAAAAA	59.9	349	INTERGENIC	--	--	--
Ca6_P4_21401312	Ca6	21401312	C/CT	AGCTCAGTCCTCACAACCGT	TTAGATCGGACAAGAAACCGA	59.9	344	INTERGENIC	--	--	--
Ca6_P4_21470474	Ca6	21470474	CAAAAAAAAAA/CAAAAAAAAAAA	TTTCACATATCCTATCAAACACATTTT	TTCGTTGCTCCAAAGACTCC	59.2	367	INTERGENIC	--	--	--
Ca6_P4_21485595	Ca6	21485595	GTTTTTTT/GTTTTTTTT	TGCGGGCTAGCTCACTTAAT	TCAAAACAAATAGGCAGGGG	60.0	115	INTERGENIC	--	--	--
Ca6_P4_21485767	Ca6	21485767	ACCCC/ACCCCC	TGCGGGCTAGCTCACTTAAT	TTTGATTCTCTGGCCGAGTT	60.0	263	INTERGENIC	--	--	--
Ca6_P4_21605985	Ca6	21605985	CTATATATATATATATATA/CTATATATATATATATATATA	GATTTTCGCAGTGAACGGAT	TCCCCTTCTATTTCCCTTGAA	60.1	437	INTERGENIC	--	--	--
Ca6_P4_216120	Ca6	216120	ATTTTTTTTTTTTT/ATTTTTTTTTTT	TGCTGACGATGTGATGGAAT	GTGGTCGTCGTAGGGAAAAA	60.1	553	INTERGENIC	--	--	--
Ca6_P4_2164101	Ca6	2164101	GTATATATATATATATATATATATATATATA/GTATATATATATATATATATATATATATA	CTCCTGCTAGATCACGCCTC	TGACTTTGCAGAGAAGGAAAAA	60.1	557	INTERGENIC	--	--	--
Ca6_P4_21739441	Ca6	21739441	AATATATATATATATATATATATATATATATATATAT/AATATATATATATATATATATATATATATATATATATATATATAT	TGAAGAACCATGGTCATATTAGACA	AAACAAGGCTTCAACCCTGA	59.8	569	INTERGENIC	--	--	--
Ca6_P4_21769271	Ca6	21769271	TTTATTAAATTATTAAATTATTAA/TTTATTAAATTATTAA	AATTTCCTTTGGAGGTTCGC	CCAAGCCCGCATATGATAGT	60.4	814	INTERGENIC	--	--	--

**INDEL marker IDs**	**Chromosomes/ unanchored scaffolds**	**Physical positions (bp)**	**InDels (*Kabuli* reference genome-CDC Frontier/PII)**	**Forward primers (5'-3')**	**Reverse primers (5'-3')**	**Anneling temperature (0C)**	**Expected amplified product size (bp)**	**Structural annotation**		**Functional annotation**	
								**Sequence components of *kabuli* genome**	***Kabuli* gene accession IDs**	**NCBI-KOG**	**TFs**
Ca6_P4_21780461	Ca6	21780461	T/TCG	GGTCGTTGATATGCGAATTTT	AAAACAAATGACCAAATCGAAA	59.0	556	INTERGENIC	--	--	--
Ca6_P4_21782816	Ca6	21782816	TTTTATTT/TTTT	TAATGAGTTGGGACGGGAGA	ATGCAAACGCTACCCAATTC	60.5	782	INTERGENIC	--	--	--
Ca6_P4_2179226	Ca6	2179226	CTTAGGGTTAGGGTTAGGGTT/CTTAGGGTTAGGGTTAGGGTTAGGGTT	GGGCAAAAAGGTGAGTGAAA	TAAACCGTGCTTGCTCTTGA	60.1	368	INTERGENIC	--	--	--
Ca6_P4_21797693	Ca6	21797693	ATTTAA/ATTTAATTTAATTTTAA	TTGCTAATATACTTAACCCAATCTCAA	AAAGATGACACAAATCGGGG	58.7	166	INTERGENIC	--	--	--
Ca6_P4_21799195	Ca6	21799195	GAAAAAA/GAAAAA	CAAAAGCATGTGCTCAAAGG	AATGGCAATCCAAATGCTTC	59.5	399	INTERGENIC	--	--	--
Ca6_P4_21801686	Ca6	21801686	GA/GAA	TCTTGAACCGATACCCTTTTT	GACGGATGAGCATCGGTTAT	57.7	311	INTERGENIC	--	--	--
Ca6_P4_21812932	Ca6	21812932	TGA/TGAGA	GATGACACGGACCAAACCTT	TTCAGCAGCAAAACCATCAG	59.8	154	INTERGENIC	--	--	--
Ca6_P4_21822725	Ca6	21822725	ATTTTTTT/ATTTTTT	CGAAGGTTTGTGCATCTGAA	TGAATTCACATGATTTTGAGCA	59.8	241	INTERGENIC	--	--	--
Ca6_P4_21901902	Ca6	21901902	TAAAAAAAAAAAAA/TAAAAAAAAAAAA	CACCAGAAACAGTGGCAAAA	CCCGGATCTATGACCTGCTA	59.7	265	INTERGENIC	--	--	--
Ca6_P4_21927352	Ca6	21927352	TCACACACACACACACACACACA/TCACACACACACACACACACACACACA	AAGGGAGGAGGAGAAAGCTG	CAAACAAATTTATTACCGCTCAAA	60.0	232	INTERGENIC	--	--	--

INDEL marker IDs	Chromosomes/ unanchored scaffolds	Physical positions (bp)	InDels (*Kabuli* reference genome-CDC Frontier/PII)	Forward primers (5'-3')	Reverse primers (5'-3')	Anneling temperature (0C)	Expected amplified product size (bp)	Structural annotation		Functional annotation	
								Sequence components of *kabuli* genome	*Kabuli* gene accession IDs	NCBI-KOG	TFs
Ca6_P4_21988310	Ca6	21988310	GAATAATAATAATAATAATAATAATAATAATA/GAATAATAATAATAATAATAATAATAATA	TGAAATTCGTACCTCATGCTT	AATGAGATAACCCGTGCGTC	57.4	391	INTERGENIC	--	--	--
Ca6_P4_22034371	Ca6	22034371	AATTATTATTATTATTATTATTATTATTATTATTATTATTATTATTATTATTATTATTATTATTA/AATTATTATTATTATTATTATTATTATTATTATTATTATTATTATTATTATTATTATTATTATTA	CGCCCAATTTCGTAAGTAGC	CTGGCCCAAGTGACCTCTTA	59.7	378	INTERGENIC	--	--	--
Ca6_P4_22082431	Ca6	22082431	TTATATATATATATATATATATA/TTATATATATATATATATATATATA	TATGAATTTTGCGTTGCGAG	GAGTTTGGCGAAGAATTCACA	59.8	509	INTERGENIC	--	--	--
Ca6_P4_22099449	Ca6	22099449	ATTTTTTTTT/ATTTTTTTTTTTT	TGTGTTGCTTCTTGGCTTTG	ACGTGTGGGTTTCAGGTCTC	60.0	620	INTERGENIC	--	--	--
Ca6_P4_22124013	Ca6	22124013	TTCTCTCTCTCTCTCTCTCTCT/TTCTCTCTCTCTCTCTCT	ATTCCAACGATCCAAGATGG	TGCTCCAATGATGTTAGAAACG	59.8	692	INTERGENIC	--	--	--
Ca6_P4_22128416	Ca6	22128416	CTTTTTTTTTT/CTTTTTTTTT	CACACACACACAAAGAGGGG	ATAGGACCTGCTGCCTGAGA	60.0	557	INTERGENIC	--	--	--
Ca6_P4_22410012	Ca6	22410012	ATTT/ACACAAACATGACTCTTT	TTTCATGTATCGGGCCTAGC	CTCCTGGATCAAAGGGTCAA	60.1	688	INTERGENIC	--	--	--
Ca6_P4_22477290	Ca6	22477290	GTATATATATATATATATATAT/GTATATATATATATATATATAT	AATTGAGTGAGTGGAATGAACA	GGTCTTCCACCAACCTTTGA	57.2	805	INTERGENIC	--	--	--
Ca6_P4_23064745	Ca6	23064745	CA/C	TGGGTTGATTTTCCCACATT	TCTCGAGCAAATCACAAGTTAAA	60.0	571	INTERGENIC	--	--	--
Ca6_P4_23065041	Ca6	23065041	TGG/TG	TTTCGAATGCCTATCTAATTGGT	AAAAATCCGCAGGAAAGGTT	59.1	522	INTERGENIC	--	--	--

INDEL marker IDs	Chromosomes/ unanchored scaffolds	Physical positions (bp)	InDels (*Kabuli* reference genome-CDC Frontier/PII)	Forward primers (5'-3')	Reverse primers (5'-3')	Anneling temperature (0C)	Expected amplified product size (bp)	Structural annotation		Functional annotation	
								Sequence components of *kabuli* genome	*Kabuli* gene accession IDs	NCBI-KOG	TFs
Ca6_P4_23078754	Ca6	23078754	TGG/TGGG	TGGACAATCGCCCAAATTAT	TCTCGAGCAAATCACGAGTTA	60.2	716	INTERGENIC	--	--	--
Ca6_P4_23078938	Ca6	23078938	T/TC	TGGACAATCGCCCAAATTAT	TCTCGAGCAAATCACGAGTTA	60.2	716	INTERGENIC	--	--	--
Ca6_P4_23090578	Ca6	23090578	AC/A	GCCAAGCTTGTTTGTTGGTT	TGTGTGCACATGTTGTTCGT	60.2	490	INTERGENIC	--	--	--
Ca6_P4_23365057	Ca6	23365057	ATTTTTTTTTTT/ATTTTTTTTTT	CCATATGCGAGAAGGACGAT	GCAAGCCTTTTGGCTATGAC	60.1	407	INTERGENIC	--	--	--
Ca6_P4_233746	Ca6	233746	TTATTTATTTATTTATTTATTTATTTATTTAT/TTATTTATTTATTTATATATTTATTTATTTATTTATTTATTTATTTAT	CGGATCCCACCACTTGATAA	TTGATCGTTGGGTTGATGAAT	60.7	640	INTERGENIC	--	--	--
Ca6_P4_23409253	Ca6	23409253	CTTTTT/CTTTTTT	TGCCGTATAAAATGGGCTTC	CTTGCTCTTCCTTCGTTGCT	59.9	533	INTERGENIC	--	--	--
Ca6_P4_23655687	Ca6	23655687	AATATATATATATATATATATA/AATATATATATATATATATA	CAAGTTGAATTCATTAAGACGCC	GATTGATCGCAGGGACAAGT	60.0	294	INTERGENIC	--	--	--
Ca6_P4_23741801	Ca6	23741801	GTATATATATATATATATATAT/GTATATATATATATATATATATATATATATATATAT	TAGTGCTCTGCATTTGTCCG	TGAAGGACGATGAATTCCTAAAA	60.0	768	INTERGENIC	--	--	--
Ca6_P4_2382805	Ca6	2382805	ATTTTTTTTT/ATTTTTTTT	TTACGGCCACCTATCGAATC	AAAATGGAGCGTTGTTGGAC	59.9	932	INTERGENIC	--	--	--
Ca6_P4_2384865	Ca6	2384865	TTAATAATAATAATAATAATAATAATAATAATAATAA/TTAATAATAATAATAATAATAATAATAATAATAATAA	AGATTGCCCCACTTCAAAAA	TAACCTGCCAAAGAGGAGGA	59.5	866	INTERGENIC	--	--	--

INDEL marker IDs	Chromosomes/ unanchored scaffolds	Physical positions (bp)	InDels (*Kabuli* reference genome-CDC Frontier/PII)	Forward primers (5'-3')	Reverse primers (5'-3')	Anneling temperature (0C)	Expected amplified product size (bp)	Structural annotation		Functional annotation	
								Sequence components of *kabuli* genome	*Kabuli* gene accession IDs	NCBI-KOG	TFs
Ca6_P4_23873936	Ca6	23873936	CAT/CATCCTTTAAT	GAGCATAGTTCCTTTTCACGG	AATTTTGGTCCCCAAACAGA	58.8	441	INTERGENIC	--	--	--
Ca6_P4_24186567	Ca6	24186567	AATATATATATATATATATATAT/AATATATATATATATATATATAT	CCGTTGGGAGTTTGGAAGTA	CCCTAACCCTAACCTAAACCA	60.0	844	INTERGENIC	--	--	--
Ca6_P4_24232847	Ca6	24232847	TTATATATATATATATATAT/TTATATATATATATATAT	TGAGGTGCTGTCCAAATTGT	ATAAATTCCGCAACCGATCA	59.1	521	INTERGENIC	--	--	--
Ca6_P4_24252379	Ca6	24252379	AATATATATATATATATATATATAT/AATATATATATATATATATATATATAT	TTGCCTGGCATTTTTATATGAG	TTCGGGTCGTGTTGATTGTA	59.1	598	INTERGENIC	--	--	--
Ca6_P4_24296638	Ca6	24296638	CATATATATATATATATATATATA/CATATATATATATATATATATATA	GAGAAGGAGGGAAAAATGGC	ATGTTTTGGATTTTGGCTCG	60.0	421	INTERGENIC	--	--	--
Ca6_P4_24305629	Ca6	24305629	AATATATATATATATATATATATATATATA/AATATATATATATATATATATATATA	CGTCATTTTCAAACAAACCTCA	TGACACCGTCGTAACCTCAA	60.0	648	INTERGENIC	--	--	--
Ca6_P4_24309703	Ca6	24309703	AATATATATATATATAT/AATATATATATATATAT	TGATGTGGTTGACCTGTGTG	CTAGGCCCATGTGAAGGAAA	58.9	441	INTERGENIC	--	--	--
Ca6_P4_24637049	Ca6	24637049	TTTCTTCTTCTTCTTCTTCTTCTTCTTCT/TTTCTTCTTCTTCTTCTTCTTCTTCT	TGTTTTTGGTGAAACGTGGA	AGGATGAATGTGGTGTTGAAGA	60.0	376	INTERGENIC	--	--	--
Ca6_P4_24744388	Ca6	24744388	TACACACACA/TACACA	TTTGGATTTTGACTCGGAGG	AATGGAGGAAGGAGGGAGAA	60.0	384	INTERGENIC	--	--	--
Ca6_P4_24872220	Ca6	24872220	TAAAAAAAAA/TAAAAAAAAAA	TCCGATGCATCTAAAATTCG	GGGAAAAATTTCGTTCAAATCA	58.7	535	INTERGENIC	--	--	--

INDEL marker IDs	Chromosomes/ unanchored scaffolds	Physical positions (bp)	InDels (*Kabuli* reference genome-CDC Frontier/PII)	Forward primers (5'-3')	Reverse primers (5'-3')	Anneling temperature (0C)	Expected amplified product size (bp)	Structural annotation		Functional annotation	
								Sequence components of *kabuli* genome	*Kabuli* gene accession IDs	NCBI-KOG	TFs
Ca6_P4_25002657	Ca6	25002657	AATATATATATATATATAT/AATATATATATATATATATATAT	CTACATATGCAAAAGCGGCA	GGATGTGTTTTCCCCTTCAA	59.9	400	INTERGENIC	--	--	--
Ca6_P4_2501071	Ca6	2501071	TAACAACAACAACAACAACAA/TAACAACAACAACAACAA	CCCACCAACCCAAATTAAAA	TCACCATCGTGTGATGGACT	59.5	305	INTERGENIC	--	--	--
Ca6_P4_2525217	Ca6	2525217	TT/TTGCAT	AGTAACCGACCAGCCAATCA	CAAATTTTGGTCACATTCCG	60.5	741	INTERGENIC	--	--	--
Ca6_P4_2525428	Ca6	2525428	TAAAAAAAAAA/TAAAAAAAAAAAA	CCTGAAATTTGATTCTGCGT	CAAATTTTGGTCACATTCCG	57.8	578	INTERGENIC	--	--	--
Ca6_P4_2528953	Ca6	2528953	AATAT/AAT	TGGGTCAATGTTAAGAGGGC	CGTCAACGTCGTGCATTTAC	59.9	515	INTERGENIC	--	--	--
Ca6_P4_25356142	Ca6	25356142	TA/TAAA	GCCGCTTCCAAATTAGTTCA	TCGATTTGTCACAGGGATCA	60.2	609	INTERGENIC	--	--	--
Ca6_P4_25495130	Ca6	25495130	AGGTTTTAG/AGGTTTTAGGGTTTTAG	TTCGTTCAAAAATGCAAAAGTC	CCAAAAACCAAAATGACCAAA	59.3	529	INTERGENIC	--	--	--
Ca6_P4_25583382	Ca6	25583382	TAGGGTTCAGGGTTCAGGGTTCAGGGTT/TAGGGTTCAGGGTTCAGGGTT	GGGTTTAGGGTTTTGGGATT	AAAAACCAAAATGACCCAACA	59.0	377	INTERGENIC	--	--	--
Ca6_P4_25692899	Ca6	25692899	AT/ATATCT	ATTCCAAACCAAAACCCTCC	AAATTGGTCCACTAGCACCG	60.0	883	INTERGENIC	--	--	--
Ca6_P4_257739	Ca6	257739	CATATATATATATATATATATATATA/CATATATATATATATATATATATATATA	CGACACATGTGCTATTTAGCCT	ATGGGTTGCAGTTAGCTTGG	59.3	799	INTERGENIC	--	--	--

INDEL marker IDs	Chromosomes/ unanchored scaffolds	Physical positions (bp)	InDels (*Kabuli* reference genome-CDC Frontier/PII)	Forward primers (5'-3')	Reverse primers (5'-3')	Anneling temperature (0C)	Expected amplified product size (bp)	Structural annotation		Functional annotation	
								Sequence components of *kabuli* genome	*Kabuli* gene accession IDs	NCBI-KOG	TFs
Ca6_P4_2598910	Ca6	2598910	ATAATAATAA/ATAATAATAATAATAATAGTAATAATAA	CAGTCCCAACGAAAAAGCAT	ATGGGACAAAACACCAGAGC	60.1	430	INTERGENIC	--	--	--
Ca6_P4_26044857	Ca6	26044857	GTTTTTTTTT/GTTTTTTTT	CAATGGAAAGTGTGGTTCCC	AAAAGGCGCGCTATAAAATG	60.2	929	INTERGENIC	--	--	--
Ca6_P4_26084259	Ca6	26084259	AATATATATATATATATATATATATATATATATATATATA/AATATATATATATATATATATATATATATATATATATATATATATATATATA	TTGGGTCTTGTGAAAAGAGAA	CATTGTGCGGCTACTCTTCA	57.4	184	INTERGENIC	--	--	--
Ca6_P4_26152782	Ca6	26152782	G/GA	TCTTGCGATTTCACTGTTGC	CAGGTTTGGTCCCCCTATTT	60.0	703	INTERGENIC	--	--	--
Ca6_P4_26263542	Ca6	26263542	ATTTTTTTTTT/ATTTTTTTTTTT	TTAGGGGGCGAGTGTGAATA	CGCATACCAATTTACATGATGC	60.5	409	INTERGENIC	--	--	--
Ca6_P4_26277672	Ca6	26277672	T/TG	TTAGGTGCGCTTGACTGATG	GTTTTGGCGTTGGTTTCACT	60.0	558	INTERGENIC	--	--	--
Ca6_P4_26382209	Ca6	26382209	A/AGC	GGCCTTCTCCTCTCAATCCT	TGGTTGGGATTTTTGGGTTA	59.8	870	INTERGENIC	--	--	--
Ca6_P4_26391440	Ca6	26391440	CTATATATATATATATATATATATATAT/CTATATATATATATATATATATATATAT	GAGAAATGCATTGATGTATCGAG	TTGGTCGCAATGCTTATTTTT	58.7	428	INTERGENIC	--	--	--
Ca6_P4_2639909	Ca6	2639909	CTT/CT	AGCTGAAGCAATTTTCGCAT	GCAATGCAGCAGCAATAAAA	60.0	479	INTERGENIC	--	--	--
Ca6_P4_26433995	Ca6	26433995	AATATATATATATATATATATAT/AATATATATATATATATATAT	AGCCTAAATGTAGGTGCATGG	CACAAATGAGCCATGGTGAG	59.1	610	INTERGENIC	--	--	--

INDEL marker IDs	Chromosomes/ unanchored scaffolds	Physical positions (bp)	InDels (*Kabuli* reference genome-CDC Frontier/PII)	Forward primers (5'-3')	Reverse primers (5'-3')	Anneling temperature (0C)	Expected amplified product size (bp)	Structural annotation		Functional annotation	
								Sequence components of *kabuli* genome	*Kabuli* gene accession IDs	NCBI-KOG	TFs
Ca6_P4_26438751	Ca6	26438751	ATTTTTTTTT/ATTTTTTTTTTT	TCAGACACTACCCAGCATCAA	CTTAGGCCGCCACAAAATA	59.3	960	INTERGENIC	--	--	--
Ca6_P4_26498886	Ca6	26498886	TTATATATATATATATATAT/TTATATATATATATATAT	AAATTCCATCTTTATTGGTTTGAGA	TCAGCACATCACAATCATCTG	59.3	343	INTERGENIC	--	--	--
Ca6_P4_26545231	Ca6	26545231	AATATATATATATATATATATATATATA/AATATATATATATATATATATATATA	TTTGTGATCCTTCCATTTTTAGG	TGGTGAAATAAACATTATCATATCGTG	59.4	753	INTERGENIC	--	--	--
Ca6_P4_26559767	Ca6	26559767	CCATGATGAGCAGCCTCA/CCA	AGGAAAATCTGACGGTGGTG	TGCTGCTGCTGGTCATATTC	60.0	240	INTERGENIC	--	--	--
Ca6_P4_26560475	Ca6	26560475	CTA/CGTATA	GTCTTTTGAGCATCCCGTGT	AGATGTTTGTTTTGCCGTCC	60.1	331	INTERGENIC	--	--	--
Ca6_P4_2662582	Ca6	2662582	AAGAGAGAGAGAGAGAGAGAGAGAGAGAGAGAGAG/AAGAGAGAGAGAGAGAGAGAGAGAGAGAGAGAG	TTGGGTGCAGCAACAAGTAG	CCATACTTTAACCCTCGCCA	59.9	257	INTERGENIC	--	--	--
Ca6_P4_26686344	Ca6	26686344	T/TATA	CTAGTGCGAATGGCAGACAA	GATTATGGCCCAGCTTTTGA	60.0	358	INTERGENIC	--	--	--
Ca6_P4_26790864	Ca6	26790864	GAAAAAAAA/GAAAAAAA	CCGAAAATTTGAAAATCCGA	CAAGTACAGGTGGAGTGGCA	59.9	728	INTERGENIC	--	--	--
Ca6_P4_26804449	Ca6	26804449	AATTATTATTATTATTATTATTATTATTATTATTATTATTATTATTATTATTATTATTATTATTATTAT/AATTATTATTATTATTATTATTATTATTATTATTATTATTATTATTATTATTATTATTATTATTAT	CCACCTCCACCATCAGTTTT	CTACCACCAAACGGACCATA	59.8	959	INTERGENIC	--	--	--
Ca6_P4_26834295	Ca6	26834295	ATTTTTTTTTTT/ATTTTTTTTTT	GAAATTAAGTTAAGGGACCGCA	TGGTTTTGGGCTATAATGGG	59.5	428	INTERGENIC	--	--	--

INDEL marker IDs	Chromosomes/ unanchored scaffolds	Physical positions (bp)	InDels (*Kabuli* reference genome-CDC Frontier/PII)	Forward primers (5'-3')	Reverse primers (5'-3')	Anneling temperature (0C)	Expected amplified product size (bp)	Structural annotation		Functional annotation	
								Sequence components of *kabuli* genome	*Kabuli* gene accession IDs	NCBI-KOG	TFs
Ca6_P4_26931819	Ca6	26931819	TTATATATATATATATAT/TTATATATATATATATATATAT	GTACCACATTGCCGAGGTTT	GCTTGCCAAAATAAATGGCT	59.9	580	INTERGENIC	--	--	--
Ca6_P4_2695274	Ca6	2695274	ATTTTTTTTTT/ATTTTTTTTT	TTCAAAAACATATACCGCACG	TTTAACACGGTTTGTGCATGA	58.6	250	INTERGENIC	--	--	--
Ca6_P4_27009865	Ca6	27009865	TT/TGTTATAT	CGTGTGAGAAAACCTCGACA	ATTTTGCAAACCCAAGTTCG	59.9	314	INTERGENIC	--	--	--
Ca6_P4_27037809	Ca6	27037809	TTTATTATTATTATTATTATT/TTTATTATTATTATTATT	CGTCCAGACTCGCCTTTCTA	ATTTTTCATCTGCGCGATTT	60.5	643	INTERGENIC	--	--	--
Ca6_P4_27165011	Ca6	27165011	ATATTATTATTATTATTATTATTATTATT/ATATTATTATTATTATTATTATTATT	CATTGTTTGATCCATGTCGC	CCATCATTGACACTCGAAAGAA	59.9	675	INTERGENIC	--	--	--
Ca6_P4_27264510	Ca6	27264510	ATTTTTTTTT/ATTTTTTTT	AACGTCCCATTAAGTTCGCA	TGACAAATTGCCTTTGCTTG	60.5	465	INTERGENIC	--	--	--
Ca6_P4_27289106	Ca6	27289106	GATATATAT/GATATATATAT	CAATTCCCACAGGTACGCTT	AATTTCAAGGATTGGCGAAG	60.0	612	INTERGENIC	--	--	--
Ca6_P4_27315680	Ca6	27315680	GAGTTTTCAATTTTAGTTTTCAATTTTA/GAGTTTTCAATTTTA	CATGCATCGGAAGTTGTGTT	TCACTGTCAGGCAGATTTCG	59.6	496	INTERGENIC	--	--	--
Ca6_P4_27348230	Ca6	27348230	ATTTTTTTTTT/ATTTTTTTTTTT	TTGATCCCATATCCTCCCAA	GACACCCAAATCAAACCCAC	60.1	595	INTERGENIC	--	--	--
Ca6_P4_27421945	Ca6	27421945	TAAAAAA/TAAAAAAA	CATCTCCAGATGCAACCTTCT	TAGAATGGGGTGTTTGCGAC	59.3	179	INTERGENIC	--	--	--

INDEL marker IDs	Chromosomes/ unanchored scaffolds	Physical positions (bp)	InDels (*Kabuli* reference genome-CDC Frontier/PII)	Forward primers (5'-3')	Reverse primers (5'-3')	Anneling temperature (0C)	Expected amplified product size (bp)	Structural annotation		Functional annotation	
								Sequence components of *kabuli* genome	*Kabuli* gene accession IDs	NCBI-KOG	TFs
Ca6_P4_27447919	Ca6	27447919	ATTTTTTTT/ATTTTTTTTT	CACCATGTGCCACTTCATTC	TGGGCGGCTATGTTAAAACT	60.0	359	INTERGENIC	--	--	--
Ca6_P4_27448301	Ca6	27448301	TTATATATATATATATATATATATATATATATAT/TTATATATATATATATATATATATATATATATATAT	CACCATGTGCCACTTCATTC	CTAGTGCAAGGTCGCTCCTC	60.0	629	INTERGENIC	--	--	--
Ca6_P4_2763643	Ca6	2763643	TA/T	ATCGCGTTCAATGTGTTTGA	TGCCATGAATATTTGATAAATGTGA	60.1	358	INTERGENIC	--	--	--
Ca6_P4_27691258	Ca6	27691258	ATTTTTTTTTTT/ATTTTTTTTTTTT	CACTCGGTTGAGACGTGAGA	AGGTCGGCGTCATAAACATC	60.0	290	INTERGENIC	--	--	--
Ca6_P4_27970080	Ca6	27970080	C/CAAA	GCATAGTGGGAAGAAGAGCG	CGAATGCTATTAGGGGCGTA	60.0	904	INTERGENIC	--	--	--
Ca6_P4_28188243	Ca6	28188243	AATATATATATATATATATATAT/AATATATATATATATATATATATATAT	ATCCAACCTCAACCTCACCA	TTTGATGAATTAAAATGATGCGA	60.4	922	INTERGENIC	--	--	--
Ca6_P4_28189675	Ca6	28189675	TAAAAAAA/TAAAAAAAAA	TTCATACTTCCCATTATATTGACTGC	TGAATTGCATAGGGTTTAGGG	59.7	553	INTERGENIC	--	--	--
Ca6_P4_28190647	Ca6	28190647	AA/AATAATAATAATA	AGGTCTCATACCAGAGATGCG	TGTGGTTGAGTAAATGTGCCA	59.3	580	INTERGENIC	--	--	--
Ca6_P4_28190835	Ca6	28190835	ATATTATTATTATTATT/ATATTATTATTATTATTATT	AGGTCTCATACCAGAGATGCG	AAGTTAAGGGACCGCAAATG	59.3	871	INTERGENIC	--	--	--
Ca6_P4_28193559	Ca6	28193559	AGGG/AGG	AAGGACTAAGTCGGGAAGAAAAA	GATGGTTTTTGTCCATTGAATTT	59.7	131	INTERGENIC	--	--	--

INDEL marker IDs	Chromosomes/ unanchored scaffolds	Physical positions (bp)	InDels (*Kabuli* reference genome-CDC Frontier/PII)	Forward primers (5'-3')	Reverse primers (5'-3')	Anneling temperature (0C)	Expected amplified product size (bp)	Structural annotation		Functional annotation	
								Sequence components of ***kabuli*** **genome**	***Kabuli*** **gene accession IDs**	NCBI-KOG	TFs
Ca6_P4_28194024	Ca6	28194024	ATTTTTT/ATTTTTTT	GGACCACGAATGTAAT TTAGCC	TGCGAGCATTAGAAGT TGTTG	59.7	737	INTERGENIC	--	--	--
Ca6_P4_28222145	Ca6	28222145	TTTTCT/TT	CAAGAAGACAAAGGGG GACA	TGCTCGTACGTTGTCG GATA	60.1	597	INTERGENIC	--	--	--
Ca6_P4_28222721	Ca6	28222721	ATATTATTATTATTATTATTATTATTATTA TTATTATTATTATTATTATTATTATTATTA TTATTATTAT/ATATTATTATTATTAT TATTATTATTATTATTATTATTATTAT TATTATTATTAT	TATCCGACAACGTACG AGCA	CCCTACCACGTCCGTT AAAA	60.3	536	INTERGENIC	--	--	--
Ca6_P4_28264967	Ca6	28264967	AATATATATATATATATATATA/AATA TATATATATATATATA	GAAAATAGATGAAGCG GCCA	GACCTTTACCGACCAC CGTA	60.2	606	INTERGENIC	--	--	--
Ca6_P4_28332319	Ca6	28332319	ATTTT/ATTTTT	TTTCAAAAAGTCCCAAT CGC	GGTTTGGAGAAGAAGA CGAAA	60.1	751	INTERGENIC	--	--	--
Ca6_P4_28332406	Ca6	28332406	GTTTT/GTTT	TTTCAAAAAGTCCCAAT CGC	GGTTTGGAGAAGAAGA CGAAA	60.1	751	INTERGENIC	--	--	--
Ca6_P4_28332579	Ca6	28332579	A/AG	GTCGGAGCTCTGCCAT AAAA	GGTTTGGAGAAGAAGA CGAAA	60.4	599	INTERGENIC	--	--	--
Ca6_P4_28332651	Ca6	28332651	C/CT	GTCGGAGCTCTGCCAT AAAA	GGTTTGGAGAAGAAGA CGAAA	60.4	599	INTERGENIC	--	--	--
Ca6_P4_28332758	Ca6	28332758	TAAA/TAAAA	TTTCGTCTTCTTCTCCA AACC	CCCAACATATTACCTGT TACCCA	58.4	502	INTERGENIC	--	--	--
Ca6_P4_28332973	Ca6	28332973	A/AT	TTTCGTCTTCTTCTCCA AACC	CGTGCAAAGTCGAAGC GTAT	58.4	669	INTERGENIC	--	--	--

INDEL marker IDs	Chromosomes/ unanchored scaffolds	Physical positions (bp)	InDels (*Kabuli* reference genome-CDC Frontier/PII)	Forward primers (5'-3')	Reverse primers (5'-3')	Anneling temperature (0C)	Expected amplified product size (bp)	Structural annotation		Functional annotation	
								Sequence components of *kabuli* genome	*Kabuli* gene accession IDs	NCBI-KOG	TFs
Ca6_P4_28336699	Ca6	28336699	ATTTT/ATTT	TCTCCCATTTTTACCATGCC	TTTGGCTTTGGTTGATTCCT	59.8	980	INTERGENIC	--	--	--
Ca6_P4_28354937	Ca6	28354937	GTAT/GT	TTTTATGTGACCCGCTTTGC	AGAACGGCATCCAGAGAAGA	61.0	600	INTERGENIC	--	--	--
Ca6_P4_28388573	Ca6	28388573	CA/CAACTTTA	GTTGGCAACGATTTTGTCCT	GGAGAACGTTAAGGCAGCAG	60.0	640	INTERGENIC	--	--	--
Ca6_P4_28407890	Ca6	28407890	ATTTTTTTTTTT/ATTTTTTTTTT	GGTCCCTTGTGTAATGTCTCG	TTGAATGCCAAAATAATCGTT	59.5	724	INTERGENIC	--	--	--
Ca6_P4_28443583	Ca6	28443583	CTCTTTTGTTGTTA/CTCTTTTGTTGTTATATCTTTTGTTGTTA	AAAAACACCAAAAGCCCCTC	CATAAAATGCCATTGATCCCT	60.3	499	INTERGENIC	--	--	--
Ca6_P4_28444744	Ca6	28444744	GATAATAATAATAATAATAA/GATAATAATAATAATAATAATAATAATAA	TCCCAAACAGCCTAACAACC	CAAACGCAATCTGAACAAAA	60.0	509	INTERGENIC	--	--	--
Ca6_P4_28496437	Ca6	28496437	TTGT/TTGTCGTTTATTTATGT	ATGGGGAGAACAACAGCAAC	GAAACTGCAGACTCAAGGGG	60.0	427	INTERGENIC	--	--	--
Ca6_P4_28513941	Ca6	28513941	ANNNNNNNNNN/ANNNNN	TGAAGAAAATTTAAAGGGACGAA	GTGTTGCATCTCATTGGTGG	59.2	569	INTERGENIC	--	--	--
Ca6_P4_28520924	Ca6	28520924	CAA/CAATTAAA	TTAGAAACAGGGACCCCTCC	TTGTCTGTGAACCGAAGCTG	60.3	577	INTERGENIC	--	--	--
Ca6_P4_28520971	Ca6	28520971	TCTTGCCTTGCCTTG/TCTTGCCTTG	TTAGAAACAGGGACCCCTCC	TTGTCTGTGAACCGAAGCTG	60.3	577	INTERGENIC	--	--	--

INDEL marker IDs	Chromosomes/ unanchored scaffolds	Physical positions (bp)	InDels (*Kabuli* reference genome-CDC Frontier/PII)	Forward primers (5'-3')	Reverse primers (5'-3')	Anneling temperature (0C)	Expected amplified product size (bp)	Structural annotation		Functional annotation	
								Sequence components of *kabuli* genome	*Kabuli* gene accession IDs	NCBI-KOG	TFs
Ca6_P4_28521027	Ca6	28521027	A/ACATAG	TCTCATTCTTGCCTTGCCTT	TTGTCTGTGAACCGAAGCTG	60.0	172	INTERGENIC	--	--	--
Ca6_P4_28521076	Ca6	28521076	AACTAT/A	TCTCATTCTTGCCTTGCCTT	TTGTCTGTGAACCGAAGCTG	60.0	172	INTERGENIC	--	--	--
Ca6_P4_28851395	Ca6	28851395	TAAAAAAAAAAA/TAAAAAAAAAA	AATTAGCTTGGGAGCGACAA	CGAAAATGACACATCAAAAGGA	59.8	374	INTERGENIC	--	--	--
Ca6_P4_28878487	Ca6	28878487	T/TCA	CTCCGTTTTCTGCACATCAA	TGATGTTGCCTCTGTTTTGC	59.8	396	INTERGENIC	--	--	--
Ca6_P4_28977511	Ca6	28977511	ATTTTTTTTTTT/ATTTTTTTTTT	TCCAAAATGTTTAAGACGATCC	TGATTCCACCCTTGGATTTT	58.1	598	INTERGENIC	--	--	--
Ca6_P4_29231459	Ca6	29231459	AAAGTCTCAAGTCTCAAGTCTCAA/AAAGTCTCAAGTCTCAAGTCTCAAGTCTCAA	ACTCCCACACACCTCAAAGG	TTTCAAGTCTAGTCGAAGTTCATCA	60.0	834	INTERGENIC	--	--	--
Ca6_P4_29244002	Ca6	29244002	ATTTTTT/ATTTTTTT	TGGCCATTATCCCTCATTTG	ATTTTGTCTCCTTCACCCCC	60.7	603	INTERGENIC	--	--	--
Ca6_P4_29259303	Ca6	29259303	AATATATATATATATATATATATATATATATATATATAT/AATATATATATATATATATATATATATATATATATAT	GGAATCCAAATTCCCAACAA	TGTCCACATGCTTCGCTTAG	59.6	685	INTERGENIC	--	--	--
Ca6_P4_29314280	Ca6	29314280	TTATATATATATATATATATATATAT/TTATATATATATATATATATATAT	ATGTACCAATGCGTCGTCAA	CCACCCAGATTTCGCTCTTA	60.0	435	INTERGENIC	--	--	--
Ca6_P4_29475109	Ca6	29475109	CATATATATATATATATATATAT/CATATATATATATATATATATATAT	AAATGAATGGAGATGAAGGCA	CACGCATCTATCCGTCAGC	59.5	603	INTERGENIC	--	--	--

INDEL marker IDs	Chromosomes/ unanchored scaffolds	Physical positions (bp)	InDels (*Kabuli* reference genome-CDC Frontier/PII)	Forward primers (5'-3')	Reverse primers (5'-3')	Anneling temperature (0C)	Expected amplified product size (bp)	Structural annotation		Functional annotation	
								Sequence components of *kabuli* genome	*Kabuli* gene accession IDs	NCBI-KOG	TFs
Ca6_P4_29669016	Ca6	29669016	T/TC	GCCCTCAGATGAGAGTCAGG	AATGGCCAGATGAAATCGAG	59.9	596	INTERGENIC	--	--	--
Ca6_P4_29815012	Ca6	29815012	GAAAAAAAAAA/GAAAAAAAAAAA	TGACAGTCAGAGTGCGATCC	TGCAGATAAATGGCCAAACA	60.0	807	INTERGENIC	--	--	--
Ca6_P4_29832986	Ca6	29832986	CATATATATATATATATATATATAT/CATATATATATATATATATAT	ACGCTCCATGGTAGTTCCAA	TTGTGAGGGTTTTATTCGGC	60.5	153	INTERGENIC	--	--	--
Ca6_P4_29915132	Ca6	29915132	AATATATATATATATATATATAT/AATATATATATATATATATAT	AAATGTGCAATTTGTTGGCA	TCATTCCAACCTGCAGTTACC	60.0	632	INTERGENIC	--	--	--
Ca6_P4_29955539	Ca6	29955539	ATTAT/ATTATTTTAT	TCAGCCAGAAAGCTGATGTG	TTGTGCAGAGAATATTTATGTCGAT	60.1	488	INTERGENIC	--	--	--
Ca6_P4_29969559	Ca6	29969559	TAAAAAAAAAA/TAAAAAAAAA	CTGTCGTGTCCAGTTTGTCC	TGTTTCACCGTCACGTTCAT	59.2	583	INTERGENIC	--	--	--
Ca6_P4_30102387	Ca6	30102387	ATTTTTTTTTTTT/ATTTTTTTTTTT	TTGGGAGATGCATGTATTGG	TTAGACGTAACCCACCTTGGA	59.4	596	INTERGENIC	--	--	--
Ca6_P4_30125014	Ca6	30125014	TTATATATATATATATATATATATATATATATATATATATATATA/TTATATATATATATATATATATATATATATATATATATATATATA	GGAAAAGGAAGGGAATCACG	TGCATGCACAAATTAGACACTTT	60.8	854	INTERGENIC	--	--	--
Ca6_P4_30186365	Ca6	30186365	TATTGATTGA/TATTGA	CCGTTGTCAACCTCAAATCC	ATCCCGCGACTCAAAATTC	60.4	811	INTERGENIC	--	--	--
Ca6_P4_30358500	Ca6	30358500	A/AT	GCAATAAAGCACATGCAGGA	CCCTGTGAGGACTCAACCAT	59.8	665	INTERGENIC	--	--	--

INDEL marker IDs	Chromosomes/ unanchored scaffolds	Physical positions (bp)	InDels (*Kabuli* reference genome-CDC Frontier/PII)	Forward primers (5'-3')	Reverse primers (5'-3')	Anneling temperature (0C)	Expected amplified product size (bp)	Structural annotation		Functional annotation	
								Sequence components of *kabuli* genome	*Kabuli* gene accession IDs	NCBI-KOG	TFs
Ca6_P4_30456247	Ca6	30456247	AATTAT/AAT	GCGTTTCGACACAATTTTCC	AAAATAACGGGCAAGCCTTT	60.5	882	INTERGENIC	--	--	--
Ca6_P4_30621500	Ca6	30621500	ATGAAAGTTTTGAAAGTTTTGAAA/ATGAAAGTTTTGAAA	ATTTTCGAACCTTTCGCCTT	TTTGAAAGTGTTGATAAAATCCCA	60.1	522	INTERGENIC	--	--	--
Ca6_P4_30646917	Ca6	30646917	CTTTTT/CTTTTTT	TGTTTGCATCAAGATGGGTT	GCGACTTCCCACGTGATATT	59.0	592	INTERGENIC	--	--	--
Ca6_P4_30698837	Ca6	30698837	GTTATTTATTTATTTATTTATTTATTTATTTATT/GTTATTTATTTATTTATTTATTTATTTATTTATTTATTTATT	CGTTTTCGGTTTCAGGGTAA	CCCATACCTATTACCCACGC	60.0	949	INTERGENIC	--	--	--
Ca6_P4_30724309	Ca6	30724309	AATATATATATATATATATATATAT/AATATATATATATATATATATATATAT	CCGTTGGGAGTTTGGAAGTA	TGCGTATGCTTCAACGTCAT	60.0	587	INTERGENIC	--	--	--
Ca6_P4_3073674	Ca6	3073674	AATATATATATATATATATATATATA/AATATATATATATATATATATATATATATATATATA	CACCCATTTTGCTTTGGTCT	CCTTTCCTTCCAGCCATACA	60.0	164	INTERGENIC	--	--	--
Ca6_P4_30832584	Ca6	30832584	AATATATATATATATATATATATATATATAT/AATATATATATATATATATATATATATAT	TTTTTGAATTGGTGATCCAGA	TTCGTAGATTTACCGCATGTGT	58.1	664	INTERGENIC	--	--	--
Ca6_P4_3093515	Ca6	3093515	ATT/AT	GCGGCAAGCTTTTCTTTATG	TCGAACCCGTTCATTACCTC	60.0	447	INTERGENIC	--	--	--
Ca6_P4_31051947	Ca6	31051947	TTAATAATAATAATAATA/TTAATAATAATAATA	ACCCATTTACTCTTGCACGG	TCTCCAATGATTTGTTTTGTGG	60.0	425	INTERGENIC	--	--	--
Ca6_P4_3108108	Ca6	3108108	AT/ATAATAATAATATT	CACCAGTGTCCAATCACCCT	CGTCTCATGTTCCTCCAGGT	60.8	508	INTERGENIC	--	--	--

INDEL marker IDs	Chromosomes/ unanchored scaffolds	Physical positions (bp)	InDels (*Kabuli* reference genome-CDC Frontier/PII)	Forward primers (5'-3')	Reverse primers (5'-3')	Anneling temperature (0C)	Expected amplified product size (bp)	Structural annotation		Functional annotation	
								Sequence components of *kabuli* genome	*Kabuli* gene accession IDs	NCBI-KOG	TFs
Ca6_P4_31082373	Ca6	31082373	GATAATAATAATAATAATAATAATAATAATAATAATAATAATAATAATAATAATAATAATAATA/GATAATAATAATAATAATAATAATAATAATAATAATAATAATAATAATAATAATAATAATAATAATAATAATAATAATA	ATTATCGCCTACAACGTCCG	TCATTCATTCATTCATTCAAAACA	60.0	444	INTERGENIC	--	--	--
Ca6_P4_31097072	Ca6	31097072	TA/T	AAGAGTCAATGACCACCTTCAAA	CTTTGCTTGGACCAAACTGG	60.0	318	INTERGENIC	--	--	--
Ca6_P4_31097180	Ca6	31097180	ATTT/ATTTTT	TCCCAATTGAGTGACATCTTTC	CTTATAGCCGAATGGCCAAA	59.0	587	INTERGENIC	--	--	--
Ca6_P4_31097254	Ca6	31097254	ATT/ATTTTT	CAACAATTTCCCAAACAAAACA	CTTATAGCCGAATGGCCAAA	59.8	492	INTERGENIC	--	--	--
Ca6_P4_31099504	Ca6	31099504	TA/T	TGGGATGATGTTGGGAAAGT	TTGCAAAGAAAACCTCGCTT	60.2	129	INTERGENIC	--	--	--
Ca6_P4_31146150	Ca6	31146150	TAAA/TAAAA	TGCACGAGATGATTTGTTTGA	TCAATGGTGGCTTGTGGTTA	60.3	374	INTERGENIC	--	--	--
Ca6_P4_31288919	Ca6	31288919	AATATATATATATATATATATTTATATATATATATATTTATATATATATATATATATATA/AATATATATATATATATATATTTATATATATATATATTTATATATATATATATATATATA	TGATTCTTGAAAGTTAGCAAACACA	ATCGACAAACCAACATGCAA	60.2	460	INTERGENIC	--	--	--
Ca6_P4_31416514	Ca6	31416514	TA/TAATTAGTTTTGAA	GGGGAAATTTTATTGACCTCG	AATTGCATTCACCTCCTCCA	59.7	434	INTERGENIC	--	--	--
Ca6_P4_31427404	Ca6	31427404	TTATATATATATATATATATATATATAT/TTATATATATATATATATATATATATATAT	CCTGATTTCAAGAAAATGGTCG	CATATCCCCTTCCAACATGG	60.8	202	INTERGENIC	--	--	--
Ca6_P4_31471169	Ca6	31471169	TTATATATATATATATATAT/TTATATATATATATATATATATAT	CAACGTCGTCGAAGACAAGA	CCTTCGGCAAACTTCTTCAG	60.0	731	INTERGENIC	--	--	--

INDEL marker IDs	Chromosomes/ unanchored scaffolds	Physical positions (bp)	InDels (*Kabuli* reference genome-CDC Frontier/PII)	Forward primers (5'-3')	Reverse primers (5'-3')	Anneling temperature (0C)	Expected amplified product size (bp)	Structural annotation		Functional annotation	
								Sequence components of *kabuli* genome	*Kabuli* gene accession IDs	NCBI-KOG	TFs
Ca6_P4_31551380	Ca6	31551380	A/ATTG	ATGGCATCGTTGTCACAAAA	CAACTAAACTGCGCCACCTT	60.0	699	INTERGENIC	--	--	--
Ca6_P4_31604532	Ca6	31604532	AATATATATATATATATATATATATATATATATATATATA/AATATATATATATATATATATATATATATATATATATATATATATATA	TGCGGTCGAAAAATTATAATCAC	TGTAAATTCTTTTACAACAACAGTGTG	60.2	523	INTERGENIC	--	--	--
Ca6_P4_31607874	Ca6	31607874	TTATATATATATATATATATAT/TTATATATATATATATATATATATAT	TAAAGATTCGGGAGAGGGGT	CTCCAGTTTTGCATCCGATT	59.9	322	INTERGENIC	--	--	--
Ca6_P4_31631825	Ca6	31631825	TAAA/TAA	TCAGGGTTTCATACTCGCAA	CGTCGCCGTTTTATCGTTAC	59.3	727	INTERGENIC	--	--	--
Ca6_P4_3166147	Ca6	3166147	TATCGGTTCGGCCATTTCCATCGGTTCGGCC/TATCGGTTCGGCC	TCAGATTCTATCGGTTCGGC	ATATGCTGCACCTTGGTTCC	60.2	202	INTERGENIC	--	--	--
Ca6_P4_31681100	Ca6	31681100	ATTTTTTTTT/ATTTTTTTTTT	GAGCCCCGATCCATATACCT	GTCACACAATCACTGACCGC	60.1	635	INTERGENIC	--	--	--
Ca6_P4_31727074	Ca6	31727074	CTATATATATATATATATATATATATA/CTATATATATATATATATATATATATATA	CGGGTACGCATGTTATTGTG	TCGCTTTTCAGGACAATGCT	59.9	515	INTERGENIC	--	--	--
Ca6_P4_31734338	Ca6	31734338	ATTTTTTTTTTT/ATTTTTTTTTT	GCGGACAAAATTAGGTGTCAA	TGAATAGGCTTGCCTGTCAT	60.0	549	INTERGENIC	--	--	--
Ca6_P4_3176026	Ca6	3176026	AGG/AGGG	GCAATGGAAGGTGAAAATGG	GCATAGGAGCGTGGAAATTG	60.3	522	INTERGENIC	--	--	--
Ca6_P4_3192535	Ca6	3192535	CAATAATAATAATAATAATAA/CAATAATAATAATAATAA	ATTTAACGACGGATAGGCCC	ACTAACCTTGACAATGGCGG	60.2	217	INTERGENIC	--	--	--

INDEL marker IDs	Chromosomes/ unanchored scaffolds	Physical positions (bp)	InDels (*Kabuli* reference genome-CDC Frontier/PII)	Forward primers (5'-3')	Reverse primers (5'-3')	Anneling temperature (0C)	Expected amplified product size (bp)	Structural annotation		Functional annotation	
								Sequence components of *kabuli* genome	*Kabuli* gene accession IDs	NCBI-KOG	TFs
Ca6_P4_31939689	Ca6	31939689	G/GAT	CTGCGGTTCTTGCTGATGTA	AGGCAGCTTGATTTCTTGGA	60.0	382	INTERGENIC	--	--	--
Ca6_P4_31944698	Ca6	31944698	TAAAAAA/TAAAAAAA	GATCATCGAGTTTCCCGTGT	ACCATGCAAAGACATCCACA	59.9	549	INTERGENIC	--	--	--
Ca6_P4_32308001	Ca6	32308001	CTTATTTATTTATTTATTTATTTATTTATTTATTTATTTATTTATTTATTTATTTAT/CTTATTTATTTATTTATTTATTTATTTATTTATTTATTTATTTATTTATTTATTTATTTAT	ATCCTTTGCACAATGGTTCC	TTTTCCCTTCTTAGGCAGCA	59.8	451	INTERGENIC	--	--	--
Ca6_P4_32309196	Ca6	32309196	AATATATATATATATATATATATATATATAT/AATATATATATATATATATATATATAT	CGTTATCGCGAACCAACAAT	TGTGGGTGATAAGGGGTTGT	60.9	520	INTERGENIC	--	--	--
Ca6_P4_32350907	Ca6	32350907	AATTATTATTATTATTATTATTAT/AATTATTATTATTATTATTAT	TTGATATTTTCTACAATTGATCATACG	GAATGCCCTACTTTTTGGCA	57.8	710	INTERGENIC	--	--	--
Ca6_P4_32371063	Ca6	32371063	TGAT/TGATCGAT	TGAAATCCATGGAGCAAACA	CCTTTGTTCCCACAAATTGC	60.0	485	INTERGENIC	--	--	--
Ca6_P4_32372827	Ca6	32372827	CAA/CA	GAATACGCGGCTGATTCATT	CGCCCCGTTTTAACTCATTA	60.1	613	INTERGENIC	--	--	--
Ca6_P4_32372986	Ca6	32372986	ATTTTT/ATTT	GAATACGCGGCTGATTCATT	CGCCCCGTTTTAACTCATTA	60.1	613	INTERGENIC	--	--	--
Ca6_P4_32373522	Ca6	32373522	AATA/ATATA	TAATGAGTTAAAACGGGGCG	TTGGTCCAACACGAAGAGA	60.0	382	INTERGENIC	--	--	--
Ca6_P4_32391786	Ca6	32391786	AATATATATATATATA/AATATATATATATATATA	CGCTATGCCCCATTAGATGT	TTTGATTGACTCATTGCGTG	59.9	294	INTERGENIC	--	--	--

INDEL marker IDs	Chromosomes/ unanchored scaffolds	Physical positions (bp)	InDels (*Kabuli* reference genome-CDC Frontier/PII)	Forward primers (5'-3')	Reverse primers (5'-3')	Anneling temperature (0C)	Expected amplified product size (bp)	Structural annotation		Functional annotation	
								Sequence components of *kabuli* genome	*Kabuli* gene accession IDs	NCBI-KOG	TFs
Ca6_P4_32476687	Ca6	32476687	TAGAAG/TAGAAGAAG	TGCCTCAAGATCTCATGGTG	CCGCTTCCTTTGTCTTTGTC	59.8	557	INTERGENIC	--	--	--
Ca6_P4_32519592	Ca6	32519592	AG/A	CGCATGCCACTCACTCTAAA	CGCTGCCACTTTCTACATCA	60.0	611	INTERGENIC	--	--	--
Ca6_P4_32521477	Ca6	32521477	GACATGATAC/GAC	TTGCCAACGTGTCAATCACT	TTTTCCCTACTATTTTGCAGCC	60.2	259	INTERGENIC	--	--	--
Ca6_P4_32522263	Ca6	32522263	CA/CAA	TTGTTCATTTTTGGTCGTTCA	CCTTGGCGGATATAGGGAAT	59.0	347	INTERGENIC	--	--	--
Ca6_P4_32539826	Ca6	32539826	TAAAAAAAAAA/TAAAAAAAAA	TATGAGCCTCTGCCAGCTTT	GTCCGCGTCCATTGATAAAC	60.1	562	INTERGENIC	--	--	--
Ca6_P4_32540172	Ca6	32540172	TTATATATATATATAT/TTATATATATATATATATAT	GTTTATCAATGGACGCGGAC	TTCCCCTAAAAACGGTTGTG	60.3	341	INTERGENIC	--	--	--
Ca6_P4_32540257	Ca6	32540257	TACATAA/TACATAAGAAACATAA	GTTTATCAATGGACGCGGAC	TTCCCCTAAAAACGGTTGTG	60.3	341	INTERGENIC	--	--	--
Ca6_P4_32540480	Ca6	32540480	GTTTTTTTT/GTTTTTTT	CACAACCGTTTTTAGGGGAA	GAGGATTGCTATGGACAACCA	59.8	214	INTERGENIC	--	--	--
Ca6_P4_32540585	Ca6	32540585	AATATATAT/AATATAT	CACAACCGTTTTTAGGGGAA	AAGGAGAACCAGTCAACCACA	59.8	278	INTERGENIC	--	--	--
Ca6_P4_3255826	Ca6	3255826	AATATATATATATATAT/AATATATATATATATAT	TGGCTTGTTTGATTTAGGACA	ATTCGAATGATCAGTTGCCC	58.3	547	INTERGENIC	--	--	--

INDEL marker IDs	Chromosomes/ unanchored scaffolds	Physical positions (bp)	InDels (*Kabuli* reference genome-CDC Frontier/PII)	Forward primers (5'-3')	Reverse primers (5'-3')	Anneling temperature (0C)	Expected amplified product size (bp)	Structural annotation		Functional annotation	
								Sequence components of *kabuli* genome	*Kabuli* gene accession IDs	NCBI-KOG	TFs
Ca6_P4_32617100	Ca6	32617100	AAA/AAATAA	GAACGGTCCATGCAAAAGTT	CCCCCGATCCTTATACGACT	60.0	554	INTERGENIC	--	--	--
Ca6_P4_32629991	Ca6	32629991	TAATGCA/TA	CGCTCTCAATCGTGCAATAA	TAGTGATTGGAAAGGACGGC	60.0	719	INTERGENIC	--	--	--
Ca6_P4_32630101	Ca6	32630101	TTAAA/TTAAATAAA	TTTGAGGGAGGGAATTAGGG	TAGTGATTGGAAAGGACGGC	60.3	583	INTERGENIC	--	--	--
Ca6_P4_32630376	Ca6	32630376	AGGGG/AGGGGGG	GCCGTCCTTTCCAATCACTA	ATCGGGAAGAGGTTAGGGAA	60.1	123	INTERGENIC	--	--	--
Ca6_P4_32929483	Ca6	32929483	TTTATTATTATTATTATTATTATTATTATTATTATTATT/TTTATTATTATTATTATTATTATTATTATTATTATTATTATTAT T	TTCAGTGCCAACAATACCCA	ACTAGATGGTCGCATCCTGG	60.0	765	INTERGENIC	--	--	--
Ca6_P4_32971894	Ca6	32971894	CTTTTTTTTTTT/CTTTTTTTTTT	TTAACCTACCAATTCGAATACCA	TTAATGCATCTTGATGGTGG	57.7	257	INTERGENIC	--	--	--
Ca6_P4_329954	Ca6	329954	C/CGT	GCAGTTTGATCCTTAGGCTGAT	TTGACCACACAACCATAAAGAAA	59.8	642	INTERGENIC	--	--	--
Ca6_P4_33094621	Ca6	33094621	ATTT/ATT	TCAAAATGTAGCGGGTTTCC	AAGCCTTCCTCAAAATCCAA	59.9	398	INTERGENIC	--	--	--
Ca6_P4_33094845	Ca6	33094845	CAAAAA/CAAAAAA	TCAAAATGTAGCGGGTTTCC	AAGTTGGACAAAATTGGGGT	59.9	950	INTERGENIC	--	--	--
Ca6_P4_33094956	Ca6	33094956	ATTTTTTT/ATTTTTT	AATTGGATTTTGAGGAAGGC	AAGTTGGACAAAATTGGGGT	58.1	574	INTERGENIC	--	--	--

INDEL marker IDs	Chromosomes/ unanchored scaffolds	Physical positions (bp)	InDels (*Kabuli* reference genome-CDC Frontier/PII)	Forward primers (5'-3')	Reverse primers (5'-3')	Anneling temperature (0C)	Expected amplified product size (bp)	Structural annotation		Functional annotation	
								Sequence components of *kabuli* genome	*Kabuli* gene accession IDs	NCBI-KOG	TFs
Ca6_P4_33113807	Ca6	33113807	TTTATTATTATTATTATTATTATTATTATTATTATTATTATTATTATTATT/TTTATTATTATTATTATTATTATTATTATTATTATTATTATTATTATT	AGAGGCAAACAAGAACCGAA	TTGTGGCATTTTCCACTTCA	59.9	433	INTERGENIC	--	--	--
Ca6_P4_33210738	Ca6	33210738	AACGTAC/AAC	CATGCAGTCCTTTGTTGCAT	TTTTTCCGATGTCAACTTTCG	59.7	595	INTERGENIC	--	--	--
Ca6_P4_33229272	Ca6	33229272	GAAAA/GAAA	TGTTGACGGTGTGTTTTTAAGG	AAATTTTAGACCGCGACACG	59.9	477	INTERGENIC	--	--	--
Ca6_P4_33229296	Ca6	33229296	TG/TGG	CGGTGTGTTTTTAAGGATTGG	AAATTTTAGACCGCGACACG	59.4	471	INTERGENIC	--	--	--
Ca6_P4_33229523	Ca6	33229523	AAAAACGAAAA/AAAAA	CGGTGTGTTTTTAAGGATTGG	GCGGATATCGTAGGGTGCTA	59.4	644	INTERGENIC	--	--	--
Ca6_P4_33229803	Ca6	33229803	TAAAAAAAA/TAAAAAA	AAGCAAGCCAAAAACGAAAA	GCGGATATCGTAGGGTGCTA	59.9	382	INTERGENIC	--	--	--
Ca6_P4_33229847	Ca6	33229847	T/TC	AAGCAAGCCAAAAACGAAAA	GCGGATATCGTAGGGTGCTA	59.9	382	INTERGENIC	--	--	--
Ca6_P4_33231576	Ca6	33231576	AT/ATT	TGGTGTATTGGGTGCTGATG	GACCCATCGATTTGAAGCAT	60.4	975	INTERGENIC	--	--	--
Ca6_P4_33233620	Ca6	33233620	G/GAA	CCGCCCACTAAAAAGTTGAA	AATCGCCATAACTTGAAATGC	60.1	935	INTERGENIC	--	--	--
Ca6_P4_33240261	Ca6	33240261	A/AAAGAG	GTTTGGTATTGGGCTCCCTT	CACAATTCCTCCCTCTCCAA	60.2	314	INTERGENIC	--	--	--

INDEL marker IDs	Chromosomes/ unanchored scaffolds	Physical positions (bp)	InDels (*Kabuli* reference genome-CDC Frontier/PII)	Forward primers (5'-3')	Reverse primers (5'-3')	Anneling temperature (0C)	Expected amplified product size (bp)	Structural annotation		Functional annotation	
								Sequence components of *kabuli* genome	*Kabuli* gene accession IDs	NCBI-KOG	TFs
Ca6_P4_33240380	Ca6	33240380	A/AG	GTTTGGTATTGGGCTCCCTT	CGAAACTCACCCCTTGTTGT	60.2	750	INTERGENIC	--	--	--
Ca6_P4_33240742	Ca6	33240742	AA/AATATA	TTGGAGAGGGAGGAATTGTG	CGAAACTCACCCCTTGTTGT	60.0	456	INTERGENIC	--	--	--
Ca6_P4_33285610	Ca6	33285610	CATA/CATATA	CAAAATGTCAATTGTTGCACG	ATTGTTTCCTCGTGCCTTTG	60.0	637	INTERGENIC	--	--	--
Ca6_P4_33293827	Ca6	33293827	TGGG/TGGGG	CCAAGGCTATGAGACCTATCTGA	TGAAAACCCTTTGAAGGTGC	59.8	510	INTERGENIC	--	--	--
Ca6_P4_3330798	Ca6	3330798	AATTATTATTATTATTATTATTATTATTATTATTATT/AATTATTATTATTATTATTATTATTATTATTATT	TCAATTGATTTTCGGTATCCTTC	GACCTCCCAAAGGCATTTTT	59.3	406	INTERGENIC	--	--	--
Ca6_P4_3331064	Ca6	3331064	TAAAAAAA/TAAAAAA	CAAATTGAAAAGTGGGGCAT	TTAAAGTTGGGAGACCACCA	59.8	417	INTERGENIC	--	--	--
Ca6_P4_33334768	Ca6	33334768	AATATATATATATATATATATATATA/AATATATATATATATATATATATATA	TTTTGTGTGCAGAGGCTATGA	GGGTTCAAGCTAATGGCAGA	59.5	534	INTERGENIC	--	--	--
Ca6_P4_3340164	Ca6	3340164	ATTTTTTTTT/ATTTTTTTTTT	AGGACAATGGGCAAAGACAC	TCATCACCTCAACCAATCCA	60.0	267	INTERGENIC	--	--	--
Ca6_P4_3346050	Ca6	3346050	AATATATATATATATATATATATATATATAT/AATATATATATATATATATATATATATATATATATATAT	ATAAATCACGGCCCAACAAC	CCATTGCACATTTGGTTTTC	59.7	611	INTERGENIC	--	--	--
Ca6_P4_33506970	Ca6	33506970	AAAAGTTAA/AAA	CCATTTGATCTGCAACATTGA	GAAAAGGGGTGATTTGGGAT	59.5	273	INTERGENIC	--	--	--

INDEL marker IDs	Chromosomes/ unanchored scaffolds	Physical positions (bp)	InDels (*Kabuli* reference genome-CDC Frontier/PII)	Forward primers (5'-3')	Reverse primers (5'-3')	Anneling temperature (0C)	Expected amplified product size (bp)	Structural annotation		Functional annotation	
								Sequence components of *kabuli* genome	*Kabuli* gene accession IDs	NCBI-KOG	TFs
Ca6_P4_3351662	Ca6	3351662	GATATAT/GATATATATAT	CGAAAATGGAAAACACCACC	TCGAGTTGCGTAACTGACATAG	60.2	687	INTERGENIC	--	--	--
Ca6_P4_3351712	Ca6	3351712	ATTTTTTTT/ATTTTTT	GGTTGATTTAGGCTTCACGG	TCGAGTTGCGTAACTGACATAG	59.6	580	INTERGENIC	--	--	--
Ca6_P4_3351745	Ca6	3351745	CATATTATATATA/CATA	GGTTGATTTAGGCTTCACGG	TCGAGTTGCGTAACTGACATAG	59.6	580	INTERGENIC	--	--	--
Ca6_P4_3352912	Ca6	3352912	AATTA/AA	AAAGTGGGTGGCAATTAACG	TTATCCAATTCCGTTGCACA	59.9	247	INTERGENIC	--	--	--
Ca6_P4_33535351	Ca6	33535351	AATATATATATATATATATATATATATATATAT/AATATATATATATATATATATATATATATATATAT	CTTGCTCTTGGTCACATCCA	TTGGAAAGAAGCCATCCAAG	59.8	954	INTERGENIC	--	--	--
Ca6_P4_33539008	Ca6	33539008	AAAAATAAAATAAAATAAAATAAAATAAAATAAAATAAAATAAAATAAAATA/AAAAATAAAATAAAATAAAATAAAATAAAATAAAATAAAATAAAATA	GTGGGAACGTTAAGCCAAAA	GGAGTCAAGTTTCGAGGACG	60.0	424	INTERGENIC	--	--	--
Ca6_P4_3355103	Ca6	3355103	GCTATTGATCAATCTATTGAT/GCTATTGAT	TGAATAGATGTGCGCGAGAC	TCCACGGGAGTGAGAGAGAT	60.0	401	INTERGENIC	--	--	--
Ca6_P4_3355875	Ca6	3355875	CTTT/CTTTT	AATTTGGCCCTATTGGCTTT	AGAAACGGAATGAGTCACGG	59.8	709	INTERGENIC	--	--	--
Ca6_P4_3374594	Ca6	3374594	TTAA/TTAAATAA	GAAACTTCAGCAAAAACCGC	TGTGATATCCCAATCCGCTT	59.9	806	INTERGENIC	--	--	--
Ca6_P4_3375268	Ca6	3375268	TAAA/TA	GATCAGTTGACCCGCGTAGT	CCATTGAACATTTGGTTTTCA	60.1	680	INTERGENIC	--	--	--

INDEL marker IDs	Chromosomes/ unanchored scaffolds	Physical positions (bp)	InDels (*Kabuli* reference genome-CDC Frontier/PII)	Forward primers (5'-3')	Reverse primers (5'-3')	Anneling temperature (0C)	Expected amplified product size (bp)	Structural annotation		Functional annotation	
								Sequence components of *kabuli* genome	*Kabuli* gene accession IDs	NCBI-KOG	TFs
Ca6_P4_3377584	Ca6	3377584	CTTTTT/CTT	ATGCGGTGATGGTGATATTG	TATGGCGAATATGGATGGGT	59.2	745	INTERGENIC	--	--	--
Ca6_P4_3378118	Ca6	3378118	GAGAA/GA	ACCCATCCATATTCGCCATA	TTTGCCATCCTCTCTTTTTCA	60.0	653	INTERGENIC	--	--	--
Ca6_P4_3382708	Ca6	3382708	AA/AAGGGTTGGA	ATCACCGACCACTCACCTTC	TCAATTTTTCTTCGTTGACGG	60.0	787	INTERGENIC	--	--	--
Ca6_P4_3382789	Ca6	3382789	CTTTTT/CTTT	ATCACCGACCACTCACCTTC	GTTCAATCAGTGCAGCGTGT	60.0	810	INTERGENIC	--	--	--
Ca6_P4_3382946	Ca6	3382946	CAAAAA/CAAAA	TAACCACCAACATTCTGCCA	TGTTCCGTCCCACTCATACA	60.0	927	INTERGENIC	--	--	--
Ca6_P4_3387211	Ca6	3387211	CTG/CTGGTGTAGTG	ACCCCTGAACGATGTAAACG	CGCTCTTATCCTGTTGGCTC	59.9	547	INTERGENIC	--	--	--
Ca6_P4_3387853	Ca6	3387853	AGTACGTA/AGTA	GAGCCAACAGGATAAGAGCG	GAAAATTGAGTTCCATGACGTTT	60.0	477	INTERGENIC	--	--	--
Ca6_P4_3387937	Ca6	3387937	A/AG	GAGCCAACAGGATAAGAGCG	GAAAATTGAGTTCCATGACGTTT	60.0	477	INTERGENIC	--	--	--
Ca6_P4_3388065	Ca6	3388065	GATATATATAT/GATATATATATAT	GAGCCAACAGGATAAGAGCG	TTGTCTTTCACTTCTTTTTCCG	60.0	841	INTERGENIC	--	--	--
Ca6_P4_3392451	Ca6	3392451	T/TGTAAG	TGATTGCAAAGGTGTTCTCG	ATAATGAGTTGCGGGAAGGC	59.8	378	INTERGENIC	--	--	--

INDEL marker IDs	Chromosomes/ unanchored scaffolds	Physical positions (bp)	InDels (*Kabuli* reference genome-CDC Frontier/PII)	Forward primers (5'-3')	Reverse primers (5'-3')	Anneling temperature (0C)	Expected amplified product size (bp)	Structural annotation		Functional annotation	
								Sequence components of *kabuli* genome	*Kabuli* gene accession IDs	NCBI-KOG	TFs
Ca6_P4_3392779	Ca6	3392779	CTTTT/CTTT	AGATTACTCTGCCTTCCCGC	TGACGAAAAAGCTTGGAGATG	60.7	349	INTERGENIC	--	--	--
Ca6_P4_3393664	Ca6	3393664	CAAAAAAA/CCAAAAAAAAA	CATTTTGGCCATCTAAAACGA	TGGAAAAATAAACGTTGCCC	60.0	603	INTERGENIC	--	--	--
Ca6_P4_3394112	Ca6	3394112	GT/GTAT	CCATCCAGAGCACCTGAAAT	TCAATTGCCTTGCATGTTGT	60.1	371	INTERGENIC	--	--	--
Ca6_P4_3394137	Ca6	3394137	AT/ATT	CCATCCAGAGCACCTGAAAT	TCAATTGCCTTGCATGTTGT	60.1	371	INTERGENIC	--	--	--
Ca6_P4_33981637	Ca6	33981637	ACTACATTTATATCCCTA/ACTA	CTTTGATCTCTGCCACCCAT	TCCCAATAAAGAGCTGCCTC	60.1	609	INTERGENIC	--	--	--
Ca6_P4_34004073	Ca6	34004073	TAATAAATAAAAATAAATAAAAA/TAATAAATAAAAATAAATAAAAATAAATAAAAA	TGTGTAATCTGATGGGCGAA	AAATGATCTGCACCCAAAGC	60.1	696	INTERGENIC	--	--	--
Ca6_P4_34039542	Ca6	34039542	AATATATATATATATATAT/AATATATATATATATAT	TTTGATGCGCATGATAAGAAA	CTTAACATCAGTCGAGCGCA	59.3	411	INTERGENIC	--	--	--
Ca6_P4_34132247	Ca6	34132247	C/CT	AATTATCGATCATCGGTGCC	CCGAGCCAAAATCCAAAATA	59.8	700	INTERGENIC	--	--	--
Ca6_P4_34162137	Ca6	34162137	A/AGC	ATGGAAAGTTTGGTGCCTTG	AAAAACGCACGCGTTAAAAT	60.0	376	INTERGENIC	--	--	--
Ca6_P4_34164737	Ca6	34164737	CCCACCAC/CCCAC	CCACTTTGTTGGGAAAAACC	AGAAGAACGGTTGCCAAAGA	59.3	504	INTERGENIC	--	--	--

INDEL marker IDs	Chromosomes/ unanchored scaffolds	Physical positions (bp)	InDels (*Kabuli* reference genome-CDC Frontier/PII)	Forward primers (5'-3')	Reverse primers (5'-3')	Anneling temperature (0C)	Expected amplified product size (bp)	Structural annotation		Functional annotation	
								Sequence components of *kabuli* genome	*Kabuli* gene accession IDs	NCBI-KOG	TFs
Ca6_P4_34177096	Ca6	34177096	AATATATATATATATATATATATATATAT A/AATATATATATATATATATATATATAT A	TCCTTGGGTATTGTGA AACCA	TCATCAACACCAAATAT TTTCCA	60.2	434	INTERGENIC	--	--	--
Ca6_P4_3420698	Ca6	3420698	TTATATATATATATATATATATATATATA T/TTATATATATATATATATATAT	TAGCCGACAACATTGG AACA	TGAACTCGCACATTTG AGGA	60.1	386	INTERGENIC	--	--	--
Ca6_P4_34235790	Ca6	34235790	TAGAGAGAGAGAGAGAGAGAGAGAGA GAGAGAGAGA/TAGAGAGAGAGAG AGAGAGAGAGAGAGAGA	TGTTGCTGCTGAGGTA GGTG	TTCATTCGGGTTTTCGA TTC	60.0	863	INTERGENIC	--	--	--
Ca6_P4_34320971	Ca6	34320971	CATATATATATAT/CATATATATATAT AT	AGACGTTTGGTGTGTC TTGC	GCACATCATGTCAAGA ACCAA	58.8	889	INTERGENIC	--	--	--
Ca6_P4_34540623	Ca6	34540623	ATATTATTATTATTATTATTATTATTATTA TTATTATTATTATTATTATTATTATTATTA TTATT/ATATTATTATTATTATTATTA TTATTATTATTATTATTATTATTATTATTA TTATTATTATTATT	GTCAAACTGCCTCAAC CCAT	GTTTTCACCGGAGAGT TGGA	60.0	513	INTERGENIC	--	--	--
Ca6_P4_34684404	Ca6	34684404	GAAAAA/GAAAAAA	CCCTGAGACTCACGAA GAGG	CCCCCTTTTTGCATAAG TGA	60.0	699	INTERGENIC	--	--	--
Ca6_P4_34728984	Ca6	34728984	TTATATATATATATATATATAT/TTAT ATATATATATATATATATAT	TAGGCACATCAAGTCA ACCG	TCAGACCATCCGATAG AGGC	59.7	570	INTERGENIC	--	--	--
Ca6_P4_34748246	Ca6	34748246	ATTTTTTTTT/ATTTTTTTT	GCCTGCTTGAAACAAA CACA	ATAATGGTGACCCGAG TTGC	59.9	227	INTERGENIC	--	--	--
Ca6_P4_34816135	Ca6	34816135	ATTTT/ATTTTT	ATGCAATGCGTATATG CCAA	GTGTTTTGGTGTGGAT CGTG	59.9	872	INTERGENIC	--	--	--
Ca6_P4_34827878	Ca6	34827878	CATATATATATATATATATATATATATA TATATA/CATATATATATATATATATA TATATATATA	AAGTTTCGGGGTAGAG CGAT	TGTAGCATTCTCATGG CGAG	60.1	645	INTERGENIC	--	--	--

INDEL marker IDs	Chromosomes/ unanchored scaffolds	Physical positions (bp)	InDels (*Kabuli* reference genome-CDC Frontier/PII)	Forward primers (5'-3')	Reverse primers (5'-3')	Anneling temperature (0C)	Expected amplified product size (bp)	Structural annotation		Functional annotation	
								Sequence components of *kabuli* genome	*Kabuli* gene accession IDs	NCBI-KOG	TFs
Ca6_P4_34831881	Ca6	34831881	AATATATATATATATATATATATATATATA/AATATATATATATATATATATATATATATATATATATA	GAGGAACCAAAGAACCAATTTT	TCACTTATGCAATTCACGTTCC	58.5	353	INTERGENIC	--	--	--
Ca6_P4_34838549	Ca6	34838549	CAATAATAATAATAATAATAATAATAATAATAATAATAATAATAATAA/CAATAATAATAATAATAATAATAATAATAATAATAATAATAATAA	CACGTTTGATGTTGGATCAGA	ATGTGGATGGAACGTGGTTT	59.6	759	INTERGENIC	--	--	--
Ca6_P4_34859064	Ca6	34859064	GATATATATATATATATATATATATA/GATATATATATATATATATATATATATA	GAAAGAGTTTTGAGCCACGG	AAGTCCAATCTCGGTCAAACTT	59.9	625	INTERGENIC	--	--	--
Ca6_P4_34893967	Ca6	34893967	GAATCAAACACATAGAAATCAAACACATAGAAATCAAACA/GAATCAAACACATAGAAATCAAACA	TCAGACCGAACCAATGGAAT	TATCACTTGACTCCCCCTGC	60.3	804	INTERGENIC	--	--	--
Ca6_P4_34982866	Ca6	34982866	ATT/AT	ATCACTGCATCACCGAAACA	TCAGTGTGTTCCTTGCCAAA	60.1	751	INTERGENIC	--	--	--
Ca6_P4_35048353	Ca6	35048353	CTAATAATAATAATAATAATAATAATAATAATAATAATAATAATAATAATAATAA/CTAATAATAATAATAATAATAATAATAATAATAATAATAATAATAATAATAATAATAATAATAATAA	CATTGCTGCCCAAAGGTATT	AGCGAGAGTCACGGAGAAAG	60.0	555	INTERGENIC	--	--	--
Ca6_P4_35093227	Ca6	35093227	GAGTGTTGACTTTTTCA/GAGTGTTGACTTTTTCATAGTGTTGACTTTTTCA	AACCGCTATTGGGCTAGGTT	CTCTGATATGCGTGGCAAGA	60.0	646	INTERGENIC	--	--	--
Ca6_P4_35161798	Ca6	35161798	TATA/TAAATATAAAATA	AGGAAGAGTGGAGTTGCGAA	CGGTTGAGAACCAAGGATGT	60.0	649	INTERGENIC	--	--	--
Ca6_P4_35169658	Ca6	35169658	GTATATATATATATATATATATATATA/GTATATATATATATATATATATATATATATATATATA	AGGAAGAGTGGAGTTGCGAA	TATTTTGGATTTTGGCTCGG	60.0	524	INTERGENIC	--	--	--
Ca6_P4_35246739	Ca6	35246739	CAAAAA/CAAAA	GCCTCTCGAGGCTCTGAGTAT	AAAGCTCGCAATGCAAAGAT	60.1	881	INTERGENIC	--	--	--

INDEL marker IDs	Chromosomes/ unanchored scaffolds	Physical positions (bp)	InDels (*Kabuli* reference genome-CDC Frontier/PII)	Forward primers (5'-3')	Reverse primers (5'-3')	Anneling temperature (0C)	Expected amplified product size (bp)	Structural annotation		Functional annotation	
								Sequence components of *kabuli* genome	*Kabuli* gene accession IDs	NCBI-KOG	TFs
Ca6_P4_3539767	Ca6	3539767	AAATAATAATAATAATAATAATAATAATAATAATAATAATAA/AAATAATAATAATAATAATAATAATAATAATAATAATAATAA	AACCCCTTGACCATCTTTCC	CTAGGTGGGAGACAAAACGC	60.2	339	INTERGENIC	--	--	--
Ca6_P4_35399219	Ca6	35399219	GGA/GGAGA	GAAGCTCAGATGGCACACAA	AAACACCTAGTGTGGGCTGG	60.0	302	INTERGENIC	--	--	--
Ca6_P4_35399356	Ca6	35399356	GTTT/GAATTT	TTGAAGCTCAGATGGCACAC	CTCCCCCTTTTTGGCATAAT	60.0	718	INTERGENIC	--	--	--
Ca6_P4_35616799	Ca6	35616799	C/CCTT	GTTTCTGAGGCAAAATCCCA	TGCATTTGTCTTCCCATTGA	60.1	537	INTERGENIC	--	--	--
Ca6_P4_35800993	Ca6	35800993	CTTTTTTTTTTT/CTTTTTTTTTT	CTTCCATAAGCCGCATTGTT	GGGCCAACGACCTCTAAGTA	60.1	234	INTERGENIC	--	--	--
Ca6_P4_35802672	Ca6	35802672	TTTCT/TT	GAGCCTTAGAGTTGGGAGCA	ACCCAACTTGTCGAACCAAC	59.6	778	INTERGENIC	--	--	--
Ca6_P4_35810337	Ca6	35810337	C/CT	TCATCGATTGCAACTTGCTT	CCCTAAAATTTTGTGGGCTT	59.4	672	INTERGENIC	--	--	--
Ca6_P4_35832130	Ca6	35832130	TTCTCT/TTCT	TCGCCTTCTTCGAAATTAAGTC	AAAAGATTGGTTGGATTAAAAAGA	59.9	147	INTERGENIC	--	--	--
Ca6_P4_3589483	Ca6	3589483	CG/CGG	CTAATCAGCCACCCTTCTCG	GGATGAAGATCCTCGGTTGA	59.8	334	INTERGENIC	--	--	--
Ca6_P4_3589752	Ca6	3589752	AC/ACC	ATGCAACAGCAATGAGCATC	TTTAGTCAAGCTCCGCCACT	59.8	524	INTERGENIC	--	--	--

INDEL marker IDs	Chromosomes/ unanchored scaffolds	Physical positions (bp)	InDels (*Kabuli* reference genome-CDC Frontier/PII)	Forward primers (5'-3')	Reverse primers (5'-3')	Anneling temperature (0C)	Expected amplified product size (bp)	Structural annotation		Functional annotation	
								Sequence components of *kabuli* genome	*Kabuli* gene accession IDs	NCBI-KOG	TFs
Ca6_P4_35922388	Ca6	35922388	AT/ATT	TCTCCGTTTCATTCTCCACC	CCTGGGGGCTGTTTTACATA	60.1	450	INTERGENIC	--	--	--
Ca6_P4_36029920	Ca6	36029920	CTTT/CTTTT	TTTGGAACCAAAAACTTCTTCAA	GGAGAAAAAGAATTAAAATCCTCTAAA	60.0	520	INTERGENIC	--	--	--
Ca6_P4_36070342	Ca6	36070342	TAA/TAAA	GGCATTCGGGTCTGTTTTTA	TCTCCATTCTCCCCTCTCAA	59.9	274	INTERGENIC	--	--	--
Ca6_P4_36150824	Ca6	36150824	ATT/ATTATTT	TTGCAAAGTTTTGAACCGAA	AAGCCTCCTAAATGACTCATGC	59.3	398	INTERGENIC	--	--	--
Ca6_P4_36200184	Ca6	36200184	GATATATATATATATATATATATATATATATATATATATATATAT/GATATATATATATATATATATATATATATATATATATATAT	TCTCGCCTTCGTAAATCGTT	TCCACACATCAAACCCTTCA	59.8	663	INTERGENIC	--	--	--
Ca6_P4_36237842	Ca6	36237842	CATTATTATTATTATTATTATTATTATTATTATTATTATTATTATTATTATTATTAT/CATTATTATTATTATTATTATTATTATTATTATTATTATTATTATTATTATTATTATTAT	TCTACCCCCAATCCCTCTCT	ATTTGCCCTGATGTTTTTGG	59.9	573	INTERGENIC	--	--	--
Ca6_P4_36607738	Ca6	36607738	TTTTATTTATTTATTTATTTATTTATTT/TTTTATTTATTTATTTATTTATTTATTTATTTATTT	GGATTAGCACCCCTCTCCTC	TAGGCTGACCAATCCCAATC	60.0	515	INTERGENIC	--	--	--
Ca6_P4_36644370	Ca6	36644370	CATTATTAT/CATTATTATTAT	TGGTGGCATTCACATTTGTT	AGCGATGCATGTCTCAAGTG	59.8	482	INTERGENIC	--	--	--
Ca6_P4_36722414	Ca6	36722414	ACCC/ACC	CTTAGGTTTGCACTCAGCCC	TCGCGGATCTGCTCTTAAAT	59.9	685	INTERGENIC	--	--	--
Ca6_P4_36722630	Ca6	36722630	TA/TAA	TTGCTCCTACGTATCCCCAG	TCGCGGATCTGCTCTTAAAT	60.1	295	INTERGENIC	--	--	--

INDEL marker IDs	Chromosomes/ unanchored scaffolds	Physical positions (bp)	InDels (*Kabuli* reference genome-CDC Frontier/PII)	Forward primers (5'-3')	Reverse primers (5'-3')	Anneling temperature (0C)	Expected amplified product size (bp)	Structural annotation		Functional annotation	
								Sequence components of *kabuli* genome	*Kabuli* gene accession IDs	NCBI-KOG	TFs
Ca6_P4_36789650	Ca6	36789650	C/CT	CACAAGAGGGCAACAAGGAT	TGATCCGAGAGTTGCACTTG	60.1	201	INTERGENIC	--	--	--
Ca6_P4_36898169	Ca6	36898169	AATATATATATAT/AATATATATATATAT	AAGAGAGGTCACTTTGGCGA	CAATTCCCACAGGTACGCTT	60.0	692	INTERGENIC	--	--	--
Ca6_P4_37110970	Ca6	37110970	ATAAATTAAAGT/ATAAATTAAAGTAAATTAAAGT	GCTGGCCTCGTACTCATTTT	ATCTTGACACTTCCGATGCC	59.3	652	INTERGENIC	--	--	--
Ca6_P4_37202118	Ca6	37202118	CATATATATATATATATATATATATA/CATATATATATATATATATATATATA	GCGAAAATGGAAATGGAAGA	TTTGGTTTTGAACTCGGAGG	60.0	521	INTERGENIC	--	--	--
Ca6_P4_37221968	Ca6	37221968	CCTAAT/CCTAATCTAAT	AACCCATTTGAGGTGACTGC	CCGATCTTCCAATCGTTGAT	60.0	420	INTERGENIC	--	--	--
Ca6_P4_3742101	Ca6	3742101	A/ATTATTATT	TGAAAGTTTAGCGGCAATCA	AATTGTTGGCACGCTTTTTC	59.4	609	INTERGENIC	--	--	--
Ca6_P4_37640608	Ca6	37640608	CAAAATTTAAAAATTTAAAAATTTAAAA/CAAAATTTAAAAATTTAAAAATTTAAAAATTTAAAA	TTTGGCGTAGTGTGACTTGC	CTCGTGAGGGGAAGCATAAG	59.9	308	INTERGENIC	--	--	--
Ca6_P4_37741039	Ca6	37741039	CAAA/CAA	AGGGTGAGTGATCCGTTGTC	CCACGAGTTCAGGGTTTCAT	60.0	558	INTERGENIC	--	--	--
Ca6_P4_37798672	Ca6	37798672	TTATATATATATATATATATATATATATAT/TTATATATATATATATATATATATATATATATATAT	GGACGTGAACAACGAGTCAA	GATGCAGTGGCCAAAGAAAT	59.7	684	INTERGENIC	--	--	--
Ca6_P4_3780129	Ca6	3780129	CTTTTTT/CTTTTTTT	GCGTTGTCAAAATTACAAATTCA	TTATTTGAATCCCCCAAAACA	59.1	693	INTERGENIC	--	--	--

INDEL marker IDs	Chromosomes/ unanchored scaffolds	Physical positions (bp)	InDels (*Kabuli* reference genome-CDC Frontier/PII)	Forward primers (5'-3')	Reverse primers (5'-3')	Anneling temperature (0C)	Expected amplified product size (bp)	Structural annotation		Functional annotation	
								Sequence components of *kabuli* genome	*Kabuli* gene accession IDs	NCBI-KOG	TFs
Ca6_P4_37902107	Ca6	37902107	TT/TTGATAT	GTTTTGACAGGCCCGAATAA	ATGGTGGTTCGGTCACATTT	59.9	462	INTERGENIC	--	--	--
Ca6_P4_37981298	Ca6	37981298	A/AATGTC	GCTGCTCCTTTTGAACTTTCC	TATTGCCTCAAAGTGGCTGA	60.4	402	INTERGENIC	--	--	--
Ca6_P4_37981463	Ca6	37981463	GAAA/GA	CCAATTGGAACAAGGTTGGA	TATTGCCTCAAAGTGGCTGA	60.7	243	INTERGENIC	--	--	--
Ca6_P4_37981858	Ca6	37981858	TAAA/TAA	TGCAATGACCTTGAAATTCG	CTTTTAGCCTTCATCGCCAG	59.7	817	INTERGENIC	--	--	--
Ca6_P4_37982172	Ca6	37982172	AATGAT/AAT	GGTACACTTTCCCATCAGCAA	CTTTTAGCCTTCATCGCCAG	60.0	468	INTERGENIC	--	--	--
Ca6_P4_38201434	Ca6	38201434	GTTTTTTT/GTTTTTTTT	GGTTCGTAATGGATGGGATG	AAGGCCTTTTACAACGCTCA	60.0	609	INTERGENIC	--	--	--
Ca6_P4_38202225	Ca6	38202225	CT/CTGAT	AAGCTCAACATGCAAAACCC	CAAATGTGAATGCCACCAAG	60.1	691	INTERGENIC	--	--	--
Ca6_P4_38220530	Ca6	38220530	TTATATATATATATATATAT/TTATATATATATATATATATAT	GGGTGGGAAAAAGCTAGACC	GATAGTTTTTCGACCGCGAG	59.9	628	INTERGENIC	--	--	--
Ca6_P4_38385351	Ca6	38385351	CC/CCCTCTTGTCCAGATCTC	CGAAGCCTTGTCTCCTCAAC	GGAAAACAATGCCTCCAAGA	60.0	524	INTERGENIC	--	--	--
Ca6_P4_3855737	Ca6	3855737	GATATATATATATATATATATATATATATATATATATATATAT/GATATATATATATATATATATATATATATATATATATAT	GTTTCCAAGTTCCATGGTCG	TTTTTCAGTGTCGCAAAACAA	60.4	770	INTERGENIC	--	--	--

INDEL marker IDs	Chromosomes/ unanchored scaffolds	Physical positions (bp)	InDels (*Kabuli* reference genome-CDC Frontier/PII)	Forward primers (5'-3')	Reverse primers (5'-3')	Anneling temperature (0C)	Expected amplified product size (bp)	Structural annotation		Functional annotation	
								Sequence components of *kabuli* genome	*Kabuli* gene accession IDs	NCBI-KOG	TFs
Ca6_P4_38677967	Ca6	38677967	TA/TATACACACA	AACACCCCAAAACGACACTC	TCGAAGAGAAGGTTGCCACT	59.9	948	INTERGENIC	--	--	--
Ca6_P4_38718082	Ca6	38718082	GT/GTATCAGTAT	TTGAGGATTTCCAGAAACGG	CCCCAATTCACACCAAATTC	60.0	291	INTERGENIC	--	--	--
Ca6_P4_38908988	Ca6	38908988	AT/A	CGGTGTCTCCATGAATGTTG	CCATCTCTTCCTTTTGCTCG	60.0	359	INTERGENIC	--	--	--
Ca6_P4_39127532	Ca6	39127532	GAGTATA/GA	CGGTCAAATCTGAAGCAACA	ACCAACTGGTTGGCTACGTC	59.8	615	INTERGENIC	--	--	--
Ca6_P4_39250014	Ca6	39250014	ANNNNNNNNNNNNN/ANNNNNNNNNNNN	CTCGAGGGGCGTAAATCATA	GGGTGGTTCCATGAGTTGAT	60.1	172	INTERGENIC	--	--	--
Ca6_P4_3937441	Ca6	3937441	ATTTTTTTTTTT/ATTTTTTTTTT	TGAAGGAAGATGATCCACGA	CAAATTTCAGCAGCACTGGA	59.2	733	INTERGENIC	--	--	--
Ca6_P4_39446229	Ca6	39446229	GAA/GAAA	CTTCACCTTGAGTCCCCTTG	TTGGCCAGACCACTTTTACC	59.7	815	INTERGENIC	--	--	--
Ca6_P4_39450071	Ca6	39450071	CTTTTTT/CTTTTT	TTTCAGAACACATATAGTAAACGTTGG	TGATTTCACTTTTGGTGAGTAATGA	59.8	313	INTERGENIC	--	--	--
Ca6_P4_39465065	Ca6	39465065	ATTTTTTTT/ATTTTTTTTT	TAAGGTACGGGATTTTCCCC	TCACCAATTCCCCAAGACTC	60.0	416	INTERGENIC	--	--	--
Ca6_P4_39467723	Ca6	39467723	TAAA/TAA	GGGAATCAAACCCGGTAACT	TCCTAAACACATGCGTCCAA	60.1	626	INTERGENIC	--	--	--

INDEL marker IDs	Chromosomes/ unanchored scaffolds	Physical positions (bp)	InDels (*Kabuli* reference genome-CDC Frontier/PII)	Forward primers (5'-3')	Reverse primers (5'-3')	Anneling temperature (0C)	Expected amplified product size (bp)	Structural annotation		Functional annotation	
								Sequence components of *kabuli* genome	*Kabuli* gene accession IDs	NCBI-KOG	TFs
Ca6_P4_3977548	Ca6	3977548	TC/T	AACTCAGAAGGCCGAGTTGA	TGATGCGGCTTTGTACTCTG	60.0	547	INTERGENIC	--	--	--
Ca6_P4_3977816	Ca6	3977816	ATTTTTTTTTTT/ATTTTTTTTTT	AACTCAGAAGGCCGAGTTGA	TGATGCGGCTTTGTACTCTG	60.0	547	INTERGENIC	--	--	--
Ca6_P4_39791831	Ca6	39791831	T/TC	CTCGTGTTCGTTCGTGAGAA	CAGGGTACGAACCGGTAAGA	60.0	869	INTERGENIC	--	--	--
Ca6_P4_39811005	Ca6	39811005	AATACATAC/AATAC	ATATGACACGTGGCAGTCCA	GAGTCGGTCCAACAAATTCAA	60.0	463	INTERGENIC	--	--	--
Ca6_P4_39829036	Ca6	39829036	CTTTC/CTTTCCCTCTTTTC	CCCACAATCATATTCCCCTG	CGCTCCAAGAGTCAATGTCA	60.0	465	INTERGENIC	--	--	--
Ca6_P4_39831963	Ca6	39831963	CATATATATATATATATATATATATATATATATATAT/CATATATATATATATATATATATATATATATATATATATATATATAT	TTGGAAGGAGCCCTTACCTT	ATGTCAATTCAGTTGCGACG	60.1	492	INTERGENIC	--	--	--
Ca6_P4_39879844	Ca6	39879844	GA/GAA	ATCAACCCGCCCTTTACTTT	CGGCGGCTCTTTATTTAGTG	59.8	497	INTERGENIC	--	--	--
Ca6_P4_39931353	Ca6	39931353	ATTT/A	TGGTGCTCCACTTATCCCTC	GCATGTTCCATGGTGTGAAT	60.1	249	INTERGENIC	--	--	--
Ca6_P4_40031841	Ca6	40031841	T/TA	TGAGAAACTTTTCCGCTGCT	CTATTAGCGTTGGCTCTCGG	60.1	411	INTERGENIC	--	--	--
Ca6_P4_40508760	Ca6	40508760	GAAAA/GAAAAA	AATTGACGTCGAACCCTGTC	CGTAGCGAAGTCGGATGAAT	60.0	364	INTERGENIC	--	--	--

INDEL marker IDs	Chromosomes/ unanchored scaffolds	Physical positions (bp)	InDels (*Kabuli* reference genome-CDC Frontier/PII)	Forward primers (5'-3')	Reverse primers (5'-3')	Anneling temperature (0C)	Expected amplified product size (bp)	Structural annotation		Functional annotation	
								Sequence components of *kabuli* genome	*Kabuli* gene accession IDs	NCBI-KOG	TFs
Ca6_P4_40594605	Ca6	40594605	AC/A	TCTGATCAGCTCTATGCCTCC	GGATGGTTGTTTGAGTGGCT	59.5	158	INTERGENIC	--	--	--
Ca6_P4_40699134	Ca6	40699134	GATATATATATATATATATATAT/GATATATATATATATATAT	TGGATTTCAGCCTCGTAACC	AAGCACGTGTCAAATCGTGA	60.1	743	INTERGENIC	--	--	--
Ca6_P4_40730901	Ca6	40730901	AATATATATATATATATATA/AATATATATATATATATATATA	TCCCGAATTCTTTCATTGTTG	TAGCGATCCATTCCCAAAAC	59.9	476	INTERGENIC	--	--	--
Ca6_P4_40803495	Ca6	40803495	ATTT/ATTGTTT	ATGAATGGAAGACGTGACGA	TGGACAAATTGCGTATTGACA	59.1	862	INTERGENIC	--	--	--
Ca6_P4_41128605	Ca6	41128605	TAAAAAAAAAAA/TAAAAAAA	AATGGGCGCTGTTAAATGTC	GGGAGCTGATACCACAGCAT	60.0	702	INTERGENIC	--	--	--
Ca6_P4_41168319	Ca6	41168319	ATATTATTATTATTATTATTATTATTATTATTATTATTATTATTATTATT/ATATTATTATTATTATTATTATTATTATTATTATTATTATTATT	TTGAATCGTGAGGGTGTCAG	TGATTTCAAAAGCAATGTCAAAA	59.7	600	INTERGENIC	--	--	--
Ca6_P4_41197274	Ca6	41197274	TAAAAAAAAA/TAAAAAAAAAA	AGCATTGGTGGTGTAGGAGG	TGCAACGAAACAGATTGGAG	60.0	489	INTERGENIC	--	--	--
Ca6_P4_41464281	Ca6	41464281	CTTTGATTTGA/CTTTGA	AAGCAGAAGGTAGTGGCGAA	GTACGTGGTCCTTCCCTGAA	60.0	395	INTERGENIC	--	--	--
Ca6_P4_41464328	Ca6	41464328	GAAA/GAAAA	AAGCAGAAGGTAGTGGCGAA	GTACGTGGTCCTTCCCTGAA	60.0	395	INTERGENIC	--	--	--
Ca6_P4_41498708	Ca6	41498708	AATATATATATATATATATATATATATATATATATATATATAT/AATATATATATATATATATATATATATATATATATATAT	TTCTCCCTTGAGAACCATGC	TGTCGCAAGTAATTTCCAAGC	60.2	504	INTERGENIC	--	--	--

**INDEL marker IDs**	**Chromosomes/ unanchored scaffolds**	**Physical positions (bp)**	**InDels (*Kabuli* reference genome- CDC Frontier/PII)**	**Forward primers (5'-3')**	**Reverse primers (5'-3')**	**Anneling temperature (0C)**	**Expected amplified product size (bp)**	**Structural annotation**		**Functional annotation**	
								**Sequence components of *kabuli* genome**	***Kabuli* gene accession IDs**	**NCBI-KOG**	**TFs**
Ca6_P4_41574227	Ca6	41574227	CATAATAATAATAATAAT/CATAATAATAATAATAATAAT	TGATTAATCGCAAATTGTCCA	TTTTTGGGGTCGTTTTATGC	59.0	471	INTERGENIC	--	--	--
Ca6_P4_41574282	Ca6	41574282	TTTATTATTATTATTATT/TTTATTATTATTATTATTATT	TGATTAATCGCAAATTGTCCA	TTTTTGGGGTCGTTTTATGC	59.0	471	INTERGENIC	--	--	--
Ca6_P4_41659483	Ca6	41659483	CAAAA/CAAAAA	CCATCAGAAACTGGGATGGT	TGGCCCTTCATTTTTCAATC	59.8	495	INTERGENIC	--	--	--
Ca6_P4_41668275	Ca6	41668275	AATATATATATATATATATATA/AATATATATATATATATATATATATATATATATA	TCAAAACAGAACAGATCATGTGG	GTAAAATGAAGCGCTTTCGC	60.0	480	INTERGENIC	--	--	--
Ca6_P4_41669222	Ca6	41669222	AA/AAAGGACTTGGA	AATGGGCGCTGTTAAATGTC	AGGGGTGATGAAGAAATTCG	60.0	265	INTERGENIC	--	--	--
Ca6_P4_41669872	Ca6	41669872	ATT/ATTT	TGTAGAATCCACCAATGTCCA	ACCAATCATGTTTCGTGGAA	58.8	651	INTERGENIC	--	--	--
Ca6_P4_41777270	Ca6	41777270	GT/GTT	CTTGGGTTGACTTGGGACTC	TTGAACAACATGGGAGAGGA	59.5	696	INTERGENIC	--	--	--
Ca6_P4_418262	Ca6	418262	GTTTTTTTT/GTTTTTTTTT	AACGCTCCTCTTCCTTCAAA	TCTTCACTTGTGGGTTCCAA	59.1	631	INTERGENIC	--	--	--
Ca6_P4_41874600	Ca6	41874600	AAGA/AA	TGTAGTTACGTTTTCTTTTTGAATTT	CATTTAAGATTTGCGGCATTT	57.0	347	INTERGENIC	--	--	--
Ca6_P4_41903041	Ca6	41903041	TTATATATATATATATA/TTATATATATATATATATA	TCGGTATTTCGAACTTGGGT	AGCCCCAAAAAGTGGCTATT	59.4	658	INTERGENIC	--	--	--

INDEL marker IDs	Chromosomes/ unanchored scaffolds	Physical positions (bp)	InDels (*Kabuli* reference genome-CDC Frontier/PII)	Forward primers (5'-3')	Reverse primers (5'-3')	Anneling temperature (0C)	Expected amplified product size (bp)	Structural annotation		Functional annotation	
								Sequence components of *kabuli* genome	*Kabuli* gene accession IDs	NCBI-KOG	TFs
Ca6_P4_41903127	Ca6	41903127	TTTGGATT/TTT	TCGGTATTTCGAACTTGGGT	AGCCCCAAAAAGTGGCTATT	59.4	658	INTERGENIC	--	--	--
Ca6_P4_42130307	Ca6	42130307	GAAAA/GAAAAA	CGAATACACTTGGCCACTGA	CCGAAGCGACTGGAAATTAG	59.7	656	INTERGENIC	--	--	--
Ca6_P4_42269920	Ca6	42269920	AC/A	CCAAGAAATTAGAATTCGACATCA	TTTCACGTGGGACAACAAAA	59.5	570	INTERGENIC	--	--	--
Ca6_P4_42308665	Ca6	42308665	CTTATTATTATTATTATTATTATTATTATTATTATTACTATTATTATTGTTATTATTATTATTATTATTATTATTATTATT/CTTATTATTATTATTATTATTATTATTATTATTATTATTACTATTATTATTGT[illegible]	CTGAATCGCGAAAGAAGTCC	ATGGAGATGCGACAATGTGA	60.0	595	INTERGENIC	--	--	--
Ca6_P4_42360169	Ca6	42360169	TC/TCCCAAAAAGC	AGAACAGAGGGAACGCAGAA	CAGCGCTTATTTGGGAAAAG	60.0	625	INTERGENIC	--	--	--
Ca6_P4_42398678	Ca6	42398678	ATTTTTTT/ATTTTTT	GGTTTGGGAGAGAAAAAGGAA	TGGTTAAAACTCATTGGTCCC	59.6	900	INTERGENIC	--	--	--
Ca6_P4_42418850	Ca6	42418850	ATATTATTATTATTATTATT/ATATTATTATTATTATT	CCGACGTCTCTCATTCTCAA	AACGACAACTTTCAACCCATTT	59.0	686	INTERGENIC	--	--	--
Ca6_P4_42464964	Ca6	42464964	A/AT	GGTGAGTTTTCCACAAGCGT	GCTTCAATGAAATTTGGCGT	60.2	982	INTERGENIC	--	--	--
Ca6_P4_42480728	Ca6	42480728	A/AT	CACAAACATCACGTCGAACC	GATATGGGCAGACAGGGTAAA	60.0	885	INTERGENIC	--	--	--
Ca6_P4_42608436	Ca6	42608436	ATTT/ATTTT	CCTCGGATGCATTCCTCTAA	TGGGAACGTGTATTCGACAA	60.2	515	INTERGENIC	--	--	--

INDEL marker IDs	Chromosomes/ unanchored scaffolds	Physical positions (bp)	InDels (*Kabuli* reference genome-CDC Frontier/PII)	Forward primers (5'-3')	Reverse primers (5'-3')	Anneling temperature (0C)	Expected amplified product size (bp)	Structural annotation		Functional annotation	
								Sequence components of *kabuli* genome	*Kabuli* gene accession IDs	NCBI-KOG	TFs
Ca6_P4_43017644	Ca6	43017644	ACATCAT/ACATCATCAT	GGAAGGATTTTGAAGAAACCA	GCCGTGCCTTTTAAATGTGT	58.1	529	INTERGENIC	--	--	--
Ca6_P4_43043105	Ca6	43043105	CA/C	TGATTTGGTCGCAACAGCTA	AAAAATTGTGCCAAAGTTATGTGA	60.4	556	INTERGENIC	--	--	--
Ca6_P4_43064353	Ca6	43064353	A/AACTG	CGCCACAACTCCTACTCCTT	GAGGTGCGACAACAGATGAA	59.4	785	INTERGENIC	--	--	--
Ca6_P4_43064766	Ca6	43064766	ACC/ACCC	GCACCAAGGTTTTACGTTCA	GCCGATGACACATCAAAATG	58.7	719	INTERGENIC	--	--	--
Ca6_P4_43138332	Ca6	43138332	GAAAA/GAAA	GTTCCCTTTGGAATGCTTGA	TCTTTGGGCCAGTGAAAATC	60.1	741	INTERGENIC	--	--	--
Ca6_P4_438370	Ca6	438370	A/AG	AACCATTGCTTTCCATTCGT	ATGGACCCCATACAAGACCA	59.4	850	INTERGENIC	--	--	--
Ca6_P4_43909185	Ca6	43909185	TTGAAG/TTGAAGCAAGTACAACTCATGAAG	TGATTGGGTTTGGGTTCATT	ACATAGATGCCGCCTTCATT	60.0	445	INTERGENIC	--	--	--
Ca6_P4_43909507	Ca6	43909507	CTTTTTTTTTT/CTTTTT	GAAGGCGGCATCTATGTGTT	GGCTTTTGAATCACCGAAAA	60.1	447	INTERGENIC	--	--	--
Ca6_P4_43921995	Ca6	43921995	TAAA/TAA	GGTGTGTCTGTGTGTTGCGT	GCTTAATTCTAGGGAGGCGG	60.7	578	INTERGENIC	--	--	--
Ca6_P4_44134697	Ca6	44134697	GGTGGCC/GGTGGCCGTGGCC	GAGGCTGGATGGAAGAGAGA	AGGTTGCTGTTGTGCAGATG	59.5	197	INTERGENIC	--	--	--

INDEL marker IDs	Chromosomes/ unanchored scaffolds	Physical positions (bp)	InDels (*Kabuli* reference genome-CDC Frontier/PII)	Forward primers (5'-3')	Reverse primers (5'-3')	Anneling temperature (0C)	Expected amplified product size (bp)	Structural annotation		Functional annotation	
								Sequence components of *kabuli* genome	*Kabuli* gene accession IDs	NCBI-KOG	TFs
Ca6_P4_44263063	Ca6	44263063	TTATATATATATATATATATATATAT/TTATATATATATATATATATATAT	CCCTTCCTCCACCATCACTA	TTTAAAATTCGGCCGTGAAG	59.9	643	INTERGENIC	--	--	--
Ca6_P4_44292405	Ca6	44292405	TAA/TAAA	AACACTAGAAACTCGCGCAAA	CCAATCTACATGATGCACGG	60.1	304	INTERGENIC	--	--	--
Ca6_P4_44301191	Ca6	44301191	CTTTTT/CTTTTTT	TGCACATTTTACAGCGCTTC	TGAAATTATGGGTGGGCATT	60.0	254	INTERGENIC	--	--	--
Ca6_P4_4433303	Ca6	4433303	TAAAAAAAAAAA/TAAAAAAAAAA	TGGAGAGACTTCGGTTGCTT	CCACTCGTACTCCCTGAGATTC	60.0	911	INTERGENIC	--	--	--
Ca6_P4_443925	Ca6	443925	TTATATATATATATATATATATA/TTATATATATATATATATA	GGACTTTGAGGAGAATCGAGG	TGCTCACAAATATGCATTCCA	60.2	500	INTERGENIC	--	--	--
Ca6_P4_44636710	Ca6	44636710	CTATATATATATATATATATATATATAT/CTATATATATATATATATATATAT	CGCAGTCGTGCTTTCAATAA	TGTCCAGAGCAATCTTCGACT	60.0	637	INTERGENIC	--	--	--
Ca6_P4_44637030	Ca6	44637030	GATATATATATATATATATA/GATATATATATATATATATATA	AATCCAAAAGTTGAACCCGA	CAATAAGTGGTTTTTCCCGC	59.4	577	INTERGENIC	--	--	--
Ca6_P4_44845775	Ca6	44845775	GAAA/GAAAA	GTGTTGCCTGATGAATGTGG	ATCGTGTGATTGGGAGGAAG	60.0	641	INTERGENIC	--	--	--
Ca6_P4_449509	Ca6	449509	TAAAAAAA/TAAAAAA	ATGGTGGTTGGGAACACATT	GTCATCCGACCCCTATGCTA	60.0	468	INTERGENIC	--	--	--
Ca6_P4_44977370	Ca6	44977370	CCTTTCTATTTC/CCTTTCTATTTCTTTTCTTTCTATTTC	GTTGGTTCCCTAGCACCGTA	TGGATATTGGCTCAGAGGCT	60.0	623	INTERGENIC	--	--	--

**INDEL marker IDs**	**Chromosomes/ unanchored scaffolds**	**Physical positions (bp)**	**InDels (*Kabuli* reference genome-CDC Frontier/PII)**	**Forward primers (5'-3')**	**Reverse primers (5'-3')**	**Anneling temperature (0C)**	**Expected amplified product size (bp)**	**Structural annotation**		**Functional annotation**	
								**Sequence components of *kabuli* genome**	***Kabuli* gene accession IDs**	**NCBI-KOG**	**TFs**
Ca6_P4_45282323	Ca6	45282323	TCCTTTTTAGAAAGTAC/TC	TGTTGGAGAGGCTCTGCTTT	CGTTTTTCTGCACTCTGCAA	60.1	214	INTERGENIC	--	--	--
Ca6_P4_45290500	Ca6	45290500	AT/A	GGCACGCTATGGAAAGAGTC	TGAAATTCCATAGGAAGCGG	59.8	589	INTERGENIC	--	--	--
Ca6_P4_45329060	Ca6	45329060	T/TTTAAAA	TTTTTGCTATTTTATTCTCGTGG	TCGATTATGCGTAGGGAAGG	57.6	762	INTERGENIC	--	--	--
Ca6_P4_45329314	Ca6	45329314	TC/T	GGGAGATACGTAGGAGCACG	TCGATTATGCGTAGGGAAGG	59.7	242	INTERGENIC	--	--	--
Ca6_P4_45585418	Ca6	45585418	G/GCTCGGT	AAAAAGGGGAGCTCAACGAT	CTTCGAATGGTGTTGGTCCT	60.1	566	INTERGENIC	--	--	--
Ca6_P4_4559660	Ca6	4559660	AATATATATATATATATATA/AATATATATATATATATATATA	GCCAGGTTGCTGATATTTTCAT	ATCACGCCAATATCGCTGTA	60.3	935	INTERGENIC	--	--	--
Ca6_P4_45895264	Ca6	45895264	TTATATATATATATATATA/TTATATATATATATATA	TTGAAAGAACCAAACGAGGG	TTTGAGAAATCCCCATTGGA	60.1	470	INTERGENIC	--	--	--
Ca6_P4_45900035	Ca6	45900035	TTATATATATATATATATATAT/TTATATATATATATATATATATAT	TCCGACACATTGCACAAAAT	TCCAAGTTTCAAATGGGGAT	60.0	600	INTERGENIC	--	--	--
Ca6_P4_45908144	Ca6	45908144	CATATATATATATATATATATATATATATAT/CATATATATATATATATATATATATAT	AAAGGAGCTGGCATATCGAA	GATGTGGGTACGACGGATCT	59.8	501	INTERGENIC	--	--	--
Ca6_P4_46305635	Ca6	46305635	CTTTTTTTTTT/CTTTTTTTTTTT	AATGAAGGGAAGGGCATTTT	TGGTCGTTTTTCGTTTGTTG	59.8	399	INTERGENIC	--	--	--

INDEL marker IDs	Chromosomes/ unanchored scaffolds	Physical positions (bp)	InDels (*Kabuli* reference genome-CDC Frontier/PII)	Forward primers (5'-3')	Reverse primers (5'-3')	Anneling temperature (0C)	Expected amplified product size (bp)	Structural annotation		Functional annotation	
								Sequence components of *kabuli* genome	*Kabuli* gene accession IDs	NCBI-KOG	TFs
Ca6_P4_46346578	Ca6	46346578	TTATGAATAT/TTAT	TAACCCAAAAATCCCAACCA	ACTTGGGAACCCGAAGAAGT	60.0	466	INTERGENIC	--	--	--
Ca6_P4_4635371	Ca6	4635371	GT/GAGAAAAATTCGGTT	CCAAAGCCAACGGCTATAAT	ATGAATTGAGGGGGTAAGGG	59.1	302	INTERGENIC	--	--	--
Ca6_P4_46399842	Ca6	46399842	T/TCA	TAACCTTGCATGGGGAAAAG	TTCATGGTGTTTTCTTGGCA	59.9	619	INTERGENIC	--	--	--
Ca6_P4_4649762	Ca6	4649762	AATAAGT/AATAAGTATAAGT	GGTCGAACCACTTGAGGTGT	AATCCACTCCCACTCCACTG	60.0	389	INTERGENIC	--	--	--
Ca6_P4_465128	Ca6	465128	TTTTATTTATTTATTTATTTATT/TTTTATTTATTTATTTATT	TGAGCGAAAAATCATGAAAGG	TTTGGGTTTTGTTTTCCTCG	60.2	986	INTERGENIC	--	--	--
Ca6_P4_46525735	Ca6	46525735	TTTATTATTATTATTATTATTATTATTATTGTTATTATTATTATTATTATTATT/TTTATTATTATTATTATTATTATTATTATTATTGTTATTATTATTATTATTATTATT	AGAAGAGATGGTTGCTGTCCA	GCATAATTGATCCTACGGTCC	59.9	574	INTERGENIC	--	--	--
Ca6_P4_46772986	Ca6	46772986	A/ATT	GATTATTCTGCCTCCCGTCA	AAACGCAATCCTTGAGTTCG	60.0	633	INTERGENIC	--	--	--
Ca6_P4_46790016	Ca6	46790016	TATTCATT/TATT	AAAGCTGTCATACTCAGCCCA	TTGCTCCTACGTATCTCCCG	59.9	895	INTERGENIC	--	--	--
Ca6_P4_46827577	Ca6	46827577	AATATATATATATATATATATATA/AATATATATATATATATATATATATA	TGCATCGTTACCCCCATTAT	GGGGACCACTCTATCTTTTCG	60.0	517	INTERGENIC	--	--	--
Ca6_P4_4699610	Ca6	4699610	CG/CGG	TTCTCTTCAAAGCGTGAGCA	TTCCTTGTCCCATCTCCATC	59.9	304	INTERGENIC	--	--	--

INDEL marker IDs	Chromosomes/ unanchored scaffolds	Physical positions (bp)	InDels (*Kabuli* reference genome-CDC Frontier/PII)	Forward primers (5'-3')	Reverse primers (5'-3')	Anneling temperature (0C)	Expected amplified product size (bp)	Structural annotation		Functional annotation	
								Sequence components of *kabuli* genome	*Kabuli* gene accession IDs	NCBI-KOG	TFs
Ca6_P4_47001052	Ca6	47001052	A/AG	GAAGCGGTACTTCCCATTGA	GGTTACAATGTGGGGACGAG	60.1	417	INTERGENIC	--	--	--
Ca6_P4_47036359	Ca6	47036359	TTGTGTGT/TTGTGT	TCTTGATCGTGTGAGGCATT	TGTGTGGGGCATTACTTTGA	59.2	384	INTERGENIC	--	--	--
Ca6_P4_47040606	Ca6	47040606	TTTATTATTATTATTATTATTATTATTATTATTATTATTATTAT/TTTATTATTATTATTATTATTATTATTATTATTATTAT	TATCATCACCTCACCCCCAT	TCCAGTCAATGTGCAAAAACA	60.0	550	INTERGENIC	--	--	--
Ca6_P4_4713516	Ca6	4713516	CAAAAAAAAA/CAAAAAAAA	TGGAGAGGTGTGTGCAAGAG	GAGGCAAGAGCGACGAATAG	60.0	376	INTERGENIC	--	--	--
Ca6_P4_47141352	Ca6	47141352	CAAAA/CAAA	TATGTCAGAATCCCGATCCC	AAACATTTGGCTACAAGGCG	59.7	439	INTERGENIC	--	--	--
Ca6_P4_47299841	Ca6	47299841	AACATATTCA/AACATATTCATATACATATTCA	TTGCGCGCTTGTTATACTTG	GCATGCTGCAAGGGATAGTT	60.0	497	INTERGENIC	--	--	--
Ca6_P4_47312521	Ca6	47312521	GTT/GTTT	TATCTGTGGTCGTGTGGGAA	TCACGGGTCAACACGATTTA	60.0	822	INTERGENIC	--	--	--
Ca6_P4_47527023	Ca6	47527023	GAAAAAAAAAAA/GAAAAAAAAAA	AACGAGAAAACGGAGAGCAA	CCCACAAAACATGACTGCAC	60.0	504	INTERGENIC	--	--	--
Ca6_P4_47615563	Ca6	47615563	GAAAAAA/GAAAAAAA	GGATTATTAATTCGGCGGGT	AAATCTGGTCGCTGAATTGG	60.0	627	INTERGENIC	--	--	--
Ca6_P4_47701039	Ca6	47701039	CAAA/CAAAA	TGTCCCTGAGTATTCCGGTC	ATGTGACAAACCTTTTCGGG	59.9	645	INTERGENIC	--	--	--

INDEL marker IDs	Chromosomes/ unanchored scaffolds	Physical positions (bp)	InDels (*Kabuli* reference genome-CDC Frontier/PII)	Forward primers (5'-3')	Reverse primers (5'-3')	Anneling temperature (0C)	Expected amplified product size (bp)	Structural annotation		Functional annotation	
								Sequence components of *kabuli* genome	*Kabuli* gene accession IDs	NCBI-KOG	TFs
Ca6_P4_4771733	Ca6	4771733	TTATATATATATATATATATAT/TTATATATATATATATATAT	GGGCCACATGTATTTGTGTTT	GGAGGCAAGGCAATAAATCA	59.6	532	INTERGENIC	--	--	--
Ca6_P4_47787286	Ca6	47787286	TT/TTAT	AGGAAACAAACGAAGGCAGA	CCGCTTTGCATCCTCATAAT	59.9	226	INTERGENIC	--	--	--
Ca6_P4_47790346	Ca6	47790346	TTATATATATATATATATATATAT/TTATATATATATATATATAT	GCAAGATCGCATTCTTGTCA	CGGGTATAAGGCTCCACTCA	60.0	591	INTERGENIC	--	--	--
Ca6_P4_4781527	Ca6	4781527	TA/TACTCTTCCGA	GATTTCCTCGCTTCCCTTCT	CCTTGTCCAGTTGTTCCCAT	59.8	523	INTERGENIC	--	--	--
Ca6_P4_48011269	Ca6	48011269	GATATATATATATAT/GATATATATATATATAT	TTTGTTGCGTAAATGTTGTTGA	TAGCTGTTGCGACCAAATCA	59.2	665	INTERGENIC	--	--	--
Ca6_P4_4801698	Ca6	4801698	TGG/TGGG	ATAACCGACACAGACGAGGC	GTTGTTGCTTCTGCTCCTCC	60.1	278	INTERGENIC	--	--	--
Ca6_P4_48063867	Ca6	48063867	AAGA/AA	CGTTTTATTTCGGGACTGGA	CTGCGAGGAACATTTCAACA	59.9	325	INTERGENIC	--	--	--
Ca6_P4_48143669	Ca6	48143669	ATTTT/AT	TAAAACGGATGGAGGGATCA	TGAACGAGGATTTTGGAAGG	60.3	586	INTERGENIC	--	--	--
Ca6_P4_4823285	Ca6	4823285	GTATATATATATATATATATAT/GTATATATATATATATATAT	CATCTTGCAAAACAAACAAATG	TCCACTAACAAGTACTCCAAAACG	58.3	644	INTERGENIC	--	--	--
Ca6_P4_4823723	Ca6	4823723	TAA/TAAA	CGTTTTGGAGTACTTGTTAGTGGA	ACAACCATCAAACCGATTCC	59.6	432	INTERGENIC	--	--	--

INDEL marker IDs	Chromosomes/ unanchored scaffolds	Physical positions (bp)	InDels (*Kabuli* reference genome-CDC Frontier/PII)	Forward primers (5'-3')	Reverse primers (5'-3')	Anneling temperature (0C)	Expected amplified product size (bp)	Structural annotation		Functional annotation	
								Sequence components of *kabuli* genome	*Kabuli* gene accession IDs	NCBI-KOG	TFs
Ca6_P4_48260819	Ca6	48260819	GAAAATTAATAAAATA/GAAAATTAATAAAATAAAATTAATAAAATA	GTGGATGGTGGGGAATAGTG	CTGAGGTCCCAAGCTACGTC	60.1	504	INTERGENIC	--	--	--
Ca6_P4_48267774	Ca6	48267774	ATTTTTTTTTT/ATTTTTTTTT	GACATAAGAATGGAGGCGGA	TTTCTTCGCGTTTGACTAATG	60.0	674	INTERGENIC	--	--	--
Ca6_P4_48271447	Ca6	48271447	TTTATTATTATTATTATTATTATTATTATTATTATTATTATTATTATTATTATTATTAT/TTTATTATTATTATTATTATTATTATTATTATTATTATTATTATTATTATTATTAT	TGACTAATTGGTCGCCCTTC	GGATGCGATCATGTGAAGAA	60.1	685	INTERGENIC	--	--	--
Ca6_P4_48279466	Ca6	48279466	G/GA	AACCAGATACTAACCCGCGA	TCTGGAAGTTTCTTGACCGTG	59.6	620	INTERGENIC	--	--	--
Ca6_P4_48279626	Ca6	48279626	ATTTTTTT/ATTTTTT	AACCAGATACTAACCCGCGA	TCTGGAAGTTTCTTGACCGTG	59.6	620	INTERGENIC	--	--	--
Ca6_P4_48280097	Ca6	48280097	GAAAAAAA/GAAAAAAAA	GACACGGTCAAGAAACTTCCA	TGCGTTGACTCTTCAATACACA	60.1	376	INTERGENIC	--	--	--
Ca6_P4_48280225	Ca6	48280225	GTAT/GT	GACACGGTCAAGAAACTTCCA	TGTGGCACATTCCAAATTTAT	60.1	601	INTERGENIC	--	--	--
Ca6_P4_48280286	Ca6	48280286	ATTTTTTTTTTTTTTT/ATTTTTTTTTTTT	GACACGGTCAAGAAACTTCCA	TGTGGCACATTCCAAATTTAT	60.1	601	INTERGENIC	--	--	--
Ca6_P4_48280545	Ca6	48280545	ATT/ATTT	TGTGTATTGAAGAGTCAACGCAT	AAAAGTTGTGCCAAAGTTATGTGA	59.7	732	INTERGENIC	--	--	--
Ca6_P4_48280766	Ca6	48280766	TCTA/TCTAAAACTA	TGTATTGATGAGTCGTTGAATGAT	GGAGACGTTAAAGTCCCACA	58.5	902	INTERGENIC	--	--	--

INDEL marker IDs	Chromosomes/ unanchored scaffolds	Physical positions (bp)	InDels (*Kabuli* reference genome-CDC Frontier/PII)	Forward primers (5'-3')	Reverse primers (5'-3')	Anneling temperature (0C)	Expected amplified product size (bp)	Structural annotation		Functional annotation	
								Sequence components of *kabuli* genome	*Kabuli* gene accession IDs	NCBI-KOG	TFs
Ca6_P4_48281397	Ca6	48281397	TAAAAAAAA/TGAAAAAAAAAA	GGGACTTTAACGTCTCCAATACA	CTTGAGAACGATCTGCCACA	59.4	473	INTERGENIC	--	--	--
Ca6_P4_48281570	Ca6	48281570	TA/TAATTAAA	TAGGGCAATTGTGCACATGA	CTCCAGGTAACGGTTGTGGT	61.1	335	INTERGENIC	--	--	--
Ca6_P4_48373786	Ca6	48373786	GTTTCCACCACTCT/GT	TCACACGACGTTCACCATTT	TCCATCAAGGTAGGCCATTC	60.0	706	INTERGENIC	--	--	--
Ca6_P4_48385345	Ca6	48385345	G/GA	GGCTTTTGCAATGGTTGTTT	TGCACTCACTCGCCATTTAC	60.0	586	INTERGENIC	--	--	--
Ca6_P4_4843264	Ca6	4843264	A/ATGCAAT	CACACATCAATCGACCCAAG	ACACGGTATTTCCAATTCGC	60.0	748	INTERGENIC	--	--	--
Ca6_P4_48821438	Ca6	48821438	AATATATATATATATATATATATATATATATATATAT/AATATATATATATATATATATATATATATATATATATATATAT	CTACGTTCCGTCGATTCCAT	CCAGTATGCGACAAGCTGAA	60.0	640	INTERGENIC	--	--	--
Ca6_P4_48850339	Ca6	48850339	CTT/CT	TTGAGGGGTGTGATTAGCAA	TGGGGACCATAACTGAGGAG	59.1	846	INTERGENIC	--	--	--
Ca6_P4_48891611	Ca6	48891611	TAAA/TAAAA	TGTGTCCCAAAACTCAAGGA	GCTGCCTCCCCTCTCTAACT	59.1	773	INTERGENIC	--	--	--
Ca6_P4_48905387	Ca6	48905387	GAAAAAA/GAAAAAAAA	CACGGTCCCTAGAGCCAATA	ATCGACTCCCGAACTCAAAA	60.1	666	INTERGENIC	--	--	--
Ca6_P4_49079894	Ca6	49079894	TTAATAATAATAATAATAATAATAATAATAATAATAATA/TTAATAATAATAATAATAATAATAATAATAATAATAATAATA	AATACCTGGACGACTGTGGC	CCTCCATCTTTTCTGATCAACC	60.0	618	INTERGENIC	--	--	--

INDEL marker IDs	Chromosomes/ unanchored scaffolds	Physical positions (bp)	InDels (*Kabuli* reference genome-CDC Frontier/PII)	Forward primers (5'-3')	Reverse primers (5'-3')	Anneling temperature (0C)	Expected amplified product size (bp)	Structural annotation		Functional annotation	
								Sequence components of *kabuli* genome	*Kabuli* gene accession IDs	NCBI-KOG	TFs
Ca6_P4_49218883	Ca6	49218883	TAAAAAA/TAAAAAAAA	CCCCTTCATATGCTTCCAGA	GTGCTTGCTTTTACTTCGCC	60.0	446	INTERGENIC	--	--	--
Ca6_P4_49222205	Ca6	49222205	T/TG	CCTGCGGAATTTCCTAATGA	AGGTAACACGGCAGGTAACG	60.0	197	INTERGENIC	--	--	--
Ca6_P4_4941032	Ca6	4941032	GATAATAATAATAATAATAATAATAATAATAATAATAATAATAATAATAATAATAAT/GATAATAATAATAATAATAATAATAATAATAATAATAATAATAATAATAATAATAAT	TATGACCCGAGATCCGCTAC	GGTTCTCGCACACAACAAAA	60.1	318	INTERGENIC	--	--	--
Ca6_P4_49437012	Ca6	49437012	TG/T	CCGTTGAGAAGTTAATGGCG	TTGAAGCGGTAGTTGACGAA	60.6	248	INTERGENIC	--	--	--
Ca6_P4_49497029	Ca6	49497029	AATATATATATATATATATATATAT/AATATATATATATATATATATAT	GTTTTTGGCACGCAATTTGT	TGACCTTCAACTGATCGCAC	60.9	637	INTERGENIC	--	--	--
Ca6_P4_49611302	Ca6	49611302	AAG/AAGCTAG	CATTGAAGGCAACCAATGTG	CTGTAGGCCACACAAAATGC	60.0	951	INTERGENIC	--	--	--
Ca6_P4_49619550	Ca6	49619550	ATTTTTTTTTTTTTT/ATTTTTTTTTTTTTTT	CCGATGAAGGTGGTTTATTTTT	AAACCCACTGAATTGAAACCT	59.3	816	INTERGENIC	--	--	--
Ca6_P4_4964508	Ca6	4964508	TAAAAAAAAAA/TAAAAAAAAA	GACAGAAGCGACATGGTTGA	AGTTGCTTAGTTGGCAACCC	59.8	767	INTERGENIC	--	--	--
Ca6_P4_49686510	Ca6	49686510	T/TCAA	CATGTCGTTATTGCGACCAC	AAGCCGTGAAATGGTTTTTG	60.0	519	INTERGENIC	--	--	--
Ca6_P4_49733827	Ca6	49733827	TCC/TC	TTGCAATCTCATCAACTCGG	GTAAAATGAAGCGCTTTCGC	59.8	810	INTERGENIC	--	--	--

INDEL marker IDs	Chromosomes/ unanchored scaffolds	Physical positions (bp)	InDels (*Kabuli* reference genome-CDC Frontier/PII)	Forward primers (5'-3')	Reverse primers (5'-3')	Anneling temperature (0C)	Expected amplified product size (bp)	Structural annotation		Functional annotation	
								Sequence components of *kabuli* genome	*Kabuli* gene accession IDs	NCBI-KOG	TFs
Ca6_P4_49749941	Ca6	49749941	TATAATAATAATAATAATAATAATAAAAATAATAATAATAATAATAATAATAATAA/TATAATAATAATAATAATAATAATAATAATAAAAATAATAATAATAATAATAATAATAA	GACGCACGGGTTACACACTA	ATGTGGATGGTGCAGTACGA	59.6	299	INTERGENIC	--	--	--
Ca6_P4_49863050	Ca6	49863050	TAAAAAA/TAAAAAAA	ATCTTCCCTCTCCCGAGTTC	TTGGATTGTCACTTCACGACA	59.6	727	INTERGENIC	--	--	--
Ca6_P4_49920252	Ca6	49920252	AC/A	AAGCGAGGAGCAAAAACGTA	TCAACACGTAGACTCGACCG	60.0	326	INTERGENIC	--	--	--
Ca6_P4_49998830	Ca6	49998830	GTTTTTTT/GTTTTT	AAGAGAGGTCACTTTGGCGA	TTGCATTCCTCCAAAACACA	60.0	519	INTERGENIC	--	--	--
Ca6_P4_50002097	Ca6	50002097	CT/CTGTTGAAAATT	CGATCACCATGTTTGTCGTC	CGAACCCCAGATGAATTGTT	60.0	337	INTERGENIC	--	--	--
Ca6_P4_5025748	Ca6	5025748	T/TATATA	TGTGGATCATGACTCGGAAA	CACTTTCGGTCTAAGCCATTG	60.0	707	INTERGENIC	--	--	--
Ca6_P4_5031791	Ca6	5031791	ATTTTTTT/ATTTTTT	TGATGTCGCATGGGTATTGT	AAAAAGTTGAGGCCCAAAGG	59.8	459	INTERGENIC	--	--	--
Ca6_P4_50349238	Ca6	50349238	GAA/GAATTGTTTATTTAAA	CATCACAAAATCCCACACACT	GCAATTGCGCAAATACAAAA	58.3	386	INTERGENIC	--	--	--
Ca6_P4_50467177	Ca6	50467177	TTATATATATATATATATATATATATATAT/TTATATATATATATATATATATATATATAT	TGATGTGGCACGTCTAACAAA	ATGCTTGCTAAATGGCACAA	60.2	280	INTERGENIC	--	--	--
Ca6_P4_50528740	Ca6	50528740	TAAAAA/TAAAA	TGGGTTTATCCCTTCTTTGC	TGGATCCAACTTTGAGACACC	59.0	704	INTERGENIC	--	--	--

INDEL marker IDs	Chromosomes/ unanchored scaffolds	Physical positions (bp)	InDels (*Kabuli* reference genome-CDC Frontier/PII)	Forward primers (5'-3')	Reverse primers (5'-3')	Anneling temperature (0C)	Expected amplified product size (bp)	Structural annotation		Functional annotation	
								Sequence components of *kabuli* genome	*Kabuli* gene accession IDs	NCBI-KOG	TFs
Ca6_P4_50797014	Ca6	50797014	ATTTTTTTTTTTTTTTTT/ATTTTTTTTTTTTTT	GGAGAATGCGATAGCTGGAG	TGGCGTTCTTTTTAGCCTGT	59.9	827	INTERGENIC	--	--	--
Ca6_P4_50938274	Ca6	50938274	TTTATTATTATTATTA/TTTATTATTATTATTATTA	ACACGTTTTGTTTGTGGCAA	AGTTGGCTTTTGTACCCCCT	60.1	396	INTERGENIC	--	--	--
Ca6_P4_50968923	Ca6	50968923	TAAAAAAAAAAAA/TAAAAAAAAAAA	TCCCTTTCAGTCCCTTCTCA	CACTGCACCGTTGCTTAAAA	59.8	557	INTERGENIC	--	--	--
Ca6_P4_51039137	Ca6	51039137	ATGTGTGTGTGTGTGTGTGTGTGT/ATGTGTGTGTGTGTGTGTGTGTGTGTGT	TAAATGGATGGTGGGGATGT	AATTTTGCAATTGTGTGCGA	59.9	740	INTERGENIC	--	--	--
Ca6_P4_51142833	Ca6	51142833	CTTTATTTATTTATTTATTTATTT/CTTTATTTATTTATTTATTTATTTATTT	TTGCTTTGCTTTGAAAACCC	CCACTTGTGTCATAACAACTCAAA	60.2	637	INTERGENIC	--	--	--
Ca6_P4_51270485	Ca6	51270485	AATATATATATATATATATAT/AATATATATATATATATATATAT	AAAACCGAATATGTGGCTGC	TTTCCCTCCAAACACACACA	60.0	534	INTERGENIC	--	--	--
Ca6_P4_51350130	Ca6	51350130	GATAATAAT/GATAAT	CAATCCAATCAACGGTGCTA	TTTTAAAGTCACGAGGAACCAAA	59.5	543	INTERGENIC	--	--	--
Ca6_P4_51391817	Ca6	51391817	CTATATATATATATATATATAT/CTATATATATATATATATATAT	TATTTTTATGTCGGCCACCC	AAACAAAATCGCAAAAACGG	59.7	340	INTERGENIC	--	--	--
Ca6_P4_51433841	Ca6	51433841	AAATAATAATAATAATAATAATAATAATAATA/AAATAATAATAATAATAATAATAATAATAATAATA	GCGAAAGAGCAGCAAAAGAC	TGCATGGATGTAAATCAAACAA	60.3	771	INTERGENIC	--	--	--
Ca6_P4_51482572	Ca6	51482572	TAAAAAAAA/TAAAAAAAAA	TGCACAATTGTTTATGATGTTTTT	ACACTCCCAAAATCGTCCAG	58.5	552	INTERGENIC	--	--	--

INDEL marker IDs	Chromosomes/ unanchored scaffolds	Physical positions (bp)	InDels (*Kabuli* reference genome-CDC Frontier/PII)	Forward primers (5'-3')	Reverse primers (5'-3')	Anneling temperature (0C)	Expected amplified product size (bp)	Structural annotation		Functional annotation	
								Sequence components of *kabuli* genome	*Kabuli* gene accession IDs	NCBI-KOG	TFs
Ca6_P4_51482605	Ca6	51482605	TAAAAAAA/TAAAAAA	TGCACAATTGTTTATGATGTTTTT	ACACTCCCAAAATCGTCCAG	58.5	552	INTERGENIC	--	--	--
Ca6_P4_51746046	Ca6	51746046	TTAATAATAATAATAATAATAATAATAATAATAATAATAATAAT/TTAATAATAATAATAATAATAATAATAATAATAATAATAATAATAAT	GACGAAATTCTTTTAAGAGGGG	TTTATGGCACAACACCATCG	58.3	412	INTERGENIC	--	--	--
Ca6_P4_51772881	Ca6	51772881	ATTTTTTTTT/ATTTTTTTT	CTGGGGATACGTAGGAGCAA	CTTAGGTTTGCACTCAGCCC	60.1	509	INTERGENIC	--	--	--
Ca6_P4_51775849	Ca6	51775849	GATTTTTTATTT/GATTT	TGATGTGCTACCCATACCGA	GAGAGCATGCGTTTGTTTCA	59.9	205	INTERGENIC	--	--	--
Ca6_P4_51785881	Ca6	51785881	GTTTTTTTTTTT/GTTTTTTTTTT	AACATTGTTGCGGCCTAAAA	CAGAAGATCACTCGTGCCAA	60.5	382	INTERGENIC	--	--	--
Ca6_P4_51816063	Ca6	51816063	TTATATATATATATATATATATATATATA/TTATATATATATATATATATATATATATATATATA	ATTGGAGACAACGGGAACAG	GCACAGGCACCTAACCAAAT	60.0	526	INTERGENIC	--	--	--
Ca6_P4_5191855	Ca6	5191855	TTATATATATATATATATA/TTATATATATATATATA	ATCAACAATTCAAGGCCAGG	CAAGTCATGCAATTTGACGG	59.9	807	INTERGENIC	--	--	--
Ca6_P4_51978058	Ca6	51978058	TTATATATATATATATATAT/TTATATATATATATATATAT	AAAAACCTTTGGGAAATCGG	CCAAATTTCAAATGGCGACT	60.2	211	INTERGENIC	--	--	--
Ca6_P4_52087568	Ca6	52087568	GT/GTTTTAATCT	GCCAGCCATTCGATAACACT	GAAGCAAGGATTACCGACCA	60.1	244	INTERGENIC	--	--	--
Ca6_P4_52107130	Ca6	52107130	CAA/CAAA	TGGCCCTGTCCTCATAAATC	AAAGGCTATTTGCTCGTGGA	59.9	911	INTERGENIC	--	--	--

INDEL marker IDs	Chromosomes/ unanchored scaffolds	Physical positions (bp)	InDels (*Kabuli* reference genome-CDC Frontier/PII)	Forward primers (5'-3')	Reverse primers (5'-3')	Anneling temperature (0C)	Expected amplified product size (bp)	Structural annotation		Functional annotation	
								Sequence components of *kabuli* genome	*Kabuli* gene accession IDs	NCBI-KOG	TFs
Ca6_P4_52329133	Ca6	52329133	CTTATTTATTTATTTATTTATTTATT/ CTTATTTATTTATTTATT	TCACCCCATAATCAAG GTGAA	TTGCGTTCTCTTACGCA TCA	60.2	990	INTERGENIC	--	--	--
Ca6_P4_52486383	Ca6	52486383	GTTTTTTTTTTTTTTTTTT/GTTTTTTT TTT	GTTCCATGCTGCCTTT TCAT	AGAGGAAGCAGCCACT GTGT	60.1	271	INTERGENIC	--	--	--
Ca6_P4_52486696	Ca6	52486696	ATTTTTTTTTTT/ATTTTTTTT	AGGCCATCACATTCCT TTTG	CACTTTGGGGACGTCA AGTT	59.9	449	INTERGENIC	--	--	--
Ca6_P4_52657541	Ca6	52657541	AATATATATATATATATATATA/AATA TATATATATATATATATA	TGGTTAGCATGTGTTT GGGA	TTGTGGTGCATTCTTCA TCC	60.0	412	INTERGENIC	--	--	--
Ca6_P4_52774565	Ca6	52774565	AATATATATATATATATATATAT/AAT ATATATATATATATAT	CGGTGAATCCTTTACG GAGA	GCGAGTCAATCCGGTA AAAA	60.1	569	INTERGENIC	--	--	--
Ca6_P4_52787964	Ca6	52787964	CG/C	TTCTCACGAACGAACA CGAG	GTGGCACCTCGGTAAA CAGT	60.0	667	INTERGENIC	--	--	--
Ca6_P4_52848807	Ca6	52848807	ACTCGCT/A	CTGCCAAATACTGCGA CTCA	TCAATTTTGCTTGTAGG ATGGA	60.0	381	INTERGENIC	--	--	--
Ca6_P4_52850074	Ca6	52850074	ACTAATTTCTAATT/ACTAATTTCTAA TTTCTAATT	TCTGATTCGTTAATGAC AACCTTT	CGTGGGAGCAATTATG TCCT	59.1	218	INTERGENIC	--	--	--
Ca6_P4_52850184	Ca6	52850184	TA/T	AGGACATAATTGCTCC CACG	TCCATCTCCAACAACAT CAAA	60.0	305	INTERGENIC	--	--	--
Ca6_P4_52854746	Ca6	52854746	CATATATATAT/CAATATATATAT	TGAGAATAAAGAAATTT GTTGCTAAA	AGGTCAACGTACAACA TTTGC	57.9	543	INTERGENIC	--	--	--

INDEL marker IDs	Chromosomes/ unanchored scaffolds	Physical positions (bp)	InDels (*Kabuli* reference genome-CDC Frontier/PII)	Forward primers (5'-3')	Reverse primers (5'-3')	Anneling temperature (0C)	Expected amplified product size (bp)	Structural annotation		Functional annotation	
								Sequence components of *kabuli* genome	*Kabuli* gene accession IDs	NCBI-KOG	TFs
Ca6_P4_52856262	Ca6	52856262	TG/T	CCGGACCTAACACGTCCTAA	TTCAGTGACAACTCCATGTGC	60.0	389	INTERGENIC	--	--	--
Ca6_P4_52858291	Ca6	52858291	AAAATAA/AAA	CACATGCTGCAAAATTGAATG	CCACAAAAATGGTGTTGCAG	60.1	190	INTERGENIC	--	--	--
Ca6_P4_52858643	Ca6	52858643	GTATCTATCTATCTAT/GTATCTATCTATCTATCTAT	CTGCAACACCATTTTTGTGG	TTTGCTGAAAGCATGTTTGTTT	60.0	689	INTERGENIC	--	--	--
Ca6_P4_52859170	Ca6	52859170	CTTTCTTTTT/CTTT	AAGTGAATTCCACCTTCCCA	CGAGCCTCCAACTTCTCTTG	59.4	560	INTERGENIC	--	--	--
Ca6_P4_52859293	Ca6	52859293	ATATATTATATT/ATATATT	AAGTGAATTCCACCTTCCCA	CGAGCCTCCAACTTCTCTTG	59.4	560	INTERGENIC	--	--	--
Ca6_P4_52862596	Ca6	52862596	T/TTATAA	ACATGAAGGCTTGGGTTGAC	GAGGAGCATGATAGCAAGGC	60.0	193	INTERGENIC	--	--	--
Ca6_P4_5287896	Ca6	5287896	TAAA/TA	CCGAAGAAAAGAAAGCAACG	AGTGCGGAGCGAGGATACTA	60.0	518	INTERGENIC	--	--	--
Ca6_P4_52889876	Ca6	52889876	ATT/ATTT	TAAAGGACCAAGTCGGGAAA	TGCTCATGGAGTTAGAAGCAA	59.5	471	INTERGENIC	--	--	--
Ca6_P4_52890200	Ca6	52890200	TTATATATATATATATATATATATAT/TTATATATATATATATATATATAT	TAAAGGACCAAGTCGGGAAA	TTATTTGGGTGCATGAGCAA	59.5	863	INTERGENIC	--	--	--
Ca6_P4_52935026	Ca6	52935026	CATATATATATATATATATATATAT/CATATATATATATATATATATATATATAT	CTTTGTGTGCTTCCCATCAA	TGCACGAACTTGTGTATGAAAA	59.7	888	INTERGENIC	--	--	--

INDEL marker IDs	Chromosomes/ unanchored scaffolds	Physical positions (bp)	InDels (*Kabuli* reference genome-CDC Frontier/PII)	Forward primers (5'-3')	Reverse primers (5'-3')	Anneling temperature (0C)	Expected amplified product size (bp)	Structural annotation		Functional annotation	
								Sequence components of *kabuli* genome	*Kabuli* gene accession IDs	NCBI-KOG	TFs
Ca6_P4_5295925	Ca6	5295925	TTTATTATTTATTA/TTTATTATTTATTATTATTTATTA	TTGACATTTCAAACCGGTCTAT	TGGAAAAGACTCCTGAATATGGA	58.5	359	INTERGENIC	--	--	--
Ca6_P4_52960258	Ca6	52960258	A/ACCTT	TTGTCCCCAAGCAACAATTA	GTTTGGGGTGGTGATTCTCA	59.0	607	INTERGENIC	--	--	--
Ca6_P4_52997933	Ca6	52997933	G/GCC	CCAAGGAAGGACGAAAATGA	GAGACCATTTGAACAATTATGCC	60.0	471	INTERGENIC	--	--	--
Ca6_P4_53017362	Ca6	53017362	GATATATATATATATAT/GATATATATATAT	TTCTCTTCAGTCCCCTTCTCC	GTCACTTGTTCGTTGACCCA	59.8	508	INTERGENIC	--	--	--
Ca6_P4_53311252	Ca6	53311252	AT/ATT	GATGGTGGCCATTGAAGAGT	CAACAGTGTATGCACCTGCC	59.9	721	INTERGENIC	--	--	--
Ca6_P4_53312302	Ca6	53312302	AAAAATAAAATAAAATAAAATAAAA/AAAAATAAAATAAAATAAAATAAAATAAAA	ATAGCAATTGGTGCATCCCT	ATGACCAAGGCATTGAGGAC	59.4	761	INTERGENIC	--	--	--
Ca6_P4_53359449	Ca6	53359449	TAAAAAAAAAAAAAAA/TAAAAAAA	GTTCGAATCTCTCAGGCGAC	ACGCGAGAAAGAGGAGATGA	60.0	239	INTERGENIC	--	--	--
Ca6_P4_53363665	Ca6	53363665	TAAAAAA/TAAAAAAA	AGGCTTCTGTGGGATACAAA	TTGGAGATTGGGACTTGGAG	57.3	297	INTERGENIC	--	--	--
Ca6_P4_53413835	Ca6	53413835	ATT/ATTT	CGTCATTTGGGGGACTAAAA	GAGTTCGGGAGTAGGGAAGG	59.8	978	INTERGENIC	--	--	--
Ca6_P4_53493467	Ca6	53493467	CATATATATATATATATA/CATATATATATATATATATA	TCAACAGCAGAACCTTAGCG	TTGTTAGCTTTTCAATTGTCGC	59.2	117	INTERGENIC	--	--	--

INDEL marker IDs	Chromosomes/ unanchored scaffolds	Physical positions (bp)	InDels (*Kabuli* reference genome-CDC Frontier/PII)	Forward primers (5'-3')	Reverse primers (5'-3')	Anneling temperature (0C)	Expected amplified product size (bp)	Structural annotation		Functional annotation	
								Sequence components of *kabuli* genome	*Kabuli* gene accession IDs	NCBI-KOG	TFs
Ca6_P4_53650549	Ca6	53650549	GTATATATATATATATATATATATATAT/GTATATATATATATATATATATATATATATATATATATATATAT	TTTTTCTCCAGCAATCAACAA	TTGAGATGTACCATGCAAGGAG	58.4	740	INTERGENIC	--	--	--
Ca6_P4_53666018	Ca6	53666018	TTATTATATTATATTATATTATATTATATTATATTATATTATATTATATTATATTATATTATATTATATTATATTATATTATAT/TTATTATATTATATTATATTATATTATATTATATTATATTATATTATATTATATT[illegible]	TCAGTGAGTTAGCGGAGCCT	CGGACCCATGACCATTAAAC	60.2	702	INTERGENIC	--	--	--
Ca6_P4_53729112	Ca6	53729112	AATATATATATATATATATATATATATAT/AATATATATATATATATATATATATATATATATATATATAT	ATGTGACACCCTAAACCCCA	CCTTTTGATTTTTGAGAAACGTG	60.1	166	INTERGENIC	--	--	--
Ca6_P4_53780329	Ca6	53780329	GATTAATTAATTAATTAATTAATTAATTAATTAATTAATTAATT/GATTAATTAATTAATTAATTAATTAATTAATTAATTAATTAATT	CACACCCTCTCAAGGTGGAT	TCGAATGGTTGATAAAATTAACG	60.0	723	INTERGENIC	--	--	--
Ca6_P4_53799025	Ca6	53799025	ATTT/ATTTT	GGCCCAATATTTGAAACCCT	CAACTTGCAAAGACCCATCA	60.0	704	INTERGENIC	--	--	--
Ca6_P4_54068748	Ca6	54068748	CCATCAT/CCAT	GTAAGTGAAAGCGGGAGTGC	ACCACCAACTTGCTTGCTCT	59.9	527	INTERGENIC	--	--	--
Ca6_P4_54068855	Ca6	54068855	AATAAAATTATAAA/AATAAAATTATAAAATTATAAA	GATCCCAACCATCATTGTCC	ACCACCAACTTGCTTGCTCT	60.0	258	INTERGENIC	--	--	--
Ca6_P4_54068881	Ca6	54068881	TAATATAAAAA/TAATATAAAAATATAAAAA	GATCCCAACCATCATTGTCC	ACCACCAACTTGCTTGCTCT	60.0	258	INTERGENIC	--	--	--
Ca6_P4_54068968	Ca6	54068968	GA/GATA	GATCCCAACCATCATTGTCC	ACCACCAACTTGCTTGCTCT	60.0	258	INTERGENIC	--	--	--
Ca6_P4_54069131	Ca6	54069131	ATTTT/ATTTTTT	GATCCCAACCATCATTGTCC	AATTACCACGGCCTCTTGTG	60.0	824	INTERGENIC	--	--	--

INDEL marker IDs	Chromosomes/ unanchored scaffolds	Physical positions (bp)	InDels (*Kabuli* reference genome-CDC Frontier/PII)	Forward primers (5'-3')	Reverse primers (5'-3')	Anneling temperature (0C)	Expected amplified product size (bp)	Structural annotation		Functional annotation	
								Sequence components of *kabuli* genome	*Kabuli* gene accession IDs	NCBI-KOG	TFs
Ca6_P4_54069955	Ca6	54069955	TAAAAA/TAAAA	ACAAGAGGCCGTGGTAATTG	TTTTGGTGGGTTCCACTTTT	60.0	751	INTERGENIC	--	--	--
Ca6_P4_54070088	Ca6	54070088	ATT/ATTT	GCAATTGTCGTGATTTTATTTTTG	TTTTGGTGGGTTCCACTTTT	59.8	379	INTERGENIC	--	--	--
Ca6_P4_54070236	Ca6	54070236	TAAAA/TAA	TTTCTTTCCTCATGCTTTAGAGG	TTTTGGTGGGTTCCACTTTT	59.1	123	INTERGENIC	--	--	--
Ca6_P4_5410706	Ca6	5410706	ATTTTTTTT/ATTTTTTTTT	TTTGAAAAAGTGAGGGGTGG	CTTTGGTGGTGGTCAGAGGT	59.9	956	INTERGENIC	--	--	--
Ca6_P4_54108101	Ca6	54108101	TAATAAAATAAAATAAAATAAAAT/TAATAAAATAAAATAAAAT	TTTGTGCTACATGGGTGAGTG	AATCCGTGCATTTAGAGCATT	59.6	838	INTERGENIC	--	--	--
Ca6_P4_54143290	Ca6	54143290	ATTTTTTTTTTTTTTT/ATTTTTTTTTTT	GGGATCGAAAATCACGAGAA	GAAATTAAGGGATGTATTAAGTCCAAA	60.0	680	INTERGENIC	--	--	--
Ca6_P4_54143384	Ca6	54143384	T/TAA	CCAAGAGATATACGGGGCAA	TGAAAGTCTGATCCGATCTAATAGT	59.9	623	INTERGENIC	--	--	--
Ca6_P4_54143529	Ca6	54143529	CTTTTTTT/CTTTTTTTTT	CCAAGAGATATACGGGGCAA	GCGGCCGATAAGTAAGACAT	59.9	772	INTERGENIC	--	--	--
Ca6_P4_54143698	Ca6	54143698	CAAATAAAATAAATAAA/CAAATAAA	CCAAGAGATATACGGGGCAA	TATGTCCACCCGACAAAACA	59.9	917	INTERGENIC	--	--	--
Ca6_P4_54144071	Ca6	54144071	ATTTT/ATTT	GCCGCCGAATCTTATATTACC	CGTGCATTAACATCGACAGG	59.8	580	INTERGENIC	--	--	--

INDEL marker IDs	Chromosomes/ unanchored scaffolds	Physical positions (bp)	InDels (*Kabuli* reference genome-CDC Frontier/PII)	Forward primers (5'-3')	Reverse primers (5'-3')	Anneling temperature (0C)	Expected amplified product size (bp)	Structural annotation		Functional annotation	
								Sequence components of *kabuli* genome	*Kabuli* gene accession IDs	NCBI-KOG	TFs
Ca6_P4_54144443	Ca6	54144443	ATAAACTAA/ATAAACTAAACTAA	TGTTTTGTCGGGTGGACATA	CGTGCATTAACATCGACAGG	59.8	450	INTERGENIC	--	--	--
Ca6_P4_54160745	Ca6	54160745	ATTGCTTG/ATTG	GCGAGTTAGAAGATATGAGGGG	CGAAAAAGTTCCAAAGATGGAC	59.2	818	INTERGENIC	--	--	--
Ca6_P4_54160778	Ca6	54160778	GA/GACATTAA	GCGAGTTAGAAGATATGAGGGG	CGAAAAAGTTCCAAAGATGGAC	59.2	818	INTERGENIC	--	--	--
Ca6_P4_54161011	Ca6	54161011	TGAGAGA/TGAGA	TTGATTTGACAATTTTCATTGCTT	TCAGTTTTCCGGTTTCTTGC	59.9	703	INTERGENIC	--	--	--
Ca6_P4_54163818	Ca6	54163818	CTTTTTTTTT/CTTTTTTTT	CACACGTTGGTAGTCGTTCG	TTTGTTGGCGTGTGTTTGTT	60.2	809	INTERGENIC	--	--	--
Ca6_P4_54192734	Ca6	54192734	ATTTTTTTT/ATTTTTTTTT	GGACGGCGAAAATAAAACAA	TTGAAAATGGCTAAGGCAAAA	59.9	642	INTERGENIC	--	--	--
Ca6_P4_54193024	Ca6	54193024	GTATATATATATATATATATA/GTATATATATATATATATA	GGACGGCGAAAATAAAACAA	TGGAGAGCGTTGAAGATGAA	59.9	918	INTERGENIC	--	--	--
Ca6_P4_54320493	Ca6	54320493	CTTTTTTT/CTTTTTTTT	CTCCAATATTCCTCTCGGCA	TGACACGAGAAATGTCCCAA	60.2	724	INTERGENIC	--	--	--
Ca6_P4_54343914	Ca6	54343914	CTTTTT/CTTTT	ACGTCGTCGCTTTTGAGAGT	GCCGTTTTCCATTGTGACTT	60.1	214	INTERGENIC	--	--	--
Ca6_P4_54359904	Ca6	54359904	AC/A	CCTTGAACCCATTTTCTGGA	CCATTCCAACGACCACTCTT	59.9	418	INTERGENIC	--	--	--

INDEL marker IDs	Chromosomes/ unanchored scaffolds	Physical positions (bp)	InDels (*Kabuli* reference genome-CDC Frontier/PII)	Forward primers (5'-3')	Reverse primers (5'-3')	Anneling temperature (0C)	Expected amplified product size (bp)	Structural annotation		Functional annotation	
								Sequence components of *kabuli* genome	*Kabuli* gene accession IDs	NCBI-KOG	TFs
Ca6_P4_54364730	Ca6	54364730	TAAAAAAAAAAAAA/TAAAAAAAAA	TGGTCCAATTTAGGGAGAACA	ATTTTCCTCGATCCCTTGCT	59.4	395	INTERGENIC	--	--	--
Ca6_P4_54666936	Ca6	54666936	GTATATATATATATATATATAT/GTATATATATATATATATAT	GTGCGCATGTGTTTGTCTTT	GGGGAGAGAGAGGGACAGAG	59.8	672	INTERGENIC	--	--	--
Ca6_P4_54683653	Ca6	54683653	TTAATAATAATAATAATAATAATAATAATAATAATAATAATAATAATAATAATAATAA/TTAATAATAATAATAATAATAATAATAATAATAATAATAATAATAATAATAA	TAGTCTCATGCTGACACGCC	TGCGAGAGAGAAGAAGGAGG	60.0	219	INTERGENIC	--	--	--
Ca6_P4_5469396	Ca6	5469396	CATATATATATATATATATAT/CATATATATATATATAT	GGAATAACGGCTGGCATAGA	CAAAATGCAGCGCCTAAAA	60.1	335	INTERGENIC	--	--	--
Ca6_P4_54754837	Ca6	54754837	GCCAAATCGATTT/GCCAAATCGATTTCCAAATCGATTT	GTAAAGGCCACCAATCGAAA	TTGTCCGTGAGAGACTGCAC	59.9	939	INTERGENIC	--	--	--
Ca6_P4_54952283	Ca6	54952283	TTATATATATATATATATATATATATATAT/TTATATATATATATATATATATATAT	CCATTGTAACACCCCAAATTTTA	TTTCCTCCATTTCTTCGTGG	59.9	937	INTERGENIC	--	--	--
Ca6_P4_55143076	Ca6	55143076	A/AAAGAGTGTAAGGTAAT	ATAAAGCCTCGCGATGAAGA	CACCCAATACGGGTTACGTC	59.9	466	INTERGENIC	--	--	--
Ca6_P4_55445634	Ca6	55445634	TAAAAAAAAAAAA/TAAAAAAAAA	AATCACGCGAATCCACAAAT	AATTCCTACCCTCCCCACAC	60.3	582	INTERGENIC	--	--	--
Ca6_P4_55446083	Ca6	55446083	AG/A	TGAGAGTGTGGGGAGGGTAG	TTCATGTGAATAAATGACGTTGC	60.1	523	INTERGENIC	--	--	--
Ca6_P4_55446463	Ca6	55446463	AACA/AACACA	AAGGTCCTAACACCTGCCTT	TCGGTGAAAACTATAGGGCG	57.8	285	INTERGENIC	--	--	--

INDEL marker IDs	Chromosomes/ unanchored scaffolds	Physical positions (bp)	InDels (*Kabuli* reference genome-CDC Frontier/PII)	Forward primers (5'-3')	Reverse primers (5'-3')	Anneling temperature (0C)	Expected amplified product size (bp)	Structural annotation		Functional annotation	
								Sequence components of *kabuli* genome	*Kabuli* gene accession IDs	NCBI-KOG	TFs
Ca6_P4_55471469	Ca6	55471469	AAGA/AA	CTTTCACTTTGCAGCT CGTG	GAAATTGTCCTTGCAT GGCT	59.8	475	INTERGENIC	--	--	--
Ca6_P4_55490592	Ca6	55490592	A/AC	CAAAAGAATGTGAGGG GACAA	CTCCAATGTGCGTCTC TTCA	60.0	179	INTERGENIC	--	--	--
Ca6_P4_55490894	Ca6	55490894	T/TTAATAC	TGAAGAGACGCACATT GGAG	TGTCGGTTACTAAACAT GATGGA	60.0	542	INTERGENIC	--	--	--
Ca6_P4_5551268	Ca6	5551268	TAAAAAAAAAA/TAAAAAAAAAAA	ACGTCACATTACCCAT GCAA	TAACAAACGGGCTCTT GACC	59.8	413	INTERGENIC	--	--	--
Ca6_P4_55769150	Ca6	55769150	C/CT	TCGATTATGCGTAGGG AAGG	TTTTTCCTTTGTAGGCC CAA	60.1	943	INTERGENIC	--	--	--
Ca6_P4_55771133	Ca6	55771133	GTC/GTCTTC	GGAAACGTGTGAACGG AAGT	GGTAACCAATCCGAAG ACGA	60.0	575	INTERGENIC	--	--	--
Ca6_P4_55771190	Ca6	55771190	TAT/TATCGTCTTCGGAT	TTACCGATGCATTGAT CGAC	GGTAACCAATCCGAAG ACGA	59.5	143	INTERGENIC	--	--	--
Ca6_P4_55771649	Ca6	55771649	TAAAAAAAAA/TAAAAAAAAAAAAAAA A	GGATTGATTATCCGAT GCGT	GAACGCGAGATATTGG GTGT	59.8	544	INTERGENIC	--	--	--
Ca6_P4_55847701	Ca6	55847701	ATTT/ATT	AGCCCATACGGATGCT CTAA	TGGGAAGATTGACTTG GAGG	59.7	487	INTERGENIC	--	--	--
Ca6_P4_55867824	Ca6	55867824	GTTATTATTATTATTATTATTATTATT ATTA/GTTATTATTATTATTATTATTA TTATTATTATTA	GGATAGATACCCATCC AATCCA	CTTGGCAACCACCCCT ATTA	59.9	275	INTERGENIC	--	--	--

INDEL marker IDs	Chromosomes/ unanchored scaffolds	Physical positions (bp)	InDels (*Kabuli* reference genome-CDC Frontier/PII)	Forward primers (5'-3')	Reverse primers (5'-3')	Anneling temperature (0C)	Expected amplified product size (bp)	Structural annotation		Functional annotation	
								Sequence components of *kabuli* genome	*Kabuli* gene accession IDs	NCBI-KOG	TFs
Ca6_P4_55987809	Ca6	55987809	AATATATATATATATATATATATATATATATATAT/AATATATATATATATATATATATATATATATATATATATAT	TTCAAGGTTCATTAACATCAACG	AAAGTTGACTCATATCAAAACCCA	59.0	390	INTERGENIC	--	--	--
Ca6_P4_56061377	Ca6	56061377	AATATATATATATATATATATATATATATA/AATATATATATATATATATATATATATATATATATATATA	TTTGGTTGACGCATGTGTTT	ACCAACGCCATTTTCATTGT	60.0	737	INTERGENIC	--	--	--
Ca6_P4_56087656	Ca6	56087656	CTTT/CTTTT	TTGTGAGCATGAGAAGTTGTTG	TGAAAAATTATTGAAAAAGTTACTTCC	59.0	737	INTERGENIC	--	--	--
Ca6_P4_56139090	Ca6	56139090	A/AAACACAAATT	ACCCTCTCACCCCTCTTGAT	TCCTCATCATTCCCTCCTTG	59.9	387	INTERGENIC	--	--	--
Ca6_P4_56191156	Ca6	56191156	CAAATAAATAAATAAATAAATAAATAAA/CAAATAAATAAATAAATAAATAAA	TCGATTATGCGTAGGGAAGG	TCCTTGAGTTTTGGGACACA	60.1	482	INTERGENIC	--	--	--
Ca6_P4_56304604	Ca6	56304604	AC/A	TTGTCGTGCTTCCATCAGAG	CAACTCAGAACTGCCGTCAA	60.0	431	INTERGENIC	--	--	--
Ca6_P4_56410876	Ca6	56410876	ATTGTTTTGTTTTGTTTTGTTTT/ATTGTTTTGTTTTGTTTT	CGTTTCTCGCCATTTTTCAT	TCAATTTTGGCATTTCTTTCA	60.1	745	INTERGENIC	--	--	--
Ca6_P4_5645292	Ca6	5645292	CTATATATATATATATATATAT/CTATATATATATATATATATAT	GTAAACCCAAGAGAGGTGCG	TTCTAAATGGCGACTTTCCA	59.7	923	INTERGENIC	--	--	--
Ca6_P4_56503513	Ca6	56503513	CAAAAA/CAAAAAA	CTCACAAACATTTGGGGGAC	GACCATCCGACTTTGCATTT	60.2	568	INTERGENIC	--	--	--
Ca6_P4_56503742	Ca6	56503742	C/CCACTTATCT	GTTGGCGCAAACTCTTCTTC	CGGGTAAACATGACCCTTTG	60.0	273	INTERGENIC	--	--	--

INDEL marker IDs	Chromosomes/ unanchored scaffolds	Physical positions (bp)	InDels (*Kabuli* reference genome-CDC Frontier/PII)	Forward primers (5'-3')	Reverse primers (5'-3')	Anneling temperature (0C)	Expected amplified product size (bp)	Structural annotation		Functional annotation	
								Sequence components of *kabuli* genome	*Kabuli* gene accession IDs	NCBI-KOG	TFs
Ca6_P4_56503979	Ca6	56503979	AAACAA/AAA	GTTGGCGCAAACTCTTCTTC	TGCAACTTTCAGGTCCCTTT	60.0	583	INTERGENIC	--	--	--
Ca6_P4_56504001	Ca6	56504001	ATTTTT/ATTTT	GTTGGCGCAAACTCTTCTTC	TGCAACTTTCAGGTCCCTTT	60.0	583	INTERGENIC	--	--	--
Ca6_P4_56504140	Ca6	56504140	AAAAGAAA/AAAA	AAATGCAAAGTCGGATGGTC	TGCGAAGAAGAATGATCACG	59.9	896	INTERGENIC	--	--	--
Ca6_P4_56504753	Ca6	56504753	ATTTTTTTTTTTTT/ATTTTTTTTTT	CGTGATCATTCTTCTTCGCA	TCGGTCACCAAGTTACCACA	59.9	614	INTERGENIC	--	--	--
Ca6_P4_5650524	Ca6	5650524	AAATAATAATAATAATAATAATAATAATAATAATAATAATAA/AAATAATAATAATAATAATAATAATAATAATAATAATAATAA	CCCCACCTTTATGCAGTACC	TGTGGACTTTTTACCAAATTCTTG	59.3	655	INTERGENIC	--	--	--
Ca6_P4_56505598	Ca6	56505598	AC/A	TGGTAACTTGGTGACCGACA	AATTTTTGCAGAAGAAAGAAAAAG	60.0	485	INTERGENIC	--	--	--
Ca6_P4_56506826	Ca6	56506826	TAAAAAAA/TAAAAAAAAA	GGGCACTTGTTAGCATTTCC	TGCGAAGAAGAATGATCACG	59.6	495	INTERGENIC	--	--	--
Ca6_P4_56507452	Ca6	56507452	TATCA/TA	CGTGATCATTCTTCTTCGCA	ATCCAAACATTGGCCATTTC	59.9	685	INTERGENIC	--	--	--
Ca6_P4_56507723	Ca6	56507723	GTTTTTTTTTTTTTT/GTTTTTTTTTTTTTTTT	CGTGATCATTCTTCTTCGCA	CTCCCAATTTGCAGTGTCCT	59.9	966	INTERGENIC	--	--	--
Ca6_P4_56507798	Ca6	56507798	CA/CAA	AAATTCAAGTCTCGACGCAAA	CTCCCAATTTGCAGTGTCCT	59.9	511	INTERGENIC	--	--	--

INDEL marker IDs	Chromosomes/ unanchored scaffolds	Physical positions (bp)	InDels (*Kabuli* reference genome-CDC Frontier/PII)	Forward primers (5'-3')	Reverse primers (5'-3')	Anneling temperature (0C)	Expected amplified product size (bp)	Structural annotation		Functional annotation	
								Sequence components of *kabuli* genome	*Kabuli* gene accession IDs	NCBI-KOG	TFs
Ca6_P4_56578763	Ca6	56578763	AATATATATATATATATATATATA/AATATATATATATATATATATATATA	GAATGCCCTATGTGACACGA	GTAAAGCAAAACGGGTCGAA	59.5	855	INTERGENIC	--	--	--
Ca6_P4_56713561	Ca6	56713561	ATT/ATATTT	TTCACGTTGGAACTTGCTTG	TTTTTGTGATTCAGGTGGCA	59.9	833	INTERGENIC	--	--	--
Ca6_P4_56727392	Ca6	56727392	TAA/TAAA	AGGCAAAACATGTTCCCTCA	AATGTTAGTGCACCGTGTCG	60.5	706	INTERGENIC	--	--	--
Ca6_P4_56748962	Ca6	56748962	ATTTTTTTTTTT/ATTTTTTTTTT	TTTCGTGCTGTGATTGAAGC	TTTTTCAAGTGCAAGCCAAT	60.0	725	INTERGENIC	--	--	--
Ca6_P4_56767873	Ca6	56767873	TAAAAAAAAAAAA/TAAAAAAAAAAAAA	TCATGGATTCAACCAGATGC	TTCATCTTTCAAACTTGAGACTCG	59.5	188	INTERGENIC	--	--	--
Ca6_P4_57039572	Ca6	57039572	TAAAAAAAA/TAAAAAAAAA	GGTGTAGTGGATCGGTCTTGA	ATAGTGCCGAAGGAAGCAAA	60.0	334	INTERGENIC	--	--	--
Ca6_P4_57117976	Ca6	57117976	AATATATATATATATATATATATATATATATATA/AATATATATATATATATATATATATATA	TTTCATGTGCGTGTGAGTGA	TCAACCAATTCAACAATTTGACA	59.9	519	INTERGENIC	--	--	--
Ca6_P4_57460927	Ca6	57460927	CAAAAAAAAAAA/CAAAAAAAAA	GTTGTGGGATGAACTGACCC	GTTGGAATTCCTTTTGGCAC	60.2	848	INTERGENIC	--	--	--
Ca6_P4_5746619	Ca6	5746619	TTATATATATATATATATATATAT/TTATATATATATATATATATAT	TTGCTAGTATGCGCTGATGG	GGATCAATTAATGCCCATCCT	60.0	575	INTERGENIC	--	--	--
Ca6_P4_57648762	Ca6	57648762	CATATATATATATATATATATATAT/CATATATATATATATATATATATATATATAT	AACATTTGTTGAGAGTGTGACAA	CAGTTAACGCAACTAATAGTGTTTCA	57.3	320	INTERGENIC	--	--	--

INDEL marker IDs	Chromosomes/ unanchored scaffolds	Physical positions (bp)	InDels (*Kabuli* reference genome-CDC Frontier/PII)	Forward primers (5'-3')	Reverse primers (5'-3')	Anneling temperature (0C)	Expected amplified product size (bp)	Structural annotation		Functional annotation	
								Sequence components of *kabuli* genome	*Kabuli* gene accession IDs	NCBI-KOG	TFs
Ca6_P4_5765272	Ca6	5765272	TTTTTTATTTTTATTTTTATTTTTATT/ TTTTTTATTTTTATTTTTATT	CTCTTGGACATGTGGTGGTG	TCCCTTTCGTTTTGGTTGAC	60.0	541	INTERGENIC	--	--	--
Ca6_P4_57935713	Ca6	57935713	CATATATATATATATATATATATATA/ CATATATATATATATATATATATATATATA	AACACTGCATTCCTCCAAAAA	GTGGAGAAAGCCATTGGAGA	59.6	736	INTERGENIC	--	--	--
Ca6_P4_58204126	Ca6	58204126	GAAAAAAAAAA/GAAAAAAAAAAA	GGGACATTTACTTCCCTCCC	TTCAACCACGTTATAATTCCAAAA	59.6	627	INTERGENIC	--	--	--
Ca6_P4_58296268	Ca6	58296268	ATATTTAT/ATAT	ACGGTTGTCTGTGTTCACCA	TTGGATCTGAACACCTTCCC	60.0	599	INTERGENIC	--	--	--
Ca6_P4_58313547	Ca6	58313547	TNNNNNNNNNN/TNNN	TACAAGCCTGAGCCTGACCT	TTAACGTTGTGCTTGTTCGC	60.0	340	INTERGENIC	--	--	--
Ca6_P4_58373671	Ca6	58373671	GATATATATATATATATATATATATA/GATATATATATATATATATATATATA	TCACGCAAAAGATTCATAAAAGA	AGTTTGTAAGCTGTGCGTCG	58.9	500	INTERGENIC	--	--	--
Ca6_P4_5846506	Ca6	5846506	TAAAAAAAAAAAAA/TAAAAAAAAAA	TGATTAGGCCTGAATTGTGGT	GCAAGTTGCCAAGAAAAAGC	59.4	461	INTERGENIC	--	--	--
Ca6_P4_5852510	Ca6	5852510	TAATAATAATAA/TAATAATAATAAATAATAATAA	TGACGTATGTTGCACTTTGGA	CCAATTCATGAAATCCGTCC	60.2	564	INTERGENIC	--	--	--
Ca6_P4_5853411	Ca6	5853411	GTATTATTATTATTA/GTATTATTATTATTATTATTA	TCGAACATAACACCTCGCAG	TCCGGTTACATTCCTTCTTTTG	59.9	467	INTERGENIC	--	--	--
Ca6_P4_58539579	Ca6	58539579	CATATATATATATATATATATATATATATATATATAT/CATATATATATATATATATATATATATATATATATATAT	CGAGCCTAATAGTCGACCCA	AACGGGTCAACCAATCACAT	60.2	258	INTERGENIC	--	--	--

INDEL marker IDs	Chromosomes/ unanchored scaffolds	Physical positions (bp)	InDels (*Kabuli* reference genome-CDC Frontier/PII)	Forward primers (5'-3')	Reverse primers (5'-3')	Anneling temperature (0C)	Expected amplified product size (bp)	Structural annotation		Functional annotation	
								Sequence components of *kabuli* genome	*Kabuli* gene accession IDs	NCBI-KOG	TFs
Ca6_P4_5854308	Ca6	5854308	TAAAAAAAAA/TAAAAAAAAAA	CACGTGGTCATACTTCCATCA	TGTCAATTCCAAATGCATCAA	59.4	623	INTERGENIC	--	--	--
Ca6_P4_58615933	Ca6	58615933	GTAGTT/G	CAAATTGTCCGTGCAATCAC	GAAGAATGGTAGGCGGAACA	60.0	227	INTERGENIC	--	--	--
Ca6_P4_58627027	Ca6	58627027	AATATATATATATATATATATATATATATA/AATATATATATATATATATATATATATATATATATATA	CCAAAATCGAGCCTTCACAT	TGTGTGTAGCACGGTTCTCTC	60.1	403	INTERGENIC	--	--	--
Ca6_P4_58758402	Ca6	58758402	ATTTTTTTTTT/ATTTTTTTTTTT	TGGTCTGGCCTATTCTCACC	GATCCGTCAAGTCCCTCGTA	60.1	449	INTERGENIC	--	--	--
Ca6_P4_58781669	Ca6	58781669	T/TG	GGACCTCATTTAGTCGGAGG	TGTTGGGATTGTAGGGTGGT	58.6	453	INTERGENIC	--	--	--
Ca6_P4_58782759	Ca6	58782759	TC/T	TTGAAATCGTGATTTTGGCTT	CGTTGGCAATTTTTGGTCTT	59.6	225	INTERGENIC	--	--	--
Ca6_P4_58782804	Ca6	58782804	T/TC	TTGAAATCGTGATTTTGGCTT	CGTTGGCAATTTTTGGTCTT	59.6	225	INTERGENIC	--	--	--
Ca6_P4_58783805	Ca6	58783805	ATTTTTTT/ATTTTTT	TCGGTATAAGTGCTTTGCCA	AAACAAAACATGGGACAAACAT	59.3	218	INTERGENIC	--	--	--
Ca6_P4_58784094	Ca6	58784094	A/AT	TCGGTATAAGTGCTTTGCCA	AAGCATCGGAAAAAGTACAAAAA	59.3	887	INTERGENIC	--	--	--
Ca6_P4_58784343	Ca6	58784343	ATTT/ATT	CAAATGTTTGTCCCATGTTTTG	GCTCTCTTCAATATTAAGCATCGG	60.1	709	INTERGENIC	--	--	--

INDEL marker IDs	Chromosomes/ unanchored scaffolds	Physical positions (bp)	InDels (*Kabuli* reference genome-CDC Frontier/PII)	Forward primers (5'-3')	Reverse primers (5'-3')	Anneling temperature (0C)	Expected amplified product size (bp)	Structural annotation		Functional annotation	
								Sequence components of *kabuli* genome	*Kabuli* gene accession IDs	NCBI-KOG	TFs
Ca6_P4_58784829	Ca6	58784829	ATTTTTTT/ATTTTTTTT	TTTTTGTACTTTTTCCGATGCTT	CCGTAATTTTGCTATGGAGC	59.2	795	INTERGENIC	--	--	--
Ca6_P4_58790530	Ca6	58790530	GTTTTTTTT/GTTTTTTT	GGCGACGTGTTTTTCATTTT	TTTTGGCGGTGTGCAAAT	60.0	420	INTERGENIC	--	--	--
Ca6_P4_58791052	Ca6	58791052	ATAA/ATAAGTAA	TTTTAAATTTGCACACCGCC	GAGCCGTCTTCATTTCAATCTT	60.8	594	INTERGENIC	--	--	--
Ca6_P4_58805367	Ca6	58805367	AAATTAATT/AAATT	AAGTCGAACAAAGGGAGGGT	AAAATGAAATTTGGTGGCGA	60.0	116	INTERGENIC	--	--	--
Ca6_P4_58806728	Ca6	58806728	AAA/AAATTAA	GGCTCGAAAGATGGTTGAAG	ATTTATATGAGTGCGGGGCA	59.8	885	INTERGENIC	--	--	--
Ca6_P4_58808278	Ca6	58808278	T/TC	TGGTGCGTCCACTATTCAAA	ATTTTCCCACATGGACTGGT	60.1	622	INTERGENIC	--	--	--
Ca6_P4_58815507	Ca6	58815507	CT/C	ATTCCTCGACCAGTTGAAAA	CATCAATCATGTTGCATGGC	57.2	242	INTERGENIC	--	--	--
Ca6_P4_58817752	Ca6	58817752	AATAT/AATATTATAT	AACGGATATGATTGATTGATTGTG	CTCCTAACGGATTCGTGGAT	60.0	700	INTERGENIC	--	--	--
Ca6_P4_58818339	Ca6	58818339	A/AT	ACGAAGCCGGAAATTAAACA	TCAATTTTTGTCGGCTCAAC	59.6	446	INTERGENIC	--	--	--
Ca6_P4_58851448	Ca6	58851448	AG/A	CTTGATTTGCGGATGGATTT	TTAAAGCGCTCCCATCAACT	59.9	896	INTERGENIC	--	--	--

INDEL marker IDs	Chromosomes/ unanchored scaffolds	Physical positions (bp)	InDels (*Kabuli* reference genome-CDC Frontier/PII)	Forward primers (5'-3')	Reverse primers (5'-3')	Anneling temperature (0C)	Expected amplified product size (bp)	Structural annotation		Functional annotation	
								Sequence components of *kabuli* genome	*Kabuli* gene accession IDs	NCBI-KOG	TFs
Ca6_P4_58872310	Ca6	58872310	GTTTTTTTTT/GTTTTTTTT	CCGATTGGTTGGTTGAGTTT	AAAATGGTGATGAAGTGTTAATTTTT	59.8	828	INTERGENIC	--	--	--
Ca6_P4_58872639	Ca6	58872639	TAAAAAAAAA/TAAAAAAAAAA	CGAACTCAAGTACAACTTTTTGGA	TGCATTCTCTCTTGACTTTCAAT	59.7	865	INTERGENIC	--	--	--
Ca6_P4_58873196	Ca6	58873196	GATATATATATATATATATATATATATATA/GATATATATATATATATATATATATATATA	TTGAGCGAGAATGTAGAGAGAATG	AGAGCAAGCTTAAGCCGACA	60.0	233	INTERGENIC	--	--	--
Ca6_P4_58874492	Ca6	58874492	GTATATATATATATATATATATATATA/GTATATATATATATATATATATA	TCTCAATGTGATTTTAATGTTCCC	GCCTCCTCATGCTGATTCAC	59.3	671	INTERGENIC	--	--	--
Ca6_P4_59059948	Ca6	59059948	CT/CTTT	ATAGGACGCAAGAAAAGCCA	TCCACAACAACCTACGTCCA	59.8	263	INTERGENIC	--	--	--
Ca6_P4_59066187	Ca6	59066187	TAAAAAAAAA/TAAAAAAAAAAA	TGTTTGTCACACTTTCAAATCCA	TGTGGGCTGATGTTGGTTTA	60.4	608	INTERGENIC	--	--	--
Ca6_P4_59072971	Ca6	59072971	CTT/CTTT	TGCGAGTCTTGTTTCTCCCT	ATTTGGTCACTCGAGGTTGC	60.0	869	INTERGENIC	--	--	--
Ca6_P4_59074556	Ca6	59074556	TTTC/TTTCTTC	TGGAAATGATGAGCAATGGA	CGTTGTCAAGAGAATTCACGC	60.0	723	INTERGENIC	--	--	--
Ca6_P4_59093194	Ca6	59093194	ATTTTTTTTTTT/ATTTTTTTTTT	GGAGGCAACCCAACTCTATG	TCGCATAATTGGAACGTCAA	59.6	271	INTERGENIC	--	--	--
Ca6_P4_59133166	Ca6	59133166	GTATATATATATATATAT/GTATATATATATATAT	TAATCATTTTCGGCGGCTAC	GCATGCTCAATTTTTGGTGA	60.1	656	INTERGENIC	--	--	--

INDEL marker IDs	Chromosomes/ unanchored scaffolds	Physical positions (bp)	InDels (*Kabuli* reference genome-CDC Frontier/PII)	Forward primers (5'-3')	Reverse primers (5'-3')	Anneling temperature (0C)	Expected amplified product size (bp)	Structural annotation		Functional annotation	
								Sequence components of *kabuli* genome	*Kabuli* gene accession IDs	NCBI-KOG	TFs
Ca6_P4_59190908	Ca6	59190908	TTATATATATATATATATATATATATAT/TTATATATATATATATATATATAT	TTAATTATCGTCTATCCTTCAATTTTT	TCGGACTTGGTTTAATTGCC	57.3	932	INTERGENIC	--	--	--
Ca6_P4_59273311	Ca6	59273311	TA/T	TTTTTGGGCTGAAGCCATAC	GGTGGAGAATGAAACGGAGA	60.1	359	INTERGENIC	--	--	--
Ca6_P4_59349983	Ca6	59349983	AATATATATATATATATATATATATATATA/AATATATATATATATATATATATATATATATATA	TTTCCATTGCACTCACTCCA	CCTATCATGGCCTGACCTGT	60.2	777	INTERGENIC	--	--	--
Ca6_P4_59384361	Ca6	59384361	AATATATATATATATATA/AATATATATATATATATATA	CGGCCTTATCCTAAAGACCC	AAAATAGGGAGACCAATTTCG	59.9	393	INTERGENIC	--	--	--
Ca6_P4_59406741	Ca6	59406741	ACTTCTTCTTCTTCTTCTTCTTCTTCTTCTTCTTCTTCTTCTTCTTC/ACTTCTTCTTCTTCTTCTTCTTCTTCTTCTTCTTCTTCTTCTTC	AATCAAAACCCATCCAACCA	TTGGCGAGGAAGTTGAAATC	60.0	254	INTERGENIC	--	--	--
Ca6_P4_59434628	Ca6	59434628	CAAAAA/CAAAAAA	ACAGCAGCCTTGCATTCTCT	TGTTGCCTCTGTTTTTGCAG	60.2	374	INTERGENIC	--	--	--
Ca6_P4_6082681	Ca6	6082681	TTATATATATATATATATATAT/TTATATATATATATATATATATATAT	CGCACTAGACTAAATGCGTGC	GCCCTTTGACGTCCAACTAA	61.0	442	INTERGENIC	--	--	--
Ca6_P4_6089953	Ca6	6089953	ATTTTTTTTT/ATTTTTTTTTT	CGAATATCAAGCACGCACAT	GCAACGGCAAGGATTGTTAT	59.7	921	INTERGENIC	--	--	--
Ca6_P4_6149697	Ca6	6149697	AATATATATATATATATATA/AATATATATATATATATATATA	CGAAAACCAAAGTGGAGGAA	TGAATTGTTTTTGGTTGGCA	60.1	550	INTERGENIC	--	--	--
Ca6_P4_6152979	Ca6	6152979	AATATATATATATATATATATATAT/AATATATATATATATATATATATATATAT	CCATTAGTTGACCATCGCCT	GAGGACTAGAGGGAGTGCCC	60.0	155	INTERGENIC	--	--	--

INDEL marker IDs	Chromosomes/ unanchored scaffolds	Physical positions (bp)	InDels (*Kabuli* reference genome-CDC Frontier/PII)	Forward primers (5'-3')	Reverse primers (5'-3')	Anneling temperature (0C)	Expected amplified product size (bp)	Structural annotation		Functional annotation	
								Sequence components of *kabuli* genome	*Kabuli* gene accession IDs	NCBI-KOG	TFs
Ca6_P4_6158112	Ca6	6158112	GTATATATATATATAT/GTATATATATATATATAT	GATGAGTGGAAGGAGCCAAA	TGAAAGATAATCCAATTTTAAATGAGT	60.2	418	INTERGENIC	--	--	--
Ca6_P4_6303863	Ca6	6303863	CAAAAAAAAAAA/CAAAAAAAAAAAA	TCTCATTTTACGGATAGATGTTCCT	GTGGATGTGCTTGCTTCAAA	59.5	293	INTERGENIC	--	--	--
Ca6_P4_6321313	Ca6	6321313	A/ACCTTTTAAACG	CATTTAAGGGCTCGTGTTAAAGA	GGAAAATGGGTTTCAATTCG	59.7	303	INTERGENIC	--	--	--
Ca6_P4_6332755	Ca6	6332755	AG/A	GCAACAGTTTCCCATGGAGT	AGGAAGTTGCGACAAAGCAT	60.0	189	INTERGENIC	--	--	--
Ca6_P4_6341110	Ca6	6341110	AA/AATAATA	CAAGTCGTTTGTTTTGGCAG	TATGGAAGTACCACCCGGAA	59.4	648	INTERGENIC	--	--	--
Ca6_P4_6409101	Ca6	6409101	ATT/ATTT	TTTTGTGGGTGAATGATATTGC	ATAACACAAGAGGCCGTGGA	59.7	449	INTERGENIC	--	--	--
Ca6_P4_6411336	Ca6	6411336	AAT/AATAT	ATCTCGTGGTGCAACAATCA	TTTGGGAAGGGTCAAGAAAA	60.1	628	INTERGENIC	--	--	--
Ca6_P4_6411394	Ca6	6411394	TTATTTAT/TTATTTATATATTTAT	ATCTCGTGGTGCAACAATCA	TTTGGGAAGGGTCAAGAAAA	60.1	628	INTERGENIC	--	--	--
Ca6_P4_6415040	Ca6	6415040	TAAAAATTAAAAAAATTAAAA/TAAAAATTAAAA	AATTTTCATTTTAGTTTGGATTGAA	GGTCGCATTTTTATCGTGTG	57.4	359	INTERGENIC	--	--	--
Ca6_P4_6469039	Ca6	6469039	ATT/ATTT	ATGGTTGAAGGATCGTGGTC	ATCATTCATGTCCATCGGGT	59.8	495	INTERGENIC	--	--	--

INDEL marker IDs	Chromosomes/ unanchored scaffolds	Physical positions (bp)	InDels (*Kabuli* reference genome-CDC Frontier/PII)	Forward primers (5'-3')	Reverse primers (5'-3')	Anneling temperature (0C)	Expected amplified product size (bp)	Structural annotation		Functional annotation	
								Sequence components of *kabuli* genome	*Kabuli* gene accession IDs	NCBI-KOG	TFs
Ca6_P4_6484232	Ca6	6484232	ATTTTTTTTTTT/ATTTTTTTTTTTT	AGGAATGCATGGAACAAACA	TGAAAAACTAAATATGCGGGG	59.0	416	INTERGENIC	--	--	--
Ca6_P4_6489760	Ca6	6489760	TAAAAAAAA/TAAAAAAAAA	AACTGTTTTACAATCGGGCG	ATTTTAAATGCGCGTTTTGG	60.0	520	INTERGENIC	--	--	--
Ca6_P4_6490812	Ca6	6490812	TG/TGG	GCACAAAATCAAAGGGTTGC	TATTCCCTGGCCAAACAAAG	60.5	281	INTERGENIC	--	--	--
Ca6_P4_660659	Ca6	660659	A/AC	CAGGAGTGGGGAACGTTAAA	AGGGTCCCGTAAAGAGGAAA	60.0	633	INTERGENIC	--	--	--
Ca6_P4_672553	Ca6	672553	AATATATATATATATATATATATATATATATATATATATATATATAT/AATATATATATATATATATATATATATATATATATATATATATAT	TCCATTGTGAATACATCCATCC	CGATCAAAGCAACCAATCAA	59.5	259	INTERGENIC	--	--	--
Ca6_P4_6730589	Ca6	6730589	GTATATATATATATATATATATAT/GTATATATATATATATATAT	TTGCAGAACCAAAACAGAACC	TGGGGTTTAGGGTGTCACAT	60.1	561	INTERGENIC	--	--	--
Ca6_P4_6748873	Ca6	6748873	TTATA/TTATATA	TCAACACGCACCGTTGTTAT	GTGCAACACGATTGATCTGG	60.0	574	INTERGENIC	--	--	--
Ca6_P4_6862212	Ca6	6862212	A/AGAATGAGTTAT	TGATGATGAGAGGCGAGACA	GGTGGAGCTGTTTATTGGGA	60.5	198	INTERGENIC	--	--	--
Ca6_P4_6938647	Ca6	6938647	AAT/AATTATTATTAT	TGCAAATTTCATCAAATCCA	AGTTGGTTTCCGGAATGAAT	57.5	767	INTERGENIC	--	--	--
Ca6_P4_6958058	Ca6	6958058	A/ATATAT	TCGTAACATGAAAACGTCGC	CAAATTTGTTGCGGTTCCTT	59.7	223	INTERGENIC	--	--	--

INDEL marker IDs	Chromosomes/ unanchored scaffolds	Physical positions (bp)	InDels (*Kabuli* reference genome-CDC Frontier/PII)	Forward primers (5'-3')	Reverse primers (5'-3')	Anneling temperature (0C)	Expected amplified product size (bp)	Structural annotation		Functional annotation	
								Sequence components of *kabuli* genome	*Kabuli* gene accession IDs	NCBI-KOG	TFs
Ca6_P4_697724	Ca6	697724	CATATATATATATATATATATATATATAT/CATATATATATATATATATATATATATATATATATATATATAT	GACTTGCACATATTGGGCCT	TGACAAAAGAGTGAAAAAGGAAAA	60.0	586	INTERGENIC	--	--	--
Ca6_P4_7192124	Ca6	7192124	TA/TGATATAAGGGAAA	GCATGGCAACGTTGTAAGAA	GAGAAAGACAAAATGCGGCT	59.7	362	INTERGENIC	--	--	--
Ca6_P4_7318576	Ca6	7318576	TAAAAAAAAAA/TAAAAAAAAAAAA	GGCATGTAACATCGCAACTG	CGAGAAAGTGTTGGGGAAAA	60.1	739	INTERGENIC	--	--	--
Ca6_P4_7319973	Ca6	7319973	AC/ACC	TTTATGAGCTCGATGGGTCC	CGTCTGTAGAAGCAGGGGAG	60.0	613	INTERGENIC	--	--	--
Ca6_P4_7353319	Ca6	7353319	CAAAAAAAAAAA/CAAAAAAAAAA	AAGCTTGTGGAGGAGCACAT	CCGGTGAGACCTTTGGATAA	59.9	520	INTERGENIC	--	--	--
Ca6_P4_7360320	Ca6	7360320	ATTTTTT/ATTTTTTT	CACCAAATGTGGGTTACATCA	ATTCACCCCCTCTTAGGTGC	59.2	614	INTERGENIC	--	--	--
Ca6_P4_7505796	Ca6	7505796	CATATATATATATATATAT/CATATATATATATAT	AGGGCATTTGACGAACACAT	CATTAAGTCTATCACGAGTTTAACCA	60.4	508	INTERGENIC	--	--	--
Ca6_P4_7587726	Ca6	7587726	TC/T	GTGTGATGAAGGATTCGCCT	GATGAGACTTTTCCCTAGCCTT	60.1	842	INTERGENIC	--	--	--
Ca6_P4_7588189	Ca6	7588189	TT/TTGCAT	AAGGCTAGGGAAAAGTCTCATC	GCTCTCAGTTGAATAGCACGG	58.1	504	INTERGENIC	--	--	--
Ca6_P4_7588870	Ca6	7588870	AAAG/A	TATGAGCACCGCCAGTTGTA	CCAAAAATCTTCCCGAACAG	60.3	227	INTERGENIC	--	--	--

INDEL marker IDs	Chromosomes/ unanchored scaffolds	Physical positions (bp)	InDels (*Kabuli* reference genome-CDC Frontier/PII)	Forward primers (5'-3')	Reverse primers (5'-3')	Anneling temperature (0C)	Expected amplified product size (bp)	Structural annotation		Functional annotation	
								Sequence components of *kabuli* genome	*Kabuli* gene accession IDs	NCBI-KOG	TFs
Ca6_P4_7594302	Ca6	7594302	A/AT	CACCAATTATTGCAAGTCTCG	TGAAACGAGTTTTGATTGCC	58.3	738	INTERGENIC	--	--	--
Ca6_P4_7594709	Ca6	7594709	TA/T	GGCAATCAAAACTCGTTTCA	TTCGCTCTACGTTTATGGGG	58.7	299	INTERGENIC	--	--	--
Ca6_P4_7595372	Ca6	7595372	TTATAT/TTAT	CATGGGCATATTACACCACG	ACGGTGTTGGTGTTTCTGGT	59.7	847	INTERGENIC	--	--	--
Ca6_P4_7612067	Ca6	7612067	TTATATATA/TTATATATATA	AATGTTTTAACGACGGCGAC	TGTGGAAGATGATGTACAACGG	60.0	861	INTERGENIC	--	--	--
Ca6_P4_7612342	Ca6	7612342	AAAATAAATAAATAAATAAATAAA/AAAATAAATAAATAAA	TGGCTGTAAGTTTGCGTCTG	AACACACCATCCATGCAAAA	60.1	762	INTERGENIC	--	--	--
Ca6_P4_7612818	Ca6	7612818	A/ACTATT	TTTTGCATGGATGGTGTGTT	GCAGTTTCTTTGGCATCCTC	59.8	525	INTERGENIC	--	--	--
Ca6_P4_7631953	Ca6	7631953	TATTTATTTTTAATTTATTTTT/TATTTATTTTTAATTTATTTTTAATTTATTTTT	TGCTTGCCTCTAGTTGTTCG	TGACAAAATCTTACTGCATGCTT	59.2	653	INTERGENIC	--	--	--
Ca6_P4_7634031	Ca6	7634031	TT/TTCT	ACGAGGCACCGACATGTAAT	AATTTGCACATATCACGCCA	60.4	506	INTERGENIC	--	--	--
Ca6_P4_7636237	Ca6	7636237	AG/A	CATGAGATACCGACATGCGA	TGGGCGTCTTTTAGCTATGC	60.7	171	INTERGENIC	--	--	--
Ca6_P4_7638969	Ca6	7638969	CATAAAAAATAAAAAATAA/CATAAAAAATAA	AACCACATTTGTTGTCGCTG	TTGGATGTCAACAATGTACACG	59.6	469	INTERGENIC	--	--	--

INDEL marker IDs	Chromosomes/ unanchored scaffolds	Physical positions (bp)	InDels (*Kabuli* reference genome-CDC Frontier/PII)	Forward primers (5'-3')	Reverse primers (5'-3')	Anneling temperature (0C)	Expected amplified product size (bp)	Structural annotation		Functional annotation	
								Sequence components of *kabuli* genome	*Kabuli* gene accession IDs	NCBI-KOG	TFs
Ca6_P4_7641189	Ca6	7641189	CT/C	TCTGTGTCGTGCTCTCAACC	AAGGGCCATTTGAAGAGAAAA	60.0	808	INTERGENIC	--	--	--
Ca6_P4_7641770	Ca6	7641770	G/GA	AGCGAATCACTCGAGCAAAA	AAGAACGAAGAAAGGTCGCA	61.1	865	INTERGENIC	--	--	--
Ca6_P4_7643275	Ca6	7643275	TAAAAA/TAAAA	TTGCATTCCATAAACACGGA	TGATCAGATAGATATGTTGACCGA	59.9	695	INTERGENIC	--	--	--
Ca6_P4_7643356	Ca6	7643356	ATTTTTT/ATTTTT	TTGCATTCCATAAACACGGA	TGATCAGATAGATATGTTGACCGA	59.9	695	INTERGENIC	--	--	--
Ca6_P4_7643618	Ca6	7643618	ATGT/AT	CAATTGTCAACATGACCCTTAAA	TCATTGATTCAACCTCTGGTCTT	58.9	411	INTERGENIC	--	--	--
Ca6_P4_7644876	Ca6	7644876	ATGTGTGTGTGTGTGT/ATGTGTGTGTGTGTGTGT	GACTTCGTTCGTGCTTCCTC	GCCACCGTTGTGTTCCTTAT	60.0	495	INTERGENIC	--	--	--
Ca6_P4_7660694	Ca6	7660694	GAATT/GAATTCATTCTAACAATT	AGGACGAAGAGTTGAAGCCA	AATGTCGTAAGGGGCACAAA	60.0	766	INTERGENIC	--	--	--
Ca6_P4_7660929	Ca6	7660929	AATATATATATATATATATATATATA/AATATATATATATATATATATATATATATATA	GCCCAAAAGAGCCAATACAA	ACGAAAACGCCACTTACCTG	60.1	788	INTERGENIC	--	--	--
Ca6_P4_7670210	Ca6	7670210	GTTTTTTTT/GTTTTTTTTTTTT	TCCTTCAAACTCGTCCCTTG	CTCCTCATGCGCTAACACAA	60.2	264	INTERGENIC	--	--	--
Ca6_P4_7670257	Ca6	7670257	TTATATATATATATATATATA/TTATATATATATATATATATA	TCCTTCAAACTCGTCCCTTG	CTCCTCATGCGCTAACACAA	60.2	264	INTERGENIC	--	--	--

INDEL marker IDs	Chromosomes/ unanchored scaffolds	Physical positions (bp)	InDels (*Kabuli* reference genome-CDC Frontier/PII)	Forward primers (5'-3')	Reverse primers (5'-3')	Anneling temperature (0C)	Expected amplified product size (bp)	Structural annotation		Functional annotation	
								Sequence components of *kabuli* genome	*Kabuli* gene accession IDs	NCBI-KOG	TFs
Ca6_P4_7689057	Ca6	7689057	TC/T	GGTGAGACAACCCATTTGCT	AACCCTCCACCGAAACTTCT	60.0	415	INTERGENIC	--	--	--
Ca6_P4_7689459	Ca6	7689459	TA/T	AGAAGTTTCGGTGGAGGGTT	TCCTTTGCAGGATGGGTAAG	60.0	733	INTERGENIC	--	--	--
Ca6_P4_7691192	Ca6	7691192	TTGTGT/TTGT	TGAAAATGGGAGGAAGGTTG	TTTGAGTGTGTTGCATGGGT	59.9	658	INTERGENIC	--	--	--
Ca6_P4_7708179	Ca6	7708179	CTATA/CTA	CGTGTGGACATTGGTACGAG	TAAATCGCAATGGAACACGA	60.0	149	INTERGENIC	--	--	--
Ca6_P4_7708884	Ca6	7708884	AAGAG/AAG	CTCATCACACTGGGCAAATG	TGTTCATTTCCAATGCCAAA	60.1	983	INTERGENIC	--	--	--
Ca6_P4_7711932	Ca6	7711932	ATTTTTTTTTTT/ATTTTTTTTT	ATTAAGCGGGTGCAGGTATG	TCAGTGCATGTACTCTCTTGGG	60.0	610	INTERGENIC	--	--	--
Ca6_P4_7712192	Ca6	7712192	CA/C	CCCCACAATGGGTATGGTAA	CCTGTCCTCATACCTGCGAT	60.3	715	INTERGENIC	--	--	--
Ca6_P4_7713878	Ca6	7713878	TTATATATATAT/TTATATATAT	CAAATCGTTTTGTCCCATTG	TACCGCACTTAGACGACGAG	58.9	816	INTERGENIC	--	--	--
Ca6_P4_7718474	Ca6	7718474	A/AGT	ACATGTTGCATTTCACTGCC	TTTGAAGGGGTGAATCGAAG	59.6	775	INTERGENIC	--	--	--
Ca6_P4_7732809	Ca6	7732809	CTAT/CTATAT	TGGGCAGAACCCACTAATTT	CCTCCACATGTTAATGTCCTAGC	59.4	298	INTERGENIC	--	--	--

INDEL marker IDs	Chromosomes/ unanchored scaffolds	Physical positions (bp)	InDels (*Kabuli* reference genome-CDC Frontier/PII)	Forward primers (5'-3')	Reverse primers (5'-3')	Anneling temperature (0C)	Expected amplified product size (bp)	Structural annotation		Functional annotation	
								Sequence components of *kabuli* genome	*Kabuli* gene accession IDs	NCBI-KOG	TFs
Ca6_P4_7733001	Ca6	7733001	TATATA/T	AAGCGATGGTTTAAGTGGCA	TGGTGAACATATGGGGTCTG	60.6	740	INTERGENIC	--	--	--
Ca6_P4_7734369	Ca6	7734369	GTTTTTTT/GTTTTTT	TCTCGAGGCATCTCAATCCT	TCAATAAAATCACCGACTTGTGA	59.9	212	INTERGENIC	--	--	--
Ca6_P4_7735386	Ca6	7735386	ATATATTATATTATATTATATT/ATATATTATATTATATT	TGATGAGTTCCACCCTGTCA	AAATTTCCATTGGCCAACAA	60.1	494	INTERGENIC	--	--	--
Ca6_P4_7737928	Ca6	7737928	TGA/T	AAATCCCATCAAACAATGGC	TTCGCTTGAAATAACAACAAAAA	59.6	517	INTERGENIC	--	--	--
Ca6_P4_7773242	Ca6	7773242	ATCTCTCCTTCTCTC/ATCTCTCCTTCTCTCCTTCTCTC	CCTTTCCAACATCCAAGCAT	AAGCACAGGGAGAACAAAGC	59.9	353	INTERGENIC	--	--	--
Ca6_P4_7775563	Ca6	7775563	ATTTTTTT/ATTTTTTTT	CACAATTGCTCATGTGGTCC	TGGTCCCTTGACTTAATTTCAGA	60.0	372	INTERGENIC	--	--	--
Ca6_P4_7781946	Ca6	7781946	A/AATTT	GGGTAAGGGACGTGTCATTG	CAAGTGCCCTACTTGTTAAGGA	60.2	591	INTERGENIC	--	--	--
Ca6_P4_7799591	Ca6	7799591	TTATCTATCTAT/TTATCTATCTATCTAT	ACAACTTAAATGGTGGCTTCA	GACAATCTCAGGGGTCTCCA	57.3	531	INTERGENIC	--	--	--
Ca6_P4_7801585	Ca6	7801585	AT/ATT	TGAGGGATGCATCAACATTC	CATTGAAGAGAGGAACCCCA	59.5	578	INTERGENIC	--	--	--
Ca6_P4_7802935	Ca6	7802935	ACA/ACATGCA	ATGTGGGCCCCTTGACTATT	TGCTAAAGGCTTCCCTTCAA	60.6	524	INTERGENIC	--	--	--

INDEL marker IDs	Chromosomes/ unanchored scaffolds	Physical positions (bp)	InDels (*Kabuli* reference genome-CDC Frontier/PII)	Forward primers (5'-3')	Reverse primers (5'-3')	Anneling temperature (0C)	Expected amplified product size (bp)	Structural annotation		Functional annotation	
								Sequence components of *kabuli* genome	*Kabuli* gene accession IDs	NCBI-KOG	TFs
Ca6_P4_7806679	Ca6	7806679	TA/TACA	TCAAGGGATCCCCATAACAA	TTCACATTCCCCATTAAGCC	60.1	742	INTERGENIC	--	--	--
Ca6_P4_7806865	Ca6	7806865	TC/TCC	TTCATCGCAGGTTTCAGAGA	TTCACATTCCCCATTAAGCC	59.5	174	INTERGENIC	--	--	--
Ca6_P4_7812857	Ca6	7812857	GTA/GTAATGCTA	CATTGCCCAAAATCCTGAGT	CCCACTCGTTTTCGCTATTT	59.9	798	INTERGENIC	--	--	--
Ca6_P4_7814183	Ca6	7814183	ATTTTTTT/ATTTTTTTT	TGTGTTTGGCTGTGAACAGAA	TACACCGCCAATGCATTTTA	60.3	556	INTERGENIC	--	--	--
Ca6_P4_7819596	Ca6	7819596	GCACAC/GCACACAC	TGACCATGCTTTACAGGGGT	AAGGGGGCAAGGATACAAGT	60.4	710	INTERGENIC	--	--	--
Ca6_P4_7859686	Ca6	7859686	ACTC/ACTCTC	CAAAAGCAACAAATTAAAGCG	TGGCGTCTTAATGGTCACTG	57.7	468	INTERGENIC	--	--	--
Ca6_P4_7860074	Ca6	7860074	AC/ACC	CGACTCACACAGTGCAACAA	GAGACTCTCCACCCTCGTGT	59.5	609	INTERGENIC	--	--	--
Ca6_P4_7861129	Ca6	7861129	TACCAC/TAC	CAAATATTCCAACTGCAACCAA	ACGCCTCCGCTTTTATTGAT	59.9	556	INTERGENIC	--	--	--
Ca6_P4_7869663	Ca6	7869663	ATTTT/ATTTTT	TGAACCATCGCAACATTCAT	TCCATAAGTGGTTTCCGACA	59.9	551	INTERGENIC	--	--	--
Ca6_P4_7870049	Ca6	7870049	GAAAAA/GAAAA	TGTCGGAAACCACTTATGGA	CCAGAAATGACTCCACCGTT	59.0	608	INTERGENIC	--	--	--

INDEL marker IDs	Chromosomes/ unanchored scaffolds	Physical positions (bp)	InDels (*Kabuli* reference genome-CDC Frontier/PII)	Forward primers (5'-3')	Reverse primers (5'-3')	Anneling temperature (0C)	Expected amplified product size (bp)	Structural annotation		Functional annotation	
								Sequence components of *kabuli* genome	*Kabuli* gene accession IDs	NCBI-KOG	TFs
Ca6_P4_7881252	Ca6	7881252	CTTTATTTTATTTTAT/CTTTATTTTATTTTATTTTAT	TTTTCCGATCGTTGACGTTT	AACGGGGTTCACCCTAACAT	60.5	951	INTERGENIC	--	--	--
Ca6_P4_7881359	Ca6	7881359	TTTAAT/TTTAATTAAT	CCACACCTCTCTCTCTCTCTCC	AACGGGGTTCACCCTAACAT	59.6	848	INTERGENIC	--	--	--
Ca6_P4_7881668	Ca6	7881668	ATATTATT/ATATT	TTGGATGCCCCTTAACTCTTT	GTACCAATGATGGTGGGAGG	60.0	346	INTERGENIC	--	--	--
Ca6_P4_7883319	Ca6	7883319	ATTTGTTTGTT/ATTTGTT	AAAAATGAATGAAAAGGGGTG	GAAAGAGGCATTGGGATTCA	58.0	710	INTERGENIC	--	--	--
Ca6_P4_7883626	Ca6	7883626	CT/CTTT	TGAATCCCAATGCCTCTTTC	TGAGAATTTTTGTGCATCGC	60.0	160	INTERGENIC	--	--	--
Ca6_P4_7884565	Ca6	7884565	AACACAC/AACAC	CACCAAAACCTGCAATTCCT	GTTGAAAACCGACCCTGAAA	60.0	687	INTERGENIC	--	--	--
Ca6_P4_7899205	Ca6	7899205	ATTATTTTATTTTATTTT/ATTATTTTATTTTATTTTATTTTATTTT	GCACGGGCACCTTATTAGTTA	CCGGATCACCGTATTGAATC	59.2	604	INTERGENIC	--	--	--
Ca6_P4_7899665	Ca6	7899665	TAAAAAA/TAAAAAAA	ATTAGGGTGGTGGTTGACGA	TTCGCATGTTCTAAATTGGTTG	60.2	723	INTERGENIC	--	--	--
Ca6_P4_7903885	Ca6	7903885	A/AAT	TAGGACCTCCCCACGTTATG	ACACCACAATCAATGGAGCA	59.8	877	INTERGENIC	--	--	--
Ca6_P4_7911705	Ca6	7911705	TAAAA/TAAAAA	CCTCAACCTTAAGTGCTCCG	GCATTTGATTTGGAACGTGA	59.9	376	INTERGENIC	--	--	--

INDEL marker IDs	Chromosomes/ unanchored scaffolds	Physical positions (bp)	InDels (*Kabuli* reference genome-CDC Frontier/PII)	Forward primers (5'-3')	Reverse primers (5'-3')	Anneling temperature (0C)	Expected amplified product size (bp)	Structural annotation		Functional annotation	
								Sequence components of *kabuli* genome	*Kabuli* gene accession IDs	NCBI-KOG	TFs
Ca6_P4_7921239	Ca6	7921239	CTAATTTAATTTAATTTA/CTAATTTAATTTA	TGGAGTCGTTACCGTCCTTC	AAAAGTTTGCCCATTATCAATTTT	60.1	477	INTERGENIC	--	--	--
Ca6_P4_7922258	Ca6	7922258	TT/TTTAT	TATCAACCAAATGCGTCCAA	AAAAGGATTTAACATAAACCGAGTC	59.9	566	INTERGENIC	--	--	--
Ca6_P4_7935468	Ca6	7935468	ATTTT/ATTTTT	TTGTTGTCATTTGCAGTAAAGC	TTGTGCACACTCGTTAAGTTGA	58.0	428	INTERGENIC	--	--	--
Ca6_P4_7936124	Ca6	7936124	TC/TCC	GGACAATTGCCCAATTTCTTT	GCGTTGTGTTTGTTCGTCAT	60.2	421	INTERGENIC	--	--	--
Ca6_P4_7942841	Ca6	7942841	ATT/AT	TCAAAGCTGGAATTTGGACC	TCAACAGAAGCCGGAACTCT	60.1	508	INTERGENIC	--	--	--
Ca6_P4_7948620	Ca6	7948620	CTT/CTTTTTT	GCGCGTTAAACTACTCACCC	TCATTGCAATTCTCAGCCTCT	59.8	430	INTERGENIC	--	--	--
Ca6_P4_7948820	Ca6	7948820	AATATATATATATATATAT/AATATATATATATATATATATATATATATATAT	ATCCACCATGATAGCCTCCA	GGAGGGACACTGTTGAAAGG	60.3	765	INTERGENIC	--	--	--
Ca6_P4_7953411	Ca6	7953411	AAAAA/AAAAAGAAAA	CGCTTCTCCAAAGAGCGATA	ACTCAATGGTGCCGAGTTGT	60.6	635	INTERGENIC	--	--	--
Ca6_P4_7954309	Ca6	7954309	TAAAAAAA/TAAAAAA	CTAACGCGTATACATCGGCA	CACGGTACCACATTGACGAC	59.7	580	INTERGENIC	--	--	--
Ca6_P4_7954796	Ca6	7954796	A/ATTT	TTTCGTGACCTCGAAAGGAT	TTTTCTCCGGTATCTCACGG	59.7	577	INTERGENIC	--	--	--

INDEL marker IDs	Chromosomes/ unanchored scaffolds	Physical positions (bp)	InDels (*Kabuli* reference genome-CDC Frontier/PII)	Forward primers (5'-3')	Reverse primers (5'-3')	Anneling temperature (0C)	Expected amplified product size (bp)	Structural annotation		Functional annotation	
								Sequence components of *kabuli* genome	*Kabuli* gene accession IDs	NCBI-KOG	TFs
Ca6_P4_7960922	Ca6	7960922	ATTTT/ATTTTT	ATGACATGGAGCACAAGCAA	GCGTGCTGAATGAGAATGAA	60.3	432	INTERGENIC	--	--	--
Ca6_P4_7960958	Ca6	7960958	T/TA	ATGACATGGAGCACAAGCAA	GCGTGCTGAATGAGAATGAA	60.3	432	INTERGENIC	--	--	--
Ca6_P4_7961982	Ca6	7961982	A/AAT	CGTTGCAGCTGCTTTGTTTA	TTGGGGAAGAAAGTTGGAGA	60.2	841	INTERGENIC	--	--	--
Ca6_P4_7965961	Ca6	7965961	T/TA	GATGGTTTCTGTGGCACCTT	GTTTGACCCATTTGAGGCAT	60.0	505	INTERGENIC	--	--	--
Ca6_P4_7966465	Ca6	7966465	ATT/ATTT	ATGCCTCAAATGGGTCAAAC	ACCCACCCGCTAAACTTTTT	59.8	735	INTERGENIC	--	--	--
Ca6_P4_7967823	Ca6	7967823	A/AT	CCCTGGCCCCTATGTTTATT	CTTTGAAGTGGCAAAGGCAT	60.0	838	INTERGENIC	--	--	--
Ca6_P4_7967989	Ca6	7967989	ACT/ACTCT	AGTGGGAGTGGCCTTTAAGC	CTTTGAAGTGGCAAAGGCAT	60.6	603	INTERGENIC	--	--	--
Ca6_P4_7969855	Ca6	7969855	TTATATATATATATATATATATATA/TTATATATATATATATATATATATATATATATATATA	GTGTGTTGGTGGCCAAAAGT	GGCCTTTGAATCTAAACTGGC	60.9	891	INTERGENIC	--	--	--
Ca6_P4_7970165	Ca6	7970165	ATTT/ATT	TGATGGTCCTGTCAATTAGCTG	TGCAAGTGGATGTGGAAAAA	60.1	374	INTERGENIC	--	--	--
Ca6_P4_7970612	Ca6	7970612	CA/C	AAGGCCTTGGTTTGTTTGTG	GAGCAAAAGTTGGCAGGAAG	60.0	782	INTERGENIC	--	--	--

INDEL marker IDs	Chromosomes/ unanchored scaffolds	Physical positions (bp)	InDels (*Kabuli* reference genome-CDC Frontier/PII)	Forward primers (5'-3')	Reverse primers (5'-3')	Anneling temperature (0C)	Expected amplified product size (bp)	Structural annotation		Functional annotation	
								Sequence components of *kabuli* genome	*Kabuli* gene accession IDs	NCBI-KOG	TFs
Ca6_P4_7970811	Ca6	7970811	CTCTTCTTCTTCT/CTCTTCTTCTTCTTCT	GGGCACGGATCAGTTCTTTA	GAGCAAAAGTTGGCAGGAAG	60.1	311	INTERGENIC	--	--	--
Ca6_P4_7972071	Ca6	7972071	AT/ATT	TAGTTTCTGACTCGGCGACA	ACGGATCCCAAGTCAATGAG	59.6	786	INTERGENIC	--	--	--
Ca6_P4_7972712	Ca6	7972712	TAAAAAAAAA/TAAAAAAAAAAAA	CTCATTGACTTGGGATCCGT	TCATTGCTTATGTGTAATTTGTGC	59.9	559	INTERGENIC	--	--	--
Ca6_P4_7972862	Ca6	7972862	GTATATATATAT/GTATATATATATATAT	CTCATTGACTTGGGATCCGT	GAGAAAAGGTGGATCCTACATACC	59.9	921	INTERGENIC	--	--	--
Ca6_P4_7973691	Ca6	7973691	TTATAT/TTATATAT	TGCCCATCTTTTCCTTCATC	CGCCGTCGTCCTACTCTCTA	60.0	438	INTERGENIC	--	--	--
Ca6_P4_7976984	Ca6	7976984	TAAAAA/TAAAA	AGGCCAATCCTCCATTTGTA	ATATGGATTTGGGGTAGGGC	59.4	230	INTERGENIC	--	--	--
Ca6_P4_7989945	Ca6	7989945	ATTT/ATT	CCACAAGGGGTGGTGTAGTC	GTGGAGACTTTTGGGACCCT	60.3	523	INTERGENIC	--	--	--
Ca6_P4_7990379	Ca6	7990379	ATTATGT/AT	AGGGTCCCAAAAGTCTCCAC	TGAGCCTTGAGTGCAAAAGA	60.3	542	INTERGENIC	--	--	--
Ca6_P4_7990494	Ca6	7990494	CAAAAA/CAAAAAAA	AGGGTCCCAAAAGTCTCCAC	TGAGCCTTGAGTGCAAAAGA	60.3	542	INTERGENIC	--	--	--
Ca6_P4_7991799	Ca6	7991799	T/TC	CCCGTGCAACACACTACTTC	TTGTGCGGATCGTTTATTTG	59.2	727	INTERGENIC	--	--	--

INDEL marker IDs	Chromosomes/ unanchored scaffolds	Physical positions (bp)	InDels (*Kabuli* reference genome-CDC Frontier/PII)	Forward primers (5'-3')	Reverse primers (5'-3')	Anneling temperature (0C)	Expected amplified product size (bp)	Structural annotation		Functional annotation	
								Sequence components of *kabuli* genome	*Kabuli* gene accession IDs	NCBI-KOG	TFs
Ca6_P4_7995646	Ca6	7995646	TTT/TTTGTT	TAACAATTGCGGCAACAAAA	AAAAGAACTGGCCGTCACAC	60.1	254	INTERGENIC	--	--	--
Ca6_P4_7995932	Ca6	7995932	TTT/TTTGTT	TAACAATTGCGGCAACAAAA	CCTGACGGATTCATTTCTCAA	60.1	699	INTERGENIC	--	--	--
Ca6_P4_7997168	Ca6	7997168	AAAAAATTAT/AAAAAATTATGAAAAATTAT	GCTTGCATTCAGTCAGACCA	GACACATGGTTGCACGATTC	60.0	625	INTERGENIC	--	--	--
Ca6_P4_8005951	Ca6	8005951	T/TTA	TCACCGACCTTCATTAGCAA	AGGTTGACCTGGTTCATTCG	59.3	981	INTERGENIC	--	--	--
Ca6_P4_8006058	Ca6	8006058	AATATATATATATATATATATA/AATATATATATATATATA	TTTGTTTTTGATGGAGTTATTTTCTTT	AGGTTGACCTGGTTCATTCG	59.8	748	INTERGENIC	--	--	--
Ca6_P4_8022121	Ca6	8022121	TAATTAAAAATTAAAAAT/TAATTAAAAATTAAAAATTAAAAAT	TTTGTTTGCGATGGCTCATA	TGTTTTTCCGAGCGTACACA	60.2	818	INTERGENIC	--	--	--
Ca6_P4_8033661	Ca6	8033661	ATAAATGTCGCT/AT	AAAAATTGATGCCCTCACCA	CAGCGATAAATATTTGCCCA	60.3	558	INTERGENIC	--	--	--
Ca6_P4_8035204	Ca6	8035204	GAAA/GAA	ACACCCCACCAATTGTGTTT	GCATATGTGTCTTGGCCACC	60.0	694	INTERGENIC	--	--	--
Ca6_P4_8035617	Ca6	8035617	AATATATATA/AATATATATATA	CAAGCTTTATCTGTAACCGCTG	TGAGGTGTGCGTGTAATCGT	59.1	393	INTERGENIC	--	--	--
Ca6_P4_8044596	Ca6	8044596	G/GAA	TAGATGATTACGCGGGAACC	CCCTTATTTTGGGAGGGAAA	59.9	494	INTERGENIC	--	--	--

INDEL marker IDs	Chromosomes/ unanchored scaffolds	Physical positions (bp)	InDels (*Kabuli* reference genome-CDC Frontier/PII)	Forward primers (5'-3')	Reverse primers (5'-3')	Anneling temperature (0C)	Expected amplified product size (bp)	Structural annotation		Functional annotation	
								Sequence components of *kabuli* genome	*Kabuli* gene accession IDs	NCBI-KOG	TFs
Ca6_P4_8048922	Ca6	8048922	GTTTTTATTTTTTA/GTTTTTATTTTTTATTTTTTA	TGCTAGAACATGCTGCTGCT	CATTCTCAGCCCAGGAAAAC	59.9	438	INTERGENIC	--	--	--
Ca6_P4_8064946	Ca6	8064946	ATTTTTTT/ATTTTTTTT	ATCACGTTATTCTTTCTACGATGA	CCCGTGACATACACATGGAA	57.1	563	INTERGENIC	--	--	--
Ca6_P4_8098915	Ca6	8098915	CATA/C	TTCCGGTTGTTATCCGTTTT	CAAGTGAAAATCCTCGTTGACA	59.3	772	INTERGENIC	--	--	--
Ca6_P4_8099378	Ca6	8099378	ATTTTTT/ATTTTT	TGTCAACGAGGATTTTCACTTG	TGCCGCTTAACTTCACCTTT	60.1	167	INTERGENIC	--	--	--
Ca6_P4_8100368	Ca6	8100368	AAAAATAAAA/AAAAATAAAATAAAA	AGGTAGGAACTAATTGCCTATTTTT	TTCACATGGCAAAATCTTTCA	57.3	467	INTERGENIC	--	--	--
Ca6_P4_8122786	Ca6	8122786	TTTAAAGATTAAA/TTTAAAGATTAAAGATTAAA	CACACGCGGTCCTTTAATTT	GATTCAAGTGCCTTGCCATT	60.0	755	INTERGENIC	--	--	--
Ca6_P4_8123635	Ca6	8123635	TC/T	TGGTCATTCGGAAAGAAAGAA	TTGCAATCAAAAGCTCTTAAAAA	59.7	286	INTERGENIC	--	--	--
Ca6_P4_8131261	Ca6	8131261	TACA/T	ATCCCAATTTCCCAAAGGTG	CTCGGGGTTTTGTTGATGTT	60.9	239	INTERGENIC	--	--	--
Ca6_P4_8131551	Ca6	8131551	CTTTTTTTTTTTTTTTTTTTT/CTTTTTTTTTTTTTTT	AACATCAACAAAACCCCGAG	TTGCGTAACGTTTTGGACAC	59.8	558	INTERGENIC	--	--	--
Ca6_P4_8156312	Ca6	8156312	AAA/AAATTTCGTTAA	GTCTCCAGCAGAGCAAAACC	CGTTTGAACACGAAGCTGAA	60.0	874	INTERGENIC	--	--	--

INDEL marker IDs	Chromosomes/ unanchored scaffolds	Physical positions (bp)	InDels (*Kabuli* reference genome-CDC Frontier/PII)	Forward primers (5'-3')	Reverse primers (5'-3')	Anneling temperature (0C)	Expected amplified product size (bp)	Structural annotation		Functional annotation	
								Sequence components of *kabuli* genome	*Kabuli* gene accession IDs	NCBI-KOG	TFs
Ca6_P4_8164226	Ca6	8164226	GAAAAAA/GAAAAAAA	CCTCCGCTCTACCAACTGAG	TCGAATTTTCTTCGAATGGG	60.0	324	INTERGENIC	--	--	--
Ca6_P4_8177320	Ca6	8177320	G/GTATT	TTGTATAACTCGGCTGACGG	AACCCTCATTTGAGTCCGTG	58.8	408	INTERGENIC	--	--	--
Ca6_P4_8178292	Ca6	8178292	GTATA/GTA	AGTTGACGAACCAACATGTGA	CCATGACTTCGAAATGCTCA	59.0	971	INTERGENIC	--	--	--
Ca6_P4_8181367	Ca6	8181367	CAAAA/CAAA	GGTGCACAATAGTGGTGTCG	AGCCTCTCTCACCCCAAAAT	60.0	400	INTERGENIC	--	--	--
Ca6_P4_8182365	Ca6	8182365	ATTTT/ATTTTT	TGCTGATACAAATGTGGGAAA	ACACTGAAACCAACCCTTCG	59.0	520	INTERGENIC	--	--	--
Ca6_P4_8200088	Ca6	8200088	CTTTAAATTTAA/CTTTAAATTTAAATTTAA	AAGACACGGCGGTTATTACG	AAATGGAATACGTCGAGTTCAAA	60.0	684	INTERGENIC	--	--	--
Ca6_P4_8312848	Ca6	8312848	ATTTTTTTTTTTTTTTTT/ATTTTTTTTTTTT	GGATCTTTTTGCCGAAATCA	TTTGTTTATGCACATTTAGTCAATCA	60.0	753	INTERGENIC	--	--	--
Ca6_P4_8410500	Ca6	8410500	CAA/CA	AATCGATTGATTCAGCCCAG	TAATGCCCCGTTTTTCAAAG	60.0	795	INTERGENIC	--	--	--
Ca6_P4_850916	Ca6	850916	TCC/TCCC	AATCAAGTGCAAAAGTGGGG	ACCACACCCATATCCAGCAT	60.0	704	INTERGENIC	--	--	--
Ca6_P4_8624865	Ca6	8624865	AATATATATATATATATATA/AATATATATATATATATATATATATA	ATTGGGTTGATTGCGAAGAG	TCCAAATCCAACTCCACGTA	60.1	846	INTERGENIC	--	--	--

INDEL marker IDs	Chromosomes/ unanchored scaffolds	Physical positions (bp)	InDels (*Kabuli* reference genome-CDC Frontier/PII)	Forward primers (5'-3')	Reverse primers (5'-3')	Anneling temperature (0C)	Expected amplified product size (bp)	Structural annotation		Functional annotation	
								Sequence components of *kabuli* genome	*Kabuli* gene accession IDs	NCBI-KOG	TFs
Ca6_P4_8640916	Ca6	8640916	ATTTTTTTTTTT/ATTTTTTTTTTTTTT	CGAAAAAGTTAAACTCCTGCGA	TTTTTCCCGACTTGGTCTTTT	60.7	667	INTERGENIC	--	--	--
Ca6_P4_8672001	Ca6	8672001	AATATATATATATATATATATATATA/AATATATATATATATATATATATATATA	TCATAGTTTGCCGCTAGGCT	AGAACTTTGATGCCATTTTGTG	60.0	115	INTERGENIC	--	--	--
Ca6_P4_8705473	Ca6	8705473	AAGAGAGAGAGAG/AAGAGAGAGAG	GCTTGTGGGGATGTTAAACC	GAAGGGAATGAAATGCGAAA	59.3	492	INTERGENIC	--	--	--
Ca6_P4_8771580	Ca6	8771580	A/ATTCAATACAGTTACAAT	TGACACCACCTCCAAAAACA	GTCGCAAAATCGGAGAAAAA	60.0	733	INTERGENIC	--	--	--
Ca6_P4_8834153	Ca6	8834153	ATTTTTTTTT/ATTTTTTTT	TTGCAGATTTTTGAGAAGTTCA	TTTGGTGGGTGATAGGCAAT	57.7	401	INTERGENIC	--	--	--
Ca6_P4_8849732	Ca6	8849732	CTATATATATATAT/CTATATATATATATAT	AACTGGCCGAGATTCAAAGA	TCAAGAAATGATGGACAAACTCA	59.8	440	INTERGENIC	--	--	--
Ca6_P4_8904663	Ca6	8904663	GATATATATATATATATATATATATATATATATAT/GATATATATATATATATATATATATATATATAT	TGAATTGCTTCGAACCTGTG	TGCATTTCAAGCTCTCATGC	59.8	465	INTERGENIC	--	--	--
Ca6_P4_89501	Ca6	89501	TAATATA/TAATATAAATATA	TCCGATAAAATCTCCGTTCG	GCATTCTACTGCAATGCCAA	60.0	820	INTERGENIC	--	--	--
Ca6_P4_9014214	Ca6	9014214	CA/CAA	TACTGGGGTGTCTGGTGTGA	AGCTGCACGCAGACTGTAAA	60.0	441	INTERGENIC	--	--	--
Ca6_P4_9015636	Ca6	9015636	AAGT/AAGTAGT	TGAAACAAGTGCTGGCAAAG	ATCAACCCCAACAATCTCCA	60.0	410	INTERGENIC	--	--	--

INDEL marker IDs	Chromosomes/ unanchored scaffolds	Physical positions (bp)	InDels (*Kabuli* reference genome-CDC Frontier/PII)	Forward primers (5'-3')	Reverse primers (5'-3')	Anneling temperature (0C)	Expected amplified product size (bp)	Structural annotation		Functional annotation	
								Sequence components of *kabuli* genome	*Kabuli* gene accession IDs	NCBI-KOG	TFs
Ca6_P4_9018972	Ca6	9018972	TTAAATAAATAAATAAATAAATAA/TTAAATAAATAAATAA	GTCTGTAATTTCGTGCGTCG	ATTTCCAGCGAGGAGAAAAA	59.3	649	INTERGENIC	--	--	--
Ca6_P4_9239871	Ca6	9239871	GTCTCT/GTCT	GAGGGGAACCCCAAAAAGTA	ATGGTCTCACCTTTATGCCG	60.2	529	INTERGENIC	--	--	--
Ca6_P4_925681	Ca6	925681	AATATATATATATATATATATATATATATAT/AATATATATATATATATATATATATAT	CCGAGTTGGGTGAACCAATA	AACTTGTGGTTTTTGCGACC	60.7	580	INTERGENIC	--	--	--
Ca6_P4_9335470	Ca6	9335470	AATATATATATATATATATATATAT/AATATATATATATATATATATATATATATAT	GTGTGTGCTTAAATTGGCCC	TGAAATTCCAAACCACATGC	60.4	776	INTERGENIC	--	--	--
Ca6_P4_9483877	Ca6	9483877	GTTATTATTATTATTATTATTATTATTATTATTATTATTGTTGTTATTGTTATTGTTATTGTTATTGTTATTATTATTATTATTATTATTATTATTGTTATTGTTATTATTATTATTCTTATTATTATT[illegible]	CAACTTCCTACGTTTTGGCA	CCCAATTGTTCATCCCAATC	58.8	717	INTERGENIC	--	--	--
Ca6_P4_9932385	Ca6	9932385	CATATATATATATATAT/CATATATATATATAT	CCCATCATTGACCGAAGAAT	CTTGTGGGGGTATGAGGAAA	59.8	553	INTERGENIC	--	--	--
Ca6_P4_9947644	Ca6	9947644	AAA/AAAGAGAA	ACATGACCATCACATCACGG	CGAACTTTTTGGAATGCAAC	60.3	519	INTERGENIC	--	--	--
Ca6_P4_9998146	Ca6	9998146	AATATATATATATATATA/AATATATATATATATATATA	AGTGCATGTTCCATGACGTG	CTCACTTACCCTCGCTCCTG	60.6	116	INTERGENIC	--	--	--
Ca7_P4_10060696	Ca7	10060696	CAAAAAAAAAAA/CAAAAAAAAAA	GCTGACTCTACGAGGAAAGGG	CAGATGACTAATGGTCGACAACA	60.4	429	INTERGENIC	--	--	--
Ca7_P4_10063869	Ca7	10063869	CATATATATATATATATATATATATAT/CATATATATATATATATATATATATATATATAT	CCATCCTCTCGCTGTCATTT	TTGTATTTGGTTGCTGATCTCG	60.2	204	INTERGENIC	--	--	--

INDEL marker IDs	Chromosomes/ unanchored scaffolds	Physical positions (bp)	InDels (*Kabuli* reference genome-CDC Frontier/PII)	Forward primers (5'-3')	Reverse primers (5'-3')	Anneling temperature (0C)	Expected amplified product size (bp)	Structural annotation		Functional annotation	
								Sequence components of *kabuli* genome	*Kabuli* gene accession IDs	NCBI-KOG	TFs
Ca7_P4_10168743	Ca7	10168743	CTTTTTTTTTTTTT/CTTTTTTTTTTTT	CGCGAAACAACGTATGATCT	GCCACCAGAATGAAATATACAAAA	58.8	344	INTERGENIC	--	--	--
Ca7_P4_1018745	Ca7	1018745	C/CT	TTGCAAACATGATAGCTGCC	TTGTTGGCATTGTTGTGGTT	59.8	467	INTERGENIC	--	--	--
Ca7_P4_1018777	Ca7	1018777	ATTGCAT/ATTGCATATTTGCAT	TTGTTCCCGTAAAAGATCGC	TTGTTGGCATTGTTGTGGTT	60.1	233	INTERGENIC	--	--	--
Ca7_P4_10192729	Ca7	10192729	TTTTTTTCATATA/TTTTTTTCATATAATGTTTTTTCATATA	CCCATTCCGAATACATTGCT	GAGTTGGGTGCACGATTTTT	59.8	624	INTERGENIC	--	--	--
Ca7_P4_10222115	Ca7	10222115	TTATATATATATATATATAT/TTATATATATATATATATATATAT	TTTCTTCGATCCCCACTTTTT	TTGTTTTCGTTGACATGTGATT	59.9	588	INTERGENIC	--	--	--
Ca7_P4_10240117	Ca7	10240117	ATTTTTTT/ATTTTTTTT	CCTCAGGATGCGACCATCTA	CCAGCTTGAACAATGTTTCTT	61.2	687	INTERGENIC	--	--	--
Ca7_P4_10240420	Ca7	10240420	CTTTTTTTTT/CTTTTTTTTTT	CGTGTTCCTTATATTTTGCCG	AATCTCAAAATGCCCCACTT	59.5	629	INTERGENIC	--	--	--
Ca7_P4_10268860	Ca7	10268860	TTATATATATATATATATATATATATATATATATATATATA/TTATATATATATATATATATATATATATATATA	CCACTCCTTTCATCCCTTGA	AAAATGATGCGGCGTTTTAC	60.0	549	INTERGENIC	--	--	--
Ca7_P4_10374231	Ca7	10374231	TTATATATATATATATATATATATATAT/TTATATATATATATATATATATATATATATAT	AATCGGCATTTTGAACTTGG	AAACGGCGACTTACCAAAGA	59.9	520	INTERGENIC	--	--	--
Ca7_P4_10442254	Ca7	10442254	AATATATATATATATATATATATATATATAT/AATATATATATATATATATATATATATATAT	ATTGGTGGGTCGGGTACATA	ATTCATCCAACAATGCCACA	59.9	377	INTERGENIC	--	--	--

**INDEL marker IDs**	**Chromosomes/ unanchored scaffolds**	**Physical positions (bp)**	**InDels (*Kabuli* reference genome-CDC Frontier/PII)**	**Forward primers (5'-3')**	**Reverse primers (5'-3')**	**Anneling temperature (0C)**	**Expected amplified product size (bp)**	**Structural annotation**		**Functional annotation**	
								**Sequence components of *kabuli* genome**	***Kabuli* gene accession IDs**	**NCBI-KOG**	**TFs**
Ca7_P4_10478608	Ca7	10478608	ATTTTTTTTTT/ATTTTTTTTT	CCGATTCAAAAACTTCGTCC	CCGTTCCTAGAAGCGTGAAG	59.5	844	INTERGENIC	--	--	--
Ca7_P4_10568493	Ca7	10568493	TTATATATATATATATATATATATATATAT/TTATATATATATATATATATATATAT	ACGATGGAGGCCAAAGTATG	AAAATAAGGCATGGTGTTGGA	60.0	487	INTERGENIC	--	--	--
Ca7_P4_10599617	Ca7	10599617	ATTTTTTTTTT/ATTTTTTTTTTT	GGAAGGGGTCCCTAGAACTG	GACGTCACATCCAAGTCGAA	59.9	651	INTERGENIC	--	--	--
Ca7_P4_10663412	Ca7	10663412	ATTTTTTTTTTT/ATTTTTTTTTT	TTATCCATCTCGCAAATGTCA	TCGAGGAGATGGAAGCCTTA	59.1	449	INTERGENIC	--	--	--
Ca7_P4_10667777	Ca7	10667777	TA/T	AAACGTCATAGCAAATAGATGAAA	ACTCACTAGGCAACCGCAAT	57.2	713	INTERGENIC	--	--	--
Ca7_P4_10740094	Ca7	10740094	TAAAAATTT/TAAAAATTTTAAAAAAATTT	TTTTTGCATTGCCACTTCTG	TTCTGATTTAAATTCCAACTTGTGAG	59.8	492	INTERGENIC	--	--	--
Ca7_P4_10764812	Ca7	10764812	GTGTCATGTCATATAAAGTTGTCATGTCA/GTGTCATGTCATATAAAGTTGTCATGTCATATAAAGTTGTCATGTCA	TGGGGAGATACGGATTTCAA	TAAATTGCGTTGGTTGGTGA	60.3	483	INTERGENIC	--	--	--
Ca7_P4_10781110	Ca7	10781110	TTATATATATATATATATATATATATATATATATATATATA/TTATATATATATATATATATATATATATATA	ATCTGGTTGGTTCAAGTGGC	TGTAATTGGATACTCGGGCA	60.0	572	INTERGENIC	--	--	--
Ca7_P4_10783305	Ca7	10783305	GAAAAAA/GAAAAAAA	GCATAACTGCAACGAGTTGGT	GTCCATTTTCCAGTGGCTGT	60.2	847	INTERGENIC	--	--	--
Ca7_P4_10785011	Ca7	10785011	GAAAAAAAAAAAA/GAAAAAAAAAAA	TTTGCGTTACGAATTTGATGA	TTGAAGCTTACCGACAACCC	59.2	294	INTERGENIC	--	--	--

INDEL marker IDs	Chromosomes/ unanchored scaffolds	Physical positions (bp)	InDels (*Kabuli* reference genome-CDC Frontier/PII)	Forward primers (5'-3')	Reverse primers (5'-3')	Anneling temperature (0C)	Expected amplified product size (bp)	Structural annotation		Functional annotation	
								Sequence components of *kabuli* genome	*Kabuli* gene accession IDs	NCBI-KOG	TFs
Ca7_P4_10790569	Ca7	10790569	ATT/ATTTT	CCAGAAATTTTGTTGGGGAA	TTCCGGAATTTTAGGGAAAAA	59.8	480	INTERGENIC	--	--	--
Ca7_P4_10791606	Ca7	10791606	TGGGG/TGGGGG	GGTGCGTACCGGAAATCTT	GTTTTTATGGGGTAGCCGGT	59.9	464	INTERGENIC	--	--	--
Ca7_P4_10806849	Ca7	10806849	ATTTTTTTTTT/ATTTTTTTTTTT	GTTCGCCGTTCATTCATTTT	CGATGAAGGCAGACCAATTT	59.9	824	INTERGENIC	--	--	--
Ca7_P4_10821468	Ca7	10821468	AAATAATAATAATAATAATAATAATAATAATAATAAT/AAATAATAATAATAATAATAATAATAATAATAATAATAAT	ACGTGACGTGGATGGGTTAT	TTTCGTCGTCTCAAATCGAA	60.1	816	INTERGENIC	--	--	--
Ca7_P4_10825347	Ca7	10825347	ATTTTTTTT/ATTTTTTT	CACTCAACGGGAACAGGTTT	TTGGATCCGACAGTGATGAA	60.0	843	INTERGENIC	--	--	--
Ca7_P4_10863846	Ca7	10863846	TGGGGGGGG/TGGGGGGG	GGACAGGGAAGAGGAAAAGG	ACAACCTACAACAGTCCCCG	60.0	466	INTERGENIC	--	--	--
Ca7_P4_10911713	Ca7	10911713	TAAAAAAAAAAA/TAAAAAAAAAA	ATGAGTACCGGACAGCGAGT	CACCACTTGTTCAACATGCC	59.8	718	INTERGENIC	--	--	--
Ca7_P4_10942267	Ca7	10942267	TAAAAAAAAA/TAAAAAAAA	GCATGTATGGATTGAACGCA	CATCCCCACCACCAATTAAG	60.5	898	INTERGENIC	--	--	--
Ca7_P4_11032853	Ca7	11032853	TATAATAATAATAATAATAATAATAATAATAATAAT/TATAATAATAATAATAATAATAATAATAAT	GAAATTCAGTTGAGGGACCG	CTTTCTCGAGGAGCGACCT	59.5	294	INTERGENIC	--	--	--
Ca7_P4_11034499	Ca7	11034499	AATATATATATATATATATATAT/AATATATATATATATATATATATAT	TGTCAATGTCAACCCATGCT	TCAAAAGGCCACCTCTTCAT	60.0	270	INTERGENIC	--	--	--

INDEL marker IDs	Chromosomes/ unanchored scaffolds	Physical positions (bp)	InDels (*Kabuli* reference genome-CDC Frontier/PII)	Forward primers (5'-3')	Reverse primers (5'-3')	Anneling temperature (0C)	Expected amplified product size (bp)	Structural annotation		Functional annotation	
								Sequence components of *kabuli* genome	*Kabuli* gene accession IDs	NCBI-KOG	TFs
Ca7_P4_11040579	Ca7	11040579	GAGATAGA/GAGA	AAGCTGTTGTGAATTGGGCT	CAACAAATGCATGTCCCAAG	59.7	335	INTERGENIC	--	--	--
Ca7_P4_11073789	Ca7	11073789	CTTTTTTTTTTT/CTTTTTTTTTT	TTGCAGCATCAACTAGGCAC	CCTCTCCCAAATCGCTACAA	60.0	649	INTERGENIC	--	--	--
Ca7_P4_11074091	Ca7	11074091	TAAAAAAAAAA/TAAAAAAAAA	TACCGATTGCCATCCTTAGC	ATTACCTTTCATATTTCGTTTTTCTAA	60.1	576	INTERGENIC	--	--	--
Ca7_P4_11083612	Ca7	11083612	TAAAAAAAAAAA/TAAAAAAAAAAAA	ACTGGTTTGTCATCAACCTGA	TTTTTAGGCAGGTGGACATATT	58.0	702	INTERGENIC	--	--	--
Ca7_P4_11332314	Ca7	11332314	AATATATATATATATATA/AATATATATATATATATATATA	AACCCAAGTTCCAAATGACG	ACCCAAGAGAGGTGCGACTA	59.8	569	INTERGENIC	--	--	--
Ca7_P4_11382209	Ca7	11382209	ATTTTTTTTTT/ATTTTTTTTTTT	TGTGCTACTTGCCATGCTCT	CGACCGACTCACTTTTCCAT	59.6	826	INTERGENIC	--	--	--
Ca7_P4_11430729	Ca7	11430729	CCTACNNN/C	AAGGTGGTGGTGGAGTTGAG	TCCTGTGAATGTCCACCAAA	60.0	392	INTERGENIC	--	--	--
Ca7_P4_11513709	Ca7	11513709	CATATATATATATATATATATATA/CATATATATATATATATATATATA	GTGTGACGTCGGTGAATACG	ACTCAACGAGCCTGCAATTT	60.0	709	INTERGENIC	--	--	--
Ca7_P4_11572510	Ca7	11572510	TTATATATATATATATATATATATATATATATATATATATA/TTATATATATATATATATATATATATATA	GAAAGGTGAAAGGCGAAATG	ATCAGCGGACAGGGATTACA	59.7	191	INTERGENIC	--	--	--
Ca7_P4_11647837	Ca7	11647837	GATTTTATTTT/GATTTTATTTTAATTTTATTTT	GCACCAAACAAGAAGGGAAA	GGAGATTCGTGCGCATATTT	60.1	166	INTERGENIC	--	--	--

INDEL marker IDs	Chromosomes/ unanchored scaffolds	Physical positions (bp)	InDels (*Kabuli* reference genome-CDC Frontier/PII)	Forward primers (5'-3')	Reverse primers (5'-3')	Anneling temperature (0C)	Expected amplified product size (bp)	Structural annotation		Functional annotation	
								Sequence components of *kabuli* genome	*Kabuli* gene accession IDs	NCBI-KOG	TFs
Ca7_P4_11960847	Ca7	11960847	ATAT/ATATGTATATGTATGGATGTAT	TGATGGCACATCATTGTCCT	AACCCCATCCCCAAAATAAG	59.9	221	INTERGENIC	--	--	--
Ca7_P4_11977859	Ca7	11977859	GTTATTATTATTATTATTATTATTATTATTATTATTATTATTATTATT/GTTATTATTATTATTATTATTATTATTATTATTATTATTATTATTATT	AGGGTGTGGTTATTGATGCC	TAACGGAATTGGTGGCCTTA	59.7	808	INTERGENIC	--	--	--
Ca7_P4_12003043	Ca7	12003043	TT/TTTAT	GATGGCAAAGACACACATGG	CGGTTGTGAGCATTGGACTA	60.0	884	INTERGENIC	--	--	--
Ca7_P4_12010139	Ca7	12010139	TAAAAAAAA/TAAAAAAA	AACACGGGAAAAACACGAAG	TTTGTGAGGGCTTTATTCGG	60.0	440	INTERGENIC	--	--	--
Ca7_P4_12062688	Ca7	12062688	GTTTTTTT/GTTTTTTTT	CCTTTTACAGCGCTTTTTCC	TGTTCATGCACTATGCGAAA	59.0	213	INTERGENIC	--	--	--
Ca7_P4_12082631	Ca7	12082631	TA/TAATATAATAATAATAA	CGGATTGCATTTGCTTTTCT	TCAAGGGATTGTCCCCAATA	60.2	250	INTERGENIC	--	--	--
Ca7_P4_12183467	Ca7	12183467	CTGTAGCGT/CT	CAGGCGTTGAAAACCTTTGT	CCACATTGAGAGGGAAGGAA	60.1	535	INTERGENIC	--	--	--
Ca7_P4_12204671	Ca7	12204671	ATTTTTTTTTTT/ATTTTTTTTTT	GCTTGGTTGGATAGCGAGAG	CACCTCAGCTACCGTTCACA	60.0	730	INTERGENIC	--	--	--
Ca7_P4_12261342	Ca7	12261342	AA/ATCTA	CTCAATTTCTGTTTGGGGGA	AATATGTTTGCCATGTCGGC	59.9	526	INTERGENIC	--	--	--
Ca7_P4_12261371	Ca7	12261371	ATA/ATATCTA	CTCAATTTCTGTTTGGGGGA	AATATGTTTGCCATGTCGGC	59.9	526	INTERGENIC	--	--	--

INDEL marker IDs	Chromosomes/ unanchored scaffolds	Physical positions (bp)	InDels (*Kabuli* reference genome-CDC Frontier/PII)	Forward primers (5'-3')	Reverse primers (5'-3')	Anneling temperature (0C)	Expected amplified product size (bp)	Structural annotation		Functional annotation	
								Sequence components of *kabuli* genome	*Kabuli* gene accession IDs	NCBI-KOG	TFs
Ca7_P4_12281757	Ca7	12281757	GAAAAAA/GAAAAAAA	TGGTTCCACGCTACACATTT	CTACCACTTCAATGGGGCAC	59.1	811	INTERGENIC	--	--	--
Ca7_P4_12447118	Ca7	12447118	TAAAAAAAA/TAAAAAAAAA	TTTGTCCCGAGTTCGAATGT	ATAAGGGGCAGCCTTAGGAA	60.5	301	INTERGENIC	--	--	--
Ca7_P4_12448298	Ca7	12448298	TTATATATATATATATATATATATATATATATATAT/TTATATATATATATATATATATATATATAT	AAGGCCAACCAAATTTGACA	TGTGATTTCTTGCTCACACCA	60.3	508	INTERGENIC	--	--	--
Ca7_P4_1245279	Ca7	1245279	A/AATTTCAT	TGCACAAAACACACAACCCT	TCGCTCCTGTGTCAGTTTTG	60.0	468	INTERGENIC	--	--	--
Ca7_P4_12486216	Ca7	12486216	CATAATAATAATAATAATAATAATAATAATAATAA/CATAATAATAATAATAATAATAATAATAATAA	TTTGATAAAGGGCAAAACAATAA	TGATGAATGCAACTAGTTTTGAGA	57.8	387	INTERGENIC	--	--	--
Ca7_P4_12509201	Ca7	12509201	CTCATCAT/CTCAT	TGACCTTGAGAATGCAACTGA	TGTGGACTGTGGTGTCGTCT	59.4	410	INTERGENIC	--	--	--
Ca7_P4_12539752	Ca7	12539752	TTTTATTT/TTTTATTTTATTTATTT	TCCTACTGAAGTCGTGGCCT	TGCATTTTCACCAACATCGT	59.9	596	INTERGENIC	--	--	--
Ca7_P4_12581086	Ca7	12581086	A/ATAT	TCTAAATGGCGACTTCCCAC	GTCAAAATGCCGTACGTGTG	60.1	808	INTERGENIC	--	--	--
Ca7_P4_12586098	Ca7	12586098	CCTCTCTCTCTCTCTCTCTCTCTCTCTCTCTCTCT/CCTCTCTCTCTCTCTCTCTCTCTCTCTCTCTCTCTCTCTCTCTCT	TTTCGCTGCAACAAAACAAC	AACGTAAGGAACGATGACGG	59.9	255	INTERGENIC	--	--	--
Ca7_P4_12651541	Ca7	12651541	AATATATATATATATATATATATATATATATATATA/AATATATATATATATATATATATATATATATA	AAGGGTGTCGGTAAGGTTGA	CCTTTCTCCACGTTCCATTC	59.4	835	INTERGENIC	--	--	--

INDEL marker IDs	Chromosomes/ unanchored scaffolds	Physical positions (bp)	InDels (*Kabuli* reference genome-CDC Frontier/PII)	Forward primers (5'-3')	Reverse primers (5'-3')	Anneling temperature (0C)	Expected amplified product size (bp)	Structural annotation		Functional annotation	
								Sequence components of *kabuli* genome	*Kabuli* gene accession IDs	NCBI-KOG	TFs
Ca7_P4_12691360	Ca7	12691360	CAAAAATCATTTT/CAAAAATCATTTTATTAAAAAAAATCATTTT	GTCAAGGGCCTATGGTCAGA	TTTGGTCATGGGATGTGCTA	60.1	909	INTERGENIC	--	--	--
Ca7_P4_12733352	Ca7	12733352	TT/TTATTAT	ATAAGATTTGGGGTGGGGAG	GGTTGCGTCTAGCGAAAGTT	60.0	374	INTERGENIC	--	--	--
Ca7_P4_12856742	Ca7	12856742	CAAA/CAA	CAATCACCAAACATCATGACAA	TGCCATAATTTCCTCTTCCG	59.3	565	INTERGENIC	--	--	--
Ca7_P4_12923739	Ca7	12923739	TGGAC/T	GAAGTGTAGCCTTTCAGGCG	CCTGGACTCGACAAAAGGAA	60.0	556	INTERGENIC	--	--	--
Ca7_P4_1295880	Ca7	1295880	TTATATATATATATATATATATATAT/TTATATATATATATATATATATATATATATATAT	TTCGATTTATCGATGAGGCA	TGAATTTAATTGGGGCGGTA	59.2	538	INTERGENIC	--	--	--
Ca7_P4_13090405	Ca7	13090405	ATTTTTTTT/ATTTTTTTTTTT	GGACAAAACGAGGCATTTGT	TTCATCACCGTGCCATCTAA	60.0	156	INTERGENIC	--	--	--
Ca7_P4_13183128	Ca7	13183128	TGACCCAG/TG	AGTATTTGAATTCCGACGAAATAA	GGGGTCTCCAACTGTTTCAA	58.2	100	INTERGENIC	--	--	--
Ca7_P4_1326824	Ca7	1326824	T/TA	TCCAAAAATGCACTCAAATCA	ATGAGTGGGAGAACATTGCC	59.1	715	INTERGENIC	--	--	--
Ca7_P4_13300743	Ca7	13300743	AAA/AAATTGCAATTAA	TGCAGCAAACATAAGCATGA	AGTAACCCGAACTCGGTCCT	59.0	494	INTERGENIC	--	--	--
Ca7_P4_1339137	Ca7	1339137	ATTTTTTTTT/ATTTTTTTTTT	CTTCACAACCAGAGCACCAA	TGCATTAAATTTTACGGAGAAACA	59.9	638	INTERGENIC	--	--	--

INDEL marker IDs	Chromosomes/ unanchored scaffolds	Physical positions (bp)	InDels (*Kabuli* reference genome-CDC Frontier/PII)	Forward primers (5'-3')	Reverse primers (5'-3')	Anneling temperature (0C)	Expected amplified product size (bp)	Structural annotation		Functional annotation	
								Sequence components of *kabuli* genome	*Kabuli* gene accession IDs	NCBI-KOG	TFs
Ca7_P4_13397822	Ca7	13397822	ATTTTTTTT/ATTTTTTTTT	GGCTTACACCAATGCTTAAAATG	TTCGAAAATACACAATCCAAAAA	59.9	628	INTERGENIC	--	--	--
Ca7_P4_13526291	Ca7	13526291	TATAAATAAATAAATAAATAAATAAATAAATAAATAAA/TATAAATAAATAAATAAATAAATAAATAAATAAATAAATAAA	AAATTGGTCATCGGTGCTTC	TTCCACACCACACTTGATCC	59.9	713	INTERGENIC	--	--	--
Ca7_P4_13559247	Ca7	13559247	TACA/TA	TTCAACTTTCTCCAATCCACA	TGAAAAGACGGAGGGTTGTT	58.2	470	INTERGENIC	--	--	--
Ca7_P4_13588964	Ca7	13588964	AATATATATATATATAT/AATATATATATATATATAT	AAATCGATGAATGGGTGGAG	AGAAACGTGCGGTGTCTCTT	59.8	561	INTERGENIC	--	--	--
Ca7_P4_13609089	Ca7	13609089	AAATAATAATAATAATAATAATAATAAT/AAATAATAATAATAATAATAATAATAATAATAAT	CCATTTCACGCGGAACTAAT	AGTAGCGTTGTCGGAAGCAT	60.0	699	INTERGENIC	--	--	--
Ca7_P4_1368308	Ca7	1368308	TAAAAAAAAA/TAAAAAAAAAA	GGGTTTTGTTTTTGGGGTTT	AGGGACACATTTTGGCATGT	59.9	854	INTERGENIC	--	--	--
Ca7_P4_13745061	Ca7	13745061	CC/CCCCGCCGGGAC	CGGCAATGTGGTTTGTTATG	GTGTAAATGGCGTCTGGGTT	59.8	593	INTERGENIC	--	--	--
Ca7_P4_13866056	Ca7	13866056	ATTTTTTTTTT/ATTTTTTTTT	TTGCAGACTGGGACTTCCTT	ATTGTGTGGGAGCCTCGTAT	59.8	513	INTERGENIC	--	--	--
Ca7_P4_13891702	Ca7	13891702	CTATATATATATATATAT/CTATATATATATATATATAT	TGCCTTTTACTCTTGGTGGG	GAGTGGGATTTTGGAGGTGA	60.1	596	INTERGENIC	--	--	--
Ca7_P4_13912935	Ca7	13912935	TTATATATATATATATATATAT/TTATATATATATATATATATATAT	ACAATCCTCAAATGCGGAGA	TACTCCAGGCCATTGACTCC	60.6	759	INTERGENIC	--	--	--

INDEL marker IDs	Chromosomes/ unanchored scaffolds	Physical positions (bp)	InDels (*Kabuli* reference genome-CDC Frontier/PII)	Forward primers (5'-3')	Reverse primers (5'-3')	Anneling temperature (0C)	Expected amplified product size (bp)	Structural annotation		Functional annotation	
								Sequence components of *kabuli* genome	*Kabuli* gene accession IDs	NCBI-KOG	TFs
Ca7_P4_14071479	Ca7	14071479	TTATTA/TTATTAATATTA	CAATGCGTTTTCCCAACTTT	CCGTTTTAGCGAACGACGTA	60.0	384	INTERGENIC	--	--	--
Ca7_P4_14071498	Ca7	14071498	T/TAATA	CAATGCGTTTTCCCAACTTT	CCGTTTTAGCGAACGACGTA	60.0	384	INTERGENIC	--	--	--
Ca7_P4_14075293	Ca7	14075293	CATATATATATATATAT/CATATATATATATAT	TTTGTTTAGCCACAACTTTTGA	CGTGCTCGGGATTTATTTGT	57.6	862	INTERGENIC	--	--	--
Ca7_P4_14083390	Ca7	14083390	AGACTTTTT/A	TCCCTACAAACACCGTCCTC	TTTTACAGCGCTTTTGGACA	60.0	291	INTERGENIC	--	--	--
Ca7_P4_1418041	Ca7	1418041	AG/A	CGTTGTGGTGGGTGTAGTTG	ACACCATGCGTCAGTTGAAA	59.9	188	INTERGENIC	--	--	--
Ca7_P4_1418185	Ca7	1418185	CAAAAAAAA/CAAAAAA	TTTCAACTGACGCATGGTGT	CCACGTGTCAGCCAAATAGA	60.2	324	INTERGENIC	--	--	--
Ca7_P4_1418248	Ca7	1418248	CA/CCATTAA	TTTCAACTGACGCATGGTGT	CCACGTGTCAGCCAAATAGA	60.2	324	INTERGENIC	--	--	--
Ca7_P4_1424328	Ca7	1424328	GAAAAAAAAA/GAAAAAAAA	TTCCAGCTCCAATTTTCACC	ACAGAGAGAGGCTGGATGGA	60.1	598	INTERGENIC	--	--	--
Ca7_P4_14244076	Ca7	14244076	GAAAAAAA/GAAAAAAAA	GCAATTTTGGTCCCCTCTTT	TTAAAAATTAACCACCACCATATTT	60.3	536	INTERGENIC	--	--	--
Ca7_P4_14404415	Ca7	14404415	CGAGAG/CGAGAGAG	TCTCCCTAGAAGCTGCAACC	GCCAGAATCATCCCAACCTA	59.6	129	INTERGENIC	--	--	--

INDEL marker IDs	Chromosomes/ unanchored scaffolds	Physical positions (bp)	InDels (*Kabuli* reference genome-CDC Frontier/PII)	Forward primers (5'-3')	Reverse primers (5'-3')	Anneling temperature (0C)	Expected amplified product size (bp)	Structural annotation		Functional annotation	
								Sequence components of *kabuli* genome	*Kabuli* gene accession IDs	NCBI-KOG	TFs
Ca7_P4_14456818	Ca7	14456818	CTTA/CTTAATTTA	GACGAACCAAGGGATGGTAA	TTTCTTCTTCTCGATTTGGTCC	59.8	671	INTERGENIC	--	--	--
Ca7_P4_14483644	Ca7	14483644	ATAT/ATATTTAT	TTAATAGGGCCGACGAATTTT	TAAAGGACCAAGTCGGGAAA	59.8	615	INTERGENIC	--	--	--
Ca7_P4_14657537	Ca7	14657537	TTTTTACT/TTTTTACTTTTACT	AGAGCATCTCCAAGGGTGAA	AGCCAAAATGAGTTTGTCGG	59.8	607	INTERGENIC	--	--	--
Ca7_P4_14664323	Ca7	14664323	CTTTTTTTTTTT/CTTTTTTTTTT	AATTGAAATGTTGAGGCTGTG	GGTCCTAACAACGCCAGGTA	57.7	634	INTERGENIC	--	--	--
Ca7_P4_14669638	Ca7	14669638	AA/AAAGAGACACCTCTGA	CATCGAGCGTTGTCAAGGTA	AATCTTTCTTCCAACACGGC	59.9	114	INTERGENIC	--	--	--
Ca7_P4_14831868	Ca7	14831868	AATATATATATATATATATATATATATATATATA/AATATATATATATATATATATATATATATATATATATATA	GGCCTATGGCCTGACCTACT	TGCAACAACCTTCCCATAAA	60.5	796	INTERGENIC	--	--	--
Ca7_P4_15217322	Ca7	15217322	AATATATATATATATATAT/AATATATATATATATATATATAT	ACCAATGTCCCTTGCATGAT	TCGCAATTACTCTTGCGATG	60.2	381	INTERGENIC	--	--	--
Ca7_P4_15305196	Ca7	15305196	AG/A	CTGCCCCCACTATTTCTCAA	ATACAGATCCGTGGAGGCAC	60.1	756	INTERGENIC	--	--	--
Ca7_P4_1531607	Ca7	1531607	AAATAATAATAATAATAATAATAATAATAATAATAATAATAA/AAATAATAATAATAATAATAATAATAATAATAATAATAATAATAATAA	TCGCAAGATCCAATGTGATG	CCACAATGATCGCCACTTTT	60.6	402	INTERGENIC	--	--	--
Ca7_P4_15328060	Ca7	15328060	CTGATTTG/CTG	TGAACTGAGAGAGGGGAGGA	CCTAACCCTTAATAAAATGAAAGCC	59.9	137	INTERGENIC	--	--	--

INDEL marker IDs	Chromosomes/ unanchored scaffolds	Physical positions (bp)	InDels (*Kabuli* reference genome-CDC Frontier/PII)	Forward primers (5'-3')	Reverse primers (5'-3')	Anneling temperature (0C)	Expected amplified product size (bp)	Structural annotation		Functional annotation	
								Sequence components of *kabuli* genome	*Kabuli* gene accession IDs	NCBI-KOG	TFs
Ca7_P4_15467222	Ca7	15467222	TTTATTATTATTATTATTATTATTATTATTATTATTATTATTATTAT/TTTATTATTATTATTATTATTATTATTATTATTATTATTATTAT	TGGGAAACGGTGCTAAACTT	TCCATTTACGGCAAATTCAA	59.6	716	INTERGENIC	--	--	--
Ca7_P4_15507878	Ca7	15507878	TATAATAATAATAATAATAATAATAATAATAATAATAATAATAATAATAATAATAATAAT/TATAATAATAATAATAATAATAATAATAATAATAATAATAATAATAATAAT	AACACACTCCCTCCCTCCAT	CCACCAGCCTTCCCTAATTT	60.8	653	INTERGENIC	--	--	--
Ca7_P4_15562093	Ca7	15562093	GAAAATAA/GAA	AATACACCACCTTCCACCCA	TTTTTAAGGGATCCGACGTG	60.1	161	INTERGENIC	--	--	--
Ca7_P4_15644224	Ca7	15644224	ATAT/ATATTTAT	CCGATTTCCCAAAGGTTTTT	TTCTGCAGCACTTCCACCTA	60.2	367	INTERGENIC	--	--	--
Ca7_P4_15653714	Ca7	15653714	CTTTTTT/CTTTTTTT	TTTCTTGCAAGGGTAATTTGC	GCTTGTGTGGCCGACTTAAT	59.2	641	INTERGENIC	--	--	--
Ca7_P4_15667150	Ca7	15667150	CTTTTTTTTTT/CTTTTTTTTTTT	TTTCAAGCTCCATTTTCTTCG	CGGAAACATTTGTAAATCAATCA	59.5	177	INTERGENIC	--	--	--
Ca7_P4_15688080	Ca7	15688080	ATTTTTTTTTT/ATTTTTTTTTTT	CCATTTGCCTGATCGTTCTT	TTTAACTTGTGCTTTTGAGTGTGTT	60.1	417	INTERGENIC	--	--	--
Ca7_P4_15688440	Ca7	15688440	AATATATATATATATATATATATATAT/AATATATATATATATATATATATATATATATATAT	TCATGGAGAACATTCTTCGTTTT	TTTCCGTCTTGATGCAATGT	60.0	865	INTERGENIC	--	--	--
Ca7_P4_15693431	Ca7	15693431	CTTTTTTTT/CTTTTTTT	TGAATTAGTCGGACAAAACTGAA	CGTAAAACCGAATTGCTCAAA	58.8	907	INTERGENIC	--	--	--
Ca7_P4_15735780	Ca7	15735780	CTATATATATATATATAT/CTATATATATATATATATAT	CATCAACACAAGGTCGCATC	CTTCCTCCATTTTTGCATGTT	60.1	363	INTERGENIC	--	--	--

INDEL marker IDs	Chromosomes/ unanchored scaffolds	Physical positions (bp)	InDels (*Kabuli* reference genome-CDC Frontier/PII)	Forward primers (5'-3')	Reverse primers (5'-3')	Anneling temperature (0C)	Expected amplified product size (bp)	Structural annotation		Functional annotation	
								Sequence components of *kabuli* genome	*Kabuli* gene accession IDs	NCBI-KOG	TFs
Ca7_P4_15774270	Ca7	15774270	TCC/TC	AAGTGTCGACCATGACTCTCAA	GCGCATTTCTTCCAAAATGT	59.8	654	INTERGENIC	--	--	--
Ca7_P4_158561	Ca7	158561	TCACACA/TCACA	ACTAGACATGACAACGGGGC	GCTCTAATTAGGTGCGGCTG	60.0	399	INTERGENIC	--	--	--
Ca7_P4_15932194	Ca7	15932194	ATATTATTATTATTATTATTATTATTATTATTATT/ATATTATTATTATTATTATTATTATTATTATT	AAGGCACATCATCGAAAACC	AAATTACAAACATAGGCAAGCCA	59.9	486	INTERGENIC	--	--	--
Ca7_P4_16001405	Ca7	16001405	AATTATTATTATTATTATTATTATTAT/AATTATTATTATTATTATTATTATTATTAT	AAAACACAACGTGCAAACGA	GGGTCACCAAGCACAGTTTT	60.2	679	INTERGENIC	--	--	--
Ca7_P4_16051093	Ca7	16051093	TAA/TAAA	TGGTTCGTCGGATATGTGAA	TTGAGTTCACGTACGCGTTT	59.9	600	INTERGENIC	--	--	--
Ca7_P4_1608740	Ca7	1608740	ATTTTTTTT/ATTTTTTT	TTCACGTTGCCAACTACTCG	CATTCGCGTTCATTTTGTTG	59.9	566	INTERGENIC	--	--	--
Ca7_P4_16126431	Ca7	16126431	TAAAAAAAAAAA/TAAAAAAAAAAAA	TCACTATTTCATGCAACCGC	TTTGAAGATTTGCGTTACGA	59.7	494	INTERGENIC	--	--	--
Ca7_P4_16127650	Ca7	16127650	AATGATTCT/AATGATTCTCCCATGATTCT	TCACCGAAATCAAGCATTCA	TATGGATTCCGGGTTGTTGT	60.2	706	INTERGENIC	--	--	--
Ca7_P4_16251371	Ca7	16251371	TAAAAAAA/TAAAAAA	GGAGTCGATTATGCGTAGGG	CGCACTGGAAGATACGTAGGA	59.6	264	INTERGENIC	--	--	--
Ca7_P4_16298175	Ca7	16298175	ATTTTTTTTTTTTTT/ATTTTTTTTTTTTTTTT	TGCGCTTCTGTATTTAGAGTTTT	CCTTCACCAGATGGAAGAACA	57.5	366	INTERGENIC	--	--	--

INDEL marker IDs	Chromosomes/ unanchored scaffolds	Physical positions (bp)	InDels (*Kabuli* reference genome-CDC Frontier/PII)	Forward primers (5'-3')	Reverse primers (5'-3')	Anneling temperature (0C)	Expected amplified product size (bp)	Structural annotation		Functional annotation	
								Sequence components of *kabuli* genome	*Kabuli* gene accession IDs	NCBI-KOG	TFs
Ca7_P4_16320388	Ca7	16320388	TTTATTATTATTATTATTATTATTATTATTATTATTATTATTATTATTA/TTTATTATTATTATTATTATTATTATTATTATTATTATTATTATTATTATTATTATTATTA	TTTGCGTTACGAATTTGATGA	TTGGTCCTAAACAAGATGCAAA	59.2	607	INTERGENIC	--	--	--
Ca7_P4_16349610	Ca7	16349610	CTTTTTTTTTTT/CTTTTTTTTTT	GCAGTGCTGAATTTTGGTGA	TTGGTTTCATGACTTGCATCT	59.8	740	INTERGENIC	--	--	--
Ca7_P4_164757	Ca7	164757	TTATATATATATATATATATATATAT/TTATATATATATATATATATAT	CGTTGCCACAATGTTATTGG	CATGAGTTTTTGGTCTGCCA	59.8	671	INTERGENIC	--	--	--
Ca7_P4_1649022	Ca7	1649022	ATTTTTT/ATTTT	CCTTAGTGATTTGGAGGGCA	CGAGAAGGTCGTGAGGTAGC	60.1	564	INTERGENIC	--	--	--
Ca7_P4_16560646	Ca7	16560646	TAAAAAAAAAAAA/TAAAAAAAAAAA	ATTTTCGCATGAAGGGAGTG	CGCAAAGGAGAAACGAGAAT	60.1	724	INTERGENIC	--	--	--
Ca7_P4_16610320	Ca7	16610320	TAAAAAAA/TAAAAAAAA	CCACATCTCGTCTCCCTCAT	TTGCAGGTCATGTGGCTAAC	60.1	247	INTERGENIC	--	--	--
Ca7_P4_16687553	Ca7	16687553	ATTGATTTG/ATTGATTTGATTTG	CGTCTTGGGACCAACTCAAT	TCGATTCACAACTGCACTGA	60.0	470	INTERGENIC	--	--	--
Ca7_P4_1686051	Ca7	1686051	ATTTTTT/ATTTTTTT	TCAAAACTTCACATTTGTGCG	CCTTTTTATTTTTGTGGGGGA	59.8	697	INTERGENIC	--	--	--
Ca7_P4_16899888	Ca7	16899888	AATATATATATATATATATA/AATATATATATATATATATATA	TCATCAAATTCGTAACGCAAA	TCCTCTAATTTATCCTTCGTCCA	59.2	620	INTERGENIC	--	--	--
Ca7_P4_16941588	Ca7	16941588	TAAA/TAAAA	TGCCGTAGTATTCACCACCA	TGCCATGAAATTCTGCCATA	60.0	852	INTERGENIC	--	--	--

INDEL marker IDs	Chromosomes/ unanchored scaffolds	Physical positions (bp)	InDels (*Kabuli* reference genome-CDC Frontier/PII)	Forward primers (5'-3')	Reverse primers (5'-3')	Anneling temperature (0C)	Expected amplified product size (bp)	Structural annotation		Functional annotation	
								Sequence components of *kabuli* genome	*Kabuli* gene accession IDs	NCBI-KOG	TFs
Ca7_P4_16970696	Ca7	16970696	AT/A	CAAAGGAACATTGTGATCGAA	CAAAGCCAAAACCAACATCA	58.6	742	INTERGENIC	--	--	--
Ca7_P4_17073516	Ca7	17073516	CATAT/CAT	TCCTTAGCCAAAGAGCCAAA	GTGTGGGAACCTTGTTGCTT	60.0	726	INTERGENIC	--	--	--
Ca7_P4_17133790	Ca7	17133790	ATTTTTTT/ATTTTTT	GACACTACCCGGCATCAAGT	CCAACCCAAATAAATGAAAATCA	60.0	530	INTERGENIC	--	--	--
Ca7_P4_17155284	Ca7	17155284	CATATATATATA/CATATATATA	AGTCCATACTTGGCAGGGTG	ACTACATGTTTGCAATGGCG	60.0	957	INTERGENIC	--	--	--
Ca7_P4_17172621	Ca7	17172621	AATCATAT/AATCATATCATAT	GGCTAAACCTTGTCTTTGGC	AGACTCTGCTTGACCGGAAA	58.8	541	INTERGENIC	--	--	--
Ca7_P4_17352977	Ca7	17352977	AATATATATATA/AATATATATATATA	ATTGCGGTCGAACACACATA	TCTCTTCCGTTTCCACCATC	60.0	508	INTERGENIC	--	--	--
Ca7_P4_17357820	Ca7	17357820	TTTTATTTATTTATTTATTTATTTAT/TTTTATTTATTTATTTATTTAT	TGGATCTTATGTCAAACGCCT	TGAAATGCAAATAGAGCCCC	59.6	698	INTERGENIC	--	--	--
Ca7_P4_17374590	Ca7	17374590	CTTTTTTTTTTTT/CTTTTTTTTT	GCATCTGTAAAACAAGACCCG	ACATCGATAAGAGGCAGCGT	59.6	525	INTERGENIC	--	--	--
Ca7_P4_174141	Ca7	174141	CATATATATATATATATATAT/CATATATATATATATATATATAT	CATGTTTGCCATGGGATCTA	TCTTTATTCGGTTTGGCCTG	59.4	693	INTERGENIC	--	--	--
Ca7_P4_174353	Ca7	174353	TAAAAAAAAAA/TAAAAAAAAAAA	ATTGCGCTTGATAGACCGAC	CTTTATTCGGTTTGGCCTGA	60.2	333	INTERGENIC	--	--	--

INDEL marker IDs	Chromosomes/ unanchored scaffolds	Physical positions (bp)	InDels (*Kabuli* reference genome-CDC Frontier/PII)	Forward primers (5'-3')	Reverse primers (5'-3')	Anneling temperature (0C)	Expected amplified product size (bp)	Structural annotation		Functional annotation	
								Sequence components of *kabuli* genome	*Kabuli* gene accession IDs	NCBI-KOG	TFs
Ca7_P4_17552678	Ca7	17552678	GTTTTTTT/GTTTTTTTTT	GCCACCAAAGTTTATG TCGG	TTGTTCCATGTCCCTTT TGC	60.4	135	INTERGENIC	--	--	--
Ca7_P4_17576147	Ca7	17576147	AAAAATAAAATAAAAT/AAAAATAAAA T	CATCAAAATCATGCAT GTCCA	CCTTCGCATTGGAACT GTTT	60.3	543	INTERGENIC	--	--	--
Ca7_P4_17685877	Ca7	17685877	TAAAAA/TAAAAAA	CTCATCCCCCTTCATCT TCA	TGTTGAAGCAAAGGTA AAACG	60.0	532	INTERGENIC	--	--	--
Ca7_P4_1776003	Ca7	1776003	AACTT/AACTTACTT	ATGTCTAGCATGGTTT CGCC	TGTTTCCATTATTGGAT TGTGC	60.1	646	INTERGENIC	--	--	--
Ca7_P4_17841305	Ca7	17841305	TG/T	TTTATGTCCATGTGCG TGCT	CCACGGTTAAATTTGAT GTTGA	60.1	346	INTERGENIC	--	--	--
Ca7_P4_17961701	Ca7	17961701	GAAAAAAAAAA/GAAAAAAAAA	AAGACCCCACAAATTG GAAA	GAAAGCGCTTCCGTTT ACAG	59.3	479	INTERGENIC	--	--	--
Ca7_P4_18007228	Ca7	18007228	TTAATAATAATAATAATAATAA/TTAA TAATAATAATAATAATAA	GGCGACACTTCAATGG ACTT	TGCATTGCCATTTCCA GTTA	60.1	627	INTERGENIC	--	--	--
Ca7_P4_18007668	Ca7	18007668	CTTATTATTATTATTATTAT/CTTATT ATTATTATTAT	CTCGTGTTTCTCGGTG GTTT	AAGTCCATTGAAGTGT CGCC	60.1	264	INTERGENIC	--	--	--
Ca7_P4_180427	Ca7	180427	C/CAGAGGCA	TCAACAGCAGCTTTGC ATTC	TTTCGCAGCTGGTATG TGAG	60.1	280	INTERGENIC	--	--	--
Ca7_P4_18045160	Ca7	18045160	ATTTTTTT/ATTTTTTTT	AGGTTTTCAACCTAGC GGGT	GAGGAGTCGGAATGGG TACA	60.0	407	INTERGENIC	--	--	--

INDEL marker IDs	Chromosomes/ unanchored scaffolds	Physical positions (bp)	InDels (*Kabuli* reference genome-CDC Frontier/PII)	Forward primers (5'-3')	Reverse primers (5'-3')	Anneling temperature (0C)	Expected amplified product size (bp)	Structural annotation		Functional annotation	
								Sequence components of *kabuli* genome	*Kabuli* gene accession IDs	NCBI-KOG	TFs
Ca7_P4_18126020	Ca7	18126020	TAAAAAAA/TATAAAAAAAA	GTTTTGGCGTTGGTTTCACT	TTAAATCTGTCCGGGTTTGG	60.0	695	INTERGENIC	--	--	--
Ca7_P4_18188416	Ca7	18188416	CTTT/CTTTT	TGTCTTGAGCATGCCTTGTC	AAATGCAAACCCAAATGCTC	60.0	525	INTERGENIC	--	--	--
Ca7_P4_18333235	Ca7	18333235	A/ATTAT	GCAAGTGTGTTTGTGGCACT	TTCAAGCACATAATGCAAGTCC	59.8	113	INTERGENIC	--	--	--
Ca7_P4_18347733	Ca7	18347733	TTCTCTCTCTCTCTCTCTCTCTCTCTCTCTCTCT/TTCTCTCTCTCTCTCTCTCTCTCTCTCTCT	CTCAACGACAACTTGCCAAA	TTTGATACCAATTGGAGGTGC	59.9	452	INTERGENIC	--	--	--
Ca7_P4_1835089	Ca7	1835089	CAA/CAAA	AACAAAAGTAGCGTGCGACA	ATTTGTCAACTCGGGGTTTG	59.5	632	INTERGENIC	--	--	--
Ca7_P4_18437525	Ca7	18437525	CTATATATATATATATATATATATATATAT/CTATATATATATATATATATATATATATATATATAT	GTTGGTTCCCTAGCACCGTA	TTGCTGAAAATTGTGCCAAA	60.0	570	INTERGENIC	--	--	--
Ca7_P4_18439833	Ca7	18439833	TAGAG/TAG	TATTTGGAGCAGCGGGTTAG	CCCACAACCACACACTCATC	60.2	813	INTERGENIC	--	--	--
Ca7_P4_18587623	Ca7	18587623	AAAGGCGGAAGGCGG/AAAGGCGG	CGATGATGACTGATTGGGGT	TGTTTGTGTTTTGAGGCGTT	60.7	374	INTERGENIC	--	--	--
Ca7_P4_18588675	Ca7	18588675	TTATAT/TTATATAT	CCAAGACTCCTTGTAACACCC	GCACATATATACACGTAAAATCGCA	58.6	299	INTERGENIC	--	--	--
Ca7_P4_18588937	Ca7	18588937	ACTATATAT/AATATAT	CCAAGACTCCTTGTAACACCC	TTCTCCGTTTTCGTAAGACCA	58.6	792	INTERGENIC	--	--	--

INDEL marker IDs	Chromosomes/ unanchored scaffolds	Physical positions (bp)	InDels (*Kabuli* reference genome-CDC Frontier/PII)	Forward primers (5'-3')	Reverse primers (5'-3')	Anneling temperature (0C)	Expected amplified product size (bp)	Structural annotation		Functional annotation	
								Sequence components of *kabuli* genome	*Kabuli* gene accession IDs	NCBI-KOG	TFs
Ca7_P4_18756922	Ca7	18756922	CTTTTTTTTT/CTTTTTTTTTT	CAAAAACAAAAGCTTGCGAA	TGAGACGTCTACCTTGGGGT	59.1	418	INTERGENIC	--	--	--
Ca7_P4_18769932	Ca7	18769932	TTTATTATTATTATTATTATTATTATTATTATTATTATTATTATTATTAT/TTTATTATTATTATTATTATTATTATTATTATTATTAT	AACAAAATCGGGAATAGGGTT	TGAAATTTGTGCAACTCGGA	58.7	773	INTERGENIC	--	--	--
Ca7_P4_18770611	Ca7	18770611	GTTTTTTTTT/GTTTTTTTT	TCCGAGTTGCACAAATTTCA	TTCAATTTTGGAAAAACGTTCA	60.2	662	INTERGENIC	--	--	--
Ca7_P4_18774607	Ca7	18774607	CTTTTTTTTTT/CTTTTTTTTTTT	CATGTTTCATTTCTGCACGG	CGACATATTTAGTCCCTCTTTGG	60.1	429	INTERGENIC	--	--	--
Ca7_P4_18780433	Ca7	18780433	TTATATATATATATATAT/TTATATATATATATAT	TTTTTGTGCGTTTGACTGATATT	CCTCTTTGCTTTTACGGCTG	58.7	618	INTERGENIC	--	--	--
Ca7_P4_18793363	Ca7	18793363	TTATATATATATATATATATATATATATAT/TTATATATATATATATATATATATATAT	GCCAACCCACAAATCTGTCT	TGTCTTCTCGCTGTTTCATTG	60.0	699	INTERGENIC	--	--	--
Ca7_P4_18917406	Ca7	18917406	TTTATTATTATTATTATTATTATTATTATTATTATTAT/TTTATTATTATTATTATTATTATTATTATTATTATTATTATTAT	CAAACCCAAAAATAAGATGGAAA	AAAACCCACCGAATCACTCA	59.3	517	INTERGENIC	--	--	--
Ca7_P4_18928675	Ca7	18928675	TAAAAAAAAAAAA/TAAAAAAAAAAAAA	AGCTCACAGAAAAACACGGG	TGTCCCGTGCGATTTTTAAT	60.3	392	INTERGENIC	--	--	--
Ca7_P4_18984652	Ca7	18984652	C/CTT	AGTGGTTGCGCGTTATCTTT	AAAGAGGGAAACCACCTTTGA	59.8	173	INTERGENIC	--	--	--
Ca7_P4_19008569	Ca7	19008569	ATTTT/ATTTTTCTTTT	TTCTGGATCGGATCTTGGAC	TGAAAGTTCGATCTCAATCATCA	60.0	432	INTERGENIC	--	--	--

INDEL marker IDs	Chromosomes/ unanchored scaffolds	Physical positions (bp)	InDels (*Kabuli* reference genome-CDC Frontier/PII)	Forward primers (5'-3')	Reverse primers (5'-3')	Anneling temperature (0C)	Expected amplified product size (bp)	Structural annotation		Functional annotation	
								Sequence components of *kabuli* genome	*Kabuli* gene accession IDs	NCBI-KOG	TFs
Ca7_P4_1902358	Ca7	1902358	GTTTTAATAATA/G	CAAGTTTATCTCTTCAATCGCGT	TCGCATATTGCTCTCCCTCT	59.8	318	INTERGENIC	--	--	--
Ca7_P4_19077612	Ca7	19077612	C/CTTTCCCTAATGTCTTAGT	ACTCGAGCCTTGGAAGATCA	GTTGGGAGACTTGTGCCCTA	60.0	861	INTERGENIC	--	--	--
Ca7_P4_19095896	Ca7	19095896	GTTTTTTTT/GTTTTTTT	GTTTTGCTTCCCACTTCCAA	CTCCTTGCAGATATAGCGCC	60.1	580	INTERGENIC	--	--	--
Ca7_P4_19128824	Ca7	19128824	AG/A	TAAGACTCCGTCCAACTCGG	CCCAAACAACCCCTAAAACA	60.2	428	INTERGENIC	--	--	--
Ca7_P4_19175066	Ca7	19175066	ACC/ACCC	ATCAGCATGGTTAGCCCAAG	TGCGTTTGTGTCATTGGATT	60.1	571	INTERGENIC	--	--	--
Ca7_P4_19263812	Ca7	19263812	TTTGTTGTTGTTGTTGTT/TTTGTTGTTGTTGTTGTTGTTGTT	CCTTCTATCCAGCCATCCAA	TTCCCCCTCCTTTTATTTAATTG	60.0	713	INTERGENIC	--	--	--
Ca7_P4_19359942	Ca7	19359942	TA/TATGCCATTGA	ACGAGTGCGGGTATCAATGT	GAGAAATGGAGGCAATGGAA	60.4	456	INTERGENIC	--	--	--
Ca7_P4_19444691	Ca7	19444691	TA/T	AAGCGGTGTCGACTTTTGTT	ATAAACGTGTGCCGGAAAAT	59.8	560	INTERGENIC	--	--	--
Ca7_P4_19554104	Ca7	19554104	GTTTTTTTTTTTT/GTTTTTTTTTTTTTT	TGAACCCCATGAAAGAGGAA	TTTGGAGGGTGAAGGTTCTG	60.4	530	INTERGENIC	--	--	--
Ca7_P4_19562455	Ca7	19562455	TAAAAAAAAA/TAAAAAAAAAA	CCTTTCGACTTGGTTTGGAT	TTATTCGACTCCACACGCAC	59.0	432	INTERGENIC	--	--	--

INDEL marker IDs	Chromosomes/ unanchored scaffolds	Physical positions (bp)	InDels (*Kabuli* reference genome-CDC Frontier/PII)	Forward primers (5'-3')	Reverse primers (5'-3')	Anneling temperature (0C)	Expected amplified product size (bp)	Structural annotation		Functional annotation	
								Sequence components of *kabuli* genome	*Kabuli* gene accession IDs	NCBI-KOG	TFs
Ca7_P4_19581964	Ca7	19581964	G/GCCTAGGGAAAGAAAAAA	ACGTGCATACGATGCAGAAA	AAGTCGCCATTTGGAACTTG	60.3	341	INTERGENIC	--	--	--
Ca7_P4_19589014	Ca7	19589014	AATTTATCTATTTATCTATTTAT/AATTTATCTATTTATCTATTTATCTATTTAT	GGTCGTCCAAATAGTACCCG	CCGATTTCCCAAAGGTTTTT	59.3	358	INTERGENIC	--	--	--
Ca7_P4_19589201	Ca7	19589201	TTATATATATATATATATATATATAT/TTATATATATATATATATATATATATAT	GGTCGTCCAAATAGTACCCG	AGGAAGCCCAAGTTCCAAAT	59.3	828	INTERGENIC	--	--	--
Ca7_P4_19608417	Ca7	19608417	TTATATATATATATATATATATATA/TTATATATATATATATATATATATATATATA	TCATATAGCGAGCCCAATCC	AAGACCTTGCCTCTTTGGCT	60.0	524	INTERGENIC	--	--	--
Ca7_P4_1961188	Ca7	1961188	CATATATATATATATATAT/CATATATATATATATATATAT	TTAAGATGCCGTGTCATGGA	AATACAAAGCAAAATGCCCG	60.1	593	INTERGENIC	--	--	--
Ca7_P4_19707176	Ca7	19707176	TAAAAAAAAAAA/TAAAAAAAAAA	GCCGAGGGGGAGAGATATAG	CCGTGCCATTTTCACACTAA	60.0	523	INTERGENIC	--	--	--
Ca7_P4_19718791	Ca7	19718791	GTAATAATAATAATAATAATAATAATAATAATAATAATAATAATAA/GTAATAATAATAATAATAATAATAATAATAATAATAATAATAATAATAA	TTAGGGAGCTTACATCGCGT	TAGCTGTGAGTTGACGACGG	59.9	488	INTERGENIC	--	--	--
Ca7_P4_19731364	Ca7	19731364	ATTTTTTTTTT/ATTTTTTTTTTT	CACTTGCACGCAAAACTGAT	AATGGAACTCGGTTCGTTTG	59.9	250	INTERGENIC	--	--	--
Ca7_P4_19786359	Ca7	19786359	AATATATATATATATATATATATATATATA/AATATATATATATATATATATATATATATATA	AAATTAAAGTTTCAAACCACGG	TCAGCTCCACTCACACCATC	57.3	679	INTERGENIC	--	--	--
Ca7_P4_19786465	Ca7	19786465	TACA/TA	AAATTAAAGTTTCAAACCACGG	TCAGCTCCACTCACACCATC	57.3	679	INTERGENIC	--	--	--

INDEL marker IDs	Chromosomes/ unanchored scaffolds	Physical positions (bp)	InDels (*Kabuli* reference genome-CDC Frontier/PII)	Forward primers (5'-3')	Reverse primers (5'-3')	Anneling temperature (0C)	Expected amplified product size (bp)	Structural annotation		Functional annotation	
								Sequence components of *kabuli* genome	*Kabuli* gene accession IDs	NCBI-KOG	TFs
Ca7_P4_19857139	Ca7	19857139	AAATAATAATAATAATAATAATAATAATAATAATAA/AAATAATAATAATAATAATAATAATAATAATAATAA	CCATATGTCATAGTGCGGTCA	AGAGGCAAACAAGAACCGAA	59.4	314	INTERGENIC	--	--	--
Ca7_P4_19857337	Ca7	19857337	GAAAAAAAA/GAAAAAAAAA	TTCGGTTCTTGTTTGCCTCT	ACGAATTCATGTCCCCTTTG	59.9	394	INTERGENIC	--	--	--
Ca7_P4_19860378	Ca7	19860378	GT/G	CCTCAATATGTTGGGACATGC	TGATTCTCAGGGAGGGTGTC	60.2	464	INTERGENIC	--	--	--
Ca7_P4_19993135	Ca7	19993135	ATTTTTTTTTTT/ATTTTTTTTTTTT	TTAGGGTTGGACAGTGGGTC	GGAGACAATAGCCAAGTGGG	59.8	366	INTERGENIC	--	--	--
Ca7_P4_20032652	Ca7	20032652	TC/T	AGTGCCCATCCAAACTGAAC	TGAAGGAATGCAAGGAAACC	60.0	568	INTERGENIC	--	--	--
Ca7_P4_20032669	Ca7	20032669	TAAAAAAA/TAAAAATAAAATAAAAAAAA	AGTGCCCATCCAAACTGAAC	TGAAGGAATGCAAGGAAACC	60.0	568	INTERGENIC	--	--	--
Ca7_P4_20058919	Ca7	20058919	AATATATATATATATATATATATATATATATA/AATATATATATATATATATATATATATATATATA	CTCATTTGGTACGACTTGTGG	TAATACGGGATGACCCAACC	58.1	840	INTERGENIC	--	--	--
Ca7_P4_20059991	Ca7	20059991	TTGT/TTGTGT	TGATTCGCATCAAACCAATAA	TCATCATTTGAGTCATCTTGCC	59.0	487	INTERGENIC	--	--	--
Ca7_P4_20154075	Ca7	20154075	GTTTTTTT/GTTTTTTTT	GAGGACTGGACGTAACCCAA	TGGCGATTTACAAGGAAGGA	60.0	290	INTERGENIC	--	--	--
Ca7_P4_20164032	Ca7	20164032	AATATATATATATATATATATATATATATATATATAT/AATATATATATATATATATATATATATATATATATAT	CCTCGTCTCCAAATTTCCAA	GAAGCCCACCCTGTAAATGA	60.0	415	INTERGENIC	--	--	--

**INDEL marker IDs**	**Chromosomes/ unanchored scaffolds**	**Physical positions (bp)**	**InDels (*Kabuli* reference genome-CDC Frontier/PII)**	**Forward primers (5'-3')**	**Reverse primers (5'-3')**	**Anneling temperature (0C)**	**Expected amplified product size (bp)**	**Structural annotation**		**Functional annotation**	
								**Sequence components of *kabuli* genome**	***Kabuli* gene accession IDs**	**NCBI-KOG**	**TFs**
Ca7_P4_20332113	Ca7	20332113	ATTCTTCTTCTTCTTCTTCTT/ATTCTTCTTCTTCTTCTTCTTCTTCTT	TGCAGATTTTCCCCGATTAG	ATCACATTTACGAGGCACCC	60.0	355	INTERGENIC	--	--	--
Ca7_P4_20356212	Ca7	20356212	GATCCGAAGGAAT/GAT	AAGGCTAAACGGGGATCAAG	CTCCATAAGCCTTTTGCTCG	60.4	605	INTERGENIC	--	--	--
Ca7_P4_20485643	Ca7	20485643	ATTTTTTTTTTT/ATTTTTTTTTT	GGCTAAATTACATTTGTGGTCCC	TTGTTGATATTGTGTCGTAGAGCA	60.8	621	INTERGENIC	--	--	--
Ca7_P4_20501733	Ca7	20501733	TGTA/TGTAGTA	TAATGCACCTTCCGTGTCAA	TTATAGGAAAAAGAACGAAGGAAA	60.1	636	INTERGENIC	--	--	--
Ca7_P4_20502470	Ca7	20502470	CAAAAAA/CAAAAAAA	TTCCGATTTACTTCCTCTAAAATGA	GCTTCCTCCTATTCATGCGT	59.6	456	INTERGENIC	--	--	--
Ca7_P4_20505830	Ca7	20505830	TAAAAAAA/TAAAAAAAA	GGCATGGAAAATTCTGAAAAA	AAGAAGCGACGAAGAGAATTTG	59.0	732	INTERGENIC	--	--	--
Ca7_P4_20505889	Ca7	20505889	ATA/ATAGAAATTA	GGCATGGAAAATTCTGAAAAA	TTCATTTCTCGATTGAGGGC	59.0	803	INTERGENIC	--	--	--
Ca7_P4_20505916	Ca7	20505916	GGA/G	GGCATGGAAAATTCTGAAAAA	TTCATTTCTCGATTGAGGGC	59.0	803	INTERGENIC	--	--	--
Ca7_P4_20509106	Ca7	20509106	ATAACTAA/ATAA	TTTCCGAGCAAACAAAACCT	TCGCAATTGGTGATGAGTTC	59.7	516	INTERGENIC	--	--	--
Ca7_P4_20510218	Ca7	20510218	GTAAT/GTAATAAT	TTGAACAATGCAAGAGCCAC	CTGCTCATGTTTCCCGTTTT	59.8	933	INTERGENIC	--	--	--

INDEL marker IDs	Chromosomes/ unanchored scaffolds	Physical positions (bp)	InDels (*Kabuli* reference genome-CDC Frontier/PII)	Forward primers (5'-3')	Reverse primers (5'-3')	Anneling temperature (0C)	Expected amplified product size (bp)	Structural annotation		Functional annotation	
								Sequence components of *kabuli* genome	*Kabuli* gene accession IDs	NCBI-KOG	TFs
Ca7_P4_20510427	Ca7	20510427	TTT/TTTATTAGTGTATTAAAATT	TGCATTGCTCCCTAGACACA	GGCACTGCTTTATGCACTCA	60.4	503	INTERGENIC	--	--	--
Ca7_P4_20510596	Ca7	20510596	TAAAAATAAA/TAAAAATAAAAAAATAAAAAAAATAAA	TCGTCGGCTAAATCCAACTC	GGCACTGCTTTATGCACTCA	60.2	366	INTERGENIC	--	--	--
Ca7_P4_20519746	Ca7	20519746	CTTTTTTT/CTTTTTTTT	TGGGGATGTGGGAAAAATAA	AGTGGCATTCTTTGTGAATGA	60.0	626	INTERGENIC	--	--	--
Ca7_P4_20537234	Ca7	20537234	CAAAAA/CAAAAAA	TCGCACAGGATGAGAACTTG	TCAGCCTCTCCATCAAACCT	60.0	179	INTERGENIC	--	--	--
Ca7_P4_20559674	Ca7	20559674	ATGT/ATGTGT	TTTTGCGTTTTGCTTTTGTG	TTCCTGCAAATTTCAAATGC	59.9	476	INTERGENIC	--	--	--
Ca7_P4_20566181	Ca7	20566181	CATATATA/CATATA	ATTGTTCCAGCTTTGCCAGT	CATTCAAATGTTTGCAACAACC	59.7	188	INTERGENIC	--	--	--
Ca7_P4_20566222	Ca7	20566222	T/TC	ATTGTTCCAGCTTTGCCAGT	CATTCAAATGTTTGCAACAACC	59.7	188	INTERGENIC	--	--	--
Ca7_P4_20566359	Ca7	20566359	T/TA	GGTGACTGGTGGGATTGTTC	TGAAAGTTCTCTTTAATCTGTCACG	60.2	370	INTERGENIC	--	--	--
Ca7_P4_20566739	Ca7	20566739	ATTT/ATT	TGTAAAATGTTTAGGAGTTTTCAACG	CACCTTCCTTGTGTTCCGAT	59.9	509	INTERGENIC	--	--	--
Ca7_P4_20566755	Ca7	20566755	AT/A	TGTAAAATGTTTAGGAGTTTTCAACG	CACCTTCCTTGTGTTCCGAT	59.9	509	INTERGENIC	--	--	--

INDEL marker IDs	Chromosomes/ unanchored scaffolds	Physical positions (bp)	InDels (*Kabuli* reference genome-CDC Frontier/PII)	Forward primers (5'-3')	Reverse primers (5'-3')	Anneling temperature (0C)	Expected amplified product size (bp)	Structural annotation		Functional annotation	
								Sequence components of *kabuli* genome	*Kabuli* gene accession IDs	NCBI-KOG	TFs
Ca7_P4_20567011	Ca7	20567011	TAAAAAAA/TAAAAAA	TGGAATTCATGTGTCTTTGGA	CACCTTCCTTGTGTTCCGAT	59.0	281	INTERGENIC	--	--	--
Ca7_P4_20574766	Ca7	20574766	GTTT/GTTTT	GGGAGATCGGAATGCATAGA	ATGCGTGACGGTGCATAATA	60.0	276	INTERGENIC	--	--	--
Ca7_P4_20617780	Ca7	20617780	C/CAAT	GGCGAGGGATGGAATTTTAG	GAATTGGGACTTCTGCCGTA	60.8	777	INTERGENIC	--	--	--
Ca7_P4_20623367	Ca7	20623367	ATGTCTAAGGTGTC/ATGTC	CTTTGAAAATGGCCACATGA	ATTTAGATGACCGTCGCACC	59.5	486	INTERGENIC	--	--	--
Ca7_P4_20623409	Ca7	20623409	TAACAA/TAA	AGGTGTCGAAGGATCACCAC	CTCGAGGAGACGAGAAATGC	60.0	212	INTERGENIC	--	--	--
Ca7_P4_20624207	Ca7	20624207	TATTTAATTTAATTTAAT/TATTTAATTTAATTTAATTTAAT	ATTCCCCGTGCACAAAAATA	AATCCCGTTAACCCCGTAAC	60.2	390	INTERGENIC	--	--	--
Ca7_P4_20626314	Ca7	20626314	AAAA/AAAATAAA	TCAGTTATCGTCGTTGGTCG	TTCATCGAACCGACATCATC	59.7	361	INTERGENIC	--	--	--
Ca7_P4_20626429	Ca7	20626429	CTAATA/CTAATAATA	TCAGTTATCGTCGTTGGTCG	CGTGCAACATTTTTCTTCCA	59.7	623	INTERGENIC	--	--	--
Ca7_P4_20630370	Ca7	20630370	TAAAAAAA/TAAAAAA	ATCAGGCTGGTTTGATTTGG	CCAAAATGTGTGGTGTTCCA	59.9	527	INTERGENIC	--	--	--
Ca7_P4_2066755	Ca7	2066755	CAAAAAAAAAAA/CAAAAAAAAAAAA	TCGTTGGAGAGTTGGTGATG	TGGGATGGTAAGCATTTGAA	59.7	563	INTERGENIC	--	--	--

INDEL marker IDs	Chromosomes/ unanchored scaffolds	Physical positions (bp)	InDels (*Kabuli* reference genome-CDC Frontier/PII)	Forward primers (5'-3')	Reverse primers (5'-3')	Anneling temperature (0C)	Expected amplified product size (bp)	Structural annotation		Functional annotation	
								Sequence components of *kabuli* genome	*Kabuli* gene accession IDs	NCBI-KOG	TFs
Ca7_P4_20969765	Ca7	20969765	CTT/CT	TGAGTAATTGCCGCTCAGTG	TTGTCCACCATTATCGACCTC	60.0	103	INTERGENIC	--	--	--
Ca7_P4_21099731	Ca7	21099731	T/TGCGCAA	GAGAAATCAAAATTGCCCCA	AGTGCCTCCTGTTCCTGAGA	59.9	500	INTERGENIC	--	--	--
Ca7_P4_21151037	Ca7	21151037	AAATAATAATAATAATAATAATAATAATAATAATAA/AAATAATAATAATAATAATAATAATAATAATAA	CCCGGAGATGCGAGTACTAA	TTGGCACATCTACTCAACCG	60.2	566	INTERGENIC	--	--	--
Ca7_P4_21171384	Ca7	21171384	ATTTTTTTT/ATTTTTTTTT	GTTCGCCGTTTTATGTCGTT	TGTGGCTGTAAACCTGCAAA	60.0	581	INTERGENIC	--	--	--
Ca7_P4_21207750	Ca7	21207750	GCATCCCCA/GCATCCCCATCCCCA	TTTTATCCACCAAAAATTCAAAAA	GAATGAGCAAGAAAGACGGC	58.9	505	INTERGENIC	--	--	--
Ca7_P4_21264825	Ca7	21264825	TTATATATATATATATATATATATA/TTATATATATATATATATATATATATATATATA	GTCATTTGGAATGGCGACTT	TTGTCCCAATGTTACAGCCA	59.9	489	INTERGENIC	--	--	--
Ca7_P4_2128379	Ca7	2128379	TTCTCTCTCTCT/TTCTCTCTCT	TGCTTCATGCGTTTCAAGAC	CTCGTGCACAAAACCTTTGA	60.0	616	INTERGENIC	--	--	--
Ca7_P4_21372221	Ca7	21372221	GCC/GC	ACTTCTTTCATGTTTGGCCG	GCAACACGCACGAGTTACAC	60.1	476	INTERGENIC	--	--	--
Ca7_P4_21373697	Ca7	21373697	AATATATATATATATATATATA/AATATATATATATATATATA	TTTAAATTTGCATGTTAGTCGTCA	CCCGTATATGGAACAATGGG	58.8	620	INTERGENIC	--	--	--
Ca7_P4_21434053	Ca7	21434053	ATTTTTTTT/ATTTTTTTTT	TTTTTGTGGGCTAAGTGGAG	GAAGCACAAAGAATAAAAGCAACA	57.9	939	INTERGENIC	--	--	--

INDEL marker IDs	Chromosomes/ unanchored scaffolds	Physical positions (bp)	InDels (*Kabuli* reference genome-CDC Frontier/PII)	Forward primers (5'-3')	Reverse primers (5'-3')	Anneling temperature (0C)	Expected amplified product size (bp)	Structural annotation		Functional annotation	
								Sequence components of *kabuli* genome	*Kabuli* gene accession IDs	NCBI-KOG	TFs
Ca7_P4_21448520	Ca7	21448520	T/TATAATTATTA	CATGCAGTGCAATGTCCTTT	TGGTGCAGTGGCAATAAAAA	59.7	358	INTERGENIC	--	--	--
Ca7_P4_21598915	Ca7	21598915	TTATATATATATATATAT/TTATATATATATATAT	TTTCATGGTTCGCATCACAT	AACCCCATGTGTGAAATGCT	59.9	242	INTERGENIC	--	--	--
Ca7_P4_21670841	Ca7	21670841	A/AAGAT	TGACAAATCTTTGCAACCCA	ATTGCTCATTTCATGCCCTC	60.1	217	INTERGENIC	--	--	--
Ca7_P4_21675094	Ca7	21675094	AAA/AAACAA	TTCCCTCTTGGATCACAACC	CAACCTCTTCACGCATCAAA	59.9	644	INTERGENIC	--	--	--
Ca7_P4_21678026	Ca7	21678026	TTAAAATTTTAATATAAAATAAAATTTTA/TTAAAATTTTA	GAGCTTTGTTGGAACATGGA	ATAACCGTGCCAATAGGTGG	58.7	850	INTERGENIC	--	--	--
Ca7_P4_21685577	Ca7	21685577	TT/TTAT	TTTATAAATTGGCCCCATCG	TAGGGGGACCAAGAAACTGG	59.6	501	INTERGENIC	--	--	--
Ca7_P4_21728387	Ca7	21728387	CAAAA/CAAA	ATCTCTGCGCAATATCGCTT	AGCCGACTTTTCCTACCACA	60.0	674	INTERGENIC	--	--	--
Ca7_P4_21729097	Ca7	21729097	TAAAAA/TAAAA	TGCTCAAAAGGGGAAGAAAA	GCCACGTGGTTGAAAGAAGT	59.8	240	INTERGENIC	--	--	--
Ca7_P4_21729303	Ca7	21729303	T/TC	TTGCTCAAAAGGGGAAGAAA	TTGTTATTTTCTTTATGCCGTTG	59.8	426	INTERGENIC	--	--	--
Ca7_P4_21731192	Ca7	21731192	ATTTTTTTTT/ATTTTTTTT	TGATGGATACCCTTTTGAACG	ACCGCTCCAGCTGAGAATAA	59.8	838	INTERGENIC	--	--	--

INDEL marker IDs	Chromosomes/ unanchored scaffolds	Physical positions (bp)	InDels (*Kabuli* reference genome-CDC Frontier/PII)	Forward primers (5'-3')	Reverse primers (5'-3')	Anneling temperature (0C)	Expected amplified product size (bp)	Structural annotation		Functional annotation	
								Sequence components of *kabuli* genome	*Kabuli* gene accession IDs	NCBI-KOG	TFs
Ca7_P4_21772693	Ca7	21772693	AATATATATATATATATATATATA/AATATATATATATATATATATATA	CAAACATGTCCTCATGTCCA	TGATGGCTTGTGTCATTGGT	57.9	720	INTERGENIC	--	--	--
Ca7_P4_21786856	Ca7	21786856	CAAA/CAAAA	TGAAAAACGTGTTGCAGCTAA	TAGAAGGCTGCACAACTCCA	59.5	648	INTERGENIC	--	--	--
Ca7_P4_21801971	Ca7	21801971	GTTTTATTTTATTTTATTTTATTTTATTTTATTTTATTTTATTTTA/GTTTTATTTTATTTTATTTTATTTTATTTTATTTTATTTTA	GCTTCATTTGGAGCTTTTGC	TTGGGGATTTCCATTTGAGA	60.0	675	INTERGENIC	--	--	--
Ca7_P4_22015790	Ca7	22015790	T/TTCGG	ACCTCCGTCATCATTTTTCG	TTCCCCGAACACAGAAACTC	59.9	646	INTERGENIC	--	--	--
Ca7_P4_22036269	Ca7	22036269	GCTC/G	GCTCAGCCTTGAAATCGAAC	TTCATCATCATCTTCAAACAAAA	60.0	627	INTERGENIC	--	--	--
Ca7_P4_22036958	Ca7	22036958	GAAAAAAAAA/GAAAAAAAAAA	TGAAACATCAAATTGGGAAAAA	TTGATGGCAATGCTATTGGA	59.3	504	INTERGENIC	--	--	--
Ca7_P4_22038011	Ca7	22038011	C/CT	CTCCAAATTTCCAGCCAAAA	ACACGGACGGGTCTTGTATC	60.0	822	INTERGENIC	--	--	--
Ca7_P4_22039243	Ca7	22039243	ACC/AC	CGTTATTGCAACCACAACCA	CAATTCAACTCCGACGTGTG	60.4	622	INTERGENIC	--	--	--
Ca7_P4_22043317	Ca7	22043317	T/TG	TGGTTCAAATGCTGTAATCTTCTC	ATTCGCTCAATACACGGGTC	59.7	884	INTERGENIC	--	--	--
Ca7_P4_22045478	Ca7	22045478	ATTTTTTTT/ATTTTTTT	TGTGGTGTTCCCAAAAGACA	CAAAGCGAAGTGCAAAATGA	60.0	246	INTERGENIC	--	--	--

INDEL marker IDs	Chromosomes/ unanchored scaffolds	Physical positions (bp)	InDels (*Kabuli* reference genome-CDC Frontier/PII)	Forward primers (5'-3')	Reverse primers (5'-3')	Anneling temperature (0C)	Expected amplified product size (bp)	Structural annotation		Functional annotation	
								Sequence components of *kabuli* genome	*Kabuli* gene accession IDs	NCBI-KOG	TFs
Ca7_P4_22046891	Ca7	22046891	CATAATAATAATAATAATAATAATAATAATAATAATAATAATAAT/CATAATAATAATAATAATAATAATAATAATAATAATAATAATAATAAT	ATAATTTGCCTTTGCGGTTG	TTCTGTGAGCAAGGCTGATG	60.0	319	INTERGENIC	--	--	--
Ca7_P4_22046957	Ca7	22046957	CTATATATATATATATATATATAT/CTATATATATATATATATATAT	ATAATTTGCCTTTGCGGTTG	TTCTGTGAGCAAGGCTGATG	60.0	319	INTERGENIC	--	--	--
Ca7_P4_22046996	Ca7	22046996	TGCGCGC/TGCGC	ATAATTTGCCTTTGCGGTTG	ACCTGGTCGTAGATGCCACT	60.0	414	INTERGENIC	--	--	--
Ca7_P4_22091542	Ca7	22091542	GAAAAAAAAAAA/GAAAAAAAAAA	CCCCAACCCTCCATTCTATT	AATTGCAAGATGAGGGCAAC	60.0	347	INTERGENIC	--	--	--
Ca7_P4_22101651	Ca7	22101651	CTTTATT/CT	AACCCAAGTTCCAAATGACG	GAAGCCCAAGTTCCAAATGA	59.8	672	INTERGENIC	--	--	--
Ca7_P4_22126044	Ca7	22126044	TAAAAAAAAA/TAAAAAAAAAA	TGAAGCAACAAAGCTGATGAA	TTGTTGCAACCACTTCCAAA	59.6	518	INTERGENIC	--	--	--
Ca7_P4_22315085	Ca7	22315085	ATTTTTTTTTT/ATTTTTTTTT	TTTAAGCCCATTTTCCACCA	AGGTGCAAGTGTGTTCCTCC	60.3	836	INTERGENIC	--	--	--
Ca7_P4_22325946	Ca7	22325946	CACTCCGGACTGCGCGACTCCGGACTGC/CACTCCGGACTGC	CTTCAACATGCGGGTCATC	AAGATGCACGTGGGGTAGAC	60.1	413	INTERGENIC	--	--	--
Ca7_P4_22523165	Ca7	22523165	CCGG/CCGGACGG	ACCTTGTGGATGGCAGTTTC	TGGCATTGATGATGGCTTTA	60.0	328	INTERGENIC	--	--	--
Ca7_P4_22523183	Ca7	22523183	GGCG/GG	ACCTTGTGGATGGCAGTTTC	TGGCATTGATGATGGCTTTA	60.0	328	INTERGENIC	--	--	--

INDEL marker IDs	Chromosomes/ unanchored scaffolds	Physical positions (bp)	InDels (*Kabuli* reference genome-CDC Frontier/PII)	Forward primers (5'-3')	Reverse primers (5'-3')	Anneling temperature (0C)	Expected amplified product size (bp)	Structural annotation		Functional annotation	
								Sequence components of *kabuli* genome	*Kabuli* gene accession IDs	NCBI-KOG	TFs
Ca7_P4_22561436	Ca7	22561436	CAAAAAAAAAA/CAAAAAAAAAAA	AGGTTGTTGCATGGAGAAGG	AAAAGTCATACCCCGTGATCC	60.1	364	INTERGENIC	--	--	--
Ca7_P4_22569920	Ca7	22569920	ATATATT/A	TCTCTCGAAGTGTTTGGGCT	TGGACTGGCATGATTGGATA	60.0	811	INTERGENIC	--	--	--
Ca7_P4_22587401	Ca7	22587401	TATATATATATATATATATATATATAT/TATATATATATATATATATATATATATATAT	AAGGAACAAAACGTCATTTTCAA	AGTCAGACGCTACTCGGCAT	59.9	519	INTERGENIC	--	--	--
Ca7_P4_22960245	Ca7	22960245	AATATATATATATATATATATATATATATATATATATAT/AATATATATATATATATATATATATATATATATATATATAT	TTGGTATTGAACTCGGAGGC	CTTTCACGTTCGGTTACGGT	60.1	700	INTERGENIC	--	--	--
Ca7_P4_22969908	Ca7	22969908	CTT/CTTT	AATGTGCTGATCTGTGAGCG	CCTTGGAGTATTCTCGGCAG	60.0	894	INTERGENIC	--	--	--
Ca7_P4_23037556	Ca7	23037556	ATTTTTTTT/ATTTTT	TTGAGTTTCAAAACTGCCAAG	GGCTTGGGGCTCCTTACTAC	58.1	564	INTERGENIC	--	--	--
Ca7_P4_23075370	Ca7	23075370	AAATAATAATAATAATAATAATAATAATA/AAATAATAATAATAATAATAATAATAATAATAATAATA	AACGGATGACCCTCTCCTCT	TTTTGGTTGGGATTTTTGGA	60.1	812	INTERGENIC	--	--	--
Ca7_P4_23184353	Ca7	23184353	CTATATATATATATATAT/CTATATATATATATATAT	AGTGGTTGATTGTGGTTCGG	TTTTTCGGGACACTCCTTTG	60.8	596	INTERGENIC	--	--	--
Ca7_P4_23195329	Ca7	23195329	GATATATATATATATATATATATA/GATATATATATATATATATATATATATA	GCATGGCTTGATCTGATTTG	TAAATCGCGGGTATGAGGAC	59.2	547	INTERGENIC	--	--	--
Ca7_P4_23264424	Ca7	23264424	AATATATATATATATATATATAT/AATATATATATATATATAT	GCGACCCACTTGAAACAAAT	CCACCGATGCCTAACTCATT	60.0	169	INTERGENIC	--	--	--

INDEL marker IDs	Chromosomes/ unanchored scaffolds	Physical positions (bp)	InDels (*Kabuli* reference genome-CDC Frontier/PII)	Forward primers (5'-3')	Reverse primers (5'-3')	Anneling temperature (0C)	Expected amplified product size (bp)	Structural annotation		Functional annotation	
								Sequence components of *kabuli* genome	*Kabuli* gene accession IDs	NCBI-KOG	TFs
Ca7_P4_2327043	Ca7	2327043	CACACCCCAGACACCCCAG/CACACCCCAG	CTAAACCGGCAACCAGAGAA	TGTGTGGTGATGTGTCGATG	60.2	486	INTERGENIC	--	--	--
Ca7_P4_23425466	Ca7	23425466	AATATATATATATATATATATATATATATATATATATATATAT/AATATATATATATATATATATATATATATATATATATATATATATATATATATAT	GACCGAGTTGAGAAAAGATATGAGA	CGTAATTTTTCACGTTCCCTC	60.1	519	INTERGENIC	--	--	--
Ca7_P4_2349174	Ca7	2349174	ATTTTTTTTTTT/ATTTTTTTTTT	TGCCAAGTGGAATCACTTTTT	TCTTCGGATATGCAACGTGA	59.6	727	INTERGENIC	--	--	--
Ca7_P4_23780060	Ca7	23780060	TTATATATATATATAT/TTATATATATATAT	CGCTCGTCTATGCGATTGTA	TAAAATCTCCGCTCGCAACT	60.0	946	INTERGENIC	--	--	--
Ca7_P4_23797195	Ca7	23797195	AATATATATATATATATATATATATATA/AATATATATATATATATATATATATATATATA	AAGAAATCGCGAGTCACAGG	TTAATAGAACGCGCCAAAGG	60.4	652	INTERGENIC	--	--	--
Ca7_P4_23803981	Ca7	23803981	GTTTT/GTTT	GCACCGTGAGTAAATTCGGT	ATGTTTTGGATTTTGGCTCG	60.0	704	INTERGENIC	--	--	--
Ca7_P4_2380600	Ca7	2380600	ATTTAATTTGTTTAA/ATTTAATTTGTTTAATTTGTTTAA	CCACCATTTTTGATGACGTG	TGGAAAAACCACAAATATTACATTG	59.8	593	INTERGENIC	--	--	--
Ca7_P4_23868384	Ca7	23868384	GAAAAAAAAA/GAAAAAAAAAA	ATCAAGCCACTGGAGACACC	TTTTGAATGGAAGACGTCCC	60.1	375	INTERGENIC	--	--	--
Ca7_P4_23882859	Ca7	23882859	AATATATATATATATATATA/AATATATATATATATATATATATA	TATTTTGGATTTTGGCTCGG	ATGGGTTTCGCGTAGACAAC	59.9	400	INTERGENIC	--	--	--
Ca7_P4_23903739	Ca7	23903739	TAAATAA/TAAATAAAAATAA	TGAGCATGGAATTTGGTTGT	TGAATTCATGGCTAAGAAGCCT	59.0	592	INTERGENIC	--	--	--

INDEL marker IDs	Chromosomes/ unanchored scaffolds	Physical positions (bp)	InDels (*Kabuli* reference genome-CDC Frontier/PII)	Forward primers (5'-3')	Reverse primers (5'-3')	Anneling temperature (0C)	Expected amplified product size (bp)	Structural annotation		Functional annotation	
								Sequence components of *kabuli* genome	*Kabuli* gene accession IDs	NCBI-KOG	TFs
Ca7_P4_23907440	Ca7	23907440	AATATATATAT/AATATATATATAT	TTGGAGAATTCAAAAT AAGGCA	CGTGACAAAGCACGAA AGAA	60.0	513	INTERGENIC	--	--	--
Ca7_P4_24027767	Ca7	24027767	GATAATAATAATAATAATAATAATAA/ GATAATAATAATAATAATAATAATAA TAA	ACAATGGATGCTCACG ACAC	GTTTCCCATCCTCCCTT TGT	59.6	633	INTERGENIC	--	--	--
Ca7_P4_24037474	Ca7	24037474	ATT/ATTT	ATGTTGAAGTCGGGAA AACG	TCTTCTTGTGGCCGAC TCTT	60.0	741	INTERGENIC	--	--	--
Ca7_P4_24211988	Ca7	24211988	CATTATTATTATTATTATTATTATT/C ATTATTATTATTATTATTATTATTATT	ACTGGGGATGCAATTT TCTG	AATGGGTCAAAAGAGG AGGAA	59.9	650	INTERGENIC	--	--	--
Ca7_P4_24267624	Ca7	24267624	CTTTTTTTTTT/CTTTTTTTTTTT	TTCTCCTTGAAGCTGT CGGT	AACCAATCCCACATAAC CCA	60.0	639	INTERGENIC	--	--	--
Ca7_P4_24274526	Ca7	24274526	GAAAAAAAAAAA/GAAAAAAAAAA	AAGAGAGAGGGATGCA TGAGA	ATCATCCACCCTAGGA ACCC	59.0	869	INTERGENIC	--	--	--
Ca7_P4_24282647	Ca7	24282647	TAAAAAAAA/TAAAAAAA	GGAACGAGGGACATAT CAAGA	CGGTTTAGGCCCCTAC ATTT	59.0	592	INTERGENIC	--	--	--
Ca7_P4_2430437	Ca7	2430437	AATTATTATTATTATTATTATTATTAT TATTATTATTATTA/AATTATTATTAT TATTATTATTATTATTATTATTATTA	GCGGTTCGAATTTTGT GAAG	GAAGATCGAATCCCAC GATG	60.6	571	INTERGENIC	--	--	--
Ca7_P4_24347972	Ca7	24347972	TAAAA/TAAAAA	CTCTCATCTGAGGGCA AAGC	CGATTGAATTAAGCCC AGGA	60.1	356	INTERGENIC	--	--	--
Ca7_P4_24348355	Ca7	24348355	TAA/TAAA	TCCTGGGCTTAATTCA ATCG	CGTTCCTGAGGGAATT GTGT	60.0	378	INTERGENIC	--	--	--

INDEL marker IDs	Chromosomes/ unanchored scaffolds	Physical positions (bp)	InDels (*Kabuli* reference genome-CDC Frontier/PII)	Forward primers (5'-3')	Reverse primers (5'-3')	Anneling temperature (0C)	Expected amplified product size (bp)	Structural annotation		Functional annotation	
								Sequence components of *kabuli* genome	*Kabuli* gene accession IDs	NCBI-KOG	TFs
Ca7_P4_24348515	Ca7	24348515	CAA/CAAA	TCCTGGGCTTAATTCAATCG	CGTTCCTGAGGGAATTGTGT	60.0	378	INTERGENIC	--	--	--
Ca7_P4_24354887	Ca7	24354887	AATATATATAT/AATATATATATAT	CAATTCCCACAGGTACGCTT	GGAAGACCCTTGCAAAATCA	60.0	283	INTERGENIC	--	--	--
Ca7_P4_2438236	Ca7	2438236	TTATATATATATATATATATAT/TTATATATATATATATATATAT	CATGTTTGATACCATAATGGCG	TGGGGATTGTTTATCTAGAAGTGTC	60.1	341	INTERGENIC	--	--	--
Ca7_P4_24421160	Ca7	24421160	AATATATATATATATATATATA/AATATATATATATATA	AGGAAGCCCAAGTTCCAAAT	ACAGCCAACAACCACATCAA	59.9	469	INTERGENIC	--	--	--
Ca7_P4_24436102	Ca7	24436102	GTTATTATTATTATTATTATTATTATTATTATTATTATTATTAT/GTTATTATTATTATTATTATTATTATTATTATTATTAT	TGCGACGCTTGATCGTTAAT	AGTTTCGAAGCAATCCGAGA	61.7	465	INTERGENIC	--	--	--
Ca7_P4_24450388	Ca7	24450388	TTATATATATATATATATAT/TTATATATATATATATATAT	TTGTTAGTTTGACGAGGGCA	TGGGTGCTATTTGCAATCAA	59.3	380	INTERGENIC	--	--	--
Ca7_P4_24477362	Ca7	24477362	ATATATATATATATGT/AT	CAATTGAGTTGCGACGAGAG	TGAAGTTGGGAAATCCTTGC	59.6	434	INTERGENIC	--	--	--
Ca7_P4_24485265	Ca7	24485265	C/CAACTTT	GTCCCACCCTAGGAGCTTTC	CGGACCAAAAATTAAAGCGA	60.1	986	INTERGENIC	--	--	--
Ca7_P4_24485905	Ca7	24485905	GAAAAA/GAAAA	ATTCAATATTTCCCCCAGCC	TCACCCCAATTCCATTCATT	60.0	496	INTERGENIC	--	--	--
Ca7_P4_24486242	Ca7	24486242	CTTTTTTTTT/CTTTTTTTT	ATGAATGGAATTGGGGTGAA	GGGCATGGCAACCTAAAGTA	60.0	341	INTERGENIC	--	--	--

INDEL marker IDs	Chromosomes/ unanchored scaffolds	Physical positions (bp)	InDels (*Kabuli* reference genome-CDC Frontier/PII)	Forward primers (5'-3')	Reverse primers (5'-3')	Anneling temperature (0C)	Expected amplified product size (bp)	Structural annotation		Functional annotation	
								Sequence components of *kabuli* genome	*Kabuli* gene accession IDs	NCBI-KOG	TFs
Ca7_P4_24502290	Ca7	24502290	T/TAAGCA	AATTTGGCAGAACAGGGATG	AGAAAAGCAGGGGAAGCAAT	59.9	575	INTERGENIC	--	--	--
Ca7_P4_24535021	Ca7	24535021	GAATAATAATAATAATAATAATAATAATAATAATAATA/GAATAATAATAATAATAATAATAATAATAATAATAATA	CCGAGTCGGATCTATCAAAA	GCGATAGAAATGGGACCTGA	57.8	852	INTERGENIC	--	--	--
Ca7_P4_24651390	Ca7	24651390	TAAAAAAAAAA/TAAAAAAAAAAA	CAACCGTTTTGATGTTTTATTCA	GCTGATGGTTCTTTTGGGAA	58.9	847	INTERGENIC	--	--	--
Ca7_P4_24861630	Ca7	24861630	GACTT/GACTTACTT	TTCCATTTCCTCCACAAAGC	TCATTCCAGAAGGAGATGGG	60.1	548	INTERGENIC	--	--	--
Ca7_P4_25060530	Ca7	25060530	GTTTTTTTT/GTTTTTTTTT	TTGTCGATTTGGCACTTCAG	TGAATGTGGATCCCCTCAAT	59.8	327	INTERGENIC	--	--	--
Ca7_P4_2512120	Ca7	2512120	T/TAA	TCCCTAAAAATCAAAACAGGAA	ATCGACAAGATCACAAGGGC	57.8	770	INTERGENIC	--	--	--
Ca7_P4_25131144	Ca7	25131144	ATATTATTATTATTATTATTATTATTATTA/ATATTATTATTATTATTATTATTATTATTA	TTGTTAAAAACGTGTGCCGA	CGAAGACGATCATCCTTGGT	60.1	465	INTERGENIC	--	--	--
Ca7_P4_25170286	Ca7	25170286	TAAAAAA/TAAAAAAA	GTTTGTGGGAGCAGAGCTTC	CCCTTTCAACGCATCCTTTA	60.0	629	INTERGENIC	--	--	--
Ca7_P4_25219421	Ca7	25219421	CAAA/CAA	TGGGATATGGAAGGGTTGAG	CAGATTCCTACGGGAAACGA	59.7	202	INTERGENIC	--	--	--
Ca7_P4_2525335	Ca7	2525335	CAATACAATTGATTAATA/CAATACAATTGATTAATACAATTGATTAATA	TGGTGGAAAAATAGCATTTGG	CCTCTCCCGAATGACTCAAG	59.8	868	INTERGENIC	--	--	--

INDEL marker IDs	Chromosomes/ unanchored scaffolds	Physical positions (bp)	InDels (*Kabuli* reference genome-CDC Frontier/PII)	Forward primers (5'-3')	Reverse primers (5'-3')	Anneling temperature (0C)	Expected amplified product size (bp)	Structural annotation		Functional annotation	
								Sequence components of *kabuli* genome	*Kabuli* gene accession IDs	NCBI-KOG	TFs
Ca7_P4_25342809	Ca7	25342809	TAAAAAAAA/TAAAAAAAAA	CATTCAACAGCCACATGACC	CGGTACCGCATTCGAGTATT	60.0	642	INTERGENIC	--	--	--
Ca7_P4_25411773	Ca7	25411773	CTATATATATATATATATATATATATA/CTATATATATATATATATATATATATATATATA	GAGGATGCAGTTCCAGAAGG	GCTTGCTAATCGTTTATCACCA	59.8	642	INTERGENIC	--	--	--
Ca7_P4_25436582	Ca7	25436582	GAAAAAAA/GAAAAAAAA	ATTTTTGAGAGCCAAGCCAA	GAACGTGTTCGGTAAGGGTG	59.8	786	INTERGENIC	--	--	--
Ca7_P4_25540307	Ca7	25540307	CTTTT/CTTT	CCTCAGATGAGAGCCAGGAC	GAGCGCTATTACAGTTCGGC	59.9	472	INTERGENIC	--	--	--
Ca7_P4_25556203	Ca7	25556203	TAAA/TAA	TGCATCAAAACTCCAATCCA	ATTGAATGGAAGATGGTGGG	60.0	236	INTERGENIC	--	--	--
Ca7_P4_25626892	Ca7	25626892	CGG/CGGG	TTGGCCAAGTCCCTGTTTAC	CCCGTGATAGGCAGTACGTT	60.0	420	INTERGENIC	--	--	--
Ca7_P4_25717539	Ca7	25717539	TCAACCTATCAGGCCTACAACCTATCAGGCCTA/TCAACCTATCAGGCCTA	GCCTGAGCCTGACCTACAAC	TAGGGGCCTATGACCTGATG	59.9	543	INTERGENIC	--	--	--
Ca7_P4_25916920	Ca7	25916920	TAAAAAAAAAA/TAAAAAAAAA	GGAGGCATTGTTTCCTCAAA	TACCGATTAGTACCCTGGCG	60.1	732	INTERGENIC	--	--	--
Ca7_P4_26035041	Ca7	26035041	TAAAAAAAA/TAAAAAAAAA	TAAAGAGATTCGCAGCGGTT	CATGTTTTCGAAGCTCACCA	60.0	407	INTERGENIC	--	--	--
Ca7_P4_26075420	Ca7	26075420	TTATATATATAT/TTATATATATATAT	TATTTTGGATTTTGGCTCGG	TTTGGATCAAGGAGAAACGG	59.9	584	INTERGENIC	--	--	--

INDEL marker IDs	Chromosomes/ unanchored scaffolds	Physical positions (bp)	InDels (*Kabuli* reference genome-CDC Frontier/PII)	Forward primers (5'-3')	Reverse primers (5'-3')	Anneling temperature (0C)	Expected amplified product size (bp)	Structural annotation		Functional annotation	
								Sequence components of *kabuli* genome	*Kabuli* gene accession IDs	NCBI-KOG	TFs
Ca7_P4_26086416	Ca7	26086416	GTATATATATATATATATATATATAT/ GTATATATATATATATATATAT	TGAAAAGACGAGCAACAAGG	CTGCCAAAAGCCCTCATAAA	59.0	267	INTERGENIC	--	--	--
Ca7_P4_26095745	Ca7	26095745	GAAAAAAAAA/GAAAAAAAAAA	GTGGCGTCTCACTGTTGAAA	TGCTTTACGGAAGAAACAATCA	59.9	500	INTERGENIC	--	--	--
Ca7_P4_26158210	Ca7	26158210	GTATATATATATATATATATATATATATAT/GTATATATATATATATATAT	TTGTTGACACCGAGTTAGCG	TTTTCGCAAAAACTCAAAGGA	59.9	174	INTERGENIC	--	--	--
Ca7_P4_26160184	Ca7	26160184	AG/A	GTGGCTACCTTCTTGGGTGA	TTTCATCGCCGAAACCTATC	60.1	141	INTERGENIC	--	--	--
Ca7_P4_26221720	Ca7	26221720	AATATATATATATATATATAT/AATATATATATATAT	CTTGGGAATTGGAGAGCAAG	GGGTTTCTTTCCTTGAAGCC	59.8	671	INTERGENIC	--	--	--
Ca7_P4_26228410	Ca7	26228410	GAAAAAAAAAA/GAAAAAAAAAAAA	ATGGTGCAAAATGGTGACAA	GAAAGTCGGTGCATGGAAGT	59.8	304	INTERGENIC	--	--	--
Ca7_P4_26233274	Ca7	26233274	TAAAAAAA/TAAAAAAAA	TACGCCATCCAAAAATGTGA	CCCTCATCCGAAAAGATGAA	59.9	945	INTERGENIC	--	--	--
Ca7_P4_26235537	Ca7	26235537	TTATATATATATA/TTATATATATATATATA	TGCGACCTCAGTTTCTCAAA	GGGGTCATAGCATCCCCTAT	59.6	157	INTERGENIC	--	--	--
Ca7_P4_26277638	Ca7	26277638	AT/A	GCCCTCAGATGAGAGTCAGG	GGAAGGCTTGTCGACTTGAG	59.9	555	INTERGENIC	--	--	--
Ca7_P4_26331505	Ca7	26331505	AATATATATAT/AATATATATATATATATATATATAT	TCTTTAGTAGCCCGCCAAGA	CGTTTTGGGTCGTTGTTACC	60.0	534	INTERGENIC	--	--	--

INDEL marker IDs	Chromosomes/ unanchored scaffolds	Physical positions (bp)	InDels (*Kabuli* reference genome-CDC Frontier/PII)	Forward primers (5'-3')	Reverse primers (5'-3')	Anneling temperature (0C)	Expected amplified product size (bp)	Structural annotation		Functional annotation	
								Sequence components of ***kabuli*** genome	***Kabuli*** gene accession IDs	NCBI-KOG	TFs
Ca7_P4_26428559	Ca7	26428559	GAAAAAAAAAAA/GAAAAAAAAAA	CGACCAATAAAATTGCCTCAA	AAGATGCGTTGAGAAGCGAT	60.0	827	INTERGENIC	--	--	--
Ca7_P4_26662956	Ca7	26662956	CAA/CA	TCCAAGCTTCCTTCAATGGT	CTCAAAAGCGCTCCAATTTC	59.7	542	INTERGENIC	--	--	--
Ca7_P4_26696023	Ca7	26696023	T/TAAGGGG	CGTACACATTCCGACGATTG	CCGGCTTAGCTACGATCTTG	60.0	635	INTERGENIC	--	--	--
Ca7_P4_26703143	Ca7	26703143	AGG/AG	CGACTCCAACTACCGTCCAT	TTGGAGAGCGCAGTACAATG	60.0	483	INTERGENIC	--	--	--
Ca7_P4_26758487	Ca7	26758487	TGGGG/TGGGGG	TGGAGTGGGGTACCTAGTGG	CATCAAACCCACACACAACC	59.8	196	INTERGENIC	--	--	--
Ca7_P4_26795611	Ca7	26795611	AA/AAATAATCA	TCAGCAAAATTGTAACACCCC	CTTGGGCTTTACCCATGAAA	59.9	690	INTERGENIC	--	--	--
Ca7_P4_26835592	Ca7	26835592	CG/CGCTCACAG	GAGCAGCACAAGTCAAACGA	GATGGAGACCAGAAACGCAT	60.2	512	INTERGENIC	--	--	--
Ca7_P4_26962939	Ca7	26962939	ATTTTTTTT/ATTTTTTT	TCGAAACTTTCAAATGCAACC	CCATTCTGATTTCACACCCC	60.1	232	INTERGENIC	--	--	--
Ca7_P4_27031473	Ca7	27031473	TTCTCTCTCTCTCTCTCTCTCTCTCTCTCTCTCTCTCTC/TTCTCTCTCTCTCTCTCTCTCTCTCTCTCTCTCTCTCTCTCTCTC	TCTTCCTTCCTTGCTCTCCA	GACTTGGCATGTCATGATGG	60.1	551	INTERGENIC	--	--	--
Ca7_P4_27039821	Ca7	27039821	TTATATATATATATATATATATATAT/TTATATATATATATATATATATAT	CTTCTGCAGCACTTCCTCCT	ACCCAAGTTTTAAATGGGCA	59.7	732	INTERGENIC	--	--	--

INDEL marker IDs	Chromosomes/ unanchored scaffolds	Physical positions (bp)	InDels (*Kabuli* reference genome-CDC Frontier/PII)	Forward primers (5'-3')	Reverse primers (5'-3')	Anneling temperature (0C)	Expected amplified product size (bp)	Structural annotation		Functional annotation	
								Sequence components of *kabuli* genome	*Kabuli* gene accession IDs	NCBI-KOG	TFs
Ca7_P4_27043217	Ca7	27043217	T/TC	TGCGCATTCAGACTCAAAAA	TCATATGCCCAGATTGATCG	60.5	774	INTERGENIC	--	--	--
Ca7_P4_27108973	Ca7	27108973	TTATATATATATATATATATA/TTATATATATATATATATATATATA	GAAACAATCGATTTTGATCCC	CATTGGTTTTGTGAGAGGGAA	58.4	494	INTERGENIC	--	--	--
Ca7_P4_27341260	Ca7	27341260	A/AG	CACGATCCACACCAAAACAC	TCTCCCTCCTTCTCCCATTT	59.9	382	INTERGENIC	--	--	--
Ca7_P4_27346895	Ca7	27346895	TTATATATATATATATATATATAT/TTATATATATATATATATATATATATAT	TGCTTGATTGCTTGAGTTCC	CGCGCAGATTGTCAAATAGA	59.0	641	INTERGENIC	--	--	--
Ca7_P4_27365034	Ca7	27365034	CTTT/CTTTT	TATGGTGCTGTGAAAAGGCA	GCAAGAGATGGAAAATGGGA	60.3	693	INTERGENIC	--	--	--
Ca7_P4_27396861	Ca7	27396861	TATAATAATAATAATACTAATAATAATAATAATAATAATA/TATAATAATAATAATAATACTAATAATAATAATAATAATAATA	AACAATTGATTAGTGGCGCA	TGCTCTTTTAGCGTCGGTTT	59.2	427	INTERGENIC	--	--	--
Ca7_P4_27521328	Ca7	27521328	CAAA/CAA	GCCAAATTCCCTCTTTCTCC	CCTTTCCACACAACCACAGA	60.0	393	INTERGENIC	--	--	--
Ca7_P4_27745203	Ca7	27745203	TTATATATATATATATATATATATATATATAT/TTATATATATATATATATATATATATATATATAT	TCAAAATGCCCTACACTCAAAA	GTCACTTCCTGGACCTACGG	59.6	894	INTERGENIC	--	--	--
Ca7_P4_27761690	Ca7	27761690	TTATATATATATATATATATATATAT/TTATATATATATATATATATATAT	AAATTCGCAGCACTTACGCT	GTTTGTGAGTAAGCTCGCCC	60.0	873	INTERGENIC	--	--	--
Ca7_P4_27818786	Ca7	27818786	GAAAAAAAAAA/GAAAAAAAAAAA	CAAAAGGGCAACATGCCTAT	GCAAGATCGCATTCTTGTCA	60.0	454	INTERGENIC	--	--	--

INDEL marker IDs	Chromosomes/ unanchored scaffolds	Physical positions (bp)	InDels (*Kabuli* reference genome-CDC Frontier/PII)	Forward primers (5'-3')	Reverse primers (5'-3')	Anneling temperature (0C)	Expected amplified product size (bp)	Structural annotation		Functional annotation	
								Sequence components of *kabuli* genome	*Kabuli* gene accession IDs	NCBI-KOG	TFs
Ca7_P4_27833544	Ca7	27833544	C/CCGATCCCTT	ATGACGGTGATTTCGGTTTC	GTTTGCTTTCCTTTTGGTGC	59.8	706	INTERGENIC	--	--	--
Ca7_P4_27834486	Ca7	27834486	TAAAAAAAAA/TAAAAAAAAAA	ATTAAAACGGAAAAGGCGCT	TCCAACGATCGATGTCTACG	60.1	330	INTERGENIC	--	--	--
Ca7_P4_27875250	Ca7	27875250	GAAAAAAAA/GAAAAAAAAA	GGTTGATTATCGTCCGCTGT	CCTATTTCTCTGCTTTGGCG	60.0	420	INTERGENIC	--	--	--
Ca7_P4_27875295	Ca7	27875295	TTCT/TTCTCT	GGTTGATTATCGTCCGCTGT	CCTATTTCTCTGCTTTGGCG	60.0	420	INTERGENIC	--	--	--
Ca7_P4_2789203	Ca7	2789203	TTATATATATATATATATATATA/TTATATATATATATATATATATATA	GAATTAGGCCGCTATGTTCA	CCATGCTTCAAAAGCTGACA	57.9	842	INTERGENIC	--	--	--
Ca7_P4_27979863	Ca7	27979863	TGGG/TGGGG	ACCCAATTTGTCGTGGTGAT	TGATCCCCTCACTTAATGCC	60.1	251	INTERGENIC	--	--	--
Ca7_P4_2808679	Ca7	2808679	GAAAAAAAAAA/GAAAAAAAAAAAA	ACTTCCCTGTGGAACGACAC	ATATGCACGGAACGAAAACC	60.0	431	INTERGENIC	--	--	--
Ca7_P4_28089294	Ca7	28089294	GAAAAA/GAAAAAA	CGTAAAGTCCACTTCCGTTTC	CAACAGATGTTGTGCGTGTG	58.8	500	INTERGENIC	--	--	--
Ca7_P4_28129563	Ca7	28129563	CAAAAAAAAAAAAA/CAAAAAAAAAAAAAA	TCTTCACCAAAACAAACCCA	GCATCGTAAAGTTGCACATCA	59.0	835	INTERGENIC	--	--	--
Ca7_P4_28189950	Ca7	28189950	CTTT/CTTTT	CTCAACATGCATGCAAAACC	TCCCAACCCAAATAAACCAA	60.1	602	INTERGENIC	--	--	--
[truncated: 411,883 more chars]
